# Supplementary material for: Systematic review with meta-analysis of the epidemiological evidence in the 1900s relating smoking to lung cancer
Source: BMC Cancer. 2012 Sep 3;12:385. doi: 10.1186/1471-2407-12-385 (PMC3505152; doi:10.1186/1471-2407-12-385)
Supplement: Additional file 5 — Detailed Analysis Tables (Individual file names as described in Additional file 1: Methods, Table1). [file 1471-2407-12-385-S5.zip › PDF/2C.pdf]

Table 2C1 -

IESLC - Meta-anal of Ever Smoking (or Current if Ever not available), Any prod (or Cigs if Any not avail)
Squamous

This analysis is restricted to results for:
1) Non-dose-response data
2) Results complete enough for use in metaanalysis

Within each study, results are then selected (in the following order of preference, within each sex) for:
3) SMKSTA: ever smokers, current smokers
4) PRODUCT: all/unspec, cigarettes regardless of other products, cigarettes only
5) CIGTYPE: all/unspecified, MC regardless of HR, MC only
6) DENOM: never smoked anything, never smoked cigarettes, (never +1 = +long term ex, +2 = +amount unknown, +3 = never cigs+long term ex)
7) Followup period (YF, prospective studies): whole study (coded as 0) or longest available
8) LCtype: squamous or nearest available, but not adeno. (q = squamous, s = small, a = adeno, KI = Kreyberg I, u = undifferentiated)
9) Race: all or nearest available, otherwise by race (wh or w = white, bl or b = black, hi = hispanic ch = chinese, jap = japanese, haw = hawaiian, w+o = white + oriental, sca = scandinavian, as = asian)
10) For overlapping studies: principal rather than subsidiary studies
Finally by Age: whole study (coded as 0) if available, otherwise by widest available age group
and then for single sex results (m, f) in preference to combined sex results (c).

Results adjusted (AD) for the most potential confounders are then chosen in Sections -1 to -3
and results adjusted for the least confounders in Sections -4 to -6. (Those least adjusted results which
actually differ from the most adjusted as marked 'x' in column X in Section -4)
(Results adjusted for an unknown number of confounder(s) are coded as 20.)

Section -7 shows excluded studies, together with the stage (as above) at which no qualifying
results were found.

Section -8 lists the potentially overlapping studies which have been included (1=principal, 2=subsidiary).

Section -9 lists any results which would have been included in preference except that they had data not complete
enough for use in meta-analysis, with their significance (yes/no), if known, and any further comment as entered
on the database.

In addition to those mentioned above, the following fields, levels and abbreviations are used:
* or nk = not known, n = no, y = yes, ot = other
ev = ever, cu = current, nev = never
all/unspec = all or unspecified, cig+/-ot = cigarettes irrespective of other products (cigar, pipe etc)
MC = manufactured cigarettes, HR = hand-rolled cigarettes
REF: 6-character study reference
NRR: number of the RR on the database within the study
ST : study type (CC = case control, pr or prosp = prospective)
NLC: number of lung cancer cases in whole study
R : risky occupational population (n = no, m = mining, o = other risky)
VB : national cigarette type (V = at least 75% Virginia, bl = at least 75% blended, ot = other)
P : any proxy use
H : full histological confirmation
De : derivation of RR/CI (or = original, st = standard method, ot = other method of estimation)

Table 2C1 - 1

IESLC - Meta-anal of Ever Smoking (or Current if Ever not available), Any prod (or Cigs if Any not avail)
Squamous
Most adjusted

REF	NRR	SEX	AGEL	AGEH	RACE	YF	LC TYPE	LOC	START	ST	NLC	R	VB	P	H	AD	SM	PRODUCT	DENOM	De
ABRAHA	1	m	0	0	all	0	q	Eu:est	1975	pr	571	n	bl	n	n	0	ev	all/unsp	nev any	ot
ABRAHA	4	f	0	0	all	0	q	Eu:est	1975	pr	571	n	bl	n	n	0	ev	all/unsp	nev any	ot
ALDERS	52	m	0	0	all	-	q	Eu:UK	1977	CC	1448	n	V	n	n	2	ev	all/unsp	nev any	or
ALDERS	55	f	0	0	all	-	q	Eu:UK	1977	CC	1448	n	V	n	n	2	ev	all/unsp	nev any	or
ANDERS	10	f	0	0	all	0	q	NAmer	1986	pr	343	n	bl	n	n	0	ev	cig+/-ot	nev cigs	st
BAND	5	m	0	0	all	-	q	NAmer	1983	CC	2831	n	V	y	y	2	ev	cig only	nev any	ot
BARBON	127	m	0	0	all	-	q	Eu:wst	1979	CC	755	n	bl	y	y	3	ev	all/unsp	nev any	ot
BECHER	11	f	0	0	all	-	q+s	Eu:Ger	1985	CC	194	n	bl	n	y	1	ev	all/unsp	nev any	or
BOUCOT	141	m	0	0	all	0	q	NAmer	1951	pr	121	n	bl	n	n	2	cu	cig only	nev any	ot
BRESLO	36	c	0	0	all	-	not a	NAmer	1949	CC	518	n	bl	n	y	0	ev	all/unsp	nev+1	st
BROWN2	6	m	0	0	wh	-	q	NAmer	1984	CC	14596	n	bl	n	y	2	ev	cig+/-ot	nev cigs	or
BROWN2	5	f	0	0	wh	-	q	NAmer	1984	CC	14596	n	bl	n	y	2	ev	cig+/-ot	nev cigs	or
BUFFLE	49	m	0	0	wh	-	q	NAmer	1976	CC	943	n	bl	y	n	0	ev	cig+/-ot	nev cigs	ot
BUFFLE	62	f	0	0	w-hi	-	q	NAmer	1976	CC	943	n	bl	y	n	0	ev	cig+/-ot	nev cigs	st
BYERS1	1	m	0	0	wh	-	q	NAmer	1957	CC	1002	n	bl	n	n	0	ev	cig+/-ot	nev cigs	st
CHAN	11	m	0	0	all	-	q+s	As:HK	1976	CC	397	n	bl	n	n	0	ev	all/unsp	nev any	st
CHAN	15	f	0	0	all	-	q+s	As:HK	1976	CC	397	n	bl	n	n	0	ev	all/unsp	nev any	st
CHOI	62	m	0	0	all	-	q	As:oth	1985	CC	375	n	bl	n	n	0	ev	cig+/-ot	nev cigs	st
CHOI	64	f	0	0	all	-	q	As:oth	1985	CC	375	n	bl	n	n	0	ev	cig+/-ot	nev cigs	st
COMSTO	66	m	0	0	all	-	q	NAmer	1975	ot	258	n	bl	n	n	0	ev	cig+/-ot	nev cigs	st
COMSTO	78	f	0	0	all	-	q	NAmer	1975	ot	258	n	bl	n	n	0	ev	cig+/-ot	nev cigs	ot
CORREA	35	c	0	0	all	-	q+s	NAmer	1979	CC	1359	n	bl	y	n	1	ev	cig+/-ot	nev cigs	or
CPSI	403	m	0	0	all	2	q	NAmer	1959	pr	5138	n	bl	n	n	1	cu	cig only	nev any	ot
CPSI	405	f	0	0	all	2	q	NAmer	1959	pr	5138	n	bl	n	n	1	cu	cig only	nev any	ot
CPSII	114	m	0	0	all	2	q	NAmer	1982	pr	3229	n	bl	n	n	1	cu	cig only	nev any	ot
CPSII	117	f	0	0	all	2	q	NAmer	1982	pr	3229	n	bl	n	n	1	cu	cig+/-ot	nev cigs	ot
DAMBER	33	m	0	0	all	-	q	Eu:Sca	1972	CC	579	n	bl	y	n	1	ev	all/unsp	nev any	or
DESTE2	16	m	0	0	all	-	q	SCAmer	1993	CC	463	n	bl	n	n	2	ev	all/unsp	nev any	or
DOLL	86	m	0	0	all	-	KI	Eu:UK	1948	CC	1465	n	V	n	n	1	ev	all/unsp	nev any	ot
DOLL	88	f	0	0	all	-	KI	Eu:UK	1948	CC	1465	n	V	n	n	1	ev	all/unsp	nev any	ot
DORGAN	113	m	0	0	wh	-	q	NAmer	1980	CC	2026	n	bl	y	y	2	ev	cig+/-ot	nev any	or
DORGAN	98	f	0	0	all	-	q	NAmer	1980	CC	2026	n	bl	y	y	3	ev	cig+/-ot	nev any	or
DORN	338	m	0	0	wh	8	q	NAmer	1954	pr	5097	n	bl	n	n	1	cu	cig only	nev any	ot
DOSEME	3	m	0	0	all	-	q	Eu:bal	1979	CC	1210	n	bl	n	n	2	ev	cig+/-ot	nev cigs	or
ENGELA	62	m	0	0	all	0	q	Eu:Sca	1964	pr	435	n	bl	n	n	7	ev	cig+/-ot	nev cigs	ot
FAN	3	c	0	0	all	-	q	As:Chi	1990	CC	403	n	ot	y	n	0	ev	cig+/-ot	nev cigs	ot
GAO	2	m	0	0	all	-	q	As:Chi	1984	CC	1405	n	ot	n	n	2	ev	cig+/-ot	nev cigs	or
GAO	12	f	0	0	all	-	q	As:Chi	1984	CC	1405	n	ot	n	n	2	ev	cig+/-ot	nev cigs	or
GER	13	c	0	0	all	-	q+s	As:oth	1990	CC	141	n	ot	y	n	10	ev	all/unsp	nev any	ot
HAENSZ	1	f	0	0	all	-	q+u	NAmer	1955	CC	158	n	bl	n	y	2	ev	all/unsp	nev any	ot
HAMMON	60	m	0	0	wh	0	not a	NAmer	1952	pr	448	n	bl	n	n	1	ev	all/unsp	nev any	ot
HEGMAN	2	c	0	0	all	-	q	NAmer	1989	CC	282	n	bl	y	y	0	ev	all/unsp	nev any	st
HINDS	23	f	0	0	o	-	q+s	NAmer	1968	CC	292	n	bl	n	n	3	ev	all/unsp	nev any	st
ISHIMA	6	c	0	0	all	-	q	As:Jap	1961	CC	180	n	bl	y	y	5	ev	all/unsp	nev any	st
JAHN	46	m	0	0	all	-	q	Eu:Ger	1988	CC	1004	n	bl	n	n	0	ev	all/unsp	nev any	st
JAIN	48	m	0	0	all	-	q	NAmer	1981	CC	845	n	V	y	n	2	ev	cig+/-ot	nev cigs	or
JAIN	43	f	0	0	all	-	q	NAmer	1981	CC	845	n	V	y	n	2	ev	cig+/-ot	nev cigs	or
JEDRYC	54	m	0	0	all	-	q	Eu:est	1980	CC	1630	n	bl	y	n	3	ev	cig+/-ot	nev any	ot
JOLY	54	m	0	0	all	-	q	SCAmer	1978	CC	826	n	bl	n	n	0	ev	cig+/-ot	nev any	st
JOLY	52	f	0	0	all	-	q	SCAmer	1978	CC	826	n	bl	n	n	0	ev	cig+/-ot	nev any	st
JUSSAW	23	m	0	0	all	-	KI	As:Ind	1964	CC	792	n	V	n	n	0	ev	all/unsp	nev any	st
KATSOU	37	f	0	0	all	-	KI	Eu:bal	1987	CC	101	n	bl	n	n	1	ev	all/unsp	nev any	ot
KHUDER	24	m	0	0	all	-	q	NAmer	1985	CC	482	n	bl	n	y	0	ev	cig+/-ot	nev cigs	ot
KIHARA	26	c	0	0	jap	-	q	As:Jap	1991	CC	440	n	bl	n	n	0	ev	all/unsp	nev any	st
KOO	6	f	0	0	all	-	q+s	As:HK	1981	CC	200	n	bl	n	n	0	ev	all/unsp	nev any	st
KREYBE	4	m	0	0	all	-	KI	Eu:Sca	1948	CC	300	n	bl	n	y	1	ev	all/unsp	nev any	ot
KREYBE	25	f	0	0	all	-	KI	Eu:Sca	1948	CC	300	n	bl	n	y	1	ev	all/unsp	nev any	ot
LAMTH	1	f	0	0	ch	-	q	As:HK	1983	CC	445	n	bl	n	n	0	ev	all/unsp	nev any	or
LAMWK	2	f	0	0	ch	-	q	As:HK	1981	CC	163	n	bl	n	n	0	ev	all/unsp	nev any	st
LAMWK2	1	m	0	0	all	-	q	As:HK	1976	CC	480	n	bl	n	n	0	ev	all/unsp	nev any	st
LAMWK2	5	f	0	0	all	-	q	As:HK	1976	CC	480	n	bl	n	n	0	ev	all/unsp	nev any	st
LOMBA2	2	f	0	0	all	-	q+u	NAmer	1960	CC	225	n	bl	n	n	0	ev	cig+/-ot	nev cigs	st
LUBIN	33	m	0	0	all	-	KI	As:Chi	1984	CC	427	m	ot	y	n	0	ev	all/unsp	nev any	st
LUBIN2	145	m	0	0	all	-	q	Eu:mul	1976	CC	7804	n	bl	n	y	0	ev	cig+/-ot	nev any	st
LUBIN2	165	f	0	0	all	-	q	Eu:mul	1976	CC	7804	n	bl	n	y	0	ev	cig+/-ot	nev any	st
LUO	8	c	0	0	all	-	q	As:Chi	1990	CC	102	n	ot	n	y	20	ev	cig+/-ot	nev cigs	or
MATOS	67	m	0	0	all	-	q	SCAmer	1994	CC	200	n	bl	n	n	2	ev	cig+/-ot	nev any	ot
MATSUD	11	m	0	0	all	-	q	As:Jap	1965	CC	179	n	bl	n	n	0	ev	cig+/-ot	nev cigs	st
NOU	1	m	0	0	all	-	q	Eu:Sca	1971	CC	273	n	bl	y	n	0	ev	all/unsp	nev any	st
NOU	6	f	0	0	all	-	q	Eu:Sca	1971	CC	273	n	bl	y	n	0	ev	all/unsp	nev any	st
ORMOS	8	m	0	0	all	-	q	Eu:est	1947	CC	119	n	bl	y	y	0	ev	cig+/-ot	nev any	st
OSANN	43	m	0	0	all	-	q	NAmer	1984	CC	1986	n	bl	n	n	2	ev	cig+/-ot	nev cigs	or

Table 2C1 - 1

IESLC - Meta-anal of Ever Smoking (or Current if Ever not available), Any prod (or Cigs if Any not avail)
Squamous
Most adjusted

REF	NRR	SEX	AGEL	AGEH	RACE	YF	LC TYPE	LOC	START	ST	NLC	R	VB	P	H	AD	SM	PRODUCT	DENOM	De
OSANN	44	f	0	0	all	-	q	NAmer	1984	CC	1986	n	bl	n	n	2	ev	cig+/-ot	nev cigs	or
OSANN2	25	f	0	0	all	-	KI	NAmer	1964	ot	217	n	bl	n	y	1	ev	cig+/-ot	nev cigs	or
PEZZOT	6	m	0	0	all	-	q	SCAmer	1987	CC	215	n	bl	n	y	0	ev	cig only	nev cigs	ot
SCHWAR	10	m	40	54	wh	-	q	NAmer	1984	CC	5588	n	bl	y	y	0	ev	cig+/-ot	nev cigs	st
SCHWAR	9	m	40	54	bl	-	q	NAmer	1984	CC	5588	n	bl	y	y	0	ev	cig+/-ot	nev cigs	st
SCHWAR	18	f	40	54	wh	-	q	NAmer	1984	CC	5588	n	bl	y	y	0	ev	cig+/-ot	nev cigs	ot
SCHWAR	17	f	40	54	bl	-	q	NAmer	1984	CC	5588	n	bl	y	y	0	ev	cig+/-ot	nev cigs	ot
SEOW	3	f	0	0	ch	-	q	As:oth	1997	CC	153	n	bl	n	y	0	ev	cig+/-ot	nev cigs	st
SIEMIA	7	m	0	0	all	-	q	NAmer	1979	CC	857	n	V	y	y	7	ev	cig+/-ot	nev cigs	or
SOBUE	97	m	0	0	all	-	q	As:Jap	1986	CC	1376	n	bl	n	y	1	ev	cig+/-ot	nev cigs	ot
SOBUE	107	f	0	0	all	-	q	As:Jap	1986	CC	1376	n	bl	n	y	1	ev	cig+/-ot	nev cigs	ot
SOBUE2	1	m	0	0	all	-	q	As:Jap	1965	CC	2083	n	bl	n	n	2	cu	cig+/-ot	nev any	or
SOBUE2	5	f	0	0	all	-	q	As:Jap	1965	CC	2083	n	bl	n	n	2	cu	cig+/-ot	nev any	or
STASZE	12	m	0	0	all	-	q	Eu:est	1954	CC	281	n	bl	n	y	0	ev	all/unsp	nev any	ot
STASZE	38	f	0	0	all	-	q	Eu:est	1954	CC	281	n	bl	n	y	0	ev	all/unsp	nev any	ot
STAYNE	3	m	0	0	all	-	q	NAmer	1969	CC	420	n	bl	n	n	0	ev	all/unsp	nev any	st
SUZUK2	15	c	0	0	all	-	q	SCAmer	1991	CC	123	n	bl	n	y	3	ev	all/unsp	nev any	or
SVENSS	72	f	0	0	all	-	q	Eu:Sca	1983	CC	210	n	bl	n	n	1	ev	all/unsp	nev any	ot
TIZZAN	18	c	0	0	all	-	q+u	Eu:wst	1959	CC	1358	n	bl	n	n	0	ev	all/unsp	nev any	st
TOKARS	10	c	0	0	all	-	q	Eu:est	1966	ot	162	o	bl	n	y	3	ev	all/unsp	nev any	or
TSUGAN	13	m	0	0	all	-	q	As:Jap	1976	CC	134	n	bl	n	y	0	ev	all/unsp	nev any	ot
WAKAI	74	m	0	0	all	-	q	As:Jap	1988	CC	333	n	bl	n	y	1	ev	all/unsp	nev any	ot
WAKAI	80	f	0	0	all	-	q	As:Jap	1988	CC	333	n	bl	n	y	1	ev	all/unsp	nev any	ot
WU	32	f	0	0	wh	-	q	NAmer	1981	CC	220	n	bl	n	y	2	ev	all/unsp	nev any	ot
WUWILL	9	f	0	0	all	-	q	As:Chi	1985	CC	965	n	ot	n	n	3	ev	cig+/-ot	nev cigs	or
WYNDE2	7	m	0	0	all	-	KI	NAmer	1962	CC	404	n	bl	n	y	0	ev	all/unsp	nev any	st
WYNDE3	9	m	0	0	all	-	KI	NAmer	1966	CC	350	n	bl	n	y	0	ev	all/unsp	nev any	st
WYNDE3	132	f	0	0	all	-	KI	NAmer	1966	CC	350	n	bl	n	y	0	ev	all/unsp	nev any	st
WYNDE4	68	m	0	0	all	-	not a	NAmer	1948	CC	684	n	bl	y	n	2	ev	all/unsp	nev any	ot
WYNDE4	54	f	0	0	all	-	not a	NAmer	1948	CC	684	n	bl	y	n	2	ev	all/unsp	nev any	ot
WYNDE6	66	m	0	0	all	-	KI	NAmer	1969	CC	4423	n	bl	n	y	0	ev	all/unsp	nev any	st
WYNDE6	412	f	0	0	wh	-	q	NAmer	1969	CC	4423	n	bl	n	y	1	ev	cig+/-ot	nev cigs	ot
XU3	20	m	0	0	all	-	KI	As:Chi	1981	CC	135	n	ot	n	n	1	ev	all/unsp	nev any	ot
XU3	24	f	0	0	all	-	KI	As:Chi	1981	CC	135	n	ot	n	n	1	ev	all/unsp	nev any	ot
ZHENG	5	m	0	0	all	-	q	As:Chi	1982	CC	540	n	ot	*	y	0	ev	cig+/-ot	nev cigs	st
ZHENG	18	f	0	0	all	-	q	As:Chi	1982	CC	540	n	ot	*	y	0	ev	cig+/-ot	nev cigs	st
ZHOU	8	m	0	0	all	-	q	As:Chi	1978	CC	1360	n	ot	n	n	0	ev	all/unsp	nev any	st
ZHOU	9	f	0	0	all	-	q	As:Chi	1978	CC	1360	n	ot	n	n	0	ev	all/unsp	nev any	st

Cigarette type is all/unspec for all RRs

Table 2C1 - 2

IESLC - Meta-anal of Ever Smoking (or Current if Ever not available), Any prod (or Cigs if Any not avail)
Squamous
Most adjusted

REF	NRR	SEX	AD	Number Exposed Case	Cont	Non-exposed Case	Cont	RR	95.00%CI
*ABRAHA	1	m	0	142	10351	0	3365	92.66~	(5.77-1488.21)
*ABRAHA	4	f	0	17	5256	7	11589	5.35	(2.22- 12.90)
Subtotal ABRAHA								6.95	(3.00- 16.06)
ALDERS	52	m	2	-	-	-	-	14.70	(3.40- 63.64)
ALDERS	55	f	2	-	-	-	-	6.09	(2.68- 13.82)
Subtotal ALDERS								7.52	(3.67- 15.37)
*ANDERS	10	f	0	63	96164	5	195158	25.57	(10.29- 63.56)
BAND	5	m	2	-	-	-	-	37.45	(17.62- 79.58)
BARBON	127	m	3	-	-	-	-	14.52	(6.35- 33.20)
BECHER	11	f	1	-	-	-	-	10.69	(2.43- 47.00)
*BOUCOT	141	m	2	-	-	-	-	27.54	(1.69- 448.37)
BRESLO	36	c	0	457	462	15	56	3.69	(2.06- 6.62)
BROWN2	6	m	2	-	-	-	-	11.10	(9.50- 12.90)
BROWN2	5	f	2	-	-	-	-	20.10	(16.40- 24.80)
Subtotal BROWN2								13.69	(12.11- 15.49)
BUFFLE	49	m	0	-	-	-	-	14.03	(4.73- 41.61)
BUFFLE	62	f	0	58	166	3	112	13.04	(3.99- 42.66)
Subtotal BUFFLE								13.57	(6.09- 30.24)
BYERS1	1	m	0	299	695	22	424	8.29	(5.29- 13.00)
CHAN	11	m	0	114	161	2	43	15.22	(3.61- 64.12)
CHAN	15	f	0	44	50	19	139	6.44	(3.44- 12.06)
Subtotal CHAN								7.39	(4.16- 13.13)
CHOI	62	m	0	160	465	6	95	5.45	(2.34- 12.67)
CHOI	64	f	0	11	26	10	164	6.94	(2.68- 17.96)
Subtotal CHOI								6.06	(3.22- 11.40)
COMSTO	66	m	0	44	229	2	84	8.07	(1.91- 34.02)
COMSTO	78	f	0	17	87	0	115	46.20~	(2.74- 778.83)
Subtotal COMSTO								11.56	(3.21- 41.67)
CORREA	35	c	1	-	-	-	-	28.30	(18.60- 43.20)
*CPSI	403	m	1	-	-	-	-	29.35	(4.02- 214.28)
*CPSI	405	f	1	-	-	-	-	4.25	(1.23- 14.68)
Subtotal CPSI								7.30	(2.55- 20.90)
*CPSII	114	m	1	-	-	-	-	39.26	(10.38- 148.55)
*CPSII	117	f	1	-	-	-	-	78.91	(15.83- 393.37)
Subtotal CPSII								52.16	(18.72- 145.32)
DAMBER	33	m	1	-	-	-	-	11.80	(6.40- 23.00)
DESTE2	16	m	2	-	-	-	-	13.20	(4.70- 37.10)
DOLL	86	m	1	-	-	-	-	13.17	(4.12- 42.10)
DOLL	88	f	1	-	-	-	-	2.13	(1.06- 4.27)
Subtotal DOLL								3.45	(1.90- 6.27)
DORGAN	113	m	2	-	-	-	-	18.90	(7.00- 51.30)
DORGAN	98	f	3	-	-	-	-	11.10	(7.20- 17.10)
Subtotal DORGAN								12.08	(8.12- 17.96)
*DORN	338	m	1	-	-	-	-	17.09	(8.96- 32.60)
DOSEME	3	m	2	-	-	-	-	3.60	(2.60- 5.00)
*ENGELA	62	m	7	-	-	-	-	6.45	(1.97- 21.11)
FAN	3	c	0	75	595	6	556	11.68	(5.04- 27.04)
GAO	2	m	2	-	-	-	-	8.40	(4.70- 15.00)
GAO	12	f	2	-	-	-	-	7.20	(4.60- 11.10)
Subtotal GAO								7.62	(5.36- 10.82)
GER	13	c	10	-	-	-	-	3.19	(1.08- 9.42)
HAENSZ	1	f	2	-	-	-	-	3.00	(1.90- 4.73)
*HAMMON	60	m	1	-	-	-	-	16.88	(6.29- 45.29)
HEGMAN	2	c	0	89	1202	5	2080	30.80	(12.48- 76.03)
HINDS	23	f	3	-	-	-	-	16.13	(7.66- 33.97)
ISHIMA	6	c	5	-	-	-	-	21.00	(3.38- 868.40)
JAHN	46	m	0	351	701	3	138	23.03	(7.29- 72.81)
JAIN	48	m	2	-	-	-	-	18.00	(5.50- 111.00)
JAIN	43	f	2	-	-	-	-	25.50	(7.93- 156.00)
Subtotal JAIN								21.46	(7.45- 61.79)
JEDRYC	54	m	3	-	-	-	-	12.84	(5.58- 29.55)
JOLY	54	m	0	203	709	2	218	31.21	(7.69- 126.68)
JOLY	52	f	0	48	122	6	283	18.56	(7.74- 44.51)
Subtotal JOLY								21.47	(10.22- 45.09)
JUSSAW	23	m	0	89	168	13	624	25.43	(13.87- 46.63)
KATSOU	37	f	1	-	-	-	-	6.11	(2.69- 13.87)
KHUDER	24	m	0	176	-	9	-	7.82	(3.87- 15.77)
KIHARA	26	c	0	132	232	5	237	26.97	(10.84- 67.08)
KOO	6	f	0	61	63	32	137	4.15	(2.46- 6.98)
KREYBE	4	m	1	-	-	-	-	10.87	(3.47- 34.04)
KREYBE	25	f	1	-	-	-	-	2.29	(0.89- 5.88)

Table 2C1 - 2

IESLC - Meta-anal of Ever Smoking (or Current if Ever not available), Any prod (or Cigs if Any not avail)
Squamous
Most adjusted

REF	NRR	SEX	AD	Number Exposed Case	Number Exposed Cont	Non-exposed Case	Non-exposed Cont	RR	95.00%CI
Subtotal KREYBE								4.31	(2.08- 8.92)
LAMTH	1	f	0	63	20	28	72	8.10	(4.16- 15.77)
LAMWK	2	f	0	21	41	7	144	10.54	(4.19- 26.52)
LAMWK2	1	m	0	129	161	5	43	6.89	(2.65- 17.90)
LAMWK2	5	f	0	35	50	15	139	6.49	(3.27- 12.88)
Subtotal LAMWK2								6.62	(3.79- 11.56)
LOMBA2	2	f	0	94	353	15	239	4.24	(2.40- 7.50)
LUBIN	33	m	0	330	939	4	72	6.33	(2.29- 17.45)
LUBIN2	145	m	0	3587	10433	54	2616	16.66	(12.69- 21.86)
LUBIN2	165	f	0	200	567	72	1180	5.78	(4.34- 7.71)
Subtotal LUBIN2								10.10	(8.29- 12.31)
LUO	8	c	20	-	-	-	-	10.90	(2.50- 47.90)
MATOS	67	m	2	-	-	-	-	8.08	(2.59- 25.20)
MATSUD	11	m	0	103	3314	1	1255	39.01	(5.44- 279.84)
NOU	1	m	0	110	247	2	122	27.17	(6.60- 111.85)
NOU	6	f	0	5	92	2	261	7.09	(1.35- 37.19)
Subtotal NOU								15.42	(5.26- 45.22)
ORMOS	8	m	0	27	1034	2	777	10.14	(2.41- 42.79)
OSANN	43	m	2	-	-	-	-	36.10	(17.80- 73.30)
OSANN	44	f	2	-	-	-	-	26.40	(14.50- 48.10)
Subtotal OSANN								30.09	(19.04- 47.54)
OSANN2	25	f	1	-	-	-	-	35.10	(4.80- 256.00)
PEZZOT	6	m	0	85	317	0	116	62.74~	(3.86-1019.50)
SCHWAR	10	m	0	80	178	1	73	32.81	(4.48- 240.23)
SCHWAR	9	m	0	41	39	4	7	1.84	(0.50- 6.78)
SCHWAR	18	f	0	29	108	0	79	43.23~	(2.60- 718.15)
SCHWAR	17	f	0	21	28	0	41	62.61~	(3.64-1076.10)
Subtotal SCHWAR								7.71	(2.96- 20.10)
SEOW	3	f	0	21	15	10	125	17.50	(6.95- 44.09)
SIEMIA	7	m	7	-	-	-	-	22.70	(6.90- 75.20)
SOBUE	97	m	1	-	-	-	-	17.88	(7.82- 40.87)
SOBUE	107	f	1	-	-	-	-	8.74	(5.09- 15.02)
Subtotal SOBUE								10.83	(6.89- 17.03)
SOBUE2	1	m	2	-	-	-	-	5.20	(4.20- 6.50)
SOBUE2	5	f	2	-	-	-	-	7.20	(4.80- 10.80)
Subtotal SOBUE2								5.59	(4.62- 6.78)
STASZE	12	m	0	137	754	0	158	57.77~	(3.58- 933.17)
STASZE	38	f	0	1	153	0	1660	32.45~	(1.32- 800.04)
Subtotal STASZE								45.09	(5.52- 368.55)
STAYNE	3	m	0	130	567	22	333	3.47	(2.17- 5.56)
SUZUK2	15	c	3	-	-	-	-	31.00	(4.20- 227.00)
SVENSS	72	f	1	-	-	-	-	12.62	(3.97- 40.14)
TIZZAN	18	c	0	333	939	55	419	2.70	(1.99- 3.67)
TOKARS	10	c	3	-	-	-	-	6.80	(1.20- 38.70)
TSUGAN	13	m	0	20	15	0	5	14.55~	(0.75- 283.37)
WAKAI	74	m	1	-	-	-	-	8.61	(2.08- 35.72)
WAKAI	80	f	1	-	-	-	-	25.23	(6.87- 92.66)
Subtotal WAKAI								15.46	(5.92- 40.36)
WU	32	f	2	-	-	-	-	24.29	(3.40- 173.76)
WUWILL	9	f	3	-	-	-	-	4.20	(3.00- 5.90)
WYNDE2	7	m	0	347	616	3	105	19.72	(6.21- 62.59)
WYNDE3	9	m	0	207	332	3	88	18.29	(5.71- 58.56)
WYNDE3	132	f	0	25	56	5	76	6.79	(2.45- 18.82)
Subtotal WYNDE3								10.44	(4.85- 22.49)
WYNDE4	68	m	2	-	-	-	-	12.79	(6.19- 26.41)
WYNDE4	54	f	2	-	-	-	-	5.82	(2.55- 13.31)
Subtotal WYNDE4								9.08	(5.26- 15.66)
WYNDE6	66	m	0	1744	1996	29	617	18.59	(12.74- 27.13)
WYNDE6	412	f	1	-	-	-	-	32.37	(17.66- 59.35)
Subtotal WYNDE6								21.71	(15.76- 29.92)
XU3	20	m	1	-	-	-	-	5.90	(1.69- 20.57)
XU3	24	f	1	-	-	-	-	25.67	(4.99- 131.94)
Subtotal XU3								10.14	(3.75- 27.37)
ZHENG	5	m	0	156	218	4	94	16.82	(6.05- 46.71)
ZHENG	18	f	0	43	44	33	184	5.45	(3.11- 9.54)
Subtotal ZHENG								7.07	(4.33- 11.56)
ZHOU	8	m	0	343	41	96	36	3.14	(1.90- 5.18)
ZHOU	9	f	0	35	7	42	32	3.81	(1.50- 9.68)
Subtotal ZHOU								3.28	(2.11- 5.10)
Partial Totals				11152	141578	686	226785		

*prospective study ~ With 0.5 adjustment for zero

Table 2C1 - 2

IESLC - Meta-anal of Ever Smoking (or Current if Ever not available), Any prod (or Cigs if Any not avail)
Squamous
Most adjusted

REF	NRR	SEX	AD	Ys	Ws	Qs	Ps
*ABRAHA	1	m	0	4.53	0.50	2.67	0.0014
*ABRAHA	4	f	0	1.68	4.97	1.43	0.0002
Subtotal ABRAHA				1.94	5.46	4.10	
ALDERS	52	m	2	2.69	1.79	0.40	0.0003
ALDERS	55	f	2	1.81	5.71	0.95	0.0000
Subtotal ALDERS				2.02	7.50	1.35	
*ANDERS	10	f	0	3.24	4.63	4.89	0.0000
BAND	5	m	2	3.62	6.76	13.42	0.0000
BARBON	127	m	3	2.68	5.62	1.20	0.0000
BECHER	11	f	1	2.37	1.75	0.04	0.0017
*BOUCOT	141	m	2	3.32	0.49	0.60	0.0199
BRESLO	36	c	0	1.31	11.25	9.26	0.0000
BROWN2	6	m	2	2.41	164.17	6.13	0.0000
BROWN2	5	f	2	3.00	89.84	55.64	0.0000
Subtotal BROWN2				2.62	254.01	61.76	
BUFFLE	49	m	0	2.64	3.25	0.59	0.0000
BUFFLE	62	f	0	2.57	2.74	0.34	0.0000
Subtotal BUFFLE				2.61	5.99	0.94	
BYERS1	1	m	0	2.12	19.01	0.18	0.0000
CHAN	11	m	0	2.72	1.86	0.48	0.0002
CHAN	15	f	0	1.86	9.75	1.21	0.0000
Subtotal CHAN				2.00	11.61	1.69	
CHOI	62	m	0	1.70	5.39	1.45	0.0001
CHOI	64	f	0	1.94	4.25	0.33	0.0001
Subtotal CHOI				1.80	9.63	1.77	
COMSTO	66	m	0	2.09	1.86	0.03	0.0045
COMSTO	78	f	0	3.83	0.48	1.26	0.0078
Subtotal COMSTO				2.45	2.34	1.29	
CORREA	35	c	1	3.34	21.64	27.59	0.0000
*CPSI	403	m	1	3.38	0.97	1.32	0.0009
*CPSI	405	f	1	1.45	2.50	1.47	0.0222
Subtotal CPSI				1.99	3.47	2.79	
*CPSII	114	m	1	3.67	2.17	4.60	0.0000
*CPSII	117	f	1	4.37	1.49	6.91	0.0000
Subtotal CPSII				3.95	3.66	11.51	
DAMBER	33	m	1	2.47	9.39	0.61	0.0000
DESTE2	16	m	2	2.58	3.60	0.48	0.0000
DOLL	86	m	1	2.58	2.84	0.38	0.0000
DOLL	88	f	1	0.76	7.91	16.82	0.0334
Subtotal DOLL				1.24	10.76	17.19	
DORGAN	113	m	2	2.94	3.87	2.04	0.0000
DORGAN	98	f	3	2.41	20.54	0.77	0.0000
Subtotal DORGAN				2.49	24.41	2.80	
*DORN	338	m	1	2.84	9.21	3.60	0.0000
DOSEME	3	m	2	1.28	35.93	31.27	0.0000
*ENGELA	62	m	7	1.86	2.73	0.33	0.0021
FAN	3	c	0	2.46	5.45	0.32	0.0000
GAO	2	m	2	2.13	11.41	0.08	0.0000
GAO	12	f	2	1.97	19.80	1.14	0.0000
Subtotal GAO				2.03	31.21	1.22	
GER	13	c	10	1.16	3.28	3.64	0.0358
HAENSZ	1	f	2	1.10	18.47	22.97	0.0000
*HAMMON	60	m	1	2.83	3.94	1.48	0.0000
HEGMAN	2	c	0	3.43	4.70	6.93	0.0000
HINDS	23	f	3	2.78	6.93	2.23	0.0000
ISHIMA	6	c	5	3.04	0.50	0.34	0.0315
JAHN	46	m	0	3.14	2.90	2.47	0.0000
JAIN	48	m	2	2.89	1.70	0.78	0.0002
JAIN	43	f	2	3.24	1.73	1.82	0.0000
Subtotal JAIN				3.07	3.43	2.60	
JEDRYC	54	m	3	2.55	5.53	0.63	0.0000
JOLY	54	m	0	3.44	1.96	2.95	0.0000
JOLY	52	f	0	2.92	5.02	2.51	0.0000
Subtotal JOLY				3.07	6.98	5.46	
JUSSAW	23	m	0	3.24	10.45	10.91	0.0000
KATSOU	37	f	1	1.81	5.71	0.93	0.0000
KHUDER	24	m	0	2.06	7.79	0.19	0.0000
KIHARA	26	c	0	3.29	4.63	5.41	0.0000
KOO	6	f	0	1.42	14.12	8.85	0.0000
KREYBE	4	m	1	2.39	2.95	0.09	0.0000
KREYBE	25	f	1	0.83	4.31	8.27	0.0854

Table 2C1 - 2

IESLC - Meta-anal of Ever Smoking (or Current if Ever not available), Any prod (or Cigs if Any not avail)
Squamous
Most adjusted

REF	NRR	SEX	AD	Ys	Ws	Qs	Ps
Subtotal KREYBE				1.46	7.26	8.36	
LAMTH	1	f	0	2.09	8.66	0.13	0.0000
LAMWK	2	f	0	2.35	4.51	0.09	0.0000
LAMWK2	1	m	0	1.93	4.22	0.34	0.0001
LAMWK2	5	f	0	1.87	8.17	0.97	0.0000
Subtotal LAMWK2				1.89	12.38	1.31	
LOMBA2	2	f	0	1.45	11.86	7.00	0.0000
LUBIN	33	m	0	1.84	3.73	0.51	0.0004
LUBIN2	145	m	0	2.81	51.88	18.61	0.0000
LUBIN2	165	f	0	1.75	46.51	9.81	0.0000
Subtotal LUBIN2				2.31	98.39	28.42	
LUO	8	c	20	2.39	1.76	0.05	0.0015
MATOS	67	m	2	2.09	2.97	0.05	0.0003
MATSUD	11	m	0	3.66	0.99	2.08	0.0003
NOU	1	m	0	3.30	1.92	2.27	0.0000
NOU	6	f	0	1.96	1.40	0.09	0.0205
Subtotal NOU				2.74	3.32	2.36	
ORMOS	8	m	0	2.32	1.85	0.02	0.0016
OSANN	43	m	2	3.59	7.67	14.45	0.0000
OSANN	44	f	2	3.27	10.69	12.00	0.0000
Subtotal OSANN				3.40	18.36	26.45	
OSANN2	25	f	1	3.56	0.97	1.76	0.0005
PEZZOT	6	m	0	4.14	0.49	1.83	0.0036
SCHWAR	10	m	0	3.49	0.97	1.58	0.0006
SCHWAR	9	m	0	0.61	2.26	5.81	0.3596
SCHWAR	18	f	0	3.77	0.49	1.17	0.0086
SCHWAR	17	f	0	4.14	0.47	1.76	0.0044
Subtotal SCHWAR				2.04	4.19	10.32	
SEOW	3	f	0	2.86	4.50	1.89	0.0000
SIEMIA	7	m	7	3.12	2.69	2.22	0.0000
SOBUE	97	m	1	2.88	5.62	2.52	0.0000
SOBUE	107	f	1	2.17	13.12	0.03	0.0000
Subtotal SOBUE				2.38	18.74	2.55	
SOBUE2	1	m	2	1.65	80.57	25.73	0.0000
SOBUE2	5	f	2	1.97	23.37	1.34	0.0000
Subtotal SOBUE2				1.72	103.93	27.07	
STASZE	12	m	0	4.06	0.50	1.69	0.0043
STASZE	38	f	0	3.48	0.37	0.60	0.0333
Subtotal STASZE				3.81	0.87	2.28	
STAYNE	3	m	0	1.24	17.27	16.23	0.0000
SUZUK2	15	c	3	3.43	0.97	1.44	0.0007
SVENSS	72	f	1	2.54	2.87	0.30	0.0000
TIZZAN	18	c	0	0.99	40.59	60.41	0.0000
TOKARS	10	c	3	1.92	1.27	0.11	0.0305
TSUGAN	13	m	0	2.68	0.44	0.09	0.0772
WAKAI	74	m	1	2.15	1.90	0.01	0.0030
WAKAI	80	f	1	3.23	2.27	2.34	0.0000
Subtotal WAKAI				2.74	4.17	2.34	
WU	32	f	2	3.19	0.99	0.95	0.0015
WUWILL	9	f	3	1.44	33.59	20.37	0.0000
WYNDE2	7	m	0	2.98	2.88	1.70	0.0000
WYNDE3	9	m	0	2.91	2.84	1.36	0.0000
WYNDE3	132	f	0	1.91	3.69	0.33	0.0002
Subtotal WYNDE3				2.35	6.53	1.69	
WYNDE4	68	m	2	2.55	7.30	0.82	0.0000
WYNDE4	54	f	2	1.76	5.63	1.15	0.0000
Subtotal WYNDE4				2.21	12.93	1.97	
WYNDE6	66	m	0	2.92	26.90	13.51	0.0000
WYNDE6	412	f	1	3.48	10.46	16.69	0.0000
Subtotal WYNDE6				3.08	37.36	30.21	
XU3	20	m	1	1.77	2.46	0.47	0.0054
XU3	24	f	1	3.25	1.43	1.52	0.0001
Subtotal XU3				2.32	3.89	2.00	
ZHENG	5	m	0	2.82	3.68	1.36	0.0000
ZHENG	18	f	0	1.70	12.24	3.29	0.0000
Subtotal ZHENG				1.96	15.92	4.65	
ZHOU	8	m	0	1.14	15.27	17.49	0.0000
ZHOU	9	f	0	1.34	4.42	3.39	0.0049
Subtotal ZHOU				1.19	19.68	20.88	

Table 2C1 - 2

IESLC - Meta-anal of Ever Smoking (or Current if Ever not available), Any prod (or Cigs if Any not avail)
Squamous
Most adjusted

	N	110
	NS	78
	Wt	1120.65
Het	Chi	569.36
Het	df	109
Het	P	***
Fixed	RR	9.15
	RRl	8.63
	RRu	9.70
	P	+++
Random	RR	10.58
	RRl	9.04
	RRu	12.37
	P	+++
Asymm	P	(*)

Table 2C1 - 3

IESLC - Meta-anal of Ever Smoking (or Current if Ever not available), Any prod (or Cigs if Any not avail)
Squamous
Most adjusted

	Sex			
	combined	male	female	Total
N	11	54	45	110
NS	11	53	44	108
Wt	96.04	579.29	445.32	1120.65
Het Chi	108.90	220.97	229.02	569.36
Het df	10	53	44	109
Het P	***	***	***	***
Fixed RR	7.04	9.85	8.79	9.15
RRl	5.76	9.08	8.01	8.63
RRu	8.60	10.69	9.65	9.70
P	+++	+++	+++	+++
Random RR	10.70	12.14	9.03	10.58
RRl	4.89	9.86	7.08	9.04
RRu	23.40	14.94	11.52	12.37
P	+++	+++	+++	+++
Between Chi				10.47
Between df				2
Between P				**
Btwn(F) P				N.S.
Btwn(R) P				N.S.

	Lung cancer type					
	q	q+s	q+u	KI	not a	Total
N	82	7	3	14	4	110
NS	57	6	3	10	3	79
Wt	883.21	59.32	70.92	79.08	28.12	1120.65
Het Chi	359.49	41.80	1.87	55.77	10.50	569.36
Het df	81	6	2	13	3	109
Het P	***	***	N.S.	***	*	***
Fixed RR	9.80	11.11	2.99	11.04	6.91	9.15
RRl	9.18	8.61	2.37	8.86	4.78	8.63
RRu	10.47	14.33	3.78	13.77	10.00	9.70
P	+++	+++	+++	+++	+++	+++
Random RR	11.62	9.39	2.99	9.71	7.85	10.58
RRl	9.80	4.47	2.37	5.85	3.84	9.04
RRu	13.78	19.74	3.78	16.11	16.05	12.37
P	+++	+++	+++	+++	+++	+++
Between Chi						99.94
Between df						4
Between P						***
Btwn(F) P						***
Btwn(R) P						***

	Location								
	NAmer	UK	Scand	othEur	China	Japan	othAs	other	Total
N	44	4	7	15	12	10	12	6	110
NS	30	2	5	12	8	7	9	5	78
Wt	528.16	18.26	25.57	205.89	115.24	133.40	79.14	15.00	1120.65
Het Chi	216.85	10.69	11.88	109.09	23.25	28.64	28.49	3.82	569.36
Het df	43	3	6	14	11	9	11	5	109
Het P	***	*	(*)	***	*	***	**	N.S.	***
Fixed RR	12.84	4.75	8.67	6.56	5.65	6.84	7.87	16.70	9.15
RRl	11.79	3.00	5.88	5.72	4.71	5.78	6.31	10.07	8.63
RRu	13.99	7.51	12.78	7.52	6.78	8.11	9.81	27.70	9.70
P	+++	+++	+++	+++	+++	+++	+++	+++	+++
Random RR	13.99	6.27	8.62	8.87	6.39	11.19	8.02	16.70	10.58
RRl	11.03	2.49	4.81	5.50	4.73	7.15	5.54	10.07	9.04
RRu	17.74	15.83	15.43	14.31	8.63	17.51	11.60	27.70	12.37
P	+++	+++	+++	+++	+++	+++	+++	+++	+++
Between Chi									136.64
Between df									7
Between P									***
Btwn(F) P									***
Btwn(R) P									**

Table 2C1 - 3

IESLC - Meta-anal of Ever Smoking (or Current if Ever not available), Any prod (or Cigs if Any not avail)

Squamous

Most adjusted

	Detailed Country in "other Europe"					
	multi	Germany	othWest	East	Balkans	Total
N	2	2	2	7	2	15
NS	1	2	2	5	2	12
Wt	98.39	4.65	46.21	14.99	41.65	205.89
Het Chi	27.46	0.64	13.95	6.98	1.38	109.09
Het df	1	1	1	6	1	14
Het P	***	N.S.	***	N.S.	N.S.	***
Fixed RR	10.10	17.25	3.31	10.16	3.87	6.56
RRl	8.29	6.95	2.48	6.12	2.86	5.72
RRu	12.31	42.81	4.42	16.85	5.24	7.52
P	+++	+++	+++	+++	+++	+++
Random RR	9.82	17.25	5.98	10.63	4.08	8.87
RRl	3.48	6.95	1.15	5.91	2.63	5.50
RRu	27.71	42.81	31.02	19.11	6.34	14.31
P	+++	+++	+	+++	+++	+++
Between Chi						58.67
Between df						4
Between P						***
Btwn(F) P						(*)
Btwn(R) P						*

	Detailed Country in "other Asia"			
	India	HongKong	other	Total
N	1	7	4	12
NS	1	5	3	9
Wt	10.45	51.28	17.41	79.14
Het Chi	0.00	5.68	6.14	28.49
Het df	0	6	3	11
Het P	N.S.	N.S.	N.S.	**
Fixed RR	25.43	6.43	7.06	7.87
RRl	13.87	4.89	4.42	6.31
RRu	46.63	8.45	11.30	9.81
P	+++	+++	+++	+++
Random RR	25.43	6.43	6.94	8.02
RRl	13.87	4.89	3.53	5.54
RRu	46.63	8.45	13.65	11.60
P	+++	+++	+++	+++
Between Chi				16.67
Between df				2
Between P				***
Btwn(F) P				*
Btwn(R) P				***

	Detailed other continent			
	SCAmer	Auslia	Africa	Total
N	6			6
NS	5			5
Wt	15.00			15.00
Het Chi	3.82			3.82
Het df	5			5
Het P	N.S.			N.S.
Fixed RR	16.70			16.70
RRl	10.07			10.07
RRu	27.70			27.70
P	+++			+++
Random RR	16.70			16.70
RRl	10.07			10.07
RRu	27.70			27.70
P	+++			+++
Between Chi				
Between df				
Between P				N.S.
Btwn(F) P				N.S.
Btwn(R) P				N.S.

Table 2C1 - 3

IESLC - Meta-anal of Ever Smoking (or Current if Ever not available), Any prod (or Cigs if Any not avail)
Squamous
Most adjusted

	Start year of study <1960	1960-69	1970-79	1980-89	1990+	Total
N	18	16	26	42	8	110
NS	13	13	16	28	8	78
Wt	140.11	203.66	249.35	500.37	27.15	1120.65
Het Chi	74.89	98.55	130.30	165.37	10.78	569.36
Het df	17	15	25	41	7	109
Het P	***	***	***	***	N.S.	***
Fixed RR	4.86	8.08	8.76	11.55	12.39	9.15
RRl	4.12	7.05	7.74	10.58	8.51	8.63
RRu	5.73	9.28	9.92	12.61	18.05	9.70
P	+++	+++	+++	+++	+++	+++
Random RR	6.86	11.21	10.08	12.31	12.21	10.58
RRl	4.56	7.30	7.21	9.86	7.56	9.04
RRu	10.32	17.22	14.09	15.37	19.72	12.37
P	+++	+++	+++	+++	+++	+++
Between Chi						89.47
Between df						4
Between P						***
Btwn(F) P						**
Btwn(R) P						N.S.

	Study type (1) CC	other	Total
N	95	15	110
NS	67	11	78
Wt	1082.46	38.19	1120.65
Het Chi	536.60	23.91	569.36
Het df	94	14	109
Het P	***	*	***
Fixed RR	9.00	14.69	9.15
RRl	8.48	10.70	8.63
RRu	9.55	20.17	9.70
P	+++	+++	+++
Random RR	10.12	15.50	10.58
RRl	8.58	9.83	9.04
RRu	11.94	24.44	12.37
P	+++	+++	+++
Between Chi			8.85
Between df			1
Between P			**
Btwn(F) P			N.S.
Btwn(R) P			(*)

	Study type (2) CC	prosp	other	Total
N	95	11	4	110
NS	67	8	3	78
Wt	1082.46	33.61	4.58	1120.65
Het Chi	536.60	21.10	2.69	569.36
Het df	94	10	3	109
Het P	***	*	N.S.	***
Fixed RR	9.00	14.99	12.62	9.15
RRl	8.48	10.69	5.05	8.63
RRu	9.55	21.02	31.54	9.70
P	+++	+++	+++	+++
Random RR	10.12	16.24	12.62	10.58
RRl	8.58	9.47	5.05	9.04
RRu	11.94	27.87	31.54	12.37
P	+++	+++	+++	+++
Between Chi				8.97
Between df				2
Between P				*
Btwn(F) P				N.S.
Btwn(R) P				N.S.

Table 2C1 - 3

IESLC - Meta-anal of Ever Smoking (or Current if Ever not available), Any prod (or Cigs if Any not avail)

Squamous

Most adjusted

	Study size (number of LC cases)				
	100-249	250-499	500-999	1000+	Total
N	23	31	18	38	110
NS	22	22	12	22	78
Wt	84.66	135.04	123.69	777.25	1120.65
Het Chi	42.77	63.82	62.97	380.71	569.36
Het df	22	30	17	37	109
Het P	**	***	***	***	***
Fixed RR	6.07	8.89	8.26	9.78	9.15
RRl	4.91	7.51	6.92	9.11	8.63
RRu	7.51	10.52	9.85	10.49	9.70
P	+++	+++	+++	+++	+++
Random RR	8.53	10.17	11.35	11.09	10.58
RRl	6.02	7.81	7.79	8.56	9.04
RRu	12.08	13.22	16.53	14.37	12.37
P	+++	+++	+++	+++	+++
Between Chi					19.08
Between df					3
Between P					***
Btwn(F) P					N.S.
Btwn(R) P					N.S.

	Risky occupational population			
	no	mining	othRisky	Total
N	108	1	1	110
NS	76	1	1	78
Wt	1115.64	3.73	1.27	1120.65
Het Chi	568.73	0.00	0.00	569.36
Het df	107	0	0	109
Het P	***	N.S.	N.S.	***
Fixed RR	9.16	6.33	6.80	9.15
RRl	8.64	2.29	1.20	8.63
RRu	9.72	17.45	38.62	9.70
P	+++	+++	+	+++
Random RR	10.66	6.33	6.80	10.58
RRl	9.10	2.29	1.20	9.04
RRu	12.48	17.45	38.62	12.37
P	+++	+++	+	+++
Between Chi				0.62
Between df				2
Between P				N.S.
Btwn(F) P				N.S.
Btwn(R) P				N.S.

	National cigarette tobacco type			
	Virginia	blended	other	Total
N	9	88	13	110
NS	6	63	9	78
Wt	41.59	960.54	118.52	1120.65
Het Chi	43.26	465.86	24.29	569.36
Het df	8	87	12	109
Het P	***	***	*	***
Fixed RR	12.69	9.59	5.56	9.15
RRl	9.36	9.01	4.65	8.63
RRu	17.20	10.22	6.66	9.70
P	+++	+++	+++	+++
Random RR	13.80	11.17	6.15	10.58
RRl	6.53	9.38	4.60	9.04
RRu	29.17	13.30	8.23	12.37
P	+++	+++	+++	+++
Between Chi				35.95
Between df				2
Between P				***
Btwn(F) P				*
Btwn(R) P				**

Table 2C1 - 3

IESLC - Meta-anal of Ever Smoking (or Current if Ever not available), Any prod (or Cigs if Any not avail)
Squamous
Most adjusted

	Any proxy use No/nk	Yes	Total
N	84	26	110
NS	60	18	78
Wt	995.24	125.41	1120.65
Het Chi	487.42	50.62	569.36
Het df	83	25	109
Het P	***	**	***
Fixed RR	8.62	14.65	9.15
RRl	8.10	12.30	8.63
RRu	9.18	17.46	9.70
P	+++	+++	+++
Random RR	9.83	13.82	10.58
RRl	8.24	10.45	9.04
RRu	11.73	18.27	12.37
P	+++	+++	+++
Between Chi			31.31
Between df			1
Between P			***
Btwn(F) P			*
Btwn(R) P			*

	Full histological confirmation No	Yes	Total
N	67	43	110
NS	48	30	78
Wt	573.15	547.50	1120.65
Het Chi	309.69	182.20	569.36
Het df	66	42	109
Het P	***	***	***
Fixed RR	7.08	11.97	9.15
RRl	6.52	11.01	8.63
RRu	7.68	13.02	9.70
P	+++	+++	+++
Random RR	9.62	12.32	10.58
RRl	7.89	9.78	9.04
RRu	11.73	15.52	12.37
P	+++	+++	+++
Between Chi			77.47
Between df			1
Between P			***
Btwn(F) P			***
Btwn(R) P			N.S.

	Number of adjustment variables (1) 0	1	2+/+nk	Total
N	53	23	34	110
NS	37	16	26	79
Wt	412.88	117.90	589.87	1120.65
Het Chi	233.67	83.49	227.14	569.36
Het df	52	22	33	109
Het P	***	***	***	***
Fixed RR	7.93	13.26	9.39	9.15
RRl	7.20	11.07	8.66	8.63
RRu	8.74	15.89	10.18	9.70
P	+++	+++	+++	+++
Random RR	9.86	12.69	10.52	10.58
RRl	7.79	8.65	8.12	9.04
RRu	12.49	18.61	13.64	12.37
P	+++	+++	+++	+++
Between Chi				25.06
Between df				2
Between P				***
Btwn(F) P				(*)
Btwn(R) P				N.S.

Table 2C1 - 3

IESLC - Meta-anal of Ever Smoking (or Current if Ever not available), Any prod (or Cigs if Any not avail)
Squamous
Most adjusted

	Number of adjustment variables (2) 0	1	2	3-5	6+/+nk	Total
N	53	23	22	8	4	110
NS	37	16	15	8	4	80
Wt	412.88	117.90	504.47	74.94	10.46	1120.65
Het Chi	233.67	83.49	192.48	24.97	5.99	569.36
Het df	52	22	21	7	3	109
Het P	***	***	***	***	N.S.	***
Fixed RR	7.93	13.26	9.70	7.74	7.81	9.15
RRl	7.20	11.07	8.89	6.17	4.26	8.63
RRu	8.74	15.89	10.58	9.70	14.32	9.70
P	+++	+++	+++	+++	+++	+++
Random RR	9.86	12.69	10.85	10.71	8.13	10.58
RRl	7.79	8.65	7.87	6.25	3.43	9.04
RRu	12.49	18.61	14.97	18.36	19.30	12.37
P	+++	+++	+++	+++	+++	+++
Between Chi						28.76
Between df						4
Between P						***
Btwn(F) P						N.S.
Btwn(R) P						N.S.

	Product all/unsp	cig+/-ot	cig only	Total
N	54	49	7	110
NS	41	33	6	80
Wt	324.67	773.38	22.60	1120.65
Het Chi	213.86	294.64	10.61	569.36
Het df	53	48	6	109
Het P	***	***	N.S.	***
Fixed RR	6.86	10.07	21.35	9.15
RRl	6.16	9.39	14.14	8.63
RRu	7.65	10.81	32.25	9.70
P	+++	+++	+++	+++
Random RR	8.94	11.66	21.38	10.58
RRl	7.02	9.45	11.24	9.04
RRu	11.39	14.39	40.65	12.37
P	+++	+++	+++	+++
Between Chi				50.24
Between df				2
Between P				***
Btwn(F) P				**
Btwn(R) P				*

	Denominator nev any	nev cigs	Total
N	71	39	110
NS	53	27	80
Wt	590.84	529.81	1120.65
Het Chi	312.53	219.43	569.36
Het df	70	38	109
Het P	***	***	***
Fixed RR	7.70	11.10	9.15
RRl	7.10	10.19	8.63
RRu	8.34	12.08	9.70
P	+++	+++	+++
Random RR	9.70	12.30	10.58
RRl	7.97	9.53	9.04
RRu	11.79	15.87	12.37
P	+++	+++	+++
Between Chi			37.40
Between df			1
Between P			***
Btwn(F) P			**
Btwn(R) P			N.S.

Table 2C1 - 3

IESLC - Meta-anal of Ever Smoking (or Current if Ever not available), Any prod (or Cigs if Any not avail)

Squamous

Most adjusted

	Derivation of RR/CI			
	Orig	StdCalc	Other	Total
N	26	42	42	110
NS	19	32	31	82
Wt	565.09	386.46	169.11	1120.65
Het Chi	205.21	222.05	128.47	569.36
Het df	25	41	41	109
Het P	***	***	***	***
Fixed RR	9.96	7.86	9.73	9.15
RRl	9.18	7.12	8.37	8.63
RRu	10.82	8.69	11.31	9.70
P	+++	+++	+++	+++
Random RR	11.42	9.46	11.68	10.58
RRl	8.57	7.30	8.69	9.04
RRu	15.22	12.26	15.70	12.37
P	+++	+++	+++	+++
Between Chi				13.61
Between df				2
Between P				**
Btwn(F) P				N.S.
Btwn(R) P				N.S.

	Smoking status		
	ever	current	Total
N	102	8	110
NS	73	5	78
Wt	999.88	120.77	1120.65
Het Chi	522.25	32.81	569.36
Het df	101	7	109
Het P	***	***	***
Fixed RR	9.52	6.61	9.15
RRl	8.94	5.53	8.63
RRu	10.13	7.90	9.70
P	+++	+++	+++
Random RR	10.47	12.44	10.58
RRl	8.88	6.84	9.04
RRu	12.33	22.61	12.37
P	+++	+++	+++
Between Chi			14.30
Between df			1
Between P			***
Btwn(F) P			(*)
Btwn(R) P			N.S.

Table 2C1 - 4

IESLC - Meta-anal of Ever Smoking (or Current if Ever not available), Any prod (or Cigs if Any not avail)
Squamous
Least adjusted

REF	NRR	X	SEX	AGEL	AGEH	RACE	YF	LC TYPE	LOC	START	ST	NLC	R	VB	P	H	AD	SM	PRODUCT	DENOM	De
ABRAHA	1		m	0	0	all	0	q	Eu:est	1975	pr	571	n	bl	n	n	0	ev	all/unsp	nev any	ot
ABRAHA	4		f	0	0	all	0	q	Eu:est	1975	pr	571	n	bl	n	n	0	ev	all/unsp	nev any	ot
ALDERS	52		m	0	0	all	-	q	Eu:UK	1977	CC	1448	n	V	n	n	2	ev	all/unsp	nev any	or
ALDERS	55		f	0	0	all	-	q	Eu:UK	1977	CC	1448	n	V	n	n	2	ev	all/unsp	nev any	or
ANDERS	10		f	0	0	all	0	q	NAmer	1986	pr	343	n	bl	n	n	0	ev	cig+/-ot	nev cigs	st
BAND	5		m	0	0	all	-	q	NAmer	1983	CC	2831	n	V	y	y	2	ev	cig only	nev any	ot
BARBON	110	x	m	0	0	all	-	q	Eu:wst	1979	CC	755	n	bl	y	y	0	ev	all/unsp	nev any	st
BECHER	11		f	0	0	all	-	q+s	Eu:Ger	1985	CC	194	n	bl	n	y	1	ev	all/unsp	nev any	or
BOUCOT	70	x	m	0	0	all	0	q	NAmer	1951	pr	121	n	bl	n	n	0	cu	cig only	nev any	ot
BRESLO	36		c	0	0	all	-	not a	NAmer	1949	CC	518	n	bl	n	y	0	ev	all/unsp	nev+1	st
BROWN2	6		m	0	0	wh	-	q	NAmer	1984	CC	14596	n	bl	n	y	2	ev	cig+/-ot	nev cigs	or
BROWN2	5		f	0	0	wh	-	q	NAmer	1984	CC	14596	n	bl	n	y	2	ev	cig+/-ot	nev cigs	or
BUFFLE	49		m	0	0	wh	-	q	NAmer	1976	CC	943	n	bl	y	n	0	ev	cig+/-ot	nev cigs	ot
BUFFLE	62		f	0	0	w-hi	-	q	NAmer	1976	CC	943	n	bl	y	n	0	ev	cig+/-ot	nev cigs	st
BYERS1	1		m	0	0	wh	-	q	NAmer	1957	CC	1002	n	bl	n	n	0	ev	cig+/-ot	nev cigs	st
CHAN	11		m	0	0	all	-	q+s	As:HK	1976	CC	397	n	bl	n	n	0	ev	all/unsp	nev any	st
CHAN	15		f	0	0	all	-	q+s	As:HK	1976	CC	397	n	bl	n	n	0	ev	all/unsp	nev any	st
CHOI	62		m	0	0	all	-	q	As:oth	1985	CC	375	n	bl	n	n	0	ev	cig+/-ot	nev cigs	st
CHOI	64		f	0	0	all	-	q	As:oth	1985	CC	375	n	bl	n	n	0	ev	cig+/-ot	nev cigs	st
COMSTO	66		m	0	0	all	-	q	NAmer	1975	ot	258	n	bl	n	n	0	ev	cig+/-ot	nev cigs	st
COMSTO	78		f	0	0	all	-	q	NAmer	1975	ot	258	n	bl	n	n	0	ev	cig+/-ot	nev cigs	ot
CORREA	35		c	0	0	all	-	q+s	NAmer	1979	CC	1359	n	bl	y	n	1	ev	cig+/-ot	nev cigs	or
CPSI	403		m	0	0	all	2	q	NAmer	1959	pr	5138	n	bl	n	n	1	cu	cig only	nev any	ot
CPSI	405		f	0	0	all	2	q	NAmer	1959	pr	5138	n	bl	n	n	1	cu	cig only	nev any	ot
CPSII	114		m	0	0	all	2	q	NAmer	1982	pr	3229	n	bl	n	n	1	cu	cig only	nev any	ot
CPSII	117		f	0	0	all	2	q	NAmer	1982	pr	3229	n	bl	n	n	1	cu	cig+/-ot	nev cigs	ot
DAMBER	12	x	m	0	0	all	-	q	Eu:Sca	1972	CC	579	n	bl	y	n	0	ev	all/unsp	nev any	st
DESTE2	16		m	0	0	all	-	q	SCAmer	1993	CC	463	n	bl	n	n	2	ev	all/unsp	nev any	or
DOLL	82	x	m	0	0	all	-	KI	Eu:UK	1948	CC	1465	n	V	n	n	0	ev	all/unsp	nev any	st
DOLL	84	x	f	0	0	all	-	KI	Eu:UK	1948	CC	1465	n	V	n	n	0	ev	all/unsp	nev any	st
DORGAN	113		m	0	0	wh	-	q	NAmer	1980	CC	2026	n	bl	y	y	2	ev	cig+/-ot	nev any	or
DORGAN	98		f	0	0	all	-	q	NAmer	1980	CC	2026	n	bl	y	y	3	ev	cig+/-ot	nev any	or
DORN	338		m	0	0	wh	8	q	NAmer	1954	pr	5097	n	bl	n	n	1	cu	cig only	nev any	ot
DOSEME	19	x	m	0	0	all	-	q	Eu:bal	1979	CC	1210	n	bl	n	n	0	ev	cig+/-ot	nev cigs	st
ENGELA	62		m	0	0	all	0	q	Eu:Sca	1964	pr	435	n	bl	n	n	7	ev	cig+/-ot	nev cigs	ot
FAN	3		c	0	0	all	-	q	As:Chi	1990	CC	403	n	ot	y	n	0	ev	cig+/-ot	nev cigs	ot
GAO	7	x	m	0	0	all	-	q	As:Chi	1984	CC	1405	n	ot	n	n	0	ev	cig+/-ot	nev cigs	st
GAO	17	x	f	0	0	all	-	q	As:Chi	1984	CC	1405	n	ot	n	n	0	ev	cig+/-ot	nev cigs	st
GER	5	x	c	0	0	all	-	q+s	As:oth	1990	CC	141	n	ot	y	n	0	ev	all/unsp	nev any	st
HAENSZ	16	x	f	0	0	all	-	q+u	NAmer	1955	CC	158	n	bl	n	y	0	ev	all/unsp	nev any	or
HAMMON	73	x	m	0	0	wh	0	not a	NAmer	1952	pr	448	n	bl	n	n	0	ev	all/unsp	nev any	st
HEGMAN	2		c	0	0	all	-	q	NAmer	1989	CC	282	n	bl	y	y	0	ev	all/unsp	nev any	st
HINDS	23		f	0	0	o	-	q+s	NAmer	1968	CC	292	n	bl	n	n	3	ev	all/unsp	nev any	st
ISHIMA	1	x	c	0	0	all	-	q	As:Jap	1961	CC	180	n	bl	y	y	0	ev	all/unsp	nev any	st
JAHN	46		m	0	0	all	-	q	Eu:Ger	1988	CC	1004	n	bl	n	n	0	ev	all/unsp	nev any	st
JAIN	8	x	m	0	0	all	-	q	NAmer	1981	CC	845	n	V	y	n	0	ev	cig+/-ot	nev cigs	st
JAIN	3	x	f	0	0	all	-	q	NAmer	1981	CC	845	n	V	y	n	0	ev	cig+/-ot	nev cigs	st
JEDRYC	7	x	m	0	0	all	-	q	Eu:est	1980	CC	1630	n	bl	y	n	0	ev	cig+/-ot	nev any	st
JOLY	54		m	0	0	all	-	q	SCAmer	1978	CC	826	n	bl	n	n	0	ev	cig+/-ot	nev any	st
JOLY	52		f	0	0	all	-	q	SCAmer	1978	CC	826	n	bl	n	n	0	ev	cig+/-ot	nev any	st
JUSSAW	23		m	0	0	all	-	KI	As:Ind	1964	CC	792	n	V	n	n	0	ev	all/unsp	nev any	st
KATSOU	35	x	f	0	0	all	-	KI	Eu:bal	1987	CC	101	n	bl	n	n	0	ev	all/unsp	nev any	st
KHUDER	24		m	0	0	all	-	q	NAmer	1985	CC	482	n	bl	n	y	0	ev	cig+/-ot	nev cigs	ot
KIHARA	26		c	0	0	jap	-	q	As:Jap	1991	CC	440	n	bl	n	n	0	ev	all/unsp	nev any	st
KOO	6		f	0	0	all	-	q+s	As:HK	1981	CC	200	n	bl	n	n	0	ev	all/unsp	nev any	st
KREYBE	16	x	m	0	0	all	-	KI	Eu:Sca	1948	CC	300	n	bl	n	y	0	ev	all/unsp	nev any	st
KREYBE	33	x	f	0	0	all	-	KI	Eu:Sca	1948	CC	300	n	bl	n	y	0	ev	all/unsp	nev any	st
LAMTH	1		f	0	0	ch	-	q	As:HK	1983	CC	445	n	bl	n	n	0	ev	all/unsp	nev any	or
LAMWK	2		f	0	0	ch	-	q	As:HK	1981	CC	163	n	bl	n	n	0	ev	all/unsp	nev any	st
LAMWK2	1		m	0	0	all	-	q	As:HK	1976	CC	480	n	bl	n	n	0	ev	all/unsp	nev any	st
LAMWK2	5		f	0	0	all	-	q	As:HK	1976	CC	480	n	bl	n	n	0	ev	all/unsp	nev any	st
LOMBA2	2		f	0	0	all	-	q+u	NAmer	1960	CC	225	n	bl	n	n	0	ev	cig+/-ot	nev cigs	st
LUBIN	33		m	0	0	all	-	KI	As:Chi	1984	CC	427	m	ot	y	n	0	ev	all/unsp	nev any	st
LUBIN2	145		m	0	0	all	-	q	Eu:mul	1976	CC	7804	n	bl	n	y	0	ev	cig+/-ot	nev any	st
LUBIN2	165		f	0	0	all	-	q	Eu:mul	1976	CC	7804	n	bl	n	y	0	ev	cig+/-ot	nev any	st
LUO	2	x	c	0	0	all	-	q	As:Chi	1990	CC	102	n	ot	n	y	0	ev	cig+/-ot	nev cigs	st
MATOS	66	x	m	0	0	all	-	q	SCAmer	1994	CC	200	n	bl	n	n	0	ev	cig+/-ot	nev any	st
MATSUD	11		m	0	0	all	-	q	As:Jap	1965	CC	179	n	bl	n	n	0	ev	cig+/-ot	nev cigs	st
NOU	1		m	0	0	all	-	q	Eu:Sca	1971	CC	273	n	bl	y	n	0	ev	all/unsp	nev any	st
NOU	6		f	0	0	all	-	q	Eu:Sca	1971	CC	273	n	bl	y	n	0	ev	all/unsp	nev any	st
ORMOS	8		m	0	0	all	-	q	Eu:est	1947	CC	119	n	bl	y	y	0	ev	cig+/-ot	nev any	st
OSANN	18	x	m	0	0	all	-	q	NAmer	1984	CC	1986	n	bl	n	n	0	ev	cig+/-ot	nev cigs	st

Table 2C1 - 4

IESLC - Meta-anal of Ever Smoking (or Current if Ever not available), Any prod (or Cigs if Any not avail)
Squamous
Least adjusted

REF	NRR	X	SEX	AGEL	AGEH	RACE	YF	LC TYPE	LOC	START	ST	NLC	R	VB	P	H	AD	SM	PRODUCT	DENOM	De
OSANN	22	x	f	0	0	all	-	q	NAmer	1984	CC	1986	n	bl	n	n	0	ev	cig+/-ot	nev cigs	st
OSANN2	7	x	f	0	0	all	-	KI	NAmer	1964	ot	217	n	bl	n	y	0	ev	cig+/-ot	nev cigs	st
PEZZOT	6		m	0	0	all	-	q	SCAmer	1987	CC	215	n	bl	n	y	0	ev	cig only	nev cigs	ot
SCHWAR	10		m	40	54	wh	-	q	NAmer	1984	CC	5588	n	bl	y	y	0	ev	cig+/-ot	nev cigs	st
SCHWAR	9		m	40	54	bl	-	q	NAmer	1984	CC	5588	n	bl	y	y	0	ev	cig+/-ot	nev cigs	st
SCHWAR	18		f	40	54	wh	-	q	NAmer	1984	CC	5588	n	bl	y	y	0	ev	cig+/-ot	nev cigs	ot
SCHWAR	17		f	40	54	bl	-	q	NAmer	1984	CC	5588	n	bl	y	y	0	ev	cig+/-ot	nev cigs	ot
SEOW	3		f	0	0	ch	-	q	As:oth	1997	CC	153	n	bl	n	y	0	ev	cig+/-ot	nev cigs	st
SIEMIA	11	x	m	0	0	all	-	q	NAmer	1979	CC	857	n	V	y	y	0	ev	cig+/-ot	nev cigs	st
SOBUE	3	x	m	0	0	all	-	q	As:Jap	1986	CC	1376	n	bl	n	y	0	ev	cig+/-ot	nev cigs	st
SOBUE	19	x	f	0	0	all	-	q	As:Jap	1986	CC	1376	n	bl	n	y	0	ev	cig+/-ot	nev cigs	st
SOBUE2	1		m	0	0	all	-	q	As:Jap	1965	CC	2083	n	bl	n	n	2	cu	cig+/-ot	nev any	or
SOBUE2	5		f	0	0	all	-	q	As:Jap	1965	CC	2083	n	bl	n	n	2	cu	cig+/-ot	nev any	or
STASZE	12		m	0	0	all	-	q	Eu:est	1954	CC	281	n	bl	n	y	0	ev	all/unsp	nev any	ot
STASZE	38		f	0	0	all	-	q	Eu:est	1954	CC	281	n	bl	n	y	0	ev	all/unsp	nev any	ot
STAYNE	3		m	0	0	all	-	q	NAmer	1969	CC	420	n	bl	n	n	0	ev	all/unsp	nev any	st
SUZUK2	12	x	c	0	0	all	-	q	SCAmer	1991	CC	123	n	bl	n	y	0	ev	all/unsp	nev any	st
SVENSS	57	x	f	0	0	all	-	q	Eu:Sca	1983	CC	210	n	bl	n	n	0	ev	all/unsp	nev any	st
TIZZAN	18		c	0	0	all	-	q+u	Eu:wst	1959	CC	1358	n	bl	n	n	0	ev	all/unsp	nev any	st
TOKARS	9	x	c	0	0	all	-	q	Eu:est	1966	ot	162	o	bl	n	y	0	ev	all/unsp	nev any	st
TSUGAN	13		m	0	0	all	-	q	As:Jap	1976	CC	134	n	bl	n	y	0	ev	all/unsp	nev any	ot
WAKAI	15	x	m	0	0	all	-	q	As:Jap	1988	CC	333	n	bl	n	y	0	ev	all/unsp	nev any	st
WAKAI	33	x	f	0	0	all	-	q	As:Jap	1988	CC	333	n	bl	n	y	0	ev	all/unsp	nev any	st
WU	14	x	f	0	0	wh	-	q	NAmer	1981	CC	220	n	bl	n	y	0	ev	all/unsp	nev any	st
WUWILL	9		f	0	0	all	-	q	As:Chi	1985	CC	965	n	ot	n	n	3	ev	cig+/-ot	nev cigs	or
WYNDE2	7		m	0	0	all	-	KI	NAmer	1962	CC	404	n	bl	n	y	0	ev	all/unsp	nev any	st
WYNDE3	9		m	0	0	all	-	KI	NAmer	1966	CC	350	n	bl	n	y	0	ev	all/unsp	nev any	st
WYNDE3	132		f	0	0	all	-	KI	NAmer	1966	CC	350	n	bl	n	y	0	ev	all/unsp	nev any	st
WYNDE4	35	x	m	0	0	all	-	not a	NAmer	1948	CC	684	n	bl	y	n	0	ev	all/unsp	nev any	st
WYNDE4	54		f	0	0	all	-	not a	NAmer	1948	CC	684	n	bl	y	n	2	ev	all/unsp	nev any	ot
WYNDE6	66		m	0	0	all	-	KI	NAmer	1969	CC	4423	n	bl	n	y	0	ev	all/unsp	nev any	st
WYNDE6	411	x	f	0	0	wh	-	q	NAmer	1969	CC	4423	n	bl	n	y	0	ev	cig+/-ot	nev cigs	st
XU3	19	x	m	0	0	all	-	KI	As:Chi	1981	CC	135	n	ot	n	n	0	ev	all/unsp	nev any	st
XU3	23	x	f	0	0	all	-	KI	As:Chi	1981	CC	135	n	ot	n	n	0	ev	all/unsp	nev any	st
ZHENG	5		m	0	0	all	-	q	As:Chi	1982	CC	540	n	ot	*	y	0	ev	cig+/-ot	nev cigs	st
ZHENG	18		f	0	0	all	-	q	As:Chi	1982	CC	540	n	ot	*	y	0	ev	cig+/-ot	nev cigs	st
ZHOU	8		m	0	0	all	-	q	As:Chi	1978	CC	1360	n	ot	n	n	0	ev	all/unsp	nev any	st
ZHOU	9		f	0	0	all	-	q	As:Chi	1978	CC	1360	n	ot	n	n	0	ev	all/unsp	nev any	st

Cigarette type is all/unspec for all RRs

Table 2C1 - 5

IESLC - Meta-anal of Ever Smoking (or Current if Ever not available), Any prod (or Cigs if Any not avail)
Squamous
Least adjusted

REF	NRR	SEX	AD	Number Exposed Case	Number Exposed Cont	Non-exposed Case	Non-exposed Cont	RR	95.00%CI
*ABRAHA	1	m	0	142	10351	0	3365	92.66~	(5.77-1488.21)
*ABRAHA	4	f	0	17	5256	7	11589	5.35	(2.22- 12.90)
Subtotal ABRAHA								6.95	(3.00- 16.06)
ALDERS	52	m	2	-	-	-	-	14.70	(3.40- 63.64)
ALDERS	55	f	2	-	-	-	-	6.09	(2.68- 13.82)
Subtotal ALDERS								7.52	(3.67- 15.37)
*ANDERS	10	f	0	63	96164	5	195158	25.57	(10.29- 63.56)
BAND	5	m	2	-	-	-	-	37.45	(17.62- 79.58)
BARBON	110	m	0	261	567	6	188	14.42	(6.31- 32.94)
BECHER	11	f	1	-	-	-	-	10.69	(2.43- 47.00)
*BOUCOT	70	m	0	38	22177	0	7551	26.22~	(1.61- 426.71)
BRESLO	36	c	0	457	462	15	56	3.69	(2.06- 6.62)
BROWN2	6	m	2	-	-	-	-	11.10	(9.50- 12.90)
BROWN2	5	f	2	-	-	-	-	20.10	(16.40- 24.80)
Subtotal BROWN2								13.69	(12.11- 15.49)
BUFFLE	49	m	0	-	-	-	-	14.03	(4.73- 41.61)
BUFFLE	62	f	0	58	166	3	112	13.04	(3.99- 42.66)
Subtotal BUFFLE								13.57	(6.09- 30.24)
BYERS1	1	m	0	299	695	22	424	8.29	(5.29- 13.00)
CHAN	11	m	0	114	161	2	43	15.22	(3.61- 64.12)
CHAN	15	f	0	44	50	19	139	6.44	(3.44- 12.06)
Subtotal CHAN								7.39	(4.16- 13.13)
CHOI	62	m	0	160	465	6	95	5.45	(2.34- 12.67)
CHOI	64	f	0	11	26	10	164	6.94	(2.68- 17.96)
Subtotal CHOI								6.06	(3.22- 11.40)
COMSTO	66	m	0	44	229	2	84	8.07	(1.91- 34.02)
COMSTO	78	f	0	17	87	0	115	46.20~	(2.74- 778.83)
Subtotal COMSTO								11.56	(3.21- 41.67)
CORREA	35	c	1	-	-	-	-	28.30	(18.60- 43.20)
*CPSI	403	m	1	-	-	-	-	29.35	(4.02- 214.28)
*CPSI	405	f	1	-	-	-	-	4.25	(1.23- 14.68)
Subtotal CPSI								7.30	(2.55- 20.90)
*CPSII	114	m	1	-	-	-	-	39.26	(10.38- 148.55)
*CPSII	117	f	1	-	-	-	-	78.91	(15.83- 393.37)
Subtotal CPSII								52.16	(18.72- 145.32)
DAMBER	12	m	0	271	169	14	103	11.80	(6.54- 21.29)
DESTE2	16	m	2	-	-	-	-	13.20	(4.70- 37.10)
DOLL	82	m	0	829	1296	3	61	13.01	(4.07- 41.59)
DOLL	84	f	0	32	49	16	59	2.41	(1.18- 4.90)
Subtotal DOLL								3.81	(2.08- 6.98)
DORGAN	113	m	2	-	-	-	-	18.90	(7.00- 51.30)
DORGAN	98	f	3	-	-	-	-	11.10	(7.20- 17.10)
Subtotal DORGAN								12.08	(8.12- 17.96)
*DORN	338	m	1	-	-	-	-	17.09	(8.96- 32.60)
DOSEME	19	m	0	434	536	58	293	4.09	(3.00- 5.57)
*ENGELA	62	m	7	-	-	-	-	6.45	(1.97- 21.11)
FAN	3	c	0	75	595	6	556	11.68	(5.04- 27.04)
GAO	7	m	0	314	558	13	202	8.74	(4.91- 15.58)
GAO	17	f	0	66	130	53	605	5.80	(3.85- 8.72)
Subtotal GAO								6.65	(4.76- 9.28)
GER	5	c	0	48	156	11	80	2.24	(1.10- 4.54)
HAENSZ	16	f	0	58	103	44	236	3.02	(1.92- 4.76)
*HAMMON	73	m	0	286	510108	4	115884	16.24	(6.05- 43.57)
HEGMAN	2	c	0	89	1202	5	2080	30.80	(12.48- 76.03)
HINDS	23	f	3	-	-	-	-	16.13	(7.66- 33.97)
ISHIMA	1	c	0	53	33	5	25	8.03	(2.80- 23.04)
JAHN	46	m	0	351	701	3	138	23.03	(7.29- 72.81)
JAIN	8	m	0	154	277	2	85	23.63	(5.73- 97.35)
JAIN	3	f	0	103	196	6	214	18.74	(8.05- 43.66)
Subtotal JAIN								19.92	(9.64- 41.17)
JEDRYC	7	m	0	337	1054	6	289	15.40	(6.80- 34.89)
JOLY	54	m	0	203	709	2	218	31.21	(7.69- 126.68)
JOLY	52	f	0	48	122	6	283	18.56	(7.74- 44.51)
Subtotal JOLY								21.47	(10.22- 45.09)
JUSSAW	23	m	0	89	168	13	624	25.43	(13.87- 46.63)
KATSOU	35	f	0	28	22	14	67	6.09	(2.73- 13.59)
KHUDER	24	m	0	176	-	9	-	7.82	(3.87- 15.77)
KIHARA	26	c	0	132	232	5	237	26.97	(10.84- 67.08)
KOO	6	f	0	61	63	32	137	4.15	(2.46- 6.98)
KREYBE	16	m	0	210	3514	3	644	12.83	(4.09- 40.22)
KREYBE	33	f	0	2	328	3	657	1.34	(0.22- 8.03)

Table 2C1 - 5

IESLC - Meta-anal of Ever Smoking (or Current if Ever not available), Any prod (or Cigs if Any not avail)
Squamous
Least adjusted

REF	NRR	SEX	AD	Number Exposed Case	Number Exposed Cont	Non-exposed Case	Non-exposed Cont	RR	95.00%CI
Subtotal KREYBE								6.68	(2.55- 17.51)
LAMTH	1	f	0	63	20	28	72	8.10	(4.16- 15.77)
LAMWK	2	f	0	21	41	7	144	10.54	(4.19- 26.52)
LAMWK2	1	m	0	129	161	5	43	6.89	(2.65- 17.90)
LAMWK2	5	f	0	35	50	15	139	6.49	(3.27- 12.88)
Subtotal LAMWK2								6.62	(3.79- 11.56)
LOMBA2	2	f	0	94	353	15	239	4.24	(2.40- 7.50)
LUBIN	33	m	0	330	939	4	72	6.33	(2.29- 17.45)
LUBIN2	145	m	0	3587	10433	54	2616	16.66	(12.69- 21.86)
LUBIN2	165	f	0	200	567	72	1180	5.78	(4.34- 7.71)
Subtotal LUBIN2								10.10	(8.29- 12.31)
LUO	2	c	0	34	146	5	160	7.45	(2.84- 19.56)
MATOS	66	m	0	47	283	3	110	6.09	(1.86- 19.97)
MATSUD	11	m	0	103	3314	1	1255	39.01	(5.44- 279.84)
NOU	1	m	0	110	247	2	122	27.17	(6.60- 111.85)
NOU	6	f	0	5	92	2	261	7.09	(1.35- 37.19)
Subtotal NOU								15.42	(5.26- 45.22)
ORMOS	8	m	0	27	1034	2	777	10.14	(2.41- 42.79)
OSANN	18	m	0	352	1018	8	833	36.00	(17.76- 72.99)
OSANN	22	f	0	159	563	12	1093	25.72	(14.18- 46.66)
Subtotal OSANN								29.58	(18.76- 46.64)
OSANN2	7	f	0	112	61	7	58	15.21	(6.54- 35.38)
PEZZOT	6	m	0	85	317	0	116	62.74~	(3.86-1019.50)
SCHWAR	10	m	0	80	178	1	73	32.81	(4.48- 240.23)
SCHWAR	9	m	0	41	39	4	7	1.84	(0.50- 6.78)
SCHWAR	18	f	0	29	108	0	79	43.23~	(2.60- 718.15)
SCHWAR	17	f	0	21	28	0	41	62.61~	(3.64-1076.10)
Subtotal SCHWAR								7.71	(2.96- 20.10)
SEOW	3	f	0	21	15	10	125	17.50	(6.95- 44.09)
SIEMIA	11	m	0	356	428	3	105	29.11	(9.16- 92.52)
SOBUE	3	m	0	422	1013	3	128	17.77	(5.63- 56.16)
SOBUE	19	f	0	36	232	14	857	9.50	(5.04- 17.91)
Subtotal SOBUE								10.99	(6.31- 19.15)
SOBUE2	1	m	2	-	-	-	-	5.20	(4.20- 6.50)
SOBUE2	5	f	2	-	-	-	-	7.20	(4.80- 10.80)
Subtotal SOBUE2								5.59	(4.62- 6.78)
STASZE	12	m	0	137	754	0	158	57.77~	(3.58- 933.17)
STASZE	38	f	0	1	153	0	1660	32.45~	(1.32- 800.04)
Subtotal STASZE								45.09	(5.52- 368.55)
STAYNE	3	m	0	130	567	22	333	3.47	(2.17- 5.56)
SUZUK2	12	c	0	75	36	5	44	18.33	(6.70- 50.17)
SVENSS	57	f	0	48	89	5	120	12.94	(4.95- 33.84)
TIZZAN	18	c	0	333	939	55	419	2.70	(1.99- 3.67)
TOKARS	9	c	0	45	77	2	19	5.55	(1.24- 24.95)
TSUGAN	13	m	0	20	15	0	5	14.55~	(0.75- 283.37)
WAKAI	15	m	0	113	424	2	65	8.66	(2.09- 35.92)
WAKAI	33	f	0	16	31	3	145	24.95	(6.85- 90.87)
Subtotal WAKAI								15.46	(5.94- 40.24)
WU	14	f	0	69	41	2	30	25.24	(5.73- 111.19)
WUWILL	9	f	3	-	-	-	-	4.20	(3.00- 5.90)
WYNDE2	7	m	0	347	616	3	105	19.72	(6.21- 62.59)
WYNDE3	9	m	0	207	332	3	88	18.29	(5.71- 58.56)
WYNDE3	132	f	0	25	56	5	76	6.79	(2.45- 18.82)
Subtotal WYNDE3								10.44	(4.85- 22.49)
WYNDE4	35	m	0	597	665	8	115	12.91	(6.25- 26.65)
WYNDE4	54	f	2	-	-	-	-	5.82	(2.55- 13.31)
Subtotal WYNDE4								9.13	(5.29- 15.74)
WYNDE6	66	m	0	1744	1996	29	617	18.59	(12.74- 27.13)
WYNDE6	411	f	0	153	275	12	673	31.20	(17.05- 57.09)
Subtotal WYNDE6								21.51	(15.61- 29.63)
XU3	19	m	0	39	68	3	31	5.93	(1.70- 20.66)
XU3	23	f	0	15	11	2	25	17.05	(3.32- 87.61)
Subtotal XU3								8.74	(3.24- 23.59)
ZHENG	5	m	0	156	218	4	94	16.82	(6.05- 46.71)
ZHENG	18	f	0	43	44	33	184	5.45	(3.11- 9.54)
Subtotal ZHENG								7.07	(4.33- 11.56)
ZHOU	8	m	0	343	41	96	36	3.14	(1.90- 5.18)
ZHOU	9	f	0	35	7	42	32	3.81	(1.50- 9.68)
Subtotal ZHOU								3.28	(2.11- 5.10)
Partial Totals				17364	688312	1046	358639		

*prospective study ~ With 0.5 adjustment for zero

Table 2C1 - 5

IESLC - Meta-anal of Ever Smoking (or Current if Ever not available), Any prod (or Cigs if Any not avail)
Squamous
Least adjusted

REF	NRR	SEX	AD	Ys	Ws	Qs	Ps
*ABRAHA	1	m	0	4.53	0.50	2.68	0.0014
*ABRAHA	4	f	0	1.68	4.97	1.42	0.0002
Subtotal ABRAHA				1.94	5.46	4.09	
ALDERS	52	m	2	2.69	1.79	0.41	0.0003
ALDERS	55	f	2	1.81	5.71	0.94	0.0000
Subtotal ALDERS				2.02	7.50	1.34	
*ANDERS	10	f	0	3.24	4.63	4.91	0.0000
BAND	5	m	2	3.62	6.76	13.46	0.0000
BARBON	110	m	0	2.67	5.63	1.18	0.0000
BECHER	11	f	1	2.37	1.75	0.04	0.0017
*BOUCOT	70	m	0	3.27	0.49	0.55	0.0217
BRESLO	36	c	0	1.31	11.25	9.23	0.0000
BROWN2	6	m	2	2.41	164.17	6.24	0.0000
BROWN2	5	f	2	3.00	89.84	55.89	0.0000
Subtotal BROWN2				2.62	254.01	62.13	
BUFFLE	49	m	0	2.64	3.25	0.60	0.0000
BUFFLE	62	f	0	2.57	2.74	0.35	0.0000
Subtotal BUFFLE				2.61	5.99	0.95	
BYERS1	1	m	0	2.12	19.01	0.18	0.0000
CHAN	11	m	0	2.72	1.86	0.48	0.0002
CHAN	15	f	0	1.86	9.75	1.19	0.0000
Subtotal CHAN				2.00	11.61	1.68	
CHOI	62	m	0	1.70	5.39	1.44	0.0001
CHOI	64	f	0	1.94	4.25	0.32	0.0001
Subtotal CHOI				1.80	9.63	1.76	
COMSTO	66	m	0	2.09	1.86	0.03	0.0045
COMSTO	78	f	0	3.83	0.48	1.26	0.0078
Subtotal COMSTO				2.45	2.34	1.29	
CORREA	35	c	1	3.34	21.64	27.67	0.0000
*CPSI	403	m	1	3.38	0.97	1.32	0.0009
*CPSI	405	f	1	1.45	2.50	1.46	0.0222
Subtotal CPSI				1.99	3.47	2.79	
*CPSII	114	m	1	3.67	2.17	4.61	0.0000
*CPSII	117	f	1	4.37	1.49	6.92	0.0000
Subtotal CPSII				3.95	3.66	11.54	
DAMBER	12	m	0	2.47	11.02	0.72	0.0000
DESTE2	16	m	2	2.58	3.60	0.49	0.0000
DOLL	82	m	0	2.57	2.84	0.36	0.0000
DOLL	84	f	0	0.88	7.63	13.56	0.0152
Subtotal DOLL				1.34	10.47	13.91	
DORGAN	113	m	2	2.94	3.87	2.05	0.0000
DORGAN	98	f	3	2.41	20.54	0.78	0.0000
Subtotal DORGAN				2.49	24.41	2.83	
*DORN	338	m	1	2.84	9.21	3.62	0.0000
DOSEME	19	m	0	1.41	40.28	26.00	0.0000
*ENGELA	62	m	7	1.86	2.73	0.33	0.0021
FAN	3	c	0	2.46	5.45	0.33	0.0000
GAO	7	m	0	2.17	11.51	0.02	0.0000
GAO	17	f	0	1.76	23.06	4.77	0.0000
Subtotal GAO				1.89	34.57	4.79	
GER	5	c	0	0.81	7.65	15.14	0.0259
HAENSZ	16	f	0	1.11	18.55	22.71	0.0000
*HAMMON	73	m	0	2.79	3.94	1.31	0.0000
HEGMAN	2	c	0	3.43	4.70	6.95	0.0000
HINDS	23	f	3	2.78	6.93	2.24	0.0000
ISHIMA	1	c	0	2.08	3.46	0.06	0.0001
JAHN	46	m	0	3.14	2.90	2.48	0.0000
JAIN	8	m	0	3.16	1.92	1.73	0.0000
JAIN	3	f	0	2.93	5.37	2.78	0.0000
Subtotal JAIN				2.99	7.29	4.51	
JEDRYC	7	m	0	2.73	5.75	1.57	0.0000
JOLY	54	m	0	3.44	1.96	2.95	0.0000
JOLY	52	f	0	2.92	5.02	2.52	0.0000
Subtotal JOLY				3.07	6.98	5.48	
JUSSAW	23	m	0	3.24	10.45	10.95	0.0000
KATSOU	35	f	0	1.81	5.97	0.98	0.0000
KHUDER	24	m	0	2.06	7.79	0.19	0.0000
KIHARA	26	c	0	3.29	4.63	5.42	0.0000
KOO	6	f	0	1.42	14.12	8.81	0.0000
KREYBE	16	m	0	2.55	2.94	0.34	0.0000
KREYBE	33	f	0	0.29	1.19	4.41	0.7520

Table 2C1 - 5

IESLC - Meta-anal of Ever Smoking (or Current if Ever not available), Any prod (or Cigs if Any not avail)
Squamous
Least adjusted

REF	NRR	SEX	AD	Ys	Ws	Qs	Ps
Subtotal KREYBE				1.90	4.14	4.75	
LAMTH	1	f	0	2.09	8.66	0.12	0.0000
LAMWK	2	f	0	2.35	4.51	0.09	0.0000
LAMWK2	1	m	0	1.93	4.22	0.33	0.0001
LAMWK2	5	f	0	1.87	8.17	0.96	0.0000
Subtotal LAMWK2				1.89	12.38	1.29	
LOMBA2	2	f	0	1.45	11.86	6.97	0.0000
LUBIN	33	m	0	1.84	3.73	0.50	0.0004
LUBIN2	145	m	0	2.81	51.88	18.72	0.0000
LUBIN2	165	f	0	1.75	46.51	9.73	0.0000
Subtotal LUBIN2				2.31	98.39	28.46	
LUO	2	c	0	2.01	4.12	0.17	0.0000
MATOS	66	m	0	1.81	2.72	0.45	0.0029
MATSUD	11	m	0	3.66	0.99	2.08	0.0003
NOU	1	m	0	3.30	1.92	2.28	0.0000
NOU	6	f	0	1.96	1.40	0.09	0.0205
Subtotal NOU				2.74	3.32	2.37	
ORMOS	8	m	0	2.32	1.85	0.02	0.0016
OSANN	18	m	0	3.58	7.69	14.47	0.0000
OSANN	22	f	0	3.25	10.83	11.61	0.0000
Subtotal OSANN				3.39	18.52	26.08	
OSANN2	7	f	0	2.72	5.39	1.40	0.0000
PEZZOT	6	m	0	4.14	0.49	1.84	0.0036
SCHWAR	10	m	0	3.49	0.97	1.58	0.0006
SCHWAR	9	m	0	0.61	2.26	5.80	0.3596
SCHWAR	18	f	0	3.77	0.49	1.18	0.0086
SCHWAR	17	f	0	4.14	0.47	1.76	0.0044
Subtotal SCHWAR				2.04	4.19	10.32	
SEOW	3	f	0	2.86	4.50	1.90	0.0000
SIEMIA	11	m	0	3.37	2.87	3.86	0.0000
SOBUE	3	m	0	2.88	2.90	1.29	0.0000
SOBUE	19	f	0	2.25	9.55	0.01	0.0000
Subtotal SOBUE				2.40	12.46	1.30	
SOBUE2	1	m	2	1.65	80.57	25.57	0.0000
SOBUE2	5	f	2	1.97	23.37	1.32	0.0000
Subtotal SOBUE2				1.72	103.93	26.89	
STASZE	12	m	0	4.06	0.50	1.69	0.0043
STASZE	38	f	0	3.48	0.37	0.60	0.0333
Subtotal STASZE				3.81	0.87	2.29	
STAYNE	3	m	0	1.24	17.27	16.17	0.0000
SUZUK2	12	c	0	2.91	3.79	1.84	0.0000
SVENSS	57	f	0	2.56	4.16	0.51	0.0000
TIZZAN	18	c	0	0.99	40.59	60.23	0.0000
TOKARS	9	c	0	1.71	1.70	0.42	0.0254
TSUGAN	13	m	0	2.68	0.44	0.09	0.0772
WAKAI	15	m	0	2.16	1.90	0.01	0.0029
WAKAI	33	f	0	3.22	2.30	2.32	0.0000
Subtotal WAKAI				2.74	4.20	2.33	
WU	14	f	0	3.23	1.75	1.81	0.0000
WUWILL	9	f	3	1.44	33.59	20.28	0.0000
WYNDE2	7	m	0	2.98	2.88	1.70	0.0000
WYNDE3	9	m	0	2.91	2.84	1.37	0.0000
WYNDE3	132	f	0	1.91	3.69	0.33	0.0002
Subtotal WYNDE3				2.35	6.53	1.69	
WYNDE4	35	m	0	2.56	7.31	0.87	0.0000
WYNDE4	54	f	2	1.76	5.63	1.14	0.0000
Subtotal WYNDE4				2.21	12.93	2.02	
WYNDE6	66	m	0	2.92	26.90	13.58	0.0000
WYNDE6	411	f	0	3.44	10.53	15.89	0.0000
Subtotal WYNDE6				3.07	37.42	29.47	
XU3	19	m	0	1.78	2.46	0.46	0.0052
XU3	23	f	0	2.84	1.43	0.56	0.0007
Subtotal XU3				2.17	3.90	1.02	
ZHENG	5	m	0	2.82	3.68	1.37	0.0000
ZHENG	18	f	0	1.70	12.24	3.27	0.0000
Subtotal ZHENG				1.96	15.92	4.64	
ZHOU	8	m	0	1.14	15.27	17.44	0.0000
ZHOU	9	f	0	1.34	4.42	3.38	0.0049
Subtotal ZHOU				1.19	19.68	20.81	

Table 2C1 - 5

IESLC - Meta-anal of Ever Smoking (or Current if Ever not available), Any prod (or Cigs if Any not avail)
Squamous
Least adjusted

	N	110
	NS	78
	Wt	1144.34
Het	Chi	574.79
Het	df	109
Het	P	***
Fixed	RR	9.13
	RRl	8.62
	RRu	9.68
	P	+++
Random	RR	10.48
	RRl	8.99
	RRu	12.23
	P	+++
Asymm	P	(*)

Table 2C1 - 6

IESLC - Meta-anal of Ever Smoking (or Current if Ever not available), Any prod (or Cigs if Any not avail)
Squamous
Least adjusted

		Sex		
	combined	male	female	Total
N	11	54	45	110
NS	11	53	44	108
Wt	108.99	583.06	452.29	1144.34
Het Chi	117.26	218.15	225.13	574.79
Het df	10	53	44	109
Het P	***	***	***	***
Fixed RR	6.73	9.89	8.88	9.13
RRl	5.57	9.12	8.10	8.62
RRu	8.12	10.72	9.73	9.68
P	+++	+++	+++	+++
Random RR	9.05	12.27	9.08	10.48
RRl	4.46	9.98	7.16	8.99
RRu	18.35	15.09	11.52	12.23
P	+++	+++	+++	+++
Between Chi				14.24
Between df				2
Between P				***
Btwn(F) P				N.S.
Btwn(R) P				N.S.

		Lung cancer type				
	q	q+s	q+u	KI	not a	Total
N	82	7	3	14	4	110
NS	57	6	3	10	3	79
Wt	901.17	63.70	71.00	80.35	28.13	1144.34
Het Chi	362.04	55.30	1.87	44.89	10.32	574.79
Het df	81	6	2	13	3	109
Het P	***	***	N.S.	***	*	***
Fixed RR	9.78	9.77	3.00	11.90	6.89	9.13
RRl	9.16	7.64	2.38	9.56	4.76	8.62
RRu	10.44	12.49	3.78	14.80	9.97	9.68
P	+++	+++	+++	+++	+++	+++
Random RR	11.53	8.69	3.00	9.86	7.80	10.48
RRl	9.75	3.88	2.38	6.27	3.84	8.99
RRu	13.62	19.47	3.78	15.49	15.84	12.23
P	+++	+++	+++	+++	+++	+++
Between Chi						100.37
Between df						4
Between P						***
Btwn(F) P						***
Btwn(R) P						***

					Location				
	NAmer	UK	Scand	othEur	China	Japan	othAs	other	Total
N	44	4	7	15	12	10	12	6	110
NS	30	2	5	12	8	7	9	5	78
Wt	537.69	17.97	25.36	211.15	120.97	130.10	83.52	17.58	1144.34
Het Chi	216.64	8.97	8.12	106.77	20.37	25.74	37.39	4.65	574.79
Het df	43	3	6	14	11	9	11	5	109
Het P	***	*	N.S.	***	*	**	***	N.S.	***
Fixed RR	12.89	5.06	10.59	6.66	5.45	6.70	7.27	15.93	9.13
RRl	11.85	3.18	7.18	5.82	4.56	5.65	5.86	9.98	8.62
RRu	14.03	8.03	15.63	7.63	6.52	7.96	9.01	25.42	9.68
P	+++	+++	+++	+++	+++	+++	+++	+++	+++
Random RR	13.99	6.38	10.14	8.94	6.02	10.55	7.61	15.93	10.48
RRl	11.10	2.72	6.21	5.60	4.59	6.88	5.06	9.98	8.99
RRu	17.64	14.96	16.57	14.26	7.90	16.17	11.45	25.42	12.23
P	+++	+++	+++	+++	+++	+++	+++	+++	+++
Between Chi									146.13
Between df									7
Between P									***
Btwn(F) P									***
Btwn(R) P									***

Table 2C1 - 6

IESLC - Meta-anal of Ever Smoking (or Current if Ever not available), Any prod (or Cigs if Any not avail)
Squamous
Least adjusted

	Detailed Country in "other Europe"					
	multi	Germany	othWest	East	Balkans	Total
N	2	2	2	7	2	15
NS	1	2	2	5	2	12
Wt	98.39	4.65	46.22	15.63	46.25	211.15
Het Chi	27.46	0.64	13.87	8.07	0.82	106.77
Het df	1	1	1	6	1	14
Het P	***	N.S.	***	N.S.	N.S.	***
Fixed RR	10.10	17.25	3.31	10.54	4.31	6.66
RRl	8.29	6.95	2.48	6.42	3.23	5.82
RRu	12.31	42.81	4.42	17.31	5.74	7.63
P	+++	+++	+++	+++	+++	+++
Random RR	9.82	17.25	5.96	11.20	4.31	8.94
RRl	3.48	6.95	1.16	5.91	3.23	5.60
RRu	27.71	42.81	30.71	21.22	5.74	14.26
P	+++	+++	+	+++	+++	+++
Between Chi						55.90
Between df						4
Between P						***
Btwn(F) P						(*)
Btwn(R) P						**

	Detailed Country in "other Asia"			
	India	HongKong	other	Total
N	1	7	4	12
NS	1	5	3	9
Wt	10.45	51.28	21.79	83.52
Het Chi	0.00	5.68	12.42	37.39
Het df	0	6	3	11
Het P	N.S.	N.S.	**	***
Fixed RR	25.43	6.43	5.32	7.27
RRl	13.87	4.89	3.49	5.86
RRu	46.63	8.45	8.09	9.01
P	+++	+++	+++	+++
Random RR	25.43	6.43	6.00	7.61
RRl	13.87	4.89	2.53	5.06
RRu	46.63	8.45	14.26	11.45
P	+++	+++	+++	+++
Between Chi				19.29
Between df				2
Between P				***
Btwn(F) P				*
Btwn(R) P				***

	Detailed other continent			
	SCAmer	Auslia	Africa	Total
N	6			6
NS	5			5
Wt	17.58			17.58
Het Chi	4.65			4.65
Het df	5			5
Het P	N.S.			N.S.
Fixed RR	15.93			15.93
RRl	9.98			9.98
RRu	25.42			25.42
P	+++			+++
Random RR	15.93			15.93
RRl	9.98			9.98
RRu	25.42			25.42
P	+++			+++
Between Chi				
Between df				
Between P				N.S.
Btwn(F) P				N.S.
Btwn(R) P				N.S.

Table 2C1 - 6

IESLC - Meta-anal of Ever Smoking (or Current if Ever not available), Any prod (or Cigs if Any not avail)
Squamous
Least adjusted

	Start year of study <1960	1960-69	1970-79	1980-89	1990+	Total
N	18	16	26	42	8	110
NS	13	13	16	28	8	78
Wt	136.79	211.54	255.53	504.02	36.47	1144.34
Het Chi	73.11	97.43	127.03	171.24	25.74	574.79
Het df	17	15	25	41	7	109
Het P	***	***	***	***	***	***
Fixed RR	4.98	8.11	8.85	11.49	9.27	9.13
RRl	4.21	7.09	7.83	10.53	6.70	8.62
RRu	5.88	9.29	10.01	12.54	12.82	9.68
P	+++	+++	+++	+++	+++	+++
Random RR	7.06	10.58	10.21	12.36	10.21	10.48
RRl	4.67	7.08	7.35	9.89	5.44	8.99
RRu	10.69	15.80	14.17	15.45	19.18	12.23
P	+++	+++	+++	+++	+++	+++
Between Chi						80.24
Between df						4
Between P						***
Btwn(F) P						**
Btwn(R) P						N.S.

	Study type (1) CC	other	Total
N	95	15	110
NS	67	11	78
Wt	1101.30	43.04	1144.34
Het Chi	542.22	23.91	574.79
Het df	94	14	109
Het P	***	*	***
Fixed RR	8.98	14.18	9.13
RRl	8.46	10.52	8.62
RRu	9.52	19.12	9.68
P	+++	+++	+++
Random RR	10.08	14.57	10.48
RRl	8.56	9.53	8.99
RRu	11.86	22.28	12.23
P	+++	+++	+++
Between Chi			8.66
Between df			1
Between P			**
Btwn(F) P			N.S.
Btwn(R) P			N.S.

	Study type (2) CC	prosp	other	Total
N	95	11	4	110
NS	67	8	3	78
Wt	1101.30	33.61	9.43	1144.34
Het Chi	542.22	21.04	2.48	574.79
Het df	94	10	3	109
Het P	***	*	N.S.	***
Fixed RR	8.98	14.92	11.85	9.13
RRl	8.46	10.64	6.26	8.62
RRu	9.52	20.92	22.43	9.68
P	+++	+++	+++	+++
Random RR	10.08	16.14	11.85	10.48
RRl	8.56	9.41	6.26	8.99
RRu	11.86	27.67	22.43	12.23
P	+++	+++	+++	+++
Between Chi				9.05
Between df				2
Between P				*
Btwn(F) P				N.S.
Btwn(R) P				N.S.

Table 2C1 - 6

IESLC - Meta-anal of Ever Smoking (or Current if Ever not available), Any prod (or Cigs if Any not avail)
Squamous
Least adjusted

	Study size (number of LC cases)				
	100-249	250-499	500-999	1000+	Total
N	23	31	18	38	110
NS	22	22	12	22	78
Wt	104.17	131.95	129.38	778.84	1144.34
Het Chi	52.31	60.12	67.34	374.69	574.79
Het df	22	30	17	37	109
Het P	***	***	***	***	***
Fixed RR	6.13	9.16	8.56	9.74	9.13
RRl	5.06	7.72	7.20	9.08	8.62
RRu	7.42	10.86	10.17	10.44	9.68
P	+++	+++	+++	+++	+++
Random RR	8.08	10.39	11.66	11.13	10.48
RRl	5.80	8.01	8.01	8.61	8.99
RRu	11.26	13.46	16.98	14.40	12.23
P	+++	+++	+++	+++	+++
Between Chi					20.33
Between df					3
Between P					***
Btwn(F) P					N.S.
Btwn(R) P					N.S.

	Risky occupational population			
	no	mining	othRisky	Total
N	108	1	1	110
NS	76	1	1	78
Wt	1138.91	3.73	1.70	1144.34
Het Chi	573.86	0.00	0.00	574.79
Het df	107	0	0	109
Het P	***	N.S.	N.S.	***
Fixed RR	9.15	6.33	5.55	9.13
RRl	8.64	2.29	1.24	8.62
RRu	9.70	17.45	24.95	9.68
P	+++	+++	+	+++
Random RR	10.58	6.33	5.55	10.48
RRl	9.05	2.29	1.24	8.99
RRu	12.36	17.45	24.95	12.23
P	+++	+++	+	+++
Between Chi				0.93
Between df				2
Between P				N.S.
Btwn(F) P				N.S.
Btwn(R) P				N.S.

	National cigarette tobacco type			
	Virginia	blended	other	Total
N	9	88	13	110
NS	6	63	9	78
Wt	45.34	970.38	128.62	1144.34
Het Chi	40.38	456.01	26.08	574.79
Het df	8	87	12	109
Het P	***	***	*	***
Fixed RR	13.77	9.66	5.17	9.13
RRl	10.29	9.07	4.35	8.62
RRu	18.43	10.29	6.15	9.68
P	+++	+++	+++	+++
Random RR	14.45	11.16	5.64	10.48
RRl	7.30	9.40	4.24	8.99
RRu	28.60	13.24	7.49	12.23
P	+++	+++	+++	+++
Between Chi				52.31
Between df				2
Between P				***
Btwn(F) P				**
Btwn(R) P				***

Table 2C1 - 6

IESLC - Meta-anal of Ever Smoking (or Current if Ever not available), Any prod (or Cigs if Any not avail)
Squamous
Least adjusted

	Any proxy use		
	No/nk	Yes	Total
N	84	26	110
NS	60	18	78
Wt	1005.70	138.64	1144.34
Het Chi	477.19	71.59	574.79
Het df	83	25	109
Het P	***	***	***
Fixed RR	8.64	13.71	9.13
RRl	8.12	11.61	8.62
RRu	9.19	16.19	9.68
P	+++	+++	+++
Random RR	9.78	13.26	10.48
RRl	8.22	9.73	8.99
RRu	11.63	18.08	12.23
P	+++	+++	+++
Between Chi			26.01
Between df			1
Between P			***
Btwn(F) P			*
Btwn(R) P			(*)

	Full histological confirmation		
	No	Yes	Total
N	67	43	110
NS	48	30	78
Wt	592.13	552.21	1144.34
Het Chi	315.99	176.48	574.79
Het df	66	42	109
Het P	***	***	***
Fixed RR	7.05	12.06	9.13
RRl	6.50	11.09	8.62
RRu	7.64	13.11	9.68
P	+++	+++	+++
Random RR	9.53	12.26	10.48
RRl	7.83	9.82	8.99
RRu	11.59	15.30	12.23
P	+++	+++	+++
Between Chi			82.31
Between df			1
Between P			***
Btwn(F) P			***
Btwn(R) P			(*)

	Number of adjustment variables (1)			
	0	1	2+/+nk	Total
N	89	7	14	110
NS	64	5	10	79
Wt	655.52	39.73	449.09	1144.34
Het Chi	388.58	12.83	127.10	574.79
Het df	88	6	13	109
Het P	***	*	***	***
Fixed RR	8.10	22.67	10.04	9.13
RRl	7.50	16.61	9.15	8.62
RRu	8.75	30.93	11.01	9.68
P	+++	+++	+++	+++
Random RR	10.12	20.52	10.16	10.48
RRl	8.45	11.57	7.16	8.99
RRu	12.11	36.40	14.41	12.23
P	+++	+++	+++	+++
Between Chi				46.27
Between df				2
Between P				***
Btwn(F) P				*
Btwn(R) P				(*)

Table 2C1 - 6

IESLC - Meta-anal of Ever Smoking (or Current if Ever not available), Any prod (or Cigs if Any not avail)
Squamous
Least adjusted

	Number of adjustment variables (2) 0	1	2	3-5	6+/+nk	Total
N	89	7	10	3	1	110
NS	64	5	7	3	1	80
Wt	655.52	39.73	385.31	61.05	2.73	1144.34
Het Chi	388.58	12.83	97.65	17.90	0.00	574.79
Het df	88	6	9	2	0	109
Het P	***	*	***	***	N.S.	***
Fixed RR	8.10	22.67	10.72	6.78	6.45	9.13
RRl	7.50	16.61	9.70	5.28	1.97	8.62
RRu	8.75	30.93	11.84	8.72	21.11	9.68
P	+++	+++	+++	+++	++	+++
Random RR	10.12	20.52	11.07	8.72	6.45	10.48
RRl	8.45	11.57	7.32	3.84	1.97	8.99
RRu	12.11	36.40	16.74	19.80	21.11	12.23
P	+++	+++	+++	+++	++	+++
Between Chi						57.83
Between df						4
Between P						***
Btwn(F) P						*
Btwn(R) P						N.S.

	Product all/unsp	cig+/-ot	cig only	Total
N	54	49	7	110
NS	41	33	6	80
Wt	335.91	785.83	22.60	1144.34
Het Chi	219.32	295.50	10.60	574.79
Het df	53	48	6	109
Het P	***	***	N.S.	***
Fixed RR	6.91	10.04	21.33	9.13
RRl	6.21	9.36	14.12	8.62
RRu	7.69	10.77	32.21	9.68
P	+++	+++	+++	+++
Random RR	8.86	11.58	21.33	10.48
RRl	6.98	9.42	11.22	8.99
RRu	11.24	14.24	40.53	12.23
P	+++	+++	+++	+++
Between Chi				49.37
Between df				2
Between P				***
Btwn(F) P				**
Btwn(R) P				*

	Denominator nev any	nev cigs	Total
N	71	39	110
NS	53	27	80
Wt	602.05	542.29	1144.34
Het Chi	318.97	219.68	574.79
Het df	70	38	109
Het P	***	***	***
Fixed RR	7.72	11.01	9.13
RRl	7.12	10.13	8.62
RRu	8.36	11.98	9.68
P	+++	+++	+++
Random RR	9.62	12.16	10.48
RRl	7.93	9.49	8.99
RRu	11.68	15.59	12.23
P	+++	+++	+++
Between Chi			36.14
Between df			1
Between P			***
Btwn(F) P			**
Btwn(R) P			N.S.

Table 2C1 - 6

IESLC - Meta-anal of Ever Smoking (or Current if Ever not available), Any prod (or Cigs if Any not avail)

Squamous

Least adjusted

	Derivation of RR/CI Orig	StdCalc	Other	Total
N	14	75	21	110
NS	10	56	16	82
Wt	477.64	609.55	57.15	1144.34
Het Chi	161.84	357.59	37.82	574.79
Het df	13	74	20	109
Het P	***	***	**	***
Fixed RR	9.84	8.30	13.65	9.13
RRl	9.00	7.66	10.53	8.62
RRu	10.77	8.98	17.69	9.68
P	+++	+++	+++	+++
Random RR	9.33	10.04	15.57	10.48
RRl	6.44	8.29	10.33	8.99
RRu	13.50	12.15	23.45	12.23
P	+++	+++	+++	+++
Between Chi				17.54
Between df				2
Between P				***
Btwn(F) P				N.S.
Btwn(R) P				N.S.

	Smoking status ever	current	Total
N	102	8	110
NS	73	5	78
Wt	1023.57	120.77	1144.34
Het Chi	527.92	32.75	574.79
Het df	101	7	109
Het P	***	***	***
Fixed RR	9.49	6.61	9.13
RRl	8.93	5.53	8.62
RRu	10.09	7.90	9.68
P	+++	+++	+++
Random RR	10.37	12.41	10.48
RRl	8.83	6.83	8.99
RRu	12.18	22.55	12.23
P	+++	+++	+++
Between Chi			14.12
Between df			1
Between P			***
Btwn(F) P			N.S.
Btwn(R) P			N.S.

Table 2C1 - 7

IESLC - Meta-anal of Ever Smoking (or Current if Ever not available), Any prod (or Cigs if Any not avail)
Squamous
Excluded studies (and stage at which they were excluded)

Stage	Studies
1	BOUCHA BUELL LAURIL MZILEN
2	BERRIN CHEN RESTRE TAO ULMER
5	RIMING TANG2 WYNDE5 WYNDE8
6	BLOT1 BLOT2 BLOT3 BYERS2 HIRAOK HIRAY2 JONES LICKIN MOLLO MRFIT MURATA NOTANI PERSHA SAARIK SCHWA2 SHIMIZ SITAS VANDER WARSIN WATSON WYNDER
8	ABELIN AGUDO AKIBA AMANDU AMES ARCHER ARMADA AUSTIN AUVINE AXELSO AXELSS BENSHL BEST BLOHMK BLOT4 BOFFET BRETT BROCKM BROSS BROWN1 CARPEN CASCO2 CASCOR CEDERL CHANG CHATZI CHEN2 CHEN3 CHIAZZ CHOW CHYOU COOKSO DARBY DAVEYS DEAN DEAN2 DEAN3 DEKLER DESTEF DOCKER DOLL2 DORANT DROSTE DU DUNN EBELIN ENSTRO ESAKI GAO2 GARCIA GARDIN GARSHI GENG GILLIS GODLEY GOLLED GOODMA GRAHAM GREGOR GSELL HAMMO2 HANSEN HEIN HENNEK HIRAYA HITOSU HOLE HOROWI HORWIT HU HU2 HUANG HUMBLE JARUP JARVHO JIANG KAISE2 KAISER KANELL KAUFMA KELLER KINLEN KJUUS KNEKT KO KOHLME KOULUM KREUZE KUBIK LANGE LAUSSM LEI LEMARC LETOUR LEVIN LIAW LIDDEL LIU LIU2 LIU3 LIU4 LIU5 LOMBAR MACLEN MAGNUS MARSH MARSH2 MARTIS MASTRA MCCONN MCDUFF MCLAUG MIGRAN MILLER MILLS MRFITR NAM NOTAN2 ODRISC PARKIN PASTOR PAWLEG PERNU PERSH2 PETO PEZZO2 PIKE PISANI POFFIJ POLEDN PRESCO QIAO QIAO2 RACHTA RADZIK RANDIG REN RONCO ROOTS ROTHSC SADOWS SANKAR SEGI SEGI2 SHAW SIMARA SIMONA SPEIZE SPITZ STOCKS STOCKW STUCKE SUN SUZUKI TANG TENKAN TOUSEY TULINI TVERDA VEIERO WALD WANG WANG2 WANG3 WANG4 WICKLU WIGLE WILKIN WU2 WUNSCH XIANGZ XU XU2 XU4 YAMAGU YONG YUAN ZHANG
10	BENHAM VUTUC WYNDE7

Table 2C1 - 8

Potentially overlapping studies

REF	REFGP	PRINC	.	OVERLAP/LINK
LUBIN2	LUBIN2	1		Lubin-combined
CHAN	CHAN	1		Chan/LamWK2
LAMWK2	LAMWK2	1		Chan/LamWK2
LAMTH	LAMTH	1		KOO/LAMTH/LAMWK
KOO	KOO	1		KOO/LAMTH/LAMWK
LAMWK	LAMWK	1		KOO/LAMTH/LAMWK
ISHIMA	AKIBA	2		AKIBA/ISHIMA
BYERS1	BYERS1	2		GRAHAM/BROSS/BYERS1
OSANN2	KAISER	2		KAISER/OSANN2
WYNDE6	WYNDE6	1		WYNDE5/6/7/8
CPSI	CPSI	1		CPSI overall
SOBUE2	SOBUE2	1		SOBUE2/MATSUD
MATSUD	MATSUD	1		SOBUE2/MATSUD
JAHN	BOFFET	2		Subset of BOFFET
LUBIN	XIANGZ	2		LUBIN/XIANGZ/QIAO

Table 2C1 - 9

Most adjusted - insufficient data for metaanalysis

REF	NRR	SEX	AGEL	AGEH	RACE	YF	LC TYPE	LOC	START	ST	NLC	R	VB	P	H	AD	SM	PRODUCT	DENOM	De
BUFFLE	47	f	0	0	wh	-	q	NAmer	1976	CC	943	n	bl	y	n	0	ev	cig+/-ot	nev cigs	or
CHEN	1	c	0	0	all	-	q	As:oth	1987	CC	323	n	ot	n	y	2	ev	cig+/-ot	nev cigs	ot
CHYOU	5	m	0	0	jap	0	q+s	NAmer	1965	pr	227	n	bl	n	y	1	cu	cig+/-ot	nev cigs	or
JIANG	3	m	0	0	all	-	q	As:Chi	1984	CC	125	n	ot	n	n	0	ev	all/unsp	nev any	or
JIANG	4	f	0	0	all	-	q	As:Chi	1984	CC	125	n	ot	n	n	0	ev	all/unsp	nev any	ot
KOO	2	f	0	0	all	-	q	As:HK	1981	CC	200	n	bl	n	n	0	ev	all/unsp	nev any	or
WANG	3	c	0	0	all	-	q	As:Chi	1990	CC	390	n	ot	*	y	0	ev	all/unsp	nev any	ot

REF	NRR	RR	SIG	RRDATA comment
BUFFLE	47	8.30		0
CHEN	1	6.66	y	P < 0.001
CHYOU	5	16.00		0
JIANG	3	12.78		0
JIANG	4	*	n	0
KOO	2	3.62		0
WANG	3	4.66	y	p<0.01

Table 2C2 -

IESLC - Meta-anal of Current Smoking (or Ever if Current not available), Any prod (or Cigs if Any not avail)
Squamous

This analysis is restricted to results for:
1) Non-dose-response data
2) Results complete enough for use in metaanalysis

Within each study, results are then selected (in the following order of preference, within each sex) for:
3) SMKSTA: current smokers, ever smokers
4) PRODUCT: all/unspec, cigarettes regardless of other products, cigarettes only
5) CIGTYPE: all/unspecified, MC regardless of HR, MC only
6) DENOM: never smoked anything, never smoked cigarettes, (never +1 = +long term ex, +2 = +amount unknown,
+3 = never cigs+long term ex)
7) Followup period (YF, prospective studies): whole study (coded as 0) or longest available
8) LCtype: squamous or nearest available, but not adeno. (q = squamous, s = small,
a = adeno, KI = Kreyberg I, u = undifferentiated)
9) Race: all or nearest available, otherwise by race (wh or w = white, bl or b = black, hi = hispanic
ch = chinese, jap = japanese, haw = hawaiian, w+o = white + oriental, sca = scandinavian, as = asian)
10) For overlapping studies: principal rather than subsidiary studies
Finally by Age: whole study (coded as 0) if available, otherwise by widest available age group
and then for single sex results (m, f) in preference to combined sex results (c).

Results adjusted (AD) for the most potential confounders are then chosen in Sections -1 to -3
(and those which actually differ from the adjusted results in Table 2C1 - 1 are marked 'x' in Section -1)
and results adjusted for the least confounders in Sections -4 to -6. (Those least adjusted results which
actually differ from the most adjusted as marked 'x' in column X in Section -4)
(Results adjusted for an unknown number of confounder(s) are coded as 20.)

Section -7 shows excluded studies, together with the stage (as above) at which no qualifying
results were found.

Section -8 lists the potentially overlapping studies which have been included (1=principal, 2=subsidiary).

Section -9 lists any results which would have been included in preference except that they had data not complete
enough for use in meta-analysis, with their significance (yes/no), if known, and any further comment as entered
on the database.

In addition to those mentioned above, the following fields, levels and abbreviations are used:
* or nk = not known, n = no, y = yes, ot = other
ev = ever, cu = current, nev = never
all/unspec = all or unspecified, cig+/-ot = cigarettes irrespective of other products (cigar, pipe etc)
MC = manufactured cigarettes, HR = hand-rolled cigarettes
REF: 6-character study reference
NRR: number of the RR on the database within the study
ST : study type (CC = case control, pr or prosp = prospective)
NLC: number of lung cancer cases in whole study
R : risky occupational population (n = no, m = mining, o = other risky)
VB : national cigarette type (V = at least 75% Virginia, bl = at least 75% blended, ot = other)
P : any proxy use
H : full histological confirmation
De : derivation of RR/CI (or = original, st = standard method, ot = other method of estimation)

Table 2C2 - 1

IESLC - Meta-anal of Current Smoking (or Ever if Current not available), Any prod (or Cigs if Any not avail)
Squamous
Most adjusted

REF	NRR	2C1	SEX	AGEL	AGEH	RACE	YF	LC TYPE	LOC	START	ST	NLC	R	VB	P	H	AD	SM	PRODUCT	DENOM	De
ABRAHA	1		m	0	0	all	0	q	Eu:est	1975	pr	571	n	bl	n	n	0	ev	all/unsp	nev any	ot
ABRAHA	4		f	0	0	all	0	q	Eu:est	1975	pr	571	n	bl	n	n	0	ev	all/unsp	nev any	ot
ALDERS	52		m	0	0	all	-	q	Eu:UK	1977	CC	1448	n	V	n	n	2	ev	all/unsp	nev any	or
ALDERS	55		f	0	0	all	-	q	Eu:UK	1977	CC	1448	n	V	n	n	2	ev	all/unsp	nev any	or
ANDERS	10		f	0	0	all	0	q	NAmer	1986	pr	343	n	bl	n	n	0	ev	cig+/-ot	nev cigs	st
BAND	5		m	0	0	all	-	q	NAmer	1983	CC	2831	n	V	y	y	2	ev	cig only	nev any	ot
BARBON	18	x	m	0	0	all	-	q	Eu:wst	1979	CC	755	n	bl	y	y	1	cu	all/unsp	nev any	or
BECHER	11		f	0	0	all	-	q+s	Eu:Ger	1985	CC	194	n	bl	n	y	1	ev	all/unsp	nev any	or
BOUCOT	141		m	0	0	all	0	q	NAmer	1951	pr	121	n	bl	n	n	2	cu	cig only	nev any	ot
BRESLO	36		c	0	0	all	-	not a	NAmer	1949	CC	518	n	bl	n	y	0	ev	all/unsp	nev+1	st
BROWN2	16	x	m	0	0	wh	-	q	NAmer	1984	CC	14596	n	bl	n	y	2	cu	cig+/-ot	nev cigs	or
BROWN2	15	x	f	0	0	wh	-	q	NAmer	1984	CC	14596	n	bl	n	y	2	cu	cig+/-ot	nev cigs	or
BUFFLE	49		m	0	0	wh	-	q	NAmer	1976	CC	943	n	bl	y	n	0	ev	cig+/-ot	nev cigs	ot
BUFFLE	63	x	f	0	0	w-hi	-	q	NAmer	1976	CC	943	n	bl	y	n	0	cu	cig+/-ot	nev cigs	st
BYERS1	1		m	0	0	wh	-	q	NAmer	1957	CC	1002	n	bl	n	n	0	ev	cig+/-ot	nev cigs	st
CHAN	11		m	0	0	all	-	q+s	As:HK	1976	CC	397	n	bl	n	n	0	ev	all/unsp	nev any	st
CHAN	15		f	0	0	all	-	q+s	As:HK	1976	CC	397	n	bl	n	n	0	ev	all/unsp	nev any	st
CHOI	62		m	0	0	all	-	q	As:oth	1985	CC	375	n	bl	n	n	0	ev	cig+/-ot	nev cigs	st
CHOI	64		f	0	0	all	-	q	As:oth	1985	CC	375	n	bl	n	n	0	ev	cig+/-ot	nev cigs	st
COMSTO	23	x	m	0	0	all	-	q	NAmer	1975	ot	258	n	bl	n	n	0	cu	cig+/-ot	nev cigs	st
COMSTO	30	x	f	0	0	all	-	q	NAmer	1975	ot	258	n	bl	n	n	0	cu	cig+/-ot	nev cigs	st
CORREA	43	x	c	0	0	all	-	q+s	NAmer	1979	CC	1359	n	bl	y	n	1	cu	cig+/-ot	nev cigs	or
CPSI	403		m	0	0	all	2	q	NAmer	1959	pr	5138	n	bl	n	n	1	cu	cig only	nev any	ot
CPSI	405		f	0	0	all	2	q	NAmer	1959	pr	5138	n	bl	n	n	1	cu	cig only	nev any	ot
CPSII	114		m	0	0	all	2	q	NAmer	1982	pr	3229	n	bl	n	n	1	cu	cig only	nev any	ot
CPSII	117		f	0	0	all	2	q	NAmer	1982	pr	3229	n	bl	n	n	1	cu	cig+/-ot	nev cigs	ot
DAMBER	33		m	0	0	all	-	q	Eu:Sca	1972	CC	579	n	bl	y	n	1	ev	all/unsp	nev any	or
DESTE2	16		m	0	0	all	-	q	SCAmer	1993	CC	463	n	bl	n	n	2	ev	all/unsp	nev any	or
DOLL	86		m	0	0	all	-	KI	Eu:UK	1948	CC	1465	n	V	n	n	1	ev	all/unsp	nev any	ot
DOLL	88		f	0	0	all	-	KI	Eu:UK	1948	CC	1465	n	V	n	n	1	ev	all/unsp	nev any	ot
DORGAN	113		m	0	0	wh	-	q	NAmer	1980	CC	2026	n	bl	y	y	2	ev	cig+/-ot	nev any	or
DORGAN	98		f	0	0	all	-	q	NAmer	1980	CC	2026	n	bl	y	y	3	ev	cig+/-ot	nev any	or
DORN	338		m	0	0	wh	8	q	NAmer	1954	pr	5097	n	bl	n	n	1	cu	cig only	nev any	ot
DOSEME	3		m	0	0	all	-	q	Eu:bal	1979	CC	1210	n	bl	n	n	2	ev	cig+/-ot	nev cigs	or
ENGELA	56	x	m	0	0	all	0	q	Eu:Sca	1964	pr	435	n	bl	n	n	7	cu	cig+/-ot	nev cigs	ot
FAN	3		c	0	0	all	-	q	As:Chi	1990	CC	403	n	ot	y	n	0	ev	cig+/-ot	nev cigs	ot
GAO	2		m	0	0	all	-	q	As:Chi	1984	CC	1405	n	ot	n	n	2	ev	cig+/-ot	nev cigs	or
GAO	12		f	0	0	all	-	q	As:Chi	1984	CC	1405	n	ot	n	n	2	ev	cig+/-ot	nev cigs	or
GER	13		c	0	0	all	-	q+s	As:oth	1990	CC	141	n	ot	y	n	10	ev	all/unsp	nev any	ot
HAENSZ	20	x	f	0	0	all	-	q+u	NAmer	1955	CC	158	n	bl	n	y	0	cu	cig+/-ot	nev any	st
HAMMON	102	x	m	0	0	wh	0	not a	NAmer	1952	pr	448	n	bl	n	n	1	cu	cig only	nev any	ot
HEGMAN	2		c	0	0	all	-	q	NAmer	1989	CC	282	n	bl	y	y	0	ev	all/unsp	nev any	st
HINDS	23		f	0	0	o	-	q+s	NAmer	1968	CC	292	n	bl	n	n	3	ev	all/unsp	nev any	st
ISHIMA	6		c	0	0	all	-	q	As:Jap	1961	CC	180	n	bl	y	y	5	ev	all/unsp	nev any	st
JAHN	7	x	m	0	0	all	-	q	Eu:Ger	1988	CC	1004	n	bl	n	n	0	cu	cig+/-ot	nev any	st
JAIN	18	x	m	0	0	all	-	q	NAmer	1981	CC	845	n	V	y	n	0	cu	cig+/-ot	nev cigs	st
JAIN	13	x	f	0	0	all	-	q	NAmer	1981	CC	845	n	V	y	n	0	cu	cig+/-ot	nev cigs	st
JEDRYC	22	x	m	0	0	all	-	q	Eu:est	1980	CC	1630	n	bl	y	n	0	cu	cig+/-ot	nev any	st
JOLY	54		m	0	0	all	-	q	SCAmer	1978	CC	826	n	bl	n	n	0	ev	cig+/-ot	nev any	st
JOLY	52		f	0	0	all	-	q	SCAmer	1978	CC	826	n	bl	n	n	0	ev	cig+/-ot	nev any	st
JUSSAW	23		m	0	0	all	-	KI	As:Ind	1964	CC	792	n	V	n	n	0	ev	all/unsp	nev any	st
KATSOU	20	x	f	0	0	all	-	KI	Eu:bal	1987	CC	101	n	bl	n	n	1	cu	all/unsp	nev any	or
KHUDER	14	x	m	0	0	all	-	q	NAmer	1985	CC	482	n	bl	n	y	0	cu	cig+/-ot	nev cigs	or
KIHARA	2	x	c	0	0	jap	-	q	As:Jap	1991	CC	440	n	bl	n	n	0	cu	all/unsp	nev any	st
KOO	6		f	0	0	all	-	q+s	As:HK	1981	CC	200	n	bl	n	n	0	ev	all/unsp	nev any	st
KREYBE	4		m	0	0	all	-	KI	Eu:Sca	1948	CC	300	n	bl	n	y	1	ev	all/unsp	nev any	ot
KREYBE	25		f	0	0	all	-	KI	Eu:Sca	1948	CC	300	n	bl	n	y	1	ev	all/unsp	nev any	ot
LAMTH	1		f	0	0	ch	-	q	As:HK	1983	CC	445	n	bl	n	n	0	ev	all/unsp	nev any	or
LAMWK	2		f	0	0	ch	-	q	As:HK	1981	CC	163	n	bl	n	n	0	ev	all/unsp	nev any	st
LAMWK2	1		m	0	0	all	-	q	As:HK	1976	CC	480	n	bl	n	n	0	ev	all/unsp	nev any	st
LAMWK2	5		f	0	0	all	-	q	As:HK	1976	CC	480	n	bl	n	n	0	ev	all/unsp	nev any	st
LOMBA2	2		f	0	0	all	-	q+u	NAmer	1960	CC	225	n	bl	n	n	0	ev	cig+/-ot	nev cigs	st
LUBIN	33		m	0	0	all	-	KI	As:Chi	1984	CC	427	m	ot	y	n	0	ev	all/unsp	nev any	st
LUBIN2	249	x	m	0	0	all	-	q	Eu:mul	1976	CC	7804	n	bl	n	y	0	cu	cig+/-ot	nev any	st
LUBIN2	261	x	f	0	0	all	-	q	Eu:mul	1976	CC	7804	n	bl	n	y	0	cu	cig+/-ot	nev any	st
LUO	8		c	0	0	all	-	q	As:Chi	1990	CC	102	n	ot	n	y	20	ev	cig+/-ot	nev cigs	or
MATOS	39	x	m	0	0	all	-	q	SCAmer	1994	CC	200	n	bl	n	n	2	cu	cig+/-ot	nev any	or
MATSUD	11		m	0	0	all	-	q	As:Jap	1965	CC	179	n	bl	n	n	0	ev	cig+/-ot	nev cigs	st
NOU	1		m	0	0	all	-	q	Eu:Sca	1971	CC	273	n	bl	y	n	0	ev	all/unsp	nev any	st
NOU	6		f	0	0	all	-	q	Eu:Sca	1971	CC	273	n	bl	y	n	0	ev	all/unsp	nev any	st
ORMOS	8		m	0	0	all	-	q	Eu:est	1947	CC	119	n	bl	y	y	0	ev	cig+/-ot	nev any	st
OSANN	35	x	m	0	0	all	-	q	NAmer	1984	CC	1986	n	bl	n	n	2	cu	cig+/-ot	nev cigs	or

Table 2C2 - 1

IESLC - Meta-anal of Current Smoking (or Ever if Current not available), Any prod (or Cigs if Any not avail)
Squamous
Most adjusted

REF	NRR	2C1	SEX	AGEL	AGEH	RACE	YF	LC TYPE	LOC	START	ST	NLC	R	VB	P	H	AD	SM	PRODUCT	DENOM	De
OSANN	36	x	f	0	0	all	-	q	NAmer	1984	CC	1986	n	bl	n	n	2	cu	cig+/-ot	nev cigs	or
OSANN2	26	x	f	0	0	all	-	KI	NAmer	1964	ot	217	n	bl	n	y	1	cu	cig+/-ot	nev cigs	or
PEZZOT	6		m	0	0	all	-	q	SCAmer	1987	CC	215	n	bl	n	y	0	ev	cig only	nev cigs	ot
SCHWAR	10		m	40	54	wh	-	q	NAmer	1984	CC	5588	n	bl	y	y	0	ev	cig+/-ot	nev cigs	st
SCHWAR	9		m	40	54	bl	-	q	NAmer	1984	CC	5588	n	bl	y	y	0	ev	cig+/-ot	nev cigs	st
SCHWAR	18		f	40	54	wh	-	q	NAmer	1984	CC	5588	n	bl	y	y	0	ev	cig+/-ot	nev cigs	ot
SCHWAR	17		f	40	54	bl	-	q	NAmer	1984	CC	5588	n	bl	y	y	0	ev	cig+/-ot	nev cigs	ot
SEOW	3		f	0	0	ch	-	q	As:oth	1997	CC	153	n	bl	n	y	0	ev	cig+/-ot	nev cigs	st
SIEMIA	7		m	0	0	all	-	q	NAmer	1979	CC	857	n	V	y	y	7	ev	cig+/-ot	nev cigs	or
SOBUE	34	x	m	0	0	all	-	q	As:Jap	1986	CC	1376	n	bl	n	y	1	cu	cig+/-ot	nev cigs	or
SOBUE	44	x	f	0	0	all	-	q	As:Jap	1986	CC	1376	n	bl	n	y	1	cu	cig+/-ot	nev cigs	or
SOBUE2	1		m	0	0	all	-	q	As:Jap	1965	CC	2083	n	bl	n	n	2	cu	cig+/-ot	nev any	or
SOBUE2	5		f	0	0	all	-	q	As:Jap	1965	CC	2083	n	bl	n	n	2	cu	cig+/-ot	nev any	or
STASZE	12		m	0	0	all	-	q	Eu:est	1954	CC	281	n	bl	n	y	0	ev	all/unsp	nev any	ot
STASZE	38		f	0	0	all	-	q	Eu:est	1954	CC	281	n	bl	n	y	0	ev	all/unsp	nev any	ot
STAYNE	3		m	0	0	all	-	q	NAmer	1969	CC	420	n	bl	n	n	0	ev	all/unsp	nev any	st
SUZUK2	15		c	0	0	all	-	q	SCAmer	1991	CC	123	n	bl	n	y	3	ev	all/unsp	nev any	or
SVENSS	97	x	f	0	0	all	-	q	Eu:Sca	1983	CC	210	n	bl	n	n	1	cu	all/unsp	nev any	ot
TIZZAN	18		c	0	0	all	-	q+u	Eu:wst	1959	CC	1358	n	bl	n	n	0	ev	all/unsp	nev any	st
TOKARS	10		c	0	0	all	-	q	Eu:est	1966	ot	162	o	bl	n	y	3	ev	all/unsp	nev any	or
TSUGAN	14	x	m	0	0	all	-	q	As:Jap	1976	CC	134	n	bl	n	y	0	cu	all/unsp	nev any	ot
WAKAI	10	x	m	0	0	all	-	q	As:Jap	1988	CC	333	n	bl	n	y	1	cu	all/unsp	nev any	or
WAKAI	28	x	f	0	0	all	-	q	As:Jap	1988	CC	333	n	bl	n	y	1	cu	all/unsp	nev any	or
WU	16	x	f	0	0	wh	-	q	NAmer	1981	CC	220	n	bl	n	y	2	cu	all/unsp	nev any	or
WUWILL	9		f	0	0	all	-	q	As:Chi	1985	CC	965	n	ot	n	n	3	ev	cig+/-ot	nev cigs	or
WYNDE2	7		m	0	0	all	-	KI	NAmer	1962	CC	404	n	bl	n	y	0	ev	all/unsp	nev any	st
WYNDE3	10	x	m	0	0	all	-	KI	NAmer	1966	CC	350	n	bl	n	y	0	cu	all/unsp	nev any	st
WYNDE3	132		f	0	0	all	-	KI	NAmer	1966	CC	350	n	bl	n	y	0	ev	all/unsp	nev any	st
WYNDE4	68		m	0	0	all	-	not a	NAmer	1948	CC	684	n	bl	y	n	2	ev	all/unsp	nev any	ot
WYNDE4	54		f	0	0	all	-	not a	NAmer	1948	CC	684	n	bl	y	n	2	ev	all/unsp	nev any	ot
WYNDE6	12	x	m	0	0	all	-	KI	NAmer	1969	CC	4423	n	bl	n	y	0	cu	cig+/-ot	nev any	st
WYNDE6	201	x	f	0	0	all	-	KI	NAmer	1969	CC	4423	n	bl	n	y	0	cu	cig+/-ot	nev cigs	st
XU3	20		m	0	0	all	-	KI	As:Chi	1981	CC	135	n	ot	n	n	1	ev	all/unsp	nev any	ot
XU3	24		f	0	0	all	-	KI	As:Chi	1981	CC	135	n	ot	n	n	1	ev	all/unsp	nev any	ot
ZHENG	5		m	0	0	all	-	q	As:Chi	1982	CC	540	n	ot	*	y	0	ev	cig+/-ot	nev cigs	st
ZHENG	18		f	0	0	all	-	q	As:Chi	1982	CC	540	n	ot	*	y	0	ev	cig+/-ot	nev cigs	st
ZHOU	8		m	0	0	all	-	q	As:Chi	1978	CC	1360	n	ot	n	n	0	ev	all/unsp	nev any	st
ZHOU	9		f	0	0	all	-	q	As:Chi	1978	CC	1360	n	ot	n	n	0	ev	all/unsp	nev any	st

Cigarette type is all/unspec for all RRs

Table 2C2 - 2

IESLC - Meta-anal of Current Smoking (or Ever if Current not available), Any prod (or Cigs if Any not avail)
Squamous
Most adjusted

				Number Exposed		Non-exposed			
REF	NRR	SEX	AD	Case	Cont	Case	Cont	RR	95.00%CI
*ABRAHA	1	m	0	142	10351	0	3365	92.66~	(5.77-1488.21)
*ABRAHA	4	f	0	17	5256	7	11589	5.35	(2.22- 12.90)
Subtotal ABRAHA								6.95	(3.00- 16.06)
ALDERS	52	m	2	-	-	-	-	14.70	(3.40- 63.64)
ALDERS	55	f	2	-	-	-	-	6.09	(2.68- 13.82)
Subtotal ALDERS								7.52	(3.67- 15.37)
*ANDERS	10	f	0	63	96164	5	195158	25.57	(10.29- 63.56)
BAND	5	m	2	-	-	-	-	37.45	(17.62- 79.58)
BARBON	18	m	1	-	-	-	-	18.80	(8.20- 43.40)
BECHER	11	f	1	-	-	-	-	10.69	(2.43- 47.00)
*BOUCOT	141	m	2	-	-	-	-	27.54	(1.69- 448.37)
BRESLO	36	c	0	457	462	15	56	3.69	(2.06- 6.62)
BROWN2	16	m	2	-	-	-	-	13.70	(11.70- 16.10)
BROWN2	15	f	2	-	-	-	-	20.60	(16.60- 25.60)
Subtotal BROWN2								15.81	(13.91- 17.98)
BUFFLE	49	m	0	-	-	-	-	14.03	(4.73- 41.61)
BUFFLE	63	f	0	39	110	3	112	13.24	(3.97- 44.10)
Subtotal BUFFLE								13.67	(6.10- 30.62)
BYERS1	1	m	0	299	695	22	424	8.29	(5.29- 13.00)
CHAN	11	m	0	114	161	2	43	15.22	(3.61- 64.12)
CHAN	15	f	0	44	50	19	139	6.44	(3.44- 12.06)
Subtotal CHAN								7.39	(4.16- 13.13)
CHOI	62	m	0	160	465	6	95	5.45	(2.34- 12.67)
CHOI	64	f	0	11	26	10	164	6.94	(2.68- 17.96)
Subtotal CHOI								6.06	(3.22- 11.40)
COMSTO	23	m	0	27	100	2	84	11.34	(2.62- 49.09)
COMSTO	30	f	0	16	52	0	115	72.60~	(4.27-1233.12)
Subtotal COMSTO								16.78	(4.57- 61.67)
CORREA	43	c	1	-	-	-	-	34.60	(22.80- 52.40)
*CPSI	403	m	1	-	-	-	-	29.35	(4.02- 214.28)
*CPSI	405	f	1	-	-	-	-	4.25	(1.23- 14.68)
Subtotal CPSI								7.30	(2.55- 20.90)
*CPSII	114	m	1	-	-	-	-	39.26	(10.38- 148.55)
*CPSII	117	f	1	-	-	-	-	78.91	(15.83- 393.37)
Subtotal CPSII								52.16	(18.72- 145.32)
DAMBER	33	m	1	-	-	-	-	11.80	(6.40- 23.00)
DESTE2	16	m	2	-	-	-	-	13.20	(4.70- 37.10)
DOLL	86	m	1	-	-	-	-	13.17	(4.12- 42.10)
DOLL	88	f	1	-	-	-	-	2.13	(1.06- 4.27)
Subtotal DOLL								3.45	(1.90- 6.27)
DORGAN	113	m	2	-	-	-	-	18.90	(7.00- 51.30)
DORGAN	98	f	3	-	-	-	-	11.10	(7.20- 17.10)
Subtotal DORGAN								12.08	(8.12- 17.96)
*DORN	338	m	1	-	-	-	-	17.09	(8.96- 32.60)
DOSEME	3	m	2	-	-	-	-	3.60	(2.60- 5.00)
*ENGELA	56	m	7	-	-	-	-	10.89	(3.25- 36.43)
FAN	3	c	0	75	595	6	556	11.68	(5.04- 27.04)
GAO	2	m	2	-	-	-	-	8.40	(4.70- 15.00)
GAO	12	f	2	-	-	-	-	7.20	(4.60- 11.10)
Subtotal GAO								7.62	(5.36- 10.82)
GER	13	c	10	-	-	-	-	3.19	(1.08- 9.42)
HAENSZ	20	f	0	53	94	44	236	3.02	(1.90- 4.82)
*HAMMON	102	m	1	-	-	-	-	26.42	(9.78- 71.34)
HEGMAN	2	c	0	89	1202	5	2080	30.80	(12.48- 76.03)
HINDS	23	f	3	-	-	-	-	16.13	(7.66- 33.97)
ISHIMA	6	c	5	-	-	-	-	21.00	(3.38- 868.40)
JAHN	7	m	0	153	269	3	138	26.16	(8.19- 83.54)
JAIN	18	m	0	107	118	2	85	38.54	(9.26- 160.45)
JAIN	13	f	0	81	99	6	214	29.18	(12.31- 69.15)
Subtotal JAIN								31.44	(15.03- 65.78)
JEDRYC	22	m	0	235	516	6	289	21.94	(9.63- 49.96)
JOLY	54	m	0	203	709	2	218	31.21	(7.69- 126.68)
JOLY	52	f	0	48	122	6	283	18.56	(7.74- 44.51)
Subtotal JOLY								21.47	(10.22- 45.09)
JUSSAW	23	m	0	89	168	13	624	25.43	(13.87- 46.63)
KATSOU	20	f	1	-	-	-	-	6.45	(2.73- 15.25)
KHUDER	14	m	0	112	-	9	-	8.60	(4.20- 17.60)
KIHARA	2	c	0	111	162	5	237	32.48	(12.97- 81.34)
KOO	6	f	0	61	63	32	137	4.15	(2.46- 6.98)
KREYBE	4	m	1	-	-	-	-	10.87	(3.47- 34.04)
KREYBE	25	f	1	-	-	-	-	2.29	(0.89- 5.88)

Table 2C2 - 2

IESLC - Meta-anal of Current Smoking (or Ever if Current not available), Any prod (or Cigs if Any not avail)
Squamous
Most adjusted

REF	NRR	SEX	AD	Number Exposed Case	Number Exposed Cont	Non-exposed Case	Non-exposed Cont	RR	95.00%CI
Subtotal KREYBE								4.31	(2.08- 8.92)
LAMTH	1	f	0	63	20	28	72	8.10	(4.16- 15.77)
LAMWK	2	f	0	21	41	7	144	10.54	(4.19- 26.52)
LAMWK2	1	m	0	129	161	5	43	6.89	(2.65- 17.90)
LAMWK2	5	f	0	35	50	15	139	6.49	(3.27- 12.88)
Subtotal LAMWK2								6.62	(3.79- 11.56)
LOMBA2	2	f	0	94	353	15	239	4.24	(2.40- 7.50)
LUBIN	33	m	0	330	939	4	72	6.33	(2.29- 17.45)
LUBIN2	249	m	0	2518	6209	54	2616	19.65	(14.95- 25.82)
LUBIN2	261	f	0	154	410	72	1180	6.16	(4.55- 8.32)
Subtotal LUBIN2								11.64	(9.50- 14.25)
LUO	8	c	20	-	-	-	-	10.90	(2.50- 47.90)
MATOS	39	m	2	-	-	-	-	10.40	(3.60- 35.50)
MATSUD	11	m	0	103	3314	1	1255	39.01	(5.44- 279.84)
NOU	1	m	0	110	247	2	122	27.17	(6.60- 111.85)
NOU	6	f	0	5	92	2	261	7.09	(1.35- 37.19)
Subtotal NOU								15.42	(5.26- 45.22)
ORMOS	8	m	0	27	1034	2	777	10.14	(2.41- 42.79)
OSANN	35	m	2	-	-	-	-	49.30	(24.10- 101.00)
OSANN	36	f	2	-	-	-	-	35.20	(19.10- 65.10)
Subtotal OSANN								40.59	(25.47- 64.67)
OSANN2	26	f	1	-	-	-	-	39.00	(5.30- 287.00)
PEZZOT	6	m	0	85	317	0	116	62.74~	(3.86-1019.50)
SCHWAR	10	m	0	80	178	1	73	32.81	(4.48- 240.23)
SCHWAR	9	m	0	41	39	4	7	1.84	(0.50- 6.78)
SCHWAR	18	f	0	29	108	0	79	43.23~	(2.60- 718.15)
SCHWAR	17	f	0	21	28	0	41	62.61~	(3.64-1076.10)
Subtotal SCHWAR								7.71	(2.96- 20.10)
SEOW	3	f	0	21	15	10	125	17.50	(6.95- 44.09)
SIEMIA	7	m	7	-	-	-	-	22.70	(6.90- 75.20)
SOBUE	34	m	1	-	-	-	-	18.10	(7.90- 41.30)
SOBUE	44	f	1	-	-	-	-	9.70	(5.50- 16.80)
Subtotal SOBUE								11.79	(7.42- 18.73)
SOBUE2	1	m	2	-	-	-	-	5.20	(4.20- 6.50)
SOBUE2	5	f	2	-	-	-	-	7.20	(4.80- 10.80)
Subtotal SOBUE2								5.59	(4.62- 6.78)
STASZE	12	m	0	137	754	0	158	57.77~	(3.58- 933.17)
STASZE	38	f	0	1	153	0	1660	32.45~	(1.32- 800.04)
Subtotal STASZE								45.09	(5.52- 368.55)
STAYNE	3	m	0	130	567	22	333	3.47	(2.17- 5.56)
SUZUK2	15	c	3	-	-	-	-	31.00	(4.20- 227.00)
SVENSS	97	f	1	-	-	-	-	21.92	(6.63- 72.51)
TIZZAN	18	c	0	333	939	55	419	2.70	(1.99- 3.67)
TOKARS	10	c	3	-	-	-	-	6.80	(1.20- 38.70)
TSUGAN	14	m	0	18	13	0	5	15.07~	(0.77- 296.43)
WAKAI	10	m	1	-	-	-	-	9.82	(2.36- 41.00)
WAKAI	28	f	1	-	-	-	-	28.20	(7.55- 105.00)
Subtotal WAKAI								17.37	(6.60- 45.71)
WU	16	f	2	-	-	-	-	35.30	(4.70- 267.30)
WUWILL	9	f	3	-	-	-	-	4.20	(3.00- 5.90)
WYNDE2	7	m	0	347	616	3	105	19.72	(6.21- 62.59)
WYNDE3	10	m	0	171	207	3	88	24.23	(7.53- 77.95)
WYNDE3	132	f	0	25	56	5	76	6.79	(2.45- 18.82)
Subtotal WYNDE3								11.77	(5.46- 25.38)
WYNDE4	68	m	2	-	-	-	-	12.79	(6.19- 26.41)
WYNDE4	54	f	2	-	-	-	-	5.82	(2.55- 13.31)
Subtotal WYNDE4								9.08	(5.26- 15.66)
WYNDE6	12	m	0	1026	741	29	617	29.46	(20.06- 43.26)
WYNDE6	201	f	0	550	376	40	856	31.30	(22.21- 44.12)
Subtotal WYNDE6								30.47	(23.59- 39.36)
XU3	20	m	1	-	-	-	-	5.90	(1.69- 20.57)
XU3	24	f	1	-	-	-	-	25.67	(4.99- 131.94)
Subtotal XU3								10.14	(3.75- 27.37)
ZHENG	5	m	0	156	218	4	94	16.82	(6.05- 46.71)
ZHENG	18	f	0	43	44	33	184	5.45	(3.11- 9.54)
Subtotal ZHENG								7.07	(4.33- 11.56)
ZHOU	8	m	0	343	41	96	36	3.14	(1.90- 5.18)
ZHOU	9	f	0	35	7	42	32	3.81	(1.50- 9.68)
Subtotal ZHOU								3.28	(2.11- 5.10)
Partial Totals				9987	136296	784	228465		

*prospective study ~ With 0.5 adjustment for zero

Table 2C2 - 2

IESLC - Meta-anal of Current Smoking (or Ever if Current not available), Any prod (or Cigs if Any not avail)
Squamous
Most adjusted

REF	NRR	SEX	AD	Ys	Ws	Qs	Ps
*ABRAHA	1	m	0	4.53	0.50	2.45	0.0014
*ABRAHA	4	f	0	1.68	4.97	1.99	0.0002
Subtotal ABRAHA				1.94	5.46	4.44	
ALDERS	52	m	2	2.69	1.79	0.25	0.0003
ALDERS	55	f	2	1.81	5.71	1.45	0.0000
Subtotal ALDERS				2.02	7.50	1.71	
*ANDERS	10	f	0	3.24	4.63	4.01	0.0000
BAND	5	m	2	3.62	6.76	11.64	0.0000
BARBON	18	m	1	2.93	5.53	2.15	0.0000
BECHER	11	f	1	2.37	1.75	0.01	0.0017
*BOUCOT	141	m	2	3.32	0.49	0.50	0.0199
BRESLO	36	c	0	1.31	11.25	11.35	0.0000
BROWN2	16	m	2	2.62	150.78	14.17	0.0000
BROWN2	15	f	2	3.03	81.88	41.80	0.0000
Subtotal BROWN2				2.76	232.67	55.97	
BUFFLE	49	m	0	2.64	3.25	0.35	0.0000
BUFFLE	63	f	0	2.58	2.65	0.20	0.0000
Subtotal BUFFLE				2.62	5.90	0.55	
BYERS1	1	m	0	2.12	19.01	0.73	0.0000
CHAN	11	m	0	2.72	1.86	0.32	0.0002
CHAN	15	f	0	1.86	9.75	1.96	0.0000
Subtotal CHAN				2.00	11.61	2.28	
CHOI	62	m	0	1.70	5.39	2.04	0.0001
CHOI	64	f	0	1.94	4.25	0.59	0.0001
Subtotal CHOI				1.80	9.63	2.63	
COMSTO	23	m	0	2.43	1.79	0.02	0.0012
COMSTO	30	f	0	4.28	0.48	1.87	0.0030
Subtotal COMSTO				2.82	2.27	1.89	
CORREA	43	c	1	3.54	22.19	33.74	0.0000
*CPSI	403	m	1	3.38	0.97	1.11	0.0009
*CPSI	405	f	1	1.45	2.50	1.87	0.0222
Subtotal CPSI				1.99	3.47	2.98	
*CPSII	114	m	1	3.67	2.17	4.01	0.0000
*CPSII	117	f	1	4.37	1.49	6.30	0.0000
Subtotal CPSII				3.95	3.66	10.31	
DAMBER	33	m	1	2.47	9.39	0.23	0.0000
DESTE2	16	m	2	2.58	3.60	0.26	0.0000
DOLL	86	m	1	2.58	2.84	0.20	0.0000
DOLL	88	f	1	0.76	7.91	19.13	0.0334
Subtotal DOLL				1.24	10.76	19.33	
DORGAN	113	m	2	2.94	3.87	1.53	0.0000
DORGAN	98	f	3	2.41	20.54	0.19	0.0000
Subtotal DORGAN				2.49	24.41	1.72	
*DORN	338	m	1	2.84	9.21	2.56	0.0000
DOSEME	3	m	2	1.28	35.93	38.11	0.0000
*ENGELA	56	m	7	2.39	2.63	0.02	0.0001
FAN	3	c	0	2.46	5.45	0.12	0.0000
GAO	2	m	2	2.13	11.41	0.38	0.0000
GAO	12	f	2	1.97	19.80	2.25	0.0000
Subtotal GAO				2.03	31.21	2.63	
GER	13	c	10	1.16	3.28	4.34	0.0358
HAENSZ	20	f	0	1.11	17.71	25.68	0.0000
*HAMMON	102	m	1	3.27	3.89	3.61	0.0000
HEGMAN	2	c	0	3.43	4.70	5.87	0.0000
HINDS	23	f	3	2.78	6.93	1.53	0.0000
ISHIMA	6	c	5	3.04	0.50	0.27	0.0315
JAHN	7	m	0	3.26	2.85	2.59	0.0000
JAIN	18	m	0	3.65	1.89	3.39	0.0000
JAIN	13	f	0	3.37	5.16	5.83	0.0000
Subtotal JAIN				3.45	7.05	9.22	
JEDRYC	22	m	0	3.09	5.67	3.43	0.0000
JOLY	54	m	0	3.44	1.96	2.50	0.0000
JOLY	52	f	0	2.92	5.02	1.87	0.0000
Subtotal JOLY				3.07	6.98	4.37	
JUSSAW	23	m	0	3.24	10.45	8.94	0.0000
KATSOU	20	f	1	1.86	5.19	1.04	0.0000
KHUDER	14	m	0	2.15	7.48	0.19	0.0000
KIHARA	2	c	0	3.48	4.56	6.24	0.0000
KOO	6	f	0	1.42	14.12	11.16	0.0000
KREYBE	4	m	1	2.39	2.95	0.02	0.0000
KREYBE	25	f	1	0.83	4.31	9.47	0.0854

Table 2C2 - 2

IESLC - Meta-anal of Current Smoking (or Ever if Current not available), Any prod (or Cigs if Any not avail)
Squamous
Most adjusted

REF	NRR	SEX	AD	Ys	Ws	Qs	Ps
Subtotal KREYBE				1.46	7.26	9.49	
LAMTH	1	f	0	2.09	8.66	0.42	0.0000
LAMWK	2	f	0	2.35	4.51	0.01	0.0000
LAMWK2	1	m	0	1.93	4.22	0.61	0.0001
LAMWK2	5	f	0	1.87	8.17	1.59	0.0000
Subtotal LAMWK2				1.89	12.38	2.20	
LOMBA2	2	f	0	1.45	11.86	8.89	0.0000
LUBIN	33	m	0	1.84	3.73	0.81	0.0004
LUBIN2	249	m	0	2.98	51.39	22.87	0.0000
LUBIN2	261	f	0	1.82	42.25	10.29	0.0000
Subtotal LUBIN2				2.45	93.64	33.15	
LUO	8	c	20	2.39	1.76	0.01	0.0015
MATOS	39	m	2	2.34	2.93	0.00	0.0001
MATSUD	11	m	0	3.66	0.99	1.81	0.0003
NOU	1	m	0	3.30	1.92	1.88	0.0000
NOU	6	f	0	1.96	1.40	0.17	0.0205
Subtotal NOU				2.74	3.32	2.06	
ORMOS	8	m	0	2.32	1.85	0.00	0.0016
OSANN	35	m	2	3.90	7.48	18.85	0.0000
OSANN	36	f	2	3.56	10.22	15.97	0.0000
Subtotal OSANN				3.70	17.70	34.82	
OSANN2	26	f	1	3.66	0.96	1.76	0.0003
PEZZOT	6	m	0	4.14	0.49	1.65	0.0036
SCHWAR	10	m	0	3.49	0.97	1.35	0.0006
SCHWAR	9	m	0	0.61	2.26	6.53	0.3596
SCHWAR	18	f	0	3.77	0.49	1.03	0.0086
SCHWAR	17	f	0	4.14	0.47	1.58	0.0044
Subtotal SCHWAR				2.04	4.19	10.50	
SEOW	3	f	0	2.86	4.50	1.37	0.0000
SIEMIA	7	m	7	3.12	2.69	1.77	0.0000
SOBUE	34	m	1	2.90	5.62	1.92	0.0000
SOBUE	44	f	1	2.27	12.32	0.02	0.0000
Subtotal SOBUE				2.47	17.94	1.94	
SOBUE2	1	m	2	1.65	80.57	35.32	0.0000
SOBUE2	5	f	2	1.97	23.37	2.65	0.0000
Subtotal SOBUE2				1.72	103.93	37.97	
STASZE	12	m	0	4.06	0.50	1.51	0.0043
STASZE	38	f	0	3.48	0.37	0.51	0.0333
Subtotal STASZE				3.81	0.87	2.02	
STAYNE	3	m	0	1.24	17.27	19.64	0.0000
SUZUK2	15	c	3	3.43	0.97	1.22	0.0007
SVENSS	97	f	1	3.09	2.69	1.62	0.0000
TIZZAN	18	c	0	0.99	40.59	70.40	0.0000
TOKARS	10	c	3	1.92	1.27	0.20	0.0305
TSUGAN	14	m	0	2.71	0.43	0.07	0.0743
WAKAI	10	m	1	2.28	1.89	0.00	0.0017
WAKAI	28	f	1	3.34	2.22	2.35	0.0000
Subtotal WAKAI				2.85	4.10	2.35	
WU	16	f	2	3.56	0.94	1.48	0.0005
WUWILL	9	f	3	1.44	33.59	25.76	0.0000
WYNDE2	7	m	0	2.98	2.88	1.29	0.0000
WYNDE3	10	m	0	3.19	2.81	2.16	0.0000
WYNDE3	132	f	0	1.91	3.69	0.58	0.0002
Subtotal WYNDE3				2.47	6.50	2.74	
WYNDE4	68	m	2	2.55	7.30	0.41	0.0000
WYNDE4	54	f	2	1.76	5.63	1.70	0.0000
Subtotal WYNDE4				2.21	12.93	2.11	
WYNDE6	12	m	0	3.38	26.02	29.92	0.0000
WYNDE6	201	f	0	3.44	32.63	41.88	0.0000
Subtotal WYNDE6				3.42	58.65	71.80	
XU3	20	m	1	1.77	2.46	0.71	0.0054
XU3	24	f	1	3.25	1.43	1.25	0.0001
Subtotal XU3				2.32	3.89	1.96	
ZHENG	5	m	0	2.82	3.68	0.96	0.0000
ZHENG	18	f	0	1.70	12.24	4.63	0.0000
Subtotal ZHENG				1.96	15.92	5.60	
ZHOU	8	m	0	1.14	15.27	20.81	0.0000
ZHOU	9	f	0	1.34	4.42	4.18	0.0049
Subtotal ZHOU				1.19	19.68	24.99	

Table 2C2 - 2

IESLC - Meta-anal of Current Smoking (or Ever if Current not available), Any prod (or Cigs if Any not avail)
Squamous
Most adjusted

	N	110
	NS	78
	Wt	1116.24
Het	Chi	686.20
Het	df	109
Het	P	***
Fixed	RR	10.08
	RRl	9.51
	RRu	10.69
	P	+++
Random	RR	11.53
	RRl	9.73
	RRu	13.66
	P	+++
Asymm	P	N.S.

Table 2C2 - 3

IESLC - Meta-anal of Current Smoking (or Ever if Current not available), Any prod (or Cigs if Any not avail)

Squamous

Most adjusted

		combined	Sex male	female	Total
	N	11	54	45	110
	NS	11	53	44	108
	Wt	96.52	563.95	455.77	1116.24
Het	Chi	125.21	275.88	270.71	686.20
Het	df	10	53	44	109
Het	P	***	***	***	***
Fixed	RR	7.49	11.05	9.59	10.08
	RRl	6.14	10.17	8.75	9.51
	RRu	9.14	12.00	10.51	10.69
	P	+++	+++	+++	+++
Random	RR	11.20	13.62	9.60	11.53
	RRl	4.87	10.82	7.42	9.73
	RRu	25.74	17.14	12.43	13.66
	P	+++	+++	+++	+++
Between	Chi				14.40
Between	df				2
Between	P				***
Btwn(F)	P				N.S.
Btwn(R)	P				N.S.

		Smoking status ever	current	Total
	N	69	41	110
	NS	50	30	80
	Wt	470.32	645.92	1116.24
Het	Chi	248.57	288.68	686.20
Het	df	68	40	109
Het	P	***	***	***
Fixed	RR	6.57	13.77	10.08
	RRl	6.00	12.75	9.51
	RRu	7.19	14.87	10.69
	P	+++	+++	+++
Random	RR	8.82	16.91	11.53
	RRl	7.26	13.14	9.73
	RRu	10.71	21.76	13.66
	P	+++	+++	+++
Between	Chi			148.95
Between	df			1
Between	P			***
Btwn(F)	P			***
Btwn(R)	P			***

Table 2C2 - 4

IESLC - Meta-anal of Current Smoking (or Ever if Current not available), Any prod (or Cigs if Any not avail)
Squamous
Least adjusted

REF	NRR	X	SEX	AGEL	AGEH	RACE	YF	LC TYPE	LOC	START	ST	NLC	R	VB	P	H	AD	SM	PRODUCT	DENOM	De
ABRAHA	1		m	0	0	all	0	q	Eu:est	1975	pr	571	n	bl	n	n	0	ev	all/unsp	nev any	ot
ABRAHA	4		f	0	0	all	0	q	Eu:est	1975	pr	571	n	bl	n	n	0	ev	all/unsp	nev any	ot
ALDERS	52		m	0	0	all	-	q	Eu:UK	1977	CC	1448	n	V	n	n	2	ev	all/unsp	nev any	or
ALDERS	55		f	0	0	all	-	q	Eu:UK	1977	CC	1448	n	V	n	n	2	ev	all/unsp	nev any	or
ANDERS	10		f	0	0	all	0	q	NAmer	1986	pr	343	n	bl	n	n	0	ev	cig+/-ot	nev cigs	st
BAND	5		m	0	0	all	-	q	NAmer	1983	CC	2831	n	V	y	y	2	ev	cig only	nev any	ot
BARBON	17	x	m	0	0	all	-	q	Eu:wst	1979	CC	755	n	bl	y	y	0	cu	all/unsp	nev any	st
BECHER	11		f	0	0	all	-	q+s	Eu:Ger	1985	CC	194	n	bl	n	y	1	ev	all/unsp	nev any	or
BOUCOT	70	x	m	0	0	all	0	q	NAmer	1951	pr	121	n	bl	n	n	0	cu	cig only	nev any	ot
BRESLO	36		c	0	0	all	-	not a	NAmer	1949	CC	518	n	bl	n	y	0	ev	all/unsp	nev+1	st
BROWN2	16		m	0	0	wh	-	q	NAmer	1984	CC	14596	n	bl	n	y	2	cu	cig+/-ot	nev cigs	or
BROWN2	15		f	0	0	wh	-	q	NAmer	1984	CC	14596	n	bl	n	y	2	cu	cig+/-ot	nev cigs	or
BUFFLE	49		m	0	0	wh	-	q	NAmer	1976	CC	943	n	bl	y	n	0	ev	cig+/-ot	nev cigs	ot
BUFFLE	63		f	0	0	w-hi	-	q	NAmer	1976	CC	943	n	bl	y	n	0	cu	cig+/-ot	nev cigs	st
BYERS1	1		m	0	0	wh	-	q	NAmer	1957	CC	1002	n	bl	n	n	0	ev	cig+/-ot	nev cigs	st
CHAN	11		m	0	0	all	-	q+s	As:HK	1976	CC	397	n	bl	n	n	0	ev	all/unsp	nev any	st
CHAN	15		f	0	0	all	-	q+s	As:HK	1976	CC	397	n	bl	n	n	0	ev	all/unsp	nev any	st
CHOI	62		m	0	0	all	-	q	As:oth	1985	CC	375	n	bl	n	n	0	ev	cig+/-ot	nev cigs	st
CHOI	64		f	0	0	all	-	q	As:oth	1985	CC	375	n	bl	n	n	0	ev	cig+/-ot	nev cigs	st
COMSTO	23		m	0	0	all	-	q	NAmer	1975	ot	258	n	bl	n	n	0	cu	cig+/-ot	nev cigs	st
COMSTO	30		f	0	0	all	-	q	NAmer	1975	ot	258	n	bl	n	n	0	cu	cig+/-ot	nev cigs	ot
CORREA	43		c	0	0	all	-	q+s	NAmer	1979	CC	1359	n	bl	y	n	1	cu	cig+/-ot	nev cigs	or
CPSI	403		m	0	0	all	2	q	NAmer	1959	pr	5138	n	bl	n	n	1	cu	cig only	nev any	ot
CPSI	405		f	0	0	all	2	q	NAmer	1959	pr	5138	n	bl	n	n	1	cu	cig only	nev any	ot
CPSII	114		m	0	0	all	2	q	NAmer	1982	pr	3229	n	bl	n	n	1	cu	cig only	nev any	ot
CPSII	117		f	0	0	all	2	q	NAmer	1982	pr	3229	n	bl	n	n	1	cu	cig+/-ot	nev cigs	ot
DAMBER	12	x	m	0	0	all	-	q	Eu:Sca	1972	CC	579	n	bl	y	n	0	ev	all/unsp	nev any	st
DESTE2	16		m	0	0	all	-	q	SCAmer	1993	CC	463	n	bl	n	n	2	ev	all/unsp	nev any	or
DOLL	82	x	m	0	0	all	-	KI	Eu:UK	1948	CC	1465	n	V	n	n	0	ev	all/unsp	nev any	st
DOLL	84	x	f	0	0	all	-	KI	Eu:UK	1948	CC	1465	n	V	n	n	0	ev	all/unsp	nev any	st
DORGAN	113		m	0	0	wh	-	q	NAmer	1980	CC	2026	n	bl	y	y	2	ev	cig+/-ot	nev any	or
DORGAN	98		f	0	0	all	-	q	NAmer	1980	CC	2026	n	bl	y	y	3	ev	cig+/-ot	nev any	or
DORN	338		m	0	0	wh	8	q	NAmer	1954	pr	5097	n	bl	n	n	1	cu	cig only	nev any	ot
DOSEME	19	x	m	0	0	all	-	q	Eu:bal	1979	CC	1210	n	bl	n	n	0	ev	cig+/-ot	nev cigs	st
ENGELA	56		m	0	0	all	0	q	Eu:Sca	1964	pr	435	n	bl	n	n	7	cu	cig+/-ot	nev cigs	ot
FAN	3		c	0	0	all	-	q	As:Chi	1990	CC	403	n	ot	y	n	0	ev	cig+/-ot	nev cigs	ot
GAO	7	x	m	0	0	all	-	q	As:Chi	1984	CC	1405	n	ot	n	n	0	ev	cig+/-ot	nev cigs	st
GAO	17	x	f	0	0	all	-	q	As:Chi	1984	CC	1405	n	ot	n	n	0	ev	cig+/-ot	nev cigs	st
GER	5	x	c	0	0	all	-	q+s	As:oth	1990	CC	141	n	ot	y	n	0	ev	all/unsp	nev any	st
HAENSZ	20		f	0	0	all	-	q+u	NAmer	1955	CC	158	n	bl	n	y	0	cu	cig+/-ot	nev any	st
HAMMON	102		m	0	0	wh	0	not a	NAmer	1952	pr	448	n	bl	n	n	1	cu	cig only	nev any	ot
HEGMAN	2		c	0	0	all	-	q	NAmer	1989	CC	282	n	bl	y	y	0	ev	all/unsp	nev any	st
HINDS	23		f	0	0	o	-	q+s	NAmer	1968	CC	292	n	bl	n	n	3	ev	all/unsp	nev any	st
ISHIMA	1	x	c	0	0	all	-	q	As:Jap	1961	CC	180	n	bl	y	y	0	ev	all/unsp	nev any	st
JAHN	7		m	0	0	all	-	q	Eu:Ger	1988	CC	1004	n	bl	n	n	0	cu	cig+/-ot	nev any	st
JAIN	18		m	0	0	all	-	q	NAmer	1981	CC	845	n	V	y	n	0	cu	cig+/-ot	nev cigs	st
JAIN	13		f	0	0	all	-	q	NAmer	1981	CC	845	n	V	y	n	0	cu	cig+/-ot	nev cigs	st
JEDRYC	22		m	0	0	all	-	q	Eu:est	1980	CC	1630	n	bl	y	n	0	cu	cig+/-ot	nev any	st
JOLY	54		m	0	0	all	-	q	SCAmer	1978	CC	826	n	bl	n	n	0	ev	cig+/-ot	nev any	st
JOLY	52		f	0	0	all	-	q	SCAmer	1978	CC	826	n	bl	n	n	0	ev	cig+/-ot	nev any	st
JUSSAW	23		m	0	0	all	-	KI	As:Ind	1964	CC	792	n	V	n	n	0	ev	all/unsp	nev any	st
KATSOU	24	x	f	0	0	all	-	KI	Eu:bal	1987	CC	101	n	bl	n	n	0	cu	all/unsp	nev any	st
KHUDER	14		m	0	0	all	-	q	NAmer	1985	CC	482	n	bl	n	y	0	cu	cig+/-ot	nev cigs	or
KIHARA	2		c	0	0	jap	-	q	As:Jap	1991	CC	440	n	bl	n	n	0	cu	all/unsp	nev any	st
KOO	6		f	0	0	all	-	q+s	As:HK	1981	CC	200	n	bl	n	n	0	ev	all/unsp	nev any	st
KREYBE	16	x	m	0	0	all	-	KI	Eu:Sca	1948	CC	300	n	bl	n	y	0	ev	all/unsp	nev any	st
KREYBE	33	x	f	0	0	all	-	KI	Eu:Sca	1948	CC	300	n	bl	n	y	0	ev	all/unsp	nev any	st
LAMTH	1		f	0	0	ch	-	q	As:HK	1983	CC	445	n	bl	n	n	0	ev	all/unsp	nev any	or
LAMWK	2		f	0	0	ch	-	q	As:HK	1981	CC	163	n	bl	n	n	0	ev	all/unsp	nev any	st
LAMWK2	1		m	0	0	all	-	q	As:HK	1976	CC	480	n	bl	n	n	0	ev	all/unsp	nev any	st
LAMWK2	5		f	0	0	all	-	q	As:HK	1976	CC	480	n	bl	n	n	0	ev	all/unsp	nev any	st
LOMBA2	2		f	0	0	all	-	q+u	NAmer	1960	CC	225	n	bl	n	n	0	ev	cig+/-ot	nev cigs	st
LUBIN	33		m	0	0	all	-	KI	As:Chi	1984	CC	427	m	ot	y	n	0	ev	all/unsp	nev any	st
LUBIN2	249		m	0	0	all	-	q	Eu:mul	1976	CC	7804	n	bl	n	y	0	cu	cig+/-ot	nev any	st
LUBIN2	261		f	0	0	all	-	q	Eu:mul	1976	CC	7804	n	bl	n	y	0	cu	cig+/-ot	nev any	st
LUO	2	x	c	0	0	all	-	q	As:Chi	1990	CC	102	n	ot	n	y	0	ev	cig+/-ot	nev cigs	st
MATOS	38	x	m	0	0	all	-	q	SCAmer	1994	CC	200	n	bl	n	n	0	cu	cig+/-ot	nev any	st
MATSUD	11		m	0	0	all	-	q	As:Jap	1965	CC	179	n	bl	n	n	0	ev	cig+/-ot	nev cigs	st
NOU	1		m	0	0	all	-	q	Eu:Sca	1971	CC	273	n	bl	y	n	0	ev	all/unsp	nev any	st
NOU	6		f	0	0	all	-	q	Eu:Sca	1971	CC	273	n	bl	y	n	0	ev	all/unsp	nev any	st
ORMOS	8		m	0	0	all	-	q	Eu:est	1947	CC	119	n	bl	y	y	0	ev	cig+/-ot	nev any	st
OSANN	10	x	m	0	0	all	-	q	NAmer	1984	CC	1986	n	bl	n	n	0	cu	cig+/-ot	nev cigs	st

Table 2C2 - 4

IESLC - Meta-anal of Current Smoking (or Ever if Current not available), Any prod (or Cigs if Any not avail)
Squamous
Least adjusted

REF	NRR	X	SEX	AGEL	AGEH	RACE	YF	LC TYPE	LOC	START	ST	NLC	R	VB	P	H	AD	SM	PRODUCT	DENOM	De
OSANN	14	x	f	0	0	all	-	q	NAmer	1984	CC	1986	n	bl	n	n	0	cu	cig+/-ot	nev cigs	st
OSANN2	8	x	f	0	0	all	-	KI	NAmer	1964	ot	217	n	bl	n	y	0	cu	cig+/-ot	nev cigs	st
PEZZOT	6		m	0	0	all	-	q	SCAmer	1987	CC	215	n	bl	n	y	0	ev	cig only	nev cigs	ot
SCHWAR	10		m	40	54	wh	-	q	NAmer	1984	CC	5588	n	bl	y	y	0	ev	cig+/-ot	nev cigs	st
SCHWAR	9		m	40	54	bl	-	q	NAmer	1984	CC	5588	n	bl	y	y	0	ev	cig+/-ot	nev cigs	st
SCHWAR	18		f	40	54	wh	-	q	NAmer	1984	CC	5588	n	bl	y	y	0	ev	cig+/-ot	nev cigs	ot
SCHWAR	17		f	40	54	bl	-	q	NAmer	1984	CC	5588	n	bl	y	y	0	ev	cig+/-ot	nev cigs	ot
SEOW	3		f	0	0	ch	-	q	As:oth	1997	CC	153	n	bl	n	y	0	ev	cig+/-ot	nev cigs	st
SIEMIA	11	x	m	0	0	all	-	q	NAmer	1979	CC	857	n	V	y	y	0	ev	cig+/-ot	nev cigs	st
SOBUE	2	x	m	0	0	all	-	q	As:Jap	1986	CC	1376	n	bl	n	y	0	cu	cig+/-ot	nev cigs	st
SOBUE	18	x	f	0	0	all	-	q	As:Jap	1986	CC	1376	n	bl	n	y	0	cu	cig+/-ot	nev cigs	st
SOBUE2	1		m	0	0	all	-	q	As:Jap	1965	CC	2083	n	bl	n	n	2	cu	cig+/-ot	nev any	or
SOBUE2	5		f	0	0	all	-	q	As:Jap	1965	CC	2083	n	bl	n	n	2	cu	cig+/-ot	nev any	or
STASZE	12		m	0	0	all	-	q	Eu:est	1954	CC	281	n	bl	n	y	0	ev	all/unsp	nev any	ot
STASZE	38		f	0	0	all	-	q	Eu:est	1954	CC	281	n	bl	n	y	0	ev	all/unsp	nev any	ot
STAYNE	3		m	0	0	all	-	q	NAmer	1969	CC	420	n	bl	n	n	0	ev	all/unsp	nev any	st
SUZUK2	12	x	c	0	0	all	-	q	SCAmer	1991	CC	123	n	bl	n	y	0	ev	all/unsp	nev any	st
SVENSS	62	x	f	0	0	all	-	q	Eu:Sca	1983	CC	210	n	bl	n	n	0	cu	all/unsp	nev any	st
TIZZAN	18		c	0	0	all	-	q+u	Eu:wst	1959	CC	1358	n	bl	n	n	0	ev	all/unsp	nev any	st
TOKARS	9	x	c	0	0	all	-	q	Eu:est	1966	ot	162	o	bl	n	y	0	ev	all/unsp	nev any	st
TSUGAN	14		m	0	0	all	-	q	As:Jap	1976	CC	134	n	bl	n	y	0	cu	all/unsp	nev any	ot
WAKAI	4	x	m	0	0	all	-	q	As:Jap	1988	CC	333	n	bl	n	y	0	cu	all/unsp	nev any	st
WAKAI	22	x	f	0	0	all	-	q	As:Jap	1988	CC	333	n	bl	n	y	0	cu	all/unsp	nev any	st
WU	11	x	f	0	0	wh	-	q	NAmer	1981	CC	220	n	bl	n	y	0	cu	all/unsp	nev any	st
WUWILL	9		f	0	0	all	-	q	As:Chi	1985	CC	965	n	ot	n	n	3	ev	cig+/-ot	nev cigs	or
WYNDE2	7		m	0	0	all	-	KI	NAmer	1962	CC	404	n	bl	n	y	0	ev	all/unsp	nev any	st
WYNDE3	10		m	0	0	all	-	KI	NAmer	1966	CC	350	n	bl	n	y	0	cu	all/unsp	nev any	st
WYNDE3	132		f	0	0	all	-	KI	NAmer	1966	CC	350	n	bl	n	y	0	ev	all/unsp	nev any	st
WYNDE4	35	x	m	0	0	all	-	not a	NAmer	1948	CC	684	n	bl	y	n	0	ev	all/unsp	nev any	st
WYNDE4	54		f	0	0	all	-	not a	NAmer	1948	CC	684	n	bl	y	n	2	ev	all/unsp	nev any	ot
WYNDE6	12		m	0	0	all	-	KI	NAmer	1969	CC	4423	n	bl	n	y	0	cu	cig+/-ot	nev any	st
WYNDE6	201		f	0	0	all	-	KI	NAmer	1969	CC	4423	n	bl	n	y	0	cu	cig+/-ot	nev cigs	st
XU3	19	x	m	0	0	all	-	KI	As:Chi	1981	CC	135	n	ot	n	n	0	ev	all/unsp	nev any	st
XU3	23	x	f	0	0	all	-	KI	As:Chi	1981	CC	135	n	ot	n	n	0	ev	all/unsp	nev any	st
ZHENG	5		m	0	0	all	-	q	As:Chi	1982	CC	540	n	ot	*	y	0	ev	cig+/-ot	nev cigs	st
ZHENG	18		f	0	0	all	-	q	As:Chi	1982	CC	540	n	ot	*	y	0	ev	cig+/-ot	nev cigs	st
ZHOU	8		m	0	0	all	-	q	As:Chi	1978	CC	1360	n	ot	n	n	0	ev	all/unsp	nev any	st
ZHOU	9		f	0	0	all	-	q	As:Chi	1978	CC	1360	n	ot	n	n	0	ev	all/unsp	nev any	st

Cigarette type is all/unspec for all RRs

Table 2C2 - 5

IESLC - Meta-anal of Current Smoking (or Ever if Current not available), Any prod (or Cigs if Any not avail)
Squamous
Least adjusted

REF	NRR	SEX	AD	Number Exposed Case	Number Exposed Cont	Non-exposed Case	Non-exposed Cont	RR	95.00%CI
*ABRAHA	1	m	0	142	10351	0	3365	92.66~	(5.77-1488.21)
*ABRAHA	4	f	0	17	5256	7	11589	5.35	(2.22- 12.90)
Subtotal ABRAHA								6.95	(3.00- 16.06)
ALDERS	52	m	2	-	-	-	-	14.70	(3.40- 63.64)
ALDERS	55	f	2	-	-	-	-	6.09	(2.68- 13.82)
Subtotal ALDERS								7.52	(3.67- 15.37)
*ANDERS	10	f	0	63	96164	5	195158	25.57	(10.29- 63.56)
BAND	5	m	2	-	-	-	-	37.45	(17.62- 79.58)
BARBON	17	m	0	203	362	6	188	17.57	(7.66- 40.33)
BECHER	11	f	1	-	-	-	-	10.69	(2.43- 47.00)
*BOUCOT	70	m	0	38	22177	0	7551	26.22~	(1.61- 426.71)
BRESLO	36	c	0	457	462	15	56	3.69	(2.06- 6.62)
BROWN2	16	m	2	-	-	-	-	13.70	(11.70- 16.10)
BROWN2	15	f	2	-	-	-	-	20.60	(16.60- 25.60)
Subtotal BROWN2								15.81	(13.91- 17.98)
BUFFLE	49	m	0	-	-	-	-	14.03	(4.73- 41.61)
BUFFLE	63	f	0	39	110	3	112	13.24	(3.97- 44.10)
Subtotal BUFFLE								13.67	(6.10- 30.62)
BYERS1	1	m	0	299	695	22	424	8.29	(5.29- 13.00)
CHAN	11	m	0	114	161	2	43	15.22	(3.61- 64.12)
CHAN	15	f	0	44	50	19	139	6.44	(3.44- 12.06)
Subtotal CHAN								7.39	(4.16- 13.13)
CHOI	62	m	0	160	465	6	95	5.45	(2.34- 12.67)
CHOI	64	f	0	11	26	10	164	6.94	(2.68- 17.96)
Subtotal CHOI								6.06	(3.22- 11.40)
COMSTO	23	m	0	27	100	2	84	11.34	(2.62- 49.09)
COMSTO	30	f	0	16	52	0	115	72.60~	(4.27-1233.12)
Subtotal COMSTO								16.78	(4.57- 61.67)
CORREA	43	c	1	-	-	-	-	34.60	(22.80- 52.40)
*CPSI	403	m	1	-	-	-	-	29.35	(4.02- 214.28)
*CPSI	405	f	1	-	-	-	-	4.25	(1.23- 14.68)
Subtotal CPSI								7.30	(2.55- 20.90)
*CPSII	114	m	1	-	-	-	-	39.26	(10.38- 148.55)
*CPSII	117	f	1	-	-	-	-	78.91	(15.83- 393.37)
Subtotal CPSII								52.16	(18.72- 145.32)
DAMBER	12	m	0	271	169	14	103	11.80	(6.54- 21.29)
DESTE2	16	m	2	-	-	-	-	13.20	(4.70- 37.10)
DOLL	82	m	0	829	1296	3	61	13.01	(4.07- 41.59)
DOLL	84	f	0	32	49	16	59	2.41	(1.18- 4.90)
Subtotal DOLL								3.81	(2.08- 6.98)
DORGAN	113	m	2	-	-	-	-	18.90	(7.00- 51.30)
DORGAN	98	f	3	-	-	-	-	11.10	(7.20- 17.10)
Subtotal DORGAN								12.08	(8.12- 17.96)
*DORN	338	m	1	-	-	-	-	17.09	(8.96- 32.60)
DOSEME	19	m	0	434	536	58	293	4.09	(3.00- 5.57)
*ENGELA	56	m	7	-	-	-	-	10.89	(3.25- 36.43)
FAN	3	c	0	75	595	6	556	11.68	(5.04- 27.04)
GAO	7	m	0	314	558	13	202	8.74	(4.91- 15.58)
GAO	17	f	0	66	130	53	605	5.80	(3.85- 8.72)
Subtotal GAO								6.65	(4.76- 9.28)
GER	5	c	0	48	156	11	80	2.24	(1.10- 4.54)
HAENSZ	20	f	0	53	94	44	236	3.02	(1.90- 4.82)
*HAMMON	102	m	1	-	-	-	-	26.42	(9.78- 71.34)
HEGMAN	2	c	0	89	1202	5	2080	30.80	(12.48- 76.03)
HINDS	23	f	3	-	-	-	-	16.13	(7.66- 33.97)
ISHIMA	1	c	0	53	33	5	25	8.03	(2.80- 23.04)
JAHN	7	m	0	153	269	3	138	26.16	(8.19- 83.54)
JAIN	18	m	0	107	118	2	85	38.54	(9.26- 160.45)
JAIN	13	f	0	81	99	6	214	29.18	(12.31- 69.15)
Subtotal JAIN								31.44	(15.03- 65.78)
JEDRYC	22	m	0	235	516	6	289	21.94	(9.63- 49.96)
JOLY	54	m	0	203	709	2	218	31.21	(7.69- 126.68)
JOLY	52	f	0	48	122	6	283	18.56	(7.74- 44.51)
Subtotal JOLY								21.47	(10.22- 45.09)
JUSSAW	23	m	0	89	168	13	624	25.43	(13.87- 46.63)
KATSOU	24	f	0	24	18	14	67	6.38	(2.76- 14.78)
KHUDER	14	m	0	112	-	9	-	8.60	(4.20- 17.60)
KIHARA	2	c	0	111	162	5	237	32.48	(12.97- 81.34)
KOO	6	f	0	61	63	32	137	4.15	(2.46- 6.98)
KREYBE	16	m	0	210	3514	3	644	12.83	(4.09- 40.22)
KREYBE	33	f	0	2	328	3	657	1.34	(0.22- 8.03)

Table 2C2 - 5

IESLC - Meta-anal of Current Smoking (or Ever if Current not available), Any prod (or Cigs if Any not avail)
Squamous
Least adjusted

REF	NRR	SEX	AD	Number Exposed Case	Number Exposed Cont	Non-exposed Case	Non-exposed Cont	RR	95.00%CI
Subtotal KREYBE								6.68	(2.55- 17.51)
LAMTH	1	f	0	63	20	28	72	8.10	(4.16- 15.77)
LAMWK	2	f	0	21	41	7	144	10.54	(4.19- 26.52)
LAMWK2	1	m	0	129	161	5	43	6.89	(2.65- 17.90)
LAMWK2	5	f	0	35	50	15	139	6.49	(3.27- 12.88)
Subtotal LAMWK2								6.62	(3.79- 11.56)
LOMBA2	2	f	0	94	353	15	239	4.24	(2.40- 7.50)
LUBIN	33	m	0	330	939	4	72	6.33	(2.29- 17.45)
LUBIN2	249	m	0	2518	6209	54	2616	19.65	(14.95- 25.82)
LUBIN2	261	f	0	154	410	72	1180	6.16	(4.55- 8.32)
Subtotal LUBIN2								11.64	(9.50- 14.25)
LUO	2	c	0	34	146	5	160	7.45	(2.84- 19.56)
MATOS	38	m	0	33	132	3	110	9.17	(2.74- 30.70)
MATSUD	11	m	0	103	3314	1	1255	39.01	(5.44- 279.84)
NOU	1	m	0	110	247	2	122	27.17	(6.60- 111.85)
NOU	6	f	0	5	92	2	261	7.09	(1.35- 37.19)
Subtotal NOU								15.42	(5.26- 45.22)
ORMOS	8	m	0	27	1034	2	777	10.14	(2.41- 42.79)
OSANN	10	m	0	247	541	8	833	47.54	(23.32- 96.92)
OSANN	14	f	0	130	367	12	1093	32.26	(17.65- 58.97)
Subtotal OSANN								37.93	(23.94- 60.10)
OSANN2	8	f	0	105	42	7	58	20.71	(8.75- 49.05)
PEZZOT	6	m	0	85	317	0	116	62.74~	(3.86-1019.50)
SCHWAR	10	m	0	80	178	1	73	32.81	(4.48- 240.23)
SCHWAR	9	m	0	41	39	4	7	1.84	(0.50- 6.78)
SCHWAR	18	f	0	29	108	0	79	43.23~	(2.60- 718.15)
SCHWAR	17	f	0	21	28	0	41	62.61~	(3.64-1076.10)
Subtotal SCHWAR								7.71	(2.96- 20.10)
SEOW	3	f	0	21	15	10	125	17.50	(6.95- 44.09)
SIEMIA	11	m	0	356	428	3	105	29.11	(9.16- 92.52)
SOBUE	2	m	0	301	650	3	128	19.76	(6.24- 62.58)
SOBUE	18	f	0	29	168	14	857	10.57	(5.47- 20.42)
Subtotal SOBUE								12.33	(6.96- 21.84)
SOBUE2	1	m	2	-	-	-	-	5.20	(4.20- 6.50)
SOBUE2	5	f	2	-	-	-	-	7.20	(4.80- 10.80)
Subtotal SOBUE2								5.59	(4.62- 6.78)
STASZE	12	m	0	137	754	0	158	57.77~	(3.58- 933.17)
STASZE	38	f	0	1	153	0	1660	32.45~	(1.32- 800.04)
Subtotal STASZE								45.09	(5.52- 368.55)
STAYNE	3	m	0	130	567	22	333	3.47	(2.17- 5.56)
SUZUK2	12	c	0	75	36	5	44	18.33	(6.70- 50.17)
SVENSS	62	f	0	42	53	5	120	19.02	(7.12- 50.77)
TIZZAN	18	c	0	333	939	55	419	2.70	(1.99- 3.67)
TOKARS	9	c	0	45	77	2	19	5.55	(1.24- 24.95)
TSUGAN	14	m	0	18	13	0	5	15.07~	(0.77- 296.43)
WAKAI	4	m	0	86	284	2	65	9.84	(2.36- 41.03)
WAKAI	22	f	0	15	26	3	145	27.88	(7.54- 103.14)
Subtotal WAKAI								17.34	(6.61- 45.48)
WU	11	f	0	61	23	2	30	39.78	(8.79- 180.01)
WUWILL	9	f	3	-	-	-	-	4.20	(3.00- 5.90)
WYNDE2	7	m	0	347	616	3	105	19.72	(6.21- 62.59)
WYNDE3	10	m	0	171	207	3	88	24.23	(7.53- 77.95)
WYNDE3	132	f	0	25	56	5	76	6.79	(2.45- 18.82)
Subtotal WYNDE3								11.77	(5.46- 25.38)
WYNDE4	35	m	0	597	665	8	115	12.91	(6.25- 26.65)
WYNDE4	54	f	2	-	-	-	-	5.82	(2.55- 13.31)
Subtotal WYNDE4								9.13	(5.29- 15.74)
WYNDE6	12	m	0	1026	741	29	617	29.46	(20.06- 43.26)
WYNDE6	201	f	0	550	376	40	856	31.30	(22.21- 44.12)
Subtotal WYNDE6								30.47	(23.59- 39.36)
XU3	19	m	0	39	68	3	31	5.93	(1.70- 20.66)
XU3	23	f	0	15	11	2	25	17.05	(3.32- 87.61)
Subtotal XU3								8.74	(3.24- 23.59)
ZHENG	5	m	0	156	218	4	94	16.82	(6.05- 46.71)
ZHENG	18	f	0	43	44	33	184	5.45	(3.11- 9.54)
Subtotal ZHENG								7.07	(4.33- 11.56)
ZHOU	8	m	0	343	41	96	36	3.14	(1.90- 5.18)
ZHOU	9	f	0	35	7	42	32	3.81	(1.50- 9.68)
Subtotal ZHOU								3.28	(2.11- 5.10)
Partial Totals				14721	169339	1070	242938		

*prospective study ~ With 0.5 adjustment for zero

Table 2C2 - 5

IESLC - Meta-anal of Current Smoking (or Ever if Current not available), Any prod (or Cigs if Any not avail)
Squamous
Least adjusted

REF	NRR	SEX	AD	Ys	Ws	Qs	Ps
*ABRAHA	1	m	0	4.53	0.50	2.46	0.0014
*ABRAHA	4	f	0	1.68	4.97	1.95	0.0002
Subtotal ABRAHA				1.94	5.46	4.42	
ALDERS	52	m	2	2.69	1.79	0.26	0.0003
ALDERS	55	f	2	1.81	5.71	1.42	0.0000
Subtotal ALDERS				2.02	7.50	1.68	
*ANDERS	10	f	0	3.24	4.63	4.06	0.0000
BAND	5	m	2	3.62	6.76	11.74	0.0000
BARBON	17	m	0	2.87	5.57	1.75	0.0000
BECHER	11	f	1	2.37	1.75	0.01	0.0017
*BOUCOT	70	m	0	3.27	0.49	0.46	0.0217
BRESLO	36	c	0	1.31	11.25	11.23	0.0000
BROWN2	16	m	2	2.62	150.78	14.68	0.0000
BROWN2	15	f	2	3.03	81.88	42.44	0.0000
Subtotal BROWN2				2.76	232.67	57.12	
BUFFLE	49	m	0	2.64	3.25	0.37	0.0000
BUFFLE	63	f	0	2.58	2.65	0.20	0.0000
Subtotal BUFFLE				2.62	5.90	0.57	
BYERS1	1	m	0	2.12	19.01	0.69	0.0000
CHAN	11	m	0	2.72	1.86	0.32	0.0002
CHAN	15	f	0	1.86	9.75	1.92	0.0000
Subtotal CHAN				2.00	11.61	2.24	
CHOI	62	m	0	1.70	5.39	2.01	0.0001
CHOI	64	f	0	1.94	4.25	0.58	0.0001
Subtotal CHOI				1.80	9.63	2.58	
COMSTO	23	m	0	2.43	1.79	0.03	0.0012
COMSTO	30	f	0	4.28	0.48	1.88	0.0030
Subtotal COMSTO				2.82	2.27	1.90	
CORREA	43	c	1	3.54	22.19	34.04	0.0000
*CPSI	403	m	1	3.38	0.97	1.12	0.0009
*CPSI	405	f	1	1.45	2.50	1.84	0.0222
Subtotal CPSI				1.99	3.47	2.96	
*CPSII	114	m	1	3.67	2.17	4.04	0.0000
*CPSII	117	f	1	4.37	1.49	6.33	0.0000
Subtotal CPSII				3.95	3.66	10.38	
DAMBER	12	m	0	2.47	11.02	0.29	0.0000
DESTE2	16	m	2	2.58	3.60	0.27	0.0000
DOLL	82	m	0	2.57	2.84	0.19	0.0000
DOLL	84	f	0	0.88	7.63	15.52	0.0152
Subtotal DOLL				1.34	10.47	15.71	
DORGAN	113	m	2	2.94	3.87	1.56	0.0000
DORGAN	98	f	3	2.41	20.54	0.21	0.0000
Subtotal DORGAN				2.49	24.41	1.77	
*DORN	338	m	1	2.84	9.21	2.62	0.0000
DOSEME	19	m	0	1.41	40.28	32.39	0.0000
*ENGELA	56	m	7	2.39	2.63	0.02	0.0001
FAN	3	c	0	2.46	5.45	0.13	0.0000
GAO	7	m	0	2.17	11.51	0.22	0.0000
GAO	17	f	0	1.76	23.06	6.93	0.0000
Subtotal GAO				1.89	34.57	7.15	
GER	5	c	0	0.81	7.65	17.22	0.0259
HAENSZ	20	f	0	1.11	17.71	25.45	0.0000
*HAMMON	102	m	1	3.27	3.89	3.65	0.0000
HEGMAN	2	c	0	3.43	4.70	5.92	0.0000
HINDS	23	f	3	2.78	6.93	1.56	0.0000
ISHIMA	1	c	0	2.08	3.46	0.17	0.0001
JAHN	7	m	0	3.26	2.85	2.62	0.0000
JAIN	18	m	0	3.65	1.89	3.42	0.0000
JAIN	13	f	0	3.37	5.16	5.89	0.0000
Subtotal JAIN				3.45	7.05	9.31	
JEDRYC	22	m	0	3.09	5.67	3.48	0.0000
JOLY	54	m	0	3.44	1.96	2.52	0.0000
JOLY	52	f	0	2.92	5.02	1.90	0.0000
Subtotal JOLY				3.07	6.98	4.42	
JUSSAW	23	m	0	3.24	10.45	9.05	0.0000
KATSOU	24	f	0	1.85	5.45	1.11	0.0000
KHUDER	14	m	0	2.15	7.48	0.18	0.0000
KIHARA	2	c	0	3.48	4.56	6.29	0.0000
KOO	6	f	0	1.42	14.12	11.02	0.0000
KREYBE	16	m	0	2.55	2.94	0.18	0.0000
KREYBE	33	f	0	0.29	1.19	4.85	0.7520

Table 2C2 - 5

IESLC - Meta-anal of Current Smoking (or Ever if Current not available), Any prod (or Cigs if Any not avail)
Squamous
Least adjusted

REF	NRR	SEX	AD	Ys	Ws	Qs	Ps
Subtotal KREYBE				1.90	4.14	5.03	
LAMTH	1	f	0	2.09	8.66	0.39	0.0000
LAMWK	2	f	0	2.35	4.51	0.01	0.0000
LAMWK2	1	m	0	1.93	4.22	0.59	0.0001
LAMWK2	5	f	0	1.87	8.17	1.55	0.0000
Subtotal LAMWK2				1.89	12.38	2.14	
LOMBA2	2	f	0	1.45	11.86	8.77	0.0000
LUBIN	33	m	0	1.84	3.73	0.79	0.0004
LUBIN2	249	m	0	2.98	51.39	23.24	0.0000
LUBIN2	261	f	0	1.82	42.25	10.06	0.0000
Subtotal LUBIN2				2.45	93.64	33.30	
LUO	2	c	0	2.01	4.12	0.36	0.0000
MATOS	38	m	0	2.22	2.63	0.02	0.0003
MATSUD	11	m	0	3.66	0.99	1.83	0.0003
NOU	1	m	0	3.30	1.92	1.91	0.0000
NOU	6	f	0	1.96	1.40	0.17	0.0205
Subtotal NOU				2.74	3.32	2.07	
ORMOS	8	m	0	2.32	1.85	0.00	0.0016
OSANN	10	m	0	3.86	7.57	18.33	0.0000
OSANN	14	f	0	3.47	10.56	14.42	0.0000
Subtotal OSANN				3.64	18.13	32.76	
OSANN2	8	f	0	3.03	5.17	2.72	0.0000
PEZZOT	6	m	0	4.14	0.49	1.66	0.0036
SCHWAR	10	m	0	3.49	0.97	1.36	0.0006
SCHWAR	9	m	0	0.61	2.26	6.49	0.3596
SCHWAR	18	f	0	3.77	0.49	1.04	0.0086
SCHWAR	17	f	0	4.14	0.47	1.59	0.0044
Subtotal SCHWAR				2.04	4.19	10.49	
SEOW	3	f	0	2.86	4.50	1.39	0.0000
SIEMIA	11	m	0	3.37	2.87	3.26	0.0000
SOBUE	2	m	0	2.98	2.89	1.33	0.0000
SOBUE	18	f	0	2.36	8.85	0.02	0.0000
Subtotal SOBUE				2.51	11.74	1.35	
SOBUE2	1	m	2	1.65	80.57	34.75	0.0000
SOBUE2	5	f	2	1.97	23.37	2.56	0.0000
Subtotal SOBUE2				1.72	103.93	37.31	
STASZE	12	m	0	4.06	0.50	1.52	0.0043
STASZE	38	f	0	3.48	0.37	0.52	0.0333
Subtotal STASZE				3.81	0.87	2.04	
STAYNE	3	m	0	1.24	17.27	19.44	0.0000
SUZUK2	12	c	0	2.91	3.79	1.38	0.0000
SVENSS	62	f	0	2.95	3.98	1.63	0.0000
TIZZAN	18	c	0	0.99	40.59	69.82	0.0000
TOKARS	9	c	0	1.71	1.70	0.59	0.0254
TSUGAN	14	m	0	2.71	0.43	0.07	0.0743
WAKAI	4	m	0	2.29	1.88	0.00	0.0017
WAKAI	22	f	0	3.33	2.25	2.35	0.0000
Subtotal WAKAI				2.85	4.13	2.35	
WU	11	f	0	3.68	1.69	3.20	0.0000
WUWILL	9	f	3	1.44	33.59	25.44	0.0000
WYNDE2	7	m	0	2.98	2.88	1.32	0.0000
WYNDE3	10	m	0	3.19	2.81	2.19	0.0000
WYNDE3	132	f	0	1.91	3.69	0.56	0.0002
Subtotal WYNDE3				2.47	6.50	2.75	
WYNDE4	35	m	0	2.56	7.31	0.46	0.0000
WYNDE4	54	f	2	1.76	5.63	1.67	0.0000
Subtotal WYNDE4				2.21	12.93	2.13	
WYNDE6	12	m	0	3.38	26.02	30.22	0.0000
WYNDE6	201	f	0	3.44	32.63	42.28	0.0000
Subtotal WYNDE6				3.42	58.65	72.50	
XU3	19	m	0	1.78	2.46	0.68	0.0052
XU3	23	f	0	2.84	1.43	0.40	0.0007
Subtotal XU3				2.17	3.90	1.08	
ZHENG	5	m	0	2.82	3.68	0.98	0.0000
ZHENG	18	f	0	1.70	12.24	4.55	0.0000
Subtotal ZHENG				1.96	15.92	5.54	
ZHOU	8	m	0	1.14	15.27	20.62	0.0000
ZHOU	9	f	0	1.34	4.42	4.14	0.0049
Subtotal ZHOU				1.19	19.68	24.75	

Table 2C2 - 5

IESLC - Meta-anal of Current Smoking (or Ever if Current not available), Any prod (or Cigs if Any not avail)
Squamous
Least adjusted

	N	110
	NS	78
	Wt	1135.80
Het	Chi	691.35
Het	df	109
Het	P	***
Fixed	RR	10.03
	RRl	9.46
	RRu	10.63
	P	+++
Random	RR	11.39
	RRl	9.63
	RRu	13.46
	P	+++
Asymm	P	N.S.

Table 2C2 - 6

IESLC - Meta-anal of Current Smoking (or Ever if Current not available), Any prod (or Cigs if Any not avail)
Squamous
Least adjusted

		Sex			
		combined	male	female	Total
	N	11	54	45	110
	NS	11	53	44	108
	Wt	109.47	567.30	459.03	1135.80
Het	Chi	134.16	270.27	267.88	691.35
Het	df	10	53	44	109
Het	P	***	***	***	***
Fixed	RR	7.11	11.05	9.65	10.03
	RRl	5.89	10.18	8.81	9.46
	RRu	8.57	12.00	10.58	10.63
	P	+++	+++	+++	+++
Random	RR	9.40	13.70	9.67	11.39
	RRl	4.43	10.91	7.50	9.63
	RRu	19.96	17.21	12.48	13.46
	P	+++	+++	+++	+++
Between	Chi				19.04
Between	df				2
Between	P				***
Btwn(F)	P				N.S.
Btwn(R)	P				N.S.

		Smoking status		
		ever	current	Total
	N	69	41	110
	NS	50	30	80
	Wt	489.39	646.41	1135.80
Het	Chi	250.94	286.73	691.35
Het	df	68	40	109
Het	P	***	***	***
Fixed	RR	6.57	13.81	10.03
	RRl	6.01	12.79	9.46
	RRu	7.18	14.92	10.63
	P	+++	+++	+++
Random	RR	8.70	16.83	11.39
	RRl	7.20	13.12	9.63
	RRu	10.53	21.60	13.46
	P	+++	+++	+++
Between	Chi			153.67
Between	df			1
Between	P			***
Btwn(F)	P			***
Btwn(R)	P			***

Table 2C2 - 7

IESLC - Meta-anal of Current Smoking (or Ever if Current not available), Any prod (or Cigs if Any not avail)
Squamous
Excluded studies (and stage at which they were excluded)

1 BOUCHA BUELL LAURIL MZILEN

2 BERRIN CHEN RESTRE TAO ULMER

5 RIMING TANG2 WYNDE5 WYNDE8

6 BLOT1 BLOT2 BLOT3 BYERS2 HIRAOK HIRAY2 JONES LICKIN MOLLO MRFIT MURATA NOTANI PERSHA SAARIK SCHWA2 SHIMIZ SITAS VANDER WARSIN WATSON WYNDER

8 ABELIN AGUDO AKIBA AMANDU AMES ARCHER ARMADA AUSTIN AUVINE AXELSO AXELSS BENSHL BEST BLOHMK BLOT4 BOFFET BRETT BROCKM BROSS BROWN1 CARPEN CASCO2 CASCOR CEDERL CHANG CHATZI CHEN2 CHEN3 CHIAZZ CHOW CHYOU COOKSO DARBY DAVEYS DEAN DEAN2 DEAN3 DEKLER DESTEF DOCKER DOLL2 DORANT DROSTE DU DUNN EBELIN ENSTRO ESAKI GAO2 GARCIA GARDIN GARSHI GENG GILLIS GODLEY GOLLED GOODMA GRAHAM GREGOR GSELL HAMMO2 HANSEN HEIN HENNEK HIRAYA HITOSU HOLE HOROWI HORWIT HU HU2 HUANG HUMBLE JARUP JARVHO JIANG KAISE2 KAISER KANELL KAUFMA KELLER KINLEN KJUUS KNEKT KO KOHLME KOULUM KREUZE KUBIK LANGE LAUSSM LEI LEMARC LETOUR LEVIN LIAW LIDDEL LIU LIU2 LIU3 LIU4 LIU5 LOMBAR MACLEN MAGNUS MARSH MARSH2 MARTIS MASTRA MCCONN MCDUFF MCLAUG MIGRAN MILLER MILLS MRFITR NAM NOTAN2 ODRISC PARKIN PASTOR PAWLEG PERNU PERSH2 PETO PEZZO2 PIKE PISANI POFFIJ POLEDN PRESCO QIAO QIAO2 RACHTA RADZIK RANDIG REN RONCO ROOTS ROTHSC SADOWS SANKAR SEGI SEGI2 SHAW SIMARA SIMONA SPEIZE SPITZ STOCKS STOCKW STUCKE SUN SUZUKI TANG TENKAN TOUSEY TULINI TVERDA VEIERO WALD WANG WANG2 WANG3 WANG4 WICKLU WIGLE WILKIN WU2 WUNSCH XIANGZ XU XU2 XU4 YAMAGU YONG YUAN ZHANG

10 BENHAM VUTUC WYNDE7

Table 2C2 - 8

Potentially overlapping studies

REF	REFGP	PRINC	. OVERLAP/LINK
LUBIN2	LUBIN2	1	Lubin-combined
CHAN	CHAN	1	Chan/LamWK2
LAMWK2	LAMWK2	1	Chan/LamWK2
LAMTH	LAMTH	1	KOO/LAMTH/LAMWK
KOO	KOO	1	KOO/LAMTH/LAMWK
LAMWK	LAMWK	1	KOO/LAMTH/LAMWK
ISHIMA	AKIBA	2	AKIBA/ISHIMA
BYERS1	BYERS1	2	GRAHAM/BROSS/BYERS1
OSANN2	KAISER	2	KAISER/OSANN2
WYNDE6	WYNDE6	1	WYNDE5/6/7/8
CPSI	CPSI	1	CPSI overall
SOBUE2	SOBUE2	1	SOBUE2/MATSUD
MATSUD	MATSUD	1	SOBUE2/MATSUD
JAHN	BOFFET	2	Subset of BOFFET
LUBIN	XIANGZ	2	LUBIN/XIANGZ/QIAO

Table 2C2 - 9

Most adjusted - insufficient data for metaanalysis

REF	NRR	SEX	AGEL	AGEH	RACE	YF	LC TYPE	LOC	START	ST	NLC	R	VB	P	H	AD	SM	PRODUCT	DENOM	De
CHEN	1	c	0	0	all	-	q	As:oth	1987	CC	323	n	ot	n	y	2	ev	cig+/-ot	nev cigs	ot
CHYOU	5	m	0	0	jap	0	q+s	NAmer	1965	pr	227	n	bl	n	y	1	cu	cig+/-ot	nev cigs	or
JIANG	3	m	0	0	all	-	q	As:Chi	1984	CC	125	n	ot	n	n	0	ev	all/unsp	nev any	or
JIANG	4	f	0	0	all	-	q	As:Chi	1984	CC	125	n	ot	n	n	0	ev	all/unsp	nev any	or
KOO	2	f	0	0	all	-	q	As:HK	1981	CC	200	n	bl	n	n	0	ev	all/unsp	nev any	or
WANG	3	c	0	0	all	-	q	As:Chi	1990	CC	390	n	ot	*	y	0	ev	all/unsp	nev any	ot

REF	NRR	RR	SIG	RRDATA comment
CHEN	1	6.66	y	P < 0.001
CHYOU	5	16.00		0
JIANG	3	12.78		0
JIANG	4	*	n	0
KOO	2	3.62		0
WANG	3	4.66	y	p<0.01

Table 2C3 -

IESLC - Meta-anal of Ever Smoking (or Current if Ever not available), Cigs (or Any Prod if Cigs not avail)
Squamous

This analysis is restricted to results for:
1) Non-dose-response data
2) Results complete enough for use in metaanalysis

Within each study, results are then selected (in the following order of preference, within each sex) for:
3) SMKSTA: ever smokers, current smokers
4) PRODUCT: cigarettes regardless of other products, cigarettes only, all/unspec
5) CIGTYPE: all/unspecified, MC regardless of HR, MC only
6) DENOM: never smoked anything, never smoked cigarettes, (never +1 = +long term ex, +2 = +amount unknown,
+3 = never cigs+long term ex)
7) Followup period (YF, prospective studies): whole study (coded as 0) or longest available
8) LCtype: squamous or nearest available, but not adeno. (q = squamous, s = small,
a = adeno, KI = Kreyberg I, u = undifferentiated)
9) Race: all or nearest available, otherwise by race (wh or w = white, bl or b = black, hi = hispanic
ch = chinese, jap = japanese, haw = hawaiian, w+o = white + oriental, sca = scandinavian, as = asian)
10) For overlapping studies: principal rather than subsidiary studies
Finally by Age: whole study (coded as 0) if available, otherwise by widest available age group
and then for single sex results (m, f) in preference to combined sex results (c).

Results adjusted (AD) for the most potential confounders are then chosen in Sections -1 to -3
(and those which actually differ from the adjusted results in Table 2C1 - 1 are marked 'x' in Section -1)
and results adjusted for the least confounders in Sections -4 to -6. (Those least adjusted results which
actually differ from the most adjusted as marked 'x' in column X in Section -4)
(Results adjusted for an unknown number of confounder(s) are coded as 20.)

Section -7 shows excluded studies, together with the stage (as above) at which no qualifying
results were found.

Section -8 lists the potentially overlapping studies which have been included (1=principal, 2=subsidiary).

Section -9 lists any results which would have been included in preference except that they had data not complete
enough for use in meta-analysis, with their significance (yes/no), if known, and any further comment as entered
on the database.

In addition to those mentioned above, the following fields, levels and abbreviations are used:
* or nk = not known, n = no, y = yes, ot = other
ev = ever, cu = current, nev = never
all/unspec = all or unspecified, cig+/-ot = cigarettes irrespective of other products (cigar, pipe etc)
MC = manufactured cigarettes, HR = hand-rolled cigarettes
REF: 6-character study reference
NRR: number of the RR on the database within the study
ST : study type (CC = case control, pr or prosp = prospective)
NLC: number of lung cancer cases in whole study
R : risky occupational population (n = no, m = mining, o = other risky)
VB : national cigarette type (V = at least 75% Virginia, bl = at least 75% blended, ot = other)
P : any proxy use
H : full histological confirmation
De : derivation of RR/CI (or = original, st = standard method, ot = other method of estimation)

Table 2C3 - 1

IESLC - Meta-anal of Ever Smoking (or Current if Ever not available), Cigs (or Any Prod if Cigs not avail)
Squamous
Most adjusted

REF	NRR	2C1	SEX	AGEL	AGEH	RACE	YF	LC TYPE	LOC	START	ST	NLC	R	VB	P	H	AD	SM	PRODUCT	DENOM	De
ABRAHA	1		m	0	0	all	0	q	Eu:est	1975	pr	571	n	bl	n	n	0	ev	all/unsp	nev any	ot
ABRAHA	4		f	0	0	all	0	q	Eu:est	1975	pr	571	n	bl	n	n	0	ev	all/unsp	nev any	ot
ALDERS	75	x	m	0	0	all	-	q+s	Eu:UK	1977	CC	1448	n	V	n	n	1	ev	cig+/-ot	nev any	ot
ALDERS	33	x	f	0	0	all	-	q+s	Eu:UK	1977	CC	1448	n	V	n	n	1	ev	cig only	nev any	ot
ANDERS	10		f	0	0	all	0	q	NAmer	1986	pr	343	n	bl	n	n	0	ev	cig+/-ot	nev cigs	st
BAND	5		m	0	0	all	-	q	NAmer	1983	CC	2831	n	V	y	y	2	ev	cig only	nev any	ot
BARBON	127		m	0	0	all	-	q	Eu:wst	1979	CC	755	n	bl	y	y	3	ev	all/unsp	nev any	ot
BECHER	11		f	0	0	all	-	q+s	Eu:Ger	1985	CC	194	n	bl	n	y	1	ev	all/unsp	nev any	or
BOUCOT	141		m	0	0	all	0	q	NAmer	1951	pr	121	n	bl	n	n	2	cu	cig only	nev any	ot
BRESLO	7	x	c	0	0	all	-	not a	NAmer	1949	CC	518	n	bl	n	y	0	ev	cig+/-ot	nev+1	st
BROWN2	6		m	0	0	wh	-	q	NAmer	1984	CC	14596	n	bl	n	y	2	ev	cig+/-ot	nev cigs	or
BROWN2	5		f	0	0	wh	-	q	NAmer	1984	CC	14596	n	bl	n	y	2	ev	cig+/-ot	nev cigs	or
BUFFLE	49		m	0	0	wh	-	q	NAmer	1976	CC	943	n	bl	y	n	0	ev	cig+/-ot	nev cigs	ot
BUFFLE	62		f	0	0	w-hi	-	q	NAmer	1976	CC	943	n	bl	y	n	0	ev	cig+/-ot	nev cigs	st
BYERS1	1		m	0	0	wh	-	q	NAmer	1957	CC	1002	n	bl	n	n	0	ev	cig+/-ot	nev cigs	st
CHAN	18	x	m	0	0	all	-	q+s	As:HK	1976	CC	397	n	bl	n	n	0	ev	cig+/-ot	nev any	st
CHAN	22	x	f	0	0	all	-	q+s	As:HK	1976	CC	397	n	bl	n	n	0	ev	cig+/-ot	nev any	st
CHOI	62		m	0	0	all	-	q	As:oth	1985	CC	375	n	bl	n	n	0	ev	cig+/-ot	nev cigs	st
CHOI	64		f	0	0	all	-	q	As:oth	1985	CC	375	n	bl	n	n	0	ev	cig+/-ot	nev cigs	st
COMSTO	66		m	0	0	all	-	q	NAmer	1975	ot	258	n	bl	n	n	0	ev	cig+/-ot	nev cigs	st
COMSTO	78		f	0	0	all	-	q	NAmer	1975	ot	258	n	bl	n	n	0	ev	cig+/-ot	nev cigs	ot
CORREA	35		c	0	0	all	-	q+s	NAmer	1979	CC	1359	n	bl	y	n	1	ev	cig+/-ot	nev cigs	or
CPSI	403		m	0	0	all	2	q	NAmer	1959	pr	5138	n	bl	n	n	1	cu	cig only	nev any	ot
CPSI	405		f	0	0	all	2	q	NAmer	1959	pr	5138	n	bl	n	n	1	cu	cig only	nev any	ot
CPSII	114		m	0	0	all	2	q	NAmer	1982	pr	3229	n	bl	n	n	1	cu	cig only	nev any	ot
CPSII	117		f	0	0	all	2	q	NAmer	1982	pr	3229	n	bl	n	n	1	cu	cig+/-ot	nev cigs	ot
DAMBER	33		m	0	0	all	-	q	Eu:Sca	1972	CC	579	n	bl	y	n	1	ev	all/unsp	nev any	or
DESTE2	16		m	0	0	all	-	q	SCAmer	1993	CC	463	n	bl	n	n	2	ev	all/unsp	nev any	or
DOLL	86		m	0	0	all	-	KI	Eu:UK	1948	CC	1465	n	V	n	n	1	ev	all/unsp	nev any	ot
DOLL	88		f	0	0	all	-	KI	Eu:UK	1948	CC	1465	n	V	n	n	1	ev	all/unsp	nev any	ot
DORGAN	113		m	0	0	wh	-	q	NAmer	1980	CC	2026	n	bl	y	y	2	ev	cig+/-ot	nev any	or
DORGAN	98		f	0	0	all	-	q	NAmer	1980	CC	2026	n	bl	y	y	3	ev	cig+/-ot	nev any	or
DORN	338		m	0	0	wh	8	q	NAmer	1954	pr	5097	n	bl	n	n	1	cu	cig only	nev any	ot
DOSEME	3		m	0	0	all	-	q	Eu:bal	1979	CC	1210	n	bl	n	n	2	ev	cig+/-ot	nev cigs	or
ENGELA	62		m	0	0	all	0	q	Eu:Sca	1964	pr	435	n	bl	n	n	7	ev	cig+/-ot	nev cigs	ot
FAN	3		c	0	0	all	-	q	As:Chi	1990	CC	403	n	ot	y	n	0	ev	cig+/-ot	nev cigs	ot
GAO	2		m	0	0	all	-	q	As:Chi	1984	CC	1405	n	ot	n	n	2	ev	cig+/-ot	nev cigs	or
GAO	12		f	0	0	all	-	q	As:Chi	1984	CC	1405	n	ot	n	n	2	ev	cig+/-ot	nev cigs	or
GER	13		c	0	0	all	-	q+s	As:oth	1990	CC	141	n	ot	y	n	10	ev	all/unsp	nev any	ot
HAENSZ	22	x	f	0	0	all	-	q+u	NAmer	1955	CC	158	n	bl	n	y	0	ev	cig+/-ot	nev any	st
HAMMON	59	x	m	0	0	wh	0	not a	NAmer	1952	pr	448	n	bl	n	n	1	ev	cig+/-ot	nev any	ot
HEGMAN	2		c	0	0	all	-	q	NAmer	1989	CC	282	n	bl	y	y	0	ev	all/unsp	nev any	st
HINDS	23		f	0	0	o	-	q+s	NAmer	1968	CC	292	n	bl	n	n	3	ev	all/unsp	nev any	st
ISHIMA	6		c	0	0	all	-	q	As:Jap	1961	CC	180	n	bl	y	y	5	ev	all/unsp	nev any	st
JAHN	42	x	m	0	0	all	-	q	Eu:Ger	1988	CC	1004	n	bl	n	n	0	ev	cig+/-ot	nev any	st
JAIN	48		m	0	0	all	-	q	NAmer	1981	CC	845	n	V	y	n	2	ev	cig+/-ot	nev cigs	or
JAIN	43		f	0	0	all	-	q	NAmer	1981	CC	845	n	V	y	n	2	ev	cig+/-ot	nev cigs	or
JEDRYC	54		m	0	0	all	-	q	Eu:est	1980	CC	1630	n	bl	y	n	3	ev	cig+/-ot	nev any	ot
JOLY	54		m	0	0	all	-	q	SCAmer	1978	CC	826	n	bl	n	n	0	ev	cig+/-ot	nev any	st
JOLY	52		f	0	0	all	-	q	SCAmer	1978	CC	826	n	bl	n	n	0	ev	cig+/-ot	nev any	st
JUSSAW	25	x	m	0	0	all	-	KI	As:Ind	1964	CC	792	n	V	n	n	0	ev	cig only	nev any	st
KATSOU	37		f	0	0	all	-	KI	Eu:bal	1987	CC	101	n	bl	n	n	1	ev	all/unsp	nev any	ot
KHUDER	24		m	0	0	all	-	q	NAmer	1985	CC	482	n	bl	n	y	0	ev	cig+/-ot	nev cigs	ot
KIHARA	26		c	0	0	jap	-	q	As:Jap	1991	CC	440	n	bl	n	n	0	ev	all/unsp	nev any	st
KOO	6		f	0	0	all	-	q+s	As:HK	1981	CC	200	n	bl	n	n	0	ev	all/unsp	nev any	st
KREYBE	4		m	0	0	all	-	KI	Eu:Sca	1948	CC	300	n	bl	n	y	1	ev	all/unsp	nev any	ot
KREYBE	25		f	0	0	all	-	KI	Eu:Sca	1948	CC	300	n	bl	n	y	1	ev	all/unsp	nev any	ot
LAMTH	1		f	0	0	ch	-	q	As:HK	1983	CC	445	n	bl	n	n	0	ev	all/unsp	nev any	or
LAMWK	2		f	0	0	ch	-	q	As:HK	1981	CC	163	n	bl	n	n	0	ev	all/unsp	nev any	st
LAMWK2	1		m	0	0	all	-	q	As:HK	1976	CC	480	n	bl	n	n	0	ev	all/unsp	nev any	st
LAMWK2	5		f	0	0	all	-	q	As:HK	1976	CC	480	n	bl	n	n	0	ev	all/unsp	nev any	st
LOMBA2	2		f	0	0	all	-	q+u	NAmer	1960	CC	225	n	bl	n	n	0	ev	cig+/-ot	nev cigs	st
LUBIN	34	x	m	0	0	all	-	KI	As:Chi	1984	CC	427	m	ot	y	n	0	ev	cig+/-ot	nev any	st
LUBIN2	145		m	0	0	all	-	q	Eu:mul	1976	CC	7804	n	bl	n	y	0	ev	cig+/-ot	nev any	st
LUBIN2	165		f	0	0	all	-	q	Eu:mul	1976	CC	7804	n	bl	n	y	0	ev	cig+/-ot	nev any	st
LUO	8		c	0	0	all	-	q	As:Chi	1990	CC	102	n	ot	n	y	20	ev	cig+/-ot	nev cigs	or
MATOS	67		m	0	0	all	-	q	SCAmer	1994	CC	200	n	bl	n	n	2	ev	cig+/-ot	nev any	ot
MATSUD	11		m	0	0	all	-	q	As:Jap	1965	CC	179	n	bl	n	n	0	ev	cig+/-ot	nev cigs	st
NOU	1		m	0	0	all	-	q	Eu:Sca	1971	CC	273	n	bl	y	n	0	ev	all/unsp	nev any	st
NOU	6		f	0	0	all	-	q	Eu:Sca	1971	CC	273	n	bl	y	n	0	ev	all/unsp	nev any	st
ORMOS	8		m	0	0	all	-	q	Eu:est	1947	CC	119	n	bl	y	y	0	ev	cig+/-ot	nev any	st
OSANN	43		m	0	0	all	-	q	NAmer	1984	CC	1986	n	bl	n	n	2	ev	cig+/-ot	nev cigs	or

Table 2C3 - 1

IESLC - Meta-anal of Ever Smoking (or Current if Ever not available), Cigs (or Any Prod if Cigs not avail)
Squamous
Most adjusted

REF	NRR	2C1	SEX	AGEL	AGEH	RACE	YF	LC TYPE	LOC	START	ST	NLC	R	VB	P	H	AD	SM	PRODUCT	DENOM	De
OSANN	44		f	0	0	all	-	q	NAmer	1984	CC	1986	n	bl	n	n	2	ev	cig+/-ot	nev cigs	or
OSANN2	25		f	0	0	all	-	KI	NAmer	1964	ot	217	n	bl	n	y	1	ev	cig+/-ot	nev cigs	or
PEZZOT	6		m	0	0	all	-	q	SCAmer	1987	CC	215	n	bl	n	y	0	ev	cig only	nev cigs	ot
SCHWAR	10		m	40	54	wh	-	q	NAmer	1984	CC	5588	n	bl	y	y	0	ev	cig+/-ot	nev cigs	st
SCHWAR	9		m	40	54	bl	-	q	NAmer	1984	CC	5588	n	bl	y	y	0	ev	cig+/-ot	nev cigs	st
SCHWAR	18		f	40	54	wh	-	q	NAmer	1984	CC	5588	n	bl	y	y	0	ev	cig+/-ot	nev cigs	ot
SCHWAR	17		f	40	54	bl	-	q	NAmer	1984	CC	5588	n	bl	y	y	0	ev	cig+/-ot	nev cigs	ot
SEOW	3		f	0	0	ch	-	q	As:oth	1997	CC	153	n	bl	n	y	0	ev	cig+/-ot	nev cigs	st
SIEMIA	7		m	0	0	all	-	q	NAmer	1979	CC	857	n	V	y	y	7	ev	cig+/-ot	nev cigs	or
SOBUE	97		m	0	0	all	-	q	As:Jap	1986	CC	1376	n	bl	n	y	1	ev	cig+/-ot	nev cigs	ot
SOBUE	107		f	0	0	all	-	q	As:Jap	1986	CC	1376	n	bl	n	y	1	ev	cig+/-ot	nev cigs	ot
SOBUE2	1		m	0	0	all	-	q	As:Jap	1965	CC	2083	n	bl	n	n	2	cu	cig+/-ot	nev any	or
SOBUE2	5		f	0	0	all	-	q	As:Jap	1965	CC	2083	n	bl	n	n	2	cu	cig+/-ot	nev any	or
STASZE	16	x	m	0	0	all	-	q	Eu:est	1954	CC	281	n	bl	n	y	0	ev	cig+/-ot	nev any	ot
STASZE	38		f	0	0	all	-	q	Eu:est	1954	CC	281	n	bl	n	y	0	ev	all/unsp	nev any	ot
STAYNE	3		m	0	0	all	-	q	NAmer	1969	CC	420	n	bl	n	n	0	ev	all/unsp	nev any	st
SUZUK2	15		c	0	0	all	-	q	SCAmer	1991	CC	123	n	bl	n	y	3	ev	all/unsp	nev any	or
SVENSS	72		f	0	0	all	-	q	Eu:Sca	1983	CC	210	n	bl	n	n	1	ev	all/unsp	nev any	ot
TIZZAN	18		c	0	0	all	-	q+u	Eu:wst	1959	CC	1358	n	bl	n	n	0	ev	all/unsp	nev any	st
TOKARS	10		c	0	0	all	-	q	Eu:est	1966	ot	162	o	bl	n	y	3	ev	all/unsp	nev any	or
TSUGAN	13		m	0	0	all	-	q	As:Jap	1976	CC	134	n	bl	n	y	0	ev	all/unsp	nev any	ot
WAKAI	74		m	0	0	all	-	q	As:Jap	1988	CC	333	n	bl	n	y	1	ev	all/unsp	nev any	ot
WAKAI	80		f	0	0	all	-	q	As:Jap	1988	CC	333	n	bl	n	y	1	ev	all/unsp	nev any	ot
WU	32		f	0	0	wh	-	q	NAmer	1981	CC	220	n	bl	n	y	2	ev	all/unsp	nev any	ot
WUWILL	9		f	0	0	all	-	q	As:Chi	1985	CC	965	n	ot	n	n	3	ev	cig+/-ot	nev cigs	or
WYNDE2	2	x	m	0	0	all	-	KI	NAmer	1962	CC	404	n	bl	n	y	0	ev	cig+/-ot	nev any	st
WYNDE3	8	x	m	0	0	all	-	KI	NAmer	1966	CC	350	n	bl	n	y	0	ev	cig+/-ot	nev any	st
WYNDE3	67	x	f	0	0	all	-	KI	NAmer	1966	CC	350	n	bl	n	y	0	ev	cig+/-ot	nev any	st
WYNDE4	69	x	m	0	0	all	-	not a	NAmer	1948	CC	684	n	bl	y	n	2	ev	cig+/-ot	nev any	ot
WYNDE4	54		f	0	0	all	-	not a	NAmer	1948	CC	684	n	bl	y	n	2	ev	all/unsp	nev any	ot
WYNDE6	75	x	m	0	0	all	-	KI	NAmer	1969	CC	4423	n	bl	n	y	0	ev	cig+/-ot	nev any	st
WYNDE6	412		f	0	0	wh	-	q	NAmer	1969	CC	4423	n	bl	n	y	1	ev	cig+/-ot	nev cigs	ot
XU3	20		m	0	0	all	-	KI	As:Chi	1981	CC	135	n	ot	n	n	1	ev	all/unsp	nev any	ot
XU3	24		f	0	0	all	-	KI	As:Chi	1981	CC	135	n	ot	n	n	1	ev	all/unsp	nev any	ot
ZHENG	5		m	0	0	all	-	q	As:Chi	1982	CC	540	n	ot	*	y	0	ev	cig+/-ot	nev cigs	st
ZHENG	18		f	0	0	all	-	q	As:Chi	1982	CC	540	n	ot	*	y	0	ev	cig+/-ot	nev cigs	st
ZHOU	8		m	0	0	all	-	q	As:Chi	1978	CC	1360	n	ot	n	n	0	ev	all/unsp	nev any	st
ZHOU	9		f	0	0	all	-	q	As:Chi	1978	CC	1360	n	ot	n	n	0	ev	all/unsp	nev any	st

Cigarette type is all/unspec for all RRs

except for the following:

REF	NRR	CIGTYPE
ALDERS	33	MC only
CHAN	18	MC+-HR
CHAN	22	MC+-HR
JUSSAW	25	MC only

Table 2C3 - 2

IESLC - Meta-anal of Ever Smoking (or Current if Ever not available), Cigs (or Any Prod if Cigs not avail)
Squamous
Most adjusted

				Number Exposed		Non-exposed			
REF	NRR	SEX	AD	Case	Cont	Case	Cont	RR	95.00%CI
*ABRAHA	1	m	0	142	10351	0	3365	92.66~	(5.77-1488.21)
*ABRAHA	4	f	0	17	5256	7	11589	5.35	(2.22- 12.90)
Subtotal ABRAHA								6.95	(3.00- 16.06)
ALDERS	75	m	1	-	-	-	-	10.23	(3.78- 27.72)
ALDERS	33	f	1	-	-	-	-	6.70	(3.92- 11.46)
Subtotal ALDERS								7.37	(4.59- 11.82)
*ANDERS	10	f	0	63	96164	5	195158	25.57	(10.29- 63.56)
BAND	5	m	2	-	-	-	-	37.45	(17.62- 79.58)
BARBON	127	m	3	-	-	-	-	14.52	(6.35- 33.20)
BECHER	11	f	1	-	-	-	-	10.69	(2.43- 47.00)
*BOUCOT	141	m	2	-	-	-	-	27.54	(1.69- 448.37)
BRESLO	7	c	0	444	394	15	56	4.21	(2.34- 7.56)
BROWN2	6	m	2	-	-	-	-	11.10	(9.50- 12.90)
BROWN2	5	f	2	-	-	-	-	20.10	(16.40- 24.80)
Subtotal BROWN2								13.69	(12.11- 15.49)
BUFFLE	49	m	0	-	-	-	-	14.03	(4.73- 41.61)
BUFFLE	62	f	0	58	166	3	112	13.04	(3.99- 42.66)
Subtotal BUFFLE								13.57	(6.09- 30.24)
BYERS1	1	m	0	299	695	22	424	8.29	(5.29- 13.00)
CHAN	18	m	0	112	160	2	43	15.05	(3.57- 63.41)
CHAN	22	f	0	37	38	19	139	7.12	(3.68- 13.77)
Subtotal CHAN								8.11	(4.45- 14.77)
CHOI	62	m	0	160	465	6	95	5.45	(2.34- 12.67)
CHOI	64	f	0	11	26	10	164	6.94	(2.68- 17.96)
Subtotal CHOI								6.06	(3.22- 11.40)
COMSTO	66	m	0	44	229	2	84	8.07	(1.91- 34.02)
COMSTO	78	f	0	17	87	0	115	46.20~	(2.74- 778.83)
Subtotal COMSTO								11.56	(3.21- 41.67)
CORREA	35	c	1	-	-	-	-	28.30	(18.60- 43.20)
*CPSI	403	m	1	-	-	-	-	29.35	(4.02- 214.28)
*CPSI	405	f	1	-	-	-	-	4.25	(1.23- 14.68)
Subtotal CPSI								7.30	(2.55- 20.90)
*CPSII	114	m	1	-	-	-	-	39.26	(10.38- 148.55)
*CPSII	117	f	1	-	-	-	-	78.91	(15.83- 393.37)
Subtotal CPSII								52.16	(18.72- 145.32)
DAMBER	33	m	1	-	-	-	-	11.80	(6.40- 23.00)
DESTE2	16	m	2	-	-	-	-	13.20	(4.70- 37.10)
DOLL	86	m	1	-	-	-	-	13.17	(4.12- 42.10)
DOLL	88	f	1	-	-	-	-	2.13	(1.06- 4.27)
Subtotal DOLL								3.45	(1.90- 6.27)
DORGAN	113	m	2	-	-	-	-	18.90	(7.00- 51.30)
DORGAN	98	f	3	-	-	-	-	11.10	(7.20- 17.10)
Subtotal DORGAN								12.08	(8.12- 17.96)
*DORN	338	m	1	-	-	-	-	17.09	(8.96- 32.60)
DOSEME	3	m	2	-	-	-	-	3.60	(2.60- 5.00)
*ENGELA	62	m	7	-	-	-	-	6.45	(1.97- 21.11)
FAN	3	c	0	75	595	6	556	11.68	(5.04- 27.04)
GAO	2	m	2	-	-	-	-	8.40	(4.70- 15.00)
GAO	12	f	2	-	-	-	-	7.20	(4.60- 11.10)
Subtotal GAO								7.62	(5.36- 10.82)
GER	13	c	10	-	-	-	-	3.19	(1.08- 9.42)
HAENSZ	22	f	0	56	103	44	236	2.92	(1.85- 4.61)
*HAMMON	59	m	1	-	-	-	-	21.66	(8.07- 58.14)
HEGMAN	2	c	0	89	1202	5	2080	30.80	(12.48- 76.03)
HINDS	23	f	3	-	-	-	-	16.13	(7.66- 33.97)
ISHIMA	6	c	5	-	-	-	-	21.00	(3.38- 868.40)
JAHN	42	m	0	343	671	3	138	23.51	(7.44- 74.35)
JAIN	48	m	2	-	-	-	-	18.00	(5.50- 111.00)
JAIN	43	f	2	-	-	-	-	25.50	(7.93- 156.00)
Subtotal JAIN								21.46	(7.45- 61.79)
JEDRYC	54	m	3	-	-	-	-	12.84	(5.58- 29.55)
JOLY	54	m	0	203	709	2	218	31.21	(7.69- 126.68)
JOLY	52	f	0	48	122	6	283	18.56	(7.74- 44.51)
Subtotal JOLY								21.47	(10.22- 45.09)
JUSSAW	25	m	0	17	77	13	624	10.60	(4.96- 22.66)
KATSOU	37	f	1	-	-	-	-	6.11	(2.69- 13.87)
KHUDER	24	m	0	176	-	9	-	7.82	(3.87- 15.77)
KIHARA	26	c	0	132	232	5	237	26.97	(10.84- 67.08)
KOO	6	f	0	61	63	32	137	4.15	(2.46- 6.98)
KREYBE	4	m	1	-	-	-	-	10.87	(3.47- 34.04)
KREYBE	25	f	1	-	-	-	-	2.29	(0.89- 5.88)

Table 2C3 - 2

IESLC - Meta-anal of Ever Smoking (or Current if Ever not available), Cigs (or Any Prod if Cigs not avail)
Squamous
Most adjusted

REF	NRR	SEX	AD	Number Exposed Case	Number Exposed Cont	Non-exposed Case	Non-exposed Cont	RR	95.00%CI
Subtotal KREYBE								4.31	(2.08- 8.92)
LAMTH	1	f	0	63	20	28	72	8.10	(4.16- 15.77)
LAMWK	2	f	0	21	41	7	144	10.54	(4.19- 26.52)
LAMWK2	1	m	0	129	161	5	43	6.89	(2.65- 17.90)
LAMWK2	5	f	0	35	50	15	139	6.49	(3.27- 12.88)
Subtotal LAMWK2								6.62	(3.79- 11.56)
LOMBA2	2	f	0	94	353	15	239	4.24	(2.40- 7.50)
LUBIN	34	m	0	291	788	4	72	6.65	(2.41- 18.36)
LUBIN2	145	m	0	3587	10433	54	2616	16.66	(12.69- 21.86)
LUBIN2	165	f	0	200	567	72	1180	5.78	(4.34- 7.71)
Subtotal LUBIN2								10.10	(8.29- 12.31)
LUO	8	c	20	-	-	-	-	10.90	(2.50- 47.90)
MATOS	67	m	2	-	-	-	-	8.08	(2.59- 25.20)
MATSUD	11	m	0	103	3314	1	1255	39.01	(5.44- 279.84)
NOU	1	m	0	110	247	2	122	27.17	(6.60- 111.85)
NOU	6	f	0	5	92	2	261	7.09	(1.35- 37.19)
Subtotal NOU								15.42	(5.26- 45.22)
ORMOS	8	m	0	27	1034	2	777	10.14	(2.41- 42.79)
OSANN	43	m	2	-	-	-	-	36.10	(17.80- 73.30)
OSANN	44	f	2	-	-	-	-	26.40	(14.50- 48.10)
Subtotal OSANN								30.09	(19.04- 47.54)
OSANN2	25	f	1	-	-	-	-	35.10	(4.80- 256.00)
PEZZOT	6	m	0	85	317	0	116	62.74~	(3.86-1019.50)
SCHWAR	10	m	0	80	178	1	73	32.81	(4.48- 240.23)
SCHWAR	9	m	0	41	39	4	7	1.84	(0.50- 6.78)
SCHWAR	18	f	0	29	108	0	79	43.23~	(2.60- 718.15)
SCHWAR	17	f	0	21	28	0	41	62.61~	(3.64-1076.10)
Subtotal SCHWAR								7.71	(2.96- 20.10)
SEOW	3	f	0	21	15	10	125	17.50	(6.95- 44.09)
SIEMIA	7	m	7	-	-	-	-	22.70	(6.90- 75.20)
SOBUE	97	m	1	-	-	-	-	17.88	(7.82- 40.87)
SOBUE	107	f	1	-	-	-	-	8.74	(5.09- 15.02)
Subtotal SOBUE								10.83	(6.89- 17.03)
SOBUE2	1	m	2	-	-	-	-	5.20	(4.20- 6.50)
SOBUE2	5	f	2	-	-	-	-	7.20	(4.80- 10.80)
Subtotal SOBUE2								5.59	(4.62- 6.78)
STASZE	16	m	0	135	653	0	158	65.73~	(4.07-1061.95)
STASZE	38	f	0	1	153	0	1660	32.45~	(1.32- 800.04)
Subtotal STASZE								48.53	(5.94- 396.73)
STAYNE	3	m	0	130	567	22	333	3.47	(2.17- 5.56)
SUZUK2	15	c	3	-	-	-	-	31.00	(4.20- 227.00)
SVENSS	72	f	1	-	-	-	-	12.62	(3.97- 40.14)
TIZZAN	18	c	0	333	939	55	419	2.70	(1.99- 3.67)
TOKARS	10	c	3	-	-	-	-	6.80	(1.20- 38.70)
TSUGAN	13	m	0	20	15	0	5	14.55~	(0.75- 283.37)
WAKAI	74	m	1	-	-	-	-	8.61	(2.08- 35.72)
WAKAI	80	f	1	-	-	-	-	25.23	(6.87- 92.66)
Subtotal WAKAI								15.46	(5.92- 40.36)
WU	32	f	2	-	-	-	-	24.29	(3.40- 173.76)
WUWILL	9	f	3	-	-	-	-	4.20	(3.00- 5.90)
WYNDE2	2	m	0	336	512	3	105	22.97	(7.23- 72.97)
WYNDE3	8	m	0	197	264	3	88	21.89	(6.82- 70.20)
WYNDE3	67	f	0	25	56	5	76	6.79	(2.45- 18.82)
Subtotal WYNDE3								11.28	(5.23- 24.31)
WYNDE4	69	m	2	-	-	-	-	15.45	(7.47- 31.96)
WYNDE4	54	f	2	-	-	-	-	5.82	(2.55- 13.31)
Subtotal WYNDE4								10.09	(5.85- 17.42)
WYNDE6	75	m	0	1706	1797	29	617	20.20	(13.84- 29.48)
WYNDE6	412	f	1	-	-	-	-	32.37	(17.66- 59.35)
Subtotal WYNDE6								23.05	(16.73- 31.78)
XU3	20	m	1	-	-	-	-	5.90	(1.69- 20.57)
XU3	24	f	1	-	-	-	-	25.67	(4.99- 131.94)
Subtotal XU3								10.14	(3.75- 27.37)
ZHENG	5	m	0	156	218	4	94	16.82	(6.05- 46.71)
ZHENG	18	f	0	43	44	33	184	5.45	(3.11- 9.54)
Subtotal ZHENG								7.07	(4.33- 11.56)
ZHOU	8	m	0	343	41	96	36	3.14	(1.90- 5.18)
ZHOU	9	f	0	35	7	42	32	3.81	(1.50- 9.68)
Subtotal ZHOU								3.28	(2.11- 5.10)
Partial Totals				11006	140856	730	227021		

*prospective study ~ With 0.5 adjustment for zero

Table 2C3 - 2

IESLC - Meta-anal of Ever Smoking (or Current if Ever not available), Cigs (or Any Prod if Cigs not avail)
Squamous
Most adjusted

REF	NRR	SEX	AD	Ys	Ws	Qs	Ps
*ABRAHA	1	m	0	4.53	0.50	2.68	0.0014
*ABRAHA	4	f	0	1.68	4.97	1.41	0.0002
Subtotal ABRAHA				1.94	5.46	4.09	
ALDERS	75	m	1	2.33	3.87	0.05	0.0000
ALDERS	33	f	1	1.90	13.35	1.27	0.0000
Subtotal ALDERS				2.00	17.22	1.32	
*ANDERS	10	f	0	3.24	4.63	4.93	0.0000
BAND	5	m	2	3.62	6.76	13.49	0.0000
BARBON	127	m	3	2.68	5.62	1.22	0.0000
BECHER	11	f	1	2.37	1.75	0.04	0.0017
*BOUCOT	141	m	2	3.32	0.49	0.60	0.0199
BRESLO	7	c	0	1.44	11.20	6.70	0.0000
BROWN2	6	m	2	2.41	164.17	6.34	0.0000
BROWN2	5	f	2	3.00	89.84	56.12	0.0000
Subtotal BROWN2				2.62	254.01	62.46	
BUFFLE	49	m	0	2.64	3.25	0.60	0.0000
BUFFLE	62	f	0	2.57	2.74	0.35	0.0000
Subtotal BUFFLE				2.61	5.99	0.95	
BYERS1	1	m	0	2.12	19.01	0.17	0.0000
CHAN	18	m	0	2.71	1.86	0.47	0.0002
CHAN	22	f	0	1.96	8.84	0.54	0.0000
Subtotal CHAN				2.09	10.69	1.01	
CHOI	62	m	0	1.70	5.39	1.43	0.0001
CHOI	64	f	0	1.94	4.25	0.32	0.0001
Subtotal CHOI				1.80	9.63	1.75	
COMSTO	66	m	0	2.09	1.86	0.03	0.0045
COMSTO	78	f	0	3.83	0.48	1.27	0.0078
Subtotal COMSTO				2.45	2.34	1.30	
CORREA	35	c	1	3.34	21.64	27.75	0.0000
*CPSI	403	m	1	3.38	0.97	1.33	0.0009
*CPSI	405	f	1	1.45	2.50	1.46	0.0222
Subtotal CPSI				1.99	3.47	2.78	
*CPSII	114	m	1	3.67	2.17	4.62	0.0000
*CPSII	117	f	1	4.37	1.49	6.93	0.0000
Subtotal CPSII				3.95	3.66	11.56	
DAMBER	33	m	1	2.47	9.39	0.62	0.0000
DESTE2	16	m	2	2.58	3.60	0.49	0.0000
DOLL	86	m	1	2.58	2.84	0.38	0.0000
DOLL	88	f	1	0.76	7.91	16.74	0.0334
Subtotal DOLL				1.24	10.76	17.12	
DORGAN	113	m	2	2.94	3.87	2.06	0.0000
DORGAN	98	f	3	2.41	20.54	0.79	0.0000
Subtotal DORGAN				2.49	24.41	2.85	
*DORN	338	m	1	2.84	9.21	3.63	0.0000
DOSEME	3	m	2	1.28	35.93	31.04	0.0000
*ENGELA	62	m	7	1.86	2.73	0.33	0.0021
FAN	3	c	0	2.46	5.45	0.33	0.0000
GAO	2	m	2	2.13	11.41	0.08	0.0000
GAO	12	f	2	1.97	19.80	1.11	0.0000
Subtotal GAO				2.03	31.21	1.18	
GER	13	c	10	1.16	3.28	3.61	0.0358
HAENSZ	22	f	0	1.07	18.34	23.84	0.0000
*HAMMON	59	m	1	3.08	3.94	2.95	0.0000
HEGMAN	2	c	0	3.43	4.70	6.97	0.0000
HINDS	23	f	3	2.78	6.93	2.25	0.0000
ISHIMA	6	c	5	3.04	0.50	0.35	0.0315
JAHN	42	m	0	3.16	2.90	2.60	0.0000
JAIN	48	m	2	2.89	1.70	0.79	0.0002
JAIN	43	f	2	3.24	1.73	1.83	0.0000
Subtotal JAIN				3.07	3.43	2.62	
JEDRYC	54	m	3	2.55	5.53	0.65	0.0000
JOLY	54	m	0	3.44	1.96	2.96	0.0000
JOLY	52	f	0	2.92	5.02	2.53	0.0000
Subtotal JOLY				3.07	6.98	5.50	
JUSSAW	25	m	0	2.36	6.65	0.15	0.0000
KATSOU	37	f	1	1.81	5.71	0.92	0.0000
KHUDER	24	m	0	2.06	7.79	0.18	0.0000
KIHARA	26	c	0	3.29	4.63	5.44	0.0000
KOO	6	f	0	1.42	14.12	8.78	0.0000
KREYBE	4	m	1	2.39	2.95	0.09	0.0000
KREYBE	25	f	1	0.83	4.31	8.23	0.0854

Table 2C3 - 2

IESLC - Meta-anal of Ever Smoking (or Current if Ever not available), Cigs (or Any Prod if Cigs not avail)
Squamous
Most adjusted

REF	NRR	SEX	AD	Ys	Ws	Qs	Ps
Subtotal KREYBE				1.46	7.26	8.32	
LAMTH	1	f	0	2.09	8.66	0.12	0.0000
LAMWK	2	f	0	2.35	4.51	0.09	0.0000
LAMWK2	1	m	0	1.93	4.22	0.33	0.0001
LAMWK2	5	f	0	1.87	8.17	0.95	0.0000
Subtotal LAMWK2				1.89	12.38	1.28	
LOMBA2	2	f	0	1.45	11.86	6.94	0.0000
LUBIN	34	m	0	1.89	3.72	0.37	0.0003
LUBIN2	145	m	0	2.81	51.88	18.83	0.0000
LUBIN2	165	f	0	1.75	46.51	9.66	0.0000
Subtotal LUBIN2				2.31	98.39	28.49	
LUO	8	c	20	2.39	1.76	0.06	0.0015
MATOS	67	m	2	2.09	2.97	0.04	0.0003
MATSUD	11	m	0	3.66	0.99	2.09	0.0003
NOU	1	m	0	3.30	1.92	2.29	0.0000
NOU	6	f	0	1.96	1.40	0.09	0.0205
Subtotal NOU				2.74	3.32	2.37	
ORMOS	8	m	0	2.32	1.85	0.02	0.0016
OSANN	43	m	2	3.59	7.67	14.52	0.0000
OSANN	44	f	2	3.27	10.69	12.07	0.0000
Subtotal OSANN				3.40	18.36	26.60	
OSANN2	25	f	1	3.56	0.97	1.77	0.0005
PEZZOT	6	m	0	4.14	0.49	1.84	0.0036
SCHWAR	10	m	0	3.49	0.97	1.59	0.0006
SCHWAR	9	m	0	0.61	2.26	5.79	0.3596
SCHWAR	18	f	0	3.77	0.49	1.18	0.0086
SCHWAR	17	f	0	4.14	0.47	1.76	0.0044
Subtotal SCHWAR				2.04	4.19	10.32	
SEOW	3	f	0	2.86	4.50	1.91	0.0000
SIEMIA	7	m	7	3.12	2.69	2.24	0.0000
SOBUE	97	m	1	2.88	5.62	2.55	0.0000
SOBUE	107	f	1	2.17	13.12	0.02	0.0000
Subtotal SOBUE				2.38	18.74	2.57	
SOBUE2	1	m	2	1.65	80.57	25.42	0.0000
SOBUE2	5	f	2	1.97	23.37	1.30	0.0000
Subtotal SOBUE2				1.72	103.93	26.73	
STASZE	16	m	0	4.19	0.50	1.94	0.0032
STASZE	38	f	0	3.48	0.37	0.60	0.0333
Subtotal STASZE				3.88	0.87	2.54	
STAYNE	3	m	0	1.24	17.27	16.12	0.0000
SUZUK2	15	c	3	3.43	0.97	1.45	0.0007
SVENSS	72	f	1	2.54	2.87	0.30	0.0000
TIZZAN	18	c	0	0.99	40.59	60.07	0.0000
TOKARS	10	c	3	1.92	1.27	0.11	0.0305
TSUGAN	13	m	0	2.68	0.44	0.10	0.0772
WAKAI	74	m	1	2.15	1.90	0.01	0.0030
WAKAI	80	f	1	3.23	2.27	2.35	0.0000
Subtotal WAKAI				2.74	4.17	2.36	
WU	32	f	2	3.19	0.99	0.95	0.0015
WUWILL	9	f	3	1.44	33.59	20.19	0.0000
WYNDE2	2	m	0	3.13	2.88	2.45	0.0000
WYNDE3	8	m	0	3.09	2.83	2.17	0.0000
WYNDE3	67	f	0	1.91	3.69	0.32	0.0002
Subtotal WYNDE3				2.42	6.52	2.49	
WYNDE4	69	m	2	2.74	7.27	2.02	0.0000
WYNDE4	54	f	2	1.76	5.63	1.13	0.0000
Subtotal WYNDE4				2.31	12.90	3.16	
WYNDE6	75	m	0	3.01	26.85	16.98	0.0000
WYNDE6	412	f	1	3.48	10.46	16.78	0.0000
Subtotal WYNDE6				3.14	37.31	33.76	
XU3	20	m	1	1.77	2.46	0.47	0.0054
XU3	24	f	1	3.25	1.43	1.53	0.0001
Subtotal XU3				2.32	3.89	2.00	
ZHENG	5	m	0	2.82	3.68	1.38	0.0000
ZHENG	18	f	0	1.70	12.24	3.24	0.0000
Subtotal ZHENG				1.96	15.92	4.62	
ZHOU	8	m	0	1.14	15.27	17.38	0.0000
ZHOU	9	f	0	1.34	4.42	3.36	0.0049
Subtotal ZHOU				1.19	19.68	20.75	

Table 2C3 - 2

IESLC - Meta-anal of Ever Smoking (or Current if Ever not available), Cigs (or Any Prod if Cigs not avail)
Squamous
Most adjusted

	N	110
	NS	78
	Wt	1125.37
Het	Chi	564.10
Het	df	109
Het	P	***
Fixed	RR	9.12
	RRl	8.60
	RRu	9.67
	P	+++
Random	RR	10.55
	RRl	9.03
	RRu	12.33
	P	+++
Asymm	P	(*)

Table 2C3 - 3

IESLC - Meta-anal of Ever Smoking (or Current if Ever not available), Cigs (or Any Prod if Cigs not avail)
Squamous
Most adjusted

	Sex			
	combined	male	female	Total
N	11	54	45	110
NS	11	53	44	108
Wt	95.98	577.47	451.91	1125.37
Het Chi	107.16	218.12	229.66	564.10
Het df	10	53	44	109
Het P	***	***	***	***
Fixed RR	7.15	9.78	8.78	9.12
RRl	5.85	9.01	8.01	8.60
RRu	8.73	10.61	9.63	9.67
P	+++	+++	+++	+++
Random RR	10.84	12.01	9.05	10.55
RRl	4.98	9.77	7.11	9.03
RRu	23.59	14.77	11.53	12.33
P	+++	+++	+++	+++
Between Chi				9.16
Between df				2
Between P				*
Btwn(F) P				N.S.
Btwn(R) P				N.S.

	Lung cancer type					
	q	q+s	q+u	KI	not a	Total
N	80	9	3	14	4	110
NS	56	7	3	10	3	79
Wt	875.71	75.63	70.79	75.21	28.04	1125.37
Het Chi	358.24	43.64	1.88	51.55	12.25	564.10
Het df	79	8	2	13	3	109
Het P	***	***	N.S.	***	**	***
Fixed RR	9.83	10.30	2.97	10.24	7.92	9.12
RRl	9.20	8.22	2.35	8.17	5.47	8.60
RRu	10.50	12.91	3.75	12.84	11.47	9.67
P	+++	+++	+++	+++	+++	+++
Random RR	11.73	9.19	2.97	9.26	9.10	10.55
RRl	9.86	5.18	2.35	5.62	4.20	9.03
RRu	13.94	16.31	3.75	15.26	19.69	12.33
P	+++	+++	+++	+++	+++	+++
Between Chi						96.54
Between df						4
Between P						***
Btwn(F) P						***
Btwn(R) P						***

	Location								
	NAmer	UK	Scand	othEur	China	Japan	othAs	other	Total
N	44	4	7	15	12	10	12	6	110
NS	30	2	5	12	8	7	9	5	78
Wt	527.88	27.98	25.57	205.88	115.23	133.40	74.43	15.00	1125.37
Het Chi	218.23	11.30	11.88	109.52	23.30	28.64	13.23	3.82	564.10
Het df	43	3	6	14	11	9	11	5	109
Het P	***	*	(*)	***	*	***	N.S.	N.S.	***
Fixed RR	13.01	5.50	8.67	6.56	5.66	6.84	6.95	16.70	9.12
RRl	11.94	3.80	5.88	5.73	4.72	5.78	5.54	10.07	8.60
RRu	14.16	7.97	12.78	7.52	6.79	8.11	8.73	27.70	9.67
P	+++	+++	+++	+++	+++	+++	+++	+++	+++
Random RR	14.33	6.14	8.62	8.91	6.41	11.19	7.10	16.70	10.55
RRl	11.29	2.82	4.81	5.52	4.74	7.15	5.50	10.07	9.03
RRu	18.19	13.39	15.43	14.39	8.66	17.51	9.15	27.70	12.33
P	+++	+++	+++	+++	+++	+++	+++	+++	+++
Between Chi									144.16
Between df									7
Between P									***
Btwn(F) P									***
Btwn(R) P									***

Table 2C3 - 3

IESLC - Meta-anal of Ever Smoking (or Current if Ever not available), Cigs (or Any Prod if Cigs not avail)
Squamous
Most adjusted

	Detailed Country in "other Europe" multi	Germany	othWest	East	Balkans	Total
N	2	2	2	7	2	15
NS	1	2	2	5	2	12
Wt	98.39	4.65	46.21	14.99	41.65	205.88
Het Chi	27.46	0.68	13.95	7.21	1.38	109.52
Het df	1	1	1	6	1	14
Het P	***	N.S.	***	N.S.	N.S.	***
Fixed RR	10.10	17.47	3.31	10.20	3.87	6.56
RRl	8.29	7.04	2.48	6.15	2.86	5.73
RRu	12.31	43.37	4.42	16.93	5.24	7.52
P	+++	+++	+++	+++	+++	+++
Random RR	9.82	17.47	5.98	10.79	4.08	8.91
RRl	3.48	7.04	1.15	5.91	2.63	5.52
RRu	27.71	43.37	31.02	19.72	6.34	14.39
P	+++	+++	+	+++	+++	+++
Between Chi						58.84
Between df						4
Between P						***
Btwn(F) P						(*)
Btwn(R) P						*

	Detailed Country in "other Asia" India	HongKong	other	Total
N	1	7	4	12
NS	1	5	3	9
Wt	6.65	50.37	17.41	74.43
Het Chi	0.00	5.72	6.14	13.23
Het df	0	6	3	11
Het P	N.S.	N.S.	N.S.	N.S.
Fixed RR	10.60	6.54	7.06	6.95
RRl	4.96	4.96	4.42	5.54
RRu	22.66	8.62	11.30	8.73
P	+++	+++	+++	+++
Random RR	10.60	6.54	6.94	7.10
RRl	4.96	4.96	3.53	5.50
RRu	22.66	8.62	13.65	9.15
P	+++	+++	+++	+++
Between Chi				1.37
Between df				2
Between P				N.S.
Btwn(F) P				N.S.
Btwn(R) P				N.S.

	Detailed other continent SCAmer	Auslia	Africa	Total
N	6			6
NS	5			5
Wt	15.00			15.00
Het Chi	3.82			3.82
Het df	5			5
Het P	N.S.			N.S.
Fixed RR	16.70			16.70
RRl	10.07			10.07
RRu	27.70			27.70
P	+++			+++
Random RR	16.70			16.70
RRl	10.07			10.07
RRu	27.70			27.70
P	+++			+++
Between Chi				
Between df				
Between P				N.S.
Btwn(F) P				N.S.
Btwn(R) P				N.S.

Table 2C3 - 3

IESLC - Meta-anal of Ever Smoking (or Current if Ever not available), Cigs (or Any Prod if Cigs not avail)
Squamous
Most adjusted

	Start year of study <1960	1960-69	1970-79	1980-89	1990+	Total
N	18	16	26	42	8	110
NS	13	13	16	28	8	78
Wt	139.90	199.80	258.16	500.36	27.15	1125.37
Het Chi	80.59	90.69	129.54	165.23	10.78	564.10
Het df	17	15	25	41	7	109
Het P	***	***	***	***	N.S.	***
Fixed RR	4.98	7.80	8.74	11.56	12.39	9.12
RRl	4.22	6.79	7.73	10.59	8.51	8.60
RRu	5.87	8.97	9.87	12.61	18.05	9.67
P	+++	+++	+++	+++	+++	+++
Random RR	7.19	10.69	10.01	12.33	12.21	10.55
RRl	4.71	7.03	7.22	9.88	7.56	9.03
RRu	10.98	16.26	13.87	15.39	19.72	12.33
P	+++	+++	+++	+++	+++	+++
Between Chi						87.26
Between df						4
Between P						***
Btwn(F) P						**
Btwn(R) P						N.S.

	Study type (1) CC	other	Total
N	95	15	110
NS	67	11	78
Wt	1087.18	38.19	1125.37
Het Chi	529.73	24.40	564.10
Het df	94	14	109
Het P	***	*	***
Fixed RR	8.96	15.07	9.12
RRl	8.44	10.97	8.60
RRu	9.51	20.69	9.67
P	+++	+++	+++
Random RR	10.07	15.92	10.55
RRl	8.55	10.04	9.03
RRu	11.86	25.24	12.33
P	+++	+++	+++
Between Chi			9.97
Between df			1
Between P			**
Btwn(F) P			N.S.
Btwn(R) P			(*)

	Study type (2) CC	prosp	other	Total
N	95	11	4	110
NS	67	8	3	78
Wt	1087.18	33.60	4.58	1125.37
Het Chi	529.73	21.55	2.69	564.10
Het df	94	10	3	109
Het P	***	*	N.S.	***
Fixed RR	8.96	15.44	12.62	9.12
RRl	8.44	11.01	5.05	8.60
RRu	9.51	21.65	31.54	9.67
P	+++	+++	+++	+++
Random RR	10.07	16.77	12.62	10.55
RRl	8.55	9.71	5.05	9.03
RRu	11.86	28.95	31.54	12.33
P	+++	+++	+++	+++
Between Chi				10.14
Between df				2
Between P				**
Btwn(F) P				N.S.
Btwn(R) P				N.S.

Table 2C3 - 3

IESLC - Meta-anal of Ever Smoking (or Current if Ever not available), Cigs (or Any Prod if Cigs not avail)

Squamous

Most adjusted

	Study size (number of LC cases)				
	100-249	250-499	500-999	1000+	Total
N	23	31	18	38	110
NS	22	22	12	22	78
Wt	84.53	134.11	119.82	786.92	1125.37
Het Chi	43.45	66.35	48.99	384.18	564.10
Het df	22	30	17	37	109
Het P	**	***	***	***	***
Fixed RR	6.04	9.11	7.77	9.77	9.12
RRl	4.88	7.70	6.50	9.11	8.60
RRu	7.47	10.80	9.30	10.47	9.67
P	+++	+++	+++	+++	+++
Random RR	8.54	10.48	10.60	11.05	10.55
RRl	6.01	8.00	7.55	8.55	9.03
RRu	12.14	13.71	14.87	14.29	12.33
P	+++	+++	+++	+++	+++
Between Chi					21.13
Between df					3
Between P					***
Btwn(F) P					N.S.
Btwn(R) P					N.S.

	Risky occupational population			
	no	mining	othRisky	Total
N	108	1	1	110
NS	76	1	1	78
Wt	1120.37	3.72	1.27	1125.37
Het Chi	563.62	0.00	0.00	564.10
Het df	107	0	0	109
Het P	***	N.S.	N.S.	***
Fixed RR	9.13	6.65	6.80	9.12
RRl	8.61	2.41	1.20	8.60
RRu	9.68	18.36	38.62	9.67
P	+++	+++	+	+++
Random RR	10.63	6.65	6.80	10.55
RRl	9.08	2.41	1.20	9.03
RRu	12.43	18.36	38.62	12.33
P	+++	+++	+	+++
Between Chi				0.48
Between df				2
Between P				N.S.
Btwn(F) P				N.S.
Btwn(R) P				N.S.

	National cigarette tobacco type			
	Virginia	blended	other	Total
N	9	88	13	110
NS	6	63	9	78
Wt	47.52	959.34	118.51	1125.37
Het Chi	36.87	470.70	24.35	564.10
Het df	8	87	12	109
Het P	***	***	*	***
Fixed RR	9.47	9.67	5.57	9.12
RRl	7.13	9.08	4.65	8.60
RRu	12.59	10.31	6.67	9.67
P	+++	+++	+++	+++
Random RR	11.73	11.34	6.17	10.55
RRl	6.13	9.51	4.61	9.03
RRu	22.47	13.52	8.26	12.33
P	+++	+++	+++	+++
Between Chi				32.18
Between df				2
Between P				***
Btwn(F) P				*
Btwn(R) P				**

Table 2C3 - 3

IESLC - Meta-anal of Ever Smoking (or Current if Ever not available), Cigs (or Any Prod if Cigs not avail)
Squamous
Most adjusted

	Any proxy use No/nk	Yes	Total
N	84	26	110
NS	60	18	78
Wt	1000.00	125.37	1125.37
Het Chi	480.48	50.18	564.10
Het df	83	25	109
Het P	***	**	***
Fixed RR	8.58	14.84	9.12
RRl	8.06	12.46	8.60
RRu	9.13	17.68	9.67
P	+++	+++	+++
Random RR	9.77	14.00	10.55
RRl	8.20	10.60	9.03
RRu	11.64	18.48	12.33
P	+++	+++	+++
Between Chi			33.44
Between df			1
Between P			***
Btwn(F) P			*
Btwn(R) P			*

	Full histological confirmation No	Yes	Total
N	67	43	110
NS	48	30	78
Wt	578.12	547.25	1125.37
Het Chi	296.89	183.41	564.10
Het df	66	42	109
Het P	***	***	***
Fixed RR	6.99	12.07	9.12
RRl	6.45	11.10	8.60
RRu	7.59	13.13	9.67
P	+++	+++	+++
Random RR	9.49	12.51	10.55
RRl	7.82	9.93	9.03
RRu	11.51	15.77	12.33
P	+++	+++	+++
Between Chi			83.80
Between df			1
Between P			***
Btwn(F) P			***
Btwn(R) P			(*)

	Number of adjustment variables (1) 0	1	2+/+nk	Total
N	54	25	31	110
NS	38	17	24	79
Wt	426.38	135.12	563.87	1125.37
Het Chi	240.85	90.08	201.74	564.10
Het df	53	24	30	109
Het P	***	***	***	***
Fixed RR	7.52	12.40	9.80	9.12
RRl	6.84	10.47	9.02	8.60
RRu	8.27	14.67	10.64	9.67
P	+++	+++	+++	+++
Random RR	9.48	12.25	11.31	10.55
RRl	7.51	8.59	8.67	9.03
RRu	11.97	17.47	14.76	12.33
P	+++	+++	+++	+++
Between Chi				31.43
Between df				2
Between P				***
Btwn(F) P				*
Btwn(R) P				N.S.

Table 2C3 - 3

IESLC - Meta-anal of Ever Smoking (or Current if Ever not available), Cigs (or Any Prod if Cigs not avail)

Squamous

Most adjusted

	Number of adjustment variables (2)					
	0	1	2	3-5	6+/+nk	Total
N	54	25	19	8	4	110
NS	38	17	13	8	4	80
Wt	426.38	135.12	478.47	74.94	10.46	1125.37
Het Chi	240.85	90.08	165.21	24.97	5.99	564.10
Het df	53	24	18	7	3	109
Het P	***	***	***	***	N.S.	***
Fixed RR	7.52	12.40	10.22	7.74	7.81	9.12
RRl	6.84	10.47	9.34	6.17	4.26	8.60
RRu	8.27	14.67	11.18	9.70	14.32	9.67
P	+++	+++	+++	+++	+++	+++
Random RR	9.48	12.25	12.15	10.71	8.13	10.55
RRl	7.51	8.59	8.69	6.25	3.43	9.03
RRu	11.97	17.47	16.98	18.36	19.30	12.33
P	+++	+++	+++	+++	+++	+++
Between Chi						37.00
Between df						4
Between P						***
Btwn(F) P						N.S.
Btwn(R) P						N.S.

	Product			
	all/unsp	cig+/-ot	cig only	Total
N	38	63	9	110
NS	30	44	8	82
Wt	210.72	872.05	42.60	1125.37
Het Chi	124.61	358.03	22.30	564.10
Het df	37	62	8	109
Het P	***	***	**	***
Fixed RR	5.73	10.01	13.31	9.12
RRl	5.01	9.37	9.86	8.60
RRu	6.56	10.70	17.97	9.67
P	+++	+++	+++	+++
Random RR	8.14	11.49	15.64	10.55
RRl	6.15	9.51	8.73	9.03
RRu	10.75	13.88	28.03	12.33
P	+++	+++	+++	+++
Between Chi				59.16
Between df				2
Between P				***
Btwn(F) P				**
Btwn(R) P				(*)

	Denominator		
	nev any	nev cigs	Total
N	71	39	110
NS	53	27	80
Wt	595.56	529.81	1125.37
Het Chi	306.08	219.43	564.10
Het df	70	38	109
Het P	***	***	***
Fixed RR	7.66	11.10	9.12
RRl	7.07	10.19	8.60
RRu	8.30	12.08	9.67
P	+++	+++	+++
Random RR	9.66	12.30	10.55
RRl	7.97	9.53	9.03
RRu	11.71	15.87	12.33
P	+++	+++	+++
Between Chi			38.59
Between df			1
Between P			***
Btwn(F) P			**
Btwn(R) P			N.S.

Table 2C3 - 3

IESLC - Meta-anal of Ever Smoking (or Current if Ever not available), Cigs (or Any Prod if Cigs not avail)

Squamous
Most adjusted

	Derivation of RR/CI			
	Orig	StdCalc	Other	Total
N	24	43	43	110
NS	18	33	31	82
Wt	557.59	399.96	167.83	1125.37
Het Chi	203.55	228.51	105.11	564.10
Het df	23	42	42	109
Het P	***	***	***	***
Fixed RR	10.00	7.43	10.92	9.12
RRl	9.21	6.74	9.39	8.60
RRu	10.87	8.20	12.70	9.67
P	+++	+++	+++	+++
Random RR	11.66	9.05	11.91	10.55
RRl	8.65	7.02	9.15	9.03
RRu	15.72	11.67	15.51	12.33
P	+++	+++	+++	+++
Between Chi				26.93
Between df				2
Between P				***
Btwn(F) P				(*)
Btwn(R) P				N.S.

	Smoking status		
	ever	current	Total
N	102	8	110
NS	73	5	78
Wt	1004.60	120.77	1125.37
Het Chi	517.30	32.81	564.10
Het df	101	7	109
Het P	***	***	***
Fixed RR	9.48	6.61	9.12
RRl	8.91	5.53	8.60
RRu	10.08	7.90	9.67
P	+++	+++	+++
Random RR	10.44	12.44	10.55
RRl	8.88	6.84	9.03
RRu	12.29	22.61	12.33
P	+++	+++	+++
Between Chi			13.99
Between df			1
Between P			***
Btwn(F) P			N.S.
Btwn(R) P			N.S.

Table 2C3 - 4

IESLC - Meta-anal of Ever Smoking (or Current if Ever not available), Cigs (or Any Prod if Cigs not avail)
Squamous
Least adjusted

REF	NRR	X	SEX	AGEL	AGEH	RACE	YF	LC TYPE	LOC	START	ST	NLC	R	VB	P	H	AD	SM	PRODUCT	DENOM	De
ABRAHA	1		m	0	0	all	0	q	Eu:est	1975	pr	571	n	bl	n	n	0	ev	all/unsp	nev any	ot
ABRAHA	4		f	0	0	all	0	q	Eu:est	1975	pr	571	n	bl	n	n	0	ev	all/unsp	nev any	ot
ALDERS	88	x	m	0	0	all	-	q+s	Eu:UK	1977	CC	1448	n	V	n	n	0	ev	cig+/-ot	nev any	st
ALDERS	84	x	f	0	0	all	-	q+s	Eu:UK	1977	CC	1448	n	V	n	n	0	ev	cig only	nev any	st
ANDERS	10		f	0	0	all	0	q	NAmer	1986	pr	343	n	bl	n	n	0	ev	cig+/-ot	nev cigs	st
BAND	5		m	0	0	all	-	q	NAmer	1983	CC	2831	n	V	y	y	2	ev	cig only	nev any	ot
BARBON	110	x	m	0	0	all	-	q	Eu:wst	1979	CC	755	n	bl	y	y	0	ev	all/unsp	nev any	st
BECHER	11		f	0	0	all	-	q+s	Eu:Ger	1985	CC	194	n	bl	n	y	1	ev	all/unsp	nev any	or
BOUCOT	70	x	m	0	0	all	0	q	NAmer	1951	pr	121	n	bl	n	n	0	cu	cig only	nev any	ot
BRESLO	7		c	0	0	all	-	not a	NAmer	1949	CC	518	n	bl	n	y	0	ev	cig+/-ot	nev+1	st
BROWN2	6		m	0	0	wh	-	q	NAmer	1984	CC	14596	n	bl	n	y	2	ev	cig+/-ot	nev cigs	or
BROWN2	5		f	0	0	wh	-	q	NAmer	1984	CC	14596	n	bl	n	y	2	ev	cig+/-ot	nev cigs	or
BUFFLE	49		m	0	0	wh	-	q	NAmer	1976	CC	943	n	bl	y	n	0	ev	cig+/-ot	nev cigs	ot
BUFFLE	62		f	0	0	w-hi	-	q	NAmer	1976	CC	943	n	bl	y	n	0	ev	cig+/-ot	nev cigs	st
BYERS1	1		m	0	0	wh	-	q	NAmer	1957	CC	1002	n	bl	n	n	0	ev	cig+/-ot	nev cigs	st
CHAN	18		m	0	0	all	-	q+s	As:HK	1976	CC	397	n	bl	n	n	0	ev	cig+/-ot	nev any	st
CHAN	22		f	0	0	all	-	q+s	As:HK	1976	CC	397	n	bl	n	n	0	ev	cig+/-ot	nev any	st
CHOI	62		m	0	0	all	-	q	As:oth	1985	CC	375	n	bl	n	n	0	ev	cig+/-ot	nev cigs	st
CHOI	64		f	0	0	all	-	q	As:oth	1985	CC	375	n	bl	n	n	0	ev	cig+/-ot	nev cigs	st
COMSTO	66		m	0	0	all	-	q	NAmer	1975	ot	258	n	bl	n	n	0	ev	cig+/-ot	nev cigs	st
COMSTO	78		f	0	0	all	-	q	NAmer	1975	ot	258	n	bl	n	n	0	ev	cig+/-ot	nev cigs	ot
CORREA	35		c	0	0	all	-	q+s	NAmer	1979	CC	1359	n	bl	y	n	1	ev	cig+/-ot	nev cigs	or
CPSI	403		m	0	0	all	2	q	NAmer	1959	pr	5138	n	bl	n	n	1	cu	cig only	nev any	ot
CPSI	405		f	0	0	all	2	q	NAmer	1959	pr	5138	n	bl	n	n	1	cu	cig only	nev any	ot
CPSII	114		m	0	0	all	2	q	NAmer	1982	pr	3229	n	bl	n	n	1	cu	cig only	nev any	ot
CPSII	117		f	0	0	all	2	q	NAmer	1982	pr	3229	n	bl	n	n	1	cu	cig+/-ot	nev cigs	ot
DAMBER	12	x	m	0	0	all	-	q	Eu:Sca	1972	CC	579	n	bl	y	n	0	ev	all/unsp	nev any	st
DESTE2	16		m	0	0	all	-	q	SCAmer	1993	CC	463	n	bl	n	n	2	ev	all/unsp	nev any	or
DOLL	82	x	m	0	0	all	-	KI	Eu:UK	1948	CC	1465	n	V	n	n	0	ev	all/unsp	nev any	st
DOLL	84	x	f	0	0	all	-	KI	Eu:UK	1948	CC	1465	n	V	n	n	0	ev	all/unsp	nev any	st
DORGAN	113		m	0	0	wh	-	q	NAmer	1980	CC	2026	n	bl	y	y	2	ev	cig+/-ot	nev any	or
DORGAN	98		f	0	0	all	-	q	NAmer	1980	CC	2026	n	bl	y	y	3	ev	cig+/-ot	nev any	or
DORN	338		m	0	0	wh	8	q	NAmer	1954	pr	5097	n	bl	n	n	1	cu	cig only	nev any	ot
DOSEME	19	x	m	0	0	all	-	q	Eu:bal	1979	CC	1210	n	bl	n	n	0	ev	cig+/-ot	nev cigs	st
ENGELA	62		m	0	0	all	0	q	Eu:Sca	1964	pr	435	n	bl	n	n	7	ev	cig+/-ot	nev cigs	ot
FAN	3		c	0	0	all	-	q	As:Chi	1990	CC	403	n	ot	y	n	0	ev	cig+/-ot	nev cigs	ot
GAO	7	x	m	0	0	all	-	q	As:Chi	1984	CC	1405	n	ot	n	n	0	ev	cig+/-ot	nev cigs	st
GAO	17	x	f	0	0	all	-	q	As:Chi	1984	CC	1405	n	ot	n	n	0	ev	cig+/-ot	nev cigs	st
GER	5	x	c	0	0	all	-	q+s	As:oth	1990	CC	141	n	ot	y	n	0	ev	all/unsp	nev any	st
HAENSZ	22		f	0	0	all	-	q+u	NAmer	1955	CC	158	n	bl	n	y	0	ev	cig+/-ot	nev any	st
HAMMON	72	x	m	0	0	wh	0	not a	NAmer	1952	pr	448	n	bl	n	n	0	ev	cig+/-ot	nev any	st
HEGMAN	2		c	0	0	all	-	q	NAmer	1989	CC	282	n	bl	y	y	0	ev	all/unsp	nev any	st
HINDS	23		f	0	0	o	-	q+s	NAmer	1968	CC	292	n	bl	n	n	3	ev	all/unsp	nev any	st
ISHIMA	1	x	c	0	0	all	-	q	As:Jap	1961	CC	180	n	bl	y	y	0	ev	all/unsp	nev any	st
JAHN	42		m	0	0	all	-	q	Eu:Ger	1988	CC	1004	n	bl	n	n	0	ev	cig+/-ot	nev any	st
JAIN	8	x	m	0	0	all	-	q	NAmer	1981	CC	845	n	V	y	n	0	ev	cig+/-ot	nev cigs	st
JAIN	3	x	f	0	0	all	-	q	NAmer	1981	CC	845	n	V	y	n	0	ev	cig+/-ot	nev cigs	st
JEDRYC	7	x	m	0	0	all	-	q	Eu:est	1980	CC	1630	n	bl	y	n	0	ev	cig+/-ot	nev any	st
JOLY	54		m	0	0	all	-	q	SCAmer	1978	CC	826	n	bl	n	n	0	ev	cig+/-ot	nev any	st
JOLY	52		f	0	0	all	-	q	SCAmer	1978	CC	826	n	bl	n	n	0	ev	cig+/-ot	nev any	st
JUSSAW	25		m	0	0	all	-	KI	As:Ind	1964	CC	792	n	V	n	n	0	ev	cig only	nev any	st
KATSOU	35	x	f	0	0	all	-	KI	Eu:bal	1987	CC	101	n	bl	n	n	0	ev	all/unsp	nev any	st
KHUDER	24		m	0	0	all	-	q	NAmer	1985	CC	482	n	bl	n	y	0	ev	cig+/-ot	nev cigs	ot
KIHARA	26		c	0	0	jap	-	q	As:Jap	1991	CC	440	n	bl	n	n	0	ev	all/unsp	nev any	st
KOO	6		f	0	0	all	-	q+s	As:HK	1981	CC	200	n	bl	n	n	0	ev	all/unsp	nev any	st
KREYBE	16	x	m	0	0	all	-	KI	Eu:Sca	1948	CC	300	n	bl	n	y	0	ev	all/unsp	nev any	st
KREYBE	33	x	f	0	0	all	-	KI	Eu:Sca	1948	CC	300	n	bl	n	y	0	ev	all/unsp	nev any	st
LAMTH	1		f	0	0	ch	-	q	As:HK	1983	CC	445	n	bl	n	n	0	ev	all/unsp	nev any	or
LAMWK	2		f	0	0	ch	-	q	As:HK	1981	CC	163	n	bl	n	n	0	ev	all/unsp	nev any	st
LAMWK2	1		m	0	0	all	-	q	As:HK	1976	CC	480	n	bl	n	n	0	ev	all/unsp	nev any	st
LAMWK2	5		f	0	0	all	-	q	As:HK	1976	CC	480	n	bl	n	n	0	ev	all/unsp	nev any	st
LOMBA2	2		f	0	0	all	-	q+u	NAmer	1960	CC	225	n	bl	n	n	0	ev	cig+/-ot	nev cigs	st
LUBIN	34		m	0	0	all	-	KI	As:Chi	1984	CC	427	m	ot	y	n	0	ev	cig+/-ot	nev any	st
LUBIN2	145		m	0	0	all	-	q	Eu:mul	1976	CC	7804	n	bl	n	y	0	ev	cig+/-ot	nev any	st
LUBIN2	165		f	0	0	all	-	q	Eu:mul	1976	CC	7804	n	bl	n	y	0	ev	cig+/-ot	nev any	st
LUO	2	x	c	0	0	all	-	q	As:Chi	1990	CC	102	n	ot	n	y	0	ev	cig+/-ot	nev cigs	st
MATOS	66	x	m	0	0	all	-	q	SCAmer	1994	CC	200	n	bl	n	n	0	ev	cig+/-ot	nev any	st
MATSUD	11		m	0	0	all	-	q	As:Jap	1965	CC	179	n	bl	n	n	0	ev	cig+/-ot	nev cigs	st
NOU	1		m	0	0	all	-	q	Eu:Sca	1971	CC	273	n	bl	y	n	0	ev	all/unsp	nev any	st
NOU	6		f	0	0	all	-	q	Eu:Sca	1971	CC	273	n	bl	y	n	0	ev	all/unsp	nev any	st
ORMOS	8		m	0	0	all	-	q	Eu:est	1947	CC	119	n	bl	y	y	0	ev	cig+/-ot	nev any	st
OSANN	18	x	m	0	0	all	-	q	NAmer	1984	CC	1986	n	bl	n	n	0	ev	cig+/-ot	nev cigs	st

Table 2C3 - 4

IESLC - Meta-anal of Ever Smoking (or Current if Ever not available), Cigs (or Any Prod if Cigs not avail)
Squamous
Least adjusted

REF	NRR	X	SEX	AGEL	AGEH	RACE	YF	LC TYPE	LOC	START	ST	NLC	R	VB	P	H	AD	SM	PRODUCT	DENOM	De
OSANN	22	x	f	0	0	all	-	q	NAmer	1984	CC	1986	n	bl	n	n	0	ev	cig+/-ot	nev cigs	st
OSANN2	7	x	f	0	0	all	-	KI	NAmer	1964	ot	217	n	bl	n	y	0	ev	cig+/-ot	nev cigs	st
PEZZOT	6		m	0	0	all	-	q	SCAmer	1987	CC	215	n	bl	n	y	0	ev	cig only	nev cigs	ot
SCHWAR	10		m	40	54	wh	-	q	NAmer	1984	CC	5588	n	bl	y	y	0	ev	cig+/-ot	nev cigs	st
SCHWAR	9		m	40	54	bl	-	q	NAmer	1984	CC	5588	n	bl	y	y	0	ev	cig+/-ot	nev cigs	st
SCHWAR	18		f	40	54	wh	-	q	NAmer	1984	CC	5588	n	bl	y	y	0	ev	cig+/-ot	nev cigs	ot
SCHWAR	17		f	40	54	bl	-	q	NAmer	1984	CC	5588	n	bl	y	y	0	ev	cig+/-ot	nev cigs	ot
SEOW	3		f	0	0	ch	-	q	As:oth	1997	CC	153	n	bl	n	y	0	ev	cig+/-ot	nev cigs	st
SIEMIA	11	x	m	0	0	all	-	q	NAmer	1979	CC	857	n	V	y	y	0	ev	cig+/-ot	nev cigs	st
SOBUE	3	x	m	0	0	all	-	q	As:Jap	1986	CC	1376	n	bl	n	y	0	ev	cig+/-ot	nev cigs	st
SOBUE	19	x	f	0	0	all	-	q	As:Jap	1986	CC	1376	n	bl	n	y	0	ev	cig+/-ot	nev cigs	st
SOBUE2	1		m	0	0	all	-	q	As:Jap	1965	CC	2083	n	bl	n	n	2	cu	cig+/-ot	nev any	or
SOBUE2	5		f	0	0	all	-	q	As:Jap	1965	CC	2083	n	bl	n	n	2	cu	cig+/-ot	nev any	or
STASZE	16		m	0	0	all	-	q	Eu:est	1954	CC	281	n	bl	n	y	0	ev	cig+/-ot	nev any	ot
STASZE	38		f	0	0	all	-	q	Eu:est	1954	CC	281	n	bl	n	y	0	ev	all/unsp	nev any	ot
STAYNE	3		m	0	0	all	-	q	NAmer	1969	CC	420	n	bl	n	n	0	ev	all/unsp	nev any	st
SUZUK2	12	x	c	0	0	all	-	q	SCAmer	1991	CC	123	n	bl	n	y	0	ev	all/unsp	nev any	st
SVENSS	57	x	f	0	0	all	-	q	Eu:Sca	1983	CC	210	n	bl	n	n	0	ev	all/unsp	nev any	st
TIZZAN	18		c	0	0	all	-	q+u	Eu:wst	1959	CC	1358	n	bl	n	n	0	ev	all/unsp	nev any	st
TOKARS	9	x	c	0	0	all	-	q	Eu:est	1966	ot	162	o	bl	n	y	0	ev	all/unsp	nev any	st
TSUGAN	13		m	0	0	all	-	q	As:Jap	1976	CC	134	n	bl	n	y	0	ev	all/unsp	nev any	ot
WAKAI	15	x	m	0	0	all	-	q	As:Jap	1988	CC	333	n	bl	n	y	0	ev	all/unsp	nev any	st
WAKAI	33	x	f	0	0	all	-	q	As:Jap	1988	CC	333	n	bl	n	y	0	ev	all/unsp	nev any	st
WU	14	x	f	0	0	wh	-	q	NAmer	1981	CC	220	n	bl	n	y	0	ev	all/unsp	nev any	st
WUWILL	9		f	0	0	all	-	q	As:Chi	1985	CC	965	n	ot	n	n	3	ev	cig+/-ot	nev cigs	or
WYNDE2	2		m	0	0	all	-	KI	NAmer	1962	CC	404	n	bl	n	y	0	ev	cig+/-ot	nev any	st
WYNDE3	8		m	0	0	all	-	KI	NAmer	1966	CC	350	n	bl	n	y	0	ev	cig+/-ot	nev any	st
WYNDE3	67		f	0	0	all	-	KI	NAmer	1966	CC	350	n	bl	n	y	0	ev	cig+/-ot	nev any	st
WYNDE4	69		m	0	0	all	-	not a	NAmer	1948	CC	684	n	bl	y	n	2	ev	cig+/-ot	nev any	ot
WYNDE4	54		f	0	0	all	-	not a	NAmer	1948	CC	684	n	bl	y	n	2	ev	all/unsp	nev any	ot
WYNDE6	75		m	0	0	all	-	KI	NAmer	1969	CC	4423	n	bl	n	y	0	ev	cig+/-ot	nev any	st
WYNDE6	411	x	f	0	0	wh	-	q	NAmer	1969	CC	4423	n	bl	n	y	0	ev	cig+/-ot	nev cigs	st
XU3	19	x	m	0	0	all	-	KI	As:Chi	1981	CC	135	n	ot	n	n	0	ev	all/unsp	nev any	st
XU3	23	x	f	0	0	all	-	KI	As:Chi	1981	CC	135	n	ot	n	n	0	ev	all/unsp	nev any	st
ZHENG	5		m	0	0	all	-	q	As:Chi	1982	CC	540	n	ot	*	y	0	ev	cig+/-ot	nev cigs	st
ZHENG	18		f	0	0	all	-	q	As:Chi	1982	CC	540	n	ot	*	y	0	ev	cig+/-ot	nev cigs	st
ZHOU	8		m	0	0	all	-	q	As:Chi	1978	CC	1360	n	ot	n	n	0	ev	all/unsp	nev any	st
ZHOU	9		f	0	0	all	-	q	As:Chi	1978	CC	1360	n	ot	n	n	0	ev	all/unsp	nev any	st

Cigarette type is all/unspec for all RRs

except for the following:

REF	NRR	CIGTYPE
ALDERS	84	MC only
CHAN	18	MC+-HR
CHAN	22	MC+-HR
JUSSAW	25	MC only

Table 2C3 - 5

IESLC - Meta-anal of Ever Smoking (or Current if Ever not available), Cigs (or Any Prod if Cigs not avail)
Squamous
Least adjusted

REF	NRR	SEX	AD	Number Exposed Case	Number Exposed Cont	Non-exposed Case	Non-exposed Cont	RR	95.00%CI
*ABRAHA	1	m	0	142	10351	0	3365	92.66~	(5.77-1488.21)
*ABRAHA	4	f	0	17	5256	7	11589	5.35	(2.22- 12.90)
Subtotal ABRAHA								6.95	(3.00- 16.06)
ALDERS	88	m	0	277	641	4	133	14.37	(5.26- 39.24)
ALDERS	84	f	0	176	371	16	243	7.20	(4.21- 12.32)
Subtotal ALDERS								8.40	(5.23- 13.48)
*ANDERS	10	f	0	63	96164	5	195158	25.57	(10.29- 63.56)
BAND	5	m	2	-	-	-	-	37.45	(17.62- 79.58)
BARBON	110	m	0	261	567	6	188	14.42	(6.31- 32.94)
BECHER	11	f	1	-	-	-	-	10.69	(2.43- 47.00)
*BOUCOT	70	m	0	38	22177	0	7551	26.22~	(1.61- 426.71)
BRESLO	7	c	0	444	394	15	56	4.21	(2.34- 7.56)
BROWN2	6	m	2	-	-	-	-	11.10	(9.50- 12.90)
BROWN2	5	f	2	-	-	-	-	20.10	(16.40- 24.80)
Subtotal BROWN2								13.69	(12.11- 15.49)
BUFFLE	49	m	0	-	-	-	-	14.03	(4.73- 41.61)
BUFFLE	62	f	0	58	166	3	112	13.04	(3.99- 42.66)
Subtotal BUFFLE								13.57	(6.09- 30.24)
BYERS1	1	m	0	299	695	22	424	8.29	(5.29- 13.00)
CHAN	18	m	0	112	160	2	43	15.05	(3.57- 63.41)
CHAN	22	f	0	37	38	19	139	7.12	(3.68- 13.77)
Subtotal CHAN								8.11	(4.45- 14.77)
CHOI	62	m	0	160	465	6	95	5.45	(2.34- 12.67)
CHOI	64	f	0	11	26	10	164	6.94	(2.68- 17.96)
Subtotal CHOI								6.06	(3.22- 11.40)
COMSTO	66	m	0	44	229	2	84	8.07	(1.91- 34.02)
COMSTO	78	f	0	17	87	0	115	46.20~	(2.74- 778.83)
Subtotal COMSTO								11.56	(3.21- 41.67)
CORREA	35	c	1	-	-	-	-	28.30	(18.60- 43.20)
*CPSI	403	m	1	-	-	-	-	29.35	(4.02- 214.28)
*CPSI	405	f	1	-	-	-	-	4.25	(1.23- 14.68)
Subtotal CPSI								7.30	(2.55- 20.90)
*CPSII	114	m	1	-	-	-	-	39.26	(10.38- 148.55)
*CPSII	117	f	1	-	-	-	-	78.91	(15.83- 393.37)
Subtotal CPSII								52.16	(18.72- 145.32)
DAMBER	12	m	0	271	169	14	103	11.80	(6.54- 21.29)
DESTE2	16	m	2	-	-	-	-	13.20	(4.70- 37.10)
DOLL	82	m	0	829	1296	3	61	13.01	(4.07- 41.59)
DOLL	84	f	0	32	49	16	59	2.41	(1.18- 4.90)
Subtotal DOLL								3.81	(2.08- 6.98)
DORGAN	113	m	2	-	-	-	-	18.90	(7.00- 51.30)
DORGAN	98	f	3	-	-	-	-	11.10	(7.20- 17.10)
Subtotal DORGAN								12.08	(8.12- 17.96)
*DORN	338	m	1	-	-	-	-	17.09	(8.96- 32.60)
DOSEME	19	m	0	434	536	58	293	4.09	(3.00- 5.57)
*ENGELA	62	m	7	-	-	-	-	6.45	(1.97- 21.11)
FAN	3	c	0	75	595	6	556	11.68	(5.04- 27.04)
GAO	7	m	0	314	558	13	202	8.74	(4.91- 15.58)
GAO	17	f	0	66	130	53	605	5.80	(3.85- 8.72)
Subtotal GAO								6.65	(4.76- 9.28)
GER	5	c	0	48	156	11	80	2.24	(1.10- 4.54)
HAENSZ	22	f	0	56	103	44	236	2.92	(1.85- 4.61)
*HAMMON	72	m	0	265	382338	4	115884	20.08	(7.48- 53.90)
HEGMAN	2	c	0	89	1202	5	2080	30.80	(12.48- 76.03)
HINDS	23	f	3	-	-	-	-	16.13	(7.66- 33.97)
ISHIMA	1	c	0	53	33	5	25	8.03	(2.80- 23.04)
JAHN	42	m	0	343	671	3	138	23.51	(7.44- 74.35)
JAIN	8	m	0	154	277	2	85	23.63	(5.73- 97.35)
JAIN	3	f	0	103	196	6	214	18.74	(8.05- 43.66)
Subtotal JAIN								19.92	(9.64- 41.17)
JEDRYC	7	m	0	337	1054	6	289	15.40	(6.80- 34.89)
JOLY	54	m	0	203	709	2	218	31.21	(7.69- 126.68)
JOLY	52	f	0	48	122	6	283	18.56	(7.74- 44.51)
Subtotal JOLY								21.47	(10.22- 45.09)
JUSSAW	25	m	0	17	77	13	624	10.60	(4.96- 22.66)
KATSOU	35	f	0	28	22	14	67	6.09	(2.73- 13.59)
KHUDER	24	m	0	176	-	9	-	7.82	(3.87- 15.77)
KIHARA	26	c	0	132	232	5	237	26.97	(10.84- 67.08)
KOO	6	f	0	61	63	32	137	4.15	(2.46- 6.98)
KREYBE	16	m	0	210	3514	3	644	12.83	(4.09- 40.22)
KREYBE	33	f	0	2	328	3	657	1.34	(0.22- 8.03)

Table 2C3 - 5

IESLC - Meta-anal of Ever Smoking (or Current if Ever not available), Cigs (or Any Prod if Cigs not avail)
Squamous
Least adjusted

REF	NRR	SEX	AD	Number Exposed Case	Number Exposed Cont	Non-exposed Case	Non-exposed Cont	RR	95.00%CI
Subtotal KREYBE								6.68	(2.55- 17.51)
LAMTH	1	f	0	63	20	28	72	8.10	(4.16- 15.77)
LAMWK	2	f	0	21	41	7	144	10.54	(4.19- 26.52)
LAMWK2	1	m	0	129	161	5	43	6.89	(2.65- 17.90)
LAMWK2	5	f	0	35	50	15	139	6.49	(3.27- 12.88)
Subtotal LAMWK2								6.62	(3.79- 11.56)
LOMBA2	2	f	0	94	353	15	239	4.24	(2.40- 7.50)
LUBIN	34	m	0	291	788	4	72	6.65	(2.41- 18.36)
LUBIN2	145	m	0	3587	10433	54	2616	16.66	(12.69- 21.86)
LUBIN2	165	f	0	200	567	72	1180	5.78	(4.34- 7.71)
Subtotal LUBIN2								10.10	(8.29- 12.31)
LUO	2	c	0	34	146	5	160	7.45	(2.84- 19.56)
MATOS	66	m	0	47	283	3	110	6.09	(1.86- 19.97)
MATSUD	11	m	0	103	3314	1	1255	39.01	(5.44- 279.84)
NOU	1	m	0	110	247	2	122	27.17	(6.60- 111.85)
NOU	6	f	0	5	92	2	261	7.09	(1.35- 37.19)
Subtotal NOU								15.42	(5.26- 45.22)
ORMOS	8	m	0	27	1034	2	777	10.14	(2.41- 42.79)
OSANN	18	m	0	352	1018	8	833	36.00	(17.76- 72.99)
OSANN	22	f	0	159	563	12	1093	25.72	(14.18- 46.66)
Subtotal OSANN								29.58	(18.76- 46.64)
OSANN2	7	f	0	112	61	7	58	15.21	(6.54- 35.38)
PEZZOT	6	m	0	85	317	0	116	62.74~	(3.86-1019.50)
SCHWAR	10	m	0	80	178	1	73	32.81	(4.48- 240.23)
SCHWAR	9	m	0	41	39	4	7	1.84	(0.50- 6.78)
SCHWAR	18	f	0	29	108	0	79	43.23~	(2.60- 718.15)
SCHWAR	17	f	0	21	28	0	41	62.61~	(3.64-1076.10)
Subtotal SCHWAR								7.71	(2.96- 20.10)
SEOW	3	f	0	21	15	10	125	17.50	(6.95- 44.09)
SIEMIA	11	m	0	356	428	3	105	29.11	(9.16- 92.52)
SOBUE	3	m	0	422	1013	3	128	17.77	(5.63- 56.16)
SOBUE	19	f	0	36	232	14	857	9.50	(5.04- 17.91)
Subtotal SOBUE								10.99	(6.31- 19.15)
SOBUE2	1	m	2	-	-	-	-	5.20	(4.20- 6.50)
SOBUE2	5	f	2	-	-	-	-	7.20	(4.80- 10.80)
Subtotal SOBUE2								5.59	(4.62- 6.78)
STASZE	16	m	0	135	653	0	158	65.73~	(4.07-1061.95)
STASZE	38	f	0	1	153	0	1660	32.45~	(1.32- 800.04)
Subtotal STASZE								48.53	(5.94- 396.73)
STAYNE	3	m	0	130	567	22	333	3.47	(2.17- 5.56)
SUZUK2	12	c	0	75	36	5	44	18.33	(6.70- 50.17)
SVENSS	57	f	0	48	89	5	120	12.94	(4.95- 33.84)
TIZZAN	18	c	0	333	939	55	419	2.70	(1.99- 3.67)
TOKARS	9	c	0	45	77	2	19	5.55	(1.24- 24.95)
TSUGAN	13	m	0	20	15	0	5	14.55~	(0.75- 283.37)
WAKAI	15	m	0	113	424	2	65	8.66	(2.09- 35.92)
WAKAI	33	f	0	16	31	3	145	24.95	(6.85- 90.87)
Subtotal WAKAI								15.46	(5.94- 40.24)
WU	14	f	0	69	41	2	30	25.24	(5.73- 111.19)
WUWILL	9	f	3	-	-	-	-	4.20	(3.00- 5.90)
WYNDE2	2	m	0	336	512	3	105	22.97	(7.23- 72.97)
WYNDE3	8	m	0	197	264	3	88	21.89	(6.82- 70.20)
WYNDE3	67	f	0	25	56	5	76	6.79	(2.45- 18.82)
Subtotal WYNDE3								11.28	(5.23- 24.31)
WYNDE4	69	m	2	-	-	-	-	15.45	(7.47- 31.96)
WYNDE4	54	f	2	-	-	-	-	5.82	(2.55- 13.31)
Subtotal WYNDE4								10.09	(5.85- 17.42)
WYNDE6	75	m	0	1706	1797	29	617	20.20	(13.84- 29.48)
WYNDE6	411	f	0	153	275	12	673	31.20	(17.05- 57.09)
Subtotal WYNDE6								22.83	(16.57- 31.46)
XU3	19	m	0	39	68	3	31	5.93	(1.70- 20.66)
XU3	23	f	0	15	11	2	25	17.05	(3.32- 87.61)
Subtotal XU3								8.74	(3.24- 23.59)
ZHENG	5	m	0	156	218	4	94	16.82	(6.05- 46.71)
ZHENG	18	f	0	43	44	33	184	5.45	(3.11- 9.54)
Subtotal ZHENG								7.07	(4.33- 11.56)
ZHOU	8	m	0	343	41	96	36	3.14	(1.90- 5.18)
ZHOU	9	f	0	35	7	42	32	3.81	(1.50- 9.68)
Subtotal ZHOU								3.28	(2.11- 5.10)
Partial Totals				16995	560064	1058	358900		

*prospective study ~ With 0.5 adjustment for zero

Table 2C3 - 5

IESLC - Meta-anal of Ever Smoking (or Current if Ever not available), Cigs (or Any Prod if Cigs not avail)
Squamous
Least adjusted

REF	NRR	SEX	AD	Ys	Ws	Qs	Ps
*ABRAHA	1	m	0	4.53	0.50	2.68	0.0014
*ABRAHA	4	f	0	1.68	4.97	1.41	0.0002
Subtotal ABRAHA				1.94	5.46	4.09	
ALDERS	88	m	0	2.67	3.81	0.79	0.0000
ALDERS	84	f	0	1.97	13.33	0.74	0.0000
Subtotal ALDERS				2.13	17.14	1.53	
*ANDERS	10	f	0	3.24	4.63	4.92	0.0000
BAND	5	m	2	3.62	6.76	13.49	0.0000
BARBON	110	m	0	2.67	5.63	1.18	0.0000
BECHER	11	f	1	2.37	1.75	0.04	0.0017
*BOUCOT	70	m	0	3.27	0.49	0.55	0.0217
BRESLO	7	c	0	1.44	11.20	6.70	0.0000
BROWN2	6	m	2	2.41	164.17	6.34	0.0000
BROWN2	5	f	2	3.00	89.84	56.11	0.0000
Subtotal BROWN2				2.62	254.01	62.45	
BUFFLE	49	m	0	2.64	3.25	0.60	0.0000
BUFFLE	62	f	0	2.57	2.74	0.35	0.0000
Subtotal BUFFLE				2.61	5.99	0.95	
BYERS1	1	m	0	2.12	19.01	0.17	0.0000
CHAN	18	m	0	2.71	1.86	0.47	0.0002
CHAN	22	f	0	1.96	8.84	0.54	0.0000
Subtotal CHAN				2.09	10.69	1.01	
CHOI	62	m	0	1.70	5.39	1.43	0.0001
CHOI	64	f	0	1.94	4.25	0.32	0.0001
Subtotal CHOI				1.80	9.63	1.75	
COMSTO	66	m	0	2.09	1.86	0.03	0.0045
COMSTO	78	f	0	3.83	0.48	1.27	0.0078
Subtotal COMSTO				2.45	2.34	1.30	
CORREA	35	c	1	3.34	21.64	27.75	0.0000
*CPSI	403	m	1	3.38	0.97	1.33	0.0009
*CPSI	405	f	1	1.45	2.50	1.46	0.0222
Subtotal CPSI				1.99	3.47	2.78	
*CPSII	114	m	1	3.67	2.17	4.62	0.0000
*CPSII	117	f	1	4.37	1.49	6.93	0.0000
Subtotal CPSII				3.95	3.66	11.56	
DAMBER	12	m	0	2.47	11.02	0.73	0.0000
DESTE2	16	m	2	2.58	3.60	0.49	0.0000
DOLL	82	m	0	2.57	2.84	0.36	0.0000
DOLL	84	f	0	0.88	7.63	13.52	0.0152
Subtotal DOLL				1.34	10.47	13.88	
DORGAN	113	m	2	2.94	3.87	2.06	0.0000
DORGAN	98	f	3	2.41	20.54	0.79	0.0000
Subtotal DORGAN				2.49	24.41	2.85	
*DORN	338	m	1	2.84	9.21	3.63	0.0000
DOSEME	19	m	0	1.41	40.28	25.90	0.0000
*ENGELA	62	m	7	1.86	2.73	0.33	0.0021
FAN	3	c	0	2.46	5.45	0.33	0.0000
GAO	7	m	0	2.17	11.51	0.02	0.0000
GAO	17	f	0	1.76	23.06	4.74	0.0000
Subtotal GAO				1.89	34.57	4.76	
GER	5	c	0	0.81	7.65	15.11	0.0259
HAENSZ	22	f	0	1.07	18.34	23.84	0.0000
*HAMMON	72	m	0	3.00	3.94	2.46	0.0000
HEGMAN	2	c	0	3.43	4.70	6.97	0.0000
HINDS	23	f	3	2.78	6.93	2.25	0.0000
ISHIMA	1	c	0	2.08	3.46	0.06	0.0001
JAHN	42	m	0	3.16	2.90	2.60	0.0000
JAIN	8	m	0	3.16	1.92	1.74	0.0000
JAIN	3	f	0	2.93	5.37	2.79	0.0000
Subtotal JAIN				2.99	7.29	4.52	
JEDRYC	7	m	0	2.73	5.75	1.58	0.0000
JOLY	54	m	0	3.44	1.96	2.96	0.0000
JOLY	52	f	0	2.92	5.02	2.53	0.0000
Subtotal JOLY				3.07	6.98	5.50	
JUSSAW	25	m	0	2.36	6.65	0.15	0.0000
KATSOU	35	f	0	1.81	5.97	0.97	0.0000
KHUDER	24	m	0	2.06	7.79	0.18	0.0000
KIHARA	26	c	0	3.29	4.63	5.44	0.0000
KOO	6	f	0	1.42	14.12	8.78	0.0000
KREYBE	16	m	0	2.55	2.94	0.34	0.0000
KREYBE	33	f	0	0.29	1.19	4.41	0.7520

Table 2C3 - 5

IESLC - Meta-anal of Ever Smoking (or Current if Ever not available), Cigs (or Any Prod if Cigs not avail)
Squamous
Least adjusted

REF	NRR	SEX	AD	Ys	Ws	Qs	Ps
Subtotal KREYBE				1.90	4.14	4.75	
LAMTH	1	f	0	2.09	8.66	0.12	0.0000
LAMWK	2	f	0	2.35	4.51	0.09	0.0000
LAMWK2	1	m	0	1.93	4.22	0.33	0.0001
LAMWK2	5	f	0	1.87	8.17	0.95	0.0000
Subtotal LAMWK2				1.89	12.38	1.28	
LOMBA2	2	f	0	1.45	11.86	6.94	0.0000
LUBIN	34	m	0	1.89	3.72	0.37	0.0003
LUBIN2	145	m	0	2.81	51.88	18.82	0.0000
LUBIN2	165	f	0	1.75	46.51	9.67	0.0000
Subtotal LUBIN2				2.31	98.39	28.49	
LUO	2	c	0	2.01	4.12	0.17	0.0000
MATOS	66	m	0	1.81	2.72	0.44	0.0029
MATSUD	11	m	0	3.66	0.99	2.09	0.0003
NOU	1	m	0	3.30	1.92	2.29	0.0000
NOU	6	f	0	1.96	1.40	0.09	0.0205
Subtotal NOU				2.74	3.32	2.37	
ORMOS	8	m	0	2.32	1.85	0.02	0.0016
OSANN	18	m	0	3.58	7.69	14.50	0.0000
OSANN	22	f	0	3.25	10.83	11.65	0.0000
Subtotal OSANN				3.39	18.52	26.15	
OSANN2	7	f	0	2.72	5.39	1.41	0.0000
PEZZOT	6	m	0	4.14	0.49	1.84	0.0036
SCHWAR	10	m	0	3.49	0.97	1.59	0.0006
SCHWAR	9	m	0	0.61	2.26	5.79	0.3596
SCHWAR	18	f	0	3.77	0.49	1.18	0.0086
SCHWAR	17	f	0	4.14	0.47	1.76	0.0044
Subtotal SCHWAR				2.04	4.19	10.32	
SEOW	3	f	0	2.86	4.50	1.91	0.0000
SIEMIA	11	m	0	3.37	2.87	3.87	0.0000
SOBUE	3	m	0	2.88	2.90	1.29	0.0000
SOBUE	19	f	0	2.25	9.55	0.02	0.0000
Subtotal SOBUE				2.40	12.46	1.31	
SOBUE2	1	m	2	1.65	80.57	25.42	0.0000
SOBUE2	5	f	2	1.97	23.37	1.31	0.0000
Subtotal SOBUE2				1.72	103.93	26.73	
STASZE	16	m	0	4.19	0.50	1.94	0.0032
STASZE	38	f	0	3.48	0.37	0.60	0.0333
Subtotal STASZE				3.88	0.87	2.54	
STAYNE	3	m	0	1.24	17.27	16.12	0.0000
SUZUK2	12	c	0	2.91	3.79	1.85	0.0000
SVENSS	57	f	0	2.56	4.16	0.51	0.0000
TIZZAN	18	c	0	0.99	40.59	60.07	0.0000
TOKARS	9	c	0	1.71	1.70	0.42	0.0254
TSUGAN	13	m	0	2.68	0.44	0.10	0.0772
WAKAI	15	m	0	2.16	1.90	0.01	0.0029
WAKAI	33	f	0	3.22	2.30	2.33	0.0000
Subtotal WAKAI				2.74	4.20	2.33	
WU	14	f	0	3.23	1.75	1.81	0.0000
WUWILL	9	f	3	1.44	33.59	20.19	0.0000
WYNDE2	2	m	0	3.13	2.88	2.45	0.0000
WYNDE3	8	m	0	3.09	2.83	2.17	0.0000
WYNDE3	67	f	0	1.91	3.69	0.32	0.0002
Subtotal WYNDE3				2.42	6.52	2.49	
WYNDE4	69	m	2	2.74	7.27	2.02	0.0000
WYNDE4	54	f	2	1.76	5.63	1.14	0.0000
Subtotal WYNDE4				2.31	12.90	3.16	
WYNDE6	75	m	0	3.01	26.85	16.98	0.0000
WYNDE6	411	f	0	3.44	10.53	15.93	0.0000
Subtotal WYNDE6				3.13	37.38	32.91	
XU3	19	m	0	1.78	2.46	0.46	0.0052
XU3	23	f	0	2.84	1.43	0.56	0.0007
Subtotal XU3				2.17	3.90	1.02	
ZHENG	5	m	0	2.82	3.68	1.38	0.0000
ZHENG	18	f	0	1.70	12.24	3.25	0.0000
Subtotal ZHENG				1.96	15.92	4.62	
ZHOU	8	m	0	1.14	15.27	17.38	0.0000
ZHOU	9	f	0	1.34	4.42	3.36	0.0049
Subtotal ZHOU				1.19	19.68	20.75	

Table 2C3 - 5

IESLC - Meta-anal of Ever Smoking (or Current if Ever not available), Cigs (or Any Prod if Cigs not avail)
Squamous
Least adjusted

	N	110
	NS	78
	Wt	1148.90
Het	Chi	569.55
Het	df	109
Het	P	***
Fixed	RR	9.12
	RRl	8.61
	RRu	9.66
	P	+++
Random	RR	10.50
	RRl	9.01
	RRu	12.23
	P	+++
Asymm	P	(*)

Table 2C3 - 6

IESLC - Meta-anal of Ever Smoking (or Current if Ever not available), Cigs (or Any Prod if Cigs not avail)

Squamous

Least adjusted

		Sex			
		combined	male	female	Total
	N	11	54	45	110
	NS	11	53	44	108
	Wt	108.93	581.17	458.79	1148.90
Het	Chi	115.66	215.56	225.49	569.55
Het	df	10	53	44	109
Het	P	***	***	***	***
Fixed	RR	6.82	9.83	8.88	9.12
	RRl	5.65	9.07	8.10	8.61
	RRu	8.23	10.67	9.73	9.66
	P	+++	+++	+++	+++
Random	RR	9.17	12.22	9.12	10.50
	RRl	4.54	9.95	7.20	9.01
	RRu	18.50	15.01	11.55	12.23
	P	+++	+++	+++	+++
Between	Chi				12.84
Between	df				2
Between	P				**
Btwn(F)	P				N.S.
Btwn(R)	P				N.S.

Table 2C3 - 7

IESLC - Meta-anal of Ever Smoking (or Current if Ever not available), Cigs (or Any Prod if Cigs not avail)
Squamous
Excluded studies (and stage at which they were excluded)

1 BOUCHA BUELL LAURIL MZILEN

2 BERRIN CHEN RESTRE TAO ULMER

5 RIMING TANG2 WYNDE5 WYNDE8

6 BLOT1 BLOT2 BLOT3 BYERS2 HIRAOK HIRAY2 JONES LICKIN MOLLO MRFIT MURATA NOTANI PERSHA SAARIK SCHWA2 SHIMIZ SITAS VANDER WARSIN WATSON WYNDER

8 ABELIN AGUDO AKIBA AMANDU AMES ARCHER ARMADA AUSTIN AUVINE AXELSO AXELSS BENSHL BEST BLOHMK BLOT4 BOFFET BRETT BROCKM BROSS BROWN1 CARPEN CASCO2 CASCOR CEDERL CHANG CHATZI CHEN2 CHEN3 CHIAZZ CHOW CHYOU COOKSO DARBY DAVEYS DEAN DEAN2 DEAN3 DEKLER DESTEF DOCKER DOLL2 DORANT DROSTE DU DUNN EBELIN ENSTRO ESAKI GAO2 GARCIA GARDIN GARSHI GENG GILLIS GODLEY GOLLED GOODMA GRAHAM GREGOR GSELL HAMMO2 HANSEN HEIN HENNEK HIRAYA HITOSU HOLE HOROWI HORWIT HU HU2 HUANG HUMBLE JARUP JARVHO JIANG KAISE2 KAISER KANELL KAUFMA KELLER KINLEN KJUUS KNEKT KO KOHLME KOULUM KREUZE KUBIK LANGE LAUSSM LEI LEMARC LETOUR LEVIN LIAW LIDDEL LIU LIU2 LIU3 LIU4 LIU5 LOMBAR MACLEN MAGNUS MARSH MARSH2 MARTIS MASTRA MCCONN MCDUFF MCLAUG MIGRAN MILLER MILLS MRFITR NAM NOTAN2 ODRISC PARKIN PASTOR PAWLEG PERNU PERSH2 PETO PEZZO2 PIKE PISANI POFFIJ POLEDN PRESCO QIAO QIAO2 RACHTA RADZIK RANDIG REN RONCO ROOTS ROTHSC SADOWS SANKAR SEGI SEGI2 SHAW SIMARA SIMONA SPEIZE SPITZ STOCKS STOCKW STUCKE SUN SUZUKI TANG TENKAN TOUSEY TULINI TVERDA VEIERO WALD WANG WANG2 WANG3 WANG4 WICKLU WIGLE WILKIN WU2 WUNSCH XIANGZ XU XU2 XU4 YAMAGU YONG YUAN ZHANG

10 BENHAM VUTUC WYNDE7

Table 2C3 - 8

Potentially overlapping studies

REF	REFGP	PRINC	.	OVERLAP/LINK
LUBIN2	LUBIN2	1		Lubin-combined
CHAN	CHAN	1		Chan/LamWK2
LAMWK2	LAMWK2	1		Chan/LamWK2
LAMTH	LAMTH	1		KOO/LAMTH/LAMWK
KOO	KOO	1		KOO/LAMTH/LAMWK
LAMWK	LAMWK	1		KOO/LAMTH/LAMWK
ISHIMA	AKIBA	2		AKIBA/ISHIMA
BYERS1	BYERS1	2		GRAHAM/BROSS/BYERS1
OSANN2	KAISER	2		KAISER/OSANN2
WYNDE6	WYNDE6	1		WYNDE5/6/7/8
CPSI	CPSI	1		CPSI overall
SOBUE2	SOBUE2	1		SOBUE2/MATSUD
MATSUD	MATSUD	1		SOBUE2/MATSUD
JAHN	BOFFET	2		Subset of BOFFET
LUBIN	XIANGZ	2		LUBIN/XIANGZ/QIAO

Table 2C3 - 9

Most adjusted - insufficient data for metaanalysis

REF	NRR	SEX	AGEL	AGEH	RACE	YF	LC TYPE	LOC	START	ST	NLC	R	VB	P	H	AD	SM	PRODUCT	DENOM	De
BUFFLE	47	f	0	0	wh	-	q	NAmer	1976	CC	943	n	bl	y	n	0	ev	cig+/-ot	nev cigs	or
CHEN	1	c	0	0	all	-	q	As:oth	1987	CC	323	n	ot	n	y	2	ev	cig+/-ot	nev cigs	ot
CHYOU	5	m	0	0	jap	0	q+s	NAmer	1965	pr	227	n	bl	n	y	1	cu	cig+/-ot	nev cigs	or
JIANG	3	m	0	0	all	-	q	As:Chi	1984	CC	125	n	ot	n	n	0	ev	all/unsp	nev any	or
JIANG	4	f	0	0	all	-	q	As:Chi	1984	CC	125	n	ot	n	n	0	ev	all/unsp	nev any	ot
KOO	2	f	0	0	all	-	q	As:HK	1981	CC	200	n	bl	n	n	0	ev	all/unsp	nev any	or
WANG	3	c	0	0	all	-	q	As:Chi	1990	CC	390	n	ot	*	y	0	ev	all/unsp	nev any	ot

REF	NRR	RR	SIG	RRDATA comment
BUFFLE	47	8.30		0
CHEN	1	6.66	y	P < 0.001
CHYOU	5	16.00		0
JIANG	3	12.78		0
JIANG	4	*	n	0
KOO	2	3.62		0
WANG	3	4.66	y	p<0.01

Table 2C4 -

IESLC - Meta-anal of Current Smoking (or Ever if Current not available), Cigs (or Any Prod if Cigs not avail)
Squamous

This analysis is restricted to results for:
1) Non-dose-response data
2) Results complete enough for use in metaanalysis

Within each study, results are then selected (in the following order of preference, within each sex) for:
3) SMKSTA: current smokers, ever smokers
4) PRODUCT: cigarettes regardless of other products, cigarettes only, all/unspec
5) CIGTYPE: all/unspecified, MC regardless of HR, MC only
6) DENOM: never smoked anything, never smoked cigarettes, (never +1 = +long term ex, +2 = +amount unknown, +3 = never cigs+long term ex)
7) Followup period (YF, prospective studies): whole study (coded as 0) or longest available
8) LCtype: squamous or nearest available, but not adeno. (q = squamous, s = small, a = adeno, KI = Kreyberg I, u = undifferentiated)
9) Race: all or nearest available, otherwise by race (wh or w = white, bl or b = black, hi = hispanic ch = chinese, jap = japanese, haw = hawaiian, w+o = white + oriental, sca = scandinavian, as = asian)
10) For overlapping studies: principal rather than subsidiary studies
Finally by Age: whole study (coded as 0) if available, otherwise by widest available age group and then for single sex results (m, f) in preference to combined sex results (c).

Results adjusted (AD) for the most potential confounders are then chosen in Sections -1 to -3 (and those which actually differ from the adjusted results in Table 2C3 - 1 are marked 'x' in Section -1) and results adjusted for the least confounders in Sections -4 to -6. (Those least adjusted results which actually differ from the most adjusted as marked 'x' in column X in Section -4) (Results adjusted for an unknown number of confounder(s) are coded as 20.)

Section -7 shows excluded studies, together with the stage (as above) at which no qualifying results were found.

Section -8 lists the potentially overlapping studies which have been included (1=principal, 2=subsidiary).

Section -9 lists any results which would have been included in preference except that they had data not complete enough for use in meta-analysis, with their significance (yes/no), if known, and any further comment as entered on the database.

In addition to those mentioned above, the following fields, levels and abbreviations are used:
* or nk = not known, n = no, y = yes, ot = other
ev = ever, cu = current, nev = never
all/unspec = all or unspecified, cig+/-ot = cigarettes irrespective of other products (cigar, pipe etc)
MC = manufactured cigarettes, HR = hand-rolled cigarettes
REF: 6-character study reference
NRR: number of the RR on the database within the study
ST : study type (CC = case control, pr or prosp = prospective)
NLC: number of lung cancer cases in whole study
R : risky occupational population (n = no, m = mining, o = other risky)
VB : national cigarette type (V = at least 75% Virginia, bl = at least 75% blended, ot = other)
P : any proxy use
H : full histological confirmation
De : derivation of RR/CI (or = original, st = standard method, ot = other method of estimation)

Table 2C4 - 1

IESLC - Meta-anal of Current Smoking (or Ever if Current not available), Cigs (or Any Prod if Cigs not avail)
Squamous
Most adjusted

REF	NRR	2C3	SEX	AGEL	AGEH	RACE	YF	LC TYPE	LOC	START	ST	NLC	R	VB	P	H	AD	SM	PRODUCT	DENOM	De
ABRAHA	1		m	0	0	all	0	q	Eu:est	1975	pr	571	n	bl	n	n	0	ev	all/unsp	nev any	ot
ABRAHA	4		f	0	0	all	0	q	Eu:est	1975	pr	571	n	bl	n	n	0	ev	all/unsp	nev any	ot
ALDERS	75		m	0	0	all	-	q+s	Eu:UK	1977	CC	1448	n	V	n	n	1	ev	cig+/-ot	nev any	ot
ALDERS	33		f	0	0	all	-	q+s	Eu:UK	1977	CC	1448	n	V	n	n	1	ev	cig only	nev any	ot
ANDERS	10		f	0	0	all	0	q	NAmer	1986	pr	343	n	bl	n	n	0	ev	cig+/-ot	nev cigs	st
BAND	5		m	0	0	all	-	q	NAmer	1983	CC	2831	n	V	y	y	2	ev	cig only	nev any	ot
BARBON	18	x	m	0	0	all	-	q	Eu:wst	1979	CC	755	n	bl	y	y	1	cu	all/unsp	nev any	or
BECHER	11		f	0	0	all	-	q+s	Eu:Ger	1985	CC	194	n	bl	n	y	1	ev	all/unsp	nev any	or
BOUCOT	141		m	0	0	all	0	q	NAmer	1951	pr	121	n	bl	n	n	2	cu	cig only	nev any	ot
BRESLO	7		c	0	0	all	-	not a	NAmer	1949	CC	518	n	bl	n	y	0	ev	cig+/-ot	nev+1	st
BROWN2	16	x	m	0	0	wh	-	q	NAmer	1984	CC	14596	n	bl	n	y	2	cu	cig+/-ot	nev cigs	or
BROWN2	15	x	f	0	0	wh	-	q	NAmer	1984	CC	14596	n	bl	n	y	2	cu	cig+/-ot	nev cigs	or
BUFFLE	49		m	0	0	wh	-	q	NAmer	1976	CC	943	n	bl	y	n	0	ev	cig+/-ot	nev cigs	ot
BUFFLE	63	x	f	0	0	w-hi	-	q	NAmer	1976	CC	943	n	bl	y	n	0	cu	cig+/-ot	nev cigs	st
BYERS1	1		m	0	0	wh	-	q	NAmer	1957	CC	1002	n	bl	n	n	0	ev	cig+/-ot	nev cigs	st
CHAN	18		m	0	0	all	-	q+s	As:HK	1976	CC	397	n	bl	n	n	0	ev	cig+/-ot	nev any	st
CHAN	22		f	0	0	all	-	q+s	As:HK	1976	CC	397	n	bl	n	n	0	ev	cig+/-ot	nev any	st
CHOI	62		m	0	0	all	-	q	As:oth	1985	CC	375	n	bl	n	n	0	ev	cig+/-ot	nev cigs	st
CHOI	64		f	0	0	all	-	q	As:oth	1985	CC	375	n	bl	n	n	0	ev	cig+/-ot	nev cigs	st
COMSTO	23	x	m	0	0	all	-	q	NAmer	1975	ot	258	n	bl	n	n	0	cu	cig+/-ot	nev cigs	st
COMSTO	30	x	f	0	0	all	-	q	NAmer	1975	ot	258	n	bl	n	n	0	cu	cig+/-ot	nev cigs	st
CORREA	43	x	c	0	0	all	-	q+s	NAmer	1979	CC	1359	n	bl	y	n	1	cu	cig+/-ot	nev cigs	or
CPSI	403		m	0	0	all	2	q	NAmer	1959	pr	5138	n	bl	n	n	1	cu	cig only	nev any	ot
CPSI	405		f	0	0	all	2	q	NAmer	1959	pr	5138	n	bl	n	n	1	cu	cig only	nev any	ot
CPSII	114		m	0	0	all	2	q	NAmer	1982	pr	3229	n	bl	n	n	1	cu	cig only	nev any	ot
CPSII	117		f	0	0	all	2	q	NAmer	1982	pr	3229	n	bl	n	n	1	cu	cig+/-ot	nev cigs	ot
DAMBER	33		m	0	0	all	-	q	Eu:Sca	1972	CC	579	n	bl	y	n	1	ev	all/unsp	nev any	or
DESTE2	16		m	0	0	all	-	q	SCAmer	1993	CC	463	n	bl	n	n	2	ev	all/unsp	nev any	or
DOLL	86		m	0	0	all	-	KI	Eu:UK	1948	CC	1465	n	V	n	n	1	ev	all/unsp	nev any	ot
DOLL	88		f	0	0	all	-	KI	Eu:UK	1948	CC	1465	n	V	n	n	1	ev	all/unsp	nev any	ot
DORGAN	113		m	0	0	wh	-	q	NAmer	1980	CC	2026	n	bl	y	y	2	ev	cig+/-ot	nev any	or
DORGAN	98		f	0	0	all	-	q	NAmer	1980	CC	2026	n	bl	y	y	3	ev	cig+/-ot	nev any	or
DORN	338		m	0	0	wh	8	q	NAmer	1954	pr	5097	n	bl	n	n	1	cu	cig only	nev any	ot
DOSEME	3		m	0	0	all	-	q	Eu:bal	1979	CC	1210	n	bl	n	n	2	ev	cig+/-ot	nev cigs	or
ENGELA	56	x	m	0	0	all	0	q	Eu:Sca	1964	pr	435	n	bl	n	n	7	cu	cig+/-ot	nev cigs	ot
FAN	3		c	0	0	all	-	q	As:Chi	1990	CC	403	n	ot	y	n	0	ev	cig+/-ot	nev cigs	ot
GAO	2		m	0	0	all	-	q	As:Chi	1984	CC	1405	n	ot	n	n	2	ev	cig+/-ot	nev cigs	or
GAO	12		f	0	0	all	-	q	As:Chi	1984	CC	1405	n	ot	n	n	2	ev	cig+/-ot	nev cigs	or
GER	13		c	0	0	all	-	q+s	As:oth	1990	CC	141	n	ot	y	n	10	ev	all/unsp	nev any	ot
HAENSZ	20	x	f	0	0	all	-	q+u	NAmer	1955	CC	158	n	bl	n	y	0	cu	cig+/-ot	nev any	st
HAMMON	102	x	m	0	0	wh	0	not a	NAmer	1952	pr	448	n	bl	n	n	1	cu	cig only	nev any	ot
HEGMAN	2		c	0	0	all	-	q	NAmer	1989	CC	282	n	bl	y	y	0	ev	all/unsp	nev any	st
HINDS	23		f	0	0	o	-	q+s	NAmer	1968	CC	292	n	bl	n	n	3	ev	all/unsp	nev any	st
ISHIMA	6		c	0	0	all	-	q	As:Jap	1961	CC	180	n	bl	y	y	5	ev	all/unsp	nev any	st
JAHN	7	x	m	0	0	all	-	q	Eu:Ger	1988	CC	1004	n	bl	n	n	0	cu	cig+/-ot	nev any	st
JAIN	18	x	m	0	0	all	-	q	NAmer	1981	CC	845	n	V	y	n	0	cu	cig+/-ot	nev cigs	st
JAIN	13	x	f	0	0	all	-	q	NAmer	1981	CC	845	n	V	y	n	0	cu	cig+/-ot	nev cigs	st
JEDRYC	22	x	m	0	0	all	-	q	Eu:est	1980	CC	1630	n	bl	y	n	0	cu	cig+/-ot	nev any	st
JOLY	54		m	0	0	all	-	q	SCAmer	1978	CC	826	n	bl	n	n	0	ev	cig+/-ot	nev any	st
JOLY	52		f	0	0	all	-	q	SCAmer	1978	CC	826	n	bl	n	n	0	ev	cig+/-ot	nev any	st
JUSSAW	25		m	0	0	all	-	KI	As:Ind	1964	CC	792	n	V	n	n	0	ev	cig only	nev any	st
KATSOU	20	x	f	0	0	all	-	KI	Eu:bal	1987	CC	101	n	bl	n	n	1	cu	all/unsp	nev any	or
KHUDER	14	x	m	0	0	all	-	q	NAmer	1985	CC	482	n	bl	n	y	0	cu	cig+/-ot	nev cigs	or
KIHARA	2	x	c	0	0	jap	-	q	As:Jap	1991	CC	440	n	bl	n	n	0	cu	all/unsp	nev any	st
KOO	6		f	0	0	all	-	q+s	As:HK	1981	CC	200	n	bl	n	n	0	ev	all/unsp	nev any	st
KREYBE	4		m	0	0	all	-	KI	Eu:Sca	1948	CC	300	n	bl	n	y	1	ev	all/unsp	nev any	ot
KREYBE	25		f	0	0	all	-	KI	Eu:Sca	1948	CC	300	n	bl	n	y	1	ev	all/unsp	nev any	ot
LAMTH	1		f	0	0	ch	-	q	As:HK	1983	CC	445	n	bl	n	n	0	ev	all/unsp	nev any	or
LAMWK	2		f	0	0	ch	-	q	As:HK	1981	CC	163	n	bl	n	n	0	ev	all/unsp	nev any	st
LAMWK2	1		m	0	0	all	-	q	As:HK	1976	CC	480	n	bl	n	n	0	ev	all/unsp	nev any	st
LAMWK2	5		f	0	0	all	-	q	As:HK	1976	CC	480	n	bl	n	n	0	ev	all/unsp	nev any	st
LOMBA2	2		f	0	0	all	-	q+u	NAmer	1960	CC	225	n	bl	n	n	0	ev	cig+/-ot	nev cigs	st
LUBIN	34		m	0	0	all	-	KI	As:Chi	1984	CC	427	m	ot	y	n	0	ev	cig+/-ot	nev any	st
LUBIN2	249	x	m	0	0	all	-	q	Eu:mul	1976	CC	7804	n	bl	n	y	0	cu	cig+/-ot	nev any	st
LUBIN2	261	x	f	0	0	all	-	q	Eu:mul	1976	CC	7804	n	bl	n	y	0	cu	cig+/-ot	nev any	st
LUO	8		c	0	0	all	-	q	As:Chi	1990	CC	102	n	ot	n	y	20	ev	cig+/-ot	nev cigs	or
MATOS	39	x	m	0	0	all	-	q	SCAmer	1994	CC	200	n	bl	n	n	2	cu	cig+/-ot	nev any	or
MATSUD	11		m	0	0	all	-	q	As:Jap	1965	CC	179	n	bl	n	n	0	ev	cig+/-ot	nev cigs	st
NOU	1		m	0	0	all	-	q	Eu:Sca	1971	CC	273	n	bl	y	n	0	ev	all/unsp	nev any	st
NOU	6		f	0	0	all	-	q	Eu:Sca	1971	CC	273	n	bl	y	n	0	ev	all/unsp	nev any	st
ORMOS	8		m	0	0	all	-	q	Eu:est	1947	CC	119	n	bl	y	y	0	ev	cig+/-ot	nev any	st
OSANN	35	x	m	0	0	all	-	q	NAmer	1984	CC	1986	n	bl	n	n	2	cu	cig+/-ot	nev cigs	or

Table 2C4 - 1

IESLC - Meta-anal of Current Smoking (or Ever if Current not available), Cigs (or Any Prod if Cigs not avail)
Squamous
Most adjusted

REF	NRR	2C3	SEX	AGEL	AGEH	RACE	YF	LC TYPE	LOC	START	ST	NLC	R	VB	P	H	AD	SM	PRODUCT	DENOM	De
OSANN	36	x	f	0	0	all	-	q	NAmer	1984	CC	1986	n	bl	n	n	2	cu	cig+/-ot	nev cigs	or
OSANN2	26	x	f	0	0	all	-	KI	NAmer	1964	ot	217	n	bl	n	y	1	cu	cig+/-ot	nev cigs	or
PEZZOT	6		m	0	0	all	-	q	SCAmer	1987	CC	215	n	bl	n	y	0	ev	cig only	nev cigs	ot
SCHWAR	10		m	40	54	wh	-	q	NAmer	1984	CC	5588	n	bl	y	y	0	ev	cig+/-ot	nev cigs	st
SCHWAR	9		m	40	54	bl	-	q	NAmer	1984	CC	5588	n	bl	y	y	0	ev	cig+/-ot	nev cigs	st
SCHWAR	18		f	40	54	wh	-	q	NAmer	1984	CC	5588	n	bl	y	y	0	ev	cig+/-ot	nev cigs	ot
SCHWAR	17		f	40	54	bl	-	q	NAmer	1984	CC	5588	n	bl	y	y	0	ev	cig+/-ot	nev cigs	ot
SEOW	3		f	0	0	ch	-	q	As:oth	1997	CC	153	n	bl	n	y	0	ev	cig+/-ot	nev cigs	st
SIEMIA	7		m	0	0	all	-	q	NAmer	1979	CC	857	n	V	y	y	7	ev	cig+/-ot	nev cigs	or
SOBUE	34	x	m	0	0	all	-	q	As:Jap	1986	CC	1376	n	bl	n	y	1	cu	cig+/-ot	nev cigs	or
SOBUE	44	x	f	0	0	all	-	q	As:Jap	1986	CC	1376	n	bl	n	y	1	cu	cig+/-ot	nev cigs	or
SOBUE2	1		m	0	0	all	-	q	As:Jap	1965	CC	2083	n	bl	n	n	2	cu	cig+/-ot	nev any	or
SOBUE2	5		f	0	0	all	-	q	As:Jap	1965	CC	2083	n	bl	n	n	2	cu	cig+/-ot	nev any	or
STASZE	16		m	0	0	all	-	q	Eu:est	1954	CC	281	n	bl	n	y	0	ev	cig+/-ot	nev any	ot
STASZE	38		f	0	0	all	-	q	Eu:est	1954	CC	281	n	bl	n	y	0	ev	all/unsp	nev any	ot
STAYNE	3		m	0	0	all	-	q	NAmer	1969	CC	420	n	bl	n	n	0	ev	all/unsp	nev any	st
SUZUK2	15		c	0	0	all	-	q	SCAmer	1991	CC	123	n	bl	n	y	3	ev	all/unsp	nev any	or
SVENSS	97	x	f	0	0	all	-	q	Eu:Sca	1983	CC	210	n	bl	n	n	1	cu	all/unsp	nev any	ot
TIZZAN	18		c	0	0	all	-	q+u	Eu:wst	1959	CC	1358	n	bl	n	n	0	ev	all/unsp	nev any	st
TOKARS	10		c	0	0	all	-	q	Eu:est	1966	ot	162	o	bl	n	y	3	ev	all/unsp	nev any	or
TSUGAN	14	x	m	0	0	all	-	q	As:Jap	1976	CC	134	n	bl	n	y	0	cu	all/unsp	nev any	ot
WAKAI	10	x	m	0	0	all	-	q	As:Jap	1988	CC	333	n	bl	n	y	1	cu	all/unsp	nev any	or
WAKAI	28	x	f	0	0	all	-	q	As:Jap	1988	CC	333	n	bl	n	y	1	cu	all/unsp	nev any	or
WU	16	x	f	0	0	wh	-	q	NAmer	1981	CC	220	n	bl	n	y	2	cu	all/unsp	nev any	or
WUWILL	9		f	0	0	all	-	q	As:Chi	1985	CC	965	n	ot	n	n	3	ev	cig+/-ot	nev cigs	or
WYNDE2	2		m	0	0	all	-	KI	NAmer	1962	CC	404	n	bl	n	y	0	ev	cig+/-ot	nev any	st
WYNDE3	10	x	m	0	0	all	-	KI	NAmer	1966	CC	350	n	bl	n	y	0	cu	all/unsp	nev any	st
WYNDE3	67		f	0	0	all	-	KI	NAmer	1966	CC	350	n	bl	n	y	0	ev	cig+/-ot	nev any	st
WYNDE4	69		m	0	0	all	-	not a	NAmer	1948	CC	684	n	bl	y	n	2	ev	cig+/-ot	nev any	ot
WYNDE4	54		f	0	0	all	-	not a	NAmer	1948	CC	684	n	bl	y	n	2	ev	all/unsp	nev any	ot
WYNDE6	12	x	m	0	0	all	-	KI	NAmer	1969	CC	4423	n	bl	n	y	0	cu	cig+/-ot	nev any	st
WYNDE6	201	x	f	0	0	all	-	KI	NAmer	1969	CC	4423	n	bl	n	y	0	cu	cig+/-ot	nev cigs	st
XU3	20		m	0	0	all	-	KI	As:Chi	1981	CC	135	n	ot	n	n	1	ev	all/unsp	nev any	ot
XU3	24		f	0	0	all	-	KI	As:Chi	1981	CC	135	n	ot	n	n	1	ev	all/unsp	nev any	ot
ZHENG	5		m	0	0	all	-	q	As:Chi	1982	CC	540	n	ot	*	y	0	ev	cig+/-ot	nev cigs	st
ZHENG	18		f	0	0	all	-	q	As:Chi	1982	CC	540	n	ot	*	y	0	ev	cig+/-ot	nev cigs	st
ZHOU	8		m	0	0	all	-	q	As:Chi	1978	CC	1360	n	ot	n	n	0	ev	all/unsp	nev any	st
ZHOU	9		f	0	0	all	-	q	As:Chi	1978	CC	1360	n	ot	n	n	0	ev	all/unsp	nev any	st

Cigarette type is all/unspec for all RRs

except for the following:

REF	NRR	CIGTYPE
ALDERS	33	MC only
CHAN	18	MC+-HR
CHAN	22	MC+-HR
JUSSAW	25	MC only

Table 2C4 - 2

IESLC - Meta-anal of Current Smoking (or Ever if Current not available), Cigs (or Any Prod if Cigs not avail)
Squamous
Most adjusted

REF	NRR	SEX	AD	Number Exposed Case	Number Exposed Cont	Non-exposed Case	Non-exposed Cont	RR	95.00%CI
*ABRAHA	1	m	0	142	10351	0	3365	92.66~	(5.77-1488.21)
*ABRAHA	4	f	0	17	5256	7	11589	5.35	(2.22- 12.90)
Subtotal ABRAHA								6.95	(3.00- 16.06)
ALDERS	75	m	1	-	-	-	-	10.23	(3.78- 27.72)
ALDERS	33	f	1	-	-	-	-	6.70	(3.92- 11.46)
Subtotal ALDERS								7.37	(4.59- 11.82)
*ANDERS	10	f	0	63	96164	5	195158	25.57	(10.29- 63.56)
BAND	5	m	2	-	-	-	-	37.45	(17.62- 79.58)
BARBON	18	m	1	-	-	-	-	18.80	(8.20- 43.40)
BECHER	11	f	1	-	-	-	-	10.69	(2.43- 47.00)
*BOUCOT	141	m	2	-	-	-	-	27.54	(1.69- 448.37)
BRESLO	7	c	0	444	394	15	56	4.21	(2.34- 7.56)
BROWN2	16	m	2	-	-	-	-	13.70	(11.70- 16.10)
BROWN2	15	f	2	-	-	-	-	20.60	(16.60- 25.60)
Subtotal BROWN2								15.81	(13.91- 17.98)
BUFFLE	49	m	0	-	-	-	-	14.03	(4.73- 41.61)
BUFFLE	63	f	0	39	110	3	112	13.24	(3.97- 44.10)
Subtotal BUFFLE								13.67	(6.10- 30.62)
BYERS1	1	m	0	299	695	22	424	8.29	(5.29- 13.00)
CHAN	18	m	0	112	160	2	43	15.05	(3.57- 63.41)
CHAN	22	f	0	37	38	19	139	7.12	(3.68- 13.77)
Subtotal CHAN								8.11	(4.45- 14.77)
CHOI	62	m	0	160	465	6	95	5.45	(2.34- 12.67)
CHOI	64	f	0	11	26	10	164	6.94	(2.68- 17.96)
Subtotal CHOI								6.06	(3.22- 11.40)
COMSTO	23	m	0	27	100	2	84	11.34	(2.62- 49.09)
COMSTO	30	f	0	16	52	0	115	72.60~	(4.27-1233.12)
Subtotal COMSTO								16.78	(4.57- 61.67)
CORREA	43	c	1	-	-	-	-	34.60	(22.80- 52.40)
*CPSI	403	m	1	-	-	-	-	29.35	(4.02- 214.28)
*CPSI	405	f	1	-	-	-	-	4.25	(1.23- 14.68)
Subtotal CPSI								7.30	(2.55- 20.90)
*CPSII	114	m	1	-	-	-	-	39.26	(10.38- 148.55)
*CPSII	117	f	1	-	-	-	-	78.91	(15.83- 393.37)
Subtotal CPSII								52.16	(18.72- 145.32)
DAMBER	33	m	1	-	-	-	-	11.80	(6.40- 23.00)
DESTE2	16	m	2	-	-	-	-	13.20	(4.70- 37.10)
DOLL	86	m	1	-	-	-	-	13.17	(4.12- 42.10)
DOLL	88	f	1	-	-	-	-	2.13	(1.06- 4.27)
Subtotal DOLL								3.45	(1.90- 6.27)
DORGAN	113	m	2	-	-	-	-	18.90	(7.00- 51.30)
DORGAN	98	f	3	-	-	-	-	11.10	(7.20- 17.10)
Subtotal DORGAN								12.08	(8.12- 17.96)
*DORN	338	m	1	-	-	-	-	17.09	(8.96- 32.60)
DOSEME	3	m	2	-	-	-	-	3.60	(2.60- 5.00)
*ENGELA	56	m	7	-	-	-	-	10.89	(3.25- 36.43)
FAN	3	c	0	75	595	6	556	11.68	(5.04- 27.04)
GAO	2	m	2	-	-	-	-	8.40	(4.70- 15.00)
GAO	12	f	2	-	-	-	-	7.20	(4.60- 11.10)
Subtotal GAO								7.62	(5.36- 10.82)
GER	13	c	10	-	-	-	-	3.19	(1.08- 9.42)
HAENSZ	20	f	0	53	94	44	236	3.02	(1.90- 4.82)
*HAMMON	102	m	1	-	-	-	-	26.42	(9.78- 71.34)
HEGMAN	2	c	0	89	1202	5	2080	30.80	(12.48- 76.03)
HINDS	23	f	3	-	-	-	-	16.13	(7.66- 33.97)
ISHIMA	6	c	5	-	-	-	-	21.00	(3.38- 868.40)
JAHN	7	m	0	153	269	3	138	26.16	(8.19- 83.54)
JAIN	18	m	0	107	118	2	85	38.54	(9.26- 160.45)
JAIN	13	f	0	81	99	6	214	29.18	(12.31- 69.15)
Subtotal JAIN								31.44	(15.03- 65.78)
JEDRYC	22	m	0	235	516	6	289	21.94	(9.63- 49.96)
JOLY	54	m	0	203	709	2	218	31.21	(7.69- 126.68)
JOLY	52	f	0	48	122	6	283	18.56	(7.74- 44.51)
Subtotal JOLY								21.47	(10.22- 45.09)
JUSSAW	25	m	0	17	77	13	624	10.60	(4.96- 22.66)
KATSOU	20	f	1	-	-	-	-	6.45	(2.73- 15.25)
KHUDER	14	m	0	112	-	9	-	8.60	(4.20- 17.60)
KIHARA	2	c	0	111	162	5	237	32.48	(12.97- 81.34)
KOO	6	f	0	61	63	32	137	4.15	(2.46- 6.98)
KREYBE	4	m	1	-	-	-	-	10.87	(3.47- 34.04)
KREYBE	25	f	1	-	-	-	-	2.29	(0.89- 5.88)

Table 2C4 - 2

IESLC - Meta-anal of Current Smoking (or Ever if Current not available), Cigs (or Any Prod if Cigs not avail)
Squamous
Most adjusted

REF	NRR	SEX	AD	Number Exposed Case	Number Exposed Cont	Non-exposed Case	Non-exposed Cont	RR	95.00%CI
Subtotal KREYBE								4.31	(2.08- 8.92)
LAMTH	1	f	0	63	20	28	72	8.10	(4.16- 15.77)
LAMWK	2	f	0	21	41	7	144	10.54	(4.19- 26.52)
LAMWK2	1	m	0	129	161	5	43	6.89	(2.65- 17.90)
LAMWK2	5	f	0	35	50	15	139	6.49	(3.27- 12.88)
Subtotal LAMWK2								6.62	(3.79- 11.56)
LOMBA2	2	f	0	94	353	15	239	4.24	(2.40- 7.50)
LUBIN	34	m	0	291	788	4	72	6.65	(2.41- 18.36)
LUBIN2	249	m	0	2518	6209	54	2616	19.65	(14.95- 25.82)
LUBIN2	261	f	0	154	410	72	1180	6.16	(4.55- 8.32)
Subtotal LUBIN2								11.64	(9.50- 14.25)
LUO	8	c	20	-	-	-	-	10.90	(2.50- 47.90)
MATOS	39	m	2	-	-	-	-	10.40	(3.60- 35.50)
MATSUD	11	m	0	103	3314	1	1255	39.01	(5.44- 279.84)
NOU	1	m	0	110	247	2	122	27.17	(6.60- 111.85)
NOU	6	f	0	5	92	2	261	7.09	(1.35- 37.19)
Subtotal NOU								15.42	(5.26- 45.22)
ORMOS	8	m	0	27	1034	2	777	10.14	(2.41- 42.79)
OSANN	35	m	2	-	-	-	-	49.30	(24.10- 101.00)
OSANN	36	f	2	-	-	-	-	35.20	(19.10- 65.10)
Subtotal OSANN								40.59	(25.47- 64.67)
OSANN2	26	f	1	-	-	-	-	39.00	(5.30- 287.00)
PEZZOT	6	m	0	85	317	0	116	62.74~	(3.86-1019.50)
SCHWAR	10	m	0	80	178	1	73	32.81	(4.48- 240.23)
SCHWAR	9	m	0	41	39	4	7	1.84	(0.50- 6.78)
SCHWAR	18	f	0	29	108	0	79	43.23~	(2.60- 718.15)
SCHWAR	17	f	0	21	28	0	41	62.61~	(3.64-1076.10)
Subtotal SCHWAR								7.71	(2.96- 20.10)
SEOW	3	f	0	21	15	10	125	17.50	(6.95- 44.09)
SIEMIA	7	m	7	-	-	-	-	22.70	(6.90- 75.20)
SOBUE	34	m	1	-	-	-	-	18.10	(7.90- 41.30)
SOBUE	44	f	1	-	-	-	-	9.70	(5.50- 16.80)
Subtotal SOBUE								11.79	(7.42- 18.73)
SOBUE2	1	m	2	-	-	-	-	5.20	(4.20- 6.50)
SOBUE2	5	f	2	-	-	-	-	7.20	(4.80- 10.80)
Subtotal SOBUE2								5.59	(4.62- 6.78)
STASZE	16	m	0	135	653	0	158	65.73~	(4.07-1061.95)
STASZE	38	f	0	1	153	0	1660	32.45~	(1.32- 800.04)
Subtotal STASZE								48.53	(5.94- 396.73)
STAYNE	3	m	0	130	567	22	333	3.47	(2.17- 5.56)
SUZUK2	15	c	3	-	-	-	-	31.00	(4.20- 227.00)
SVENSS	97	f	1	-	-	-	-	21.92	(6.63- 72.51)
TIZZAN	18	c	0	333	939	55	419	2.70	(1.99- 3.67)
TOKARS	10	c	3	-	-	-	-	6.80	(1.20- 38.70)
TSUGAN	14	m	0	18	13	0	5	15.07~	(0.77- 296.43)
WAKAI	10	m	1	-	-	-	-	9.82	(2.36- 41.00)
WAKAI	28	f	1	-	-	-	-	28.20	(7.55- 105.00)
Subtotal WAKAI								17.37	(6.60- 45.71)
WU	16	f	2	-	-	-	-	35.30	(4.70- 267.30)
WUWILL	9	f	3	-	-	-	-	4.20	(3.00- 5.90)
WYNDE2	2	m	0	336	512	3	105	22.97	(7.23- 72.97)
WYNDE3	10	m	0	171	207	3	88	24.23	(7.53- 77.95)
WYNDE3	67	f	0	25	56	5	76	6.79	(2.45- 18.82)
Subtotal WYNDE3								11.77	(5.46- 25.38)
WYNDE4	69	m	2	-	-	-	-	15.45	(7.47- 31.96)
WYNDE4	54	f	2	-	-	-	-	5.82	(2.55- 13.31)
Subtotal WYNDE4								10.09	(5.85- 17.42)
WYNDE6	12	m	0	1026	741	29	617	29.46	(20.06- 43.26)
WYNDE6	201	f	0	550	376	40	856	31.30	(22.21- 44.12)
Subtotal WYNDE6								30.47	(23.59- 39.36)
XU3	20	m	1	-	-	-	-	5.90	(1.69- 20.57)
XU3	24	f	1	-	-	-	-	25.67	(4.99- 131.94)
Subtotal XU3								10.14	(3.75- 27.37)
ZHENG	5	m	0	156	218	4	94	16.82	(6.05- 46.71)
ZHENG	18	f	0	43	44	33	184	5.45	(3.11- 9.54)
Subtotal ZHENG								7.07	(4.33- 11.56)
ZHOU	8	m	0	343	41	96	36	3.14	(1.90- 5.18)
ZHOU	9	f	0	35	7	42	32	3.81	(1.50- 9.68)
Subtotal ZHOU								3.28	(2.11- 5.10)
Partial Totals				9841	135768	784	228465		

*prospective study ~ With 0.5 adjustment for zero

Table 2C4 - 2

IESLC - Meta-anal of Current Smoking (or Ever if Current not available), Cigs (or Any Prod if Cigs not avail)
Squamous
Most adjusted

REF	NRR	SEX	AD	Ys	Ws	Qs	Ps
*ABRAHA	1	m	0	4.53	0.50	2.47	0.0014
*ABRAHA	4	f	0	1.68	4.97	1.95	0.0002
Subtotal ABRAHA				1.94	5.46	4.41	
ALDERS	75	m	1	2.33	3.87	0.00	0.0000
ALDERS	33	f	1	1.90	13.35	2.16	0.0000
Subtotal ALDERS				2.00	17.22	2.16	
*ANDERS	10	f	0	3.24	4.63	4.07	0.0000
BAND	5	m	2	3.62	6.76	11.76	0.0000
BARBON	18	m	1	2.93	5.53	2.20	0.0000
BECHER	11	f	1	2.37	1.75	0.01	0.0017
*BOUCOT	141	m	2	3.32	0.49	0.50	0.0199
BRESLO	7	c	0	1.44	11.20	8.42	0.0000
BROWN2	16	m	2	2.62	150.78	14.81	0.0000
BROWN2	15	f	2	3.03	81.88	42.61	0.0000
Subtotal BROWN2				2.76	232.67	57.42	
BUFFLE	49	m	0	2.64	3.25	0.37	0.0000
BUFFLE	63	f	0	2.58	2.65	0.21	0.0000
Subtotal BUFFLE				2.62	5.90	0.58	
BYERS1	1	m	0	2.12	19.01	0.68	0.0000
CHAN	18	m	0	2.71	1.86	0.31	0.0002
CHAN	22	f	0	1.96	8.84	1.03	0.0000
Subtotal CHAN				2.09	10.69	1.33	
CHOI	62	m	0	1.70	5.39	2.00	0.0001
CHOI	64	f	0	1.94	4.25	0.57	0.0001
Subtotal CHOI				1.80	9.63	2.57	
COMSTO	23	m	0	2.43	1.79	0.03	0.0012
COMSTO	30	f	0	4.28	0.48	1.88	0.0030
Subtotal COMSTO				2.82	2.27	1.91	
CORREA	43	c	1	3.54	22.19	34.11	0.0000
*CPSI	403	m	1	3.38	0.97	1.12	0.0009
*CPSI	405	f	1	1.45	2.50	1.84	0.0222
Subtotal CPSI				1.99	3.47	2.96	
*CPSII	114	m	1	3.67	2.17	4.05	0.0000
*CPSII	117	f	1	4.37	1.49	6.34	0.0000
Subtotal CPSII				3.95	3.66	10.39	
DAMBER	33	m	1	2.47	9.39	0.25	0.0000
DESTE2	16	m	2	2.58	3.60	0.27	0.0000
DOLL	86	m	1	2.58	2.84	0.21	0.0000
DOLL	88	f	1	0.76	7.91	18.96	0.0334
Subtotal DOLL				1.24	10.76	19.18	
DORGAN	113	m	2	2.94	3.87	1.56	0.0000
DORGAN	98	f	3	2.41	20.54	0.22	0.0000
Subtotal DORGAN				2.49	24.41	1.78	
*DORN	338	m	1	2.84	9.21	2.63	0.0000
DOSEME	3	m	2	1.28	35.93	37.61	0.0000
*ENGELA	56	m	7	2.39	2.63	0.02	0.0001
FAN	3	c	0	2.46	5.45	0.13	0.0000
GAO	2	m	2	2.13	11.41	0.35	0.0000
GAO	12	f	2	1.97	19.80	2.15	0.0000
Subtotal GAO				2.03	31.21	2.51	
GER	13	c	10	1.16	3.28	4.29	0.0358
HAENSZ	20	f	0	1.11	17.71	25.39	0.0000
*HAMMON	102	m	1	3.27	3.89	3.66	0.0000
HEGMAN	2	c	0	3.43	4.70	5.94	0.0000
HINDS	23	f	3	2.78	6.93	1.57	0.0000
ISHIMA	6	c	5	3.04	0.50	0.27	0.0315
JAHN	7	m	0	3.26	2.85	2.63	0.0000
JAIN	18	m	0	3.65	1.89	3.43	0.0000
JAIN	13	f	0	3.37	5.16	5.90	0.0000
Subtotal JAIN				3.45	7.05	9.33	
JEDRYC	22	m	0	3.09	5.67	3.49	0.0000
JOLY	54	m	0	3.44	1.96	2.53	0.0000
JOLY	52	f	0	2.92	5.02	1.91	0.0000
Subtotal JOLY				3.07	6.98	4.44	
JUSSAW	25	m	0	2.36	6.65	0.02	0.0000
KATSOU	20	f	1	1.86	5.19	1.00	0.0000
KHUDER	14	m	0	2.15	7.48	0.17	0.0000
KIHARA	2	c	0	3.48	4.56	6.31	0.0000
KOO	6	f	0	1.42	14.12	10.98	0.0000
KREYBE	4	m	1	2.39	2.95	0.02	0.0000
KREYBE	25	f	1	0.83	4.31	9.38	0.0854

Table 2C4 - 2

IESLC - Meta-anal of Current Smoking (or Ever if Current not available), Cigs (or Any Prod if Cigs not avail)
Squamous
Most adjusted

REF	NRR	SEX	AD	Ys	Ws	Qs	Ps
Subtotal KREYBE				1.46	7.26	9.40	
LAMTH	1	f	0	2.09	8.66	0.39	0.0000
LAMWK	2	f	0	2.35	4.51	0.01	0.0000
LAMWK2	1	m	0	1.93	4.22	0.59	0.0001
LAMWK2	5	f	0	1.87	8.17	1.54	0.0000
Subtotal LAMWK2				1.89	12.38	2.13	
LOMBA2	2	f	0	1.45	11.86	8.75	0.0000
LUBIN	34	m	0	1.89	3.72	0.63	0.0003
LUBIN2	249	m	0	2.98	51.39	23.34	0.0000
LUBIN2	261	f	0	1.82	42.25	10.00	0.0000
Subtotal LUBIN2				2.45	93.64	33.34	
LUO	8	c	20	2.39	1.76	0.01	0.0015
MATOS	39	m	2	2.34	2.93	0.00	0.0001
MATSUD	11	m	0	3.66	0.99	1.83	0.0003
NOU	1	m	0	3.30	1.92	1.91	0.0000
NOU	6	f	0	1.96	1.40	0.17	0.0205
Subtotal NOU				2.74	3.32	2.08	
ORMOS	8	m	0	2.32	1.85	0.00	0.0016
OSANN	35	m	2	3.90	7.48	19.01	0.0000
OSANN	36	f	2	3.56	10.22	16.15	0.0000
Subtotal OSANN				3.70	17.70	35.16	
OSANN2	26	f	1	3.66	0.96	1.78	0.0003
PEZZOT	6	m	0	4.14	0.49	1.66	0.0036
SCHWAR	10	m	0	3.49	0.97	1.36	0.0006
SCHWAR	9	m	0	0.61	2.26	6.48	0.3596
SCHWAR	18	f	0	3.77	0.49	1.04	0.0086
SCHWAR	17	f	0	4.14	0.47	1.60	0.0044
Subtotal SCHWAR				2.04	4.19	10.48	
SEOW	3	f	0	2.86	4.50	1.40	0.0000
SIEMIA	7	m	7	3.12	2.69	1.80	0.0000
SOBUE	34	m	1	2.90	5.62	1.97	0.0000
SOBUE	44	f	1	2.27	12.32	0.01	0.0000
Subtotal SOBUE				2.47	17.94	1.98	
SOBUE2	1	m	2	1.65	80.57	34.60	0.0000
SOBUE2	5	f	2	1.97	23.37	2.54	0.0000
Subtotal SOBUE2				1.72	103.93	37.14	
STASZE	16	m	0	4.19	0.50	1.76	0.0032
STASZE	38	f	0	3.48	0.37	0.52	0.0333
Subtotal STASZE				3.88	0.87	2.27	
STAYNE	3	m	0	1.24	17.27	19.39	0.0000
SUZUK2	15	c	3	3.43	0.97	1.23	0.0007
SVENSS	97	f	1	3.09	2.69	1.65	0.0000
TIZZAN	18	c	0	0.99	40.59	69.67	0.0000
TOKARS	10	c	3	1.92	1.27	0.19	0.0305
TSUGAN	14	m	0	2.71	0.43	0.07	0.0743
WAKAI	10	m	1	2.28	1.89	0.00	0.0017
WAKAI	28	f	1	3.34	2.22	2.38	0.0000
Subtotal WAKAI				2.85	4.10	2.38	
WU	16	f	2	3.56	0.94	1.49	0.0005
WUWILL	9	f	3	1.44	33.59	25.36	0.0000
WYNDE2	2	m	0	3.13	2.88	1.98	0.0000
WYNDE3	10	m	0	3.19	2.81	2.20	0.0000
WYNDE3	67	f	0	1.91	3.69	0.56	0.0002
Subtotal WYNDE3				2.47	6.50	2.76	
WYNDE4	69	m	2	2.74	7.27	1.37	0.0000
WYNDE4	54	f	2	1.76	5.63	1.66	0.0000
Subtotal WYNDE4				2.31	12.90	3.02	
WYNDE6	12	m	0	3.38	26.02	30.30	0.0000
WYNDE6	201	f	0	3.44	32.63	42.39	0.0000
Subtotal WYNDE6				3.42	58.65	72.69	
XU3	20	m	1	1.77	2.46	0.69	0.0054
XU3	24	f	1	3.25	1.43	1.27	0.0001
Subtotal XU3				2.32	3.89	1.96	
ZHENG	5	m	0	2.82	3.68	0.99	0.0000
ZHENG	18	f	0	1.70	12.24	4.53	0.0000
Subtotal ZHENG				1.96	15.92	5.52	
ZHOU	8	m	0	1.14	15.27	20.57	0.0000
ZHOU	9	f	0	1.34	4.42	4.12	0.0049
Subtotal ZHOU				1.19	19.68	24.69	

Table 2C4 - 2

IESLC - Meta-anal of Current Smoking (or Ever if Current not available), Cigs (or Any Prod if Cigs not avail)
Squamous
Most adjusted

	N	110
	NS	78
	Wt	1121.16
Het	Chi	675.68
Het	df	109
Het	P	***
Fixed	RR	10.01
	RRl	9.44
	RRu	10.62
	P	+++
Random	RR	11.44
	RRl	9.67
	RRu	13.53
	P	+++
Asymm	P	N.S.

Table 2C4 - 3

IESLC - Meta-anal of Current Smoking (or Ever if Current not available), Cigs (or Any Prod if Cigs not avail)

Squamous

Most adjusted

		Sex			
		combined	male	female	Total
	N	11	54	45	110
	NS	11	53	44	108
	Wt	96.47	562.19	462.50	1121.16
Het	Chi	123.29	269.65	270.48	675.68
Het	df	10	53	44	109
Het	P	***	***	***	***
Fixed	RR	7.61	10.90	9.57	10.01
	RRl	6.23	10.03	8.74	9.44
	RRu	9.29	11.84	10.48	10.62
	P	+++	+++	+++	+++
Random	RR	11.35	13.34	9.63	11.44
	RRl	4.97	10.62	7.45	9.67
	RRu	25.94	16.75	12.44	13.53
	P	+++	+++	+++	+++
Between	Chi				12.26
Between	df				2
Between	P				**
Btwn(F)	P				N.S.
Btwn(R)	P				N.S.

		Smoking status		
		ever	current	Total
	N	69	41	110
	NS	50	30	80
	Wt	475.23	645.92	1121.16
Het	Chi	232.36	288.68	675.68
Het	df	68	40	109
Het	P	***	***	***
Fixed	RR	6.49	13.77	10.01
	RRl	5.94	12.75	9.44
	RRu	7.11	14.87	10.62
	P	+++	+++	+++
Random	RR	8.67	16.91	11.44
	RRl	7.19	13.14	9.67
	RRu	10.45	21.76	13.53
	P	+++	+++	+++
Between	Chi			154.64
Between	df			1
Between	P			***
Btwn(F)	P			***
Btwn(R)	P			***

Table 2C4 - 4

IESLC - Meta-anal of Current Smoking (or Ever if Current not available), Cigs (or Any Prod if Cigs not avail)
Squamous
Least adjusted

REF	NRR	X	SEX	AGEL	AGEH	RACE	YF	LC TYPE	LOC	START	ST	NLC	R	VB	P	H	AD	SM	PRODUCT	DENOM	De
ABRAHA	1		m	0	0	all	0	q	Eu:est	1975	pr	571	n	bl	n	n	0	ev	all/unsp	nev any	ot
ABRAHA	4		f	0	0	all	0	q	Eu:est	1975	pr	571	n	bl	n	n	0	ev	all/unsp	nev any	ot
ALDERS	88	x	m	0	0	all	-	q+s	Eu:UK	1977	CC	1448	n	V	n	n	0	ev	cig+/-ot	nev any	st
ALDERS	84	x	f	0	0	all	-	q+s	Eu:UK	1977	CC	1448	n	V	n	n	0	ev	cig only	nev any	st
ANDERS	10		f	0	0	all	0	q	NAmer	1986	pr	343	n	bl	n	n	0	ev	cig+/-ot	nev cigs	st
BAND	5		m	0	0	all	-	q	NAmer	1983	CC	2831	n	V	y	y	2	ev	cig only	nev any	ot
BARBON	17	x	m	0	0	all	-	q	Eu:wst	1979	CC	755	n	bl	y	y	0	cu	all/unsp	nev any	st
BECHER	11		f	0	0	all	-	q+s	Eu:Ger	1985	CC	194	n	bl	n	y	1	ev	all/unsp	nev any	or
BOUCOT	70	x	m	0	0	all	0	q	NAmer	1951	pr	121	n	bl	n	n	0	cu	cig only	nev any	ot
BRESLO	7		c	0	0	all	-	not a	NAmer	1949	CC	518	n	bl	n	y	0	ev	cig+/-ot	nev+1	st
BROWN2	16		m	0	0	wh	-	q	NAmer	1984	CC	14596	n	bl	n	y	2	cu	cig+/-ot	nev cigs	or
BROWN2	15		f	0	0	wh	-	q	NAmer	1984	CC	14596	n	bl	n	y	2	cu	cig+/-ot	nev cigs	or
BUFFLE	49		m	0	0	wh	-	q	NAmer	1976	CC	943	n	bl	y	n	0	ev	cig+/-ot	nev cigs	ot
BUFFLE	63		f	0	0	w-hi	-	q	NAmer	1976	CC	943	n	bl	y	n	0	cu	cig+/-ot	nev cigs	st
BYERS1	1		m	0	0	wh	-	q	NAmer	1957	CC	1002	n	bl	n	n	0	ev	cig+/-ot	nev cigs	st
CHAN	18		m	0	0	all	-	q+s	As:HK	1976	CC	397	n	bl	n	n	0	ev	cig+/-ot	nev any	st
CHAN	22		f	0	0	all	-	q+s	As:HK	1976	CC	397	n	bl	n	n	0	ev	cig+/-ot	nev any	st
CHOI	62		m	0	0	all	-	q	As:oth	1985	CC	375	n	bl	n	n	0	ev	cig+/-ot	nev cigs	st
CHOI	64		f	0	0	all	-	q	As:oth	1985	CC	375	n	bl	n	n	0	ev	cig+/-ot	nev cigs	st
COMSTO	23		m	0	0	all	-	q	NAmer	1975	ot	258	n	bl	n	n	0	cu	cig+/-ot	nev cigs	st
COMSTO	30		f	0	0	all	-	q	NAmer	1975	ot	258	n	bl	n	n	0	cu	cig+/-ot	nev cigs	ot
CORREA	43		c	0	0	all	-	q+s	NAmer	1979	CC	1359	n	bl	y	n	1	cu	cig+/-ot	nev cigs	or
CPSI	403		m	0	0	all	2	q	NAmer	1959	pr	5138	n	bl	n	n	1	cu	cig only	nev any	ot
CPSI	405		f	0	0	all	2	q	NAmer	1959	pr	5138	n	bl	n	n	1	cu	cig only	nev any	ot
CPSII	114		m	0	0	all	2	q	NAmer	1982	pr	3229	n	bl	n	n	1	cu	cig only	nev any	ot
CPSII	117		f	0	0	all	2	q	NAmer	1982	pr	3229	n	bl	n	n	1	cu	cig+/-ot	nev cigs	ot
DAMBER	12	x	m	0	0	all	-	q	Eu:Sca	1972	CC	579	n	bl	y	n	0	ev	all/unsp	nev any	st
DESTE2	16		m	0	0	all	-	q	SCAmer	1993	CC	463	n	bl	n	n	2	ev	all/unsp	nev any	or
DOLL	82	x	m	0	0	all	-	KI	Eu:UK	1948	CC	1465	n	V	n	n	0	ev	all/unsp	nev any	st
DOLL	84	x	f	0	0	all	-	KI	Eu:UK	1948	CC	1465	n	V	n	n	0	ev	all/unsp	nev any	st
DORGAN	113		m	0	0	wh	-	q	NAmer	1980	CC	2026	n	bl	y	y	2	ev	cig+/-ot	nev any	or
DORGAN	98		f	0	0	all	-	q	NAmer	1980	CC	2026	n	bl	y	y	3	ev	cig+/-ot	nev any	or
DORN	338		m	0	0	wh	8	q	NAmer	1954	pr	5097	n	bl	n	n	1	cu	cig only	nev any	ot
DOSEME	19	x	m	0	0	all	-	q	Eu:bal	1979	CC	1210	n	bl	n	n	0	ev	cig+/-ot	nev cigs	st
ENGELA	56		m	0	0	all	0	q	Eu:Sca	1964	pr	435	n	bl	n	n	7	cu	cig+/-ot	nev cigs	ot
FAN	3		c	0	0	all	-	q	As:Chi	1990	CC	403	n	ot	y	n	0	ev	cig+/-ot	nev cigs	ot
GAO	7	x	m	0	0	all	-	q	As:Chi	1984	CC	1405	n	ot	n	n	0	ev	cig+/-ot	nev cigs	st
GAO	17	x	f	0	0	all	-	q	As:Chi	1984	CC	1405	n	ot	n	n	0	ev	cig+/-ot	nev cigs	st
GER	5	x	c	0	0	all	-	q+s	As:oth	1990	CC	141	n	ot	y	n	0	ev	all/unsp	nev any	st
HAENSZ	20		f	0	0	all	-	q+u	NAmer	1955	CC	158	n	bl	n	y	0	cu	cig+/-ot	nev any	st
HAMMON	102		m	0	0	wh	0	not a	NAmer	1952	pr	448	n	bl	n	n	1	cu	cig only	nev any	ot
HEGMAN	2		c	0	0	all	-	q	NAmer	1989	CC	282	n	bl	y	y	0	ev	all/unsp	nev any	st
HINDS	23		f	0	0	o	-	q+s	NAmer	1968	CC	292	n	bl	n	n	3	ev	all/unsp	nev any	st
ISHIMA	1	x	c	0	0	all	-	q	As:Jap	1961	CC	180	n	bl	y	y	0	ev	all/unsp	nev any	st
JAHN	7		m	0	0	all	-	q	Eu:Ger	1988	CC	1004	n	bl	n	n	0	cu	cig+/-ot	nev any	st
JAIN	18		m	0	0	all	-	q	NAmer	1981	CC	845	n	V	y	n	0	cu	cig+/-ot	nev cigs	st
JAIN	13		f	0	0	all	-	q	NAmer	1981	CC	845	n	V	y	n	0	cu	cig+/-ot	nev cigs	st
JEDRYC	22		m	0	0	all	-	q	Eu:est	1980	CC	1630	n	bl	y	n	0	cu	cig+/-ot	nev any	st
JOLY	54		m	0	0	all	-	q	SCAmer	1978	CC	826	n	bl	n	n	0	ev	cig+/-ot	nev any	st
JOLY	52		f	0	0	all	-	q	SCAmer	1978	CC	826	n	bl	n	n	0	ev	cig+/-ot	nev any	st
JUSSAW	25		m	0	0	all	-	KI	As:Ind	1964	CC	792	n	V	n	n	0	ev	cig only	nev any	st
KATSOU	24	x	f	0	0	all	-	KI	Eu:bal	1987	CC	101	n	bl	n	n	0	cu	all/unsp	nev any	st
KHUDER	14		m	0	0	all	-	q	NAmer	1985	CC	482	n	bl	n	y	0	cu	cig+/-ot	nev cigs	or
KIHARA	2		c	0	0	jap	-	q	As:Jap	1991	CC	440	n	bl	n	n	0	cu	all/unsp	nev any	st
KOO	6		f	0	0	all	-	q+s	As:HK	1981	CC	200	n	bl	n	n	0	ev	all/unsp	nev any	st
KREYBE	16	x	m	0	0	all	-	KI	Eu:Sca	1948	CC	300	n	bl	n	y	0	ev	all/unsp	nev any	st
KREYBE	33	x	f	0	0	all	-	KI	Eu:Sca	1948	CC	300	n	bl	n	y	0	ev	all/unsp	nev any	st
LAMTH	1		f	0	0	ch	-	q	As:HK	1983	CC	445	n	bl	n	n	0	ev	all/unsp	nev any	or
LAMWK	2		f	0	0	ch	-	q	As:HK	1981	CC	163	n	bl	n	n	0	ev	all/unsp	nev any	st
LAMWK2	1		m	0	0	all	-	q	As:HK	1976	CC	480	n	bl	n	n	0	ev	all/unsp	nev any	st
LAMWK2	5		f	0	0	all	-	q	As:HK	1976	CC	480	n	bl	n	n	0	ev	all/unsp	nev any	st
LOMBA2	2		f	0	0	all	-	q+u	NAmer	1960	CC	225	n	bl	n	n	0	ev	cig+/-ot	nev cigs	st
LUBIN	34		m	0	0	all	-	KI	As:Chi	1984	CC	427	m	ot	y	n	0	ev	cig+/-ot	nev any	st
LUBIN2	249		m	0	0	all	-	q	Eu:mul	1976	CC	7804	n	bl	n	y	0	cu	cig+/-ot	nev any	st
LUBIN2	261		f	0	0	all	-	q	Eu:mul	1976	CC	7804	n	bl	n	y	0	cu	cig+/-ot	nev any	st
LUO	2	x	c	0	0	all	-	q	As:Chi	1990	CC	102	n	ot	n	y	0	ev	cig+/-ot	nev cigs	st
MATOS	38	x	m	0	0	all	-	q	SCAmer	1994	CC	200	n	bl	n	n	0	cu	cig+/-ot	nev any	st
MATSUD	11		m	0	0	all	-	q	As:Jap	1965	CC	179	n	bl	n	n	0	ev	cig+/-ot	nev cigs	st
NOU	1		m	0	0	all	-	q	Eu:Sca	1971	CC	273	n	bl	y	n	0	ev	all/unsp	nev any	st
NOU	6		f	0	0	all	-	q	Eu:Sca	1971	CC	273	n	bl	y	n	0	ev	all/unsp	nev any	st
ORMOS	8		m	0	0	all	-	q	Eu:est	1947	CC	119	n	bl	y	y	0	ev	cig+/-ot	nev any	st
OSANN	10	x	m	0	0	all	-	q	NAmer	1984	CC	1986	n	bl	n	n	0	cu	cig+/-ot	nev cigs	st

Table 2C4 - 4

IESLC - Meta-anal of Current Smoking (or Ever if Current not available), Cigs (or Any Prod if Cigs not avail)
Squamous
Least adjusted

REF	NRR	X	SEX	AGEL	AGEH	RACE	YF	LC TYPE	LOC	START	ST	NLC	R	VB	P	H	AD	SM	PRODUCT	DENOM	De
OSANN	14	x	f	0	0	all	-	q	NAmer	1984	CC	1986	n	bl	n	n	0	cu	cig+/-ot	nev cigs	st
OSANN2	8	x	f	0	0	all	-	KI	NAmer	1964	ot	217	n	bl	n	y	0	cu	cig+/-ot	nev cigs	st
PEZZOT	6		m	0	0	all	-	q	SCAmer	1987	CC	215	n	bl	n	y	0	ev	cig only	nev cigs	ot
SCHWAR	10		m	40	54	wh	-	q	NAmer	1984	CC	5588	n	bl	y	y	0	ev	cig+/-ot	nev cigs	st
SCHWAR	9		m	40	54	bl	-	q	NAmer	1984	CC	5588	n	bl	y	y	0	ev	cig+/-ot	nev cigs	st
SCHWAR	18		f	40	54	wh	-	q	NAmer	1984	CC	5588	n	bl	y	y	0	ev	cig+/-ot	nev cigs	ot
SCHWAR	17		f	40	54	bl	-	q	NAmer	1984	CC	5588	n	bl	y	y	0	ev	cig+/-ot	nev cigs	ot
SEOW	3		f	0	0	ch	-	q	As:oth	1997	CC	153	n	bl	n	y	0	ev	cig+/-ot	nev cigs	st
SIEMIA	11	x	m	0	0	all	-	q	NAmer	1979	CC	857	n	V	y	y	0	ev	cig+/-ot	nev cigs	st
SOBUE	2	x	m	0	0	all	-	q	As:Jap	1986	CC	1376	n	bl	n	y	0	cu	cig+/-ot	nev cigs	st
SOBUE	18	x	f	0	0	all	-	q	As:Jap	1986	CC	1376	n	bl	n	y	0	cu	cig+/-ot	nev cigs	st
SOBUE2	1		m	0	0	all	-	q	As:Jap	1965	CC	2083	n	bl	n	n	2	cu	cig+/-ot	nev any	or
SOBUE2	5		f	0	0	all	-	q	As:Jap	1965	CC	2083	n	bl	n	n	2	cu	cig+/-ot	nev any	or
STASZE	16		m	0	0	all	-	q	Eu:est	1954	CC	281	n	bl	n	y	0	ev	cig+/-ot	nev any	ot
STASZE	38		f	0	0	all	-	q	Eu:est	1954	CC	281	n	bl	n	y	0	ev	all/unsp	nev any	ot
STAYNE	3		m	0	0	all	-	q	NAmer	1969	CC	420	n	bl	n	n	0	ev	all/unsp	nev any	st
SUZUK2	12	x	c	0	0	all	-	q	SCAmer	1991	CC	123	n	bl	n	y	0	ev	all/unsp	nev any	st
SVENSS	62	x	f	0	0	all	-	q	Eu:Sca	1983	CC	210	n	bl	n	n	0	cu	all/unsp	nev any	st
TIZZAN	18		c	0	0	all	-	q+u	Eu:wst	1959	CC	1358	n	bl	n	n	0	ev	all/unsp	nev any	st
TOKARS	9	x	c	0	0	all	-	q	Eu:est	1966	ot	162	o	bl	n	y	0	ev	all/unsp	nev any	st
TSUGAN	14		m	0	0	all	-	q	As:Jap	1976	CC	134	n	bl	n	y	0	cu	all/unsp	nev any	ot
WAKAI	4	x	m	0	0	all	-	q	As:Jap	1988	CC	333	n	bl	n	y	0	cu	all/unsp	nev any	st
WAKAI	22	x	f	0	0	all	-	q	As:Jap	1988	CC	333	n	bl	n	y	0	cu	all/unsp	nev any	st
WU	11	x	f	0	0	wh	-	q	NAmer	1981	CC	220	n	bl	n	y	0	cu	all/unsp	nev any	st
WUWILL	9		f	0	0	all	-	q	As:Chi	1985	CC	965	n	ot	n	n	3	ev	cig+/-ot	nev cigs	or
WYNDE2	2		m	0	0	all	-	KI	NAmer	1962	CC	404	n	bl	n	y	0	ev	cig+/-ot	nev any	st
WYNDE3	10		m	0	0	all	-	KI	NAmer	1966	CC	350	n	bl	n	y	0	cu	all/unsp	nev any	st
WYNDE3	67		f	0	0	all	-	KI	NAmer	1966	CC	350	n	bl	n	y	0	ev	cig+/-ot	nev any	st
WYNDE4	69		m	0	0	all	-	not a	NAmer	1948	CC	684	n	bl	y	n	2	ev	cig+/-ot	nev any	ot
WYNDE4	54		f	0	0	all	-	not a	NAmer	1948	CC	684	n	bl	y	n	2	ev	all/unsp	nev any	ot
WYNDE6	12		m	0	0	all	-	KI	NAmer	1969	CC	4423	n	bl	n	y	0	cu	cig+/-ot	nev any	st
WYNDE6	201		f	0	0	all	-	KI	NAmer	1969	CC	4423	n	bl	n	y	0	cu	cig+/-ot	nev cigs	st
XU3	19	x	m	0	0	all	-	KI	As:Chi	1981	CC	135	n	ot	n	n	0	ev	all/unsp	nev any	st
XU3	23	x	f	0	0	all	-	KI	As:Chi	1981	CC	135	n	ot	n	n	0	ev	all/unsp	nev any	st
ZHENG	5		m	0	0	all	-	q	As:Chi	1982	CC	540	n	ot	*	y	0	ev	cig+/-ot	nev cigs	st
ZHENG	18		f	0	0	all	-	q	As:Chi	1982	CC	540	n	ot	*	y	0	ev	cig+/-ot	nev cigs	st
ZHOU	8		m	0	0	all	-	q	As:Chi	1978	CC	1360	n	ot	n	n	0	ev	all/unsp	nev any	st
ZHOU	9		f	0	0	all	-	q	As:Chi	1978	CC	1360	n	ot	n	n	0	ev	all/unsp	nev any	st

Cigarette type is all/unspec for all RRs

except for the following:

REF	NRR	CIGTYPE
ALDERS	84	MC only
CHAN	18	MC+-HR
CHAN	22	MC+-HR
JUSSAW	25	MC only

Table 2C4 - 5

IESLC - Meta-anal of Current Smoking (or Ever if Current not available), Cigs (or Any Prod if Cigs not avail)
Squamous
Least adjusted

REF	NRR	SEX	AD	Number Exposed Case	Number Exposed Cont	Non-exposed Case	Non-exposed Cont	RR	95.00%CI
*ABRAHA	1	m	0	142	10351	0	3365	92.66~	(5.77-1488.21)
*ABRAHA	4	f	0	17	5256	7	11589	5.35	(2.22- 12.90)
Subtotal ABRAHA								6.95	(3.00- 16.06)
ALDERS	88	m	0	277	641	4	133	14.37	(5.26- 39.24)
ALDERS	84	f	0	176	371	16	243	7.20	(4.21- 12.32)
Subtotal ALDERS								8.40	(5.23- 13.48)
*ANDERS	10	f	0	63	96164	5	195158	25.57	(10.29- 63.56)
BAND	5	m	2	-	-	-	-	37.45	(17.62- 79.58)
BARBON	17	m	0	203	362	6	188	17.57	(7.66- 40.33)
BECHER	11	f	1	-	-	-	-	10.69	(2.43- 47.00)
*BOUCOT	70	m	0	38	22177	0	7551	26.22~	(1.61- 426.71)
BRESLO	7	c	0	444	394	15	56	4.21	(2.34- 7.56)
BROWN2	16	m	2	-	-	-	-	13.70	(11.70- 16.10)
BROWN2	15	f	2	-	-	-	-	20.60	(16.60- 25.60)
Subtotal BROWN2								15.81	(13.91- 17.98)
BUFFLE	49	m	0	-	-	-	-	14.03	(4.73- 41.61)
BUFFLE	63	f	0	39	110	3	112	13.24	(3.97- 44.10)
Subtotal BUFFLE								13.67	(6.10- 30.62)
BYERS1	1	m	0	299	695	22	424	8.29	(5.29- 13.00)
CHAN	18	m	0	112	160	2	43	15.05	(3.57- 63.41)
CHAN	22	f	0	37	38	19	139	7.12	(3.68- 13.77)
Subtotal CHAN								8.11	(4.45- 14.77)
CHOI	62	m	0	160	465	6	95	5.45	(2.34- 12.67)
CHOI	64	f	0	11	26	10	164	6.94	(2.68- 17.96)
Subtotal CHOI								6.06	(3.22- 11.40)
COMSTO	23	m	0	27	100	2	84	11.34	(2.62- 49.09)
COMSTO	30	f	0	16	52	0	115	72.60~	(4.27-1233.12)
Subtotal COMSTO								16.78	(4.57- 61.67)
CORREA	43	c	1	-	-	-	-	34.60	(22.80- 52.40)
*CPSI	403	m	1	-	-	-	-	29.35	(4.02- 214.28)
*CPSI	405	f	1	-	-	-	-	4.25	(1.23- 14.68)
Subtotal CPSI								7.30	(2.55- 20.90)
*CPSII	114	m	1	-	-	-	-	39.26	(10.38- 148.55)
*CPSII	117	f	1	-	-	-	-	78.91	(15.83- 393.37)
Subtotal CPSII								52.16	(18.72- 145.32)
DAMBER	12	m	0	271	169	14	103	11.80	(6.54- 21.29)
DESTE2	16	m	2	-	-	-	-	13.20	(4.70- 37.10)
DOLL	82	m	0	829	1296	3	61	13.01	(4.07- 41.59)
DOLL	84	f	0	32	49	16	59	2.41	(1.18- 4.90)
Subtotal DOLL								3.81	(2.08- 6.98)
DORGAN	113	m	2	-	-	-	-	18.90	(7.00- 51.30)
DORGAN	98	f	3	-	-	-	-	11.10	(7.20- 17.10)
Subtotal DORGAN								12.08	(8.12- 17.96)
*DORN	338	m	1	-	-	-	-	17.09	(8.96- 32.60)
DOSEME	19	m	0	434	536	58	293	4.09	(3.00- 5.57)
*ENGELA	56	m	7	-	-	-	-	10.89	(3.25- 36.43)
FAN	3	c	0	75	595	6	556	11.68	(5.04- 27.04)
GAO	7	m	0	314	558	13	202	8.74	(4.91- 15.58)
GAO	17	f	0	66	130	53	605	5.80	(3.85- 8.72)
Subtotal GAO								6.65	(4.76- 9.28)
GER	5	c	0	48	156	11	80	2.24	(1.10- 4.54)
HAENSZ	20	f	0	53	94	44	236	3.02	(1.90- 4.82)
*HAMMON	102	m	1	-	-	-	-	26.42	(9.78- 71.34)
HEGMAN	2	c	0	89	1202	5	2080	30.80	(12.48- 76.03)
HINDS	23	f	3	-	-	-	-	16.13	(7.66- 33.97)
ISHIMA	1	c	0	53	33	5	25	8.03	(2.80- 23.04)
JAHN	7	m	0	153	269	3	138	26.16	(8.19- 83.54)
JAIN	18	m	0	107	118	2	85	38.54	(9.26- 160.45)
JAIN	13	f	0	81	99	6	214	29.18	(12.31- 69.15)
Subtotal JAIN								31.44	(15.03- 65.78)
JEDRYC	22	m	0	235	516	6	289	21.94	(9.63- 49.96)
JOLY	54	m	0	203	709	2	218	31.21	(7.69- 126.68)
JOLY	52	f	0	48	122	6	283	18.56	(7.74- 44.51)
Subtotal JOLY								21.47	(10.22- 45.09)
JUSSAW	25	m	0	17	77	13	624	10.60	(4.96- 22.66)
KATSOU	24	f	0	24	18	14	67	6.38	(2.76- 14.78)
KHUDER	14	m	0	112	-	9	-	8.60	(4.20- 17.60)
KIHARA	2	c	0	111	162	5	237	32.48	(12.97- 81.34)
KOO	6	f	0	61	63	32	137	4.15	(2.46- 6.98)
KREYBE	16	m	0	210	3514	3	644	12.83	(4.09- 40.22)
KREYBE	33	f	0	2	328	3	657	1.34	(0.22- 8.03)

Table 2C4 - 5

IESLC - Meta-anal of Current Smoking (or Ever if Current not available), Cigs (or Any Prod if Cigs not avail)
Squamous
Least adjusted

REF	NRR	SEX	AD	Number Exposed Case	Number Exposed Cont	Non-exposed Case	Non-exposed Cont	RR	95.00%CI
Subtotal KREYBE								6.68	(2.55- 17.51)
LAMTH	1	f	0	63	20	28	72	8.10	(4.16- 15.77)
LAMWK	2	f	0	21	41	7	144	10.54	(4.19- 26.52)
LAMWK2	1	m	0	129	161	5	43	6.89	(2.65- 17.90)
LAMWK2	5	f	0	35	50	15	139	6.49	(3.27- 12.88)
Subtotal LAMWK2								6.62	(3.79- 11.56)
LOMBA2	2	f	0	94	353	15	239	4.24	(2.40- 7.50)
LUBIN	34	m	0	291	788	4	72	6.65	(2.41- 18.36)
LUBIN2	249	m	0	2518	6209	54	2616	19.65	(14.95- 25.82)
LUBIN2	261	f	0	154	410	72	1180	6.16	(4.55- 8.32)
Subtotal LUBIN2								11.64	(9.50- 14.25)
LUO	2	c	0	34	146	5	160	7.45	(2.84- 19.56)
MATOS	38	m	0	33	132	3	110	9.17	(2.74- 30.70)
MATSUD	11	m	0	103	3314	1	1255	39.01	(5.44- 279.84)
NOU	1	m	0	110	247	2	122	27.17	(6.60- 111.85)
NOU	6	f	0	5	92	2	261	7.09	(1.35- 37.19)
Subtotal NOU								15.42	(5.26- 45.22)
ORMOS	8	m	0	27	1034	2	777	10.14	(2.41- 42.79)
OSANN	10	m	0	247	541	8	833	47.54	(23.32- 96.92)
OSANN	14	f	0	130	367	12	1093	32.26	(17.65- 58.97)
Subtotal OSANN								37.93	(23.94- 60.10)
OSANN2	8	f	0	105	42	7	58	20.71	(8.75- 49.05)
PEZZOT	6	m	0	85	317	0	116	62.74~	(3.86-1019.50)
SCHWAR	10	m	0	80	178	1	73	32.81	(4.48- 240.23)
SCHWAR	9	m	0	41	39	4	7	1.84	(0.50- 6.78)
SCHWAR	18	f	0	29	108	0	79	43.23~	(2.60- 718.15)
SCHWAR	17	f	0	21	28	0	41	62.61~	(3.64-1076.10)
Subtotal SCHWAR								7.71	(2.96- 20.10)
SEOW	3	f	0	21	15	10	125	17.50	(6.95- 44.09)
SIEMIA	11	m	0	356	428	3	105	29.11	(9.16- 92.52)
SOBUE	2	m	0	301	650	3	128	19.76	(6.24- 62.58)
SOBUE	18	f	0	29	168	14	857	10.57	(5.47- 20.42)
Subtotal SOBUE								12.33	(6.96- 21.84)
SOBUE2	1	m	2	-	-	-	-	5.20	(4.20- 6.50)
SOBUE2	5	f	2	-	-	-	-	7.20	(4.80- 10.80)
Subtotal SOBUE2								5.59	(4.62- 6.78)
STASZE	16	m	0	135	653	0	158	65.73~	(4.07-1061.95)
STASZE	38	f	0	1	153	0	1660	32.45~	(1.32- 800.04)
Subtotal STASZE								48.53	(5.94- 396.73)
STAYNE	3	m	0	130	567	22	333	3.47	(2.17- 5.56)
SUZUK2	12	c	0	75	36	5	44	18.33	(6.70- 50.17)
SVENSS	62	f	0	42	53	5	120	19.02	(7.12- 50.77)
TIZZAN	18	c	0	333	939	55	419	2.70	(1.99- 3.67)
TOKARS	9	c	0	45	77	2	19	5.55	(1.24- 24.95)
TSUGAN	14	m	0	18	13	0	5	15.07~	(0.77- 296.43)
WAKAI	4	m	0	86	284	2	65	9.84	(2.36- 41.03)
WAKAI	22	f	0	15	26	3	145	27.88	(7.54- 103.14)
Subtotal WAKAI								17.34	(6.61- 45.48)
WU	11	f	0	61	23	2	30	39.78	(8.79- 180.01)
WUWILL	9	f	3	-	-	-	-	4.20	(3.00- 5.90)
WYNDE2	2	m	0	336	512	3	105	22.97	(7.23- 72.97)
WYNDE3	10	m	0	171	207	3	88	24.23	(7.53- 77.95)
WYNDE3	67	f	0	25	56	5	76	6.79	(2.45- 18.82)
Subtotal WYNDE3								11.77	(5.46- 25.38)
WYNDE4	69	m	2	-	-	-	-	15.45	(7.47- 31.96)
WYNDE4	54	f	2	-	-	-	-	5.82	(2.55- 13.31)
Subtotal WYNDE4								10.09	(5.85- 17.42)
WYNDE6	12	m	0	1026	741	29	617	29.46	(20.06- 43.26)
WYNDE6	201	f	0	550	376	40	856	31.30	(22.21- 44.12)
Subtotal WYNDE6								30.47	(23.59- 39.36)
XU3	19	m	0	39	68	3	31	5.93	(1.70- 20.66)
XU3	23	f	0	15	11	2	25	17.05	(3.32- 87.61)
Subtotal XU3								8.74	(3.24- 23.59)
ZHENG	5	m	0	156	218	4	94	16.82	(6.05- 46.71)
ZHENG	18	f	0	43	44	33	184	5.45	(3.11- 9.54)
Subtotal ZHENG								7.07	(4.33- 11.56)
ZHOU	8	m	0	343	41	96	36	3.14	(1.90- 5.18)
ZHOU	9	f	0	35	7	42	32	3.81	(1.50- 9.68)
Subtotal ZHOU								3.28	(2.11- 5.10)
Partial Totals				14431	169158	1082	243199		

*prospective study ~ With 0.5 adjustment for zero

Table 2C4 - 5

IESLC - Meta-anal of Current Smoking (or Ever if Current not available), Cigs (or Any Prod if Cigs not avail)
Squamous
Least adjusted

REF	NRR	SEX	AD	Ys	Ws	Qs	Ps
*ABRAHA	1	m	0	4.53	0.50	2.47	0.0014
*ABRAHA	4	f	0	1.68	4.97	1.92	0.0002
Subtotal ABRAHA				1.94	5.46	4.40	
ALDERS	88	m	0	2.67	3.81	0.51	0.0000
ALDERS	84	f	0	1.97	13.33	1.42	0.0000
Subtotal ALDERS				2.13	17.14	1.92	
*ANDERS	10	f	0	3.24	4.63	4.10	0.0000
BAND	5	m	2	3.62	6.76	11.82	0.0000
BARBON	17	m	0	2.87	5.57	1.78	0.0000
BECHER	11	f	1	2.37	1.75	0.01	0.0017
*BOUCOT	70	m	0	3.27	0.49	0.46	0.0217
BRESLO	7	c	0	1.44	11.20	8.35	0.0000
BROWN2	16	m	2	2.62	150.78	15.13	0.0000
BROWN2	15	f	2	3.03	81.88	43.00	0.0000
Subtotal BROWN2				2.76	232.67	58.13	
BUFFLE	49	m	0	2.64	3.25	0.38	0.0000
BUFFLE	63	f	0	2.58	2.65	0.21	0.0000
Subtotal BUFFLE				2.62	5.90	0.59	
BYERS1	1	m	0	2.12	19.01	0.65	0.0000
CHAN	18	m	0	2.71	1.86	0.31	0.0002
CHAN	22	f	0	1.96	8.84	1.00	0.0000
Subtotal CHAN				2.09	10.69	1.32	
CHOI	62	m	0	1.70	5.39	1.97	0.0001
CHOI	64	f	0	1.94	4.25	0.56	0.0001
Subtotal CHOI				1.80	9.63	2.54	
COMSTO	23	m	0	2.43	1.79	0.03	0.0012
COMSTO	30	f	0	4.28	0.48	1.89	0.0030
Subtotal COMSTO				2.82	2.27	1.91	
CORREA	43	c	1	3.54	22.19	34.30	0.0000
*CPSI	403	m	1	3.38	0.97	1.13	0.0009
*CPSI	405	f	1	1.45	2.50	1.82	0.0222
Subtotal CPSI				1.99	3.47	2.95	
*CPSII	114	m	1	3.67	2.17	4.07	0.0000
*CPSII	117	f	1	4.37	1.49	6.36	0.0000
Subtotal CPSII				3.95	3.66	10.43	
DAMBER	12	m	0	2.47	11.02	0.31	0.0000
DESTE2	16	m	2	2.58	3.60	0.28	0.0000
DOLL	82	m	0	2.57	2.84	0.20	0.0000
DOLL	84	f	0	0.88	7.63	15.42	0.0152
Subtotal DOLL				1.34	10.47	15.62	
DORGAN	113	m	2	2.94	3.87	1.58	0.0000
DORGAN	98	f	3	2.41	20.54	0.23	0.0000
Subtotal DORGAN				2.49	24.41	1.81	
*DORN	338	m	1	2.84	9.21	2.67	0.0000
DOSEME	19	m	0	1.41	40.28	32.05	0.0000
*ENGELA	56	m	7	2.39	2.63	0.02	0.0001
FAN	3	c	0	2.46	5.45	0.13	0.0000
GAO	7	m	0	2.17	11.51	0.20	0.0000
GAO	17	f	0	1.76	23.06	6.81	0.0000
Subtotal GAO				1.89	34.57	7.01	
GER	5	c	0	0.81	7.65	17.11	0.0259
HAENSZ	20	f	0	1.11	17.71	25.24	0.0000
*HAMMON	102	m	1	3.27	3.89	3.69	0.0000
HEGMAN	2	c	0	3.43	4.70	5.98	0.0000
HINDS	23	f	3	2.78	6.93	1.60	0.0000
ISHIMA	1	c	0	2.08	3.46	0.16	0.0001
JAHN	7	m	0	3.26	2.85	2.65	0.0000
JAIN	18	m	0	3.65	1.89	3.45	0.0000
JAIN	13	f	0	3.37	5.16	5.94	0.0000
Subtotal JAIN				3.45	7.05	9.39	
JEDRYC	22	m	0	3.09	5.67	3.52	0.0000
JOLY	54	m	0	3.44	1.96	2.54	0.0000
JOLY	52	f	0	2.92	5.02	1.93	0.0000
Subtotal JOLY				3.07	6.98	4.48	
JUSSAW	25	m	0	2.36	6.65	0.02	0.0000
KATSOU	24	f	0	1.85	5.45	1.09	0.0000
KHUDER	14	m	0	2.15	7.48	0.17	0.0000
KIHARA	2	c	0	3.48	4.56	6.35	0.0000
KOO	6	f	0	1.42	14.12	10.90	0.0000
KREYBE	16	m	0	2.55	2.94	0.19	0.0000
KREYBE	33	f	0	0.29	1.19	4.83	0.7520

Table 2C4 - 5

IESLC - Meta-anal of Current Smoking (or Ever if Current not available), Cigs (or Any Prod if Cigs not avail)
Squamous
Least adjusted

REF	NRR	SEX	AD	Ys	Ws	Qs	Ps
Subtotal KREYBE				1.90	4.14	5.01	
LAMTH	1	f	0	2.09	8.66	0.38	0.0000
LAMWK	2	f	0	2.35	4.51	0.01	0.0000
LAMWK2	1	m	0	1.93	4.22	0.58	0.0001
LAMWK2	5	f	0	1.87	8.17	1.52	0.0000
Subtotal LAMWK2				1.89	12.38	2.09	
LOMBA2	2	f	0	1.45	11.86	8.68	0.0000
LUBIN	34	m	0	1.89	3.72	0.61	0.0003
LUBIN2	249	m	0	2.98	51.39	23.57	0.0000
LUBIN2	261	f	0	1.82	42.25	9.86	0.0000
Subtotal LUBIN2				2.45	93.64	33.44	
LUO	2	c	0	2.01	4.12	0.35	0.0000
MATOS	38	m	0	2.22	2.63	0.02	0.0003
MATSUD	11	m	0	3.66	0.99	1.84	0.0003
NOU	1	m	0	3.30	1.92	1.92	0.0000
NOU	6	f	0	1.96	1.40	0.16	0.0205
Subtotal NOU				2.74	3.32	2.09	
ORMOS	8	m	0	2.32	1.85	0.00	0.0016
OSANN	10	m	0	3.86	7.57	18.45	0.0000
OSANN	14	f	0	3.47	10.56	14.54	0.0000
Subtotal OSANN				3.64	18.13	32.99	
OSANN2	8	f	0	3.03	5.17	2.76	0.0000
PEZZOT	6	m	0	4.14	0.49	1.67	0.0036
SCHWAR	10	m	0	3.49	0.97	1.37	0.0006
SCHWAR	9	m	0	0.61	2.26	6.46	0.3596
SCHWAR	18	f	0	3.77	0.49	1.05	0.0086
SCHWAR	17	f	0	4.14	0.47	1.60	0.0044
Subtotal SCHWAR				2.04	4.19	10.48	
SEOW	3	f	0	2.86	4.50	1.42	0.0000
SIEMIA	11	m	0	3.37	2.87	3.29	0.0000
SOBUE	2	m	0	2.98	2.89	1.35	0.0000
SOBUE	18	f	0	2.36	8.85	0.03	0.0000
Subtotal SOBUE				2.51	11.74	1.38	
SOBUE2	1	m	2	1.65	80.57	34.24	0.0000
SOBUE2	5	f	2	1.97	23.37	2.49	0.0000
Subtotal SOBUE2				1.72	103.93	36.73	
STASZE	16	m	0	4.19	0.50	1.76	0.0032
STASZE	38	f	0	3.48	0.37	0.52	0.0333
Subtotal STASZE				3.88	0.87	2.28	
STAYNE	3	m	0	1.24	17.27	19.27	0.0000
SUZUK2	12	c	0	2.91	3.79	1.40	0.0000
SVENSS	62	f	0	2.95	3.98	1.66	0.0000
TIZZAN	18	c	0	0.99	40.59	69.31	0.0000
TOKARS	9	c	0	1.71	1.70	0.59	0.0254
TSUGAN	14	m	0	2.71	0.43	0.07	0.0743
WAKAI	4	m	0	2.29	1.88	0.00	0.0017
WAKAI	22	f	0	3.33	2.25	2.37	0.0000
Subtotal WAKAI				2.85	4.13	2.37	
WU	11	f	0	3.68	1.69	3.22	0.0000
WUWILL	9	f	3	1.44	33.59	25.16	0.0000
WYNDE2	2	m	0	3.13	2.88	2.00	0.0000
WYNDE3	10	m	0	3.19	2.81	2.21	0.0000
WYNDE3	67	f	0	1.91	3.69	0.55	0.0002
Subtotal WYNDE3				2.47	6.50	2.76	
WYNDE4	69	m	2	2.74	7.27	1.39	0.0000
WYNDE4	54	f	2	1.76	5.63	1.64	0.0000
Subtotal WYNDE4				2.31	12.90	3.03	
WYNDE6	12	m	0	3.38	26.02	30.49	0.0000
WYNDE6	201	f	0	3.44	32.63	42.64	0.0000
Subtotal WYNDE6				3.42	58.65	73.13	
XU3	19	m	0	1.78	2.46	0.67	0.0052
XU3	23	f	0	2.84	1.43	0.41	0.0007
Subtotal XU3				2.17	3.90	1.08	
ZHENG	5	m	0	2.82	3.68	1.00	0.0000
ZHENG	18	f	0	1.70	12.24	4.48	0.0000
Subtotal ZHENG				1.96	15.92	5.48	
ZHOU	8	m	0	1.14	15.27	20.45	0.0000
ZHOU	9	f	0	1.34	4.42	4.10	0.0049
Subtotal ZHOU				1.19	19.68	24.54	

Table 2C4 - 5

IESLC - Meta-anal of Current Smoking (or Ever if Current not available), Cigs (or Any Prod if Cigs not avail)
Squamous
Least adjusted

	N	110
	NS	78
	Wt	1140.63
Het	Chi	680.51
Het	df	109
Het	P	***
Fixed	RR	9.98
	RRl	9.42
	RRu	10.58
	P	+++
Random	RR	11.35
	RRl	9.62
	RRu	13.40
	P	+++
Asymm	P	N.S.

Table 2C4 - 6

IESLC - Meta-anal of Current Smoking (or Ever if Current not available), Cigs (or Any Prod if Cigs not avail)

Squamous

Least adjusted

	Sex: combined	male	female	Total
N	11	54	45	110
NS	11	53	44	108
Wt	109.42	565.48	465.74	1140.63
Het Chi	132.40	264.29	267.02	680.51
Het df	10	53	44	109
Het P	***	***	***	***
Fixed RR	7.20	10.93	9.65	9.98
RRl	5.97	10.06	8.81	9.42
RRu	8.69	11.87	10.57	10.58
P	+++	+++	+++	+++
Random RR	9.52	13.50	9.72	11.35
RRl	4.51	10.78	7.55	9.62
RRu	20.12	16.92	12.51	13.40
P	+++	+++	+++	+++
Between Chi				16.79
Between df				2
Between P				***
Btwn(F) P				N.S.
Btwn(R) P				N.S.

Table 2C4 - 7

IESLC - Meta-anal of Current Smoking (or Ever if Current not available), Cigs (or Any Prod if Cigs not avail)
Squamous
Excluded studies (and stage at which they were excluded)

1	BOUCHA BUELL LAURIL MZILEN
2	BERRIN CHEN RESTRE TAO ULMER
5	RIMING TANG2 WYNDE5 WYNDE8
6	BLOT1 BLOT2 BLOT3 BYERS2 HIRAOK HIRAY2 JONES LICKIN MOLLO MRFIT MURATA NOTANI PERSHA SAARIK SCHWA2 SHIMIZ SITAS VANDER WARSIN WATSON WYNDER
8	ABELIN AGUDO AKIBA AMANDU AMES ARCHER ARMADA AUSTIN AUVINE AXELSO AXELSS BENSHL BEST BLOHMK BLOT4 BOFFET BRETT BROCKM BROSS BROWN1 CARPEN CASCO2 CASCOR CEDERL CHANG CHATZI CHEN2 CHEN3 CHIAZZ CHOW CHYOU COOKSO DARBY DAVEYS DEAN DEAN2 DEAN3 DEKLER DESTEF DOCKER DOLL2 DORANT DROSTE DU DUNN EBELIN ENSTRO ESAKI GAO2 GARCIA GARDIN GARSHI GENG GILLIS GODLEY GOLLED GOODMA GRAHAM GREGOR GSELL HAMMO2 HANSEN HEIN HENNEK HIRAYA HITOSU HOLE HOROWI HORWIT HU HU2 HUANG HUMBLE JARUP JARVHO JIANG KAISE2 KAISER KANELL KAUFMA KELLER KINLEN KJUUS KNEKT KO KOHLME KOULUM KREUZE KUBIK LANGE LAUSSM LEI LEMARC LETOUR LEVIN LIAW LIDDEL LIU LIU2 LIU3 LIU4 LIU5 LOMBAR MACLEN MAGNUS MARSH MARSH2 MARTIS MASTRA MCCONN MCDUFF MCLAUG MIGRAN MILLER MILLS MRFITR NAM NOTAN2 ODRISC PARKIN PASTOR PAWLEG PERNU PERSH2 PETO PEZZO2 PIKE PISANI POFFIJ POLEDN PRESCO QIAO QIAO2 RACHTA RADZIK RANDIG REN RONCO ROOTS ROTHSC SADOWS SANKAR SEGI SEGI2 SHAW SIMARA SIMONA SPEIZE SPITZ STOCKS STOCKW STUCKE SUN SUZUKI TANG TENKAN TOUSEY TULINI TVERDA VEIERO WALD WANG WANG2 WANG3 WANG4 WICKLU WIGLE WILKIN WU2 WUNSCH XIANGZ XU XU2 XU4 YAMAGU YONG YUAN ZHANG
10	BENHAM VUTUC WYNDE7

Table 2C4 - 8

Potentially overlapping studies

REF	REFGP	PRINC	.	OVERLAP/LINK
LUBIN2	LUBIN2	1		Lubin-combined
CHAN	CHAN	1		Chan/LamWK2
LAMWK2	LAMWK2	1		Chan/LamWK2
LAMTH	LAMTH	1		KOO/LAMTH/LAMWK
KOO	KOO	1		KOO/LAMTH/LAMWK
LAMWK	LAMWK	1		KOO/LAMTH/LAMWK
ISHIMA	AKIBA	2		AKIBA/ISHIMA
BYERS1	BYERS1	2		GRAHAM/BROSS/BYERS1
OSANN2	KAISER	2		KAISER/OSANN2
WYNDE6	WYNDE6	1		WYNDE5/6/7/8
CPSI	CPSI	1		CPSI overall
SOBUE2	SOBUE2	1		SOBUE2/MATSUD
MATSUD	MATSUD	1		SOBUE2/MATSUD
JAHN	BOFFET	2		Subset of BOFFET
LUBIN	XIANGZ	2		LUBIN/XIANGZ/QIAO

Table 2C4 - 9

Most adjusted - insufficient data for metaanalysis

REF	NRR	SEX	AGEL	AGEH	RACE	YF	LC TYPE	LOC	START	ST	NLC	R	VB	P	H	AD	SM	PRODUCT	DENOM	De
CHEN	1	c	0	0	all	-	q	As:oth	1987	CC	323	n	ot	n	y	2	ev	cig+/-ot	nev cigs	ot
CHYOU	5	m	0	0	jap	0	q+s	NAmer	1965	pr	227	n	bl	n	y	1	cu	cig+/-ot	nev cigs	or
JIANG	3	m	0	0	all	-	q	As:Chi	1984	CC	125	n	ot	n	n	0	ev	all/unsp	nev any	or
JIANG	4	f	0	0	all	-	q	As:Chi	1984	CC	125	n	ot	n	n	0	ev	all/unsp	nev any	or
KOO	2	f	0	0	all	-	q	As:HK	1981	CC	200	n	bl	n	n	0	ev	all/unsp	nev any	or
WANG	3	c	0	0	all	-	q	As:Chi	1990	CC	390	n	ot	*	y	0	ev	all/unsp	nev any	ot

REF	NRR	RR	SIG	RRDATA comment
CHEN	1	6.66	y	P < 0.001
CHYOU	5	16.00		0
JIANG	3	12.78		0
JIANG	4	*	n	0
KOO	2	3.62		0
WANG	3	4.66	y	p<0.01

Table 2C5 -

IESLC - Meta-anal of Ever Smoking (or Current if Ever not available), Cigarettes only
Squamous

This analysis is restricted to results for:
1) Non-dose-response data
2) Results complete enough for use in metaanalysis

Within each study, results are then selected (in the following order of preference, within each sex) for:
3) SMKSTA: ever smokers, current smokers
4) PRODUCT: cigarettes only
5) CIGTYPE: all/unspecified, MC regardless of HR, MC only
6) DENOM: never smoked anything, never smoked cigarettes, (never +1 = +long term ex, +2 = +amount unknown,
+3 = never cigs+long term ex)
7) Followup period (YF, prospective studies): whole study (coded as 0) or longest available
8) LCtype: squamous or nearest available, but not adeno. (q = squamous, s = small,
a = adeno, KI = Kreyberg I, u = undifferentiated)
9) Race: all or nearest available, otherwise by race (wh or w = white, bl or b = black, hi = hispanic
ch = chinese, jap = japanese, haw = hawaiian, w+o = white + oriental, sca = scandinavian, as = asian)
10) For overlapping studies: principal rather than subsidiary studies
Finally by Age: whole study (coded as 0) if available, otherwise by widest available age group
and then for single sex results (m, f) in preference to combined sex results (c).

Results adjusted (AD) for the most potential confounders are then chosen in Sections -1 to -3
(and those which actually differ from the adjusted results in Table 2C1 - 1 are marked 'x' in Section -1)
and results adjusted for the least confounders in Sections -4 to -6. (Those least adjusted results which
actually differ from the most adjusted as marked 'x' in column X in Section -4)
(Results adjusted for an unknown number of confounder(s) are coded as 20.)

Section -7 shows excluded studies, together with the stage (as above) at which no qualifying
results were found.

Section -8 lists the potentially overlapping studies which have been included (1=principal, 2=subsidiary).

Section -9 lists any results which would have been included in preference except that they had data not complete
enough for use in meta-analysis, with their significance (yes/no), if known, and any further comment as entered
on the database.

In addition to those mentioned above, the following fields, levels and abbreviations are used:
* or nk = not known, n = no, y = yes, ot = other
ev = ever, cu = current, nev = never
all/unspec = all or unspecified, MC = manufactured cigarettes, HR = hand-rolled cigarettes
REF: 6-character study reference
NRR: number of the RR on the database within the study
ST : study type (CC = case control, pr or prosp = prospective)
NLC: number of lung cancer cases in whole study
R : risky occupational population (n = no, m = mining, o = other risky)
VB : national cigarette type (V = at least 75% Virginia, bl = at least 75% blended, ot = other)
P : any proxy use
H : full histological confirmation
De : derivation of RR/CI (or = original, st = standard method, ot = other method of estimation)

Table 2C5 - 1

IESLC - Meta-anal of Ever Smoking (or Current if Ever not available), Cigarettes only
Squamous
Most adjusted

REF	NRR	2C1	SEX	AGEL	AGEH	RACE	YF	LC TYPE	LOC	START	ST	NLC	R	VB	P	H	AD	SM	PRODUCT	DENOM	De
ALDERS	74	x	m	0	0	all	-	q+s	Eu:UK	1977	CC	1448	n	V	n	n	1	ev	cig only	nev any	ot
ALDERS	33	x	f	0	0	all	-	q+s	Eu:UK	1977	CC	1448	n	V	n	n	1	ev	cig only	nev any	ot
BAND	5		m	0	0	all	-	q	NAmer	1983	CC	2831	n	V	y	y	2	ev	cig only	nev any	ot
BOUCOT	141		m	0	0	all	0	q	NAmer	1951	pr	121	n	bl	n	n	2	cu	cig only	nev any	ot
BRESLO	8	x	c	0	0	all	-	not a	NAmer	1949	CC	518	n	bl	n	y	0	ev	cig only	nev+1	st
CPSI	403		m	0	0	all	2	q	NAmer	1959	pr	5138	n	bl	n	n	1	cu	cig only	nev any	ot
CPSI	405		f	0	0	all	2	q	NAmer	1959	pr	5138	n	bl	n	n	1	cu	cig only	nev any	ot
CPSII	114		m	0	0	all	2	q	NAmer	1982	pr	3229	n	bl	n	n	1	cu	cig only	nev any	ot
DORN	338		m	0	0	wh	8	q	NAmer	1954	pr	5097	n	bl	n	n	1	cu	cig only	nev any	ot
HAMMON	58	x	m	0	0	wh	0	not a	NAmer	1952	pr	448	n	bl	n	n	1	ev	cig only	nev any	ot
JUSSAW	25	x	m	0	0	all	-	KI	As:Ind	1964	CC	792	n	V	n	n	0	ev	cig only	nev any	st
LUBIN	15	x	m	0	0	all	-	KI	As:Chi	1984	CC	427	m	ot	y	n	0	ev	cig only	nev any	st
LUBIN2	141	x	m	0	0	all	-	q	Eu:mul	1976	CC	7804	n	bl	n	y	0	ev	cig only	nev any	st
PEZZOT	6		m	0	0	all	-	q	SCAmer	1987	CC	215	n	bl	n	y	0	ev	cig only	nev cigs	ot
STASZE	13	x	m	0	0	all	-	q	Eu:est	1954	CC	281	n	bl	n	y	0	ev	cig only	nev any	ot
WYNDE7	59	x	m	0	0	all	-	KI	NAmer	1977	CC	2085	n	bl	n	y	0	ev	cig only	nev any	st

Cigarette type is all/unspec for all RRs

except for the following:

REF	NRR	CIGTYPE
ALDERS	33	MC only
JUSSAW	25	MC only

Table 2C5 - 2

IESLC - Meta-anal of Ever Smoking (or Current if Ever not available), Cigarettes only
Squamous
Most adjusted

REF	NRR	SEX	AD	Number Exposed Case	Number Exposed Cont	Non-exposed Case	Non-exposed Cont	RR	95.00%CI
ALDERS	74	m	1	-	-	-	-	11.58	(4.25- 31.54)
ALDERS	33	f	1	-	-	-	-	6.70	(3.92- 11.46)
Subtotal ALDERS								7.57	(4.72- 12.14)
BAND	5	m	2	-	-	-	-	37.45	(17.62- 79.58)
*BOUCOT	141	m	2	-	-	-	-	27.54	(1.69- 448.37)
BRESLO	8	c	0	298	240	15	56	4.64	(2.56- 8.40)
*CPSI	403	m	1	-	-	-	-	29.35	(4.02- 214.28)
*CPSI	405	f	1	-	-	-	-	4.25	(1.23- 14.68)
Subtotal CPSI								7.30	(2.55- 20.90)
*CPSII	114	m	1	-	-	-	-	39.26	(10.38- 148.55)
*DORN	338	m	1	-	-	-	-	17.09	(8.96- 32.60)
*HAMMON	58	m	1	-	-	-	-	23.12	(8.57- 62.34)
JUSSAW	25	m	0	17	77	13	624	10.60	(4.96- 22.66)
LUBIN	15	m	0	42	191	4	72	3.96	(1.37- 11.43)
LUBIN2	141	m	0	3474	9345	127	2616	7.66	(6.38- 9.19)
PEZZOT	6	m	0	85	317	0	116	62.74~	(3.86-1019.50)
STASZE	13	m	0	117	552	0	158	67.42~	(4.17-1090.28)
WYNDE7	59	m	0	1000	2108	22	918	19.79	(12.88- 30.41)
Partial Totals				5033	12830	181	4560		

*prospective study ~ With 0.5 adjustment for zero

REF	NRR	SEX	AD	Ys	Ws	Qs	Ps
ALDERS	74	m	1	2.45	3.82	0.16	0.0000
ALDERS	33	f	1	1.90	13.35	1.59	0.0000
Subtotal ALDERS				2.02	17.18	1.75	
BAND	5	m	2	3.62	6.76	12.80	0.0000
*BOUCOT	141	m	2	3.32	0.49	0.56	0.0199
BRESLO	8	c	0	1.53	10.86	5.53	0.0000
*CPSI	403	m	1	3.38	0.97	1.25	0.0009
*CPSI	405	f	1	1.45	2.50	1.60	0.0222
Subtotal CPSI				1.99	3.47	2.85	
*CPSII	114	m	1	3.67	2.17	4.39	0.0000
*DORN	338	m	1	2.84	9.21	3.22	0.0000
*HAMMON	58	m	1	3.14	3.90	3.12	0.0000
JUSSAW	25	m	0	2.36	6.65	0.09	0.0000
LUBIN	15	m	0	1.38	3.41	2.59	0.0110
LUBIN2	141	m	0	2.04	115.59	5.17	0.0000
PEZZOT	6	m	0	4.14	0.49	1.77	0.0036
STASZE	13	m	0	4.21	0.50	1.91	0.0030
WYNDE7	59	m	0	2.99	20.83	11.35	0.0000

	N	16
	NS	14
	Wt	201.52
Het	Chi	57.09
Het	df	15
Het	P	***
Fixed	RR	9.46
	RRl	8.24
	RRu	10.86
	P	+++
Random	RR	12.46
	RRl	8.54
	RRu	18.18
	P	+++
Asymm	P	(*)

Table 2C5 - 3

IESLC - Meta-anal of Ever Smoking (or Current if Ever not available), Cigarettes only
Squamous
Most adjusted

		combined	Sex<br>male	female	Total
	N	1	13	2	16
	NS	1	13	2	16
	Wt	10.86	174.81	15.85	201.52
Het	Chi	0.00	47.19	0.44	57.09
Het	df	0	12	1	15
Het	P	N.S.	***	N.S.	***
Fixed	RR	4.64	10.27	6.24	9.46
	RRl	2.56	8.85	3.81	8.24
	RRu	8.40	11.91	10.20	10.86
	P	+++	+++	+++	+++
Random	RR	4.64	16.38	6.24	12.46
	RRl	2.56	10.42	3.81	8.54
	RRu	8.40	25.75	10.20	18.18
	P	+++	+++	+++	+++
Between	Chi				9.46
Between	df				2
Between	P				**
Btwn(F)	P				N.S.
Btwn(R)	P				**

		q	Lung cancer type<br>q+s	q+u	KI	not a	Total
	N	9	2		3	2	16
	NS	8	1		3	2	14
	Wt	138.69	17.18		30.89	14.77	201.52
Het	Chi	32.49	0.89		8.43	7.41	57.09
Het	df	8	1		2	1	15
Het	P	***	N.S.		*	**	***
Fixed	RR	9.12	7.57		14.48	7.09	9.46
	RRl	7.72	4.72		10.18	4.26	8.24
	RRu	10.77	12.14		20.61	11.80	10.86
	P	+++	+++		+++	+++	+++
Random	RR	18.51	7.57		10.49	9.84	12.46
	RRl	9.32	4.72		4.45	2.04	8.54
	RRu	36.76	12.14		24.73	47.36	18.18
	P	+++	+++		+++	++	+++
Between	Chi						7.87
Between	df						3
Between	P						*
Btwn(F)	P						N.S.
Btwn(R)	P						N.S.

		NAmer	UK	Scand	Location<br>othEur	China	Japan	othAs	other	Total
	N	9	2		2	1		1	1	16
	NS	8	1		2	1		1	1	14
	Wt	57.70	17.18		116.09	3.41		6.65	0.49	201.52
Het	Chi	29.67	0.89		2.34	0.00		0.00	0.00	57.09
Het	df	8	1		1	0		0	0	15
Het	P	***	N.S.		N.S.	N.S.		N.S.	N.S.	***
Fixed	RR	15.52	7.57		7.73	3.96		10.60	62.74	9.46
	RRl	11.99	4.72		6.44	1.37		4.96	3.86	8.24
	RRu	20.09	12.14		9.27	11.43		22.66	1019.50	10.86
	P	+++	+++		+++	+		+++	++	+++
Random	RR	16.38	7.57		14.32	3.96		10.60	62.74	12.46
	RRl	9.21	4.72		2.08	1.37		4.96	3.86	8.54
	RRu	29.12	12.14		98.68	11.43		22.66	1019.50	18.18
	P	+++	+++		++	+		+++	++	+++
Between	Chi									24.20
Between	df									5
Between	P									***
Btwn(F)	P									N.S.
Btwn(R)	P									N.S.

Table 2C5 - 3

IESLC - Meta-anal of Ever Smoking (or Current if Ever not available), Cigarettes only
Squamous
Most adjusted

Detailed Country in "other Europe"

	multi	Germany	othWest	East	Balkans	Total
N	1			1		2
NS	1			1		2
Wt	115.59			0.50		116.09
Het Chi	0.00			0.00		2.34
Het df	0			0		1
Het P	N.S.			N.S.		N.S.
Fixed RR	7.66			67.42		7.73
RRl	6.38			4.17		6.44
RRu	9.19			1090.28		9.27
P	+++			++		+++
Random RR	7.66			67.42		14.32
RRl	6.38			4.17		2.08
RRu	9.19			1090.28		98.68
P	+++			++		++
Between Chi						2.34
Between df						1
Between P						N.S.
Btwn(F) P						N.S.
Btwn(R) P						N.S.

Detailed Country in "other Asia"

	India	HongKong	other	Total
N	1			1
NS	1			1
Wt	6.65			6.65
Het Chi	0.00			0.00
Het df	0			0
Het P	N.S.			N.S.
Fixed RR	10.60			10.60
RRl	4.96			4.96
RRu	22.66			22.66
P	+++			+++
Random RR	10.60			10.60
RRl	4.96			4.96
RRu	22.66			22.66
P	+++			+++
Between Chi				
Between df				
Between P				N.S.
Btwn(F) P				N.S.
Btwn(R) P				N.S.

Detailed other continent

	SCAmer	Auslia	Africa	Total
N	1			1
NS	1			1
Wt	0.49			0.49
Het Chi	0.00			0.00
Het df	0			0
Het P	N.S.			N.S.
Fixed RR	62.74			62.74
RRl	3.86			3.86
RRu	1019.50			1019.50
P	++			++
Random RR	62.74			62.74
RRl	3.86			3.86
RRu	1019.50			1019.50
P	++			++
Between Chi				
Between df				
Between P				N.S.
Btwn(F) P				N.S.
Btwn(R) P				N.S.

Table 2C5 - 3

IESLC - Meta-anal of Ever Smoking (or Current if Ever not available), Cigarettes only
Squamous
Most adjusted

	Start year of study <1960	1960-69	1970-79	1980-89	1990+	Total
N	7	1	4	4		16
NS	6	1	3	4		14
Wt	28.44	6.65	153.59	12.84		201.52
Het Chi	17.08	0.00	17.18	13.21		57.09
Het df	6	0	3	3		15
Het P	**	N.S.	***	**		***
Fixed RR	10.07	10.60	8.70	21.18		9.46
RRl	6.98	4.96	7.43	12.26		8.24
RRu	14.55	22.66	10.19	36.61		10.86
P	+++	+++	+++	+++		+++
Random RR	12.59	10.60	10.25	21.33		12.46
RRl	5.96	4.96	5.98	5.72		8.54
RRu	26.61	22.66	17.56	79.61		18.18
P	+++	+++	+++	+++		+++
Between Chi						9.62
Between df						3
Between P						*
Btwn(F) P						N.S.
Btwn(R) P						N.S.

	Study type (1) CC	other	Total
N	10	6	16
NS	9	5	14
Wt	182.27	19.25	201.52
Het Chi	42.20	7.09	57.09
Het df	9	5	15
Het P	***	N.S.	***
Fixed RR	8.87	17.33	9.46
RRl	7.68	11.09	8.24
RRu	10.26	27.09	10.86
P	+++	+++	+++
Random RR	10.78	17.49	12.46
RRl	6.90	9.67	8.54
RRu	16.85	31.64	18.18
P	+++	+++	+++
Between Chi			7.80
Between df			1
Between P			**
Btwn(F) P			N.S.
Btwn(R) P			N.S.

	Study type (2) CC	prosp	other	Total
N	10	6		16
NS	9	5		14
Wt	182.27	19.25		201.52
Het Chi	42.20	7.09		57.09
Het df	9	5		15
Het P	***	N.S.		***
Fixed RR	8.87	17.33		9.46
RRl	7.68	11.09		8.24
RRu	10.26	27.09		10.86
P	+++	+++		+++
Random RR	10.78	17.49		12.46
RRl	6.90	9.67		8.54
RRu	16.85	31.64		18.18
P	+++	+++		+++
Between Chi				7.80
Between df				1
Between P				**
Btwn(F) P				N.S.
Btwn(R) P				N.S.

Table 2C5 - 3

IESLC - Meta-anal of Ever Smoking (or Current if Ever not available), Cigarettes only
Squamous
Most adjusted

	Study size (number of LC cases) 100-249	250-499	500-999	1000+	Total
N	2	3	2	9	16
NS	2	3	2	7	14
Wt	0.99	7.81	17.52	175.21	201.52
Het Chi	0.17	7.34	2.82	41.43	57.09
Het df	1	2	1	8	15
Het P	N.S.	*	(*)	***	***
Fixed RR	41.58	11.44	6.35	9.68	9.46
RRl	5.79	5.68	3.97	8.35	8.24
RRu	298.87	23.07	10.14	11.23	10.86
P	+++	+++	+++	+++	+++
Random RR	41.58	13.93	6.77	13.82	12.46
RRl	5.79	3.01	3.02	8.55	8.54
RRu	298.87	64.45	15.17	22.34	18.18
P	+++	+++	+++	+++	+++
Between Chi					5.33
Between df					3
Between P					N.S.
Btwn(F) P					N.S.
Btwn(R) P					N.S.

	Risky occupational population no	mining	othRisky	Total
N	15	1		16
NS	13	1		14
Wt	198.11	3.41		201.52
Het Chi	54.45	0.00		57.09
Het df	14	0		15
Het P	***	N.S.		***
Fixed RR	9.60	3.96		9.46
RRl	8.35	1.37		8.24
RRu	11.04	11.43		10.86
P	+++	+		+++
Random RR	13.41	3.96		12.46
RRl	9.09	1.37		8.54
RRu	19.78	11.43		18.18
P	+++	+		+++
Between Chi				2.64
Between df				1
Between P				N.S.
Btwn(F) P				N.S.
Btwn(R) P				*

	National cigarette tobacco type Virginia	blended	other	Total
N	4	11	1	16
NS	3	10	1	14
Wt	30.59	167.52	3.41	201.52
Het Chi	13.36	39.81	0.00	57.09
Het df	3	10	0	15
Het P	**	***	N.S.	***
Fixed RR	11.59	9.28	3.96	9.46
RRl	8.13	7.97	1.37	8.24
RRu	16.53	10.80	11.43	10.86
P	+++	+++	+	+++
Random RR	13.02	13.81	3.96	12.46
RRl	5.96	8.43	1.37	8.54
RRu	28.48	22.61	11.43	18.18
P	+++	+++	+	+++
Between Chi				3.92
Between df				2
Between P				N.S.
Btwn(F) P				N.S.
Btwn(R) P				N.S.

Table 2C5 - 3

IESLC - Meta-anal of Ever Smoking (or Current if Ever not available), Cigarettes only
Squamous
Most adjusted

	Any proxy use No/nk	Yes	Total
N	14	2	16
NS	12	2	14
Wt	191.35	10.17	201.52
Het Chi	41.49	11.45	57.09
Het df	13	1	15
Het P	***	***	***
Fixed RR	9.15	17.62	9.46
RRl	7.94	9.53	8.24
RRu	10.55	32.57	10.86
P	+++	+++	+++
Random RR	11.93	12.57	12.46
RRl	8.23	1.39	8.54
RRu	17.29	113.64	18.18
P	+++	+	+++
Between Chi			4.14
Between df			1
Between P			*
Btwn(F) P			N.S.
Btwn(R) P			N.S.

	Full histological confirmation No	Yes	Total
N	10	6	16
NS	8	6	14
Wt	46.49	155.03	201.52
Het Chi	17.89	38.32	57.09
Het df	9	5	15
Het P	*	***	***
Fixed RR	10.67	9.12	9.46
RRl	8.00	7.80	8.24
RRu	14.22	10.68	10.86
P	+++	+++	+++
Random RR	11.45	14.63	12.46
RRl	7.32	7.20	8.54
RRu	17.92	29.70	18.18
P	+++	+++	+++
Between Chi			0.88
Between df			1
Between P			N.S.
Btwn(F) P			N.S.
Btwn(R) P			N.S.

	Number of adjustment variables (1) 0	1	2+/+nk	Total
N	7	7	2	16
NS	7	5	2	14
Wt	158.34	35.93	7.25	201.52
Het Chi	26.55	13.85	0.04	57.09
Het df	6	6	1	15
Het P	***	*	N.S.	***
Fixed RR	8.49	11.59	36.68	9.46
RRl	7.27	8.36	17.71	8.24
RRu	9.92	16.07	75.93	10.86
P	+++	+++	+++	+++
Random RR	9.49	13.18	36.68	12.46
RRl	5.59	7.58	17.71	8.54
RRu	16.13	22.93	75.93	18.18
P	+++	+++	+++	+++
Between Chi				16.65
Between df				2
Between P				***
Btwn(F) P				N.S.
Btwn(R) P				*

Table 2C5 - 3

IESLC - Meta-anal of Ever Smoking (or Current if Ever not available), Cigarettes only
Squamous
Most adjusted

	Number of adjustment variables (2) 0	1	2	3-5	6+/+nk	Total
N	7	7	2			16
NS	7	5	2			14
Wt	158.34	35.93	7.25			201.52
Het Chi	26.55	13.85	0.04			57.09
Het df	6	6	1			15
Het P	***	*	N.S.			***
Fixed RR	8.49	11.59	36.68			9.46
RRl	7.27	8.36	17.71			8.24
RRu	9.92	16.07	75.93			10.86
P	+++	+++	+++			+++
Random RR	9.49	13.18	36.68			12.46
RRl	5.59	7.58	17.71			8.54
RRu	16.13	22.93	75.93			18.18
P	+++	+++	+++			+++
Between Chi						16.65
Between df						2
Between P						***
Btwn(F) P						N.S.
Btwn(R) P						*

	Product all/unsp	cig+/-ot	cig only	Total
N			16	16
NS			14	14
Wt			201.52	201.52
Het Chi			57.09	57.09
Het df			15	15
Het P			***	***
Fixed RR			9.46	9.46
RRl			8.24	8.24
RRu			10.86	10.86
P			+++	+++
Random RR			12.46	12.46
RRl			8.54	8.54
RRu			18.18	18.18
P			+++	+++
Between Chi				
Between df				
Between P				N.S.
Btwn(F) P				N.S.
Btwn(R) P				N.S.

	Denominator nev any	nev cigs	Total
N	15	1	16
NS	13	1	14
Wt	201.03	0.49	201.52
Het Chi	55.32	0.00	57.09
Het df	14	0	15
Het P	***	N.S.	***
Fixed RR	9.42	62.74	9.46
RRl	8.20	3.86	8.24
RRu	10.81	1019.50	10.86
P	+++	++	+++
Random RR	12.13	62.74	12.46
RRl	8.31	3.86	8.54
RRu	17.72	1019.50	18.18
P	+++	++	+++
Between Chi			1.77
Between df			1
Between P			N.S.
Btwn(F) P			N.S.
Btwn(R) P			N.S.

Table 2C5 - 3

IESLC - Meta-anal of Ever Smoking (or Current if Ever not available), Cigarettes only
Squamous
Most adjusted

	Derivation of RR/CI			
	Orig	StdCalc	Other	Total
N		5	11	16
NS		5	9	14
Wt		157.35	44.17	201.52
Het Chi		22.42	24.17	57.09
Het df		4	10	15
Het P		***	**	***
Fixed RR		8.38	14.55	9.46
RRl		7.17	10.84	8.24
RRu		9.80	19.54	10.86
P		+++	+++	+++
Random RR		8.36	17.37	12.46
RRl		4.93	10.18	8.54
RRu		14.20	29.64	18.18
P		+++	+++	+++
Between Chi				10.50
Between df				1
Between P				**
Btwn(F) P				(*)
Btwn(R) P				(*)

	Smoking status		
	ever	current	Total
N	11	5	16
NS	10	4	14
Wt	186.18	15.35	201.52
Het Chi	45.71	6.68	57.09
Het df	10	4	15
Het P	***	N.S.	***
Fixed RR	9.05	16.10	9.46
RRl	7.84	9.76	8.24
RRu	10.45	26.56	10.86
P	+++	+++	+++
Random RR	11.50	16.35	12.46
RRl	7.47	7.53	8.54
RRu	17.69	35.47	18.18
P	+++	+++	+++
Between Chi			4.70
Between df			1
Between P			*
Btwn(F) P			N.S.
Btwn(R) P			N.S.

Table 2C5 - 4

IESLC - Meta-anal of Ever Smoking (or Current if Ever not available), Cigarettes only
Squamous
Least adjusted

REF	NRR	X	SEX	AGEL	AGEH	RACE	YF	LC TYPE	LOC	START	ST	NLC	R	VB	P	H	AD	SM	PRODUCT	DENOM	De
ALDERS	87	x	m	0	0	all	-	q+s	Eu:UK	1977	CC	1448	n	V	n	n	0	ev	cig only	nev any	st
ALDERS	84	x	f	0	0	all	-	q+s	Eu:UK	1977	CC	1448	n	V	n	n	0	ev	cig only	nev any	st
BAND	5		m	0	0	all	-	q	NAmer	1983	CC	2831	n	V	y	y	2	ev	cig only	nev any	ot
BOUCOT	70	x	m	0	0	all	0	q	NAmer	1951	pr	121	n	bl	n	n	0	cu	cig only	nev any	ot
BRESLO	8		c	0	0	all	-	not a	NAmer	1949	CC	518	n	bl	n	y	0	ev	cig only	nev+1	st
CPSI	403		m	0	0	all	2	q	NAmer	1959	pr	5138	n	bl	n	n	1	cu	cig only	nev any	ot
CPSI	405		f	0	0	all	2	q	NAmer	1959	pr	5138	n	bl	n	n	1	cu	cig only	nev any	ot
CPSII	114		m	0	0	all	2	q	NAmer	1982	pr	3229	n	bl	n	n	1	cu	cig only	nev any	ot
DORN	338		m	0	0	wh	8	q	NAmer	1954	pr	5097	n	bl	n	n	1	cu	cig only	nev any	ot
HAMMON	71	x	m	0	0	wh	0	not a	NAmer	1952	pr	448	n	bl	n	n	0	ev	cig only	nev any	st
JUSSAW	25		m	0	0	all	-	KI	As:Ind	1964	CC	792	n	V	n	n	0	ev	cig only	nev any	st
LUBIN	15		m	0	0	all	-	KI	As:Chi	1984	CC	427	m	ot	y	n	0	ev	cig only	nev any	st
LUBIN2	141		m	0	0	all	-	q	Eu:mul	1976	CC	7804	n	bl	n	y	0	ev	cig only	nev any	st
PEZZOT	6		m	0	0	all	-	q	SCAmer	1987	CC	215	n	bl	n	y	0	ev	cig only	nev cigs	ot
STASZE	13		m	0	0	all	-	q	Eu:est	1954	CC	281	n	bl	n	y	0	ev	cig only	nev any	ot
WYNDE7	59		m	0	0	all	-	KI	NAmer	1977	CC	2085	n	bl	n	y	0	ev	cig only	nev any	st

Cigarette type is all/unspec for all RRs

except for the following:

REF	NRR	CIGTYPE
ALDERS	84	MC only
JUSSAW	25	MC only

Table 2C5 - 5

IESLC - Meta-anal of Ever Smoking (or Current if Ever not available), Cigarettes only
Squamous
Least adjusted

REF	NRR	SEX	AD	Number Exposed Case	Number Exposed Cont	Non-exposed Case	Non-exposed Cont	RR	95.00%CI
ALDERS	87	m	0	207	462	4	133	14.90	(5.44- 40.82)
ALDERS	84	f	0	176	371	16	243	7.20	(4.21- 12.32)
Subtotal ALDERS								8.46	(5.27- 13.59)
BAND	5	m	2	-	-	-	-	37.45	(17.62- 79.58)
*BOUCOT	70	m	0	38	22177	0	7551	26.22~	(1.61- 426.71)
BRESLO	8	c	0	298	240	15	56	4.64	(2.56- 8.40)
*CPSI	403	m	1	-	-	-	-	29.35	(4.02- 214.28)
*CPSI	405	f	1	-	-	-	-	4.25	(1.23- 14.68)
Subtotal CPSI								7.30	(2.55- 20.90)
*CPSII	114	m	1	-	-	-	-	39.26	(10.38- 148.55)
*DORN	338	m	1	-	-	-	-	17.09	(8.96- 32.60)
*HAMMON	71	m	0	162	225565	4	115884	20.81	(7.72- 56.11)
JUSSAW	25	m	0	17	77	13	624	10.60	(4.96- 22.66)
LUBIN	15	m	0	42	191	4	72	3.96	(1.37- 11.43)
LUBIN2	141	m	0	3474	9345	127	2616	7.66	(6.38- 9.19)
PEZZOT	6	m	0	85	317	0	116	62.74~	(3.86-1019.50)
STASZE	13	m	0	117	552	0	158	67.42~	(4.17-1090.28)
WYNDE7	59	m	0	1000	2108	22	918	19.79	(12.88- 30.41)
Partial Totals				5616	261405	205	128371		

*prospective study ~ With 0.5 adjustment for zero

REF	NRR	SEX	AD	Ys	Ws	Qs	Ps
ALDERS	87	m	0	2.70	3.78	0.75	0.0000
ALDERS	84	f	0	1.97	13.33	1.04	0.0000
Subtotal ALDERS				2.14	17.12	1.80	
BAND	5	m	2	3.62	6.76	12.66	0.0000
*BOUCOT	70	m	0	3.27	0.49	0.51	0.0217
BRESLO	8	c	0	1.53	10.86	5.64	0.0000
*CPSI	403	m	1	3.38	0.97	1.23	0.0009
*CPSI	405	f	1	1.45	2.50	1.63	0.0222
Subtotal CPSI				1.99	3.47	2.86	
*CPSII	114	m	1	3.67	2.17	4.35	0.0000
*DORN	338	m	1	2.84	9.21	3.14	0.0000
*HAMMON	71	m	0	3.04	3.90	2.38	0.0000
JUSSAW	25	m	0	2.36	6.65	0.07	0.0000
LUBIN	15	m	0	1.38	3.41	2.64	0.0110
LUBIN2	141	m	0	2.04	115.59	5.53	0.0000
PEZZOT	6	m	0	4.14	0.49	1.76	0.0036
STASZE	13	m	0	4.21	0.50	1.90	0.0030
WYNDE7	59	m	0	2.99	20.83	11.13	0.0000

	N	16
	NS	14
	Wt	201.46
Het	Chi	56.36
Het	df	15
Het	P	***
Fixed	RR	9.53
	RRl	8.30
	RRu	10.94
	P	+++
Random	RR	12.64
	RRl	8.68
	RRu	18.40
	P	+++
Asymm	P	(*)

Table 2C5 - 6

IESLC - Meta-anal of Ever Smoking (or Current if Ever not available), Cigarettes only
Squamous
Least adjusted

	Sex			
	combined	male	female	Total
N	1	13	2	16
NS	1	13	2	16
Wt	10.86	174.76	15.83	201.46
Het Chi	0.00	46.99	0.59	56.36
Het df	0	12	1	15
Het P	N.S.	***	N.S.	***
Fixed RR	4.64	10.30	6.63	9.53
RRl	2.56	8.88	4.05	8.30
RRu	8.40	11.95	10.85	10.94
P	+++	+++	+++	+++
Random RR	4.64	16.56	6.63	12.64
RRl	2.56	10.54	4.05	8.68
RRu	8.40	26.01	10.85	18.40
P	+++	+++	+++	+++
Between Chi				8.78
Between df				2
Between P				*
Btwn(F) P				N.S.
Btwn(R) P				**

Table 2C5 - 7

IESLC - Meta-anal of Ever Smoking (or Current if Ever not available), Cigarettes only
Squamous
Excluded studies (and stage at which they were excluded)

1	BOUCHA BUELL LAURIL MZILEN
2	BERRIN CHEN RESTRE TAO ULMER
4	ABRAHA AKIBA AMANDU AMES ANDERS ARCHER AUSTIN AUVINE AXELSO AXELSS BARBON BECHER BENSHL BLOHMK BLOT1 BLOT2 BLOT3 BLOT4 BRETT BROCKM BROSS BROWN1 BROWN2 BUFFLE BYERS1 BYERS2 CARPEN CASCO2 CASCOR CHAN CHANG CHATZI CHEN2 CHEN3 CHIAZZ CHOI CHYOU COMSTO COOKSO CORREA DARBY DAVEYS DEKLER DESTE2 DESTEF DOCKER DORANT DORGAN DOSEME DROSTE DU DUNN EBELIN ESAKI FAN GAO GAO2 GARCIA GARSHI GENG GER GILLIS GODLEY GOODMA GREGOR GSELL HAENSZ HAMMO2 HANSEN HEGMAN HENNEK HINDS HIRAOK HIRAYA HITOSU HOLE HOROWI HORWIT HU HU2 HUANG HUMBLE ISHIMA JAHN JAIN JARUP JARVHO JEDRYC JIANG JONES KAISER KANELL KATSOU KAUFMA KELLER KHUDER KIHARA KINLEN KNEKT KO KOHLME KOO KREUZE KREYBE KUBIK LAMTH LAMWK LAMWK2 LAUSSM LEI LEMARC LETOUR LEVIN LIAW LICKIN LIDDEL LIU LIU2 LIU3 LIU5 LOMBA2 LUO MACLEN MAGNUS MARSH MARSH2 MARTIS MASTRA MATOS MATSUD MCDUFF MCLAUG MILLER MOLLO MRFIT MRFITR MURATA NAM NOTANI NOU ODRISC ORMOS OSANN OSANN2 PARKIN PASTOR PAWLEG PERSH2 PERSHA PETO PEZZO2 PIKE PISANI POFFIJ POLEDN PRESCO QIAO2 RACHTA RADZIK RANDIG REN ROOTS ROTHSC SAARIK SANKAR SCHWAR SEGI SEGI2 SEOW SHAW SHIMIZ SIEMIA SIMARA SIMONA SITAS SOBUE SOBUE2 SPEIZE SPITZ STAYNE STOCKS STOCKW STUCKE SUN SUZUKI SVENSS TANG TENKAN TOKARS TOUSEY TSUGAN TULINI VANDER VUTUC WAKAI WANG WANG2 WANG3 WANG4 WARSIN WATSON WICKLU WILKIN WU WU2 WUNSCH WUWILL WYNDE2 WYNDE3 WYNDE4 WYNDE5 WYNDE6 WYNDE8 XU XU2 XU3 XU4 YAMAGU YONG YUAN ZHANG ZHENG ZHOU
5	LANGE RIMING TANG2
6	HIRAY2 SCHWA2 WYNDER
8	ABELIN AGUDO ARMADA BEST BOFFET CEDERL CHOW DAMBER DEAN DEAN2 DEAN3 DOLL DOLL2 ENGELA ENSTRO GARDIN GOLLED GRAHAM HEIN JOLY KAISE2 KJUUS KOULUM LIU4 LOMBAR MCCONN MIGRAN MILLS NOTAN2 PERNU QIAO RONCO SADOWS SUZUK2 TIZZAN TVERDA VEIERO WALD WIGLE XIANGZ
10	BENHAM

Table 2C5 - 8

Potentially overlapping studies

REF	REFGP	PRINC	OVERLAP/LINK
LUBIN2	LUBIN2	1	Lubin-combined
WYNDE7	WYNDE6	2	WYNDE5/6/7/8
CPSI	CPSI	1	CPSI overall
LUBIN	XIANGZ	2	LUBIN/XIANGZ/QIAO

Table 2C6 -

IESLC - Meta-anal of Current Smoking (or Ever if Current not available), Cigarettes only
Squamous

This analysis is restricted to results for:
1) Non-dose-response data
2) Results complete enough for use in metaanalysis

Within each study, results are then selected (in the following order of preference, within each sex) for:
3) SMKSTA: current smokers, ever smokers
4) PRODUCT: cigarettes only
5) CIGTYPE: all/unspecified, MC regardless of HR, MC only
6) DENOM: never smoked anything, never smoked cigarettes, (never +1 = +long term ex, +2 = +amount unknown, +3 = never cigs+long term ex)
7) Followup period (YF, prospective studies): whole study (coded as 0) or longest available
8) LCtype: squamous or nearest available, but not adeno. (q = squamous, s = small, a = adeno, KI = Kreyberg I, u = undifferentiated)
9) Race: all or nearest available, otherwise by race (wh or w = white, bl or b = black, hi = hispanic ch = chinese, jap = japanese, haw = hawaiian, w+o = white + oriental, sca = scandinavian, as = asian)
10) For overlapping studies: principal rather than subsidiary studies
Finally by Age: whole study (coded as 0) if available, otherwise by widest available age group and then for single sex results (m, f) in preference to combined sex results (c).

Results adjusted (AD) for the most potential confounders are then chosen in Sections -1 to -3 (and those which actually differ from the adjusted results in Table 2C5 - 1 are marked 'x' in Section -1) and results adjusted for the least confounders in Sections -4 to -6. (Those least adjusted results which actually differ from the most adjusted as marked 'x' in column X in Section -4) (Results adjusted for an unknown number of confounder(s) are coded as 20.)

Section -7 shows excluded studies, together with the stage (as above) at which no qualifying results were found.

Section -8 lists the potentially overlapping studies which have been included (1=principal, 2=subsidiary).

Section -9 lists any results which would have been included in preference except that they had data not complete enough for use in meta-analysis, with their significance (yes/no), if known, and any further comment as entered on the database.

In addition to those mentioned above, the following fields, levels and abbreviations are used:
* or nk = not known, n = no, y = yes, ot = other
ev = ever, cu = current, nev = never
all/unspec = all or unspecified, MC = manufactured cigarettes, HR = hand-rolled cigarettes
REF: 6-character study reference
NRR: number of the RR on the database within the study
ST : study type (CC = case control, pr or prosp = prospective)
NLC: number of lung cancer cases in whole study
R : risky occupational population (n = no, m = mining, o = other risky)
VB : national cigarette type (V = at least 75% Virginia, bl = at least 75% blended, ot = other)
P : any proxy use
H : full histological confirmation
De : derivation of RR/CI (or = original, st = standard method, ot = other method of estimation)

Table 2C6 - 1

IESLC - Meta-anal of Current Smoking (or Ever if Current not available), Cigarettes only
Squamous
Most adjusted

REF	NRR	2C5	SEX	AGEL	AGEH	RACE	YF	LC TYPE	LOC	START	ST	NLC	R	VB	P	H	AD	SM	PRODUCT	DENOM	De
ALDERS	74		m	0	0	all	-	q+s	Eu:UK	1977	CC	1448	n	V	n	n	1	ev	cig only	nev any	ot
ALDERS	33		f	0	0	all	-	q+s	Eu:UK	1977	CC	1448	n	V	n	n	1	ev	cig only	nev any	ot
BAND	5		m	0	0	all	-	q	NAmer	1983	CC	2831	n	V	y	y	2	ev	cig only	nev any	ot
BOUCOT	141		m	0	0	all	0	q	NAmer	1951	pr	121	n	bl	n	n	2	cu	cig only	nev any	ot
BRESLO	8		c	0	0	all	-	not a	NAmer	1949	CC	518	n	bl	n	y	0	ev	cig only	nev+1	st
CPSI	403		m	0	0	all	2	q	NAmer	1959	pr	5138	n	bl	n	n	1	cu	cig only	nev any	ot
CPSI	405		f	0	0	all	2	q	NAmer	1959	pr	5138	n	bl	n	n	1	cu	cig only	nev any	ot
CPSII	114		m	0	0	all	2	q	NAmer	1982	pr	3229	n	bl	n	n	1	cu	cig only	nev any	ot
DORN	338		m	0	0	wh	8	q	NAmer	1954	pr	5097	n	bl	n	n	1	cu	cig only	nev any	ot
HAMMON	102	x	m	0	0	wh	0	not a	NAmer	1952	pr	448	n	bl	n	n	1	cu	cig only	nev any	ot
JUSSAW	25		m	0	0	all	-	KI	As:Ind	1964	CC	792	n	V	n	n	0	ev	cig only	nev any	st
LUBIN	15		m	0	0	all	-	KI	As:Chi	1984	CC	427	m	ot	y	n	0	ev	cig only	nev any	st
LUBIN2	141		m	0	0	all	-	q	Eu:mul	1976	CC	7804	n	bl	n	y	0	ev	cig only	nev any	st
PEZZOT	6		m	0	0	all	-	q	SCAmer	1987	CC	215	n	bl	n	y	0	ev	cig only	nev cigs	ot
STASZE	13		m	0	0	all	-	q	Eu:est	1954	CC	281	n	bl	n	y	0	ev	cig only	nev any	ot
WYNDE7	22	x	m	0	0	all	-	KI	NAmer	1977	CC	2085	n	bl	n	y	0	cu	cig only	nev any	st

Cigarette type is all/unspec for all RRs

except for the following:

REF	NRR	CIGTYPE
ALDERS	33	MC only
JUSSAW	25	MC only

Table 2C6 - 2

IESLC - Meta-anal of Current Smoking (or Ever if Current not available), Cigarettes only
Squamous
Most adjusted

REF	NRR	SEX	AD	Number Exposed Case	Number Exposed Cont	Non-exposed Case	Non-exposed Cont	RR	95.00%CI
ALDERS	74	m	1	-	-	-	-	11.58	(4.25- 31.54)
ALDERS	33	f	1	-	-	-	-	6.70	(3.92- 11.46)
Subtotal ALDERS								7.57	(4.72- 12.14)
BAND	5	m	2	-	-	-	-	37.45	(17.62- 79.58)
*BOUCOT	141	m	2	-	-	-	-	27.54	(1.69- 448.37)
BRESLO	8	c	0	298	240	15	56	4.64	(2.56- 8.40)
*CPSI	403	m	1	-	-	-	-	29.35	(4.02- 214.28)
*CPSI	405	f	1	-	-	-	-	4.25	(1.23- 14.68)
Subtotal CPSI								7.30	(2.55- 20.90)
*CPSII	114	m	1	-	-	-	-	39.26	(10.38- 148.55)
*DORN	338	m	1	-	-	-	-	17.09	(8.96- 32.60)
*HAMMON	102	m	1	-	-	-	-	26.42	(9.78- 71.34)
JUSSAW	25	m	0	17	77	13	624	10.60	(4.96- 22.66)
LUBIN	15	m	0	42	191	4	72	3.96	(1.37- 11.43)
LUBIN2	141	m	0	3474	9345	127	2616	7.66	(6.38- 9.19)
PEZZOT	6	m	0	85	317	0	116	62.74~	(3.86-1019.50)
STASZE	13	m	0	117	552	0	158	67.42~	(4.17-1090.28)
WYNDE7	22	m	0	666	993	22	918	27.99	(18.13- 43.20)
Partial Totals				4699	11715	181	4560		

*prospective study ~ With 0.5 adjustment for zero

REF	NRR	SEX	AD	Ys	Ws	Qs	Ps
ALDERS	74	m	1	2.45	3.82	0.11	0.0000
ALDERS	33	f	1	1.90	13.35	1.94	0.0000
Subtotal ALDERS				2.02	17.18	2.04	
BAND	5	m	2	3.62	6.76	12.14	0.0000
*BOUCOT	141	m	2	3.32	0.49	0.53	0.0199
BRESLO	8	c	0	1.53	10.86	6.10	0.0000
*CPSI	403	m	1	3.38	0.97	1.17	0.0009
*CPSI	405	f	1	1.45	2.50	1.75	0.0222
Subtotal CPSI				1.99	3.47	2.92	
*CPSII	114	m	1	3.67	2.17	4.18	0.0000
*DORN	338	m	1	2.84	9.21	2.84	0.0000
*HAMMON	102	m	1	3.27	3.89	3.82	0.0000
JUSSAW	25	m	0	2.36	6.65	0.04	0.0000
LUBIN	15	m	0	1.38	3.41	2.81	0.0110
LUBIN2	141	m	0	2.04	115.59	7.08	0.0000
PEZZOT	6	m	0	4.14	0.49	1.70	0.0036
STASZE	13	m	0	4.21	0.50	1.84	0.0030
WYNDE7	22	m	0	3.33	20.39	22.42	0.0000

	N	16
	NS	14
	Wt	201.07
Het	Chi	70.45
Het	df	15
Het	P	***
Fixed	RR	9.81
	RRl	8.54
	RRu	11.26
	P	+++
Random	RR	13.22
	RRl	8.72
	RRu	20.04
	P	+++
Asymm	P	N.S.

Table 2C6 - 3

IESLC - Meta-anal of Current Smoking (or Ever if Current not available), Cigarettes only
Squamous
Most adjusted

	Sex combined	male	female	Total
N	1	13	2	16
NS	1	13	2	16
Wt	10.86	174.36	15.85	201.07
Het Chi	0.00	59.32	0.44	70.45
Het df	0	12	1	15
Het P	N.S.	***	N.S.	***
Fixed RR	4.64	10.71	6.24	9.81
RRl	2.56	9.23	3.81	8.54
RRu	8.40	12.42	10.20	11.26
P	+++	+++	+++	+++
Random RR	4.64	17.65	6.24	13.22
RRl	2.56	10.68	3.81	8.72
RRu	8.40	29.16	10.20	20.04
P	+++	+++	+++	+++
Between Chi				10.70
Between df				2
Between P				**
Btwn(F) P				N.S.
Btwn(R) P				**

	Smoking status ever	current	Total
N	9	7	16
NS	8	6	14
Wt	161.45	39.62	201.07
Het Chi	27.07	9.47	70.45
Het df	8	6	15
Het P	***	N.S.	***
Fixed RR	8.00	22.47	9.81
RRl	6.86	16.46	8.54
RRu	9.34	30.67	11.26
P	+++	+++	+++
Random RR	9.59	20.74	13.22
RRl	6.16	12.92	8.72
RRu	14.94	33.30	20.04
P	+++	+++	+++
Between Chi			33.91
Between df			1
Between P			***
Btwn(F) P			**
Btwn(R) P			*

Table 2C6 - 4

IESLC - Meta-anal of Current Smoking (or Ever if Current not available), Cigarettes only
Squamous
Least adjusted

REF	NRR	X	SEX	AGEL	AGEH	RACE	YF	LC TYPE	LOC	START	ST	NLC	R	VB	P	H	AD	SM	PRODUCT	DENOM	De
ALDERS	87	x	m	0	0	all	-	q+s	Eu:UK	1977	CC	1448	n	V	n	n	0	ev	cig only	nev any	st
ALDERS	84	x	f	0	0	all	-	q+s	Eu:UK	1977	CC	1448	n	V	n	n	0	ev	cig only	nev any	st
BAND	5		m	0	0	all	-	q	NAmer	1983	CC	2831	n	V	y	y	2	ev	cig only	nev any	ot
BOUCOT	70	x	m	0	0	all	0	q	NAmer	1951	pr	121	n	bl	n	n	0	cu	cig only	nev any	ot
BRESLO	8		c	0	0	all	-	not a	NAmer	1949	CC	518	n	bl	n	y	0	ev	cig only	nev+1	st
CPSI	403		m	0	0	all	2	q	NAmer	1959	pr	5138	n	bl	n	n	1	cu	cig only	nev any	ot
CPSI	405		f	0	0	all	2	q	NAmer	1959	pr	5138	n	bl	n	n	1	cu	cig only	nev any	ot
CPSII	114		m	0	0	all	2	q	NAmer	1982	pr	3229	n	bl	n	n	1	cu	cig only	nev any	ot
DORN	338		m	0	0	wh	8	q	NAmer	1954	pr	5097	n	bl	n	n	1	cu	cig only	nev any	ot
HAMMON	102		m	0	0	wh	0	not a	NAmer	1952	pr	448	n	bl	n	n	1	cu	cig only	nev any	ot
JUSSAW	25		m	0	0	all	-	KI	As:Ind	1964	CC	792	n	V	n	n	0	ev	cig only	nev any	st
LUBIN	15		m	0	0	all	-	KI	As:Chi	1984	CC	427	m	ot	y	n	0	ev	cig only	nev any	st
LUBIN2	141		m	0	0	all	-	q	Eu:mul	1976	CC	7804	n	bl	n	y	0	ev	cig only	nev any	st
PEZZOT	6		m	0	0	all	-	q	SCAmer	1987	CC	215	n	bl	n	y	0	ev	cig only	nev cigs	ot
STASZE	13		m	0	0	all	-	q	Eu:est	1954	CC	281	n	bl	n	y	0	ev	cig only	nev any	ot
WYNDE7	22		m	0	0	all	-	KI	NAmer	1977	CC	2085	n	bl	n	y	0	cu	cig only	nev any	st

Cigarette type is all/unspec for all RRs

except for the following:

REF	NRR	CIGTYPE
ALDERS	84	MC only
JUSSAW	25	MC only

Table 2C6 - 5

IESLC - Meta-anal of Current Smoking (or Ever if Current not available), Cigarettes only
Squamous
Least adjusted

REF	NRR	SEX	AD	Number Exposed Case	Number Exposed Cont	Non-exposed Case	Non-exposed Cont	RR	95.00%CI
ALDERS	87	m	0	207	462	4	133	14.90	(5.44- 40.82)
ALDERS	84	f	0	176	371	16	243	7.20	(4.21- 12.32)
Subtotal ALDERS								8.46	(5.27- 13.59)
BAND	5	m	2	-	-	-	-	37.45	(17.62- 79.58)
*BOUCOT	70	m	0	38	22177	0	7551	26.22~	(1.61- 426.71)
BRESLO	8	c	0	298	240	15	56	4.64	(2.56- 8.40)
*CPSI	403	m	1	-	-	-	-	29.35	(4.02- 214.28)
*CPSI	405	f	1	-	-	-	-	4.25	(1.23- 14.68)
Subtotal CPSI								7.30	(2.55- 20.90)
*CPSII	114	m	1	-	-	-	-	39.26	(10.38- 148.55)
*DORN	338	m	1	-	-	-	-	17.09	(8.96- 32.60)
*HAMMON	102	m	1	-	-	-	-	26.42	(9.78- 71.34)
JUSSAW	25	m	0	17	77	13	624	10.60	(4.96- 22.66)
LUBIN	15	m	0	42	191	4	72	3.96	(1.37- 11.43)
LUBIN2	141	m	0	3474	9345	127	2616	7.66	(6.38- 9.19)
PEZZOT	6	m	0	85	317	0	116	62.74~	(3.86-1019.50)
STASZE	13	m	0	117	552	0	158	67.42~	(4.17-1090.28)
WYNDE7	22	m	0	666	993	22	918	27.99	(18.13- 43.20)
Partial Totals				5120	34725	201	12487		

*prospective study ~ With 0.5 adjustment for zero

REF	NRR	SEX	AD	Ys	Ws	Qs	Ps
ALDERS	87	m	0	2.70	3.78	0.63	0.0000
ALDERS	84	f	0	1.97	13.33	1.35	0.0000
Subtotal ALDERS				2.14	17.12	1.98	
BAND	5	m	2	3.62	6.76	11.97	0.0000
*BOUCOT	70	m	0	3.27	0.49	0.47	0.0217
BRESLO	8	c	0	1.53	10.86	6.26	0.0000
*CPSI	403	m	1	3.38	0.97	1.15	0.0009
*CPSI	405	f	1	1.45	2.50	1.79	0.0222
Subtotal CPSI				1.99	3.47	2.94	
*CPSII	114	m	1	3.67	2.17	4.12	0.0000
*DORN	338	m	1	2.84	9.21	2.75	0.0000
*HAMMON	102	m	1	3.27	3.89	3.75	0.0000
JUSSAW	25	m	0	2.36	6.65	0.03	0.0000
LUBIN	15	m	0	1.38	3.41	2.87	0.0110
LUBIN2	141	m	0	2.04	115.59	7.63	0.0000
PEZZOT	6	m	0	4.14	0.49	1.69	0.0036
STASZE	13	m	0	4.21	0.50	1.82	0.0030
WYNDE7	22	m	0	3.33	20.39	22.01	0.0000

	N	16
	NS	14
	Wt	201.01
Het	Chi	70.27
Het	df	15
Het	P	***
Fixed	RR	9.90
	RRl	8.62
	RRu	11.37
	P	+++
Random	RR	13.52
	RRl	8.92
	RRu	20.48
	P	+++
Asymm	P	(*)

Table 2C6 - 6

IESLC - Meta-anal of Current Smoking (or Ever if Current not available), Cigarettes only
Squamous
Least adjusted

		Sex			
		combined	male	female	Total
	N	1	13	2	16
	NS	1	13	2	16
	Wt	10.86	174.31	15.83	201.01
Het	Chi	0.00	59.66	0.59	70.27
Het	df	0	12	1	15
Het	P	N.S.	***	N.S.	***
Fixed	RR	4.64	10.76	6.63	9.90
	RRl	2.56	9.28	4.05	8.62
	RRu	8.40	12.49	10.85	11.37
	P	+++	+++	+++	+++
Random	RR	4.64	18.02	6.63	13.52
	RRl	2.56	10.89	4.05	8.92
	RRu	8.40	29.82	10.85	20.48
	P	+++	+++	+++	+++
Between	Chi				10.02
Between	df				2
Between	P				**
Btwn(F)	P				N.S.
Btwn(R)	P				**

Table 2C6 - 7

IESLC - Meta-anal of Current Smoking (or Ever if Current not available), Cigarettes only
Squamous
Excluded studies (and stage at which they were excluded)

1 BOUCHA BUELL LAURIL MZILEN

2 BERRIN CHEN RESTRE TAO ULMER

4 ABRAHA AKIBA AMANDU AMES ANDERS ARCHER AUSTIN AUVINE AXELSO AXELSS BARBON BECHER BENSHL BLOHMK BLOT1 BLOT2
BLOT3 BLOT4 BRETT BROCKM BROSS BROWN1 BROWN2 BUFFLE BYERS1 BYERS2 CARPEN CASCO2 CASCOR CHAN CHANG CHATZI
CHEN2 CHEN3 CHIAZZ CHOI CHYOU COMSTO COOKSO CORREA DARBY DAVEYS DEKLER DESTE2 DESTEF DOCKER DORANT DORGAN
DOSEME DROSTE DU DUNN EBELIN ESAKI FAN GAO GAO2 GARCIA GARSHI GENG GER GILLIS GODLEY GOODMA
GREGOR GSELL HAENSZ HAMMO2 HANSEN HEGMAN HENNEK HINDS HIRAOK HIRAYA HITOSU HOLE HOROWI HORWIT HU HU2
HUANG HUMBLE ISHIMA JAHN JAIN JARUP JARVHO JEDRYC JIANG JONES KAISER KANELL KATSOU KAUFMA KELLER KHUDER
KIHARA KINLEN KNEKT KO KOHLME KOO KREUZE KREYBE KUBIK LAMTH LAMWK LAMWK2 LAUSSM LEI LEMARC LETOUR
LEVIN LIAW LICKIN LIDDEL LIU LIU2 LIU3 LIU5 LOMBA2 LUO MACLEN MAGNUS MARSH MARSH2 MARTIS MASTRA
MATOS MATSUD MCDUFF MCLAUG MILLER MOLLO MRFIT MRFITR MURATA NAM NOTANI NOU ODRISC ORMOS OSANN OSANN2
PARKIN PASTOR PAWLEG PERSH2 PERSHA PETO PEZZO2 PIKE PISANI POFFIJ POLEDN PRESCO QIAO2 RACHTA RADZIK RANDIG
REN ROOTS ROTHSC SAARIK SANKAR SCHWAR SEGI SEGI2 SEOW SHAW SHIMIZ SIEMIA SIMARA SIMONA SITAS SOBUE
SOBUE2 SPEIZE SPITZ STAYNE STOCKS STOCKW STUCKE SUN SUZUKI SVENSS TANG TENKAN TOKARS TOUSEY TSUGAN TULINI
VANDER VUTUC WAKAI WANG WANG2 WANG3 WANG4 WARSIN WATSON WICKLU WILKIN WU WU2 WUNSCH WUWILL WYNDE2
WYNDE3 WYNDE4 WYNDE5 WYNDE6 WYNDE8 XU XU2 XU3 XU4 YAMAGU YONG YUAN ZHANG ZHENG ZHOU

5 LANGE RIMING TANG2

6 HIRAY2 SCHWA2 WYNDER

8 ABELIN AGUDO ARMADA BEST BOFFET CEDERL CHOW DAMBER DEAN DEAN2 DEAN3 DOLL DOLL2 ENGELA ENSTRO GARDIN
GOLLED GRAHAM HEIN JOLY KAISE2 KJUUS KOULUM LIU4 LOMBAR MCCONN MIGRAN MILLS NOTAN2 PERNU QIAO RONCO
SADOWS SUZUK2 TIZZAN TVERDA VEIERO WALD WIGLE XIANGZ

10 BENHAM

Table 2C6 - 8
Potentially overlapping studies

REF	REFGP	PRINC	.	OVERLAP/LINK
LUBIN2	LUBIN2	1		Lubin-combined
WYNDE7	WYNDE6	2		WYNDE5/6/7/8
CPSI	CPSI	1		CPSI overall
LUBIN	XIANGZ	2		LUBIN/XIANGZ/QIAO
