# Supplementary material for: Systematic review with meta-analysis of the epidemiological evidence in the 1900s relating smoking to lung cancer
Source: BMC Cancer. 2012 Sep 3;12:385. doi: 10.1186/1471-2407-12-385 (PMC3505152; doi:10.1186/1471-2407-12-385)
Supplement: Additional file 5 — Detailed Analysis Tables (Individual file names as described in Additional file 1: Methods, Table1). [file 1471-2407-12-385-S5.zip › PDF/2B.pdf]

Table 2B1 -

IESLC - Meta-analysis of Current Smoking (vs never smoking), Any product (or Cigarettes if Any not available)
Squamous

This analysis is restricted to results for:
1) Non-dose-response data
2) Current smokers
3) Results complete enough for use in metaanalysis

Within each study, results are then selected (in the following order of preference, within each sex) for:
4) PRODUCT: all/unspec, cigarettes regardless of other products, cigarettes only
5) CIGTYPE: all/unspecified, MC regardless of HR, MC only
6) DENOM: never smoked anything, never smoked cigarettes, (never +1 = +long term ex, +2 = +amount unknown,
+3 = never cigs+long term ex)
7) Followup period (YF, prospective studies): whole study (coded as 0) or longest available
8) LCtype: squamous or nearest available, but not adeno. (q = squamous, s = small,
a = adeno, KI = Kreyberg I, u = undifferentiated)
9) Race: all or nearest available, otherwise by race (wh or w = white, bl or b = black, hi = hispanic
ch = chinese, jap = japanese, haw = hawaiian, w+o = white + oriental, sca = scandinavian, as = asian)
10) For overlapping studies: principal rather than subsidiary studies
Finally by Age: whole study (coded as 0) if available, otherwise by widest available age group
and then for single sex results (m, f) in preference to combined sex results (c).

Results adjusted (AD) for the most potential confounders are then chosen in Sections -1 to -3
and results adjusted for the least confounders in Sections -4 to -6. (Those least adjusted results which
actually differ from the most adjusted as marked 'x' in column X in Section -4)
(Results adjusted for an unknown number of confounder(s) are coded as 20.)

Section -7 shows excluded studies, together with the stage (as above) at which no qualifying
results were found.

Section -8 lists the potentially overlapping studies which have been included (1=principal, 2=subsidiary).

Section -9 lists any results which would have been included in preference except that they had data not complete
enough for use in meta-analysis, with their significance (yes/no), if known, and any further comment as entered
on the database.

In addition to those mentioned above, the following fields, levels and abbreviations are used:
* or nk = not known, n = no, y = yes, ot = other
nev = never
all/unspec = all or unspecified, cig+/-ot = cigarettes irrespective of other products (cigar, pipe etc)
MC = manufactured cigarettes, HR = hand-rolled cigarettes
REF: 6-character study reference
NRR: number of the RR on the database within the study
ST : study type (CC = case control, pr or prosp = prospective)
NLC: number of lung cancer cases in whole study
R : risky occupational population (n = no, m = mining, o = other risky)
VB : national cigarette type (V = at least 75% Virginia, bl = at least 75% blended, ot = other)
P : any proxy use
H : full histological confirmation
De : derivation of RR/CI (or = original, st = standard method, ot = other method of estimation)

Table 2B1 - 1

IESLC - Meta-analysis of Current Smoking (vs never smoking), Any product (or Cigarettes if Any not available)
Squamous
Most adjusted

REF	NRR	SEX	AGEL	AGEH	RACE	YF	LC TYPE	LOC	START	ST	NLC	R	VB	P	H	AD	PRODUCT	DENOM	De
BARBON	18	m	0	0	all	-	q	Eu:wst	1979	CC	755	n	bl	y	y	1	all/unsp	nev any	or
BOUCOT	141	m	0	0	all	0	q	NAmer	1951	pr	121	n	bl	n	n	2	cig only	nev any	ot
BROWN2	16	m	0	0	wh	-	q	NAmer	1984	CC	14596	n	bl	n	y	2	cig+/-ot	nev cigs	or
BROWN2	15	f	0	0	wh	-	q	NAmer	1984	CC	14596	n	bl	n	y	2	cig+/-ot	nev cigs	or
BUFFLE	63	f	0	0	w-hi	-	q	NAmer	1976	CC	943	n	bl	y	n	0	cig+/-ot	nev cigs	st
COMSTO	23	m	0	0	all	-	q	NAmer	1975	ot	258	n	bl	n	n	0	cig+/-ot	nev cigs	st
COMSTO	30	f	0	0	all	-	q	NAmer	1975	ot	258	n	bl	n	n	0	cig+/-ot	nev cigs	ot
CORREA	43	c	0	0	all	-	q+s	NAmer	1979	CC	1359	n	bl	y	n	1	cig+/-ot	nev cigs	or
CPSI	403	m	0	0	all	2	q	NAmer	1959	pr	5138	n	bl	n	n	1	cig only	nev any	ot
CPSI	405	f	0	0	all	2	q	NAmer	1959	pr	5138	n	bl	n	n	1	cig only	nev any	ot
CPSII	114	m	0	0	all	2	q	NAmer	1982	pr	3229	n	bl	n	n	1	cig only	nev any	ot
CPSII	117	f	0	0	all	2	q	NAmer	1982	pr	3229	n	bl	n	n	1	cig+/-ot	nev cigs	ot
DORN	338	m	0	0	wh	8	q	NAmer	1954	pr	5097	n	bl	n	n	1	cig only	nev any	ot
ENGELA	56	m	0	0	all	0	q	Eu:Sca	1964	pr	435	n	bl	n	n	7	cig+/-ot	nev cigs	ot
HAENSZ	20	f	0	0	all	-	q+u	NAmer	1955	CC	158	n	bl	n	y	0	cig+/-ot	nev any	st
HAMMON	102	m	0	0	wh	0	not a	NAmer	1952	pr	448	n	bl	n	n	1	cig only	nev any	ot
JAHN	7	m	0	0	all	-	q	Eu:Ger	1988	CC	1004	n	bl	n	n	0	cig+/-ot	nev any	st
JAIN	18	m	0	0	all	-	q	NAmer	1981	CC	845	n	V	y	n	0	cig+/-ot	nev cigs	st
JAIN	13	f	0	0	all	-	q	NAmer	1981	CC	845	n	V	y	n	0	cig+/-ot	nev cigs	st
JEDRYC	22	m	0	0	all	-	q	Eu:est	1980	CC	1630	n	bl	y	n	0	cig+/-ot	nev any	st
KATSOU	20	f	0	0	all	-	KI	Eu:bal	1987	CC	101	n	bl	n	n	1	all/unsp	nev any	or
KHUDER	14	m	0	0	all	-	q	NAmer	1985	CC	482	n	bl	n	y	0	cig+/-ot	nev cigs	or
KIHARA	2	c	0	0	jap	-	q	As:Jap	1991	CC	440	n	bl	n	n	0	all/unsp	nev any	st
LUBIN2	249	m	0	0	all	-	q	Eu:mul	1976	CC	7804	n	bl	n	y	0	cig+/-ot	nev any	st
LUBIN2	261	f	0	0	all	-	q	Eu:mul	1976	CC	7804	n	bl	n	y	0	cig+/-ot	nev any	st
MATOS	39	m	0	0	all	-	q	SCAmer	1994	CC	200	n	bl	n	n	2	cig+/-ot	nev any	or
OSANN	35	m	0	0	all	-	q	NAmer	1984	CC	1986	n	bl	n	n	2	cig+/-ot	nev cigs	or
OSANN	36	f	0	0	all	-	q	NAmer	1984	CC	1986	n	bl	n	n	2	cig+/-ot	nev cigs	or
OSANN2	26	f	0	0	all	-	KI	NAmer	1964	ot	217	n	bl	n	y	1	cig+/-ot	nev cigs	or
SOBUE	34	m	0	0	all	-	q	As:Jap	1986	CC	1376	n	bl	n	y	1	cig+/-ot	nev cigs	or
SOBUE	44	f	0	0	all	-	q	As:Jap	1986	CC	1376	n	bl	n	y	1	cig+/-ot	nev cigs	or
SOBUE2	1	m	0	0	all	-	q	As:Jap	1965	CC	2083	n	bl	n	n	2	cig+/-ot	nev any	or
SOBUE2	5	f	0	0	all	-	q	As:Jap	1965	CC	2083	n	bl	n	n	2	cig+/-ot	nev any	or
SVENSS	97	f	0	0	all	-	q	Eu:Sca	1983	CC	210	n	bl	n	n	1	all/unsp	nev any	ot
TSUGAN	14	m	0	0	all	-	q	As:Jap	1976	CC	134	n	bl	n	y	0	all/unsp	nev any	ot
WAKAI	10	m	0	0	all	-	q	As:Jap	1988	CC	333	n	bl	n	y	1	all/unsp	nev any	or
WAKAI	28	f	0	0	all	-	q	As:Jap	1988	CC	333	n	bl	n	y	1	all/unsp	nev any	or
WU	16	f	0	0	wh	-	q	NAmer	1981	CC	220	n	bl	n	y	2	all/unsp	nev any	or
WYNDE3	10	m	0	0	all	-	KI	NAmer	1966	CC	350	n	bl	n	y	0	all/unsp	nev any	st
WYNDE6	12	m	0	0	all	-	KI	NAmer	1969	CC	4423	n	bl	n	y	0	cig+/-ot	nev any	st
WYNDE6	201	f	0	0	all	-	KI	NAmer	1969	CC	4423	n	bl	n	y	0	cig+/-ot	nev cigs	st

Cigarette type is all/unspec for all RRs

Table 2B1 - 2

IESLC - Meta-analysis of Current Smoking (vs never smoking), Any product (or Cigarettes if Any not available)
Squamous
Most adjusted

REF	NRR	SEX	AD	Number Exposed Case	Number Exposed Cont	Non-exposed Case	Non-exposed Cont	RR	95.00%CI
BARBON	18	m	1	-	-	-	-	18.80	(8.20- 43.40)
*BOUCOT	141	m	2	-	-	-	-	27.54	(1.69- 448.37)
BROWN2	16	m	2	-	-	-	-	13.70	(11.70- 16.10)
BROWN2	15	f	2	-	-	-	-	20.60	(16.60- 25.60)
Subtotal BROWN2								15.81	(13.91- 17.98)
BUFFLE	63	f	0	39	110	3	112	13.24	(3.97- 44.10)
COMSTO	23	m	0	27	100	2	84	11.34	(2.62- 49.09)
COMSTO	30	f	0	16	52	0	115	72.60~	(4.27-1233.12)
Subtotal COMSTO								16.78	(4.57- 61.67)
CORREA	43	c	1	-	-	-	-	34.60	(22.80- 52.40)
*CPSI	403	m	1	-	-	-	-	29.35	(4.02- 214.28)
*CPSI	405	f	1	-	-	-	-	4.25	(1.23- 14.68)
Subtotal CPSI								7.30	(2.55- 20.90)
*CPSII	114	m	1	-	-	-	-	39.26	(10.38- 148.55)
*CPSII	117	f	1	-	-	-	-	78.91	(15.83- 393.37)
Subtotal CPSII								52.16	(18.72- 145.32)
*DORN	338	m	1	-	-	-	-	17.09	(8.96- 32.60)
*ENGELA	56	m	7	-	-	-	-	10.89	(3.25- 36.43)
HAENSZ	20	f	0	53	94	44	236	3.02	(1.90- 4.82)
*HAMMON	102	m	1	-	-	-	-	26.42	(9.78- 71.34)
JAHN	7	m	0	153	269	3	138	26.16	(8.19- 83.54)
JAIN	18	m	0	107	118	2	85	38.54	(9.26- 160.45)
JAIN	13	f	0	81	99	6	214	29.18	(12.31- 69.15)
Subtotal JAIN								31.44	(15.03- 65.78)
JEDRYC	22	m	0	235	516	6	289	21.94	(9.63- 49.96)
KATSOU	20	f	1	-	-	-	-	6.45	(2.73- 15.25)
KHUDER	14	m	0	112	-	9	-	8.60	(4.20- 17.60)
KIHARA	2	c	0	111	162	5	237	32.48	(12.97- 81.34)
LUBIN2	249	m	0	2518	6209	54	2616	19.65	(14.95- 25.82)
LUBIN2	261	f	0	154	410	72	1180	6.16	(4.55- 8.32)
Subtotal LUBIN2								11.64	(9.50- 14.25)
MATOS	39	m	2	-	-	-	-	10.40	(3.60- 35.50)
OSANN	35	m	2	-	-	-	-	49.30	(24.10- 101.00)
OSANN	36	f	2	-	-	-	-	35.20	(19.10- 65.10)
Subtotal OSANN								40.59	(25.47- 64.67)
OSANN2	26	f	1	-	-	-	-	39.00	(5.30- 287.00)
SOBUE	34	m	1	-	-	-	-	18.10	(7.90- 41.30)
SOBUE	44	f	1	-	-	-	-	9.70	(5.50- 16.80)
Subtotal SOBUE								11.79	(7.42- 18.73)
SOBUE2	1	m	2	-	-	-	-	5.20	(4.20- 6.50)
SOBUE2	5	f	2	-	-	-	-	7.20	(4.80- 10.80)
Subtotal SOBUE2								5.59	(4.62- 6.78)
SVENSS	97	f	1	-	-	-	-	21.92	(6.63- 72.51)
TSUGAN	14	m	0	18	13	0	5	15.07~	(0.77- 296.43)
WAKAI	10	m	1	-	-	-	-	9.82	(2.36- 41.00)
WAKAI	28	f	1	-	-	-	-	28.20	(7.55- 105.00)
Subtotal WAKAI								17.37	(6.60- 45.71)
WU	16	f	2	-	-	-	-	35.30	(4.70- 267.30)
WYNDE3	10	m	0	171	207	3	88	24.23	(7.53- 77.95)
WYNDE6	12	m	0	1026	741	29	617	29.46	(20.06- 43.26)
WYNDE6	201	f	0	550	376	40	856	31.30	(22.21- 44.12)
Subtotal WYNDE6								30.47	(23.59- 39.36)
Partial Totals				5371	9476	278	6872		

*prospective study ~ With 0.5 adjustment for zero

Table 2B1 - 2

IESLC - Meta-analysis of Current Smoking (vs never smoking), Any product (or Cigarettes if Any not available)
Squamous
Most adjusted

REF	NRR	SEX	AD	Ys	Ws	Qs	Ps
BARBON	18	m	1	2.93	5.53	0.54	0.0000
*BOUCOT	141	m	2	3.32	0.49	0.24	0.0199
BROWN2	16	m	2	2.62	150.78	0.00	0.0000
BROWN2	15	f	2	3.03	81.88	13.28	0.0000
Subtotal BROWN2				2.76	232.67	13.29	
BUFFLE	63	f	0	2.58	2.65	0.00	0.0000
COMSTO	23	m	0	2.43	1.79	0.07	0.0012
COMSTO	30	f	0	4.28	0.48	1.32	0.0030
Subtotal COMSTO				2.82	2.27	1.39	
CORREA	43	c	1	3.54	22.19	18.84	0.0000
*CPSI	403	m	1	3.38	0.97	0.56	0.0009
*CPSI	405	f	1	1.45	2.50	3.45	0.0222
Subtotal CPSI				1.99	3.47	4.01	
*CPSII	114	m	1	3.67	2.17	2.38	0.0000
*CPSII	117	f	1	4.37	1.49	4.54	0.0000
Subtotal CPSII				3.95	3.66	6.92	
*DORN	338	m	1	2.84	9.21	0.43	0.0000
*ENGELA	56	m	7	2.39	2.63	0.14	0.0001
HAENSZ	20	f	0	1.11	17.71	40.69	0.0000
*HAMMON	102	m	1	3.27	3.89	1.65	0.0000
JAHN	7	m	0	3.26	2.85	1.17	0.0000
JAIN	18	m	0	3.65	1.89	2.00	0.0000
JAIN	13	f	0	3.37	5.16	2.91	0.0000
Subtotal JAIN				3.45	7.05	4.91	
JEDRYC	22	m	0	3.09	5.67	1.23	0.0000
KATSOU	20	f	1	1.86	5.19	2.99	0.0000
KHUDER	14	m	0	2.15	7.48	1.66	0.0000
KIHARA	2	c	0	3.48	4.56	3.36	0.0000
LUBIN2	249	m	0	2.98	51.39	6.49	0.0000
LUBIN2	261	f	0	1.82	42.25	27.39	0.0000
Subtotal LUBIN2				2.45	93.64	33.88	
MATOS	39	m	2	2.34	2.93	0.23	0.0001
OSANN	35	m	2	3.90	7.48	12.17	0.0000
OSANN	36	f	2	3.56	10.22	9.00	0.0000
Subtotal OSANN				3.70	17.70	21.17	
OSANN2	26	f	1	3.66	0.96	1.05	0.0003
SOBUE	34	m	1	2.90	5.62	0.42	0.0000
SOBUE	44	f	1	2.27	12.32	1.51	0.0000
Subtotal SOBUE				2.47	17.94	1.93	
SOBUE2	1	m	2	1.65	80.57	76.41	0.0000
SOBUE2	5	f	2	1.97	23.37	9.82	0.0000
Subtotal SOBUE2				1.72	103.93	86.24	
SVENSS	97	f	1	3.09	2.69	0.58	0.0000
TSUGAN	14	m	0	2.71	0.43	0.00	0.0743
WAKAI	10	m	1	2.28	1.89	0.22	0.0017
WAKAI	28	f	1	3.34	2.22	1.14	0.0000
Subtotal WAKAI				2.85	4.10	1.35	
WU	16	f	2	3.56	0.94	0.83	0.0005
WYNDE3	10	m	0	3.19	2.81	0.90	0.0000
WYNDE6	12	m	0	3.38	26.02	15.05	0.0000
WYNDE6	201	f	0	3.44	32.63	22.00	0.0000
Subtotal WYNDE6				3.42	58.65	37.05	

Table 2B1 - 2

IESLC - Meta-analysis of Current Smoking (vs never smoking), Any product (or Cigarettes if Any not available)
Squamous
Most adjusted

	N	41
	NS	30
	Wt	645.92
Het	Chi	288.68
Het	df	40
Het	P	***
Fixed	RR	13.77
	RRl	12.75
	RRu	14.87
	P	+++
Random	RR	16.91
	RRl	13.14
	RRu	21.76
	P	+++
Asymm	P	N.S.

Table 2B1 - 3

IESLC - Meta-analysis of Current Smoking (vs never smoking), Any product (or Cigarettes if Any not available)
Squamous
Most adjusted

	Sex			
	combined	male	female	Total
N	2	22	17	41
NS	2	22	17	41
Wt	26.75	374.51	244.66	645.92
Het Chi	0.02	123.06	142.38	288.68
Het df	1	21	16	40
Het P	N.S.	***	***	***
Fixed RR	34.23	13.11	13.44	13.77
RRl	23.43	11.85	11.86	12.75
RRu	50.00	14.51	15.23	14.87
P	+++	+++	+++	+++
Random RR	34.23	17.73	14.77	16.91
RRl	23.43	12.78	9.42	13.14
RRu	50.00	24.59	23.17	21.76
P	+++	+++	+++	+++
Between Chi				23.23
Between df				2
Between P				***
Btwn(F) P				N.S.
Btwn(R) P				**

	Lung cancer type					
	q	q+s	q+u	KI	not a	Total
N	33	1	1	5	1	41
NS	23	1	1	4	1	30
Wt	534.51	22.19	17.71	67.62	3.89	645.92
Het Chi	182.31	0.00	0.00	11.72	0.00	288.68
Het df	32	0	0	4	0	40
Het P	***	N.S.	N.S.	*	N.S.	***
Fixed RR	12.74	34.60	3.02	26.88	26.42	13.77
RRl	11.71	22.82	1.90	21.18	9.78	12.75
RRu	13.87	52.45	4.82	34.12	71.36	14.87
P	+++	+++	+++	+++	+++	+++
Random RR	16.43	34.60	3.02	22.51	26.42	16.91
RRl	12.66	22.82	1.90	13.42	9.78	13.14
RRu	21.32	52.45	4.82	37.78	71.36	21.76
P	+++	+++	+++	+++	+++	+++
Between Chi						94.65
Between df						4
Between P						***
Btwn(F) P						**
Btwn(R) P						***

	Location								
	NAmer	UK	Scand	othEur	China	Japan	othAs	other	Total
N	24		2	6		8		1	41
NS	17		2	5		5		1	30
Wt	393.82		5.32	112.89		130.97		2.93	645.92
Het Chi	126.94		0.65	38.19		28.96		0.00	288.68
Het df	23		1	5		7		0	40
Het P	***		N.S.	***		***		N.S.	***
Fixed RR	17.99		15.51	12.22		6.85		10.40	13.77
RRl	16.29		6.63	10.16		5.77		3.31	12.75
RRu	19.85		36.28	14.69		8.13		32.66	14.87
P	+++		+++	+++		+++		+++	+++
Random RR	20.95		15.51	13.66		11.25		10.40	16.91
RRl	15.41		6.63	7.38		6.89		3.31	13.14
RRu	28.48		36.28	25.30		18.35		32.66	21.76
P	+++		+++	+++		+++		+++	+++
Between Chi									93.93
Between df									4
Between P									***
Btwn(F) P									**
Btwn(R) P									N.S.

Table 2B1 - 3

IESLC - Meta-analysis of Current Smoking (vs never smoking), Any product (or Cigarettes if Any not available)
Squamous
Most adjusted

	Detailed Country in "other Europe"					
	multi	Germany	othWest	East	Balkans	Total
N	2	1	1	1	1	6
NS	1	1	1	1	1	5
Wt	93.64	2.85	5.53	5.67	5.19	112.89
Het Chi	31.23	0.00	0.00	0.00	0.00	38.19
Het df	1	0	0	0	0	5
Het P	***	N.S.	N.S.	N.S.	N.S.	***
Fixed RR	11.64	26.16	18.80	21.94	6.45	12.22
RRl	9.50	8.19	8.17	9.63	2.73	10.16
RRu	14.25	83.54	43.25	49.96	15.24	14.69
P	+++	+++	+++	+++	+++	+++
Random RR	11.02	26.16	18.80	21.94	6.45	13.66
RRl	3.53	8.19	8.17	9.63	2.73	7.38
RRu	34.35	83.54	43.25	49.96	15.24	25.30
P	+++	+++	+++	+++	+++	+++
Between Chi						6.96
Between df						4
Between P						N.S.
Btwn(F) P						N.S.
Btwn(R) P						N.S.

	Detailed Country in "other Asia"			
	India	HongKong	other	Total
N				
NS				
Wt				
Het Chi				
Het df				
Het P				N.S.
Fixed RR				
RRl				
RRu				
P				+++
Random RR				
RRl				
RRu				
P				+++
Between Chi				
Between df				
Between P				N.S.
Btwn(F) P				N.S.
Btwn(R) P				N.S.

	Detailed other continent			
	SCAmer	Auslia	Africa	Total
N	1			1
NS	1			1
Wt	2.93			2.93
Het Chi	0.00			0.00
Het df	0			0
Het P	N.S.			N.S.
Fixed RR	10.40			10.40
RRl	3.31			3.31
RRu	32.66			32.66
P	+++			+++
Random RR	10.40			10.40
RRl	3.31			3.31
RRu	32.66			32.66
P	+++			+++
Between Chi				
Between df				
Between P				N.S.
Btwn(F) P				N.S.
Btwn(R) P				N.S.

Table 2B1 - 3

IESLC - Meta-analysis of Current Smoking (vs never smoking), Any product (or Cigarettes if Any not available)
Squamous
Most adjusted

	<1960	1960-69	1970-79	1980-89	1990+	Total
	Start year of study					
N	6	7	8	18	2	41
NS	5	5	6	12	2	30
Wt	34.78	169.00	126.72	307.94	7.49	645.92
Het Chi	30.21	113.37	54.28	46.61	2.31	288.68
Het df	5	6	7	17	1	40
Het P	***	***	***	***	N.S.	***
Fixed RR	6.87	10.55	14.53	16.70	20.79	13.77
RRl	4.93	9.07	12.21	14.93	10.16	12.75
RRu	9.58	12.26	17.29	18.67	42.55	14.87
P	+++	+++	+++	+++	+++	+++
Random RR	11.05	15.48	16.43	19.69	19.39	16.91
RRl	4.07	7.08	8.67	15.08	6.38	13.14
RRu	30.00	33.83	31.14	25.71	58.88	21.76
P	+++	+++	+++	+++	+++	+++
Between Chi						41.89
Between df						4
Between P						***
Btwn(F) P						N.S.
Btwn(R) P						N.S.

	Study type (1)		
	CC	other	Total
N	30	11	41
NS	22	8	30
Wt	619.33	26.59	645.92
Het Chi	272.73	13.19	288.68
Het df	29	10	40
Het P	***	N.S.	***
Fixed RR	13.59	18.87	13.77
RRl	12.56	12.90	12.75
RRu	14.70	27.59	14.87
P	+++	+++	+++
Random RR	16.21	19.63	16.91
RRl	12.23	12.21	13.14
RRu	21.50	31.56	21.76
P	+++	+++	+++
Between Chi			2.75
Between df			1
Between P			(*)
Btwn(F) P			N.S.
Btwn(R) P			N.S.

	Study type (2)			
	CC	prosp	other	Total
N	30	8	3	41
NS	22	6	2	30
Wt	619.33	23.36	3.23	645.92
Het Chi	272.73	11.34	1.78	288.68
Het df	29	7	2	40
Het P	***	N.S.	N.S.	***
Fixed RR	13.59	18.52	21.58	13.77
RRl	12.56	12.34	7.26	12.75
RRu	14.70	27.78	64.20	14.87
P	+++	+++	+++	+++
Random RR	16.21	19.36	21.58	16.91
RRl	12.23	10.95	7.26	13.14
RRu	21.50	34.24	64.20	21.76
P	+++	+++	+++	+++
Between Chi				2.82
Between df				2
Between P				N.S.
Btwn(F) P				N.S.
Btwn(R) P				N.S.

Table 2B1 - 3

IESLC - Meta-analysis of Current Smoking (vs never smoking), Any product (or Cigarettes if Any not available)

Squamous

Most adjusted

	Study size (number of LC cases) 100-249	250-499	500-999	1000+	Total
N	8	9	4	20	41
NS	8	7	3	12	30
Wt	31.35	27.75	15.24	571.59	645.92
Het Chi	21.45	9.44	1.82	225.75	288.68
Het df	7	8	3	19	40
Het P	**	N.S.	N.S.	***	***
Fixed RR	5.62	16.67	22.44	14.15	13.77
RRl	3.96	11.49	13.58	13.03	12.75
RRu	7.98	24.18	37.07	15.35	14.87
P	+++	+++	+++	+++	+++
Random RR	11.28	17.09	22.44	17.87	16.91
RRl	5.08	11.28	13.58	12.92	13.14
RRu	25.04	25.91	37.07	24.70	21.76
P	+++	+++	+++	+++	+++
Between Chi					30.21
Between df					3
Between P					***
Btwn(F) P					N.S.
Btwn(R) P					N.S.

	Risky occupational population no	mining	othRisky	Total
N	41			41
NS	30			30
Wt	645.92			645.92
Het Chi	288.68			288.68
Het df	40			40
Het P	***			***
Fixed RR	13.77			13.77
RRl	12.75			12.75
RRu	14.87			14.87
P	+++			+++
Random RR	16.91			16.91
RRl	13.14			13.14
RRu	21.76			21.76
P	+++			+++
Between Chi				
Between df				
Between P				N.S.
Btwn(F) P				N.S.
Btwn(R) P				N.S.

	National cigarette tobacco type Virginia	blended	other	Total
N	2	39		41
NS	1	29		30
Wt	7.05	638.87		645.92
Het Chi	0.11	283.71		288.68
Het df	1	38		40
Het P	N.S.	***		***
Fixed RR	31.44	13.65		13.77
RRl	15.03	12.63		12.75
RRu	65.78	14.75		14.87
P	+++	+++		+++
Random RR	31.44	16.41		16.91
RRl	15.03	12.68		13.14
RRu	65.78	21.24		21.76
P	+++	+++		+++
Between Chi				4.86
Between df				1
Between P				*
Btwn(F) P				N.S.
Btwn(R) P				N.S.

Table 2B1 - 3

IESLC - Meta-analysis of Current Smoking (vs never smoking), Any product (or Cigarettes if Any not available)
Squamous
Most adjusted

	Any proxy use No/nk	Yes	Total
N	35	6	41
NS	25	5	30
Wt	602.83	43.10	645.92
Het Chi	261.62	3.90	288.68
Het df	34	5	40
Het P	***	N.S.	***
Fixed RR	13.09	27.96	13.77
RRl	12.09	20.74	12.75
RRu	14.18	37.69	14.87
P	+++	+++	+++
Random RR	15.78	27.96	16.91
RRl	12.00	20.74	13.14
RRu	20.74	37.69	21.76
P	+++	+++	+++
Between Chi			23.16
Between df			1
Between P			***
Btwn(F) P			(*)
Btwn(R) P			**

	Full histological confirmation No	Yes	Total
N	24	17	41
NS	18	12	30
Wt	203.04	442.88	645.92
Het Chi	149.09	130.23	288.68
Het df	23	16	40
Het P	***	***	***
Fixed RR	11.53	14.94	13.77
RRl	10.05	13.61	12.75
RRu	13.23	16.40	14.87
P	+++	+++	+++
Random RR	19.20	14.77	16.91
RRl	12.62	10.61	13.14
RRu	29.22	20.57	21.76
P	+++	+++	+++
Between Chi			9.35
Between df			1
Between P			**
Btwn(F) P			N.S.
Btwn(R) P			N.S.

	Number of adjustment variables (1) 0	1	2+/+nk	Total
N	16	15	10	41
NS	12	11	7	30
Wt	205.78	78.84	361.30	645.92
Het Chi	124.90	31.81	117.15	288.68
Het df	15	14	9	40
Het P	***	**	***	***
Fixed RR	14.93	19.11	12.24	13.77
RRl	13.02	15.33	11.04	12.75
RRu	17.12	23.83	13.57	14.87
P	+++	+++	+++	+++
Random RR	17.15	18.27	15.20	16.91
RRl	10.78	12.55	9.40	13.14
RRu	27.27	26.60	24.58	21.76
P	+++	+++	+++	+++
Between Chi				14.81
Between df				2
Between P				***
Btwn(F) P				N.S.
Btwn(R) P				N.S.

Table 2B1 - 3

IESLC - Meta-analysis of Current Smoking (vs never smoking), Any product (or Cigarettes if Any not available)
Squamous
Most adjusted

	Number of adjustment variables (2) 0	1	2	3-5	6+/+nk	Total
N	16	15	9		1	41
NS	12	11	6		1	30
Wt	205.78	78.84	358.67		2.63	645.92
Het Chi	124.90	31.81	117.11		0.00	288.68
Het df	15	14	8		0	40
Het P	***	**	***		N.S.	***
Fixed RR	14.93	19.11	12.25		10.89	13.77
RRl	13.02	15.33	11.05		3.25	12.75
RRu	17.12	23.83	13.59		36.46	14.87
P	+++	+++	+++		+++	+++
Random RR	17.15	18.27	15.64		10.89	16.91
RRl	10.78	12.55	9.45		3.25	13.14
RRu	27.27	26.60	25.90		36.46	21.76
P	+++	+++	+++		+++	+++
Between Chi						14.85
Between df						3
Between P						**
Btwn(F) P						N.S.
Btwn(R) P						N.S.

	Product all/unsp	cig+/-ot	cig only	Total
N	9	26	6	41
NS	8	18	5	31
Wt	26.26	600.43	19.24	645.92
Het Chi	8.98	269.20	7.44	288.68
Het df	8	25	5	40
Het P	N.S.	***	N.S.	***
Fixed RR	17.58	13.51	17.80	13.77
RRl	11.99	12.47	11.39	12.75
RRu	25.77	14.64	27.83	14.87
P	+++	+++	+++	+++
Random RR	17.76	16.43	18.04	16.91
RRl	11.75	12.08	9.79	13.14
RRu	26.86	22.33	33.27	21.76
P	+++	+++	+++	+++
Between Chi				3.05
Between df				2
Between P				N.S.
Btwn(F) P				N.S.
Btwn(R) P				N.S.

	Denominator nev any	nev cigs	Total
N	24	17	41
NS	20	12	32
Wt	298.26	347.67	645.92
Het Chi	163.26	61.34	288.68
Het df	23	16	40
Het P	***	***	***
Fixed RR	9.80	18.44	13.77
RRl	8.75	16.60	12.75
RRu	10.98	20.48	14.87
P	+++	+++	+++
Random RR	14.05	21.71	16.91
RRl	9.75	16.38	13.14
RRu	20.23	28.76	21.76
P	+++	+++	+++
Between Chi			64.08
Between df			1
Between P			***
Btwn(F) P			**
Btwn(R) P			(*)

Table 2B1 - 3

IESLC - Meta-analysis of Current Smoking (vs never smoking), Any product (or Cigarettes if Any not available)
Squamous
Most adjusted

	Derivation of RR/CI			
	Orig	StdCalc	Other	Total
N	17	13	11	41
NS	12	10	9	31
Wt	421.58	197.39	26.95	645.92
Het Chi	148.21	121.37	12.29	288.68
Het df	16	12	10	40
Het P	***	***	N.S.	***
Fixed RR	12.88	15.19	19.23	13.77
RRl	11.70	13.21	13.18	12.75
RRu	14.17	17.46	28.05	14.87
P	+++	+++	+++	+++
Random RR	15.26	17.63	19.81	16.91
RRl	10.67	10.66	12.62	13.14
RRu	21.84	29.15	31.08	21.76
P	+++	+++	+++	+++
Between Chi				6.80
Between df				2
Between P				*
Btwn(F) P				N.S.
Btwn(R) P				N.S.

Table 2B1 - 4

IESLC - Meta-analysis of Current Smoking (vs never smoking), Any product (or Cigarettes if Any not available)
Squamous
Least adjusted

REF	NRR	X	SEX	AGEL	AGEH	RACE	YF	LC TYPE	LOC	START	ST	NLC	R	VB	P	H	AD	PRODUCT	DENOM	De
BARBON	17	x	m	0	0	all	-	q	Eu:wst	1979	CC	755	n	bl	y	y	0	all/unsp	nev any	st
BOUCOT	70	x	m	0	0	all	0	q	NAmer	1951	pr	121	n	bl	n	n	0	cig only	nev any	ot
BROWN2	16		m	0	0	wh	-	q	NAmer	1984	CC	14596	n	bl	n	y	2	cig+/-ot	nev cigs	or
BROWN2	15		f	0	0	wh	-	q	NAmer	1984	CC	14596	n	bl	n	y	2	cig+/-ot	nev cigs	or
BUFFLE	63		f	0	0	w-hi	-	q	NAmer	1976	CC	943	n	bl	y	n	0	cig+/-ot	nev cigs	st
COMSTO	23		m	0	0	all	-	q	NAmer	1975	ot	258	n	bl	n	n	0	cig+/-ot	nev cigs	st
COMSTO	30		f	0	0	all	-	q	NAmer	1975	ot	258	n	bl	n	n	0	cig+/-ot	nev cigs	ot
CORREA	43		c	0	0	all	-	q+s	NAmer	1979	CC	1359	n	bl	y	n	1	cig+/-ot	nev cigs	or
CPSI	403		m	0	0	all	2	q	NAmer	1959	pr	5138	n	bl	n	n	1	cig only	nev any	ot
CPSI	405		f	0	0	all	2	q	NAmer	1959	pr	5138	n	bl	n	n	1	cig only	nev any	ot
CPSII	114		m	0	0	all	2	q	NAmer	1982	pr	3229	n	bl	n	n	1	cig only	nev any	ot
CPSII	117		f	0	0	all	2	q	NAmer	1982	pr	3229	n	bl	n	n	1	cig+/-ot	nev cigs	ot
DORN	338		m	0	0	wh	8	q	NAmer	1954	pr	5097	n	bl	n	n	1	cig only	nev any	ot
ENGELA	56		m	0	0	all	0	q	Eu:Sca	1964	pr	435	n	bl	n	n	7	cig+/-ot	nev cigs	ot
HAENSZ	20		f	0	0	all	-	q+u	NAmer	1955	CC	158	n	bl	n	y	0	cig+/-ot	nev any	st
HAMMON	102		m	0	0	wh	0	not a	NAmer	1952	pr	448	n	bl	n	n	1	cig only	nev any	ot
JAHN	7		m	0	0	all	-	q	Eu:Ger	1988	CC	1004	n	bl	n	n	0	cig+/-ot	nev any	st
JAIN	18		m	0	0	all	-	q	NAmer	1981	CC	845	n	V	y	n	0	cig+/-ot	nev cigs	st
JAIN	13		f	0	0	all	-	q	NAmer	1981	CC	845	n	V	y	n	0	cig+/-ot	nev cigs	st
JEDRYC	22		m	0	0	all	-	q	Eu:est	1980	CC	1630	n	bl	y	n	0	cig+/-ot	nev any	st
KATSOU	24	x	f	0	0	all	-	KI	Eu:bal	1987	CC	101	n	bl	n	n	0	all/unsp	nev any	st
KHUDER	14		m	0	0	all	-	q	NAmer	1985	CC	482	n	bl	n	y	0	cig+/-ot	nev cigs	or
KIHARA	2		c	0	0	jap	-	q	As:Jap	1991	CC	440	n	bl	n	n	0	all/unsp	nev any	st
LUBIN2	249		m	0	0	all	-	q	Eu:mul	1976	CC	7804	n	bl	n	y	0	cig+/-ot	nev any	st
LUBIN2	261		f	0	0	all	-	q	Eu:mul	1976	CC	7804	n	bl	n	y	0	cig+/-ot	nev any	st
MATOS	38	x	m	0	0	all	-	q	SCAmer	1994	CC	200	n	bl	n	n	0	cig+/-ot	nev any	st
OSANN	10	x	m	0	0	all	-	q	NAmer	1984	CC	1986	n	bl	n	n	0	cig+/-ot	nev cigs	st
OSANN	14	x	f	0	0	all	-	q	NAmer	1984	CC	1986	n	bl	n	n	0	cig+/-ot	nev cigs	st
OSANN2	8	x	f	0	0	all	-	KI	NAmer	1964	ot	217	n	bl	n	y	0	cig+/-ot	nev cigs	st
SOBUE	2	x	m	0	0	all	-	q	As:Jap	1986	CC	1376	n	bl	n	y	0	cig+/-ot	nev cigs	st
SOBUE	18	x	f	0	0	all	-	q	As:Jap	1986	CC	1376	n	bl	n	y	0	cig+/-ot	nev cigs	st
SOBUE2	1		m	0	0	all	-	q	As:Jap	1965	CC	2083	n	bl	n	n	2	cig+/-ot	nev any	or
SOBUE2	5		f	0	0	all	-	q	As:Jap	1965	CC	2083	n	bl	n	n	2	cig+/-ot	nev any	or
SVENSS	62	x	f	0	0	all	-	q	Eu:Sca	1983	CC	210	n	bl	n	n	0	all/unsp	nev any	st
TSUGAN	14		m	0	0	all	-	q	As:Jap	1976	CC	134	n	bl	n	y	0	all/unsp	nev any	ot
WAKAI	4	x	m	0	0	all	-	q	As:Jap	1988	CC	333	n	bl	n	y	0	all/unsp	nev any	st
WAKAI	22	x	f	0	0	all	-	q	As:Jap	1988	CC	333	n	bl	n	y	0	all/unsp	nev any	st
WU	11	x	f	0	0	wh	-	q	NAmer	1981	CC	220	n	bl	n	y	0	all/unsp	nev any	st
WYNDE3	10		m	0	0	all	-	KI	NAmer	1966	CC	350	n	bl	n	y	0	all/unsp	nev any	st
WYNDE6	12		m	0	0	all	-	KI	NAmer	1969	CC	4423	n	bl	n	y	0	cig+/-ot	nev any	st
WYNDE6	201		f	0	0	all	-	KI	NAmer	1969	CC	4423	n	bl	n	y	0	cig+/-ot	nev cigs	st

Cigarette type is all/unspec for all RRs

Table 2B1 - 5

IESLC - Meta-analysis of Current Smoking (vs never smoking), Any product (or Cigarettes if Any not available)
Squamous
Least adjusted

REF	NRR	SEX	AD	Number Exposed Case	Number Exposed Cont	Non-exposed Case	Non-exposed Cont	RR	95.00%CI
BARBON	17	m	0	203	362	6	188	17.57	(7.66- 40.33)
*BOUCOT	70	m	0	38	22177	0	7551	26.22~	(1.61- 426.71)
BROWN2	16	m	2	-	-	-	-	13.70	(11.70- 16.10)
BROWN2	15	f	2	-	-	-	-	20.60	(16.60- 25.60)
Subtotal BROWN2								15.81	(13.91- 17.98)
BUFFLE	63	f	0	39	110	3	112	13.24	(3.97- 44.10)
COMSTO	23	m	0	27	100	2	84	11.34	(2.62- 49.09)
COMSTO	30	f	0	16	52	0	115	72.60~	(4.27-1233.12)
Subtotal COMSTO								16.78	(4.57- 61.67)
CORREA	43	c	1	-	-	-	-	34.60	(22.80- 52.40)
*CPSI	403	m	1	-	-	-	-	29.35	(4.02- 214.28)
*CPSI	405	f	1	-	-	-	-	4.25	(1.23- 14.68)
Subtotal CPSI								7.30	(2.55- 20.90)
*CPSII	114	m	1	-	-	-	-	39.26	(10.38- 148.55)
*CPSII	117	f	1	-	-	-	-	78.91	(15.83- 393.37)
Subtotal CPSII								52.16	(18.72- 145.32)
*DORN	338	m	1	-	-	-	-	17.09	(8.96- 32.60)
*ENGELA	56	m	7	-	-	-	-	10.89	(3.25- 36.43)
HAENSZ	20	f	0	53	94	44	236	3.02	(1.90- 4.82)
*HAMMON	102	m	1	-	-	-	-	26.42	(9.78- 71.34)
JAHN	7	m	0	153	269	3	138	26.16	(8.19- 83.54)
JAIN	18	m	0	107	118	2	85	38.54	(9.26- 160.45)
JAIN	13	f	0	81	99	6	214	29.18	(12.31- 69.15)
Subtotal JAIN								31.44	(15.03- 65.78)
JEDRYC	22	m	0	235	516	6	289	21.94	(9.63- 49.96)
KATSOU	24	f	0	24	18	14	67	6.38	(2.76- 14.78)
KHUDER	14	m	0	112	-	9	-	8.60	(4.20- 17.60)
KIHARA	2	c	0	111	162	5	237	32.48	(12.97- 81.34)
LUBIN2	249	m	0	2518	6209	54	2616	19.65	(14.95- 25.82)
LUBIN2	261	f	0	154	410	72	1180	6.16	(4.55- 8.32)
Subtotal LUBIN2								11.64	(9.50- 14.25)
MATOS	38	m	0	33	132	3	110	9.17	(2.74- 30.70)
OSANN	10	m	0	247	541	8	833	47.54	(23.32- 96.92)
OSANN	14	f	0	130	367	12	1093	32.26	(17.65- 58.97)
Subtotal OSANN								37.93	(23.94- 60.10)
OSANN2	8	f	0	105	42	7	58	20.71	(8.75- 49.05)
SOBUE	2	m	0	301	650	3	128	19.76	(6.24- 62.58)
SOBUE	18	f	0	29	168	14	857	10.57	(5.47- 20.42)
Subtotal SOBUE								12.33	(6.96- 21.84)
SOBUE2	1	m	2	-	-	-	-	5.20	(4.20- 6.50)
SOBUE2	5	f	2	-	-	-	-	7.20	(4.80- 10.80)
Subtotal SOBUE2								5.59	(4.62- 6.78)
SVENSS	62	f	0	42	53	5	120	19.02	(7.12- 50.77)
TSUGAN	14	m	0	18	13	0	5	15.07~	(0.77- 296.43)
WAKAI	4	m	0	86	284	2	65	9.84	(2.36- 41.03)
WAKAI	22	f	0	15	26	3	145	27.88	(7.54- 103.14)
Subtotal WAKAI								17.34	(6.61- 45.48)
WU	11	f	0	61	23	2	30	39.78	(8.79- 180.01)
WYNDE3	10	m	0	171	207	3	88	24.23	(7.53- 77.95)
WYNDE6	12	m	0	1026	741	29	617	29.46	(20.06- 43.26)
WYNDE6	201	f	0	550	376	40	856	31.30	(22.21- 44.12)
Subtotal WYNDE6								30.47	(23.59- 39.36)
Partial Totals				6685	34319	357	18117		

*prospective study ~ With 0.5 adjustment for zero

Table 2B1 - 5

IESLC - Meta-analysis of Current Smoking (vs never smoking), Any product (or Cigarettes if Any not available)
Squamous
Least adjusted

REF	NRR	SEX	AD	Ys	Ws	Qs	Ps
BARBON	17	m	0	2.87	5.57	0.32	0.0000
*BOUCOT	70	m	0	3.27	0.49	0.20	0.0217
BROWN2	16	m	2	2.62	150.78	0.01	0.0000
BROWN2	15	f	2	3.03	81.88	13.09	0.0000
Subtotal BROWN2				2.76	232.67	13.10	
BUFFLE	63	f	0	2.58	2.65	0.00	0.0000
COMSTO	23	m	0	2.43	1.79	0.07	0.0012
COMSTO	30	f	0	4.28	0.48	1.32	0.0030
Subtotal COMSTO				2.82	2.27	1.39	
CORREA	43	c	1	3.54	22.19	18.72	0.0000
*CPSI	403	m	1	3.38	0.97	0.55	0.0009
*CPSI	405	f	1	1.45	2.50	3.47	0.0222
Subtotal CPSI				1.99	3.47	4.02	
*CPSII	114	m	1	3.67	2.17	2.37	0.0000
*CPSII	117	f	1	4.37	1.49	4.52	0.0000
Subtotal CPSII				3.95	3.66	6.89	
*DORN	338	m	1	2.84	9.21	0.42	0.0000
*ENGELA	56	m	7	2.39	2.63	0.15	0.0001
HAENSZ	20	f	0	1.11	17.71	40.85	0.0000
*HAMMON	102	m	1	3.27	3.89	1.64	0.0000
JAHN	7	m	0	3.26	2.85	1.16	0.0000
JAIN	18	m	0	3.65	1.89	1.99	0.0000
JAIN	13	f	0	3.37	5.16	2.89	0.0000
Subtotal JAIN				3.45	7.05	4.88	
JEDRYC	22	m	0	3.09	5.67	1.21	0.0000
KATSOU	24	f	0	1.85	5.45	3.25	0.0000
KHUDER	14	m	0	2.15	7.48	1.68	0.0000
KIHARA	2	c	0	3.48	4.56	3.33	0.0000
LUBIN2	249	m	0	2.98	51.39	6.38	0.0000
LUBIN2	261	f	0	1.82	42.25	27.59	0.0000
Subtotal LUBIN2				2.45	93.64	33.97	
MATOS	38	m	0	2.22	2.63	0.44	0.0003
OSANN	10	m	0	3.86	7.57	11.57	0.0000
OSANN	14	f	0	3.47	10.56	7.61	0.0000
Subtotal OSANN				3.64	18.13	19.17	
OSANN2	8	f	0	3.03	5.17	0.85	0.0000
SOBUE	2	m	0	2.98	2.89	0.37	0.0000
SOBUE	18	f	0	2.36	8.85	0.63	0.0000
Subtotal SOBUE				2.51	11.74	1.00	
SOBUE2	1	m	2	1.65	80.57	76.87	0.0000
SOBUE2	5	f	2	1.97	23.37	9.91	0.0000
Subtotal SOBUE2				1.72	103.93	86.78	
SVENSS	62	f	0	2.95	3.98	0.41	0.0000
TSUGAN	14	m	0	2.71	0.43	0.00	0.0743
WAKAI	4	m	0	2.29	1.88	0.22	0.0017
WAKAI	22	f	0	3.33	2.25	1.11	0.0000
Subtotal WAKAI				2.85	4.13	1.32	
WU	11	f	0	3.68	1.69	1.89	0.0000
WYNDE3	10	m	0	3.19	2.81	0.89	0.0000
WYNDE6	12	m	0	3.38	26.02	14.93	0.0000
WYNDE6	201	f	0	3.44	32.63	21.85	0.0000
Subtotal WYNDE6				3.42	58.65	36.78	

Table 2B1 - 5

IESLC - Meta-analysis of Current Smoking (vs never smoking), Any product (or Cigarettes if Any not available)
Squamous
Least adjusted

	N	41
	NS	30
	Wt	646.41
Het	Chi	286.73
Het	df	40
Het	P	***
Fixed	RR	13.81
	RRl	12.79
	RRu	14.92
	P	+++
Random	RR	16.83
	RRl	13.12
	RRu	21.60
	P	+++
Asymm	P	N.S.

Table 2B1 - 6

IESLC - Meta-analysis of Current Smoking (vs never smoking), Any product (or Cigarettes if Any not available)
Squamous
Least adjusted

	Sex			
	combined	male	female	Total
N	2	22	17	41
NS	2	22	17	41
Wt	26.75	371.60	248.06	646.41
Het Chi	0.02	122.29	141.18	286.73
Het df	1	21	16	40
Het P	N.S.	***	***	***
Fixed RR	34.23	13.06	13.62	13.81
RRl	23.43	11.80	12.02	12.79
RRu	50.00	14.46	15.42	14.92
P	+++	+++	+++	+++
Random RR	34.23	17.62	14.75	16.83
RRl	23.43	12.66	9.56	13.12
RRu	50.00	24.51	22.77	21.60
P	+++	+++	+++	+++
Between Chi				23.25
Between df				2
Between P				***
Btwn(F) P				N.S.
Btwn(R) P				**

	Lung cancer type					
	q	q+s	q+u	KI	not a	Total
N	33	1	1	5	1	41
NS	23	1	1	4	1	30
Wt	530.54	22.19	17.71	72.08	3.89	646.41
Het Chi	180.45	0.00	0.00	12.56	0.00	286.73
Het df	32	0	0	4	0	40
Het P	***	N.S.	N.S.	*	N.S.	***
Fixed RR	12.76	34.60	3.02	26.10	26.42	13.81
RRl	11.72	22.82	1.90	20.72	9.78	12.79
RRu	13.90	52.45	4.82	32.88	71.36	14.92
P	+++	+++	+++	+++	+++	+++
Random RR	16.44	34.60	3.02	21.49	26.42	16.83
RRl	12.67	22.82	1.90	13.27	9.78	13.12
RRu	21.33	52.45	4.82	34.83	71.36	21.60
P	+++	+++	+++	+++	+++	+++
Between Chi						93.72
Between df						4
Between P						***
Btwn(F) P						**
Btwn(R) P						***

	Location								
	NAmer	UK	Scand	othEur	China	Japan	othAs	other	Total
N	24		2	6		8		1	41
NS	17		2	5		5		1	30
Wt	399.20		6.61	113.17		124.79		2.63	646.41
Het Chi	125.62		0.49	38.07		27.00		0.00	286.73
Het df	23		1	5		7		0	40
Het P	***		N.S.	***		***		N.S.	***
Fixed RR	17.98		15.24	12.16		6.69		9.17	13.81
RRl	16.30		7.11	10.11		5.62		2.74	12.79
RRu	19.83		32.65	14.61		7.98		30.70	14.92
P	+++		+++	+++		+++		+++	+++
Random RR	20.71		15.24	13.47		11.35		9.17	16.83
RRl	15.37		7.11	7.30		6.85		2.74	13.12
RRu	27.90		32.65	24.88		18.81		30.70	21.60
P	+++		+++	+++		+++		+++	+++
Between Chi									95.55
Between df									4
Between P									***
Btwn(F) P									**
Btwn(R) P									N.S.

Table 2B1 - 6

IESLC - Meta-analysis of Current Smoking (vs never smoking), Any product (or Cigarettes if Any not available)
Squamous
Least adjusted

	Detailed Country in "other Europe" multi	Germany	othWest	East	Balkans	Total
N	2	1	1	1	1	6
NS	1	1	1	1	1	5
Wt	93.64	2.85	5.57	5.67	5.45	113.17
Het Chi	31.23	0.00	0.00	0.00	0.00	38.07
Het df	1	0	0	0	0	5
Het P	***	N.S.	N.S.	N.S.	N.S.	***
Fixed RR	11.64	26.16	17.57	21.94	6.38	12.16
RRl	9.50	8.19	7.66	9.63	2.76	10.11
RRu	14.25	83.54	40.33	49.96	14.78	14.61
P	+++	+++	+++	+++	+++	+++
Random RR	11.02	26.16	17.57	21.94	6.38	13.47
RRl	3.53	8.19	7.66	9.63	2.76	7.30
RRu	34.35	83.54	40.33	49.96	14.78	24.88
P	+++	+++	+++	+++	+++	+++
Between Chi						6.85
Between df						4
Between P						N.S.
Btwn(F) P						N.S.
Btwn(R) P						N.S.

	Detailed Country in "other Asia" India	HongKong	other	Total
N				
NS				
Wt				
Het Chi				
Het df				
Het P				N.S.
Fixed RR				
RRl				
RRu				
P				+++
Random RR				
RRl				
RRu				
P				+++
Between Chi				
Between df				
Between P				N.S.
Btwn(F) P				N.S.
Btwn(R) P				N.S.

	Detailed other continent SCAmer	Auslia	Africa	Total
N	1			1
NS	1			1
Wt	2.63			2.63
Het Chi	0.00			0.00
Het df	0			0
Het P	N.S.			N.S.
Fixed RR	9.17			9.17
RRl	2.74			2.74
RRu	30.70			30.70
P	+++			+++
Random RR	9.17			9.17
RRl	2.74			2.74
RRu	30.70			30.70
P	+++			+++
Between Chi				
Between df				
Between P				N.S.
Btwn(F) P				N.S.
Btwn(R) P				N.S.

Table 2B1 - 6

IESLC - Meta-analysis of Current Smoking (vs never smoking), Any product (or Cigarettes if Any not available)
Squamous
Least adjusted

	Start year of study <1960	1960-69	1970-79	1980-89	1990+	Total
N	6	7	8	18	2	41
NS	5	5	6	12	2	30
Wt	34.78	173.20	126.75	304.50	7.19	646.41
Het Chi	30.14	114.05	54.12	44.20	2.67	286.73
Het df	5	6	7	17	1	40
Het P	***	***	***	***	N.S.	***
Fixed RR	6.87	10.68	14.49	16.81	20.45	13.81
RRl	4.92	9.21	12.17	15.03	9.84	12.79
RRu	9.57	12.40	17.24	18.81	42.47	14.92
P	+++	+++	+++	+++	+++	+++
Random RR	11.00	14.99	16.26	19.86	18.39	16.83
RRl	4.06	7.11	8.59	15.27	5.36	13.12
RRu	29.84	31.61	30.80	25.83	63.12	21.60
P	+++	+++	+++	+++	+++	+++
Between Chi						41.56
Between df						4
Between P						***
Btwn(F) P						N.S.
Btwn(R) P						N.S.

	Study type (1) CC	other	Total
N	30	11	41
NS	22	8	30
Wt	615.62	30.80	646.41
Het Chi	271.03	12.71	286.73
Het df	29	10	40
Het P	***	N.S.	***
Fixed RR	13.60	18.72	13.81
RRl	12.57	13.15	12.79
RRu	14.72	26.65	14.92
P	+++	+++	+++
Random RR	16.22	19.07	16.83
RRl	12.23	12.46	13.12
RRu	21.50	29.18	21.60
P	+++	+++	+++
Between Chi			2.99
Between df			1
Between P			(*)
Btwn(F) P			N.S.
Btwn(R) P			N.S.

	Study type (2) CC	prosp	other	Total
N	30	8	3	41
NS	22	6	2	30
Wt	615.62	23.36	7.44	646.41
Het Chi	271.03	11.33	1.37	286.73
Het df	29	7	2	40
Het P	***	N.S.	N.S.	***
Fixed RR	13.60	18.50	19.43	13.81
RRl	12.57	12.33	9.47	12.79
RRu	14.72	27.75	39.86	14.92
P	+++	+++	+++	+++
Random RR	16.22	19.33	19.43	16.83
RRl	12.23	10.94	9.47	13.12
RRu	21.50	34.15	39.86	21.60
P	+++	+++	+++	+++
Between Chi				3.00
Between df				2
Between P				N.S.
Btwn(F) P				N.S.
Btwn(R) P				N.S.

Table 2B1 - 6

IESLC - Meta-analysis of Current Smoking (vs never smoking), Any product (or Cigarettes if Any not available)

Squamous

Least adjusted

	Study size (number of LC cases)				
	100-249	250-499	500-999	1000+	Total
N	8	9	4	20	41
NS	8	7	3	12	30
Wt	37.55	27.78	15.27	565.82	646.41
Het Chi	28.95	9.42	1.97	222.87	286.73
Het df	7	8	3	19	40
Het P	***	N.S.	N.S.	***	***
Fixed RR	6.79	16.66	21.88	14.17	13.81
RRl	4.93	11.49	13.25	13.05	12.79
RRu	9.35	24.17	36.13	15.38	14.92
P	+++	+++	+++	+++	+++
Random RR	11.50	17.08	21.88	17.92	16.83
RRl	5.27	11.28	13.25	12.94	13.12
RRu	25.09	25.87	36.13	24.83	21.60
P	+++	+++	+++	+++	+++
Between Chi					23.52
Between df					3
Between P					***
Btwn(F) P					N.S.
Btwn(R) P					N.S.

	Risky occupational population			
	no	mining	othRisky	Total
N	41			41
NS	30			30
Wt	646.41			646.41
Het Chi	286.73			286.73
Het df	40			40
Het P	***			***
Fixed RR	13.81			13.81
RRl	12.79			12.79
RRu	14.92			14.92
P	+++			+++
Random RR	16.83			16.83
RRl	13.12			13.12
RRu	21.60			21.60
P	+++			+++
Between Chi				
Between df				
Between P				N.S.
Btwn(F) P				N.S.
Btwn(R) P				N.S.

	National cigarette tobacco type			
	Virginia	blended	other	Total
N	2	39		41
NS	1	29		30
Wt	7.05	639.36		646.41
Het Chi	0.11	281.80		286.73
Het df	1	38		40
Het P	N.S.	***		***
Fixed RR	31.44	13.69		13.81
RRl	15.03	12.67		12.79
RRu	65.78	14.79		14.92
P	+++	+++		+++
Random RR	31.44	16.34		16.83
RRl	15.03	12.66		13.12
RRu	65.78	21.09		21.60
P	+++	+++		+++
Between Chi				4.82
Between df				1
Between P				*
Btwn(F) P				N.S.
Btwn(R) P				N.S.

Table 2B1 - 6

IESLC - Meta-analysis of Current Smoking (vs never smoking), Any product (or Cigarettes if Any not available)
Squamous
Least adjusted

	Any proxy use No/nk	Yes	Total
N	35	6	41
NS	25	5	30
Wt	603.28	43.13	646.41
Het Chi	260.10	4.23	286.73
Het df	34	5	40
Het P	***	N.S.	***
Fixed RR	13.14	27.71	13.81
RRl	12.13	20.56	12.79
RRu	14.23	37.34	14.92
P	+++	+++	+++
Random RR	15.75	27.71	16.83
RRl	12.01	20.56	13.12
RRu	20.63	37.34	21.60
P	+++	+++	+++
Between Chi			22.40
Between df			1
Between P			***
Btwn(F) P			(*)
Btwn(R) P			**

	Full histological confirmation No	Yes	Total
N	24	17	41
NS	18	12	30
Wt	204.72	441.69	646.41
Het Chi	147.04	129.41	286.73
Het df	23	16	40
Het P	***	***	***
Fixed RR	11.48	15.05	13.81
RRl	10.01	13.71	12.79
RRu	13.16	16.52	14.92
P	+++	+++	+++
Random RR	18.84	15.01	16.83
RRl	12.45	10.83	13.12
RRu	28.52	20.80	21.60
P	+++	+++	+++
Between Chi			10.28
Between df			1
Between P			**
Btwn(F) P			N.S.
Btwn(R) P			N.S.

	Number of adjustment variables (1) 0	1	2+/+nk	Total
N	29	7	5	41
NS	22	5	3	30
Wt	264.76	42.42	339.23	646.41
Het Chi	150.26	13.84	88.33	286.73
Het df	28	6	4	40
Het P	***	*	***	***
Fixed RR	15.79	26.42	11.47	13.81
RRl	14.00	19.55	10.31	12.79
RRu	17.81	35.69	12.76	14.92
P	+++	+++	+++	+++
Random RR	17.63	23.91	10.29	16.83
RRl	12.77	13.63	5.82	13.12
RRu	24.34	41.93	18.20	21.60
P	+++	+++	+++	+++
Between Chi				34.31
Between df				2
Between P				***
Btwn(F) P				(*)
Btwn(R) P				N.S.

Table 2B1 - 6

IESLC - Meta-analysis of Current Smoking (vs never smoking), Any product (or Cigarettes if Any not available)
Squamous
Least adjusted

	Number of adjustment variables (2) 0	1	2	3-5	6+/+nk	Total
N	29	7	4		1	41
NS	22	5	2		1	30
Wt	264.76	42.42	336.60		2.63	646.41
Het Chi	150.26	13.84	88.32		0.00	286.73
Het df	28	6	3		0	40
Het P	***	*	***		N.S.	***
Fixed RR	15.79	26.42	11.47		10.89	13.81
RRl	14.00	19.55	10.31		3.25	12.79
RRu	17.81	35.69	12.77		36.46	14.92
P	+++	+++	+++		+++	+++
Random RR	17.63	23.91	10.22		10.89	16.83
RRl	12.77	13.63	5.53		3.25	13.12
RRu	24.34	41.93	18.87		36.46	21.60
P	+++	+++	+++		+++	+++
Between Chi						34.32
Between df						3
Between P						***
Btwn(F) P						N.S.
Btwn(R) P						N.S.

	Product all/unsp	cig+/-ot	cig only	Total
N	9	26	6	41
NS	8	18	5	31
Wt	28.62	598.55	19.24	646.41
Het Chi	9.87	266.45	7.42	286.73
Het df	8	25	5	40
Het P	N.S.	***	N.S.	***
Fixed RR	17.42	13.55	17.78	13.81
RRl	12.08	12.50	11.37	12.79
RRu	25.13	14.68	27.79	14.92
P	+++	+++	+++	+++
Random RR	17.77	16.31	18.00	16.83
RRl	11.70	12.03	9.78	13.12
RRu	27.00	22.12	33.16	21.60
P	+++	+++	+++	+++
Between Chi				2.99
Between df				2
Between P				N.S.
Btwn(F) P				N.S.
Btwn(R) P				N.S.

	Denominator nev any	nev cigs	Total
N	24	17	41
NS	20	12	32
Wt	300.31	346.10	646.41
Het Chi	164.61	57.11	286.73
Het df	23	16	40
Het P	***	***	***
Fixed RR	9.83	18.56	13.81
RRl	8.77	16.70	12.79
RRu	11.00	20.62	14.92
P	+++	+++	+++
Random RR	14.03	21.67	16.83
RRl	9.77	16.51	13.12
RRu	20.16	28.44	21.60
P	+++	+++	+++
Between Chi			65.02
Between df			1
Between P			***
Btwn(F) P			**
Btwn(R) P			(*)

Table 2B1 - 6

IESLC - Meta-analysis of Current Smoking (vs never smoking), Any product (or Cigarettes if Any not available)
Squamous
Least adjusted

	Derivation of RR/CI			
	Orig	StdCalc	Other	Total
N	6	25	10	41
NS	4	19	8	31
Wt	366.27	255.87	24.27	646.41
Het Chi	114.61	146.20	12.23	286.73
Het df	5	24	9	40
Het P	***	***	N.S.	***
Fixed RR	12.20	16.01	18.94	13.81
RRl	11.01	14.17	12.72	12.79
RRu	13.51	18.10	28.19	14.92
P	+++	+++	+++	+++
Random RR	12.18	17.89	19.80	16.83
RRl	7.12	12.73	11.83	13.12
RRu	20.86	25.13	33.14	21.60
P	+++	+++	+++	+++
Between Chi				13.69
Between df				2
Between P				**
Btwn(F) P				N.S.
Btwn(R) P				N.S.

Table 2B1 - 7

IESLC - Meta-analysis of Current Smoking (vs never smoking), Any product (or Cigarettes if Any not available)
Squamous
Excluded studies (and stage at which they were excluded)

1	BOUCHA BUELL LAURIL MZILEN
2	ABELIN ABRAHA AUVINE AXELSO BAND BERRIN BLOT1 BLOT2 BLOT3 BLOT4 BRESLO BROCKM BROWN1 BYERS1 CASCO2 CASCOR CHAN CHATZI CHEN CHEN2 CHEN3 CHIAZZ COOKSO DAVEYS DEAN DOSEME DU DUNN EBELIN ESAKI FAN GENG GER GODLEY GOLLED GSELL HANSEN HEGMAN HINDS HIRAOK HOROWI HORWIT HU HU2 HUANG ISHIMA JARUP JIANG JONES JUSSAW KO KOHLME KOULUM KREYBE LAMTH LAMWK LAMWK2 LAUSSM LEI LETOUR LEVIN LIU LIU2 LIU3 LIU4 LIU5 LOMBA2 LUO MAGNUS MARSH MARSH2 MARTIS MASTRA MATSUD MCCONN MCDUFF MCLAUG MILLER MILLS MOLLO NOTAN2 NOU ORMOS PASTOR PAWLEG PERNU PERSHA PIKE POFFIJ POLEDN PRESCO QIAO RADZIK RANDIG REN RONCO ROOTS ROTHSC SADOWS SANKAR SCHWA2 SEGI SEOW SIEMIA SIMARA STASZE STOCKS SUN TAO ULMER VANDER WANG WANG3 WANG4 WICKLU WILKIN WUWILL WYNDE2 WYNDE4 XIANGZ XU XU2 XU3 XU4 YUAN ZHANG ZHENG ZHOU
3	RESTRE
4	BOFFET
5	RIMING TANG2 WYNDE5 WYNDE8
6	BYERS2 HIRAY2 LICKIN MRFIT MURATA NOTANI SAARIK SHIMIZ SITAS STAYNE WARSIN WATSON WYNDER
8	AGUDO AKIBA ALDERS AMANDU AMES ANDERS ARCHER ARMADA AUSTIN AXELSS BECHER BENSHL BEST BLOHMK BRETT BROSS CARPEN CEDERL CHANG CHOI CHOW CHYOU DAMBER DARBY DEAN2 DEAN3 DEKLER DESTE2 DESTEF DOCKER DOLL DOLL2 DORANT DORGAN DROSTE ENSTRO GAO GAO2 GARCIA GARDIN GARSHI GILLIS GOODMA GRAHAM GREGOR HAMMO2 HEIN HENNEK HIRAYA HITOSU HOLE HUMBLE JARVHO JOLY KAISE2 KAISER KANELL KAUFMA KELLER KINLEN KJUUS KNEKT KOO KREUZE KUBIK LANGE LEMARC LIAW LIDDEL LOMBAR LUBIN MACLEN MIGRAN MRFITR NAM ODRISC PARKIN PERSH2 PETO PEZZO2 PEZZOT PISANI QIAO2 RACHTA SCHWAR SEGI2 SHAW SIMONA SPEIZE SPITZ STOCKW STUCKE SUZUK2 SUZUKI TANG TENKAN TIZZAN TOKARS TOUSEY TULINI TVERDA VEIERO WALD WANG2 WIGLE WU2 WUNSCH YAMAGU YONG
10	BENHAM VUTUC WYNDE7

Table 2B1 - 8

Potentially overlapping studies

REF	REFGP	PRINC	. OVERLAP/LINK
LUBIN2	LUBIN2	1	Lubin-combined
OSANN2	KAISER	2	KAISER/OSANN2
WYNDE6	WYNDE6	1	WYNDE5/6/7/8
CPSI	CPSI	1	CPSI overall
SOBUE2	SOBUE2	1	SOBUE2/MATSUD
JAHN	BOFFET	2	Subset of BOFFET

Table 2B1 - 9

Most adjusted - insufficient data for metaanalysis

REF	NRR	SEX	AGEL	AGEH	RACE	YF	LC TYPE	LOC	START	ST	NLC	R	VB	P	H	AD	PRODUCT	DENOM	De
CHYOU	5	m	0	0	jap	0	q+s	NAmer	1965	pr	227	n	bl	n	y	1	cig+/-ot	nev cigs or	

REF	NRR	RR	SIG	RRDATA comment
CHYOU	5	16.00		0

Table 2B2 -

IESLC - Meta-analysis of Current Smoking (vs never smoking), Cigarettes (or Any Product if Cigarettes not available)
Squamous

This analysis is restricted to results for:
1) Non-dose-response data
2) Current smokers
3) Results complete enough for use in metaanalysis

Within each study, results are then selected (in the following order of preference, within each sex) for:
4) PRODUCT: cigarettes regardless of other products, cigarettes only, all/unspec
5) CIGTYPE: all/unspecified, MC regardless of HR, MC only
6) DENOM: never smoked anything, never smoked cigarettes, (never +1 = +long term ex, +2 = +amount unknown,
+3 = never cigs+long term ex)
7) Followup period (YF, prospective studies): whole study (coded as 0) or longest available
8) LCtype: squamous or nearest available, but not adeno. (q = squamous, s = small,
a = adeno, KI = Kreyberg I, u = undifferentiated)
9) Race: all or nearest available, otherwise by race (wh or w = white, bl or b = black, hi = hispanic
ch = chinese, jap = japanese, haw = hawaiian, w+o = white + oriental, sca = scandinavian, as = asian)
10) For overlapping studies: principal rather than subsidiary studies
Finally by Age: whole study (coded as 0) if available, otherwise by widest available age group
and then for single sex results (m, f) in preference to combined sex results (c).

Results adjusted (AD) for the most potential confounders are then chosen in Sections -1 to -3
(and those which actually differ from the adjusted results in Table 2B1 - 1 are marked 'x' in Section -1)
and results adjusted for the least confounders in Sections -4 to -6. (Those least adjusted results which
actually differ from the most adjusted as marked 'x' in column X in Section -4)
(Results adjusted for an unknown number of confounder(s) are coded as 20.)

Section -7 shows excluded studies, together with the stage (as above) at which no qualifying
results were found.

Section -8 lists the potentially overlapping studies which have been included (1=principal, 2=subsidiary).

Section -9 lists any results which would have been included in preference except that they had data not complete
enough for use in meta-analysis, with their significance (yes/no), if known, and any further comment as entered
on the database.

In addition to those mentioned above, the following fields, levels and abbreviations are used:
* or nk = not known, n = no, y = yes, ot = other
nev = never
all/unspec = all or unspecified, cig+/-ot = cigarettes irrespective of other products (cigar, pipe etc)
MC = manufactured cigarettes, HR = hand-rolled cigarettes
REF: 6-character study reference
NRR: number of the RR on the database within the study
ST : study type (CC = case control, pr or prosp = prospective)
NLC: number of lung cancer cases in whole study
R : risky occupational population (n = no, m = mining, o = other risky)
VB : national cigarette type (V = at least 75% Virginia, bl = at least 75% blended, ot = other)
P : any proxy use
H : full histological confirmation
De : derivation of RR/CI (or = original, st = standard method, ot = other method of estimation)

Table 2B2 - 1

IESLC - Meta-analysis of Current Smoking (vs never smoking), Cigarettes (or Any Product if Cigarettes not available)
Squamous
Most adjusted

REF	NRR	2B1	SEX	AGEL	AGEH	RACE	YF	LC TYPE	LOC	START	ST	NLC	R	VB	P	H	AD	PRODUCT	DENOM	De
BARBON	18		m	0	0	all	-	q	Eu:wst	1979	CC	755	n	bl	y	y	1	all/unsp	nev any	or
BOUCOT	141		m	0	0	all	0	q	NAmer	1951	pr	121	n	bl	n	n	2	cig only	nev any	ot
BROWN2	16		m	0	0	wh	-	q	NAmer	1984	CC	14596	n	bl	n	y	2	cig+/-ot	nev cigs	or
BROWN2	15		f	0	0	wh	-	q	NAmer	1984	CC	14596	n	bl	n	y	2	cig+/-ot	nev cigs	or
BUFFLE	63		f	0	0	w-hi	-	q	NAmer	1976	CC	943	n	bl	y	n	0	cig+/-ot	nev cigs	st
COMSTO	23		m	0	0	all	-	q	NAmer	1975	ot	258	n	bl	n	n	0	cig+/-ot	nev cigs	st
COMSTO	30		f	0	0	all	-	q	NAmer	1975	ot	258	n	bl	n	n	0	cig+/-ot	nev cigs	ot
CORREA	43		c	0	0	all	-	q+s	NAmer	1979	CC	1359	n	bl	y	n	1	cig+/-ot	nev cigs	or
CPSI	403		m	0	0	all	2	q	NAmer	1959	pr	5138	n	bl	n	n	1	cig only	nev any	ot
CPSI	405		f	0	0	all	2	q	NAmer	1959	pr	5138	n	bl	n	n	1	cig only	nev any	ot
CPSII	114		m	0	0	all	2	q	NAmer	1982	pr	3229	n	bl	n	n	1	cig only	nev any	ot
CPSII	117		f	0	0	all	2	q	NAmer	1982	pr	3229	n	bl	n	n	1	cig+/-ot	nev cigs	ot
DORN	338		m	0	0	wh	8	q	NAmer	1954	pr	5097	n	bl	n	n	1	cig only	nev any	ot
ENGELA	56		m	0	0	all	0	q	Eu:Sca	1964	pr	435	n	bl	n	n	7	cig+/-ot	nev cigs	ot
HAENSZ	20		f	0	0	all	-	q+u	NAmer	1955	CC	158	n	bl	n	y	0	cig+/-ot	nev any	st
HAMMON	102		m	0	0	wh	0	not a	NAmer	1952	pr	448	n	bl	n	n	1	cig only	nev any	ot
JAHN	7		m	0	0	all	-	q	Eu:Ger	1988	CC	1004	n	bl	n	n	0	cig+/-ot	nev any	st
JAIN	18		m	0	0	all	-	q	NAmer	1981	CC	845	n	V	y	n	0	cig+/-ot	nev cigs	st
JAIN	13		f	0	0	all	-	q	NAmer	1981	CC	845	n	V	y	n	0	cig+/-ot	nev cigs	st
JEDRYC	22		m	0	0	all	-	q	Eu:est	1980	CC	1630	n	bl	y	n	0	cig+/-ot	nev any	st
KATSOU	20		f	0	0	all	-	KI	Eu:bal	1987	CC	101	n	bl	n	n	1	all/unsp	nev any	or
KHUDER	14		m	0	0	all	-	q	NAmer	1985	CC	482	n	bl	n	y	0	cig+/-ot	nev cigs	or
KIHARA	2		c	0	0	jap	-	q	As:Jap	1991	CC	440	n	bl	n	n	0	all/unsp	nev any	st
LUBIN2	249		m	0	0	all	-	q	Eu:mul	1976	CC	7804	n	bl	n	y	0	cig+/-ot	nev any	st
LUBIN2	261		f	0	0	all	-	q	Eu:mul	1976	CC	7804	n	bl	n	y	0	cig+/-ot	nev any	st
MATOS	39		m	0	0	all	-	q	SCAmer	1994	CC	200	n	bl	n	n	2	cig+/-ot	nev any	or
OSANN	35		m	0	0	all	-	q	NAmer	1984	CC	1986	n	bl	n	n	2	cig+/-ot	nev cigs	or
OSANN	36		f	0	0	all	-	q	NAmer	1984	CC	1986	n	bl	n	n	2	cig+/-ot	nev cigs	or
OSANN2	26		f	0	0	all	-	KI	NAmer	1964	ot	217	n	bl	n	y	1	cig+/-ot	nev cigs	or
SOBUE	34		m	0	0	all	-	q	As:Jap	1986	CC	1376	n	bl	n	y	1	cig+/-ot	nev cigs	or
SOBUE	44		f	0	0	all	-	q	As:Jap	1986	CC	1376	n	bl	n	y	1	cig+/-ot	nev cigs	or
SOBUE2	1		m	0	0	all	-	q	As:Jap	1965	CC	2083	n	bl	n	n	2	cig+/-ot	nev any	or
SOBUE2	5		f	0	0	all	-	q	As:Jap	1965	CC	2083	n	bl	n	n	2	cig+/-ot	nev any	or
SVENSS	97		f	0	0	all	-	q	Eu:Sca	1983	CC	210	n	bl	n	n	1	all/unsp	nev any	ot
TSUGAN	14		m	0	0	all	-	q	As:Jap	1976	CC	134	n	bl	n	y	0	all/unsp	nev any	ot
WAKAI	10		m	0	0	all	-	q	As:Jap	1988	CC	333	n	bl	n	y	1	all/unsp	nev any	or
WAKAI	28		f	0	0	all	-	q	As:Jap	1988	CC	333	n	bl	n	y	1	all/unsp	nev any	or
WU	16		f	0	0	wh	-	q	NAmer	1981	CC	220	n	bl	n	y	2	all/unsp	nev any	or
WYNDE3	10		m	0	0	all	-	KI	NAmer	1966	CC	350	n	bl	n	y	0	all/unsp	nev any	st
WYNDE6	12		m	0	0	all	-	KI	NAmer	1969	CC	4423	n	bl	n	y	0	cig+/-ot	nev any	st
WYNDE6	201		f	0	0	all	-	KI	NAmer	1969	CC	4423	n	bl	n	y	0	cig+/-ot	nev cigs	st

Cigarette type is all/unspec for all RRs

Table 2B2 - 2

IESLC - Meta-analysis of Current Smoking (vs never smoking), Cigarettes (or Any Product if Cigarettes not available)
Squamous
Most adjusted

REF	NRR	SEX	AD	Number Exposed Case	Number Exposed Cont	Non-exposed Case	Non-exposed Cont	RR	95.00%CI
BARBON	18	m	1	-	-	-	-	18.80	(8.20- 43.40)
*BOUCOT	141	m	2	-	-	-	-	27.54	(1.69- 448.37)
BROWN2	16	m	2	-	-	-	-	13.70	(11.70- 16.10)
BROWN2	15	f	2	-	-	-	-	20.60	(16.60- 25.60)
Subtotal BROWN2								15.81	(13.91- 17.98)
BUFFLE	63	f	0	39	110	3	112	13.24	(3.97- 44.10)
COMSTO	23	m	0	27	100	2	84	11.34	(2.62- 49.09)
COMSTO	30	f	0	16	52	0	115	72.60~	(4.27-1233.12)
Subtotal COMSTO								16.78	(4.57- 61.67)
CORREA	43	c	1	-	-	-	-	34.60	(22.80- 52.40)
*CPSI	403	m	1	-	-	-	-	29.35	(4.02- 214.28)
*CPSI	405	f	1	-	-	-	-	4.25	(1.23- 14.68)
Subtotal CPSI								7.30	(2.55- 20.90)
*CPSII	114	m	1	-	-	-	-	39.26	(10.38- 148.55)
*CPSII	117	f	1	-	-	-	-	78.91	(15.83- 393.37)
Subtotal CPSII								52.16	(18.72- 145.32)
*DORN	338	m	1	-	-	-	-	17.09	(8.96- 32.60)
*ENGELA	56	m	7	-	-	-	-	10.89	(3.25- 36.43)
HAENSZ	20	f	0	53	94	44	236	3.02	(1.90- 4.82)
*HAMMON	102	m	1	-	-	-	-	26.42	(9.78- 71.34)
JAHN	7	m	0	153	269	3	138	26.16	(8.19- 83.54)
JAIN	18	m	0	107	118	2	85	38.54	(9.26- 160.45)
JAIN	13	f	0	81	99	6	214	29.18	(12.31- 69.15)
Subtotal JAIN								31.44	(15.03- 65.78)
JEDRYC	22	m	0	235	516	6	289	21.94	(9.63- 49.96)
KATSOU	20	f	1	-	-	-	-	6.45	(2.73- 15.25)
KHUDER	14	m	0	112	-	9	-	8.60	(4.20- 17.60)
KIHARA	2	c	0	111	162	5	237	32.48	(12.97- 81.34)
LUBIN2	249	m	0	2518	6209	54	2616	19.65	(14.95- 25.82)
LUBIN2	261	f	0	154	410	72	1180	6.16	(4.55- 8.32)
Subtotal LUBIN2								11.64	(9.50- 14.25)
MATOS	39	m	2	-	-	-	-	10.40	(3.60- 35.50)
OSANN	35	m	2	-	-	-	-	49.30	(24.10- 101.00)
OSANN	36	f	2	-	-	-	-	35.20	(19.10- 65.10)
Subtotal OSANN								40.59	(25.47- 64.67)
OSANN2	26	f	1	-	-	-	-	39.00	(5.30- 287.00)
SOBUE	34	m	1	-	-	-	-	18.10	(7.90- 41.30)
SOBUE	44	f	1	-	-	-	-	9.70	(5.50- 16.80)
Subtotal SOBUE								11.79	(7.42- 18.73)
SOBUE2	1	m	2	-	-	-	-	5.20	(4.20- 6.50)
SOBUE2	5	f	2	-	-	-	-	7.20	(4.80- 10.80)
Subtotal SOBUE2								5.59	(4.62- 6.78)
SVENSS	97	f	1	-	-	-	-	21.92	(6.63- 72.51)
TSUGAN	14	m	0	18	13	0	5	15.07~	(0.77- 296.43)
WAKAI	10	m	1	-	-	-	-	9.82	(2.36- 41.00)
WAKAI	28	f	1	-	-	-	-	28.20	(7.55- 105.00)
Subtotal WAKAI								17.37	(6.60- 45.71)
WU	16	f	2	-	-	-	-	35.30	(4.70- 267.30)
WYNDE3	10	m	0	171	207	3	88	24.23	(7.53- 77.95)
WYNDE6	12	m	0	1026	741	29	617	29.46	(20.06- 43.26)
WYNDE6	201	f	0	550	376	40	856	31.30	(22.21- 44.12)
Subtotal WYNDE6								30.47	(23.59- 39.36)
Partial Totals				5371	9476	278	6872		

*prospective study ~ With 0.5 adjustment for zero

Table 2B2 - 2

IESLC - Meta-analysis of Current Smoking (vs never smoking), Cigarettes (or Any Product if Cigarettes not available)
Squamous
Most adjusted

REF	NRR	SEX	AD	Ys	Ws	Qs	Ps
BARBON	18	m	1	2.93	5.53	0.54	0.0000
*BOUCOT	141	m	2	3.32	0.49	0.24	0.0199
BROWN2	16	m	2	2.62	150.78	0.00	0.0000
BROWN2	15	f	2	3.03	81.88	13.28	0.0000
Subtotal BROWN2				2.76	232.67	13.29	
BUFFLE	63	f	0	2.58	2.65	0.00	0.0000
COMSTO	23	m	0	2.43	1.79	0.07	0.0012
COMSTO	30	f	0	4.28	0.48	1.32	0.0030
Subtotal COMSTO				2.82	2.27	1.39	
CORREA	43	c	1	3.54	22.19	18.84	0.0000
*CPSI	403	m	1	3.38	0.97	0.56	0.0009
*CPSI	405	f	1	1.45	2.50	3.45	0.0222
Subtotal CPSI				1.99	3.47	4.01	
*CPSII	114	m	1	3.67	2.17	2.38	0.0000
*CPSII	117	f	1	4.37	1.49	4.54	0.0000
Subtotal CPSII				3.95	3.66	6.92	
*DORN	338	m	1	2.84	9.21	0.43	0.0000
*ENGELA	56	m	7	2.39	2.63	0.14	0.0001
HAENSZ	20	f	0	1.11	17.71	40.69	0.0000
*HAMMON	102	m	1	3.27	3.89	1.65	0.0000
JAHN	7	m	0	3.26	2.85	1.17	0.0000
JAIN	18	m	0	3.65	1.89	2.00	0.0000
JAIN	13	f	0	3.37	5.16	2.91	0.0000
Subtotal JAIN				3.45	7.05	4.91	
JEDRYC	22	m	0	3.09	5.67	1.23	0.0000
KATSOU	20	f	1	1.86	5.19	2.99	0.0000
KHUDER	14	m	0	2.15	7.48	1.66	0.0000
KIHARA	2	c	0	3.48	4.56	3.36	0.0000
LUBIN2	249	m	0	2.98	51.39	6.49	0.0000
LUBIN2	261	f	0	1.82	42.25	27.39	0.0000
Subtotal LUBIN2				2.45	93.64	33.88	
MATOS	39	m	2	2.34	2.93	0.23	0.0001
OSANN	35	m	2	3.90	7.48	12.17	0.0000
OSANN	36	f	2	3.56	10.22	9.00	0.0000
Subtotal OSANN				3.70	17.70	21.17	
OSANN2	26	f	1	3.66	0.96	1.05	0.0003
SOBUE	34	m	1	2.90	5.62	0.42	0.0000
SOBUE	44	f	1	2.27	12.32	1.51	0.0000
Subtotal SOBUE				2.47	17.94	1.93	
SOBUE2	1	m	2	1.65	80.57	76.41	0.0000
SOBUE2	5	f	2	1.97	23.37	9.82	0.0000
Subtotal SOBUE2				1.72	103.93	86.24	
SVENSS	97	f	1	3.09	2.69	0.58	0.0000
TSUGAN	14	m	0	2.71	0.43	0.00	0.0743
WAKAI	10	m	1	2.28	1.89	0.22	0.0017
WAKAI	28	f	1	3.34	2.22	1.14	0.0000
Subtotal WAKAI				2.85	4.10	1.35	
WU	16	f	2	3.56	0.94	0.83	0.0005
WYNDE3	10	m	0	3.19	2.81	0.90	0.0000
WYNDE6	12	m	0	3.38	26.02	15.05	0.0000
WYNDE6	201	f	0	3.44	32.63	22.00	0.0000
Subtotal WYNDE6				3.42	58.65	37.05	

Table 2B2 - 2

IESLC - Meta-analysis of Current Smoking (vs never smoking), Cigarettes (or Any Product if Cigarettes not available)
Squamous
Most adjusted

	N	41
	NS	30
	Wt	645.92
Het	Chi	288.68
Het	df	40
Het	P	***
Fixed	RR	13.77
	RRl	12.75
	RRu	14.87
	P	+++
Random	RR	16.91
	RRl	13.14
	RRu	21.76
	P	+++
Asymm	P	N.S.

Table 2B2 - 3

IESLC - Meta-analysis of Current Smoking (vs never smoking), Cigarettes (or Any Product if Cigarettes not available)
Squamous
Most adjusted

		Sex		
	combined	male	female	Total
N	2	22	17	41
NS	2	22	17	41
Wt	26.75	374.51	244.66	645.92
Het Chi	0.02	123.06	142.38	288.68
Het df	1	21	16	40
Het P	N.S.	***	***	***
Fixed RR	34.23	13.11	13.44	13.77
RRl	23.43	11.85	11.86	12.75
RRu	50.00	14.51	15.23	14.87
P	+++	+++	+++	+++
Random RR	34.23	17.73	14.77	16.91
RRl	23.43	12.78	9.42	13.14
RRu	50.00	24.59	23.17	21.76
P	+++	+++	+++	+++
Between Chi				23.23
Between df				2
Between P				***
Btwn(F) P				N.S.
Btwn(R) P				**

		Lung cancer type				
	q	q+s	q+u	KI	not a	Total
N	33	1	1	5	1	41
NS	23	1	1	4	1	30
Wt	534.51	22.19	17.71	67.62	3.89	645.92
Het Chi	182.31	0.00	0.00	11.72	0.00	288.68
Het df	32	0	0	4	0	40
Het P	***	N.S.	N.S.	*	N.S.	***
Fixed RR	12.74	34.60	3.02	26.88	26.42	13.77
RRl	11.71	22.82	1.90	21.18	9.78	12.75
RRu	13.87	52.45	4.82	34.12	71.36	14.87
P	+++	+++	+++	+++	+++	+++
Random RR	16.43	34.60	3.02	22.51	26.42	16.91
RRl	12.66	22.82	1.90	13.42	9.78	13.14
RRu	21.32	52.45	4.82	37.78	71.36	21.76
P	+++	+++	+++	+++	+++	+++
Between Chi						94.65
Between df						4
Between P						***
Btwn(F) P						**
Btwn(R) P						***

				Location					
	NAmer	UK	Scand	othEur	China	Japan	othAs	other	Total
N	24		2	6		8		1	41
NS	17		2	5		5		1	30
Wt	393.82		5.32	112.89		130.97		2.93	645.92
Het Chi	126.94		0.65	38.19		28.96		0.00	288.68
Het df	23		1	5		7		0	40
Het P	***		N.S.	***		***		N.S.	***
Fixed RR	17.99		15.51	12.22		6.85		10.40	13.77
RRl	16.29		6.63	10.16		5.77		3.31	12.75
RRu	19.85		36.28	14.69		8.13		32.66	14.87
P	+++		+++	+++		+++		+++	+++
Random RR	20.95		15.51	13.66		11.25		10.40	16.91
RRl	15.41		6.63	7.38		6.89		3.31	13.14
RRu	28.48		36.28	25.30		18.35		32.66	21.76
P	+++		+++	+++		+++		+++	+++
Between Chi									93.93
Between df									4
Between P									***
Btwn(F) P									**
Btwn(R) P									N.S.

Table 2B2 - 3

IESLC - Meta-analysis of Current Smoking (vs never smoking), Cigarettes (or Any Product if Cigarettes not available)
Squamous
Most adjusted

	Detailed Country in "other Europe"					
	multi	Germany	othWest	East	Balkans	Total
N	2	1	1	1	1	6
NS	1	1	1	1	1	5
Wt	93.64	2.85	5.53	5.67	5.19	112.89
Het Chi	31.23	0.00	0.00	0.00	0.00	38.19
Het df	1	0	0	0	0	5
Het P	***	N.S.	N.S.	N.S.	N.S.	***
Fixed RR	11.64	26.16	18.80	21.94	6.45	12.22
RRl	9.50	8.19	8.17	9.63	2.73	10.16
RRu	14.25	83.54	43.25	49.96	15.24	14.69
P	+++	+++	+++	+++	+++	+++
Random RR	11.02	26.16	18.80	21.94	6.45	13.66
RRl	3.53	8.19	8.17	9.63	2.73	7.38
RRu	34.35	83.54	43.25	49.96	15.24	25.30
P	+++	+++	+++	+++	+++	+++
Between Chi						6.96
Between df						4
Between P						N.S.
Btwn(F) P						N.S.
Btwn(R) P						N.S.

	Detailed Country in "other Asia"			
	India	HongKong	other	Total
N				
NS				
Wt				
Het Chi				
Het df				
Het P				N.S.
Fixed RR				
RRl				
RRu				
P				+++
Random RR				
RRl				
RRu				
P				+++
Between Chi				
Between df				
Between P				N.S.
Btwn(F) P				N.S.
Btwn(R) P				N.S.

	Detailed other continent			
	SCAmer	Auslia	Africa	Total
N	1			1
NS	1			1
Wt	2.93			2.93
Het Chi	0.00			0.00
Het df	0			0
Het P	N.S.			N.S.
Fixed RR	10.40			10.40
RRl	3.31			3.31
RRu	32.66			32.66
P	+++			+++
Random RR	10.40			10.40
RRl	3.31			3.31
RRu	32.66			32.66
P	+++			+++
Between Chi				
Between df				
Between P				N.S.
Btwn(F) P				N.S.
Btwn(R) P				N.S.

Table 2B2 - 3

IESLC - Meta-analysis of Current Smoking (vs never smoking), Cigarettes (or Any Product if Cigarettes not available)
Squamous
Most adjusted

	Start year of study <1960	1960-69	1970-79	1980-89	1990+	Total
N	6	7	8	18	2	41
NS	5	5	6	12	2	30
Wt	34.78	169.00	126.72	307.94	7.49	645.92
Het Chi	30.21	113.37	54.28	46.61	2.31	288.68
Het df	5	6	7	17	1	40
Het P	***	***	***	***	N.S.	***
Fixed RR	6.87	10.55	14.53	16.70	20.79	13.77
RRl	4.93	9.07	12.21	14.93	10.16	12.75
RRu	9.58	12.26	17.29	18.67	42.55	14.87
P	+++	+++	+++	+++	+++	+++
Random RR	11.05	15.48	16.43	19.69	19.39	16.91
RRl	4.07	7.08	8.67	15.08	6.38	13.14
RRu	30.00	33.83	31.14	25.71	58.88	21.76
P	+++	+++	+++	+++	+++	+++
Between Chi						41.89
Between df						4
Between P						***
Btwn(F) P						N.S.
Btwn(R) P						N.S.

	Study type (1) CC	other	Total
N	30	11	41
NS	22	8	30
Wt	619.33	26.59	645.92
Het Chi	272.73	13.19	288.68
Het df	29	10	40
Het P	***	N.S.	***
Fixed RR	13.59	18.87	13.77
RRl	12.56	12.90	12.75
RRu	14.70	27.59	14.87
P	+++	+++	+++
Random RR	16.21	19.63	16.91
RRl	12.23	12.21	13.14
RRu	21.50	31.56	21.76
P	+++	+++	+++
Between Chi			2.75
Between df			1
Between P			(*)
Btwn(F) P			N.S.
Btwn(R) P			N.S.

	Study type (2) CC	prosp	other	Total
N	30	8	3	41
NS	22	6	2	30
Wt	619.33	23.36	3.23	645.92
Het Chi	272.73	11.34	1.78	288.68
Het df	29	7	2	40
Het P	***	N.S.	N.S.	***
Fixed RR	13.59	18.52	21.58	13.77
RRl	12.56	12.34	7.26	12.75
RRu	14.70	27.78	64.20	14.87
P	+++	+++	+++	+++
Random RR	16.21	19.36	21.58	16.91
RRl	12.23	10.95	7.26	13.14
RRu	21.50	34.24	64.20	21.76
P	+++	+++	+++	+++
Between Chi				2.82
Between df				2
Between P				N.S.
Btwn(F) P				N.S.
Btwn(R) P				N.S.

Table 2B2 - 3

IESLC - Meta-analysis of Current Smoking (vs never smoking), Cigarettes (or Any Product if Cigarettes not available)
Squamous
Most adjusted

	Study size (number of LC cases) 100-249	250-499	500-999	1000+	Total
N	8	9	4	20	41
NS	8	7	3	12	30
Wt	31.35	27.75	15.24	571.59	645.92
Het Chi	21.45	9.44	1.82	225.75	288.68
Het df	7	8	3	19	40
Het P	**	N.S.	N.S.	***	***
Fixed RR	5.62	16.67	22.44	14.15	13.77
RRl	3.96	11.49	13.58	13.03	12.75
RRu	7.98	24.18	37.07	15.35	14.87
P	+++	+++	+++	+++	+++
Random RR	11.28	17.09	22.44	17.87	16.91
RRl	5.08	11.28	13.58	12.92	13.14
RRu	25.04	25.91	37.07	24.70	21.76
P	+++	+++	+++	+++	+++
Between Chi					30.21
Between df					3
Between P					***
Btwn(F) P					N.S.
Btwn(R) P					N.S.

	Risky occupational population no	mining	othRisky	Total
N	41			41
NS	30			30
Wt	645.92			645.92
Het Chi	288.68			288.68
Het df	40			40
Het P	***			***
Fixed RR	13.77			13.77
RRl	12.75			12.75
RRu	14.87			14.87
P	+++			+++
Random RR	16.91			16.91
RRl	13.14			13.14
RRu	21.76			21.76
P	+++			+++
Between Chi				
Between df				
Between P				N.S.
Btwn(F) P				N.S.
Btwn(R) P				N.S.

	National cigarette tobacco type Virginia	blended	other	Total
N	2	39		41
NS	1	29		30
Wt	7.05	638.87		645.92
Het Chi	0.11	283.71		288.68
Het df	1	38		40
Het P	N.S.	***		***
Fixed RR	31.44	13.65		13.77
RRl	15.03	12.63		12.75
RRu	65.78	14.75		14.87
P	+++	+++		+++
Random RR	31.44	16.41		16.91
RRl	15.03	12.68		13.14
RRu	65.78	21.24		21.76
P	+++	+++		+++
Between Chi				4.86
Between df				1
Between P				*
Btwn(F) P				N.S.
Btwn(R) P				N.S.

Table 2B2 - 3

IESLC - Meta-analysis of Current Smoking (vs never smoking), Cigarettes (or Any Product if Cigarettes not available)
Squamous
Most adjusted

	Any proxy use: No/nk	Yes	Total
N	35	6	41
NS	25	5	30
Wt	602.83	43.10	645.92
Het Chi	261.62	3.90	288.68
Het df	34	5	40
Het P	***	N.S.	***
Fixed RR	13.09	27.96	13.77
RRl	12.09	20.74	12.75
RRu	14.18	37.69	14.87
P	+++	+++	+++
Random RR	15.78	27.96	16.91
RRl	12.00	20.74	13.14
RRu	20.74	37.69	21.76
P	+++	+++	+++
Between Chi			23.16
Between df			1
Between P			***
Btwn(F) P			(*)
Btwn(R) P			**

	Full histological confirmation: No	Yes	Total
N	24	17	41
NS	18	12	30
Wt	203.04	442.88	645.92
Het Chi	149.09	130.23	288.68
Het df	23	16	40
Het P	***	***	***
Fixed RR	11.53	14.94	13.77
RRl	10.05	13.61	12.75
RRu	13.23	16.40	14.87
P	+++	+++	+++
Random RR	19.20	14.77	16.91
RRl	12.62	10.61	13.14
RRu	29.22	20.57	21.76
P	+++	+++	+++
Between Chi			9.35
Between df			1
Between P			**
Btwn(F) P			N.S.
Btwn(R) P			N.S.

	Number of adjustment variables (1): 0	1	2+/+nk	Total
N	16	15	10	41
NS	12	11	7	30
Wt	205.78	78.84	361.30	645.92
Het Chi	124.90	31.81	117.15	288.68
Het df	15	14	9	40
Het P	***	**	***	***
Fixed RR	14.93	19.11	12.24	13.77
RRl	13.02	15.33	11.04	12.75
RRu	17.12	23.83	13.57	14.87
P	+++	+++	+++	+++
Random RR	17.15	18.27	15.20	16.91
RRl	10.78	12.55	9.40	13.14
RRu	27.27	26.60	24.58	21.76
P	+++	+++	+++	+++
Between Chi				14.81
Between df				2
Between P				***
Btwn(F) P				N.S.
Btwn(R) P				N.S.

Table 2B2 - 3

IESLC - Meta-analysis of Current Smoking (vs never smoking), Cigarettes (or Any Product if Cigarettes not available)

Squamous

Most adjusted

	Number of adjustment variables (2) 0	1	2	3-5	6+/+nk	Total
N	16	15	9		1	41
NS	12	11	6		1	30
Wt	205.78	78.84	358.67		2.63	645.92
Het Chi	124.90	31.81	117.11		0.00	288.68
Het df	15	14	8		0	40
Het P	***	**	***		N.S.	***
Fixed RR	14.93	19.11	12.25		10.89	13.77
RRl	13.02	15.33	11.05		3.25	12.75
RRu	17.12	23.83	13.59		36.46	14.87
P	+++	+++	+++		+++	+++
Random RR	17.15	18.27	15.64		10.89	16.91
RRl	10.78	12.55	9.45		3.25	13.14
RRu	27.27	26.60	25.90		36.46	21.76
P	+++	+++	+++		+++	+++
Between Chi						14.85
Between df						3
Between P						**
Btwn(F) P						N.S.
Btwn(R) P						N.S.

	Product all/unsp	cig+/-ot	cig only	Total
N	9	26	6	41
NS	8	18	5	31
Wt	26.26	600.43	19.24	645.92
Het Chi	8.98	269.20	7.44	288.68
Het df	8	25	5	40
Het P	N.S.	***	N.S.	***
Fixed RR	17.58	13.51	17.80	13.77
RRl	11.99	12.47	11.39	12.75
RRu	25.77	14.64	27.83	14.87
P	+++	+++	+++	+++
Random RR	17.76	16.43	18.04	16.91
RRl	11.75	12.08	9.79	13.14
RRu	26.86	22.33	33.27	21.76
P	+++	+++	+++	+++
Between Chi				3.05
Between df				2
Between P				N.S.
Btwn(F) P				N.S.
Btwn(R) P				N.S.

	Denominator nev any	nev cigs	Total
N	24	17	41
NS	20	12	32
Wt	298.26	347.67	645.92
Het Chi	163.26	61.34	288.68
Het df	23	16	40
Het P	***	***	***
Fixed RR	9.80	18.44	13.77
RRl	8.75	16.60	12.75
RRu	10.98	20.48	14.87
P	+++	+++	+++
Random RR	14.05	21.71	16.91
RRl	9.75	16.38	13.14
RRu	20.23	28.76	21.76
P	+++	+++	+++
Between Chi			64.08
Between df			1
Between P			***
Btwn(F) P			**
Btwn(R) P			(*)

Table 2B2 - 3

IESLC - Meta-analysis of Current Smoking (vs never smoking), Cigarettes (or Any Product if Cigarettes not available)

Squamous
Most adjusted

	Derivation of RR/CI			
	Orig	StdCalc	Other	Total
N	17	13	11	41
NS	12	10	9	31
Wt	421.58	197.39	26.95	645.92
Het Chi	148.21	121.37	12.29	288.68
Het df	16	12	10	40
Het P	***	***	N.S.	***
Fixed RR	12.88	15.19	19.23	13.77
RRl	11.70	13.21	13.18	12.75
RRu	14.17	17.46	28.05	14.87
P	+++	+++	+++	+++
Random RR	15.26	17.63	19.81	16.91
RRl	10.67	10.66	12.62	13.14
RRu	21.84	29.15	31.08	21.76
P	+++	+++	+++	+++
Between Chi				6.80
Between df				2
Between P				*
Btwn(F) P				N.S.
Btwn(R) P				N.S.

Table 2B2 - 4

IESLC - Meta-analysis of Current Smoking (vs never smoking), Cigarettes (or Any Product if Cigarettes not available)
Squamous
Least adjusted

REF	NRR	X	SEX	AGEL	AGEH	RACE	YF	LC TYPE	LOC	START	ST	NLC	R	VB	P	H	AD	PRODUCT	DENOM	De
BARBON	17	x	m	0	0	all	-	q	Eu:wst	1979	CC	755	n	bl	y	y	0	all/unsp	nev any	st
BOUCOT	70	x	m	0	0	all	0	q	NAmer	1951	pr	121	n	bl	n	n	0	cig only	nev any	ot
BROWN2	16		m	0	0	wh	-	q	NAmer	1984	CC	14596	n	bl	n	y	2	cig+/-ot	nev cigs	or
BROWN2	15		f	0	0	wh	-	q	NAmer	1984	CC	14596	n	bl	n	y	2	cig+/-ot	nev cigs	or
BUFFLE	63		f	0	0	w-hi	-	q	NAmer	1976	CC	943	n	bl	y	n	0	cig+/-ot	nev cigs	st
COMSTO	23		m	0	0	all	-	q	NAmer	1975	ot	258	n	bl	n	n	0	cig+/-ot	nev cigs	st
COMSTO	30		f	0	0	all	-	q	NAmer	1975	ot	258	n	bl	n	n	0	cig+/-ot	nev cigs	ot
CORREA	43		c	0	0	all	-	q+s	NAmer	1979	CC	1359	n	bl	y	n	1	cig+/-ot	nev cigs	or
CPSI	403		m	0	0	all	2	q	NAmer	1959	pr	5138	n	bl	n	n	1	cig only	nev any	ot
CPSI	405		f	0	0	all	2	q	NAmer	1959	pr	5138	n	bl	n	n	1	cig only	nev any	ot
CPSII	114		m	0	0	all	2	q	NAmer	1982	pr	3229	n	bl	n	n	1	cig only	nev any	ot
CPSII	117		f	0	0	all	2	q	NAmer	1982	pr	3229	n	bl	n	n	1	cig+/-ot	nev cigs	ot
DORN	338		m	0	0	wh	8	q	NAmer	1954	pr	5097	n	bl	n	n	1	cig only	nev any	ot
ENGELA	56		m	0	0	all	0	q	Eu:Sca	1964	pr	435	n	bl	n	n	7	cig+/-ot	nev cigs	ot
HAENSZ	20		f	0	0	all	-	q+u	NAmer	1955	CC	158	n	bl	n	y	0	cig+/-ot	nev any	st
HAMMON	102		m	0	0	wh	0	not a	NAmer	1952	pr	448	n	bl	n	n	1	cig only	nev any	ot
JAHN	7		m	0	0	all	-	q	Eu:Ger	1988	CC	1004	n	bl	n	n	0	cig+/-ot	nev any	st
JAIN	18		m	0	0	all	-	q	NAmer	1981	CC	845	n	V	y	n	0	cig+/-ot	nev cigs	st
JAIN	13		f	0	0	all	-	q	NAmer	1981	CC	845	n	V	y	n	0	cig+/-ot	nev cigs	st
JEDRYC	22		m	0	0	all	-	q	Eu:est	1980	CC	1630	n	bl	y	n	0	cig+/-ot	nev any	st
KATSOU	24	x	f	0	0	all	-	KI	Eu:bal	1987	CC	101	n	bl	n	n	0	all/unsp	nev any	st
KHUDER	14		m	0	0	all	-	q	NAmer	1985	CC	482	n	bl	n	y	0	cig+/-ot	nev cigs	or
KIHARA	2		c	0	0	jap	-	q	As:Jap	1991	CC	440	n	bl	n	n	0	all/unsp	nev any	st
LUBIN2	249		m	0	0	all	-	q	Eu:mul	1976	CC	7804	n	bl	n	y	0	cig+/-ot	nev any	st
LUBIN2	261		f	0	0	all	-	q	Eu:mul	1976	CC	7804	n	bl	n	y	0	cig+/-ot	nev any	st
MATOS	38	x	m	0	0	all	-	q	SCAmer	1994	CC	200	n	bl	n	n	0	cig+/-ot	nev any	st
OSANN	10	x	m	0	0	all	-	q	NAmer	1984	CC	1986	n	bl	n	n	0	cig+/-ot	nev cigs	st
OSANN	14	x	f	0	0	all	-	q	NAmer	1984	CC	1986	n	bl	n	n	0	cig+/-ot	nev cigs	st
OSANN2	8	x	f	0	0	all	-	KI	NAmer	1964	ot	217	n	bl	n	y	0	cig+/-ot	nev cigs	st
SOBUE	2	x	m	0	0	all	-	q	As:Jap	1986	CC	1376	n	bl	n	y	0	cig+/-ot	nev cigs	st
SOBUE	18	x	f	0	0	all	-	q	As:Jap	1986	CC	1376	n	bl	n	y	0	cig+/-ot	nev cigs	st
SOBUE2	1		m	0	0	all	-	q	As:Jap	1965	CC	2083	n	bl	n	n	2	cig+/-ot	nev any	or
SOBUE2	5		f	0	0	all	-	q	As:Jap	1965	CC	2083	n	bl	n	n	2	cig+/-ot	nev any	or
SVENSS	62	x	f	0	0	all	-	q	Eu:Sca	1983	CC	210	n	bl	n	n	0	all/unsp	nev any	st
TSUGAN	14		m	0	0	all	-	q	As:Jap	1976	CC	134	n	bl	n	y	0	all/unsp	nev any	ot
WAKAI	4	x	m	0	0	all	-	q	As:Jap	1988	CC	333	n	bl	n	y	0	all/unsp	nev any	st
WAKAI	22	x	f	0	0	all	-	q	As:Jap	1988	CC	333	n	bl	n	y	0	all/unsp	nev any	st
WU	11	x	f	0	0	wh	-	q	NAmer	1981	CC	220	n	bl	n	y	0	all/unsp	nev any	st
WYNDE3	10		m	0	0	all	-	KI	NAmer	1966	CC	350	n	bl	n	y	0	all/unsp	nev any	st
WYNDE6	12		m	0	0	all	-	KI	NAmer	1969	CC	4423	n	bl	n	y	0	cig+/-ot	nev any	st
WYNDE6	201		f	0	0	all	-	KI	NAmer	1969	CC	4423	n	bl	n	y	0	cig+/-ot	nev cigs	st

Cigarette type is all/unspec for all RRs

Table 2B2 - 5

IESLC - Meta-analysis of Current Smoking (vs never smoking), Cigarettes (or Any Product if Cigarettes not available)
Squamous
Least adjusted

REF	NRR	SEX	AD	Number Exposed Case	Number Exposed Cont	Non-exposed Case	Non-exposed Cont	RR	95.00%CI
BARBON	17	m	0	203	362	6	188	17.57	(7.66- 40.33)
*BOUCOT	70	m	0	38	22177	0	7551	26.22~	(1.61- 426.71)
BROWN2	16	m	2	-	-	-	-	13.70	(11.70- 16.10)
BROWN2	15	f	2	-	-	-	-	20.60	(16.60- 25.60)
Subtotal BROWN2								15.81	(13.91- 17.98)
BUFFLE	63	f	0	39	110	3	112	13.24	(3.97- 44.10)
COMSTO	23	m	0	27	100	2	84	11.34	(2.62- 49.09)
COMSTO	30	f	0	16	52	0	115	72.60~	(4.27-1233.12)
Subtotal COMSTO								16.78	(4.57- 61.67)
CORREA	43	c	1	-	-	-	-	34.60	(22.80- 52.40)
*CPSI	403	m	1	-	-	-	-	29.35	(4.02- 214.28)
*CPSI	405	f	1	-	-	-	-	4.25	(1.23- 14.68)
Subtotal CPSI								7.30	(2.55- 20.90)
*CPSII	114	m	1	-	-	-	-	39.26	(10.38- 148.55)
*CPSII	117	f	1	-	-	-	-	78.91	(15.83- 393.37)
Subtotal CPSII								52.16	(18.72- 145.32)
*DORN	338	m	1	-	-	-	-	17.09	(8.96- 32.60)
*ENGELA	56	m	7	-	-	-	-	10.89	(3.25- 36.43)
HAENSZ	20	f	0	53	94	44	236	3.02	(1.90- 4.82)
*HAMMON	102	m	1	-	-	-	-	26.42	(9.78- 71.34)
JAHN	7	m	0	153	269	3	138	26.16	(8.19- 83.54)
JAIN	18	m	0	107	118	2	85	38.54	(9.26- 160.45)
JAIN	13	f	0	81	99	6	214	29.18	(12.31- 69.15)
Subtotal JAIN								31.44	(15.03- 65.78)
JEDRYC	22	m	0	235	516	6	289	21.94	(9.63- 49.96)
KATSOU	24	f	0	24	18	14	67	6.38	(2.76- 14.78)
KHUDER	14	m	0	112	-	9	-	8.60	(4.20- 17.60)
KIHARA	2	c	0	111	162	5	237	32.48	(12.97- 81.34)
LUBIN2	249	m	0	2518	6209	54	2616	19.65	(14.95- 25.82)
LUBIN2	261	f	0	154	410	72	1180	6.16	(4.55- 8.32)
Subtotal LUBIN2								11.64	(9.50- 14.25)
MATOS	38	m	0	33	132	3	110	9.17	(2.74- 30.70)
OSANN	10	m	0	247	541	8	833	47.54	(23.32- 96.92)
OSANN	14	f	0	130	367	12	1093	32.26	(17.65- 58.97)
Subtotal OSANN								37.93	(23.94- 60.10)
OSANN2	8	f	0	105	42	7	58	20.71	(8.75- 49.05)
SOBUE	2	m	0	301	650	3	128	19.76	(6.24- 62.58)
SOBUE	18	f	0	29	168	14	857	10.57	(5.47- 20.42)
Subtotal SOBUE								12.33	(6.96- 21.84)
SOBUE2	1	m	2	-	-	-	-	5.20	(4.20- 6.50)
SOBUE2	5	f	2	-	-	-	-	7.20	(4.80- 10.80)
Subtotal SOBUE2								5.59	(4.62- 6.78)
SVENSS	62	f	0	42	53	5	120	19.02	(7.12- 50.77)
TSUGAN	14	m	0	18	13	0	5	15.07~	(0.77- 296.43)
WAKAI	4	m	0	86	284	2	65	9.84	(2.36- 41.03)
WAKAI	22	f	0	15	26	3	145	27.88	(7.54- 103.14)
Subtotal WAKAI								17.34	(6.61- 45.48)
WU	11	f	0	61	23	2	30	39.78	(8.79- 180.01)
WYNDE3	10	m	0	171	207	3	88	24.23	(7.53- 77.95)
WYNDE6	12	m	0	1026	741	29	617	29.46	(20.06- 43.26)
WYNDE6	201	f	0	550	376	40	856	31.30	(22.21- 44.12)
Subtotal WYNDE6								30.47	(23.59- 39.36)
Partial Totals				6685	34319	357	18117		

*prospective study ~ With 0.5 adjustment for zero

Table 2B2 - 5

IESLC - Meta-analysis of Current Smoking (vs never smoking), Cigarettes (or Any Product if Cigarettes not available)
Squamous
Least adjusted

REF	NRR	SEX	AD	Ys	Ws	Qs	Ps
BARBON	17	m	0	2.87	5.57	0.32	0.0000
*BOUCOT	70	m	0	3.27	0.49	0.20	0.0217
BROWN2	16	m	2	2.62	150.78	0.01	0.0000
BROWN2	15	f	2	3.03	81.88	13.09	0.0000
Subtotal BROWN2				2.76	232.67	13.10	
BUFFLE	63	f	0	2.58	2.65	0.00	0.0000
COMSTO	23	m	0	2.43	1.79	0.07	0.0012
COMSTO	30	f	0	4.28	0.48	1.32	0.0030
Subtotal COMSTO				2.82	2.27	1.39	
CORREA	43	c	1	3.54	22.19	18.72	0.0000
*CPSI	403	m	1	3.38	0.97	0.55	0.0009
*CPSI	405	f	1	1.45	2.50	3.47	0.0222
Subtotal CPSI				1.99	3.47	4.02	
*CPSII	114	m	1	3.67	2.17	2.37	0.0000
*CPSII	117	f	1	4.37	1.49	4.52	0.0000
Subtotal CPSII				3.95	3.66	6.89	
*DORN	338	m	1	2.84	9.21	0.42	0.0000
*ENGELA	56	m	7	2.39	2.63	0.15	0.0001
HAENSZ	20	f	0	1.11	17.71	40.85	0.0000
*HAMMON	102	m	1	3.27	3.89	1.64	0.0000
JAHN	7	m	0	3.26	2.85	1.16	0.0000
JAIN	18	m	0	3.65	1.89	1.99	0.0000
JAIN	13	f	0	3.37	5.16	2.89	0.0000
Subtotal JAIN				3.45	7.05	4.88	
JEDRYC	22	m	0	3.09	5.67	1.21	0.0000
KATSOU	24	f	0	1.85	5.45	3.25	0.0000
KHUDER	14	m	0	2.15	7.48	1.68	0.0000
KIHARA	2	c	0	3.48	4.56	3.33	0.0000
LUBIN2	249	m	0	2.98	51.39	6.38	0.0000
LUBIN2	261	f	0	1.82	42.25	27.59	0.0000
Subtotal LUBIN2				2.45	93.64	33.97	
MATOS	38	m	0	2.22	2.63	0.44	0.0003
OSANN	10	m	0	3.86	7.57	11.57	0.0000
OSANN	14	f	0	3.47	10.56	7.61	0.0000
Subtotal OSANN				3.64	18.13	19.17	
OSANN2	8	f	0	3.03	5.17	0.85	0.0000
SOBUE	2	m	0	2.98	2.89	0.37	0.0000
SOBUE	18	f	0	2.36	8.85	0.63	0.0000
Subtotal SOBUE				2.51	11.74	1.00	
SOBUE2	1	m	2	1.65	80.57	76.87	0.0000
SOBUE2	5	f	2	1.97	23.37	9.91	0.0000
Subtotal SOBUE2				1.72	103.93	86.78	
SVENSS	62	f	0	2.95	3.98	0.41	0.0000
TSUGAN	14	m	0	2.71	0.43	0.00	0.0743
WAKAI	4	m	0	2.29	1.88	0.22	0.0017
WAKAI	22	f	0	3.33	2.25	1.11	0.0000
Subtotal WAKAI				2.85	4.13	1.32	
WU	11	f	0	3.68	1.69	1.89	0.0000
WYNDE3	10	m	0	3.19	2.81	0.89	0.0000
WYNDE6	12	m	0	3.38	26.02	14.93	0.0000
WYNDE6	201	f	0	3.44	32.63	21.85	0.0000
Subtotal WYNDE6				3.42	58.65	36.78	

Table 2B2 - 5

IESLC - Meta-analysis of Current Smoking (vs never smoking), Cigarettes (or Any Product if Cigarettes not available)
Squamous
Least adjusted

	N	41
	NS	30
	Wt	646.41
Het	Chi	286.73
Het	df	40
Het	P	***
Fixed	RR	13.81
	RRl	12.79
	RRu	14.92
	P	+++
Random	RR	16.83
	RRl	13.12
	RRu	21.60
	P	+++
Asymm	P	N.S.

Table 2B2 - 6

IESLC - Meta-analysis of Current Smoking (vs never smoking), Cigarettes (or Any Product if Cigarettes not available)
Squamous
Least adjusted

	Sex			
	combined	male	female	Total
N	2	22	17	41
NS	2	22	17	41
Wt	26.75	371.60	248.06	646.41
Het Chi	0.02	122.29	141.18	286.73
Het df	1	21	16	40
Het P	N.S.	***	***	***
Fixed RR	34.23	13.06	13.62	13.81
RRl	23.43	11.80	12.02	12.79
RRu	50.00	14.46	15.42	14.92
P	+++	+++	+++	+++
Random RR	34.23	17.62	14.75	16.83
RRl	23.43	12.66	9.56	13.12
RRu	50.00	24.51	22.77	21.60
P	+++	+++	+++	+++
Between Chi				23.25
Between df				2
Between P				***
Btwn(F) P				N.S.
Btwn(R) P				**

Table 2B2 - 7

IESLC - Meta-analysis of Current Smoking (vs never smoking), Cigarettes (or Any Product if Cigarettes not available)
Squamous
Excluded studies (and stage at which they were excluded)

1	BOUCHA BUELL LAURIL MZILEN
2	ABELIN ABRAHA AUVINE AXELSO BAND BERRIN BLOT1 BLOT2 BLOT3 BLOT4 BRESLO BROCKM BROWN1 BYERS1 CASCO2 CASCOR CHAN CHATZI CHEN CHEN2 CHEN3 CHIAZZ COOKSO DAVEYS DEAN DOSEME DU DUNN EBELIN ESAKI FAN GENG GER GODLEY GOLLED GSELL HANSEN HEGMAN HINDS HIRAOK HOROWI HORWIT HU HU2 HUANG ISHIMA JARUP JIANG JONES JUSSAW KO KOHLME KOULUM KREYBE LAMTH LAMWK LAMWK2 LAUSSM LEI LETOUR LEVIN LIU LIU2 LIU3 LIU4 LIU5 LOMBA2 LUO MAGNUS MARSH MARSH2 MARTIS MASTRA MATSUD MCCONN MCDUFF MCLAUG MILLER MILLS MOLLO NOTAN2 NOU ORMOS PASTOR PAWLEG PERNU PERSHA PIKE POFFIJ POLEDN PRESCO QIAO RADZIK RANDIG REN RONCO ROOTS ROTHSC SADOWS SANKAR SCHWA2 SEGI SEOW SIEMIA SIMARA STASZE STOCKS SUN TAO ULMER VANDER WANG WANG3 WANG4 WICKLU WILKIN WUWILL WYNDE2 WYNDE4 XIANGZ XU XU2 XU3 XU4 YUAN ZHANG ZHENG ZHOU
3	RESTRE
4	BOFFET
5	RIMING TANG2 WYNDE5 WYNDE8
6	BYERS2 HIRAY2 LICKIN MRFIT MURATA NOTANI SAARIK SHIMIZ SITAS STAYNE WARSIN WATSON WYNDER
8	AGUDO AKIBA ALDERS AMANDU AMES ANDERS ARCHER ARMADA AUSTIN AXELSS BECHER BENSHL BEST BLOHMK BRETT BROSS CARPEN CEDERL CHANG CHOI CHOW CHYOU DAMBER DARBY DEAN2 DEAN3 DEKLER DESTE2 DESTEF DOCKER DOLL DOLL2 DORANT DORGAN DROSTE ENSTRO GAO GAO2 GARCIA GARDIN GARSHI GILLIS GOODMA GRAHAM GREGOR HAMMO2 HEIN HENNEK HIRAYA HITOSU HOLE HUMBLE JARVHO JOLY KAISE2 KAISER KANELL KAUFMA KELLER KINLEN KJUUS KNEKT KOO KREUZE KUBIK LANGE LEMARC LIAW LIDDEL LOMBAR LUBIN MACLEN MIGRAN MRFITR NAM ODRISC PARKIN PERSH2 PETO PEZZO2 PEZZOT PISANI QIAO2 RACHTA SCHWAR SEGI2 SHAW SIMONA SPEIZE SPITZ STOCKW STUCKE SUZUK2 SUZUKI TANG TENKAN TIZZAN TOKARS TOUSEY TULINI TVERDA VEIERO WALD WANG2 WIGLE WU2 WUNSCH YAMAGU YONG
10	BENHAM VUTUC WYNDE7

Table 2B2 - 8
Potentially overlapping studies

REF	REFGP	PRINC	OVERLAP/LINK
LUBIN2	LUBIN2	1	Lubin-combined
OSANN2	KAISER	2	KAISER/OSANN2
WYNDE6	WYNDE6	1	WYNDE5/6/7/8
CPSI	CPSI	1	CPSI overall
SOBUE2	SOBUE2	1	SOBUE2/MATSUD
JAHN	BOFFET	2	Subset of BOFFET

Table 2B2 - 9
Most adjusted - insufficient data for metaanalysis

REF	NRR	SEX	AGEL	AGEH	RACE	YF	LC TYPE	LOC	START	ST	NLC	R	VB	P	H	AD	PRODUCT	DENOM	De
CHYOU	5	m	0	0	jap	0	q+s	NAmer	1965	pr	227	n	bl	n	y	1	cig+/-ot	nev cigs	or

REF	NRR	RR	SIG	RRDATA comment
CHYOU	5	16.00		0

Table 2B3 -

IESLC - Meta-analysis of Current Smoking (vs never smoking), Cigarettes only
Squamous

This analysis is restricted to results for:
1) Non-dose-response data
2) Current smokers
3) Results complete enough for use in metaanalysis

Within each study, results are then selected (in the following order of preference, within each sex) for:
4) PRODUCT: cigarettes only
5) CIGTYPE: all/unspecified, MC regardless of HR, MC only
6) DENOM: never smoked anything, never smoked cigarettes, (never +1 = +long term ex, +2 = +amount unknown,
+3 = never cigs+long term ex)
7) Followup period (YF, prospective studies): whole study (coded as 0) or longest available
8) LCtype: squamous or nearest available, but not adeno. (q = squamous, s = small,
a = adeno, KI = Kreyberg I, u = undifferentiated)
9) Race: all or nearest available, otherwise by race (wh or w = white, bl or b = black, hi = hispanic
ch = chinese, jap = japanese, haw = hawaiian, w+o = white + oriental, sca = scandinavian, as = asian)
10) For overlapping studies: principal rather than subsidiary studies
Finally by Age: whole study (coded as 0) if available, otherwise by widest available age group
and then for single sex results (m, f) in preference to combined sex results (c).

Results adjusted (AD) for the most potential confounders are then chosen in Sections -1 to -3
(and those which actually differ from the adjusted results in Table 2B1 - 1 are marked 'x' in Section -1)
and results adjusted for the least confounders in Sections -4 to -6. (Those least adjusted results which
actually differ from the most adjusted as marked 'x' in column X in Section -4)
(Results adjusted for an unknown number of confounder(s) are coded as 20.)

Section -7 shows excluded studies, together with the stage (as above) at which no qualifying
results were found.

Section -8 lists the potentially overlapping studies which have been included (1=principal, 2=subsidiary).

Section -9 lists any results which would have been included in preference except that they had data not complete
enough for use in meta-analysis, with their significance (yes/no), if known, and any further comment as entered
on the database.

In addition to those mentioned above, the following fields, levels and abbreviations are used:
* or nk = not known, n = no, y = yes, ot = other
nev = never
all/unspec = all or unspecified, MC = manufactured cigarettes, HR = hand-rolled cigarettes
REF: 6-character study reference
NRR: number of the RR on the database within the study
ST : study type (CC = case control, pr or prosp = prospective)
NLC: number of lung cancer cases in whole study
R : risky occupational population (n = no, m = mining, o = other risky)
VB : national cigarette type (V = at least 75% Virginia, bl = at least 75% blended, ot = other)
P : any proxy use
H : full histological confirmation
De : derivation of RR/CI (or = original, st = standard method, ot = other method of estimation)

Table 2B3 - 1

IESLC - Meta-analysis of Current Smoking (vs never smoking), Cigarettes only
Squamous
Most adjusted

REF	NRR	2B1	SEX	AGEL	AGEH	RACE	YF	LC TYPE	LOC	START	ST	NLC	R	VB	P	H	AD	PRODUCT	DENOM	De
BENHAM	20	x	m	0	0	all	-	KI	Eu:wst	1976	CC	1625	n	bl	n	y	0	cig only	nev any	st
BOUCOT	141		m	0	0	all	0	q	NAmer	1951	pr	121	n	bl	n	n	2	cig only	nev any	ot
CPSI	403		m	0	0	all	2	q	NAmer	1959	pr	5138	n	bl	n	n	1	cig only	nev any	ot
CPSI	405		f	0	0	all	2	q	NAmer	1959	pr	5138	n	bl	n	n	1	cig only	nev any	ot
CPSII	114		m	0	0	all	2	q	NAmer	1982	pr	3229	n	bl	n	n	1	cig only	nev any	ot
DORN	338		m	0	0	wh	8	q	NAmer	1954	pr	5097	n	bl	n	n	1	cig only	nev any	ot
HAMMON	102		m	0	0	wh	0	not a	NAmer	1952	pr	448	n	bl	n	n	1	cig only	nev any	ot
WYNDE7	22	x	m	0	0	all	-	KI	NAmer	1977	CC	2085	n	bl	n	y	0	cig only	nev any	st

Cigarette type is all/unspec for all RRs

Table 2B3 - 2

IESLC - Meta-analysis of Current Smoking (vs never smoking), Cigarettes only
Squamous
Most adjusted

REF	NRR	SEX	AD	Number Exposed Case	Number Exposed Cont	Non-exposed Case	Non-exposed Cont	RR	95.00%CI
BENHAM	20	m	0	789	798	24	481	19.82	(13.00- 30.21)
*BOUCOT	141	m	2	-	-	-	-	27.54	(1.69- 448.37)
*CPSI	403	m	1	-	-	-	-	29.35	(4.02- 214.28)
*CPSI	405	f	1	-	-	-	-	4.25	(1.23- 14.68)
Subtotal CPSI								7.30	(2.55- 20.90)
*CPSII	114	m	1	-	-	-	-	39.26	(10.38- 148.55)
*DORN	338	m	1	-	-	-	-	17.09	(8.96- 32.60)
*HAMMON	102	m	1	-	-	-	-	26.42	(9.78- 71.34)
WYNDE7	22	m	0	666	993	22	918	27.99	(18.13- 43.20)
Partial Totals				1455	1791	46	1399		

*prospective study

REF	NRR	SEX	AD	Ys	Ws	Qs	Ps
BENHAM	20	m	0	2.99	21.61	0.14	0.0000
*BOUCOT	141	m	2	3.32	0.49	0.03	0.0199
*CPSI	403	m	1	3.38	0.97	0.09	0.0009
*CPSI	405	f	1	1.45	2.50	6.57	0.0222
Subtotal CPSI				1.99	3.47	6.66	
*CPSII	114	m	1	3.67	2.17	0.79	0.0000
*DORN	338	m	1	2.84	9.21	0.48	0.0000
*HAMMON	102	m	1	3.27	3.89	0.17	0.0000
WYNDE7	22	m	0	3.33	20.39	1.42	0.0000

	N	8
	NS	7
	Wt	61.24
Het	Chi	9.69
Het	df	7
Het	P	N.S.
Fixed	RR	21.49
	RRl	16.73
	RRu	27.61
	P	+++
Random	RR	20.85
	RRl	14.84
	RRu	29.29
	P	+++
Asymm	P	N.S.

Table 2B3 - 3

IESLC - Meta-analysis of Current Smoking (vs never smoking), Cigarettes only
Squamous
Most adjusted

		Sex		
	combined	male	female	Total
N		7	1	8
NS		7	1	8
Wt		58.74	2.50	61.24
Het Chi		2.85	0.00	9.69
Het df		6	0	7
Het P		N.S.	N.S.	N.S.
Fixed RR		23.03	4.25	21.49
RRl		17.83	1.23	16.73
RRu		29.74	14.68	27.61
P		+++	+	+++
Random RR		23.03	4.25	20.85
RRl		17.83	1.23	14.84
RRu		29.74	14.68	29.29
P		+++	+	+++
Between Chi				6.85
Between df				1
Between P				**
Btwn(F) P				**
Btwn(R) P				**

Too few RRs for analysis by factor

Table 2B3 - 4

IESLC - Meta-analysis of Current Smoking (vs never smoking), Cigarettes only
Squamous
Least adjusted

REF	NRR	X	SEX	AGEL	AGEH	RACE	YF	LC TYPE	LOC	START	ST	NLC	R	VB	P	H	AD	PRODUCT	DENOM	De
BENHAM	20		m	0	0	all	-	KI	Eu:wst	1976	CC	1625	n	bl	n	y	0	cig only	nev any	st
BOUCOT	70	x	m	0	0	all	0	q	NAmer	1951	pr	121	n	bl	n	n	0	cig only	nev any	ot
CPSI	403		m	0	0	all	2	q	NAmer	1959	pr	5138	n	bl	n	n	1	cig only	nev any	ot
CPSI	405		f	0	0	all	2	q	NAmer	1959	pr	5138	n	bl	n	n	1	cig only	nev any	ot
CPSII	114		m	0	0	all	2	q	NAmer	1982	pr	3229	n	bl	n	n	1	cig only	nev any	ot
DORN	338		m	0	0	wh	8	q	NAmer	1954	pr	5097	n	bl	n	n	1	cig only	nev any	ot
HAMMON	102		m	0	0	wh	0	not a	NAmer	1952	pr	448	n	bl	n	n	1	cig only	nev any	ot
WYNDE7	22		m	0	0	all	-	KI	NAmer	1977	CC	2085	n	bl	n	y	0	cig only	nev any	st

Cigarette type is all/unspec for all RRs

Table 2B3 - 5

IESLC - Meta-analysis of Current Smoking (vs never smoking), Cigarettes only
Squamous
Least adjusted

REF	NRR	SEX	AD	Number Exposed Case	Number Exposed Cont	Non-exposed Case	Non-exposed Cont	RR	95.00%CI
BENHAM	20	m	0	789	798	24	481	19.82	(13.00- 30.21)
*BOUCOT	70	m	0	38	22177	0	7551	26.22~	(1.61- 426.71)
*CPSI	403	m	1	-	-	-	-	29.35	(4.02- 214.28)
*CPSI	405	f	1	-	-	-	-	4.25	(1.23- 14.68)
Subtotal CPSI								7.30	(2.55- 20.90)
*CPSII	114	m	1	-	-	-	-	39.26	(10.38- 148.55)
*DORN	338	m	1	-	-	-	-	17.09	(8.96- 32.60)
*HAMMON	102	m	1	-	-	-	-	26.42	(9.78- 71.34)
WYNDE7	22	m	0	666	993	22	918	27.99	(18.13- 43.20)
Partial Totals				1493	23968	46	8950		

*prospective study ~ With 0.5 adjustment for zero

REF	NRR	SEX	AD	Ys	Ws	Qs	Ps
BENHAM	20	m	0	2.99	21.61	0.14	0.0000
*BOUCOT	70	m	0	3.27	0.49	0.02	0.0217
*CPSI	403	m	1	3.38	0.97	0.09	0.0009
*CPSI	405	f	1	1.45	2.50	6.56	0.0222
Subtotal CPSI				1.99	3.47	6.66	
*CPSII	114	m	1	3.67	2.17	0.79	0.0000
*DORN	338	m	1	2.84	9.21	0.48	0.0000
*HAMMON	102	m	1	3.27	3.89	0.17	0.0000
WYNDE7	22	m	0	3.33	20.39	1.43	0.0000

	N	8
	NS	7
	Wt	61.24
Het	Chi	9.68
Het	df	7
Het	P	N.S.
Fixed	RR	21.48
	RRl	16.72
	RRu	27.60
	P	+++
Random	RR	20.83
	RRl	14.83
	RRu	29.27
	P	+++
Asymm	P	N.S.

Table 2B3 - 6

IESLC - Meta-analysis of Current Smoking (vs never smoking), Cigarettes only
Squamous
Least adjusted

	Sex combined	male	female	Total
N		7	1	8
NS		7	1	8
Wt		58.74	2.50	61.24
Het Chi		2.84	0.00	9.68
Het df		6	0	7
Het P		N.S.	N.S.	N.S.
Fixed RR		23.02	4.25	21.48
RRl		17.82	1.23	16.72
RRu		29.72	14.68	27.60
P		+++	+	+++
Random RR		23.02	4.25	20.83
RRl		17.82	1.23	14.83
RRu		29.72	14.68	29.27
P		+++	+	+++
Between Chi				6.84
Between df				1
Between P				**
Btwn(F) P				**
Btwn(R) P				**

Table 2B3 - 7

IESLC - Meta-analysis of Current Smoking (vs never smoking), Cigarettes only
Squamous
Excluded studies (and stage at which they were excluded)

1	BOUCHA	BUELL	LAURIL	MZILEN												
2	ABELIN	ABRAHA	AUVINE	AXELSO	BAND	BERRIN	BLOT1	BLOT2	BLOT3	BLOT4	BRESLO	BROCKM	BROWN1	BYERS1	CASCO2	CASCOR
	CHAN	CHATZI	CHEN	CHEN2	CHEN3	CHIAZZ	COOKSO	DAVEYS	DEAN	DOSEME	DU	DUNN	EBELIN	ESAKI	FAN	GENG
	GER	GODLEY	GOLLED	GSELL	HANSEN	HEGMAN	HINDS	HIRAOK	HOROWI	HORWIT	HU	HU2	HUANG	ISHIMA	JARUP	JIANG
	JONES	JUSSAW	KO	KOHLME	KOULUM	KREYBE	LAMTH	LAMWK	LAMWK2	LAUSSM	LEI	LETOUR	LEVIN	LIU	LIU2	LIU3
	LIU4	LIU5	LOMBA2	LUO	MAGNUS	MARSH	MARSH2	MARTIS	MASTRA	MATSUD	MCCONN	MCDUFF	MCLAUG	MILLER	MILLS	MOLLO
	NOTAN2	NOU	ORMOS	PASTOR	PAWLEG	PERNU	PERSHA	PIKE	POFFIJ	POLEDN	PRESCO	QIAO	RADZIK	RANDIG	REN	RONCO
	ROOTS	ROTHSC	SADOWS	SANKAR	SCHWA2	SEGI	SEOW	SIEMIA	SIMARA	STASZE	STOCKS	SUN	TAO	ULMER	VANDER	WANG
	WANG3	WANG4	WICKLU	WILKIN	WUWILL	WYNDE2	WYNDE4	XIANGZ	XU	XU2	XU3	XU4	YUAN	ZHANG	ZHENG	ZHOU
3	RESTRE															
4	AKIBA	AMANDU	AMES	ANDERS	ARCHER	ARMADA	AUSTIN	AXELSS	BARBON	BECHER	BENSHL	BLOHMK	BOFFET	BRETT	BROSS	BROWN2
	BUFFLE	BYERS2	CARPEN	CHANG	CHOI	CHYOU	COMSTO	CORREA	DARBY	DEAN2	DEKLER	DESTE2	DESTEF	DOCKER	DOLL	DORANT
	DORGAN	DROSTE	GAO	GAO2	GARCIA	GARSHI	GILLIS	GOODMA	GREGOR	HAENSZ	HAMMO2	HENNEK	HIRAYA	HITOSU	HOLE	HUMBLE
	JAHN	JAIN	JARVHO	JEDRYC	JOLY	KAISER	KANELL	KATSOU	KAUFMA	KELLER	KHUDER	KIHARA	KINLEN	KJUUS	KNEKT	KOO
	KREUZE	KUBIK	LEMARC	LIAW	LICKIN	LIDDEL	LUBIN	MACLEN	MATOS	MRFIT	MRFITR	MURATA	NAM	NOTANI	ODRISC	OSANN
	OSANN2	PARKIN	PERSH2	PETO	PEZZO2	PISANI	QIAO2	RACHTA	SAARIK	SCHWAR	SEGI2	SHAW	SHIMIZ	SIMONA	SITAS	SOBUE
	SOBUE2	SPEIZE	SPITZ	STAYNE	STOCKW	STUCKE	SUZUK2	SUZUKI	SVENSS	TANG	TENKAN	TIZZAN	TOKARS	TOUSEY	TSUGAN	TULINI
	VUTUC	WAKAI	WANG2	WARSIN	WATSON	WU	WU2	WUNSCH	WYNDE3	WYNDE5	WYNDE6	WYNDE8	YAMAGU	YONG		
5	LANGE	RIMING	TANG2													
6	HIRAY2	WYNDER														
8	AGUDO	ALDERS	BEST	CEDERL	CHOW	DAMBER	DEAN3	DOLL2	ENGELA	ENSTRO	GARDIN	GRAHAM	HEIN	KAISE2	LOMBAR	LUBIN2
	MIGRAN	PEZZOT	TVERDA	VEIERO	WALD	WIGLE										

Table 2B3 - 8
Potentially overlapping studies

REF	REFGP	PRINC	OVERLAP/LINK
BENHAM	LUBIN2	2	Subset of Lubin2
WYNDE7	WYNDE6	2	WYNDE5/6/7/8
CPSI	CPSI	1	CPSI overall

Table 2B4 -

IESLC - Meta-analysis of Current Smoking, Any product (or Cigarettes if Any not available), Age <56
Squamous

This analysis is restricted to results for:
1) Non-dose-response data
2) Current smokers
3) Age <56
4) Results complete enough for use in metaanalysis

Within each study, results are then selected (in the following order of preference, within each sex) for:
5) PRODUCT: all/unspec, cigarettes regardless of other products, cigarettes only
6) CIGTYPE: all/unspecified, MC regardless of HR, MC only
7) DENOM: never smoked anything, never smoked cigarettes, (never +1 = +long term ex, +2 = +amount unknown, +3 = never cigs+long term ex)
8) Followup period (YF, prospective studies): whole study (coded as 0) or longest available
9) LCtype: all or nearest available, at least Squamous and Adeno. (q = squamous, s = small, a = adeno, KI = Kreyberg I, u = undifferentiated)
10) Race: all or nearest available, otherwise by race (wh or w = white, bl or b = black, hi = hispanic ch = chinese, jap = japanese, haw = hawaiian, w+o = white + oriental, sca = scandinavian, as = asian)
11) For overlapping studies: principal rather than subsidiary studies
Finally by Age: whole study (actual age shown) if available, otherwise by widest available age group and then for single sex results (m, f) in preference to combined sex results (c).

Results adjusted (AD) for the most potential confounders are then chosen in Sections -1 to -3 (and those which actually differ from the adjusted results in Table 2B1 - 1 are marked 'x' in Section -1) and results adjusted for the least confounders in Sections -4 to -6. (Those least adjusted results which actually differ from the most adjusted as marked 'x' in column X in Section -4)
(Results adjusted for an unknown number of confounder(s) are coded as 20.)

Section -7 shows excluded studies, together with the stage (as above) at which no qualifying results were found.

Section -8 lists the potentially overlapping studies which have been included (1=principal, 2=subsidiary).

Section -9 lists any results which would have been included in preference except that they had data not complete enough for use in meta-analysis, with their significance (yes/no), if known, and any further comment as entered on the database.

In addition to those mentioned above, the following fields, levels and abbreviations are used:
* or nk = not known, n = no, y = yes, ot = other
nev = never
all/unspec = all or unspecified, cig+/-ot = cigarettes irrespective of other products (cigar, pipe etc)
MC = manufactured cigarettes, HR = hand-rolled cigarettes
REF: 6-character study reference
NRR: number of the RR on the database within the study
ST : study type (CC = case control, pr or prosp = prospective)
NLC: number of lung cancer cases in whole study
R : risky occupational population (n = no, m = mining, o = other risky)
VB : national cigarette type (V = at least 75% Virginia, bl = at least 75% blended, ot = other)
P : any proxy use
H : full histological confirmation
De : derivation of RR/CI (or = original, st = standard method, ot = other method of estimation)

Table 2B4 - 1

IESLC - Meta-analysis of Current Smoking, Any product (or Cigarettes if Any not available), Age <56
Squamous
Most adjusted

REF	NRR	2B1	SEX	AGEL	AGEH	RACE	YF	LC TYPE	LOC	START	ST	NLC	R	VB	P	H	AD	PRODUCT	DENOM	De
TSUGAN	14		m	30	49	all	-	q	As:Jap	1976	CC	134	n	bl	n	y	0	all/unsp	nev any	ot
VUTUC	21	x	m	41	50	all	-	KI	Eu:wst	1976	CC	1877	n	bl	n	n	0	cig+/-ot	nev cigs	st

Cigarette type is all/unspec for all RRs

Table 2B4 - 2

IESLC - Meta-analysis of Current Smoking, Any product (or Cigarettes if Any not available), Age <56
Squamous
Most adjusted

REF	NRR	SEX	AD	Number Exposed Case	Number Exposed Cont	Non-exposed Case	Non-exposed Cont	RR	95.00%CI
TSUGAN	14	m	0	18	13	0	5	15.07~	(0.77- 296.43)
VUTUC	21	m	0	69	104	7	64	6.07	(2.63- 14.02)
Totals				87	117	7	69		

*prospective study

~ With 0.5 adjustment for zero

REF	NRR	SEX	AD	Ys	Ws	Qs	Ps
TSUGAN	14	m	0	2.71	0.43	0.31	0.0743
VUTUC	21	m	0	1.80	5.48	0.02	0.0000

	N	2
	NS	2
	Wt	5.91
Het	Chi	0.33
Het	df	1
Het	P	N.S.
Fixed	RR	6.48
	RRl	2.90
	RRu	14.52
	P	+++
Random	RR	6.48
	RRl	2.90
	RRu	14.52
	P	+++
Asymm	P	

Table 2B4 - 3

IESLC - Meta-analysis of Current Smoking, Any product (or Cigarettes if Any not available), Age <56
Squamous
Most adjusted

	Sex combined	male	female	Total
N		2		2
NS		2		2
Wt		5.91		5.91
Het Chi		0.33		0.33
Het df		1		1
Het P		N.S.		N.S.
Fixed RR		6.48		6.48
RRl		2.90		2.90
RRu		14.52		14.52
P		+++		+++
Random RR		6.48		6.48
RRl		2.90		2.90
RRu		14.52		14.52
P		+++		+++
Between Chi				
Between df				
Between P				N.S.
Btwn(F) P				N.S.
Btwn(R) P				N.S.

Too few RRs for analysis by factor

Table 2B4 - 4

IESLC - Meta-analysis of Current Smoking, Any product (or Cigarettes if Any not available), Age <56
Squamous
Least adjusted

REF	NRR	X	SEX	AGEL	AGEH	RACE	YF	LC TYPE	LOC	START	ST	NLC	R	VB	P	H	AD	PRODUCT	DENOM	De
TSUGAN	14		m	30	49	all	-	q	As:Jap	1976	CC	134	n	bl	n	y	0	all/unsp	nev any	ot
VUTUC	21		m	41	50	all	-	KI	Eu:wst	1976	CC	1877	n	bl	n	n	0	cig+/-ot	nev cigs	st

Cigarette type is all/unspec for all RRs

Table 2B4 - 5

IESLC - Meta-analysis of Current Smoking, Any product (or Cigarettes if Any not available), Age <56
Squamous
Least adjusted

REF	NRR	SEX	AD	Number Exposed Case	Number Exposed Cont	Non-exposed Case	Non-exposed Cont	RR	95.00%CI
TSUGAN	14	m	0	18	13	0	5	15.07~	(0.77- 296.43)
VUTUC	21	m	0	69	104	7	64	6.07	(2.63- 14.02)
Totals				87	117	7	69		

*prospective study

~ With 0.5 adjustment for zero

REF	NRR	SEX	AD	Ys	Ws	Qs	Ps
TSUGAN	14	m	0	2.71	0.43	0.31	0.0743
VUTUC	21	m	0	1.80	5.48	0.02	0.0000

	N	2
	NS	2
	Wt	5.91
Het	Chi	0.33
Het	df	1
Het	P	N.S.
Fixed	RR	6.48
	RRl	2.90
	RRu	14.52
	P	+++
Random	RR	6.48
	RRl	2.90
	RRu	14.52
	P	+++
Asymm	P	

Table 2B4 - 6

IESLC - Meta-analysis of Current Smoking, Any product (or Cigarettes if Any not available), Age <56
Squamous
Least adjusted

	Sex combined	male	female	Total
N		2		2
NS		2		2
Wt		5.91		5.91
Het Chi		0.33		0.33
Het df		1		1
Het P		N.S.		N.S.
Fixed RR		6.48		6.48
RRl		2.90		2.90
RRu		14.52		14.52
P		+++		+++
Random RR		6.48		6.48
RRl		2.90		2.90
RRu		14.52		14.52
P		+++		+++
Between Chi				
Between df				
Between P				N.S.
Btwn(F) P				N.S.
Btwn(R) P				N.S.

Table 2B4 - 7

IESLC - Meta-analysis of Current Smoking, Any product (or Cigarettes if Any not available), Age <56
Squamous
Excluded studies (and stage at which they were excluded)

1 BOUCHA BUELL LAURIL MZILEN

2 ABELIN ABRAHA AUVINE AXELSO BAND BERRIN BLOT1 BLOT2 BLOT3 BLOT4 BRESLO BROCKM BROWN1 BYERS1 CASCO2 CASCOR
CHAN CHATZI CHEN CHEN2 CHEN3 CHIAZZ COOKSO DAVEYS DEAN DOSEME DU DUNN EBELIN ESAKI FAN GENG
GER GODLEY GOLLED GSELL HANSEN HEGMAN HINDS HIRAOK HOROWI HORWIT HU HU2 HUANG ISHIMA JARUP JIANG
JONES JUSSAW KO KOHLME KOULUM KREYBE LAMTH LAMWK LAMWK2 LAUSSM LEI LETOUR LEVIN LIU LIU2 LIU3
LIU4 LIU5 LOMBA2 LUO MAGNUS MARSH MARSH2 MARTIS MASTRA MATSUD MCCONN MCDUFF MCLAUG MILLER MILLS MOLLO
NOTAN2 NOU ORMOS PASTOR PAWLEG PERNU PERSHA PIKE POFFIJ POLEDN PRESCO QIAO RADZIK RANDIG REN RONCO
ROOTS ROTHSC SADOWS SANKAR SCHWA2 SEGI SEOW SIEMIA SIMARA STASZE STOCKS SUN TAO ULMER VANDER WANG
WANG3 WANG4 WICKLU WILKIN WUWILL WYNDE2 WYNDE4 XIANGZ XU XU2 XU3 XU4 YUAN ZHANG ZHENG ZHOU

3 AGUDO AKIBA ALDERS AMANDU AMES ANDERS ARCHER ARMADA AUSTIN AXELSS BARBON BECHER BENHAM BENSHL BLOHMK BOFFET
BOUCOT BRETT BROSS BROWN2 BUFFLE BYERS2 CARPEN CEDERL CHANG CHOI CHOW CHYOU CORREA DAMBER DARBY DEAN2
DEKLER DESTE2 DESTEF DOCKER DOLL DOLL2 DORANT DORGAN DORN DROSTE ENGELA ENSTRO GAO GAO2 GARCIA GARDIN
GARSHI GILLIS GOODMA GRAHAM GREGOR HAENSZ HAMMO2 HAMMON HEIN HENNEK HIRAY2 HUMBLE JAHN JAIN JARVHO JEDRYC
JOLY KAISER KATSOU KAUFMA KELLER KHUDER KIHARA KINLEN KJUUS KNEKT KOO LANGE LEMARC LIAW LICKIN LIDDEL
LOMBAR LUBIN LUBIN2 MACLEN MATOS MIGRAN MRFIT MRFITR MURATA NOTANI ODRISC OSANN OSANN2 PARKIN PERSH2 PETO
PEZZO2 PEZZOT PISANI QIAO2 RACHTA RESTRE RIMING SAARIK SCHWAR SHAW SHIMIZ SIMONA SITAS SOBUE SOBUE2 SPITZ
STAYNE STOCKW STUCKE SUZUK2 SUZUKI SVENSS TANG TANG2 TENKAN TIZZAN TOKARS TOUSEY TULINI VEIERO WAKAI WALD
WANG2 WARSIN WATSON WIGLE WU WU2 WUNSCH WYNDE3 WYNDE5 WYNDE6 WYNDE7 WYNDE8 WYNDER YAMAGU YONG

4 HIRAYA

6 KAISE2

7 HOLE

9 BEST COMSTO CPSI CPSII DEAN3 HITOSU KANELL KREUZE KUBIK NAM SEGI2 SPEIZE TVERDA

Table 2B4 - 8

Potentially overlapping studies

REF	REFGP	PRINC	. OVERLAP/LINK
VUTUC	LUBIN2	2	Subset of Lubin2

Table 2B5 -

IESLC - Meta-analysis of Current Smoking, Any product (or Cigarettes if Any not available), Age 50-70
Squamous

This analysis is restricted to results for:
1) Non-dose-response data
2) Current smokers
3) Maximum age range 50-70
4) Results complete enough for use in metaanalysis

Within each study, results are then selected (in the following order of preference, within each sex) for:
5) PRODUCT: all/unspec, cigarettes regardless of other products, cigarettes only
6) CIGTYPE: all/unspecified, MC regardless of HR, MC only
7) DENOM: never smoked anything, never smoked cigarettes, (never +1 = +long term ex, +2 = +amount unknown,
+3 = never cigs+long term ex)
8) Followup period (YF, prospective studies): whole study (coded as 0) or longest available
9) LCtype: all or nearest available, at least Squamous and Adeno. (q = squamous, s = small,
a = adeno, KI = Kreyberg I, u = undifferentiated)
10) Race: all or nearest available, otherwise by race (wh or w = white, bl or b = black, hi = hispanic
ch = chinese, jap = japanese, haw = hawaiian, w+o = white + oriental, sca = scandinavian, as = asian)
11) For overlapping studies: principal rather than subsidiary studies
Finally by Age: whole study (actual age shown) if available, otherwise by widest available age group
and then for single sex results (m, f) in preference to combined sex results (c).

Results adjusted (AD) for the most potential confounders are then chosen in Sections -1 to -3
(and those which actually differ from the adjusted results in Table 2B1 - 1 are marked 'x' in Section -1)
and results adjusted for the least confounders in Sections -4 to -6. (Those least adjusted results which
actually differ from the most adjusted as marked 'x' in column X in Section -4)
(Results adjusted for an unknown number of confounder(s) are coded as 20.)

Section -7 shows excluded studies, together with the stage (as above) at which no qualifying
results were found.

Section -8 lists the potentially overlapping studies which have been included (1=principal, 2=subsidiary).

Section -9 lists any results which would have been included in preference except that they had data not complete
enough for use in meta-analysis, with their significance (yes/no), if known, and any further comment as entered
on the database.

In addition to those mentioned above, the following fields, levels and abbreviations are used:
* or nk = not known, n = no, y = yes, ot = other
nev = never
all/unspec = all or unspecified, cig+/-ot = cigarettes irrespective of other products (cigar, pipe etc)
MC = manufactured cigarettes, HR = hand-rolled cigarettes
REF: 6-character study reference
NRR: number of the RR on the database within the study
ST : study type (CC = case control, pr or prosp = prospective)
NLC: number of lung cancer cases in whole study
R : risky occupational population (n = no, m = mining, o = other risky)
VB : national cigarette type (V = at least 75% Virginia, bl = at least 75% blended, ot = other)
P : any proxy use
H : full histological confirmation
De : derivation of RR/CI (or = original, st = standard method, ot = other method of estimation)

Table 2B5 - 1

IESLC - Meta-analysis of Current Smoking, Any product (or Cigarettes if Any not available), Age 50-70
Squamous
Most adjusted

REF	NRR	2B1	SEX	AGEL	AGEH	RACE	YF	LC TYPE	LOC	START	ST	NLC	R	VB	P	H	AD	PRODUCT	DENOM	De
HAMMON	102		m	50	69	wh	0	not a	NAmer	1952	pr	448	n	bl	n	n	1	cig only	nev any	ot

Cigarette type is all/unspec for all RRs

Table 2B5 - 2

IESLC - Meta-analysis of Current Smoking, Any product (or Cigarettes if Any not available), Age 50-70
Squamous
Most adjusted

REF	NRR	SEX	AD	Number Exposed Case	Cont	Non-exposed Case	Cont	RR	95.00%CI
*HAMMON	102	m	1	-	-	-	-	26.42	(9.78- 71.34)
Partial Totals				0	0	0	0		

*prospective study

REF	NRR	SEX	AD	Ys	Ws	Qs	Ps
*HAMMON	102	m	1	3.27	3.89	0.00	0.0000

	N	1
	NS	1
	Wt	3.89
Het	Chi	0.00
Het	df	0
Het	P	N.S.
Fixed	RR	26.42
	RRl	9.78
	RRu	71.36
	P	+++
Random	RR	26.42
	RRl	9.78
	RRu	71.36
	P	+++
Asymm	P	

Table 2B5 - 3

IESLC - Meta-analysis of Current Smoking, Any product (or Cigarettes if Any not available), Age 50-70
Squamous
Most adjusted

	Sex combined	male	female	Total
N		1		1
NS		1		1
Wt		3.89		3.89
Het Chi		0.00		0.00
Het df		0		0
Het P		N.S.		N.S.
Fixed RR		26.42		26.42
RRl		9.78		9.78
RRu		71.36		71.36
P		+++		+++
Random RR		26.42		26.42
RRl		9.78		9.78
RRu		71.36		71.36
P		+++		+++
Between Chi				
Between df				
Between P				N.S.
Btwn(F) P				N.S.
Btwn(R) P				N.S.

Too few RRs for analysis by factor

Table 2B5 - 4

IESLC - Meta-analysis of Current Smoking, Any product (or Cigarettes if Any not available), Age 50-70
Squamous
Least adjusted

REF	NRR	X	SEX	AGEL	AGEH	RACE	YF	LC TYPE	LOC	START	ST	NLC	R	VB	P	H	AD	PRODUCT	DENOM	De
HAMMON	102		m	50	69	wh	0	not a	NAmer	1952	pr	448	n	bl	n	n	1	cig only	nev any	ot

Cigarette type is all/unspec for all RRs

Table 2B5 - 5

IESLC - Meta-analysis of Current Smoking, Any product (or Cigarettes if Any not available), Age 50-70
Squamous
Least adjusted

REF	NRR	SEX	AD	Number Exposed Case	Number Exposed Cont	Non-exposed Case	Non-exposed Cont	RR	95.00%CI
*HAMMON	102	m	1	-	-	-	-	26.42	(9.78- 71.34)
Partial Totals				0	0	0	0		

*prospective study

REF	NRR	SEX	AD	Ys	Ws	Qs	Ps
*HAMMON	102	m	1	3.27	3.89	0.00	0.0000

	N	1
	NS	1
	Wt	3.89
Het	Chi	0.00
Het	df	0
Het	P	N.S.
Fixed	RR	26.42
	RRl	9.78
	RRu	71.36
	P	+++
Random	RR	26.42
	RRl	9.78
	RRu	71.36
	P	+++
Asymm	P	

Table 2B5 - 6

IESLC - Meta-analysis of Current Smoking, Any product (or Cigarettes if Any not available), Age 50-70
Squamous
Least adjusted

	combined	Sex male	female	Total
N		1		1
NS		1		1
Wt		3.89		3.89
Het Chi		0.00		0.00
Het df		0		0
Het P		N.S.		N.S.
Fixed RR		26.42		26.42
RRl		9.78		9.78
RRu		71.36		71.36
P		+++		+++
Random RR		26.42		26.42
RRl		9.78		9.78
RRu		71.36		71.36
P		+++		+++
Between Chi				
Between df				
Between P				N.S.
Btwn(F) P				N.S.
Btwn(R) P				N.S.

Table 2B5 - 7

IESLC - Meta-analysis of Current Smoking, Any product (or Cigarettes if Any not available), Age 50-70
Squamous
Excluded studies (and stage at which they were excluded)

1	BOUCHA	BUELL	LAURIL	MZILEN												
2	ABELIN	ABRAHA	AUVINE	AXELSO	BAND	BERRIN	BLOT1	BLOT2	BLOT3	BLOT4	BRESLO	BROCKM	BROWN1	BYERS1	CASCO2	CASCOR
	CHAN	CHATZI	CHEN	CHEN2	CHEN3	CHIAZZ	COOKSO	DAVEYS	DEAN	DOSEME	DU	DUNN	EBELIN	ESAKI	FAN	GENG
	GER	GODLEY	GOLLED	GSELL	HANSEN	HEGMAN	HINDS	HIRAOK	HOROWI	HORWIT	HU	HU2	HUANG	ISHIMA	JARUP	JIANG
	JONES	JUSSAW	KO	KOHLME	KOULUM	KREYBE	LAMTH	LAMWK	LAMWK2	LAUSSM	LEI	LETOUR	LEVIN	LIU	LIU2	LIU3
	LIU4	LIU5	LOMBA2	LUO	MAGNUS	MARSH	MARSH2	MARTIS	MASTRA	MATSUD	MCCONN	MCDUFF	MCLAUG	MILLER	MILLS	MOLLO
	NOTAN2	NOU	ORMOS	PASTOR	PAWLEG	PERNU	PERSHA	PIKE	POFFIJ	POLEDN	PRESCO	QIAO	RADZIK	RANDIG	REN	RONCO
	ROOTS	ROTHSC	SADOWS	SANKAR	SCHWA2	SEGI	SEOW	SIEMIA	SIMARA	STASZE	STOCKS	SUN	TAO	ULMER	VANDER	WANG
	WANG3	WANG4	WICKLU	WILKIN	WUWILL	WYNDE2	WYNDE4	XIANGZ	XU	XU2	XU3	XU4	YUAN	ZHANG	ZHENG	ZHOU
3	AGUDO	AKIBA	ALDERS	AMANDU	AMES	ARCHER	ARMADA	AUSTIN	AXELSS	BARBON	BECHER	BENHAM	BENSHL	BLOHMK	BOFFET	BOUCOT
	BRETT	BROSS	BROWN2	BUFFLE	BYERS2	CARPEN	CHANG	CHOI	CHOW	CHYOU	COMSTO	CORREA	DAMBER	DARBY	DEAN2	DEKLER
	DESTE2	DESTEF	DOCKER	DOLL	DOLL2	DORGAN	DROSTE	ENGELA	ENSTRO	GAO	GAO2	GARCIA	GARDIN	GARSHI	GILLIS	GOODMA
	GRAHAM	GREGOR	HAENSZ	HAMMO2	HEIN	HENNEK	HIRAY2	HUMBLE	JAHN	JAIN	JARVHO	JEDRYC	JOLY	KAISER	KATSOU	KAUFMA
	KELLER	KHUDER	KIHARA	KINLEN	KJUUS	KNEKT	KOO	LANGE	LEMARC	LIAW	LICKIN	LIDDEL	LOMBAR	LUBIN	LUBIN2	MACLEN
	MATOS	MIGRAN	MRFIT	MRFITR	MURATA	NOTANI	ODRISC	OSANN	OSANN2	PARKIN	PERSH2	PETO	PEZZO2	PEZZOT	PISANI	QIAO2
	RACHTA	RESTRE	RIMING	SAARIK	SCHWAR	SHAW	SHIMIZ	SIMONA	SITAS	SOBUE	SOBUE2	SPEIZE	SPITZ	STAYNE	STOCKW	STUCKE
	SUZUK2	SUZUKI	SVENSS	TANG	TANG2	TENKAN	TIZZAN	TOKARS	TOUSEY	TSUGAN	TULINI	TVERDA	VEIERO	VUTUC	WAKAI	WALD
	WANG2	WARSIN	WATSON	WIGLE	WU	WU2	WUNSCH	WYNDE3	WYNDE5	WYNDE6	WYNDE7	WYNDE8	WYNDER	YAMAGU	YONG	
4	HIRAYA															
6	KAISE2															
7	HOLE															
9	ANDERS	BEST	CEDERL	CPSI	CPSII	DEAN3	DORANT	DORN	HITOSU	KANELL	KREUZE	KUBIK	NAM	SEGI2		

Table 2B6 -

IESLC - Meta-analysis of Current Smoking, Any product (or Cigarettes if Any not available), Age 65+
Squamous

This analysis is restricted to results for:
1) Non-dose-response data
2) Current smokers
3) Age 65+
4) Results complete enough for use in metaanalysis

Within each study, results are then selected (in the following order of preference, within each sex) for:
5) PRODUCT: all/unspec, cigarettes regardless of other products, cigarettes only
6) CIGTYPE: all/unspecified, MC regardless of HR, MC only
7) DENOM: never smoked anything, never smoked cigarettes, (never +1 = +long term ex, +2 = +amount unknown,
+3 = never cigs+long term ex)
8) Followup period (YF, prospective studies): whole study (coded as 0) or longest available
9) LCtype: all or nearest available, at least Squamous and Adeno. (q = squamous, s = small,
a = adeno, KI = Kreyberg I, u = undifferentiated)
10) Race: all or nearest available, otherwise by race (wh or w = white, bl or b = black, hi = hispanic
ch = chinese, jap = japanese, haw = hawaiian, w+o = white + oriental, sca = scandinavian, as = asian)
11) For overlapping studies: principal rather than subsidiary studies
Finally by Age: whole study (actual age shown) if available, otherwise by widest available age group
and then for single sex results (m, f) in preference to combined sex results (c).

Results adjusted (AD) for the most potential confounders are then chosen in Sections -1 to -3
(and those which actually differ from the adjusted results in Table 2B1 - 1 are marked 'x' in Section -1)
and results adjusted for the least confounders in Sections -4 to -6. (Those least adjusted results which
actually differ from the most adjusted as marked 'x' in column X in Section -4)
(Results adjusted for an unknown number of confounder(s) are coded as 20.)

Section -7 shows excluded studies, together with the stage (as above) at which no qualifying
results were found.

Section -8 lists the potentially overlapping studies which have been included (1=principal, 2=subsidiary).

Section -9 lists any results which would have been included in preference except that they had data not complete
enough for use in meta-analysis, with their significance (yes/no), if known, and any further comment as entered
on the database.

In addition to those mentioned above, the following fields, levels and abbreviations are used:
* or nk = not known, n = no, y = yes, ot = other
nev = never
all/unspec = all or unspecified, cig+/-ot = cigarettes irrespective of other products (cigar, pipe etc)
MC = manufactured cigarettes, HR = hand-rolled cigarettes
REF: 6-character study reference
NRR: number of the RR on the database within the study
ST : study type (CC = case control, pr or prosp = prospective)
NLC: number of lung cancer cases in whole study
R : risky occupational population (n = no, m = mining, o = other risky)
VB : national cigarette type (V = at least 75% Virginia, bl = at least 75% blended, ot = other)
P : any proxy use
H : full histological confirmation
De : derivation of RR/CI (or = original, st = standard method, ot = other method of estimation)

Table 2B6 - 0

No RRs selected for this analysis

Table 2B6 - 7

IESLC - Meta-analysis of Current Smoking, Any product (or Cigarettes if Any not available), Age 65+
Squamous
Excluded studies (and stage at which they were excluded)

1	BOUCHA	BUELL	LAURIL	MZILEN													
2	ABELIN	ABRAHA	AUVINE	AXELSO	BAND	BERRIN	BLOT1	BLOT2	BLOT3	BLOT4	BRESLO	BROCKM	BROWN1	BYERS1	CASCO2	CASCOR	CHAN
	CHATZI	CHEN	CHEN2	CHEN3	CHIAZZ	COOKSO	DAVEYS	DEAN	DOSEME	DU	DUNN	EBELIN	ESAKI	FAN	GENG	GER	GODLEY
	GOLLED	GSELL	HANSEN	HEGMAN	HINDS	HIRAOK	HOROWI	HORWIT	HU	HU2	HUANG	ISHIMA	JARUP	JIANG	JONES	JUSSAW	KO
	KOHLME	KOULUM	KREYBE	LAMTH	LAMWK	LAMWK2	LAUSSM	LEI	LETOUR	LEVIN	LIU	LIU2	LIU3	LIU4	LIU5	LOMBA2	LUO
	MAGNUS	MARSH	MARSH2	MARTIS	MASTRA	MATSUD	MCCONN	MCDUFF	MCLAUG	MILLER	MILLS	MOLLO	NOTAN2	NOU	ORMOS	PASTOR	PAWLEG
	PERNU	PERSHA	PIKE	POFFIJ	POLEDN	PRESCO	QIAO	RADZIK	RANDIG	REN	RONCO	ROOTS	ROTHSC	SADOWS	SANKAR	SCHWA2	SEGI
	SEOW	SIEMIA	SIMARA	STASZE	STOCKS	SUN	TAO	ULMER	VANDER	WANG	WANG3	WANG4	WICKLU	WILKIN	WUWILL	WYNDE2	WYNDE4
	XIANGZ	XU	XU2	XU3	XU4	YUAN	ZHANG	ZHENG	ZHOU								
3	AGUDO	AKIBA	ALDERS	AMANDU	AMES	ANDERS	ARCHER	ARMADA	AUSTIN	AXELSS	BARBON	BECHER	BENHAM	BENSHL	BLOHMK	BOFFET	BOUCOT
	BRETT	BROSS	BROWN2	BUFFLE	BYERS2	CARPEN	CEDERL	CHANG	CHOI	CHOW	CHYOU	CORREA	DAMBER	DARBY	DEAN2	DEKLER	DESTE2
	DESTEF	DOCKER	DOLL	DOLL2	DORANT	DORGAN	DROSTE	ENGELA	ENSTRO	GAO	GAO2	GARCIA	GARDIN	GILLIS	GOODMA	GRAHAM	GREGOR
	HAENSZ	HAMMO2	HAMMON	HEIN	HENNEK	HIRAY2	HITOSU	HOLE	JAHN	JAIN	JARVHO	JEDRYC	JOLY	KAISER	KATSOU	KAUFMA	KELLER
	KHUDER	KIHARA	KINLEN	KJUUS	KNEKT	KOO	KREUZE	KUBIK	LANGE	LEMARC	LIAW	LICKIN	LIDDEL	LOMBAR	LUBIN	LUBIN2	MACLEN
	MATOS	MIGRAN	MRFIT	MRFITR	MURATA	NOTANI	ODRISC	OSANN	OSANN2	PARKIN	PERSH2	PETO	PEZZO2	PEZZOT	PISANI	QIAO2	RACHTA
	RESTRE	RIMING	SAARIK	SCHWAR	SHAW	SHIMIZ	SIMONA	SITAS	SOBUE	SOBUE2	SPEIZE	SPITZ	STAYNE	STOCKW	STUCKE	SUZUK2	SUZUKI
	SVENSS	TANG	TANG2	TIZZAN	TOKARS	TOUSEY	TSUGAN	TULINI	TVERDA	VEIERO	VUTUC	WAKAI	WALD	WANG2	WARSIN	WATSON	WIGLE
	WU	WU2	WUNSCH	WYNDE3	WYNDE5	WYNDE6	WYNDE7	WYNDE8	WYNDER	YAMAGU	YONG						
4	HIRAYA																
7	TENKAN																
9	BEST	COMSTO	CPSI	CPSII	DEAN3	DORN	GARSHI	HUMBLE	KAISE2	KANELL	NAM	SEGI2					

Table 2B7 -

IESLC - Meta-analysis of Current Smoking, Cigarettes (or Any Product if Cigarettes not available), Age <56
Squamous

This analysis is restricted to results for:
1) Non-dose-response data
2) Current smokers
3) Age <56
4) Results complete enough for use in metaanalysis

Within each study, results are then selected (in the following order of preference, within each sex) for:
5) PRODUCT: cigarettes regardless of other products, cigarettes only, all/unspec
6) CIGTYPE: all/unspecified, MC regardless of HR, MC only
7) DENOM: never smoked anything, never smoked cigarettes, (never +1 = +long term ex, +2 = +amount unknown, +3 = never cigs+long term ex)
8) Followup period (YF, prospective studies): whole study (coded as 0) or longest available
9) LCtype: all or nearest available, at least Squamous and Adeno. (q = squamous, s = small, a = adeno, KI = Kreyberg I, u = undifferentiated)
10) Race: all or nearest available, otherwise by race (wh or w = white, bl or b = black, hi = hispanic ch = chinese, jap = japanese, haw = hawaiian, w+o = white + oriental, sca = scandinavian, as = asian)
11) For overlapping studies: principal rather than subsidiary studies
Finally by Age: whole study (actual age shown) if available, otherwise by widest available age group and then for single sex results (m, f) in preference to combined sex results (c).

Results adjusted (AD) for the most potential confounders are then chosen in Sections -1 to -3 (and those which actually differ from the adjusted results in Table 1B2 - 1 are marked 'x' in Section -1) and results adjusted for the least confounders in Sections -4 to -6. (Those least adjusted results which actually differ from the most adjusted as marked 'x' in column X in Section -4) (Results adjusted for an unknown number of confounder(s) are coded as 20.)

Section -7 shows excluded studies, together with the stage (as above) at which no qualifying results were found.

Section -8 lists the potentially overlapping studies which have been included (1=principal, 2=subsidiary).

Section -9 lists any results which would have been included in preference except that they had data not complete enough for use in meta-analysis, with their significance (yes/no), if known, and any further comment as entered on the database.

In addition to those mentioned above, the following fields, levels and abbreviations are used:
* or nk = not known, n = no, y = yes, ot = other
nev = never
all/unspec = all or unspecified, cig+/-ot = cigarettes irrespective of other products (cigar, pipe etc)
MC = manufactured cigarettes, HR = hand-rolled cigarettes
REF: 6-character study reference
NRR: number of the RR on the database within the study
ST : study type (CC = case control, pr or prosp = prospective)
NLC: number of lung cancer cases in whole study
R : risky occupational population (n = no, m = mining, o = other risky)
VB : national cigarette type (V = at least 75% Virginia, bl = at least 75% blended, ot = other)
P : any proxy use
H : full histological confirmation
De : derivation of RR/CI (or = original, st = standard method, ot = other method of estimation)

Table 2B7 - 1

IESLC - Meta-analysis of Current Smoking, Cigarettes (or Any Product if Cigarettes not available), Age <56
Squamous
Most adjusted

REF	NRR	2B2	SEX	AGEL	AGEH	RACE	YF	LC TYPE	LOC	START	ST	NLC	R	VB	P	H	AD	PRODUCT	DENOM	De
TSUGAN	14		m	30	49	all	-	q	As:Jap	1976	CC	134	n	bl	n	y	0	all/unsp	nev any	ot
VUTUC	21	x	m	41	50	all	-	KI	Eu:wst	1976	CC	1877	n	bl	n	n	0	cig+/-ot	nev cigs	st

Cigarette type is all/unspec for all RRs

Table 2B7 - 2

IESLC - Meta-analysis of Current Smoking, Cigarettes (or Any Product if Cigarettes not available), Age <56
Squamous
Most adjusted

REF	NRR	SEX	AD	Number Exposed Case	Number Exposed Cont	Non-exposed Case	Non-exposed Cont	RR	95.00%CI
TSUGAN	14	m	0	18	13	0	5	15.07~	(0.77- 296.43)
VUTUC	21	m	0	69	104	7	64	6.07	(2.63- 14.02)
Totals				87	117	7	69		

*prospective study

~ With 0.5 adjustment for zero

REF	NRR	SEX	AD	Ys	Ws	Qs	Ps
TSUGAN	14	m	0	2.71	0.43	0.31	0.0743
VUTUC	21	m	0	1.80	5.48	0.02	0.0000

	N	2
	NS	2
	Wt	5.91
Het	Chi	0.33
Het	df	1
Het	P	N.S.
Fixed	RR	6.48
	RRl	2.90
	RRu	14.52
	P	+++
Random	RR	6.48
	RRl	2.90
	RRu	14.52
	P	+++
Asymm	P	

Table 2B7 - 3

IESLC - Meta-analysis of Current Smoking, Cigarettes (or Any Product if Cigarettes not available), Age <56
Squamous
Most adjusted

	Sex combined	male	female	Total
N		2		2
NS		2		2
Wt		5.91		5.91
Het Chi		0.33		0.33
Het df		1		1
Het P		N.S.		N.S.
Fixed RR		6.48		6.48
RRl		2.90		2.90
RRu		14.52		14.52
P		+++		+++
Random RR		6.48		6.48
RRl		2.90		2.90
RRu		14.52		14.52
P		+++		+++
Between Chi				
Between df				
Between P				N.S.
Btwn(F) P				N.S.
Btwn(R) P				N.S.

Too few RRs for analysis by factor

Table 2B7 - 4

IESLC - Meta-analysis of Current Smoking, Cigarettes (or Any Product if Cigarettes not available), Age <56
Squamous
Least adjusted

REF	NRR	X	SEX	AGEL	AGEH	RACE	YF	LC TYPE	LOC	START	ST	NLC	R	VB	P	H	AD	PRODUCT	DENOM	De
TSUGAN	14		m	30	49	all	-	q	As:Jap	1976	CC	134	n	bl	n	y	0	all/unsp	nev any	ot
VUTUC	21		m	41	50	all	-	KI	Eu:wst	1976	CC	1877	n	bl	n	n	0	cig+/-ot	nev cigs	st

Cigarette type is all/unspec for all RRs

Table 2B7 - 5

IESLC - Meta-analysis of Current Smoking, Cigarettes (or Any Product if Cigarettes not available), Age <56
Squamous
Least adjusted

REF	NRR	SEX	AD	Number Exposed Case	Cont	Non-exposed Case	Cont	RR	95.00%CI
TSUGAN	14	m	0	18	13	0	5	15.07~	(0.77- 296.43)
VUTUC	21	m	0	69	104	7	64	6.07	(2.63- 14.02)
Totals				87	117	7	69		

*prospective study

~ With 0.5 adjustment for zero

REF	NRR	SEX	AD	Ys	Ws	Qs	Ps
TSUGAN	14	m	0	2.71	0.43	0.31	0.0743
VUTUC	21	m	0	1.80	5.48	0.02	0.0000

	N	2
	NS	2
	Wt	5.91
Het	Chi	0.33
Het	df	1
Het	P	N.S.
Fixed	RR	6.48
	RRl	2.90
	RRu	14.52
	P	+++
Random	RR	6.48
	RRl	2.90
	RRu	14.52
	P	+++
Asymm	P	

Table 2B7 - 6

IESLC - Meta-analysis of Current Smoking, Cigarettes (or Any Product if Cigarettes not available), Age <56

Squamous

Least adjusted

	Sex: combined	male	female	Total
N		2		2
NS		2		2
Wt		5.91		5.91
Het Chi		0.33		0.33
Het df		1		1
Het P		N.S.		N.S.
Fixed RR		6.48		6.48
RRl		2.90		2.90
RRu		14.52		14.52
P		+++		+++
Random RR		6.48		6.48
RRl		2.90		2.90
RRu		14.52		14.52
P		+++		+++
Between Chi				
Between df				
Between P				N.S.
Btwn(F) P				N.S.
Btwn(R) P				N.S.

Table 2B7 - 7

IESLC - Meta-analysis of Current Smoking, Cigarettes (or Any Product if Cigarettes not available), Age <56
Squamous
Excluded studies (and stage at which they were excluded)

1 BOUCHA BUELL LAURIL MZILEN

2 ABELIN ABRAHA AUVINE AXELSO BAND BERRIN BLOT1 BLOT2 BLOT3 BLOT4 BRESLO BROCKM BROWN1 BYERS1 CASCO2 CASCOR CHAN CHATZI CHEN CHEN2 CHEN3 CHIAZZ COOKSO DAVEYS DEAN DOSEME DU DUNN EBELIN ESAKI FAN GENG GER GODLEY GOLLED GSELL HANSEN HEGMAN HINDS HIRAOK HOROWI HORWIT HU HU2 HUANG ISHIMA JARUP JIANG JONES JUSSAW KO KOHLME KOULUM KREYBE LAMTH LAMWK LAMWK2 LAUSSM LEI LETOUR LEVIN LIU LIU2 LIU3 LIU4 LIU5 LOMBA2 LUO MAGNUS MARSH MARSH2 MARTIS MASTRA MATSUD MCCONN MCDUFF MCLAUG MILLER MILLS MOLLO NOTAN2 NOU ORMOS PASTOR PAWLEG PERNU PERSHA PIKE POFFIJ POLEDN PRESCO QIAO RADZIK RANDIG REN RONCO ROOTS ROTHSC SADOWS SANKAR SCHWA2 SEGI SEOW SIEMIA SIMARA STASZE STOCKS SUN TAO ULMER VANDER WANG WANG3 WANG4 WICKLU WILKIN WUWILL WYNDE2 WYNDE4 XIANGZ XU XU2 XU3 XU4 YUAN ZHANG ZHENG ZHOU

3 AGUDO AKIBA ALDERS AMANDU AMES ANDERS ARCHER ARMADA AUSTIN AXELSS BARBON BECHER BENHAM BENSHL BLOHMK BOFFET BOUCOT BRETT BROSS BROWN2 BUFFLE BYERS2 CARPEN CEDERL CHANG CHOI CHOW CHYOU CORREA DAMBER DARBY DEAN2 DEKLER DESTE2 DESTEF DOCKER DOLL DOLL2 DORANT DORGAN DORN DROSTE ENGELA ENSTRO GAO GAO2 GARCIA GARDIN GARSHI GILLIS GOODMA GRAHAM GREGOR HAENSZ HAMMO2 HAMMON HEIN HENNEK HIRAY2 HUMBLE JAHN JAIN JARVHO JEDRYC JOLY KAISER KATSOU KAUFMA KELLER KHUDER KIHARA KINLEN KJUUS KNEKT KOO LANGE LEMARC LIAW LICKIN LIDDEL LOMBAR LUBIN LUBIN2 MACLEN MATOS MIGRAN MRFIT MRFITR MURATA NOTANI ODRISC OSANN OSANN2 PARKIN PERSH2 PETO PEZZO2 PEZZOT PISANI QIAO2 RACHTA RESTRE RIMING SAARIK SCHWAR SHAW SHIMIZ SIMONA SITAS SOBUE SOBUE2 SPITZ STAYNE STOCKW STUCKE SUZUK2 SUZUKI SVENSS TANG TANG2 TENKAN TIZZAN TOKARS TOUSEY TULINI VEIERO WAKAI WALD WANG2 WARSIN WATSON WIGLE WU WU2 WUNSCH WYNDE3 WYNDE5 WYNDE6 WYNDE7 WYNDE8 WYNDER YAMAGU YONG

4 HIRAYA

6 KAISE2

7 HOLE

9 BEST COMSTO CPSI CPSII DEAN3 HITOSU KANELL KREUZE KUBIK NAM SEGI2 SPEIZE TVERDA

Table 2B7 - 8

Potentially overlapping studies

REF	REFGP	PRINC	. OVERLAP/LINK
VUTUC	LUBIN2	2	Subset of Lubin2

Table 2B8 -

IESLC - Meta-analysis of Current Smoking, Cigarettes (or Any Product if Cigarettes not available), Age 50-70
Squamous

This analysis is restricted to results for:
1) Non-dose-response data
2) Current smokers
3) Maximum age range 50-70
4) Results complete enough for use in metaanalysis

Within each study, results are then selected (in the following order of preference, within each sex) for:
5) PRODUCT: cigarettes regardless of other products, cigarettes only, all/unspec
6) CIGTYPE: all/unspecified, MC regardless of HR, MC only
7) DENOM: never smoked anything, never smoked cigarettes, (never +1 = +long term ex, +2 = +amount unknown,
+3 = never cigs+long term ex)
8) Followup period (YF, prospective studies): whole study (coded as 0) or longest available
9) LCtype: all or nearest available, at least Squamous and Adeno. (q = squamous, s = small,
a = adeno, KI = Kreyberg I, u = undifferentiated)
10) Race: all or nearest available, otherwise by race (wh or w = white, bl or b = black, hi = hispanic
ch = chinese, jap = japanese, haw = hawaiian, w+o = white + oriental, sca = scandinavian, as = asian)
11) For overlapping studies: principal rather than subsidiary studies
Finally by Age: whole study (actual age shown) if available, otherwise by widest available age group
and then for single sex results (m, f) in preference to combined sex results (c).

Results adjusted (AD) for the most potential confounders are then chosen in Sections -1 to -3
(and those which actually differ from the adjusted results in Table 1B2 - 1 are marked 'x' in Section -1)
and results adjusted for the least confounders in Sections -4 to -6. (Those least adjusted results which
actually differ from the most adjusted as marked 'x' in column X in Section -4)
(Results adjusted for an unknown number of confounder(s) are coded as 20.)

Section -7 shows excluded studies, together with the stage (as above) at which no qualifying
results were found.

Section -8 lists the potentially overlapping studies which have been included (1=principal, 2=subsidiary).

Section -9 lists any results which would have been included in preference except that they had data not complete
enough for use in meta-analysis, with their significance (yes/no), if known, and any further comment as entered
on the database.

In addition to those mentioned above, the following fields, levels and abbreviations are used:
* or nk = not known, n = no, y = yes, ot = other
nev = never
all/unspec = all or unspecified, cig+/-ot = cigarettes irrespective of other products (cigar, pipe etc)
MC = manufactured cigarettes, HR = hand-rolled cigarettes
REF: 6-character study reference
NRR: number of the RR on the database within the study
ST : study type (CC = case control, pr or prosp = prospective)
NLC: number of lung cancer cases in whole study
R : risky occupational population (n = no, m = mining, o = other risky)
VB : national cigarette type (V = at least 75% Virginia, bl = at least 75% blended, ot = other)
P : any proxy use
H : full histological confirmation
De : derivation of RR/CI (or = original, st = standard method, ot = other method of estimation)

Table 2B8 - 1

IESLC - Meta-analysis of Current Smoking, Cigarettes (or Any Product if Cigarettes not available), Age 50-70
Squamous
Most adjusted

REF	NRR	2B2	SEX	AGEL	AGEH	RACE	YF	LC TYPE	LOC	START	ST	NLC	R	VB	P	H	AD	PRODUCT	DENOM	De
HAMMON	102		m	50	69	wh	0	not a	NAmer	1952	pr	448	n	bl	n	n	1	cig only	nev any	ot

Cigarette type is all/unspec for all RRs

Table 2B8 - 2

IESLC - Meta-analysis of Current Smoking, Cigarettes (or Any Product if Cigarettes not available), Age 50-70
Squamous
Most adjusted

REF	NRR	SEX	AD	Number Exposed Case	Number Exposed Cont	Non-exposed Case	Non-exposed Cont	RR	95.00%CI
*HAMMON	102	m	1	-	-	-	-	26.42	(9.78- 71.34)
Partial Totals				0	0	0	0		

*prospective study

REF	NRR	SEX	AD	Ys	Ws	Qs	Ps
*HAMMON	102	m	1	3.27	3.89	0.00	0.0000

	N	1
	NS	1
	Wt	3.89
Het	Chi	0.00
Het	df	0
Het	P	N.S.
Fixed	RR	26.42
	RRl	9.78
	RRu	71.36
	P	+++
Random	RR	26.42
	RRl	9.78
	RRu	71.36
	P	+++
Asymm	P	

Table 2B8 - 3

IESLC - Meta-analysis of Current Smoking, Cigarettes (or Any Product if Cigarettes not available), Age 50-70
Squamous
Most adjusted

	combined	Sex<br>male	female	Total
N		1		1
NS		1		1
Wt		3.89		3.89
Het Chi		0.00		0.00
Het df		0		0
Het P		N.S.		N.S.
Fixed RR		26.42		26.42
RRl		9.78		9.78
RRu		71.36		71.36
P		+++		+++
Random RR		26.42		26.42
RRl		9.78		9.78
RRu		71.36		71.36
P		+++		+++
Between Chi				
Between df				
Between P				N.S.
Btwn(F) P				N.S.
Btwn(R) P				N.S.

Too few RRs for analysis by factor

Table 2B8 - 4

IESLC - Meta-analysis of Current Smoking, Cigarettes (or Any Product if Cigarettes not available), Age 50-70
Squamous
Least adjusted

REF	NRR	X	SEX	AGEL	AGEH	RACE	YF	LC TYPE	LOC	START	ST	NLC	R	VB	P	H	AD	PRODUCT	DENOM	De
HAMMON	102		m	50	69	wh	0	not a	NAmer	1952	pr	448	n	bl	n	n	1	cig only	nev any	ot

Cigarette type is all/unspec for all RRs

Table 2B8 - 5

IESLC - Meta-analysis of Current Smoking, Cigarettes (or Any Product if Cigarettes not available), Age 50-70
Squamous
Least adjusted

REF	NRR	SEX	AD	Number Exposed Case	Cont	Non-exposed Case	Cont	RR	95.00%CI
*HAMMON	102	m	1	-	-	-	-	26.42	(9.78- 71.34)
Partial Totals				0	0	0	0		

*prospective study

REF	NRR	SEX	AD	Ys	Ws	Qs	Ps
*HAMMON	102	m	1	3.27	3.89	0.00	0.0000

	N	1
	NS	1
	Wt	3.89
Het	Chi	0.00
Het	df	0
Het	P	N.S.
Fixed	RR	26.42
	RRl	9.78
	RRu	71.36
	P	+++
Random	RR	26.42
	RRl	9.78
	RRu	71.36
	P	+++
Asymm	P	

Table 2B8 - 6

IESLC - Meta-analysis of Current Smoking, Cigarettes (or Any Product if Cigarettes not available), Age 50-70

Squamous

Least adjusted

	Sex combined	male	female	Total
N		1		1
NS		1		1
Wt		3.89		3.89
Het Chi		0.00		0.00
Het df		0		0
Het P		N.S.		N.S.
Fixed RR		26.42		26.42
RRl		9.78		9.78
RRu		71.36		71.36
P		+++		+++
Random RR		26.42		26.42
RRl		9.78		9.78
RRu		71.36		71.36
P		+++		+++
Between Chi				
Between df				
Between P				N.S.
Btwn(F) P				N.S.
Btwn(R) P				N.S.

Table 2B8 - 7

IESLC - Meta-analysis of Current Smoking, Cigarettes (or Any Product if Cigarettes not available), Age 50-70
Squamous
Excluded studies (and stage at which they were excluded)

1	BOUCHA	BUELL	LAURIL	MZILEN												
2	ABELIN	ABRAHA	AUVINE	AXELSO	BAND	BERRIN	BLOT1	BLOT2	BLOT3	BLOT4	BRESLO	BROCKM	BROWN1	BYERS1	CASCO2	CASCOR
	CHAN	CHATZI	CHEN	CHEN2	CHEN3	CHIAZZ	COOKSO	DAVEYS	DEAN	DOSEME	DU	DUNN	EBELIN	ESAKI	FAN	GENG
	GER	GODLEY	GOLLED	GSELL	HANSEN	HEGMAN	HINDS	HIRAOK	HOROWI	HORWIT	HU	HU2	HUANG	ISHIMA	JARUP	JIANG
	JONES	JUSSAW	KO	KOHLME	KOULUM	KREYBE	LAMTH	LAMWK	LAMWK2	LAUSSM	LEI	LETOUR	LEVIN	LIU	LIU2	LIU3
	LIU4	LIU5	LOMBA2	LUO	MAGNUS	MARSH	MARSH2	MARTIS	MASTRA	MATSUD	MCCONN	MCDUFF	MCLAUG	MILLER	MILLS	MOLLO
	NOTAN2	NOU	ORMOS	PASTOR	PAWLEG	PERNU	PERSHA	PIKE	POFFIJ	POLEDN	PRESCO	QIAO	RADZIK	RANDIG	REN	RONCO
	ROOTS	ROTHSC	SADOWS	SANKAR	SCHWA2	SEGI	SEOW	SIEMIA	SIMARA	STASZE	STOCKS	SUN	TAO	ULMER	VANDER	WANG
	WANG3	WANG4	WICKLU	WILKIN	WUWILL	WYNDE2	WYNDE4	XIANGZ	XU	XU2	XU3	XU4	YUAN	ZHANG	ZHENG	ZHOU
3	AGUDO	AKIBA	ALDERS	AMANDU	AMES	ARCHER	ARMADA	AUSTIN	AXELSS	BARBON	BECHER	BENHAM	BENSHL	BLOHMK	BOFFET	BOUCOT
	BRETT	BROSS	BROWN2	BUFFLE	BYERS2	CARPEN	CHANG	CHOI	CHOW	CHYOU	COMSTO	CORREA	DAMBER	DARBY	DEAN2	DEKLER
	DESTE2	DESTEF	DOCKER	DOLL	DOLL2	DORGAN	DROSTE	ENGELA	ENSTRO	GAO	GAO2	GARCIA	GARDIN	GARSHI	GILLIS	GOODMA
	GRAHAM	GREGOR	HAENSZ	HAMMO2	HEIN	HENNEK	HIRAY2	HUMBLE	JAHN	JAIN	JARVHO	JEDRYC	JOLY	KAISER	KATSOU	KAUFMA
	KELLER	KHUDER	KIHARA	KINLEN	KJUUS	KNEKT	KOO	LANGE	LEMARC	LIAW	LICKIN	LIDDEL	LOMBAR	LUBIN	LUBIN2	MACLEN
	MATOS	MIGRAN	MRFIT	MRFITR	MURATA	NOTANI	ODRISC	OSANN	OSANN2	PARKIN	PERSH2	PETO	PEZZO2	PEZZOT	PISANI	QIAO2
	RACHTA	RESTRE	RIMING	SAARIK	SCHWAR	SHAW	SHIMIZ	SIMONA	SITAS	SOBUE	SOBUE2	SPEIZE	SPITZ	STAYNE	STOCKW	STUCKE
	SUZUK2	SUZUKI	SVENSS	TANG	TANG2	TENKAN	TIZZAN	TOKARS	TOUSEY	TSUGAN	TULINI	TVERDA	VEIERO	VUTUC	WAKAI	WALD
	WANG2	WARSIN	WATSON	WIGLE	WU	WU2	WUNSCH	WYNDE3	WYNDE5	WYNDE6	WYNDE7	WYNDE8	WYNDER	YAMAGU	YONG	
4	HIRAYA															
6	KAISE2															
7	HOLE															
9	ANDERS	BEST	CEDERL	CPSI	CPSII	DEAN3	DORANT	DORN	HITOSU	KANELL	KREUZE	KUBIK	NAM	SEGI2		

Table 2B9 -

IESLC - Meta-analysis of Current Smoking, Cigarettes (or Any Product if Cigarettes not available), Age 65+
Squamous

This analysis is restricted to results for:
1) Non-dose-response data
2) Current smokers
3) Age 65+
4) Results complete enough for use in metaanalysis

Within each study, results are then selected (in the following order of preference, within each sex) for:
5) PRODUCT: cigarettes regardless of other products, cigarettes only, all/unspec
6) CIGTYPE: all/unspecified, MC regardless of HR, MC only
7) DENOM: never smoked anything, never smoked cigarettes, (never +1 = +long term ex, +2 = +amount unknown,
+3 = never cigs+long term ex)
8) Followup period (YF, prospective studies): whole study (coded as 0) or longest available
9) LCtype: all or nearest available, at least Squamous and Adeno. (q = squamous, s = small,
a = adeno, KI = Kreyberg I, u = undifferentiated)
10) Race: all or nearest available, otherwise by race (wh or w = white, bl or b = black, hi = hispanic
ch = chinese, jap = japanese, haw = hawaiian, w+o = white + oriental, sca = scandinavian, as = asian)
11) For overlapping studies: principal rather than subsidiary studies
Finally by Age: whole study (actual age shown) if available, otherwise by widest available age group
and then for single sex results (m, f) in preference to combined sex results (c).

Results adjusted (AD) for the most potential confounders are then chosen in Sections -1 to -3
(and those which actually differ from the adjusted results in Table 1B2 - 1 are marked 'x' in Section -1)
and results adjusted for the least confounders in Sections -4 to -6. (Those least adjusted results which
actually differ from the most adjusted as marked 'x' in column X in Section -4)
(Results adjusted for an unknown number of confounder(s) are coded as 20.)

Section -7 shows excluded studies, together with the stage (as above) at which no qualifying
results were found.

Section -8 lists the potentially overlapping studies which have been included (1=principal, 2=subsidiary).

Section -9 lists any results which would have been included in preference except that they had data not complete
enough for use in meta-analysis, with their significance (yes/no), if known, and any further comment as entered
on the database.

In addition to those mentioned above, the following fields, levels and abbreviations are used:
* or nk = not known, n = no, y = yes, ot = other
nev = never
all/unspec = all or unspecified, cig+/-ot = cigarettes irrespective of other products (cigar, pipe etc)
MC = manufactured cigarettes, HR = hand-rolled cigarettes
REF: 6-character study reference
NRR: number of the RR on the database within the study
ST : study type (CC = case control, pr or prosp = prospective)
NLC: number of lung cancer cases in whole study
R : risky occupational population (n = no, m = mining, o = other risky)
VB : national cigarette type (V = at least 75% Virginia, bl = at least 75% blended, ot = other)
P : any proxy use
H : full histological confirmation
De : derivation of RR/CI (or = original, st = standard method, ot = other method of estimation)

Table 2B9 - 0

No RRs selected for this analysis

Table 2B9 - 7

IESLC - Meta-analysis of Current Smoking, Cigarettes (or Any Product if Cigarettes not available), Age 65+
Squamous
Excluded studies (and stage at which they were excluded)

1 BOUCHA BUELL LAURIL MZILEN

2 ABELIN ABRAHA AUVINE AXELSO BAND BERRIN BLOT1 BLOT2 BLOT3 BLOT4 BRESLO BROCKM BROWN1 BYERS1 CASCO2 CASCOR CHAN
CHATZI CHEN CHEN2 CHEN3 CHIAZZ COOKSO DAVEYS DEAN DOSEME DU DUNN EBELIN ESAKI FAN GENG GER GODLEY
GOLLED GSELL HANSEN HEGMAN HINDS HIRAOK HOROWI HORWIT HU HU2 HUANG ISHIMA JARUP JIANG JONES JUSSAW KO
KOHLME KOULUM KREYBE LAMTH LAMWK LAMWK2 LAUSSM LEI LETOUR LEVIN LIU LIU2 LIU3 LIU4 LIU5 LOMBA2 LUO
MAGNUS MARSH MARSH2 MARTIS MASTRA MATSUD MCCONN MCDUFF MCLAUG MILLER MILLS MOLLO NOTAN2 NOU ORMOS PASTOR PAWLEG
PERNU PERSHA PIKE POFFIJ POLEDN PRESCO QIAO RADZIK RANDIG REN RONCO ROOTS ROTHSC SADOWS SANKAR SCHWA2 SEGI
SEOW SIEMIA SIMARA STASZE STOCKS SUN TAO ULMER VANDER WANG WANG3 WANG4 WICKLU WILKIN WUWILL WYNDE2 WYNDE4
XIANGZ XU XU2 XU3 XU4 YUAN ZHANG ZHENG ZHOU

3 AGUDO AKIBA ALDERS AMANDU AMES ANDERS ARCHER ARMADA AUSTIN AXELSS BARBON BECHER BENHAM BENSHL BLOHMK BOFFET BOUCOT
BRETT BROSS BROWN2 BUFFLE BYERS2 CARPEN CEDERL CHANG CHOI CHOW CHYOU CORREA DAMBER DARBY DEAN2 DEKLER DESTE2
DESTEF DOCKER DOLL DOLL2 DORANT DORGAN DROSTE ENGELA ENSTRO GAO GAO2 GARCIA GARDIN GILLIS GOODMA GRAHAM GREGOR
HAENSZ HAMMO2 HAMMON HEIN HENNEK HIRAY2 HITOSU HOLE JAHN JAIN JARVHO JEDRYC JOLY KAISER KATSOU KAUFMA KELLER
KHUDER KIHARA KINLEN KJUUS KNEKT KOO KREUZE KUBIK LANGE LEMARC LIAW LICKIN LIDDEL LOMBAR LUBIN LUBIN2 MACLEN
MATOS MIGRAN MRFIT MRFITR MURATA NOTANI ODRISC OSANN OSANN2 PARKIN PERSH2 PETO PEZZO2 PEZZOT PISANI QIAO2 RACHTA
RESTRE RIMING SAARIK SCHWAR SHAW SHIMIZ SIMONA SITAS SOBUE SOBUE2 SPEIZE SPITZ STAYNE STOCKW STUCKE SUZUK2 SUZUKI
SVENSS TANG TANG2 TIZZAN TOKARS TOUSEY TSUGAN TULINI TVERDA VEIERO VUTUC WAKAI WALD WANG2 WARSIN WATSON WIGLE
WU WU2 WUNSCH WYNDE3 WYNDE5 WYNDE6 WYNDE7 WYNDE8 WYNDER YAMAGU YONG

4 HIRAYA

7 TENKAN

9 BEST COMSTO CPSI CPSII DEAN3 DORN GARSHI HUMBLE KAISE2 KANELL NAM SEGI2

Table 2B10 -

IESLC - Meta-analysis of Current Smoking, Cigarettes only, Age <56
Squamous

This analysis is restricted to results for:
1) Non-dose-response data
2) Current smokers
3) Age <56
4) Results complete enough for use in metaanalysis

Within each study, results are then selected (in the following order of preference, within each sex) for:
5) PRODUCT: cigarettes only
6) CIGTYPE: all/unspecified, MC regardless of HR, MC only
7) DENOM: never smoked anything, never smoked cigarettes, (never +1 = +long term ex, +2 = +amount unknown,
+3 = never cigs+long term ex)
8) Followup period (YF, prospective studies): whole study (coded as 0) or longest available
9) LCtype: all or nearest available, at least Squamous and Adeno. (q = squamous, s = small,
a = adeno, KI = Kreyberg I, u = undifferentiated)
10) Race: all or nearest available, otherwise by race (wh or w = white, bl or b = black, hi = hispanic
ch = chinese, jap = japanese, haw = hawaiian, w+o = white + oriental, sca = scandinavian, as = asian)
11) For overlapping studies: principal rather than subsidiary studies
Finally by Age: whole study (actual age shown) if available, otherwise by widest available age group
and then for single sex results (m, f) in preference to combined sex results (c).

Results adjusted (AD) for the most potential confounders are then chosen in Sections -1 to -3
(and those which actually differ from the adjusted results in Table 2B3 - 1 are marked 'x' in Section -1)
and results adjusted for the least confounders in Sections -4 to -6. (Those least adjusted results which
actually differ from the most adjusted as marked 'x' in column X in Section -4)
(Results adjusted for an unknown number of confounder(s) are coded as 20.)

Section -7 shows excluded studies, together with the stage (as above) at which no qualifying
results were found.

Section -8 lists the potentially overlapping studies which have been included (1=principal, 2=subsidiary).

Section -9 lists any results which would have been included in preference except that they had data not complete
enough for use in meta-analysis, with their significance (yes/no), if known, and any further comment as entered
on the database.

In addition to those mentioned above, the following fields, levels and abbreviations are used:
* or nk = not known, n = no, y = yes, ot = other
nev = never
all/unspec = all or unspecified, MC = manufactured cigarettes, HR = hand-rolled cigarettes
REF: 6-character study reference
NRR: number of the RR on the database within the study
ST : study type (CC = case control, pr or prosp = prospective)
NLC: number of lung cancer cases in whole study
R : risky occupational population (n = no, m = mining, o = other risky)
VB : national cigarette type (V = at least 75% Virginia, bl = at least 75% blended, ot = other)
P : any proxy use
H : full histological confirmation
De : derivation of RR/CI (or = original, st = standard method, ot = other method of estimation)

Table 2B10 - 0

No RRs selected for this analysis

Table 2B10 - 7

IESLC - Meta-analysis of Current Smoking, Cigarettes only, Age <56
Squamous
Excluded studies (and stage at which they were excluded)

1 BOUCHA BUELL LAURIL MZILEN

2 ABELIN ABRAHA AUVINE AXELSO BAND BERRIN BLOT1 BLOT2 BLOT3 BLOT4 BRESLO BROCKM BROWN1 BYERS1 CASCO2 CASCOR CHAN
CHATZI CHEN CHEN2 CHEN3 CHIAZZ COOKSO DAVEYS DEAN DOSEME DU DUNN EBELIN ESAKI FAN GENG GER GODLEY
GOLLED GSELL HANSEN HEGMAN HINDS HIRAOK HOROWI HORWIT HU HU2 HUANG ISHIMA JARUP JIANG JONES JUSSAW KO
KOHLME KOULUM KREYBE LAMTH LAMWK LAMWK2 LAUSSM LEI LETOUR LEVIN LIU LIU2 LIU3 LIU4 LIU5 LOMBA2 LUO
MAGNUS MARSH MARSH2 MARTIS MASTRA MATSUD MCCONN MCDUFF MCLAUG MILLER MILLS MOLLO NOTAN2 NOU ORMOS PASTOR PAWLEG
PERNU PERSHA PIKE POFFIJ POLEDN PRESCO QIAO RADZIK RANDIG REN RONCO ROOTS ROTHSC SADOWS SANKAR SCHWA2 SEGI
SEOW SIEMIA SIMARA STASZE STOCKS SUN TAO ULMER VANDER WANG WANG3 WANG4 WICKLU WILKIN WUWILL WYNDE2 WYNDE4
XIANGZ XU XU2 XU3 XU4 YUAN ZHANG ZHENG ZHOU

3 AGUDO AKIBA ALDERS AMANDU AMES ANDERS ARCHER ARMADA AUSTIN AXELSS BARBON BECHER BENHAM BENSHL BLOHMK BOFFET BOUCOT
BRETT BROSS BROWN2 BUFFLE BYERS2 CARPEN CEDERL CHANG CHOI CHOW CHYOU CORREA DAMBER DARBY DEAN2 DEKLER DESTE2
DESTEF DOCKER DOLL DOLL2 DORANT DORGAN DORN DROSTE ENGELA ENSTRO GAO GAO2 GARCIA GARDIN GARSHI GILLIS GOODMA
GRAHAM GREGOR HAENSZ HAMMO2 HAMMON HEIN HENNEK HIRAY2 HUMBLE JAHN JAIN JARVHO JEDRYC JOLY KAISER KATSOU KAUFMA
KELLER KHUDER KIHARA KINLEN KJUUS KNEKT KOO LANGE LEMARC LIAW LICKIN LIDDEL LOMBAR LUBIN LUBIN2 MACLEN MATOS
MIGRAN MRFIT MRFITR MURATA NOTANI ODRISC OSANN OSANN2 PARKIN PERSH2 PETO PEZZO2 PEZZOT PISANI QIAO2 RACHTA RESTRE
RIMING SAARIK SCHWAR SHAW SHIMIZ SIMONA SITAS SOBUE SOBUE2 SPITZ STAYNE STOCKW STUCKE SUZUK2 SUZUKI SVENSS TANG
TANG2 TENKAN TIZZAN TOKARS TOUSEY TULINI VEIERO WAKAI WALD WANG2 WARSIN WATSON WIGLE WU WU2 WUNSCH WYNDE3
WYNDE5 WYNDE6 WYNDE7 WYNDE8 WYNDER YAMAGU YONG

4 HIRAYA

5 COMSTO HITOSU HOLE KANELL KREUZE KUBIK NAM SEGI2 SPEIZE TSUGAN VUTUC

6 KAISE2

9 BEST CPSI CPSII DEAN3 TVERDA

Table 2B11 -

IESLC - Meta-analysis of Current Smoking, Cigarettes only, Age 50-70
Squamous

This analysis is restricted to results for:
1) Non-dose-response data
2) Current smokers
3) Maximum age range 50-70
4) Results complete enough for use in metaanalysis

Within each study, results are then selected (in the following order of preference, within each sex) for:
5) PRODUCT: cigarettes only
6) CIGTYPE: all/unspecified, MC regardless of HR, MC only
7) DENOM: never smoked anything, never smoked cigarettes, (never +1 = +long term ex, +2 = +amount unknown,
+3 = never cigs+long term ex)
8) Followup period (YF, prospective studies): whole study (coded as 0) or longest available
9) LCtype: all or nearest available, at least Squamous and Adeno. (q = squamous, s = small,
a = adeno, KI = Kreyberg I, u = undifferentiated)
10) Race: all or nearest available, otherwise by race (wh or w = white, bl or b = black, hi = hispanic
ch = chinese, jap = japanese, haw = hawaiian, w+o = white + oriental, sca = scandinavian, as = asian)
11) For overlapping studies: principal rather than subsidiary studies
Finally by Age: whole study (actual age shown) if available, otherwise by widest available age group
and then for single sex results (m, f) in preference to combined sex results (c).

Results adjusted (AD) for the most potential confounders are then chosen in Sections -1 to -3
(and those which actually differ from the adjusted results in Table 2B3 - 1 are marked 'x' in Section -1)
and results adjusted for the least confounders in Sections -4 to -6. (Those least adjusted results which
actually differ from the most adjusted as marked 'x' in column X in Section -4)
(Results adjusted for an unknown number of confounder(s) are coded as 20.)

Section -7 shows excluded studies, together with the stage (as above) at which no qualifying
results were found.

Section -8 lists the potentially overlapping studies which have been included (1=principal, 2=subsidiary).

Section -9 lists any results which would have been included in preference except that they had data not complete
enough for use in meta-analysis, with their significance (yes/no), if known, and any further comment as entered
on the database.

In addition to those mentioned above, the following fields, levels and abbreviations are used:
* or nk = not known, n = no, y = yes, ot = other
nev = never
all/unspec = all or unspecified, MC = manufactured cigarettes, HR = hand-rolled cigarettes
REF: 6-character study reference
NRR: number of the RR on the database within the study
ST : study type (CC = case control, pr or prosp = prospective)
NLC: number of lung cancer cases in whole study
R : risky occupational population (n = no, m = mining, o = other risky)
VB : national cigarette type (V = at least 75% Virginia, bl = at least 75% blended, ot = other)
P : any proxy use
H : full histological confirmation
De : derivation of RR/CI (or = original, st = standard method, ot = other method of estimation)

Table 2B11 - 1

IESLC - Meta-analysis of Current Smoking, Cigarettes only, Age 50-70
Squamous
Most adjusted

REF	NRR	2B3	SEX	AGEL	AGEH	RACE	YF	LC TYPE	LOC	START	ST	NLC	R	VB	P	H	AD	PRODUCT	DENOM	De
HAMMON	102		m	50	69	wh	0	not a	NAmer	1952	pr	448	n	bl	n	n	1	cig only	nev any	ot

Cigarette type is all/unspec for all RRs

Table 2B11 - 2

IESLC - Meta-analysis of Current Smoking, Cigarettes only, Age 50-70
Squamous
Most adjusted

REF	NRR	SEX	AD	Number Exposed Case	Number Exposed Cont	Non-exposed Case	Non-exposed Cont	RR	95.00%CI
*HAMMON	102	m	1	-	-	-	-	26.42	(9.78- 71.34)
Partial Totals				0	0	0	0		

*prospective study

REF	NRR	SEX	AD	Ys	Ws	Qs	Ps
*HAMMON	102	m	1	3.27	3.89	0.00	0.0000

	N	1
	NS	1
	Wt	3.89
Het	Chi	0.00
Het	df	0
Het	P	N.S.
Fixed	RR	26.42
	RRl	9.78
	RRu	71.36
	P	+++
Random	RR	26.42
	RRl	9.78
	RRu	71.36
	P	+++
Asymm	P	

Table 2B11 - 3

IESLC - Meta-analysis of Current Smoking, Cigarettes only, Age 50-70
Squamous
Most adjusted

	Sex combined	male	female	Total
N		1		1
NS		1		1
Wt		3.89		3.89
Het Chi		0.00		0.00
Het df		0		0
Het P		N.S.		N.S.
Fixed RR		26.42		26.42
RRl		9.78		9.78
RRu		71.36		71.36
P		+++		+++
Random RR		26.42		26.42
RRl		9.78		9.78
RRu		71.36		71.36
P		+++		+++
Between Chi				
Between df				
Between P				N.S.
Btwn(F) P				N.S.
Btwn(R) P				N.S.

Too few RRs for analysis by factor

Table 2B11 - 4

IESLC - Meta-analysis of Current Smoking, Cigarettes only, Age 50-70
Squamous
Least adjusted

REF	NRR	X	SEX	AGEL	AGEH	RACE	YF	LC TYPE	LOC	START	ST	NLC	R	VB	P	H	AD	PRODUCT	DENOM	De
HAMMON	102		m	50	69	wh	0	not a	NAmer	1952	pr	448	n	bl	n	n	1	cig only	nev any	ot

Cigarette type is all/unspec for all RRs

Table 2B11 - 5

IESLC - Meta-analysis of Current Smoking, Cigarettes only, Age 50-70
Squamous
Least adjusted

REF	NRR	SEX	AD	Number Case	Exposed Cont	Non-exposed Case	Cont	RR	95.00%CI
*HAMMON	102	m	1	-	-	-	-	26.42	(9.78- 71.34)
Partial Totals				0	0	0	0		

*prospective study

REF	NRR	SEX	AD	Ys	Ws	Qs	Ps
*HAMMON	102	m	1	3.27	3.89	0.00	0.0000

	N	1
	NS	1
	Wt	3.89
Het	Chi	0.00
Het	df	0
Het	P	N.S.
Fixed	RR	26.42
	RRl	9.78
	RRu	71.36
	P	+++
Random	RR	26.42
	RRl	9.78
	RRu	71.36
	P	+++
Asymm	P	

Table 2B11 - 6

IESLC - Meta-analysis of Current Smoking, Cigarettes only, Age 50-70
Squamous
Least adjusted

	combined	Sex male	female	Total
N		1		1
NS		1		1
Wt		3.89		3.89
Het Chi		0.00		0.00
Het df		0		0
Het P		N.S.		N.S.
Fixed RR		26.42		26.42
RRl		9.78		9.78
RRu		71.36		71.36
P		+++		+++
Random RR		26.42		26.42
RRl		9.78		9.78
RRu		71.36		71.36
P		+++		+++
Between Chi				
Between df				
Between P				N.S.
Btwn(F) P				N.S.
Btwn(R) P				N.S.

Table 2B11 - 7

IESLC - Meta-analysis of Current Smoking, Cigarettes only, Age 50-70
Squamous
Excluded studies (and stage at which they were excluded)

Stage	Excluded studies
1	BOUCHA BUELL LAURIL MZILEN
2	ABELIN ABRAHA AUVINE AXELSO BAND BERRIN BLOT1 BLOT2 BLOT3 BLOT4 BRESLO BROCKM BROWN1 BYERS1 CASCO2 CASCOR CHAN CHATZI CHEN CHEN2 CHEN3 CHIAZZ COOKSO DAVEYS DEAN DOSEME DU DUNN EBELIN ESAKI FAN GENG GER GODLEY GOLLED GSELL HANSEN HEGMAN HINDS HIRAOK HOROWI HORWIT HU HU2 HUANG ISHIMA JARUP JIANG JONES JUSSAW KO KOHLME KOULUM KREYBE LAMTH LAMWK LAMWK2 LAUSSM LEI LETOUR LEVIN LIU LIU2 LIU3 LIU4 LIU5 LOMBA2 LUO MAGNUS MARSH MARSH2 MARTIS MASTRA MATSUD MCCONN MCDUFF MCLAUG MILLER MILLS MOLLO NOTAN2 NOU ORMOS PASTOR PAWLEG PERNU PERSHA PIKE POFFIJ POLEDN PRESCO QIAO RADZIK RANDIG REN RONCO ROOTS ROTHSC SADOWS SANKAR SCHWA2 SEGI SEOW SIEMIA SIMARA STASZE STOCKS SUN TAO ULMER VANDER WANG WANG3 WANG4 WICKLU WILKIN WUWILL WYNDE2 WYNDE4 XIANGZ XU XU2 XU3 XU4 YUAN ZHANG ZHENG ZHOU
3	AGUDO AKIBA ALDERS AMANDU AMES ARCHER ARMADA AUSTIN AXELSS BARBON BECHER BENHAM BENSHL BLOHMK BOFFET BOUCOT BRETT BROSS BROWN2 BUFFLE BYERS2 CARPEN CHANG CHOI CHOW CHYOU COMSTO CORREA DAMBER DARBY DEAN2 DEKLER DESTE2 DESTEF DOCKER DOLL DOLL2 DORGAN DROSTE ENGELA ENSTRO GAO GAO2 GARCIA GARDIN GARSHI GILLIS GOODMA GRAHAM GREGOR HAENSZ HAMMO2 HEIN HENNEK HIRAY2 HUMBLE JAHN JAIN JARVHO JEDRYC JOLY KAISER KATSOU KAUFMA KELLER KHUDER KIHARA KINLEN KJUUS KNEKT KOO LANGE LEMARC LIAW LICKIN LIDDEL LOMBAR LUBIN LUBIN2 MACLEN MATOS MIGRAN MRFIT MRFITR MURATA NOTANI ODRISC OSANN OSANN2 PARKIN PERSH2 PETO PEZZO2 PEZZOT PISANI QIAO2 RACHTA RESTRE RIMING SAARIK SCHWAR SHAW SHIMIZ SIMONA SITAS SOBUE SOBUE2 SPEIZE SPITZ STAYNE STOCKW STUCKE SUZUK2 SUZUKI SVENSS TANG TANG2 TENKAN TIZZAN TOKARS TOUSEY TSUGAN TULINI TVERDA VEIERO VUTUC WAKAI WALD WANG2 WARSIN WATSON WIGLE WU WU2 WUNSCH WYNDE3 WYNDE5 WYNDE6 WYNDE7 WYNDE8 WYNDER YAMAGU YONG
4	HIRAYA
5	ANDERS DORANT DORN HITOSU HOLE KANELL KREUZE KUBIK NAM SEGI2
6	KAISE2
9	BEST CEDERL CPSI CPSII DEAN3

Table 2B12 -

IESLC - Meta-analysis of Current Smoking, Cigarettes only, Age 65+
Squamous

This analysis is restricted to results for:
1) Non-dose-response data
2) Current smokers
3) Age 65+
4) Results complete enough for use in metaanalysis

Within each study, results are then selected (in the following order of preference, within each sex) for:
5) PRODUCT: cigarettes only
6) CIGTYPE: all/unspecified, MC regardless of HR, MC only
7) DENOM: never smoked anything, never smoked cigarettes, (never +1 = +long term ex, +2 = +amount unknown,
+3 = never cigs+long term ex)
8) Followup period (YF, prospective studies): whole study (coded as 0) or longest available
9) LCtype: all or nearest available, at least Squamous and Adeno. (q = squamous, s = small,
a = adeno, KI = Kreyberg I, u = undifferentiated)
10) Race: all or nearest available, otherwise by race (wh or w = white, bl or b = black, hi = hispanic
ch = chinese, jap = japanese, haw = hawaiian, w+o = white + oriental, sca = scandinavian, as = asian)
11) For overlapping studies: principal rather than subsidiary studies
Finally by Age: whole study (actual age shown) if available, otherwise by widest available age group
and then for single sex results (m, f) in preference to combined sex results (c).

Results adjusted (AD) for the most potential confounders are then chosen in Sections -1 to -3
(and those which actually differ from the adjusted results in Table 2B3 - 1 are marked 'x' in Section -1)
and results adjusted for the least confounders in Sections -4 to -6. (Those least adjusted results which
actually differ from the most adjusted as marked 'x' in column X in Section -4)
(Results adjusted for an unknown number of confounder(s) are coded as 20.)

Section -7 shows excluded studies, together with the stage (as above) at which no qualifying
results were found.

Section -8 lists the potentially overlapping studies which have been included (1=principal, 2=subsidiary).

Section -9 lists any results which would have been included in preference except that they had data not complete
enough for use in meta-analysis, with their significance (yes/no), if known, and any further comment as entered
on the database.

In addition to those mentioned above, the following fields, levels and abbreviations are used:
* or nk = not known, n = no, y = yes, ot = other
nev = never
all/unspec = all or unspecified, MC = manufactured cigarettes, HR = hand-rolled cigarettes
REF: 6-character study reference
NRR: number of the RR on the database within the study
ST : study type (CC = case control, pr or prosp = prospective)
NLC: number of lung cancer cases in whole study
R : risky occupational population (n = no, m = mining, o = other risky)
VB : national cigarette type (V = at least 75% Virginia, bl = at least 75% blended, ot = other)
P : any proxy use
H : full histological confirmation
De : derivation of RR/CI (or = original, st = standard method, ot = other method of estimation)

Table 2B12 - 0

No RRs selected for this analysis

Table 2B12 - 7

IESLC - Meta-analysis of Current Smoking, Cigarettes only, Age 65+
Squamous
Excluded studies (and stage at which they were excluded)

1 BOUCHA BUELL LAURIL MZILEN

2 ABELIN ABRAHA AUVINE AXELSO BAND BERRIN BLOT1 BLOT2 BLOT3 BLOT4 BRESLO BROCKM BROWN1 BYERS1 CASCO2 CASCOR CHAN
CHATZI CHEN CHEN2 CHEN3 CHIAZZ COOKSO DAVEYS DEAN DOSEME DU DUNN EBELIN ESAKI FAN GENG GER GODLEY
GOLLED GSELL HANSEN HEGMAN HINDS HIRAOK HOROWI HORWIT HU HU2 HUANG ISHIMA JARUP JIANG JONES JUSSAW KO
KOHLME KOULUM KREYBE LAMTH LAMWK LAMWK2 LAUSSM LEI LETOUR LEVIN LIU LIU2 LIU3 LIU4 LIU5 LOMBA2 LUO
MAGNUS MARSH MARSH2 MARTIS MASTRA MATSUD MCCONN MCDUFF MCLAUG MILLER MILLS MOLLO NOTAN2 NOU ORMOS PASTOR PAWLEG
PERNU PERSHA PIKE POFFIJ POLEDN PRESCO QIAO RADZIK RANDIG REN RONCO ROOTS ROTHSC SADOWS SANKAR SCHWA2 SEGI
SEOW SIEMIA SIMARA STASZE STOCKS SUN TAO ULMER VANDER WANG WANG3 WANG4 WICKLU WILKIN WUWILL WYNDE2 WYNDE4
XIANGZ XU XU2 XU3 XU4 YUAN ZHANG ZHENG ZHOU

3 AGUDO AKIBA ALDERS AMANDU AMES ANDERS ARCHER ARMADA AUSTIN AXELSS BARBON BECHER BENHAM BENSHL BLOHMK BOFFET BOUCOT
BRETT BROSS BROWN2 BUFFLE BYERS2 CARPEN CEDERL CHANG CHOI CHOW CHYOU CORREA DAMBER DARBY DEAN2 DEKLER DESTE2
DESTEF DOCKER DOLL DOLL2 DORANT DORGAN DROSTE ENGELA ENSTRO GAO GAO2 GARCIA GARDIN GILLIS GOODMA GRAHAM GREGOR
HAENSZ HAMMO2 HAMMON HEIN HENNEK HIRAY2 HITOSU HOLE JAHN JAIN JARVHO JEDRYC JOLY KAISER KATSOU KAUFMA KELLER
KHUDER KIHARA KINLEN KJUUS KNEKT KOO KREUZE KUBIK LANGE LEMARC LIAW LICKIN LIDDEL LOMBAR LUBIN LUBIN2 MACLEN
MATOS MIGRAN MRFIT MRFITR MURATA NOTANI ODRISC OSANN OSANN2 PARKIN PERSH2 PETO PEZZO2 PEZZOT PISANI QIAO2 RACHTA
RESTRE RIMING SAARIK SCHWAR SHAW SHIMIZ SIMONA SITAS SOBUE SOBUE2 SPEIZE SPITZ STAYNE STOCKW STUCKE SUZUK2 SUZUKI
SVENSS TANG TANG2 TIZZAN TOKARS TOUSEY TSUGAN TULINI TVERDA VEIERO VUTUC WAKAI WALD WANG2 WARSIN WATSON WIGLE
WU WU2 WUNSCH WYNDE3 WYNDE5 WYNDE6 WYNDE7 WYNDE8 WYNDER YAMAGU YONG

4 HIRAYA

5 COMSTO GARSHI HUMBLE KANELL NAM SEGI2 TENKAN

9 BEST CPSI CPSII DEAN3 DORN KAISE2

Table 2B13 -

IESLC - Meta-analysis of Current Smoking (vs non-current), Any product (or Cigarettes if Any not available)
Squamous

This analysis is restricted to results for:
1) Non-dose-response data
2) Current smokers
3) Results complete enough for use in metaanalysis

Within each study, results are then selected (in the following order of preference, within each sex) for:
4) PRODUCT: all/unspec, cigarettes regardless of other products, cigarettes only
5) CIGTYPE: all/unspecified, MC regardless of HR, MC only
6) DENOM: non smoker of anything, non smoker of cigarettes
7) Followup period (YF, prospective studies): whole study (coded as 0) or longest available
8) LCtype: squamous or nearest available, but not adeno. (q = squamous, s = small,
a = adeno, KI = Kreyberg I, u = undifferentiated)
9) Race: all or nearest available, otherwise by race (wh or w = white, bl or b = black, hi = hispanic
ch = chinese, jap = japanese, haw = hawaiian, w+o = white + oriental, sca = scandinavian, as = asian)
10) For overlapping studies: principal rather than subsidiary studies
Finally by Age: whole study (coded as 0) if available, otherwise by widest available age group
and then for single sex results (m, f) in preference to combined sex results (c).

Results adjusted (AD) for the most potential confounders are then chosen in Sections -1 to -3
and results adjusted for the least confounders in Sections -4 to -6. (Those least adjusted results which
actually differ from the most adjusted as marked 'x' in column X in Section -4)
(Results adjusted for an unknown number of confounder(s) are coded as 20.)

Section -7 shows excluded studies, together with the stage (as above) at which no qualifying
results were found.

Section -8 lists the potentially overlapping studies which have been included (1=principal, 2=subsidiary).

Section -9 lists any results which would have been included in preference except that they had data not complete
enough for use in meta-analysis, with their significance (yes/no), if known, and any further comment as entered
on the database.

In addition to those mentioned above, the following fields, levels and abbreviations are used:
* or nk = not known, n = no, y = yes, ot = other
non = not current
all/unspec = all or unspecified, cig+/-ot = cigarettes irrespective of other products (cigar, pipe etc)
MC = manufactured cigarettes, HR = hand-rolled cigarettes
REF: 6-character study reference
NRR: number of the RR on the database within the study
ST : study type (CC = case control, pr or prosp = prospective)
NLC: number of lung cancer cases in whole study
R : risky occupational population (n = no, m = mining, o = other risky)
VB : national cigarette type (V = at least 75% Virginia, bl = at least 75% blended, ot = other)
P : any proxy use
H : full histological confirmation
De : derivation of RR/CI (or = original, st = standard method, ot = other method of estimation)

Table 2B13 - 1

IESLC - Meta-analysis of Current Smoking (vs non-current), Any product (or Cigarettes if Any not available)
Squamous
Most adjusted

REF	NRR	SEX	AGEL	AGEH	RACE	YF	LC TYPE	LOC	START	ST	NLC	R	VB	P	H	AD	PRODUCT	DENOM	De
BARBON	113	m	0	0	all	-	q	Eu:wst	1979	CC	755	n	bl	y	y	1	all/unsp	non any	ot
BUFFLE	65	f	0	0	w-hi	-	q	NAmer	1976	CC	943	n	bl	y	n	0	cig+/-ot	non cigs	st
COMSTO	71	m	0	0	all	-	q	NAmer	1975	ot	258	n	bl	n	n	0	cig+/-ot	non cigs	st
COMSTO	83	f	0	0	all	-	q	NAmer	1975	ot	258	n	bl	n	n	0	cig+/-ot	non cigs	st
CORREA	66	c	0	0	all	-	q+s	NAmer	1979	CC	1359	n	bl	y	n	1	cig+/-ot	non cigs	ot
ENGELA	63	m	0	0	all	0	q	Eu:Sca	1964	pr	435	n	bl	n	n	7	cig+/-ot	non cigs	ot
HAENSZ	32	f	0	0	all	-	q+u	NAmer	1955	CC	158	n	bl	n	y	0	cig+/-ot	non cigs	st
JAHN	34	m	0	0	all	-	q	Eu:Ger	1988	CC	1004	n	bl	n	n	0	cig+/-ot	non cigs	st
JAIN	38	m	0	0	all	-	q	NAmer	1981	CC	845	n	V	y	n	0	cig+/-ot	non cigs	st
JAIN	33	f	0	0	all	-	q	NAmer	1981	CC	845	n	V	y	n	0	cig+/-ot	non cigs	st
JEDRYC	51	m	0	0	all	-	q	Eu:est	1980	CC	1630	n	bl	y	n	0	cig+/-ot	non any	st
KATSOU	38	f	0	0	all	-	KI	Eu:bal	1987	CC	101	n	bl	n	n	1	all/unsp	non any	ot
KHUDER	29	m	0	0	all	-	q	NAmer	1985	CC	482	n	bl	n	y	0	cig+/-ot	non cigs	ot
KIHARA	18	c	0	0	jap	-	q	As:Jap	1991	CC	440	n	bl	n	n	0	all/unsp	non any	st
LUBIN2	253	m	0	0	all	-	q	Eu:mul	1976	CC	7804	n	bl	n	y	0	cig+/-ot	non any	st
LUBIN2	265	f	0	0	all	-	q	Eu:mul	1976	CC	7804	n	bl	n	y	0	cig+/-ot	non any	st
MATOS	87	m	0	0	all	-	q	SCAmer	1994	CC	200	n	bl	n	n	2	cig+/-ot	non any	ot
OSANN	75	m	0	0	all	-	q	NAmer	1984	CC	1986	n	bl	n	n	2	cig+/-ot	non cigs	ot
OSANN	76	f	0	0	all	-	q	NAmer	1984	CC	1986	n	bl	n	n	2	cig+/-ot	non cigs	ot
OSANN2	30	f	0	0	all	-	KI	NAmer	1964	ot	217	n	bl	n	y	1	cig+/-ot	non cigs	ot
SAARIK	2	c	0	0	wh	-	q	Eu:Sca	1988	CC	205	n	bl	n	y	0	all/unsp	non any	st
SEGI2	42	m	0	0	all	-	q	As:Jap	1962	CC	378	n	bl	n	n	1	cig+/-ot	non any	ot
SEGI2	44	f	0	0	all	-	q	As:Jap	1962	CC	378	n	bl	n	n	1	cig+/-ot	non any	ot
SHIMIZ	2	m	0	0	all	-	q	As:Jap	1977	CC	751	n	bl	y	n	2	all/unsp	non any	or
SHIMIZ	7	f	0	0	all	-	q	As:Jap	1977	CC	751	n	bl	y	n	2	all/unsp	non any	or
SOBUE	98	m	0	0	all	-	q	As:Jap	1986	CC	1376	n	bl	n	y	1	cig+/-ot	non cigs	ot
SOBUE	108	f	0	0	all	-	q	As:Jap	1986	CC	1376	n	bl	n	y	1	cig+/-ot	non cigs	ot
STAYNE	7	m	0	0	all	-	q	NAmer	1969	CC	420	n	bl	n	n	1	cig+/-ot	non cigs	st
SVENSS	92	f	0	0	all	-	q	Eu:Sca	1983	CC	210	n	bl	n	n	1	all/unsp	non any	ot
TSUGAN	18	m	0	0	all	-	q	As:Jap	1976	CC	134	n	bl	n	y	0	all/unsp	non any	st
WAKAI	75	m	0	0	all	-	q	As:Jap	1988	CC	333	n	bl	n	y	1	all/unsp	non any	ot
WAKAI	81	f	0	0	all	-	q	As:Jap	1988	CC	333	n	bl	n	y	1	all/unsp	non any	ot
WU	28	f	0	0	wh	-	q	NAmer	1981	CC	220	n	bl	n	y	2	all/unsp	non any	ot
WYNDE3	13	m	0	0	all	-	KI	NAmer	1966	CC	350	n	bl	n	y	0	all/unsp	non any	st
WYNDE6	147	m	0	0	all	-	KI	NAmer	1969	CC	4423	n	bl	n	y	0	cig+/-ot	non cigs	st
WYNDE6	255	f	0	0	all	-	KI	NAmer	1969	CC	4423	n	bl	n	y	0	cig+/-ot	non cigs	st

Cigarette type is all/unspec for all RRs

Table 2B13 - 2

IESLC - Meta-analysis of Current Smoking (vs non-current), Any product (or Cigarettes if Any not available)
Squamous
Most adjusted

REF	NRR	SEX	AD	Number Exposed Case	Number Exposed Cont	Non-exposed Case	Non-exposed Cont	RR	95.00%CI
BARBON	113	m	1	-	-	-	-	3.74	(2.73- 5.12)
BUFFLE	65	f	0	39	110	22	168	2.71	(1.52- 4.81)
COMSTO	71	m	0	27	100	19	213	3.03	(1.61- 5.70)
COMSTO	83	f	0	16	52	1	150	46.15	(5.97- 356.64)
Subtotal COMSTO								3.84	(2.10- 7.03)
CORREA	66	c	1	-	-	-	-	11.81	(9.31- 14.97)
*ENGELA	63	m	7	-	-	-	-	3.60	(2.13- 6.09)
HAENSZ	32	f	0	53	94	49	245	2.82	(1.79- 4.45)
JAHN	34	m	0	153	269	201	570	1.61	(1.25- 2.08)
JAIN	38	m	0	107	118	49	244	4.52	(3.02- 6.76)
JAIN	33	f	0	81	99	28	311	9.09	(5.59- 14.77)
Subtotal JAIN								6.01	(4.41- 8.19)
JEDRYC	51	m	0	235	516	51	601	5.37	(3.88- 7.43)
KATSOU	38	f	1	-	-	-	-	5.28	(2.33- 11.98)
KHUDER	29	m	0	112	-	73	-	2.20	(1.55- 3.12)
KIHARA	18	c	0	111	162	26	307	8.09	(5.07- 12.91)
LUBIN2	253	m	0	2518	6209	1136	6844	2.44	(2.26- 2.64)
LUBIN2	265	f	0	154	410	117	1337	4.29	(3.29- 5.59)
Subtotal LUBIN2								2.56	(2.37- 2.75)
MATOS	87	m	2	-	-	-	-	4.69	(2.52- 8.73)
OSANN	75	m	2	-	-	-	-	5.30	(4.16- 6.75)
OSANN	76	f	2	-	-	-	-	11.42	(7.93- 16.46)
Subtotal OSANN								6.70	(5.48- 8.20)
OSANN2	30	f	1	-	-	-	-	9.38	(3.17- 27.76)
SAARIK	2	c	0	47	66	45	224	3.54	(2.17- 5.80)
SEGI2	42	m	1	-	-	-	-	2.86	(1.35- 6.05)
SEGI2	44	f	1	-	-	-	-	1.70	(0.48- 5.98)
Subtotal SEGI2								2.50	(1.31- 4.76)
SHIMIZ	2	m	2	-	-	-	-	4.30	(2.80- 6.70)
SHIMIZ	7	f	2	-	-	-	-	6.40	(2.30- 17.30)
Subtotal SHIMIZ								4.58	(3.07- 6.83)
SOBUE	98	m	1	-	-	-	-	4.37	(3.02- 6.32)
SOBUE	108	f	1	-	-	-	-	7.62	(4.57- 12.69)
Subtotal SOBUE								5.29	(3.92- 7.13)
STAYNE	7	m	1	-	-	-	-	3.10	(1.97- 4.88)
SVENSS	92	f	1	-	-	-	-	11.95	(5.04- 28.31)
TSUGAN	18	m	0	18	13	2	7	4.85	(0.86- 27.22)
WAKAI	75	m	1	-	-	-	-	2.17	(1.37- 3.44)
WAKAI	81	f	1	-	-	-	-	21.82	(6.67- 71.32)
Subtotal WAKAI								2.94	(1.91- 4.51)
WU	28	f	2	-	-	-	-	10.49	(3.42- 32.17)
WYNDE3	13	m	0	171	207	39	213	4.51	(3.03- 6.71)
WYNDE6	147	m	0	1026	741	747	1872	3.47	(3.06- 3.94)
WYNDE6	255	f	0	550	376	201	1181	8.59	(7.04- 10.49)
Subtotal WYNDE6								4.51	(4.05- 5.02)
Partial Totals				5418	9542	2806	14487		

*prospective study

REF	NRR	SEX	AD	Ys	Ws	Qs	Ps
BARBON	113	m	1	1.32	38.86	0.06	0.0000
BUFFLE	65	f	0	1.00	11.61	0.93	0.0007
COMSTO	71	m	0	1.11	9.58	0.28	0.0006
COMSTO	83	f	0	3.83	0.92	5.99	0.0002
Subtotal COMSTO				1.35	10.50	6.27	
CORREA	66	c	1	2.47	68.12	96.53	0.0000
*ENGELA	63	m	7	1.28	13.92	0.00	0.0000
HAENSZ	32	f	0	1.04	18.52	1.09	0.0000
JAHN	34	m	0	0.48	58.88	37.73	0.0002
JAIN	38	m	0	1.51	23.63	1.24	0.0000
JAIN	33	f	0	2.21	16.29	14.04	0.0000
Subtotal JAIN				1.79	39.92	15.28	
JEDRYC	51	m	0	1.68	36.41	5.88	0.0000
KATSOU	38	f	1	1.66	5.73	0.85	0.0001
KHUDER	29	m	0	0.79	31.40	7.54	0.0000
KIHARA	18	c	0	2.09	17.57	11.59	0.0000
LUBIN2	253	m	0	0.89	631.08	93.63	0.0000
LUBIN2	265	f	0	1.46	54.86	1.74	0.0000
Subtotal LUBIN2				0.94	685.94	95.37	
MATOS	87	m	2	1.55	9.95	0.71	0.0000
OSANN	75	m	2	1.67	65.59	9.93	0.0000

Table 2B13 - 2

IESLC - Meta-analysis of Current Smoking (vs non-current), Any product (or Cigarettes if Any not available)
Squamous
Most adjusted

REF	NRR	SEX	AD	Ys	Ws	Qs	Ps
OSANN	76	f	2	2.44	28.81	38.56	0.0000
Subtotal OSANN				1.90	94.40	48.49	
OSANN2	30	f	1	2.24	3.26	3.01	0.0001
SAARIK	2	c	0	1.27	15.84	0.00	0.0000
SEGI2	42	m	1	1.05	6.83	0.35	0.0060
SEGI2	44	f	1	0.53	2.42	1.35	0.4096
Subtotal SEGI2				0.91	9.24	1.70	
SHIMIZ	2	m	2	1.46	20.19	0.65	0.0000
SHIMIZ	7	f	2	1.86	3.77	1.26	0.0003
Subtotal SHIMIZ				1.52	23.96	1.91	
SOBUE	98	m	1	1.47	28.18	1.09	0.0000
SOBUE	108	f	1	2.03	14.73	8.34	0.0000
Subtotal SOBUE				1.67	42.91	9.42	
STAYNE	7	m	1	1.13	18.67	0.40	0.0000
SVENSS	92	f	1	2.48	5.16	7.46	0.0000
TSUGAN	18	m	0	1.58	1.29	0.12	0.0731
WAKAI	75	m	1	0.77	18.13	4.60	0.0010
WAKAI	81	f	1	3.08	2.74	8.91	0.0000
Subtotal WAKAI				1.08	20.86	13.51	
WU	28	f	2	2.35	3.06	3.51	0.0000
WYNDE3	13	m	0	1.51	24.38	1.27	0.0000
WYNDE6	147	m	0	1.24	238.26	0.28	0.0000
WYNDE6	255	f	0	2.15	97.09	73.93	0.0000
Subtotal WYNDE6				1.51	335.35	74.22	

	N	36
	NS	27
	Wt	1645.73
Het	Chi	444.86
Het	df	35
Het	P	***
Fixed	RR	3.59
	RRl	3.42
	RRu	3.77
	P	+++
Random	RR	4.71
	RRl	3.84
	RRu	5.79
	P	+++
Asymm	P	*

Table 2B13 - 3

IESLC - Meta-analysis of Current Smoking (vs non-current), Any product (or Cigarettes if Any not available)
Squamous
Most adjusted

	Sex combined	male	female	Total
N	3	18	15	36
NS	3	18	15	36
Wt	101.53	1275.22	268.98	1645.73
Het Chi	18.95	111.15	65.08	444.86
Het df	2	17	14	35
Het P	***	***	***	***
Fixed RR	9.17	2.92	6.73	3.59
RRl	7.55	2.76	5.97	3.42
RRu	11.14	3.08	7.58	3.77
P	+++	+++	+++	+++
Random RR	7.14	3.41	6.75	4.71
RRl	3.55	2.85	4.93	3.84
RRu	14.35	4.09	9.23	5.79
P	+++	+++	+++	+++
Between Chi				249.69
Between df				2
Between P				***
Btwn(F) P				***
Btwn(R) P				***

Table 2B13 - 4

IESLC - Meta-analysis of Current Smoking (vs non-current), Any product (or Cigarettes if Any not available)
Squamous
Least adjusted

REF	NRR	X	SEX	AGEL	AGEH	RACE	YF	LC TYPE	LOC	START	ST	NLC	R	VB	P	H	AD	PRODUCT	DENOM	De
BARBON	112	x	m	0	0	all	-	q	Eu:wst	1979	CC	755	n	bl	y	y	0	all/unsp	non any	st
BUFFLE	65		f	0	0	w-hi	-	q	NAmer	1976	CC	943	n	bl	y	n	0	cig+/-ot	non cigs	st
COMSTO	71		m	0	0	all	-	q	NAmer	1975	ot	258	n	bl	n	n	0	cig+/-ot	non cigs	st
COMSTO	83		f	0	0	all	-	q	NAmer	1975	ot	258	n	bl	n	n	0	cig+/-ot	non cigs	st
CORREA	66		c	0	0	all	-	q+s	NAmer	1979	CC	1359	n	bl	y	n	1	cig+/-ot	non cigs	ot
ENGELA	63		m	0	0	all	0	q	Eu:Sca	1964	pr	435	n	bl	n	n	7	cig+/-ot	non cigs	ot
HAENSZ	32		f	0	0	all	-	q+u	NAmer	1955	CC	158	n	bl	n	y	0	cig+/-ot	non cigs	st
JAHN	34		m	0	0	all	-	q	Eu:Ger	1988	CC	1004	n	bl	n	n	0	cig+/-ot	non cigs	st
JAIN	38		m	0	0	all	-	q	NAmer	1981	CC	845	n	V	y	n	0	cig+/-ot	non cigs	st
JAIN	33		f	0	0	all	-	q	NAmer	1981	CC	845	n	V	y	n	0	cig+/-ot	non cigs	st
JEDRYC	51		m	0	0	all	-	q	Eu:est	1980	CC	1630	n	bl	y	n	0	cig+/-ot	non any	st
KATSOU	36	x	f	0	0	all	-	KI	Eu:bal	1987	CC	101	n	bl	n	n	0	all/unsp	non any	st
KHUDER	29		m	0	0	all	-	q	NAmer	1985	CC	482	n	bl	n	y	0	cig+/-ot	non cigs	ot
KIHARA	18		c	0	0	jap	-	q	As:Jap	1991	CC	440	n	bl	n	n	0	all/unsp	non any	st
LUBIN2	253		m	0	0	all	-	q	Eu:mul	1976	CC	7804	n	bl	n	y	0	cig+/-ot	non any	st
LUBIN2	265		f	0	0	all	-	q	Eu:mul	1976	CC	7804	n	bl	n	y	0	cig+/-ot	non any	st
MATOS	86	x	m	0	0	all	-	q	SCAmer	1994	CC	200	n	bl	n	n	0	cig+/-ot	non any	st
OSANN	66	x	m	0	0	all	-	q	NAmer	1984	CC	1986	n	bl	n	n	0	cig+/-ot	non cigs	st
OSANN	70	x	f	0	0	all	-	q	NAmer	1984	CC	1986	n	bl	n	n	0	cig+/-ot	non cigs	st
OSANN2	12	x	f	0	0	all	-	KI	NAmer	1964	ot	217	n	bl	n	y	0	cig+/-ot	non cigs	st
SAARIK	2		c	0	0	wh	-	q	Eu:Sca	1988	CC	205	n	bl	n	y	0	all/unsp	non any	st
SEGI2	41	x	m	0	0	all	-	q	As:Jap	1962	CC	378	n	bl	n	n	0	cig+/-ot	non any	st
SEGI2	43	x	f	0	0	all	-	q	As:Jap	1962	CC	378	n	bl	n	n	0	cig+/-ot	non any	st
SHIMIZ	2		m	0	0	all	-	q	As:Jap	1977	CC	751	n	bl	y	n	2	all/unsp	non any	or
SHIMIZ	7		f	0	0	all	-	q	As:Jap	1977	CC	751	n	bl	y	n	2	all/unsp	non any	or
SOBUE	4	x	m	0	0	all	-	q	As:Jap	1986	CC	1376	n	bl	n	y	0	cig+/-ot	non cigs	st
SOBUE	20	x	f	0	0	all	-	q	As:Jap	1986	CC	1376	n	bl	n	y	0	cig+/-ot	non cigs	st
STAYNE	7		m	0	0	all	-	q	NAmer	1969	CC	420	n	bl	n	n	1	cig+/-ot	non cigs	st
SVENSS	67	x	f	0	0	all	-	q	Eu:Sca	1983	CC	210	n	bl	n	n	0	all/unsp	non any	st
TSUGAN	18		m	0	0	all	-	q	As:Jap	1976	CC	134	n	bl	n	y	0	all/unsp	non any	st
WAKAI	16	x	m	0	0	all	-	q	As:Jap	1988	CC	333	n	bl	n	y	0	all/unsp	non any	st
WAKAI	34	x	f	0	0	all	-	q	As:Jap	1988	CC	333	n	bl	n	y	0	all/unsp	non any	st
WU	25	x	f	0	0	wh	-	q	NAmer	1981	CC	220	n	bl	n	y	0	all/unsp	non any	st
WYNDE3	13		m	0	0	all	-	KI	NAmer	1966	CC	350	n	bl	n	y	0	all/unsp	non any	st
WYNDE6	147		m	0	0	all	-	KI	NAmer	1969	CC	4423	n	bl	n	y	0	cig+/-ot	non cigs	st
WYNDE6	255		f	0	0	all	-	KI	NAmer	1969	CC	4423	n	bl	n	y	0	cig+/-ot	non cigs	st

Cigarette type is all/unspec for all RRs

Table 2B13 - 5

IESLC - Meta-analysis of Current Smoking (vs non-current), Any product (or Cigarettes if Any not available)
Squamous
Least adjusted

REF	NRR	SEX	AD	Number Exposed Case	Number Exposed Cont	Non-exposed Case	Non-exposed Cont	RR	95.00%CI
BARBON	112	m	0	203	362	64	393	3.44	(2.51- 4.72)
BUFFLE	65	f	0	39	110	22	168	2.71	(1.52- 4.81)
COMSTO	71	m	0	27	100	19	213	3.03	(1.61- 5.70)
COMSTO	83	f	0	16	52	1	150	46.15	(5.97- 356.64)
Subtotal COMSTO								3.84	(2.10- 7.03)
CORREA	66	c	1	-	-	-	-	11.81	(9.31- 14.97)
*ENGELA	63	m	7	-	-	-	-	3.60	(2.13- 6.09)
HAENSZ	32	f	0	53	94	49	245	2.82	(1.79- 4.45)
JAHN	34	m	0	153	269	201	570	1.61	(1.25- 2.08)
JAIN	38	m	0	107	118	49	244	4.52	(3.02- 6.76)
JAIN	33	f	0	81	99	28	311	9.09	(5.59- 14.77)
Subtotal JAIN								6.01	(4.41- 8.19)
JEDRYC	51	m	0	235	516	51	601	5.37	(3.88- 7.43)
KATSOU	36	f	0	24	18	28	71	3.38	(1.59- 7.17)
KHUDER	29	m	0	112	-	73	-	2.20	(1.55- 3.12)
KIHARA	18	c	0	111	162	26	307	8.09	(5.07- 12.91)
LUBIN2	253	m	0	2518	6209	1136	6844	2.44	(2.26- 2.64)
LUBIN2	265	f	0	154	410	117	1337	4.29	(3.29- 5.59)
Subtotal LUBIN2								2.56	(2.37- 2.75)
MATOS	86	m	0	33	132	17	261	3.84	(2.06- 7.15)
OSANN	66	m	0	247	541	113	1310	5.29	(4.15- 6.76)
OSANN	70	f	0	130	367	41	1289	11.14	(7.69- 16.12)
Subtotal OSANN								6.63	(5.41- 8.13)
OSANN2	12	f	0	105	42	14	77	13.75	(7.02- 26.94)
SAARIK	2	c	0	47	66	45	224	3.54	(2.17- 5.80)
SEGI2	41	m	0	105	485	8	102	2.76	(1.30- 5.84)
SEGI2	43	f	0	4	34	8	128	1.88	(0.53- 6.63)
Subtotal SEGI2								2.50	(1.31- 4.76)
SHIMIZ	2	m	2	-	-	-	-	4.30	(2.80- 6.70)
SHIMIZ	7	f	2	-	-	-	-	6.40	(2.30- 17.30)
Subtotal SHIMIZ								4.58	(3.07- 6.83)
SOBUE	4	m	0	301	650	124	491	1.83	(1.44- 2.33)
SOBUE	20	f	0	29	168	21	921	7.57	(4.22- 13.59)
Subtotal SOBUE								2.25	(1.80- 2.81)
STAYNE	7	m	1	-	-	-	-	3.10	(1.97- 4.88)
SVENSS	67	f	0	42	53	11	156	11.24	(5.40- 23.40)
TSUGAN	18	m	0	18	13	2	7	4.85	(0.86- 27.22)
WAKAI	16	m	0	86	284	29	205	2.14	(1.35- 3.38)
WAKAI	34	f	0	15	26	4	150	21.63	(6.66- 70.33)
Subtotal WAKAI								2.90	(1.89- 4.44)
WU	25	f	0	61	23	10	48	12.73	(5.53- 29.29)
WYNDE3	13	m	0	171	207	39	213	4.51	(3.03- 6.71)
WYNDE6	147	m	0	1026	741	747	1872	3.47	(3.06- 3.94)
WYNDE6	255	f	0	550	376	201	1181	8.59	(7.04- 10.49)
Subtotal WYNDE6								4.51	(4.05- 5.02)
Partial Totals				6803	12727	3298	20089		

*prospective study

REF	NRR	SEX	AD	Ys	Ws	Qs	Ps
BARBON	112	m	0	1.24	38.67	0.01	0.0000
BUFFLE	65	f	0	1.00	11.61	0.74	0.0007
COMSTO	71	m	0	1.11	9.58	0.19	0.0006
COMSTO	83	f	0	3.83	0.92	6.13	0.0002
Subtotal COMSTO				1.35	10.50	6.32	
CORREA	66	c	1	2.47	68.12	101.33	0.0000
*ENGELA	63	m	7	1.28	13.92	0.01	0.0000
HAENSZ	32	f	0	1.04	18.52	0.84	0.0000
JAHN	34	m	0	0.48	58.88	35.02	0.0002
JAIN	38	m	0	1.51	23.63	1.58	0.0000
JAIN	33	f	0	2.21	16.29	14.94	0.0000
Subtotal JAIN				1.79	39.92	16.52	
JEDRYC	51	m	0	1.68	36.41	6.76	0.0000
KATSOU	36	f	0	1.22	6.80	0.01	0.0015
KHUDER	29	m	0	0.79	31.40	6.67	0.0000
KIHARA	18	c	0	2.09	17.57	12.44	0.0000
LUBIN2	253	m	0	0.89	631.08	79.95	0.0000
LUBIN2	265	f	0	1.46	54.86	2.36	0.0000
Subtotal LUBIN2				0.94	685.94	82.31	
MATOS	86	m	0	1.35	9.95	0.09	0.0000
OSANN	66	m	0	1.67	64.47	11.22	0.0000

Table 2B13 - 5

IESLC - Meta-analysis of Current Smoking (vs non-current), Any product (or Cigarettes if Any not available)
Squamous
Least adjusted

REF	NRR	SEX	AD	Ys	Ws	Qs	Ps
OSANN	70	f	0	2.41	28.10	37.88	0.0000
Subtotal OSANN				1.89	92.58	49.10	
OSANN2	12	f	0	2.62	8.49	15.98	0.0000
SAARIK	2	c	0	1.27	15.84	0.00	0.0000
SEGI2	41	m	0	1.02	6.83	0.37	0.0080
SEGI2	43	f	0	0.63	2.43	0.92	0.3245
Subtotal SEGI2				0.92	9.26	1.30	
SHIMIZ	2	m	2	1.46	20.19	0.88	0.0000
SHIMIZ	7	f	2	1.86	3.77	1.39	0.0003
Subtotal SHIMIZ				1.52	23.96	2.28	
SOBUE	4	m	0	0.61	66.84	27.63	0.0000
SOBUE	20	f	0	2.02	11.22	6.74	0.0000
Subtotal SOBUE				0.81	78.05	34.37	
STAYNE	7	m	1	1.13	18.67	0.26	0.0000
SVENSS	67	f	0	2.42	7.14	9.78	0.0000
TSUGAN	18	m	0	1.58	1.29	0.14	0.0731
WAKAI	16	m	0	0.76	18.35	4.37	0.0011
WAKAI	34	f	0	3.07	2.76	9.21	0.0000
Subtotal WAKAI				1.06	21.11	13.58	
WU	25	f	0	2.54	5.53	9.28	0.0000
WYNDE3	13	m	0	1.51	24.38	1.62	0.0000
WYNDE6	147	m	0	1.24	238.26	0.01	0.0000
WYNDE6	255	f	0	2.15	97.09	78.97	0.0000
Subtotal WYNDE6				1.51	335.35	78.98	

	N	36
	NS	27
	Wt	1689.88
Het	Chi	485.73
Het	df	35
Het	P	***
Fixed	RR	3.49
	RRl	3.33
	RRu	3.66
	P	+++
Random	RR	4.60
	RRl	3.74
	RRu	5.67
	P	+++
Asymm	P	*

Table 2B13 - 6

IESLC - Meta-analysis of Current Smoking (vs non-current), Any product (or Cigarettes if Any not available)
Squamous
Least adjusted

	Sex			
	combined	male	female	Total
N	3	18	15	36
NS	3	18	15	36
Wt	101.53	1312.80	275.55	1689.88
Het Chi	18.95	116.07	72.08	485.73
Het df	2	17	14	35
Het P	***	***	***	***
Fixed RR	9.17	2.81	6.80	3.49
RRl	7.55	2.66	6.05	3.33
RRu	11.14	2.97	7.66	3.66
P	+++	+++	+++	+++
Random RR	7.14	3.17	6.93	4.60
RRl	3.55	2.65	5.05	3.74
RRu	14.35	3.79	9.50	5.67
P	+++	+++	+++	+++
Between Chi				278.62
Between df				2
Between P				***
Btwn(F) P				***
Btwn(R) P				***

Table 2B13 - 7

IESLC - Meta-analysis of Current Smoking (vs non-current), Any product (or Cigarettes if Any not available)
Squamous
Excluded studies (and stage at which they were excluded)

1	BOUCHA BUELL LAURIL MZILEN
2	ABELIN ABRAHA AUVINE AXELSO BAND BERRIN BLOT1 BLOT2 BLOT3 BLOT4 BRESLO BROCKM BROWN1 BYERS1 CASCO2 CASCOR CHAN CHATZI CHEN CHEN2 CHEN3 CHIAZZ COOKSO DAVEYS DEAN DOSEME DU DUNN EBELIN ESAKI FAN GENG GER GODLEY GOLLED GSELL HANSEN HEGMAN HINDS HIRAOK HOROWI HORWIT HU HU2 HUANG ISHIMA JARUP JIANG JONES JUSSAW KO KOHLME KOULUM KREYBE LAMTH LAMWK LAMWK2 LAUSSM LEI LETOUR LEVIN LIU LIU2 LIU3 LIU4 LIU5 LOMBA2 LUO MAGNUS MARSH MARSH2 MARTIS MASTRA MATSUD MCCONN MCDUFF MCLAUG MILLER MILLS MOLLO NOTAN2 NOU ORMOS PASTOR PAWLEG PERNU PERSHA PIKE POFFIJ POLEDN PRESCO QIAO RADZIK RANDIG REN RONCO ROOTS ROTHSC SADOWS SANKAR SCHWA2 SEGI SEOW SIEMIA SIMARA STASZE STOCKS SUN TAO ULMER VANDER WANG WANG3 WANG4 WICKLU WILKIN WUWILL WYNDE2 WYNDE4 XIANGZ XU XU2 XU3 XU4 YUAN ZHANG ZHENG ZHOU
3	RESTRE
4	BOFFET
5	RIMING TANG2 WYNDE5 WYNDE8
6	ALDERS BENHAM BEST BOUCOT BROWN2 DAMBER ENSTRO GILLIS GOODMA HAMMON KANELL LIAW LUBIN SOBUE2 WALD WU2 WYNDE7
8	AGUDO AKIBA AMANDU AMES ANDERS ARCHER ARMADA AUSTIN AXELSS BECHER BENSHL BLOHMK BRETT BROSS BYERS2 CARPEN CEDERL CHANG CHOI CHOW CHYOU CPSI CPSII DARBY DEAN2 DEAN3 DEKLER DESTE2 DESTEF DOCKER DOLL DOLL2 DORANT DORGAN DORN DROSTE GAO GAO2 GARCIA GARDIN GARSHI GRAHAM GREGOR HAMMO2 HEIN HENNEK HIRAY2 HIRAYA HITOSU HOLE HUMBLE JARVHO JOLY KAISE2 KAISER KAUFMA KELLER KINLEN KJUUS KNEKT KOO KREUZE KUBIK LANGE LEMARC LICKIN LIDDEL LOMBAR MACLEN MIGRAN MRFIT MRFITR MURATA NAM NOTANI ODRISC PARKIN PERSH2 PETO PEZZO2 PEZZOT PISANI QIAO2 RACHTA SCHWAR SHAW SIMONA SITAS SPEIZE SPITZ STOCKW STUCKE SUZUK2 SUZUKI TANG TENKAN TIZZAN TOKARS TOUSEY TULINI TVERDA VEIERO WANG2 WARSIN WATSON WIGLE WUNSCH WYNDER YAMAGU YONG
10	VUTUC

Table 2B13 - 8
Potentially overlapping studies

REF	REFGP	PRINC	. OVERLAP/LINK
LUBIN2	LUBIN2	1	Lubin-combined
OSANN2	KAISER	2	KAISER/OSANN2
WYNDE6	WYNDE6	1	WYNDE5/6/7/8
JAHN	BOFFET	2	Subset of BOFFET

Table 2B14 -

IESLC - Meta-analysis of Current Smoking (vs non-current), Cigarettes (or Any Product if Cigarettes not available)
Squamous

This analysis is restricted to results for:
1) Non-dose-response data
2) Current smokers
3) Results complete enough for use in metaanalysis

Within each study, results are then selected (in the following order of preference, within each sex) for:
4) PRODUCT: cigarettes regardless of other products, cigarettes only, all/unspec
5) CIGTYPE: all/unspecified, MC regardless of HR, MC only
6) DENOM: non smoker of anything, non smoker of cigarettes
7) Followup period (YF, prospective studies): whole study (coded as 0) or longest available
8) LCtype: squamous or nearest available, but not adeno. (q = squamous, s = small,
a = adeno, KI = Kreyberg I, u = undifferentiated)
9) Race: all or nearest available, otherwise by race (wh or w = white, bl or b = black, hi = hispanic
ch = chinese, jap = japanese, haw = hawaiian, w+o = white + oriental, sca = scandinavian, as = asian)
10) For overlapping studies: principal rather than subsidiary studies
Finally by Age: whole study (coded as 0) if available, otherwise by widest available age group
and then for single sex results (m, f) in preference to combined sex results (c).

Results adjusted (AD) for the most potential confounders are then chosen in Sections -1 to -3
and results adjusted for the least confounders in Sections -4 to -6. (Those least adjusted results which
actually differ from the most adjusted as marked 'x' in column X in Section -4)
(Results adjusted for an unknown number of confounder(s) are coded as 20.)

Section -7 shows excluded studies, together with the stage (as above) at which no qualifying
results were found.

Section -8 lists the potentially overlapping studies which have been included (1=principal, 2=subsidiary).

Section -9 lists any results which would have been included in preference except that they had data not complete
enough for use in meta-analysis, with their significance (yes/no), if known, and any further comment as entered
on the database.

In addition to those mentioned above, the following fields, levels and abbreviations are used:
* or nk = not known, n = no, y = yes, ot = other
non = not current
all/unspec = all or unspecified, cig+/-ot = cigarettes irrespective of other products (cigar, pipe etc)
MC = manufactured cigarettes, HR = hand-rolled cigarettes
REF: 6-character study reference
NRR: number of the RR on the database within the study
ST : study type (CC = case control, pr or prosp = prospective)
NLC: number of lung cancer cases in whole study
R : risky occupational population (n = no, m = mining, o = other risky)
VB : national cigarette type (V = at least 75% Virginia, bl = at least 75% blended, ot = other)
P : any proxy use
H : full histological confirmation
De : derivation of RR/CI (or = original, st = standard method, ot = other method of estimation)

Table 2B14 - 1

IESLC - Meta-analysis of Current Smoking (vs non-current), Cigarettes (or Any Product if Cigarettes not available)
Squamous
Most adjusted

REF	NRR	SEX	AGEL	AGEH	RACE	YF	LC TYPE	LOC	START	ST	NLC	R	VB	P	H	AD	PRODUCT	DENOM	De
BARBON	113	m	0	0	all	-	q	Eu:wst	1979	CC	755	n	bl	y	y	1	all/unsp	non any	ot
BUFFLE	65	f	0	0	w-hi	-	q	NAmer	1976	CC	943	n	bl	y	n	0	cig+/-ot	non cigs	st
COMSTO	71	m	0	0	all	-	q	NAmer	1975	ot	258	n	bl	n	n	0	cig+/-ot	non cigs	st
COMSTO	83	f	0	0	all	-	q	NAmer	1975	ot	258	n	bl	n	n	0	cig+/-ot	non cigs	st
CORREA	66	c	0	0	all	-	q+s	NAmer	1979	CC	1359	n	bl	y	n	1	cig+/-ot	non cigs	ot
ENGELA	63	m	0	0	all	0	q	Eu:Sca	1964	pr	435	n	bl	n	n	7	cig+/-ot	non cigs	ot
HAENSZ	32	f	0	0	all	-	q+u	NAmer	1955	CC	158	n	bl	n	y	0	cig+/-ot	non cigs	st
JAHN	34	m	0	0	all	-	q	Eu:Ger	1988	CC	1004	n	bl	n	n	0	cig+/-ot	non cigs	st
JAIN	38	m	0	0	all	-	q	NAmer	1981	CC	845	n	V	y	n	0	cig+/-ot	non cigs	st
JAIN	33	f	0	0	all	-	q	NAmer	1981	CC	845	n	V	y	n	0	cig+/-ot	non cigs	st
JEDRYC	51	m	0	0	all	-	q	Eu:est	1980	CC	1630	n	bl	y	n	0	cig+/-ot	non any	st
KATSOU	38	f	0	0	all	-	KI	Eu:bal	1987	CC	101	n	bl	n	n	1	all/unsp	non any	ot
KHUDER	29	m	0	0	all	-	q	NAmer	1985	CC	482	n	bl	n	y	0	cig+/-ot	non cigs	ot
KIHARA	18	c	0	0	jap	-	q	As:Jap	1991	CC	440	n	bl	n	n	0	all/unsp	non any	st
LUBIN2	253	m	0	0	all	-	q	Eu:mul	1976	CC	7804	n	bl	n	y	0	cig+/-ot	non any	st
LUBIN2	265	f	0	0	all	-	q	Eu:mul	1976	CC	7804	n	bl	n	y	0	cig+/-ot	non any	st
MATOS	87	m	0	0	all	-	q	SCAmer	1994	CC	200	n	bl	n	n	2	cig+/-ot	non any	ot
OSANN	75	m	0	0	all	-	q	NAmer	1984	CC	1986	n	bl	n	n	2	cig+/-ot	non cigs	ot
OSANN	76	f	0	0	all	-	q	NAmer	1984	CC	1986	n	bl	n	n	2	cig+/-ot	non cigs	ot
OSANN2	30	f	0	0	all	-	KI	NAmer	1964	ot	217	n	bl	n	y	1	cig+/-ot	non cigs	ot
SAARIK	2	c	0	0	wh	-	q	Eu:Sca	1988	CC	205	n	bl	n	y	0	all/unsp	non any	st
SEGI2	42	m	0	0	all	-	q	As:Jap	1962	CC	378	n	bl	n	n	1	cig+/-ot	non any	ot
SEGI2	44	f	0	0	all	-	q	As:Jap	1962	CC	378	n	bl	n	n	1	cig+/-ot	non any	ot
SHIMIZ	2	m	0	0	all	-	q	As:Jap	1977	CC	751	n	bl	y	n	2	all/unsp	non any	or
SHIMIZ	7	f	0	0	all	-	q	As:Jap	1977	CC	751	n	bl	y	n	2	all/unsp	non any	or
SOBUE	98	m	0	0	all	-	q	As:Jap	1986	CC	1376	n	bl	n	y	1	cig+/-ot	non cigs	ot
SOBUE	108	f	0	0	all	-	q	As:Jap	1986	CC	1376	n	bl	n	y	1	cig+/-ot	non cigs	ot
STAYNE	7	m	0	0	all	-	q	NAmer	1969	CC	420	n	bl	n	n	1	cig+/-ot	non cigs	st
SVENSS	92	f	0	0	all	-	q	Eu:Sca	1983	CC	210	n	bl	n	n	1	all/unsp	non any	ot
TSUGAN	18	m	0	0	all	-	q	As:Jap	1976	CC	134	n	bl	n	y	0	all/unsp	non any	st
WAKAI	75	m	0	0	all	-	q	As:Jap	1988	CC	333	n	bl	n	y	1	all/unsp	non any	ot
WAKAI	81	f	0	0	all	-	q	As:Jap	1988	CC	333	n	bl	n	y	1	all/unsp	non any	ot
WU	28	f	0	0	wh	-	q	NAmer	1981	CC	220	n	bl	n	y	2	all/unsp	non any	ot
WYNDE3	13	m	0	0	all	-	KI	NAmer	1966	CC	350	n	bl	n	y	0	all/unsp	non any	st
WYNDE6	147	m	0	0	all	-	KI	NAmer	1969	CC	4423	n	bl	n	y	0	cig+/-ot	non cigs	st
WYNDE6	255	f	0	0	all	-	KI	NAmer	1969	CC	4423	n	bl	n	y	0	cig+/-ot	non cigs	st

Cigarette type is all/unspec for all RRs

Table 2B14 - 2

IESLC - Meta-analysis of Current Smoking (vs non-current), Cigarettes (or Any Product if Cigarettes not available)
Squamous
Most adjusted

REF	NRR	SEX	AD	Number Exposed Case	Cont	Non-exposed Case	Cont	RR	95.00%CI
BARBON	113	m	1	-	-	-	-	3.74	(2.73- 5.12)
BUFFLE	65	f	0	39	110	22	168	2.71	(1.52- 4.81)
COMSTO	71	m	0	27	100	19	213	3.03	(1.61- 5.70)
COMSTO	83	f	0	16	52	1	150	46.15	(5.97- 356.64)
Subtotal COMSTO								3.84	(2.10- 7.03)
CORREA	66	c	1	-	-	-	-	11.81	(9.31- 14.97)
*ENGELA	63	m	7	-	-	-	-	3.60	(2.13- 6.09)
HAENSZ	32	f	0	53	94	49	245	2.82	(1.79- 4.45)
JAHN	34	m	0	153	269	201	570	1.61	(1.25- 2.08)
JAIN	38	m	0	107	118	49	244	4.52	(3.02- 6.76)
JAIN	33	f	0	81	99	28	311	9.09	(5.59- 14.77)
Subtotal JAIN								6.01	(4.41- 8.19)
JEDRYC	51	m	0	235	516	51	601	5.37	(3.88- 7.43)
KATSOU	38	f	1	-	-	-	-	5.28	(2.33- 11.98)
KHUDER	29	m	0	112	-	73	-	2.20	(1.55- 3.12)
KIHARA	18	c	0	111	162	26	307	8.09	(5.07- 12.91)
LUBIN2	253	m	0	2518	6209	1136	6844	2.44	(2.26- 2.64)
LUBIN2	265	f	0	154	410	117	1337	4.29	(3.29- 5.59)
Subtotal LUBIN2								2.56	(2.37- 2.75)
MATOS	87	m	2	-	-	-	-	4.69	(2.52- 8.73)
OSANN	75	m	2	-	-	-	-	5.30	(4.16- 6.75)
OSANN	76	f	2	-	-	-	-	11.42	(7.93- 16.46)
Subtotal OSANN								6.70	(5.48- 8.20)
OSANN2	30	f	1	-	-	-	-	9.38	(3.17- 27.76)
SAARIK	2	c	0	47	66	45	224	3.54	(2.17- 5.80)
SEGI2	42	m	1	-	-	-	-	2.86	(1.35- 6.05)
SEGI2	44	f	1	-	-	-	-	1.70	(0.48- 5.98)
Subtotal SEGI2								2.50	(1.31- 4.76)
SHIMIZ	2	m	2	-	-	-	-	4.30	(2.80- 6.70)
SHIMIZ	7	f	2	-	-	-	-	6.40	(2.30- 17.30)
Subtotal SHIMIZ								4.58	(3.07- 6.83)
SOBUE	98	m	1	-	-	-	-	4.37	(3.02- 6.32)
SOBUE	108	f	1	-	-	-	-	7.62	(4.57- 12.69)
Subtotal SOBUE								5.29	(3.92- 7.13)
STAYNE	7	m	1	-	-	-	-	3.10	(1.97- 4.88)
SVENSS	92	f	1	-	-	-	-	11.95	(5.04- 28.31)
TSUGAN	18	m	0	18	13	2	7	4.85	(0.86- 27.22)
WAKAI	75	m	1	-	-	-	-	2.17	(1.37- 3.44)
WAKAI	81	f	1	-	-	-	-	21.82	(6.67- 71.32)
Subtotal WAKAI								2.94	(1.91- 4.51)
WU	28	f	2	-	-	-	-	10.49	(3.42- 32.17)
WYNDE3	13	m	0	171	207	39	213	4.51	(3.03- 6.71)
WYNDE6	147	m	0	1026	741	747	1872	3.47	(3.06- 3.94)
WYNDE6	255	f	0	550	376	201	1181	8.59	(7.04- 10.49)
Subtotal WYNDE6								4.51	(4.05- 5.02)
Partial Totals				5418	9542	2806	14487		

*prospective study

REF	NRR	SEX	AD	Ys	Ws	Qs	Ps
BARBON	113	m	1	1.32	38.86	0.06	0.0000
BUFFLE	65	f	0	1.00	11.61	0.93	0.0007
COMSTO	71	m	0	1.11	9.58	0.28	0.0006
COMSTO	83	f	0	3.83	0.92	5.99	0.0002
Subtotal COMSTO				1.35	10.50	6.27	
CORREA	66	c	1	2.47	68.12	96.53	0.0000
*ENGELA	63	m	7	1.28	13.92	0.00	0.0000
HAENSZ	32	f	0	1.04	18.52	1.09	0.0000
JAHN	34	m	0	0.48	58.88	37.73	0.0002
JAIN	38	m	0	1.51	23.63	1.24	0.0000
JAIN	33	f	0	2.21	16.29	14.04	0.0000
Subtotal JAIN				1.79	39.92	15.28	
JEDRYC	51	m	0	1.68	36.41	5.88	0.0000
KATSOU	38	f	1	1.66	5.73	0.85	0.0001
KHUDER	29	m	0	0.79	31.40	7.54	0.0000
KIHARA	18	c	0	2.09	17.57	11.59	0.0000
LUBIN2	253	m	0	0.89	631.08	93.63	0.0000
LUBIN2	265	f	0	1.46	54.86	1.74	0.0000
Subtotal LUBIN2				0.94	685.94	95.37	
MATOS	87	m	2	1.55	9.95	0.71	0.0000
OSANN	75	m	2	1.67	65.59	9.93	0.0000

Table 2B14 - 2

IESLC - Meta-analysis of Current Smoking (vs non-current), Cigarettes (or Any Product if Cigarettes not available)
Squamous
Most adjusted

REF	NRR	SEX	AD	Ys	Ws	Qs	Ps
OSANN	76	f	2	2.44	28.81	38.56	0.0000
Subtotal OSANN				1.90	94.40	48.49	
OSANN2	30	f	1	2.24	3.26	3.01	0.0001
SAARIK	2	c	0	1.27	15.84	0.00	0.0000
SEGI2	42	m	1	1.05	6.83	0.35	0.0060
SEGI2	44	f	1	0.53	2.42	1.35	0.4096
Subtotal SEGI2				0.91	9.24	1.70	
SHIMIZ	2	m	2	1.46	20.19	0.65	0.0000
SHIMIZ	7	f	2	1.86	3.77	1.26	0.0003
Subtotal SHIMIZ				1.52	23.96	1.91	
SOBUE	98	m	1	1.47	28.18	1.09	0.0000
SOBUE	108	f	1	2.03	14.73	8.34	0.0000
Subtotal SOBUE				1.67	42.91	9.42	
STAYNE	7	m	1	1.13	18.67	0.40	0.0000
SVENSS	92	f	1	2.48	5.16	7.46	0.0000
TSUGAN	18	m	0	1.58	1.29	0.12	0.0731
WAKAI	75	m	1	0.77	18.13	4.60	0.0010
WAKAI	81	f	1	3.08	2.74	8.91	0.0000
Subtotal WAKAI				1.08	20.86	13.51	
WU	28	f	2	2.35	3.06	3.51	0.0000
WYNDE3	13	m	0	1.51	24.38	1.27	0.0000
WYNDE6	147	m	0	1.24	238.26	0.28	0.0000
WYNDE6	255	f	0	2.15	97.09	73.93	0.0000
Subtotal WYNDE6				1.51	335.35	74.22	

	N	36
	NS	27
	Wt	1645.73
Het	Chi	444.86
Het	df	35
Het	P	***
Fixed	RR	3.59
	RRl	3.42
	RRu	3.77
	P	+++
Random	RR	4.71
	RRl	3.84
	RRu	5.79
	P	+++
Asymm	P	*

Table 2B14 - 3

IESLC - Meta-analysis of Current Smoking (vs non-current), Cigarettes (or Any Product if Cigarettes not available)
Squamous
Most adjusted

	combined	Sex male	female	Total
N	3	18	15	36
NS	3	18	15	36
Wt	101.53	1275.22	268.98	1645.73
Het Chi	18.95	111.15	65.08	444.86
Het df	2	17	14	35
Het P	***	***	***	***
Fixed RR	9.17	2.92	6.73	3.59
RRl	7.55	2.76	5.97	3.42
RRu	11.14	3.08	7.58	3.77
P	+++	+++	+++	+++
Random RR	7.14	3.41	6.75	4.71
RRl	3.55	2.85	4.93	3.84
RRu	14.35	4.09	9.23	5.79
P	+++	+++	+++	+++
Between Chi				249.69
Between df				2
Between P				***
Btwn(F) P				***
Btwn(R) P				***

Table 2B14 - 4

IESLC - Meta-analysis of Current Smoking (vs non-current), Cigarettes (or Any Product if Cigarettes not available)
Squamous
Least adjusted

REF	NRR	X	SEX	AGEL	AGEH	RACE	YF	LC TYPE	LOC	START	ST	NLC	R	VB	P	H	AD	PRODUCT	DENOM	De
BARBON	112	x	m	0	0	all	-	q	Eu:wst	1979	CC	755	n	bl	y	y	0	all/unsp	non any	st
BUFFLE	65		f	0	0	w-hi	-	q	NAmer	1976	CC	943	n	bl	y	n	0	cig+/-ot	non cigs	st
COMSTO	71		m	0	0	all	-	q	NAmer	1975	ot	258	n	bl	n	n	0	cig+/-ot	non cigs	st
COMSTO	83		f	0	0	all	-	q	NAmer	1975	ot	258	n	bl	n	n	0	cig+/-ot	non cigs	st
CORREA	66		c	0	0	all	-	q+s	NAmer	1979	CC	1359	n	bl	y	n	1	cig+/-ot	non cigs	ot
ENGELA	63		m	0	0	all	0	q	Eu:Sca	1964	pr	435	n	bl	n	n	7	cig+/-ot	non cigs	ot
HAENSZ	32		f	0	0	all	-	q+u	NAmer	1955	CC	158	n	bl	n	y	0	cig+/-ot	non cigs	st
JAHN	34		m	0	0	all	-	q	Eu:Ger	1988	CC	1004	n	bl	n	n	0	cig+/-ot	non cigs	st
JAIN	38		m	0	0	all	-	q	NAmer	1981	CC	845	n	V	y	n	0	cig+/-ot	non cigs	st
JAIN	33		f	0	0	all	-	q	NAmer	1981	CC	845	n	V	y	n	0	cig+/-ot	non cigs	st
JEDRYC	51		m	0	0	all	-	q	Eu:est	1980	CC	1630	n	bl	y	n	0	cig+/-ot	non any	st
KATSOU	36	x	f	0	0	all	-	KI	Eu:bal	1987	CC	101	n	bl	n	n	0	all/unsp	non any	st
KHUDER	29		m	0	0	all	-	q	NAmer	1985	CC	482	n	bl	n	y	0	cig+/-ot	non cigs	ot
KIHARA	18		c	0	0	jap	-	q	As:Jap	1991	CC	440	n	bl	n	n	0	all/unsp	non any	st
LUBIN2	253		m	0	0	all	-	q	Eu:mul	1976	CC	7804	n	bl	n	y	0	cig+/-ot	non any	st
LUBIN2	265		f	0	0	all	-	q	Eu:mul	1976	CC	7804	n	bl	n	y	0	cig+/-ot	non any	st
MATOS	86	x	m	0	0	all	-	q	SCAmer	1994	CC	200	n	bl	n	n	0	cig+/-ot	non any	st
OSANN	66	x	m	0	0	all	-	q	NAmer	1984	CC	1986	n	bl	n	n	0	cig+/-ot	non cigs	st
OSANN	70	x	f	0	0	all	-	q	NAmer	1984	CC	1986	n	bl	n	n	0	cig+/-ot	non cigs	st
OSANN2	12	x	f	0	0	all	-	KI	NAmer	1964	ot	217	n	bl	n	y	0	cig+/-ot	non cigs	st
SAARIK	2		c	0	0	wh	-	q	Eu:Sca	1988	CC	205	n	bl	n	y	0	all/unsp	non any	st
SEGI2	41	x	m	0	0	all	-	q	As:Jap	1962	CC	378	n	bl	n	n	0	cig+/-ot	non any	st
SEGI2	43	x	f	0	0	all	-	q	As:Jap	1962	CC	378	n	bl	n	n	0	cig+/-ot	non any	st
SHIMIZ	2		m	0	0	all	-	q	As:Jap	1977	CC	751	n	bl	y	n	2	all/unsp	non any	or
SHIMIZ	7		f	0	0	all	-	q	As:Jap	1977	CC	751	n	bl	y	n	2	all/unsp	non any	or
SOBUE	4	x	m	0	0	all	-	q	As:Jap	1986	CC	1376	n	bl	n	y	0	cig+/-ot	non cigs	st
SOBUE	20	x	f	0	0	all	-	q	As:Jap	1986	CC	1376	n	bl	n	y	0	cig+/-ot	non cigs	st
STAYNE	7		m	0	0	all	-	q	NAmer	1969	CC	420	n	bl	n	n	1	cig+/-ot	non cigs	st
SVENSS	67	x	f	0	0	all	-	q	Eu:Sca	1983	CC	210	n	bl	n	n	0	all/unsp	non any	st
TSUGAN	18		m	0	0	all	-	q	As:Jap	1976	CC	134	n	bl	n	y	0	all/unsp	non any	st
WAKAI	16	x	m	0	0	all	-	q	As:Jap	1988	CC	333	n	bl	n	y	0	all/unsp	non any	st
WAKAI	34	x	f	0	0	all	-	q	As:Jap	1988	CC	333	n	bl	n	y	0	all/unsp	non any	st
WU	25	x	f	0	0	wh	-	q	NAmer	1981	CC	220	n	bl	n	y	0	all/unsp	non any	st
WYNDE3	13		m	0	0	all	-	KI	NAmer	1966	CC	350	n	bl	n	y	0	all/unsp	non any	st
WYNDE6	147		m	0	0	all	-	KI	NAmer	1969	CC	4423	n	bl	n	y	0	cig+/-ot	non cigs	st
WYNDE6	255		f	0	0	all	-	KI	NAmer	1969	CC	4423	n	bl	n	y	0	cig+/-ot	non cigs	st

Cigarette type is all/unspec for all RRs

Table 2B14 - 5

IESLC - Meta-analysis of Current Smoking (vs non-current), Cigarettes (or Any Product if Cigarettes not available)
Squamous
Least adjusted

REF	NRR	SEX	AD	Number Exposed Case	Number Exposed Cont	Non-exposed Case	Non-exposed Cont	RR	95.00%CI
BARBON	112	m	0	203	362	64	393	3.44	(2.51- 4.72)
BUFFLE	65	f	0	39	110	22	168	2.71	(1.52- 4.81)
COMSTO	71	m	0	27	100	19	213	3.03	(1.61- 5.70)
COMSTO	83	f	0	16	52	1	150	46.15	(5.97- 356.64)
Subtotal COMSTO								3.84	(2.10- 7.03)
CORREA	66	c	1	-	-	-	-	11.81	(9.31- 14.97)
*ENGELA	63	m	7	-	-	-	-	3.60	(2.13- 6.09)
HAENSZ	32	f	0	53	94	49	245	2.82	(1.79- 4.45)
JAHN	34	m	0	153	269	201	570	1.61	(1.25- 2.08)
JAIN	38	m	0	107	118	49	244	4.52	(3.02- 6.76)
JAIN	33	f	0	81	99	28	311	9.09	(5.59- 14.77)
Subtotal JAIN								6.01	(4.41- 8.19)
JEDRYC	51	m	0	235	516	51	601	5.37	(3.88- 7.43)
KATSOU	36	f	0	24	18	28	71	3.38	(1.59- 7.17)
KHUDER	29	m	0	112	-	73	-	2.20	(1.55- 3.12)
KIHARA	18	c	0	111	162	26	307	8.09	(5.07- 12.91)
LUBIN2	253	m	0	2518	6209	1136	6844	2.44	(2.26- 2.64)
LUBIN2	265	f	0	154	410	117	1337	4.29	(3.29- 5.59)
Subtotal LUBIN2								2.56	(2.37- 2.75)
MATOS	86	m	0	33	132	17	261	3.84	(2.06- 7.15)
OSANN	66	m	0	247	541	113	1310	5.29	(4.15- 6.76)
OSANN	70	f	0	130	367	41	1289	11.14	(7.69- 16.12)
Subtotal OSANN								6.63	(5.41- 8.13)
OSANN2	12	f	0	105	42	14	77	13.75	(7.02- 26.94)
SAARIK	2	c	0	47	66	45	224	3.54	(2.17- 5.80)
SEGI2	41	m	0	105	485	8	102	2.76	(1.30- 5.84)
SEGI2	43	f	0	4	34	8	128	1.88	(0.53- 6.63)
Subtotal SEGI2								2.50	(1.31- 4.76)
SHIMIZ	2	m	2	-	-	-	-	4.30	(2.80- 6.70)
SHIMIZ	7	f	2	-	-	-	-	6.40	(2.30- 17.30)
Subtotal SHIMIZ								4.58	(3.07- 6.83)
SOBUE	4	m	0	301	650	124	491	1.83	(1.44- 2.33)
SOBUE	20	f	0	29	168	21	921	7.57	(4.22- 13.59)
Subtotal SOBUE								2.25	(1.80- 2.81)
STAYNE	7	m	1	-	-	-	-	3.10	(1.97- 4.88)
SVENSS	67	f	0	42	53	11	156	11.24	(5.40- 23.40)
TSUGAN	18	m	0	18	13	2	7	4.85	(0.86- 27.22)
WAKAI	16	m	0	86	284	29	205	2.14	(1.35- 3.38)
WAKAI	34	f	0	15	26	4	150	21.63	(6.66- 70.33)
Subtotal WAKAI								2.90	(1.89- 4.44)
WU	25	f	0	61	23	10	48	12.73	(5.53- 29.29)
WYNDE3	13	m	0	171	207	39	213	4.51	(3.03- 6.71)
WYNDE6	147	m	0	1026	741	747	1872	3.47	(3.06- 3.94)
WYNDE6	255	f	0	550	376	201	1181	8.59	(7.04- 10.49)
Subtotal WYNDE6								4.51	(4.05- 5.02)
Partial Totals				6803	12727	3298	20089		

*prospective study

REF	NRR	SEX	AD	Ys	Ws	Qs	Ps
BARBON	112	m	0	1.24	38.67	0.01	0.0000
BUFFLE	65	f	0	1.00	11.61	0.74	0.0007
COMSTO	71	m	0	1.11	9.58	0.19	0.0006
COMSTO	83	f	0	3.83	0.92	6.13	0.0002
Subtotal COMSTO				1.35	10.50	6.32	
CORREA	66	c	1	2.47	68.12	101.33	0.0000
*ENGELA	63	m	7	1.28	13.92	0.01	0.0000
HAENSZ	32	f	0	1.04	18.52	0.84	0.0000
JAHN	34	m	0	0.48	58.88	35.02	0.0002
JAIN	38	m	0	1.51	23.63	1.58	0.0000
JAIN	33	f	0	2.21	16.29	14.94	0.0000
Subtotal JAIN				1.79	39.92	16.52	
JEDRYC	51	m	0	1.68	36.41	6.76	0.0000
KATSOU	36	f	0	1.22	6.80	0.01	0.0015
KHUDER	29	m	0	0.79	31.40	6.67	0.0000
KIHARA	18	c	0	2.09	17.57	12.44	0.0000
LUBIN2	253	m	0	0.89	631.08	79.95	0.0000
LUBIN2	265	f	0	1.46	54.86	2.36	0.0000
Subtotal LUBIN2				0.94	685.94	82.31	
MATOS	86	m	0	1.35	9.95	0.09	0.0000
OSANN	66	m	0	1.67	64.47	11.22	0.0000

Table 2B14 - 5

IESLC - Meta-analysis of Current Smoking (vs non-current), Cigarettes (or Any Product if Cigarettes not available)
Squamous
Least adjusted

REF	NRR	SEX	AD	Ys	Ws	Qs	Ps
OSANN	70	f	0	2.41	28.10	37.88	0.0000
Subtotal OSANN				1.89	92.58	49.10	
OSANN2	12	f	0	2.62	8.49	15.98	0.0000
SAARIK	2	c	0	1.27	15.84	0.00	0.0000
SEGI2	41	m	0	1.02	6.83	0.37	0.0080
SEGI2	43	f	0	0.63	2.43	0.92	0.3245
Subtotal SEGI2				0.92	9.26	1.30	
SHIMIZ	2	m	2	1.46	20.19	0.88	0.0000
SHIMIZ	7	f	2	1.86	3.77	1.39	0.0003
Subtotal SHIMIZ				1.52	23.96	2.28	
SOBUE	4	m	0	0.61	66.84	27.63	0.0000
SOBUE	20	f	0	2.02	11.22	6.74	0.0000
Subtotal SOBUE				0.81	78.05	34.37	
STAYNE	7	m	1	1.13	18.67	0.26	0.0000
SVENSS	67	f	0	2.42	7.14	9.78	0.0000
TSUGAN	18	m	0	1.58	1.29	0.14	0.0731
WAKAI	16	m	0	0.76	18.35	4.37	0.0011
WAKAI	34	f	0	3.07	2.76	9.21	0.0000
Subtotal WAKAI				1.06	21.11	13.58	
WU	25	f	0	2.54	5.53	9.28	0.0000
WYNDE3	13	m	0	1.51	24.38	1.62	0.0000
WYNDE6	147	m	0	1.24	238.26	0.01	0.0000
WYNDE6	255	f	0	2.15	97.09	78.97	0.0000
Subtotal WYNDE6				1.51	335.35	78.98	

	N	36
	NS	27
	Wt	1689.88
Het	Chi	485.73
Het	df	35
Het	P	***
Fixed	RR	3.49
	RRl	3.33
	RRu	3.66
	P	+++
Random	RR	4.60
	RRl	3.74
	RRu	5.67
	P	+++
Asymm	P	*

Table 2B14 - 6

IESLC - Meta-analysis of Current Smoking (vs non-current), Cigarettes (or Any Product if Cigarettes not available)
Squamous
Least adjusted

		Sex		
	combined	male	female	Total
N	3	18	15	36
NS	3	18	15	36
Wt	101.53	1312.80	275.55	1689.88
Het Chi	18.95	116.07	72.08	485.73
Het df	2	17	14	35
Het P	***	***	***	***
Fixed RR	9.17	2.81	6.80	3.49
RRl	7.55	2.66	6.05	3.33
RRu	11.14	2.97	7.66	3.66
P	+++	+++	+++	+++
Random RR	7.14	3.17	6.93	4.60
RRl	3.55	2.65	5.05	3.74
RRu	14.35	3.79	9.50	5.67
P	+++	+++	+++	+++
Between Chi				278.62
Between df				2
Between P				***
Btwn(F) P				***
Btwn(R) P				***

Table 2B14 - 7

IESLC - Meta-analysis of Current Smoking (vs non-current), Cigarettes (or Any Product if Cigarettes not available)
Squamous
Excluded studies (and stage at which they were excluded)

1 BOUCHA BUELL LAURIL MZILEN

2 ABELIN ABRAHA AUVINE AXELSO BAND BERRIN BLOT1 BLOT2 BLOT3 BLOT4 BRESLO BROCKM BROWN1 BYERS1 CASCO2 CASCOR CHAN CHATZI CHEN CHEN2 CHEN3 CHIAZZ COOKSO DAVEYS DEAN DOSEME DU DUNN EBELIN ESAKI FAN GENG GER GODLEY GOLLED GSELL HANSEN HEGMAN HINDS HIRAOK HOROWI HORWIT HU HU2 HUANG ISHIMA JARUP JIANG JONES JUSSAW KO KOHLME KOULUM KREYBE LAMTH LAMWK LAMWK2 LAUSSM LEI LETOUR LEVIN LIU LIU2 LIU3 LIU4 LIU5 LOMBA2 LUO MAGNUS MARSH MARSH2 MARTIS MASTRA MATSUD MCCONN MCDUFF MCLAUG MILLER MILLS MOLLO NOTAN2 NOU ORMOS PASTOR PAWLEG PERNU PERSHA PIKE POFFIJ POLEDN PRESCO QIAO RADZIK RANDIG REN RONCO ROOTS ROTHSC SADOWS SANKAR SCHWA2 SEGI SEOW SIEMIA SIMARA STASZE STOCKS SUN TAO ULMER VANDER WANG WANG3 WANG4 WICKLU WILKIN WUWILL WYNDE2 WYNDE4 XIANGZ XU XU2 XU3 XU4 YUAN ZHANG ZHENG ZHOU

3 RESTRE

4 BOFFET

5 RIMING TANG2 WYNDE5 WYNDE8

6 ALDERS BENHAM BEST BOUCOT BROWN2 DAMBER ENSTRO GILLIS GOODMA HAMMON KANELL LIAW LUBIN SOBUE2 WALD WU2 WYNDE7

8 AGUDO AKIBA AMANDU AMES ANDERS ARCHER ARMADA AUSTIN AXELSS BECHER BENSHL BLOHMK BRETT BROSS BYERS2 CARPEN CEDERL CHANG CHOI CHOW CHYOU CPSI CPSII DARBY DEAN2 DEAN3 DEKLER DESTE2 DESTEF DOCKER DOLL DOLL2 DORANT DORGAN DORN DROSTE GAO GAO2 GARCIA GARDIN GARSHI GRAHAM GREGOR HAMMO2 HEIN HENNEK HIRAY2 HIRAYA HITOSU HOLE HUMBLE JARVHO JOLY KAISE2 KAISER KAUFMA KELLER KINLEN KJUUS KNEKT KOO KREUZE KUBIK LANGE LEMARC LICKIN LIDDEL LOMBAR MACLEN MIGRAN MRFIT MRFITR MURATA NAM NOTANI ODRISC PARKIN PERSH2 PETO PEZZO2 PEZZOT PISANI QIAO2 RACHTA SCHWAR SHAW SIMONA SITAS SPEIZE SPITZ STOCKW STUCKE SUZUK2 SUZUKI TANG TENKAN TIZZAN TOKARS TOUSEY TULINI TVERDA VEIERO WANG2 WARSIN WATSON WIGLE WUNSCH WYNDER YAMAGU YONG

10 VUTUC

Table 2B14 - 8
Potentially overlapping studies

REF	REFGP	PRINC	. OVERLAP/LINK
LUBIN2	LUBIN2	1	Lubin-combined
OSANN2	KAISER	2	KAISER/OSANN2
WYNDE6	WYNDE6	1	WYNDE5/6/7/8
JAHN	BOFFET	2	Subset of BOFFET

Table 2B15 -

IESLC - Meta-analysis of Current Smoking (vs non-current), Cigarettes only
Squamous

This analysis is restricted to results for:
1) Non-dose-response data
2) Current smokers
3) Results complete enough for use in metaanalysis

Within each study, results are then selected (in the following order of preference, within each sex) for:
4) PRODUCT: cigarettes only
5) CIGTYPE: all/unspecified, MC regardless of HR, MC only
6) DENOM: non smoker of anything, non smoker of cigarettes
7) Followup period (YF, prospective studies): whole study (coded as 0) or longest available
8) LCtype: squamous or nearest available, but not adeno. (q = squamous, s = small,
a = adeno, KI = Kreyberg I, u = undifferentiated)
9) Race: all or nearest available, otherwise by race (wh or w = white, bl or b = black, hi = hispanic
ch = chinese, jap = japanese, haw = hawaiian, w+o = white + oriental, sca = scandinavian, as = asian)
10) For overlapping studies: principal rather than subsidiary studies
Finally by Age: whole study (coded as 0) if available, otherwise by widest available age group
and then for single sex results (m, f) in preference to combined sex results (c).

Results adjusted (AD) for the most potential confounders are then chosen in Sections -1 to -3
and results adjusted for the least confounders in Sections -4 to -6. (Those least adjusted results which
actually differ from the most adjusted as marked 'x' in column X in Section -4)
(Results adjusted for an unknown number of confounder(s) are coded as 20.)

Section -7 shows excluded studies, together with the stage (as above) at which no qualifying
results were found.

Section -8 lists the potentially overlapping studies which have been included (1=principal, 2=subsidiary).

Section -9 lists any results which would have been included in preference except that they had data not complete
enough for use in meta-analysis, with their significance (yes/no), if known, and any further comment as entered
on the database.

In addition to those mentioned above, the following fields, levels and abbreviations are used:
* or nk = not known, n = no, y = yes, ot = other
non = not current
all/unspec = all or unspecified, MC = manufactured cigarettes, HR = hand-rolled cigarettes
REF: 6-character study reference
NRR: number of the RR on the database within the study
ST : study type (CC = case control, pr or prosp = prospective)
NLC: number of lung cancer cases in whole study
R : risky occupational population (n = no, m = mining, o = other risky)
VB : national cigarette type (V = at least 75% Virginia, bl = at least 75% blended, ot = other)
P : any proxy use
H : full histological confirmation
De : derivation of RR/CI (or = original, st = standard method, ot = other method of estimation)

Table 2B15 - 0

No RRs selected for this analysis

Table 2B15 - 7

IESLC - Meta-analysis of Current Smoking (vs non-current), Cigarettes only
Squamous
Excluded studies (and stage at which they were excluded)

1 BOUCHA BUELL LAURIL MZILEN

2 ABELIN ABRAHA AUVINE AXELSO BAND BERRIN BLOT1 BLOT2 BLOT3 BLOT4 BRESLO BROCKM BROWN1 BYERS1 CASCO2 CASCOR CHAN CHATZI CHEN CHEN2 CHEN3 CHIAZZ COOKSO DAVEYS DEAN DOSEME DU DUNN EBELIN ESAKI FAN GENG GER GODLEY GOLLED GSELL HANSEN HEGMAN HINDS HIRAOK HOROWI HORWIT HU HU2 HUANG ISHIMA JARUP JIANG JONES JUSSAW KO KOHLME KOULUM KREYBE LAMTH LAMWK LAMWK2 LAUSSM LEI LETOUR LEVIN LIU LIU2 LIU3 LIU4 LIU5 LOMBA2 LUO MAGNUS MARSH MARSH2 MARTIS MASTRA MATSUD MCCONN MCDUFF MCLAUG MILLER MILLS MOLLO NOTAN2 NOU ORMOS PASTOR PAWLEG PERNU PERSHA PIKE POFFIJ POLEDN PRESCO QIAO RADZIK RANDIG REN RONCO ROOTS ROTHSC SADOWS SANKAR SCHWA2 SEGI SEOW SIEMIA SIMARA STASZE STOCKS SUN TAO ULMER VANDER WANG WANG3 WANG4 WICKLU WILKIN WUWILL WYNDE2 WYNDE4 XIANGZ XU XU2 XU3 XU4 YUAN ZHANG ZHENG ZHOU

3 RESTRE

4 AKIBA AMANDU AMES ANDERS ARCHER ARMADA AUSTIN AXELSS BARBON BECHER BENSHL BLOHMK BOFFET BRETT BROSS BROWN2 BUFFLE BYERS2 CARPEN CHANG CHOI CHYOU COMSTO CORREA DARBY DEAN2 DEKLER DESTE2 DESTEF DOCKER DOLL DORANT DORGAN DROSTE GAO GAO2 GARCIA GARSHI GILLIS GOODMA GREGOR HAENSZ HAMMO2 HENNEK HIRAYA HITOSU HOLE HUMBLE JAHN JAIN JARVHO JEDRYC JOLY KAISER KANELL KATSOU KAUFMA KELLER KHUDER KIHARA KINLEN KJUUS KNEKT KOO KREUZE KUBIK LEMARC LIAW LICKIN LIDDEL LUBIN MACLEN MATOS MRFIT MRFITR MURATA NAM NOTANI ODRISC OSANN OSANN2 PARKIN PERSH2 PETO PEZZO2 PISANI QIAO2 RACHTA SAARIK SCHWAR SEGI2 SHAW SHIMIZ SIMONA SITAS SOBUE SOBUE2 SPEIZE SPITZ STAYNE STOCKW STUCKE SUZUK2 SUZUKI SVENSS TANG TENKAN TIZZAN TOKARS TOUSEY TSUGAN TULINI VUTUC WAKAI WANG2 WARSIN WATSON WU WU2 WUNSCH WYNDE3 WYNDE5 WYNDE6 WYNDE8 YAMAGU YONG

5 LANGE RIMING TANG2

6 ALDERS BENHAM BEST BOUCOT CHOW DAMBER ENSTRO HAMMON WALD WYNDE7

8 AGUDO CEDERL CPSI CPSII DEAN3 DOLL2 DORN ENGELA GARDIN GRAHAM HEIN HIRAY2 KAISE2 LOMBAR LUBIN2 MIGRAN PEZZOT TVERDA VEIERO WIGLE WYNDER
