# Supplementary material for: Systematic review with meta-analysis of the epidemiological evidence in the 1900s relating smoking to lung cancer
Source: BMC Cancer. 2012 Sep 3;12:385. doi: 10.1186/1471-2407-12-385 (PMC3505152; doi:10.1186/1471-2407-12-385)
Supplement: Additional file 5 — Detailed Analysis Tables (Individual file names as described in Additional file 1: Methods, Table1). [file 1471-2407-12-385-S5.zip › PDF/1F.pdf]

Table 1F1 -

IESLC - Meta-analysis of Cigarette Smoking, only Filter vs only Plain (or nearest available)
All LC types

This analysis is restricted to results for:
1) Non-dose-response data
2) Results complete enough for use in metaanalysis

Within each study, results are then selected (in the following order of preference, within each sex) for:
3) CIGTYP: filter only/NOS, always, mainly, both, equally, ever
4) DENOM: plain only/NOS, always, mainly, ever
5) PRODUCT: cigarettes regardless of other products, cigarettes only (Note only study ALDERS has both product definitions available)
6) SMKSTA: ever, current. (Note only study MATOS has both ever and current available)
7) LCtype: all or nearest available, at least Squamous and Adeno. (q = squamous, s = small, l = large, a = adeno, mix = mixed, alv = alveolar)
8) Race: all or nearest available, otherwise by race (wh or w = white, bl or b = black, hi = hispanic ch = chinese, jap = japanese, haw = hawaiian, w+o = white + oriental, sca = scandinavian, as = asian)
9) Followup period (YF, prospective studies): whole study (coded as 0) or longest available
10) For overlapping studies: principal rather than subsidiary studies
Finally by Age: whole study (coded as 0) if available, otherwise by widest available age group
and then for single sex results (m, f) in preference to combined sex results (c).

Results adjusted (AD) for the most potential confounders are then chosen in Sections -1 to -3
and results adjusted for the least confounders in Sections -4 to -6. (Those least adjusted results which
actually differ from the most adjusted as marked 'x' in column X in Section -4)
(Results adjusted for an unknown number of confounder(s) are coded as 20.)

Section -7 shows excluded studies, together with the stage (as above) at which no qualifying
results were found.

Section -8 lists the potentially overlapping studies which have been included (1=principal, 2=subsidiary).

Section -9 lists any results which would have been included in preference except that they had data not complete
enough for use in meta-analysis, with their significance (yes/no), if known, and any further comment as entered
on the database.

In addition to those mentioned above, the following fields, levels and abbreviations are used:
* or nk = not known, n = no, y = yes, ot = other
ev = ever, cu = current, cig+/-ot = cigarettes irrespective of other products (cigar, pipe etc)
f = filter, p = plain, NOS = not otherwise specified
REF: 6-character study reference
NRR: number of the RR on the database within the study
ST : study type (CC = case control, pr or prosp = prospective)
NLC: number of lung cancer cases in whole study
R : risky occupational population (n = no, m = mining, o = other risky)
VB : national cigarette type (V = at least 75% Virginia, bl = at least 75% blended, ot = other)
P : any proxy use
H : full histological confirmation
De : derivation of RR/CI (or = original, st = standard method, ot = other method of estimation)

Table 1F1 - 1

IESLC - Meta-analysis of Cigarette Smoking, only Filter vs only Plain (or nearest available)
All LC types
Most adjusted

REF	NRR	SEX	AGEL	AGEH	RACE	YF	LC TYPE	LOC	START	ST	NLC	R	VB	P	H	AD	SM	PRODUCT	CIGTYP	DENOM	De
AGUDO	17	f	0	0	all	-	all	Eu:wst	1989	CC	103	n	bl	n	n	3	ev	cig only	only f	ever p	ot
ALDERS	169	m	0	0	all	-	all	Eu:UK	1977	CC	1448	n	V	n	n	2	cu	cig+/-ot	always f	always p	ot
ALDERS	16	f	0	0	all	-	all	Eu:UK	1977	CC	1448	n	V	n	n	2	cu	cig only	always f	always p	ot
ARMADA	19	m	0	0	all	-	all	Eu:wst	1986	CC	325	n	bl	n	y	2	ev	cig+/-ot	always f	always p	or
BECHER	10	m	0	0	all	-	all	Eu:Ger	1985	CC	194	n	bl	n	y	3	ev	cig+/-ot	always f	ever p	st
BROSS	14	m	0	0	wh	-	all	NAmer	1960	CC	974	n	bl	n	n	0	cu	cig+/-ot	only f	p NOS	st
BUFFLE	54	f	0	0	w-hi	-	all	NAmer	1976	CC	943	n	bl	y	n	0	ev	cig+/-ot	only f	p NOS	or
CHOI	30	m	0	0	all	-	all	As:oth	1985	CC	375	n	bl	n	n	0	ev	cig+/-ot	only f	always p	st
CHOI	34	f	0	0	all	-	all	As:oth	1985	CC	375	n	bl	n	n	0	ev	cig+/-ot	only f	always p	ot
CORREA	65	c	0	0	all	-	all	NAmer	1979	CC	1359	n	bl	y	n	2	ev	cig+/-ot	only f	p NOS	ot
CPSII	138	f	0	0	all	4	all	NAmer	1982	pr	3229	n	bl	n	n	2	cu	cig+/-ot	only f	mainly p	st
DEAN2	36	m	0	0	all	-	all	Eu:UK	1960	CC	954	n	V	y	n	0	ev	cig+/-ot	ever f	always p	st
DEAN2	42	f	0	0	all	-	all	Eu:UK	1960	CC	954	n	V	y	n	0	ev	cig+/-ot	ever f	always p	st
DEAN3	156	m	0	0	all	-	all	Eu:UK	1969	CC	766	n	V	y	n	2	cu	cig only	only f	p NOS	or
DEAN3	162	f	0	0	all	-	all	Eu:UK	1969	CC	766	n	V	y	n	2	cu	cig only	only f	p NOS	or
DESTE2	23	c	0	0	all	-	all	SCAmer	1993	CC	463	n	bl	n	n	7	ev	cig+/-ot	always f	ever p	ot
DESTEF	53	m	0	0	all	-	all	SCAmer	1988	CC	497	n	bl	n	y	4	ev	cig+/-ot	always f	ever p	ot
DOLL	47	m	0	0	all	-	all	Eu:UK	1948	CC	1465	n	V	n	n	0	ev	cig+/-ot	ever f	always p	st
ENGELA	129	m	0	0	all	0	all	Eu:Sca	1964	pr	435	n	bl	n	n	7	cu	cig+/-ot	only f	always p	st
ENGELA	198	f	0	0	all	0	all	Eu:Sca	1964	pr	435	n	bl	n	n	5	cu	cig+/-ot	only f	always p	st
KAISE2	55	m	30	89	all	9	all	NAmer	1979	pr	318	n	bl	n	n	5	cu	cig only	only f	p NOS	or
KAISE2	56	f	30	89	all	9	all	NAmer	1979	pr	318	n	bl	n	n	5	cu	cig only	only f	p NOS	or
KHUDER	7	m	0	0	all	-	all	NAmer	1985	CC	482	n	bl	n	y	0	ev	cig+/-ot	ever f	always p	st
LANGE	20	m	0	0	all	0	all	Eu:Sca	1976	pr	268	n	bl	n	n	2	cu	cig only	only f	p NOS	or
LANGE	19	f	0	0	all	0	all	Eu:Sca	1976	pr	268	n	bl	n	n	2	cu	cig only	only f	p NOS	or
LUBIN2	107	m	0	0	all	-	all	Eu:mul	1976	CC	7804	n	bl	n	y	3	ev	cig+/-ot	only f	always p	st
LUBIN2	115	f	0	0	all	-	all	Eu:mul	1976	CC	7804	n	bl	n	y	3	ev	cig+/-ot	only f	always p	st
MACLEN	85	f	0	0	ch	-	all	As:oth	1972	CC	233	n	bl	n	n	0	cu	cig+/-ot	ever f	always p	ot
MATOS	37	m	0	0	all	-	all	SCAmer	1994	CC	200	n	bl	n	n	4	ev	cig+/-ot	mainly f	mainly p	st
MIGRAN	100	m	0	0	all	0	all	Eu:UK	1964	pr	259	n	V	n	n	3	cu	cig only	only f	p NOS	ot
MIGRAN	104	f	0	0	all	0	all	Eu:UK	1964	pr	259	n	V	n	n	3	cu	cig only	only f	p NOS	ot
MRFITR	7	m	0	0	all	0	all	NAmer	1973	pr	119	n	bl	n	n	9	cu	cig+/-ot	only f	p NOS	ot
PEZZOT	24	m	0	0	all	-	all	SCAmer	1987	CC	215	n	bl	n	y	4	ev	cig only	only f	ever p	ot
RIMING	8	m	0	0	all	0	all	Eu:UK	1970	pr	104	n	V	n	n	1	cu	cig only	only f	p NOS	ot
SEGI2	52	m	0	0	all	-	all	As:Jap	1962	CC	378	n	bl	n	n	1	cu	cig+/-ot	only f	p NOS	ot
SOBUE	125	m	0	0	all	-	q+s+l+a	As:Jap	1986	CC	1376	n	bl	n	y	5	cu	cig+/-ot	only f	p NOS	st
TANG2	1	m	0	0	all	0	all	Eu:UK	1967	pr	836	n	V	n	n	3	cu	cig only	only f	p NOS	or
WAKAI	64	m	0	0	all	-	all	As:Jap	1988	CC	333	n	bl	n	y	5	cu	cig+/-ot	only f	p NOS	st
WYNDE5	3	m	0	0	all	-	all	NAmer	1969	CC	1365	n	bl	n	y	0	cu	cig+/-ot	ever f	always p	st
WYNDE5	6	f	0	0	all	-	all	NAmer	1969	CC	1365	n	bl	n	y	0	cu	cig+/-ot	ever f	always p	st
WYNDE6	421	m	0	0	all	-	q+a	NAmer	1969	CC	4423	n	bl	n	y	3	cu	cig+/-ot	only f	always p	ot
WYNDE6	424	f	0	0	all	-	q+a	NAmer	1969	CC	4423	n	bl	n	y	3	cu	cig+/-ot	only f	always p	ot

Table 1F1 - 2

IESLC - Meta-analysis of Cigarette Smoking, only Filter vs only Plain (or nearest available)
All LC types
Most adjusted

REF	NRR	SEX	AD	Number Exposed Case	Number Exposed Cont	Non-exposed Case	Non-exposed Cont	RR	95.00%CI
AGUDO	17	f	3	-	-	-	-	0.22	(0.04- 1.27)
ALDERS	169	m	2	-	-	-	-	0.83	(0.51- 1.34)
ALDERS	16	f	2	-	-	-	-	0.85	(0.52- 1.38)
Subtotal ALDERS								0.84	(0.60- 1.18)
ARMADA	19	m	2	-	-	-	-	0.70	(0.40- 1.20)
BECHER	10	m	3	-	-	-	-	0.41	(0.21- 0.81)
BROSS	14	m	0	65	76	200	138	0.59	(0.40- 0.88)
BUFFLE	54	f	0	129	78	47	38	1.34	(0.80- 2.23)
CHOI	30	m	0	43	103	15	2	0.06	(0.01- 0.25)
CHOI	34	f	0	5	11	1	0	0.16~	(0.01- 4.58)
Subtotal CHOI								0.07	(0.02- 0.27)
CORREA	65	c	2	-	-	-	-	0.55	(0.35- 0.85)
*CPSII	138	f	2	-	-	-	-	0.66	(0.57- 0.78)
DEAN2	36	m	0	20	17	644	529	0.97	(0.50- 1.86)
DEAN2	42	f	0	12	2	50	26	3.12	(0.65- 15.00)
Subtotal DEAN2								1.15	(0.63- 2.11)
DEAN3	156	m	2	-	-	-	-	0.54	(0.40- 0.73)
DEAN3	162	f	2	-	-	-	-	0.68	(0.42- 1.11)
Subtotal DEAN3								0.58	(0.45- 0.74)
DESTE2	23	c	7	-	-	-	-	0.73	(0.51- 1.05)
DESTEF	53	m	4	-	-	-	-	0.72	(0.54- 0.96)
DOLL	47	m	0	3	15	501	452	0.18	(0.05- 0.63)
*ENGELA	129	m	7	-	-	-	-	0.67	(0.30- 1.43)
*ENGELA	198	f	5	-	-	-	-	0.91	(0.40- 2.00)
Subtotal ENGELA								0.78	(0.44- 1.36)
*KAISE2	55	m	5	-	-	-	-	1.03	(0.61- 1.75)
*KAISE2	56	f	5	-	-	-	-	0.65	(0.32- 1.31)
Subtotal KAISE2								0.87	(0.57- 1.33)
KHUDER	7	m	0	173	440	284	334	0.46	(0.36- 0.59)
*LANGE	20	m	2	-	-	-	-	0.90	(0.60- 1.40)
*LANGE	19	f	2	-	-	-	-	0.70	(0.40- 1.40)
Subtotal LANGE								0.83	(0.59- 1.18)
LUBIN2	107	m	3	-	-	-	-	0.48	(0.40- 0.56)
LUBIN2	115	f	3	-	-	-	-	0.43	(0.22- 0.85)
Subtotal LUBIN2								0.48	(0.41- 0.56)
MACLEN	85	f	0	15	13	21	14	0.77	(0.28- 2.10)
MATOS	37	m	4	-	-	-	-	1.25	(0.67- 2.50)
*MIGRAN	100	m	3	-	-	-	-	1.23	(0.84- 1.81)
*MIGRAN	104	f	3	-	-	-	-	1.44	(0.61- 3.40)
Subtotal MIGRAN								1.26	(0.89- 1.79)
*MRFITR	7	m	9	-	-	-	-	0.53	(0.24- 1.17)
PEZZOT	24	m	4	-	-	-	-	0.29	(0.20- 0.41)
*RIMING	8	m	1	-	-	-	-	0.65	(0.44- 0.96)
SEGI2	52	m	1	-	-	-	-	0.62	(0.45- 0.85)
SOBUE	125	m	5	-	-	-	-	0.67	(0.38- 1.11)
*TANG2	1	m	3	-	-	-	-	0.94	(0.75- 1.18)
WAKAI	64	m	5	-	-	-	-	1.02	(0.31- 3.33)
WYNDE5	3	m	0	417	629	273	398	0.97	(0.79- 1.18)
WYNDE5	6	f	0	152	200	34	30	0.67	(0.39- 1.14)
Subtotal WYNDE5								0.92	(0.77- 1.11)
WYNDE6	421	m	3	-	-	-	-	0.92	(0.65- 1.29)
WYNDE6	424	f	3	-	-	-	-	0.68	(0.39- 1.19)
Subtotal WYNDE6								0.85	(0.63- 1.13)
Partial Totals				1034	1584	2070	1961		

*prospective study

~ With 0.5 adjustment for zero

Table 1F1 - 2

IESLC - Meta-analysis of Cigarette Smoking, only Filter vs only Plain (or nearest available)
All LC types
Most adjusted

REF	NRR	SEX	AD	Ys	Ws	Qs	Ps
AGUDO	17	f	3	-1.51	1.29	1.61	0.0861
ALDERS	169	m	2	-0.19	16.47	0.71	0.4496
ALDERS	16	f	2	-0.16	16.13	0.87	0.5139
Subtotal ALDERS				-0.17	32.60	1.58	
ARMADA	19	m	2	-0.36	12.73	0.02	0.2031
BECHER	10	m	3	-0.89	8.43	2.08	0.0096
BROSS	14	m	0	-0.53	24.52	0.43	0.0090
BUFFLE	54	f	0	0.29	14.67	6.88	0.2658
CHOI	30	m	0	-2.89	1.67	10.37	0.0002
CHOI	34	f	0	-1.84	0.34	0.71	0.2838
Subtotal CHOI				-2.71	2.01	11.08	
CORREA	65	c	2	-0.60	19.52	0.81	0.0083
*CPSII	138	f	2	-0.42	156.19	0.07	0.0000
DEAN2	36	m	0	-0.03	8.91	1.16	0.9187
DEAN2	42	f	0	1.14	1.56	3.66	0.1555
Subtotal DEAN2				0.14	10.47	4.81	
DEAN3	156	m	2	-0.62	42.46	2.09	0.0001
DEAN3	162	f	2	-0.39	16.27	0.00	0.1198
Subtotal DEAN3				-0.55	58.73	2.09	
DESTE2	23	c	7	-0.31	29.47	0.19	0.0876
DESTEF	53	m	4	-0.33	46.42	0.20	0.0252
DOLL	47	m	0	-1.71	2.47	4.30	0.0071
*ENGELA	129	m	7	-0.40	6.30	0.00	0.3148
*ENGELA	198	f	5	-0.09	5.93	0.53	0.8183
Subtotal ENGELA				-0.25	12.23	0.53	
*KAISE2	55	m	5	0.03	13.83	2.49	0.9125
*KAISE2	56	f	5	-0.43	7.73	0.01	0.2309
Subtotal KAISE2				-0.14	21.57	2.50	
KHUDER	7	m	0	-0.77	68.64	9.75	0.0000
*LANGE	20	m	2	-0.11	21.40	1.79	0.6259
*LANGE	19	f	2	-0.36	9.79	0.01	0.2644
Subtotal LANGE				-0.18	31.19	1.80	
LUBIN2	107	m	3	-0.73	135.72	15.65	0.0000
LUBIN2	115	f	3	-0.84	8.41	1.70	0.0144
Subtotal LUBIN2				-0.74	144.14	17.35	
MACLEN	85	f	0	-0.26	3.81	0.07	0.6087
MATOS	37	m	4	0.22	8.86	3.38	0.5065
*MIGRAN	100	m	3	0.21	26.07	9.43	0.2905
*MIGRAN	104	f	3	0.36	5.21	3.00	0.4054
Subtotal MIGRAN				0.23	31.28	12.43	
*MRFITR	7	m	9	-0.63	6.12	0.35	0.1162
PEZZOT	24	m	4	-1.24	29.82	21.21	0.0000
*RIMING	8	m	1	-0.43	25.25	0.03	0.0304
SEGI2	52	m	1	-0.48	37.99	0.27	0.0032
SOBUE	125	m	5	-0.40	13.37	0.00	0.1431
*TANG2	1	m	3	-0.06	74.81	8.27	0.5925
WAKAI	64	m	5	0.02	2.73	0.47	0.9739
WYNDE5	3	m	0	-0.03	98.39	12.78	0.7354
WYNDE5	6	f	0	-0.40	13.45	0.00	0.1427
Subtotal WYNDE5				-0.08	111.85	12.78	
WYNDE6	421	m	3	-0.08	32.71	3.16	0.6335
WYNDE6	424	f	3	-0.39	12.35	0.00	0.1754
Subtotal WYNDE6				-0.17	45.05	3.17	

Table 1F1 - 2

IESLC - Meta-analysis of Cigarette Smoking, only Filter vs only Plain (or nearest available)
All LC types
Most adjusted

	N	42
	NS	31
	Wt	1088.20
Het	Chi	130.52
Het	df	41
Het	P	***
Fixed	RR	0.67
	RRl	0.64
	RRu	0.72
	P	---
Random	RR	0.69
	RRl	0.61
	RRu	0.78
	P	---
Asymm	P	N.S.

Table 1F1 - 3

IESLC - Meta-analysis of Cigarette Smoking, only Filter vs only Plain (or nearest available)
All LC types
Most adjusted

	Sex			
	combined	male	female	Total
N	2	25	15	42
NS	2	25	15	42
Wt	48.98	766.10	273.12	1088.20
Het Chi	0.94	110.21	18.44	130.52
Het df	1	24	14	41
Het P	N.S.	***	N.S.	***
Fixed RR	0.65	0.66	0.71	0.67
RRl	0.49	0.62	0.63	0.64
RRu	0.86	0.71	0.80	0.72
P	--	---	---	---
Random RR	0.65	0.67	0.75	0.69
RRl	0.49	0.56	0.62	0.61
RRu	0.86	0.79	0.89	0.78
P	--	---	--	---
Between Chi				0.93
Between df				2
Between P				N.S.
Btwn(F) P				N.S.
Btwn(R) P				N.S.

	All LC (or nearest)		
	all	other	Total
N	39	3	42
NS	29	2	31
Wt	1029.78	58.43	1088.20
Het Chi	127.25	1.38	130.52
Het df	38	2	41
Het P	***	N.S.	***
Fixed RR	0.67	0.80	0.67
RRl	0.63	0.62	0.64
RRu	0.71	1.04	0.72
P	---	(-)	---
Random RR	0.69	0.80	0.69
RRl	0.60	0.62	0.61
RRu	0.78	1.04	0.78
P	---	(-)	---
Between Chi			1.88
Between df			1
Between P			N.S.
Btwn(F) P			N.S.
Btwn(R) P			N.S.

	Location								
	NAmer	UK	Scand	othEur	China	Japan	othAs	other	Total
N	12	11	4	5		3	3	4	42
NS	9	7	2	4		3	2	4	31
Wt	468.13	235.60	43.43	166.58		54.09	5.82	114.56	1088.20
Het Chi	35.56	25.37	0.75	2.89		0.65	8.19	23.21	130.52
Het df	11	10	3	4		2	2	3	41
Het P	***	**	N.S.	N.S.		N.S.	*	***	***
Fixed RR	0.71	0.81	0.82	0.48		0.65	0.33	0.60	0.67
RRl	0.65	0.71	0.61	0.42		0.50	0.15	0.50	0.64
RRu	0.78	0.92	1.10	0.56		0.85	0.74	0.71	0.72
P	---	--	N.S.	---		--	--	---	---
Random RR	0.72	0.82	0.82	0.48		0.65	0.21	0.64	0.69
RRl	0.60	0.65	0.61	0.42		0.50	0.03	0.37	0.61
RRu	0.87	1.03	1.10	0.56		0.85	1.55	1.09	0.78
P	---	(-)	N.S.	---		--	N.S.	N.S.	---
Between Chi									33.90
Between df									6
Between P									***
Btwn(F) P									(*)
Btwn(R) P									**

Table 1F1 - 3

IESLC - Meta-analysis of Cigarette Smoking, only Filter vs only Plain (or nearest available)
All LC types
Most adjusted

Detailed Country in "other Europe"

	multi	Germany	othWest	East	Balkans	Total
N	2	1	2			5
NS	1	1	2			4
Wt	144.14	8.43	14.02			166.58
Het Chi	0.10	0.00	1.56			2.89
Het df	1	0	1			4
Het P	N.S.	N.S.	N.S.			N.S.
Fixed RR	0.48	0.41	0.63			0.48
RRl	0.41	0.21	0.37			0.42
RRu	0.56	0.81	1.06			0.56
P	---	--	(-)			---
Random RR	0.48	0.41	0.53			0.48
RRl	0.41	0.21	0.20			0.42
RRu	0.56	0.81	1.40			0.56
P	---	--	N.S.			---
Between Chi						1.23
Between df						2
Between P						N.S.
Btwn(F) P						N.S.
Btwn(R) P						N.S.

Detailed Country in "other Asia"

	India	HongKong	other	Total
N			3	3
NS			2	2
Wt			5.82	5.82
Het Chi			8.19	8.19
Het df			2	2
Het P			*	*
Fixed RR			0.33	0.33
RRl			0.15	0.15
RRu			0.74	0.74
P			--	--
Random RR			0.21	0.21
RRl			0.03	0.03
RRu			1.55	1.55
P			N.S.	N.S.
Between Chi				
Between df				
Between P				N.S.
Btwn(F) P				N.S.
Btwn(R) P				N.S.

Detailed other continent

	SCAmer	Auslia	Africa	Total
N	4			4
NS	4			4
Wt	114.56			114.56
Het Chi	23.21			23.21
Het df	3			3
Het P	***			***
Fixed RR	0.60			0.60
RRl	0.50			0.50
RRu	0.71			0.71
P	---			---
Random RR	0.64			0.64
RRl	0.37			0.37
RRu	1.09			1.09
P	N.S.			N.S.
Between Chi				
Between df				
Between P				N.S.
Btwn(F) P				N.S.
Btwn(R) P				N.S.

Table 1F1 - 3

IESLC - Meta-analysis of Cigarette Smoking, only Filter vs only Plain (or nearest available)
All LC types
Most adjusted

	Start year of study <1960	1960-69	1970-79	1980-89	1990+	Total
N	1	15	13	11	2	42
NS	1	9	9	10	2	31
Wt	2.47	406.92	298.86	341.62	38.33	1088.20
Het Chi	0.00	28.34	29.05	35.96	1.97	130.52
Het df	0	14	12	10	1	41
Het P	N.S.	*	**	***	N.S.	***
Fixed RR	0.18	0.82	0.62	0.57	0.83	0.67
RRl	0.05	0.75	0.55	0.51	0.60	0.64
RRu	0.63	0.91	0.69	0.63	1.13	0.72
P	--	---	---	---	N.S.	---
Random RR	0.18	0.81	0.71	0.50	0.89	0.69
RRl	0.05	0.69	0.57	0.39	0.53	0.61
RRu	0.63	0.94	0.87	0.66	1.47	0.78
P	--	--	--	---	N.S.	---
Between Chi						35.21
Between df						4
Between P						***
Btwn(F) P						*
Btwn(R) P						**

	Study type (1) CC	other	Total
N	30	12	42
NS	23	8	31
Wt	729.56	358.64	1088.20
Het Chi	100.71	18.25	130.52
Het df	29	11	41
Het P	***	(*)	***
Fixed RR	0.63	0.78	0.67
RRl	0.58	0.70	0.64
RRu	0.67	0.87	0.72
P	---	---	---
Random RR	0.64	0.82	0.69
RRl	0.55	0.70	0.61
RRu	0.75	0.97	0.78
P	---	-	---
Between Chi			11.56
Between df			1
Between P			***
Btwn(F) P			(*)
Btwn(R) P			*

	Study type (2) CC	prosp	other	Total
N	30	12		42
NS	23	8		31
Wt	729.56	358.64		1088.20
Het Chi	100.71	18.25		130.52
Het df	29	11		41
Het P	***	(*)		***
Fixed RR	0.63	0.78		0.67
RRl	0.58	0.70		0.64
RRu	0.67	0.87		0.72
P	---	---		---
Random RR	0.64	0.82		0.69
RRl	0.55	0.70		0.61
RRu	0.75	0.97		0.78
P	---	-		---
Between Chi				11.56
Between df				1
Between P				***
Btwn(F) P				(*)
Btwn(R) P				*

Table 1F1 - 3

IESLC - Meta-analysis of Cigarette Smoking, only Filter vs only Plain (or nearest available)
All LC types
Most adjusted

	Study size (number of LC cases)				
	100-249	250-499	500-999	1000+	Total
N	7	16	7	12	42
NS	7	11	5	8	31
Wt	83.58	296.25	183.19	525.18	1088.20
Het Chi	19.89	39.08	18.22	39.93	130.52
Het df	6	15	6	11	41
Het P	**	***	**	***	***
Fixed RR	0.49	0.69	0.79	0.66	0.67
RRl	0.39	0.62	0.68	0.61	0.64
RRu	0.60	0.77	0.91	0.72	0.72
P	---	---	--	---	---
Random RR	0.53	0.73	0.81	0.67	0.69
RRl	0.34	0.59	0.61	0.55	0.61
RRu	0.83	0.90	1.09	0.81	0.78
P	--	--	N.S.	---	---
Between Chi					13.40
Between df					3
Between P					**
Btwn(F) P					N.S.
Btwn(R) P					N.S.

	Risky occupational population			
	no	mining	othRisky	Total
N	42			42
NS	31			31
Wt	1088.20			1088.20
Het Chi	130.52			130.52
Het df	41			41
Het P	***			***
Fixed RR	0.67			0.67
RRl	0.64			0.64
RRu	0.72			0.72
P	---			---
Random RR	0.69			0.69
RRl	0.61			0.61
RRu	0.78			0.78
P	---			---
Between Chi				
Between df				
Between P				N.S.
Btwn(F) P				N.S.
Btwn(R) P				N.S.

	National cigarette tobacco type			
	Virginia	blended	other	Total
N	11	31		42
NS	7	24		31
Wt	235.60	852.60		1088.20
Het Chi	25.37	94.75		130.52
Het df	10	30		41
Het P	**	***		***
Fixed RR	0.81	0.64		0.67
RRl	0.71	0.60		0.64
RRu	0.92	0.68		0.72
P	--	---		---
Random RR	0.82	0.65		0.69
RRl	0.65	0.57		0.61
RRu	1.03	0.75		0.78
P	(-)	---		---
Between Chi				10.40
Between df				1
Between P				**
Btwn(F) P				(*)
Btwn(R) P				N.S.

Table 1F1 - 3

IESLC - Meta-analysis of Cigarette Smoking, only Filter vs only Plain (or nearest available)
All LC types
Most adjusted

	Any proxy use No/nk	Yes	Total
N	36	6	42
NS	27	4	31
Wt	984.82	103.38	1088.20
Het Chi	115.92	14.54	130.52
Het df	35	5	41
Het P	***	*	***
Fixed RR	0.67	0.69	0.67
RRl	0.63	0.57	0.64
RRu	0.72	0.84	0.72
P	---	---	---
Random RR	0.68	0.79	0.69
RRl	0.59	0.55	0.61
RRu	0.78	1.14	0.78
P	---	N.S.	---
Between Chi			0.06
Between df			1
Between P			N.S.
Btwn(F) P			N.S.
Btwn(R) P			N.S.

	Full histological confirmation No	Yes	Total
N	29	13	42
NS	21	10	31
Wt	605.03	483.18	1088.20
Het Chi	58.53	60.81	130.52
Het df	28	12	41
Het P	***	***	***
Fixed RR	0.74	0.60	0.67
RRl	0.68	0.55	0.64
RRu	0.80	0.66	0.72
P	---	---	---
Random RR	0.75	0.60	0.69
RRl	0.66	0.48	0.61
RRu	0.86	0.75	0.78
P	---	---	---
Between Chi			11.19
Between df			1
Between P			***
Btwn(F) P			(*)
Btwn(R) P			(*)

	Number of adjustment variables (1) 0	1	2+/+nk	Total
N	11	2	29	42
NS	8	2	21	31
Wt	238.43	63.23	786.54	1088.20
Het Chi	49.22	0.03	79.98	130.52
Het df	10	1	28	41
Het P	***	N.S.	***	***
Fixed RR	0.72	0.63	0.67	0.67
RRl	0.63	0.49	0.62	0.64
RRu	0.81	0.81	0.71	0.72
P	---	---	---	---
Random RR	0.66	0.63	0.70	0.69
RRl	0.45	0.49	0.61	0.61
RRu	0.95	0.81	0.80	0.78
P	-	---	---	---
Between Chi				1.28
Between df				2
Between P				N.S.
Btwn(F) P				N.S.
Btwn(R) P				N.S.

Table 1F1 - 3

IESLC - Meta-analysis of Cigarette Smoking, only Filter vs only Plain (or nearest available)
All LC types
Most adjusted

	Number of adjustment variables (2) 0	1	2	3-5	6+/+nk	Total
N	11	2	9	17	3	42
NS	8	2	6	13	3	32
Wt	238.43	63.23	310.95	433.70	41.89	1088.20
Het Chi	49.22	0.03	6.35	73.02	0.52	130.52
Het df	10	1	8	16	2	41
Het P	***	N.S.	N.S.	***	N.S.	***
Fixed RR	0.72	0.63	0.67	0.66	0.69	0.67
RRl	0.63	0.49	0.60	0.60	0.51	0.64
RRu	0.81	0.81	0.75	0.73	0.93	0.72
P	---	---	---	---	-	---
Random RR	0.66	0.63	0.67	0.71	0.69	0.69
RRl	0.45	0.49	0.60	0.56	0.51	0.61
RRu	0.95	0.81	0.75	0.90	0.93	0.78
P	-	---	---	--	-	---
Between Chi						1.36
Between df						4
Between P						N.S.
Btwn(F) P						N.S.
Btwn(R) P						N.S.

	Smoking status ever	current	Total
N	17	25	42
NS	14	17	31
Wt	398.93	689.28	1088.20
Het Chi	62.74	34.73	130.52
Het df	16	24	41
Het P	***	(*)	***
Fixed RR	0.54	0.77	0.67
RRl	0.49	0.71	0.64
RRu	0.59	0.83	0.72
P	---	---	---
Random RR	0.57	0.77	0.69
RRl	0.45	0.70	0.61
RRu	0.72	0.85	0.78
P	---	---	---
Between Chi			33.05
Between df			1
Between P			***
Btwn(F) P			***
Btwn(R) P			*

	Product cig+/-ot	cig only	Total
N	29	13	42
NS	23	9	32
Wt	798.14	290.06	1088.20
Het Chi	79.00	48.90	130.52
Het df	28	12	41
Het P	***	***	***
Fixed RR	0.65	0.73	0.67
RRl	0.61	0.65	0.64
RRu	0.70	0.82	0.72
P	---	---	---
Random RR	0.67	0.73	0.69
RRl	0.59	0.57	0.61
RRu	0.78	0.94	0.78
P	---	-	---
Between Chi			2.62
Between df			1
Between P			N.S.
Btwn(F) P			N.S.
Btwn(R) P			N.S.

Table 1F1 - 3

IESLC - Meta-analysis of Cigarette Smoking, only Filter vs only Plain (or nearest available)
All LC types
Most adjusted

	Cigarette type						
	only f	always f	mainly f	equl p&f	both p&f	ever f	Total
N	28	6	1			7	42
NS	20	5	1			5	31
Wt	752.47	129.64	8.86			197.24	1088.20
Het Chi	90.41	3.47	0.00			30.93	130.52
Het df	27	5	0			6	41
Het P	***	N.S.	N.S.			***	***
Fixed RR	0.65	0.72	1.25			0.72	0.67
RRl	0.61	0.61	0.65			0.62	0.64
RRu	0.70	0.86	2.41			0.83	0.72
P	---	---	N.S.			---	---
Random RR	0.67	0.72	1.25			0.71	0.69
RRl	0.58	0.61	0.65			0.46	0.61
RRu	0.79	0.86	2.41			1.08	0.78
P	---	---	N.S.			N.S.	---
Between Chi							5.71
Between df							3
Between P							N.S.
Btwn(F) P							N.S.
Btwn(R) P							N.S.

	Denominator				
	ever p	mainly p	p NOS	always p	Total
N	5	2	17	18	42
NS	5	2	13	11	31
Wt	115.42	165.05	361.74	445.99	1088.20
Het Chi	19.72	3.42	30.57	64.30	130.52
Het df	4	1	16	17	41
Het P	***	(*)	*	***	***
Fixed RR	0.54	0.68	0.76	0.64	0.67
RRl	0.45	0.59	0.69	0.58	0.64
RRu	0.65	0.80	0.85	0.70	0.72
P	---	---	---	---	---
Random RR	0.49	0.84	0.76	0.66	0.69
RRl	0.30	0.46	0.66	0.53	0.61
RRu	0.77	1.53	0.89	0.83	0.78
P	--	N.S.	---	---	---
Between Chi					12.51
Between df					3
Between P					**
Btwn(F) P					N.S.
Btwn(R) P					N.S.

	Derivation of RR/CI			
	Orig	StdCalc	Other	Total
N	9	17	16	42
NS	6	13	13	32
Wt	213.71	565.56	308.94	1088.20
Het Chi	14.71	62.80	42.49	130.52
Het df	8	16	15	41
Het P	(*)	***	***	***
Fixed RR	0.81	0.62	0.69	0.67
RRl	0.71	0.57	0.62	0.64
RRu	0.92	0.68	0.77	0.72
P	--	---	---	---
Random RR	0.80	0.63	0.69	0.69
RRl	0.66	0.51	0.56	0.61
RRu	0.98	0.77	0.85	0.78
P	-	---	---	---
Between Chi				10.51
Between df				2
Between P				**
Btwn(F) P				N.S.
Btwn(R) P				N.S.

Table 1F1 - 4

IESLC - Meta-analysis of Cigarette Smoking, only Filter vs only Plain (or nearest available)
All LC types
Least adjusted

REF	NRR	X	SEX	AGEL	AGEH	RACE	YF	LC TYPE	LOC	START	ST	NLC	R	VB	P	H	AD	SM	PRODUCT	CIGTYP	DENOM	De
AGUDO	18	x	f	0	0	all	-	all	Eu:wst	1989	CC	103	n	bl	n	n	0	ev	cig only	only f	ever p	st
ALDERS	168	x	m	0	0	all	-	all	Eu:UK	1977	CC	1448	n	V	n	n	0	cu	cig+/-ot	always f	always p	st
ALDERS	15	x	f	0	0	all	-	all	Eu:UK	1977	CC	1448	n	V	n	n	0	cu	cig only	always f	always p	st
ARMADA	17	x	m	0	0	all	-	all	Eu:wst	1986	CC	325	n	bl	n	y	0	ev	cig+/-ot	always f	always p	st
BECHER	9	x	m	0	0	all	-	all	Eu:Ger	1985	CC	194	n	bl	n	y	0	ev	cig+/-ot	always f	ever p	st
BROSS	14		m	0	0	wh	-	all	NAmer	1960	CC	974	n	bl	n	n	0	cu	cig+/-ot	only f	p NOS	st
BUFFLE	54		f	0	0	w-hi	-	all	NAmer	1976	CC	943	n	bl	y	n	0	ev	cig+/-ot	only f	p NOS	or
CHOI	30		m	0	0	all	-	all	As:oth	1985	CC	375	n	bl	n	n	0	ev	cig+/-ot	only f	always p	st
CHOI	34		f	0	0	all	-	all	As:oth	1985	CC	375	n	bl	n	n	0	ev	cig+/-ot	only f	always p	ot
CORREA	65		c	0	0	all	-	all	NAmer	1979	CC	1359	n	bl	y	n	2	ev	cig+/-ot	only f	p NOS	ot
CPSII	138		f	0	0	all	4	all	NAmer	1982	pr	3229	n	bl	n	n	2	cu	cig+/-ot	only f	mainly p	st
DEAN2	36		m	0	0	all	-	all	Eu:UK	1960	CC	954	n	V	y	n	0	ev	cig+/-ot	ever f	always p	st
DEAN2	42		f	0	0	all	-	all	Eu:UK	1960	CC	954	n	V	y	n	0	ev	cig+/-ot	ever f	always p	st
DEAN3	155	x	m	0	0	all	-	all	Eu:UK	1969	CC	766	n	V	y	n	0	cu	cig only	only f	p NOS	st
DEAN3	161	x	f	0	0	all	-	all	Eu:UK	1969	CC	766	n	V	y	n	0	cu	cig only	only f	p NOS	st
DESTE2	22	x	c	0	0	all	-	all	SCAmer	1993	CC	463	n	bl	n	n	0	ev	cig+/-ot	always f	ever p	st
DESTEF	52	x	m	0	0	all	-	all	SCAmer	1988	CC	497	n	bl	n	y	0	ev	cig+/-ot	always f	ever p	st
DOLL	47		m	0	0	all	-	all	Eu:UK	1948	CC	1465	n	V	n	n	0	ev	cig+/-ot	ever f	always p	st
ENGELA	105	x	m	0	0	all	0	all	Eu:Sca	1964	pr	435	n	bl	n	n	0	cu	cig+/-ot	only f	always p	st
ENGELA	114	x	f	0	0	all	0	all	Eu:Sca	1964	pr	435	n	bl	n	n	0	cu	cig+/-ot	only f	always p	st
KAISE2	55		m	30	89	all	9	all	NAmer	1979	pr	318	n	bl	n	n	5	cu	cig only	only f	p NOS	or
KAISE2	56		f	30	89	all	9	all	NAmer	1979	pr	318	n	bl	n	n	5	cu	cig only	only f	p NOS	or
KHUDER	7		m	0	0	all	-	all	NAmer	1985	CC	482	n	bl	n	y	0	ev	cig+/-ot	ever f	always p	st
LANGE	42	x	m	0	0	all	0	all	Eu:Sca	1976	pr	268	n	bl	n	n	0	cu	cig only	only f	p NOS	st
LANGE	41	x	f	0	0	all	0	all	Eu:Sca	1976	pr	268	n	bl	n	n	0	cu	cig only	only f	p NOS	st
LUBIN2	119	x	m	0	0	all	-	all	Eu:mul	1976	CC	7804	n	bl	n	y	0	ev	cig+/-ot	only f	always p	st
LUBIN2	123	x	f	0	0	all	-	all	Eu:mul	1976	CC	7804	n	bl	n	y	0	ev	cig+/-ot	only f	always p	st
MACLEN	85		f	0	0	ch	-	all	As:oth	1972	CC	233	n	bl	n	n	0	cu	cig+/-ot	ever f	always p	ot
MATOS	72	x	m	0	0	all	-	all	SCAmer	1994	CC	200	n	bl	n	n	0	ev	cig+/-ot	mainly f	mainly p	st
MIGRAN	99	x	m	0	0	all	0	all	Eu:UK	1964	pr	259	n	V	n	n	0	cu	cig only	only f	p NOS	st
MIGRAN	103	x	f	0	0	all	0	all	Eu:UK	1964	pr	259	n	V	n	n	0	cu	cig only	only f	p NOS	st
MRFITR	7		m	0	0	all	0	all	NAmer	1973	pr	119	n	bl	n	n	9	cu	cig+/-ot	only f	p NOS	ot
PEZZOT	23	x	m	0	0	all	-	all	SCAmer	1987	CC	215	n	bl	n	y	2	ev	cig only	only f	ever p	ot
RIMING	4	x	m	0	0	all	0	all	Eu:UK	1970	pr	104	n	V	n	n	0	cu	cig only	only f	p NOS	st
SEGI2	51	x	m	0	0	all	-	all	As:Jap	1962	CC	378	n	bl	n	n	0	cu	cig+/-ot	only f	p NOS	st
SOBUE	122	x	m	0	0	all	-	q+s+l+a	As:Jap	1986	CC	1376	n	bl	n	y	0	cu	cig+/-ot	only f	p NOS	st
TANG2	1		m	0	0	all	0	all	Eu:UK	1967	pr	836	n	V	n	n	3	cu	cig only	only f	p NOS	or
WAKAI	63	x	m	0	0	all	-	all	As:Jap	1988	CC	333	n	bl	n	y	0	cu	cig+/-ot	only f	p NOS	st
WYNDE5	3		m	0	0	all	-	all	NAmer	1969	CC	1365	n	bl	n	y	0	cu	cig+/-ot	ever f	always p	st
WYNDE5	6		f	0	0	all	-	all	NAmer	1969	CC	1365	n	bl	n	y	0	cu	cig+/-ot	ever f	always p	st
WYNDE6	415	x	m	0	0	all	-	q+a	NAmer	1969	CC	4423	n	bl	n	y	0	cu	cig+/-ot	only f	always p	st
WYNDE6	418	x	f	0	0	all	-	q+a	NAmer	1969	CC	4423	n	bl	n	y	0	cu	cig+/-ot	only f	always p	st

Table 1F1 - 5

IESLC - Meta-analysis of Cigarette Smoking, only Filter vs only Plain (or nearest available)
All LC types
Least adjusted

REF	NRR	SEX	AD	Number Exposed Case	Number Exposed Cont	Non-exposed Case	Non-exposed Cont	RR	95.00%CI
AGUDO	18	f	0	14	21	8	2	0.17	(0.03- 0.90)
ALDERS	168	m	0	53	40	178	97	0.72	(0.45- 1.17)
ALDERS	15	f	0	134	100	62	37	0.80	(0.49- 1.30)
Subtotal ALDERS								0.76	(0.54- 1.07)
ARMADA	17	m	0	57	72	63	55	0.69	(0.42- 1.14)
BECHER	9	m	0	102	196	35	21	0.31	(0.17- 0.56)
BROSS	14	m	0	65	76	200	138	0.59	(0.40- 0.88)
BUFFLE	54	f	0	129	78	47	38	1.34	(0.80- 2.23)
CHOI	30	m	0	43	103	15	2	0.06	(0.01- 0.25)
CHOI	34	f	0	5	11	1	0	0.16~	(0.01- 4.58)
Subtotal CHOI								0.07	(0.02- 0.27)
CORREA	65	c	2	-	-	-	-	0.55	(0.35- 0.85)
*CPSII	138	f	2	-	-	-	-	0.66	(0.57- 0.78)
DEAN2	36	m	0	20	17	644	529	0.97	(0.50- 1.86)
DEAN2	42	f	0	12	2	50	26	3.12	(0.65- 15.00)
Subtotal DEAN2								1.15	(0.63- 2.11)
DEAN3	155	m	0	108	522	210	366	0.36	(0.28- 0.47)
DEAN3	161	f	0	66	888	30	228	0.56	(0.36- 0.89)
Subtotal DEAN3								0.40	(0.32- 0.51)
DESTE2	22	c	0	158	128	142	84	0.73	(0.51- 1.04)
DESTEF	52	m	0	178	156	292	178	0.70	(0.52- 0.92)
DOLL	47	m	0	3	15	501	452	0.18	(0.05- 0.63)
*ENGELA	105	m	0	7	5257	38	15055	0.53	(0.24- 1.18)
*ENGELA	114	f	0	11	23317	11	13521	0.58	(0.25- 1.34)
Subtotal ENGELA								0.55	(0.31- 0.99)
*KAISE2	55	m	5	-	-	-	-	1.03	(0.61- 1.75)
*KAISE2	56	f	5	-	-	-	-	0.65	(0.32- 1.31)
Subtotal KAISE2								0.87	(0.57- 1.33)
KHUDER	7	m	0	173	440	284	334	0.46	(0.36- 0.59)
*LANGE	42	m	0	25	953	65	1642	0.66	(0.42- 1.04)
*LANGE	41	f	0	19	2269	20	1344	0.56	(0.30- 1.05)
Subtotal LANGE								0.63	(0.43- 0.90)
LUBIN2	119	m	0	326	851	1737	2988	0.66	(0.57- 0.76)
LUBIN2	123	f	0	102	184	56	53	0.52	(0.34- 0.82)
Subtotal LUBIN2								0.65	(0.56- 0.74)
MACLEN	85	f	0	15	13	21	14	0.77	(0.28- 2.10)
MATOS	72	m	0	163	229	22	46	1.49	(0.86- 2.57)
*MIGRAN	99	m	0	47	1326	57	1630	1.01	(0.69- 1.48)
*MIGRAN	103	f	0	15	1773	8	871	0.92	(0.39- 2.16)
Subtotal MIGRAN								1.00	(0.71- 1.41)
*MRFITR	7	m	9	-	-	-	-	0.53	(0.24- 1.17)
PEZZOT	23	m	2	-	-	-	-	0.23	(0.16- 0.34)
*RIMING	4	m	0	45	3045	59	2393	0.60	(0.41- 0.88)
SEGI2	51	m	0	105	250	135	203	0.63	(0.46- 0.87)
SOBUE	122	m	0	560	540	49	26	0.55	(0.34- 0.90)
*TANG2	1	m	3	-	-	-	-	0.94	(0.75- 1.18)
WAKAI	63	m	0	174	271	5	9	1.16	(0.38- 3.51)
WYNDE5	3	m	0	417	629	273	398	0.97	(0.79- 1.18)
WYNDE5	6	f	0	152	200	34	30	0.67	(0.39- 1.14)
Subtotal WYNDE5								0.92	(0.77- 1.11)
WYNDE6	415	m	0	150	122	294	165	0.69	(0.51- 0.94)
WYNDE6	418	f	0	215	158	65	30	0.63	(0.39- 1.01)
Subtotal WYNDE6								0.67	(0.52- 0.87)
Partial Totals				3868	44252	5711	43005		

*prospective study

~ With 0.5 adjustment for zero

Table 1F1 - 5

IESLC - Meta-analysis of Cigarette Smoking, only Filter vs only Plain (or nearest available)
All LC types
Least adjusted

REF	NRR	SEX	AD	Ys	Ws	Qs	Ps
AGUDO	18	f	0	-1.79	1.34	2.53	0.0378
ALDERS	168	m	0	-0.33	16.72	0.15	0.1829
ALDERS	15	f	0	-0.22	16.50	0.63	0.3639
Subtotal ALDERS				-0.27	33.22	0.78	
ARMADA	17	m	0	-0.37	15.27	0.04	0.1489
BECHER	9	m	0	-1.16	10.98	6.08	0.0001
BROSS	14	m	0	-0.53	24.52	0.29	0.0090
BUFFLE	54	f	0	0.29	14.67	7.40	0.2658
CHOI	30	m	0	-2.89	1.67	10.17	0.0002
CHOI	34	f	0	-1.84	0.34	0.68	0.2838
Subtotal CHOI				-2.71	2.01	10.85	
CORREA	65	c	2	-0.60	19.52	0.62	0.0083
*CPSII	138	f	2	-0.42	156.19	0.00	0.0000
DEAN2	36	m	0	-0.03	8.91	1.32	0.9187
DEAN2	42	f	0	1.14	1.56	3.78	0.1555
Subtotal DEAN2				0.14	10.47	5.10	
DEAN3	155	m	0	-1.02	53.56	19.32	0.0000
DEAN3	161	f	0	-0.57	18.52	0.43	0.0140
Subtotal DEAN3				-0.90	72.08	19.74	
DESTE2	22	c	0	-0.31	30.22	0.33	0.0839
DESTEF	52	m	0	-0.36	47.46	0.15	0.0124
DOLL	47	m	0	-1.71	2.47	4.14	0.0071
*ENGELA	105	m	0	-0.64	5.92	0.29	0.1197
*ENGELA	114	f	0	-0.54	5.50	0.09	0.2011
Subtotal ENGELA				-0.59	11.42	0.37	
*KAISE2	55	m	5	0.03	13.83	2.79	0.9125
*KAISE2	56	f	5	-0.43	7.73	0.00	0.2309
Subtotal KAISE2				-0.14	21.57	2.79	
KHUDER	7	m	0	-0.77	68.64	8.50	0.0000
*LANGE	42	m	0	-0.41	18.61	0.00	0.0759
*LANGE	41	f	0	-0.57	9.86	0.24	0.0710
Subtotal LANGE				-0.47	28.47	0.24	
LUBIN2	119	m	0	-0.42	194.06	0.00	0.0000
LUBIN2	123	f	0	-0.65	19.24	0.98	0.0047
Subtotal LUBIN2				-0.44	213.31	0.98	
MACLEN	85	f	0	-0.26	3.81	0.09	0.6087
MATOS	72	m	0	0.40	12.87	8.59	0.1537
*MIGRAN	99	m	0	0.01	26.70	5.01	0.9443
*MIGRAN	103	f	0	-0.08	5.26	0.60	0.8504
Subtotal MIGRAN				-0.00	31.96	5.61	
*MRFITR	7	m	9	-0.63	6.12	0.28	0.1162
PEZZOT	23	m	2	-1.47	27.04	29.83	0.0000
*RIMING	4	m	0	-0.51	26.02	0.22	0.0090
SEGI2	51	m	0	-0.46	38.67	0.06	0.0043
SOBUE	122	m	0	-0.60	16.00	0.51	0.0169
*TANG2	1	m	3	-0.06	74.81	9.57	0.5925
WAKAI	63	m	0	0.14	3.12	0.99	0.7982
WYNDE5	3	m	0	-0.03	98.39	14.62	0.7354
WYNDE5	6	f	0	-0.40	13.45	0.01	0.1427
Subtotal WYNDE5				-0.08	111.85	14.62	
WYNDE6	415	m	0	-0.37	41.11	0.10	0.0174
WYNDE6	418	f	0	-0.47	16.75	0.03	0.0569
Subtotal WYNDE6				-0.40	57.86	0.13	

Table 1F1 - 5

IESLC - Meta-analysis of Cigarette Smoking, only Filter vs only Plain (or nearest available)
All LC types
Least adjusted

	N	42
	NS	31
	Wt	1193.97
Het	Chi	141.44
Het	df	41
Het	P	***
Fixed	RR	0.66
	RRl	0.62
	RRu	0.70
	P	---
Random	RR	0.64
	RRl	0.57
	RRu	0.73
	P	---
Asymm	P	N.S.

Table 1F1 - 6

IESLC - Meta-analysis of Cigarette Smoking, only Filter vs only Plain (or nearest available)
All LC types
Least adjusted

		Sex		
	combined	male	female	Total
N	2	25	15	42
NS	2	25	15	42
Wt	49.74	853.50	290.73	1193.97
Het Chi	0.95	122.95	17.33	141.44
Het df	1	24	14	41
Het P	N.S.	***	N.S.	***
Fixed RR	0.65	0.65	0.67	0.66
RRl	0.49	0.61	0.60	0.62
RRu	0.86	0.70	0.75	0.70
P	--	---	---	---
Random RR	0.65	0.62	0.68	0.64
RRl	0.49	0.52	0.58	0.57
RRu	0.86	0.74	0.80	0.73
P	--	---	---	---
Between Chi				0.21
Between df				2
Between P				N.S.
Btwn(F) P				N.S.
Btwn(R) P				N.S.

Table 1F1 - 7

IESLC - Meta-analysis of Cigarette Smoking, only Filter vs only Plain (or nearest available)
All LC types
Excluded studies (and stage at which they were excluded)

Stage	Studies
1	BOUCHA BUELL LAURIL MZILEN
2	BERRIN CHEN RESTRE TAO ULMER
3	ABELIN ABRAHA AKIBA AMANDU AMES ANDERS ARCHER AUSTIN AUVINE AXELSO AXELSS BAND BARBON BENSHL BEST BLOHMK BLOT1 BLOT2 BLOT3 BLOT4 BOFFET BOUCOT BRESLO BRETT BROCKM BROWN1 BROWN2 BYERS1 BYERS2 CARPEN CASCO2 CASCOR CEDERL CHAN CHANG CHATZI CHEN2 CHEN3 CHIAZZ CHOW CHYOU COMSTO COOKSO CPSI DAMBER DARBY DAVEYS DEAN DEKLER DOCKER DOLL2 DORANT DORGAN DORN DOSEME DROSTE DU DUNN EBELIN ENSTRO ESAKI FAN GAO GAO2 GARCIA GARDIN GARSHI GENG GER GILLIS GODLEY GOLLED GOODMA GRAHAM GREGOR GSELL HAENSZ HAMMO2 HAMMON HANSEN HEGMAN HEIN HENNEK HINDS HIRAOK HIRAY2 HIRAYA HITOSU HOLE HOROWI HORWIT HU HU2 HUANG HUMBLE ISHIMA JAHN JAIN JARUP JARVHO JEDRYC JIANG JOLY JONES JUSSAW KAISER KANELL KATSOU KAUFMA KELLER KIHARA KINLEN KJUUS KNEKT KO KOHLME KOO KOULUM KREUZE KREYBE KUBIK LAMTH LAMWK LAMWK2 LAUSSM LEI LEMARC LETOUR LEVIN LIAW LICKIN LIDDEL LIU LIU2 LIU3 LIU4 LIU5 LOMBA2 LOMBAR LUBIN LUO MAGNUS MARSH MARSH2 MARTIS MASTRA MATSUD MCCONN MCDUFF MCLAUG MILLER MILLS MOLLO MRFIT MURATA NAM NOTAN2 NOTANI NOU ODRISC ORMOS OSANN OSANN2 PARKIN PASTOR PAWLEG PERNU PERSH2 PERSHA PETO PEZZO2 PIKE PISANI POFFIJ POLEDN PRESCO QIAO QIAO2 RACHTA RADZIK RANDIG REN RONCO ROOTS ROTHSC SAARIK SADOWS SANKAR SCHWA2 SCHWAR SEGI SEOW SHAW SHIMIZ SIEMIA SIMARA SIMONA SITAS SOBUE2 SPEIZE SPITZ STASZE STAYNE STOCKS STOCKW STUCKE SUN SUZUK2 SUZUKI SVENSS TANG TENKAN TIZZAN TOKARS TOUSEY TSUGAN TULINI TVERDA VANDER VEIERO VUTUC WALD WANG WANG2 WANG3 WANG4 WARSIN WATSON WICKLU WIGLE WILKIN WU WU2 WUNSCH WUWILL WYNDE2 WYNDE4 WYNDE7 WYNDE8 WYNDER XIANGZ XU XU2 XU3 XU4 YAMAGU YONG YUAN ZHANG ZHENG ZHOU
7	WYNDE3
10	BENHAM

Table 1F1 - 8

Potentially overlapping studies

REF	REFGP	PRINC	.	OVERLAP/LINK
LUBIN2	LUBIN2	1		Lubin-combined
LANGE	PRESCO	1		Subset of PRESCO
BROSS	BYERS1	1		GRAHAM/BROSS/BYERS1
TANG2	TANG2	2		TANG2-combined
MRFITR	MRFIT	2		Subset of MRFIT
WYNDE5	WYNDE6	2		WYNDE5/6/7/8
WYNDE6	WYNDE6	1		WYNDE5/6/7/8

Table 1F1 - 9

Most adjusted - insufficient data for metaanalysis

REF	NRR	SEX	AGEL	AGEH	RACE	YF	LC TYPE	LOC	START	ST	NLC	R	VB	P	H	AD	SM	PRODUCT	CIGTYP	DENOM	De
BUFFLE	88	m	0	0	wh	-	all	NAmer	1976	CC	943	n	bl	y	n	0	ev	cig+/-ot	only f	p NOS	st
CPSII	135	m	0	0	all	6	all	NAmer	1982	pr	3229	n	bl	n	n	1	cu	cig only	mainly f	mainly p	ot
HIRAYA	155	c	0	0	all	16	all	As:Jap	1965	pr	1917	n	bl	n	n	1	ev	cig+/-ot	only f	p NOS	ot
HUMBLE	53	c	0	0	w-hi	-	not alv	NAmer	1980	CC	521	n	bl	y	n	5	cu	cig+/-ot	always f	always p	or
HUMBLE	54	c	0	0	hi	-	not alv	NAmer	1980	CC	521	n	bl	y	n	5	cu	cig+/-ot	always f	always p	or
JOLY	56	m	0	0	all	-	all	SCAmer	1978	CC	826	n	bl	n	n	0	ev	cig+/-ot	ever f	ever p	or
JOLY	57	f	0	0	all	-	all	SCAmer	1978	CC	826	n	bl	n	n	0	ev	cig+/-ot	ever f	ever p	or

REF	NRR	RR	SIG	RRDATA comment
BUFFLE	88	0.92		0
CPSII	135	0.47		0
HIRAYA	155	0.51		0
HUMBLE	53	0.80		0
HUMBLE	54	0.04		0
JOLY	56	*		No significant difference was noted between cases and controls with respect to use of filter cigarettes.
JOLY	57	*		No significant difference was noted between cases and controls with respect to use of filter cigarettes.

Table 1F2 -

IESLC - Meta-analysis of Cigarette Smoking, ever Filter vs only Plain (or nearest available)
All LC types

This analysis is restricted to results for:
1) Non-dose-response data
2) Results complete enough for use in metaanalysis

Within each study, results are then selected (in the following order of preference, within each sex) for:
3) CIGTYP: filter ever, equally, both, mainly, always, only/NOS
4) DENOM: plain only/NOS, always, mainly, ever
5) PRODUCT: cigarettes regardless of other products, cigarettes only (Note only study ALDERS has both product definitions available)
6) SMKSTA: ever, current. (Note only study MATOS has both ever and current available)
7) LCtype: all or nearest available, at least Squamous and Adeno. (q = squamous, s = small, l = large, a = adeno, mix = mixed, alv = alveolar)
8) Race: all or nearest available, otherwise by race (wh or w = white, bl or b = black, hi = hispanic ch = chinese, jap = japanese, haw = hawaiian, w+o = white + oriental, sca = scandinavian, as = asian)
9) Followup period (YF, prospective studies): whole study (coded as 0) or longest available
10) For overlapping studies: principal rather than subsidiary studies
Finally by Age: whole study (coded as 0) if available, otherwise by widest available age group
and then for single sex results (m, f) in preference to combined sex results (c).

Results adjusted (AD) for the most potential confounders are then chosen in Sections -1 to -3
(and those which actually differ from the adjusted results in Table 1F1 - 1 are marked 'x' in Section -1)
and results adjusted for the least confounders in Sections -4 to -6. (Those least adjusted results which
actually differ from the most adjusted as marked 'x' in column X in Section -4)
(Results adjusted for an unknown number of confounder(s) are coded as 20.)

Section -7 shows excluded studies, together with the stage (as above) at which no qualifying
results were found.

Section -8 lists the potentially overlapping studies which have been included (1=principal, 2=subsidiary).

Section -9 lists any results which would have been included in preference except that they had data not complete
enough for use in meta-analysis, with their significance (yes/no), if known, and any further comment as entered
on the database.

In addition to those mentioned above, the following fields, levels and abbreviations are used:
* or nk = not known, n = no, y = yes, ot = other
ev = ever, cu = current, cig+/-ot = cigarettes irrespective of other products (cigar, pipe etc)
f = filter, p = plain, NOS = not otherwise specified
REF: 6-character study reference
NRR: number of the RR on the database within the study
ST : study type (CC = case control, pr or prosp = prospective)
NLC: number of lung cancer cases in whole study
R : risky occupational population (n = no, m = mining, o = other risky)
VB : national cigarette type (V = at least 75% Virginia, bl = at least 75% blended, ot = other)
P : any proxy use
H : full histological confirmation
De : derivation of RR/CI (or = original, st = standard method, ot = other method of estimation)

Table 1F2 - 1

IESLC - Meta-analysis of Cigarette Smoking, ever Filter vs only Plain (or nearest available)
All LC types
Most adjusted

REF	NRR	1F1	SEX	AGEL	AGEH	RACE	YF	LC TYPE	LOC	START	ST	NLC	R	VB	P	H	AD	SM	PRODUCT	CIGTYP	DENOM	De
AGUDO	17		f	0	0	all	-	all	Eu:wst	1989	CC	103	n	bl	n	n	3	ev	cig only	only f	ever p	ot
ALDERS	171	x	m	0	0	all	-	all	Eu:UK	1977	CC	1448	n	V	n	n	2	cu	cig+/-ot	ever f	always p	ot
ALDERS	163	x	f	0	0	all	-	all	Eu:UK	1977	CC	1448	n	V	n	n	2	cu	cig only	ever f	always p	ot
ARMADA	23	x	m	0	0	all	-	all	Eu:wst	1986	CC	325	n	bl	n	y	1	ev	cig+/-ot	ever f	always p	ot
BECHER	10		m	0	0	all	-	all	Eu:Ger	1985	CC	194	n	bl	n	y	3	ev	cig+/-ot	always f	ever p	st
BROSS	14		m	0	0	wh	-	all	NAmer	1960	CC	974	n	bl	n	n	0	cu	cig+/-ot	only f	p NOS	st
BUFFLE	56	x	f	0	0	w-hi	-	all	NAmer	1976	CC	943	n	bl	y	n	0	ev	cig+/-ot	ever f	p NOS	st
CHOI	40	x	m	0	0	all	-	all	As:oth	1985	CC	375	n	bl	n	n	0	ev	cig+/-ot	ever f	always p	st
CHOI	44	x	f	0	0	all	-	all	As:oth	1985	CC	375	n	bl	n	n	0	ev	cig+/-ot	ever f	always p	ot
CORREA	65		c	0	0	all	-	all	NAmer	1979	CC	1359	n	bl	y	n	2	ev	cig+/-ot	only f	p NOS	ot
CPSII	138		f	0	0	all	4	all	NAmer	1982	pr	3229	n	bl	n	n	2	cu	cig+/-ot	only f	mainly p	st
DEAN2	36		m	0	0	all	-	all	Eu:UK	1960	CC	954	n	V	y	n	0	ev	cig+/-ot	ever f	always p	st
DEAN2	42		f	0	0	all	-	all	Eu:UK	1960	CC	954	n	V	y	n	0	ev	cig+/-ot	ever f	always p	st
DEAN3	168	x	m	0	0	all	-	all	Eu:UK	1969	CC	766	n	V	y	n	2	cu	cig only	ever f	always p	ot
DEAN3	172	x	f	0	0	all	-	all	Eu:UK	1969	CC	766	n	V	y	n	2	cu	cig only	ever f	always p	ot
DESTE2	23		c	0	0	all	-	all	SCAmer	1993	CC	463	n	bl	n	n	7	ev	cig+/-ot	always f	ever p	ot
DESTEF	53		m	0	0	all	-	all	SCAmer	1988	CC	497	n	bl	n	y	4	ev	cig+/-ot	always f	ever p	ot
DOLL	47		m	0	0	all	-	all	Eu:UK	1948	CC	1465	n	V	n	n	0	ev	cig+/-ot	ever f	always p	st
ENGELA	116	x	m	0	0	all	0	all	Eu:Sca	1964	pr	435	n	bl	n	n	0	cu	cig+/-ot	ever f	always p	st
ENGELA	200	x	f	0	0	all	0	all	Eu:Sca	1964	pr	435	n	bl	n	n	5	cu	cig+/-ot	ever f	always p	ot
KAISE2	55		m	30	89	all	9	all	NAmer	1979	pr	318	n	bl	n	n	5	cu	cig only	only f	p NOS	or
KAISE2	56		f	30	89	all	9	all	NAmer	1979	pr	318	n	bl	n	n	5	cu	cig only	only f	p NOS	or
KHUDER	7		m	0	0	all	-	all	NAmer	1985	CC	482	n	bl	n	y	0	ev	cig+/-ot	ever f	always p	st
LANGE	20		m	0	0	all	0	all	Eu:Sca	1976	pr	268	n	bl	n	n	2	cu	cig only	only f	p NOS	or
LANGE	19		f	0	0	all	0	all	Eu:Sca	1976	pr	268	n	bl	n	n	2	cu	cig only	only f	p NOS	or
LUBIN2	109	x	m	0	0	all	-	all	Eu:mul	1976	CC	7804	n	bl	n	y	3	ev	cig+/-ot	ever f	always p	ot
LUBIN2	117	x	f	0	0	all	-	all	Eu:mul	1976	CC	7804	n	bl	n	y	3	ev	cig+/-ot	ever f	always p	ot
MACLEN	85		f	0	0	ch	-	all	As:oth	1972	CC	233	n	bl	n	n	0	cu	cig+/-ot	ever f	always p	ot
MATOS	37		m	0	0	all	-	all	SCAmer	1994	CC	200	n	bl	n	n	4	ev	cig+/-ot	mainly f	mainly p	st
MIGRAN	100		m	0	0	all	0	all	Eu:UK	1964	pr	259	n	V	n	n	3	cu	cig only	only f	p NOS	ot
MIGRAN	104		f	0	0	all	0	all	Eu:UK	1964	pr	259	n	V	n	n	3	cu	cig only	only f	p NOS	ot
MRFITR	7		m	0	0	all	0	all	NAmer	1973	pr	119	n	bl	n	n	9	cu	cig+/-ot	only f	p NOS	ot
PEZZOT	24		m	0	0	all	-	all	SCAmer	1987	CC	215	n	bl	n	y	4	ev	cig only	only f	ever p	ot
RIMING	8		m	0	0	all	0	all	Eu:UK	1970	pr	104	n	V	n	n	1	cu	cig only	only f	p NOS	ot
SEGI2	52		m	0	0	all	-	all	As:Jap	1962	CC	378	n	bl	n	n	1	cu	cig+/-ot	only f	p NOS	ot
SOBUE	125		m	0	0	all	-	q+s+l+a	As:Jap	1986	CC	1376	n	bl	n	y	5	cu	cig+/-ot	only f	p NOS	st
TANG2	1		m	0	0	all	0	all	Eu:UK	1967	pr	836	n	V	n	n	3	cu	cig only	only f	p NOS	or
WAKAI	64		m	0	0	all	-	all	As:Jap	1988	CC	333	n	bl	n	y	5	cu	cig+/-ot	only f	p NOS	st
WYNDE5	3		m	0	0	all	-	all	NAmer	1969	CC	1365	n	bl	n	y	0	cu	cig+/-ot	ever f	always p	st
WYNDE5	6		f	0	0	all	-	all	NAmer	1969	CC	1365	n	bl	n	y	0	cu	cig+/-ot	ever f	always p	st
WYNDE6	423	x	m	0	0	all	-	q+a	NAmer	1969	CC	4423	n	bl	n	y	3	cu	cig+/-ot	ever f	always p	ot
WYNDE6	426	x	f	0	0	all	-	q+a	NAmer	1969	CC	4423	n	bl	n	y	3	cu	cig+/-ot	ever f	always p	ot

Table 1F2 - 2

IESLC - Meta-analysis of Cigarette Smoking, ever Filter vs only Plain (or nearest available)
All LC types
Most adjusted

REF	NRR	SEX	AD	Number Exposed Case	Number Exposed Cont	Non-exposed Case	Non-exposed Cont	RR	95.00%CI
AGUDO	17	f	3	-	-	-	-	0.22	(0.04- 1.27)
ALDERS	171	m	2	-	-	-	-	0.85	(0.63- 1.15)
ALDERS	163	f	2	-	-	-	-	1.09	(0.70- 1.70)
Subtotal ALDERS								0.92	(0.72- 1.18)
ARMADA	23	m	1	-	-	-	-	0.88	(0.56- 1.40)
BECHER	10	m	3	-	-	-	-	0.41	(0.21- 0.81)
BROSS	14	m	0	65	76	200	138	0.59	(0.40- 0.88)
BUFFLE	56	f	0	193	123	47	38	1.27	(0.78- 2.06)
CHOI	40	m	0	254	463	15	2	0.07	(0.02- 0.32)
CHOI	44	f	0	18	26	1	0	0.23~	(0.01- 6.03)
Subtotal CHOI								0.09	(0.02- 0.34)
CORREA	65	c	2	-	-	-	-	0.55	(0.35- 0.85)
*CPSII	138	f	2	-	-	-	-	0.66	(0.57- 0.78)
DEAN2	36	m	0	20	17	644	529	0.97	(0.50- 1.86)
DEAN2	42	f	0	12	2	50	26	3.12	(0.65- 15.00)
Subtotal DEAN2								1.15	(0.63- 2.11)
DEAN3	168	m	2	-	-	-	-	0.46	(0.34- 0.63)
DEAN3	172	f	2	-	-	-	-	0.66	(0.44- 0.97)
Subtotal DEAN3								0.53	(0.41- 0.67)
DESTE2	23	c	7	-	-	-	-	0.73	(0.51- 1.05)
DESTEF	53	m	4	-	-	-	-	0.72	(0.54- 0.96)
DOLL	47	m	0	3	15	501	452	0.18	(0.05- 0.63)
*ENGELA	116	m	0	7	6306	38	15055	0.44	(0.20- 0.98)
*ENGELA	200	f	5	-	-	-	-	0.99	(0.46- 2.15)
Subtotal ENGELA								0.67	(0.38- 1.17)
*KAISE2	55	m	5	-	-	-	-	1.03	(0.61- 1.75)
*KAISE2	56	f	5	-	-	-	-	0.65	(0.32- 1.31)
Subtotal KAISE2								0.87	(0.57- 1.33)
KHUDER	7	m	0	173	440	284	334	0.46	(0.36- 0.59)
*LANGE	20	m	2	-	-	-	-	0.90	(0.60- 1.40)
*LANGE	19	f	2	-	-	-	-	0.70	(0.40- 1.40)
Subtotal LANGE								0.83	(0.59- 1.18)
LUBIN2	109	m	3	-	-	-	-	0.95	(0.87- 1.04)
LUBIN2	117	f	3	-	-	-	-	0.81	(0.43- 1.50)
Subtotal LUBIN2								0.95	(0.87- 1.03)
MACLEN	85	f	0	15	13	21	14	0.77	(0.28- 2.10)
MATOS	37	m	4	-	-	-	-	1.25	(0.67- 2.50)
*MIGRAN	100	m	3	-	-	-	-	1.23	(0.84- 1.81)
*MIGRAN	104	f	3	-	-	-	-	1.44	(0.61- 3.40)
Subtotal MIGRAN								1.26	(0.89- 1.79)
*MRFITR	7	m	9	-	-	-	-	0.53	(0.24- 1.17)
PEZZOT	24	m	4	-	-	-	-	0.29	(0.20- 0.41)
*RIMING	8	m	1	-	-	-	-	0.65	(0.44- 0.96)
SEGI2	52	m	1	-	-	-	-	0.62	(0.45- 0.85)
SOBUE	125	m	5	-	-	-	-	0.67	(0.38- 1.11)
*TANG2	1	m	3	-	-	-	-	0.94	(0.75- 1.18)
WAKAI	64	m	5	-	-	-	-	1.02	(0.31- 3.33)
WYNDE5	3	m	0	417	629	273	398	0.97	(0.79- 1.18)
WYNDE5	6	f	0	152	200	34	30	0.67	(0.39- 1.14)
Subtotal WYNDE5								0.92	(0.77- 1.11)
WYNDE6	423	m	3	-	-	-	-	0.96	(0.77- 1.18)
WYNDE6	426	f	3	-	-	-	-	0.91	(0.56- 1.46)
Subtotal WYNDE6								0.95	(0.78- 1.16)
Partial Totals				1329	8310	2108	17016		

*prospective study

~ With 0.5 adjustment for zero

Table 1F2 - 2

IESLC - Meta-analysis of Cigarette Smoking, ever Filter vs only Plain (or nearest available)
All LC types
Most adjusted

REF	NRR	SEX	AD	Ys	Ws	Qs	Ps
AGUDO	17	f	3	-1.51	1.29	2.11	0.0861
ALDERS	171	m	2	-0.16	42.43	0.20	0.2898
ALDERS	163	f	2	0.09	19.52	1.97	0.7034
Subtotal ALDERS				-0.08	61.95	2.17	
ARMADA	23	m	1	-0.13	18.30	0.20	0.5845
BECHER	10	m	3	-0.89	8.43	3.68	0.0096
BROSS	14	m	0	-0.53	24.52	2.15	0.0090
BUFFLE	56	f	0	0.24	16.42	3.62	0.3350
CHOI	40	m	0	-2.62	1.75	9.92	0.0005
CHOI	44	f	0	-1.46	0.36	0.55	0.3800
Subtotal CHOI				-2.42	2.11	10.47	
CORREA	65	c	2	-0.60	19.52	2.62	0.0083
*CPSII	138	f	2	-0.42	156.19	5.30	0.0000
DEAN2	36	m	0	-0.03	8.91	0.35	0.9187
DEAN2	42	f	0	1.14	1.56	2.92	0.1555
Subtotal DEAN2				0.14	10.47	3.27	
DEAN3	168	m	2	-0.78	40.39	12.01	0.0000
DEAN3	172	f	2	-0.42	24.59	0.83	0.0394
Subtotal DEAN3				-0.64	64.98	12.84	
DESTE2	23	c	7	-0.31	29.47	0.20	0.0876
DESTEF	53	m	4	-0.33	46.42	0.44	0.0252
DOLL	47	m	0	-1.71	2.47	5.43	0.0071
*ENGELA	116	m	0	-0.82	5.92	2.06	0.0457
*ENGELA	200	f	5	-0.01	6.46	0.32	0.9796
Subtotal ENGELA				-0.40	12.38	2.38	
*KAISE2	55	m	5	0.03	13.83	0.94	0.9125
*KAISE2	56	f	5	-0.43	7.73	0.31	0.2309
Subtotal KAISE2				-0.14	21.57	1.25	
KHUDER	7	m	0	-0.77	68.64	20.02	0.0000
*LANGE	20	m	2	-0.11	21.40	0.34	0.6259
*LANGE	19	f	2	-0.36	9.79	0.15	0.2644
Subtotal LANGE				-0.18	31.19	0.49	
LUBIN2	109	m	3	-0.05	482.35	15.63	0.2599
LUBIN2	117	f	3	-0.21	9.84	0.00	0.5085
Subtotal LUBIN2				-0.05	492.19	15.64	
MACLEN	85	f	0	-0.26	3.81	0.00	0.6087
MATOS	37	m	4	0.22	8.86	1.83	0.5065
*MIGRAN	100	m	3	0.21	26.07	5.01	0.2905
*MIGRAN	104	f	3	0.36	5.21	1.85	0.4054
Subtotal MIGRAN				0.23	31.28	6.86	
*MRFITR	7	m	9	-0.63	6.12	1.00	0.1162
PEZZOT	24	m	4	-1.24	29.82	30.21	0.0000
*RIMING	8	m	1	-0.43	25.25	1.00	0.0304
SEGI2	52	m	1	-0.48	37.99	2.31	0.0032
SOBUE	125	m	5	-0.40	13.37	0.38	0.1431
*TANG2	1	m	3	-0.06	74.81	2.15	0.5925
WAKAI	64	m	5	0.02	2.73	0.17	0.9739
WYNDE5	3	m	0	-0.03	98.39	3.83	0.7354
WYNDE5	6	f	0	-0.40	13.45	0.38	0.1427
Subtotal WYNDE5				-0.08	111.85	4.21	
WYNDE6	423	m	3	-0.04	84.32	3.06	0.7078
WYNDE6	426	f	3	-0.09	16.73	0.31	0.6996
Subtotal WYNDE6				-0.05	101.06	3.37	

Table 1F2 - 2

IESLC - Meta-analysis of Cigarette Smoking, ever Filter vs only Plain (or nearest available)
All LC types
Most adjusted

	N	42
	NS	31
	Wt	1535.44
Het	Chi	147.76
Het	df	41
Het	P	***
Fixed	RR	0.79
	RRl	0.75
	RRu	0.83
	P	---
Random	RR	0.73
	RRl	0.65
	RRu	0.82
	P	---
Asymm	P	(*)

Table 1F2 - 3

IESLC - Meta-analysis of Cigarette Smoking, ever Filter vs only Plain (or nearest available)
All LC types
Most adjusted

	Sex: combined	male	female	Total
N	2	25	15	42
NS	2	25	15	42
Wt	48.98	1193.50	292.95	1535.44
Het Chi	0.94	123.70	19.58	147.76
Het df	1	24	14	41
Het P	N.S.	***	N.S.	***
Fixed RR	0.65	0.81	0.75	0.79
RRl	0.49	0.77	0.67	0.75
RRu	0.86	0.86	0.84	0.83
P	--	---	---	---
Random RR	0.65	0.70	0.81	0.73
RRl	0.49	0.60	0.68	0.65
RRu	0.86	0.82	0.97	0.82
P	--	---	-	---
Between Chi				3.54
Between df				2
Between P				N.S.
Btwn(F) P				N.S.
Btwn(R) P				N.S.

	All LC (or nearest): all	other	Total
N	39	3	42
NS	29	2	31
Wt	1421.01	114.43	1535.44
Het Chi	143.83	1.49	147.76
Het df	38	2	41
Het P	***	N.S.	***
Fixed RR	0.78	0.91	0.79
RRl	0.74	0.76	0.75
RRu	0.83	1.10	0.83
P	---	N.S.	---
Random RR	0.72	0.91	0.73
RRl	0.64	0.76	0.65
RRu	0.82	1.10	0.82
P	---	N.S.	---
Between Chi			2.45
Between df			1
Between P			N.S.
Btwn(F) P			N.S.
Btwn(R) P			*

	Location: NAmer	UK	Scand	othEur	China	Japan	othAs	other	Total
N	12	11	4	5		3	3	4	42
NS	9	7	2	4		3	2	4	31
Wt	525.88	271.20	43.58	520.21		54.09	5.92	114.56	1535.44
Het Chi	40.87	33.62	2.86	8.79		0.65	6.70	23.21	147.76
Het df	11	10	3	4		2	2	3	41
Het P	***	***	N.S.	(*)		N.S.	*	***	***
Fixed RR	0.74	0.81	0.78	0.93		0.65	0.36	0.60	0.79
RRl	0.68	0.72	0.58	0.85		0.50	0.16	0.50	0.75
RRu	0.80	0.91	1.05	1.01		0.85	0.80	0.71	0.83
P	---	---	N.S.	(-)		--	-	---	---
Random RR	0.74	0.82	0.78	0.76		0.65	0.25	0.64	0.73
RRl	0.61	0.64	0.58	0.55		0.50	0.04	0.37	0.65
RRu	0.90	1.05	1.05	1.06		0.85	1.50	1.09	0.82
P	--	N.S.	N.S.	N.S.		--	N.S.	N.S.	---
Between Chi									31.07
Between df									6
Between P									***
Btwn(F) P									N.S.
Btwn(R) P									N.S.

Table 1F2 - 3

IESLC - Meta-analysis of Cigarette Smoking, ever Filter vs only Plain (or nearest available)
All LC types
Most adjusted

	Detailed Country in "other Europe"					
	multi	Germany	othWest	East	Balkans	Total
N	2	1	2			5
NS	1	1	2			4
Wt	492.19	8.43	19.59			520.21
Het Chi	0.25	0.00	2.31			8.79
Het df	1	0	1			4
Het P	N.S.	N.S.	N.S.			(*)
Fixed RR	0.95	0.41	0.80			0.93
RRl	0.87	0.21	0.52			0.85
RRu	1.03	0.81	1.25			1.01
P	N.S.	--	N.S.			(-)
Random RR	0.95	0.41	0.57			0.76
RRl	0.87	0.21	0.16			0.55
RRu	1.03	0.81	2.01			1.06
P	N.S.	--	N.S.			N.S.
Between Chi						6.23
Between df						2
Between P						*
Btwn(F) P						N.S.
Btwn(R) P						*

	Detailed Country in "other Asia"			
	India	HongKong	other	Total
N			3	3
NS			2	2
Wt			5.92	5.92
Het Chi			6.70	6.70
Het df			2	2
Het P			*	*
Fixed RR			0.36	0.36
RRl			0.16	0.16
RRu			0.80	0.80
P			-	-
Random RR			0.25	0.25
RRl			0.04	0.04
RRu			1.50	1.50
P			N.S.	N.S.
Between Chi				
Between df				
Between P				N.S.
Btwn(F) P				N.S.
Btwn(R) P				N.S.

	Detailed other continent			
	SCAmer	Auslia	Africa	Total
N	4			4
NS	4			4
Wt	114.56			114.56
Het Chi	23.21			23.21
Het df	3			3
Het P	***			***
Fixed RR	0.60			0.60
RRl	0.50			0.50
RRu	0.71			0.71
P	---			---
Random RR	0.64			0.64
RRl	0.37			0.37
RRu	1.09			1.09
P	N.S.			N.S.
Between Chi				
Between df				
Between P				N.S.
Btwn(F) P				N.S.
Btwn(R) P				N.S.

Table 1F2 - 3

IESLC - Meta-analysis of Cigarette Smoking, ever Filter vs only Plain (or nearest available)
All LC types
Most adjusted

		Start year of study				
	<1960	1960-69	1970-79	1980-89	1990+	Total
N	1	15	13	11	2	42
NS	1	9	9	10	2	31
Wt	2.47	469.33	678.01	347.29	38.33	1535.44
Het Chi	0.00	38.57	15.14	36.94	1.97	147.76
Het df	0	14	12	10	1	41
Het P	N.S.	***	N.S.	***	N.S.	***
Fixed RR	0.18	0.83	0.91	0.57	0.83	0.79
RRl	0.05	0.76	0.84	0.52	0.60	0.75
RRu	0.63	0.91	0.98	0.64	1.13	0.83
P	--	---	-	---	N.S.	---
Random RR	0.18	0.81	0.86	0.52	0.89	0.73
RRl	0.05	0.68	0.76	0.40	0.53	0.65
RRu	0.63	0.96	0.97	0.69	1.47	0.82
P	--	-	-	---	N.S.	---
Between Chi						55.15
Between df						4
Between P						***
Btwn(F) P						**
Btwn(R) P						**

	Study type (1)		
	CC	other	Total
N	30	12	42
NS	23	8	31
Wt	1176.64	358.79	1535.44
Het Chi	127.29	20.27	147.76
Het df	29	11	41
Het P	***	*	***
Fixed RR	0.80	0.78	0.79
RRl	0.75	0.70	0.75
RRu	0.85	0.86	0.83
P	---	---	---
Random RR	0.70	0.81	0.73
RRl	0.60	0.68	0.65
RRu	0.81	0.97	0.82
P	---	-	---
Between Chi			0.21
Between df			1
Between P			N.S.
Btwn(F) P			N.S.
Btwn(R) P			N.S.

	Study type (2)			
	CC	prosp	other	Total
N	30	12		42
NS	23	8		31
Wt	1176.64	358.79		1535.44
Het Chi	127.29	20.27		147.76
Het df	29	11		41
Het P	***	*		***
Fixed RR	0.80	0.78		0.79
RRl	0.75	0.70		0.75
RRu	0.85	0.86		0.83
P	---	---		---
Random RR	0.70	0.81		0.73
RRl	0.60	0.68		0.65
RRu	0.81	0.97		0.82
P	---	-		---
Between Chi				0.21
Between df				1
Between P				N.S.
Btwn(F) P				N.S.
Btwn(R) P				N.S.

Table 1F2 - 3

IESLC - Meta-analysis of Cigarette Smoking, ever Filter vs only Plain (or nearest available)
All LC types
Most adjusted

	Study size (number of LC cases)				
	100-249	250-499	500-999	1000+	Total
N	7	16	7	12	42
NS	7	11	5	8	31
Wt	83.58	302.07	191.20	958.59	1535.44
Het Chi	19.89	39.64	23.55	30.71	147.76
Het df	6	15	6	11	41
Het P	**	***	***	**	***
Fixed RR	0.49	0.70	0.75	0.87	0.79
RRl	0.39	0.62	0.66	0.82	0.75
RRu	0.60	0.78	0.87	0.93	0.83
P	---	---	---	---	---
Random RR	0.53	0.74	0.79	0.82	0.73
RRl	0.34	0.60	0.57	0.71	0.65
RRu	0.83	0.91	1.08	0.94	0.82
P	--	--	N.S.	--	---
Between Chi					33.98
Between df					3
Between P					***
Btwn(F) P					*
Btwn(R) P					N.S.

	Risky occupational population			
	no	mining	othRisky	Total
N	42			42
NS	31			31
Wt	1535.44			1535.44
Het Chi	147.76			147.76
Het df	41			41
Het P	***			***
Fixed RR	0.79			0.79
RRl	0.75			0.75
RRu	0.83			0.83
P	---			---
Random RR	0.73			0.73
RRl	0.65			0.65
RRu	0.82			0.82
P	---			---
Between Chi				
Between df				
Between P				N.S.
Btwn(F) P				N.S.
Btwn(R) P				N.S.

	National cigarette tobacco type			
	Virginia	blended	other	Total
N	11	31		42
NS	7	24		31
Wt	271.20	1264.23		1535.44
Het Chi	33.62	114.03		147.76
Het df	10	30		41
Het P	***	***		***
Fixed RR	0.81	0.79		0.79
RRl	0.72	0.75		0.75
RRu	0.91	0.84		0.83
P	---	---		---
Random RR	0.82	0.70		0.73
RRl	0.64	0.61		0.65
RRu	1.05	0.81		0.82
P	N.S.	---		---
Between Chi				0.11
Between df				1
Between P				N.S.
Btwn(F) P				N.S.
Btwn(R) P				N.S.

Table 1F2 - 3

IESLC - Meta-analysis of Cigarette Smoking, ever Filter vs only Plain (or nearest available)
All LC types
Most adjusted

	Any proxy use No/nk	Yes	Total
N	36	6	42
NS	27	4	31
Wt	1424.05	111.38	1535.44
Het Chi	125.08	17.96	147.76
Het df	35	5	41
Het P	***	**	***
Fixed RR	0.81	0.65	0.79
RRl	0.77	0.54	0.75
RRu	0.85	0.78	0.83
P	---	---	---
Random RR	0.73	0.76	0.73
RRl	0.65	0.51	0.65
RRu	0.83	1.12	0.82
P	---	N.S.	---
Between Chi			4.73
Between df			1
Between P			*
Btwn(F) P			N.S.
Btwn(R) P			N.S.

	Full histological confirmation No	Yes	Total
N	29	13	42
NS	21	10	31
Wt	642.63	892.81	1535.44
Het Chi	66.20	75.97	147.76
Het df	28	12	41
Het P	***	***	***
Fixed RR	0.74	0.84	0.79
RRl	0.68	0.78	0.75
RRu	0.80	0.89	0.83
P	---	---	---
Random RR	0.75	0.70	0.73
RRl	0.65	0.57	0.65
RRu	0.86	0.87	0.82
P	---	--	---
Between Chi			5.59
Between df			1
Between P			*
Btwn(F) P			N.S.
Btwn(R) P			N.S.

	Number of adjustment variables (1) 0	1	2+/+nk	Total
N	12	3	27	42
NS	9	3	20	32
Wt	246.20	81.54	1207.70	1535.44
Het Chi	48.15	1.59	91.71	147.76
Het df	11	2	26	41
Het P	***	N.S.	***	***
Fixed RR	0.71	0.68	0.82	0.79
RRl	0.63	0.55	0.78	0.75
RRu	0.80	0.85	0.87	0.83
P	---	---	---	---
Random RR	0.64	0.68	0.76	0.73
RRl	0.46	0.55	0.66	0.65
RRu	0.90	0.85	0.87	0.82
P	-	---	---	---
Between Chi				6.31
Between df				2
Between P				*
Btwn(F) P				N.S.
Btwn(R) P				N.S.

Table 1F2 - 3

IESLC - Meta-analysis of Cigarette Smoking, ever Filter vs only Plain (or nearest available)
All LC types
Most adjusted

	Number of adjustment variables (2) 0	1	2	3-5	6+/+nk	Total
N	12	3	8	17	2	42
NS	9	3	5	13	2	32
Wt	246.20	81.54	333.82	838.29	35.59	1535.44
Het Chi	48.15	1.59	15.36	57.23	0.52	147.76
Het df	11	2	7	16	1	41
Het P	***	N.S.	*	***	N.S.	***
Fixed RR	0.71	0.68	0.68	0.89	0.69	0.79
RRl	0.63	0.55	0.61	0.83	0.50	0.75
RRu	0.80	0.85	0.76	0.95	0.96	0.83
P	---	---	---	---	-	---
Random RR	0.64	0.68	0.70	0.81	0.69	0.73
RRl	0.46	0.55	0.58	0.67	0.50	0.65
RRu	0.90	0.85	0.84	0.97	0.96	0.82
P	-	---	---	-	-	---
Between Chi						24.91
Between df						4
Between P						***
Btwn(F) P						N.S.
Btwn(R) P						N.S.

	Smoking status ever	current	Total
N	17	25	42
NS	14	17	31
Wt	754.40	781.03	1535.44
Het Chi	99.64	47.96	147.76
Het df	16	24	41
Het P	***	**	***
Fixed RR	0.80	0.79	0.79
RRl	0.75	0.73	0.75
RRu	0.86	0.84	0.83
P	---	---	---
Random RR	0.64	0.78	0.73
RRl	0.49	0.70	0.65
RRu	0.83	0.88	0.82
P	---	---	---
Between Chi			0.16
Between df			1
Between P			N.S.
Btwn(F) P			N.S.
Btwn(R) P			N.S.

	Product cig+/-ot	cig only	Total
N	29	13	42
NS	23	9	32
Wt	1235.73	299.70	1535.44
Het Chi	88.32	56.58	147.76
Het df	28	12	41
Het P	***	***	***
Fixed RR	0.81	0.73	0.79
RRl	0.77	0.65	0.75
RRu	0.86	0.81	0.83
P	---	---	---
Random RR	0.74	0.73	0.73
RRl	0.65	0.56	0.65
RRu	0.84	0.96	0.82
P	---	-	---
Between Chi			2.87
Between df			1
Between P			(*)
Btwn(F) P			N.S.
Btwn(R) P			N.S.

Table 1F2 - 3

IESLC - Meta-analysis of Cigarette Smoking, ever Filter vs only Plain (or nearest available)
All LC types
Most adjusted

	Cigarette type						
	only f	always f	mainly f	equl p&f	both p&f	ever f	Total
N	17	3	1			21	42
NS	14	3	1			13	31
Wt	475.64	84.31	8.86			966.62	1535.44
Het Chi	49.79	2.46	0.00			78.38	147.76
Het df	16	2	0			20	41
Het P	***	N.S.	N.S.			***	***
Fixed RR	0.70	0.68	1.25			0.85	0.79
RRl	0.64	0.55	0.65			0.80	0.75
RRu	0.76	0.85	2.41			0.91	0.83
P	---	---	N.S.			---	---
Random RR	0.70	0.67	1.25			0.77	0.73
RRl	0.58	0.53	0.65			0.65	0.65
RRu	0.84	0.86	2.41			0.91	0.82
P	---	--	N.S.			--	---
Between Chi							17.15
Between df							3
Between P							***
Btwn(F) P							N.S.
Btwn(R) P							N.S.

	Denominator				
	ever p	mainly p	p NOS	always p	Total
N	5	2	15	20	42
NS	5	2	12	12	31
Wt	115.42	165.05	304.77	950.20	1535.44
Het Chi	19.72	3.42	23.90	75.76	147.76
Het df	4	1	14	19	41
Het P	***	(*)	*	***	***
Fixed RR	0.54	0.68	0.81	0.85	0.79
RRl	0.45	0.59	0.72	0.80	0.75
RRu	0.65	0.80	0.90	0.90	0.83
P	---	---	---	---	---
Random RR	0.49	0.84	0.80	0.75	0.73
RRl	0.30	0.46	0.68	0.63	0.65
RRu	0.77	1.53	0.94	0.89	0.82
P	--	N.S.	--	--	---
Between Chi					24.96
Between df					3
Between P					***
Btwn(F) P					(*)
Btwn(R) P					N.S.

	Derivation of RR/CI			
	Orig	StdCalc	Other	Total
N	5	15	22	42
NS	3	13	17	33
Wt	127.58	431.61	976.25	1535.44
Het Chi	1.83	54.02	79.99	147.76
Het df	4	14	21	41
Het P	N.S.	***	***	***
Fixed RR	0.90	0.69	0.83	0.79
RRl	0.76	0.63	0.78	0.75
RRu	1.07	0.76	0.88	0.83
P	N.S.	---	---	---
Random RR	0.90	0.68	0.74	0.73
RRl	0.76	0.53	0.63	0.65
RRu	1.07	0.86	0.86	0.82
P	N.S.	--	---	---
Between Chi				11.93
Between df				2
Between P				**
Btwn(F) P				N.S.
Btwn(R) P				N.S.

Table 1F2 - 4

IESLC - Meta-analysis of Cigarette Smoking, ever Filter vs only Plain (or nearest available)
All LC types
Least adjusted

REF	NRR	X	SEX	AGEL	AGEH	RACE	YF	LC TYPE	LOC	START	ST	NLC	R	VB	P	H	AD	SM	PRODUCT	CIGTYP	DENOM	De
AGUDO	18	x	f	0	0	all	-	all	Eu:wst	1989	CC	103	n	bl	n	n	0	ev	cig only	only f	ever p	st
ALDERS	170	x	m	0	0	all	-	all	Eu:UK	1977	CC	1448	n	V	n	n	0	cu	cig+/-ot	ever f	always p	st
ALDERS	162	x	f	0	0	all	-	all	Eu:UK	1977	CC	1448	n	V	n	n	0	cu	cig only	ever f	always p	st
ARMADA	21	x	m	0	0	all	-	all	Eu:wst	1986	CC	325	n	bl	n	y	0	ev	cig+/-ot	ever f	always p	st
BECHER	9	x	m	0	0	all	-	all	Eu:Ger	1985	CC	194	n	bl	n	y	0	ev	cig+/-ot	always f	ever p	st
BROSS	14		m	0	0	wh	-	all	NAmer	1960	CC	974	n	bl	n	n	0	cu	cig+/-ot	only f	p NOS	st
BUFFLE	56		f	0	0	w-hi	-	all	NAmer	1976	CC	943	n	bl	y	n	0	ev	cig+/-ot	ever f	p NOS	st
CHOI	40		m	0	0	all	-	all	As:oth	1985	CC	375	n	bl	n	n	0	ev	cig+/-ot	ever f	always p	st
CHOI	44		f	0	0	all	-	all	As:oth	1985	CC	375	n	bl	n	n	0	ev	cig+/-ot	ever f	always p	ot
CORREA	65		c	0	0	all	-	all	NAmer	1979	CC	1359	n	bl	y	n	2	ev	cig+/-ot	only f	p NOS	ot
CPSII	138		f	0	0	all	4	all	NAmer	1982	pr	3229	n	bl	n	n	2	cu	cig+/-ot	only f	mainly p	st
DEAN2	36		m	0	0	all	-	all	Eu:UK	1960	CC	954	n	V	y	n	0	ev	cig+/-ot	ever f	always p	st
DEAN2	42		f	0	0	all	-	all	Eu:UK	1960	CC	954	n	V	y	n	0	ev	cig+/-ot	ever f	always p	st
DEAN3	167	x	m	0	0	all	-	all	Eu:UK	1969	CC	766	n	V	y	n	0	cu	cig only	ever f	always p	st
DEAN3	171	x	f	0	0	all	-	all	Eu:UK	1969	CC	766	n	V	y	n	0	cu	cig only	ever f	always p	st
DESTE2	22	x	c	0	0	all	-	all	SCAmer	1993	CC	463	n	bl	n	n	0	ev	cig+/-ot	always f	ever p	st
DESTEF	52	x	m	0	0	all	-	all	SCAmer	1988	CC	497	n	bl	n	y	0	ev	cig+/-ot	always f	ever p	st
DOLL	47		m	0	0	all	-	all	Eu:UK	1948	CC	1465	n	V	n	n	0	ev	cig+/-ot	ever f	always p	st
ENGELA	116		m	0	0	all	0	all	Eu:Sca	1964	pr	435	n	bl	n	n	0	cu	cig+/-ot	ever f	always p	st
ENGELA	123	x	f	0	0	all	0	all	Eu:Sca	1964	pr	435	n	bl	n	n	0	cu	cig+/-ot	ever f	always p	st
KAISE2	55		m	30	89	all	9	all	NAmer	1979	pr	318	n	bl	n	n	5	cu	cig only	only f	p NOS	or
KAISE2	56		f	30	89	all	9	all	NAmer	1979	pr	318	n	bl	n	n	5	cu	cig only	only f	p NOS	or
KHUDER	7		m	0	0	all	-	all	NAmer	1985	CC	482	n	bl	n	y	0	ev	cig+/-ot	ever f	always p	st
LANGE	42	x	m	0	0	all	0	all	Eu:Sca	1976	pr	268	n	bl	n	n	0	cu	cig only	only f	p NOS	st
LANGE	41	x	f	0	0	all	0	all	Eu:Sca	1976	pr	268	n	bl	n	n	0	cu	cig only	only f	p NOS	st
LUBIN2	121	x	m	0	0	all	-	all	Eu:mul	1976	CC	7804	n	bl	n	y	0	ev	cig+/-ot	ever f	always p	st
LUBIN2	125	x	f	0	0	all	-	all	Eu:mul	1976	CC	7804	n	bl	n	y	0	ev	cig+/-ot	ever f	always p	st
MACLEN	85		f	0	0	ch	-	all	As:oth	1972	CC	233	n	bl	n	n	0	cu	cig+/-ot	ever f	always p	ot
MATOS	72	x	m	0	0	all	-	all	SCAmer	1994	CC	200	n	bl	n	n	0	ev	cig+/-ot	mainly f	mainly p	st
MIGRAN	99	x	m	0	0	all	0	all	Eu:UK	1964	pr	259	n	V	n	n	0	cu	cig only	only f	p NOS	st
MIGRAN	103	x	f	0	0	all	0	all	Eu:UK	1964	pr	259	n	V	n	n	0	cu	cig only	only f	p NOS	st
MRFITR	7		m	0	0	all	0	all	NAmer	1973	pr	119	n	bl	n	n	9	cu	cig+/-ot	only f	p NOS	ot
PEZZOT	23	x	m	0	0	all	-	all	SCAmer	1987	CC	215	n	bl	n	y	2	ev	cig only	only f	ever p	ot
RIMING	4	x	m	0	0	all	0	all	Eu:UK	1970	pr	104	n	V	n	n	0	cu	cig only	only f	p NOS	st
SEGI2	51	x	m	0	0	all	-	all	As:Jap	1962	CC	378	n	bl	n	n	0	cu	cig+/-ot	only f	p NOS	st
SOBUE	122	x	m	0	0	all	-	q+s+l+a	As:Jap	1986	CC	1376	n	bl	n	y	0	cu	cig+/-ot	only f	p NOS	st
TANG2	1		m	0	0	all	0	all	Eu:UK	1967	pr	836	n	V	n	n	3	cu	cig only	only f	p NOS	or
WAKAI	63	x	m	0	0	all	-	all	As:Jap	1988	CC	333	n	bl	n	y	0	cu	cig+/-ot	only f	p NOS	st
WYNDE5	3		m	0	0	all	-	all	NAmer	1969	CC	1365	n	bl	n	y	0	cu	cig+/-ot	ever f	always p	st
WYNDE5	6		f	0	0	all	-	all	NAmer	1969	CC	1365	n	bl	n	y	0	cu	cig+/-ot	ever f	always p	st
WYNDE6	417	x	m	0	0	all	-	q+a	NAmer	1969	CC	4423	n	bl	n	y	0	cu	cig+/-ot	ever f	always p	st
WYNDE6	420	x	f	0	0	all	-	q+a	NAmer	1969	CC	4423	n	bl	n	y	0	cu	cig+/-ot	ever f	always p	st

Table 1F2 - 5

IESLC - Meta-analysis of Cigarette Smoking, ever Filter vs only Plain (or nearest available)
All LC types
Least adjusted

REF	NRR	SEX	AD	Number Exposed Case	Number Exposed Cont	Non-exposed Case	Non-exposed Cont	RR	95.00%CI
AGUDO	18	f	0	14	21	8	2	0.17	(0.03- 0.90)
ALDERS	170	m	0	341	225	178	97	0.83	(0.61- 1.11)
ALDERS	162	f	0	348	192	62	37	1.08	(0.69- 1.69)
Subtotal ALDERS								0.90	(0.70- 1.15)
ARMADA	21	m	0	254	199	63	55	1.11	(0.74- 1.67)
BECHER	9	m	0	102	196	35	21	0.31	(0.17- 0.56)
BROSS	14	m	0	65	76	200	138	0.59	(0.40- 0.88)
BUFFLE	56	f	0	193	123	47	38	1.27	(0.78- 2.06)
CHOI	40	m	0	254	463	15	2	0.07	(0.02- 0.32)
CHOI	44	f	0	18	26	1	0	0.23~	(0.01- 6.03)
Subtotal CHOI								0.09	(0.02- 0.34)
CORREA	65	c	2	-	-	-	-	0.55	(0.35- 0.85)
*CPSII	138	f	2	-	-	-	-	0.66	(0.57- 0.78)
DEAN2	36	m	0	20	17	644	529	0.97	(0.50- 1.86)
DEAN2	42	f	0	12	2	50	26	3.12	(0.65- 15.00)
Subtotal DEAN2								1.15	(0.63- 2.11)
DEAN3	167	m	0	78	407	194	322	0.32	(0.24- 0.43)
DEAN3	171	f	0	55	646	27	170	0.54	(0.33- 0.88)
Subtotal DEAN3								0.37	(0.28- 0.47)
DESTE2	22	c	0	158	128	142	84	0.73	(0.51- 1.04)
DESTEF	52	m	0	178	156	292	178	0.70	(0.52- 0.92)
DOLL	47	m	0	3	15	501	452	0.18	(0.05- 0.63)
*ENGELA	116	m	0	7	6306	38	15055	0.44	(0.20- 0.98)
*ENGELA	123	f	0	13	24819	11	13521	0.64	(0.29- 1.44)
Subtotal ENGELA								0.53	(0.30- 0.94)
*KAISE2	55	m	5	-	-	-	-	1.03	(0.61- 1.75)
*KAISE2	56	f	5	-	-	-	-	0.65	(0.32- 1.31)
Subtotal KAISE2								0.87	(0.57- 1.33)
KHUDER	7	m	0	173	440	284	334	0.46	(0.36- 0.59)
*LANGE	42	m	0	25	953	65	1642	0.66	(0.42- 1.04)
*LANGE	41	f	0	19	2269	20	1344	0.56	(0.30- 1.05)
Subtotal LANGE								0.63	(0.43- 0.90)
LUBIN2	121	m	0	4889	7446	1737	2988	1.13	(1.05- 1.21)
LUBIN2	125	f	0	495	514	56	53	0.91	(0.61- 1.35)
Subtotal LUBIN2								1.12	(1.05- 1.20)
MACLEN	85	f	0	15	13	21	14	0.77	(0.28- 2.10)
MATOS	72	m	0	163	229	22	46	1.49	(0.86- 2.57)
*MIGRAN	99	m	0	47	1326	57	1630	1.01	(0.69- 1.48)
*MIGRAN	103	f	0	15	1773	8	871	0.92	(0.39- 2.16)
Subtotal MIGRAN								1.00	(0.71- 1.41)
*MRFITR	7	m	9	-	-	-	-	0.53	(0.24- 1.17)
PEZZOT	23	m	2	-	-	-	-	0.23	(0.16- 0.34)
*RIMING	4	m	0	45	3045	59	2393	0.60	(0.41- 0.88)
SEGI2	51	m	0	105	250	135	203	0.63	(0.46- 0.87)
SOBUE	122	m	0	560	540	49	26	0.55	(0.34- 0.90)
*TANG2	1	m	3	-	-	-	-	0.94	(0.75- 1.18)
WAKAI	63	m	0	174	271	5	9	1.16	(0.38- 3.51)
WYNDE5	3	m	0	417	629	273	398	0.97	(0.79- 1.18)
WYNDE5	6	f	0	152	200	34	30	0.67	(0.39- 1.14)
Subtotal WYNDE5								0.92	(0.77- 1.11)
WYNDE6	417	m	0	1148	711	294	165	0.91	(0.73- 1.12)
WYNDE6	420	f	0	785	437	65	30	0.83	(0.53- 1.30)
Subtotal WYNDE6								0.89	(0.74- 1.08)
Partial Totals				11340	55063	5692	42903		

*prospective study

~ With 0.5 adjustment for zero

Table 1F2 - 5

IESLC - Meta-analysis of Cigarette Smoking, ever Filter vs only Plain (or nearest available)
All LC types
Least adjusted

REF	NRR	SEX	AD	Ys	Ws	Qs	Ps
AGUDO	18	f	0	-1.79	1.34	3.62	0.0378
ALDERS	170	m	0	-0.19	42.91	0.07	0.2102
ALDERS	162	f	0	0.08	19.52	1.03	0.7288
Subtotal ALDERS				-0.11	62.43	1.10	
ARMADA	21	m	0	0.11	23.25	1.57	0.6018
BECHER	9	m	0	-1.16	10.98	11.25	0.0001
BROSS	14	m	0	-0.53	24.52	3.46	0.0090
BUFFLE	56	f	0	0.24	16.42	2.49	0.3350
CHOI	40	m	0	-2.62	1.75	10.60	0.0005
CHOI	44	f	0	-1.46	0.36	0.62	0.3800
Subtotal CHOI				-2.42	2.11	11.22	
CORREA	65	c	2	-0.60	19.52	3.89	0.0083
*CPSII	138	f	2	-0.42	156.19	10.88	0.0000
DEAN2	36	m	0	-0.03	8.91	0.12	0.9187
DEAN2	42	f	0	1.14	1.56	2.59	0.1555
Subtotal DEAN2				0.14	10.47	2.71	
DEAN3	167	m	0	-1.15	42.48	41.96	0.0000
DEAN3	171	f	0	-0.62	15.96	3.55	0.0127
Subtotal DEAN3				-1.00	58.45	45.51	
DESTE2	22	c	0	-0.31	30.22	0.80	0.0839
DESTEF	52	m	0	-0.36	47.46	2.12	0.0124
DOLL	47	m	0	-1.71	2.47	6.03	0.0071
*ENGELA	116	m	0	-0.82	5.92	2.66	0.0457
*ENGELA	123	f	0	-0.44	5.96	0.50	0.2823
Subtotal ENGELA				-0.63	11.88	3.15	
*KAISE2	55	m	5	0.03	13.83	0.45	0.9125
*KAISE2	56	f	5	-0.43	7.73	0.60	0.2309
Subtotal KAISE2				-0.14	21.57	1.06	
KHUDER	7	m	0	-0.77	68.64	26.36	0.0000
*LANGE	42	m	0	-0.41	18.61	1.26	0.0759
*LANGE	41	f	0	-0.57	9.86	1.77	0.0710
Subtotal LANGE				-0.47	28.47	3.02	
LUBIN2	121	m	0	0.12	800.50	59.83	0.0006
LUBIN2	125	f	0	-0.09	24.58	0.09	0.6458
Subtotal LUBIN2				0.12	825.08	59.91	
MACLEN	85	f	0	-0.26	3.81	0.05	0.6087
MATOS	72	m	0	0.40	12.87	3.88	0.1537
*MIGRAN	99	m	0	0.01	26.70	0.73	0.9443
*MIGRAN	103	f	0	-0.08	5.26	0.03	0.8504
Subtotal MIGRAN				-0.00	31.96	0.75	
*MRFITR	7	m	9	-0.63	6.12	1.43	0.1162
PEZZOT	23	m	2	-1.47	27.04	46.98	0.0000
*RIMING	4	m	0	-0.51	26.02	3.38	0.0090
SEGI2	51	m	0	-0.46	38.67	3.67	0.0043
SOBUE	122	m	0	-0.60	16.00	3.18	0.0169
*TANG2	1	m	3	-0.06	74.81	0.60	0.5925
WAKAI	63	m	0	0.14	3.12	0.27	0.7982
WYNDE5	3	m	0	-0.03	98.39	1.36	0.7354
WYNDE5	6	f	0	-0.40	13.45	0.83	0.1427
Subtotal WYNDE5				-0.08	111.85	2.19	
WYNDE6	417	m	0	-0.10	85.18	0.24	0.3632
WYNDE6	420	f	0	-0.19	19.13	0.02	0.4123
Subtotal WYNDE6				-0.11	104.31	0.26	

Table 1F2 - 5

IESLC - Meta-analysis of Cigarette Smoking, ever Filter vs only Plain (or nearest available)
All LC types
Least adjusted

	N	42
	NS	31
	Wt	1878.05
Het	Chi	266.80
Het	df	41
Het	P	***
Fixed	RR	0.86
	RRl	0.82
	RRu	0.90
	P	---
Random	RR	0.69
	RRl	0.60
	RRu	0.80
	P	---
Asymm	P	***

Table 1F2 - 6

IESLC - Meta-analysis of Cigarette Smoking, ever Filter vs only Plain (or nearest available)
All LC types
Least adjusted

	combined	Sex male	female	Total
N	2	25	15	42
NS	2	25	15	42
Wt	49.74	1527.17	301.13	1878.05
Het Chi	0.95	230.87	20.61	266.80
Het df	1	24	14	41
Het P	N.S.	***	N.S.	***
Fixed RR	0.65	0.90	0.73	0.86
RRl	0.49	0.85	0.65	0.82
RRu	0.86	0.94	0.82	0.90
P	--	---	---	---
Random RR	0.65	0.66	0.77	0.69
RRl	0.49	0.54	0.64	0.60
RRu	0.86	0.80	0.91	0.80
P	--	---	--	---
Between Chi				14.36
Between df				2
Between P				***
Btwn(F) P				N.S.
Btwn(R) P				N.S.

Table 1F2 - 7

IESLC - Meta-analysis of Cigarette Smoking, ever Filter vs only Plain (or nearest available)

All LC types

Excluded studies (and stage at which they were excluded)

1	BOUCHA BUELL LAURIL MZILEN
2	BERRIN CHEN RESTRE TAO ULMER
3	ABELIN ABRAHA AKIBA AMANDU AMES ANDERS ARCHER AUSTIN AUVINE AXELSO AXELSS BAND BARBON BENSHL BEST BLOHMK BLOT1 BLOT2 BLOT3 BLOT4 BOFFET BOUCOT BRESLO BRETT BROCKM BROWN1 BROWN2 BYERS1 BYERS2 CARPEN CASCO2 CASCOR CEDERL CHAN CHANG CHATZI CHEN2 CHEN3 CHIAZZ CHOW CHYOU COMSTO COOKSO CPSI DAMBER DARBY DAVEYS DEAN DEKLER DOCKER DOLL2 DORANT DORGAN DORN DOSEME DROSTE DU DUNN EBELIN ENSTRO ESAKI FAN GAO GAO2 GARCIA GARDIN GARSHI GENG GER GILLIS GODLEY GOLLED GOODMA GRAHAM GREGOR GSELL HAENSZ HAMMO2 HAMMON HANSEN HEGMAN HEIN HENNEK HINDS HIRAOK HIRAY2 HIRAYA HITOSU HOLE HOROWI HORWIT HU HU2 HUANG HUMBLE ISHIMA JAHN JAIN JARUP JARVHO JEDRYC JIANG JOLY JONES JUSSAW KAISER KANELL KATSOU KAUFMA KELLER KIHARA KINLEN KJUUS KNEKT KO KOHLME KOO KOULUM KREUZE KREYBE KUBIK LAMTH LAMWK LAMWK2 LAUSSM LEI LEMARC LETOUR LEVIN LIAW LICKIN LIDDEL LIU LIU2 LIU3 LIU4 LIU5 LOMBA2 LOMBAR LUBIN LUO MAGNUS MARSH MARSH2 MARTIS MASTRA MATSUD MCCONN MCDUFF MCLAUG MILLER MILLS MOLLO MRFIT MURATA NAM NOTAN2 NOTANI NOU ODRISC ORMOS OSANN OSANN2 PARKIN PASTOR PAWLEG PERNU PERSH2 PERSHA PETO PEZZO2 PIKE PISANI POFFIJ POLEDN PRESCO QIAO QIAO2 RACHTA RADZIK RANDIG REN RONCO ROOTS ROTHSC SAARIK SADOWS SANKAR SCHWA2 SCHWAR SEGI SEOW SHAW SHIMIZ SIEMIA SIMARA SIMONA SITAS SOBUE2 SPEIZE SPITZ STASZE STAYNE STOCKS STOCKW STUCKE SUN SUZUK2 SUZUKI SVENSS TANG TENKAN TIZZAN TOKARS TOUSEY TSUGAN TULINI TVERDA VANDER VEIERO VUTUC WALD WANG WANG2 WANG3 WANG4 WARSIN WATSON WICKLU WIGLE WILKIN WU WU2 WUNSCH WUWILL WYNDE2 WYNDE4 WYNDE7 WYNDE8 WYNDER XIANGZ XU XU2 XU3 XU4 YAMAGU YONG YUAN ZHANG ZHENG ZHOU
7	WYNDE3
10	BENHAM

Table 1F2 - 8

Potentially overlapping studies

REF	REFGP	PRINC	.	OVERLAP/LINK
LUBIN2	LUBIN2	1		Lubin-combined
LANGE	PRESCO	1		Subset of PRESCO
BROSS	BYERS1	1		GRAHAM/BROSS/BYERS1
TANG2	TANG2	2		TANG2-combined
MRFITR	MRFIT	2		Subset of MRFIT
WYNDE5	WYNDE6	2		WYNDE5/6/7/8
WYNDE6	WYNDE6	1		WYNDE5/6/7/8

Table 1F2 - 9

Most adjusted - insufficient data for metaanalysis

REF	NRR	SEX	AGEL	AGEH	RACE	YF	LC TYPE	LOC	START	ST	NLC	R	VB	P	H	AD	SM	PRODUCT	CIGTYP	DENOM	De
BUFFLE	89	m	0	0	wh	-	all	NAmer	1976	CC	943	n	bl	y	n	0	ev	cig+/-ot	both p&f	p NOS	st
CPSII	134	m	0	0	all	6	all	NAmer	1982	pr	3229	n	bl	n	n	1	cu	cig only	both p&f	mainly p	ot
CPSII	136	f	0	0	all	6	all	NAmer	1982	pr	3229	n	bl	n	n	1	cu	cig+/-ot	both p&f	mainly p	ot
HIRAYA	155	c	0	0	all	16	all	As:Jap	1965	pr	1917	n	bl	n	n	1	ev	cig+/-ot	only f	p NOS	ot
HUMBLE	53	c	0	0	w-hi	-	not alv	NAmer	1980	CC	521	n	bl	y	n	5	cu	cig+/-ot	always f	always p	or
HUMBLE	54	c	0	0	hi	-	not alv	NAmer	1980	CC	521	n	bl	y	n	5	cu	cig+/-ot	always f	always p	or
JOLY	56	m	0	0	all	-	all	SCAmer	1978	CC	826	n	bl	n	n	0	ev	cig+/-ot	ever f	ever p	or
JOLY	57	f	0	0	all	-	all	SCAmer	1978	CC	826	n	bl	n	n	0	ev	cig+/-ot	ever f	ever p	or

REF	NRR	RR	SIG	RRDATA comment
BUFFLE	89	1.20		0
CPSII	134	0.79		0
CPSII	136	0.71		0
HIRAYA	155	0.51		0
HUMBLE	53	0.80		0
HUMBLE	54	0.04		0
JOLY	56	*		No significant difference was noted between cases and controls with respect to use of filter cigarettes.
JOLY	57	*		No significant difference was noted between cases and controls with respect to use of filter cigarettes.

Table 1F3 -

IESLC - Meta-analysis of Cigarette Smoking, only Filter vs ever Plain (or nearest available)
All LC types

This analysis is restricted to results for:
1) Non-dose-response data
2) Results complete enough for use in metaanalysis

Within each study, results are then selected (in the following order of preference, within each sex) for:
3) CIGTYP: filter only/NOS, always, mainly, both, equally, ever
4) DENOM: plain ever, mainly, always, only/NOS
5) PRODUCT: cigarettes regardless of other products, cigarettes only (Note only study ALDERS has both product definitions available)
6) SMKSTA: ever, current (Note only study MATOS has both ever and current available)
7) LCtype: all or nearest available, at least Squamous and Adeno. (q = squamous, s = small, l = large, a = adeno, mix = mixed, alv = alveolar)
8) Race: all or nearest available, otherwise by race (wh or w = white, bl or b = black, hi = hispanic ch = chinese, jap = japanese, haw = hawaiian, w+o = white + oriental, sca = scandinavian, as = asian)
9) Followup period (YF, prospective studies): whole study (coded as 0) or longest available
10) For overlapping studies: principal rather than subsidiary studies
Finally by Age: whole study (coded as 0) if available, otherwise by widest available age group
and then for single sex results (m, f) in preference to combined sex results (c).

Results adjusted (AD) for the most potential confounders are then chosen in Sections -1 to -3
(and those which actually differ from the adjusted results in Table 1F1 - 1 are marked 'x' in Section -1)
and results adjusted for the least confounders in Sections -4 to -6. (Those least adjusted results which
actually differ from the most adjusted as marked 'x' in column X in Section -4)
(Results adjusted for an unknown number of confounder(s) are coded as 20.)

Section -7 shows excluded studies, together with the stage (as above) at which no qualifying
results were found.

Section -8 lists the potentially overlapping studies which have been included (1=principal, 2=subsidiary).

Section -9 lists any results which would have been included in preference except that they had data not complete
enough for use in meta-analysis, with their significance (yes/no), if known, and any further comment as entered
on the database.

In addition to those mentioned above, the following fields, levels and abbreviations are used:
* or nk = not known, n = no, y = yes, ot = other
ev = ever, cu = current, cig+/-ot = cigarettes irrespective of other products (cigar, pipe etc)
f = filter, p = plain, NOS = not otherwise specified
REF: 6-character study reference
NRR: number of the RR on the database within the study
ST : study type (CC = case control, pr or prosp = prospective)
NLC: number of lung cancer cases in whole study
R : risky occupational population (n = no, m = mining, o = other risky)
VB : national cigarette type (V = at least 75% Virginia, bl = at least 75% blended, ot = other)
P : any proxy use
H : full histological confirmation
De : derivation of RR/CI (or = original, st = standard method, ot = other method of estimation)

Table 1F3 - 1

IESLC - Meta-analysis of Cigarette Smoking, only Filter vs ever Plain (or nearest available)
All LC types
Most adjusted

REF	NRR	1F1	SEX	AGEL	AGEH	RACE	YF	LC TYPE	LOC	START	ST	NLC	R	VB	P	H	AD	SM	PRODUCT	CIGTYP	DENOM	De
AGUDO	17		f	0	0	all	-	all	Eu:wst	1989	CC	103	n	bl	n	n	3	ev	cig only	only f	ever p	ot
ALDERS	173	x	m	0	0	all	-	all	Eu:UK	1977	CC	1448	n	V	n	n	2	cu	cig+/-ot	always f	ever p	ot
ALDERS	167	x	f	0	0	all	-	all	Eu:UK	1977	CC	1448	n	V	n	n	2	cu	cig only	always f	ever p	ot
ARMADA	22	x	m	0	0	all	-	all	Eu:wst	1986	CC	325	n	bl	n	y	1	ev	cig+/-ot	always f	ever p	ot
BECHER	10		m	0	0	all	-	all	Eu:Ger	1985	CC	194	n	bl	n	y	3	ev	cig+/-ot	always f	ever p	st
BROSS	14		m	0	0	wh	-	all	NAmer	1960	CC	974	n	bl	n	n	0	cu	cig+/-ot	only f	p NOS	st
BUFFLE	57	x	f	0	0	w-hi	-	all	NAmer	1976	CC	943	n	bl	y	n	0	ev	cig+/-ot	only f	ever p	st
CHOI	41	x	m	0	0	all	-	all	As:oth	1985	CC	375	n	bl	n	n	0	ev	cig+/-ot	only f	ever p	st
CHOI	45	x	f	0	0	all	-	all	As:oth	1985	CC	375	n	bl	n	n	0	ev	cig+/-ot	only f	ever p	st
CORREA	65		c	0	0	all	-	all	NAmer	1979	CC	1359	n	bl	y	n	2	ev	cig+/-ot	only f	p NOS	ot
CPSII	138		f	0	0	all	4	all	NAmer	1982	pr	3229	n	bl	n	n	2	cu	cig+/-ot	only f	mainly p	st
DEAN2	36		m	0	0	all	-	all	Eu:UK	1960	CC	954	n	V	y	n	0	ev	cig+/-ot	ever f	always p	st
DEAN2	42		f	0	0	all	-	all	Eu:UK	1960	CC	954	n	V	y	n	0	ev	cig+/-ot	ever f	always p	st
DEAN3	156		m	0	0	all	-	all	Eu:UK	1969	CC	766	n	V	y	n	2	cu	cig only	only f	p NOS	or
DEAN3	162		f	0	0	all	-	all	Eu:UK	1969	CC	766	n	V	y	n	2	cu	cig only	only f	p NOS	or
DESTE2	23		c	0	0	all	-	all	SCAmer	1993	CC	463	n	bl	n	n	7	ev	cig+/-ot	always f	ever p	ot
DESTEF	53		m	0	0	all	-	all	SCAmer	1988	CC	497	n	bl	n	y	4	ev	cig+/-ot	always f	ever p	ot
DOLL	47		m	0	0	all	-	all	Eu:UK	1948	CC	1465	n	V	n	n	0	ev	cig+/-ot	ever f	always p	st
ENGELA	115	x	m	0	0	all	0	all	Eu:Sca	1964	pr	435	n	bl	n	n	0	cu	cig+/-ot	only f	ever p	st
ENGELA	199	x	f	0	0	all	0	all	Eu:Sca	1964	pr	435	n	bl	n	n	5	cu	cig+/-ot	only f	ever p	ot
KAISE2	55		m	30	89	all	9	all	NAmer	1979	pr	318	n	bl	n	n	5	cu	cig only	only f	p NOS	or
KAISE2	56		f	30	89	all	9	all	NAmer	1979	pr	318	n	bl	n	n	5	cu	cig only	only f	p NOS	or
KHUDER	7		m	0	0	all	-	all	NAmer	1985	CC	482	n	bl	n	y	0	ev	cig+/-ot	ever f	always p	st
LANGE	20		m	0	0	all	0	all	Eu:Sca	1976	pr	268	n	bl	n	n	2	cu	cig only	only f	p NOS	or
LANGE	19		f	0	0	all	0	all	Eu:Sca	1976	pr	268	n	bl	n	n	2	cu	cig only	only f	p NOS	or
LUBIN2	110	x	m	0	0	all	-	all	Eu:mul	1976	CC	7804	n	bl	n	y	3	ev	cig+/-ot	only f	ever p	ot
LUBIN2	118	x	f	0	0	all	-	all	Eu:mul	1976	CC	7804	n	bl	n	y	3	ev	cig+/-ot	only f	ever p	ot
MACLEN	85		f	0	0	ch	-	all	As:oth	1972	CC	233	n	bl	n	n	0	cu	cig+/-ot	ever f	always p	ot
MATOS	37		m	0	0	all	-	all	SCAmer	1994	CC	200	n	bl	n	n	4	ev	cig+/-ot	mainly f	mainly p	st
MIGRAN	100		m	0	0	all	0	all	Eu:UK	1964	pr	259	n	V	n	n	3	cu	cig only	only f	p NOS	ot
MIGRAN	104		f	0	0	all	0	all	Eu:UK	1964	pr	259	n	V	n	n	3	cu	cig only	only f	p NOS	ot
MRFITR	7		m	0	0	all	0	all	NAmer	1973	pr	119	n	bl	n	n	9	cu	cig+/-ot	only f	p NOS	ot
PEZZOT	24		m	0	0	all	-	all	SCAmer	1987	CC	215	n	bl	n	y	4	ev	cig only	only f	ever p	ot
RIMING	8		m	0	0	all	0	all	Eu:UK	1970	pr	104	n	V	n	n	1	cu	cig only	only f	p NOS	ot
SEGI2	52		m	0	0	all	-	all	As:Jap	1962	CC	378	n	bl	n	n	1	cu	cig+/-ot	only f	p NOS	ot
SOBUE	125		m	0	0	all	-	q+s+l+a	As:Jap	1986	CC	1376	n	bl	n	y	5	cu	cig+/-ot	only f	p NOS	st
TANG2	1		m	0	0	all	0	all	Eu:UK	1967	pr	836	n	V	n	n	3	cu	cig only	only f	p NOS	or
WAKAI	64		m	0	0	all	-	all	As:Jap	1988	CC	333	n	bl	n	y	5	cu	cig+/-ot	only f	p NOS	st
WYNDE5	3		m	0	0	all	-	all	NAmer	1969	CC	1365	n	bl	n	y	0	cu	cig+/-ot	ever f	always p	st
WYNDE5	6		f	0	0	all	-	all	NAmer	1969	CC	1365	n	bl	n	y	0	cu	cig+/-ot	ever f	always p	st
WYNDE6	422	x	m	0	0	all	-	q+a	NAmer	1969	CC	4423	n	bl	n	y	3	cu	cig+/-ot	only f	ever p	ot
WYNDE6	425	x	f	0	0	all	-	q+a	NAmer	1969	CC	4423	n	bl	n	y	3	cu	cig+/-ot	only f	ever p	ot

Table 1F3 - 2

IESLC - Meta-analysis of Cigarette Smoking, only Filter vs ever Plain (or nearest available)
All LC types
Most adjusted

REF	NRR	SEX	AD	Number Exposed Case	Number Exposed Cont	Non-exposed Case	Non-exposed Cont	RR	95.00%CI
AGUDO	17	f	3	-	-	-	-	0.22	(0.04- 1.27)
ALDERS	173	m	2	-	-	-	-	1.03	(0.67- 1.59)
ALDERS	167	f	2	-	-	-	-	0.66	(0.47- 0.92)
Subtotal ALDERS								0.78	(0.60- 1.02)
ARMADA	22	m	1	-	-	-	-	0.70	(0.45- 1.08)
BECHER	10	m	3	-	-	-	-	0.41	(0.21- 0.81)
BROSS	14	m	0	65	76	200	138	0.59	(0.40- 0.88)
BUFFLE	57	f	0	129	78	111	83	1.24	(0.83- 1.84)
CHOI	41	m	0	43	103	226	362	0.67	(0.45- 0.99)
CHOI	45	f	0	5	11	14	15	0.49	(0.13- 1.76)
Subtotal CHOI								0.65	(0.45- 0.95)
CORREA	65	c	2	-	-	-	-	0.55	(0.35- 0.85)
*CPSII	138	f	2	-	-	-	-	0.66	(0.57- 0.78)
DEAN2	36	m	0	20	17	644	529	0.97	(0.50- 1.86)
DEAN2	42	f	0	12	2	50	26	3.12	(0.65- 15.00)
Subtotal DEAN2								1.15	(0.63- 2.11)
DEAN3	156	m	2	-	-	-	-	0.54	(0.40- 0.73)
DEAN3	162	f	2	-	-	-	-	0.68	(0.42- 1.11)
Subtotal DEAN3								0.58	(0.45- 0.74)
DESTE2	23	c	7	-	-	-	-	0.73	(0.51- 1.05)
DESTEF	53	m	4	-	-	-	-	0.72	(0.54- 0.96)
DOLL	47	m	0	3	15	501	452	0.18	(0.05- 0.63)
*ENGELA	115	m	0	7	5257	38	16104	0.56	(0.25- 1.26)
*ENGELA	199	f	5	-	-	-	-	0.84	(0.39- 1.83)
Subtotal ENGELA								0.69	(0.40- 1.21)
*KAISE2	55	m	5	-	-	-	-	1.03	(0.61- 1.75)
*KAISE2	56	f	5	-	-	-	-	0.65	(0.32- 1.31)
Subtotal KAISE2								0.87	(0.57- 1.33)
KHUDER	7	m	0	173	440	284	334	0.46	(0.36- 0.59)
*LANGE	20	m	2	-	-	-	-	0.90	(0.60- 1.40)
*LANGE	19	f	2	-	-	-	-	0.70	(0.40- 1.40)
Subtotal LANGE								0.83	(0.59- 1.18)
LUBIN2	110	m	3	-	-	-	-	0.48	(0.41- 0.55)
LUBIN2	118	f	3	-	-	-	-	0.43	(0.31- 0.61)
Subtotal LUBIN2								0.47	(0.41- 0.54)
MACLEN	85	f	0	15	13	21	14	0.77	(0.28- 2.10)
MATOS	37	m	4	-	-	-	-	1.25	(0.67- 2.50)
*MIGRAN	100	m	3	-	-	-	-	1.23	(0.84- 1.81)
*MIGRAN	104	f	3	-	-	-	-	1.44	(0.61- 3.40)
Subtotal MIGRAN								1.26	(0.89- 1.79)
*MRFITR	7	m	9	-	-	-	-	0.53	(0.24- 1.17)
PEZZOT	24	m	4	-	-	-	-	0.29	(0.20- 0.41)
*RIMING	8	m	1	-	-	-	-	0.65	(0.44- 0.96)
SEGI2	52	m	1	-	-	-	-	0.62	(0.45- 0.85)
SOBUE	125	m	5	-	-	-	-	0.67	(0.38- 1.11)
*TANG2	1	m	3	-	-	-	-	0.94	(0.75- 1.18)
WAKAI	64	m	5	-	-	-	-	1.02	(0.31- 3.33)
WYNDE5	3	m	0	417	629	273	398	0.97	(0.79- 1.18)
WYNDE5	6	f	0	152	200	34	30	0.67	(0.39- 1.14)
Subtotal WYNDE5								0.92	(0.77- 1.11)
WYNDE6	422	m	3	-	-	-	-	0.95	(0.70- 1.28)
WYNDE6	425	f	3	-	-	-	-	0.71	(0.51- 0.99)
Subtotal WYNDE6								0.83	(0.67- 1.04)
Partial Totals				1041	6841	2396	18485		

*prospective study

Table 1F3 - 2

IESLC - Meta-analysis of Cigarette Smoking, only Filter vs ever Plain (or nearest available)
All LC types
Most adjusted

REF	NRR	SEX	AD	Ys	Ws	Qs	Ps
AGUDO	17	f	3	-1.51	1.29	1.58	0.0861
ALDERS	173	m	2	0.03	20.57	3.86	0.8933
ALDERS	167	f	2	-0.42	34.06	0.00	0.0153
Subtotal ALDERS				-0.25	54.64	3.87	
ARMADA	22	m	1	-0.36	20.05	0.04	0.1103
BECHER	10	m	3	-0.89	8.43	2.01	0.0096
BROSS	14	m	0	-0.53	24.52	0.37	0.0090
BUFFLE	57	f	0	0.21	24.02	9.12	0.2978
CHOI	41	m	0	-0.40	24.91	0.00	0.0446
CHOI	45	f	0	-0.72	2.33	0.23	0.2720
Subtotal CHOI				-0.43	27.24	0.23	
CORREA	65	c	2	-0.60	19.52	0.73	0.0083
*CPSII	138	f	2	-0.42	156.19	0.02	0.0000
DEAN2	36	m	0	-0.03	8.91	1.22	0.9187
DEAN2	42	f	0	1.14	1.56	3.70	0.1555
Subtotal DEAN2				0.14	10.47	4.92	
DEAN3	156	m	2	-0.62	42.46	1.92	0.0001
DEAN3	162	f	2	-0.39	16.27	0.01	0.1198
Subtotal DEAN3				-0.55	58.73	1.92	
DESTE2	23	c	7	-0.31	29.47	0.23	0.0876
DESTEF	53	m	4	-0.33	46.42	0.26	0.0252
DOLL	47	m	0	-1.71	2.47	4.24	0.0071
*ENGELA	115	m	0	-0.57	5.92	0.17	0.1639
*ENGELA	199	f	5	-0.17	6.43	0.34	0.6584
Subtotal ENGELA				-0.37	12.35	0.51	
*KAISE2	55	m	5	0.03	13.83	2.60	0.9125
*KAISE2	56	f	5	-0.43	7.73	0.01	0.2309
Subtotal KAISE2				-0.14	21.57	2.60	
KHUDER	7	m	0	-0.77	68.64	9.27	0.0000
*LANGE	20	m	2	-0.11	21.40	1.91	0.6259
*LANGE	19	f	2	-0.36	9.79	0.02	0.2644
Subtotal LANGE				-0.18	31.19	1.93	
LUBIN2	110	m	3	-0.73	178.06	19.41	0.0000
LUBIN2	118	f	3	-0.84	33.54	6.50	0.0000
Subtotal LUBIN2				-0.75	211.60	25.91	
MACLEN	85	f	0	-0.26	3.81	0.08	0.6087
MATOS	37	m	4	0.22	8.86	3.48	0.5065
*MIGRAN	100	m	3	0.21	26.07	9.73	0.2905
*MIGRAN	104	f	3	0.36	5.21	3.07	0.4054
Subtotal MIGRAN				0.23	31.28	12.80	
*MRFITR	7	m	9	-0.63	6.12	0.33	0.1162
PEZZOT	24	m	4	-1.24	29.82	20.75	0.0000
*RIMING	8	m	1	-0.43	25.25	0.02	0.0304
SEGI2	52	m	1	-0.48	37.99	0.21	0.0032
SOBUE	125	m	5	-0.40	13.37	0.00	0.1431
*TANG2	1	m	3	-0.06	74.81	8.75	0.5925
WAKAI	64	m	5	0.02	2.73	0.49	0.9739
WYNDE5	3	m	0	-0.03	98.39	13.45	0.7354
WYNDE5	6	f	0	-0.40	13.45	0.00	0.1427
Subtotal WYNDE5				-0.08	111.85	13.45	
WYNDE6	422	m	3	-0.05	42.18	5.24	0.7390
WYNDE6	425	f	3	-0.34	34.93	0.13	0.0430
Subtotal WYNDE6				-0.18	77.11	5.37	

Table 1F3 - 2

IESLC - Meta-analysis of Cigarette Smoking, only Filter vs ever Plain (or nearest available)
All LC types
Most adjusted

	N	42
	NS	31
	Wt	1251.78
Het	Chi	135.50
Het	df	41
Het	P	***
Fixed	RR	0.67
	RRl	0.63
	RRu	0.71
	P	---
Random	RR	0.70
	RRl	0.62
	RRu	0.78
	P	---
Asymm	P	N.S.

Table 1F3 - 3

IESLC - Meta-analysis of Cigarette Smoking, only Filter vs ever Plain (or nearest available)
All LC types
Most adjusted

	Sex			
	combined	male	female	Total
N	2	25	15	42
NS	2	25	15	42
Wt	48.98	852.19	350.60	1251.78
Het Chi	0.94	109.68	24.69	135.50
Het df	1	24	14	41
Het P	N.S.	***	*	***
Fixed RR	0.65	0.66	0.68	0.67
RRl	0.49	0.62	0.61	0.63
RRu	0.86	0.71	0.76	0.71
P	--	---	---	---
Random RR	0.65	0.69	0.71	0.70
RRl	0.49	0.59	0.59	0.62
RRu	0.86	0.81	0.84	0.78
P	--	---	---	---
Between Chi				0.20
Between df				2
Between P				N.S.
Btwn(F) P				N.S.
Btwn(R) P				N.S.

	All LC (or nearest)		
	all	other	Total
N	39	3	42
NS	29	2	31
Wt	1161.29	90.48	1251.78
Het Chi	129.88	2.16	135.50
Het df	38	2	41
Het P	***	N.S.	***
Fixed RR	0.66	0.81	0.67
RRl	0.62	0.66	0.63
RRu	0.70	0.99	0.71
P	---	-	---
Random RR	0.69	0.80	0.70
RRl	0.61	0.65	0.62
RRu	0.78	1.00	0.78
P	---	-	---
Between Chi			3.46
Between df			1
Between P			(*)
Btwn(F) P			N.S.
Btwn(R) P			N.S.

	Location								
	NAmer	UK	Scand	othEur	China	Japan	othAs	other	Total
N	12	11	4	5		3	3	4	42
NS	9	7	2	4		3	2	4	31
Wt	509.53	257.64	43.54	241.36		54.09	31.04	114.56	1251.78
Het Chi	38.33	27.91	1.20	4.24		0.65	0.31	23.21	135.50
Het df	11	10	3	4		2	2	3	41
Het P	***	**	N.S.	N.S.		N.S.	N.S.	***	***
Fixed RR	0.72	0.80	0.79	0.48		0.65	0.66	0.60	0.67
RRl	0.66	0.71	0.59	0.43		0.50	0.47	0.50	0.63
RRu	0.79	0.91	1.06	0.55		0.85	0.94	0.71	0.71
P	---	---	N.S.	---		--	-	---	---
Random RR	0.73	0.81	0.79	0.48		0.65	0.66	0.64	0.70
RRl	0.61	0.64	0.59	0.42		0.50	0.47	0.37	0.62
RRu	0.87	1.02	1.06	0.56		0.85	0.94	1.09	0.78
P	---	(-)	N.S.	---		--	-	N.S.	---
Between Chi									39.65
Between df									6
Between P									***
Btwn(F) P									*
Btwn(R) P									**

Table 1F3 - 3

IESLC - Meta-analysis of Cigarette Smoking, only Filter vs ever Plain (or nearest available)
All LC types
Most adjusted

	Detailed Country in "other Europe"					
	multi	Germany	othWest	East	Balkans	Total
N	2	1	2			5
NS	1	1	2			4
Wt	211.60	8.43	21.33			241.36
Het Chi	0.34	0.00	1.62			4.24
Het df	1	0	1			4
Het P	N.S.	N.S.	N.S.			N.S.
Fixed RR	0.47	0.41	0.65			0.48
RRl	0.41	0.21	0.43			0.43
RRu	0.54	0.81	1.00			0.55
P	---	--	-			---
Random RR	0.47	0.41	0.54			0.48
RRl	0.41	0.21	0.21			0.42
RRu	0.54	0.81	1.39			0.56
P	---	--	N.S.			---
Between Chi						2.28
Between df						2
Between P						N.S.
Btwn(F) P						N.S.
Btwn(R) P						N.S.

	Detailed Country in "other Asia"			
	India	HongKong	other	Total
N			3	3
NS			2	2
Wt			31.04	31.04
Het Chi			0.31	0.31
Het df			2	2
Het P			N.S.	N.S.
Fixed RR			0.66	0.66
RRl			0.47	0.47
RRu			0.94	0.94
P			-	-
Random RR			0.66	0.66
RRl			0.47	0.47
RRu			0.94	0.94
P			-	-
Between Chi				
Between df				
Between P				N.S.
Btwn(F) P				N.S.
Btwn(R) P				N.S.

	Detailed other continent			
	SCAmer	Auslia	Africa	Total
N	4			4
NS	4			4
Wt	114.56			114.56
Het Chi	23.21			23.21
Het df	3			3
Het P	***			***
Fixed RR	0.60			0.60
RRl	0.50			0.50
RRu	0.71			0.71
P	---			---
Random RR	0.64			0.64
RRl	0.37			0.37
RRu	1.09			1.09
P	N.S.			N.S.
Between Chi				
Between df				
Between P				N.S.
Btwn(F) P				N.S.
Btwn(R) P				N.S.

Table 1F3 - 3

IESLC - Meta-analysis of Cigarette Smoking, only Filter vs ever Plain (or nearest available)
All LC types
Most adjusted

	<1960	1960-69	1970-79	1980-89	1990+	Total
	Start year of study					
N	1	15	13	11	2	42
NS	1	9	9	10	2	31
Wt	2.47	439.10	397.71	374.17	38.33	1251.78
Het Chi	0.00	29.63	39.95	27.28	1.97	135.50
Het df	0	14	12	10	1	41
Het P	N.S.	**	***	**	N.S.	***
Fixed RR	0.18	0.82	0.60	0.58	0.83	0.67
RRl	0.05	0.75	0.54	0.52	0.60	0.63
RRu	0.63	0.90	0.66	0.64	1.13	0.71
P	--	---	---	---	N.S.	---
Random RR	0.18	0.80	0.70	0.56	0.89	0.70
RRl	0.05	0.69	0.56	0.45	0.53	0.62
RRu	0.63	0.94	0.86	0.68	1.47	0.78
P	--	--	---	---	N.S.	---
Between Chi						36.67
Between df						4
Between P						***
Btwn(F) P						*
Btwn(R) P						*

	CC	other	Total
Study type (1)			
N	30	12	42
NS	23	8	31
Wt	893.01	358.76	1251.78
Het Chi	105.20	18.61	135.50
Het df	29	11	41
Het P	***	(*)	***
Fixed RR	0.63	0.78	0.67
RRl	0.59	0.70	0.63
RRu	0.67	0.86	0.71
P	---	---	---
Random RR	0.65	0.81	0.70
RRl	0.57	0.69	0.62
RRu	0.75	0.96	0.78
P	---	-	---
Between Chi			11.69
Between df			1
Between P			***
Btwn(F) P			(*)
Btwn(R) P			(*)

	CC	prosp	other	Total
Study type (2)				
N	30	12		42
NS	23	8		31
Wt	893.01	358.76		1251.78
Het Chi	105.20	18.61		135.50
Het df	29	11		41
Het P	***	(*)		***
Fixed RR	0.63	0.78		0.67
RRl	0.59	0.70		0.63
RRu	0.67	0.86		0.71
P	---	---		---
Random RR	0.65	0.81		0.70
RRl	0.57	0.69		0.62
RRu	0.75	0.96		0.78
P	---	-		---
Between Chi				11.69
Between df				1
Between P				***
Btwn(F) P				(*)
Btwn(R) P				(*)

Table 1F3 - 3

IESLC - Meta-analysis of Cigarette Smoking, only Filter vs ever Plain (or nearest available)
All LC types
Most adjusted

	Study size (number of LC cases)				
	100-249	250-499	500-999	1000+	Total
N	7	16	7	12	42
NS	7	11	5	8	31
Wt	83.58	328.92	192.54	646.74	1251.78
Het Chi	19.89	28.11	18.96	52.95	135.50
Het df	6	15	6	11	41
Het P	**	*	**	***	***
Fixed RR	0.49	0.69	0.80	0.65	0.67
RRl	0.39	0.62	0.69	0.60	0.63
RRu	0.60	0.77	0.92	0.70	0.71
P	---	---	--	---	---
Random RR	0.53	0.74	0.81	0.66	0.70
RRl	0.34	0.63	0.61	0.55	0.62
RRu	0.83	0.87	1.08	0.80	0.78
P	--	---	N.S.	---	---
Between Chi					15.59
Between df					3
Between P					**
Btwn(F) P					N.S.
Btwn(R) P					N.S.

	Risky occupational population			
	no	mining	othRisky	Total
N	42			42
NS	31			31
Wt	1251.78			1251.78
Het Chi	135.50			135.50
Het df	41			41
Het P	***			***
Fixed RR	0.67			0.67
RRl	0.63			0.63
RRu	0.71			0.71
P	---			---
Random RR	0.70			0.70
RRl	0.62			0.62
RRu	0.78			0.78
P	---			---
Between Chi				
Between df				
Between P				N.S.
Btwn(F) P				N.S.
Btwn(R) P				N.S.

	National cigarette tobacco type			
	Virginia	blended	other	Total
N	11	31		42
NS	7	24		31
Wt	257.64	994.13		1251.78
Het Chi	27.91	96.76		135.50
Het df	10	30		41
Het P	**	***		***
Fixed RR	0.80	0.64		0.67
RRl	0.71	0.60		0.63
RRu	0.91	0.68		0.71
P	---	---		---
Random RR	0.81	0.66		0.70
RRl	0.64	0.58		0.62
RRu	1.02	0.75		0.78
P	(-)	---		---
Between Chi				10.83
Between df				1
Between P				***
Btwn(F) P				(*)
Btwn(R) P				N.S.

Table 1F3 - 3

IESLC - Meta-analysis of Cigarette Smoking, only Filter vs ever Plain (or nearest available)
All LC types
Most adjusted

	Any proxy use No/nk	Yes	Total
N	36	6	42
NS	27	4	31
Wt	1139.04	112.73	1251.78
Het Chi	118.75	16.13	135.50
Het df	35	5	41
Het P	***	**	***
Fixed RR	0.66	0.72	0.67
RRl	0.63	0.60	0.63
RRu	0.70	0.86	0.71
P	---	---	---
Random RR	0.68	0.79	0.70
RRl	0.60	0.55	0.62
RRu	0.77	1.14	0.78
P	---	N.S.	---
Between Chi			0.62
Between df			1
Between P			N.S.
Btwn(F) P			N.S.
Btwn(R) P			N.S.

	Full histological confirmation No	Yes	Total
N	29	13	42
NS	21	10	31
Wt	661.77	590.01	1251.78
Het Chi	50.82	69.56	135.50
Het df	28	12	41
Het P	**	***	***
Fixed RR	0.74	0.59	0.67
RRl	0.69	0.55	0.63
RRu	0.80	0.64	0.71
P	---	---	---
Random RR	0.76	0.60	0.70
RRl	0.67	0.48	0.62
RRu	0.85	0.75	0.78
P	---	---	---
Between Chi			15.13
Between df			1
Between P			***
Btwn(F) P			*
Btwn(R) P			(*)

	Number of adjustment variables (1) 0	1	2+/+nk	Total
N	12	3	27	42
NS	9	3	20	32
Wt	278.93	83.28	889.56	1251.78
Het Chi	39.74	0.19	92.85	135.50
Het df	11	2	26	41
Het P	***	N.S.	***	***
Fixed RR	0.73	0.65	0.65	0.67
RRl	0.65	0.52	0.61	0.63
RRu	0.82	0.80	0.70	0.71
P	---	---	---	---
Random RR	0.71	0.65	0.69	0.70
RRl	0.54	0.52	0.60	0.62
RRu	0.94	0.80	0.80	0.78
P	-	---	---	---
Between Chi				2.72
Between df				2
Between P				N.S.
Btwn(F) P				N.S.
Btwn(R) P				N.S.

Table 1F3 - 3

IESLC - Meta-analysis of Cigarette Smoking, only Filter vs ever Plain (or nearest available)
All LC types
Most adjusted

	Number of adjustment variables (2) 0	1	2	3-5	6+/+nk	Total
N	12	3	8	17	2	42
NS	9	3	5	13	2	32
Wt	278.93	83.28	320.26	533.71	35.59	1251.78
Het Chi	39.74	0.19	8.47	83.27	0.52	135.50
Het df	11	2	7	16	1	41
Het P	***	N.S.	N.S.	***	N.S.	***
Fixed RR	0.73	0.65	0.67	0.64	0.69	0.67
RRl	0.65	0.52	0.60	0.59	0.50	0.63
RRu	0.82	0.80	0.75	0.70	0.96	0.71
P	---	---	---	---	-	---
Random RR	0.71	0.65	0.68	0.71	0.69	0.70
RRl	0.54	0.52	0.59	0.56	0.50	0.62
RRu	0.94	0.80	0.77	0.88	0.96	0.78
P	-	---	---	--	-	---
Between Chi						3.31
Between df						4
Between P						N.S.
Btwn(F) P						N.S.
Btwn(R) P						N.S.

	Smoking status ever	current	Total
N	17	25	42
NS	14	17	31
Wt	508.28	743.49	1251.78
Het Chi	61.59	38.22	135.50
Het df	16	24	41
Het P	***	*	***
Fixed RR	0.54	0.77	0.67
RRl	0.50	0.71	0.63
RRu	0.59	0.83	0.71
P	---	---	---
Random RR	0.60	0.77	0.70
RRl	0.49	0.69	0.62
RRu	0.73	0.85	0.78
P	---	---	---
Between Chi			35.70
Between df			1
Between P			***
Btwn(F) P			***
Btwn(R) P			*

	Product cig+/-ot	cig only	Total
N	29	13	42
NS	23	9	32
Wt	943.78	308.00	1251.78
Het Chi	84.63	48.78	135.50
Het df	28	12	41
Het P	***	***	***
Fixed RR	0.65	0.72	0.67
RRl	0.61	0.64	0.63
RRu	0.70	0.80	0.71
P	---	---	---
Random RR	0.69	0.71	0.70
RRl	0.60	0.56	0.62
RRu	0.78	0.91	0.78
P	---	--	---
Between Chi			2.09
Between df			1
Between P			N.S.
Btwn(F) P			N.S.
Btwn(R) P			N.S.

Table 1F3 - 3

IESLC - Meta-analysis of Cigarette Smoking, only Filter vs ever Plain (or nearest available)
All LC types
Most adjusted

	Cigarette type						
	only f	always f	mainly f	equl p&f	both p&f	ever f	Total
N	28	6	1			7	42
NS	20	5	1			5	31
Wt	886.68	159.00	8.86			197.24	1251.78
Het Chi	92.55	5.59	0.00			30.93	135.50
Het df	27	5	0			6	41
Het P	***	N.S.	N.S.			***	***
Fixed RR	0.64	0.72	1.25			0.72	0.67
RRl	0.60	0.61	0.65			0.62	0.63
RRu	0.69	0.84	2.41			0.83	0.71
P	---	---	N.S.			---	---
Random RR	0.68	0.72	1.25			0.71	0.70
RRl	0.59	0.61	0.65			0.46	0.62
RRu	0.78	0.85	2.41			1.08	0.78
P	---	---	N.S.			N.S.	---
Between Chi							6.43
Between df							3
Between P							(*)
Btwn(F) P							N.S.
Btwn(R) P							N.S.

	Denominator				
	ever p	mainly p	p NOS	always p	Total
N	17	2	16	7	42
NS	12	2	12	5	31
Wt	542.42	165.05	347.07	197.24	1251.78
Het Chi	63.94	3.42	25.80	30.93	135.50
Het df	16	1	15	6	41
Het P	***	(*)	*	***	***
Fixed RR	0.60	0.68	0.75	0.72	0.67
RRl	0.55	0.59	0.67	0.62	0.63
RRu	0.65	0.80	0.83	0.83	0.71
P	---	---	---	---	---
Random RR	0.64	0.84	0.74	0.71	0.70
RRl	0.53	0.46	0.64	0.46	0.62
RRu	0.78	1.53	0.86	1.08	0.78
P	---	N.S.	---	N.S.	---
Between Chi					11.41
Between df					3
Between P					**
Btwn(F) P					N.S.
Btwn(R) P					N.S.

	Derivation of RR/CI			
	Orig	StdCalc	Other	Total
N	7	16	19	42
NS	4	13	15	32
Wt	186.30	464.70	600.77	1251.78
Het Chi	10.53	46.48	67.67	135.50
Het df	6	15	18	41
Het P	N.S.	***	***	***
Fixed RR	0.78	0.70	0.61	0.67
RRl	0.68	0.64	0.56	0.63
RRu	0.90	0.77	0.66	0.71
P	---	---	---	---
Random RR	0.77	0.71	0.66	0.70
RRl	0.62	0.58	0.56	0.62
RRu	0.95	0.87	0.79	0.78
P	-	---	---	---
Between Chi				10.82
Between df				2
Between P				**
Btwn(F) P				N.S.
Btwn(R) P				N.S.

Table 1F3 - 4

IESLC - Meta-analysis of Cigarette Smoking, only Filter vs ever Plain (or nearest available)
All LC types
Least adjusted

REF	NRR	X	SEX	AGEL	AGEH	RACE	YF	LC TYPE	LOC	START	ST	NLC	R	VB	P	H	AD	SM	PRODUCT	CIGTYP	DENOM	De
AGUDO	18	x	f	0	0	all	-	all	Eu:wst	1989	CC	103	n	bl	n	n	0	ev	cig only	only f	ever p	st
ALDERS	172	x	m	0	0	all	-	all	Eu:UK	1977	CC	1448	n	V	n	n	0	cu	cig+/-ot	always f	ever p	st
ALDERS	166	x	f	0	0	all	-	all	Eu:UK	1977	CC	1448	n	V	n	n	0	cu	cig only	always f	ever p	st
ARMADA	20	x	m	0	0	all	-	all	Eu:wst	1986	CC	325	n	bl	n	y	0	ev	cig+/-ot	always f	ever p	st
BECHER	9	x	m	0	0	all	-	all	Eu:Ger	1985	CC	194	n	bl	n	y	0	ev	cig+/-ot	always f	ever p	st
BROSS	14		m	0	0	wh	-	all	NAmer	1960	CC	974	n	bl	n	n	0	cu	cig+/-ot	only f	p NOS	st
BUFFLE	57		f	0	0	w-hi	-	all	NAmer	1976	CC	943	n	bl	y	n	0	ev	cig+/-ot	only f	ever p	st
CHOI	41		m	0	0	all	-	all	As:oth	1985	CC	375	n	bl	n	n	0	ev	cig+/-ot	only f	ever p	st
CHOI	45		f	0	0	all	-	all	As:oth	1985	CC	375	n	bl	n	n	0	ev	cig+/-ot	only f	ever p	st
CORREA	65		c	0	0	all	-	all	NAmer	1979	CC	1359	n	bl	y	n	2	ev	cig+/-ot	only f	p NOS	ot
CPSII	138		f	0	0	all	4	all	NAmer	1982	pr	3229	n	bl	n	n	2	cu	cig+/-ot	only f	mainly p	st
DEAN2	36		m	0	0	all	-	all	Eu:UK	1960	CC	954	n	V	y	n	0	ev	cig+/-ot	ever f	always p	st
DEAN2	42		f	0	0	all	-	all	Eu:UK	1960	CC	954	n	V	y	n	0	ev	cig+/-ot	ever f	always p	st
DEAN3	155	x	m	0	0	all	-	all	Eu:UK	1969	CC	766	n	V	y	n	0	cu	cig only	only f	p NOS	st
DEAN3	161	x	f	0	0	all	-	all	Eu:UK	1969	CC	766	n	V	y	n	0	cu	cig only	only f	p NOS	st
DESTE2	22	x	c	0	0	all	-	all	SCAmer	1993	CC	463	n	bl	n	n	0	ev	cig+/-ot	always f	ever p	st
DESTEF	52	x	m	0	0	all	-	all	SCAmer	1988	CC	497	n	bl	n	y	0	ev	cig+/-ot	always f	ever p	st
DOLL	47		m	0	0	all	-	all	Eu:UK	1948	CC	1465	n	V	n	n	0	ev	cig+/-ot	ever f	always p	st
ENGELA	115		m	0	0	all	0	all	Eu:Sca	1964	pr	435	n	bl	n	n	0	cu	cig+/-ot	only f	ever p	st
ENGELA	122	x	f	0	0	all	0	all	Eu:Sca	1964	pr	435	n	bl	n	n	0	cu	cig+/-ot	only f	ever p	st
KAISE2	55		m	30	89	all	9	all	NAmer	1979	pr	318	n	bl	n	n	5	cu	cig only	only f	p NOS	or
KAISE2	56		f	30	89	all	9	all	NAmer	1979	pr	318	n	bl	n	n	5	cu	cig only	only f	p NOS	or
KHUDER	7		m	0	0	all	-	all	NAmer	1985	CC	482	n	bl	n	y	0	ev	cig+/-ot	ever f	always p	st
LANGE	42	x	m	0	0	all	0	all	Eu:Sca	1976	pr	268	n	bl	n	n	0	cu	cig only	only f	p NOS	st
LANGE	41	x	f	0	0	all	0	all	Eu:Sca	1976	pr	268	n	bl	n	n	0	cu	cig only	only f	p NOS	st
LUBIN2	122	x	m	0	0	all	-	all	Eu:mul	1976	CC	7804	n	bl	n	y	0	ev	cig+/-ot	only f	ever p	st
LUBIN2	126	x	f	0	0	all	-	all	Eu:mul	1976	CC	7804	n	bl	n	y	0	ev	cig+/-ot	only f	ever p	st
MACLEN	85		f	0	0	ch	-	all	As:oth	1972	CC	233	n	bl	n	n	0	cu	cig+/-ot	ever f	always p	ot
MATOS	72	x	m	0	0	all	-	all	SCAmer	1994	CC	200	n	bl	n	n	0	ev	cig+/-ot	mainly f	mainly p	st
MIGRAN	99	x	m	0	0	all	0	all	Eu:UK	1964	pr	259	n	V	n	n	0	cu	cig only	only f	p NOS	st
MIGRAN	103	x	f	0	0	all	0	all	Eu:UK	1964	pr	259	n	V	n	n	0	cu	cig only	only f	p NOS	st
MRFITR	7		m	0	0	all	0	all	NAmer	1973	pr	119	n	bl	n	n	9	cu	cig+/-ot	only f	p NOS	ot
PEZZOT	23	x	m	0	0	all	-	all	SCAmer	1987	CC	215	n	bl	n	y	2	ev	cig only	only f	ever p	ot
RIMING	4	x	m	0	0	all	0	all	Eu:UK	1970	pr	104	n	V	n	n	0	cu	cig only	only f	p NOS	st
SEGI2	51	x	m	0	0	all	-	all	As:Jap	1962	CC	378	n	bl	n	n	0	cu	cig+/-ot	only f	p NOS	st
SOBUE	122	x	m	0	0	all	-	q+s+l+a	As:Jap	1986	CC	1376	n	bl	n	y	0	cu	cig+/-ot	only f	p NOS	st
TANG2	1		m	0	0	all	0	all	Eu:UK	1967	pr	836	n	V	n	n	3	cu	cig only	only f	p NOS	or
WAKAI	63	x	m	0	0	all	-	all	As:Jap	1988	CC	333	n	bl	n	y	0	cu	cig+/-ot	only f	p NOS	st
WYNDE5	3		m	0	0	all	-	all	NAmer	1969	CC	1365	n	bl	n	y	0	cu	cig+/-ot	ever f	always p	st
WYNDE5	6		f	0	0	all	-	all	NAmer	1969	CC	1365	n	bl	n	y	0	cu	cig+/-ot	ever f	always p	st
WYNDE6	416	x	m	0	0	all	-	q+a	NAmer	1969	CC	4423	n	bl	n	y	0	cu	cig+/-ot	only f	ever p	st
WYNDE6	419	x	f	0	0	all	-	q+a	NAmer	1969	CC	4423	n	bl	n	y	0	cu	cig+/-ot	only f	ever p	st

Table 1F3 - 5

IESLC - Meta-analysis of Cigarette Smoking, only Filter vs ever Plain (or nearest available)
All LC types
Least adjusted

REF	NRR	SEX	AD	Number Exposed Case	Number Exposed Cont	Non-exposed Case	Non-exposed Cont	RR	95.00%CI
AGUDO	18	f	0	14	21	8	2	0.17	(0.03- 0.90)
ALDERS	172	m	0	53	40	466	282	0.80	(0.52- 1.24)
ALDERS	166	f	0	134	100	276	129	0.63	(0.45- 0.87)
Subtotal ALDERS								0.69	(0.53- 0.89)
ARMADA	20	m	0	57	72	260	182	0.55	(0.37- 0.82)
BECHER	9	m	0	102	196	35	21	0.31	(0.17- 0.56)
BROSS	14	m	0	65	76	200	138	0.59	(0.40- 0.88)
BUFFLE	57	f	0	129	78	111	83	1.24	(0.83- 1.84)
CHOI	41	m	0	43	103	226	362	0.67	(0.45- 0.99)
CHOI	45	f	0	5	11	14	15	0.49	(0.13- 1.76)
Subtotal CHOI								0.65	(0.45- 0.95)
CORREA	65	c	2	-	-	-	-	0.55	(0.35- 0.85)
*CPSII	138	f	2	-	-	-	-	0.66	(0.57- 0.78)
DEAN2	36	m	0	20	17	644	529	0.97	(0.50- 1.86)
DEAN2	42	f	0	12	2	50	26	3.12	(0.65- 15.00)
Subtotal DEAN2								1.15	(0.63- 2.11)
DEAN3	155	m	0	108	522	210	366	0.36	(0.28- 0.47)
DEAN3	161	f	0	66	888	30	228	0.56	(0.36- 0.89)
Subtotal DEAN3								0.40	(0.32- 0.51)
DESTE2	22	c	0	158	128	142	84	0.73	(0.51- 1.04)
DESTEF	52	m	0	178	156	292	178	0.70	(0.52- 0.92)
DOLL	47	m	0	3	15	501	452	0.18	(0.05- 0.63)
*ENGELA	115	m	0	7	5257	38	16104	0.56	(0.25- 1.26)
*ENGELA	122	f	0	11	23317	13	15023	0.55	(0.24- 1.22)
Subtotal ENGELA								0.55	(0.31- 0.98)
*KAISE2	55	m	5	-	-	-	-	1.03	(0.61- 1.75)
*KAISE2	56	f	5	-	-	-	-	0.65	(0.32- 1.31)
Subtotal KAISE2								0.87	(0.57- 1.33)
KHUDER	7	m	0	173	440	284	334	0.46	(0.36- 0.59)
*LANGE	42	m	0	25	953	65	1642	0.66	(0.42- 1.04)
*LANGE	41	f	0	19	2269	20	1344	0.56	(0.30- 1.05)
Subtotal LANGE								0.63	(0.43- 0.90)
LUBIN2	122	m	0	326	851	6300	9583	0.58	(0.51- 0.66)
LUBIN2	126	f	0	102	184	449	383	0.47	(0.36- 0.62)
Subtotal LUBIN2								0.56	(0.50- 0.63)
MACLEN	85	f	0	15	13	21	14	0.77	(0.28- 2.10)
MATOS	72	m	0	163	229	22	46	1.49	(0.86- 2.57)
*MIGRAN	99	m	0	47	1326	57	1630	1.01	(0.69- 1.48)
*MIGRAN	103	f	0	15	1773	8	871	0.92	(0.39- 2.16)
Subtotal MIGRAN								1.00	(0.71- 1.41)
*MRFITR	7	m	9	-	-	-	-	0.53	(0.24- 1.17)
PEZZOT	23	m	2	-	-	-	-	0.23	(0.16- 0.34)
*RIMING	4	m	0	45	3045	59	2393	0.60	(0.41- 0.88)
SEGI2	51	m	0	105	250	135	203	0.63	(0.46- 0.87)
SOBUE	122	m	0	560	540	49	26	0.55	(0.34- 0.90)
*TANG2	1	m	3	-	-	-	-	0.94	(0.75- 1.18)
WAKAI	63	m	0	174	271	5	9	1.16	(0.38- 3.51)
WYNDE5	3	m	0	417	629	273	398	0.97	(0.79- 1.18)
WYNDE5	6	f	0	152	200	34	30	0.67	(0.39- 1.14)
Subtotal WYNDE5								0.92	(0.77- 1.11)
WYNDE6	416	m	0	150	122	1292	754	0.72	(0.56- 0.93)
WYNDE6	419	f	0	215	158	635	309	0.66	(0.52- 0.85)
Subtotal WYNDE6								0.69	(0.58- 0.82)
Partial Totals				3868	44252	13224	54173		

*prospective study

Table 1F3 - 5

IESLC - Meta-analysis of Cigarette Smoking, only Filter vs ever Plain (or nearest available)
All LC types
Least adjusted

REF	NRR	SEX	AD	Ys	Ws	Qs	Ps
AGUDO	18	f	0	-1.79	1.34	2.44	0.0378
ALDERS	172	m	0	-0.22	20.18	1.00	0.3211
ALDERS	166	f	0	-0.47	34.68	0.02	0.0059
Subtotal ALDERS				-0.38	54.85	1.02	
ARMADA	20	m	0	-0.59	24.53	0.53	0.0035
BECHER	9	m	0	-1.16	10.98	5.70	0.0001
BROSS	14	m	0	-0.53	24.52	0.17	0.0090
BUFFLE	57	f	0	0.21	24.02	10.34	0.2978
CHOI	41	m	0	-0.40	24.91	0.04	0.0446
CHOI	45	f	0	-0.72	2.33	0.18	0.2720
Subtotal CHOI				-0.43	27.24	0.22	
CORREA	65	c	2	-0.60	19.52	0.46	0.0083
*CPSII	138	f	2	-0.42	156.19	0.12	0.0000
DEAN2	36	m	0	-0.03	8.91	1.49	0.9187
DEAN2	42	f	0	1.14	1.56	3.90	0.1555
Subtotal DEAN2				0.14	10.47	5.39	
DEAN3	155	m	0	-1.02	53.56	17.80	0.0000
DEAN3	161	f	0	-0.57	18.52	0.30	0.0140
Subtotal DEAN3				-0.90	72.08	18.10	
DESTE2	22	c	0	-0.31	30.22	0.50	0.0839
DESTEF	52	m	0	-0.36	47.46	0.31	0.0124
DOLL	47	m	0	-1.71	2.47	3.98	0.0071
*ENGELA	115	m	0	-0.57	5.92	0.10	0.1639
*ENGELA	122	f	0	-0.61	5.96	0.16	0.1385
Subtotal ENGELA				-0.59	11.88	0.26	
*KAISE2	55	m	5	0.03	13.83	3.10	0.9125
*KAISE2	56	f	5	-0.43	7.73	0.00	0.2309
Subtotal KAISE2				-0.14	21.57	3.10	
KHUDER	7	m	0	-0.77	68.64	7.37	0.0000
*LANGE	42	m	0	-0.41	18.61	0.02	0.0759
*LANGE	41	f	0	-0.57	9.86	0.17	0.0710
Subtotal LANGE				-0.47	28.47	0.19	
LUBIN2	122	m	0	-0.54	221.94	2.07	0.0000
LUBIN2	126	f	0	-0.75	49.81	4.65	0.0000
Subtotal LUBIN2				-0.58	271.75	6.71	
MACLEN	85	f	0	-0.26	3.81	0.13	0.6087
MATOS	72	m	0	0.40	12.87	9.11	0.1537
*MIGRAN	99	m	0	0.01	26.70	5.58	0.9443
*MIGRAN	103	f	0	-0.08	5.26	0.69	0.8504
Subtotal MIGRAN				-0.00	31.96	6.27	
*MRFITR	7	m	9	-0.63	6.12	0.22	0.1162
PEZZOT	23	m	2	-1.47	27.04	28.48	0.0000
*RIMING	4	m	0	-0.51	26.02	0.12	0.0090
SEGI2	51	m	0	-0.46	38.67	0.01	0.0043
SOBUE	122	m	0	-0.60	16.00	0.38	0.0169
*TANG2	1	m	3	-0.06	74.81	10.90	0.5925
WAKAI	63	m	0	0.14	3.12	1.08	0.7982
WYNDE5	3	m	0	-0.03	98.39	16.50	0.7354
WYNDE5	6	f	0	-0.40	13.45	0.03	0.1427
Subtotal WYNDE5				-0.08	111.85	16.52	
WYNDE6	416	m	0	-0.33	58.95	0.73	0.0108
WYNDE6	419	f	0	-0.41	63.33	0.06	0.0010
Subtotal WYNDE6				-0.37	122.28	0.80	

Table 1F3 - 5

IESLC - Meta-analysis of Cigarette Smoking, only Filter vs ever Plain (or nearest available)
All LC types
Least adjusted

	N	42
	NS	31
	Wt	1382.76
Het	Chi	140.93
Het	df	41
Het	P	***
Fixed	RR	0.64
	RRl	0.61
	RRu	0.68
	P	---
Random	RR	0.64
	RRl	0.57
	RRu	0.72
	P	---
Asymm	P	N.S.

Table 1F3 - 6

IESLC - Meta-analysis of Cigarette Smoking, only Filter vs ever Plain (or nearest available)
All LC types
Least adjusted

		Sex		
	combined	male	female	Total
N	2	25	15	42
NS	2	25	15	42
Wt	49.74	935.17	397.85	1382.76
Het Chi	0.95	116.75	23.12	140.93
Het df	1	24	14	41
Het P	N.S.	***	(*)	***
Fixed RR	0.65	0.64	0.65	0.64
RRl	0.49	0.60	0.59	0.61
RRu	0.86	0.68	0.72	0.68
P	--	---	---	---
Random RR	0.65	0.63	0.66	0.64
RRl	0.49	0.54	0.56	0.57
RRu	0.86	0.74	0.77	0.72
P	--	---	---	---
Between Chi				0.11
Between df				2
Between P				N.S.
Btwn(F) P				N.S.
Btwn(R) P				N.S.

Table 1F3 - 7

IESLC - Meta-analysis of Cigarette Smoking, only Filter vs ever Plain (or nearest available)
All LC types
Excluded studies (and stage at which they were excluded)

1	BOUCHA BUELL LAURIL MZILEN
2	BERRIN CHEN RESTRE TAO ULMER
3	ABELIN ABRAHA AKIBA AMANDU AMES ANDERS ARCHER AUSTIN AUVINE AXELSO AXELSS BAND BARBON BENSHL BEST BLOHMK BLOT1 BLOT2 BLOT3 BLOT4 BOFFET BOUCOT BRESLO BRETT BROCKM BROWN1 BROWN2 BYERS1 BYERS2 CARPEN CASCO2 CASCOR CEDERL CHAN CHANG CHATZI CHEN2 CHEN3 CHIAZZ CHOW CHYOU COMSTO COOKSO CPSI DAMBER DARBY DAVEYS DEAN DEKLER DOCKER DOLL2 DORANT DORGAN DORN DOSEME DROSTE DU DUNN EBELIN ENSTRO ESAKI FAN GAO GAO2 GARCIA GARDIN GARSHI GENG GER GILLIS GODLEY GOLLED GOODMA GRAHAM GREGOR GSELL HAENSZ HAMMO2 HAMMON HANSEN HEGMAN HEIN HENNEK HINDS HIRAOK HIRAY2 HIRAYA HITOSU HOLE HOROWI HORWIT HU HU2 HUANG HUMBLE ISHIMA JAHN JAIN JARUP JARVHO JEDRYC JIANG JOLY JONES JUSSAW KAISER KANELL KATSOU KAUFMA KELLER KIHARA KINLEN KJUUS KNEKT KO KOHLME KOO KOULUM KREUZE KREYBE KUBIK LAMTH LAMWK LAMWK2 LAUSSM LEI LEMARC LETOUR LEVIN LIAW LICKIN LIDDEL LIU LIU2 LIU3 LIU4 LIU5 LOMBA2 LOMBAR LUBIN LUO MAGNUS MARSH MARSH2 MARTIS MASTRA MATSUD MCCONN MCDUFF MCLAUG MILLER MILLS MOLLO MRFIT MURATA NAM NOTAN2 NOTANI NOU ODRISC ORMOS OSANN OSANN2 PARKIN PASTOR PAWLEG PERNU PERSH2 PERSHA PETO PEZZO2 PIKE PISANI POFFIJ POLEDN PRESCO QIAO QIAO2 RACHTA RADZIK RANDIG REN RONCO ROOTS ROTHSC SAARIK SADOWS SANKAR SCHWA2 SCHWAR SEGI SEOW SHAW SHIMIZ SIEMIA SIMARA SIMONA SITAS SOBUE2 SPEIZE SPITZ STASZE STAYNE STOCKS STOCKW STUCKE SUN SUZUK2 SUZUKI SVENSS TANG TENKAN TIZZAN TOKARS TOUSEY TSUGAN TULINI TVERDA VANDER VEIERO VUTUC WALD WANG WANG2 WANG3 WANG4 WARSIN WATSON WICKLU WIGLE WILKIN WU WU2 WUNSCH WUWILL WYNDE2 WYNDE4 WYNDE7 WYNDE8 WYNDER XIANGZ XU XU2 XU3 XU4 YAMAGU YONG YUAN ZHANG ZHENG ZHOU
7	WYNDE3
10	BENHAM

Table 1F3 - 8

Potentially overlapping studies

REF	REFGP	PRINC	.	OVERLAP/LINK
LUBIN2	LUBIN2	1		Lubin-combined
LANGE	PRESCO	1		Subset of PRESCO
BROSS	BYERS1	1		GRAHAM/BROSS/BYERS1
TANG2	TANG2	2		TANG2-combined
MRFITR	MRFIT	2		Subset of MRFIT
WYNDE5	WYNDE6	2		WYNDE5/6/7/8
WYNDE6	WYNDE6	1		WYNDE5/6/7/8

Table 1F3 - 9

Most adjusted - insufficient data for metaanalysis

REF	NRR	SEX	AGEL	AGEH	RACE	YF	LC TYPE	LOC	START	ST	NLC	R	VB	P	H	AD	SM	PRODUCT	CIGTYP	DENOM	De
BUFFLE	88	m	0	0	wh	-	all	NAmer	1976	CC	943	n	bl	y	n	0	ev	cig+/-ot	only f	p NOS	st
CPSII	135	m	0	0	all	6	all	NAmer	1982	pr	3229	n	bl	n	n	1	cu	cig only	mainly f	mainly p	ot
HIRAYA	155	c	0	0	all	16	all	As:Jap	1965	pr	1917	n	bl	n	n	1	ev	cig+/-ot	only f	p NOS	ot
HUMBLE	53	c	0	0	w-hi	-	not alv	NAmer	1980	CC	521	n	bl	y	n	5	cu	cig+/-ot	always f	always p	or
HUMBLE	54	c	0	0	hi	-	not alv	NAmer	1980	CC	521	n	bl	y	n	5	cu	cig+/-ot	always f	always p	or
JOLY	56	m	0	0	all	-	all	SCAmer	1978	CC	826	n	bl	n	n	0	ev	cig+/-ot	ever f	ever p	or
JOLY	57	f	0	0	all	-	all	SCAmer	1978	CC	826	n	bl	n	n	0	ev	cig+/-ot	ever f	ever p	or

REF	NRR	RR	SIG	RRDATA comment
BUFFLE	88	0.92		0
CPSII	135	0.47		0
HIRAYA	155	0.51		RR originally given as % reduction in rate
HUMBLE	53	0.80		0
HUMBLE	54	0.04		0
JOLY	56	*		No significant difference was noted between cases and controls with respect to use of filter cigarettes.
JOLY	57	*		No significant difference was noted between cases and controls with respect to use of filter cigarettes.

Table 1F4 -

IESLC - Meta-analysis of Cigarette Smoking, Hand-rolled vs Manufactured
All LC types

This analysis is restricted to results for:
1) Non-dose-response data
2) Results complete enough for use in metaanalysis

Within each study, results are then selected (in the following order of preference, within each sex) for:
3) CIGTYP: hand-rolled any, both, mainly, only
4) DENOM: manufactured only ever, only current, any, ever
5) PRODUCT: cigarettes regardless of other products, cigarettes only
6) SMKSTA: ever, current
7) LCtype: all or nearest available, at least Squamous and Adeno. (q = squamous, s = small,
l = large, a = adeno, mix = mixed, alv = alveolar)
8) Race: all or nearest available, otherwise by race (wh or w = white, bl or b = black, hi = hispanic
ch = chinese, jap = japanese, haw = hawaiian, w+o = white + oriental, sca = scandinavian, as = asian)
9) Followup period (YF, prospective studies): whole study (coded as 0) or longest available
10) For overlapping studies: principal rather than subsidiary studies
Finally by Age: whole study (coded as 0) if available, otherwise by widest available age group
and then for single sex results (m, f) in preference to combined sex results (c).

Results adjusted (AD) for the most potential confounders are then chosen in Sections -1 to -3
and results adjusted for the least confounders in Sections -4 to -6. (Those least adjusted results which
actually differ from the most adjusted as marked 'x' in column X in Section -4)
(Results adjusted for an unknown number of confounder(s) are coded as 20.)

Section -7 shows excluded studies, together with the stage (as above) at which no qualifying
results were found.

Section -8 lists the potentially overlapping studies which have been included (1=principal, 2=subsidiary).

Section -9 lists any results which would have been included in preference except that they had data not complete
enough for use in meta-analysis, with their significance (yes/no), if known, and any further comment as entered
on the database.

In addition to those mentioned above, the following fields, levels and abbreviations are used:
* or nk = not known, n = no, y = yes, ot = other
ev = ever, cu = current, cig+/-ot = cigarettes irrespective of other products (cigar, pipe etc)
m or mc = manufactured cigarettes, h or hr = hand-rolled cigarettes
REF: 6-character study reference
NRR: number of the RR on the database within the study
ST : study type (CC = case control, pr or prosp = prospective)
NLC: number of lung cancer cases in whole study
R : risky occupational population (n = no, m = mining, o = other risky)
VB : national cigarette type (V = at least 75% Virginia, bl = at least 75% blended, ot = other)
P : any proxy use
H : full histological confirmation
De : derivation of RR/CI (or = original, st = standard method, ot = other method of estimation)

Table 1F4 - 1

IESLC - Meta-analysis of Cigarette Smoking, Hand-rolled vs Manufactured
All LC types
Most adjusted

REF	NRR	SEX	AGEL	AGEH	RACE	YF	LC TYPE	LOC	START	ST	NLC	R	VB	P	H	AD	SM	PRODUCT	CIGTYP	DENOM	De
ALDERS	119	m	0	0	all	-	all	Eu:UK	1977	CC	1448	n	V	n	n	1	ev	cig only	both m&h	only mc	ot
BENHAM	109	m	0	0	all	-	all	Eu:wst	1976	CC	1625	n	bl	n	y	2	cu	cig only	any hr	only mc	or
CHAN	25	m	0	0	all	-	all	As:HK	1976	CC	397	n	bl	n	n	0	ev	cig+/-ot	both m&h	only mc	st
CHAN	26	f	0	0	all	-	all	As:HK	1976	CC	397	n	bl	n	n	0	ev	cig+/-ot	both m&h	only mc	st
DESTE2	21	c	0	0	all	-	all	SCAmer	1993	CC	463	n	bl	n	n	7	ev	cig+/-ot	both m&h	only mc	ot
DESTEF	39	m	0	0	all	-	all	SCAmer	1988	CC	497	n	bl	n	y	6	ev	cig+/-ot	both m&h	only mc	or
ENGELA	132	m	0	0	all	0	all	Eu:Sca	1964	pr	435	n	bl	n	n	7	cu	cig+/-ot	both m&h	only mc	ot
ENGELA	204	f	0	0	all	0	all	Eu:Sca	1964	pr	435	n	bl	n	n	5	cu	cig+/-ot	both m&h	only mc	ot
GARDIN	13	c	0	0	all	-	all	Eu:UK	1988	CC	143	n	V	y	n	0	cu	cig only	only hr	cu onlym	st
HU	21	m	0	0	all	-	all	As:Chi	1985	CC	227	n	ot	n	y	0	ev	cig+/-ot	both m&h	only mc	st
HU	22	f	0	0	all	-	all	As:Chi	1985	CC	227	n	ot	n	y	0	ev	cig+/-ot	both m&h	only mc	st
HU2	11	c	0	0	all	-	all	As:Chi	1977	CC	523	n	ot	y	n	1	ev	cig+/-ot	only hr	cu onlym	st
JUSSAW	41	m	0	0	all	-	all	As:Ind	1964	CC	792	n	V	n	n	3	ev	cig only	only hr	only mc	ot
MACLEN	76	m	0	0	ch	-	all	As:oth	1972	CC	233	n	bl	n	n	0	ev	cig+/-ot	both m&h	only mc	st
MACLEN	80	f	0	0	ch	-	all	As:oth	1972	CC	233	n	bl	n	n	0	ev	cig+/-ot	both m&h	only mc	st
MIGRAN	102	m	0	0	all	0	all	Eu:UK	1964	pr	259	n	V	n	n	3	cu	cig only	only hr	cu onlym	ot
NOTAN2	21	m	0	0	all	-	all	As:Ind	1963	CC	683	n	V	n	n	2	ev	cig only	only hr	only mc	ot
PERNU	10	m	0	0	all	-	all	Eu:Sca	1944	CC	1606	n	bl	n	n	0	ev	cig only	only hr	only mc	st
PERNU	6	f	0	0	all	-	all	Eu:Sca	1944	CC	1606	n	bl	n	n	0	ev	cig only	only hr	only mc	st
SUZUK2	11	c	0	0	all	-	all	SCAmer	1991	CC	123	n	bl	n	y	4	ev	cig only	any hr	only mc	or

Table 1F4 - 2

IESLC - Meta-analysis of Cigarette Smoking, Hand-rolled vs Manufactured
All LC types
Most adjusted

REF	NRR	SEX	AD	Number Exposed Case	Cont	Non-exposed Case	Cont	RR	95.00%CI
ALDERS	119	m	1	-	-	-	-	1.46	(1.10- 1.93)
BENHAM	109	m	2	-	-	-	-	1.20	(0.90- 1.60)
CHAN	25	m	0	39	23	167	138	1.40	(0.80- 2.46)
CHAN	26	f	0	63	38	42	12	0.47	(0.22- 1.01)
Subtotal CHAN								0.95	(0.61- 1.50)
DESTE2	21	c	7	-	-	-	-	2.00	(1.28- 3.12)
DESTEF	39	m	6	-	-	-	-	1.60	(1.20- 2.30)
*ENGELA	132	m	7	-	-	-	-	1.06	(0.79- 1.43)
*ENGELA	204	f	5	-	-	-	-	1.56	(0.91- 2.69)
Subtotal ENGELA								1.16	(0.89- 1.50)
GARDIN	13	c	0	11	8	72	39	0.74	(0.28- 2.01)
HU	21	m	0	61	43	57	51	1.27	(0.74- 2.18)
HU	22	f	0	18	8	7	9	2.89	(0.79- 10.53)
Subtotal HU								1.44	(0.87- 2.37)
HU2	11	c	1	-	-	-	-	1.21	(0.82- 1.79)
JUSSAW	41	m	3	-	-	-	-	2.54	(1.12- 5.77)
MACLEN	76	m	0	52	31	90	88	1.64	(0.96- 2.79)
MACLEN	80	f	0	18	28	27	29	0.69	(0.31- 1.52)
Subtotal MACLEN								1.25	(0.80- 1.95)
*MIGRAN	102	m	3	-	-	-	-	1.31	(0.83- 2.08)
NOTAN2	21	m	2	-	-	-	-	1.43	(1.05- 1.95)
PERNU	10	m	0	432	137	706	216	0.96	(0.75- 1.23)
PERNU	6	f	0	10	63	7	24	0.54	(0.19- 1.59)
Subtotal PERNU								0.94	(0.74- 1.19)
SUZUK2	11	c	4	-	-	-	-	2.80	(1.00- 7.70)
Partial Totals				704	379	1175	606		

*prospective study

REF	NRR	SEX	AD	Ys	Ws	Qs	Ps
ALDERS	119	m	1	0.38	48.61	0.92	0.0083
BENHAM	109	m	2	0.18	46.42	0.16	0.2142
CHAN	25	m	0	0.34	12.14	0.11	0.2398
CHAN	26	f	0	-0.75	6.70	6.54	0.0532
Subtotal CHAN				-0.05	18.84	6.65	
DESTE2	21	c	7	0.69	19.36	3.96	0.0023
DESTEF	39	m	6	0.47	36.30	1.90	0.0046
*ENGELA	132	m	7	0.06	43.64	1.46	0.7003
*ENGELA	204	f	5	0.44	13.08	0.54	0.1078
Subtotal ENGELA				0.15	56.72	2.00	
GARDIN	13	c	0	-0.29	3.91	1.12	0.5599
HU	21	m	0	0.24	13.02	0.00	0.3896
HU	22	f	0	1.06	2.30	1.55	0.1071
Subtotal HU				0.36	15.32	1.55	
HU2	11	c	1	0.19	25.21	0.06	0.3385
JUSSAW	41	m	3	0.93	5.72	2.73	0.0258
MACLEN	76	m	0	0.49	13.52	0.87	0.0689
MACLEN	80	f	0	-0.37	6.14	2.30	0.3586
Subtotal MACLEN				0.22	19.66	3.17	
*MIGRAN	102	m	3	0.27	18.21	0.02	0.2493
NOTAN2	21	m	2	0.36	40.10	0.55	0.0235
PERNU	10	m	0	-0.04	63.86	4.89	0.7743
PERNU	6	f	0	-0.61	3.33	2.40	0.2670
Subtotal PERNU				-0.06	67.19	7.30	
SUZUK2	11	c	4	1.03	3.69	2.29	0.0480

Table 1F4 - 2

IESLC - Meta-analysis of Cigarette Smoking, Hand-rolled vs Manufactured
All LC types
Most adjusted

	N	20
	NS	15
	Wt	425.25
Het	Chi	34.38
Het	df	19
Het	P	*
Fixed	RR	1.27
	RRl	1.16
	RRu	1.40
	P	+++
Random	RR	1.29
	RRl	1.12
	RRu	1.49
	P	+++
Asymm	P	N.S.

Table 1F4 - 3

IESLC - Meta-analysis of Cigarette Smoking, Hand-rolled vs Manufactured
All LC types
Most adjusted

	Sex			
	combined	male	female	Total
N	4	11	5	20
NS	4	11	5	20
Wt	52.17	341.53	31.55	425.25
Het Chi	6.12	13.61	10.96	34.38
Het df	3	10	4	19
Het P	N.S.	N.S.	*	*
Fixed RR	1.49	1.27	0.97	1.27
RRl	1.14	1.15	0.68	1.16
RRu	1.96	1.42	1.37	1.40
P	++	+++	N.S.	+++
Random RR	1.51	1.30	0.92	1.29
RRl	0.97	1.14	0.49	1.12
RRu	2.34	1.47	1.71	1.49
P	(+)	+++	N.S.	+++
Between Chi				3.69
Between df				2
Between P				N.S.
Btwn(F) P				N.S.
Btwn(R) P				N.S.

	All LC (or nearest)		
	all	other	Total
N	20		20
NS	15		15
Wt	425.25		425.25
Het Chi	34.38		34.38
Het df	19		19
Het P	*		*
Fixed RR	1.27		1.27
RRl	1.16		1.16
RRu	1.40		1.40
P	+++		+++
Random RR	1.29		1.29
RRl	1.12		1.12
RRu	1.49		1.49
P	+++		+++
Between Chi			
Between df			
Between P			N.S.
Btwn(F) P			N.S.
Btwn(R) P			N.S.

	Location								
	NAmer	UK	Scand	othEur	China	Japan	othAs	other	Total
N		3	4	1	3		6	3	20
NS		3	2	1	2		4	3	15
Wt		70.73	123.90	46.42	40.54		84.32	59.35	425.25
Het Chi		1.69	3.92	0.00	1.61		13.00	1.43	34.38
Het df		2	3	0	2		5	2	19
Het P		N.S.	N.S.	N.S.	N.S.		*	N.S.	*
Fixed RR		1.37	1.03	1.20	1.29		1.32	1.78	1.27
RRl		1.08	0.87	0.90	0.95		1.06	1.38	1.16
RRu		1.73	1.23	1.60	1.76		1.63	2.30	1.40
P		++	N.S.	N.S.	N.S.		+	+++	+++
Random RR		1.37	1.04	1.20	1.29		1.23	1.78	1.29
RRl		1.08	0.84	0.90	0.95		0.84	1.38	1.12
RRu		1.73	1.30	1.60	1.76		1.81	2.30	1.49
P		++	N.S.	N.S.	N.S.		N.S.	+++	+++
Between Chi									12.74
Between df									5
Between P									*
Btwn(F) P									N.S.
Btwn(R) P									(*)

Table 1F4 - 3

IESLC - Meta-analysis of Cigarette Smoking, Hand-rolled vs Manufactured
All LC types
Most adjusted

	Detailed Country in "other Europe"					
	multi	Germany	othWest	East	Balkans	Total
N			1			1
NS			1			1
Wt			46.42			46.42
Het Chi			0.00			0.00
Het df			0			0
Het P			N.S.			N.S.
Fixed RR			1.20			1.20
RRl			0.90			0.90
RRu			1.60			1.60
P			N.S.			N.S.
Random RR			1.20			1.20
RRl			0.90			0.90
RRu			1.60			1.60
P			N.S.			N.S.
Between Chi						
Between df						
Between P						N.S.
Btwn(F) P						N.S.
Btwn(R) P						N.S.

	Detailed Country in "other Asia"			
	India	HongKong	other	Total
N	2	2	2	6
NS	2	1	1	4
Wt	45.82	18.84	19.66	84.32
Het Chi	1.65	5.08	3.16	13.00
Het df	1	1	1	5
Het P	N.S.	*	(*)	*
Fixed RR	1.54	0.95	1.25	1.32
RRl	1.15	0.61	0.80	1.06
RRu	2.05	1.50	1.95	1.63
P	++	N.S.	N.S.	+
Random RR	1.67	0.84	1.12	1.23
RRl	1.01	0.29	0.48	0.84
RRu	2.76	2.43	2.60	1.81
P	+	N.S.	N.S.	N.S.
Between Chi				3.11
Between df				2
Between P				N.S.
Btwn(F) P				N.S.
Btwn(R) P				N.S.

	Detailed other continent			
	SCAmer	Auslia	Africa	Total
N	3			3
NS	3			3
Wt	59.35			59.35
Het Chi	1.43			1.43
Het df	2			2
Het P	N.S.			N.S.
Fixed RR	1.78			1.78
RRl	1.38			1.38
RRu	2.30			2.30
P	+++			+++
Random RR	1.78			1.78
RRl	1.38			1.38
RRu	2.30			2.30
P	+++			+++
Between Chi				
Between df				
Between P				N.S.
Btwn(F) P				N.S.
Btwn(R) P				N.S.

Table 1F4 - 3

IESLC - Meta-analysis of Cigarette Smoking, Hand-rolled vs Manufactured
All LC types
Most adjusted

	<1960	1960-69	1970-79	1980-89	1990+	Total
		Start year of study				
N	2	5	7	4	2	20
NS	1	4	5	3	2	15
Wt	67.19	120.74	158.75	55.54	23.04	425.25
Het Chi	1.04	5.17	10.90	3.41	0.35	34.38
Het df	1	4	6	3	1	19
Het P	N.S.	N.S.	(*)	N.S.	N.S.	*
Fixed RR	0.94	1.31	1.25	1.47	2.11	1.27
RRl	0.74	1.10	1.07	1.13	1.40	1.16
RRu	1.19	1.57	1.46	1.91	3.17	1.40
P	N.S.	++	++	++	+++	+++
Random RR	0.93	1.34	1.20	1.45	2.11	1.29
RRl	0.70	1.08	0.95	1.07	1.40	1.12
RRu	1.23	1.66	1.50	1.97	3.17	1.49
P	N.S.	++	N.S.	+	+++	+++
Between Chi						13.52
Between df						4
Between P						**
Btwn(F) P						(*)
Btwn(R) P						*

Study type (1)

	CC	other	Total
N	17	3	20
NS	13	2	15
Wt	350.33	74.92	425.25
Het Chi	32.30	1.71	34.38
Het df	16	2	19
Het P	**	N.S.	*
Fixed RR	1.29	1.19	1.27
RRl	1.16	0.95	1.16
RRu	1.43	1.50	1.40
P	+++	N.S.	+++
Random RR	1.30	1.19	1.29
RRl	1.10	0.95	1.12
RRu	1.54	1.50	1.49
P	++	N.S.	+++
Between Chi			0.37
Between df			1
Between P			N.S.
Btwn(F) P			N.S.
Btwn(R) P			N.S.

Study type (2)

	CC	prosp	other	Total
N	17	3		20
NS	13	2		15
Wt	350.33	74.92		425.25
Het Chi	32.30	1.71		34.38
Het df	16	2		19
Het P	**	N.S.		*
Fixed RR	1.29	1.19		1.27
RRl	1.16	0.95		1.16
RRu	1.43	1.50		1.40
P	+++	N.S.		+++
Random RR	1.30	1.19		1.29
RRl	1.10	0.95		1.12
RRu	1.54	1.50		1.49
P	++	N.S.		+++
Between Chi				0.37
Between df				1
Between P				N.S.
Btwn(F) P				N.S.
Btwn(R) P				N.S.

Table 1F4 - 3

IESLC - Meta-analysis of Cigarette Smoking, Hand-rolled vs Manufactured
All LC types
Most adjusted

	Study size (number of LC cases) 100-249	250-499	500-999	1000+	Total
N	6	7	3	4	20
NS	4	5	3	3	15
Wt	42.59	149.42	71.03	162.22	425.25
Het Chi	8.01	14.22	2.58	6.69	34.38
Het df	5	6	2	3	19
Het P	N.S.	*	N.S.	(*)	*
Fixed RR	1.34	1.33	1.41	1.15	1.27
RRl	1.00	1.13	1.12	0.99	1.16
RRu	1.82	1.56	1.78	1.34	1.40
P	(+)	+++	++	(+)	+++
Random RR	1.35	1.32	1.43	1.14	1.29
RRl	0.90	1.01	1.08	0.88	1.12
RRu	2.02	1.71	1.89	1.47	1.49
P	N.S.	+	+	N.S.	+++
Between Chi					2.89
Between df					3
Between P					N.S.
Btwn(F) P					N.S.
Btwn(R) P					N.S.

	Risky occupational population no	mining	othRisky	Total
N	20			20
NS	15			15
Wt	425.25			425.25
Het Chi	34.38			34.38
Het df	19			19
Het P	*			*
Fixed RR	1.27			1.27
RRl	1.16			1.16
RRu	1.40			1.40
P	+++			+++
Random RR	1.29			1.29
RRl	1.12			1.12
RRu	1.49			1.49
P	+++			+++
Between Chi				
Between df				
Between P				N.S.
Btwn(F) P				N.S.
Btwn(R) P				N.S.

	National cigarette tobacco type Virginia	blended	other	Total
N	5	12	3	20
NS	5	8	2	15
Wt	116.55	268.17	40.54	425.25
Het Chi	3.71	26.66	1.61	34.38
Het df	4	11	2	19
Het P	N.S.	**	N.S.	*
Fixed RR	1.43	1.21	1.29	1.27
RRl	1.19	1.07	0.95	1.16
RRu	1.72	1.36	1.76	1.40
P	+++	++	N.S.	+++
Random RR	1.43	1.23	1.29	1.29
RRl	1.19	1.00	0.95	1.12
RRu	1.72	1.51	1.76	1.49
P	+++	(+)	N.S.	+++
Between Chi				2.40
Between df				2
Between P				N.S.
Btwn(F) P				N.S.
Btwn(R) P				N.S.

Table 1F4 - 3

IESLC - Meta-analysis of Cigarette Smoking, Hand-rolled vs Manufactured
All LC types
Most adjusted

	Any proxy use No/nk	Yes	Total
N	18	2	20
NS	13	2	15
Wt	396.13	29.13	425.25
Het Chi	33.17	0.80	34.38
Het df	17	1	19
Het P	*	N.S.	*
Fixed RR	1.28	1.13	1.27
RRl	1.16	0.79	1.16
RRu	1.42	1.63	1.40
P	+++	N.S.	+++
Random RR	1.31	1.13	1.29
RRl	1.13	0.79	1.12
RRu	1.53	1.63	1.49
P	+++	N.S.	+++
Between Chi			0.42
Between df			1
Between P			N.S.
Btwn(F) P			N.S.
Btwn(R) P			N.S.

	Full histological confirmation No	Yes	Total
N	15	5	20
NS	11	4	15
Wt	323.53	101.73	425.25
Het Chi	28.14	4.86	34.38
Het df	14	4	19
Het P	*	N.S.	*
Fixed RR	1.23	1.41	1.27
RRl	1.11	1.16	1.16
RRu	1.37	1.71	1.40
P	+++	+++	+++
Random RR	1.24	1.44	1.29
RRl	1.04	1.14	1.12
RRu	1.47	1.81	1.49
P	+	++	+++
Between Chi			1.39
Between df			1
Between P			N.S.
Btwn(F) P			N.S.
Btwn(R) P			N.S.

	Number of adjustment variables (1) 0	1	2+/+nk	Total
N	9	2	9	20
NS	5	2	8	15
Wt	124.92	73.83	226.50	425.25
Het Chi	14.16	0.59	11.64	34.38
Het df	8	1	8	19
Het P	(*)	N.S.	N.S.	*
Fixed RR	1.03	1.37	1.40	1.27
RRl	0.86	1.09	1.23	1.16
RRu	1.23	1.72	1.59	1.40
P	N.S.	++	+++	+++
Random RR	1.03	1.37	1.44	1.29
RRl	0.78	1.09	1.22	1.12
RRu	1.36	1.72	1.70	1.49
P	N.S.	++	+++	+++
Between Chi				7.99
Between df				2
Between P				*
Btwn(F) P				N.S.
Btwn(R) P				N.S.

Table 1F4 - 3

IESLC - Meta-analysis of Cigarette Smoking, Hand-rolled vs Manufactured
All LC types
Most adjusted

	Number of adjustment variables (2) 0	1	2	3-5	6+/+nk	Total
N	9	2	2	4	3	20
NS	5	2	2	4	3	16
Wt	124.92	73.83	86.51	40.69	99.30	425.25
Het Chi	14.16	0.59	0.66	3.10	6.49	34.38
Het df	8	1	1	3	2	19
Het P	(*)	N.S.	N.S.	N.S.	*	*
Fixed RR	1.03	1.37	1.30	1.63	1.39	1.27
RRl	0.86	1.09	1.05	1.20	1.15	1.16
RRu	1.23	1.72	1.61	2.22	1.70	1.40
P	N.S.	++	+	++	+++	+++
Random RR	1.03	1.37	1.30	1.64	1.46	1.29
RRl	0.78	1.09	1.05	1.20	1.02	1.12
RRu	1.36	1.72	1.61	2.24	2.11	1.49
P	N.S.	++	+	++	+	+++
Between Chi						9.39
Between df						4
Between P						(*)
Btwn(F) P						N.S.
Btwn(R) P						N.S.

	Smoking status ever	current	Total
N	15	5	20
NS	11	4	15
Wt	300.00	125.26	425.25
Het Chi	30.78	2.56	34.38
Het df	14	4	19
Het P	**	N.S.	*
Fixed RR	1.31	1.18	1.27
RRl	1.17	0.99	1.16
RRu	1.47	1.40	1.40
P	+++	(+)	+++
Random RR	1.33	1.18	1.29
RRl	1.10	0.99	1.12
RRu	1.61	1.40	1.49
P	++	(+)	+++
Between Chi			1.04
Between df			1
Between P			N.S.
Btwn(F) P			N.S.
Btwn(R) P			N.S.

	Product cig+/-ot	cig only	Total
N	11	9	20
NS	7	8	15
Wt	191.42	233.84	425.25
Het Chi	19.07	14.90	34.38
Het df	10	8	19
Het P	*	(*)	*
Fixed RR	1.32	1.24	1.27
RRl	1.14	1.09	1.16
RRu	1.52	1.41	1.40
P	+++	++	+++
Random RR	1.31	1.27	1.29
RRl	1.06	1.04	1.12
RRu	1.61	1.55	1.49
P	+	+	+++
Between Chi			0.41
Between df			1
Between P			N.S.
Btwn(F) P			N.S.
Btwn(R) P			N.S.

Table 1F4 - 3

IESLC - Meta-analysis of Cigarette Smoking, Hand-rolled vs Manufactured
All LC types
Most adjusted

	Cigarette type				
	only hr	mainly hr	both m&h	any hr	Total
N	7		11	2	20
NS	6		7	2	15
Wt	160.33		214.82	50.10	425.25
Het Chi	10.43		19.17	2.45	34.38
Het df	6		10	1	19
Het P	N.S.		*	N.S.	*
Fixed RR	1.16		1.36	1.28	1.27
RRl	0.99		1.19	0.97	1.16
RRu	1.36		1.56	1.68	1.40
P	(+)		+++	(+)	+++
Random RR	1.19		1.34	1.58	1.29
RRl	0.94		1.10	0.73	1.12
RRu	1.50		1.64	3.45	1.49
P	N.S.		++	N.S.	+++
Between Chi					2.33
Between df					2
Between P					N.S.
Btwn(F) P					N.S.
Btwn(R) P					N.S.

	Denominator				
	ever mc	any mc	cu onlym	only mc	Total
N			3	17	20
NS			3	12	15
Wt			47.33	377.92	425.25
Het Chi			1.03	33.16	34.38
Het df			2	16	19
Het P			N.S.	**	*
Fixed RR			1.20	1.28	1.27
RRl			0.90	1.16	1.16
RRu			1.59	1.42	1.40
P			N.S.	+++	+++
Random RR			1.20	1.31	1.29
RRl			0.90	1.12	1.12
RRu			1.59	1.55	1.49
P			N.S.	+++	+++
Between Chi					0.19
Between df					1
Between P					N.S.
Btwn(F) P					N.S.
Btwn(R) P					N.S.

	Derivation of RR/CI			
	Orig	StdCalc	Other	Total
N	3	10	7	20
NS	3	6	6	15
Wt	86.41	150.14	188.71	425.25
Het Chi	3.52	14.71	8.20	34.38
Het df	2	9	6	19
Het P	N.S.	(*)	N.S.	*
Fixed RR	1.40	1.06	1.41	1.27
RRl	1.14	0.90	1.22	1.16
RRu	1.73	1.24	1.63	1.40
P	++	N.S.	+++	+++
Random RR	1.46	1.06	1.43	1.29
RRl	1.06	0.84	1.21	1.12
RRu	2.01	1.35	1.71	1.49
P	+	N.S.	+++	+++
Between Chi				7.95
Between df				2
Between P				*
Btwn(F) P				N.S.
Btwn(R) P				N.S.

Table 1F4 - 4

IESLC - Meta-analysis of Cigarette Smoking, Hand-rolled vs Manufactured
All LC types
Least adjusted

REF	NRR	X	SEX	AGEL	AGEH	RACE	YF	LC TYPE	LOC	START	ST	NLC	R	VB	P	H	AD	SM	PRODUCT	CIGTYP	DENOM	De
ALDERS	127	x	m	0	0	all	-	all	Eu:UK	1977	CC	1448	n	V	n	n	0	ev	cig only	both m&h	only mc	st
BENHAM	109		m	0	0	all	-	all	Eu:wst	1976	CC	1625	n	bl	n	y	2	cu	cig only	any hr	only mc	or
CHAN	25		m	0	0	all	-	all	As:HK	1976	CC	397	n	bl	n	n	0	ev	cig+/-ot	both m&h	only mc	st
CHAN	26		f	0	0	all	-	all	As:HK	1976	CC	397	n	bl	n	n	0	ev	cig+/-ot	both m&h	only mc	st
DESTE2	20	x	c	0	0	all	-	all	SCAmer	1993	CC	463	n	bl	n	n	0	ev	cig+/-ot	both m&h	only mc	st
DESTEF	50	x	m	0	0	all	-	all	SCAmer	1988	CC	497	n	bl	n	y	0	ev	cig+/-ot	both m&h	only mc	st
ENGELA	120	x	m	0	0	all	0	all	Eu:Sca	1964	pr	435	n	bl	n	n	0	cu	cig+/-ot	both m&h	only mc	st
ENGELA	127	x	f	0	0	all	0	all	Eu:Sca	1964	pr	435	n	bl	n	n	0	cu	cig+/-ot	both m&h	only mc	st
GARDIN	13		c	0	0	all	-	all	Eu:UK	1988	CC	143	n	V	y	n	0	cu	cig only	only hr	cu onlym	st
HU	21		m	0	0	all	-	all	As:Chi	1985	CC	227	n	ot	n	y	0	ev	cig+/-ot	both m&h	only mc	st
HU	22		f	0	0	all	-	all	As:Chi	1985	CC	227	n	ot	n	y	0	ev	cig+/-ot	both m&h	only mc	st
HU2	8	x	c	0	0	all	-	all	As:Chi	1977	CC	523	n	ot	y	n	0	ev	cig+/-ot	only hr	cu onlym	st
JUSSAW	16	x	m	0	0	all	-	all	As:Ind	1964	CC	792	n	V	n	n	0	ev	cig only	only hr	only mc	st
MACLEN	76		m	0	0	ch	-	all	As:oth	1972	CC	233	n	bl	n	n	0	ev	cig+/-ot	both m&h	only mc	st
MACLEN	80		f	0	0	ch	-	all	As:oth	1972	CC	233	n	bl	n	n	0	ev	cig+/-ot	both m&h	only mc	st
MIGRAN	101	x	m	0	0	all	0	all	Eu:UK	1964	pr	259	n	V	n	n	0	cu	cig only	only hr	cu onlym	st
NOTAN2	11	x	m	0	0	all	-	all	As:Ind	1963	CC	683	n	V	n	n	0	ev	cig only	only hr	only mc	st
PERNU	10		m	0	0	all	-	all	Eu:Sca	1944	CC	1606	n	bl	n	n	0	ev	cig only	only hr	only mc	st
PERNU	6		f	0	0	all	-	all	Eu:Sca	1944	CC	1606	n	bl	n	n	0	ev	cig only	only hr	only mc	st
SUZUK2	9	x	c	0	0	all	-	all	SCAmer	1991	CC	123	n	bl	n	y	0	ev	cig only	any hr	only mc	st

Table 1F4 - 5

IESLC - Meta-analysis of Cigarette Smoking, Hand-rolled vs Manufactured
All LC types
Least adjusted

REF	NRR	SEX	AD	Number Exposed Case	Number Exposed Cont	Non-exposed Case	Non-exposed Cont	RR	95.00%CI
ALDERS	127	m	0	191	113	385	349	1.53	(1.16- 2.02)
BENHAM	109	m	2	-	-	-	-	1.20	(0.90- 1.60)
CHAN	25	m	0	39	23	167	138	1.40	(0.80- 2.46)
CHAN	26	f	0	63	38	42	12	0.47	(0.22- 1.01)
Subtotal CHAN								0.95	(0.61- 1.50)
DESTE2	20	c	0	232	133	68	79	2.03	(1.37- 2.99)
DESTEF	50	m	0	363	221	108	113	1.72	(1.26- 2.35)
*ENGELA	120	m	0	199	75803	45	21361	1.25	(0.90- 1.72)
*ENGELA	127	f	0	39	37142	24	38340	1.68	(1.01- 2.79)
Subtotal ENGELA								1.36	(1.03- 1.78)
GARDIN	13	c	0	11	8	72	39	0.74	(0.28- 2.01)
HU	21	m	0	61	43	57	51	1.27	(0.74- 2.18)
HU	22	f	0	18	8	7	9	2.89	(0.79- 10.53)
Subtotal HU								1.44	(0.87- 2.37)
HU2	8	c	0	83	60	217	192	1.22	(0.83- 1.80)
JUSSAW	16	m	0	451	85	126	77	3.24	(2.25- 4.68)
MACLEN	76	m	0	52	31	90	88	1.64	(0.96- 2.79)
MACLEN	80	f	0	18	28	27	29	0.69	(0.31- 1.52)
Subtotal MACLEN								1.25	(0.80- 1.95)
*MIGRAN	101	m	0	21	496	115	3165	1.17	(0.74- 1.84)
NOTAN2	11	m	0	435	521	78	129	1.38	(1.01- 1.88)
PERNU	10	m	0	432	137	706	216	0.96	(0.75- 1.23)
PERNU	6	f	0	10	63	7	24	0.54	(0.19- 1.59)
Subtotal PERNU								0.94	(0.74- 1.19)
SUZUK2	9	c	0	30	6	82	63	3.84	(1.51- 9.79)
Partial Totals				2748	114959	2423	64474		

*prospective study

REF	NRR	SEX	AD	Ys	Ws	Qs	Ps
ALDERS	127	m	0	0.43	51.16	0.50	0.0023
BENHAM	109	m	2	0.18	46.42	0.98	0.2142
CHAN	25	m	0	0.34	12.14	0.00	0.2398
CHAN	26	f	0	-0.75	6.70	7.74	0.0532
Subtotal CHAN				-0.05	18.84	7.74	
DESTE2	20	c	0	0.71	25.51	3.66	0.0004
DESTEF	50	m	0	0.54	39.39	1.80	0.0007
*ENGELA	120	m	0	0.22	36.78	0.43	0.1820
*ENGELA	127	f	0	0.52	14.87	0.53	0.0461
Subtotal ENGELA				0.31	51.65	0.96	
GARDIN	13	c	0	-0.29	3.91	1.52	0.5599
HU	21	m	0	0.24	13.02	0.10	0.3896
HU	22	f	0	1.06	2.30	1.24	0.1071
Subtotal HU				0.36	15.32	1.35	
HU2	8	c	0	0.20	25.95	0.41	0.3032
JUSSAW	16	m	0	1.18	28.65	20.63	0.0000
MACLEN	76	m	0	0.49	13.52	0.38	0.0689
MACLEN	80	f	0	-0.37	6.14	2.99	0.3586
Subtotal MACLEN				0.22	19.66	3.37	
*MIGRAN	101	m	0	0.15	18.52	0.57	0.5104
NOTAN2	11	m	0	0.32	40.34	0.00	0.0404
PERNU	10	m	0	-0.04	63.86	8.44	0.7743
PERNU	6	f	0	-0.61	3.33	2.92	0.2670
Subtotal PERNU				-0.06	67.19	11.36	
SUZUK2	9	c	0	1.35	4.38	4.55	0.0048

Table 1F4 - 5

IESLC - Meta-analysis of Cigarette Smoking, Hand-rolled vs Manufactured
All LC types
Least adjusted

	N	20
	NS	15
	Wt	456.90
Het	Chi	59.39
Het	df	19
Het	P	***
Fixed	RR	1.39
	RRl	1.27
	RRu	1.52
	P	+++
Random	RR	1.38
	RRl	1.16
	RRu	1.65
	P	+++
Asymm	P	N.S.

Table 1F4 - 6

IESLC - Meta-analysis of Cigarette Smoking, Hand-rolled vs Manufactured
All LC types
Least adjusted

		Sex		
	combined	male	female	Total
N	4	11	5	20
NS	4	11	5	20
Wt	59.77	363.79	33.34	456.90
Het Chi	8.94	33.82	12.37	59.39
Het df	3	10	4	19
Het P	*	***	*	***
Fixed RR	1.60	1.39	1.03	1.39
RRl	1.24	1.26	0.73	1.27
RRu	2.06	1.54	1.44	1.52
P	+++	+++	N.S.	+++
Random RR	1.63	1.44	0.94	1.38
RRl	0.98	1.18	0.49	1.16
RRu	2.70	1.75	1.80	1.65
P	(+)	+++	N.S.	+++
Between Chi				4.25
Between df				2
Between P				N.S.
Btwn(F) P				N.S.
Btwn(R) P				N.S.

Table 1F4 - 7

IESLC - Meta-analysis of Cigarette Smoking, Hand-rolled vs Manufactured
All LC types
Excluded studies (and stage at which they were excluded)

1	BOUCHA	BUELL	LAURIL	MZILEN												
2	BERRIN	CHEN	RESTRE	TAO	ULMER											
3	ABELIN	ABRAHA	AGUDO	AKIBA	AMANDU	AMES	ANDERS	ARCHER	ARMADA	AUSTIN	AUVINE	AXELSO	AXELSS	BAND	BARBON	BECHER
	BENSHL	BEST	BLOHMK	BLOT1	BLOT2	BLOT3	BLOT4	BOFFET	BOUCOT	BRESLO	BRETT	BROCKM	BROSS	BROWN1	BROWN2	BYERS1
	BYERS2	CARPEN	CASCO2	CASCOR	CEDERL	CHANG	CHATZI	CHEN2	CHEN3	CHIAZZ	CHOI	CHOW	CHYOU	COMSTO	COOKSO	CORREA
	CPSI	CPSII	DAMBER	DARBY	DAVEYS	DEAN	DEAN2	DEKLER	DOCKER	DOLL	DOLL2	DORANT	DORGAN	DORN	DOSEME	DROSTE
	DU	DUNN	EBELIN	ENSTRO	ESAKI	FAN	GAO	GAO2	GARCIA	GARSHI	GENG	GER	GILLIS	GODLEY	GOLLED	GOODMA
	GRAHAM	GREGOR	GSELL	HAENSZ	HAMMO2	HAMMON	HANSEN	HEGMAN	HEIN	HENNEK	HINDS	HIRAOK	HIRAY2	HIRAYA	HITOSU	HOLE
	HOROWI	HORWIT	HUANG	HUMBLE	ISHIMA	JAHN	JAIN	JARUP	JARVHO	JEDRYC	JIANG	JOLY	JONES	KAISE2	KAISER	KANELL
	KATSOU	KAUFMA	KELLER	KHUDER	KIHARA	KINLEN	KJUUS	KNEKT	KO	KOHLME	KOO	KOULUM	KREUZE	KREYBE	KUBIK	LAMTH
	LAMWK	LAMWK2	LANGE	LAUSSM	LEI	LEMARC	LETOUR	LEVIN	LIAW	LICKIN	LIDDEL	LIU	LIU2	LIU3	LIU4	LIU5
	LOMBA2	LOMBAR	LUBIN	LUBIN2	LUO	MAGNUS	MARSH	MARSH2	MARTIS	MASTRA	MATOS	MATSUD	MCCONN	MCDUFF	MCLAUG	MILLER
	MILLS	MOLLO	MRFIT	MRFITR	MURATA	NAM	NOTANI	NOU	ODRISC	ORMOS	OSANN	OSANN2	PARKIN	PASTOR	PAWLEG	PERSH2
	PERSHA	PETO	PEZZO2	PEZZOT	PIKE	PISANI	POFFIJ	POLEDN	PRESCO	QIAO	QIAO2	RACHTA	RADZIK	RANDIG	REN	RIMING
	RONCO	ROOTS	ROTHSC	SAARIK	SADOWS	SANKAR	SCHWA2	SCHWAR	SEGI	SEGI2	SEOW	SHAW	SHIMIZ	SIEMIA	SIMARA	SIMONA
	SITAS	SOBUE	SOBUE2	SPEIZE	SPITZ	STASZE	STAYNE	STOCKS	STOCKW	STUCKE	SUN	SUZUKI	SVENSS	TANG	TANG2	TENKAN
	TIZZAN	TOKARS	TOUSEY	TSUGAN	TULINI	TVERDA	VANDER	VEIERO	VUTUC	WAKAI	WALD	WANG	WANG2	WANG3	WANG4	WARSIN
	WATSON	WICKLU	WIGLE	WILKIN	WU	WU2	WUNSCH	WUWILL	WYNDE2	WYNDE3	WYNDE4	WYNDE5	WYNDE6	WYNDE7	WYNDE8	WYNDER
	XIANGZ	XU	XU2	XU3	XU4	YAMAGU	YONG	YUAN	ZHANG	ZHENG	ZHOU					
4	BUFFLE	DEAN3														

Table 1F4 - 8
Potentially overlapping studies

REF	REFGP	PRINC	OVERLAP/LINK
BENHAM	LUBIN2	2	Subset of Lubin2
CHAN	CHAN	1	Chan/LamWK2

Table 1F5 -

IESLC - Meta-analysis of Cigarette Smoking, Menthol vs non-menthol
All LC types

This analysis is restricted to results for:
1) Non-dose-response data
2) Results complete enough for use in metaanalysis

Within each study, results are then selected (in the following order of preference, within each sex) for:
3) Cigarette type: menthol
4) Denominator: non-menthol
5) PRODUCT: cigarettes regardless of other products, cigarettes only
6) SMKSTA: ever, current
7) LCtype: all or nearest available, at least Squamous and Adeno. (q = squamous, s = small,
l = large, a = adeno, mix = mixed, alv = alveolar)
8) Race: all or nearest available, otherwise by race (wh or w = white, bl or b = black, hi = hispanic
ch = chinese, jap = japanese, haw = hawaiian, w+o = white + oriental, sca = scandinavian, as = asian)
9) Followup period (YF, prospective studies): whole study (coded as 0) or longest available
10) For overlapping studies: principal rather than subsidiary studies
Finally by Age: whole study (coded as 0) if available, otherwise by widest available age group
and then for single sex results (m, f) in preference to combined sex results (c).

Results adjusted (AD) for the most potential confounders are then chosen in Sections -1 to -3
and results adjusted for the least confounders in Sections -4 to -6. (Those least adjusted results which
actually differ from the most adjusted as marked 'x' in column X in Section -4)
(Results adjusted for an unknown number of confounder(s) are coded as 20.)

Section -7 shows excluded studies, together with the stage (as above) at which no qualifying
results were found.

Section -8 lists the potentially overlapping studies which have been included (1=principal, 2=subsidiary).

Section -9 lists any results which would have been included in preference except that they had data not complete
enough for use in meta-analysis, with their significance (yes/no), if known, and any further comment as entered
on the database.

In addition to those mentioned above, the following fields, levels and abbreviations are used:
* or nk = not known, n = no, y = yes, ot = other
ev = ever, cu = current, cig+/-ot = cigarettes irrespective of other products (cigar, pipe etc)
REF: 6-character study reference
NRR: number of the RR on the database within the study
ST : study type (CC = case control, pr or prosp = prospective)
NLC: number of lung cancer cases in whole study
R : risky occupational population (n = no, m = mining, o = other risky)
VB : national cigarette type (V = at least 75% Virginia, bl = at least 75% blended, ot = other)
P : any proxy use
H : full histological confirmation
De : derivation of RR/CI (or = original, st = standard method, ot = other method of estimation)

Table 1F5 - 1

IESLC - Meta-analysis of Cigarette Smoking, Menthol vs non-menthol
All LC types
Most adjusted

REF	NRR	SEX	AGEL	AGEH	RACE	YF	LC TYPE	LOC	START	ST	NLC	R	VB	P	H	AD	SM	PRODUCT	De
CARPEN	18	m	0	0	w+b	-	all	NAmer	1991	CC	356	n	bl	n	n	4	ev	cig+/-ot	ot
CARPEN	14	f	0	0	w+b	-	all	NAmer	1991	CC	356	n	bl	n	n	4	ev	cig+/-ot	ot
KAISE2	81	m	30	89	all	0	all	NAmer	1979	pr	318	n	bl	n	n	5	cu	cig only	or
KAISE2	82	f	30	89	all	0	all	NAmer	1979	pr	318	n	bl	n	n	5	cu	cig only	or
WYNDE8	3	m	0	0	all	-	all	NAmer	1985	CC	1044	n	bl	n	y	7	cu	cig+/-ot	ot
WYNDE8	4	f	0	0	all	-	all	NAmer	1985	CC	1044	n	bl	n	y	7	cu	cig+/-ot	ot

Table 1F5 - 2

IESLC - Meta-analysis of Cigarette Smoking, Menthol vs non-menthol
All LC types
Most adjusted

REF	NRR	SEX	AD	Number Exposed Case	Number Exposed Cont	Non-exposed Case	Non-exposed Cont	RR	95.00%CI
CARPEN	18	m	4	-	-	-	-	1.00	(0.68- 1.48)
CARPEN	14	f	4	-	-	-	-	0.88	(0.50- 1.57)
Subtotal CARPEN								0.96	(0.70- 1.32)
*KAISE2	81	m	5	-	-	-	-	1.45	(1.03- 2.02)
*KAISE2	82	f	5	-	-	-	-	0.75	(0.51- 1.11)
Subtotal KAISE2								1.09	(0.85- 1.41)
WYNDE8	3	m	7	-	-	-	-	1.06	(0.82- 1.37)
WYNDE8	4	f	7	-	-	-	-	0.78	(0.57- 1.08)
Subtotal WYNDE8								0.94	(0.77- 1.15)
Partial Totals				0	0	0	0		

*prospective study

REF	NRR	SEX	AD	Ys	Ws	Qs	Ps
CARPEN	18	m	4	0.00	25.41	0.00	1.0000
CARPEN	14	f	4	-0.13	11.74	0.16	0.6614
Subtotal CARPEN				-0.04	37.14	0.16	
*KAISE2	81	m	5	0.37	33.87	4.96	0.0306
*KAISE2	82	f	5	-0.29	25.41	1.94	0.1471
Subtotal KAISE2				0.09	59.28	6.91	
WYNDE8	3	m	7	0.06	58.33	0.28	0.6563
WYNDE8	4	f	7	-0.25	37.62	2.12	0.1275
Subtotal WYNDE8				-0.06	95.95	2.40	

	N	6
	NS	3
	Wt	192.37
Het	Chi	9.47
Het	df	5
Het	P	(*)
Fixed	RR	0.99
	RRl	0.86
	RRu	1.14
	P	N.S.
Random	RR	0.98
	RRl	0.80
	RRu	1.20
	P	N.S.
Asymm	P	N.S.

Table 1F5 - 3

IESLC - Meta-analysis of Cigarette Smoking, Menthol vs non-menthol
All LC types
Most adjusted

	combined	Sex male	female	Total
N		3	3	6
NS		3	3	6
Wt		117.60	74.76	192.37
Het Chi		2.70	0.21	9.47
Het df		2	2	5
Het P		N.S.	N.S.	(*)
Fixed RR		1.15	0.78	0.99
RRl		0.96	0.63	0.86
RRu		1.37	0.98	1.14
P		N.S.	-	N.S.
Random RR		1.15	0.78	0.98
RRl		0.93	0.63	0.80
RRu		1.43	0.98	1.20
P		N.S.	-	N.S.
Between Chi				6.56
Between df				1
Between P				*
Btwn(F) P				*
Btwn(R) P				*

Too few RRs for analysis by factor

Table 1F5 - 4

IESLC - Meta-analysis of Cigarette Smoking, Menthol vs non-menthol
All LC types
Least adjusted

REF	NRR	X	SEX	AGEL	AGEH	RACE	YF	LC TYPE	LOC	START	ST	NLC	R	VB	P	H	AD	SM	PRODUCT	De
CARPEN	20	x	m	0	0	w+b	-	all	NAmer	1991	CC	356	n	bl	n	n	0	ev	cig+/-ot	st
CARPEN	19	x	f	0	0	w+b	-	all	NAmer	1991	CC	356	n	bl	n	n	0	ev	cig+/-ot	st
KAISE2	83	x	m	30	89	all	0	all	NAmer	1979	pr	318	n	bl	n	n	1	cu	cig only	st
KAISE2	84	x	f	30	89	all	0	all	NAmer	1979	pr	318	n	bl	n	n	1	cu	cig only	st
WYNDE8	1	x	m	0	0	all	-	all	NAmer	1985	CC	1044	n	bl	n	y	0	cu	cig+/-ot	st
WYNDE8	2	x	f	0	0	all	-	all	NAmer	1985	CC	1044	n	bl	n	y	0	cu	cig+/-ot	st

Table 1F5 - 5

IESLC - Meta-analysis of Cigarette Smoking, Menthol vs non-menthol
All LC types
Least adjusted

REF	NRR	SEX	AD	Number Exposed Case	Number Exposed Cont	Non-exposed Case	Non-exposed Cont	RR	95.00%CI
CARPEN	20	m	0	88	159	114	190	0.92	(0.65- 1.31)
CARPEN	19	f	0	63	64	72	65	0.89	(0.55- 1.44)
Subtotal CARPEN								0.91	(0.69- 1.21)
*KAISE2	83	m	1	-	-	-	-	1.40	(1.01- 1.94)
*KAISE2	84	f	1	-	-	-	-	0.71	(0.50- 1.00)
Subtotal KAISE2								1.02	(0.80- 1.29)
WYNDE8	1	m	0	148	244	440	670	0.92	(0.73- 1.17)
WYNDE8	2	f	0	110	149	346	261	0.56	(0.41- 0.75)
Subtotal WYNDE8								0.76	(0.63- 0.91)
Partial Totals				409	616	972	1186		

*prospective study

REF	NRR	SEX	AD	Ys	Ws	Qs	Ps
CARPEN	20	m	0	-0.08	31.56	0.16	0.6501
CARPEN	19	f	0	-0.12	16.46	0.02	0.6321
Subtotal CARPEN				-0.09	48.01	0.18	
*KAISE2	83	m	1	0.34	36.06	8.60	0.0433
*KAISE2	84	f	1	-0.34	31.98	1.16	0.0528
Subtotal KAISE2				0.02	68.05	9.76	
WYNDE8	1	m	0	-0.08	68.40	0.36	0.5111
WYNDE8	2	f	0	-0.59	44.40	8.35	0.0001
Subtotal WYNDE8				-0.28	112.79	8.71	

	N	6
	NS	3
	Wt	228.85
Het	Chi	18.64
Het	df	5
Het	P	**
Fixed	RR	0.86
	RRl	0.75
	RRu	0.98
	P	-
Random	RR	0.86
	RRl	0.67
	RRu	1.12
	P	N.S.
Asymm	P	N.S.

Table 1F5 - 6

IESLC - Meta-analysis of Cigarette Smoking, Menthol vs non-menthol
All LC types
Least adjusted

	Sex			
	combined	male	female	Total
N		3	3	6
NS		3	3	6
Wt		136.02	92.83	228.85
Het Chi		4.59	2.91	18.64
Het df		2	2	5
Het P		N.S.	N.S.	**
Fixed RR		1.03	0.66	0.86
RRl		0.87	0.54	0.75
RRu		1.22	0.81	0.98
P		N.S.	---	-
Random RR		1.05	0.67	0.86
RRl		0.81	0.52	0.67
RRu		1.37	0.86	1.12
P		N.S.	--	N.S.
Between Chi				11.14
Between df				1
Between P				***
Btwn(F) P				(*)
Btwn(R) P				*

Table 1F5 - 7

IESLC - Meta-analysis of Cigarette Smoking, Menthol vs non-menthol
All LC types
Excluded studies (and stage at which they were excluded)

1 BOUCHA BUELL LAURIL MZILEN

2 BERRIN CHEN RESTRE TAO ULMER

3 ABELIN ABRAHA AGUDO AKIBA ALDERS AMANDU AMES ANDERS ARCHER ARMADA AUSTIN AUVINE AXELSO AXELSS BAND BARBON
BECHER BENHAM BENSHL BEST BLOHMK BLOT1 BLOT2 BLOT3 BLOT4 BOFFET BOUCOT BRESLO BRETT BROCKM BROWN1 BROWN2
BUFFLE BYERS1 BYERS2 CASCO2 CASCOR CEDERL CHAN CHANG CHATZI CHEN2 CHEN3 CHIAZZ CHOI CHOW CHYOU COMSTO
COOKSO CORREA CPSI CPSII DAMBER DARBY DAVEYS DEAN DEAN2 DEAN3 DEKLER DESTE2 DESTEF DOCKER DOLL DOLL2
DORANT DORGAN DORN DOSEME DROSTE DU DUNN EBELIN ENGELA ENSTRO ESAKI FAN GAO GAO2 GARCIA GARDIN
GARSHI GENG GER GILLIS GODLEY GOLLED GOODMA GRAHAM GREGOR GSELL HAENSZ HAMMO2 HAMMON HANSEN HEGMAN HEIN
HENNEK HINDS HIRAOK HIRAY2 HIRAYA HITOSU HOLE HOROWI HORWIT HU HU2 HUANG HUMBLE ISHIMA JAHN JAIN
JARUP JARVHO JEDRYC JIANG JOLY JONES JUSSAW KAISER KANELL KATSOU KAUFMA KELLER KHUDER KIHARA KINLEN KJUUS
KNEKT KO KOHLME KOO KOULUM KREUZE KREYBE KUBIK LAMTH LAMWK LAMWK2 LANGE LAUSSM LEI LEMARC LETOUR
LEVIN LIAW LICKIN LIDDEL LIU LIU2 LIU3 LIU4 LIU5 LOMBA2 LOMBAR LUBIN LUBIN2 LUO MACLEN MAGNUS
MARSH MARSH2 MARTIS MASTRA MATOS MATSUD MCCONN MCDUFF MCLAUG MIGRAN MILLER MILLS MOLLO MRFIT MRFITR MURATA
NAM NOTAN2 NOTANI NOU ODRISC ORMOS OSANN OSANN2 PARKIN PASTOR PAWLEG PERNU PERSH2 PERSHA PETO PEZZO2
PEZZOT PIKE PISANI POFFIJ POLEDN PRESCO QIAO QIAO2 RACHTA RADZIK RANDIG REN RIMING RONCO ROOTS ROTHSC
SAARIK SADOWS SANKAR SCHWA2 SCHWAR SEGI SEGI2 SEOW SHAW SHIMIZ SIEMIA SIMARA SIMONA SITAS SOBUE SOBUE2
SPEIZE SPITZ STASZE STAYNE STOCKS STOCKW STUCKE SUN SUZUK2 SUZUKI SVENSS TANG TANG2 TENKAN TIZZAN TOKARS
TOUSEY TSUGAN TULINI TVERDA VANDER VEIERO VUTUC WAKAI WALD WANG WANG2 WANG3 WANG4 WARSIN WATSON WICKLU
WIGLE WILKIN WU WU2 WUNSCH WUWILL WYNDE2 WYNDE3 WYNDE4 WYNDE5 WYNDE6 WYNDE7 WYNDER XIANGZ XU XU2
XU3 XU4 YAMAGU YONG YUAN ZHANG ZHENG ZHOU

4 BROSS

Table 1F5 - 8

Potentially overlapping studies

REF	REFGP	PRINC	.	OVERLAP/LINK
WYNDE8	WYNDE6	2		WYNDE5/6/7/8
