# Supplementary material for: Systematic review with meta-analysis of the epidemiological evidence in the 1900s relating smoking to lung cancer
Source: BMC Cancer. 2012 Sep 3;12:385. doi: 10.1186/1471-2407-12-385 (PMC3505152; doi:10.1186/1471-2407-12-385)
Supplement: Additional file 5 — Detailed Analysis Tables (Individual file names as described in Additional file 1: Methods, Table1). [file 1471-2407-12-385-S5.zip › PDF/1BR.pdf]

Table 1B1R - 1

IESLC - Meta-regression of current smoking, any product (or cigs if any not available)
Multiple regression of data from Table 1B1
All LC types

Stepwise allowing only characteristics from the fixed model

Log Relative risk
WEIGHTED on Weight

		Deviance	(DF)				
Model 1		2669.216	(194)				
		Estimate	S.E.	P	RR	95%CIl	95%CIu
Constant		2.229	0.012	+++	9.291	9.072	9.515
		Deviance	(DF)	Drop Dev	P		
Model 2		1257.714	(187)	1411.501	***		
		Estimate	S.E.	P	RR	95%CIl	95%CIu
Constant		2.521	0.015	+++	12.447	12.089	12.816
Location							
NAmer	84	Aliased			12.447	11.540	13.426
UK	25	-0.590	0.065	---	6.903	5.011	9.509
Scand	21	-0.422	0.059	---	8.159	6.108	10.900
othEur	23	-0.749	0.044	---	5.883	4.769	7.258
China	5	-1.400	0.094	---	3.070	1.912	4.928
Japan	18	-1.219	0.039	---	3.680	3.057	4.431
othAs	7	-1.452	0.131	---	2.915	1.502	5.654
other	12	-0.563	0.078	---	7.089	4.802	10.466
		Deviance	(DF)	Drop Dev	P		
Model 3		918.536	(183)	339.178	***		
		Estimate	S.E.	P	RR	95%CIl	95%CIu
Constant		1.937	0.038	+++	6.936	6.437	7.473
Location							
NAmer	84	Aliased			12.214	11.370	13.121
UK	25	-0.433	0.068	---	7.922	5.957	10.535
Scand	21	-0.418	0.061	---	8.040	6.244	10.353
othEur	23	-0.605	0.052	---	6.671	5.408	8.230
China	5	-1.534	0.095	---	2.634	1.744	3.978
Japan	18	-1.177	0.052	---	3.764	3.074	4.608
othAs	7	-1.495	0.132	---	2.738	1.542	4.863
other	12	-0.627	0.083	---	6.524	4.591	9.270
Start year of study							
<1960	22	Aliased			5.276	4.472	6.224
1960-69	40	0.490	0.056	+++	8.613	7.289	10.177
1970-79	41	0.451	0.054	+++	8.279	6.936	9.882
1980-89	70	0.696	0.041	+++	10.583	9.781	11.450
1990+	22	0.973	0.077	+++	13.965	10.395	18.760
		Deviance	(DF)	Drop Dev	P		
Model 4		869.479	(181)	49.057	**		
		Estimate	S.E.	P	RR	95%CIl	95%CIu
Constant		2.024	0.040	+++	7.569	6.995	8.189
Location							
NAmer	84	Aliased			12.296	11.461	13.192
UK	25	-0.436	0.068	---	7.952	6.016	10.511
Scand	21	-0.435	0.061	---	7.962	6.211	10.207
othEur	23	-0.655	0.053	---	6.388	5.183	7.874
China	5	-1.540	0.095	---	2.637	1.758	3.955
Japan	18	-1.178	0.052	---	3.787	3.107	4.617
othAs	7	-1.487	0.132	---	2.779	1.582	4.882
other	12	-0.682	0.084	---	6.214	4.397	8.782
Start year of study							
<1960	22	Aliased			5.222	4.428	6.159
1960-69	40	0.493	0.056	+++	8.549	7.235	10.100
1970-79	41	0.479	0.054	+++	8.431	7.086	10.033
1980-89	70	0.706	0.043	+++	10.578	9.779	11.442
1990+	22	0.997	0.080	+++	14.157	10.563	18.974
Sex(RR)							
Male	108	Aliased			10.174	9.336	11.089
Female	68	-0.197	0.028	---	8.358	7.665	9.114
Combined	19	-0.078	0.036	-	9.407	8.345	10.604

Table 1B1R - 1

IESLC - Meta-regression of current smoking, any product (or cigs if any not available)
Multiple regression of data from Table 1B1
All LC types

Fixed model

Log Relative risk
WEIGHTED on Weight

		Deviance	(DF)				
Model 7		819.856	(175)				
		Estimate	S.E.	P	RR	95%CIl	95%CIu
Constant		1.566	0.081	+++	4.788	4.084	5.614
Sex(RR)							
Male	108	Aliased			10.121	9.277	11.042
Female	68	-0.204	0.029	---	8.249	7.566	8.995
Combined	19	-0.043	0.039	N.S.	9.698	8.529	11.028
Location							
NAmer	84	Aliased			12.126	11.291	13.023
UK	25	-0.345	0.071	---	8.587	6.457	11.421
Scand	21	-0.417	0.066	---	7.994	6.116	10.450
othEur	23	-0.602	0.057	---	6.639	5.316	8.293
China	5	-1.528	0.097	---	2.631	1.754	3.946
Japan	18	-1.174	0.056	---	3.749	3.039	4.625
othAs	7	-1.221	0.139	---	3.576	1.989	6.430
other	12	-0.593	0.088	---	6.698	4.683	9.582
Start year of study							
<1960	22	Aliased			4.862	4.028	5.868
1960-69	40	0.538	0.063	+++	8.323	6.962	9.949
1970-79	41	0.571	0.058	+++	8.609	7.186	10.314
1980-89	70	0.788	0.051	+++	10.696	9.833	11.634
1990+	22	1.169	0.089	+++	15.643	11.401	21.464
Study type (1)							
CC	128	Aliased			8.921	8.333	9.551
other	67	0.182	0.047	+++	10.698	9.089	12.591
Study size (number of LC cases)							
100-249	56	Aliased			6.849	5.344	8.777
250-499	48	0.222	0.071	++	8.551	6.910	10.583
500-999	38	0.266	0.070	+++	8.939	7.473	10.692
1000+	53	0.340	0.062	+++	9.622	9.029	10.254
Number of adjustment variables (1)							
0	86	Aliased			9.093	8.286	9.978
1	62	0.002	0.047	N.S.	9.106	7.849	10.566
2+/+nk	47	0.069	0.034	+	9.745	8.772	10.826

Table 1B1R - 2

IESLC - Meta-regression of current smoking, any product (or cigs if any not available)
Multiple regression of data from Table 1B1
All LC types
Effect of removing characteristics

Log Relative risk
WEIGHTED on Weight

		Deviance	(DF)				
Model 7		819.856	(175)				
		Estimate	S.E.	P	RR	95%CIl	95%CIu
Constant		1.566	0.081	+++	4.788	4.084	5.614
Sex(RR)							
Male	108	Aliased			10.121	9.277	11.042
Female	68	-0.204	0.029	---	8.249	7.566	8.995
Combined	19	-0.043	0.039	N.S.	9.698	8.529	11.028
Location							
NAmer	84	Aliased			12.126	11.291	13.023
UK	25	-0.345	0.071	---	8.587	6.457	11.421
Scand	21	-0.417	0.066	---	7.994	6.116	10.450
othEur	23	-0.602	0.057	---	6.639	5.316	8.293
China	5	-1.528	0.097	---	2.631	1.754	3.946
Japan	18	-1.174	0.056	---	3.749	3.039	4.625
othAs	7	-1.221	0.139	---	3.576	1.989	6.430
other	12	-0.593	0.088	---	6.698	4.683	9.582
Start year of study							
<1960	22	Aliased			4.862	4.028	5.868
1960-69	40	0.538	0.063	+++	8.323	6.962	9.949
1970-79	41	0.571	0.058	+++	8.609	7.186	10.314
1980-89	70	0.788	0.051	+++	10.696	9.833	11.634
1990+	22	1.169	0.089	+++	15.643	11.401	21.464
Study type (1)							
CC	128	Aliased			8.921	8.333	9.551
other	67	0.182	0.047	+++	10.698	9.089	12.591
Study size (number of LC cases)							
100-249	56	Aliased			6.849	5.344	8.777
250-499	48	0.222	0.071	++	8.551	6.910	10.583
500-999	38	0.266	0.070	+++	8.939	7.473	10.692
1000+	53	0.340	0.062	+++	9.622	9.029	10.254
Number of adjustment variables (1)							
0	86	Aliased			9.093	8.286	9.978
1	62	0.002	0.047	N.S.	9.106	7.849	10.566
2+/+nk	47	0.069	0.034	+	9.745	8.772	10.826

Omit Sex		Deviance	(DF)	Drop Dev	P		
Model 8		875.738	(177)	-55.883	**		
		Estimate	S.E.	P	RR	95%CIl	95%CIu
Constant		1.517	0.080	+++	4.558	3.897	5.332
Number of adjustment variables (1)							
0	86	Aliased			9.134	8.362	9.977
1	62	-0.010	0.046	N.S.	9.044	7.813	10.470
2+/+nk	47	0.063	0.032	+	9.733	8.791	10.775
Location							
NAmer	84	Aliased			12.079	11.253	12.966
UK	25	-0.357	0.071	---	8.457	6.374	11.220
Scand	21	-0.396	0.065	---	8.131	6.262	10.557
othEur	23	-0.572	0.055	---	6.820	5.507	8.446
China	5	-1.534	0.097	---	2.606	1.742	3.898
Japan	18	-1.173	0.056	---	3.739	3.037	4.604
othAs	7	-1.254	0.139	---	3.448	1.927	6.169
other	12	-0.558	0.088	---	6.916	4.849	9.864
Start year of study							
<1960	22	Aliased			4.969	4.126	5.984
1960-69	40	0.517	0.063	+++	8.330	6.997	9.918
1970-79	41	0.531	0.057	+++	8.454	7.066	10.115
1980-89	70	0.767	0.050	+++	10.698	9.845	11.624
1990+	22	1.134	0.087	+++	15.441	11.332	21.038
Study type (1)							
CC	128	Aliased			8.973	8.386	9.602
other	67	0.155	0.047	++	10.482	8.919	12.320

Table 1B1R - 2

IESLC - Meta-regression of current smoking, any product (or cigs if any not available)
Multiple regression of data from Table 1B1
All LC types
Effect of removing characteristics

Log Relative risk
WEIGHTED on Weight

		Estimate	S.E.	P	RR	95%CIl	95%CIu
Study size (number of LC cases)							
100-249	56	Aliased			6.904	5.397	8.832
250-499	48	0.231	0.071	++	8.699	7.046	10.740
500-999	38	0.264	0.070	+++	8.992	7.525	10.745
1000+	53	0.329	0.062	+++	9.591	9.005	10.216

Omit Location		Deviance	(DF)	Drop Dev	P		
Model 8		1594.215	(182)	-774.360	***		
		Estimate	S.E.	P	RR	95%CIl	95%CIu
Constant		1.187	0.075	+++	3.278	2.832	3.795
Number of adjustment variables (1)							
0	86	Aliased			10.270	9.427	11.189
1	62	-0.260	0.044	---	7.919	6.892	9.100
2+/+nk	47	-0.122	0.032	---	9.089	8.226	10.044
Sex(RR)							
Male	108	Aliased			9.873	9.082	10.733
Female	68	-0.181	0.028	---	8.237	7.570	8.962
Combined	19	0.028	0.037	N.S.	10.154	8.999	11.458
Start year of study							
<1960	22	Aliased			5.304	4.438	6.338
1960-69	40	-0.015	0.053	N.S.	5.225	4.567	5.979
1970-79	41	0.364	0.056	+++	7.630	6.529	8.917
1980-89	70	0.862	0.048	+++	12.558	11.669	13.516
1990+	22	0.866	0.086	+++	12.615	9.408	16.916
Study type (1)							
CC	128	Aliased			8.335	7.806	8.899
other	67	0.485	0.045	+++	13.541	11.621	15.778
Study size (number of LC cases)							
100-249	56	Aliased			5.449	4.333	6.853
250-499	48	0.364	0.070	+++	7.842	6.445	9.543
500-999	38	0.643	0.067	+++	10.366	8.788	12.228
1000+	53	0.572	0.058	+++	9.654	9.089	10.255

Omit Start year		Deviance	(DF)	Drop Dev	P		
Model 8		1109.973	(179)	-290.117	***		
		Estimate	S.E.	P	RR	95%CIl	95%CIu
Constant		2.348	0.065	+++	10.468	9.214	11.893
Number of adjustment variables (1)							
0	86	Aliased			9.106	8.320	9.967
1	62	-0.060	0.044	N.S.	8.573	7.474	9.834
2+/+nk	47	0.113	0.034	+++	10.198	9.207	11.296
Sex(RR)							
Male	108	Aliased			9.607	8.828	10.455
Female	68	-0.148	0.028	---	8.285	7.607	9.022
Combined	19	0.094	0.038	+	10.553	9.329	11.937
Location							
NAmer	84	Aliased			12.416	11.630	13.255
UK	25	-0.494	0.069	---	7.573	5.734	10.002
Scand	21	-0.470	0.065	---	7.760	5.985	10.060
othEur	23	-0.746	0.047	---	5.890	4.901	7.078
China	5	-1.442	0.097	---	2.936	1.972	4.371
Japan	18	-1.211	0.042	---	3.700	3.153	4.341
othAs	7	-1.250	0.139	---	3.556	1.999	6.327
other	12	-0.606	0.085	---	6.770	4.788	9.573
Study type (1)							
CC	128	Aliased			9.465	8.869	10.100
other	67	-0.083	0.043	(-)	8.712	7.509	10.108
Study size (number of LC cases)							
100-249	56	Aliased			7.587	5.968	9.646
250-499	48	0.297	0.070	+++	10.208	8.399	12.407
500-999	38	0.135	0.068	(+)	8.682	7.342	10.267
1000+	53	0.216	0.060	+++	9.419	8.859	10.014

Table 1B1R - 2

IESLC - Meta-regression of current smoking, any product (or cigs if any not available)
Multiple regression of data from Table 1B1
All LC types
Effect of removing characteristics

Log Relative risk
WEIGHTED on Weight

Omit Study type		Deviance	(DF)	Drop Dev	P		
Model 8		834.831	(176)	-14.975	(*)		
		Estimate	S.E.	P	RR	95%CIl	95%CIu
Constant		1.659	0.078	+++	5.252	4.511	6.115
Number of adjustment variables (1)							
0	86	Aliased			8.838	8.099	9.644
1	62	0.104	0.039	++	9.806	8.661	11.103
2+/+nk	47	0.081	0.034	+	9.586	8.645	10.630
Sex(RR)							
Male	108	Aliased			10.107	9.266	11.024
Female	68	-0.198	0.028	---	8.292	7.609	9.038
Combined	19	-0.047	0.039	N.S.	9.640	8.482	10.955
Location							
NAmer	84	Aliased			12.266	11.437	13.156
UK	25	-0.369	0.071	---	8.482	6.385	11.268
Scand	21	-0.415	0.066	---	8.101	6.205	10.578
othEur	23	-0.642	0.056	---	6.454	5.182	8.039
China	5	-1.540	0.097	---	2.631	1.756	3.942
Japan	18	-1.222	0.054	---	3.615	2.943	4.441
othAs	7	-1.246	0.139	---	3.528	1.966	6.331
other	12	-0.655	0.087	---	6.374	4.479	9.070
Start year of study							
<1960	22	Aliased			5.057	4.213	6.070
1960-69	40	0.542	0.063	+++	8.694	7.324	10.320
1970-79	41	0.534	0.057	+++	8.625	7.203	10.327
1980-89	70	0.730	0.048	+++	10.490	9.673	11.376
1990+	22	1.105	0.087	+++	15.271	11.152	20.912
Study size (number of LC cases)							
100-249	56	Aliased			7.062	5.527	9.024
250-499	48	0.225	0.071	++	8.848	7.177	10.907
500-999	38	0.232	0.070	++	8.905	7.448	10.646
1000+	53	0.304	0.061	+++	9.573	8.987	10.197

Omit Study size		Deviance	(DF)	Drop Dev	P		
Model 8		850.683	(178)	-30.827	(*)		
		Estimate	S.E.	P	RR	95%CIl	95%CIu
Constant		1.894	0.054	+++	6.649	5.977	7.396
Number of adjustment variables (1)							
0	86	Aliased			9.014	8.228	9.875
1	62	0.017	0.047	N.S.	9.167	7.917	10.614
2+/+nk	47	0.086	0.034	+	9.820	8.850	10.896
Sex(RR)							
Male	108	Aliased			10.047	9.219	10.949
Female	68	-0.196	0.028	---	8.258	7.580	8.997
Combined	19	-0.024	0.039	N.S.	9.807	8.638	11.133
Location							
NAmer	84	Aliased			12.243	11.412	13.133
UK	25	-0.397	0.069	---	8.230	6.245	10.846
Scand	21	-0.494	0.063	---	7.467	5.804	9.605
othEur	23	-0.589	0.057	---	6.796	5.462	8.456
China	5	-1.569	0.097	---	2.550	1.707	3.808
Japan	18	-1.182	0.055	---	3.753	3.059	4.604
othAs	7	-1.457	0.133	---	2.852	1.638	4.966
other	12	-0.656	0.086	---	6.353	4.505	8.960
Start year of study							
<1960	22	Aliased			4.961	4.123	5.970
1960-69	40	0.520	0.062	+++	8.346	7.027	9.913
1970-79	41	0.516	0.056	+++	8.311	6.982	9.894
1980-89	70	0.777	0.051	+++	10.791	9.945	11.708
1990+	22	1.050	0.082	+++	14.182	10.642	18.901
Study type (1)							
CC	128	Aliased			9.007	8.424	9.630
other	67	0.139	0.046	++	10.348	8.835	12.121

Table 1B1R - 2

IESLC - Meta-regression of current smoking, any product (or cigs if any not available)
Multiple regression of data from Table 1B1
All LC types
Effect of removing characteristics

Log Relative risk
WEIGHTED on Weight

Omit N adjustment vars		Deviance	(DF)	Drop Dev	P		
Model 8		824.786	(177)	-4.930	N.S.		
		Estimate	S.E.	P	RR	95%CIl	95%CIu
Constant		1.573	0.080	+++	4.820	4.123	5.635
Study size (number of LC cases)							
100-249	56	Aliased			6.797	5.316	8.692
250-499	48	0.220	0.071	++	8.473	6.866	10.457
500-999	38	0.276	0.070	+++	8.955	7.500	10.693
1000+	53	0.349	0.062	+++	9.634	9.044	10.262
Sex(RR)							
Male	108	Aliased			10.197	9.367	11.099
Female	68	-0.209	0.028	---	8.273	7.594	9.013
Combined	19	-0.068	0.037	(-)	9.527	8.449	10.743
Location							
NAmer	84	Aliased			12.082	11.262	12.961
UK	25	-0.351	0.071	---	8.509	6.420	11.279
Scand	21	-0.381	0.064	---	8.254	6.373	10.690
othEur	23	-0.603	0.056	---	6.612	5.317	8.222
China	5	-1.494	0.096	---	2.713	1.821	4.043
Japan	18	-1.162	0.053	---	3.780	3.103	4.604
othAs	7	-1.234	0.139	---	3.516	1.965	6.292
other	12	-0.575	0.088	---	6.796	4.768	9.687
Start year of study							
<1960	22	Aliased			4.792	3.996	5.747
1960-69	40	0.561	0.059	+++	8.397	7.091	9.943
1970-79	41	0.579	0.058	+++	8.552	7.159	10.216
1980-89	70	0.804	0.050	+++	10.713	9.854	11.645
1990+	22	1.183	0.089	+++	15.639	11.425	21.406
Study type (1)							
CC	128	Aliased			8.950	8.406	9.529
other	67	0.167	0.038	+++	10.577	9.241	12.107

Table 1B1R - 3

IESLC - Meta-regression of current smoking, any product (or cigs if any not available)
Multiple regression of data from Table 1B1
All LC types
Study outliers

Study Ref	NRR	LOGRR	FITVAL	SEFITV	STDRES
BROWN2	12	2.425	2.764	0.108	-3.145
TIZZAN	5	0.640	1.304	0.236	-2.815
CPSI	279	1.163	1.885	0.258	-2.795
BLOHMK	1	1.162	1.801	0.280	-2.279
LIDDEL	4	1.484	2.543	0.511	-2.070
BROSS	11	1.577	2.370	0.405	-1.956
GARSHI	31	2.041	2.696	0.376	-1.743
HIRAYA	3	0.850	1.248	0.246	-1.620
PARKIN	26	1.406	1.846	0.274	-1.606
GREGOR	2	0.255	1.792	0.986	-1.559
LUBIN2	317	1.345	1.671	0.209	-1.558
DEAN2	2	1.334	2.025	0.444	-1.555
HOLE	31	0.425	1.771	0.965	-1.395
HAENSZ	54	0.760	1.362	0.442	-1.361
QIAO2	14	0.464	1.390	0.699	-1.325
KAISER	9	1.876	2.416	0.411	-1.315
DEAN2	6	1.064	1.820	0.580	-1.304
CPSII	133	2.466	2.673	0.165	-1.252
CHOI	7	0.199	1.151	0.801	-1.189
TVERDA	5	1.409	1.972	0.477	-1.180
KREUZE	42	1.769	2.268	0.435	-1.148
NAM	76	2.160	2.696	0.470	-1.142
WIGLE	30	1.649	2.201	0.487	-1.134
WU	42	1.581	2.219	0.625	-1.022
KIHARA	7	1.401	1.740	0.332	-1.021
SCHWAR	26	2.266	2.695	0.424	-1.012
DOLL	93	0.736	1.357	0.624	-0.996
KHUDER	19	2.092	2.577	0.492	-0.985
CEDERL	75	1.573	1.956	0.391	-0.978
TSUGAN	28	0.201	0.964	0.787	-0.970
PERSH2	10	2.115	2.305	0.209	-0.908
MIGRAN	20	1.369	2.232	1.090	-0.792
SOBUE	52	1.030	1.318	0.368	-0.783
KELLER	1	2.578	2.695	0.155	-0.752
WUNSCH	5	1.886	2.365	0.667	-0.718
KAISE2	68	2.084	2.543	0.662	-0.692
AMES	1	1.517	1.970	0.671	-0.674
SEGI2	28	0.501	0.949	0.666	-0.672
BRETT	4	1.367	1.940	0.899	-0.638
CHANG	11	1.639	2.115	0.753	-0.631
LEMARC	2	2.602	2.914	0.522	-0.598
WUNSCH	11	1.788	2.160	0.670	-0.555
YONG	15	1.649	2.116	0.861	-0.543
TANG	1	2.199	2.692	0.955	-0.517
ALDERS	176	1.758	1.928	0.336	-0.506
KATSOU	2	1.224	1.549	0.730	-0.446
LANGE	35	1.613	1.922	0.759	-0.406
DEAN3	42	1.905	2.094	0.497	-0.381
MIGRAN	136	1.607	2.027	1.146	-0.366
AGUDO	3	1.284	1.617	0.917	-0.363
LANGE	38	1.740	2.126	1.084	-0.356
GARCIA	2	2.716	2.914	0.588	-0.337
DESTE2	4	2.208	2.390	0.614	-0.296
DEAN3	119	1.753	1.890	0.473	-0.290
SOBUE	42	1.411	1.522	0.411	-0.271
NAM	92	2.383	2.492	0.442	-0.245
CEDERL	106	2.044	2.160	0.476	-0.244
DOCKER	1	2.079	2.346	1.092	-0.244
JAIN	52	2.518	2.690	0.781	-0.221
BUFFLE	7	2.111	2.199	0.422	-0.208
HAMMO2	8	2.316	2.509	0.974	-0.198
CHANG	5	2.127	2.319	1.022	-0.188

Table 1B1R - 3

IESLC - Meta-regression of current smoking, any product (or cigs if any not available)
Multiple regression of data from Table 1B1
All LC types
Study outliers

Study Ref	NRR	LOGRR	FITVAL	SEFITV	STDRES
GOODMA	6	2.267	2.372	0.660	-0.159
ENGELA	164	1.754	1.888	0.846	-0.158
ENGELA	158	1.997	2.092	0.844	-0.112
WIGLE	25	2.342	2.405	0.594	-0.107
KELLER	13	2.455	2.490	0.367	-0.096
MATOS	3	2.140	2.211	0.745	-0.095
BENSHL	16	2.102	2.164	0.897	-0.069
GRAHAM	25	1.802	1.834	0.519	-0.062
DORGAN	79	2.436	2.490	1.117	-0.048
RACHTA	9	1.913	1.929	0.661	-0.026
STOCKW	7	2.651	2.652	0.062	-0.020
LIAW	1	1.308	1.317	0.626	-0.013
BUFFLE	3	2.401	2.404	1.035	-0.003
KOO	9	0.936	0.929	0.656	0.011
WAKAI	8	1.482	1.472	0.769	0.013
DORGAN	56	2.498	2.490	0.334	0.025
WANG2	18	0.875	0.853	0.817	0.027
SCHWAR	25	2.705	2.695	0.261	0.041
KNEKT	86	2.182	2.137	0.911	0.049
AMANDU	5	1.878	1.817	1.052	0.058
WYNDE3	50	2.372	2.326	0.784	0.060
DORN	51	2.108	2.089	0.252	0.073
KELLER	9	2.727	2.695	0.436	0.075
ARCHER	5	1.817	1.748	0.904	0.076
HUMBLE	15	2.759	2.622	1.683	0.081
HITOSU	34	1.026	0.931	0.864	0.110
GAO	34	1.065	1.031	0.300	0.111
LIAW	2	1.281	1.112	1.368	0.123
HOLE	33	2.092	1.976	0.832	0.140
HIRAYA	1	1.493	1.453	0.234	0.171
SEGI2	20	1.319	1.153	0.837	0.198
TULINI	37	2.302	2.160	0.673	0.210
DORGAN	9	2.829	2.695	0.632	0.212
DORANT	2	2.381	2.200	0.845	0.215
CHYOU	2	2.434	2.287	0.622	0.236
CARPEN	11	3.137	2.983	0.632	0.243
MACLEN	32	0.865	0.712	0.601	0.255
KREUZE	39	2.726	2.472	0.968	0.262
DROSTE	6	2.674	2.424	0.918	0.273
WAKAI	26	1.475	1.267	0.749	0.277
SOBUE2	12	1.188	1.135	0.180	0.295
COMSTO	3	2.897	2.541	1.150	0.309
LUBIN	40	1.293	1.048	0.775	0.315
COMSTO	8	2.573	2.337	0.741	0.318
ENSTRO	2	1.939	1.885	0.161	0.336
KINLEN	20	2.556	2.276	0.816	0.344
AKIBA	14	1.361	1.243	0.332	0.357
BECHER	14	1.887	1.548	0.936	0.363
GAO	33	1.361	1.236	0.342	0.366
MACLEN	19	1.336	0.916	1.141	0.368
PETO	4	1.970	1.403	1.535	0.370
JAHN	5	2.306	2.092	0.569	0.375
AKIBA	10	1.629	1.447	0.481	0.379
GOODMA	2	2.858	2.577	0.741	0.380
HUMBLE	13	2.994	2.622	0.972	0.382
HENNEK	2	2.733	2.536	0.509	0.387
CHOW	25	2.644	2.285	0.897	0.400
SPITZ	2	3.052	2.692	0.894	0.403
KREUZE	40	2.718	2.268	1.089	0.413
TIZZAN	13	1.460	1.099	0.873	0.414
CHOI	3	1.640	1.356	0.660	0.430
HEIN	5	2.849	1.902	2.161	0.438

Table 1B1R - 3

IESLC - Meta-regression of current smoking, any product (or cigs if any not available)
Multiple regression of data from Table 1B1
All LC types
Study outliers

Study Ref	NRR	LOGRR	FITVAL	SEFITV	STDRES
HUMBLE	17	2.817	2.418	0.893	0.447
BROWN2	11	2.610	2.559	0.113	0.448
KAISE2	60	2.673	2.338	0.729	0.459
GREGOR	6	2.666	1.588	2.306	0.467
TVERDA	15	2.402	1.767	1.320	0.481
MRFITR	2	3.878	2.319	3.060	0.509
HUMBLE	19	3.157	2.418	1.369	0.540
JAIN	51	2.821	2.486	0.620	0.541
CPSII	126	3.008	2.878	0.235	0.556
DOLL2	63	2.158	1.466	1.192	0.580
SPEIZE	10	2.541	2.382	0.266	0.595
GARDIN	2	2.618	1.967	1.082	0.602
YAMAGU	10	1.589	1.139	0.720	0.624
DEKLER	8	3.137	1.761	2.163	0.636
DAMBER	14	2.262	1.989	0.421	0.648
YONG	12	3.357	2.321	1.559	0.665
DESTEF	41	2.389	2.052	0.499	0.674
GAO2	8	1.889	1.404	0.709	0.683
SUZUK2	6	3.091	2.168	1.350	0.684
HITOSU	59	1.128	0.727	0.586	0.686
AUSTIN	6	2.976	2.164	1.179	0.688
SHAW	6	3.057	2.534	0.734	0.713
CORREA	42	2.653	2.436	0.302	0.717
BECHER	13	2.701	1.752	1.313	0.723
JARVHO	6	2.741	1.733	1.347	0.748
BOUCOT	114	4.132	1.817	3.067	0.755
DORGAN	33	3.712	2.695	1.339	0.760
TOUSEY	15	3.408	2.866	0.702	0.772
JARVHO	2	3.696	1.938	2.275	0.773
SVENSS	96	2.204	1.735	0.589	0.797
DOLL	90	2.253	1.561	0.865	0.800
LOMBAR	9	2.414	1.906	0.624	0.814
AXELSS	10	2.477	1.955	0.636	0.819
KJUUS	1	3.046	1.721	1.618	0.819
HAMMON	139	2.444	1.971	0.576	0.821
SCHWAR	28	2.866	2.490	0.454	0.828
PEZZO2	2	3.133	2.363	0.925	0.832
KAISER	12	2.976	2.621	0.426	0.833
WALD	4	2.797	1.976	0.972	0.846
ALDERS	177	2.660	2.132	0.607	0.868
AXELSS	2	2.703	2.160	0.616	0.881
TOKARS	1	3.615	1.683	2.192	0.881
BEST	2	2.702	1.971	0.825	0.885
TOUSEY	12	4.081	3.070	1.141	0.886
KAUFMA	16	3.027	2.647	0.413	0.918
JOLY	15	2.013	1.606	0.435	0.935
STUCKE	2	4.649	1.752	3.089	0.938
KANELL	30	1.597	1.232	0.389	0.941
TENKAN	24	2.822	1.870	0.930	1.024
SOBUE2	10	1.497	1.339	0.154	1.030
ARMADA	27	3.205	1.974	1.140	1.080
SCHWAR	27	2.750	2.490	0.226	1.153
KUBIK	12	3.496	1.683	1.539	1.178
JOLY	18	2.588	1.810	0.652	1.193
WYNDE6	18	2.776	2.444	0.265	1.252
TULINI	43	2.787	1.956	0.648	1.284
KELLER	5	2.677	2.490	0.141	1.321
DOLL2	54	2.397	1.671	0.502	1.447
OSANN	33	3.277	2.764	0.355	1.448
OSANN	34	2.976	2.559	0.279	1.492
PEZZOT	5	3.484	1.761	1.126	1.530
BARBON	4	2.595	1.803	0.509	1.558

Table 1B1R - 3

IESLC - Meta-regression of current smoking, any product (or cigs if any not available)
Multiple regression of data from Table 1B1
All LC types
Study outliers

Study Ref	NRR	LOGRR	FITVAL	SEFITV	STDRES
CPSI	220	2.480	2.089	0.250	1.564
ODRISC	1	3.993	2.569	0.897	1.588
ANDERS	6	3.154	2.555	0.353	1.698
DARBY	4	4.511	2.276	1.260	1.773
DARBY	11	3.029	2.071	0.506	1.891
WYNDE6	207	2.683	2.239	0.228	1.951
ENSTRO	1	2.564	2.089	0.239	1.987
LUBIN2	26	2.367	1.944	0.171	2.476
KREUZE	41	3.734	2.472	0.476	2.652

Table 1B1R - 4

IESLC - Meta-regression of current smoking, any product (or cigs if any not available)
Multiple regression of data from Table 1B1
All LC types
Effect of additional characteristics

WEIGHTED on Weight

		Deviance	(DF)				
Log Relative risk							
Model 7		819.856	(175)				
		Estimate	S.E.	P	RR	95%CIl	95%CIu
Constant		1.566	0.081	+++	4.788	4.084	5.614
Sex(RR)							
Male	108	Aliased			10.121	9.277	11.042
Female	68	-0.204	0.029	---	8.249	7.566	8.995
Combined	19	-0.043	0.039	N.S.	9.698	8.529	11.028
Location							
NAmer	84	Aliased			12.126	11.291	13.023
UK	25	-0.345	0.071	---	8.587	6.457	11.421
Scand	21	-0.417	0.066	---	7.994	6.116	10.450
othEur	23	-0.602	0.057	---	6.639	5.316	8.293
China	5	-1.528	0.097	---	2.631	1.754	3.946
Japan	18	-1.174	0.056	---	3.749	3.039	4.625
othAs	7	-1.221	0.139	---	3.576	1.989	6.430
other	12	-0.593	0.088	---	6.698	4.683	9.582
Start year of study							
<1960	22	Aliased			4.862	4.028	5.868
1960-69	40	0.538	0.063	+++	8.323	6.962	9.949
1970-79	41	0.571	0.058	+++	8.609	7.186	10.314
1980-89	70	0.788	0.051	+++	10.696	9.833	11.634
1990+	22	1.169	0.089	+++	15.643	11.401	21.464
Study type (1)							
CC	128	Aliased			8.921	8.333	9.551
other	67	0.182	0.047	+++	10.698	9.089	12.591
Study size (number of LC cases)							
100-249	56	Aliased			6.849	5.344	8.777
250-499	48	0.222	0.071	++	8.551	6.910	10.583
500-999	38	0.266	0.070	+++	8.939	7.473	10.692
1000+	53	0.340	0.062	+++	9.622	9.029	10.254
Number of adjustment variables (1)							
0	86	Aliased			9.093	8.286	9.978
1	62	0.002	0.047	N.S.	9.106	7.849	10.566
2+/+nk	47	0.069	0.034	+	9.745	8.772	10.826

		Deviance	(DF)	Drop Dev	P		
Model 8		799.214	(171)	20.642	N.S.		
		Estimate	S.E.	P	RR	95%CIl	95%CIu
Constant		1.613	0.085	+++	5.017	4.248	5.925
Sex(RR)							
Male	108	Aliased			10.206	9.350	11.140
Female	68	-0.215	0.029	---	8.230	7.548	8.973
Combined	19	-0.062	0.040	N.S.	9.593	8.428	10.920
Location							
NAmer	84	Aliased			11.456	10.725	12.237
UK	25	-0.347	0.071	---	8.095	6.068	10.800
Scand	21	-0.388	0.067	---	7.769	5.939	10.163
othEur	23	Aliased			11.456	10.725	12.237
China	5	-1.516	0.097	---	2.517	1.678	3.774
Japan	18	-1.146	0.056	---	3.641	2.947	4.498
othAs	7	-1.214	0.140	---	3.403	1.892	6.122
other	12	-0.594	0.089	---	6.328	4.404	9.092
Start year of study							
<1960	22	Aliased			5.034	4.131	6.134
1960-69	40	0.487	0.065	+++	8.195	6.850	9.804
1970-79	41	0.481	0.068	+++	8.145	6.614	10.030
1980-89	70	0.762	0.052	+++	10.788	9.907	11.747
1990+	22	1.148	0.097	+++	15.870	11.340	22.209
Study type (1)							
CC	128	Aliased			8.909	8.314	9.546
other	67	0.188	0.048	+++	10.749	9.090	12.710

Table 1B1R - 4

IESLC - Meta-regression of current smoking, any product (or cigs if any not available)
Multiple regression of data from Table 1B1
All LC types
Effect of additional characteristics

WEIGHTED on Weight

		Estimate	S.E.	P	RR	95%CIl	95%CIu
Study size (number of LC cases)							
100-249	56	Aliased			6.804	5.262	8.799
250-499	48	0.229	0.073	++	8.558	6.895	10.622
500-999	38	0.310	0.072	+++	9.277	7.646	11.256
1000+	53	0.342	0.065	+++	9.574	8.970	10.220
Number of adjustment variables (1)							
0	86	Aliased			9.253	8.412	10.178
1	62	-0.031	0.049	N.S.	8.975	7.692	10.471
2+/+nk	47	0.037	0.035	N.S.	9.606	8.624	10.700
Detailed Country in othEur							
not o E	172	Aliased			9.775	9.244	10.335
multi	2	-0.414	0.086	---	6.463	4.540	9.199
Germany	8	-0.716	0.101	---	4.776	3.144	7.255
othWest	8	-0.887	0.097	---	4.028	2.686	6.040
East	3	-0.203	0.286	N.S.	7.979	2.379	26.766
Balkans	2	-0.429	0.174	-	6.363	3.052	13.268

		Deviance	(DF)	Drop Dev	P		
Model 8		819.855	(174)	0.001	N.S.		
		Estimate	S.E.	P	RR	95%CIl	95%CIu
Constant		1.566	0.081	+++	4.787	4.082	5.614
Sex(RR)							
Male	108	Aliased			10.121	9.274	11.045
Female	68	-0.205	0.029	---	8.249	7.563	8.997
Combined	19	-0.043	0.039	N.S.	9.698	8.526	11.032
Location							
NAmer	84	Aliased			11.998	11.175	12.881
UK	25	-0.345	0.071	---	8.497	6.382	11.312
Scand	21	-0.417	0.066	---	7.910	6.044	10.351
othEur	23	-0.602	0.057	---	6.569	5.255	8.211
China	5	-1.528	0.097	---	2.603	1.733	3.909
Japan	18	-1.174	0.056	---	3.709	3.005	4.579
othAs	7	Aliased			11.998	11.175	12.881
other	12	-0.594	0.088	---	6.627	4.627	9.492
Start year of study							
<1960	22	Aliased			4.862	4.026	5.871
1960-69	40	0.538	0.063	+++	8.323	6.959	9.954
1970-79	41	0.571	0.058	+++	8.609	7.181	10.322
1980-89	70	0.788	0.051	+++	10.696	9.831	11.637
1990+	22	1.169	0.089	+++	15.643	11.391	21.483
Study type (1)							
CC	128	Aliased			8.921	8.331	9.552
other	67	0.182	0.047	+++	10.698	9.085	12.597
Study size (number of LC cases)							
100-249	56	Aliased			6.848	5.337	8.787
250-499	48	0.222	0.071	++	8.552	6.905	10.591
500-999	38	0.266	0.070	+++	8.938	7.468	10.698
1000+	53	0.340	0.062	+++	9.622	9.028	10.256
Number of adjustment variables (1)							
0	86	Aliased			9.093	8.284	9.980
1	62	0.002	0.047	N.S.	9.107	7.845	10.570
2+/+nk	47	0.069	0.034	+	9.745	8.769	10.830
Detailed Country in othAsia							
not o A	188	Aliased			9.390	8.914	9.892
India	0	Aliased			9.390	8.914	9.892
HongKong	1	-1.214	0.313	---	2.790	0.738	10.546
othAsia	6	-1.223	0.152	---	2.765	1.454	5.261

		Deviance	(DF)	Drop Dev	P		
Model 8		819.750	(174)	0.106	N.S.		
		Estimate	S.E.	P	RR	95%CIl	95%CIu
Constant		1.574	0.085	+++	4.825	4.088	5.694

Table 1B1R - 4

IESLC - Meta-regression of current smoking, any product (or cigs if any not available)
Multiple regression of data from Table 1B1
All LC types
Effect of additional characteristics

WEIGHTED on Weight

		Estimate	S.E.	P	RR	95%CIl	95%CIu
Sex(RR)							
Male	108	Aliased			10.122	9.275	11.046
Female	68	-0.204	0.029	---	8.252	7.566	9.002
Combined	19	-0.044	0.040	N.S.	9.691	8.516	11.027
Location							
NAmer	84	Aliased			12.114	11.266	13.027
UK	25	-0.346	0.071	---	8.569	6.430	11.421
Scand	21	-0.417	0.066	---	7.986	6.102	10.450
othEur	23	-0.605	0.057	---	6.614	5.263	8.312
China	5	-1.530	0.097	---	2.623	1.744	3.946
Japan	18	-1.162	0.067	---	3.790	2.937	4.890
othAs	7	-1.223	0.140	---	3.567	1.979	6.430
other	12	-0.595	0.088	---	6.683	4.662	9.581
Start year of study							
<1960	22	Aliased			4.878	4.020	5.920
1960-69	40	0.535	0.064	+++	8.330	6.962	9.967
1970-79	41	0.569	0.058	+++	8.619	7.186	10.337
1980-89	70	0.784	0.053	+++	10.685	9.810	11.637
1990+	22	1.164	0.090	+++	15.621	11.368	21.464
Study type (1)							
CC	128	Aliased			8.930	8.328	9.576
other	67	0.177	0.049	+++	10.658	8.988	12.639
Study size (number of LC cases)							
100-249	56	Aliased			6.870	5.340	8.838
250-499	48	0.218	0.072	++	8.543	6.896	10.583
500-999	38	0.262	0.072	+++	8.923	7.445	10.694
1000+	53	0.337	0.063	+++	9.624	9.029	10.258
Number of adjustment variables (1)							
0	86	Aliased			9.088	8.277	9.978
1	62	0.002	0.047	N.S.	9.102	7.840	10.566
2+/+nk	47	0.071	0.034	+	9.757	8.770	10.854
All LC (or nearest)							
all	187	Aliased			9.306	8.799	9.842
other	8	-0.024	0.075	N.S.	9.082	6.716	12.281

		Deviance	(DF)	Drop Dev	P		
Model 8		819.750	(174)	0.106	N.S.		
		Estimate	S.E.	P	RR	95%CIl	95%CIu
Constant		1.574	0.085	+++	4.825	4.088	5.694
Sex(RR)							
Male	108	Aliased			10.122	9.275	11.046
Female	68	-0.204	0.029	---	8.252	7.566	9.002
Combined	19	-0.044	0.040	N.S.	9.691	8.516	11.027
Location							
NAmer	84	Aliased			12.114	11.266	13.027
UK	25	-0.346	0.071	---	8.569	6.430	11.421
Scand	21	-0.417	0.066	---	7.986	6.102	10.450
othEur	23	-0.605	0.057	---	6.614	5.263	8.312
China	5	-1.530	0.097	---	2.623	1.744	3.946
Japan	18	-1.162	0.067	---	3.790	2.937	4.890
othAs	7	-1.223	0.140	---	3.567	1.979	6.430
other	12	-0.595	0.088	---	6.683	4.662	9.581
Start year of study							
<1960	22	Aliased			4.878	4.020	5.920
1960-69	40	0.535	0.064	+++	8.330	6.962	9.967
1970-79	41	0.569	0.058	+++	8.619	7.186	10.337
1980-89	70	0.784	0.053	+++	10.685	9.810	11.637
1990+	22	1.164	0.090	+++	15.621	11.368	21.464
Study type (1)							
CC	128	Aliased			8.930	8.328	9.576
other	67	0.177	0.049	+++	10.658	8.988	12.639

Table 1B1R - 4

IESLC - Meta-regression of current smoking, any product (or cigs if any not available)
Multiple regression of data from Table 1B1
All LC types
Effect of additional characteristics

WEIGHTED on Weight

		Estimate	S.E.	P	RR	95%CIl	95%CIu
Study size (number of LC cases)							
100-249	56	Aliased			6.870	5.340	8.838
250-499	48	0.218	0.072	++	8.543	6.896	10.583
500-999	38	0.262	0.072	+++	8.923	7.445	10.694
1000+	53	0.337	0.063	+++	9.624	9.029	10.258
Number of adjustment variables (1)							
0	86	Aliased			9.088	8.277	9.978
1	62	0.002	0.047	N.S.	9.102	7.840	10.566
2+/+nk	47	0.071	0.034	+	9.757	8.770	10.854
All LC (or nearest)							
all	187	Aliased			9.306	8.799	9.842
other	8	-0.024	0.075	N.S.	9.082	6.716	12.281

		Deviance	(DF)	Drop Dev	P		
Model 8		819.757	(174)	0.098	N.S.		
		Estimate	S.E.	P	RR	95%CIl	95%CIu
Constant		1.565	0.081	+++	4.781	4.077	5.607
Sex(RR)							
Male	108	Aliased			10.119	9.272	11.043
Female	68	-0.204	0.029	---	8.250	7.564	8.997
Combined	19	-0.042	0.040	N.S.	9.701	8.528	11.036
Location							
NAmer	84	Aliased			12.126	11.289	13.025
UK	25	-0.344	0.071	---	8.595	6.456	11.443
Scand	21	-0.415	0.067	---	8.005	6.116	10.479
othEur	23	-0.603	0.057	---	6.637	5.310	8.295
China	5	-1.528	0.097	---	2.630	1.752	3.950
Japan	18	-1.175	0.056	---	3.745	3.033	4.625
othAs	7	-1.219	0.140	---	3.583	1.989	6.454
other	12	-0.593	0.088	---	6.701	4.679	9.595
Start year of study							
<1960	22	Aliased			4.859	4.023	5.869
1960-69	40	0.539	0.064	+++	8.332	6.962	9.972
1970-79	41	0.571	0.058	+++	8.604	7.176	10.315
1980-89	70	0.789	0.051	+++	10.694	9.829	11.635
1990+	22	1.171	0.089	+++	15.667	11.401	21.530
		Estimate					
Study type (1)							
CC	128	Aliased					
other	67	Aliased					
		Estimate	S.E.	P	RR	95%CIl	95%CIu
Study size (number of LC cases)							
100-249	56	Aliased			6.849	5.340	8.784
250-499	48	0.219	0.072	++	8.529	6.867	10.593
500-999	38	0.266	0.070	+++	8.933	7.463	10.693
1000+	53	0.340	0.062	+++	9.626	9.029	10.261
Number of adjustment variables (1)							
0	86	Aliased			9.083	8.267	9.980
1	62	0.005	0.048	N.S.	9.126	7.840	10.622
2+/+nk	47	0.070	0.034	+	9.744	8.768	10.828
Study type (2)							
CC	128	Aliased			8.924	8.333	9.558
prosp	62	0.179	0.048	+++	10.671	9.031	12.609
other	5	0.239	0.190	N.S.	11.338	5.066	25.377

		Deviance	(DF)	Drop Dev	P		
Model 8		813.532	(173)	6.324	N.S.		
		Estimate	S.E.	P	RR	95%CIl	95%CIu
Constant		1.563	0.082	+++	4.775	4.070	5.602
Sex(RR)							
Male	108	Aliased			10.094	9.237	11.030
Female	68	-0.206	0.029	---	8.218	7.532	8.966
Combined	19	-0.029	0.042	N.S.	9.804	8.564	11.225

Table 1B1R - 4

IESLC - Meta-regression of current smoking, any product (or cigs if any not available)
Multiple regression of data from Table 1B1
All LC types
Effect of additional characteristics

WEIGHTED on Weight

		Estimate	S.E.	P	RR	95%CIl	95%CIu
Location							
NAmer	84	Aliased			12.125	11.274	13.040
UK	25	-0.359	0.072	---	8.470	6.350	11.298
Scand	21	-0.439	0.071	---	7.814	5.873	10.395
othEur	23	-0.596	0.057	---	6.679	5.340	8.354
China	5	-1.532	0.097	---	2.620	1.744	3.934
Japan	18	-1.191	0.056	---	3.685	2.980	4.557
othAs	7	-1.214	0.140	---	3.603	2.001	6.487
other	12	-0.475	0.103	---	7.541	4.955	11.475
Start year of study							
<1960	22	Aliased			4.867	4.030	5.877
1960-69	40	0.551	0.064	+++	8.440	7.047	10.109
1970-79	41	0.559	0.059	+++	8.516	7.075	10.249
1980-89	70	0.786	0.051	+++	10.685	9.820	11.626
1990+	22	1.151	0.089	+++	15.383	11.185	21.157
Study type (1)							
CC	128	Aliased			8.932	8.339	9.566
other	67	0.176	0.047	+++	10.653	9.035	12.561
Study size (number of LC cases)							
100-249	56	Aliased			6.852	5.344	8.786
250-499	48	0.221	0.071	++	8.546	6.901	10.582
500-999	38	0.284	0.072	+++	9.100	7.523	11.008
1000+	53	0.337	0.062	+++	9.598	9.001	10.234
		Estimate					
Number of adjustment variables (1)							
0	86	Aliased					
1	62	Aliased					
2+/+nk	47	Aliased					
		Estimate	S.E.	P	RR	95%CIl	95%CIu
Number of adjustment variables (2)							
0	86	Aliased			9.068	8.261	9.954
1	62	0.012	0.047	N.S.	9.173	7.900	10.652
2	27	0.081	0.037	+	9.836	8.716	11.100
3-5	17	0.111	0.072	N.S.	10.128	7.605	13.489
6+/+nk	3	-0.229	0.129	(-)	7.214	4.191	12.418

		Deviance	(DF)	Drop Dev	P		
Model 8		817.218	(174)	2.638	N.S.		
		Estimate	S.E.	P	RR	95%CIl	95%CIu
Constant		1.529	0.084	+++	4.616	3.913	5.444
Sex(RR)							
Male	108	Aliased			10.154	9.302	11.084
Female	68	-0.199	0.029	---	8.320	7.608	9.099
Combined	19	-0.066	0.042	N.S.	9.509	8.279	10.922
Location							
NAmer	84	Aliased			12.102	11.265	13.000
UK	25	-0.331	0.071	---	8.688	6.520	11.578
Scand	21	-0.406	0.067	---	8.066	6.162	10.559
othEur	23	-0.584	0.058	---	6.747	5.379	8.463
China	5	-1.516	0.097	---	2.658	1.769	3.992
Japan	18	-1.184	0.056	---	3.703	2.993	4.581
othAs	7	-1.182	0.142	---	3.712	2.047	6.731
other	12	-0.593	0.088	---	6.686	4.672	9.568
Start year of study							
<1960	22	Aliased			4.930	4.070	5.973
1960-69	40	0.538	0.063	+++	8.448	7.036	10.143
1970-79	41	0.567	0.058	+++	8.693	7.242	10.436
1980-89	70	0.765	0.053	+++	10.594	9.704	11.566
1990+	22	1.160	0.089	+++	15.731	11.457	21.600
Study type (1)							
CC	128	Aliased			8.954	8.357	9.593
other	67	0.165	0.048	+++	10.562	8.942	12.476

Table 1B1R - 4

IESLC - Meta-regression of current smoking, any product (or cigs if any not available)
Multiple regression of data from Table 1B1
All LC types
Effect of additional characteristics

WEIGHTED on Weight

		Estimate	S.E.	P	RR	95%CIl	95%CIu
Study size (number of LC cases)							
100-249	56	Aliased			6.839	5.334	8.767
250-499	48	0.235	0.072	++	8.653	6.975	10.735
500-999	38	0.270	0.070	+++	8.955	7.484	10.715
1000+	53	0.340	0.062	+++	9.609	9.015	10.242
Number of adjustment variables (1)							
0	86	Aliased			8.844	7.860	9.951
1	62	0.054	0.057	N.S.	9.336	7.936	10.982
2+/+nk	47	0.118	0.045	++	9.956	8.836	11.216
RR adjusted for or study matched on age							
Yes	168	Aliased			9.049	8.303	9.863
No	27	0.087	0.054	N.S.	9.875	8.351	11.678

		Deviance	(DF)	Drop Dev	P		
Model 8		819.798	(174)	0.057	N.S.		
		Estimate	S.E.	P	RR	95%CIl	95%CIu
Constant		1.576	0.091	+++	4.836	4.045	5.782
Sex(RR)							
Male	108	Aliased			10.118	9.270	11.043
Female	68	-0.205	0.029	---	8.243	7.551	8.999
Combined	19	-0.041	0.040	N.S.	9.714	8.511	11.088
Location							
NAmer	84	Aliased			12.124	11.287	13.024
UK	25	-0.347	0.071	---	8.570	6.425	11.432
Scand	21	-0.417	0.067	---	7.987	6.102	10.454
othEur	23	-0.602	0.057	---	6.637	5.310	8.296
China	5	-1.529	0.097	---	2.629	1.750	3.949
Japan	18	-1.171	0.057	---	3.758	3.030	4.662
othAs	7	-1.227	0.142	---	3.553	1.951	6.471
other	12	-0.593	0.088	---	6.700	4.679	9.595
Start year of study							
<1960	22	Aliased			4.867	4.026	5.884
1960-69	40	0.534	0.065	+++	8.301	6.899	9.987
1970-79	41	0.567	0.061	+++	8.581	7.097	10.376
1980-89	70	0.789	0.051	+++	10.711	9.809	11.696
1990+	22	1.165	0.090	+++	15.598	11.310	21.511
Study type (1)							
CC	128	Aliased			8.913	8.310	9.560
other	67	0.185	0.050	+++	10.730	9.036	12.741
Study size (number of LC cases)							
100-249	56	Aliased			6.858	5.341	8.807
250-499	48	0.221	0.071	++	8.550	6.905	10.588
500-999	38	0.264	0.071	+++	8.934	7.463	10.695
1000+	53	0.339	0.062	+++	9.622	9.027	10.256
Number of adjustment variables (1)							
0	86	Aliased			9.115	8.226	10.100
1	62	0.001	0.047	N.S.	9.127	7.821	10.652
2+/+nk	47	0.061	0.047	N.S.	9.693	8.406	11.177
RR adjusted for or matched on factor other than sex, age							
Yes	102	Aliased			9.349	8.271	10.568
No	93	-0.011	0.048	N.S.	9.243	8.315	10.274

		Deviance	(DF)	Drop Dev	P		
Model 8		800.487	(173)	19.368	N.S.		
		Estimate	S.E.	P	RR	95%CIl	95%CIu
Constant		1.521	0.082	+++	4.575	3.893	5.376
Sex(RR)							
Male	108	Aliased			10.197	9.328	11.147
Female	68	-0.216	0.029	---	8.217	7.539	8.955
Combined	19	-0.057	0.041	N.S.	9.633	8.445	10.988

Table 1B1R - 4

IESLC - Meta-regression of current smoking, any product (or cigs if any not available)
Multiple regression of data from Table 1B1
All LC types
Effect of additional characteristics

WEIGHTED on Weight

		Estimate	S.E.	P	RR	95%CIl	95%CIu
Location							
NAmer	84	Aliased			12.055	11.211	12.961
UK	25	-0.391	0.073	---	8.153	6.092	10.912
Scand	21	-0.368	0.073	---	8.346	6.242	11.160
othEur	23	-0.562	0.059	---	6.874	5.468	8.641
China	5	-1.523	0.097	---	2.629	1.755	3.939
Japan	18	-1.157	0.056	---	3.790	3.074	4.673
othAs	7	-1.219	0.140	---	3.562	1.989	6.381
other	12	-0.598	0.089	---	6.626	4.636	9.470
Start year of study							
<1960	22	Aliased			4.647	3.824	5.648
1960-69	40	0.592	0.066	+++	8.402	7.030	10.043
1970-79	41	0.597	0.060	+++	8.439	7.036	10.122
1980-89	70	0.843	0.053	+++	10.800	9.929	11.747
1990+	22	1.220	0.090	+++	15.734	11.477	21.568
Study type (1)							
CC	128	Aliased			8.999	8.404	9.635
other	67	0.143	0.048	++	10.380	8.803	12.240
Study size (number of LC cases)							
100-249	56	Aliased			7.015	5.475	8.988
250-499	48	0.207	0.071	++	8.625	6.975	10.666
500-999	38	0.275	0.070	+++	9.238	7.702	11.080
1000+	53	0.309	0.063	+++	9.552	8.962	10.181
Number of adjustment variables (1)							
0	86	Aliased			9.189	8.369	10.088
1	62	-0.021	0.047	N.S.	8.995	7.755	10.433
2+/+nk	47	0.053	0.035	N.S.	9.689	8.711	10.777
Product							
all/unsp	85	Aliased			8.881	7.874	10.017
cig+/-ot	95	0.039	0.036	N.S.	9.238	8.611	9.911
cig only	15	0.276	0.064	+++	11.708	9.266	14.795

		Deviance	(DF)	Drop Dev	P		
Model 8		809.704	(174)	10.151	N.S.		
		Estimate	S.E.	P	RR	95%CIl	95%CIu
Constant		1.575	0.081	+++	4.829	4.118	5.662
Sex(RR)							
Male	108	Aliased			10.129	9.287	11.048
Female	68	-0.189	0.029	---	8.387	7.673	9.167
Combined	19	-0.072	0.041	(-)	9.428	8.250	10.773
Location							
NAmer	84	Aliased			12.317	11.438	13.264
UK	25	-0.390	0.072	---	8.341	6.261	11.111
Scand	21	-0.480	0.069	---	7.624	5.795	10.030
othEur	23	-0.653	0.059	---	6.413	5.114	8.042
China	5	-1.524	0.097	---	2.682	1.789	4.020
Japan	18	-1.218	0.057	---	3.642	2.944	4.506
othAs	7	-1.219	0.139	---	3.642	2.029	6.537
other	12	-0.667	0.091	---	6.324	4.391	9.109
Start year of study							
<1960	22	Aliased			4.693	3.868	5.693
1960-69	40	0.556	0.064	+++	8.182	6.839	9.789
1970-79	41	0.610	0.059	+++	8.633	7.210	10.337
1980-89	70	0.834	0.053	+++	10.802	9.924	11.759
1990+	22	1.226	0.091	+++	15.995	11.654	21.954
Study type (1)							
CC	128	Aliased			8.917	8.331	9.544
other	67	0.183	0.047	+++	10.713	9.107	12.602
Study size (number of LC cases)							
100-249	56	Aliased			6.949	5.422	8.905
250-499	48	0.211	0.071	++	8.579	6.937	10.610
500-999	38	0.264	0.070	+++	9.048	7.563	10.825
1000+	53	0.322	0.062	+++	9.593	9.002	10.222

Table 1B1R - 4

IESLC - Meta-regression of current smoking, any product (or cigs if any not available)
Multiple regression of data from Table 1B1
All LC types
Effect of additional characteristics

WEIGHTED on Weight

		Estimate	S.E.	P	RR	95%CIl	95%CIu
Number of adjustment variables (1)							
0	86	Aliased			8.879	8.051	9.791
1	62	0.030	0.048	N.S.	9.146	7.885	10.607
2+/+nk	47	0.125	0.038	++	10.060	8.985	11.264
Denominator							
nev any	134	Aliased			9.644	8.979	10.358
nev cigs	61	-0.116	0.036	--	8.586	7.640	9.649

		Deviance	(DF)	Drop Dev	P		
Model 8		801.744	(173)	18.111	N.S.		
		Estimate	S.E.	P	RR	95%CIl	95%CIu
Constant		1.169	0.124	+++	3.220	2.526	4.103
Sex(RR)							
Male	108	Aliased			10.129	9.288	11.045
Female	68	-0.209	0.029	---	8.218	7.539	8.958
Combined	19	-0.039	0.039	N.S.	9.745	8.575	11.075
Location							
NAmer	84	Aliased			12.019	11.135	12.974
UK	25	0.026	0.112	N.S.	12.338	7.806	19.502
Scand	21	-0.448	0.067	---	7.677	5.855	10.066
othEur	23	-0.621	0.057	---	6.458	5.166	8.072
China	5	-1.576	0.325	---	2.486	0.648	9.539
Japan	18	-1.205	0.056	---	3.601	2.905	4.463
othAs	7	-1.246	0.158	---	3.456	1.802	6.629
other	12	-0.491	0.092	---	7.359	5.091	10.637
Start year of study							
<1960	22	Aliased			4.863	4.031	5.867
1960-69	40	0.557	0.064	+++	8.485	7.097	10.143
1970-79	41	0.581	0.058	+++	8.698	7.261	10.419
1980-89	70	0.783	0.051	+++	10.639	9.782	11.571
1990+	22	1.128	0.089	+++	15.022	10.939	20.629
Study type (1)							
CC	128	Aliased			8.973	8.382	9.606
other	67	0.156	0.047	++	10.483	8.900	12.348
Study size (number of LC cases)							
100-249	56	Aliased			6.862	5.359	8.786
250-499	48	0.244	0.072	+++	8.759	7.073	10.847
500-999	38	0.303	0.071	+++	9.290	7.742	11.147
1000+	53	0.330	0.062	+++	9.544	8.954	10.172
Number of adjustment variables (1)							
0	86	Aliased			8.994	8.193	9.872
1	62	0.029	0.048	N.S.	9.260	7.978	10.749
2+/+nk	47	0.083	0.034	+	9.774	8.802	10.853
National cigarette tobacco type							
Virginia	34	Aliased			6.317	4.293	9.295
blended	154	0.409	0.096	+++	9.512	8.940	10.121
other	7	0.449	0.326	N.S.	9.900	2.728	35.927

		Deviance	(DF)	Drop Dev	P		
Model 8		817.317	(174)	2.539	N.S.		
		Estimate	S.E.	P	RR	95%CIl	95%CIu
Constant		1.570	0.081	+++	4.806	4.099	5.636
Sex(RR)							
Male	108	Aliased			10.140	9.292	11.066
Female	68	-0.205	0.029	---	8.260	7.574	9.008
Combined	19	-0.050	0.040	N.S.	9.645	8.474	10.978

Table 1B1R - 4

IESLC - Meta-regression of current smoking, any product (or cigs if any not available)
Multiple regression of data from Table 1B1
All LC types
Effect of additional characteristics

WEIGHTED on Weight

		Estimate	S.E.	P	RR	95%CIl	95%CIu
Location							
NAmer	84	Aliased			12.130	11.294	13.029
UK	25	-0.337	0.071	---	8.663	6.505	11.536
Scand	21	-0.387	0.069	---	8.242	6.227	10.907
othEur	23	-0.614	0.057	---	6.564	5.243	8.218
China	5	-1.531	0.097	---	2.624	1.748	3.938
Japan	18	-1.185	0.056	---	3.708	2.998	4.585
othAs	7	-1.227	0.140	---	3.556	1.976	6.400
other	12	-0.580	0.089	---	6.792	4.737	9.739
Start year of study							
<1960	22	Aliased			4.832	3.999	5.837
1960-69	40	0.549	0.064	+++	8.363	6.991	10.005
1970-79	41	0.576	0.058	+++	8.592	7.170	10.297
1980-89	70	0.795	0.051	+++	10.694	9.831	11.633
1990+	22	1.180	0.089	+++	15.720	11.449	21.584
Study type (1)							
CC	128	Aliased			8.977	8.368	9.630
other	67	0.154	0.050	++	10.469	8.805	12.447
Study size (number of LC cases)							
100-249	56	Aliased			6.824	5.322	8.750
250-499	48	0.219	0.071	++	8.491	6.853	10.520
500-999	38	0.283	0.071	+++	9.054	7.543	10.868
1000+	53	0.343	0.062	+++	9.614	9.020	10.247
Number of adjustment variables (1)							
0	86	Aliased			9.061	8.252	9.949
1	62	0.021	0.049	N.S.	9.251	7.925	10.798
2+/+nk	47	0.066	0.034	(+)	9.675	8.693	10.769
Any proxy use							
No/nk	156	Aliased			9.372	8.856	9.918
Yes	39	-0.070	0.044	N.S.	8.742	7.373	10.365

		Deviance	(DF)	Drop Dev	P		
Model 8		819.385	(174)	0.470	N.S.		
		Estimate	S.E.	P	RR	95%CIl	95%CIu
Constant		1.577	0.083	+++	4.840	4.116	5.691
Sex(RR)							
Male	108	Aliased			10.143	9.285	11.080
Female	68	-0.204	0.029	---	8.271	7.572	9.033
Combined	19	-0.053	0.042	N.S.	9.621	8.380	11.045
Location							
NAmer	84	Aliased			12.156	11.298	13.078
UK	25	-0.355	0.072	---	8.526	6.384	11.386
Scand	21	-0.428	0.069	---	7.920	6.017	10.424
othEur	23	-0.601	0.057	---	6.664	5.326	8.339
China	5	-1.545	0.100	---	2.593	1.710	3.932
Japan	18	-1.183	0.057	---	3.726	3.008	4.615
othAs	7	-1.237	0.141	---	3.529	1.949	6.391
other	12	-0.605	0.090	---	6.638	4.615	9.546
Start year of study							
<1960	22	Aliased			4.837	3.996	5.857
1960-69	40	0.541	0.064	+++	8.311	6.948	9.942
1970-79	41	0.581	0.060	+++	8.651	7.200	10.394
1980-89	70	0.794	0.051	+++	10.699	9.834	11.639
1990+	22	1.175	0.089	+++	15.671	11.410	21.524
Study type (1)							
CC	128	Aliased			8.936	8.339	9.576
other	67	0.174	0.048	+++	10.635	8.996	12.573
Study size (number of LC cases)							
100-249	56	Aliased			6.881	5.356	8.839
250-499	48	0.221	0.071	++	8.587	6.924	10.649
500-999	38	0.259	0.071	+++	8.915	7.444	10.677
1000+	53	0.335	0.062	+++	9.619	9.024	10.252

Table 1B1R - 4

IESLC - Meta-regression of current smoking, any product (or cigs if any not available)
Multiple regression of data from Table 1B1
All LC types
Effect of additional characteristics

WEIGHTED on Weight

		Estimate	S.E.	P	RR	95%CIl	95%CIu
Number of adjustment variables (1)							
0	86	Aliased			9.072	8.257	9.968
1	62	0.002	0.047	N.S.	9.092	7.831	10.557
2+/+nk	47	0.076	0.035	+	9.790	8.777	10.920
Full histological confirmation							
No	144	Aliased			9.357	8.742	10.015
Yes	51	-0.025	0.036	N.S.	9.127	8.081	10.309

		Deviance	(DF)	Drop Dev	P		
Model 8		794.487	(173)	25.369	(*)		
		Estimate	S.E.	P	RR	95%CIl	95%CIu
Constant		1.627	0.082	+++	5.086	4.328	5.978
Sex(RR)							
Male	108	Aliased			10.237	9.386	11.165
Female	68	-0.225	0.029	---	8.172	7.499	8.906
Combined	19	-0.059	0.040	N.S.	9.652	8.499	10.962
Location							
NAmer	84	Aliased			12.203	11.368	13.099
UK	25	-0.367	0.071	---	8.455	6.373	11.217
Scand	21	-0.452	0.067	---	7.769	5.952	10.140
othEur	23	-0.623	0.057	---	6.542	5.246	8.158
China	5	-1.450	0.099	---	2.861	1.897	4.317
Japan	18	-1.194	0.056	---	3.699	3.001	4.558
othAs	7	-1.276	0.140	---	3.406	1.902	6.097
other	12	-0.623	0.088	---	6.546	4.590	9.336
Start year of study							
<1960	22	Aliased			4.858	4.029	5.858
1960-69	40	0.542	0.064	+++	8.354	6.996	9.976
1970-79	41	0.585	0.058	+++	8.719	7.285	10.435
1980-89	70	0.786	0.051	+++	10.660	9.808	11.587
1990+	22	1.164	0.089	+++	15.565	11.379	21.290
Study type (1)							
CC	128	Aliased			8.922	8.336	9.550
other	67	0.181	0.048	+++	10.693	9.080	12.593
Study size (number of LC cases)							
100-249	56	Aliased			7.106	5.547	9.104
250-499	48	0.229	0.071	++	8.935	7.208	11.075
500-999	38	0.221	0.071	++	8.861	7.420	10.583
1000+	53	0.297	0.063	+++	9.566	8.980	10.190
Number of adjustment variables (1)							
0	86	Aliased			9.081	8.275	9.965
1	62	0.012	0.048	N.S.	9.192	7.906	10.688
2+/+nk	47	0.065	0.034	(+)	9.692	8.731	10.759
Risky occupational population							
No	184	Aliased			9.365	8.894	9.860
Mining	7	-0.601	0.145	---	5.137	2.800	9.423
Other risky	4	-0.461	0.159	--	5.905	3.032	11.501

		Deviance	(DF)	Drop Dev	P		
Model 8		815.683	(172)	4.173	N.S.		
		Estimate	S.E.	P	RR	95%CIl	95%CIu
Constant		1.560	0.081	+++	4.757	4.057	5.579
Sex(RR)							
Male	108	Aliased			10.140	9.287	11.071
Female	68	-0.204	0.029	---	8.268	7.578	9.022
Combined	19	-0.052	0.040	N.S.	9.630	8.450	10.975

Table 1B1R - 4

IESLC - Meta-regression of current smoking, any product (or cigs if any not available)
Multiple regression of data from Table 1B1
All LC types
Effect of additional characteristics

WEIGHTED on Weight

		Estimate	S.E.	P	RR	95%CIl	95%CIu
Location							
NAmer	84	Aliased			12.112	11.271	13.015
UK	25	-0.322	0.073	---	8.774	6.525	11.798
Scand	21	-0.401	0.068	---	8.108	6.151	10.689
othEur	23	-0.611	0.058	---	6.573	5.231	8.259
China	5	-1.491	0.102	---	2.728	1.778	4.186
Japan	18	-1.174	0.056	---	3.742	3.020	4.638
othAs	7	-1.217	0.140	---	3.585	1.987	6.468
other	12	-0.603	0.089	---	6.627	4.617	9.513
Start year of study							
<1960	22	Aliased			4.838	3.992	5.863
1960-69	40	0.535	0.064	+++	8.259	6.892	9.897
1970-79	41	0.578	0.058	+++	8.622	7.176	10.359
1980-89	70	0.795	0.052	+++	10.710	9.837	11.660
1990+	22	1.199	0.091	+++	16.043	11.591	22.203
Study type (1)							
CC	128	Aliased			8.870	8.263	9.521
other	67	0.207	0.050	+++	10.912	9.159	13.000
Study size (number of LC cases)							
100-249	56	Aliased			6.769	5.252	8.724
250-499	48	0.227	0.071	++	8.492	6.842	10.540
500-999	38	0.279	0.072	+++	8.947	7.470	10.718
1000+	53	0.353	0.063	+++	9.636	9.036	10.274
Number of adjustment variables (1)							
0	86	Aliased			9.073	8.247	9.981
1	62	0.016	0.049	N.S.	9.220	7.896	10.766
2+/+nk	47	0.065	0.035	(+)	9.681	8.694	10.780
Lowest age in RR							
<25/unlim	106	Aliased			9.454	8.786	10.172
25-39	55	-0.052	0.044	N.S.	8.979	7.735	10.422
40+	33	-0.061	0.047	N.S.	8.894	7.452	10.614
unknown	1	1.373	1.011	N.S.	37.323	0.499	2793.293

		Deviance	(DF)	Drop Dev	P		
Model 8		805.546	(171)	14.310	N.S.		
		Estimate	S.E.	P	RR	95%CIl	95%CIu
Constant		1.612	0.123	+++	5.014	3.936	6.386
Sex(RR)							
Male	108	Aliased			9.985	9.131	10.919
Female	68	-0.192	0.029	---	8.242	7.555	8.991
Combined	19	-0.004	0.041	N.S.	9.945	8.709	11.357
Location							
NAmer	84	Aliased			12.076	11.225	12.993
UK	25	-0.331	0.075	---	8.677	6.426	11.718
Scand	21	-0.439	0.073	---	7.783	5.797	10.449
othEur	23	-0.567	0.058	---	6.850	5.458	8.597
China	5	-1.526	0.105	---	2.625	1.695	4.066
Japan	18	-1.169	0.056	---	3.753	3.037	4.638
othAs	7	-1.165	0.141	---	3.767	2.084	6.810
other	12	-0.578	0.089	---	6.778	4.721	9.732
Start year of study							
<1960	22	Aliased			4.826	3.987	5.842
1960-69	40	0.567	0.064	+++	8.506	7.092	10.203
1970-79	41	0.569	0.060	+++	8.525	7.065	10.287
1980-89	70	0.794	0.051	+++	10.679	9.815	11.619
1990+	22	1.153	0.090	+++	15.282	11.086	21.065
Study type (1)							
CC	128	Aliased			8.946	8.346	9.589
other	67	0.169	0.049	+++	10.595	8.952	12.539

Table 1B1R - 4

IESLC - Meta-regression of current smoking, any product (or cigs if any not available)
Multiple regression of data from Table 1B1
All LC types
Effect of additional characteristics

WEIGHTED on Weight

		Estimate	S.E.	P	RR	95%CIl	95%CIu
Study size (number of LC cases)							
100-249	56	Aliased			6.737	5.225	8.685
250-499	48	0.221	0.073	++	8.400	6.763	10.434
500-999	38	0.269	0.071	+++	8.815	7.352	10.570
1000+	53	0.361	0.063	+++	9.670	9.070	10.311
Number of adjustment variables (1)							
0	86	Aliased			9.001	8.194	9.888
1	62	0.020	0.047	N.S.	9.186	7.910	10.668
2+/+nk	47	0.087	0.034	+	9.824	8.832	10.927
Highest age in RR							
<65	16	Aliased			10.215	6.810	15.323
65-74	29	-0.100	0.105	N.S.	9.240	7.426	11.498
75-84	36	0.039	0.104	N.S.	10.625	8.890	12.699
85+/unlim	113	-0.114	0.098	N.S.	9.117	8.575	9.692
unknown	1	1.279	1.015	N.S.	36.695	0.497	2707.686
		Deviance	(DF)	Drop Dev	P		
Model 8		806.252	(174)	13.604	(*)		
		Estimate	S.E.	P	RR	95%CIl	95%CIu
Constant		2.217	0.194	+++	9.179	6.273	13.433
Sex(RR)							
Male	108	Aliased			10.161	9.317	11.082
Female	68	-0.209	0.029	---	8.242	7.563	8.983
Combined	19	-0.052	0.040	N.S.	9.643	8.485	10.959
Location							
NAmer	84	Aliased			12.161	11.327	13.056
UK	25	-0.339	0.071	---	8.662	6.522	11.503
Scand	21	-0.495	0.070	---	7.412	5.601	9.808
othEur	23	-0.591	0.057	---	6.732	5.393	8.403
China	5	-1.578	0.098	---	2.510	1.671	3.770
Japan	18	-1.177	0.056	---	3.746	3.040	4.617
othAs	7	-1.170	0.140	---	3.776	2.100	6.788
other	12	-0.581	0.088	---	6.800	4.761	9.712
Start year of study							
<1960	22	Aliased			4.766	3.947	5.754
1960-69	40	0.573	0.064	+++	8.449	7.069	10.099
1970-79	41	0.562	0.058	+++	8.362	6.965	10.038
1980-89	70	0.813	0.051	+++	10.747	9.883	11.686
1990+	22	1.201	0.089	+++	15.830	11.554	21.689
Study type (1)							
CC	128	Aliased			8.873	8.289	9.499
other	67	0.206	0.047	+++	10.898	9.255	12.833
Study size (number of LC cases)							
100-249	56	Aliased			6.673	5.204	8.555
250-499	48	0.224	0.071	++	8.347	6.740	10.336
500-999	38	0.267	0.070	+++	8.715	7.276	10.439
1000+	53	0.374	0.063	+++	9.699	9.099	10.339
Number of adjustment variables (1)							
0	86	Aliased			8.999	8.198	9.877
1	62	0.042	0.048	N.S.	9.385	8.063	10.923
2+/+nk	47	0.071	0.034	+	9.664	8.700	10.734
Midpoint age in RR		-0.012	0.003	---	18.590	8.395	41.169
		Deviance	(DF)	Drop Dev	P		
Model 8		810.405	(174)	9.450	N.S.		
		Estimate	S.E.	P	RR	95%CIl	95%CIu
Constant		1.538	0.082	+++	4.657	3.968	5.465
Sex(RR)							
Male	108	Aliased			10.233	9.369	11.176
Female	68	-0.202	0.029	---	8.358	7.653	9.128
Combined	19	-0.094	0.043	-	9.313	8.098	10.709

Table 1B1R - 4

IESLC - Meta-regression of current smoking, any product (or cigs if any not available)
Multiple regression of data from Table 1B1
All LC types
Effect of additional characteristics

WEIGHTED on Weight

		Estimate	S.E.	P	RR	95%CIl	95%CIu
Location							
NAmer	84	Aliased			12.173	11.335	13.073
UK	25	-0.351	0.071	---	8.570	6.449	11.388
Scand	21	-0.440	0.067	---	7.841	5.995	10.255
othEur	23	-0.616	0.057	---	6.576	5.266	8.211
China	5	-1.521	0.097	---	2.660	1.775	3.986
Japan	18	-1.185	0.056	---	3.722	3.018	4.591
othAs	7	-1.204	0.140	---	3.651	2.033	6.556
other	12	-0.613	0.089	---	6.596	4.613	9.431
Start year of study							
<1960	22	Aliased			4.949	4.096	5.979
1960-69	40	0.480	0.066	+++	8.002	6.644	9.638
1970-79	41	0.534	0.059	+++	8.445	7.039	10.131
1980-89	70	0.782	0.051	+++	10.815	9.931	11.776
1990+	22	1.171	0.089	+++	15.955	11.625	21.896
Study type (1)							
CC	128	Aliased			8.965	8.373	9.599
other	67	0.160	0.047	+++	10.516	8.924	12.393
Study size (number of LC cases)							
100-249	56	Aliased			6.881	5.373	8.813
250-499	48	0.248	0.072	+++	8.814	7.098	10.945
500-999	38	0.262	0.070	+++	8.942	7.480	10.691
1000+	53	0.332	0.062	+++	9.588	8.997	10.218
Number of adjustment variables (1)							
0	86	Aliased			9.010	8.206	9.893
1	62	0.006	0.047	N.S.	9.065	7.815	10.514
2+/+nk	47	0.096	0.035	++	9.913	8.903	11.039
Derivation of RR/CI							
Orig/2x2	80	Aliased			8.742	7.923	9.645
Other	115	0.100	0.032	++	9.657	8.968	10.398

		Deviance	(DF)	Drop Dev	P		
Model 8		815.802	(173)	4.053	N.S.		
		Estimate	S.E.	P	RR	95%CIl	95%CIu
Constant		1.688	0.103	+++	5.406	4.416	6.619
Sex(RR)							
Male	108	Aliased			10.117	9.271	11.042
Female	68	-0.206	0.029	---	8.238	7.545	8.994
Combined	19	-0.040	0.040	N.S.	9.725	8.537	11.079
Location							
NAmer	84	Aliased			12.074	11.226	12.987
UK	25	-0.342	0.071	---	8.573	6.438	11.415
Scand	21	-0.416	0.069	---	7.968	6.045	10.502
othEur	23	-0.584	0.058	---	6.737	5.372	8.448
China	5	-1.523	0.097	---	2.632	1.750	3.959
Japan	18	-1.154	0.057	---	3.809	3.075	4.717
othAs	7	-1.221	0.140	---	3.561	1.977	6.415
other	12	-0.586	0.088	---	6.719	4.691	9.624
Start year of study							
<1960	22	Aliased			4.782	3.942	5.800
1960-69	40	0.568	0.067	+++	8.440	7.006	10.167
1970-79	41	0.580	0.058	+++	8.540	7.116	10.251
1980-89	70	0.806	0.052	+++	10.707	9.836	11.656
1990+	22	1.180	0.089	+++	15.555	11.310	21.393
Study type (1)							
CC	128	Aliased			8.922	8.330	9.556
other	67	0.181	0.047	+++	10.694	9.072	12.605
Study size (number of LC cases)							
100-249	56	Aliased			6.883	5.361	8.838
250-499	48	0.206	0.072	++	8.456	6.801	10.513
500-999	38	0.258	0.071	+++	8.910	7.439	10.671
1000+	53	0.336	0.062	+++	9.635	9.038	10.271

Table 1B1R - 4

IESLC - Meta-regression of current smoking, any product (or cigs if any not available)
Multiple regression of data from Table 1B1
All LC types
Effect of additional characteristics

WEIGHTED on Weight

		Estimate	S.E.	P	RR	95%CIl	95%CIu
Number of adjustment variables (1)							
0	86	Aliased			9.748	8.168	11.634
1	62	-0.107	0.072	N.S.	8.760	7.390	10.385
2+/+nk	47	-0.073	0.080	N.S.	9.065	7.491	10.970
Derivation of RR/CI							
Orig	44	Aliased			9.944	8.234	12.008
StcCalc	91	-0.138	0.076	(-)	8.660	7.384	10.155
Other	60	-0.001	0.041	N.S.	9.932	8.435	11.696
