# Supplementary material for: Systematic review with meta-analysis of the epidemiological evidence in the 1900s relating smoking to lung cancer
Source: BMC Cancer. 2012 Sep 3;12:385. doi: 10.1186/1471-2407-12-385 (PMC3505152; doi:10.1186/1471-2407-12-385)
Supplement: Additional file 5 — Detailed Analysis Tables (Individual file names as described in Additional file 1: Methods, Table1). [file 1471-2407-12-385-S5.zip › PDF/1M.pdf]

Table 1M1 -
IESLC - Meta-analysis of Ever Smoking, Butt length or Fraction smoked, "Highest vs lowest"
All LC types, Cigarettes (or Any Product if Cigarettes not available)

This analysis is restricted to results for:
1) Ever smokers
2) Results by Butt length or Fraction smoked
3) Categorical results by Butt length or Fraction smoked
4) Denominator (unexposed) = "low"
5) All LC types (or near equivalent)
6) Results complete enough for use in metaanalysis

Within each study, results are then selected (in the following order of preference, within each sex) for:
7) (not applicable)
8) PRODUCT: cigarettes regardless of other products, cigarettes only, all/unspec
9) CIGTYPE: all/unspecified, MC regardless of HR, MC only
10) Results with least adjustment for other aspects of smoking (ADOS)
11) The highest vs lowest category
12) Followup period (YF, prospective studies): whole study (coded as 0) or longest available
13) LCtype: all or nearest available, at least Squamous and Adeno. (q = squamous, s = small,
l = large, a = adeno, mix = mixed, alv = alveolar)
14) Race: all or nearest available, otherwise by race (wh or w = white, bl or b = black, hi = hispanic
ch = chinese, jap = japanese, haw = hawaiian, w+o = white + oriental, sca = scandinavian, as = asian)
15) For overlapping studies: principal rather than subsidiary studies
Finally by Age: whole study (coded as 0) if available, otherwise by widest available age group
and then for single sex results (m, f) in preference to results for both sexes combined (c).

Results adjusted (AD) for the most potential confounders are then chosen in Sections -1 to -3
and results adjusted for the least confounders in Sections -4 to -6. (Those least adjusted results which
actually differ from the most adjusted are marked 'x' in column X in Section -4)

Section -7 shows excluded studies, together with the stage (as above) at which no qualifying
results were found.

Section -8 lists the potentially overlapping studies which have been included (1=principal, 2=subsidiary).

Section -9 lists any results which would have been included in preference except that they had data not complete
enough for use in meta-analysis, with their significance (yes/no), if known, and any further comment as entered
on the database. It also lists as "gap" any categories for which no data were presented by the original authors.

In addition to those mentioned above, the following fields, levels and abbreviations are used:
* or nk = not known, n = no, y = yes, ot = other
all/unspec = all or unspecified, cig+/-ot = cigarettes irrespective of other products (cigar, pipe etc)
MC = manufactured cigarettes, HR = hand-rolled cigarettes
exL, exH = range of exposure (low and high) in the "highest" group, in terms of Butt length or Fraction smoked
unexL, unexH = range of exposure (low and high) in the "lowest" group, in terms of Butt length or Fraction smoked
REF: 6-character study reference
NRR: number of the RR on the database within the study
ST : study type (CC = case control, pr or prosp = prospective)
NLC: number of lung cancer cases in whole study
R : risky occupational population (n = no, m = mining, o = other risky)
VB : national cigarette type (V = at least 75% Virginia, bl = at least 75% blended, ot = other)
P : any proxy use
H : full histological confirmation
De : derivation of RR/CI (or = original, st = standard method, ot = other method of estimation)

Table 1M1 - 1

IESLC - Meta-analysis of Ever Smoking, Butt length or Fraction smoked, "Highest vs lowest"
All LC types, Cigarettes (or Any Product if Cigarettes not available)
Most adjusted

REF	NRR	SEX	AGEL	AGEH	RACE	YF	LC TYPE	LOC	START	ST	NLC	R	VB	P	H	AD	ADOS	PRODUCT	exL	exH	unexL	unexH	De
CHOI	519	m	0	0	all	-	all	As:oth	1985	CC	375	n	bl	n	n	0	0	cig+/-ot	51	100	1	50	st
CHOI	522	f	0	0	all	-	all	As:oth	1985	CC	375	n	bl	n	n	0	0	cig+/-ot	51	100	1	50	st
KOO	503	f	0	0	all	-	all	As:HK	1981	CC	200	n	bl	n	n	0	0	all/unsp	76	100	1	75	st
TIZZAN	515	m	0	0	all	-	all	Eu:wst	1959	CC	1358	n	bl	n	n	0	0	cig only	903	903#	901	901	st
TIZZAN	528	f	0	0	all	-	all	Eu:wst	1959	CC	1358	n	bl	n	n	0	0	cig only	903	903#	901	901	st
WYNDE6	758	m	0	0	wh	-	q+s+a	NAmer	1969	CC	4423	n	bl	n	y	0	0	cig+/-ot	50	100	1	49	st
WYNDE6	766	f	0	0	wh	-	q+s+a	NAmer	1969	CC	4423	n	bl	n	y	0	0	cig+/-ot	50	100	1	49	st

Comments on values in listings

Cigarette type is all/unspec for all RRs

exL, exH, unexL, unexH refer to fraction of cigarette smoked (%) for all RRs except

TIZZAN Butt length described as short vs long
TIZZAN Butt length described as short vs long

Table 1M1 - 2

IESLC - Meta-analysis of Ever Smoking, Butt length or Fraction smoked, "Highest vs lowest"
All LC types, Cigarettes (or Any Product if Cigarettes not available)
Most adjusted

REF	NRR	SEX	AD	Number Exposed Case	Cont	Non-exposed Case	Cont	RR	95.00%CI
CHOI	519	m	0	246	355	21	110	3.63	(2.21- 5.95)
CHOI	522	f	0	13	19	6	7	0.80	(0.22- 2.93)
Subtotal CHOI								3.00	(1.89- 4.76)
KOO	503	f	0	47	27	17	8	0.82	(0.31- 2.15)
TIZZAN	515	m	0	443	306	80	78	1.41	(1.00- 1.99)
TIZZAN	528	f	0	4	3	6	4	0.89	(0.13- 6.31)
Subtotal TIZZAN								1.39	(0.99- 1.95)
WYNDE6	758	m	0	310	138	263	161	1.38	(1.04- 1.82)
WYNDE6	766	f	0	191	81	178	101	1.34	(0.94- 1.91)
Subtotal WYNDE6								1.36	(1.09- 1.70)
Totals				1254	929	571	469		

*prospective study

REF	NRR	SEX	AD	Ys	Ws	Qs	Ps
CHOI	519	m	0	1.29	15.73	12.33	0.0000
CHOI	522	f	0	-0.23	2.28	0.90	0.7338
Subtotal CHOI				1.10	18.00	13.23	
KOO	503	f	0	-0.20	4.13	1.50	0.6852
TIZZAN	515	m	0	0.34	32.42	0.11	0.0497
TIZZAN	528	f	0	-0.12	1.00	0.27	0.9062
Subtotal TIZZAN				0.33	33.42	0.39	
WYNDE6	758	m	0	0.32	48.81	0.35	0.0260
WYNDE6	766	f	0	0.29	30.21	0.38	0.1095
Subtotal WYNDE6				0.31	79.03	0.74	

	N	7
	NS	4
	Wt	134.58
Het	Chi	15.85
Het	df	6
Het	P	*
Fixed	RR	1.50
	RRl	1.26
	RRu	1.77
	P	+++
Random	RR	1.50
	RRl	1.08
	RRu	2.08
	P	+
Asymm	P	N.S.

Table 1M1 - 3

IESLC - Meta-analysis of Ever Smoking, Butt length or Fraction smoked, "Highest vs lowest"
All LC types, Cigarettes (or Any Product if Cigarettes not available)
Most adjusted

	combined	Sex male	female	Total
N		3	4	7
NS		3	4	7
Wt		96.96	37.62	134.58
Het Chi		12.16	1.42	15.85
Het df		2	3	6
Het P		**	N.S.	*
Fixed RR		1.62	1.22	1.50
RRl		1.33	0.88	1.26
RRu		1.98	1.67	1.77
P		+++	N.S.	+++
Random RR		1.85	1.22	1.50
RRl		1.10	0.88	1.08
RRu		3.10	1.67	2.08
P		+	N.S.	+
Between Chi				2.27
Between df				1
Between P				N.S.
Btwn(F) P				N.S.
Btwn(R) P				N.S.

Too few RRs for analysis by factor

Table 1M1 - 4

IESLC - Meta-analysis of Ever Smoking, Butt length or Fraction smoked, "Highest vs lowest"
All LC types, Cigarettes (or Any Product if Cigarettes not available)
Least adjusted

REF	NRR	X	SEX	AGEL	AGEH	RACE	YF	LC TYPE	LOC	START	ST	NLC	R	VB	P	H	AD	ADOS	PRODUCT	exL	exH	unexL	unexH	De
CHOI	519		m	0	0	all	-	all	As:oth	1985	CC	375	n	bl	n	n	0	0	cig+/-ot	51	100	1	50	st
CHOI	522		f	0	0	all	-	all	As:oth	1985	CC	375	n	bl	n	n	0	0	cig+/-ot	51	100	1	50	st
KOO	503		f	0	0	all	-	all	As:HK	1981	CC	200	n	bl	n	n	0	0	all/unsp	76	100	1	75	st
TIZZAN	515		m	0	0	all	-	all	Eu:wst	1959	CC	1358	n	bl	n	n	0	0	cig only	903	903#	901	901	st
TIZZAN	528		f	0	0	all	-	all	Eu:wst	1959	CC	1358	n	bl	n	n	0	0	cig only	903	903#	901	901	st
WYNDE6	758		m	0	0	wh	-	q+s+a	NAmer	1969	CC	4423	n	bl	n	y	0	0	cig+/-ot	50	100	1	49	st
WYNDE6	766		f	0	0	wh	-	q+s+a	NAmer	1969	CC	4423	n	bl	n	y	0	0	cig+/-ot	50	100	1	49	st

Comments on values in lsiting

Cigarette type is all/unspec for all RRs

exL, exH, unexL, unexH refer to fraction of cigarette smoked (%) for all RRs except

TIZZAN Butt length described as short vs long
TIZZAN Butt length described as short vs long

Table 1M1 - 5

IESLC - Meta-analysis of Ever Smoking, Butt length or Fraction smoked, "Highest vs lowest"
All LC types, Cigarettes (or Any Product if Cigarettes not available)
Least adjusted

REF	NRR	SEX	AD	Number Exposed Case	Number Exposed Cont	Non-exposed Case	Non-exposed Cont	RR	95.00%CI
CHOI	519	m	0	246	355	21	110	3.63	(2.21- 5.95)
CHOI	522	f	0	13	19	6	7	0.80	(0.22- 2.93)
Subtotal CHOI								3.00	(1.89- 4.76)
KOO	503	f	0	47	27	17	8	0.82	(0.31- 2.15)
TIZZAN	515	m	0	443	306	80	78	1.41	(1.00- 1.99)
TIZZAN	528	f	0	4	3	6	4	0.89	(0.13- 6.31)
Subtotal TIZZAN								1.39	(0.99- 1.95)
WYNDE6	758	m	0	310	138	263	161	1.38	(1.04- 1.82)
WYNDE6	766	f	0	191	81	178	101	1.34	(0.94- 1.91)
Subtotal WYNDE6								1.36	(1.09- 1.70)
Totals				1254	929	571	469		

*prospective study

REF	NRR	SEX	AD	Ys	Ws	Qs	Ps
CHOI	519	m	0	1.29	15.73	12.33	0.0000
CHOI	522	f	0	-0.23	2.28	0.90	0.7338
Subtotal CHOI				1.10	18.00	13.23	
KOO	503	f	0	-0.20	4.13	1.50	0.6852
TIZZAN	515	m	0	0.34	32.42	0.11	0.0497
TIZZAN	528	f	0	-0.12	1.00	0.27	0.9062
Subtotal TIZZAN				0.33	33.42	0.39	
WYNDE6	758	m	0	0.32	48.81	0.35	0.0260
WYNDE6	766	f	0	0.29	30.21	0.38	0.1095
Subtotal WYNDE6				0.31	79.03	0.74	

	N	7
	NS	4
	Wt	134.58
Het	Chi	15.85
Het	df	6
Het	P	*
Fixed	RR	1.50
	RRl	1.26
	RRu	1.77
	P	+++
Random	RR	1.50
	RRl	1.08
	RRu	2.08
	P	+
Asymm	P	N.S.

Table 1M1 - 6

IESLC - Meta-analysis of Ever Smoking, Butt length or Fraction smoked, "Highest vs lowest"
All LC types, Cigarettes (or Any Product if Cigarettes not available)
Least adjusted

	Sex		
combined	male	female	Total
N	3	4	7
NS	3	4	7
Wt	96.96	37.62	134.58
Het Chi	12.16	1.42	15.85
Het df	2	3	6
Het P	**	N.S.	*
Fixed RR	1.62	1.22	1.50
RRl	1.33	0.88	1.26
RRu	1.98	1.67	1.77
P	+++	N.S.	+++
Random RR	1.85	1.22	1.50
RRl	1.10	0.88	1.08
RRu	3.10	1.67	2.08
P	+	N.S.	+
Between Chi			2.27
Between df			1
Between P			N.S.
Btwn(F) P			N.S.
Btwn(R) P			N.S.

Table 1M1 - 7

IESLC - Meta-analysis of Ever Smoking, Butt length or Fraction smoked, "Highest vs lowest"
All LC types, Cigarettes (or Any Product if Cigarettes not available)
Excluded studies (and stage at which they were excluded)

1 AKIBA AMANDU AMES BECHER BENSHL BEST BLOT1 BROSS BROWN3 CARPEN CEDERL CHYOU CPSI CPSII DARBY DEAN2 DEAN3 DOLL2 ENGELA GAO2 GARCIA GILLIS GRAHAM GURSEL HAMMO2 HIRAYA HOLE HUMBLE JAHN JAIN KAISE2 KATSOU KAUFMA LAUSSM LIAW MCDUFF MIGRAN MRFITR PEZZO2 PISANI PRESCO QIAO SEGI2 SPEIZE SVENSS TVERDA WAKAI WATSON WIGLE WU WYNDE3 WYNDE8

2 AGUDO ALDERS ARMADA AUVINE AXELSS BARBON BENHAM BOFFET BOUCHA BOUCOT BRESLO BUFFLE CHEN CHEN2 CHIAZZ CORREA DAMBER DESTEF DOLL DORGAN DORN DOSEME FAN GAO GARSHI GENG GER GUO HAENSZ HAMMON HEGMAN HU HU2 JEDRYC JUSSAW KHUDER KOULUM KREUZE LETOUR LEVIN LIU3 LIU4 LIU5 LUBIN LUO MATOS MCCONN NOTAN2 OSANN2 PERNU PEZZOT QIAO2 RACHTA SADOWS SOBUE SPITZ STASZE SUZUK2 VUTUC WANG2 WU2 WUWILL WYNDE7 XU YUAN ZHANG ZHENG ZHOU

3 JOLY

5 LUBIN2 WYNDE2

6 RESTRE

Table 1M1 - 8
Potentially overlapping studies

REF	REFGP	PRINC	.	OVERLAP/LINK
KOO	KOO	1		KOO/LAMTH/LAMWK
WYNDE6	WYNDE6	1		WYNDE5/6/7/8

Table 1M1 - 9
Most adjusted - insufficient data for meta-analysis

REF	NRR	SEX	AGEL	AGEH	RACE	YF	LC TYPE	LOC	START	ST	NLC	R	VB	P	H	AD	ADOS	PRODUCT	exL	exH	unexL	unexH	De
RESTRE	509	c	0	0	all	-	all	SCAmer	1978	CC	102	n	bl	n	n	2	0	cig+/-ot	76	100	25	50	st

REF	NRR	RR	SIG	RRDATA comment
RESTRE	509	1.76		0

Table 1M2 -

IESLC - Meta-analysis of Current Smoking, Butt length or Fraction smoked, "Highest vs lowest"
All LC types, Cigarettes (or Any Product if Cigarettes not available)

This analysis is restricted to results for:
1) Current smokers
2) Results by Butt length or Fraction smoked
3) Categorical results by Butt length or Fraction smoked
4) Denominator (unexposed) = "low"
5) All LC types (or near equivalent)
6) Results complete enough for use in metaanalysis

Within each study, results are then selected (in the following order of preference, within each sex) for:
7) (not applicable)
8) PRODUCT: cigarettes regardless of other products, cigarettes only, all/unspec
9) CIGTYPE: all/unspecified, MC regardless of HR, MC only
10) Results with least adjustment for other aspects of smoking (ADOS)
11) The highest vs lowest category
12) Followup period (YF, prospective studies): whole study (coded as 0) or longest available
13) LCtype: all or nearest available, at least Squamous and Adeno. (q = squamous, s = small,
l = large, a = adeno, mix = mixed, alv = alveolar)
14) Race: all or nearest available, otherwise by race (wh or w = white, bl or b = black, hi = hispanic
ch = chinese, jap = japanese, haw = hawaiian, w+o = white + oriental, sca = scandinavian, as = asian)
15) For overlapping studies: principal rather than subsidiary studies
Finally by Age: whole study (coded as 0) if available, otherwise by widest available age group
and then for single sex results (m, f) in preference to results for both sexes combined (c).

Results adjusted (AD) for the most potential confounders are then chosen in Sections -1 to -3
and results adjusted for the least confounders in Sections -4 to -6. (Those least adjusted results which
actually differ from the most adjusted are marked 'x' in column X in Section -4)

Section -7 shows excluded studies, together with the stage (as above) at which no qualifying
results were found.

Section -8 lists the potentially overlapping studies which have been included (1=principal, 2=subsidiary).

Section -9 lists any results which would have been included in preference except that they had data not complete
enough for use in meta-analysis, with their significance (yes/no), if known, and any further comment as entered
on the database. It also lists as "gap" any categories for which no data were presented by the original authors.

In addition to those mentioned above, the following fields, levels and abbreviations are used:
* or nk = not known, n = no, y = yes, ot = other
all/unspec = all or unspecified, cig+/-ot = cigarettes irrespective of other products (cigar, pipe etc)
MC = manufactured cigarettes, HR = hand-rolled cigarettes
exL, exH = range of exposure (low and high) in the "highest" group, in terms of Butt length or Fraction smoked
unexL, unexH = range of exposure (low and high) in the "lowest" group, in terms of Butt length or Fraction smoked
REF: 6-character study reference
NRR: number of the RR on the database within the study
ST : study type (CC = case control, pr or prosp = prospective)
NLC: number of lung cancer cases in whole study
R : risky occupational population (n = no, m = mining, o = other risky)
VB : national cigarette type (V = at least 75% Virginia, bl = at least 75% blended, ot = other)
P : any proxy use
H : full histological confirmation
De : derivation of RR/CI (or = original, st = standard method, ot = other method of estimation)

Table 1M2 - 1

IESLC - Meta-analysis of Current Smoking, Butt length or Fraction smoked, "Highest vs lowest"
All LC types, Cigarettes (or Any Product if Cigarettes not available)
Most adjusted

REF	NRR	SEX	AGEL	AGEH	RACE	YF	LC TYPE	LOC	START	ST	NLC	R	VB	P	H	AD	ADOS	PRODUCT	exL	exH	unexL	unexH	De
KAISE2	665	m	0	0	all	9	all	NAmer	1979	pr	318	n	bl	n	n	5	2	#cig only	76	100	1	75	or
KAISE2	666	f	0	0	all	9	all	NAmer	1979	pr	318	n	bl	n	n	5	2	#cig only	76	100	1	75	or
SOBUE	604	m	0	0	all	-	q+s+l+a	As:Jap	1986	CC	1376	n	bl	n	y	0	0	cig+/-ot	67	100	1	49	st
WAKAI	583	m	0	0	all	-	all	As:Jap	1988	CC	333	n	bl	n	y	0	0	cig+/-ot	76	100	1	49	st

Comments on values in listings

KAISE2 ADOS Cigs/day and years of smoking
KAISE2 ADOS Cigs/day and years of smoking

Cigarette type is all/unspec for all RRs

exL, exH, unexL, unexH refer to fraction of cigarette smoked (%) for all RRs

Table 1M2 - 2

IESLC - Meta-analysis of Current Smoking, Butt length or Fraction smoked, "Highest vs lowest"
All LC types, Cigarettes (or Any Product if Cigarettes not available)
Most adjusted

REF	NRR	SEX	AD	Number Exposed Case	Cont	Non-exposed Case	Cont	RR	95.00%CI
*KAISE2	665	m	5	37	-	58	-	0.98	(0.64- 1.50)
*KAISE2	666	f	5	29	-	50	-	1.10	(0.68- 1.79)
Subtotal KAISE2								1.03	(0.75- 1.42)
SOBUE	604	m	0	138	87	122	148	1.92	(1.34- 2.76)
WAKAI	583	m	0	63	91	13	26	1.38	(0.66- 2.90)
Partial Totals				267	178	243	174		

*prospective study

REF	NRR	SEX	AD	Ys	Ws	Qs	Ps
*KAISE2	665	m	5	-0.02	21.18	2.28	0.9259
*KAISE2	666	f	5	0.10	16.40	0.74	0.6995
Subtotal KAISE2				0.03	37.58	3.01	
SOBUE	604	m	0	0.65	29.68	3.57	0.0004
WAKAI	583	m	0	0.33	7.03	0.00	0.3882

	N	4
	NS	3
	Wt	74.29
Het	Chi	6.59
Het	df	3
Het	P	(*)
Fixed	RR	1.36
	RRl	1.08
	RRu	1.71
	P	++
Random	RR	1.32
	RRl	0.93
	RRu	1.87
	P	N.S.
Asymm	P	N.S.

Table 1M2 - 3

IESLC - Meta-analysis of Current Smoking, Butt length or Fraction smoked, "Highest vs lowest"
All LC types, Cigarettes (or Any Product if Cigarettes not available)
Most adjusted

	Sex combined	male	female	Total
N		3	1	4
NS		3	1	4
Wt		57.89	16.40	74.29
Het Chi		5.64	0.00	6.59
Het df		2	0	3
Het P		(*)	N.S.	(*)
Fixed RR		1.44	1.10	1.36
RRl		1.12	0.68	1.08
RRu		1.87	1.78	1.71
P		++	N.S.	++
Random RR		1.39	1.10	1.32
RRl		0.87	0.68	0.93
RRu		2.22	1.78	1.87
P		N.S.	N.S.	N.S.
Between Chi				0.95
Between df				1
Between P				N.S.
Btwn(F) P				N.S.
Btwn(R) P				N.S.

Too few RRs for analysis by factor

Table 1M2 - 4

IESLC - Meta-analysis of Current Smoking, Butt length or Fraction smoked, "Highest vs lowest"
All LC types, Cigarettes (or Any Product if Cigarettes not available)
Least adjusted

REF	NRR	X	SEX	AGEL	AGEH	RACE	YF	LC TYPE	LOC	START	ST	NLC	R	VB	P	H	AD	ADOS	PRODUCT	exL	exH	unexL	unexH	De
KAISE2	665		m	0	0	all	9	all	NAmer	1979	pr	318	n	bl	n	n	5	2#	cig only	76	100	1	75	or
KAISE2	666		f	0	0	all	9	all	NAmer	1979	pr	318	n	bl	n	n	5	2#	cig only	76	100	1	75	or
SOBUE	604		m	0	0	all	-	q+s+l+a	As:Jap	1986	CC	1376	n	bl	n	y	0	0	cig+/-ot	67	100	1	49	st
WAKAI	583		m	0	0	all	-	all	As:Jap	1988	CC	333	n	bl	n	y	0	0	cig+/-ot	76	100	1	49	st

Comments on values in listings

KAISE2 ADOS Cigs/day and years of smoking
KAISE2 ADOS Cigs/day and years of smoking

Cigarette type is all/unspec for all RRs

exL, exH, unexL, unexH refer to fraction of cigarette smoked (%) for all RRs

Table 1M2 - 5

IESLC - Meta-analysis of Current Smoking, Butt length or Fraction smoked, "Highest vs lowest"
All LC types, Cigarettes (or Any Product if Cigarettes not available)
Least adjusted

REF	NRR	SEX	AD	Number Exposed Case	Exposed Cont	Non-exposed Case	Non-exposed Cont	RR	95.00%CI
*KAISE2	665	m	5	37	-	58	-	0.98	(0.64- 1.50)
*KAISE2	666	f	5	29	-	50	-	1.10	(0.68- 1.79)
Subtotal KAISE2								1.03	(0.75- 1.42)
SOBUE	604	m	0	138	87	122	148	1.92	(1.34- 2.76)
WAKAI	583	m	0	63	91	13	26	1.38	(0.66- 2.90)
Partial Totals				267	178	243	174		

*prospective study

REF	NRR	SEX	AD	Ys	Ws	Qs	Ps
*KAISE2	665	m	5	-0.02	21.18	2.28	0.9259
*KAISE2	666	f	5	0.10	16.40	0.74	0.6995
Subtotal KAISE2				0.03	37.58	3.01	
SOBUE	604	m	0	0.65	29.68	3.57	0.0004
WAKAI	583	m	0	0.33	7.03	0.00	0.3882

	N	4
	NS	3
	Wt	74.29
Het	Chi	6.59
Het	df	3
Het	P	(*)
Fixed	RR	1.36
	RRl	1.08
	RRu	1.71
	P	++
Random	RR	1.32
	RRl	0.93
	RRu	1.87
	P	N.S.
Asymm	P	N.S.

Table 1M2 - 6

IESLC - Meta-analysis of Current Smoking, Butt length or Fraction smoked, "Highest vs lowest"
All LC types, Cigarettes (or Any Product if Cigarettes not available)
Least adjusted

	Sex combined	male	female	Total
N		3	1	4
NS		3	1	4
Wt		57.89	16.40	74.29
Het Chi		5.64	0.00	6.59
Het df		2	0	3
Het P		(*)	N.S.	(*)
Fixed RR		1.44	1.10	1.36
RRl		1.12	0.68	1.08
RRu		1.87	1.78	1.71
P		++	N.S.	++
Random RR		1.39	1.10	1.32
RRl		0.87	0.68	0.93
RRu		2.22	1.78	1.87
P		N.S.	N.S.	N.S.
Between Chi				0.95
Between df				1
Between P				N.S.
Btwn(F) P				N.S.
Btwn(R) P				N.S.

Table 1M2 - 7

IESLC - Meta-analysis of Current Smoking, Butt length or Fraction smoked, "Highest vs lowest"
All LC types, Cigarettes (or Any Product if Cigarettes not available)
Excluded studies (and stage at which they were excluded)

1	AGUDO	ALDERS	ARMADA	AUVINE	AXELSS	BARBON	BECHER	BENHAM	BLOT1	BOFFET	BOUCHA	BRESLO	BROWN3	CARPEN	CHEN	CHEN2
	CHIAZZ	CHOI	CHYOU	CORREA	DAMBER	DARBY	DESTEF	DOLL	DOLL2	DORGAN	DOSEME	FAN	GAO	GARCIA	GARSHI	GENG
	GER	GRAHAM	GUO	GURSEL	HAENSZ	HAMMO2	HAMMON	HEGMAN	HU	HU2	JAHN	JAIN	JEDRYC	JOLY	JUSSAW	KHUDER
	KOO	KOULUM	KREUZE	LAUSSM	LETOUR	LEVIN	LIU3	LIU4	LIU5	LUBIN	LUBIN2	LUO	MCCONN	NOTAN2	OSANN2	PERNU
	PEZZOT	PRESCO	QIAO	QIAO2	RACHTA	RESTRE	SADOWS	STASZE	SUZUK2	TIZZAN	TVERDA	VUTUC	WANG2	WIGLE	WU2	WUWILL
	WYNDE2	WYNDE3	XU	YUAN	ZHANG	ZHENG	ZHOU									
2	AKIBA	AMANDU	AMES	BENSHL	BEST	BOUCOT	BROSS	BUFFLE	CEDERL	CPSI	CPSII	DEAN2	DEAN3	DORN	ENGELA	GAO2
	GILLIS	HIRAYA	HOLE	HUMBLE	KATSOU	KAUFMA	LIAW	MATOS	MCDUFF	MIGRAN	MRFITR	PEZZO2	PISANI	SEGI2	SPEIZE	SPITZ
	SVENSS	WATSON	WU	WYNDE6	WYNDE7	WYNDE8										

Table 1M3 -

IESLC - Meta-analysis of Ever/current Smoking, Butt length or Fraction smoked, "Highest vs lowest"
All LC types, Cigarettes (or Any Product if Cigarettes not available)

This analysis is restricted to results for:
1) Ever/current smokers
2) Results by Butt length or Fraction smoked
3) Categorical results by Butt length or Fraction smoked
4) Denominator (unexposed) = "low"
5) All LC types (or near equivalent)
6) Results complete enough for use in metaanalysis

Within each study, results are then selected (in the following order of preference, within each sex) for:
7) SMKSTA: ever, current
8) PRODUCT: cigarettes regardless of other products, cigarettes only, all/unspec
9) CIGTYPE: all/unspecified, MC regardless of HR, MC only
10) Results with least adjustment for other aspects of smoking (ADOS)
11) The highest vs lowest category
12) Followup period (YF, prospective studies): whole study (coded as 0) or longest available
13) LCtype: all or nearest available, at least Squamous and Adeno. (q = squamous, s = small,
l = large, a = adeno, mix = mixed, alv = alveolar)
14) Race: all or nearest available, otherwise by race (wh or w = white, bl or b = black, hi = hispanic
ch = chinese, jap = japanese, haw = hawaiian, w+o = white + oriental, sca = scandinavian, as = asian)
15) For overlapping studies: principal rather than subsidiary studies
Finally by Age: whole study (coded as 0) if available, otherwise by widest available age group
and then for single sex results (m, f) in preference to results for both sexes combined (c).

Results adjusted (AD) for the most potential confounders are then chosen in Sections -1 to -3
and results adjusted for the least confounders in Sections -4 to -6. (Those least adjusted results which
actually differ from the most adjusted are marked 'x' in column X in Section -4)

Section -7 shows excluded studies, together with the stage (as above) at which no qualifying
results were found.

Section -8 lists the potentially overlapping studies which have been included (1=principal, 2=subsidiary).

Section -9 lists any results which would have been included in preference except that they had data not complete
enough for use in meta-analysis, with their significance (yes/no), if known, and any further comment as entered
on the database. It also lists as "gap" any categories for which no data were presented by the original authors.

In addition to those mentioned above, the following fields, levels and abbreviations are used:
* or nk = not known, n = no, y = yes, ot = other
all/unspec = all or unspecified, cig+/-ot = cigarettes irrespective of other products (cigar, pipe etc)
MC = manufactured cigarettes, HR = hand-rolled cigarettes
exL, exH = range of exposure (low and high) in the "highest" group, in terms of Butt length or Fraction smoked
unexL, unexH = range of exposure (low and high) in the "lowest" group, in terms of Butt length or Fraction smoked
REF: 6-character study reference
NRR: number of the RR on the database within the study
ST : study type (CC = case control, pr or prosp = prospective)
NLC: number of lung cancer cases in whole study
R : risky occupational population (n = no, m = mining, o = other risky)
VB : national cigarette type (V = at least 75% Virginia, bl = at least 75% blended, ot = other)
P : any proxy use
H : full histological confirmation
De : derivation of RR/CI (or = original, st = standard method, ot = other method of estimation)

Table 1M3 - 1

IESLC - Meta-analysis of Ever/current Smoking, Butt length or Fraction smoked, "Highest vs lowest"
All LC types, Cigarettes (or Any Product if Cigarettes not available)
Most adjusted

REF	NRR	SEX	AGEL	AGEH	RACE	YF	LC TYPE	LOC	START	ST	NLC	R	VB	P	H	AD	ADOS	SM	PRODUCT	exL	exH	unexL	unexH	De
CHOI	519	m	0	0	all	-	all	As:oth	1985	CC	375	n	bl	n	n	0	0	ev	cig+/-ot	51	100	1	50	st
CHOI	522	f	0	0	all	-	all	As:oth	1985	CC	375	n	bl	n	n	0	0	ev	cig+/-ot	51	100	1	50	st
KAISE2	665	m	0	0	all	9	all	NAmer	1979	pr	318	n	bl	n	n	5	2#	cu	cig only	76	100	1	75	or
KAISE2	666	f	0	0	all	9	all	NAmer	1979	pr	318	n	bl	n	n	5	2#	cu	cig only	76	100	1	75	or
KOO	503	f	0	0	all	-	all	As:HK	1981	CC	200	n	bl	n	n	0	0	ev	all/unsp	76	100	1	75	st
SOBUE	604	m	0	0	all	-	q+s+l+a	As:Jap	1986	CC	1376	n	bl	n	y	0	0	cu	cig+/-ot	67	100	1	49	st
TIZZAN	515	m	0	0	all	-	all	Eu:wst	1959	CC	1358	n	bl	n	n	0	0	ev	cig only	903	903#	901	901	st
TIZZAN	528	f	0	0	all	-	all	Eu:wst	1959	CC	1358	n	bl	n	n	0	0	ev	cig only	903	903#	901	901	st
WAKAI	583	m	0	0	all	-	all	As:Jap	1988	CC	333	n	bl	n	y	0	0	cu	cig+/-ot	76	100	1	49	st
WYNDE6	758	m	0	0	wh	-	q+s+a	NAmer	1969	CC	4423	n	bl	n	y	0	0	ev	cig+/-ot	50	100	1	49	st
WYNDE6	766	f	0	0	wh	-	q+s+a	NAmer	1969	CC	4423	n	bl	n	y	0	0	ev	cig+/-ot	50	100	1	49	st

Comments on values in listings

KAISE2 ADOS Cigs/day and years of smoking
KAISE2 ADOS Cigs/day and years of smoking

Cigarette type is all/unspec for all RRs

exL, exH, unexL, unexH refer to fraction of cigarette smoked (%) for all RRs except

TIZZAN Butt length described as short vs long
TIZZAN Butt length described as short vs long

Table 1M3 - 2

IESLC - Meta-analysis of Ever/current Smoking, Butt length or Fraction smoked, "Highest vs lowest"
All LC types, Cigarettes (or Any Product if Cigarettes not available)
Most adjusted

REF	NRR	SEX	AD	Number Exposed Case	Number Exposed Cont	Non-exposed Case	Non-exposed Cont	RR	95.00%CI
CHOI	519	m	0	246	355	21	110	3.63	(2.21- 5.95)
CHOI	522	f	0	13	19	6	7	0.80	(0.22- 2.93)
Subtotal CHOI								3.00	(1.89- 4.76)
*KAISE2	665	m	5	37	-	58	-	0.98	(0.64- 1.50)
*KAISE2	666	f	5	29	-	50	-	1.10	(0.68- 1.79)
Subtotal KAISE2								1.03	(0.75- 1.42)
KOO	503	f	0	47	27	17	8	0.82	(0.31- 2.15)
SOBUE	604	m	0	138	87	122	148	1.92	(1.34- 2.76)
TIZZAN	515	m	0	443	306	80	78	1.41	(1.00- 1.99)
TIZZAN	528	f	0	4	3	6	4	0.89	(0.13- 6.31)
Subtotal TIZZAN								1.39	(0.99- 1.95)
WAKAI	583	m	0	63	91	13	26	1.38	(0.66- 2.90)
WYNDE6	758	m	0	310	138	263	161	1.38	(1.04- 1.82)
WYNDE6	766	f	0	191	81	178	101	1.34	(0.94- 1.91)
Subtotal WYNDE6								1.36	(1.09- 1.70)
Partial Totals				1521	1107	814	643		

*prospective study

REF	NRR	SEX	AD	Ys	Ws	Qs	Ps
CHOI	519	m	0	1.29	15.73	13.30	0.0000
CHOI	522	f	0	-0.23	2.28	0.81	0.7338
Subtotal CHOI				1.10	18.00	14.10	
*KAISE2	665	m	5	-0.02	21.18	3.22	0.9259
*KAISE2	666	f	5	0.10	16.40	1.23	0.6995
Subtotal KAISE2				0.03	37.58	4.45	
KOO	503	f	0	-0.20	4.13	1.34	0.6852
SOBUE	604	m	0	0.65	29.68	2.41	0.0004
TIZZAN	515	m	0	0.34	32.42	0.02	0.0497
TIZZAN	528	f	0	-0.12	1.00	0.24	0.9062
Subtotal TIZZAN				0.33	33.42	0.26	
WAKAI	583	m	0	0.33	7.03	0.01	0.3882
WYNDE6	758	m	0	0.32	48.81	0.13	0.0260
WYNDE6	766	f	0	0.29	30.21	0.19	0.1095
Subtotal WYNDE6				0.31	79.03	0.31	

	N	11
	NS	7
	Wt	208.87
Het	Chi	22.89
Het	df	10
Het	P	*
Fixed	RR	1.45
	RRl	1.26
	RRu	1.66
	P	+++
Random	RR	1.43
	RRl	1.14
	RRu	1.79
	P	++
Asymm	P	N.S.

Table 1M3 - 3

IESLC - Meta-analysis of Ever/current Smoking, Butt length or Fraction smoked, "Highest vs lowest"
All LC types, Cigarettes (or Any Product if Cigarettes not available)
Most adjusted

	Sex			
	combined	male	female	Total
N		6	5	11
NS		6	5	11
Wt		154.85	54.02	208.87
Het Chi		18.30	1.54	22.89
Het df		5	4	10
Het P		**	N.S.	*
Fixed RR		1.55	1.18	1.45
RRl		1.33	0.90	1.26
RRu		1.82	1.54	1.66
P		+++	N.S.	+++
Random RR		1.61	1.18	1.43
RRl		1.17	0.90	1.14
RRu		2.21	1.54	1.79
P		++	N.S.	++
Between Chi				3.05
Between df				1
Between P				(*)
Btwn(F) P				N.S.
Btwn(R) P				N.S.

	Lung cancer type		
	all	other	Total
N	8	3	11
NS	5	2	7
Wt	100.16	108.70	208.87
Het Chi	20.03	2.60	22.89
Het df	7	2	10
Het P	**	N.S.	*
Fixed RR	1.40	1.50	1.45
RRl	1.15	1.24	1.26
RRu	1.70	1.81	1.66
P	+++	+++	+++
Random RR	1.34	1.50	1.43
RRl	0.92	1.21	1.14
RRu	1.95	1.87	1.79
P	N.S.	+++	++
Between Chi			0.25
Between df			1
Between P			N.S.
Btwn(F) P			N.S.
Btwn(R) P			N.S.

	Location								
	NAmer	UK	Scand	othEur	China	Japan	othAs	other	Total
N	4			2		2	3		11
NS	2			1		2	2		7
Wt	116.61			33.42		36.71	22.13		208.87
Het Chi	2.10			0.21		0.62	10.21		22.89
Het df	3			1		1	2		10
Het P	N.S.			N.S.		N.S.	**		*
Fixed RR	1.24			1.39		1.81	2.35		1.45
RRl	1.04			0.99		1.31	1.55		1.26
RRu	1.49			1.95		2.50	3.57		1.66
P	+			(+)		+++	+++		+++
Random RR	1.24			1.39		1.81	1.46		1.43
RRl	1.04			0.99		1.31	0.46		1.14
RRu	1.49			1.95		2.50	4.67		1.79
P	+			(+)		+++	N.S.		++
Between Chi									9.75
Between df									3
Between P									*
Btwn(F) P									N.S.
Btwn(R) P									N.S.

Table 1M3 - 3

IESLC - Meta-analysis of Ever/current Smoking, Butt length or Fraction smoked, "Highest vs lowest"
All LC types, Cigarettes (or Any Product if Cigarettes not available)
Most adjusted

Detailed Country in "other Europe"

	multi	Germany	othWest	East	Balkans	Total
N			2			2
NS			1			1
Wt			33.42			33.42
Het Chi			0.21			0.21
Het df			1			1
Het P			N.S.			N.S.
Fixed RR			1.39			1.39
RRl			0.99			0.99
RRu			1.95			1.95
P			(+)			(+)
Random RR			1.39			1.39
RRl			0.99			0.99
RRu			1.95			1.95
P			(+)			(+)
Between Chi						
Between df						
Between P						N.S.
Btwn(F) P						N.S.
Btwn(R) P						N.S.

Detailed Country in "other Asia"

	India	HongKong	other	Total
N		1	2	3
NS		1	1	2
Wt		4.13	18.00	22.13
Het Chi		0.00	4.56	10.21
Het df		0	1	2
Het P		N.S.	*	**
Fixed RR		0.82	3.00	2.35
RRl		0.31	1.89	1.55
RRu		2.15	4.76	3.57
P		N.S.	+++	+++
Random RR		0.82	1.93	1.46
RRl		0.31	0.45	0.46
RRu		2.15	8.33	4.67
P		N.S.	N.S.	N.S.
Between Chi				5.65
Between df				1
Between P				*
Btwn(F) P				N.S.
Btwn(R) P				N.S.

Detailed other continent

	SCAmer	Total
N		
NS		
Wt		
Het Chi		
Het df		
Het P		N.S.
Fixed RR		
RRl		
RRu		
P		N.S.
Random RR		
RRl		
RRu		
P		N.S.
Between Chi		
Between df		
Between P		N.S.
Btwn(F) P		N.S.
Btwn(R) P		N.S.

Table 1M3 - 3

IESLC - Meta-analysis of Ever/current Smoking, Butt length or Fraction smoked, "Highest vs lowest"
All LC types, Cigarettes (or Any Product if Cigarettes not available)
Most adjusted

	Start year of study					
	<1960	1960-69	1970-79	1980-89	1990+	Total
N	2	2	2	5		11
NS	1	1	1	4		7
Wt	33.42	79.03	37.58	58.84		208.87
Het Chi	0.21	0.01	0.12	11.79		22.89
Het df	1	1	1	4		10
Het P	N.S.	N.S.	N.S.	*		*
Fixed RR	1.39	1.36	1.03	2.00		1.45
RRl	0.99	1.09	0.75	1.55		1.26
RRu	1.95	1.70	1.42	2.58		1.66
P	(+)	++	N.S.	+++		+++
Random RR	1.39	1.36	1.03	1.70		1.43
RRl	0.99	1.09	0.75	1.02		1.14
RRu	1.95	1.70	1.42	2.83		1.79
P	(+)	++	N.S.	+		++
Between Chi						10.75
Between df						3
Between P						*
Btwn(F) P						N.S.
Btwn(R) P						N.S.

	Study type (1)		
	CC	other	Total
N	9	2	11
NS	6	1	7
Wt	171.29	37.58	208.87
Het Chi	17.49	0.12	22.89
Het df	8	1	10
Het P	*	N.S.	*
Fixed RR	1.56	1.03	1.45
RRl	1.34	0.75	1.26
RRu	1.81	1.42	1.66
P	+++	N.S.	+++
Random RR	1.56	1.03	1.43
RRl	1.21	0.75	1.14
RRu	2.01	1.42	1.79
P	+++	N.S.	++
Between Chi			5.28
Between df			1
Between P			*
Btwn(F) P			N.S.
Btwn(R) P			*

	Study type (2)			
	CC	prosp	other	Total
N	9	2		11
NS	6	1		7
Wt	171.29	37.58		208.87
Het Chi	17.49	0.12		22.89
Het df	8	1		10
Het P	*	N.S.		*
Fixed RR	1.56	1.03		1.45
RRl	1.34	0.75		1.26
RRu	1.81	1.42		1.66
P	+++	N.S.		+++
Random RR	1.56	1.03		1.43
RRl	1.21	0.75		1.14
RRu	2.01	1.42		1.79
P	+++	N.S.		++
Between Chi				5.28
Between df				1
Between P				*
Btwn(F) P				N.S.
Btwn(R) P				*

Table 1M3 - 3

IESLC - Meta-analysis of Ever/current Smoking, Butt length or Fraction smoked, "Highest vs lowest"
All LC types, Cigarettes (or Any Product if Cigarettes not available)
Most adjusted

	Study size (number of LC cases) 100-249	250-499	500-999	1000+	Total
N	1	5		5	11
NS	1	3		3	7
Wt	4.13	62.62		142.12	208.87
Het Chi	0.00	18.57		2.94	22.89
Het df	0	4		4	10
Het P	N.S.	***		N.S.	*
Fixed RR	0.82	1.45		1.47	1.45
RRl	0.31	1.13		1.25	1.26
RRu	2.15	1.86		1.73	1.66
P	N.S.	++		+++	+++
Random RR	0.82	1.41		1.47	1.43
RRl	0.31	0.80		1.25	1.14
RRu	2.15	2.52		1.73	1.79
P	N.S.	N.S.		+++	++
Between Chi					1.37
Between df					2
Between P					N.S.
Btwn(F) P					N.S.
Btwn(R) P					N.S.

	Risky occupational population no	mining	othRisky	Total
N	11			11
NS	7			7
Wt	208.87			208.87
Het Chi	22.89			22.89
Het df	10			10
Het P	*			*
Fixed RR	1.45			1.45
RRl	1.26			1.26
RRu	1.66			1.66
P	+++			+++
Random RR	1.43			1.43
RRl	1.14			1.14
RRu	1.79			1.79
P	++			++
Between Chi				
Between df				
Between P				N.S.
Btwn(F) P				N.S.
Btwn(R) P				N.S.

	National cigarette tobacco type Virginia	blended	other	Total
N		11		11
NS		7		7
Wt		208.87		208.87
Het Chi		22.89		22.89
Het df		10		10
Het P		*		*
Fixed RR		1.45		1.45
RRl		1.26		1.26
RRu		1.66		1.66
P		+++		+++
Random RR		1.43		1.43
RRl		1.14		1.14
RRu		1.79		1.79
P		++		++
Between Chi				
Between df				
Between P				N.S.
Btwn(F) P				N.S.
Btwn(R) P				N.S.

Table 1M3 - 3

IESLC - Meta-analysis of Ever/current Smoking, Butt length or Fraction smoked, "Highest vs lowest"
All LC types, Cigarettes (or Any Product if Cigarettes not available)
Most adjusted

	Any proxy use No/nk	Yes	Total
N	11		11
NS	7		7
Wt	208.87		208.87
Het Chi	22.89		22.89
Het df	10		10
Het P	*		*
Fixed RR	1.45		1.45
RRl	1.26		1.26
RRu	1.66		1.66
P	+++		+++
Random RR	1.43		1.43
RRl	1.14		1.14
RRu	1.79		1.79
P	++		++
Between Chi			
Between df			
Between P			N.S.
Btwn(F) P			N.S.
Btwn(R) P			N.S.

	Full histological confirmation No	Yes	Total
N	7	4	11
NS	4	3	7
Wt	93.13	115.73	208.87
Het Chi	20.03	2.64	22.89
Het df	6	3	10
Het P	**	N.S.	*
Fixed RR	1.40	1.49	1.45
RRl	1.14	1.24	1.26
RRu	1.71	1.79	1.66
P	++	+++	+++
Random RR	1.32	1.49	1.43
RRl	0.86	1.24	1.14
RRu	2.03	1.79	1.79
P	N.S.	+++	++
Between Chi			0.21
Between df			1
Between P			N.S.
Btwn(F) P			N.S.
Btwn(R) P			N.S.

	Number of adjustment variables (1) 0	1	2+/+nk	Total
N	9		2	11
NS	6		1	7
Wt	171.29		37.58	208.87
Het Chi	17.49		0.12	22.89
Het df	8		1	10
Het P	*		N.S.	*
Fixed RR	1.56		1.03	1.45
RRl	1.34		0.75	1.26
RRu	1.81		1.42	1.66
P	+++		N.S.	+++
Random RR	1.56		1.03	1.43
RRl	1.21		0.75	1.14
RRu	2.01		1.42	1.79
P	+++		N.S.	++
Between Chi				5.28
Between df				1
Between P				*
Btwn(F) P				N.S.
Btwn(R) P				*

Table 1M3 - 3

IESLC - Meta-analysis of Ever/current Smoking, Butt length or Fraction smoked, "Highest vs lowest"
All LC types, Cigarettes (or Any Product if Cigarettes not available)
Most adjusted

	Number of adjustment variables (2) 0	1	2	3-5	6+/+nk	Total
N	9			2		11
NS	6			1		7
Wt	171.29			37.58		208.87
Het Chi	17.49			0.12		22.89
Het df	8			1		10
Het P	*			N.S.		*
Fixed RR	1.56			1.03		1.45
RRl	1.34			0.75		1.26
RRu	1.81			1.42		1.66
P	+++			N.S.		+++
Random RR	1.56			1.03		1.43
RRl	1.21			0.75		1.14
RRu	2.01			1.42		1.79
P	+++			N.S.		++
Between Chi						5.28
Between df						1
Between P						*
Btwn(F) P						N.S.
Btwn(R) P						*

	Smoking status ever	current	Total
N	7	4	11
NS	4	3	7
Wt	134.58	74.29	208.87
Het Chi	15.85	6.59	22.89
Het df	6	3	10
Het P	*	(*)	*
Fixed RR	1.50	1.36	1.45
RRl	1.26	1.08	1.26
RRu	1.77	1.71	1.66
P	+++	++	+++
Random RR	1.50	1.32	1.43
RRl	1.08	0.93	1.14
RRu	2.08	1.87	1.79
P	+	N.S.	++
Between Chi			0.44
Between df			1
Between P			N.S.
Btwn(F) P			N.S.
Btwn(R) P			N.S.

	Product all/unsp	cig+/-ot	cig only	Total
N	1	6	4	11
NS	1	4	2	7
Wt	4.13	133.74	71.00	208.87
Het Chi	0.00	14.83	1.93	22.89
Het df	0	5	3	10
Het P	N.S.	*	N.S.	*
Fixed RR	0.82	1.64	1.19	1.45
RRl	0.31	1.38	0.94	1.26
RRu	2.15	1.94	1.50	1.66
P	N.S.	+++	N.S.	+++
Random RR	0.82	1.69	1.19	1.43
RRl	0.31	1.22	0.94	1.14
RRu	2.15	2.34	1.50	1.79
P	N.S.	++	N.S.	++
Between Chi				6.13
Between df				2
Between P				*
Btwn(F) P				N.S.
Btwn(R) P				N.S.

Table 1M3 - 3

IESLC - Meta-analysis of Ever/current Smoking, Butt length or Fraction smoked, "Highest vs lowest"
All LC types, Cigarettes (or Any Product if Cigarettes not available)
Most adjusted

	Derivation of RR/CI			
	Orig	StdCalc	Other	Total
N	2	9		11
NS	1	6		7
Wt	37.58	171.29		208.87
Het Chi	0.12	17.49		22.89
Het df	1	8		10
Het P	N.S.	*		*
Fixed RR	1.03	1.56		1.45
RRl	0.75	1.34		1.26
RRu	1.42	1.81		1.66
P	N.S.	+++		+++
Random RR	1.03	1.56		1.43
RRl	0.75	1.21		1.14
RRu	1.42	2.01		1.79
P	N.S.	+++		++
Between Chi				5.28
Between df				1
Between P				*
Btwn(F) P				N.S.
Btwn(R) P				*

Table 1M3 - 4

IESLC - Meta-analysis of Ever/current Smoking, Butt length or Fraction smoked, "Highest vs lowest"
All LC types, Cigarettes (or Any Product if Cigarettes not available)
Least adjusted

REF	NRR	X	SEX	AGEL	AGEH	RACE	YF	LC TYPE	LOC	START	ST	NLC	R	VB	P	H	AD	ADOS	SM	PRODUCT	exL	exH	unexL	unexH	De
CHOI	519		m	0	0	all	-	all	As:oth	1985	CC	375	n	bl	n	n	0	0	ev	cig+/-ot	51	100	1	50	st
CHOI	522		f	0	0	all	-	all	As:oth	1985	CC	375	n	bl	n	n	0	0	ev	cig+/-ot	51	100	1	50	st
KAISE2	665		m	0	0	all	9	all	NAmer	1979	pr	318	n	bl	n	n	5	2#	cu	cig only	76	100	1	75	or
KAISE2	666		f	0	0	all	9	all	NAmer	1979	pr	318	n	bl	n	n	5	2#	cu	cig only	76	100	1	75	or
KOO	503		f	0	0	all	-	all	As:HK	1981	CC	200	n	bl	n	n	0	0	ev	all/unsp	76	100	1	75	st
SOBUE	604		m	0	0	all	-	q+s+l+a	As:Jap	1986	CC	1376	n	bl	n	y	0	0	cu	cig+/-ot	67	100	1	49	st
TIZZAN	515		m	0	0	all	-	all	Eu:wst	1959	CC	1358	n	bl	n	n	0	0	ev	cig only	903	903#	901	901	st
TIZZAN	528		f	0	0	all	-	all	Eu:wst	1959	CC	1358	n	bl	n	n	0	0	ev	cig only	903	903#	901	901	st
WAKAI	583		m	0	0	all	-	all	As:Jap	1988	CC	333	n	bl	n	y	0	0	cu	cig+/-ot	76	100	1	49	st
WYNDE6	758		m	0	0	wh	-	q+s+a	NAmer	1969	CC	4423	n	bl	n	y	0	0	ev	cig+/-ot	50	100	1	49	st
WYNDE6	766		f	0	0	wh	-	q+s+a	NAmer	1969	CC	4423	n	bl	n	y	0	0	ev	cig+/-ot	50	100	1	49	st

Comments on values in listings

KAISE2 ADOS Cigs/day and years of smoking
KAISE2 ADOS Cigs/day and years of smoking

Cigarette type is all/unspec for all RRs

exL, exH, unexL, unexH refer to fraction of cigarette smoked (%) for all RRs except

TIZZAN Butt length described as short vs long
TIZZAN Butt length described as short vs long

Table 1M3 - 5

IESLC - Meta-analysis of Ever/current Smoking, Butt length or Fraction smoked, "Highest vs lowest"
All LC types, Cigarettes (or Any Product if Cigarettes not available)
Least adjusted

REF	NRR	SEX	AD	Number Exposed Case	Number Exposed Cont	Non-exposed Case	Non-exposed Cont	RR	95.00%CI
CHOI	519	m	0	246	355	21	110	3.63	(2.21- 5.95)
CHOI	522	f	0	13	19	6	7	0.80	(0.22- 2.93)
Subtotal CHOI								3.00	(1.89- 4.76)
*KAISE2	665	m	5	37	-	58	-	0.98	(0.64- 1.50)
*KAISE2	666	f	5	29	-	50	-	1.10	(0.68- 1.79)
Subtotal KAISE2								1.03	(0.75- 1.42)
KOO	503	f	0	47	27	17	8	0.82	(0.31- 2.15)
SOBUE	604	m	0	138	87	122	148	1.92	(1.34- 2.76)
TIZZAN	515	m	0	443	306	80	78	1.41	(1.00- 1.99)
TIZZAN	528	f	0	4	3	6	4	0.89	(0.13- 6.31)
Subtotal TIZZAN								1.39	(0.99- 1.95)
WAKAI	583	m	0	63	91	13	26	1.38	(0.66- 2.90)
WYNDE6	758	m	0	310	138	263	161	1.38	(1.04- 1.82)
WYNDE6	766	f	0	191	81	178	101	1.34	(0.94- 1.91)
Subtotal WYNDE6								1.36	(1.09- 1.70)
Partial Totals				1521	1107	814	643		

*prospective study

REF	NRR	SEX	AD	Ys	Ws	Qs	Ps
CHOI	519	m	0	1.29	15.73	13.30	0.0000
CHOI	522	f	0	-0.23	2.28	0.81	0.7338
Subtotal CHOI				1.10	18.00	14.10	
*KAISE2	665	m	5	-0.02	21.18	3.22	0.9259
*KAISE2	666	f	5	0.10	16.40	1.23	0.6995
Subtotal KAISE2				0.03	37.58	4.45	
KOO	503	f	0	-0.20	4.13	1.34	0.6852
SOBUE	604	m	0	0.65	29.68	2.41	0.0004
TIZZAN	515	m	0	0.34	32.42	0.02	0.0497
TIZZAN	528	f	0	-0.12	1.00	0.24	0.9062
Subtotal TIZZAN				0.33	33.42	0.26	
WAKAI	583	m	0	0.33	7.03	0.01	0.3882
WYNDE6	758	m	0	0.32	48.81	0.13	0.0260
WYNDE6	766	f	0	0.29	30.21	0.19	0.1095
Subtotal WYNDE6				0.31	79.03	0.31	

	N	11
	NS	7
	Wt	208.87
Het	Chi	22.89
Het	df	10
Het	P	*
Fixed	RR	1.45
	RRl	1.26
	RRu	1.66
	P	+++
Random	RR	1.43
	RRl	1.14
	RRu	1.79
	P	++
Asymm	P	N.S.

Table 1M3 - 6

IESLC - Meta-analysis of Ever/current Smoking, Butt length or Fraction smoked, "Highest vs lowest"
All LC types, Cigarettes (or Any Product if Cigarettes not available)
Least adjusted

	Sex combined	male	female	Total
N		6	5	11
NS		6	5	11
Wt		154.85	54.02	208.87
Het Chi		18.30	1.54	22.89
Het df		5	4	10
Het P		**	N.S.	*
Fixed RR		1.55	1.18	1.45
RRl		1.33	0.90	1.26
RRu		1.82	1.54	1.66
P		+++	N.S.	+++
Random RR		1.61	1.18	1.43
RRl		1.17	0.90	1.14
RRu		2.21	1.54	1.79
P		++	N.S.	++
Between Chi				3.05
Between df				1
Between P				(*)
Btwn(F) P				N.S.
Btwn(R) P				N.S.

Table 1M3 - 7

IESLC - Meta-analysis of Ever/current Smoking, Butt length or Fraction smoked, "Highest vs lowest"
All LC types, Cigarettes (or Any Product if Cigarettes not available)
Excluded studies (and stage at which they were excluded)

1 BECHER BLOT1 BROWN3 CARPEN CHYOU DARBY DOLL2 GARCIA GRAHAM GURSEL HAMMO2 JAHN JAIN LAUSSM PRESCO QIAO
TVERDA WIGLE WYNDE3

2 AGUDO AKIBA ALDERS AMANDU AMES ARMADA AUVINE AXELSS BARBON BENHAM BENSHL BEST BOFFET BOUCHA BOUCOT BRESLO
BROSS BUFFLE CEDERL CHEN CHEN2 CHIAZZ CORREA CPSI CPSII DAMBER DEAN2 DEAN3 DESTEF DOLL DORGAN DORN
DOSEME ENGELA FAN GAO GAO2 GARSHI GENG GER GILLIS GUO HAENSZ HAMMON HEGMAN HIRAYA HOLE HU
HU2 HUMBLE JEDRYC JUSSAW KATSOU KAUFMA KHUDER KOULUM KREUZE LETOUR LEVIN LIAW LIU3 LIU4 LIU5 LUBIN
LUO MATOS MCCONN MCDUFF MIGRAN MRFITR NOTAN2 OSANN2 PERNU PEZZO2 PEZZOT PISANI QIAO2 RACHTA SADOWS SEGI2
SPEIZE SPITZ STASZE SUZUK2 SVENSS VUTUC WANG2 WATSON WU WU2 WUWILL WYNDE7 WYNDE8 XU YUAN ZHANG
ZHENG ZHOU

3 JOLY

5 LUBIN2 WYNDE2

6 RESTRE

Table 1M3 - 8

Potentially overlapping studies

REF	REFGP	PRINC	.	OVERLAP/LINK
KOO	KOO	1		KOO/LAMTH/LAMWK
WYNDE6	WYNDE6	1		WYNDE5/6/7/8

Table 1M3 - 9

Most adjusted - insufficient data for meta-analysis

REF	NRR	SEX	AGEL	AGEH	RACE	YF	LC TYPE	LOC	START	ST	NLC	R	VB	P	H	AD	ADOS	SM	PRODUCT	exL	exH	unexL	unexH	De
RESTRE	509	c	0	0	all	-	all	SCAmer	1978	CC	102	n	bl	n	n	2	0	ev	cig+/-ot	76	100	25	50	st

REF	NRR	RR	SIG	RRDATA comment
RESTRE	509	1.76		0

Table 1M4 -

IESLC - Meta-analysis of Ever Smoking, Butt length or Fraction smoked, "Highest vs lowest"
All LC types, Cigarettes only

This analysis is restricted to results for:
1) Ever smokers
2) Results by Butt length or Fraction smoked
3) Categorical results by Butt length or Fraction smoked
4) Denominator (unexposed) = "low"
5) All LC types (or near equivalent)
6) Results complete enough for use in metaanalysis

Within each study, results are then selected (in the following order of preference, within each sex) for:
7) (not applicable)
8) PRODUCT: cigarettes only
9) CIGTYPE: all/unspecified, MC regardless of HR, MC only
10) Results with least adjustment for other aspects of smoking (ADOS)
11) The highest vs lowest category
12) Followup period (YF, prospective studies): whole study (coded as 0) or longest available
13) LCtype: all or nearest available, at least Squamous and Adeno. (q = squamous, s = small,
l = large, a = adeno, mix = mixed, alv = alveolar)
14) Race: all or nearest available, otherwise by race (wh or w = white, bl or b = black, hi = hispanic
ch = chinese, jap = japanese, haw = hawaiian, w+o = white + oriental, sca = scandinavian, as = asian)
15) For overlapping studies: principal rather than subsidiary studies
Finally by Age: whole study (coded as 0) if available, otherwise by widest available age group
and then for single sex results (m, f) in preference to results for both sexes combined (c).

Results adjusted (AD) for the most potential confounders are then chosen in Sections -1 to -3
and results adjusted for the least confounders in Sections -4 to -6. (Those least adjusted results which
actually differ from the most adjusted are marked 'x' in column X in Section -4)

Section -7 shows excluded studies, together with the stage (as above) at which no qualifying
results were found.

Section -8 lists the potentially overlapping studies which have been included (1=principal, 2=subsidiary).

Section -9 lists any results which would have been included in preference except that they had data not complete
enough for use in meta-analysis, with their significance (yes/no), if known, and any further comment as entered
on the database. It also lists as "gap" any categories for which no data were presented by the original authors.

In addition to those mentioned above, the following fields, levels and abbreviations are used:
* or nk = not known, n = no, y = yes, ot = other
all/unspec = all or unspecified, MC = manufactured cigarettes, HR = hand-rolled cigarettes
exL, exH = range of exposure (low and high) in the "highest" group, in terms of Butt length or Fraction smoked
unexL, unexH = range of exposure (low and high) in the "lowest" group, in terms of Butt length or Fraction smoked
REF: 6-character study reference
NRR: number of the RR on the database within the study
ST : study type (CC = case control, pr or prosp = prospective)
NLC: number of lung cancer cases in whole study
R : risky occupational population (n = no, m = mining, o = other risky)
VB : national cigarette type (V = at least 75% Virginia, bl = at least 75% blended, ot = other)
P : any proxy use
H : full histological confirmation
De : derivation of RR/CI (or = original, st = standard method, ot = other method of estimation)

Table 1M4 - 1

IESLC - Meta-analysis of Ever Smoking, Butt length or Fraction smoked, "Highest vs lowest"
All LC types, Cigarettes only
Most adjusted

REF	NRR	SEX	AGEL	AGEH	RACE	YF	LC TYPE	LOC	START	ST	NLC	R	VB	P	H	AD	ADOS	PRODUCT	exL	exH	unexL	unexH	De
TIZZAN	515	m	0	0	all	-	all	Eu:wst	1959	CC	1358	n	bl	n	n	0	0	cig only	903	903#	901	901	st
TIZZAN	528	f	0	0	all	-	all	Eu:wst	1959	CC	1358	n	bl	n	n	0	0	cig only	903	903#	901	901	st

Comments on values in listing

Cigarette type is all/unspec for all RRs

exL, exH, unexL, unexH refer to fraction of cigarette smoked (%) for all RRs except

TIZZAN Butt length described as short vs long
TIZZAN Butt length described as short vs long

Table 1M4 - 2

IESLC - Meta-analysis of Ever Smoking, Butt length or Fraction smoked, "Highest vs lowest"
All LC types, Cigarettes only
Most adjusted

REF	NRR	SEX	AD	Number Exposed Case	Cont	Non-exposed Case	Cont	RR	95.00%CI
TIZZAN	515	m	0	443	306	80	78	1.41	(1.00- 1.99)
TIZZAN	528	f	0	4	3	6	4	0.89	(0.13- 6.31)
Subtotal TIZZAN								1.39	(0.99- 1.95)
Totals				447	309	86	82		

*prospective study

REF	NRR	SEX	AD	Ys	Ws	Qs	Ps
TIZZAN	515	m	0	0.34	32.42	0.01	0.0497
TIZZAN	528	f	0	-0.12	1.00	0.20	0.9062
Subtotal TIZZAN				0.33	33.42	0.21	

	N	2
	NS	1
	Wt	33.42
Het	Chi	0.21
Het	df	1
Het	P	N.S.
Fixed	RR	1.39
	RRl	0.99
	RRu	1.95
	P	(+)
Random	RR	1.39
	RRl	0.99
	RRu	1.95
	P	(+)
Asymm	P	

Table 1M4 - 3

IESLC - Meta-analysis of Ever Smoking, Butt length or Fraction smoked, "Highest vs lowest"
All LC types, Cigarettes only
Most adjusted

	combined	Sex male	female	Total
N		1	1	2
NS		1	1	1
Wt		32.42	1.00	33.42
Het Chi		0.00	0.00	0.21
Het df		0	0	1
Het P		N.S.	N.S.	N.S.
Fixed RR		1.41	0.89	1.39
RRl		1.00	0.13	0.99
RRu		1.99	6.31	1.95
P		+	N.S.	(+)
Random RR		1.41	0.89	1.39
RRl		1.00	0.13	0.99
RRu		1.99	6.31	1.95
P		+	N.S.	(+)
Between Chi				0.21
Between df				1
Between P				N.S.
Btwn(F) P				N.S.
Btwn(R) P				N.S.

Too few RRs for analysis by factor

Table 1M4 - 4

IESLC - Meta-analysis of Ever Smoking, Butt length or Fraction smoked, "Highest vs lowest"
All LC types, Cigarettes only
Least adjusted

REF	NRR	X	SEX	AGEL	AGEH	RACE	YF	LC TYPE	LOC	START	ST	NLC	R	VB	P	H	AD	ADOS	PRODUCT	exL	exH	unexL	unexH	De
TIZZAN	515		m	0	0	all	-	all	Eu:wst	1959	CC	1358	n	bl	n	n	0	0	cig only	903	903#	901	901	st
TIZZAN	528		f	0	0	all	-	all	Eu:wst	1959	CC	1358	n	bl	n	n	0	0	cig only	903	903#	901	901	st

Comments on values in listing

Cigarette type is all/unspec for all RRs

exL, exH, unexL, unexH refer to fraction of cigarette smoked (%) for all RRs except

TIZZAN Butt length described as short vs long
TIZZAN Butt length described as short vs long

Table 1M4 - 5

IESLC - Meta-analysis of Ever Smoking, Butt length or Fraction smoked, "Highest vs lowest"
All LC types, Cigarettes only
Least adjusted

REF	NRR	SEX	AD	Number Exposed Case	Cont	Non-exposed Case	Cont	RR	95.00%CI
TIZZAN	515	m	0	443	306	80	78	1.41	(1.00- 1.99)
TIZZAN	528	f	0	4	3	6	4	0.89	(0.13- 6.31)
Subtotal TIZZAN								1.39	(0.99- 1.95)
Totals				447	309	86	82		

*prospective study

REF	NRR	SEX	AD	Ys	Ws	Qs	Ps
TIZZAN	515	m	0	0.34	32.42	0.01	0.0497
TIZZAN	528	f	0	-0.12	1.00	0.20	0.9062
Subtotal TIZZAN				0.33	33.42	0.21	

	N	2
	NS	1
	Wt	33.42
Het	Chi	0.21
Het	df	1
Het	P	N.S.
Fixed	RR	1.39
	RRl	0.99
	RRu	1.95
	P	(+)
Random	RR	1.39
	RRl	0.99
	RRu	1.95
	P	(+)
Asymm	P	

Table 1M4 - 6

IESLC - Meta-analysis of Ever Smoking, Butt length or Fraction smoked, "Highest vs lowest"
All LC types, Cigarettes only
Least adjusted

	Sex combined	male	female	Total
N		1	1	2
NS		1	1	1
Wt		32.42	1.00	33.42
Het Chi		0.00	0.00	0.21
Het df		0	0	1
Het P		N.S.	N.S.	N.S.
Fixed RR		1.41	0.89	1.39
RRl		1.00	0.13	0.99
RRu		1.99	6.31	1.95
P		+	N.S.	(+)
Random RR		1.41	0.89	1.39
RRl		1.00	0.13	0.99
RRu		1.99	6.31	1.95
P		+	N.S.	(+)
Between Chi				0.21
Between df				1
Between P				N.S.
Btwn(F) P				N.S.
Btwn(R) P				N.S.

Table 1M4 - 7

IESLC - Meta-analysis of Ever Smoking, Butt length or Fraction smoked, "Highest vs lowest"
All LC types, Cigarettes only
Excluded studies (and stage at which they were excluded)

1	AKIBA AMANDU AMES BECHER BENSHL BEST BLOT1 BROSS BROWN3 CARPEN CEDERL CHYOU CPSI CPSII DARBY DEAN2 DEAN3 DOLL2 ENGELA GAO2 GARCIA GILLIS GRAHAM GURSEL HAMMO2 HIRAYA HOLE HUMBLE JAHN JAIN KAISE2 KATSOU KAUFMA LAUSSM LIAW MCDUFF MIGRAN MRFITR PEZZO2 PISANI PRESCO QIAO SEGI2 SPEIZE SVENSS TVERDA WAKAI WATSON WIGLE WU WYNDE3 WYNDE8
2	AGUDO ALDERS ARMADA AUVINE AXELSS BARBON BENHAM BOFFET BOUCHA BOUCOT BRESLO BUFFLE CHEN CHEN2 CHIAZZ CORREA DAMBER DESTEF DOLL DORGAN DORN DOSEME FAN GAO GARSHI GENG GER GUO HAENSZ HAMMON HEGMAN HU HU2 JEDRYC JUSSAW KHUDER KOULUM KREUZE LETOUR LEVIN LIU3 LIU4 LIU5 LUBIN LUO MATOS MCCONN NOTAN2 OSANN2 PERNU PEZZOT QIAO2 RACHTA SADOWS SOBUE SPITZ STASZE SUZUK2 VUTUC WANG2 WU2 WUWILL WYNDE7 XU YUAN ZHANG ZHENG ZHOU
3	JOLY
5	LUBIN2 WYNDE2
6	RESTRE
8	CHOI KOO WYNDE6

Table 1M5 -

IESLC - Meta-analysis of Current Smoking, Butt length or Fraction smoked, "Highest vs lowest"
All LC types, Cigarettes only

This analysis is restricted to results for:
1) Current smokers
2) Results by Butt length or Fraction smoked
3) Categorical results by Butt length or Fraction smoked
4) Denominator (unexposed) = "low"
5) All LC types (or near equivalent)
6) Results complete enough for use in metaanalysis

Within each study, results are then selected (in the following order of preference, within each sex) for:
7) (not applicable)
8) PRODUCT: cigarettes only
9) CIGTYPE: all/unspecified, MC regardless of HR, MC only
10) Results with least adjustment for other aspects of smoking (ADOS)
11) The highest vs lowest category
12) Followup period (YF, prospective studies): whole study (coded as 0) or longest available
13) LCtype: all or nearest available, at least Squamous and Adeno. (q = squamous, s = small, l = large, a = adeno, mix = mixed, alv = alveolar)
14) Race: all or nearest available, otherwise by race (wh or w = white, bl or b = black, hi = hispanic ch = chinese, jap = japanese, haw = hawaiian, w+o = white + oriental, sca = scandinavian, as = asian)
15) For overlapping studies: principal rather than subsidiary studies
Finally by Age: whole study (coded as 0) if available, otherwise by widest available age group and then for single sex results (m, f) in preference to results for both sexes combined (c).

Results adjusted (AD) for the most potential confounders are then chosen in Sections -1 to -3 and results adjusted for the least confounders in Sections -4 to -6. (Those least adjusted results which actually differ from the most adjusted are marked 'x' in column X in Section -4)

Section -7 shows excluded studies, together with the stage (as above) at which no qualifying results were found.

Section -8 lists the potentially overlapping studies which have been included (1=principal, 2=subsidiary).

Section -9 lists any results which would have been included in preference except that they had data not complete enough for use in meta-analysis, with their significance (yes/no), if known, and any further comment as entered on the database. It also lists as "gap" any categories for which no data were presented by the original authors.

In addition to those mentioned above, the following fields, levels and abbreviations are used:
* or nk = not known, n = no, y = yes, ot = other
all/unspec = all or unspecified, MC = manufactured cigarettes, HR = hand-rolled cigarettes
exL, exH = range of exposure (low and high) in the "highest" group, in terms of Butt length or Fraction smoked
unexL, unexH = range of exposure (low and high) in the "lowest" group, in terms of Butt length or Fraction smoked
REF: 6-character study reference
NRR: number of the RR on the database within the study
ST : study type (CC = case control, pr or prosp = prospective)
NLC: number of lung cancer cases in whole study
R : risky occupational population (n = no, m = mining, o = other risky)
VB : national cigarette type (V = at least 75% Virginia, bl = at least 75% blended, ot = other)
P : any proxy use
H : full histological confirmation
De : derivation of RR/CI (or = original, st = standard method, ot = other method of estimation)

Table 1M5 - 1

IESLC - Meta-analysis of Current Smoking, Butt length or Fraction smoked, "Highest vs lowest"
All LC types, Cigarettes only
Most adjusted

REF	NRR	SEX	AGEL	AGEH	RACE	YF	LC TYPE	LOC	START	ST	NLC	R	VB	P	H	AD	ADOS	PRODUCT	exL	exH	unexL	unexH	De
KAISE2	665	m	0	0	all	9	all	NAmer	1979	pr	318	n	bl	n	n	5		2#cig only	76	100	1	75	or
KAISE2	666	f	0	0	all	9	all	NAmer	1979	pr	318	n	bl	n	n	5		2#cig only	76	100	1	75	or

Comments on values in listings

KAISE2 ADOS Cigs/day and years of smoking
KAISE2 ADOS Cigs/day and years of smoking

Cigarette type is all/unspec for all RRs

exL, exH, unexL, unexH refer to fraction of cigarette smoked (%) for all RRs

Table 1M5 - 2

IESLC - Meta-analysis of Current Smoking, Butt length or Fraction smoked, "Highest vs lowest"
All LC types, Cigarettes only
Most adjusted

REF	NRR	SEX	AD	Number Exposed Case	Cont	Non-exposed Case	Cont	RR	95.00%CI
*KAISE2	665	m	5	37	-	58	-	0.98	(0.64- 1.50)
*KAISE2	666	f	5	29	-	50	-	1.10	(0.68- 1.79)
Subtotal KAISE2								1.03	(0.75- 1.42)
Partial Totals				66	0	108	0		

*prospective study

REF	NRR	SEX	AD	Ys	Ws	Qs	Ps
*KAISE2	665	m	5	-0.02	21.18	0.05	0.9259
*KAISE2	666	f	5	0.10	16.40	0.07	0.6995
Subtotal KAISE2				0.03	37.58	0.12	

	N	2
	NS	1
	Wt	37.58
Het	Chi	0.12
Het	df	1
Het	P	N.S.
Fixed	RR	1.03
	RRl	0.75
	RRu	1.42
	P	N.S.
Random	RR	1.03
	RRl	0.75
	RRu	1.42
	P	N.S.
Asymm	P	

Table 1M5 - 3

IESLC - Meta-analysis of Current Smoking, Butt length or Fraction smoked, "Highest vs lowest"
All LC types, Cigarettes only
Most adjusted

	combined	Sex male	female	Total
N		1	1	2
NS		1	1	1
Wt		21.18	16.40	37.58
Het Chi		0.00	0.00	0.12
Het df		0	0	1
Het P		N.S.	N.S.	N.S.
Fixed RR		0.98	1.10	1.03
RRl		0.64	0.68	0.75
RRu		1.50	1.78	1.42
P		N.S.	N.S.	N.S.
Random RR		0.98	1.10	1.03
RRl		0.64	0.68	0.75
RRu		1.50	1.78	1.42
P		N.S.	N.S.	N.S.
Between Chi				0.12
Between df				1
Between P				N.S.
Btwn(F) P				N.S.
Btwn(R) P				N.S.

Too few RRs for analysis by factor

Table 1M5 - 4

IESLC - Meta-analysis of Current Smoking, Butt length or Fraction smoked, "Highest vs lowest"
All LC types, Cigarettes only
Least adjusted

REF	NRR	X	SEX	AGEL	AGEH	RACE	YF	LC TYPE	LOC	START	ST	NLC	R	VB	P	H	AD	ADOS	PRODUCT	exL	exH	unexL	unexH	De
KAISE2	665		m	0	0	all	9	all	NAmer	1979	pr	318	n	bl	n	n	5		2#cig only	76	100	1	75	or
KAISE2	666		f	0	0	all	9	all	NAmer	1979	pr	318	n	bl	n	n	5		2#cig only	76	100	1	75	or

Comments on values in listings

KAISE2 ADOS Cigs/day and years of smoking
KAISE2 ADOS Cigs/day and years of smoking

Cigarette type is all/unspec for all RRs

exL, exH, unexL, unexH refer to fraction of cigarette smoked (%) for all RRs

Table 1M5 - 5

IESLC - Meta-analysis of Current Smoking, Butt length or Fraction smoked, "Highest vs lowest"
All LC types, Cigarettes only
Least adjusted

REF	NRR	SEX	AD	Number Exposed Case	Cont	Non-exposed Case	Cont	RR	95.00%CI
*KAISE2	665	m	5	37	-	58	-	0.98	(0.64- 1.50)
*KAISE2	666	f	5	29	-	50	-	1.10	(0.68- 1.79)
Subtotal KAISE2								1.03	(0.75- 1.42)
Partial Totals				66	0	108	0		

*prospective study

REF	NRR	SEX	AD	Ys	Ws	Qs	Ps
*KAISE2	665	m	5	-0.02	21.18	0.05	0.9259
*KAISE2	666	f	5	0.10	16.40	0.07	0.6995
Subtotal KAISE2				0.03	37.58	0.12	

	N	2
	NS	1
	Wt	37.58
Het	Chi	0.12
Het	df	1
Het	P	N.S.
Fixed	RR	1.03
	RRl	0.75
	RRu	1.42
	P	N.S.
Random	RR	1.03
	RRl	0.75
	RRu	1.42
	P	N.S.
Asymm	P	

Table 1M5 - 6

IESLC - Meta-analysis of Current Smoking, Butt length or Fraction smoked, "Highest vs lowest"
All LC types, Cigarettes only
Least adjusted

	Sex combined	male	female	Total
N		1	1	2
NS		1	1	1
Wt		21.18	16.40	37.58
Het Chi		0.00	0.00	0.12
Het df		0	0	1
Het P		N.S.	N.S.	N.S.
Fixed RR		0.98	1.10	1.03
RRl		0.64	0.68	0.75
RRu		1.50	1.78	1.42
P		N.S.	N.S.	N.S.
Random RR		0.98	1.10	1.03
RRl		0.64	0.68	0.75
RRu		1.50	1.78	1.42
P		N.S.	N.S.	N.S.
Between Chi				0.12
Between df				1
Between P				N.S.
Btwn(F) P				N.S.
Btwn(R) P				N.S.

Table 1M5 - 7

IESLC - Meta-analysis of Current Smoking, Butt length or Fraction smoked, "Highest vs lowest"
All LC types, Cigarettes only
Excluded studies (and stage at which they were excluded)

1	AGUDO ALDERS ARMADA AUVINE AXELSS BARBON BECHER BENHAM BLOT1 BOFFET BOUCHA BRESLO BROWN3 CARPEN CHEN CHEN2 CHIAZZ CHOI CHYOU CORREA DAMBER DARBY DESTEF DOLL DOLL2 DORGAN DOSEME FAN GAO GARCIA GARSHI GENG GER GRAHAM GUO GURSEL HAENSZ HAMMO2 HAMMON HEGMAN HU HU2 JAHN JAIN JEDRYC JOLY JUSSAW KHUDER KOO KOULUM KREUZE LAUSSM LETOUR LEVIN LIU3 LIU4 LIU5 LUBIN LUBIN2 LUO MCCONN NOTAN2 OSANN2 PERNU PEZZOT PRESCO QIAO QIAO2 RACHTA RESTRE SADOWS STASZE SUZUK2 TIZZAN TVERDA VUTUC WANG2 WIGLE WU2 WUWILL WYNDE2 WYNDE3 XU YUAN ZHANG ZHENG ZHOU
2	AKIBA AMANDU AMES BENSHL BEST BOUCOT BROSS BUFFLE CEDERL CPSI CPSII DEAN2 DEAN3 DORN ENGELA GAO2 GILLIS HIRAYA HOLE HUMBLE KATSOU KAUFMA LIAW MATOS MCDUFF MIGRAN MRFITR PEZZO2 PISANI SEGI2 SPEIZE SPITZ SVENSS WATSON WU WYNDE6 WYNDE7 WYNDE8
8	SOBUE WAKAI

Table 1M6 -

IESLC - Meta-analysis of Ever/current Smoking, Butt length or Fraction smoked, "Highest vs lowest"
All LC types, Cigarettes only

This analysis is restricted to results for:
1) Ever/current smokers
2) Results by Butt length or Fraction smoked
3) Categorical results by Butt length or Fraction smoked
4) Denominator (unexposed) = "low"
5) All LC types (or near equivalent)
6) Results complete enough for use in metaanalysis

Within each study, results are then selected (in the following order of preference, within each sex) for:
7) SMKSTA: ever, current
8) PRODUCT: cigarettes only
9) CIGTYPE: all/unspecified, MC regardless of HR, MC only
10) Results with least adjustment for other aspects of smoking (ADOS)
11) The highest vs lowest category
12) Followup period (YF, prospective studies): whole study (coded as 0) or longest available
13) LCtype: all or nearest available, at least Squamous and Adeno. (q = squamous, s = small,
l = large, a = adeno, mix = mixed, alv = alveolar)
14) Race: all or nearest available, otherwise by race (wh or w = white, bl or b = black, hi = hispanic
ch = chinese, jap = japanese, haw = hawaiian, w+o = white + oriental, sca = scandinavian, as = asian)
15) For overlapping studies: principal rather than subsidiary studies
Finally by Age: whole study (coded as 0) if available, otherwise by widest available age group
and then for single sex results (m, f) in preference to results for both sexes combined (c).

Results adjusted (AD) for the most potential confounders are then chosen in Sections -1 to -3
(and those which actually differ from the adjusted results in Table 1M3 - 1 are marked 'x' in Section -1)
and results adjusted for the least confounders in Sections -4 to -6. (Those least adjusted results which
actually differ from the most adjusted are marked 'x' in column X in Section -4)

Section -7 shows excluded studies, together with the stage (as above) at which no qualifying
results were found.

Section -8 lists the potentially overlapping studies which have been included (1=principal, 2=subsidiary).

Section -9 lists any results which would have been included in preference except that they had data not complete
enough for use in meta-analysis, with their significance (yes/no), if known, and any further comment as entered
on the database. It also lists as "gap" any categories for which no data were presented by the original authors.

In addition to those mentioned above, the following fields, levels and abbreviations are used:
* or nk = not known, n = no, y = yes, ot = other
all/unspec = all or unspecified, MC = manufactured cigarettes, HR = hand-rolled cigarettes
exL, exH = range of exposure (low and high) in the "highest" group, in terms of Butt length or Fraction smoked
unexL, unexH = range of exposure (low and high) in the "lowest" group, in terms of Butt length or Fraction smoked
REF: 6-character study reference
NRR: number of the RR on the database within the study
ST : study type (CC = case control, pr or prosp = prospective)
NLC: number of lung cancer cases in whole study
R : risky occupational population (n = no, m = mining, o = other risky)
VB : national cigarette type (V = at least 75% Virginia, bl = at least 75% blended, ot = other)
P : any proxy use
H : full histological confirmation
De : derivation of RR/CI (or = original, st = standard method, ot = other method of estimation)

Table 1M6 - 1

IESLC - Meta-analysis of Ever/current Smoking, Butt length or Fraction smoked, "Highest vs lowest"
All LC types, Cigarettes only
Most adjusted

REF	NRR	1M3	SEX	AGEL	AGEH	RACE	YF	LC TYPE	LOC	START	ST	NLC	R	VB	P	H	AD	ADOS	SM	PRODUCT	exL	exH	unexL	unexH	De
KAISE2	665		m	0	0	all	9	all	NAmer	1979	pr	318	n	bl	n	n	5	2#	cu	cig only	76	100	1	75	or
KAISE2	666		f	0	0	all	9	all	NAmer	1979	pr	318	n	bl	n	n	5	2#	cu	cig only	76	100	1	75	or
TIZZAN	515		m	0	0	all	-	all	Eu:wst	1959	CC	1358	n	bl	n	n	0	0	ev	cig only	903	903#	901	901	st
TIZZAN	528		f	0	0	all	-	all	Eu:wst	1959	CC	1358	n	bl	n	n	0	0	ev	cig only	903	903#	901	901	st

Comments on values in listings

KAISE2 ADOS Cigs/day and years of smoking
KAISE2 ADOS Cigs/day and years of smoking

Cigarette type is all/unspec for all RRs

exL, exH, unexL, unexH refer to fraction of cigarette smoked (%) for all RRs except

TIZZAN Butt length described as short vs long
TIZZAN Butt length described as short vs long

Table 1M6 - 2

IESLC - Meta-analysis of Ever/current Smoking, Butt length or Fraction smoked, "Highest vs lowest"
All LC types, Cigarettes only
Most adjusted

REF	NRR	SEX	AD	Number Exposed Case	Number Exposed Cont	Non-exposed Case	Non-exposed Cont	RR	95.00%CI
*KAISE2	665	m	5	37	-	58	-	0.98	(0.64- 1.50)
*KAISE2	666	f	5	29	-	50	-	1.10	(0.68- 1.79)
Subtotal KAISE2								1.03	(0.75- 1.42)
TIZZAN	515	m	0	443	306	80	78	1.41	(1.00- 1.99)
TIZZAN	528	f	0	4	3	6	4	0.89	(0.13- 6.31)
Subtotal TIZZAN								1.39	(0.99- 1.95)
Partial Totals				513	309	194	82		

*prospective study

REF	NRR	SEX	AD	Ys	Ws	Qs	Ps
*KAISE2	665	m	5	-0.02	21.18	0.78	0.9259
*KAISE2	666	f	5	0.10	16.40	0.10	0.6995
Subtotal KAISE2				0.03	37.58	0.88	
TIZZAN	515	m	0	0.34	32.42	0.97	0.0497
TIZZAN	528	f	0	-0.12	1.00	0.08	0.9062
Subtotal TIZZAN				0.33	33.42	1.05	

	N	4
	NS	2
	Wt	71.00
Het	Chi	1.93
Het	df	3
Het	P	N.S.
Fixed	RR	1.19
	RRl	0.94
	RRu	1.50
	P	N.S.
Random	RR	1.19
	RRl	0.94
	RRu	1.50
	P	N.S.
Asymm	P	N.S.

Table 1M6 - 3

IESLC - Meta-analysis of Ever/current Smoking, Butt length or Fraction smoked, "Highest vs lowest"
All LC types, Cigarettes only
Most adjusted

	Sex combined	male	female	Total
N		2	2	4
NS		2	2	4
Wt		53.60	17.40	71.00
Het Chi		1.71	0.04	1.93
Het df		1	1	3
Het P		N.S.	N.S.	N.S.
Fixed RR		1.22	1.09	1.19
RRl		0.93	0.68	0.94
RRu		1.60	1.74	1.50
P		N.S.	N.S.	N.S.
Random RR		1.20	1.09	1.19
RRl		0.84	0.68	0.94
RRu		1.72	1.74	1.50
P		N.S.	N.S.	N.S.
Between Chi				0.18
Between df				1
Between P				N.S.
Btwn(F) P				N.S.
Btwn(R) P				N.S.

Too few RRs for analysis by factor

Table 1M6 - 4

IESLC - Meta-analysis of Ever/current Smoking, Butt length or Fraction smoked, "Highest vs lowest"
All LC types, Cigarettes only
Least adjusted

REF	NRR	X	SEX	AGEL	AGEH	RACE	YF	LC TYPE	LOC	START	ST	NLC	R	VB	P	H	AD	ADOS	SM	PRODUCT	exL	exH	unexL	unexH	De
KAISE2	665		m	0	0	all	9	all	NAmer	1979	pr	318	n	bl	n	n	5	2#	cu	cig only	76	100	1	75	or
KAISE2	666		f	0	0	all	9	all	NAmer	1979	pr	318	n	bl	n	n	5	2#	cu	cig only	76	100	1	75	or
TIZZAN	515		m	0	0	all	-	all	Eu:wst	1959	CC	1358	n	bl	n	n	0	0	ev	cig only	903	903#	901	901	st
TIZZAN	528		f	0	0	all	-	all	Eu:wst	1959	CC	1358	n	bl	n	n	0	0	ev	cig only	903	903#	901	901	st

Comments on values in listings

KAISE2 ADOS Cigs/day and years of smoking
KAISE2 ADOS Cigs/day and years of smoking

Cigarette type is all/unspec for all RRs

exL, exH, unexL, unexH refer to fraction of cigarette smoked (%) for all RRs except

TIZZAN Butt length described as short vs long
TIZZAN Butt length described as short vs long

Table 1M6 - 5

IESLC - Meta-analysis of Ever/current Smoking, Butt length or Fraction smoked, "Highest vs lowest"
All LC types, Cigarettes only
Least adjusted

REF	NRR	SEX	AD	Number Exposed Case	Number Exposed Cont	Non-exposed Case	Non-exposed Cont	RR	95.00%CI
*KAISE2	665	m	5	37	-	58	-	0.98	(0.64- 1.50)
*KAISE2	666	f	5	29	-	50	-	1.10	(0.68- 1.79)
Subtotal KAISE2								1.03	(0.75- 1.42)
TIZZAN	515	m	0	443	306	80	78	1.41	(1.00- 1.99)
TIZZAN	528	f	0	4	3	6	4	0.89	(0.13- 6.31)
Subtotal TIZZAN								1.39	(0.99- 1.95)
Partial Totals				513	309	194	82		

*prospective study

REF	NRR	SEX	AD	Ys	Ws	Qs	Ps
*KAISE2	665	m	5	-0.02	21.18	0.78	0.9259
*KAISE2	666	f	5	0.10	16.40	0.10	0.6995
Subtotal KAISE2				0.03	37.58	0.88	
TIZZAN	515	m	0	0.34	32.42	0.97	0.0497
TIZZAN	528	f	0	-0.12	1.00	0.08	0.9062
Subtotal TIZZAN				0.33	33.42	1.05	

	N	4
	NS	2
	Wt	71.00
Het	Chi	1.93
Het	df	3
Het	P	N.S.
Fixed	RR	1.19
	RRl	0.94
	RRu	1.50
	P	N.S.
Random	RR	1.19
	RRl	0.94
	RRu	1.50
	P	N.S.
Asymm	P	N.S.

Table 1M6 - 6

IESLC - Meta-analysis of Ever/current Smoking, Butt length or Fraction smoked, "Highest vs lowest"
All LC types, Cigarettes only
Least adjusted

	Sex: combined	male	female	Total
N		2	2	4
NS		2	2	4
Wt		53.60	17.40	71.00
Het Chi		1.71	0.04	1.93
Het df		1	1	3
Het P		N.S.	N.S.	N.S.
Fixed RR		1.22	1.09	1.19
RRl		0.93	0.68	0.94
RRu		1.60	1.74	1.50
P		N.S.	N.S.	N.S.
Random RR		1.20	1.09	1.19
RRl		0.84	0.68	0.94
RRu		1.72	1.74	1.50
P		N.S.	N.S.	N.S.
Between Chi				0.18
Between df				1
Between P				N.S.
Btwn(F) P				N.S.
Btwn(R) P				N.S.

Table 1M6 - 7

IESLC - Meta-analysis of Ever/current Smoking, Butt length or Fraction smoked, "Highest vs lowest"
All LC types, Cigarettes only
Excluded studies (and stage at which they were excluded)

1	BECHER BLOT1 BROWN3 CARPEN CHYOU DARBY DOLL2 GARCIA GRAHAM GURSEL HAMMO2 JAHN JAIN LAUSSM PRESCO QIAO TVERDA WIGLE WYNDE3
2	AGUDO AKIBA ALDERS AMANDU AMES ARMADA AUVINE AXELSS BARBON BENHAM BENSHL BEST BOFFET BOUCHA BOUCOT BRESLO BROSS BUFFLE CEDERL CHEN CHEN2 CHIAZZ CORREA CPSI CPSII DAMBER DEAN2 DEAN3 DESTEF DOLL DORGAN DORN DOSEME ENGELA FAN GAO GAO2 GARSHI GENG GER GILLIS GUO HAENSZ HAMMON HEGMAN HIRAYA HOLE HU HU2 HUMBLE JEDRYC JUSSAW KATSOU KAUFMA KHUDER KOULUM KREUZE LETOUR LEVIN LIAW LIU3 LIU4 LIU5 LUBIN LUO MATOS MCCONN MCDUFF MIGRAN MRFITR NOTAN2 OSANN2 PERNU PEZZO2 PEZZOT PISANI QIAO2 RACHTA SADOWS SEGI2 SPEIZE SPITZ STASZE SUZUK2 SVENSS VUTUC WANG2 WATSON WU WU2 WUWILL WYNDE7 WYNDE8 XU YUAN ZHANG ZHENG ZHOU
3	JOLY
5	LUBIN2 WYNDE2
6	RESTRE
8	CHOI KOO SOBUE WAKAI WYNDE6
