# Supplementary material for: Systematic review with meta-analysis of the epidemiological evidence in the 1900s relating smoking to lung cancer
Source: BMC Cancer. 2012 Sep 3;12:385. doi: 10.1186/1471-2407-12-385 (PMC3505152; doi:10.1186/1471-2407-12-385)
Supplement: Additional file 5 — Detailed Analysis Tables (Individual file names as described in Additional file 1: Methods, Table1). [file 1471-2407-12-385-S5.zip › PDF/2L.pdf]

Table 2L1 -
IESLC - Meta-analysis of Ever Smoking, Tar, "Highest vs lowest"
Squamous, Cigarettes (or Any Product if Cigarettes not available)

This analysis is restricted to results for:
1) Ever smokers
2) Results by Tar
3) Categorical results by Tar
4) Denominator (unexposed) = "low"
5) Squamous (or near equivalent)
6) Results complete enough for use in metaanalysis

Within each study, results are then selected (in the following order of preference, within each sex) for:
7) (not applicable)
8) PRODUCT: cigarettes regardless of other products, cigarettes only, all/unspec
9) CIGTYPE: all/unspecified, MC regardless of HR, MC only
10) Results with least adjustment for other aspects of smoking (ADOS)
11) The highest vs lowest category
12) Followup period (YF, prospective studies): whole study (coded as 0) or longest available
13) LCtype: squamous or nearest available, but not adeno. (q = squamous, s = small,
a = adeno, KI = Kreyberg I, u = undifferentiated)
14) Race: all or nearest available, otherwise by race (wh or w = white, bl or b = black, hi = hispanic
ch = chinese, jap = japanese, haw = hawaiian, w+o = white + oriental, sca = scandinavian, as = asian)
15) For overlapping studies: principal rather than subsidiary studies
Finally by Age: whole study (coded as 0) if available, otherwise by widest available age group
and then for single sex results (m, f) in preference to results for both sexes combined (c).

Results adjusted (AD) for the most potential confounders are then chosen in Sections -1 to -3
and results adjusted for the least confounders in Sections -4 to -6. (Those least adjusted results which
actually differ from the most adjusted are marked 'x' in column X in Section -4)

Section -7 shows excluded studies, together with the stage (as above) at which no qualifying
results were found.

Section -8 lists the potentially overlapping studies which have been included (1=principal, 2=subsidiary).

Section -9 lists any results which would have been included in preference except that they had data not complete
enough for use in meta-analysis, with their significance (yes/no), if known, and any further comment as entered
on the database. It also lists as "gap" any categories for which no data were presented by the original authors.

In addition to those mentioned above, the following fields, levels and abbreviations are used:
* or nk = not known, n = no, y = yes, ot = other
all/unspec = all or unspecified, cig+/-ot = cigarettes irrespective of other products (cigar, pipe etc)
MC = manufactured cigarettes, HR = hand-rolled cigarettes
exL, exH = range of exposure (low and high) in the "highest" group, in terms of Tar
unexL, unexH = range of exposure (low and high) in the "lowest" group, in terms of Tar
REF: 6-character study reference
NRR: number of the RR on the database within the study
ST : study type (CC = case control, pr or prosp = prospective)
NLC: number of lung cancer cases in whole study
R : risky occupational population (n = no, m = mining, o = other risky)
VB : national cigarette type (V = at least 75% Virginia, bl = at least 75% blended, ot = other)
P : any proxy use
H : full histological confirmation
De : derivation of RR/CI (or = original, st = standard method, ot = other method of estimation)

Table 2L1 - 0

No RRs selected for this analysis

Table 2L1 - 7

IESLC - Meta-analysis of Ever Smoking, Tar, "Highest vs lowest"
Squamous, Cigarettes (or Any Product if Cigarettes not available)
Excluded studies (and stage at which they were excluded)

1 AKIBA AMANDU AMES BECHER BENSHL BEST BLOT1 BROSS BROWN3 CARPEN CEDERL CHYOU CPSI CPSII DARBY DEAN2 DEAN3 DOLL2 ENGELA GAO2 GARCIA GILLIS GRAHAM GURSEL HAMMO2 HIRAYA HOLE HUMBLE JAHN JAIN KAISE2 KATSOU KAUFMA LAUSSM LIAW MCDUFF MIGRAN MRFITR PEZZO2 PISANI PRESCO QIAO SEGI2 SPEIZE SVENSS TVERDA WAKAI WATSON WIGLE WU WYNDE3 WYNDE8

2 AGUDO ARMADA AUVINE AXELSS BARBON BENHAM BOFFET BOUCHA BOUCOT BRESLO BUFFLE CHEN CHEN2 CHIAZZ CHOI CORREA DAMBER DESTEF DOLL DORN DOSEME FAN GAO GARSHI GENG GER GUO HAENSZ HAMMON HEGMAN HU HU2 JEDRYC JOLY JUSSAW KHUDER KOO KOULUM KREUZE LETOUR LEVIN LIU3 LIU4 LIU5 LUBIN LUO MATOS MCCONN NOTAN2 OSANN2 PERNU PEZZOT QIAO2 RACHTA RESTRE SADOWS SOBUE SPITZ STASZE SUZUK2 TIZZAN WANG2 WU2 WUWILL WYNDE2 WYNDE7 XU YUAN ZHANG ZHENG ZHOU

3 WYNDE6

5 DORGAN LUBIN2 VUTUC

6 ALDERS

Table 2L1 - 8

Table 2L1 - 9

Most adjusted - insufficient data for meta-analysis

REF	NRR	SEX	AGEL	AGEH	RACE	YF	LC TYPE	LOC	START	ST	NLC	R	VB	P	H	AD	ADOS	PRODUCT	exL	exH	unexL	unexH	De
ALDERS	559	m	0	0	all	-	q+s	Eu:UK	1977	CC	1448	n	V	n	n	2		1#cig only	17	22	1	16	ot
ALDERS	560	f	0	0	all	-	q+s	Eu:UK	1977	CC	1448	n	V	n	n	2		1#cig only	17	22	1	16	ot

Comments on values in listings

ALDERS ADOS Number of cigs/day
ALDERS ADOS Number of cigs/day

REF	NRR	RR	SIG	RRDATA comment
ALDERS	559	1.05	n	0
ALDERS	560	0.82	n	0

Table 2L2 -

IESLC - Meta-analysis of Current Smoking, Tar, "Highest vs lowest"
Squamous, Cigarettes (or Any Product if Cigarettes not available)

This analysis is restricted to results for:
1) Current smokers
2) Results by Tar
3) Categorical results by Tar
4) Denominator (unexposed) = "low"
5) Squamous (or near equivalent)
6) Results complete enough for use in metaanalysis

Within each study, results are then selected (in the following order of preference, within each sex) for:
7) (not applicable)
8) PRODUCT: cigarettes regardless of other products, cigarettes only, all/unspec
9) CIGTYPE: all/unspecified, MC regardless of HR, MC only
10) Results with least adjustment for other aspects of smoking (ADOS)
11) The highest vs lowest category
12) Followup period (YF, prospective studies): whole study (coded as 0) or longest available
13) LCtype: squamous or nearest available, but not adeno. (q = squamous, s = small,
a = adeno, KI = Kreyberg I, u = undifferentiated)
14) Race: all or nearest available, otherwise by race (wh or w = white, bl or b = black, hi = hispanic
ch = chinese, jap = japanese, haw = hawaiian, w+o = white + oriental, sca = scandinavian, as = asian)
15) For overlapping studies: principal rather than subsidiary studies
Finally by Age: whole study (coded as 0) if available, otherwise by widest available age group
and then for single sex results (m, f) in preference to results for both sexes combined (c).

Results adjusted (AD) for the most potential confounders are then chosen in Sections -1 to -3
and results adjusted for the least confounders in Sections -4 to -6. (Those least adjusted results which
actually differ from the most adjusted are marked 'x' in column X in Section -4)

Section -7 shows excluded studies, together with the stage (as above) at which no qualifying
results were found.

Section -8 lists the potentially overlapping studies which have been included (1=principal, 2=subsidiary).

Section -9 lists any results which would have been included in preference except that they had data not complete
enough for use in meta-analysis, with their significance (yes/no), if known, and any further comment as entered
on the database. It also lists as "gap" any categories for which no data were presented by the original authors.

In addition to those mentioned above, the following fields, levels and abbreviations are used:
* or nk = not known, n = no, y = yes, ot = other
all/unspec = all or unspecified, cig+/-ot = cigarettes irrespective of other products (cigar, pipe etc)
MC = manufactured cigarettes, HR = hand-rolled cigarettes
exL, exH = range of exposure (low and high) in the "highest" group, in terms of Tar
unexL, unexH = range of exposure (low and high) in the "lowest" group, in terms of Tar
REF: 6-character study reference
NRR: number of the RR on the database within the study
ST : study type (CC = case control, pr or prosp = prospective)
NLC: number of lung cancer cases in whole study
R : risky occupational population (n = no, m = mining, o = other risky)
VB : national cigarette type (V = at least 75% Virginia, bl = at least 75% blended, ot = other)
P : any proxy use
H : full histological confirmation
De : derivation of RR/CI (or = original, st = standard method, ot = other method of estimation)

Table 2L2 - 0

No RRs selected for this analysis

Table 2L2 - 7

IESLC - Meta-analysis of Current Smoking, Tar, "Highest vs lowest"
Squamous, Cigarettes (or Any Product if Cigarettes not available)
Excluded studies (and stage at which they were excluded)

1	AGUDO ALDERS ARMADA AUVINE AXELSS BARBON BECHER BENHAM BLOT1 BOFFET BOUCHA BRESLO BROWN3 CARPEN CHEN CHEN2 CHIAZZ CHOI CHYOU CORREA DAMBER DARBY DESTEF DOLL DOLL2 DORGAN DOSEME FAN GAO GARCIA GARSHI GENG GER GRAHAM GUO GURSEL HAENSZ HAMMO2 HAMMON HEGMAN HU HU2 JAHN JAIN JEDRYC JOLY JUSSAW KHUDER KOO KOULUM KREUZE LAUSSM LETOUR LEVIN LIU3 LIU4 LIU5 LUBIN LUBIN2 LUO MCCONN NOTAN2 OSANN2 PERNU PEZZOT PRESCO QIAO QIAO2 RACHTA RESTRE SADOWS STASZE SUZUK2 TIZZAN TVERDA VUTUC WANG2 WIGLE WU2 WUWILL WYNDE2 WYNDE3 XU YUAN ZHANG ZHENG ZHOU
2	AKIBA AMANDU AMES BEST BOUCOT BROSS BUFFLE CEDERL DEAN2 DEAN3 DORN ENGELA GAO2 HIRAYA HOLE HUMBLE KATSOU LIAW MATOS MCDUFF MIGRAN PEZZO2 PISANI SEGI2 SOBUE SPITZ SVENSS WAKAI WATSON WU WYNDE7 WYNDE8
3	CPSII
5	BENSHL CPSI GILLIS KAISE2 KAUFMA MRFITR SPEIZE
9	WYNDE6

Table 2L3 -

IESLC - Meta-analysis of Ever/current Smoking, Tar, "Highest vs lowest"
Squamous, Cigarettes (or Any Product if Cigarettes not available)

This analysis is restricted to results for:
1) Ever/current smokers
2) Results by Tar
3) Categorical results by Tar
4) Denominator (unexposed) = "low"
5) Squamous (or near equivalent)
6) Results complete enough for use in metaanalysis

Within each study, results are then selected (in the following order of preference, within each sex) for:
7) SMKSTA: ever, current
8) PRODUCT: cigarettes regardless of other products, cigarettes only, all/unspec
9) CIGTYPE: all/unspecified, MC regardless of HR, MC only
10) Results with least adjustment for other aspects of smoking (ADOS)
11) The highest vs lowest category
12) Followup period (YF, prospective studies): whole study (coded as 0) or longest available
13) LCtype: squamous or nearest available, but not adeno. (q = squamous, s = small,
a = adeno, KI = Kreyberg I, u = undifferentiated)
14) Race: all or nearest available, otherwise by race (wh or w = white, bl or b = black, hi = hispanic
ch = chinese, jap = japanese, haw = hawaiian, w+o = white + oriental, sca = scandinavian, as = asian)
15) For overlapping studies: principal rather than subsidiary studies
Finally by Age: whole study (coded as 0) if available, otherwise by widest available age group
and then for single sex results (m, f) in preference to results for both sexes combined (c).

Results adjusted (AD) for the most potential confounders are then chosen in Sections -1 to -3
and results adjusted for the least confounders in Sections -4 to -6. (Those least adjusted results which
actually differ from the most adjusted are marked 'x' in column X in Section -4)

Section -7 shows excluded studies, together with the stage (as above) at which no qualifying
results were found.

Section -8 lists the potentially overlapping studies which have been included (1=principal, 2=subsidiary).

Section -9 lists any results which would have been included in preference except that they had data not complete
enough for use in meta-analysis, with their significance (yes/no), if known, and any further comment as entered
on the database. It also lists as "gap" any categories for which no data were presented by the original authors.

In addition to those mentioned above, the following fields, levels and abbreviations are used:
* or nk = not known, n = no, y = yes, ot = other
all/unspec = all or unspecified, cig+/-ot = cigarettes irrespective of other products (cigar, pipe etc)
MC = manufactured cigarettes, HR = hand-rolled cigarettes
exL, exH = range of exposure (low and high) in the "highest" group, in terms of Tar
unexL, unexH = range of exposure (low and high) in the "lowest" group, in terms of Tar
REF: 6-character study reference
NRR: number of the RR on the database within the study
ST : study type (CC = case control, pr or prosp = prospective)
NLC: number of lung cancer cases in whole study
R : risky occupational population (n = no, m = mining, o = other risky)
VB : national cigarette type (V = at least 75% Virginia, bl = at least 75% blended, ot = other)
P : any proxy use
H : full histological confirmation
De : derivation of RR/CI (or = original, st = standard method, ot = other method of estimation)

Table 2L3 - 0

No RRs selected for this analysis

Table 2L3 - 7

IESLC - Meta-analysis of Ever/current Smoking, Tar, "Highest vs lowest"
Squamous, Cigarettes (or Any Product if Cigarettes not available)
Excluded studies (and stage at which they were excluded)

1 BECHER BLOT1 BROWN3 CARPEN CHYOU DARBY DOLL2 GARCIA GRAHAM GURSEL HAMMO2 JAHN JAIN LAUSSM PRESCO QIAO TVERDA WIGLE WYNDE3

2 AGUDO AKIBA AMANDU AMES ARMADA AUVINE AXELSS BARBON BENHAM BEST BOFFET BOUCHA BOUCOT BRESLO BROSS BUFFLE CEDERL CHEN CHEN2 CHIAZZ CHOI CORREA DAMBER DEAN2 DEAN3 DESTEF DOLL DORN DOSEME ENGELA FAN GAO GAO2 GARSHI GENG GER GUO HAENSZ HAMMON HEGMAN HIRAYA HOLE HU HU2 HUMBLE JEDRYC JOLY JUSSAW KATSOU KHUDER KOO KOULUM KREUZE LETOUR LEVIN LIAW LIU3 LIU4 LIU5 LUBIN LUO MATOS MCCONN MCDUFF MIGRAN NOTAN2 OSANN2 PERNU PEZZO2 PEZZOT PISANI QIAO2 RACHTA RESTRE SADOWS SEGI2 SOBUE SPITZ STASZE SUZUK2 SVENSS TIZZAN WAKAI WANG2 WATSON WU WU2 WUWILL WYNDE2 WYNDE7 WYNDE8 XU YUAN ZHANG ZHENG ZHOU

3 CPSII

5 BENSHL CPSI DORGAN GILLIS KAISE2 KAUFMA LUBIN2 MRFITR SPEIZE VUTUC

6 ALDERS

9 WYNDE6

Table 2L3 - 8

Table 2L3 - 9

Most adjusted - insufficient data for meta-analysis

REF	NRR	SEX	AGEL	AGEH	RACE	YF	LC TYPE	LOC	START	ST	NLC	R	VB	P	H	AD	ADOS	SM	PRODUCT	exL	exH	unexL	unexH	De
ALDERS	559	m	0	0	all	-	q+s	Eu:UK	1977	CC	1448	n	V	n	n	2		1#ev	cig only	17	22	1	16	ot
ALDERS	560	f	0	0	all	-	q+s	Eu:UK	1977	CC	1448	n	V	n	n	2		1#ev	cig only	17	22	1	16	ot

Comments on values in listings

ALDERS ADOS Number of cigs/day
ALDERA ADOS Number of cigs/day

REF	NRR	RR	SIG	RRDATA comment
ALDERS	559	1.05	n	0
ALDERS	560	0.82	n	0

Table 2L4 -

IESLC - Meta-analysis of Ever Smoking, Tar, "Highest vs lowest"
Squamous, Cigarettes only

This analysis is restricted to results for:
1) Ever smokers
2) Results by Tar
3) Categorical results by Tar
4) Denominator (unexposed) = "low"
5) Squamous (or near equivalent)
6) Results complete enough for use in metaanalysis

Within each study, results are then selected (in the following order of preference, within each sex) for:
7) (not applicable)
8) PRODUCT: cigarettes only
9) CIGTYPE: all/unspecified, MC regardless of HR, MC only
10) Results with least adjustment for other aspects of smoking (ADOS)
11) The highest vs lowest category
12) Followup period (YF, prospective studies): whole study (coded as 0) or longest available
13) LCtype: squamous or nearest available, but not adeno. (q = squamous, s = small,
a = adeno, KI = Kreyberg I, u = undifferentiated)
14) Race: all or nearest available, otherwise by race (wh or w = white, bl or b = black, hi = hispanic
ch = chinese, jap = japanese, haw = hawaiian, w+o = white + oriental, sca = scandinavian, as = asian)
15) For overlapping studies: principal rather than subsidiary studies
Finally by Age: whole study (coded as 0) if available, otherwise by widest available age group
and then for single sex results (m, f) in preference to results for both sexes combined (c).

Results adjusted (AD) for the most potential confounders are then chosen in Sections -1 to -3
and results adjusted for the least confounders in Sections -4 to -6. (Those least adjusted results which
actually differ from the most adjusted are marked 'x' in column X in Section -4)

Section -7 shows excluded studies, together with the stage (as above) at which no qualifying
results were found.

Section -8 lists the potentially overlapping studies which have been included (1=principal, 2=subsidiary).

Section -9 lists any results which would have been included in preference except that they had data not complete
enough for use in meta-analysis, with their significance (yes/no), if known, and any further comment as entered
on the database. It also lists as "gap" any categories for which no data were presented by the original authors.

In addition to those mentioned above, the following fields, levels and abbreviations are used:
* or nk = not known, n = no, y = yes, ot = other
all/unspec = all or unspecified, MC = manufactured cigarettes, HR = hand-rolled cigarettes
exL, exH = range of exposure (low and high) in the "highest" group, in terms of Tar
unexL, unexH = range of exposure (low and high) in the "lowest" group, in terms of Tar
REF: 6-character study reference
NRR: number of the RR on the database within the study
ST : study type (CC = case control, pr or prosp = prospective)
NLC: number of lung cancer cases in whole study
R : risky occupational population (n = no, m = mining, o = other risky)
VB : national cigarette type (V = at least 75% Virginia, bl = at least 75% blended, ot = other)
P : any proxy use
H : full histological confirmation
De : derivation of RR/CI (or = original, st = standard method, ot = other method of estimation)

Table 2L4 - 0

No RRs selected for this analysis

Table 2L4 - 7

IESLC - Meta-analysis of Ever Smoking, Tar, "Highest vs lowest"
Squamous, Cigarettes only
Excluded studies (and stage at which they were excluded)

1 AKIBA AMANDU AMES BECHER BENSHL BEST BLOT1 BROSS BROWN3 CARPEN CEDERL CHYOU CPSI CPSII DARBY DEAN2 DEAN3 DOLL2 ENGELA GAO2 GARCIA GILLIS GRAHAM GURSEL HAMMO2 HIRAYA HOLE HUMBLE JAHN JAIN KAISE2 KATSOU KAUFMA LAUSSM LIAW MCDUFF MIGRAN MRFITR PEZZO2 PISANI PRESCO QIAO SEGI2 SPEIZE SVENSS TVERDA WAKAI WATSON WIGLE WU WYNDE3 WYNDE8

2 AGUDO ARMADA AUVINE AXELSS BARBON BENHAM BOFFET BOUCHA BOUCOT BRESLO BUFFLE CHEN CHEN2 CHIAZZ CHOI CORREA DAMBER DESTEF DOLL DORN DOSEME FAN GAO GARSHI GENG GER GUO HAENSZ HAMMON HEGMAN HU HU2 JEDRYC JOLY JUSSAW KHUDER KOO KOULUM KREUZE LETOUR LEVIN LIU3 LIU4 LIU5 LUBIN LUO MATOS MCCONN NOTAN2 OSANN2 PERNU PEZZOT QIAO2 RACHTA RESTRE SADOWS SOBUE SPITZ STASZE SUZUK2 TIZZAN WANG2 WU2 WUWILL WYNDE2 WYNDE7 XU YUAN ZHANG ZHENG ZHOU

3 WYNDE6

5 DORGAN LUBIN2 VUTUC

6 ALDERS

Table 2L4 - 8

Table 2L4 - 9

Most adjusted - insufficient data for meta-analysis

REF	NRR	SEX	AGEL	AGEH	RACE	YF	LC TYPE	LOC	START	ST	NLC	R	VB	P	H	AD	ADOS	PRODUCT	exL	exH	unexL	unexH	De
ALDERS	559	m	0	0	all	-	q+s	Eu:UK	1977	CC	1448	n	V	n	n	2	1#cig	only	17	22	1	16	ot
ALDERS	560	f	0	0	all	-	q+s	Eu:UK	1977	CC	1448	n	V	n	n	2	1#cig	only	17	22	1	16	ot

Comments on values in listings

ALDERS ADOS Number of cigs/day
ALDERS ADOS Number of cigs/day

REF	NRR	RR	SIG	RRDATA comment
ALDERS	559	1.05	n	0
ALDERS	560	0.82	n	0

Table 2L5 -

IESLC - Meta-analysis of Current Smoking, Tar, "Highest vs lowest"
Squamous, Cigarettes only

This analysis is restricted to results for:
1) Current smokers
2) Results by Tar
3) Categorical results by Tar
4) Denominator (unexposed) = "low"
5) Squamous (or near equivalent)
6) Results complete enough for use in metaanalysis

Within each study, results are then selected (in the following order of preference, within each sex) for:
7) (not applicable)
8) PRODUCT: cigarettes only
9) CIGTYPE: all/unspecified, MC regardless of HR, MC only
10) Results with least adjustment for other aspects of smoking (ADOS)
11) The highest vs lowest category
12) Followup period (YF, prospective studies): whole study (coded as 0) or longest available
13) LCtype: squamous or nearest available, but not adeno. (q = squamous, s = small,
a = adeno, KI = Kreyberg I, u = undifferentiated)
14) Race: all or nearest available, otherwise by race (wh or w = white, bl or b = black, hi = hispanic
ch = chinese, jap = japanese, haw = hawaiian, w+o = white + oriental, sca = scandinavian, as = asian)
15) For overlapping studies: principal rather than subsidiary studies
Finally by Age: whole study (coded as 0) if available, otherwise by widest available age group
and then for single sex results (m, f) in preference to results for both sexes combined (c).

Results adjusted (AD) for the most potential confounders are then chosen in Sections -1 to -3
and results adjusted for the least confounders in Sections -4 to -6. (Those least adjusted results which
actually differ from the most adjusted are marked 'x' in column X in Section -4)

Section -7 shows excluded studies, together with the stage (as above) at which no qualifying
results were found.

Section -8 lists the potentially overlapping studies which have been included (1=principal, 2=subsidiary).

Section -9 lists any results which would have been included in preference except that they had data not complete
enough for use in meta-analysis, with their significance (yes/no), if known, and any further comment as entered
on the database. It also lists as "gap" any categories for which no data were presented by the original authors.

In addition to those mentioned above, the following fields, levels and abbreviations are used:
* or nk = not known, n = no, y = yes, ot = other
all/unspec = all or unspecified, MC = manufactured cigarettes, HR = hand-rolled cigarettes
exL, exH = range of exposure (low and high) in the "highest" group, in terms of Tar
unexL, unexH = range of exposure (low and high) in the "lowest" group, in terms of Tar
REF: 6-character study reference
NRR: number of the RR on the database within the study
ST : study type (CC = case control, pr or prosp = prospective)
NLC: number of lung cancer cases in whole study
R : risky occupational population (n = no, m = mining, o = other risky)
VB : national cigarette type (V = at least 75% Virginia, bl = at least 75% blended, ot = other)
P : any proxy use
H : full histological confirmation
De : derivation of RR/CI (or = original, st = standard method, ot = other method of estimation)

Table 2L5 - 0

No RRs selected for this analysis

Table 2L5 - 7

IESLC - Meta-analysis of Current Smoking, Tar, "Highest vs lowest"
Squamous, Cigarettes only
Excluded studies (and stage at which they were excluded)

1 AGUDO ALDERS ARMADA AUVINE AXELSS BARBON BECHER BENHAM BLOT1 BOFFET BOUCHA BRESLO BROWN3 CARPEN CHEN CHEN2 CHIAZZ
CHOI CHYOU CORREA DAMBER DARBY DESTEF DOLL DOLL2 DORGAN DOSEME FAN GAO GARCIA GARSHI GENG GER GRAHAM
GUO GURSEL HAENSZ HAMMO2 HAMMON HEGMAN HU HU2 JAHN JAIN JEDRYC JOLY JUSSAW KHUDER KOO KOULUM KREUZE
LAUSSM LETOUR LEVIN LIU3 LIU4 LIU5 LUBIN LUBIN2 LUO MCCONN NOTAN2 OSANN2 PERNU PEZZOT PRESCO QIAO QIAO2
RACHTA RESTRE SADOWS STASZE SUZUK2 TIZZAN TVERDA VUTUC WANG2 WIGLE WU2 WUWILL WYNDE2 WYNDE3 XU YUAN ZHANG
ZHENG ZHOU

2 AKIBA AMANDU AMES BEST BOUCOT BROSS BUFFLE CEDERL DEAN2 DEAN3 DORN ENGELA GAO2 HIRAYA HOLE HUMBLE KATSOU
LIAW MATOS MCDUFF MIGRAN PEZZO2 PISANI SEGI2 SOBUE SPITZ SVENSS WAKAI WATSON WU WYNDE7 WYNDE8

3 CPSII

5 BENSHL CPSI GILLIS KAISE2 KAUFMA MRFITR SPEIZE

9 WYNDE6

Table 2L6 -

IESLC - Meta-analysis of Ever/current Smoking, Tar, "Highest vs lowest"
Squamous, Cigarettes only

This analysis is restricted to results for:
1) Ever/current smokers
2) Results by Tar
3) Categorical results by Tar
4) Denominator (unexposed) = "low"
5) Squamous (or near equivalent)
6) Results complete enough for use in metaanalysis

Within each study, results are then selected (in the following order of preference, within each sex) for:
7) SMKSTA: ever, current
8) PRODUCT: cigarettes only
9) CIGTYPE: all/unspecified, MC regardless of HR, MC only
10) Results with least adjustment for other aspects of smoking (ADOS)
11) The highest vs lowest category
12) Followup period (YF, prospective studies): whole study (coded as 0) or longest available
13) LCtype: squamous or nearest available, but not adeno. (q = squamous, s = small,
a = adeno, KI = Kreyberg I, u = undifferentiated)
14) Race: all or nearest available, otherwise by race (wh or w = white, bl or b = black, hi = hispanic
ch = chinese, jap = japanese, haw = hawaiian, w+o = white + oriental, sca = scandinavian, as = asian)
15) For overlapping studies: principal rather than subsidiary studies
Finally by Age: whole study (coded as 0) if available, otherwise by widest available age group
and then for single sex results (m, f) in preference to results for both sexes combined (c).

Results adjusted (AD) for the most potential confounders are then chosen in Sections -1 to -3
(and those which actually differ from the adjusted results in Table 2L3 - 1 are marked 'x' in Section -1)
and results adjusted for the least confounders in Sections -4 to -6. (Those least adjusted results which
actually differ from the most adjusted are marked 'x' in column X in Section -4)

Section -7 shows excluded studies, together with the stage (as above) at which no qualifying
results were found.

Section -8 lists the potentially overlapping studies which have been included (1=principal, 2=subsidiary).

Section -9 lists any results which would have been included in preference except that they had data not complete
enough for use in meta-analysis, with their significance (yes/no), if known, and any further comment as entered
on the database. It also lists as "gap" any categories for which no data were presented by the original authors.

In addition to those mentioned above, the following fields, levels and abbreviations are used:
* or nk = not known, n = no, y = yes, ot = other
all/unspec = all or unspecified, MC = manufactured cigarettes, HR = hand-rolled cigarettes
exL, exH = range of exposure (low and high) in the "highest" group, in terms of Tar
unexL, unexH = range of exposure (low and high) in the "lowest" group, in terms of Tar
REF: 6-character study reference
NRR: number of the RR on the database within the study
ST : study type (CC = case control, pr or prosp = prospective)
NLC: number of lung cancer cases in whole study
R : risky occupational population (n = no, m = mining, o = other risky)
VB : national cigarette type (V = at least 75% Virginia, bl = at least 75% blended, ot = other)
P : any proxy use
H : full histological confirmation
De : derivation of RR/CI (or = original, st = standard method, ot = other method of estimation)

Table 2L6 - 0

No RRs selected for this analysis

Table 2L6 - 7

IESLC - Meta-analysis of Ever/current Smoking, Tar, "Highest vs lowest"
Squamous, Cigarettes only
Excluded studies (and stage at which they were excluded)

1 BECHER BLOT1 BROWN3 CARPEN CHYOU DARBY DOLL2 GARCIA GRAHAM GURSEL HAMMO2 JAHN JAIN LAUSSM PRESCO QIAO TVERDA
WIGLE WYNDE3

2 AGUDO AKIBA AMANDU AMES ARMADA AUVINE AXELSS BARBON BENHAM BEST BOFFET BOUCHA BOUCOT BRESLO BROSS BUFFLE CEDERL
CHEN CHEN2 CHIAZZ CHOI CORREA DAMBER DEAN2 DEAN3 DESTEF DOLL DORN DOSEME ENGELA FAN GAO GAO2 GARSHI
GENG GER GUO HAENSZ HAMMON HEGMAN HIRAYA HOLE HU HU2 HUMBLE JEDRYC JOLY JUSSAW KATSOU KHUDER KOO
KOULUM KREUZE LETOUR LEVIN LIAW LIU3 LIU4 LIU5 LUBIN LUO MATOS MCCONN MCDUFF MIGRAN NOTAN2 OSANN2 PERNU
PEZZO2 PEZZOT PISANI QIAO2 RACHTA RESTRE SADOWS SEGI2 SOBUE SPITZ STASZE SUZUK2 SVENSS TIZZAN WAKAI WANG2 WATSON
WU WU2 WUWILL WYNDE2 WYNDE7 WYNDE8 XU YUAN ZHANG ZHENG ZHOU

3 CPSII

5 BENSHL CPSI DORGAN GILLIS KAISE2 KAUFMA LUBIN2 MRFITR SPEIZE VUTUC

6 ALDERS

9 WYNDE6

Table 2L6 - 8

Table 2L6 - 9

Most adjusted - insufficient data for meta-analysis

REF	NRR	SEX	AGEL	AGEH	RACE	YF	LC TYPE	LOC	START	ST	NLC	R	VB	P	H	AD	ADOS	SM	PRODUCT	exL	exH	unexL	unexH	De
ALDERS	559	m	0	0	all	-	q+s	Eu:UK	1977	CC	1448	n	V	n	n	2	1#	ev	cig only	17	22	1	16	ot
ALDERS	560	f	0	0	all	-	q+s	Eu:UK	1977	CC	1448	n	V	n	n	2	1#	ev	cig only	17	22	1	16	ot

Comments on values in listings

ALDERS ADOS Number of cigs/day
ALDERS ADOS Number of cigs/day

REF	NRR	RR	SIG	RRDATA comment
ALDERS	559	1.05	n	0
ALDERS	560	0.82	n	0
