# Supplementary material for: Systematic review with meta-analysis of the epidemiological evidence in the 1900s relating smoking to lung cancer
Source: BMC Cancer. 2012 Sep 3;12:385. doi: 10.1186/1471-2407-12-385 (PMC3505152; doi:10.1186/1471-2407-12-385)
Supplement: Additional file 5 — Detailed Analysis Tables (Individual file names as described in Additional file 1: Methods, Table1). [file 1471-2407-12-385-S5.zip › PDF/3A.pdf]

Table 3A1 -

IESLC - Meta-analysis of Ever Smoking, Any product (or Cigarettes if Any not available)
Adenocarcinoma

This analysis is restricted to results for:
1) Non-dose-response data
2) Ever smokers
3) Results complete enough for use in metaanalysis

Within each study, results are then selected (in the following order of preference, within each sex) for:
4) PRODUCT: all/unspec, cigarettes regardless of other products, cigarettes only
5) CIGTYPE: all/unspecified, MC regardless of HR, MC only
6) DENOM: never smoked anything, never smoked cigarettes, (never +1 = +long term ex, +2 = +amount unknown, +3 = never cigs+long term ex)
7) Followup period (YF, prospective studies): whole study (coded as 0) or longest available
8) LCtype: adeno or nearest available, but not squamous. (q = squamous, s = small, a = adeno, l = large, KII = Kreyberg II, al = alveolar, br = bronchiolar, u = undifferentiated)
9) Race: all or nearest available, otherwise by race (wh or w = white, bl or b = black, hi = hispanic ch = chinese, jap = japanese, haw = hawaiian, w+o = white + oriental, sca = scandinavian, as = asian)
10) For overlapping studies: principal rather than subsidiary studies
Finally by Age: whole study (coded as 0) if available, otherwise by widest available age group
and then for single sex results (m, f) in preference to combined sex results (c).

Results adjusted (AD) for the most potential confounders are then chosen in Sections -1 to -3
and results adjusted for the least confounders in Sections -4 to -6. (Those least adjusted results which
actually differ from the most adjusted as marked 'x' in column X in Section -4)
(Results adjusted for an unknown number of confounder(s) are coded as 20.)

Section -7 shows excluded studies, together with the stage (as above) at which no qualifying
results were found.

Section -8 lists the potentially overlapping studies which have been included (1=principal, 2=subsidiary).

Section -9 lists any results which would have been included in preference except that they had data not complete
enough for use in meta-analysis, with their significance (yes/no), if known, and any further comment as entered
on the database.

In addition to those mentioned above, the following fields, levels and abbreviations are used:
* or nk = not known, n = no, y = yes, ot = other
nev = never
all/unspec = all or unspecified, cig+/-ot = cigarettes irrespective of other products (cigar, pipe etc)
MC = manufactured cigarettes, HR = hand-rolled cigarettes
REF: 6-character study reference
NRR: number of the RR on the database within the study
ST : study type (CC = case control, pr or prosp = prospective)
NLC: number of lung cancer cases in whole study
R : risky occupational population (n = no, m = mining, o = other risky)
VB : national cigarette type (V = at least 75% Virginia, bl = at least 75% blended, ot = other)
P : any proxy use
H : full histological confirmation
De : derivation of RR/CI (or = original, st = standard method, ot = other method of estimation)

Table 3A1 - 1

IESLC - Meta-analysis of Ever Smoking, Any product (or Cigarettes if Any not available)
Adenocarcinoma
Most adjusted

REF	NRR	SEX	AGEL	AGEH	RACE	YF	LC TYPE	LOC	START	ST	NLC	R	VB	P	H	AD	PRODUCT	DENOM	De
ABRAHA	2	m	0	0	all	0	a	Eu:est	1975	pr	571	n	bl	n	n	0	all/unsp	nev any	ot
ABRAHA	5	f	0	0	all	0	a	Eu:est	1975	pr	571	n	bl	n	n	0	all/unsp	nev any	ot
ALDERS	54	m	0	0	all	-	a	Eu:UK	1977	CC	1448	n	V	n	n	2	all/unsp	nev any	or
ALDERS	57	f	0	0	all	-	a	Eu:UK	1977	CC	1448	n	V	n	n	1	all/unsp	nev any	or
ANDERS	12	f	0	0	all	0	a	NAmer	1986	pr	343	n	bl	n	n	0	cig+/-ot	nev cigs	st
BAND	2	m	0	0	all	-	a	NAmer	1983	CC	2831	n	V	y	y	2	cig only	nev any	ot
BARBON	130	m	0	0	all	-	a	Eu:wst	1979	CC	755	n	bl	y	y	3	all/unsp	nev any	ot
BECHER	12	f	0	0	all	-	not q+s	Eu:Ger	1985	CC	194	n	bl	n	y	1	all/unsp	nev any	or
BRESLO	35	c	0	0	all	-	a	NAmer	1949	CC	518	n	bl	n	y	0	all/unsp	nev+1	st
BROWN1	3	m	0	0	wh	-	a	NAmer	1979	CC	102	n	bl	y	y	1	cig+/-ot	nev cigs	or
BROWN1	4	f	0	0	wh	-	a	NAmer	1979	CC	102	n	bl	y	y	1	cig+/-ot	nev cigs	or
BROWN2	4	m	0	0	wh	-	a	NAmer	1984	CC	14596	n	bl	n	y	2	cig+/-ot	nev cigs	or
BROWN2	3	f	0	0	wh	-	a	NAmer	1984	CC	14596	n	bl	n	y	2	cig+/-ot	nev cigs	or
BUFFLE	50	m	0	0	wh	-	a	NAmer	1976	CC	943	n	bl	y	n	0	cig+/-ot	nev cigs	ot
BUFFLE	45	f	0	0	wh	-	a	NAmer	1976	CC	943	n	bl	y	n	0	cig+/-ot	nev cigs	ot
BYERS1	3	m	0	0	wh	-	a	NAmer	1957	CC	1002	n	bl	n	n	0	cig+/-ot	nev cigs	st
CHAN	12	m	0	0	all	-	a+l	As:HK	1976	CC	397	n	bl	n	n	0	all/unsp	nev any	ot
CHAN	16	f	0	0	all	-	a+l	As:HK	1976	CC	397	n	bl	n	n	0	all/unsp	nev any	st
CHOI	63	m	0	0	all	-	a	As:oth	1985	CC	375	n	bl	n	n	0	cig+/-ot	nev cigs	st
CHOI	65	f	0	0	all	-	a	As:oth	1985	CC	375	n	bl	n	n	0	cig+/-ot	nev cigs	st
COMSTO	67	m	0	0	all	-	a	NAmer	1975	ot	258	n	bl	n	n	0	cig+/-ot	nev cigs	st
COMSTO	79	f	0	0	all	-	a	NAmer	1975	ot	258	n	bl	n	n	0	cig+/-ot	nev cigs	st
CORREA	36	c	0	0	all	-	a	NAmer	1979	CC	1359	n	bl	y	n	1	cig+/-ot	nev cigs	or
DAMBER	32	m	0	0	all	-	a+al+br	Eu:Sca	1972	CC	579	n	bl	y	n	1	all/unsp	nev any	or
DESTE2	17	m	0	0	all	-	a	SCAmer	1993	CC	463	n	bl	n	n	2	all/unsp	nev any	or
DOLL	87	m	0	0	all	-	KII	Eu:UK	1948	CC	1465	n	V	n	n	1	all/unsp	nev any	ot
DOLL	89	f	0	0	all	-	KII	Eu:UK	1948	CC	1465	n	V	n	n	1	all/unsp	nev any	ot
DORGAN	125	m	0	0	wh	-	a	NAmer	1980	CC	2026	n	bl	y	y	2	cig+/-ot	nev any	or
DORGAN	104	f	0	0	all	-	a	NAmer	1980	CC	2026	n	bl	y	y	3	cig+/-ot	nev any	or
DOSEME	4	m	0	0	all	-	not q+s	Eu:bal	1979	CC	1210	n	bl	n	n	2	cig+/-ot	nev cigs	or
ENGELA	76	m	0	0	all	0	a	Eu:Sca	1964	pr	435	n	bl	n	n	7	cig+/-ot	nev cigs	ot
FAN	4	c	0	0	all	-	a	As:Chi	1990	CC	403	n	ot	y	n	0	cig+/-ot	nev cigs	ot
GAO	3	m	0	0	all	-	a	As:Chi	1984	CC	1405	n	ot	n	n	2	cig+/-ot	nev cigs	or
GAO	13	f	0	0	all	-	a	As:Chi	1984	CC	1405	n	ot	n	n	2	cig+/-ot	nev cigs	or
GER	9	c	0	0	all	-	a	As:oth	1990	CC	141	n	ot	y	n	8	all/unsp	nev any	ot
HAENSZ	5	f	0	0	all	-	a	NAmer	1955	CC	158	n	bl	n	y	2	all/unsp	nev any	ot
HAMMON	92	m	0	0	wh	0	a	NAmer	1952	pr	448	n	bl	n	n	0	all/unsp	nev any	st
HEGMAN	4	c	0	0	all	-	a	NAmer	1989	CC	282	n	bl	y	y	0	all/unsp	nev any	st
HINDS	24	f	0	0	o	-	a	NAmer	1968	CC	292	n	bl	n	n	3	all/unsp	nev any	st
ISHIMA	8	c	0	0	all	-	a	As:Jap	1961	CC	180	n	bl	y	y	5	all/unsp	nev any	st
JAHN	47	m	0	0	all	-	a	Eu:Ger	1988	CC	1004	n	bl	n	n	0	all/unsp	nev any	st
JAIN	47	m	0	0	all	-	a	NAmer	1981	CC	845	n	V	y	n	2	cig+/-ot	nev cigs	or
JAIN	42	f	0	0	all	-	a	NAmer	1981	CC	845	n	V	y	n	2	cig+/-ot	nev cigs	or
JEDRYC	56	m	0	0	all	-	a	Eu:est	1980	CC	1630	n	bl	y	n	3	cig+/-ot	nev any	ot
JOLY	51	m	0	0	all	-	a	SCAmer	1978	CC	826	n	bl	n	n	0	cig+/-ot	nev any	st
JOLY	50	f	0	0	all	-	a	SCAmer	1978	CC	826	n	bl	n	n	0	cig+/-ot	nev any	st
JUSSAW	26	m	0	0	all	-	KII	As:Ind	1964	CC	792	n	V	n	n	0	all/unsp	nev any	st
KATSOU	33	f	0	0	all	-	a	Eu:bal	1987	CC	101	n	bl	n	n	1	all/unsp	nev any	ot
KHUDER	27	m	0	0	all	-	a	NAmer	1985	CC	482	n	bl	n	y	0	cig+/-ot	nev cigs	ot
KIHARA	29	c	0	0	jap	-	a	As:Jap	1991	CC	440	n	bl	n	n	0	all/unsp	nev any	st
KOO	7	f	0	0	all	-	a+l	As:HK	1981	CC	200	n	bl	n	n	0	all/unsp	nev any	st
KREYBE	8	m	0	0	all	-	KII	Eu:Sca	1948	CC	300	n	bl	n	y	1	all/unsp	nev any	ot
KREYBE	27	f	0	0	all	-	KII	Eu:Sca	1948	CC	300	n	bl	n	y	1	all/unsp	nev any	ot
LAMTH	3	f	0	0	ch	-	a	As:HK	1983	CC	445	n	bl	n	n	0	all/unsp	nev any	or
LAMWK	4	f	0	0	ch	-	a	As:HK	1981	CC	163	n	bl	n	n	0	all/unsp	nev any	st
LAMWK2	3	m	0	0	all	-	a	As:HK	1976	CC	480	n	bl	n	n	0	all/unsp	nev any	st
LAMWK2	7	f	0	0	all	-	a	As:HK	1976	CC	480	n	bl	n	n	0	all/unsp	nev any	st
LOMBA2	3	f	0	0	all	-	not q+u	NAmer	1960	CC	225	n	bl	n	n	0	cig+/-ot	nev cigs	st
LUBIN	36	m	0	0	all	-	KII	As:Chi	1984	CC	427	m	ot	y	n	0	all/unsp	nev any	st
LUBIN2	148	m	0	0	all	-	a	Eu:mul	1976	CC	7804	n	bl	n	y	0	cig+/-ot	nev any	st
LUBIN2	168	f	0	0	all	-	a	Eu:mul	1976	CC	7804	n	bl	n	y	0	cig+/-ot	nev any	st
LUO	9	c	0	0	all	-	a	As:Chi	1990	CC	102	n	ot	n	y	20	cig+/-ot	nev cigs	or
MATOS	69	m	0	0	all	-	a	SCAmer	1994	CC	200	n	bl	n	n	2	cig+/-ot	nev any	ot
MATSUD	12	m	0	0	all	-	a	As:Jap	1965	CC	179	n	bl	n	n	0	cig+/-ot	nev cigs	ot
NOU	3	m	0	0	all	-	a	Eu:Sca	1971	CC	273	n	bl	y	n	0	all/unsp	nev any	st
NOU	8	f	0	0	all	-	a	Eu:Sca	1971	CC	273	n	bl	y	n	0	all/unsp	nev any	st
ORMOS	21	m	0	0	all	-	a	Eu:est	1947	CC	119	n	bl	y	y	0	cig+/-ot	nev any	ot
OSANN	47	m	0	0	all	-	a	NAmer	1984	CC	1986	n	bl	n	n	2	cig+/-ot	nev cigs	or
OSANN	48	f	0	0	all	-	a	NAmer	1984	CC	1986	n	bl	n	n	2	cig+/-ot	nev cigs	or
OSANN2	31	f	0	0	all	-	KII	NAmer	1964	ot	217	n	bl	n	y	1	cig+/-ot	nev cigs	or
PEZZOT	7	m	0	0	all	-	a	SCAmer	1987	CC	215	n	bl	n	y	0	cig only	nev cigs	st
SCHWAR	8	m	40	54	wh	-	a	NAmer	1984	CC	5588	n	bl	y	y	0	cig+/-ot	nev cigs	st

Table 3A1 - 1

IESLC - Meta-analysis of Ever Smoking, Any product (or Cigarettes if Any not available)
Adenocarcinoma
Most adjusted

REF	NRR	SEX	AGEL	AGEH	RACE	YF	LC TYPE	LOC	START	ST	NLC	R	VB	P	H	AD	PRODUCT	DENOM	De
SCHWAR	7	m	40	54	bl	-	a	NAmer	1984	CC	5588	n	bl	y	y	0	cig+/-ot	nev cigs	st
SCHWAR	16	f	40	54	wh	-	a	NAmer	1984	CC	5588	n	bl	y	y	0	cig+/-ot	nev cigs	st
SCHWAR	15	f	40	54	bl	-	a	NAmer	1984	CC	5588	n	bl	y	y	0	cig+/-ot	nev cigs	st
SEOW	2	f	0	0	ch	-	a	As:oth	1997	CC	153	n	bl	n	y	0	cig+/-ot	nev cigs	st
SIEMIA	8	m	0	0	all	-	a	NAmer	1979	CC	857	n	V	y	y	7	cig+/-ot	nev cigs	or
SOBUE	99	m	0	0	all	-	a	As:Jap	1986	CC	1376	n	bl	n	y	1	cig+/-ot	nev cigs	ot
SOBUE	109	f	0	0	all	-	a	As:Jap	1986	CC	1376	n	bl	n	y	1	cig+/-ot	nev cigs	ot
STASZE	21	m	0	0	all	-	a	Eu:est	1954	CC	281	n	bl	n	y	0	all/unsp	nev any	ot
STASZE	4	f	0	0	all	-	a	Eu:est	1954	CC	281	n	bl	n	y	0	all/unsp	nev any	st
STAYNE	4	m	0	0	all	-	a	NAmer	1969	CC	420	n	bl	n	n	0	all/unsp	nev any	st
SUZUK2	16	c	0	0	all	-	a	SCAmer	1991	CC	123	n	bl	n	y	3	all/unsp	nev any	or
SUZUKI	11	m	0	0	all	-	a	As:Jap	1978	CC	238	n	bl	n	y	2	cig+/-ot	nev any	ot
SUZUKI	15	f	0	0	all	-	a	As:Jap	1978	CC	238	n	bl	n	y	2	cig+/-ot	nev any	ot
SVENSS	74	f	0	0	all	-	a	Eu:Sca	1983	CC	210	n	bl	n	n	1	all/unsp	nev any	ot
TIZZAN	19	c	0	0	all	-	a	Eu:wst	1959	CC	1358	n	bl	n	n	0	all/unsp	nev any	st
TOKARS	8	c	0	0	all	-	a	Eu:est	1966	ot	162	o	bl	n	y	3	all/unsp	nev any	or
TSUGAN	10	m	0	0	all	-	a	As:Jap	1976	CC	134	n	bl	n	y	3	all/unsp	nev any	ot
TSUGAN	11	f	0	0	all	-	a	As:Jap	1976	CC	134	n	bl	n	y	3	all/unsp	nev any	ot
WAKAI	76	m	0	0	all	-	a	As:Jap	1988	CC	333	n	bl	n	y	1	all/unsp	nev any	ot
WAKAI	82	f	0	0	all	-	a	As:Jap	1988	CC	333	n	bl	n	y	1	all/unsp	nev any	ot
WU	31	f	0	0	wh	-	a	NAmer	1981	CC	220	n	bl	n	y	2	all/unsp	nev any	ot
WUWILL	11	f	0	0	all	-	a	As:Chi	1985	CC	965	n	ot	n	n	3	cig+/-ot	nev cigs	or
WYNDE2	14	m	0	0	all	-	KII	NAmer	1962	CC	404	n	bl	n	y	0	all/unsp	nev any	st
WYNDE3	29	m	0	0	all	-	KII	NAmer	1966	CC	350	n	bl	n	y	0	all/unsp	nev any	st
WYNDE3	135	f	0	0	all	-	KII	NAmer	1966	CC	350	n	bl	n	y	0	all/unsp	nev any	st
WYNDE4	42	m	0	0	all	-	a	NAmer	1948	CC	684	n	bl	y	n	0	all/unsp	nev any	st
WYNDE4	56	f	0	0	all	-	a	NAmer	1948	CC	684	n	bl	y	n	2	all/unsp	nev any	ot
WYNDE6	69	m	0	0	all	-	KII	NAmer	1969	CC	4423	n	bl	n	y	0	all/unsp	nev any	st
WYNDE6	414	f	0	0	wh	-	a	NAmer	1969	CC	4423	n	bl	n	y	1	cig+/-ot	nev cigs	ot
XU3	22	m	0	0	all	-	KII	As:Chi	1981	CC	135	n	ot	n	n	1	all/unsp	nev any	ot
XU3	26	f	0	0	all	-	KII	As:Chi	1981	CC	135	n	ot	n	n	1	all/unsp	nev any	ot
ZHENG	10	m	0	0	all	-	a	As:Chi	1982	CC	540	n	ot	*	y	0	cig+/-ot	nev cigs	st
ZHENG	21	f	0	0	all	-	a	As:Chi	1982	CC	540	n	ot	*	y	0	cig+/-ot	nev cigs	st
ZHOU	26	m	0	0	all	-	a	As:Chi	1978	CC	1360	n	ot	n	n	0	all/unsp	nev any	st
ZHOU	27	f	0	0	all	-	a	As:Chi	1978	CC	1360	n	ot	n	n	0	all/unsp	nev any	st

Cigarette type is all/unspec for all RRs

Table 3A1 - 2

IESLC - Meta-analysis of Ever Smoking, Any product (or Cigarettes if Any not available)
Adenocarcinoma
Most adjusted

REF	NRR	SEX	AD	Number Exposed Case	Number Exposed Cont	Non-exposed Case	Non-exposed Cont	RR	95.00%CI
*ABRAHA	2	m	0	59	10351	8	3365	2.40	(1.15- 5.01)
*ABRAHA	5	f	0	19	5256	16	11589	2.62	(1.35- 5.09)
Subtotal ABRAHA								2.52	(1.54- 4.12)
ALDERS	54	m	2	-	-	-	-	7.11	(1.49- 33.85)
ALDERS	57	f	1	-	-	-	-	3.58	(1.48- 8.65)
Subtotal ALDERS								4.23	(1.96- 9.12)
*ANDERS	12	f	0	99	96164	33	195158	6.09	(4.11- 9.03)
BAND	2	m	2	-	-	-	-	4.10	(3.01- 5.59)
BARBON	130	m	3	-	-	-	-	7.02	(3.24- 15.22)
BECHER	12	f	1	-	-	-	-	10.83	(1.32- 88.70)
BRESLO	35	c	0	42	462	4	56	1.27	(0.44- 3.68)
BROWN1	3	m	1	-	-	-	-	4.49	(1.44- 13.98)
BROWN1	4	f	1	-	-	-	-	3.95	(1.76- 8.80)
Subtotal BROWN1								4.12	(2.14- 7.95)
BROWN2	4	m	2	-	-	-	-	8.20	(6.90- 9.70)
BROWN2	3	f	2	-	-	-	-	6.90	(6.10- 7.90)
Subtotal BROWN2								7.35	(6.63- 8.15)
BUFFLE	50	m	0	-	-	-	-	4.50	(1.85- 10.95)
BUFFLE	45	f	0	-	-	-	-	4.02	(2.42- 6.67)
Subtotal BUFFLE								4.13	(2.66- 6.42)
BYERS1	3	m	0	47	695	7	424	4.10	(1.83- 9.15)
CHAN	12	m	0	56	161	0	43	30.44~	(1.84- 502.58)
CHAN	16	f	0	28	50	40	139	1.95	(1.09- 3.48)
Subtotal CHAN								2.18	(1.23- 3.85)
CHOI	63	m	0	46	465	7	95	1.34	(0.59- 3.06)
CHOI	65	f	0	5	26	49	164	0.64	(0.23- 1.77)
Subtotal CHOI								1.00	(0.53- 1.89)
COMSTO	67	m	0	43	229	2	84	7.89	(1.87- 33.27)
COMSTO	79	f	0	29	87	8	115	4.79	(2.09- 11.00)
Subtotal COMSTO								5.43	(2.64- 11.14)
CORREA	36	c	1	-	-	-	-	5.60	(3.60- 8.80)
DAMBER	32	m	1	-	-	-	-	2.40	(1.10- 5.30)
DESTE2	17	m	2	-	-	-	-	4.30	(1.60- 11.40)
DOLL	87	m	1	-	-	-	-	0.95	(0.22- 4.02)
DOLL	89	f	1	-	-	-	-	1.97	(0.60- 6.46)
Subtotal DOLL								1.47	(0.59- 3.69)
DORGAN	125	m	2	-	-	-	-	4.80	(1.90- 12.00)
DORGAN	104	f	3	-	-	-	-	3.90	(2.80- 5.40)
Subtotal DORGAN								3.99	(2.93- 5.44)
DOSEME	4	m	2	-	-	-	-	2.60	(1.70- 4.20)
*ENGELA	76	m	7	-	-	-	-	2.33	(0.92- 5.89)
FAN	4	c	0	67	595	45	556	1.39	(0.94- 2.07)
GAO	3	m	2	-	-	-	-	1.60	(1.10- 2.40)
GAO	13	f	2	-	-	-	-	1.50	(1.00- 2.10)
Subtotal GAO								1.55	(1.18- 2.02)
GER	9	c	8	-	-	-	-	1.10	(0.55- 2.19)
HAENSZ	5	f	2	-	-	-	-	1.19	(0.65- 2.19)
*HAMMON	92	m	0	29	510108	2	115884	3.29	(0.79- 13.80)
HEGMAN	4	c	0	83	1202	15	2080	9.58	(5.50- 16.67)
HINDS	24	f	3	-	-	-	-	3.89	(2.49- 6.07)
ISHIMA	8	c	5	-	-	-	-	15.00	(2.31- 631.48)
JAHN	47	m	0	204	701	8	138	5.02	(2.42- 10.41)
JAIN	47	m	2	-	-	-	-	8.00	(2.28- 50.60)
JAIN	42	f	2	-	-	-	-	3.45	(1.83- 7.10)
Subtotal JAIN								3.95	(2.12- 7.35)
JEDRYC	56	m	3	-	-	-	-	3.44	(1.52- 7.78)
JOLY	51	m	0	72	709	5	218	4.43	(1.77- 11.10)
JOLY	50	f	0	33	122	25	283	3.06	(1.75- 5.37)
Subtotal JOLY								3.38	(2.10- 5.46)
JUSSAW	26	m	0	34	168	13	624	9.71	(5.01- 18.82)
KATSOU	33	f	1	-	-	-	-	1.72	(0.80- 3.71)
KHUDER	27	m	0	155	-	7	-	8.11	(3.67- 17.93)
KIHARA	29	c	0	130	232	78	237	1.70	(1.22- 2.38)
KOO	7	f	0	34	63	46	137	1.61	(0.94- 2.74)
KREYBE	8	m	1	-	-	-	-	2.44	(0.76- 7.86)
KREYBE	27	f	1	-	-	-	-	1.28	(0.60- 2.74)
Subtotal KREYBE								1.55	(0.82- 2.93)
LAMTH	3	f	0	79	51	131	158	1.87	(1.23- 2.85)
LAMWK	4	f	0	36	41	60	144	2.11	(1.23- 3.61)
LAMWK2	3	m	0	52	161	15	43	0.93	(0.48- 1.80)
LAMWK2	7	f	0	26	50	41	139	1.76	(0.98- 3.17)

Table 3A1 - 2

IESLC - Meta-analysis of Ever Smoking, Any product (or Cigarettes if Any not available)
Adenocarcinoma
Most adjusted

REF	NRR	SEX	AD	Number Exposed Case	Cont	Non-exposed Case	Cont	RR	95.00%CI
Subtotal LAMWK2								1.33	(0.86- 2.07)
LOMBA2	3	f	0	42	353	54	239	0.53	(0.34- 0.81)
LUBIN	36	m	0	37	939	4	72	0.71	(0.25- 2.05)
LUBIN2	148	m	0	655	10433	57	2616	2.88	(2.19- 3.79)
LUBIN2	168	f	0	85	567	138	1180	1.28	(0.96- 1.71)
Subtotal LUBIN2								1.96	(1.61- 2.39)
LUO	9	c	20	-	-	-	-	1.50	(0.70- 3.00)
MATOS	69	m	2	-	-	-	-	6.21	(2.42- 15.96)
MATSUD	12	m	0	23	3314	0	1255	17.80~	(1.08- 293.32)
NOU	3	m	0	36	247	4	122	4.45	(1.55- 12.77)
NOU	8	f	0	9	92	29	261	0.88	(0.40- 1.93)
Subtotal NOU								1.57	(0.83- 2.94)
ORMOS	21	m	0	4	1034	0	777	6.76~	(0.36- 125.82)
OSANN	47	m	2	-	-	-	-	17.90	(10.40- 31.00)
OSANN	48	f	2	-	-	-	-	9.50	(6.80- 13.80)
Subtotal OSANN								11.46	(8.51- 15.42)
OSANN2	31	f	1	-	-	-	-	2.50	(1.30- 5.10)
PEZZOT	7	m	0	60	317	3	116	7.32	(2.25- 23.79)
SCHWAR	8	m	0	84	178	1	73	34.45	(4.71- 252.10)
SCHWAR	7	m	0	45	39	1	7	8.08	(0.95- 68.56)
SCHWAR	16	f	0	92	108	10	79	6.73	(3.29- 13.75)
SCHWAR	15	f	0	20	28	3	41	9.76	(2.65- 36.00)
Subtotal SCHWAR								8.40	(4.73- 14.94)
SEOW	2	f	0	19	15	67	125	2.36	(1.13- 4.95)
SIEMIA	8	m	7	-	-	-	-	6.30	(2.50- 16.20)
SOBUE	99	m	1	-	-	-	-	1.83	(1.21- 2.77)
SOBUE	109	f	1	-	-	-	-	1.41	(1.00- 1.99)
Subtotal SOBUE								1.57	(1.20- 2.04)
STASZE	21	m	0	20	754	0	158	8.61~	(0.52- 143.15)
STASZE	4	f	0	1	153	10	1660	1.08	(0.14- 8.53)
Subtotal STASZE								2.24	(0.42- 11.81)
STAYNE	4	m	0	43	567	7	333	3.61	(1.60- 8.11)
SUZUK2	16	c	3	-	-	-	-	6.00	(0.70- 50.00)
SUZUKI	11	m	2	-	-	-	-	4.53	(2.48- 8.29)
SUZUKI	15	f	2	-	-	-	-	2.19	(1.16- 4.14)
Subtotal SUZUKI								3.21	(2.07- 4.97)
SVENSS	74	f	1	-	-	-	-	2.91	(1.56- 5.42)
TIZZAN	19	c	0	88	939	25	419	1.57	(0.99- 2.49)
TOKARS	8	c	3	-	-	-	-	4.30	(1.90- 9.90)
TSUGAN	10	m	3	-	-	-	-	0.93	(0.43- 1.99)
TSUGAN	11	f	3	-	-	-	-	0.67	(0.22- 2.07)
Subtotal TSUGAN								0.84	(0.45- 1.58)
WAKAI	76	m	1	-	-	-	-	1.93	(0.90- 4.18)
WAKAI	82	f	1	-	-	-	-	1.39	(0.66- 2.90)
Subtotal WAKAI								1.63	(0.96- 2.77)
WU	31	f	2	-	-	-	-	2.60	(1.53- 4.44)
WUWILL	11	f	3	-	-	-	-	1.50	(1.10- 1.90)
WYNDE2	14	m	0	49	616	5	105	1.67	(0.65- 4.29)
WYNDE3	29	m	0	68	332	6	88	3.00	(1.26- 7.15)
WYNDE3	135	f	0	21	56	15	76	1.90	(0.90- 4.01)
Subtotal WYNDE3								2.31	(1.31- 4.07)
WYNDE4	42	m	0	35	665	4	115	1.51	(0.53- 4.34)
WYNDE4	56	f	2	-	-	-	-	0.60	(0.13- 2.69)
Subtotal WYNDE4								1.12	(0.47- 2.66)
WYNDE6	69	m	0	1079	1996	58	617	5.75	(4.35- 7.60)
WYNDE6	414	f	1	-	-	-	-	13.99	(10.18- 19.23)
Subtotal WYNDE6								8.47	(6.87- 10.44)
XU3	22	m	1	-	-	-	-	4.84	(1.37- 17.10)
XU3	26	f	1	-	-	-	-	1.09	(0.26- 4.50)
Subtotal XU3								2.51	(0.98- 6.47)
ZHENG	10	m	0	123	218	29	94	1.83	(1.14- 2.93)
ZHENG	21	f	0	33	44	119	184	1.16	(0.70- 1.93)
Subtotal ZHENG								1.48	(1.05- 2.09)
ZHOU	26	m	0	131	41	88	36	1.31	(0.77- 2.20)
ZHOU	27	f	0	30	7	96	32	1.43	(0.57- 3.57)
Subtotal ZHOU								1.34	(0.85- 2.10)
Partial Totals				4439	652201	1498	342753		

*prospective study ~ With 0.5 adjustment for zero

Table 3A1 - 2

IESLC - Meta-analysis of Ever Smoking, Any product (or Cigarettes if Any not available)
Adenocarcinoma
Most adjusted

REF	NRR	SEX	AD	Ys	Ws	Qs	Ps
*ABRAHA	2	m	0	0.87	7.06	0.91	0.0201
*ABRAHA	5	f	0	0.96	8.71	0.64	0.0045
Subtotal ABRAHA				0.92	15.77	1.56	
ALDERS	54	m	2	1.96	1.58	0.83	0.0138
ALDERS	57	f	1	1.28	4.93	0.01	0.0046
Subtotal ALDERS				1.44	6.50	0.84	
*ANDERS	12	f	0	1.81	24.76	8.10	0.0000
BAND	2	m	2	1.41	40.10	1.25	0.0000
BARBON	130	m	3	1.95	6.42	3.28	0.0000
BECHER	12	f	1	2.38	0.87	1.14	0.0265
BRESLO	35	c	0	0.24	3.40	3.36	0.6564
BROWN1	3	m	1	1.50	2.97	0.21	0.0096
BROWN1	4	f	1	1.37	5.93	0.12	0.0008
Subtotal BROWN1				1.42	8.91	0.33	
BROWN2	4	m	2	2.10	132.45	100.21	0.0000
BROWN2	3	f	2	1.93	229.82	111.71	0.0000
Subtotal BROWN2				1.99	362.27	211.93	
BUFFLE	50	m	0	1.50	4.86	0.35	0.0009
BUFFLE	45	f	0	1.39	14.95	0.37	0.0000
Subtotal BUFFLE				1.42	19.81	0.72	
BYERS1	3	m	0	1.41	5.95	0.18	0.0006
CHAN	12	m	0	3.42	0.49	2.32	0.0170
CHAN	16	f	0	0.67	11.38	3.68	0.0247
Subtotal CHAN				0.78	11.86	6.00	
CHOI	63	m	0	0.29	5.64	4.98	0.4842
CHOI	65	f	0	-0.44	3.77	10.59	0.3920
Subtotal CHOI				-0.00	9.42	15.57	
COMSTO	67	m	0	2.07	1.85	1.28	0.0049
COMSTO	79	f	0	1.57	5.57	0.62	0.0002
Subtotal COMSTO				1.69	7.42	1.89	
CORREA	36	c	1	1.72	19.23	4.59	0.0000
DAMBER	32	m	1	0.88	6.21	0.80	0.0291
DESTE2	17	m	2	1.46	3.99	0.20	0.0036
DOLL	87	m	1	-0.05	1.82	3.01	0.9448
DOLL	89	f	1	0.68	2.72	0.84	0.2634
Subtotal DOLL				0.39	4.54	3.85	
DORGAN	125	m	2	1.57	4.52	0.51	0.0008
DORGAN	104	f	3	1.36	35.62	0.57	0.0000
Subtotal DORGAN				1.38	40.15	1.08	
DOSEME	4	m	2	0.96	18.78	1.46	0.0000
*ENGELA	76	m	7	0.85	4.46	0.67	0.0741
FAN	4	c	0	0.33	24.61	20.12	0.1013
GAO	3	m	2	0.47	25.25	14.75	0.0182
GAO	13	f	2	0.41	27.91	19.18	0.0322
Subtotal GAO				0.44	53.16	33.92	
GER	9	c	8	0.10	8.05	10.44	0.7869
HAENSZ	5	f	2	0.17	10.41	11.71	0.5745
*HAMMON	92	m	0	1.19	1.87	0.00	0.1030
HEGMAN	4	c	0	2.26	12.50	13.12	0.0000
HINDS	24	f	3	1.36	19.35	0.30	0.0000
ISHIMA	8	c	5	2.71	0.49	1.06	0.0585
JAHN	47	m	0	1.61	7.22	1.04	0.0000
JAIN	47	m	2	2.08	1.60	1.14	0.0085
JAIN	42	f	2	1.24	8.36	0.00	0.0003
Subtotal JAIN				1.37	9.96	1.14	
JEDRYC	56	m	3	1.24	5.76	0.00	0.0030
JOLY	51	m	0	1.49	4.55	0.29	0.0015
JOLY	50	f	0	1.12	12.19	0.16	0.0001
Subtotal JOLY				1.22	16.74	0.45	
JUSSAW	26	m	0	2.27	8.78	9.48	0.0000
KATSOU	33	f	1	0.54	6.53	3.13	0.1658
KHUDER	27	m	0	2.09	6.11	4.50	0.0000
KIHARA	29	c	0	0.53	34.43	16.98	0.0018
KOO	7	f	0	0.47	13.45	7.77	0.0817
KREYBE	8	m	1	0.89	2.82	0.33	0.1345
KREYBE	27	f	1	0.25	6.66	6.50	0.5240
Subtotal KREYBE				0.44	9.48	6.83	
LAMTH	3	f	0	0.63	21.63	8.03	0.0036
LAMWK	4	f	0	0.75	13.20	3.15	0.0068
LAMWK2	3	m	0	-0.08	8.67	14.91	0.8206
LAMWK2	7	f	0	0.57	11.11	4.95	0.0588

Table 3A1 - 2

IESLC - Meta-analysis of Ever Smoking, Any product (or Cigarettes if Any not available)
Adenocarcinoma
Most adjusted

REF	NRR	SEX	AD	Ys	Ws	Qs	Ps
Subtotal LAMWK2				0.28	19.77	19.85	
LOMBA2	3	f	0	-0.64	20.27	71.29	0.0039
LUBIN	36	m	0	-0.34	3.42	8.53	0.5249
LUBIN2	148	m	0	1.06	51.15	1.59	0.0000
LUBIN2	168	f	0	0.25	46.25	44.96	0.0913
Subtotal LUBIN2				0.67	97.40	46.55	
LUO	9	c	20	0.41	7.26	4.98	0.2748
MATOS	69	m	2	1.83	4.32	1.51	0.0001
MATSUD	12	m	0	2.88	0.49	1.32	0.0440
NOU	3	m	0	1.49	3.45	0.23	0.0056
NOU	8	f	0	-0.13	6.24	11.57	0.7504
Subtotal NOU				0.45	9.69	11.80	
ORMOS	21	m	0	1.91	0.45	0.21	0.1999
OSANN	47	m	2	2.88	12.88	35.09	0.0000
OSANN	48	f	2	2.25	30.68	31.73	0.0000
Subtotal OSANN				2.44	43.56	66.82	
OSANN2	31	f	1	0.92	8.22	0.83	0.0086
PEZZOT	7	m	0	1.99	2.76	1.58	0.0009
SCHWAR	8	m	0	3.54	0.97	5.15	0.0005
SCHWAR	7	m	0	2.09	0.84	0.61	0.0556
SCHWAR	16	f	0	1.91	7.53	3.40	0.0000
SCHWAR	15	f	0	2.28	2.26	2.46	0.0006
Subtotal SCHWAR				2.13	11.60	11.63	
SEOW	2	f	0	0.86	7.03	0.99	0.0226
SIEMIA	8	m	7	1.84	4.40	1.62	0.0001
SOBUE	99	m	1	0.60	22.40	8.89	0.0042
SOBUE	109	f	1	0.34	32.45	25.75	0.0503
Subtotal SOBUE				0.45	54.85	34.64	
STASZE	21	m	0	2.15	0.49	0.41	0.1332
STASZE	4	f	0	0.08	0.90	1.20	0.9382
Subtotal STASZE				0.81	1.39	1.61	
STAYNE	4	m	0	1.28	5.85	0.01	0.0019
SUZUK2	16	c	3	1.79	0.84	0.26	0.0999
SUZUKI	11	m	2	1.51	10.55	0.81	0.0000
SUZUKI	15	f	2	0.78	9.49	1.93	0.0157
Subtotal SUZUKI				1.17	20.04	2.73	
SVENSS	74	f	1	1.07	9.91	0.27	0.0008
TIZZAN	19	c	0	0.45	18.24	11.18	0.0538
TOKARS	8	c	3	1.46	5.64	0.28	0.0005
TSUGAN	10	m	3	-0.07	6.55	11.18	0.8527
TSUGAN	11	f	3	-0.40	3.06	8.17	0.4837
Subtotal TSUGAN				-0.18	9.60	19.35	
WAKAI	76	m	1	0.66	6.52	2.17	0.0933
WAKAI	82	f	1	0.33	7.01	5.74	0.3832
Subtotal WAKAI				0.49	13.53	7.91	
WU	31	f	2	0.96	13.54	1.05	0.0004
WUWILL	11	f	3	0.41	51.44	35.34	0.0036
WYNDE2	14	m	0	0.51	4.32	2.25	0.2863
WYNDE3	29	m	0	1.10	5.11	0.09	0.0129
WYNDE3	135	f	0	0.64	6.88	2.42	0.0922
Subtotal WYNDE3				0.84	11.99	2.51	
WYNDE4	42	m	0	0.41	3.46	2.33	0.4408
WYNDE4	56	f	2	-0.51	1.67	5.10	0.5087
Subtotal WYNDE4				0.11	5.14	7.43	
WYNDE6	69	m	0	1.75	49.29	13.07	0.0000
WYNDE6	414	f	1	2.64	37.98	74.87	0.0000
Subtotal WYNDE6				2.14	87.27	87.95	
XU3	22	m	1	1.58	2.41	0.28	0.0143
XU3	26	f	1	0.09	1.89	2.49	0.9057
Subtotal XU3				0.92	4.30	2.77	
ZHENG	10	m	0	0.60	17.29	6.88	0.0121
ZHENG	21	f	0	0.15	14.95	17.64	0.5668
Subtotal ZHENG				0.39	32.24	24.52	
ZHOU	26	m	0	0.27	14.05	13.13	0.3154
ZHOU	27	f	0	0.36	4.59	3.54	0.4448
Subtotal ZHOU				0.29	18.64	16.66	

Table 3A1 - 2

IESLC - Meta-analysis of Ever Smoking, Any product (or Cigarettes if Any not available)
Adenocarcinoma
Most adjusted

	N	107
	NS	75
	Wt	1513.97
Het	Chi	930.22
Het	df	106
Het	P	***
Fixed	RR	3.44
	RRl	3.27
	RRu	3.61
	P	+++
Random	RR	2.84
	RRl	2.41
	RRu	3.35
	P	+++
Asymm	P	**

Table 3A1 - 3

IESLC - Meta-analysis of Ever Smoking, Any product (or Cigarettes if Any not available)
Adenocarcinoma
Most adjusted

		Sex		
	combined	male	female	Total
N	11	51	45	107
NS	11	50	44	105
Wt	134.70	555.20	824.07	1513.97
Het Chi	64.23	271.20	553.90	930.22
Het df	10	50	44	106
Het P	***	***	***	***
Fixed RR	2.29	4.09	3.27	3.44
RRl	1.93	3.76	3.05	3.27
RRu	2.71	4.44	3.50	3.61
P	+++	+++	+++	+++
Random RR	2.52	3.55	2.32	2.84
RRl	1.56	2.83	1.78	2.41
RRu	4.08	4.45	3.02	3.35
P	+++	+++	+++	+++
Between Chi				40.89
Between df				2
Between P				***
Btwn(F) P				(*)
Btwn(R) P				*

			Lung cancer type				
	a	a+l	a+al+br	KII	not q+u	not q+s	Total
N	87	3	1	13	1	2	107
NS	61	2	1	9	1	2	76
Wt	1338.17	25.32	6.21	104.34	20.27	19.65	1513.97
Het Chi	789.44	4.13	0.00	49.79	0.00	1.69	930.22
Het df	86	2	0	12	0	1	106
Het P	***	N.S.	N.S.	***	N.S.	N.S.	***
Fixed RR	3.58	1.85	2.40	3.63	0.53	2.77	3.44
RRl	3.39	1.26	1.09	3.00	0.34	1.78	3.27
RRu	3.78	2.74	5.27	4.40	0.81	4.31	3.61
P	+++	++	+	+++	--	+++	+++
Random RR	2.99	2.05	2.40	2.39	0.53	3.61	2.84
RRl	2.49	1.05	1.09	1.51	0.34	1.11	2.41
RRu	3.58	3.99	5.27	3.79	0.81	11.71	3.35
P	+++	+	+	+++	--	+	+++
Between Chi							85.18
Between df							5
Between P							***
Btwn(F) P							(*)
Btwn(R) P							***

					Location				
	NAmer	UK	Scand	othEur	China	Japan	othAs	other	Total
N	40	4	7	15	12	11	12	6	107
NS	27	2	5	12	8	7	9	5	75
Wt	808.34	11.05	39.74	184.48	195.08	133.44	113.20	28.65	1513.97
Het Chi	303.91	4.12	9.53	41.55	7.56	22.36	40.04	2.85	930.22
Het df	39	3	6	14	11	10	11	5	106
Het P	***	N.S.	N.S.	***	N.S.	*	***	N.S.	***
Fixed RR	5.75	2.74	2.04	2.30	1.48	1.74	1.88	4.20	3.44
RRl	5.36	1.52	1.49	1.99	1.28	1.47	1.56	2.91	3.27
RRu	6.16	4.94	2.78	2.65	1.70	2.06	2.26	6.06	3.61
P	+++	+++	+++	+++	+++	+++	+++	+++	+++
Random RR	4.37	2.68	2.05	2.72	1.48	1.80	1.87	4.20	2.84
RRl	3.48	1.31	1.37	2.01	1.28	1.34	1.29	2.91	2.41
RRu	5.48	5.47	3.06	3.68	1.70	2.41	2.71	6.06	3.35
P	+++	++	+++	+++	+++	+++	+++	+++	+++
Between Chi									498.30
Between df									7
Between P									***
Btwn(F) P									***
Btwn(R) P									***

Table 3A1 - 3

IESLC - Meta-analysis of Ever Smoking, Any product (or Cigarettes if Any not available)
Adenocarcinoma
Most adjusted

Detailed Country in "other Europe"

	multi	Germany	othWest	East	Balkans	Total
N	2	2	2	7	2	15
NS	1	2	2	5	2	12
Wt	97.40	8.08	24.66	29.01	25.31	184.48
Het Chi	15.93	0.46	10.65	3.13	0.83	41.55
Het df	1	1	1	6	1	14
Het P	***	N.S.	**	N.S.	N.S.	***
Fixed RR	1.96	5.45	2.32	3.00	2.34	2.30
RRl	1.61	2.74	1.56	2.09	1.58	1.99
RRu	2.39	10.86	3.44	4.32	3.45	2.65
P	+++	+++	+++	+++	+++	+++
Random RR	1.92	5.45	3.21	3.00	2.34	2.72
RRl	0.87	2.74	0.74	2.09	1.58	2.01
RRu	4.26	10.86	13.90	4.32	3.45	3.68
P	N.S.	+++	N.S.	+++	+++	+++
Between Chi						10.55
Between df						4
Between P						*
Btwn(F) P						N.S.
Btwn(R) P						N.S.

Detailed Country in "other Asia"

	India	HongKong	other	Total
N	1	7	4	12
NS	1	5	3	9
Wt	8.78	79.92	24.49	113.20
Het Chi	0.00	8.27	4.60	40.04
Het df	0	6	3	11
Het P	N.S.	N.S.	N.S.	***
Fixed RR	9.71	1.75	1.32	1.88
RRl	5.01	1.40	0.89	1.56
RRu	18.82	2.18	1.96	2.26
P	+++	+++	N.S.	+++
Random RR	9.71	1.74	1.29	1.87
RRl	5.01	1.33	0.79	1.29
RRu	18.82	2.28	2.13	2.71
P	+++	+++	N.S.	+++
Between Chi				27.16
Between df				2
Between P				***
Btwn(F) P				**
Btwn(R) P				***

Detailed other continent

	SCAmer	Auslia	Africa	Total
N	6			6
NS	5			5
Wt	28.65			28.65
Het Chi	2.85			2.85
Het df	5			5
Het P	N.S.			N.S.
Fixed RR	4.20			4.20
RRl	2.91			2.91
RRu	6.06			6.06
P	+++			+++
Random RR	4.20			4.20
RRl	2.91			2.91
RRu	6.06			6.06
P	+++			+++
Between Chi				
Between df				
Between P				N.S.
Btwn(F) P				N.S.
Btwn(R) P				N.S.

Table 3A1 - 3

IESLC - Meta-analysis of Ever Smoking, Any product (or Cigarettes if Any not available)
Adenocarcinoma
Most adjusted

	Start year of study					
	<1960	1960-69	1970-79	1980-89	1990+	Total
N	14	14	31	40	8	107
NS	10	12	18	27	8	75
Wt	60.88	177.13	317.22	868.22	90.53	1513.97
Het Chi	12.82	167.49	105.81	477.70	15.29	930.22
Het df	13	13	30	39	7	106
Het P	N.S.	***	***	***	*	***
Fixed RR	1.64	4.38	2.47	4.17	1.76	3.44
RRl	1.27	3.78	2.21	3.90	1.44	3.27
RRu	2.10	5.08	2.76	4.45	2.17	3.61
P	+++	+++	+++	+++	+++	+++
Random RR	1.64	3.62	2.69	3.23	1.99	2.84
RRl	1.27	2.00	2.14	2.48	1.40	2.41
RRu	2.10	6.54	3.37	4.20	2.83	3.35
P	+++	+++	+++	+++	+++	+++
Between Chi						151.12
Between df						4
Between P						***
Btwn(F) P						**
Btwn(R) P						**

	Study type (1)		
	CC	other	Total
N	98	9	107
NS	68	7	75
Wt	1445.83	68.14	1513.97
Het Chi	916.81	12.03	930.22
Het df	97	8	106
Het P	***	N.S.	***
Fixed RR	3.41	3.95	3.44
RRl	3.24	3.11	3.27
RRu	3.59	5.01	3.61
P	+++	+++	+++
Random RR	2.78	3.66	2.84
RRl	2.33	2.67	2.41
RRu	3.32	5.03	3.35
P	+++	+++	+++
Between Chi			1.38
Between df			1
Between P			N.S.
Btwn(F) P			N.S.
Btwn(R) P			N.S.

	Study type (2)			
	CC	prosp	other	Total
N	98	5	4	107
NS	68	4	3	75
Wt	1445.83	46.86	21.28	1513.97
Het Chi	916.81	9.15	2.81	930.22
Het df	97	4	3	106
Het P	***	(*)	N.S.	***
Fixed RR	3.41	4.03	3.78	3.44
RRl	3.24	3.02	2.47	3.27
RRu	3.59	5.36	5.78	3.61
P	+++	+++	+++	+++
Random RR	2.78	3.34	3.78	2.84
RRl	2.33	2.03	2.47	2.41
RRu	3.32	5.50	5.78	3.35
P	+++	+++	+++	+++
Between Chi				1.44
Between df				2
Between P				N.S.
Btwn(F) P				N.S.
Btwn(R) P				N.S.

Table 3A1 - 3

IESLC - Meta-analysis of Ever Smoking, Any product (or Cigarettes if Any not available)
Adenocarcinoma
Most adjusted

	Study size (number of LC cases) 100-249	250-499	500-999	1000+	Total
N	27	31	18	31	107
NS	23	22	12	18	75
Wt	176.58	261.85	180.32	895.23	1513.97
Het Chi	89.81	120.73	66.34	457.95	930.22
Het df	26	30	17	30	106
Het P	***	***	***	***	***
Fixed RR	1.93	2.37	2.40	4.61	3.44
RRl	1.67	2.10	2.07	4.32	3.27
RRu	2.24	2.68	2.77	4.92	3.61
P	+++	+++	+++	+++	+++
Random RR	2.31	2.37	2.86	3.77	2.84
RRl	1.71	1.81	2.08	2.82	2.41
RRu	3.12	3.09	3.92	5.02	3.35
P	+++	+++	+++	+++	+++
Between Chi					195.39
Between df					3
Between P					***
Btwn(F) P					***
Btwn(R) P					(*)

	Risky occupational population no	mining	othRisky	Total
N	105	1	1	107
NS	73	1	1	75
Wt	1504.91	3.42	5.64	1513.97
Het Chi	921.40	0.00	0.00	930.22
Het df	104	0	0	106
Het P	***	N.S.	N.S.	***
Fixed RR	3.45	0.71	4.30	3.44
RRl	3.28	0.25	1.88	3.27
RRu	3.62	2.05	9.82	3.61
P	+++	N.S.	+++	+++
Random RR	2.86	0.71	4.30	2.84
RRl	2.42	0.25	1.88	2.41
RRu	3.38	2.05	9.82	3.35
P	+++	N.S.	+++	+++
Between Chi				8.82
Between df				2
Between P				*
Btwn(F) P				N.S.
Btwn(R) P				*

	National cigarette tobacco type Virginia	blended	other	Total
N	9	85	13	107
NS	6	60	9	75
Wt	74.28	1236.56	203.13	1513.97
Het Chi	13.94	735.00	8.23	930.22
Het df	8	84	12	106
Het P	(*)	***	N.S.	***
Fixed RR	4.36	3.90	1.46	3.44
RRl	3.48	3.69	1.27	3.27
RRu	5.48	4.12	1.67	3.61
P	+++	+++	+++	+++
Random RR	4.37	3.07	1.46	2.84
RRl	3.01	2.55	1.27	2.41
RRu	6.34	3.69	1.67	3.35
P	+++	+++	+++	+++
Between Chi				173.05
Between df				2
Between P				***
Btwn(F) P				***
Btwn(R) P				***

Table 3A1 - 3

IESLC - Meta-analysis of Ever Smoking, Any product (or Cigarettes if Any not available)
Adenocarcinoma
Most adjusted

	Any proxy use No/nk	Yes	Total
N	79	28	107
NS	56	19	75
Wt	1277.08	236.89	1513.97
Het Chi	831.03	98.85	930.22
Het df	78	27	106
Het P	***	***	***
Fixed RR	3.41	3.56	3.44
RRl	3.23	3.13	3.27
RRu	3.61	4.04	3.61
P	+++	+++	+++
Random RR	2.63	3.61	2.84
RRl	2.15	2.73	2.41
RRu	3.20	4.76	3.35
P	+++	+++	+++
Between Chi			0.34
Between df			1
Between P			N.S.
Btwn(F) P			N.S.
Btwn(R) P			(*)

	Full histological confirmation No	Yes	Total
N	59	48	107
NS	43	32	75
Wt	618.50	895.47	1513.97
Het Chi	342.36	461.34	930.22
Het df	58	47	106
Het P	***	***	***
Fixed RR	2.43	4.37	3.44
RRl	2.24	4.09	3.27
RRu	2.63	4.66	3.61
P	+++	+++	+++
Random RR	2.56	3.22	2.84
RRl	2.09	2.53	2.41
RRu	3.15	4.10	3.35
P	+++	+++	+++
Between Chi			126.52
Between df			1
Between P			***
Btwn(F) P			***
Btwn(R) P			N.S.

	Number of adjustment variables (1) 0	1	2+/+nk	Total
N	54	20	33	107
NS	38	14	26	78
Wt	577.24	189.49	747.23	1513.97
Het Chi	295.04	141.30	354.00	930.22
Het df	53	19	32	106
Het P	***	***	***	***
Fixed RR	2.40	3.24	4.61	3.44
RRl	2.21	2.81	4.29	3.27
RRu	2.60	3.73	4.95	3.61
P	+++	+++	+++	+++
Random RR	2.61	2.66	3.30	2.84
RRl	2.11	1.74	2.50	2.41
RRu	3.23	4.06	4.36	3.35
P	+++	+++	+++	+++
Between Chi				139.88
Between df				2
Between P				***
Btwn(F) P				***
Btwn(R) P				N.S.

Table 3A1 - 3

IESLC - Meta-analysis of Ever Smoking, Any product (or Cigarettes if Any not available)
Adenocarcinoma
Most adjusted

	Number of adjustment variables (2) 0	1	2	3-5	6+/+nk	Total
N	54	20	19	10	4	107
NS	38	14	14	9	4	79
Wt	577.24	189.49	587.90	135.17	24.16	1513.97
Het Chi	295.04	141.30	210.87	47.25	9.32	930.22
Het df	53	19	18	9	3	106
Het P	***	***	***	***	*	***
Fixed RR	2.40	3.24	5.49	2.51	1.91	3.44
RRl	2.21	2.81	5.07	2.12	1.28	3.27
RRu	2.60	3.73	5.95	2.98	2.84	3.61
P	+++	+++	+++	+++	++	+++
Random RR	2.61	2.66	3.88	2.79	2.11	2.84
RRl	2.11	1.74	2.78	1.74	1.03	2.41
RRu	3.23	4.06	5.40	4.47	4.31	3.35
P	+++	+++	+++	+++	+	+++
Between Chi						226.45
Between df						4
Between P						***
Btwn(F) P						***
Btwn(R) P						N.S.

	Product all/unsp	cig+/-ot	cig only	Total
N	55	50	2	107
NS	41	33	2	76
Wt	432.06	1039.04	42.86	1513.97
Het Chi	187.77	667.03	0.87	930.22
Het df	54	49	1	106
Het P	***	***	N.S.	***
Fixed RR	2.42	3.94	4.26	3.44
RRl	2.20	3.71	3.15	3.27
RRu	2.66	4.19	5.74	3.61
P	+++	+++	+++	+++
Random RR	2.27	3.46	4.26	2.84
RRl	1.87	2.70	3.15	2.41
RRu	2.76	4.44	5.74	3.35
P	+++	+++	+++	+++
Between Chi				74.56
Between df				2
Between P				***
Btwn(F) P				*
Btwn(R) P				***

	Denominator nev any	nev cigs	Total
N	67	40	107
NS	49	27	76
Wt	657.02	856.95	1513.97
Het Chi	238.71	592.68	930.22
Het df	66	39	106
Het P	***	***	***
Fixed RR	2.57	4.30	3.44
RRl	2.38	4.02	3.27
RRu	2.77	4.60	3.61
P	+++	+++	+++
Random RR	2.46	3.56	2.84
RRl	2.09	2.66	2.41
RRu	2.89	4.78	3.35
P	+++	+++	+++
Between Chi			98.83
Between df			1
Between P			***
Btwn(F) P			***
Btwn(R) P			*

Table 3A1 - 3

IESLC - Meta-analysis of Ever Smoking, Any product (or Cigarettes if Any not available)
Adenocarcinoma
Most adjusted

	Derivation of RR/CI			
	Orig	StdCalc	Other	Total
N	26	45	36	107
NS	19	33	27	79
Wt	673.02	507.24	333.71	1513.97
Het Chi	290.28	271.03	210.40	930.22
Het df	25	44	35	106
Het P	***	***	***	***
Fixed RR	4.91	2.43	2.84	3.44
RRl	4.55	2.23	2.55	3.27
RRu	5.29	2.65	3.16	3.61
P	+++	+++	+++	+++
Random RR	3.93	2.53	2.53	2.84
RRl	2.90	2.00	1.90	2.41
RRu	5.34	3.20	3.39	3.35
P	+++	+++	+++	+++
Between Chi				158.50
Between df				2
Between P				***
Btwn(F) P				***
Btwn(R) P				(*)

Table 3A1 - 4

IESLC - Meta-analysis of Ever Smoking, Any product (or Cigarettes if Any not available)
Adenocarcinoma
Least adjusted

REF	NRR	X	SEX	AGEL	AGEH	RACE	YF	LC TYPE	LOC	START	ST	NLC	R	VB	P	H	AD	PRODUCT	DENOM	De
ABRAHA	2		m	0	0	all	0	a	Eu:est	1975	pr	571	n	bl	n	n	0	all/unsp	nev any	ot
ABRAHA	5		f	0	0	all	0	a	Eu:est	1975	pr	571	n	bl	n	n	0	all/unsp	nev any	ot
ALDERS	54		m	0	0	all	-	a	Eu:UK	1977	CC	1448	n	V	n	n	2	all/unsp	nev any	or
ALDERS	57		f	0	0	all	-	a	Eu:UK	1977	CC	1448	n	V	n	n	1	all/unsp	nev any	or
ANDERS	12		f	0	0	all	0	a	NAmer	1986	pr	343	n	bl	n	n	0	cig+/-ot	nev cigs	st
BAND	2		m	0	0	all	-	a	NAmer	1983	CC	2831	n	V	y	y	2	cig only	nev any	ot
BARBON	122	x	m	0	0	all	-	a	Eu:wst	1979	CC	755	n	bl	y	y	0	all/unsp	nev any	st
BECHER	12		f	0	0	all	-	not q+s	Eu:Ger	1985	CC	194	n	bl	n	y	1	all/unsp	nev any	or
BRESLO	35		c	0	0	all	-	a	NAmer	1949	CC	518	n	bl	n	y	0	all/unsp	nev+1	st
BROWN1	1	x	m	0	0	wh	-	a	NAmer	1979	CC	102	n	bl	y	y	0	cig+/-ot	nev cigs	st
BROWN1	2	x	f	0	0	wh	-	a	NAmer	1979	CC	102	n	bl	y	y	0	cig+/-ot	nev cigs	st
BROWN2	4		m	0	0	wh	-	a	NAmer	1984	CC	14596	n	bl	n	y	2	cig+/-ot	nev cigs	or
BROWN2	3		f	0	0	wh	-	a	NAmer	1984	CC	14596	n	bl	n	y	2	cig+/-ot	nev cigs	or
BUFFLE	50		m	0	0	wh	-	a	NAmer	1976	CC	943	n	bl	y	n	0	cig+/-ot	nev cigs	ot
BUFFLE	45		f	0	0	wh	-	a	NAmer	1976	CC	943	n	bl	y	n	0	cig+/-ot	nev cigs	ot
BYERS1	3		m	0	0	wh	-	a	NAmer	1957	CC	1002	n	bl	n	n	0	cig+/-ot	nev cigs	st
CHAN	12		m	0	0	all	-	a+l	As:HK	1976	CC	397	n	bl	n	n	0	all/unsp	nev any	ot
CHAN	16		f	0	0	all	-	a+l	As:HK	1976	CC	397	n	bl	n	n	0	all/unsp	nev any	st
CHOI	63		m	0	0	all	-	a	As:oth	1985	CC	375	n	bl	n	n	0	cig+/-ot	nev cigs	st
CHOI	65		f	0	0	all	-	a	As:oth	1985	CC	375	n	bl	n	n	0	cig+/-ot	nev cigs	st
COMSTO	67		m	0	0	all	-	a	NAmer	1975	ot	258	n	bl	n	n	0	cig+/-ot	nev cigs	st
COMSTO	79		f	0	0	all	-	a	NAmer	1975	ot	258	n	bl	n	n	0	cig+/-ot	nev cigs	st
CORREA	36		c	0	0	all	-	a	NAmer	1979	CC	1359	n	bl	y	n	1	cig+/-ot	nev cigs	or
DAMBER	11	x	m	0	0	all	-	a+al+br	Eu:Sca	1972	CC	579	n	bl	y	n	0	all/unsp	nev any	st
DESTE2	17		m	0	0	all	-	a	SCAmer	1993	CC	463	n	bl	n	n	2	all/unsp	nev any	or
DOLL	83	x	m	0	0	all	-	KII	Eu:UK	1948	CC	1465	n	V	n	n	0	all/unsp	nev any	st
DOLL	85	x	f	0	0	all	-	KII	Eu:UK	1948	CC	1465	n	V	n	n	0	all/unsp	nev any	st
DORGAN	125		m	0	0	wh	-	a	NAmer	1980	CC	2026	n	bl	y	y	2	cig+/-ot	nev any	or
DORGAN	104		f	0	0	all	-	a	NAmer	1980	CC	2026	n	bl	y	y	3	cig+/-ot	nev any	or
DOSEME	20	x	m	0	0	all	-	not q+s	Eu:bal	1979	CC	1210	n	bl	n	n	0	cig+/-ot	nev cigs	st
ENGELA	76		m	0	0	all	0	a	Eu:Sca	1964	pr	435	n	bl	n	n	7	cig+/-ot	nev cigs	ot
FAN	4		c	0	0	all	-	a	As:Chi	1990	CC	403	n	ot	y	n	0	cig+/-ot	nev cigs	ot
GAO	8	x	m	0	0	all	-	a	As:Chi	1984	CC	1405	n	ot	n	n	0	cig+/-ot	nev cigs	st
GAO	18	x	f	0	0	all	-	a	As:Chi	1984	CC	1405	n	ot	n	n	0	cig+/-ot	nev cigs	st
GER	1	x	c	0	0	all	-	a	As:oth	1990	CC	141	n	ot	y	n	0	all/unsp	nev any	st
HAENSZ	33	x	f	0	0	all	-	a	NAmer	1955	CC	158	n	bl	n	y	0	all/unsp	nev any	or
HAMMON	92		m	0	0	wh	0	a	NAmer	1952	pr	448	n	bl	n	n	0	all/unsp	nev any	st
HEGMAN	4		c	0	0	all	-	a	NAmer	1989	CC	282	n	bl	y	y	0	all/unsp	nev any	st
HINDS	24		f	0	0	o	-	a	NAmer	1968	CC	292	n	bl	n	n	3	all/unsp	nev any	st
ISHIMA	3	x	c	0	0	all	-	a	As:Jap	1961	CC	180	n	bl	y	y	0	all/unsp	nev any	st
JAHN	47		m	0	0	all	-	a	Eu:Ger	1988	CC	1004	n	bl	n	n	0	all/unsp	nev any	st
JAIN	7	x	m	0	0	all	-	a	NAmer	1981	CC	845	n	V	y	n	0	cig+/-ot	nev cigs	st
JAIN	2	x	f	0	0	all	-	a	NAmer	1981	CC	845	n	V	y	n	0	cig+/-ot	nev cigs	st
JEDRYC	21	x	m	0	0	all	-	a	Eu:est	1980	CC	1630	n	bl	y	n	0	cig+/-ot	nev any	st
JOLY	51		m	0	0	all	-	a	SCAmer	1978	CC	826	n	bl	n	n	0	cig+/-ot	nev any	st
JOLY	50		f	0	0	all	-	a	SCAmer	1978	CC	826	n	bl	n	n	0	cig+/-ot	nev any	st
JUSSAW	26		m	0	0	all	-	KII	As:Ind	1964	CC	792	n	V	n	n	0	all/unsp	nev any	st
KATSOU	31	x	f	0	0	all	-	a	Eu:bal	1987	CC	101	n	bl	n	n	0	all/unsp	nev any	st
KHUDER	27		m	0	0	all	-	a	NAmer	1985	CC	482	n	bl	n	y	0	cig+/-ot	nev cigs	ot
KIHARA	29		c	0	0	jap	-	a	As:Jap	1991	CC	440	n	bl	n	n	0	all/unsp	nev any	st
KOO	7		f	0	0	all	-	a+l	As:HK	1981	CC	200	n	bl	n	n	0	all/unsp	nev any	st
KREYBE	20	x	m	0	0	all	-	KII	Eu:Sca	1948	CC	300	n	bl	n	y	0	all/unsp	nev any	st
KREYBE	36	x	f	0	0	all	-	KII	Eu:Sca	1948	CC	300	n	bl	n	y	0	all/unsp	nev any	st
LAMTH	3		f	0	0	ch	-	a	As:HK	1983	CC	445	n	bl	n	n	0	all/unsp	nev any	or
LAMWK	4		f	0	0	ch	-	a	As:HK	1981	CC	163	n	bl	n	n	0	all/unsp	nev any	st
LAMWK2	3		m	0	0	all	-	a	As:HK	1976	CC	480	n	bl	n	n	0	all/unsp	nev any	st
LAMWK2	7		f	0	0	all	-	a	As:HK	1976	CC	480	n	bl	n	n	0	all/unsp	nev any	st
LOMBA2	3		f	0	0	all	-	not q+u	NAmer	1960	CC	225	n	bl	n	n	0	cig+/-ot	nev cigs	st
LUBIN	36		m	0	0	all	-	KII	As:Chi	1984	CC	427	m	ot	y	n	0	all/unsp	nev any	st
LUBIN2	148		m	0	0	all	-	a	Eu:mul	1976	CC	7804	n	bl	n	y	0	cig+/-ot	nev any	st
LUBIN2	168		f	0	0	all	-	a	Eu:mul	1976	CC	7804	n	bl	n	y	0	cig+/-ot	nev any	st
LUO	3	x	c	0	0	all	-	a	As:Chi	1990	CC	102	n	ot	n	y	0	cig+/-ot	nev cigs	st
MATOS	68	x	m	0	0	all	-	a	SCAmer	1994	CC	200	n	bl	n	n	0	cig+/-ot	nev any	st
MATSUD	12		m	0	0	all	-	a	As:Jap	1965	CC	179	n	bl	n	n	0	cig+/-ot	nev cigs	ot
NOU	3		m	0	0	all	-	a	Eu:Sca	1971	CC	273	n	bl	y	n	0	all/unsp	nev any	st
NOU	8		f	0	0	all	-	a	Eu:Sca	1971	CC	273	n	bl	y	n	0	all/unsp	nev any	st
ORMOS	21		m	0	0	all	-	a	Eu:est	1947	CC	119	n	bl	y	y	0	cig+/-ot	nev any	ot
OSANN	19	x	m	0	0	all	-	a	NAmer	1984	CC	1986	n	bl	n	n	0	cig+/-ot	nev cigs	st
OSANN	23	x	f	0	0	all	-	a	NAmer	1984	CC	1986	n	bl	n	n	0	cig+/-ot	nev cigs	st
OSANN2	13	x	f	0	0	all	-	KII	NAmer	1964	ot	217	n	bl	n	y	0	cig+/-ot	nev cigs	st
PEZZOT	7		m	0	0	all	-	a	SCAmer	1987	CC	215	n	bl	n	y	0	cig only	nev cigs	st
SCHWAR	8		m	40	54	wh	-	a	NAmer	1984	CC	5588	n	bl	y	y	0	cig+/-ot	nev cigs	st

Table 3A1 - 4

IESLC - Meta-analysis of Ever Smoking, Any product (or Cigarettes if Any not available)
Adenocarcinoma
Least adjusted

REF	NRR	X	SEX	AGEL	AGEH	RACE	YF	LC TYPE	LOC	START	ST	NLC	R	VB	P	H	AD	PRODUCT	DENOM	De
SCHWAR	7		m	40	54	bl	-	a	NAmer	1984	CC	5588	n	bl	y	y	0	cig+/-ot	nev cigs	st
SCHWAR	16		f	40	54	wh	-	a	NAmer	1984	CC	5588	n	bl	y	y	0	cig+/-ot	nev cigs	st
SCHWAR	15		f	40	54	bl	-	a	NAmer	1984	CC	5588	n	bl	y	y	0	cig+/-ot	nev cigs	st
SEOW	2		f	0	0	ch	-	a	As:oth	1997	CC	153	n	bl	n	y	0	cig+/-ot	nev cigs	st
SIEMIA	12	x	m	0	0	all	-	a	NAmer	1979	CC	857	n	V	y	y	0	cig+/-ot	nev cigs	st
SOBUE	7	x	m	0	0	all	-	a	As:Jap	1986	CC	1376	n	bl	n	y	0	cig+/-ot	nev cigs	st
SOBUE	23	x	f	0	0	all	-	a	As:Jap	1986	CC	1376	n	bl	n	y	0	cig+/-ot	nev cigs	st
STASZE	21		m	0	0	all	-	a	Eu:est	1954	CC	281	n	bl	n	y	0	all/unsp	nev any	ot
STASZE	4		f	0	0	all	-	a	Eu:est	1954	CC	281	n	bl	n	y	0	all/unsp	nev any	st
STAYNE	4		m	0	0	all	-	a	NAmer	1969	CC	420	n	bl	n	n	0	all/unsp	nev any	st
SUZUK2	13	x	c	0	0	all	-	a	SCAmer	1991	CC	123	n	bl	n	y	0	all/unsp	nev any	st
SUZUKI	3	x	m	0	0	all	-	a	As:Jap	1978	CC	238	n	bl	n	y	0	cig+/-ot	nev any	st
SUZUKI	7	x	f	0	0	all	-	a	As:Jap	1978	CC	238	n	bl	n	y	0	cig+/-ot	nev any	st
SVENSS	59	x	f	0	0	all	-	a	Eu:Sca	1983	CC	210	n	bl	n	n	0	all/unsp	nev any	st
TIZZAN	19		c	0	0	all	-	a	Eu:wst	1959	CC	1358	n	bl	n	n	0	all/unsp	nev any	st
TOKARS	7	x	c	0	0	all	-	a	Eu:est	1966	ot	162	o	bl	n	y	0	all/unsp	nev any	st
TSUGAN	2	x	m	0	0	all	-	a	As:Jap	1976	CC	134	n	bl	n	y	0	all/unsp	nev any	st
TSUGAN	8	x	f	0	0	all	-	a	As:Jap	1976	CC	134	n	bl	n	y	0	all/unsp	nev any	or
WAKAI	17	x	m	0	0	all	-	a	As:Jap	1988	CC	333	n	bl	n	y	0	all/unsp	nev any	st
WAKAI	35	x	f	0	0	all	-	a	As:Jap	1988	CC	333	n	bl	n	y	0	all/unsp	nev any	st
WU	5	x	f	0	0	wh	-	a	NAmer	1981	CC	220	n	bl	n	y	0	all/unsp	nev any	st
WUWILL	25	x	f	0	0	all	-	a	As:Chi	1985	CC	965	n	ot	n	n	0	cig+/-ot	nev cigs	st
WYNDE2	14		m	0	0	all	-	KII	NAmer	1962	CC	404	n	bl	n	y	0	all/unsp	nev any	st
WYNDE3	29		m	0	0	all	-	KII	NAmer	1966	CC	350	n	bl	n	y	0	all/unsp	nev any	st
WYNDE3	135		f	0	0	all	-	KII	NAmer	1966	CC	350	n	bl	n	y	0	all/unsp	nev any	st
WYNDE4	42		m	0	0	all	-	a	NAmer	1948	CC	684	n	bl	y	n	0	all/unsp	nev any	st
WYNDE4	56		f	0	0	all	-	a	NAmer	1948	CC	684	n	bl	y	n	2	all/unsp	nev any	ot
WYNDE6	69		m	0	0	all	-	KII	NAmer	1969	CC	4423	n	bl	n	y	0	all/unsp	nev any	st
WYNDE6	413	x	f	0	0	wh	-	a	NAmer	1969	CC	4423	n	bl	n	y	0	cig+/-ot	nev cigs	st
XU3	21	x	m	0	0	all	-	KII	As:Chi	1981	CC	135	n	ot	n	n	0	all/unsp	nev any	st
XU3	25	x	f	0	0	all	-	KII	As:Chi	1981	CC	135	n	ot	n	n	0	all/unsp	nev any	st
ZHENG	10		m	0	0	all	-	a	As:Chi	1982	CC	540	n	ot	*	y	0	cig+/-ot	nev cigs	st
ZHENG	21		f	0	0	all	-	a	As:Chi	1982	CC	540	n	ot	*	y	0	cig+/-ot	nev cigs	st
ZHOU	26		m	0	0	all	-	a	As:Chi	1978	CC	1360	n	ot	n	n	0	all/unsp	nev any	st
ZHOU	27		f	0	0	all	-	a	As:Chi	1978	CC	1360	n	ot	n	n	0	all/unsp	nev any	st

Cigarette type is all/unspec for all RRs

Table 3A1 - 5

IESLC - Meta-analysis of Ever Smoking, Any product (or Cigarettes if Any not available)
Adenocarcinoma
Least adjusted

REF	NRR	SEX	AD	Number Exposed Case	Cont	Non-exposed Case	Cont	RR	95.00%CI
*ABRAHA	2	m	0	59	10351	8	3365	2.40	(1.15- 5.01)
*ABRAHA	5	f	0	19	5256	16	11589	2.62	(1.35- 5.09)
Subtotal ABRAHA								2.52	(1.54- 4.12)
ALDERS	54	m	2	-	-	-	-	7.11	(1.49- 33.85)
ALDERS	57	f	1	-	-	-	-	3.58	(1.48- 8.65)
Subtotal ALDERS								4.23	(1.96- 9.12)
*ANDERS	12	f	0	99	96164	33	195158	6.09	(4.11- 9.03)
BAND	2	m	2	-	-	-	-	4.10	(3.01- 5.59)
BARBON	122	m	0	151	567	7	188	7.15	(3.29- 15.53)
BECHER	12	f	1	-	-	-	-	10.83	(1.32- 88.70)
BRESLO	35	c	0	42	462	4	56	1.27	(0.44- 3.68)
BROWN1	1	m	0	46	46	4	19	4.75	(1.50- 15.05)
BROWN1	2	f	0	33	19	19	47	4.30	(1.98- 9.34)
Subtotal BROWN1								4.43	(2.33- 8.44)
BROWN2	4	m	2	-	-	-	-	8.20	(6.90- 9.70)
BROWN2	3	f	2	-	-	-	-	6.90	(6.10- 7.90)
Subtotal BROWN2								7.35	(6.63- 8.15)
BUFFLE	50	m	0	-	-	-	-	4.50	(1.85- 10.95)
BUFFLE	45	f	0	-	-	-	-	4.02	(2.42- 6.67)
Subtotal BUFFLE								4.13	(2.66- 6.42)
BYERS1	3	m	0	47	695	7	424	4.10	(1.83- 9.15)
CHAN	12	m	0	56	161	0	43	30.44~	(1.84- 502.58)
CHAN	16	f	0	28	50	40	139	1.95	(1.09- 3.48)
Subtotal CHAN								2.18	(1.23- 3.85)
CHOI	63	m	0	46	465	7	95	1.34	(0.59- 3.06)
CHOI	65	f	0	5	26	49	164	0.64	(0.23- 1.77)
Subtotal CHOI								1.00	(0.53- 1.89)
COMSTO	67	m	0	43	229	2	84	7.89	(1.87- 33.27)
COMSTO	79	f	0	29	87	8	115	4.79	(2.09- 11.00)
Subtotal COMSTO								5.43	(2.64- 11.14)
CORREA	36	c	1	-	-	-	-	5.60	(3.60- 8.80)
DAMBER	11	m	0	65	49	16	29	2.40	(1.18- 4.91)
DESTE2	17	m	2	-	-	-	-	4.30	(1.60- 11.40)
DOLL	83	m	0	38	1296	2	61	0.89	(0.21- 3.79)
DOLL	85	f	0	8	49	5	59	1.93	(0.59- 6.27)
Subtotal DOLL								1.42	(0.57- 3.53)
DORGAN	125	m	2	-	-	-	-	4.80	(1.90- 12.00)
DORGAN	104	f	3	-	-	-	-	3.90	(2.80- 5.40)
Subtotal DORGAN								3.99	(2.93- 5.44)
DOSEME	20	m	0	142	536	24	293	3.23	(2.05- 5.10)
*ENGELA	76	m	7	-	-	-	-	2.33	(0.92- 5.89)
FAN	4	c	0	67	595	45	556	1.39	(0.94- 2.07)
GAO	8	m	0	180	558	42	202	1.55	(1.07- 2.25)
GAO	18	f	0	62	130	266	605	1.08	(0.78- 1.52)
Subtotal GAO								1.27	(0.99- 1.63)
GER	1	c	0	35	139	37	149	1.01	(0.60- 1.70)
HAENSZ	33	f	0	18	103	37	236	1.11	(0.61- 2.05)
*HAMMON	92	m	0	29	510108	2	115884	3.29	(0.79- 13.80)
HEGMAN	4	c	0	83	1202	15	2080	9.58	(5.50- 16.67)
HINDS	24	f	3	-	-	-	-	3.89	(2.49- 6.07)
ISHIMA	3	c	0	39	25	13	27	3.24	(1.41- 7.43)
JAHN	47	m	0	204	701	8	138	5.02	(2.42- 10.41)
JAIN	7	m	0	90	277	4	85	6.90	(2.46- 19.35)
JAIN	2	f	0	86	196	24	214	3.91	(2.39- 6.40)
Subtotal JAIN								4.35	(2.79- 6.78)
JEDRYC	21	m	0	99	1054	7	289	3.88	(1.78- 8.44)
JOLY	51	m	0	72	709	5	218	4.43	(1.77- 11.10)
JOLY	50	f	0	33	122	25	283	3.06	(1.75- 5.37)
Subtotal JOLY								3.38	(2.10- 5.46)
JUSSAW	26	m	0	34	168	13	624	9.71	(5.01- 18.82)
KATSOU	31	f	0	18	22	30	67	1.83	(0.86- 3.90)
KHUDER	27	m	0	155	-	7	-	8.11	(3.67- 17.93)
KIHARA	29	c	0	130	232	78	237	1.70	(1.22- 2.38)
KOO	7	f	0	34	63	46	137	1.61	(0.94- 2.74)
KREYBE	20	m	0	42	3514	3	644	2.57	(0.79- 8.30)
KREYBE	36	f	0	10	328	27	657	0.74	(0.35- 1.55)
Subtotal KREYBE								1.05	(0.56- 1.97)
LAMTH	3	f	0	79	51	131	158	1.87	(1.23- 2.85)
LAMWK	4	f	0	36	41	60	144	2.11	(1.23- 3.61)
LAMWK2	3	m	0	52	161	15	43	0.93	(0.48- 1.80)
LAMWK2	7	f	0	26	50	41	139	1.76	(0.98- 3.17)

Table 3A1 - 5

IESLC - Meta-analysis of Ever Smoking, Any product (or Cigarettes if Any not available)
Adenocarcinoma
Least adjusted

REF	NRR	SEX	AD	Number Exposed Case	Number Exposed Cont	Non-exposed Case	Non-exposed Cont	RR	95.00%CI
Subtotal LAMWK2								1.33	(0.86- 2.07)
LOMBA2	3	f	0	42	353	54	239	0.53	(0.34- 0.81)
LUBIN	36	m	0	37	939	4	72	0.71	(0.25- 2.05)
LUBIN2	148	m	0	655	10433	57	2616	2.88	(2.19- 3.79)
LUBIN2	168	f	0	85	567	138	1180	1.28	(0.96- 1.71)
Subtotal LUBIN2								1.96	(1.61- 2.39)
LUO	3	c	0	28	146	29	160	1.06	(0.60- 1.86)
MATOS	68	m	0	79	283	5	110	6.14	(2.42- 15.57)
MATSUD	12	m	0	23	3314	0	1255	17.80~	(1.08- 293.32)
NOU	3	m	0	36	247	4	122	4.45	(1.55- 12.77)
NOU	8	f	0	9	92	29	261	0.88	(0.40- 1.93)
Subtotal NOU								1.57	(0.83- 2.94)
ORMOS	21	m	0	4	1034	0	777	6.76~	(0.36- 125.82)
OSANN	19	m	0	319	1018	14	833	18.64	(10.83- 32.09)
OSANN	23	f	0	243	563	47	1093	10.04	(7.23- 13.94)
Subtotal OSANN								11.85	(8.95- 15.69)
OSANN2	13	f	0	61	40	22	43	2.98	(1.56- 5.71)
PEZZOT	7	m	0	60	317	3	116	7.32	(2.25- 23.79)
SCHWAR	8	m	0	84	178	1	73	34.45	(4.71- 252.10)
SCHWAR	7	m	0	45	39	1	7	8.08	(0.95- 68.56)
SCHWAR	16	f	0	92	108	10	79	6.73	(3.29- 13.75)
SCHWAR	15	f	0	20	28	3	41	9.76	(2.65- 36.00)
Subtotal SCHWAR								8.40	(4.73- 14.94)
SEOW	2	f	0	19	15	67	125	2.36	(1.13- 4.95)
SIEMIA	12	m	0	162	428	5	105	7.95	(3.18- 19.85)
SOBUE	7	m	0	393	1013	27	128	1.84	(1.20- 2.83)
SOBUE	23	f	0	58	232	137	857	1.56	(1.11- 2.20)
Subtotal SOBUE								1.66	(1.27- 2.17)
STASZE	21	m	0	20	754	0	158	8.61~	(0.52- 143.15)
STASZE	4	f	0	1	153	10	1660	1.08	(0.14- 8.53)
Subtotal STASZE								2.24	(0.42- 11.81)
STAYNE	4	m	0	43	567	7	333	3.61	(1.60- 8.11)
SUZUK2	13	c	0	20	10	5	15	6.00	(1.69- 21.26)
SUZUKI	3	m	0	144	217	14	99	4.69	(2.58- 8.53)
SUZUKI	7	f	0	25	27	55	133	2.24	(1.19- 4.20)
Subtotal SUZUKI								3.30	(2.14- 5.09)
SVENSS	59	f	0	50	89	22	120	3.06	(1.73- 5.43)
TIZZAN	19	c	0	88	939	25	419	1.57	(0.99- 2.49)
TOKARS	7	c	0	68	112	10	54	3.28	(1.57- 6.86)
TSUGAN	2	m	0	53	56	18	17	0.89	(0.42- 1.91)
TSUGAN	8	f	0	6	10	33	30	0.55	(0.18- 1.68)
Subtotal TSUGAN								0.77	(0.41- 1.44)
WAKAI	17	m	0	98	424	8	65	1.88	(0.87- 4.04)
WAKAI	35	f	0	13	31	46	145	1.32	(0.64- 2.74)
Subtotal WAKAI								1.56	(0.92- 2.65)
WU	5	f	0	120	87	29	62	2.95	(1.75- 4.96)
WUWILL	25	f	0	138	351	172	601	1.37	(1.06- 1.78)
WYNDE2	14	m	0	49	616	5	105	1.67	(0.65- 4.29)
WYNDE3	29	m	0	68	332	6	88	3.00	(1.26- 7.15)
WYNDE3	135	f	0	21	56	15	76	1.90	(0.90- 4.01)
Subtotal WYNDE3								2.31	(1.31- 4.07)
WYNDE4	42	m	0	35	665	4	115	1.51	(0.53- 4.34)
WYNDE4	56	f	2	-	-	-	-	0.60	(0.13- 2.69)
Subtotal WYNDE4								1.12	(0.47- 2.66)
WYNDE6	69	m	0	1079	1996	58	617	5.75	(4.35- 7.60)
WYNDE6	413	f	0	326	275	58	673	13.76	(10.06- 18.80)
Subtotal WYNDE6								8.47	(6.88- 10.43)
XU3	21	m	0	29	68	3	31	4.41	(1.25- 15.57)
XU3	25	f	0	4	11	7	25	1.30	(0.31- 5.36)
Subtotal XU3								2.57	(1.00- 6.59)
ZHENG	10	m	0	123	218	29	94	1.83	(1.14- 2.93)
ZHENG	21	f	0	33	44	119	184	1.16	(0.70- 1.93)
Subtotal ZHENG								1.48	(1.05- 2.09)
ZHOU	26	m	0	131	41	88	36	1.31	(0.77- 2.20)
ZHOU	27	f	0	30	7	96	32	1.43	(0.57- 3.57)
Subtotal ZHOU								1.34	(0.85- 2.10)
Partial Totals				8038	666600	2831	352023		

*prospective study ~ With 0.5 adjustment for zero

Table 3A1 - 5

IESLC - Meta-analysis of Ever Smoking, Any product (or Cigarettes if Any not available)
Adenocarcinoma
Least adjusted

REF	NRR	SEX	AD	Ys	Ws	Qs	Ps
*ABRAHA	2	m	0	0.87	7.06	0.83	0.0201
*ABRAHA	5	f	0	0.96	8.71	0.56	0.0045
Subtotal ABRAHA				0.92	15.77	1.39	
ALDERS	54	m	2	1.96	1.58	0.87	0.0138
ALDERS	57	f	1	1.28	4.93	0.02	0.0046
Subtotal ALDERS				1.44	6.50	0.89	
*ANDERS	12	f	0	1.81	24.76	8.59	0.0000
BAND	2	m	2	1.41	40.10	1.51	0.0000
BARBON	122	m	0	1.97	6.39	3.60	0.0000
BECHER	12	f	1	2.38	0.87	1.18	0.0265
BRESLO	35	c	0	0.24	3.40	3.24	0.6564
BROWN1	1	m	0	1.56	2.89	0.34	0.0081
BROWN1	2	f	0	1.46	6.38	0.37	0.0002
Subtotal BROWN1				1.49	9.27	0.70	
BROWN2	4	m	2	2.10	132.45	104.19	0.0000
BROWN2	3	f	2	1.93	229.82	117.27	0.0000
Subtotal BROWN2				1.99	362.27	221.46	
BUFFLE	50	m	0	1.50	4.86	0.40	0.0009
BUFFLE	45	f	0	1.39	14.95	0.45	0.0000
Subtotal BUFFLE				1.42	19.81	0.85	
BYERS1	3	m	0	1.41	5.95	0.22	0.0006
CHAN	12	m	0	3.42	0.49	2.36	0.0170
CHAN	16	f	0	0.67	11.38	3.46	0.0247
Subtotal CHAN				0.78	11.86	5.82	
CHOI	63	m	0	0.29	5.64	4.80	0.4842
CHOI	65	f	0	-0.44	3.77	10.37	0.3920
Subtotal CHOI				-0.00	9.42	15.17	
COMSTO	67	m	0	2.07	1.85	1.33	0.0049
COMSTO	79	f	0	1.57	5.57	0.68	0.0002
Subtotal COMSTO				1.69	7.42	2.01	
CORREA	36	c	1	1.72	19.23	4.92	0.0000
DAMBER	11	m	0	0.88	7.53	0.87	0.0161
DESTE2	17	m	2	1.46	3.99	0.23	0.0036
DOLL	83	m	0	-0.11	1.84	3.25	0.8795
DOLL	85	f	0	0.66	2.76	0.87	0.2760
Subtotal DOLL				0.35	4.60	4.12	
DORGAN	125	m	2	1.57	4.52	0.56	0.0008
DORGAN	104	f	3	1.36	35.62	0.74	0.0000
Subtotal DORGAN				1.38	40.15	1.30	
DOSEME	20	m	0	1.17	18.52	0.03	0.0000
*ENGELA	76	m	7	0.85	4.46	0.61	0.0741
FAN	4	c	0	0.33	24.61	19.36	0.1013
GAO	8	m	0	0.44	27.69	16.76	0.0208
GAO	18	f	0	0.08	34.21	44.13	0.6343
Subtotal GAO				0.24	61.90	60.90	
GER	1	c	0	0.01	14.39	20.83	0.9579
HAENSZ	33	f	0	0.11	10.36	12.73	0.7268
*HAMMON	92	m	0	1.19	1.87	0.00	0.1030
HEGMAN	4	c	0	2.26	12.50	13.57	0.0000
HINDS	24	f	3	1.36	19.35	0.39	0.0000
ISHIMA	3	c	0	1.18	5.57	0.01	0.0055
JAHN	47	m	0	1.61	7.22	1.13	0.0000
JAIN	7	m	0	1.93	3.62	1.85	0.0002
JAIN	2	f	0	1.36	15.86	0.34	0.0000
Subtotal JAIN				1.47	19.47	2.19	
JEDRYC	21	m	0	1.36	6.35	0.12	0.0006
JOLY	51	m	0	1.49	4.55	0.33	0.0015
JOLY	50	f	0	1.12	12.19	0.12	0.0001
Subtotal JOLY				1.22	16.74	0.45	
JUSSAW	26	m	0	2.27	8.78	9.80	0.0000
KATSOU	31	f	0	0.60	6.70	2.53	0.1187
KHUDER	27	m	0	2.09	6.11	4.68	0.0000
KIHARA	29	c	0	0.53	34.43	16.16	0.0018
KOO	7	f	0	0.47	13.45	7.42	0.0817
KREYBE	20	m	0	0.94	2.79	0.21	0.1158
KREYBE	36	f	0	-0.30	7.06	16.23	0.4275
Subtotal KREYBE				0.05	9.85	16.44	
LAMTH	3	f	0	0.63	21.63	7.59	0.0036
LAMWK	4	f	0	0.75	13.20	2.94	0.0068
LAMWK2	3	m	0	-0.08	8.67	14.52	0.8206
LAMWK2	7	f	0	0.57	11.11	4.70	0.0588

Table 3A1 - 5

IESLC - Meta-analysis of Ever Smoking, Any product (or Cigarettes if Any not available)
Adenocarcinoma
Least adjusted

REF	NRR	SEX	AD	Ys	Ws	Qs	Ps
Subtotal LAMWK2				0.28	19.77	19.21	
LOMBA2	3	f	0	-0.64	20.27	70.00	0.0039
LUBIN	36	m	0	-0.34	3.42	8.34	0.5249
LUBIN2	148	m	0	1.06	51.15	1.29	0.0000
LUBIN2	168	f	0	0.25	46.25	43.42	0.0913
Subtotal LUBIN2				0.67	97.40	44.71	
LUO	3	c	0	0.06	12.01	16.17	0.8449
MATOS	68	m	0	1.82	4.44	1.59	0.0001
MATSUD	12	m	0	2.88	0.49	1.35	0.0440
NOU	3	m	0	1.49	3.45	0.26	0.0056
NOU	8	f	0	-0.13	6.24	11.28	0.7504
Subtotal NOU				0.45	9.69	11.54	
ORMOS	21	m	0	1.91	0.45	0.22	0.1999
OSANN	19	m	0	2.93	13.03	38.03	0.0000
OSANN	23	f	0	2.31	35.61	42.24	0.0000
Subtotal OSANN				2.47	48.64	80.27	
OSANN2	13	f	0	1.09	9.08	0.14	0.0010
PEZZOT	7	m	0	1.99	2.76	1.65	0.0009
SCHWAR	8	m	0	3.54	0.97	5.23	0.0005
SCHWAR	7	m	0	2.09	0.84	0.64	0.0556
SCHWAR	16	f	0	1.91	7.53	3.58	0.0000
SCHWAR	15	f	0	2.28	2.26	2.54	0.0006
Subtotal SCHWAR				2.13	11.60	11.99	
SEOW	2	f	0	0.86	7.03	0.90	0.0226
SIEMIA	12	m	0	2.07	4.59	3.36	0.0000
SOBUE	7	m	0	0.61	20.67	7.64	0.0056
SOBUE	23	f	0	0.45	33.31	19.75	0.0099
Subtotal SOBUE				0.51	53.98	27.39	
STASZE	21	m	0	2.15	0.49	0.43	0.1332
STASZE	4	f	0	0.08	0.90	1.16	0.9382
Subtotal STASZE				0.81	1.39	1.59	
STAYNE	4	m	0	1.28	5.85	0.03	0.0019
SUZUK2	13	c	0	1.79	2.40	0.79	0.0055
SUZUKI	3	m	0	1.55	10.74	1.16	0.0000
SUZUKI	7	f	0	0.81	9.73	1.65	0.0119
Subtotal SUZUKI				1.19	20.48	2.81	
SVENSS	59	f	0	1.12	11.76	0.11	0.0001
TIZZAN	19	c	0	0.45	18.24	10.70	0.0538
TOKARS	7	c	0	1.19	7.03	0.01	0.0016
TSUGAN	2	m	0	-0.11	6.62	11.70	0.7728
TSUGAN	8	f	0	-0.61	3.03	10.07	0.2916
Subtotal TSUGAN				-0.27	9.65	21.76	
WAKAI	17	m	0	0.63	6.54	2.25	0.1071
WAKAI	35	f	0	0.28	7.26	6.39	0.4522
Subtotal WAKAI				0.45	13.79	8.64	
WU	5	f	0	1.08	14.20	0.26	0.0000
WUWILL	25	f	0	0.32	56.90	46.06	0.0166
WYNDE2	14	m	0	0.51	4.32	2.14	0.2863
WYNDE3	29	m	0	1.10	5.11	0.07	0.0129
WYNDE3	135	f	0	0.64	6.88	2.28	0.0922
Subtotal WYNDE3				0.84	11.99	2.35	
WYNDE4	42	m	0	0.41	3.46	2.23	0.4408
WYNDE4	56	f	2	-0.51	1.67	5.00	0.5087
Subtotal WYNDE4				0.11	5.14	7.23	
WYNDE6	69	m	0	1.75	49.29	13.96	0.0000
WYNDE6	413	f	0	2.62	39.32	77.54	0.0000
Subtotal WYNDE6				2.14	88.61	91.49	
XU3	21	m	0	1.48	2.41	0.17	0.0213
XU3	25	f	0	0.26	1.91	1.74	0.7180
Subtotal XU3				0.94	4.32	1.91	
ZHENG	10	m	0	0.60	17.29	6.51	0.0121
ZHENG	21	f	0	0.15	14.95	17.09	0.5668
Subtotal ZHENG				0.39	32.24	23.60	
ZHOU	26	m	0	0.27	14.05	12.67	0.3154
ZHOU	27	f	0	0.36	4.59	3.40	0.4448
Subtotal ZHOU				0.29	18.64	16.06	

Table 3A1 - 5

IESLC - Meta-analysis of Ever Smoking, Any product (or Cigarettes if Any not available)
Adenocarcinoma
Least adjusted

	N	107
	NS	75
	Wt	1569.32
Het	Chi	1014.33
Het	df	106
Het	P	***
Fixed	RR	3.38
	RRl	3.21
	RRu	3.55
	P	+++
Random	RR	2.83
	RRl	2.39
	RRu	3.34
	P	+++
Asymm	P	**

Table 3A1 - 6

IESLC - Meta-analysis of Ever Smoking, Any product (or Cigarettes if Any not available)
Adenocarcinoma
Least adjusted

	Sex			
	combined	male	female	Total
N	11	51	45	107
NS	11	50	44	105
Wt	153.82	560.20	855.31	1569.32
Het Chi	73.65	276.06	608.24	1014.33
Het df	10	50	44	106
Het P	***	***	***	***
Fixed RR	2.14	4.12	3.22	3.38
RRl	1.83	3.80	3.01	3.21
RRu	2.51	4.48	3.44	3.55
P	+++	+++	+++	+++
Random RR	2.36	3.60	2.30	2.83
RRl	1.50	2.88	1.75	2.39
RRu	3.71	4.52	3.02	3.34
P	+++	+++	+++	+++
Between Chi				56.37
Between df				2
Between P				***
Btwn(F) P				(*)
Btwn(R) P				*

	Lung cancer type						
	a	a+l	a+al+br	KII	not q+u	not q+s	Total
N	87	3	1	13	1	2	107
NS	61	2	1	9	1	2	76
Wt	1391.16	25.32	7.53	105.65	20.27	19.39	1569.32
Het Chi	867.98	4.13	0.00	58.98	0.00	1.21	1014.33
Het df	86	2	0	12	0	1	106
Het P	***	N.S.	N.S.	***	N.S.	N.S.	***
Fixed RR	3.50	1.85	2.40	3.53	0.53	3.41	3.38
RRl	3.32	1.26	1.18	2.92	0.34	2.19	3.21
RRu	3.69	2.74	4.91	4.28	0.81	5.33	3.55
P	+++	++	+	+++	--	+++	+++
Random RR	2.97	2.05	2.40	2.29	0.53	3.76	2.83
RRl	2.47	1.05	1.18	1.40	0.34	1.72	2.39
RRu	3.57	3.99	4.91	3.76	0.81	8.20	3.34
P	+++	+	+	++	--	+++	+++
Between Chi							82.03
Between df							5
Between P							***
Btwn(F) P							N.S.
Btwn(R) P							***

	Location								
	NAmer	UK	Scand	othEur	China	Japan	othAs	other	Total
N	40	4	7	15	12	11	12	6	107
NS	27	2	5	12	8	7	9	5	75
Wt	826.29	11.10	43.28	186.34	214.06	138.39	119.53	30.33	1569.32
Het Chi	306.73	4.43	15.96	42.83	9.68	23.86	43.03	3.02	1014.33
Het df	39	3	6	14	11	10	11	5	106
Het P	***	N.S.	*	***	N.S.	**	***	N.S.	***
Fixed RR	5.80	2.69	1.93	2.35	1.34	1.81	1.81	4.28	3.38
RRl	5.41	1.49	1.43	2.04	1.17	1.53	1.51	3.00	3.21
RRu	6.20	4.84	2.60	2.72	1.53	2.13	2.16	6.11	3.55
P	+++	+++	+++	+++	+++	+++	+++	+++	+++
Random RR	4.47	2.61	1.93	2.78	1.34	1.84	1.85	4.28	2.83
RRl	3.57	1.25	1.17	2.05	1.17	1.38	1.27	3.00	2.39
RRu	5.58	5.49	3.20	3.77	1.53	2.46	2.68	6.11	3.34
P	+++	+	+	+++	+++	+++	++	+++	+++
Between Chi									564.79
Between df									7
Between P									***
Btwn(F) P									***
Btwn(R) P									***

Table 3A1 - 6

IESLC - Meta-analysis of Ever Smoking, Any product (or Cigarettes if Any not available)
Adenocarcinoma
Least adjusted

	Detailed Country in "other Europe"					
	multi	Germany	othWest	East	Balkans	Total
N	2	2	2	7	2	15
NS	1	2	2	5	2	12
Wt	97.40	8.08	24.63	31.00	25.22	186.34
Het Chi	15.93	0.46	10.87	2.75	1.60	42.83
Het df	1	1	1	6	1	14
Het P	***	N.S.	***	N.S.	N.S.	***
Fixed RR	1.96	5.45	2.33	2.95	2.78	2.35
RRl	1.61	2.74	1.57	2.07	1.88	2.04
RRu	2.39	10.86	3.45	4.19	4.11	2.72
P	+++	+++	+++	+++	+++	+++
Random RR	1.92	5.45	3.24	2.95	2.64	2.78
RRl	0.87	2.74	0.73	2.07	1.55	2.05
RRu	4.26	10.86	14.30	4.19	4.51	3.77
P	N.S.	+++	N.S.	+++	+++	+++
Between Chi						11.21
Between df						4
Between P						*
Btwn(F) P						N.S.
Btwn(R) P						N.S.

	Detailed Country in "other Asia"			
	India	HongKong	other	Total
N	1	7	4	12
NS	1	5	3	9
Wt	8.78	79.92	30.83	119.53
Het Chi	0.00	8.27	5.16	43.03
Het df	0	6	3	11
Het P	N.S.	N.S.	N.S.	***
Fixed RR	9.71	1.75	1.22	1.81
RRl	5.01	1.40	0.86	1.51
RRu	18.82	2.18	1.74	2.16
P	+++	+++	N.S.	+++
Random RR	9.71	1.74	1.24	1.85
RRl	5.01	1.33	0.76	1.27
RRu	18.82	2.28	2.01	2.68
P	+++	+++	N.S.	++
Between Chi				29.60
Between df				2
Between P				***
Btwn(F) P				**
Btwn(R) P				***

	Detailed other continent			
	SCAmer	Auslia	Africa	Total
N	6			6
NS	5			5
Wt	30.33			30.33
Het Chi	3.02			3.02
Het df	5			5
Het P	N.S.			N.S.
Fixed RR	4.28			4.28
RRl	3.00			3.00
RRu	6.11			6.11
P	+++			+++
Random RR	4.28			4.28
RRl	3.00			3.00
RRu	6.11			6.11
P	+++			+++
Between Chi				
Between df				
Between P				N.S.
Btwn(F) P				N.S.
Btwn(R) P				N.S.

Table 3A1 - 6

IESLC - Meta-analysis of Ever Smoking, Any product (or Cigarettes if Any not available)
Adenocarcinoma
Least adjusted

	<1960	1960-69	1970-79	1980-89	1990+	Total
	Start year of study					
N	14	14	31	40	8	107
NS	10	12	18	27	8	75
Wt	61.25	185.80	319.26	899.71	103.29	1569.32
Het Chi	17.18	166.85	113.24	529.91	22.76	1014.33
Het df	13	13	30	39	7	106
Het P	N.S.	***	***	***	**	***
Fixed RR	1.52	4.33	2.52	4.09	1.65	3.38
RRl	1.18	3.75	2.25	3.83	1.36	3.21
RRu	1.95	5.00	2.81	4.36	2.00	3.55
P	++	+++	+++	+++	+++	+++
Random RR	1.55	3.42	2.74	3.25	1.96	2.83
RRl	1.13	1.95	2.17	2.48	1.33	2.39
RRu	2.13	6.00	3.46	4.26	2.90	3.34
P	++	+++	+++	+++	+++	+++
Between Chi						164.40
Between df						4
Between P						***
Btwn(F) P						**
Btwn(R) P						**

	CC	other	Total
	Study type (1)		
N	98	9	107
NS	68	7	75
Wt	1498.93	70.40	1569.32
Het Chi	1001.49	11.22	1014.33
Het df	97	8	106
Het P	***	N.S.	***
Fixed RR	3.35	3.92	3.38
RRl	3.19	3.10	3.21
RRu	3.53	4.95	3.55
P	+++	+++	+++
Random RR	2.77	3.66	2.83
RRl	2.32	2.72	2.39
RRu	3.31	4.93	3.34
P	+++	+++	+++
Between Chi			1.61
Between df			1
Between P			N.S.
Btwn(F) P			N.S.
Btwn(R) P			N.S.

	CC	prosp	other	Total
	Study type (2)			
N	98	5	4	107
NS	68	4	3	75
Wt	1498.93	46.86	23.54	1569.32
Het Chi	1001.49	9.15	1.96	1014.33
Het df	97	4	3	106
Het P	***	(*)	N.S.	***
Fixed RR	3.35	4.03	3.70	3.38
RRl	3.19	3.02	2.47	3.21
RRu	3.53	5.36	5.55	3.55
P	+++	+++	+++	+++
Random RR	2.77	3.34	3.70	2.83
RRl	2.32	2.03	2.47	2.39
RRu	3.31	5.50	5.55	3.34
P	+++	+++	+++	+++
Between Chi				1.72
Between df				2
Between P				N.S.
Btwn(F) P				N.S.
Btwn(R) P				N.S.

Table 3A1 - 6

IESLC - Meta-analysis of Ever Smoking, Any product (or Cigarettes if Any not available)
Adenocarcinoma
Least adjusted

	Study size (number of LC cases)				
	100-249	250-499	500-999	1000+	Total
N	27	31	18	31	107
NS	23	22	12	18	75
Wt	200.16	262.48	196.76	909.91	1569.32
Het Chi	104.18	128.22	79.80	494.49	1014.33
Het df	26	30	17	30	106
Het P	***	***	***	***	***
Fixed RR	1.92	2.33	2.40	4.58	3.38
RRl	1.67	2.06	2.09	4.29	3.21
RRu	2.21	2.63	2.76	4.89	3.55
P	+++	+++	+++	+++	+++
Random RR	2.30	2.32	2.93	3.78	2.83
RRl	1.71	1.76	2.11	2.81	2.39
RRu	3.10	3.05	4.08	5.08	3.34
P	+++	+++	+++	+++	+++
Between Chi					207.64
Between df					3
Between P					***
Btwn(F) P					***
Btwn(R) P					(*)

	Risky occupational population			
	no	mining	othRisky	Total
N	105	1	1	107
NS	73	1	1	75
Wt	1558.86	3.42	7.03	1569.32
Het Chi	1005.96	0.00	0.00	1014.33
Het df	104	0	0	106
Het P	***	N.S.	N.S.	***
Fixed RR	3.39	0.71	3.28	3.38
RRl	3.23	0.25	1.57	3.21
RRu	3.56	2.05	6.86	3.55
P	+++	N.S.	++	+++
Random RR	2.86	0.71	3.28	2.83
RRl	2.41	0.25	1.57	2.39
RRu	3.38	2.05	6.86	3.34
P	+++	N.S.	++	+++
Between Chi				8.37
Between df				2
Between P				*
Btwn(F) P				N.S.
Btwn(R) P				*

	National cigarette tobacco type			
	Virginia	blended	other	Total
N	9	85	13	107
NS	6	60	9	75
Wt	84.04	1256.83	228.45	1569.32
Het Chi	15.38	749.97	10.73	1014.33
Het df	8	84	12	106
Het P	(*)	***	N.S.	***
Fixed RR	4.46	3.93	1.32	3.38
RRl	3.60	3.72	1.16	3.21
RRu	5.52	4.16	1.50	3.55
P	+++	+++	+++	+++
Random RR	4.55	3.06	1.32	2.83
RRl	3.18	2.55	1.16	2.39
RRu	6.52	3.68	1.50	3.34
P	+++	+++	+++	+++
Between Chi				238.25
Between df				2
Between P				***
Btwn(F) P				***
Btwn(R) P				***

Table 3A1 - 6

IESLC - Meta-analysis of Ever Smoking, Any product (or Cigarettes if Any not available)
Adenocarcinoma
Least adjusted

	Any proxy use No/nk	Yes	Total
N	79	28	107
NS	56	19	75
Wt	1309.08	260.24	1569.32
Het Chi	901.77	112.21	1014.33
Het df	78	27	106
Het P	***	***	***
Fixed RR	3.36	3.49	3.38
RRl	3.18	3.09	3.21
RRu	3.54	3.94	3.55
P	+++	+++	+++
Random RR	2.60	3.62	2.83
RRl	2.12	2.75	2.39
RRu	3.18	4.77	3.34
P	+++	+++	+++
Between Chi			0.35
Between df			1
Between P			N.S.
Btwn(F) P			N.S.
Btwn(R) P			(*)

	Full histological confirmation No	Yes	Total
N	59	48	107
NS	43	32	75
Wt	657.51	911.81	1569.32
Het Chi	394.96	486.86	1014.33
Het df	58	47	106
Het P	***	***	***
Fixed RR	2.40	4.32	3.38
RRl	2.22	4.05	3.21
RRu	2.59	4.61	3.55
P	+++	+++	+++
Random RR	2.58	3.16	2.83
RRl	2.08	2.48	2.39
RRu	3.19	4.02	3.34
P	+++	+++	+++
Between Chi			132.51
Between df			1
Between P			***
Btwn(F) P			***
Btwn(R) P			N.S.

	Number of adjustment variables (1) 0	1	2+/+nk	Total
N	94	3	10	107
NS	66	3	8	77
Wt	1070.73	25.03	473.56	1569.32
Het Chi	688.57	1.26	45.80	1014.33
Het df	93	2	9	106
Het P	***	N.S.	***	***
Fixed RR	2.53	5.25	6.32	3.38
RRl	2.39	3.55	5.77	3.21
RRu	2.69	7.76	6.91	3.55
P	+++	+++	+++	+++
Random RR	2.66	5.25	4.67	2.83
RRl	2.23	3.55	3.52	2.39
RRu	3.17	7.76	6.21	3.34
P	+++	+++	+++	+++
Between Chi				278.71
Between df				2
Between P				***
Btwn(F) P				***
Btwn(R) P				***

Table 3A1 - 6

IESLC - Meta-analysis of Ever Smoking, Any product (or Cigarettes if Any not available)
Adenocarcinoma
Least adjusted

	Number of adjustment variables (2) 0	1	2	3-5	6+/+nk	Total
N	94	3	7	2	1	107
NS	66	3	6	2	1	78
Wt	1070.73	25.03	414.13	54.97	4.46	1569.32
Het Chi	688.57	1.26	26.21	0.00	0.00	1014.33
Het df	93	2	6	1	0	106
Het P	***	N.S.	***	N.S.	N.S.	***
Fixed RR	2.53	5.25	6.81	3.90	2.33	3.38
RRl	2.39	3.55	6.18	2.99	0.92	3.21
RRu	2.69	7.76	7.50	5.08	5.90	3.55
P	+++	+++	+++	+++	(+)	+++
Random RR	2.66	5.25	5.52	3.90	2.33	2.83
RRl	2.23	3.55	4.06	2.99	0.92	2.39
RRu	3.17	7.76	7.51	5.08	5.90	3.34
P	+++	+++	+++	+++	(+)	+++
Between Chi						298.30
Between df						4
Between P						***
Btwn(F) P						***
Btwn(R) P						***

	Product all/unsp	cig+/-ot	cig only	Total
N	55	50	2	107
NS	41	33	2	76
Wt	451.10	1075.36	42.86	1569.32
Het Chi	203.71	731.10	0.87	1014.33
Het df	54	49	1	106
Het P	***	***	N.S.	***
Fixed RR	2.38	3.88	4.26	3.38
RRl	2.17	3.65	3.15	3.21
RRu	2.61	4.12	5.74	3.55
P	+++	+++	+++	+++
Random RR	2.24	3.50	4.26	2.83
RRl	1.84	2.71	3.15	2.39
RRu	2.72	4.51	5.74	3.34
P	+++	+++	+++	+++
Between Chi				78.66
Between df				2
Between P				***
Btwn(F) P				*
Btwn(R) P				***

	Denominator nev any	nev cigs	Total
N	67	40	107
NS	49	27	76
Wt	677.21	892.11	1569.32
Het Chi	256.79	660.08	1014.33
Het df	66	39	106
Het P	***	***	***
Fixed RR	2.54	4.20	3.38
RRl	2.35	3.93	3.21
RRu	2.74	4.48	3.55
P	+++	+++	+++
Random RR	2.43	3.59	2.83
RRl	2.07	2.66	2.39
RRu	2.87	4.85	3.34
P	+++	+++	+++
Between Chi			97.46
Between df			1
Between P			***
Btwn(F) P			**
Btwn(R) P			*

Table 3A1 - 6

IESLC - Meta-analysis of Ever Smoking, Any product (or Cigarettes if Any not available)
Adenocarcinoma
Least adjusted

	Derivation of RR/CI			
	Orig	StdCalc	Other	Total
N	12	82	13	107
NS	9	59	11	79
Wt	468.03	986.85	114.44	1569.32
Het Chi	102.63	647.47	36.67	1014.33
Het df	11	81	12	106
Het P	***	***	***	***
Fixed RR	6.04	2.59	3.06	3.38
RRl	5.51	2.44	2.55	3.21
RRu	6.61	2.76	3.68	3.55
P	+++	+++	+++	+++
Random RR	3.71	2.66	3.25	2.83
RRl	2.55	2.21	2.17	2.39
RRu	5.40	3.21	4.85	3.34
P	+++	+++	+++	+++
Between Chi				227.56
Between df				2
Between P				***
Btwn(F) P				***
Btwn(R) P				N.S.

Table 3A1 - 7

IESLC - Meta-analysis of Ever Smoking, Any product (or Cigarettes if Any not available)
Adenocarcinoma
Excluded studies (and stage at which they were excluded)

1	BOUCHA	BUELL	LAURIL	MZILEN												
2	BYERS2	ENSTRO	HIRAY2	KANELL	LIAW	LICKIN	MRFIT	MURATA	NOTANI	RESTRE	RIMING	SAARIK	SEGI2	SHIMIZ	SITAS	SOBUE2
	TANG2	WALD	WARSIN	WATSON	WU2	WYNDE5	WYNDE8	WYNDER								
3	BERRIN	CHEN	TAO	ULMER												
6	BLOT1	BLOT2	BLOT3	HIRAOK	JONES	MOLLO	PERSHA	SCHWA2	VANDER							
8	ABELIN	AGUDO	AKIBA	AMANDU	AMES	ARCHER	ARMADA	AUSTIN	AUVINE	AXELSO	AXELSS	BENSHL	BEST	BLOHMK	BLOT4	BOFFET
	BOUCOT	BRETT	BROCKM	BROSS	CARPEN	CASCO2	CASCOR	CEDERL	CHANG	CHATZI	CHEN2	CHEN3	CHIAZZ	CHOW	CHYOU	COOKSO
	CPSI	CPSII	DARBY	DAVEYS	DEAN	DEAN2	DEAN3	DEKLER	DESTEF	DOCKER	DOLL2	DORANT	DORN	DROSTE	DU	DUNN
	EBELIN	ESAKI	GAO2	GARCIA	GARDIN	GARSHI	GENG	GILLIS	GODLEY	GOLLED	GOODMA	GRAHAM	GREGOR	GSELL	HAMMO2	HANSEN
	HEIN	HENNEK	HIRAYA	HITOSU	HOLE	HOROWI	HORWIT	HU	HU2	HUANG	HUMBLE	JARUP	JARVHO	JIANG	KAISE2	KAISER
	KAUFMA	KELLER	KINLEN	KJUUS	KNEKT	KO	KOHLME	KOULUM	KREUZE	KUBIK	LANGE	LAUSSM	LEI	LEMARC	LETOUR	LEVIN
	LIDDEL	LIU	LIU2	LIU3	LIU4	LIU5	LOMBAR	MACLEN	MAGNUS	MARSH	MARSH2	MARTIS	MASTRA	MCCONN	MCDUFF	MCLAUG
	MIGRAN	MILLER	MILLS	MRFITR	NAM	NOTAN2	ODRISC	PARKIN	PASTOR	PAWLEG	PERNU	PERSH2	PETO	PEZZO2	PIKE	PISANI
	POFFIJ	POLEDN	PRESCO	QIAO	QIAO2	RACHTA	RADZIK	RANDIG	REN	RONCO	ROOTS	ROTHSC	SADOWS	SANKAR	SEGI	SHAW
	SIMARA	SIMONA	SPEIZE	SPITZ	STOCKS	STOCKW	STUCKE	SUN	TANG	TENKAN	TOUSEY	TULINI	TVERDA	VEIERO	WANG	WANG2
	WANG3	WANG4	WICKLU	WIGLE	WILKIN	WUNSCH	XIANGZ	XU	XU2	XU4	YAMAGU	YONG	YUAN	ZHANG		
10	BENHAM	VUTUC	WYNDE7													

Table 3A1 - 8

Potentially overlapping studies

REF	REFGP	PRINC	.	OVERLAP/LINK
LUBIN2	LUBIN2	1		Lubin-combined
CHAN	CHAN	1		Chan/LamWK2
LAMWK2	LAMWK2	1		Chan/LamWK2
LAMTH	LAMTH	1		KOO/LAMTH/LAMWK
KOO	KOO	1		KOO/LAMTH/LAMWK
LAMWK	LAMWK	1		KOO/LAMTH/LAMWK
ISHIMA	AKIBA	2		AKIBA/ISHIMA
BYERS1	BYERS1	2		GRAHAM/BROSS/BYERS1
OSANN2	KAISER	2		KAISER/OSANN2
WYNDE6	WYNDE6	1		WYNDE5/6/7/8
MATSUD	MATSUD	1		SOBUE2/MATSUD
JAHN	BOFFET	2		Subset of BOFFET
LUBIN	XIANGZ	2		LUBIN/XIANGZ/QIAO

Table 3A1 - 9

Most adjusted - insufficient data for metaanalysis

REF	NRR	SEX	AGEL	AGEH	RACE	YF	LC TYPE	LOC	START	ST	NLC	R	VB	P	H	AD	PRODUCT	DENOM	De
CHEN	11	c	0	0	all	-	a	As:oth	1987	CC	323	n	ot	n	y	2	cig+/-ot	nev cigs	ot
JIANG	5	m	0	0	all	-	a	As:Chi	1984	CC	125	n	ot	n	n	0	all/unsp	nev any	ot
JIANG	6	f	0	0	all	-	a	As:Chi	1984	CC	125	n	ot	n	n	0	all/unsp	nev any	ot
KOO	4	f	0	0	all	-	a	As:HK	1981	CC	200	n	bl	n	n	0	all/unsp	nev any	or
WANG	4	c	0	0	all	-	a	As:Chi	1990	CC	390	n	ot	*	y	0	all/unsp	nev any	ot

REF	NRR	RR	SIG	RRDATA comment
CHEN	11	2.08	y	P < 0.001
JIANG	5	*	n	0
JIANG	6	*	n	0
KOO	4	1.88		0
WANG	4	2.22	n	P>0.05

Table 3A2 -

IESLC - Meta-analysis of Ever Smoking, Cigarettes (or Any Product if Cigarettes not available)
Adenocarcinoma

This analysis is restricted to results for:
1) Non-dose-response data
2) Ever smokers
3) Results complete enough for use in metaanalysis

Within each study, results are then selected (in the following order of preference, within each sex) for:
4) PRODUCT: cigarettes regardless of other products, cigarettes only, all/unspec
5) CIGTYPE: all/unspecified, MC regardless of HR, MC only
6) DENOM: never smoked anything, never smoked cigarettes, (never +1 = +long term ex, +2 = +amount unknown,
+3 = never cigs+long term ex)
7) Followup period (YF, prospective studies): whole study (coded as 0) or longest available
8) LCtype: adeno or nearest available, but not squamous. (q = squamous, s = small,
a = adeno, l = large, KII = Kreyberg II, al = alveolar, br = bronchiolar, u = undifferentiated)
9) Race: all or nearest available, otherwise by race (wh or w = white, bl or b = black, hi = hispanic
ch = chinese, jap = japanese, haw = hawaiian, w+o = white + oriental, sca = scandinavian, as = asian)
10) For overlapping studies: principal rather than subsidiary studies
Finally by Age: whole study (coded as 0) if available, otherwise by widest available age group
and then for single sex results (m, f) in preference to combined sex results (c).

Results adjusted (AD) for the most potential confounders are then chosen in Sections -1 to -3
(and those which actually differ from the adjusted results in Table 3A1 - 1 are marked 'x' in Section -1)
and results adjusted for the least confounders in Sections -4 to -6. (Those least adjusted results which
actually differ from the most adjusted as marked 'x' in column X in Section -4)
(Results adjusted for an unknown number of confounder(s) are coded as 20.)

Section -7 shows excluded studies, together with the stage (as above) at which no qualifying
results were found.

Section -8 lists the potentially overlapping studies which have been included (1=principal, 2=subsidiary).

Section -9 lists any results which would have been included in preference except that they had data not complete
enough for use in meta-analysis, with their significance (yes/no), if known, and any further comment as entered
on the database.

In addition to those mentioned above, the following fields, levels and abbreviations are used:
* or nk = not known, n = no, y = yes, ot = other
nev = never
all/unspec = all or unspecified, cig+/-ot = cigarettes irrespective of other products (cigar, pipe etc)
MC = manufactured cigarettes, HR = hand-rolled cigarettes
REF: 6-character study reference
NRR: number of the RR on the database within the study
ST : study type (CC = case control, pr or prosp = prospective)
NLC: number of lung cancer cases in whole study
R : risky occupational population (n = no, m = mining, o = other risky)
VB : national cigarette type (V = at least 75% Virginia, bl = at least 75% blended, ot = other)
P : any proxy use
H : full histological confirmation
De : derivation of RR/CI (or = original, st = standard method, ot = other method of estimation)

Table 3A2 - 1

IESLC - Meta-analysis of Ever Smoking, Cigarettes (or Any Product if Cigarettes not available)
Adenocarcinoma
Most adjusted

REF	NRR	3A1	SEX	AGEL	AGEH	RACE	YF	LC TYPE	LOC	START	ST	NLC	R	VB	P	H	AD	PRODUCT	DENOM	De
ABRAHA	2		m	0	0	all	0	a	Eu:est	1975	pr	571	n	bl	n	n	0	all/unsp	nev any	ot
ABRAHA	5		f	0	0	all	0	a	Eu:est	1975	pr	571	n	bl	n	n	0	all/unsp	nev any	ot
ALDERS	95	x	m	0	0	all	-	not q+s	Eu:UK	1977	CC	1448	n	V	n	n	1	cig+/-ot	nev any	ot
ALDERS	45	x	f	0	0	all	-	not q+s	Eu:UK	1977	CC	1448	n	V	n	n	1	cig only	nev any	ot
ANDERS	12		f	0	0	all	0	a	NAmer	1986	pr	343	n	bl	n	n	0	cig+/-ot	nev cigs	st
BAND	2		m	0	0	all	-	a	NAmer	1983	CC	2831	n	V	y	y	2	cig only	nev any	ot
BARBON	130		m	0	0	all	-	a	Eu:wst	1979	CC	755	n	bl	y	y	3	all/unsp	nev any	ot
BECHER	12		f	0	0	all	-	not q+s	Eu:Ger	1985	CC	194	n	bl	n	y	1	all/unsp	nev any	or
BRESLO	1	x	c	0	0	all	-	a	NAmer	1949	CC	518	n	bl	n	y	0	cig+/-ot	nev+1	st
BROWN1	3		m	0	0	wh	-	a	NAmer	1979	CC	102	n	bl	y	y	1	cig+/-ot	nev cigs	or
BROWN1	4		f	0	0	wh	-	a	NAmer	1979	CC	102	n	bl	y	y	1	cig+/-ot	nev cigs	or
BROWN2	4		m	0	0	wh	-	a	NAmer	1984	CC	14596	n	bl	n	y	2	cig+/-ot	nev cigs	or
BROWN2	3		f	0	0	wh	-	a	NAmer	1984	CC	14596	n	bl	n	y	2	cig+/-ot	nev cigs	or
BUFFLE	50		m	0	0	wh	-	a	NAmer	1976	CC	943	n	bl	y	n	0	cig+/-ot	nev cigs	ot
BUFFLE	45		f	0	0	wh	-	a	NAmer	1976	CC	943	n	bl	y	n	0	cig+/-ot	nev cigs	ot
BYERS1	3		m	0	0	wh	-	a	NAmer	1957	CC	1002	n	bl	n	n	0	cig+/-ot	nev cigs	st
CHAN	19	x	m	0	0	all	-	a+l	As:HK	1976	CC	397	n	bl	n	n	0	cig+/-ot	nev any	ot
CHAN	23	x	f	0	0	all	-	a+l	As:HK	1976	CC	397	n	bl	n	n	0	cig+/-ot	nev any	st
CHOI	63		m	0	0	all	-	a	As:oth	1985	CC	375	n	bl	n	n	0	cig+/-ot	nev cigs	st
CHOI	65		f	0	0	all	-	a	As:oth	1985	CC	375	n	bl	n	n	0	cig+/-ot	nev cigs	st
COMSTO	67		m	0	0	all	-	a	NAmer	1975	ot	258	n	bl	n	n	0	cig+/-ot	nev cigs	st
COMSTO	79		f	0	0	all	-	a	NAmer	1975	ot	258	n	bl	n	n	0	cig+/-ot	nev cigs	st
CORREA	36		c	0	0	all	-	a	NAmer	1979	CC	1359	n	bl	y	n	1	cig+/-ot	nev cigs	or
DAMBER	32		m	0	0	all	-	a+al+br	Eu:Sca	1972	CC	579	n	bl	y	n	1	all/unsp	nev any	or
DESTE2	17		m	0	0	all	-	a	SCAmer	1993	CC	463	n	bl	n	n	2	all/unsp	nev any	or
DOLL	87		m	0	0	all	-	KII	Eu:UK	1948	CC	1465	n	V	n	n	1	all/unsp	nev any	ot
DOLL	89		f	0	0	all	-	KII	Eu:UK	1948	CC	1465	n	V	n	n	1	all/unsp	nev any	ot
DORGAN	125		m	0	0	wh	-	a	NAmer	1980	CC	2026	n	bl	y	y	2	cig+/-ot	nev any	or
DORGAN	104		f	0	0	all	-	a	NAmer	1980	CC	2026	n	bl	y	y	3	cig+/-ot	nev any	or
DOSEME	4		m	0	0	all	-	not q+s	Eu:bal	1979	CC	1210	n	bl	n	n	2	cig+/-ot	nev cigs	or
ENGELA	76		m	0	0	all	0	a	Eu:Sca	1964	pr	435	n	bl	n	n	7	cig+/-ot	nev cigs	ot
FAN	4		c	0	0	all	-	a	As:Chi	1990	CC	403	n	ot	y	n	0	cig+/-ot	nev cigs	ot
GAO	3		m	0	0	all	-	a	As:Chi	1984	CC	1405	n	ot	n	n	2	cig+/-ot	nev cigs	or
GAO	13		f	0	0	all	-	a	As:Chi	1984	CC	1405	n	ot	n	n	2	cig+/-ot	nev cigs	or
GER	9		c	0	0	all	-	a	As:oth	1990	CC	141	n	ot	y	n	8	all/unsp	nev any	ot
HAENSZ	39	x	f	0	0	all	-	a	NAmer	1955	CC	158	n	bl	n	y	0	cig+/-ot	nev any	st
HAMMON	84	x	m	0	0	wh	0	a	NAmer	1952	pr	448	n	bl	n	n	1	cig+/-ot	nev any	ot
HEGMAN	4		c	0	0	all	-	a	NAmer	1989	CC	282	n	bl	y	y	0	all/unsp	nev any	st
HINDS	24		f	0	0	o	-	a	NAmer	1968	CC	292	n	bl	n	n	3	all/unsp	nev any	st
ISHIMA	8		c	0	0	all	-	a	As:Jap	1961	CC	180	n	bl	y	y	5	all/unsp	nev any	st
JAHN	43	x	m	0	0	all	-	a	Eu:Ger	1988	CC	1004	n	bl	n	n	0	cig+/-ot	nev any	st
JAIN	47		m	0	0	all	-	a	NAmer	1981	CC	845	n	V	y	n	2	cig+/-ot	nev cigs	or
JAIN	42		f	0	0	all	-	a	NAmer	1981	CC	845	n	V	y	n	2	cig+/-ot	nev cigs	or
JEDRYC	56		m	0	0	all	-	a	Eu:est	1980	CC	1630	n	bl	y	n	3	cig+/-ot	nev any	ot
JOLY	51		m	0	0	all	-	a	SCAmer	1978	CC	826	n	bl	n	n	0	cig+/-ot	nev any	st
JOLY	50		f	0	0	all	-	a	SCAmer	1978	CC	826	n	bl	n	n	0	cig+/-ot	nev any	st
JUSSAW	28	x	m	0	0	all	-	KII	As:Ind	1964	CC	792	n	V	n	n	0	cig only	nev any	st
KATSOU	33		f	0	0	all	-	a	Eu:bal	1987	CC	101	n	bl	n	n	1	all/unsp	nev any	ot
KHUDER	27		m	0	0	all	-	a	NAmer	1985	CC	482	n	bl	n	y	0	cig+/-ot	nev cigs	ot
KIHARA	29		c	0	0	jap	-	a	As:Jap	1991	CC	440	n	bl	n	n	0	all/unsp	nev any	st
KOO	7		f	0	0	all	-	a+l	As:HK	1981	CC	200	n	bl	n	n	0	all/unsp	nev any	st
KREYBE	8		m	0	0	all	-	KII	Eu:Sca	1948	CC	300	n	bl	n	y	1	all/unsp	nev any	ot
KREYBE	27		f	0	0	all	-	KII	Eu:Sca	1948	CC	300	n	bl	n	y	1	all/unsp	nev any	ot
LAMTH	3		f	0	0	ch	-	a	As:HK	1983	CC	445	n	bl	n	n	0	all/unsp	nev any	or
LAMWK	4		f	0	0	ch	-	a	As:HK	1981	CC	163	n	bl	n	n	0	all/unsp	nev any	st
LAMWK2	3		m	0	0	all	-	a	As:HK	1976	CC	480	n	bl	n	n	0	all/unsp	nev any	st
LAMWK2	7		f	0	0	all	-	a	As:HK	1976	CC	480	n	bl	n	n	0	all/unsp	nev any	st
LOMBA2	3		f	0	0	all	-	not q+u	NAmer	1960	CC	225	n	bl	n	n	0	cig+/-ot	nev cigs	st
LUBIN	37	x	m	0	0	all	-	KII	As:Chi	1984	CC	427	m	ot	y	n	0	cig+/-ot	nev any	st
LUBIN2	148		m	0	0	all	-	a	Eu:mul	1976	CC	7804	n	bl	n	y	0	cig+/-ot	nev any	st
LUBIN2	168		f	0	0	all	-	a	Eu:mul	1976	CC	7804	n	bl	n	y	0	cig+/-ot	nev any	st
LUO	9		c	0	0	all	-	a	As:Chi	1990	CC	102	n	ot	n	y	20	cig+/-ot	nev cigs	or
MATOS	69		m	0	0	all	-	a	SCAmer	1994	CC	200	n	bl	n	n	2	cig+/-ot	nev any	ot
MATSUD	12		m	0	0	all	-	a	As:Jap	1965	CC	179	n	bl	n	n	0	cig+/-ot	nev cigs	ot
NOU	3		m	0	0	all	-	a	Eu:Sca	1971	CC	273	n	bl	y	n	0	all/unsp	nev any	st
NOU	8		f	0	0	all	-	a	Eu:Sca	1971	CC	273	n	bl	y	n	0	all/unsp	nev any	st
ORMOS	21		m	0	0	all	-	a	Eu:est	1947	CC	119	n	bl	y	y	0	cig+/-ot	nev any	ot
OSANN	47		m	0	0	all	-	a	NAmer	1984	CC	1986	n	bl	n	n	2	cig+/-ot	nev cigs	or
OSANN	48		f	0	0	all	-	a	NAmer	1984	CC	1986	n	bl	n	n	2	cig+/-ot	nev cigs	or
OSANN2	31		f	0	0	all	-	KII	NAmer	1964	ot	217	n	bl	n	y	1	cig+/-ot	nev cigs	or
PEZZOT	7		m	0	0	all	-	a	SCAmer	1987	CC	215	n	bl	n	y	0	cig only	nev cigs	st
SCHWAR	8		m	40	54	wh	-	a	NAmer	1984	CC	5588	n	bl	y	y	0	cig+/-ot	nev cigs	st

Table 3A2 - 1

IESLC - Meta-analysis of Ever Smoking, Cigarettes (or Any Product if Cigarettes not available)
Adenocarcinoma
Most adjusted

REF	NRR	3A1	SEX	AGEL	AGEH	RACE	YF	LC TYPE	LOC	START	ST	NLC	R	VB	P	H	AD	PRODUCT	DENOM	De
SCHWAR	7		m	40	54	bl	-	a	NAmer	1984	CC	5588	n	bl	y	y	0	cig+/-ot	nev cigs	st
SCHWAR	16		f	40	54	wh	-	a	NAmer	1984	CC	5588	n	bl	y	y	0	cig+/-ot	nev cigs	st
SCHWAR	15		f	40	54	bl	-	a	NAmer	1984	CC	5588	n	bl	y	y	0	cig+/-ot	nev cigs	st
SEOW	2		f	0	0	ch	-	a	As:oth	1997	CC	153	n	bl	n	y	0	cig+/-ot	nev cigs	st
SIEMIA	8		m	0	0	all	-	a	NAmer	1979	CC	857	n	V	y	y	7	cig+/-ot	nev cigs	or
SOBUE	99		m	0	0	all	-	a	As:Jap	1986	CC	1376	n	bl	n	y	1	cig+/-ot	nev cigs	ot
SOBUE	109		f	0	0	all	-	a	As:Jap	1986	CC	1376	n	bl	n	y	1	cig+/-ot	nev cigs	ot
STASZE	24	x	m	0	0	all	-	a	Eu:est	1954	CC	281	n	bl	n	y	0	cig+/-ot	nev any	ot
STASZE	4		f	0	0	all	-	a	Eu:est	1954	CC	281	n	bl	n	y	0	all/unsp	nev any	st
STAYNE	4		m	0	0	all	-	a	NAmer	1969	CC	420	n	bl	n	n	0	all/unsp	nev any	st
SUZUK2	16		c	0	0	all	-	a	SCAmer	1991	CC	123	n	bl	n	y	3	all/unsp	nev any	or
SUZUKI	11		m	0	0	all	-	a	As:Jap	1978	CC	238	n	bl	n	y	2	cig+/-ot	nev any	ot
SUZUKI	15		f	0	0	all	-	a	As:Jap	1978	CC	238	n	bl	n	y	2	cig+/-ot	nev any	ot
SVENSS	74		f	0	0	all	-	a	Eu:Sca	1983	CC	210	n	bl	n	n	1	all/unsp	nev any	ot
TIZZAN	19		c	0	0	all	-	a	Eu:wst	1959	CC	1358	n	bl	n	n	0	all/unsp	nev any	st
TOKARS	8		c	0	0	all	-	a	Eu:est	1966	ot	162	o	bl	n	y	3	all/unsp	nev any	or
TSUGAN	10		m	0	0	all	-	a	As:Jap	1976	CC	134	n	bl	n	y	3	all/unsp	nev any	ot
TSUGAN	11		f	0	0	all	-	a	As:Jap	1976	CC	134	n	bl	n	y	3	all/unsp	nev any	ot
WAKAI	76		m	0	0	all	-	a	As:Jap	1988	CC	333	n	bl	n	y	1	all/unsp	nev any	ot
WAKAI	82		f	0	0	all	-	a	As:Jap	1988	CC	333	n	bl	n	y	1	all/unsp	nev any	ot
WU	31		f	0	0	wh	-	a	NAmer	1981	CC	220	n	bl	n	y	2	all/unsp	nev any	ot
WUWILL	11		f	0	0	all	-	a	As:Chi	1985	CC	965	n	ot	n	n	3	cig+/-ot	nev cigs	or
WYNDE2	9	x	m	0	0	all	-	KII	NAmer	1962	CC	404	n	bl	n	y	0	cig+/-ot	nev any	st
WYNDE3	28	x	m	0	0	all	-	KII	NAmer	1966	CC	350	n	bl	n	y	0	cig+/-ot	nev any	st
WYNDE3	75	x	f	0	0	all	-	KII	NAmer	1966	CC	350	n	bl	n	y	0	cig+/-ot	nev any	st
WYNDE4	42		m	0	0	all	-	a	NAmer	1948	CC	684	n	bl	y	n	0	all/unsp	nev any	st
WYNDE4	56		f	0	0	all	-	a	NAmer	1948	CC	684	n	bl	y	n	2	all/unsp	nev any	ot
WYNDE6	78	x	m	0	0	all	-	KII	NAmer	1969	CC	4423	n	bl	n	y	0	cig+/-ot	nev any	st
WYNDE6	414		f	0	0	wh	-	a	NAmer	1969	CC	4423	n	bl	n	y	1	cig+/-ot	nev cigs	ot
XU3	22		m	0	0	all	-	KII	As:Chi	1981	CC	135	n	ot	n	n	1	all/unsp	nev any	ot
XU3	26		f	0	0	all	-	KII	As:Chi	1981	CC	135	n	ot	n	n	1	all/unsp	nev any	ot
ZHENG	10		m	0	0	all	-	a	As:Chi	1982	CC	540	n	ot	*	y	0	cig+/-ot	nev cigs	st
ZHENG	21		f	0	0	all	-	a	As:Chi	1982	CC	540	n	ot	*	y	0	cig+/-ot	nev cigs	st
ZHOU	26		m	0	0	all	-	a	As:Chi	1978	CC	1360	n	ot	n	n	0	all/unsp	nev any	st
ZHOU	27		f	0	0	all	-	a	As:Chi	1978	CC	1360	n	ot	n	n	0	all/unsp	nev any	st

Cigarette type is all/unspec for all RRs

except for the following:

REF	NRR	CIGTYPE
ALDERS	45	MC only
CHAN	19	MC+-HR
CHAN	23	MC+-HR
JUSSAW	28	MC only

Table 3A2 - 2

IESLC - Meta-analysis of Ever Smoking, Cigarettes (or Any Product if Cigarettes not available)
Adenocarcinoma
Most adjusted

REF	NRR	SEX	AD	Number Exposed Case	Number Exposed Cont	Non-exposed Case	Non-exposed Cont	RR	95.00%CI
*ABRAHA	2	m	0	59	10351	8	3365	2.40	(1.15- 5.01)
*ABRAHA	5	f	0	19	5256	16	11589	2.62	(1.35- 5.09)
Subtotal ABRAHA								2.52	(1.54- 4.12)
ALDERS	95	m	1	-	-	-	-	4.41	(1.92- 10.13)
ALDERS	45	f	1	-	-	-	-	3.69	(2.32- 5.88)
Subtotal ALDERS								3.85	(2.57- 5.78)
*ANDERS	12	f	0	99	96164	33	195158	6.09	(4.11- 9.03)
BAND	2	m	2	-	-	-	-	4.10	(3.01- 5.59)
BARBON	130	m	3	-	-	-	-	7.02	(3.24- 15.22)
BECHER	12	f	1	-	-	-	-	10.83	(1.32- 88.70)
BRESLO	1	c	0	40	394	4	56	1.42	(0.49- 4.12)
BROWN1	3	m	1	-	-	-	-	4.49	(1.44- 13.98)
BROWN1	4	f	1	-	-	-	-	3.95	(1.76- 8.80)
Subtotal BROWN1								4.12	(2.14- 7.95)
BROWN2	4	m	2	-	-	-	-	8.20	(6.90- 9.70)
BROWN2	3	f	2	-	-	-	-	6.90	(6.10- 7.90)
Subtotal BROWN2								7.35	(6.63- 8.15)
BUFFLE	50	m	0	-	-	-	-	4.50	(1.85- 10.95)
BUFFLE	45	f	0	-	-	-	-	4.02	(2.42- 6.67)
Subtotal BUFFLE								4.13	(2.66- 6.42)
BYERS1	3	m	0	47	695	7	424	4.10	(1.83- 9.15)
CHAN	19	m	0	56	160	0	43	30.63~	(1.85- 505.72)
CHAN	23	f	0	24	38	40	139	2.19	(1.18- 4.08)
Subtotal CHAN								2.48	(1.35- 4.55)
CHOI	63	m	0	46	465	7	95	1.34	(0.59- 3.06)
CHOI	65	f	0	5	26	49	164	0.64	(0.23- 1.77)
Subtotal CHOI								1.00	(0.53- 1.89)
COMSTO	67	m	0	43	229	2	84	7.89	(1.87- 33.27)
COMSTO	79	f	0	29	87	8	115	4.79	(2.09- 11.00)
Subtotal COMSTO								5.43	(2.64- 11.14)
CORREA	36	c	1	-	-	-	-	5.60	(3.60- 8.80)
DAMBER	32	m	1	-	-	-	-	2.40	(1.10- 5.30)
DESTE2	17	m	2	-	-	-	-	4.30	(1.60- 11.40)
DOLL	87	m	1	-	-	-	-	0.95	(0.22- 4.02)
DOLL	89	f	1	-	-	-	-	1.97	(0.60- 6.46)
Subtotal DOLL								1.47	(0.59- 3.69)
DORGAN	125	m	2	-	-	-	-	4.80	(1.90- 12.00)
DORGAN	104	f	3	-	-	-	-	3.90	(2.80- 5.40)
Subtotal DORGAN								3.99	(2.93- 5.44)
DOSEME	4	m	2	-	-	-	-	2.60	(1.70- 4.20)
*ENGELA	76	m	7	-	-	-	-	2.33	(0.92- 5.89)
FAN	4	c	0	67	595	45	556	1.39	(0.94- 2.07)
GAO	3	m	2	-	-	-	-	1.60	(1.10- 2.40)
GAO	13	f	2	-	-	-	-	1.50	(1.00- 2.10)
Subtotal GAO								1.55	(1.18- 2.02)
GER	9	c	8	-	-	-	-	1.10	(0.55- 2.19)
HAENSZ	39	f	0	18	103	37	236	1.11	(0.61- 2.05)
*HAMMON	84	m	1	-	-	-	-	3.67	(0.87- 15.45)
HEGMAN	4	c	0	83	1202	15	2080	9.58	(5.50- 16.67)
HINDS	24	f	3	-	-	-	-	3.89	(2.49- 6.07)
ISHIMA	8	c	5	-	-	-	-	15.00	(2.31- 631.48)
JAHN	43	m	0	202	671	8	138	5.19	(2.50- 10.77)
JAIN	47	m	2	-	-	-	-	8.00	(2.28- 50.60)
JAIN	42	f	2	-	-	-	-	3.45	(1.83- 7.10)
Subtotal JAIN								3.95	(2.12- 7.35)
JEDRYC	56	m	3	-	-	-	-	3.44	(1.52- 7.78)
JOLY	51	m	0	72	709	5	218	4.43	(1.77- 11.10)
JOLY	50	f	0	33	122	25	283	3.06	(1.75- 5.37)
Subtotal JOLY								3.38	(2.10- 5.46)
JUSSAW	28	m	0	3	77	13	624	1.87	(0.52- 6.71)
KATSOU	33	f	1	-	-	-	-	1.72	(0.80- 3.71)
KHUDER	27	m	0	155	-	7	-	8.11	(3.67- 17.93)
KIHARA	29	c	0	130	232	78	237	1.70	(1.22- 2.38)
KOO	7	f	0	34	63	46	137	1.61	(0.94- 2.74)
KREYBE	8	m	1	-	-	-	-	2.44	(0.76- 7.86)
KREYBE	27	f	1	-	-	-	-	1.28	(0.60- 2.74)
Subtotal KREYBE								1.55	(0.82- 2.93)
LAMTH	3	f	0	79	51	131	158	1.87	(1.23- 2.85)
LAMWK	4	f	0	36	41	60	144	2.11	(1.23- 3.61)
LAMWK2	3	m	0	52	161	15	43	0.93	(0.48- 1.80)
LAMWK2	7	f	0	26	50	41	139	1.76	(0.98- 3.17)

Table 3A2 - 2

IESLC - Meta-analysis of Ever Smoking, Cigarettes (or Any Product if Cigarettes not available)
Adenocarcinoma
Most adjusted

REF	NRR	SEX	AD	Number Exposed Case	Number Exposed Cont	Non-exposed Case	Non-exposed Cont	RR	95.00%CI
Subtotal LAMWK2								1.33	(0.86- 2.07)
LOMBA2	3	f	0	42	353	54	239	0.53	(0.34- 0.81)
LUBIN	37	m	0	32	788	4	72	0.73	(0.25- 2.12)
LUBIN2	148	m	0	655	10433	57	2616	2.88	(2.19- 3.79)
LUBIN2	168	f	0	85	567	138	1180	1.28	(0.96- 1.71)
Subtotal LUBIN2								1.96	(1.61- 2.39)
LUO	9	c	20	-	-	-	-	1.50	(0.70- 3.00)
MATOS	69	m	2	-	-	-	-	6.21	(2.42- 15.96)
MATSUD	12	m	0	23	3314	0	1255	17.80~	(1.08- 293.32)
NOU	3	m	0	36	247	4	122	4.45	(1.55- 12.77)
NOU	8	f	0	9	92	29	261	0.88	(0.40- 1.93)
Subtotal NOU								1.57	(0.83- 2.94)
ORMOS	21	m	0	4	1034	0	777	6.76~	(0.36- 125.82)
OSANN	47	m	2	-	-	-	-	17.90	(10.40- 31.00)
OSANN	48	f	2	-	-	-	-	9.50	(6.80- 13.80)
Subtotal OSANN								11.46	(8.51- 15.42)
OSANN2	31	f	1	-	-	-	-	2.50	(1.30- 5.10)
PEZZOT	7	m	0	60	317	3	116	7.32	(2.25- 23.79)
SCHWAR	8	m	0	84	178	1	73	34.45	(4.71- 252.10)
SCHWAR	7	m	0	45	39	1	7	8.08	(0.95- 68.56)
SCHWAR	16	f	0	92	108	10	79	6.73	(3.29- 13.75)
SCHWAR	15	f	0	20	28	3	41	9.76	(2.65- 36.00)
Subtotal SCHWAR								8.40	(4.73- 14.94)
SEOW	2	f	0	19	15	67	125	2.36	(1.13- 4.95)
SIEMIA	8	m	7	-	-	-	-	6.30	(2.50- 16.20)
SOBUE	99	m	1	-	-	-	-	1.83	(1.21- 2.77)
SOBUE	109	f	1	-	-	-	-	1.41	(1.00- 1.99)
Subtotal SOBUE								1.57	(1.20- 2.04)
STASZE	24	m	0	20	653	0	158	9.94~	(0.60- 165.30)
STASZE	4	f	0	1	153	10	1660	1.08	(0.14- 8.53)
Subtotal STASZE								2.36	(0.45- 12.42)
STAYNE	4	m	0	43	567	7	333	3.61	(1.60- 8.11)
SUZUK2	16	c	3	-	-	-	-	6.00	(0.70- 50.00)
SUZUKI	11	m	2	-	-	-	-	4.53	(2.48- 8.29)
SUZUKI	15	f	2	-	-	-	-	2.19	(1.16- 4.14)
Subtotal SUZUKI								3.21	(2.07- 4.97)
SVENSS	74	f	1	-	-	-	-	2.91	(1.56- 5.42)
TIZZAN	19	c	0	88	939	25	419	1.57	(0.99- 2.49)
TOKARS	8	c	3	-	-	-	-	4.30	(1.90- 9.90)
TSUGAN	10	m	3	-	-	-	-	0.93	(0.43- 1.99)
TSUGAN	11	f	3	-	-	-	-	0.67	(0.22- 2.07)
Subtotal TSUGAN								0.84	(0.45- 1.58)
WAKAI	76	m	1	-	-	-	-	1.93	(0.90- 4.18)
WAKAI	82	f	1	-	-	-	-	1.39	(0.66- 2.90)
Subtotal WAKAI								1.63	(0.96- 2.77)
WU	31	f	2	-	-	-	-	2.60	(1.53- 4.44)
WUWILL	11	f	3	-	-	-	-	1.50	(1.10- 1.90)
WYNDE2	9	m	0	46	512	5	105	1.89	(0.73- 4.86)
WYNDE3	28	m	0	64	264	6	88	3.56	(1.49- 8.49)
WYNDE3	75	f	0	21	56	15	76	1.90	(0.90- 4.01)
Subtotal WYNDE3								2.48	(1.41- 4.37)
WYNDE4	42	m	0	35	665	4	115	1.51	(0.53- 4.34)
WYNDE4	56	f	2	-	-	-	-	0.60	(0.13- 2.69)
Subtotal WYNDE4								1.12	(0.47- 2.66)
WYNDE6	78	m	0	1059	1797	58	617	6.27	(4.74- 8.29)
WYNDE6	414	f	1	-	-	-	-	13.99	(10.18- 19.23)
Subtotal WYNDE6								8.90	(7.21- 10.98)
XU3	22	m	1	-	-	-	-	4.84	(1.37- 17.10)
XU3	26	f	1	-	-	-	-	1.09	(0.26- 4.50)
Subtotal XU3								2.51	(0.98- 6.47)
ZHENG	10	m	0	123	218	29	94	1.83	(1.14- 2.93)
ZHENG	21	f	0	33	44	119	184	1.16	(0.70- 1.93)
Subtotal ZHENG								1.48	(1.05- 2.09)
ZHOU	26	m	0	131	41	88	36	1.31	(0.77- 2.20)
ZHOU	27	f	0	30	7	96	32	1.43	(0.57- 3.57)
Subtotal ZHOU								1.34	(0.85- 2.10)
Partial Totals				4357	141371	1533	227105		

*prospective study

~ With 0.5 adjustment for zero

Table 3A2 - 2

IESLC - Meta-analysis of Ever Smoking, Cigarettes (or Any Product if Cigarettes not available)
Adenocarcinoma
Most adjusted

REF	NRR	SEX	AD	Ys	Ws	Qs	Ps
*ABRAHA	2	m	0	0.87	7.06	0.91	0.0201
*ABRAHA	5	f	0	0.96	8.71	0.64	0.0045
Subtotal ABRAHA				0.92	15.77	1.55	
ALDERS	95	m	1	1.48	5.55	0.35	0.0005
ALDERS	45	f	1	1.31	17.77	0.09	0.0000
Subtotal ALDERS				1.35	23.32	0.44	
*ANDERS	12	f	0	1.81	24.76	8.13	0.0000
BAND	2	m	2	1.41	40.10	1.26	0.0000
BARBON	130	m	3	1.95	6.42	3.28	0.0000
BECHER	12	f	1	2.38	0.87	1.15	0.0265
BRESLO	1	c	0	0.35	3.39	2.63	0.5177
BROWN1	3	m	1	1.50	2.97	0.21	0.0096
BROWN1	4	f	1	1.37	5.93	0.12	0.0008
Subtotal BROWN1				1.42	8.91	0.33	
BROWN2	4	m	2	2.10	132.45	100.40	0.0000
BROWN2	3	f	2	1.93	229.82	111.98	0.0000
Subtotal BROWN2				1.99	362.27	212.37	
BUFFLE	50	m	0	1.50	4.86	0.36	0.0009
BUFFLE	45	f	0	1.39	14.95	0.37	0.0000
Subtotal BUFFLE				1.42	19.81	0.73	
BYERS1	3	m	0	1.41	5.95	0.19	0.0006
CHAN	19	m	0	3.42	0.49	2.34	0.0168
CHAN	23	f	0	0.79	9.98	2.00	0.0130
Subtotal CHAN				0.91	10.47	4.34	
CHOI	63	m	0	0.29	5.64	4.97	0.4842
CHOI	65	f	0	-0.44	3.77	10.58	0.3920
Subtotal CHOI				-0.00	9.42	15.55	
COMSTO	67	m	0	2.07	1.85	1.28	0.0049
COMSTO	79	f	0	1.57	5.57	0.62	0.0002
Subtotal COMSTO				1.69	7.42	1.90	
CORREA	36	c	1	1.72	19.23	4.60	0.0000
DAMBER	32	m	1	0.88	6.21	0.80	0.0291
DESTE2	17	m	2	1.46	3.99	0.20	0.0036
DOLL	87	m	1	-0.05	1.82	3.00	0.9448
DOLL	89	f	1	0.68	2.72	0.84	0.2634
Subtotal DOLL				0.39	4.54	3.84	
DORGAN	125	m	2	1.57	4.52	0.51	0.0008
DORGAN	104	f	3	1.36	35.62	0.58	0.0000
Subtotal DORGAN				1.38	40.15	1.09	
DOSEME	4	m	2	0.96	18.78	1.45	0.0000
*ENGELA	76	m	7	0.85	4.46	0.67	0.0741
FAN	4	c	0	0.33	24.61	20.08	0.1013
GAO	3	m	2	0.47	25.25	14.72	0.0182
GAO	13	f	2	0.41	27.91	19.14	0.0322
Subtotal GAO				0.44	53.16	33.86	
GER	9	c	8	0.10	8.05	10.43	0.7869
HAENSZ	39	f	0	0.11	10.36	13.11	0.7268
*HAMMON	84	m	1	1.30	1.86	0.01	0.0765
HEGMAN	4	c	0	2.26	12.50	13.15	0.0000
HINDS	24	f	3	1.36	19.35	0.30	0.0000
ISHIMA	8	c	5	2.71	0.49	1.06	0.0585
JAHN	43	m	0	1.65	7.21	1.23	0.0000
JAIN	47	m	2	2.08	1.60	1.14	0.0085
JAIN	42	f	2	1.24	8.36	0.00	0.0003
Subtotal JAIN				1.37	9.96	1.14	
JEDRYC	56	m	3	1.24	5.76	0.00	0.0030
JOLY	51	m	0	1.49	4.55	0.29	0.0015
JOLY	50	f	0	1.12	12.19	0.16	0.0001
Subtotal JOLY				1.22	16.74	0.45	
JUSSAW	28	m	0	0.63	2.35	0.87	0.3368
KATSOU	33	f	1	0.54	6.53	3.12	0.1658
KHUDER	27	m	0	2.09	6.11	4.51	0.0000
KIHARA	29	c	0	0.53	34.43	16.94	0.0018
KOO	7	f	0	0.47	13.45	7.75	0.0817
KREYBE	8	m	1	0.89	2.82	0.33	0.1345
KREYBE	27	f	1	0.25	6.66	6.48	0.5240
Subtotal KREYBE				0.44	9.48	6.81	
LAMTH	3	f	0	0.63	21.63	8.01	0.0036
LAMWK	4	f	0	0.75	13.20	3.14	0.0068
LAMWK2	3	m	0	-0.08	8.67	14.89	0.8206
LAMWK2	7	f	0	0.57	11.11	4.93	0.0588

Table 3A2 - 2

IESLC - Meta-analysis of Ever Smoking, Cigarettes (or Any Product if Cigarettes not available)
Adenocarcinoma
Most adjusted

REF	NRR	SEX	AD	Ys	Ws	Qs	Ps
Subtotal LAMWK2				0.28	19.77	19.82	
LOMBA2	3	f	0	-0.64	20.27	71.23	0.0039
LUBIN	37	m	0	-0.31	3.37	8.07	0.5649
LUBIN2	148	m	0	1.06	51.15	1.57	0.0000
LUBIN2	168	f	0	0.25	46.25	44.89	0.0913
Subtotal LUBIN2				0.67	97.40	46.46	
LUO	9	c	20	0.41	7.26	4.97	0.2748
MATOS	69	m	2	1.83	4.32	1.52	0.0001
MATSUD	12	m	0	2.88	0.49	1.33	0.0440
NOU	3	m	0	1.49	3.45	0.23	0.0056
NOU	8	f	0	-0.13	6.24	11.55	0.7504
Subtotal NOU				0.45	9.69	11.78	
ORMOS	21	m	0	1.91	0.45	0.21	0.1999
OSANN	47	m	2	2.88	12.88	35.13	0.0000
OSANN	48	f	2	2.25	30.68	31.78	0.0000
Subtotal OSANN				2.44	43.56	66.90	
OSANN2	31	f	1	0.92	8.22	0.83	0.0086
PEZZOT	7	m	0	1.99	2.76	1.58	0.0009
SCHWAR	8	m	0	3.54	0.97	5.16	0.0005
SCHWAR	7	m	0	2.09	0.84	0.61	0.0556
SCHWAR	16	f	0	1.91	7.53	3.41	0.0000
SCHWAR	15	f	0	2.28	2.26	2.46	0.0006
Subtotal SCHWAR				2.13	11.60	11.64	
SEOW	2	f	0	0.86	7.03	0.98	0.0226
SIEMIA	8	m	7	1.84	4.40	1.62	0.0001
SOBUE	99	m	1	0.60	22.40	8.87	0.0042
SOBUE	109	f	1	0.34	32.45	25.70	0.0503
Subtotal SOBUE				0.45	54.85	34.57	
STASZE	24	m	0	2.30	0.49	0.55	0.1092
STASZE	4	f	0	0.08	0.90	1.20	0.9382
Subtotal STASZE				0.86	1.39	1.75	
STAYNE	4	m	0	1.28	5.85	0.01	0.0019
SUZUK2	16	c	3	1.79	0.84	0.26	0.0999
SUZUKI	11	m	2	1.51	10.55	0.81	0.0000
SUZUKI	15	f	2	0.78	9.49	1.92	0.0157
Subtotal SUZUKI				1.17	20.04	2.73	
SVENSS	74	f	1	1.07	9.91	0.27	0.0008
TIZZAN	19	c	0	0.45	18.24	11.16	0.0538
TOKARS	8	c	3	1.46	5.64	0.29	0.0005
TSUGAN	10	m	3	-0.07	6.55	11.17	0.8527
TSUGAN	11	f	3	-0.40	3.06	8.16	0.4837
Subtotal TSUGAN				-0.18	9.60	19.33	
WAKAI	76	m	1	0.66	6.52	2.16	0.0933
WAKAI	82	f	1	0.33	7.01	5.73	0.3832
Subtotal WAKAI				0.49	13.53	7.90	
WU	31	f	2	0.96	13.54	1.05	0.0004
WUWILL	11	f	3	0.41	51.44	35.27	0.0036
WYNDE2	9	m	0	0.63	4.29	1.54	0.1887
WYNDE3	28	m	0	1.27	5.06	0.01	0.0043
WYNDE3	75	f	0	0.64	6.88	2.41	0.0922
Subtotal WYNDE3				0.91	11.95	2.42	
WYNDE4	42	m	0	0.41	3.46	2.32	0.4408
WYNDE4	56	f	2	-0.51	1.67	5.09	0.5087
Subtotal WYNDE4				0.11	5.14	7.42	
WYNDE6	78	m	0	1.84	49.11	17.80	0.0000
WYNDE6	414	f	1	2.64	37.98	74.96	0.0000
Subtotal WYNDE6				2.19	87.09	92.77	
XU3	22	m	1	1.58	2.41	0.28	0.0143
XU3	26	f	1	0.09	1.89	2.49	0.9057
Subtotal XU3				0.92	4.30	2.77	
ZHENG	10	m	0	0.60	17.29	6.86	0.0121
ZHENG	21	f	0	0.15	14.95	17.62	0.5668
Subtotal ZHENG				0.39	32.24	24.48	
ZHOU	26	m	0	0.27	14.05	13.10	0.3154
ZHOU	27	f	0	0.36	4.59	3.53	0.4448
Subtotal ZHOU				0.29	18.64	16.63	

Table 3A2 - 2

IESLC - Meta-analysis of Ever Smoking, Cigarettes (or Any Product if Cigarettes not available)
Adenocarcinoma
Most adjusted

	N	107
	NS	75
	Wt	1522.57
Het	Chi	924.03
Het	df	106
Het	P	***
Fixed	RR	3.43
	RRl	3.27
	RRu	3.61
	P	+++
Random	RR	2.82
	RRl	2.39
	RRu	3.32
	P	+++
Asymm	P	**

Table 3A2 - 3

IESLC - Meta-analysis of Ever Smoking, Cigarettes (or Any Product if Cigarettes not available)
Adenocarcinoma
Most adjusted

	Sex			
	combined	male	female	Total
N	11	51	45	107
NS	11	50	44	105
Wt	134.68	552.43	835.46	1522.57
Het Chi	63.82	267.20	554.00	924.03
Het df	10	50	44	106
Het P	***	***	***	***
Fixed RR	2.30	4.06	3.28	3.43
RRl	1.94	3.73	3.06	3.27
RRu	2.72	4.41	3.51	3.61
P	+++	+++	+++	+++
Random RR	2.55	3.46	2.33	2.82
RRl	1.58	2.76	1.79	2.39
RRu	4.11	4.33	3.03	3.32
P	+++	+++	+++	+++
Between Chi				39.01
Between df				2
Between P				***
Btwn(F) P				N.S.
Btwn(R) P				(*)

	Lung cancer type						
	a	a+l	a+al+br	KII	not q+u	not q+s	Total
N	85	3	1	13	1	4	107
NS	60	2	1	9	1	3	76
Wt	1331.58	23.93	6.21	97.62	20.27	42.97	1522.57
Het Chi	789.71	4.35	0.00	44.94	0.00	2.98	924.03
Het df	84	2	0	12	0	3	106
Het P	***	N.S.	N.S.	***	N.S.	N.S.	***
Fixed RR	3.58	1.94	2.40	3.47	0.53	3.31	3.43
RRl	3.39	1.30	1.09	2.85	0.34	2.46	3.27
RRu	3.77	2.90	5.27	4.24	0.81	4.47	3.61
P	+++	++	+	+++	--	+++	+++
Random RR	2.97	2.20	2.40	2.14	0.53	3.31	2.82
RRl	2.47	1.08	1.09	1.34	0.34	2.46	2.39
RRu	3.56	4.47	5.27	3.40	0.81	4.47	3.32
P	+++	+	+	++	--	+++	+++
Between Chi							82.05
Between df							5
Between P							***
Btwn(F) P							(*)
Btwn(R) P							***

	Location								
	NAmer	UK	Scand	othEur	China	Japan	othAs	other	Total
N	40	4	7	15	12	11	12	6	107
NS	27	2	5	12	8	7	9	5	75
Wt	808.00	27.86	39.74	184.47	195.03	133.44	105.38	28.65	1522.57
Het Chi	302.69	4.24	9.53	42.13	7.39	22.36	14.91	2.85	924.03
Het df	39	3	6	14	11	10	11	5	106
Het P	***	N.S.	N.S.	***	N.S.	*	N.S.	N.S.	***
Fixed RR	5.79	3.29	2.04	2.30	1.48	1.74	1.66	4.20	3.43
RRl	5.40	2.27	1.49	1.99	1.28	1.47	1.37	2.91	3.27
RRu	6.20	4.77	2.78	2.66	1.70	2.06	2.00	6.06	3.61
P	+++	+++	+++	+++	+++	+++	+++	+++	+++
Random RR	4.42	3.04	2.05	2.74	1.48	1.80	1.63	4.20	2.82
RRl	3.53	1.83	1.37	2.02	1.28	1.34	1.29	2.91	2.39
RRu	5.54	5.06	3.06	3.71	1.70	2.41	2.06	6.06	3.32
P	+++	+++	+++	+++	+++	+++	+++	+++	+++
Between Chi									517.93
Between df									7
Between P									***
Btwn(F) P									***
Btwn(R) P									***

Table 3A2 - 3

IESLC - Meta-analysis of Ever Smoking, Cigarettes (or Any Product if Cigarettes not available)
Adenocarcinoma
Most adjusted

	Detailed Country in "other Europe"					
	multi	Germany	othWest	East	Balkans	Total
N	2	2	2	7	2	15
NS	1	2	2	5	2	12
Wt	97.40	8.08	24.66	29.01	25.31	184.47
Het Chi	15.93	0.42	10.65	3.28	0.83	42.13
Het df	1	1	1	6	1	14
Het P	***	N.S.	**	N.S.	N.S.	***
Fixed RR	1.96	5.62	2.32	3.01	2.34	2.30
RRl	1.61	2.82	1.56	2.09	1.58	1.99
RRu	2.39	11.20	3.44	4.33	3.45	2.66
P	+++	+++	+++	+++	+++	+++
Random RR	1.92	5.62	3.21	3.01	2.34	2.74
RRl	0.87	2.82	0.74	2.09	1.58	2.02
RRu	4.26	11.20	13.90	4.33	3.45	3.71
P	N.S.	+++	N.S.	+++	+++	+++
Between Chi						11.02
Between df						4
Between P						*
Btwn(F) P						N.S.
Btwn(R) P						N.S.

	Detailed Country in "other Asia"			
	India	HongKong	other	Total
N	1	7	4	12
NS	1	5	3	9
Wt	2.35	78.53	24.49	105.38
Het Chi	0.00	8.66	4.60	14.91
Het df	0	6	3	11
Het P	N.S.	N.S.	N.S.	N.S.
Fixed RR	1.87	1.77	1.32	1.66
RRl	0.52	1.42	0.89	1.37
RRu	6.71	2.21	1.96	2.00
P	N.S.	+++	N.S.	+++
Random RR	1.87	1.77	1.29	1.63
RRl	0.52	1.34	0.79	1.29
RRu	6.71	2.33	2.13	2.06
P	N.S.	+++	N.S.	+++
Between Chi				1.64
Between df				2
Between P				N.S.
Btwn(F) P				N.S.
Btwn(R) P				N.S.

	Detailed other continent			
	SCAmer	Auslia	Africa	Total
N	6			6
NS	5			5
Wt	28.65			28.65
Het Chi	2.85			2.85
Het df	5			5
Het P	N.S.			N.S.
Fixed RR	4.20			4.20
RRl	2.91			2.91
RRu	6.06			6.06
P	+++			+++
Random RR	4.20			4.20
RRl	2.91			2.91
RRu	6.06			6.06
P	+++			+++
Between Chi				
Between df				
Between P				N.S.
Btwn(F) P				N.S.
Btwn(R) P				N.S.

Table 3A2 - 3

IESLC - Meta-analysis of Ever Smoking, Cigarettes (or Any Product if Cigarettes not available)
Adenocarcinoma
Most adjusted

	Start year of study					
	<1960	1960-69	1970-79	1980-89	1990+	Total
N	14	14	31	40	8	107
NS	10	12	18	27	8	75
Wt	60.79	170.45	332.64	868.16	90.53	1522.57
Het Chi	13.68	164.74	107.41	477.28	15.29	924.03
Het df	13	13	30	39	7	106
Het P	N.S.	***	***	***	*	***
Fixed RR	1.64	4.30	2.53	4.17	1.76	3.43
RRl	1.27	3.70	2.27	3.90	1.44	3.27
RRu	2.10	4.99	2.82	4.46	2.17	3.61
P	+++	+++	+++	+++	+++	+++
Random RR	1.65	3.31	2.71	3.23	1.99	2.82
RRl	1.26	1.81	2.18	2.48	1.40	2.39
RRu	2.15	6.05	3.38	4.21	2.83	3.32
P	+++	+++	+++	+++	+++	+++
Between Chi						145.64
Between df						4
Between P						***
Btwn(F) P						**
Btwn(R) P						**

	Study type (1)		
	CC	other	Total
N	98	9	107
NS	68	7	75
Wt	1454.44	68.13	1522.57
Het Chi	910.59	11.97	924.03
Het df	97	8	106
Het P	***	N.S.	***
Fixed RR	3.41	3.96	3.43
RRl	3.24	3.12	3.27
RRu	3.59	5.02	3.61
P	+++	+++	+++
Random RR	2.75	3.68	2.82
RRl	2.31	2.69	2.39
RRu	3.28	5.05	3.32
P	+++	+++	+++
Between Chi			1.46
Between df			1
Between P			N.S.
Btwn(F) P			N.S.
Btwn(R) P			N.S.

	Study type (2)			
	CC	prosp	other	Total
N	98	5	4	107
NS	68	4	3	75
Wt	1454.44	46.84	21.28	1522.57
Het Chi	910.59	9.09	2.81	924.03
Het df	97	4	3	106
Het P	***	(*)	N.S.	***
Fixed RR	3.41	4.05	3.78	3.43
RRl	3.24	3.04	2.47	3.27
RRu	3.59	5.39	5.78	3.61
P	+++	+++	+++	+++
Random RR	2.75	3.38	3.78	2.82
RRl	2.31	2.06	2.47	2.39
RRu	3.28	5.55	5.78	3.32
P	+++	+++	+++	+++
Between Chi				1.52
Between df				2
Between P				N.S.
Btwn(F) P				N.S.
Btwn(R) P				N.S.

Table 3A2 - 3

IESLC - Meta-analysis of Ever Smoking, Cigarettes (or Any Product if Cigarettes not available)
Adenocarcinoma
Most adjusted

	Study size (number of LC cases)				
	100-249	250-499	500-999	1000+	Total
N	27	31	18	31	107
NS	23	22	12	18	75
Wt	176.52	260.31	173.87	911.86	1522.57
Het Chi	90.50	120.58	47.96	460.51	924.03
Het df	26	30	17	30	106
Het P	***	***	***	***	***
Fixed RR	1.93	2.40	2.23	4.62	3.43
RRl	1.66	2.13	1.92	4.33	3.27
RRu	2.23	2.71	2.59	4.93	3.61
P	+++	+++	+++	+++	+++
Random RR	2.31	2.41	2.60	3.76	2.82
RRl	1.71	1.85	1.96	2.83	2.39
RRu	3.12	3.15	3.44	4.99	3.32
P	+++	+++	+++	+++	+++
Between Chi					204.47
Between df					3
Between P					***
Btwn(F) P					***
Btwn(R) P					(*)

	Risky occupational population			
	no	mining	othRisky	Total
N	105	1	1	107
NS	73	1	1	75
Wt	1513.56	3.37	5.64	1522.57
Het Chi	915.66	0.00	0.00	924.03
Het df	104	0	0	106
Het P	***	N.S.	N.S.	***
Fixed RR	3.44	0.73	4.30	3.43
RRl	3.27	0.25	1.88	3.27
RRu	3.62	2.12	9.82	3.61
P	+++	N.S.	+++	+++
Random RR	2.84	0.73	4.30	2.82
RRl	2.40	0.25	1.88	2.39
RRu	3.35	2.12	9.82	3.32
P	+++	N.S.	+++	+++
Between Chi				8.37
Between df				2
Between P				*
Btwn(F) P				N.S.
Btwn(R) P				*

	National cigarette tobacco type			
	Virginia	blended	other	Total
N	9	85	13	107
NS	6	60	9	75
Wt	84.67	1234.82	203.08	1522.57
Het Chi	8.31	736.21	8.06	924.03
Het df	8	84	12	106
Het P	N.S.	***	N.S.	***
Fixed RR	3.80	3.92	1.46	3.43
RRl	3.07	3.71	1.27	3.27
RRu	4.70	4.15	1.68	3.61
P	+++	+++	+++	+++
Random RR	3.78	3.09	1.46	2.82
RRl	3.01	2.57	1.27	2.39
RRu	4.73	3.72	1.68	3.32
P	+++	+++	+++	+++
Between Chi				171.44
Between df				2
Between P				***
Btwn(F) P				***
Btwn(R) P				***

Table 3A2 - 3

IESLC - Meta-analysis of Ever Smoking, Cigarettes (or Any Product if Cigarettes not available)
Adenocarcinoma
Most adjusted

	Any proxy use No/nk	Yes	Total
N	79	28	107
NS	56	19	75
Wt	1285.73	236.84	1522.57
Het Chi	825.27	98.39	924.03
Het df	78	27	106
Het P	***	***	***
Fixed RR	3.41	3.56	3.43
RRl	3.23	3.13	3.27
RRu	3.60	4.04	3.61
P	+++	+++	+++
Random RR	2.59	3.61	2.82
RRl	2.13	2.74	2.39
RRu	3.15	4.76	3.32
P	+++	+++	+++
Between Chi			0.37
Between df			1
Between P			N.S.
Btwn(F) P			N.S.
Btwn(R) P			(*)

	Full histological confirmation No	Yes	Total
N	59	48	107
NS	43	32	75
Wt	627.42	895.15	1522.57
Het Chi	327.88	463.43	924.03
Het df	58	47	106
Het P	***	***	***
Fixed RR	2.41	4.40	3.43
RRl	2.23	4.12	3.27
RRu	2.61	4.69	3.61
P	+++	+++	+++
Random RR	2.50	3.26	2.82
RRl	2.05	2.56	2.39
RRu	3.06	4.14	3.32
P	+++	+++	+++
Between Chi			132.72
Between df			1
Between P			***
Btwn(F) P			***
Btwn(R) P			N.S.

	Number of adjustment variables (1) 0	1	2+/+nk	Total
N	54	22	31	107
NS	38	15	24	77
Wt	577.59	209.74	735.24	1522.57
Het Chi	290.81	142.04	334.37	924.03
Het df	53	21	30	106
Het P	***	***	***	***
Fixed RR	2.34	3.30	4.69	3.43
RRl	2.16	2.88	4.37	3.27
RRu	2.54	3.77	5.04	3.61
P	+++	+++	+++	+++
Random RR	2.51	2.76	3.37	2.82
RRl	2.03	1.88	2.54	2.39
RRu	3.10	4.06	4.47	3.32
P	+++	+++	+++	+++
Between Chi				156.80
Between df				2
Between P				***
Btwn(F) P				***
Btwn(R) P				N.S.

Table 3A2 - 3

IESLC - Meta-analysis of Ever Smoking, Cigarettes (or Any Product if Cigarettes not available)
Adenocarcinoma
Most adjusted

	Number of adjustment variables (2) 0	1	2	3-5	6+/+nk	Total
N	54	22	17	10	4	107
NS	38	15	12	9	4	78
Wt	577.59	209.74	575.91	135.17	24.16	1522.57
Het Chi	290.81	142.04	185.98	47.25	9.32	924.03
Het df	53	21	16	9	3	106
Het P	***	***	***	***	*	***
Fixed RR	2.34	3.30	5.64	2.51	1.91	3.43
RRl	2.16	2.88	5.20	2.12	1.28	3.27
RRu	2.54	3.77	6.12	2.98	2.84	3.61
P	+++	+++	+++	+++	++	+++
Random RR	2.51	2.76	4.10	2.79	2.11	2.82
RRl	2.03	1.88	2.93	1.74	1.03	2.39
RRu	3.10	4.06	5.72	4.47	4.31	3.32
P	+++	+++	+++	+++	+	+++
Between Chi						248.62
Between df						4
Between P						***
Btwn(F) P						***
Btwn(R) P						N.S.

	Product all/unsp	cig+/-ot	cig only	Total
N	40	63	4	107
NS	30	43	4	77
Wt	312.50	1147.09	62.98	1522.57
Het Chi	102.47	719.70	2.50	924.03
Het df	39	62	3	106
Het P	***	***	N.S.	***
Fixed RR	2.08	3.91	3.96	3.43
RRl	1.86	3.69	3.10	3.27
RRu	2.32	4.14	5.08	3.61
P	+++	+++	+++	+++
Random RR	2.08	3.31	3.96	2.82
RRl	1.71	2.66	3.10	2.39
RRu	2.53	4.13	5.08	3.32
P	+++	+++	+++	+++
Between Chi				99.35
Between df				2
Between P				***
Btwn(F) P				**
Btwn(R) P				***

	Denominator nev any	nev cigs	Total
N	67	40	107
NS	49	27	76
Wt	665.62	856.95	1522.57
Het Chi	232.53	592.68	924.03
Het df	66	39	106
Het P	***	***	***
Fixed RR	2.57	4.30	3.43
RRl	2.38	4.02	3.27
RRu	2.77	4.60	3.61
P	+++	+++	+++
Random RR	2.43	3.56	2.82
RRl	2.07	2.66	2.39
RRu	2.85	4.78	3.32
P	+++	+++	+++
Between Chi			98.82
Between df			1
Between P			***
Btwn(F) P			***
Btwn(R) P			*

Table 3A2 - 3

IESLC - Meta-analysis of Ever Smoking, Cigarettes (or Any Product if Cigarettes not available)

Adenocarcinoma

Most adjusted

	Derivation of RR/CI			
	Orig	StdCalc	Other	Total
N	24	45	38	107
NS	18	33	28	79
Wt	666.52	507.58	348.47	1522.57
Het Chi	289.58	266.91	204.32	924.03
Het df	23	44	37	106
Het P	***	***	***	***
Fixed RR	4.91	2.36	2.98	3.43
RRl	4.55	2.17	2.68	3.27
RRu	5.30	2.58	3.31	3.61
P	+++	+++	+++	+++
Random RR	3.90	2.41	2.69	2.82
RRl	2.84	1.91	2.05	2.39
RRu	5.35	3.05	3.54	3.32
P	+++	+++	+++	+++
Between Chi				163.22
Between df				2
Between P				***
Btwn(F) P				***
Btwn(R) P				(*)

Table 3A2 - 4

IESLC - Meta-analysis of Ever Smoking, Cigarettes (or Any Product if Cigarettes not available)
Adenocarcinoma
Least adjusted

REF	NRR	X	SEX	AGEL	AGEH	RACE	YF	LC TYPE	LOC	START	ST	NLC	R	VB	P	H	AD	PRODUCT	DENOM	De
ABRAHA	2		m	0	0	all	0	a	Eu:est	1975	pr	571	n	bl	n	n	0	all/unsp	nev any	ot
ABRAHA	5		f	0	0	all	0	a	Eu:est	1975	pr	571	n	bl	n	n	0	all/unsp	nev any	ot
ALDERS	108	x	m	0	0	all	-	not q+s	Eu:UK	1977	CC	1448	n	V	n	n	0	cig+/-ot	nev any	st
ALDERS	104	x	f	0	0	all	-	not q+s	Eu:UK	1977	CC	1448	n	V	n	n	0	cig only	nev any	st
ANDERS	12		f	0	0	all	0	a	NAmer	1986	pr	343	n	bl	n	n	0	cig+/-ot	nev cigs	st
BAND	2		m	0	0	all	-	a	NAmer	1983	CC	2831	n	V	y	y	2	cig only	nev any	ot
BARBON	122	x	m	0	0	all	-	a	Eu:wst	1979	CC	755	n	bl	y	y	0	all/unsp	nev any	st
BECHER	12		f	0	0	all	-	not q+s	Eu:Ger	1985	CC	194	n	bl	n	y	1	all/unsp	nev any	or
BRESLO	1		c	0	0	all	-	a	NAmer	1949	CC	518	n	bl	n	y	0	cig+/-ot	nev+1	st
BROWN1	1	x	m	0	0	wh	-	a	NAmer	1979	CC	102	n	bl	y	y	0	cig+/-ot	nev cigs	st
BROWN1	2	x	f	0	0	wh	-	a	NAmer	1979	CC	102	n	bl	y	y	0	cig+/-ot	nev cigs	st
BROWN2	4		m	0	0	wh	-	a	NAmer	1984	CC	14596	n	bl	n	y	2	cig+/-ot	nev cigs	or
BROWN2	3		f	0	0	wh	-	a	NAmer	1984	CC	14596	n	bl	n	y	2	cig+/-ot	nev cigs	or
BUFFLE	50		m	0	0	wh	-	a	NAmer	1976	CC	943	n	bl	y	n	0	cig+/-ot	nev cigs	ot
BUFFLE	45		f	0	0	wh	-	a	NAmer	1976	CC	943	n	bl	y	n	0	cig+/-ot	nev cigs	ot
BYERS1	3		m	0	0	wh	-	a	NAmer	1957	CC	1002	n	bl	n	n	0	cig+/-ot	nev cigs	st
CHAN	19		m	0	0	all	-	a+l	As:HK	1976	CC	397	n	bl	n	n	0	cig+/-ot	nev any	ot
CHAN	23		f	0	0	all	-	a+l	As:HK	1976	CC	397	n	bl	n	n	0	cig+/-ot	nev any	st
CHOI	63		m	0	0	all	-	a	As:oth	1985	CC	375	n	bl	n	n	0	cig+/-ot	nev cigs	st
CHOI	65		f	0	0	all	-	a	As:oth	1985	CC	375	n	bl	n	n	0	cig+/-ot	nev cigs	st
COMSTO	67		m	0	0	all	-	a	NAmer	1975	ot	258	n	bl	n	n	0	cig+/-ot	nev cigs	st
COMSTO	79		f	0	0	all	-	a	NAmer	1975	ot	258	n	bl	n	n	0	cig+/-ot	nev cigs	st
CORREA	36		c	0	0	all	-	a	NAmer	1979	CC	1359	n	bl	y	n	1	cig+/-ot	nev cigs	or
DAMBER	11	x	m	0	0	all	-	a+al+br	Eu:Sca	1972	CC	579	n	bl	y	n	0	all/unsp	nev any	st
DESTE2	17		m	0	0	all	-	a	SCAmer	1993	CC	463	n	bl	n	n	2	all/unsp	nev any	or
DOLL	83	x	m	0	0	all	-	KII	Eu:UK	1948	CC	1465	n	V	n	n	0	all/unsp	nev any	st
DOLL	85	x	f	0	0	all	-	KII	Eu:UK	1948	CC	1465	n	V	n	n	0	all/unsp	nev any	st
DORGAN	125		m	0	0	wh	-	a	NAmer	1980	CC	2026	n	bl	y	y	2	cig+/-ot	nev any	or
DORGAN	104		f	0	0	all	-	a	NAmer	1980	CC	2026	n	bl	y	y	3	cig+/-ot	nev any	or
DOSEME	20	x	m	0	0	all	-	not q+s	Eu:bal	1979	CC	1210	n	bl	n	n	0	cig+/-ot	nev cigs	st
ENGELA	76		m	0	0	all	0	a	Eu:Sca	1964	pr	435	n	bl	n	n	7	cig+/-ot	nev cigs	ot
FAN	4		c	0	0	all	-	a	As:Chi	1990	CC	403	n	ot	y	n	0	cig+/-ot	nev cigs	ot
GAO	8	x	m	0	0	all	-	a	As:Chi	1984	CC	1405	n	ot	n	n	0	cig+/-ot	nev cigs	st
GAO	18	x	f	0	0	all	-	a	As:Chi	1984	CC	1405	n	ot	n	n	0	cig+/-ot	nev cigs	st
GER	1	x	c	0	0	all	-	a	As:oth	1990	CC	141	n	ot	y	n	0	all/unsp	nev any	st
HAENSZ	39		f	0	0	all	-	a	NAmer	1955	CC	158	n	bl	n	y	0	cig+/-ot	nev any	st
HAMMON	91	x	m	0	0	wh	0	a	NAmer	1952	pr	448	n	bl	n	n	0	cig+/-ot	nev any	st
HEGMAN	4		c	0	0	all	-	a	NAmer	1989	CC	282	n	bl	y	y	0	all/unsp	nev any	st
HINDS	24		f	0	0	o	-	a	NAmer	1968	CC	292	n	bl	n	n	3	all/unsp	nev any	st
ISHIMA	3	x	c	0	0	all	-	a	As:Jap	1961	CC	180	n	bl	y	y	0	all/unsp	nev any	st
JAHN	43		m	0	0	all	-	a	Eu:Ger	1988	CC	1004	n	bl	n	n	0	cig+/-ot	nev any	st
JAIN	7	x	m	0	0	all	-	a	NAmer	1981	CC	845	n	V	y	n	0	cig+/-ot	nev cigs	st
JAIN	2	x	f	0	0	all	-	a	NAmer	1981	CC	845	n	V	y	n	0	cig+/-ot	nev cigs	st
JEDRYC	21	x	m	0	0	all	-	a	Eu:est	1980	CC	1630	n	bl	y	n	0	cig+/-ot	nev any	st
JOLY	51		m	0	0	all	-	a	SCAmer	1978	CC	826	n	bl	n	n	0	cig+/-ot	nev any	st
JOLY	50		f	0	0	all	-	a	SCAmer	1978	CC	826	n	bl	n	n	0	cig+/-ot	nev any	st
JUSSAW	28		m	0	0	all	-	KII	As:Ind	1964	CC	792	n	V	n	n	0	cig only	nev any	st
KATSOU	31	x	f	0	0	all	-	a	Eu:bal	1987	CC	101	n	bl	n	n	0	all/unsp	nev any	st
KHUDER	27		m	0	0	all	-	a	NAmer	1985	CC	482	n	bl	n	y	0	cig+/-ot	nev cigs	ot
KIHARA	29		c	0	0	jap	-	a	As:Jap	1991	CC	440	n	bl	n	n	0	all/unsp	nev any	st
KOO	7		f	0	0	all	-	a+l	As:HK	1981	CC	200	n	bl	n	n	0	all/unsp	nev any	st
KREYBE	20	x	m	0	0	all	-	KII	Eu:Sca	1948	CC	300	n	bl	n	y	0	all/unsp	nev any	st
KREYBE	36	x	f	0	0	all	-	KII	Eu:Sca	1948	CC	300	n	bl	n	y	0	all/unsp	nev any	st
LAMTH	3		f	0	0	ch	-	a	As:HK	1983	CC	445	n	bl	n	n	0	all/unsp	nev any	or
LAMWK	4		f	0	0	ch	-	a	As:HK	1981	CC	163	n	bl	n	n	0	all/unsp	nev any	st
LAMWK2	3		m	0	0	all	-	a	As:HK	1976	CC	480	n	bl	n	n	0	all/unsp	nev any	st
LAMWK2	7		f	0	0	all	-	a	As:HK	1976	CC	480	n	bl	n	n	0	all/unsp	nev any	st
LOMBA2	3		f	0	0	all	-	not q+u	NAmer	1960	CC	225	n	bl	n	n	0	cig+/-ot	nev cigs	st
LUBIN	37		m	0	0	all	-	KII	As:Chi	1984	CC	427	m	ot	y	n	0	cig+/-ot	nev any	st
LUBIN2	148		m	0	0	all	-	a	Eu:mul	1976	CC	7804	n	bl	n	y	0	cig+/-ot	nev any	st
LUBIN2	168		f	0	0	all	-	a	Eu:mul	1976	CC	7804	n	bl	n	y	0	cig+/-ot	nev any	st
LUO	3	x	c	0	0	all	-	a	As:Chi	1990	CC	102	n	ot	n	y	0	cig+/-ot	nev cigs	st
MATOS	68	x	m	0	0	all	-	a	SCAmer	1994	CC	200	n	bl	n	n	0	cig+/-ot	nev any	st
MATSUD	12		m	0	0	all	-	a	As:Jap	1965	CC	179	n	bl	n	n	0	cig+/-ot	nev cigs	ot
NOU	3		m	0	0	all	-	a	Eu:Sca	1971	CC	273	n	bl	y	n	0	all/unsp	nev any	st
NOU	8		f	0	0	all	-	a	Eu:Sca	1971	CC	273	n	bl	y	n	0	all/unsp	nev any	st
ORMOS	21		m	0	0	all	-	a	Eu:est	1947	CC	119	n	bl	y	y	0	cig+/-ot	nev any	ot
OSANN	19	x	m	0	0	all	-	a	NAmer	1984	CC	1986	n	bl	n	n	0	cig+/-ot	nev cigs	st
OSANN	23	x	f	0	0	all	-	a	NAmer	1984	CC	1986	n	bl	n	n	0	cig+/-ot	nev cigs	st
OSANN2	13	x	f	0	0	all	-	KII	NAmer	1964	ot	217	n	bl	n	y	0	cig+/-ot	nev cigs	st
PEZZOT	7		m	0	0	all	-	a	SCAmer	1987	CC	215	n	bl	n	y	0	cig only	nev cigs	st
SCHWAR	8		m	40	54	wh	-	a	NAmer	1984	CC	5588	n	bl	y	y	0	cig+/-ot	nev cigs	st

Table 3A2 - 4

IESLC - Meta-analysis of Ever Smoking, Cigarettes (or Any Product if Cigarettes not available)
Adenocarcinoma
Least adjusted

REF	NRR	X	SEX	AGEL	AGEH	RACE	YF	LC TYPE	LOC	START	ST	NLC	R	VB	P	H	AD	PRODUCT	DENOM	De
SCHWAR	7		m	40	54	bl	-	a	NAmer	1984	CC	5588	n	bl	y	y	0	cig+/-ot	nev cigs	st
SCHWAR	16		f	40	54	wh	-	a	NAmer	1984	CC	5588	n	bl	y	y	0	cig+/-ot	nev cigs	st
SCHWAR	15		f	40	54	bl	-	a	NAmer	1984	CC	5588	n	bl	y	y	0	cig+/-ot	nev cigs	st
SEOW	2		f	0	0	ch	-	a	As:oth	1997	CC	153	n	bl	n	y	0	cig+/-ot	nev cigs	st
SIEMIA	12	x	m	0	0	all	-	a	NAmer	1979	CC	857	n	V	y	y	0	cig+/-ot	nev cigs	st
SOBUE	7	x	m	0	0	all	-	a	As:Jap	1986	CC	1376	n	bl	n	y	0	cig+/-ot	nev cigs	st
SOBUE	23	x	f	0	0	all	-	a	As:Jap	1986	CC	1376	n	bl	n	y	0	cig+/-ot	nev cigs	st
STASZE	24		m	0	0	all	-	a	Eu:est	1954	CC	281	n	bl	n	y	0	cig+/-ot	nev any	ot
STASZE	4		f	0	0	all	-	a	Eu:est	1954	CC	281	n	bl	n	y	0	all/unsp	nev any	st
STAYNE	4		m	0	0	all	-	a	NAmer	1969	CC	420	n	bl	n	n	0	all/unsp	nev any	st
SUZUK2	13	x	c	0	0	all	-	a	SCAmer	1991	CC	123	n	bl	n	y	0	all/unsp	nev any	st
SUZUKI	3	x	m	0	0	all	-	a	As:Jap	1978	CC	238	n	bl	n	y	0	cig+/-ot	nev any	st
SUZUKI	7	x	f	0	0	all	-	a	As:Jap	1978	CC	238	n	bl	n	y	0	cig+/-ot	nev any	st
SVENSS	59	x	f	0	0	all	-	a	Eu:Sca	1983	CC	210	n	bl	n	n	0	all/unsp	nev any	st
TIZZAN	19		c	0	0	all	-	a	Eu:wst	1959	CC	1358	n	bl	n	n	0	all/unsp	nev any	st
TOKARS	7	x	c	0	0	all	-	a	Eu:est	1966	ot	162	o	bl	n	y	0	all/unsp	nev any	st
TSUGAN	2	x	m	0	0	all	-	a	As:Jap	1976	CC	134	n	bl	n	y	0	all/unsp	nev any	st
TSUGAN	8	x	f	0	0	all	-	a	As:Jap	1976	CC	134	n	bl	n	y	0	all/unsp	nev any	or
WAKAI	17	x	m	0	0	all	-	a	As:Jap	1988	CC	333	n	bl	n	y	0	all/unsp	nev any	st
WAKAI	35	x	f	0	0	all	-	a	As:Jap	1988	CC	333	n	bl	n	y	0	all/unsp	nev any	st
WU	5	x	f	0	0	wh	-	a	NAmer	1981	CC	220	n	bl	n	y	0	all/unsp	nev any	st
WUWILL	25	x	f	0	0	all	-	a	As:Chi	1985	CC	965	n	ot	n	n	0	cig+/-ot	nev cigs	st
WYNDE2	9		m	0	0	all	-	KII	NAmer	1962	CC	404	n	bl	n	y	0	cig+/-ot	nev any	st
WYNDE3	28		m	0	0	all	-	KII	NAmer	1966	CC	350	n	bl	n	y	0	cig+/-ot	nev any	st
WYNDE3	75		f	0	0	all	-	KII	NAmer	1966	CC	350	n	bl	n	y	0	cig+/-ot	nev any	st
WYNDE4	42		m	0	0	all	-	a	NAmer	1948	CC	684	n	bl	y	n	0	all/unsp	nev any	st
WYNDE4	56		f	0	0	all	-	a	NAmer	1948	CC	684	n	bl	y	n	2	all/unsp	nev any	ot
WYNDE6	78		m	0	0	all	-	KII	NAmer	1969	CC	4423	n	bl	n	y	0	cig+/-ot	nev any	st
WYNDE6	413	x	f	0	0	wh	-	a	NAmer	1969	CC	4423	n	bl	n	y	0	cig+/-ot	nev cigs	st
XU3	21	x	m	0	0	all	-	KII	As:Chi	1981	CC	135	n	ot	n	n	0	all/unsp	nev any	st
XU3	25	x	f	0	0	all	-	KII	As:Chi	1981	CC	135	n	ot	n	n	0	all/unsp	nev any	st
ZHENG	10		m	0	0	all	-	a	As:Chi	1982	CC	540	n	ot	*	y	0	cig+/-ot	nev cigs	st
ZHENG	21		f	0	0	all	-	a	As:Chi	1982	CC	540	n	ot	*	y	0	cig+/-ot	nev cigs	st
ZHOU	26		m	0	0	all	-	a	As:Chi	1978	CC	1360	n	ot	n	n	0	all/unsp	nev any	st
ZHOU	27		f	0	0	all	-	a	As:Chi	1978	CC	1360	n	ot	n	n	0	all/unsp	nev any	st

Cigarette type is all/unspec for all RRs

except for the following:

REF	NRR	CIGTYPE
ALDERS	104	MC only
CHAN	19	MC+-HR
CHAN	23	MC+-HR
JUSSAW	28	MC only

Table 3A2 - 5

IESLC - Meta-analysis of Ever Smoking, Cigarettes (or Any Product if Cigarettes not available)
Adenocarcinoma
Least adjusted

REF	NRR	SEX	AD	Number Exposed Case	Number Exposed Cont	Non-exposed Case	Non-exposed Cont	RR	95.00%CI
*ABRAHA	2	m	0	59	10351	8	3365	2.40	(1.15- 5.01)
*ABRAHA	5	f	0	19	5256	16	11589	2.62	(1.35- 5.09)
Subtotal ABRAHA								2.52	(1.54- 4.12)
ALDERS	108	m	0	141	641	6	133	4.88	(2.11- 11.27)
ALDERS	104	f	0	104	371	25	243	2.72	(1.71- 4.34)
Subtotal ALDERS								3.13	(2.08- 4.70)
*ANDERS	12	f	0	99	96164	33	195158	6.09	(4.11- 9.03)
BAND	2	m	2	-	-	-	-	4.10	(3.01- 5.59)
BARBON	122	m	0	151	567	7	188	7.15	(3.29- 15.53)
BECHER	12	f	1	-	-	-	-	10.83	(1.32- 88.70)
BRESLO	1	c	0	40	394	4	56	1.42	(0.49- 4.12)
BROWN1	1	m	0	46	46	4	19	4.75	(1.50- 15.05)
BROWN1	2	f	0	33	19	19	47	4.30	(1.98- 9.34)
Subtotal BROWN1								4.43	(2.33- 8.44)
BROWN2	4	m	2	-	-	-	-	8.20	(6.90- 9.70)
BROWN2	3	f	2	-	-	-	-	6.90	(6.10- 7.90)
Subtotal BROWN2								7.35	(6.63- 8.15)
BUFFLE	50	m	0	-	-	-	-	4.50	(1.85- 10.95)
BUFFLE	45	f	0	-	-	-	-	4.02	(2.42- 6.67)
Subtotal BUFFLE								4.13	(2.66- 6.42)
BYERS1	3	m	0	47	695	7	424	4.10	(1.83- 9.15)
CHAN	19	m	0	56	160	0	43	30.63~	(1.85- 505.72)
CHAN	23	f	0	24	38	40	139	2.19	(1.18- 4.08)
Subtotal CHAN								2.48	(1.35- 4.55)
CHOI	63	m	0	46	465	7	95	1.34	(0.59- 3.06)
CHOI	65	f	0	5	26	49	164	0.64	(0.23- 1.77)
Subtotal CHOI								1.00	(0.53- 1.89)
COMSTO	67	m	0	43	229	2	84	7.89	(1.87- 33.27)
COMSTO	79	f	0	29	87	8	115	4.79	(2.09- 11.00)
Subtotal COMSTO								5.43	(2.64- 11.14)
CORREA	36	c	1	-	-	-	-	5.60	(3.60- 8.80)
DAMBER	11	m	0	65	49	16	29	2.40	(1.18- 4.91)
DESTE2	17	m	2	-	-	-	-	4.30	(1.60- 11.40)
DOLL	83	m	0	38	1296	2	61	0.89	(0.21- 3.79)
DOLL	85	f	0	8	49	5	59	1.93	(0.59- 6.27)
Subtotal DOLL								1.42	(0.57- 3.53)
DORGAN	125	m	2	-	-	-	-	4.80	(1.90- 12.00)
DORGAN	104	f	3	-	-	-	-	3.90	(2.80- 5.40)
Subtotal DORGAN								3.99	(2.93- 5.44)
DOSEME	20	m	0	142	536	24	293	3.23	(2.05- 5.10)
*ENGELA	76	m	7	-	-	-	-	2.33	(0.92- 5.89)
FAN	4	c	0	67	595	45	556	1.39	(0.94- 2.07)
GAO	8	m	0	180	558	42	202	1.55	(1.07- 2.25)
GAO	18	f	0	62	130	266	605	1.08	(0.78- 1.52)
Subtotal GAO								1.27	(0.99- 1.63)
GER	1	c	0	35	139	37	149	1.01	(0.60- 1.70)
HAENSZ	39	f	0	18	103	37	236	1.11	(0.61- 2.05)
*HAMMON	91	m	0	26	382338	2	115884	3.94	(0.94- 16.60)
HEGMAN	4	c	0	83	1202	15	2080	9.58	(5.50- 16.67)
HINDS	24	f	3	-	-	-	-	3.89	(2.49- 6.07)
ISHIMA	3	c	0	39	25	13	27	3.24	(1.41- 7.43)
JAHN	43	m	0	202	671	8	138	5.19	(2.50- 10.77)
JAIN	7	m	0	90	277	4	85	6.90	(2.46- 19.35)
JAIN	2	f	0	86	196	24	214	3.91	(2.39- 6.40)
Subtotal JAIN								4.35	(2.79- 6.78)
JEDRYC	21	m	0	99	1054	7	289	3.88	(1.78- 8.44)
JOLY	51	m	0	72	709	5	218	4.43	(1.77- 11.10)
JOLY	50	f	0	33	122	25	283	3.06	(1.75- 5.37)
Subtotal JOLY								3.38	(2.10- 5.46)
JUSSAW	28	m	0	3	77	13	624	1.87	(0.52- 6.71)
KATSOU	31	f	0	18	22	30	67	1.83	(0.86- 3.90)
KHUDER	27	m	0	155	-	7	-	8.11	(3.67- 17.93)
KIHARA	29	c	0	130	232	78	237	1.70	(1.22- 2.38)
KOO	7	f	0	34	63	46	137	1.61	(0.94- 2.74)
KREYBE	20	m	0	42	3514	3	644	2.57	(0.79- 8.30)
KREYBE	36	f	0	10	328	27	657	0.74	(0.35- 1.55)
Subtotal KREYBE								1.05	(0.56- 1.97)
LAMTH	3	f	0	79	51	131	158	1.87	(1.23- 2.85)
LAMWK	4	f	0	36	41	60	144	2.11	(1.23- 3.61)
LAMWK2	3	m	0	52	161	15	43	0.93	(0.48- 1.80)
LAMWK2	7	f	0	26	50	41	139	1.76	(0.98- 3.17)

Table 3A2 - 5

IESLC - Meta-analysis of Ever Smoking, Cigarettes (or Any Product if Cigarettes not available)
Adenocarcinoma
Least adjusted

REF	NRR	SEX	AD	Number Exposed Case	Number Exposed Cont	Non-exposed Case	Non-exposed Cont	RR	95.00%CI
Subtotal LAMWK2								1.33	(0.86- 2.07)
LOMBA2	3	f	0	42	353	54	239	0.53	(0.34- 0.81)
LUBIN	37	m	0	32	788	4	72	0.73	(0.25- 2.12)
LUBIN2	148	m	0	655	10433	57	2616	2.88	(2.19- 3.79)
LUBIN2	168	f	0	85	567	138	1180	1.28	(0.96- 1.71)
Subtotal LUBIN2								1.96	(1.61- 2.39)
LUO	3	c	0	28	146	29	160	1.06	(0.60- 1.86)
MATOS	68	m	0	79	283	5	110	6.14	(2.42- 15.57)
MATSUD	12	m	0	23	3314	0	1255	17.80~	(1.08- 293.32)
NOU	3	m	0	36	247	4	122	4.45	(1.55- 12.77)
NOU	8	f	0	9	92	29	261	0.88	(0.40- 1.93)
Subtotal NOU								1.57	(0.83- 2.94)
ORMOS	21	m	0	4	1034	0	777	6.76~	(0.36- 125.82)
OSANN	19	m	0	319	1018	14	833	18.64	(10.83- 32.09)
OSANN	23	f	0	243	563	47	1093	10.04	(7.23- 13.94)
Subtotal OSANN								11.85	(8.95- 15.69)
OSANN2	13	f	0	61	40	22	43	2.98	(1.56- 5.71)
PEZZOT	7	m	0	60	317	3	116	7.32	(2.25- 23.79)
SCHWAR	8	m	0	84	178	1	73	34.45	(4.71- 252.10)
SCHWAR	7	m	0	45	39	1	7	8.08	(0.95- 68.56)
SCHWAR	16	f	0	92	108	10	79	6.73	(3.29- 13.75)
SCHWAR	15	f	0	20	28	3	41	9.76	(2.65- 36.00)
Subtotal SCHWAR								8.40	(4.73- 14.94)
SEOW	2	f	0	19	15	67	125	2.36	(1.13- 4.95)
SIEMIA	12	m	0	162	428	5	105	7.95	(3.18- 19.85)
SOBUE	7	m	0	393	1013	27	128	1.84	(1.20- 2.83)
SOBUE	23	f	0	58	232	137	857	1.56	(1.11- 2.20)
Subtotal SOBUE								1.66	(1.27- 2.17)
STASZE	24	m	0	20	653	0	158	9.94~	(0.60- 165.30)
STASZE	4	f	0	1	153	10	1660	1.08	(0.14- 8.53)
Subtotal STASZE								2.36	(0.45- 12.42)
STAYNE	4	m	0	43	567	7	333	3.61	(1.60- 8.11)
SUZUK2	13	c	0	20	10	5	15	6.00	(1.69- 21.26)
SUZUKI	3	m	0	144	217	14	99	4.69	(2.58- 8.53)
SUZUKI	7	f	0	25	27	55	133	2.24	(1.19- 4.20)
Subtotal SUZUKI								3.30	(2.14- 5.09)
SVENSS	59	f	0	50	89	22	120	3.06	(1.73- 5.43)
TIZZAN	19	c	0	88	939	25	419	1.57	(0.99- 2.49)
TOKARS	7	c	0	68	112	10	54	3.28	(1.57- 6.86)
TSUGAN	2	m	0	53	56	18	17	0.89	(0.42- 1.91)
TSUGAN	8	f	0	6	10	33	30	0.55	(0.18- 1.68)
Subtotal TSUGAN								0.77	(0.41- 1.44)
WAKAI	17	m	0	98	424	8	65	1.88	(0.87- 4.04)
WAKAI	35	f	0	13	31	46	145	1.32	(0.64- 2.74)
Subtotal WAKAI								1.56	(0.92- 2.65)
WU	5	f	0	120	87	29	62	2.95	(1.75- 4.96)
WUWILL	25	f	0	138	351	172	601	1.37	(1.06- 1.78)
WYNDE2	9	m	0	46	512	5	105	1.89	(0.73- 4.86)
WYNDE3	28	m	0	64	264	6	88	3.56	(1.49- 8.49)
WYNDE3	75	f	0	21	56	15	76	1.90	(0.90- 4.01)
Subtotal WYNDE3								2.48	(1.41- 4.37)
WYNDE4	42	m	0	35	665	4	115	1.51	(0.53- 4.34)
WYNDE4	56	f	2	-	-	-	-	0.60	(0.13- 2.69)
Subtotal WYNDE4								1.12	(0.47- 2.66)
WYNDE6	78	m	0	1059	1797	58	617	6.27	(4.74- 8.29)
WYNDE6	413	f	0	326	275	58	673	13.76	(10.06- 18.80)
Subtotal WYNDE6								8.89	(7.22- 10.95)
XU3	21	m	0	29	68	3	31	4.41	(1.25- 15.57)
XU3	25	f	0	4	11	7	25	1.30	(0.31- 5.36)
Subtotal XU3								2.57	(1.00- 6.59)
ZHENG	10	m	0	123	218	29	94	1.83	(1.14- 2.93)
ZHENG	21	f	0	33	44	119	184	1.16	(0.70- 1.93)
Subtotal ZHENG								1.48	(1.05- 2.09)
ZHOU	26	m	0	131	41	88	36	1.31	(0.77- 2.20)
ZHOU	27	f	0	30	7	96	32	1.43	(0.57- 3.57)
Subtotal ZHOU								1.34	(0.85- 2.10)
Partial Totals				8209	539017	2862	352399		

*prospective study ~ With 0.5 adjustment for zero

Table 3A2 - 5

IESLC - Meta-analysis of Ever Smoking, Cigarettes (or Any Product if Cigarettes not available)
Adenocarcinoma
Least adjusted

REF	NRR	SEX	AD	Ys	Ws	Qs	Ps
*ABRAHA	2	m	0	0.87	7.06	0.81	0.0201
*ABRAHA	5	f	0	0.96	8.71	0.55	0.0045
Subtotal ABRAHA				0.92	15.77	1.36	
ALDERS	108	m	0	1.58	5.47	0.75	0.0002
ALDERS	104	f	0	1.00	17.72	0.79	0.0000
Subtotal ALDERS				1.14	23.19	1.54	
*ANDERS	12	f	0	1.81	24.76	8.69	0.0000
BAND	2	m	2	1.41	40.10	1.56	0.0000
BARBON	122	m	0	1.97	6.39	3.63	0.0000
BECHER	12	f	1	2.38	0.87	1.18	0.0265
BRESLO	1	c	0	0.35	3.39	2.52	0.5177
BROWN1	1	m	0	1.56	2.89	0.34	0.0081
BROWN1	2	f	0	1.46	6.38	0.38	0.0002
Subtotal BROWN1				1.49	9.27	0.72	
BROWN2	4	m	2	2.10	132.45	104.97	0.0000
BROWN2	3	f	2	1.93	229.82	118.35	0.0000
Subtotal BROWN2				1.99	362.27	223.32	
BUFFLE	50	m	0	1.50	4.86	0.41	0.0009
BUFFLE	45	f	0	1.39	14.95	0.47	0.0000
Subtotal BUFFLE				1.42	19.81	0.88	
BYERS1	3	m	0	1.41	5.95	0.23	0.0006
CHAN	19	m	0	3.42	0.49	2.38	0.0168
CHAN	23	f	0	0.79	9.98	1.83	0.0130
Subtotal CHAN				0.91	10.47	4.21	
CHOI	63	m	0	0.29	5.64	4.77	0.4842
CHOI	65	f	0	-0.44	3.77	10.33	0.3920
Subtotal CHOI				-0.00	9.42	15.10	
COMSTO	67	m	0	2.07	1.85	1.34	0.0049
COMSTO	79	f	0	1.57	5.57	0.69	0.0002
Subtotal COMSTO				1.69	7.42	2.04	
CORREA	36	c	1	1.72	19.23	4.98	0.0000
DAMBER	11	m	0	0.88	7.53	0.85	0.0161
DESTE2	17	m	2	1.46	3.99	0.24	0.0036
DOLL	83	m	0	-0.11	1.84	3.23	0.8795
DOLL	85	f	0	0.66	2.76	0.86	0.2760
Subtotal DOLL				0.35	4.60	4.09	
DORGAN	125	m	2	1.57	4.52	0.57	0.0008
DORGAN	104	f	3	1.36	35.62	0.77	0.0000
Subtotal DORGAN				1.38	40.15	1.34	
DOSEME	20	m	0	1.17	18.52	0.03	0.0000
*ENGELA	76	m	7	0.85	4.46	0.60	0.0741
FAN	4	c	0	0.33	24.61	19.22	0.1013
GAO	8	m	0	0.44	27.69	16.62	0.0208
GAO	18	f	0	0.08	34.21	43.88	0.6343
Subtotal GAO				0.24	61.90	60.50	
GER	1	c	0	0.01	14.39	20.72	0.9579
HAENSZ	39	f	0	0.11	10.36	12.66	0.7268
*HAMMON	91	m	0	1.37	1.86	0.05	0.0617
HEGMAN	4	c	0	2.26	12.50	13.65	0.0000
HINDS	24	f	3	1.36	19.35	0.40	0.0000
ISHIMA	3	c	0	1.18	5.57	0.01	0.0055
JAHN	43	m	0	1.65	7.21	1.35	0.0000
JAIN	7	m	0	1.93	3.62	1.87	0.0002
JAIN	2	f	0	1.36	15.86	0.36	0.0000
Subtotal JAIN				1.47	19.47	2.22	
JEDRYC	21	m	0	1.36	6.35	0.13	0.0006
JOLY	51	m	0	1.49	4.55	0.34	0.0015
JOLY	50	f	0	1.12	12.19	0.11	0.0001
Subtotal JOLY				1.22	16.74	0.45	
JUSSAW	28	m	0	0.63	2.35	0.81	0.3368
KATSOU	31	f	0	0.60	6.70	2.50	0.1187
KHUDER	27	m	0	2.09	6.11	4.72	0.0000
KIHARA	29	c	0	0.53	34.43	16.00	0.0018
KOO	7	f	0	0.47	13.45	7.35	0.0817
KREYBE	20	m	0	0.94	2.79	0.21	0.1158
KREYBE	36	f	0	-0.30	7.06	16.15	0.4275
Subtotal KREYBE				0.05	9.85	16.36	
LAMTH	3	f	0	0.63	21.63	7.50	0.0036
LAMWK	4	f	0	0.75	13.20	2.90	0.0068
LAMWK2	3	m	0	-0.08	8.67	14.45	0.8206
LAMWK2	7	f	0	0.57	11.11	4.65	0.0588

Table 3A2 - 5

IESLC - Meta-analysis of Ever Smoking, Cigarettes (or Any Product if Cigarettes not available)
Adenocarcinoma
Least adjusted

REF	NRR	SEX	AD	Ys	Ws	Qs	Ps
Subtotal LAMWK2				0.28	19.77	19.09	
LOMBA2	3	f	0	-0.64	20.27	69.75	0.0039
LUBIN	37	m	0	-0.31	3.37	7.87	0.5649
LUBIN2	148	m	0	1.06	51.15	1.24	0.0000
LUBIN2	168	f	0	0.25	46.25	43.12	0.0913
Subtotal LUBIN2				0.67	97.40	44.36	
LUO	3	c	0	0.06	12.01	16.08	0.8449
MATOS	68	m	0	1.82	4.44	1.60	0.0001
MATSUD	12	m	0	2.88	0.49	1.36	0.0440
NOU	3	m	0	1.49	3.45	0.27	0.0056
NOU	8	f	0	-0.13	6.24	11.22	0.7504
Subtotal NOU				0.45	9.69	11.49	
ORMOS	21	m	0	1.91	0.45	0.22	0.1999
OSANN	19	m	0	2.93	13.03	38.18	0.0000
OSANN	23	f	0	2.31	35.61	42.49	0.0000
Subtotal OSANN				2.47	48.64	80.67	
OSANN2	13	f	0	1.09	9.08	0.13	0.0010
PEZZOT	7	m	0	1.99	2.76	1.67	0.0009
SCHWAR	8	m	0	3.54	0.97	5.24	0.0005
SCHWAR	7	m	0	2.09	0.84	0.64	0.0556
SCHWAR	16	f	0	1.91	7.53	3.61	0.0000
SCHWAR	15	f	0	2.28	2.26	2.56	0.0006
Subtotal SCHWAR				2.13	11.60	12.06	
SEOW	2	f	0	0.86	7.03	0.88	0.0226
SIEMIA	12	m	0	2.07	4.59	3.38	0.0000
SOBUE	7	m	0	0.61	20.67	7.55	0.0056
SOBUE	23	f	0	0.45	33.31	19.59	0.0099
Subtotal SOBUE				0.51	53.98	27.14	
STASZE	24	m	0	2.30	0.49	0.57	0.1092
STASZE	4	f	0	0.08	0.90	1.16	0.9382
Subtotal STASZE				0.86	1.39	1.73	
STAYNE	4	m	0	1.28	5.85	0.03	0.0019
SUZUK2	13	c	0	1.79	2.40	0.80	0.0055
SUZUKI	3	m	0	1.55	10.74	1.18	0.0000
SUZUKI	7	f	0	0.81	9.73	1.62	0.0119
Subtotal SUZUKI				1.19	20.48	2.80	
SVENSS	59	f	0	1.12	11.76	0.10	0.0001
TIZZAN	19	c	0	0.45	18.24	10.60	0.0538
TOKARS	7	c	0	1.19	7.03	0.00	0.0016
TSUGAN	2	m	0	-0.11	6.62	11.64	0.7728
TSUGAN	8	f	0	-0.61	3.03	10.03	0.2916
Subtotal TSUGAN				-0.27	9.65	21.67	
WAKAI	17	m	0	0.63	6.54	2.23	0.1071
WAKAI	35	f	0	0.28	7.26	6.34	0.4522
Subtotal WAKAI				0.45	13.79	8.57	
WU	5	f	0	1.08	14.20	0.25	0.0000
WUWILL	25	f	0	0.32	56.90	45.72	0.0166
WYNDE2	9	m	0	0.63	4.29	1.44	0.1887
WYNDE3	28	m	0	1.27	5.06	0.02	0.0043
WYNDE3	75	f	0	0.64	6.88	2.25	0.0922
Subtotal WYNDE3				0.91	11.95	2.27	
WYNDE4	42	m	0	0.41	3.46	2.21	0.4408
WYNDE4	56	f	2	-0.51	1.67	4.98	0.5087
Subtotal WYNDE4				0.11	5.14	7.19	
WYNDE6	78	m	0	1.84	49.11	18.98	0.0000
WYNDE6	413	f	0	2.62	39.32	77.90	0.0000
Subtotal WYNDE6				2.19	88.43	96.88	
XU3	21	m	0	1.48	2.41	0.17	0.0213
XU3	25	f	0	0.26	1.91	1.73	0.7180
Subtotal XU3				0.94	4.32	1.91	
ZHENG	10	m	0	0.60	17.29	6.44	0.0121
ZHENG	21	f	0	0.15	14.95	16.99	0.5668
Subtotal ZHENG				0.39	32.24	23.43	
ZHOU	26	m	0	0.27	14.05	12.58	0.3154
ZHOU	27	f	0	0.36	4.59	3.37	0.4448
Subtotal ZHOU				0.29	18.64	15.95	

Table 3A2 - 5

IESLC - Meta-analysis of Ever Smoking, Cigarettes (or Any Product if Cigarettes not available)
Adenocarcinoma
Least adjusted

	N	107
	NS	75
	Wt	1577.85
Het	Chi	1007.74
Het	df	106
Het	P	***
Fixed	RR	3.37
	RRl	3.20
	RRu	3.54
	P	+++
Random	RR	2.80
	RRl	2.37
	RRu	3.31
	P	+++
Asymm	P	**

Table 3A2 - 6

IESLC - Meta-analysis of Ever Smoking, Cigarettes (or Any Product if Cigarettes not available)
Adenocarcinoma
Least adjusted

		Sex			
		combined	male	female	Total
	N	11	51	45	107
	NS	11	50	44	105
	Wt	153.80	557.34	866.71	1577.85
Het	Chi	73.30	272.24	607.26	1007.74
Het	df	10	50	44	106
Het	P	***	***	***	***
Fixed	RR	2.14	4.10	3.21	3.37
	RRl	1.83	3.77	3.01	3.20
	RRu	2.51	4.46	3.43	3.54
	P	+++	+++	+++	+++
Random	RR	2.38	3.52	2.29	2.80
	RRl	1.51	2.81	1.75	2.37
	RRu	3.74	4.41	3.00	3.31
	P	+++	+++	+++	+++
Between	Chi				54.94
Between	df				2
Between	P				***
Btwn(F)	P				(*)
Btwn(R)	P				*

Table 3A2 - 7

IESLC - Meta-analysis of Ever Smoking, Cigarettes (or Any Product if Cigarettes not available)
Adenocarcinoma
Excluded studies (and stage at which they were excluded)

1	BOUCHA BUELL LAURIL MZILEN
2	BYERS2 ENSTRO HIRAY2 KANELL LIAW LICKIN MRFIT MURATA NOTANI RESTRE RIMING SAARIK SEGI2 SHIMIZ SITAS SOBUE2 TANG2 WALD WARSIN WATSON WU2 WYNDE5 WYNDE8 WYNDER
3	BERRIN CHEN TAO ULMER
6	BLOT1 BLOT2 BLOT3 HIRAOK JONES MOLLO PERSHA SCHWA2 VANDER
8	ABELIN AGUDO AKIBA AMANDU AMES ARCHER ARMADA AUSTIN AUVINE AXELSO AXELSS BENSHL BEST BLOHMK BLOT4 BOFFET BOUCOT BRETT BROCKM BROSS CARPEN CASCO2 CASCOR CEDERL CHANG CHATZI CHEN2 CHEN3 CHIAZZ CHOW CHYOU COOKSO CPSI CPSII DARBY DAVEYS DEAN DEAN2 DEAN3 DEKLER DESTEF DOCKER DOLL2 DORANT DORN DROSTE DU DUNN EBELIN ESAKI GAO2 GARCIA GARDIN GARSHI GENG GILLIS GODLEY GOLLED GOODMA GRAHAM GREGOR GSELL HAMMO2 HANSEN HEIN HENNEK HIRAYA HITOSU HOLE HOROWI HORWIT HU HU2 HUANG HUMBLE JARUP JARVHO JIANG KAISE2 KAISER KAUFMA KELLER KINLEN KJUUS KNEKT KO KOHLME KOULUM KREUZE KUBIK LANGE LAUSSM LEI LEMARC LETOUR LEVIN LIDDEL LIU LIU2 LIU3 LIU4 LIU5 LOMBAR MACLEN MAGNUS MARSH MARSH2 MARTIS MASTRA MCCONN MCDUFF MCLAUG MIGRAN MILLER MILLS MRFITR NAM NOTAN2 ODRISC PARKIN PASTOR PAWLEG PERNU PERSH2 PETO PEZZO2 PIKE PISANI POFFIJ POLEDN PRESCO QIAO QIAO2 RACHTA RADZIK RANDIG REN RONCO ROOTS ROTHSC SADOWS SANKAR SEGI SHAW SIMARA SIMONA SPEIZE SPITZ STOCKS STOCKW STUCKE SUN TANG TENKAN TOUSEY TULINI TVERDA VEIERO WANG WANG2 WANG3 WANG4 WICKLU WIGLE WILKIN WUNSCH XIANGZ XU XU2 XU4 YAMAGU YONG YUAN ZHANG
10	BENHAM VUTUC WYNDE7

Table 3A2 - 8
Potentially overlapping studies

REF	REFGP	PRINC	OVERLAP/LINK
LUBIN2	LUBIN2	1	Lubin-combined
CHAN	CHAN	1	Chan/LamWK2
LAMWK2	LAMWK2	1	Chan/LamWK2
LAMTH	LAMTH	1	KOO/LAMTH/LAMWK
KOO	KOO	1	KOO/LAMTH/LAMWK
LAMWK	LAMWK	1	KOO/LAMTH/LAMWK
ISHIMA	AKIBA	2	AKIBA/ISHIMA
BYERS1	BYERS1	2	GRAHAM/BROSS/BYERS1
OSANN2	KAISER	2	KAISER/OSANN2
WYNDE6	WYNDE6	1	WYNDE5/6/7/8
MATSUD	MATSUD	1	SOBUE2/MATSUD
JAHN	BOFFET	2	Subset of BOFFET
LUBIN	XIANGZ	2	LUBIN/XIANGZ/QIAO

Table 3A2 - 9
Most adjusted - insufficient data for metaanalysis

REF	NRR	SEX	AGEL	AGEH	RACE	YF	LC TYPE	LOC	START	ST	NLC	R	VB	P	H	AD	PRODUCT	DENOM	De
CHEN	11	c	0	0	all	-	a	As:oth	1987	CC	323	n	ot	n	y	2	cig+/-ot	nev cigs	ot
JIANG	5	m	0	0	all	-	a	As:Chi	1984	CC	125	n	ot	n	n	0	all/unsp	nev any	ot
JIANG	6	f	0	0	all	-	a	As:Chi	1984	CC	125	n	ot	n	n	0	all/unsp	nev any	ot
KOO	4	f	0	0	all	-	a	As:HK	1981	CC	200	n	bl	n	n	0	all/unsp	nev any	or
WANG	4	c	0	0	all	-	a	As:Chi	1990	CC	390	n	ot	*	y	0	all/unsp	nev any	ot

REF	NRR	RR	SIG	RRDATA comment
CHEN	11	2.08	y	P < 0.001
JIANG	5	*	n	0
JIANG	6	*	n	0
KOO	4	1.88		0
WANG	4	2.22	n	P>0.05

Table 3A3 -

IESLC - Meta-analysis of Ever Smoking, Cigarettes only
Adenocarcinoma

This analysis is restricted to results for:
1) Non-dose-response data
2) Ever smokers
3) Results complete enough for use in metaanalysis

Within each study, results are then selected (in the following order of preference, within each sex) for:
4) PRODUCT: cigarettes only
5) CIGTYPE: all/unspecified, MC regardless of HR, MC only
6) DENOM: never smoked anything, never smoked cigarettes, (never +1 = +long term ex, +2 = +amount unknown,
+3 = never cigs+long term ex)
7) Followup period (YF, prospective studies): whole study (coded as 0) or longest available
8) LCtype: adeno or nearest available, but not squamous. (q = squamous, s = small,
a = adeno, l = large, KII = Kreyberg II, al = alveolar, br = bronchiolar, u = undifferentiated)
9) Race: all or nearest available, otherwise by race (wh or w = white, bl or b = black, hi = hispanic
ch = chinese, jap = japanese, haw = hawaiian, w+o = white + oriental, sca = scandinavian, as = asian)
10) For overlapping studies: principal rather than subsidiary studies
Finally by Age: whole study (coded as 0) if available, otherwise by widest available age group
and then for single sex results (m, f) in preference to combined sex results (c).

Results adjusted (AD) for the most potential confounders are then chosen in Sections -1 to -3
(and those which actually differ from the adjusted results in Table 3A1 - 1 are marked 'x' in Section -1)
and results adjusted for the least confounders in Sections -4 to -6. (Those least adjusted results which
actually differ from the most adjusted as marked 'x' in column X in Section -4)
(Results adjusted for an unknown number of confounder(s) are coded as 20.)

Section -7 shows excluded studies, together with the stage (as above) at which no qualifying
results were found.

Section -8 lists the potentially overlapping studies which have been included (1=principal, 2=subsidiary).

Section -9 lists any results which would have been included in preference except that they had data not complete
enough for use in meta-analysis, with their significance (yes/no), if known, and any further comment as entered
on the database.

In addition to those mentioned above, the following fields, levels and abbreviations are used:
* or nk = not known, n = no, y = yes, ot = other
nev = never
all/unspec = all or unspecified, MC = manufactured cigarettes, HR = hand-rolled cigarettes
REF: 6-character study reference
NRR: number of the RR on the database within the study
ST : study type (CC = case control, pr or prosp = prospective)
NLC: number of lung cancer cases in whole study
R : risky occupational population (n = no, m = mining, o = other risky)
VB : national cigarette type (V = at least 75% Virginia, bl = at least 75% blended, ot = other)
P : any proxy use
H : full histological confirmation
De : derivation of RR/CI (or = original, st = standard method, ot = other method of estimation)

Table 3A3 - 1

IESLC - Meta-analysis of Ever Smoking, Cigarettes only
Adenocarcinoma
Most adjusted

REF	NRR	3A1	SEX	AGEL	AGEH	RACE	YF	LC TYPE	LOC	START	ST	NLC	R	VB	P	H	AD	PRODUCT	DENOM	De
ALDERS	94	x	m	0	0	all	-	not q+s	Eu:UK	1977	CC	1448	n	V	n	n	1	cig only	nev any	ot
ALDERS	45	x	f	0	0	all	-	not q+s	Eu:UK	1977	CC	1448	n	V	n	n	1	cig only	nev any	ot
BAND	2		m	0	0	all	-	a	NAmer	1983	CC	2831	n	V	y	y	2	cig only	nev any	ot
BRESLO	2	x	c	0	0	all	-	a	NAmer	1949	CC	518	n	bl	n	y	0	cig only	nev+1	st
HAMMON	83	x	m	0	0	wh	0	a	NAmer	1952	pr	448	n	bl	n	n	1	cig only	nev any	ot
JUSSAW	28	x	m	0	0	all	-	KII	As:Ind	1964	CC	792	n	V	n	n	0	cig only	nev any	st
LUBIN	18	x	m	0	0	all	-	KII	As:Chi	1984	CC	427	m	ot	y	n	0	cig only	nev any	st
LUBIN2	144	x	m	0	0	all	-	a	Eu:mul	1976	CC	7804	n	bl	n	y	0	cig only	nev any	st
PEZZOT	7		m	0	0	all	-	a	SCAmer	1987	CC	215	n	bl	n	y	0	cig only	nev cigs	st
STASZE	22	x	m	0	0	all	-	a	Eu:est	1954	CC	281	n	bl	n	y	0	cig only	nev any	ot
WYNDE7	64	x	m	0	0	all	-	KII	NAmer	1977	CC	2085	n	bl	n	y	0	cig only	nev any	st

Cigarette type is all/unspec for all RRs

except for the following:

REF	NRR	CIGTYPE
ALDERS	45	MC only
JUSSAW	28	MC only

Table 3A3 - 2

IESLC - Meta-analysis of Ever Smoking, Cigarettes only
Adenocarcinoma
Most adjusted

REF	NRR	SEX	AD	Number Exposed Case	Cont	Non-exposed Case	Cont	RR	95.00%CI
ALDERS	94	m	1	-	-	-	-	4.53	(1.96- 10.50)
ALDERS	45	f	1	-	-	-	-	3.69	(2.32- 5.88)
Subtotal ALDERS								3.87	(2.58- 5.82)
BAND	2	m	2	-	-	-	-	4.10	(3.01- 5.59)
BRESLO	2	c	0	31	240	4	56	1.81	(0.61- 5.33)
*HAMMON	83	m	1	-	-	-	-	3.39	(0.77- 14.91)
JUSSAW	28	m	0	3	77	13	624	1.87	(0.52- 6.71)
LUBIN	18	m	0	5	191	4	72	0.47	(0.12- 1.80)
LUBIN2	144	m	0	686	9345	195	2616	0.98	(0.84- 1.16)
PEZZOT	7	m	0	60	317	3	116	7.32	(2.25- 23.79)
STASZE	22	m	0	15	552	0	158	8.89~	(0.53- 149.45)
WYNDE7	64	m	0	645	2108	42	918	6.69	(4.85- 9.22)
Partial Totals				1445	12830	261	4560		

*prospective study ~ With 0.5 adjustment for zero

REF	NRR	SEX	AD	Ys	Ws	Qs	Ps
ALDERS	94	m	1	1.51	5.45	3.99	0.0004
ALDERS	45	f	1	1.31	17.77	7.50	0.0000
Subtotal ALDERS				1.35	23.22	11.49	
BAND	2	m	2	1.41	40.10	22.87	0.0000
BRESLO	2	c	0	0.59	3.29	0.01	0.2828
*HAMMON	83	m	1	1.22	1.75	0.56	0.1063
JUSSAW	28	m	0	0.63	2.35	0.00	0.3368
LUBIN	18	m	0	-0.75	2.13	4.23	0.2719
LUBIN2	144	m	0	-0.02	141.34	63.65	0.8555
PEZZOT	7	m	0	1.99	2.76	4.92	0.0009
STASZE	22	m	0	2.19	0.48	1.13	0.1290
WYNDE7	64	m	0	1.90	37.14	57.53	0.0000

	N	11
	NS	10
	Wt	254.57
Het	Chi	166.39
Het	df	10
Het	P	***
Fixed	RR	1.93
	RRl	1.70
	RRu	2.18
	P	+++
Random	RR	2.87
	RRl	1.49
	RRu	5.55
	P	++
Asymm	P	N.S.

Table 3A3 - 3

IESLC - Meta-analysis of Ever Smoking, Cigarettes only
Adenocarcinoma
Most adjusted

	Sex			
	combined	male	female	Total
N	1	9	1	11
NS	1	9	1	11
Wt	3.29	233.51	17.77	254.57
Het Chi	0.00	158.33	0.00	166.39
Het df	0	8	0	10
Het P	N.S.	***	N.S.	***
Fixed RR	1.81	1.84	3.69	1.93
RRl	0.61	1.61	2.32	1.70
RRu	5.33	2.09	5.87	2.18
P	N.S.	+++	+++	+++
Random RR	1.81	2.93	3.69	2.87
RRl	0.61	1.34	2.32	1.49
RRu	5.33	6.39	5.87	5.55
P	N.S.	++	+++	++
Between Chi				8.07
Between df				2
Between P				*
Btwn(F) P				N.S.
Btwn(R) P				N.S.

	Lung cancer type						
	a	a+l	a+al+br	KII	not q+u	not q+s	Total
N	6			3		2	11
NS	6			3		1	10
Wt	189.72			41.63		23.22	254.57
Het Chi	74.57			17.02		0.18	166.39
Het df	5			2		1	10
Het P	***			***		N.S.	***
Fixed RR	1.41			5.43		3.87	1.93
RRl	1.22			4.01		2.58	1.70
RRu	1.62			7.36		5.82	2.18
P	+++			+++		+++	+++
Random RR	2.91			1.99		3.87	2.87
RRl	1.18			0.38		2.58	1.49
RRu	7.15			10.29		5.82	5.55
P	+			N.S.		+++	++
Between Chi							74.62
Between df							2
Between P							***
Btwn(F) P							(*)
Btwn(R) P							N.S.

	Location								
	NAmer	UK	Scand	othEur	China	Japan	othAs	other	Total
N	4	2		2	1		1	1	11
NS	4	1		2	1		1	1	10
Wt	82.28	23.22		141.82	2.13		2.35	2.76	254.57
Het Chi	8.37	0.18		2.33	0.00		0.00	0.00	166.39
Het df	3	1		1	0		0	0	10
Het P	*	N.S.		N.S.	N.S.		N.S.	N.S.	***
Fixed RR	4.93	3.87		0.99	0.47		1.87	7.32	1.93
RRl	3.97	2.58		0.84	0.12		0.52	2.25	1.70
RRu	6.12	5.82		1.17	1.80		6.71	23.79	2.18
P	+++	+++		N.S.	N.S.		N.S.	+++	+++
Random RR	4.38	3.87		1.85	0.47		1.87	7.32	2.87
RRl	2.75	2.58		0.26	0.12		0.52	2.25	1.49
RRu	6.97	5.82		13.02	1.80		6.71	23.79	5.55
P	+++	+++		N.S.	N.S.		N.S.	+++	++
Between Chi									155.52
Between df									5
Between P									***
Btwn(F) P									**
Btwn(R) P									*

Table 3A3 - 3

IESLC - Meta-analysis of Ever Smoking, Cigarettes only
Adenocarcinoma
Most adjusted

Detailed Country in "other Europe"

	multi	Germany	othWest	East	Balkans	Total
N	1			1		2
NS	1			1		2
Wt	141.34			0.48		141.82
Het Chi	0.00			0.00		2.33
Het df	0			0		1
Het P	N.S.			N.S.		N.S.
Fixed RR	0.98			8.89		0.99
RRl	0.84			0.53		0.84
RRu	1.16			149.45		1.17
P	N.S.			N.S.		N.S.
Random RR	0.98			8.89		1.85
RRl	0.84			0.53		0.26
RRu	1.16			149.45		13.02
P	N.S.			N.S.		N.S.
Between Chi						2.33
Between df						1
Between P						N.S.
Btwn(F) P						N.S.
Btwn(R) P						N.S.

Detailed Country in "other Asia"

	India	HongKong	other	Total
N	1			1
NS	1			1
Wt	2.35			2.35
Het Chi	0.00			0.00
Het df	0			0
Het P	N.S.			N.S.
Fixed RR	1.87			1.87
RRl	0.52			0.52
RRu	6.71			6.71
P	N.S.			N.S.
Random RR	1.87			1.87
RRl	0.52			0.52
RRu	6.71			6.71
P	N.S.			N.S.
Between Chi				
Between df				
Between P				N.S.
Btwn(F) P				N.S.
Btwn(R) P				N.S.

Detailed other continent

	SCAmer	Auslia	Africa	Total
N	1			1
NS	1			1
Wt	2.76			2.76
Het Chi	0.00			0.00
Het df	0			0
Het P	N.S.			N.S.
Fixed RR	7.32			7.32
RRl	2.25			2.25
RRu	23.79			23.79
P	+++			+++
Random RR	7.32			7.32
RRl	2.25			2.25
RRu	23.79			23.79
P	+++			+++
Between Chi				
Between df				
Between P				N.S.
Btwn(F) P				N.S.
Btwn(R) P				N.S.

Table 3A3 - 3

IESLC - Meta-analysis of Ever Smoking, Cigarettes only
Adenocarcinoma
Most adjusted

	Start year of study					
	<1960	1960-69	1970-79	1980-89	1990+	Total
N	3	1	4	3		11
NS	3	1	3	3		10
Wt	5.52	2.35	201.70	44.99		254.57
Het Chi	1.28	0.00	127.46	10.70		166.39
Het df	2	0	3	2		10
Het P	N.S.	N.S.	***	**		***
Fixed RR	2.54	1.87	1.64	3.83		1.93
RRl	1.10	0.52	1.43	2.86		1.70
RRu	5.84	6.71	1.88	5.14		2.18
P	+	N.S.	+++	+++		+++
Random RR	2.54	1.87	3.19	2.65		2.87
RRl	1.10	0.52	1.01	0.76		1.49
RRu	5.84	6.71	10.13	9.27		5.55
P	+	N.S.	+	N.S.		++
Between Chi						26.95
Between df						3
Between P						***
Btwn(F) P						N.S.
Btwn(R) P						N.S.

	Study type (1)		
	CC	other	Total
N	10	1	11
NS	9	1	10
Wt	252.82	1.75	254.57
Het Chi	165.83	0.00	166.39
Het df	9	0	10
Het P	***	N.S.	***
Fixed RR	1.92	3.39	1.93
RRl	1.70	0.77	1.70
RRu	2.17	14.92	2.18
P	+++	N.S.	+++
Random RR	2.84	3.39	2.87
RRl	1.42	0.77	1.49
RRu	5.65	14.92	5.55
P	++	N.S.	++
Between Chi			0.56
Between df			1
Between P			N.S.
Btwn(F) P			N.S.
Btwn(R) P			N.S.

	Study type (2)			
	CC	prosp	other	Total
N	10	1		11
NS	9	1		10
Wt	252.82	1.75		254.57
Het Chi	165.83	0.00		166.39
Het df	9	0		10
Het P	***	N.S.		***
Fixed RR	1.92	3.39		1.93
RRl	1.70	0.77		1.70
RRu	2.17	14.92		2.18
P	+++	N.S.		+++
Random RR	2.84	3.39		2.87
RRl	1.42	0.77		1.49
RRu	5.65	14.92		5.55
P	++	N.S.		++
Between Chi				0.56
Between df				1
Between P				N.S.
Btwn(F) P				N.S.
Btwn(R) P				N.S.

Table 3A3 - 3

IESLC - Meta-analysis of Ever Smoking, Cigarettes only
Adenocarcinoma
Most adjusted

	Study size (number of LC cases)				
	100-249	250-499	500-999	1000+	Total
N	1	3	2	5	11
NS	1	3	2	4	10
Wt	2.76	4.36	5.64	241.80	254.57
Het Chi	0.00	5.54	0.00	155.52	166.39
Het df	0	2	1	4	10
Het P	N.S.	(*)	N.S.	***	***
Fixed RR	7.32	1.44	1.83	1.91	1.93
RRl	2.25	0.56	0.80	1.68	1.70
RRu	23.79	3.68	4.19	2.17	2.18
P	+++	N.S.	N.S.	+++	+++
Random RR	7.32	1.89	1.83	3.35	2.87
RRl	2.25	0.35	0.80	1.34	1.49
RRu	23.79	10.23	4.19	8.40	5.55
P	+++	N.S.	N.S.	++	++
Between Chi					5.33
Between df					3
Between P					N.S.
Btwn(F) P					N.S.
Btwn(R) P					N.S.

	Risky occupational population			
	no	mining	othRisky	Total
N	10	1		11
NS	9	1		10
Wt	252.44	2.13		254.57
Het Chi	162.13	0.00		166.39
Het df	9	0		10
Het P	***	N.S.		***
Fixed RR	1.95	0.47		1.93
RRl	1.72	0.12		1.70
RRu	2.21	1.80		2.18
P	+++	N.S.		+++
Random RR	3.35	0.47		2.87
RRl	1.69	0.12		1.49
RRu	6.65	1.80		5.55
P	+++	N.S.		++
Between Chi				4.26
Between df				1
Between P				*
Btwn(F) P				N.S.
Btwn(R) P				*

	National cigarette tobacco type			
	Virginia	blended	other	Total
N	4	6	1	11
NS	3	6	1	10
Wt	65.67	186.76	2.13	254.57
Het Chi	1.55	117.71	0.00	166.39
Het df	3	5	0	10
Het P	N.S.	***	N.S.	***
Fixed RR	3.91	1.53	0.47	1.93
RRl	3.07	1.32	0.12	1.70
RRu	4.98	1.76	1.80	2.18
P	+++	+++	N.S.	+++
Random RR	3.91	3.35	0.47	2.87
RRl	3.07	1.10	0.12	1.49
RRu	4.98	10.27	1.80	5.55
P	+++	+	N.S.	++
Between Chi				47.13
Between df				2
Between P				***
Btwn(F) P				N.S.
Btwn(R) P				**

Table 3A3 - 3

IESLC - Meta-analysis of Ever Smoking, Cigarettes only
Adenocarcinoma
Most adjusted

	Any proxy use No/nk	Yes	Total
N	9	2	11
NS	8	2	10
Wt	212.34	42.23	254.57
Het Chi	135.79	9.47	166.39
Het df	8	1	10
Het P	***	**	***
Fixed RR	1.69	3.68	1.93
RRl	1.48	2.72	1.70
RRu	1.94	4.97	2.18
P	+++	+++	+++
Random RR	3.28	1.54	2.87
RRl	1.49	0.19	1.49
RRu	7.25	12.71	5.55
P	++	N.S.	++
Between Chi			21.13
Between df			1
Between P			***
Btwn(F) P			N.S.
Btwn(R) P			N.S.

	Full histological confirmation No	Yes	Total
N	5	6	11
NS	4	6	10
Wt	29.46	225.11	254.57
Het Chi	9.50	149.23	166.39
Het df	4	5	10
Het P	*	***	***
Fixed RR	3.11	1.81	1.93
RRl	2.17	1.59	1.70
RRu	4.47	2.06	2.18
P	+++	+++	+++
Random RR	2.52	3.43	2.87
RRl	1.28	1.31	1.49
RRu	4.95	8.98	5.55
P	++	+	++
Between Chi			7.66
Between df			1
Between P			**
Btwn(F) P			N.S.
Btwn(R) P			N.S.

	Number of adjustment variables (1) 0	1	2+/+nk	Total
N	7	3	1	11
NS	7	2	1	10
Wt	189.50	24.97	40.10	254.57
Het Chi	119.58	0.20	0.00	166.39
Het df	6	2	0	10
Het P	***	N.S.	N.S.	***
Fixed RR	1.50	3.84	4.10	1.93
RRl	1.30	2.59	3.01	1.70
RRu	1.73	5.68	5.59	2.18
P	+++	+++	+++	+++
Random RR	2.36	3.84	4.10	2.87
RRl	0.86	2.59	3.01	1.49
RRu	6.48	5.68	5.59	5.55
P	(+)	+++	+++	++
Between Chi				46.61
Between df				2
Between P				***
Btwn(F) P				N.S.
Btwn(R) P				N.S.

Table 3A3 - 3

IESLC - Meta-analysis of Ever Smoking, Cigarettes only
Adenocarcinoma
Most adjusted

	Number of adjustment variables (2) 0	1	2	3-5	6+/+nk	Total
N	7	3	1			11
NS	7	2	1			10
Wt	189.50	24.97	40.10			254.57
Het Chi	119.58	0.20	0.00			166.39
Het df	6	2	0			10
Het P	***	N.S.	N.S.			***
Fixed RR	1.50	3.84	4.10			1.93
RRl	1.30	2.59	3.01			1.70
RRu	1.73	5.68	5.59			2.18
P	+++	+++	+++			+++
Random RR	2.36	3.84	4.10			2.87
RRl	0.86	2.59	3.01			1.49
RRu	6.48	5.68	5.59			5.55
P	(+)	+++	+++			++
Between Chi						46.61
Between df						2
Between P						***
Btwn(F) P						N.S.
Btwn(R) P						N.S.

	Product all/unsp	cig+/-ot	cig only	Total
N			11	11
NS			10	10
Wt			254.57	254.57
Het Chi			166.39	166.39
Het df			10	10
Het P			***	***
Fixed RR			1.93	1.93
RRl			1.70	1.70
RRu			2.18	2.18
P			+++	+++
Random RR			2.87	2.87
RRl			1.49	1.49
RRu			5.55	5.55
P			++	++
Between Chi				
Between df				
Between P				N.S.
Btwn(F) P				N.S.
Btwn(R) P				N.S.

	Denominator nev any	nev cigs	Total
N	10	1	11
NS	9	1	10
Wt	251.80	2.76	254.57
Het Chi	161.41	0.00	166.39
Het df	9	0	10
Het P	***	N.S.	***
Fixed RR	1.90	7.32	1.93
RRl	1.68	2.25	1.70
RRu	2.15	23.79	2.18
P	+++	+++	+++
Random RR	2.63	7.32	2.87
RRl	1.32	2.25	1.49
RRu	5.24	23.79	5.55
P	++	+++	++
Between Chi			4.98
Between df			1
Between P			*
Btwn(F) P			N.S.
Btwn(R) P			N.S.

Table 3A3 - 3

IESLC - Meta-analysis of Ever Smoking, Cigarettes only
Adenocarcinoma
Most adjusted

	Derivation of RR/CI Orig	StdCalc	Other	Total
N		6	5	11
NS		6	4	10
Wt		189.02	65.55	254.57
Het Chi		118.05	0.58	166.39
Het df		5	4	10
Het P		***	N.S.	***
Fixed RR		1.49	4.02	1.93
RRl		1.29	3.16	1.70
RRu		1.72	5.12	2.18
P		+++	+++	+++
Random RR		2.12	4.02	2.87
RRl		0.74	3.16	1.49
RRu		6.07	5.12	5.55
P		N.S.	+++	++
Between Chi				47.77
Between df				1
Between P				***
Btwn(F) P				(*)
Btwn(R) P				N.S.

Table 3A3 - 4

IESLC - Meta-analysis of Ever Smoking, Cigarettes only
Adenocarcinoma
Least adjusted

REF	NRR	X	SEX	AGEL	AGEH	RACE	YF	LC TYPE	LOC	START	ST	NLC	R	VB	P	H	AD	PRODUCT	DENOM	De
ALDERS	107	x	m	0	0	all	-	not q+s	Eu:UK	1977	CC	1448	n	V	n	n	0	cig only	nev any	st
ALDERS	104	x	f	0	0	all	-	not q+s	Eu:UK	1977	CC	1448	n	V	n	n	0	cig only	nev any	st
BAND	2		m	0	0	all	-	a	NAmer	1983	CC	2831	n	V	y	y	2	cig only	nev any	ot
BRESLO	2		c	0	0	all	-	a	NAmer	1949	CC	518	n	bl	n	y	0	cig only	nev+1	st
HAMMON	90	x	m	0	0	wh	0	a	NAmer	1952	pr	448	n	bl	n	n	0	cig only	nev any	st
JUSSAW	28		m	0	0	all	-	KII	As:Ind	1964	CC	792	n	V	n	n	0	cig only	nev any	st
LUBIN	18		m	0	0	all	-	KII	As:Chi	1984	CC	427	m	ot	y	n	0	cig only	nev any	st
LUBIN2	144		m	0	0	all	-	a	Eu:mul	1976	CC	7804	n	bl	n	y	0	cig only	nev any	st
PEZZOT	7		m	0	0	all	-	a	SCAmer	1987	CC	215	n	bl	n	y	0	cig only	nev cigs	st
STASZE	22		m	0	0	all	-	a	Eu:est	1954	CC	281	n	bl	n	y	0	cig only	nev any	ot
WYNDE7	64		m	0	0	all	-	KII	NAmer	1977	CC	2085	n	bl	n	y	0	cig only	nev any	st

Cigarette type is all/unspec for all RRs

except for the following:

REF	NRR	CIGTYPE
ALDERS	104	MC only
JUSSAW	28	MC only

Table 3A3 - 5

IESLC - Meta-analysis of Ever Smoking, Cigarettes only
Adenocarcinoma
Least adjusted

REF	NRR	SEX	AD	Number Exposed Case	Number Exposed Cont	Non-exposed Case	Non-exposed Cont	RR	95.00%CI
ALDERS	107	m	0	102	462	6	133	4.89	(2.10- 11.40)
ALDERS	104	f	0	104	371	25	243	2.72	(1.71- 4.34)
Subtotal ALDERS								3.12	(2.08- 4.69)
BAND	2	m	2	-	-	-	-	4.10	(3.01- 5.59)
BRESLO	2	c	0	31	240	4	56	1.81	(0.61- 5.33)
*HAMMON	90	m	0	14	225565	2	115884	3.60	(0.82- 15.82)
JUSSAW	28	m	0	3	77	13	624	1.87	(0.52- 6.71)
LUBIN	18	m	0	5	191	4	72	0.47	(0.12- 1.80)
LUBIN2	144	m	0	686	9345	195	2616	0.98	(0.84- 1.16)
PEZZOT	7	m	0	60	317	3	116	7.32	(2.25- 23.79)
STASZE	22	m	0	15	552	0	158	8.89~	(0.53- 149.45)
WYNDE7	64	m	0	645	2108	42	918	6.69	(4.85- 9.22)
Partial Totals				1665	239228	294	120820		

*prospective study ~ With 0.5 adjustment for zero

REF	NRR	SEX	AD	Ys	Ws	Qs	Ps
ALDERS	107	m	0	1.59	5.37	4.87	0.0002
ALDERS	104	f	0	1.00	17.72	2.38	0.0000
Subtotal ALDERS				1.14	23.09	7.24	
BAND	2	m	2	1.41	40.10	24.06	0.0000
BRESLO	2	c	0	0.59	3.29	0.01	0.2828
*HAMMON	90	m	0	1.28	1.75	0.72	0.0904
JUSSAW	28	m	0	0.63	2.35	0.00	0.3368
LUBIN	18	m	0	-0.75	2.13	4.11	0.2719
LUBIN2	144	m	0	-0.02	141.34	60.01	0.8555
PEZZOT	7	m	0	1.99	2.76	5.07	0.0009
STASZE	22	m	0	2.19	0.48	1.16	0.1290
WYNDE7	64	m	0	1.90	37.14	59.34	0.0000

	N	11
	NS	10
	Wt	254.44
Het	Chi	161.73
Het	df	10
Het	P	***
Fixed	RR	1.89
	RRl	1.67
	RRu	2.14
	P	+++
Random	RR	2.81
	RRl	1.47
	RRu	5.39
	P	++
Asymm	P	N.S.

Table 3A3 - 6

IESLC - Meta-analysis of Ever Smoking, Cigarettes only
Adenocarcinoma
Least adjusted

	combined	Sex: male	female	Total
N	1	9	1	11
NS	1	9	1	11
Wt	3.29	233.43	17.72	254.44
Het Chi	0.00	159.17	0.00	161.73
Het df	0	8	0	10
Het P	N.S.	***	N.S.	***
Fixed RR	1.81	1.84	2.72	1.89
RRl	0.61	1.62	1.71	1.67
RRu	5.33	2.09	4.34	2.14
P	N.S.	+++	+++	+++
Random RR	1.81	2.97	2.72	2.81
RRl	0.61	1.36	1.71	1.47
RRu	5.33	6.50	4.34	5.39
P	N.S.	++	+++	++
Between Chi				2.55
Between df				2
Between P				N.S.
Btwn(F) P				N.S.
Btwn(R) P				N.S.

Table 3A3 - 7

IESLC - Meta-analysis of Ever Smoking, Cigarettes only
Adenocarcinoma
Excluded studies (and stage at which they were excluded)

1	BOUCHA BUELL LAURIL MZILEN
2	BYERS2 ENSTRO HIRAY2 KANELL LIAW LICKIN MRFIT MURATA NOTANI RESTRE RIMING SAARIK SEGI2 SHIMIZ SITAS SOBUE2 TANG2 WALD WARSIN WATSON WU2 WYNDE5 WYNDE8 WYNDER
3	BERRIN CHEN TAO ULMER
4	ABRAHA AKIBA AMANDU AMES ANDERS ARCHER AUSTIN AUVINE AXELSO AXELSS BARBON BECHER BENSHL BLOHMK BLOT1 BLOT2 BLOT3 BLOT4 BRETT BROCKM BROSS BROWN1 BROWN2 BUFFLE BYERS1 CARPEN CASCO2 CASCOR CEDERL CHAN CHANG CHATZI CHEN2 CHEN3 CHIAZZ CHOI CHYOU COMSTO COOKSO CORREA DARBY DAVEYS DEKLER DESTE2 DESTEF DOCKER DORANT DORGAN DOSEME DROSTE DU DUNN EBELIN ENGELA ESAKI FAN GAO GAO2 GARCIA GARDIN GARSHI GENG GER GILLIS GODLEY GOODMA GREGOR GSELL HAENSZ HAMMO2 HANSEN HEGMAN HEIN HENNEK HINDS HIRAOK HIRAYA HITOSU HOLE HOROWI HORWIT HU HU2 HUANG HUMBLE ISHIMA JAHN JAIN JARUP JARVHO JEDRYC JIANG JONES KAISER KATSOU KAUFMA KELLER KHUDER KIHARA KINLEN KNEKT KO KOHLME KOO KREUZE KREYBE KUBIK LAMTH LAMWK LAMWK2 LANGE LAUSSM LEI LEMARC LETOUR LEVIN LIDDEL LIU LIU2 LIU3 LIU5 LOMBA2 LUO MACLEN MAGNUS MARSH MARSH2 MARTIS MASTRA MATOS MATSUD MCDUFF MCLAUG MIGRAN MILLER MOLLO MRFITR NAM NOU ODRISC ORMOS OSANN OSANN2 PARKIN PASTOR PAWLEG PERSH2 PERSHA PETO PEZZO2 PIKE PISANI POFFIJ POLEDN PRESCO QIAO2 RACHTA RADZIK RANDIG REN ROOTS ROTHSC SANKAR SCHWAR SEGI SEOW SHAW SIEMIA SIMARA SIMONA SOBUE SPEIZE SPITZ STAYNE STOCKS STOCKW STUCKE SUN SUZUKI SVENSS TANG TENKAN TOKARS TOUSEY TSUGAN TULINI TVERDA VANDER VEIERO VUTUC WAKAI WANG WANG2 WANG3 WANG4 WICKLU WILKIN WU WUNSCH WUWILL WYNDE2 WYNDE3 WYNDE4 WYNDE6 XU XU2 XU3 XU4 YAMAGU YONG YUAN ZHANG ZHENG ZHOU
6	SCHWA2
8	ABELIN AGUDO ARMADA BEST BOFFET BOUCOT CHOW CPSI CPSII DAMBER DEAN DEAN2 DEAN3 DOLL DOLL2 DORN GOLLED GRAHAM JOLY KAISE2 KJUUS KOULUM LIU4 LOMBAR MCCONN MILLS NOTAN2 PERNU QIAO RONCO SADOWS SUZUK2 TIZZAN WIGLE XIANGZ
10	BENHAM

Table 3A3 - 8
Potentially overlapping studies

REF	REFGP	PRINC	. OVERLAP/LINK
LUBIN2	LUBIN2	1	Lubin-combined
WYNDE7	WYNDE6	2	WYNDE5/6/7/8
LUBIN	XIANGZ	2	LUBIN/XIANGZ/QIAO

Table 3A4 -

IESLC - Meta-analysis of Ever Smoking, Any product (or Cigarettes if Any not available), Age <56
Adenocarcinoma

This analysis is restricted to results for:
1) Non-dose-response data
2) Ever smokers
3) Age <56
4) Results complete enough for use in metaanalysis

Within each study, results are then selected (in the following order of preference, within each sex) for:
5) PRODUCT: all/unspec, cigarettes regardless of other products, cigarettes only
6) CIGTYPE: all/unspecified, MC regardless of HR, MC only
7) DENOM: never smoked anything, never smoked cigarettes, (never +1 = +long term ex, +2 = +amount unknown, +3 = never cigs+long term ex)
8) Followup period (YF, prospective studies): whole study (coded as 0) or longest available
9) LCtype: all or nearest available, at least Squamous and Adeno. (q = squamous, s = small, a = adeno, l = large, KII = Kreyberg II, al = alveolar, br = bronchiolar, u = undifferentiated)
10) Race: all or nearest available, otherwise by race (wh or w = white, bl or b = black, hi = hispanic ch = chinese, jap = japanese, haw = hawaiian, w+o = white + oriental, sca = scandinavian, as = asian)
11) For overlapping studies: principal rather than subsidiary studies
Finally by Age: whole study (actual age shown) if available, otherwise by widest available age group and then for single sex results (m, f) in preference to combined sex results (c).

Results adjusted (AD) for the most potential confounders are then chosen in Sections -1 to -3 (and those which actually differ from the adjusted results in Table 3A1 - 1 are marked 'x' in Section -1) and results adjusted for the least confounders in Sections -4 to -6. (Those least adjusted results which actually differ from the most adjusted as marked 'x' in column X in Section -4) (Results adjusted for an unknown number of confounder(s) are coded as 20.)

Section -7 shows excluded studies, together with the stage (as above) at which no qualifying results were found.

Section -8 lists the potentially overlapping studies which have been included (1=principal, 2=subsidiary).

Section -9 lists any results which would have been included in preference except that they had data not complete enough for use in meta-analysis, with their significance (yes/no), if known, and any further comment as entered on the database.

In addition to those mentioned above, the following fields, levels and abbreviations are used:
* or nk = not known, n = no, y = yes, ot = other
nev = never
all/unspec = all or unspecified, cig+/-ot = cigarettes irrespective of other products (cigar, pipe etc)
MC = manufactured cigarettes, HR = hand-rolled cigarettes
REF: 6-character study reference
NRR: number of the RR on the database within the study
ST : study type (CC = case control, pr or prosp = prospective)
NLC: number of lung cancer cases in whole study
R : risky occupational population (n = no, m = mining, o = other risky)
VB : national cigarette type (V = at least 75% Virginia, bl = at least 75% blended, ot = other)
P : any proxy use
H : full histological confirmation
De : derivation of RR/CI (or = original, st = standard method, ot = other method of estimation)

Table 3A4 - 1

IESLC - Meta-analysis of Ever Smoking, Any product (or Cigarettes if Any not available), Age <56
Adenocarcinoma
Most adjusted

REF	NRR	3A1	SEX	AGEL	AGEH	RACE	YF	LC TYPE	LOC	START	ST	NLC	R	VB	P	H	AD	PRODUCT	DENOM	De
BENHAM	7	x	m	1	49	all	-	KII	Eu:wst	1976	CC	1625	n	bl	n	y	0	cig only	nev any	st
SCHWAR	8		m	40	54	wh	-	a	NAmer	1984	CC	5588	n	bl	y	y	0	cig+/-ot	nev cigs	st
SCHWAR	7		m	40	54	bl	-	a	NAmer	1984	CC	5588	n	bl	y	y	0	cig+/-ot	nev cigs	st
SCHWAR	16		f	40	54	wh	-	a	NAmer	1984	CC	5588	n	bl	y	y	0	cig+/-ot	nev cigs	st
SCHWAR	15		f	40	54	bl	-	a	NAmer	1984	CC	5588	n	bl	y	y	0	cig+/-ot	nev cigs	st
TSUGAN	10		m	30	49	all	-	a	As:Jap	1976	CC	134	n	bl	n	y	3	all/unsp	nev any	ot
TSUGAN	11		f	30	49	all	-	a	As:Jap	1976	CC	134	n	bl	n	y	3	all/unsp	nev any	ot
VUTUC	22	x	m	41	50	all	-	KII	Eu:wst	1976	CC	1877	n	bl	n	n	0	cig+/-ot	nev cigs	st
WYNDE6	324	x	m	1	54	wh	-	a	NAmer	1969	CC	4423	n	bl	n	y	0	cig+/-ot	nev cigs	st
WYNDE6	328	x	f	1	54	wh	-	a	NAmer	1969	CC	4423	n	bl	n	y	0	cig+/-ot	nev cigs	st

Cigarette type is all/unspec for all RRs

Table 3A4 - 2

IESLC - Meta-analysis of Ever Smoking, Any product (or Cigarettes if Any not available), Age <56
Adenocarcinoma
Most adjusted

REF	NRR	SEX	AD	Number Exposed Case	Number Exposed Cont	Non-exposed Case	Non-exposed Cont	RR	95.00%CI
BENHAM	7	m	0	27	40	4	14	2.36	(0.70- 7.95)
SCHWAR	8	m	0	84	178	1	73	34.45	(4.71- 252.10)
SCHWAR	7	m	0	45	39	1	7	8.08	(0.95- 68.56)
SCHWAR	16	f	0	92	108	10	79	6.73	(3.29- 13.75)
SCHWAR	15	f	0	20	28	3	41	9.76	(2.65- 36.00)
Subtotal SCHWAR								8.40	(4.73- 14.94)
TSUGAN	10	m	3	-	-	-	-	0.93	(0.43- 1.99)
TSUGAN	11	f	3	-	-	-	-	0.67	(0.22- 2.07)
Subtotal TSUGAN								0.84	(0.45- 1.58)
VUTUC	22	m	0	8	157	4	64	0.82	(0.24- 2.80)
WYNDE6	324	m	0	146	177	16	150	7.73	(4.42- 13.54)
WYNDE6	328	f	0	130	115	12	183	17.24	(9.13- 32.55)
Subtotal WYNDE6								10.98	(7.21- 16.71)
Partial Totals				552	842	51	611		

*prospective study

REF	NRR	SEX	AD	Ys	Ws	Qs	Ps
BENHAM	7	m	0	0.86	2.61	1.42	0.1650
SCHWAR	8	m	0	3.54	0.97	3.66	0.0005
SCHWAR	7	m	0	2.09	0.84	0.20	0.0556
SCHWAR	16	f	0	1.91	7.53	0.72	0.0000
SCHWAR	15	f	0	2.28	2.26	1.04	0.0006
Subtotal SCHWAR				2.13	11.60	5.62	
TSUGAN	10	m	3	-0.07	6.55	18.27	0.8527
TSUGAN	11	f	3	-0.40	3.06	12.21	0.4837
Subtotal TSUGAN				-0.18	9.60	30.48	
VUTUC	22	m	0	-0.20	2.52	8.18	0.7459
WYNDE6	324	m	0	2.05	12.25	2.45	0.0000
WYNDE6	328	f	0	2.85	9.51	14.84	0.0000
Subtotal WYNDE6				2.40	21.75	17.29	

	N	10
	NS	5
	Wt	48.08
Het	Chi	62.99
Het	df	9
Het	P	***
Fixed	RR	4.94
	RRl	3.73
	RRu	6.56
	P	+++
Random	RR	4.17
	RRl	1.86
	RRu	9.35
	P	+++
Asymm	P	N.S.

Table 3A4 - 3

IESLC - Meta-analysis of Ever Smoking, Any product (or Cigarettes if Any not available), Age <56

Adenocarcinoma

Most adjusted

	Sex combined	male	female	Total
N		6	4	10
NS		5	3	8
Wt		25.73	22.35	48.08
Het Chi		30.58	24.67	62.99
Het df		5	3	9
Het P		***	***	***
Fixed RR		3.40	7.60	4.94
RRl		2.31	5.02	3.73
RRu		5.00	11.51	6.56
P		+++	+++	+++
Random RR		3.42	5.46	4.17
RRl		1.13	1.54	1.86
RRu		10.32	19.39	9.35
P		+	++	+++
Between Chi				7.74
Between df				1
Between P				**
Btwn(F) P				N.S.
Btwn(R) P				N.S.

Table 3A4 - 4

IESLC - Meta-analysis of Ever Smoking, Any product (or Cigarettes if Any not available), Age <56
Adenocarcinoma
Least adjusted

REF	NRR	X	SEX	AGEL	AGEH	RACE	YF	LC TYPE	LOC	START	ST	NLC	R	VB	P	H	AD	PRODUCT	DENOM	De
BENHAM	7		m	1	49	all	-	KII	Eu:wst	1976	CC	1625	n	bl	n	y	0	cig only	nev any	st
SCHWAR	8		m	40	54	wh	-	a	NAmer	1984	CC	5588	n	bl	y	y	0	cig+/-ot	nev cigs	st
SCHWAR	7		m	40	54	bl	-	a	NAmer	1984	CC	5588	n	bl	y	y	0	cig+/-ot	nev cigs	st
SCHWAR	16		f	40	54	wh	-	a	NAmer	1984	CC	5588	n	bl	y	y	0	cig+/-ot	nev cigs	st
SCHWAR	15		f	40	54	bl	-	a	NAmer	1984	CC	5588	n	bl	y	y	0	cig+/-ot	nev cigs	st
TSUGAN	2	x	m	30	49	all	-	a	As:Jap	1976	CC	134	n	bl	n	y	0	all/unsp	nev any	st
TSUGAN	8	x	f	30	49	all	-	a	As:Jap	1976	CC	134	n	bl	n	y	0	all/unsp	nev any	or
VUTUC	22		m	41	50	all	-	KII	Eu:wst	1976	CC	1877	n	bl	n	n	0	cig+/-ot	nev cigs	st
WYNDE6	324		m	1	54	wh	-	a	NAmer	1969	CC	4423	n	bl	n	y	0	cig+/-ot	nev cigs	st
WYNDE6	328		f	1	54	wh	-	a	NAmer	1969	CC	4423	n	bl	n	y	0	cig+/-ot	nev cigs	st

Cigarette type is all/unspec for all RRs

Table 3A4 - 5

IESLC - Meta-analysis of Ever Smoking, Any product (or Cigarettes if Any not available), Age <56
Adenocarcinoma
Least adjusted

REF	NRR	SEX	AD	Number Exposed Case	Number Exposed Cont	Non-exposed Case	Non-exposed Cont	RR	95.00%CI
BENHAM	7	m	0	27	40	4	14	2.36	(0.70- 7.95)
SCHWAR	8	m	0	84	178	1	73	34.45	(4.71- 252.10)
SCHWAR	7	m	0	45	39	1	7	8.08	(0.95- 68.56)
SCHWAR	16	f	0	92	108	10	79	6.73	(3.29- 13.75)
SCHWAR	15	f	0	20	28	3	41	9.76	(2.65- 36.00)
Subtotal SCHWAR								8.40	(4.73- 14.94)
TSUGAN	2	m	0	53	56	18	17	0.89	(0.42- 1.91)
TSUGAN	8	f	0	6	10	33	30	0.55	(0.18- 1.68)
Subtotal TSUGAN								0.77	(0.41- 1.44)
VUTUC	22	m	0	8	157	4	64	0.82	(0.24- 2.80)
WYNDE6	324	m	0	146	177	16	150	7.73	(4.42- 13.54)
WYNDE6	328	f	0	130	115	12	183	17.24	(9.13- 32.55)
Subtotal WYNDE6								10.98	(7.21- 16.71)
Totals				611	908	102	658		

*prospective study

REF	NRR	SEX	AD	Ys	Ws	Qs	Ps
BENHAM	7	m	0	0.86	2.61	1.35	0.1650
SCHWAR	8	m	0	3.54	0.97	3.73	0.0005
SCHWAR	7	m	0	2.09	0.84	0.22	0.0556
SCHWAR	16	f	0	1.91	7.53	0.81	0.0000
SCHWAR	15	f	0	2.28	2.26	1.11	0.0006
Subtotal SCHWAR				2.13	11.60	5.87	
TSUGAN	2	m	0	-0.11	6.62	18.91	0.7728
TSUGAN	8	f	0	-0.61	3.03	14.45	0.2916
Subtotal TSUGAN				-0.27	9.65	33.36	
VUTUC	22	m	0	-0.20	2.52	8.00	0.7459
WYNDE6	324	m	0	2.05	12.25	2.67	0.0000
WYNDE6	328	f	0	2.85	9.51	15.31	0.0000
Subtotal WYNDE6				2.40	21.75	17.98	

	N	10
	NS	5
	Wt	48.12
Het	Chi	66.56
Het	df	9
Het	P	***
Fixed	RR	4.85
	RRl	3.65
	RRu	6.43
	P	+++
Random	RR	4.07
	RRl	1.78
	RRu	9.32
	P	+++
Asymm	P	N.S.

Table 3A4 - 6

IESLC - Meta-analysis of Ever Smoking, Any product (or Cigarettes if Any not available), Age <56
Adenocarcinoma
Least adjusted

	Sex combined	male	female	Total
N		6	4	10
NS		5	3	8
Wt		25.80	22.32	48.12
Het Chi		31.39	27.63	66.56
Het df		5	3	9
Het P		***	***	***
Fixed RR		3.35	7.42	4.85
RRl		2.28	4.90	3.65
RRu		4.93	11.23	6.43
P		+++	+++	+++
Random RR		3.40	5.18	4.07
RRl		1.11	1.35	1.78
RRu		10.38	19.80	9.32
P		+	+	+++
Between Chi				7.54
Between df				1
Between P				**
Btwn(F) P				N.S.
Btwn(R) P				N.S.

Table 3A4 - 7

IESLC - Meta-analysis of Ever Smoking, Any product (or Cigarettes if Any not available), Age <56
Adenocarcinoma
Excluded studies (and stage at which they were excluded)

1	BOUCHA BUELL LAURIL MZILEN
2	BYERS2 ENSTRO HIRAY2 KANELL LIAW LICKIN MRFIT MURATA NOTANI RESTRE RIMING SAARIK SEGI2 SHIMIZ SITAS SOBUE2 TANG2 WALD WARSIN WATSON WU2 WYNDE5 WYNDE8 WYNDER
3	ABELIN ABRAHA AGUDO AKIBA ALDERS AMANDU AMES ANDERS ARCHER ARMADA AUSTIN AUVINE AXELSO AXELSS BAND BARBON BECHER BENSHL BERRIN BEST BLOHMK BLOT1 BLOT2 BLOT3 BLOT4 BOFFET BOUCOT BRESLO BRETT BROCKM BROSS BROWN1 BROWN2 BUFFLE BYERS1 CARPEN CASCO2 CASCOR CEDERL CHAN CHANG CHATZI CHEN CHEN2 CHEN3 CHIAZZ CHOI CHOW CHYOU COOKSO CORREA CPSII DAMBER DARBY DAVEYS DEAN DEAN2 DEKLER DESTE2 DESTEF DOCKER DOLL DOLL2 DORANT DORGAN DORN DOSEME DROSTE DU DUNN EBELIN ENGELA ESAKI FAN GAO GAO2 GARCIA GARDIN GARSHI GENG GER GILLIS GODLEY GOLLED GOODMA GRAHAM GREGOR GSELL HAENSZ HAMMO2 HANSEN HEGMAN HEIN HENNEK HIRAOK HIRAYA HOLE HOROWI HORWIT HU HU2 HUANG HUMBLE ISHIMA JAHN JAIN JARUP JARVHO JEDRYC JIANG JOLY JONES JUSSAW KAISE2 KAISER KATSOU KAUFMA KELLER KHUDER KIHARA KINLEN KJUUS KNEKT KO KOHLME KOO KOULUM KREYBE LAMTH LAMWK LAMWK2 LANGE LAUSSM LEI LEMARC LETOUR LIDDEL LIU LIU2 LIU3 LIU4 LIU5 LOMBA2 LOMBAR LUBIN LUBIN2 LUO MACLEN MAGNUS MARSH MARSH2 MARTIS MASTRA MATOS MATSUD MCCONN MCDUFF MCLAUG MIGRAN MILLER MILLS MOLLO MRFITR NOTAN2 NOU ODRISC ORMOS OSANN OSANN2 PARKIN PASTOR PAWLEG PERNU PERSH2 PERSHA PETO PEZZO2 PEZZOT PIKE PISANI POFFIJ POLEDN QIAO QIAO2 RACHTA RADZIK RANDIG REN RONCO ROOTS ROTHSC SANKAR SCHWA2 SEGI SEOW SHAW SIEMIA SIMARA SIMONA SOBUE SPITZ STASZE STAYNE STOCKW STUCKE SUN SUZUK2 SUZUKI SVENSS TANG TAO TENKAN TIZZAN TOKARS TOUSEY TULINI ULMER VANDER VEIERO WAKAI WANG WANG2 WANG3 WANG4 WICKLU WIGLE WILKIN WU WUNSCH WYNDE2 WYNDE3 WYNDE7 XIANGZ XU XU2 XU3 XU4 YAMAGU YONG YUAN ZHANG ZHENG ZHOU
4	SADOWS
9	COMSTO CPSI DEAN3 HAMMON HINDS HITOSU KREUZE KUBIK LEVIN NAM PRESCO SPEIZE STOCKS TVERDA WUWILL WYNDE4

Table 3A4 - 8

Potentially overlapping studies

REF	REFGP	PRINC	OVERLAP/LINK
VUTUC	LUBIN2	2	Subset of Lubin2
BENHAM	LUBIN2	2	Subset of Lubin2
WYNDE6	WYNDE6	1	WYNDE5/6/7/8

Table 3A5 -

IESLC - Meta-analysis of Ever Smoking, Any product (or Cigarettes if Any not available), Age 50-70
Adenocarcinoma

This analysis is restricted to results for:
1) Non-dose-response data
2) Ever smokers
3) Maximum age range 50-70
4) Results complete enough for use in metaanalysis

Within each study, results are then selected (in the following order of preference, within each sex) for:
5) PRODUCT: all/unspec, cigarettes regardless of other products, cigarettes only
6) CIGTYPE: all/unspecified, MC regardless of HR, MC only
7) DENOM: never smoked anything, never smoked cigarettes, (never +1 = +long term ex, +2 = +amount unknown, +3 = never cigs+long term ex)
8) Followup period (YF, prospective studies): whole study (coded as 0) or longest available
9) LCtype: all or nearest available, at least Squamous and Adeno. (q = squamous, s = small, a = adeno, l = large, KII = Kreyberg II, al = alveolar, br = bronchiolar, u = undifferentiated)
10) Race: all or nearest available, otherwise by race (wh or w = white, bl or b = black, hi = hispanic ch = chinese, jap = japanese, haw = hawaiian, w+o = white + oriental, sca = scandinavian, as = asian)
11) For overlapping studies: principal rather than subsidiary studies
Finally by Age: whole study (actual age shown) if available, otherwise by widest available age group and then for single sex results (m, f) in preference to combined sex results (c).

Results adjusted (AD) for the most potential confounders are then chosen in Sections -1 to -3 (and those which actually differ from the adjusted results in Table 3A1 - 1 are marked 'x' in Section -1) and results adjusted for the least confounders in Sections -4 to -6. (Those least adjusted results which actually differ from the most adjusted as marked 'x' in column X in Section -4) (Results adjusted for an unknown number of confounder(s) are coded as 20.)

Section -7 shows excluded studies, together with the stage (as above) at which no qualifying results were found.

Section -8 lists the potentially overlapping studies which have been included (1=principal, 2=subsidiary).

Section -9 lists any results which would have been included in preference except that they had data not complete enough for use in meta-analysis, with their significance (yes/no), if known, and any further comment as entered on the database.

In addition to those mentioned above, the following fields, levels and abbreviations are used:
* or nk = not known, n = no, y = yes, ot = other
nev = never
all/unspec = all or unspecified, cig+/-ot = cigarettes irrespective of other products (cigar, pipe etc)
MC = manufactured cigarettes, HR = hand-rolled cigarettes
REF: 6-character study reference
NRR: number of the RR on the database within the study
ST : study type (CC = case control, pr or prosp = prospective)
NLC: number of lung cancer cases in whole study
R : risky occupational population (n = no, m = mining, o = other risky)
VB : national cigarette type (V = at least 75% Virginia, bl = at least 75% blended, ot = other)
P : any proxy use
H : full histological confirmation
De : derivation of RR/CI (or = original, st = standard method, ot = other method of estimation)

Table 3A5 - 1

IESLC - Meta-analysis of Ever Smoking, Any product (or Cigarettes if Any not available), Age 50-70
Adenocarcinoma
Most adjusted

REF	NRR	3A1	SEX	AGEL	AGEH	RACE	YF	LC TYPE	LOC	START	ST	NLC	R	VB	P	H	AD	PRODUCT	DENOM	De
ANDERS	12		f	55	69	all	0	a	NAmer	1986	pr	343	n	bl	n	n	0	cig+/-ot	nev cigs	st
BENHAM	8	x	m	50	59	all	-	KII	Eu:wst	1976	CC	1625	n	bl	n	y	0	cig only	nev any	st
BENHAM	9	x	m	60	69	all	-	KII	Eu:wst	1976	CC	1625	n	bl	n	y	0	cig only	nev any	ot
HAMMON	92		m	50	69	wh	0	a	NAmer	1952	pr	448	n	bl	n	n	0	all/unsp	nev any	st

Cigarette type is all/unspec for all RRs

Table 3A5 - 2

IESLC - Meta-analysis of Ever Smoking, Any product (or Cigarettes if Any not available), Age 50-70
Adenocarcinoma
Most adjusted

REF	NRR	SEX	AD	Number Exposed Case	Number Exposed Cont	Non-exposed Case	Non-exposed Cont	RR	95.00%CI
*ANDERS	12	f	0	99	96164	33	195158	6.09	(4.11- 9.03)
BENHAM	8	m	0	42	62	3	11	2.48	(0.65- 9.44)
BENHAM	9	m	0	36	28	0	11	29.46~	(1.66- 521.34)
Subtotal BENHAM								3.85	(1.15- 12.94)
*HAMMON	92	m	0	29	510108	2	115884	3.29	(0.79- 13.80)
Totals				206	606362	38	311064		

*prospective study ~ With 0.5 adjustment for zero

REF	NRR	SEX	AD	Ys	Ws	Qs	Ps
*ANDERS	12	f	0	1.81	24.76	0.16	0.0000
BENHAM	8	m	0	0.91	2.15	1.44	0.1817
BENHAM	9	m	0	3.38	0.47	1.28	0.0210
Subtotal BENHAM				1.35	2.62	2.71	
*HAMMON	92	m	0	1.19	1.87	0.53	0.1030

	N	4
	NS	3
	Wt	29.25
Het	Chi	3.41
Het	df	3
Het	P	N.S.
Fixed	RR	5.62
	RRl	3.91
	RRu	8.07
	P	+++
Random	RR	5.31
	RRl	3.20
	RRu	8.79
	P	+++
Asymm	P	N.S.

Table 3A5 - 3

IESLC - Meta-analysis of Ever Smoking, Any product (or Cigarettes if Any not available), Age 50-70
Adenocarcinoma
Most adjusted

	Sex combined	male	female	Total
N		3	1	4
NS		2	1	3
Wt		4.49	24.76	29.25
Het Chi		2.37	0.00	3.41
Het df		2	0	3
Het P		N.S.	N.S.	N.S.
Fixed RR		3.61	6.09	5.62
RRl		1.43	4.11	3.91
RRu		9.10	9.03	8.07
P		++	+++	+++
Random RR		3.78	6.09	5.31
RRl		1.34	4.11	3.20
RRu		10.65	9.03	8.79
P		+	+++	+++
Between Chi				1.04
Between df				1
Between P				N.S.
Btwn(F) P				N.S.
Btwn(R) P				N.S.

Too few RRs for analysis by factor

Table 3A5 - 4

IESLC - Meta-analysis of Ever Smoking, Any product (or Cigarettes if Any not available), Age 50-70
Adenocarcinoma
Least adjusted

REF	NRR	X	SEX	AGEL	AGEH	RACE	YF	LC TYPE	LOC	START	ST	NLC	R	VB	P	H	AD	PRODUCT	DENOM	De
ANDERS	12		f	55	69	all	0	a	NAmer	1986	pr	343	n	bl	n	n	0	cig+/-ot	nev cigs	st
BENHAM	8		m	50	59	all	-	KII	Eu:wst	1976	CC	1625	n	bl	n	y	0	cig only	nev any	st
BENHAM	9		m	60	69	all	-	KII	Eu:wst	1976	CC	1625	n	bl	n	y	0	cig only	nev any	ot
HAMMON	92		m	50	69	wh	0	a	NAmer	1952	pr	448	n	bl	n	n	0	all/unsp	nev any	st

Cigarette type is all/unspec for all RRs

Table 3A5 - 5

IESLC - Meta-analysis of Ever Smoking, Any product (or Cigarettes if Any not available), Age 50-70
Adenocarcinoma
Least adjusted

REF	NRR	SEX	AD	Number Exposed Case	Number Exposed Cont	Non-exposed Case	Non-exposed Cont	RR	95.00%CI
*ANDERS	12	f	0	99	96164	33	195158	6.09	(4.11- 9.03)
BENHAM	8	m	0	42	62	3	11	2.48	(0.65- 9.44)
BENHAM	9	m	0	36	28	0	11	29.46~	(1.66- 521.34)
Subtotal BENHAM								3.85	(1.15- 12.94)
*HAMMON	92	m	0	29	510108	2	115884	3.29	(0.79- 13.80)
Totals				206	606362	38	311064		

*prospective study ~ With 0.5 adjustment for zero

REF	NRR	SEX	AD	Ys	Ws	Qs	Ps
*ANDERS	12	f	0	1.81	24.76	0.16	0.0000
BENHAM	8	m	0	0.91	2.15	1.44	0.1817
BENHAM	9	m	0	3.38	0.47	1.28	0.0210
Subtotal BENHAM				1.35	2.62	2.71	
*HAMMON	92	m	0	1.19	1.87	0.53	0.1030

	N	4
	NS	3
	Wt	29.25
Het	Chi	3.41
Het	df	3
Het	P	N.S.
Fixed	RR	5.62
	RRl	3.91
	RRu	8.07
	P	+++
Random	RR	5.31
	RRl	3.20
	RRu	8.79
	P	+++
Asymm	P	N.S.

Table 3A5 - 6

IESLC - Meta-analysis of Ever Smoking, Any product (or Cigarettes if Any not available), Age 50-70
Adenocarcinoma
Least adjusted

	combined	Sex male	female	Total
N		3	1	4
NS		2	1	3
Wt		4.49	24.76	29.25
Het Chi		2.37	0.00	3.41
Het df		2	0	3
Het P		N.S.	N.S.	N.S.
Fixed RR		3.61	6.09	5.62
RRl		1.43	4.11	3.91
RRu		9.10	9.03	8.07
P		++	+++	+++
Random RR		3.78	6.09	5.31
RRl		1.34	4.11	3.20
RRu		10.65	9.03	8.79
P		+	+++	+++
Between Chi				1.04
Between df				1
Between P				N.S.
Btwn(F) P				N.S.
Btwn(R) P				N.S.

Table 3A5 - 7

IESLC - Meta-analysis of Ever Smoking, Any product (or Cigarettes if Any not available), Age 50-70
Adenocarcinoma
Excluded studies (and stage at which they were excluded)

1 BOUCHA BUELL LAURIL MZILEN

2 BYERS2 ENSTRO HIRAY2 KANELL LIAW LICKIN MRFIT MURATA NOTANI RESTRE RIMING SAARIK SEGI2 SHIMIZ SITAS SOBUE2
TANG2 WALD WARSIN WATSON WU2 WYNDE5 WYNDE8 WYNDER

3 ABELIN ABRAHA AGUDO AKIBA ALDERS AMANDU AMES ARCHER ARMADA AUSTIN AUVINE AXELSO AXELSS BAND BARBON BECHER
BENSHL BERRIN BEST BLOHMK BLOT1 BLOT2 BLOT3 BLOT4 BOFFET BOUCOT BRESLO BRETT BROCKM BROSS BROWN1 BYERS1
CARPEN CASCO2 CASCOR CEDERL CHAN CHANG CHATZI CHEN CHEN2 CHEN3 CHIAZZ CHOI CHOW CHYOU COMSTO COOKSO
CORREA CPSII DARBY DAVEYS DEAN DEAN2 DEKLER DESTE2 DESTEF DOCKER DOLL DOLL2 DORGAN DOSEME DROSTE DU
DUNN EBELIN ENGELA ESAKI FAN GAO GAO2 GARCIA GARDIN GARSHI GENG GER GILLIS GODLEY GOLLED GOODMA
GRAHAM GREGOR GSELL HAENSZ HAMMO2 HANSEN HEGMAN HEIN HENNEK HIRAOK HIRAYA HOLE HOROWI HORWIT HU HU2
HUANG HUMBLE ISHIMA JAHN JAIN JARUP JARVHO JEDRYC JIANG JOLY JONES JUSSAW KAISE2 KAISER KATSOU KAUFMA
KELLER KHUDER KINLEN KJUUS KNEKT KO KOHLME KOO KOULUM KREYBE LAMTH LAMWK LAMWK2 LANGE LAUSSM LEI
LEMARC LETOUR LIDDEL LIU LIU2 LIU3 LIU4 LIU5 LOMBA2 LOMBAR LUBIN LUBIN2 LUO MACLEN MAGNUS MARSH
MARSH2 MARTIS MASTRA MATOS MATSUD MCCONN MCDUFF MCLAUG MIGRAN MILLER MILLS MOLLO MRFITR NOTAN2 NOU ODRISC
ORMOS OSANN OSANN2 PARKIN PASTOR PAWLEG PERNU PERSH2 PERSHA PETO PEZZO2 PEZZOT PIKE PISANI POFFIJ POLEDN
QIAO QIAO2 RACHTA RADZIK RANDIG REN RONCO ROOTS ROTHSC SANKAR SCHWA2 SCHWAR SEGI SEOW SHAW SIEMIA
SIMARA SIMONA SOBUE SPEIZE SPITZ STASZE STAYNE STOCKW STUCKE SUN SUZUK2 SUZUKI SVENSS TANG TAO TENKAN
TIZZAN TOKARS TOUSEY TSUGAN TULINI TVERDA ULMER VANDER VEIERO WAKAI WANG WANG2 WANG3 WANG4 WICKLU WIGLE
WILKIN WU WUNSCH WYNDE2 WYNDE3 WYNDE6 WYNDE7 XIANGZ XU XU2 XU3 XU4 YAMAGU YONG YUAN ZHANG
ZHENG ZHOU

4 BROWN2 SADOWS

9 BUFFLE CPSI DAMBER DEAN3 DORANT DORN HINDS HITOSU KIHARA KREUZE KUBIK LEVIN NAM PRESCO STOCKS VUTUC
WUWILL WYNDE4

Table 3A5 - 8

Potentially overlapping studies

REF	REFGP	PRINC	OVERLAP/LINK
BENHAM	LUBIN2	2	Subset of Lubin2

Table 3A6 -

IESLC - Meta-analysis of Ever Smoking, Any product (or Cigarettes if Any not available), Age 65+
Adenocarcinoma

This analysis is restricted to results for:
1) Non-dose-response data
2) Ever smokers
3) Age 65+
4) Results complete enough for use in metaanalysis

Within each study, results are then selected (in the following order of preference, within each sex) for:
5) PRODUCT: all/unspec, cigarettes regardless of other products, cigarettes only
6) CIGTYPE: all/unspecified, MC regardless of HR, MC only
7) DENOM: never smoked anything, never smoked cigarettes, (never +1 = +long term ex, +2 = +amount unknown, +3 = never cigs+long term ex)
8) Followup period (YF, prospective studies): whole study (coded as 0) or longest available
9) LCtype: all or nearest available, at least Squamous and Adeno. (q = squamous, s = small, a = adeno, l = large, KII = Kreyberg II, al = alveolar, br = bronchiolar, u = undifferentiated)
10) Race: all or nearest available, otherwise by race (wh or w = white, bl or b = black, hi = hispanic ch = chinese, jap = japanese, haw = hawaiian, w+o = white + oriental, sca = scandinavian, as = asian)
11) For overlapping studies: principal rather than subsidiary studies
Finally by Age: whole study (actual age shown) if available, otherwise by widest available age group and then for single sex results (m, f) in preference to combined sex results (c).

Results adjusted (AD) for the most potential confounders are then chosen in Sections -1 to -3 (and those which actually differ from the adjusted results in Table 3A1 - 1 are marked 'x' in Section -1) and results adjusted for the least confounders in Sections -4 to -6. (Those least adjusted results which actually differ from the most adjusted as marked 'x' in column X in Section -4) (Results adjusted for an unknown number of confounder(s) are coded as 20.)

Section -7 shows excluded studies, together with the stage (as above) at which no qualifying results were found.

Section -8 lists the potentially overlapping studies which have been included (1=principal, 2=subsidiary).

Section -9 lists any results which would have been included in preference except that they had data not complete enough for use in meta-analysis, with their significance (yes/no), if known, and any further comment as entered on the database.

In addition to those mentioned above, the following fields, levels and abbreviations are used:
* or nk = not known, n = no, y = yes, ot = other
nev = never
all/unspec = all or unspecified, cig+/-ot = cigarettes irrespective of other products (cigar, pipe etc)
MC = manufactured cigarettes, HR = hand-rolled cigarettes
REF: 6-character study reference
NRR: number of the RR on the database within the study
ST : study type (CC = case control, pr or prosp = prospective)
NLC: number of lung cancer cases in whole study
R : risky occupational population (n = no, m = mining, o = other risky)
VB : national cigarette type (V = at least 75% Virginia, bl = at least 75% blended, ot = other)
P : any proxy use
H : full histological confirmation
De : derivation of RR/CI (or = original, st = standard method, ot = other method of estimation)

Table 3A6 - 1

IESLC - Meta-analysis of Ever Smoking, Any product (or Cigarettes if Any not available), Age 65+
Adenocarcinoma
Most adjusted

REF	NRR	3A1	SEX	AGEL	AGEH	RACE	YF	LC TYPE	LOC	START	ST	NLC	R	VB	P	H	AD	PRODUCT	DENOM	De
BENHAM	10	x	m	70	99	all	-	KII	Eu:wst	1976	CC	1625	n	bl	n	y	0	cig only	nev any	st
DORGAN	134	x	f	72	99	all	-	a	NAmer	1980	CC	2026	n	bl	y	y	2	cig+/-ot	nev any	or

Cigarette type is all/unspec for all RRs

Table 3A6 - 2

IESLC - Meta-analysis of Ever Smoking, Any product (or Cigarettes if Any not available), Age 65+
Adenocarcinoma
Most adjusted

REF	NRR	SEX	AD	Number Exposed Case	Number Exposed Cont	Non-exposed Case	Non-exposed Cont	RR	95.00%CI
BENHAM	10	m	0	5	13	2	6	1.15	(0.17- 7.74)
DORGAN	134	f	2	-	-	-	-	1.80	(1.00- 3.20)
Partial Totals				5	13	2	6		

*prospective study

REF	NRR	SEX	AD	Ys	Ws	Qs	Ps
BENHAM	10	m	0	0.14	1.06	0.18	0.8829
DORGAN	134	f	2	0.59	11.36	0.02	0.0476

	N	2
	NS	2
	Wt	12.42
Het	Chi	0.19
Het	df	1
Het	P	N.S.
Fixed	RR	1.73
	RRl	0.99
	RRu	3.02
	P	(+)
Random	RR	1.73
	RRl	0.99
	RRu	3.02
	P	(+)
Asymm	P	

Table 3A6 - 3

IESLC - Meta-analysis of Ever Smoking, Any product (or Cigarettes if Any not available), Age 65+
Adenocarcinoma
Most adjusted

	combined	Sex male	female	Total
N		1	1	2
NS		1	1	2
Wt		1.06	11.36	12.42
Het Chi		0.00	0.00	0.19
Het df		0	0	1
Het P		N.S.	N.S.	N.S.
Fixed RR		1.15	1.80	1.73
RRl		0.17	1.01	0.99
RRu		7.74	3.22	3.02
P		N.S.	+	(+)
Random RR		1.15	1.80	1.73
RRl		0.17	1.01	0.99
RRu		7.74	3.22	3.02
P		N.S.	+	(+)
Between Chi				0.19
Between df				1
Between P				N.S.
Btwn(F) P				N.S.
Btwn(R) P				N.S.

Too few RRs for analysis by factor

Table 3A6 - 4

IESLC - Meta-analysis of Ever Smoking, Any product (or Cigarettes if Any not available), Age 65+
Adenocarcinoma
Least adjusted

REF	NRR	X	SEX	AGEL	AGEH	RACE	YF	LC TYPE	LOC	START	ST	NLC	R	VB	P	H	AD	PRODUCT	DENOM	De
BENHAM	10		m	70	99	all	-	KII	Eu:wst	1976	CC	1625	n	bl	n	y	0	cig only	nev any	st
DORGAN	134		f	72	99	all	-	a	NAmer	1980	CC	2026	n	bl	y	y	2	cig+/-ot	nev any	or

Cigarette type is all/unspec for all RRs

Table 3A6 - 5

IESLC - Meta-analysis of Ever Smoking, Any product (or Cigarettes if Any not available), Age 65+
Adenocarcinoma
Least adjusted

REF	NRR	SEX	AD	Number Exposed Case	Number Exposed Cont	Non-exposed Case	Non-exposed Cont	RR	95.00%CI
BENHAM	10	m	0	5	13	2	6	1.15	(0.17- 7.74)
DORGAN	134	f	2	-	-	-	-	1.80	(1.00- 3.20)
Partial Totals				5	13	2	6		

*prospective study

REF	NRR	SEX	AD	Ys	Ws	Qs	Ps
BENHAM	10	m	0	0.14	1.06	0.18	0.8829
DORGAN	134	f	2	0.59	11.36	0.02	0.0476

	N	2
	NS	2
	Wt	12.42
Het	Chi	0.19
Het	df	1
Het	P	N.S.
Fixed	RR	1.73
	RRl	0.99
	RRu	3.02
	P	(+)
Random	RR	1.73
	RRl	0.99
	RRu	3.02
	P	(+)
Asymm	P	

Table 3A6 - 6

IESLC - Meta-analysis of Ever Smoking, Any product (or Cigarettes if Any not available), Age 65+
Adenocarcinoma
Least adjusted

	combined	Sex male	female	Total
N		1	1	2
NS		1	1	2
Wt		1.06	11.36	12.42
Het Chi		0.00	0.00	0.19
Het df		0	0	1
Het P		N.S.	N.S.	N.S.
Fixed RR		1.15	1.80	1.73
RRl		0.17	1.01	0.99
RRu		7.74	3.22	3.02
P		N.S.	+	(+)
Random RR		1.15	1.80	1.73
RRl		0.17	1.01	0.99
RRu		7.74	3.22	3.02
P		N.S.	+	(+)
Between Chi				0.19
Between df				1
Between P				N.S.
Btwn(F) P				N.S.
Btwn(R) P				N.S.

Table 3A6 - 7

IESLC - Meta-analysis of Ever Smoking, Any product (or Cigarettes if Any not available), Age 65+
Adenocarcinoma
Excluded studies (and stage at which they were excluded)

1 BOUCHA BUELL LAURIL MZILEN

2 BYERS2 ENSTRO HIRAY2 KANELL LIAW LICKIN MRFIT MURATA NOTANI RESTRE RIMING SAARIK SEGI2 SHIMIZ SITAS SOBUE2 TANG2 WALD WARSIN WATSON WU2 WYNDE5 WYNDE8 WYNDER

3 ABELIN ABRAHA AGUDO AKIBA ALDERS AMANDU AMES ANDERS ARCHER ARMADA AUSTIN AUVINE AXELSO AXELSS BAND BARBON BECHER BENSHL BERRIN BEST BLOHMK BLOT1 BLOT2 BLOT3 BLOT4 BOFFET BOUCOT BRESLO BRETT BROCKM BROSS BROWN1 BYERS1 CARPEN CASCO2 CASCOR CEDERL CHAN CHANG CHATZI CHEN CHEN2 CHEN3 CHIAZZ CHOI CHOW CHYOU COOKSO CORREA CPSII DARBY DAVEYS DEAN DEAN2 DEKLER DESTE2 DESTEF DOCKER DOLL DOLL2 DORANT DOSEME DROSTE DU DUNN EBELIN ENGELA ESAKI FAN GAO GAO2 GARCIA GARDIN GENG GER GILLIS GOLLED GOODMA GRAHAM GREGOR GSELL HAENSZ HAMMO2 HANSEN HEGMAN HEIN HENNEK HIRAOK HIRAYA HITOSU HOLE HOROWI HORWIT HU HU2 HUANG ISHIMA JAHN JAIN JARUP JARVHO JEDRYC JIANG JOLY JONES JUSSAW KAISER KATSOU KAUFMA KELLER KHUDER KIHARA KINLEN KJUUS KNEKT KO KOHLME KOO KOULUM KREUZE KREYBE KUBIK LAMTH LAMWK LAMWK2 LANGE LAUSSM LEI LEMARC LETOUR LIDDEL LIU LIU2 LIU3 LIU5 LOMBA2 LOMBAR LUBIN LUBIN2 LUO MACLEN MAGNUS MARSH MARSH2 MARTIS MASTRA MATOS MATSUD MCCONN MCDUFF MCLAUG MIGRAN MILLER MILLS MOLLO MRFITR NOTAN2 NOU ODRISC ORMOS OSANN OSANN2 PARKIN PASTOR PAWLEG PERNU PERSH2 PETO PEZZO2 PEZZOT PIKE PISANI POFFIJ POLEDN QIAO QIAO2 RACHTA RADZIK RANDIG REN RONCO ROOTS ROTHSC SANKAR SCHWA2 SCHWAR SEGI SEOW SHAW SIEMIA SIMARA SIMONA SOBUE SPEIZE SPITZ STASZE STAYNE STOCKW STUCKE SUN SUZUK2 SUZUKI SVENSS TANG TAO TENKAN TIZZAN TOKARS TOUSEY TSUGAN TULINI TVERDA ULMER VANDER VEIERO VUTUC WAKAI WANG WANG2 WANG3 WANG4 WICKLU WIGLE WILKIN WU WUNSCH WYNDE2 WYNDE3 WYNDE6 WYNDE7 XIANGZ XU XU2 XU3 XU4 YAMAGU YONG YUAN ZHANG ZHENG ZHOU

4 BROWN2 SADOWS

7 PERSHA

9 BUFFLE COMSTO CPSI DAMBER DEAN3 DORN GARSHI GODLEY HAMMON HINDS HUMBLE KAISE2 LEVIN LIU4 NAM PRESCO STOCKS WUWILL WYNDE4

Table 3A6 - 8

Potentially overlapping studies

REF	REFGP	PRINC	. OVERLAP/LINK
BENHAM	LUBIN2	2	Subset of Lubin2

Table 3A7 -

IESLC - Meta-analysis of Ever Smoking, Cigarettes (or Any Product if Cigarettes not available), Age <56
Adenocarcinoma

This analysis is restricted to results for:
1) Non-dose-response data
2) Ever smokers
3) Age <56
4) Results complete enough for use in metaanalysis

Within each study, results are then selected (in the following order of preference, within each sex) for:
5) PRODUCT: cigarettes regardless of other products, cigarettes only, all/unspec
6) CIGTYPE: all/unspecified, MC regardless of HR, MC only
7) DENOM: never smoked anything, never smoked cigarettes, (never +1 = +long term ex, +2 = +amount unknown,
+3 = never cigs+long term ex)
8) Followup period (YF, prospective studies): whole study (coded as 0) or longest available
9) LCtype: all or nearest available, at least Squamous and Adeno. (q = squamous, s = small,
a = adeno, l = large, KII = Kreyberg II, al = alveolar, br = bronchiolar, u = undifferentiated)
10) Race: all or nearest available, otherwise by race (wh or w = white, bl or b = black, hi = hispanic
ch = chinese, jap = japanese, haw = hawaiian, w+o = white + oriental, sca = scandinavian, as = asian)
11) For overlapping studies: principal rather than subsidiary studies
Finally by Age: whole study (actual age shown) if available, otherwise by widest available age group
and then for single sex results (m, f) in preference to combined sex results (c).

Results adjusted (AD) for the most potential confounders are then chosen in Sections -1 to -3
(and those which actually differ from the adjusted results in Table 3A2 - 1 are marked 'x' in Section -1)
and results adjusted for the least confounders in Sections -4 to -6. (Those least adjusted results which
actually differ from the most adjusted as marked 'x' in column X in Section -4)
(Results adjusted for an unknown number of confounder(s) are coded as 20.)

Section -7 shows excluded studies, together with the stage (as above) at which no qualifying
results were found.

Section -8 lists the potentially overlapping studies which have been included (1=principal, 2=subsidiary).

Section -9 lists any results which would have been included in preference except that they had data not complete
enough for use in meta-analysis, with their significance (yes/no), if known, and any further comment as entered
on the database.

In addition to those mentioned above, the following fields, levels and abbreviations are used:
* or nk = not known, n = no, y = yes, ot = other
nev = never
all/unspec = all or unspecified, cig+/-ot = cigarettes irrespective of other products (cigar, pipe etc)
MC = manufactured cigarettes, HR = hand-rolled cigarettes
REF: 6-character study reference
NRR: number of the RR on the database within the study
ST : study type (CC = case control, pr or prosp = prospective)
NLC: number of lung cancer cases in whole study
R : risky occupational population (n = no, m = mining, o = other risky)
VB : national cigarette type (V = at least 75% Virginia, bl = at least 75% blended, ot = other)
P : any proxy use
H : full histological confirmation
De : derivation of RR/CI (or = original, st = standard method, ot = other method of estimation)

Table 3A7 - 1

IESLC - Meta-analysis of Ever Smoking, Cigarettes (or Any Product if Cigarettes not available), Age <56
Adenocarcinoma
Most adjusted

REF	NRR	3A2	SEX	AGEL	AGEH	RACE	YF	LC TYPE	LOC	START	ST	NLC	R	VB	P	H	AD	PRODUCT	DENOM	De
BENHAM	7	x	m	1	49	all	-	KII	Eu:wst	1976	CC	1625	n	bl	n	y	0	cig only	nev any	st
SCHWAR	8		m	40	54	wh	-	a	NAmer	1984	CC	5588	n	bl	y	y	0	cig+/-ot	nev cigs	st
SCHWAR	7		m	40	54	bl	-	a	NAmer	1984	CC	5588	n	bl	y	y	0	cig+/-ot	nev cigs	st
SCHWAR	16		f	40	54	wh	-	a	NAmer	1984	CC	5588	n	bl	y	y	0	cig+/-ot	nev cigs	st
SCHWAR	15		f	40	54	bl	-	a	NAmer	1984	CC	5588	n	bl	y	y	0	cig+/-ot	nev cigs	st
TSUGAN	10		m	30	49	all	-	a	As:Jap	1976	CC	134	n	bl	n	y	3	all/unsp	nev any	ot
TSUGAN	11		f	30	49	all	-	a	As:Jap	1976	CC	134	n	bl	n	y	3	all/unsp	nev any	ot
VUTUC	22	x	m	41	50	all	-	KII	Eu:wst	1976	CC	1877	n	bl	n	n	0	cig+/-ot	nev cigs	st
WYNDE6	324	x	m	1	54	wh	-	a	NAmer	1969	CC	4423	n	bl	n	y	0	cig+/-ot	nev cigs	st
WYNDE6	328	x	f	1	54	wh	-	a	NAmer	1969	CC	4423	n	bl	n	y	0	cig+/-ot	nev cigs	st

Cigarette type is all/unspec for all RRs

Table 3A7 - 2

IESLC - Meta-analysis of Ever Smoking, Cigarettes (or Any Product if Cigarettes not available), Age <56
Adenocarcinoma
Most adjusted

REF	NRR	SEX	AD	Number Exposed Case	Number Exposed Cont	Non-exposed Case	Non-exposed Cont	RR	95.00%CI
BENHAM	7	m	0	27	40	4	14	2.36	(0.70- 7.95)
SCHWAR	8	m	0	84	178	1	73	34.45	(4.71- 252.10)
SCHWAR	7	m	0	45	39	1	7	8.08	(0.95- 68.56)
SCHWAR	16	f	0	92	108	10	79	6.73	(3.29- 13.75)
SCHWAR	15	f	0	20	28	3	41	9.76	(2.65- 36.00)
Subtotal SCHWAR								8.40	(4.73- 14.94)
TSUGAN	10	m	3	-	-	-	-	0.93	(0.43- 1.99)
TSUGAN	11	f	3	-	-	-	-	0.67	(0.22- 2.07)
Subtotal TSUGAN								0.84	(0.45- 1.58)
VUTUC	22	m	0	8	157	4	64	0.82	(0.24- 2.80)
WYNDE6	324	m	0	146	177	16	150	7.73	(4.42- 13.54)
WYNDE6	328	f	0	130	115	12	183	17.24	(9.13- 32.55)
Subtotal WYNDE6								10.98	(7.21- 16.71)
Partial Totals				552	842	51	611		

*prospective study

REF	NRR	SEX	AD	Ys	Ws	Qs	Ps
BENHAM	7	m	0	0.86	2.61	1.42	0.1650
SCHWAR	8	m	0	3.54	0.97	3.66	0.0005
SCHWAR	7	m	0	2.09	0.84	0.20	0.0556
SCHWAR	16	f	0	1.91	7.53	0.72	0.0000
SCHWAR	15	f	0	2.28	2.26	1.04	0.0006
Subtotal SCHWAR				2.13	11.60	5.62	
TSUGAN	10	m	3	-0.07	6.55	18.27	0.8527
TSUGAN	11	f	3	-0.40	3.06	12.21	0.4837
Subtotal TSUGAN				-0.18	9.60	30.48	
VUTUC	22	m	0	-0.20	2.52	8.18	0.7459
WYNDE6	324	m	0	2.05	12.25	2.45	0.0000
WYNDE6	328	f	0	2.85	9.51	14.84	0.0000
Subtotal WYNDE6				2.40	21.75	17.29	

	N	10
	NS	5
	Wt	48.08
Het	Chi	62.99
Het	df	9
Het	P	***
Fixed	RR	4.94
	RRl	3.73
	RRu	6.56
	P	+++
Random	RR	4.17
	RRl	1.86
	RRu	9.35
	P	+++
Asymm	P	N.S.

Table 3A7 - 3

IESLC - Meta-analysis of Ever Smoking, Cigarettes (or Any Product if Cigarettes not available), Age <56
Adenocarcinoma
Most adjusted

	Sex combined	male	female	Total
N		6	4	10
NS		5	3	8
Wt		25.73	22.35	48.08
Het Chi		30.58	24.67	62.99
Het df		5	3	9
Het P		***	***	***
Fixed RR		3.40	7.60	4.94
RRl		2.31	5.02	3.73
RRu		5.00	11.51	6.56
P		+++	+++	+++
Random RR		3.42	5.46	4.17
RRl		1.13	1.54	1.86
RRu		10.32	19.39	9.35
P		+	++	+++
Between Chi				7.74
Between df				1
Between P				**
Btwn(F) P				N.S.
Btwn(R) P				N.S.

Table 3A7 - 4

IESLC - Meta-analysis of Ever Smoking, Cigarettes (or Any Product if Cigarettes not available), Age <56
Adenocarcinoma
Least adjusted

REF	NRR	X	SEX	AGEL	AGEH	RACE	YF	LC TYPE	LOC	START	ST	NLC	R	VB	P	H	AD	PRODUCT	DENOM	De
BENHAM	7		m	1	49	all	-	KII	Eu:wst	1976	CC	1625	n	bl	n	y	0	cig only	nev any	st
SCHWAR	8		m	40	54	wh	-	a	NAmer	1984	CC	5588	n	bl	y	y	0	cig+/-ot	nev cigs	st
SCHWAR	7		m	40	54	bl	-	a	NAmer	1984	CC	5588	n	bl	y	y	0	cig+/-ot	nev cigs	st
SCHWAR	16		f	40	54	wh	-	a	NAmer	1984	CC	5588	n	bl	y	y	0	cig+/-ot	nev cigs	st
SCHWAR	15		f	40	54	bl	-	a	NAmer	1984	CC	5588	n	bl	y	y	0	cig+/-ot	nev cigs	st
TSUGAN	2	x	m	30	49	all	-	a	As:Jap	1976	CC	134	n	bl	n	y	0	all/unsp	nev any	st
TSUGAN	8	x	f	30	49	all	-	a	As:Jap	1976	CC	134	n	bl	n	y	0	all/unsp	nev any	or
VUTUC	22		m	41	50	all	-	KII	Eu:wst	1976	CC	1877	n	bl	n	n	0	cig+/-ot	nev cigs	st
WYNDE6	324		m	1	54	wh	-	a	NAmer	1969	CC	4423	n	bl	n	y	0	cig+/-ot	nev cigs	st
WYNDE6	328		f	1	54	wh	-	a	NAmer	1969	CC	4423	n	bl	n	y	0	cig+/-ot	nev cigs	st

Cigarette type is all/unspec for all RRs

Table 3A7 - 5

IESLC - Meta-analysis of Ever Smoking, Cigarettes (or Any Product if Cigarettes not available), Age <56
Adenocarcinoma
Least adjusted

REF	NRR	SEX	AD	Number Exposed Case	Number Exposed Cont	Non-exposed Case	Non-exposed Cont	RR	95.00%CI
BENHAM	7	m	0	27	40	4	14	2.36	(0.70- 7.95)
SCHWAR	8	m	0	84	178	1	73	34.45	(4.71- 252.10)
SCHWAR	7	m	0	45	39	1	7	8.08	(0.95- 68.56)
SCHWAR	16	f	0	92	108	10	79	6.73	(3.29- 13.75)
SCHWAR	15	f	0	20	28	3	41	9.76	(2.65- 36.00)
Subtotal SCHWAR								8.40	(4.73- 14.94)
TSUGAN	2	m	0	53	56	18	17	0.89	(0.42- 1.91)
TSUGAN	8	f	0	6	10	33	30	0.55	(0.18- 1.68)
Subtotal TSUGAN								0.77	(0.41- 1.44)
VUTUC	22	m	0	8	157	4	64	0.82	(0.24- 2.80)
WYNDE6	324	m	0	146	177	16	150	7.73	(4.42- 13.54)
WYNDE6	328	f	0	130	115	12	183	17.24	(9.13- 32.55)
Subtotal WYNDE6								10.98	(7.21- 16.71)
Totals				611	908	102	658		

*prospective study

REF	NRR	SEX	AD	Ys	Ws	Qs	Ps
BENHAM	7	m	0	0.86	2.61	1.35	0.1650
SCHWAR	8	m	0	3.54	0.97	3.73	0.0005
SCHWAR	7	m	0	2.09	0.84	0.22	0.0556
SCHWAR	16	f	0	1.91	7.53	0.81	0.0000
SCHWAR	15	f	0	2.28	2.26	1.11	0.0006
Subtotal SCHWAR				2.13	11.60	5.87	
TSUGAN	2	m	0	-0.11	6.62	18.91	0.7728
TSUGAN	8	f	0	-0.61	3.03	14.45	0.2916
Subtotal TSUGAN				-0.27	9.65	33.36	
VUTUC	22	m	0	-0.20	2.52	8.00	0.7459
WYNDE6	324	m	0	2.05	12.25	2.67	0.0000
WYNDE6	328	f	0	2.85	9.51	15.31	0.0000
Subtotal WYNDE6				2.40	21.75	17.98	

	N	10
	NS	5
	Wt	48.12
Het	Chi	66.56
Het	df	9
Het	P	***
Fixed	RR	4.85
	RRl	3.65
	RRu	6.43
	P	+++
Random	RR	4.07
	RRl	1.78
	RRu	9.32
	P	+++
Asymm	P	N.S.

Table 3A7 - 6

IESLC - Meta-analysis of Ever Smoking, Cigarettes (or Any Product if Cigarettes not available), Age <56
Adenocarcinoma
Least adjusted

	Sex			
	combined	male	female	Total
N		6	4	10
NS		5	3	8
Wt		25.80	22.32	48.12
Het Chi		31.39	27.63	66.56
Het df		5	3	9
Het P		***	***	***
Fixed RR		3.35	7.42	4.85
RRl		2.28	4.90	3.65
RRu		4.93	11.23	6.43
P		+++	+++	+++
Random RR		3.40	5.18	4.07
RRl		1.11	1.35	1.78
RRu		10.38	19.80	9.32
P		+	+	+++
Between Chi				7.54
Between df				1
Between P				**
Btwn(F) P				N.S.
Btwn(R) P				N.S.

Table 3A7 - 7

IESLC - Meta-analysis of Ever Smoking, Cigarettes (or Any Product if Cigarettes not available), Age <56
Adenocarcinoma
Excluded studies (and stage at which they were excluded)

Stage	Studies
1	BOUCHA BUELL LAURIL MZILEN
2	BYERS2 ENSTRO HIRAY2 KANELL LIAW LICKIN MRFIT MURATA NOTANI RESTRE RIMING SAARIK SEGI2 SHIMIZ SITAS SOBUE2 TANG2 WALD WARSIN WATSON WU2 WYNDE5 WYNDE8 WYNDER
3	ABELIN ABRAHA AGUDO AKIBA ALDERS AMANDU AMES ANDERS ARCHER ARMADA AUSTIN AUVINE AXELSO AXELSS BAND BARBON BECHER BENSHL BERRIN BEST BLOHMK BLOT1 BLOT2 BLOT3 BLOT4 BOFFET BOUCOT BRESLO BRETT BROCKM BROSS BROWN1 BROWN2 BUFFLE BYERS1 CARPEN CASCO2 CASCOR CEDERL CHAN CHANG CHATZI CHEN CHEN2 CHEN3 CHIAZZ CHOI CHOW CHYOU COOKSO CORREA CPSII DAMBER DARBY DAVEYS DEAN DEAN2 DEKLER DESTE2 DESTEF DOCKER DOLL DOLL2 DORANT DORGAN DORN DOSEME DROSTE DU DUNN EBELIN ENGELA ESAKI FAN GAO GAO2 GARCIA GARDIN GARSHI GENG GER GILLIS GODLEY GOLLED GOODMA GRAHAM GREGOR GSELL HAENSZ HAMMO2 HANSEN HEGMAN HEIN HENNEK HIRAOK HIRAYA HOLE HOROWI HORWIT HU HU2 HUANG HUMBLE ISHIMA JAHN JAIN JARUP JARVHO JEDRYC JIANG JOLY JONES JUSSAW KAISE2 KAISER KATSOU KAUFMA KELLER KHUDER KIHARA KINLEN KJUUS KNEKT KO KOHLME KOO KOULUM KREYBE LAMTH LAMWK LAMWK2 LANGE LAUSSM LEI LEMARC LETOUR LIDDEL LIU LIU2 LIU3 LIU4 LIU5 LOMBA2 LOMBAR LUBIN LUBIN2 LUO MACLEN MAGNUS MARSH MARSH2 MARTIS MASTRA MATOS MATSUD MCCONN MCDUFF MCLAUG MIGRAN MILLER MILLS MOLLO MRFITR NOTAN2 NOU ODRISC ORMOS OSANN OSANN2 PARKIN PASTOR PAWLEG PERNU PERSH2 PERSHA PETO PEZZO2 PEZZOT PIKE PISANI POFFIJ POLEDN QIAO QIAO2 RACHTA RADZIK RANDIG REN RONCO ROOTS ROTHSC SANKAR SCHWA2 SEGI SEOW SHAW SIEMIA SIMARA SIMONA SOBUE SPITZ STASZE STAYNE STOCKW STUCKE SUN SUZUK2 SUZUKI SVENSS TANG TAO TENKAN TIZZAN TOKARS TOUSEY TULINI ULMER VANDER VEIERO WAKAI WANG WANG2 WANG3 WANG4 WICKLU WIGLE WILKIN WU WUNSCH WYNDE2 WYNDE3 WYNDE7 XIANGZ XU XU2 XU3 XU4 YAMAGU YONG YUAN ZHANG ZHENG ZHOU
4	SADOWS
9	COMSTO CPSI DEAN3 HAMMON HINDS HITOSU KREUZE KUBIK LEVIN NAM PRESCO SPEIZE STOCKS TVERDA WUWILL WYNDE4

Table 3A7 - 8
Potentially overlapping studies

REF	REFGP	PRINC	. OVERLAP/LINK
VUTUC	LUBIN2	2	Subset of Lubin2
BENHAM	LUBIN2	2	Subset of Lubin2
WYNDE6	WYNDE6	1	WYNDE5/6/7/8

Table 3A8 -

IESLC - Meta-analysis of Ever Smoking, Cigarettes (or Any Product if Cigarettes not available), Age 50-70
Adenocarcinoma

This analysis is restricted to results for:
1) Non-dose-response data
2) Ever smokers
3) Maximum age range 50-70
4) Results complete enough for use in metaanalysis

Within each study, results are then selected (in the following order of preference, within each sex) for:
5) PRODUCT: cigarettes regardless of other products, cigarettes only, all/unspec
6) CIGTYPE: all/unspecified, MC regardless of HR, MC only
7) DENOM: never smoked anything, never smoked cigarettes, (never +1 = +long term ex, +2 = +amount unknown,
+3 = never cigs+long term ex)
8) Followup period (YF, prospective studies): whole study (coded as 0) or longest available
9) LCtype: all or nearest available, at least Squamous and Adeno. (q = squamous, s = small,
a = adeno, l = large, KII = Kreyberg II, al = alveolar, br = bronchiolar, u = undifferentiated)
10) Race: all or nearest available, otherwise by race (wh or w = white, bl or b = black, hi = hispanic
ch = chinese, jap = japanese, haw = hawaiian, w+o = white + oriental, sca = scandinavian, as = asian)
11) For overlapping studies: principal rather than subsidiary studies
Finally by Age: whole study (actual age shown) if available, otherwise by widest available age group
and then for single sex results (m, f) in preference to combined sex results (c).

Results adjusted (AD) for the most potential confounders are then chosen in Sections -1 to -3
(and those which actually differ from the adjusted results in Table 3A2 - 1 are marked 'x' in Section -1)
and results adjusted for the least confounders in Sections -4 to -6. (Those least adjusted results which
actually differ from the most adjusted as marked 'x' in column X in Section -4)
(Results adjusted for an unknown number of confounder(s) are coded as 20.)

Section -7 shows excluded studies, together with the stage (as above) at which no qualifying
results were found.

Section -8 lists the potentially overlapping studies which have been included (1=principal, 2=subsidiary).

Section -9 lists any results which would have been included in preference except that they had data not complete
enough for use in meta-analysis, with their significance (yes/no), if known, and any further comment as entered
on the database.

In addition to those mentioned above, the following fields, levels and abbreviations are used:
* or nk = not known, n = no, y = yes, ot = other
nev = never
all/unspec = all or unspecified, cig+/-ot = cigarettes irrespective of other products (cigar, pipe etc)
MC = manufactured cigarettes, HR = hand-rolled cigarettes
REF: 6-character study reference
NRR: number of the RR on the database within the study
ST : study type (CC = case control, pr or prosp = prospective)
NLC: number of lung cancer cases in whole study
R : risky occupational population (n = no, m = mining, o = other risky)
VB : national cigarette type (V = at least 75% Virginia, bl = at least 75% blended, ot = other)
P : any proxy use
H : full histological confirmation
De : derivation of RR/CI (or = original, st = standard method, ot = other method of estimation)

Table 3A8 - 1

IESLC - Meta-analysis of Ever Smoking, Cigarettes (or Any Product if Cigarettes not available), Age 50-70
Adenocarcinoma
Most adjusted

REF	NRR	3A2	SEX	AGEL	AGEH	RACE	YF	LC TYPE	LOC	START	ST	NLC	R	VB	P	H	AD	PRODUCT	DENOM	De
ANDERS	12		f	55	69	all	0	a	NAmer	1986	pr	343	n	bl	n	n	0	cig+/-ot	nev cigs	st
BENHAM	8	x	m	50	59	all	-	KII	Eu:wst	1976	CC	1625	n	bl	n	y	0	cig only	nev any	st
BENHAM	9	x	m	60	69	all	-	KII	Eu:wst	1976	CC	1625	n	bl	n	y	0	cig only	nev any	ot
HAMMON	84		m	50	69	wh	0	a	NAmer	1952	pr	448	n	bl	n	n	1	cig+/-ot	nev any	ot

Cigarette type is all/unspec for all RRs

Table 3A8 - 2

IESLC - Meta-analysis of Ever Smoking, Cigarettes (or Any Product if Cigarettes not available), Age 50-70
Adenocarcinoma
Most adjusted

REF	NRR	SEX	AD	Number Case	Exposed Cont	Non-exposed Case	Cont	RR	95.00%CI
*ANDERS	12	f	0	99	96164	33	195158	6.09	(4.11- 9.03)
BENHAM	8	m	0	42	62	3	11	2.48	(0.65- 9.44)
BENHAM	9	m	0	36	28	0	11	29.46~	(1.66- 521.34)
Subtotal BENHAM								3.85	(1.15- 12.94)
*HAMMON	84	m	1	-	-	-	-	3.67	(0.87- 15.45)
Partial Totals				177	96254	36	195180		

*prospective study ~ With 0.5 adjustment for zero

REF	NRR	SEX	AD	Ys	Ws	Qs	Ps
*ANDERS	12	f	0	1.81	24.76	0.13	0.0000
BENHAM	8	m	0	0.91	2.15	1.46	0.1817
BENHAM	9	m	0	3.38	0.47	1.27	0.0210
Subtotal BENHAM				1.35	2.62	2.73	
*HAMMON	84	m	1	1.30	1.86	0.35	0.0765

	N	4
	NS	3
	Wt	29.24
Het	Chi	3.21
Het	df	3
Het	P	N.S.
Fixed	RR	5.66
	RRl	3.94
	RRu	8.13
	P	+++
Random	RR	5.49
	RRl	3.52
	RRu	8.57
	P	+++
Asymm	P	N.S.

Table 3A8 - 3

IESLC - Meta-analysis of Ever Smoking, Cigarettes (or Any Product if Cigarettes not available), Age 50-70
Adenocarcinoma
Most adjusted

	Sex			
	combined	male	female	Total
N		3	1	4
NS		2	1	3
Wt		4.48	24.76	29.24
Het Chi		2.34	0.00	3.21
Het df		2	0	3
Het P		N.S.	N.S.	N.S.
Fixed RR		3.78	6.09	5.66
RRl		1.50	4.11	3.94
RRu		9.54	9.03	8.13
P		++	+++	+++
Random RR		3.94	6.09	5.49
RRl		1.41	4.11	3.52
RRu		11.05	9.03	8.57
P		++	+++	+++
Between Chi				0.86
Between df				1
Between P				N.S.
Btwn(F) P				N.S.
Btwn(R) P				N.S.

Too few RRs for analysis by factor

Table 3A8 - 4

IESLC - Meta-analysis of Ever Smoking, Cigarettes (or Any Product if Cigarettes not available), Age 50-70
Adenocarcinoma
Least adjusted

REF	NRR	X	SEX	AGEL	AGEH	RACE	YF	LC TYPE	LOC	START	ST	NLC	R	VB	P	H	AD	PRODUCT	DENOM	De
ANDERS	12		f	55	69	all	0	a	NAmer	1986	pr	343	n	bl	n	n	0	cig+/-ot	nev cigs	st
BENHAM	8		m	50	59	all	-	KII	Eu:wst	1976	CC	1625	n	bl	n	y	0	cig only	nev any	st
BENHAM	9		m	60	69	all	-	KII	Eu:wst	1976	CC	1625	n	bl	n	y	0	cig only	nev any	ot
HAMMON	91	x	m	50	69	wh	0	a	NAmer	1952	pr	448	n	bl	n	n	0	cig+/-ot	nev any	st

Cigarette type is all/unspec for all RRs

Table 3A8 - 5

IESLC - Meta-analysis of Ever Smoking, Cigarettes (or Any Product if Cigarettes not available), Age 50-70
Adenocarcinoma
Least adjusted

REF	NRR	SEX	AD	Number Exposed Case	Cont	Non-exposed Case	Cont	RR	95.00%CI
*ANDERS	12	f	0	99	96164	33	195158	6.09	(4.11- 9.03)
BENHAM	8	m	0	42	62	3	11	2.48	(0.65- 9.44)
BENHAM	9	m	0	36	28	0	11	29.46~	(1.66- 521.34)
Subtotal BENHAM								3.85	(1.15- 12.94)
*HAMMON	91	m	0	26	382338	2	115884	3.94	(0.94- 16.60)
Totals				203	478592	38	311064		

*prospective study ~ With 0.5 adjustment for zero

REF	NRR	SEX	AD	Ys	Ws	Qs	Ps
*ANDERS	12	f	0	1.81	24.76	0.12	0.0000
BENHAM	8	m	0	0.91	2.15	1.48	0.1817
BENHAM	9	m	0	3.38	0.47	1.26	0.0210
Subtotal BENHAM				1.35	2.62	2.74	
*HAMMON	91	m	0	1.37	1.86	0.25	0.0617

	N	4
	NS	3
	Wt	29.24
Het	Chi	3.10
Het	df	3
Het	P	N.S.
Fixed	RR	5.68
	RRl	3.96
	RRu	8.17
	P	+++
Random	RR	5.60
	RRl	3.73
	RRu	8.41
	P	+++
Asymm	P	N.S.

Table 3A8 - 6

IESLC - Meta-analysis of Ever Smoking, Cigarettes (or Any Product if Cigarettes not available), Age 50-70
Adenocarcinoma
Least adjusted

	Sex combined	male	female	Total
N		3	1	4
NS		2	1	3
Wt		4.48	24.76	29.24
Het Chi		2.34	0.00	3.10
Het df		2	0	3
Het P		N.S.	N.S.	N.S.
Fixed RR		3.89	6.09	5.68
RRl		1.54	4.11	3.96
RRu		9.82	9.03	8.17
P		++	+++	+++
Random RR		4.06	6.09	5.60
RRl		1.45	4.11	3.73
RRu		11.37	9.03	8.41
P		++	+++	+++
Between Chi				0.76
Between df				1
Between P				N.S.
Btwn(F) P				N.S.
Btwn(R) P				N.S.

Table 3A8 - 7

IESLC - Meta-analysis of Ever Smoking, Cigarettes (or Any Product if Cigarettes not available), Age 50-70
Adenocarcinoma
Excluded studies (and stage at which they were excluded)

Stage	Excluded studies
1	BOUCHA BUELL LAURIL MZILEN
2	BYERS2 ENSTRO HIRAY2 KANELL LIAW LICKIN MRFIT MURATA NOTANI RESTRE RIMING SAARIK SEGI2 SHIMIZ SITAS SOBUE2 TANG2 WALD WARSIN WATSON WU2 WYNDE5 WYNDE8 WYNDER
3	ABELIN ABRAHA AGUDO AKIBA ALDERS AMANDU AMES ARCHER ARMADA AUSTIN AUVINE AXELSO AXELSS BAND BARBON BECHER BENSHL BERRIN BEST BLOHMK BLOT1 BLOT2 BLOT3 BLOT4 BOFFET BOUCOT BRESLO BRETT BROCKM BROSS BROWN1 BYERS1 CARPEN CASCO2 CASCOR CEDERL CHAN CHANG CHATZI CHEN CHEN2 CHEN3 CHIAZZ CHOI CHOW CHYOU COMSTO COOKSO CORREA CPSII DARBY DAVEYS DEAN DEAN2 DEKLER DESTE2 DESTEF DOCKER DOLL DOLL2 DORGAN DOSEME DROSTE DU DUNN EBELIN ENGELA ESAKI FAN GAO GAO2 GARCIA GARDIN GARSHI GENG GER GILLIS GODLEY GOLLED GOODMA GRAHAM GREGOR GSELL HAENSZ HAMMO2 HANSEN HEGMAN HEIN HENNEK HIRAOK HIRAYA HOLE HOROWI HORWIT HU HU2 HUANG HUMBLE ISHIMA JAHN JAIN JARUP JARVHO JEDRYC JIANG JOLY JONES JUSSAW KAISE2 KAISER KATSOU KAUFMA KELLER KHUDER KINLEN KJUUS KNEKT KO KOHLME KOO KOULUM KREYBE LAMTH LAMWK LAMWK2 LANGE LAUSSM LEI LEMARC LETOUR LIDDEL LIU LIU2 LIU3 LIU4 LIU5 LOMBA2 LOMBAR LUBIN LUBIN2 LUO MACLEN MAGNUS MARSH MARSH2 MARTIS MASTRA MATOS MATSUD MCCONN MCDUFF MCLAUG MIGRAN MILLER MILLS MOLLO MRFITR NOTAN2 NOU ODRISC ORMOS OSANN OSANN2 PARKIN PASTOR PAWLEG PERNU PERSH2 PERSHA PETO PEZZO2 PEZZOT PIKE PISANI POFFIJ POLEDN QIAO QIAO2 RACHTA RADZIK RANDIG REN RONCO ROOTS ROTHSC SANKAR SCHWA2 SCHWAR SEGI SEOW SHAW SIEMIA SIMARA SIMONA SOBUE SPEIZE SPITZ STASZE STAYNE STOCKW STUCKE SUN SUZUK2 SUZUKI SVENSS TANG TAO TENKAN TIZZAN TOKARS TOUSEY TSUGAN TULINI TVERDA ULMER VANDER VEIERO WAKAI WANG WANG2 WANG3 WANG4 WICKLU WIGLE WILKIN WU WUNSCH WYNDE2 WYNDE3 WYNDE6 WYNDE7 XIANGZ XU XU2 XU3 XU4 YAMAGU YONG YUAN ZHANG ZHENG ZHOU
4	BROWN2 SADOWS
9	BUFFLE CPSI DAMBER DEAN3 DORANT DORN HINDS HITOSU KIHARA KREUZE KUBIK LEVIN NAM PRESCO STOCKS VUTUC WUWILL WYNDE4

Table 3A8 - 8
Potentially overlapping studies

REF	REFGP	PRINC	.	OVERLAP/LINK
BENHAM	LUBIN2	2		Subset of Lubin2

Table 3A9 -

IESLC - Meta-analysis of Ever Smoking, Cigarettes (or Any Product if Cigarettes not available), Age 65+
Adenocarcinoma

This analysis is restricted to results for:
1) Non-dose-response data
2) Ever smokers
3) Age 65+
4) Results complete enough for use in metaanalysis

Within each study, results are then selected (in the following order of preference, within each sex) for:
5) PRODUCT: cigarettes regardless of other products, cigarettes only, all/unspec
6) CIGTYPE: all/unspecified, MC regardless of HR, MC only
7) DENOM: never smoked anything, never smoked cigarettes, (never +1 = +long term ex, +2 = +amount unknown,
+3 = never cigs+long term ex)
8) Followup period (YF, prospective studies): whole study (coded as 0) or longest available
9) LCtype: all or nearest available, at least Squamous and Adeno. (q = squamous, s = small,
a = adeno, l = large, KII = Kreyberg II, al = alveolar, br = bronchiolar, u = undifferentiated)
10) Race: all or nearest available, otherwise by race (wh or w = white, bl or b = black, hi = hispanic
ch = chinese, jap = japanese, haw = hawaiian, w+o = white + oriental, sca = scandinavian, as = asian)
11) For overlapping studies: principal rather than subsidiary studies
Finally by Age: whole study (actual age shown) if available, otherwise by widest available age group
and then for single sex results (m, f) in preference to combined sex results (c).

Results adjusted (AD) for the most potential confounders are then chosen in Sections -1 to -3
(and those which actually differ from the adjusted results in Table 3A2 - 1 are marked 'x' in Section -1)
and results adjusted for the least confounders in Sections -4 to -6. (Those least adjusted results which
actually differ from the most adjusted as marked 'x' in column X in Section -4)
(Results adjusted for an unknown number of confounder(s) are coded as 20.)

Section -7 shows excluded studies, together with the stage (as above) at which no qualifying
results were found.

Section -8 lists the potentially overlapping studies which have been included (1=principal, 2=subsidiary).

Section -9 lists any results which would have been included in preference except that they had data not complete
enough for use in meta-analysis, with their significance (yes/no), if known, and any further comment as entered
on the database.

In addition to those mentioned above, the following fields, levels and abbreviations are used:
* or nk = not known, n = no, y = yes, ot = other
nev = never
all/unspec = all or unspecified, cig+/-ot = cigarettes irrespective of other products (cigar, pipe etc)
MC = manufactured cigarettes, HR = hand-rolled cigarettes
REF: 6-character study reference
NRR: number of the RR on the database within the study
ST : study type (CC = case control, pr or prosp = prospective)
NLC: number of lung cancer cases in whole study
R : risky occupational population (n = no, m = mining, o = other risky)
VB : national cigarette type (V = at least 75% Virginia, bl = at least 75% blended, ot = other)
P : any proxy use
H : full histological confirmation
De : derivation of RR/CI (or = original, st = standard method, ot = other method of estimation)

Table 3A9 - 1

IESLC - Meta-analysis of Ever Smoking, Cigarettes (or Any Product if Cigarettes not available), Age 65+
Adenocarcinoma
Most adjusted

REF	NRR	3A2	SEX	AGEL	AGEH	RACE	YF	LC TYPE	LOC	START	ST	NLC	R	VB	P	H	AD	PRODUCT	DENOM	De
BENHAM	10	x	m	70	99	all	-	KII	Eu:wst	1976	CC	1625	n	bl	n	y	0	cig only	nev any	st
DORGAN	134	x	f	72	99	all	-	a	NAmer	1980	CC	2026	n	bl	y	y	2	cig+/-ot	nev any	or

Cigarette type is all/unspec for all RRs

Table 3A9 - 2

IESLC - Meta-analysis of Ever Smoking, Cigarettes (or Any Product if Cigarettes not available), Age 65+
Adenocarcinoma
Most adjusted

REF	NRR	SEX	AD	Number Exposed Case	Cont	Non-exposed Case	Cont	RR	95.00%CI
BENHAM	10	m	0	5	13	2	6	1.15	(0.17- 7.74)
DORGAN	134	f	2	-	-	-	-	1.80	(1.00- 3.20)
Partial Totals				5	13	2	6		

*prospective study

REF	NRR	SEX	AD	Ys	Ws	Qs	Ps
BENHAM	10	m	0	0.14	1.06	0.18	0.8829
DORGAN	134	f	2	0.59	11.36	0.02	0.0476

	N	2
	NS	2
	Wt	12.42
Het	Chi	0.19
Het	df	1
Het	P	N.S.
Fixed	RR	1.73
	RRl	0.99
	RRu	3.02
	P	(+)
Random	RR	1.73
	RRl	0.99
	RRu	3.02
	P	(+)
Asymm	P	

Table 3A9 - 3

IESLC - Meta-analysis of Ever Smoking, Cigarettes (or Any Product if Cigarettes not available), Age 65+
Adenocarcinoma
Most adjusted

	Sex			
	combined	male	female	Total
N		1	1	2
NS		1	1	2
Wt		1.06	11.36	12.42
Het Chi		0.00	0.00	0.19
Het df		0	0	1
Het P		N.S.	N.S.	N.S.
Fixed RR		1.15	1.80	1.73
RRl		0.17	1.01	0.99
RRu		7.74	3.22	3.02
P		N.S.	+	(+)
Random RR		1.15	1.80	1.73
RRl		0.17	1.01	0.99
RRu		7.74	3.22	3.02
P		N.S.	+	(+)
Between Chi				0.19
Between df				1
Between P				N.S.
Btwn(F) P				N.S.
Btwn(R) P				N.S.

Too few RRs for analysis by factor

Table 3A9 - 4

IESLC - Meta-analysis of Ever Smoking, Cigarettes (or Any Product if Cigarettes not available), Age 65+
Adenocarcinoma
Least adjusted

REF	NRR	X	SEX	AGEL	AGEH	RACE	YF	LC TYPE	LOC	START	ST	NLC	R	VB	P	H	AD	PRODUCT	DENOM	De
BENHAM	10		m	70	99	all	-	KII	Eu:wst	1976	CC	1625	n	bl	n	y	0	cig only	nev any	st
DORGAN	134		f	72	99	all	-	a	NAmer	1980	CC	2026	n	bl	y	y	2	cig+/-ot	nev any	or

Cigarette type is all/unspec for all RRs

Table 3A9 - 5

IESLC - Meta-analysis of Ever Smoking, Cigarettes (or Any Product if Cigarettes not available), Age 65+
Adenocarcinoma
Least adjusted

				Number Exposed		Non-exposed			
REF	NRR	SEX	AD	Case	Cont	Case	Cont	RR	95.00%CI
BENHAM	10	m	0	5	13	2	6	1.15	(0.17- 7.74)
DORGAN	134	f	2	-	-	-	-	1.80	(1.00- 3.20)
Partial Totals				5	13	2	6		

*prospective study

REF	NRR	SEX	AD	Ys	Ws	Qs	Ps
BENHAM	10	m	0	0.14	1.06	0.18	0.8829
DORGAN	134	f	2	0.59	11.36	0.02	0.0476

	N	2
	NS	2
	Wt	12.42
Het	Chi	0.19
Het	df	1
Het	P	N.S.
Fixed	RR	1.73
	RRl	0.99
	RRu	3.02
	P	(+)
Random	RR	1.73
	RRl	0.99
	RRu	3.02
	P	(+)
Asymm	P	

Table 3A9 - 6

IESLC - Meta-analysis of Ever Smoking, Cigarettes (or Any Product if Cigarettes not available), Age 65+
Adenocarcinoma
Least adjusted

	Sex combined	male	female	Total
N		1	1	2
NS		1	1	2
Wt		1.06	11.36	12.42
Het Chi		0.00	0.00	0.19
Het df		0	0	1
Het P		N.S.	N.S.	N.S.
Fixed RR		1.15	1.80	1.73
RRl		0.17	1.01	0.99
RRu		7.74	3.22	3.02
P		N.S.	+	(+)
Random RR		1.15	1.80	1.73
RRl		0.17	1.01	0.99
RRu		7.74	3.22	3.02
P		N.S.	+	(+)
Between Chi				0.19
Between df				1
Between P				N.S.
Btwn(F) P				N.S.
Btwn(R) P				N.S.

Table 3A9 - 7

IESLC - Meta-analysis of Ever Smoking, Cigarettes (or Any Product if Cigarettes not available), Age 65+
Adenocarcinoma
Excluded studies (and stage at which they were excluded)

1 BOUCHA BUELL LAURIL MZILEN

2 BYERS2 ENSTRO HIRAY2 KANELL LIAW LICKIN MRFIT MURATA NOTANI RESTRE RIMING SAARIK SEGI2 SHIMIZ SITAS SOBUE2
TANG2 WALD WARSIN WATSON WU2 WYNDE5 WYNDE8 WYNDER

3 ABELIN ABRAHA AGUDO AKIBA ALDERS AMANDU AMES ANDERS ARCHER ARMADA AUSTIN AUVINE AXELSO AXELSS BAND BARBON
BECHER BENSHL BERRIN BEST BLOHMK BLOT1 BLOT2 BLOT3 BLOT4 BOFFET BOUCOT BRESLO BRETT BROCKM BROSS BROWN1
BYERS1 CARPEN CASCO2 CASCOR CEDERL CHAN CHANG CHATZI CHEN CHEN2 CHEN3 CHIAZZ CHOI CHOW CHYOU COOKSO
CORREA CPSII DARBY DAVEYS DEAN DEAN2 DEKLER DESTE2 DESTEF DOCKER DOLL DOLL2 DORANT DOSEME DROSTE DU
DUNN EBELIN ENGELA ESAKI FAN GAO GAO2 GARCIA GARDIN GENG GER GILLIS GOLLED GOODMA GRAHAM GREGOR
GSELL HAENSZ HAMMO2 HANSEN HEGMAN HEIN HENNEK HIRAOK HIRAYA HITOSU HOLE HOROWI HORWIT HU HU2 HUANG
ISHIMA JAHN JAIN JARUP JARVHO JEDRYC JIANG JOLY JONES JUSSAW KAISER KATSOU KAUFMA KELLER KHUDER KIHARA
KINLEN KJUUS KNEKT KO KOHLME KOO KOULUM KREUZE KREYBE KUBIK LAMTH LAMWK LAMWK2 LANGE LAUSSM LEI
LEMARC LETOUR LIDDEL LIU LIU2 LIU3 LIU5 LOMBA2 LOMBAR LUBIN LUBIN2 LUO MACLEN MAGNUS MARSH MARSH2
MARTIS MASTRA MATOS MATSUD MCCONN MCDUFF MCLAUG MIGRAN MILLER MILLS MOLLO MRFITR NOTAN2 NOU ODRISC ORMOS
OSANN OSANN2 PARKIN PASTOR PAWLEG PERNU PERSH2 PETO PEZZO2 PEZZOT PIKE PISANI POFFIJ POLEDN QIAO QIAO2
RACHTA RADZIK RANDIG REN RONCO ROOTS ROTHSC SANKAR SCHWA2 SCHWAR SEGI SEOW SHAW SIEMIA SIMARA SIMONA
SOBUE SPEIZE SPITZ STASZE STAYNE STOCKW STUCKE SUN SUZUK2 SUZUKI SVENSS TANG TAO TENKAN TIZZAN TOKARS
TOUSEY TSUGAN TULINI TVERDA ULMER VANDER VEIERO VUTUC WAKAI WANG WANG2 WANG3 WANG4 WICKLU WIGLE WILKIN
WU WUNSCH WYNDE2 WYNDE3 WYNDE6 WYNDE7 XIANGZ XU XU2 XU3 XU4 YAMAGU YONG YUAN ZHANG ZHENG
ZHOU

4 BROWN2 SADOWS

7 PERSHA

9 BUFFLE COMSTO CPSI DAMBER DEAN3 DORN GARSHI GODLEY HAMMON HINDS HUMBLE KAISE2 LEVIN LIU4 NAM PRESCO
STOCKS WUWILL WYNDE4

Table 3A9 - 8

Potentially overlapping studies

REF	REFGP	PRINC	. OVERLAP/LINK
BENHAM	LUBIN2	2	Subset of Lubin2

Table 3A10 -

IESLC - Meta-analysis of Ever Smoking, Cigarettes only, Age <56
Adenocarcinoma

This analysis is restricted to results for:
1) Non-dose-response data
2) Ever smokers
3) Age <56
4) Results complete enough for use in metaanalysis

Within each study, results are then selected (in the following order of preference, within each sex) for:
5) PRODUCT: cigarettes only
6) CIGTYPE: all/unspecified, MC regardless of HR, MC only
7) DENOM: never smoked anything, never smoked cigarettes, (never +1 = +long term ex, +2 = +amount unknown, +3 = never cigs+long term ex)
8) Followup period (YF, prospective studies): whole study (coded as 0) or longest available
9) LCtype: all or nearest available, at least Squamous and Adeno. (q = squamous, s = small, a = adeno, l = large, KII = Kreyberg II, al = alveolar, br = bronchiolar, u = undifferentiated)
10) Race: all or nearest available, otherwise by race (wh or w = white, bl or b = black, hi = hispanic ch = chinese, jap = japanese, haw = hawaiian, w+o = white + oriental, sca = scandinavian, as = asian)
11) For overlapping studies: principal rather than subsidiary studies
Finally by Age: whole study (actual age shown) if available, otherwise by widest available age group and then for single sex results (m, f) in preference to combined sex results (c).

Results adjusted (AD) for the most potential confounders are then chosen in Sections -1 to -3 (and those which actually differ from the adjusted results in Table 3A3 - 1 are marked 'x' in Section -1) and results adjusted for the least confounders in Sections -4 to -6. (Those least adjusted results which actually differ from the most adjusted as marked 'x' in column X in Section -4) (Results adjusted for an unknown number of confounder(s) are coded as 20.)

Section -7 shows excluded studies, together with the stage (as above) at which no qualifying results were found.

Section -8 lists the potentially overlapping studies which have been included (1=principal, 2=subsidiary).

Section -9 lists any results which would have been included in preference except that they had data not complete enough for use in meta-analysis, with their significance (yes/no), if known, and any further comment as entered on the database.

In addition to those mentioned above, the following fields, levels and abbreviations are used:
* or nk = not known, n = no, y = yes, ot = other
nev = never
all/unspec = all or unspecified, MC = manufactured cigarettes, HR = hand-rolled cigarettes
REF: 6-character study reference
NRR: number of the RR on the database within the study
ST : study type (CC = case control, pr or prosp = prospective)
NLC: number of lung cancer cases in whole study
R : risky occupational population (n = no, m = mining, o = other risky)
VB : national cigarette type (V = at least 75% Virginia, bl = at least 75% blended, ot = other)
P : any proxy use
H : full histological confirmation
De : derivation of RR/CI (or = original, st = standard method, ot = other method of estimation)

Table 3A10 - 1

IESLC - Meta-analysis of Ever Smoking, Cigarettes only, Age <56
Adenocarcinoma
Most adjusted

REF	NRR	3A3	SEX	AGEL	AGEH	RACE	YF	LC TYPE	LOC	START	ST	NLC	R	VB	P	H	AD	PRODUCT	DENOM	De
BENHAM	7	x	m	1	49	all	-	KII	Eu:wst	1976	CC	1625	n	bl	n	y	0	cig only	nev any	st

Cigarette type is all/unspec for all RRs

Table 3A10 - 2

IESLC - Meta-analysis of Ever Smoking, Cigarettes only, Age <56
Adenocarcinoma
Most adjusted

REF	NRR	SEX	AD	Number Exposed Case	Cont	Non-exposed Case	Cont	RR	95.00%CI
BENHAM	7	m	0	27	40	4	14	2.36	(0.70- 7.95)
Totals				27	40	4	14		

*prospective study

REF	NRR	SEX	AD	Ys	Ws	Qs	Ps
BENHAM	7	m	0	0.86	2.61	0.00	0.1650

	N	1
	NS	1
	Wt	2.61
Het	Chi	0.00
Het	df	0
Het	P	N.S.
Fixed	RR	2.36
	RRl	0.70
	RRu	7.95
	P	N.S.
Random	RR	2.36
	RRl	0.70
	RRu	7.95
	P	N.S.
Asymm	P	

Table 3A10 - 3

IESLC - Meta-analysis of Ever Smoking, Cigarettes only, Age <56
Adenocarcinoma
Most adjusted

	Sex combined	male	female	Total
N		1		1
NS		1		1
Wt		2.61		2.61
Het Chi		0.00		0.00
Het df		0		0
Het P		N.S.		N.S.
Fixed RR		2.36		2.36
RRl		0.70		0.70
RRu		7.95		7.95
P		N.S.		N.S.
Random RR		2.36		2.36
RRl		0.70		0.70
RRu		7.95		7.95
P		N.S.		N.S.
Between Chi				
Between df				
Between P				N.S.
Btwn(F) P				N.S.
Btwn(R) P				N.S.

Too few RRs for analysis by factor

Table 3A10 - 4

IESLC - Meta-analysis of Ever Smoking, Cigarettes only, Age <56
Adenocarcinoma
Least adjusted

REF	NRR	X	SEX	AGEL	AGEH	RACE	YF	LC TYPE	LOC	START	ST	NLC	R	VB	P	H	AD	PRODUCT	DENOM	De
BENHAM	7		m	1	49	all	-	KII	Eu:wst	1976	CC	1625	n	bl	n	y	0	cig only	nev any	st

Cigarette type is all/unspec for all RRs

Table 3A10 - 5

IESLC - Meta-analysis of Ever Smoking, Cigarettes only, Age <56
Adenocarcinoma
Least adjusted

REF	NRR	SEX	AD	Number Exposed Case	Number Exposed Cont	Non-exposed Case	Non-exposed Cont	RR	95.00%CI
BENHAM	7	m	0	27	40	4	14	2.36	(0.70- 7.95)
Totals				27	40	4	14		

*prospective study

REF	NRR	SEX	AD	Ys	Ws	Qs	Ps
BENHAM	7	m	0	0.86	2.61	0.00	0.1650

	N	1
	NS	1
	Wt	2.61
Het	Chi	0.00
Het	df	0
Het	P	N.S.
Fixed	RR	2.36
	RRl	0.70
	RRu	7.95
	P	N.S.
Random	RR	2.36
	RRl	0.70
	RRu	7.95
	P	N.S.
Asymm	P	

Table 3A10 - 6

IESLC - Meta-analysis of Ever Smoking, Cigarettes only, Age <56
Adenocarcinoma
Least adjusted

	Sex combined	male	female	Total
N		1		1
NS		1		1
Wt		2.61		2.61
Het Chi		0.00		0.00
Het df		0		0
Het P		N.S.		N.S.
Fixed RR		2.36		2.36
RRl		0.70		0.70
RRu		7.95		7.95
P		N.S.		N.S.
Random RR		2.36		2.36
RRl		0.70		0.70
RRu		7.95		7.95
P		N.S.		N.S.
Between Chi				
Between df				
Between P				N.S.
Btwn(F) P				N.S.
Btwn(R) P				N.S.

Table 3A10 - 7

IESLC - Meta-analysis of Ever Smoking, Cigarettes only, Age <56
Adenocarcinoma
Excluded studies (and stage at which they were excluded)

Stage	Studies
1	BOUCHA BUELL LAURIL MZILEN
2	BYERS2 ENSTRO HIRAY2 KANELL LIAW LICKIN MRFIT MURATA NOTANI RESTRE RIMING SAARIK SEGI2 SHIMIZ SITAS SOBUE2 TANG2 WALD WARSIN WATSON WU2 WYNDE5 WYNDE8 WYNDER
3	ABELIN ABRAHA AGUDO AKIBA ALDERS AMANDU AMES ANDERS ARCHER ARMADA AUSTIN AUVINE AXELSO AXELSS BAND BARBON BECHER BENSHL BERRIN BEST BLOHMK BLOT1 BLOT2 BLOT3 BLOT4 BOFFET BOUCOT BRESLO BRETT BROCKM BROSS BROWN1 BROWN2 BUFFLE BYERS1 CARPEN CASCO2 CASCOR CEDERL CHAN CHANG CHATZI CHEN CHEN2 CHEN3 CHIAZZ CHOI CHOW CHYOU COOKSO CORREA CPSII DAMBER DARBY DAVEYS DEAN DEAN2 DEKLER DESTE2 DESTEF DOCKER DOLL DOLL2 DORANT DORGAN DORN DOSEME DROSTE DU DUNN EBELIN ENGELA ESAKI FAN GAO GAO2 GARCIA GARDIN GARSHI GENG GER GILLIS GODLEY GOLLED GOODMA GRAHAM GREGOR GSELL HAENSZ HAMMO2 HANSEN HEGMAN HEIN HENNEK HIRAOK HIRAYA HOLE HOROWI HORWIT HU HU2 HUANG HUMBLE ISHIMA JAHN JAIN JARUP JARVHO JEDRYC JIANG JOLY JONES JUSSAW KAISE2 KAISER KATSOU KAUFMA KELLER KHUDER KIHARA KINLEN KJUUS KNEKT KO KOHLME KOO KOULUM KREYBE LAMTH LAMWK LAMWK2 LANGE LAUSSM LEI LEMARC LETOUR LIDDEL LIU LIU2 LIU3 LIU4 LIU5 LOMBA2 LOMBAR LUBIN LUBIN2 LUO MACLEN MAGNUS MARSH MARSH2 MARTIS MASTRA MATOS MATSUD MCCONN MCDUFF MCLAUG MIGRAN MILLER MILLS MOLLO MRFITR NOTAN2 NOU ODRISC ORMOS OSANN OSANN2 PARKIN PASTOR PAWLEG PERNU PERSH2 PERSHA PETO PEZZO2 PEZZOT PIKE PISANI POFFIJ POLEDN QIAO QIAO2 RACHTA RADZIK RANDIG REN RONCO ROOTS ROTHSC SANKAR SCHWA2 SEGI SEOW SHAW SIEMIA SIMARA SIMONA SOBUE SPITZ STASZE STAYNE STOCKW STUCKE SUN SUZUK2 SUZUKI SVENSS TANG TAO TENKAN TIZZAN TOKARS TOUSEY TULINI ULMER VANDER VEIERO WAKAI WANG WANG2 WANG3 WANG4 WICKLU WIGLE WILKIN WU WUNSCH WYNDE2 WYNDE3 WYNDE7 XIANGZ XU XU2 XU3 XU4 YAMAGU YONG YUAN ZHANG ZHENG ZHOU
4	SADOWS
5	COMSTO HINDS HITOSU KREUZE KUBIK LEVIN NAM PRESCO SCHWAR SPEIZE STOCKS TSUGAN TVERDA VUTUC WUWILL WYNDE4 WYNDE6
9	CPSI DEAN3 HAMMON

Table 3A10 - 8

Potentially overlapping studies

REF	REFGP	PRINC	.	OVERLAP/LINK
BENHAM	LUBIN2	2		Subset of Lubin2

Table 3A11 -

IESLC - Meta-analysis of Ever Smoking, Cigarettes only, Age 50-70
Adenocarcinoma

This analysis is restricted to results for:
1) Non-dose-response data
2) Ever smokers
3) Maximum age range 50-70
4) Results complete enough for use in metaanalysis

Within each study, results are then selected (in the following order of preference, within each sex) for:
5) PRODUCT: cigarettes only
6) CIGTYPE: all/unspecified, MC regardless of HR, MC only
7) DENOM: never smoked anything, never smoked cigarettes, (never +1 = +long term ex, +2 = +amount unknown, +3 = never cigs+long term ex)
8) Followup period (YF, prospective studies): whole study (coded as 0) or longest available
9) LCtype: all or nearest available, at least Squamous and Adeno. (q = squamous, s = small, a = adeno, l = large, KII = Kreyberg II, al = alveolar, br = bronchiolar, u = undifferentiated)
10) Race: all or nearest available, otherwise by race (wh or w = white, bl or b = black, hi = hispanic ch = chinese, jap = japanese, haw = hawaiian, w+o = white + oriental, sca = scandinavian, as = asian)
11) For overlapping studies: principal rather than subsidiary studies
Finally by Age: whole study (actual age shown) if available, otherwise by widest available age group and then for single sex results (m, f) in preference to combined sex results (c).

Results adjusted (AD) for the most potential confounders are then chosen in Sections -1 to -3 (and those which actually differ from the adjusted results in Table 3A3 - 1 are marked 'x' in Section -1) and results adjusted for the least confounders in Sections -4 to -6. (Those least adjusted results which actually differ from the most adjusted as marked 'x' in column X in Section -4)
(Results adjusted for an unknown number of confounder(s) are coded as 20.)

Section -7 shows excluded studies, together with the stage (as above) at which no qualifying results were found.

Section -8 lists the potentially overlapping studies which have been included (1=principal, 2=subsidiary).

Section -9 lists any results which would have been included in preference except that they had data not complete enough for use in meta-analysis, with their significance (yes/no), if known, and any further comment as entered on the database.

In addition to those mentioned above, the following fields, levels and abbreviations are used:
* or nk = not known, n = no, y = yes, ot = other
nev = never
all/unspec = all or unspecified, MC = manufactured cigarettes, HR = hand-rolled cigarettes
REF: 6-character study reference
NRR: number of the RR on the database within the study
ST : study type (CC = case control, pr or prosp = prospective)
NLC: number of lung cancer cases in whole study
R : risky occupational population (n = no, m = mining, o = other risky)
VB : national cigarette type (V = at least 75% Virginia, bl = at least 75% blended, ot = other)
P : any proxy use
H : full histological confirmation
De : derivation of RR/CI (or = original, st = standard method, ot = other method of estimation)

Table 3A11 - 1

IESLC - Meta-analysis of Ever Smoking, Cigarettes only, Age 50-70
Adenocarcinoma
Most adjusted

REF	NRR	3A3	SEX	AGEL	AGEH	RACE	YF	LC TYPE	LOC	START	ST	NLC	R	VB	P	H	AD	PRODUCT	DENOM	De
BENHAM	8	x	m	50	59	all	-	KII	Eu:wst	1976	CC	1625	n	bl	n	y	0	cig only	nev any	st
BENHAM	9	x	m	60	69	all	-	KII	Eu:wst	1976	CC	1625	n	bl	n	y	0	cig only	nev any	ot
HAMMON	83		m	50	69	wh	0	a	NAmer	1952	pr	448	n	bl	n	n	1	cig only	nev any	ot

Cigarette type is all/unspec for all RRs

Table 3A11 - 2

IESLC - Meta-analysis of Ever Smoking, Cigarettes only, Age 50-70
Adenocarcinoma
Most adjusted

REF	NRR	SEX	AD	Number Exposed Case	Number Exposed Cont	Non-exposed Case	Non-exposed Cont	RR	95.00%CI
BENHAM	8	m	0	42	62	3	11	2.48	(0.65- 9.44)
BENHAM	9	m	0	36	28	0	11	29.46~	(1.66- 521.34)
Subtotal BENHAM								3.85	(1.15- 12.94)
*HAMMON	83	m	1	-	-	-	-	3.39	(0.77- 14.91)
Partial Totals				78	90	3	22		

*prospective study

~ With 0.5 adjustment for zero

REF	NRR	SEX	AD	Ys	Ws	Qs	Ps
BENHAM	8	m	0	0.91	2.15	0.32	0.1817
BENHAM	9	m	0	3.38	0.47	2.02	0.0210
Subtotal BENHAM				1.35	2.62	2.35	
*HAMMON	83	m	1	1.22	1.75	0.01	0.1063

	N	3
	NS	2
	Wt	4.37
Het	Chi	2.36
Het	df	2
Het	P	N.S.
Fixed	RR	3.66
	RRl	1.43
	RRu	9.35
	P	++
Random	RR	3.83
	RRl	1.34
	RRu	10.93
	P	+
Asymm	P	*

Table 3A11 - 3

IESLC - Meta-analysis of Ever Smoking, Cigarettes only, Age 50-70
Adenocarcinoma
Most adjusted

	Sex combined	male	female	Total
N		3		3
NS		2		2
Wt		4.37		4.37
Het Chi		2.36		2.36
Het df		2		2
Het P		N.S.		N.S.
Fixed RR		3.66		3.66
RRl		1.43		1.43
RRu		9.35		9.35
P		++		++
Random RR		3.83		3.83
RRl		1.34		1.34
RRu		10.93		10.93
P		+		+
Between Chi				
Between df				
Between P				N.S.
Btwn(F) P				N.S.
Btwn(R) P				N.S.

Too few RRs for analysis by factor

Table 3A11 - 4

IESLC - Meta-analysis of Ever Smoking, Cigarettes only, Age 50-70
Adenocarcinoma
Least adjusted

REF	NRR	X	SEX	AGEL	AGEH	RACE	YF	LC TYPE	LOC	START	ST	NLC	R	VB	P	H	AD	PRODUCT	DENOM	De
BENHAM	8		m	50	59	all	-	KII	Eu:wst	1976	CC	1625	n	bl	n	y	0	cig only	nev any	st
BENHAM	9		m	60	69	all	-	KII	Eu:wst	1976	CC	1625	n	bl	n	y	0	cig only	nev any	ot
HAMMON	90	x	m	50	69	wh	0	a	NAmer	1952	pr	448	n	bl	n	n	0	cig only	nev any	st

Cigarette type is all/unspec for all RRs

Table 3A11 - 5

IESLC - Meta-analysis of Ever Smoking, Cigarettes only, Age 50-70
Adenocarcinoma
Least adjusted

REF	NRR	SEX	AD	Number Exposed Case	Cont	Non-exposed Case	Cont	RR	95.00%CI
BENHAM	8	m	0	42	62	3	11	2.48	(0.65- 9.44)
BENHAM	9	m	0	36	28	0	11	29.46~	(1.66- 521.34)
Subtotal BENHAM								3.85	(1.15- 12.94)
*HAMMON	90	m	0	14	225565	2	115884	3.60	(0.82- 15.82)
Totals				92	225655	5	115906		

*prospective study

~ With 0.5 adjustment for zero

REF	NRR	SEX	AD	Ys	Ws	Qs	Ps
BENHAM	8	m	0	0.91	2.15	0.36	0.1817
BENHAM	9	m	0	3.38	0.47	1.98	0.0210
Subtotal BENHAM				1.35	2.62	2.34	
*HAMMON	90	m	0	1.28	1.75	0.00	0.0904

	N	3
	NS	2
	Wt	4.37
Het	Chi	2.35
Het	df	2
Het	P	N.S.
Fixed	RR	3.75
	RRl	1.47
	RRu	9.57
	P	++
Random	RR	3.92
	RRl	1.38
	RRu	11.14
	P	+
Asymm	P	(*)

Table 3A11 - 6

IESLC - Meta-analysis of Ever Smoking, Cigarettes only, Age 50-70
Adenocarcinoma
Least adjusted

	Sex: combined	male	female	Total
N		3		3
NS		2		2
Wt		4.37		4.37
Het Chi		2.35		2.35
Het df		2		2
Het P		N.S.		N.S.
Fixed RR		3.75		3.75
RRl		1.47		1.47
RRu		9.57		9.57
P		++		++
Random RR		3.92		3.92
RRl		1.38		1.38
RRu		11.14		11.14
P		+		+
Between Chi				
Between df				
Between P				N.S.
Btwn(F) P				N.S.
Btwn(R) P				N.S.

Table 3A11 - 7

IESLC - Meta-analysis of Ever Smoking, Cigarettes only, Age 50-70
Adenocarcinoma
Excluded studies (and stage at which they were excluded)

1 BOUCHA BUELL LAURIL MZILEN

2 BYERS2 ENSTRO HIRAY2 KANELL LIAW LICKIN MRFIT MURATA NOTANI RESTRE RIMING SAARIK SEGI2 SHIMIZ SITAS SOBUE2 TANG2 WALD WARSIN WATSON WU2 WYNDE5 WYNDE8 WYNDER

3 ABELIN ABRAHA AGUDO AKIBA ALDERS AMANDU AMES ARCHER ARMADA AUSTIN AUVINE AXELSO AXELSS BAND BARBON BECHER BENSHL BERRIN BEST BLOHMK BLOT1 BLOT2 BLOT3 BLOT4 BOFFET BOUCOT BRESLO BRETT BROCKM BROSS BROWN1 BYERS1 CARPEN CASCO2 CASCOR CEDERL CHAN CHANG CHATZI CHEN CHEN2 CHEN3 CHIAZZ CHOI CHOW CHYOU COMSTO COOKSO CORREA CPSII DARBY DAVEYS DEAN DEAN2 DEKLER DESTE2 DESTEF DOCKER DOLL DOLL2 DORGAN DOSEME DROSTE DU DUNN EBELIN ENGELA ESAKI FAN GAO GAO2 GARCIA GARDIN GARSHI GENG GER GILLIS GODLEY GOLLED GOODMA GRAHAM GREGOR GSELL HAENSZ HAMMO2 HANSEN HEGMAN HEIN HENNEK HIRAOK HIRAYA HOLE HOROWI HORWIT HU HU2 HUANG HUMBLE ISHIMA JAHN JAIN JARUP JARVHO JEDRYC JIANG JOLY JONES JUSSAW KAISE2 KAISER KATSOU KAUFMA KELLER KHUDER KINLEN KJUUS KNEKT KO KOHLME KOO KOULUM KREYBE LAMTH LAMWK LAMWK2 LANGE LAUSSM LEI LEMARC LETOUR LIDDEL LIU LIU2 LIU3 LIU4 LIU5 LOMBA2 LOMBAR LUBIN LUBIN2 LUO MACLEN MAGNUS MARSH MARSH2 MARTIS MASTRA MATOS MATSUD MCCONN MCDUFF MCLAUG MIGRAN MILLER MILLS MOLLO MRFITR NOTAN2 NOU ODRISC ORMOS OSANN OSANN2 PARKIN PASTOR PAWLEG PERNU PERSH2 PERSHA PETO PEZZO2 PEZZOT PIKE PISANI POFFIJ POLEDN QIAO QIAO2 RACHTA RADZIK RANDIG REN RONCO ROOTS ROTHSC SANKAR SCHWA2 SCHWAR SEGI SEOW SHAW SIEMIA SIMARA SIMONA SOBUE SPEIZE SPITZ STASZE STAYNE STOCKW STUCKE SUN SUZUK2 SUZUKI SVENSS TANG TAO TENKAN TIZZAN TOKARS TOUSEY TSUGAN TULINI TVERDA ULMER VANDER VEIERO WAKAI WANG WANG2 WANG3 WANG4 WICKLU WIGLE WILKIN WU WUNSCH WYNDE2 WYNDE3 WYNDE6 WYNDE7 XIANGZ XU XU2 XU3 XU4 YAMAGU YONG YUAN ZHANG ZHENG ZHOU

4 BROWN2 SADOWS

5 ANDERS BUFFLE DAMBER DORANT DORN HINDS HITOSU KIHARA KREUZE KUBIK LEVIN NAM PRESCO STOCKS VUTUC WUWILL WYNDE4

9 CPSI DEAN3

Table 3A11 - 8
Potentially overlapping studies

REF	REFGP	PRINC	.	OVERLAP/LINK
BENHAM	LUBIN2	2		Subset of Lubin2

Table 3A12 -

IESLC - Meta-analysis of Ever Smoking, Cigarettes only, Age 65+
Adenocarcinoma

This analysis is restricted to results for:
1) Non-dose-response data
2) Ever smokers
3) Age 65+
4) Results complete enough for use in metaanalysis

Within each study, results are then selected (in the following order of preference, within each sex) for:
5) PRODUCT: cigarettes only
6) CIGTYPE: all/unspecified, MC regardless of HR, MC only
7) DENOM: never smoked anything, never smoked cigarettes, (never +1 = +long term ex, +2 = +amount unknown,
+3 = never cigs+long term ex)
8) Followup period (YF, prospective studies): whole study (coded as 0) or longest available
9) LCtype: all or nearest available, at least Squamous and Adeno. (q = squamous, s = small,
a = adeno, l = large, KII = Kreyberg II, al = alveolar, br = bronchiolar, u = undifferentiated)
10) Race: all or nearest available, otherwise by race (wh or w = white, bl or b = black, hi = hispanic
ch = chinese, jap = japanese, haw = hawaiian, w+o = white + oriental, sca = scandinavian, as = asian)
11) For overlapping studies: principal rather than subsidiary studies
Finally by Age: whole study (actual age shown) if available, otherwise by widest available age group
and then for single sex results (m, f) in preference to combined sex results (c).

Results adjusted (AD) for the most potential confounders are then chosen in Sections -1 to -3
(and those which actually differ from the adjusted results in Table 3A3 - 1 are marked 'x' in Section -1)
and results adjusted for the least confounders in Sections -4 to -6. (Those least adjusted results which
actually differ from the most adjusted as marked 'x' in column X in Section -4)
(Results adjusted for an unknown number of confounder(s) are coded as 20.)

Section -7 shows excluded studies, together with the stage (as above) at which no qualifying
results were found.

Section -8 lists the potentially overlapping studies which have been included (1=principal, 2=subsidiary).

Section -9 lists any results which would have been included in preference except that they had data not complete
enough for use in meta-analysis, with their significance (yes/no), if known, and any further comment as entered
on the database.

In addition to those mentioned above, the following fields, levels and abbreviations are used:
* or nk = not known, n = no, y = yes, ot = other
nev = never
all/unspec = all or unspecified, MC = manufactured cigarettes, HR = hand-rolled cigarettes
REF: 6-character study reference
NRR: number of the RR on the database within the study
ST : study type (CC = case control, pr or prosp = prospective)
NLC: number of lung cancer cases in whole study
R : risky occupational population (n = no, m = mining, o = other risky)
VB : national cigarette type (V = at least 75% Virginia, bl = at least 75% blended, ot = other)
P : any proxy use
H : full histological confirmation
De : derivation of RR/CI (or = original, st = standard method, ot = other method of estimation)

Table 3A12 - 1

IESLC - Meta-analysis of Ever Smoking, Cigarettes only, Age 65+
Adenocarcinoma
Most adjusted

REF	NRR	3A3	SEX	AGEL	AGEH	RACE	YF	LC TYPE	LOC	START	ST	NLC	R	VB	P	H	AD	PRODUCT	DENOM	De
BENHAM	10	x	m	70	99	all	-		KII	Eu:wst	1976	CC	1625	n	bl	n	y	0 cig only	nev	any st

Cigarette type is all/unspec for all RRs

Table 3A12 - 2

IESLC - Meta-analysis of Ever Smoking, Cigarettes only, Age 65+
Adenocarcinoma
Most adjusted

REF	NRR	SEX	AD	Number Case	Exposed Cont	Non-exposed Case	Cont	RR	95.00%CI
BENHAM	10	m	0	5	13	2	6	1.15	(0.17- 7.74)
Totals				5	13	2	6		

*prospective study

REF	NRR	SEX	AD	Ys	Ws	Qs	Ps
BENHAM	10	m	0	0.14	1.06	0.00	0.8829

	N	1
	NS	1
	Wt	1.06
Het	Chi	0.00
Het	df	0
Het	P	N.S.
Fixed	RR	1.15
	RRl	0.17
	RRu	7.74
	P	N.S.
Random	RR	1.15
	RRl	0.17
	RRu	7.74
	P	N.S.
Asymm	P	

Table 3A12 - 3

IESLC - Meta-analysis of Ever Smoking, Cigarettes only, Age 65+
Adenocarcinoma
Most adjusted

	Sex combined	male	female	Total
N		1		1
NS		1		1
Wt		1.06		1.06
Het Chi		0.00		0.00
Het df		0		0
Het P		N.S.		N.S.
Fixed RR		1.15		1.15
RRl		0.17		0.17
RRu		7.74		7.74
P		N.S.		N.S.
Random RR		1.15		1.15
RRl		0.17		0.17
RRu		7.74		7.74
P		N.S.		N.S.
Between Chi				
Between df				
Between P				N.S.
Btwn(F) P				N.S.
Btwn(R) P				N.S.

Too few RRs for analysis by factor

Table 3A12 - 4

IESLC - Meta-analysis of Ever Smoking, Cigarettes only, Age 65+
Adenocarcinoma
Least adjusted

REF	NRR	X	SEX	AGEL	AGEH	RACE	YF	LC TYPE	LOC	START	ST	NLC	R	VB	P	H	AD	PRODUCT	DENOM	De
BENHAM	10		m	70	99	all	-	KII	Eu:wst	1976	CC	1625	n	bl	n	y	0	cig only	nev any	st

Cigarette type is all/unspec for all RRs

Table 3A12 - 5

IESLC - Meta-analysis of Ever Smoking, Cigarettes only, Age 65+
Adenocarcinoma
Least adjusted

REF	NRR	SEX	AD	Number Exposed Case	Number Exposed Cont	Non-exposed Case	Non-exposed Cont	RR	95.00%CI
BENHAM	10	m	0	5	13	2	6	1.15	(0.17- 7.74)
Totals				5	13	2	6		

*prospective study

REF	NRR	SEX	AD	Ys	Ws	Qs	Ps
BENHAM	10	m	0	0.14	1.06	0.00	0.8829

	N	1
	NS	1
	Wt	1.06
Het	Chi	0.00
Het	df	0
Het	P	N.S.
Fixed	RR	1.15
	RRl	0.17
	RRu	7.74
	P	N.S.
Random	RR	1.15
	RRl	0.17
	RRu	7.74
	P	N.S.
Asymm	P	

Table 3A12 - 6

IESLC - Meta-analysis of Ever Smoking, Cigarettes only, Age 65+
Adenocarcinoma
Least adjusted

	Sex combined	male	female	Total
N		1		1
NS		1		1
Wt		1.06		1.06
Het Chi		0.00		0.00
Het df		0		0
Het P		N.S.		N.S.
Fixed RR		1.15		1.15
RRl		0.17		0.17
RRu		7.74		7.74
P		N.S.		N.S.
Random RR		1.15		1.15
RRl		0.17		0.17
RRu		7.74		7.74
P		N.S.		N.S.
Between Chi				
Between df				
Between P				N.S.
Btwn(F) P				N.S.
Btwn(R) P				N.S.

Table 3A12 - 7

IESLC - Meta-analysis of Ever Smoking, Cigarettes only, Age 65+
Adenocarcinoma
Excluded studies (and stage at which they were excluded)

1 BOUCHA BUELL LAURIL MZILEN

2 BYERS2 ENSTRO HIRAY2 KANELL LIAW LICKIN MRFIT MURATA NOTANI RESTRE RIMING SAARIK SEGI2 SHIMIZ SITAS SOBUE2
TANG2 WALD WARSIN WATSON WU2 WYNDE5 WYNDE8 WYNDER

3 ABELIN ABRAHA AGUDO AKIBA ALDERS AMANDU AMES ANDERS ARCHER ARMADA AUSTIN AUVINE AXELSO AXELSS BAND BARBON
BECHER BENSHL BERRIN BEST BLOHMK BLOT1 BLOT2 BLOT3 BLOT4 BOFFET BOUCOT BRESLO BRETT BROCKM BROSS BROWN1
BYERS1 CARPEN CASCO2 CASCOR CEDERL CHAN CHANG CHATZI CHEN CHEN2 CHEN3 CHIAZZ CHOI CHOW CHYOU COOKSO
CORREA CPSII DARBY DAVEYS DEAN DEAN2 DEKLER DESTE2 DESTEF DOCKER DOLL DOLL2 DORANT DOSEME DROSTE DU
DUNN EBELIN ENGELA ESAKI FAN GAO GAO2 GARCIA GARDIN GENG GER GILLIS GOLLED GOODMA GRAHAM GREGOR
GSELL HAENSZ HAMMO2 HANSEN HEGMAN HEIN HENNEK HIRAOK HIRAYA HITOSU HOLE HOROWI HORWIT HU HU2 HUANG
ISHIMA JAHN JAIN JARUP JARVHO JEDRYC JIANG JOLY JONES JUSSAW KAISER KATSOU KAUFMA KELLER KHUDER KIHARA
KINLEN KJUUS KNEKT KO KOHLME KOO KOULUM KREUZE KREYBE KUBIK LAMTH LAMWK LAMWK2 LANGE LAUSSM LEI
LEMARC LETOUR LIDDEL LIU LIU2 LIU3 LIU5 LOMBA2 LOMBAR LUBIN LUBIN2 LUO MACLEN MAGNUS MARSH MARSH2
MARTIS MASTRA MATOS MATSUD MCCONN MCDUFF MCLAUG MIGRAN MILLER MILLS MOLLO MRFITR NOTAN2 NOU ODRISC ORMOS
OSANN OSANN2 PARKIN PASTOR PAWLEG PERNU PERSH2 PETO PEZZO2 PEZZOT PIKE PISANI POFFIJ POLEDN QIAO QIAO2
RACHTA RADZIK RANDIG REN RONCO ROOTS ROTHSC SANKAR SCHWA2 SCHWAR SEGI SEOW SHAW SIEMIA SIMARA SIMONA
SOBUE SPEIZE SPITZ STASZE STAYNE STOCKW STUCKE SUN SUZUK2 SUZUKI SVENSS TANG TAO TENKAN TIZZAN TOKARS
TOUSEY TSUGAN TULINI TVERDA ULMER VANDER VEIERO VUTUC WAKAI WANG WANG2 WANG3 WANG4 WICKLU WIGLE WILKIN
WU WUNSCH WYNDE2 WYNDE3 WYNDE6 WYNDE7 XIANGZ XU XU2 XU3 XU4 YAMAGU YONG YUAN ZHANG ZHENG
ZHOU

4 BROWN2 SADOWS

5 BUFFLE COMSTO DAMBER DORGAN DORN GARSHI GODLEY HINDS HUMBLE LEVIN LIU4 NAM PERSHA PRESCO STOCKS WUWILL
WYNDE4

9 CPSI DEAN3 HAMMON KAISE2

Table 3A12 - 8

Potentially overlapping studies

REF	REFGP	PRINC	. OVERLAP/LINK
BENHAM	LUBIN2	2	Subset of Lubin2
