# Supplementary material for: Systematic review with meta-analysis of the epidemiological evidence in the 1900s relating smoking to lung cancer
Source: BMC Cancer. 2012 Sep 3;12:385. doi: 10.1186/1471-2407-12-385 (PMC3505152; doi:10.1186/1471-2407-12-385)
Supplement: Additional file 5 — Detailed Analysis Tables (Individual file names as described in Additional file 1: Methods, Table1). [file 1471-2407-12-385-S5.zip › PDF/1K.pdf]

Table 1K1 -

IESLC - Meta-analysis of Ex Smoking by Years quit (vs current), Overview
All LC types, Any Product (or Cigarettes if Any not available)

This analysis is restricted to results for:
1) Ex smokers
2) Results by Years quit (vs current)
3) Categorical results by Years quit (vs current)
Results by Years quit (vs current) are grouped under 2 schemes (S1, S2). Each scheme has a set of "key values". An interval is allocated to the category whose key value it includes, and intervals which include none or more than one of the key values are excluded. (Open-ended intervals are coded as 999)

S1	key value	maximum range
1	3	1-6
2	7	4-11
3	12	8+

S2	key value	maximum range
1	3	1-11
2	12	4-19
3	20	13+

4) All LC types (or near equivalent)
5) Results complete enough for use in metaanalysis

Within each study, results are then selected (in the following order of preference, within each sex) for:
6) (not applicable)
7) PRODUCT: all/unspec, cigarettes regardless of other products, cigarettes only
8) CIGTYPE: all/unspecified, MC regardless of HR, MC only
9) Results with least adjustment for other aspects of smoking (ADOS)
10) DENOM: current smokers, current + recent smokers (up to number of m=months or y=years, max 2 years)
11) Followup period (YF, prospective studies): whole study (coded as 0) or longest available
12) LCtype: all or nearest available, at least Squamous and Adeno. (q = squamous, s = small,
l = large, a = adeno, mix = mixed, alv = alveolar)
13) Race: all or nearest available, otherwise by race (wh or w = white, bl or b = black, hi = hispanic
ch = chinese, jap = japanese, haw = hawaiian, w+o = white + oriental, sca = scandinavian, as = asian)
14) For overlapping studies: principal rather than subsidiary studies
Finally by Age: whole study (coded as 0) if available, otherwise by widest available age group
and then for single sex results (m, f) in preference to results for both sexes combined (c).

Results adjusted (AD) for the most potential confounders are then chosen in Sections -1 to -3
and results adjusted for the least confounders in Sections -4 to -6. (Those least adjusted results which actually differ from the most adjusted are marked 'x' in column X in Section -4)

Section -7 shows excluded studies, together with the stage (as above) at which no qualifying results were found.

Section -8 lists the potentially overlapping studies which have been included (1=principal, 2=subsidiary).

Section -9 lists any results which would have been included in preference except that they had data not complete enough for use in meta-analysis, with their significance (yes/no), if known, and any further comment as entered on the database. It also lists as "gap" any categories for which no data were presented by the original authors.

In addition to those mentioned above, the following fields, levels and abbreviations are used:
* or nk = not known, n = no, y = yes, ot = other
nev = never
all/unspec = all or unspecified, cig+/-ot = cigarettes irrespective of other products (cigar, pipe etc)
MC = manufactured cigarettes, HR = hand-rolled cigarettes
exL, exH = range of exposure (low and high) in the smoking group, in terms of Years quit (vs current)
REF: 6-character study reference
NRR: number of the RR on the database within the study
ST : study type (CC = case control, pr or prosp = prospective)
NLC: number of lung cancer cases in whole study
R : risky occupational population (n = no, m = mining, o = other risky)
VB : national cigarette type (V = at least 75% Virginia, bl = at least 75% blended, ot = other)
P : any proxy use
H : full histological confirmation
De : derivation of RR/CI (or = original, st = standard method, ot = other method of estimation)

Table 1K1 - 1

IESLC - Meta-analysis of Ex Smoking by Years quit (vs current), Overview
All LC types, Any Product (or Cigarettes if Any not available)
Most adjusted

REF	NRR	SEX	AGEL	AGEH	RACE	YF	LC TYPE	LOC	START	ST	NLC	R	VB	P	H	AD	ADOS	PRODUCT	exL	exH	S1	S2	DENOM	De
ALDERS	513	m	0	0	all	-	all	Eu:UK	1977	CC	1448	n	V	n	n	1	0	cig only	0.1	2	0	0	current	ot
ALDERS	514	m	0	0	all	-	all	Eu:UK	1977	CC	1448	n	V	n	n	1	0	cig only	3	9	0	1	current	ot
ALDERS	515	m	0	0	all	-	all	Eu:UK	1977	CC	1448	n	V	n	n	1	0	cig only	10	999	3	0	current	ot
ALDERS	524	f	0	0	all	-	all	Eu:UK	1977	CC	1448	n	V	n	n	1	0	cig only	0.1	2	0	0	current	ot
ALDERS	525	f	0	0	all	-	all	Eu:UK	1977	CC	1448	n	V	n	n	1	0	cig only	3	9	0	1	current	ot
ALDERS	526	f	0	0	all	-	all	Eu:UK	1977	CC	1448	n	V	n	n	1	0	cig only	10	999	3	0	current	ot
ARMADA	518	m	0	0	all	-	all	Eu:wst	1986	CC	325	n	bl	n	y	0	0	cig+/-ot	1.0	5	1	1	cur+1y	st
ARMADA	519	m	0	0	all	-	all	Eu:wst	1986	CC	325	n	bl	n	y	0	0	cig+/-ot	6	999	0	0	cur+1y	st
BARBON	548	m	0	0	all	-	all	Eu:wst	1979	CC	755	n	bl	y	y	1	0	all/unsp	0.1	4	1	1	current	ot
BARBON	549	m	0	0	all	-	all	Eu:wst	1979	CC	755	n	bl	y	y	1	0	all/unsp	5	14	0	2	current	ot
BARBON	550	m	0	0	all	-	all	Eu:wst	1979	CC	755	n	bl	y	y	1	0	all/unsp	15	24	0	3	current	ot
BARBON	551	m	0	0	all	-	all	Eu:wst	1979	CC	755	n	bl	y	y	1	0	all/unsp	25	999	0	0	current	ot
BECHER	506	m	0	0	all	-	all	Eu:Ger	1985	CC	194	n	bl	n	y	0	0	all/unsp	2	4	1	1	cur+1y	st
BECHER	507	m	0	0	all	-	all	Eu:Ger	1985	CC	194	n	bl	n	y	0	0	all/unsp	5	9	2	0	cur+1y	st
BECHER	508	m	0	0	all	-	all	Eu:Ger	1985	CC	194	n	bl	n	y	0	0	all/unsp	10	999	3	0	cur+1y	st
BECHER	516	f	0	0	all	-	all	Eu:Ger	1985	CC	194	n	bl	n	y	0	0	all/unsp	2	4	1	1	cur+1y	st
BECHER	517	f	0	0	all	-	all	Eu:Ger	1985	CC	194	n	bl	n	y	0	0	all/unsp	5	9	2	0	cur+1y	st
BECHER	518	f	0	0	all	-	all	Eu:Ger	1985	CC	194	n	bl	n	y	0	0	all/unsp	10	999	3	0	cur+1y	st
BROSS	518	m	0	0	wh	-	all	NAmer	1960	CC	974	n	bl	n	n	0	0	cig+/-ot	0.1	5	1	1	current	st
BROSS	519	m	0	0	wh	-	all	NAmer	1960	CC	974	n	bl	n	n	0	0	cig+/-ot	6	999	0	0	current	st
CARPEN	508	c	0	0	w+b	-	all	NAmer	1991	CC	356	n	bl	n	n	0	0	cig+/-ot	0.1	4	1	1	current	st
CARPEN	509	c	0	0	w+b	-	all	NAmer	1991	CC	356	n	bl	n	n	0	0	cig+/-ot	5	9	2	0	current	st
CARPEN	510	c	0	0	w+b	-	all	NAmer	1991	CC	356	n	bl	n	n	0	0	cig+/-ot	10	14	3	2	current	st
CARPEN	511	c	0	0	w+b	-	all	NAmer	1991	CC	356	n	bl	n	n	0	0	cig+/-ot	15	999	0	3	current	st
CEDERL	538	m	40	69	all	10	all	Eu:Sca	1963	pr	491	n	bl	n	n	1	0	all/unsp	0.1	9	0	1	current	ot
CEDERL	539	m	40	69	all	10	all	Eu:Sca	1963	pr	491	n	bl	n	n	1	0	all/unsp	10	999	3	0	current	ot
CHOI	543	m	0	0	all	-	all	As:oth	1985	CC	375	n	bl	n	n	0	0	cig+/-ot	0.1	4	1	1	current	st
CHOI	544	m	0	0	all	-	all	As:oth	1985	CC	375	n	bl	n	n	0	0	cig+/-ot	5	9	2	0	current	st
CHOI	545	m	0	0	all	-	all	As:oth	1985	CC	375	n	bl	n	n	0	0	cig+/-ot	10	14	3	2	current	st
CHOI	546	m	0	0	all	-	all	As:oth	1985	CC	375	n	bl	n	n	0	0	cig+/-ot	15	999	0	3	current	st
CHOI	556	f	0	0	all	-	all	As:oth	1985	CC	375	n	bl	n	n	0	0	cig+/-ot	0.1	4	1	1	current	st
CHOI	557	f	0	0	all	-	all	As:oth	1985	CC	375	n	bl	n	n	0	0	cig+/-ot	5	999	0	0	current	ot
CHYOU	510	m	0	0	jap	21	all	NAmer	1965	pr	227	n	bl	n	y	2	0	cig+/-ot	0.1	14	0	0	current	ot
CHYOU	511	m	0	0	jap	21	all	NAmer	1965	pr	227	n	bl	n	y	2	0	cig+/-ot	15	999	0	3	current	ot
CPSI	815	m	50	74	all	6	all	NAmer	1959	pr	5138	n	bl	n	n	1	0	cig only	0.1	0.9	0	0	current	ot
CPSI	816	m	50	74	all	6	all	NAmer	1959	pr	5138	n	bl	n	n	1	0	cig only	1.0	4	1	1	current	ot
CPSI	817	m	50	74	all	6	all	NAmer	1959	pr	5138	n	bl	n	n	1	0	cig only	5	9	2	0	current	ot
CPSI	818	m	50	74	all	6	all	NAmer	1959	pr	5138	n	bl	n	n	1	0	cig only	10	999	3	0	current	ot
CPSII	664	m	35	99	all	4	all	NAmer	1982	pr	3229	n	bl	n	n	1	0	cig only	0.1	0.9	0	0	current	ot
CPSII	665	m	35	99	all	4	all	NAmer	1982	pr	3229	n	bl	n	n	1	0	cig only	1.0	2	0	0	current	ot
CPSII	666	m	35	99	all	4	all	NAmer	1982	pr	3229	n	bl	n	n	1	0	cig only	3	5	1	1	current	ot
CPSII	667	m	35	99	all	4	all	NAmer	1982	pr	3229	n	bl	n	n	1	0	cig only	6	10	2	0	current	ot
CPSII	668	m	35	99	all	4	all	NAmer	1982	pr	3229	n	bl	n	n	1	0	cig only	11	15	3	2	current	ot
CPSII	669	m	35	99	all	4	all	NAmer	1982	pr	3229	n	bl	n	n	1	0	cig only	16	999	0	3	current	ot
CPSII	642	f	0	0	all	4	all	NAmer	1982	pr	3229	n	bl	n	n	1	0	cig+/-ot	0.1	2	0	0	current	ot
CPSII	643	f	0	0	all	4	all	NAmer	1982	pr	3229	n	bl	n	n	1	0	cig+/-ot	3	5	1	1	current	ot
CPSII	644	f	0	0	all	4	all	NAmer	1982	pr	3229	n	bl	n	n	1	0	cig+/-ot	6	10	2	0	current	ot
CPSII	645	f	0	0	all	4	all	NAmer	1982	pr	3229	n	bl	n	n	1	0	cig+/-ot	11	15	3	2	current	ot
CPSII	646	f	0	0	all	4	all	NAmer	1982	pr	3229	n	bl	n	n	1	0	cig+/-ot	16	999	0	3	current	ot
DAMBER	528	m	0	0	all	-	all	Eu:Sca	1972	CC	579	n	bl	y	n	1	0	all/unsp	0.1	5	1	1	current	ot
DAMBER	529	m	0	0	all	-	all	Eu:Sca	1972	CC	579	n	bl	y	n	1	0	all/unsp	6	10	2	0	current	ot
DAMBER	530	m	0	0	all	-	all	Eu:Sca	1972	CC	579	n	bl	y	n	1	0	all/unsp	11	999	3	0	current	ot
DARBY	507	m	0	0	wh	-	all	Eu:UK	1988	CC	982	n	V	n	n	0	0	all/unsp	0.1	9	0	1	current	st
DARBY	508	m	0	0	wh	-	all	Eu:UK	1988	CC	982	n	V	n	n	0	0	all/unsp	10	999	3	0	current	st
DARBY	516	f	0	0	wh	-	all	Eu:UK	1988	CC	982	n	V	n	n	0	0	all/unsp	0.1	9	0	1	current	st
DARBY	517	f	0	0	wh	-	all	Eu:UK	1988	CC	982	n	V	n	n	0	0	all/unsp	10	999	3	0	current	st
DEAN3	636	m	0	0	all	-	all	Eu:UK	1969	CC	766	n	V	y	n	1	0	all/unsp	3	4	1	1	cur+2y	ot
DEAN3	637	m	0	0	all	-	all	Eu:UK	1969	CC	766	n	V	y	n	1	0	all/unsp	5	8	2	0	cur+2y	ot
DEAN3	638	m	0	0	all	-	all	Eu:UK	1969	CC	766	n	V	y	n	1	0	all/unsp	9	999	3	0	cur+2y	ot
DEAN3	559	f	0	0	all	-	all	Eu:UK	1969	CC	766	n	V	y	n	1	0	all/unsp	3	4	1	1	cur+2y	ot
DEAN3	560	f	0	0	all	-	all	Eu:UK	1969	CC	766	n	V	y	n	1	0	all/unsp	5	8	2	0	cur+2y	ot
DEAN3	561	f	0	0	all	-	all	Eu:UK	1969	CC	766	n	V	y	n	1	0	all/unsp	9	999	3	0	cur+2y	ot
DESTEF	530	m	0	0	all	-	all	SCAmer	1988	CC	497	n	bl	n	y	4	0	all/unsp	0.1	4	1	1	current	ot
DESTEF	531	m	0	0	all	-	all	SCAmer	1988	CC	497	n	bl	n	y	4	0	all/unsp	5	9	2	0	current	ot
DESTEF	532	m	0	0	all	-	all	SCAmer	1988	CC	497	n	bl	n	y	4	0	all/unsp	10	999	3	0	current	ot
DOLL	537	m	0	0	all	-	all	Eu:UK	1948	CC	1465	n	V	n	n	0	0	all/unsp	0.1	9	0	1	current	st
DOLL	538	m	0	0	all	-	all	Eu:UK	1948	CC	1465	n	V	n	n	0	0	all/unsp	10	19	3	2	current	st
DOLL	539	m	0	0	all	-	all	Eu:UK	1948	CC	1465	n	V	n	n	0	0	all/unsp	20	999	0	3	current	st
DOLL	548	f	0	0	all	-	all	Eu:UK	1948	CC	1465	n	V	n	n	0	0	all/unsp	0.1	9	0	1	current	st
DOLL	549	f	0	0	all	-	all	Eu:UK	1948	CC	1465	n	V	n	n	0	0	all/unsp	10	999	3	0	current	st
DOLL2	509	m	0	0	all	20	all	Eu:UK	1951	pr	920	n	V	n	n	1	0	cig only	0.1	4	1	1	current	ot
DOLL2	510	m	0	0	all	20	all	Eu:UK	1951	pr	920	n	V	n	n	1	0	cig only	5	9	2	0	current	ot

Table 1K1 - 1

IESLC - Meta-analysis of Ex Smoking by Years quit (vs current), Overview
All LC types, Any Product (or Cigarettes if Any not available)
Most adjusted

REF	NRR	SEX	AGEL	AGEH	RACE	YF	LC TYPE	LOC	START	ST	NLC	R	VB	P	H	AD	ADOS	PRODUCT	exL	exH	S1	S2	DENOM	De
DOLL2	511	m	0	0	all	20	all	Eu:UK	1951	pr	920	n	V	n	n	1	0	cig only	10	14	3	2	current	ot
DOLL2	512	m	0	0	all	20	all	Eu:UK	1951	pr	920	n	V	n	n	1	0	cig only	15	999	0	3	current	ot
DORGAN	514	m	0	0	wh	-	all	NAmer	1980	CC	2026	n	bl	y	y	0	0	cig+/-ot	1.1	5	1	1	cur+1y	st
DORGAN	515	m	0	0	wh	-	all	NAmer	1980	CC	2026	n	bl	y	y	0	0	cig+/-ot	6	9	2	0	cur+1y	st
DORGAN	516	m	0	0	wh	-	all	NAmer	1980	CC	2026	n	bl	y	y	0	0	cig+/-ot	10	999	3	0	cur+1y	st
DORGAN	559	f	0	0	all	-	all	NAmer	1980	CC	2026	n	bl	y	y	0	0	cig+/-ot	1.1	9	0	1	cur+1y	st
DORGAN	560	f	0	0	all	-	all	NAmer	1980	CC	2026	n	bl	y	y	0	0	cig+/-ot	10	999	3	0	cur+1y	st
DORN	823	m	55	64	wh	8	all	NAmer	1954	pr	5097	n	bl	n	n	0	0	cig+/-ot	0.1	4	1	1	current	st
DORN	824	m	55	64	wh	8	all	NAmer	1954	pr	5097	n	bl	n	n	0	0	cig+/-ot	5	9	2	0	current	st
DORN	825	m	55	64	wh	8	all	NAmer	1954	pr	5097	n	bl	n	n	0	0	cig+/-ot	10	14	3	2	current	st
DORN	826	m	55	64	wh	8	all	NAmer	1954	pr	5097	n	bl	n	n	0	0	cig+/-ot	15	999	0	3	current	st
DORN	827	m	65	74	wh	8	all	NAmer	1954	pr	5097	n	bl	n	n	0	0	cig+/-ot	0.1	4	1	1	current	st
DORN	828	m	65	74	wh	8	all	NAmer	1954	pr	5097	n	bl	n	n	0	0	cig+/-ot	5	9	2	0	current	st
DORN	829	m	65	74	wh	8	all	NAmer	1954	pr	5097	n	bl	n	n	0	0	cig+/-ot	10	14	3	2	current	st
DORN	830	m	65	74	wh	8	all	NAmer	1954	pr	5097	n	bl	n	n	0	0	cig+/-ot	15	999	0	3	current	st
GAO	536	m	0	0	all	-	all	As:Chi	1984	CC	1405	n	ot	n	n	2	0	cig+/-ot	0.1	4	1	1	current	ot
GAO	537	m	0	0	all	-	all	As:Chi	1984	CC	1405	n	ot	n	n	2	0	cig+/-ot	5	9	2	0	current	ot
GAO	538	m	0	0	all	-	all	As:Chi	1984	CC	1405	n	ot	n	n	2	0	cig+/-ot	10	999	3	0	current	ot
GAO	556	f	0	0	all	-	all	As:Chi	1984	CC	1405	n	ot	n	n	2	0	cig+/-ot	0.1	4	1	1	current	ot
GAO	557	f	0	0	all	-	all	As:Chi	1984	CC	1405	n	ot	n	n	2	0	cig+/-ot	5	9	2	0	current	ot
GAO	558	f	0	0	all	-	all	As:Chi	1984	CC	1405	n	ot	n	n	2	0	cig+/-ot	10	999	3	0	current	ot
GAO2	518	m	0	0	all	-	all	As:Jap	1988	CC	282	n	bl	n	n	0	0	cig+/-ot	1.0	4	1	1	cur+1y	st
GAO2	519	m	0	0	all	-	all	As:Jap	1988	CC	282	n	bl	n	n	0	0	cig+/-ot	5	9	2	0	cur+1y	st
GAO2	520	m	0	0	all	-	all	As:Jap	1988	CC	282	n	bl	n	n	0	0	cig+/-ot	10	14	3	2	cur+1y	st
GAO2	521	m	0	0	all	-	all	As:Jap	1988	CC	282	n	bl	n	n	0	0	cig+/-ot	15	19	0	0	cur+1y	st
GAO2	522	m	0	0	all	-	all	As:Jap	1988	CC	282	n	bl	n	n	0	0	cig+/-ot	20	999	0	3	cur+1y	st
GARCIA	508	c	0	0	all	-	all	NAmer	1992	CC	416	n	bl	n	y	0	0	cig+/-ot	1.0	4	1	1	cur+1y	st
GARCIA	509	c	0	0	all	-	all	NAmer	1992	CC	416	n	bl	n	y	0	0	cig+/-ot	5	14	0	2	cur+1y	st
GARCIA	510	c	0	0	all	-	all	NAmer	1992	CC	416	n	bl	n	y	0	0	cig+/-ot	15	29	0	3	cur+1y	st
GARCIA	511	c	0	0	all	-	all	NAmer	1992	CC	416	n	bl	n	y	0	0	cig+/-ot	30	999	0	0	cur+1y	st
GRAHAM	540	m	0	0	wh	-	all	NAmer	1956	CC	685	n	bl	n	n	1	0	cig+/-ot	0.1	1.0	0	0	current	ot
GRAHAM	541	m	0	0	wh	-	all	NAmer	1956	CC	685	n	bl	n	n	1	0	cig+/-ot	1.1	5	1	1	current	ot
GRAHAM	542	m	0	0	wh	-	all	NAmer	1956	CC	685	n	bl	n	n	1	0	cig+/-ot	5	999	0	0	current	ot
HAMMO2	510	m	0	0	all	0	all	NAmer	1967	pr	450	o	bl	n	n	1	0	cig+/-ot	0.1	4	1	1	current	ot
HAMMO2	511	m	0	0	all	0	all	NAmer	1967	pr	450	o	bl	n	n	1	0	cig+/-ot	5	9	2	0	current	ot
HAMMO2	512	m	0	0	all	0	all	NAmer	1967	pr	450	o	bl	n	n	1	0	cig+/-ot	10	999	3	0	current	ot
HIRAYA	513	m	0	0	all	0	all	As:Jap	1965	pr	1917	n	bl	n	n	1	0	cig+/-ot	0.1	4	1	1	current	ot
HIRAYA	514	m	0	0	all	0	all	As:Jap	1965	pr	1917	n	bl	n	n	1	0	cig+/-ot	5	9	2	0	current	ot
HIRAYA	515	m	0	0	all	0	all	As:Jap	1965	pr	1917	n	bl	n	n	1	0	cig+/-ot	10	999	3	0	current	ot
HIRAYA	524	f	0	0	all	0	all	As:Jap	1965	pr	1917	n	bl	n	n	1	0	cig+/-ot	0.1	4	1	1	current	ot
HIRAYA	525	f	0	0	all	0	all	As:Jap	1965	pr	1917	n	bl	n	n	1	0	cig+/-ot	5	9	2	0	current	ot
HIRAYA	526	f	0	0	all	0	all	As:Jap	1965	pr	1917	n	bl	n	n	1	0	cig+/-ot	10	999	3	0	current	ot
JAHN	513	m	0	0	all	-	all	Eu:Ger	1988	CC	1004	n	bl	n	n	0	0	cig+/-ot	0.1	0.9	0	0	current	st
JAHN	514	m	0	0	all	-	all	Eu:Ger	1988	CC	1004	n	bl	n	n	0	0	cig+/-ot	1.0	1.9	0	0	current	st
JAHN	515	m	0	0	all	-	all	Eu:Ger	1988	CC	1004	n	bl	n	n	0	0	cig+/-ot	2	5	1	1	current	st
JAHN	516	m	0	0	all	-	all	Eu:Ger	1988	CC	1004	n	bl	n	n	0	0	cig+/-ot	6	10	2	0	current	st
JAHN	517	m	0	0	all	-	all	Eu:Ger	1988	CC	1004	n	bl	n	n	0	0	cig+/-ot	11	20	3	0	current	st
JAHN	518	m	0	0	all	-	all	Eu:Ger	1988	CC	1004	n	bl	n	n	0	0	cig+/-ot	21	999	0	0	current	st
JAIN	570	m	0	0	all	-	all	NAmer	1981	CC	845	n	V	y	n	0	0	cig+/-ot	2	9	0	1	cur+2y	st
JAIN	571	m	0	0	all	-	all	NAmer	1981	CC	845	n	V	y	n	0	0	cig+/-ot	10	999	3	0	cur+2y	st
JAIN	534	f	0	0	all	-	all	NAmer	1981	CC	845	n	V	y	n	0	0	cig+/-ot	2	9	0	1	cur+2y	st
JAIN	535	f	0	0	all	-	all	NAmer	1981	CC	845	n	V	y	n	0	0	cig+/-ot	10	999	3	0	cur+2y	st
JOLY	571	m	0	0	all	-	all	SCAmer	1978	CC	826	n	bl	n	n	0	0	cig+/-ot	1.0	4	1	1	cur+1y	st
JOLY	572	m	0	0	all	-	all	SCAmer	1978	CC	826	n	bl	n	n	0	0	cig+/-ot	5	999	0	0	cur+1y	st
JOLY	558	f	0	0	all	-	all	SCAmer	1978	CC	826	n	bl	n	n	0	0	cig+/-ot	1.0	4	1	1	cur+1y	st
JOLY	559	f	0	0	all	-	all	SCAmer	1978	CC	826	n	bl	n	n	0	0	cig+/-ot	5	999	0	0	cur+1y	st
KAISE2	655	m	0	0	all	9	all	NAmer	1979	pr	318	n	bl	n	n	1	0	cig only	2	10	0	1	cur+2y	st
KAISE2	656	m	0	0	all	9	all	NAmer	1979	pr	318	n	bl	n	n	1	0	cig only	11	20	3	0	cur+2y	ot
KAISE2	657	m	0	0	all	9	all	NAmer	1979	pr	318	n	bl	n	n	1	0	cig only	21	999	0	0	cur+2y	st
KAISE2	575	f	0	0	all	9	all	NAmer	1979	pr	318	n	bl	n	n	1	0	cig only	2	10	0	1	cur+2y	st
KAISE2	576	f	0	0	all	9	all	NAmer	1979	pr	318	n	bl	n	n	1	0	cig only	11	20	3	0	cur+2y	ot
KAISE2	577	f	0	0	all	9	all	NAmer	1979	pr	318	n	bl	n	n	1	0	cig only	21	999	0	0	cur+2y	ot
KHUDER	516	m	0	0	all	-	all	NAmer	1985	CC	482	n	bl	n	y	0	0	cig+/-ot	0.1	4	1	1	current	st
KHUDER	517	m	0	0	all	-	all	NAmer	1985	CC	482	n	bl	n	y	0	0	cig+/-ot	5	14	0	2	current	st
KHUDER	518	m	0	0	all	-	all	NAmer	1985	CC	482	n	bl	n	y	0	0	cig+/-ot	15	999	0	3	current	st
LUBIN	592	m	0	0	all	-	all	As:Chi	1984	CC	427	m	ot	y	n	0	0	cig+/-ot	3	4	1	1	cur+2y	st
LUBIN	593	m	0	0	all	-	all	As:Chi	1984	CC	427	m	ot	y	n	0	0	cig+/-ot	5	9	2	0	cur+2y	st
LUBIN	594	m	0	0	all	-	all	As:Chi	1984	CC	427	m	ot	y	n	0	0	cig+/-ot	10	999	3	0	cur+2y	st
LUBIN2	1081	m	0	0	all	-	all	Eu:mul	1976	CC	7804	n	bl	n	y	0	0	cig+/-ot	0.1	4	1	1	current	st
LUBIN2	1082	m	0	0	all	-	all	Eu:mul	1976	CC	7804	n	bl	n	y	0	0	cig+/-ot	5	9	2	0	current	st
LUBIN2	1083	m	0	0	all	-	all	Eu:mul	1976	CC	7804	n	bl	n	y	0	0	cig+/-ot	10	14	3	2	current	st
LUBIN2	1084	m	0	0	all	-	all	Eu:mul	1976	CC	7804	n	bl	n	y	0	0	cig+/-ot	15	19	0	0	current	st

Table 1K1 - 1

IESLC - Meta-analysis of Ex Smoking by Years quit (vs current), Overview
All LC types, Any Product (or Cigarettes if Any not available)
Most adjusted

REF	NRR	SEX	AGEL	AGEH	RACE	YF	LC TYPE	LOC	START	ST	NLC	R	VB	P	H	AD	ADOS	PRODUCT	exL	exH	S1	S2	DENOM	De
LUBIN2	1085	m	0	0	all	-	all	Eu:mul	1976	CC	7804	n	bl	n	y	0	0	cig+/-ot	20	24	0	3	current	st
LUBIN2	1086	m	0	0	all	-	all	Eu:mul	1976	CC	7804	n	bl	n	y	0	0	cig+/-ot	25	999	0	0	current	st
LUBIN2	1120	f	0	0	all	-	all	Eu:mul	1976	CC	7804	n	bl	n	y	0	0	cig+/-ot	0.1	4	1	1	current	st
LUBIN2	1121	f	0	0	all	-	all	Eu:mul	1976	CC	7804	n	bl	n	y	0	0	cig+/-ot	5	9	2	0	current	st
LUBIN2	1122	f	0	0	all	-	all	Eu:mul	1976	CC	7804	n	bl	n	y	0	0	cig+/-ot	10	14	3	2	current	st
LUBIN2	1123	f	0	0	all	-	all	Eu:mul	1976	CC	7804	n	bl	n	y	0	0	cig+/-ot	15	19	0	0	current	st
LUBIN2	1124	f	0	0	all	-	all	Eu:mul	1976	CC	7804	n	bl	n	y	0	0	cig+/-ot	20	24	0	3	current	st
LUBIN2	1125	f	0	0	all	-	all	Eu:mul	1976	CC	7804	n	bl	n	y	0	0	cig+/-ot	25	999	0	0	current	st
MATOS	596	m	0	0	all	-	all	SCAmer	1994	CC	200	n	bl	n	n	2	0	cig+/-ot	1.0	5	1	1	cur+1y	or
MATOS	597	m	0	0	all	-	all	SCAmer	1994	CC	200	n	bl	n	n	2	0	cig+/-ot	6	10	2	0	cur+1y	or
MATOS	598	m	0	0	all	-	all	SCAmer	1994	CC	200	n	bl	n	n	2	0	cig+/-ot	11	999	3	0	cur+1y	or
PEZZO2	504	m	0	0	all	-	all	SCAmer	1992	CC	367	n	bl	n	y	0	0	cig+/-ot	1.0	10	0	1	cur+1y	st
PEZZO2	505	m	0	0	all	-	all	SCAmer	1992	CC	367	n	bl	n	y	0	0	cig+/-ot	11	999	3	0	cur+1y	st
PEZZOT	504	m	0	0	all	-	all	SCAmer	1987	CC	215	n	bl	n	y	0	0	cig only	1.0	10	0	1	cur+1y	st
PEZZOT	505	m	0	0	all	-	all	SCAmer	1987	CC	215	n	bl	n	y	0	0	cig only	11	999	3	0	cur+1y	st
SOBUE	728	m	0	0	all	-	all	As:Jap	1986	CC	1376	n	bl	n	y	0	0	cig+/-ot	1.0	4	1	1	cur+1y	st
SOBUE	729	m	0	0	all	-	all	As:Jap	1986	CC	1376	n	bl	n	y	0	0	cig+/-ot	5	9	2	0	cur+1y	st
SOBUE	730	m	0	0	all	-	all	As:Jap	1986	CC	1376	n	bl	n	y	0	0	cig+/-ot	10	14	3	2	cur+1y	st
SOBUE	731	m	0	0	all	-	all	As:Jap	1986	CC	1376	n	bl	n	y	0	0	cig+/-ot	15	19	0	0	cur+1y	st
SOBUE	732	m	0	0	all	-	all	As:Jap	1986	CC	1376	n	bl	n	y	0	0	cig+/-ot	20	24	0	3	cur+1y	st
SOBUE	733	m	0	0	all	-	all	As:Jap	1986	CC	1376	n	bl	n	y	0	0	cig+/-ot	25	999	0	0	cur+1y	st
SPEIZE	511	f	0	0	all	0	all	NAmer	1976	pr	593	n	bl	n	y	2	0	cig+/-ot	0.1	1.9	0	0	current	or
SPEIZE	512	f	0	0	all	0	all	NAmer	1976	pr	593	n	bl	n	y	2	0	cig+/-ot	2	5	1	1	current	or
SPEIZE	513	f	0	0	all	0	all	NAmer	1976	pr	593	n	bl	n	y	2	0	cig+/-ot	5	10	2	0	current	or
SPEIZE	514	f	0	0	all	0	all	NAmer	1976	pr	593	n	bl	n	y	2	0	cig+/-ot	10	15	3	2	current	or
SPEIZE	515	f	0	0	all	0	all	NAmer	1976	pr	593	n	bl	n	y	2	0	cig+/-ot	15	999	0	3	current	or
SUZUK2	524	c	0	0	all	-	all	SCAmer	1991	CC	123	n	bl	n	y	3	0	all/unsp	0.1	5	1	1	current	or
SUZUK2	525	c	0	0	all	-	all	SCAmer	1991	CC	123	n	bl	n	y	3	0	all/unsp	6	10	2	0	current	or
SUZUK2	526	c	0	0	all	-	all	SCAmer	1991	CC	123	n	bl	n	y	3	0	all/unsp	11	999	3	0	current	or
SVENSS	554	f	0	0	all	-	all	Eu:Sca	1983	CC	210	n	bl	n	n	0	0	all/unsp	3	10	0	1	cur+2y	st
SVENSS	555	f	0	0	all	-	all	Eu:Sca	1983	CC	210	n	bl	n	n	0	0	all/unsp	11	999	3	0	cur+2y	st
TVERDA	506	m	0	0	all	0	all	Eu:Sca	1972	pr	238	n	bl	n	n	2	0	cig only	0.1	0.9	0	0	current	ot
TVERDA	507	m	0	0	all	0	all	Eu:Sca	1972	pr	238	n	bl	n	n	2	0	cig only	1.0	5	1	1	current	ot
TVERDA	508	m	0	0	all	0	all	Eu:Sca	1972	pr	238	n	bl	n	n	2	0	cig only	5	999	0	0	current	ot
WANG2	515	c	0	0	all	-	all	As:Chi	1980	CC	103	n	ot	n	n	0	0	cig+/-ot	0.1	3	1	1	current	st
WANG2	516	c	0	0	all	-	all	As:Chi	1980	CC	103	n	ot	n	n	0	0	cig+/-ot	4	999	0	0	current	st
WYNDE3	545	m	0	0	all	-	all	NAmer	1966	CC	350	n	bl	n	y	0	0	all/unsp	1.0	3	1	1	cur+1y	st
WYNDE3	546	m	0	0	all	-	all	NAmer	1966	CC	350	n	bl	n	y	0	0	all/unsp	4	6	0	0	cur+1y	st
WYNDE3	547	m	0	0	all	-	all	NAmer	1966	CC	350	n	bl	n	y	0	0	all/unsp	7	12	0	2	cur+1y	st
WYNDE3	548	m	0	0	all	-	all	NAmer	1966	CC	350	n	bl	n	y	0	0	all/unsp	13	999	0	3	cur+1y	st
WYNDE6	719	m	0	0	wh	-	all	NAmer	1969	CC	4423	n	bl	n	y	5	1#	cig+/-ot	1.0	10	0	1	cur+1y	or
WYNDE6	720	m	0	0	wh	-	all	NAmer	1969	CC	4423	n	bl	n	y	5	1#	cig+/-ot	11	19	3	2	cur+1y	or
WYNDE6	721	m	0	0	wh	-	all	NAmer	1969	CC	4423	n	bl	n	y	5	1#	cig+/-ot	20	999	0	3	cur+1y	or
WYNDE6	726	m	0	0	bl	-	all	NAmer	1969	CC	4423	n	bl	n	y	5	1#	cig+/-ot	1.0	10	0	1	cur+1y	or
WYNDE6	727	m	0	0	bl	-	all	NAmer	1969	CC	4423	n	bl	n	y	5	1#	cig+/-ot	11	19	3	2	cur+1y	or
WYNDE6	728	m	0	0	bl	-	all	NAmer	1969	CC	4423	n	bl	n	y	5	1#	cig+/-ot	20	999	0	3	cur+1y	or
WYNDE6	733	f	0	0	wh	-	all	NAmer	1969	CC	4423	n	bl	n	y	5	1#	cig+/-ot	1.0	10	0	1	cur+1y	or
WYNDE6	734	f	0	0	wh	-	all	NAmer	1969	CC	4423	n	bl	n	y	5	1#	cig+/-ot	11	999	3	0	cur+1y	or
WYNDE6	737	f	0	0	bl	-	all	NAmer	1969	CC	4423	n	bl	n	y	5	1#	cig+/-ot	1.0	10	0	1	cur+1y	or
WYNDE6	738	f	0	0	bl	-	all	NAmer	1969	CC	4423	n	bl	n	y	5	1#	cig+/-ot	11	999	3	0	cur+1y	or

Comments on values in listings

WYNDE6 ADOS Number of cigs/day
WYNDE6 ADOS Number of cigs/day
WYNDE6 ADOS Number of cigs/day
WYNDE6 ADOS Number of cigs/day
WYNDE6 ADOS Number of cigs/day
WYNDE6 ADOS Number of cigs/day
WYNDE6 ADOS Number of cigs/day
WYNDE6 ADOS Number of cigs/day
WYNDE6 ADOS Number of cigs/day
WYNDE6 ADOS Number of cigs/day

Cigarette type is all/unspec for all RRs
except for the following:

REF	NRR	CIGTYPE
ALDERS	513	MC only

Table 1K1 - 1

IESLC - Meta-analysis of Ex Smoking by Years quit (vs current), Overview
All LC types, Any Product (or Cigarettes if Any not available)
Most adjusted

REF	NRR	CIGTYPE
ALDERS	514	MC only
ALDERS	515	MC only
ALDERS	524	MC only
ALDERS	525	MC only
ALDERS	526	MC only

In this overview table, subtotals and Qs values may be invalid and should be ignored

Table 1K1 - 2

IESLC - Meta-analysis of Ex Smoking by Years quit (vs current), Overview
All LC types, Any Product (or Cigarettes if Any not available)
Most adjusted

REF	NRR	SEX	AD	Number Case	Exposed Cont	Non-exposed Case	Cont	RR	95.00%CI
ALDERS	513	m	1	121	-	207	-	1.81	(1.24- 2.64)
ALDERS	514	m	1	28	-	207	-	0.43	(0.26- 0.71)
ALDERS	515	m	1	29	-	207	-	0.32	(0.20- 0.51)
ALDERS	524	f	1	206	-	244	-	2.08	(1.49- 2.90)
ALDERS	525	f	1	54	-	244	-	0.65	(0.43- 0.99)
ALDERS	526	f	1	26	-	244	-	0.28	(0.17- 0.46)
Subtotal ALDERS								0.85	(0.72- 1.01)
ARMADA	518	m	0	79	45	188	122	1.14	(0.74- 1.75)
ARMADA	519	m	0	50	87	188	122	0.37	(0.25- 0.57)
Subtotal ARMADA								0.64	(0.47- 0.86)
BARBON	548	m	1	32	-	562	-	1.01	(0.57- 1.79)
BARBON	549	m	1	89	-	562	-	0.66	(0.47- 0.92)
BARBON	550	m	1	33	-	562	-	0.49	(0.31- 0.79)
BARBON	551	m	1	15	-	562	-	0.15	(0.08- 0.28)
Subtotal BARBON								0.54	(0.43- 0.68)
BECHER	506	m	0	10	12	101	122	1.01	(0.42- 2.43)
BECHER	507	m	0	16	32	101	122	0.60	(0.31- 1.16)
BECHER	508	m	0	16	72	101	122	0.27	(0.15- 0.49)
BECHER	516	f	0	2	3	33	26	0.53	(0.08- 3.38)
BECHER	517	f	0	2	5	33	26	0.32	(0.06- 1.76)
BECHER	518	f	0	1	10	33	26	0.08	(0.01- 0.66)
Subtotal BECHER								0.44	(0.30- 0.64)
BROSS	518	m	0	169	67	565	427	1.91	(1.40- 2.60)
BROSS	519	m	0	43	79	565	427	0.41	(0.28- 0.61)
Subtotal BROSS								1.06	(0.83- 1.35)
CARPEN	508	c	0	28	46	228	164	0.44	(0.26- 0.73)
CARPEN	509	c	0	31	52	228	164	0.43	(0.26- 0.70)
CARPEN	510	c	0	13	58	228	164	0.16	(0.09- 0.30)
CARPEN	511	c	0	37	158	228	164	0.17	(0.11- 0.25)
Subtotal CARPEN								0.27	(0.21- 0.34)
*CEDERL	538	m	1	12	-	97	-	0.78	(0.43- 1.41)
*CEDERL	539	m	1	3	-	97	-	0.14	(0.04- 0.45)
Subtotal CEDERL								0.56	(0.33- 0.95)
CHOI	543	m	0	25	64	231	329	0.56	(0.34- 0.91)
CHOI	544	m	0	5	30	231	329	0.24	(0.09- 0.62)
CHOI	545	m	0	4	23	231	329	0.25	(0.08- 0.73)
CHOI	546	m	0	4	19	231	329	0.30	(0.10- 0.89)
CHOI	556	f	0	3	2	13	25	2.88	(0.43- 19.49)
CHOI	557	f	0	2	0	13	25	9.44~	(0.42- 211.16)
Subtotal CHOI								0.46	(0.32- 0.67)
*CHYOU	510	m	2	21	-	138	-	0.37	(0.24- 0.57)
*CHYOU	511	m	2	5	-	138	-	0.27	(0.11- 0.66)
Subtotal CHYOU								0.35	(0.24- 0.51)
*CPSI	815	m	1	37	-	844	-	1.07	(0.77- 1.49)
*CPSI	816	m	1	49	-	844	-	0.59	(0.44- 0.78)
*CPSI	817	m	1	32	-	844	-	0.37	(0.26- 0.53)
*CPSI	818	m	1	15	-	844	-	0.09	(0.06- 0.15)
Subtotal CPSI								0.48	(0.40- 0.57)
*CPSII	664	m	1	97	-	1159	-	1.77	(1.44- 2.17)
*CPSII	665	m	1	188	-	1159	-	1.28	(1.10- 1.49)
*CPSII	666	m	1	178	-	1159	-	0.85	(0.72- 0.99)
*CPSII	667	m	1	186	-	1159	-	0.52	(0.45- 0.61)
*CPSII	668	m	1	164	-	1159	-	0.39	(0.33- 0.46)
*CPSII	669	m	1	256	-	1159	-	0.17	(0.15- 0.20)
*CPSII	642	f	1	91	-	530	-	1.38	(1.10- 1.72)
*CPSII	643	f	1	56	-	530	-	0.85	(0.65- 1.12)
*CPSII	644	f	1	37	-	530	-	0.40	(0.28- 0.55)
*CPSII	645	f	1	28	-	530	-	0.31	(0.21- 0.46)
*CPSII	646	f	1	50	-	530	-	0.14	(0.11- 0.19)
Subtotal CPSII								0.57	(0.54- 0.60)
DAMBER	528	m	1	-	-	-	-	0.80	(0.50- 1.28)
DAMBER	529	m	1	-	-	-	-	0.45	(0.26- 0.78)
DAMBER	530	m	1	42	-	-	-	0.27	(0.18- 0.40)
Subtotal DAMBER								0.43	(0.33- 0.56)
DARBY	507	m	0	146	339	379	618	0.70	(0.56- 0.89)
DARBY	508	m	0	139	767	379	618	0.30	(0.24- 0.37)
DARBY	516	f	0	68	93	198	231	0.85	(0.59- 1.23)
DARBY	517	f	0	26	224	198	231	0.14	(0.09- 0.21)
Subtotal DARBY								0.44	(0.38- 0.50)
DEAN3	636	m	1	42	-	502	-	0.80	(0.56- 1.15)
DEAN3	637	m	1	15	-	502	-	0.55	(0.31- 0.97)

Table 1K1 - 2

IESLC - Meta-analysis of Ex Smoking by Years quit (vs current), Overview
All LC types, Any Product (or Cigarettes if Any not available)
Most adjusted

REF	NRR	SEX	AD	Number Exposed Case	Cont	Non-exposed Case	Cont	RR	95.00%CI
DEAN3	638	m	1	32	-	502	-	0.34	(0.23- 0.50)
DEAN3	559	f	1	4	-	102	-	0.29	(0.11- 0.81)
DEAN3	560	f	1	1	-	102	-	0.20	(0.03- 1.42)
DEAN3	561	f	1	2	-	102	-	0.13	(0.03- 0.53)
Subtotal DEAN3								0.50	(0.40- 0.63)
DESTEF	530	m	4	64	-	362	-	0.83	(0.55- 1.24)
DESTEF	531	m	4	27	-	362	-	0.57	(0.33- 0.98)
DESTEF	532	m	4	17	-	362	-	0.26	(0.14- 0.46)
Subtotal DESTEF								0.57	(0.43- 0.76)
DOLL	537	m	0	56	75	1280	1172	0.68	(0.48- 0.98)
DOLL	538	m	0	6	26	1280	1172	0.21	(0.09- 0.52)
DOLL	539	m	0	8	23	1280	1172	0.32	(0.14- 0.71)
DOLL	548	f	0	9	6	58	41	1.06	(0.35- 3.21)
DOLL	549	f	0	1	2	58	41	0.35	(0.03- 4.03)
Subtotal DOLL								0.56	(0.42- 0.75)
*DOLL2	509	m	1	15	-	236	-	1.02	(0.61- 1.72)
*DOLL2	510	m	1	12	-	236	-	0.35	(0.20- 0.63)
*DOLL2	511	m	1	9	-	236	-	0.28	(0.14- 0.54)
*DOLL2	512	m	1	7	-	236	-	0.11	(0.05- 0.23)
Subtotal DOLL2								0.40	(0.30- 0.55)
DORGAN	514	m	0	59	51	465	303	0.75	(0.50- 1.13)
DORGAN	515	m	0	49	38	465	303	0.84	(0.54- 1.31)
DORGAN	516	m	0	134	255	465	303	0.34	(0.27- 0.44)
DORGAN	559	f	0	49	27	289	112	0.70	(0.42- 1.18)
DORGAN	560	f	0	34	50	289	112	0.26	(0.16- 0.43)
Subtotal DORGAN								0.47	(0.40- 0.56)
*DORN	823	m	0	34	22086	528	334175	0.97	(0.69- 1.38)
*DORN	824	m	0	32	34566	528	334175	0.59	(0.41- 0.84)
*DORN	825	m	0	12	23682	528	334175	0.32	(0.18- 0.57)
*DORN	826	m	0	16	58370	528	334175	0.17	(0.11- 0.29)
*DORN	827	m	0	14	6195	537	207895	0.87	(0.51- 1.49)
*DORN	828	m	0	41	24089	537	207895	0.66	(0.48- 0.90)
*DORN	829	m	0	29	20056	537	207895	0.56	(0.39- 0.81)
*DORN	830	m	0	34	51243	537	207895	0.26	(0.18- 0.36)
Subtotal DORN								0.51	(0.45- 0.59)
GAO	536	m	2	105	-	529	-	1.77	(1.22- 2.56)
GAO	537	m	2	24	-	529	-	0.79	(0.45- 1.40)
GAO	538	m	2	13	-	529	-	0.28	(0.14- 0.57)
GAO	556	f	2	37	-	170	-	2.48	(1.15- 5.38)
GAO	557	f	2	14	-	170	-	1.34	(0.51- 3.53)
GAO	558	f	2	16	-	170	-	0.76	(0.34- 1.67)
Subtotal GAO								1.14	(0.90- 1.46)
GAO2	518	m	0	31	26	184	117	0.76	(0.43- 1.34)
GAO2	519	m	0	21	26	184	117	0.51	(0.28- 0.95)
GAO2	520	m	0	16	18	184	117	0.57	(0.28- 1.15)
GAO2	521	m	0	7	9	184	117	0.49	(0.18- 1.36)
GAO2	522	m	0	8	25	184	117	0.20	(0.09- 0.47)
Subtotal GAO2								0.51	(0.37- 0.70)
GARCIA	508	c	0	33	11	77	42	1.64	(0.75- 3.57)
GARCIA	509	c	0	43	36	77	42	0.65	(0.36- 1.16)
GARCIA	510	c	0	32	67	77	42	0.26	(0.15- 0.46)
GARCIA	511	c	0	10	37	77	42	0.15	(0.07- 0.33)
Subtotal GARCIA								0.44	(0.32- 0.61)
GRAHAM	540	m	1	113	-	453	-	4.93	(3.53- 6.88)
GRAHAM	541	m	1	24	-	453	-	1.17	(0.70- 1.94)
GRAHAM	542	m	1	13	-	453	-	0.36	(0.19- 0.68)
Subtotal GRAHAM								2.25	(1.74- 2.91)
*HAMMO2	510	m	1	59	-	209	-	1.08	(0.82- 1.43)
*HAMMO2	511	m	1	11	-	209	-	0.39	(0.22- 0.71)
*HAMMO2	512	m	1	20	-	209	-	0.34	(0.22- 0.53)
Subtotal HAMMO2								0.71	(0.57- 0.88)
*HIRAYA	513	m	1	-	-	-	-	0.46	(0.26- 0.82)
*HIRAYA	514	m	1	-	-	-	-	0.36	(0.15- 0.86)
*HIRAYA	515	m	1	-	-	-	-	0.31	(0.14- 0.69)
*HIRAYA	524	f	1	-	-	-	-	1.59	(0.47- 5.37)
*HIRAYA	525	f	1	-	-	-	-	1.41	(0.23- 8.48)
*HIRAYA	526	f	1	-	-	-	-	0.41	(0.01- 14.37)
Subtotal HIRAYA								0.48	(0.33- 0.69)
JAHN	513	m	0	166	8	352	269	15.86	(7.66- 32.81)
JAHN	514	m	0	60	9	352	269	5.09	(2.48- 10.45)
JAHN	515	m	0	77	46	352	269	1.28	(0.86- 1.90)

Table 1K1 - 2

IESLC - Meta-analysis of Ex Smoking by Years quit (vs current), Overview
All LC types, Any Product (or Cigarettes if Any not available)
Most adjusted

REF	NRR	SEX	AD	Number Exposed Case	Cont	Non-exposed Case	Cont	RR	95.00%CI
JAHN	516	m	0	59	63	352	269	0.72	(0.49- 1.06)
JAHN	517	m	0	64	130	352	269	0.38	(0.27- 0.53)
JAHN	518	m	0	29	146	352	269	0.15	(0.10- 0.23)
Subtotal JAHN								0.70	(0.58- 0.84)
JAIN	570	m	0	74	46	265	118	0.72	(0.47- 1.10)
JAIN	571	m	0	52	113	265	118	0.20	(0.14- 0.30)
JAIN	534	f	0	66	36	305	99	0.60	(0.37- 0.95)
JAIN	535	f	0	19	61	305	99	0.10	(0.06- 0.18)
Subtotal JAIN								0.33	(0.27- 0.42)
JOLY	571	m	0	38	36	451	524	1.23	(0.76- 1.97)
JOLY	572	m	0	63	149	451	524	0.49	(0.36- 0.68)
JOLY	558	f	0	19	8	132	96	1.73	(0.73- 4.11)
JOLY	559	f	0	15	19	132	96	0.57	(0.28- 1.19)
Subtotal JOLY								0.70	(0.55- 0.88)
*KAISE2	655	m	1	12	-	51	-	1.00	(0.53- 1.88)
*KAISE2	656	m	1	8	-	51	-	0.43	(0.20- 0.92)
*KAISE2	657	m	1	6	-	51	-	0.26	(0.10- 0.67)
*KAISE2	575	f	1	6	-	50	-	0.53	(0.23- 1.23)
*KAISE2	576	f	1	4	-	50	-	0.25	(0.09- 0.70)
*KAISE2	577	f	1	4	-	50	-	0.34	(0.13- 0.91)
Subtotal KAISE2								0.49	(0.35- 0.68)
KHUDER	516	m	0	88	123	245	316	0.92	(0.67- 1.27)
KHUDER	517	m	0	63	133	245	316	0.61	(0.43- 0.86)
KHUDER	518	m	0	63	213	245	316	0.38	(0.28- 0.53)
Subtotal KHUDER								0.60	(0.50- 0.73)
LUBIN	592	m	0	33	18	296	650	4.03	(2.23- 7.27)
LUBIN	593	m	0	20	48	296	650	0.91	(0.53- 1.57)
LUBIN	594	m	0	17	73	296	650	0.51	(0.30- 0.88)
Subtotal LUBIN								1.16	(0.84- 1.60)
LUBIN2	1081	m	0	866	1047	4684	6211	1.10	(0.99- 1.21)
LUBIN2	1082	m	0	466	822	4684	6211	0.75	(0.67- 0.85)
LUBIN2	1083	m	0	270	693	4684	6211	0.52	(0.45- 0.60)
LUBIN2	1084	m	0	130	478	4684	6211	0.36	(0.30- 0.44)
LUBIN2	1085	m	0	106	413	4684	6211	0.34	(0.27- 0.42)
LUBIN2	1086	m	0	109	715	4684	6211	0.20	(0.16- 0.25)
LUBIN2	1120	f	0	60	55	440	410	1.02	(0.69- 1.50)
LUBIN2	1121	f	0	30	40	440	410	0.70	(0.43- 1.14)
LUBIN2	1122	f	0	10	26	440	410	0.36	(0.17- 0.75)
LUBIN2	1123	f	0	3	7	440	410	0.40	(0.10- 1.55)
LUBIN2	1124	f	0	4	9	440	410	0.41	(0.13- 1.36)
LUBIN2	1125	f	0	4	20	440	410	0.19	(0.06- 0.55)
Subtotal LUBIN2								0.65	(0.61- 0.68)
MATOS	596	m	2	28	-	112	-	1.40	(0.80- 2.60)
MATOS	597	m	2	21	-	112	-	0.90	(0.40- 1.60)
MATOS	598	m	2	27	-	112	-	0.30	(0.20- 0.60)
Subtotal MATOS								0.68	(0.48- 0.96)
PEZZO2	504	m	0	85	110	233	198	0.66	(0.47- 0.92)
PEZZO2	505	m	0	43	161	233	198	0.23	(0.15- 0.33)
Subtotal PEZZO2								0.41	(0.32- 0.53)
PEZZOT	504	m	0	46	82	145	129	0.50	(0.32- 0.77)
PEZZOT	505	m	0	20	106	145	129	0.17	(0.10- 0.29)
Subtotal PEZZOT								0.32	(0.23- 0.45)
SOBUE	728	m	0	128	116	737	633	0.95	(0.72- 1.24)
SOBUE	729	m	0	67	92	737	633	0.63	(0.45- 0.87)
SOBUE	730	m	0	35	50	737	633	0.60	(0.39- 0.94)
SOBUE	731	m	0	24	31	737	633	0.66	(0.39- 1.14)
SOBUE	732	m	0	15	23	737	633	0.56	(0.29- 1.08)
SOBUE	733	m	0	17	40	737	633	0.37	(0.20- 0.65)
Subtotal SOBUE								0.69	(0.59- 0.82)
*SPEIZE	511	f	2	24	-	319	-	0.60	(0.40- 0.90)
*SPEIZE	512	f	2	34	-	319	-	0.60	(0.40- 0.80)
*SPEIZE	513	f	2	41	-	319	-	0.50	(0.40- 0.70)
*SPEIZE	514	f	2	17	-	319	-	0.20	(0.10- 0.40)
*SPEIZE	515	f	2	28	-	319	-	0.10	(0.10- 0.40)
Subtotal SPEIZE								0.46	(0.38- 0.55)
SUZUK2	524	c	3	15	-	77	-	0.60	(0.20- 1.50)
SUZUK2	525	c	3	10	-	77	-	0.50	(0.20- 1.40)
SUZUK2	526	c	3	9	-	77	-	0.10	(0.10- 0.40)
Subtotal SUZUK2								0.23	(0.14- 0.38)
SVENSS	554	f	0	16	13	142	53	0.46	(0.21- 1.02)
SVENSS	555	f	0	14	24	142	53	0.22	(0.10- 0.45)

Table 1K1 - 2

IESLC - Meta-analysis of Ex Smoking by Years quit (vs current), Overview
All LC types, Any Product (or Cigarettes if Any not available)
Most adjusted

REF	NRR	SEX	AD	Number Exposed Case	Cont	Non-exposed Case	Cont	RR	95.00%CI
Subtotal SVENSS								0.31	(0.18- 0.52)
*TVERDA	506	m	2	2	-	144	-	0.17	(0.04- 0.70)
*TVERDA	507	m	2	5	-	144	-	0.18	(0.07- 0.43)
*TVERDA	508	m	2	4	-	144	-	0.08	(0.03- 0.23)
Subtotal TVERDA								0.13	(0.07- 0.25)
WANG2	515	c	0	6	10	49	78	0.96	(0.33- 2.79)
WANG2	516	c	0	5	11	49	78	0.72	(0.24- 2.21)
Subtotal WANG2								0.84	(0.39- 1.81)
WYNDE3	545	m	0	21	22	227	207	0.87	(0.47- 1.63)
WYNDE3	546	m	0	11	17	227	207	0.59	(0.27- 1.29)
WYNDE3	547	m	0	11	31	227	207	0.32	(0.16- 0.66)
WYNDE3	548	m	0	5	55	227	207	0.08	(0.03- 0.21)
Subtotal WYNDE3								0.42	(0.29- 0.61)
WYNDE6	719	m	5	-	-	-	-	0.60	(0.50- 0.70)
WYNDE6	720	m	5	-	-	-	-	0.30	(0.20- 0.40)
WYNDE6	721	m	5	-	-	-	-	0.20	(0.10- 0.20)
WYNDE6	726	m	5	-	-	-	-	0.70	(0.40- 1.10)
WYNDE6	727	m	5	-	-	-	-	0.20	(0.10- 0.50)
WYNDE6	728	m	5	-	-	-	-	0.30	(0.10- 0.60)
WYNDE6	733	f	5	-	-	-	-	0.50	(0.40- 0.60)
WYNDE6	734	f	5	-	-	-	-	0.20	(0.20- 0.30)
WYNDE6	737	f	5	-	-	-	-	0.60	(0.30- 1.30)
WYNDE6	738	f	5	-	-	-	-	0.40	(0.10- 1.10)
Subtotal WYNDE6								0.39	(0.35- 0.43)
Partial Totals				8630	250179	87467	2230480		

*prospective study ~ With 0.5 adjustment for zero

REF	NRR	SEX	AD	Ys	Ws	Qs	Ps
ALDERS	513	m	1	0.59	26.91	36.99	0.0021
ALDERS	514	m	1	-0.84	15.23	1.07	0.0010
ALDERS	515	m	1	-1.14	17.54	5.50	0.0000
ALDERS	524	f	1	0.73	34.65	59.60	0.0000
ALDERS	525	f	1	-0.43	22.10	0.49	0.0429
ALDERS	526	f	1	-1.27	15.51	7.46	0.0000
Subtotal ALDERS				-0.16	131.92	111.12	
ARMADA	518	m	0	0.13	20.66	10.40	0.5535
ARMADA	519	m	0	-0.99	22.22	3.68	0.0000
Subtotal ARMADA				-0.45	42.88	14.09	
BARBON	548	m	1	0.01	11.73	4.07	0.9728
BARBON	549	m	1	-0.42	34.06	0.91	0.0153
BARBON	550	m	1	-0.71	17.56	0.32	0.0028
BARBON	551	m	1	-1.90	9.79	17.01	0.0000
Subtotal BARBON				-0.62	73.15	22.31	
BECHER	506	m	0	0.01	4.96	1.70	0.9883
BECHER	507	m	0	-0.50	8.94	0.05	0.1316
BECHER	508	m	0	-1.32	10.58	5.73	0.0000
BECHER	516	f	0	-0.64	1.11	0.00	0.4978
BECHER	517	f	0	-1.15	1.30	0.43	0.1879
BECHER	518	f	0	-2.54	0.86	3.29	0.0188
Subtotal BECHER				-0.82	27.75	11.22	
BROSS	518	m	0	0.65	40.07	60.07	0.0000
BROSS	519	m	0	-0.89	24.98	2.39	0.0000
Subtotal BROSS				0.06	65.06	62.46	
CARPEN	508	c	0	-0.83	14.72	0.90	0.0015
CARPEN	509	c	0	-0.85	16.14	1.16	0.0007
CARPEN	510	c	0	-1.82	9.56	14.83	0.0000
CARPEN	511	c	0	-1.78	22.81	32.96	0.0000
Subtotal CARPEN				-1.33	63.22	49.84	
*CEDERL	538	m	1	-0.25	10.90	1.19	0.4121
*CEDERL	539	m	1	-1.97	2.62	5.05	0.0015
Subtotal CEDERL				-0.58	13.52	6.24	
CHOI	543	m	0	-0.59	15.87	0.00	0.0195
CHOI	544	m	0	-1.44	4.15	3.07	0.0034
CHOI	545	m	0	-1.40	3.32	2.22	0.0109
CHOI	546	m	0	-1.20	3.23	1.26	0.0305
CHOI	556	f	0	1.06	1.05	2.83	0.2771
CHOI	557	f	0	2.25	0.40	3.17	0.1567
Subtotal CHOI				-0.78	28.03	12.54	
*CHYOU	510	m	2	-0.99	20.54	3.54	0.0000
*CHYOU	511	m	2	-1.31	4.79	2.55	0.0042

Table 1K1 - 2

IESLC - Meta-analysis of Ex Smoking by Years quit (vs current), Overview
All LC types, Any Product (or Cigarettes if Any not available)
Most adjusted

REF	NRR	SEX	AD	Ys	Ws	Qs	Ps
Subtotal CHYOU				-1.05	25.32	6.09	
*CPSI	815	m	1	0.07	35.26	14.75	0.6879
*CPSI	816	m	1	-0.53	46.88	0.12	0.0003
*CPSI	817	m	1	-0.99	30.29	5.22	0.0000
*CPSI	818	m	1	-2.41	18.30	61.21	0.0000
Subtotal CPSI				-0.74	130.73	81.30	
*CPSII	664	m	1	0.57	91.37	120.87	0.0000
*CPSII	665	m	1	0.25	166.85	113.85	0.0014
*CPSII	666	m	1	-0.16	151.52	26.30	0.0454
*CPSII	667	m	1	-0.65	166.04	0.93	0.0000
*CPSII	668	m	1	-0.94	139.29	18.30	0.0000
*CPSII	669	m	1	-1.77	185.67	264.15	0.0000
*CPSII	642	f	1	0.32	76.90	62.46	0.0047
*CPSII	643	f	1	-0.16	51.90	9.01	0.2417
*CPSII	644	f	1	-0.92	33.71	3.83	0.0000
*CPSII	645	f	1	-1.17	24.99	8.76	0.0000
*CPSII	646	f	1	-1.97	51.44	98.95	0.0000
Subtotal CPSII				-0.57	1139.68	727.41	
DAMBER	528	m	1	-0.22	17.39	2.20	0.3521
DAMBER	529	m	1	-0.80	12.73	0.61	0.0044
DAMBER	530	m	1	-1.31	24.10	12.85	0.0000
Subtotal DAMBER				-0.84	54.22	15.66	
DARBY	507	m	0	-0.35	71.14	3.62	0.0029
DARBY	508	m	0	-1.22	78.40	32.10	0.0000
DARBY	516	f	0	-0.16	28.70	5.07	0.3945
DARBY	517	f	0	-2.00	19.12	38.56	0.0000
Subtotal DARBY				-0.83	197.37	79.36	
DEAN3	636	m	1	-0.22	29.68	3.76	0.2241
DEAN3	637	m	1	-0.60	11.81	0.00	0.0399
DEAN3	638	m	1	-1.08	25.48	6.36	0.0000
DEAN3	559	f	1	-1.24	3.85	1.67	0.0151
DEAN3	560	f	1	-1.61	1.03	1.10	0.1019
DEAN3	561	f	1	-2.04	1.86	3.98	0.0054
Subtotal DEAN3				-0.70	73.72	16.87	
DESTEF	530	m	4	-0.19	23.25	3.59	0.3689
DESTEF	531	m	4	-0.56	12.97	0.00	0.0429
DESTEF	532	m	4	-1.35	10.86	6.40	0.0000
Subtotal DESTEF				-0.56	47.08	9.99	
DOLL	537	m	0	-0.38	30.46	1.21	0.0358
DOLL	538	m	0	-1.55	4.84	4.60	0.0006
DOLL	539	m	0	-1.14	5.88	1.88	0.0055
DOLL	548	f	0	0.06	3.13	1.27	0.9174
DOLL	549	f	0	-1.04	0.65	0.14	0.4022
Subtotal DOLL				-0.59	44.96	9.09	
*DOLL2	509	m	1	0.02	14.30	5.13	0.9403
*DOLL2	510	m	1	-1.05	11.67	2.59	0.0003
*DOLL2	511	m	1	-1.27	8.43	4.06	0.0002
*DOLL2	512	m	1	-2.21	6.60	17.49	0.0000
Subtotal DOLL2				-0.91	41.00	29.26	
DORGAN	514	m	0	-0.28	23.81	2.09	0.1680
DORGAN	515	m	0	-0.17	19.17	3.15	0.4460
DORGAN	516	m	0	-1.07	59.40	14.41	0.0000
DORGAN	559	f	0	-0.35	14.32	0.74	0.1829
DORGAN	560	f	0	-1.33	16.18	9.21	0.0000
Subtotal DORGAN				-0.76	132.87	29.60	
*DORN	823	m	0	-0.03	31.99	9.79	0.8830
*DORN	824	m	0	-0.53	30.20	0.06	0.0033
*DORN	825	m	0	-1.14	11.74	3.66	0.0001
*DORN	826	m	0	-1.75	15.53	21.36	0.0000
*DORN	827	m	0	-0.13	13.68	2.71	0.6211
*DORN	828	m	0	-0.42	38.16	1.00	0.0100
*DORN	829	m	0	-0.58	27.56	0.00	0.0023
*DORN	830	m	0	-1.36	32.00	19.47	0.0000
Subtotal DORN				-0.67	200.86	58.05	
GAO	536	m	2	0.57	27.97	37.00	0.0025
GAO	537	m	2	-0.24	11.93	1.41	0.4156
GAO	538	m	2	-1.27	7.80	3.75	0.0004
GAO	556	f	2	0.91	6.45	14.28	0.0210
GAO	557	f	2	0.29	4.11	3.12	0.5532
GAO	558	f	2	-0.27	6.07	0.56	0.4991
Subtotal GAO				0.13	64.32	60.13	

Table 1K1 - 2

IESLC - Meta-analysis of Ex Smoking by Years quit (vs current), Overview
All LC types, Any Product (or Cigarettes if Any not available)
Most adjusted

REF	NRR	SEX	AD	Ys	Ws	Qs	Ps
GAO2	518	m	0	-0.28	11.81	1.08	0.3414
GAO2	519	m	0	-0.67	9.99	0.08	0.0352
GAO2	520	m	0	-0.57	7.57	0.00	0.1164
GAO2	521	m	0	-0.70	3.73	0.06	0.1738
GAO2	522	m	0	-1.59	5.59	5.73	0.0002
Subtotal GAO2				-0.67	38.69	6.95	
GARCIA	508	c	0	0.49	6.33	7.27	0.2154
GARCIA	509	c	0	-0.43	11.39	0.26	0.1483
GARCIA	510	c	0	-1.35	12.05	7.07	0.0000
GARCIA	511	c	0	-1.91	6.10	10.88	0.0000
Subtotal GARCIA				-0.83	35.87	25.48	
GRAHAM	540	m	1	1.60	34.51	163.16	0.0000
GRAHAM	541	m	1	0.16	14.79	8.01	0.5460
GRAHAM	542	m	1	-1.02	9.45	1.85	0.0017
Subtotal GRAHAM				0.81	58.74	173.02	
*HAMMO2	510	m	1	0.08	49.68	21.39	0.5875
*HAMMO2	511	m	1	-0.94	11.19	1.47	0.0016
*HAMMO2	512	m	1	-1.08	19.88	4.96	0.0000
Subtotal HAMMO2				-0.35	80.75	27.82	
*HIRAYA	513	m	1	-0.78	11.65	0.45	0.0080
*HIRAYA	514	m	1	-1.02	5.04	0.99	0.0218
*HIRAYA	515	m	1	-1.17	6.04	2.12	0.0040
*HIRAYA	524	f	1	0.46	2.59	2.82	0.4555
*HIRAYA	525	f	1	0.34	1.18	1.01	0.7089
*HIRAYA	526	f	1	-0.89	0.29	0.03	0.6307
Subtotal HIRAYA				-0.74	26.79	7.41	
JAHN	513	m	0	2.76	7.27	81.22	0.0000
JAHN	514	m	0	1.63	7.44	36.27	0.0000
JAHN	515	m	0	0.25	24.22	16.50	0.2255
JAHN	516	m	0	-0.33	25.39	1.52	0.0919
JAHN	517	m	0	-0.98	33.47	5.31	0.0000
JAHN	518	m	0	-1.89	20.88	35.62	0.0000
Subtotal JAHN				-0.36	118.68	176.44	
JAIN	570	m	0	-0.33	21.05	1.27	0.1258
JAIN	571	m	0	-1.59	24.80	25.10	0.0000
JAIN	534	f	0	-0.52	17.76	0.06	0.0287
JAIN	535	f	0	-2.29	12.14	35.59	0.0000
Subtotal JAIN				-1.10	75.74	62.02	
JOLY	571	m	0	0.20	17.18	10.54	0.3976
JOLY	572	m	0	-0.71	37.44	0.65	0.0000
JOLY	558	f	0	0.55	5.11	6.48	0.2166
JOLY	559	f	0	-0.55	7.28	0.00	0.1343
Subtotal JOLY				-0.36	67.01	17.67	
*KAISE2	655	m	1	0.00	9.58	3.22	1.0000
*KAISE2	656	m	1	-0.84	6.60	0.46	0.0302
*KAISE2	657	m	1	-1.35	4.25	2.50	0.0055
*KAISE2	575	f	1	-0.63	5.47	0.02	0.1377
*KAISE2	576	f	1	-1.39	3.65	2.38	0.0081
*KAISE2	577	f	1	-1.08	4.06	1.01	0.0298
Subtotal KAISE2				-0.72	33.61	9.59	
KHUDER	516	m	0	-0.08	37.40	9.30	0.6231
KHUDER	517	m	0	-0.49	32.64	0.24	0.0049
KHUDER	518	m	0	-0.96	35.95	5.32	0.0000
Subtotal KHUDER				-0.51	105.99	14.86	
LUBIN	592	m	0	1.39	11.02	42.84	0.0000
LUBIN	593	m	0	-0.09	13.20	3.17	0.7468
LUBIN	594	m	0	-0.67	12.91	0.11	0.0160
Subtotal LUBIN				0.15	37.13	46.12	
LUBIN2	1081	m	0	0.09	402.52	181.52	0.0639
LUBIN2	1082	m	0	-0.29	267.60	23.10	0.0000
LUBIN2	1083	m	0	-0.66	181.12	1.20	0.0000
LUBIN2	1084	m	0	-1.02	98.44	19.12	0.0000
LUBIN2	1085	m	0	-1.08	81.77	20.33	0.0000
LUBIN2	1086	m	0	-1.60	91.35	94.96	0.0000
LUBIN2	1120	f	0	0.02	25.28	8.97	0.9343
LUBIN2	1121	f	0	-0.36	15.86	0.77	0.1536
LUBIN2	1122	f	0	-1.03	6.98	1.40	0.0067
LUBIN2	1123	f	0	-0.92	2.08	0.24	0.1856
LUBIN2	1124	f	0	-0.88	2.73	0.25	0.1450
LUBIN2	1125	f	0	-1.68	3.28	3.98	0.0023
Subtotal LUBIN2				-0.44	1179.01	355.83	

Table 1K1 - 2

IESLC - Meta-analysis of Ex Smoking by Years quit (vs current), Overview
All LC types, Any Product (or Cigarettes if Any not available)
Most adjusted

REF	NRR	SEX	AD	Ys	Ws	Qs	Ps
MATOS	596	m	2	0.34	11.06	9.27	0.2631
MATOS	597	m	2	-0.11	8.00	1.79	0.7658
MATOS	598	m	2	-1.20	12.73	4.97	0.0000
Subtotal MATOS				-0.39	31.79	16.04	
PEZZO2	504	m	0	-0.42	33.11	0.83	0.0155
PEZZO2	505	m	0	-1.48	25.77	21.05	0.0000
Subtotal PEZZO2				-0.89	58.88	21.88	
PEZZOT	504	m	0	-0.69	20.58	0.28	0.0016
PEZZOT	505	m	0	-1.78	13.50	19.62	0.0000
Subtotal PEZZOT				-1.13	34.08	19.89	
SOBUE	728	m	0	-0.05	51.63	14.26	0.6997
SOBUE	729	m	0	-0.47	34.80	0.42	0.0056
SOBUE	730	m	0	-0.51	19.41	0.10	0.0250
SOBUE	731	m	0	-0.41	13.01	0.38	0.1411
SOBUE	732	m	0	-0.58	8.84	0.00	0.0848
SOBUE	733	m	0	-1.01	11.53	2.12	0.0006
Subtotal SOBUE				-0.37	139.23	17.27	
*SPEIZE	511	f	2	-0.51	23.37	0.11	0.0135
*SPEIZE	512	f	2	-0.51	31.98	0.15	0.0039
*SPEIZE	513	f	2	-0.69	49.07	0.64	0.0000
*SPEIZE	514	f	2	-1.61	8.00	8.49	0.0000
*SPEIZE	515	f	2	-2.30	8.00	23.75	0.0000
Subtotal SPEIZE				-0.78	120.40	33.13	
SUZUK2	524	c	3	-0.51	3.78	0.02	0.3203
SUZUK2	525	c	3	-0.69	4.06	0.05	0.1626
SUZUK2	526	c	3	-2.30	8.00	23.75	0.0000
Subtotal SUZUK2				-1.46	15.84	23.82	
SVENSS	554	f	0	-0.78	6.05	0.24	0.0557
SVENSS	555	f	0	-1.52	7.19	6.43	0.0000
Subtotal SVENSS				-1.18	13.24	6.67	
*TVERDA	506	m	2	-1.77	1.88	2.67	0.0152
*TVERDA	507	m	2	-1.71	4.66	6.01	0.0002
*TVERDA	508	m	2	-2.53	3.70	14.03	0.0000
Subtotal TVERDA				-2.02	10.24	22.72	
WANG2	515	c	0	-0.05	3.33	0.95	0.9331
WANG2	516	c	0	-0.32	3.09	0.20	0.5698
Subtotal WANG2				-0.18	6.42	1.15	
WYNDE3	545	m	0	-0.14	9.77	1.90	0.6644
WYNDE3	546	m	0	-0.53	6.29	0.02	0.1858
WYNDE3	547	m	0	-1.13	7.55	2.28	0.0019
WYNDE3	548	m	0	-2.49	4.40	16.06	0.0000
Subtotal WYNDE3				-0.86	28.01	20.25	
WYNDE6	719	m	5	-0.51	135.72	0.63	0.0000
WYNDE6	720	m	5	-1.20	31.98	12.49	0.0000
WYNDE6	721	m	5	-1.61	31.98	33.95	0.0000
WYNDE6	726	m	5	-0.36	15.02	0.74	0.1669
WYNDE6	727	m	5	-1.61	5.93	6.30	0.0001
WYNDE6	728	m	5	-1.20	4.79	1.87	0.0084
WYNDE6	733	f	5	-0.69	93.46	1.21	0.0000
WYNDE6	734	f	5	-1.61	93.46	99.21	0.0000
WYNDE6	737	f	5	-0.51	7.15	0.03	0.1721
WYNDE6	738	f	5	-0.92	2.67	0.30	0.1342
Subtotal WYNDE6				-0.95	422.17	156.74	

N	194
NS	43

Table 1K1 - 3

IESLC - Meta-analysis of Ex Smoking by Years quit (vs current), Overview
All LC types, Any Product (or Cigarettes if Any not available)
Most adjusted

	Sex			
	combined	male	female	Total
N	13	129	52	194
NS	4	37	17	58

In this overview table, other than the "N" rows, entries in the "absent" and "Total" columns may be invalid and should be ignored

	Years quit vs current (lower focus)				
	absent	1-6k3	4-11k7	8+k12	Total
N	76	41	29	48	194
NS	33	32	22	32	119
Wt	2098.46	1288.65	859.73	1089.15	5335.99
Het Chi	1498.82	141.92	58.59	198.19	2675.39
Het df	75	40	28	47	193
Het P	***	***	***	***	***
Fixed RR	0.52	0.98	0.60	0.32	0.56
RRl	0.50	0.93	0.56	0.30	0.55
RRu	0.54	1.04	0.64	0.34	0.58
P	---	N.S.	---	---	---
Random RR	0.48	0.95	0.57	0.28	0.50
RRl	0.39	0.84	0.50	0.24	0.45
RRu	0.59	1.08	0.64	0.32	0.55
P	---	N.S.	---	---	---

	Years quit vs current (higher focus)				
	absent	1-11k3	4-19k12	13+k20	Total
N	95	60	19	20	194
NS	42	42	15	16	115
Wt	2368.43	1849.59	576.37	541.60	5335.99
Het Chi	1433.44	241.03	55.17	87.33	2675.39
Het df	94	59	18	19	193
Het P	***	***	***	***	***
Fixed RR	0.53	0.85	0.44	0.22	0.56
RRl	0.51	0.82	0.40	0.20	0.55
RRu	0.55	0.89	0.48	0.24	0.58
P	---	---	---	---	---
Random RR	0.44	0.84	0.39	0.23	0.50
RRl	0.37	0.75	0.33	0.19	0.45
RRu	0.52	0.93	0.47	0.29	0.55
P	---	---	---	---	---

Table 1K1 - 3

IESLC - Meta-analysis of Ex Smoking by Years quit (vs current), Overview
All LC types, Any Product (or Cigarettes if Any not available)
Most adjusted

MALES

		Years quit vs current (lower focus)				
		absent	1-6k3	4-11k7	8+k12	Total
	N	51	28	20	30	129
	NS	27	27	19	28	101
	Wt	1630.70	1131.15	733.28	851.98	4347.10
Het	Chi	1182.64	105.07	42.95	111.27	2020.92
Het	df	50	27	19	29	128
Het	P	***	***	**	***	***
Fixed	RR	0.51	1.01	0.62	0.35	0.59
	RRl	0.49	0.95	0.58	0.33	0.57
	RRu	0.54	1.07	0.67	0.38	0.60
	P	---	N.S.	---	---	---
Random	RR	0.48	0.98	0.58	0.31	0.52
	RRl	0.37	0.85	0.51	0.27	0.46
	RRu	0.61	1.12	0.67	0.36	0.59
	P	---	N.S.	---	---	---

		Years quit vs current (higher focus)				
		absent	1-11k3	4-19k12	13+k20	Total
	N	62	38	14	15	129
	NS	34	36	12	13	95
	Wt	1893.13	1493.95	515.46	444.56	4347.10
Het	Chi	1055.19	169.15	34.75	67.54	2020.92
Het	df	61	37	13	14	128
Het	P	***	***	***	***	***
Fixed	RR	0.55	0.90	0.46	0.23	0.59
	RRl	0.53	0.86	0.42	0.21	0.57
	RRu	0.58	0.95	0.50	0.26	0.60
	P	---	---	---	---	---
Random	RR	0.47	0.87	0.43	0.25	0.52
	RRl	0.38	0.77	0.36	0.20	0.46
	RRu	0.57	0.99	0.51	0.33	0.59
	P	---	-	---	---	---

FEMALES

		Years quit vs current (lower focus)				
		absent	1-6k3	4-11k7	8+k12	Total
	N	20	9	7	16	52
	NS	13	9	7	15	44
	Wt	412.32	129.33	106.26	219.62	867.53
Het	Chi	259.64	21.93	9.84	30.79	527.20
Het	df	19	8	6	15	51
Het	P	***	**	N.S.	**	***
Fixed	RR	0.61	0.86	0.51	0.22	0.48
	RRl	0.55	0.73	0.42	0.19	0.45
	RRu	0.67	1.03	0.61	0.25	0.52
	P	---	(-)	---	---	---
Random	RR	0.54	0.97	0.54	0.23	0.48
	RRl	0.36	0.67	0.40	0.18	0.37
	RRu	0.81	1.39	0.74	0.29	0.60
	P	--	N.S.	---	---	---

Table 1K1 - 3

IESLC - Meta-analysis of Ex Smoking by Years quit (vs current), Overview
All LC types, Any Product (or Cigarettes if Any not available)
Most adjusted

FEMALES

	Years quit vs current (higher focus) absent	1-11k3	4-19k12	13+k20	Total
N	28	18	3	3	52
NS	17	17	3	3	40
Wt	437.92	327.47	39.97	62.17	867.53
Het Chi	333.97	42.03	1.53	4.12	527.20
Het df	27	17	2	2	51
Het P	***	***	N.S.	N.S.	***
Fixed RR	0.47	0.69	0.29	0.14	0.48
RRl	0.42	0.62	0.21	0.11	0.45
RRu	0.51	0.76	0.40	0.18	0.52
P	---	---	---	---	---
Random RR	0.40	0.75	0.29	0.15	0.48
RRl	0.27	0.61	0.21	0.09	0.37
RRu	0.59	0.92	0.40	0.25	0.60
P	---	--	---	---	---

Table 1K1 - 4

IESLC - Meta-analysis of Ex Smoking by Years quit (vs current), Overview
All LC types, Any Product (or Cigarettes if Any not available)
Least adjusted

REF	NRR	X	SEX	AGEL	AGEH	RACE	YF	LC TYPE	LOC	START	ST	NLC	R	VB	P	H	AD	ADOS	PRODUCT	exL	exH	S1	S2	DENOM	De
ALDERS	513		m	0	0	all	-	all	Eu:UK	1977	CC	1448	n	V	n	n	1	0	cig only	0.1	2	0	0	current	ot
ALDERS	514		m	0	0	all	-	all	Eu:UK	1977	CC	1448	n	V	n	n	1	0	cig only	3	9	0	1	current	ot
ALDERS	515		m	0	0	all	-	all	Eu:UK	1977	CC	1448	n	V	n	n	1	0	cig only	10	999	3	0	current	ot
ALDERS	524		f	0	0	all	-	all	Eu:UK	1977	CC	1448	n	V	n	n	1	0	cig only	0.1	2	0	0	current	ot
ALDERS	525		f	0	0	all	-	all	Eu:UK	1977	CC	1448	n	V	n	n	1	0	cig only	3	9	0	1	current	ot
ALDERS	526		f	0	0	all	-	all	Eu:UK	1977	CC	1448	n	V	n	n	1	0	cig only	10	999	3	0	current	ot
ARMADA	518		m	0	0	all	-	all	Eu:wst	1986	CC	325	n	bl	n	y	0	0	cig+/-ot	1.0	5	1	1	cur+1y	st
ARMADA	519		m	0	0	all	-	all	Eu:wst	1986	CC	325	n	bl	n	y	0	0	cig+/-ot	6	999	0	0	cur+1y	st
BARBON	533	x	m	0	0	all	-	all	Eu:wst	1979	CC	755	n	bl	y	y	0	0	all/unsp	0.1	4	1	1	current	st
BARBON	534	x	m	0	0	all	-	all	Eu:wst	1979	CC	755	n	bl	y	y	0	0	all/unsp	5	14	0	2	current	st
BARBON	535	x	m	0	0	all	-	all	Eu:wst	1979	CC	755	n	bl	y	y	0	0	all/unsp	15	24	0	3	current	st
BARBON	536	x	m	0	0	all	-	all	Eu:wst	1979	CC	755	n	bl	y	y	0	0	all/unsp	25	999	0	0	current	st
BECHER	506		m	0	0	all	-	all	Eu:Ger	1985	CC	194	n	bl	n	y	0	0	all/unsp	2	4	1	1	cur+1y	st
BECHER	507		m	0	0	all	-	all	Eu:Ger	1985	CC	194	n	bl	n	y	0	0	all/unsp	5	9	2	0	cur+1y	st
BECHER	508		m	0	0	all	-	all	Eu:Ger	1985	CC	194	n	bl	n	y	0	0	all/unsp	10	999	3	0	cur+1y	st
BECHER	516		f	0	0	all	-	all	Eu:Ger	1985	CC	194	n	bl	n	y	0	0	all/unsp	2	4	1	1	cur+1y	st
BECHER	517		f	0	0	all	-	all	Eu:Ger	1985	CC	194	n	bl	n	y	0	0	all/unsp	5	9	2	0	cur+1y	st
BECHER	518		f	0	0	all	-	all	Eu:Ger	1985	CC	194	n	bl	n	y	0	0	all/unsp	10	999	3	0	cur+1y	st
BROSS	518		m	0	0	wh	-	all	NAmer	1960	CC	974	n	bl	n	n	0	0	cig+/-ot	0.1	5	1	1	current	st
BROSS	519		m	0	0	wh	-	all	NAmer	1960	CC	974	n	bl	n	n	0	0	cig+/-ot	6	999	0	0	current	st
CARPEN	508		c	0	0	w+b	-	all	NAmer	1991	CC	356	n	bl	n	n	0	0	cig+/-ot	0.1	4	1	1	current	st
CARPEN	509		c	0	0	w+b	-	all	NAmer	1991	CC	356	n	bl	n	n	0	0	cig+/-ot	5	9	2	0	current	st
CARPEN	510		c	0	0	w+b	-	all	NAmer	1991	CC	356	n	bl	n	n	0	0	cig+/-ot	10	14	3	2	current	st
CARPEN	511		c	0	0	w+b	-	all	NAmer	1991	CC	356	n	bl	n	n	0	0	cig+/-ot	15	999	0	3	current	st
CEDERL	538		m	40	69	all	10	all	Eu:Sca	1963	pr	491	n	bl	n	n	1	0	all/unsp	0.1	9	0	1	current	ot
CEDERL	539		m	40	69	all	10	all	Eu:Sca	1963	pr	491	n	bl	n	n	1	0	all/unsp	10	999	3	0	current	ot
CHOI	543		m	0	0	all	-	all	As:oth	1985	CC	375	n	bl	n	n	0	0	cig+/-ot	0.1	4	1	1	current	st
CHOI	544		m	0	0	all	-	all	As:oth	1985	CC	375	n	bl	n	n	0	0	cig+/-ot	5	9	2	0	current	st
CHOI	545		m	0	0	all	-	all	As:oth	1985	CC	375	n	bl	n	n	0	0	cig+/-ot	10	14	3	2	current	st
CHOI	546		m	0	0	all	-	all	As:oth	1985	CC	375	n	bl	n	n	0	0	cig+/-ot	15	999	0	3	current	st
CHOI	556		f	0	0	all	-	all	As:oth	1985	CC	375	n	bl	n	n	0	0	cig+/-ot	0.1	4	1	1	current	st
CHOI	557		f	0	0	all	-	all	As:oth	1985	CC	375	n	bl	n	n	0	0	cig+/-ot	5	999	0	0	current	ot
CHYOU	504	x	m	0	0	jap	21	all	NAmer	1965	pr	227	n	bl	n	y	1	0	cig+/-ot	0.1	14	0	0	current	ot
CHYOU	505	x	m	0	0	jap	21	all	NAmer	1965	pr	227	n	bl	n	y	1	0	cig+/-ot	15	999	0	3	current	ot
CPSI	815		m	50	74	all	6	all	NAmer	1959	pr	5138	n	bl	n	n	1	0	cig only	0.1	0.9	0	0	current	ot
CPSI	816		m	50	74	all	6	all	NAmer	1959	pr	5138	n	bl	n	n	1	0	cig only	1.0	4	1	1	current	ot
CPSI	817		m	50	74	all	6	all	NAmer	1959	pr	5138	n	bl	n	n	1	0	cig only	5	9	2	0	current	ot
CPSI	818		m	50	74	all	6	all	NAmer	1959	pr	5138	n	bl	n	n	1	0	cig only	10	999	3	0	current	ot
CPSII	664		m	35	99	all	4	all	NAmer	1982	pr	3229	n	bl	n	n	1	0	cig only	0.1	0.9	0	0	current	ot
CPSII	665		m	35	99	all	4	all	NAmer	1982	pr	3229	n	bl	n	n	1	0	cig only	1.0	2	0	0	current	ot
CPSII	666		m	35	99	all	4	all	NAmer	1982	pr	3229	n	bl	n	n	1	0	cig only	3	5	1	1	current	ot
CPSII	667		m	35	99	all	4	all	NAmer	1982	pr	3229	n	bl	n	n	1	0	cig only	6	10	2	0	current	ot
CPSII	668		m	35	99	all	4	all	NAmer	1982	pr	3229	n	bl	n	n	1	0	cig only	11	15	3	2	current	ot
CPSII	669		m	35	99	all	4	all	NAmer	1982	pr	3229	n	bl	n	n	1	0	cig only	16	999	0	3	current	ot
CPSII	642		f	0	0	all	4	all	NAmer	1982	pr	3229	n	bl	n	n	1	0	cig+/-ot	0.1	2	0	0	current	ot
CPSII	643		f	0	0	all	4	all	NAmer	1982	pr	3229	n	bl	n	n	1	0	cig+/-ot	3	5	1	1	current	ot
CPSII	644		f	0	0	all	4	all	NAmer	1982	pr	3229	n	bl	n	n	1	0	cig+/-ot	6	10	2	0	current	ot
CPSII	645		f	0	0	all	4	all	NAmer	1982	pr	3229	n	bl	n	n	1	0	cig+/-ot	11	15	3	2	current	ot
CPSII	646		f	0	0	all	4	all	NAmer	1982	pr	3229	n	bl	n	n	1	0	cig+/-ot	16	999	0	3	current	ot
DAMBER	528		m	0	0	all	-	all	Eu:Sca	1972	CC	579	n	bl	y	n	1	0	all/unsp	0.1	5	1	1	current	ot
DAMBER	529		m	0	0	all	-	all	Eu:Sca	1972	CC	579	n	bl	y	n	1	0	all/unsp	6	10	2	0	current	ot
DAMBER	530		m	0	0	all	-	all	Eu:Sca	1972	CC	579	n	bl	y	n	1	0	all/unsp	11	999	3	0	current	ot
DARBY	507		m	0	0	wh	-	all	Eu:UK	1988	CC	982	n	V	n	n	0	0	all/unsp	0.1	9	0	1	current	st
DARBY	508		m	0	0	wh	-	all	Eu:UK	1988	CC	982	n	V	n	n	0	0	all/unsp	10	999	3	0	current	st
DARBY	516		f	0	0	wh	-	all	Eu:UK	1988	CC	982	n	V	n	n	0	0	all/unsp	0.1	9	0	1	current	st
DARBY	517		f	0	0	wh	-	all	Eu:UK	1988	CC	982	n	V	n	n	0	0	all/unsp	10	999	3	0	current	st
DEAN3	537	x	m	0	0	all	-	all	Eu:UK	1969	CC	766	n	V	y	n	0	0	all/unsp	3	4	1	1	cur+2y	st
DEAN3	538	x	m	0	0	all	-	all	Eu:UK	1969	CC	766	n	V	y	n	0	0	all/unsp	5	8	2	0	cur+2y	st
DEAN3	539	x	m	0	0	all	-	all	Eu:UK	1969	CC	766	n	V	y	n	0	0	all/unsp	9	999	3	0	cur+2y	st
DEAN3	548	x	f	0	0	all	-	all	Eu:UK	1969	CC	766	n	V	y	n	0	0	all/unsp	3	4	1	1	cur+2y	st
DEAN3	549	x	f	0	0	all	-	all	Eu:UK	1969	CC	766	n	V	y	n	0	0	all/unsp	5	8	2	0	cur+2y	st
DEAN3	550	x	f	0	0	all	-	all	Eu:UK	1969	CC	766	n	V	y	n	0	0	all/unsp	9	999	3	0	cur+2y	st
DESTEF	520	x	m	0	0	all	-	all	SCAmer	1988	CC	497	n	bl	n	y	0	0	all/unsp	0.1	4	1	1	current	st
DESTEF	521	x	m	0	0	all	-	all	SCAmer	1988	CC	497	n	bl	n	y	0	0	all/unsp	5	9	2	0	current	st
DESTEF	522	x	m	0	0	all	-	all	SCAmer	1988	CC	497	n	bl	n	y	0	0	all/unsp	10	999	3	0	current	st
DOLL	537		m	0	0	all	-	all	Eu:UK	1948	CC	1465	n	V	n	n	0	0	all/unsp	0.1	9	0	1	current	st
DOLL	538		m	0	0	all	-	all	Eu:UK	1948	CC	1465	n	V	n	n	0	0	all/unsp	10	19	3	2	current	st
DOLL	539		m	0	0	all	-	all	Eu:UK	1948	CC	1465	n	V	n	n	0	0	all/unsp	20	999	0	3	current	st
DOLL	548		f	0	0	all	-	all	Eu:UK	1948	CC	1465	n	V	n	n	0	0	all/unsp	0.1	9	0	1	current	st
DOLL	549		f	0	0	all	-	all	Eu:UK	1948	CC	1465	n	V	n	n	0	0	all/unsp	10	999	3	0	current	st
DOLL2	509		m	0	0	all	20	all	Eu:UK	1951	pr	920	n	V	n	n	1	0	cig only	0.1	4	1	1	current	ot
DOLL2	510		m	0	0	all	20	all	Eu:UK	1951	pr	920	n	V	n	n	1	0	cig only	5	9	2	0	current	ot

Table 1K1 - 4

IESLC - Meta-analysis of Ex Smoking by Years quit (vs current), Overview
All LC types, Any Product (or Cigarettes if Any not available)
Least adjusted

REF	NRR	X	SEX	AGEL	AGEH	RACE	YF	LC TYPE	LOC	START	ST	NLC	R	VB	P	H	AD	ADOS	PRODUCT	exL	exH	S1	S2	DENOM	De
DOLL2	511		m	0	0	all	20	all	Eu:UK	1951	pr	920	n	V	n	n	1	0	cig only	10	14	3	2	current	ot
DOLL2	512		m	0	0	all	20	all	Eu:UK	1951	pr	920	n	V	n	n	1	0	cig only	15	999	0	3	current	ot
DORGAN	514		m	0	0	wh	-	all	NAmer	1980	CC	2026	n	bl	y	y	0	0	cig+/-ot	1.1	5	1	1	cur+1y	st
DORGAN	515		m	0	0	wh	-	all	NAmer	1980	CC	2026	n	bl	y	y	0	0	cig+/-ot	6	9	2	0	cur+1y	st
DORGAN	516		m	0	0	wh	-	all	NAmer	1980	CC	2026	n	bl	y	y	0	0	cig+/-ot	10	999	3	0	cur+1y	st
DORGAN	559		f	0	0	all	-	all	NAmer	1980	CC	2026	n	bl	y	y	0	0	cig+/-ot	1.1	9	0	1	cur+1y	st
DORGAN	560		f	0	0	all	-	all	NAmer	1980	CC	2026	n	bl	y	y	0	0	cig+/-ot	10	999	3	0	cur+1y	st
DORN	823		m	55	64	wh	8	all	NAmer	1954	pr	5097	n	bl	n	n	0	0	cig+/-ot	0.1	4	1	1	current	st
DORN	824		m	55	64	wh	8	all	NAmer	1954	pr	5097	n	bl	n	n	0	0	cig+/-ot	5	9	2	0	current	st
DORN	825		m	55	64	wh	8	all	NAmer	1954	pr	5097	n	bl	n	n	0	0	cig+/-ot	10	14	3	2	current	st
DORN	826		m	55	64	wh	8	all	NAmer	1954	pr	5097	n	bl	n	n	0	0	cig+/-ot	15	999	0	3	current	st
DORN	827		m	65	74	wh	8	all	NAmer	1954	pr	5097	n	bl	n	n	0	0	cig+/-ot	0.1	4	1	1	current	st
DORN	828		m	65	74	wh	8	all	NAmer	1954	pr	5097	n	bl	n	n	0	0	cig+/-ot	5	9	2	0	current	st
DORN	829		m	65	74	wh	8	all	NAmer	1954	pr	5097	n	bl	n	n	0	0	cig+/-ot	10	14	3	2	current	st
DORN	830		m	65	74	wh	8	all	NAmer	1954	pr	5097	n	bl	n	n	0	0	cig+/-ot	15	999	0	3	current	st
GAO	526	x	m	0	0	all	-	all	As:Chi	1984	CC	1405	n	ot	n	n	0	0	cig+/-ot	0.1	4	1	1	current	st
GAO	527	x	m	0	0	all	-	all	As:Chi	1984	CC	1405	n	ot	n	n	0	0	cig+/-ot	5	9	2	0	current	st
GAO	528	x	m	0	0	all	-	all	As:Chi	1984	CC	1405	n	ot	n	n	0	0	cig+/-ot	10	999	3	0	current	st
GAO	546	x	f	0	0	all	-	all	As:Chi	1984	CC	1405	n	ot	n	n	0	0	cig+/-ot	0.1	4	1	1	current	st
GAO	547	x	f	0	0	all	-	all	As:Chi	1984	CC	1405	n	ot	n	n	0	0	cig+/-ot	5	9	2	0	current	st
GAO	548	x	f	0	0	all	-	all	As:Chi	1984	CC	1405	n	ot	n	n	0	0	cig+/-ot	10	999	3	0	current	st
GAO2	518		m	0	0	all	-	all	As:Jap	1988	CC	282	n	bl	n	n	0	0	cig+/-ot	1.0	4	1	1	cur+1y	st
GAO2	519		m	0	0	all	-	all	As:Jap	1988	CC	282	n	bl	n	n	0	0	cig+/-ot	5	9	2	0	cur+1y	st
GAO2	520		m	0	0	all	-	all	As:Jap	1988	CC	282	n	bl	n	n	0	0	cig+/-ot	10	14	3	2	cur+1y	st
GAO2	521		m	0	0	all	-	all	As:Jap	1988	CC	282	n	bl	n	n	0	0	cig+/-ot	15	19	0	0	cur+1y	st
GAO2	522		m	0	0	all	-	all	As:Jap	1988	CC	282	n	bl	n	n	0	0	cig+/-ot	20	999	0	3	cur+1y	st
GARCIA	508		c	0	0	all	-	all	NAmer	1992	CC	416	n	bl	n	y	0	0	cig+/-ot	1.0	4	1	1	cur+1y	st
GARCIA	509		c	0	0	all	-	all	NAmer	1992	CC	416	n	bl	n	y	0	0	cig+/-ot	5	14	0	2	cur+1y	st
GARCIA	510		c	0	0	all	-	all	NAmer	1992	CC	416	n	bl	n	y	0	0	cig+/-ot	15	29	0	3	cur+1y	st
GARCIA	511		c	0	0	all	-	all	NAmer	1992	CC	416	n	bl	n	y	0	0	cig+/-ot	30	999	0	0	cur+1y	st
GRAHAM	530	x	m	0	0	wh	-	all	NAmer	1956	CC	685	n	bl	n	n	0	0	cig+/-ot	0.1	1.0	0	0	current	st
GRAHAM	531	x	m	0	0	wh	-	all	NAmer	1956	CC	685	n	bl	n	n	0	0	cig+/-ot	1.1	5	1	1	current	st
GRAHAM	532	x	m	0	0	wh	-	all	NAmer	1956	CC	685	n	bl	n	n	0	0	cig+/-ot	5	999	0	0	current	st
HAMMO2	510		m	0	0	all	0	all	NAmer	1967	pr	450	o	bl	n	n	1	0	cig+/-ot	0.1	4	1	1	current	ot
HAMMO2	511		m	0	0	all	0	all	NAmer	1967	pr	450	o	bl	n	n	1	0	cig+/-ot	5	9	2	0	current	ot
HAMMO2	512		m	0	0	all	0	all	NAmer	1967	pr	450	o	bl	n	n	1	0	cig+/-ot	10	999	3	0	current	ot
HIRAYA	513		m	0	0	all	0	all	As:Jap	1965	pr	1917	n	bl	n	n	1	0	cig+/-ot	0.1	4	1	1	current	ot
HIRAYA	514		m	0	0	all	0	all	As:Jap	1965	pr	1917	n	bl	n	n	1	0	cig+/-ot	5	9	2	0	current	ot
HIRAYA	515		m	0	0	all	0	all	As:Jap	1965	pr	1917	n	bl	n	n	1	0	cig+/-ot	10	999	3	0	current	ot
HIRAYA	524		f	0	0	all	0	all	As:Jap	1965	pr	1917	n	bl	n	n	1	0	cig+/-ot	0.1	4	1	1	current	ot
HIRAYA	525		f	0	0	all	0	all	As:Jap	1965	pr	1917	n	bl	n	n	1	0	cig+/-ot	5	9	2	0	current	ot
HIRAYA	526		f	0	0	all	0	all	As:Jap	1965	pr	1917	n	bl	n	n	1	0	cig+/-ot	10	999	3	0	current	ot
JAHN	513		m	0	0	all	-	all	Eu:Ger	1988	CC	1004	n	bl	n	n	0	0	cig+/-ot	0.1	0.9	0	0	current	st
JAHN	514		m	0	0	all	-	all	Eu:Ger	1988	CC	1004	n	bl	n	n	0	0	cig+/-ot	1.0	1.9	0	0	current	st
JAHN	515		m	0	0	all	-	all	Eu:Ger	1988	CC	1004	n	bl	n	n	0	0	cig+/-ot	2	5	1	1	current	st
JAHN	516		m	0	0	all	-	all	Eu:Ger	1988	CC	1004	n	bl	n	n	0	0	cig+/-ot	6	10	2	0	current	st
JAHN	517		m	0	0	all	-	all	Eu:Ger	1988	CC	1004	n	bl	n	n	0	0	cig+/-ot	11	20	3	0	current	st
JAHN	518		m	0	0	all	-	all	Eu:Ger	1988	CC	1004	n	bl	n	n	0	0	cig+/-ot	21	999	0	0	current	st
JAIN	570		m	0	0	all	-	all	NAmer	1981	CC	845	n	V	y	n	0	0	cig+/-ot	2	9	0	1	cur+2y	st
JAIN	571		m	0	0	all	-	all	NAmer	1981	CC	845	n	V	y	n	0	0	cig+/-ot	10	999	3	0	cur+2y	st
JAIN	534		f	0	0	all	-	all	NAmer	1981	CC	845	n	V	y	n	0	0	cig+/-ot	2	9	0	1	cur+2y	st
JAIN	535		f	0	0	all	-	all	NAmer	1981	CC	845	n	V	y	n	0	0	cig+/-ot	10	999	3	0	cur+2y	st
JOLY	571		m	0	0	all	-	all	SCAmer	1978	CC	826	n	bl	n	n	0	0	cig+/-ot	1.0	4	1	1	cur+1y	st
JOLY	572		m	0	0	all	-	all	SCAmer	1978	CC	826	n	bl	n	n	0	0	cig+/-ot	5	999	0	0	cur+1y	st
JOLY	558		f	0	0	all	-	all	SCAmer	1978	CC	826	n	bl	n	n	0	0	cig+/-ot	1.0	4	1	1	cur+1y	st
JOLY	559		f	0	0	all	-	all	SCAmer	1978	CC	826	n	bl	n	n	0	0	cig+/-ot	5	999	0	0	cur+1y	st
KAISE2	655		m	0	0	all	9	all	NAmer	1979	pr	318	n	bl	n	n	1	0	cig only	2	10	0	1	cur+2y	st
KAISE2	656		m	0	0	all	9	all	NAmer	1979	pr	318	n	bl	n	n	1	0	cig only	11	20	3	0	cur+2y	ot
KAISE2	657		m	0	0	all	9	all	NAmer	1979	pr	318	n	bl	n	n	1	0	cig only	21	999	0	0	cur+2y	ot
KAISE2	575		f	0	0	all	9	all	NAmer	1979	pr	318	n	bl	n	n	1	0	cig only	2	10	0	1	cur+2y	st
KAISE2	576		f	0	0	all	9	all	NAmer	1979	pr	318	n	bl	n	n	1	0	cig only	11	20	3	0	cur+2y	st
KAISE2	577		f	0	0	all	9	all	NAmer	1979	pr	318	n	bl	n	n	1	0	cig only	21	999	0	0	cur+2y	ot
KHUDER	516		m	0	0	all	-	all	NAmer	1985	CC	482	n	bl	n	y	0	0	cig+/-ot	0.1	4	1	1	current	st
KHUDER	517		m	0	0	all	-	all	NAmer	1985	CC	482	n	bl	n	y	0	0	cig+/-ot	5	14	0	2	current	st
KHUDER	518		m	0	0	all	-	all	NAmer	1985	CC	482	n	bl	n	y	0	0	cig+/-ot	15	999	0	3	current	st
LUBIN	592		m	0	0	all	-	all	As:Chi	1984	CC	427	m	ot	y	n	0	0	cig+/-ot	3	4	1	1	cur+2y	st
LUBIN	593		m	0	0	all	-	all	As:Chi	1984	CC	427	m	ot	y	n	0	0	cig+/-ot	5	9	2	0	cur+2y	st
LUBIN	594		m	0	0	all	-	all	As:Chi	1984	CC	427	m	ot	y	n	0	0	cig+/-ot	10	999	3	0	cur+2y	st
LUBIN2	1081		m	0	0	all	-	all	Eu:mul	1976	CC	7804	n	bl	n	y	0	0	cig+/-ot	0.1	4	1	1	current	st
LUBIN2	1082		m	0	0	all	-	all	Eu:mul	1976	CC	7804	n	bl	n	y	0	0	cig+/-ot	5	9	2	0	current	st
LUBIN2	1083		m	0	0	all	-	all	Eu:mul	1976	CC	7804	n	bl	n	y	0	0	cig+/-ot	10	14	3	2	current	st
LUBIN2	1084		m	0	0	all	-	all	Eu:mul	1976	CC	7804	n	bl	n	y	0	0	cig+/-ot	15	19	0	0	current	st

Table 1K1 - 4

IESLC - Meta-analysis of Ex Smoking by Years quit (vs current), Overview
All LC types, Any Product (or Cigarettes if Any not available)
Least adjusted

REF	NRR	X	SEX	AGEL	AGEH	RACE	YF	LC TYPE	LOC	START	ST	NLC	R	VB	P	H	AD	ADOS	PRODUCT	exL	exH	S1	S2	DENOM	De
LUBIN2	1085		m	0	0	all	-	all	Eu:mul	1976	CC	7804	n	bl	n	y	0	0	cig+/-ot	20	24	0	3	current	st
LUBIN2	1086		m	0	0	all	-	all	Eu:mul	1976	CC	7804	n	bl	n	y	0	0	cig+/-ot	25	999	0	0	current	st
LUBIN2	1120		f	0	0	all	-	all	Eu:mul	1976	CC	7804	n	bl	n	y	0	0	cig+/-ot	0.1	4	1	1	current	st
LUBIN2	1121		f	0	0	all	-	all	Eu:mul	1976	CC	7804	n	bl	n	y	0	0	cig+/-ot	5	9	2	0	current	st
LUBIN2	1122		f	0	0	all	-	all	Eu:mul	1976	CC	7804	n	bl	n	y	0	0	cig+/-ot	10	14	3	2	current	st
LUBIN2	1123		f	0	0	all	-	all	Eu:mul	1976	CC	7804	n	bl	n	y	0	0	cig+/-ot	15	19	0	0	current	st
LUBIN2	1124		f	0	0	all	-	all	Eu:mul	1976	CC	7804	n	bl	n	y	0	0	cig+/-ot	20	24	0	3	current	st
LUBIN2	1125		f	0	0	all	-	all	Eu:mul	1976	CC	7804	n	bl	n	y	0	0	cig+/-ot	25	999	0	0	current	st
MATOS	586	x	m	0	0	all	-	all	SCAmer	1994	CC	200	n	bl	n	n	0	0	cig+/-ot	1.0	5	1	1	cur+1y	st
MATOS	587	x	m	0	0	all	-	all	SCAmer	1994	CC	200	n	bl	n	n	0	0	cig+/-ot	6	10	2	0	cur+1y	st
MATOS	588	x	m	0	0	all	-	all	SCAmer	1994	CC	200	n	bl	n	n	0	0	cig+/-ot	11	999	3	0	cur+1y	st
PEZZO2	504		m	0	0	all	-	all	SCAmer	1992	CC	367	n	bl	n	y	0	0	cig+/-ot	1.0	10	0	1	cur+1y	st
PEZZO2	505		m	0	0	all	-	all	SCAmer	1992	CC	367	n	bl	n	y	0	0	cig+/-ot	11	999	3	0	cur+1y	st
PEZZOT	504		m	0	0	all	-	all	SCAmer	1987	CC	215	n	bl	n	y	0	0	cig only	1.0	10	0	1	cur+1y	st
PEZZOT	505		m	0	0	all	-	all	SCAmer	1987	CC	215	n	bl	n	y	0	0	cig only	11	999	3	0	cur+1y	st
SOBUE	728		m	0	0	all	-	all	As:Jap	1986	CC	1376	n	bl	n	y	0	0	cig+/-ot	1.0	4	1	1	cur+1y	st
SOBUE	729		m	0	0	all	-	all	As:Jap	1986	CC	1376	n	bl	n	y	0	0	cig+/-ot	5	9	2	0	cur+1y	st
SOBUE	730		m	0	0	all	-	all	As:Jap	1986	CC	1376	n	bl	n	y	0	0	cig+/-ot	10	14	3	2	cur+1y	st
SOBUE	731		m	0	0	all	-	all	As:Jap	1986	CC	1376	n	bl	n	y	0	0	cig+/-ot	15	19	0	0	cur+1y	st
SOBUE	732		m	0	0	all	-	all	As:Jap	1986	CC	1376	n	bl	n	y	0	0	cig+/-ot	20	24	0	3	cur+1y	st
SOBUE	733		m	0	0	all	-	all	As:Jap	1986	CC	1376	n	bl	n	y	0	0	cig+/-ot	25	999	0	0	cur+1y	st
SPEIZE	511		f	0	0	all	0	all	NAmer	1976	pr	593	n	bl	n	y	2	0	cig+/-ot	0.1	1.9	0	0	current	or
SPEIZE	512		f	0	0	all	0	all	NAmer	1976	pr	593	n	bl	n	y	2	0	cig+/-ot	2	5	1	1	current	or
SPEIZE	513		f	0	0	all	0	all	NAmer	1976	pr	593	n	bl	n	y	2	0	cig+/-ot	5	10	2	0	current	or
SPEIZE	514		f	0	0	all	0	all	NAmer	1976	pr	593	n	bl	n	y	2	0	cig+/-ot	10	15	3	2	current	or
SPEIZE	515		f	0	0	all	0	all	NAmer	1976	pr	593	n	bl	n	y	2	0	cig+/-ot	15	999	0	3	current	or
SUZUK2	513	x	c	0	0	all	-	all	SCAmer	1991	CC	123	n	bl	n	y	0	0	all/unsp	0.1	5	1	1	current	st
SUZUK2	514	x	c	0	0	all	-	all	SCAmer	1991	CC	123	n	bl	n	y	0	0	all/unsp	6	10	2	0	current	st
SUZUK2	515	x	c	0	0	all	-	all	SCAmer	1991	CC	123	n	bl	n	y	0	0	all/unsp	11	999	3	0	current	st
SVENSS	554		f	0	0	all	-	all	Eu:Sca	1983	CC	210	n	bl	n	n	0	0	all/unsp	3	10	0	1	cur+2y	st
SVENSS	555		f	0	0	all	-	all	Eu:Sca	1983	CC	210	n	bl	n	n	0	0	all/unsp	11	999	3	0	cur+2y	st
TVERDA	506		m	0	0	all	0	all	Eu:Sca	1972	pr	238	n	bl	n	n	2	0	cig only	0.1	0.9	0	0	current	ot
TVERDA	507		m	0	0	all	0	all	Eu:Sca	1972	pr	238	n	bl	n	n	2	0	cig only	1.0	5	1	1	current	ot
TVERDA	508		m	0	0	all	0	all	Eu:Sca	1972	pr	238	n	bl	n	n	2	0	cig only	5	999	0	0	current	ot
WANG2	515		c	0	0	all	-	all	As:Chi	1980	CC	103	n	ot	n	n	0	0	cig+/-ot	0.1	3	1	1	current	st
WANG2	516		c	0	0	all	-	all	As:Chi	1980	CC	103	n	ot	n	n	0	0	cig+/-ot	4	999	0	0	current	st
WYNDE3	545		m	0	0	all	-	all	NAmer	1966	CC	350	n	bl	n	y	0	0	all/unsp	1.0	3	1	1	cur+1y	st
WYNDE3	546		m	0	0	all	-	all	NAmer	1966	CC	350	n	bl	n	y	0	0	all/unsp	4	6	0	0	cur+1y	st
WYNDE3	547		m	0	0	all	-	all	NAmer	1966	CC	350	n	bl	n	y	0	0	all/unsp	7	12	0	2	cur+1y	st
WYNDE3	548		m	0	0	all	-	all	NAmer	1966	CC	350	n	bl	n	y	0	0	all/unsp	13	999	0	3	cur+1y	st
WYNDE6	719		m	0	0	wh	-	all	NAmer	1969	CC	4423	n	bl	n	y	5	1#	cig+/-ot	1.0	10	0	1	cur+1y	or
WYNDE6	720		m	0	0	wh	-	all	NAmer	1969	CC	4423	n	bl	n	y	5	1#	cig+/-ot	11	19	3	2	cur+1y	or
WYNDE6	721		m	0	0	wh	-	all	NAmer	1969	CC	4423	n	bl	n	y	5	1#	cig+/-ot	20	999	0	3	cur+1y	or
WYNDE6	726		m	0	0	bl	-	all	NAmer	1969	CC	4423	n	bl	n	y	5	1#	cig+/-ot	1.0	10	0	1	cur+1y	or
WYNDE6	727		m	0	0	bl	-	all	NAmer	1969	CC	4423	n	bl	n	y	5	1#	cig+/-ot	11	19	3	2	cur+1y	or
WYNDE6	728		m	0	0	bl	-	all	NAmer	1969	CC	4423	n	bl	n	y	5	1#	cig+/-ot	20	999	0	3	cur+1y	or
WYNDE6	733		f	0	0	wh	-	all	NAmer	1969	CC	4423	n	bl	n	y	5	1#	cig+/-ot	1.0	10	0	1	cur+1y	or
WYNDE6	734		f	0	0	wh	-	all	NAmer	1969	CC	4423	n	bl	n	y	5	1#	cig+/-ot	11	999	3	0	cur+1y	or
WYNDE6	737		f	0	0	bl	-	all	NAmer	1969	CC	4423	n	bl	n	y	5	1#	cig+/-ot	1.0	10	0	1	cur+1y	or
WYNDE6	738		f	0	0	bl	-	all	NAmer	1969	CC	4423	n	bl	n	y	5	1#	cig+/-ot	11	999	3	0	cur+1y	or

Comments on values in listings

WYNDE6 ADOS Number of cigs/day
WYNDE6 ADOS Number of cigs/day
WYNDE6 ADOS Number of cigs/day
WYNDE6 ADOS Number of cigs/day
WYNDE6 ADOS Number of cigs/day
WYNDE6 ADOS Number of cigs/day
WYNDE6 ADOS Number of cigs/day
WYNDE6 ADOS Number of cigs/day
WYNDE6 ADOS Number of cigs/day
WYNDE6 ADOS Number of cigs/day

Cigarette type is all/unspec for all RRs
except for the following:

REF	NRR	CIGTYPE
ALDERS	513	MC only

Table 1K1 - 4

IESLC - Meta-analysis of Ex Smoking by Years quit (vs current), Overview
All LC types, Any Product (or Cigarettes if Any not available)
Least adjusted

REF	NRR	CIGTYPE
ALDERS	514	MC only
ALDERS	515	MC only
ALDERS	524	MC only
ALDERS	525	MC only
ALDERS	526	MC only

In this overview table, subtotals and Qs values may be invalid and should be ignored

Table 1K1 - 5

IESLC - Meta-analysis of Ex Smoking by Years quit (vs current), Overview
All LC types, Any Product (or Cigarettes if Any not available)
Least adjusted

REF	NRR	SEX	AD	Number Exposed Case	Cont	Non-exposed Case	Cont	RR	95.00%CI
ALDERS	513	m	1	121	-	207	-	1.81	(1.24- 2.64)
ALDERS	514	m	1	28	-	207	-	0.43	(0.26- 0.71)
ALDERS	515	m	1	29	-	207	-	0.32	(0.20- 0.51)
ALDERS	524	f	1	206	-	244	-	2.08	(1.49- 2.90)
ALDERS	525	f	1	54	-	244	-	0.65	(0.43- 0.99)
ALDERS	526	f	1	26	-	244	-	0.28	(0.17- 0.46)
Subtotal ALDERS								0.85	(0.72- 1.01)
ARMADA	518	m	0	79	45	188	122	1.14	(0.74- 1.75)
ARMADA	519	m	0	50	87	188	122	0.37	(0.25- 0.57)
Subtotal ARMADA								0.64	(0.47- 0.86)
BARBON	533	m	0	32	20	562	362	1.03	(0.58- 1.83)
BARBON	534	m	0	89	85	562	362	0.67	(0.49- 0.93)
BARBON	535	m	0	33	41	562	362	0.52	(0.32- 0.84)
BARBON	536	m	0	15	59	562	362	0.16	(0.09- 0.29)
Subtotal BARBON								0.55	(0.44- 0.69)
BECHER	506	m	0	10	12	101	122	1.01	(0.42- 2.43)
BECHER	507	m	0	16	32	101	122	0.60	(0.31- 1.16)
BECHER	508	m	0	16	72	101	122	0.27	(0.15- 0.49)
BECHER	516	f	0	2	3	33	26	0.53	(0.08- 3.38)
BECHER	517	f	0	2	5	33	26	0.32	(0.06- 1.76)
BECHER	518	f	0	1	10	33	26	0.08	(0.01- 0.66)
Subtotal BECHER								0.44	(0.30- 0.64)
BROSS	518	m	0	169	67	565	427	1.91	(1.40- 2.60)
BROSS	519	m	0	43	79	565	427	0.41	(0.28- 0.61)
Subtotal BROSS								1.06	(0.83- 1.35)
CARPEN	508	c	0	28	46	228	164	0.44	(0.26- 0.73)
CARPEN	509	c	0	31	52	228	164	0.43	(0.26- 0.70)
CARPEN	510	c	0	13	58	228	164	0.16	(0.09- 0.30)
CARPEN	511	c	0	37	158	228	164	0.17	(0.11- 0.25)
Subtotal CARPEN								0.27	(0.21- 0.34)
*CEDERL	538	m	1	12	-	97	-	0.78	(0.43- 1.41)
*CEDERL	539	m	1	3	-	97	-	0.14	(0.04- 0.45)
Subtotal CEDERL								0.56	(0.33- 0.95)
CHOI	543	m	0	25	64	231	329	0.56	(0.34- 0.91)
CHOI	544	m	0	5	30	231	329	0.24	(0.09- 0.62)
CHOI	545	m	0	4	23	231	329	0.25	(0.08- 0.73)
CHOI	546	m	0	4	19	231	329	0.30	(0.10- 0.89)
CHOI	556	f	0	3	2	13	25	2.88	(0.43- 19.49)
CHOI	557	f	0	2	0	13	25	9.44~	(0.42- 211.16)
Subtotal CHOI								0.46	(0.32- 0.67)
*CHYOU	504	m	1	21	-	138	-	0.32	(0.20- 0.50)
*CHYOU	505	m	1	5	-	138	-	0.23	(0.09- 0.56)
Subtotal CHYOU								0.30	(0.20- 0.45)
*CPSI	815	m	1	37	-	844	-	1.07	(0.77- 1.49)
*CPSI	816	m	1	49	-	844	-	0.59	(0.44- 0.78)
*CPSI	817	m	1	32	-	844	-	0.37	(0.26- 0.53)
*CPSI	818	m	1	15	-	844	-	0.09	(0.06- 0.15)
Subtotal CPSI								0.48	(0.40- 0.57)
*CPSII	664	m	1	97	-	1159	-	1.77	(1.44- 2.17)
*CPSII	665	m	1	188	-	1159	-	1.28	(1.10- 1.49)
*CPSII	666	m	1	178	-	1159	-	0.85	(0.72- 0.99)
*CPSII	667	m	1	186	-	1159	-	0.52	(0.45- 0.61)
*CPSII	668	m	1	164	-	1159	-	0.39	(0.33- 0.46)
*CPSII	669	m	1	256	-	1159	-	0.17	(0.15- 0.20)
*CPSII	642	f	1	91	-	530	-	1.38	(1.10- 1.72)
*CPSII	643	f	1	56	-	530	-	0.85	(0.65- 1.12)
*CPSII	644	f	1	37	-	530	-	0.40	(0.28- 0.55)
*CPSII	645	f	1	28	-	530	-	0.31	(0.21- 0.46)
*CPSII	646	f	1	50	-	530	-	0.14	(0.11- 0.19)
Subtotal CPSII								0.57	(0.54- 0.60)
DAMBER	528	m	1	-	-	-	-	0.80	(0.50- 1.28)
DAMBER	529	m	1	-	-	-	-	0.45	(0.26- 0.78)
DAMBER	530	m	1	42	-	-	-	0.27	(0.18- 0.40)
Subtotal DAMBER								0.43	(0.33- 0.56)
DARBY	507	m	0	146	339	379	618	0.70	(0.56- 0.89)
DARBY	508	m	0	139	767	379	618	0.30	(0.24- 0.37)
DARBY	516	f	0	68	93	198	231	0.85	(0.59- 1.23)
DARBY	517	f	0	26	224	198	231	0.14	(0.09- 0.21)
Subtotal DARBY								0.44	(0.38- 0.50)
DEAN3	537	m	0	42	147	502	1636	0.93	(0.65- 1.33)
DEAN3	538	m	0	15	67	502	1636	0.73	(0.41- 1.29)

Table 1K1 - 5

IESLC - Meta-analysis of Ex Smoking by Years quit (vs current), Overview
All LC types, Any Product (or Cigarettes if Any not available)
Least adjusted

REF	NRR	SEX	AD	Number Exposed Case	Cont	Non-exposed Case	Cont	RR	95.00%CI
DEAN3	539	m	0	32	204	502	1636	0.51	(0.35- 0.75)
DEAN3	548	f	0	4	110	102	1158	0.41	(0.15- 1.14)
DEAN3	549	f	0	1	38	102	1158	0.30	(0.04- 2.20)
DEAN3	550	f	0	2	114	102	1158	0.20	(0.05- 0.82)
Subtotal DEAN3								0.66	(0.53- 0.83)
DESTEF	520	m	0	64	45	362	226	0.89	(0.59- 1.35)
DESTEF	521	m	0	27	27	362	226	0.62	(0.36- 1.09)
DESTEF	522	m	0	17	36	362	226	0.29	(0.16- 0.54)
Subtotal DESTEF								0.62	(0.46- 0.83)
DOLL	537	m	0	56	75	1280	1172	0.68	(0.48- 0.98)
DOLL	538	m	0	6	26	1280	1172	0.21	(0.09- 0.52)
DOLL	539	m	0	8	23	1280	1172	0.32	(0.14- 0.71)
DOLL	548	f	0	9	6	58	41	1.06	(0.35- 3.21)
DOLL	549	f	0	1	2	58	41	0.35	(0.03- 4.03)
Subtotal DOLL								0.56	(0.42- 0.75)
*DOLL2	509	m	1	15	-	236	-	1.02	(0.61- 1.72)
*DOLL2	510	m	1	12	-	236	-	0.35	(0.20- 0.63)
*DOLL2	511	m	1	9	-	236	-	0.28	(0.14- 0.54)
*DOLL2	512	m	1	7	-	236	-	0.11	(0.05- 0.23)
Subtotal DOLL2								0.40	(0.30- 0.55)
DORGAN	514	m	0	59	51	465	303	0.75	(0.50- 1.13)
DORGAN	515	m	0	49	38	465	303	0.84	(0.54- 1.31)
DORGAN	516	m	0	134	255	465	303	0.34	(0.27- 0.44)
DORGAN	559	f	0	49	27	289	112	0.70	(0.42- 1.18)
DORGAN	560	f	0	34	50	289	112	0.26	(0.16- 0.43)
Subtotal DORGAN								0.47	(0.40- 0.56)
*DORN	823	m	0	34	22086	528	334175	0.97	(0.69- 1.38)
*DORN	824	m	0	32	34566	528	334175	0.59	(0.41- 0.84)
*DORN	825	m	0	12	23682	528	334175	0.32	(0.18- 0.57)
*DORN	826	m	0	16	58370	528	334175	0.17	(0.11- 0.29)
*DORN	827	m	0	14	6195	537	207895	0.87	(0.51- 1.49)
*DORN	828	m	0	41	24089	537	207895	0.66	(0.48- 0.90)
*DORN	829	m	0	29	20056	537	207895	0.56	(0.39- 0.81)
*DORN	830	m	0	34	51243	537	207895	0.26	(0.18- 0.36)
Subtotal DORN								0.51	(0.45- 0.59)
GAO	526	m	0	105	52	529	438	1.67	(1.17- 2.39)
GAO	527	m	0	24	27	529	438	0.74	(0.42- 1.29)
GAO	528	m	0	13	41	529	438	0.26	(0.14- 0.50)
GAO	546	f	0	37	9	170	100	2.42	(1.12- 5.22)
GAO	547	f	0	14	7	170	100	1.18	(0.46- 3.01)
GAO	548	f	0	16	14	170	100	0.67	(0.31- 1.44)
Subtotal GAO								1.04	(0.83- 1.32)
GAO2	518	m	0	31	26	184	117	0.76	(0.43- 1.34)
GAO2	519	m	0	21	26	184	117	0.51	(0.28- 0.95)
GAO2	520	m	0	16	18	184	117	0.57	(0.28- 1.15)
GAO2	521	m	0	7	9	184	117	0.49	(0.18- 1.36)
GAO2	522	m	0	8	25	184	117	0.20	(0.09- 0.47)
Subtotal GAO2								0.51	(0.37- 0.70)
GARCIA	508	c	0	33	11	77	42	1.64	(0.75- 3.57)
GARCIA	509	c	0	43	36	77	42	0.65	(0.36- 1.16)
GARCIA	510	c	0	32	67	77	42	0.26	(0.15- 0.46)
GARCIA	511	c	0	10	37	77	42	0.15	(0.07- 0.33)
Subtotal GARCIA								0.44	(0.32- 0.61)
GRAHAM	530	m	0	113	59	453	1075	4.55	(3.26- 6.34)
GRAHAM	531	m	0	24	48	453	1075	1.19	(0.72- 1.96)
GRAHAM	532	m	0	13	71	453	1075	0.43	(0.24- 0.79)
Subtotal GRAHAM								2.14	(1.67- 2.76)
*HAMMO2	510	m	1	59	-	209	-	1.08	(0.82- 1.43)
*HAMMO2	511	m	1	11	-	209	-	0.39	(0.22- 0.71)
*HAMMO2	512	m	1	20	-	209	-	0.34	(0.22- 0.53)
Subtotal HAMMO2								0.71	(0.57- 0.88)
*HIRAYA	513	m	1	-	-	-	-	0.46	(0.26- 0.82)
*HIRAYA	514	m	1	-	-	-	-	0.36	(0.15- 0.86)
*HIRAYA	515	m	1	-	-	-	-	0.31	(0.14- 0.69)
*HIRAYA	524	f	1	-	-	-	-	1.59	(0.47- 5.37)
*HIRAYA	525	f	1	-	-	-	-	1.41	(0.23- 8.48)
*HIRAYA	526	f	1	-	-	-	-	0.41	(0.01- 14.37)
Subtotal HIRAYA								0.48	(0.33- 0.69)
JAHN	513	m	0	166	8	352	269	15.86	(7.66- 32.81)
JAHN	514	m	0	60	9	352	269	5.09	(2.48- 10.45)
JAHN	515	m	0	77	46	352	269	1.28	(0.86- 1.90)

Table 1K1 - 5

IESLC - Meta-analysis of Ex Smoking by Years quit (vs current), Overview
All LC types, Any Product (or Cigarettes if Any not available)
Least adjusted

REF	NRR	SEX	AD	Number Exposed Case	Cont	Non-exposed Case	Cont	RR	95.00%CI
JAHN	516	m	0	59	63	352	269	0.72	(0.49- 1.06)
JAHN	517	m	0	64	130	352	269	0.38	(0.27- 0.53)
JAHN	518	m	0	29	146	352	269	0.15	(0.10- 0.23)
Subtotal JAHN								0.70	(0.58- 0.84)
JAIN	570	m	0	74	46	265	118	0.72	(0.47- 1.10)
JAIN	571	m	0	52	113	265	118	0.20	(0.14- 0.30)
JAIN	534	f	0	66	36	305	99	0.60	(0.37- 0.95)
JAIN	535	f	0	19	61	305	99	0.10	(0.06- 0.18)
Subtotal JAIN								0.33	(0.27- 0.42)
JOLY	571	m	0	38	36	451	524	1.23	(0.76- 1.97)
JOLY	572	m	0	63	149	451	524	0.49	(0.36- 0.68)
JOLY	558	f	0	19	8	132	96	1.73	(0.73- 4.11)
JOLY	559	f	0	15	19	132	96	0.57	(0.28- 1.19)
Subtotal JOLY								0.70	(0.55- 0.88)
*KAISE2	655	m	1	12	-	51	-	1.00	(0.53- 1.88)
*KAISE2	656	m	1	8	-	51	-	0.43	(0.20- 0.92)
*KAISE2	657	m	1	6	-	51	-	0.26	(0.10- 0.67)
*KAISE2	575	f	1	6	-	50	-	0.53	(0.23- 1.23)
*KAISE2	576	f	1	4	-	50	-	0.25	(0.09- 0.70)
*KAISE2	577	f	1	4	-	50	-	0.34	(0.13- 0.91)
Subtotal KAISE2								0.49	(0.35- 0.68)
KHUDER	516	m	0	88	123	245	316	0.92	(0.67- 1.27)
KHUDER	517	m	0	63	133	245	316	0.61	(0.43- 0.86)
KHUDER	518	m	0	63	213	245	316	0.38	(0.28- 0.53)
Subtotal KHUDER								0.60	(0.50- 0.73)
LUBIN	592	m	0	33	18	296	650	4.03	(2.23- 7.27)
LUBIN	593	m	0	20	48	296	650	0.91	(0.53- 1.57)
LUBIN	594	m	0	17	73	296	650	0.51	(0.30- 0.88)
Subtotal LUBIN								1.16	(0.84- 1.60)
LUBIN2	1081	m	0	866	1047	4684	6211	1.10	(0.99- 1.21)
LUBIN2	1082	m	0	466	822	4684	6211	0.75	(0.67- 0.85)
LUBIN2	1083	m	0	270	693	4684	6211	0.52	(0.45- 0.60)
LUBIN2	1084	m	0	130	478	4684	6211	0.36	(0.30- 0.44)
LUBIN2	1085	m	0	106	413	4684	6211	0.34	(0.27- 0.42)
LUBIN2	1086	m	0	109	715	4684	6211	0.20	(0.16- 0.25)
LUBIN2	1120	f	0	60	55	440	410	1.02	(0.69- 1.50)
LUBIN2	1121	f	0	30	40	440	410	0.70	(0.43- 1.14)
LUBIN2	1122	f	0	10	26	440	410	0.36	(0.17- 0.75)
LUBIN2	1123	f	0	3	7	440	410	0.40	(0.10- 1.55)
LUBIN2	1124	f	0	4	9	440	410	0.41	(0.13- 1.36)
LUBIN2	1125	f	0	4	20	440	410	0.19	(0.06- 0.55)
Subtotal LUBIN2								0.65	(0.61- 0.68)
MATOS	586	m	0	28	23	112	132	1.43	(0.78- 2.63)
MATOS	587	m	0	21	27	112	132	0.92	(0.49- 1.71)
MATOS	588	m	0	27	101	112	132	0.32	(0.19- 0.52)
Subtotal MATOS								0.65	(0.47- 0.91)
PEZZO2	504	m	0	85	110	233	198	0.66	(0.47- 0.92)
PEZZO2	505	m	0	43	161	233	198	0.23	(0.15- 0.33)
Subtotal PEZZO2								0.41	(0.32- 0.53)
PEZZOT	504	m	0	46	82	145	129	0.50	(0.32- 0.77)
PEZZOT	505	m	0	20	106	145	129	0.17	(0.10- 0.29)
Subtotal PEZZOT								0.32	(0.23- 0.45)
SOBUE	728	m	0	128	116	737	633	0.95	(0.72- 1.24)
SOBUE	729	m	0	67	92	737	633	0.63	(0.45- 0.87)
SOBUE	730	m	0	35	50	737	633	0.60	(0.39- 0.94)
SOBUE	731	m	0	24	31	737	633	0.66	(0.39- 1.14)
SOBUE	732	m	0	15	23	737	633	0.56	(0.29- 1.08)
SOBUE	733	m	0	17	40	737	633	0.37	(0.20- 0.65)
Subtotal SOBUE								0.69	(0.59- 0.82)
*SPEIZE	511	f	2	24	-	319	-	0.60	(0.40- 0.90)
*SPEIZE	512	f	2	34	-	319	-	0.60	(0.40- 0.80)
*SPEIZE	513	f	2	41	-	319	-	0.50	(0.40- 0.70)
*SPEIZE	514	f	2	17	-	319	-	0.20	(0.10- 0.40)
*SPEIZE	515	f	2	28	-	319	-	0.10	(0.10- 0.40)
Subtotal SPEIZE								0.46	(0.38- 0.55)
SUZUK2	513	c	0	15	10	77	30	0.58	(0.24- 1.44)
SUZUK2	514	c	0	10	8	77	30	0.49	(0.18- 1.35)
SUZUK2	515	c	0	9	22	77	30	0.16	(0.07- 0.39)
Subtotal SUZUK2								0.34	(0.20- 0.59)
SVENSS	554	f	0	16	13	142	53	0.46	(0.21- 1.02)
SVENSS	555	f	0	14	24	142	53	0.22	(0.10- 0.45)

Table 1K1 - 5

IESLC - Meta-analysis of Ex Smoking by Years quit (vs current), Overview
All LC types, Any Product (or Cigarettes if Any not available)
Least adjusted

REF	NRR	SEX	AD	Number Exposed Case	Cont	Non-exposed Case	Cont	RR	95.00%CI
Subtotal SVENSS								0.31	(0.18- 0.52)
*TVERDA	506	m	2	2	-	144	-	0.17	(0.04- 0.70)
*TVERDA	507	m	2	5	-	144	-	0.18	(0.07- 0.43)
*TVERDA	508	m	2	4	-	144	-	0.08	(0.03- 0.23)
Subtotal TVERDA								0.13	(0.07- 0.25)
WANG2	515	c	0	6	10	49	78	0.96	(0.33- 2.79)
WANG2	516	c	0	5	11	49	78	0.72	(0.24- 2.21)
Subtotal WANG2								0.84	(0.39- 1.81)
WYNDE3	545	m	0	21	22	227	207	0.87	(0.47- 1.63)
WYNDE3	546	m	0	11	17	227	207	0.59	(0.27- 1.29)
WYNDE3	547	m	0	11	31	227	207	0.32	(0.16- 0.66)
WYNDE3	548	m	0	5	55	227	207	0.08	(0.03- 0.21)
Subtotal WYNDE3								0.42	(0.29- 0.61)
WYNDE6	719	m	5	-	-	-	-	0.60	(0.50- 0.70)
WYNDE6	720	m	5	-	-	-	-	0.30	(0.20- 0.40)
WYNDE6	721	m	5	-	-	-	-	0.20	(0.10- 0.20)
WYNDE6	726	m	5	-	-	-	-	0.70	(0.40- 1.10)
WYNDE6	727	m	5	-	-	-	-	0.20	(0.10- 0.50)
WYNDE6	728	m	5	-	-	-	-	0.30	(0.10- 0.60)
WYNDE6	733	f	5	-	-	-	-	0.50	(0.40- 0.60)
WYNDE6	734	f	5	-	-	-	-	0.20	(0.20- 0.30)
WYNDE6	737	f	5	-	-	-	-	0.60	(0.30- 1.30)
WYNDE6	738	f	5	-	-	-	-	0.40	(0.10- 1.10)
Subtotal WYNDE6								0.39	(0.35- 0.43)
Partial Totals				8630	251691	87467	2246313		

*prospective study ~ With 0.5 adjustment for zero

REF	NRR	SEX	AD	Ys	Ws	Qs	Ps
ALDERS	513	m	1	0.59	26.91	36.67	0.0021
ALDERS	514	m	1	-0.84	15.23	1.11	0.0010
ALDERS	515	m	1	-1.14	17.54	5.61	0.0000
ALDERS	524	f	1	0.73	34.65	59.13	0.0000
ALDERS	525	f	1	-0.43	22.10	0.45	0.0429
ALDERS	526	f	1	-1.27	15.51	7.58	0.0000
Subtotal ALDERS				-0.16	131.92	110.55	
ARMADA	518	m	0	0.13	20.66	10.25	0.5535
ARMADA	519	m	0	-0.99	22.22	3.78	0.0000
Subtotal ARMADA				-0.45	42.88	14.03	
BARBON	533	m	0	0.03	11.66	4.25	0.9180
BARBON	534	m	0	-0.39	36.31	1.18	0.0176
BARBON	535	m	0	-0.66	16.88	0.12	0.0070
BARBON	536	m	0	-1.81	11.34	17.31	0.0000
Subtotal BARBON				-0.60	76.19	22.86	
BECHER	506	m	0	0.01	4.96	1.67	0.9883
BECHER	507	m	0	-0.50	8.94	0.04	0.1316
BECHER	508	m	0	-1.32	10.58	5.81	0.0000
BECHER	516	f	0	-0.64	1.11	0.01	0.4978
BECHER	517	f	0	-1.15	1.30	0.44	0.1879
BECHER	518	f	0	-2.54	0.86	3.31	0.0188
Subtotal BECHER				-0.82	27.75	11.29	
BROSS	518	m	0	0.65	40.07	59.56	0.0000
BROSS	519	m	0	-0.89	24.98	2.47	0.0000
Subtotal BROSS				0.06	65.06	62.03	
CARPEN	508	c	0	-0.83	14.72	0.93	0.0015
CARPEN	509	c	0	-0.85	16.14	1.20	0.0007
CARPEN	510	c	0	-1.82	9.56	14.95	0.0000
CARPEN	511	c	0	-1.78	22.81	33.24	0.0000
Subtotal CARPEN				-1.33	63.22	50.33	
*CEDERL	538	m	1	-0.25	10.90	1.15	0.4121
*CEDERL	539	m	1	-1.97	2.62	5.08	0.0015
Subtotal CEDERL				-0.58	13.52	6.24	
CHOI	543	m	0	-0.59	15.87	0.00	0.0195
CHOI	544	m	0	-1.44	4.15	3.10	0.0034
CHOI	545	m	0	-1.40	3.32	2.24	0.0109
CHOI	546	m	0	-1.20	3.23	1.28	0.0305
CHOI	556	f	0	1.06	1.05	2.81	0.2771
CHOI	557	f	0	2.25	0.40	3.16	0.1567
Subtotal CHOI				-0.78	28.03	12.60	
*CHYOU	504	m	1	-1.14	18.30	5.85	0.0000
*CHYOU	505	m	1	-1.47	4.60	3.69	0.0016

Table 1K1 - 5

IESLC - Meta-analysis of Ex Smoking by Years quit (vs current), Overview
All LC types, Any Product (or Cigarettes if Any not available)
Least adjusted

REF	NRR	SEX	AD	Ys	Ws	Qs	Ps
Subtotal CHYOU				-1.21	22.90	9.54	
*CPSI	815	m	1	0.07	35.26	14.52	0.6879
*CPSI	816	m	1	-0.53	46.88	0.10	0.0003
*CPSI	817	m	1	-0.99	30.29	5.35	0.0000
*CPSI	818	m	1	-2.41	18.30	61.55	0.0000
Subtotal CPSI				-0.74	130.73	81.52	
*CPSII	664	m	1	0.57	91.37	119.79	0.0000
*CPSII	665	m	1	0.25	166.85	112.43	0.0014
*CPSII	666	m	1	-0.16	151.52	25.66	0.0454
*CPSII	667	m	1	-0.65	166.04	1.06	0.0000
*CPSII	668	m	1	-0.94	139.29	18.82	0.0000
*CPSII	669	m	1	-1.77	185.67	266.44	0.0000
*CPSII	642	f	1	0.32	76.90	61.75	0.0047
*CPSII	643	f	1	-0.16	51.90	8.79	0.2417
*CPSII	644	f	1	-0.92	33.71	3.95	0.0000
*CPSII	645	f	1	-1.17	24.99	8.91	0.0000
*CPSII	646	f	1	-1.97	51.44	99.69	0.0000
Subtotal CPSII				-0.57	1139.68	727.29	
DAMBER	528	m	1	-0.22	17.39	2.14	0.3521
DAMBER	529	m	1	-0.80	12.73	0.64	0.0044
DAMBER	530	m	1	-1.31	24.10	13.03	0.0000
Subtotal DAMBER				-0.84	54.22	15.81	
DARBY	507	m	0	-0.35	71.14	3.46	0.0029
DARBY	508	m	0	-1.22	78.40	32.62	0.0000
DARBY	516	f	0	-0.16	28.70	4.95	0.3945
DARBY	517	f	0	-2.00	19.12	38.84	0.0000
Subtotal DARBY				-0.83	197.37	79.87	
DEAN3	537	m	0	-0.07	30.11	7.61	0.6954
DEAN3	538	m	0	-0.32	11.88	0.80	0.2773
DEAN3	539	m	0	-0.67	25.80	0.24	0.0007
DEAN3	548	f	0	-0.88	3.71	0.36	0.0885
DEAN3	549	f	0	-1.21	0.96	0.39	0.2355
DEAN3	550	f	0	-1.61	1.93	2.08	0.0252
Subtotal DEAN3				-0.41	74.38	11.47	
DESTEF	520	m	0	-0.12	22.21	4.60	0.5753
DESTEF	521	m	0	-0.47	12.31	0.13	0.0984
DESTEF	522	m	0	-1.22	10.66	4.47	0.0001
Subtotal DESTEF				-0.48	45.17	9.20	
DOLL	537	m	0	-0.38	30.46	1.14	0.0358
DOLL	538	m	0	-1.55	4.84	4.65	0.0006
DOLL	539	m	0	-1.14	5.88	1.91	0.0055
DOLL	548	f	0	0.06	3.13	1.25	0.9174
DOLL	549	f	0	-1.04	0.65	0.14	0.4022
Subtotal DOLL				-0.59	44.96	9.10	
*DOLL2	509	m	1	0.02	14.30	5.04	0.9403
*DOLL2	510	m	1	-1.05	11.67	2.64	0.0003
*DOLL2	511	m	1	-1.27	8.43	4.12	0.0002
*DOLL2	512	m	1	-2.21	6.60	17.60	0.0000
Subtotal DOLL2				-0.91	41.00	29.40	
DORGAN	514	m	0	-0.28	23.81	2.02	0.1680
DORGAN	515	m	0	-0.17	19.17	3.07	0.4460
DORGAN	516	m	0	-1.07	59.40	14.71	0.0000
DORGAN	559	f	0	-0.35	14.32	0.71	0.1829
DORGAN	560	f	0	-1.33	16.18	9.34	0.0000
Subtotal DORGAN				-0.76	132.87	29.84	
*DORN	823	m	0	-0.03	31.99	9.61	0.8830
*DORN	824	m	0	-0.53	30.20	0.05	0.0033
*DORN	825	m	0	-1.14	11.74	3.72	0.0001
*DORN	826	m	0	-1.75	15.53	21.54	0.0000
*DORN	827	m	0	-0.13	13.68	2.65	0.6211
*DORN	828	m	0	-0.42	38.16	0.94	0.0100
*DORN	829	m	0	-0.58	27.56	0.00	0.0023
*DORN	830	m	0	-1.36	32.00	19.73	0.0000
Subtotal DORN				-0.67	200.86	58.24	
GAO	526	m	0	0.51	30.37	35.95	0.0046
GAO	527	m	0	-0.31	12.07	0.86	0.2869
GAO	528	m	0	-1.34	9.48	5.52	0.0000
GAO	546	f	0	0.88	6.49	13.78	0.0244
GAO	547	f	0	0.16	4.34	2.36	0.7348
GAO	548	f	0	-0.40	6.68	0.21	0.3049
Subtotal GAO				0.04	69.43	58.68	

Table 1K1 - 5

IESLC - Meta-analysis of Ex Smoking by Years quit (vs current), Overview
All LC types, Any Product (or Cigarettes if Any not available)
Least adjusted

REF	NRR	SEX	AD	Ys	Ws	Qs	Ps
GAO2	518	m	0	-0.28	11.81	1.04	0.3414
GAO2	519	m	0	-0.67	9.99	0.09	0.0352
GAO2	520	m	0	-0.57	7.57	0.00	0.1164
GAO2	521	m	0	-0.70	3.73	0.06	0.1738
GAO2	522	m	0	-1.59	5.59	5.79	0.0002
Subtotal GAO2				-0.67	38.69	6.98	
GARCIA	508	c	0	0.49	6.33	7.20	0.2154
GARCIA	509	c	0	-0.43	11.39	0.24	0.1483
GARCIA	510	c	0	-1.35	12.05	7.17	0.0000
GARCIA	511	c	0	-1.91	6.10	10.97	0.0000
Subtotal GARCIA				-0.83	35.87	25.57	
GRAHAM	530	m	0	1.51	34.56	150.67	0.0000
GRAHAM	531	m	0	0.17	15.24	8.46	0.5044
GRAHAM	532	m	0	-0.83	10.62	0.72	0.0066
Subtotal GRAHAM				0.76	60.42	159.85	
*HAMMO2	510	m	1	0.08	49.68	21.05	0.5875
*HAMMO2	511	m	1	-0.94	11.19	1.51	0.0016
*HAMMO2	512	m	1	-1.08	19.88	5.06	0.0000
Subtotal HAMMO2				-0.35	80.75	27.63	
*HIRAYA	513	m	1	-0.78	11.65	0.48	0.0080
*HIRAYA	514	m	1	-1.02	5.04	1.01	0.0218
*HIRAYA	515	m	1	-1.17	6.04	2.15	0.0040
*HIRAYA	524	f	1	0.46	2.59	2.79	0.4555
*HIRAYA	525	f	1	0.34	1.18	0.99	0.7089
*HIRAYA	526	f	1	-0.89	0.29	0.03	0.6307
Subtotal HIRAYA				-0.74	26.79	7.45	
JAHN	513	m	0	2.76	7.27	80.97	0.0000
JAHN	514	m	0	1.63	7.44	36.10	0.0000
JAHN	515	m	0	0.25	24.22	16.30	0.2255
JAHN	516	m	0	-0.33	25.39	1.46	0.0919
JAHN	517	m	0	-0.98	33.47	5.45	0.0000
JAHN	518	m	0	-1.89	20.88	35.90	0.0000
Subtotal JAHN				-0.36	118.68	176.18	
JAIN	570	m	0	-0.33	21.05	1.22	0.1258
JAIN	571	m	0	-1.59	24.80	25.35	0.0000
JAIN	534	f	0	-0.52	17.76	0.05	0.0287
JAIN	535	f	0	-2.29	12.14	35.80	0.0000
Subtotal JAIN				-1.10	75.74	62.42	
JOLY	571	m	0	0.20	17.18	10.40	0.3976
JOLY	572	m	0	-0.71	37.44	0.70	0.0000
JOLY	558	f	0	0.55	5.11	6.42	0.2166
JOLY	559	f	0	-0.55	7.28	0.00	0.1343
Subtotal JOLY				-0.36	67.01	17.52	
*KAISE2	655	m	1	0.00	9.58	3.16	1.0000
*KAISE2	656	m	1	-0.84	6.60	0.48	0.0302
*KAISE2	657	m	1	-1.35	4.25	2.54	0.0055
*KAISE2	575	f	1	-0.63	5.47	0.02	0.1377
*KAISE2	576	f	1	-1.39	3.65	2.41	0.0081
*KAISE2	577	f	1	-1.08	4.06	1.03	0.0298
Subtotal KAISE2				-0.72	33.61	9.64	
KHUDER	516	m	0	-0.08	37.40	9.11	0.6231
KHUDER	517	m	0	-0.49	32.64	0.22	0.0049
KHUDER	518	m	0	-0.96	35.95	5.46	0.0000
Subtotal KHUDER				-0.51	105.99	14.79	
LUBIN	592	m	0	1.39	11.02	42.61	0.0000
LUBIN	593	m	0	-0.09	13.20	3.11	0.7468
LUBIN	594	m	0	-0.67	12.91	0.12	0.0160
Subtotal LUBIN				0.15	37.13	45.84	
LUBIN2	1081	m	0	0.09	402.52	178.75	0.0639
LUBIN2	1082	m	0	-0.29	267.60	22.29	0.0000
LUBIN2	1083	m	0	-0.66	181.12	1.35	0.0000
LUBIN2	1084	m	0	-1.02	98.44	19.57	0.0000
LUBIN2	1085	m	0	-1.08	81.77	20.76	0.0000
LUBIN2	1086	m	0	-1.60	91.35	95.92	0.0000
LUBIN2	1120	f	0	0.02	25.28	8.81	0.9343
LUBIN2	1121	f	0	-0.36	15.86	0.74	0.1536
LUBIN2	1122	f	0	-1.03	6.98	1.43	0.0067
LUBIN2	1123	f	0	-0.92	2.08	0.25	0.1856
LUBIN2	1124	f	0	-0.88	2.73	0.26	0.1450
LUBIN2	1125	f	0	-1.68	3.28	4.01	0.0023
Subtotal LUBIN2				-0.44	1179.01	354.14	

Table 1K1 - 5

IESLC - Meta-analysis of Ex Smoking by Years quit (vs current), Overview
All LC types, Any Product (or Cigarettes if Any not available)
Least adjusted

REF	NRR	SEX	AD	Ys	Ws	Qs	Ps
MATOS	586	m	0	0.36	10.45	9.14	0.2432
MATOS	587	m	0	-0.09	9.89	2.34	0.7844
MATOS	588	m	0	-1.15	15.76	5.32	0.0000
Subtotal MATOS				-0.42	36.10	16.80	
PEZZO2	504	m	0	-0.42	33.11	0.78	0.0155
PEZZO2	505	m	0	-1.48	25.77	21.29	0.0000
Subtotal PEZZO2				-0.89	58.88	22.07	
PEZZOT	504	m	0	-0.69	20.58	0.30	0.0016
PEZZOT	505	m	0	-1.78	13.50	19.78	0.0000
Subtotal PEZZOT				-1.13	34.08	20.08	
SOBUE	728	m	0	-0.05	51.63	13.98	0.6997
SOBUE	729	m	0	-0.47	34.80	0.38	0.0056
SOBUE	730	m	0	-0.51	19.41	0.08	0.0250
SOBUE	731	m	0	-0.41	13.01	0.36	0.1411
SOBUE	732	m	0	-0.58	8.84	0.00	0.0848
SOBUE	733	m	0	-1.01	11.53	2.17	0.0006
Subtotal SOBUE				-0.37	139.23	16.97	
*SPEIZE	511	f	2	-0.51	23.37	0.09	0.0135
*SPEIZE	512	f	2	-0.51	31.98	0.13	0.0039
*SPEIZE	513	f	2	-0.69	49.07	0.70	0.0000
*SPEIZE	514	f	2	-1.61	8.00	8.57	0.0000
*SPEIZE	515	f	2	-2.30	8.00	23.89	0.0000
Subtotal SPEIZE				-0.78	120.40	33.38	
SUZUK2	513	c	0	-0.54	4.70	0.01	0.2445
SUZUK2	514	c	0	-0.72	3.69	0.08	0.1672
SUZUK2	515	c	0	-1.84	4.93	7.86	0.0000
Subtotal SUZUK2				-1.07	13.31	7.94	
SVENSS	554	f	0	-0.78	6.05	0.25	0.0557
SVENSS	555	f	0	-1.52	7.19	6.50	0.0000
Subtotal SVENSS				-1.18	13.24	6.75	
*TVERDA	506	m	2	-1.77	1.88	2.69	0.0152
*TVERDA	507	m	2	-1.71	4.66	6.07	0.0002
*TVERDA	508	m	2	-2.53	3.70	14.11	0.0000
Subtotal TVERDA				-2.02	10.24	22.87	
WANG2	515	c	0	-0.05	3.33	0.93	0.9331
WANG2	516	c	0	-0.32	3.09	0.19	0.5698
Subtotal WANG2				-0.18	6.42	1.12	
WYNDE3	545	m	0	-0.14	9.77	1.85	0.6644
WYNDE3	546	m	0	-0.53	6.29	0.01	0.1858
WYNDE3	547	m	0	-1.13	7.55	2.32	0.0019
WYNDE3	548	m	0	-2.49	4.40	16.14	0.0000
Subtotal WYNDE3				-0.86	28.01	20.33	
WYNDE6	719	m	5	-0.51	135.72	0.54	0.0000
WYNDE6	720	m	5	-1.20	31.98	12.69	0.0000
WYNDE6	721	m	5	-1.61	31.98	34.29	0.0000
WYNDE6	726	m	5	-0.36	15.02	0.71	0.1669
WYNDE6	727	m	5	-1.61	5.93	6.36	0.0001
WYNDE6	728	m	5	-1.20	4.79	1.90	0.0084
WYNDE6	733	f	5	-0.69	93.46	1.33	0.0000
WYNDE6	734	f	5	-1.61	93.46	100.20	0.0000
WYNDE6	737	f	5	-0.51	7.15	0.03	0.1721
WYNDE6	738	f	5	-0.92	2.67	0.31	0.1342
Subtotal WYNDE6				-0.95	422.17	158.36	

N	194
NS	43

Table 1K1 - 6

IESLC - Meta-analysis of Ex Smoking by Years quit (vs current), Overview
All LC types, Any Product (or Cigarettes if Any not available)
Least adjusted

	Sex			
	combined	male	female	Total
N	13	129	52	194
NS	4	37	17	58

In this overview table, other than the "N" rows, entries in the "absent" and "Total" columns may be invalid and should be ignored

	Years quit vs current (lower focus)				
	absent	1-6k3	4-11k7	8+k12	Total
N	76	41	29	48	194
NS	33	32	22	32	119
Wt	2100.38	1290.99	860.96	1091.59	5343.92
Het Chi	1489.14	136.53	57.54	193.30	2644.59
Het df	75	40	28	47	193
Het P	***	***	***	***	***
Fixed RR	0.52	0.99	0.60	0.32	0.56
RRl	0.50	0.93	0.56	0.30	0.55
RRu	0.54	1.04	0.65	0.34	0.58
P	---	N.S.	---	---	---
Random RR	0.48	0.96	0.57	0.28	0.50
RRl	0.39	0.85	0.51	0.25	0.45
RRu	0.59	1.09	0.65	0.33	0.56
P	---	N.S.	---	---	---

	Years quit vs current (higher focus)				
	absent	1-11k3	4-19k12	13+k20	Total
N	95	60	19	20	194
NS	42	42	15	16	115
Wt	2372.65	1851.93	578.62	540.73	5343.92
Het Chi	1399.84	237.69	56.20	88.27	2644.59
Het df	94	59	18	19	193
Het P	***	***	***	***	***
Fixed RR	0.54	0.86	0.44	0.22	0.56
RRl	0.51	0.82	0.41	0.20	0.55
RRu	0.56	0.90	0.48	0.24	0.58
P	---	---	---	---	---
Random RR	0.45	0.84	0.39	0.23	0.50
RRl	0.38	0.76	0.33	0.19	0.45
RRu	0.53	0.94	0.47	0.29	0.56
P	---	--	---	---	---

Table 1K1 - 6

IESLC - Meta-analysis of Ex Smoking by Years quit (vs current), Overview
All LC types, Any Product (or Cigarettes if Any not available)
Least adjusted

MALES

	Years quit vs current (lower focus) absent	1-6k3	4-11k7	8+k12	Total
N	51	28	20	30	129
NS	27	27	19	28	101
Wt	1632.62	1132.69	734.71	856.82	4356.84
Het Chi	1172.88	102.24	43.02	114.15	2009.93
Het df	50	27	19	29	128
Het P	***	***	**	***	***
Fixed RR	0.51	1.01	0.62	0.36	0.59
RRl	0.49	0.95	0.58	0.34	0.57
RRu	0.53	1.07	0.67	0.38	0.61
P	---	N.S.	---	---	---
Random RR	0.48	0.99	0.59	0.32	0.52
RRl	0.37	0.86	0.52	0.27	0.46
RRu	0.61	1.13	0.68	0.37	0.59
P	---	N.S.	---	---	---

	Years quit vs current (higher focus) absent	1-11k3	4-19k12	13+k20	Total
N	62	38	14	15	129
NS	34	36	12	13	95
Wt	1899.94	1495.49	517.70	443.70	4356.84
Het Chi	1039.66	167.75	35.65	68.53	2009.93
Het df	61	37	13	14	128
Het P	***	***	***	***	***
Fixed RR	0.56	0.90	0.46	0.23	0.59
RRl	0.53	0.86	0.42	0.21	0.57
RRu	0.58	0.95	0.50	0.25	0.61
P	---	---	---	---	---
Random RR	0.47	0.88	0.43	0.25	0.52
RRl	0.39	0.78	0.36	0.20	0.46
RRu	0.58	0.99	0.52	0.33	0.59
P	---	-	---	---	---

FEMALES

	Years quit vs current (lower focus) absent	1-6k3	4-11k7	8+k12	Total
N	20	9	7	16	52
NS	13	9	7	15	44
Wt	412.32	129.22	106.43	220.29	868.26
Het Chi	259.64	19.04	8.41	29.31	522.22
Het df	19	8	6	15	51
Het P	***	*	N.S.	*	***
Fixed RR	0.61	0.87	0.51	0.22	0.49
RRl	0.55	0.73	0.42	0.19	0.45
RRu	0.67	1.04	0.61	0.25	0.52
P	---	N.S.	---	---	---
Random RR	0.54	0.98	0.53	0.23	0.48
RRl	0.36	0.70	0.41	0.18	0.38
RRu	0.81	1.38	0.70	0.29	0.61
P	--	N.S.	---	---	---

Table 1K1 - 6

IESLC - Meta-analysis of Ex Smoking by Years quit (vs current), Overview
All LC types, Any Product (or Cigarettes if Any not available)
Least adjusted

FEMALES

	Years quit vs current (higher focus) absent	1-11k3	4-19k12	13+k20	Total
N	28	18	3	3	52
NS	17	17	3	3	40
Wt	438.76	327.36	39.97	62.17	868.26
Het Chi	330.37	39.77	1.53	4.12	522.22
Het df	27	17	2	2	51
Het P	***	**	N.S.	N.S.	***
Fixed RR	0.47	0.69	0.29	0.14	0.49
RRl	0.43	0.62	0.21	0.11	0.45
RRu	0.51	0.77	0.40	0.18	0.52
P	---	---	---	---	---
Random RR	0.41	0.75	0.29	0.15	0.48
RRl	0.28	0.62	0.21	0.09	0.38
RRu	0.60	0.92	0.40	0.25	0.61
P	---	--	---	---	---

Table 1K1 - 7

IESLC - Meta-analysis of Ex Smoking by Years quit (vs current), Overview
All LC types, Any Product (or Cigarettes if Any not available)
Excluded studies (and stage at which they were excluded)

1	AGUDO AKIBA AMANDU AMES AXELSS BEST BOUCHA BOUCOT BRESLO CHEN CHEN2 CHIAZZ DEAN2 DOSEME ENGELA FAN
	GENG GER GUO HAENSZ HEGMAN HOLE HU HU2 JUSSAW KATSOU KAUFMA KOO KOULUM KREUZE LETOUR LEVIN
	LIAW LIU3 LIU4 LIU5 MCCONN MIGRAN MRFITR NOTAN2 OSANN2 PERNU QIAO2 RACHTA RESTRE SADOWS SEGI2 STASZE
	TIZZAN VUTUC WATSON WU WUWILL WYNDE2 WYNDE8 XU YUAN ZHANG ZHENG ZHOU
2	AUVINE BENSHL BLOT1 BROWN3 BUFFLE GURSEL LAUSSM LUO MCDUFF PISANI PRESCO SPITZ WU2 WYNDE7
4	HAMMON
5	CORREA GILLIS HUMBLE QIAO WIGLE
7	BOFFET
10	GARSHI JEDRYC WAKAI
14	BENHAM

Table 1K1 - 8

Potentially overlapping studies

REF	REFGP	PRINC	OVERLAP/LINK
LUBIN2	LUBIN2	1	Lubin-combined
TVERDA	TVERDA	1	VEIERO/TVERDAL
BROSS	BYERS1	1	GRAHAM/BROSS/BYERS1
GRAHAM	BYERS1	1	GRAHAM/BROSS/BYERS1
CHYOU	CHYOU	1	GOODMA/CHYOU
WYNDE6	WYNDE6	1	WYNDE5/6/7/8
CPSI	CPSI	1	CPSI overall
JAHN	BOFFET	2	Subset of BOFFET
LUBIN	XIANGZ	2	LUBIN/XIANGZ/QIAO

Table 1K1 - 9

Most adjusted - insufficient data for meta-analysis

REF	NRR	SEX	AGEL	AGEH	RACE	YF	LC TYPE	LOC	START	ST	NLC	R	VB	P	H	AD	ADOS	PRODUCT	exL	exH	S1	S2	DENOM	De
CORREA	550	c	0	0	all	-	all	NAmer	1979	CC	1359	n	bl	y	n	2	0	cig+/-ot	3	5	1	1	cur+2y	st
CORREA	551	c	0	0	all	-	all	NAmer	1979	CC	1359	n	bl	y	n	2	0	cig+/-ot	6	20	0	0	cur+2y	st
CORREA	552	c	0	0	all	-	all	NAmer	1979	CC	1359	n	bl	y	n	2	0	cig+/-ot	21	999	0	0	cur+2y	st
HUMBLE	549	c	0	0	wh	-	not alv	NAmer	1980	CC	521	n	bl	y	n	2	2#	cig+/-ot	2	5	1	1	cur+1y	or
HUMBLE	550	c	0	0	wh	-	not alv	NAmer	1980	CC	521	n	bl	y	n	2	2#	cig+/-ot	6	15	0	2	cur+1y	or
HUMBLE	551	c	0	0	wh	-	not alv	NAmer	1980	CC	521	n	bl	y	n	2	2#	cig+/-ot	16	30	0	3	cur+1y	or
WIGLE	501	m	0	0	all	-	all	NAmer	1971	CC	728	n	V	n	n	2	1#	cig+/-ot	0.1	1.0	0	0	current	ot
WIGLE	502	m	0	0	all	-	all	NAmer	1971	CC	728	n	V	n	n	2	1#	cig+/-ot	2	9	0	1	current	ot
WIGLE	503	m	0	0	all	-	all	NAmer	1971	CC	728	n	V	n	n	2	1#	cig+/-ot	10	14	3	2	current	ot
WIGLE	504	m	0	0	all	-	all	NAmer	1971	CC	728	n	V	n	n	2	1#	cig+/-ot	15	999	0	3	current	ot
WIGLE	505	f	0	0	all	-	all	NAmer	1971	CC	728	n	V	n	n	2	1#	cig+/-ot	0.1	1.0	0	0	current	ot
WIGLE	506	f	0	0	all	-	all	NAmer	1971	CC	728	n	V	n	n	2	1#	cig+/-ot	2	9	0	1	current	ot
WIGLE	507	f	0	0	all	-	all	NAmer	1971	CC	728	n	V	n	n	2	1#	cig+/-ot	10	14	3	2	current	ot
WIGLE	508	f	0	0	all	-	all	NAmer	1971	CC	728	n	V	n	n	2	1#	cig+/-ot	15	999	0	3	current	ot

Comments on values in listings

HUMBLE	ADOS	Number of cigarettes and duration
HUMBLE	ADOS	Number of cigarettes and duration
HUMBLE	ADOS	Number of cigarettes and duration
WIGLE	ADOS	Cumulative exposure
WIGLE	ADOS	Cumulative exposure
WIGLE	ADOS	Cumulative exposure
WIGLE	ADOS	Cumulative exposure
WIGLE	ADOS	Cumulative exposure
WIGLE	ADOS	Cumulative exposure
WIGLE	ADOS	Cumulative exposure
WIGLE	ADOS	Cumulative exposure

REF	NRR	RR	SIG	RRDATA comment
CORREA	550	0.61		0
CORREA	551	0.56		0
CORREA	552	0.31		0
HUMBLE	549	0.69		0
HUMBLE	550	0.33		0

Table 1K1 - 9

IESLC - Meta-analysis of Ex Smoking by Years quit (vs current), Overview
All LC types, Any Product (or Cigarettes if Any not available)
Most adjusted - insufficient data for meta-analysis

REF	NRR	RR	SIG	RRDATA comment
HUMBLE	551	0.11		0
WIGLE	501	2.40	n	0
WIGLE	502	0.70	n	0
WIGLE	503	0.70	n	0
WIGLE	504	0.20	y	p<0.01
WIGLE	505	0.90	n	0
WIGLE	506	0.50	n	0
WIGLE	507	0.50	n	0
WIGLE	508	0.40	n	0

Table 1K2 -

IESLC - Meta-analysis of Ex Smoking, Years quit (vs current), "Low"
All LC types, Any Product (or Cigarettes if Any not available)

This analysis is restricted to results for:
1) Ex smokers
2) Results by Years quit (vs current)
3) Categorical results by Years quit (vs current)
4) All LC types (or near equivalent)
5) Results complete enough for use in metaanalysis

Within each study, results are then selected (in the following order of preference, within each sex) for:
6) (not applicable)
7) PRODUCT: all/unspec, cigarettes regardless of other products, cigarettes only
8) CIGTYPE: all/unspecified, MC regardless of HR, MC only
9) Results with least adjustment for other aspects of smoking (ADOS)
10) DENOM: current smokers, current + recent smokers (up to number of m=months or y=years, max 2 years)
11) Followup period (YF, prospective studies): whole study (coded as 0) or longest available
12) LCtype: all or nearest available, at least Squamous and Adeno. (q = squamous, s = small,
l = large, a = adeno, mix = mixed, alv = alveolar)
13) Race: all or nearest available, otherwise by race (wh or w = white, bl or b = black, hi = hispanic
ch = chinese, jap = japanese, haw = hawaiian, w+o = white + oriental, sca = scandinavian, as = asian)
14) Years quit (vs current) "low" in key scheme 1 (key value 3, maximum range 1-6)
15) For overlapping studies: principal rather than subsidiary studies
Finally by Age: whole study (coded as 0) if available, otherwise by widest available age group
and then for single sex results (m, f) in preference to results for both sexes combined (c).

Results adjusted (AD) for the most potential confounders are then chosen in Sections -1 to -3
and results adjusted for the least confounders in Sections -4 to -6. (Those least adjusted results which
actually differ from the most adjusted are marked 'x' in column X in Section -4)

Section -7 shows excluded studies, together with the stage (as above) at which no qualifying
results were found.

Section -8 lists the potentially overlapping studies which have been included (1=principal, 2=subsidiary).

Section -9 lists any results which would have been included in preference except that they had data not complete
enough for use in meta-analysis, with their significance (yes/no), if known, and any further comment as entered
on the database. It also lists as "gap" any categories for which no data were presented by the original authors.

In addition to those mentioned above, the following fields, levels and abbreviations are used:
* or nk = not known, n = no, y = yes, ot = other
nev = never
all/unspec = all or unspecified, cig+/-ot = cigarettes irrespective of other products (cigar, pipe etc)
MC = manufactured cigarettes, HR = hand-rolled cigarettes
exL, exH = range of exposure (low and high) in the smoking group, in terms of Years quit (vs current)
REF: 6-character study reference
NRR: number of the RR on the database within the study
ST : study type (CC = case control, pr or prosp = prospective)
NLC: number of lung cancer cases in whole study
R : risky occupational population (n = no, m = mining, o = other risky)
VB : national cigarette type (V = at least 75% Virginia, bl = at least 75% blended, ot = other)
P : any proxy use
H : full histological confirmation
De : derivation of RR/CI (or = original, st = standard method, ot = other method of estimation)

Table 1K2 - 1

IESLC - Meta-analysis of Ex Smoking, Years quit (vs current), "Low"
All LC types, Any Product (or Cigarettes if Any not available)
Most adjusted

REF	NRR	SEX	AGEL	AGEH	RACE	YF	LC TYPE	LOC	START	ST	NLC	R	VB	P	H	AD	ADOS	PRODUCT	exL	exH	DENOM	De
ARMADA	518	m	0	0	all	-	all	Eu:wst	1986	CC	325	n	bl	n	y	0	0	cig+/-ot	1.0	5	cur+1y	st
BARBON	548	m	0	0	all	-	all	Eu:wst	1979	CC	755	n	bl	y	y	1	0	all/unsp	0.1	4	current	ot
BECHER	506	m	0	0	all	-	all	Eu:Ger	1985	CC	194	n	bl	n	y	0	0	all/unsp	2	4	cur+1y	st
BECHER	516	f	0	0	all	-	all	Eu:Ger	1985	CC	194	n	bl	n	y	0	0	all/unsp	2	4	cur+1y	st
BROSS	518	m	0	0	wh	-	all	NAmer	1960	CC	974	n	bl	n	n	0	0	cig+/-ot	0.1	5	current	st
CARPEN	508	c	0	0	w+b	-	all	NAmer	1991	CC	356	n	bl	n	n	0	0	cig+/-ot	0.1	4	current	st
CHOI	543	m	0	0	all	-	all	As:oth	1985	CC	375	n	bl	n	n	0	0	cig+/-ot	0.1	4	current	st
CHOI	556	f	0	0	all	-	all	As:oth	1985	CC	375	n	bl	n	n	0	0	cig+/-ot	0.1	4	current	st
CPSI	816	m	50	74	all	6	all	NAmer	1959	pr	5138	n	bl	n	n	1	0	cig only	1.0	4	current	ot
CPSII	666	m	35	99	all	4	all	NAmer	1982	pr	3229	n	bl	n	n	1	0	cig only	3	5	current	ot
CPSII	643	f	0	0	all	4	all	NAmer	1982	pr	3229	n	bl	n	n	1	0	cig+/-ot	3	5	current	ot
DAMBER	528	m	0	0	all	-	all	Eu:Sca	1972	CC	579	n	bl	y	n	1	0	all/unsp	0.1	5	current	ot
DEAN3	636	m	0	0	all	-	all	Eu:UK	1969	CC	766	n	V	y	n	1	0	all/unsp	3	4	cur+2y	ot
DEAN3	559	f	0	0	all	-	all	Eu:UK	1969	CC	766	n	V	y	n	1	0	all/unsp	3	4	cur+2y	ot
DESTEF	530	m	0	0	all	-	all	SCAmer	1988	CC	497	n	bl	n	y	4	0	all/unsp	0.1	4	current	ot
DOLL2	509	m	0	0	all	20	all	Eu:UK	1951	pr	920	n	V	n	n	1	0	cig only	0.1	4	current	ot
DORGAN	514	m	0	0	wh	-	all	NAmer	1980	CC	2026	n	bl	y	y	0	0	cig+/-ot	1.1	5	cur+1y	st
DORN	823	m	55	64	wh	8	all	NAmer	1954	pr	5097	n	bl	n	n	0	0	cig+/-ot	0.1	4	current	st
DORN	827	m	65	74	wh	8	all	NAmer	1954	pr	5097	n	bl	n	n	0	0	cig+/-ot	0.1	4	current	st
GAO	536	m	0	0	all	-	all	As:Chi	1984	CC	1405	n	ot	n	n	2	0	cig+/-ot	0.1	4	current	ot
GAO	556	f	0	0	all	-	all	As:Chi	1984	CC	1405	n	ot	n	n	2	0	cig+/-ot	0.1	4	current	ot
GAO2	518	m	0	0	all	-	all	As:Jap	1988	CC	282	n	bl	n	n	0	0	cig+/-ot	1.0	4	cur+1y	st
GARCIA	508	c	0	0	all	-	all	NAmer	1992	CC	416	n	bl	n	y	0	0	cig+/-ot	1.0	4	cur+1y	st
GRAHAM	541	m	0	0	wh	-	all	NAmer	1956	CC	685	n	bl	n	n	1	0	cig+/-ot	1.1	5	current	ot
HAMMO2	510	m	0	0	all	0	all	NAmer	1967	pr	450	o	bl	n	n	1	0	cig+/-ot	0.1	4	current	ot
HIRAYA	513	m	0	0	all	0	all	As:Jap	1965	pr	1917	n	bl	n	n	1	0	cig+/-ot	0.1	4	current	ot
HIRAYA	524	f	0	0	all	0	all	As:Jap	1965	pr	1917	n	bl	n	n	1	0	cig+/-ot	0.1	4	current	ot
JAHN	515	m	0	0	all	-	all	Eu:Ger	1988	CC	1004	n	bl	n	n	0	0	cig+/-ot	2	5	current	st
JOLY	571	m	0	0	all	-	all	SCAmer	1978	CC	826	n	bl	n	n	0	0	cig+/-ot	1.0	4	cur+1y	st
JOLY	558	f	0	0	all	-	all	SCAmer	1978	CC	826	n	bl	n	n	0	0	cig+/-ot	1.0	4	cur+1y	st
KHUDER	516	m	0	0	all	-	all	NAmer	1985	CC	482	n	bl	n	y	0	0	cig+/-ot	0.1	4	current	st
LUBIN	592	m	0	0	all	-	all	As:Chi	1984	CC	427	m	ot	y	n	0	0	cig+/-ot	3	4	cur+2y	st
LUBIN2	1081	m	0	0	all	-	all	Eu:mul	1976	CC	7804	n	bl	n	y	0	0	cig+/-ot	0.1	4	current	st
LUBIN2	1120	f	0	0	all	-	all	Eu:mul	1976	CC	7804	n	bl	n	y	0	0	cig+/-ot	0.1	4	current	st
MATOS	596	m	0	0	all	-	all	SCAmer	1994	CC	200	n	bl	n	n	2	0	cig+/-ot	1.0	5	cur+1y	or
SOBUE	728	m	0	0	all	-	all	As:Jap	1986	CC	1376	n	bl	n	y	0	0	cig+/-ot	1.0	4	cur+1y	st
SPEIZE	512	f	0	0	all	0	all	NAmer	1976	pr	593	n	bl	n	y	2	0	cig+/-ot	2	5	current	or
SUZUK2	524	c	0	0	all	-	all	SCAmer	1991	CC	123	n	bl	n	y	3	0	all/unsp	0.1	5	current	or
TVERDA	507	m	0	0	all	0	all	Eu:Sca	1972	pr	238	n	bl	n	n	2	0	cig only	1.0	5	current	ot
WANG2	515	c	0	0	all	-	all	As:Chi	1980	CC	103	n	ot	n	n	0	0	cig+/-ot	0.1	3	current	st
WYNDE3	545	m	0	0	all	-	all	NAmer	1966	CC	350	n	bl	n	y	0	0	all/unsp	1.0	3	cur+1y	st
WYNDE6	513	m	0	0	all	-	all	NAmer	1969	CC	4423	n	bl	n	y	0	0	cig only	1.0	4	cur+1y	st
WYNDE6	534	f	0	0	all	-	all	NAmer	1969	CC	4423	n	bl	n	y	0	0	cig only	1.0	4	cur+1y	st

Cigarette type is all/unspec for all RRs

Table 1K2 - 2

IESLC - Meta-analysis of Ex Smoking, Years quit (vs current), "Low"
All LC types, Any Product (or Cigarettes if Any not available)
Most adjusted

REF	NRR	SEX	AD	Number Exposed Case	Number Exposed Cont	Non-exposed Case	Non-exposed Cont	RR	95.00%CI
ARMADA	518	m	0	79	45	188	122	1.14	(0.74- 1.75)
BARBON	548	m	1	32	-	562	-	1.01	(0.57- 1.79)
BECHER	506	m	0	10	12	101	122	1.01	(0.42- 2.43)
BECHER	516	f	0	2	3	33	26	0.53	(0.08- 3.38)
Subtotal BECHER								0.89	(0.40- 1.98)
BROSS	518	m	0	169	67	565	427	1.91	(1.40- 2.60)
CARPEN	508	c	0	28	46	228	164	0.44	(0.26- 0.73)
CHOI	543	m	0	25	64	231	329	0.56	(0.34- 0.91)
CHOI	556	f	0	3	2	13	25	2.88	(0.43- 19.49)
Subtotal CHOI								0.62	(0.38- 0.99)
*CPSI	816	m	1	49	-	844	-	0.59	(0.44- 0.78)
*CPSII	666	m	1	178	-	1159	-	0.85	(0.72- 0.99)
*CPSII	643	f	1	56	-	530	-	0.85	(0.65- 1.12)
Subtotal CPSII								0.85	(0.74- 0.98)
DAMBER	528	m	1	-	-	-	-	0.80	(0.50- 1.28)
DEAN3	636	m	1	42	-	502	-	0.80	(0.56- 1.15)
DEAN3	559	f	1	4	-	102	-	0.29	(0.11- 0.81)
Subtotal DEAN3								0.71	(0.51- 1.00)
DESTEF	530	m	4	64	-	362	-	0.83	(0.55- 1.24)
*DOLL2	509	m	1	15	-	236	-	1.02	(0.61- 1.72)
DORGAN	514	m	0	59	51	465	303	0.75	(0.50- 1.13)
*DORN	823	m	0	34	22086	528	334175	0.97	(0.69- 1.38)
*DORN	827	m	0	14	6195	537	207895	0.87	(0.51- 1.49)
Subtotal DORN								0.94	(0.71- 1.26)
GAO	536	m	2	105	-	529	-	1.77	(1.22- 2.56)
GAO	556	f	2	37	-	170	-	2.48	(1.15- 5.38)
Subtotal GAO								1.89	(1.35- 2.63)
GAO2	518	m	0	31	26	184	117	0.76	(0.43- 1.34)
GARCIA	508	c	0	33	11	77	42	1.64	(0.75- 3.57)
GRAHAM	541	m	1	24	-	453	-	1.17	(0.70- 1.94)
*HAMMO2	510	m	1	59	-	209	-	1.08	(0.82- 1.43)
*HIRAYA	513	m	1	-	-	-	-	0.46	(0.26- 0.82)
*HIRAYA	524	f	1	-	-	-	-	1.59	(0.47- 5.37)
Subtotal HIRAYA								0.58	(0.34- 0.97)
JAHN	515	m	0	77	46	352	269	1.28	(0.86- 1.90)
JOLY	571	m	0	38	36	451	524	1.23	(0.76- 1.97)
JOLY	558	f	0	19	8	132	96	1.73	(0.73- 4.11)
Subtotal JOLY								1.33	(0.88- 2.01)
KHUDER	516	m	0	88	123	245	316	0.92	(0.67- 1.27)
LUBIN	592	m	0	33	18	296	650	4.03	(2.23- 7.27)
LUBIN2	1081	m	0	866	1047	4684	6211	1.10	(0.99- 1.21)
LUBIN2	1120	f	0	60	55	440	410	1.02	(0.69- 1.50)
Subtotal LUBIN2								1.09	(0.99- 1.20)
MATOS	596	m	2	28	-	112	-	1.40	(0.80- 2.60)
SOBUE	728	m	0	128	116	737	633	0.95	(0.72- 1.24)
*SPEIZE	512	f	2	34	-	319	-	0.60	(0.40- 0.80)
SUZUK2	524	c	3	15	-	77	-	0.60	(0.20- 1.50)
*TVERDA	507	m	2	5	-	144	-	0.18	(0.07- 0.43)
WANG2	515	c	0	6	10	49	78	0.96	(0.33- 2.79)
WYNDE3	545	m	0	21	22	227	207	0.87	(0.47- 1.63)
WYNDE6	513	m	0	201	166	1107	993	1.09	(0.87- 1.36)
WYNDE6	534	f	0	82	70	683	496	0.85	(0.61- 1.19)
Subtotal WYNDE6								1.01	(0.84- 1.22)
Partial Totals				2853	30325	18863	554630		

*prospective study

Table 1K2 - 2

IESLC - Meta-analysis of Ex Smoking, Years quit (vs current), "Low"
All LC types, Any Product (or Cigarettes if Any not available)
Most adjusted

REF	NRR	SEX	AD	Ys	Ws	Qs	Ps
ARMADA	518	m	0	0.13	20.66	0.44	0.5535
BARBON	548	m	1	0.01	11.73	0.01	0.9728
BECHER	506	m	0	0.01	4.96	0.00	0.9883
BECHER	516	f	0	-0.64	1.11	0.44	0.4978
Subtotal BECHER				-0.11	6.07	0.44	
BROSS	518	m	0	0.65	40.07	17.52	0.0000
CARPEN	508	c	0	-0.83	14.72	9.65	0.0015
CHOI	543	m	0	-0.59	15.87	5.16	0.0195
CHOI	556	f	0	1.06	1.05	1.22	0.2771
Subtotal CHOI				-0.48	16.93	6.38	
*CPSI	816	m	1	-0.53	46.88	12.27	0.0003
*CPSII	666	m	1	-0.16	151.52	3.25	0.0454
*CPSII	643	f	1	-0.16	51.90	1.11	0.2417
Subtotal CPSII				-0.16	203.42	4.36	
DAMBER	528	m	1	-0.22	17.39	0.75	0.3521
DEAN3	636	m	1	-0.22	29.68	1.27	0.2241
DEAN3	559	f	1	-1.24	3.85	5.75	0.0151
Subtotal DEAN3				-0.34	33.53	7.03	
DESTEF	530	m	4	-0.19	23.25	0.67	0.3689
*DOLL2	509	m	1	0.02	14.30	0.02	0.9403
DORGAN	514	m	0	-0.28	23.81	1.69	0.1680
*DORN	823	m	0	-0.03	31.99	0.00	0.8830
*DORN	827	m	0	-0.13	13.68	0.19	0.6211
Subtotal DORN				-0.06	45.67	0.19	
GAO	536	m	2	0.57	27.97	9.64	0.0025
GAO	556	f	2	0.91	6.45	5.51	0.0210
Subtotal GAO				0.63	34.43	15.16	
GAO2	518	m	0	-0.28	11.81	0.80	0.3414
GARCIA	508	c	0	0.49	6.33	1.64	0.2154
GRAHAM	541	m	1	0.16	14.79	0.44	0.5460
*HAMMO2	510	m	1	0.08	49.68	0.43	0.5875
*HIRAYA	513	m	1	-0.78	11.65	6.73	0.0080
*HIRAYA	524	f	1	0.46	2.59	0.60	0.4555
Subtotal HIRAYA				-0.55	14.24	7.33	
JAHN	515	m	0	0.25	24.22	1.67	0.2255
JOLY	571	m	0	0.20	17.18	0.83	0.3976
JOLY	558	f	0	0.55	5.11	1.62	0.2166
Subtotal JOLY				0.28	22.29	2.45	
KHUDER	516	m	0	-0.08	37.40	0.15	0.6231
LUBIN	592	m	0	1.39	11.02	21.87	0.0000
LUBIN2	1081	m	0	0.09	402.52	4.73	0.0639
LUBIN2	1120	f	0	0.02	25.28	0.03	0.9343
Subtotal LUBIN2				0.09	427.80	4.76	
MATOS	596	m	2	0.34	11.06	1.37	0.2631
SOBUE	728	m	0	-0.05	51.63	0.07	0.6997
*SPEIZE	512	f	2	-0.51	31.98	7.83	0.0039
SUZUK2	524	c	3	-0.51	3.78	0.93	0.3203
*TVERDA	507	m	2	-1.71	4.66	13.46	0.0002
WANG2	515	c	0	-0.05	3.33	0.00	0.9331
WYNDE3	545	m	0	-0.14	9.77	0.15	0.6644
WYNDE6	513	m	0	0.08	77.46	0.75	0.4670
WYNDE6	534	f	0	-0.16	33.38	0.71	0.3502
Subtotal WYNDE6				0.01	110.84	1.46	

Table 1K2 - 2

IESLC - Meta-analysis of Ex Smoking, Years quit (vs current), "Low"
All LC types, Any Product (or Cigarettes if Any not available)
Most adjusted

	N	43
	NS	33
	Wt	1399.49
Het	Chi	143.39
Het	df	42
Het	P	***
Fixed	RR	0.98
	RRl	0.93
	RRu	1.04
	P	N.S.
Random	RR	0.96
	RRl	0.85
	RRu	1.07
	P	N.S.
Asymm	P	N.S.

Table 1K2 - 3

IESLC - Meta-analysis of Ex Smoking, Years quit (vs current), "Low"
All LC types, Any Product (or Cigarettes if Any not available)
Most adjusted

	combined	Sex male	female	Total
N	4	29	10	43
NS	4	28	10	42
Wt	28.17	1208.61	162.71	1399.49
Het Chi	8.17	105.49	21.93	143.39
Het df	3	28	9	42
Het P	*	***	**	***
Fixed RR	0.67	1.01	0.86	0.98
RRl	0.47	0.96	0.74	0.93
RRu	0.97	1.07	1.00	1.04
P	-	N.S.	(-)	N.S.
Random RR	0.77	0.98	0.93	0.96
RRl	0.40	0.86	0.70	0.85
RRu	1.50	1.12	1.25	1.07
P	N.S.	N.S.	N.S.	N.S.
Between Chi				7.79
Between df				2
Between P				*
Btwn(F) P				N.S.
Btwn(R) P				N.S.

	Lung cancer type all	other	Total
N	43		43
NS	33		33
Wt	1399.49		1399.49
Het Chi	143.39		143.39
Het df	42		42
Het P	***		***
Fixed RR	0.98		0.98
RRl	0.93		0.93
RRu	1.04		1.04
P	N.S.		N.S.
Random RR	0.96		0.96
RRl	0.85		0.85
RRu	1.07		1.07
P	N.S.		N.S.
Between Chi			
Between df			
Between P			N.S.
Btwn(F) P			N.S.
Btwn(R) P			N.S.

	Location NAmer	UK	Scand	othEur	China	Japan	othAs	other	Total
N	16	3	2	7	4	4	2	5	43
NS	13	2	2	5	3	3	1	4	33
Wt	635.35	47.83	22.05	490.49	48.78	77.67	16.93	60.38	1399.49
Het Chi	53.25	4.81	8.18	1.47	7.72	6.15	2.67	5.06	143.39
Het df	15	2	1	6	3	3	1	4	42
Het P	***	(*)	**	N.S.	(*)	N.S.	N.S.	N.S.	***
Fixed RR	0.90	0.79	0.58	1.10	2.14	0.84	0.62	1.06	0.98
RRl	0.84	0.60	0.38	1.00	1.61	0.67	0.38	0.83	0.93
RRu	0.98	1.05	0.89	1.20	2.83	1.04	0.99	1.37	1.04
P	-	N.S.	-	+	+++	N.S.	-	N.S.	N.S.
Random RR	0.90	0.73	0.40	1.10	2.18	0.78	0.97	1.08	0.96
RRl	0.77	0.44	0.09	1.00	1.30	0.53	0.21	0.80	0.85
RRu	1.05	1.21	1.72	1.20	3.66	1.17	4.44	1.45	1.07
P	N.S.	N.S.	N.S.	+	++	N.S.	N.S.	N.S.	N.S.
Between Chi									54.08
Between df									7
Between P									***
Btwn(F) P									*
Btwn(R) P									*

Table 1K2 - 3

IESLC - Meta-analysis of Ex Smoking, Years quit (vs current), "Low"
All LC types, Any Product (or Cigarettes if Any not available)
Most adjusted

Detailed Country in "other Europe"

	multi	Germany	othWest	East	Balkans	Total
N	2	3	2			7
NS	1	2	2			5
Wt	427.80	30.30	32.40			490.49
Het Chi	0.14	1.01	0.11			1.47
Het df	1	2	1			6
Het P	N.S.	N.S.	N.S.			N.S.
Fixed RR	1.09	1.19	1.09			1.10
RRl	0.99	0.83	0.77			1.00
RRu	1.20	1.70	1.54			1.20
P	(+)	N.S.	N.S.			+
Random RR	1.09	1.19	1.09			1.10
RRl	0.99	0.83	0.77			1.00
RRu	1.20	1.70	1.54			1.20
P	(+)	N.S.	N.S.			+
Between Chi						0.21
Between df						2
Between P						N.S.
Btwn(F) P						N.S.
Btwn(R) P						N.S.

Detailed Country in "other Asia"

	India	HongKong	other	Total
N			2	2
NS			1	1
Wt			16.93	16.93
Het Chi			2.67	2.67
Het df			1	1
Het P			N.S.	N.S.
Fixed RR			0.62	0.62
RRl			0.38	0.38
RRu			0.99	0.99
P			-	-
Random RR			0.97	0.97
RRl			0.21	0.21
RRu			4.44	4.44
P			N.S.	N.S.
Between Chi				
Between df				
Between P				N.S.
Btwn(F) P				N.S.
Btwn(R) P				N.S.

Detailed other continent

	SCAmer	Total
N	5	5
NS	4	4
Wt	60.38	60.38
Het Chi	5.06	5.06
Het df	4	4
Het P	N.S.	N.S.
Fixed RR	1.06	1.06
RRl	0.83	0.83
RRu	1.37	1.37
P	N.S.	N.S.
Random RR	1.08	1.08
RRl	0.80	0.80
RRu	1.45	1.45
P	N.S.	N.S.
Between Chi		
Between df		
Between P		N.S.
Btwn(F) P		N.S.
Btwn(R) P		N.S.

Table 1K2 - 3

IESLC - Meta-analysis of Ex Smoking, Years quit (vs current), "Low"
All LC types, Any Product (or Cigarettes if Any not available)
Most adjusted

	Start year of study <1960	1960-69	1970-79	1980-89	1990+	Total
N	5	9	8	17	4	43
NS	4	6	6	13	4	33
Wt	121.63	258.14	515.86	467.97	35.89	1399.49
Het Chi	8.63	33.13	28.12	53.54	12.35	143.39
Het df	4	8	7	16	3	42
Het P	(*)	***	***	***	**	***
Fixed RR	0.82	1.04	1.03	0.97	0.82	0.98
RRl	0.68	0.92	0.95	0.88	0.59	0.93
RRu	0.97	1.17	1.12	1.06	1.13	1.04
P	-	N.S.	N.S.	N.S.	N.S.	N.S.
Random RR	0.87	0.93	0.87	1.06	0.88	0.96
RRl	0.66	0.70	0.66	0.87	0.44	0.85
RRu	1.14	1.23	1.16	1.28	1.77	1.07
P	N.S.	N.S.	N.S.	N.S.	N.S.	N.S.
Between Chi						7.61
Between df						4
Between P						N.S.
Btwn(F) P						N.S.
Btwn(R) P						N.S.

	Study type (1) CC	other	Total
N	32	11	43
NS	25	8	33
Wt	988.66	410.83	1399.49
Het Chi	90.72	29.55	143.39
Het df	31	10	42
Het P	***	**	***
Fixed RR	1.07	0.81	0.98
RRl	1.00	0.73	0.93
RRu	1.14	0.89	1.04
P	+	---	N.S.
Random RR	1.05	0.77	0.96
RRl	0.92	0.63	0.85
RRu	1.20	0.93	1.07
P	N.S.	--	N.S.
Between Chi			23.12
Between df			1
Between P			***
Btwn(F) P			**
Btwn(R) P			**

	Study type (2) CC	prosp	other	Total
N	32	11		43
NS	25	8		33
Wt	988.66	410.83		1399.49
Het Chi	90.72	29.55		143.39
Het df	31	10		42
Het P	***	**		***
Fixed RR	1.07	0.81		0.98
RRl	1.00	0.73		0.93
RRu	1.14	0.89		1.04
P	+	---		N.S.
Random RR	1.05	0.77		0.96
RRl	0.92	0.63		0.85
RRu	1.20	0.93		1.07
P	N.S.	--		N.S.
Between Chi				23.12
Between df				1
Between P				***
Btwn(F) P				**
Btwn(R) P				**

Table 1K2 - 3

IESLC - Meta-analysis of Ex Smoking, Years quit (vs current), "Low"
All LC types, Any Product (or Cigarettes if Any not available)
Most adjusted

	Study size (number of LC cases)				
	100-249	250-499	500-999	1000+	Total
N	6	11	10	16	43
NS	5	10	8	10	33
Wt	28.92	201.57	186.08	982.92	1399.49
Het Chi	14.70	42.10	35.83	48.94	143.39
Het df	5	10	9	15	42
Het P	*	***	***	***	***
Fixed RR	0.78	0.96	1.02	0.99	0.98
RRl	0.54	0.84	0.88	0.93	0.93
RRu	1.13	1.11	1.17	1.05	1.04
P	N.S.	N.S.	N.S.	N.S.	N.S.
Random RR	0.68	1.00	0.98	0.96	0.96
RRl	0.35	0.74	0.72	0.84	0.85
RRu	1.34	1.36	1.32	1.10	1.07
P	N.S.	N.S.	N.S.	N.S.	N.S.
Between Chi					1.81
Between df					3
Between P					N.S.
Btwn(F) P					N.S.
Btwn(R) P					N.S.

	Risky occupational population			
	no	mining	othRisky	Total
N	41	1	1	43
NS	31	1	1	33
Wt	1338.79	11.02	49.68	1399.49
Het Chi	120.79	0.00	0.00	143.39
Het df	40	0	0	42
Het P	***	N.S.	N.S.	***
Fixed RR	0.97	4.03	1.08	0.98
RRl	0.92	2.23	0.82	0.93
RRu	1.02	7.27	1.43	1.04
P	N.S.	+++	N.S.	N.S.
Random RR	0.92	4.03	1.08	0.96
RRl	0.83	2.23	0.82	0.85
RRu	1.03	7.27	1.43	1.07
P	N.S.	+++	N.S.	N.S.
Between Chi				22.60
Between df				2
Between P				***
Btwn(F) P				*
Btwn(R) P				***

	National cigarette tobacco type			
	Virginia	blended	other	Total
N	3	36	4	43
NS	2	28	3	33
Wt	47.83	1302.88	48.78	1399.49
Het Chi	4.81	98.74	7.72	143.39
Het df	2	35	3	42
Het P	(*)	***	(*)	***
Fixed RR	0.79	0.96	2.14	0.98
RRl	0.60	0.91	1.61	0.93
RRu	1.05	1.02	2.83	1.04
P	N.S.	N.S.	+++	N.S.
Random RR	0.73	0.91	2.18	0.96
RRl	0.44	0.82	1.30	0.85
RRu	1.21	1.02	3.66	1.07
P	N.S.	(-)	++	N.S.
Between Chi				32.12
Between df				2
Between P				***
Btwn(F) P				**
Btwn(R) P				**

Table 1K2 - 3

IESLC - Meta-analysis of Ex Smoking, Years quit (vs current), "Low"
All LC types, Any Product (or Cigarettes if Any not available)
Most adjusted

	Any proxy use No/nk	Yes	Total
N	37	6	43
NS	28	5	33
Wt	1302.01	97.48	1399.49
Het Chi	112.04	31.08	143.39
Het df	36	5	42
Het P	***	***	***
Fixed RR	0.99	0.94	0.98
RRl	0.94	0.77	0.93
RRu	1.04	1.14	1.04
P	N.S.	N.S.	N.S.
Random RR	0.95	0.95	0.96
RRl	0.85	0.56	0.85
RRu	1.07	1.60	1.07
P	N.S.	N.S.	N.S.
Between Chi			0.27
Between df			1
Between P			N.S.
Btwn(F) P			N.S.
Btwn(R) P			N.S.

	Full histological confirmation No	Yes	Total
N	27	16	43
NS	20	13	33
Wt	634.43	765.06	1399.49
Het Chi	122.54	19.74	143.39
Het df	26	15	42
Het P	***	N.S.	***
Fixed RR	0.95	1.01	0.98
RRl	0.88	0.94	0.93
RRu	1.03	1.08	1.04
P	N.S.	N.S.	N.S.
Random RR	0.97	0.96	0.96
RRl	0.81	0.86	0.85
RRu	1.18	1.06	1.07
P	N.S.	N.S.	N.S.
Between Chi			1.12
Between df			1
Between P			N.S.
Btwn(F) P			N.S.
Btwn(R) P			N.S.

	Number of adjustment variables (1) 0	1	2+/+nk	Total
N	24	12	7	43
NS	18	9	6	33
Wt	884.36	405.96	109.17	1399.49
Het Chi	65.63	21.30	39.32	143.39
Het df	23	11	6	42
Het P	***	*	***	***
Fixed RR	1.07	0.83	0.95	0.98
RRl	1.00	0.76	0.79	0.93
RRu	1.14	0.92	1.15	1.04
P	(+)	---	N.S.	N.S.
Random RR	1.04	0.82	0.90	0.96
RRl	0.90	0.70	0.53	0.85
RRu	1.20	0.96	1.51	1.07
P	N.S.	-	N.S.	N.S.
Between Chi				17.15
Between df				2
Between P				***
Btwn(F) P				(*)
Btwn(R) P				(*)

Table 1K2 - 3

IESLC - Meta-analysis of Ex Smoking, Years quit (vs current), "Low"
All LC types, Any Product (or Cigarettes if Any not available)
Most adjusted

	Number of adjustment variables (2) 0	1	2	3-5	6+/+nk	Total
N	24	12	5	2		43
NS	18	9	4	2		33
Wt	884.36	405.96	82.13	27.04		1399.49
Het Chi	65.63	21.30	37.74	0.34		143.39
Het df	23	11	4	1		42
Het P	***	*	***	N.S.		***
Fixed RR	1.07	0.83	1.01	0.79		0.98
RRl	1.00	0.76	0.82	0.54		0.93
RRu	1.14	0.92	1.26	1.16		1.04
P	(+)	---	N.S.	N.S.		N.S.
Random RR	1.04	0.82	0.96	0.79		0.96
RRl	0.90	0.70	0.46	0.54		0.85
RRu	1.20	0.96	1.97	1.16		1.07
P	N.S.	-	N.S.	N.S.		N.S.
Between Chi						18.39
Between df						3
Between P						***
Btwn(F) P						N.S.
Btwn(R) P						N.S.

	Product all/unsp	cig+/-ot	cig only	Total
N	9	28	6	43
NS	7	22	5	34
Wt	105.54	965.75	328.20	1399.49
Het Chi	5.48	97.52	22.59	143.39
Het df	8	27	5	42
Het P	N.S.	***	***	***
Fixed RR	0.80	1.06	0.84	0.98
RRl	0.66	1.00	0.76	0.93
RRu	0.97	1.13	0.94	1.04
P	-	(+)	--	N.S.
Random RR	0.80	1.06	0.77	0.96
RRl	0.66	0.92	0.59	0.85
RRu	0.97	1.23	1.01	1.07
P	-	N.S.	(-)	N.S.
Between Chi				17.80
Between df				2
Between P				***
Btwn(F) P				(*)
Btwn(R) P				*

	Denominator current	cur+rec	Total
N	27	16	43
NS	21	12	33
Wt	1080.68	318.81	1399.49
Het Chi	103.86	39.03	143.39
Het df	26	15	42
Het P	***	***	***
Fixed RR	0.97	1.02	0.98
RRl	0.92	0.91	0.93
RRu	1.03	1.14	1.04
P	N.S.	N.S.	N.S.
Random RR	0.91	1.05	0.96
RRl	0.79	0.86	0.85
RRu	1.05	1.28	1.07
P	N.S.	N.S.	N.S.
Between Chi			0.50
Between df			1
Between P			N.S.
Btwn(F) P			N.S.
Btwn(R) P			N.S.

Table 1K2 - 3

IESLC - Meta-analysis of Ex Smoking, Years quit (vs current), "Low"
All LC types, Any Product (or Cigarettes if Any not available)
Most adjusted

	Derivation of RR/CI			
	Orig	StdCalc	Other	Total
N	3	24	16	43
NS	3	18	12	33
Wt	46.83	884.36	468.30	1399.49
Het Chi	6.07	65.63	54.90	143.39
Het df	2	23	15	42
Het P	*	***	***	***
Fixed RR	0.73	1.07	0.87	0.98
RRl	0.55	1.00	0.80	0.93
RRu	0.98	1.14	0.95	1.04
P	-	(+)	--	N.S.
Random RR	0.80	1.04	0.86	0.96
RRl	0.44	0.90	0.71	0.85
RRu	1.47	1.20	1.05	1.07
P	N.S.	N.S.	N.S.	N.S.
Between Chi				16.80
Between df				2
Between P				***
Btwn(F) P				(*)
Btwn(R) P				N.S.

Table 1K2 - 4

IESLC - Meta-analysis of Ex Smoking, Years quit (vs current), "Low"
All LC types, Any Product (or Cigarettes if Any not available)
Least adjusted

REF	NRR	X	SEX	AGEL	AGEH	RACE	YF	LC TYPE	LOC	START	ST	NLC	R	VB	P	H	AD	ADOS	PRODUCT	exL	exH	DENOM	De
ARMADA	518		m	0	0	all	-	all	Eu:wst	1986	CC	325	n	bl	n	y	0	0	cig+/-ot	1.0	5	cur+1y	st
BARBON	533	x	m	0	0	all	-	all	Eu:wst	1979	CC	755	n	bl	y	y	0	0	all/unsp	0.1	4	current	st
BECHER	506		m	0	0	all	-	all	Eu:Ger	1985	CC	194	n	bl	n	y	0	0	all/unsp	2	4	cur+1y	st
BECHER	516		f	0	0	all	-	all	Eu:Ger	1985	CC	194	n	bl	n	y	0	0	all/unsp	2	4	cur+1y	st
BROSS	518		m	0	0	wh	-	all	NAmer	1960	CC	974	n	bl	n	n	0	0	cig+/-ot	0.1	5	current	st
CARPEN	508		c	0	0	w+b	-	all	NAmer	1991	CC	356	n	bl	n	n	0	0	cig+/-ot	0.1	4	current	st
CHOI	543		m	0	0	all	-	all	As:oth	1985	CC	375	n	bl	n	n	0	0	cig+/-ot	0.1	4	current	st
CHOI	556		f	0	0	all	-	all	As:oth	1985	CC	375	n	bl	n	n	0	0	cig+/-ot	0.1	4	current	st
CPSI	816		m	50	74	all	6	all	NAmer	1959	pr	5138	n	bl	n	n	1	0	cig only	1.0	4	current	ot
CPSII	666		m	35	99	all	4	all	NAmer	1982	pr	3229	n	bl	n	n	1	0	cig only	3	5	current	ot
CPSII	643		f	0	0	all	4	all	NAmer	1982	pr	3229	n	bl	n	n	1	0	cig+/-ot	3	5	current	ot
DAMBER	528		m	0	0	all	-	all	Eu:Sca	1972	CC	579	n	bl	y	n	1	0	all/unsp	0.1	5	current	ot
DEAN3	537	x	m	0	0	all	-	all	Eu:UK	1969	CC	766	n	V	y	n	0	0	all/unsp	3	4	cur+2y	st
DEAN3	548	x	f	0	0	all	-	all	Eu:UK	1969	CC	766	n	V	y	n	0	0	all/unsp	3	4	cur+2y	st
DESTEF	520	x	m	0	0	all	-	all	SCAmer	1988	CC	497	n	bl	n	y	0	0	all/unsp	0.1	4	current	st
DOLL2	509		m	0	0	all	20	all	Eu:UK	1951	pr	920	n	V	n	n	1	0	cig only	0.1	4	current	ot
DORGAN	514		m	0	0	wh	-	all	NAmer	1980	CC	2026	n	bl	y	y	0	0	cig+/-ot	1.1	5	cur+1y	st
DORN	823		m	55	64	wh	8	all	NAmer	1954	pr	5097	n	bl	n	n	0	0	cig+/-ot	0.1	4	current	st
DORN	827		m	65	74	wh	8	all	NAmer	1954	pr	5097	n	bl	n	n	0	0	cig+/-ot	0.1	4	current	st
GAO	526	x	m	0	0	all	-	all	As:Chi	1984	CC	1405	n	ot	n	n	0	0	cig+/-ot	0.1	4	current	st
GAO	546	x	f	0	0	all	-	all	As:Chi	1984	CC	1405	n	ot	n	n	0	0	cig+/-ot	0.1	4	current	st
GAO2	518		m	0	0	all	-	all	As:Jap	1988	CC	282	n	bl	n	n	0	0	cig+/-ot	1.0	4	cur+1y	st
GARCIA	508		c	0	0	all	-	all	NAmer	1992	CC	416	n	bl	n	y	0	0	cig+/-ot	1.0	4	cur+1y	st
GRAHAM	531	x	m	0	0	wh	-	all	NAmer	1956	CC	685	n	bl	n	n	0	0	cig+/-ot	1.1	5	current	st
HAMMO2	510		m	0	0	all	0	all	NAmer	1967	pr	450	o	bl	n	n	1	0	cig+/-ot	0.1	4	current	ot
HIRAYA	513		m	0	0	all	0	all	As:Jap	1965	pr	1917	n	bl	n	n	1	0	cig+/-ot	0.1	4	current	ot
HIRAYA	524		f	0	0	all	0	all	As:Jap	1965	pr	1917	n	bl	n	n	1	0	cig+/-ot	0.1	4	current	ot
JAHN	515		m	0	0	all	-	all	Eu:Ger	1988	CC	1004	n	bl	n	n	0	0	cig+/-ot	2	5	current	st
JOLY	571		m	0	0	all	-	all	SCAmer	1978	CC	826	n	bl	n	n	0	0	cig+/-ot	1.0	4	cur+1y	st
JOLY	558		f	0	0	all	-	all	SCAmer	1978	CC	826	n	bl	n	n	0	0	cig+/-ot	1.0	4	cur+1y	st
KHUDER	516		m	0	0	all	-	all	NAmer	1985	CC	482	n	bl	n	y	0	0	cig+/-ot	0.1	4	current	st
LUBIN	592		m	0	0	all	-	all	As:Chi	1984	CC	427	m	ot	y	n	0	0	cig+/-ot	3	4	cur+2y	st
LUBIN2	1081		m	0	0	all	-	all	Eu:mul	1976	CC	7804	n	bl	n	y	0	0	cig+/-ot	0.1	4	current	st
LUBIN2	1120		f	0	0	all	-	all	Eu:mul	1976	CC	7804	n	bl	n	y	0	0	cig+/-ot	0.1	4	current	st
MATOS	586	x	m	0	0	all	-	all	SCAmer	1994	CC	200	n	bl	n	n	0	0	cig+/-ot	1.0	5	cur+1y	st
SOBUE	728		m	0	0	all	-	all	As:Jap	1986	CC	1376	n	bl	n	y	0	0	cig+/-ot	1.0	4	cur+1y	st
SPEIZE	512		f	0	0	all	0	all	NAmer	1976	pr	593	n	bl	n	y	2	0	cig+/-ot	2	5	current	or
SUZUK2	513	x	c	0	0	all	-	all	SCAmer	1991	CC	123	n	bl	n	y	0	0	all/unsp	0.1	5	current	st
TVERDA	507		m	0	0	all	0	all	Eu:Sca	1972	pr	238	n	bl	n	n	2	0	cig only	1.0	5	current	ot
WANG2	515		c	0	0	all	-	all	As:Chi	1980	CC	103	n	ot	n	n	0	0	cig+/-ot	0.1	3	current	st
WYNDE3	545		m	0	0	all	-	all	NAmer	1966	CC	350	n	bl	n	y	0	0	all/unsp	1.0	3	cur+1y	st
WYNDE6	513		m	0	0	all	-	all	NAmer	1969	CC	4423	n	bl	n	y	0	0	cig only	1.0	4	cur+1y	st
WYNDE6	534		f	0	0	all	-	all	NAmer	1969	CC	4423	n	bl	n	y	0	0	cig only	1.0	4	cur+1y	st

Cigarette type is all/unspec for all RRs

Table 1K2 - 5

IESLC - Meta-analysis of Ex Smoking, Years quit (vs current), "Low"
All LC types, Any Product (or Cigarettes if Any not available)
Least adjusted

REF	NRR	SEX	AD	Number Exposed Case	Cont	Non-exposed Case	Cont	RR	95.00%CI
ARMADA	518	m	0	79	45	188	122	1.14	(0.74- 1.75)
BARBON	533	m	0	32	20	562	362	1.03	(0.58- 1.83)
BECHER	506	m	0	10	12	101	122	1.01	(0.42- 2.43)
BECHER	516	f	0	2	3	33	26	0.53	(0.08- 3.38)
Subtotal BECHER								0.89	(0.40- 1.98)
BROSS	518	m	0	169	67	565	427	1.91	(1.40- 2.60)
CARPEN	508	c	0	28	46	228	164	0.44	(0.26- 0.73)
CHOI	543	m	0	25	64	231	329	0.56	(0.34- 0.91)
CHOI	556	f	0	3	2	13	25	2.88	(0.43- 19.49)
Subtotal CHOI								0.62	(0.38- 0.99)
*CPSI	816	m	1	49	-	844	-	0.59	(0.44- 0.78)
*CPSII	666	m	1	178	-	1159	-	0.85	(0.72- 0.99)
*CPSII	643	f	1	56	-	530	-	0.85	(0.65- 1.12)
Subtotal CPSII								0.85	(0.74- 0.98)
DAMBER	528	m	1	-	-	-	-	0.80	(0.50- 1.28)
DEAN3	537	m	0	42	147	502	1636	0.93	(0.65- 1.33)
DEAN3	548	f	0	4	110	102	1158	0.41	(0.15- 1.14)
Subtotal DEAN3								0.85	(0.61- 1.19)
DESTEF	520	m	0	64	45	362	226	0.89	(0.59- 1.35)
*DOLL2	509	m	1	15	-	236	-	1.02	(0.61- 1.72)
DORGAN	514	m	0	59	51	465	303	0.75	(0.50- 1.13)
*DORN	823	m	0	34	22086	528	334175	0.97	(0.69- 1.38)
*DORN	827	m	0	14	6195	537	207895	0.87	(0.51- 1.49)
Subtotal DORN								0.94	(0.71- 1.26)
GAO	526	m	0	105	52	529	438	1.67	(1.17- 2.39)
GAO	546	f	0	37	9	170	100	2.42	(1.12- 5.22)
Subtotal GAO								1.78	(1.29- 2.46)
GAO2	518	m	0	31	26	184	117	0.76	(0.43- 1.34)
GARCIA	508	c	0	33	11	77	42	1.64	(0.75- 3.57)
GRAHAM	531	m	0	24	48	453	1075	1.19	(0.72- 1.96)
*HAMMO2	510	m	1	59	-	209	-	1.08	(0.82- 1.43)
*HIRAYA	513	m	1	-	-	-	-	0.46	(0.26- 0.82)
*HIRAYA	524	f	1	-	-	-	-	1.59	(0.47- 5.37)
Subtotal HIRAYA								0.58	(0.34- 0.97)
JAHN	515	m	0	77	46	352	269	1.28	(0.86- 1.90)
JOLY	571	m	0	38	36	451	524	1.23	(0.76- 1.97)
JOLY	558	f	0	19	8	132	96	1.73	(0.73- 4.11)
Subtotal JOLY								1.33	(0.88- 2.01)
KHUDER	516	m	0	88	123	245	316	0.92	(0.67- 1.27)
LUBIN	592	m	0	33	18	296	650	4.03	(2.23- 7.27)
LUBIN2	1081	m	0	866	1047	4684	6211	1.10	(0.99- 1.21)
LUBIN2	1120	f	0	60	55	440	410	1.02	(0.69- 1.50)
Subtotal LUBIN2								1.09	(0.99- 1.20)
MATOS	586	m	0	28	23	112	132	1.43	(0.78- 2.63)
SOBUE	728	m	0	128	116	737	633	0.95	(0.72- 1.24)
*SPEIZE	512	f	2	34	-	319	-	0.60	(0.40- 0.80)
SUZUK2	513	c	0	15	10	77	30	0.58	(0.24- 1.44)
*TVERDA	507	m	2	5	-	144	-	0.18	(0.07- 0.43)
WANG2	515	c	0	6	10	49	78	0.96	(0.33- 2.79)
WYNDE3	545	m	0	21	22	227	207	0.87	(0.47- 1.63)
WYNDE6	513	m	0	201	166	1107	993	1.09	(0.87- 1.36)
WYNDE6	534	f	0	82	70	683	496	0.85	(0.61- 1.19)
Subtotal WYNDE6								1.01	(0.84- 1.22)
Partial Totals				2853	30789	18863	559787		

*prospective study

Table 1K2 - 5

IESLC - Meta-analysis of Ex Smoking, Years quit (vs current), "Low"
All LC types, Any Product (or Cigarettes if Any not available)
Least adjusted

REF	NRR	SEX	AD	Ys	Ws	Qs	Ps
ARMADA	518	m	0	0.13	20.66	0.41	0.5535
BARBON	533	m	0	0.03	11.66	0.02	0.9180
BECHER	506	m	0	0.01	4.96	0.00	0.9883
BECHER	516	f	0	-0.64	1.11	0.44	0.4978
Subtotal BECHER				-0.11	6.07	0.45	
BROSS	518	m	0	0.65	40.07	17.25	0.0000
CARPEN	508	c	0	-0.83	14.72	9.78	0.0015
CHOI	543	m	0	-0.59	15.87	5.26	0.0195
CHOI	556	f	0	1.06	1.05	1.21	0.2771
Subtotal CHOI				-0.48	16.93	6.46	
*CPSI	816	m	1	-0.53	46.88	12.51	0.0003
*CPSII	666	m	1	-0.16	151.52	3.48	0.0454
*CPSII	643	f	1	-0.16	51.90	1.19	0.2417
Subtotal CPSII				-0.16	203.42	4.67	
DAMBER	528	m	1	-0.22	17.39	0.78	0.3521
DEAN3	537	m	0	-0.07	30.11	0.11	0.6954
DEAN3	548	f	0	-0.88	3.71	2.83	0.0885
Subtotal DEAN3				-0.16	33.81	2.94	
DESTEF	520	m	0	-0.12	22.21	0.26	0.5753
*DOLL2	509	m	1	0.02	14.30	0.01	0.9403
DORGAN	514	m	0	-0.28	23.81	1.76	0.1680
*DORN	823	m	0	-0.03	31.99	0.01	0.8830
*DORN	827	m	0	-0.13	13.68	0.21	0.6211
Subtotal DORN				-0.06	45.67	0.21	
GAO	526	m	0	0.51	30.37	8.37	0.0046
GAO	546	f	0	0.88	6.49	5.19	0.0244
Subtotal GAO				0.58	36.86	13.56	
GAO2	518	m	0	-0.28	11.81	0.83	0.3414
GARCIA	508	c	0	0.49	6.33	1.60	0.2154
GRAHAM	531	m	0	0.17	15.24	0.50	0.5044
*HAMMO2	510	m	1	0.08	49.68	0.38	0.5875
*HIRAYA	513	m	1	-0.78	11.65	6.83	0.0080
*HIRAYA	524	f	1	0.46	2.59	0.58	0.4555
Subtotal HIRAYA				-0.55	14.24	7.41	
JAHN	515	m	0	0.25	24.22	1.60	0.2255
JOLY	571	m	0	0.20	17.18	0.79	0.3976
JOLY	558	f	0	0.55	5.11	1.59	0.2166
Subtotal JOLY				0.28	22.29	2.38	
KHUDER	516	m	0	-0.08	37.40	0.18	0.6231
LUBIN	592	m	0	1.39	11.02	21.71	0.0000
LUBIN2	1081	m	0	0.09	402.52	4.30	0.0639
LUBIN2	1120	f	0	0.02	25.28	0.02	0.9343
Subtotal LUBIN2				0.09	427.80	4.32	
MATOS	586	m	0	0.36	10.45	1.45	0.2432
SOBUE	728	m	0	-0.05	51.63	0.09	0.6997
*SPEIZE	512	f	2	-0.51	31.98	7.99	0.0039
SUZUK2	513	c	0	-0.54	4.70	1.30	0.2445
*TVERDA	507	m	2	-1.71	4.66	13.54	0.0002
WANG2	515	c	0	-0.05	3.33	0.00	0.9331
WYNDE3	545	m	0	-0.14	9.77	0.16	0.6644
WYNDE6	513	m	0	0.08	77.46	0.68	0.4670
WYNDE6	534	f	0	-0.16	33.38	0.76	0.3502
Subtotal WYNDE6				0.01	110.84	1.44	

Table 1K2 - 5

IESLC - Meta-analysis of Ex Smoking, Years quit (vs current), "Low"
All LC types, Any Product (or Cigarettes if Any not available)
Least adjusted

	N	43
	NS	33
	Wt	1401.83
Het	Chi	137.97
Het	df	42
Het	P	***
Fixed	RR	0.99
	RRl	0.94
	RRu	1.04
	P	N.S.
Random	RR	0.96
	RRl	0.86
	RRu	1.08
	P	N.S.
Asymm	P	N.S.

Table 1K2 - 6

IESLC - Meta-analysis of Ex Smoking, Years quit (vs current), "Low"
All LC types, Any Product (or Cigarettes if Any not available)
Least adjusted

	Sex combined	male	female	Total
N	4	29	10	43
NS	4	28	10	42
Wt	29.08	1210.15	162.60	1401.83
Het Chi	8.22	102.61	19.06	137.97
Het df	3	28	9	42
Het P	*	***	*	***
Fixed RR	0.67	1.02	0.87	0.99
RRl	0.46	0.96	0.74	0.94
RRu	0.96	1.07	1.01	1.04
P	-	N.S.	(-)	N.S.
Random RR	0.76	0.99	0.94	0.96
RRl	0.40	0.87	0.72	0.86
RRu	1.46	1.12	1.24	1.08
P	N.S.	N.S.	N.S.	N.S.
Between Chi				8.08
Between df				2
Between P				*
Btwn(F) P				N.S.
Btwn(R) P				N.S.

Table 1K2 - 7

IESLC - Meta-analysis of Ex Smoking, Years quit (vs current), "Low"
All LC types, Any Product (or Cigarettes if Any not available)
Excluded studies (and stage at which they were excluded)

1	AGUDO AKIBA AMANDU AMES AXELSS BEST BOUCHA BOUCOT BRESLO CHEN CHEN2 CHIAZZ DEAN2 DOSEME ENGELA FAN GENG GER GUO HAENSZ HEGMAN HOLE HU HU2 JUSSAW KATSOU KAUFMA KOO KOULUM KREUZE LETOUR LEVIN LIAW LIU3 LIU4 LIU5 MCCONN MIGRAN MRFITR NOTAN2 OSANN2 PERNU QIAO2 RACHTA RESTRE SADOWS SEGI2 STASZE TIZZAN VUTUC WATSON WU WUWILL WYNDE2 WYNDE8 XU YUAN ZHANG ZHENG ZHOU
2	AUVINE BENSHL BLOT1 BROWN3 BUFFLE GURSEL LAUSSM LUO MCDUFF PISANI PRESCO SPITZ WU2 WYNDE7
4	HAMMON
5	CORREA GILLIS HUMBLE QIAO WIGLE
7	BOFFET
10	GARSHI JEDRYC WAKAI
14	ALDERS CEDERL CHYOU DARBY DOLL JAIN KAISE2 PEZZO2 PEZZOT SVENSS
15	BENHAM

Table 1K2 - 8
Potentially overlapping studies

REF	REFGP	PRINC	.	OVERLAP/LINK
LUBIN2	LUBIN2	1		Lubin-combined
TVERDA	TVERDA	1		VEIERO/TVERDAL
BROSS	BYERS1	1		GRAHAM/BROSS/BYERS1
GRAHAM	BYERS1	1		GRAHAM/BROSS/BYERS1
WYNDE6	WYNDE6	1		WYNDE5/6/7/8
CPSI	CPSI	1		CPSI overall
JAHN	BOFFET	2		Subset of BOFFET
LUBIN	XIANGZ	2		LUBIN/XIANGZ/QIAO

Table 1K2 - 9
Most adjusted - insufficient data for meta-analysis

REF	NRR	SEX	AGEL	AGEH	RACE	YF	LC TYPE	LOC	START	ST	NLC	R	VB	P	H	AD	ADOS	PRODUCT	exL	exH	DENOM	De
CORREA	550	c	0	0	all	-	all	NAmer	1979	CC	1359	n	bl	y	n	2	0	cig+/-ot	3	5	cur+2y	st
HUMBLE	549	c	0	0	wh	-	not alv	NAmer	1980	CC	521	n	bl	y	n	2	2#	cig+/-ot	2	5	cur+1y	or

Comments on values in listings

HUMBLE ADOS Number of cigarettes and duration

REF	NRR	RR	SIG	RRDATA comment
CORREA	550	0.61		0
HUMBLE	549	0.69		0

Table 1K3 -

IESLC - Meta-analysis of Ex Smoking, Years quit (vs current), "Mid"
All LC types, Any Product (or Cigarettes if Any not available)

This analysis is restricted to results for:
1) Ex smokers
2) Results by Years quit (vs current)
3) Categorical results by Years quit (vs current)
4) All LC types (or near equivalent)
5) Results complete enough for use in metaanalysis

Within each study, results are then selected (in the following order of preference, within each sex) for:
6) (not applicable)
7) PRODUCT: all/unspec, cigarettes regardless of other products, cigarettes only
8) CIGTYPE: all/unspecified, MC regardless of HR, MC only
9) Results with least adjustment for other aspects of smoking (ADOS)
10) DENOM: current smokers, current + recent smokers (up to number of m=months or y=years, max 2 years)
11) Followup period (YF, prospective studies): whole study (coded as 0) or longest available
12) LCtype: all or nearest available, at least Squamous and Adeno. (q = squamous, s = small,
l = large, a = adeno, mix = mixed, alv = alveolar)
13) Race: all or nearest available, otherwise by race (wh or w = white, bl or b = black, hi = hispanic
ch = chinese, jap = japanese, haw = hawaiian, w+o = white + oriental, sca = scandinavian, as = asian)
14) Years quit (vs current) "mid" in key scheme 1 (key value 7, maximum range 4-11)
15) For overlapping studies: principal rather than subsidiary studies
Finally by Age: whole study (coded as 0) if available, otherwise by widest available age group
and then for single sex results (m, f) in preference to results for both sexes combined (c).

Results adjusted (AD) for the most potential confounders are then chosen in Sections -1 to -3
and results adjusted for the least confounders in Sections -4 to -6. (Those least adjusted results which
actually differ from the most adjusted are marked 'x' in column X in Section -4)

Section -7 shows excluded studies, together with the stage (as above) at which no qualifying
results were found.

Section -8 lists the potentially overlapping studies which have been included (1=principal, 2=subsidiary).

Section -9 lists any results which would have been included in preference except that they had data not complete
enough for use in meta-analysis, with their significance (yes/no), if known, and any further comment as entered
on the database. It also lists as "gap" any categories for which no data were presented by the original authors.

In addition to those mentioned above, the following fields, levels and abbreviations are used:
* or nk = not known, n = no, y = yes, ot = other
nev = never
all/unspec = all or unspecified, cig+/-ot = cigarettes irrespective of other products (cigar, pipe etc)
MC = manufactured cigarettes, HR = hand-rolled cigarettes
exL, exH = range of exposure (low and high) in the smoking group, in terms of Years quit (vs current)
REF: 6-character study reference
NRR: number of the RR on the database within the study
ST : study type (CC = case control, pr or prosp = prospective)
NLC: number of lung cancer cases in whole study
R : risky occupational population (n = no, m = mining, o = other risky)
VB : national cigarette type (V = at least 75% Virginia, bl = at least 75% blended, ot = other)
P : any proxy use
H : full histological confirmation
De : derivation of RR/CI (or = original, st = standard method, ot = other method of estimation)

Table 1K3 - 1

IESLC - Meta-analysis of Ex Smoking, Years quit (vs current), "Mid"
All LC types, Any Product (or Cigarettes if Any not available)
Most adjusted

REF	NRR	SEX	AGEL	AGEH	RACE	YF	LC TYPE	LOC	START	ST	NLC	R	VB	P	H	AD	ADOS	PRODUCT	exL	exH	DENOM	De
BECHER	507	m	0	0	all	-	all	Eu:Ger	1985	CC	194	n	bl	n	y	0	0	all/unsp	5	9	cur+1y	st
BECHER	517	f	0	0	all	-	all	Eu:Ger	1985	CC	194	n	bl	n	y	0	0	all/unsp	5	9	cur+1y	st
CARPEN	509	c	0	0	w+b	-	all	NAmer	1991	CC	356	n	bl	n	n	0	0	cig+/-ot	5	9	current	st
CHOI	544	m	0	0	all	-	all	As:oth	1985	CC	375	n	bl	n	n	0	0	cig+/-ot	5	9	current	st
CPSI	817	m	50	74	all	6	all	NAmer	1959	pr	5138	n	bl	n	n	1	0	cig only	5	9	current	ot
CPSII	667	m	35	99	all	4	all	NAmer	1982	pr	3229	n	bl	n	n	1	0	cig only	6	10	current	ot
CPSII	644	f	0	0	all	4	all	NAmer	1982	pr	3229	n	bl	n	n	1	0	cig+/-ot	6	10	current	ot
DAMBER	529	m	0	0	all	-	all	Eu:Sca	1972	CC	579	n	bl	y	n	1	0	all/unsp	6	10	current	ot
DEAN3	637	m	0	0	all	-	all	Eu:UK	1969	CC	766	n	V	y	n	1	0	all/unsp	5	8	cur+2y	ot
DEAN3	560	f	0	0	all	-	all	Eu:UK	1969	CC	766	n	V	y	n	1	0	all/unsp	5	8	cur+2y	ot
DESTEF	531	m	0	0	all	-	all	SCAmer	1988	CC	497	n	bl	n	y	4	0	all/unsp	5	9	current	ot
DOLL2	510	m	0	0	all	20	all	Eu:UK	1951	pr	920	n	V	n	n	1	0	cig only	5	9	current	ot
DORGAN	515	m	0	0	wh	-	all	NAmer	1980	CC	2026	n	bl	y	y	0	0	cig+/-ot	6	9	cur+1y	st
DORN	824	m	55	64	wh	8	all	NAmer	1954	pr	5097	n	bl	n	n	0	0	cig+/-ot	5	9	current	st
DORN	828	m	65	74	wh	8	all	NAmer	1954	pr	5097	n	bl	n	n	0	0	cig+/-ot	5	9	current	st
GAO	537	m	0	0	all	-	all	As:Chi	1984	CC	1405	n	ot	n	n	2	0	cig+/-ot	5	9	current	ot
GAO	557	f	0	0	all	-	all	As:Chi	1984	CC	1405	n	ot	n	n	2	0	cig+/-ot	5	9	current	ot
GAO2	519	m	0	0	all	-	all	As:Jap	1988	CC	282	n	bl	n	n	0	0	cig+/-ot	5	9	cur+1y	st
GRAHAM	510	m	0	0	wh	-	all	NAmer	1956	CC	685	n	bl	n	n	0	0	cig only	3	10	current	st
HAMMO2	511	m	0	0	all	0	all	NAmer	1967	pr	450	o	bl	n	n	1	0	cig+/-ot	5	9	current	ot
HIRAYA	514	m	0	0	all	0	all	As:Jap	1965	pr	1917	n	bl	n	n	1	0	cig+/-ot	5	9	current	ot
HIRAYA	525	f	0	0	all	0	all	As:Jap	1965	pr	1917	n	bl	n	n	1	0	cig+/-ot	5	9	current	ot
JAHN	516	m	0	0	all	-	all	Eu:Ger	1988	CC	1004	n	bl	n	n	0	0	cig+/-ot	6	10	current	st
LUBIN	593	m	0	0	all	-	all	As:Chi	1984	CC	427	m	ot	y	n	0	0	cig+/-ot	5	9	cur+2y	st
LUBIN2	1082	m	0	0	all	-	all	Eu:mul	1976	CC	7804	n	bl	n	y	0	0	cig+/-ot	5	9	current	st
LUBIN2	1121	f	0	0	all	-	all	Eu:mul	1976	CC	7804	n	bl	n	y	0	0	cig+/-ot	5	9	current	st
MATOS	597	m	0	0	all	-	all	SCAmer	1994	CC	200	n	bl	n	n	2	0	cig+/-ot	6	10	cur+1y	or
SOBUE	729	m	0	0	all	-	all	As:Jap	1986	CC	1376	n	bl	n	y	0	0	cig+/-ot	5	9	cur+1y	st
SPEIZE	513	f	0	0	all	0	all	NAmer	1976	pr	593	n	bl	n	y	2	0	cig+/-ot	5	10	current	or
SUZUK2	525	c	0	0	all	-	all	SCAmer	1991	CC	123	n	bl	n	y	3	0	all/unsp	6	10	current	or
WYNDE6	514	m	0	0	all	-	all	NAmer	1969	CC	4423	n	bl	n	y	0	0	cig only	5	9	cur+1y	st
WYNDE6	535	f	0	0	all	-	all	NAmer	1969	CC	4423	n	bl	n	y	0	0	cig only	5	9	cur+1y	st

Cigarette type is all/unspec for all RRs

Table 1K3 - 2

IESLC - Meta-analysis of Ex Smoking, Years quit (vs current), "Mid"
All LC types, Any Product (or Cigarettes if Any not available)
Most adjusted

REF	NRR	SEX	AD	Number Exposed Case	Cont	Non-exposed Case	Cont	RR	95.00%CI
BECHER	507	m	0	16	32	101	122	0.60	(0.31- 1.16)
BECHER	517	f	0	2	5	33	26	0.32	(0.06- 1.76)
Subtotal BECHER								0.56	(0.30- 1.03)
CARPEN	509	c	0	31	52	228	164	0.43	(0.26- 0.70)
CHOI	544	m	0	5	30	231	329	0.24	(0.09- 0.62)
*CPSI	817	m	1	32	-	844	-	0.37	(0.26- 0.53)
*CPSII	667	m	1	186	-	1159	-	0.52	(0.45- 0.61)
*CPSII	644	f	1	37	-	530	-	0.40	(0.28- 0.55)
Subtotal CPSII								0.50	(0.43- 0.57)
DAMBER	529	m	1	-	-	-	-	0.45	(0.26- 0.78)
DEAN3	637	m	1	15	-	502	-	0.55	(0.31- 0.97)
DEAN3	560	f	1	1	-	102	-	0.20	(0.03- 1.42)
Subtotal DEAN3								0.51	(0.29- 0.88)
DESTEF	531	m	4	27	-	362	-	0.57	(0.33- 0.98)
*DOLL2	510	m	1	12	-	236	-	0.35	(0.20- 0.63)
DORGAN	515	m	0	49	38	465	303	0.84	(0.54- 1.31)
*DORN	824	m	0	32	34566	528	334175	0.59	(0.41- 0.84)
*DORN	828	m	0	41	24089	537	207895	0.66	(0.48- 0.90)
Subtotal DORN								0.63	(0.49- 0.79)
GAO	537	m	2	24	-	529	-	0.79	(0.45- 1.40)
GAO	557	f	2	14	-	170	-	1.34	(0.51- 3.53)
Subtotal GAO								0.90	(0.55- 1.48)
GAO2	519	m	0	21	26	184	117	0.51	(0.28- 0.95)
GRAHAM	510	m	0	5	29	371	821	0.38	(0.15- 0.99)
*HAMMO2	511	m	1	11	-	209	-	0.39	(0.22- 0.71)
*HIRAYA	514	m	1	-	-	-	-	0.36	(0.15- 0.86)
*HIRAYA	525	f	1	-	-	-	-	1.41	(0.23- 8.48)
Subtotal HIRAYA								0.47	(0.21- 1.02)
JAHN	516	m	0	59	63	352	269	0.72	(0.49- 1.06)
LUBIN	593	m	0	20	48	296	650	0.91	(0.53- 1.57)
LUBIN2	1082	m	0	466	822	4684	6211	0.75	(0.67- 0.85)
LUBIN2	1121	f	0	30	40	440	410	0.70	(0.43- 1.14)
Subtotal LUBIN2								0.75	(0.67- 0.84)
MATOS	597	m	2	21	-	112	-	0.90	(0.40- 1.60)
SOBUE	729	m	0	67	92	737	633	0.63	(0.45- 0.87)
*SPEIZE	513	f	2	41	-	319	-	0.50	(0.40- 0.70)
SUZUK2	525	c	3	10	-	77	-	0.50	(0.20- 1.40)
WYNDE6	514	m	0	98	194	1107	993	0.45	(0.35- 0.59)
WYNDE6	535	f	0	51	84	683	496	0.44	(0.31- 0.64)
Subtotal WYNDE6								0.45	(0.36- 0.55)
Partial Totals				1424	60210	16128	553614		

*prospective study

REF	NRR	SEX	AD	Ys	Ws	Qs	Ps
BECHER	507	m	0	-0.50	8.94	0.01	0.1316
BECHER	517	f	0	-1.15	1.30	0.49	0.1879
Subtotal BECHER				-0.59	10.24	0.50	
CARPEN	509	c	0	-0.85	16.14	1.54	0.0007
CHOI	544	m	0	-1.44	4.15	3.36	0.0034
*CPSI	817	m	1	-0.99	30.29	6.30	0.0000
*CPSII	667	m	1	-0.65	166.04	2.22	0.0000
*CPSII	644	f	1	-0.92	33.71	4.82	0.0000
Subtotal CPSII				-0.70	199.75	7.04	
DAMBER	529	m	1	-0.80	12.73	0.86	0.0044
DEAN3	637	m	1	-0.60	11.81	0.04	0.0399
DEAN3	560	f	1	-1.61	1.03	1.18	0.1019
Subtotal DEAN3				-0.68	12.84	1.23	
DESTEF	531	m	4	-0.56	12.97	0.01	0.0429
*DOLL2	510	m	1	-1.05	11.67	3.05	0.0003
DORGAN	515	m	0	-0.17	19.17	2.54	0.4460
*DORN	824	m	0	-0.53	30.20	0.00	0.0033
*DORN	828	m	0	-0.42	38.16	0.56	0.0100
Subtotal DORN				-0.47	68.36	0.56	
GAO	537	m	2	-0.24	11.93	1.09	0.4156
GAO	557	f	2	0.29	4.11	2.83	0.5532
Subtotal GAO				-0.10	16.03	3.93	
GAO2	519	m	0	-0.67	9.99	0.16	0.0352
GRAHAM	510	m	0	-0.96	4.19	0.76	0.0484
*HAMMO2	511	m	1	-0.94	11.19	1.82	0.0016
*HIRAYA	514	m	1	-1.02	5.04	1.18	0.0218

Table 1K3 - 2

IESLC - Meta-analysis of Ex Smoking, Years quit (vs current), "Mid"
All LC types, Any Product (or Cigarettes if Any not available)
Most adjusted

REF	NRR	SEX	AD	Ys	Ws	Qs	Ps
*HIRAYA	525	f	1	0.34	1.18	0.92	0.7089
Subtotal HIRAYA				-0.76	6.22	2.10	
JAHN	516	m	0	-0.33	25.39	1.05	0.0919
LUBIN	593	m	0	-0.09	13.20	2.67	0.7468
LUBIN2	1082	m	0	-0.29	267.60	17.12	0.0000
LUBIN2	1121	f	0	-0.36	15.86	0.51	0.1536
Subtotal LUBIN2				-0.29	283.46	17.63	
MATOS	597	m	2	-0.11	8.00	1.50	0.7658
SOBUE	729	m	0	-0.47	34.80	0.17	0.0056
*SPEIZE	513	f	2	-0.69	49.07	1.18	0.0000
SUZUK2	525	c	3	-0.69	4.06	0.10	0.1626
WYNDE6	514	m	0	-0.79	57.91	3.71	0.0000
WYNDE6	535	f	0	-0.82	28.58	2.25	0.0000
Subtotal WYNDE6				-0.80	86.48	5.96	

	N	32
	NS	24
	Wt	950.41
Het	Chi	66.01
Het	df	31
Het	P	***
Fixed	RR	0.58
	RRl	0.55
	RRu	0.62
	P	---
Random	RR	0.55
	RRl	0.49
	RRu	0.62
	P	---
Asymm	P	N.S.

Table 1K3 - 3

IESLC - Meta-analysis of Ex Smoking, Years quit (vs current), "Mid"
All LC types, Any Product (or Cigarettes if Any not available)
Most adjusted

	combined	Sex<br>male	female	Total
N	2	22	8	32
NS	2	21	8	31
Wt	20.19	795.38	134.84	950.41
Het Chi	0.08	49.16	10.28	66.01
Het df	1	21	7	31
Het P	N.S.	***	N.S.	***
Fixed RR	0.44	0.61	0.49	0.58
RRl	0.29	0.56	0.42	0.55
RRu	0.68	0.65	0.58	0.62
P	---	---	---	---
Random RR	0.44	0.57	0.51	0.55
RRl	0.29	0.50	0.40	0.49
RRu	0.68	0.65	0.65	0.62
P	---	---	---	---
Between Chi				6.49
Between df				2
Between P				*
Btwn(F) P				N.S.
Btwn(R) P				N.S.

	Lung cancer type<br>all	other	Total
N	32		32
NS	24		24
Wt	950.41		950.41
Het Chi	66.01		66.01
Het df	31		31
Het P	***		***
Fixed RR	0.58		0.58
RRl	0.55		0.55
RRu	0.62		0.62
P	---		---
Random RR	0.55		0.55
RRl	0.49		0.49
RRu	0.62		0.62
P	---		---
Between Chi			
Between df			
Between P			N.S.
Btwn(F) P			N.S.
Btwn(R) P			N.S.

				Location					
	NAmer	UK	Scand	othEur	China	Japan	othAs	other	Total
N	12	3	1	5	3	4	1	3	32
NS	9	2	1	3	2	3	1	3	24
Wt	484.64	24.51	12.73	319.09	29.23	51.02	4.15	25.02	950.41
Het Chi	15.90	1.81	0.00	1.46	0.85	2.42	0.00	1.35	66.01
Het df	11	2	0	4	2	3	0	2	31
Het P	N.S.	N.S.	N.S.	N.S.	N.S.	N.S.	N.S.	N.S.	***
Fixed RR	0.50	0.43	0.45	0.74	0.91	0.58	0.24	0.65	0.58
RRl	0.46	0.29	0.26	0.66	0.63	0.44	0.09	0.44	0.55
RRu	0.55	0.63	0.78	0.82	1.31	0.76	0.62	0.96	0.62
P	---	---	--	---	N.S.	---	--	-	---
Random RR	0.50	0.43	0.45	0.74	0.91	0.58	0.24	0.65	0.55
RRl	0.44	0.29	0.26	0.66	0.63	0.44	0.09	0.44	0.49
RRu	0.56	0.63	0.78	0.82	1.31	0.76	0.62	0.96	0.62
P	---	---	--	---	N.S.	---	--	-	---
Between Chi									42.21
Between df									7
Between P									***
Btwn(F) P									***
Btwn(R) P									***

Table 1K3 - 3

IESLC - Meta-analysis of Ex Smoking, Years quit (vs current), "Mid"
All LC types, Any Product (or Cigarettes if Any not available)
Most adjusted

Detailed Country in "other Europe"

	multi	Germany	othWest	East	Balkans	Total
N	2	3				5
NS	1	2				3
Wt	283.46	35.63				319.09
Het Chi	0.08	0.95				1.46
Het df	1	2				4
Het P	N.S.	N.S.				N.S.
Fixed RR	0.75	0.67				0.74
RRl	0.67	0.48				0.66
RRu	0.84	0.92				0.82
P	---	-				---
Random RR	0.75	0.67				0.74
RRl	0.67	0.48				0.66
RRu	0.84	0.92				0.82
P	---	-				---
Between Chi						0.44
Between df						1
Between P						N.S.
Btwn(F) P						N.S.
Btwn(R) P						N.S.

Detailed Country in "other Asia"

	India	HongKong	other	Total
N			1	1
NS			1	1
Wt			4.15	4.15
Het Chi			0.00	0.00
Het df			0	0
Het P			N.S.	N.S.
Fixed RR			0.24	0.24
RRl			0.09	0.09
RRu			0.62	0.62
P			--	--
Random RR			0.24	0.24
RRl			0.09	0.09
RRu			0.62	0.62
P			--	--
Between Chi				
Between df				
Between P				N.S.
Btwn(F) P				N.S.
Btwn(R) P				N.S.

Detailed other continent

	SCAmer	Total
N	3	3
NS	3	3
Wt	25.02	25.02
Het Chi	1.35	1.35
Het df	2	2
Het P	N.S.	N.S.
Fixed RR	0.65	0.65
RRl	0.44	0.44
RRu	0.96	0.96
P	-	-
Random RR	0.65	0.65
RRl	0.44	0.44
RRu	0.96	0.96
P	-	-
Between Chi		
Between df		
Between P		N.S.
Btwn(F) P		N.S.
Btwn(R) P		N.S.

Table 1K3 - 3

IESLC - Meta-analysis of Ex Smoking, Years quit (vs current), "Mid"
All LC types, Any Product (or Cigarettes if Any not available)
Most adjusted

	Start year of study					
	<1960	1960-69	1970-79	1980-89	1990+	Total
N	5	7	4	13	3	32
NS	4	4	3	10	3	24
Wt	114.52	116.74	345.26	345.71	28.19	950.41
Het Chi	8.20	3.19	9.37	21.13	2.97	66.01
Het df	4	6	3	12	2	31
Het P	(*)	N.S.	*	*	N.S.	***
Fixed RR	0.50	0.45	0.69	0.57	0.54	0.58
RRl	0.42	0.38	0.62	0.51	0.37	0.55
RRu	0.61	0.54	0.77	0.63	0.78	0.62
P	---	---	---	---	--	---
Random RR	0.48	0.45	0.61	0.60	0.56	0.55
RRl	0.36	0.38	0.46	0.51	0.35	0.49
RRu	0.64	0.54	0.80	0.72	0.90	0.62
P	---	---	---	---	-	---
Between Chi						21.16
Between df						4
Between P						***
Btwn(F) P						*
Btwn(R) P						N.S.

	Study type (1)		
	CC	other	Total
N	22	10	32
NS	17	7	24
Wt	573.86	376.55	950.41
Het Chi	37.59	12.32	66.01
Het df	21	9	31
Het P	*	N.S.	***
Fixed RR	0.65	0.50	0.58
RRl	0.60	0.45	0.55
RRu	0.70	0.55	0.62
P	---	---	---
Random RR	0.60	0.49	0.55
RRl	0.52	0.43	0.49
RRu	0.69	0.56	0.62
P	---	---	---
Between Chi			16.09
Between df			1
Between P			***
Btwn(F) P			**
Btwn(R) P			*

	Study type (2)			
	CC	prosp	other	Total
N	22	10		32
NS	17	7		24
Wt	573.86	376.55		950.41
Het Chi	37.59	12.32		66.01
Het df	21	9		31
Het P	*	N.S.		***
Fixed RR	0.65	0.50		0.58
RRl	0.60	0.45		0.55
RRu	0.70	0.55		0.62
P	---	---		---
Random RR	0.60	0.49		0.55
RRl	0.52	0.43		0.49
RRu	0.69	0.56		0.62
P	---	---		---
Between Chi				16.09
Between df				1
Between P				***
Btwn(F) P				**
Btwn(R) P				*

Table 1K3 - 3

IESLC - Meta-analysis of Ex Smoking, Years quit (vs current), "Mid"
All LC types, Any Product (or Cigarettes if Any not available)
Most adjusted

	Study size (number of LC cases)				
	100-249	250-499	500-999	1000+	Total
N	4	6	6	16	32
NS	3	6	5	10	24
Wt	22.29	67.65	90.50	769.96	950.41
Het Chi	1.86	8.39	2.45	46.33	66.01
Het df	3	5	5	15	31
Het P	N.S.	N.S.	N.S.	***	***
Fixed RR	0.65	0.51	0.47	0.60	0.58
RRl	0.43	0.40	0.38	0.56	0.55
RRu	0.98	0.65	0.57	0.65	0.62
P	-	---	---	---	---
Random RR	0.65	0.50	0.47	0.58	0.55
RRl	0.43	0.37	0.38	0.50	0.49
RRu	0.98	0.69	0.57	0.68	0.62
P	-	---	---	---	---
Between Chi					6.99
Between df					3
Between P					(*)
Btwn(F) P					N.S.
Btwn(R) P					N.S.

	Risky occupational population			
	no	mining	othRisky	Total
N	30	1	1	32
NS	22	1	1	24
Wt	926.02	13.20	11.19	950.41
Het Chi	61.52	0.00	0.00	66.01
Het df	29	0	0	31
Het P	***	N.S.	N.S.	***
Fixed RR	0.58	0.91	0.39	0.58
RRl	0.55	0.53	0.22	0.55
RRu	0.62	1.57	0.70	0.62
P	---	N.S.	--	---
Random RR	0.55	0.91	0.39	0.55
RRl	0.49	0.53	0.22	0.49
RRu	0.61	1.57	0.70	0.62
P	---	N.S.	--	---
Between Chi				4.49
Between df				2
Between P				N.S.
Btwn(F) P				N.S.
Btwn(R) P				(*)

	National cigarette tobacco type			
	Virginia	blended	other	Total
N	3	26	3	32
NS	2	20	2	24
Wt	24.51	896.66	29.23	950.41
Het Chi	1.81	55.11	0.85	66.01
Het df	2	25	2	31
Het P	N.S.	***	N.S.	***
Fixed RR	0.43	0.58	0.91	0.58
RRl	0.29	0.54	0.63	0.55
RRu	0.63	0.62	1.31	0.62
P	---	---	N.S.	---
Random RR	0.43	0.54	0.91	0.55
RRl	0.29	0.48	0.63	0.49
RRu	0.63	0.61	1.31	0.62
P	---	---	N.S.	---
Between Chi				8.24
Between df				2
Between P				*
Btwn(F) P				N.S.
Btwn(R) P				*

Table 1K3 - 3

IESLC - Meta-analysis of Ex Smoking, Years quit (vs current), "Mid"
All LC types, Any Product (or Cigarettes if Any not available)
Most adjusted

	Any proxy use No/nk	Yes	Total
N	27	5	32
NS	20	4	24
Wt	892.47	57.94	950.41
Het Chi	58.65	6.25	66.01
Het df	26	4	31
Het P	***	N.S.	***
Fixed RR	0.58	0.67	0.58
RRl	0.54	0.52	0.55
RRu	0.62	0.86	0.62
P	---	--	---
Random RR	0.54	0.65	0.55
RRl	0.48	0.46	0.49
RRu	0.61	0.91	0.62
P	---	-	---
Between Chi			1.12
Between df			1
Between P			N.S.
Btwn(F) P			N.S.
Btwn(R) P			N.S.

	Full histological confirmation No	Yes	Total
N	21	11	32
NS	16	8	24
Wt	450.16	500.25	950.41
Het Chi	32.76	23.45	66.01
Het df	20	10	31
Het P	*	**	***
Fixed RR	0.52	0.64	0.58
RRl	0.48	0.59	0.55
RRu	0.58	0.70	0.62
P	---	---	---
Random RR	0.53	0.59	0.55
RRl	0.46	0.49	0.49
RRu	0.61	0.70	0.62
P	---	---	---
Between Chi			9.81
Between df			1
Between P			**
Btwn(F) P			*
Btwn(R) P			N.S.

	Number of adjustment variables (1) 0	1	2+/+nk	Total
N	16	10	6	32
NS	12	7	5	24
Wt	575.59	284.70	90.12	950.41
Het Chi	30.49	8.56	6.66	66.01
Het df	15	9	5	31
Het P	*	N.S.	N.S.	***
Fixed RR	0.65	0.47	0.60	0.58
RRl	0.60	0.42	0.49	0.55
RRu	0.70	0.53	0.73	0.62
P	---	---	---	---
Random RR	0.60	0.47	0.64	0.55
RRl	0.52	0.42	0.49	0.49
RRu	0.69	0.53	0.83	0.62
P	---	---	--	---
Between Chi				20.30
Between df				2
Between P				***
Btwn(F) P				**
Btwn(R) P				*

Table 1K3 - 3

IESLC - Meta-analysis of Ex Smoking, Years quit (vs current), "Mid"
All LC types, Any Product (or Cigarettes if Any not available)
Most adjusted

	Number of adjustment variables (2) 0	1	2	3-5	6+/+nk	Total
N	16	10	4	2		32
NS	12	7	3	2		24
Wt	575.59	284.70	73.09	17.03		950.41
Het Chi	30.49	8.56	6.49	0.05		66.01
Het df	15	9	3	1		31
Het P	*	N.S.	(*)	N.S.		***
Fixed RR	0.65	0.47	0.61	0.55		0.58
RRl	0.60	0.42	0.48	0.34		0.55
RRu	0.70	0.53	0.76	0.89		0.62
P	---	---	---	-		---
Random RR	0.60	0.47	0.73	0.55		0.55
RRl	0.52	0.42	0.48	0.34		0.49
RRu	0.69	0.53	1.10	0.89		0.62
P	---	---	N.S.	-		---
Between Chi						20.42
Between df						3
Between P						***
Btwn(F) P						*
Btwn(R) P						*

	Product all/unsp	cig+/-ot	cig only	Total
N	7	19	6	32
NS	5	15	5	25
Wt	52.84	598.89	298.68	950.41
Het Chi	1.88	36.84	4.82	66.01
Het df	6	18	5	31
Het P	N.S.	**	N.S.	***
Fixed RR	0.52	0.66	0.47	0.58
RRl	0.39	0.61	0.42	0.55
RRu	0.68	0.71	0.53	0.62
P	---	---	---	---
Random RR	0.52	0.61	0.47	0.55
RRl	0.39	0.53	0.42	0.49
RRu	0.68	0.71	0.53	0.62
P	---	---	---	---
Between Chi				22.47
Between df				2
Between P				***
Btwn(F) P				**
Btwn(R) P				*

	Denominator current	cur+rec	Total
N	21	11	32
NS	16	8	24
Wt	755.68	194.73	950.41
Het Chi	51.20	14.45	66.01
Het df	20	10	31
Het P	***	N.S.	***
Fixed RR	0.59	0.56	0.58
RRl	0.55	0.49	0.55
RRu	0.63	0.65	0.62
P	---	---	---
Random RR	0.53	0.58	0.55
RRl	0.46	0.48	0.49
RRu	0.62	0.70	0.62
P	---	---	---
Between Chi			0.36
Between df			1
Between P			N.S.
Btwn(F) P			N.S.
Btwn(R) P			N.S.

Table 1K3 - 3

IESLC - Meta-analysis of Ex Smoking, Years quit (vs current), "Mid"
All LC types, Any Product (or Cigarettes if Any not available)
Most adjusted

		Derivation of RR/CI			
		Orig	StdCalc	Other	Total
	N	3	16	13	32
	NS	3	12	9	24
	Wt	61.12	575.59	313.70	950.41
Het	Chi	2.40	30.49	16.32	66.01
Het	df	2	15	12	31
Het	P	N.S.	*	N.S.	***
Fixed	RR	0.54	0.65	0.49	0.58
	RRl	0.42	0.60	0.44	0.55
	RRu	0.69	0.70	0.55	0.62
	P	---	---	---	---
Random	RR	0.56	0.60	0.48	0.55
	RRl	0.40	0.52	0.41	0.49
	RRu	0.78	0.69	0.56	0.62
	P	---	---	---	---
Between	Chi				16.81
Between	df				2
Between	P				***
Btwn(F)	P				*
Btwn(R)	P				N.S.

Table 1K3 - 4

IESLC - Meta-analysis of Ex Smoking, Years quit (vs current), "Mid"
All LC types, Any Product (or Cigarettes if Any not available)
Least adjusted

REF	NRR	X	SEX	AGEL	AGEH	RACE	YF	LC TYPE	LOC	START	ST	NLC	R	VB	P	H	AD	ADOS	PRODUCT	exL	exH	DENOM	De
BECHER	507		m	0	0	all	-	all	Eu:Ger	1985	CC	194	n	bl	n	y	0	0	all/unsp	5	9	cur+1y	st
BECHER	517		f	0	0	all	-	all	Eu:Ger	1985	CC	194	n	bl	n	y	0	0	all/unsp	5	9	cur+1y	st
CARPEN	509		c	0	0	w+b	-	all	NAmer	1991	CC	356	n	bl	n	n	0	0	cig+/-ot	5	9	current	st
CHOI	544		m	0	0	all	-	all	As:oth	1985	CC	375	n	bl	n	n	0	0	cig+/-ot	5	9	current	st
CPSI	817		m	50	74	all	6	all	NAmer	1959	pr	5138	n	bl	n	n	1	0	cig only	5	9	current	ot
CPSII	667		m	35	99	all	4	all	NAmer	1982	pr	3229	n	bl	n	n	1	0	cig only	6	10	current	ot
CPSII	644		f	0	0	all	4	all	NAmer	1982	pr	3229	n	bl	n	n	1	0	cig+/-ot	6	10	current	ot
DAMBER	529		m	0	0	all	-	all	Eu:Sca	1972	CC	579	n	bl	y	n	1	0	all/unsp	6	10	current	ot
DEAN3	538	x	m	0	0	all	-	all	Eu:UK	1969	CC	766	n	V	y	n	0	0	all/unsp	5	8	cur+2y	st
DEAN3	549	x	f	0	0	all	-	all	Eu:UK	1969	CC	766	n	V	y	n	0	0	all/unsp	5	8	cur+2y	st
DESTEF	521	x	m	0	0	all	-	all	SCAmer	1988	CC	497	n	bl	n	y	0	0	all/unsp	5	9	current	st
DOLL2	510		m	0	0	all	20	all	Eu:UK	1951	pr	920	n	V	n	n	1	0	cig only	5	9	current	ot
DORGAN	515		m	0	0	wh	-	all	NAmer	1980	CC	2026	n	bl	y	y	0	0	cig+/-ot	6	9	cur+1y	st
DORN	824		m	55	64	wh	8	all	NAmer	1954	pr	5097	n	bl	n	n	0	0	cig+/-ot	5	9	current	st
DORN	828		m	65	74	wh	8	all	NAmer	1954	pr	5097	n	bl	n	n	0	0	cig+/-ot	5	9	current	st
GAO	527	x	m	0	0	all	-	all	As:Chi	1984	CC	1405	n	ot	n	n	0	0	cig+/-ot	5	9	current	st
GAO	547	x	f	0	0	all	-	all	As:Chi	1984	CC	1405	n	ot	n	n	0	0	cig+/-ot	5	9	current	st
GAO2	519		m	0	0	all	-	all	As:Jap	1988	CC	282	n	bl	n	n	0	0	cig+/-ot	5	9	cur+1y	st
GRAHAM	510		m	0	0	wh	-	all	NAmer	1956	CC	685	n	bl	n	n	0	0	cig only	3	10	current	st
HAMMO2	511		m	0	0	all	0	all	NAmer	1967	pr	450	o	bl	n	n	1	0	cig+/-ot	5	9	current	ot
HIRAYA	514		m	0	0	all	0	all	As:Jap	1965	pr	1917	n	bl	n	n	1	0	cig+/-ot	5	9	current	ot
HIRAYA	525		f	0	0	all	0	all	As:Jap	1965	pr	1917	n	bl	n	n	1	0	cig+/-ot	5	9	current	ot
JAHN	516		m	0	0	all	-	all	Eu:Ger	1988	CC	1004	n	bl	n	n	0	0	cig+/-ot	6	10	current	st
LUBIN	593		m	0	0	all	-	all	As:Chi	1984	CC	427	m	ot	y	n	0	0	cig+/-ot	5	9	cur+2y	st
LUBIN2	1082		m	0	0	all	-	all	Eu:mul	1976	CC	7804	n	bl	n	y	0	0	cig+/-ot	5	9	current	st
LUBIN2	1121		f	0	0	all	-	all	Eu:mul	1976	CC	7804	n	bl	n	y	0	0	cig+/-ot	5	9	current	st
MATOS	587	x	m	0	0	all	-	all	SCAmer	1994	CC	200	n	bl	n	n	0	0	cig+/-ot	6	10	cur+1y	st
SOBUE	729		m	0	0	all	-	all	As:Jap	1986	CC	1376	n	bl	n	y	0	0	cig+/-ot	5	9	cur+1y	st
SPEIZE	513		f	0	0	all	0	all	NAmer	1976	pr	593	n	bl	n	y	2	0	cig+/-ot	5	10	current	or
SUZUK2	514	x	c	0	0	all	-	all	SCAmer	1991	CC	123	n	bl	n	y	0	0	all/unsp	6	10	current	st
WYNDE6	514		m	0	0	all	-	all	NAmer	1969	CC	4423	n	bl	n	y	0	0	cig only	5	9	cur+1y	st
WYNDE6	535		f	0	0	all	-	all	NAmer	1969	CC	4423	n	bl	n	y	0	0	cig only	5	9	cur+1y	st

Cigarette type is all/unspec for all RRs

Table 1K3 - 5

IESLC - Meta-analysis of Ex Smoking, Years quit (vs current), "Mid"
All LC types, Any Product (or Cigarettes if Any not available)
Least adjusted

REF	NRR	SEX	AD	Number Exposed Case	Cont	Non-exposed Case	Cont	RR	95.00%CI
BECHER	507	m	0	16	32	101	122	0.60	(0.31- 1.16)
BECHER	517	f	0	2	5	33	26	0.32	(0.06- 1.76)
Subtotal BECHER								0.56	(0.30- 1.03)
CARPEN	509	c	0	31	52	228	164	0.43	(0.26- 0.70)
CHOI	544	m	0	5	30	231	329	0.24	(0.09- 0.62)
*CPSI	817	m	1	32	-	844	-	0.37	(0.26- 0.53)
*CPSII	667	m	1	186	-	1159	-	0.52	(0.45- 0.61)
*CPSII	644	f	1	37	-	530	-	0.40	(0.28- 0.55)
Subtotal CPSII								0.50	(0.43- 0.57)
DAMBER	529	m	1	-	-	-	-	0.45	(0.26- 0.78)
DEAN3	538	m	0	15	67	502	1636	0.73	(0.41- 1.29)
DEAN3	549	f	0	1	38	102	1158	0.30	(0.04- 2.20)
Subtotal DEAN3								0.68	(0.39- 1.18)
DESTEF	521	m	0	27	27	362	226	0.62	(0.36- 1.09)
*DOLL2	510	m	1	12	-	236	-	0.35	(0.20- 0.63)
DORGAN	515	m	0	49	38	465	303	0.84	(0.54- 1.31)
*DORN	824	m	0	32	34566	528	334175	0.59	(0.41- 0.84)
*DORN	828	m	0	41	24089	537	207895	0.66	(0.48- 0.90)
Subtotal DORN								0.63	(0.49- 0.79)
GAO	527	m	0	24	27	529	438	0.74	(0.42- 1.29)
GAO	547	f	0	14	7	170	100	1.18	(0.46- 3.01)
Subtotal GAO								0.83	(0.51- 1.35)
GAO2	519	m	0	21	26	184	117	0.51	(0.28- 0.95)
GRAHAM	510	m	0	5	29	371	821	0.38	(0.15- 0.99)
*HAMMO2	511	m	1	11	-	209	-	0.39	(0.22- 0.71)
*HIRAYA	514	m	1	-	-	-	-	0.36	(0.15- 0.86)
*HIRAYA	525	f	1	-	-	-	-	1.41	(0.23- 8.48)
Subtotal HIRAYA								0.47	(0.21- 1.02)
JAHN	516	m	0	59	63	352	269	0.72	(0.49- 1.06)
LUBIN	593	m	0	20	48	296	650	0.91	(0.53- 1.57)
LUBIN2	1082	m	0	466	822	4684	6211	0.75	(0.67- 0.85)
LUBIN2	1121	f	0	30	40	440	410	0.70	(0.43- 1.14)
Subtotal LUBIN2								0.75	(0.67- 0.84)
MATOS	587	m	0	21	27	112	132	0.92	(0.49- 1.71)
SOBUE	729	m	0	67	92	737	633	0.63	(0.45- 0.87)
*SPEIZE	513	f	2	41	-	319	-	0.50	(0.40- 0.70)
SUZUK2	514	c	0	10	8	77	30	0.49	(0.18- 1.35)
WYNDE6	514	m	0	98	194	1107	993	0.45	(0.35- 0.59)
WYNDE6	535	f	0	51	84	683	496	0.44	(0.31- 0.64)
Subtotal WYNDE6								0.45	(0.36- 0.55)
Partial Totals				1424	60411	16128	557334		

*prospective study

REF	NRR	SEX	AD	Ys	Ws	Qs	Ps
BECHER	507	m	0	-0.50	8.94	0.01	0.1316
BECHER	517	f	0	-1.15	1.30	0.50	0.1879
Subtotal BECHER				-0.59	10.24	0.51	
CARPEN	509	c	0	-0.85	16.14	1.58	0.0007
CHOI	544	m	0	-1.44	4.15	3.40	0.0034
*CPSI	817	m	1	-0.99	30.29	6.44	0.0000
*CPSII	667	m	1	-0.65	166.04	2.41	0.0000
*CPSII	644	f	1	-0.92	33.71	4.94	0.0000
Subtotal CPSII				-0.70	199.75	7.36	
DAMBER	529	m	1	-0.80	12.73	0.90	0.0044
DEAN3	538	m	0	-0.32	11.88	0.56	0.2773
DEAN3	549	f	0	-1.21	0.96	0.44	0.2355
Subtotal DEAN3				-0.38	12.84	1.00	
DESTEF	521	m	0	-0.47	12.31	0.05	0.0984
*DOLL2	510	m	1	-1.05	11.67	3.11	0.0003
DORGAN	515	m	0	-0.17	19.17	2.47	0.4460
*DORN	824	m	0	-0.53	30.20	0.00	0.0033
*DORN	828	m	0	-0.42	38.16	0.52	0.0100
Subtotal DORN				-0.47	68.36	0.52	
GAO	527	m	0	-0.31	12.07	0.62	0.2869
GAO	547	f	0	0.16	4.34	2.10	0.7348
Subtotal GAO				-0.18	16.41	2.72	
GAO2	519	m	0	-0.67	9.99	0.18	0.0352
GRAHAM	510	m	0	-0.96	4.19	0.78	0.0484
*HAMMO2	511	m	1	-0.94	11.19	1.87	0.0016
*HIRAYA	514	m	1	-1.02	5.04	1.20	0.0218

Table 1K3 - 5

IESLC - Meta-analysis of Ex Smoking, Years quit (vs current), "Mid"
All LC types, Any Product (or Cigarettes if Any not available)
Least adjusted

REF	NRR	SEX	AD	Ys	Ws	Qs	Ps
*HIRAYA	525	f	1	0.34	1.18	0.91	0.7089
Subtotal HIRAYA				-0.76	6.22	2.11	
JAHN	516	m	0	-0.33	25.39	1.00	0.0919
LUBIN	593	m	0	-0.09	13.20	2.61	0.7468
LUBIN2	1082	m	0	-0.29	267.60	16.45	0.0000
LUBIN2	1121	f	0	-0.36	15.86	0.49	0.1536
Subtotal LUBIN2				-0.29	283.46	16.94	
MATOS	587	m	0	-0.09	9.89	1.97	0.7844
SOBUE	729	m	0	-0.47	34.80	0.14	0.0056
*SPEIZE	513	f	2	-0.69	49.07	1.25	0.0000
SUZUK2	514	c	0	-0.72	3.69	0.13	0.1672
WYNDE6	514	m	0	-0.79	57.91	3.86	0.0000
WYNDE6	535	f	0	-0.82	28.58	2.33	0.0000
Subtotal WYNDE6				-0.80	86.48	6.19	

	N	32
	NS	24
	Wt	951.64
Het	Chi	65.23
Het	df	31
Het	P	***
Fixed	RR	0.59
	RRl	0.55
	RRu	0.63
	P	---
Random	RR	0.56
	RRl	0.50
	RRu	0.62
	P	---
Asymm	P	N.S.

Table 1K3 - 6

IESLC - Meta-analysis of Ex Smoking, Years quit (vs current), "Mid"
All LC types, Any Product (or Cigarettes if Any not available)
Least adjusted

	Sex combined	male	female	Total
N	2	22	8	32
NS	2	21	8	31
Wt	19.82	796.81	135.01	951.64
Het Chi	0.05	49.47	8.86	65.23
Het df	1	21	7	31
Het P	N.S.	***	N.S.	***
Fixed RR	0.44	0.61	0.49	0.59
RRl	0.28	0.57	0.42	0.55
RRu	0.68	0.65	0.58	0.63
P	---	---	---	---
Random RR	0.44	0.57	0.50	0.56
RRl	0.28	0.50	0.41	0.50
RRu	0.68	0.66	0.62	0.62
P	---	---	---	---
Between Chi				6.84
Between df				2
Between P				*
Btwn(F) P				N.S.
Btwn(R) P				N.S.

Table 1K3 - 7

IESLC - Meta-analysis of Ex Smoking, Years quit (vs current), "Mid"
All LC types, Any Product (or Cigarettes if Any not available)
Excluded studies (and stage at which they were excluded)

1	AGUDO AKIBA AMANDU AMES AXELSS BEST BOUCHA BOUCOT BRESLO CHEN CHEN2 CHIAZZ DEAN2 DOSEME ENGELA FAN GENG GER GUO HAENSZ HEGMAN HOLE HU HU2 JUSSAW KATSOU KAUFMA KOO KOULUM KREUZE LETOUR LEVIN LIAW LIU3 LIU4 LIU5 MCCONN MIGRAN MRFITR NOTAN2 OSANN2 PERNU QIAO2 RACHTA RESTRE SADOWS SEGI2 STASZE TIZZAN VUTUC WATSON WU WUWILL WYNDE2 WYNDE8 XU YUAN ZHANG ZHENG ZHOU
2	AUVINE BENSHL BLOT1 BROWN3 BUFFLE GURSEL LAUSSM LUO MCDUFF PISANI PRESCO SPITZ WU2 WYNDE7
4	HAMMON
5	CORREA GILLIS HUMBLE QIAO WIGLE
7	BOFFET
10	GARSHI JEDRYC WAKAI
14	ALDERS ARMADA BARBON BROSS CEDERL CHYOU DARBY DOLL GARCIA JAIN JOLY KAISE2 KHUDER PEZZO2 PEZZOT SVENSS TVERDA WANG2 WYNDE3
15	BENHAM

Table 1K3 - 8
Potentially overlapping studies

REF	REFGP	PRINC	.	OVERLAP/LINK
LUBIN2	LUBIN2	1		Lubin-combined
GRAHAM	BYERS1	1		GRAHAM/BROSS/BYERS1
WYNDE6	WYNDE6	1		WYNDE5/6/7/8
CPSI	CPSI	1		CPSI overall
JAHN	BOFFET	2		Subset of BOFFET
LUBIN	XIANGZ	2		LUBIN/XIANGZ/QIAO

Table 1K3 - 9
Most adjusted - insufficient data for meta-analysis

REF	NRR	SEX	AGEL	AGEH	RACE	YF	LC TYPE	LOC	START	ST	NLC	R	VB	P	H	AD	ADOS	PRODUCT	exL	exH	DENOM	De
HUMBLE	560	c	25	64	wh	-	not alv	NAmer	1980	CC	521	n	bl	y	n	2		2#cig+/-ot	6	10	cur+1y	or
HUMBLE	555	c	65	84	wh	-	not alv	NAmer	1980	CC	521	n	bl	y	n	2		2#cig+/-ot	6	10	cur+1y	or

Comments on values in listings

HUMBLE ADOS Number of cigarettes and duration
HUMBLE ADOS Number of cigarettes and duration

REF	NRR	RR	SIG	RRDATA comment
HUMBLE	560	0.24		0
HUMBLE	555	0.54		0

Table 1K4 -

IESLC - Meta-analysis of Ex Smoking, Years quit (vs current), "High"
All LC types, Any Product (or Cigarettes if Any not available)

This analysis is restricted to results for:
1) Ex smokers
2) Results by Years quit (vs current)
3) Categorical results by Years quit (vs current)
4) All LC types (or near equivalent)
5) Results complete enough for use in metaanalysis

Within each study, results are then selected (in the following order of preference, within each sex) for:
6) PRODUCT: all/unspec, cigarettes regardless of other products, cigarettes only
7) CIGTYPE: all/unspecified, MC regardless of HR, MC only
8) Results with least adjustment for other aspects of smoking (ADOS)
9) DENOM: current smokers, current + recent smokers (up to number of m=months or y=years, max 2 years)
10) Followup period (YF, prospective studies): whole study (coded as 0) or longest available
11) LCtype: all or nearest available, at least Squamous and Adeno. (q = squamous, s = small,
l = large, a = adeno, mix = mixed, alv = alveolar)
12) Race: all or nearest available, otherwise by race (wh or w = white, bl or b = black, hi = hispanic
ch = chinese, jap = japanese, haw = hawaiian, w+o = white + oriental, sca = scandinavian, as = asian)
13) Years quit (vs current) "high" in key scheme 1 (key value 12, maximum range 8+)
14) For overlapping studies: principal rather than subsidiary studies
Finally by Age: whole study (coded as 0) if available, otherwise by widest available age group
and then for single sex results (m, f) in preference to results for both sexes combined (c).

Results adjusted (AD) for the most potential confounders are then chosen in Sections -1 to -3
and results adjusted for the least confounders in Sections -4 to -6. (Those least adjusted results which
actually differ from the most adjusted are marked 'x' in column X in Section -4)

Section -7 shows excluded studies, together with the stage (as above) at which no qualifying
results were found.

Section -8 lists the potentially overlapping studies which have been included (1=principal, 2=subsidiary).

Section -9 lists any results which would have been included in preference except that they had data not complete
enough for use in meta-analysis, with their significance (yes/no), if known, and any further comment as entered
on the database. It also lists as "gap" any categories for which no data were presented by the original authors.

In addition to those mentioned above, the following fields, levels and abbreviations are used:
* or nk = not known, n = no, y = yes, ot = other
nev = never
all/unspec = all or unspecified, cig+/-ot = cigarettes irrespective of other products (cigar, pipe etc)
MC = manufactured cigarettes, HR = hand-rolled cigarettes
exL, exH = range of exposure (low and high) in the smoking group, in terms of Years quit (vs current)
REF: 6-character study reference
NRR: number of the RR on the database within the study
ST : study type (CC = case control, pr or prosp = prospective)
NLC: number of lung cancer cases in whole study
R : risky occupational population (n = no, m = mining, o = other risky)
VB : national cigarette type (V = at least 75% Virginia, bl = at least 75% blended, ot = other)
P : any proxy use
H : full histological confirmation
De : derivation of RR/CI (or = original, st = standard method, ot = other method of estimation)

Table 1K4 - 1

IESLC - Meta-analysis of Ex Smoking, Years quit (vs current), "High"
All LC types, Any Product (or Cigarettes if Any not available)
Most adjusted

REF	NRR	SEX	AGEL	AGEH	RACE	YF	LC TYPE	LOC	START	ST	NLC	R	VB	P	H	AD	ADOS	PRODUCT	exL	exH	DENOM	De
ALDERS	515	m	0	0	all	-	all	Eu:UK	1977	CC	1448	n	V	n	n	1	0	cig only	10	999	current	ot
ALDERS	526	f	0	0	all	-	all	Eu:UK	1977	CC	1448	n	V	n	n	1	0	cig only	10	999	current	ot
BECHER	508	m	0	0	all	-	all	Eu:Ger	1985	CC	194	n	bl	n	y	0	0	all/unsp	10	999	cur+1y	st
BECHER	518	f	0	0	all	-	all	Eu:Ger	1985	CC	194	n	bl	n	y	0	0	all/unsp	10	999	cur+1y	st
CARPEN	510	c	0	0	w+b	-	all	NAmer	1991	CC	356	n	bl	n	n	0	0	cig+/-ot	10	14	current	st
CEDERL	539	m	40	69	all	10	all	Eu:Sca	1963	pr	491	n	bl	n	n	1	0	all/unsp	10	999	current	ot
CHOI	545	m	0	0	all	-	all	As:oth	1985	CC	375	n	bl	n	n	0	0	cig+/-ot	10	14	current	st
CPSI	818	m	50	74	all	6	all	NAmer	1959	pr	5138	n	bl	n	n	1	0	cig only	10	999	current	ot
CPSII	668	m	35	99	all	4	all	NAmer	1982	pr	3229	n	bl	n	n	1	0	cig only	11	15	current	ot
CPSII	645	f	0	0	all	4	all	NAmer	1982	pr	3229	n	bl	n	n	1	0	cig+/-ot	11	15	current	ot
DAMBER	530	m	0	0	all	-	all	Eu:Sca	1972	CC	579	n	bl	y	n	1	0	all/unsp	11	999	current	ot
DARBY	508	m	0	0	wh	-	all	Eu:UK	1988	CC	982	n	V	n	n	0	0	all/unsp	10	999	current	st
DARBY	517	f	0	0	wh	-	all	Eu:UK	1988	CC	982	n	V	n	n	0	0	all/unsp	10	999	current	st
DEAN3	638	m	0	0	all	-	all	Eu:UK	1969	CC	766	n	V	y	n	1	0	all/unsp	9	999	cur+2y	ot
DEAN3	561	f	0	0	all	-	all	Eu:UK	1969	CC	766	n	V	y	n	1	0	all/unsp	9	999	cur+2y	ot
DESTEF	532	m	0	0	all	-	all	SCAmer	1988	CC	497	n	bl	n	y	4	0	all/unsp	10	999	current	ot
DOLL	538	m	0	0	all	-	all	Eu:UK	1948	CC	1465	n	V	n	n	0	0	all/unsp	10	19	current	st
DOLL	549	f	0	0	all	-	all	Eu:UK	1948	CC	1465	n	V	n	n	0	0	all/unsp	10	999	current	st
DOLL2	511	m	0	0	all	20	all	Eu:UK	1951	pr	920	n	V	n	n	1	0	cig only	10	14	current	ot
DORGAN	516	m	0	0	wh	-	all	NAmer	1980	CC	2026	n	bl	y	y	0	0	cig+/-ot	10	999	cur+1y	st
DORGAN	560	f	0	0	all	-	all	NAmer	1980	CC	2026	n	bl	y	y	0	0	cig+/-ot	10	999	cur+1y	st
DORN	825	m	55	64	wh	8	all	NAmer	1954	pr	5097	n	bl	n	n	0	0	cig+/-ot	10	14	current	st
DORN	829	m	65	74	wh	8	all	NAmer	1954	pr	5097	n	bl	n	n	0	0	cig+/-ot	10	14	current	st
GAO	538	m	0	0	all	-	all	As:Chi	1984	CC	1405	n	ot	n	n	2	0	cig+/-ot	10	999	current	ot
GAO	558	f	0	0	all	-	all	As:Chi	1984	CC	1405	n	ot	n	n	2	0	cig+/-ot	10	999	current	ot
GAO2	520	m	0	0	all	-	all	As:Jap	1988	CC	282	n	bl	n	n	0	0	cig+/-ot	10	14	cur+1y	st
GRAHAM	511	m	0	0	wh	-	all	NAmer	1956	CC	685	n	bl	n	n	0	0	cig only	10	999	current	st
HAMMO2	512	m	0	0	all	0	all	NAmer	1967	pr	450	o	bl	n	n	1	0	cig+/-ot	10	999	current	ot
HIRAYA	515	m	0	0	all	0	all	As:Jap	1965	pr	1917	n	bl	n	n	1	0	cig+/-ot	10	999	current	ot
HIRAYA	526	f	0	0	all	0	all	As:Jap	1965	pr	1917	n	bl	n	n	1	0	cig+/-ot	10	999	current	ot
JAHN	517	m	0	0	all	-	all	Eu:Ger	1988	CC	1004	n	bl	n	n	0	0	cig+/-ot	11	20	current	st
JAIN	571	m	0	0	all	-	all	NAmer	1981	CC	845	n	V	y	n	0	0	cig+/-ot	10	999	cur+2y	st
JAIN	535	f	0	0	all	-	all	NAmer	1981	CC	845	n	V	y	n	0	0	cig+/-ot	10	999	cur+2y	st
KAISE2	656	m	0	0	all	9	all	NAmer	1979	pr	318	n	bl	n	n	1	0	cig only	11	20	cur+2y	ot
KAISE2	576	f	0	0	all	9	all	NAmer	1979	pr	318	n	bl	n	n	1	0	cig only	11	20	cur+2y	st
LUBIN	594	m	0	0	all	-	all	As:Chi	1984	CC	427	m	ot	y	n	0	0	cig+/-ot	10	999	cur+2y	st
LUBIN2	1083	m	0	0	all	-	all	Eu:mul	1976	CC	7804	n	bl	n	y	0	0	cig+/-ot	10	14	current	st
LUBIN2	1122	f	0	0	all	-	all	Eu:mul	1976	CC	7804	n	bl	n	y	0	0	cig+/-ot	10	14	current	st
MATOS	598	m	0	0	all	-	all	SCAmer	1994	CC	200	n	bl	n	n	2	0	cig+/-ot	11	999	cur+1y	or
PEZZO2	505	m	0	0	all	-	all	SCAmer	1992	CC	367	n	bl	n	y	0	0	cig+/-ot	11	999	cur+1y	st
PEZZOT	505	m	0	0	all	-	all	SCAmer	1987	CC	215	n	bl	n	y	0	0	cig only	11	999	cur+1y	st
SOBUE	730	m	0	0	all	-	all	As:Jap	1986	CC	1376	n	bl	n	y	0	0	cig+/-ot	10	14	cur+1y	st
SPEIZE	514	f	0	0	all	0	all	NAmer	1976	pr	593	n	bl	n	y	2	0	cig+/-ot	10	15	current	or
SUZUK2	526	c	0	0	all	-	all	SCAmer	1991	CC	123	n	bl	n	y	3	0	all/unsp	11	999	current	or
SVENSS	555	f	0	0	all	-	all	Eu:Sca	1983	CC	210	n	bl	n	n	0	0	all/unsp	11	999	cur+2y	st
WYNDE6	720	m	0	0	wh	-	all	NAmer	1969	CC	4423	n	bl	n	y	5	1#	cig+/-ot	11	19	cur+1y	or
WYNDE6	727	m	0	0	bl	-	all	NAmer	1969	CC	4423	n	bl	n	y	5	1#	cig+/-ot	11	19	cur+1y	or
WYNDE6	734	f	0	0	wh	-	all	NAmer	1969	CC	4423	n	bl	n	y	5	1#	cig+/-ot	11	999	cur+1y	or
WYNDE6	738	f	0	0	bl	-	all	NAmer	1969	CC	4423	n	bl	n	y	5	1#	cig+/-ot	11	999	cur+1y	or

Comments on values in listings

WYNDE6	ADOS	Number of cigs/day
WYNDE6	ADOS	Number of cigs/day
WYNDE6	ADOS	Number of cigs/day
WYNDE6	ADOS	Number of cigs/day

Cigarette type is all/unspec for all RRs
except for the following:

REF	NRR	CIGTYPE
ALDERS	515	MC only
ALDERS	526	MC only

Table 1K4 - 2

IESLC - Meta-analysis of Ex Smoking, Years quit (vs current), "High"
All LC types, Any Product (or Cigarettes if Any not available)
Most adjusted

REF	NRR	SEX	AD	Number Exposed Case	Cont	Non-exposed Case	Cont	RR	95.00%CI
ALDERS	515	m	1	29	-	207	-	0.32	(0.20- 0.51)
ALDERS	526	f	1	26	-	244	-	0.28	(0.17- 0.46)
Subtotal ALDERS								0.30	(0.21- 0.42)
BECHER	508	m	0	16	72	101	122	0.27	(0.15- 0.49)
BECHER	518	f	0	1	10	33	26	0.08	(0.01- 0.66)
Subtotal BECHER								0.24	(0.14- 0.44)
CARPEN	510	c	0	13	58	228	164	0.16	(0.09- 0.30)
*CEDERL	539	m	1	3	-	97	-	0.14	(0.04- 0.45)
CHOI	545	m	0	4	23	231	329	0.25	(0.08- 0.73)
*CPSI	818	m	1	15	-	844	-	0.09	(0.06- 0.15)
*CPSII	668	m	1	164	-	1159	-	0.39	(0.33- 0.46)
*CPSII	645	f	1	28	-	530	-	0.31	(0.21- 0.46)
Subtotal CPSII								0.38	(0.32- 0.44)
DAMBER	530	m	1	42	-	-	-	0.27	(0.18- 0.40)
DARBY	508	m	0	139	767	379	618	0.30	(0.24- 0.37)
DARBY	517	f	0	26	224	198	231	0.14	(0.09- 0.21)
Subtotal DARBY								0.25	(0.21- 0.31)
DEAN3	638	m	1	32	-	502	-	0.34	(0.23- 0.50)
DEAN3	561	f	1	2	-	102	-	0.13	(0.03- 0.53)
Subtotal DEAN3								0.32	(0.22- 0.46)
DESTEF	532	m	4	17	-	362	-	0.26	(0.14- 0.46)
DOLL	538	m	0	6	26	1280	1172	0.21	(0.09- 0.52)
DOLL	549	f	0	1	2	58	41	0.35	(0.03- 4.03)
Subtotal DOLL								0.22	(0.10- 0.52)
*DOLL2	511	m	1	9	-	236	-	0.28	(0.14- 0.54)
DORGAN	516	m	0	134	255	465	303	0.34	(0.27- 0.44)
DORGAN	560	f	0	34	50	289	112	0.26	(0.16- 0.43)
Subtotal DORGAN								0.32	(0.26- 0.41)
*DORN	825	m	0	12	23682	528	334175	0.32	(0.18- 0.57)
*DORN	829	m	0	29	20056	537	207895	0.56	(0.39- 0.81)
Subtotal DORN								0.47	(0.35- 0.65)
GAO	538	m	2	13	-	529	-	0.28	(0.14- 0.57)
GAO	558	f	2	16	-	170	-	0.76	(0.34- 1.67)
Subtotal GAO								0.43	(0.26- 0.73)
GAO2	520	m	0	16	18	184	117	0.57	(0.28- 1.15)
GRAHAM	511	m	0	2	30	371	821	0.15	(0.04- 0.62)
*HAMMO2	512	m	1	20	-	209	-	0.34	(0.22- 0.53)
*HIRAYA	515	m	1	-	-	-	-	0.31	(0.14- 0.69)
*HIRAYA	526	f	1	-	-	-	-	0.41	(0.01- 14.37)
Subtotal HIRAYA								0.31	(0.14- 0.68)
JAHN	517	m	0	64	130	352	269	0.38	(0.27- 0.53)
JAIN	571	m	0	52	113	265	118	0.20	(0.14- 0.30)
JAIN	535	f	0	19	61	305	99	0.10	(0.06- 0.18)
Subtotal JAIN								0.16	(0.12- 0.22)
*KAISE2	656	m	1	8	-	51	-	0.43	(0.20- 0.92)
*KAISE2	576	f	1	4	-	50	-	0.25	(0.09- 0.70)
Subtotal KAISE2								0.35	(0.19- 0.65)
LUBIN	594	m	0	17	73	296	650	0.51	(0.30- 0.88)
LUBIN2	1083	m	0	270	693	4684	6211	0.52	(0.45- 0.60)
LUBIN2	1122	f	0	10	26	440	410	0.36	(0.17- 0.75)
Subtotal LUBIN2								0.51	(0.44- 0.59)
MATOS	598	m	2	27	-	112	-	0.30	(0.20- 0.60)
PEZZO2	505	m	0	43	161	233	198	0.23	(0.15- 0.33)
PEZZOT	505	m	0	20	106	145	129	0.17	(0.10- 0.29)
SOBUE	730	m	0	35	50	737	633	0.60	(0.39- 0.94)
*SPEIZE	514	f	2	17	-	319	-	0.20	(0.10- 0.40)
SUZUK2	526	c	3	9	-	77	-	0.10	(0.10- 0.40)
SVENSS	555	f	0	14	24	142	53	0.22	(0.10- 0.45)
WYNDE6	720	m	5	-	-	-	-	0.30	(0.20- 0.40)
WYNDE6	727	m	5	-	-	-	-	0.20	(0.10- 0.50)
WYNDE6	734	f	5	-	-	-	-	0.20	(0.20- 0.30)
WYNDE6	738	f	5	-	-	-	-	0.40	(0.10- 1.10)
Subtotal WYNDE6								0.22	(0.19- 0.26)
Partial Totals				1458	46710	18281	554896		

*prospective study

Table 1K4 - 2

IESLC - Meta-analysis of Ex Smoking, Years quit (vs current), "High"
All LC types, Any Product (or Cigarettes if Any not available)
Most adjusted

REF	NRR	SEX	AD	Ys	Ws	Qs	Ps
ALDERS	515	m	1	-1.14	17.54	0.00	0.0000
ALDERS	526	f	1	-1.27	15.51	0.23	0.0000
Subtotal ALDERS				-1.20	33.04	0.23	
BECHER	508	m	0	-1.32	10.58	0.28	0.0000
BECHER	518	f	0	-2.54	0.86	1.65	0.0188
Subtotal BECHER				-1.41	11.44	1.93	
CARPEN	510	c	0	-1.82	9.56	4.32	0.0000
*CEDERL	539	m	1	-1.97	2.62	1.74	0.0015
CHOI	545	m	0	-1.40	3.32	0.20	0.0109
*CPSI	818	m	1	-2.41	18.30	28.86	0.0000
*CPSII	668	m	1	-0.94	139.29	6.18	0.0000
*CPSII	645	f	1	-1.17	24.99	0.01	0.0000
Subtotal CPSII				-0.98	164.28	6.19	
DAMBER	530	m	1	-1.31	24.10	0.59	0.0000
DARBY	508	m	0	-1.22	78.40	0.35	0.0000
DARBY	517	f	0	-2.00	19.12	13.72	0.0000
Subtotal DARBY				-1.37	97.52	14.07	
DEAN3	638	m	1	-1.08	25.48	0.14	0.0000
DEAN3	561	f	1	-2.04	1.86	1.47	0.0054
Subtotal DEAN3				-1.14	27.35	1.61	
DESTEF	532	m	4	-1.35	10.86	0.41	0.0000
DOLL	538	m	0	-1.55	4.84	0.78	0.0006
DOLL	549	f	0	-1.04	0.65	0.01	0.4022
Subtotal DOLL				-1.49	5.49	0.79	
*DOLL2	511	m	1	-1.27	8.43	0.12	0.0002
DORGAN	516	m	0	-1.07	59.40	0.39	0.0000
DORGAN	560	f	0	-1.33	16.18	0.53	0.0000
Subtotal DORGAN				-1.13	75.58	0.92	
*DORN	825	m	0	-1.14	11.74	0.00	0.0001
*DORN	829	m	0	-0.58	27.56	9.02	0.0023
Subtotal DORN				-0.75	39.30	9.02	
GAO	538	m	2	-1.27	7.80	0.11	0.0004
GAO	558	f	2	-0.27	6.07	4.67	0.4991
Subtotal GAO				-0.84	13.86	4.79	
GAO2	520	m	0	-0.57	7.57	2.56	0.1164
GRAHAM	511	m	0	-1.91	1.86	1.08	0.0090
*HAMMO2	512	m	1	-1.08	19.88	0.11	0.0000
*HIRAYA	515	m	1	-1.17	6.04	0.00	0.0040
*HIRAYA	526	f	1	-0.89	0.29	0.02	0.6307
Subtotal HIRAYA				-1.16	6.33	0.02	
JAHN	517	m	0	-0.98	33.47	1.02	0.0000
JAIN	571	m	0	-1.59	24.80	4.65	0.0000
JAIN	535	f	0	-2.29	12.14	15.75	0.0000
Subtotal JAIN				-1.82	36.93	20.40	
*KAISE2	656	m	1	-0.84	6.60	0.63	0.0302
*KAISE2	576	f	1	-1.39	3.65	0.20	0.0081
Subtotal KAISE2				-1.04	10.25	0.83	
LUBIN	594	m	0	-0.67	12.91	3.00	0.0160
LUBIN2	1083	m	0	-0.66	181.12	43.82	0.0000
LUBIN2	1122	f	0	-1.03	6.98	0.11	0.0067
Subtotal LUBIN2				-0.67	188.10	43.93	
MATOS	598	m	2	-1.20	12.73	0.03	0.0000
PEZZO2	505	m	0	-1.48	25.77	2.82	0.0000
PEZZOT	505	m	0	-1.78	13.50	5.40	0.0000
SOBUE	730	m	0	-0.51	19.41	8.04	0.0250
*SPEIZE	514	f	2	-1.61	8.00	1.67	0.0000
SUZUK2	526	c	3	-2.30	8.00	10.58	0.0000
SVENSS	555	f	0	-1.52	7.19	1.00	0.0000
WYNDE6	720	m	5	-1.20	31.98	0.09	0.0000
WYNDE6	727	m	5	-1.61	5.93	1.24	0.0001
WYNDE6	734	f	5	-1.61	93.46	19.53	0.0000
WYNDE6	738	f	5	-0.92	2.67	0.15	0.1342
Subtotal WYNDE6				-1.50	134.05	21.01	

Table 1K4 - 2

IESLC - Meta-analysis of Ex Smoking, Years quit (vs current), "High"
All LC types, Any Product (or Cigarettes if Any not available)
Most adjusted

	N	49
	NS	33
	Wt	1091.01
Het	Chi	199.27
Het	df	48
Het	P	***
Fixed	RR	0.32
	RRl	0.30
	RRu	0.34
	P	---
Random	RR	0.28
	RRl	0.24
	RRu	0.32
	P	---
Asymm	P	*

Table 1K4 - 3

IESLC - Meta-analysis of Ex Smoking, Years quit (vs current), "High"
All LC types, Any Product (or Cigarettes if Any not available)
Most adjusted

	Sex: combined	male	female	Total
N	2	31	16	49
NS	2	29	15	46
Wt	17.55	853.84	219.62	1091.01
Het Chi	0.99	112.70	30.79	199.27
Het df	1	30	15	48
Het P	N.S.	***	**	***
Fixed RR	0.13	0.35	0.22	0.32
RRl	0.08	0.33	0.19	0.30
RRu	0.21	0.38	0.25	0.34
P	---	---	---	---
Random RR	0.13	0.31	0.23	0.28
RRl	0.08	0.27	0.18	0.24
RRu	0.21	0.36	0.29	0.32
P	---	---	---	---
Between Chi				54.79
Between df				2
Between P				***
Btwn(F) P				***
Btwn(R) P				***

	Lung cancer type: all	other	Total
N	49		49
NS	33		33
Wt	1091.01		1091.01
Het Chi	199.27		199.27
Het df	48		48
Het P	***		***
Fixed RR	0.32		0.32
RRl	0.30		0.30
RRu	0.34		0.34
P	---		---
Random RR	0.28		0.28
RRl	0.24		0.24
RRu	0.32		0.32
P	---		---
Between Chi			
Between df			
Between P			N.S.
Btwn(F) P			N.S.
Btwn(R) P			N.S.

	Location: NAmer	UK	Scand	othEur	China	Japan	othAs	other	Total
N	19	9	3	5	3	4	1	5	49
NS	11	5	3	3	2	3	1	5	33
Wt	517.98	171.83	33.92	233.02	26.77	33.32	3.32	70.85	1091.01
Het Chi	88.30	12.96	1.15	9.84	3.58	2.09	0.00	7.35	199.27
Het df	18	8	2	4	2	3	0	4	48
Het P	***	N.S.	N.S.	*	N.S.	N.S.	N.S.	N.S.	***
Fixed RR	0.28	0.27	0.25	0.47	0.47	0.52	0.25	0.21	0.32
RRl	0.26	0.23	0.18	0.41	0.32	0.37	0.08	0.17	0.30
RRu	0.31	0.32	0.34	0.54	0.69	0.74	0.73	0.26	0.34
P	---	---	---	---	---	---	-	---	---
Random RR	0.25	0.26	0.25	0.38	0.47	0.52	0.25	0.20	0.28
RRl	0.20	0.21	0.18	0.28	0.28	0.37	0.08	0.15	0.24
RRu	0.32	0.33	0.34	0.53	0.79	0.74	0.73	0.28	0.32
P	---	---	---	---	--	---	-	---	---
Between Chi									73.99
Between df									7
Between P									***
Btwn(F) P									**
Btwn(R) P									***

Table 1K4 - 3

IESLC - Meta-analysis of Ex Smoking, Years quit (vs current), "High"
All LC types, Any Product (or Cigarettes if Any not available)
Most adjusted

Detailed Country in "other Europe"

	multi	Germany	othWest	East	Balkans	Total
N	2	3				5
NS	1	2				3
Wt	188.10	44.91				233.02
Het Chi	0.90	2.76				9.84
Het df	1	2				4
Het P	N.S.	N.S.				*
Fixed RR	0.51	0.34				0.47
RRl	0.44	0.25				0.41
RRu	0.59	0.45				0.54
P	---	---				---
Random RR	0.51	0.32				0.38
RRl	0.44	0.21				0.28
RRu	0.59	0.48				0.53
P	---	---				---
Between Chi						6.18
Between df						1
Between P						*
Btwn(F) P						N.S.
Btwn(R) P						*

Detailed Country in "other Asia"

	India	HongKong	other	Total
N			1	1
NS			1	1
Wt			3.32	3.32
Het Chi			0.00	0.00
Het df			0	0
Het P			N.S.	N.S.
Fixed RR			0.25	0.25
RRl			0.08	0.08
RRu			0.73	0.73
P			-	-
Random RR			0.25	0.25
RRl			0.08	0.08
RRu			0.73	0.73
P			-	-
Between Chi				
Between df				
Between P				N.S.
Btwn(F) P				N.S.
Btwn(R) P				N.S.

Detailed other continent

	SCAmer	Total
N	5	5
NS	5	5
Wt	70.85	70.85
Het Chi	7.35	7.35
Het df	4	4
Het P	N.S.	N.S.
Fixed RR	0.21	0.21
RRl	0.17	0.17
RRu	0.26	0.26
P	---	---
Random RR	0.20	0.20
RRl	0.15	0.15
RRu	0.28	0.28
P	---	---
Between Chi		
Between df		
Between P		N.S.
Btwn(F) P		N.S.
Btwn(R) P		N.S.

Table 1K4 - 3

IESLC - Meta-analysis of Ex Smoking, Years quit (vs current), "High"
All LC types, Any Product (or Cigarettes if Any not available)
Most adjusted

	Start year of study					
	<1960	1960-69	1970-79	1980-89	1990+	Total
N	7	10	8	20	4	49
NS	5	5	5	14	4	33
Wt	73.38	190.23	263.49	507.87	56.05	1091.01
Het Chi	38.11	12.89	21.69	69.92	6.78	199.27
Het df	6	9	7	19	3	48
Het P	***	N.S.	**	***	(*)	***
Fixed RR	0.27	0.25	0.43	0.32	0.20	0.32
RRl	0.22	0.21	0.38	0.29	0.16	0.30
RRu	0.34	0.28	0.49	0.35	0.26	0.34
P	---	---	---	---	---	---
Random RR	0.24	0.26	0.33	0.29	0.19	0.28
RRl	0.12	0.21	0.24	0.24	0.13	0.24
RRu	0.47	0.33	0.45	0.35	0.29	0.32
P	---	---	---	---	---	---
Between Chi						49.88
Between df						4
Between P						***
Btwn(F) P						*
Btwn(R) P						N.S.

	Study type (1)		
	CC	other	Total
N	36	13	49
NS	24	9	33
Wt	813.62	277.39	1091.01
Het Chi	150.33	47.44	199.27
Het df	35	12	48
Het P	***	***	***
Fixed RR	0.31	0.34	0.32
RRl	0.29	0.30	0.30
RRu	0.33	0.38	0.34
P	---	---	---
Random RR	0.27	0.29	0.28
RRl	0.23	0.21	0.24
RRu	0.32	0.39	0.32
P	---	---	---
Between Chi			1.50
Between df			1
Between P			N.S.
Btwn(F) P			N.S.
Btwn(R) P			N.S.

	Study type (2)			
	CC	prosp	other	Total
N	36	13		49
NS	24	9		33
Wt	813.62	277.39		1091.01
Het Chi	150.33	47.44		199.27
Het df	35	12		48
Het P	***	***		***
Fixed RR	0.31	0.34		0.32
RRl	0.29	0.30		0.30
RRu	0.33	0.38		0.34
P	---	---		---
Random RR	0.27	0.29		0.28
RRl	0.23	0.21		0.24
RRu	0.32	0.39		0.32
P	---	---		---
Between Chi				1.50
Between df				1
Between P				N.S.
Btwn(F) P				N.S.
Btwn(R) P				N.S.

Table 1K4 - 3

IESLC - Meta-analysis of Ex Smoking, Years quit (vs current), "High"
All LC types, Any Product (or Cigarettes if Any not available)
Most adjusted

	Study size (number of LC cases) 100-249	250-499	500-999	1000+	Total
N	6	10	10	23	49
NS	5	9	7	12	33
Wt	52.86	102.74	204.19	731.22	1091.01
Het Chi	8.06	15.54	24.38	114.61	199.27
Het df	5	9	9	22	48
Het P	N.S.	(*)	**	***	***
Fixed RR	0.20	0.30	0.24	0.36	0.32
RRl	0.15	0.24	0.21	0.33	0.30
RRu	0.26	0.36	0.28	0.38	0.34
P	---	---	---	---	---
Random RR	0.20	0.30	0.21	0.32	0.28
RRl	0.14	0.23	0.16	0.27	0.24
RRu	0.28	0.39	0.28	0.39	0.32
P	---	---	---	---	---
Between Chi					36.69
Between df					3
Between P					***
Btwn(F) P					*
Btwn(R) P					*

	Risky occupational population no	mining	othRisky	Total
N	47	1	1	49
NS	31	1	1	33
Wt	1058.22	12.91	19.88	1091.01
Het Chi	196.11	0.00	0.00	199.27
Het df	46	0	0	48
Het P	***	N.S.	N.S.	***
Fixed RR	0.31	0.51	0.34	0.32
RRl	0.30	0.30	0.22	0.30
RRu	0.33	0.88	0.53	0.34
P	---	-	---	---
Random RR	0.27	0.51	0.34	0.28
RRl	0.23	0.30	0.22	0.24
RRu	0.31	0.88	0.53	0.32
P	---	-	---	---
Between Chi				3.16
Between df				2
Between P				N.S.
Btwn(F) P				N.S.
Btwn(R) P				(*)

	National cigarette tobacco type Virginia	blended	other	Total
N	11	35	3	49
NS	6	25	2	33
Wt	208.76	855.48	26.77	1091.01
Het Chi	25.09	152.43	3.58	199.27
Het df	10	34	2	48
Het P	**	***	N.S.	***
Fixed RR	0.25	0.33	0.47	0.32
RRl	0.22	0.31	0.32	0.30
RRu	0.28	0.35	0.69	0.34
P	---	---	---	---
Random RR	0.23	0.28	0.47	0.28
RRl	0.18	0.24	0.28	0.24
RRu	0.29	0.33	0.79	0.32
P	---	---	--	---
Between Chi				18.17
Between df				2
Between P				***
Btwn(F) P				N.S.
Btwn(R) P				*

Table 1K4 - 3

IESLC - Meta-analysis of Ex Smoking, Years quit (vs current), "High"
All LC types, Any Product (or Cigarettes if Any not available)
Most adjusted

	Any proxy use No/nk	Yes	Total
N	41	8	49
NS	28	5	33
Wt	914.14	176.87	1091.01
Het Chi	172.32	24.24	199.27
Het df	40	7	48
Het P	***	**	***
Fixed RR	0.32	0.28	0.32
RRl	0.30	0.24	0.30
RRu	0.34	0.33	0.34
P	---	---	---
Random RR	0.28	0.26	0.28
RRl	0.24	0.19	0.24
RRu	0.33	0.35	0.32
P	---	---	---
Between Chi			2.71
Between df			1
Between P			(*)
Btwn(F) P			N.S.
Btwn(R) P			N.S.

	Full histological confirmation No	Yes	Total
N	33	16	49
NS	23	10	33
Wt	596.30	494.70	1091.01
Het Chi	101.69	95.64	199.27
Het df	32	15	48
Het P	***	***	***
Fixed RR	0.30	0.33	0.32
RRl	0.28	0.30	0.30
RRu	0.33	0.36	0.34
P	---	---	---
Random RR	0.28	0.27	0.28
RRl	0.24	0.21	0.24
RRu	0.33	0.35	0.32
P	---	---	---
Between Chi			1.94
Between df			1
Between P			N.S.
Btwn(F) P			N.S.
Btwn(R) P			N.S.

	Number of adjustment variables (1) 0	1	2+/+nk	Total
N	24	15	10	49
NS	17	10	6	33
Wt	588.93	314.58	187.49	1091.01
Het Chi	114.86	40.29	20.73	199.27
Het df	23	14	9	48
Het P	***	***	*	***
Fixed RR	0.35	0.32	0.23	0.32
RRl	0.32	0.28	0.20	0.30
RRu	0.38	0.35	0.27	0.34
P	---	---	---	---
Random RR	0.28	0.28	0.25	0.28
RRl	0.23	0.22	0.19	0.24
RRu	0.35	0.35	0.33	0.32
P	---	---	---	---
Between Chi				23.38
Between df				2
Between P				***
Btwn(F) P				(*)
Btwn(R) P				N.S.

Table 1K4 - 3

IESLC - Meta-analysis of Ex Smoking, Years quit (vs current), "High"
All LC types, Any Product (or Cigarettes if Any not available)
Most adjusted

	Number of adjustment variables (2)					
	0	1	2	3-5	6+/+nk	Total
N	24	15	4	6		49
NS	17	10	3	3		33
Wt	588.93	314.58	34.59	152.91		1091.01
Het Chi	114.86	40.29	6.49	10.17		199.27
Het df	23	14	3	5		48
Het P	***	***	(*)	(*)		***
Fixed RR	0.35	0.32	0.32	0.22		0.32
RRl	0.32	0.28	0.23	0.18		0.30
RRu	0.38	0.35	0.44	0.25		0.34
P	---	---	---	---		---
Random RR	0.28	0.28	0.33	0.22		0.28
RRl	0.23	0.22	0.20	0.16		0.24
RRu	0.35	0.35	0.54	0.29		0.32
P	---	---	---	---		---
Between Chi						27.46
Between df						3
Between P						***
Btwn(F) P						(*)
Btwn(R) P						N.S.

	Product			
	all/unsp	cig+/-ot	cig only	Total
N	13	27	9	49
NS	9	18	7	34
Wt	194.56	671.77	224.68	1091.01
Het Chi	21.89	119.99	42.58	199.27
Het df	12	26	8	48
Het P	*	***	***	***
Fixed RR	0.25	0.34	0.31	0.32
RRl	0.22	0.32	0.27	0.30
RRu	0.29	0.37	0.35	0.34
P	---	---	---	---
Random RR	0.22	0.31	0.24	0.28
RRl	0.18	0.26	0.16	0.24
RRu	0.28	0.38	0.36	0.32
P	---	---	---	---
Between Chi				14.81
Between df				2
Between P				***
Btwn(F) P				N.S.
Btwn(R) P				(*)

	Denominator		
	current	cur+rec	Total
N	28	21	49
NS	20	13	33
Wt	696.32	394.69	1091.01
Het Chi	123.11	57.83	199.27
Het df	27	20	48
Het P	***	***	***
Fixed RR	0.35	0.27	0.32
RRl	0.32	0.24	0.30
RRu	0.38	0.29	0.34
P	---	---	---
Random RR	0.28	0.27	0.28
RRl	0.23	0.23	0.24
RRu	0.33	0.33	0.32
P	---	---	---
Between Chi			18.33
Between df			1
Between P			***
Btwn(F) P			*
Btwn(R) P			N.S.

Table 1K4 - 3

IESLC - Meta-analysis of Ex Smoking, Years quit (vs current), "High"
All LC types, Any Product (or Cigarettes if Any not available)
Most adjusted

	Derivation of RR/CI			
	Orig	StdCalc	Other	Total
N	7	25	17	49
NS	4	18	12	34
Wt	162.77	592.58	335.65	1091.01
Het Chi	11.20	115.26	45.26	199.27
Het df	6	24	16	48
Het P	(*)	***	***	***
Fixed RR	0.22	0.35	0.32	0.32
RRl	0.19	0.32	0.29	0.30
RRu	0.25	0.38	0.36	0.34
P	---	---	---	---
Random RR	0.22	0.28	0.29	0.28
RRl	0.17	0.23	0.23	0.24
RRu	0.29	0.35	0.36	0.32
P	---	---	---	---
Between Chi				27.55
Between df				2
Between P				***
Btwn(F) P				*
Btwn(R) P				N.S.

Table 1K4 - 4

IESLC - Meta-analysis of Ex Smoking, Years quit (vs current), "High"
All LC types, Any Product (or Cigarettes if Any not available)
Least adjusted

REF	NRR	X	SEX	AGEL	AGEH	RACE	YF	LC TYPE	LOC	START	ST	NLC	R	VB	P	H	AD	ADOS	PRODUCT	exL	exH	DENOM	De
ALDERS	515		m	0	0	all	-	all	Eu:UK	1977	CC	1448	n	V	n	n	1	0	cig only	10	999	current	ot
ALDERS	526		f	0	0	all	-	all	Eu:UK	1977	CC	1448	n	V	n	n	1	0	cig only	10	999	current	ot
BECHER	508		m	0	0	all	-	all	Eu:Ger	1985	CC	194	n	bl	n	y	0	0	all/unsp	10	999	cur+1y	st
BECHER	518		f	0	0	all	-	all	Eu:Ger	1985	CC	194	n	bl	n	y	0	0	all/unsp	10	999	cur+1y	st
CARPEN	510		c	0	0	w+b	-	all	NAmer	1991	CC	356	n	bl	n	n	0	0	cig+/-ot	10	14	current	st
CEDERL	539		m	40	69	all	10	all	Eu:Sca	1963	pr	491	n	bl	n	n	1	0	all/unsp	10	999	current	ot
CHOI	545		m	0	0	all	-	all	As:oth	1985	CC	375	n	bl	n	n	0	0	cig+/-ot	10	14	current	st
CPSI	818		m	50	74	all	6	all	NAmer	1959	pr	5138	n	bl	n	n	1	0	cig only	10	999	current	ot
CPSII	668		m	35	99	all	4	all	NAmer	1982	pr	3229	n	bl	n	n	1	0	cig only	11	15	current	ot
CPSII	645		f	0	0	all	4	all	NAmer	1982	pr	3229	n	bl	n	n	1	0	cig+/-ot	11	15	current	ot
DAMBER	530		m	0	0	all	-	all	Eu:Sca	1972	CC	579	n	bl	y	n	1	0	all/unsp	11	999	current	ot
DARBY	508		m	0	0	wh	-	all	Eu:UK	1988	CC	982	n	V	n	n	0	0	all/unsp	10	999	current	st
DARBY	517		f	0	0	wh	-	all	Eu:UK	1988	CC	982	n	V	n	n	0	0	all/unsp	10	999	current	st
DEAN3	539	x	m	0	0	all	-	all	Eu:UK	1969	CC	766	n	V	y	n	0	0	all/unsp	9	999	cur+2y	st
DEAN3	550	x	f	0	0	all	-	all	Eu:UK	1969	CC	766	n	V	y	n	0	0	all/unsp	9	999	cur+2y	st
DESTEF	522	x	m	0	0	all	-	all	SCAmer	1988	CC	497	n	bl	n	y	0	0	all/unsp	10	999	current	st
DOLL	538		m	0	0	all	-	all	Eu:UK	1948	CC	1465	n	V	n	n	0	0	all/unsp	10	19	current	st
DOLL	549		f	0	0	all	-	all	Eu:UK	1948	CC	1465	n	V	n	n	0	0	all/unsp	10	999	current	st
DOLL2	511		m	0	0	all	20	all	Eu:UK	1951	pr	920	n	V	n	n	1	0	cig only	10	14	current	ot
DORGAN	516		m	0	0	wh	-	all	NAmer	1980	CC	2026	n	bl	y	y	0	0	cig+/-ot	10	999	cur+1y	st
DORGAN	560		f	0	0	all	-	all	NAmer	1980	CC	2026	n	bl	y	y	0	0	cig+/-ot	10	999	cur+1y	st
DORN	825		m	55	64	wh	8	all	NAmer	1954	pr	5097	n	bl	n	n	0	0	cig+/-ot	10	14	current	st
DORN	829		m	65	74	wh	8	all	NAmer	1954	pr	5097	n	bl	n	n	0	0	cig+/-ot	10	14	current	st
GAO	528	x	m	0	0	all	-	all	As:Chi	1984	CC	1405	n	ot	n	n	0	0	cig+/-ot	10	999	current	st
GAO	548	x	f	0	0	all	-	all	As:Chi	1984	CC	1405	n	ot	n	n	0	0	cig+/-ot	10	999	current	st
GAO2	520		m	0	0	all	-	all	As:Jap	1988	CC	282	n	bl	n	n	0	0	cig+/-ot	10	14	cur+1y	st
GRAHAM	511		m	0	0	wh	-	all	NAmer	1956	CC	685	n	bl	n	n	0	0	cig only	10	999	current	st
HAMMO2	512		m	0	0	all	0	all	NAmer	1967	pr	450	o	bl	n	n	1	0	cig+/-ot	10	999	current	ot
HIRAYA	515		m	0	0	all	0	all	As:Jap	1965	pr	1917	n	bl	n	n	1	0	cig+/-ot	10	999	current	ot
HIRAYA	526		f	0	0	all	0	all	As:Jap	1965	pr	1917	n	bl	n	n	1	0	cig+/-ot	10	999	current	ot
JAHN	517		m	0	0	all	-	all	Eu:Ger	1988	CC	1004	n	bl	n	n	0	0	cig+/-ot	11	20	current	st
JAIN	571		m	0	0	all	-	all	NAmer	1981	CC	845	n	V	y	n	0	0	cig+/-ot	10	999	cur+2y	st
JAIN	535		f	0	0	all	-	all	NAmer	1981	CC	845	n	V	y	n	0	0	cig+/-ot	10	999	cur+2y	st
KAISE2	656		m	0	0	all	9	all	NAmer	1979	pr	318	n	bl	n	n	1	0	cig only	11	20	cur+2y	ot
KAISE2	576		f	0	0	all	9	all	NAmer	1979	pr	318	n	bl	n	n	1	0	cig only	11	20	cur+2y	st
LUBIN	594		m	0	0	all	-	all	As:Chi	1984	CC	427	m	ot	y	n	0	0	cig+/-ot	10	999	cur+2y	st
LUBIN2	1083		m	0	0	all	-	all	Eu:mul	1976	CC	7804	n	bl	n	y	0	0	cig+/-ot	10	14	current	st
LUBIN2	1122		f	0	0	all	-	all	Eu:mul	1976	CC	7804	n	bl	n	y	0	0	cig+/-ot	10	14	current	st
MATOS	588	x	m	0	0	all	-	all	SCAmer	1994	CC	200	n	bl	n	n	0	0	cig+/-ot	11	999	cur+1y	st
PEZZO2	505		m	0	0	all	-	all	SCAmer	1992	CC	367	n	bl	n	y	0	0	cig+/-ot	11	999	cur+1y	st
PEZZOT	505		m	0	0	all	-	all	SCAmer	1987	CC	215	n	bl	n	y	0	0	cig only	11	999	cur+1y	st
SOBUE	730		m	0	0	all	-	all	As:Jap	1986	CC	1376	n	bl	n	y	0	0	cig+/-ot	10	14	cur+1y	st
SPEIZE	514		f	0	0	all	0	all	NAmer	1976	pr	593	n	bl	n	y	2	0	cig+/-ot	10	15	current	or
SUZUK2	515	x	c	0	0	all	-	all	SCAmer	1991	CC	123	n	bl	n	y	0	0	all/unsp	11	999	current	st
SVENSS	555		f	0	0	all	-	all	Eu:Sca	1983	CC	210	n	bl	n	n	0	0	all/unsp	11	999	cur+2y	st
WYNDE6	720		m	0	0	wh	-	all	NAmer	1969	CC	4423	n	bl	n	y	5	1#	cig+/-ot	11	19	cur+1y	or
WYNDE6	727		m	0	0	bl	-	all	NAmer	1969	CC	4423	n	bl	n	y	5	1#	cig+/-ot	11	19	cur+1y	or
WYNDE6	734		f	0	0	wh	-	all	NAmer	1969	CC	4423	n	bl	n	y	5	1#	cig+/-ot	11	999	cur+1y	or
WYNDE6	738		f	0	0	bl	-	all	NAmer	1969	CC	4423	n	bl	n	y	5	1#	cig+/-ot	11	999	cur+1y	or

Comments on values in listings

WYNDE6 ADOS Number of cigs/day
WYNDE6 ADOS Number of cigs/day
WYNDE6 ADOS Number of cigs/day
WYNDE6 ADOS Number of cigs/day

Cigarette type is all/unspec for all RRs
except for the following:

REF	NRR	CIGTYPE
ALDERS	515	MC only
ALDERS	526	MC only

Table 1K4 - 5

IESLC - Meta-analysis of Ex Smoking, Years quit (vs current), "High"
All LC types, Any Product (or Cigarettes if Any not available)
Least adjusted

REF	NRR	SEX	AD	Number Exposed Case	Cont	Non-exposed Case	Cont	RR	95.00%CI
ALDERS	515	m	1	29	-	207	-	0.32	(0.20- 0.51)
ALDERS	526	f	1	26	-	244	-	0.28	(0.17- 0.46)
Subtotal ALDERS								0.30	(0.21- 0.42)
BECHER	508	m	0	16	72	101	122	0.27	(0.15- 0.49)
BECHER	518	f	0	1	10	33	26	0.08	(0.01- 0.66)
Subtotal BECHER								0.24	(0.14- 0.44)
CARPEN	510	c	0	13	58	228	164	0.16	(0.09- 0.30)
*CEDERL	539	m	1	3	-	97	-	0.14	(0.04- 0.45)
CHOI	545	m	0	4	23	231	329	0.25	(0.08- 0.73)
*CPSI	818	m	1	15	-	844	-	0.09	(0.06- 0.15)
*CPSII	668	m	1	164	-	1159	-	0.39	(0.33- 0.46)
*CPSII	645	f	1	28	-	530	-	0.31	(0.21- 0.46)
Subtotal CPSII								0.38	(0.32- 0.44)
DAMBER	530	m	1	42	-	-	-	0.27	(0.18- 0.40)
DARBY	508	m	0	139	767	379	618	0.30	(0.24- 0.37)
DARBY	517	f	0	26	224	198	231	0.14	(0.09- 0.21)
Subtotal DARBY								0.25	(0.21- 0.31)
DEAN3	539	m	0	32	204	502	1636	0.51	(0.35- 0.75)
DEAN3	550	f	0	2	114	102	1158	0.20	(0.05- 0.82)
Subtotal DEAN3								0.48	(0.33- 0.69)
DESTEF	522	m	0	17	36	362	226	0.29	(0.16- 0.54)
DOLL	538	m	0	6	26	1280	1172	0.21	(0.09- 0.52)
DOLL	549	f	0	1	2	58	41	0.35	(0.03- 4.03)
Subtotal DOLL								0.22	(0.10- 0.52)
*DOLL2	511	m	1	9	-	236	-	0.28	(0.14- 0.54)
DORGAN	516	m	0	134	255	465	303	0.34	(0.27- 0.44)
DORGAN	560	f	0	34	50	289	112	0.26	(0.16- 0.43)
Subtotal DORGAN								0.32	(0.26- 0.41)
*DORN	825	m	0	12	23682	528	334175	0.32	(0.18- 0.57)
*DORN	829	m	0	29	20056	537	207895	0.56	(0.39- 0.81)
Subtotal DORN								0.47	(0.35- 0.65)
GAO	528	m	0	13	41	529	438	0.26	(0.14- 0.50)
GAO	548	f	0	16	14	170	100	0.67	(0.31- 1.44)
Subtotal GAO								0.39	(0.24- 0.63)
GAO2	520	m	0	16	18	184	117	0.57	(0.28- 1.15)
GRAHAM	511	m	0	2	30	371	821	0.15	(0.04- 0.62)
*HAMMO2	512	m	1	20	-	209	-	0.34	(0.22- 0.53)
*HIRAYA	515	m	1	-	-	-	-	0.31	(0.14- 0.69)
*HIRAYA	526	f	1	-	-	-	-	0.41	(0.01- 14.37)
Subtotal HIRAYA								0.31	(0.14- 0.68)
JAHN	517	m	0	64	130	352	269	0.38	(0.27- 0.53)
JAIN	571	m	0	52	113	265	118	0.20	(0.14- 0.30)
JAIN	535	f	0	19	61	305	99	0.10	(0.06- 0.18)
Subtotal JAIN								0.16	(0.12- 0.22)
*KAISE2	656	m	1	8	-	51	-	0.43	(0.20- 0.92)
*KAISE2	576	f	1	4	-	50	-	0.25	(0.09- 0.70)
Subtotal KAISE2								0.35	(0.19- 0.65)
LUBIN	594	m	0	17	73	296	650	0.51	(0.30- 0.88)
LUBIN2	1083	m	0	270	693	4684	6211	0.52	(0.45- 0.60)
LUBIN2	1122	f	0	10	26	440	410	0.36	(0.17- 0.75)
Subtotal LUBIN2								0.51	(0.44- 0.59)
MATOS	588	m	0	27	101	112	132	0.32	(0.19- 0.52)
PEZZO2	505	m	0	43	161	233	198	0.23	(0.15- 0.33)
PEZZOT	505	m	0	20	106	145	129	0.17	(0.10- 0.29)
SOBUE	730	m	0	35	50	737	633	0.60	(0.39- 0.94)
*SPEIZE	514	f	2	17	-	319	-	0.20	(0.10- 0.40)
SUZUK2	515	c	0	9	22	77	30	0.16	(0.07- 0.39)
SVENSS	555	f	0	14	24	142	53	0.22	(0.10- 0.45)
WYNDE6	720	m	5	-	-	-	-	0.30	(0.20- 0.40)
WYNDE6	727	m	5	-	-	-	-	0.20	(0.10- 0.50)
WYNDE6	734	f	5	-	-	-	-	0.20	(0.20- 0.30)
WYNDE6	738	f	5	-	-	-	-	0.40	(0.10- 1.10)
Subtotal WYNDE6								0.22	(0.19- 0.26)
Partial Totals				1458	47242	18281	558616		

*prospective study

Table 1K4 - 5

IESLC - Meta-analysis of Ex Smoking, Years quit (vs current), "High"
All LC types, Any Product (or Cigarettes if Any not available)
Least adjusted

REF	NRR	SEX	AD	Ys	Ws	Qs	Ps
ALDERS	515	m	1	-1.14	17.54	0.00	0.0000
ALDERS	526	f	1	-1.27	15.51	0.29	0.0000
Subtotal ALDERS				-1.20	33.04	0.29	
BECHER	508	m	0	-1.32	10.58	0.34	0.0000
BECHER	518	f	0	-2.54	0.86	1.69	0.0188
Subtotal BECHER				-1.41	11.44	2.03	
CARPEN	510	c	0	-1.82	9.56	4.54	0.0000
*CEDERL	539	m	1	-1.97	2.62	1.81	0.0015
CHOI	545	m	0	-1.40	3.32	0.22	0.0109
*CPSI	818	m	1	-2.41	18.30	29.62	0.0000
*CPSII	668	m	1	-0.94	139.29	5.25	0.0000
*CPSII	645	f	1	-1.17	24.99	0.03	0.0000
Subtotal CPSII				-0.98	164.28	5.28	
DAMBER	530	m	1	-1.31	24.10	0.73	0.0000
DARBY	508	m	0	-1.22	78.40	0.54	0.0000
DARBY	517	f	0	-2.00	19.12	14.26	0.0000
Subtotal DARBY				-1.37	97.52	14.80	
DEAN3	539	m	0	-0.67	25.80	5.58	0.0007
DEAN3	550	f	0	-1.61	1.93	0.44	0.0252
Subtotal DEAN3				-0.74	27.73	6.01	
DESTEF	522	m	0	-1.22	10.66	0.08	0.0001
DOLL	538	m	0	-1.55	4.84	0.85	0.0006
DOLL	549	f	0	-1.04	0.65	0.01	0.4022
Subtotal DOLL				-1.49	5.49	0.85	
*DOLL2	511	m	1	-1.27	8.43	0.16	0.0002
DORGAN	516	m	0	-1.07	59.40	0.24	0.0000
DORGAN	560	f	0	-1.33	16.18	0.63	0.0000
Subtotal DORGAN				-1.13	75.58	0.88	
*DORN	825	m	0	-1.14	11.74	0.00	0.0001
*DORN	829	m	0	-0.58	27.56	8.51	0.0023
Subtotal DORN				-0.75	39.30	8.51	
GAO	528	m	0	-1.34	9.48	0.39	0.0000
GAO	548	f	0	-0.40	6.68	3.64	0.3049
Subtotal GAO				-0.95	16.15	4.03	
GAO2	520	m	0	-0.57	7.57	2.42	0.1164
GRAHAM	511	m	0	-1.91	1.86	1.13	0.0090
*HAMMO2	512	m	1	-1.08	19.88	0.06	0.0000
*HIRAYA	515	m	1	-1.17	6.04	0.01	0.0040
*HIRAYA	526	f	1	-0.89	0.29	0.02	0.6307
Subtotal HIRAYA				-1.16	6.33	0.02	
JAHN	517	m	0	-0.98	33.47	0.84	0.0000
JAIN	571	m	0	-1.59	24.80	5.01	0.0000
JAIN	535	f	0	-2.29	12.14	16.21	0.0000
Subtotal JAIN				-1.82	36.93	21.22	
*KAISE2	656	m	1	-0.84	6.60	0.56	0.0302
*KAISE2	576	f	1	-1.39	3.65	0.23	0.0081
Subtotal KAISE2				-1.04	10.25	0.79	
LUBIN	594	m	0	-0.67	12.91	2.79	0.0160
LUBIN2	1083	m	0	-0.66	181.12	40.93	0.0000
LUBIN2	1122	f	0	-1.03	6.98	0.08	0.0067
Subtotal LUBIN2				-0.67	188.10	41.01	
MATOS	588	m	0	-1.15	15.76	0.01	0.0000
PEZZO2	505	m	0	-1.48	25.77	3.11	0.0000
PEZZOT	505	m	0	-1.78	13.50	5.68	0.0000
SOBUE	730	m	0	-0.51	19.41	7.63	0.0250
*SPEIZE	514	f	2	-1.61	8.00	1.79	0.0000
SUZUK2	515	c	0	-1.84	4.93	2.42	0.0000
SVENSS	555	f	0	-1.52	7.19	1.09	0.0000
WYNDE6	720	m	5	-1.20	31.98	0.15	0.0000
WYNDE6	727	m	5	-1.61	5.93	1.33	0.0001
WYNDE6	734	f	5	-1.61	93.46	20.97	0.0000
WYNDE6	738	f	5	-0.92	2.67	0.13	0.1342
Subtotal WYNDE6				-1.50	134.05	22.57	

Table 1K4 - 5

IESLC - Meta-analysis of Ex Smoking, Years quit (vs current), "High"
All LC types, Any Product (or Cigarettes if Any not available)
Least adjusted

	N	49
	NS	33
	Wt	1093.45
Het	Chi	194.43
Het	df	48
Het	P	***
Fixed	RR	0.32
	RRl	0.30
	RRu	0.34
	P	---
Random	RR	0.28
	RRl	0.25
	RRu	0.32
	P	---
Asymm	P	*

Table 1K4 - 6

IESLC - Meta-analysis of Ex Smoking, Years quit (vs current), "High"
All LC types, Any Product (or Cigarettes if Any not available)
Least adjusted

		Sex			
		combined	male	female	Total
	N	2	31	16	49
	NS	2	29	15	46
	Wt	14.48	858.68	220.29	1093.45
Het	Chi	0.00	115.62	29.31	194.43
Het	df	1	30	15	48
Het	P	N.S.	***	*	***
Fixed	RR	0.16	0.36	0.22	0.32
	RRl	0.10	0.34	0.19	0.30
	RRu	0.27	0.38	0.25	0.34
	P	---	---	---	---
Random	RR	0.16	0.31	0.23	0.28
	RRl	0.10	0.27	0.18	0.25
	RRu	0.27	0.36	0.29	0.32
	P	---	---	---	---
Between	Chi				49.50
Between	df				2
Between	P				***
Btwn(F)	P				**
Btwn(R)	P				*

Table 1K4 - 7

IESLC - Meta-analysis of Ex Smoking, Years quit (vs current), "High"
All LC types, Any Product (or Cigarettes if Any not available)
Excluded studies (and stage at which they were excluded)

1	AGUDO AKIBA AMANDU AMES AXELSS BEST BOUCHA BOUCOT BRESLO CHEN CHEN2 CHIAZZ DEAN2 DOSEME ENGELA FAN GENG GER GUO HAENSZ HEGMAN HOLE HU HU2 JUSSAW KATSOU KAUFMA KOO KOULUM KREUZE LETOUR LEVIN LIAW LIU3 LIU4 LIU5 MCCONN MIGRAN MRFITR NOTAN2 OSANN2 PERNU QIAO2 RACHTA RESTRE SADOWS SEGI2 STASZE TIZZAN VUTUC WATSON WU WUWILL WYNDE2 WYNDE8 XU YUAN ZHANG ZHENG ZHOU
2	AUVINE BENSHL BLOT1 BROWN3 BUFFLE GURSEL LAUSSM LUO MCDUFF PISANI PRESCO SPITZ WU2 WYNDE7
4	HAMMON
5	CORREA GILLIS HUMBLE QIAO WIGLE
7	BOFFET
10	GARSHI JEDRYC WAKAI
14	ARMADA BARBON BROSS CHYOU GARCIA JOLY KHUDER TVERDA WANG2 WYNDE3
15	BENHAM

Table 1K4 - 8
Potentially overlapping studies

REF	REFGP	PRINC	. OVERLAP/LINK
LUBIN2	LUBIN2	1	Lubin-combined
GRAHAM	BYERS1	1	GRAHAM/BROSS/BYERS1
WYNDE6	WYNDE6	1	WYNDE5/6/7/8
CPSI	CPSI	1	CPSI overall
JAHN	BOFFET	2	Subset of BOFFET
LUBIN	XIANGZ	2	LUBIN/XIANGZ/QIAO

Table 1K4 - 9
Most adjusted - insufficient data for meta-analysis

REF	NRR	SEX	AGEL	AGEH	RACE	YF	LC TYPE	LOC	START	ST	NLC	R	VB	P	H	AD	ADOS	PRODUCT	exL	exH	DENOM	De
HUMBLE	561	c	25	64	wh	-	not alv	NAmer	1980	CC	521	n	bl	y	n	2		2#cig+/-ot	11	20	cur+1y	or
HUMBLE	556	c	65	84	wh	-	not alv	NAmer	1980	CC	521	n	bl	y	n	2		2#cig+/-ot	11	20	cur+1y	or
WIGLE	503	m	0	0	all	-	all	NAmer	1971	CC	728	n	V	n	n	2		1#cig+/-ot	10	14	current	ot
WIGLE	507	f	0	0	all	-	all	NAmer	1971	CC	728	n	V	n	n	2		1#cig+/-ot	10	14	current	ot

Comments on values in listings

HUMBLE	ADOS	Number of cigarettes and duration
HUMBLE	ADOS	Number of cigarettes and duration
WIGLE	ADOS	Cumulative exposure
WIGLE	ADOS	Cumulative exposure

REF	NRR	RR	SIG	RRDATA comment
HUMBLE	561	0.06		0
HUMBLE	556	0.29		0
WIGLE	503	0.70	n	0
WIGLE	507	0.50	n	0

Table 1K5 -

IESLC - Meta-analysis of Ex Smoking, Years quit (vs current), "Highest vs lowest"
All LC types, Any Product (or Cigarettes if Any not available)

This analysis is restricted to results for:
1) Ex smokers
2) Results by Years quit (vs current)
3) Categorical results by Years quit (vs current)
4) Denominator (unexposed) = "low"
5) All LC types (or near equivalent)
6) Results complete enough for use in metaanalysis

Within each study, results are then selected (in the following order of preference, within each sex) for:
7) (not applicable)
8) PRODUCT: all/unspec, cigarettes regardless of other products, cigarettes only
9) CIGTYPE: all/unspecified, MC regardless of HR, MC only
10) Results with least adjustment for other aspects of smoking (ADOS)
11) The highest vs lowest category
12) Followup period (YF, prospective studies): whole study (coded as 0) or longest available
13) LCtype: all or nearest available, at least Squamous and Adeno. (q = squamous, s = small,
l = large, a = adeno, mix = mixed, alv = alveolar)
14) Race: all or nearest available, otherwise by race (wh or w = white, bl or b = black, hi = hispanic
ch = chinese, jap = japanese, haw = hawaiian, w+o = white + oriental, sca = scandinavian, as = asian)
15) For overlapping studies: principal rather than subsidiary studies
Finally by Age: whole study (coded as 0) if available, otherwise by widest available age group
and then for single sex results (m, f) in preference to results for both sexes combined (c).

Results adjusted (AD) for the most potential confounders are then chosen in Sections -1 to -3
and results adjusted for the least confounders in Sections -4 to -6. (Those least adjusted results which
actually differ from the most adjusted are marked 'x' in column X in Section -4)

Section -7 shows excluded studies, together with the stage (as above) at which no qualifying
results were found.

Section -8 lists the potentially overlapping studies which have been included (1=principal, 2=subsidiary).

Section -9 lists any results which would have been included in preference except that they had data not complete
enough for use in meta-analysis, with their significance (yes/no), if known, and any further comment as entered
on the database. It also lists as "gap" any categories for which no data were presented by the original authors.

In addition to those mentioned above, the following fields, levels and abbreviations are used:
* or nk = not known, n = no, y = yes, ot = other
all/unspec = all or unspecified, cig+/-ot = cigarettes irrespective of other products (cigar, pipe etc)
MC = manufactured cigarettes, HR = hand-rolled cigarettes
exL, exH = range of exposure (low and high) in the "highest" group, in terms of Years quit (vs current)
unexL, unexH = range of exposure (low and high) in the "lowest" group, in terms of Years quit (vs current)
REF: 6-character study reference
NRR: number of the RR on the database within the study
ST : study type (CC = case control, pr or prosp = prospective)
NLC: number of lung cancer cases in whole study
R : risky occupational population (n = no, m = mining, o = other risky)
VB : national cigarette type (V = at least 75% Virginia, bl = at least 75% blended, ot = other)
P : any proxy use
H : full histological confirmation
De : derivation of RR/CI (or = original, st = standard method, ot = other method of estimation)

Table 1K5 - 1

IESLC - Meta-analysis of Ex Smoking, Years quit (vs current), "Highest vs lowest"
All LC types, Any Product (or Cigarettes if Any not available)
Most adjusted

REF	NRR	SEX	AGEL	AGEH	RACE	YF	LC TYPE	LOC	START	ST	NLC	R	VB	P	H	AD	ADOS	PRODUCT	exL	exH	unexL	unexH	De
ALDERS	517	m	0	0	all	-	all	Eu:UK	1977	CC	1448	n	V	n	n	1	0	cig only	10	999	0.1	2	ot
ALDERS	528	f	0	0	all	-	all	Eu:UK	1977	CC	1448	n	V	n	n	1	0	cig only	10	999	0.1	2	ot
ARMADA	520	m	0	0	all	-	all	Eu:wst	1986	CC	325	n	bl	n	y	0	0	cig+/-ot	6	999	1.0	5	st
BARBON	554	m	0	0	all	-	all	Eu:wst	1979	CC	755	n	bl	y	y	1	0	all/unsp	25	999	0.1	4	ot
BECHER	510	m	0	0	all	-	all	Eu:Ger	1985	CC	194	n	bl	n	y	0	0	all/unsp	10	999	2	4	st
BECHER	520	f	0	0	all	-	all	Eu:Ger	1985	CC	194	n	bl	n	y	0	0	all/unsp	10	999	2	4	st
BROSS	520	m	0	0	wh	-	all	NAmer	1960	CC	974	n	bl	n	n	0	0	cig+/-ot	6	999	0.1	5	st
CARPEN	514	c	0	0	w+b	-	all	NAmer	1991	CC	356	n	bl	n	n	0	0	cig+/-ot	15	999	0.1	4	st
CEDERL	540	m	40	69	all	10	all	Eu:Sca	1963	pr	491	n	bl	n	n	1	0	all/unsp	10	999	0.1	9	ot
CHOI	549	m	0	0	all	-	all	As:oth	1985	CC	375	n	bl	n	n	0	0	cig+/-ot	15	999	0.1	4	st
CHOI	558	f	0	0	all	-	all	As:oth	1985	CC	375	n	bl	n	n	0	0	cig+/-ot	5	999	0.1	4	ot
CHYOU	512	m	0	0	jap	21	all	NAmer	1965	pr	227	n	bl	n	y	2	0	cig+/-ot	15	999	0.1	14	ot
CPSI	821	m	50	74	all	6	all	NAmer	1959	pr	5138	n	bl	n	n	1	0	cig only	10	999	0.1	0.9	ot
CPSII	674	m	35	99	all	4	all	NAmer	1982	pr	3229	n	bl	n	n	1	0	cig only	16	999	0.1	0.9	ot
CPSII	650	f	0	0	all	4	all	NAmer	1982	pr	3229	n	bl	n	n	1	0	cig+/-ot	16	999	0.1	2	ot
DAMBER	532	m	0	0	all	-	all	Eu:Sca	1972	CC	579	n	bl	y	n	1	0	all/unsp	11	999	0.1	5	ot
DARBY	506	m	0	0	wh	-	all	Eu:UK	1988	CC	982	n	V	n	n	0	0	all/unsp	10	999	0.1	9	st
DARBY	515	f	0	0	wh	-	all	Eu:UK	1988	CC	982	n	V	n	n	0	0	all/unsp	10	999	0.1	9	st
DEAN3	640	m	0	0	all	-	all	Eu:UK	1969	CC	766	n	V	y	n	1	0	all/unsp	9	999	3	4	ot
DEAN3	563	f	0	0	all	-	all	Eu:UK	1969	CC	766	n	V	y	n	1	0	all/unsp	9	999	3	4	ot
DESTEF	534	m	0	0	all	-	all	SCAmer	1988	CC	497	n	bl	n	y	4	0	all/unsp	10	999	0.1	4	ot
DOLL	541	m	0	0	all	-	all	Eu:UK	1948	CC	1465	n	V	n	n	0	0	all/unsp	20	999	0.1	9	st
DOLL	550	f	0	0	all	-	all	Eu:UK	1948	CC	1465	n	V	n	n	0	0	all/unsp	10	999	0.1	9	st
DOLL2	515	m	0	0	all	20	all	Eu:UK	1951	pr	920	n	V	n	n	1	0	cig only	15	999	0.1	4	ot
DORGAN	518	m	0	0	wh	-	all	NAmer	1980	CC	2026	n	bl	y	y	0	0	cig+/-ot	10	999	1	5	st
DORGAN	561	f	0	0	all	-	all	NAmer	1980	CC	2026	n	bl	y	y	0	0	cig+/-ot	10	999	1	9	st
DORN	667	m	55	64	wh	8	all	NAmer	1954	pr	5097	n	bl	n	n	0	0	cig+/-ot	15	999	0.1	4	st
DORN	690	m	65	74	wh	8	all	NAmer	1954	pr	5097	n	bl	n	n	0	0	cig+/-ot	15	999	0.1	4	st
GAO	540	m	0	0	all	-	all	As:Chi	1984	CC	1405	n	ot	n	n	2	0	cig+/-ot	10	999	0.1	4	ot
GAO	560	f	0	0	all	-	all	As:Chi	1984	CC	1405	n	ot	n	n	2	0	cig+/-ot	10	999	0.1	4	ot
GAO2	526	m	0	0	all	-	all	As:Jap	1988	CC	282	n	bl	n	n	0	0	cig+/-ot	20	999	1.0	4	st
GARCIA	514	c	0	0	all	-	all	NAmer	1992	CC	416	n	bl	n	y	0	0	cig+/-ot	30	999	1.0	4	st
GRAHAM	544	m	0	0	wh	-	all	NAmer	1956	CC	685	n	bl	n	n	1	0	cig+/-ot	5	999	0.1	1.0	ot
HAMMO2	514	m	0	0	all	0	all	NAmer	1967	pr	450	o	bl	n	n	1	0	cig+/-ot	10	999	0.1	4	ot
HIRAYA	517	m	0	0	all	0	all	As:Jap	1965	pr	1917	n	bl	n	n	1	0	cig+/-ot	10	999	0.1	4	ot
HIRAYA	528	f	0	0	all	0	all	As:Jap	1965	pr	1917	n	bl	n	n	1	0	cig+/-ot	10	999	0.1	4	ot
JAHN	523	m	0	0	all	-	all	Eu:Ger	1988	CC	1004	n	bl	n	n	0	0	cig+/-ot	21	999	0.1	0.9	st
JAIN	572	m	0	0	all	-	all	NAmer	1981	CC	845	n	V	y	n	0	0	cig+/-ot	10	999	2	9	st
JAIN	536	f	0	0	all	-	all	NAmer	1981	CC	845	n	V	y	n	0	0	cig+/-ot	10	999	2	9	st
JOLY	573	m	0	0	all	-	all	SCAmer	1978	CC	826	n	bl	n	n	0	0	cig+/-ot	5	999	1.0	4	st
JOLY	560	f	0	0	all	-	all	SCAmer	1978	CC	826	n	bl	n	n	0	0	cig+/-ot	5	999	1.0	4	st
KAISE2	660	m	0	0	all	9	all	NAmer	1979	pr	318	n	bl	n	n	1	0	cig only	21	999	2	10	st
KAISE2	580	f	0	0	all	9	all	NAmer	1979	pr	318	n	bl	n	n	1	0	cig only	21	999	2	10	ot
KHUDER	520	m	0	0	all	-	all	NAmer	1985	CC	482	n	bl	n	y	0	0	cig+/-ot	15	999	0.1	4	st
LUBIN	596	m	0	0	all	-	all	As:Chi	1984	CC	427	m	ot	y	n	0	0	cig+/-ot	10	999	3	4	st
LUBIN2	1091	m	0	0	all	-	all	Eu:mul	1976	CC	7804	n	bl	n	y	0	0	cig+/-ot	25	999	0.1	4	st
LUBIN2	1130	f	0	0	all	-	all	Eu:mul	1976	CC	7804	n	bl	n	y	0	0	cig+/-ot	25	999	0.1	4	st
MATOS	600	m	0	0	all	-	all	SCAmer	1994	CC	200	n	bl	n	n	2	0	cig+/-ot	11	999	1.0	5	ot
PEZZO2	506	m	0	0	all	-	all	SCAmer	1992	CC	367	n	bl	n	y	0	0	cig+/-ot	11	999	1.0	10	st
PEZZOT	506	m	0	0	all	-	all	SCAmer	1987	CC	215	n	bl	n	y	0	0	cig only	11	999	1.0	10	st
SOBUE	738	m	0	0	all	-	all	As:Jap	1986	CC	1376	n	bl	n	y	0	0	cig+/-ot	25	999	1.0	4	st
SPEIZE	519	f	0	0	all	0	all	NAmer	1976	pr	593	n	bl	n	y	2	0	cig+/-ot	15	999	0.1	2	ot
SUZUK2	528	c	0	0	all	-	all	SCAmer	1991	CC	123	n	bl	n	y	3	0	all/unsp	11	999	0.1	5	ot
SVENSS	556	f	0	0	all	-	all	Eu:Sca	1983	CC	210	n	bl	n	n	0	0	all/unsp	11	999	3	10	st
TVERDA	510	m	0	0	all	0	all	Eu:Sca	1972	pr	238	n	bl	n	n	2	0	cig only	5	999	0.1	0.9	ot
WANG2	517	c	0	0	all	-	all	As:Chi	1980	CC	103	n	ot	n	n	0	0	cig+/-ot	4	999	0.1	3	st
WYNDE3	551	m	0	0	all	-	all	NAmer	1966	CC	350	n	bl	n	y	0	0	all/unsp	13	999	1.0	3	st
WYNDE6	725	m	0	0	wh	-	all	NAmer	1969	CC	4423	n	bl	n	y	5	1#	cig+/-ot	20	999	1.0	10	ot
WYNDE6	732	m	0	0	bl	-	all	NAmer	1969	CC	4423	n	bl	n	y	5	1#	cig+/-ot	20	999	1.0	10	ot
WYNDE6	736	f	0	0	wh	-	all	NAmer	1969	CC	4423	n	bl	n	y	5	1#	cig+/-ot	11	999	1.0	10	ot
WYNDE6	740	f	0	0	bl	-	all	NAmer	1969	CC	4423	n	bl	n	y	5	1#	cig+/-ot	11	999	1.0	10	ot

Comments on values in listings

WYNDE6 ADOS Number of cigs/day
WYNDE6 ADOS Number of cigs/day
WYNDE6 ADOS Number of cigs/day
WYNDE6 ADOS Number of cigs/day

Cigarette type is all/unspec for all RRs

Table 1K5 - 1

IESLC - Meta-analysis of Ex Smoking, Years quit (vs current), "Highest vs lowest"
All LC types, Any Product (or Cigarettes if Any not available)
Most adjusted

except for the following:

REF	NRR	CIGTYPE
ALDERS	517	MC only
ALDERS	528	MC only

Table 1K5 - 2

IESLC - Meta-analysis of Ex Smoking, Years quit (vs current), "Highest vs lowest"
All LC types, Any Product (or Cigarettes if Any not available)
Most adjusted

REF	NRR	SEX	AD	Number Exposed Case	Exposed Cont	Non-exposed Case	Non-exposed Cont	RR	95.00%CI
ALDERS	517	m	1	29	-	121	-	0.18	(0.10- 0.30)
ALDERS	528	f	1	26	-	206	-	0.13	(0.08- 0.23)
Subtotal ALDERS								0.15	(0.10- 0.22)
ARMADA	520	m	0	50	87	79	45	0.33	(0.20- 0.54)
BARBON	554	m	1	15	-	32	-	0.15	(0.07- 0.34)
BECHER	510	m	0	16	72	10	12	0.27	(0.10- 0.72)
BECHER	520	f	0	1	10	2	3	0.15	(0.01- 2.29)
Subtotal BECHER								0.25	(0.10- 0.64)
BROSS	520	m	0	43	79	169	67	0.22	(0.14- 0.34)
CARPEN	514	c	0	37	158	28	46	0.38	(0.21- 0.69)
*CEDERL	540	m	1	3	-	12	-	0.18	(0.05- 0.64)
CHOI	549	m	0	4	19	25	64	0.54	(0.17- 1.74)
CHOI	558	f	0	2	0	3	2	3.57~	(0.11- 111.71)
Subtotal CHOI								0.66	(0.22- 1.99)
*CHYOU	512	m	2	5	-	21	-	0.74	(0.28- 1.94)
*CPSI	821	m	1	15	-	37	-	0.09	(0.05- 0.16)
*CPSII	674	m	1	256	-	97	-	0.10	(0.08- 0.12)
*CPSII	650	f	1	50	-	91	-	0.10	(0.07- 0.14)
Subtotal CPSII								0.10	(0.08- 0.12)
DAMBER	532	m	1	42	-	-	-	0.34	(0.19- 0.59)
DARBY	506	m	0	139	767	146	339	0.42	(0.32- 0.55)
DARBY	515	f	0	26	224	68	93	0.16	(0.10- 0.27)
Subtotal DARBY								0.34	(0.27- 0.43)
DEAN3	640	m	1	32	-	42	-	0.42	(0.25- 0.71)
DEAN3	563	f	1	2	-	4	-	0.44	(0.08- 2.45)
Subtotal DEAN3								0.42	(0.26- 0.69)
DESTEF	534	m	4	17	-	64	-	0.31	(0.16- 0.61)
DOLL	541	m	0	8	23	56	75	0.47	(0.19- 1.12)
DOLL	550	f	0	1	2	9	6	0.33	(0.02- 4.55)
Subtotal DOLL								0.45	(0.20- 1.03)
*DOLL2	515	m	1	7	-	15	-	0.13	(0.05- 0.31)
DORGAN	518	m	0	134	255	59	51	0.45	(0.30- 0.70)
DORGAN	561	f	0	34	50	49	27	0.37	(0.20- 0.71)
Subtotal DORGAN								0.43	(0.30- 0.61)
*DORN	667	m	0	16	58370	34	22086	0.18	(0.10- 0.32)
*DORN	690	m	0	34	51243	14	6195	0.29	(0.16- 0.55)
Subtotal DORN								0.23	(0.15- 0.35)
GAO	540	m	2	13	-	105	-	0.16	(0.07- 0.34)
GAO	560	f	2	16	-	37	-	0.31	(0.11- 0.87)
Subtotal GAO								0.20	(0.11- 0.38)
GAO2	526	m	0	8	25	31	26	0.27	(0.10- 0.70)
GARCIA	514	c	0	10	37	33	11	0.09	(0.03- 0.24)
GRAHAM	544	m	1	13	-	113	-	0.07	(0.04- 0.15)
*HAMMO2	514	m	1	20	-	59	-	0.31	(0.19- 0.52)
*HIRAYA	517	m	1	-	-	-	-	0.68	(0.25- 1.87)
*HIRAYA	528	f	1	-	-	-	-	0.26	(0.01- 11.52)
Subtotal HIRAYA								0.63	(0.24- 1.66)
JAHN	523	m	0	29	146	166	8	0.01	(0.00- 0.02)
JAIN	572	m	0	52	113	74	46	0.29	(0.17- 0.47)
JAIN	536	f	0	19	61	66	36	0.17	(0.09- 0.33)
Subtotal JAIN								0.24	(0.16- 0.35)
JOLY	573	m	0	63	149	38	36	0.40	(0.23- 0.69)
JOLY	560	f	0	15	19	19	8	0.33	(0.11- 0.97)
Subtotal JOLY								0.39	(0.24- 0.63)
*KAISE2	660	m	1	6	-	12	-	0.21	(0.07- 0.62)
*KAISE2	580	f	1	4	-	6	-	0.58	(0.15- 2.22)
Subtotal KAISE2								0.31	(0.13- 0.73)
KHUDER	520	m	0	63	213	88	123	0.41	(0.28- 0.61)
LUBIN	596	m	0	17	73	33	18	0.13	(0.06- 0.28)
LUBIN2	1091	m	0	109	715	866	1047	0.18	(0.15- 0.23)
LUBIN2	1130	f	0	4	20	60	55	0.18	(0.06- 0.57)
Subtotal LUBIN2								0.18	(0.15- 0.23)
MATOS	600	m	2	27	-	28	-	0.21	(0.10- 0.44)
PEZZO2	506	m	0	43	161	85	110	0.35	(0.22- 0.54)
PEZZOT	506	m	0	20	106	46	82	0.34	(0.18- 0.61)
SOBUE	738	m	0	17	40	128	116	0.39	(0.21- 0.72)
*SPEIZE	519	f	2	28	-	24	-	0.17	(0.08- 0.37)
SUZUK2	528	c	3	9	-	15	-	0.17	(0.05- 0.51)
SVENSS	556	f	0	14	24	16	13	0.47	(0.18- 1.27)
*TVERDA	510	m	2	4	-	2	-	0.48	(0.09- 2.66)
WANG2	517	c	0	5	11	6	10	0.76	(0.18- 3.27)

Table 1K5 - 2

IESLC - Meta-analysis of Ex Smoking, Years quit (vs current), "Highest vs lowest"
All LC types, Any Product (or Cigarettes if Any not available)
Most adjusted

REF	NRR	SEX	AD	Number Case	Exposed Cont	Non-exposed Case	Cont	RR	95.00%CI
WYNDE3	551	m	0	5	55	21	22	0.10	(0.03- 0.28)
WYNDE6	725	m	5	-	-	-	-	0.33	(0.23- 0.47)
WYNDE6	732	m	5	-	-	-	-	0.43	(0.16- 1.15)
WYNDE6	736	f	5	-	-	-	-	0.40	(0.31- 0.52)
WYNDE6	740	f	5	-	-	-	-	0.67	(0.18- 2.49)
Subtotal WYNDE6								0.38	(0.31- 0.47)
Partial Totals				1677	113327	3670	30878		

*prospective study ~ With 0.5 adjustment for zero

REF	NRR	SEX	AD	Ys	Ws	Qs	Ps
ALDERS	517	m	1	-1.71	12.73	0.73	0.0000
ALDERS	528	f	1	-2.04	13.78	4.41	0.0000
Subtotal ALDERS				-1.88	26.51	5.14	
ARMADA	520	m	0	-1.12	15.07	1.93	0.0000
BARBON	554	m	1	-1.90	6.15	1.10	0.0000
BECHER	510	m	0	-1.32	3.85	0.09	0.0095
BECHER	520	f	0	-1.90	0.52	0.09	0.1724
Subtotal BECHER				-1.39	4.37	0.18	
BROSS	520	m	0	-1.53	17.62	0.06	0.0000
CARPEN	514	c	0	-0.96	11.01	2.97	0.0015
*CEDERL	540	m	1	-1.71	2.36	0.14	0.0084
CHOI	549	m	0	-0.62	2.79	2.05	0.3017
CHOI	558	f	0	1.27	0.32	2.45	0.4687
Subtotal CHOI				-0.42	3.12	4.49	
*CHYOU	512	m	2	-0.30	4.10	5.65	0.5420
*CPSI	821	m	1	-2.41	11.36	9.89	0.0000
*CPSII	674	m	1	-2.30	93.46	64.07	0.0000
*CPSII	650	f	1	-2.30	31.98	21.92	0.0000
Subtotal CPSII				-2.30	125.45	86.00	
DAMBER	532	m	1	-1.08	11.97	1.88	0.0002
DARBY	506	m	0	-0.87	54.65	20.27	0.0000
DARBY	515	f	0	-1.84	14.62	1.96	0.0000
Subtotal DARBY				-1.07	69.28	22.23	
DEAN3	640	m	1	-0.87	14.10	5.20	0.0011
DEAN3	563	f	1	-0.82	1.31	0.56	0.3470
Subtotal DEAN3				-0.86	15.42	5.76	
DESTEF	534	m	4	-1.17	8.58	0.79	0.0006
DOLL	541	m	0	-0.76	5.01	2.53	0.0873
DOLL	550	f	0	-1.10	0.56	0.08	0.4100
Subtotal DOLL				-0.80	5.57	2.61	
*DOLL2	515	m	1	-2.04	4.62	1.48	0.0000
DORGAN	518	m	0	-0.79	20.86	9.80	0.0003
DORGAN	561	f	0	-0.98	9.36	2.27	0.0027
Subtotal DORGAN				-0.85	30.22	12.08	
*DORN	667	m	0	-1.73	10.89	0.69	0.0000
*DORN	690	m	0	-1.23	9.93	0.62	0.0001
Subtotal DORN				-1.49	20.82	1.30	
GAO	540	m	2	-1.83	6.15	0.79	0.0000
GAO	560	f	2	-1.17	3.59	0.33	0.0264
Subtotal GAO				-1.59	9.74	1.12	
GAO2	526	m	0	-1.32	4.24	0.11	0.0067
GARCIA	514	c	0	-2.41	4.03	3.50	0.0000
GRAHAM	544	m	1	-2.66	8.80	12.34	0.0000
*HAMMO2	514	m	1	-1.17	15.16	1.40	0.0000
*HIRAYA	517	m	1	-0.39	3.79	4.50	0.4525
*HIRAYA	528	f	1	-1.35	0.31	0.01	0.4538
Subtotal HIRAYA				-0.46	4.10	4.51	
JAHN	523	m	0	-4.65	5.80	58.46	0.0000
JAIN	572	m	0	-1.25	15.79	0.79	0.0000
JAIN	536	f	0	-1.77	8.93	0.79	0.0000
Subtotal JAIN				-1.44	24.72	1.58	
JOLY	573	m	0	-0.91	13.04	4.09	0.0010
JOLY	560	f	0	-1.10	3.37	0.47	0.0433
Subtotal JOLY				-0.95	16.41	4.56	
*KAISE2	660	m	1	-1.56	3.23	0.02	0.0050
*KAISE2	580	f	1	-0.54	2.12	1.83	0.4281
Subtotal KAISE2				-1.16	5.35	1.85	
KHUDER	520	m	0	-0.88	24.96	8.73	0.0000
LUBIN	596	m	0	-2.06	6.31	2.19	0.0000
LUBIN2	1091	m	0	-1.69	78.85	3.70	0.0000

Table 1K5 - 2

IESLC - Meta-analysis of Ex Smoking, Years quit (vs current), "Highest vs lowest"
All LC types, Any Product (or Cigarettes if Any not available)
Most adjusted

REF	NRR	SEX	AD	Ys	Ws	Qs	Ps
LUBIN2	1130	f	0	-1.70	2.99	0.15	0.0034
Subtotal LUBIN2				-1.69	81.83	3.84	
MATOS	600	m	2	-1.56	7.00	0.05	0.0000
PEZZO2	506	m	0	-1.06	19.87	3.38	0.0000
PEZZOT	506	m	0	-1.09	10.71	1.59	0.0004
SOBUE	738	m	0	-0.95	9.97	2.70	0.0026
*SPEIZE	519	f	2	-1.77	6.55	0.58	0.0000
SUZUK2	528	c	3	-1.77	2.85	0.25	0.0028
SVENSS	556	f	0	-0.75	3.96	2.10	0.1373
*TVERDA	510	m	2	-0.73	1.34	0.74	0.3955
WANG2	517	c	0	-0.28	1.79	2.57	0.7100
WYNDE3	551	m	0	-2.35	3.21	2.47	0.0000
WYNDE6	725	m	5	-1.11	30.09	4.03	0.0000
WYNDE6	732	m	5	-0.84	3.95	1.57	0.0935
WYNDE6	736	f	5	-0.92	57.43	17.90	0.0000
WYNDE6	740	f	5	-0.40	2.23	2.57	0.5501
Subtotal WYNDE6				-0.96	93.69	26.07	

	N	61
	NS	43
	Wt	765.99
Het	Chi	312.34
Het	df	60
Het	P	***
Fixed	RR	0.23
	RRl	0.21
	RRu	0.25
	P	---
Random	RR	0.24
	RRl	0.20
	RRu	0.29
	P	---
Asymm	P	N.S.

Table 1K5 - 3

IESLC - Meta-analysis of Ex Smoking, Years quit (vs current), "Highest vs lowest"
All LC types, Any Product (or Cigarettes if Any not available)
Most adjusted

	Sex			
	combined	male	female	Total
N	4	39	18	61
NS	4	37	17	58
Wt	19.68	582.38	163.93	765.99
Het Chi	8.75	242.51	60.41	312.34
Het df	3	38	17	60
Het P	*	***	***	***
Fixed RR	0.27	0.23	0.23	0.23
RRl	0.17	0.21	0.20	0.21
RRu	0.42	0.25	0.27	0.25
P	---	---	---	---
Random RR	0.25	0.24	0.25	0.24
RRl	0.11	0.19	0.18	0.20
RRu	0.58	0.30	0.36	0.29
P	--	---	---	---
Between Chi				0.67
Between df				2
Between P				N.S.
Btwn(F) P				N.S.
Btwn(R) P				N.S.

	Lung cancer type		
	all	other	Total
N	61		61
NS	43		43
Wt	765.99		765.99
Het Chi	312.34		312.34
Het df	60		60
Het P	***		***
Fixed RR	0.23		0.23
RRl	0.21		0.21
RRu	0.25		0.25
P	---		---
Random RR	0.24		0.24
RRl	0.20		0.20
RRu	0.29		0.29
P	---		---
Between Chi			
Between df			
Between P			N.S.
Btwn(F) P			N.S.
Btwn(R) P			N.S.

	Location								
	NAmer	UK	Scand	othEur	China	Japan	othAs	other	Total
N	24	9	4	7	4	4	2	7	61
NS	16	5	4	5	3	3	1	6	43
Wt	407.04	121.39	19.63	113.22	17.85	18.32	3.12	65.42	765.99
Het Chi	173.39	30.91	1.55	55.90	5.47	1.81	1.04	3.20	312.34
Het df	23	8	3	6	3	3	1	6	60
Het P	***	***	N.S.	***	N.S.	N.S.	N.S.	N.S.	***
Fixed RR	0.21	0.29	0.34	0.17	0.20	0.40	0.66	0.32	0.23
RRl	0.19	0.24	0.22	0.14	0.12	0.25	0.22	0.25	0.21
RRu	0.23	0.34	0.54	0.21	0.31	0.63	1.99	0.41	0.25
P	---	---	---	---	---	---	N.S.	---	---
Random RR	0.23	0.25	0.34	0.13	0.22	0.40	0.67	0.32	0.24
RRl	0.18	0.16	0.22	0.06	0.11	0.25	0.20	0.25	0.20
RRu	0.31	0.38	0.54	0.29	0.42	0.63	2.24	0.41	0.29
P	---	---	---	---	---	---	N.S.	---	---
Between Chi									39.06
Between df									7
Between P									***
Btwn(F) P									N.S.
Btwn(R) P									(*)

Table 1K5 - 3

IESLC - Meta-analysis of Ex Smoking, Years quit (vs current), "Highest vs lowest"
All LC types, Any Product (or Cigarettes if Any not available)
Most adjusted

Detailed Country in "other Europe"

	multi	Germany	othWest	East	Balkans	Total
N	2	3	2			7
NS	1	2	2			5
Wt	81.83	10.17	21.22			113.22
Het Chi	0.00	26.62	2.66			55.90
Het df	1	2	1			6
Het P	N.S.	***	N.S.			***
Fixed RR	0.18	0.04	0.26			0.17
RRl	0.15	0.02	0.17			0.14
RRu	0.23	0.07	0.40			0.21
P	---	---	---			---
Random RR	0.18	0.07	0.24			0.13
RRl	0.15	0.01	0.11			0.06
RRu	0.23	0.90	0.50			0.29
P	---	-	---			---
Between Chi						26.63
Between df						2
Between P						***
Btwn(F) P						N.S.
Btwn(R) P						N.S.

Detailed Country in "other Asia"

	India	HongKong	other	Total
N			2	2
NS			1	1
Wt			3.12	3.12
Het Chi			1.04	1.04
Het df			1	1
Het P			N.S.	N.S.
Fixed RR			0.66	0.66
RRl			0.22	0.22
RRu			1.99	1.99
P			N.S.	N.S.
Random RR			0.67	0.67
RRl			0.20	0.20
RRu			2.24	2.24
P			N.S.	N.S.
Between Chi				
Between df				
Between P				N.S.
Btwn(F) P				N.S.
Btwn(R) P				N.S.

Detailed other continent

	SCAmer	Total
N	7	7
NS	6	6
Wt	65.42	65.42
Het Chi	3.20	3.20
Het df	6	6
Het P	N.S.	N.S.
Fixed RR	0.32	0.32
RRl	0.25	0.25
RRu	0.41	0.41
P	---	---
Random RR	0.32	0.32
RRl	0.25	0.25
RRu	0.41	0.41
P	---	---
Between Chi		
Between df		
Between P		N.S.
Btwn(F) P		N.S.
Btwn(R) P		N.S.

Table 1K5 - 3

IESLC - Meta-analysis of Ex Smoking, Years quit (vs current), "Highest vs lowest"
All LC types, Any Product (or Cigarettes if Any not available)
Most adjusted

	Start year of study					
	<1960	1960-69	1970-79	1980-89	1990+	Total
N	7	13	12	24	5	61
NS	5	8	8	17	5	43
Wt	51.16	155.67	156.11	358.29	44.76	765.99
Het Chi	19.71	17.60	17.80	204.81	8.46	312.34
Het df	6	12	11	23	4	60
Het P	**	N.S.	(*)	***	(*)	***
Fixed RR	0.15	0.35	0.21	0.21	0.28	0.23
RRl	0.12	0.30	0.18	0.19	0.21	0.21
RRu	0.20	0.41	0.24	0.23	0.37	0.25
P	---	---	---	---	---	---
Random RR	0.16	0.35	0.22	0.24	0.24	0.24
RRl	0.10	0.28	0.17	0.17	0.15	0.20
RRu	0.28	0.43	0.28	0.34	0.39	0.29
P	---	---	---	---	---	---
Between Chi						43.96
Between df						4
Between P						***
Btwn(F) P						(*)
Btwn(R) P						*

	Study type (1)		
	CC	other	Total
N	46	15	61
NS	32	11	43
Wt	564.79	201.21	765.99
Het Chi	179.82	60.20	312.34
Het df	45	14	60
Het P	***	***	***
Fixed RR	0.27	0.14	0.23
RRl	0.25	0.12	0.21
RRu	0.30	0.16	0.25
P	---	---	---
Random RR	0.26	0.21	0.24
RRl	0.21	0.14	0.20
RRu	0.31	0.30	0.29
P	---	---	---
Between Chi			72.32
Between df			1
Between P			***
Btwn(F) P			***
Btwn(R) P			N.S.

	Study type (2)			
	CC	prosp	other	Total
N	46	15		61
NS	32	11		43
Wt	564.79	201.21		765.99
Het Chi	179.82	60.20		312.34
Het df	45	14		60
Het P	***	***		***
Fixed RR	0.27	0.14		0.23
RRl	0.25	0.12		0.21
RRu	0.30	0.16		0.25
P	---	---		---
Random RR	0.26	0.21		0.24
RRl	0.21	0.14		0.20
RRu	0.31	0.30		0.29
P	---	---		---
Between Chi				72.32
Between df				1
Between P				***
Btwn(F) P				***
Btwn(R) P				N.S.

Table 1K5 - 3

IESLC - Meta-analysis of Ex Smoking, Years quit (vs current), "Highest vs lowest"
All LC types, Any Product (or Cigarettes if Any not available)
Most adjusted

	Study size (number of LC cases) 100-249	250-499	500-999	1000+	Total
N	9	15	14	23	61
NS	8	13	10	12	43
Wt	36.12	123.27	181.52	425.08	765.99
Het Chi	7.79	23.40	45.59	197.58	312.34
Het df	8	14	13	22	60
Het P	N.S.	(*)	***	***	***
Fixed RR	0.34	0.31	0.27	0.19	0.23
RRl	0.24	0.26	0.24	0.17	0.21
RRu	0.47	0.37	0.32	0.21	0.25
P	---	---	---	---	---
Random RR	0.34	0.29	0.23	0.21	0.24
RRl	0.24	0.22	0.17	0.15	0.20
RRu	0.47	0.37	0.32	0.29	0.29
P	---	---	---	---	---
Between Chi					37.98
Between df					3
Between P					***
Btwn(F) P					(*)
Btwn(R) P					N.S.

	Risky occupational population no	mining	othRisky	Total
N	59	1	1	61
NS	41	1	1	43
Wt	744.52	6.31	15.16	765.99
Het Chi	308.76	0.00	0.00	312.34
Het df	58	0	0	60
Het P	***	N.S.	N.S.	***
Fixed RR	0.23	0.13	0.31	0.23
RRl	0.21	0.06	0.19	0.21
RRu	0.25	0.28	0.51	0.25
P	---	---	---	---
Random RR	0.25	0.13	0.31	0.24
RRl	0.20	0.06	0.19	0.20
RRu	0.30	0.28	0.51	0.29
P	---	---	---	---
Between Chi				3.59
Between df				2
Between P				N.S.
Btwn(F) P				N.S.
Btwn(R) P				N.S.

	National cigarette tobacco type Virginia	blended	other	Total
N	11	46	4	61
NS	6	34	3	43
Wt	146.11	602.03	17.85	765.99
Het Chi	33.22	266.56	5.47	312.34
Het df	10	45	3	60
Het P	***	***	N.S.	***
Fixed RR	0.28	0.22	0.20	0.23
RRl	0.24	0.20	0.12	0.21
RRu	0.33	0.24	0.31	0.25
P	---	---	---	---
Random RR	0.24	0.25	0.22	0.24
RRl	0.17	0.20	0.11	0.20
RRu	0.34	0.31	0.42	0.29
P	---	---	---	---
Between Chi				7.08
Between df				2
Between P				*
Btwn(F) P				N.S.
Btwn(R) P				N.S.

Table 1K5 - 3

IESLC - Meta-analysis of Ex Smoking, Years quit (vs current), "Highest vs lowest"
All LC types, Any Product (or Cigarettes if Any not available)
Most adjusted

	Any proxy use No/nk	Yes	Total
N	52	9	61
NS	37	6	43
Wt	671.21	94.79	765.99
Het Chi	286.63	16.53	312.34
Het df	51	8	60
Het P	***	*	***
Fixed RR	0.22	0.31	0.23
RRl	0.20	0.25	0.21
RRu	0.24	0.37	0.25
P	---	---	---
Random RR	0.24	0.29	0.24
RRl	0.19	0.21	0.20
RRu	0.29	0.39	0.29
P	---	---	---
Between Chi			9.18
Between df			1
Between P			**
Btwn(F) P			N.S.
Btwn(R) P			N.S.

	Full histological confirmation No	Yes	Total
N	39	22	61
NS	27	16	43
Wt	439.82	326.17	765.99
Het Chi	221.99	53.91	312.34
Het df	38	21	60
Het P	***	***	***
Fixed RR	0.19	0.29	0.23
RRl	0.17	0.26	0.21
RRu	0.21	0.33	0.25
P	---	---	---
Random RR	0.22	0.30	0.24
RRl	0.17	0.24	0.20
RRu	0.29	0.36	0.29
P	---	---	---
Between Chi			36.44
Between df			1
Between P			***
Btwn(F) P			**
Btwn(R) P			(*)

	Number of adjustment variables (1) 0	1	2+/+nk	Total
N	32	17	12	61
NS	23	12	8	43
Wt	394.90	237.23	133.86	765.99
Het Chi	134.91	73.88	15.16	312.34
Het df	31	16	11	60
Het P	***	***	N.S.	***
Fixed RR	0.27	0.14	0.34	0.23
RRl	0.24	0.12	0.28	0.21
RRu	0.30	0.16	0.40	0.25
P	---	---	---	---
Random RR	0.26	0.18	0.32	0.24
RRl	0.20	0.13	0.25	0.20
RRu	0.32	0.26	0.40	0.29
P	---	---	---	---
Between Chi				88.40
Between df				2
Between P				***
Btwn(F) P				***
Btwn(R) P				*

Table 1K5 - 3

IESLC - Meta-analysis of Ex Smoking, Years quit (vs current), "Highest vs lowest"
All LC types, Any Product (or Cigarettes if Any not available)
Most adjusted

	Number of adjustment variables (2) 0	1	2	3-5	6+/+nk	Total
N	32	17	6	6		61
NS	23	12	5	3		43
Wt	394.90	237.23	28.74	105.12		765.99
Het Chi	134.91	73.88	7.97	3.60		312.34
Het df	31	16	5	5		60
Het P	***	***	N.S.	N.S.		***
Fixed RR	0.27	0.14	0.25	0.37		0.23
RRl	0.24	0.12	0.17	0.30		0.21
RRu	0.30	0.16	0.36	0.44		0.25
P	---	---	---	---		---
Random RR	0.26	0.18	0.26	0.37		0.24
RRl	0.20	0.13	0.16	0.30		0.20
RRu	0.32	0.26	0.42	0.44		0.29
P	---	---	---	---		---
Between Chi						91.98
Between df						3
Between P						***
Btwn(F) P						***
Btwn(R) P						**

	Product all/unsp	cig+/-ot	cig only	Total
N	15	37	9	61
NS	11	26	7	44
Wt	133.72	478.93	153.34	765.99
Het Chi	24.43	182.92	26.35	312.34
Het df	14	36	8	60
Het P	*	***	***	***
Fixed RR	0.32	0.25	0.12	0.23
RRl	0.27	0.23	0.11	0.21
RRu	0.38	0.28	0.14	0.25
P	---	---	---	---
Random RR	0.28	0.25	0.17	0.24
RRl	0.22	0.20	0.11	0.20
RRu	0.37	0.32	0.25	0.29
P	---	---	---	---
Between Chi				78.65
Between df				2
Between P				***
Btwn(F) P				***
Btwn(R) P				(*)

	Derivation of RR/CI Orig	StdCalc	Other	Total
N		32	29	61
NS		24	21	45
Wt		397.81	368.19	765.99
Het Chi		132.94	158.07	312.34
Het df		31	28	60
Het P		***	***	***
Fixed RR		0.27	0.19	0.23
RRl		0.24	0.17	0.21
RRu		0.30	0.21	0.25
P		---	---	---
Random RR		0.25	0.23	0.24
RRl		0.20	0.18	0.20
RRu		0.32	0.31	0.29
P		---	---	---
Between Chi				21.33
Between df				1
Between P				***
Btwn(F) P				*
Btwn(R) P				N.S.

Table 1K5 - 4

IESLC - Meta-analysis of Ex Smoking, Years quit (vs current), "Highest vs lowest"
All LC types, Any Product (or Cigarettes if Any not available)
Least adjusted

REF	NRR	X	SEX	AGEL	AGEH	RACE	YF	LC TYPE	LOC	START	ST	NLC	R	VB	P	H	AD	ADOS	PRODUCT	exL	exH	unexL	unexH	De
ALDERS	517		m	0	0	all	-	all	Eu:UK	1977	CC	1448	n	V	n	n	1	0	cig only	10	999	0.1	2	ot
ALDERS	528		f	0	0	all	-	all	Eu:UK	1977	CC	1448	n	V	n	n	1	0	cig only	10	999	0.1	2	ot
ARMADA	520		m	0	0	all	-	all	Eu:wst	1986	CC	325	n	bl	n	y	0	0	cig+/-ot	6	999	1.0	5	st
BARBON	539	x	m	0	0	all	-	all	Eu:wst	1979	CC	755	n	bl	y	y	0	0	all/unsp	25	999	0.1	4	st
BECHER	510		m	0	0	all	-	all	Eu:Ger	1985	CC	194	n	bl	n	y	0	0	all/unsp	10	999	2	4	st
BECHER	520		f	0	0	all	-	all	Eu:Ger	1985	CC	194	n	bl	n	y	0	0	all/unsp	10	999	2	4	st
BROSS	520		m	0	0	wh	-	all	NAmer	1960	CC	974	n	bl	n	n	0	0	cig+/-ot	6	999	0.1	5	st
CARPEN	514		c	0	0	w+b	-	all	NAmer	1991	CC	356	n	bl	n	n	0	0	cig+/-ot	15	999	0.1	4	st
CEDERL	540		m	40	69	all	10	all	Eu:Sca	1963	pr	491	n	bl	n	n	1	0	all/unsp	10	999	0.1	9	ot
CHOI	549		m	0	0	all	-	all	As:oth	1985	CC	375	n	bl	n	n	0	0	cig+/-ot	15	999	0.1	4	st
CHOI	558		f	0	0	all	-	all	As:oth	1985	CC	375	n	bl	n	n	0	0	cig+/-ot	5	999	0.1	4	ot
CHYOU	506	x	m	0	0	jap	21	all	NAmer	1965	pr	227	n	bl	n	y	1	0	cig+/-ot	15	999	0.1	14	ot
CPSI	821		m	50	74	all	6	all	NAmer	1959	pr	5138	n	bl	n	n	1	0	cig only	10	999	0.1	0.9	ot
CPSII	674		m	35	99	all	4	all	NAmer	1982	pr	3229	n	bl	n	n	1	0	cig only	16	999	0.1	0.9	ot
CPSII	650		f	0	0	all	4	all	NAmer	1982	pr	3229	n	bl	n	n	1	0	cig+/-ot	16	999	0.1	2	ot
DAMBER	532		m	0	0	all	-	all	Eu:Sca	1972	CC	579	n	bl	y	n	1	0	all/unsp	11	999	0.1	5	ot
DARBY	506		m	0	0	wh	-	all	Eu:UK	1988	CC	982	n	V	n	n	0	0	all/unsp	10	999	0.1	9	st
DARBY	515		f	0	0	wh	-	all	Eu:UK	1988	CC	982	n	V	n	n	0	0	all/unsp	10	999	0.1	9	st
DEAN3	541	x	m	0	0	all	-	all	Eu:UK	1969	CC	766	n	V	y	n	0	0	all/unsp	9	999	3	4	st
DEAN3	552	x	f	0	0	all	-	all	Eu:UK	1969	CC	766	n	V	y	n	0	0	all/unsp	9	999	3	4	st
DESTEF	524	x	m	0	0	all	-	all	SCAmer	1988	CC	497	n	bl	n	y	0	0	all/unsp	10	999	0.1	4	st
DOLL	541		m	0	0	all	-	all	Eu:UK	1948	CC	1465	n	V	n	n	0	0	all/unsp	20	999	0.1	9	st
DOLL	550		f	0	0	all	-	all	Eu:UK	1948	CC	1465	n	V	n	n	0	0	all/unsp	10	999	0.1	9	st
DOLL2	515		m	0	0	all	20	all	Eu:UK	1951	pr	920	n	V	n	n	1	0	cig only	15	999	0.1	4	ot
DORGAN	518		m	0	0	wh	-	all	NAmer	1980	CC	2026	n	bl	y	y	0	0	cig+/-ot	10	999	1	5	st
DORGAN	561		f	0	0	all	-	all	NAmer	1980	CC	2026	n	bl	y	y	0	0	cig+/-ot	10	999	1	9	st
DORN	667		m	55	64	wh	8	all	NAmer	1954	pr	5097	n	bl	n	n	0	0	cig+/-ot	15	999	0.1	4	st
DORN	690		m	65	74	wh	8	all	NAmer	1954	pr	5097	n	bl	n	n	0	0	cig+/-ot	15	999	0.1	4	st
GAO	530	x	m	0	0	all	-	all	As:Chi	1984	CC	1405	n	ot	n	n	0	0	cig+/-ot	10	999	0.1	4	st
GAO	550	x	f	0	0	all	-	all	As:Chi	1984	CC	1405	n	ot	n	n	0	0	cig+/-ot	10	999	0.1	4	st
GAO2	526		m	0	0	all	-	all	As:Jap	1988	CC	282	n	bl	n	n	0	0	cig+/-ot	20	999	1.0	4	st
GARCIA	514		c	0	0	all	-	all	NAmer	1992	CC	416	n	bl	n	y	0	0	cig+/-ot	30	999	1.0	4	st
GRAHAM	534	x	m	0	0	wh	-	all	NAmer	1956	CC	685	n	bl	n	n	0	0	cig+/-ot	5	999	0.1	1.0	st
HAMMO2	514		m	0	0	all	0	all	NAmer	1967	pr	450	o	bl	n	n	1	0	cig+/-ot	10	999	0.1	4	ot
HIRAYA	517		m	0	0	all	0	all	As:Jap	1965	pr	1917	n	bl	n	n	1	0	cig+/-ot	10	999	0.1	4	ot
HIRAYA	528		f	0	0	all	0	all	As:Jap	1965	pr	1917	n	bl	n	n	1	0	cig+/-ot	10	999	0.1	4	ot
JAHN	523		m	0	0	all	-	all	Eu:Ger	1988	CC	1004	n	bl	n	n	0	0	cig+/-ot	21	999	0.1	0.9	st
JAIN	572		m	0	0	all	-	all	NAmer	1981	CC	845	n	V	y	n	0	0	cig+/-ot	10	999	2	9	st
JAIN	536		f	0	0	all	-	all	NAmer	1981	CC	845	n	V	y	n	0	0	cig+/-ot	10	999	2	9	st
JOLY	573		m	0	0	all	-	all	SCAmer	1978	CC	826	n	bl	n	n	0	0	cig+/-ot	5	999	1.0	4	st
JOLY	560		f	0	0	all	-	all	SCAmer	1978	CC	826	n	bl	n	n	0	0	cig+/-ot	5	999	1.0	4	st
KAISE2	660		m	0	0	all	9	all	NAmer	1979	pr	318	n	bl	n	n	1	0	cig only	21	999	2	10	st
KAISE2	580		f	0	0	all	9	all	NAmer	1979	pr	318	n	bl	n	n	1	0	cig only	21	999	2	10	ot
KHUDER	520		m	0	0	all	-	all	NAmer	1985	CC	482	n	bl	n	y	0	0	cig+/-ot	15	999	0.1	4	st
LUBIN	596		m	0	0	all	-	all	As:Chi	1984	CC	427	m	ot	y	n	0	0	cig+/-ot	10	999	3	4	st
LUBIN2	1091		m	0	0	all	-	all	Eu:mul	1976	CC	7804	n	bl	n	y	0	0	cig+/-ot	25	999	0.1	4	st
LUBIN2	1130		f	0	0	all	-	all	Eu:mul	1976	CC	7804	n	bl	n	y	0	0	cig+/-ot	25	999	0.1	4	st
MATOS	590	x	m	0	0	all	-	all	SCAmer	1994	CC	200	n	bl	n	n	0	0	cig+/-ot	11	999	1.0	5	st
PEZZO2	506		m	0	0	all	-	all	SCAmer	1992	CC	367	n	bl	n	y	0	0	cig+/-ot	11	999	1.0	10	st
PEZZOT	506		m	0	0	all	-	all	SCAmer	1987	CC	215	n	bl	n	y	0	0	cig only	11	999	1.0	10	st
SOBUE	738		m	0	0	all	-	all	As:Jap	1986	CC	1376	n	bl	n	y	0	0	cig+/-ot	25	999	1.0	4	st
SPEIZE	519		f	0	0	all	0	all	NAmer	1976	pr	593	n	bl	n	y	2	0	cig+/-ot	15	999	0.1	2	ot
SUZUK2	517	x	c	0	0	all	-	all	SCAmer	1991	CC	123	n	bl	n	y	0	0	all/unsp	11	999	0.1	5	st
SVENSS	556		f	0	0	all	-	all	Eu:Sca	1983	CC	210	n	bl	n	n	0	0	all/unsp	11	999	3	10	st
TVERDA	510		m	0	0	all	0	all	Eu:Sca	1972	pr	238	n	bl	n	n	2	0	cig only	5	999	0.1	0.9	ot
WANG2	517		c	0	0	all	-	all	As:Chi	1980	CC	103	n	ot	n	n	0	0	cig+/-ot	4	999	0.1	3	st
WYNDE3	551		m	0	0	all	-	all	NAmer	1966	CC	350	n	bl	n	y	0	0	all/unsp	13	999	1.0	3	st
WYNDE6	725		m	0	0	wh	-	all	NAmer	1969	CC	4423	n	bl	n	y	5	1#	cig+/-ot	20	999	1.0	10	ot
WYNDE6	732		m	0	0	bl	-	all	NAmer	1969	CC	4423	n	bl	n	y	5	1#	cig+/-ot	20	999	1.0	10	ot
WYNDE6	736		f	0	0	wh	-	all	NAmer	1969	CC	4423	n	bl	n	y	5	1#	cig+/-ot	11	999	1.0	10	ot
WYNDE6	740		f	0	0	bl	-	all	NAmer	1969	CC	4423	n	bl	n	y	5	1#	cig+/-ot	11	999	1.0	10	ot

Comments on values in listings

WYNDE6 ADOS Number of cigs/day
WYNDE6 ADOS Number of cigs/day
WYNDE6 ADOS Number of cigs/day
WYNDE6 ADOS Number of cigs/day

Table 1K5 - 4

IESLC - Meta-analysis of Ex Smoking, Years quit (vs current), "Highest vs lowest"
All LC types, Any Product (or Cigarettes if Any not available)
Least adjusted

Cigarette type is all/unspec for all RRs
except for the following:

REF	NRR	CIGTYPE
ALDERS	517	MC only
ALDERS	528	MC only

Table 1K5 - 5

IESLC - Meta-analysis of Ex Smoking, Years quit (vs current), "Highest vs lowest"
All LC types, Any Product (or Cigarettes if Any not available)
Least adjusted

REF	NRR	SEX	AD	Number Exposed Case	Cont	Non-exposed Case	Cont	RR	95.00%CI
ALDERS	517	m	1	29	-	121	-	0.18	(0.10- 0.30)
ALDERS	528	f	1	26	-	206	-	0.13	(0.08- 0.23)
Subtotal ALDERS								0.15	(0.10- 0.22)
ARMADA	520	m	0	50	87	79	45	0.33	(0.20- 0.54)
BARBON	539	m	0	15	59	32	20	0.16	(0.07- 0.35)
BECHER	510	m	0	16	72	10	12	0.27	(0.10- 0.72)
BECHER	520	f	0	1	10	2	3	0.15	(0.01- 2.29)
Subtotal BECHER								0.25	(0.10- 0.64)
BROSS	520	m	0	43	79	169	67	0.22	(0.14- 0.34)
CARPEN	514	c	0	37	158	28	46	0.38	(0.21- 0.69)
*CEDERL	540	m	1	3	-	12	-	0.18	(0.05- 0.64)
CHOI	549	m	0	4	19	25	64	0.54	(0.17- 1.74)
CHOI	558	f	0	2	0	3	2	3.57~	(0.11- 111.71)
Subtotal CHOI								0.66	(0.22- 1.99)
*CHYOU	506	m	1	5	-	21	-	0.72	(0.27- 1.91)
*CPSI	821	m	1	15	-	37	-	0.09	(0.05- 0.16)
*CPSII	674	m	1	256	-	97	-	0.10	(0.08- 0.12)
*CPSII	650	f	1	50	-	91	-	0.10	(0.07- 0.14)
Subtotal CPSII								0.10	(0.08- 0.12)
DAMBER	532	m	1	42	-	-	-	0.34	(0.19- 0.59)
DARBY	506	m	0	139	767	146	339	0.42	(0.32- 0.55)
DARBY	515	f	0	26	224	68	93	0.16	(0.10- 0.27)
Subtotal DARBY								0.34	(0.27- 0.43)
DEAN3	541	m	0	32	204	42	147	0.55	(0.33- 0.91)
DEAN3	552	f	0	2	114	4	110	0.48	(0.09- 2.69)
Subtotal DEAN3								0.54	(0.33- 0.88)
DESTEF	524	m	0	17	36	64	45	0.33	(0.17- 0.66)
DOLL	541	m	0	8	23	56	75	0.47	(0.19- 1.12)
DOLL	550	f	0	1	2	9	6	0.33	(0.02- 4.55)
Subtotal DOLL								0.45	(0.20- 1.03)
*DOLL2	515	m	1	7	-	15	-	0.13	(0.05- 0.31)
DORGAN	518	m	0	134	255	59	51	0.45	(0.30- 0.70)
DORGAN	561	f	0	34	50	49	27	0.37	(0.20- 0.71)
Subtotal DORGAN								0.43	(0.30- 0.61)
*DORN	667	m	0	16	58370	34	22086	0.18	(0.10- 0.32)
*DORN	690	m	0	34	51243	14	6195	0.29	(0.16- 0.55)
Subtotal DORN								0.23	(0.15- 0.35)
GAO	530	m	0	13	41	105	52	0.16	(0.08- 0.32)
GAO	550	f	0	16	14	37	9	0.28	(0.10- 0.77)
Subtotal GAO								0.19	(0.11- 0.34)
GAO2	526	m	0	8	25	31	26	0.27	(0.10- 0.70)
GARCIA	514	c	0	10	37	33	11	0.09	(0.03- 0.24)
GRAHAM	534	m	0	13	71	113	59	0.10	(0.05- 0.19)
*HAMMO2	514	m	1	20	-	59	-	0.31	(0.19- 0.52)
*HIRAYA	517	m	1	-	-	-	-	0.68	(0.25- 1.87)
*HIRAYA	528	f	1	-	-	-	-	0.26	(0.01- 11.52)
Subtotal HIRAYA								0.63	(0.24- 1.66)
JAHN	523	m	0	29	146	166	8	0.01	(0.00- 0.02)
JAIN	572	m	0	52	113	74	46	0.29	(0.17- 0.47)
JAIN	536	f	0	19	61	66	36	0.17	(0.09- 0.33)
Subtotal JAIN								0.24	(0.16- 0.35)
JOLY	573	m	0	63	149	38	36	0.40	(0.23- 0.69)
JOLY	560	f	0	15	19	19	8	0.33	(0.11- 0.97)
Subtotal JOLY								0.39	(0.24- 0.63)
*KAISE2	660	m	1	6	-	12	-	0.21	(0.07- 0.62)
*KAISE2	580	f	1	4	-	6	-	0.58	(0.15- 2.22)
Subtotal KAISE2								0.31	(0.13- 0.73)
KHUDER	520	m	0	63	213	88	123	0.41	(0.28- 0.61)
LUBIN	596	m	0	17	73	33	18	0.13	(0.06- 0.28)
LUBIN2	1091	m	0	109	715	866	1047	0.18	(0.15- 0.23)
LUBIN2	1130	f	0	4	20	60	55	0.18	(0.06- 0.57)
Subtotal LUBIN2								0.18	(0.15- 0.23)
MATOS	590	m	0	27	101	28	23	0.22	(0.11- 0.44)
PEZZO2	506	m	0	43	161	85	110	0.35	(0.22- 0.54)
PEZZOT	506	m	0	20	106	46	82	0.34	(0.18- 0.61)
SOBUE	738	m	0	17	40	128	116	0.39	(0.21- 0.72)
*SPEIZE	519	f	2	28	-	24	-	0.17	(0.08- 0.37)
SUZUK2	517	c	0	9	22	15	10	0.27	(0.09- 0.83)
SVENSS	556	f	0	14	24	16	13	0.47	(0.18- 1.27)
*TVERDA	510	m	2	4	-	2	-	0.48	(0.09- 2.66)
WANG2	517	c	0	5	11	6	10	0.76	(0.18- 3.27)

Table 1K5 - 5

IESLC - Meta-analysis of Ex Smoking, Years quit (vs current), "Highest vs lowest"
All LC types, Any Product (or Cigarettes if Any not available)
Least adjusted

REF	NRR	SEX	AD	Number Exposed Case	Cont	Non-exposed Case	Cont	RR	95.00%CI
WYNDE3	551	m	0	5	55	21	22	0.10	(0.03- 0.28)
WYNDE6	725	m	5	-	-	-	-	0.33	(0.23- 0.47)
WYNDE6	732	m	5	-	-	-	-	0.43	(0.16- 1.15)
WYNDE6	736	f	5	-	-	-	-	0.40	(0.31- 0.52)
WYNDE6	740	f	5	-	-	-	-	0.67	(0.18- 2.49)
Subtotal WYNDE6								0.38	(0.31- 0.47)
Partial Totals				1677	113989	3670	31353		

*prospective study ~ With 0.5 adjustment for zero

REF	NRR	SEX	AD	Ys	Ws	Qs	Ps
ALDERS	517	m	1	-1.71	12.73	0.81	0.0000
ALDERS	528	f	1	-2.04	13.78	4.59	0.0000
Subtotal ALDERS				-1.88	26.51	5.39	
ARMADA	520	m	0	-1.12	15.07	1.81	0.0000
BARBON	539	m	0	-1.84	6.07	0.86	0.0000
BECHER	510	m	0	-1.32	3.85	0.08	0.0095
BECHER	520	f	0	-1.90	0.52	0.10	0.1724
Subtotal BECHER				-1.39	4.37	0.17	
BROSS	520	m	0	-1.53	17.62	0.09	0.0000
CARPEN	514	c	0	-0.96	11.01	2.84	0.0015
*CEDERL	540	m	1	-1.71	2.36	0.15	0.0084
CHOI	549	m	0	-0.62	2.79	1.99	0.3017
CHOI	558	f	0	1.27	0.32	2.43	0.4687
Subtotal CHOI				-0.42	3.12	4.42	
*CHYOU	506	m	1	-0.33	4.01	5.17	0.5104
*CPSI	821	m	1	-2.41	11.36	10.14	0.0000
*CPSII	674	m	1	-2.30	93.46	65.85	0.0000
*CPSII	650	f	1	-2.30	31.98	22.53	0.0000
Subtotal CPSII				-2.30	125.45	88.38	
DAMBER	532	m	1	-1.08	11.97	1.77	0.0002
DARBY	506	m	0	-0.87	54.65	19.52	0.0000
DARBY	515	f	0	-1.84	14.62	2.08	0.0000
Subtotal DARBY				-1.07	69.28	21.60	
DEAN3	541	m	0	-0.60	14.98	11.17	0.0203
DEAN3	552	f	0	-0.73	1.30	0.70	0.4055
Subtotal DEAN3				-0.61	16.28	11.87	
DESTEF	524	m	0	-1.10	8.04	1.05	0.0018
DOLL	541	m	0	-0.76	5.01	2.45	0.0873
DOLL	550	f	0	-1.10	0.56	0.07	0.4100
Subtotal DOLL				-0.80	5.57	2.52	
*DOLL2	515	m	1	-2.04	4.62	1.54	0.0000
DORGAN	518	m	0	-0.79	20.86	9.48	0.0003
DORGAN	561	f	0	-0.98	9.36	2.17	0.0027
Subtotal DORGAN				-0.85	30.22	11.65	
*DORN	667	m	0	-1.73	10.89	0.75	0.0000
*DORN	690	m	0	-1.23	9.93	0.56	0.0001
Subtotal DORN				-1.49	20.82	1.31	
GAO	530	m	0	-1.85	7.69	1.16	0.0000
GAO	550	f	0	-1.28	3.68	0.12	0.0141
Subtotal GAO				-1.67	11.36	1.28	
GAO2	526	m	0	-1.32	4.24	0.09	0.0067
GARCIA	514	c	0	-2.41	4.03	3.59	0.0000
GRAHAM	534	m	0	-2.35	8.56	6.70	0.0000
*HAMMO2	514	m	1	-1.17	15.16	1.29	0.0000
*HIRAYA	517	m	1	-0.39	3.79	4.41	0.4525
*HIRAYA	528	f	1	-1.35	0.31	0.00	0.4538
Subtotal HIRAYA				-0.46	4.10	4.41	
JAHN	523	m	0	-4.65	5.80	58.88	0.0000
JAIN	572	m	0	-1.25	15.79	0.71	0.0000
JAIN	536	f	0	-1.77	8.93	0.85	0.0000
Subtotal JAIN				-1.44	24.72	1.56	
JOLY	573	m	0	-0.91	13.04	3.92	0.0010
JOLY	560	f	0	-1.10	3.37	0.44	0.0433
Subtotal JOLY				-0.95	16.41	4.36	
*KAISE2	660	m	1	-1.56	3.23	0.03	0.0050
*KAISE2	580	f	1	-0.54	2.12	1.79	0.4281
Subtotal KAISE2				-1.16	5.35	1.82	
KHUDER	520	m	0	-0.88	24.96	8.39	0.0000
LUBIN	596	m	0	-2.06	6.31	2.27	0.0000
LUBIN2	1091	m	0	-1.69	78.85	4.10	0.0000

Table 1K5 - 5

IESLC - Meta-analysis of Ex Smoking, Years quit (vs current), "Highest vs lowest"
All LC types, Any Product (or Cigarettes if Any not available)
Least adjusted

REF	NRR	SEX	AD	Ys	Ws	Qs	Ps
LUBIN2	1130	f	0	-1.70	2.99	0.16	0.0034
Subtotal LUBIN2				-1.69	81.83	4.26	
MATOS	590	m	0	-1.52	7.93	0.02	0.0000
PEZZO2	506	m	0	-1.06	19.87	3.19	0.0000
PEZZOT	506	m	0	-1.09	10.71	1.49	0.0004
SOBUE	738	m	0	-0.95	9.97	2.59	0.0026
*SPEIZE	519	f	2	-1.77	6.55	0.62	0.0000
SUZUK2	517	c	0	-1.30	3.09	0.08	0.0223
SVENSS	556	f	0	-0.75	3.96	2.03	0.1373
*TVERDA	510	m	2	-0.73	1.34	0.71	0.3955
WANG2	517	c	0	-0.28	1.79	2.52	0.7100
WYNDE3	551	m	0	-2.35	3.21	2.53	0.0000
WYNDE6	725	m	5	-1.11	30.09	3.78	0.0000
WYNDE6	732	m	5	-0.84	3.95	1.51	0.0935
WYNDE6	736	f	5	-0.92	57.43	17.18	0.0000
WYNDE6	740	f	5	-0.40	2.23	2.51	0.5501
Subtotal WYNDE6				-0.96	93.69	24.99	

	N	61
	NS	43
	Wt	768.70
Het	Chi	312.43
Het	df	60
Het	P	***
Fixed	RR	0.23
	RRl	0.22
	RRu	0.25
	P	---
Random	RR	0.25
	RRl	0.21
	RRu	0.30
	P	---
Asymm	P	N.S.

Table 1K5 - 6

IESLC - Meta-analysis of Ex Smoking, Years quit (vs current), "Highest vs lowest"
All LC types, Any Product (or Cigarettes if Any not available)
Least adjusted

		Sex		
	combined	male	female	Total
N	4	39	18	61
NS	4	37	17	58
Wt	19.93	584.77	164.00	768.70
Het Chi	8.05	242.95	60.39	312.43
Het df	3	38	17	60
Het P	*	***	***	***
Fixed RR	0.29	0.23	0.23	0.23
RRl	0.19	0.21	0.20	0.22
RRu	0.45	0.25	0.27	0.25
P	---	---	---	---
Random RR	0.28	0.24	0.25	0.25
RRl	0.12	0.20	0.17	0.21
RRu	0.62	0.31	0.36	0.30
P	--	---	---	---
Between Chi				1.04
Between df				2
Between P				N.S.
Btwn(F) P				N.S.
Btwn(R) P				N.S.

Table 1K5 - 7

IESLC - Meta-analysis of Ex Smoking, Years quit (vs current), "Highest vs lowest"
All LC types, Any Product (or Cigarettes if Any not available)
Excluded studies (and stage at which they were excluded)

1	AGUDO AKIBA AMANDU AMES AXELSS BEST BOUCHA BOUCOT BRESLO CHEN CHEN2 CHIAZZ DEAN2 DOSEME ENGELA FAN GENG GER GUO HAENSZ HEGMAN HOLE HU HU2 JUSSAW KATSOU KAUFMA KOO KOULUM KREUZE LETOUR LEVIN LIAW LIU3 LIU4 LIU5 MCCONN MIGRAN MRFITR NOTAN2 OSANN2 PERNU QIAO2 RACHTA RESTRE SADOWS SEGI2 STASZE TIZZAN VUTUC WATSON WU WUWILL WYNDE2 WYNDE8 XU YUAN ZHANG ZHENG ZHOU
2	AUVINE BENSHL BLOT1 BROWN3 BUFFLE GURSEL LAUSSM LUO MCDUFF PISANI PRESCO SPITZ WU2 WYNDE7
4	GARSHI JEDRYC WAKAI
5	CORREA HAMMON
6	GILLIS HUMBLE QIAO WIGLE
8	BOFFET
15	BENHAM

Table 1K5 - 8
Potentially overlapping studies

REF	REFGP	PRINC	. OVERLAP/LINK
LUBIN2	LUBIN2	1	Lubin-combined
TVERDA	TVERDA	1	VEIERO/TVERDAL
BROSS	BYERS1	1	GRAHAM/BROSS/BYERS1
GRAHAM	BYERS1	1	GRAHAM/BROSS/BYERS1
CHYOU	CHYOU	1	GOODMA/CHYOU
WYNDE6	WYNDE6	1	WYNDE5/6/7/8
CPSI	CPSI	1	CPSI overall
JAHN	BOFFET	2	Subset of BOFFET
LUBIN	XIANGZ	2	LUBIN/XIANGZ/QIAO

Table 1K5 - 9
Most adjusted - insufficient data for meta-analysis

REF	NRR	SEX	AGEL	AGEH	RACE	YF	LC TYPE	LOC	START	ST	NLC	R	VB	P	H	AD	ADOS	PRODUCT	exL	exH	unexL	unexH	De
CPSI	717	m	0	0	wh	0	all	NAmer	1959	pr	5138	n	bl	n	n	1	0	cig only	35	39	2	4	st
CPSI	733	f	0	0	wh	0	all	NAmer	1959	pr	5138	n	bl	n	n	1	0	cig only	25	29	2	4	st
HUMBLE	553	c	0	0	wh	-	not alv	NAmer	1980	CC	521	n	bl	y	n	2	2#	cig+/-ot	16	30	2	5	st
WIGLE	518	m	0	0	all	-	all	NAmer	1971	CC	728	n	V	n	n	2	1#	cig+/-ot	15	999	0.1	1.0	st
WIGLE	528	f	0	0	all	-	all	NAmer	1971	CC	728	n	V	n	n	2	1#	cig+/-ot	15	999	0.1	1.0	st

Comments on values in listings

HUMBLE	ADOS	Number of cigarettes and duration
WIGLE	ADOS	Cumulative exposure
WIGLE	ADOS	Cumulative exposure

REF	NRR	RR	SIG	RRDATA comment
CPSI	717	0.24		0
CPSI	733	0.92		0
HUMBLE	553	0.16		0
WIGLE	518	0.08		0
WIGLE	528	0.44		0

Table 1K6 -

IESLC - Meta-analysis of Ex Smoking by Years quit (vs current), Overview
All LC types, Cigarettes (or Any Product if Cigarettes not available)

This analysis is restricted to results for:
1) Ex smokers
2) Results by Years quit (vs current)
3) Categorical results by Years quit (vs current)
Results by Years quit (vs current) are grouped under 2 schemes (S1, S2). Each scheme has a set of "key values". An interval is allocated to the category whose key value it includes, and intervals which include none or more than one of the key values are excluded. (Open-ended intervals are coded as 999)

S1	key value	maximum range
1	3	1-6
2	7	4-11
3	12	8+

S2	key value	maximum range
1	3	1-11
2	12	4-19
3	20	13+

4) All LC types (or near equivalent)
5) Results complete enough for use in metaanalysis

Within each study, results are then selected (in the following order of preference, within each sex) for:
6) (not applicable)
7) PRODUCT: cigarettes regardless of other products, cigarettes only, all/unspec
8) CIGTYPE: all/unspecified, MC regardless of HR, MC only
9) Results with least adjustment for other aspects of smoking (ADOS)
10) DENOM: current smokers, current + recent smokers (up to number of m=months or y=years, max 2 years)
11) Followup period (YF, prospective studies): whole study (coded as 0) or longest available
12) LCtype: all or nearest available, at least Squamous and Adeno. (q = squamous, s = small,
l = large, a = adeno, mix = mixed, alv = alveolar)
13) Race: all or nearest available, otherwise by race (wh or w = white, bl or b = black, hi = hispanic
ch = chinese, jap = japanese, haw = hawaiian, w+o = white + oriental, sca = scandinavian, as = asian)
14) For overlapping studies: principal rather than subsidiary studies
Finally by Age: whole study (coded as 0) if available, otherwise by widest available age group
and then for single sex results (m, f) in preference to results for both sexes combined (c).

Results adjusted (AD) for the most potential confounders are then chosen in Sections -1 to -3
(and those which actually differ from the adjusted results in Table 1K1 - 1 are marked 'x' in Section -1)
and results adjusted for the least confounders in Sections -4 to -6. (Those least adjusted results which
actually differ from the most adjusted are marked 'x' in column X in Section -4)

Section -7 shows excluded studies, together with the stage (as above) at which no qualifying
results were found.

Section -8 lists the potentially overlapping studies which have been included (1=principal, 2=subsidiary).

Section -9 lists any results which would have been included in preference except that they had data not complete
enough for use in meta-analysis, with their significance (yes/no), if known, and any further comment as entered
on the database. It also lists as "gap" any categories for which no data were presented by the original authors.

In addition to those mentioned above, the following fields, levels and abbreviations are used:
* or nk = not known, n = no, y = yes, ot = other
nev = never
all/unspec = all or unspecified, cig+/-ot = cigarettes irrespective of other products (cigar, pipe etc)
MC = manufactured cigarettes, HR = hand-rolled cigarettes
exL, exH = range of exposure (low and high) in the smoking group, in terms of Years quit (vs current)
REF: 6-character study reference
NRR: number of the RR on the database within the study
ST : study type (CC = case control, pr or prosp = prospective)
NLC: number of lung cancer cases in whole study
R : risky occupational population (n = no, m = mining, o = other risky)
VB : national cigarette type (V = at least 75% Virginia, bl = at least 75% blended, ot = other)
P : any proxy use
H : full histological confirmation
De : derivation of RR/CI (or = original, st = standard method, ot = other method of estimation)

Table 1K6 - 1

IESLC - Meta-analysis of Ex Smoking by Years quit (vs current), Overview
All LC types, Cigarettes (or Any Product if Cigarettes not available)
Most adjusted

REF	NRR	1K1	SEX	AGEL	AGEH	RACE	YF	LC TYPE	LOC	START	ST	NLC	R	VB	P	H	AD	ADOS	PRODUCT	exL	exH	S1	S2	DENOM	De
ALDERS	513		m	0	0	all	-	all	Eu:UK	1977	CC	1448	n	V	n	n	1	0	cig only	0.1	2	0	0	current	ot
ALDERS	514		m	0	0	all	-	all	Eu:UK	1977	CC	1448	n	V	n	n	1	0	cig only	3	9	0	1	current	ot
ALDERS	515		m	0	0	all	-	all	Eu:UK	1977	CC	1448	n	V	n	n	1	0	cig only	10	999	3	0	current	ot
ALDERS	524		f	0	0	all	-	all	Eu:UK	1977	CC	1448	n	V	n	n	1	0	cig only	0.1	2	0	0	current	ot
ALDERS	525		f	0	0	all	-	all	Eu:UK	1977	CC	1448	n	V	n	n	1	0	cig only	3	9	0	1	current	ot
ALDERS	526		f	0	0	all	-	all	Eu:UK	1977	CC	1448	n	V	n	n	1	0	cig only	10	999	3	0	current	ot
ARMADA	518		m	0	0	all	-	all	Eu:wst	1986	CC	325	n	bl	n	y	0	0	cig+/-ot	1.0	5	1	1	cur+1y	st
ARMADA	519		m	0	0	all	-	all	Eu:wst	1986	CC	325	n	bl	n	y	0	0	cig+/-ot	6	999	0	0	cur+1y	st
BARBON	548		m	0	0	all	-	all	Eu:wst	1979	CC	755	n	bl	y	y	1	0	all/unsp	0.1	4	1	1	current	ot
BARBON	549		m	0	0	all	-	all	Eu:wst	1979	CC	755	n	bl	y	y	1	0	all/unsp	5	14	0	2	current	ot
BARBON	550		m	0	0	all	-	all	Eu:wst	1979	CC	755	n	bl	y	y	1	0	all/unsp	15	24	0	3	current	ot
BARBON	551		m	0	0	all	-	all	Eu:wst	1979	CC	755	n	bl	y	y	1	0	all/unsp	25	999	0	0	current	ot
BECHER	506		m	0	0	all	-	all	Eu:Ger	1985	CC	194	n	bl	n	y	0	0	all/unsp	2	4	1	1	cur+1y	st
BECHER	507		m	0	0	all	-	all	Eu:Ger	1985	CC	194	n	bl	n	y	0	0	all/unsp	5	9	2	0	cur+1y	st
BECHER	508		m	0	0	all	-	all	Eu:Ger	1985	CC	194	n	bl	n	y	0	0	all/unsp	10	999	3	0	cur+1y	st
BECHER	516		f	0	0	all	-	all	Eu:Ger	1985	CC	194	n	bl	n	y	0	0	all/unsp	2	4	1	1	cur+1y	st
BECHER	517		f	0	0	all	-	all	Eu:Ger	1985	CC	194	n	bl	n	y	0	0	all/unsp	5	9	2	0	cur+1y	st
BECHER	518		f	0	0	all	-	all	Eu:Ger	1985	CC	194	n	bl	n	y	0	0	all/unsp	10	999	3	0	cur+1y	st
BROSS	518		m	0	0	wh	-	all	NAmer	1960	CC	974	n	bl	n	n	0	0	cig+/-ot	0.1	5	1	1	current	st
BROSS	519		m	0	0	wh	-	all	NAmer	1960	CC	974	n	bl	n	n	0	0	cig+/-ot	6	999	0	0	current	st
CARPEN	508		c	0	0	w+b	-	all	NAmer	1991	CC	356	n	bl	n	n	0	0	cig+/-ot	0.1	4	1	1	current	st
CARPEN	509		c	0	0	w+b	-	all	NAmer	1991	CC	356	n	bl	n	n	0	0	cig+/-ot	5	9	2	0	current	st
CARPEN	510		c	0	0	w+b	-	all	NAmer	1991	CC	356	n	bl	n	n	0	0	cig+/-ot	10	14	3	2	current	st
CARPEN	511		c	0	0	w+b	-	all	NAmer	1991	CC	356	n	bl	n	n	0	0	cig+/-ot	15	999	0	3	current	st
CEDERL	538		m	40	69	all	10	all	Eu:Sca	1963	pr	491	n	bl	n	n	1	0	all/unsp	0.1	9	0	1	current	ot
CEDERL	539		m	40	69	all	10	all	Eu:Sca	1963	pr	491	n	bl	n	n	1	0	all/unsp	10	999	3	0	current	ot
CHOI	543		m	0	0	all	-	all	As:oth	1985	CC	375	n	bl	n	n	0	0	cig+/-ot	0.1	4	1	1	current	st
CHOI	544		m	0	0	all	-	all	As:oth	1985	CC	375	n	bl	n	n	0	0	cig+/-ot	5	9	2	0	current	st
CHOI	545		m	0	0	all	-	all	As:oth	1985	CC	375	n	bl	n	n	0	0	cig+/-ot	10	14	3	2	current	st
CHOI	546		m	0	0	all	-	all	As:oth	1985	CC	375	n	bl	n	n	0	0	cig+/-ot	15	999	0	3	current	st
CHOI	556		f	0	0	all	-	all	As:oth	1985	CC	375	n	bl	n	n	0	0	cig+/-ot	0.1	4	1	1	current	st
CHOI	557		f	0	0	all	-	all	As:oth	1985	CC	375	n	bl	n	n	0	0	cig+/-ot	5	999	0	0	current	ot
CHYOU	510		m	0	0	jap	21	all	NAmer	1965	pr	227	n	bl	n	y	2	0	cig+/-ot	0.1	14	0	0	current	ot
CHYOU	511		m	0	0	jap	21	all	NAmer	1965	pr	227	n	bl	n	y	2	0	cig+/-ot	15	999	0	3	current	ot
CPSI	815		m	50	74	all	6	all	NAmer	1959	pr	5138	n	bl	n	n	1	0	cig only	0.1	0.9	0	0	current	ot
CPSI	816		m	50	74	all	6	all	NAmer	1959	pr	5138	n	bl	n	n	1	0	cig only	1.0	4	1	1	current	ot
CPSI	817		m	50	74	all	6	all	NAmer	1959	pr	5138	n	bl	n	n	1	0	cig only	5	9	2	0	current	ot
CPSI	818		m	50	74	all	6	all	NAmer	1959	pr	5138	n	bl	n	n	1	0	cig only	10	999	3	0	current	ot
CPSII	664		m	35	99	all	4	all	NAmer	1982	pr	3229	n	bl	n	n	1	0	cig only	0.1	0.9	0	0	current	ot
CPSII	665		m	35	99	all	4	all	NAmer	1982	pr	3229	n	bl	n	n	1	0	cig only	1.0	2	0	0	current	ot
CPSII	666		m	35	99	all	4	all	NAmer	1982	pr	3229	n	bl	n	n	1	0	cig only	3	5	1	1	current	ot
CPSII	667		m	35	99	all	4	all	NAmer	1982	pr	3229	n	bl	n	n	1	0	cig only	6	10	2	0	current	ot
CPSII	668		m	35	99	all	4	all	NAmer	1982	pr	3229	n	bl	n	n	1	0	cig only	11	15	3	2	current	ot
CPSII	669		m	35	99	all	4	all	NAmer	1982	pr	3229	n	bl	n	n	1	0	cig only	16	999	0	3	current	ot
CPSII	642		f	0	0	all	4	all	NAmer	1982	pr	3229	n	bl	n	n	1	0	cig+/-ot	0.1	2	0	0	current	ot
CPSII	643		f	0	0	all	4	all	NAmer	1982	pr	3229	n	bl	n	n	1	0	cig+/-ot	3	5	1	1	current	ot
CPSII	644		f	0	0	all	4	all	NAmer	1982	pr	3229	n	bl	n	n	1	0	cig+/-ot	6	10	2	0	current	ot
CPSII	645		f	0	0	all	4	all	NAmer	1982	pr	3229	n	bl	n	n	1	0	cig+/-ot	11	15	3	2	current	ot
CPSII	646		f	0	0	all	4	all	NAmer	1982	pr	3229	n	bl	n	n	1	0	cig+/-ot	16	999	0	3	current	ot
DAMBER	557	x	m	0	0	all	-	all	Eu:Sca	1972	CC	579	n	bl	y	n	1	0	cig only	0.1	10	0	1	current	ot
DAMBER	558	x	m	0	0	all	-	all	Eu:Sca	1972	CC	579	n	bl	y	n	1	0	cig only	11	999	3	0	current	ot
DARBY	507		m	0	0	wh	-	all	Eu:UK	1988	CC	982	n	V	n	n	0	0	all/unsp	0.1	9	0	1	current	st
DARBY	508		m	0	0	wh	-	all	Eu:UK	1988	CC	982	n	V	n	n	0	0	all/unsp	10	999	3	0	current	st
DARBY	516		f	0	0	wh	-	all	Eu:UK	1988	CC	982	n	V	n	n	0	0	all/unsp	0.1	9	0	1	current	st
DARBY	517		f	0	0	wh	-	all	Eu:UK	1988	CC	982	n	V	n	n	0	0	all/unsp	10	999	3	0	current	st
DEAN3	524	x	m	0	0	all	-	all	Eu:UK	1969	CC	766	n	V	y	n	1	0	cig only	3	4	1	1	cur+2y	ot
DEAN3	525	x	m	0	0	all	-	all	Eu:UK	1969	CC	766	n	V	y	n	1	0	cig only	5	8	2	0	cur+2y	ot
DEAN3	526	x	m	0	0	all	-	all	Eu:UK	1969	CC	766	n	V	y	n	1	0	cig only	9	18	3	2	cur+2y	ot
DEAN3	527	x	m	0	0	all	-	all	Eu:UK	1969	CC	766	n	V	y	n	1	0	cig only	19	999	0	3	cur+2y	ot
DEAN3	559		f	0	0	all	-	all	Eu:UK	1969	CC	766	n	V	y	n	1	0	all/unsp	3	4	1	1	cur+2y	ot
DEAN3	560		f	0	0	all	-	all	Eu:UK	1969	CC	766	n	V	y	n	1	0	all/unsp	5	8	2	0	cur+2y	ot
DEAN3	561		f	0	0	all	-	all	Eu:UK	1969	CC	766	n	V	y	n	1	0	all/unsp	9	999	3	0	cur+2y	ot
DESTEF	543	x	m	0	0	all	-	all	SCAmer	1988	CC	497	n	bl	n	y	0	0	cig+/-ot	0.1	4	1	1	current	st
DESTEF	544	x	m	0	0	all	-	all	SCAmer	1988	CC	497	n	bl	n	y	0	0	cig+/-ot	5	9	2	0	current	st
DESTEF	545	x	m	0	0	all	-	all	SCAmer	1988	CC	497	n	bl	n	y	0	0	cig+/-ot	10	999	3	0	current	st
DOLL	537		m	0	0	all	-	all	Eu:UK	1948	CC	1465	n	V	n	n	0	0	all/unsp	0.1	9	0	1	current	st
DOLL	538		m	0	0	all	-	all	Eu:UK	1948	CC	1465	n	V	n	n	0	0	all/unsp	10	19	3	2	current	st
DOLL	539		m	0	0	all	-	all	Eu:UK	1948	CC	1465	n	V	n	n	0	0	all/unsp	20	999	0	3	current	st
DOLL	548		f	0	0	all	-	all	Eu:UK	1948	CC	1465	n	V	n	n	0	0	all/unsp	0.1	9	0	1	current	st
DOLL	549		f	0	0	all	-	all	Eu:UK	1948	CC	1465	n	V	n	n	0	0	all/unsp	10	999	3	0	current	st
DOLL2	509		m	0	0	all	20	all	Eu:UK	1951	pr	920	n	V	n	n	1	0	cig only	0.1	4	1	1	current	ot
DOLL2	510		m	0	0	all	20	all	Eu:UK	1951	pr	920	n	V	n	n	1	0	cig only	5	9	2	0	current	ot

Table 1K6 - 1

IESLC - Meta-analysis of Ex Smoking by Years quit (vs current), Overview
All LC types, Cigarettes (or Any Product if Cigarettes not available)
Most adjusted

REF	NRR	1K1	SEX	AGEL	AGEH	RACE	YF	LC TYPE	LOC	START	ST	NLC	R	VB	P	H	AD	ADOS	PRODUCT	exL	exH	S1	S2	DENOM	De
DOLL2	511		m	0	0	all	20	all	Eu:UK	1951	pr	920	n	V	n	n	1	0	cig only	10	14	3	2	current	ot
DOLL2	512		m	0	0	all	20	all	Eu:UK	1951	pr	920	n	V	n	n	1	0	cig only	15	999	0	3	current	ot
DORGAN	514		m	0	0	wh	-	all	NAmer	1980	CC	2026	n	bl	y	y	0	0	cig+/-ot	1.1	5	1	1	cur+1y	st
DORGAN	515		m	0	0	wh	-	all	NAmer	1980	CC	2026	n	bl	y	y	0	0	cig+/-ot	6	9	2	0	cur+1y	st
DORGAN	516		m	0	0	wh	-	all	NAmer	1980	CC	2026	n	bl	y	y	0	0	cig+/-ot	10	999	3	0	cur+1y	st
DORGAN	559		f	0	0	all	-	all	NAmer	1980	CC	2026	n	bl	y	y	0	0	cig+/-ot	1.1	9	0	1	cur+1y	st
DORGAN	560		f	0	0	all	-	all	NAmer	1980	CC	2026	n	bl	y	y	0	0	cig+/-ot	10	999	3	0	cur+1y	st
DORN	823		m	55	64	wh	8	all	NAmer	1954	pr	5097	n	bl	n	n	0	0	cig+/-ot	0.1	4	1	1	current	st
DORN	824		m	55	64	wh	8	all	NAmer	1954	pr	5097	n	bl	n	n	0	0	cig+/-ot	5	9	2	0	current	st
DORN	825		m	55	64	wh	8	all	NAmer	1954	pr	5097	n	bl	n	n	0	0	cig+/-ot	10	14	3	2	current	st
DORN	826		m	55	64	wh	8	all	NAmer	1954	pr	5097	n	bl	n	n	0	0	cig+/-ot	15	999	0	3	current	st
DORN	827		m	65	74	wh	8	all	NAmer	1954	pr	5097	n	bl	n	n	0	0	cig+/-ot	0.1	4	1	1	current	st
DORN	828		m	65	74	wh	8	all	NAmer	1954	pr	5097	n	bl	n	n	0	0	cig+/-ot	5	9	2	0	current	st
DORN	829		m	65	74	wh	8	all	NAmer	1954	pr	5097	n	bl	n	n	0	0	cig+/-ot	10	14	3	2	current	st
DORN	830		m	65	74	wh	8	all	NAmer	1954	pr	5097	n	bl	n	n	0	0	cig+/-ot	15	999	0	3	current	st
GAO	536		m	0	0	all	-	all	As:Chi	1984	CC	1405	n	ot	n	n	2	0	cig+/-ot	0.1	4	1	1	current	ot
GAO	537		m	0	0	all	-	all	As:Chi	1984	CC	1405	n	ot	n	n	2	0	cig+/-ot	5	9	2	0	current	ot
GAO	538		m	0	0	all	-	all	As:Chi	1984	CC	1405	n	ot	n	n	2	0	cig+/-ot	10	999	3	0	current	ot
GAO	556		f	0	0	all	-	all	As:Chi	1984	CC	1405	n	ot	n	n	2	0	cig+/-ot	0.1	4	1	1	current	ot
GAO	557		f	0	0	all	-	all	As:Chi	1984	CC	1405	n	ot	n	n	2	0	cig+/-ot	5	9	2	0	current	ot
GAO	558		f	0	0	all	-	all	As:Chi	1984	CC	1405	n	ot	n	n	2	0	cig+/-ot	10	999	3	0	current	ot
GAO2	518		m	0	0	all	-	all	As:Jap	1988	CC	282	n	bl	n	n	0	0	cig+/-ot	1.0	4	1	1	cur+1y	st
GAO2	519		m	0	0	all	-	all	As:Jap	1988	CC	282	n	bl	n	n	0	0	cig+/-ot	5	9	2	0	cur+1y	st
GAO2	520		m	0	0	all	-	all	As:Jap	1988	CC	282	n	bl	n	n	0	0	cig+/-ot	10	14	3	2	cur+1y	st
GAO2	521		m	0	0	all	-	all	As:Jap	1988	CC	282	n	bl	n	n	0	0	cig+/-ot	15	19	0	0	cur+1y	st
GAO2	522		m	0	0	all	-	all	As:Jap	1988	CC	282	n	bl	n	n	0	0	cig+/-ot	20	999	0	3	cur+1y	st
GARCIA	508		c	0	0	all	-	all	NAmer	1992	CC	416	n	bl	n	y	0	0	cig+/-ot	1.0	4	1	1	cur+1y	st
GARCIA	509		c	0	0	all	-	all	NAmer	1992	CC	416	n	bl	n	y	0	0	cig+/-ot	5	14	0	2	cur+1y	st
GARCIA	510		c	0	0	all	-	all	NAmer	1992	CC	416	n	bl	n	y	0	0	cig+/-ot	15	29	0	3	cur+1y	st
GARCIA	511		c	0	0	all	-	all	NAmer	1992	CC	416	n	bl	n	y	0	0	cig+/-ot	30	999	0	0	cur+1y	st
GRAHAM	540		m	0	0	wh	-	all	NAmer	1956	CC	685	n	bl	n	n	1	0	cig+/-ot	0.1	1.0	0	0	current	ot
GRAHAM	541		m	0	0	wh	-	all	NAmer	1956	CC	685	n	bl	n	n	1	0	cig+/-ot	1.1	5	1	1	current	ot
GRAHAM	542		m	0	0	wh	-	all	NAmer	1956	CC	685	n	bl	n	n	1	0	cig+/-ot	5	999	0	0	current	ot
HAMMO2	510		m	0	0	all	0	all	NAmer	1967	pr	450	o	bl	n	n	1	0	cig+/-ot	0.1	4	1	1	current	ot
HAMMO2	511		m	0	0	all	0	all	NAmer	1967	pr	450	o	bl	n	n	1	0	cig+/-ot	5	9	2	0	current	ot
HAMMO2	512		m	0	0	all	0	all	NAmer	1967	pr	450	o	bl	n	n	1	0	cig+/-ot	10	999	3	0	current	ot
HIRAYA	513		m	0	0	all	0	all	As:Jap	1965	pr	1917	n	bl	n	n	1	0	cig+/-ot	0.1	4	1	1	current	ot
HIRAYA	514		m	0	0	all	0	all	As:Jap	1965	pr	1917	n	bl	n	n	1	0	cig+/-ot	5	9	2	0	current	ot
HIRAYA	515		m	0	0	all	0	all	As:Jap	1965	pr	1917	n	bl	n	n	1	0	cig+/-ot	10	999	3	0	current	ot
HIRAYA	524		f	0	0	all	0	all	As:Jap	1965	pr	1917	n	bl	n	n	1	0	cig+/-ot	0.1	4	1	1	current	ot
HIRAYA	525		f	0	0	all	0	all	As:Jap	1965	pr	1917	n	bl	n	n	1	0	cig+/-ot	5	9	2	0	current	ot
HIRAYA	526		f	0	0	all	0	all	As:Jap	1965	pr	1917	n	bl	n	n	1	0	cig+/-ot	10	999	3	0	current	ot
JAHN	513		m	0	0	all	-	all	Eu:Ger	1988	CC	1004	n	bl	n	n	0	0	cig+/-ot	0.1	0.9	0	0	current	st
JAHN	514		m	0	0	all	-	all	Eu:Ger	1988	CC	1004	n	bl	n	n	0	0	cig+/-ot	1.0	1.9	0	0	current	st
JAHN	515		m	0	0	all	-	all	Eu:Ger	1988	CC	1004	n	bl	n	n	0	0	cig+/-ot	2	5	1	1	current	st
JAHN	516		m	0	0	all	-	all	Eu:Ger	1988	CC	1004	n	bl	n	n	0	0	cig+/-ot	6	10	2	0	current	st
JAHN	517		m	0	0	all	-	all	Eu:Ger	1988	CC	1004	n	bl	n	n	0	0	cig+/-ot	11	20	3	0	current	st
JAHN	518		m	0	0	all	-	all	Eu:Ger	1988	CC	1004	n	bl	n	n	0	0	cig+/-ot	21	999	0	0	current	st
JAIN	570		m	0	0	all	-	all	NAmer	1981	CC	845	n	V	y	n	0	0	cig+/-ot	2	9	0	1	cur+2y	st
JAIN	571		m	0	0	all	-	all	NAmer	1981	CC	845	n	V	y	n	0	0	cig+/-ot	10	999	3	0	cur+2y	st
JAIN	534		f	0	0	all	-	all	NAmer	1981	CC	845	n	V	y	n	0	0	cig+/-ot	2	9	0	1	cur+2y	st
JAIN	535		f	0	0	all	-	all	NAmer	1981	CC	845	n	V	y	n	0	0	cig+/-ot	10	999	3	0	cur+2y	st
JOLY	571		m	0	0	all	-	all	SCAmer	1978	CC	826	n	bl	n	n	0	0	cig+/-ot	1.0	4	1	1	cur+1y	st
JOLY	572		m	0	0	all	-	all	SCAmer	1978	CC	826	n	bl	n	n	0	0	cig+/-ot	5	999	0	0	cur+1y	st
JOLY	558		f	0	0	all	-	all	SCAmer	1978	CC	826	n	bl	n	n	0	0	cig+/-ot	1.0	4	1	1	cur+1y	st
JOLY	559		f	0	0	all	-	all	SCAmer	1978	CC	826	n	bl	n	n	0	0	cig+/-ot	5	999	0	0	cur+1y	st
KAISE2	655		m	0	0	all	9	all	NAmer	1979	pr	318	n	bl	n	n	1	0	cig only	2	10	0	1	cur+2y	st
KAISE2	656		m	0	0	all	9	all	NAmer	1979	pr	318	n	bl	n	n	1	0	cig only	11	20	3	0	cur+2y	ot
KAISE2	657		m	0	0	all	9	all	NAmer	1979	pr	318	n	bl	n	n	1	0	cig only	21	999	0	0	cur+2y	st
KAISE2	575		f	0	0	all	9	all	NAmer	1979	pr	318	n	bl	n	n	1	0	cig only	2	10	0	1	cur+2y	st
KAISE2	576		f	0	0	all	9	all	NAmer	1979	pr	318	n	bl	n	n	1	0	cig only	11	20	3	0	cur+2y	st
KAISE2	577		f	0	0	all	9	all	NAmer	1979	pr	318	n	bl	n	n	1	0	cig only	21	999	0	0	cur+2y	ot
KHUDER	516		m	0	0	all	-	all	NAmer	1985	CC	482	n	bl	n	y	0	0	cig+/-ot	0.1	4	1	1	current	st
KHUDER	517		m	0	0	all	-	all	NAmer	1985	CC	482	n	bl	n	y	0	0	cig+/-ot	5	14	0	2	current	st
KHUDER	518		m	0	0	all	-	all	NAmer	1985	CC	482	n	bl	n	y	0	0	cig+/-ot	15	999	0	3	current	st
LUBIN	592		m	0	0	all	-	all	As:Chi	1984	CC	427	m	ot	y	n	0	0	cig+/-ot	3	4	1	1	cur+2y	st
LUBIN	593		m	0	0	all	-	all	As:Chi	1984	CC	427	m	ot	y	n	0	0	cig+/-ot	5	9	2	0	cur+2y	st
LUBIN	594		m	0	0	all	-	all	As:Chi	1984	CC	427	m	ot	y	n	0	0	cig+/-ot	10	999	3	0	cur+2y	st
LUBIN2	1081		m	0	0	all	-	all	Eu:mul	1976	CC	7804	n	bl	n	y	0	0	cig+/-ot	0.1	4	1	1	current	st
LUBIN2	1082		m	0	0	all	-	all	Eu:mul	1976	CC	7804	n	bl	n	y	0	0	cig+/-ot	5	9	2	0	current	st
LUBIN2	1083		m	0	0	all	-	all	Eu:mul	1976	CC	7804	n	bl	n	y	0	0	cig+/-ot	10	14	3	2	current	st
LUBIN2	1084		m	0	0	all	-	all	Eu:mul	1976	CC	7804	n	bl	n	y	0	0	cig+/-ot	15	19	0	0	current	st

Table 1K6 - 1

IESLC - Meta-analysis of Ex Smoking by Years quit (vs current), Overview
All LC types, Cigarettes (or Any Product if Cigarettes not available)
Most adjusted

REF	NRR	1K1	SEX	AGEL	AGEH	RACE	YF	LC TYPE	LOC	START	ST	NLC	R	VB	P	H	AD	ADOS	PRODUCT	exL	exH	S1	S2	DENOM	De
LUBIN2	1085		m	0	0	all	-	all	Eu:mul	1976	CC	7804	n	bl	n	y	0	0	cig+/-ot	20	24	0	3	current	st
LUBIN2	1086		m	0	0	all	-	all	Eu:mul	1976	CC	7804	n	bl	n	y	0	0	cig+/-ot	25	999	0	0	current	st
LUBIN2	1120		f	0	0	all	-	all	Eu:mul	1976	CC	7804	n	bl	n	y	0	0	cig+/-ot	0.1	4	1	1	current	st
LUBIN2	1121		f	0	0	all	-	all	Eu:mul	1976	CC	7804	n	bl	n	y	0	0	cig+/-ot	5	9	2	0	current	st
LUBIN2	1122		f	0	0	all	-	all	Eu:mul	1976	CC	7804	n	bl	n	y	0	0	cig+/-ot	10	14	3	2	current	st
LUBIN2	1123		f	0	0	all	-	all	Eu:mul	1976	CC	7804	n	bl	n	y	0	0	cig+/-ot	15	19	0	0	current	st
LUBIN2	1124		f	0	0	all	-	all	Eu:mul	1976	CC	7804	n	bl	n	y	0	0	cig+/-ot	20	24	0	3	current	st
LUBIN2	1125		f	0	0	all	-	all	Eu:mul	1976	CC	7804	n	bl	n	y	0	0	cig+/-ot	25	999	0	0	current	st
MATOS	596		m	0	0	all	-	all	SCAmer	1994	CC	200	n	bl	n	n	2	0	cig+/-ot	1.0	5	1	1	cur+1y	or
MATOS	597		m	0	0	all	-	all	SCAmer	1994	CC	200	n	bl	n	n	2	0	cig+/-ot	6	10	2	0	cur+1y	or
MATOS	598		m	0	0	all	-	all	SCAmer	1994	CC	200	n	bl	n	n	2	0	cig+/-ot	11	999	3	0	cur+1y	or
PEZZO2	504		m	0	0	all	-	all	SCAmer	1992	CC	367	n	bl	n	y	0	0	cig+/-ot	1.0	10	0	1	cur+1y	st
PEZZO2	505		m	0	0	all	-	all	SCAmer	1992	CC	367	n	bl	n	y	0	0	cig+/-ot	11	999	3	0	cur+1y	st
PEZZOT	504		m	0	0	all	-	all	SCAmer	1987	CC	215	n	bl	n	y	0	0	cig only	1.0	10	0	1	cur+1y	st
PEZZOT	505		m	0	0	all	-	all	SCAmer	1987	CC	215	n	bl	n	y	0	0	cig only	11	999	3	0	cur+1y	st
SOBUE	728		m	0	0	all	-	all	As:Jap	1986	CC	1376	n	bl	n	y	0	0	cig+/-ot	1.0	4	1	1	cur+1y	st
SOBUE	729		m	0	0	all	-	all	As:Jap	1986	CC	1376	n	bl	n	y	0	0	cig+/-ot	5	9	2	0	cur+1y	st
SOBUE	730		m	0	0	all	-	all	As:Jap	1986	CC	1376	n	bl	n	y	0	0	cig+/-ot	10	14	3	2	cur+1y	st
SOBUE	731		m	0	0	all	-	all	As:Jap	1986	CC	1376	n	bl	n	y	0	0	cig+/-ot	15	19	0	0	cur+1y	st
SOBUE	732		m	0	0	all	-	all	As:Jap	1986	CC	1376	n	bl	n	y	0	0	cig+/-ot	20	24	0	3	cur+1y	st
SOBUE	733		m	0	0	all	-	all	As:Jap	1986	CC	1376	n	bl	n	y	0	0	cig+/-ot	25	999	0	0	cur+1y	st
SPEIZE	511		f	0	0	all	0	all	NAmer	1976	pr	593	n	bl	n	y	2	0	cig+/-ot	0.1	1.9	0	0	current	or
SPEIZE	512		f	0	0	all	0	all	NAmer	1976	pr	593	n	bl	n	y	2	0	cig+/-ot	2	5	1	1	current	or
SPEIZE	513		f	0	0	all	0	all	NAmer	1976	pr	593	n	bl	n	y	2	0	cig+/-ot	5	10	2	0	current	or
SPEIZE	514		f	0	0	all	0	all	NAmer	1976	pr	593	n	bl	n	y	2	0	cig+/-ot	10	15	3	2	current	or
SPEIZE	515		f	0	0	all	0	all	NAmer	1976	pr	593	n	bl	n	y	2	0	cig+/-ot	15	999	0	3	current	or
SUZUK2	524		c	0	0	all	-	all	SCAmer	1991	CC	123	n	bl	n	y	3	0	all/unsp	0.1	5	1	1	current	or
SUZUK2	525		c	0	0	all	-	all	SCAmer	1991	CC	123	n	bl	n	y	3	0	all/unsp	6	10	2	0	current	or
SUZUK2	526		c	0	0	all	-	all	SCAmer	1991	CC	123	n	bl	n	y	3	0	all/unsp	11	999	3	0	current	or
SVENSS	554		f	0	0	all	-	all	Eu:Sca	1983	CC	210	n	bl	n	n	0	0	all/unsp	3	10	0	1	cur+2y	st
SVENSS	555		f	0	0	all	-	all	Eu:Sca	1983	CC	210	n	bl	n	n	0	0	all/unsp	11	999	3	0	cur+2y	st
TVERDA	506		m	0	0	all	0	all	Eu:Sca	1972	pr	238	n	bl	n	n	2	0	cig only	0.1	0.9	0	0	current	ot
TVERDA	507		m	0	0	all	0	all	Eu:Sca	1972	pr	238	n	bl	n	n	2	0	cig only	1.0	5	1	1	current	ot
TVERDA	508		m	0	0	all	0	all	Eu:Sca	1972	pr	238	n	bl	n	n	2	0	cig only	5	999	0	0	current	ot
WANG2	515		c	0	0	all	-	all	As:Chi	1980	CC	103	n	ot	n	n	0	0	cig+/-ot	0.1	3	1	1	current	st
WANG2	516		c	0	0	all	-	all	As:Chi	1980	CC	103	n	ot	n	n	0	0	cig+/-ot	4	999	0	0	current	st
WYNDE3	545		m	0	0	all	-	all	NAmer	1966	CC	350	n	bl	n	y	0	0	all/unsp	1.0	3	1	1	cur+1y	st
WYNDE3	546		m	0	0	all	-	all	NAmer	1966	CC	350	n	bl	n	y	0	0	all/unsp	4	6	0	0	cur+1y	st
WYNDE3	547		m	0	0	all	-	all	NAmer	1966	CC	350	n	bl	n	y	0	0	all/unsp	7	12	0	2	cur+1y	st
WYNDE3	548		m	0	0	all	-	all	NAmer	1966	CC	350	n	bl	n	y	0	0	all/unsp	13	999	0	3	cur+1y	st
WYNDE6	719		m	0	0	wh	-	all	NAmer	1969	CC	4423	n	bl	n	y	5	1#	cig+/-ot	1.0	10	0	1	cur+1y	or
WYNDE6	720		m	0	0	wh	-	all	NAmer	1969	CC	4423	n	bl	n	y	5	1#	cig+/-ot	11	19	3	2	cur+1y	or
WYNDE6	721		m	0	0	wh	-	all	NAmer	1969	CC	4423	n	bl	n	y	5	1#	cig+/-ot	20	999	0	3	cur+1y	or
WYNDE6	726		m	0	0	bl	-	all	NAmer	1969	CC	4423	n	bl	n	y	5	1#	cig+/-ot	1.0	10	0	1	cur+1y	or
WYNDE6	727		m	0	0	bl	-	all	NAmer	1969	CC	4423	n	bl	n	y	5	1#	cig+/-ot	11	19	3	2	cur+1y	or
WYNDE6	728		m	0	0	bl	-	all	NAmer	1969	CC	4423	n	bl	n	y	5	1#	cig+/-ot	20	999	0	3	cur+1y	or
WYNDE6	733		f	0	0	wh	-	all	NAmer	1969	CC	4423	n	bl	n	y	5	1#	cig+/-ot	1.0	10	0	1	cur+1y	or
WYNDE6	734		f	0	0	wh	-	all	NAmer	1969	CC	4423	n	bl	n	y	5	1#	cig+/-ot	11	999	3	0	cur+1y	or
WYNDE6	737		f	0	0	bl	-	all	NAmer	1969	CC	4423	n	bl	n	y	5	1#	cig+/-ot	1.0	10	0	1	cur+1y	or
WYNDE6	738		f	0	0	bl	-	all	NAmer	1969	CC	4423	n	bl	n	y	5	1#	cig+/-ot	11	999	3	0	cur+1y	or

Comments on values in listings

WYNDE6	ADOS	Number of cigs/day
WYNDE6	ADOS	Number of cigs/day
WYNDE6	ADOS	Number of cigs/day
WYNDE6	ADOS	Number of cigs/day
WYNDE6	ADOS	Number of cigs/day
WYNDE6	ADOS	Number of cigs/day
WYNDE6	ADOS	Number of cigs/day
WYNDE6	ADOS	Number of cigs/day
WYNDE6	ADOS	Number of cigs/day
WYNDE6	ADOS	Number of cigs/day

Cigarette type is all/unspec for all RRs
except for the following:

REF	NRR	CIGTYPE
ALDERS	513	MC only

Table 1K6 - 1

IESLC - Meta-analysis of Ex Smoking by Years quit (vs current), Overview
All LC types, Cigarettes (or Any Product if Cigarettes not available)
Most adjusted

REF	NRR	CIGTYPE
ALDERS	514	MC only
ALDERS	515	MC only
ALDERS	524	MC only
ALDERS	525	MC only
ALDERS	526	MC only
DEAN3	524	MC only
DEAN3	525	MC only
DEAN3	526	MC only
DEAN3	527	MC only
DESTEF	543	MC only
DESTEF	544	MC only
DESTEF	545	MC only

In this overview table, subtotals and Qs values may be invalid and should be ignored

Table 1K6 - 2

IESLC - Meta-analysis of Ex Smoking by Years quit (vs current), Overview
All LC types, Cigarettes (or Any Product if Cigarettes not available)
Most adjusted

REF	NRR	SEX	AD	Number Exposed Case	Cont	Non-exposed Case	Cont	RR	95.00%CI
ALDERS	513	m	1	121	-	207	-	1.81 (	1.24- 2.64)
ALDERS	514	m	1	28	-	207	-	0.43 (	0.26- 0.71)
ALDERS	515	m	1	29	-	207	-	0.32 (	0.20- 0.51)
ALDERS	524	f	1	206	-	244	-	2.08 (	1.49- 2.90)
ALDERS	525	f	1	54	-	244	-	0.65 (	0.43- 0.99)
ALDERS	526	f	1	26	-	244	-	0.28 (	0.17- 0.46)
Subtotal ALDERS								0.85 (	0.72- 1.01)
ARMADA	518	m	0	79	45	188	122	1.14 (	0.74- 1.75)
ARMADA	519	m	0	50	87	188	122	0.37 (	0.25- 0.57)
Subtotal ARMADA								0.64 (	0.47- 0.86)
BARBON	548	m	1	32	-	562	-	1.01 (	0.57- 1.79)
BARBON	549	m	1	89	-	562	-	0.66 (	0.47- 0.92)
BARBON	550	m	1	33	-	562	-	0.49 (	0.31- 0.79)
BARBON	551	m	1	15	-	562	-	0.15 (	0.08- 0.28)
Subtotal BARBON								0.54 (	0.43- 0.68)
BECHER	506	m	0	10	12	101	122	1.01 (	0.42- 2.43)
BECHER	507	m	0	16	32	101	122	0.60 (	0.31- 1.16)
BECHER	508	m	0	16	72	101	122	0.27 (	0.15- 0.49)
BECHER	516	f	0	2	3	33	26	0.53 (	0.08- 3.38)
BECHER	517	f	0	2	5	33	26	0.32 (	0.06- 1.76)
BECHER	518	f	0	1	10	33	26	0.08 (	0.01- 0.66)
Subtotal BECHER								0.44 (	0.30- 0.64)
BROSS	518	m	0	169	67	565	427	1.91 (	1.40- 2.60)
BROSS	519	m	0	43	79	565	427	0.41 (	0.28- 0.61)
Subtotal BROSS								1.06 (	0.83- 1.35)
CARPEN	508	c	0	28	46	228	164	0.44 (	0.26- 0.73)
CARPEN	509	c	0	31	52	228	164	0.43 (	0.26- 0.70)
CARPEN	510	c	0	13	58	228	164	0.16 (	0.09- 0.30)
CARPEN	511	c	0	37	158	228	164	0.17 (	0.11- 0.25)
Subtotal CARPEN								0.27 (	0.21- 0.34)
*CEDERL	538	m	1	12	-	97	-	0.78 (	0.43- 1.41)
*CEDERL	539	m	1	3	-	97	-	0.14 (	0.04- 0.45)
Subtotal CEDERL								0.56 (	0.33- 0.95)
CHOI	543	m	0	25	64	231	329	0.56 (	0.34- 0.91)
CHOI	544	m	0	5	30	231	329	0.24 (	0.09- 0.62)
CHOI	545	m	0	4	23	231	329	0.25 (	0.08- 0.73)
CHOI	546	m	0	4	19	231	329	0.30 (	0.10- 0.89)
CHOI	556	f	0	3	2	13	25	2.88 (	0.43- 19.49)
CHOI	557	f	0	2	0	13	25	9.44~(	0.42- 211.16)
Subtotal CHOI								0.46 (	0.32- 0.67)
*CHYOU	510	m	2	21	-	138	-	0.37 (	0.24- 0.57)
*CHYOU	511	m	2	5	-	138	-	0.27 (	0.11- 0.66)
Subtotal CHYOU								0.35 (	0.24- 0.51)
*CPSI	815	m	1	37	-	844	-	1.07 (	0.77- 1.49)
*CPSI	816	m	1	49	-	844	-	0.59 (	0.44- 0.78)
*CPSI	817	m	1	32	-	844	-	0.37 (	0.26- 0.53)
*CPSI	818	m	1	15	-	844	-	0.09 (	0.06- 0.15)
Subtotal CPSI								0.48 (	0.40- 0.57)
*CPSII	664	m	1	97	-	1159	-	1.77 (	1.44- 2.17)
*CPSII	665	m	1	188	-	1159	-	1.28 (	1.10- 1.49)
*CPSII	666	m	1	178	-	1159	-	0.85 (	0.72- 0.99)
*CPSII	667	m	1	186	-	1159	-	0.52 (	0.45- 0.61)
*CPSII	668	m	1	164	-	1159	-	0.39 (	0.33- 0.46)
*CPSII	669	m	1	256	-	1159	-	0.17 (	0.15- 0.20)
*CPSII	642	f	1	91	-	530	-	1.38 (	1.10- 1.72)
*CPSII	643	f	1	56	-	530	-	0.85 (	0.65- 1.12)
*CPSII	644	f	1	37	-	530	-	0.40 (	0.28- 0.55)
*CPSII	645	f	1	28	-	530	-	0.31 (	0.21- 0.46)
*CPSII	646	f	1	50	-	530	-	0.14 (	0.11- 0.19)
Subtotal CPSII								0.57 (	0.54- 0.60)
DAMBER	557	m	1	-	-	-	-	0.56 (	0.29- 1.08)
DAMBER	558	m	1	-	-	-	-	0.16 (	0.08- 0.35)
Subtotal DAMBER								0.32 (	0.20- 0.53)
DARBY	507	m	0	146	339	379	618	0.70 (	0.56- 0.89)
DARBY	508	m	0	139	767	379	618	0.30 (	0.24- 0.37)
DARBY	516	f	0	68	93	198	231	0.85 (	0.59- 1.23)
DARBY	517	f	0	26	224	198	231	0.14 (	0.09- 0.21)
Subtotal DARBY								0.44 (	0.38- 0.50)
DEAN3	524	m	1	28	-	337	-	0.64 (	0.41- 1.00)
DEAN3	525	m	1	11	-	337	-	0.57 (	0.29- 1.12)
DEAN3	526	m	1	15	-	337	-	0.41 (	0.23- 0.72)

Table 1K6 - 2

IESLC - Meta-analysis of Ex Smoking by Years quit (vs current), Overview
All LC types, Cigarettes (or Any Product if Cigarettes not available)
Most adjusted

REF	NRR	SEX	AD	Number Exposed Case	Cont	Non-exposed Case	Cont	RR	95.00%CI
DEAN3	527	m	1	8	-	337	-	0.18	(0.08- 0.38)
DEAN3	559	f	1	4	-	102	-	0.29	(0.11- 0.81)
DEAN3	560	f	1	1	-	102	-	0.20	(0.03- 1.42)
DEAN3	561	f	1	2	-	102	-	0.13	(0.03- 0.53)
Subtotal DEAN3								0.43	(0.32- 0.56)
DESTEF	543	m	0	10	19	78	109	0.74	(0.32- 1.67)
DESTEF	544	m	0	9	15	78	109	0.84	(0.35- 2.01)
DESTEF	545	m	0	10	41	78	109	0.34	(0.16- 0.72)
Subtotal DESTEF								0.57	(0.35- 0.90)
DOLL	537	m	0	56	75	1280	1172	0.68	(0.48- 0.98)
DOLL	538	m	0	6	26	1280	1172	0.21	(0.09- 0.52)
DOLL	539	m	0	8	23	1280	1172	0.32	(0.14- 0.71)
DOLL	548	f	0	9	6	58	41	1.06	(0.35- 3.21)
DOLL	549	f	0	1	2	58	41	0.35	(0.03- 4.03)
Subtotal DOLL								0.56	(0.42- 0.75)
*DOLL2	509	m	1	15	-	236	-	1.02	(0.61- 1.72)
*DOLL2	510	m	1	12	-	236	-	0.35	(0.20- 0.63)
*DOLL2	511	m	1	9	-	236	-	0.28	(0.14- 0.54)
*DOLL2	512	m	1	7	-	236	-	0.11	(0.05- 0.23)
Subtotal DOLL2								0.40	(0.30- 0.55)
DORGAN	514	m	0	59	51	465	303	0.75	(0.50- 1.13)
DORGAN	515	m	0	49	38	465	303	0.84	(0.54- 1.31)
DORGAN	516	m	0	134	255	465	303	0.34	(0.27- 0.44)
DORGAN	559	f	0	49	27	289	112	0.70	(0.42- 1.18)
DORGAN	560	f	0	34	50	289	112	0.26	(0.16- 0.43)
Subtotal DORGAN								0.47	(0.40- 0.56)
*DORN	823	m	0	34	22086	528	334175	0.97	(0.69- 1.38)
*DORN	824	m	0	32	34566	528	334175	0.59	(0.41- 0.84)
*DORN	825	m	0	12	23682	528	334175	0.32	(0.18- 0.57)
*DORN	826	m	0	16	58370	528	334175	0.17	(0.11- 0.29)
*DORN	827	m	0	14	6195	537	207895	0.87	(0.51- 1.49)
*DORN	828	m	0	41	24089	537	207895	0.66	(0.48- 0.90)
*DORN	829	m	0	29	20056	537	207895	0.56	(0.39- 0.81)
*DORN	830	m	0	34	51243	537	207895	0.26	(0.18- 0.36)
Subtotal DORN								0.51	(0.45- 0.59)
GAO	536	m	2	105	-	529	-	1.77	(1.22- 2.56)
GAO	537	m	2	24	-	529	-	0.79	(0.45- 1.40)
GAO	538	m	2	13	-	529	-	0.28	(0.14- 0.57)
GAO	556	f	2	37	-	170	-	2.48	(1.15- 5.38)
GAO	557	f	2	14	-	170	-	1.34	(0.51- 3.53)
GAO	558	f	2	16	-	170	-	0.76	(0.34- 1.67)
Subtotal GAO								1.14	(0.90- 1.46)
GAO2	518	m	0	31	26	184	117	0.76	(0.43- 1.34)
GAO2	519	m	0	21	26	184	117	0.51	(0.28- 0.95)
GAO2	520	m	0	16	18	184	117	0.57	(0.28- 1.15)
GAO2	521	m	0	7	9	184	117	0.49	(0.18- 1.36)
GAO2	522	m	0	8	25	184	117	0.20	(0.09- 0.47)
Subtotal GAO2								0.51	(0.37- 0.70)
GARCIA	508	c	0	33	11	77	42	1.64	(0.75- 3.57)
GARCIA	509	c	0	43	36	77	42	0.65	(0.36- 1.16)
GARCIA	510	c	0	32	67	77	42	0.26	(0.15- 0.46)
GARCIA	511	c	0	10	37	77	42	0.15	(0.07- 0.33)
Subtotal GARCIA								0.44	(0.32- 0.61)
GRAHAM	540	m	1	113	-	453	-	4.93	(3.53- 6.88)
GRAHAM	541	m	1	24	-	453	-	1.17	(0.70- 1.94)
GRAHAM	542	m	1	13	-	453	-	0.36	(0.19- 0.68)
Subtotal GRAHAM								2.25	(1.74- 2.91)
*HAMMO2	510	m	1	59	-	209	-	1.08	(0.82- 1.43)
*HAMMO2	511	m	1	11	-	209	-	0.39	(0.22- 0.71)
*HAMMO2	512	m	1	20	-	209	-	0.34	(0.22- 0.53)
Subtotal HAMMO2								0.71	(0.57- 0.88)
*HIRAYA	513	m	1	-	-	-	-	0.46	(0.26- 0.82)
*HIRAYA	514	m	1	-	-	-	-	0.36	(0.15- 0.86)
*HIRAYA	515	m	1	-	-	-	-	0.31	(0.14- 0.69)
*HIRAYA	524	f	1	-	-	-	-	1.59	(0.47- 5.37)
*HIRAYA	525	f	1	-	-	-	-	1.41	(0.23- 8.48)
*HIRAYA	526	f	1	-	-	-	-	0.41	(0.01- 14.37)
Subtotal HIRAYA								0.48	(0.33- 0.69)
JAHN	513	m	0	166	8	352	269	15.86	(7.66- 32.81)
JAHN	514	m	0	60	9	352	269	5.09	(2.48- 10.45)
JAHN	515	m	0	77	46	352	269	1.28	(0.86- 1.90)

Table 1K6 - 2

IESLC - Meta-analysis of Ex Smoking by Years quit (vs current), Overview
All LC types, Cigarettes (or Any Product if Cigarettes not available)
Most adjusted

REF	NRR	SEX	AD	Number Exposed Case	Number Exposed Cont	Non-exposed Case	Non-exposed Cont	RR	95.00%CI
JAHN	516	m	0	59	63	352	269	0.72	(0.49- 1.06)
JAHN	517	m	0	64	130	352	269	0.38	(0.27- 0.53)
JAHN	518	m	0	29	146	352	269	0.15	(0.10- 0.23)
Subtotal JAHN								0.70	(0.58- 0.84)
JAIN	570	m	0	74	46	265	118	0.72	(0.47- 1.10)
JAIN	571	m	0	52	113	265	118	0.20	(0.14- 0.30)
JAIN	534	f	0	66	36	305	99	0.60	(0.37- 0.95)
JAIN	535	f	0	19	61	305	99	0.10	(0.06- 0.18)
Subtotal JAIN								0.33	(0.27- 0.42)
JOLY	571	m	0	38	36	451	524	1.23	(0.76- 1.97)
JOLY	572	m	0	63	149	451	524	0.49	(0.36- 0.68)
JOLY	558	f	0	19	8	132	96	1.73	(0.73- 4.11)
JOLY	559	f	0	15	19	132	96	0.57	(0.28- 1.19)
Subtotal JOLY								0.70	(0.55- 0.88)
*KAISE2	655	m	1	12	-	51	-	1.00	(0.53- 1.88)
*KAISE2	656	m	1	8	-	51	-	0.43	(0.20- 0.92)
*KAISE2	657	m	1	6	-	51	-	0.26	(0.10- 0.67)
*KAISE2	575	f	1	6	-	50	-	0.53	(0.23- 1.23)
*KAISE2	576	f	1	4	-	50	-	0.25	(0.09- 0.70)
*KAISE2	577	f	1	4	-	50	-	0.34	(0.13- 0.91)
Subtotal KAISE2								0.49	(0.35- 0.68)
KHUDER	516	m	0	88	123	245	316	0.92	(0.67- 1.27)
KHUDER	517	m	0	63	133	245	316	0.61	(0.43- 0.86)
KHUDER	518	m	0	63	213	245	316	0.38	(0.28- 0.53)
Subtotal KHUDER								0.60	(0.50- 0.73)
LUBIN	592	m	0	33	18	296	650	4.03	(2.23- 7.27)
LUBIN	593	m	0	20	48	296	650	0.91	(0.53- 1.57)
LUBIN	594	m	0	17	73	296	650	0.51	(0.30- 0.88)
Subtotal LUBIN								1.16	(0.84- 1.60)
LUBIN2	1081	m	0	866	1047	4684	6211	1.10	(0.99- 1.21)
LUBIN2	1082	m	0	466	822	4684	6211	0.75	(0.67- 0.85)
LUBIN2	1083	m	0	270	693	4684	6211	0.52	(0.45- 0.60)
LUBIN2	1084	m	0	130	478	4684	6211	0.36	(0.30- 0.44)
LUBIN2	1085	m	0	106	413	4684	6211	0.34	(0.27- 0.42)
LUBIN2	1086	m	0	109	715	4684	6211	0.20	(0.16- 0.25)
LUBIN2	1120	f	0	60	55	440	410	1.02	(0.69- 1.50)
LUBIN2	1121	f	0	30	40	440	410	0.70	(0.43- 1.14)
LUBIN2	1122	f	0	10	26	440	410	0.36	(0.17- 0.75)
LUBIN2	1123	f	0	3	7	440	410	0.40	(0.10- 1.55)
LUBIN2	1124	f	0	4	9	440	410	0.41	(0.13- 1.36)
LUBIN2	1125	f	0	4	20	440	410	0.19	(0.06- 0.55)
Subtotal LUBIN2								0.65	(0.61- 0.68)
MATOS	596	m	2	28	-	112	-	1.40	(0.80- 2.60)
MATOS	597	m	2	21	-	112	-	0.90	(0.40- 1.60)
MATOS	598	m	2	27	-	112	-	0.30	(0.20- 0.60)
Subtotal MATOS								0.68	(0.48- 0.96)
PEZZO2	504	m	0	85	110	233	198	0.66	(0.47- 0.92)
PEZZO2	505	m	0	43	161	233	198	0.23	(0.15- 0.33)
Subtotal PEZZO2								0.41	(0.32- 0.53)
PEZZOT	504	m	0	46	82	145	129	0.50	(0.32- 0.77)
PEZZOT	505	m	0	20	106	145	129	0.17	(0.10- 0.29)
Subtotal PEZZOT								0.32	(0.23- 0.45)
SOBUE	728	m	0	128	116	737	633	0.95	(0.72- 1.24)
SOBUE	729	m	0	67	92	737	633	0.63	(0.45- 0.87)
SOBUE	730	m	0	35	50	737	633	0.60	(0.39- 0.94)
SOBUE	731	m	0	24	31	737	633	0.66	(0.39- 1.14)
SOBUE	732	m	0	15	23	737	633	0.56	(0.29- 1.08)
SOBUE	733	m	0	17	40	737	633	0.37	(0.20- 0.65)
Subtotal SOBUE								0.69	(0.59- 0.82)
*SPEIZE	511	f	2	24	-	319	-	0.60	(0.40- 0.90)
*SPEIZE	512	f	2	34	-	319	-	0.60	(0.40- 0.80)
*SPEIZE	513	f	2	41	-	319	-	0.50	(0.40- 0.70)
*SPEIZE	514	f	2	17	-	319	-	0.20	(0.10- 0.40)
*SPEIZE	515	f	2	28	-	319	-	0.10	(0.10- 0.40)
Subtotal SPEIZE								0.46	(0.38- 0.55)
SUZUK2	524	c	3	15	-	77	-	0.60	(0.20- 1.50)
SUZUK2	525	c	3	10	-	77	-	0.50	(0.20- 1.40)
SUZUK2	526	c	3	9	-	77	-	0.10	(0.10- 0.40)
Subtotal SUZUK2								0.23	(0.14- 0.38)
SVENSS	554	f	0	16	13	142	53	0.46	(0.21- 1.02)
SVENSS	555	f	0	14	24	142	53	0.22	(0.10- 0.45)

Table 1K6 - 2

IESLC - Meta-analysis of Ex Smoking by Years quit (vs current), Overview
All LC types, Cigarettes (or Any Product if Cigarettes not available)
Most adjusted

REF	NRR	SEX	AD	Number Exposed Case	Cont	Non-exposed Case	Cont	RR	95.00%CI
Subtotal SVENSS								0.31	(0.18- 0.52)
*TVERDA	506	m	2	2	-	144	-	0.17	(0.04- 0.70)
*TVERDA	507	m	2	5	-	144	-	0.18	(0.07- 0.43)
*TVERDA	508	m	2	4	-	144	-	0.08	(0.03- 0.23)
Subtotal TVERDA								0.13	(0.07- 0.25)
WANG2	515	c	0	6	10	49	78	0.96	(0.33- 2.79)
WANG2	516	c	0	5	11	49	78	0.72	(0.24- 2.21)
Subtotal WANG2								0.84	(0.39- 1.81)
WYNDE3	545	m	0	21	22	227	207	0.87	(0.47- 1.63)
WYNDE3	546	m	0	11	17	227	207	0.59	(0.27- 1.29)
WYNDE3	547	m	0	11	31	227	207	0.32	(0.16- 0.66)
WYNDE3	548	m	0	5	55	227	207	0.08	(0.03- 0.21)
Subtotal WYNDE3								0.42	(0.29- 0.61)
WYNDE6	719	m	5	-	-	-	-	0.60	(0.50- 0.70)
WYNDE6	720	m	5	-	-	-	-	0.30	(0.20- 0.40)
WYNDE6	721	m	5	-	-	-	-	0.20	(0.10- 0.20)
WYNDE6	726	m	5	-	-	-	-	0.70	(0.40- 1.10)
WYNDE6	727	m	5	-	-	-	-	0.20	(0.10- 0.50)
WYNDE6	728	m	5	-	-	-	-	0.30	(0.10- 0.60)
WYNDE6	733	f	5	-	-	-	-	0.50	(0.40- 0.60)
WYNDE6	734	f	5	-	-	-	-	0.20	(0.20- 0.30)
WYNDE6	737	f	5	-	-	-	-	0.60	(0.30- 1.30)
WYNDE6	738	f	5	-	-	-	-	0.40	(0.10- 1.10)
Subtotal WYNDE6								0.39	(0.35- 0.43)
Partial Totals				8482	250254	86457	2230807		

*prospective study ~ With 0.5 adjustment for zero

REF	NRR	SEX	AD	Ys	Ws	Qs	Ps
ALDERS	513	m	1	0.59	26.91	37.01	0.0021
ALDERS	514	m	1	-0.84	15.23	1.07	0.0010
ALDERS	515	m	1	-1.14	17.54	5.50	0.0000
ALDERS	524	f	1	0.73	34.65	59.62	0.0000
ALDERS	525	f	1	-0.43	22.10	0.49	0.0429
ALDERS	526	f	1	-1.27	15.51	7.46	0.0000
Subtotal ALDERS				-0.16	131.92	111.14	
ARMADA	518	m	0	0.13	20.66	10.41	0.5535
ARMADA	519	m	0	-0.99	22.22	3.68	0.0000
Subtotal ARMADA				-0.45	42.88	14.09	
BARBON	548	m	1	0.01	11.73	4.08	0.9728
BARBON	549	m	1	-0.42	34.06	0.91	0.0153
BARBON	550	m	1	-0.71	17.56	0.32	0.0028
BARBON	551	m	1	-1.90	9.79	17.00	0.0000
Subtotal BARBON				-0.62	73.15	22.31	
BECHER	506	m	0	0.01	4.96	1.70	0.9883
BECHER	507	m	0	-0.50	8.94	0.05	0.1316
BECHER	508	m	0	-1.32	10.58	5.73	0.0000
BECHER	516	f	0	-0.64	1.11	0.00	0.4978
BECHER	517	f	0	-1.15	1.30	0.43	0.1879
BECHER	518	f	0	-2.54	0.86	3.29	0.0188
Subtotal BECHER				-0.82	27.75	11.21	
BROSS	518	m	0	0.65	40.07	60.09	0.0000
BROSS	519	m	0	-0.89	24.98	2.38	0.0000
Subtotal BROSS				0.06	65.06	62.48	
CARPEN	508	c	0	-0.83	14.72	0.89	0.0015
CARPEN	509	c	0	-0.85	16.14	1.15	0.0007
CARPEN	510	c	0	-1.82	9.56	14.83	0.0000
CARPEN	511	c	0	-1.78	22.81	32.94	0.0000
Subtotal CARPEN				-1.33	63.22	49.82	
*CEDERL	538	m	1	-0.25	10.90	1.19	0.4121
*CEDERL	539	m	1	-1.97	2.62	5.04	0.0015
Subtotal CEDERL				-0.58	13.52	6.24	
CHOI	543	m	0	-0.59	15.87	0.00	0.0195
CHOI	544	m	0	-1.44	4.15	3.06	0.0034
CHOI	545	m	0	-1.40	3.32	2.21	0.0109
CHOI	546	m	0	-1.20	3.23	1.26	0.0305
CHOI	556	f	0	1.06	1.05	2.83	0.2771
CHOI	557	f	0	2.25	0.40	3.17	0.1567
Subtotal CHOI				-0.78	28.03	12.54	
*CHYOU	510	m	2	-0.99	20.54	3.53	0.0000
*CHYOU	511	m	2	-1.31	4.79	2.55	0.0042

Table 1K6 - 2

IESLC - Meta-analysis of Ex Smoking by Years quit (vs current), Overview
All LC types, Cigarettes (or Any Product if Cigarettes not available)
Most adjusted

REF	NRR	SEX	AD	Ys	Ws	Qs	Ps
Subtotal CHYOU				-1.05	25.32	6.08	
*CPSI	815	m	1	0.07	35.26	14.76	0.6879
*CPSI	816	m	1	-0.53	46.88	0.13	0.0003
*CPSI	817	m	1	-0.99	30.29	5.21	0.0000
*CPSI	818	m	1	-2.41	18.30	61.19	0.0000
Subtotal CPSI				-0.74	130.73	81.29	
*CPSII	664	m	1	0.57	91.37	120.92	0.0000
*CPSII	665	m	1	0.25	166.85	113.91	0.0014
*CPSII	666	m	1	-0.16	151.52	26.33	0.0454
*CPSII	667	m	1	-0.65	166.04	0.92	0.0000
*CPSII	668	m	1	-0.94	139.29	18.27	0.0000
*CPSII	669	m	1	-1.77	185.67	264.05	0.0000
*CPSII	642	f	1	0.32	76.90	62.49	0.0047
*CPSII	643	f	1	-0.16	51.90	9.02	0.2417
*CPSII	644	f	1	-0.92	33.71	3.83	0.0000
*CPSII	645	f	1	-1.17	24.99	8.75	0.0000
*CPSII	646	f	1	-1.97	51.44	98.92	0.0000
Subtotal CPSII				-0.57	1139.68	727.42	
DAMBER	557	m	1	-0.58	8.89	0.00	0.0839
DAMBER	558	m	1	-1.83	7.05	11.08	0.0000
Subtotal DAMBER				-1.13	15.94	11.08	
DARBY	507	m	0	-0.35	71.14	3.63	0.0029
DARBY	508	m	0	-1.22	78.40	32.08	0.0000
DARBY	516	f	0	-0.16	28.70	5.07	0.3945
DARBY	517	f	0	-2.00	19.12	38.55	0.0000
Subtotal DARBY				-0.83	197.37	79.34	
DEAN3	524	m	1	-0.45	19.33	0.34	0.0497
DEAN3	525	m	1	-0.56	8.42	0.00	0.1029
DEAN3	526	m	1	-0.89	11.80	1.15	0.0022
DEAN3	527	m	1	-1.71	6.33	8.16	0.0000
DEAN3	559	f	1	-1.24	3.85	1.67	0.0151
DEAN3	560	f	1	-1.61	1.03	1.10	0.1019
DEAN3	561	f	1	-2.04	1.86	3.98	0.0054
Subtotal DEAN3				-0.85	52.62	16.40	
DESTEF	543	m	0	-0.31	5.73	0.42	0.4622
DESTEF	544	m	0	-0.18	5.01	0.81	0.6934
DESTEF	545	m	0	-1.08	6.83	1.69	0.0049
Subtotal DESTEF				-0.57	17.56	2.93	
DOLL	537	m	0	-0.38	30.46	1.21	0.0358
DOLL	538	m	0	-1.55	4.84	4.60	0.0006
DOLL	539	m	0	-1.14	5.88	1.88	0.0055
DOLL	548	f	0	0.06	3.13	1.27	0.9174
DOLL	549	f	0	-1.04	0.65	0.14	0.4022
Subtotal DOLL				-0.59	44.96	9.09	
*DOLL2	509	m	1	0.02	14.30	5.13	0.9403
*DOLL2	510	m	1	-1.05	11.67	2.58	0.0003
*DOLL2	511	m	1	-1.27	8.43	4.06	0.0002
*DOLL2	512	m	1	-2.21	6.60	17.48	0.0000
Subtotal DOLL2				-0.91	41.00	29.26	
DORGAN	514	m	0	-0.28	23.81	2.10	0.1680
DORGAN	515	m	0	-0.17	19.17	3.15	0.4460
DORGAN	516	m	0	-1.07	59.40	14.40	0.0000
DORGAN	559	f	0	-0.35	14.32	0.74	0.1829
DORGAN	560	f	0	-1.33	16.18	9.20	0.0000
Subtotal DORGAN				-0.76	132.87	29.59	
*DORN	823	m	0	-0.03	31.99	9.80	0.8830
*DORN	824	m	0	-0.53	30.20	0.06	0.0033
*DORN	825	m	0	-1.14	11.74	3.65	0.0001
*DORN	826	m	0	-1.75	15.53	21.35	0.0000
*DORN	827	m	0	-0.13	13.68	2.72	0.6211
*DORN	828	m	0	-0.42	38.16	1.00	0.0100
*DORN	829	m	0	-0.58	27.56	0.00	0.0023
*DORN	830	m	0	-1.36	32.00	19.46	0.0000
Subtotal DORN				-0.67	200.86	58.04	
GAO	536	m	2	0.57	27.97	37.02	0.0025
GAO	537	m	2	-0.24	11.93	1.41	0.4156
GAO	538	m	2	-1.27	7.80	3.75	0.0004
GAO	556	f	2	0.91	6.45	14.28	0.0210
GAO	557	f	2	0.29	4.11	3.12	0.5532
GAO	558	f	2	-0.27	6.07	0.56	0.4991
Subtotal GAO				0.13	64.32	60.15	

Table 1K6 - 2

IESLC - Meta-analysis of Ex Smoking by Years quit (vs current), Overview
All LC types, Cigarettes (or Any Product if Cigarettes not available)
Most adjusted

REF	NRR	SEX	AD	Ys	Ws	Qs	Ps
GAO2	518	m	0	-0.28	11.81	1.08	0.3414
GAO2	519	m	0	-0.67	9.99	0.08	0.0352
GAO2	520	m	0	-0.57	7.57	0.00	0.1164
GAO2	521	m	0	-0.70	3.73	0.06	0.1738
GAO2	522	m	0	-1.59	5.59	5.73	0.0002
Subtotal GAO2				-0.67	38.69	6.95	
GARCIA	508	c	0	0.49	6.33	7.27	0.2154
GARCIA	509	c	0	-0.43	11.39	0.26	0.1483
GARCIA	510	c	0	-1.35	12.05	7.07	0.0000
GARCIA	511	c	0	-1.91	6.10	10.88	0.0000
Subtotal GARCIA				-0.83	35.87	25.48	
GRAHAM	540	m	1	1.60	34.51	163.19	0.0000
GRAHAM	541	m	1	0.16	14.79	8.02	0.5460
GRAHAM	542	m	1	-1.02	9.45	1.85	0.0017
Subtotal GRAHAM				0.81	58.74	173.06	
*HAMMO2	510	m	1	0.08	49.68	21.40	0.5875
*HAMMO2	511	m	1	-0.94	11.19	1.47	0.0016
*HAMMO2	512	m	1	-1.08	19.88	4.96	0.0000
Subtotal HAMMO2				-0.35	80.75	27.83	
*HIRAYA	513	m	1	-0.78	11.65	0.45	0.0080
*HIRAYA	514	m	1	-1.02	5.04	0.99	0.0218
*HIRAYA	515	m	1	-1.17	6.04	2.12	0.0040
*HIRAYA	524	f	1	0.46	2.59	2.82	0.4555
*HIRAYA	525	f	1	0.34	1.18	1.01	0.7089
*HIRAYA	526	f	1	-0.89	0.29	0.03	0.6307
Subtotal HIRAYA				-0.74	26.79	7.41	
JAHN	513	m	0	2.76	7.27	81.23	0.0000
JAHN	514	m	0	1.63	7.44	36.28	0.0000
JAHN	515	m	0	0.25	24.22	16.51	0.2255
JAHN	516	m	0	-0.33	25.39	1.52	0.0919
JAHN	517	m	0	-0.98	33.47	5.31	0.0000
JAHN	518	m	0	-1.89	20.88	35.61	0.0000
Subtotal JAHN				-0.36	118.68	176.46	
JAIN	570	m	0	-0.33	21.05	1.27	0.1258
JAIN	571	m	0	-1.59	24.80	25.08	0.0000
JAIN	534	f	0	-0.52	17.76	0.06	0.0287
JAIN	535	f	0	-2.29	12.14	35.58	0.0000
Subtotal JAIN				-1.10	75.74	62.00	
JOLY	571	m	0	0.20	17.18	10.54	0.3976
JOLY	572	m	0	-0.71	37.44	0.65	0.0000
JOLY	558	f	0	0.55	5.11	6.48	0.2166
JOLY	559	f	0	-0.55	7.28	0.00	0.1343
Subtotal JOLY				-0.36	67.01	17.68	
*KAISE2	655	m	1	0.00	9.58	3.22	1.0000
*KAISE2	656	m	1	-0.84	6.60	0.46	0.0302
*KAISE2	657	m	1	-1.35	4.25	2.50	0.0055
*KAISE2	575	f	1	-0.63	5.47	0.02	0.1377
*KAISE2	576	f	1	-1.39	3.65	2.38	0.0081
*KAISE2	577	f	1	-1.08	4.06	1.01	0.0298
Subtotal KAISE2				-0.72	33.61	9.59	
KHUDER	516	m	0	-0.08	37.40	9.31	0.6231
KHUDER	517	m	0	-0.49	32.64	0.25	0.0049
KHUDER	518	m	0	-0.96	35.95	5.31	0.0000
Subtotal KHUDER				-0.51	105.99	14.87	
LUBIN	592	m	0	1.39	11.02	42.85	0.0000
LUBIN	593	m	0	-0.09	13.20	3.18	0.7468
LUBIN	594	m	0	-0.67	12.91	0.11	0.0160
Subtotal LUBIN				0.15	37.13	46.13	
LUBIN2	1081	m	0	0.09	402.52	181.65	0.0639
LUBIN2	1082	m	0	-0.29	267.60	23.13	0.0000
LUBIN2	1083	m	0	-0.66	181.12	1.19	0.0000
LUBIN2	1084	m	0	-1.02	98.44	19.10	0.0000
LUBIN2	1085	m	0	-1.08	81.77	20.31	0.0000
LUBIN2	1086	m	0	-1.60	91.35	94.92	0.0000
LUBIN2	1120	f	0	0.02	25.28	8.97	0.9343
LUBIN2	1121	f	0	-0.36	15.86	0.78	0.1536
LUBIN2	1122	f	0	-1.03	6.98	1.39	0.0067
LUBIN2	1123	f	0	-0.92	2.08	0.24	0.1856
LUBIN2	1124	f	0	-0.88	2.73	0.25	0.1450
LUBIN2	1125	f	0	-1.68	3.28	3.98	0.0023
Subtotal LUBIN2				-0.44	1179.01	355.91	

Table 1K6 - 2

IESLC - Meta-analysis of Ex Smoking by Years quit (vs current), Overview
All LC types, Cigarettes (or Any Product if Cigarettes not available)
Most adjusted

REF	NRR	SEX	AD	Ys	Ws	Qs	Ps
MATOS	596	m	2	0.34	11.06	9.28	0.2631
MATOS	597	m	2	-0.11	8.00	1.80	0.7658
MATOS	598	m	2	-1.20	12.73	4.97	0.0000
Subtotal MATOS				-0.39	31.79	16.04	
PEZZO2	504	m	0	-0.42	33.11	0.84	0.0155
PEZZO2	505	m	0	-1.48	25.77	21.04	0.0000
Subtotal PEZZO2				-0.89	58.88	21.87	
PEZZOT	504	m	0	-0.69	20.58	0.28	0.0016
PEZZOT	505	m	0	-1.78	13.50	19.61	0.0000
Subtotal PEZZOT				-1.13	34.08	19.88	
SOBUE	728	m	0	-0.05	51.63	14.27	0.6997
SOBUE	729	m	0	-0.47	34.80	0.42	0.0056
SOBUE	730	m	0	-0.51	19.41	0.10	0.0250
SOBUE	731	m	0	-0.41	13.01	0.38	0.1411
SOBUE	732	m	0	-0.58	8.84	0.00	0.0848
SOBUE	733	m	0	-1.01	11.53	2.12	0.0006
Subtotal SOBUE				-0.37	139.23	17.29	
*SPEIZE	511	f	2	-0.51	23.37	0.11	0.0135
*SPEIZE	512	f	2	-0.51	31.98	0.15	0.0039
*SPEIZE	513	f	2	-0.69	49.07	0.63	0.0000
*SPEIZE	514	f	2	-1.61	8.00	8.48	0.0000
*SPEIZE	515	f	2	-2.30	8.00	23.74	0.0000
Subtotal SPEIZE				-0.78	120.40	33.12	
SUZUK2	524	c	3	-0.51	3.78	0.02	0.3203
SUZUK2	525	c	3	-0.69	4.06	0.05	0.1626
SUZUK2	526	c	3	-2.30	8.00	23.74	0.0000
Subtotal SUZUK2				-1.46	15.84	23.81	
SVENSS	554	f	0	-0.78	6.05	0.24	0.0557
SVENSS	555	f	0	-1.52	7.19	6.43	0.0000
Subtotal SVENSS				-1.18	13.24	6.66	
*TVERDA	506	m	2	-1.77	1.88	2.67	0.0152
*TVERDA	507	m	2	-1.71	4.66	6.01	0.0002
*TVERDA	508	m	2	-2.53	3.70	14.03	0.0000
Subtotal TVERDA				-2.02	10.24	22.71	
WANG2	515	c	0	-0.05	3.33	0.95	0.9331
WANG2	516	c	0	-0.32	3.09	0.20	0.5698
Subtotal WANG2				-0.18	6.42	1.15	
WYNDE3	545	m	0	-0.14	9.77	1.90	0.6644
WYNDE3	546	m	0	-0.53	6.29	0.02	0.1858
WYNDE3	547	m	0	-1.13	7.55	2.28	0.0019
WYNDE3	548	m	0	-2.49	4.40	16.05	0.0000
Subtotal WYNDE3				-0.86	28.01	20.24	
WYNDE6	719	m	5	-0.51	135.72	0.64	0.0000
WYNDE6	720	m	5	-1.20	31.98	12.48	0.0000
WYNDE6	721	m	5	-1.61	31.98	33.93	0.0000
WYNDE6	726	m	5	-0.36	15.02	0.74	0.1669
WYNDE6	727	m	5	-1.61	5.93	6.29	0.0001
WYNDE6	728	m	5	-1.20	4.79	1.87	0.0084
WYNDE6	733	f	5	-0.69	93.46	1.21	0.0000
WYNDE6	734	f	5	-1.61	93.46	99.16	0.0000
WYNDE6	737	f	5	-0.51	7.15	0.03	0.1721
WYNDE6	738	f	5	-0.92	2.67	0.30	0.1342
Subtotal WYNDE6				-0.95	422.17	156.66	

N	194
NS	43

Table 1K6 - 3

IESLC - Meta-analysis of Ex Smoking by Years quit (vs current), Overview
All LC types, Cigarettes (or Any Product if Cigarettes not available)
Most adjusted

	Sex			
	combined	male	female	Total
N	13	129	52	194
NS	4	37	17	58

In this overview table, other than the "N" rows, entries in the "absent" and "Total" columns may be invalid and should be ignored

	Years quit vs current (lower focus)				
	absent	1-6k3	4-11k7	8+k12	Total
N	78	40	28	48	194
NS	35	31	21	32	119
Wt	2113.68	1243.39	835.64	1054.39	5247.10
Het Chi	1505.96	143.30	57.93	201.14	2663.27
Het df	77	39	27	47	193
Het P	***	***	***	***	***
Fixed RR	0.52	0.98	0.61	0.32	0.56
RRl	0.50	0.93	0.57	0.30	0.55
RRu	0.54	1.04	0.65	0.34	0.58
P	---	N.S.	---	---	---
Random RR	0.48	0.95	0.57	0.28	0.49
RRl	0.39	0.84	0.51	0.24	0.44
RRu	0.58	1.08	0.65	0.32	0.55
P	---	N.S.	---	---	---

	Years quit vs current (higher focus)				
	absent	1-11k3	4-19k12	13+k20	Total
N	93	60	20	21	194
NS	42	42	16	17	117
Wt	2297.79	1813.22	588.17	547.93	5247.10
Het Chi	1423.58	244.12	55.22	87.55	2663.27
Het df	92	59	19	20	193
Het P	***	***	***	***	***
Fixed RR	0.54	0.85	0.44	0.22	0.56
RRl	0.52	0.81	0.40	0.20	0.55
RRu	0.56	0.89	0.47	0.24	0.58
P	---	---	---	---	---
Random RR	0.44	0.83	0.39	0.23	0.49
RRl	0.37	0.74	0.33	0.19	0.44
RRu	0.52	0.92	0.47	0.28	0.55
P	---	---	---	---	---

Table 1K6 - 3

IESLC - Meta-analysis of Ex Smoking by Years quit (vs current), Overview
All LC types, Cigarettes (or Any Product if Cigarettes not available)
Most adjusted

MALES

		Years quit vs current (lower focus)				
		absent	1-6k3	4-11k7	8+k12	Total
	N	53	27	19	30	129
	NS	29	26	18	28	101
	Wt	1645.92	1085.89	709.19	817.22	4258.22
Het	Chi	1189.57	106.23	41.81	113.07	2008.73
Het	df	52	26	18	29	128
Het	P	***	***	**	***	***
Fixed	RR	0.51	1.01	0.63	0.36	0.59
	RRl	0.48	0.95	0.58	0.33	0.57
	RRu	0.53	1.07	0.68	0.38	0.60
	P	---	N.S.	---	---	---
Random	RR	0.47	0.98	0.59	0.31	0.51
	RRl	0.37	0.85	0.52	0.27	0.45
	RRu	0.60	1.13	0.69	0.36	0.58
	P	---	N.S.	---	---	---

		Years quit vs current (higher focus)				
		absent	1-11k3	4-19k12	13+k20	Total
	N	60	38	15	16	129
	NS	34	36	13	14	97
	Wt	1822.49	1457.58	527.26	450.89	4258.22
Het	Chi	1042.66	172.82	34.89	67.95	2008.73
Het	df	59	37	14	15	128
Het	P	***	***	**	***	***
Fixed	RR	0.56	0.90	0.46	0.23	0.59
	RRl	0.54	0.85	0.42	0.21	0.57
	RRu	0.59	0.95	0.50	0.25	0.60
	P	---	---	---	---	---
Random	RR	0.47	0.86	0.43	0.25	0.51
	RRl	0.38	0.76	0.36	0.20	0.45
	RRu	0.58	0.98	0.51	0.32	0.58
	P	---	-	---	---	---

FEMALES

		Years quit vs current (lower focus)				
		absent	1-6k3	4-11k7	8+k12	Total
	N	20	9	7	16	52
	NS	13	9	7	15	44
	Wt	412.32	129.33	106.26	219.62	867.53
Het	Chi	259.64	21.93	9.84	30.79	527.20
Het	df	19	8	6	15	51
Het	P	***	**	N.S.	**	***
Fixed	RR	0.61	0.86	0.51	0.22	0.48
	RRl	0.55	0.73	0.42	0.19	0.45
	RRu	0.67	1.03	0.61	0.25	0.52
	P	---	(-)	---	---	---
Random	RR	0.54	0.97	0.54	0.23	0.48
	RRl	0.36	0.67	0.40	0.18	0.37
	RRu	0.81	1.39	0.74	0.29	0.60
	P	--	N.S.	---	---	---

Table 1K6 - 3

IESLC - Meta-analysis of Ex Smoking by Years quit (vs current), Overview
All LC types, Cigarettes (or Any Product if Cigarettes not available)
Most adjusted

FEMALES

	Years quit vs current (higher focus) absent	1-11k3	4-19k12	13+k20	Total
N	28	18	3	3	52
NS	17	17	3	3	40
Wt	437.92	327.47	39.97	62.17	867.53
Het Chi	333.97	42.03	1.53	4.12	527.20
Het df	27	17	2	2	51
Het P	***	***	N.S.	N.S.	***
Fixed RR	0.47	0.69	0.29	0.14	0.48
RRl	0.42	0.62	0.21	0.11	0.45
RRu	0.51	0.76	0.40	0.18	0.52
P	---	---	---	---	---
Random RR	0.40	0.75	0.29	0.15	0.48
RRl	0.27	0.61	0.21	0.09	0.37
RRu	0.59	0.92	0.40	0.25	0.60
P	---	--	---	---	---

Table 1K6 - 4

IESLC - Meta-analysis of Ex Smoking by Years quit (vs current), Overview
All LC types, Cigarettes (or Any Product if Cigarettes not available)
Least adjusted

REF	NRR	X	SEX	AGEL	AGEH	RACE	YF	LC TYPE	LOC	START	ST	NLC	R	VB	P	H	AD	ADOS	PRODUCT	exL	exH	S1	S2	DENOM	De
ALDERS	513		m	0	0	all	-	all	Eu:UK	1977	CC	1448	n	V	n	n	1	0	cig only	0.1	2	0	0	current	ot
ALDERS	514		m	0	0	all	-	all	Eu:UK	1977	CC	1448	n	V	n	n	1	0	cig only	3	9	0	1	current	ot
ALDERS	515		m	0	0	all	-	all	Eu:UK	1977	CC	1448	n	V	n	n	1	0	cig only	10	999	3	0	current	ot
ALDERS	524		f	0	0	all	-	all	Eu:UK	1977	CC	1448	n	V	n	n	1	0	cig only	0.1	2	0	0	current	ot
ALDERS	525		f	0	0	all	-	all	Eu:UK	1977	CC	1448	n	V	n	n	1	0	cig only	3	9	0	1	current	ot
ALDERS	526		f	0	0	all	-	all	Eu:UK	1977	CC	1448	n	V	n	n	1	0	cig only	10	999	3	0	current	ot
ARMADA	518		m	0	0	all	-	all	Eu:wst	1986	CC	325	n	bl	n	y	0	0	cig+/-ot	1.0	5	1	1	cur+1y	st
ARMADA	519		m	0	0	all	-	all	Eu:wst	1986	CC	325	n	bl	n	y	0	0	cig+/-ot	6	999	0	0	cur+1y	st
BARBON	533	x	m	0	0	all	-	all	Eu:wst	1979	CC	755	n	bl	y	y	0	0	all/unsp	0.1	4	1	1	current	st
BARBON	534	x	m	0	0	all	-	all	Eu:wst	1979	CC	755	n	bl	y	y	0	0	all/unsp	5	14	0	2	current	st
BARBON	535	x	m	0	0	all	-	all	Eu:wst	1979	CC	755	n	bl	y	y	0	0	all/unsp	15	24	0	3	current	st
BARBON	536	x	m	0	0	all	-	all	Eu:wst	1979	CC	755	n	bl	y	y	0	0	all/unsp	25	999	0	0	current	st
BECHER	506		m	0	0	all	-	all	Eu:Ger	1985	CC	194	n	bl	n	y	0	0	all/unsp	2	4	1	1	cur+1y	st
BECHER	507		m	0	0	all	-	all	Eu:Ger	1985	CC	194	n	bl	n	y	0	0	all/unsp	5	9	2	0	cur+1y	st
BECHER	508		m	0	0	all	-	all	Eu:Ger	1985	CC	194	n	bl	n	y	0	0	all/unsp	10	999	3	0	cur+1y	st
BECHER	516		f	0	0	all	-	all	Eu:Ger	1985	CC	194	n	bl	n	y	0	0	all/unsp	2	4	1	1	cur+1y	st
BECHER	517		f	0	0	all	-	all	Eu:Ger	1985	CC	194	n	bl	n	y	0	0	all/unsp	5	9	2	0	cur+1y	st
BECHER	518		f	0	0	all	-	all	Eu:Ger	1985	CC	194	n	bl	n	y	0	0	all/unsp	10	999	3	0	cur+1y	st
BROSS	518		m	0	0	wh	-	all	NAmer	1960	CC	974	n	bl	n	n	0	0	cig+/-ot	0.1	5	1	1	current	st
BROSS	519		m	0	0	wh	-	all	NAmer	1960	CC	974	n	bl	n	n	0	0	cig+/-ot	6	999	0	0	current	st
CARPEN	508		c	0	0	w+b	-	all	NAmer	1991	CC	356	n	bl	n	n	0	0	cig+/-ot	0.1	4	1	1	current	st
CARPEN	509		c	0	0	w+b	-	all	NAmer	1991	CC	356	n	bl	n	n	0	0	cig+/-ot	5	9	2	0	current	st
CARPEN	510		c	0	0	w+b	-	all	NAmer	1991	CC	356	n	bl	n	n	0	0	cig+/-ot	10	14	3	2	current	st
CARPEN	511		c	0	0	w+b	-	all	NAmer	1991	CC	356	n	bl	n	n	0	0	cig+/-ot	15	999	0	3	current	st
CEDERL	538		m	40	69	all	10	all	Eu:Sca	1963	pr	491	n	bl	n	n	1	0	all/unsp	0.1	9	0	1	current	ot
CEDERL	539		m	40	69	all	10	all	Eu:Sca	1963	pr	491	n	bl	n	n	1	0	all/unsp	10	999	3	0	current	ot
CHOI	543		m	0	0	all	-	all	As:oth	1985	CC	375	n	bl	n	n	0	0	cig+/-ot	0.1	4	1	1	current	st
CHOI	544		m	0	0	all	-	all	As:oth	1985	CC	375	n	bl	n	n	0	0	cig+/-ot	5	9	2	0	current	st
CHOI	545		m	0	0	all	-	all	As:oth	1985	CC	375	n	bl	n	n	0	0	cig+/-ot	10	14	3	2	current	st
CHOI	546		m	0	0	all	-	all	As:oth	1985	CC	375	n	bl	n	n	0	0	cig+/-ot	15	999	0	3	current	st
CHOI	556		f	0	0	all	-	all	As:oth	1985	CC	375	n	bl	n	n	0	0	cig+/-ot	0.1	4	1	1	current	st
CHOI	557		f	0	0	all	-	all	As:oth	1985	CC	375	n	bl	n	n	0	0	cig+/-ot	5	999	0	0	current	ot
CHYOU	504	x	m	0	0	jap	21	all	NAmer	1965	pr	227	n	bl	n	y	1	0	cig+/-ot	0.1	14	0	0	current	ot
CHYOU	505	x	m	0	0	jap	21	all	NAmer	1965	pr	227	n	bl	n	y	1	0	cig+/-ot	15	999	0	3	current	ot
CPSI	815		m	50	74	all	6	all	NAmer	1959	pr	5138	n	bl	n	n	1	0	cig only	0.1	0.9	0	0	current	ot
CPSI	816		m	50	74	all	6	all	NAmer	1959	pr	5138	n	bl	n	n	1	0	cig only	1.0	4	1	1	current	ot
CPSI	817		m	50	74	all	6	all	NAmer	1959	pr	5138	n	bl	n	n	1	0	cig only	5	9	2	0	current	ot
CPSI	818		m	50	74	all	6	all	NAmer	1959	pr	5138	n	bl	n	n	1	0	cig only	10	999	3	0	current	ot
CPSII	664		m	35	99	all	4	all	NAmer	1982	pr	3229	n	bl	n	n	1	0	cig only	0.1	0.9	0	0	current	ot
CPSII	665		m	35	99	all	4	all	NAmer	1982	pr	3229	n	bl	n	n	1	0	cig only	1.0	2	0	0	current	ot
CPSII	666		m	35	99	all	4	all	NAmer	1982	pr	3229	n	bl	n	n	1	0	cig only	3	5	1	1	current	ot
CPSII	667		m	35	99	all	4	all	NAmer	1982	pr	3229	n	bl	n	n	1	0	cig only	6	10	2	0	current	ot
CPSII	668		m	35	99	all	4	all	NAmer	1982	pr	3229	n	bl	n	n	1	0	cig only	11	15	3	2	current	ot
CPSII	669		m	35	99	all	4	all	NAmer	1982	pr	3229	n	bl	n	n	1	0	cig only	16	999	0	3	current	ot
CPSII	642		f	0	0	all	4	all	NAmer	1982	pr	3229	n	bl	n	n	1	0	cig+/-ot	0.1	2	0	0	current	ot
CPSII	643		f	0	0	all	4	all	NAmer	1982	pr	3229	n	bl	n	n	1	0	cig+/-ot	3	5	1	1	current	ot
CPSII	644		f	0	0	all	4	all	NAmer	1982	pr	3229	n	bl	n	n	1	0	cig+/-ot	6	10	2	0	current	ot
CPSII	645		f	0	0	all	4	all	NAmer	1982	pr	3229	n	bl	n	n	1	0	cig+/-ot	11	15	3	2	current	ot
CPSII	646		f	0	0	all	4	all	NAmer	1982	pr	3229	n	bl	n	n	1	0	cig+/-ot	16	999	0	3	current	ot
DAMBER	557		m	0	0	all	-	all	Eu:Sca	1972	CC	579	n	bl	y	n	1	0	cig only	0.1	10	0	1	current	ot
DAMBER	558		m	0	0	all	-	all	Eu:Sca	1972	CC	579	n	bl	y	n	1	0	cig only	11	999	3	0	current	ot
DARBY	507		m	0	0	wh	-	all	Eu:UK	1988	CC	982	n	V	n	n	0	0	all/unsp	0.1	9	0	1	current	st
DARBY	508		m	0	0	wh	-	all	Eu:UK	1988	CC	982	n	V	n	n	0	0	all/unsp	10	999	3	0	current	st
DARBY	516		f	0	0	wh	-	all	Eu:UK	1988	CC	982	n	V	n	n	0	0	all/unsp	0.1	9	0	1	current	st
DARBY	517		f	0	0	wh	-	all	Eu:UK	1988	CC	982	n	V	n	n	0	0	all/unsp	10	999	3	0	current	st
DEAN3	509	x	m	0	0	all	-	all	Eu:UK	1969	CC	766	n	V	y	n	0	0	cig only	3	4	1	1	cur+2y	st
DEAN3	510	x	m	0	0	all	-	all	Eu:UK	1969	CC	766	n	V	y	n	0	0	cig only	5	8	2	0	cur+2y	st
DEAN3	511	x	m	0	0	all	-	all	Eu:UK	1969	CC	766	n	V	y	n	0	0	cig only	9	18	3	2	cur+2y	st
DEAN3	512	x	m	0	0	all	-	all	Eu:UK	1969	CC	766	n	V	y	n	0	0	cig only	19	999	0	3	cur+2y	st
DEAN3	548	x	f	0	0	all	-	all	Eu:UK	1969	CC	766	n	V	y	n	0	0	all/unsp	3	4	1	1	cur+2y	st
DEAN3	549	x	f	0	0	all	-	all	Eu:UK	1969	CC	766	n	V	y	n	0	0	all/unsp	5	8	2	0	cur+2y	st
DEAN3	550	x	f	0	0	all	-	all	Eu:UK	1969	CC	766	n	V	y	n	0	0	all/unsp	9	999	3	0	cur+2y	st
DESTEF	543		m	0	0	all	-	all	SCAmer	1988	CC	497	n	bl	n	y	0	0	cig+/-ot	0.1	4	1	1	current	st
DESTEF	544		m	0	0	all	-	all	SCAmer	1988	CC	497	n	bl	n	y	0	0	cig+/-ot	5	9	2	0	current	st
DESTEF	545		m	0	0	all	-	all	SCAmer	1988	CC	497	n	bl	n	y	0	0	cig+/-ot	10	999	3	0	current	st
DOLL	537		m	0	0	all	-	all	Eu:UK	1948	CC	1465	n	V	n	n	0	0	all/unsp	0.1	9	0	1	current	st
DOLL	538		m	0	0	all	-	all	Eu:UK	1948	CC	1465	n	V	n	n	0	0	all/unsp	10	19	3	2	current	st
DOLL	539		m	0	0	all	-	all	Eu:UK	1948	CC	1465	n	V	n	n	0	0	all/unsp	20	999	0	3	current	st
DOLL	548		f	0	0	all	-	all	Eu:UK	1948	CC	1465	n	V	n	n	0	0	all/unsp	0.1	9	0	1	current	st
DOLL	549		f	0	0	all	-	all	Eu:UK	1948	CC	1465	n	V	n	n	0	0	all/unsp	10	999	3	0	current	st
DOLL2	509		m	0	0	all	20	all	Eu:UK	1951	pr	920	n	V	n	n	1	0	cig only	0.1	4	1	1	current	ot
DOLL2	510		m	0	0	all	20	all	Eu:UK	1951	pr	920	n	V	n	n	1	0	cig only	5	9	2	0	current	ot

Table 1K6 - 4

IESLC - Meta-analysis of Ex Smoking by Years quit (vs current), Overview
All LC types, Cigarettes (or Any Product if Cigarettes not available)
Least adjusted

REF	NRR	X	SEX	AGEL	AGEH	RACE	YF	LC TYPE	LOC	START	ST	NLC	R	VB	P	H	AD	ADOS	PRODUCT	exL	exH	S1	S2	DENOM	De
DOLL2	511		m	0	0	all	20	all	Eu:UK	1951	pr	920	n	V	n	n	1	0	cig only	10	14	3	2	current	ot
DOLL2	512		m	0	0	all	20	all	Eu:UK	1951	pr	920	n	V	n	n	1	0	cig only	15	999	0	3	current	ot
DORGAN	514		m	0	0	wh	-	all	NAmer	1980	CC	2026	n	bl	y	y	0	0	cig+/-ot	1.1	5	1	1	cur+1y	st
DORGAN	515		m	0	0	wh	-	all	NAmer	1980	CC	2026	n	bl	y	y	0	0	cig+/-ot	6	9	2	0	cur+1y	st
DORGAN	516		m	0	0	wh	-	all	NAmer	1980	CC	2026	n	bl	y	y	0	0	cig+/-ot	10	999	3	0	cur+1y	st
DORGAN	559		f	0	0	all	-	all	NAmer	1980	CC	2026	n	bl	y	y	0	0	cig+/-ot	1.1	9	0	1	cur+1y	st
DORGAN	560		f	0	0	all	-	all	NAmer	1980	CC	2026	n	bl	y	y	0	0	cig+/-ot	10	999	3	0	cur+1y	st
DORN	823		m	55	64	wh	8	all	NAmer	1954	pr	5097	n	bl	n	n	0	0	cig+/-ot	0.1	4	1	1	current	st
DORN	824		m	55	64	wh	8	all	NAmer	1954	pr	5097	n	bl	n	n	0	0	cig+/-ot	5	9	2	0	current	st
DORN	825		m	55	64	wh	8	all	NAmer	1954	pr	5097	n	bl	n	n	0	0	cig+/-ot	10	14	3	2	current	st
DORN	826		m	55	64	wh	8	all	NAmer	1954	pr	5097	n	bl	n	n	0	0	cig+/-ot	15	999	0	3	current	st
DORN	827		m	65	74	wh	8	all	NAmer	1954	pr	5097	n	bl	n	n	0	0	cig+/-ot	0.1	4	1	1	current	st
DORN	828		m	65	74	wh	8	all	NAmer	1954	pr	5097	n	bl	n	n	0	0	cig+/-ot	5	9	2	0	current	st
DORN	829		m	65	74	wh	8	all	NAmer	1954	pr	5097	n	bl	n	n	0	0	cig+/-ot	10	14	3	2	current	st
DORN	830		m	65	74	wh	8	all	NAmer	1954	pr	5097	n	bl	n	n	0	0	cig+/-ot	15	999	0	3	current	st
GAO	526	x	m	0	0	all	-	all	As:Chi	1984	CC	1405	n	ot	n	n	0	0	cig+/-ot	0.1	4	1	1	current	st
GAO	527	x	m	0	0	all	-	all	As:Chi	1984	CC	1405	n	ot	n	n	0	0	cig+/-ot	5	9	2	0	current	st
GAO	528	x	m	0	0	all	-	all	As:Chi	1984	CC	1405	n	ot	n	n	0	0	cig+/-ot	10	999	3	0	current	st
GAO	546	x	f	0	0	all	-	all	As:Chi	1984	CC	1405	n	ot	n	n	0	0	cig+/-ot	0.1	4	1	1	current	st
GAO	547	x	f	0	0	all	-	all	As:Chi	1984	CC	1405	n	ot	n	n	0	0	cig+/-ot	5	9	2	0	current	st
GAO	548	x	f	0	0	all	-	all	As:Chi	1984	CC	1405	n	ot	n	n	0	0	cig+/-ot	10	999	3	0	current	st
GAO2	518		m	0	0	all	-	all	As:Jap	1988	CC	282	n	bl	n	n	0	0	cig+/-ot	1.0	4	1	1	cur+1y	st
GAO2	519		m	0	0	all	-	all	As:Jap	1988	CC	282	n	bl	n	n	0	0	cig+/-ot	5	9	2	0	cur+1y	st
GAO2	520		m	0	0	all	-	all	As:Jap	1988	CC	282	n	bl	n	n	0	0	cig+/-ot	10	14	3	2	cur+1y	st
GAO2	521		m	0	0	all	-	all	As:Jap	1988	CC	282	n	bl	n	n	0	0	cig+/-ot	15	19	0	0	cur+1y	st
GAO2	522		m	0	0	all	-	all	As:Jap	1988	CC	282	n	bl	n	n	0	0	cig+/-ot	20	999	0	3	cur+1y	st
GARCIA	508		c	0	0	all	-	all	NAmer	1992	CC	416	n	bl	n	y	0	0	cig+/-ot	1.0	4	1	1	cur+1y	st
GARCIA	509		c	0	0	all	-	all	NAmer	1992	CC	416	n	bl	n	y	0	0	cig+/-ot	5	14	0	2	cur+1y	st
GARCIA	510		c	0	0	all	-	all	NAmer	1992	CC	416	n	bl	n	y	0	0	cig+/-ot	15	29	0	3	cur+1y	st
GARCIA	511		c	0	0	all	-	all	NAmer	1992	CC	416	n	bl	n	y	0	0	cig+/-ot	30	999	0	0	cur+1y	st
GRAHAM	530	x	m	0	0	wh	-	all	NAmer	1956	CC	685	n	bl	n	n	0	0	cig+/-ot	0.1	1.0	0	0	current	st
GRAHAM	531	x	m	0	0	wh	-	all	NAmer	1956	CC	685	n	bl	n	n	0	0	cig+/-ot	1.1	5	1	1	current	st
GRAHAM	532	x	m	0	0	wh	-	all	NAmer	1956	CC	685	n	bl	n	n	0	0	cig+/-ot	5	999	0	0	current	st
HAMMO2	510		m	0	0	all	0	all	NAmer	1967	pr	450	o	bl	n	n	1	0	cig+/-ot	0.1	4	1	1	current	ot
HAMMO2	511		m	0	0	all	0	all	NAmer	1967	pr	450	o	bl	n	n	1	0	cig+/-ot	5	9	2	0	current	ot
HAMMO2	512		m	0	0	all	0	all	NAmer	1967	pr	450	o	bl	n	n	1	0	cig+/-ot	10	999	3	0	current	ot
HIRAYA	513		m	0	0	all	0	all	As:Jap	1965	pr	1917	n	bl	n	n	1	0	cig+/-ot	0.1	4	1	1	current	ot
HIRAYA	514		m	0	0	all	0	all	As:Jap	1965	pr	1917	n	bl	n	n	1	0	cig+/-ot	5	9	2	0	current	ot
HIRAYA	515		m	0	0	all	0	all	As:Jap	1965	pr	1917	n	bl	n	n	1	0	cig+/-ot	10	999	3	0	current	ot
HIRAYA	524		f	0	0	all	0	all	As:Jap	1965	pr	1917	n	bl	n	n	1	0	cig+/-ot	0.1	4	1	1	current	ot
HIRAYA	525		f	0	0	all	0	all	As:Jap	1965	pr	1917	n	bl	n	n	1	0	cig+/-ot	5	9	2	0	current	ot
HIRAYA	526		f	0	0	all	0	all	As:Jap	1965	pr	1917	n	bl	n	n	1	0	cig+/-ot	10	999	3	0	current	ot
JAHN	513		m	0	0	all	-	all	Eu:Ger	1988	CC	1004	n	bl	n	n	0	0	cig+/-ot	0.1	0.9	0	0	current	st
JAHN	514		m	0	0	all	-	all	Eu:Ger	1988	CC	1004	n	bl	n	n	0	0	cig+/-ot	1.0	1.9	0	0	current	st
JAHN	515		m	0	0	all	-	all	Eu:Ger	1988	CC	1004	n	bl	n	n	0	0	cig+/-ot	2	5	1	1	current	st
JAHN	516		m	0	0	all	-	all	Eu:Ger	1988	CC	1004	n	bl	n	n	0	0	cig+/-ot	6	10	2	0	current	st
JAHN	517		m	0	0	all	-	all	Eu:Ger	1988	CC	1004	n	bl	n	n	0	0	cig+/-ot	11	20	3	0	current	st
JAHN	518		m	0	0	all	-	all	Eu:Ger	1988	CC	1004	n	bl	n	n	0	0	cig+/-ot	21	999	0	0	current	st
JAIN	570		m	0	0	all	-	all	NAmer	1981	CC	845	n	V	y	n	0	0	cig+/-ot	2	9	0	1	cur+2y	st
JAIN	571		m	0	0	all	-	all	NAmer	1981	CC	845	n	V	y	n	0	0	cig+/-ot	10	999	3	0	cur+2y	st
JAIN	534		f	0	0	all	-	all	NAmer	1981	CC	845	n	V	y	n	0	0	cig+/-ot	2	9	0	1	cur+2y	st
JAIN	535		f	0	0	all	-	all	NAmer	1981	CC	845	n	V	y	n	0	0	cig+/-ot	10	999	3	0	cur+2y	st
JOLY	571		m	0	0	all	-	all	SCAmer	1978	CC	826	n	bl	n	n	0	0	cig+/-ot	1.0	4	1	1	cur+1y	st
JOLY	572		m	0	0	all	-	all	SCAmer	1978	CC	826	n	bl	n	n	0	0	cig+/-ot	5	999	0	0	cur+1y	st
JOLY	558		f	0	0	all	-	all	SCAmer	1978	CC	826	n	bl	n	n	0	0	cig+/-ot	1.0	4	1	1	cur+1y	st
JOLY	559		f	0	0	all	-	all	SCAmer	1978	CC	826	n	bl	n	n	0	0	cig+/-ot	5	999	0	0	cur+1y	st
KAISE2	655		m	0	0	all	9	all	NAmer	1979	pr	318	n	bl	n	n	1	0	cig only	2	10	0	1	cur+2y	st
KAISE2	656		m	0	0	all	9	all	NAmer	1979	pr	318	n	bl	n	n	1	0	cig only	11	20	3	0	cur+2y	ot
KAISE2	657		m	0	0	all	9	all	NAmer	1979	pr	318	n	bl	n	n	1	0	cig only	21	999	0	0	cur+2y	ot
KAISE2	575		f	0	0	all	9	all	NAmer	1979	pr	318	n	bl	n	n	1	0	cig only	2	10	0	1	cur+2y	st
KAISE2	576		f	0	0	all	9	all	NAmer	1979	pr	318	n	bl	n	n	1	0	cig only	11	20	3	0	cur+2y	st
KAISE2	577		f	0	0	all	9	all	NAmer	1979	pr	318	n	bl	n	n	1	0	cig only	21	999	0	0	cur+2y	ot
KHUDER	516		m	0	0	all	-	all	NAmer	1985	CC	482	n	bl	n	y	0	0	cig+/-ot	0.1	4	1	1	current	st
KHUDER	517		m	0	0	all	-	all	NAmer	1985	CC	482	n	bl	n	y	0	0	cig+/-ot	5	14	0	2	current	st
KHUDER	518		m	0	0	all	-	all	NAmer	1985	CC	482	n	bl	n	y	0	0	cig+/-ot	15	999	0	3	current	st
LUBIN	592		m	0	0	all	-	all	As:Chi	1984	CC	427	m	ot	y	n	0	0	cig+/-ot	3	4	1	1	cur+2y	st
LUBIN	593		m	0	0	all	-	all	As:Chi	1984	CC	427	m	ot	y	n	0	0	cig+/-ot	5	9	2	0	cur+2y	st
LUBIN	594		m	0	0	all	-	all	As:Chi	1984	CC	427	m	ot	y	n	0	0	cig+/-ot	10	999	3	0	cur+2y	st
LUBIN2	1081		m	0	0	all	-	all	Eu:mul	1976	CC	7804	n	bl	n	y	0	0	cig+/-ot	0.1	4	1	1	current	st
LUBIN2	1082		m	0	0	all	-	all	Eu:mul	1976	CC	7804	n	bl	n	y	0	0	cig+/-ot	5	9	2	0	current	st
LUBIN2	1083		m	0	0	all	-	all	Eu:mul	1976	CC	7804	n	bl	n	y	0	0	cig+/-ot	10	14	3	2	current	st
LUBIN2	1084		m	0	0	all	-	all	Eu:mul	1976	CC	7804	n	bl	n	y	0	0	cig+/-ot	15	19	0	0	current	st

Table 1K6 - 4

IESLC - Meta-analysis of Ex Smoking by Years quit (vs current), Overview
All LC types, Cigarettes (or Any Product if Cigarettes not available)
Least adjusted

REF	NRR	X	SEX	AGEL	AGEH	RACE	YF	LC TYPE	LOC	START	ST	NLC	R	VB	P	H	AD	ADOS	PRODUCT	exL	exH	S1	S2	DENOM	De
LUBIN2	1085		m	0	0	all	-	all	Eu:mul	1976	CC	7804	n	bl	n	y	0	0	cig+/-ot	20	24	0	3	current	st
LUBIN2	1086		m	0	0	all	-	all	Eu:mul	1976	CC	7804	n	bl	n	y	0	0	cig+/-ot	25	999	0	0	current	st
LUBIN2	1120		f	0	0	all	-	all	Eu:mul	1976	CC	7804	n	bl	n	y	0	0	cig+/-ot	0.1	4	1	1	current	st
LUBIN2	1121		f	0	0	all	-	all	Eu:mul	1976	CC	7804	n	bl	n	y	0	0	cig+/-ot	5	9	2	0	current	st
LUBIN2	1122		f	0	0	all	-	all	Eu:mul	1976	CC	7804	n	bl	n	y	0	0	cig+/-ot	10	14	3	2	current	st
LUBIN2	1123		f	0	0	all	-	all	Eu:mul	1976	CC	7804	n	bl	n	y	0	0	cig+/-ot	15	19	0	0	current	st
LUBIN2	1124		f	0	0	all	-	all	Eu:mul	1976	CC	7804	n	bl	n	y	0	0	cig+/-ot	20	24	0	3	current	st
LUBIN2	1125		f	0	0	all	-	all	Eu:mul	1976	CC	7804	n	bl	n	y	0	0	cig+/-ot	25	999	0	0	current	st
MATOS	586	x	m	0	0	all	-	all	SCAmer	1994	CC	200	n	bl	n	n	0	0	cig+/-ot	1.0	5	1	1	cur+1y	st
MATOS	587	x	m	0	0	all	-	all	SCAmer	1994	CC	200	n	bl	n	n	0	0	cig+/-ot	6	10	2	0	cur+1y	st
MATOS	588	x	m	0	0	all	-	all	SCAmer	1994	CC	200	n	bl	n	n	0	0	cig+/-ot	11	999	3	0	cur+1y	st
PEZZO2	504		m	0	0	all	-	all	SCAmer	1992	CC	367	n	bl	n	y	0	0	cig+/-ot	1.0	10	0	1	cur+1y	st
PEZZO2	505		m	0	0	all	-	all	SCAmer	1992	CC	367	n	bl	n	y	0	0	cig+/-ot	11	999	3	0	cur+1y	st
PEZZOT	504		m	0	0	all	-	all	SCAmer	1987	CC	215	n	bl	n	y	0	0	cig only	1.0	10	0	1	cur+1y	st
PEZZOT	505		m	0	0	all	-	all	SCAmer	1987	CC	215	n	bl	n	y	0	0	cig only	11	999	3	0	cur+1y	st
SOBUE	728		m	0	0	all	-	all	As:Jap	1986	CC	1376	n	bl	n	y	0	0	cig+/-ot	1.0	4	1	1	cur+1y	st
SOBUE	729		m	0	0	all	-	all	As:Jap	1986	CC	1376	n	bl	n	y	0	0	cig+/-ot	5	9	2	0	cur+1y	st
SOBUE	730		m	0	0	all	-	all	As:Jap	1986	CC	1376	n	bl	n	y	0	0	cig+/-ot	10	14	3	2	cur+1y	st
SOBUE	731		m	0	0	all	-	all	As:Jap	1986	CC	1376	n	bl	n	y	0	0	cig+/-ot	15	19	0	0	cur+1y	st
SOBUE	732		m	0	0	all	-	all	As:Jap	1986	CC	1376	n	bl	n	y	0	0	cig+/-ot	20	24	0	3	cur+1y	st
SOBUE	733		m	0	0	all	-	all	As:Jap	1986	CC	1376	n	bl	n	y	0	0	cig+/-ot	25	999	0	0	cur+1y	st
SPEIZE	511		f	0	0	all	0	all	NAmer	1976	pr	593	n	bl	n	y	2	0	cig+/-ot	0.1	1.9	0	0	current	or
SPEIZE	512		f	0	0	all	0	all	NAmer	1976	pr	593	n	bl	n	y	2	0	cig+/-ot	2	5	1	1	current	or
SPEIZE	513		f	0	0	all	0	all	NAmer	1976	pr	593	n	bl	n	y	2	0	cig+/-ot	5	10	2	0	current	or
SPEIZE	514		f	0	0	all	0	all	NAmer	1976	pr	593	n	bl	n	y	2	0	cig+/-ot	10	15	3	2	current	or
SPEIZE	515		f	0	0	all	0	all	NAmer	1976	pr	593	n	bl	n	y	2	0	cig+/-ot	15	999	0	3	current	or
SUZUK2	513	x	c	0	0	all	-	all	SCAmer	1991	CC	123	n	bl	n	y	0	0	all/unsp	0.1	5	1	1	current	st
SUZUK2	514	x	c	0	0	all	-	all	SCAmer	1991	CC	123	n	bl	n	y	0	0	all/unsp	6	10	2	0	current	st
SUZUK2	515	x	c	0	0	all	-	all	SCAmer	1991	CC	123	n	bl	n	y	0	0	all/unsp	11	999	3	0	current	st
SVENSS	554		f	0	0	all	-	all	Eu:Sca	1983	CC	210	n	bl	n	n	0	0	all/unsp	3	10	0	1	cur+2y	st
SVENSS	555		f	0	0	all	-	all	Eu:Sca	1983	CC	210	n	bl	n	n	0	0	all/unsp	11	999	3	0	cur+2y	st
TVERDA	506		m	0	0	all	0	all	Eu:Sca	1972	pr	238	n	bl	n	n	2	0	cig only	0.1	0.9	0	0	current	ot
TVERDA	507		m	0	0	all	0	all	Eu:Sca	1972	pr	238	n	bl	n	n	2	0	cig only	1.0	5	1	1	current	ot
TVERDA	508		m	0	0	all	0	all	Eu:Sca	1972	pr	238	n	bl	n	n	2	0	cig only	5	999	0	0	current	ot
WANG2	515		c	0	0	all	-	all	As:Chi	1980	CC	103	n	ot	n	n	0	0	cig+/-ot	0.1	3	1	1	current	st
WANG2	516		c	0	0	all	-	all	As:Chi	1980	CC	103	n	ot	n	n	0	0	cig+/-ot	4	999	0	0	current	st
WYNDE3	545		m	0	0	all	-	all	NAmer	1966	CC	350	n	bl	n	y	0	0	all/unsp	1.0	3	1	1	cur+1y	st
WYNDE3	546		m	0	0	all	-	all	NAmer	1966	CC	350	n	bl	n	y	0	0	all/unsp	4	6	0	0	cur+1y	st
WYNDE3	547		m	0	0	all	-	all	NAmer	1966	CC	350	n	bl	n	y	0	0	all/unsp	7	12	0	2	cur+1y	st
WYNDE3	548		m	0	0	all	-	all	NAmer	1966	CC	350	n	bl	n	y	0	0	all/unsp	13	999	0	3	cur+1y	st
WYNDE6	719		m	0	0	wh	-	all	NAmer	1969	CC	4423	n	bl	n	y	5	1#	cig+/-ot	1.0	10	0	1	cur+1y	or
WYNDE6	720		m	0	0	wh	-	all	NAmer	1969	CC	4423	n	bl	n	y	5	1#	cig+/-ot	11	19	3	2	cur+1y	or
WYNDE6	721		m	0	0	wh	-	all	NAmer	1969	CC	4423	n	bl	n	y	5	1#	cig+/-ot	20	999	0	3	cur+1y	or
WYNDE6	726		m	0	0	bl	-	all	NAmer	1969	CC	4423	n	bl	n	y	5	1#	cig+/-ot	1.0	10	0	1	cur+1y	or
WYNDE6	727		m	0	0	bl	-	all	NAmer	1969	CC	4423	n	bl	n	y	5	1#	cig+/-ot	11	19	3	2	cur+1y	or
WYNDE6	728		m	0	0	bl	-	all	NAmer	1969	CC	4423	n	bl	n	y	5	1#	cig+/-ot	20	999	0	3	cur+1y	or
WYNDE6	733		f	0	0	wh	-	all	NAmer	1969	CC	4423	n	bl	n	y	5	1#	cig+/-ot	1.0	10	0	1	cur+1y	or
WYNDE6	734		f	0	0	wh	-	all	NAmer	1969	CC	4423	n	bl	n	y	5	1#	cig+/-ot	11	999	3	0	cur+1y	or
WYNDE6	737		f	0	0	bl	-	all	NAmer	1969	CC	4423	n	bl	n	y	5	1#	cig+/-ot	1.0	10	0	1	cur+1y	or
WYNDE6	738		f	0	0	bl	-	all	NAmer	1969	CC	4423	n	bl	n	y	5	1#	cig+/-ot	11	999	3	0	cur+1y	or

Comments on values in listings

WYNDE6 ADOS Number of cigs/day
WYNDE6 ADOS Number of cigs/day
WYNDE6 ADOS Number of cigs/day
WYNDE6 ADOS Number of cigs/day
WYNDE6 ADOS Number of cigs/day
WYNDE6 ADOS Number of cigs/day
WYNDE6 ADOS Number of cigs/day
WYNDE6 ADOS Number of cigs/day
WYNDE6 ADOS Number of cigs/day
WYNDE6 ADOS Number of cigs/day

Cigarette type is all/unspec for all RRs
except for the following:

REF	NRR	CIGTYPE
ALDERS	513	MC only

Table 1K6 - 4

IESLC - Meta-analysis of Ex Smoking by Years quit (vs current), Overview
All LC types, Cigarettes (or Any Product if Cigarettes not available)
Least adjusted

REF	NRR	CIGTYPE
ALDERS	514	MC only
ALDERS	515	MC only
ALDERS	524	MC only
ALDERS	525	MC only
ALDERS	526	MC only
DEAN3	509	MC only
DEAN3	510	MC only
DEAN3	511	MC only
DEAN3	512	MC only
DESTEF	543	MC only
DESTEF	544	MC only
DESTEF	545	MC only

In this overview table, subtotals and Qs values may be invalid and should be ignored

Table 1K6 - 5

IESLC - Meta-analysis of Ex Smoking by Years quit (vs current), Overview
All LC types, Cigarettes (or Any Product if Cigarettes not available)
Least adjusted

REF	NRR	SEX	AD	Number Case	Exposed Cont	Non-exposed Case	Cont	RR	95.00%CI
ALDERS	513	m	1	121	-	207	-	1.81	(1.24- 2.64)
ALDERS	514	m	1	28	-	207	-	0.43	(0.26- 0.71)
ALDERS	515	m	1	29	-	207	-	0.32	(0.20- 0.51)
ALDERS	524	f	1	206	-	244	-	2.08	(1.49- 2.90)
ALDERS	525	f	1	54	-	244	-	0.65	(0.43- 0.99)
ALDERS	526	f	1	26	-	244	-	0.28	(0.17- 0.46)
Subtotal ALDERS								0.85	(0.72- 1.01)
ARMADA	518	m	0	79	45	188	122	1.14	(0.74- 1.75)
ARMADA	519	m	0	50	87	188	122	0.37	(0.25- 0.57)
Subtotal ARMADA								0.64	(0.47- 0.86)
BARBON	533	m	0	32	20	562	362	1.03	(0.58- 1.83)
BARBON	534	m	0	89	85	562	362	0.67	(0.49- 0.93)
BARBON	535	m	0	33	41	562	362	0.52	(0.32- 0.84)
BARBON	536	m	0	15	59	562	362	0.16	(0.09- 0.29)
Subtotal BARBON								0.55	(0.44- 0.69)
BECHER	506	m	0	10	12	101	122	1.01	(0.42- 2.43)
BECHER	507	m	0	16	32	101	122	0.60	(0.31- 1.16)
BECHER	508	m	0	16	72	101	122	0.27	(0.15- 0.49)
BECHER	516	f	0	2	3	33	26	0.53	(0.08- 3.38)
BECHER	517	f	0	2	5	33	26	0.32	(0.06- 1.76)
BECHER	518	f	0	1	10	33	26	0.08	(0.01- 0.66)
Subtotal BECHER								0.44	(0.30- 0.64)
BROSS	518	m	0	169	67	565	427	1.91	(1.40- 2.60)
BROSS	519	m	0	43	79	565	427	0.41	(0.28- 0.61)
Subtotal BROSS								1.06	(0.83- 1.35)
CARPEN	508	c	0	28	46	228	164	0.44	(0.26- 0.73)
CARPEN	509	c	0	31	52	228	164	0.43	(0.26- 0.70)
CARPEN	510	c	0	13	58	228	164	0.16	(0.09- 0.30)
CARPEN	511	c	0	37	158	228	164	0.17	(0.11- 0.25)
Subtotal CARPEN								0.27	(0.21- 0.34)
*CEDERL	538	m	1	12	-	97	-	0.78	(0.43- 1.41)
*CEDERL	539	m	1	3	-	97	-	0.14	(0.04- 0.45)
Subtotal CEDERL								0.56	(0.33- 0.95)
CHOI	543	m	0	25	64	231	329	0.56	(0.34- 0.91)
CHOI	544	m	0	5	30	231	329	0.24	(0.09- 0.62)
CHOI	545	m	0	4	23	231	329	0.25	(0.08- 0.73)
CHOI	546	m	0	4	19	231	329	0.30	(0.10- 0.89)
CHOI	556	f	0	3	2	13	25	2.88	(0.43- 19.49)
CHOI	557	f	0	2	0	13	25	9.44~	(0.42- 211.16)
Subtotal CHOI								0.46	(0.32- 0.67)
*CHYOU	504	m	1	21	-	138	-	0.32	(0.20- 0.50)
*CHYOU	505	m	1	5	-	138	-	0.23	(0.09- 0.56)
Subtotal CHYOU								0.30	(0.20- 0.45)
*CPSI	815	m	1	37	-	844	-	1.07	(0.77- 1.49)
*CPSI	816	m	1	49	-	844	-	0.59	(0.44- 0.78)
*CPSI	817	m	1	32	-	844	-	0.37	(0.26- 0.53)
*CPSI	818	m	1	15	-	844	-	0.09	(0.06- 0.15)
Subtotal CPSI								0.48	(0.40- 0.57)
*CPSII	664	m	1	97	-	1159	-	1.77	(1.44- 2.17)
*CPSII	665	m	1	188	-	1159	-	1.28	(1.10- 1.49)
*CPSII	666	m	1	178	-	1159	-	0.85	(0.72- 0.99)
*CPSII	667	m	1	186	-	1159	-	0.52	(0.45- 0.61)
*CPSII	668	m	1	164	-	1159	-	0.39	(0.33- 0.46)
*CPSII	669	m	1	256	-	1159	-	0.17	(0.15- 0.20)
*CPSII	642	f	1	91	-	530	-	1.38	(1.10- 1.72)
*CPSII	643	f	1	56	-	530	-	0.85	(0.65- 1.12)
*CPSII	644	f	1	37	-	530	-	0.40	(0.28- 0.55)
*CPSII	645	f	1	28	-	530	-	0.31	(0.21- 0.46)
*CPSII	646	f	1	50	-	530	-	0.14	(0.11- 0.19)
Subtotal CPSII								0.57	(0.54- 0.60)
DAMBER	557	m	1	-	-	-	-	0.56	(0.29- 1.08)
DAMBER	558	m	1	-	-	-	-	0.16	(0.08- 0.35)
Subtotal DAMBER								0.32	(0.20- 0.53)
DARBY	507	m	0	146	339	379	618	0.70	(0.56- 0.89)
DARBY	508	m	0	139	767	379	618	0.30	(0.24- 0.37)
DARBY	516	f	0	68	93	198	231	0.85	(0.59- 1.23)
DARBY	517	f	0	26	224	198	231	0.14	(0.09- 0.21)
Subtotal DARBY								0.44	(0.38- 0.50)
DEAN3	509	m	0	28	102	337	930	0.76	(0.49- 1.17)
DEAN3	510	m	0	11	43	337	930	0.71	(0.36- 1.38)
DEAN3	511	m	0	15	86	337	930	0.48	(0.27- 0.84)

Table 1K6 - 5

IESLC - Meta-analysis of Ex Smoking by Years quit (vs current), Overview
All LC types, Cigarettes (or Any Product if Cigarettes not available)
Least adjusted

				Number Exposed		Non-exposed			
REF	NRR	SEX	AD	Case	Cont	Case	Cont	RR	95.00%CI
DEAN3	512	m	0	8	66	337	930	0.33	(0.16- 0.70)
DEAN3	548	f	0	4	110	102	1158	0.41	(0.15- 1.14)
DEAN3	549	f	0	1	38	102	1158	0.30	(0.04- 2.20)
DEAN3	550	f	0	2	114	102	1158	0.20	(0.05- 0.82)
Subtotal DEAN3								0.55	(0.42- 0.72)
DESTEF	543	m	0	10	19	78	109	0.74	(0.32- 1.67)
DESTEF	544	m	0	9	15	78	109	0.84	(0.35- 2.01)
DESTEF	545	m	0	10	41	78	109	0.34	(0.16- 0.72)
Subtotal DESTEF								0.57	(0.35- 0.90)
DOLL	537	m	0	56	75	1280	1172	0.68	(0.48- 0.98)
DOLL	538	m	0	6	26	1280	1172	0.21	(0.09- 0.52)
DOLL	539	m	0	8	23	1280	1172	0.32	(0.14- 0.71)
DOLL	548	f	0	9	6	58	41	1.06	(0.35- 3.21)
DOLL	549	f	0	1	2	58	41	0.35	(0.03- 4.03)
Subtotal DOLL								0.56	(0.42- 0.75)
*DOLL2	509	m	1	15	-	236	-	1.02	(0.61- 1.72)
*DOLL2	510	m	1	12	-	236	-	0.35	(0.20- 0.63)
*DOLL2	511	m	1	9	-	236	-	0.28	(0.14- 0.54)
*DOLL2	512	m	1	7	-	236	-	0.11	(0.05- 0.23)
Subtotal DOLL2								0.40	(0.30- 0.55)
DORGAN	514	m	0	59	51	465	303	0.75	(0.50- 1.13)
DORGAN	515	m	0	49	38	465	303	0.84	(0.54- 1.31)
DORGAN	516	m	0	134	255	465	303	0.34	(0.27- 0.44)
DORGAN	559	f	0	49	27	289	112	0.70	(0.42- 1.18)
DORGAN	560	f	0	34	50	289	112	0.26	(0.16- 0.43)
Subtotal DORGAN								0.47	(0.40- 0.56)
*DORN	823	m	0	34	22086	528	334175	0.97	(0.69- 1.38)
*DORN	824	m	0	32	34566	528	334175	0.59	(0.41- 0.84)
*DORN	825	m	0	12	23682	528	334175	0.32	(0.18- 0.57)
*DORN	826	m	0	16	58370	528	334175	0.17	(0.11- 0.29)
*DORN	827	m	0	14	6195	537	207895	0.87	(0.51- 1.49)
*DORN	828	m	0	41	24089	537	207895	0.66	(0.48- 0.90)
*DORN	829	m	0	29	20056	537	207895	0.56	(0.39- 0.81)
*DORN	830	m	0	34	51243	537	207895	0.26	(0.18- 0.36)
Subtotal DORN								0.51	(0.45- 0.59)
GAO	526	m	0	105	52	529	438	1.67	(1.17- 2.39)
GAO	527	m	0	24	27	529	438	0.74	(0.42- 1.29)
GAO	528	m	0	13	41	529	438	0.26	(0.14- 0.50)
GAO	546	f	0	37	9	170	100	2.42	(1.12- 5.22)
GAO	547	f	0	14	7	170	100	1.18	(0.46- 3.01)
GAO	548	f	0	16	14	170	100	0.67	(0.31- 1.44)
Subtotal GAO								1.04	(0.83- 1.32)
GAO2	518	m	0	31	26	184	117	0.76	(0.43- 1.34)
GAO2	519	m	0	21	26	184	117	0.51	(0.28- 0.95)
GAO2	520	m	0	16	18	184	117	0.57	(0.28- 1.15)
GAO2	521	m	0	7	9	184	117	0.49	(0.18- 1.36)
GAO2	522	m	0	8	25	184	117	0.20	(0.09- 0.47)
Subtotal GAO2								0.51	(0.37- 0.70)
GARCIA	508	c	0	33	11	77	42	1.64	(0.75- 3.57)
GARCIA	509	c	0	43	36	77	42	0.65	(0.36- 1.16)
GARCIA	510	c	0	32	67	77	42	0.26	(0.15- 0.46)
GARCIA	511	c	0	10	37	77	42	0.15	(0.07- 0.33)
Subtotal GARCIA								0.44	(0.32- 0.61)
GRAHAM	530	m	0	113	59	453	1075	4.55	(3.26- 6.34)
GRAHAM	531	m	0	24	48	453	1075	1.19	(0.72- 1.96)
GRAHAM	532	m	0	13	71	453	1075	0.43	(0.24- 0.79)
Subtotal GRAHAM								2.14	(1.67- 2.76)
*HAMMO2	510	m	1	59	-	209	-	1.08	(0.82- 1.43)
*HAMMO2	511	m	1	11	-	209	-	0.39	(0.22- 0.71)
*HAMMO2	512	m	1	20	-	209	-	0.34	(0.22- 0.53)
Subtotal HAMMO2								0.71	(0.57- 0.88)
*HIRAYA	513	m	1	-	-	-	-	0.46	(0.26- 0.82)
*HIRAYA	514	m	1	-	-	-	-	0.36	(0.15- 0.86)
*HIRAYA	515	m	1	-	-	-	-	0.31	(0.14- 0.69)
*HIRAYA	524	f	1	-	-	-	-	1.59	(0.47- 5.37)
*HIRAYA	525	f	1	-	-	-	-	1.41	(0.23- 8.48)
*HIRAYA	526	f	1	-	-	-	-	0.41	(0.01- 14.37)
Subtotal HIRAYA								0.48	(0.33- 0.69)
JAHN	513	m	0	166	8	352	269	15.86	(7.66- 32.81)
JAHN	514	m	0	60	9	352	269	5.09	(2.48- 10.45)
JAHN	515	m	0	77	46	352	269	1.28	(0.86- 1.90)

Table 1K6 - 5

IESLC - Meta-analysis of Ex Smoking by Years quit (vs current), Overview
All LC types, Cigarettes (or Any Product if Cigarettes not available)
Least adjusted

				Number Exposed		Non-exposed			
REF	NRR	SEX	AD	Case	Cont	Case	Cont	RR	95.00%CI
JAHN	516	m	0	59	63	352	269	0.72	(0.49- 1.06)
JAHN	517	m	0	64	130	352	269	0.38	(0.27- 0.53)
JAHN	518	m	0	29	146	352	269	0.15	(0.10- 0.23)
Subtotal JAHN								0.70	(0.58- 0.84)
JAIN	570	m	0	74	46	265	118	0.72	(0.47- 1.10)
JAIN	571	m	0	52	113	265	118	0.20	(0.14- 0.30)
JAIN	534	f	0	66	36	305	99	0.60	(0.37- 0.95)
JAIN	535	f	0	19	61	305	99	0.10	(0.06- 0.18)
Subtotal JAIN								0.33	(0.27- 0.42)
JOLY	571	m	0	38	36	451	524	1.23	(0.76- 1.97)
JOLY	572	m	0	63	149	451	524	0.49	(0.36- 0.68)
JOLY	558	f	0	19	8	132	96	1.73	(0.73- 4.11)
JOLY	559	f	0	15	19	132	96	0.57	(0.28- 1.19)
Subtotal JOLY								0.70	(0.55- 0.88)
*KAISE2	655	m	1	12	-	51	-	1.00	(0.53- 1.88)
*KAISE2	656	m	1	8	-	51	-	0.43	(0.20- 0.92)
*KAISE2	657	m	1	6	-	51	-	0.26	(0.10- 0.67)
*KAISE2	575	f	1	6	-	50	-	0.53	(0.23- 1.23)
*KAISE2	576	f	1	4	-	50	-	0.25	(0.09- 0.70)
*KAISE2	577	f	1	4	-	50	-	0.34	(0.13- 0.91)
Subtotal KAISE2								0.49	(0.35- 0.68)
KHUDER	516	m	0	88	123	245	316	0.92	(0.67- 1.27)
KHUDER	517	m	0	63	133	245	316	0.61	(0.43- 0.86)
KHUDER	518	m	0	63	213	245	316	0.38	(0.28- 0.53)
Subtotal KHUDER								0.60	(0.50- 0.73)
LUBIN	592	m	0	33	18	296	650	4.03	(2.23- 7.27)
LUBIN	593	m	0	20	48	296	650	0.91	(0.53- 1.57)
LUBIN	594	m	0	17	73	296	650	0.51	(0.30- 0.88)
Subtotal LUBIN								1.16	(0.84- 1.60)
LUBIN2	1081	m	0	866	1047	4684	6211	1.10	(0.99- 1.21)
LUBIN2	1082	m	0	466	822	4684	6211	0.75	(0.67- 0.85)
LUBIN2	1083	m	0	270	693	4684	6211	0.52	(0.45- 0.60)
LUBIN2	1084	m	0	130	478	4684	6211	0.36	(0.30- 0.44)
LUBIN2	1085	m	0	106	413	4684	6211	0.34	(0.27- 0.42)
LUBIN2	1086	m	0	109	715	4684	6211	0.20	(0.16- 0.25)
LUBIN2	1120	f	0	60	55	440	410	1.02	(0.69- 1.50)
LUBIN2	1121	f	0	30	40	440	410	0.70	(0.43- 1.14)
LUBIN2	1122	f	0	10	26	440	410	0.36	(0.17- 0.75)
LUBIN2	1123	f	0	3	7	440	410	0.40	(0.10- 1.55)
LUBIN2	1124	f	0	4	9	440	410	0.41	(0.13- 1.36)
LUBIN2	1125	f	0	4	20	440	410	0.19	(0.06- 0.55)
Subtotal LUBIN2								0.65	(0.61- 0.68)
MATOS	586	m	0	28	23	112	132	1.43	(0.78- 2.63)
MATOS	587	m	0	21	27	112	132	0.92	(0.49- 1.71)
MATOS	588	m	0	27	101	112	132	0.32	(0.19- 0.52)
Subtotal MATOS								0.65	(0.47- 0.91)
PEZZO2	504	m	0	85	110	233	198	0.66	(0.47- 0.92)
PEZZO2	505	m	0	43	161	233	198	0.23	(0.15- 0.33)
Subtotal PEZZO2								0.41	(0.32- 0.53)
PEZZOT	504	m	0	46	82	145	129	0.50	(0.32- 0.77)
PEZZOT	505	m	0	20	106	145	129	0.17	(0.10- 0.29)
Subtotal PEZZOT								0.32	(0.23- 0.45)
SOBUE	728	m	0	128	116	737	633	0.95	(0.72- 1.24)
SOBUE	729	m	0	67	92	737	633	0.63	(0.45- 0.87)
SOBUE	730	m	0	35	50	737	633	0.60	(0.39- 0.94)
SOBUE	731	m	0	24	31	737	633	0.66	(0.39- 1.14)
SOBUE	732	m	0	15	23	737	633	0.56	(0.29- 1.08)
SOBUE	733	m	0	17	40	737	633	0.37	(0.20- 0.65)
Subtotal SOBUE								0.69	(0.59- 0.82)
*SPEIZE	511	f	2	24	-	319	-	0.60	(0.40- 0.90)
*SPEIZE	512	f	2	34	-	319	-	0.60	(0.40- 0.80)
*SPEIZE	513	f	2	41	-	319	-	0.50	(0.40- 0.70)
*SPEIZE	514	f	2	17	-	319	-	0.20	(0.10- 0.40)
*SPEIZE	515	f	2	28	-	319	-	0.10	(0.10- 0.40)
Subtotal SPEIZE								0.46	(0.38- 0.55)
SUZUK2	513	c	0	15	10	77	30	0.58	(0.24- 1.44)
SUZUK2	514	c	0	10	8	77	30	0.49	(0.18- 1.35)
SUZUK2	515	c	0	9	22	77	30	0.16	(0.07- 0.39)
Subtotal SUZUK2								0.34	(0.20- 0.59)
SVENSS	554	f	0	16	13	142	53	0.46	(0.21- 1.02)
SVENSS	555	f	0	14	24	142	53	0.22	(0.10- 0.45)

Table 1K6 - 5

IESLC - Meta-analysis of Ex Smoking by Years quit (vs current), Overview
All LC types, Cigarettes (or Any Product if Cigarettes not available)
Least adjusted

REF	NRR	SEX	AD	Number Exposed Case	Number Exposed Cont	Non-exposed Case	Non-exposed Cont	RR	95.00%CI
Subtotal SVENSS								0.31	(0.18- 0.52)
*TVERDA	506	m	2	2	-	144	-	0.17	(0.04- 0.70)
*TVERDA	507	m	2	5	-	144	-	0.18	(0.07- 0.43)
*TVERDA	508	m	2	4	-	144	-	0.08	(0.03- 0.23)
Subtotal TVERDA								0.13	(0.07- 0.25)
WANG2	515	c	0	6	10	49	78	0.96	(0.33- 2.79)
WANG2	516	c	0	5	11	49	78	0.72	(0.24- 2.21)
Subtotal WANG2								0.84	(0.39- 1.81)
WYNDE3	545	m	0	21	22	227	207	0.87	(0.47- 1.63)
WYNDE3	546	m	0	11	17	227	207	0.59	(0.27- 1.29)
WYNDE3	547	m	0	11	31	227	207	0.32	(0.16- 0.66)
WYNDE3	548	m	0	5	55	227	207	0.08	(0.03- 0.21)
Subtotal WYNDE3								0.42	(0.29- 0.61)
WYNDE6	719	m	5	-	-	-	-	0.60	(0.50- 0.70)
WYNDE6	720	m	5	-	-	-	-	0.30	(0.20- 0.40)
WYNDE6	721	m	5	-	-	-	-	0.20	(0.10- 0.20)
WYNDE6	726	m	5	-	-	-	-	0.70	(0.40- 1.10)
WYNDE6	727	m	5	-	-	-	-	0.20	(0.10- 0.50)
WYNDE6	728	m	5	-	-	-	-	0.30	(0.10- 0.60)
WYNDE6	733	f	5	-	-	-	-	0.50	(0.40- 0.60)
WYNDE6	734	f	5	-	-	-	-	0.20	(0.20- 0.30)
WYNDE6	737	f	5	-	-	-	-	0.60	(0.30- 1.30)
WYNDE6	738	f	5	-	-	-	-	0.40	(0.10- 1.10)
Subtotal WYNDE6								0.39	(0.35- 0.43)
Partial Totals				8482	251537	86457	2244774		

*prospective study ~ With 0.5 adjustment for zero

REF	NRR	SEX	AD	Ys	Ws	Qs	Ps
ALDERS	513	m	1	0.59	26.91	36.81	0.0021
ALDERS	514	m	1	-0.84	15.23	1.09	0.0010
ALDERS	515	m	1	-1.14	17.54	5.56	0.0000
ALDERS	524	f	1	0.73	34.65	59.34	0.0000
ALDERS	525	f	1	-0.43	22.10	0.47	0.0429
ALDERS	526	f	1	-1.27	15.51	7.53	0.0000
Subtotal ALDERS				-0.16	131.92	110.80	
ARMADA	518	m	0	0.13	20.66	10.32	0.5535
ARMADA	519	m	0	-0.99	22.22	3.73	0.0000
Subtotal ARMADA				-0.45	42.88	14.05	
BARBON	533	m	0	0.03	11.66	4.29	0.9180
BARBON	534	m	0	-0.39	36.31	1.21	0.0176
BARBON	535	m	0	-0.66	16.88	0.11	0.0070
BARBON	536	m	0	-1.81	11.34	17.25	0.0000
Subtotal BARBON				-0.60	76.19	22.85	
BECHER	506	m	0	0.01	4.96	1.69	0.9883
BECHER	507	m	0	-0.50	8.94	0.05	0.1316
BECHER	508	m	0	-1.32	10.58	5.78	0.0000
BECHER	516	f	0	-0.64	1.11	0.01	0.4978
BECHER	517	f	0	-1.15	1.30	0.44	0.1879
BECHER	518	f	0	-2.54	0.86	3.30	0.0188
Subtotal BECHER				-0.82	27.75	11.25	
BROSS	518	m	0	0.65	40.07	59.79	0.0000
BROSS	519	m	0	-0.89	24.98	2.43	0.0000
Subtotal BROSS				0.06	65.06	62.22	
CARPEN	508	c	0	-0.83	14.72	0.92	0.0015
CARPEN	509	c	0	-0.85	16.14	1.18	0.0007
CARPEN	510	c	0	-1.82	9.56	14.90	0.0000
CARPEN	511	c	0	-1.78	22.81	33.11	0.0000
Subtotal CARPEN				-1.33	63.22	50.11	
*CEDERL	538	m	1	-0.25	10.90	1.17	0.4121
*CEDERL	539	m	1	-1.97	2.62	5.07	0.0015
Subtotal CEDERL				-0.58	13.52	6.24	
CHOI	543	m	0	-0.59	15.87	0.00	0.0195
CHOI	544	m	0	-1.44	4.15	3.09	0.0034
CHOI	545	m	0	-1.40	3.32	2.23	0.0109
CHOI	546	m	0	-1.20	3.23	1.27	0.0305
CHOI	556	f	0	1.06	1.05	2.82	0.2771
CHOI	557	f	0	2.25	0.40	3.17	0.1567
Subtotal CHOI				-0.78	28.03	12.57	
*CHYOU	504	m	1	-1.14	18.30	5.80	0.0000
*CHYOU	505	m	1	-1.47	4.60	3.67	0.0016

Table 1K6 - 5

IESLC - Meta-analysis of Ex Smoking by Years quit (vs current), Overview
All LC types, Cigarettes (or Any Product if Cigarettes not available)
Least adjusted

REF	NRR	SEX	AD	Ys	Ws	Qs	Ps
Subtotal CHYOU				-1.21	22.90	9.47	
*CPSI	815	m	1	0.07	35.26	14.62	0.6879
*CPSI	816	m	1	-0.53	46.88	0.11	0.0003
*CPSI	817	m	1	-0.99	30.29	5.29	0.0000
*CPSI	818	m	1	-2.41	18.30	61.40	0.0000
Subtotal CPSI				-0.74	130.73	81.42	
*CPSII	664	m	1	0.57	91.37	120.27	0.0000
*CPSII	665	m	1	0.25	166.85	113.07	0.0014
*CPSII	666	m	1	-0.16	151.52	25.95	0.0454
*CPSII	667	m	1	-0.65	166.04	1.00	0.0000
*CPSII	668	m	1	-0.94	139.29	18.59	0.0000
*CPSII	669	m	1	-1.77	185.67	265.41	0.0000
*CPSII	642	f	1	0.32	76.90	62.07	0.0047
*CPSII	643	f	1	-0.16	51.90	8.89	0.2417
*CPSII	644	f	1	-0.92	33.71	3.90	0.0000
*CPSII	645	f	1	-1.17	24.99	8.84	0.0000
*CPSII	646	f	1	-1.97	51.44	99.36	0.0000
Subtotal CPSII				-0.57	1139.68	727.34	
DAMBER	557	m	1	-0.58	8.89	0.00	0.0839
DAMBER	558	m	1	-1.83	7.05	11.13	0.0000
Subtotal DAMBER				-1.13	15.94	11.13	
DARBY	507	m	0	-0.35	71.14	3.53	0.0029
DARBY	508	m	0	-1.22	78.40	32.39	0.0000
DARBY	516	f	0	-0.16	28.70	5.00	0.3945
DARBY	517	f	0	-2.00	19.12	38.72	0.0000
Subtotal DARBY				-0.83	197.37	79.64	
DEAN3	509	m	0	-0.28	20.18	1.80	0.2123
DEAN3	510	m	0	-0.35	8.46	0.44	0.3112
DEAN3	511	m	0	-0.73	12.15	0.29	0.0108
DEAN3	512	m	0	-1.10	6.94	1.87	0.0039
DEAN3	548	f	0	-0.88	3.71	0.35	0.0885
DEAN3	549	f	0	-1.21	0.96	0.38	0.2355
DEAN3	550	f	0	-1.61	1.93	2.07	0.0252
Subtotal DEAN3				-0.60	54.31	7.21	
DESTEF	543	m	0	-0.31	5.73	0.41	0.4622
DESTEF	544	m	0	-0.18	5.01	0.80	0.6934
DESTEF	545	m	0	-1.08	6.83	1.71	0.0049
Subtotal DESTEF				-0.57	17.56	2.92	
DOLL	537	m	0	-0.38	30.46	1.17	0.0358
DOLL	538	m	0	-1.55	4.84	4.63	0.0006
DOLL	539	m	0	-1.14	5.88	1.90	0.0055
DOLL	548	f	0	0.06	3.13	1.26	0.9174
DOLL	549	f	0	-1.04	0.65	0.14	0.4022
Subtotal DOLL				-0.59	44.96	9.10	
*DOLL2	509	m	1	0.02	14.30	5.08	0.9403
*DOLL2	510	m	1	-1.05	11.67	2.62	0.0003
*DOLL2	511	m	1	-1.27	8.43	4.09	0.0002
*DOLL2	512	m	1	-2.21	6.60	17.55	0.0000
Subtotal DOLL2				-0.91	41.00	29.34	
DORGAN	514	m	0	-0.28	23.81	2.05	0.1680
DORGAN	515	m	0	-0.17	19.17	3.10	0.4460
DORGAN	516	m	0	-1.07	59.40	14.58	0.0000
DORGAN	559	f	0	-0.35	14.32	0.72	0.1829
DORGAN	560	f	0	-1.33	16.18	9.28	0.0000
Subtotal DORGAN				-0.76	132.87	29.73	
*DORN	823	m	0	-0.03	31.99	9.69	0.8830
*DORN	824	m	0	-0.53	30.20	0.05	0.0033
*DORN	825	m	0	-1.14	11.74	3.69	0.0001
*DORN	826	m	0	-1.75	15.53	21.46	0.0000
*DORN	827	m	0	-0.13	13.68	2.68	0.6211
*DORN	828	m	0	-0.42	38.16	0.97	0.0100
*DORN	829	m	0	-0.58	27.56	0.00	0.0023
*DORN	830	m	0	-1.36	32.00	19.61	0.0000
Subtotal DORN				-0.67	200.86	58.15	
GAO	526	m	0	0.51	30.37	36.10	0.0046
GAO	527	m	0	-0.31	12.07	0.88	0.2869
GAO	528	m	0	-1.34	9.48	5.49	0.0000
GAO	546	f	0	0.88	6.49	13.83	0.0244
GAO	547	f	0	0.16	4.34	2.37	0.7348
GAO	548	f	0	-0.40	6.68	0.21	0.3049
Subtotal GAO				0.04	69.43	58.88	

Table 1K6 - 5

IESLC - Meta-analysis of Ex Smoking by Years quit (vs current), Overview
All LC types, Cigarettes (or Any Product if Cigarettes not available)
Least adjusted

REF	NRR	SEX	AD	Ys	Ws	Qs	Ps
GAO2	518	m	0	-0.28	11.81	1.06	0.3414
GAO2	519	m	0	-0.67	9.99	0.08	0.0352
GAO2	520	m	0	-0.57	7.57	0.00	0.1164
GAO2	521	m	0	-0.70	3.73	0.06	0.1738
GAO2	522	m	0	-1.59	5.59	5.77	0.0002
Subtotal GAO2				-0.67	38.69	6.97	
GARCIA	508	c	0	0.49	6.33	7.23	0.2154
GARCIA	509	c	0	-0.43	11.39	0.25	0.1483
GARCIA	510	c	0	-1.35	12.05	7.12	0.0000
GARCIA	511	c	0	-1.91	6.10	10.93	0.0000
Subtotal GARCIA				-0.83	35.87	25.53	
GRAHAM	530	m	0	1.51	34.56	151.01	0.0000
GRAHAM	531	m	0	0.17	15.24	8.51	0.5044
GRAHAM	532	m	0	-0.83	10.62	0.70	0.0066
Subtotal GRAHAM				0.76	60.42	160.22	
*HAMMO2	510	m	1	0.08	49.68	21.20	0.5875
*HAMMO2	511	m	1	-0.94	11.19	1.49	0.0016
*HAMMO2	512	m	1	-1.08	19.88	5.02	0.0000
Subtotal HAMMO2				-0.35	80.75	27.72	
*HIRAYA	513	m	1	-0.78	11.65	0.47	0.0080
*HIRAYA	514	m	1	-1.02	5.04	1.00	0.0218
*HIRAYA	515	m	1	-1.17	6.04	2.14	0.0040
*HIRAYA	524	f	1	0.46	2.59	2.80	0.4555
*HIRAYA	525	f	1	0.34	1.18	1.00	0.7089
*HIRAYA	526	f	1	-0.89	0.29	0.03	0.6307
Subtotal HIRAYA				-0.74	26.79	7.43	
JAHN	513	m	0	2.76	7.27	81.08	0.0000
JAHN	514	m	0	1.63	7.44	36.18	0.0000
JAHN	515	m	0	0.25	24.22	16.39	0.2255
JAHN	516	m	0	-0.33	25.39	1.48	0.0919
JAHN	517	m	0	-0.98	33.47	5.39	0.0000
JAHN	518	m	0	-1.89	20.88	35.77	0.0000
Subtotal JAHN				-0.36	118.68	176.30	
JAIN	570	m	0	-0.33	21.05	1.24	0.1258
JAIN	571	m	0	-1.59	24.80	25.24	0.0000
JAIN	534	f	0	-0.52	17.76	0.06	0.0287
JAIN	535	f	0	-2.29	12.14	35.70	0.0000
Subtotal JAIN				-1.10	75.74	62.24	
JOLY	571	m	0	0.20	17.18	10.46	0.3976
JOLY	572	m	0	-0.71	37.44	0.68	0.0000
JOLY	558	f	0	0.55	5.11	6.45	0.2166
JOLY	559	f	0	-0.55	7.28	0.00	0.1343
Subtotal JOLY				-0.36	67.01	17.59	
*KAISE2	655	m	1	0.00	9.58	3.18	1.0000
*KAISE2	656	m	1	-0.84	6.60	0.47	0.0302
*KAISE2	657	m	1	-1.35	4.25	2.52	0.0055
*KAISE2	575	f	1	-0.63	5.47	0.02	0.1377
*KAISE2	576	f	1	-1.39	3.65	2.40	0.0081
*KAISE2	577	f	1	-1.08	4.06	1.02	0.0298
Subtotal KAISE2				-0.72	33.61	9.62	
KHUDER	516	m	0	-0.08	37.40	9.20	0.6231
KHUDER	517	m	0	-0.49	32.64	0.23	0.0049
KHUDER	518	m	0	-0.96	35.95	5.39	0.0000
Subtotal KHUDER				-0.51	105.99	14.82	
LUBIN	592	m	0	1.39	11.02	42.71	0.0000
LUBIN	593	m	0	-0.09	13.20	3.14	0.7468
LUBIN	594	m	0	-0.67	12.91	0.11	0.0160
Subtotal LUBIN				0.15	37.13	45.96	
LUBIN2	1081	m	0	0.09	402.52	179.99	0.0639
LUBIN2	1082	m	0	-0.29	267.60	22.65	0.0000
LUBIN2	1083	m	0	-0.66	181.12	1.28	0.0000
LUBIN2	1084	m	0	-1.02	98.44	19.37	0.0000
LUBIN2	1085	m	0	-1.08	81.77	20.57	0.0000
LUBIN2	1086	m	0	-1.60	91.35	95.49	0.0000
LUBIN2	1120	f	0	0.02	25.28	8.88	0.9343
LUBIN2	1121	f	0	-0.36	15.86	0.75	0.1536
LUBIN2	1122	f	0	-1.03	6.98	1.41	0.0067
LUBIN2	1123	f	0	-0.92	2.08	0.24	0.1856
LUBIN2	1124	f	0	-0.88	2.73	0.25	0.1450
LUBIN2	1125	f	0	-1.68	3.28	4.00	0.0023
Subtotal LUBIN2				-0.44	1179.01	354.89	

Table 1K6 - 5

IESLC - Meta-analysis of Ex Smoking by Years quit (vs current), Overview
All LC types, Cigarettes (or Any Product if Cigarettes not available)
Least adjusted

REF	NRR	SEX	AD	Ys	Ws	Qs	Ps
MATOS	586	m	0	0.36	10.45	9.18	0.2432
MATOS	587	m	0	-0.09	9.89	2.37	0.7844
MATOS	588	m	0	-1.15	15.76	5.28	0.0000
Subtotal MATOS				-0.42	36.10	16.83	
PEZZO2	504	m	0	-0.42	33.11	0.80	0.0155
PEZZO2	505	m	0	-1.48	25.77	21.18	0.0000
Subtotal PEZZO2				-0.89	58.88	21.98	
PEZZOT	504	m	0	-0.69	20.58	0.29	0.0016
PEZZOT	505	m	0	-1.78	13.50	19.71	0.0000
Subtotal PEZZOT				-1.13	34.08	20.00	
SOBUE	728	m	0	-0.05	51.63	14.10	0.6997
SOBUE	729	m	0	-0.47	34.80	0.40	0.0056
SOBUE	730	m	0	-0.51	19.41	0.09	0.0250
SOBUE	731	m	0	-0.41	13.01	0.37	0.1411
SOBUE	732	m	0	-0.58	8.84	0.00	0.0848
SOBUE	733	m	0	-1.01	11.53	2.15	0.0006
Subtotal SOBUE				-0.37	139.23	17.10	
*SPEIZE	511	f	2	-0.51	23.37	0.10	0.0135
*SPEIZE	512	f	2	-0.51	31.98	0.14	0.0039
*SPEIZE	513	f	2	-0.69	49.07	0.67	0.0000
*SPEIZE	514	f	2	-1.61	8.00	8.53	0.0000
*SPEIZE	515	f	2	-2.30	8.00	23.83	0.0000
Subtotal SPEIZE				-0.78	120.40	33.27	
SUZUK2	513	c	0	-0.54	4.70	0.01	0.2445
SUZUK2	514	c	0	-0.72	3.69	0.08	0.1672
SUZUK2	515	c	0	-1.84	4.93	7.83	0.0000
Subtotal SUZUK2				-1.07	13.31	7.91	
SVENSS	554	f	0	-0.78	6.05	0.25	0.0557
SVENSS	555	f	0	-1.52	7.19	6.47	0.0000
Subtotal SVENSS				-1.18	13.24	6.71	
*TVERDA	506	m	2	-1.77	1.88	2.68	0.0152
*TVERDA	507	m	2	-1.71	4.66	6.04	0.0002
*TVERDA	508	m	2	-2.53	3.70	14.07	0.0000
Subtotal TVERDA				-2.02	10.24	22.80	
WANG2	515	c	0	-0.05	3.33	0.94	0.9331
WANG2	516	c	0	-0.32	3.09	0.20	0.5698
Subtotal WANG2				-0.18	6.42	1.14	
WYNDE3	545	m	0	-0.14	9.77	1.87	0.6644
WYNDE3	546	m	0	-0.53	6.29	0.01	0.1858
WYNDE3	547	m	0	-1.13	7.55	2.30	0.0019
WYNDE3	548	m	0	-2.49	4.40	16.11	0.0000
Subtotal WYNDE3				-0.86	28.01	20.29	
WYNDE6	719	m	5	-0.51	135.72	0.58	0.0000
WYNDE6	720	m	5	-1.20	31.98	12.60	0.0000
WYNDE6	721	m	5	-1.61	31.98	34.13	0.0000
WYNDE6	726	m	5	-0.36	15.02	0.72	0.1669
WYNDE6	727	m	5	-1.61	5.93	6.33	0.0001
WYNDE6	728	m	5	-1.20	4.79	1.89	0.0084
WYNDE6	733	f	5	-0.69	93.46	1.28	0.0000
WYNDE6	734	f	5	-1.61	93.46	99.76	0.0000
WYNDE6	737	f	5	-0.51	7.15	0.03	0.1721
WYNDE6	738	f	5	-0.92	2.67	0.31	0.1342
Subtotal WYNDE6				-0.95	422.17	157.63	

N	194
NS	43

Table 1K6 - 6

IESLC - Meta-analysis of Ex Smoking by Years quit (vs current), Overview
All LC types, Cigarettes (or Any Product if Cigarettes not available)
Least adjusted

	Sex			
	combined	male	female	Total
N	13	129	52	194
NS	4	37	17	58

In this overview table, other than the "N" rows, entries in the "absent" and "Total" columns may be invalid and should be ignored

	Years quit vs current (lower focus)				
	absent	1-6k3	4-11k7	8+k12	Total
N	78	40	28	48	194
NS	35	31	21	32	119
Wt	2116.20	1247.19	837.51	1057.06	5257.97
Het Chi	1490.53	137.32	56.72	192.34	2629.39
Het df	77	39	27	47	193
Het P	***	***	***	***	***
Fixed RR	0.52	0.99	0.61	0.32	0.56
RRl	0.50	0.93	0.57	0.30	0.55
RRu	0.54	1.04	0.65	0.34	0.58
P	---	N.S.	---	---	---
Random RR	0.48	0.96	0.58	0.28	0.50
RRl	0.39	0.85	0.51	0.24	0.45
RRu	0.58	1.09	0.65	0.32	0.56
P	---	N.S.	---	---	---

	Years quit vs current (higher focus)				
	absent	1-11k3	4-19k12	13+k20	Total
N	93	60	20	21	194
NS	42	42	16	17	117
Wt	2302.52	1817.02	590.76	547.67	5257.97
Het Chi	1396.13	239.40	56.30	89.56	2629.39
Het df	92	59	19	20	193
Het P	***	***	***	***	***
Fixed RR	0.54	0.85	0.44	0.22	0.56
RRl	0.52	0.82	0.41	0.20	0.55
RRu	0.56	0.89	0.48	0.24	0.58
P	---	---	---	---	---
Random RR	0.45	0.83	0.40	0.23	0.50
RRl	0.37	0.75	0.34	0.19	0.45
RRu	0.53	0.93	0.47	0.29	0.56
P	---	---	---	---	---

Table 1K6 - 6

IESLC - Meta-analysis of Ex Smoking by Years quit (vs current), Overview
All LC types, Cigarettes (or Any Product if Cigarettes not available)
Least adjusted

MALES

		Years quit vs current (lower focus)				
		absent	1-6k3	4-11k7	8+k12	Total
	N	53	27	19	30	129
	NS	29	26	18	28	101
	Wt	1648.45	1088.89	711.26	822.28	4270.88
Het	Chi	1174.19	102.97	41.90	114.22	1995.63
Het	df	52	26	18	29	128
Het	P	***	***	**	***	***
Fixed	RR	0.51	1.01	0.63	0.36	0.59
	RRl	0.48	0.95	0.58	0.33	0.57
	RRu	0.53	1.08	0.68	0.38	0.61
	P	---	N.S.	---	---	---
Random	RR	0.48	0.98	0.60	0.31	0.52
	RRl	0.37	0.85	0.52	0.27	0.46
	RRu	0.61	1.13	0.69	0.36	0.59
	P	---	N.S.	---	---	---

		Years quit vs current (higher focus)				
		absent	1-11k3	4-19k12	13+k20	Total
	N	60	38	15	16	129
	NS	34	36	13	14	97
	Wt	1829.82	1460.58	529.85	450.63	4270.88
Het	Chi	1034.67	170.35	35.68	69.44	1995.63
Het	df	59	37	14	15	128
Het	P	***	***	**	***	***
Fixed	RR	0.56	0.90	0.46	0.23	0.59
	RRl	0.54	0.86	0.42	0.21	0.57
	RRu	0.59	0.95	0.50	0.26	0.61
	P	---	---	---	---	---
Random	RR	0.47	0.87	0.44	0.26	0.52
	RRl	0.39	0.76	0.37	0.20	0.46
	RRu	0.58	0.98	0.52	0.33	0.59
	P	---	-	---	---	---

FEMALES

		Years quit vs current (lower focus)				
		absent	1-6k3	4-11k7	8+k12	Total
	N	20	9	7	16	52
	NS	13	9	7	15	44
	Wt	412.32	129.22	106.43	220.29	868.26
Het	Chi	259.64	19.04	8.41	29.31	522.22
Het	df	19	8	6	15	51
Het	P	***	*	N.S.	*	***
Fixed	RR	0.61	0.87	0.51	0.22	0.49
	RRl	0.55	0.73	0.42	0.19	0.45
	RRu	0.67	1.04	0.61	0.25	0.52
	P	---	N.S.	---	---	---
Random	RR	0.54	0.98	0.53	0.23	0.48
	RRl	0.36	0.70	0.41	0.18	0.38
	RRu	0.81	1.38	0.70	0.29	0.61
	P	--	N.S.	---	---	---

Table 1K6 - 6

IESLC - Meta-analysis of Ex Smoking by Years quit (vs current), Overview
All LC types, Cigarettes (or Any Product if Cigarettes not available)
Least adjusted

FEMALES

	Years quit vs current (higher focus) absent	1-11k3	4-19k12	13+k20	Total
N	28	18	3	3	52
NS	17	17	3	3	40
Wt	438.76	327.36	39.97	62.17	868.26
Het Chi	330.37	39.77	1.53	4.12	522.22
Het df	27	17	2	2	51
Het P	***	**	N.S.	N.S.	***
Fixed RR	0.47	0.69	0.29	0.14	0.49
RRl	0.43	0.62	0.21	0.11	0.45
RRu	0.51	0.77	0.40	0.18	0.52
P	---	---	---	---	---
Random RR	0.41	0.75	0.29	0.15	0.48
RRl	0.28	0.62	0.21	0.09	0.38
RRu	0.60	0.92	0.40	0.25	0.61
P	---	--	---	---	---

Table 1K6 - 7

IESLC - Meta-analysis of Ex Smoking by Years quit (vs current), Overview
All LC types, Cigarettes (or Any Product if Cigarettes not available)
Excluded studies (and stage at which they were excluded)

1	AGUDO	AKIBA	AMANDU	AMES	AXELSS	BEST	BOUCHA	BOUCOT	BRESLO	CHEN	CHEN2	CHIAZZ	DEAN2	DOSEME	ENGELA	FAN
	GENG	GER	GUO	HAENSZ	HEGMAN	HOLE	HU	HU2	JUSSAW	KATSOU	KAUFMA	KOO	KOULUM	KREUZE	LETOUR	LEVIN
	LIAW	LIU3	LIU4	LIU5	MCCONN	MIGRAN	MRFITR	NOTAN2	OSANN2	PERNU	QIAO2	RACHTA	RESTRE	SADOWS	SEGI2	STASZE
	TIZZAN	VUTUC	WATSON	WU	WUWILL	WYNDE2	WYNDE8	XU	YUAN	ZHANG	ZHENG	ZHOU				
2	AUVINE	BENSHL	BLOT1	BROWN3	BUFFLE	GURSEL	LAUSSM	LUO	MCDUFF	PISANI	PRESCO	SPITZ	WU2	WYNDE7		
4	HAMMON															
5	CORREA	GILLIS	HUMBLE	QIAO	WIGLE											
7	BOFFET															
10	GARSHI	JEDRYC	WAKAI													
14	BENHAM															

Table 1K6 - 8
Potentially overlapping studies

REF	REFGP	PRINC	. OVERLAP/LINK
LUBIN2	LUBIN2	1	Lubin-combined
TVERDA	TVERDA	1	VEIERO/TVERDAL
BROSS	BYERS1	1	GRAHAM/BROSS/BYERS1
GRAHAM	BYERS1	1	GRAHAM/BROSS/BYERS1
CHYOU	CHYOU	1	GOODMA/CHYOU
WYNDE6	WYNDE6	1	WYNDE5/6/7/8
CPSI	CPSI	1	CPSI overall
JAHN	BOFFET	2	Subset of BOFFET
LUBIN	XIANGZ	2	LUBIN/XIANGZ/QIAO

Table 1K6 - 9
Most adjusted - insufficient data for meta-analysis

REF	NRR	SEX	AGEL	AGEH	RACE	YF	LC TYPE	LOC	START	ST	NLC	R	VB	P	H	AD	ADOS	PRODUCT	exL	exH	S1	S2	DENOM	De
CORREA	550	c	0	0	all	-	all	NAmer	1979	CC	1359	n	bl	y	n	2	0	cig+/-ot	3	5	1	1	cur+2y	st
CORREA	551	c	0	0	all	-	all	NAmer	1979	CC	1359	n	bl	y	n	2	0	cig+/-ot	6	20	0	0	cur+2y	st
CORREA	552	c	0	0	all	-	all	NAmer	1979	CC	1359	n	bl	y	n	2	0	cig+/-ot	21	999	0	0	cur+2y	st
HUMBLE	549	c	0	0	wh	-	not alv	NAmer	1980	CC	521	n	bl	y	n	2	2	#cig+/-ot	2	5	1	1	cur+1y	or
HUMBLE	550	c	0	0	wh	-	not alv	NAmer	1980	CC	521	n	bl	y	n	2	2	#cig+/-ot	6	15	0	2	cur+1y	or
HUMBLE	551	c	0	0	wh	-	not alv	NAmer	1980	CC	521	n	bl	y	n	2	2	#cig+/-ot	16	30	0	3	cur+1y	or
WIGLE	501	m	0	0	all	-	all	NAmer	1971	CC	728	n	V	n	n	2	1	#cig+/-ot	0.1	1.0	0	0	current	ot
WIGLE	502	m	0	0	all	-	all	NAmer	1971	CC	728	n	V	n	n	2	1	#cig+/-ot	2	9	0	1	current	ot
WIGLE	503	m	0	0	all	-	all	NAmer	1971	CC	728	n	V	n	n	2	1	#cig+/-ot	10	14	3	2	current	ot
WIGLE	504	m	0	0	all	-	all	NAmer	1971	CC	728	n	V	n	n	2	1	#cig+/-ot	15	999	0	3	current	ot
WIGLE	505	f	0	0	all	-	all	NAmer	1971	CC	728	n	V	n	n	2	1	#cig+/-ot	0.1	1.0	0	0	current	ot
WIGLE	506	f	0	0	all	-	all	NAmer	1971	CC	728	n	V	n	n	2	1	#cig+/-ot	2	9	0	1	current	ot
WIGLE	507	f	0	0	all	-	all	NAmer	1971	CC	728	n	V	n	n	2	1	#cig+/-ot	10	14	3	2	current	ot
WIGLE	508	f	0	0	all	-	all	NAmer	1971	CC	728	n	V	n	n	2	1	#cig+/-ot	15	999	0	3	current	ot

Comments on values in listings

HUMBLE	ADOS	Number of cigarettes and duration
HUMBLE	ADOS	Number of cigarettes and duration
HUMBLE	ADOS	Number of cigarettes and duration
WIGLE	ADOS	Cumulative exposure
WIGLE	ADOS	Cumulative exposure
WIGLE	ADOS	Cumulative exposure
WIGLE	ADOS	Cumulative exposure
WIGLE	ADOS	Cumulative exposure
WIGLE	ADOS	Cumulative exposure
WIGLE	ADOS	Cumulative exposure
WIGLE	ADOS	Cumulative exposure

REF	NRR	RR	SIG	RRDATA comment
CORREA	550	0.61		0
CORREA	551	0.56		0
CORREA	552	0.31		0
HUMBLE	549	0.69		0
HUMBLE	550	0.33		0

Table 1K6 - 9

IESLC - Meta-analysis of Ex Smoking by Years quit (vs current), Overview
All LC types, Cigarettes (or Any Product if Cigarettes not available)
Most adjusted - insufficient data for meta-analysis

REF	NRR	RR	SIG	RRDATA comment
HUMBLE	551	0.11		0
WIGLE	501	2.40	n	0
WIGLE	502	0.70	n	0
WIGLE	503	0.70	n	0
WIGLE	504	0.20	y	p<0.01
WIGLE	505	0.90	n	0
WIGLE	506	0.50	n	0
WIGLE	507	0.50	n	0
WIGLE	508	0.40	n	0

Table 1K7 -

IESLC - Meta-analysis of Ex Smoking, Years quit (vs current), "Low"
All LC types, Cigarettes (or Any Product if Cigarettes not available)

This analysis is restricted to results for:
1) Ex smokers
2) Results by Years quit (vs current)
3) Categorical results by Years quit (vs current)
4) All LC types (or near equivalent)
5) Results complete enough for use in metaanalysis

Within each study, results are then selected (in the following order of preference, within each sex) for:
6) (not applicable)
7) PRODUCT: cigarettes regardless of other products, cigarettes only, all/unspec
8) CIGTYPE: all/unspecified, MC regardless of HR, MC only
9) Results with least adjustment for other aspects of smoking (ADOS)
10) DENOM: current smokers, current + recent smokers (up to number of m=months or y=years, max 2 years)
11) Followup period (YF, prospective studies): whole study (coded as 0) or longest available
12) LCtype: all or nearest available, at least Squamous and Adeno. (q = squamous, s = small,
l = large, a = adeno, mix = mixed, alv = alveolar)
13) Race: all or nearest available, otherwise by race (wh or w = white, bl or b = black, hi = hispanic
ch = chinese, jap = japanese, haw = hawaiian, w+o = white + oriental, sca = scandinavian, as = asian)
14) Years quit (vs current) "low" in key scheme 1 (key value 3, maximum range 1-6)
15) For overlapping studies: principal rather than subsidiary studies
Finally by Age: whole study (coded as 0) if available, otherwise by widest available age group
and then for single sex results (m, f) in preference to results for both sexes combined (c).

Results adjusted (AD) for the most potential confounders are then chosen in Sections -1 to -3
(and those which actually differ from the adjusted results in Table 1K2 - 1 are marked 'x' in Section -1)
and results adjusted for the least confounders in Sections -4 to -6. (Those least adjusted results which
actually differ from the most adjusted are marked 'x' in column X in Section -4)

Section -7 shows excluded studies, together with the stage (as above) at which no qualifying
results were found.

Section -8 lists the potentially overlapping studies which have been included (1=principal, 2=subsidiary).

Section -9 lists any results which would have been included in preference except that they had data not complete
enough for use in meta-analysis, with their significance (yes/no), if known, and any further comment as entered
on the database. It also lists as "gap" any categories for which no data were presented by the original authors.

In addition to those mentioned above, the following fields, levels and abbreviations are used:
* or nk = not known, n = no, y = yes, ot = other
nev = never
all/unspec = all or unspecified, cig+/-ot = cigarettes irrespective of other products (cigar, pipe etc)
MC = manufactured cigarettes, HR = hand-rolled cigarettes
exL, exH = range of exposure (low and high) in the smoking group, in terms of Years quit (vs current)
REF: 6-character study reference
NRR: number of the RR on the database within the study
ST : study type (CC = case control, pr or prosp = prospective)
NLC: number of lung cancer cases in whole study
R : risky occupational population (n = no, m = mining, o = other risky)
VB : national cigarette type (V = at least 75% Virginia, bl = at least 75% blended, ot = other)
P : any proxy use
H : full histological confirmation
De : derivation of RR/CI (or = original, st = standard method, ot = other method of estimation)

Table 1K7 - 1

IESLC - Meta-analysis of Ex Smoking, Years quit (vs current), "Low"
All LC types, Cigarettes (or Any Product if Cigarettes not available)
Most adjusted

REF	NRR	1K2	SEX	AGEL	AGEH	RACE	YF	LC TYPE	LOC	START	ST	NLC	R	VB	P	H	AD	ADOS	PRODUCT	exL	exH	DENOM	De
ARMADA	518		m	0	0	all	-	all	Eu:wst	1986	CC	325	n	bl	n	y	0	0	cig+/-ot	1.0	5	cur+1y	st
BARBON	548		m	0	0	all	-	all	Eu:wst	1979	CC	755	n	bl	y	y	1	0	all/unsp	0.1	4	current	ot
BECHER	506		m	0	0	all	-	all	Eu:Ger	1985	CC	194	n	bl	n	y	0	0	all/unsp	2	4	cur+1y	st
BECHER	516		f	0	0	all	-	all	Eu:Ger	1985	CC	194	n	bl	n	y	0	0	all/unsp	2	4	cur+1y	st
BROSS	518		m	0	0	wh	-	all	NAmer	1960	CC	974	n	bl	n	n	0	0	cig+/-ot	0.1	5	current	st
CARPEN	508		c	0	0	w+b	-	all	NAmer	1991	CC	356	n	bl	n	n	0	0	cig+/-ot	0.1	4	current	st
CHOI	543		m	0	0	all	-	all	As:oth	1985	CC	375	n	bl	n	n	0	0	cig+/-ot	0.1	4	current	st
CHOI	556		f	0	0	all	-	all	As:oth	1985	CC	375	n	bl	n	n	0	0	cig+/-ot	0.1	4	current	st
CPSI	816		m	50	74	all	6	all	NAmer	1959	pr	5138	n	bl	n	n	1	0	cig only	1.0	4	current	ot
CPSII	666		m	35	99	all	4	all	NAmer	1982	pr	3229	n	bl	n	n	1	0	cig only	3	5	current	ot
CPSII	643		f	0	0	all	4	all	NAmer	1982	pr	3229	n	bl	n	n	1	0	cig+/-ot	3	5	current	ot
DAMBER	528		m	0	0	all	-	all	Eu:Sca	1972	CC	579	n	bl	y	n	1	0	all/unsp	0.1	5	current	ot
DEAN3	524	x	m	0	0	all	-	all	Eu:UK	1969	CC	766	n	V	y	n	1	0	cig only	3	4	cur+2y	ot
DEAN3	559		f	0	0	all	-	all	Eu:UK	1969	CC	766	n	V	y	n	1	0	all/unsp	3	4	cur+2y	ot
DESTEF	543	x	m	0	0	all	-	all	SCAmer	1988	CC	497	n	bl	n	y	0	0	cig+/-ot	0.1	4	current	st
DOLL2	509		m	0	0	all	20	all	Eu:UK	1951	pr	920	n	V	n	n	1	0	cig only	0.1	4	current	ot
DORGAN	514		m	0	0	wh	-	all	NAmer	1980	CC	2026	n	bl	y	y	0	0	cig+/-ot	1.1	5	cur+1y	st
DORN	823		m	55	64	wh	8	all	NAmer	1954	pr	5097	n	bl	n	n	0	0	cig+/-ot	0.1	4	current	st
DORN	827		m	65	74	wh	8	all	NAmer	1954	pr	5097	n	bl	n	n	0	0	cig+/-ot	0.1	4	current	st
GAO	536		m	0	0	all	-	all	As:Chi	1984	CC	1405	n	ot	n	n	2	0	cig+/-ot	0.1	4	current	ot
GAO	556		f	0	0	all	-	all	As:Chi	1984	CC	1405	n	ot	n	n	2	0	cig+/-ot	0.1	4	current	ot
GAO2	518		m	0	0	all	-	all	As:Jap	1988	CC	282	n	bl	n	n	0	0	cig+/-ot	1.0	4	cur+1y	st
GARCIA	508		c	0	0	all	-	all	NAmer	1992	CC	416	n	bl	n	y	0	0	cig+/-ot	1.0	4	cur+1y	st
GRAHAM	541		m	0	0	wh	-	all	NAmer	1956	CC	685	n	bl	n	n	1	0	cig+/-ot	1.1	5	current	ot
HAMMO2	510		m	0	0	all	0	all	NAmer	1967	pr	450	o	bl	n	n	1	0	cig+/-ot	0.1	4	current	ot
HIRAYA	513		m	0	0	all	0	all	As:Jap	1965	pr	1917	n	bl	n	n	1	0	cig+/-ot	0.1	4	current	ot
HIRAYA	524		f	0	0	all	0	all	As:Jap	1965	pr	1917	n	bl	n	n	1	0	cig+/-ot	0.1	4	current	ot
JAHN	515		m	0	0	all	-	all	Eu:Ger	1988	CC	1004	n	bl	n	n	0	0	cig+/-ot	2	5	current	st
JOLY	571		m	0	0	all	-	all	SCAmer	1978	CC	826	n	bl	n	n	0	0	cig+/-ot	1.0	4	cur+1y	st
JOLY	558		f	0	0	all	-	all	SCAmer	1978	CC	826	n	bl	n	n	0	0	cig+/-ot	1.0	4	cur+1y	st
KHUDER	516		m	0	0	all	-	all	NAmer	1985	CC	482	n	bl	n	y	0	0	cig+/-ot	0.1	4	current	st
LUBIN	592		m	0	0	all	-	all	As:Chi	1984	CC	427	m	ot	y	n	0	0	cig+/-ot	3	4	cur+2y	st
LUBIN2	1081		m	0	0	all	-	all	Eu:mul	1976	CC	7804	n	bl	n	y	0	0	cig+/-ot	0.1	4	current	st
LUBIN2	1120		f	0	0	all	-	all	Eu:mul	1976	CC	7804	n	bl	n	y	0	0	cig+/-ot	0.1	4	current	st
MATOS	596		m	0	0	all	-	all	SCAmer	1994	CC	200	n	bl	n	n	2	0	cig+/-ot	1.0	5	cur+1y	or
SOBUE	728		m	0	0	all	-	all	As:Jap	1986	CC	1376	n	bl	n	y	0	0	cig+/-ot	1.0	4	cur+1y	st
SPEIZE	512		f	0	0	all	0	all	NAmer	1976	pr	593	n	bl	n	y	2	0	cig+/-ot	2	5	current	or
SUZUK2	524		c	0	0	all	-	all	SCAmer	1991	CC	123	n	bl	n	y	3	0	all/unsp	0.1	5	current	or
TVERDA	507		m	0	0	all	0	all	Eu:Sca	1972	pr	238	n	bl	n	n	2	0	cig only	1.0	5	current	ot
WANG2	515		c	0	0	all	-	all	As:Chi	1980	CC	103	n	ot	n	n	0	0	cig+/-ot	0.1	3	current	st
WYNDE3	545		m	0	0	all	-	all	NAmer	1966	CC	350	n	bl	n	y	0	0	all/unsp	1.0	3	cur+1y	st
WYNDE6	513		m	0	0	all	-	all	NAmer	1969	CC	4423	n	bl	n	y	0	0	cig only	1.0	4	cur+1y	st
WYNDE6	534		f	0	0	all	-	all	NAmer	1969	CC	4423	n	bl	n	y	0	0	cig only	1.0	4	cur+1y	st

Cigarette type is all/unspec for all RRs
except for the following:

REF	NRR	CIGTYPE
DEAN3	524	MC only
DESTEF	543	MC only

Table 1K7 - 2

IESLC - Meta-analysis of Ex Smoking, Years quit (vs current), "Low"
All LC types, Cigarettes (or Any Product if Cigarettes not available)
Most adjusted

REF	NRR	SEX	AD	Number Exposed Case	Number Exposed Cont	Non-exposed Case	Non-exposed Cont	RR	95.00%CI
ARMADA	518	m	0	79	45	188	122	1.14	(0.74- 1.75)
BARBON	548	m	1	32	-	562	-	1.01	(0.57- 1.79)
BECHER	506	m	0	10	12	101	122	1.01	(0.42- 2.43)
BECHER	516	f	0	2	3	33	26	0.53	(0.08- 3.38)
Subtotal BECHER								0.89	(0.40- 1.98)
BROSS	518	m	0	169	67	565	427	1.91	(1.40- 2.60)
CARPEN	508	c	0	28	46	228	164	0.44	(0.26- 0.73)
CHOI	543	m	0	25	64	231	329	0.56	(0.34- 0.91)
CHOI	556	f	0	3	2	13	25	2.88	(0.43- 19.49)
Subtotal CHOI								0.62	(0.38- 0.99)
*CPSI	816	m	1	49	-	844	-	0.59	(0.44- 0.78)
*CPSII	666	m	1	178	-	1159	-	0.85	(0.72- 0.99)
*CPSII	643	f	1	56	-	530	-	0.85	(0.65- 1.12)
Subtotal CPSII								0.85	(0.74- 0.98)
DAMBER	528	m	1	-	-	-	-	0.80	(0.50- 1.28)
DEAN3	524	m	1	28	-	337	-	0.64	(0.41- 1.00)
DEAN3	559	f	1	4	-	102	-	0.29	(0.11- 0.81)
Subtotal DEAN3								0.56	(0.37- 0.84)
DESTEF	543	m	0	10	19	78	109	0.74	(0.32- 1.67)
*DOLL2	509	m	1	15	-	236	-	1.02	(0.61- 1.72)
DORGAN	514	m	0	59	51	465	303	0.75	(0.50- 1.13)
*DORN	823	m	0	34	22086	528	334175	0.97	(0.69- 1.38)
*DORN	827	m	0	14	6195	537	207895	0.87	(0.51- 1.49)
Subtotal DORN								0.94	(0.71- 1.26)
GAO	536	m	2	105	-	529	-	1.77	(1.22- 2.56)
GAO	556	f	2	37	-	170	-	2.48	(1.15- 5.38)
Subtotal GAO								1.89	(1.35- 2.63)
GAO2	518	m	0	31	26	184	117	0.76	(0.43- 1.34)
GARCIA	508	c	0	33	11	77	42	1.64	(0.75- 3.57)
GRAHAM	541	m	1	24	-	453	-	1.17	(0.70- 1.94)
*HAMMO2	510	m	1	59	-	209	-	1.08	(0.82- 1.43)
*HIRAYA	513	m	1	-	-	-	-	0.46	(0.26- 0.82)
*HIRAYA	524	f	1	-	-	-	-	1.59	(0.47- 5.37)
Subtotal HIRAYA								0.58	(0.34- 0.97)
JAHN	515	m	0	77	46	352	269	1.28	(0.86- 1.90)
JOLY	571	m	0	38	36	451	524	1.23	(0.76- 1.97)
JOLY	558	f	0	19	8	132	96	1.73	(0.73- 4.11)
Subtotal JOLY								1.33	(0.88- 2.01)
KHUDER	516	m	0	88	123	245	316	0.92	(0.67- 1.27)
LUBIN	592	m	0	33	18	296	650	4.03	(2.23- 7.27)
LUBIN2	1081	m	0	866	1047	4684	6211	1.10	(0.99- 1.21)
LUBIN2	1120	f	0	60	55	440	410	1.02	(0.69- 1.50)
Subtotal LUBIN2								1.09	(0.99- 1.20)
MATOS	596	m	2	28	-	112	-	1.40	(0.80- 2.60)
SOBUE	728	m	0	128	116	737	633	0.95	(0.72- 1.24)
*SPEIZE	512	f	2	34	-	319	-	0.60	(0.40- 0.80)
SUZUK2	524	c	3	15	-	77	-	0.60	(0.20- 1.50)
*TVERDA	507	m	2	5	-	144	-	0.18	(0.07- 0.43)
WANG2	515	c	0	6	10	49	78	0.96	(0.33- 2.79)
WYNDE3	545	m	0	21	22	227	207	0.87	(0.47- 1.63)
WYNDE6	513	m	0	201	166	1107	993	1.09	(0.87- 1.36)
WYNDE6	534	f	0	82	70	683	496	0.85	(0.61- 1.19)
Subtotal WYNDE6								1.01	(0.84- 1.22)
Partial Totals				2785	30344	18414	554739		

*prospective study

Table 1K7 - 2

IESLC - Meta-analysis of Ex Smoking, Years quit (vs current), "Low"
All LC types, Cigarettes (or Any Product if Cigarettes not available)
Most adjusted

REF	NRR	SEX	AD	Ys	Ws	Qs	Ps
ARMADA	518	m	0	0.13	20.66	0.44	0.5535
BARBON	548	m	1	0.01	11.73	0.01	0.9728
BECHER	506	m	0	0.01	4.96	0.00	0.9883
BECHER	516	f	0	-0.64	1.11	0.44	0.4978
Subtotal BECHER				-0.11	6.07	0.44	
BROSS	518	m	0	0.65	40.07	17.52	0.0000
CARPEN	508	c	0	-0.83	14.72	9.66	0.0015
CHOI	543	m	0	-0.59	15.87	5.16	0.0195
CHOI	556	f	0	1.06	1.05	1.22	0.2771
Subtotal CHOI				-0.48	16.93	6.38	
*CPSI	816	m	1	-0.53	46.88	12.27	0.0003
*CPSII	666	m	1	-0.16	151.52	3.25	0.0454
*CPSII	643	f	1	-0.16	51.90	1.11	0.2417
Subtotal CPSII				-0.16	203.42	4.37	
DAMBER	528	m	1	-0.22	17.39	0.75	0.3521
DEAN3	524	m	1	-0.45	19.33	3.58	0.0497
DEAN3	559	f	1	-1.24	3.85	5.76	0.0151
Subtotal DEAN3				-0.58	23.18	9.33	
DESTEF	543	m	0	-0.31	5.73	0.49	0.4622
*DOLL2	509	m	1	0.02	14.30	0.02	0.9403
DORGAN	514	m	0	-0.28	23.81	1.69	0.1680
*DORN	823	m	0	-0.03	31.99	0.00	0.8830
*DORN	827	m	0	-0.13	13.68	0.19	0.6211
Subtotal DORN				-0.06	45.67	0.19	
GAO	536	m	2	0.57	27.97	9.64	0.0025
GAO	556	f	2	0.91	6.45	5.51	0.0210
Subtotal GAO				0.63	34.43	15.15	
GAO2	518	m	0	-0.28	11.81	0.80	0.3414
GARCIA	508	c	0	0.49	6.33	1.64	0.2154
GRAHAM	541	m	1	0.16	14.79	0.44	0.5460
*HAMMO2	510	m	1	0.08	49.68	0.43	0.5875
*HIRAYA	513	m	1	-0.78	11.65	6.74	0.0080
*HIRAYA	524	f	1	0.46	2.59	0.60	0.4555
Subtotal HIRAYA				-0.55	14.24	7.33	
JAHN	515	m	0	0.25	24.22	1.67	0.2255
JOLY	571	m	0	0.20	17.18	0.83	0.3976
JOLY	558	f	0	0.55	5.11	1.62	0.2166
Subtotal JOLY				0.28	22.29	2.45	
KHUDER	516	m	0	-0.08	37.40	0.15	0.6231
LUBIN	592	m	0	1.39	11.02	21.86	0.0000
LUBIN2	1081	m	0	0.09	402.52	4.73	0.0639
LUBIN2	1120	f	0	0.02	25.28	0.03	0.9343
Subtotal LUBIN2				0.09	427.80	4.75	
MATOS	596	m	2	0.34	11.06	1.37	0.2631
SOBUE	728	m	0	-0.05	51.63	0.07	0.6997
*SPEIZE	512	f	2	-0.51	31.98	7.83	0.0039
SUZUK2	524	c	3	-0.51	3.78	0.93	0.3203
*TVERDA	507	m	2	-1.71	4.66	13.46	0.0002
WANG2	515	c	0	-0.05	3.33	0.00	0.9331
WYNDE3	545	m	0	-0.14	9.77	0.15	0.6644
WYNDE6	513	m	0	0.08	77.46	0.75	0.4670
WYNDE6	534	f	0	-0.16	33.38	0.71	0.3502
Subtotal WYNDE6				0.01	110.84	1.46	

Table 1K7 - 2

IESLC - Meta-analysis of Ex Smoking, Years quit (vs current), "Low"
All LC types, Cigarettes (or Any Product if Cigarettes not available)
Most adjusted

	N	43
	NS	33
	Wt	1371.62
Het	Chi	145.51
Het	df	42
Het	P	***
Fixed	RR	0.98
	RRl	0.93
	RRu	1.04
	P	N.S.
Random	RR	0.95
	RRl	0.85
	RRu	1.07
	P	N.S.
Asymm	P	N.S.

Table 1K7 - 3

IESLC - Meta-analysis of Ex Smoking, Years quit (vs current), "Low"
All LC types, Cigarettes (or Any Product if Cigarettes not available)
Most adjusted

	Sex			
	combined	male	female	Total
N	4	29	10	43
NS	4	28	10	42
Wt	28.17	1180.74	162.71	1371.62
Het Chi	8.17	107.58	21.93	145.51
Het df	3	28	9	42
Het P	*	***	**	***
Fixed RR	0.67	1.01	0.86	0.98
RRl	0.47	0.96	0.74	0.93
RRu	0.97	1.07	1.00	1.04
P	-	N.S.	(-)	N.S.
Random RR	0.77	0.98	0.93	0.95
RRl	0.40	0.86	0.70	0.85
RRu	1.50	1.11	1.25	1.07
P	N.S.	N.S.	N.S.	N.S.
Between Chi				7.82
Between df				2
Between P				*
Btwn(F) P				N.S.
Btwn(R) P				N.S.

	Lung cancer type		
	all	other	Total
N	43		43
NS	33		33
Wt	1371.62		1371.62
Het Chi	145.51		145.51
Het df	42		42
Het P	***		***
Fixed RR	0.98		0.98
RRl	0.93		0.93
RRu	1.04		1.04
P	N.S.		N.S.
Random RR	0.95		0.95
RRl	0.85		0.85
RRu	1.07		1.07
P	N.S.		N.S.
Between Chi			
Between df			
Between P			N.S.
Btwn(F) P			N.S.
Btwn(R) P			N.S.

	Location								
	NAmer	UK	Scand	othEur	China	Japan	othAs	other	Total
N	16	3	2	7	4	4	2	5	43
NS	13	2	2	5	3	3	1	4	33
Wt	635.35	37.48	22.05	490.49	48.78	77.67	16.93	42.86	1371.62
Het Chi	53.25	5.17	8.18	1.47	7.72	6.15	2.67	4.09	145.51
Het df	15	2	1	6	3	3	1	4	42
Het P	***	(*)	**	N.S.	(*)	N.S.	N.S.	N.S.	***
Fixed RR	0.90	0.70	0.58	1.10	2.14	0.84	0.62	1.16	0.98
RRl	0.84	0.51	0.38	1.00	1.61	0.67	0.38	0.86	0.93
RRu	0.98	0.97	0.89	1.20	2.83	1.04	0.99	1.56	1.04
P	-	-	-	+	+++	N.S.	-	N.S.	N.S.
Random RR	0.90	0.65	0.40	1.10	2.18	0.78	0.97	1.16	0.95
RRl	0.77	0.37	0.09	1.00	1.30	0.53	0.21	0.85	0.85
RRu	1.05	1.14	1.72	1.20	3.66	1.17	4.44	1.57	1.07
P	N.S.	N.S.	N.S.	+	++	N.S.	N.S.	N.S.	N.S.
Between Chi									56.80
Between df									7
Between P									***
Btwn(F) P									**
Btwn(R) P									**

Table 1K7 - 3

IESLC - Meta-analysis of Ex Smoking, Years quit (vs current), "Low"
All LC types, Cigarettes (or Any Product if Cigarettes not available)
Most adjusted

Detailed Country in "other Europe"

	multi	Germany	othWest	East	Balkans	Total
N	2	3	2			7
NS	1	2	2			5
Wt	427.80	30.30	32.40			490.49
Het Chi	0.14	1.01	0.11			1.47
Het df	1	2	1			6
Het P	N.S.	N.S.	N.S.			N.S.
Fixed RR	1.09	1.19	1.09			1.10
RRl	0.99	0.83	0.77			1.00
RRu	1.20	1.70	1.54			1.20
P	(+)	N.S.	N.S.			+
Random RR	1.09	1.19	1.09			1.10
RRl	0.99	0.83	0.77			1.00
RRu	1.20	1.70	1.54			1.20
P	(+)	N.S.	N.S.			+
Between Chi						0.21
Between df						2
Between P						N.S.
Btwn(F) P						N.S.
Btwn(R) P						N.S.

Detailed Country in "other Asia"

	India	HongKong	other	Total
N			2	2
NS			1	1
Wt			16.93	16.93
Het Chi			2.67	2.67
Het df			1	1
Het P			N.S.	N.S.
Fixed RR			0.62	0.62
RRl			0.38	0.38
RRu			0.99	0.99
P			-	-
Random RR			0.97	0.97
RRl			0.21	0.21
RRu			4.44	4.44
P			N.S.	N.S.
Between Chi				
Between df				
Between P				N.S.
Btwn(F) P				N.S.
Btwn(R) P				N.S.

Detailed other continent

	SCAmer	Total
N	5	5
NS	4	4
Wt	42.86	42.86
Het Chi	4.09	4.09
Het df	4	4
Het P	N.S.	N.S.
Fixed RR	1.16	1.16
RRl	0.86	0.86
RRu	1.56	1.56
P	N.S.	N.S.
Random RR	1.16	1.16
RRl	0.85	0.85
RRu	1.57	1.57
P	N.S.	N.S.
Between Chi		
Between df		
Between P		N.S.
Btwn(F) P		N.S.
Btwn(R) P		N.S.

Table 1K7 - 3

IESLC - Meta-analysis of Ex Smoking, Years quit (vs current), "Low"
All LC types, Cigarettes (or Any Product if Cigarettes not available)
Most adjusted

	Start year of study					
	<1960	1960-69	1970-79	1980-89	1990+	Total
N	5	9	8	17	4	43
NS	4	6	6	13	4	33
Wt	121.63	247.79	515.86	450.44	35.89	1371.62
Het Chi	8.63	35.64	28.12	53.43	12.35	145.51
Het df	4	8	7	16	3	42
Het P	(*)	***	***	***	**	***
Fixed RR	0.82	1.03	1.03	0.97	0.82	0.98
RRl	0.68	0.91	0.95	0.88	0.59	0.93
RRu	0.97	1.17	1.12	1.06	1.13	1.04
P	-	N.S.	N.S.	N.S.	N.S.	N.S.
Random RR	0.87	0.90	0.87	1.06	0.88	0.95
RRl	0.66	0.67	0.66	0.87	0.44	0.85
RRu	1.14	1.21	1.16	1.30	1.77	1.07
P	N.S.	N.S.	N.S.	N.S.	N.S.	N.S.
Between Chi						7.34
Between df						4
Between P						N.S.
Btwn(F) P						N.S.
Btwn(R) P						N.S.

	Study type (1)		
	CC	other	Total
N	32	11	43
NS	25	8	33
Wt	960.79	410.83	1371.62
Het Chi	92.62	29.55	145.51
Het df	31	10	42
Het P	***	**	***
Fixed RR	1.07	0.81	0.98
RRl	1.01	0.73	0.93
RRu	1.14	0.89	1.04
P	+	---	N.S.
Random RR	1.04	0.77	0.95
RRl	0.91	0.63	0.85
RRu	1.20	0.93	1.07
P	N.S.	--	N.S.
Between Chi			23.34
Between df			1
Between P			***
Btwn(F) P			**
Btwn(R) P			*

	Study type (2)			
	CC	prosp	other	Total
N	32	11		43
NS	25	8		33
Wt	960.79	410.83		1371.62
Het Chi	92.62	29.55		145.51
Het df	31	10		42
Het P	***	**		***
Fixed RR	1.07	0.81		0.98
RRl	1.01	0.73		0.93
RRu	1.14	0.89		1.04
P	+	---		N.S.
Random RR	1.04	0.77		0.95
RRl	0.91	0.63		0.85
RRu	1.20	0.93		1.07
P	N.S.	--		N.S.
Between Chi				23.34
Between df				1
Between P				***
Btwn(F) P				**
Btwn(R) P				*

Table 1K7 - 3

IESLC - Meta-analysis of Ex Smoking, Years quit (vs current), "Low"
All LC types, Cigarettes (or Any Product if Cigarettes not available)
Most adjusted

	Study size (number of LC cases)				
	100-249	250-499	500-999	1000+	Total
N	6	11	10	16	43
NS	5	10	8	10	33
Wt	28.92	184.04	175.74	982.92	1371.62
Het Chi	14.70	41.98	38.25	48.94	145.51
Het df	5	10	9	15	42
Het P	*	***	***	***	***
Fixed RR	0.78	0.97	1.01	0.99	0.98
RRl	0.54	0.84	0.87	0.93	0.93
RRu	1.13	1.13	1.17	1.05	1.04
P	N.S.	N.S.	N.S.	N.S.	N.S.
Random RR	0.68	1.00	0.95	0.96	0.95
RRl	0.35	0.72	0.69	0.84	0.85
RRu	1.34	1.39	1.31	1.10	1.07
P	N.S.	N.S.	N.S.	N.S.	N.S.
Between Chi					1.63
Between df					3
Between P					N.S.
Btwn(F) P					N.S.
Btwn(R) P					N.S.

	Risky occupational population			
	no	mining	othRisky	Total
N	41	1	1	43
NS	31	1	1	33
Wt	1310.92	11.02	49.68	1371.62
Het Chi	122.91	0.00	0.00	145.51
Het df	40	0	0	42
Het P	***	N.S.	N.S.	***
Fixed RR	0.97	4.03	1.08	0.98
RRl	0.92	2.23	0.82	0.93
RRu	1.02	7.27	1.43	1.04
P	N.S.	+++	N.S.	N.S.
Random RR	0.92	4.03	1.08	0.95
RRl	0.82	2.23	0.82	0.85
RRu	1.03	7.27	1.43	1.07
P	N.S.	+++	N.S.	N.S.
Between Chi				22.60
Between df				2
Between P				***
Btwn(F) P				*
Btwn(R) P				***

	National cigarette tobacco type			
	Virginia	blended	other	Total
N	3	36	4	43
NS	2	28	3	33
Wt	37.48	1285.35	48.78	1371.62
Het Chi	5.17	98.64	7.72	145.51
Het df	2	35	3	42
Het P	(*)	***	(*)	***
Fixed RR	0.70	0.96	2.14	0.98
RRl	0.51	0.91	1.61	0.93
RRu	0.97	1.02	2.83	1.04
P	-	N.S.	+++	N.S.
Random RR	0.65	0.91	2.18	0.95
RRl	0.37	0.81	1.30	0.85
RRu	1.14	1.02	3.66	1.07
P	N.S.	(-)	++	N.S.
Between Chi				33.98
Between df				2
Between P				***
Btwn(F) P				**
Btwn(R) P				**

Table 1K7 - 3

IESLC - Meta-analysis of Ex Smoking, Years quit (vs current), "Low"
All LC types, Cigarettes (or Any Product if Cigarettes not available)
Most adjusted

	Any proxy use No/nk	Yes	Total
N	37	6	43
NS	28	5	33
Wt	1284.49	87.13	1371.62
Het Chi	111.83	33.06	145.51
Het df	36	5	42
Het P	***	***	***
Fixed RR	0.99	0.91	0.98
RRl	0.94	0.73	0.93
RRu	1.05	1.12	1.04
P	N.S.	N.S.	N.S.
Random RR	0.96	0.91	0.95
RRl	0.85	0.52	0.85
RRu	1.07	1.60	1.07
P	N.S.	N.S.	N.S.
Between Chi			0.63
Between df			1
Between P			N.S.
Btwn(F) P			N.S.
Btwn(R) P			N.S.

	Full histological confirmation No	Yes	Total
N	27	16	43
NS	20	13	33
Wt	624.08	747.53	1371.62
Het Chi	124.69	19.41	145.51
Het df	26	15	42
Het P	***	N.S.	***
Fixed RR	0.95	1.01	0.98
RRl	0.88	0.94	0.93
RRu	1.03	1.09	1.04
P	N.S.	N.S.	N.S.
Random RR	0.97	0.96	0.95
RRl	0.80	0.86	0.85
RRu	1.17	1.07	1.07
P	N.S.	N.S.	N.S.
Between Chi			1.41
Between df			1
Between P			N.S.
Btwn(F) P			N.S.
Btwn(R) P			N.S.

	Number of adjustment variables (1) 0	1	2+/+nk	Total
N	25	12	6	43
NS	19	9	5	33
Wt	890.09	395.61	85.92	1371.62
Het Chi	66.41	22.55	38.74	145.51
Het df	24	11	5	42
Het P	***	*	***	***
Fixed RR	1.06	0.82	0.99	0.98
RRl	1.00	0.75	0.80	0.93
RRu	1.14	0.91	1.23	1.04
P	(+)	---	N.S.	N.S.
Random RR	1.04	0.81	0.90	0.95
RRl	0.90	0.68	0.47	0.85
RRu	1.19	0.96	1.72	1.07
P	N.S.	-	N.S.	N.S.
Between Chi				17.81
Between df				2
Between P				***
Btwn(F) P				(*)
Btwn(R) P				(*)

Table 1K7 - 3

IESLC - Meta-analysis of Ex Smoking, Years quit (vs current), "Low"
All LC types, Cigarettes (or Any Product if Cigarettes not available)
Most adjusted

	Number of adjustment variables (2) 0	1	2	3-5	6+/+nk	Total
N	25	12	5	1		43
NS	19	9	4	1		33
Wt	890.09	395.61	82.13	3.78		1371.62
Het Chi	66.41	22.55	37.74	0.00		145.51
Het df	24	11	4	0		42
Het P	***	*	***	N.S.		***
Fixed RR	1.06	0.82	1.01	0.60		0.98
RRl	1.00	0.75	0.82	0.22		0.93
RRu	1.14	0.91	1.26	1.64		1.04
P	(+)	---	N.S.	N.S.		N.S.
Random RR	1.04	0.81	0.96	0.60		0.95
RRl	0.90	0.68	0.46	0.22		0.85
RRu	1.19	0.96	1.97	1.64		1.07
P	N.S.	-	N.S.	N.S.		N.S.
Between Chi						18.81
Between df						3
Between P						***
Btwn(F) P						N.S.
Btwn(R) P						N.S.

	Product all/unsp	cig+/-ot	cig only	Total
N	7	29	7	43
NS	6	23	6	35
Wt	52.61	971.48	347.53	1371.62
Het Chi	5.44	98.29	23.98	145.51
Het df	6	28	6	42
Het P	N.S.	***	***	***
Fixed RR	0.79	1.06	0.83	0.98
RRl	0.60	0.99	0.75	0.93
RRu	1.03	1.13	0.92	1.04
P	(-)	(+)	---	N.S.
Random RR	0.79	1.05	0.76	0.95
RRl	0.60	0.91	0.59	0.85
RRu	1.03	1.22	0.97	1.07
P	(-)	N.S.	-	N.S.
Between Chi				17.81
Between df				2
Between P				***
Btwn(F) P				(*)
Btwn(R) P				*

	Denominator current	cur+rec	Total
N	27	16	43
NS	21	12	33
Wt	1063.15	308.46	1371.62
Het Chi	103.72	41.46	145.51
Het df	26	15	42
Het P	***	***	***
Fixed RR	0.98	1.01	0.98
RRl	0.92	0.91	0.93
RRu	1.04	1.13	1.04
P	N.S.	N.S.	N.S.
Random RR	0.91	1.03	0.95
RRl	0.79	0.84	0.85
RRu	1.06	1.27	1.07
P	N.S.	N.S.	N.S.
Between Chi			0.33
Between df			1
Between P			N.S.
Btwn(F) P			N.S.
Btwn(R) P			N.S.

Table 1K7 - 3

IESLC - Meta-analysis of Ex Smoking, Years quit (vs current), "Low"
All LC types, Cigarettes (or Any Product if Cigarettes not available)
Most adjusted

	Derivation of RR/CI			
	Orig	StdCalc	Other	Total
N	3	25	15	43
NS	3	19	11	33
Wt	46.83	890.09	434.70	1371.62
Het Chi	6.07	66.41	56.45	145.51
Het df	2	24	14	42
Het P	*	***	***	***
Fixed RR	0.73	1.06	0.87	0.98
RRl	0.55	1.00	0.79	0.93
RRu	0.98	1.14	0.95	1.04
P	-	(+)	--	N.S.
Random RR	0.80	1.04	0.85	0.95
RRl	0.44	0.90	0.68	0.85
RRu	1.47	1.19	1.06	1.07
P	N.S.	N.S.	N.S.	N.S.
Between Chi				16.58
Between df				2
Between P				***
Btwn(F) P				(*)
Btwn(R) P				N.S.

Table 1K7 - 4

IESLC - Meta-analysis of Ex Smoking, Years quit (vs current), "Low"
All LC types, Cigarettes (or Any Product if Cigarettes not available)
Least adjusted

REF	NRR	X	SEX	AGEL	AGEH	RACE	YF	LC TYPE	LOC	START	ST	NLC	R	VB	P	H	AD	ADOS	PRODUCT	exL	exH	DENOM	De
ARMADA	518		m	0	0	all	-	all	Eu:wst	1986	CC	325	n	bl	n	y	0	0	cig+/-ot	1.0	5	cur+1y	st
BARBON	533	x	m	0	0	all	-	all	Eu:wst	1979	CC	755	n	bl	y	y	0	0	all/unsp	0.1	4	current	st
BECHER	506		m	0	0	all	-	all	Eu:Ger	1985	CC	194	n	bl	n	y	0	0	all/unsp	2	4	cur+1y	st
BECHER	516		f	0	0	all	-	all	Eu:Ger	1985	CC	194	n	bl	n	y	0	0	all/unsp	2	4	cur+1y	st
BROSS	518		m	0	0	wh	-	all	NAmer	1960	CC	974	n	bl	n	n	0	0	cig+/-ot	0.1	5	current	st
CARPEN	508		c	0	0	w+b	-	all	NAmer	1991	CC	356	n	bl	n	n	0	0	cig+/-ot	0.1	4	current	st
CHOI	543		m	0	0	all	-	all	As:oth	1985	CC	375	n	bl	n	n	0	0	cig+/-ot	0.1	4	current	st
CHOI	556		f	0	0	all	-	all	As:oth	1985	CC	375	n	bl	n	n	0	0	cig+/-ot	0.1	4	current	st
CPSI	816		m	50	74	all	6	all	NAmer	1959	pr	5138	n	bl	n	n	1	0	cig only	1.0	4	current	ot
CPSII	666		m	35	99	all	4	all	NAmer	1982	pr	3229	n	bl	n	n	1	0	cig only	3	5	current	ot
CPSII	643		f	0	0	all	4	all	NAmer	1982	pr	3229	n	bl	n	n	1	0	cig+/-ot	3	5	current	ot
DAMBER	528		m	0	0	all	-	all	Eu:Sca	1972	CC	579	n	bl	y	n	1	0	all/unsp	0.1	5	current	ot
DEAN3	509	x	m	0	0	all	-	all	Eu:UK	1969	CC	766	n	V	y	n	0	0	cig only	3	4	cur+2y	st
DEAN3	548	x	f	0	0	all	-	all	Eu:UK	1969	CC	766	n	V	y	n	0	0	all/unsp	3	4	cur+2y	st
DESTEF	543		m	0	0	all	-	all	SCAmer	1988	CC	497	n	bl	n	y	0	0	cig+/-ot	0.1	4	current	st
DOLL2	509		m	0	0	all	20	all	Eu:UK	1951	pr	920	n	V	n	n	1	0	cig only	0.1	4	current	ot
DORGAN	514		m	0	0	wh	-	all	NAmer	1980	CC	2026	n	bl	y	y	0	0	cig+/-ot	1.1	5	cur+1y	st
DORN	823		m	55	64	wh	8	all	NAmer	1954	pr	5097	n	bl	n	n	0	0	cig+/-ot	0.1	4	current	st
DORN	827		m	65	74	wh	8	all	NAmer	1954	pr	5097	n	bl	n	n	0	0	cig+/-ot	0.1	4	current	st
GAO	526	x	m	0	0	all	-	all	As:Chi	1984	CC	1405	n	ot	n	n	0	0	cig+/-ot	0.1	4	current	st
GAO	546	x	f	0	0	all	-	all	As:Chi	1984	CC	1405	n	ot	n	n	0	0	cig+/-ot	0.1	4	current	st
GAO2	518		m	0	0	all	-	all	As:Jap	1988	CC	282	n	bl	n	n	0	0	cig+/-ot	1.0	4	cur+1y	st
GARCIA	508		c	0	0	all	-	all	NAmer	1992	CC	416	n	bl	n	y	0	0	cig+/-ot	1.0	4	cur+1y	st
GRAHAM	531	x	m	0	0	wh	-	all	NAmer	1956	CC	685	n	bl	n	n	0	0	cig+/-ot	1.1	5	current	st
HAMMO2	510		m	0	0	all	0	all	NAmer	1967	pr	450	o	bl	n	n	1	0	cig+/-ot	0.1	4	current	ot
HIRAYA	513		m	0	0	all	0	all	As:Jap	1965	pr	1917	n	bl	n	n	1	0	cig+/-ot	0.1	4	current	ot
HIRAYA	524		f	0	0	all	0	all	As:Jap	1965	pr	1917	n	bl	n	n	1	0	cig+/-ot	0.1	4	current	ot
JAHN	515		m	0	0	all	-	all	Eu:Ger	1988	CC	1004	n	bl	n	n	0	0	cig+/-ot	2	5	current	st
JOLY	571		m	0	0	all	-	all	SCAmer	1978	CC	826	n	bl	n	n	0	0	cig+/-ot	1.0	4	cur+1y	st
JOLY	558		f	0	0	all	-	all	SCAmer	1978	CC	826	n	bl	n	n	0	0	cig+/-ot	1.0	4	cur+1y	st
KHUDER	516		m	0	0	all	-	all	NAmer	1985	CC	482	n	bl	n	y	0	0	cig+/-ot	0.1	4	current	st
LUBIN	592		m	0	0	all	-	all	As:Chi	1984	CC	427	m	ot	y	n	0	0	cig+/-ot	3	4	cur+2y	st
LUBIN2	1081		m	0	0	all	-	all	Eu:mul	1976	CC	7804	n	bl	n	y	0	0	cig+/-ot	0.1	4	current	st
LUBIN2	1120		f	0	0	all	-	all	Eu:mul	1976	CC	7804	n	bl	n	y	0	0	cig+/-ot	0.1	4	current	st
MATOS	586	x	m	0	0	all	-	all	SCAmer	1994	CC	200	n	bl	n	n	0	0	cig+/-ot	1.0	5	cur+1y	st
SOBUE	728		m	0	0	all	-	all	As:Jap	1986	CC	1376	n	bl	n	y	0	0	cig+/-ot	1.0	4	cur+1y	st
SPEIZE	512		f	0	0	all	0	all	NAmer	1976	pr	593	n	bl	n	y	2	0	cig+/-ot	2	5	current	or
SUZUK2	513	x	c	0	0	all	-	all	SCAmer	1991	CC	123	n	bl	n	y	0	0	all/unsp	0.1	5	current	st
TVERDA	507		m	0	0	all	0	all	Eu:Sca	1972	pr	238	n	bl	n	n	2	0	cig only	1.0	5	current	ot
WANG2	515		c	0	0	all	-	all	As:Chi	1980	CC	103	n	ot	n	n	0	0	cig+/-ot	0.1	3	current	st
WYNDE3	545		m	0	0	all	-	all	NAmer	1966	CC	350	n	bl	n	y	0	0	all/unsp	1.0	3	cur+1y	st
WYNDE6	513		m	0	0	all	-	all	NAmer	1969	CC	4423	n	bl	n	y	0	0	cig only	1.0	4	cur+1y	st
WYNDE6	534		f	0	0	all	-	all	NAmer	1969	CC	4423	n	bl	n	y	0	0	cig only	1.0	4	cur+1y	st

Cigarette type is all/unspec for all RRs
except for the following:

REF	NRR	CIGTYPE
DEAN3	509	MC only
DESTEF	543	MC only

Table 1K7 - 5

IESLC - Meta-analysis of Ex Smoking, Years quit (vs current), "Low"
All LC types, Cigarettes (or Any Product if Cigarettes not available)
Least adjusted

REF	NRR	SEX	AD	Number Exposed Case	Cont	Non-exposed Case	Cont	RR	95.00%CI
ARMADA	518	m	0	79	45	188	122	1.14	(0.74- 1.75)
BARBON	533	m	0	32	20	562	362	1.03	(0.58- 1.83)
BECHER	506	m	0	10	12	101	122	1.01	(0.42- 2.43)
BECHER	516	f	0	2	3	33	26	0.53	(0.08- 3.38)
Subtotal BECHER								0.89	(0.40- 1.98)
BROSS	518	m	0	169	67	565	427	1.91	(1.40- 2.60)
CARPEN	508	c	0	28	46	228	164	0.44	(0.26- 0.73)
CHOI	543	m	0	25	64	231	329	0.56	(0.34- 0.91)
CHOI	556	f	0	3	2	13	25	2.88	(0.43- 19.49)
Subtotal CHOI								0.62	(0.38- 0.99)
*CPSI	816	m	1	49	-	844	-	0.59	(0.44- 0.78)
*CPSII	666	m	1	178	-	1159	-	0.85	(0.72- 0.99)
*CPSII	643	f	1	56	-	530	-	0.85	(0.65- 1.12)
Subtotal CPSII								0.85	(0.74- 0.98)
DAMBER	528	m	1	-	-	-	-	0.80	(0.50- 1.28)
DEAN3	509	m	0	28	102	337	930	0.76	(0.49- 1.17)
DEAN3	548	f	0	4	110	102	1158	0.41	(0.15- 1.14)
Subtotal DEAN3								0.69	(0.46- 1.03)
DESTEF	543	m	0	10	19	78	109	0.74	(0.32- 1.67)
*DOLL2	509	m	1	15	-	236	-	1.02	(0.61- 1.72)
DORGAN	514	m	0	59	51	465	303	0.75	(0.50- 1.13)
*DORN	823	m	0	34	22086	528	334175	0.97	(0.69- 1.38)
*DORN	827	m	0	14	6195	537	207895	0.87	(0.51- 1.49)
Subtotal DORN								0.94	(0.71- 1.26)
GAO	526	m	0	105	52	529	438	1.67	(1.17- 2.39)
GAO	546	f	0	37	9	170	100	2.42	(1.12- 5.22)
Subtotal GAO								1.78	(1.29- 2.46)
GAO2	518	m	0	31	26	184	117	0.76	(0.43- 1.34)
GARCIA	508	c	0	33	11	77	42	1.64	(0.75- 3.57)
GRAHAM	531	m	0	24	48	453	1075	1.19	(0.72- 1.96)
*HAMMO2	510	m	1	59	-	209	-	1.08	(0.82- 1.43)
*HIRAYA	513	m	1	-	-	-	-	0.46	(0.26- 0.82)
*HIRAYA	524	f	1	-	-	-	-	1.59	(0.47- 5.37)
Subtotal HIRAYA								0.58	(0.34- 0.97)
JAHN	515	m	0	77	46	352	269	1.28	(0.86- 1.90)
JOLY	571	m	0	38	36	451	524	1.23	(0.76- 1.97)
JOLY	558	f	0	19	8	132	96	1.73	(0.73- 4.11)
Subtotal JOLY								1.33	(0.88- 2.01)
KHUDER	516	m	0	88	123	245	316	0.92	(0.67- 1.27)
LUBIN	592	m	0	33	18	296	650	4.03	(2.23- 7.27)
LUBIN2	1081	m	0	866	1047	4684	6211	1.10	(0.99- 1.21)
LUBIN2	1120	f	0	60	55	440	410	1.02	(0.69- 1.50)
Subtotal LUBIN2								1.09	(0.99- 1.20)
MATOS	586	m	0	28	23	112	132	1.43	(0.78- 2.63)
SOBUE	728	m	0	128	116	737	633	0.95	(0.72- 1.24)
*SPEIZE	512	f	2	34	-	319	-	0.60	(0.40- 0.80)
SUZUK2	513	c	0	15	10	77	30	0.58	(0.24- 1.44)
*TVERDA	507	m	2	5	-	144	-	0.18	(0.07- 0.43)
WANG2	515	c	0	6	10	49	78	0.96	(0.33- 2.79)
WYNDE3	545	m	0	21	22	227	207	0.87	(0.47- 1.63)
WYNDE6	513	m	0	201	166	1107	993	1.09	(0.87- 1.36)
WYNDE6	534	f	0	82	70	683	496	0.85	(0.61- 1.19)
Subtotal WYNDE6								1.01	(0.84- 1.22)
Partial Totals				2785	30718	18414	558964		

*prospective study

Table 1K7 - 5

IESLC - Meta-analysis of Ex Smoking, Years quit (vs current), "Low"
All LC types, Cigarettes (or Any Product if Cigarettes not available)
Least adjusted

REF	NRR	SEX	AD	Ys	Ws	Qs	Ps
ARMADA	518	m	0	0.13	20.66	0.42	0.5535
BARBON	533	m	0	0.03	11.66	0.02	0.9180
BECHER	506	m	0	0.01	4.96	0.00	0.9883
BECHER	516	f	0	-0.64	1.11	0.44	0.4978
Subtotal BECHER				-0.11	6.07	0.44	
BROSS	518	m	0	0.65	40.07	17.36	0.0000
CARPEN	508	c	0	-0.83	14.72	9.73	0.0015
CHOI	543	m	0	-0.59	15.87	5.22	0.0195
CHOI	556	f	0	1.06	1.05	1.21	0.2771
Subtotal CHOI				-0.48	16.93	6.43	
*CPSI	816	m	1	-0.53	46.88	12.41	0.0003
*CPSII	666	m	1	-0.16	151.52	3.39	0.0454
*CPSII	643	f	1	-0.16	51.90	1.16	0.2417
Subtotal CPSII				-0.16	203.42	4.55	
DAMBER	528	m	1	-0.22	17.39	0.77	0.3521
DEAN3	509	m	0	-0.28	20.18	1.41	0.2123
DEAN3	548	f	0	-0.88	3.71	2.82	0.0885
Subtotal DEAN3				-0.37	23.88	4.23	
DESTEF	543	m	0	-0.31	5.73	0.50	0.4622
*DOLL2	509	m	1	0.02	14.30	0.02	0.9403
DORGAN	514	m	0	-0.28	23.81	1.73	0.1680
*DORN	823	m	0	-0.03	31.99	0.01	0.8830
*DORN	827	m	0	-0.13	13.68	0.20	0.6211
Subtotal DORN				-0.06	45.67	0.20	
GAO	526	m	0	0.51	30.37	8.43	0.0046
GAO	546	f	0	0.88	6.49	5.21	0.0244
Subtotal GAO				0.58	36.86	13.65	
GAO2	518	m	0	-0.28	11.81	0.82	0.3414
GARCIA	508	c	0	0.49	6.33	1.62	0.2154
GRAHAM	531	m	0	0.17	15.24	0.52	0.5044
*HAMMO2	510	m	1	0.08	49.68	0.40	0.5875
*HIRAYA	513	m	1	-0.78	11.65	6.79	0.0080
*HIRAYA	524	f	1	0.46	2.59	0.59	0.4555
Subtotal HIRAYA				-0.55	14.24	7.38	
JAHN	515	m	0	0.25	24.22	1.63	0.2255
JOLY	571	m	0	0.20	17.18	0.81	0.3976
JOLY	558	f	0	0.55	5.11	1.60	0.2166
Subtotal JOLY				0.28	22.29	2.41	
KHUDER	516	m	0	-0.08	37.40	0.17	0.6231
LUBIN	592	m	0	1.39	11.02	21.77	0.0000
LUBIN2	1081	m	0	0.09	402.52	4.47	0.0639
LUBIN2	1120	f	0	0.02	25.28	0.02	0.9343
Subtotal LUBIN2				0.09	427.80	4.49	
MATOS	586	m	0	0.36	10.45	1.46	0.2432
SOBUE	728	m	0	-0.05	51.63	0.09	0.6997
*SPEIZE	512	f	2	-0.51	31.98	7.92	0.0039
SUZUK2	513	c	0	-0.54	4.70	1.29	0.2445
*TVERDA	507	m	2	-1.71	4.66	13.50	0.0002
WANG2	515	c	0	-0.05	3.33	0.00	0.9331
WYNDE3	545	m	0	-0.14	9.77	0.15	0.6644
WYNDE6	513	m	0	0.08	77.46	0.71	0.4670
WYNDE6	534	f	0	-0.16	33.38	0.74	0.3502
Subtotal WYNDE6				0.01	110.84	1.45	

Table 1K7 - 5

IESLC - Meta-analysis of Ex Smoking, Years quit (vs current), "Low"
All LC types, Cigarettes (or Any Product if Cigarettes not available)
Least adjusted

	N	43
	NS	33
	Wt	1375.42
Het	Chi	139.53
Het	df	42
Het	P	***
Fixed	RR	0.99
	RRl	0.94
	RRu	1.04
	P	N.S.
Random	RR	0.96
	RRl	0.85
	RRu	1.07
	P	N.S.
Asymm	P	N.S.

Table 1K7 - 6

IESLC - Meta-analysis of Ex Smoking, Years quit (vs current), "Low"
All LC types, Cigarettes (or Any Product if Cigarettes not available)
Least adjusted

		Sex			
		combined	male	female	Total
	N	4	29	10	43
	NS	4	28	10	42
	Wt	29.08	1183.74	162.60	1375.42
Het	Chi	8.22	104.31	19.06	139.53
Het	df	3	28	9	42
Het	P	*	***	*	***
Fixed	RR	0.67	1.01	0.87	0.99
	RRl	0.46	0.96	0.74	0.94
	RRu	0.96	1.07	1.01	1.04
	P	-	N.S.	(-)	N.S.
Random	RR	0.76	0.98	0.94	0.96
	RRl	0.40	0.86	0.72	0.85
	RRu	1.46	1.12	1.24	1.07
	P	N.S.	N.S.	N.S.	N.S.
Between	Chi				7.95
Between	df				2
Between	P				*
Btwn(F)	P				N.S.
Btwn(R)	P				N.S.

Table 1K7 - 7

IESLC - Meta-analysis of Ex Smoking, Years quit (vs current), "Low"
All LC types, Cigarettes (or Any Product if Cigarettes not available)
Excluded studies (and stage at which they were excluded)

1 AGUDO AKIBA AMANDU AMES AXELSS BEST BOUCHA BOUCOT BRESLO CHEN CHEN2 CHIAZZ DEAN2 DOSEME ENGELA FAN GENG GER GUO HAENSZ HEGMAN HOLE HU HU2 JUSSAW KATSOU KAUFMA KOO KOULUM KREUZE LETOUR LEVIN LIAW LIU3 LIU4 LIU5 MCCONN MIGRAN MRFITR NOTAN2 OSANN2 PERNU QIAO2 RACHTA RESTRE SADOWS SEGI2 STASZE TIZZAN VUTUC WATSON WU WUWILL WYNDE2 WYNDE8 XU YUAN ZHANG ZHENG ZHOU

2 AUVINE BENSHL BLOT1 BROWN3 BUFFLE GURSEL LAUSSM LUO MCDUFF PISANI PRESCO SPITZ WU2 WYNDE7

4 HAMMON

5 CORREA GILLIS HUMBLE QIAO WIGLE

7 BOFFET

10 GARSHI JEDRYC WAKAI

14 ALDERS CEDERL CHYOU DARBY DOLL JAIN KAISE2 PEZZO2 PEZZOT SVENSS

15 BENHAM

Table 1K7 - 8
Potentially overlapping studies

REF	REFGP	PRINC	OVERLAP/LINK
LUBIN2	LUBIN2	1	Lubin-combined
TVERDA	TVERDA	1	VEIERO/TVERDAL
BROSS	BYERS1	1	GRAHAM/BROSS/BYERS1
GRAHAM	BYERS1	1	GRAHAM/BROSS/BYERS1
WYNDE6	WYNDE6	1	WYNDE5/6/7/8
CPSI	CPSI	1	CPSI overall
JAHN	BOFFET	2	Subset of BOFFET
LUBIN	XIANGZ	2	LUBIN/XIANGZ/QIAO

Table 1K7 - 9
Most adjusted - insufficient data for meta-analysis

REF	NRR	SEX	AGEL	AGEH	RACE	YF	LC TYPE	LOC	START	ST	NLC	R	VB	P	H	AD	ADOS	PRODUCT	exL	exH	DENOM	De
CORREA	550	c	0	0	all	-	all	NAmer	1979	CC	1359	n	bl	y	n	2	0	cig+/-ot	3	5	cur+2y	st
HUMBLE	549	c	0	0	wh	-	not alv	NAmer	1980	CC	521	n	bl	y	n	2	2#	cig+/-ot	2	5	cur+1y	or

Comments on values in listings

HUMBLE ADOS Number of cigarettes and duration

REF	NRR	RR	SIG	RRDATA comment
CORREA	550	0.61		0
HUMBLE	549	0.69		0

Table 1K8 -

IESLC - Meta-analysis of Ex Smoking, Years quit (vs current), "Mid"
All LC types, Cigarettes (or Any Product if Cigarettes not available)

This analysis is restricted to results for:
1) Ex smokers
2) Results by Years quit (vs current)
3) Categorical results by Years quit (vs current)
4) All LC types (or near equivalent)
5) Results complete enough for use in metaanalysis

Within each study, results are then selected (in the following order of preference, within each sex) for:
6) (not applicable)
7) PRODUCT: cigarettes regardless of other products, cigarettes only, all/unspec
8) CIGTYPE: all/unspecified, MC regardless of HR, MC only
9) Results with least adjustment for other aspects of smoking (ADOS)
10) DENOM: current smokers, current + recent smokers (up to number of m=months or y=years, max 2 years)
11) Followup period (YF, prospective studies): whole study (coded as 0) or longest available
12) LCtype: all or nearest available, at least Squamous and Adeno. (q = squamous, s = small,
l = large, a = adeno, mix = mixed, alv = alveolar)
13) Race: all or nearest available, otherwise by race (wh or w = white, bl or b = black, hi = hispanic
ch = chinese, jap = japanese, haw = hawaiian, w+o = white + oriental, sca = scandinavian, as = asian)
14) Years quit (vs current) "mid" in key scheme 1 (key value 7, maximum range 4-11)
15) For overlapping studies: principal rather than subsidiary studies
Finally by Age: whole study (coded as 0) if available, otherwise by widest available age group
and then for single sex results (m, f) in preference to results for both sexes combined (c).

Results adjusted (AD) for the most potential confounders are then chosen in Sections -1 to -3
(and those which actually differ from the adjusted results in Table 1K3 - 1 are marked 'x' in Section -1)
and results adjusted for the least confounders in Sections -4 to -6. (Those least adjusted results which
actually differ from the most adjusted are marked 'x' in column X in Section -4)

Section -7 shows excluded studies, together with the stage (as above) at which no qualifying
results were found.

Section -8 lists the potentially overlapping studies which have been included (1=principal, 2=subsidiary).

Section -9 lists any results which would have been included in preference except that they had data not complete
enough for use in meta-analysis, with their significance (yes/no), if known, and any further comment as entered
on the database. It also lists as "gap" any categories for which no data were presented by the original authors.

In addition to those mentioned above, the following fields, levels and abbreviations are used:
* or nk = not known, n = no, y = yes, ot = other
nev = never
all/unspec = all or unspecified, cig+/-ot = cigarettes irrespective of other products (cigar, pipe etc)
MC = manufactured cigarettes, HR = hand-rolled cigarettes
exL, exH = range of exposure (low and high) in the smoking group, in terms of Years quit (vs current)
REF: 6-character study reference
NRR: number of the RR on the database within the study
ST : study type (CC = case control, pr or prosp = prospective)
NLC: number of lung cancer cases in whole study
R : risky occupational population (n = no, m = mining, o = other risky)
VB : national cigarette type (V = at least 75% Virginia, bl = at least 75% blended, ot = other)
P : any proxy use
H : full histological confirmation
De : derivation of RR/CI (or = original, st = standard method, ot = other method of estimation)

Table 1K8 - 1

IESLC - Meta-analysis of Ex Smoking, Years quit (vs current), "Mid"
All LC types, Cigarettes (or Any Product if Cigarettes not available)
Most adjusted

REF	NRR	1K3	SEX	AGEL	AGEH	RACE	YF	LC TYPE	LOC	START	ST	NLC	R	VB	P	H	AD	ADOS	PRODUCT	exL	exH	DENOM	De
BECHER	507		m	0	0	all	-	all	Eu:Ger	1985	CC	194	n	bl	n	y	0	0	all/unsp	5	9	cur+1y	st
BECHER	517		f	0	0	all	-	all	Eu:Ger	1985	CC	194	n	bl	n	y	0	0	all/unsp	5	9	cur+1y	st
CARPEN	509		c	0	0	w+b	-	all	NAmer	1991	CC	356	n	bl	n	n	0	0	cig+/-ot	5	9	current	st
CHOI	544		m	0	0	all	-	all	As:oth	1985	CC	375	n	bl	n	n	0	0	cig+/-ot	5	9	current	st
CPSI	817		m	50	74	all	6	all	NAmer	1959	pr	5138	n	bl	n	n	1	0	cig only	5	9	current	ot
CPSII	667		m	35	99	all	4	all	NAmer	1982	pr	3229	n	bl	n	n	1	0	cig only	6	10	current	ot
CPSII	644		f	0	0	all	4	all	NAmer	1982	pr	3229	n	bl	n	n	1	0	cig+/-ot	6	10	current	ot
DAMBER	529		m	0	0	all	-	all	Eu:Sca	1972	CC	579	n	bl	y	n	1	0	all/unsp	6	10	current	ot
DEAN3	525	x	m	0	0	all	-	all	Eu:UK	1969	CC	766	n	V	y	n	1	0	cig only	5	8	cur+2y	ot
DEAN3	560		f	0	0	all	-	all	Eu:UK	1969	CC	766	n	V	y	n	1	0	all/unsp	5	8	cur+2y	ot
DESTEF	544	x	m	0	0	all	-	all	SCAmer	1988	CC	497	n	bl	n	y	0	0	cig+/-ot	5	9	current	st
DOLL2	510		m	0	0	all	20	all	Eu:UK	1951	pr	920	n	V	n	n	1	0	cig only	5	9	current	ot
DORGAN	515		m	0	0	wh	-	all	NAmer	1980	CC	2026	n	bl	y	y	0	0	cig+/-ot	6	9	cur+1y	st
DORN	824		m	55	64	wh	8	all	NAmer	1954	pr	5097	n	bl	n	n	0	0	cig+/-ot	5	9	current	st
DORN	828		m	65	74	wh	8	all	NAmer	1954	pr	5097	n	bl	n	n	0	0	cig+/-ot	5	9	current	st
GAO	537		m	0	0	all	-	all	As:Chi	1984	CC	1405	n	ot	n	n	2	0	cig+/-ot	5	9	current	ot
GAO	557		f	0	0	all	-	all	As:Chi	1984	CC	1405	n	ot	n	n	2	0	cig+/-ot	5	9	current	ot
GAO2	519		m	0	0	all	-	all	As:Jap	1988	CC	282	n	bl	n	n	0	0	cig+/-ot	5	9	cur+1y	st
GRAHAM	510		m	0	0	wh	-	all	NAmer	1956	CC	685	n	bl	n	n	0	0	cig only	3	10	current	st
HAMMO2	511		m	0	0	all	0	all	NAmer	1967	pr	450	o	bl	n	n	1	0	cig+/-ot	5	9	current	ot
HIRAYA	514		m	0	0	all	0	all	As:Jap	1965	pr	1917	n	bl	n	n	1	0	cig+/-ot	5	9	current	ot
HIRAYA	525		f	0	0	all	0	all	As:Jap	1965	pr	1917	n	bl	n	n	1	0	cig+/-ot	5	9	current	ot
JAHN	516		m	0	0	all	-	all	Eu:Ger	1988	CC	1004	n	bl	n	n	0	0	cig+/-ot	6	10	current	st
LUBIN	593		m	0	0	all	-	all	As:Chi	1984	CC	427	m	ot	y	n	0	0	cig+/-ot	5	9	cur+2y	st
LUBIN2	1082		m	0	0	all	-	all	Eu:mul	1976	CC	7804	n	bl	n	y	0	0	cig+/-ot	5	9	current	st
LUBIN2	1121		f	0	0	all	-	all	Eu:mul	1976	CC	7804	n	bl	n	y	0	0	cig+/-ot	5	9	current	st
MATOS	597		m	0	0	all	-	all	SCAmer	1994	CC	200	n	bl	n	n	2	0	cig+/-ot	6	10	cur+1y	or
SOBUE	729		m	0	0	all	-	all	As:Jap	1986	CC	1376	n	bl	n	y	0	0	cig+/-ot	5	9	cur+1y	st
SPEIZE	513		f	0	0	all	0	all	NAmer	1976	pr	593	n	bl	n	y	2	0	cig+/-ot	5	10	current	or
SUZUK2	525		c	0	0	all	-	all	SCAmer	1991	CC	123	n	bl	n	y	3	0	all/unsp	6	10	current	or
WYNDE6	514		m	0	0	all	-	all	NAmer	1969	CC	4423	n	bl	n	y	0	0	cig only	5	9	cur+1y	st
WYNDE6	535		f	0	0	all	-	all	NAmer	1969	CC	4423	n	bl	n	y	0	0	cig only	5	9	cur+1y	st

Cigarette type is all/unspec for all RRs
except for the following:

REF	NRR	CIGTYPE
DEAN3	525	MC only
DESTEF	544	MC only

Table 1K8 - 2

IESLC - Meta-analysis of Ex Smoking, Years quit (vs current), "Mid"
All LC types, Cigarettes (or Any Product if Cigarettes not available)
Most adjusted

REF	NRR	SEX	AD	Number Exposed Case	Cont	Non-exposed Case	Cont	RR	95.00%CI
BECHER	507	m	0	16	32	101	122	0.60	(0.31- 1.16)
BECHER	517	f	0	2	5	33	26	0.32	(0.06- 1.76)
Subtotal BECHER								0.56	(0.30- 1.03)
CARPEN	509	c	0	31	52	228	164	0.43	(0.26- 0.70)
CHOI	544	m	0	5	30	231	329	0.24	(0.09- 0.62)
*CPSI	817	m	1	32	-	844	-	0.37	(0.26- 0.53)
*CPSII	667	m	1	186	-	1159	-	0.52	(0.45- 0.61)
*CPSII	644	f	1	37	-	530	-	0.40	(0.28- 0.55)
Subtotal CPSII								0.50	(0.43- 0.57)
DAMBER	529	m	1	-	-	-	-	0.45	(0.26- 0.78)
DEAN3	525	m	1	11	-	337	-	0.57	(0.29- 1.12)
DEAN3	560	f	1	1	-	102	-	0.20	(0.03- 1.42)
Subtotal DEAN3								0.51	(0.27- 0.96)
DESTEF	544	m	0	9	15	78	109	0.84	(0.35- 2.01)
*DOLL2	510	m	1	12	-	236	-	0.35	(0.20- 0.63)
DORGAN	515	m	0	49	38	465	303	0.84	(0.54- 1.31)
*DORN	824	m	0	32	34566	528	334175	0.59	(0.41- 0.84)
*DORN	828	m	0	41	24089	537	207895	0.66	(0.48- 0.90)
Subtotal DORN								0.63	(0.49- 0.79)
GAO	537	m	2	24	-	529	-	0.79	(0.45- 1.40)
GAO	557	f	2	14	-	170	-	1.34	(0.51- 3.53)
Subtotal GAO								0.90	(0.55- 1.48)
GAO2	519	m	0	21	26	184	117	0.51	(0.28- 0.95)
GRAHAM	510	m	0	5	29	371	821	0.38	(0.15- 0.99)
*HAMMO2	511	m	1	11	-	209	-	0.39	(0.22- 0.71)
*HIRAYA	514	m	1	-	-	-	-	0.36	(0.15- 0.86)
*HIRAYA	525	f	1	-	-	-	-	1.41	(0.23- 8.48)
Subtotal HIRAYA								0.47	(0.21- 1.02)
JAHN	516	m	0	59	63	352	269	0.72	(0.49- 1.06)
LUBIN	593	m	0	20	48	296	650	0.91	(0.53- 1.57)
LUBIN2	1082	m	0	466	822	4684	6211	0.75	(0.67- 0.85)
LUBIN2	1121	f	0	30	40	440	410	0.70	(0.43- 1.14)
Subtotal LUBIN2								0.75	(0.67- 0.84)
MATOS	597	m	2	21	-	112	-	0.90	(0.40- 1.60)
SOBUE	729	m	0	67	92	737	633	0.63	(0.45- 0.87)
*SPEIZE	513	f	2	41	-	319	-	0.50	(0.40- 0.70)
SUZUK2	525	c	3	10	-	77	-	0.50	(0.20- 1.40)
WYNDE6	514	m	0	98	194	1107	993	0.45	(0.35- 0.59)
WYNDE6	535	f	0	51	84	683	496	0.44	(0.31- 0.64)
Subtotal WYNDE6								0.45	(0.36- 0.55)
Partial Totals				1402	60225	15679	553723		

*prospective study

REF	NRR	SEX	AD	Ys	Ws	Qs	Ps
BECHER	507	m	0	-0.50	8.94	0.01	0.1316
BECHER	517	f	0	-1.15	1.30	0.50	0.1879
Subtotal BECHER				-0.59	10.24	0.51	
CARPEN	509	c	0	-0.85	16.14	1.56	0.0007
CHOI	544	m	0	-1.44	4.15	3.38	0.0034
*CPSI	817	m	1	-0.99	30.29	6.38	0.0000
*CPSII	667	m	1	-0.65	166.04	2.33	0.0000
*CPSII	644	f	1	-0.92	33.71	4.89	0.0000
Subtotal CPSII				-0.70	199.75	7.22	
DAMBER	529	m	1	-0.80	12.73	0.88	0.0044
DEAN3	525	m	1	-0.56	8.42	0.01	0.1029
DEAN3	560	f	1	-1.61	1.03	1.19	0.1019
Subtotal DEAN3				-0.68	9.45	1.20	
DESTEF	544	m	0	-0.18	5.01	0.65	0.6934
*DOLL2	510	m	1	-1.05	11.67	3.09	0.0003
DORGAN	515	m	0	-0.17	19.17	2.50	0.4460
*DORN	824	m	0	-0.53	30.20	0.00	0.0033
*DORN	828	m	0	-0.42	38.16	0.53	0.0100
Subtotal DORN				-0.47	68.36	0.53	
GAO	537	m	2	-0.24	11.93	1.07	0.4156
GAO	557	f	2	0.29	4.11	2.82	0.5532
Subtotal GAO				-0.10	16.03	3.89	
GAO2	519	m	0	-0.67	9.99	0.17	0.0352
GRAHAM	510	m	0	-0.96	4.19	0.77	0.0484
*HAMMO2	511	m	1	-0.94	11.19	1.85	0.0016
*HIRAYA	514	m	1	-1.02	5.04	1.19	0.0218

Table 1K8 - 2

IESLC - Meta-analysis of Ex Smoking, Years quit (vs current), "Mid"
All LC types, Cigarettes (or Any Product if Cigarettes not available)
Most adjusted

REF	NRR	SEX	AD	Ys	Ws	Qs	Ps
*HIRAYA	525	f	1	0.34	1.18	0.91	0.7089
Subtotal HIRAYA				-0.76	6.22	2.10	
JAHN	516	m	0	-0.33	25.39	1.03	0.0919
LUBIN	593	m	0	-0.09	13.20	2.63	0.7468
LUBIN2	1082	m	0	-0.29	267.60	16.74	0.0000
LUBIN2	1121	f	0	-0.36	15.86	0.50	0.1536
Subtotal LUBIN2				-0.29	283.46	17.24	
MATOS	597	m	2	-0.11	8.00	1.48	0.7658
SOBUE	729	m	0	-0.47	34.80	0.15	0.0056
*SPEIZE	513	f	2	-0.69	49.07	1.22	0.0000
SUZUK2	525	c	3	-0.69	4.06	0.10	0.1626
WYNDE6	514	m	0	-0.79	57.91	3.80	0.0000
WYNDE6	535	f	0	-0.82	28.58	2.30	0.0000
Subtotal WYNDE6				-0.80	86.48	6.09	

	N	32
	NS	24
	Wt	939.05
Het	Chi	66.62
Het	df	31
Het	P	***
Fixed	RR	0.59
	RRl	0.55
	RRu	0.62
	P	---
Random	RR	0.55
	RRl	0.49
	RRu	0.62
	P	---
Asymm	P	N.S.

Table 1K8 - 3

IESLC - Meta-analysis of Ex Smoking, Years quit (vs current), "Mid"
All LC types, Cigarettes (or Any Product if Cigarettes not available)
Most adjusted

	Sex combined	male	female	Total
N	2	22	8	32
NS	2	21	8	31
Wt	20.19	784.02	134.84	939.05
Het Chi	0.08	49.56	10.28	66.62
Het df	1	21	7	31
Het P	N.S.	***	N.S.	***
Fixed RR	0.44	0.61	0.49	0.59
RRl	0.29	0.57	0.42	0.55
RRu	0.68	0.65	0.58	0.62
P	---	---	---	---
Random RR	0.44	0.57	0.51	0.55
RRl	0.29	0.50	0.40	0.49
RRu	0.68	0.65	0.65	0.62
P	---	---	---	---
Between Chi				6.70
Between df				2
Between P				*
Btwn(F) P				N.S.
Btwn(R) P				N.S.

	Lung cancer type all	other	Total
N	32		32
NS	24		24
Wt	939.05		939.05
Het Chi	66.62		66.62
Het df	31		31
Het P	***		***
Fixed RR	0.59		0.59
RRl	0.55		0.55
RRu	0.62		0.62
P	---		---
Random RR	0.55		0.55
RRl	0.49		0.49
RRu	0.62		0.62
P	---		---
Between Chi			
Between df			
Between P			N.S.
Btwn(F) P			N.S.
Btwn(R) P			N.S.

	Location NAmer	UK	Scand	othEur	China	Japan	othAs	other	Total
N	12	3	1	5	3	4	1	3	32
NS	9	2	1	3	2	3	1	3	24
Wt	484.64	21.12	12.73	319.09	29.23	51.02	4.15	17.06	939.05
Het Chi	15.90	1.74	0.00	1.46	0.85	2.42	0.00	0.99	66.62
Het df	11	2	0	4	2	3	0	2	31
Het P	N.S.	N.S.	N.S.	N.S.	N.S.	N.S.	N.S.	N.S.	***
Fixed RR	0.50	0.41	0.45	0.74	0.91	0.58	0.24	0.77	0.59
RRl	0.46	0.27	0.26	0.66	0.63	0.44	0.09	0.48	0.55
RRu	0.55	0.63	0.78	0.82	1.31	0.76	0.62	1.23	0.62
P	---	---	--	---	N.S.	---	--	N.S.	---
Random RR	0.50	0.41	0.45	0.74	0.91	0.58	0.24	0.77	0.55
RRl	0.44	0.27	0.26	0.66	0.63	0.44	0.09	0.48	0.49
RRu	0.56	0.63	0.78	0.82	1.31	0.76	0.62	1.23	0.62
P	---	---	--	---	N.S.	---	--	N.S.	---
Between Chi									43.25
Between df									7
Between P									***
Btwn(F) P									***
Btwn(R) P									***

Table 1K8 - 3

IESLC - Meta-analysis of Ex Smoking, Years quit (vs current), "Mid"
All LC types, Cigarettes (or Any Product if Cigarettes not available)
Most adjusted

Detailed Country in "other Europe"

	multi	Germany	othWest	East	Balkans	Total
N	2	3				5
NS	1	2				3
Wt	283.46	35.63				319.09
Het Chi	0.08	0.95				1.46
Het df	1	2				4
Het P	N.S.	N.S.				N.S.
Fixed RR	0.75	0.67				0.74
RRl	0.67	0.48				0.66
RRu	0.84	0.92				0.82
P	---	-				---
Random RR	0.75	0.67				0.74
RRl	0.67	0.48				0.66
RRu	0.84	0.92				0.82
P	---	-				---
Between Chi						0.44
Between df						1
Between P						N.S.
Btwn(F) P						N.S.
Btwn(R) P						N.S.

Detailed Country in "other Asia"

	India	HongKong	other	Total
N			1	1
NS			1	1
Wt			4.15	4.15
Het Chi			0.00	0.00
Het df			0	0
Het P			N.S.	N.S.
Fixed RR			0.24	0.24
RRl			0.09	0.09
RRu			0.62	0.62
P			--	--
Random RR			0.24	0.24
RRl			0.09	0.09
RRu			0.62	0.62
P			--	--
Between Chi				
Between df				
Between P				N.S.
Btwn(F) P				N.S.
Btwn(R) P				N.S.

Detailed other continent

	SCAmer	Total
N	3	3
NS	3	3
Wt	17.06	17.06
Het Chi	0.99	0.99
Het df	2	2
Het P	N.S.	N.S.
Fixed RR	0.77	0.77
RRl	0.48	0.48
RRu	1.23	1.23
P	N.S.	N.S.
Random RR	0.77	0.77
RRl	0.48	0.48
RRu	1.23	1.23
P	N.S.	N.S.
Between Chi		
Between df		
Between P		N.S.
Btwn(F) P		N.S.
Btwn(R) P		N.S.

Table 1K8 - 3

IESLC - Meta-analysis of Ex Smoking, Years quit (vs current), "Mid"
All LC types, Cigarettes (or Any Product if Cigarettes not available)
Most adjusted

	Start year of study <1960	1960-69	1970-79	1980-89	1990+	Total
N	5	7	4	13	3	32
NS	4	4	3	10	3	24
Wt	114.52	113.35	345.26	337.74	28.19	939.05
Het Chi	8.20	3.18	9.37	21.89	2.97	66.62
Het df	4	6	3	12	2	31
Het P	(*)	N.S.	*	*	N.S.	***
Fixed RR	0.50	0.45	0.69	0.57	0.54	0.59
RRl	0.42	0.37	0.62	0.51	0.37	0.55
RRu	0.61	0.54	0.77	0.63	0.78	0.62
P	---	---	---	---	--	---
Random RR	0.48	0.45	0.61	0.61	0.56	0.55
RRl	0.36	0.37	0.46	0.51	0.35	0.49
RRu	0.64	0.54	0.80	0.74	0.90	0.62
P	---	---	---	---	-	---
Between Chi						21.01
Between df						4
Between P						***
Btwn(F) P						*
Btwn(R) P						N.S.

	Study type (1) CC	other	Total
N	22	10	32
NS	17	7	24
Wt	562.50	376.55	939.05
Het Chi	37.50	12.32	66.62
Het df	21	9	31
Het P	*	N.S.	***
Fixed RR	0.65	0.50	0.59
RRl	0.60	0.45	0.55
RRu	0.71	0.55	0.62
P	---	---	---
Random RR	0.61	0.49	0.55
RRl	0.53	0.43	0.49
RRu	0.70	0.56	0.62
P	---	---	---
Between Chi			16.79
Between df			1
Between P			***
Btwn(F) P			**
Btwn(R) P			*

	Study type (2) CC	prosp	other	Total
N	22	10		32
NS	17	7		24
Wt	562.50	376.55		939.05
Het Chi	37.50	12.32		66.62
Het df	21	9		31
Het P	*	N.S.		***
Fixed RR	0.65	0.50		0.59
RRl	0.60	0.45		0.55
RRu	0.71	0.55		0.62
P	---	---		---
Random RR	0.61	0.49		0.55
RRl	0.53	0.43		0.49
RRu	0.70	0.56		0.62
P	---	---		---
Between Chi				16.79
Between df				1
Between P				***
Btwn(F) P				**
Btwn(R) P				*

Table 1K8 - 3

IESLC - Meta-analysis of Ex Smoking, Years quit (vs current), "Mid"
All LC types, Cigarettes (or Any Product if Cigarettes not available)
Most adjusted

	Study size (number of LC cases)				
	100-249	250-499	500-999	1000+	Total
N	4	6	6	16	32
NS	3	6	5	10	24
Wt	22.29	59.69	87.11	769.96	939.05
Het Chi	1.86	9.44	2.46	46.33	66.62
Het df	3	5	5	15	31
Het P	N.S.	(*)	N.S.	***	***
Fixed RR	0.65	0.52	0.46	0.60	0.59
RRl	0.43	0.40	0.38	0.56	0.55
RRu	0.98	0.67	0.57	0.65	0.62
P	-	---	---	---	---
Random RR	0.65	0.52	0.46	0.58	0.55
RRl	0.43	0.36	0.38	0.50	0.49
RRu	0.98	0.74	0.57	0.68	0.62
P	-	---	---	---	---
Between Chi					6.53
Between df					3
Between P					(*)
Btwn(F) P					N.S.
Btwn(R) P					N.S.

	Risky occupational population			
	no	mining	othRisky	Total
N	30	1	1	32
NS	22	1	1	24
Wt	914.66	13.20	11.19	939.05
Het Chi	62.13	0.00	0.00	66.62
Het df	29	0	0	31
Het P	***	N.S.	N.S.	***
Fixed RR	0.58	0.91	0.39	0.59
RRl	0.55	0.53	0.22	0.55
RRu	0.62	1.57	0.70	0.62
P	---	N.S.	--	---
Random RR	0.55	0.91	0.39	0.55
RRl	0.49	0.53	0.22	0.49
RRu	0.62	1.57	0.70	0.62
P	---	N.S.	--	---
Between Chi				4.48
Between df				2
Between P				N.S.
Btwn(F) P				N.S.
Btwn(R) P				(*)

	National cigarette tobacco type			
	Virginia	blended	other	Total
N	3	26	3	32
NS	2	20	2	24
Wt	21.12	888.70	29.23	939.05
Het Chi	1.74	55.78	0.85	66.62
Het df	2	25	2	31
Het P	N.S.	***	N.S.	***
Fixed RR	0.41	0.58	0.91	0.59
RRl	0.27	0.54	0.63	0.55
RRu	0.63	0.62	1.31	0.62
P	---	---	N.S.	---
Random RR	0.41	0.54	0.91	0.55
RRl	0.27	0.48	0.63	0.49
RRu	0.63	0.61	1.31	0.62
P	---	---	N.S.	---
Between Chi				8.25
Between df				2
Between P				*
Btwn(F) P				N.S.
Btwn(R) P				*

Table 1K8 - 3

IESLC - Meta-analysis of Ex Smoking, Years quit (vs current), "Mid"
All LC types, Cigarettes (or Any Product if Cigarettes not available)
Most adjusted

	Any proxy use		
	No/nk	Yes	Total
N	27	5	32
NS	20	4	24
Wt	884.51	54.55	939.05
Het Chi	59.33	6.00	66.62
Het df	26	4	31
Het P	***	N.S.	***
Fixed RR	0.58	0.68	0.59
RRl	0.54	0.52	0.55
RRu	0.62	0.89	0.62
P	---	--	---
Random RR	0.54	0.66	0.55
RRl	0.48	0.47	0.49
RRu	0.61	0.93	0.62
P	---	-	---
Between Chi			1.29
Between df			1
Between P			N.S.
Btwn(F) P			N.S.
Btwn(R) P			N.S.

	Full histological confirmation		
	No	Yes	Total
N	21	11	32
NS	16	8	24
Wt	446.77	492.28	939.05
Het Chi	32.79	23.59	66.62
Het df	20	10	31
Het P	*	**	***
Fixed RR	0.52	0.65	0.59
RRl	0.48	0.59	0.55
RRu	0.58	0.71	0.62
P	---	---	---
Random RR	0.53	0.59	0.55
RRl	0.46	0.50	0.49
RRu	0.61	0.71	0.62
P	---	---	---
Between Chi			10.23
Between df			1
Between P			**
Btwn(F) P			*
Btwn(R) P			N.S.

	Number of adjustment variables (1)			
	0	1	2+/+nk	Total
N	17	10	5	32
NS	13	7	4	24
Wt	580.59	281.31	77.15	939.05
Het Chi	30.82	8.58	6.63	66.62
Het df	16	9	4	31
Het P	*	N.S.	N.S.	***
Fixed RR	0.65	0.47	0.60	0.59
RRl	0.60	0.42	0.48	0.55
RRu	0.71	0.53	0.75	0.62
P	---	---	---	---
Random RR	0.60	0.47	0.68	0.55
RRl	0.52	0.42	0.48	0.49
RRu	0.69	0.53	0.97	0.62
P	---	---	-	---
Between Chi				20.59
Between df				2
Between P				***
Btwn(F) P				**
Btwn(R) P				**

Table 1K8 - 3

IESLC - Meta-analysis of Ex Smoking, Years quit (vs current), "Mid"
All LC types, Cigarettes (or Any Product if Cigarettes not available)
Most adjusted

	Number of adjustment variables (2) 0	1	2	3-5	6+/+nk	Total
N	17	10	4	1		32
NS	13	7	3	1		24
Wt	580.59	281.31	73.09	4.06		939.05
Het Chi	30.82	8.58	6.49	0.00		66.62
Het df	16	9	3	0		31
Het P	*	N.S.	(*)	N.S.		***
Fixed RR	0.65	0.47	0.61	0.50		0.59
RRl	0.60	0.42	0.48	0.19		0.55
RRu	0.71	0.53	0.76	1.32		0.62
P	---	---	---	N.S.		---
Random RR	0.60	0.47	0.73	0.50		0.55
RRl	0.52	0.42	0.48	0.19		0.49
RRu	0.69	0.53	1.10	1.32		0.62
P	---	---	N.S.	N.S.		---
Between Chi						20.73
Between df						3
Between P						***
Btwn(F) P						*
Btwn(R) P						*

	Product all/unsp	cig+/-ot	cig only	Total
N	5	20	7	32
NS	4	16	6	26
Wt	28.06	603.89	307.10	939.05
Het Chi	1.55	37.14	5.11	66.62
Het df	4	19	6	31
Het P	N.S.	**	N.S.	***
Fixed RR	0.48	0.66	0.47	0.59
RRl	0.33	0.61	0.42	0.55
RRu	0.69	0.71	0.53	0.62
P	---	---	---	---
Random RR	0.48	0.62	0.47	0.55
RRl	0.33	0.54	0.42	0.49
RRu	0.69	0.71	0.53	0.62
P	---	---	---	---
Between Chi				22.82
Between df				2
Between P				***
Btwn(F) P				**
Btwn(R) P				*

	Denominator current	cur+rec	Total
N	21	11	32
NS	16	8	24
Wt	747.72	191.34	939.05
Het Chi	51.80	14.44	66.62
Het df	20	10	31
Het P	***	N.S.	***
Fixed RR	0.59	0.56	0.59
RRl	0.55	0.49	0.55
RRu	0.64	0.65	0.62
P	---	---	---
Random RR	0.54	0.58	0.55
RRl	0.46	0.48	0.49
RRu	0.62	0.71	0.62
P	---	---	---
Between Chi			0.37
Between df			1
Between P			N.S.
Btwn(F) P			N.S.
Btwn(R) P			N.S.

Table 1K8 - 3

IESLC - Meta-analysis of Ex Smoking, Years quit (vs current), "Mid"
All LC types, Cigarettes (or Any Product if Cigarettes not available)
Most adjusted

		Derivation of RR/CI			
		Orig	StdCalc	Other	Total
	N	3	17	12	32
	NS	3	13	8	24
	Wt	61.12	580.59	297.34	939.05
Het	Chi	2.40	30.82	16.02	66.62
Het	df	2	16	11	31
Het	P	N.S.	*	N.S.	***
Fixed	RR	0.54	0.65	0.48	0.59
	RRl	0.42	0.60	0.43	0.55
	RRu	0.69	0.71	0.54	0.62
	P	---	---	---	---
Random	RR	0.56	0.60	0.47	0.55
	RRl	0.40	0.52	0.40	0.49
	RRu	0.78	0.69	0.57	0.62
	P	---	---	---	---
Between	Chi				17.38
Between	df				2
Between	P				***
Btwn(F)	P				*
Btwn(R)	P				N.S.

Table 1K8 - 4

IESLC - Meta-analysis of Ex Smoking, Years quit (vs current), "Mid"
All LC types, Cigarettes (or Any Product if Cigarettes not available)
Least adjusted

REF	NRR	X	SEX	AGEL	AGEH	RACE	YF	LC TYPE	LOC	START	ST	NLC	R	VB	P	H	AD	ADOS	PRODUCT	exL	exH	DENOM	De
BECHER	507		m	0	0	all	-	all	Eu:Ger	1985	CC	194	n	bl	n	y	0	0	all/unsp	5	9	cur+1y	st
BECHER	517		f	0	0	all	-	all	Eu:Ger	1985	CC	194	n	bl	n	y	0	0	all/unsp	5	9	cur+1y	st
CARPEN	509		c	0	0	w+b	-	all	NAmer	1991	CC	356	n	bl	n	n	0	0	cig+/-ot	5	9	current	st
CHOI	544		m	0	0	all	-	all	As:oth	1985	CC	375	n	bl	n	n	0	0	cig+/-ot	5	9	current	st
CPSI	817		m	50	74	all	6	all	NAmer	1959	pr	5138	n	bl	n	n	1	0	cig only	5	9	current	st
CPSII	667		m	35	99	all	4	all	NAmer	1982	pr	3229	n	bl	n	n	1	0	cig only	6	10	current	ot
CPSII	644		f	0	0	all	4	all	NAmer	1982	pr	3229	n	bl	n	n	1	0	cig+/-ot	6	10	current	ot
DAMBER	529		m	0	0	all	-	all	Eu:Sca	1972	CC	579	n	bl	y	n	1	0	all/unsp	6	10	current	ot
DEAN3	510	x	m	0	0	all	-	all	Eu:UK	1969	CC	766	n	V	y	n	0	0	cig only	5	8	cur+2y	st
DEAN3	549	x	f	0	0	all	-	all	Eu:UK	1969	CC	766	n	V	y	n	0	0	all/unsp	5	8	cur+2y	st
DESTEF	544		m	0	0	all	-	all	SCAmer	1988	CC	497	n	bl	n	y	0	0	cig+/-ot	5	9	current	st
DOLL2	510		m	0	0	all	20	all	Eu:UK	1951	pr	920	n	V	n	n	1	0	cig only	5	9	current	ot
DORGAN	515		m	0	0	wh	-	all	NAmer	1980	CC	2026	n	bl	y	y	0	0	cig+/-ot	6	9	cur+1y	st
DORN	824		m	55	64	wh	8	all	NAmer	1954	pr	5097	n	bl	n	n	0	0	cig+/-ot	5	9	current	st
DORN	828		m	65	74	wh	8	all	NAmer	1954	pr	5097	n	bl	n	n	0	0	cig+/-ot	5	9	current	st
GAO	527	x	m	0	0	all	-	all	As:Chi	1984	CC	1405	n	ot	n	n	0	0	cig+/-ot	5	9	current	st
GAO	547	x	f	0	0	all	-	all	As:Chi	1984	CC	1405	n	ot	n	n	0	0	cig+/-ot	5	9	current	st
GAO2	519		m	0	0	all	-	all	As:Jap	1988	CC	282	n	bl	n	n	0	0	cig+/-ot	5	9	cur+1y	st
GRAHAM	510		m	0	0	wh	-	all	NAmer	1956	CC	685	n	bl	n	n	0	0	cig only	3	10	current	st
HAMMO2	511		m	0	0	all	0	all	NAmer	1967	pr	450	o	bl	n	n	1	0	cig+/-ot	5	9	current	ot
HIRAYA	514		m	0	0	all	0	all	As:Jap	1965	pr	1917	n	bl	n	n	1	0	cig+/-ot	5	9	current	ot
HIRAYA	525		f	0	0	all	0	all	As:Jap	1965	pr	1917	n	bl	n	n	1	0	cig+/-ot	5	9	current	ot
JAHN	516		m	0	0	all	-	all	Eu:Ger	1988	CC	1004	n	bl	n	n	0	0	cig+/-ot	6	10	current	st
LUBIN	593		m	0	0	all	-	all	As:Chi	1984	CC	427	m	ot	y	n	0	0	cig+/-ot	5	9	cur+2y	st
LUBIN2	1082		m	0	0	all	-	all	Eu:mul	1976	CC	7804	n	bl	n	y	0	0	cig+/-ot	5	9	current	st
LUBIN2	1121		f	0	0	all	-	all	Eu:mul	1976	CC	7804	n	bl	n	y	0	0	cig+/-ot	5	9	current	st
MATOS	587	x	m	0	0	all	-	all	SCAmer	1994	CC	200	n	bl	n	n	0	0	cig+/-ot	6	10	cur+1y	st
SOBUE	729		m	0	0	all	-	all	As:Jap	1986	CC	1376	n	bl	n	y	0	0	cig+/-ot	5	9	cur+1y	st
SPEIZE	513		f	0	0	all	0	all	NAmer	1976	pr	593	n	bl	n	y	2	0	cig+/-ot	5	10	current	or
SUZUK2	514	x	c	0	0	all	-	all	SCAmer	1991	CC	123	n	bl	n	y	0	0	all/unsp	6	10	current	st
WYNDE6	514		m	0	0	all	-	all	NAmer	1969	CC	4423	n	bl	n	y	0	0	cig only	5	9	cur+1y	st
WYNDE6	535		f	0	0	all	-	all	NAmer	1969	CC	4423	n	bl	n	y	0	0	cig only	5	9	cur+1y	st

Cigarette type is all/unspec for all RRs
except for the following:

REF	NRR	CIGTYPE
DEAN3	510	MC only
DESTEF	544	MC only

Table 1K8 - 5

IESLC - Meta-analysis of Ex Smoking, Years quit (vs current), "Mid"
All LC types, Cigarettes (or Any Product if Cigarettes not available)
Least adjusted

REF	NRR	SEX	AD	Number Exposed Case	Cont	Non-exposed Case	Cont	RR	95.00%CI
BECHER	507	m	0	16	32	101	122	0.60	(0.31- 1.16)
BECHER	517	f	0	2	5	33	26	0.32	(0.06- 1.76)
Subtotal BECHER								0.56	(0.30- 1.03)
CARPEN	509	c	0	31	52	228	164	0.43	(0.26- 0.70)
CHOI	544	m	0	5	30	231	329	0.24	(0.09- 0.62)
*CPSI	817	m	1	32	-	844	-	0.37	(0.26- 0.53)
*CPSII	667	m	1	186	-	1159	-	0.52	(0.45- 0.61)
*CPSII	644	f	1	37	-	530	-	0.40	(0.28- 0.55)
Subtotal CPSII								0.50	(0.43- 0.57)
DAMBER	529	m	1	-	-	-	-	0.45	(0.26- 0.78)
DEAN3	510	m	0	11	43	337	930	0.71	(0.36- 1.38)
DEAN3	549	f	0	1	38	102	1158	0.30	(0.04- 2.20)
Subtotal DEAN3								0.65	(0.34- 1.22)
DESTEF	544	m	0	9	15	78	109	0.84	(0.35- 2.01)
*DOLL2	510	m	1	12	-	236	-	0.35	(0.20- 0.63)
DORGAN	515	m	0	49	38	465	303	0.84	(0.54- 1.31)
*DORN	824	m	0	32	34566	528	334175	0.59	(0.41- 0.84)
*DORN	828	m	0	41	24089	537	207895	0.66	(0.48- 0.90)
Subtotal DORN								0.63	(0.49- 0.79)
GAO	527	m	0	24	27	529	438	0.74	(0.42- 1.29)
GAO	547	f	0	14	7	170	100	1.18	(0.46- 3.01)
Subtotal GAO								0.83	(0.51- 1.35)
GAO2	519	m	0	21	26	184	117	0.51	(0.28- 0.95)
GRAHAM	510	m	0	5	29	371	821	0.38	(0.15- 0.99)
*HAMMO2	511	m	1	11	-	209	-	0.39	(0.22- 0.71)
*HIRAYA	514	m	1	-	-	-	-	0.36	(0.15- 0.86)
*HIRAYA	525	f	1	-	-	-	-	1.41	(0.23- 8.48)
Subtotal HIRAYA								0.47	(0.21- 1.02)
JAHN	516	m	0	59	63	352	269	0.72	(0.49- 1.06)
LUBIN	593	m	0	20	48	296	650	0.91	(0.53- 1.57)
LUBIN2	1082	m	0	466	822	4684	6211	0.75	(0.67- 0.85)
LUBIN2	1121	f	0	30	40	440	410	0.70	(0.43- 1.14)
Subtotal LUBIN2								0.75	(0.67- 0.84)
MATOS	587	m	0	21	27	112	132	0.92	(0.49- 1.71)
SOBUE	729	m	0	67	92	737	633	0.63	(0.45- 0.87)
*SPEIZE	513	f	2	41	-	319	-	0.50	(0.40- 0.70)
SUZUK2	514	c	0	10	8	77	30	0.49	(0.18- 1.35)
WYNDE6	514	m	0	98	194	1107	993	0.45	(0.35- 0.59)
WYNDE6	535	f	0	51	84	683	496	0.44	(0.31- 0.64)
Subtotal WYNDE6								0.45	(0.36- 0.55)
Partial Totals				1402	60375	15679	556511		

*prospective study

REF	NRR	SEX	AD	Ys	Ws	Qs	Ps
BECHER	507	m	0	-0.50	8.94	0.01	0.1316
BECHER	517	f	0	-1.15	1.30	0.50	0.1879
Subtotal BECHER				-0.59	10.24	0.51	
CARPEN	509	c	0	-0.85	16.14	1.58	0.0007
CHOI	544	m	0	-1.44	4.15	3.40	0.0034
*CPSI	817	m	1	-0.99	30.29	6.44	0.0000
*CPSII	667	m	1	-0.65	166.04	2.41	0.0000
*CPSII	644	f	1	-0.92	33.71	4.94	0.0000
Subtotal CPSII				-0.70	199.75	7.36	
DAMBER	529	m	1	-0.80	12.73	0.90	0.0044
DEAN3	510	m	0	-0.35	8.46	0.29	0.3112
DEAN3	549	f	0	-1.21	0.96	0.44	0.2355
Subtotal DEAN3				-0.44	9.42	0.73	
DESTEF	544	m	0	-0.18	5.01	0.64	0.6934
*DOLL2	510	m	1	-1.05	11.67	3.11	0.0003
DORGAN	515	m	0	-0.17	19.17	2.47	0.4460
*DORN	824	m	0	-0.53	30.20	0.00	0.0033
*DORN	828	m	0	-0.42	38.16	0.52	0.0100
Subtotal DORN				-0.47	68.36	0.52	
GAO	527	m	0	-0.31	12.07	0.62	0.2869
GAO	547	f	0	0.16	4.34	2.10	0.7348
Subtotal GAO				-0.18	16.41	2.72	
GAO2	519	m	0	-0.67	9.99	0.18	0.0352
GRAHAM	510	m	0	-0.96	4.19	0.78	0.0484
*HAMMO2	511	m	1	-0.94	11.19	1.87	0.0016
*HIRAYA	514	m	1	-1.02	5.04	1.20	0.0218

Table 1K8 - 5

IESLC - Meta-analysis of Ex Smoking, Years quit (vs current), "Mid"
All LC types, Cigarettes (or Any Product if Cigarettes not available)
Least adjusted

REF	NRR	SEX	AD	Ys	Ws	Qs	Ps
*HIRAYA	525	f	1	0.34	1.18	0.91	0.7089
Subtotal HIRAYA				-0.76	6.22	2.11	
JAHN	516	m	0	-0.33	25.39	1.00	0.0919
LUBIN	593	m	0	-0.09	13.20	2.61	0.7468
LUBIN2	1082	m	0	-0.29	267.60	16.45	0.0000
LUBIN2	1121	f	0	-0.36	15.86	0.49	0.1536
Subtotal LUBIN2				-0.29	283.46	16.94	
MATOS	587	m	0	-0.09	9.89	1.97	0.7844
SOBUE	729	m	0	-0.47	34.80	0.14	0.0056
*SPEIZE	513	f	2	-0.69	49.07	1.25	0.0000
SUZUK2	514	c	0	-0.72	3.69	0.13	0.1672
WYNDE6	514	m	0	-0.79	57.91	3.86	0.0000
WYNDE6	535	f	0	-0.82	28.58	2.33	0.0000
Subtotal WYNDE6				-0.80	86.48	6.19	

	N	32
	NS	24
	Wt	940.92
Het	Chi	65.54
Het	df	31
Het	P	***
Fixed	RR	0.59
	RRl	0.55
	RRu	0.63
	P	---
Random	RR	0.56
	RRl	0.50
	RRu	0.62
	P	---
Asymm	P	N.S.

Table 1K8 - 6

IESLC - Meta-analysis of Ex Smoking, Years quit (vs current), "Mid"
All LC types, Cigarettes (or Any Product if Cigarettes not available)
Least adjusted

	combined	Sex male	female	Total
N	2	22	8	32
NS	2	21	8	31
Wt	19.82	786.10	135.01	940.92
Het Chi	0.05	49.77	8.86	65.54
Het df	1	21	7	31
Het P	N.S.	***	N.S.	***
Fixed RR	0.44	0.61	0.49	0.59
RRl	0.28	0.57	0.42	0.55
RRu	0.68	0.65	0.58	0.63
P	---	---	---	---
Random RR	0.44	0.58	0.50	0.56
RRl	0.28	0.50	0.41	0.50
RRu	0.68	0.66	0.62	0.62
P	---	---	---	---
Between Chi				6.86
Between df				2
Between P				*
Btwn(F) P				N.S.
Btwn(R) P				N.S.

Table 1K8 - 7

IESLC - Meta-analysis of Ex Smoking, Years quit (vs current), "Mid"
All LC types, Cigarettes (or Any Product if Cigarettes not available)
Excluded studies (and stage at which they were excluded)

1	AGUDO AKIBA AMANDU AMES AXELSS BEST BOUCHA BOUCOT BRESLO CHEN CHEN2 CHIAZZ DEAN2 DOSEME ENGELA FAN GENG GER GUO HAENSZ HEGMAN HOLE HU HU2 JUSSAW KATSOU KAUFMA KOO KOULUM KREUZE LETOUR LEVIN LIAW LIU3 LIU4 LIU5 MCCONN MIGRAN MRFITR NOTAN2 OSANN2 PERNU QIAO2 RACHTA RESTRE SADOWS SEGI2 STASZE TIZZAN VUTUC WATSON WU WUWILL WYNDE2 WYNDE8 XU YUAN ZHANG ZHENG ZHOU
2	AUVINE BENSHL BLOT1 BROWN3 BUFFLE GURSEL LAUSSM LUO MCDUFF PISANI PRESCO SPITZ WU2 WYNDE7
4	HAMMON
5	CORREA GILLIS HUMBLE QIAO WIGLE
7	BOFFET
10	GARSHI JEDRYC WAKAI
14	ALDERS ARMADA BARBON BROSS CEDERL CHYOU DARBY DOLL GARCIA JAIN JOLY KAISE2 KHUDER PEZZO2 PEZZOT SVENSS TVERDA WANG2 WYNDE3
15	BENHAM

Table 1K8 - 8
Potentially overlapping studies

REF	REFGP	PRINC	.	OVERLAP/LINK
LUBIN2	LUBIN2	1		Lubin-combined
GRAHAM	BYERS1	1		GRAHAM/BROSS/BYERS1
WYNDE6	WYNDE6	1		WYNDE5/6/7/8
CPSI	CPSI	1		CPSI overall
JAHN	BOFFET	2		Subset of BOFFET
LUBIN	XIANGZ	2		LUBIN/XIANGZ/QIAO

Table 1K8 - 9
Most adjusted - insufficient data for meta-analysis

REF	NRR	SEX	AGEL	AGEH	RACE	YF	LC TYPE	LOC	START	ST	NLC	R	VB	P	H	AD	ADOS	PRODUCT	exL	exH	DENOM	De
HUMBLE	560	c	25	64	wh	-	not alv	NAmer	1980	CC	521	n	bl	y	n	2		2#cig+/-ot	6	10	cur+1y	or
HUMBLE	555	c	65	84	wh	-	not alv	NAmer	1980	CC	521	n	bl	y	n	2		2#cig+/-ot	6	10	cur+1y	or

Comments on values in listings

HUMBLE ADOS Number of cigarettes and duration
HUMBLE ADOS Number of cigarettes and duration

REF	NRR	RR	SIG	RRDATA comment
HUMBLE	560	0.24		0
HUMBLE	555	0.54		0

Table 1K9 -

IESLC - Meta-analysis of Ex Smoking, Years quit (vs current), "High"
All LC types, Cigarettes (or Any Product if Cigarettes not available)

This analysis is restricted to results for:
1) Ex smokers
2) Results by Years quit (vs current)
3) Categorical results by Years quit (vs current)
4) All LC types (or near equivalent)
5) Results complete enough for use in metaanalysis

Within each study, results are then selected (in the following order of preference, within each sex) for:
6) PRODUCT: cigarettes regardless of other products, cigarettes only, all/unspec
7) CIGTYPE: all/unspecified, MC regardless of HR, MC only
8) Results with least adjustment for other aspects of smoking (ADOS)
9) DENOM: current smokers, current + recent smokers (up to number of m=months or y=years, max 2 years)
10) Followup period (YF, prospective studies): whole study (coded as 0) or longest available
11) LCtype: all or nearest available, at least Squamous and Adeno. (q = squamous, s = small,
l = large, a = adeno, mix = mixed, alv = alveolar)
12) Race: all or nearest available, otherwise by race (wh or w = white, bl or b = black, hi = hispanic
ch = chinese, jap = japanese, haw = hawaiian, w+o = white + oriental, sca = scandinavian, as = asian)
13) Years quit (vs current) "high" in key scheme 1 (key value 12, maximum range 8+)
14) For overlapping studies: principal rather than subsidiary studies
Finally by Age: whole study (coded as 0) if available, otherwise by widest available age group
and then for single sex results (m, f) in preference to results for both sexes combined (c).

Results adjusted (AD) for the most potential confounders are then chosen in Sections -1 to -3
(and those which actually differ from the adjusted results in Table 1K4 - 1 are marked 'x' in Section -1)
and results adjusted for the least confounders in Sections -4 to -6. (Those least adjusted results which
actually differ from the most adjusted are marked 'x' in column X in Section -4)

Section -7 shows excluded studies, together with the stage (as above) at which no qualifying
results were found.

Section -8 lists the potentially overlapping studies which have been included (1=principal, 2=subsidiary).

Section -9 lists any results which would have been included in preference except that they had data not complete
enough for use in meta-analysis, with their significance (yes/no), if known, and any further comment as entered
on the database. It also lists as "gap" any categories for which no data were presented by the original authors.

In addition to those mentioned above, the following fields, levels and abbreviations are used:
* or nk = not known, n = no, y = yes, ot = other
nev = never
all/unspec = all or unspecified, cig+/-ot = cigarettes irrespective of other products (cigar, pipe etc)
MC = manufactured cigarettes, HR = hand-rolled cigarettes
exL, exH = range of exposure (low and high) in the smoking group, in terms of Years quit (vs current)
REF: 6-character study reference
NRR: number of the RR on the database within the study
ST : study type (CC = case control, pr or prosp = prospective)
NLC: number of lung cancer cases in whole study
R : risky occupational population (n = no, m = mining, o = other risky)
VB : national cigarette type (V = at least 75% Virginia, bl = at least 75% blended, ot = other)
P : any proxy use
H : full histological confirmation
De : derivation of RR/CI (or = original, st = standard method, ot = other method of estimation)

Table 1K9 - 1

IESLC - Meta-analysis of Ex Smoking, Years quit (vs current), "High"
All LC types, Cigarettes (or Any Product if Cigarettes not available)
Most adjusted

REF	NRR	1K4	SEX	AGEL	AGEH	RACE	YF	LC TYPE	LOC	START	ST	NLC	R	VB	P	H	AD	ADOS	PRODUCT	exL	exH	DENOM	De
ALDERS	515		m	0	0	all	-	all	Eu:UK	1977	CC	1448	n	V	n	n	1	0	cig only	10	999	current	ot
ALDERS	526		f	0	0	all	-	all	Eu:UK	1977	CC	1448	n	V	n	n	1	0	cig only	10	999	current	ot
BECHER	508		m	0	0	all	-	all	Eu:Ger	1985	CC	194	n	bl	n	y	0	0	all/unsp	10	999	cur+1y	st
BECHER	518		f	0	0	all	-	all	Eu:Ger	1985	CC	194	n	bl	n	y	0	0	all/unsp	10	999	cur+1y	st
CARPEN	510		c	0	0	w+b	-	all	NAmer	1991	CC	356	n	bl	n	n	0	0	cig+/-ot	10	14	current	st
CEDERL	539		m	40	69	all	10	all	Eu:Sca	1963	pr	491	n	bl	n	n	1	0	all/unsp	10	999	current	ot
CHOI	545		m	0	0	all	-	all	As:oth	1985	CC	375	n	bl	n	n	0	0	cig+/-ot	10	14	current	st
CPSI	818		m	50	74	all	6	all	NAmer	1959	pr	5138	n	bl	n	n	1	0	cig only	10	999	current	ot
CPSII	668		m	35	99	all	4	all	NAmer	1982	pr	3229	n	bl	n	n	1	0	cig only	11	15	current	ot
CPSII	645		f	0	0	all	4	all	NAmer	1982	pr	3229	n	bl	n	n	1	0	cig+/-ot	11	15	current	ot
DAMBER	558	x	m	0	0	all	-	all	Eu:Sca	1972	CC	579	n	bl	y	n	1	0	cig only	11	999	current	ot
DARBY	508		m	0	0	wh	-	all	Eu:UK	1988	CC	982	n	V	n	n	0	0	all/unsp	10	999	current	st
DARBY	517		f	0	0	wh	-	all	Eu:UK	1988	CC	982	n	V	n	n	0	0	all/unsp	10	999	current	st
DEAN3	526	x	m	0	0	all	-	all	Eu:UK	1969	CC	766	n	V	y	n	1	0	cig only	9	18	cur+2y	ot
DEAN3	561		f	0	0	all	-	all	Eu:UK	1969	CC	766	n	V	y	n	1	0	all/unsp	9	999	cur+2y	ot
DESTEF	545	x	m	0	0	all	-	all	SCAmer	1988	CC	497	n	bl	n	y	0	0	cig+/-ot	10	999	current	st
DOLL	538		m	0	0	all	-	all	Eu:UK	1948	CC	1465	n	V	n	n	0	0	all/unsp	10	19	current	st
DOLL	549		f	0	0	all	-	all	Eu:UK	1948	CC	1465	n	V	n	n	0	0	all/unsp	10	999	current	st
DOLL2	511		m	0	0	all	20	all	Eu:UK	1951	pr	920	n	V	n	n	1	0	cig only	10	14	current	ot
DORGAN	516		m	0	0	wh	-	all	NAmer	1980	CC	2026	n	bl	y	y	0	0	cig+/-ot	10	999	cur+1y	st
DORGAN	560		f	0	0	all	-	all	NAmer	1980	CC	2026	n	bl	y	y	0	0	cig+/-ot	10	999	cur+1y	st
DORN	825		m	55	64	wh	8	all	NAmer	1954	pr	5097	n	bl	n	n	0	0	cig+/-ot	10	14	current	st
DORN	829		m	65	74	wh	8	all	NAmer	1954	pr	5097	n	bl	n	n	0	0	cig+/-ot	10	14	current	st
GAO	538		m	0	0	all	-	all	As:Chi	1984	CC	1405	n	ot	n	n	2	0	cig+/-ot	10	999	current	ot
GAO	558		f	0	0	all	-	all	As:Chi	1984	CC	1405	n	ot	n	n	2	0	cig+/-ot	10	999	current	ot
GAO2	520		m	0	0	all	-	all	As:Jap	1988	CC	282	n	bl	n	n	0	0	cig+/-ot	10	14	cur+1y	st
GRAHAM	511		m	0	0	wh	-	all	NAmer	1956	CC	685	n	bl	n	n	0	0	cig only	10	999	current	st
HAMMO2	512		m	0	0	all	0	all	NAmer	1967	pr	450	o	bl	n	n	1	0	cig+/-ot	10	999	current	ot
HIRAYA	515		m	0	0	all	0	all	As:Jap	1965	pr	1917	n	bl	n	n	1	0	cig+/-ot	10	999	current	ot
HIRAYA	526		f	0	0	all	0	all	As:Jap	1965	pr	1917	n	bl	n	n	1	0	cig+/-ot	10	999	current	ot
JAHN	517		m	0	0	all	-	all	Eu:Ger	1988	CC	1004	n	bl	n	n	0	0	cig+/-ot	11	20	current	st
JAIN	571		m	0	0	all	-	all	NAmer	1981	CC	845	n	V	y	n	0	0	cig+/-ot	10	999	cur+2y	st
JAIN	535		f	0	0	all	-	all	NAmer	1981	CC	845	n	V	y	n	0	0	cig+/-ot	10	999	cur+2y	st
KAISE2	656		m	0	0	all	9	all	NAmer	1979	pr	318	n	bl	n	n	1	0	cig only	11	20	cur+2y	ot
KAISE2	576		f	0	0	all	9	all	NAmer	1979	pr	318	n	bl	n	n	1	0	cig only	11	20	cur+2y	st
LUBIN	594		m	0	0	all	-	all	As:Chi	1984	CC	427	m	ot	y	n	0	0	cig+/-ot	10	999	cur+2y	st
LUBIN2	1083		m	0	0	all	-	all	Eu:mul	1976	CC	7804	n	bl	n	y	0	0	cig+/-ot	10	14	current	st
LUBIN2	1122		f	0	0	all	-	all	Eu:mul	1976	CC	7804	n	bl	n	y	0	0	cig+/-ot	10	14	current	st
MATOS	598		m	0	0	all	-	all	SCAmer	1994	CC	200	n	bl	n	n	2	0	cig+/-ot	11	999	cur+1y	or
PEZZO2	505		m	0	0	all	-	all	SCAmer	1992	CC	367	n	bl	n	y	0	0	cig+/-ot	11	999	cur+1y	st
PEZZOT	505		m	0	0	all	-	all	SCAmer	1987	CC	215	n	bl	n	y	0	0	cig only	11	999	cur+1y	st
SOBUE	730		m	0	0	all	-	all	As:Jap	1986	CC	1376	n	bl	n	y	0	0	cig+/-ot	10	14	cur+1y	st
SPEIZE	514		f	0	0	all	0	all	NAmer	1976	pr	593	n	bl	n	y	2	0	cig+/-ot	10	15	current	or
SUZUK2	526		c	0	0	all	-	all	SCAmer	1991	CC	123	n	bl	n	y	3	0	all/unsp	11	999	current	or
SVENSS	555		f	0	0	all	-	all	Eu:Sca	1983	CC	210	n	bl	n	n	0	0	all/unsp	11	999	cur+2y	st
WYNDE6	720		m	0	0	wh	-	all	NAmer	1969	CC	4423	n	bl	n	y	5	1#	cig+/-ot	11	19	cur+1y	or
WYNDE6	727		m	0	0	bl	-	all	NAmer	1969	CC	4423	n	bl	n	y	5	1#	cig+/-ot	11	19	cur+1y	or
WYNDE6	734		f	0	0	wh	-	all	NAmer	1969	CC	4423	n	bl	n	y	5	1#	cig+/-ot	11	999	cur+1y	or
WYNDE6	738		f	0	0	bl	-	all	NAmer	1969	CC	4423	n	bl	n	y	5	1#	cig+/-ot	11	999	cur+1y	or

Comments on values in listings

WYNDE6 ADOS Number of cigs/day
WYNDE6 ADOS Number of cigs/day
WYNDE6 ADOS Number of cigs/day
WYNDE6 ADOS Number of cigs/day

Cigarette type is all/unspec for all RRs
except for the following:

REF	NRR	CIGTYPE
ALDERS	515	MC only
ALDERS	526	MC only
DEAN3	526	MC only
DESTEF	545	MC only

Table 1K9 - 2

IESLC - Meta-analysis of Ex Smoking, Years quit (vs current), "High"
All LC types, Cigarettes (or Any Product if Cigarettes not available)
Most adjusted

REF	NRR	SEX	AD	Number Exposed Case	Cont	Non-exposed Case	Cont	RR	95.00%CI
ALDERS	515	m	1	29	-	207	-	0.32	(0.20- 0.51)
ALDERS	526	f	1	26	-	244	-	0.28	(0.17- 0.46)
Subtotal ALDERS								0.30	(0.21- 0.42)
BECHER	508	m	0	16	72	101	122	0.27	(0.15- 0.49)
BECHER	518	f	0	1	10	33	26	0.08	(0.01- 0.66)
Subtotal BECHER								0.24	(0.14- 0.44)
CARPEN	510	c	0	13	58	228	164	0.16	(0.09- 0.30)
*CEDERL	539	m	1	3	-	97	-	0.14	(0.04- 0.45)
CHOI	545	m	0	4	23	231	329	0.25	(0.08- 0.73)
*CPSI	818	m	1	15	-	844	-	0.09	(0.06- 0.15)
*CPSII	668	m	1	164	-	1159	-	0.39	(0.33- 0.46)
*CPSII	645	f	1	28	-	530	-	0.31	(0.21- 0.46)
Subtotal CPSII								0.38	(0.32- 0.44)
DAMBER	558	m	1	-	-	-	-	0.16	(0.08- 0.35)
DARBY	508	m	0	139	767	379	618	0.30	(0.24- 0.37)
DARBY	517	f	0	26	224	198	231	0.14	(0.09- 0.21)
Subtotal DARBY								0.25	(0.21- 0.31)
DEAN3	526	m	1	15	-	337	-	0.41	(0.23- 0.72)
DEAN3	561	f	1	2	-	102	-	0.13	(0.03- 0.53)
Subtotal DEAN3								0.35	(0.21- 0.60)
DESTEF	545	m	0	10	41	78	109	0.34	(0.16- 0.72)
DOLL	538	m	0	6	26	1280	1172	0.21	(0.09- 0.52)
DOLL	549	f	0	1	2	58	41	0.35	(0.03- 4.03)
Subtotal DOLL								0.22	(0.10- 0.52)
*DOLL2	511	m	1	9	-	236	-	0.28	(0.14- 0.54)
DORGAN	516	m	0	134	255	465	303	0.34	(0.27- 0.44)
DORGAN	560	f	0	34	50	289	112	0.26	(0.16- 0.43)
Subtotal DORGAN								0.32	(0.26- 0.41)
*DORN	825	m	0	12	23682	528	334175	0.32	(0.18- 0.57)
*DORN	829	m	0	29	20056	537	207895	0.56	(0.39- 0.81)
Subtotal DORN								0.47	(0.35- 0.65)
GAO	538	m	2	13	-	529	-	0.28	(0.14- 0.57)
GAO	558	f	2	16	-	170	-	0.76	(0.34- 1.67)
Subtotal GAO								0.43	(0.26- 0.73)
GAO2	520	m	0	16	18	184	117	0.57	(0.28- 1.15)
GRAHAM	511	m	0	2	30	371	821	0.15	(0.04- 0.62)
*HAMMO2	512	m	1	20	-	209	-	0.34	(0.22- 0.53)
*HIRAYA	515	m	1	-	-	-	-	0.31	(0.14- 0.69)
*HIRAYA	526	f	1	-	-	-	-	0.41	(0.01- 14.37)
Subtotal HIRAYA								0.31	(0.14- 0.68)
JAHN	517	m	0	64	130	352	269	0.38	(0.27- 0.53)
JAIN	571	m	0	52	113	265	118	0.20	(0.14- 0.30)
JAIN	535	f	0	19	61	305	99	0.10	(0.06- 0.18)
Subtotal JAIN								0.16	(0.12- 0.22)
*KAISE2	656	m	1	8	-	51	-	0.43	(0.20- 0.92)
*KAISE2	576	f	1	4	-	50	-	0.25	(0.09- 0.70)
Subtotal KAISE2								0.35	(0.19- 0.65)
LUBIN	594	m	0	17	73	296	650	0.51	(0.30- 0.88)
LUBIN2	1083	m	0	270	693	4684	6211	0.52	(0.45- 0.60)
LUBIN2	1122	f	0	10	26	440	410	0.36	(0.17- 0.75)
Subtotal LUBIN2								0.51	(0.44- 0.59)
MATOS	598	m	2	27	-	112	-	0.30	(0.20- 0.60)
PEZZO2	505	m	0	43	161	233	198	0.23	(0.15- 0.33)
PEZZOT	505	m	0	20	106	145	129	0.17	(0.10- 0.29)
SOBUE	730	m	0	35	50	737	633	0.60	(0.39- 0.94)
*SPEIZE	514	f	2	17	-	319	-	0.20	(0.10- 0.40)
SUZUK2	526	c	3	9	-	77	-	0.10	(0.10- 0.40)
SVENSS	555	f	0	14	24	142	53	0.22	(0.10- 0.45)
WYNDE6	720	m	5	-	-	-	-	0.30	(0.20- 0.40)
WYNDE6	727	m	5	-	-	-	-	0.20	(0.10- 0.50)
WYNDE6	734	f	5	-	-	-	-	0.20	(0.20- 0.30)
WYNDE6	738	f	5	-	-	-	-	0.40	(0.10- 1.10)
Subtotal WYNDE6								0.22	(0.19- 0.26)
Partial Totals				1392	46751	17832	555005		

*prospective study

Table 1K9 - 2

IESLC - Meta-analysis of Ex Smoking, Years quit (vs current), "High"
All LC types, Cigarettes (or Any Product if Cigarettes not available)
Most adjusted

REF	NRR	SEX	AD	Ys	Ws	Qs	Ps
ALDERS	515	m	1	-1.14	17.54	0.00	0.0000
ALDERS	526	f	1	-1.27	15.51	0.24	0.0000
Subtotal ALDERS				-1.20	33.04	0.24	
BECHER	508	m	0	-1.32	10.58	0.29	0.0000
BECHER	518	f	0	-2.54	0.86	1.66	0.0188
Subtotal BECHER				-1.41	11.44	1.95	
CARPEN	510	c	0	-1.82	9.56	4.36	0.0000
*CEDERL	539	m	1	-1.97	2.62	1.75	0.0015
CHOI	545	m	0	-1.40	3.32	0.20	0.0109
*CPSI	818	m	1	-2.41	18.30	28.98	0.0000
*CPSII	668	m	1	-0.94	139.29	6.03	0.0000
*CPSII	645	f	1	-1.17	24.99	0.01	0.0000
Subtotal CPSII				-0.98	164.28	6.04	
DAMBER	558	m	1	-1.83	7.05	3.29	0.0000
DARBY	508	m	0	-1.22	78.40	0.38	0.0000
DARBY	517	f	0	-2.00	19.12	13.81	0.0000
Subtotal DARBY				-1.37	97.52	14.18	
DEAN3	526	m	1	-0.89	11.80	0.79	0.0022
DEAN3	561	f	1	-2.04	1.86	1.48	0.0054
Subtotal DEAN3				-1.05	13.66	2.26	
DESTEF	545	m	0	-1.08	6.83	0.04	0.0049
DOLL	538	m	0	-1.55	4.84	0.79	0.0006
DOLL	549	f	0	-1.04	0.65	0.01	0.4022
Subtotal DOLL				-1.49	5.49	0.80	
*DOLL2	511	m	1	-1.27	8.43	0.13	0.0002
DORGAN	516	m	0	-1.07	59.40	0.36	0.0000
DORGAN	560	f	0	-1.33	16.18	0.55	0.0000
Subtotal DORGAN				-1.13	75.58	0.91	
*DORN	825	m	0	-1.14	11.74	0.00	0.0001
*DORN	829	m	0	-0.58	27.56	8.93	0.0023
Subtotal DORN				-0.75	39.30	8.94	
GAO	538	m	2	-1.27	7.80	0.12	0.0004
GAO	558	f	2	-0.27	6.07	4.65	0.4991
Subtotal GAO				-0.84	13.86	4.76	
GAO2	520	m	0	-0.57	7.57	2.54	0.1164
GRAHAM	511	m	0	-1.91	1.86	1.09	0.0090
*HAMMO2	512	m	1	-1.08	19.88	0.10	0.0000
*HIRAYA	515	m	1	-1.17	6.04	0.00	0.0040
*HIRAYA	526	f	1	-0.89	0.29	0.02	0.6307
Subtotal HIRAYA				-1.16	6.33	0.02	
JAHN	517	m	0	-0.98	33.47	0.99	0.0000
JAIN	571	m	0	-1.59	24.80	4.70	0.0000
JAIN	535	f	0	-2.29	12.14	15.83	0.0000
Subtotal JAIN				-1.82	36.93	20.53	
*KAISE2	656	m	1	-0.84	6.60	0.62	0.0302
*KAISE2	576	f	1	-1.39	3.65	0.20	0.0081
Subtotal KAISE2				-1.04	10.25	0.82	
LUBIN	594	m	0	-0.67	12.91	2.96	0.0160
LUBIN2	1083	m	0	-0.66	181.12	43.34	0.0000
LUBIN2	1122	f	0	-1.03	6.98	0.11	0.0067
Subtotal LUBIN2				-0.67	188.10	43.45	
MATOS	598	m	2	-1.20	12.73	0.04	0.0000
PEZZO2	505	m	0	-1.48	25.77	2.86	0.0000
PEZZOT	505	m	0	-1.78	13.50	5.44	0.0000
SOBUE	730	m	0	-0.51	19.41	7.97	0.0250
*SPEIZE	514	f	2	-1.61	8.00	1.69	0.0000
SUZUK2	526	c	3	-2.30	8.00	10.63	0.0000
SVENSS	555	f	0	-1.52	7.19	1.01	0.0000
WYNDE6	720	m	5	-1.20	31.98	0.09	0.0000
WYNDE6	727	m	5	-1.61	5.93	1.25	0.0001
WYNDE6	734	f	5	-1.61	93.46	19.76	0.0000
WYNDE6	738	f	5	-0.92	2.67	0.15	0.1342
Subtotal WYNDE6				-1.50	134.05	21.26	

Table 1K9 - 2

IESLC - Meta-analysis of Ex Smoking, Years quit (vs current), "High"
All LC types, Cigarettes (or Any Product if Cigarettes not available)
Most adjusted

	N	49
	NS	33
	Wt	1056.25
Het	Chi	202.23
Het	df	48
Het	P	***
Fixed	RR	0.32
	RRl	0.30
	RRu	0.34
	P	---
Random	RR	0.27
	RRl	0.24
	RRu	0.32
	P	---
Asymm	P	*

Table 1K9 - 3

IESLC - Meta-analysis of Ex Smoking, Years quit (vs current), "High"
All LC types, Cigarettes (or Any Product if Cigarettes not available)
Most adjusted

	Sex			
	combined	male	female	Total
N	2	31	16	49
NS	2	29	15	46
Wt	17.55	819.08	219.62	1056.25
Het Chi	0.99	114.52	30.79	202.23
Het df	1	30	15	48
Het P	N.S.	***	**	***
Fixed RR	0.13	0.36	0.22	0.32
RRl	0.08	0.33	0.19	0.30
RRu	0.21	0.38	0.25	0.34
P	---	---	---	---
Random RR	0.13	0.31	0.23	0.27
RRl	0.08	0.26	0.18	0.24
RRu	0.21	0.36	0.29	0.32
P	---	---	---	---
Between Chi				55.92
Between df				2
Between P				***
Btwn(F) P				***
Btwn(R) P				**

	Lung cancer type		
	all	other	Total
N	49		49
NS	33		33
Wt	1056.25		1056.25
Het Chi	202.23		202.23
Het df	48		48
Het P	***		***
Fixed RR	0.32		0.32
RRl	0.30		0.30
RRu	0.34		0.34
P	---		---
Random RR	0.27		0.27
RRl	0.24		0.24
RRu	0.32		0.32
P	---		---
Between Chi			
Between df			
Between P			N.S.
Btwn(F) P			N.S.
Btwn(R) P			N.S.

	Location								
	NAmer	UK	Scand	othEur	China	Japan	othAs	other	Total
N	19	9	3	5	3	4	1	5	49
NS	11	5	3	3	2	3	1	5	33
Wt	517.98	158.14	16.87	233.02	26.77	33.32	3.32	66.82	1056.25
Het Chi	88.30	13.67	0.52	9.84	3.58	2.09	0.00	8.44	202.23
Het df	18	8	2	4	2	3	0	4	48
Het P	***	(*)	N.S.	*	N.S.	N.S.	N.S.	(*)	***
Fixed RR	0.28	0.27	0.18	0.47	0.47	0.52	0.25	0.21	0.32
RRl	0.26	0.23	0.11	0.41	0.32	0.37	0.08	0.17	0.30
RRu	0.31	0.32	0.29	0.54	0.69	0.74	0.73	0.27	0.34
P	---	---	---	---	---	---	-	---	---
Random RR	0.25	0.26	0.18	0.38	0.47	0.52	0.25	0.21	0.27
RRl	0.20	0.20	0.11	0.28	0.28	0.37	0.08	0.15	0.24
RRu	0.32	0.33	0.29	0.53	0.79	0.74	0.73	0.30	0.32
P	---	---	---	---	--	---	-	---	---
Between Chi									75.77
Between df									7
Between P									***
Btwn(F) P									**
Btwn(R) P									***

Table 1K9 - 3

IESLC - Meta-analysis of Ex Smoking, Years quit (vs current), "High"
All LC types, Cigarettes (or Any Product if Cigarettes not available)
Most adjusted

Detailed Country in "other Europe"

	multi	Germany	othWest	East	Balkans	Total
N	2	3				5
NS	1	2				3
Wt	188.10	44.91				233.02
Het Chi	0.90	2.76				9.84
Het df	1	2				4
Het P	N.S.	N.S.				*
Fixed RR	0.51	0.34				0.47
RRl	0.44	0.25				0.41
RRu	0.59	0.45				0.54
P	---	---				---
Random RR	0.51	0.32				0.38
RRl	0.44	0.21				0.28
RRu	0.59	0.48				0.53
P	---	---				---
Between Chi						6.18
Between df						1
Between P						*
Btwn(F) P						N.S.
Btwn(R) P						*

Detailed Country in "other Asia"

	India	HongKong	other	Total
N			1	1
NS			1	1
Wt			3.32	3.32
Het Chi			0.00	0.00
Het df			0	0
Het P			N.S.	N.S.
Fixed RR			0.25	0.25
RRl			0.08	0.08
RRu			0.73	0.73
P			-	-
Random RR			0.25	0.25
RRl			0.08	0.08
RRu			0.73	0.73
P			-	-
Between Chi				
Between df				
Between P				N.S.
Btwn(F) P				N.S.
Btwn(R) P				N.S.

Detailed other continent

	SCAmer	Total
N	5	5
NS	5	5
Wt	66.82	66.82
Het Chi	8.44	8.44
Het df	4	4
Het P	(*)	(*)
Fixed RR	0.21	0.21
RRl	0.17	0.17
RRu	0.27	0.27
P	---	---
Random RR	0.21	0.21
RRl	0.15	0.15
RRu	0.30	0.30
P	---	---
Between Chi		
Between df		
Between P		N.S.
Btwn(F) P		N.S.
Btwn(R) P		N.S.

Table 1K9 - 3

IESLC - Meta-analysis of Ex Smoking, Years quit (vs current), "High"
All LC types, Cigarettes (or Any Product if Cigarettes not available)
Most adjusted

	Start year of study					
	<1960	1960-69	1970-79	1980-89	1990+	Total
N	7	10	8	20	4	49
NS	5	5	5	14	4	33
Wt	73.38	176.54	246.45	503.84	56.05	1056.25
Het Chi	38.11	13.29	23.27	69.52	6.78	202.23
Het df	6	9	7	19	3	48
Het P	***	N.S.	**	***	(*)	***
Fixed RR	0.27	0.24	0.44	0.32	0.20	0.32
RRl	0.22	0.21	0.39	0.29	0.16	0.30
RRu	0.34	0.28	0.50	0.35	0.26	0.34
P	---	---	---	---	---	---
Random RR	0.24	0.27	0.31	0.29	0.19	0.27
RRl	0.12	0.21	0.22	0.24	0.13	0.24
RRu	0.47	0.33	0.44	0.36	0.29	0.32
P	---	---	---	---	---	---
Between Chi						51.26
Between df						4
Between P						***
Btwn(F) P						*
Btwn(R) P						N.S.

	Study type (1)		
	CC	other	Total
N	36	13	49
NS	24	9	33
Wt	778.87	277.39	1056.25
Het Chi	153.40	47.44	202.23
Het df	35	12	48
Het P	***	***	***
Fixed RR	0.31	0.34	0.32
RRl	0.29	0.30	0.30
RRu	0.33	0.38	0.34
P	---	---	---
Random RR	0.27	0.29	0.27
RRl	0.23	0.21	0.24
RRu	0.32	0.39	0.32
P	---	---	---
Between Chi			1.39
Between df			1
Between P			N.S.
Btwn(F) P			N.S.
Btwn(R) P			N.S.

	Study type (2)			
	CC	prosp	other	Total
N	36	13		49
NS	24	9		33
Wt	778.87	277.39		1056.25
Het Chi	153.40	47.44		202.23
Het df	35	12		48
Het P	***	***		***
Fixed RR	0.31	0.34		0.32
RRl	0.29	0.30		0.30
RRu	0.33	0.38		0.34
P	---	---		---
Random RR	0.27	0.29		0.27
RRl	0.23	0.21		0.24
RRu	0.32	0.39		0.32
P	---	---		---
Between Chi				1.39
Between df				1
Between P				N.S.
Btwn(F) P				N.S.
Btwn(R) P				N.S.

Table 1K9 - 3

IESLC - Meta-analysis of Ex Smoking, Years quit (vs current), "High"
All LC types, Cigarettes (or Any Product if Cigarettes not available)
Most adjusted

	Study size (number of LC cases)				
	100-249	250-499	500-999	1000+	Total
N	6	10	10	23	49
NS	5	9	7	12	33
Wt	52.86	98.71	173.46	731.22	1056.25
Het Chi	8.06	15.44	25.15	114.61	202.23
Het df	5	9	9	22	48
Het P	N.S.	(*)	**	***	***
Fixed RR	0.20	0.30	0.23	0.36	0.32
RRl	0.15	0.25	0.20	0.33	0.30
RRu	0.26	0.37	0.27	0.38	0.34
P	---	---	---	---	---
Random RR	0.20	0.31	0.20	0.32	0.27
RRl	0.14	0.23	0.15	0.27	0.24
RRu	0.28	0.40	0.27	0.39	0.32
P	---	---	---	---	---
Between Chi					38.97
Between df					3
Between P					***
Btwn(F) P					*
Btwn(R) P					*

	Risky occupational population			
	no	mining	othRisky	Total
N	47	1	1	49
NS	31	1	1	33
Wt	1023.46	12.91	19.88	1056.25
Het Chi	199.11	0.00	0.00	202.23
Het df	46	0	0	48
Het P	***	N.S.	N.S.	***
Fixed RR	0.31	0.51	0.34	0.32
RRl	0.30	0.30	0.22	0.30
RRu	0.33	0.88	0.53	0.34
P	---	-	---	---
Random RR	0.27	0.51	0.34	0.27
RRl	0.23	0.30	0.22	0.24
RRu	0.31	0.88	0.53	0.32
P	---	-	---	---
Between Chi				3.12
Between df				2
Between P				N.S.
Btwn(F) P				N.S.
Btwn(R) P				(*)

	National cigarette tobacco type			
	Virginia	blended	other	Total
N	11	35	3	49
NS	6	25	2	33
Wt	195.08	834.40	26.77	1056.25
Het Chi	25.52	154.52	3.58	202.23
Het df	10	34	2	48
Het P	**	***	N.S.	***
Fixed RR	0.25	0.33	0.47	0.32
RRl	0.21	0.31	0.32	0.30
RRu	0.28	0.36	0.69	0.34
P	---	---	---	---
Random RR	0.23	0.28	0.47	0.27
RRl	0.18	0.23	0.28	0.24
RRu	0.30	0.33	0.79	0.32
P	---	---	--	---
Between Chi				18.60
Between df				2
Between P				***
Btwn(F) P				N.S.
Btwn(R) P				(*)

Table 1K9 - 3

IESLC - Meta-analysis of Ex Smoking, Years quit (vs current), "High"
All LC types, Cigarettes (or Any Product if Cigarettes not available)
Most adjusted

	Any proxy use No/nk	Yes	Total
N	41	8	49
NS	28	5	33
Wt	910.11	146.14	1056.25
Het Chi	171.82	27.15	202.23
Het df	40	7	48
Het P	***	***	***
Fixed RR	0.32	0.28	0.32
RRl	0.30	0.23	0.30
RRu	0.35	0.32	0.34
P	---	---	---
Random RR	0.28	0.25	0.27
RRl	0.24	0.17	0.24
RRu	0.33	0.36	0.32
P	---	---	---
Between Chi			3.26
Between df			1
Between P			(*)
Btwn(F) P			N.S.
Btwn(R) P			N.S.

	Full histological confirmation No	Yes	Total
N	33	16	49
NS	23	10	33
Wt	565.58	490.68	1056.25
Het Chi	104.99	95.00	202.23
Het df	32	15	48
Het P	***	***	***
Fixed RR	0.30	0.33	0.32
RRl	0.28	0.30	0.30
RRu	0.33	0.36	0.34
P	---	---	---
Random RR	0.28	0.27	0.27
RRl	0.23	0.21	0.24
RRu	0.33	0.35	0.32
P	---	---	---
Between Chi			2.24
Between df			1
Between P			N.S.
Btwn(F) P			N.S.
Btwn(R) P			N.S.

	Number of adjustment variables (1) 0	1	2+/+nk	Total
N	25	15	9	49
NS	18	10	5	33
Wt	595.76	283.86	176.63	1056.25
Het Chi	114.86	43.63	20.59	202.23
Het df	24	14	8	48
Het P	***	***	**	***
Fixed RR	0.35	0.32	0.23	0.32
RRl	0.32	0.28	0.20	0.30
RRu	0.38	0.36	0.27	0.34
P	---	---	---	---
Random RR	0.29	0.27	0.25	0.27
RRl	0.23	0.21	0.19	0.24
RRu	0.35	0.35	0.34	0.32
P	---	---	---	---
Between Chi				23.15
Between df				2
Between P				***
Btwn(F) P				(*)
Btwn(R) P				N.S.

Table 1K9 - 3

IESLC - Meta-analysis of Ex Smoking, Years quit (vs current), "High"
All LC types, Cigarettes (or Any Product if Cigarettes not available)
Most adjusted

	Number of adjustment variables (2) 0	1	2	3-5	6+/+nk	Total
N	25	15	4	5		49
NS	18	10	3	2		33
Wt	595.76	283.86	34.59	142.05		1056.25
Het Chi	114.86	43.63	6.49	9.78		202.23
Het df	24	14	3	4		48
Het P	***	***	(*)	*		***
Fixed RR	0.35	0.32	0.32	0.21		0.32
RRl	0.32	0.28	0.23	0.18		0.30
RRu	0.38	0.36	0.44	0.25		0.34
P	---	---	---	---		---
Random RR	0.29	0.27	0.33	0.21		0.27
RRl	0.23	0.21	0.20	0.15		0.24
RRu	0.35	0.35	0.54	0.30		0.32
P	---	---	---	---		---
Between Chi						27.46
Between df						3
Between P						***
Btwn(F) P						(*)
Btwn(R) P						N.S.

	Product all/unsp	cig+/-ot	cig only	Total
N	10	28	11	49
NS	7	19	9	35
Wt	134.12	678.60	243.53	1056.25
Het Chi	18.50	119.99	46.57	202.23
Het df	9	27	10	48
Het P	*	***	***	***
Fixed RR	0.23	0.34	0.31	0.32
RRl	0.20	0.32	0.27	0.30
RRu	0.27	0.37	0.35	0.34
P	---	---	---	---
Random RR	0.19	0.31	0.25	0.27
RRl	0.14	0.26	0.18	0.24
RRu	0.26	0.38	0.35	0.32
P	---	---	---	---
Between Chi				17.16
Between df				2
Between P				***
Btwn(F) P				N.S.
Btwn(R) P				*

	Denominator current	cur+rec	Total
N	28	21	49
NS	20	13	33
Wt	675.25	381.01	1056.25
Het Chi	124.87	58.50	202.23
Het df	27	20	48
Het P	***	***	***
Fixed RR	0.35	0.27	0.32
RRl	0.32	0.24	0.30
RRu	0.38	0.29	0.34
P	---	---	---
Random RR	0.27	0.27	0.27
RRl	0.23	0.23	0.24
RRu	0.33	0.33	0.32
P	---	---	---
Between Chi			18.86
Between df			1
Between P			***
Btwn(F) P			*
Btwn(R) P			N.S.

Table 1K9 - 3

IESLC - Meta-analysis of Ex Smoking, Years quit (vs current), "High"
All LC types, Cigarettes (or Any Product if Cigarettes not available)
Most adjusted

		Derivation of RR/CI			
		Orig	StdCalc	Other	Total
	N	7	26	16	49
	NS	4	19	11	34
	Wt	162.77	599.41	294.07	1056.25
Het	Chi	11.20	115.26	48.11	202.23
Het	df	6	25	15	48
Het	P	(*)	***	***	***
Fixed	RR	0.22	0.35	0.32	0.32
	RRl	0.19	0.32	0.29	0.30
	RRu	0.25	0.38	0.36	0.34
	P	---	---	---	---
Random	RR	0.22	0.28	0.28	0.27
	RRl	0.17	0.23	0.22	0.24
	RRu	0.29	0.35	0.37	0.32
	P	---	---	---	---
Between	Chi				27.66
Between	df				2
Between	P				***
Btwn(F)	P				*
Btwn(R)	P				N.S.

Table 1K9 - 4

IESLC - Meta-analysis of Ex Smoking, Years quit (vs current), "High"
All LC types, Cigarettes (or Any Product if Cigarettes not available)
Least adjusted

REF	NRR	X	SEX	AGEL	AGEH	RACE	YF	LC TYPE	LOC	START	ST	NLC	R	VB	P	H	AD	ADOS	PRODUCT	exL	exH	DENOM	De
ALDERS	515		m	0	0	all	-	all	Eu:UK	1977	CC	1448	n	V	n	n	1	0	cig only	10	999	current	ot
ALDERS	526		f	0	0	all	-	all	Eu:UK	1977	CC	1448	n	V	n	n	1	0	cig only	10	999	current	ot
BECHER	508		m	0	0	all	-	all	Eu:Ger	1985	CC	194	n	bl	n	y	0	0	all/unsp	10	999	cur+1y	st
BECHER	518		f	0	0	all	-	all	Eu:Ger	1985	CC	194	n	bl	n	y	0	0	all/unsp	10	999	cur+1y	st
CARPEN	510		c	0	0	w+b	-	all	NAmer	1991	CC	356	n	bl	n	n	0	0	cig+/-ot	10	14	current	st
CEDERL	539		m	40	69	all	10	all	Eu:Sca	1963	pr	491	n	bl	n	n	1	0	all/unsp	10	999	current	ot
CHOI	545		m	0	0	all	-	all	As:oth	1985	CC	375	n	bl	n	n	0	0	cig+/-ot	10	14	current	st
CPSI	818		m	50	74	all	6	all	NAmer	1959	pr	5138	n	bl	n	n	1	0	cig only	10	999	current	ot
CPSII	668		m	35	99	all	4	all	NAmer	1982	pr	3229	n	bl	n	n	1	0	cig only	11	15	current	ot
CPSII	645		f	0	0	all	4	all	NAmer	1982	pr	3229	n	bl	n	n	1	0	cig+/-ot	11	15	current	ot
DAMBER	558		m	0	0	all	-	all	Eu:Sca	1972	CC	579	n	bl	y	n	1	0	cig only	11	999	current	ot
DARBY	508		m	0	0	wh	-	all	Eu:UK	1988	CC	982	n	V	n	n	0	0	all/unsp	10	999	current	st
DARBY	517		f	0	0	wh	-	all	Eu:UK	1988	CC	982	n	V	n	n	0	0	all/unsp	10	999	current	st
DEAN3	511	x	m	0	0	all	-	all	Eu:UK	1969	CC	766	n	V	y	n	0	0	cig only	9	18	cur+2y	st
DEAN3	550	x	f	0	0	all	-	all	Eu:UK	1969	CC	766	n	V	y	n	0	0	all/unsp	9	999	cur+2y	st
DESTEF	545		m	0	0	all	-	all	SCAmer	1988	CC	497	n	bl	n	y	0	0	cig+/-ot	10	999	current	st
DOLL	538		m	0	0	all	-	all	Eu:UK	1948	CC	1465	n	V	n	n	0	0	all/unsp	10	19	current	st
DOLL	549		f	0	0	all	-	all	Eu:UK	1948	CC	1465	n	V	n	n	0	0	all/unsp	10	999	current	st
DOLL2	511		m	0	0	all	20	all	Eu:UK	1951	pr	920	n	V	n	n	1	0	cig only	10	14	current	ot
DORGAN	516		m	0	0	wh	-	all	NAmer	1980	CC	2026	n	bl	y	y	0	0	cig+/-ot	10	999	cur+1y	st
DORGAN	560		f	0	0	all	-	all	NAmer	1980	CC	2026	n	bl	y	y	0	0	cig+/-ot	10	999	cur+1y	st
DORN	825		m	55	64	wh	8	all	NAmer	1954	pr	5097	n	bl	n	n	0	0	cig+/-ot	10	14	current	st
DORN	829		m	65	74	wh	8	all	NAmer	1954	pr	5097	n	bl	n	n	0	0	cig+/-ot	10	14	current	st
GAO	528	x	m	0	0	all	-	all	As:Chi	1984	CC	1405	n	ot	n	n	0	0	cig+/-ot	10	999	current	st
GAO	548	x	f	0	0	all	-	all	As:Chi	1984	CC	1405	n	ot	n	n	0	0	cig+/-ot	10	999	current	st
GAO2	520		m	0	0	all	-	all	As:Jap	1988	CC	282	n	bl	n	n	0	0	cig+/-ot	10	14	cur+1y	st
GRAHAM	511		m	0	0	wh	-	all	NAmer	1956	CC	685	n	bl	n	n	0	0	cig only	10	999	current	st
HAMMO2	512		m	0	0	all	0	all	NAmer	1967	pr	450	o	bl	n	n	1	0	cig+/-ot	10	999	current	ot
HIRAYA	515		m	0	0	all	0	all	As:Jap	1965	pr	1917	n	bl	n	n	1	0	cig+/-ot	10	999	current	ot
HIRAYA	526		f	0	0	all	0	all	As:Jap	1965	pr	1917	n	bl	n	n	1	0	cig+/-ot	10	999	current	ot
JAHN	517		m	0	0	all	-	all	Eu:Ger	1988	CC	1004	n	bl	n	n	0	0	cig+/-ot	11	20	current	st
JAIN	571		m	0	0	all	-	all	NAmer	1981	CC	845	n	V	y	n	0	0	cig+/-ot	10	999	cur+2y	st
JAIN	535		f	0	0	all	-	all	NAmer	1981	CC	845	n	V	y	n	0	0	cig+/-ot	10	999	cur+2y	st
KAISE2	656		m	0	0	all	9	all	NAmer	1979	pr	318	n	bl	n	n	1	0	cig only	11	20	cur+2y	ot
KAISE2	576		f	0	0	all	9	all	NAmer	1979	pr	318	n	bl	n	n	1	0	cig only	11	20	cur+2y	st
LUBIN	594		m	0	0	all	-	all	As:Chi	1984	CC	427	m	ot	y	n	0	0	cig+/-ot	10	999	cur+2y	st
LUBIN2	1083		m	0	0	all	-	all	Eu:mul	1976	CC	7804	n	bl	n	y	0	0	cig+/-ot	10	14	current	st
LUBIN2	1122		f	0	0	all	-	all	Eu:mul	1976	CC	7804	n	bl	n	y	0	0	cig+/-ot	10	14	current	st
MATOS	588	x	m	0	0	all	-	all	SCAmer	1994	CC	200	n	bl	n	n	0	0	cig+/-ot	11	999	cur+1y	st
PEZZO2	505		m	0	0	all	-	all	SCAmer	1992	CC	367	n	bl	n	y	0	0	cig+/-ot	11	999	cur+1y	st
PEZZOT	505		m	0	0	all	-	all	SCAmer	1987	CC	215	n	bl	n	y	0	0	cig only	11	999	cur+1y	st
SOBUE	730		m	0	0	all	-	all	As:Jap	1986	CC	1376	n	bl	n	y	0	0	cig+/-ot	10	14	cur+1y	st
SPEIZE	514		f	0	0	all	0	all	NAmer	1976	pr	593	n	bl	n	y	2	0	cig+/-ot	10	15	current	or
SUZUK2	515	x	c	0	0	all	-	all	SCAmer	1991	CC	123	n	bl	n	y	0	0	all/unsp	11	999	current	st
SVENSS	555		f	0	0	all	-	all	Eu:Sca	1983	CC	210	n	bl	n	n	0	0	all/unsp	11	999	cur+2y	st
WYNDE6	720		m	0	0	wh	-	all	NAmer	1969	CC	4423	n	bl	n	y	5	1#	cig+/-ot	11	19	cur+1y	or
WYNDE6	727		m	0	0	bl	-	all	NAmer	1969	CC	4423	n	bl	n	y	5	1#	cig+/-ot	11	19	cur+1y	or
WYNDE6	734		f	0	0	wh	-	all	NAmer	1969	CC	4423	n	bl	n	y	5	1#	cig+/-ot	11	999	cur+1y	or
WYNDE6	738		f	0	0	bl	-	all	NAmer	1969	CC	4423	n	bl	n	y	5	1#	cig+/-ot	11	999	cur+1y	or

Comments on values in listings

WYNDE6 ADOS Number of cigs/day
WYNDE6 ADOS Number of cigs/day
WYNDE6 ADOS Number of cigs/day
WYNDE6 ADOS Number of cigs/day

Cigarette type is all/unspec for all RRs
except for the following:

REF	NRR	CIGTYPE
ALDERS	515	MC only
ALDERS	526	MC only
DEAN3	511	MC only
DESTEF	545	MC only

Table 1K9 - 5

IESLC - Meta-analysis of Ex Smoking, Years quit (vs current), "High"
All LC types, Cigarettes (or Any Product if Cigarettes not available)
Least adjusted

REF	NRR	SEX	AD	Number Exposed Case	Cont	Non-exposed Case	Cont	RR	95.00%CI
ALDERS	515	m	1	29	-	207	-	0.32	(0.20- 0.51)
ALDERS	526	f	1	26	-	244	-	0.28	(0.17- 0.46)
Subtotal ALDERS								0.30	(0.21- 0.42)
BECHER	508	m	0	16	72	101	122	0.27	(0.15- 0.49)
BECHER	518	f	0	1	10	33	26	0.08	(0.01- 0.66)
Subtotal BECHER								0.24	(0.14- 0.44)
CARPEN	510	c	0	13	58	228	164	0.16	(0.09- 0.30)
*CEDERL	539	m	1	3	-	97	-	0.14	(0.04- 0.45)
CHOI	545	m	0	4	23	231	329	0.25	(0.08- 0.73)
*CPSI	818	m	1	15	-	844	-	0.09	(0.06- 0.15)
*CPSII	668	m	1	164	-	1159	-	0.39	(0.33- 0.46)
*CPSII	645	f	1	28	-	530	-	0.31	(0.21- 0.46)
Subtotal CPSII								0.38	(0.32- 0.44)
DAMBER	558	m	1	-	-	-	-	0.16	(0.08- 0.35)
DARBY	508	m	0	139	767	379	618	0.30	(0.24- 0.37)
DARBY	517	f	0	26	224	198	231	0.14	(0.09- 0.21)
Subtotal DARBY								0.25	(0.21- 0.31)
DEAN3	511	m	0	15	86	337	930	0.48	(0.27- 0.84)
DEAN3	550	f	0	2	114	102	1158	0.20	(0.05- 0.82)
Subtotal DEAN3								0.43	(0.25- 0.72)
DESTEF	545	m	0	10	41	78	109	0.34	(0.16- 0.72)
DOLL	538	m	0	6	26	1280	1172	0.21	(0.09- 0.52)
DOLL	549	f	0	1	2	58	41	0.35	(0.03- 4.03)
Subtotal DOLL								0.22	(0.10- 0.52)
*DOLL2	511	m	1	9	-	236	-	0.28	(0.14- 0.54)
DORGAN	516	m	0	134	255	465	303	0.34	(0.27- 0.44)
DORGAN	560	f	0	34	50	289	112	0.26	(0.16- 0.43)
Subtotal DORGAN								0.32	(0.26- 0.41)
*DORN	825	m	0	12	23682	528	334175	0.32	(0.18- 0.57)
*DORN	829	m	0	29	20056	537	207895	0.56	(0.39- 0.81)
Subtotal DORN								0.47	(0.35- 0.65)
GAO	528	m	0	13	41	529	438	0.26	(0.14- 0.50)
GAO	548	f	0	16	14	170	100	0.67	(0.31- 1.44)
Subtotal GAO								0.39	(0.24- 0.63)
GAO2	520	m	0	16	18	184	117	0.57	(0.28- 1.15)
GRAHAM	511	m	0	2	30	371	821	0.15	(0.04- 0.62)
*HAMMO2	512	m	1	20	-	209	-	0.34	(0.22- 0.53)
*HIRAYA	515	m	1	-	-	-	-	0.31	(0.14- 0.69)
*HIRAYA	526	f	1	-	-	-	-	0.41	(0.01- 14.37)
Subtotal HIRAYA								0.31	(0.14- 0.68)
JAHN	517	m	0	64	130	352	269	0.38	(0.27- 0.53)
JAIN	571	m	0	52	113	265	118	0.20	(0.14- 0.30)
JAIN	535	f	0	19	61	305	99	0.10	(0.06- 0.18)
Subtotal JAIN								0.16	(0.12- 0.22)
*KAISE2	656	m	1	8	-	51	-	0.43	(0.20- 0.92)
*KAISE2	576	f	1	4	-	50	-	0.25	(0.09- 0.70)
Subtotal KAISE2								0.35	(0.19- 0.65)
LUBIN	594	m	0	17	73	296	650	0.51	(0.30- 0.88)
LUBIN2	1083	m	0	270	693	4684	6211	0.52	(0.45- 0.60)
LUBIN2	1122	f	0	10	26	440	410	0.36	(0.17- 0.75)
Subtotal LUBIN2								0.51	(0.44- 0.59)
MATOS	588	m	0	27	101	112	132	0.32	(0.19- 0.52)
PEZZO2	505	m	0	43	161	233	198	0.23	(0.15- 0.33)
PEZZOT	505	m	0	20	106	145	129	0.17	(0.10- 0.29)
SOBUE	730	m	0	35	50	737	633	0.60	(0.39- 0.94)
*SPEIZE	514	f	2	17	-	319	-	0.20	(0.10- 0.40)
SUZUK2	515	c	0	9	22	77	30	0.16	(0.07- 0.39)
SVENSS	555	f	0	14	24	142	53	0.22	(0.10- 0.45)
WYNDE6	720	m	5	-	-	-	-	0.30	(0.20- 0.40)
WYNDE6	727	m	5	-	-	-	-	0.20	(0.10- 0.50)
WYNDE6	734	f	5	-	-	-	-	0.20	(0.20- 0.30)
WYNDE6	738	f	5	-	-	-	-	0.40	(0.10- 1.10)
Subtotal WYNDE6								0.22	(0.19- 0.26)
Partial Totals				1392	47129	17832	557793		

*prospective study

Table 1K9 - 5

IESLC - Meta-analysis of Ex Smoking, Years quit (vs current), "High"
All LC types, Cigarettes (or Any Product if Cigarettes not available)
Least adjusted

REF	NRR	SEX	AD	Ys	Ws	Qs	Ps
ALDERS	515	m	1	-1.14	17.54	0.00	0.0000
ALDERS	526	f	1	-1.27	15.51	0.27	0.0000
Subtotal ALDERS				-1.20	33.04	0.27	
BECHER	508	m	0	-1.32	10.58	0.32	0.0000
BECHER	518	f	0	-2.54	0.86	1.67	0.0188
Subtotal BECHER				-1.41	11.44	1.99	
CARPEN	510	c	0	-1.82	9.56	4.46	0.0000
*CEDERL	539	m	1	-1.97	2.62	1.78	0.0015
CHOI	545	m	0	-1.40	3.32	0.21	0.0109
*CPSI	818	m	1	-2.41	18.30	29.33	0.0000
*CPSII	668	m	1	-0.94	139.29	5.59	0.0000
*CPSII	645	f	1	-1.17	24.99	0.02	0.0000
Subtotal CPSII				-0.98	164.28	5.61	
DAMBER	558	m	1	-1.83	7.05	3.36	0.0000
DARBY	508	m	0	-1.22	78.40	0.47	0.0000
DARBY	517	f	0	-2.00	19.12	14.06	0.0000
Subtotal DARBY				-1.37	97.52	14.52	
DEAN3	511	m	0	-0.73	12.15	2.05	0.0108
DEAN3	550	f	0	-1.61	1.93	0.43	0.0252
Subtotal DEAN3				-0.85	14.07	2.48	
DESTEF	545	m	0	-1.08	6.83	0.03	0.0049
DOLL	538	m	0	-1.55	4.84	0.82	0.0006
DOLL	549	f	0	-1.04	0.65	0.01	0.4022
Subtotal DOLL				-1.49	5.49	0.83	
*DOLL2	511	m	1	-1.27	8.43	0.14	0.0002
DORGAN	516	m	0	-1.07	59.40	0.29	0.0000
DORGAN	560	f	0	-1.33	16.18	0.59	0.0000
Subtotal DORGAN				-1.13	75.58	0.89	
*DORN	825	m	0	-1.14	11.74	0.00	0.0001
*DORN	829	m	0	-0.58	27.56	8.70	0.0023
Subtotal DORN				-0.75	39.30	8.70	
GAO	528	m	0	-1.34	9.48	0.36	0.0000
GAO	548	f	0	-0.40	6.68	3.70	0.3049
Subtotal GAO				-0.95	16.15	4.07	
GAO2	520	m	0	-0.57	7.57	2.47	0.1164
GRAHAM	511	m	0	-1.91	1.86	1.11	0.0090
*HAMMO2	512	m	1	-1.08	19.88	0.08	0.0000
*HIRAYA	515	m	1	-1.17	6.04	0.01	0.0040
*HIRAYA	526	f	1	-0.89	0.29	0.02	0.6307
Subtotal HIRAYA				-1.16	6.33	0.02	
JAHN	517	m	0	-0.98	33.47	0.90	0.0000
JAIN	571	m	0	-1.59	24.80	4.87	0.0000
JAIN	535	f	0	-2.29	12.14	16.04	0.0000
Subtotal JAIN				-1.82	36.93	20.91	
*KAISE2	656	m	1	-0.84	6.60	0.59	0.0302
*KAISE2	576	f	1	-1.39	3.65	0.22	0.0081
Subtotal KAISE2				-1.04	10.25	0.80	
LUBIN	594	m	0	-0.67	12.91	2.87	0.0160
LUBIN2	1083	m	0	-0.66	181.12	41.99	0.0000
LUBIN2	1122	f	0	-1.03	6.98	0.09	0.0067
Subtotal LUBIN2				-0.67	188.10	42.09	
MATOS	588	m	0	-1.15	15.76	0.00	0.0000
PEZZO2	505	m	0	-1.48	25.77	3.00	0.0000
PEZZOT	505	m	0	-1.78	13.50	5.58	0.0000
SOBUE	730	m	0	-0.51	19.41	7.78	0.0250
*SPEIZE	514	f	2	-1.61	8.00	1.75	0.0000
SUZUK2	515	c	0	-1.84	4.93	2.38	0.0000
SVENSS	555	f	0	-1.52	7.19	1.05	0.0000
WYNDE6	720	m	5	-1.20	31.98	0.12	0.0000
WYNDE6	727	m	5	-1.61	5.93	1.30	0.0001
WYNDE6	734	f	5	-1.61	93.46	20.43	0.0000
WYNDE6	738	f	5	-0.92	2.67	0.14	0.1342
Subtotal WYNDE6				-1.50	134.05	21.98	

Table 1K9 - 5

IESLC - Meta-analysis of Ex Smoking, Years quit (vs current), "High"
All LC types, Cigarettes (or Any Product if Cigarettes not available)
Least adjusted

	N	49
	NS	33
	Wt	1058.92
Het	Chi	193.45
Het	df	48
Het	P	***
Fixed	RR	0.32
	RRl	0.30
	RRu	0.34
	P	---
Random	RR	0.28
	RRl	0.24
	RRu	0.32
	P	---
Asymm	P	*

Table 1K9 - 6

IESLC - Meta-analysis of Ex Smoking, Years quit (vs current), "High"
All LC types, Cigarettes (or Any Product if Cigarettes not available)
Least adjusted

	Sex			
	combined	male	female	Total
N	2	31	16	49
NS	2	29	15	46
Wt	14.48	824.14	220.29	1058.92
Het Chi	0.00	115.68	29.31	193.45
Het df	1	30	15	48
Het P	N.S.	***	*	***
Fixed RR	0.16	0.36	0.22	0.32
RRl	0.10	0.33	0.19	0.30
RRu	0.27	0.38	0.25	0.34
P	---	---	---	---
Random RR	0.16	0.31	0.23	0.28
RRl	0.10	0.26	0.18	0.24
RRu	0.27	0.36	0.29	0.32
P	---	---	---	---
Between Chi				48.46
Between df				2
Between P				***
Btwn(F) P				**
Btwn(R) P				*

Table 1K9 - 7

IESLC - Meta-analysis of Ex Smoking, Years quit (vs current), "High"
All LC types, Cigarettes (or Any Product if Cigarettes not available)
Excluded studies (and stage at which they were excluded)

1	AGUDO AKIBA AMANDU AMES AXELSS BEST BOUCHA BOUCOT BRESLO CHEN CHEN2 CHIAZZ DEAN2 DOSEME ENGELA FAN GENG GER GUO HAENSZ HEGMAN HOLE HU HU2 JUSSAW KATSOU KAUFMA KOO KOULUM KREUZE LETOUR LEVIN LIAW LIU3 LIU4 LIU5 MCCONN MIGRAN MRFITR NOTAN2 OSANN2 PERNU QIAO2 RACHTA RESTRE SADOWS SEGI2 STASZE TIZZAN VUTUC WATSON WU WUWILL WYNDE2 WYNDE8 XU YUAN ZHANG ZHENG ZHOU
2	AUVINE BENSHL BLOT1 BROWN3 BUFFLE GURSEL LAUSSM LUO MCDUFF PISANI PRESCO SPITZ WU2 WYNDE7
4	HAMMON
5	CORREA GILLIS HUMBLE QIAO WIGLE
7	BOFFET
10	GARSHI JEDRYC WAKAI
14	ARMADA BARBON BROSS CHYOU GARCIA JOLY KHUDER TVERDA WANG2 WYNDE3
15	BENHAM

Table 1K9 - 8
Potentially overlapping studies

REF	REFGP	PRINC	.	OVERLAP/LINK
LUBIN2	LUBIN2	1		Lubin-combined
GRAHAM	BYERS1	1		GRAHAM/BROSS/BYERS1
WYNDE6	WYNDE6	1		WYNDE5/6/7/8
CPSI	CPSI	1		CPSI overall
JAHN	BOFFET	2		Subset of BOFFET
LUBIN	XIANGZ	2		LUBIN/XIANGZ/QIAO

Table 1K9 - 9
Most adjusted - insufficient data for meta-analysis

REF	NRR	SEX	AGEL	AGEH	RACE	YF	LC TYPE	LOC	START	ST	NLC	R	VB	P	H	AD	ADOS	PRODUCT	exL	exH	DENOM	De
HUMBLE	561	c	25	64	wh	-	not alv	NAmer	1980	CC	521	n	bl	y	n	2		2#cig+/-ot	11	20	cur+1y	or
HUMBLE	556	c	65	84	wh	-	not alv	NAmer	1980	CC	521	n	bl	y	n	2		2#cig+/-ot	11	20	cur+1y	or
WIGLE	503	m	0	0	all	-	all	NAmer	1971	CC	728	n	V	n	n	2		1#cig+/-ot	10	14	current	ot
WIGLE	507	f	0	0	all	-	all	NAmer	1971	CC	728	n	V	n	n	2		1#cig+/-ot	10	14	current	ot

Comments on values in listings

HUMBLE	ADOS	Number of cigarettes and duration
HUMBLE	ADOS	Number of cigarettes and duration
WIGLE	ADOS	Cumulative exposure
WIGLE	ADOS	Cumulative exposure

REF	NRR	RR	SIG	RRDATA comment
HUMBLE	561	0.06		0
HUMBLE	556	0.29		0
WIGLE	503	0.70	n	0
WIGLE	507	0.50	n	0

Table 1K10 -

IESLC - Meta-analysis of Ex Smoking, Years quit (vs current), "Highest vs lowest"
All LC types, Cigarettes (or Any Product if Cigarettes not available)

This analysis is restricted to results for:
1) Ex smokers
2) Results by Years quit (vs current)
3) Categorical results by Years quit (vs current)
4) Denominator (unexposed) = "low"
5) All LC types (or near equivalent)
6) Results complete enough for use in metaanalysis

Within each study, results are then selected (in the following order of preference, within each sex) for:
7) (not applicable)
8) PRODUCT: cigarettes regardless of other products, cigarettes only, all/unspec
9) CIGTYPE: all/unspecified, MC regardless of HR, MC only
10) Results with least adjustment for other aspects of smoking (ADOS)
11) The highest vs lowest category
12) Followup period (YF, prospective studies): whole study (coded as 0) or longest available
13) LCtype: all or nearest available, at least Squamous and Adeno. (q = squamous, s = small,
l = large, a = adeno, mix = mixed, alv = alveolar)
14) Race: all or nearest available, otherwise by race (wh or w = white, bl or b = black, hi = hispanic
ch = chinese, jap = japanese, haw = hawaiian, w+o = white + oriental, sca = scandinavian, as = asian)
15) For overlapping studies: principal rather than subsidiary studies
Finally by Age: whole study (coded as 0) if available, otherwise by widest available age group
and then for single sex results (m, f) in preference to results for both sexes combined (c).

Results adjusted (AD) for the most potential confounders are then chosen in Sections -1 to -3
(and those which actually differ from the adjusted results in Table 1K5 - 1 are marked 'x' in Section -1)
and results adjusted for the least confounders in Sections -4 to -6. (Those least adjusted results which
actually differ from the most adjusted are marked 'x' in column X in Section -4)

Section -7 shows excluded studies, together with the stage (as above) at which no qualifying
results were found.

Section -8 lists the potentially overlapping studies which have been included (1=principal, 2=subsidiary).

Section -9 lists any results which would have been included in preference except that they had data not complete
enough for use in meta-analysis, with their significance (yes/no), if known, and any further comment as entered
on the database. It also lists as "gap" any categories for which no data were presented by the original authors.

In addition to those mentioned above, the following fields, levels and abbreviations are used:
* or nk = not known, n = no, y = yes, ot = other
all/unspec = all or unspecified, cig+/-ot = cigarettes irrespective of other products (cigar, pipe etc)
MC = manufactured cigarettes, HR = hand-rolled cigarettes
exL, exH = range of exposure (low and high) in the "highest" group, in terms of Years quit (vs current)
unexL, unexH = range of exposure (low and high) in the "lowest" group, in terms of Years quit (vs current)
REF: 6-character study reference
NRR: number of the RR on the database within the study
ST : study type (CC = case control, pr or prosp = prospective)
NLC: number of lung cancer cases in whole study
R : risky occupational population (n = no, m = mining, o = other risky)
VB : national cigarette type (V = at least 75% Virginia, bl = at least 75% blended, ot = other)
P : any proxy use
H : full histological confirmation
De : derivation of RR/CI (or = original, st = standard method, ot = other method of estimation)

Table 1K10 - 1

IESLC - Meta-analysis of Ex Smoking, Years quit (vs current), "Highest vs lowest"
All LC types, Cigarettes (or Any Product if Cigarettes not available)
Most adjusted

REF	NRR	1K5	SEX	AGEL	AGEH	RACE	YF	LC TYPE	LOC	START	ST	NLC	R	VB	P	H	AD	ADOS	PRODUCT	exL	exH	unexL	unexH	De
ALDERS	517		m	0	0	all	-	all	Eu:UK	1977	CC	1448	n	V	n	n	1	0	cig only	10	999	0.1	2	ot
ALDERS	528		f	0	0	all	-	all	Eu:UK	1977	CC	1448	n	V	n	n	1	0	cig only	10	999	0.1	2	ot
ARMADA	520		m	0	0	all	-	all	Eu:wst	1986	CC	325	n	bl	n	y	0	0	cig+/-ot	6	999	1.0	5	st
BARBON	554		m	0	0	all	-	all	Eu:wst	1979	CC	755	n	bl	y	y	1	0	all/unsp	25	999	0.1	4	ot
BECHER	510		m	0	0	all	-	all	Eu:Ger	1985	CC	194	n	bl	n	y	0	0	all/unsp	10	999	2	4	st
BECHER	520		f	0	0	all	-	all	Eu:Ger	1985	CC	194	n	bl	n	y	0	0	all/unsp	10	999	2	4	st
BROSS	520		m	0	0	wh	-	all	NAmer	1960	CC	974	n	bl	n	n	0	0	cig+/-ot	6	999	0.1	5	st
CARPEN	514		c	0	0	w+b	-	all	NAmer	1991	CC	356	n	bl	n	n	0	0	cig+/-ot	15	999	0.1	4	st
CEDERL	540		m	40	69	all	10	all	Eu:Sca	1963	pr	491	n	bl	n	n	1	0	all/unsp	10	999	0.1	9	ot
CHOI	549		m	0	0	all	-	all	As:oth	1985	CC	375	n	bl	n	n	0	0	cig+/-ot	15	999	0.1	4	st
CHOI	558		f	0	0	all	-	all	As:oth	1985	CC	375	n	bl	n	n	0	0	cig+/-ot	5	999	0.1	4	ot
CHYOU	512		m	0	0	jap	21	all	NAmer	1965	pr	227	n	bl	n	y	2	0	cig+/-ot	15	999	0.1	14	ot
CPSI	821		m	50	74	all	6	all	NAmer	1959	pr	5138	n	bl	n	n	1	0	cig only	10	999	0.1	0.9	ot
CPSII	674		m	35	99	all	4	all	NAmer	1982	pr	3229	n	bl	n	n	1	0	cig only	16	999	0.1	0.9	ot
CPSII	650		f	0	0	all	4	all	NAmer	1982	pr	3229	n	bl	n	n	1	0	cig+/-ot	16	999	0.1	2	ot
DAMBER	559	x	m	0	0	all	-	all	Eu:Sca	1972	CC	579	n	bl	y	n	1	0	cig only	11	999	0.1	10	ot
DARBY	506		m	0	0	wh	-	all	Eu:UK	1988	CC	982	n	V	n	n	0	0	all/unsp	10	999	0.1	9	st
DARBY	515		f	0	0	wh	-	all	Eu:UK	1988	CC	982	n	V	n	n	0	0	all/unsp	10	999	0.1	9	st
DEAN3	530	x	m	0	0	all	-	all	Eu:UK	1969	CC	766	n	V	y	n	1	0	cig only	19	999	3	4	ot
DEAN3	563		f	0	0	all	-	all	Eu:UK	1969	CC	766	n	V	y	n	1	0	all/unsp	9	999	3	4	ot
DESTEF	547	x	m	0	0	all	-	all	SCAmer	1988	CC	497	n	bl	n	y	0	0	cig+/-ot	10	999	0.1	4	st
DOLL	541		m	0	0	all	-	all	Eu:UK	1948	CC	1465	n	V	n	n	0	0	all/unsp	20	999	0.1	9	st
DOLL	550		f	0	0	all	-	all	Eu:UK	1948	CC	1465	n	V	n	n	0	0	all/unsp	10	999	0.1	9	st
DOLL2	515		m	0	0	all	20	all	Eu:UK	1951	pr	920	n	V	n	n	1	0	cig only	15	999	0.1	4	ot
DORGAN	518		m	0	0	wh	-	all	NAmer	1980	CC	2026	n	bl	y	y	0	0	cig+/-ot	10	999	1	5	st
DORGAN	561		f	0	0	all	-	all	NAmer	1980	CC	2026	n	bl	y	y	0	0	cig+/-ot	10	999	1	9	st
DORN	667		m	55	64	wh	8	all	NAmer	1954	pr	5097	n	bl	n	n	0	0	cig+/-ot	15	999	0.1	4	st
DORN	690		m	65	74	wh	8	all	NAmer	1954	pr	5097	n	bl	n	n	0	0	cig+/-ot	15	999	0.1	4	st
GAO	540		m	0	0	all	-	all	As:Chi	1984	CC	1405	n	ot	n	n	2	0	cig+/-ot	10	999	0.1	4	ot
GAO	560		f	0	0	all	-	all	As:Chi	1984	CC	1405	n	ot	n	n	2	0	cig+/-ot	10	999	0.1	4	ot
GAO2	526		m	0	0	all	-	all	As:Jap	1988	CC	282	n	bl	n	n	0	0	cig+/-ot	20	999	1.0	4	st
GARCIA	514		c	0	0	all	-	all	NAmer	1992	CC	416	n	bl	n	y	0	0	cig+/-ot	30	999	1.0	4	st
GRAHAM	544		m	0	0	wh	-	all	NAmer	1956	CC	685	n	bl	n	n	1	0	cig+/-ot	5	999	0.1	1.0	ot
HAMMO2	514		m	0	0	all	0	all	NAmer	1967	pr	450	o	bl	n	n	1	0	cig+/-ot	10	999	0.1	4	ot
HIRAYA	517		m	0	0	all	0	all	As:Jap	1965	pr	1917	n	bl	n	n	1	0	cig+/-ot	10	999	0.1	4	ot
HIRAYA	528		f	0	0	all	0	all	As:Jap	1965	pr	1917	n	bl	n	n	1	0	cig+/-ot	10	999	0.1	4	ot
JAHN	523		m	0	0	all	-	all	Eu:Ger	1988	CC	1004	n	bl	n	n	0	0	cig+/-ot	21	999	0.1	0.9	st
JAIN	572		m	0	0	all	-	all	NAmer	1981	CC	845	n	V	y	n	0	0	cig+/-ot	10	999	2	9	st
JAIN	536		f	0	0	all	-	all	NAmer	1981	CC	845	n	V	y	n	0	0	cig+/-ot	10	999	2	9	st
JOLY	573		m	0	0	all	-	all	SCAmer	1978	CC	826	n	bl	n	n	0	0	cig+/-ot	5	999	1.0	4	st
JOLY	560		f	0	0	all	-	all	SCAmer	1978	CC	826	n	bl	n	n	0	0	cig+/-ot	5	999	1.0	4	st
KAISE2	660		m	0	0	all	9	all	NAmer	1979	pr	318	n	bl	n	n	1	0	cig only	21	999	2	10	st
KAISE2	580		f	0	0	all	9	all	NAmer	1979	pr	318	n	bl	n	n	1	0	cig only	21	999	2	10	ot
KHUDER	520		m	0	0	all	-	all	NAmer	1985	CC	482	n	bl	n	y	0	0	cig+/-ot	15	999	0.1	4	st
LUBIN	596		m	0	0	all	-	all	As:Chi	1984	CC	427	m	ot	y	n	0	0	cig+/-ot	10	999	3	4	st
LUBIN2	1091		m	0	0	all	-	all	Eu:mul	1976	CC	7804	n	bl	n	y	0	0	cig+/-ot	25	999	0.1	4	st
LUBIN2	1130		f	0	0	all	-	all	Eu:mul	1976	CC	7804	n	bl	n	y	0	0	cig+/-ot	25	999	0.1	4	st
MATOS	600		m	0	0	all	-	all	SCAmer	1994	CC	200	n	bl	n	n	2	0	cig+/-ot	11	999	1.0	5	ot
PEZZO2	506		m	0	0	all	-	all	SCAmer	1992	CC	367	n	bl	n	y	0	0	cig+/-ot	11	999	1.0	10	st
PEZZOT	506		m	0	0	all	-	all	SCAmer	1987	CC	215	n	bl	n	y	0	0	cig only	11	999	1.0	10	st
SOBUE	738		m	0	0	all	-	all	As:Jap	1986	CC	1376	n	bl	n	y	0	0	cig+/-ot	25	999	1.0	4	st
SPEIZE	519		f	0	0	all	0	all	NAmer	1976	pr	593	n	bl	n	y	2	0	cig+/-ot	15	999	0.1	2	ot
SUZUK2	528		c	0	0	all	-	all	SCAmer	1991	CC	123	n	bl	n	y	3	0	all/unsp	11	999	0.1	5	ot
SVENSS	556		f	0	0	all	-	all	Eu:Sca	1983	CC	210	n	bl	n	n	0	0	all/unsp	11	999	3	10	st
TVERDA	510		m	0	0	all	0	all	Eu:Sca	1972	pr	238	n	bl	n	n	2	0	cig only	5	999	0.1	0.9	ot
WANG2	517		c	0	0	all	-	all	As:Chi	1980	CC	103	n	ot	n	n	0	0	cig+/-ot	4	999	0.1	3	st
WYNDE3	551		m	0	0	all	-	all	NAmer	1966	CC	350	n	bl	n	y	0	0	all/unsp	13	999	1.0	3	st
WYNDE6	725		m	0	0	wh	-	all	NAmer	1969	CC	4423	n	bl	n	y	5	1#	cig+/-ot	20	999	1.0	10	ot
WYNDE6	732		m	0	0	bl	-	all	NAmer	1969	CC	4423	n	bl	n	y	5	1#	cig+/-ot	20	999	1.0	10	ot
WYNDE6	736		f	0	0	wh	-	all	NAmer	1969	CC	4423	n	bl	n	y	5	1#	cig+/-ot	11	999	1.0	10	ot
WYNDE6	740		f	0	0	bl	-	all	NAmer	1969	CC	4423	n	bl	n	y	5	1#	cig+/-ot	11	999	1.0	10	ot

Comments on values in listings

WYNDE6 ADOS Number of cigs/day
WYNDE6 ADOS Number of cigs/day
WYNDE6 ADOS Number of cigs/day
WYNDE6 ADOS Number of cigs/day

Cigarette type is all/unspec for all RRs

Table 1K10 - 1

IESLC - Meta-analysis of Ex Smoking, Years quit (vs current), "Highest vs lowest"
All LC types, Cigarettes (or Any Product if Cigarettes not available)
Most adjusted

except for the following:

REF	NRR	CIGTYPE
ALDERS	517	MC only
ALDERS	528	MC only
DEAN3	530	MC only
DESTEF	547	MC only

Table 1K10 - 2

IESLC - Meta-analysis of Ex Smoking, Years quit (vs current), "Highest vs lowest"
All LC types, Cigarettes (or Any Product if Cigarettes not available)
Most adjusted

REF	NRR	SEX	AD	Number Exposed Case	Cont	Non-exposed Case	Cont	RR	95.00%CI
ALDERS	517	m	1	29	-	121	-	0.18 (	0.10- 0.30)
ALDERS	528	f	1	26	-	206	-	0.13 (	0.08- 0.23)
Subtotal ALDERS								0.15 (	0.10- 0.22)
ARMADA	520	m	0	50	87	79	45	0.33 (	0.20- 0.54)
BARBON	554	m	1	15	-	32	-	0.15 (	0.07- 0.34)
BECHER	510	m	0	16	72	10	12	0.27 (	0.10- 0.72)
BECHER	520	f	0	1	10	2	3	0.15 (	0.01- 2.29)
Subtotal BECHER								0.25 (	0.10- 0.64)
BROSS	520	m	0	43	79	169	67	0.22 (	0.14- 0.34)
CARPEN	514	c	0	37	158	28	46	0.38 (	0.21- 0.69)
*CEDERL	540	m	1	3	-	12	-	0.18 (	0.05- 0.64)
CHOI	549	m	0	4	19	25	64	0.54 (	0.17- 1.74)
CHOI	558	f	0	2	0	3	2	3.57~(	0.11- 111.71)
Subtotal CHOI								0.66 (	0.22- 1.99)
*CHYOU	512	m	2	5	-	21	-	0.74 (	0.28- 1.94)
*CPSI	821	m	1	15	-	37	-	0.09 (	0.05- 0.16)
*CPSII	674	m	1	256	-	97	-	0.10 (	0.08- 0.12)
*CPSII	650	f	1	50	-	91	-	0.10 (	0.07- 0.14)
Subtotal CPSII								0.10 (	0.08- 0.12)
DAMBER	559	m	1	-	-	-	-	0.29 (	0.12- 0.73)
DARBY	506	m	0	139	767	146	339	0.42 (	0.32- 0.55)
DARBY	515	f	0	26	224	68	93	0.16 (	0.10- 0.27)
Subtotal DARBY								0.34 (	0.27- 0.43)
DEAN3	530	m	1	8	-	28	-	0.28 (	0.12- 0.66)
DEAN3	563	f	1	2	-	4	-	0.44 (	0.08- 2.45)
Subtotal DEAN3								0.31 (	0.14- 0.66)
DESTEF	547	m	0	10	41	10	19	0.46 (	0.17- 1.30)
DOLL	541	m	0	8	23	56	75	0.47 (	0.19- 1.12)
DOLL	550	f	0	1	2	9	6	0.33 (	0.02- 4.55)
Subtotal DOLL								0.45 (	0.20- 1.03)
*DOLL2	515	m	1	7	-	15	-	0.13 (	0.05- 0.31)
DORGAN	518	m	0	134	255	59	51	0.45 (	0.30- 0.70)
DORGAN	561	f	0	34	50	49	27	0.37 (	0.20- 0.71)
Subtotal DORGAN								0.43 (	0.30- 0.61)
*DORN	667	m	0	16	58370	34	22086	0.18 (	0.10- 0.32)
*DORN	690	m	0	34	51243	14	6195	0.29 (	0.16- 0.55)
Subtotal DORN								0.23 (	0.15- 0.35)
GAO	540	m	2	13	-	105	-	0.16 (	0.07- 0.34)
GAO	560	f	2	16	-	37	-	0.31 (	0.11- 0.87)
Subtotal GAO								0.20 (	0.11- 0.38)
GAO2	526	m	0	8	25	31	26	0.27 (	0.10- 0.70)
GARCIA	514	c	0	10	37	33	11	0.09 (	0.03- 0.24)
GRAHAM	544	m	1	13	-	113	-	0.07 (	0.04- 0.15)
*HAMMO2	514	m	1	20	-	59	-	0.31 (	0.19- 0.52)
*HIRAYA	517	m	1	-	-	-	-	0.68 (	0.25- 1.87)
*HIRAYA	528	f	1	-	-	-	-	0.26 (	0.01- 11.52)
Subtotal HIRAYA								0.63 (	0.24- 1.66)
JAHN	523	m	0	29	146	166	8	0.01 (	0.00- 0.02)
JAIN	572	m	0	52	113	74	46	0.29 (	0.17- 0.47)
JAIN	536	f	0	19	61	66	36	0.17 (	0.09- 0.33)
Subtotal JAIN								0.24 (	0.16- 0.35)
JOLY	573	m	0	63	149	38	36	0.40 (	0.23- 0.69)
JOLY	560	f	0	15	19	19	8	0.33 (	0.11- 0.97)
Subtotal JOLY								0.39 (	0.24- 0.63)
*KAISE2	660	m	1	6	-	12	-	0.21 (	0.07- 0.62)
*KAISE2	580	f	1	4	-	6	-	0.58 (	0.15- 2.22)
Subtotal KAISE2								0.31 (	0.13- 0.73)
KHUDER	520	m	0	63	213	88	123	0.41 (	0.28- 0.61)
LUBIN	596	m	0	17	73	33	18	0.13 (	0.06- 0.28)
LUBIN2	1091	m	0	109	715	866	1047	0.18 (	0.15- 0.23)
LUBIN2	1130	f	0	4	20	60	55	0.18 (	0.06- 0.57)
Subtotal LUBIN2								0.18 (	0.15- 0.23)
MATOS	600	m	2	27	-	28	-	0.21 (	0.10- 0.44)
PEZZO2	506	m	0	43	161	85	110	0.35 (	0.22- 0.54)
PEZZOT	506	m	0	20	106	46	82	0.34 (	0.18- 0.61)
SOBUE	738	m	0	17	40	128	116	0.39 (	0.21- 0.72)
*SPEIZE	519	f	2	28	-	24	-	0.17 (	0.08- 0.37)
SUZUK2	528	c	3	9	-	15	-	0.17 (	0.05- 0.51)
SVENSS	556	f	0	14	24	16	13	0.47 (	0.18- 1.27)
*TVERDA	510	m	2	4	-	2	-	0.48 (	0.09- 2.66)
WANG2	517	c	0	5	11	6	10	0.76 (	0.18- 3.27)

Table 1K10 - 2

IESLC - Meta-analysis of Ex Smoking, Years quit (vs current), "Highest vs lowest"
All LC types, Cigarettes (or Any Product if Cigarettes not available)
Most adjusted

REF	NRR	SEX	AD	Number Exposed Case	Number Exposed Cont	Non-exposed Case	Non-exposed Cont	RR	95.00%CI
WYNDE3	551	m	0	5	55	21	22	0.10	(0.03- 0.28)
WYNDE6	725	m	5	-	-	-	-	0.33	(0.23- 0.47)
WYNDE6	732	m	5	-	-	-	-	0.43	(0.16- 1.15)
WYNDE6	736	f	5	-	-	-	-	0.40	(0.31- 0.52)
WYNDE6	740	f	5	-	-	-	-	0.67	(0.18- 2.49)
Subtotal WYNDE6								0.38	(0.31- 0.47)
Partial Totals				1604	113368	3602	30897		

*prospective study ~ With 0.5 adjustment for zero

REF	NRR	SEX	AD	Ys	Ws	Qs	Ps
ALDERS	517	m	1	-1.71	12.73	0.65	0.0000
ALDERS	528	f	1	-2.04	13.78	4.18	0.0000
Subtotal ALDERS				-1.88	26.51	4.82	
ARMADA	520	m	0	-1.12	15.07	2.10	0.0000
BARBON	554	m	1	-1.90	6.15	1.02	0.0000
BECHER	510	m	0	-1.32	3.85	0.11	0.0095
BECHER	520	f	0	-1.90	0.52	0.09	0.1724
Subtotal BECHER				-1.39	4.37	0.19	
BROSS	520	m	0	-1.53	17.62	0.03	0.0000
CARPEN	514	c	0	-0.96	11.01	3.14	0.0015
*CEDERL	540	m	1	-1.71	2.36	0.12	0.0084
CHOI	549	m	0	-0.62	2.79	2.12	0.3017
CHOI	558	f	0	1.27	0.32	2.47	0.4687
Subtotal CHOI				-0.42	3.12	4.59	
*CHYOU	512	m	2	-0.30	4.10	5.79	0.5420
*CPSI	821	m	1	-2.41	11.36	9.58	0.0000
*CPSII	674	m	1	-2.30	93.46	61.77	0.0000
*CPSII	650	f	1	-2.30	31.98	21.14	0.0000
Subtotal CPSII				-2.30	125.45	82.91	
DAMBER	559	m	1	-1.24	4.71	0.30	0.0072
DARBY	506	m	0	-0.87	54.65	21.28	0.0000
DARBY	515	f	0	-1.84	14.62	1.80	0.0000
Subtotal DARBY				-1.07	69.28	23.08	
DEAN3	530	m	1	-1.27	5.29	0.25	0.0034
DEAN3	563	f	1	-0.82	1.31	0.59	0.3470
Subtotal DEAN3				-1.18	6.60	0.83	
DESTEF	547	m	0	-0.77	3.61	1.87	0.1439
DOLL	541	m	0	-0.76	5.01	2.64	0.0873
DOLL	550	f	0	-1.10	0.56	0.09	0.4100
Subtotal DOLL				-0.80	5.57	2.72	
*DOLL2	515	m	1	-2.04	4.62	1.40	0.0000
DORGAN	518	m	0	-0.79	20.86	10.24	0.0003
DORGAN	561	f	0	-0.98	9.36	2.41	0.0027
Subtotal DORGAN				-0.85	30.22	12.65	
*DORN	667	m	0	-1.73	10.89	0.61	0.0000
*DORN	690	m	0	-1.23	9.93	0.69	0.0001
Subtotal DORN				-1.49	20.82	1.30	
GAO	540	m	2	-1.83	6.15	0.72	0.0000
GAO	560	f	2	-1.17	3.59	0.36	0.0264
Subtotal GAO				-1.59	9.74	1.09	
GAO2	526	m	0	-1.32	4.24	0.13	0.0067
GARCIA	514	c	0	-2.41	4.03	3.39	0.0000
GRAHAM	544	m	1	-2.66	8.80	12.03	0.0000
*HAMMO2	514	m	1	-1.17	15.16	1.54	0.0000
*HIRAYA	517	m	1	-0.39	3.79	4.62	0.4525
*HIRAYA	528	f	1	-1.35	0.31	0.01	0.4538
Subtotal HIRAYA				-0.46	4.10	4.63	
JAHN	523	m	0	-4.65	5.80	57.91	0.0000
JAIN	572	m	0	-1.25	15.79	0.89	0.0000
JAIN	536	f	0	-1.77	8.93	0.72	0.0000
Subtotal JAIN				-1.44	24.72	1.61	
JOLY	573	m	0	-0.91	13.04	4.31	0.0010
JOLY	560	f	0	-1.10	3.37	0.51	0.0433
Subtotal JOLY				-0.95	16.41	4.82	
*KAISE2	660	m	1	-1.56	3.23	0.02	0.0050
*KAISE2	580	f	1	-0.54	2.12	1.89	0.4281
Subtotal KAISE2				-1.16	5.35	1.91	
KHUDER	520	m	0	-0.88	24.96	9.18	0.0000
LUBIN	596	m	0	-2.06	6.31	2.08	0.0000
LUBIN2	1091	m	0	-1.69	78.85	3.20	0.0000

Table 1K10 - 2

IESLC - Meta-analysis of Ex Smoking, Years quit (vs current), "Highest vs lowest"
All LC types, Cigarettes (or Any Product if Cigarettes not available)
Most adjusted

REF	NRR	SEX	AD	Ys	Ws	Qs	Ps
LUBIN2	1130	f	0	-1.70	2.99	0.13	0.0034
Subtotal LUBIN2				-1.69	81.83	3.33	
MATOS	600	m	2	-1.56	7.00	0.04	0.0000
PEZZO2	506	m	0	-1.06	19.87	3.63	0.0000
PEZZOT	506	m	0	-1.09	10.71	1.71	0.0004
SOBUE	738	m	0	-0.95	9.97	2.86	0.0026
*SPEIZE	519	f	2	-1.77	6.55	0.52	0.0000
SUZUK2	528	c	3	-1.77	2.85	0.23	0.0028
SVENSS	556	f	0	-0.75	3.96	2.19	0.1373
*TVERDA	510	m	2	-0.73	1.34	0.77	0.3955
WANG2	517	c	0	-0.28	1.79	2.63	0.7100
WYNDE3	551	m	0	-2.35	3.21	2.39	0.0000
WYNDE6	725	m	5	-1.11	30.09	4.37	0.0000
WYNDE6	732	m	5	-0.84	3.95	1.65	0.0935
WYNDE6	736	f	5	-0.92	57.43	18.88	0.0000
WYNDE6	740	f	5	-0.40	2.23	2.64	0.5501
Subtotal WYNDE6				-0.96	93.69	27.53	

	N	61
	NS	43
	Wt	744.95
Het	Chi	306.59
Het	df	60
Het	P	***
Fixed	RR	0.23
	RRl	0.21
	RRu	0.24
	P	---
Random	RR	0.24
	RRl	0.20
	RRu	0.29
	P	---
Asymm	P	N.S.

Table 1K10 - 3

IESLC - Meta-analysis of Ex Smoking, Years quit (vs current), "Highest vs lowest"
All LC types, Cigarettes (or Any Product if Cigarettes not available)
Most adjusted

	Sex			
	combined	male	female	Total
N	4	39	18	61
NS	4	37	17	58
Wt	19.68	561.34	163.93	744.95
Het Chi	8.75	236.44	60.41	306.59
Het df	3	38	17	60
Het P	*	***	***	***
Fixed RR	0.27	0.22	0.23	0.23
RRl	0.17	0.20	0.20	0.21
RRu	0.42	0.24	0.27	0.24
P	---	---	---	---
Random RR	0.25	0.24	0.25	0.24
RRl	0.11	0.19	0.18	0.20
RRu	0.58	0.30	0.36	0.29
P	--	---	---	---
Between Chi				0.99
Between df				2
Between P				N.S.
Btwn(F) P				N.S.
Btwn(R) P				N.S.

	Lung cancer type		
	all	other	Total
N	61		61
NS	43		43
Wt	744.95		744.95
Het Chi	306.59		306.59
Het df	60		60
Het P	***		***
Fixed RR	0.23		0.23
RRl	0.21		0.21
RRu	0.24		0.24
P	---		---
Random RR	0.24		0.24
RRl	0.20		0.20
RRu	0.29		0.29
P	---		---
Between Chi			
Between df			
Between P			N.S.
Btwn(F) P			N.S.
Btwn(R) P			N.S.

	Location								
	NAmer	UK	Scand	othEur	China	Japan	othAs	other	Total
N	24	9	4	7	4	4	2	7	61
NS	16	5	4	5	3	3	1	6	43
Wt	407.04	112.57	12.38	113.22	17.85	18.32	3.12	60.45	744.95
Het Chi	173.39	28.62	1.65	55.90	5.47	1.81	1.04	3.64	306.59
Het df	23	8	3	6	3	3	1	6	60
Het P	***	***	N.S.	***	N.S.	N.S.	N.S.	N.S.	***
Fixed RR	0.21	0.27	0.33	0.17	0.20	0.40	0.66	0.33	0.23
RRl	0.19	0.23	0.19	0.14	0.12	0.25	0.22	0.26	0.21
RRu	0.23	0.33	0.57	0.21	0.31	0.63	1.99	0.42	0.24
P	---	---	---	---	---	---	N.S.	---	---
Random RR	0.23	0.23	0.33	0.13	0.22	0.40	0.67	0.33	0.24
RRl	0.18	0.15	0.19	0.06	0.11	0.25	0.20	0.26	0.20
RRu	0.31	0.36	0.57	0.29	0.42	0.63	2.24	0.42	0.29
P	---	---	---	---	---	---	N.S.	---	---
Between Chi									35.06
Between df									7
Between P									***
Btwn(F) P									N.S.
Btwn(R) P									(*)

Table 1K10 - 3

IESLC - Meta-analysis of Ex Smoking, Years quit (vs current), "Highest vs lowest"
All LC types, Cigarettes (or Any Product if Cigarettes not available)
Most adjusted

Detailed Country in "other Europe"

	multi	Germany	othWest	East	Balkans	Total
N	2	3	2			7
NS	1	2	2			5
Wt	81.83	10.17	21.22			113.22
Het Chi	0.00	26.62	2.66			55.90
Het df	1	2	1			6
Het P	N.S.	***	N.S.			***
Fixed RR	0.18	0.04	0.26			0.17
RRl	0.15	0.02	0.17			0.14
RRu	0.23	0.07	0.40			0.21
P	---	---	---			---
Random RR	0.18	0.07	0.24			0.13
RRl	0.15	0.01	0.11			0.06
RRu	0.23	0.90	0.50			0.29
P	---	-	---			---
Between Chi						26.63
Between df						2
Between P						***
Btwn(F) P						N.S.
Btwn(R) P						N.S.

Detailed Country in "other Asia"

	India	HongKong	other	Total
N			2	2
NS			1	1
Wt			3.12	3.12
Het Chi			1.04	1.04
Het df			1	1
Het P			N.S.	N.S.
Fixed RR			0.66	0.66
RRl			0.22	0.22
RRu			1.99	1.99
P			N.S.	N.S.
Random RR			0.67	0.67
RRl			0.20	0.20
RRu			2.24	2.24
P			N.S.	N.S.
Between Chi				
Between df				
Between P				N.S.
Btwn(F) P				N.S.
Btwn(R) P				N.S.

Detailed other continent

	SCAmer	Total
N	7	7
NS	6	6
Wt	60.45	60.45
Het Chi	3.64	3.64
Het df	6	6
Het P	N.S.	N.S.
Fixed RR	0.33	0.33
RRl	0.26	0.26
RRu	0.42	0.42
P	---	---
Random RR	0.33	0.33
RRl	0.26	0.26
RRu	0.42	0.42
P	---	---
Between Chi		
Between df		
Between P		N.S.
Btwn(F) P		N.S.
Btwn(R) P		N.S.

Table 1K10 - 3

IESLC - Meta-analysis of Ex Smoking, Years quit (vs current), "Highest vs lowest"
All LC types, Cigarettes (or Any Product if Cigarettes not available)
Most adjusted

	Start year of study <1960	1960-69	1970-79	1980-89	1990+	Total
N	7	13	12	24	5	61
NS	5	8	8	17	5	43
Wt	51.16	146.85	148.85	353.32	44.76	744.95
Het Chi	19.71	17.32	15.17	205.75	8.46	306.59
Het df	6	12	11	23	4	60
Het P	**	N.S.	N.S.	***	(*)	***
Fixed RR	0.15	0.34	0.20	0.21	0.28	0.23
RRl	0.12	0.29	0.17	0.19	0.21	0.21
RRu	0.20	0.40	0.23	0.23	0.37	0.24
P	---	---	---	---	---	---
Random RR	0.16	0.33	0.21	0.24	0.24	0.24
RRl	0.10	0.26	0.17	0.17	0.15	0.20
RRu	0.28	0.42	0.27	0.34	0.39	0.29
P	---	---	---	---	---	---
Between Chi						40.17
Between df						4
Between P						***
Btwn(F) P						(*)
Btwn(R) P						*

	Study type (1) CC	other	Total
N	46	15	61
NS	32	11	43
Wt	543.75	201.21	744.95
Het Chi	177.53	60.20	306.59
Het df	45	14	60
Het P	***	***	***
Fixed RR	0.27	0.14	0.23
RRl	0.25	0.12	0.21
RRu	0.30	0.16	0.24
P	---	---	---
Random RR	0.25	0.21	0.24
RRl	0.21	0.14	0.20
RRu	0.30	0.30	0.29
P	---	---	---
Between Chi			68.86
Between df			1
Between P			***
Btwn(F) P			***
Btwn(R) P			N.S.

	Study type (2) CC	prosp	other	Total
N	46	15		61
NS	32	11		43
Wt	543.75	201.21		744.95
Het Chi	177.53	60.20		306.59
Het df	45	14		60
Het P	***	***		***
Fixed RR	0.27	0.14		0.23
RRl	0.25	0.12		0.21
RRu	0.30	0.16		0.24
P	---	---		---
Random RR	0.25	0.21		0.24
RRl	0.21	0.14		0.20
RRu	0.30	0.30		0.29
P	---	---		---
Between Chi				68.86
Between df				1
Between P				***
Btwn(F) P				***
Btwn(R) P				N.S.

Table 1K10 - 3

IESLC - Meta-analysis of Ex Smoking, Years quit (vs current), "Highest vs lowest"
All LC types, Cigarettes (or Any Product if Cigarettes not available)
Most adjusted

	Study size (number of LC cases)				
	100-249	250-499	500-999	1000+	Total
N	9	15	14	23	61
NS	8	13	10	12	43
Wt	36.12	118.30	165.45	425.08	744.95
Het Chi	7.79	24.00	42.09	197.58	306.59
Het df	8	14	13	22	60
Het P	N.S.	*	***	***	***
Fixed RR	0.34	0.31	0.26	0.19	0.23
RRl	0.24	0.26	0.22	0.17	0.21
RRu	0.47	0.37	0.30	0.21	0.24
P	---	---	---	---	---
Random RR	0.34	0.29	0.22	0.21	0.24
RRl	0.24	0.22	0.16	0.15	0.20
RRu	0.47	0.38	0.30	0.29	0.29
P	---	---	---	---	---
Between Chi					35.13
Between df					3
Between P					***
Btwn(F) P					(*)
Btwn(R) P					N.S.

	Risky occupational population			
	no	mining	othRisky	Total
N	59	1	1	61
NS	41	1	1	43
Wt	723.48	6.31	15.16	744.95
Het Chi	302.97	0.00	0.00	306.59
Het df	58	0	0	60
Het P	***	N.S.	N.S.	***
Fixed RR	0.23	0.13	0.31	0.23
RRl	0.21	0.06	0.19	0.21
RRu	0.24	0.28	0.51	0.24
P	---	---	---	---
Random RR	0.24	0.13	0.31	0.24
RRl	0.20	0.06	0.19	0.20
RRu	0.29	0.28	0.51	0.29
P	---	---	---	---
Between Chi				3.62
Between df				2
Between P				N.S.
Btwn(F) P				N.S.
Btwn(R) P				N.S.

	National cigarette tobacco type			
	Virginia	blended	other	Total
N	11	46	4	61
NS	6	34	3	43
Wt	137.29	589.81	17.85	744.95
Het Chi	30.59	265.59	5.47	306.59
Het df	10	45	3	60
Het P	***	***	N.S.	***
Fixed RR	0.27	0.22	0.20	0.23
RRl	0.23	0.20	0.12	0.21
RRu	0.32	0.24	0.31	0.24
P	---	---	---	---
Random RR	0.23	0.25	0.22	0.24
RRl	0.16	0.20	0.11	0.20
RRu	0.32	0.31	0.42	0.29
P	---	---	---	---
Between Chi				4.93
Between df				2
Between P				(*)
Btwn(F) P				N.S.
Btwn(R) P				N.S.

Table 1K10 - 3

IESLC - Meta-analysis of Ex Smoking, Years quit (vs current), "Highest vs lowest"
All LC types, Cigarettes (or Any Product if Cigarettes not available)
Most adjusted

	Any proxy use No/nk	Yes	Total
N	52	9	61
NS	37	6	43
Wt	666.24	78.72	744.95
Het Chi	287.62	14.52	306.59
Het df	51	8	60
Het P	***	(*)	***
Fixed RR	0.22	0.28	0.23
RRl	0.20	0.23	0.21
RRu	0.24	0.35	0.24
P	---	---	---
Random RR	0.24	0.26	0.24
RRl	0.19	0.19	0.20
RRu	0.29	0.36	0.29
P	---	---	---
Between Chi			4.44
Between df			1
Between P			*
Btwn(F) P			N.S.
Btwn(R) P			N.S.

	Full histological confirmation No	Yes	Total
N	39	22	61
NS	27	16	43
Wt	423.75	321.20	744.95
Het Chi	210.18	54.62	306.59
Het df	38	21	60
Het P	***	***	***
Fixed RR	0.18	0.30	0.23
RRl	0.17	0.27	0.21
RRu	0.20	0.33	0.24
P	---	---	---
Random RR	0.22	0.30	0.24
RRl	0.17	0.24	0.20
RRu	0.28	0.37	0.29
P	---	---	---
Between Chi			41.79
Between df			1
Between P			***
Btwn(F) P			**
Btwn(R) P			(*)

	Number of adjustment variables (1) 0	1	2+/+nk	Total
N	33	17	11	61
NS	24	12	7	43
Wt	398.51	221.16	125.28	744.95
Het Chi	135.96	50.79	15.09	306.59
Het df	32	16	10	60
Het P	***	***	N.S.	***
Fixed RR	0.27	0.13	0.34	0.23
RRl	0.25	0.11	0.28	0.21
RRu	0.30	0.15	0.40	0.24
P	---	---	---	---
Random RR	0.26	0.17	0.31	0.24
RRl	0.21	0.13	0.24	0.20
RRu	0.33	0.23	0.41	0.29
P	---	---	---	---
Between Chi				104.75
Between df				2
Between P				***
Btwn(F) P				***
Btwn(R) P				**

Table 1K10 - 3

IESLC - Meta-analysis of Ex Smoking, Years quit (vs current), "Highest vs lowest"
All LC types, Cigarettes (or Any Product if Cigarettes not available)
Most adjusted

	Number of adjustment variables (2) 0	1	2	3-5	6+/+nk	Total
N	33	17	6	5		61
NS	24	12	5	2		43
Wt	398.51	221.16	28.74	96.54		744.95
Het Chi	135.96	50.79	7.97	3.33		306.59
Het df	32	16	5	4		60
Het P	***	***	N.S.	N.S.		***
Fixed RR	0.27	0.13	0.25	0.37		0.23
RRl	0.25	0.11	0.17	0.31		0.21
RRu	0.30	0.15	0.36	0.46		0.24
P	---	---	---	---		---
Random RR	0.26	0.17	0.26	0.37		0.24
RRl	0.21	0.13	0.16	0.31		0.20
RRu	0.33	0.23	0.42	0.46		0.29
P	---	---	---	---		---
Between Chi						108.54
Between df						3
Between P						***
Btwn(F) P						***
Btwn(R) P						***

	Product all/unsp	cig+/-ot	cig only	Total
N	12	38	11	61
NS	9	27	9	45
Wt	99.06	482.54	163.34	744.95
Het Chi	23.15	184.21	32.90	306.59
Het df	11	37	10	60
Het P	*	***	***	***
Fixed RR	0.31	0.26	0.13	0.23
RRl	0.25	0.23	0.11	0.21
RRu	0.37	0.28	0.15	0.24
P	---	---	---	---
Random RR	0.25	0.26	0.18	0.24
RRl	0.17	0.21	0.13	0.20
RRu	0.36	0.32	0.26	0.29
P	---	---	---	---
Between Chi				66.32
Between df				2
Between P				***
Btwn(F) P				***
Btwn(R) P				N.S.

	Derivation of RR/CI Orig	StdCalc	Other	Total
N		33	28	61
NS		25	20	45
Wt		401.42	343.54	744.95
Het Chi		134.01	144.24	306.59
Het df		32	27	60
Het P		***	***	***
Fixed RR		0.27	0.18	0.23
RRl		0.24	0.16	0.21
RRu		0.30	0.20	0.24
P		---	---	---
Random RR		0.26	0.22	0.24
RRl		0.21	0.17	0.20
RRu		0.32	0.30	0.29
P		---	---	---
Between Chi				28.34
Between df				1
Between P				***
Btwn(F) P				*
Btwn(R) P				N.S.

Table 1K10 - 4

IESLC - Meta-analysis of Ex Smoking, Years quit (vs current), "Highest vs lowest"
All LC types, Cigarettes (or Any Product if Cigarettes not available)
Least adjusted

REF	NRR	X	SEX	AGEL	AGEH	RACE	YF	LC TYPE	LOC	START	ST	NLC	R	VB	P	H	AD	ADOS	PRODUCT	exL	exH	unexL	unexH	De
ALDERS	517		m	0	0	all	-	all	Eu:UK	1977	CC	1448	n	V	n	n	1	0	cig only	10	999	0.1	2	ot
ALDERS	528		f	0	0	all	-	all	Eu:UK	1977	CC	1448	n	V	n	n	1	0	cig only	10	999	0.1	2	ot
ARMADA	520		m	0	0	all	-	all	Eu:wst	1986	CC	325	n	bl	n	y	0	0	cig+/-ot	6	999	1.0	5	st
BARBON	539	x	m	0	0	all	-	all	Eu:wst	1979	CC	755	n	bl	y	y	0	0	all/unsp	25	999	0.1	4	st
BECHER	510		m	0	0	all	-	all	Eu:Ger	1985	CC	194	n	bl	n	y	0	0	all/unsp	10	999	2	4	st
BECHER	520		f	0	0	all	-	all	Eu:Ger	1985	CC	194	n	bl	n	y	0	0	all/unsp	10	999	2	4	st
BROSS	520		m	0	0	wh	-	all	NAmer	1960	CC	974	n	bl	n	n	0	0	cig+/-ot	6	999	0.1	5	st
CARPEN	514		c	0	0	w+b	-	all	NAmer	1991	CC	356	n	bl	n	n	0	0	cig+/-ot	15	999	0.1	4	st
CEDERL	540		m	40	69	all	10	all	Eu:Sca	1963	pr	491	n	bl	n	n	1	0	all/unsp	10	999	0.1	9	ot
CHOI	549		m	0	0	all	-	all	As:oth	1985	CC	375	n	bl	n	n	0	0	cig+/-ot	15	999	0.1	4	st
CHOI	558		f	0	0	all	-	all	As:oth	1985	CC	375	n	bl	n	n	0	0	cig+/-ot	5	999	0.1	4	ot
CHYOU	506	x	m	0	0	jap	21	all	NAmer	1965	pr	227	n	bl	n	y	1	0	cig+/-ot	15	999	0.1	14	ot
CPSI	821		m	50	74	all	6	all	NAmer	1959	pr	5138	n	bl	n	n	1	0	cig only	10	999	0.1	0.9	ot
CPSII	674		m	35	99	all	4	all	NAmer	1982	pr	3229	n	bl	n	n	1	0	cig only	16	999	0.1	0.9	ot
CPSII	650		f	0	0	all	4	all	NAmer	1982	pr	3229	n	bl	n	n	1	0	cig+/-ot	16	999	0.1	2	ot
DAMBER	559		m	0	0	all	-	all	Eu:Sca	1972	CC	579	n	bl	y	n	1	0	cig only	11	999	0.1	10	ot
DARBY	506		m	0	0	wh	-	all	Eu:UK	1988	CC	982	n	V	n	n	0	0	all/unsp	10	999	0.1	9	st
DARBY	515		f	0	0	wh	-	all	Eu:UK	1988	CC	982	n	V	n	n	0	0	all/unsp	10	999	0.1	9	st
DEAN3	515	x	m	0	0	all	-	all	Eu:UK	1969	CC	766	n	V	y	n	0	0	cig only	19	999	3	4	st
DEAN3	552	x	f	0	0	all	-	all	Eu:UK	1969	CC	766	n	V	y	n	0	0	all/unsp	9	999	3	4	st
DESTEF	547		m	0	0	all	-	all	SCAmer	1988	CC	497	n	bl	n	y	0	0	cig+/-ot	10	999	0.1	4	st
DOLL	541		m	0	0	all	-	all	Eu:UK	1948	CC	1465	n	V	n	n	0	0	all/unsp	20	999	0.1	9	st
DOLL	550		f	0	0	all	-	all	Eu:UK	1948	CC	1465	n	V	n	n	0	0	all/unsp	10	999	0.1	9	st
DOLL2	515		m	0	0	all	20	all	Eu:UK	1951	pr	920	n	V	n	n	1	0	cig only	15	999	0.1	4	ot
DORGAN	518		m	0	0	wh	-	all	NAmer	1980	CC	2026	n	bl	y	y	0	0	cig+/-ot	10	999	1	5	st
DORGAN	561		f	0	0	all	-	all	NAmer	1980	CC	2026	n	bl	y	y	0	0	cig+/-ot	10	999	1	9	st
DORN	667		m	55	64	wh	8	all	NAmer	1954	pr	5097	n	bl	n	n	0	0	cig+/-ot	15	999	0.1	4	st
DORN	690		m	65	74	wh	8	all	NAmer	1954	pr	5097	n	bl	n	n	0	0	cig+/-ot	15	999	0.1	4	st
GAO	530	x	m	0	0	all	-	all	As:Chi	1984	CC	1405	n	ot	n	n	0	0	cig+/-ot	10	999	0.1	4	st
GAO	550	x	f	0	0	all	-	all	As:Chi	1984	CC	1405	n	ot	n	n	0	0	cig+/-ot	10	999	0.1	4	st
GAO2	526		m	0	0	all	-	all	As:Jap	1988	CC	282	n	bl	n	n	0	0	cig+/-ot	20	999	1.0	4	st
GARCIA	514		c	0	0	all	-	all	NAmer	1992	CC	416	n	bl	n	y	0	0	cig+/-ot	30	999	1.0	4	st
GRAHAM	534	x	m	0	0	wh	-	all	NAmer	1956	CC	685	n	bl	n	n	0	0	cig+/-ot	5	999	0.1	1.0	st
HAMMO2	514		m	0	0	all	0	all	NAmer	1967	pr	450	o	bl	n	n	1	0	cig+/-ot	10	999	0.1	4	ot
HIRAYA	517		m	0	0	all	0	all	As:Jap	1965	pr	1917	n	bl	n	n	1	0	cig+/-ot	10	999	0.1	4	ot
HIRAYA	528		f	0	0	all	0	all	As:Jap	1965	pr	1917	n	bl	n	n	1	0	cig+/-ot	10	999	0.1	4	ot
JAHN	523		m	0	0	all	-	all	Eu:Ger	1988	CC	1004	n	bl	n	n	0	0	cig+/-ot	21	999	0.1	0.9	st
JAIN	572		m	0	0	all	-	all	NAmer	1981	CC	845	n	V	y	n	0	0	cig+/-ot	10	999	2	9	st
JAIN	536		f	0	0	all	-	all	NAmer	1981	CC	845	n	V	y	n	0	0	cig+/-ot	10	999	2	9	st
JOLY	573		m	0	0	all	-	all	SCAmer	1978	CC	826	n	bl	n	n	0	0	cig+/-ot	5	999	1.0	4	st
JOLY	560		f	0	0	all	-	all	SCAmer	1978	CC	826	n	bl	n	n	0	0	cig+/-ot	5	999	1.0	4	st
KAISE2	660		m	0	0	all	9	all	NAmer	1979	pr	318	n	bl	n	n	1	0	cig only	21	999	2	10	st
KAISE2	580		f	0	0	all	9	all	NAmer	1979	pr	318	n	bl	n	n	1	0	cig only	21	999	2	10	ot
KHUDER	520		m	0	0	all	-	all	NAmer	1985	CC	482	n	bl	n	y	0	0	cig+/-ot	15	999	0.1	4	st
LUBIN	596		m	0	0	all	-	all	As:Chi	1984	CC	427	m	ot	y	n	0	0	cig+/-ot	10	999	3	4	st
LUBIN2	1091		m	0	0	all	-	all	Eu:mul	1976	CC	7804	n	bl	n	y	0	0	cig+/-ot	25	999	0.1	4	st
LUBIN2	1130		f	0	0	all	-	all	Eu:mul	1976	CC	7804	n	bl	n	y	0	0	cig+/-ot	25	999	0.1	4	st
MATOS	590	x	m	0	0	all	-	all	SCAmer	1994	CC	200	n	bl	n	n	0	0	cig+/-ot	11	999	1.0	5	st
PEZZO2	506		m	0	0	all	-	all	SCAmer	1992	CC	367	n	bl	n	y	0	0	cig+/-ot	11	999	1.0	10	st
PEZZOT	506		m	0	0	all	-	all	SCAmer	1987	CC	215	n	bl	n	y	0	0	cig only	11	999	1.0	10	st
SOBUE	738		m	0	0	all	-	all	As:Jap	1986	CC	1376	n	bl	n	y	0	0	cig+/-ot	25	999	1.0	4	st
SPEIZE	519		f	0	0	all	0	all	NAmer	1976	pr	593	n	bl	n	y	2	0	cig+/-ot	15	999	0.1	2	ot
SUZUK2	517	x	c	0	0	all	-	all	SCAmer	1991	CC	123	n	bl	n	y	0	0	all/unsp	11	999	0.1	5	st
SVENSS	556		f	0	0	all	-	all	Eu:Sca	1983	CC	210	n	bl	n	n	0	0	all/unsp	11	999	3	10	st
TVERDA	510		m	0	0	all	0	all	Eu:Sca	1972	pr	238	n	bl	n	n	2	0	cig only	5	999	0.1	0.9	ot
WANG2	517		c	0	0	all	-	all	As:Chi	1980	CC	103	n	ot	n	n	0	0	cig+/-ot	4	999	0.1	3	st
WYNDE3	551		m	0	0	all	-	all	NAmer	1966	CC	350	n	bl	n	y	0	0	all/unsp	13	999	1.0	3	st
WYNDE6	725		m	0	0	wh	-	all	NAmer	1969	CC	4423	n	bl	n	y	5	1#	cig+/-ot	20	999	1.0	10	ot
WYNDE6	732		m	0	0	bl	-	all	NAmer	1969	CC	4423	n	bl	n	y	5	1#	cig+/-ot	20	999	1.0	10	ot
WYNDE6	736		f	0	0	wh	-	all	NAmer	1969	CC	4423	n	bl	n	y	5	1#	cig+/-ot	11	999	1.0	10	ot
WYNDE6	740		f	0	0	bl	-	all	NAmer	1969	CC	4423	n	bl	n	y	5	1#	cig+/-ot	11	999	1.0	10	ot

Comments on values in listings

WYNDE6 ADOS Number of cigs/day
WYNDE6 ADOS Number of cigs/day
WYNDE6 ADOS Number of cigs/day
WYNDE6 ADOS Number of cigs/day

Cigarette type is all/unspec for all RRs

Table 1K10 - 4

IESLC - Meta-analysis of Ex Smoking, Years quit (vs current), "Highest vs lowest"
All LC types, Cigarettes (or Any Product if Cigarettes not available)
Least adjusted

except for the following:

REF	NRR	CIGTYPE
ALDERS	517	MC only
ALDERS	528	MC only
DEAN3	515	MC only
DESTEF	547	MC only

Table 1K10 - 5

IESLC - Meta-analysis of Ex Smoking, Years quit (vs current), "Highest vs lowest"
All LC types, Cigarettes (or Any Product if Cigarettes not available)
Least adjusted

REF	NRR	SEX	AD	Number Exposed Case	Number Exposed Cont	Non-exposed Case	Non-exposed Cont	RR	95.00%CI
ALDERS	517	m	1	29	-	121	-	0.18	(0.10- 0.30)
ALDERS	528	f	1	26	-	206	-	0.13	(0.08- 0.23)
Subtotal ALDERS								0.15	(0.10- 0.22)
ARMADA	520	m	0	50	87	79	45	0.33	(0.20- 0.54)
BARBON	539	m	0	15	59	32	20	0.16	(0.07- 0.35)
BECHER	510	m	0	16	72	10	12	0.27	(0.10- 0.72)
BECHER	520	f	0	1	10	2	3	0.15	(0.01- 2.29)
Subtotal BECHER								0.25	(0.10- 0.64)
BROSS	520	m	0	43	79	169	67	0.22	(0.14- 0.34)
CARPEN	514	c	0	37	158	28	46	0.38	(0.21- 0.69)
*CEDERL	540	m	1	3	-	12	-	0.18	(0.05- 0.64)
CHOI	549	m	0	4	19	25	64	0.54	(0.17- 1.74)
CHOI	558	f	0	2	0	3	2	3.57~	(0.11- 111.71)
Subtotal CHOI								0.66	(0.22- 1.99)
*CHYOU	506	m	1	5	-	21	-	0.72	(0.27- 1.91)
*CPSI	821	m	1	15	-	37	-	0.09	(0.05- 0.16)
*CPSII	674	m	1	256	-	97	-	0.10	(0.08- 0.12)
*CPSII	650	f	1	50	-	91	-	0.10	(0.07- 0.14)
Subtotal CPSII								0.10	(0.08- 0.12)
DAMBER	559	m	1	-	-	-	-	0.29	(0.12- 0.73)
DARBY	506	m	0	139	767	146	339	0.42	(0.32- 0.55)
DARBY	515	f	0	26	224	68	93	0.16	(0.10- 0.27)
Subtotal DARBY								0.34	(0.27- 0.43)
DEAN3	515	m	0	8	66	28	102	0.44	(0.19- 1.03)
DEAN3	552	f	0	2	114	4	110	0.48	(0.09- 2.69)
Subtotal DEAN3								0.45	(0.21- 0.96)
DESTEF	547	m	0	10	41	10	19	0.46	(0.17- 1.30)
DOLL	541	m	0	8	23	56	75	0.47	(0.19- 1.12)
DOLL	550	f	0	1	2	9	6	0.33	(0.02- 4.55)
Subtotal DOLL								0.45	(0.20- 1.03)
*DOLL2	515	m	1	7	-	15	-	0.13	(0.05- 0.31)
DORGAN	518	m	0	134	255	59	51	0.45	(0.30- 0.70)
DORGAN	561	f	0	34	50	49	27	0.37	(0.20- 0.71)
Subtotal DORGAN								0.43	(0.30- 0.61)
*DORN	667	m	0	16	58370	34	22086	0.18	(0.10- 0.32)
*DORN	690	m	0	34	51243	14	6195	0.29	(0.16- 0.55)
Subtotal DORN								0.23	(0.15- 0.35)
GAO	530	m	0	13	41	105	52	0.16	(0.08- 0.32)
GAO	550	f	0	16	14	37	9	0.28	(0.10- 0.77)
Subtotal GAO								0.19	(0.11- 0.34)
GAO2	526	m	0	8	25	31	26	0.27	(0.10- 0.70)
GARCIA	514	c	0	10	37	33	11	0.09	(0.03- 0.24)
GRAHAM	534	m	0	13	71	113	59	0.10	(0.05- 0.19)
*HAMMO2	514	m	1	20	-	59	-	0.31	(0.19- 0.52)
*HIRAYA	517	m	1	-	-	-	-	0.68	(0.25- 1.87)
*HIRAYA	528	f	1	-	-	-	-	0.26	(0.01- 11.52)
Subtotal HIRAYA								0.63	(0.24- 1.66)
JAHN	523	m	0	29	146	166	8	0.01	(0.00- 0.02)
JAIN	572	m	0	52	113	74	46	0.29	(0.17- 0.47)
JAIN	536	f	0	19	61	66	36	0.17	(0.09- 0.33)
Subtotal JAIN								0.24	(0.16- 0.35)
JOLY	573	m	0	63	149	38	36	0.40	(0.23- 0.69)
JOLY	560	f	0	15	19	19	8	0.33	(0.11- 0.97)
Subtotal JOLY								0.39	(0.24- 0.63)
*KAISE2	660	m	1	6	-	12	-	0.21	(0.07- 0.62)
*KAISE2	580	f	1	4	-	6	-	0.58	(0.15- 2.22)
Subtotal KAISE2								0.31	(0.13- 0.73)
KHUDER	520	m	0	63	213	88	123	0.41	(0.28- 0.61)
LUBIN	596	m	0	17	73	33	18	0.13	(0.06- 0.28)
LUBIN2	1091	m	0	109	715	866	1047	0.18	(0.15- 0.23)
LUBIN2	1130	f	0	4	20	60	55	0.18	(0.06- 0.57)
Subtotal LUBIN2								0.18	(0.15- 0.23)
MATOS	590	m	0	27	101	28	23	0.22	(0.11- 0.44)
PEZZO2	506	m	0	43	161	85	110	0.35	(0.22- 0.54)
PEZZOT	506	m	0	20	106	46	82	0.34	(0.18- 0.61)
SOBUE	738	m	0	17	40	128	116	0.39	(0.21- 0.72)
*SPEIZE	519	f	2	28	-	24	-	0.17	(0.08- 0.37)
SUZUK2	517	c	0	9	22	15	10	0.27	(0.09- 0.83)
SVENSS	556	f	0	14	24	16	13	0.47	(0.18- 1.27)
*TVERDA	510	m	2	4	-	2	-	0.48	(0.09- 2.66)
WANG2	517	c	0	5	11	6	10	0.76	(0.18- 3.27)

Table 1K10 - 5

IESLC - Meta-analysis of Ex Smoking, Years quit (vs current), "Highest vs lowest"
All LC types, Cigarettes (or Any Product if Cigarettes not available)
Least adjusted

REF	NRR	SEX	AD	Number Exposed Case	Cont	Non-exposed Case	Cont	RR	95.00%CI
WYNDE3	551	m	0	5	55	21	22	0.10	(0.03- 0.28)
WYNDE6	725	m	5	-	-	-	-	0.33	(0.23- 0.47)
WYNDE6	732	m	5	-	-	-	-	0.43	(0.16- 1.15)
WYNDE6	736	f	5	-	-	-	-	0.40	(0.31- 0.52)
WYNDE6	740	f	5	-	-	-	-	0.67	(0.18- 2.49)
Subtotal WYNDE6								0.38	(0.31- 0.47)
Partial Totals				1604	113856	3602	31282		

*prospective study ~ With 0.5 adjustment for zero

REF	NRR	SEX	AD	Ys	Ws	Qs	Ps
ALDERS	517	m	1	-1.71	12.73	0.70	0.0000
ALDERS	528	f	1	-2.04	13.78	4.31	0.0000
Subtotal ALDERS				-1.88	26.51	5.00	
ARMADA	520	m	0	-1.12	15.07	2.00	0.0000
BARBON	539	m	0	-1.84	6.07	0.78	0.0000
BECHER	510	m	0	-1.32	3.85	0.10	0.0095
BECHER	520	f	0	-1.90	0.52	0.09	0.1724
Subtotal BECHER				-1.39	4.37	0.19	
BROSS	520	m	0	-1.53	17.62	0.05	0.0000
CARPEN	514	c	0	-0.96	11.01	3.05	0.0015
*CEDERL	540	m	1	-1.71	2.36	0.13	0.0084
CHOI	549	m	0	-0.62	2.79	2.08	0.3017
CHOI	558	f	0	1.27	0.32	2.46	0.4687
Subtotal CHOI				-0.42	3.12	4.54	
*CHYOU	506	m	1	-0.33	4.01	5.33	0.5104
*CPSI	821	m	1	-2.41	11.36	9.76	0.0000
*CPSII	674	m	1	-2.30	93.46	63.07	0.0000
*CPSII	650	f	1	-2.30	31.98	21.58	0.0000
Subtotal CPSII				-2.30	125.45	84.65	
DAMBER	559	m	1	-1.24	4.71	0.28	0.0072
DARBY	506	m	0	-0.87	54.65	20.71	0.0000
DARBY	515	f	0	-1.84	14.62	1.89	0.0000
Subtotal DARBY				-1.07	69.28	22.59	
DEAN3	515	m	0	-0.82	5.39	2.37	0.0578
DEAN3	552	f	0	-0.73	1.30	0.74	0.4055
Subtotal DEAN3				-0.80	6.69	3.11	
DESTEF	547	m	0	-0.77	3.61	1.83	0.1439
DOLL	541	m	0	-0.76	5.01	2.58	0.0873
DOLL	550	f	0	-1.10	0.56	0.08	0.4100
Subtotal DOLL				-0.80	5.57	2.66	
*DOLL2	515	m	1	-2.04	4.62	1.44	0.0000
DORGAN	518	m	0	-0.79	20.86	9.99	0.0003
DORGAN	561	f	0	-0.98	9.36	2.33	0.0027
Subtotal DORGAN				-0.85	30.22	12.32	
*DORN	667	m	0	-1.73	10.89	0.65	0.0000
*DORN	690	m	0	-1.23	9.93	0.65	0.0001
Subtotal DORN				-1.49	20.82	1.30	
GAO	530	m	0	-1.85	7.69	1.05	0.0000
GAO	550	f	0	-1.28	3.68	0.15	0.0141
Subtotal GAO				-1.67	11.36	1.20	
GAO2	526	m	0	-1.32	4.24	0.12	0.0067
GARCIA	514	c	0	-2.41	4.03	3.45	0.0000
GRAHAM	534	m	0	-2.35	8.56	6.43	0.0000
*HAMMO2	514	m	1	-1.17	15.16	1.46	0.0000
*HIRAYA	517	m	1	-0.39	3.79	4.55	0.4525
*HIRAYA	528	f	1	-1.35	0.31	0.01	0.4538
Subtotal HIRAYA				-0.46	4.10	4.56	
JAHN	523	m	0	-4.65	5.80	58.22	0.0000
JAIN	572	m	0	-1.25	15.79	0.83	0.0000
JAIN	536	f	0	-1.77	8.93	0.76	0.0000
Subtotal JAIN				-1.44	24.72	1.59	
JOLY	573	m	0	-0.91	13.04	4.18	0.0010
JOLY	560	f	0	-1.10	3.37	0.49	0.0433
Subtotal JOLY				-0.95	16.41	4.67	
*KAISE2	660	m	1	-1.56	3.23	0.02	0.0050
*KAISE2	580	f	1	-0.54	2.12	1.86	0.4281
Subtotal KAISE2				-1.16	5.35	1.88	
KHUDER	520	m	0	-0.88	24.96	8.92	0.0000
LUBIN	596	m	0	-2.06	6.31	2.14	0.0000
LUBIN2	1091	m	0	-1.69	78.85	3.48	0.0000

Table 1K10 - 5

IESLC - Meta-analysis of Ex Smoking, Years quit (vs current), "Highest vs lowest"
All LC types, Cigarettes (or Any Product if Cigarettes not available)
Least adjusted

REF	NRR	SEX	AD	Ys	Ws	Qs	Ps
LUBIN2	1130	f	0	-1.70	2.99	0.14	0.0034
Subtotal LUBIN2				-1.69	81.83	3.62	
MATOS	590	m	0	-1.52	7.93	0.01	0.0000
PEZZO2	506	m	0	-1.06	19.87	3.48	0.0000
PEZZOT	506	m	0	-1.09	10.71	1.64	0.0004
SOBUE	738	m	0	-0.95	9.97	2.77	0.0026
*SPEIZE	519	f	2	-1.77	6.55	0.55	0.0000
SUZUK2	517	c	0	-1.30	3.09	0.10	0.0223
SVENSS	556	f	0	-0.75	3.96	2.14	0.1373
*TVERDA	510	m	2	-0.73	1.34	0.75	0.3955
WANG2	517	c	0	-0.28	1.79	2.60	0.7100
WYNDE3	551	m	0	-2.35	3.21	2.43	0.0000
WYNDE6	725	m	5	-1.11	30.09	4.17	0.0000
WYNDE6	732	m	5	-0.84	3.95	1.60	0.0935
WYNDE6	736	f	5	-0.92	57.43	18.32	0.0000
WYNDE6	740	f	5	-0.40	2.23	2.60	0.5501
Subtotal WYNDE6				-0.96	93.69	26.70	

	N	61
	NS	43
	Wt	747.43
Het	Chi	302.43
Het	df	60
Het	P	***
Fixed	RR	0.23
	RRl	0.21
	RRu	0.24
	P	---
Random	RR	0.25
	RRl	0.21
	RRu	0.30
	P	---
Asymm	P	N.S.

Table 1K10 - 6

IESLC - Meta-analysis of Ex Smoking, Years quit (vs current), "Highest vs lowest"
All LC types, Cigarettes (or Any Product if Cigarettes not available)
Least adjusted

	Sex combined	male	female	Total
N	4	39	18	61
NS	4	37	17	58
Wt	19.93	563.50	164.00	747.43
Het Chi	8.05	232.61	60.39	302.43
Het df	3	38	17	60
Het P	*	***	***	***
Fixed RR	0.29	0.22	0.23	0.23
RRl	0.19	0.21	0.20	0.21
RRu	0.45	0.24	0.27	0.24
P	---	---	---	---
Random RR	0.28	0.24	0.25	0.25
RRl	0.12	0.19	0.17	0.21
RRu	0.62	0.30	0.36	0.30
P	--	---	---	---
Between Chi				1.38
Between df				2
Between P				N.S.
Btwn(F) P				N.S.
Btwn(R) P				N.S.

Table 1K10 - 7

IESLC - Meta-analysis of Ex Smoking, Years quit (vs current), "Highest vs lowest"
All LC types, Cigarettes (or Any Product if Cigarettes not available)
Excluded studies (and stage at which they were excluded)

1	AGUDO AKIBA AMANDU AMES AXELSS BEST BOUCHA BOUCOT BRESLO CHEN CHEN2 CHIAZZ DEAN2 DOSEME ENGELA FAN GENG GER GUO HAENSZ HEGMAN HOLE HU HU2 JUSSAW KATSOU KAUFMA KOO KOULUM KREUZE LETOUR LEVIN LIAW LIU3 LIU4 LIU5 MCCONN MIGRAN MRFITR NOTAN2 OSANN2 PERNU QIAO2 RACHTA RESTRE SADOWS SEGI2 STASZE TIZZAN VUTUC WATSON WU WUWILL WYNDE2 WYNDE8 XU YUAN ZHANG ZHENG ZHOU
2	AUVINE BENSHL BLOT1 BROWN3 BUFFLE GURSEL LAUSSM LUO MCDUFF PISANI PRESCO SPITZ WU2 WYNDE7
4	GARSHI JEDRYC WAKAI
5	CORREA HAMMON
6	GILLIS HUMBLE QIAO WIGLE
8	BOFFET
15	BENHAM

Table 1K10 - 8
Potentially overlapping studies

REF	REFGP	PRINC	OVERLAP/LINK
LUBIN2	LUBIN2	1	Lubin-combined
TVERDA	TVERDA	1	VEIERO/TVERDAL
BROSS	BYERS1	1	GRAHAM/BROSS/BYERS1
GRAHAM	BYERS1	1	GRAHAM/BROSS/BYERS1
CHYOU	CHYOU	1	GOODMA/CHYOU
WYNDE6	WYNDE6	1	WYNDE5/6/7/8
CPSI	CPSI	1	CPSI overall
JAHN	BOFFET	2	Subset of BOFFET
LUBIN	XIANGZ	2	LUBIN/XIANGZ/QIAO

Table 1K10 - 9
Most adjusted - insufficient data for meta-analysis

REF	NRR	SEX	AGEL	AGEH	RACE	YF	LC TYPE	LOC	START	ST	NLC	R	VB	P	H	AD	ADOS	PRODUCT	exL	exH	unexL	unexH	De
CPSI	717	m	0	0	wh	0	all	NAmer	1959	pr	5138	n	bl	n	n	1	0	cig only	35	39	2	4	st
CPSI	733	f	0	0	wh	0	all	NAmer	1959	pr	5138	n	bl	n	n	1	0	cig only	25	29	2	4	st
HUMBLE	553	c	0	0	wh	-	not alv	NAmer	1980	CC	521	n	bl	y	n	2	2#	cig+/-ot	16	30	2	5	st
WIGLE	518	m	0	0	all	-	all	NAmer	1971	CC	728	n	V	n	n	2	1#	cig+/-ot	15	999	0.1	1.0	st
WIGLE	528	f	0	0	all	-	all	NAmer	1971	CC	728	n	V	n	n	2	1#	cig+/-ot	15	999	0.1	1.0	st

Comments on values in listings

HUMBLE ADOS Number of cigarettes and duration
WIGLE ADOS Cumulative exposure
WIGLE ADOS Cumulative exposure

REF	NRR	RR	SIG	RRDATA comment
CPSI	717	0.24		0
CPSI	733	0.92		0
HUMBLE	553	0.16		0
WIGLE	518	0.08		0
WIGLE	528	0.44		0

Table 1K11 -

IESLC - Meta-analysis of Ex Smoking by Years quit (vs current), Overview
All LC types, Cigarettes only

This analysis is restricted to results for:
1) Ex smokers
2) Results by Years quit (vs current)
3) Categorical results by Years quit (vs current)
Results by Years quit (vs current) are grouped under 2 schemes (S1, S2). Each scheme has a set of "key values". An interval is allocated to the category whose key value it includes, and intervals which include none or more than one of the key values are excluded. (Open-ended intervals are coded as 999)

S1	key value	maximum range
1	3	1-6
2	7	4-11
3	12	8+

S2	key value	maximum range
1	3	1-11
2	12	4-19
3	20	13+

4) All LC types (or near equivalent)
5) Results complete enough for use in metaanalysis

Within each study, results are then selected (in the following order of preference, within each sex) for:
6) (not applicable)
7) PRODUCT: cigarettes only
8) CIGTYPE: all/unspecified, MC regardless of HR, MC only
9) Results with least adjustment for other aspects of smoking (ADOS)
10) DENOM: current smokers, current + recent smokers (up to number of m=months or y=years, max 2 years)
11) Followup period (YF, prospective studies): whole study (coded as 0) or longest available
12) LCtype: all or nearest available, at least Squamous and Adeno. (q = squamous, s = small, l = large, a = adeno, mix = mixed, alv = alveolar)
13) Race: all or nearest available, otherwise by race (wh or w = white, bl or b = black, hi = hispanic ch = chinese, jap = japanese, haw = hawaiian, w+o = white + oriental, sca = scandinavian, as = asian)
14) For overlapping studies: principal rather than subsidiary studies
Finally by Age: whole study (coded as 0) if available, otherwise by widest available age group and then for single sex results (m, f) in preference to results for both sexes combined (c).

Results adjusted (AD) for the most potential confounders are then chosen in Sections -1 to -3 (and those which actually differ from the adjusted results in Table 1K1 - 1 are marked 'x' in Section -1) and results adjusted for the least confounders in Sections -4 to -6. (Those least adjusted results which actually differ from the most adjusted are marked 'x' in column X in Section -4)

Section -7 shows excluded studies, together with the stage (as above) at which no qualifying results were found.

Section -8 lists the potentially overlapping studies which have been included (1=principal, 2=subsidiary).

Section -9 lists any results which would have been included in preference except that they had data not complete enough for use in meta-analysis, with their significance (yes/no), if known, and any further comment as entered on the database. It also lists as "gap" any categories for which no data were presented by the original authors.

In addition to those mentioned above, the following fields, levels and abbreviations are used:
* or nk = not known, n = no, y = yes, ot = other
nev = never
all/unspec = all or unspecified, MC = manufactured cigarettes, HR = hand-rolled cigarettes
exL, exH = range of exposure (low and high) in the smoking group, in terms of Years quit (vs current)
REF: 6-character study reference
NRR: number of the RR on the database within the study
ST : study type (CC = case control, pr or prosp = prospective)
NLC: number of lung cancer cases in whole study
R : risky occupational population (n = no, m = mining, o = other risky)
VB : national cigarette type (V = at least 75% Virginia, bl = at least 75% blended, ot = other)
P : any proxy use
H : full histological confirmation
De : derivation of RR/CI (or = original, st = standard method, ot = other method of estimation)

Table 1K11 - 1

IESLC - Meta-analysis of Ex Smoking by Years quit (vs current), Overview
All LC types, Cigarettes only
Most adjusted

REF	NRR	1K1	SEX	AGEL	AGEH	RACE	YF	LC TYPE	LOC	START	ST	NLC	R	VB	P	H	AD	ADOS	PRODUCT	exL	exH	S1	S2	DENOM	De
ALDERS	513		m	0	0	all	-	all	Eu:UK	1977	CC	1448	n	V	n	n	1	0	cig only	0.1	2	0	0	current	ot
ALDERS	514		m	0	0	all	-	all	Eu:UK	1977	CC	1448	n	V	n	n	1	0	cig only	3	9	0	1	current	ot
ALDERS	515		m	0	0	all	-	all	Eu:UK	1977	CC	1448	n	V	n	n	1	0	cig only	10	999	3	0	current	ot
ALDERS	524		f	0	0	all	-	all	Eu:UK	1977	CC	1448	n	V	n	n	1	0	cig only	0.1	2	0	0	current	ot
ALDERS	525		f	0	0	all	-	all	Eu:UK	1977	CC	1448	n	V	n	n	1	0	cig only	3	9	0	1	current	ot
ALDERS	526		f	0	0	all	-	all	Eu:UK	1977	CC	1448	n	V	n	n	1	0	cig only	10	999	3	0	current	ot
BENHAM	603	x	m	0	0	all	-	all	Eu:wst	1976	CC	1625	n	bl	n	y	0	0	cig only	1.0	4	1	1	current	st
BENHAM	604	x	m	0	0	all	-	all	Eu:wst	1976	CC	1625	n	bl	n	y	0	0	cig only	5	9	2	0	current	st
BENHAM	605	x	m	0	0	all	-	all	Eu:wst	1976	CC	1625	n	bl	n	y	0	0	cig only	10	19	3	2	current	st
BENHAM	606	x	m	0	0	all	-	all	Eu:wst	1976	CC	1625	n	bl	n	y	0	0	cig only	20	999	0	3	current	st
CPSI	815		m	50	74	all	6	all	NAmer	1959	pr	5138	n	bl	n	n	1	0	cig only	0.1	0.9	0	0	current	ot
CPSI	816		m	50	74	all	6	all	NAmer	1959	pr	5138	n	bl	n	n	1	0	cig only	1.0	4	1	1	current	ot
CPSI	817		m	50	74	all	6	all	NAmer	1959	pr	5138	n	bl	n	n	1	0	cig only	5	9	2	0	current	ot
CPSI	818		m	50	74	all	6	all	NAmer	1959	pr	5138	n	bl	n	n	1	0	cig only	10	999	3	0	current	ot
CPSII	664		m	35	99	all	4	all	NAmer	1982	pr	3229	n	bl	n	n	1	0	cig only	0.1	0.9	0	0	current	ot
CPSII	665		m	35	99	all	4	all	NAmer	1982	pr	3229	n	bl	n	n	1	0	cig only	1.0	2	0	0	current	ot
CPSII	666		m	35	99	all	4	all	NAmer	1982	pr	3229	n	bl	n	n	1	0	cig only	3	5	1	1	current	ot
CPSII	667		m	35	99	all	4	all	NAmer	1982	pr	3229	n	bl	n	n	1	0	cig only	6	10	2	0	current	ot
CPSII	668		m	35	99	all	4	all	NAmer	1982	pr	3229	n	bl	n	n	1	0	cig only	11	15	3	2	current	ot
CPSII	669		m	35	99	all	4	all	NAmer	1982	pr	3229	n	bl	n	n	1	0	cig only	16	999	0	3	current	ot
DAMBER	557	x	m	0	0	all	-	all	Eu:Sca	1972	CC	579	n	bl	y	n	1	0	cig only	0.1	10	0	1	current	ot
DAMBER	558	x	m	0	0	all	-	all	Eu:Sca	1972	CC	579	n	bl	y	n	1	0	cig only	11	999	3	0	current	ot
DEAN3	524	x	m	0	0	all	-	all	Eu:UK	1969	CC	766	n	V	y	n	1	0	cig only	3	4	1	1	cur+2y	ot
DEAN3	525	x	m	0	0	all	-	all	Eu:UK	1969	CC	766	n	V	y	n	1	0	cig only	5	8	2	0	cur+2y	ot
DEAN3	526	x	m	0	0	all	-	all	Eu:UK	1969	CC	766	n	V	y	n	1	0	cig only	9	18	3	2	cur+2y	ot
DEAN3	527	x	m	0	0	all	-	all	Eu:UK	1969	CC	766	n	V	y	n	1	0	cig only	19	999	0	3	cur+2y	ot
DOLL2	509		m	0	0	all	20	all	Eu:UK	1951	pr	920	n	V	n	n	1	0	cig only	0.1	4	1	1	current	ot
DOLL2	510		m	0	0	all	20	all	Eu:UK	1951	pr	920	n	V	n	n	1	0	cig only	5	9	2	0	current	ot
DOLL2	511		m	0	0	all	20	all	Eu:UK	1951	pr	920	n	V	n	n	1	0	cig only	10	14	3	2	current	ot
DOLL2	512		m	0	0	all	20	all	Eu:UK	1951	pr	920	n	V	n	n	1	0	cig only	15	999	0	3	current	ot
DORN	574	x	m	0	0	wh	15	all	NAmer	1954	pr	5097	n	bl	n	n	2	0	cig only	0.1	4	1	1	current	ot
DORN	575	x	m	0	0	wh	15	all	NAmer	1954	pr	5097	n	bl	n	n	2	0	cig only	5	9	2	0	current	ot
DORN	576	x	m	0	0	wh	15	all	NAmer	1954	pr	5097	n	bl	n	n	2	0	cig only	10	14	3	2	current	ot
DORN	577	x	m	0	0	wh	15	all	NAmer	1954	pr	5097	n	bl	n	n	2	0	cig only	15	19	0	0	current	ot
DORN	578	x	m	0	0	wh	15	all	NAmer	1954	pr	5097	n	bl	n	n	2	0	cig only	20	999	0	3	current	ot
GRAHAM	508	x	m	0	0	wh	-	all	NAmer	1956	CC	685	n	bl	n	n	0	0	cig only	0.1	1.0	0	0	current	st
GRAHAM	509	x	m	0	0	wh	-	all	NAmer	1956	CC	685	n	bl	n	n	0	0	cig only	1.1	3	1	1	current	st
GRAHAM	510	x	m	0	0	wh	-	all	NAmer	1956	CC	685	n	bl	n	n	0	0	cig only	3	10	2	0	current	st
GRAHAM	511	x	m	0	0	wh	-	all	NAmer	1956	CC	685	n	bl	n	n	0	0	cig only	10	999	3	0	current	st
KAISE2	655		m	0	0	all	9	all	NAmer	1979	pr	318	n	bl	n	n	1	0	cig only	2	10	0	1	cur+2y	st
KAISE2	656		m	0	0	all	9	all	NAmer	1979	pr	318	n	bl	n	n	1	0	cig only	11	20	3	0	cur+2y	ot
KAISE2	657		m	0	0	all	9	all	NAmer	1979	pr	318	n	bl	n	n	1	0	cig only	21	999	0	0	cur+2y	st
KAISE2	575		f	0	0	all	9	all	NAmer	1979	pr	318	n	bl	n	n	1	0	cig only	2	10	0	1	cur+2y	st
KAISE2	576		f	0	0	all	9	all	NAmer	1979	pr	318	n	bl	n	n	1	0	cig only	11	20	3	0	cur+2y	st
KAISE2	577		f	0	0	all	9	all	NAmer	1979	pr	318	n	bl	n	n	1	0	cig only	21	999	0	0	cur+2y	ot
PEZZOT	504		m	0	0	all	-	all	SCAmer	1987	CC	215	n	bl	n	y	0	0	cig only	1.0	10	0	1	cur+1y	st
PEZZOT	505		m	0	0	all	-	all	SCAmer	1987	CC	215	n	bl	n	y	0	0	cig only	11	999	3	0	cur+1y	st
TVERDA	506		m	0	0	all	0	all	Eu:Sca	1972	pr	238	n	bl	n	n	2	0	cig only	0.1	0.9	0	0	current	ot
TVERDA	507		m	0	0	all	0	all	Eu:Sca	1972	pr	238	n	bl	n	n	2	0	cig only	1.0	5	1	1	current	ot
TVERDA	508		m	0	0	all	0	all	Eu:Sca	1972	pr	238	n	bl	n	n	2	0	cig only	5	999	0	0	current	ot
WYNDE6	513	x	m	0	0	all	-	all	NAmer	1969	CC	4423	n	bl	n	y	0	0	cig only	1.0	4	1	1	cur+1y	st
WYNDE6	514	x	m	0	0	all	-	all	NAmer	1969	CC	4423	n	bl	n	y	0	0	cig only	5	9	2	0	cur+1y	st
WYNDE6	515	x	m	0	0	all	-	all	NAmer	1969	CC	4423	n	bl	n	y	0	0	cig only	10	19	3	2	cur+1y	st
WYNDE6	516	x	m	0	0	all	-	all	NAmer	1969	CC	4423	n	bl	n	y	0	0	cig only	20	29	0	3	cur+1y	st
WYNDE6	517	x	m	0	0	all	-	all	NAmer	1969	CC	4423	n	bl	n	y	0	0	cig only	30	999	0	0	cur+1y	st
WYNDE6	534	x	f	0	0	all	-	all	NAmer	1969	CC	4423	n	bl	n	y	0	0	cig only	1.0	4	1	1	cur+1y	st
WYNDE6	535	x	f	0	0	all	-	all	NAmer	1969	CC	4423	n	bl	n	y	0	0	cig only	5	9	2	0	cur+1y	st
WYNDE6	536	x	f	0	0	all	-	all	NAmer	1969	CC	4423	n	bl	n	y	0	0	cig only	10	19	3	2	cur+1y	st
WYNDE6	537	x	f	0	0	all	-	all	NAmer	1969	CC	4423	n	bl	n	y	0	0	cig only	20	29	0	3	cur+1y	st
WYNDE6	538	x	f	0	0	all	-	all	NAmer	1969	CC	4423	n	bl	n	y	0	0	cig only	30	999	0	0	cur+1y	st

Cigarette type is all/unspec for all RRs
except for the following:

REF	NRR	CIGTYPE
ALDERS	513	MC only
ALDERS	514	MC only
ALDERS	515	MC only
ALDERS	524	MC only

Table 1K11 - 1

IESLC - Meta-analysis of Ex Smoking by Years quit (vs current), Overview
All LC types, Cigarettes only
Most adjusted

REF	NRR	CIGTYPE
ALDERS	525	MC only
ALDERS	526	MC only
DEAN3	524	MC only
DEAN3	525	MC only
DEAN3	526	MC only
DEAN3	527	MC only

In this overview table, subtotals and Qs values may be invalid and should be ignored

Table 1K11 - 2

IESLC - Meta-analysis of Ex Smoking by Years quit (vs current), Overview
All LC types, Cigarettes only
Most adjusted

REF	NRR	SEX	AD	Number Exposed Case	Cont	Non-exposed Case	Cont	RR	95.00%CI
ALDERS	513	m	1	121	-	207	-	1.81	(1.24- 2.64)
ALDERS	514	m	1	28	-	207	-	0.43	(0.26- 0.71)
ALDERS	515	m	1	29	-	207	-	0.32	(0.20- 0.51)
ALDERS	524	f	1	206	-	244	-	2.08	(1.49- 2.90)
ALDERS	525	f	1	54	-	244	-	0.65	(0.43- 0.99)
ALDERS	526	f	1	26	-	244	-	0.28	(0.17- 0.46)
Subtotal ALDERS								0.85	(0.72- 1.01)
BENHAM	603	m	0	154	138	776	969	1.39	(1.09- 1.79)
BENHAM	604	m	0	66	120	776	969	0.69	(0.50- 0.94)
BENHAM	605	m	0	42	147	776	969	0.36	(0.25- 0.51)
BENHAM	606	m	0	19	129	776	969	0.18	(0.11- 0.30)
Subtotal BENHAM								0.70	(0.60- 0.82)
*CPSI	815	m	1	37	-	844	-	1.07	(0.77- 1.49)
*CPSI	816	m	1	49	-	844	-	0.59	(0.44- 0.78)
*CPSI	817	m	1	32	-	844	-	0.37	(0.26- 0.53)
*CPSI	818	m	1	15	-	844	-	0.09	(0.06- 0.15)
Subtotal CPSI								0.48	(0.40- 0.57)
*CPSII	664	m	1	97	-	1159	-	1.77	(1.44- 2.17)
*CPSII	665	m	1	188	-	1159	-	1.28	(1.10- 1.49)
*CPSII	666	m	1	178	-	1159	-	0.85	(0.72- 0.99)
*CPSII	667	m	1	186	-	1159	-	0.52	(0.45- 0.61)
*CPSII	668	m	1	164	-	1159	-	0.39	(0.33- 0.46)
*CPSII	669	m	1	256	-	1159	-	0.17	(0.15- 0.20)
Subtotal CPSII								0.57	(0.54- 0.61)
DAMBER	557	m	1	-	-	-	-	0.56	(0.29- 1.08)
DAMBER	558	m	1	-	-	-	-	0.16	(0.08- 0.35)
Subtotal DAMBER								0.32	(0.20- 0.53)
DEAN3	524	m	1	28	-	337	-	0.64	(0.41- 1.00)
DEAN3	525	m	1	11	-	337	-	0.57	(0.29- 1.12)
DEAN3	526	m	1	15	-	337	-	0.41	(0.23- 0.72)
DEAN3	527	m	1	8	-	337	-	0.18	(0.08- 0.38)
Subtotal DEAN3								0.47	(0.35- 0.63)
*DOLL2	509	m	1	15	-	236	-	1.02	(0.61- 1.72)
*DOLL2	510	m	1	12	-	236	-	0.35	(0.20- 0.63)
*DOLL2	511	m	1	9	-	236	-	0.28	(0.14- 0.54)
*DOLL2	512	m	1	7	-	236	-	0.11	(0.05- 0.23)
Subtotal DOLL2								0.40	(0.30- 0.55)
*DORN	574	m	2	47	-	2609	-	1.66	(1.25- 2.22)
*DORN	575	m	2	86	-	2609	-	0.67	(0.54- 0.83)
*DORN	576	m	2	100	-	2609	-	0.40	(0.33- 0.49)
*DORN	577	m	2	115	-	2609	-	0.44	(0.37- 0.53)
*DORN	578	m	2	123	-	2609	-	0.19	(0.16- 0.22)
Subtotal DORN								0.41	(0.37- 0.44)
GRAHAM	508	m	0	84	48	371	821	3.87	(2.66- 5.64)
GRAHAM	509	m	0	12	23	371	821	1.15	(0.57- 2.35)
GRAHAM	510	m	0	5	29	371	821	0.38	(0.15- 0.99)
GRAHAM	511	m	0	2	30	371	821	0.15	(0.04- 0.62)
Subtotal GRAHAM								2.10	(1.55- 2.85)
*KAISE2	655	m	1	12	-	51	-	1.00	(0.53- 1.88)
*KAISE2	656	m	1	8	-	51	-	0.43	(0.20- 0.92)
*KAISE2	657	m	1	6	-	51	-	0.26	(0.10- 0.67)
*KAISE2	575	f	1	6	-	50	-	0.53	(0.23- 1.23)
*KAISE2	576	f	1	4	-	50	-	0.25	(0.09- 0.70)
*KAISE2	577	f	1	4	-	50	-	0.34	(0.13- 0.91)
Subtotal KAISE2								0.49	(0.35- 0.68)
PEZZOT	504	m	0	46	82	145	129	0.50	(0.32- 0.77)
PEZZOT	505	m	0	20	106	145	129	0.17	(0.10- 0.29)
Subtotal PEZZOT								0.32	(0.23- 0.45)
*TVERDA	506	m	2	2	-	144	-	0.17	(0.04- 0.70)
*TVERDA	507	m	2	5	-	144	-	0.18	(0.07- 0.43)
*TVERDA	508	m	2	4	-	144	-	0.08	(0.03- 0.23)
Subtotal TVERDA								0.13	(0.07- 0.25)
WYNDE6	513	m	0	201	166	1107	993	1.09	(0.87- 1.36)
WYNDE6	514	m	0	98	194	1107	993	0.45	(0.35- 0.59)
WYNDE6	515	m	0	159	373	1107	993	0.38	(0.31- 0.47)
WYNDE6	516	m	0	55	212	1107	993	0.23	(0.17- 0.32)
WYNDE6	517	m	0	21	161	1107	993	0.12	(0.07- 0.19)
WYNDE6	534	f	0	82	70	683	496	0.85	(0.61- 1.19)
WYNDE6	535	f	0	51	84	683	496	0.44	(0.31- 0.64)
WYNDE6	536	f	0	36	132	683	496	0.20	(0.13- 0.29)
WYNDE6	537	f	0	16	77	683	496	0.15	(0.09- 0.26)

Table 1K11 - 2

IESLC - Meta-analysis of Ex Smoking by Years quit (vs current), Overview
All LC types, Cigarettes only
Most adjusted

REF	NRR	SEX	AD	Number Exposed Case	Number Exposed Cont	Non-exposed Case	Non-exposed Cont	RR	95.00%CI
WYNDE6	538	f	0	10	31	683	496	0.23	(0.11- 0.48)
Subtotal WYNDE6								0.43	(0.39- 0.48)
Partial Totals				3472	2352	41583	14863		

*prospective study

REF	NRR	SEX	AD	Ys	Ws	Qs	Ps
ALDERS	513	m	1	0.59	26.91	42.04	0.0021
ALDERS	514	m	1	-0.84	15.23	0.53	0.0010
ALDERS	515	m	1	-1.14	17.54	4.09	0.0000
ALDERS	524	f	1	0.73	34.65	66.84	0.0000
ALDERS	525	f	1	-0.43	22.10	1.13	0.0429
ALDERS	526	f	1	-1.27	15.51	5.89	0.0000
Subtotal ALDERS				-0.16	131.92	120.52	
BENHAM	603	m	0	0.33	62.26	60.82	0.0088
BENHAM	604	m	0	-0.38	38.75	3.06	0.0193
BENHAM	605	m	0	-1.03	30.36	4.25	0.0000
BENHAM	606	m	0	-1.69	15.95	17.14	0.0000
Subtotal BENHAM				-0.35	147.33	85.27	
*CPSI	815	m	1	0.07	35.26	18.49	0.6879
*CPSI	816	m	1	-0.53	46.88	0.78	0.0003
*CPSI	817	m	1	-0.99	30.29	3.45	0.0000
*CPSI	818	m	1	-2.41	18.30	56.14	0.0000
Subtotal CPSI				-0.74	130.73	78.87	
*CPSII	664	m	1	0.57	91.37	137.68	0.0000
*CPSII	665	m	1	0.25	166.85	136.18	0.0014
*CPSII	666	m	1	-0.16	151.52	36.98	0.0454
*CPSII	667	m	1	-0.65	166.04	0.00	0.0000
*CPSII	668	m	1	-0.94	139.29	11.32	0.0000
*CPSII	669	m	1	-1.77	185.67	230.99	0.0000
Subtotal CPSII				-0.56	900.74	553.15	
DAMBER	557	m	1	-0.58	8.89	0.05	0.0839
DAMBER	558	m	1	-1.83	7.05	9.76	0.0000
Subtotal DAMBER				-1.13	15.94	9.81	
DEAN3	524	m	1	-0.45	19.33	0.85	0.0497
DEAN3	525	m	1	-0.56	8.42	0.08	0.1029
DEAN3	526	m	1	-0.89	11.80	0.65	0.0022
DEAN3	527	m	1	-1.71	6.33	7.09	0.0000
Subtotal DEAN3				-0.76	45.87	8.67	
*DOLL2	509	m	1	0.02	14.30	6.54	0.9403
*DOLL2	510	m	1	-1.05	11.67	1.81	0.0003
*DOLL2	511	m	1	-1.27	8.43	3.20	0.0002
*DOLL2	512	m	1	-2.21	6.60	15.87	0.0000
Subtotal DOLL2				-0.91	41.00	27.42	
*DORN	574	m	2	0.51	46.58	63.04	0.0005
*DORN	575	m	2	-0.40	83.16	5.45	0.0003
*DORN	576	m	2	-0.92	98.33	6.63	0.0000
*DORN	577	m	2	-0.82	118.98	3.22	0.0000
*DORN	578	m	2	-1.66	151.52	152.79	0.0000
Subtotal DORN				-0.90	498.56	231.13	
GRAHAM	508	m	0	1.35	27.28	110.28	0.0000
GRAHAM	509	m	0	0.14	7.65	4.90	0.6910
GRAHAM	510	m	0	-0.96	4.19	0.40	0.0484
GRAHAM	511	m	0	-1.91	1.86	2.94	0.0090
Subtotal GRAHAM				0.74	40.99	118.52	
*KAISE2	655	m	1	0.00	9.58	4.13	1.0000
*KAISE2	656	m	1	-0.84	6.60	0.23	0.0302
*KAISE2	657	m	1	-1.35	4.25	2.03	0.0055
*KAISE2	575	f	1	-0.63	5.47	0.00	0.1377
*KAISE2	576	f	1	-1.39	3.65	1.94	0.0081
*KAISE2	577	f	1	-1.08	4.06	0.72	0.0298
Subtotal KAISE2				-0.72	33.61	9.06	
PEZZOT	504	m	0	-0.69	20.58	0.03	0.0016
PEZZOT	505	m	0	-1.78	13.50	17.18	0.0000
Subtotal PEZZOT				-1.13	34.08	17.21	
*TVERDA	506	m	2	-1.77	1.88	2.33	0.0152
*TVERDA	507	m	2	-1.71	4.66	5.22	0.0002
*TVERDA	508	m	2	-2.53	3.70	12.94	0.0000
Subtotal TVERDA				-2.02	10.24	20.50	
WYNDE6	513	m	0	0.08	77.46	42.33	0.4670
WYNDE6	514	m	0	-0.79	57.91	1.06	0.0000

Table 1K11 - 2

IESLC - Meta-analysis of Ex Smoking by Years quit (vs current), Overview
All LC types, Cigarettes only
Most adjusted

REF	NRR	SEX	AD	Ys	Ws	Qs	Ps
WYNDE6	515	m	0	-0.96	91.91	8.54	0.0000
WYNDE6	516	m	0	-1.46	40.31	25.89	0.0000
WYNDE6	517	m	0	-2.15	17.94	39.78	0.0000
WYNDE6	534	f	0	-0.16	33.38	8.17	0.3502
WYNDE6	535	f	0	-0.82	28.58	0.75	0.0000
WYNDE6	536	f	0	-1.62	25.75	23.86	0.0000
WYNDE6	537	f	0	-1.89	12.66	19.30	0.0000
WYNDE6	538	f	0	-1.45	7.37	4.65	0.0001
Subtotal WYNDE6				-0.84	393.26	174.33	

N	60
NS	13

Table 1K11 - 3

IESLC - Meta-analysis of Ex Smoking by Years quit (vs current), Overview
All LC types, Cigarettes only
Most adjusted

	Sex			
	combined	male	female	Total
N		49	11	60
NS		13	3	16

In this overview table, other than the "N" rows, entries in the "absent" and "Total" columns may be invalid and should be ignored

	Years quit vs current (lower focus)				
	absent	1-6k3	4-11k7	8+k12	Total
N	26	10	9	15	60
NS	13	9	8	12	42
Wt	1041.37	464.02	429.01	489.88	2424.28
Het Chi	1047.01	54.19	15.99	60.31	1454.44
Het df	25	9	8	14	59
Het P	***	***	*	***	***
Fixed RR	0.48	0.96	0.52	0.33	0.52
RRl	0.46	0.88	0.48	0.30	0.50
RRu	0.51	1.05	0.58	0.36	0.54
P	---	N.S.	---	---	---
Random RR	0.43	0.90	0.51	0.28	0.44
RRl	0.28	0.70	0.44	0.22	0.36
RRu	0.65	1.15	0.59	0.34	0.54
P	---	N.S.	---	---	---

	Years quit vs current (higher focus)				
	absent	1-11k3	4-19k12	13+k20	Total
N	30	16	7	7	60
NS	13	13	6	6	38
Wt	1053.51	545.86	405.87	419.03	2424.28
Het Chi	614.71	77.43	11.96	5.69	1454.44
Het df	29	15	6	6	59
Het P	***	***	(*)	N.S.	***
Fixed RR	0.68	0.89	0.37	0.18	0.52
RRl	0.64	0.82	0.34	0.16	0.50
RRu	0.72	0.97	0.41	0.20	0.54
P	---	--	---	---	---
Random RR	0.44	0.78	0.35	0.18	0.44
RRl	0.33	0.63	0.30	0.16	0.36
RRu	0.60	0.96	0.41	0.20	0.54
P	---	-	---	---	---

Table 1K11 - 3

IESLC - Meta-analysis of Ex Smoking by Years quit (vs current), Overview
All LC types, Cigarettes only
Most adjusted

MALES

Years quit vs current (lower focus)

	absent	1-6k3	4-11k7	8+k12	Total
N	20	9	8	12	49
NS	13	9	8	12	42
Wt	955.07	430.64	400.43	444.97	2231.11
Het Chi	948.37	53.68	15.07	51.95	1321.15
Het df	19	8	7	11	48
Het P	***	***	*	***	***
Fixed RR	0.47	0.97	0.53	0.35	0.52
RRl	0.44	0.88	0.48	0.32	0.50
RRu	0.50	1.06	0.59	0.38	0.54
P	---	N.S.	---	---	---
Random RR	0.41	0.90	0.52	0.29	0.45
RRl	0.26	0.68	0.44	0.22	0.35
RRu	0.67	1.19	0.61	0.36	0.56
P	---	N.S.	---	---	---

Years quit vs current (higher focus)

	absent	1-11k3	4-19k12	13+k20	Total
N	24	13	6	6	49
NS	13	13	6	6	38
Wt	959.70	484.92	380.12	406.37	2231.11
Het Chi	539.85	73.49	1.25	5.25	1321.15
Het df	23	12	5	5	48
Het P	***	***	N.S.	N.S.	***
Fixed RR	0.68	0.91	0.39	0.18	0.52
RRl	0.64	0.83	0.35	0.17	0.50
RRu	0.72	0.99	0.43	0.20	0.54
P	---	-	---	---	---
Random RR	0.44	0.79	0.39	0.18	0.45
RRl	0.32	0.62	0.35	0.16	0.35
RRu	0.62	1.02	0.43	0.20	0.56
P	---	(-)	---	---	---

FEMALES

Years quit vs current (lower focus)

	absent	1-6k3	4-11k7	8+k12	Total
N	6	1	1	3	11
NS	3	1	1	3	6
Wt	86.30	33.38	28.58	44.91	193.16
Het Chi	82.20	0.00	0.00	1.20	133.15
Het df	5	0	0	2	10
Het P	***	N.S.	N.S.	N.S.	***
Fixed RR	0.73	0.85	0.44	0.23	0.53
RRl	0.59	0.61	0.31	0.17	0.46
RRu	0.91	1.19	0.64	0.30	0.61
P	--	N.S.	---	---	---
Random RR	0.46	0.85	0.44	0.23	0.41
RRl	0.18	0.61	0.31	0.17	0.24
RRu	1.16	1.19	0.64	0.30	0.71
P	N.S.	N.S.	---	---	--

Table 1K11 - 3

IESLC - Meta-analysis of Ex Smoking by Years quit (vs current), Overview
All LC types, Cigarettes only
Most adjusted

FEMALES

		Years quit vs current (higher focus)				
		absent	1-11k3	4-19k12	13+k20	Total
	N	6	3	1	1	11
	NS	3	3	1	1	6
	Wt	93.81	60.94	25.75	12.66	193.16
Het	Chi	74.83	1.63	0.00	0.00	133.15
Het	df	5	2	0	0	10
Het	P	***	N.S.	N.S.	N.S.	***
Fixed	RR	0.67	0.74	0.20	0.15	0.53
	RRl	0.55	0.58	0.13	0.09	0.46
	RRu	0.82	0.95	0.29	0.26	0.61
	P	---	-	---	---	---
Random	RR	0.43	0.74	0.20	0.15	0.41
	RRl	0.18	0.58	0.13	0.09	0.24
	RRu	1.01	0.95	0.29	0.26	0.71
	P	(-)	-	---	---	--

Table 1K11 - 4

IESLC - Meta-analysis of Ex Smoking by Years quit (vs current), Overview
All LC types, Cigarettes only
Least adjusted

REF	NRR	X	SEX	AGEL	AGEH	RACE	YF	LC TYPE	LOC	START	ST	NLC	R	VB	P	H	AD	ADOS	PRODUCT	exL	exH	S1	S2	DENOM	De
ALDERS	513		m	0	0	all	-	all	Eu:UK	1977	CC	1448	n	V	n	n	1	0	cig only	0.1	2	0	0	current	ot
ALDERS	514		m	0	0	all	-	all	Eu:UK	1977	CC	1448	n	V	n	n	1	0	cig only	3	9	0	1	current	ot
ALDERS	515		m	0	0	all	-	all	Eu:UK	1977	CC	1448	n	V	n	n	1	0	cig only	10	999	3	0	current	ot
ALDERS	524		f	0	0	all	-	all	Eu:UK	1977	CC	1448	n	V	n	n	1	0	cig only	0.1	2	0	0	current	ot
ALDERS	525		f	0	0	all	-	all	Eu:UK	1977	CC	1448	n	V	n	n	1	0	cig only	3	9	0	1	current	ot
ALDERS	526		f	0	0	all	-	all	Eu:UK	1977	CC	1448	n	V	n	n	1	0	cig only	10	999	3	0	current	ot
BENHAM	603		m	0	0	all	-	all	Eu:wst	1976	CC	1625	n	bl	n	y	0	0	cig only	1.0	4	1	1	current	st
BENHAM	604		m	0	0	all	-	all	Eu:wst	1976	CC	1625	n	bl	n	y	0	0	cig only	5	9	2	0	current	st
BENHAM	605		m	0	0	all	-	all	Eu:wst	1976	CC	1625	n	bl	n	y	0	0	cig only	10	19	3	2	current	st
BENHAM	606		m	0	0	all	-	all	Eu:wst	1976	CC	1625	n	bl	n	y	0	0	cig only	20	999	0	3	current	st
CPSI	815		m	50	74	all	6	all	NAmer	1959	pr	5138	n	bl	n	n	1	0	cig only	0.1	0.9	0	0	current	ot
CPSI	816		m	50	74	all	6	all	NAmer	1959	pr	5138	n	bl	n	n	1	0	cig only	1.0	4	1	1	current	ot
CPSI	817		m	50	74	all	6	all	NAmer	1959	pr	5138	n	bl	n	n	1	0	cig only	5	9	2	0	current	ot
CPSI	818		m	50	74	all	6	all	NAmer	1959	pr	5138	n	bl	n	n	1	0	cig only	10	999	3	0	current	ot
CPSII	664		m	35	99	all	4	all	NAmer	1982	pr	3229	n	bl	n	n	1	0	cig only	0.1	0.9	0	0	current	ot
CPSII	665		m	35	99	all	4	all	NAmer	1982	pr	3229	n	bl	n	n	1	0	cig only	1.0	2	0	0	current	ot
CPSII	666		m	35	99	all	4	all	NAmer	1982	pr	3229	n	bl	n	n	1	0	cig only	3	5	1	1	current	ot
CPSII	667		m	35	99	all	4	all	NAmer	1982	pr	3229	n	bl	n	n	1	0	cig only	6	10	2	0	current	ot
CPSII	668		m	35	99	all	4	all	NAmer	1982	pr	3229	n	bl	n	n	1	0	cig only	11	15	3	2	current	ot
CPSII	669		m	35	99	all	4	all	NAmer	1982	pr	3229	n	bl	n	n	1	0	cig only	16	999	0	3	current	ot
DAMBER	557		m	0	0	all	-	all	Eu:Sca	1972	CC	579	n	bl	y	n	1	0	cig only	0.1	10	0	1	current	ot
DAMBER	558		m	0	0	all	-	all	Eu:Sca	1972	CC	579	n	bl	y	n	1	0	cig only	11	999	3	0	current	ot
DEAN3	509	x	m	0	0	all	-	all	Eu:UK	1969	CC	766	n	V	y	n	0	0	cig only	3	4	1	1	cur+2y	st
DEAN3	510	x	m	0	0	all	-	all	Eu:UK	1969	CC	766	n	V	y	n	0	0	cig only	5	8	2	0	cur+2y	st
DEAN3	511	x	m	0	0	all	-	all	Eu:UK	1969	CC	766	n	V	y	n	0	0	cig only	9	18	3	2	cur+2y	st
DEAN3	512	x	m	0	0	all	-	all	Eu:UK	1969	CC	766	n	V	y	n	0	0	cig only	19	999	0	3	cur+2y	st
DOLL2	509		m	0	0	all	20	all	Eu:UK	1951	pr	920	n	V	n	n	1	0	cig only	0.1	4	1	1	current	ot
DOLL2	510		m	0	0	all	20	all	Eu:UK	1951	pr	920	n	V	n	n	1	0	cig only	5	9	2	0	current	ot
DOLL2	511		m	0	0	all	20	all	Eu:UK	1951	pr	920	n	V	n	n	1	0	cig only	10	14	3	2	current	ot
DOLL2	512		m	0	0	all	20	all	Eu:UK	1951	pr	920	n	V	n	n	1	0	cig only	15	999	0	3	current	ot
DORN	574		m	0	0	wh	15	all	NAmer	1954	pr	5097	n	bl	n	n	2	0	cig only	0.1	4	1	1	current	ot
DORN	575		m	0	0	wh	15	all	NAmer	1954	pr	5097	n	bl	n	n	2	0	cig only	5	9	2	0	current	ot
DORN	576		m	0	0	wh	15	all	NAmer	1954	pr	5097	n	bl	n	n	2	0	cig only	10	14	3	2	current	ot
DORN	577		m	0	0	wh	15	all	NAmer	1954	pr	5097	n	bl	n	n	2	0	cig only	15	19	0	0	current	ot
DORN	578		m	0	0	wh	15	all	NAmer	1954	pr	5097	n	bl	n	n	2	0	cig only	20	999	0	3	current	ot
GRAHAM	508		m	0	0	wh	-	all	NAmer	1956	CC	685	n	bl	n	n	0	0	cig only	0.1	1.0	0	0	current	st
GRAHAM	509		m	0	0	wh	-	all	NAmer	1956	CC	685	n	bl	n	n	0	0	cig only	1.1	3	1	1	current	st
GRAHAM	510		m	0	0	wh	-	all	NAmer	1956	CC	685	n	bl	n	n	0	0	cig only	3	10	2	0	current	st
GRAHAM	511		m	0	0	wh	-	all	NAmer	1956	CC	685	n	bl	n	n	0	0	cig only	10	999	3	0	current	st
KAISE2	655		m	0	0	all	9	all	NAmer	1979	pr	318	n	bl	n	n	1	0	cig only	2	10	0	1	cur+2y	st
KAISE2	656		m	0	0	all	9	all	NAmer	1979	pr	318	n	bl	n	n	1	0	cig only	11	20	3	0	cur+2y	ot
KAISE2	657		m	0	0	all	9	all	NAmer	1979	pr	318	n	bl	n	n	1	0	cig only	21	999	0	0	cur+2y	st
KAISE2	575		f	0	0	all	9	all	NAmer	1979	pr	318	n	bl	n	n	1	0	cig only	2	10	0	1	cur+2y	st
KAISE2	576		f	0	0	all	9	all	NAmer	1979	pr	318	n	bl	n	n	1	0	cig only	11	20	3	0	cur+2y	st
KAISE2	577		f	0	0	all	9	all	NAmer	1979	pr	318	n	bl	n	n	1	0	cig only	21	999	0	0	cur+2y	ot
PEZZOT	504		m	0	0	all	-	all	SCAmer	1987	CC	215	n	bl	n	y	0	0	cig only	1.0	10	0	1	cur+1y	st
PEZZOT	505		m	0	0	all	-	all	SCAmer	1987	CC	215	n	bl	n	y	0	0	cig only	11	999	3	0	cur+1y	st
TVERDA	506		m	0	0	all	0	all	Eu:Sca	1972	pr	238	n	bl	n	n	2	0	cig only	0.1	0.9	0	0	current	ot
TVERDA	507		m	0	0	all	0	all	Eu:Sca	1972	pr	238	n	bl	n	n	2	0	cig only	1.0	5	1	1	current	ot
TVERDA	508		m	0	0	all	0	all	Eu:Sca	1972	pr	238	n	bl	n	n	2	0	cig only	5	999	0	0	current	ot
WYNDE6	513		m	0	0	all	-	all	NAmer	1969	CC	4423	n	bl	n	y	0	0	cig only	1.0	4	1	1	cur+1y	st
WYNDE6	514		m	0	0	all	-	all	NAmer	1969	CC	4423	n	bl	n	y	0	0	cig only	5	9	2	0	cur+1y	st
WYNDE6	515		m	0	0	all	-	all	NAmer	1969	CC	4423	n	bl	n	y	0	0	cig only	10	19	3	2	cur+1y	st
WYNDE6	516		m	0	0	all	-	all	NAmer	1969	CC	4423	n	bl	n	y	0	0	cig only	20	29	0	3	cur+1y	st
WYNDE6	517		m	0	0	all	-	all	NAmer	1969	CC	4423	n	bl	n	y	0	0	cig only	30	999	0	0	cur+1y	st
WYNDE6	534		f	0	0	all	-	all	NAmer	1969	CC	4423	n	bl	n	y	0	0	cig only	1.0	4	1	1	cur+1y	st
WYNDE6	535		f	0	0	all	-	all	NAmer	1969	CC	4423	n	bl	n	y	0	0	cig only	5	9	2	0	cur+1y	st
WYNDE6	536		f	0	0	all	-	all	NAmer	1969	CC	4423	n	bl	n	y	0	0	cig only	10	19	3	2	cur+1y	st
WYNDE6	537		f	0	0	all	-	all	NAmer	1969	CC	4423	n	bl	n	y	0	0	cig only	20	29	0	3	cur+1y	st
WYNDE6	538		f	0	0	all	-	all	NAmer	1969	CC	4423	n	bl	n	y	0	0	cig only	30	999	0	0	cur+1y	st

Cigarette type is all/unspec for all RRs
except for the following:

REF	NRR	CIGTYPE
ALDERS	513	MC only
ALDERS	514	MC only
ALDERS	515	MC only
ALDERS	524	MC only

Table 1K11 - 4

IESLC - Meta-analysis of Ex Smoking by Years quit (vs current), Overview
All LC types, Cigarettes only
Least adjusted

REF	NRR	CIGTYPE
ALDERS	525	MC only
ALDERS	526	MC only
DEAN3	509	MC only
DEAN3	510	MC only
DEAN3	511	MC only
DEAN3	512	MC only

In this overview table, subtotals and Qs values may be invalid and should be ignored

Table 1K11 - 5

IESLC - Meta-analysis of Ex Smoking by Years quit (vs current), Overview
All LC types, Cigarettes only
Least adjusted

REF	NRR	SEX	AD	Number Exposed Case	Cont	Non-exposed Case	Cont	RR	95.00%CI
ALDERS	513	m	1	121	-	207	-	1.81	(1.24- 2.64)
ALDERS	514	m	1	28	-	207	-	0.43	(0.26- 0.71)
ALDERS	515	m	1	29	-	207	-	0.32	(0.20- 0.51)
ALDERS	524	f	1	206	-	244	-	2.08	(1.49- 2.90)
ALDERS	525	f	1	54	-	244	-	0.65	(0.43- 0.99)
ALDERS	526	f	1	26	-	244	-	0.28	(0.17- 0.46)
Subtotal ALDERS								0.85	(0.72- 1.01)
BENHAM	603	m	0	154	138	776	969	1.39	(1.09- 1.79)
BENHAM	604	m	0	66	120	776	969	0.69	(0.50- 0.94)
BENHAM	605	m	0	42	147	776	969	0.36	(0.25- 0.51)
BENHAM	606	m	0	19	129	776	969	0.18	(0.11- 0.30)
Subtotal BENHAM								0.70	(0.60- 0.82)
*CPSI	815	m	1	37	-	844	-	1.07	(0.77- 1.49)
*CPSI	816	m	1	49	-	844	-	0.59	(0.44- 0.78)
*CPSI	817	m	1	32	-	844	-	0.37	(0.26- 0.53)
*CPSI	818	m	1	15	-	844	-	0.09	(0.06- 0.15)
Subtotal CPSI								0.48	(0.40- 0.57)
*CPSII	664	m	1	97	-	1159	-	1.77	(1.44- 2.17)
*CPSII	665	m	1	188	-	1159	-	1.28	(1.10- 1.49)
*CPSII	666	m	1	178	-	1159	-	0.85	(0.72- 0.99)
*CPSII	667	m	1	186	-	1159	-	0.52	(0.45- 0.61)
*CPSII	668	m	1	164	-	1159	-	0.39	(0.33- 0.46)
*CPSII	669	m	1	256	-	1159	-	0.17	(0.15- 0.20)
Subtotal CPSII								0.57	(0.54- 0.61)
DAMBER	557	m	1	-	-	-	-	0.56	(0.29- 1.08)
DAMBER	558	m	1	-	-	-	-	0.16	(0.08- 0.35)
Subtotal DAMBER								0.32	(0.20- 0.53)
DEAN3	509	m	0	28	102	337	930	0.76	(0.49- 1.17)
DEAN3	510	m	0	11	43	337	930	0.71	(0.36- 1.38)
DEAN3	511	m	0	15	86	337	930	0.48	(0.27- 0.84)
DEAN3	512	m	0	8	66	337	930	0.33	(0.16- 0.70)
Subtotal DEAN3								0.59	(0.45- 0.79)
*DOLL2	509	m	1	15	-	236	-	1.02	(0.61- 1.72)
*DOLL2	510	m	1	12	-	236	-	0.35	(0.20- 0.63)
*DOLL2	511	m	1	9	-	236	-	0.28	(0.14- 0.54)
*DOLL2	512	m	1	7	-	236	-	0.11	(0.05- 0.23)
Subtotal DOLL2								0.40	(0.30- 0.55)
*DORN	574	m	2	47	-	2609	-	1.66	(1.25- 2.22)
*DORN	575	m	2	86	-	2609	-	0.67	(0.54- 0.83)
*DORN	576	m	2	100	-	2609	-	0.40	(0.33- 0.49)
*DORN	577	m	2	115	-	2609	-	0.44	(0.37- 0.53)
*DORN	578	m	2	123	-	2609	-	0.19	(0.16- 0.22)
Subtotal DORN								0.41	(0.37- 0.44)
GRAHAM	508	m	0	84	48	371	821	3.87	(2.66- 5.64)
GRAHAM	509	m	0	12	23	371	821	1.15	(0.57- 2.35)
GRAHAM	510	m	0	5	29	371	821	0.38	(0.15- 0.99)
GRAHAM	511	m	0	2	30	371	821	0.15	(0.04- 0.62)
Subtotal GRAHAM								2.10	(1.55- 2.85)
*KAISE2	655	m	1	12	-	51	-	1.00	(0.53- 1.88)
*KAISE2	656	m	1	8	-	51	-	0.43	(0.20- 0.92)
*KAISE2	657	m	1	6	-	51	-	0.26	(0.10- 0.67)
*KAISE2	575	f	1	6	-	50	-	0.53	(0.23- 1.23)
*KAISE2	576	f	1	4	-	50	-	0.25	(0.09- 0.70)
*KAISE2	577	f	1	4	-	50	-	0.34	(0.13- 0.91)
Subtotal KAISE2								0.49	(0.35- 0.68)
PEZZOT	504	m	0	46	82	145	129	0.50	(0.32- 0.77)
PEZZOT	505	m	0	20	106	145	129	0.17	(0.10- 0.29)
Subtotal PEZZOT								0.32	(0.23- 0.45)
*TVERDA	506	m	2	2	-	144	-	0.17	(0.04- 0.70)
*TVERDA	507	m	2	5	-	144	-	0.18	(0.07- 0.43)
*TVERDA	508	m	2	4	-	144	-	0.08	(0.03- 0.23)
Subtotal TVERDA								0.13	(0.07- 0.25)
WYNDE6	513	m	0	201	166	1107	993	1.09	(0.87- 1.36)
WYNDE6	514	m	0	98	194	1107	993	0.45	(0.35- 0.59)
WYNDE6	515	m	0	159	373	1107	993	0.38	(0.31- 0.47)
WYNDE6	516	m	0	55	212	1107	993	0.23	(0.17- 0.32)
WYNDE6	517	m	0	21	161	1107	993	0.12	(0.07- 0.19)
WYNDE6	534	f	0	82	70	683	496	0.85	(0.61- 1.19)
WYNDE6	535	f	0	51	84	683	496	0.44	(0.31- 0.64)
WYNDE6	536	f	0	36	132	683	496	0.20	(0.13- 0.29)
WYNDE6	537	f	0	16	77	683	496	0.15	(0.09- 0.26)

Table 1K11 - 5

IESLC - Meta-analysis of Ex Smoking by Years quit (vs current), Overview
All LC types, Cigarettes only
Least adjusted

REF	NRR	SEX	AD	Number Exposed Case	Number Exposed Cont	Non-exposed Case	Non-exposed Cont	RR	95.00%CI
WYNDE6	538	f	0	10	31	683	496	0.23	(0.11- 0.48)
Subtotal WYNDE6								0.43	(0.39- 0.48)
Partial Totals				3472	2649	41583	18583		

*prospective study

REF	NRR	SEX	AD	Ys	Ws	Qs	Ps
ALDERS	513	m	1	0.59	26.91	41.73	0.0021
ALDERS	514	m	1	-0.84	15.23	0.56	0.0010
ALDERS	515	m	1	-1.14	17.54	4.17	0.0000
ALDERS	524	f	1	0.73	34.65	66.41	0.0000
ALDERS	525	f	1	-0.43	22.10	1.08	0.0429
ALDERS	526	f	1	-1.27	15.51	5.98	0.0000
Subtotal ALDERS				-0.16	131.92	119.93	
BENHAM	603	m	0	0.33	62.26	60.27	0.0088
BENHAM	604	m	0	-0.38	38.75	2.96	0.0193
BENHAM	605	m	0	-1.03	30.36	4.35	0.0000
BENHAM	606	m	0	-1.69	15.95	17.29	0.0000
Subtotal BENHAM				-0.35	147.33	84.87	
*CPSI	815	m	1	0.07	35.26	18.26	0.6879
*CPSI	816	m	1	-0.53	46.88	0.73	0.0003
*CPSI	817	m	1	-0.99	30.29	3.55	0.0000
*CPSI	818	m	1	-2.41	18.30	56.43	0.0000
Subtotal CPSI				-0.74	130.73	78.96	
*CPSII	664	m	1	0.57	91.37	136.67	0.0000
*CPSII	665	m	1	0.25	166.85	134.83	0.0014
*CPSII	666	m	1	-0.16	151.52	36.31	0.0454
*CPSII	667	m	1	-0.65	166.04	0.00	0.0000
*CPSII	668	m	1	-0.94	139.29	11.68	0.0000
*CPSII	669	m	1	-1.77	185.67	232.86	0.0000
Subtotal CPSII				-0.56	900.74	552.35	
DAMBER	557	m	1	-0.58	8.89	0.05	0.0839
DAMBER	558	m	1	-1.83	7.05	9.83	0.0000
Subtotal DAMBER				-1.13	15.94	9.88	
DEAN3	509	m	0	-0.28	20.18	2.83	0.2123
DEAN3	510	m	0	-0.35	8.46	0.78	0.3112
DEAN3	511	m	0	-0.73	12.15	0.08	0.0108
DEAN3	512	m	0	-1.10	6.94	1.36	0.0039
Subtotal DEAN3				-0.52	47.72	5.05	
*DOLL2	509	m	1	0.02	14.30	6.45	0.9403
*DOLL2	510	m	1	-1.05	11.67	1.85	0.0003
*DOLL2	511	m	1	-1.27	8.43	3.25	0.0002
*DOLL2	512	m	1	-2.21	6.60	15.96	0.0000
Subtotal DOLL2				-0.91	41.00	27.51	
*DORN	574	m	2	0.51	46.58	62.55	0.0005
*DORN	575	m	2	-0.40	83.16	5.26	0.0003
*DORN	576	m	2	-0.92	98.33	6.87	0.0000
*DORN	577	m	2	-0.82	118.98	3.40	0.0000
*DORN	578	m	2	-1.66	151.52	154.16	0.0000
Subtotal DORN				-0.90	498.56	232.24	
GRAHAM	508	m	0	1.35	27.28	109.79	0.0000
GRAHAM	509	m	0	0.14	7.65	4.84	0.6910
GRAHAM	510	m	0	-0.96	4.19	0.41	0.0484
GRAHAM	511	m	0	-1.91	1.86	2.96	0.0090
Subtotal GRAHAM				0.74	40.99	118.01	
*KAISE2	655	m	1	0.00	9.58	4.08	1.0000
*KAISE2	656	m	1	-0.84	6.60	0.24	0.0302
*KAISE2	657	m	1	-1.35	4.25	2.05	0.0055
*KAISE2	575	f	1	-0.63	5.47	0.00	0.1377
*KAISE2	576	f	1	-1.39	3.65	1.97	0.0081
*KAISE2	577	f	1	-1.08	4.06	0.74	0.0298
Subtotal KAISE2				-0.72	33.61	9.08	
PEZZOT	504	m	0	-0.69	20.58	0.04	0.0016
PEZZOT	505	m	0	-1.78	13.50	17.31	0.0000
Subtotal PEZZOT				-1.13	34.08	17.35	
*TVERDA	506	m	2	-1.77	1.88	2.35	0.0152
*TVERDA	507	m	2	-1.71	4.66	5.27	0.0002
*TVERDA	508	m	2	-2.53	3.70	13.00	0.0000
Subtotal TVERDA				-2.02	10.24	20.62	
WYNDE6	513	m	0	0.08	77.46	41.81	0.4670
WYNDE6	514	m	0	-0.79	57.91	1.13	0.0000

Table 1K11 - 5

IESLC - Meta-analysis of Ex Smoking by Years quit (vs current), Overview
All LC types, Cigarettes only
Least adjusted

REF	NRR	SEX	AD	Ys	Ws	Qs	Ps
WYNDE6	515	m	0	-0.96	91.91	8.79	0.0000
WYNDE6	516	m	0	-1.46	40.31	26.18	0.0000
WYNDE6	517	m	0	-2.15	17.94	40.02	0.0000
WYNDE6	534	f	0	-0.16	33.38	8.03	0.3502
WYNDE6	535	f	0	-0.82	28.58	0.80	0.0000
WYNDE6	536	f	0	-1.62	25.75	24.09	0.0000
WYNDE6	537	f	0	-1.89	12.66	19.44	0.0000
WYNDE6	538	f	0	-1.45	7.37	4.71	0.0001
Subtotal WYNDE6				-0.84	393.26	174.98	

N	60
NS	13

Table 1K11 - 6

IESLC - Meta-analysis of Ex Smoking by Years quit (vs current), Overview
All LC types, Cigarettes only
Least adjusted

	Sex combined	male	female	Total
N		49	11	60
NS		13	3	16

In this overview table, other than the "N" rows, entries in the "absent" and "Total" columns may be invalid and should be ignored

	Years quit vs current (lower focus) absent	1-6k3	4-11k7	8+k12	Total
N	26	10	9	15	60
NS	13	9	8	12	42
Wt	1041.97	464.87	429.05	490.23	2426.12
Het Chi	1041.76	52.13	16.68	61.44	1450.82
Het df	25	9	8	14	59
Het P	***	***	*	***	***
Fixed RR	0.49	0.97	0.53	0.33	0.52
RRl	0.46	0.88	0.48	0.31	0.50
RRu	0.52	1.06	0.58	0.37	0.54
P	---	N.S.	---	---	---
Random RR	0.44	0.92	0.51	0.28	0.45
RRl	0.29	0.72	0.44	0.22	0.36
RRu	0.66	1.17	0.60	0.35	0.55
P	---	N.S.	---	---	---

	Years quit vs current (higher focus) absent	1-11k3	4-19k12	13+k20	Total
N	30	16	7	7	60
NS	13	13	6	6	38
Wt	1053.55	546.71	406.22	419.63	2426.12
Het Chi	614.46	75.84	12.67	8.25	1450.82
Het df	29	15	6	6	59
Het P	***	***	*	N.S.	***
Fixed RR	0.68	0.89	0.37	0.18	0.52
RRl	0.64	0.82	0.34	0.17	0.50
RRu	0.72	0.97	0.41	0.20	0.54
P	---	--	---	---	---
Random RR	0.45	0.79	0.36	0.19	0.45
RRl	0.33	0.64	0.30	0.16	0.36
RRu	0.60	0.97	0.42	0.21	0.55
P	---	-	---	---	---

Table 1K11 - 6

IESLC - Meta-analysis of Ex Smoking by Years quit (vs current), Overview
All LC types, Cigarettes only
Least adjusted

MALES

Years quit vs current (lower focus)

		absent	1-6k3	4-11k7	8+k12	Total
	N	20	9	8	12	49
	NS	13	9	8	12	42
	Wt	955.67	431.49	400.47	445.32	2232.96
Het	Chi	943.40	51.56	15.71	52.92	1317.58
Het	df	19	8	7	11	48
Het	P	***	***	*	***	***
Fixed	RR	0.47	0.98	0.53	0.35	0.52
	RRl	0.44	0.89	0.48	0.32	0.50
	RRu	0.50	1.07	0.59	0.38	0.54
	P	---	N.S.	---	---	---
Random	RR	0.43	0.92	0.52	0.29	0.46
	RRl	0.26	0.70	0.44	0.23	0.36
	RRu	0.69	1.21	0.62	0.37	0.57
	P	---	N.S.	---	---	---

Years quit vs current (higher focus)

		absent	1-11k3	4-19k12	13+k20	Total
	N	24	13	6	6	49
	NS	13	13	6	6	38
	Wt	959.74	485.77	380.47	406.97	2232.96
Het	Chi	539.60	71.76	1.79	7.77	1317.58
Het	df	23	12	5	5	48
Het	P	***	***	N.S.	N.S.	***
Fixed	RR	0.68	0.91	0.39	0.18	0.52
	RRl	0.64	0.84	0.35	0.17	0.50
	RRu	0.73	1.00	0.43	0.20	0.54
	P	---	-	---	---	---
Random	RR	0.45	0.81	0.39	0.19	0.46
	RRl	0.32	0.63	0.35	0.16	0.36
	RRu	0.62	1.03	0.43	0.22	0.57
	P	---	(-)	---	---	---

FEMALES

Years quit vs current (lower focus)

		absent	1-6k3	4-11k7	8+k12	Total
	N	6	1	1	3	11
	NS	3	1	1	3	6
	Wt	86.30	33.38	28.58	44.91	193.16
Het	Chi	82.20	0.00	0.00	1.20	133.15
Het	df	5	0	0	2	10
Het	P	***	N.S.	N.S.	N.S.	***
Fixed	RR	0.73	0.85	0.44	0.23	0.53
	RRl	0.59	0.61	0.31	0.17	0.46
	RRu	0.91	1.19	0.64	0.30	0.61
	P	--	N.S.	---	---	---
Random	RR	0.46	0.85	0.44	0.23	0.41
	RRl	0.18	0.61	0.31	0.17	0.24
	RRu	1.16	1.19	0.64	0.30	0.71
	P	N.S.	N.S.	---	---	--

Table 1K11 - 6

IESLC - Meta-analysis of Ex Smoking by Years quit (vs current), Overview
All LC types, Cigarettes only
Least adjusted

FEMALES

	Years quit vs current (higher focus) absent	1-11k3	4-19k12	13+k20	Total
N	6	3	1	1	11
NS	3	3	1	1	6
Wt	93.81	60.94	25.75	12.66	193.16
Het Chi	74.83	1.63	0.00	0.00	133.15
Het df	5	2	0	0	10
Het P	***	N.S.	N.S.	N.S.	***
Fixed RR	0.67	0.74	0.20	0.15	0.53
RRl	0.55	0.58	0.13	0.09	0.46
RRu	0.82	0.95	0.29	0.26	0.61
P	---	-	---	---	---
Random RR	0.43	0.74	0.20	0.15	0.41
RRl	0.18	0.58	0.13	0.09	0.24
RRu	1.01	0.95	0.29	0.26	0.71
P	(-)	-	---	---	--

Table 1K11 - 7

IESLC - Meta-analysis of Ex Smoking by Years quit (vs current), Overview
All LC types, Cigarettes only
Excluded studies (and stage at which they were excluded)

1	AGUDO AKIBA AMANDU AMES AXELSS BEST BOUCHA BOUCOT BRESLO CHEN CHEN2 CHIAZZ DEAN2 DOSEME ENGELA FAN GENG GER GUO HAENSZ HEGMAN HOLE HU HU2 JUSSAW KATSOU KAUFMA KOO KOULUM KREUZE LETOUR LEVIN LIAW LIU3 LIU4 LIU5 MCCONN MIGRAN MRFITR NOTAN2 OSANN2 PERNU QIAO2 RACHTA RESTRE SADOWS SEGI2 STASZE TIZZAN VUTUC WATSON WU WUWILL WYNDE2 WYNDE8 XU YUAN ZHANG ZHENG ZHOU
2	AUVINE BENSHL BLOT1 BROWN3 BUFFLE GURSEL LAUSSM LUO MCDUFF PISANI PRESCO SPITZ WU2 WYNDE7
4	HAMMON
5	CORREA GILLIS HUMBLE QIAO WIGLE
7	ARMADA BARBON BECHER BOFFET BROSS CARPEN CEDERL CHOI CHYOU DARBY DESTEF DOLL DORGAN GAO GAO2 GARCIA GARSHI HAMMO2 HIRAYA JAHN JAIN JEDRYC JOLY KHUDER LUBIN LUBIN2 MATOS PEZZO2 SOBUE SPEIZE SUZUK2 SVENSS WAKAI WANG2 WYNDE3

Table 1K11 - 8
Potentially overlapping studies

REF	REFGP	PRINC	. OVERLAP/LINK
BENHAM	LUBIN2	2	Subset of Lubin2
TVERDA	TVERDA	1	VEIERO/TVERDAL
GRAHAM	BYERS1	1	GRAHAM/BROSS/BYERS1
WYNDE6	WYNDE6	1	WYNDE5/6/7/8
CPSI	CPSI	1	CPSI overall

Table 1K12 -

IESLC - Meta-analysis of Ex Smoking, Years quit (vs current), "Low"
All LC types, Cigarettes only

This analysis is restricted to results for:
1) Ex smokers
2) Results by Years quit (vs current)
3) Categorical results by Years quit (vs current)
4) All LC types (or near equivalent)
5) Results complete enough for use in metaanalysis

Within each study, results are then selected (in the following order of preference, within each sex) for:
6) (not applicable)
7) PRODUCT: cigarettes only
8) CIGTYPE: all/unspecified, MC regardless of HR, MC only
9) Results with least adjustment for other aspects of smoking (ADOS)
10) DENOM: current smokers, current + recent smokers (up to number of m=months or y=years, max 2 years)
11) Followup period (YF, prospective studies): whole study (coded as 0) or longest available
12) LCtype: all or nearest available, at least Squamous and Adeno. (q = squamous, s = small,
l = large, a = adeno, mix = mixed, alv = alveolar)
13) Race: all or nearest available, otherwise by race (wh or w = white, bl or b = black, hi = hispanic
ch = chinese, jap = japanese, haw = hawaiian, w+o = white + oriental, sca = scandinavian, as = asian)
14) Years quit (vs current) "low" in key scheme 1 (key value 3, maximum range 1-6)
15) For overlapping studies: principal rather than subsidiary studies
Finally by Age: whole study (coded as 0) if available, otherwise by widest available age group
and then for single sex results (m, f) in preference to results for both sexes combined (c).

Results adjusted (AD) for the most potential confounders are then chosen in Sections -1 to -3
(and those which actually differ from the adjusted results in Table 1K2 - 1 are marked 'x' in Section -1)
and results adjusted for the least confounders in Sections -4 to -6. (Those least adjusted results which
actually differ from the most adjusted are marked 'x' in column X in Section -4)

Section -7 shows excluded studies, together with the stage (as above) at which no qualifying
results were found.

Section -8 lists the potentially overlapping studies which have been included (1=principal, 2=subsidiary).

Section -9 lists any results which would have been included in preference except that they had data not complete
enough for use in meta-analysis, with their significance (yes/no), if known, and any further comment as entered
on the database. It also lists as "gap" any categories for which no data were presented by the original authors.

In addition to those mentioned above, the following fields, levels and abbreviations are used:
* or nk = not known, n = no, y = yes, ot = other
nev = never
all/unspec = all or unspecified, MC = manufactured cigarettes, HR = hand-rolled cigarettes
exL, exH = range of exposure (low and high) in the smoking group, in terms of Years quit (vs current)
REF: 6-character study reference
NRR: number of the RR on the database within the study
ST : study type (CC = case control, pr or prosp = prospective)
NLC: number of lung cancer cases in whole study
R : risky occupational population (n = no, m = mining, o = other risky)
VB : national cigarette type (V = at least 75% Virginia, bl = at least 75% blended, ot = other)
P : any proxy use
H : full histological confirmation
De : derivation of RR/CI (or = original, st = standard method, ot = other method of estimation)

Table 1K12 - 1

IESLC - Meta-analysis of Ex Smoking, Years quit (vs current), "Low"
All LC types, Cigarettes only
Most adjusted

REF	NRR	1K2	SEX	AGEL	AGEH	RACE	YF	LC TYPE	LOC	START	ST	NLC	R	VB	P	H	AD	ADOS	PRODUCT	exL	exH	DENOM	De
BENHAM	603	x	m	0	0	all	-	all	Eu:wst	1976	CC	1625	n	bl	n	y	0	0	cig only	1.0	4	current	st
CPSI	816		m	50	74	all	6	all	NAmer	1959	pr	5138	n	bl	n	n	1	0	cig only	1.0	4	current	ot
CPSII	666		m	35	99	all	4	all	NAmer	1982	pr	3229	n	bl	n	n	1	0	cig only	3	5	current	ot
DEAN3	524	x	m	0	0	all	-	all	Eu:UK	1969	CC	766	n	V	y	n	1	0	cig only	3	4	cur+2y	ot
DOLL2	509		m	0	0	all	20	all	Eu:UK	1951	pr	920	n	V	n	n	1	0	cig only	0.1	4	current	ot
DORN	574	x	m	0	0	wh	15	all	NAmer	1954	pr	5097	n	bl	n	n	2	0	cig only	0.1	4	current	ot
GRAHAM	509	x	m	0	0	wh	-	all	NAmer	1956	CC	685	n	bl	n	n	0	0	cig only	1.1	3	current	st
TVERDA	507		m	0	0	all	0	all	Eu:Sca	1972	pr	238	n	bl	n	n	2	0	cig only	1.0	5	current	ot
WYNDE6	513		m	0	0	all	-	all	NAmer	1969	CC	4423	n	bl	n	y	0	0	cig only	1.0	4	cur+1y	st
WYNDE6	534		f	0	0	all	-	all	NAmer	1969	CC	4423	n	bl	n	y	0	0	cig only	1.0	4	cur+1y	st

Cigarette type is all/unspec for all RRs
except for the following:

REF	NRR	CIGTYPE
DEAN3	524	MC only

Table 1K12 - 2

IESLC - Meta-analysis of Ex Smoking, Years quit (vs current), "Low"
All LC types, Cigarettes only
Most adjusted

REF	NRR	SEX	AD	Number Exposed Case	Cont	Non-exposed Case	Cont	RR	95.00%CI
BENHAM	603	m	0	154	138	776	969	1.39	(1.09- 1.79)
*CPSI	816	m	1	49	-	844	-	0.59	(0.44- 0.78)
*CPSII	666	m	1	178	-	1159	-	0.85	(0.72- 0.99)
DEAN3	524	m	1	28	-	337	-	0.64	(0.41- 1.00)
*DOLL2	509	m	1	15	-	236	-	1.02	(0.61- 1.72)
*DORN	574	m	2	47	-	2609	-	1.66	(1.25- 2.22)
GRAHAM	509	m	0	12	23	371	821	1.15	(0.57- 2.35)
*TVERDA	507	m	2	5	-	144	-	0.18	(0.07- 0.43)
WYNDE6	513	m	0	201	166	1107	993	1.09	(0.87- 1.36)
WYNDE6	534	f	0	82	70	683	496	0.85	(0.61- 1.19)
Subtotal WYNDE6								1.01	(0.84- 1.22)
Partial Totals				771	397	8266	3279		

*prospective study

REF	NRR	SEX	AD	Ys	Ws	Qs	Ps
BENHAM	603	m	0	0.33	62.26	8.68	0.0088
*CPSI	816	m	1	-0.53	46.88	11.07	0.0003
*CPSII	666	m	1	-0.16	151.52	2.21	0.0454
DEAN3	524	m	1	-0.45	19.33	3.16	0.0497
*DOLL2	509	m	1	0.02	14.30	0.05	0.9403
*DORN	574	m	2	0.51	46.58	14.01	0.0005
GRAHAM	509	m	0	0.14	7.65	0.26	0.6910
*TVERDA	507	m	2	-1.71	4.66	13.05	0.0002
WYNDE6	513	m	0	0.08	77.46	1.20	0.4670
WYNDE6	534	f	0	-0.16	33.38	0.48	0.3502
Subtotal WYNDE6				0.01	110.84	1.68	

	N	10
	NS	9
	Wt	464.02
Het	Chi	54.19
Het	df	9
Het	P	***
Fixed	RR	0.96
	RRl	0.88
	RRu	1.05
	P	N.S.
Random	RR	0.90
	RRl	0.70
	RRu	1.15
	P	N.S.
Asymm	P	N.S.

Table 1K12 - 3

IESLC - Meta-analysis of Ex Smoking, Years quit (vs current), "Low"
All LC types, Cigarettes only
Most adjusted

	Sex			
	combined	male	female	Total
N		9	1	10
NS		9	1	9
Wt		430.64	33.38	464.02
Het Chi		53.68	0.00	54.19
Het df		8	0	9
Het P		***	N.S.	***
Fixed RR		0.97	0.85	0.96
RRl		0.88	0.61	0.88
RRu		1.06	1.19	1.05
P		N.S.	N.S.	N.S.
Random RR		0.90	0.85	0.90
RRl		0.68	0.61	0.70
RRu		1.19	1.19	1.15
P		N.S.	N.S.	N.S.
Between Chi				0.52
Between df				1
Between P				N.S.
Btwn(F) P				N.S.
Btwn(R) P				N.S.

	Lung cancer type		
	all	other	Total
N	10		10
NS	9		9
Wt	464.02		464.02
Het Chi	54.19		54.19
Het df	9		9
Het P	***		***
Fixed RR	0.96		0.96
RRl	0.88		0.88
RRu	1.05		1.05
P	N.S.		N.S.
Random RR	0.90		0.90
RRl	0.70		0.70
RRu	1.15		1.15
P	N.S.		N.S.
Between Chi			
Between df			
Between P			N.S.
Btwn(F) P			N.S.
Btwn(R) P			N.S.

	Location								
	NAmer	UK	Scand	othEur	China	Japan	othAs	other	Total
N	6	2	1	1					10
NS	5	2	1	1					9
Wt	363.46	33.63	4.66	62.26					464.02
Het Chi	29.04	1.79	0.00	0.00					54.19
Het df	5	1	0	0					9
Het P	***	N.S.	N.S.	N.S.					***
Fixed RR	0.94	0.78	0.18	1.39					0.96
RRl	0.85	0.56	0.07	1.09					0.88
RRu	1.04	1.09	0.45	1.79					1.05
P	N.S.	N.S.	---	++					N.S.
Random RR	0.97	0.79	0.18	1.39					0.90
RRl	0.73	0.50	0.07	1.09					0.70
RRu	1.27	1.25	0.45	1.79					1.15
P	N.S.	N.S.	---	++					N.S.
Between Chi									23.37
Between df									3
Between P									***
Btwn(F) P									N.S.
Btwn(R) P									***

Table 1K12 - 3

IESLC - Meta-analysis of Ex Smoking, Years quit (vs current), "Low"
All LC types, Cigarettes only
Most adjusted

Detailed Country in "other Europe"

	multi	Germany	othWest	East	Balkans	Total
N			1			1
NS			1			1
Wt			62.26			62.26
Het Chi			0.00			0.00
Het df			0			0
Het P			N.S.			N.S.
Fixed RR			1.39			1.39
RRl			1.09			1.09
RRu			1.79			1.79
P			++			++
Random RR			1.39			1.39
RRl			1.09			1.09
RRu			1.79			1.79
P			++			++
Between Chi						
Between df						
Between P						N.S.
Btwn(F) P						N.S.
Btwn(R) P						N.S.

Detailed Country in "other Asia"

	India	HongKong	other	Total
N				
NS				
Wt				
Het Chi				
Het df				
Het P				
Fixed RR				
RRl				
RRu				
P				
Random RR				
RRl				
RRu				
P				
Between Chi				
Between df				
Between P				N.S.
Btwn(F) P				N.S.
Btwn(R) P				N.S.

Detailed other continent

	SCAmer	Total
N		
NS		
Wt		
Het Chi		
Het df		
Het P		
Fixed RR		
RRl		
RRu		
P		
Random RR		
RRl		
RRu		
P		
Between Chi		
Between df		
Between P		N.S.
Btwn(F) P		N.S.
Btwn(R) P		N.S.

Table 1K12 - 3

IESLC - Meta-analysis of Ex Smoking, Years quit (vs current), "Low"
All LC types, Cigarettes only
Most adjusted

	Start year of study					
	<1960	1960-69	1970-79	1980-89	1990+	Total
N	4	3	2	1		10
NS	4	2	2	1		9
Wt	115.41	130.17	66.93	151.52		464.02
Het Chi	25.18	4.81	18.17	0.00		54.19
Het df	3	2	1	0		9
Het P	***	(*)	***	N.S.		***
Fixed RR	1.00	0.94	1.21	0.85		0.96
RRl	0.84	0.79	0.95	0.72		0.88
RRu	1.20	1.12	1.54	1.00		1.05
P	N.S.	N.S.	N.S.	-		N.S.
Random RR	1.03	0.88	0.53	0.85		0.90
RRl	0.58	0.66	0.07	0.72		0.70
RRu	1.84	1.18	3.90	1.00		1.15
P	N.S.	N.S.	N.S.	-		N.S.
Between Chi						6.04
Between df						3
Between P						N.S.
Btwn(F) P						N.S.
Btwn(R) P						N.S.

	Study type (1)		
	CC	other	Total
N	5	5	10
NS	4	5	9
Wt	200.08	263.94	464.02
Het Chi	11.27	38.49	54.19
Het df	4	4	9
Het P	*	***	***
Fixed RR	1.07	0.88	0.96
RRl	0.93	0.78	0.88
RRu	1.23	0.99	1.05
P	N.S.	-	N.S.
Random RR	1.01	0.78	0.90
RRl	0.78	0.49	0.70
RRu	1.31	1.22	1.15
P	N.S.	N.S.	N.S.
Between Chi			4.44
Between df			1
Between P			*
Btwn(F) P			N.S.
Btwn(R) P			N.S.

	Study type (2)			
	CC	prosp	other	Total
N	5	5		10
NS	4	5		9
Wt	200.08	263.94		464.02
Het Chi	11.27	38.49		54.19
Het df	4	4		9
Het P	*	***		***
Fixed RR	1.07	0.88		0.96
RRl	0.93	0.78		0.88
RRu	1.23	0.99		1.05
P	N.S.	-		N.S.
Random RR	1.01	0.78		0.90
RRl	0.78	0.49		0.70
RRu	1.31	1.22		1.15
P	N.S.	N.S.		N.S.
Between Chi				4.44
Between df				1
Between P				*
Btwn(F) P				N.S.
Btwn(R) P				N.S.

Table 1K12 - 3

IESLC - Meta-analysis of Ex Smoking, Years quit (vs current), "Low"
All LC types, Cigarettes only
Most adjusted

	Study size (number of LC cases) 100-249	250-499	500-999	1000+	Total
N	1		3	6	10
NS	1		3	5	9
Wt	4.66		41.28	418.08	464.02
Het Chi	0.00		2.74	37.23	54.19
Het df	0		2	5	9
Het P	N.S.		N.S.	***	***
Fixed RR	0.18		0.84	0.99	0.96
RRl	0.07		0.62	0.90	0.88
RRu	0.45		1.14	1.09	1.05
P	---		N.S.	N.S.	N.S.
Random RR	0.18		0.86	1.01	0.90
RRl	0.07		0.60	0.77	0.70
RRu	0.45		1.23	1.33	1.15
P	---		N.S.	N.S.	N.S.
Between Chi					14.22
Between df					2
Between P					***
Btwn(F) P					N.S.
Btwn(R) P					**

	Risky occupational population no	mining	othRisky	Total
N	10			10
NS	9			9
Wt	464.02			464.02
Het Chi	54.19			54.19
Het df	9			9
Het P	***			***
Fixed RR	0.96			0.96
RRl	0.88			0.88
RRu	1.05			1.05
P	N.S.			N.S.
Random RR	0.90			0.90
RRl	0.70			0.70
RRu	1.15			1.15
P	N.S.			N.S.
Between Chi				
Between df				
Between P				N.S.
Btwn(F) P				N.S.
Btwn(R) P				N.S.

	National cigarette tobacco type Virginia	blended	other	Total
N	2	8		10
NS	2	7		9
Wt	33.63	430.39		464.02
Het Chi	1.79	50.86		54.19
Het df	1	7		9
Het P	N.S.	***		***
Fixed RR	0.78	0.97		0.96
RRl	0.56	0.89		0.88
RRu	1.09	1.07		1.05
P	N.S.	N.S.		N.S.
Random RR	0.79	0.92		0.90
RRl	0.50	0.69		0.70
RRu	1.25	1.22		1.15
P	N.S.	N.S.		N.S.
Between Chi				1.55
Between df				1
Between P				N.S.
Btwn(F) P				N.S.
Btwn(R) P				N.S.

Table 1K12 - 3

IESLC - Meta-analysis of Ex Smoking, Years quit (vs current), "Low"
All LC types, Cigarettes only
Most adjusted

	Any proxy use		
	No/nk	Yes	Total
N	9	1	10
NS	8	1	9
Wt	444.69	19.33	464.02
Het Chi	50.89	0.00	54.19
Het df	8	0	9
Het P	***	N.S.	***
Fixed RR	0.98	0.64	0.96
RRl	0.89	0.41	0.88
RRu	1.07	1.00	1.05
P	N.S.	-	N.S.
Random RR	0.93	0.64	0.90
RRl	0.72	0.41	0.70
RRu	1.21	1.00	1.15
P	N.S.	-	N.S.
Between Chi			3.30
Between df			1
Between P			(*)
Btwn(F) P			N.S.
Btwn(R) P			N.S.

	Full histological confirmation		
	No	Yes	Total
N	7	3	10
NS	7	2	9
Wt	290.92	173.10	464.02
Het Chi	40.97	5.55	54.19
Het df	6	2	9
Het P	***	(*)	***
Fixed RR	0.87	1.13	0.96
RRl	0.77	0.98	0.88
RRu	0.97	1.32	1.05
P	-	(+)	N.S.
Random RR	0.80	1.11	0.90
RRl	0.55	0.86	0.70
RRu	1.14	1.43	1.15
P	N.S.	N.S.	N.S.
Between Chi			7.68
Between df			1
Between P			**
Btwn(F) P			N.S.
Btwn(R) P			N.S.

	Number of adjustment variables (1)			
	0	1	2+/+nk	Total
N	4	4	2	10
NS	3	4	2	9
Wt	180.75	232.02	51.24	464.02
Het Chi	5.55	6.56	20.92	54.19
Het df	3	3	1	9
Het P	N.S.	(*)	***	***
Fixed RR	1.13	0.78	1.36	0.96
RRl	0.98	0.69	1.03	0.88
RRu	1.31	0.89	1.78	1.05
P	(+)	---	+	N.S.
Random RR	1.12	0.75	0.57	0.90
RRl	0.90	0.59	0.06	0.70
RRu	1.39	0.95	5.03	1.15
P	N.S.	-	N.S.	N.S.
Between Chi				21.17
Between df				2
Between P				***
Btwn(F) P				N.S.
Btwn(R) P				*

Table 1K12 - 3

IESLC - Meta-analysis of Ex Smoking, Years quit (vs current), "Low"
All LC types, Cigarettes only
Most adjusted

	Number of adjustment variables (2) 0	1	2	3-5	6+/+nk	Total
N	4	4	2			10
NS	3	4	2			9
Wt	180.75	232.02	51.24			464.02
Het Chi	5.55	6.56	20.92			54.19
Het df	3	3	1			9
Het P	N.S.	(*)	***			***
Fixed RR	1.13	0.78	1.36			0.96
RRl	0.98	0.69	1.03			0.88
RRu	1.31	0.89	1.78			1.05
P	(+)	---	+			N.S.
Random RR	1.12	0.75	0.57			0.90
RRl	0.90	0.59	0.06			0.70
RRu	1.39	0.95	5.03			1.15
P	N.S.	-	N.S.			N.S.
Between Chi						21.17
Between df						2
Between P						***
Btwn(F) P						N.S.
Btwn(R) P						*

	Denominator current	cur+rec	Total
N	7	3	10
NS	7	2	9
Wt	333.85	130.17	464.02
Het Chi	49.34	4.81	54.19
Het df	6	2	9
Het P	***	(*)	***
Fixed RR	0.97	0.94	0.96
RRl	0.87	0.79	0.88
RRu	1.07	1.12	1.05
P	N.S.	N.S.	N.S.
Random RR	0.90	0.88	0.90
RRl	0.63	0.66	0.70
RRu	1.29	1.18	1.15
P	N.S.	N.S.	N.S.
Between Chi			0.05
Between df			1
Between P			N.S.
Btwn(F) P			N.S.
Btwn(R) P			N.S.

	Derivation of RR/CI Orig	StdCalc	Other	Total
N		4	6	10
NS		3	6	9
Wt		180.75	283.27	464.02
Het Chi		5.55	40.33	54.19
Het df		3	5	9
Het P		N.S.	***	***
Fixed RR		1.13	0.86	0.96
RRl		0.98	0.77	0.88
RRu		1.31	0.97	1.05
P		(+)	-	N.S.
Random RR		1.12	0.76	0.90
RRl		0.90	0.51	0.70
RRu		1.39	1.12	1.15
P		N.S.	N.S.	N.S.
Between Chi				8.32
Between df				1
Between P				**
Btwn(F) P				N.S.
Btwn(R) P				(*)

Table 1K12 - 4

IESLC - Meta-analysis of Ex Smoking, Years quit (vs current), "Low"
All LC types, Cigarettes only
Least adjusted

REF	NRR	X	SEX	AGEL	AGEH	RACE	YF	LC TYPE	LOC	START	ST	NLC	R	VB	P	H	AD	ADOS	PRODUCT	exL	exH	DENOM	De
BENHAM	603		m	0	0	all	-	all	Eu:wst	1976	CC	1625	n	bl	n	y	0	0	cig only	1.0	4	current	st
CPSI	816		m	50	74	all	6	all	NAmer	1959	pr	5138	n	bl	n	n	1	0	cig only	1.0	4	current	ot
CPSII	666		m	35	99	all	4	all	NAmer	1982	pr	3229	n	bl	n	n	1	0	cig only	3	5	current	ot
DEAN3	509	x	m	0	0	all	-	all	Eu:UK	1969	CC	766	n	V	y	n	0	0	cig only	3	4	cur+2y	st
DOLL2	509		m	0	0	all	20	all	Eu:UK	1951	pr	920	n	V	n	n	1	0	cig only	0.1	4	current	ot
DORN	574		m	0	0	wh	15	all	NAmer	1954	pr	5097	n	bl	n	n	2	0	cig only	0.1	4	current	ot
GRAHAM	509		m	0	0	wh	-	all	NAmer	1956	CC	685	n	bl	n	n	0	0	cig only	1.1	3	current	st
TVERDA	507		m	0	0	all	0	all	Eu:Sca	1972	pr	238	n	bl	n	n	2	0	cig only	1.0	5	current	ot
WYNDE6	513		m	0	0	all	-	all	NAmer	1969	CC	4423	n	bl	n	y	0	0	cig only	1.0	4	cur+1y	st
WYNDE6	534		f	0	0	all	-	all	NAmer	1969	CC	4423	n	bl	n	y	0	0	cig only	1.0	4	cur+1y	st

Cigarette type is all/unspec for all RRs
except for the following:

REF	NRR	CIGTYPE
DEAN3	509	MC only

Table 1K12 - 5

IESLC - Meta-analysis of Ex Smoking, Years quit (vs current), "Low"
All LC types, Cigarettes only
Least adjusted

REF	NRR	SEX	AD	Number Exposed Case	Cont	Non-exposed Case	Cont	RR	95.00%CI
BENHAM	603	m	0	154	138	776	969	1.39	(1.09- 1.79)
*CPSI	816	m	1	49	-	844	-	0.59	(0.44- 0.78)
*CPSII	666	m	1	178	-	1159	-	0.85	(0.72- 0.99)
DEAN3	509	m	0	28	102	337	930	0.76	(0.49- 1.17)
*DOLL2	509	m	1	15	-	236	-	1.02	(0.61- 1.72)
*DORN	574	m	2	47	-	2609	-	1.66	(1.25- 2.22)
GRAHAM	509	m	0	12	23	371	821	1.15	(0.57- 2.35)
*TVERDA	507	m	2	5	-	144	-	0.18	(0.07- 0.43)
WYNDE6	513	m	0	201	166	1107	993	1.09	(0.87- 1.36)
WYNDE6	534	f	0	82	70	683	496	0.85	(0.61- 1.19)
Subtotal WYNDE6								1.01	(0.84- 1.22)
Partial Totals				771	499	8266	4209		

*prospective study

REF	NRR	SEX	AD	Ys	Ws	Qs	Ps
BENHAM	603	m	0	0.33	62.26	8.38	0.0088
*CPSI	816	m	1	-0.53	46.88	11.37	0.0003
*CPSII	666	m	1	-0.16	151.52	2.46	0.0454
DEAN3	509	m	0	-0.28	20.18	1.19	0.2123
*DOLL2	509	m	1	0.02	14.30	0.04	0.9403
*DORN	574	m	2	0.51	46.58	13.68	0.0005
GRAHAM	509	m	0	0.14	7.65	0.24	0.6910
*TVERDA	507	m	2	-1.71	4.66	13.16	0.0002
WYNDE6	513	m	0	0.08	77.46	1.07	0.4670
WYNDE6	534	f	0	-0.16	33.38	0.54	0.3502
Subtotal WYNDE6				0.01	110.84	1.61	

	N	10
	NS	9
	Wt	464.87
Het	Chi	52.13
Het	df	9
Het	P	***
Fixed	RR	0.97
	RRl	0.88
	RRu	1.06
	P	N.S.
Random	RR	0.92
	RRl	0.72
	RRu	1.17
	P	N.S.
Asymm	P	N.S.

Table 1K12 - 6

IESLC - Meta-analysis of Ex Smoking, Years quit (vs current), "Low"
All LC types, Cigarettes only
Least adjusted

	Sex combined	male	female	Total
N		9	1	10
NS		9	1	9
Wt		431.49	33.38	464.87
Het Chi		51.56	0.00	52.13
Het df		8	0	9
Het P		***	N.S.	***
Fixed RR		0.98	0.85	0.97
RRl		0.89	0.61	0.88
RRu		1.07	1.19	1.06
P		N.S.	N.S.	N.S.
Random RR		0.92	0.85	0.92
RRl		0.70	0.61	0.72
RRu		1.21	1.19	1.17
P		N.S.	N.S.	N.S.
Between Chi				0.58
Between df				1
Between P				N.S.
Btwn(F) P				N.S.
Btwn(R) P				N.S.

Table 1K12 - 7

IESLC - Meta-analysis of Ex Smoking, Years quit (vs current), "Low"
All LC types, Cigarettes only
Excluded studies (and stage at which they were excluded)

1	AGUDO AKIBA AMANDU AMES AXELSS BEST BOUCHA BOUCOT BRESLO CHEN CHEN2 CHIAZZ DEAN2 DOSEME ENGELA FAN GENG GER GUO HAENSZ HEGMAN HOLE HU HU2 JUSSAW KATSOU KAUFMA KOO KOULUM KREUZE LETOUR LEVIN LIAW LIU3 LIU4 LIU5 MCCONN MIGRAN MRFITR NOTAN2 OSANN2 PERNU QIAO2 RACHTA RESTRE SADOWS SEGI2 STASZE TIZZAN VUTUC WATSON WU WUWILL WYNDE2 WYNDE8 XU YUAN ZHANG ZHENG ZHOU
2	AUVINE BENSHL BLOT1 BROWN3 BUFFLE GURSEL LAUSSM LUO MCDUFF PISANI PRESCO SPITZ WU2 WYNDE7
4	HAMMON
5	CORREA GILLIS HUMBLE QIAO WIGLE
7	ARMADA BARBON BECHER BOFFET BROSS CARPEN CEDERL CHOI CHYOU DARBY DESTEF DOLL DORGAN GAO GAO2 GARCIA GARSHI HAMMO2 HIRAYA JAHN JAIN JEDRYC JOLY KHUDER LUBIN LUBIN2 MATOS PEZZO2 SOBUE SPEIZE SUZUK2 SVENSS WAKAI WANG2 WYNDE3
14	ALDERS DAMBER KAISE2 PEZZOT

Table 1K12 - 8
Potentially overlapping studies

REF	REFGP	PRINC	. OVERLAP/LINK
BENHAM	LUBIN2	2	Subset of Lubin2
TVERDA	TVERDA	1	VEIERO/TVERDAL
GRAHAM	BYERS1	1	GRAHAM/BROSS/BYERS1
WYNDE6	WYNDE6	1	WYNDE5/6/7/8
CPSI	CPSI	1	CPSI overall

Table 1K13 -

IESLC - Meta-analysis of Ex Smoking, Years quit (vs current), "Mid"
All LC types, Cigarettes only

This analysis is restricted to results for:
1) Ex smokers
2) Results by Years quit (vs current)
3) Categorical results by Years quit (vs current)
4) All LC types (or near equivalent)
5) Results complete enough for use in metaanalysis

Within each study, results are then selected (in the following order of preference, within each sex) for:
6) (not applicable)
7) PRODUCT: cigarettes only
8) CIGTYPE: all/unspecified, MC regardless of HR, MC only
9) Results with least adjustment for other aspects of smoking (ADOS)
10) DENOM: current smokers, current + recent smokers (up to number of m=months or y=years, max 2 years)
11) Followup period (YF, prospective studies): whole study (coded as 0) or longest available
12) LCtype: all or nearest available, at least Squamous and Adeno. (q = squamous, s = small,
l = large, a = adeno, mix = mixed, alv = alveolar)
13) Race: all or nearest available, otherwise by race (wh or w = white, bl or b = black, hi = hispanic
ch = chinese, jap = japanese, haw = hawaiian, w+o = white + oriental, sca = scandinavian, as = asian)
14) Years quit (vs current) "mid" in key scheme 1 (key value 7, maximum range 4-11)
15) For overlapping studies: principal rather than subsidiary studies
Finally by Age: whole study (coded as 0) if available, otherwise by widest available age group
and then for single sex results (m, f) in preference to results for both sexes combined (c).

Results adjusted (AD) for the most potential confounders are then chosen in Sections -1 to -3
(and those which actually differ from the adjusted results in Table 1K3 - 1 are marked 'x' in Section -1)
and results adjusted for the least confounders in Sections -4 to -6. (Those least adjusted results which
actually differ from the most adjusted are marked 'x' in column X in Section -4)

Section -7 shows excluded studies, together with the stage (as above) at which no qualifying
results were found.

Section -8 lists the potentially overlapping studies which have been included (1=principal, 2=subsidiary).

Section -9 lists any results which would have been included in preference except that they had data not complete
enough for use in meta-analysis, with their significance (yes/no), if known, and any further comment as entered
on the database. It also lists as "gap" any categories for which no data were presented by the original authors.

In addition to those mentioned above, the following fields, levels and abbreviations are used:
* or nk = not known, n = no, y = yes, ot = other
nev = never
all/unspec = all or unspecified, MC = manufactured cigarettes, HR = hand-rolled cigarettes
exL, exH = range of exposure (low and high) in the smoking group, in terms of Years quit (vs current)
REF: 6-character study reference
NRR: number of the RR on the database within the study
ST : study type (CC = case control, pr or prosp = prospective)
NLC: number of lung cancer cases in whole study
R : risky occupational population (n = no, m = mining, o = other risky)
VB : national cigarette type (V = at least 75% Virginia, bl = at least 75% blended, ot = other)
P : any proxy use
H : full histological confirmation
De : derivation of RR/CI (or = original, st = standard method, ot = other method of estimation)

Table 1K13 - 1

IESLC - Meta-analysis of Ex Smoking, Years quit (vs current), "Mid"
All LC types, Cigarettes only
Most adjusted

REF	NRR	1K3	SEX	AGEL	AGEH	RACE	YF	LC TYPE	LOC	START	ST	NLC	R	VB	P	H	AD	ADOS	PRODUCT	exL	exH	DENOM	De
BENHAM	604	x	m	0	0	all	-	all	Eu:wst	1976	CC	1625	n	bl	n	y	0	0	cig only	5	9	current	st
CPSI	817		m	50	74	all	6	all	NAmer	1959	pr	5138	n	bl	n	n	1	0	cig only	5	9	current	ot
CPSII	667		m	35	99	all	4	all	NAmer	1982	pr	3229	n	bl	n	n	1	0	cig only	6	10	current	ot
DEAN3	525	x	m	0	0	all	-	all	Eu:UK	1969	CC	766	n	V	y	n	1	0	cig only	5	8	cur+2y	ot
DOLL2	510		m	0	0	all	20	all	Eu:UK	1951	pr	920	n	V	n	n	1	0	cig only	5	9	current	ot
DORN	575	x	m	0	0	wh	15	all	NAmer	1954	pr	5097	n	bl	n	n	2	0	cig only	5	9	current	ot
GRAHAM	510		m	0	0	wh	-	all	NAmer	1956	CC	685	n	bl	n	n	0	0	cig only	3	10	current	st
WYNDE6	514		m	0	0	all	-	all	NAmer	1969	CC	4423	n	bl	n	y	0	0	cig only	5	9	cur+1y	st
WYNDE6	535		f	0	0	all	-	all	NAmer	1969	CC	4423	n	bl	n	y	0	0	cig only	5	9	cur+1y	st

Cigarette type is all/unspec for all RRs
except for the following:

REF	NRR	CIGTYPE
DEAN3	525	MC only

Table 1K13 - 2

IESLC - Meta-analysis of Ex Smoking, Years quit (vs current), "Mid"
All LC types, Cigarettes only
Most adjusted

REF	NRR	SEX	AD	Number Exposed Case	Cont	Non-exposed Case	Cont	RR	95.00%CI
BENHAM	604	m	0	66	120	776	969	0.69	(0.50- 0.94)
*CPSI	817	m	1	32	-	844	-	0.37	(0.26- 0.53)
*CPSII	667	m	1	186	-	1159	-	0.52	(0.45- 0.61)
DEAN3	525	m	1	11	-	337	-	0.57	(0.29- 1.12)
*DOLL2	510	m	1	12	-	236	-	0.35	(0.20- 0.63)
*DORN	575	m	2	86	-	2609	-	0.67	(0.54- 0.83)
GRAHAM	510	m	0	5	29	371	821	0.38	(0.15- 0.99)
WYNDE6	514	m	0	98	194	1107	993	0.45	(0.35- 0.59)
WYNDE6	535	f	0	51	84	683	496	0.44	(0.31- 0.64)
Subtotal WYNDE6								0.45	(0.36- 0.55)
Partial Totals				547	427	8122	3279		

*prospective study

REF	NRR	SEX	AD	Ys	Ws	Qs	Ps
BENHAM	604	m	0	-0.38	38.75	2.82	0.0193
*CPSI	817	m	1	-0.99	30.29	3.69	0.0000
*CPSII	667	m	1	-0.65	166.04	0.01	0.0000
DEAN3	525	m	1	-0.56	8.42	0.06	0.1029
*DOLL2	510	m	1	-1.05	11.67	1.91	0.0003
*DORN	575	m	2	-0.40	83.16	4.98	0.0003
GRAHAM	510	m	0	-0.96	4.19	0.42	0.0484
WYNDE6	514	m	0	-0.79	57.91	1.24	0.0000
WYNDE6	535	f	0	-0.82	28.58	0.86	0.0000
Subtotal WYNDE6				-0.80	86.48	2.10	

	N	9
	NS	8
	Wt	429.01
Het	Chi	15.99
Het	df	8
Het	P	*
Fixed	RR	0.52
	RRl	0.48
	RRu	0.58
	P	---
Random	RR	0.51
	RRl	0.44
	RRu	0.59
	P	---
Asymm	P	N.S.

Table 1K13 - 3

IESLC - Meta-analysis of Ex Smoking, Years quit (vs current), "Mid"
All LC types, Cigarettes only
Most adjusted

	combined	Sex male	female	Total
N		8	1	9
NS		8	1	8
Wt		400.43	28.58	429.01
Het Chi		15.07	0.00	15.99
Het df		7	0	8
Het P		*	N.S.	*
Fixed RR		0.53	0.44	0.52
RRl		0.48	0.31	0.48
RRu		0.59	0.64	0.58
P		---	---	---
Random RR		0.52	0.44	0.51
RRl		0.44	0.31	0.44
RRu		0.61	0.64	0.59
P		---	---	---
Between Chi				0.92
Between df				1
Between P				N.S.
Btwn(F) P				N.S.
Btwn(R) P				N.S.

Too few RRs for analysis by factor

Table 1K13 - 4

IESLC - Meta-analysis of Ex Smoking, Years quit (vs current), "Mid"
All LC types, Cigarettes only
Least adjusted

REF	NRR	X	SEX	AGEL	AGEH	RACE	YF	LC TYPE	LOC	START	ST	NLC	R	VB	P	H	AD	ADOS	PRODUCT	exL	exH	DENOM	De
BENHAM	604		m	0	0	all	-	all	Eu:wst	1976	CC	1625	n	bl	n	y	0	0	cig only	5	9	current	st
CPSI	817		m	50	74	all	6	all	NAmer	1959	pr	5138	n	bl	n	n	1	0	cig only	5	9	current	ot
CPSII	667		m	35	99	all	4	all	NAmer	1982	pr	3229	n	bl	n	n	1	0	cig only	6	10	current	ot
DEAN3	510	x	m	0	0	all	-	all	Eu:UK	1969	CC	766	n	V	y	n	0	0	cig only	5	8	cur+2y	st
DOLL2	510		m	0	0	all	20	all	Eu:UK	1951	pr	920	n	V	n	n	1	0	cig only	5	9	current	ot
DORN	575		m	0	0	wh	15	all	NAmer	1954	pr	5097	n	bl	n	n	2	0	cig only	5	9	current	ot
GRAHAM	510		m	0	0	wh	-	all	NAmer	1956	CC	685	n	bl	n	n	0	0	cig only	3	10	current	st
WYNDE6	514		m	0	0	all	-	all	NAmer	1969	CC	4423	n	bl	n	y	0	0	cig only	5	9	cur+1y	st
WYNDE6	535		f	0	0	all	-	all	NAmer	1969	CC	4423	n	bl	n	y	0	0	cig only	5	9	cur+1y	st

Cigarette type is all/unspec for all RRs
except for the following:

REF	NRR	CIGTYPE
DEAN3	510	MC only

Table 1K13 - 5

IESLC - Meta-analysis of Ex Smoking, Years quit (vs current), "Mid"
All LC types, Cigarettes only
Least adjusted

REF	NRR	SEX	AD	Number Exposed Case	Number Exposed Cont	Non-exposed Case	Non-exposed Cont	RR	95.00%CI
BENHAM	604	m	0	66	120	776	969	0.69	(0.50- 0.94)
*CPSI	817	m	1	32	-	844	-	0.37	(0.26- 0.53)
*CPSII	667	m	1	186	-	1159	-	0.52	(0.45- 0.61)
DEAN3	510	m	0	11	43	337	930	0.71	(0.36- 1.38)
*DOLL2	510	m	1	12	-	236	-	0.35	(0.20- 0.63)
*DORN	575	m	2	86	-	2609	-	0.67	(0.54- 0.83)
GRAHAM	510	m	0	5	29	371	821	0.38	(0.15- 0.99)
WYNDE6	514	m	0	98	194	1107	993	0.45	(0.35- 0.59)
WYNDE6	535	f	0	51	84	683	496	0.44	(0.31- 0.64)
Subtotal WYNDE6								0.45	(0.36- 0.55)
Partial Totals				547	470	8122	4209		

*prospective study

REF	NRR	SEX	AD	Ys	Ws	Qs	Ps
BENHAM	604	m	0	-0.38	38.75	2.73	0.0193
*CPSI	817	m	1	-0.99	30.29	3.78	0.0000
*CPSII	667	m	1	-0.65	166.04	0.03	0.0000
DEAN3	510	m	0	-0.35	8.46	0.73	0.3112
*DOLL2	510	m	1	-1.05	11.67	1.95	0.0003
*DORN	575	m	2	-0.40	83.16	4.81	0.0003
GRAHAM	510	m	0	-0.96	4.19	0.44	0.0484
WYNDE6	514	m	0	-0.79	57.91	1.31	0.0000
WYNDE6	535	f	0	-0.82	28.58	0.90	0.0000
Subtotal WYNDE6				-0.80	86.48	2.22	

	N	9
	NS	8
	Wt	429.05
Het	Chi	16.68
Het	df	8
Het	P	*
Fixed	RR	0.53
	RRl	0.48
	RRu	0.58
	P	---
Random	RR	0.51
	RRl	0.44
	RRu	0.60
	P	---
Asymm	P	N.S.

Table 1K13 - 6

IESLC - Meta-analysis of Ex Smoking, Years quit (vs current), "Mid"
All LC types, Cigarettes only
Least adjusted

	Sex combined	male	female	Total
N		8	1	9
NS		8	1	8
Wt		400.47	28.58	429.05
Het Chi		15.71	0.00	16.68
Het df		7	0	8
Het P		*	N.S.	*
Fixed RR		0.53	0.44	0.53
RRl		0.48	0.31	0.48
RRu		0.59	0.64	0.58
P		---	---	---
Random RR		0.52	0.44	0.51
RRl		0.44	0.31	0.44
RRu		0.62	0.64	0.60
P		---	---	---
Between Chi				0.97
Between df				1
Between P				N.S.
Btwn(F) P				N.S.
Btwn(R) P				N.S.

Table 1K13 - 7

IESLC - Meta-analysis of Ex Smoking, Years quit (vs current), "Mid"
All LC types, Cigarettes only
Excluded studies (and stage at which they were excluded)

1	AGUDO AKIBA AMANDU AMES AXELSS BEST BOUCHA BOUCOT BRESLO CHEN CHEN2 CHIAZZ DEAN2 DOSEME ENGELA FAN GENG GER GUO HAENSZ HEGMAN HOLE HU HU2 JUSSAW KATSOU KAUFMA KOO KOULUM KREUZE LETOUR LEVIN LIAW LIU3 LIU4 LIU5 MCCONN MIGRAN MRFITR NOTAN2 OSANN2 PERNU QIAO2 RACHTA RESTRE SADOWS SEGI2 STASZE TIZZAN VUTUC WATSON WU WUWILL WYNDE2 WYNDE8 XU YUAN ZHANG ZHENG ZHOU
2	AUVINE BENSHL BLOT1 BROWN3 BUFFLE GURSEL LAUSSM LUO MCDUFF PISANI PRESCO SPITZ WU2 WYNDE7
4	HAMMON
5	CORREA GILLIS HUMBLE QIAO WIGLE
7	ARMADA BARBON BECHER BOFFET BROSS CARPEN CEDERL CHOI CHYOU DARBY DESTEF DOLL DORGAN GAO GAO2 GARCIA GARSHI HAMMO2 HIRAYA JAHN JAIN JEDRYC JOLY KHUDER LUBIN LUBIN2 MATOS PEZZO2 SOBUE SPEIZE SUZUK2 SVENSS WAKAI WANG2 WYNDE3
14	ALDERS DAMBER KAISE2 PEZZOT TVERDA

Table 1K13 - 8
Potentially overlapping studies

REF	REFGP	PRINC	. OVERLAP/LINK
BENHAM	LUBIN2	2	Subset of Lubin2
GRAHAM	BYERS1	1	GRAHAM/BROSS/BYERS1
WYNDE6	WYNDE6	1	WYNDE5/6/7/8
CPSI	CPSI	1	CPSI overall

Table 1K14 -

IESLC - Meta-analysis of Ex Smoking, Years quit (vs current), "High"
All LC types, Cigarettes only

This analysis is restricted to results for:
1) Ex smokers
2) Results by Years quit (vs current)
3) Categorical results by Years quit (vs current)
4) All LC types (or near equivalent)
5) Results complete enough for use in metaanalysis

Within each study, results are then selected (in the following order of preference, within each sex) for:
6) PRODUCT: cigarettes only
7) CIGTYPE: all/unspecified, MC regardless of HR, MC only
8) Results with least adjustment for other aspects of smoking (ADOS)
9) DENOM: current smokers, current + recent smokers (up to number of m=months or y=years, max 2 years)
10) Followup period (YF, prospective studies): whole study (coded as 0) or longest available
11) LCtype: all or nearest available, at least Squamous and Adeno. (q = squamous, s = small,
l = large, a = adeno, mix = mixed, alv = alveolar)
12) Race: all or nearest available, otherwise by race (wh or w = white, bl or b = black, hi = hispanic
ch = chinese, jap = japanese, haw = hawaiian, w+o = white + oriental, sca = scandinavian, as = asian)
13) Years quit (vs current) "high" in key scheme 1 (key value 12, maximum range 8+)
14) For overlapping studies: principal rather than subsidiary studies
Finally by Age: whole study (coded as 0) if available, otherwise by widest available age group
and then for single sex results (m, f) in preference to results for both sexes combined (c).

Results adjusted (AD) for the most potential confounders are then chosen in Sections -1 to -3
(and those which actually differ from the adjusted results in Table 1K4 - 1 are marked 'x' in Section -1)
and results adjusted for the least confounders in Sections -4 to -6. (Those least adjusted results which
actually differ from the most adjusted are marked 'x' in column X in Section -4)

Section -7 shows excluded studies, together with the stage (as above) at which no qualifying
results were found.

Section -8 lists the potentially overlapping studies which have been included (1=principal, 2=subsidiary).

Section -9 lists any results which would have been included in preference except that they had data not complete
enough for use in meta-analysis, with their significance (yes/no), if known, and any further comment as entered
on the database. It also lists as "gap" any categories for which no data were presented by the original authors.

In addition to those mentioned above, the following fields, levels and abbreviations are used:
* or nk = not known, n = no, y = yes, ot = other
nev = never
all/unspec = all or unspecified, MC = manufactured cigarettes, HR = hand-rolled cigarettes
exL, exH = range of exposure (low and high) in the smoking group, in terms of Years quit (vs current)
REF: 6-character study reference
NRR: number of the RR on the database within the study
ST : study type (CC = case control, pr or prosp = prospective)
NLC: number of lung cancer cases in whole study
R : risky occupational population (n = no, m = mining, o = other risky)
VB : national cigarette type (V = at least 75% Virginia, bl = at least 75% blended, ot = other)
P : any proxy use
H : full histological confirmation
De : derivation of RR/CI (or = original, st = standard method, ot = other method of estimation)

Table 1K14 - 1

IESLC - Meta-analysis of Ex Smoking, Years quit (vs current), "High"
All LC types, Cigarettes only
Most adjusted

REF	NRR	1K4	SEX	AGEL	AGEH	RACE	YF	LC TYPE	LOC	START	ST	NLC	R	VB	P	H	AD	ADOS	PRODUCT	exL	exH	DENOM	De
ALDERS	515		m	0	0	all	-	all	Eu:UK	1977	CC	1448	n	V	n	n	1	0	cig only	10	999	current	ot
ALDERS	526		f	0	0	all	-	all	Eu:UK	1977	CC	1448	n	V	n	n	1	0	cig only	10	999	current	ot
BENHAM	605	x	m	0	0	all	-	all	Eu:wst	1976	CC	1625	n	bl	n	y	0	0	cig only	10	19	current	st
CPSI	818		m	50	74	all	6	all	NAmer	1959	pr	5138	n	bl	n	n	1	0	cig only	10	999	current	ot
CPSII	668		m	35	99	all	4	all	NAmer	1982	pr	3229	n	bl	n	n	1	0	cig only	11	15	current	ot
DAMBER	558	x	m	0	0	all	-	all	Eu:Sca	1972	CC	579	n	bl	y	n	1	0	cig only	11	999	current	ot
DEAN3	526	x	m	0	0	all	-	all	Eu:UK	1969	CC	766	n	V	y	n	1	0	cig only	9	18	cur+2y	ot
DOLL2	511		m	0	0	all	20	all	Eu:UK	1951	pr	920	n	V	n	n	1	0	cig only	10	14	current	ot
DORN	576	x	m	0	0	wh	15	all	NAmer	1954	pr	5097	n	bl	n	n	2	0	cig only	10	14	current	ot
GRAHAM	511		m	0	0	wh	-	all	NAmer	1956	CC	685	n	bl	n	n	0	0	cig only	10	999	current	st
KAISE2	656		m	0	0	all	9	all	NAmer	1979	pr	318	n	bl	n	n	1	0	cig only	11	20	cur+2y	ot
KAISE2	576		f	0	0	all	9	all	NAmer	1979	pr	318	n	bl	n	n	1	0	cig only	11	20	cur+2y	st
PEZZOT	505		m	0	0	all	-	all	SCAmer	1987	CC	215	n	bl	n	y	0	0	cig only	11	999	cur+1y	st
WYNDE6	515	x	m	0	0	all	-	all	NAmer	1969	CC	4423	n	bl	n	y	0	0	cig only	10	19	cur+1y	st
WYNDE6	536	x	f	0	0	all	-	all	NAmer	1969	CC	4423	n	bl	n	y	0	0	cig only	10	19	cur+1y	st

Cigarette type is all/unspec for all RRs
except for the following:

REF	NRR	CIGTYPE
ALDERS	515	MC only
ALDERS	526	MC only
DEAN3	526	MC only

Table 1K14 - 2

IESLC - Meta-analysis of Ex Smoking, Years quit (vs current), "High"
All LC types, Cigarettes only
Most adjusted

REF	NRR	SEX	AD	Number Case	Exposed Cont	Non-exposed Case	Cont	RR	95.00%CI
ALDERS	515	m	1	29	-	207	-	0.32	(0.20- 0.51)
ALDERS	526	f	1	26	-	244	-	0.28	(0.17- 0.46)
Subtotal ALDERS								0.30	(0.21- 0.42)
BENHAM	605	m	0	42	147	776	969	0.36	(0.25- 0.51)
*CPSI	818	m	1	15	-	844	-	0.09	(0.06- 0.15)
*CPSII	668	m	1	164	-	1159	-	0.39	(0.33- 0.46)
DAMBER	558	m	1	-	-	-	-	0.16	(0.08- 0.35)
DEAN3	526	m	1	15	-	337	-	0.41	(0.23- 0.72)
*DOLL2	511	m	1	9	-	236	-	0.28	(0.14- 0.54)
*DORN	576	m	2	100	-	2609	-	0.40	(0.33- 0.49)
GRAHAM	511	m	0	2	30	371	821	0.15	(0.04- 0.62)
*KAISE2	656	m	1	8	-	51	-	0.43	(0.20- 0.92)
*KAISE2	576	f	1	4	-	50	-	0.25	(0.09- 0.70)
Subtotal KAISE2								0.35	(0.19- 0.65)
PEZZOT	505	m	0	20	106	145	129	0.17	(0.10- 0.29)
WYNDE6	515	m	0	159	373	1107	993	0.38	(0.31- 0.47)
WYNDE6	536	f	0	36	132	683	496	0.20	(0.13- 0.29)
Subtotal WYNDE6								0.33	(0.28- 0.40)
Partial Totals				629	788	8819	3408		

*prospective study

REF	NRR	SEX	AD	Ys	Ws	Qs	Ps
ALDERS	515	m	1	-1.14	17.54	0.03	0.0000
ALDERS	526	f	1	-1.27	15.51	0.46	0.0000
Subtotal ALDERS				-1.20	33.04	0.49	
BENHAM	605	m	0	-1.03	30.36	0.15	0.0000
*CPSI	818	m	1	-2.41	18.30	31.31	0.0000
*CPSII	668	m	1	-0.94	139.29	3.49	0.0000
DAMBER	558	m	1	-1.83	7.05	3.79	0.0000
DEAN3	526	m	1	-0.89	11.80	0.51	0.0022
*DOLL2	511	m	1	-1.27	8.43	0.25	0.0002
*DORN	576	m	2	-0.92	98.33	3.32	0.0000
GRAHAM	511	m	0	-1.91	1.86	1.23	0.0090
*KAISE2	656	m	1	-0.84	6.60	0.43	0.0302
*KAISE2	576	f	1	-1.39	3.65	0.30	0.0081
Subtotal KAISE2				-1.04	10.25	0.73	
PEZZOT	505	m	0	-1.78	13.50	6.33	0.0000
WYNDE6	515	m	0	-0.96	91.91	1.77	0.0000
WYNDE6	536	f	0	-1.62	25.75	6.94	0.0000
Subtotal WYNDE6				-1.11	117.66	8.71	

	N	15
	NS	12
	Wt	489.88
Het	Chi	60.31
Het	df	14
Het	P	***
Fixed	RR	0.33
	RRl	0.30
	RRu	0.36
	P	---
Random	RR	0.28
	RRl	0.22
	RRu	0.34
	P	---
Asymm	P	*

Table 1K14 - 3

IESLC - Meta-analysis of Ex Smoking, Years quit (vs current), "High"
All LC types, Cigarettes only
Most adjusted

	Sex			
	combined	male	female	Total
N		12	3	15
NS		12	3	15
Wt		444.97	44.91	489.88
Het Chi		51.95	1.20	60.31
Het df		11	2	14
Het P		***	N.S.	***
Fixed RR		0.35	0.23	0.33
RRl		0.32	0.17	0.30
RRu		0.38	0.30	0.36
P		---	---	---
Random RR		0.29	0.23	0.28
RRl		0.22	0.17	0.22
RRu		0.36	0.30	0.34
P		---	---	---
Between Chi				7.17
Between df				1
Between P				**
Btwn(F) P				N.S.
Btwn(R) P				N.S.

	Lung cancer type		
	all	other	Total
N	15		15
NS	12		12
Wt	489.88		489.88
Het Chi	60.31		60.31
Het df	14		14
Het P	***		***
Fixed RR	0.33		0.33
RRl	0.30		0.30
RRu	0.36		0.36
P	---		---
Random RR	0.28		0.28
RRl	0.22		0.22
RRu	0.34		0.34
P	---		---
Between Chi			
Between df			
Between P			N.S.
Btwn(F) P			N.S.
Btwn(R) P			N.S.

	Location								
	NAmer	UK	Scand	othEur	China	Japan	othAs	other	Total
N	8	4	1	1				1	15
NS	6	3	1	1				1	12
Wt	385.69	53.27	7.05	30.36				13.50	489.88
Het Chi	48.24	1.15	0.00	0.00				0.00	60.31
Het df	7	3	0	0				0	14
Het P	***	N.S.	N.S.	N.S.				N.S.	***
Fixed RR	0.35	0.32	0.16	0.36				0.17	0.33
RRl	0.31	0.24	0.08	0.25				0.10	0.30
RRu	0.38	0.42	0.33	0.51				0.29	0.36
P	---	---	---	---				---	---
Random RR	0.27	0.32	0.16	0.36				0.17	0.28
RRl	0.20	0.24	0.08	0.25				0.10	0.22
RRu	0.38	0.42	0.33	0.51				0.29	0.34
P	---	---	---	---				---	---
Between Chi									10.92
Between df									4
Between P									*
Btwn(F) P									N.S.
Btwn(R) P									(*)

Table 1K14 - 3

IESLC - Meta-analysis of Ex Smoking, Years quit (vs current), "High"
All LC types, Cigarettes only
Most adjusted

Detailed Country in "other Europe"

	multi	Germany	othWest	East	Balkans	Total
N			1			1
NS			1			1
Wt			30.36			30.36
Het Chi			0.00			0.00
Het df			0			0
Het P			N.S.			N.S.
Fixed RR			0.36			0.36
RRl			0.25			0.25
RRu			0.51			0.51
P			---			---
Random RR			0.36			0.36
RRl			0.25			0.25
RRu			0.51			0.51
P			---			---
Between Chi						
Between df						
Between P						N.S.
Btwn(F) P						N.S.
Btwn(R) P						N.S.

Detailed Country in "other Asia"

	India	HongKong	other	Total
N				
NS				
Wt				
Het Chi				
Het df				
Het P				
Fixed RR				
RRl				
RRu				
P				
Random RR				
RRl				
RRu				
P				
Between Chi				
Between df				
Between P				N.S.
Btwn(F) P				N.S.
Btwn(R) P				N.S.

Detailed other continent

	SCAmer	Total
N	1	1
NS	1	1
Wt	13.50	13.50
Het Chi	0.00	0.00
Het df	0	0
Het P	N.S.	N.S.
Fixed RR	0.17	0.17
RRl	0.10	0.10
RRu	0.29	0.29
P	---	---
Random RR	0.17	0.17
RRl	0.10	0.10
RRu	0.29	0.29
P	---	---
Between Chi		
Between df		
Between P		N.S.
Btwn(F) P		N.S.
Btwn(R) P		N.S.

Table 1K14 - 3

IESLC - Meta-analysis of Ex Smoking, Years quit (vs current), "High"
All LC types, Cigarettes only
Most adjusted

	Start year of study					
	<1960	1960-69	1970-79	1980-89	1990+	Total
N	4	3	6	2		15
NS	4	2	4	2		12
Wt	126.92	129.46	80.71	152.79		489.88
Het Chi	35.50	9.19	4.74	8.75		60.31
Het df	3	2	5	1		14
Het P	***	*	N.S.	**		***
Fixed RR	0.31	0.34	0.31	0.36		0.33
RRl	0.26	0.28	0.25	0.31		0.30
RRu	0.37	0.40	0.39	0.42		0.36
P	---	---	---	---		---
Random RR	0.20	0.31	0.31	0.27		0.28
RRl	0.08	0.20	0.25	0.12		0.22
RRu	0.50	0.49	0.39	0.61		0.34
P	---	---	---	--		---
Between Chi						2.13
Between df						3
Between P						N.S.
Btwn(F) P						N.S.
Btwn(R) P						N.S.

	Study type (1)		
	CC	other	Total
N	9	6	15
NS	7	5	12
Wt	215.28	274.60	489.88
Het Chi	20.11	38.25	60.31
Het df	8	5	14
Het P	**	***	***
Fixed RR	0.31	0.35	0.33
RRl	0.27	0.31	0.30
RRu	0.35	0.40	0.36
P	---	---	---
Random RR	0.28	0.28	0.28
RRl	0.22	0.18	0.22
RRu	0.35	0.42	0.34
P	---	---	---
Between Chi			1.96
Between df			1
Between P			N.S.
Btwn(F) P			N.S.
Btwn(R) P			N.S.

	Study type (2)			
	CC	prosp	other	Total
N	9	6		15
NS	7	5		12
Wt	215.28	274.60		489.88
Het Chi	20.11	38.25		60.31
Het df	8	5		14
Het P	**	***		***
Fixed RR	0.31	0.35		0.33
RRl	0.27	0.31		0.30
RRu	0.35	0.40		0.36
P	---	---		---
Random RR	0.28	0.28		0.28
RRl	0.22	0.18		0.22
RRu	0.35	0.42		0.34
P	---	---		---
Between Chi				1.96
Between df				1
Between P				N.S.
Btwn(F) P				N.S.
Btwn(R) P				N.S.

Table 1K14 - 3

IESLC - Meta-analysis of Ex Smoking, Years quit (vs current), "High"
All LC types, Cigarettes only
Most adjusted

	Study size (number of LC cases)				
	100-249	250-499	500-999	1000+	Total
N	1	2	4	8	15
NS	1	1	4	6	12
Wt	13.50	10.25	29.15	436.99	489.88
Het Chi	0.00	0.69	4.68	47.00	60.31
Het df	0	1	3	7	14
Het P	N.S.	N.S.	N.S.	***	***
Fixed RR	0.17	0.35	0.27	0.34	0.33
RRl	0.10	0.19	0.19	0.31	0.30
RRu	0.29	0.65	0.39	0.38	0.36
P	---	---	---	---	---
Random RR	0.17	0.35	0.26	0.29	0.28
RRl	0.10	0.19	0.16	0.22	0.22
RRu	0.29	0.65	0.42	0.38	0.34
P	---	---	---	---	---
Between Chi					7.94
Between df					3
Between P					*
Btwn(F) P					N.S.
Btwn(R) P					N.S.

	Risky occupational population			
	no	mining	othRisky	Total
N	15			15
NS	12			12
Wt	489.88			489.88
Het Chi	60.31			60.31
Het df	14			14
Het P	***			***
Fixed RR	0.33			0.33
RRl	0.30			0.30
RRu	0.36			0.36
P	---			---
Random RR	0.28			0.28
RRl	0.22			0.22
RRu	0.34			0.34
P	---			---
Between Chi				
Between df				
Between P				N.S.
Btwn(F) P				N.S.
Btwn(R) P				N.S.

	National cigarette tobacco type			
	Virginia	blended	other	Total
N	4	11		15
NS	3	9		12
Wt	53.27	436.61		489.88
Het Chi	1.15	59.04		60.31
Het df	3	10		14
Het P	N.S.	***		***
Fixed RR	0.32	0.33		0.33
RRl	0.24	0.30		0.30
RRu	0.42	0.37		0.36
P	---	---		---
Random RR	0.32	0.26		0.28
RRl	0.24	0.20		0.22
RRu	0.42	0.34		0.34
P	---	---		---
Between Chi				0.12
Between df				1
Between P				N.S.
Btwn(F) P				N.S.
Btwn(R) P				N.S.

Table 1K14 - 3

IESLC - Meta-analysis of Ex Smoking, Years quit (vs current), "High"
All LC types, Cigarettes only
Most adjusted

	Any proxy use No/nk	Yes	Total
N	13	2	15
NS	10	2	12
Wt	471.03	18.85	489.88
Het Chi	56.00	3.91	60.31
Het df	12	1	14
Het P	***	*	***
Fixed RR	0.33	0.29	0.33
RRl	0.31	0.18	0.30
RRu	0.37	0.45	0.36
P	---	---	---
Random RR	0.28	0.26	0.28
RRl	0.22	0.11	0.22
RRu	0.35	0.66	0.34
P	---	--	---
Between Chi			0.40
Between df			1
Between P			N.S.
Btwn(F) P			N.S.
Btwn(R) P			N.S.

	Full histological confirmation No	Yes	Total
N	11	4	15
NS	9	3	12
Wt	328.36	161.52	489.88
Het Chi	44.94	14.81	60.31
Het df	10	3	14
Het P	***	**	***
Fixed RR	0.34	0.32	0.33
RRl	0.31	0.27	0.30
RRu	0.38	0.37	0.36
P	---	---	---
Random RR	0.28	0.27	0.28
RRl	0.21	0.18	0.22
RRu	0.37	0.40	0.34
P	---	---	---
Between Chi			0.56
Between df			1
Between P			N.S.
Btwn(F) P			N.S.
Btwn(R) P			N.S.

	Number of adjustment variables (1) 0	1	2+/+nk	Total
N	5	9	1	15
NS	4	7	1	12
Wt	163.38	228.17	98.33	489.88
Het Chi	15.89	40.24	0.00	60.31
Het df	4	8	0	14
Het P	**	***	N.S.	***
Fixed RR	0.31	0.32	0.40	0.33
RRl	0.27	0.28	0.33	0.30
RRu	0.37	0.36	0.49	0.36
P	---	---	---	---
Random RR	0.26	0.27	0.40	0.28
RRl	0.18	0.18	0.33	0.22
RRu	0.38	0.39	0.49	0.34
P	---	---	---	---
Between Chi				4.18
Between df				2
Between P				N.S.
Btwn(F) P				N.S.
Btwn(R) P				*

Table 1K14 - 3

IESLC - Meta-analysis of Ex Smoking, Years quit (vs current), "High"
All LC types, Cigarettes only
Most adjusted

	Number of adjustment variables (2) 0	1	2	3-5	6+/+nk	Total
N	5	9	1			15
NS	4	7	1			12
Wt	163.38	228.17	98.33			489.88
Het Chi	15.89	40.24	0.00			60.31
Het df	4	8	0			14
Het P	**	***	N.S.			***
Fixed RR	0.31	0.32	0.40			0.33
RRl	0.27	0.28	0.33			0.30
RRu	0.37	0.36	0.49			0.36
P	---	---	---			---
Random RR	0.26	0.27	0.40			0.28
RRl	0.18	0.18	0.33			0.22
RRu	0.38	0.39	0.49			0.34
P	---	---	---			---
Between Chi						4.18
Between df						2
Between P						N.S.
Btwn(F) P						N.S.
Btwn(R) P						*

	Denominator current	cur+rec	Total
N	9	6	15
NS	8	4	12
Wt	336.68	153.20	489.88
Het Chi	43.89	15.98	60.31
Het df	8	5	14
Het P	***	**	***
Fixed RR	0.34	0.32	0.33
RRl	0.31	0.27	0.30
RRu	0.38	0.37	0.36
P	---	---	---
Random RR	0.27	0.29	0.28
RRl	0.20	0.20	0.22
RRu	0.36	0.41	0.34
P	---	---	---
Between Chi			0.44
Between df			1
Between P			N.S.
Btwn(F) P			N.S.
Btwn(R) P			N.S.

	Derivation of RR/CI Orig	StdCalc	Other	Total
N		6	9	15
NS		5	8	13
Wt		167.03	322.85	489.88
Het Chi		16.08	43.27	60.31
Het df		5	8	14
Het P		**	***	***
Fixed RR		0.31	0.34	0.33
RRl		0.27	0.31	0.30
RRu		0.36	0.38	0.36
P		---	---	---
Random RR		0.26	0.28	0.28
RRl		0.19	0.21	0.22
RRu		0.37	0.39	0.34
P		---	---	---
Between Chi				0.97
Between df				1
Between P				N.S.
Btwn(F) P				N.S.
Btwn(R) P				N.S.

Table 1K14 - 4

IESLC - Meta-analysis of Ex Smoking, Years quit (vs current), "High"
All LC types, Cigarettes only
Least adjusted

REF	NRR	X	SEX	AGEL	AGEH	RACE	YF	LC TYPE	LOC	START	ST	NLC	R	VB	P	H	AD	ADOS	PRODUCT	exL	exH	DENOM	De
ALDERS	515		m	0	0	all	-	all	Eu:UK	1977	CC	1448	n	V	n	n	1	0	cig only	10	999	current	ot
ALDERS	526		f	0	0	all	-	all	Eu:UK	1977	CC	1448	n	V	n	n	1	0	cig only	10	999	current	ot
BENHAM	605		m	0	0	all	-	all	Eu:wst	1976	CC	1625	n	bl	n	y	0	0	cig only	10	19	current	st
CPSI	818		m	50	74	all	6	all	NAmer	1959	pr	5138	n	bl	n	n	1	0	cig only	10	999	current	ot
CPSII	668		m	35	99	all	4	all	NAmer	1982	pr	3229	n	bl	n	n	1	0	cig only	11	15	current	ot
DAMBER	558		m	0	0	all	-	all	Eu:Sca	1972	CC	579	n	bl	y	n	1	0	cig only	11	999	current	ot
DEAN3	511	x	m	0	0	all	-	all	Eu:UK	1969	CC	766	n	V	y	n	0	0	cig only	9	18	cur+2y	st
DOLL2	511		m	0	0	all	20	all	Eu:UK	1951	pr	920	n	V	n	n	1	0	cig only	10	14	current	ot
DORN	576		m	0	0	wh	15	all	NAmer	1954	pr	5097	n	bl	n	n	2	0	cig only	10	14	current	ot
GRAHAM	511		m	0	0	wh	-	all	NAmer	1956	CC	685	n	bl	n	n	0	0	cig only	10	999	current	st
KAISE2	656		m	0	0	all	9	all	NAmer	1979	pr	318	n	bl	n	n	1	0	cig only	11	20	cur+2y	ot
KAISE2	576		f	0	0	all	9	all	NAmer	1979	pr	318	n	bl	n	n	1	0	cig only	11	20	cur+2y	st
PEZZOT	505		m	0	0	all	-	all	SCAmer	1987	CC	215	n	bl	n	y	0	0	cig only	11	999	cur+1y	st
WYNDE6	515		m	0	0	all	-	all	NAmer	1969	CC	4423	n	bl	n	y	0	0	cig only	10	19	cur+1y	st
WYNDE6	536		f	0	0	all	-	all	NAmer	1969	CC	4423	n	bl	n	y	0	0	cig only	10	19	cur+1y	st

Cigarette type is all/unspec for all RRs
except for the following:

REF	NRR	CIGTYPE
ALDERS	515	MC only
ALDERS	526	MC only
DEAN3	511	MC only

Table 1K14 - 5

IESLC - Meta-analysis of Ex Smoking, Years quit (vs current), "High"
All LC types, Cigarettes only
Least adjusted

REF	NRR	SEX	AD	Number Case	Exposed Cont	Non-exposed Case	Cont	RR	95.00%CI
ALDERS	515	m	1	29	-	207	-	0.32	(0.20- 0.51)
ALDERS	526	f	1	26	-	244	-	0.28	(0.17- 0.46)
Subtotal ALDERS								0.30	(0.21- 0.42)
BENHAM	605	m	0	42	147	776	969	0.36	(0.25- 0.51)
*CPSI	818	m	1	15	-	844	-	0.09	(0.06- 0.15)
*CPSII	668	m	1	164	-	1159	-	0.39	(0.33- 0.46)
DAMBER	558	m	1	-	-	-	-	0.16	(0.08- 0.35)
DEAN3	511	m	0	15	86	337	930	0.48	(0.27- 0.84)
*DOLL2	511	m	1	9	-	236	-	0.28	(0.14- 0.54)
*DORN	576	m	2	100	-	2609	-	0.40	(0.33- 0.49)
GRAHAM	511	m	0	2	30	371	821	0.15	(0.04- 0.62)
*KAISE2	656	m	1	8	-	51	-	0.43	(0.20- 0.92)
*KAISE2	576	f	1	4	-	50	-	0.25	(0.09- 0.70)
Subtotal KAISE2								0.35	(0.19- 0.65)
PEZZOT	505	m	0	20	106	145	129	0.17	(0.10- 0.29)
WYNDE6	515	m	0	159	373	1107	993	0.38	(0.31- 0.47)
WYNDE6	536	f	0	36	132	683	496	0.20	(0.13- 0.29)
Subtotal WYNDE6								0.33	(0.28- 0.40)
Partial Totals				629	874	8819	4338		

*prospective study

REF	NRR	SEX	AD	Ys	Ws	Qs	Ps
ALDERS	515	m	1	-1.14	17.54	0.03	0.0000
ALDERS	526	f	1	-1.27	15.51	0.49	0.0000
Subtotal ALDERS				-1.20	33.04	0.52	
BENHAM	605	m	0	-1.03	30.36	0.13	0.0000
*CPSI	818	m	1	-2.41	18.30	31.51	0.0000
*CPSII	668	m	1	-0.94	139.29	3.31	0.0000
DAMBER	558	m	1	-1.83	7.05	3.83	0.0000
DEAN3	511	m	0	-0.73	12.15	1.61	0.0108
*DOLL2	511	m	1	-1.27	8.43	0.26	0.0002
*DORN	576	m	2	-0.92	98.33	3.17	0.0000
GRAHAM	511	m	0	-1.91	1.86	1.25	0.0090
*KAISE2	656	m	1	-0.84	6.60	0.42	0.0302
*KAISE2	576	f	1	-1.39	3.65	0.31	0.0081
Subtotal KAISE2				-1.04	10.25	0.73	
PEZZOT	505	m	0	-1.78	13.50	6.40	0.0000
WYNDE6	515	m	0	-0.96	91.91	1.66	0.0000
WYNDE6	536	f	0	-1.62	25.75	7.05	0.0000
Subtotal WYNDE6				-1.11	117.66	8.72	

	N	15
	NS	12
	Wt	490.23
Het	Chi	61.44
Het	df	14
Het	P	***
Fixed	RR	0.33
	RRl	0.31
	RRu	0.37
	P	---
Random	RR	0.28
	RRl	0.22
	RRu	0.35
	P	---
Asymm	P	*

Table 1K14 - 6

IESLC - Meta-analysis of Ex Smoking, Years quit (vs current), "High"
All LC types, Cigarettes only
Least adjusted

	Sex combined	male	female	Total
N		12	3	15
NS		12	3	15
Wt		445.32	44.91	490.23
Het Chi		52.92	1.20	61.44
Het df		11	2	14
Het P		***	N.S.	***
Fixed RR		0.35	0.23	0.33
RRl		0.32	0.17	0.31
RRu		0.38	0.30	0.37
P		---	---	---
Random RR		0.29	0.23	0.28
RRl		0.23	0.17	0.22
RRu		0.37	0.30	0.35
P		---	---	---
Between Chi				7.32
Between df				1
Between P				**
Btwn(F) P				N.S.
Btwn(R) P				N.S.

Table 1K14 - 7

IESLC - Meta-analysis of Ex Smoking, Years quit (vs current), "High"
All LC types, Cigarettes only
Excluded studies (and stage at which they were excluded)

1	AGUDO AKIBA AMANDU AMES AXELSS BEST BOUCHA BOUCOT BRESLO CHEN CHEN2 CHIAZZ DEAN2 DOSEME ENGELA FAN GENG GER GUO HAENSZ HEGMAN HOLE HU HU2 JUSSAW KATSOU KAUFMA KOO KOULUM KREUZE LETOUR LEVIN LIAW LIU3 LIU4 LIU5 MCCONN MIGRAN MRFITR NOTAN2 OSANN2 PERNU QIAO2 RACHTA RESTRE SADOWS SEGI2 STASZE TIZZAN VUTUC WATSON WU WUWILL WYNDE2 WYNDE8 XU YUAN ZHANG ZHENG ZHOU
2	AUVINE BENSHL BLOT1 BROWN3 BUFFLE GURSEL LAUSSM LUO MCDUFF PISANI PRESCO SPITZ WU2 WYNDE7
4	HAMMON
5	CORREA GILLIS HUMBLE QIAO WIGLE
7	ARMADA BARBON BECHER BOFFET BROSS CARPEN CEDERL CHOI CHYOU DARBY DESTEF DOLL DORGAN GAO GAO2 GARCIA GARSHI HAMMO2 HIRAYA JAHN JAIN JEDRYC JOLY KHUDER LUBIN LUBIN2 MATOS PEZZO2 SOBUE SPEIZE SUZUK2 SVENSS WAKAI WANG2 WYNDE3
14	TVERDA

Table 1K14 - 8
Potentially overlapping studies

REF	REFGP	PRINC	. OVERLAP/LINK
BENHAM	LUBIN2	2	Subset of Lubin2
GRAHAM	BYERS1	1	GRAHAM/BROSS/BYERS1
WYNDE6	WYNDE6	1	WYNDE5/6/7/8
CPSI	CPSI	1	CPSI overall

Table 1K15 -

IESLC - Meta-analysis of Ex Smoking, Years quit (vs current), "Highest vs lowest"
All LC types, Cigarettes only

This analysis is restricted to results for:
1) Ex smokers
2) Results by Years quit (vs current)
3) Categorical results by Years quit (vs current)
4) Denominator (unexposed) = "low"
5) All LC types (or near equivalent)
6) Results complete enough for use in metaanalysis

Within each study, results are then selected (in the following order of preference, within each sex) for:
7) (not applicable)
8) PRODUCT: cigarettes only
9) CIGTYPE: all/unspecified, MC regardless of HR, MC only
10) Results with least adjustment for other aspects of smoking (ADOS)
11) The highest vs lowest category
12) Followup period (YF, prospective studies): whole study (coded as 0) or longest available
13) LCtype: all or nearest available, at least Squamous and Adeno. (q = squamous, s = small,
l = large, a = adeno, mix = mixed, alv = alveolar)
14) Race: all or nearest available, otherwise by race (wh or w = white, bl or b = black, hi = hispanic
ch = chinese, jap = japanese, haw = hawaiian, w+o = white + oriental, sca = scandinavian, as = asian)
15) For overlapping studies: principal rather than subsidiary studies
Finally by Age: whole study (coded as 0) if available, otherwise by widest available age group
and then for single sex results (m, f) in preference to results for both sexes combined (c).

Results adjusted (AD) for the most potential confounders are then chosen in Sections -1 to -3
(and those which actually differ from the adjusted results in Table 1K5 - 1 are marked 'x' in Section -1)
and results adjusted for the least confounders in Sections -4 to -6. (Those least adjusted results which
actually differ from the most adjusted are marked 'x' in column X in Section -4)

Section -7 shows excluded studies, together with the stage (as above) at which no qualifying
results were found.

Section -8 lists the potentially overlapping studies which have been included (1=principal, 2=subsidiary).

Section -9 lists any results which would have been included in preference except that they had data not complete
enough for use in meta-analysis, with their significance (yes/no), if known, and any further comment as entered
on the database. It also lists as "gap" any categories for which no data were presented by the original authors.

In addition to those mentioned above, the following fields, levels and abbreviations are used:
* or nk = not known, n = no, y = yes, ot = other
all/unspec = all or unspecified, MC = manufactured cigarettes, HR = hand-rolled cigarettes
exL, exH = range of exposure (low and high) in the "highest" group, in terms of Years quit (vs current)
unexL, unexH = range of exposure (low and high) in the "lowest" group, in terms of Years quit (vs current)
REF: 6-character study reference
NRR: number of the RR on the database within the study
ST : study type (CC = case control, pr or prosp = prospective)
NLC: number of lung cancer cases in whole study
R : risky occupational population (n = no, m = mining, o = other risky)
VB : national cigarette type (V = at least 75% Virginia, bl = at least 75% blended, ot = other)
P : any proxy use
H : full histological confirmation
De : derivation of RR/CI (or = original, st = standard method, ot = other method of estimation)

Table 1K15 - 1

IESLC - Meta-analysis of Ex Smoking, Years quit (vs current), "Highest vs lowest"
All LC types, Cigarettes only
Most adjusted

REF	NRR	1K5	SEX	AGEL	AGEH	RACE	YF	LC TYPE	LOC	START	ST	NLC	R	VB	P	H	AD	ADOS	PRODUCT	exL	exH	unexL	unexH	De
ALDERS	517		m	0	0	all	-	all	Eu:UK	1977	CC	1448	n	V	n	n	1	0	cig only	10	999	0.1	2	ot
ALDERS	528		f	0	0	all	-	all	Eu:UK	1977	CC	1448	n	V	n	n	1	0	cig only	10	999	0.1	2	ot
BENHAM	627	x	m	0	0	all	-	all	Eu:wst	1976	CC	1625	n	bl	n	y	0	0	cig only	20	999	1.0	4	st
CPSI	821		m	50	74	all	6	all	NAmer	1959	pr	5138	n	bl	n	n	1	0	cig only	10	999	0.1	0.9	ot
CPSII	674		m	35	99	all	4	all	NAmer	1982	pr	3229	n	bl	n	n	1	0	cig only	16	999	0.1	0.9	ot
DAMBER	559	x	m	0	0	all	-	all	Eu:Sca	1972	CC	579	n	bl	y	n	1	0	cig only	11	999	0.1	10	ot
DEAN3	530	x	m	0	0	all	-	all	Eu:UK	1969	CC	766	n	V	y	n	1	0	cig only	19	999	3	4	ot
DOLL2	515		m	0	0	all	20	all	Eu:UK	1951	pr	920	n	V	n	n	1	0	cig only	15	999	0.1	4	ot
DORN	523	x	m	0	0	wh	0	all	NAmer	1954	pr	5097	n	bl	n	n	2	0	cig only	40	999	0.1	4	ot
GRAHAM	514	x	m	0	0	wh	-	all	NAmer	1956	CC	685	n	bl	n	n	0	0	cig only	10	999	0.1	1.0	st
KAISE2	660		m	0	0	all	9	all	NAmer	1979	pr	318	n	bl	n	n	1	0	cig only	21	999	2	10	st
KAISE2	580		f	0	0	all	9	all	NAmer	1979	pr	318	n	bl	n	n	1	0	cig only	21	999	2	10	ot
PEZZOT	506		m	0	0	all	-	all	SCAmer	1987	CC	215	n	bl	n	y	0	0	cig only	11	999	1.0	10	st
TVERDA	510		m	0	0	all	0	all	Eu:Sca	1972	pr	238	n	bl	n	n	2	0	cig only	5	999	0.1	0.9	ot
WYNDE6	521	x	m	0	0	all	-	all	NAmer	1969	CC	4423	n	bl	n	y	0	0	cig only	30	999	1.0	4	st
WYNDE6	542	x	f	0	0	all	-	all	NAmer	1969	CC	4423	n	bl	n	y	0	0	cig only	30	999	1.0	4	st

Cigarette type is all/unspec for all RRs
except for the following:

REF	NRR	CIGTYPE
ALDERS	517	MC only
ALDERS	528	MC only
DEAN3	530	MC only

Table 1K15 - 2

IESLC - Meta-analysis of Ex Smoking, Years quit (vs current), "Highest vs lowest"
All LC types, Cigarettes only
Most adjusted

REF	NRR	SEX	AD	Number Exposed Case	Number Exposed Cont	Non-exposed Case	Non-exposed Cont	RR	95.00%CI
ALDERS	517	m	1	29	-	121	-	0.18	(0.10- 0.30)
ALDERS	528	f	1	26	-	206	-	0.13	(0.08- 0.23)
Subtotal ALDERS								0.15	(0.10- 0.22)
BENHAM	627	m	0	19	129	154	138	0.13	(0.08- 0.23)
*CPSI	821	m	1	15	-	37	-	0.09	(0.05- 0.16)
*CPSII	674	m	1	256	-	97	-	0.10	(0.08- 0.12)
DAMBER	559	m	1	-	-	-	-	0.29	(0.12- 0.73)
DEAN3	530	m	1	8	-	28	-	0.28	(0.12- 0.66)
*DOLL2	515	m	1	7	-	15	-	0.13	(0.05- 0.31)
*DORN	523	m	2	49	-	56	-	0.09	(0.06- 0.15)
GRAHAM	514	m	0	2	30	84	48	0.04	(0.01- 0.17)
*KAISE2	660	m	1	6	-	12	-	0.21	(0.07- 0.62)
*KAISE2	580	f	1	4	-	6	-	0.58	(0.15- 2.22)
Subtotal KAISE2								0.31	(0.13- 0.73)
PEZZOT	506	m	0	20	106	46	82	0.34	(0.18- 0.61)
*TVERDA	510	m	2	4	-	2	-	0.48	(0.09- 2.66)
WYNDE6	521	m	0	21	161	201	166	0.11	(0.07- 0.18)
WYNDE6	542	f	0	10	31	82	70	0.28	(0.13- 0.60)
Subtotal WYNDE6								0.14	(0.09- 0.22)
Partial Totals				476	457	1147	504		

*prospective study

REF	NRR	SEX	AD	Ys	Ws	Qs	Ps
ALDERS	517	m	1	-1.71	12.73	1.62	0.0000
ALDERS	528	f	1	-2.04	13.78	0.01	0.0000
Subtotal ALDERS				-1.88	26.51	1.64	
BENHAM	627	m	0	-2.03	13.49	0.03	0.0000
*CPSI	821	m	1	-2.41	11.36	1.28	0.0000
*CPSII	674	m	1	-2.30	93.46	4.97	0.0000
DAMBER	559	m	1	-1.24	4.71	3.28	0.0072
DEAN3	530	m	1	-1.27	5.29	3.38	0.0034
*DOLL2	515	m	1	-2.04	4.62	0.00	0.0000
*DORN	523	m	2	-2.41	18.30	2.07	0.0000
GRAHAM	514	m	0	-3.27	1.77	2.53	0.0000
*KAISE2	660	m	1	-1.56	3.23	0.84	0.0050
*KAISE2	580	f	1	-0.54	2.12	4.94	0.4281
Subtotal KAISE2				-1.16	5.35	5.78	
PEZZOT	506	m	0	-1.09	10.71	10.33	0.0004
*TVERDA	510	m	2	-0.73	1.34	2.40	0.3955
WYNDE6	521	m	0	-2.23	15.43	0.38	0.0000
WYNDE6	542	f	0	-1.29	6.30	3.86	0.0012
Subtotal WYNDE6				-1.96	21.72	4.23	

	N	16
	NS	13
	Wt	218.63
Het	Chi	41.92
Het	df	15
Het	P	***
Fixed	RR	0.13
	RRl	0.11
	RRu	0.14
	P	---
Random	RR	0.16
	RRl	0.12
	RRu	0.20
	P	---
Asymm	P	*

Table 1K15 - 3

IESLC - Meta-analysis of Ex Smoking, Years quit (vs current), "Highest vs lowest"
All LC types, Cigarettes only
Most adjusted

	combined	Sex male	female	Total
N		13	3	16
NS		13	3	16
Wt		196.43	22.19	218.63
Het Chi		32.74	5.47	41.92
Het df		12	2	15
Het P		**	(*)	***
Fixed RR		0.12	0.19	0.13
RRl		0.10	0.12	0.11
RRu		0.14	0.28	0.14
P		---	---	---
Random RR		0.14	0.23	0.16
RRl		0.11	0.11	0.12
RRu		0.19	0.51	0.20
P		---	---	---
Between Chi				3.71
Between df				1
Between P				(*)
Btwn(F) P				N.S.
Btwn(R) P				N.S.

	Lung cancer type all	other	Total
N	16		16
NS	13		13
Wt	218.63		218.63
Het Chi	41.92		41.92
Het df	15		15
Het P	***		***
Fixed RR	0.13		0.13
RRl	0.11		0.11
RRu	0.14		0.14
P	---		---
Random RR	0.16		0.16
RRl	0.12		0.12
RRu	0.20		0.20
P	---		---
Between Chi			
Between df			
Between P			N.S.
Btwn(F) P			N.S.
Btwn(R) P			N.S.

	NAmer	UK	Scand	Location othEur	China	Japan	othAs	other	Total
N	8	4	2	1				1	16
NS	6	3	2	1				1	13
Wt	151.96	36.41	6.05	13.49				10.71	218.63
Het Chi	16.33	2.61	0.26	0.00				0.00	41.92
Het df	7	3	1	0				0	15
Het P	*	N.S.	N.S.	N.S.				N.S.	***
Fixed RR	0.11	0.16	0.32	0.13				0.34	0.13
RRl	0.09	0.12	0.15	0.08				0.18	0.11
RRu	0.12	0.23	0.72	0.23				0.61	0.14
P	---	---	--	---				---	---
Random RR	0.12	0.16	0.32	0.13				0.34	0.16
RRl	0.09	0.12	0.15	0.08				0.18	0.12
RRu	0.17	0.23	0.72	0.23				0.61	0.20
P	---	---	--	---				---	---
Between Chi									22.71
Between df									4
Between P									***
Btwn(F) P									(*)
Btwn(R) P									*

Table 1K15 - 3

IESLC - Meta-analysis of Ex Smoking, Years quit (vs current), "Highest vs lowest"
All LC types, Cigarettes only
Most adjusted

Detailed Country in "other Europe"

	multi	Germany	othWest	East	Balkans	Total
N			1			1
NS			1			1
Wt			13.49			13.49
Het Chi			0.00			0.00
Het df			0			0
Het P			N.S.			N.S.
Fixed RR			0.13			0.13
RRl			0.08			0.08
RRu			0.23			0.23
P			---			---
Random RR			0.13			0.13
RRl			0.08			0.08
RRu			0.23			0.23
P			---			---
Between Chi						
Between df						
Between P						N.S.
Btwn(F) P						N.S.
Btwn(R) P						N.S.

Detailed Country in "other Asia"

	India	HongKong	other	Total
N				
NS				
Wt				
Het Chi				
Het df				
Het P				
Fixed RR				
RRl				
RRu				
P				
Random RR				
RRl				
RRu				
P				
Between Chi				
Between df				
Between P				N.S.
Btwn(F) P				N.S.
Btwn(R) P				N.S.

Detailed other continent

	SCAmer	Total
N	1	1
NS	1	1
Wt	10.71	10.71
Het Chi	0.00	0.00
Het df	0	0
Het P	N.S.	N.S.
Fixed RR	0.34	0.34
RRl	0.18	0.18
RRu	0.61	0.61
P	---	---
Random RR	0.34	0.34
RRl	0.18	0.18
RRu	0.61	0.61
P	---	---
Between Chi		
Between df		
Between P		N.S.
Btwn(F) P		N.S.
Btwn(R) P		N.S.

Table 1K15 - 3

IESLC - Meta-analysis of Ex Smoking, Years quit (vs current), "Highest vs lowest"
All LC types, Cigarettes only
Most adjusted

	Start year of study					
	<1960	1960-69	1970-79	1980-89	1990+	Total
N	4	3	7	2		16
NS	4	2	5	2		13
Wt	36.04	27.01	51.40	104.18		218.63
Het Chi	1.93	5.92	8.00	14.14		41.92
Het df	3	2	6	1		15
Het P	N.S.	(*)	N.S.	***		***
Fixed RR	0.09	0.16	0.17	0.11		0.13
RRl	0.07	0.11	0.13	0.09		0.11
RRu	0.13	0.24	0.23	0.14		0.14
P	---	---	---	---		---
Random RR	0.09	0.19	0.18	0.18		0.16
RRl	0.07	0.09	0.13	0.05		0.12
RRu	0.13	0.38	0.26	0.58		0.20
P	---	---	---	--		---
Between Chi						11.92
Between df						3
Between P						**
Btwn(F) P						N.S.
Btwn(R) P						*

	Study type (1)		
	CC	other	Total
N	9	7	16
NS	7	6	13
Wt	84.20	134.43	218.63
Het Chi	18.17	11.97	41.92
Het df	8	6	15
Het P	*	(*)	***
Fixed RR	0.17	0.10	0.13
RRl	0.14	0.09	0.11
RRu	0.21	0.12	0.14
P	---	---	---
Random RR	0.18	0.12	0.16
RRl	0.13	0.09	0.12
RRu	0.25	0.17	0.20
P	---	---	---
Between Chi			11.78
Between df			1
Between P			***
Btwn(F) P			*
Btwn(R) P			N.S.

	Study type (2)			
	CC	prosp	other	Total
N	9	7		16
NS	7	6		13
Wt	84.20	134.43		218.63
Het Chi	18.17	11.97		41.92
Het df	8	6		15
Het P	*	(*)		***
Fixed RR	0.17	0.10		0.13
RRl	0.14	0.09		0.11
RRu	0.21	0.12		0.14
P	---	---		---
Random RR	0.18	0.12		0.16
RRl	0.13	0.09		0.12
RRu	0.25	0.17		0.20
P	---	---		---
Between Chi				11.78
Between df				1
Between P				***
Btwn(F) P				*
Btwn(R) P				N.S.

Table 1K15 - 3

IESLC - Meta-analysis of Ex Smoking, Years quit (vs current), "Highest vs lowest"
All LC types, Cigarettes only
Most adjusted

	Study size (number of LC cases) 100-249	250-499	500-999	1000+	Total
N	2	2	4	8	16
NS	2	1	4	6	13
Wt	12.05	5.35	16.38	184.85	218.63
Het Chi	0.15	1.32	6.85	11.26	41.92
Het df	1	1	3	7	15
Het P	N.S.	N.S.	(*)	N.S.	***
Fixed RR	0.35	0.31	0.18	0.11	0.13
RRl	0.20	0.13	0.11	0.10	0.11
RRu	0.62	0.73	0.30	0.13	0.14
P	---	--	---	---	---
Random RR	0.35	0.32	0.16	0.12	0.16
RRl	0.20	0.12	0.08	0.10	0.12
RRu	0.62	0.86	0.35	0.15	0.20
P	---	-	---	---	---
Between Chi					22.34
Between df					3
Between P					***
Btwn(F) P					*
Btwn(R) P					**

	Risky occupational population no	mining	othRisky	Total
N	16			16
NS	13			13
Wt	218.63			218.63
Het Chi	41.92			41.92
Het df	15			15
Het P	***			***
Fixed RR	0.13			0.13
RRl	0.11			0.11
RRu	0.14			0.14
P	---			---
Random RR	0.16			0.16
RRl	0.12			0.12
RRu	0.20			0.20
P	---			---
Between Chi				
Between df				
Between P				N.S.
Btwn(F) P				N.S.
Btwn(R) P				N.S.

	National cigarette tobacco type Virginia	blended	other	Total
N	4	12		16
NS	3	10		13
Wt	36.41	182.22		218.63
Het Chi	2.61	36.42		41.92
Het df	3	11		15
Het P	N.S.	***		***
Fixed RR	0.16	0.12		0.13
RRl	0.12	0.10		0.11
RRu	0.23	0.14		0.14
P	---	---		---
Random RR	0.16	0.15		0.16
RRl	0.12	0.11		0.12
RRu	0.23	0.21		0.20
P	---	---		---
Between Chi				2.88
Between df				1
Between P				(*)
Btwn(F) P				N.S.
Btwn(R) P				N.S.

Table 1K15 - 3

IESLC - Meta-analysis of Ex Smoking, Years quit (vs current), "Highest vs lowest"
All LC types, Cigarettes only
Most adjusted

	Any proxy use No/nk	Yes	Total
N	14	2	16
NS	11	2	13
Wt	208.63	10.00	218.63
Het Chi	34.95	0.00	41.92
Het df	13	1	15
Het P	***	N.S.	***
Fixed RR	0.12	0.28	0.13
RRl	0.11	0.15	0.11
RRu	0.14	0.53	0.14
P	---	---	---
Random RR	0.14	0.28	0.16
RRl	0.11	0.15	0.12
RRu	0.19	0.53	0.20
P	---	---	---
Between Chi			6.97
Between df			1
Between P			**
Btwn(F) P			N.S.
Btwn(R) P			*

	Full histological confirmation No	Yes	Total
N	12	4	16
NS	10	3	13
Wt	172.70	45.93	218.63
Het Chi	26.24	10.53	41.92
Het df	11	3	15
Het P	**	*	***
Fixed RR	0.12	0.17	0.13
RRl	0.10	0.13	0.11
RRu	0.14	0.23	0.14
P	---	---	---
Random RR	0.14	0.18	0.16
RRl	0.11	0.11	0.12
RRu	0.19	0.32	0.20
P	---	---	---
Between Chi			5.15
Between df			1
Between P			*
Btwn(F) P			N.S.
Btwn(R) P			N.S.

	Number of adjustment variables (1) 0	1	2+/+nk	Total
N	5	9	2	16
NS	4	7	2	13
Wt	47.69	151.29	19.64	218.63
Het Chi	14.32	19.99	3.50	41.92
Het df	4	8	1	15
Het P	**	*	(*)	***
Fixed RR	0.16	0.12	0.10	0.13
RRl	0.12	0.10	0.06	0.11
RRu	0.21	0.14	0.16	0.14
P	---	---	---	---
Random RR	0.16	0.16	0.17	0.16
RRl	0.09	0.11	0.03	0.12
RRu	0.28	0.22	0.83	0.20
P	---	---	-	---
Between Chi				4.11
Between df				2
Between P				N.S.
Btwn(F) P				N.S.
Btwn(R) P				N.S.

Table 1K15 - 3

IESLC - Meta-analysis of Ex Smoking, Years quit (vs current), "Highest vs lowest"
All LC types, Cigarettes only
Most adjusted

	Number of adjustment variables (2) 0	1	2	3-5	6+/+nk	Total
N	5	9	2			16
NS	4	7	2			13
Wt	47.69	151.29	19.64			218.63
Het Chi	14.32	19.99	3.50			41.92
Het df	4	8	1			15
Het P	**	*	(*)			***
Fixed RR	0.16	0.12	0.10			0.13
RRl	0.12	0.10	0.06			0.11
RRu	0.21	0.14	0.16			0.14
P	---	---	---			---
Random RR	0.16	0.16	0.17			0.16
RRl	0.09	0.11	0.03			0.12
RRu	0.28	0.22	0.83			0.20
P	---	---	-			---
Between Chi						4.11
Between df						2
Between P						N.S.
Btwn(F) P						N.S.
Btwn(R) P						N.S.

	Derivation of RR/CI Orig	StdCalc	Other	Total
N		6	10	16
NS		5	9	14
Wt		50.92	167.71	218.63
Het Chi		14.54	22.91	41.92
Het df		5	9	15
Het P		*	**	***
Fixed RR		0.16	0.12	0.13
RRl		0.12	0.10	0.11
RRu		0.21	0.14	0.14
P		---	---	---
Random RR		0.16	0.15	0.16
RRl		0.10	0.11	0.12
RRu		0.27	0.20	0.20
P		---	---	---
Between Chi				4.47
Between df				1
Between P				*
Btwn(F) P				N.S.
Btwn(R) P				N.S.

Table 1K15 - 4

IESLC - Meta-analysis of Ex Smoking, Years quit (vs current), "Highest vs lowest"
All LC types, Cigarettes only
Least adjusted

REF	NRR	X	SEX	AGEL	AGEH	RACE	YF	LC TYPE	LOC	START	ST	NLC	R	VB	P	H	AD	ADOS	PRODUCT	exL	exH	unexL	unexH	De
ALDERS	517		m	0	0	all	-	all	Eu:UK	1977	CC	1448	n	V	n	n	1	0	cig only	10	999	0.1	2	ot
ALDERS	528		f	0	0	all	-	all	Eu:UK	1977	CC	1448	n	V	n	n	1	0	cig only	10	999	0.1	2	ot
BENHAM	627		m	0	0	all	-	all	Eu:wst	1976	CC	1625	n	bl	n	y	0	0	cig only	20	999	1.0	4	st
CPSI	821		m	50	74	all	6	all	NAmer	1959	pr	5138	n	bl	n	n	1	0	cig only	10	999	0.1	0.9	ot
CPSII	674		m	35	99	all	4	all	NAmer	1982	pr	3229	n	bl	n	n	1	0	cig only	16	999	0.1	0.9	ot
DAMBER	559		m	0	0	all	-	all	Eu:Sca	1972	CC	579	n	bl	y	n	1	0	cig only	11	999	0.1	10	ot
DEAN3	515	x	m	0	0	all	-	all	Eu:UK	1969	CC	766	n	V	y	n	0	0	cig only	19	999	3	4	st
DOLL2	515		m	0	0	all	20	all	Eu:UK	1951	pr	920	n	V	n	n	1	0	cig only	15	999	0.1	4	ot
DORN	523		m	0	0	wh	0	all	NAmer	1954	pr	5097	n	bl	n	n	2	0	cig only	40	999	0.1	4	ot
GRAHAM	514		m	0	0	wh	-	all	NAmer	1956	CC	685	n	bl	n	n	0	0	cig only	10	999	0.1	1.0	st
KAISE2	660		m	0	0	all	9	all	NAmer	1979	pr	318	n	bl	n	n	1	0	cig only	21	999	2	10	st
KAISE2	580		f	0	0	all	9	all	NAmer	1979	pr	318	n	bl	n	n	1	0	cig only	21	999	2	10	ot
PEZZOT	506		m	0	0	all	-	all	SCAmer	1987	CC	215	n	bl	n	y	0	0	cig only	11	999	1.0	10	st
TVERDA	510		m	0	0	all	0	all	Eu:Sca	1972	pr	238	n	bl	n	n	2	0	cig only	5	999	0.1	0.9	ot
WYNDE6	521		m	0	0	all	-	all	NAmer	1969	CC	4423	n	bl	n	y	0	0	cig only	30	999	1.0	4	st
WYNDE6	542		f	0	0	all	-	all	NAmer	1969	CC	4423	n	bl	n	y	0	0	cig only	30	999	1.0	4	st

Cigarette type is all/unspec for all RRs
except for the following:

REF	NRR	CIGTYPE
ALDERS	517	MC only
ALDERS	528	MC only
DEAN3	515	MC only

Table 1K15 - 5

IESLC - Meta-analysis of Ex Smoking, Years quit (vs current), "Highest vs lowest"
All LC types, Cigarettes only
Least adjusted

REF	NRR	SEX	AD	Number Exposed Case	Exposed Cont	Non-exposed Case	Non-exposed Cont	RR	95.00%CI
ALDERS	517	m	1	29	-	121	-	0.18	(0.10- 0.30)
ALDERS	528	f	1	26	-	206	-	0.13	(0.08- 0.23)
Subtotal ALDERS								0.15	(0.10- 0.22)
BENHAM	627	m	0	19	129	154	138	0.13	(0.08- 0.23)
*CPSI	821	m	1	15	-	37	-	0.09	(0.05- 0.16)
*CPSII	674	m	1	256	-	97	-	0.10	(0.08- 0.12)
DAMBER	559	m	1	-	-	-	-	0.29	(0.12- 0.73)
DEAN3	515	m	0	8	66	28	102	0.44	(0.19- 1.03)
*DOLL2	515	m	1	7	-	15	-	0.13	(0.05- 0.31)
*DORN	523	m	2	49	-	56	-	0.09	(0.06- 0.15)
GRAHAM	514	m	0	2	30	84	48	0.04	(0.01- 0.17)
*KAISE2	660	m	1	6	-	12	-	0.21	(0.07- 0.62)
*KAISE2	580	f	1	4	-	6	-	0.58	(0.15- 2.22)
Subtotal KAISE2								0.31	(0.13- 0.73)
PEZZOT	506	m	0	20	106	46	82	0.34	(0.18- 0.61)
*TVERDA	510	m	2	4	-	2	-	0.48	(0.09- 2.66)
WYNDE6	521	m	0	21	161	201	166	0.11	(0.07- 0.18)
WYNDE6	542	f	0	10	31	82	70	0.28	(0.13- 0.60)
Subtotal WYNDE6								0.14	(0.09- 0.22)
Partial Totals				476	523	1147	606		

*prospective study

REF	NRR	SEX	AD	Ys	Ws	Qs	Ps
ALDERS	517	m	1	-1.71	12.73	1.52	0.0000
ALDERS	528	f	1	-2.04	13.78	0.01	0.0000
Subtotal ALDERS				-1.88	26.51	1.53	
BENHAM	627	m	0	-2.03	13.49	0.02	0.0000
*CPSI	821	m	1	-2.41	11.36	1.37	0.0000
*CPSII	674	m	1	-2.30	93.46	5.48	0.0000
DAMBER	559	m	1	-1.24	4.71	3.19	0.0072
DEAN3	515	m	0	-0.82	5.39	8.32	0.0578
*DOLL2	515	m	1	-2.04	4.62	0.00	0.0000
*DORN	523	m	2	-2.41	18.30	2.21	0.0000
GRAHAM	514	m	0	-3.27	1.77	2.57	0.0000
*KAISE2	660	m	1	-1.56	3.23	0.81	0.0050
*KAISE2	580	f	1	-0.54	2.12	4.86	0.4281
Subtotal KAISE2				-1.16	5.35	5.67	
PEZZOT	506	m	0	-1.09	10.71	10.09	0.0004
*TVERDA	510	m	2	-0.73	1.34	2.36	0.3955
WYNDE6	521	m	0	-2.23	15.43	0.43	0.0000
WYNDE6	542	f	0	-1.29	6.30	3.74	0.0012
Subtotal WYNDE6				-1.96	21.72	4.18	

	N	16
	NS	13
	Wt	218.73
Het	Chi	46.99
Het	df	15
Het	P	***
Fixed	RR	0.13
	RRl	0.11
	RRu	0.15
	P	---
Random	RR	0.16
	RRl	0.12
	RRu	0.21
	P	---
Asymm	P	*

Table 1K15 - 6

IESLC - Meta-analysis of Ex Smoking, Years quit (vs current), "Highest vs lowest"
All LC types, Cigarettes only
Least adjusted

	Sex combined	male	female	Total
N		13	3	16
NS		13	3	16
Wt		196.53	22.19	218.73
Het Chi		38.03	5.47	46.99
Het df		12	2	15
Het P		***	(*)	***
Fixed RR		0.12	0.19	0.13
RRl		0.11	0.12	0.11
RRu		0.14	0.28	0.15
P		---	---	---
Random RR		0.15	0.23	0.16
RRl		0.11	0.11	0.12
RRu		0.20	0.51	0.21
P		---	---	---
Between Chi				3.49
Between df				1
Between P				(*)
Btwn(F) P				N.S.
Btwn(R) P				N.S.

Table 1K15 - 7

IESLC - Meta-analysis of Ex Smoking, Years quit (vs current), "Highest vs lowest"
All LC types, Cigarettes only
Excluded studies (and stage at which they were excluded)

1	AGUDO AKIBA AMANDU AMES AXELSS BEST BOUCHA BOUCOT BRESLO CHEN CHEN2 CHIAZZ DEAN2 DOSEME ENGELA FAN GENG GER GUO HAENSZ HEGMAN HOLE HU HU2 JUSSAW KATSOU KAUFMA KOO KOULUM KREUZE LETOUR LEVIN LIAW LIU3 LIU4 LIU5 MCCONN MIGRAN MRFITR NOTAN2 OSANN2 PERNU QIAO2 RACHTA RESTRE SADOWS SEGI2 STASZE TIZZAN VUTUC WATSON WU WUWILL WYNDE2 WYNDE8 XU YUAN ZHANG ZHENG ZHOU
2	AUVINE BENSHL BLOT1 BROWN3 BUFFLE GURSEL LAUSSM LUO MCDUFF PISANI PRESCO SPITZ WU2 WYNDE7
4	GARSHI JEDRYC WAKAI
5	CORREA HAMMON
6	GILLIS HUMBLE QIAO WIGLE
8	ARMADA BARBON BECHER BOFFET BROSS CARPEN CEDERL CHOI CHYOU DARBY DESTEF DOLL DORGAN GAO GAO2 GARCIA HAMMO2 HIRAYA JAHN JAIN JOLY KHUDER LUBIN LUBIN2 MATOS PEZZO2 SOBUE SPEIZE SUZUK2 SVENSS WANG2 WYNDE3

Table 1K15 - 8
Potentially overlapping studies

REF	REFGP	PRINC	. OVERLAP/LINK
BENHAM	LUBIN2	2	Subset of Lubin2
TVERDA	TVERDA	1	VEIERO/TVERDAL
GRAHAM	BYERS1	1	GRAHAM/BROSS/BYERS1
WYNDE6	WYNDE6	1	WYNDE5/6/7/8
CPSI	CPSI	1	CPSI overall

Table 1K15 - 9
Most adjusted - insufficient data for meta-analysis

REF	NRR	SEX	AGEL	AGEH	RACE	YF	LC TYPE	LOC	START	ST	NLC	R	VB	P	H	AD	ADOS	PRODUCT	exL	exH	unexL	unexH	De
CPSI	717	m	0	0	wh	0	all	NAmer	1959	pr	5138	n	bl	n	n	1	0	cig only	35	39	2	4	st
CPSI	733	f	0	0	wh	0	all	NAmer	1959	pr	5138	n	bl	n	n	1	0	cig only	25	29	2	4	st

REF	NRR	RR	SIG	RRDATA comment
CPSI	717	0.24		0
CPSI	733	0.92		0

Table 1K16 -

IESLC - Meta-analysis of Ex Smoking by Years quit (vs current), Overview
All LC types, Pipes and/or cigars (not cigs)

This analysis is restricted to results for:
1) Ex smokers
2) Results by Years quit (vs current)
3) Categorical results by Years quit (vs current)
   Results by Years quit (vs current) are grouped under 2 schemes (S1, S2). Each scheme has a set of "key values". An interval is allocated to the category whose key value it includes, and intervals which include none or more than one of the key values are excluded. (Open-ended intervals are coded as 999)

S1	key value	maximum range
1	3	1-6
2	7	4-11
3	12	8+

S2	key value	maximum range
1	3	1-11
2	12	4-19
3	20	13+

4) All LC types (or near equivalent)
5) Results complete enough for use in metaanalysis
6) pipes and/or cigars (not cigs)

Within each study, results are then selected (in the following order of preference, within each sex) for:
7) Results with least adjustment for other aspects of smoking (ADOS)
8) DENOM: current smokers, current + recent smokers (up to number of m=months or y=years)
9) Followup period (YF, prospective studies): whole study (coded as 0) or longest available
10) LCtype: all or nearest available, at least Squamous and Adeno. (q = squamous, s = small, l = large, a = adeno, mix = mixed, alv = alveolar)
11) Race: all or nearest available, otherwise by race (wh or w = white, bl or b = black, hi = hispanic ch = chinese, jap = japanese, haw = hawaiian, w+o = white + oriental, sca = scandinavian, as = asian)
12) For overlapping studies: principal rather than subsidiary studies
Finally by Age: whole study (coded as 0) if available, otherwise by widest available age group and then for single sex results (m, f) in preference to results for both sexes combined (c).

Results adjusted (AD) for the most potential confounders are then chosen in Sections -1 to -3 and results adjusted for the least confounders in Sections -4 to -6. (Those least adjusted results which actually differ from the most adjusted are marked 'x' in column X in Section -4)

Section -7 shows excluded studies, together with the stage (as above) at which no qualifying results were found.

Section -8 lists the potentially overlapping studies which have been included (1=principal, 2=subsidiary).

Section -9 lists any results which would have been included in preference except that they had data not complete enough for use in meta-analysis, with their significance (yes/no), if known, and any further comment as entered on the database. It also lists as "gap" any categories for which no data were presented by the original authors.

In addition to those mentioned above, the following fields, levels and abbreviations are used:
* or nk = not known, n = no, y = yes, ot = other
nev = never
all/unspec = all or unspecified, cig+/-ot = cigarettes irrespective of other products (cigar, pipe etc)
MC = manufactured cigarettes, HR = hand-rolled cigarettes
exL, exH = range of exposure (low and high) in the smoking group, in terms of Years quit (vs current)
REF: 6-character study reference
NRR: number of the RR on the database within the study
ST : study type (CC = case control, pr or prosp = prospective)
NLC: number of lung cancer cases in whole study
R : risky occupational population (n = no, m = mining, o = other risky)
VB : national cigarette type (V = at least 75% Virginia, bl = at least 75% blended, ot = other)
P : any proxy use
H : full histological confirmation
De : derivation of RR/CI (or = original, st = standard method, ot = other method of estimation)

Table 1K16 - 0

No RRs selected for this analysis

Table 1K16 - 7

IESLC - Meta-analysis of Ex Smoking by Years quit (vs current), Overview
All LC types, Pipes and/or cigars (not cigs)
Excluded studies (and stage at which they were excluded)

1 AGUDO AKIBA AMANDU AMES AXELSS BEST BOUCHA BOUCOT BRESLO CHEN CHEN2 CHIAZZ DEAN2 DOSEME ENGELA FAN GENG GER GUO HAENSZ HEGMAN HOLE HU HU2 JUSSAW KATSOU KAUFMA KOO KOULUM KREUZE LETOUR LEVIN LIAW LIU3 LIU4 LIU5 MCCONN MIGRAN MRFITR NOTAN2 OSANN2 PERNU QIAO2 RACHTA RESTRE SADOWS SEGI2 STASZE TIZZAN VUTUC WATSON WU WUWILL WYNDE2 WYNDE8 XU YUAN ZHANG ZHENG ZHOU

2 AUVINE BENSHL BLOT1 BROWN3 BUFFLE GURSEL LAUSSM LUO MCDUFF PISANI PRESCO SPITZ WU2 WYNDE7

4 HAMMON

5 CORREA GILLIS HUMBLE QIAO WIGLE

7 ALDERS ARMADA BARBON BECHER BENHAM BOFFET BROSS CARPEN CEDERL CHOI CHYOU CPSI CPSII DAMBER DARBY DEAN3 DESTEF DOLL DOLL2 DORGAN DORN GAO GAO2 GARCIA GARSHI GRAHAM HAMMO2 HIRAYA JAHN JAIN JEDRYC JOLY KAISE2 KHUDER LUBIN LUBIN2 MATOS PEZZO2 PEZZOT SOBUE SPEIZE SUZUK2 SVENSS TVERDA WAKAI WANG2 WYNDE3 WYNDE6

Table 1K17 -

IESLC - Meta-analysis of Ex Smoking, Years quit (vs current), "Highest vs lowest"
All LC types, Pipes and/or cigars (not cigs)

This analysis is restricted to results for:
1) Ex smokers
2) Results by Years quit (vs current)
3) Categorical results by Years quit (vs current)
4) Denominator (unexposed) = "low"
5) All LC types (or near equivalent)
6) Results complete enough for use in metaanalysis
7) (not applicable)
8) PRODUCT: pipes and/or cigars (not cigs)

Within each study, results are then selected (in the following order of preference, within each sex) for:
9) Results with least adjustment for other aspects of smoking (ADOS)
10) The highest vs lowest category
11) Followup period (YF, prospective studies): whole study (coded as 0) or longest available
12) LCtype: all or nearest available, at least Squamous and Adeno. (q = squamous, s = small,
l = large, a = adeno, mix = mixed, alv = alveolar)
13) Race: all or nearest available, otherwise by race (wh or w = white, bl or b = black, hi = hispanic
ch = chinese, jap = japanese, haw = hawaiian, w+o = white + oriental, sca = scandinavian, as = asian)
14) For overlapping studies: principal rather than subsidiary studies
Finally by Age: whole study (coded as 0) if available, otherwise by widest available age group
and then for single sex results (m, f) in preference to results for both sexes combined (c).

Results adjusted (AD) for the most potential confounders are then chosen in Sections -1 to -3
and results adjusted for the least confounders in Sections -4 to -6. (Those least adjusted results which
actually differ from the most adjusted are marked 'x' in column X in Section -4)

Section -7 shows excluded studies, together with the stage (as above) at which no qualifying
results were found.

Section -8 lists the potentially overlapping studies which have been included (1=principal, 2=subsidiary).

Section -9 lists any results which would have been included in preference except that they had data not complete
enough for use in meta-analysis, with their significance (yes/no), if known, and any further comment as entered
on the database. It also lists as "gap" any categories for which no data were presented by the original authors.

In addition to those mentioned above, the following fields, levels and abbreviations are used:
* or nk = not known, n = no, y = yes, ot = other
all/unspec = all or unspecified, cig+/-ot = cigarettes irrespective of other products (cigar, pipe etc)
MC = manufactured cigarettes, HR = hand-rolled cigarettes
exL, exH = range of exposure (low and high) in the "highest" group, in terms of Years quit (vs current)
unexL, unexH = range of exposure (low and high) in the "lowest" group, in terms of Years quit (vs current)
REF: 6-character study reference
NRR: number of the RR on the database within the study
ST : study type (CC = case control, pr or prosp = prospective)
NLC: number of lung cancer cases in whole study
R : risky occupational population (n = no, m = mining, o = other risky)
VB : national cigarette type (V = at least 75% Virginia, bl = at least 75% blended, ot = other)
P : any proxy use
H : full histological confirmation
De : derivation of RR/CI (or = original, st = standard method, ot = other method of estimation)

Table 1K17 - 0

No RRs selected for this analysis

Table 1K17 - 7

IESLC - Meta-analysis of Ex Smoking, Years quit (vs current), "Highest vs lowest"
All LC types, Pipes and/or cigars (not cigs)
Excluded studies (and stage at which they were excluded)

1 AGUDO AKIBA AMANDU AMES AXELSS BEST BOUCHA BOUCOT BRESLO CHEN CHEN2 CHIAZZ DEAN2 DOSEME ENGELA FAN GENG GER GUO HAENSZ HEGMAN HOLE HU HU2 JUSSAW KATSOU KAUFMA KOO KOULUM KREUZE LETOUR LEVIN LIAW LIU3 LIU4 LIU5 MCCONN MIGRAN MRFITR NOTAN2 OSANN2 PERNU QIAO2 RACHTA RESTRE SADOWS SEGI2 STASZE TIZZAN VUTUC WATSON WU WUWILL WYNDE2 WYNDE8 XU YUAN ZHANG ZHENG ZHOU

2 AUVINE BENSHL BLOT1 BROWN3 BUFFLE GURSEL LAUSSM LUO MCDUFF PISANI PRESCO SPITZ WU2 WYNDE7

4 GARSHI JEDRYC WAKAI

5 CORREA HAMMON

6 GILLIS HUMBLE QIAO WIGLE

8 ALDERS ARMADA BARBON BECHER BENHAM BOFFET BROSS CARPEN CEDERL CHOI CHYOU CPSI CPSII DAMBER DARBY DEAN3 DESTEF DOLL DOLL2 DORGAN DORN GAO GAO2 GARCIA GRAHAM HAMMO2 HIRAYA JAHN JAIN JOLY KAISE2 KHUDER LUBIN LUBIN2 MATOS PEZZO2 PEZZOT SOBUE SPEIZE SUZUK2 SVENSS TVERDA WANG2 WYNDE3 WYNDE6

Table 1K18 -

IESLC - Meta-analysis of Ex Smoking by Years quit (vs current), Overview
All LC types, Pipes only

This analysis is restricted to results for:
1) Ex smokers
2) Results by Years quit (vs current)
3) Categorical results by Years quit (vs current)
Results by Years quit (vs current) are grouped under 2 schemes (S1, S2). Each scheme has a set of "key values". An interval is allocated to the category whose key value it includes, and intervals which include none or more than one of the key values are excluded. (Open-ended intervals are coded as 999)

S1	key value	maximum range
1	3	1-6
2	7	4-11
3	12	8+

S2	key value	maximum range
1	3	1-11
2	12	4-19
3	20	13+

4) All LC types (or near equivalent)
5) Results complete enough for use in metaanalysis
6) pipes only

Within each study, results are then selected (in the following order of preference, within each sex) for:
7) Results with least adjustment for other aspects of smoking (ADOS)
8) DENOM: current smokers, current + recent smokers (up to number of m=months or y=years)
9) Followup period (YF, prospective studies): whole study (coded as 0) or longest available
10) LCtype: all or nearest available, at least Squamous and Adeno. (q = squamous, s = small, l = large, a = adeno, mix = mixed, alv = alveolar)
11) Race: all or nearest available, otherwise by race (wh or w = white, bl or b = black, hi = hispanic ch = chinese, jap = japanese, haw = hawaiian, w+o = white + oriental, sca = scandinavian, as = asian)
12) For overlapping studies: principal rather than subsidiary studies
Finally by Age: whole study (coded as 0) if available, otherwise by widest available age group and then for single sex results (m, f) in preference to results for both sexes combined (c).

Results adjusted (AD) for the most potential confounders are then chosen in Sections -1 to -3 and results adjusted for the least confounders in Sections -4 to -6. (Those least adjusted results which actually differ from the most adjusted are marked 'x' in column X in Section -4)

Section -7 shows excluded studies, together with the stage (as above) at which no qualifying results were found.

Section -8 lists the potentially overlapping studies which have been included (1=principal, 2=subsidiary).

Section -9 lists any results which would have been included in preference except that they had data not complete enough for use in meta-analysis, with their significance (yes/no), if known, and any further comment as entered on the database. It also lists as "gap" any categories for which no data were presented by the original authors.

In addition to those mentioned above, the following fields, levels and abbreviations are used:
* or nk = not known, n = no, y = yes, ot = other
nev = never
all/unspec = all or unspecified, cig+/-ot = cigarettes irrespective of other products (cigar, pipe etc)
MC = manufactured cigarettes, HR = hand-rolled cigarettes
exL, exH = range of exposure (low and high) in the smoking group, in terms of Years quit (vs current)
REF: 6-character study reference
NRR: number of the RR on the database within the study
ST : study type (CC = case control, pr or prosp = prospective)
NLC: number of lung cancer cases in whole study
R : risky occupational population (n = no, m = mining, o = other risky)
VB : national cigarette type (V = at least 75% Virginia, bl = at least 75% blended, ot = other)
P : any proxy use
H : full histological confirmation
De : derivation of RR/CI (or = original, st = standard method, ot = other method of estimation)

Table 1K18 - 1

IESLC - Meta-analysis of Ex Smoking by Years quit (vs current), Overview
All LC types, Pipes only
Most adjusted

REF	NRR	SEX	AGEL	AGEH	RACE	YF	LC TYPE	LOC	START	ST	NLC	R	VB	P	H	AD	ADOS	exL	exH	S1	S2	DENOM	De
BOFFET	552	m	0	0	all	-	all	Eu:mul	1988	CC	5621	n	bl	y	n	2	0	0.1	14	0	0	current	ot
BOFFET	553	m	0	0	all	-	all	Eu:mul	1988	CC	5621	n	bl	y	n	2	0	15	999	0	3	current	ot
DAMBER	563	m	0	0	all	-	all	Eu:Sca	1972	CC	579	n	bl	y	n	1	0	0.1	10	0	1	current	ot
DAMBER	564	m	0	0	all	-	all	Eu:Sca	1972	CC	579	n	bl	y	n	1	0	11	999	3	0	current	ot

In this overview table, subtotals and Qs values may be invalid and should be ignored

Table 1K18 - 2

IESLC - Meta-analysis of Ex Smoking by Years quit (vs current), Overview
All LC types, Pipes only
Most adjusted

REF	NRR	SEX	AD	Number Exposed Case	Cont	Non-exposed Case	Cont	RR	95.00%CI
BOFFET	552	m	2	-	-	-	-	0.82	(0.37- 1.83)
BOFFET	553	m	2	-	-	-	-	0.11	(0.04- 0.34)
Subtotal BOFFET								0.40	(0.21- 0.76)
DAMBER	563	m	1	-	-	-	-	0.60	(0.33- 1.09)
DAMBER	564	m	1	-	-	-	-	0.54	(0.28- 1.06)
Subtotal DAMBER								0.57	(0.37- 0.89)
Partial Totals				0	0	0	0		

*prospective study

REF	NRR	SEX	AD	Ys	Ws	Qs	Ps
BOFFET	552	m	2	-0.20	6.01	1.37	0.6265
BOFFET	553	m	2	-2.21	3.36	7.88	0.0001
Subtotal BOFFET				-0.92	9.37	9.24	
DAMBER	563	m	1	-0.51	10.76	0.29	0.0938
DAMBER	564	m	1	-0.62	8.67	0.03	0.0696
Subtotal DAMBER				-0.56	19.43	0.32	

N	4
NS	2

Table 1K18 - 3

IESLC - Meta-analysis of Ex Smoking by Years quit (vs current), Overview
All LC types, Pipes only
Most adjusted

	combined	Sex male	female	Total
N		4		4
NS		2		2

In this overview table, other than the "N" rows, entries in the "absent" and "Total" columns may be invalid and should be ignored

MALES

	Years quit vs current (lower focus) absent	1-6k3	4-11k7	8+k12	Total
N	3			1	4
NS	2			1	2
Wt	20.13			8.67	28.80
Het Chi	9.52			0.00	9.56
Het df	2			0	3
Het P	**			N.S.	*
Fixed RR	0.50			0.54	0.51
RRl	0.32			0.28	0.35
RRu	0.77			1.05	0.73
P	--			(-)	---
Random RR	0.41			0.54	0.45
RRl	0.15			0.28	0.23
RRu	1.12			1.05	0.89
P	(-)			(-)	-

	Years quit vs current (higher focus) absent	1-11k3	4-19k12	13+k20	Total
N	2	1		1	4
NS	2	1		1	3
Wt	14.68	10.76		3.36	28.80
Het Chi	0.62	0.00		0.00	9.56
Het df	1	0		0	3
Het P	N.S.	N.S.		N.S.	*
Fixed RR	0.64	0.60		0.11	0.51
RRl	0.38	0.33		0.04	0.35
RRu	1.07	1.09		0.32	0.73
P	(-)	(-)		---	---
Random RR	0.64	0.60		0.11	0.45
RRl	0.38	0.33		0.04	0.23
RRu	1.07	1.09		0.32	0.89
P	(-)	(-)		---	-

Table 1K18 - 4

IESLC - Meta-analysis of Ex Smoking by Years quit (vs current), Overview
All LC types, Pipes only
Least adjusted

REF	NRR	X	SEX	AGEL	AGEH	RACE	YF	LC TYPE	LOC	START	ST	NLC	R	VB	P	H	AD	ADOS	exL	exH	S1	S2	DENOM	De
BOFFET	552		m	0	0	all	-	all	Eu:mul	1988	CC	5621	n	bl	y	n	2	0	0.1	14	0	0	current	ot
BOFFET	553		m	0	0	all	-	all	Eu:mul	1988	CC	5621	n	bl	y	n	2	0	15	999	0	3	current	ot
DAMBER	563		m	0	0	all	-	all	Eu:Sca	1972	CC	579	n	bl	y	n	1	0	0.1	10	0	1	current	ot
DAMBER	564		m	0	0	all	-	all	Eu:Sca	1972	CC	579	n	bl	y	n	1	0	11	999	3	0	current	ot

In this overview table, subtotals and Qs values may be invalid and should be ignored

Table 1K18 - 5

IESLC - Meta-analysis of Ex Smoking by Years quit (vs current), Overview
All LC types, Pipes only
Least adjusted

REF	NRR	SEX	AD	Number Exposed Case	Number Exposed Cont	Non-exposed Case	Non-exposed Cont	RR	95.00%CI
BOFFET	552	m	2	-	-	-	-	0.82	(0.37- 1.83)
BOFFET	553	m	2	-	-	-	-	0.11	(0.04- 0.34)
Subtotal BOFFET								0.40	(0.21- 0.76)
DAMBER	563	m	1	-	-	-	-	0.60	(0.33- 1.09)
DAMBER	564	m	1	-	-	-	-	0.54	(0.28- 1.06)
Subtotal DAMBER								0.57	(0.37- 0.89)
Partial Totals				0	0	0	0		

*prospective study

REF	NRR	SEX	AD	Ys	Ws	Qs	Ps
BOFFET	552	m	2	-0.20	6.01	1.37	0.6265
BOFFET	553	m	2	-2.21	3.36	7.88	0.0001
Subtotal BOFFET				-0.92	9.37	9.24	
DAMBER	563	m	1	-0.51	10.76	0.29	0.0938
DAMBER	564	m	1	-0.62	8.67	0.03	0.0696
Subtotal DAMBER				-0.56	19.43	0.32	

N	4
NS	2

Table 1K18 - 6

IESLC - Meta-analysis of Ex Smoking by Years quit (vs current), Overview
All LC types, Pipes only
Least adjusted

	Sex combined	male	female	Total
N		4		4
NS		2		2

In this overview table, other than the "N" rows, entries in the "absent" and "Total" columns may be invalid and should be ignored

MALES

	Years quit vs current (lower focus) absent	1-6k3	4-11k7	8+k12	Total
N	3			1	4
NS	2			1	2
Wt	20.13			8.67	28.80
Het Chi	9.52			0.00	9.56
Het df	2			0	3
Het P	**			N.S.	*
Fixed RR	0.50			0.54	0.51
RRl	0.32			0.28	0.35
RRu	0.77			1.05	0.73
P	--			(-)	---
Random RR	0.41			0.54	0.45
RRl	0.15			0.28	0.23
RRu	1.12			1.05	0.89
P	(-)			(-)	-

	Years quit vs current (higher focus) absent	1-11k3	4-19k12	13+k20	Total
N	2	1		1	4
NS	2	1		1	3
Wt	14.68	10.76		3.36	28.80
Het Chi	0.62	0.00		0.00	9.56
Het df	1	0		0	3
Het P	N.S.	N.S.		N.S.	*
Fixed RR	0.64	0.60		0.11	0.51
RRl	0.38	0.33		0.04	0.35
RRu	1.07	1.09		0.32	0.73
P	(-)	(-)		---	---
Random RR	0.64	0.60		0.11	0.45
RRl	0.38	0.33		0.04	0.23
RRu	1.07	1.09		0.32	0.89
P	(-)	(-)		---	-

Table 1K18 - 7

IESLC - Meta-analysis of Ex Smoking by Years quit (vs current), Overview
All LC types, Pipes only
Excluded studies (and stage at which they were excluded)

1	AGUDO AKIBA AMANDU AMES AXELSS BEST BOUCHA BOUCOT BRESLO CHEN CHEN2 CHIAZZ DEAN2 DOSEME ENGELA FAN GENG GER GUO HAENSZ HEGMAN HOLE HU HU2 JUSSAW KATSOU KAUFMA KOO KOULUM KREUZE LETOUR LEVIN LIAW LIU3 LIU4 LIU5 MCCONN MIGRAN MRFITR NOTAN2 OSANN2 PERNU QIAO2 RACHTA RESTRE SADOWS SEGI2 STASZE TIZZAN VUTUC WATSON WU WUWILL WYNDE2 WYNDE8 XU YUAN ZHANG ZHENG ZHOU
2	AUVINE BENSHL BLOT1 BROWN3 BUFFLE GURSEL LAUSSM LUO MCDUFF PISANI PRESCO SPITZ WU2 WYNDE7
4	HAMMON
5	CORREA GILLIS HUMBLE QIAO WIGLE
7	ALDERS ARMADA BARBON BECHER BENHAM BROSS CARPEN CEDERL CHOI CHYOU CPSI CPSII DARBY DEAN3 DESTEF DOLL DOLL2 DORGAN DORN GAO GAO2 GARCIA GARSHI GRAHAM HAMMO2 HIRAYA JAHN JAIN JEDRYC JOLY KAISE2 KHUDER LUBIN LUBIN2 MATOS PEZZO2 PEZZOT SOBUE SPEIZE SUZUK2 SVENSS TVERDA WAKAI WANG2 WYNDE3 WYNDE6

Table 1K18 - 8
Potentially overlapping studies

REF	REFGP	PRINC	.	OVERLAP/LINK
BOFFET	BOFFET	1		BOFFET-combined

Table 1K18 - 9
Most adjusted - insufficient data for meta-analysis

REF	NRR	SEX	AGEL	AGEH	RACE	YF	LC TYPE	LOC	START	ST	NLC	R	VB	P	H	AD	ADOS	exL	exH	S1	S2	DENOM	De
LUBIN2	1065	m	0	0	all	-	all	Eu:mul	1976	CC	7804	n	bl	n	y	2	1#	0.1	4	1	1	current	or
LUBIN2	1066	m	0	0	all	-	all	Eu:mul	1976	CC	7804	n	bl	n	y	2	1#	5	999	0	0	current	or

Comments on values in listings

LUBIN2 ADOS Duration of pipe smoking
LUBIN2 ADOS Duration of pipe smoking

REF	NRR	RR	SIG	RRDATA comment
LUBIN2	1065	2.02		0
LUBIN2	1066	0.90		0

Table 1K19 -

IESLC - Meta-analysis of Ex Smoking, Years quit (vs current), "Highest vs lowest"
All LC types, Pipes only

This analysis is restricted to results for:
1) Ex smokers
2) Results by Years quit (vs current)
3) Categorical results by Years quit (vs current)
4) Denominator (unexposed) = "low"
5) All LC types (or near equivalent)
6) Results complete enough for use in metaanalysis
7) (not applicable)
8) PRODUCT: pipes only

Within each study, results are then selected (in the following order of preference, within each sex) for:
9) Results with least adjustment for other aspects of smoking (ADOS)
10) The highest vs lowest category
11) Followup period (YF, prospective studies): whole study (coded as 0) or longest available
12) LCtype: all or nearest available, at least Squamous and Adeno. (q = squamous, s = small,
l = large, a = adeno, mix = mixed, alv = alveolar)
13) Race: all or nearest available, otherwise by race (wh or w = white, bl or b = black, hi = hispanic
ch = chinese, jap = japanese, haw = hawaiian, w+o = white + oriental, sca = scandinavian, as = asian)
14) For overlapping studies: principal rather than subsidiary studies
Finally by Age: whole study (coded as 0) if available, otherwise by widest available age group
and then for single sex results (m, f) in preference to results for both sexes combined (c).

Results adjusted (AD) for the most potential confounders are then chosen in Sections -1 to -3
and results adjusted for the least confounders in Sections -4 to -6. (Those least adjusted results which
actually differ from the most adjusted are marked 'x' in column X in Section -4)

Section -7 shows excluded studies, together with the stage (as above) at which no qualifying
results were found.

Section -8 lists the potentially overlapping studies which have been included (1=principal, 2=subsidiary).

Section -9 lists any results which would have been included in preference except that they had data not complete
enough for use in meta-analysis, with their significance (yes/no), if known, and any further comment as entered
on the database. It also lists as "gap" any categories for which no data were presented by the original authors.

In addition to those mentioned above, the following fields, levels and abbreviations are used:
* or nk = not known, n = no, y = yes, ot = other
all/unspec = all or unspecified, cig+/-ot = cigarettes irrespective of other products (cigar, pipe etc)
MC = manufactured cigarettes, HR = hand-rolled cigarettes
exL, exH = range of exposure (low and high) in the "highest" group, in terms of Years quit (vs current)
unexL, unexH = range of exposure (low and high) in the "lowest" group, in terms of Years quit (vs current)
REF: 6-character study reference
NRR: number of the RR on the database within the study
ST : study type (CC = case control, pr or prosp = prospective)
NLC: number of lung cancer cases in whole study
R : risky occupational population (n = no, m = mining, o = other risky)
VB : national cigarette type (V = at least 75% Virginia, bl = at least 75% blended, ot = other)
P : any proxy use
H : full histological confirmation
De : derivation of RR/CI (or = original, st = standard method, ot = other method of estimation)

Table 1K19 - 1

IESLC - Meta-analysis of Ex Smoking, Years quit (vs current), "Highest vs lowest"
All LC types, Pipes only
Most adjusted

REF	NRR	SEX	AGEL	AGEH	RACE	YF	LC TYPE	LOC	START	ST	NLC	R	VB	P	H	AD	ADOS	exL	exH	unexL	unexH	De
BOFFET	554	m	0	0	all	-	all	Eu:mul	1988	CC	5621	n	bl	y	n	2	0	15	999	0.1	14	ot
DAMBER	565	m	0	0	all	-	all	Eu:Sca	1972	CC	579	n	bl	y	n	1	0	11	999	0.1	10	ot

Table 1K19 - 2

IESLC - Meta-analysis of Ex Smoking, Years quit (vs current), "Highest vs lowest"
All LC types, Pipes only
Most adjusted

REF	NRR	SEX	AD	Number Exposed Case	Number Exposed Cont	Non-exposed Case	Non-exposed Cont	RR	95.00%CI
BOFFET	554	m	2	-	-	-	-	0.14	(0.04- 0.46)
DAMBER	565	m	1	-	-	-	-	0.90	(0.40- 2.00)
Partial Totals				0	0	0	0		

*prospective study

REF	NRR	SEX	AD	Ys	Ws	Qs	Ps
BOFFET	554	m	2	-1.97	2.58	4.34	0.0016
DAMBER	565	m	1	-0.11	5.93	1.88	0.7975

	N	2
	NS	2
	Wt	8.51
Het	Chi	6.22
Het	df	1
Het	P	*
Fixed	RR	0.51
	RRl	0.26
	RRu	1.00
	P	(-)
Random	RR	0.38
	RRl	0.06
	RRu	2.32
	P	N.S.
Asymm	P	

Table 1K19 - 3

IESLC - Meta-analysis of Ex Smoking, Years quit (vs current), "Highest vs lowest"
All LC types, Pipes only
Most adjusted

	Sex combined	male	female	Total
N		2		2
NS		2		2
Wt		8.51		8.51
Het Chi		6.22		6.22
Het df		1		1
Het P		*		*
Fixed RR		0.51		0.51
RRl		0.26		0.26
RRu		1.00		1.00
P		(-)		(-)
Random RR		0.38		0.38
RRl		0.06		0.06
RRu		2.32		2.32
P		N.S.		N.S.
Between Chi				
Between df				
Between P				N.S.
Btwn(F) P				N.S.
Btwn(R) P				N.S.

MALES

Too few RRs for analysis by factor

Table 1K19 - 4

IESLC - Meta-analysis of Ex Smoking, Years quit (vs current), "Highest vs lowest"
All LC types, Pipes only
Least adjusted

REF	NRR	X	SEX	AGEL	AGEH	RACE	YF	LC TYPE	LOC	START	ST	NLC	R	VB	P	H	AD	ADOS	exL	exH	unexL	unexH	De
BOFFET	554		m	0	0	all	-	all	Eu:mul	1988	CC	5621	n	bl	y	n	2	0	15	999	0.1	14	ot
DAMBER	565		m	0	0	all	-	all	Eu:Sca	1972	CC	579	n	bl	y	n	1	0	11	999	0.1	10	ot

Table 1K19 - 5

IESLC - Meta-analysis of Ex Smoking, Years quit (vs current), "Highest vs lowest"
All LC types, Pipes only
Least adjusted

REF	NRR	SEX	AD	Number Exposed Case	Number Exposed Cont	Non-exposed Case	Non-exposed Cont	RR	95.00%CI
BOFFET	554	m	2	-	-	-	-	0.14	(0.04- 0.46)
DAMBER	565	m	1	-	-	-	-	0.90	(0.40- 2.00)
Partial Totals				0	0	0	0		

*prospective study

REF	NRR	SEX	AD	Ys	Ws	Qs	Ps
BOFFET	554	m	2	-1.97	2.58	4.34	0.0016
DAMBER	565	m	1	-0.11	5.93	1.88	0.7975

	N	2
	NS	2
	Wt	8.51
Het	Chi	6.22
Het	df	1
Het	P	*
Fixed	RR	0.51
	RRl	0.26
	RRu	1.00
	P	(-)
Random	RR	0.38
	RRl	0.06
	RRu	2.32
	P	N.S.
Asymm	P	

Table 1K19 - 6

IESLC - Meta-analysis of Ex Smoking, Years quit (vs current), "Highest vs lowest"
All LC types, Pipes only
Least adjusted

	Sex combined	male	female	Total
N		2		2
NS		2		2
Wt		8.51		8.51
Het Chi		6.22		6.22
Het df		1		1
Het P		*		*
Fixed RR		0.51		0.51
RRl		0.26		0.26
RRu		1.00		1.00
P		(-)		(-)
Random RR		0.38		0.38
RRl		0.06		0.06
RRu		2.32		2.32
P		N.S.		N.S.
Between Chi				
Between df				
Between P				N.S.
Btwn(F) P				N.S.
Btwn(R) P				N.S.

Table 1K19 - 7

IESLC - Meta-analysis of Ex Smoking, Years quit (vs current), "Highest vs lowest"
All LC types, Pipes only
Excluded studies (and stage at which they were excluded)

1	AGUDO AKIBA AMANDU AMES AXELSS BEST BOUCHA BOUCOT BRESLO CHEN CHEN2 CHIAZZ DEAN2 DOSEME ENGELA FAN GENG GER GUO HAENSZ HEGMAN HOLE HU HU2 JUSSAW KATSOU KAUFMA KOO KOULUM KREUZE LETOUR LEVIN LIAW LIU3 LIU4 LIU5 MCCONN MIGRAN MRFITR NOTAN2 OSANN2 PERNU QIAO2 RACHTA RESTRE SADOWS SEGI2 STASZE TIZZAN VUTUC WATSON WU WUWILL WYNDE2 WYNDE8 XU YUAN ZHANG ZHENG ZHOU
2	AUVINE BENSHL BLOT1 BROWN3 BUFFLE GURSEL LAUSSM LUO MCDUFF PISANI PRESCO SPITZ WU2 WYNDE7
4	GARSHI JEDRYC WAKAI
5	CORREA HAMMON
6	GILLIS HUMBLE QIAO WIGLE
8	ALDERS ARMADA BARBON BECHER BENHAM BROSS CARPEN CEDERL CHOI CHYOU CPSI CPSII DARBY DEAN3 DESTEF DOLL DOLL2 DORGAN DORN GAO GAO2 GARCIA GRAHAM HAMMO2 HIRAYA JAHN JAIN JOLY KAISE2 KHUDER LUBIN LUBIN2 MATOS PEZZO2 PEZZOT SOBUE SPEIZE SUZUK2 SVENSS TVERDA WANG2 WYNDE3 WYNDE6

Table 1K19 - 8
Potentially overlapping studies

REF	REFGP	PRINC	.	OVERLAP/LINK
BOFFET	BOFFET	1		BOFFET-combined

Table 1K19 - 9
Most adjusted - insufficient data for meta-analysis

REF	NRR	SEX	AGEL	AGEH	RACE	YF	LC TYPE	LOC	START	ST	NLC	R	VB	P	H	AD	ADOS	exL	exH	unexL	unexH	De
LUBIN2	1067	m	0	0	all	-	all	Eu:mul	1976	CC	7804	n	bl	n	y	2	1#	5	999	0.1	4	st

Comments on values in listings

LUBIN2 ADOS Duration of pipe smoking

REF	NRR	RR	SIG	RRDATA comment
LUBIN2	1067	0.45		0

Table 1K20 -

IESLC - Meta-analysis of Ex Smoking by Years quit (vs current), Overview
All LC types, Cigars only

This analysis is restricted to results for:
1) Ex smokers
2) Results by Years quit (vs current)
3) Categorical results by Years quit (vs current)
Results by Years quit (vs current) are grouped under 2 schemes (S1, S2). Each scheme has a set of "key values". An interval is allocated to the category whose key value it includes, and intervals which include none or more than one of the key values are excluded. (Open-ended intervals are coded as 999)

S1	key value	maximum range
1	3	1-6
2	7	4-11
3	12	8+

S2	key value	maximum range
1	3	1-11
2	12	4-19
3	20	13+

4) All LC types (or near equivalent)
5) Results complete enough for use in metaanalysis
6) cigars only

Within each study, results are then selected (in the following order of preference, within each sex) for:
7) Results with least adjustment for other aspects of smoking (ADOS)
8) DENOM: current smokers, current + recent smokers (up to number of m=months or y=years)
9) Followup period (YF, prospective studies): whole study (coded as 0) or longest available
10) LCtype: all or nearest available, at least Squamous and Adeno. (q = squamous, s = small, l = large, a = adeno, mix = mixed, alv = alveolar)
11) Race: all or nearest available, otherwise by race (wh or w = white, bl or b = black, hi = hispanic ch = chinese, jap = japanese, haw = hawaiian, w+o = white + oriental, sca = scandinavian, as = asian)
12) For overlapping studies: principal rather than subsidiary studies
Finally by Age: whole study (coded as 0) if available, otherwise by widest available age group and then for single sex results (m, f) in preference to results for both sexes combined (c).

Results adjusted (AD) for the most potential confounders are then chosen in Sections -1 to -3 and results adjusted for the least confounders in Sections -4 to -6. (Those least adjusted results which actually differ from the most adjusted are marked 'x' in column X in Section -4)

Section -7 shows excluded studies, together with the stage (as above) at which no qualifying results were found.

Section -8 lists the potentially overlapping studies which have been included (1=principal, 2=subsidiary).

Section -9 lists any results which would have been included in preference except that they had data not complete enough for use in meta-analysis, with their significance (yes/no), if known, and any further comment as entered on the database. It also lists as "gap" any categories for which no data were presented by the original authors.

In addition to those mentioned above, the following fields, levels and abbreviations are used:
* or nk = not known, n = no, y = yes, ot = other
nev = never
all/unspec = all or unspecified, MC = manufactured cigarettes, HR = hand-rolled cigarettes
exL, exH = range of exposure (low and high) in the smoking group, in terms of Years quit (vs current)
REF: 6-character study reference
NRR: number of the RR on the database within the study
ST : study type (CC = case control, pr or prosp = prospective)
NLC: number of lung cancer cases in whole study
R : risky occupational population (n = no, m = mining, o = other risky)
VB : national cigarette type (V = at least 75% Virginia, bl = at least 75% blended, ot = other)
P : any proxy use
H : full histological confirmation
De : derivation of RR/CI (or = original, st = standard method, ot = other method of estimation)

Table 1K20 - 1

IESLC - Meta-analysis of Ex Smoking by Years quit (vs current), Overview
All LC types, Cigars only
Most adjusted

REF	NRR	SEX	AGEL	AGEH	RACE	YF	LC TYPE	LOC	START	ST	NLC	R	VB	P	H	AD	ADOS	exL	exH	S1	S2	DENOM	De
BOFFET	558	m	0	0	all	-	all	Eu:mul	1988	CC	5621	n	bl	y	n	2	0	0.1	14	0	0	current	ot
BOFFET	559	m	0	0	all	-	all	Eu:mul	1988	CC	5621	n	bl	y	n	2	0	15	999	0	3	current	ot
JOLY	689	m	0	0	all	-	all	SCAmer	1978	CC	826	n	bl	n	n	0	0	1.0	4	1	1	cur+1y	st
JOLY	690	m	0	0	all	-	all	SCAmer	1978	CC	826	n	bl	n	n	0	0	5	999	0	0	cur+1y	st

In this overview table, subtotals and Qs values may be invalid and should be ignored

Table 1K20 - 2

IESLC - Meta-analysis of Ex Smoking by Years quit (vs current), Overview
All LC types, Cigars only
Most adjusted

REF	NRR	SEX	AD	Number Exposed Case	Number Exposed Cont	Non-exposed Case	Non-exposed Cont	RR	95.00%CI
BOFFET	558	m	2	-	-	-	-	0.83	(0.32- 2.15)
BOFFET	559	m	2	-	-	-	-	0.65	(0.25- 1.68)
Subtotal BOFFET								0.73	(0.37- 1.44)
JOLY	689	m	0	2	13	36	141	0.60	(0.13- 2.79)
JOLY	690	m	0	5	25	36	141	0.78	(0.28- 2.19)
Subtotal JOLY								0.72	(0.31- 1.70)
Partial Totals				7	38	72	282		

*prospective study

REF	NRR	SEX	AD	Ys	Ws	Qs	Ps
BOFFET	558	m	2	-0.19	4.23	0.07	0.7014
BOFFET	559	m	2	-0.43	4.23	0.06	0.3754
Subtotal BOFFET				-0.31	8.47	0.13	
JOLY	689	m	0	-0.51	1.63	0.06	0.5172
JOLY	690	m	0	-0.24	3.64	0.02	0.6414
Subtotal JOLY				-0.33	5.27	0.08	

N	4
NS	2

Table 1K20 - 3

IESLC - Meta-analysis of Ex Smoking by Years quit (vs current), Overview
All LC types, Cigars only
Most adjusted

	Sex combined	male	female	Total
N		4		4
NS		2		2

In this overview table, other than the "N" rows, entries in the "absent" and "Total" columns may be invalid and should be ignored

MALES

	Years quit vs current (lower focus) absent	1-6k3	4-11k7	8+k12	Total
N	3	1			4
NS	2	1			2
Wt	12.11	1.63			13.74
Het Chi	0.14	0.00			0.21
Het df	2	0			3
Het P	N.S.	N.S.			N.S.
Fixed RR	0.75	0.60			0.73
RRl	0.43	0.13			0.43
RRu	1.32	2.79			1.24
P	N.S.	N.S.			N.S.
Random RR	0.75	0.60			0.73
RRl	0.43	0.13			0.43
RRu	1.32	2.79			1.24
P	N.S.	N.S.			N.S.

	Years quit vs current (higher focus) absent	1-11k3	4-19k12	13+k20	Total
N	2	1		1	4
NS	2	1		1	3
Wt	7.87	1.63		4.23	13.74
Het Chi	0.01	0.00		0.00	0.21
Het df	1	0		0	3
Het P	N.S.	N.S.		N.S.	N.S.
Fixed RR	0.81	0.60		0.65	0.73
RRl	0.40	0.13		0.25	0.43
RRu	1.62	2.79		1.68	1.24
P	N.S.	N.S.		N.S.	N.S.
Random RR	0.81	0.60		0.65	0.73
RRl	0.40	0.13		0.25	0.43
RRu	1.62	2.79		1.68	1.24
P	N.S.	N.S.		N.S.	N.S.

Table 1K20 - 4

IESLC - Meta-analysis of Ex Smoking by Years quit (vs current), Overview
All LC types, Cigars only
Least adjusted

REF	NRR	X	SEX	AGEL	AGEH	RACE	YF	LC TYPE	LOC	START	ST	NLC	R	VB	P	H	AD	ADOS	exL	exH	S1	S2	DENOM	De
BOFFET	558		m	0	0	all	-	all	Eu:mul	1988	CC	5621	n	bl	y	n	2	0	0.1	14	0	0	current	ot
BOFFET	559		m	0	0	all	-	all	Eu:mul	1988	CC	5621	n	bl	y	n	2	0	15	999	0	3	current	ot
JOLY	689		m	0	0	all	-	all	SCAmer	1978	CC	826	n	bl	n	n	0	0	1.0	4	1	1	cur+1y	st
JOLY	690		m	0	0	all	-	all	SCAmer	1978	CC	826	n	bl	n	n	0	0	5	999	0	0	cur+1y	st

In this overview table, subtotals and Qs values may be invalid and should be ignored

Table 1K20 - 5

IESLC - Meta-analysis of Ex Smoking by Years quit (vs current), Overview
All LC types, Cigars only
Least adjusted

REF	NRR	SEX	AD	Number Exposed Case	Cont	Non-exposed Case	Cont	RR	95.00%CI
BOFFET	558	m	2	-	-	-	-	0.83	(0.32- 2.15)
BOFFET	559	m	2	-	-	-	-	0.65	(0.25- 1.68)
Subtotal BOFFET								0.73	(0.37- 1.44)
JOLY	689	m	0	2	13	36	141	0.60	(0.13- 2.79)
JOLY	690	m	0	5	25	36	141	0.78	(0.28- 2.19)
Subtotal JOLY								0.72	(0.31- 1.70)
Partial Totals				7	38	72	282		

*prospective study

REF	NRR	SEX	AD	Ys	Ws	Qs	Ps
BOFFET	558	m	2	-0.19	4.23	0.07	0.7014
BOFFET	559	m	2	-0.43	4.23	0.06	0.3754
Subtotal BOFFET				-0.31	8.47	0.13	
JOLY	689	m	0	-0.51	1.63	0.06	0.5172
JOLY	690	m	0	-0.24	3.64	0.02	0.6414
Subtotal JOLY				-0.33	5.27	0.08	

N	4
NS	2

Table 1K20 - 6

IESLC - Meta-analysis of Ex Smoking by Years quit (vs current), Overview
All LC types, Cigars only
Least adjusted

	Sex combined	male	female	Total
N		4		4
NS		2		2

In this overview table, other than the "N" rows, entries in the "absent" and "Total" columns may be invalid and should be ignored

MALES

	Years quit vs current (lower focus) absent	1-6k3	4-11k7	8+k12	Total
N	3	1			4
NS	2	1			2
Wt	12.11	1.63			13.74
Het Chi	0.14	0.00			0.21
Het df	2	0			3
Het P	N.S.	N.S.			N.S.
Fixed RR	0.75	0.60			0.73
RRl	0.43	0.13			0.43
RRu	1.32	2.79			1.24
P	N.S.	N.S.			N.S.
Random RR	0.75	0.60			0.73
RRl	0.43	0.13			0.43
RRu	1.32	2.79			1.24
P	N.S.	N.S.			N.S.

	Years quit vs current (higher focus) absent	1-11k3	4-19k12	13+k20	Total
N	2	1		1	4
NS	2	1		1	3
Wt	7.87	1.63		4.23	13.74
Het Chi	0.01	0.00		0.00	0.21
Het df	1	0		0	3
Het P	N.S.	N.S.		N.S.	N.S.
Fixed RR	0.81	0.60		0.65	0.73
RRl	0.40	0.13		0.25	0.43
RRu	1.62	2.79		1.68	1.24
P	N.S.	N.S.		N.S.	N.S.
Random RR	0.81	0.60		0.65	0.73
RRl	0.40	0.13		0.25	0.43
RRu	1.62	2.79		1.68	1.24
P	N.S.	N.S.		N.S.	N.S.

Table 1K20 - 7

IESLC - Meta-analysis of Ex Smoking by Years quit (vs current), Overview
All LC types, Cigars only
Excluded studies (and stage at which they were excluded)

1	AGUDO AKIBA AMANDU AMES AXELSS BEST BOUCHA BOUCOT BRESLO CHEN CHEN2 CHIAZZ DEAN2 DOSEME ENGELA FAN GENG GER GUO HAENSZ HEGMAN HOLE HU HU2 JUSSAW KATSOU KAUFMA KOO KOULUM KREUZE LETOUR LEVIN LIAW LIU3 LIU4 LIU5 MCCONN MIGRAN MRFITR NOTAN2 OSANN2 PERNU QIAO2 RACHTA RESTRE SADOWS SEGI2 STASZE TIZZAN VUTUC WATSON WU WUWILL WYNDE2 WYNDE8 XU YUAN ZHANG ZHENG ZHOU
2	AUVINE BENSHL BLOT1 BROWN3 BUFFLE GURSEL LAUSSM LUO MCDUFF PISANI PRESCO SPITZ WU2 WYNDE7
4	HAMMON
5	CORREA GILLIS HUMBLE QIAO WIGLE
7	ALDERS ARMADA BARBON BECHER BENHAM BROSS CARPEN CEDERL CHOI CHYOU CPSI CPSII DAMBER DARBY DEAN3 DESTEF DOLL DOLL2 DORGAN DORN GAO GAO2 GARCIA GARSHI GRAHAM HAMMO2 HIRAYA JAHN JAIN JEDRYC KAISE2 KHUDER LUBIN LUBIN2 MATOS PEZZO2 PEZZOT SOBUE SPEIZE SUZUK2 SVENSS TVERDA WAKAI WANG2 WYNDE3 WYNDE6

Table 1K20 - 8

Potentially overlapping studies

REF	REFGP	PRINC	.	OVERLAP/LINK
BOFFET	BOFFET	1		BOFFET-combined

Table 1K20 - 9

Most adjusted - insufficient data for meta-analysis

REF	NRR	SEX	AGEL	AGEH	RACE	YF	LC TYPE	LOC	START	ST	NLC	R	VB	P	H	AD	ADOS	exL	exH	S1	S2	DENOM	De
LUBIN2	1061	m	0	0	all	-	all	Eu:mul	1976	CC	7804	n	bl	n	y	2	1#	0.1	4	1	1	current	or
LUBIN2	1062	m	0	0	all	-	all	Eu:mul	1976	CC	7804	n	bl	n	y	2	1#	5	999	0	0	current	or

Comments on values in listings

LUBIN2 ADOS Duration of cigar smoking
LUBIN2 ADOS Duration of cigar smoking

REF	NRR	RR	SIG	RRDATA comment
LUBIN2	1061	0.57		0
LUBIN2	1062	0.68		0

Table 1K21 -

IESLC - Meta-analysis of Ex Smoking, Years quit (vs current), "Highest vs lowest"
All LC types, Cigars only

This analysis is restricted to results for:
1) Ex smokers
2) Results by Years quit (vs current)
3) Categorical results by Years quit (vs current)
4) Denominator (unexposed) = "low"
5) All LC types (or near equivalent)
6) Results complete enough for use in metaanalysis
7) (not applicable)
8) PRODUCT: cigars only

Within each study, results are then selected (in the following order of preference, within each sex) for:
9) Results with least adjustment for other aspects of smoking (ADOS)
10) The highest vs lowest category
11) Followup period (YF, prospective studies): whole study (coded as 0) or longest available
12) LCtype: all or nearest available, at least Squamous and Adeno. (q = squamous, s = small,
l = large, a = adeno, mix = mixed, alv = alveolar)
13) Race: all or nearest available, otherwise by race (wh or w = white, bl or b = black, hi = hispanic
ch = chinese, jap = japanese, haw = hawaiian, w+o = white + oriental, sca = scandinavian, as = asian)
14) For overlapping studies: principal rather than subsidiary studies
Finally by Age: whole study (coded as 0) if available, otherwise by widest available age group
and then for single sex results (m, f) in preference to results for both sexes combined (c).

Results adjusted (AD) for the most potential confounders are then chosen in Sections -1 to -3
and results adjusted for the least confounders in Sections -4 to -6. (Those least adjusted results which
actually differ from the most adjusted are marked 'x' in column X in Section -4)

Section -7 shows excluded studies, together with the stage (as above) at which no qualifying
results were found.

Section -8 lists the potentially overlapping studies which have been included (1=principal, 2=subsidiary).

Section -9 lists any results which would have been included in preference except that they had data not complete
enough for use in meta-analysis, with their significance (yes/no), if known, and any further comment as entered
on the database. It also lists as "gap" any categories for which no data were presented by the original authors.

In addition to those mentioned above, the following fields, levels and abbreviations are used:
* or nk = not known, n = no, y = yes, ot = other
all/unspec = all or unspecified, MC = manufactured cigarettes, HR = hand-rolled cigarettes
exL, exH = range of exposure (low and high) in the "highest" group, in terms of Years quit (vs current)
unexL, unexH = range of exposure (low and high) in the "lowest" group, in terms of Years quit (vs current)
REF: 6-character study reference
NRR: number of the RR on the database within the study
ST : study type (CC = case control, pr or prosp = prospective)
NLC: number of lung cancer cases in whole study
R : risky occupational population (n = no, m = mining, o = other risky)
VB : national cigarette type (V = at least 75% Virginia, bl = at least 75% blended, ot = other)
P : any proxy use
H : full histological confirmation
De : derivation of RR/CI (or = original, st = standard method, ot = other method of estimation)

Table 1K21 - 1

IESLC - Meta-analysis of Ex Smoking, Years quit (vs current), "Highest vs lowest"
All LC types, Cigars only
Most adjusted

REF	NRR	SEX	AGEL	AGEH	RACE	YF	LC TYPE	LOC	START	ST	NLC	R	VB	P	H	AD	ADOS	exL	exH	unexL	unexH	De
BOFFET	560	m	0	0	all	-	all	Eu:mul	1988	CC	5621	n	bl	y	n	2	0	15	999	0.1	14	ot
JOLY	691	m	0	0	all	-	all	SCAmer	1978	CC	826	n	bl	n	n	0	0	5	999	1.0	4	st

Table 1K21 - 2

IESLC - Meta-analysis of Ex Smoking, Years quit (vs current), "Highest vs lowest"
All LC types, Cigars only
Most adjusted

REF	NRR	SEX	AD	Number Exposed Case	Number Exposed Cont	Non-exposed Case	Non-exposed Cont	RR	95.00%CI
BOFFET	560	m	2	-	-	-	-	0.78	(0.26- 2.33)
JOLY	691	m	0	5	25	2	13	1.30	(0.22- 7.64)
Partial Totals				5	25	2	13		

*prospective study

REF	NRR	SEX	AD	Ys	Ws	Qs	Ps
BOFFET	560	m	2	-0.25	3.20	0.06	0.6569
JOLY	691	m	0	0.26	1.22	0.17	0.7716

	N	2
	NS	2
	Wt	4.42
Het	Chi	0.23
Het	df	1
Het	P	N.S.
Fixed	RR	0.90
	RRl	0.35
	RRu	2.28
	P	N.S.
Random	RR	0.90
	RRl	0.35
	RRu	2.28
	P	N.S.
Asymm	P	

Table 1K21 - 3

IESLC - Meta-analysis of Ex Smoking, Years quit (vs current), "Highest vs lowest"
All LC types, Cigars only
Most adjusted

	Sex combined	male	female	Total
N		2		2
NS		2		2
Wt		4.42		4.42
Het Chi		0.23		0.23
Het df		1		1
Het P		N.S.		N.S.
Fixed RR		0.90		0.90
RRl		0.35		0.35
RRu		2.28		2.28
P		N.S.		N.S.
Random RR		0.90		0.90
RRl		0.35		0.35
RRu		2.28		2.28
P		N.S.		N.S.
Between Chi				
Between df				
Between P				N.S.
Btwn(F) P				N.S.
Btwn(R) P				N.S.

MALES

Too few RRs for analysis by factor

Table 1K21 - 4

IESLC - Meta-analysis of Ex Smoking, Years quit (vs current), "Highest vs lowest"
All LC types, Cigars only
Least adjusted

REF	NRR	X	SEX	AGEL	AGEH	RACE	YF	LC TYPE	LOC	START	ST	NLC	R	VB	P	H	AD	ADOS	exL	exH	unexL	unexH	De
BOFFET	560		m	0	0	all	-	all	Eu:mul	1988	CC	5621	n	bl	y	n	2	0	15	999	0.1	14	ot
JOLY	691		m	0	0	all	-	all	SCAmer	1978	CC	826	n	bl	n	n	0	0	5	999	1.0	4	st

Table 1K21 - 5

IESLC - Meta-analysis of Ex Smoking, Years quit (vs current), "Highest vs lowest"
All LC types, Cigars only
Least adjusted

REF	NRR	SEX	AD	Number Exposed Case	Cont	Non-exposed Case	Cont	RR	95.00%CI
BOFFET	560	m	2	-	-	-	-	0.78	(0.26- 2.33)
JOLY	691	m	0	5	25	2	13	1.30	(0.22- 7.64)
Partial Totals				5	25	2	13		

*prospective study

REF	NRR	SEX	AD	Ys	Ws	Qs	Ps
BOFFET	560	m	2	-0.25	3.20	0.06	0.6569
JOLY	691	m	0	0.26	1.22	0.17	0.7716

	N	2
	NS	2
	Wt	4.42
Het	Chi	0.23
Het	df	1
Het	P	N.S.
Fixed	RR	0.90
	RRl	0.35
	RRu	2.28
	P	N.S.
Random	RR	0.90
	RRl	0.35
	RRu	2.28
	P	N.S.
Asymm	P	

Table 1K21 - 6

IESLC - Meta-analysis of Ex Smoking, Years quit (vs current), "Highest vs lowest"
All LC types, Cigars only
Least adjusted

	Sex combined	male	female	Total
N		2		2
NS		2		2
Wt		4.42		4.42
Het Chi		0.23		0.23
Het df		1		1
Het P		N.S.		N.S.
Fixed RR		0.90		0.90
RRl		0.35		0.35
RRu		2.28		2.28
P		N.S.		N.S.
Random RR		0.90		0.90
RRl		0.35		0.35
RRu		2.28		2.28
P		N.S.		N.S.
Between Chi				
Between df				
Between P				N.S.
Btwn(F) P				N.S.
Btwn(R) P				N.S.

Table 1K21 - 7

IESLC - Meta-analysis of Ex Smoking, Years quit (vs current), "Highest vs lowest"
All LC types, Cigars only
Excluded studies (and stage at which they were excluded)

1 AGUDO AKIBA AMANDU AMES AXELSS BEST BOUCHA BOUCOT BRESLO CHEN CHEN2 CHIAZZ DEAN2 DOSEME ENGELA FAN
GENG GER GUO HAENSZ HEGMAN HOLE HU HU2 JUSSAW KATSOU KAUFMA KOO KOULUM KREUZE LETOUR LEVIN
LIAW LIU3 LIU4 LIU5 MCCONN MIGRAN MRFITR NOTAN2 OSANN2 PERNU QIAO2 RACHTA RESTRE SADOWS SEGI2 STASZE
TIZZAN VUTUC WATSON WU WUWILL WYNDE2 WYNDE8 XU YUAN ZHANG ZHENG ZHOU

2 AUVINE BENSHL BLOT1 BROWN3 BUFFLE GURSEL LAUSSM LUO MCDUFF PISANI PRESCO SPITZ WU2 WYNDE7

4 GARSHI JEDRYC WAKAI

5 CORREA HAMMON

6 GILLIS HUMBLE QIAO WIGLE

8 ALDERS ARMADA BARBON BECHER BENHAM BROSS CARPEN CEDERL CHOI CHYOU CPSI CPSII DAMBER DARBY DEAN3 DESTEF
DOLL DOLL2 DORGAN DORN GAO GAO2 GARCIA GRAHAM HAMMO2 HIRAYA JAHN JAIN KAISE2 KHUDER LUBIN LUBIN2
MATOS PEZZO2 PEZZOT SOBUE SPEIZE SUZUK2 SVENSS TVERDA WANG2 WYNDE3 WYNDE6

Table 1K21 - 8

Potentially overlapping studies

REF	REFGP	PRINC	.	OVERLAP/LINK
BOFFET	BOFFET	1		BOFFET-combined

Table 1K21 - 9

Most adjusted - insufficient data for meta-analysis

REF	NRR	SEX	AGEL	AGEH	RACE	YF	LC TYPE	LOC	START	ST	NLC	R	VB	P	H	AD	ADOS	exL	exH	unexL	unexH	De
LUBIN2	1063	m	0	0	all	-	all	Eu:mul	1976	CC	7804	n	bl	n	y	2	1#	5	999	0.1	4	st

Comments on values in listings

LUBIN2 ADOS Duration of cigar smoking

REF	NRR	RR	SIG	RRDATA comment
LUBIN2	1063	1.19		0

Table 1K22 -

IESLC - Meta-analysis of Ex Smoking by Years quit (vs current), Overview
All LC types, Mixed smokers

This analysis is restricted to results for:
1) Ex smokers
2) Results by Years quit (vs current)
3) Categorical results by Years quit (vs current)
Results by Years quit (vs current) are grouped under 2 schemes (S1, S2). Each scheme has a set of "key values". An interval is allocated to the category whose key value it includes, and intervals which include none or more than one of the key values are excluded. (Open-ended intervals are coded as 999)

S1	key value	maximum range
1	3	1-6
2	7	4-11
3	12	8+

S2	key value	maximum range
1	3	1-11
2	12	4-19
3	20	13+

4) All LC types (or near equivalent)
5) Results complete enough for use in metaanalysis
6) mixed smokers (cigarettes and pipe/cigar)

Within each study, results are then selected (in the following order of preference, within each sex) for:
7) Results with least adjustment for other aspects of smoking (ADOS)
8) DENOM: current smokers, current + recent smokers (up to number of m=months or y=years)
9) Followup period (YF, prospective studies): whole study (coded as 0) or longest available
10) LCtype: all or nearest available, at least Squamous and Adeno. (q = squamous, s = small, l = large, a = adeno, mix = mixed, alv = alveolar)
11) Race: all or nearest available, otherwise by race (wh or w = white, bl or b = black, hi = hispanic ch = chinese, jap = japanese, haw = hawaiian, w+o = white + oriental, sca = scandinavian, as = asian)
12) For overlapping studies: principal rather than subsidiary studies
Finally by Age: whole study (coded as 0) if available, otherwise by widest available age group and then for single sex results (m, f) in preference to results for both sexes combined (c).

Results adjusted (AD) for the most potential confounders are then chosen in Sections -1 to -3 and results adjusted for the least confounders in Sections -4 to -6. (Those least adjusted results which actually differ from the most adjusted are marked 'x' in column X in Section -4)

Section -7 shows excluded studies, together with the stage (as above) at which no qualifying results were found.

Section -8 lists the potentially overlapping studies which have been included (1=principal, 2=subsidiary).

Section -9 lists any results which would have been included in preference except that they had data not complete enough for use in meta-analysis, with their significance (yes/no), if known, and any further comment as entered on the database. It also lists as "gap" any categories for which no data were presented by the original authors.

In addition to those mentioned above, the following fields, levels and abbreviations are used:
* or nk = not known, n = no, y = yes, ot = other
nev = never
exL, exH = range of exposure (low and high) in the smoking group, in terms of Years quit (vs current)
REF: 6-character study reference
NRR: number of the RR on the database within the study
ST : study type (CC = case control, pr or prosp = prospective)
NLC: number of lung cancer cases in whole study
R : risky occupational population (n = no, m = mining, o = other risky)
VB : national cigarette type (V = at least 75% Virginia, bl = at least 75% blended, ot = other)
P : any proxy use
H : full histological confirmation
De : derivation of RR/CI (or = original, st = standard method, ot = other method of estimation)

Table 1K22 - 1

IESLC - Meta-analysis of Ex Smoking by Years quit (vs current), Overview
All LC types, Mixed smokers
Most adjusted

REF	NRR	SEX	AGEL	AGEH	RACE	YF	LC TYPE	LOC	START	ST	NLC	R	VB	P	H	AD	ADOS	exL	exH	S1	S2	DENOM	De
GRAHAM	520	m	0	0	wh	-	all	NAmer	1956	CC	685	n	bl	n	n	0	0	0.1	1.0	0	0	current	st
GRAHAM	521	m	0	0	wh	-	all	NAmer	1956	CC	685	n	bl	n	n	0	0	1.1	5	1	1	current	st
GRAHAM	522	m	0	0	wh	-	all	NAmer	1956	CC	685	n	bl	n	n	0	0	5	999	0	0	current	st

In this overview table, subtotals and Qs values may be invalid and should be ignored

Table 1K22 - 2

IESLC - Meta-analysis of Ex Smoking by Years quit (vs current), Overview
All LC types, Mixed smokers
Most adjusted

				Number Exposed		Non-exposed			
REF	NRR	SEX	AD	Case	Cont	Case	Cont	RR	95.00%CI
GRAHAM	520	m	0	29	11	82	254	8.17	(3.91- 17.07)
GRAHAM	521	m	0	12	9	82	254	4.13	(1.68- 10.15)
GRAHAM	522	m	0	6	28	82	254	0.66	(0.27- 1.66)
Subtotal GRAHAM								3.33	(2.05- 5.40)
Totals				47	48	246	762		

*prospective study

REF	NRR	SEX	AD	Ys	Ws	Qs	Ps
GRAHAM	520	m	0	2.10	7.07	5.70	0.0000
GRAHAM	521	m	0	1.42	4.75	0.22	0.0020
GRAHAM	522	m	0	-0.41	4.58	11.89	0.3806
Subtotal GRAHAM				1.20	16.39	17.81	

N	3
NS	1

Table 1K22 - 3

IESLC - Meta-analysis of Ex Smoking by Years quit (vs current), Overview
All LC types, Mixed smokers
Most adjusted

	combined	Sex male	female	Total
N		3		3
NS		1		1

In this overview table, other than the "N" rows, entries in the "absent" and "Total" columns may be invalid and should be ignored

MALES

	Years quit vs current (lower focus) absent	1-6k3	4-11k7	8+k12	Total
N	2	1			3
NS	1	1			1
Wt	11.64	4.75			16.39
Het Chi	17.50	0.00			17.81
Het df	1	0			2
Het P	***	N.S.			***
Fixed RR	3.04	4.13			3.33
RRl	1.71	1.68			2.05
RRu	5.41	10.15			5.40
P	+++	++			+++
Random RR	2.36	4.13			2.87
RRl	0.20	1.68			0.66
RRu	27.66	10.15			12.36
P	N.S.	++			N.S.

	Years quit vs current (higher focus) absent	1-11k3	4-19k12	13+k20	Total
N	2	1			3
NS	1	1			1
Wt	11.64	4.75			16.39
Het Chi	17.50	0.00			17.81
Het df	1	0			2
Het P	***	N.S.			***
Fixed RR	3.04	4.13			3.33
RRl	1.71	1.68			2.05
RRu	5.41	10.15			5.40
P	+++	++			+++
Random RR	2.36	4.13			2.87
RRl	0.20	1.68			0.66
RRu	27.66	10.15			12.36
P	N.S.	++			N.S.

Table 1K22 - 4

IESLC - Meta-analysis of Ex Smoking by Years quit (vs current), Overview
All LC types, Mixed smokers
Least adjusted

REF	NRR	X	SEX	AGEL	AGEH	RACE	YF	LC TYPE	LOC	START	ST	NLC	R	VB	P	H	AD	ADOS	exL	exH	S1	S2	DENOM	De
GRAHAM	520		m	0	0	wh	-	all	NAmer	1956	CC	685	n	bl	n	n	0	0	0.1	1.0	0	0	current	st
GRAHAM	521		m	0	0	wh	-	all	NAmer	1956	CC	685	n	bl	n	n	0	0	1.1	5	1	1	current	st
GRAHAM	522		m	0	0	wh	-	all	NAmer	1956	CC	685	n	bl	n	n	0	0	5	999	0	0	current	st

In this overview table, subtotals and Qs values may be invalid and should be ignored

Table 1K22 - 5

IESLC - Meta-analysis of Ex Smoking by Years quit (vs current), Overview
All LC types, Mixed smokers
Least adjusted

				Number Exposed		Non-exposed			
REF	NRR	SEX	AD	Case	Cont	Case	Cont	RR	95.00%CI
GRAHAM	520	m	0	29	11	82	254	8.17	(3.91- 17.07)
GRAHAM	521	m	0	12	9	82	254	4.13	(1.68- 10.15)
GRAHAM	522	m	0	6	28	82	254	0.66	(0.27- 1.66)
Subtotal GRAHAM								3.33	(2.05- 5.40)
Totals				47	48	246	762		

*prospective study

REF	NRR	SEX	AD	Ys	Ws	Qs	Ps
GRAHAM	520	m	0	2.10	7.07	5.70	0.0000
GRAHAM	521	m	0	1.42	4.75	0.22	0.0020
GRAHAM	522	m	0	-0.41	4.58	11.89	0.3806
Subtotal GRAHAM				1.20	16.39	17.81	

N	3
NS	1

Table 1K22 - 6

IESLC - Meta-analysis of Ex Smoking by Years quit (vs current), Overview
All LC types, Mixed smokers
Least adjusted

	Sex combined	male	female	Total
N		3		3
NS		1		1

In this overview table, other than the "N" rows, entries in the "absent" and "Total" columns may be invalid and should be ignored

MALES

Years quit vs current (lower focus)	absent	1-6k3	4-11k7	8+k12	Total
N	2	1			3
NS	1	1			1
Wt	11.64	4.75			16.39
Het Chi	17.50	0.00			17.81
Het df	1	0			2
Het P	***	N.S.			***
Fixed RR	3.04	4.13			3.33
RRl	1.71	1.68			2.05
RRu	5.41	10.15			5.40
P	+++	++			+++
Random RR	2.36	4.13			2.87
RRl	0.20	1.68			0.66
RRu	27.66	10.15			12.36
P	N.S.	++			N.S.

Years quit vs current (higher focus)	absent	1-11k3	4-19k12	13+k20	Total
N	2	1			3
NS	1	1			1
Wt	11.64	4.75			16.39
Het Chi	17.50	0.00			17.81
Het df	1	0			2
Het P	***	N.S.			***
Fixed RR	3.04	4.13			3.33
RRl	1.71	1.68			2.05
RRu	5.41	10.15			5.40
P	+++	++			+++
Random RR	2.36	4.13			2.87
RRl	0.20	1.68			0.66
RRu	27.66	10.15			12.36
P	N.S.	++			N.S.

Table 1K22 - 7

IESLC - Meta-analysis of Ex Smoking by Years quit (vs current), Overview
All LC types, Mixed smokers
Excluded studies (and stage at which they were excluded)

1	AGUDO	AKIBA	AMANDU	AMES	AXELSS	BEST	BOUCHA	BOUCOT	BRESLO	CHEN	CHEN2	CHIAZZ	DEAN2	DOSEME	ENGELA	FAN
	GENG	GER	GUO	HAENSZ	HEGMAN	HOLE	HU	HU2	JUSSAW	KATSOU	KAUFMA	KOO	KOULUM	KREUZE	LETOUR	LEVIN
	LIAW	LIU3	LIU4	LIU5	MCCONN	MIGRAN	MRFITR	NOTAN2	OSANN2	PERNU	QIAO2	RACHTA	RESTRE	SADOWS	SEGI2	STASZE
	TIZZAN	VUTUC	WATSON	WU	WUWILL	WYNDE2	WYNDE8	XU	YUAN	ZHANG	ZHENG	ZHOU				
2	AUVINE	BENSHL	BLOT1	BROWN3	BUFFLE	GURSEL	LAUSSM	LUO	MCDUFF	PISANI	PRESCO	SPITZ	WU2	WYNDE7		
4	HAMMON															
5	CORREA	GILLIS	HUMBLE	QIAO	WIGLE											
7	ALDERS	ARMADA	BARBON	BECHER	BENHAM	BOFFET	BROSS	CARPEN	CEDERL	CHOI	CHYOU	CPSI	CPSII	DAMBER	DARBY	DEAN3
	DESTEF	DOLL	DOLL2	DORGAN	DORN	GAO	GAO2	GARCIA	GARSHI	HAMMO2	HIRAYA	JAHN	JAIN	JEDRYC	JOLY	KAISE2
	KHUDER	LUBIN	LUBIN2	MATOS	PEZZO2	PEZZOT	SOBUE	SPEIZE	SUZUK2	SVENSS	TVERDA	WAKAI	WANG2	WYNDE3	WYNDE6	

Table 1K22 - 8

Potentially overlapping studies

REF	REFGP	PRINC	. OVERLAP/LINK
GRAHAM	BYERS1	1	GRAHAM/BROSS/BYERS1

Table 1K23 -

IESLC - Meta-analysis of Ex Smoking, Years quit (vs current), "Highest vs lowest"
All LC types, Mixed smokers

This analysis is restricted to results for:
1) Ex smokers
2) Results by Years quit (vs current)
3) Categorical results by Years quit (vs current)
4) Denominator (unexposed) = "low"
5) All LC types (or near equivalent)
6) Results complete enough for use in metaanalysis
7) (not applicable)
8) PRODUCT: mixed smokers (cigarettes and pipe/cigar)

Within each study, results are then selected (in the following order of preference, within each sex) for:
9) Results with least adjustment for other aspects of smoking (ADOS)
10) The highest vs lowest category
11) Followup period (YF, prospective studies): whole study (coded as 0) or longest available
12) LCtype: all or nearest available, at least Squamous and Adeno. (q = squamous, s = small,
l = large, a = adeno, mix = mixed, alv = alveolar)
13) Race: all or nearest available, otherwise by race (wh or w = white, bl or b = black, hi = hispanic
ch = chinese, jap = japanese, haw = hawaiian, w+o = white + oriental, sca = scandinavian, as = asian)
14) For overlapping studies: principal rather than subsidiary studies
Finally by Age: whole study (coded as 0) if available, otherwise by widest available age group
and then for single sex results (m, f) in preference to results for both sexes combined (c).

Results adjusted (AD) for the most potential confounders are then chosen in Sections -1 to -3
and results adjusted for the least confounders in Sections -4 to -6. (Those least adjusted results which
actually differ from the most adjusted are marked 'x' in column X in Section -4)

Section -7 shows excluded studies, together with the stage (as above) at which no qualifying
results were found.

Section -8 lists the potentially overlapping studies which have been included (1=principal, 2=subsidiary).

Section -9 lists any results which would have been included in preference except that they had data not complete
enough for use in meta-analysis, with their significance (yes/no), if known, and any further comment as entered
on the database. It also lists as "gap" any categories for which no data were presented by the original authors.

In addition to those mentioned above, the following fields, levels and abbreviations are used:
* or nk = not known, n = no, y = yes, ot = other
exL, exH = range of exposure (low and high) in the "highest" group, in terms of Years quit (vs current)
unexL, unexH = range of exposure (low and high) in the "lowest" group, in terms of Years quit (vs current)
REF: 6-character study reference
NRR: number of the RR on the database within the study
ST : study type (CC = case control, pr or prosp = prospective)
NLC: number of lung cancer cases in whole study
R : risky occupational population (n = no, m = mining, o = other risky)
VB : national cigarette type (V = at least 75% Virginia, bl = at least 75% blended, ot = other)
P : any proxy use
H : full histological confirmation
De : derivation of RR/CI (or = original, st = standard method, ot = other method of estimation)

Table 1K23 - 1

IESLC - Meta-analysis of Ex Smoking, Years quit (vs current), "Highest vs lowest"
All LC types, Mixed smokers
Most adjusted

REF	NRR	SEX	AGEL	AGEH	RACE	YF	LC TYPE	LOC	START	ST	NLC	R	VB	P	H	AD	ADOS	exL	exH	unexL	unexH	De
GRAHAM	524	m	0	0	wh	-	all	NAmer	1956	CC	685	n	bl	n	n	0	0	5	999	0.1	1.0	st

Table 1K23 - 2

IESLC - Meta-analysis of Ex Smoking, Years quit (vs current), "Highest vs lowest"
All LC types, Mixed smokers
Most adjusted

REF	NRR	SEX	AD	Number Exposed Case	Number Exposed Cont	Non-exposed Case	Non-exposed Cont	RR	95.00%CI
GRAHAM	524	m	0	6	28	29	11	0.08	(0.03- 0.25)
Totals				6	28	29	11		

*prospective study

REF	NRR	SEX	AD	Ys	Ws	Qs	Ps
GRAHAM	524	m	0	-2.51	3.05	0.00	0.0000

	N	1
	NS	1
	Wt	3.05
Het	Chi	0.00
Het	df	0
Het	P	N.S.
Fixed	RR	0.08
	RRl	0.03
	RRu	0.25
	P	---
Random	RR	0.08
	RRl	0.03
	RRu	0.25
	P	---
Asymm	P	

Table 1K23 - 3

IESLC - Meta-analysis of Ex Smoking, Years quit (vs current), "Highest vs lowest"
All LC types, Mixed smokers
Most adjusted

	Sex combined	male	female	Total
N		1		1
NS		1		1
Wt		3.05		3.05
Het Chi		0.00		0.00
Het df		0		0
Het P		N.S.		N.S.
Fixed RR		0.08		0.08
RRl		0.03		0.03
RRu		0.25		0.25
P		---		---
Random RR		0.08		0.08
RRl		0.03		0.03
RRu		0.25		0.25
P		---		---
Between Chi				
Between df				
Between P				N.S.
Btwn(F) P				N.S.
Btwn(R) P				N.S.

MALES

Too few RRs for analysis by factor

Table 1K23 - 4

IESLC - Meta-analysis of Ex Smoking, Years quit (vs current), "Highest vs lowest"
All LC types, Mixed smokers
Least adjusted

REF	NRR	X	SEX	AGEL	AGEH	RACE	YF	LC TYPE	LOC	START	ST	NLC	R	VB	P	H	AD	ADOS	exL	exH	unexL	unexH	De
GRAHAM	524		m	0	0	wh	-	all	NAmer	1956	CC	685	n	bl	n	n	0	0	5	999	0.1	1.0	st

Table 1K23 - 5

IESLC - Meta-analysis of Ex Smoking, Years quit (vs current), "Highest vs lowest"
All LC types, Mixed smokers
Least adjusted

REF	NRR	SEX	AD	Number Exposed Case	Exposed Cont	Non-exposed Case	Non-exposed Cont	RR	95.00%CI
GRAHAM	524	m	0	6	28	29	11	0.08	(0.03- 0.25)
Totals				6	28	29	11		

*prospective study

REF	NRR	SEX	AD	Ys	Ws	Qs	Ps
GRAHAM	524	m	0	-2.51	3.05	0.00	0.0000

N	1
NS	1
Wt	3.05
Het Chi	0.00
Het df	0
Het P	N.S.
Fixed RR	0.08
RRl	0.03
RRu	0.25
P	---
Random RR	0.08
RRl	0.03
RRu	0.25
P	---
Asymm P	

Table 1K23 - 6

IESLC - Meta-analysis of Ex Smoking, Years quit (vs current), "Highest vs lowest"
All LC types, Mixed smokers
Least adjusted

	Sex combined	male	female	Total
N		1		1
NS		1		1
Wt		3.05		3.05
Het Chi		0.00		0.00
Het df		0		0
Het P		N.S.		N.S.
Fixed RR		0.08		0.08
RRl		0.03		0.03
RRu		0.25		0.25
P		---		---
Random RR		0.08		0.08
RRl		0.03		0.03
RRu		0.25		0.25
P		---		---
Between Chi				
Between df				
Between P				N.S.
Btwn(F) P				N.S.
Btwn(R) P				N.S.

Table 1K23 - 7

IESLC - Meta-analysis of Ex Smoking, Years quit (vs current), "Highest vs lowest"
All LC types, Mixed smokers
Excluded studies (and stage at which they were excluded)

1	AGUDO AKIBA AMANDU AMES AXELSS BEST BOUCHA BOUCOT BRESLO CHEN CHEN2 CHIAZZ DEAN2 DOSEME ENGELA FAN GENG GER GUO HAENSZ HEGMAN HOLE HU HU2 JUSSAW KATSOU KAUFMA KOO KOULUM KREUZE LETOUR LEVIN LIAW LIU3 LIU4 LIU5 MCCONN MIGRAN MRFITR NOTAN2 OSANN2 PERNU QIAO2 RACHTA RESTRE SADOWS SEGI2 STASZE TIZZAN VUTUC WATSON WU WUWILL WYNDE2 WYNDE8 XU YUAN ZHANG ZHENG ZHOU
2	AUVINE BENSHL BLOT1 BROWN3 BUFFLE GURSEL LAUSSM LUO MCDUFF PISANI PRESCO SPITZ WU2 WYNDE7
4	GARSHI JEDRYC WAKAI
5	CORREA HAMMON
6	GILLIS HUMBLE QIAO WIGLE
8	ALDERS ARMADA BARBON BECHER BENHAM BOFFET BROSS CARPEN CEDERL CHOI CHYOU CPSI CPSII DAMBER DARBY DEAN3 DESTEF DOLL DOLL2 DORGAN DORN GAO GAO2 GARCIA HAMMO2 HIRAYA JAHN JAIN JOLY KAISE2 KHUDER LUBIN LUBIN2 MATOS PEZZO2 PEZZOT SOBUE SPEIZE SUZUK2 SVENSS TVERDA WANG2 WYNDE3 WYNDE6

Table 1K23 - 8
Potentially overlapping studies

REF	REFGP	PRINC	. OVERLAP/LINK
GRAHAM	BYERS1	1	GRAHAM/BROSS/BYERS1
