# Supplementary material for: Systematic review with meta-analysis of the epidemiological evidence in the 1900s relating smoking to lung cancer
Source: BMC Cancer. 2012 Sep 3;12:385. doi: 10.1186/1471-2407-12-385 (PMC3505152; doi:10.1186/1471-2407-12-385)
Supplement: Additional file 5 — Detailed Analysis Tables (Individual file names as described in Additional file 1: Methods, Table1). [file 1471-2407-12-385-S5.zip › PDF/1B.pdf]

Table 1B1 -

IESLC - Meta-analysis of Current Smoking (vs never smoking), Any product (or Cigarettes if Any not available)
All LC types

This analysis is restricted to results for:
1) Non-dose-response data
2) Current smokers
3) Results complete enough for use in metaanalysis

Within each study, results are then selected (in the following order of preference, within each sex) for:
4) PRODUCT: all/unspec, cigarettes regardless of other products, cigarettes only
5) CIGTYPE: all/unspecified, MC regardless of HR, MC only
6) DENOM: never smoked anything, never smoked cigarettes, (never +1 = +long term ex, +2 = +amount unknown,
+3 = never cigs+long term ex)
7) Followup period (YF, prospective studies): whole study (coded as 0) or longest available
8) LCtype: all or nearest available, at least Squamous and Adeno. (q = squamous, s = small,
l = large, a = adeno, mix = mixed, alv = alveolar)
9) Race: all or nearest available, otherwise by race (wh or w = white, bl or b = black, hi = hispanic
ch = chinese, jap = japanese, haw = hawaiian, w+o = white + oriental, sca = scandinavian, as = asian)
10) For overlapping studies: principal rather than subsidiary studies
Finally by Age: whole study (coded as 0) if available, otherwise by widest available age group
and then for single sex results (m, f) in preference to combined sex results (c).

Results adjusted (AD) for the most potential confounders are then chosen in Sections -1 to -3
and results adjusted for the least confounders in Sections -4 to -6. (Those least adjusted results which
actually differ from the most adjusted as marked 'x' in column X in Section -4)
(Results adjusted for an unknown number of confounder(s) are coded as 20.)

Section -7 shows excluded studies, together with the stage (as above) at which no qualifying
results were found.

Section -8 lists the potentially overlapping studies which have been included (1=principal, 2=subsidiary).

Section -9 lists any results which would have been included in preference except that they had data not complete
enough for use in meta-analysis, with their significance (yes/no), if known, and any further comment as entered
on the database.

In addition to those mentioned above, the following fields, levels and abbreviations are used:
* or nk = not known, n = no, y = yes, ot = other
nev = never
all/unspec = all or unspecified, cig+/-ot = cigarettes irrespective of other products (cigar, pipe etc)
MC = manufactured cigarettes, HR = hand-rolled cigarettes
REF: 6-character study reference
NRR: number of the RR on the database within the study
ST : study type (CC = case control, pr or prosp = prospective)
NLC: number of lung cancer cases in whole study
R : risky occupational population (n = no, m = mining, o = other risky)
VB : national cigarette type (V = at least 75% Virginia, bl = at least 75% blended, ot = other)
P : any proxy use
H : full histological confirmation
De : derivation of RR/CI (or = original, st = standard method, ot = other method of estimation)

Table 1B1 - 1

IESLC - Meta-analysis of Current Smoking (vs never smoking), Any product (or Cigarettes if Any not available)
All LC types
Most adjusted

REF	NRR	SEX	AGEL	AGEH	RACE	YF	LC TYPE	LOC	START	ST	NLC	R	VB	P	H	AD	PRODUCT	DENOM	De
AGUDO	3	f	0	0	all	-	all	Eu:wst	1989	CC	103	n	bl	n	n	3	cig only	nev any	or
AKIBA	10	m	0	0	all	0	all	As:Jap	1963	pr	610	n	bl	n	n	5	cig+/-ot	nev cigs	ot
AKIBA	14	f	0	0	all	0	all	As:Jap	1963	pr	610	n	bl	n	n	5	cig+/-ot	nev cigs	or
ALDERS	177	m	0	0	all	-	all	Eu:UK	1977	CC	1448	n	V	n	n	0	cig+/-ot	nev any	st
ALDERS	176	f	0	0	all	-	all	Eu:UK	1977	CC	1448	n	V	n	n	0	cig only	nev any	st
AMANDU	5	m	0	0	wh	0	all	NAmer	1959	pr	132	m	bl	n	n	2	cig+/-ot	nev cigs	ot
AMES	1	m	0	0	wh	-	all	NAmer	1959	ot	317	m	bl	n	n	0	all/unsp	nev any	or
ANDERS	6	f	0	0	all	0	all	NAmer	1986	pr	343	n	bl	n	n	1	cig+/-ot	nev cigs	or
ARCHER	5	m	0	0	wh	0	all	NAmer	1950	pr	146	m	bl	n	n	0	cig+/-ot	nev cigs	st
ARMADA	27	m	0	0	all	-	all	Eu:wst	1986	CC	325	n	bl	n	y	0	cig+/-ot	nev any	st
AUSTIN	6	c	0	0	all	-	all	NAmer	1970	CC	166	o	bl	y	n	3	cig+/-ot	nev cigs	or
AXELSS	2	m	0	0	sca	-	all	Eu:Sca	1989	CC	436	n	bl	n	n	0	all/unsp	nev any	st
AXELSS	10	f	0	0	sca	-	all	Eu:Sca	1989	CC	436	n	bl	n	n	0	all/unsp	nev any	st
BARBON	4	m	0	0	all	-	all	Eu:wst	1979	CC	755	n	bl	y	y	1	all/unsp	nev any	or
BECHER	13	m	0	0	all	-	all	Eu:Ger	1985	CC	194	n	bl	n	y	0	all/unsp	nev any	st
BECHER	14	f	0	0	all	-	all	Eu:Ger	1985	CC	194	n	bl	n	y	0	all/unsp	nev any	st
BENSHL	16	m	40	64	all	10	all	Eu:UK	1967	pr	486	n	V	n	n	1	all/unsp	nev any	ot
BEST	2	m	0	0	all	0	all	NAmer	1955	pr	381	n	V	n	n	1	cig only	nev any	ot
BLOHMK	1	m	0	0	all	-	all	Eu:Ger	1978	CC	888	n	bl	n	y	0	all/unsp	nev any	st
BOUCOT	114	m	0	0	all	0	all	NAmer	1951	pr	121	n	bl	n	n	2	cig only	nev any	ot
BRETT	4	m	0	0	all	0	all	Eu:UK	1960	pr	150	n	V	n	n	0	cig+/-ot	nev cigs	st
BROSS	11	m	0	0	wh	-	all	NAmer	1960	CC	974	n	bl	n	n	0	all/unsp	nev any	st
BROWN2	12	m	0	0	wh	-	all	NAmer	1984	CC	14596	n	bl	n	y	2	cig+/-ot	nev cigs	or
BROWN2	11	f	0	0	wh	-	all	NAmer	1984	CC	14596	n	bl	n	y	2	cig+/-ot	nev cigs	or
BUFFLE	3	m	0	0	wh	-	all	NAmer	1976	CC	943	n	bl	y	n	0	cig+/-ot	nev any	st
BUFFLE	7	f	0	0	wh	-	all	NAmer	1976	CC	943	n	bl	y	n	0	cig+/-ot	nev any	st
CARPEN	11	c	0	0	w+b	-	all	NAmer	1991	CC	356	n	bl	n	n	3	cig+/-ot	nev cigs	or
CEDERL	106	m	0	0	all	16	all	Eu:Sca	1963	pr	491	n	bl	n	n	2	all/unsp	nev any	ot
CEDERL	75	f	0	0	all	0	all	Eu:Sca	1963	pr	491	n	bl	n	n	2	all/unsp	nev any	or
CHANG	5	m	0	0	all	0	all	NAmer	1972	pr	136	n	bl	n	n	0	cig+/-ot	nev cigs	st
CHANG	11	f	0	0	all	0	all	NAmer	1972	pr	136	n	bl	n	n	0	cig+/-ot	nev cigs	st
CHOI	3	m	0	0	all	-	all	As:oth	1985	CC	375	n	bl	n	n	0	cig+/-ot	nev cigs	st
CHOI	7	f	0	0	all	-	all	As:oth	1985	CC	375	n	bl	n	n	0	cig+/-ot	nev cigs	st
CHOW	25	m	0	0	wh	0	all	NAmer	1966	pr	219	n	bl	n	n	0	all/unsp	nev any	st
CHYOU	2	m	0	0	jap	0	all	NAmer	1965	pr	227	n	bl	n	y	1	cig+/-ot	nev cigs	or
COMSTO	3	m	0	0	all	-	all	NAmer	1975	ot	258	n	bl	n	n	0	cig+/-ot	nev any	st
COMSTO	8	f	0	0	all	-	all	NAmer	1975	ot	258	n	bl	n	n	0	cig+/-ot	nev any	st
CORREA	42	c	0	0	all	-	all	NAmer	1979	CC	1359	n	bl	y	n	1	cig+/-ot	nev cigs	or
CPSI	220	m	35	84	all	6	all	NAmer	1959	pr	5138	n	bl	n	n	1	cig+/-ot	nev any	ot
CPSI	279	f	40	74	all	6	all	NAmer	1959	pr	5138	n	bl	n	n	1	cig+/-ot	nev cigs	ot
CPSII	126	m	0	0	all	6	all	NAmer	1982	pr	3229	n	bl	n	n	1	cig only	nev any	ot
CPSII	133	f	0	0	all	6	all	NAmer	1982	pr	3229	n	bl	n	n	1	cig+/-ot	nev cigs	ot
DAMBER	14	m	0	0	all	-	all	Eu:Sca	1972	CC	579	n	bl	y	n	1	all/unsp	nev any	ot
DARBY	4	m	0	0	wh	-	all	Eu:UK	1988	CC	982	n	V	n	n	0	cig+/-ot	nev any	st
DARBY	11	f	0	0	wh	-	all	Eu:UK	1988	CC	982	n	V	n	n	0	cig+/-ot	nev any	st
DEAN2	2	m	0	0	all	-	all	Eu:UK	1960	CC	954	n	V	y	n	0	all/unsp	nev any	st
DEAN2	6	f	0	0	all	-	all	Eu:UK	1960	CC	954	n	V	y	n	0	all/unsp	nev any	st
DEAN3	42	m	0	0	all	-	all	Eu:UK	1969	CC	766	n	V	y	n	3	all/unsp	nev any	ot
DEAN3	119	f	0	0	all	-	all	Eu:UK	1969	CC	766	n	V	y	n	3	cig only	nev any	ot
DEKLER	8	m	0	0	all	0	all	Auslia	1961	pr	138	m	V	n	n	2	all/unsp	nev any	ot
DESTE2	4	c	0	0	all	-	all	SCAmer	1993	CC	463	n	bl	n	n	7	all/unsp	nev any	or
DESTEF	41	m	0	0	all	-	all	SCAmer	1988	CC	497	n	bl	n	y	4	all/unsp	nev any	or
DOCKER	1	c	0	0	wh	0	all	NAmer	1974	pr	120	n	bl	n	n	4	cig+/-ot	nev cigs	or
DOLL	90	m	0	0	all	-	all	Eu:UK	1948	CC	1465	n	V	n	n	0	all/unsp	nev any	st
DOLL	93	f	0	0	all	-	all	Eu:UK	1948	CC	1465	n	V	n	n	0	all/unsp	nev any	st
DOLL2	54	m	0	0	all	0	all	Eu:UK	1951	pr	920	n	V	n	n	1	all/unsp	nev any	ot
DOLL2	63	f	0	0	all	22	all	Eu:UK	1951	pr	920	n	V	n	n	1	cig only	nev any	ot
DORANT	2	m	0	0	all	0	all	Eu:wst	1986	ot	550	n	bl	n	y	0	all/unsp	nev any	st
DORGAN	9	m	0	0	wh	-	all	NAmer	1980	CC	2026	n	bl	y	y	0	cig+/-ot	nev any	st
DORGAN	33	m	0	0	bl	-	all	NAmer	1980	CC	2026	n	bl	y	y	0	cig+/-ot	nev any	st
DORGAN	56	f	0	0	wh	-	all	NAmer	1980	CC	2026	n	bl	y	y	0	cig+/-ot	nev any	st
DORGAN	79	f	0	0	bl	-	all	NAmer	1980	CC	2026	n	bl	y	y	0	cig+/-ot	nev any	st
DORN	51	m	35	84	wh	8	all	NAmer	1954	pr	5097	n	bl	n	n	1	all/unsp	nev any	ot
DROSTE	6	m	0	0	all	-	all	Eu:wst	1995	CC	478	n	bl	n	y	4	all/unsp	nev any	or
ENGELA	158	m	0	0	all	12	all	Eu:Sca	1964	pr	435	n	bl	n	n	1	all/unsp	nev any	ot
ENGELA	164	f	0	0	all	12	all	Eu:Sca	1964	pr	435	n	bl	n	n	1	all/unsp	nev any	ot
ENSTRO	1	m	0	0	all	0	all	NAmer	1959	pr	2879	n	bl	n	n	1	cig only	nev any	or
ENSTRO	2	f	0	0	all	0	all	NAmer	1959	pr	2879	n	bl	n	n	1	cig only	nev any	or
GAO	33	m	0	0	all	-	all	As:Chi	1984	CC	1405	n	ot	n	n	2	cig+/-ot	nev cigs	or
GAO	34	f	0	0	all	-	all	As:Chi	1984	CC	1405	n	ot	n	n	2	cig+/-ot	nev cigs	or
GAO2	8	m	0	0	all	-	all	As:Jap	1988	CC	282	n	bl	n	n	1	cig+/-ot	nev cigs	or
GARCIA	2	c	0	0	all	-	all	NAmer	1992	CC	416	n	bl	n	y	0	cig+/-ot	nev cigs	st

Table 1B1 - 1

IESLC - Meta-analysis of Current Smoking (vs never smoking), Any product (or Cigarettes if Any not available)
All LC types
Most adjusted

REF	NRR	SEX	AGEL	AGEH	RACE	YF	LC TYPE	LOC	START	ST	NLC	R	VB	P	H	AD	PRODUCT	DENOM	De
GARDIN	2	c	0	0	all	-	all	Eu:UK	1988	CC	143	n	V	y	n	0	all/unsp	nev any	st
GARSHI	31	m	0	0	all	-	all	NAmer	1981	CC	1081	o	bl	y	n	1	all/unsp	nev any	st
GOODMA	2	m	0	0	w+o	-	all	NAmer	1983	CC	326	n	bl	y	y	0	cig+/-ot	nev any	st
GOODMA	6	f	0	0	w+o	-	all	NAmer	1983	CC	326	n	bl	y	y	0	cig+/-ot	nev any	st
GRAHAM	25	m	0	0	wh	-	all	NAmer	1956	CC	685	n	bl	n	n	1	all/unsp	nev any	ot
GREGOR	2	m	0	0	all	-	all	Eu:UK	1976	CC	104	n	V	n	y	0	cig+/-ot	nev cigs	st
GREGOR	6	f	0	0	all	-	all	Eu:UK	1976	CC	104	n	V	n	y	0	cig+/-ot	nev cigs	st
HAENSZ	54	f	0	0	all	-	not alv	NAmer	1955	CC	158	n	bl	n	y	0	cig+/-ot	nev any	st
HAMMO2	8	m	0	0	all	0	all	NAmer	1967	pr	450	o	bl	n	n	1	cig+/-ot	nev any	ot
HAMMON	139	m	0	0	wh	0	all	NAmer	1952	pr	448	n	bl	n	n	1	cig only	nev any	ot
HEIN	5	m	0	0	all	0	all	Eu:Sca	1970	pr	144	n	bl	n	n	0	all/unsp	nev any	st
HENNEK	2	m	0	0	all	0	all	NAmer	1982	pr	169	n	bl	n	n	0	all/unsp	nev any	st
HIRAYA	1	m	0	0	all	0	all	As:Jap	1965	pr	1917	n	bl	n	n	1	cig+/-ot	nev any	st
HIRAYA	3	f	0	0	all	0	all	As:Jap	1965	pr	1917	n	bl	n	n	1	cig+/-ot	nev any	st
HITOSU	34	m	0	0	all	-	all	As:Jap	1960	CC	216	n	bl	y	n	1	all/unsp	nev any	st
HITOSU	59	f	0	0	all	-	all	As:Jap	1960	CC	216	n	bl	y	n	1	all/unsp	nev any	st
HOLE	33	m	0	0	all	0	all	Eu:UK	1972	pr	225	n	V	n	n	1	all/unsp	nev any	ot
HOLE	31	f	0	0	all	11	all	Eu:UK	1972	pr	225	n	V	n	n	1	all/unsp	nev any	ot
HUMBLE	13	m	0	0	w-hi	-	all	NAmer	1980	CC	521	n	bl	y	n	1	cig+/-ot	nev cigs	ot
HUMBLE	15	m	0	0	hi	-	all	NAmer	1980	CC	521	n	bl	y	n	1	cig+/-ot	nev cigs	ot
HUMBLE	17	f	0	0	w-hi	-	all	NAmer	1980	CC	521	n	bl	y	n	1	cig+/-ot	nev cigs	ot
HUMBLE	19	f	0	0	hi	-	all	NAmer	1980	CC	521	n	bl	y	n	1	cig+/-ot	nev cigs	ot
JAHN	5	m	0	0	all	-	all	Eu:Ger	1988	CC	1004	n	bl	n	n	0	cig+/-ot	nev any	st
JAIN	52	m	0	0	all	-	all	NAmer	1981	CC	845	n	V	y	n	2	cig+/-ot	nev cigs	or
JAIN	51	f	0	0	all	-	all	NAmer	1981	CC	845	n	V	y	n	2	cig+/-ot	nev cigs	or
JARVHO	2	m	0	0	all	-	all	Eu:Sca	1983	CC	147	n	bl	n	n	0	all/unsp	nev any	st
JARVHO	6	f	0	0	all	-	all	Eu:Sca	1983	CC	147	n	bl	n	n	0	all/unsp	nev any	st
JOLY	18	m	0	0	all	-	all	SCAmer	1978	CC	826	n	bl	n	n	0	all/unsp	nev any	st
JOLY	15	f	0	0	all	-	all	SCAmer	1978	CC	826	n	bl	n	n	0	cig+/-ot	nev any	st
KAISE2	68	m	35	99	all	9	all	NAmer	1979	pr	318	n	bl	n	n	1	cig only	nev any	st
KAISE2	60	f	35	99	all	9	all	NAmer	1979	pr	318	n	bl	n	n	1	cig only	nev any	st
KAISER	12	m	0	0	all	0	all	NAmer	1964	pr	714	n	bl	n	n	2	cig+/-ot	nev cigs	ot
KAISER	9	f	0	0	all	0	all	NAmer	1964	pr	714	n	bl	n	n	2	cig+/-ot	nev cigs	ot
KANELL	30	m	0	0	all	-	all	Eu:bal	1950	CC	862	n	bl	n	n	1	all/unsp	nev any	st
KATSOU	2	f	0	0	all	-	all	Eu:bal	1987	CC	101	n	bl	n	n	1	all/unsp	nev any	or
KAUFMA	16	c	0	0	all	-	all	NAmer	1981	CC	881	n	bl	n	n	6	cig+/-ot	nev cigs	ot
KELLER	1	m	0	0	wh	-	all	NAmer	1985	CC	15038	n	bl	n	n	0	all/unsp	nev any	st
KELLER	9	m	0	0	nonw	-	all	NAmer	1985	CC	15038	n	bl	n	n	0	all/unsp	nev any	st
KELLER	5	f	0	0	wh	-	all	NAmer	1985	CC	15038	n	bl	n	n	0	all/unsp	nev any	st
KELLER	13	f	0	0	nonw	-	all	NAmer	1985	CC	15038	n	bl	n	n	0	all/unsp	nev any	st
KHUDER	19	m	0	0	all	-	all	NAmer	1985	CC	482	n	bl	n	y	0	cig+/-ot	nev cigs	or
KIHARA	7	c	0	0	jap	-	all	As:Jap	1991	CC	440	n	bl	n	n	0	all/unsp	nev any	st
KINLEN	20	m	0	0	all	0	all	Eu:UK	1967	pr	718	n	V	n	n	2	all/unsp	nev any	ot
KJUUS	1	m	0	0	all	-	all	Eu:Sca	1979	CC	176	n	bl	n	n	0	all/unsp	nev any	st
KNEKT	86	m	20	69	all	21	all	Eu:Sca	1966	pr	515	n	bl	n	n	1	all/unsp	nev any	ot
KOO	9	f	0	0	all	-	all	As:HK	1981	CC	200	n	bl	n	n	0	all/unsp	nev any	st
KREUZE	39	m	1	45	all	-	all	Eu:Ger	1990	CC	2260	n	bl	n	n	0	all/unsp	nev any	st
KREUZE	41	m	55	69	all	-	all	Eu:Ger	1990	CC	2260	n	bl	n	n	0	all/unsp	nev any	st
KREUZE	40	f	1	45	all	-	all	Eu:Ger	1990	CC	2260	n	bl	n	n	0	all/unsp	nev any	st
KREUZE	42	f	55	69	all	-	all	Eu:Ger	1990	CC	2260	n	bl	n	n	0	all/unsp	nev any	st
KUBIK	12	m	0	0	all	0	all	Eu:est	1965	pr	108	n	bl	n	n	0	cig+/-ot	nev any	st
LANGE	38	m	0	0	all	0	all	Eu:Sca	1976	pr	268	n	bl	n	n	1	all/unsp	nev any	ot
LANGE	35	f	0	0	all	0	all	Eu:Sca	1976	pr	268	n	bl	n	n	1	all/unsp	nev any	ot
LEMARC	2	c	0	0	w+o	-	all	NAmer	1992	CC	341	n	bl	n	y	0	all/unsp	nev any	st
LIAW	1	m	0	0	all	0	all	As:oth	1982	pr	127	n	ot	n	n	1	all/unsp	nev any	or
LIAW	2	f	0	0	all	0	all	As:oth	1982	pr	127	n	ot	n	n	1	all/unsp	nev any	or
LIDDEL	4	m	0	0	all	18	all	NAmer	1970	pr	304	m	V	n	n	1	cig+/-ot	nev cigs	ot
LOMBAR	9	m	0	0	all	-	all	NAmer	1951	CC	1040	n	bl	n	n	0	cig+/-ot	nev any	st
LUBIN	40	m	0	0	all	-	all	As:Chi	1984	CC	427	m	ot	y	n	0	cig+/-ot	nev any	st
LUBIN2	26	m	0	0	all	-	all	Eu:mul	1976	CC	7804	n	bl	n	y	2	all/unsp	nev any	ot
LUBIN2	317	f	0	0	all	-	all	Eu:mul	1976	CC	7804	n	bl	n	y	0	cig+/-ot	nev any	st
MACLEN	19	m	0	0	ch	-	all	As:oth	1972	CC	233	n	bl	n	n	0	cig+/-ot	nev cigs	st
MACLEN	32	f	0	0	ch	-	all	As:oth	1972	CC	233	n	bl	n	n	0	cig+/-ot	nev cigs	st
MATOS	3	m	0	0	all	-	all	SCAmer	1994	CC	200	n	bl	n	n	2	cig+/-ot	nev any	or
MIGRAN	20	m	0	0	all	0	all	Eu:UK	1964	pr	259	n	V	n	n	2	all/unsp	nev any	ot
MIGRAN	136	f	0	0	all	0	all	Eu:UK	1964	pr	259	n	V	n	n	2	all/unsp	nev any	ot
MRFITR	2	m	0	0	all	0	all	NAmer	1973	pr	119	n	bl	n	n	0	cig+/-ot	nev cigs	ot
NAM	76	m	0	0	all	-	all	NAmer	1986	CC	1199	n	bl	y	n	1	cig+/-ot	nev cigs	ot
NAM	92	f	0	0	all	-	all	NAmer	1986	CC	1199	n	bl	y	n	1	cig+/-ot	nev cigs	ot
ODRISC	1	c	0	0	all	-	all	Eu:UK	1992	CC	446	n	V	n	n	0	all/unsp	nev any	st
OSANN	33	m	0	0	all	-	all	NAmer	1984	CC	1986	n	bl	n	n	2	cig+/-ot	nev cigs	or
OSANN	34	f	0	0	all	-	all	NAmer	1984	CC	1986	n	bl	n	n	2	cig+/-ot	nev cigs	or

Table 1B1 - 1

IESLC - Meta-analysis of Current Smoking (vs never smoking), Any product (or Cigarettes if Any not available)
All LC types
Most adjusted

REF	NRR	SEX	AGEL	AGEH	RACE	YF	LC TYPE	LOC	START	ST	NLC	R	VB	P	H	AD	PRODUCT	DENOM	De
PARKIN	26	m	0	0	bl	-	all	Africa	1963	CC	877	n	V	y	n	6	all/unsp	nev any	ot
PERSH2	10	c	0	0	all	-	all	Eu:Sca	1980	CC	1022	n	bl	y	n	4	all/unsp	nev any	ot
PETO	4	m	0	0	all	0	all	Eu:UK	1954	pr	103	n	V	n	n	0	all/unsp	nev any	st
PEZZO2	2	m	0	0	all	-	all	SCAmer	1992	CC	367	n	bl	n	y	0	cig+/-ot	nev cigs	st
PEZZOT	5	m	0	0	all	-	all	SCAmer	1987	CC	215	n	bl	n	y	0	cig only	nev cigs	st
QIAO2	14	m	0	0	all	0	all	As:Chi	1992	pr	241	m	ot	n	n	1	all/unsp	nev any	or
RACHTA	9	f	0	0	all	-	all	Eu:est	1991	CC	118	n	bl	n	y	1	cig+/-ot	nev cigs	or
SCHWAR	25	m	0	0	wh	-	all	NAmer	1984	CC	5588	n	bl	y	y	0	cig+/-ot	nev cigs	st
SCHWAR	26	m	0	0	bl	-	all	NAmer	1984	CC	5588	n	bl	y	y	0	cig+/-ot	nev cigs	st
SCHWAR	27	f	0	0	wh	-	all	NAmer	1984	CC	5588	n	bl	y	y	0	cig+/-ot	nev cigs	st
SCHWAR	28	f	0	0	bl	-	all	NAmer	1984	CC	5588	n	bl	y	y	0	cig+/-ot	nev cigs	st
SEGI2	20	m	0	0	all	-	all	As:Jap	1962	CC	378	n	bl	n	n	1	cig+/-ot	nev any	ot
SEGI2	28	f	0	0	all	-	all	As:Jap	1962	CC	378	n	bl	n	n	1	cig+/-ot	nev any	ot
SHAW	6	c	0	0	wh	-	all	NAmer	1988	CC	335	n	V	n	y	0	all/unsp	nev any	st
SOBUE	42	m	0	0	all	-	q+s+l+a	As:Jap	1986	CC	1376	n	bl	n	y	1	cig+/-ot	nev cigs	or
SOBUE	52	f	0	0	all	-	q+s+l+a	As:Jap	1986	CC	1376	n	bl	n	y	1	cig+/-ot	nev cigs	or
SOBUE2	10	m	0	0	all	-	q+s+l+a	As:Jap	1965	CC	2083	n	bl	n	n	2	cig+/-ot	nev any	ot
SOBUE2	12	f	0	0	all	-	q+s+l+a	As:Jap	1965	CC	2083	n	bl	n	n	2	cig+/-ot	nev any	ot
SPEIZE	10	f	0	0	all	0	all	NAmer	1976	pr	593	n	bl	n	y	1	cig+/-ot	nev cigs	ot
SPITZ	2	c	0	0	b+hi	-	all	NAmer	1992	CC	177	n	bl	n	y	0	cig+/-ot	nev cigs	st
STOCKW	7	c	0	0	all	-	all	NAmer	1981	CC	22161	n	bl	n	n	0	cig+/-ot	nev any	st
STUCKE	2	m	0	0	all	-	all	Eu:wst	1989	CC	247	n	bl	n	y	0	all/unsp	nev any	ot
SUZUK2	6	c	0	0	all	-	all	SCAmer	1991	CC	123	n	bl	n	y	3	all/unsp	nev any	or
SVENSS	96	f	0	0	all	-	all	Eu:Sca	1983	CC	210	n	bl	n	n	1	all/unsp	nev any	ot
TANG	1	c	0	0	all	-	not s	NAmer	1992	CC	119	n	bl	n	y	0	cig+/-ot	nev cigs	st
TENKAN	24	m	0	0	all	17	all	Eu:Sca	1962	pr	242	n	bl	n	n	1	all/unsp	nev any	ot
TIZZAN	5	m	0	0	all	-	all	Eu:wst	1959	CC	1358	n	bl	n	n	0	all/unsp	nev any	st
TIZZAN	13	f	0	0	all	-	all	Eu:wst	1959	CC	1358	n	bl	n	n	0	all/unsp	nev any	st
TOKARS	1	m	0	0	all	-	all	Eu:est	1966	ot	162	o	bl	n	y	0	all/unsp	nev any	st
TOUSEY	12	m	0	0	all	-	all	NAmer	1993	CC	507	n	bl	y	y	3	cig+/-ot	nev any	or
TOUSEY	15	f	0	0	all	-	all	NAmer	1993	CC	507	n	bl	y	y	3	cig+/-ot	nev any	or
TSUGAN	28	m	0	0	all	-	q+a	As:Jap	1976	CC	134	n	bl	n	y	0	all/unsp	nev any	st
TULINI	37	m	0	0	all	0	all	Eu:Sca	1967	pr	472	n	bl	n	n	3	all/unsp	nev any	ot
TULINI	43	f	0	0	all	0	all	Eu:Sca	1967	pr	472	n	bl	n	n	3	all/unsp	nev any	ot
TVERDA	5	m	0	0	all	0	all	Eu:Sca	1972	pr	238	n	bl	n	n	2	cig+/-ot	nev cigs	ot
TVERDA	15	f	0	0	all	0	all	Eu:Sca	1972	pr	238	n	bl	n	n	2	cig only	nev cigs	ot
WAKAI	8	m	0	0	all	-	all	As:Jap	1988	CC	333	n	bl	n	y	2	all/unsp	nev any	or
WAKAI	26	f	0	0	all	-	all	As:Jap	1988	CC	333	n	bl	n	y	2	all/unsp	nev any	or
WALD	4	m	0	0	all	0	all	Eu:UK	1975	pr	102	n	V	n	n	1	cig only	nev any	or
WANG2	18	c	0	0	all	-	all	As:Chi	1980	CC	103	n	ot	n	n	4	cig+/-ot	nev cigs	ot
WIGLE	25	m	0	0	all	-	all	NAmer	1971	CC	728	n	V	n	n	1	all/unsp	nev any	ot
WIGLE	30	f	0	0	all	-	all	NAmer	1971	CC	728	n	V	n	n	1	all/unsp	nev any	ot
WU	42	f	0	0	wh	-	q+a	NAmer	1981	CC	220	n	bl	n	y	2	all/unsp	nev any	st
WUNSCH	5	m	0	0	all	-	all	SCAmer	1990	CC	398	n	bl	y	n	1	cig+/-ot	nev any	or
WUNSCH	11	f	0	0	all	-	all	SCAmer	1990	CC	398	n	bl	y	n	1	cig+/-ot	nev any	or
WYNDE3	50	m	0	0	all	-	all	NAmer	1966	CC	350	n	bl	n	y	0	all/unsp	nev any	st
WYNDE6	18	m	0	0	all	-	all	NAmer	1969	CC	4423	n	bl	n	y	0	cig+/-ot	nev any	st
WYNDE6	207	f	0	0	all	-	all	NAmer	1969	CC	4423	n	bl	n	y	0	cig+/-ot	nev cigs	st
YAMAGU	10	c	0	0	all	-	all	As:Jap	1989	CC	144	n	bl	n	y	1	all/unsp	nev any	ot
YONG	12	m	0	0	all	0	all	NAmer	1971	pr	216	n	bl	n	n	1	cig+/-ot	nev cigs	or
YONG	15	f	0	0	all	0	all	NAmer	1971	pr	216	n	bl	n	n	1	cig+/-ot	nev cigs	or

Cigarette type is all/unspec for all RRs
except for the following:

REF	NRR	CIGTYPE
ALDERS	177	MC+-HR
ALDERS	176	MC only
DEAN3	119	MC only

Table 1B1 - 2

IESLC - Meta-analysis of Current Smoking (vs never smoking), Any product (or Cigarettes if Any not available)
All LC types
Most adjusted

REF	NRR	SEX	AD	Number Exposed Case	Number Exposed Cont	Non-exposed Case	Non-exposed Cont	RR	95.00%CI
AGUDO	3	f	3	-	-	-	-	3.61	(1.57- 8.32)
*AKIBA	10	m	5	-	-	-	-	5.10	(3.30- 7.90)
*AKIBA	14	f	5	-	-	-	-	3.90	(2.90- 5.30)
Subtotal AKIBA								4.25	(3.32- 5.45)
ALDERS	177	m	0	519	322	15	133	14.29	(8.23- 24.81)
ALDERS	176	f	0	410	229	75	243	5.80	(4.27- 7.87)
Subtotal ALDERS								7.17	(5.49- 9.36)
*AMANDU	5	m	2	-	-	-	-	6.54	(2.52- 16.98)
AMES	1	m	0	150	136	15	62	4.56	(2.48- 8.39)
*ANDERS	6	f	1	-	-	-	-	23.43	(17.02- 32.27)
*ARCHER	5	m	0	122	32529	6	9842	6.15	(2.71- 13.96)
ARMADA	27	m	0	188	122	4	64	24.66	(8.75- 69.44)
AUSTIN	6	c	3	-	-	-	-	19.60	(6.70- 57.00)
AXELSS	2	m	0	194	130	16	160	14.92	(8.53- 26.12)
AXELSS	10	f	0	96	69	18	154	11.90	(6.68- 21.22)
Subtotal AXELSS								13.38	(8.95- 20.00)
BARBON	4	m	1	-	-	-	-	13.40	(8.50- 21.40)
BECHER	13	m	0	101	122	3	54	14.90	(4.52- 49.09)
BECHER	14	f	0	33	26	10	52	6.60	(2.82- 15.44)
Subtotal BECHER								8.68	(4.35- 17.35)
*BENSHL	16	m	1	-	-	-	-	8.18	(3.62- 18.51)
*BEST	2	m	1	-	-	-	-	14.91	(7.05- 31.52)
BLOHMK	1	m	0	419	313	126	301	3.20	(2.48- 4.12)
*BOUCOT	114	m	2	-	-	-	-	62.29	(3.86-1004.01)
*BRETT	4	m	0	135	37448	6	6530	3.92	(1.73- 8.88)
BROSS	11	m	0	690	638	38	170	4.84	(3.35- 6.99)
BROWN2	12	m	2	-	-	-	-	11.30	(10.20- 12.40)
BROWN2	11	f	2	-	-	-	-	13.60	(12.30- 15.10)
Subtotal BROWN2								12.34	(11.50- 13.25)
BUFFLE	3	m	0	257	219	5	47	11.03	(4.31- 28.22)
BUFFLE	7	f	0	313	183	41	198	8.26	(5.63- 12.11)
Subtotal BUFFLE								8.61	(6.04- 12.27)
CARPEN	11	c	3	-	-	-	-	23.03	(12.96- 40.85)
*CEDERL	106	m	2	-	-	-	-	7.72	(5.01- 11.89)
*CEDERL	75	f	2	-	-	-	-	4.82	(3.38- 6.88)
Subtotal CEDERL								5.83	(4.43- 7.67)
*CHANG	5	m	0	35	419	5	502	8.39	(3.32- 21.21)
*CHANG	11	f	0	30	603	11	1139	5.15	(2.60- 10.21)
Subtotal CHANG								6.12	(3.53- 10.60)
CHOI	3	m	0	232	329	13	95	5.15	(2.82- 9.42)
CHOI	7	f	0	13	23	76	164	1.22	(0.59- 2.54)
Subtotal CHOI								2.88	(1.81- 4.58)
*CHOW	25	m	0	167	124415	6	62913	14.07	(6.23- 31.78)
*CHYOU	2	m	1	-	-	-	-	11.40	(6.50- 20.10)
COMSTO	3	m	0	105	100	4	69	18.11	(6.37- 51.48)
COMSTO	8	f	0	77	52	13	115	13.10	(6.68- 25.67)
Subtotal COMSTO								14.40	(8.18- 25.36)
CORREA	42	c	1	-	-	-	-	14.20	(10.80- 18.70)
*CPSI	220	m	1	-	-	-	-	11.94	(9.52- 14.97)
*CPSI	279	f	1	-	-	-	-	3.20	(2.53- 4.04)
Subtotal CPSI								6.32	(5.37- 7.43)
*CPSII	126	m	1	-	-	-	-	20.25	(16.37- 25.05)
*CPSII	133	f	1	-	-	-	-	11.78	(10.14- 13.68)
Subtotal CPSII								14.10	(12.47- 15.93)
DAMBER	14	m	1	-	-	-	-	9.60	(6.60- 14.20)
DARBY	4	m	0	322	453	3	384	90.98	(28.96- 285.90)
DARBY	11	f	0	195	217	23	529	20.67	(13.05- 32.74)
Subtotal DARBY								25.40	(16.57- 38.92)
DEAN2	2	m	0	671	600	33	112	3.80	(2.54- 5.68)
DEAN2	6	f	0	59	28	88	121	2.90	(1.71- 4.91)
Subtotal DEAN2								3.43	(2.49- 4.73)
DEAN3	42	m	3	-	-	-	-	6.72	(4.28- 10.55)
DEAN3	119	f	3	-	-	-	-	5.77	(3.75- 8.86)
Subtotal DEAN3								6.20	(4.54- 8.47)
*DEKLER	8	m	2	-	-	-	-	23.03	(3.21- 164.97)
DESTE2	4	c	7	-	-	-	-	9.10	(5.20- 15.90)
DESTEF	41	m	4	-	-	-	-	10.90	(6.90- 17.10)
*DOCKER	1	c	4	-	-	-	-	8.00	(2.97- 21.60)
DOLL	90	m	0	1280	1172	7	61	9.52	(4.34- 20.89)
DOLL	93	f	0	58	41	40	59	2.09	(1.18- 3.68)
Subtotal DOLL								3.51	(2.21- 5.55)

Table 1B1 - 2

IESLC - Meta-analysis of Current Smoking (vs never smoking), Any product (or Cigarettes if Any not available)
All LC types
Most adjusted

REF	NRR	SEX	AD	Number Exposed Case	Number Exposed Cont	Non-exposed Case	Non-exposed Cont	RR	95.00%CI
*DOLL2	54	m	1	-	-	-	-	10.99	(6.97- 17.36)
*DOLL2	63	f	1	-	-	-	-	8.65	(2.93- 25.55)
Subtotal DOLL2								10.60	(6.96- 16.14)
DORANT	2	m	0	332	697	7	159	10.82	(5.02- 23.32)
DORGAN	9	m	0	464	170	15	93	16.92	(9.54- 30.01)
DORGAN	33	m	0	214	61	3	35	40.93	(12.17- 137.66)
DORGAN	56	f	0	611	119	103	244	12.16	(8.99- 16.46)
DORGAN	79	f	0	68	17	7	20	11.43	(4.16- 31.43)
Subtotal DORGAN								13.62	(10.58- 17.54)
*DORN	51	m	1	-	-	-	-	8.23	(6.55- 10.35)
DROSTE	6	m	4	-	-	-	-	14.50	(6.30- 33.40)
*ENGELA	158	m	1	-	-	-	-	7.37	(3.42- 15.86)
*ENGELA	164	f	1	-	-	-	-	5.78	(2.68- 12.46)
Subtotal ENGELA								6.53	(3.79- 11.23)
*ENSTRO	1	m	1	-	-	-	-	12.99	(10.46- 16.13)
*ENSTRO	2	f	1	-	-	-	-	6.95	(6.01- 8.04)
Subtotal ENSTRO								8.44	(7.48- 9.53)
GAO	33	m	2	-	-	-	-	3.90	(2.90- 5.40)
GAO	34	f	2	-	-	-	-	2.90	(2.20- 3.80)
Subtotal GAO								3.30	(2.69- 4.05)
GAO2	8	m	1	-	-	-	-	6.61	(3.47- 12.58)
GARCIA	2	c	0	169	74	21	139	15.12	(8.86- 25.79)
GARDIN	2	c	0	97	58	5	41	13.71	(5.13- 36.68)
GARSHI	31	m	1	-	-	-	-	7.70	(5.48- 10.83)
GOODMA	2	m	0	148	169	10	199	17.43	(8.90- 34.14)
GOODMA	6	f	0	58	56	19	177	9.65	(5.30- 17.56)
Subtotal GOODMA								12.53	(8.01- 19.60)
GRAHAM	25	m	1	-	-	-	-	6.06	(3.78- 9.70)
GREGOR	2	m	0	49	53	10	14	1.29	(0.53- 3.18)
GREGOR	6	f	0	17	26	1	22	14.38	(1.77- 116.90)
Subtotal GREGOR								1.88	(0.82- 4.30)
HAENSZ	54	f	0	69	94	81	236	2.14	(1.43- 3.19)
*HAMMO2	8	m	1	-	-	-	-	10.14	(4.19- 24.55)
*HAMMON	139	m	1	-	-	-	-	11.52	(6.83- 19.42)
*HEIN	5	m	0	132	3492	1	457	17.27	(2.42- 123.25)
*HENNEK	2	m	0	79	2438	23	10919	15.38	(9.69- 24.42)
*HIRAYA	1	m	1	-	-	-	-	4.45	(3.60- 5.50)
*HIRAYA	3	f	1	-	-	-	-	2.34	(1.87- 2.92)
Subtotal HIRAYA								3.28	(2.81- 3.82)
HITOSU	34	m	1	-	-	-	-	2.79	(1.27- 6.09)
HITOSU	59	f	1	-	-	-	-	3.09	(1.82- 5.27)
Subtotal HITOSU								2.99	(1.93- 4.65)
*HOLE	33	m	1	-	-	-	-	8.10	(3.80- 17.26)
*HOLE	31	f	1	-	-	-	-	1.53	(0.64- 3.70)
Subtotal HOLE								3.98	(2.24- 7.06)
HUMBLE	13	m	1	-	-	-	-	19.96	(8.27- 48.21)
HUMBLE	15	m	1	-	-	-	-	15.79	(3.43- 72.69)
HUMBLE	17	f	1	-	-	-	-	16.72	(7.44- 37.61)
HUMBLE	19	f	1	-	-	-	-	23.50	(6.79- 81.36)
Subtotal HUMBLE								18.65	(11.23- 30.97)
JAHN	5	m	0	352	269	18	138	10.03	(5.99- 16.81)
JAIN	52	m	2	-	-	-	-	12.40	(6.45- 26.60)
JAIN	51	f	2	-	-	-	-	16.80	(9.93- 30.60)
Subtotal JAIN								14.94	(9.61- 23.21)
JARVHO	2	m	0	73	29	1	16	40.28	(5.10- 317.77)
JARVHO	6	f	0	31	7	6	21	15.50	(4.56- 52.66)
Subtotal JARVHO								19.86	(6.93- 56.89)
JOLY	18	m	0	487	665	12	218	13.30	(7.35- 24.07)
JOLY	15	f	0	132	96	52	283	7.48	(5.04- 11.12)
Subtotal JOLY								8.94	(6.43- 12.42)
*KAISE2	68	m	1	-	-	-	-	8.04	(4.41- 14.66)
*KAISE2	60	f	1	-	-	-	-	14.48	(7.47- 28.04)
Subtotal KAISE2								10.49	(6.72- 16.36)
*KAISER	12	m	2	-	-	-	-	19.61	(13.32- 28.87)
*KAISER	9	f	2	-	-	-	-	6.53	(4.50- 9.48)
Subtotal KAISER								11.09	(8.48- 14.50)
KANELL	30	m	1	-	-	-	-	4.94	(3.47- 7.03)
KATSOU	2	f	1	-	-	-	-	3.40	(1.75- 6.61)
KAUFMA	16	c	6	-	-	-	-	20.63	(14.18- 30.01)
KELLER	1	m	0	5063	1210	323	1017	13.17	(11.45- 15.15)
KELLER	9	m	0	1053	212	38	117	15.29	(10.31- 22.69)

Table 1B1 - 2

IESLC - Meta-analysis of Current Smoking (vs never smoking), Any product (or Cigarettes if Any not available)
All LC types
Most adjusted

REF	NRR	SEX	AD	Number Exposed Case	Number Exposed Cont	Non-exposed Case	Non-exposed Cont	RR	95.00%CI
KELLER	5	f	0	2904	792	469	1860	14.54	(12.79- 16.53)
KELLER	13	f	0	454	135	67	232	11.64	(8.35- 16.24)
Subtotal KELLER								13.79	(12.62- 15.07)
KHUDER	19	m	0	275	-	23	-	8.10	(5.20- 12.70)
KIHARA	7	c	0	283	162	102	237	4.06	(3.00- 5.49)
*KINLEN	20	m	2	-	-	-	-	12.89	(6.14- 27.06)
KJUUS	1	m	0	135	77	2	24	21.04	(4.84- 91.45)
*KNEKT	86	m	1	-	-	-	-	8.86	(3.87- 20.27)
KOO	9	f	0	42	25	56	85	2.55	(1.40- 4.64)
KREUZE	39	m	0	168	99	6	54	15.27	(6.34- 36.79)
KREUZE	41	m	0	1252	524	23	403	41.86	(27.17- 64.51)
KREUZE	40	f	0	55	23	6	38	15.14	(5.63- 40.72)
KREUZE	42	f	0	170	54	95	177	5.87	(3.95- 8.70)
Subtotal KREUZE								14.48	(11.09- 18.90)
*KUBIK	12	m	0	98	6342	2	4271	33.00	(8.14- 133.74)
*LANGE	38	m	1	-	-	-	-	5.70	(2.13- 15.27)
*LANGE	35	f	1	-	-	-	-	5.02	(2.52- 10.01)
Subtotal LANGE								5.23	(2.98- 9.21)
LEMARC	2	c	0	167	65	32	168	13.49	(8.39- 21.68)
*LIAW	1	m	1	-	-	-	-	3.70	(2.10- 6.60)
*LIAW	2	f	1	-	-	-	-	3.60	(1.00- 12.20)
Subtotal LIAW								3.68	(2.19- 6.20)
*LIDDEL	4	m	1	-	-	-	-	4.41	(2.77- 7.01)
LOMBAR	9	m	0	852	610	14	112	11.17	(6.35- 19.66)
LUBIN	40	m	0	296	650	9	72	3.64	(1.80- 7.38)
LUBIN2	26	m	2	-	-	-	-	10.67	(9.14- 12.46)
LUBIN2	317	f	0	384	410	288	1180	3.84	(3.17- 4.64)
Subtotal LUBIN2								7.09	(6.28- 7.99)
MACLEN	19	m	0	137	108	5	15	3.81	(1.34- 10.80)
MACLEN	32	f	0	42	47	41	109	2.38	(1.37- 4.12)
Subtotal MACLEN								2.63	(1.62- 4.28)
MATOS	3	m	2	-	-	-	-	8.50	(4.30- 16.70)
*MIGRAN	20	m	2	-	-	-	-	3.93	(1.46- 10.60)
*MIGRAN	136	f	2	-	-	-	-	4.99	(1.76- 14.16)
Subtotal MIGRAN								4.40	(2.15- 9.03)
*MRFITR	2	m	0	106	8194	0	1859	48.33~	(3.01- 777.42)
NAM	76	m	1	-	-	-	-	8.67	(5.66- 13.26)
NAM	92	f	1	-	-	-	-	10.84	(7.26- 16.18)
Subtotal NAM								9.76	(7.29- 13.07)
ODRISC	1	c	0	293	598	6	664	54.22	(23.98- 122.60)
OSANN	33	m	2	-	-	-	-	26.50	(19.20- 36.50)
OSANN	34	f	2	-	-	-	-	19.60	(15.20- 25.20)
Subtotal OSANN								22.00	(18.03- 26.83)
PARKIN	26	m	6	-	-	-	-	4.08	(3.18- 5.23)
PERSH2	10	c	4	-	-	-	-	8.29	(6.86- 10.02)
*PETO	4	m	0	99	2036	2	295	7.17	(1.78- 28.92)
PEZZO2	2	m	0	233	198	6	117	22.95	(9.89- 53.26)
PEZZOT	5	m	0	145	129	4	116	32.60	(11.70- 90.81)
*QIAO2	14	m	1	-	-	-	-	1.59	(0.84- 3.01)
RACHTA	9	f	1	-	-	-	-	6.77	(3.71- 12.35)
SCHWAR	25	m	0	1652	349	119	376	14.96	(11.81- 18.94)
SCHWAR	26	m	0	644	139	50	104	9.64	(6.56- 14.15)
SCHWAR	27	f	0	1029	309	182	855	15.64	(12.75- 19.19)
SCHWAR	28	f	0	256	90	40	247	17.56	(11.64- 26.50)
Subtotal SCHWAR								14.70	(12.84- 16.83)
SEGI2	20	m	1	-	-	-	-	3.74	(1.75- 8.00)
SEGI2	28	f	1	-	-	-	-	1.65	(0.90- 3.02)
Subtotal SEGI2								2.27	(1.41- 3.64)
SHAW	6	c	0	212	97	11	107	21.26	(10.93- 41.36)
SOBUE	42	m	1	-	-	-	-	4.10	(2.80- 5.90)
SOBUE	52	f	1	-	-	-	-	2.80	(2.00- 3.90)
Subtotal SOBUE								3.32	(2.59- 4.26)
SOBUE2	10	m	2	-	-	-	-	4.47	(3.89- 5.14)
SOBUE2	12	f	2	-	-	-	-	3.28	(2.79- 3.87)
Subtotal SOBUE2								3.92	(3.53- 4.36)
*SPEIZE	10	f	1	-	-	-	-	12.69	(9.97- 16.16)
SPITZ	2	c	0	103	89	7	128	21.16	(9.40- 47.66)
STOCKW	7	c	0	12470	3357	2791	10641	14.16	(13.38- 14.99)
STUCKE	2	m	0	69	68	0	51	104.50~	(6.32-1727.39)
SUZUK2	6	c	3	-	-	-	-	22.00	(6.50- 76.00)
SVENSS	96	f	1	-	-	-	-	9.06	(5.31- 15.48)

Table 1B1 - 2

IESLC - Meta-analysis of Current Smoking (vs never smoking), Any product (or Cigarettes if Any not available)
All LC types
Most adjusted

REF	NRR	SEX	AD	Number Exposed Case	Number Exposed Cont	Non-exposed Case	Non-exposed Cont	RR	95.00%CI
TANG	1	c	0	52	25	9	39	9.01	(3.78- 21.46)
*TENKAN	24	m	1	-	-	-	-	16.81	(7.22- 39.14)
TIZZAN	5	m	0	693	619	180	305	1.90	(1.53- 2.35)
TIZZAN	13	f	0	17	18	25	114	4.31	(1.95- 9.51)
Subtotal TIZZAN								2.01	(1.63- 2.47)
TOKARS	1	m	0	110	157	1	53	37.13	(5.06- 272.56)
TOUSEY	12	m	3	-	-	-	-	59.20	(21.00- 167.30)
TOUSEY	15	f	3	-	-	-	-	30.20	(16.00- 57.40)
Subtotal TOUSEY								36.34	(21.09- 62.60)
TSUGAN	28	m	0	63	63	18	22	1.22	(0.60- 2.50)
*TULINI	37	m	3	-	-	-	-	9.99	(5.42- 18.40)
*TULINI	43	f	3	-	-	-	-	16.24	(9.02- 29.25)
Subtotal TULINI								12.86	(8.41- 19.64)
*TVERDA	5	m	2	-	-	-	-	4.09	(2.65- 6.31)
*TVERDA	15	f	2	-	-	-	-	11.05	(3.33- 36.71)
Subtotal TVERDA								4.59	(3.05- 6.90)
WAKAI	8	m	2	-	-	-	-	4.40	(2.19- 8.85)
WAKAI	26	f	2	-	-	-	-	4.37	(2.21- 8.62)
Subtotal WAKAI								4.38	(2.69- 7.14)
*WALD	4	m	1	-	-	-	-	16.40	(7.55- 44.20)
WANG2	18	c	4	-	-	-	-	2.40	(1.14- 5.05)
WIGLE	25	m	1	-	-	-	-	10.40	(6.07- 17.83)
WIGLE	30	f	1	-	-	-	-	5.20	(3.34- 8.08)
Subtotal WIGLE								6.87	(4.88- 9.67)
WU	42	f	2	-	-	-	-	4.86	(2.76- 8.57)
WUNSCH	5	m	1	-	-	-	-	6.59	(3.59- 12.10)
WUNSCH	11	f	1	-	-	-	-	5.98	(3.25- 11.00)
Subtotal WUNSCH								6.28	(4.08- 9.66)
WYNDE3	50	m	0	227	207	9	88	10.72	(5.26- 21.84)
WYNDE6	18	m	0	1677	741	87	617	16.05	(12.62- 20.41)
WYNDE6	207	f	0	1022	376	159	856	14.63	(11.90- 17.99)
Subtotal WYNDE6								15.22	(13.01- 17.80)
YAMAGU	10	c	1	-	-	-	-	4.90	(2.55- 9.44)
*YONG	12	m	1	-	-	-	-	28.71	(6.98- 118.16)
*YONG	15	f	1	-	-	-	-	5.20	(2.38- 11.35)
Subtotal YONG								7.75	(3.91- 15.36)
Partial Totals				45455	238932	6402	125529		

*prospective study ~ With 0.5 adjustment for zero

REF	NRR	SEX	AD	Ys	Ws	Qs	Ps
AGUDO	3	f	3	1.28	5.53	4.94	0.0025
*AKIBA	10	m	5	1.63	20.16	7.25	0.0000
*AKIBA	14	f	5	1.36	42.26	31.84	0.0000
Subtotal AKIBA				1.45	62.42	39.10	
ALDERS	177	m	0	2.66	12.62	2.34	0.0000
ALDERS	176	f	0	1.76	41.23	9.15	0.0000
Subtotal ALDERS				1.97	53.85	11.49	
*AMANDU	5	m	2	1.88	4.22	0.52	0.0001
AMES	1	m	0	1.52	10.33	5.24	0.0000
*ANDERS	6	f	1	3.15	37.54	32.12	0.0000
*ARCHER	5	m	0	1.82	5.72	0.97	0.0000
ARMADA	27	m	0	3.21	3.58	3.41	0.0000
AUSTIN	6	c	3	2.98	3.35	1.87	0.0000
AXELSS	2	m	0	2.70	12.26	2.75	0.0000
AXELSS	10	f	0	2.48	11.50	0.71	0.0000
Subtotal AXELSS				2.59	23.76	3.46	
BARBON	4	m	1	2.60	18.02	2.42	0.0000
BECHER	13	m	0	2.70	2.70	0.60	0.0000
BECHER	14	f	0	1.89	5.32	0.62	0.0000
Subtotal BECHER				2.16	8.02	1.23	
*BENSHL	16	m	1	2.10	5.77	0.09	0.0000
*BEST	2	m	1	2.70	6.85	1.53	0.0000
BLOHMK	1	m	0	1.16	59.38	67.55	0.0000
*BOUCOT	114	m	2	4.13	0.50	1.80	0.0036
*BRETT	4	m	0	1.37	5.75	4.27	0.0010
BROSS	11	m	0	1.58	28.40	12.09	0.0000
BROWN2	12	m	2	2.42	402.82	15.44	0.0000
BROWN2	11	f	2	2.61	365.29	53.03	0.0000
Subtotal BROWN2				2.51	768.12	68.47	
BUFFLE	3	m	0	2.40	4.35	0.13	0.0000

Table 1B1 - 2

IESLC - Meta-analysis of Current Smoking (vs never smoking), Any product (or Cigarettes if Any not available)
All LC types
Most adjusted

REF	NRR	SEX	AD	Ys	Ws	Qs	Ps
BUFFLE	7	f	0	2.11	26.25	0.36	0.0000
Subtotal BUFFLE				2.15	30.60	0.49	
CARPEN	11	c	3	3.14	11.66	9.61	0.0000
*CEDERL	106	m	2	2.04	20.57	0.71	0.0000
*CEDERL	75	f	2	1.57	30.42	13.10	0.0000
Subtotal CEDERL				1.76	50.99	13.81	
*CHANG	5	m	0	2.13	4.46	0.05	0.0000
*CHANG	11	f	0	1.64	8.22	2.86	0.0000
Subtotal CHANG				1.81	12.68	2.90	
CHOI	3	m	0	1.64	10.55	3.67	0.0000
CHOI	7	f	0	0.20	7.16	29.52	0.5951
Subtotal CHOI				1.06	17.71	33.19	
*CHOW	25	m	0	2.64	5.79	1.00	0.0000
*CHYOU	2	m	1	2.43	12.06	0.50	0.0000
COMSTO	3	m	0	2.90	3.52	1.57	0.0000
COMSTO	8	f	0	2.57	8.49	1.00	0.0000
Subtotal COMSTO				2.67	12.01	2.57	
CORREA	42	c	1	2.65	50.99	9.17	0.0000
*CPSI	220	m	1	2.48	74.99	4.72	0.0000
*CPSI	279	f	1	1.16	70.15	79.70	0.0000
Subtotal CPSI				1.84	145.14	84.42	
*CPSII	126	m	1	3.01	84.90	51.54	0.0000
*CPSII	133	f	1	2.47	171.36	9.65	0.0000
Subtotal CPSII				2.65	256.26	61.19	
DAMBER	14	m	1	2.26	26.18	0.03	0.0000
DARBY	4	m	0	4.51	2.93	15.26	0.0000
DARBY	11	f	0	3.03	18.15	11.60	0.0000
Subtotal DARBY				3.23	21.08	26.86	
DEAN2	2	m	0	1.33	23.59	18.91	0.0000
DEAN2	6	f	0	1.06	13.83	18.78	0.0001
Subtotal DEAN2				1.23	37.42	37.69	
DEAN3	42	m	3	1.91	18.88	1.98	0.0000
DEAN3	119	f	3	1.75	20.79	4.72	0.0000
Subtotal DEAN3				1.83	39.66	6.70	
*DEKLER	8	m	2	3.14	0.99	0.82	0.0018
DESTE2	4	c	7	2.21	12.30	0.01	0.0000
DESTEF	41	m	4	2.39	18.66	0.48	0.0000
*DOCKER	1	c	4	2.08	3.90	0.09	0.0000
DOLL	90	m	0	2.25	6.22	0.00	0.0000
DOLL	93	f	0	0.74	11.96	26.69	0.0110
Subtotal DOLL				1.25	18.18	26.69	
*DOLL2	54	m	1	2.40	18.45	0.52	0.0000
*DOLL2	63	f	1	2.16	3.28	0.02	0.0001
Subtotal DOLL2				2.36	21.73	0.54	
DORANT	2	m	0	2.38	6.51	0.15	0.0000
DORGAN	9	m	0	2.83	11.70	4.21	0.0000
DORGAN	33	m	0	3.71	2.61	5.74	0.0000
DORGAN	56	f	0	2.50	41.93	3.04	0.0000
DORGAN	79	f	0	2.44	3.75	0.16	0.0000
Subtotal DORGAN				2.61	60.00	13.15	
*DORN	51	m	1	2.11	73.41	1.08	0.0000
DROSTE	6	m	4	2.67	5.52	1.09	0.0000
*ENGELA	158	m	1	2.00	6.53	0.35	0.0000
*ENGELA	164	f	1	1.75	6.51	1.47	0.0000
Subtotal ENGELA				1.88	13.04	1.82	
*ENSTRO	1	m	1	2.56	81.91	9.20	0.0000
*ENSTRO	2	f	1	1.94	181.45	15.29	0.0000
Subtotal ENSTRO				2.13	263.36	24.49	
GAO	33	m	2	1.36	39.76	29.96	0.0000
GAO	34	f	2	1.06	51.44	69.74	0.0000
Subtotal GAO				1.19	91.20	99.70	
GAO2	8	m	1	1.89	9.26	1.07	0.0000
GARCIA	2	c	0	2.72	13.47	3.19	0.0000
GARDIN	2	c	0	2.62	3.97	0.60	0.0000
GARSHI	31	m	1	2.04	33.11	1.17	0.0000
GOODMA	2	m	0	2.86	8.50	3.36	0.0000
GOODMA	6	f	0	2.27	10.71	0.02	0.0000
Subtotal GOODMA				2.53	19.21	3.38	
GRAHAM	25	m	1	1.80	17.30	3.16	0.0000
GREGOR	2	m	0	0.26	4.75	18.44	0.5741
GREGOR	6	f	0	2.67	0.88	0.17	0.0126

Table 1B1 - 2

IESLC - Meta-analysis of Current Smoking (vs never smoking), Any product (or Cigarettes if Any not available)
All LC types
Most adjusted

REF	NRR	SEX	AD	Ys	Ws	Qs	Ps
Subtotal GREGOR				0.63	5.62	18.61	
HAENSZ	54	f	0	0.76	23.97	51.72	0.0002
*HAMMO2	8	m	1	2.32	4.92	0.04	0.0000
*HAMMON	139	m	1	2.44	14.07	0.65	0.0000
*HEIN	5	m	0	2.85	0.99	0.38	0.0045
*HENNEK	2	m	0	2.73	17.97	4.57	0.0000
*HIRAYA	1	m	1	1.49	85.55	46.36	0.0000
*HIRAYA	3	f	1	0.85	77.37	147.11	0.0000
Subtotal HIRAYA				1.19	162.92	193.47	
HITOSU	34	m	1	1.03	6.25	9.05	0.0103
HITOSU	59	f	1	1.13	13.59	16.47	0.0000
Subtotal HITOSU				1.10	19.85	25.52	
*HOLE	33	m	1	2.09	6.71	0.13	0.0000
*HOLE	31	f	1	0.43	4.99	16.24	0.3421
Subtotal HOLE				1.38	11.70	16.37	
HUMBLE	13	m	1	2.99	4.94	2.89	0.0000
HUMBLE	15	m	1	2.76	1.65	0.46	0.0004
HUMBLE	17	f	1	2.82	5.85	2.02	0.0000
HUMBLE	19	f	1	3.16	2.49	2.15	0.0000
Subtotal HUMBLE				2.93	14.94	7.52	
JAHN	5	m	0	2.31	14.42	0.08	0.0000
JAIN	52	m	2	2.52	7.65	0.64	0.0000
JAIN	51	f	2	2.82	12.13	4.26	0.0000
Subtotal JAIN				2.70	19.79	4.89	
JARVHO	2	m	0	3.70	0.90	1.94	0.0005
JARVHO	6	f	0	2.74	2.57	0.67	0.0000
Subtotal JARVHO				2.99	3.47	2.61	
JOLY	18	m	0	2.59	10.93	1.41	0.0000
JOLY	15	f	0	2.01	24.54	1.15	0.0000
Subtotal JOLY				2.19	35.47	2.56	
*KAISE2	68	m	1	2.08	10.65	0.22	0.0000
*KAISE2	60	f	1	2.67	8.78	1.73	0.0000
Subtotal KAISE2				2.35	19.43	1.95	
*KAISER	12	m	2	2.98	25.68	14.33	0.0000
*KAISER	9	f	2	1.88	27.68	3.44	0.0000
Subtotal KAISER				2.41	53.36	17.77	
KANELL	30	m	1	1.60	30.83	12.30	0.0000
KATSOU	2	f	1	1.22	8.70	8.79	0.0003
KAUFMA	16	c	6	3.03	27.34	17.40	0.0000
KELLER	1	m	0	2.58	195.95	23.90	0.0000
KELLER	9	m	0	2.73	24.67	6.13	0.0000
KELLER	5	f	0	2.68	233.82	46.92	0.0000
KELLER	13	f	0	2.45	34.67	1.77	0.0000
Subtotal KELLER				2.62	489.11	78.72	
KHUDER	19	m	0	2.09	19.27	0.36	0.0000
KIHARA	7	c	0	1.40	42.14	28.90	0.0000
*KINLEN	20	m	2	2.56	6.98	0.75	0.0000
KJUUS	1	m	0	3.05	1.78	1.19	0.0000
*KNEKT	86	m	1	2.18	5.60	0.01	0.0000
KOO	9	f	0	0.94	10.70	17.89	0.0022
KREUZE	39	m	0	2.73	4.97	1.23	0.0000
KREUZE	41	m	0	3.73	20.55	46.57	0.0000
KREUZE	40	f	0	2.72	3.93	0.94	0.0000
KREUZE	42	f	0	1.77	24.64	5.21	0.0000
Subtotal KREUZE				2.67	54.09	53.95	
*KUBIK	12	m	0	3.50	1.96	3.15	0.0000
*LANGE	38	m	1	1.74	3.96	0.95	0.0005
*LANGE	35	f	1	1.61	8.08	3.06	0.0000
Subtotal LANGE				1.66	12.04	4.01	
LEMARC	2	c	0	2.60	17.07	2.37	0.0000
*LIAW	1	m	1	1.31	11.72	9.93	0.0000
*LIAW	2	f	1	1.28	2.46	2.21	0.0447
Subtotal LIAW				1.30	14.17	12.14	
*LIDDEL	4	m	1	1.48	17.82	9.90	0.0000
LOMBAR	9	m	0	2.41	12.02	0.41	0.0000
LUBIN	40	m	0	1.29	7.70	6.75	0.0003
LUBIN2	26	m	2	2.37	160.04	3.06	0.0000
LUBIN2	317	f	0	1.34	106.80	83.51	0.0000
Subtotal LUBIN2				1.96	266.84	86.58	
MACLEN	19	m	0	1.34	3.53	2.81	0.0120
MACLEN	32	f	0	0.87	12.71	23.65	0.0020

Table 1B1 - 2

IESLC - Meta-analysis of Current Smoking (vs never smoking), Any product (or Cigarettes if Any not available)
All LC types
Most adjusted

REF	NRR	SEX	AD	Ys	Ws	Qs	Ps
Subtotal MACLEN				0.97	16.25	26.46	
MATOS	3	m	2	2.14	8.35	0.07	0.0000
*MIGRAN	20	m	2	1.37	3.91	2.89	0.0068
*MIGRAN	136	f	2	1.61	3.53	1.37	0.0025
Subtotal MIGRAN				1.48	7.44	4.26	
*MRFITR	2	m	0	3.88	0.50	1.35	0.0062
NAM	76	m	1	2.16	21.20	0.10	0.0000
NAM	92	f	1	2.38	23.93	0.57	0.0000
Subtotal NAM				2.28	45.13	0.67	
ODRISC	1	c	0	3.99	5.77	17.96	0.0000
OSANN	33	m	2	3.28	37.23	40.90	0.0000
OSANN	34	f	2	2.98	60.12	33.50	0.0000
Subtotal OSANN				3.09	97.36	74.40	
PARKIN	26	m	6	1.41	62.08	42.04	0.0000
PERSH2	10	c	4	2.12	107.04	1.39	0.0000
*PETO	4	m	0	1.97	1.98	0.13	0.0056
PEZZO2	2	m	0	3.13	5.42	4.43	0.0000
PEZZOT	5	m	0	3.48	3.66	5.77	0.0000
*QIAO2	14	m	1	0.46	9.43	29.40	0.1544
RACHTA	9	f	1	1.91	10.62	1.06	0.0000
SCHWAR	25	m	0	2.71	68.81	15.60	0.0000
SCHWAR	26	m	0	2.27	26.07	0.03	0.0000
SCHWAR	27	f	0	2.75	91.98	24.97	0.0000
SCHWAR	28	f	0	2.87	22.69	9.20	0.0000
Subtotal SCHWAR				2.69	209.54	49.80	
SEGI2	20	m	1	1.32	6.65	5.51	0.0007
SEGI2	28	f	1	0.50	10.48	31.32	0.1049
Subtotal SEGI2				0.82	17.14	36.82	
SHAW	6	c	0	3.06	8.67	5.94	0.0000
SOBUE	42	m	1	1.41	27.66	18.51	0.0000
SOBUE	52	f	1	1.03	34.45	49.57	0.0000
Subtotal SOBUE				1.20	62.11	68.08	
SOBUE2	10	m	2	1.50	197.90	105.94	0.0000
SOBUE2	12	f	2	1.19	143.51	155.59	0.0000
Subtotal SOBUE2				1.37	341.42	261.53	
*SPEIZE	10	f	1	2.54	65.88	6.40	0.0000
SPITZ	2	c	0	3.05	5.83	3.95	0.0000
STOCKW	7	c	0	2.65	1204.31	214.00	0.0000
STUCKE	2	m	0	4.65	0.49	2.86	0.0012
SUZUK2	6	c	3	3.09	2.54	1.89	0.0000
SVENSS	96	f	1	2.20	13.42	0.01	0.0000
TANG	1	c	0	2.20	5.10	0.00	0.0000
*TENKAN	24	m	1	2.82	5.38	1.89	0.0000
TIZZAN	5	m	0	0.64	84.08	212.25	0.0000
TIZZAN	13	f	0	1.46	6.13	3.62	0.0003
Subtotal TIZZAN				0.70	90.21	215.87	
TOKARS	1	m	0	3.61	0.97	1.86	0.0004
TOUSEY	12	m	3	4.08	3.57	12.24	0.0000
TOUSEY	15	f	3	3.41	9.42	13.08	0.0000
Subtotal TOUSEY				3.59	12.98	25.32	
TSUGAN	28	m	0	0.20	7.53	30.99	0.5818
*TULINI	37	m	3	2.30	10.29	0.05	0.0000
*TULINI	43	f	3	2.79	11.10	3.46	0.0000
Subtotal TULINI				2.55	21.39	3.52	
*TVERDA	5	m	2	1.41	20.41	13.74	0.0000
*TVERDA	15	f	2	2.40	2.67	0.08	0.0001
Subtotal TVERDA				1.52	23.08	13.82	
WAKAI	8	m	2	1.48	7.88	4.40	0.0000
WAKAI	26	f	2	1.47	8.29	4.72	0.0000
Subtotal WAKAI				1.48	16.17	9.12	
*WALD	4	m	1	2.80	4.92	1.59	0.0000
WANG2	18	c	4	0.88	6.94	12.71	0.0211
WIGLE	25	m	1	2.34	13.23	0.17	0.0000
WIGLE	30	f	1	1.65	19.69	6.63	0.0000
Subtotal WIGLE				1.93	32.92	6.80	
WU	42	f	2	1.58	11.97	5.03	0.0000
WUNSCH	5	m	1	1.89	10.41	1.23	0.0000
WUNSCH	11	f	1	1.79	10.34	2.01	0.0000
Subtotal WUNSCH				1.84	20.74	3.23	
WYNDE3	50	m	0	2.37	7.59	0.16	0.0000
WYNDE6	18	m	0	2.78	66.40	19.84	0.0000

Table 1B1 - 2

IESLC - Meta-analysis of Current Smoking (vs never smoking), Any product (or Cigarettes if Any not available)
All LC types
Most adjusted

REF	NRR	SEX	AD	Ys	Ws	Qs	Ps
WYNDE6	207	f	0	2.68	90.13	18.60	0.0000
Subtotal WYNDE6				2.72	156.52	38.44	
YAMAGU	10	c	1	1.59	8.97	3.67	0.0000
*YONG	12	m	1	3.36	1.92	2.44	0.0000
*YONG	15	f	1	1.65	6.30	2.12	0.0000
Subtotal YONG				2.05	8.22	4.56	

	N	195
	NS	131
	Wt	6749.50
Het	Chi	2668.89
Het	df	194
Het	P	***
Fixed	RR	9.29
	RRl	9.07
	RRu	9.52
	P	+++
Random	RR	8.43
	RRl	7.63
	RRu	9.31
	P	+++
Asymm	P	*

Table 1B1 - 3

IESLC - Meta-analysis of Current Smoking (vs never smoking), Any product (or Cigarettes if Any not available)
All LC types
Most adjusted

	Sex			
	combined	male	female	Total
N	19	108	68	195
NS	19	103	63	185
Wt	1541.37	2705.61	2502.52	6749.50
Het Chi	145.69	1087.66	1183.28	2668.89
Het df	18	107	67	194
Het P	***	***	***	***
Fixed RR	13.19	8.68	8.06	9.29
RRl	12.55	8.36	7.75	9.07
RRu	13.86	9.02	8.38	9.52
P	+++	+++	+++	+++
Random RR	12.09	9.16	6.76	8.43
RRl	9.38	8.00	5.65	7.63
RRu	15.60	10.49	8.08	9.31
P	+++	+++	+++	+++
Between Chi				252.26
Between df				2
Between P				***
Btwn(F) P				***
Btwn(R) P				***

	Lung cancer type		
	all	other	Total
N	187	8	195
NS	125	6	131
Wt	6297.39	452.11	6749.50
Het Chi	2223.98	33.50	2668.89
Het df	186	7	194
Het P	***	***	***
Fixed RR	9.93	3.70	9.29
RRl	9.68	3.37	9.07
RRu	10.17	4.05	9.52
P	+++	+++	+++
Random RR	8.79	3.38	8.43
RRl	7.97	2.63	7.63
RRu	9.68	4.35	9.31
P	+++	+++	+++
Between Chi			411.40
Between df			1
Between P			***
Btwn(F) P			***
Btwn(R) P			***

	Location								
	NAmer	UK	Scand	othEur	China	Japan	othAs	other	Total
N	84	25	21	23	5	18	7	12	195
NS	55	16	14	17	4	11	4	10	131
Wt	4509.59	251.84	308.15	585.70	115.26	749.94	58.83	170.20	6749.50
Het Chi	595.61	152.40	42.74	344.99	7.17	55.13	10.61	48.83	2668.89
Het df	83	24	20	22	4	17	6	11	194
Het P	***	***	**	***	N.S.	***	N.S.	***	***
Fixed RR	12.45	6.90	8.16	5.88	3.07	3.68	2.91	7.09	9.29
RRl	12.09	6.10	7.30	5.43	2.56	3.43	2.26	6.10	9.07
RRu	12.82	7.81	9.12	6.38	3.68	3.95	3.76	8.24	9.52
P	+++	+++	+++	+++	+++	+++	+++	+++	+++
Random RR	11.68	7.53	8.68	8.65	2.94	3.55	2.90	9.88	8.43
RRl	10.61	5.40	7.14	5.98	2.23	3.05	2.04	6.89	7.63
RRu	12.85	10.50	10.54	12.51	3.88	4.14	4.13	14.17	9.31
P	+++	+++	+++	+++	+++	+++	+++	+++	+++
Between Chi									1411.41
Between df									7
Between P									***
Btwn(F) P									***
Btwn(R) P									***

Table 1B1 - 3

IESLC - Meta-analysis of Current Smoking (vs never smoking), Any product (or Cigarettes if Any not available)
All LC types
Most adjusted

Detailed Country in "other Europe"

	multi	Germany	othWest	East	Balkans	Total
N	2	8	8	3	2	23
NS	1	4	7	3	2	17
Wt	266.84	135.91	129.87	13.55	39.53	585.70
Het Chi	66.99	111.74	103.00	6.06	0.95	344.99
Het df	1	7	7	2	1	22
Het P	***	***	***	*	N.S.	***
Fixed RR	7.09	6.98	3.45	9.61	4.55	5.88
RRl	6.28	5.90	2.90	5.65	3.33	5.43
RRu	7.99	8.26	4.09	16.37	6.21	6.38
P	+++	+++	+++	+++	+++	+++
Random RR	6.41	10.53	8.69	16.43	4.55	8.65
RRl	2.35	4.96	3.63	4.59	3.33	5.98
RRu	17.46	22.37	20.84	58.84	6.21	12.51
P	+++	+++	+++	+++	+++	+++
Between Chi						56.26
Between df						4
Between P						***
Btwn(F) P						N.S.
Btwn(R) P						(*)

Detailed Country in "other Asia"

	India	HongKong	other	Total
N		1	6	7
NS		1	3	4
Wt		10.70	48.13	58.83
Het Chi		0.00	10.38	10.61
Het df		0	5	6
Het P		N.S.	(*)	N.S.
Fixed RR		2.55	3.00	2.91
RRl		1.40	2.26	2.26
RRu		4.64	3.98	3.76
P		++	+++	+++
Random RR		2.55	2.98	2.90
RRl		1.40	1.94	2.04
RRu		4.64	4.57	4.13
P		++	+++	+++
Between Chi				0.23
Between df				1
Between P				N.S.
Btwn(F) P				N.S.
Btwn(R) P				N.S.

Detailed other continent

	SCAmer	Auslia	Africa	Total
N	10	1	1	12
NS	8	1	1	10
Wt	107.13	0.99	62.08	170.20
Het Chi	18.26	0.00	0.00	48.83
Het df	9	0	0	11
Het P	*	N.S.	N.S.	***
Fixed RR	9.66	23.03	4.08	7.09
RRl	7.99	3.21	3.18	6.10
RRu	11.67	165.10	5.23	8.24
P	+++	++	+++	+++
Random RR	10.36	23.03	4.08	9.88
RRl	7.79	3.21	3.18	6.89
RRu	13.78	165.10	5.23	14.17
P	+++	++	+++	+++
Between Chi				30.56
Between df				2
Between P				***
Btwn(F) P				*
Btwn(R) P				***

Table 1B1 - 3

IESLC - Meta-analysis of Current Smoking (vs never smoking), Any product (or Cigarettes if Any not available)
All LC types
Most adjusted

	Start year of study <1960	1960-69	1970-79	1980-89	1990+	Total
N	22	40	41	70	22	195
NS	17	27	27	43	17	131
Wt	739.82	1137.81	801.95	3826.88	243.05	6749.50
Het Chi	303.63	420.04	284.02	659.08	183.75	2668.89
Het df	21	39	40	69	21	194
Het P	***	***	***	***	***	***
Fixed RR	6.13	5.43	7.27	12.35	10.32	9.29
RRl	5.70	5.12	6.79	11.97	9.10	9.07
RRu	6.59	5.75	7.79	12.75	11.70	9.52
P	+++	+++	+++	+++	+++	+++
Random RR	6.39	6.44	7.34	10.18	12.81	8.43
RRl	4.70	5.21	5.94	9.02	8.70	7.63
RRu	8.69	7.95	9.06	11.49	18.85	9.31
P	+++	+++	+++	+++	+++	+++
Between Chi						818.38
Between df						4
Between P						***
Btwn(F) P						***
Btwn(R) P						***

	Study type (1) CC	other	Total
N	128	67	195
NS	83	48	131
Wt	5239.40	1510.10	6749.50
Het Chi	2044.88	583.57	2668.89
Het df	127	66	194
Het P	***	***	***
Fixed RR	9.69	8.04	9.29
RRl	9.43	7.65	9.07
RRu	9.95	8.46	9.52
P	+++	+++	+++
Random RR	8.56	8.15	8.43
RRl	7.58	6.87	7.63
RRu	9.68	9.67	9.31
P	+++	+++	+++
Between Chi			40.44
Between df			1
Between P			***
Btwn(F) P			(*)
Btwn(R) P			N.S.

	Study type (2) CC	prosp	other	Total
N	128	62	5	195
NS	83	44	4	131
Wt	5239.40	1480.29	29.81	6749.50
Het Chi	2044.88	573.05	9.81	2668.89
Het df	127	61	4	194
Het P	***	***	*	***
Fixed RR	9.69	8.02	9.37	9.29
RRl	9.43	7.62	6.54	9.07
RRu	9.95	8.44	13.41	9.52
P	+++	+++	+++	+++
Random RR	8.56	7.99	10.82	8.43
RRl	7.58	6.69	5.88	7.63
RRu	9.68	9.53	19.89	9.31
P	+++	+++	+++	+++
Between Chi				41.15
Between df				2
Between P				***
Btwn(F) P				N.S.
Btwn(R) P				N.S.

Table 1B1 - 3

IESLC - Meta-analysis of Current Smoking (vs never smoking), Any product (or Cigarettes if Any not available)
All LC types
Most adjusted

	Study size (number of LC cases)				
	100-249	250-499	500-999	1000+	Total
N	56	48	38	53	195
NS	46	35	24	26	131
Wt	349.51	536.45	736.87	5126.67	6749.50
Het Chi	237.69	251.88	309.30	1709.63	2668.89
Het df	55	47	37	52	194
Het P	***	***	***	***	***
Fixed RR	5.50	8.47	7.56	10.01	9.29
RRl	4.96	7.79	7.04	9.74	9.07
RRu	6.11	9.22	8.13	10.29	9.52
P	+++	+++	+++	+++	+++
Random RR	6.90	8.60	9.54	9.03	8.43
RRl	5.46	7.02	7.65	7.63	7.63
RRu	8.72	10.52	11.89	10.69	9.31
P	+++	+++	+++	+++	+++
Between Chi					160.39
Between df					3
Between P					***
Btwn(F) P					**
Btwn(R) P					N.S.

	Risky occupational population			
	no	mining	othRisky	Total
N	184	7	4	195
NS	120	7	4	131
Wt	6650.94	56.22	42.35	6749.50
Het Chi	2610.01	13.56	4.84	2668.89
Het df	183	6	3	194
Het P	***	*	N.S.	***
Fixed RR	9.36	4.00	8.87	9.29
RRl	9.14	3.08	6.57	9.07
RRu	9.59	5.19	11.99	9.52
P	+++	+++	+++	+++
Random RR	8.56	4.23	10.82	8.43
RRl	7.73	2.77	6.30	7.63
RRu	9.47	6.45	18.58	9.31
P	+++	+++	+++	+++
Between Chi				40.48
Between df				2
Between P				***
Btwn(F) P				N.S.
Btwn(R) P				**

	National cigarette tobacco type			
	Virginia	blended	other	Total
N	34	154	7	195
NS	23	103	5	131
Wt	400.96	6219.11	129.44	6749.50
Het Chi	205.44	2250.04	7.59	2668.89
Het df	33	153	6	194
Het P	***	***	N.S.	***
Fixed RR	6.75	9.70	3.13	9.29
RRl	6.12	9.46	2.64	9.07
RRu	7.44	9.95	3.72	9.52
P	+++	+++	+++	+++
Random RR	8.01	8.92	3.09	8.43
RRl	6.16	8.01	2.50	7.63
RRu	10.41	9.94	3.83	9.31
P	+++	+++	+++	+++
Between Chi				205.82
Between df				2
Between P				***
Btwn(F) P				***
Btwn(R) P				***

Table 1B1 - 3

IESLC - Meta-analysis of Current Smoking (vs never smoking), Any product (or Cigarettes if Any not available)
All LC types
Most adjusted

	Any proxy use No/nk	Yes	Total
N	156	39	195
NS	110	21	131
Wt	5907.21	842.30	6749.50
Het Chi	2424.87	242.38	2668.89
Het df	155	38	194
Het P	***	***	***
Fixed RR	9.24	9.68	9.29
RRl	9.00	9.05	9.07
RRu	9.48	10.36	9.52
P	+++	+++	+++
Random RR	8.04	10.03	8.43
RRl	7.17	8.32	7.63
RRu	9.01	12.09	9.31
P	+++	+++	+++
Between Chi			1.64
Between df			1
Between P			N.S.
Btwn(F) P			N.S.
Btwn(R) P			*

	Full histological confirmation No	Yes	Total
N	144	51	195
NS	95	36	131
Wt	4821.60	1927.91	6749.50
Het Chi	2068.86	562.90	2668.89
Het df	143	50	194
Het P	***	***	***
Fixed RR	8.87	10.45	9.29
RRl	8.62	9.99	9.07
RRu	9.12	10.92	9.52
P	+++	+++	+++
Random RR	7.91	10.08	8.43
RRl	7.00	8.46	7.63
RRu	8.93	12.01	9.31
P	+++	+++	+++
Between Chi			37.12
Between df			1
Between P			***
Btwn(F) P			N.S.
Btwn(R) P			*

	Number of adjustment variables (1) 0	1	2+/+nk	Total
N	86	62	47	195
NS	57	42	33	132
Wt	3052.34	1631.89	2065.28	6749.50
Het Chi	1166.48	631.66	731.25	2668.89
Het df	85	61	46	194
Het P	***	***	***	***
Fixed RR	10.84	7.80	8.49	9.29
RRl	10.46	7.43	8.13	9.07
RRu	11.23	8.19	8.87	9.52
P	+++	+++	+++	+++
Random RR	9.40	7.28	8.54	8.43
RRl	8.02	6.14	7.02	7.63
RRu	11.03	8.63	10.39	9.31
P	+++	+++	+++	+++
Between Chi				139.50
Between df				2
Between P				***
Btwn(F) P				**
Btwn(R) P				(*)

Table 1B1 - 3

IESLC - Meta-analysis of Current Smoking (vs never smoking), Any product (or Cigarettes if Any not available)
All LC types
Most adjusted

	Number of adjustment variables (2)					
	0	1	2	3-5	6+/+nk	Total
N	86	62	27	17	3	195
NS	57	42	17	13	3	132
Wt	3052.34	1631.89	1661.96	301.60	101.71	6749.50
Het Chi	1166.48	631.66	572.62	101.42	50.87	2668.89
Het df	85	61	26	16	2	194
Het P	***	***	***	***	***	***
Fixed RR	10.84	7.80	8.70	7.95	6.95	9.29
RRl	10.46	7.43	8.29	7.11	5.72	9.07
RRu	11.23	8.19	9.13	8.91	8.44	9.52
P	+++	+++	+++	+++	+++	+++
Random RR	9.40	7.28	7.83	9.72	9.09	8.43
RRl	8.02	6.14	6.02	7.04	3.05	7.63
RRu	11.03	8.63	10.19	13.41	27.12	9.31
P	+++	+++	+++	+++	+++	+++
Between Chi						145.84
Between df						4
Between P						***
Btwn(F) P						*
Btwn(R) P						N.S.

	Product			
	all/unsp	cig+/-ot	cig only	Total
N	85	95	15	195
NS	63	62	13	138
Wt	1854.53	4423.80	471.18	6749.50
Het Chi	802.11	1702.13	107.04	2668.89
Het df	84	94	14	194
Het P	***	***	***	***
Fixed RR	8.00	9.84	9.77	9.29
RRl	7.64	9.56	8.92	9.07
RRu	8.37	10.14	10.69	9.52
P	+++	+++	+++	+++
Random RR	7.57	8.95	10.51	8.43
RRl	6.47	7.76	7.70	7.63
RRu	8.84	10.33	14.34	9.31
P	+++	+++	+++	+++
Between Chi				57.61
Between df				2
Between P				***
Btwn(F) P				N.S.
Btwn(R) P				N.S.

	Denominator		
	nev any	nev cigs	Total
N	134	61	195
NS	93	41	134
Wt	4584.05	2165.45	6749.50
Het Chi	1890.58	735.66	2668.89
Het df	133	60	194
Het P	***	***	***
Fixed RR	8.80	10.43	9.29
RRl	8.55	10.00	9.07
RRu	9.06	10.88	9.52
P	+++	+++	+++
Random RR	8.24	8.89	8.43
RRl	7.28	7.52	7.63
RRu	9.34	10.51	9.31
P	+++	+++	+++
Between Chi			42.64
Between df			1
Between P			***
Btwn(F) P			(*)
Btwn(R) P			N.S.

Table 1B1 - 3

IESLC - Meta-analysis of Current Smoking (vs never smoking), Any product (or Cigarettes if Any not available)
All LC types
Most adjusted

	Derivation of RR/CI			
	Orig	StdCalc	Other	Total
N	44	91	60	195
NS	33	59	42	134
Wt	1709.85	3299.85	1739.80	6749.50
Het Chi	545.93	1449.02	578.35	2668.89
Het df	43	90	59	194
Het P	***	***	***	***
Fixed RR	9.82	10.04	7.60	9.29
RRl	9.36	9.70	7.25	9.07
RRu	10.29	10.39	7.96	9.52
P	+++	+++	+++	+++
Random RR	8.49	8.79	7.91	8.43
RRl	6.99	7.49	6.70	7.63
RRu	10.30	10.33	9.35	9.31
P	+++	+++	+++	+++
Between Chi				95.58
Between df				2
Between P				***
Btwn(F) P				*
Btwn(R) P				N.S.

Table 1B1 - 4

IESLC - Meta-analysis of Current Smoking (vs never smoking), Any product (or Cigarettes if Any not available)
All LC types
Least adjusted

REF	NRR	X	SEX	AGEL	AGEH	RACE	YF	LC TYPE	LOC	START	ST	NLC	R	VB	P	H	AD	PRODUCT	DENOM	De
AGUDO	10	x	f	0	0	all	-	all	Eu:wst	1989	CC	103	n	bl	n	n	0	cig only	nev any	st
AKIBA	2	x	m	0	0	all	0	all	As:Jap	1963	pr	610	n	bl	n	n	0	cig+/-ot	nev cigs	or
AKIBA	6	x	f	0	0	all	0	all	As:Jap	1963	pr	610	n	bl	n	n	0	cig+/-ot	nev cigs	or
ALDERS	177		m	0	0	all	-	all	Eu:UK	1977	CC	1448	n	V	n	n	0	cig+/-ot	nev any	st
ALDERS	176		f	0	0	all	-	all	Eu:UK	1977	CC	1448	n	V	n	n	0	cig only	nev any	st
AMANDU	1	x	m	0	0	wh	0	all	NAmer	1959	pr	132	m	bl	n	n	0	cig+/-ot	nev cigs	st
AMES	1		m	0	0	wh	-	all	NAmer	1959	ot	317	m	bl	n	n	0	all/unsp	nev any	or
ANDERS	2	x	f	0	0	all	0	all	NAmer	1986	pr	343	n	bl	n	n	0	cig+/-ot	nev cigs	st
ARCHER	5		m	0	0	wh	0	all	NAmer	1950	pr	146	m	bl	n	n	0	cig+/-ot	nev cigs	st
ARMADA	27		m	0	0	all	-	all	Eu:wst	1986	CC	325	n	bl	n	y	0	cig+/-ot	nev any	st
AUSTIN	2	x	c	0	0	all	-	all	NAmer	1970	CC	166	o	bl	y	n	0	cig+/-ot	nev cigs	st
AXELSS	2		m	0	0	sca	-	all	Eu:Sca	1989	CC	436	n	bl	n	n	0	all/unsp	nev any	st
AXELSS	10		f	0	0	sca	-	all	Eu:Sca	1989	CC	436	n	bl	n	n	0	all/unsp	nev any	st
BARBON	3	x	m	0	0	all	-	all	Eu:wst	1979	CC	755	n	bl	y	y	0	all/unsp	nev any	st
BECHER	13		m	0	0	all	-	all	Eu:Ger	1985	CC	194	n	bl	n	y	0	all/unsp	nev any	st
BECHER	14		f	0	0	all	-	all	Eu:Ger	1985	CC	194	n	bl	n	y	0	all/unsp	nev any	st
BENSHL	16		m	40	64	all	10	all	Eu:UK	1967	pr	486	n	V	n	n	1	all/unsp	nev any	ot
BEST	2		m	0	0	all	0	all	NAmer	1955	pr	381	n	V	n	n	1	cig only	nev any	ot
BLOHMK	1		m	0	0	all	-	all	Eu:Ger	1978	CC	888	n	bl	n	y	0	all/unsp	nev any	st
BOUCOT	2	x	m	0	0	all	0	all	NAmer	1951	pr	121	n	bl	n	n	0	cig only	nev any	ot
BRETT	4		m	0	0	all	0	all	Eu:UK	1960	pr	150	n	V	n	n	0	cig+/-ot	nev cigs	st
BROSS	11		m	0	0	wh	-	all	NAmer	1960	CC	974	n	bl	n	n	0	all/unsp	nev any	st
BROWN2	12		m	0	0	wh	-	all	NAmer	1984	CC	14596	n	bl	n	y	2	cig+/-ot	nev cigs	or
BROWN2	11		f	0	0	wh	-	all	NAmer	1984	CC	14596	n	bl	n	y	2	cig+/-ot	nev cigs	or
BUFFLE	3		m	0	0	wh	-	all	NAmer	1976	CC	943	n	bl	y	n	0	cig+/-ot	nev any	st
BUFFLE	7		f	0	0	wh	-	all	NAmer	1976	CC	943	n	bl	y	n	0	cig+/-ot	nev any	st
CARPEN	9	x	c	0	0	w+b	-	all	NAmer	1991	CC	356	n	bl	n	n	0	cig+/-ot	nev cigs	st
CEDERL	106		m	0	0	all	16	all	Eu:Sca	1963	pr	491	n	bl	n	n	2	all/unsp	nev any	ot
CEDERL	75		f	0	0	all	0	all	Eu:Sca	1963	pr	491	n	bl	n	n	2	all/unsp	nev any	or
CHANG	5		m	0	0	all	0	all	NAmer	1972	pr	136	n	bl	n	n	0	cig+/-ot	nev cigs	st
CHANG	11		f	0	0	all	0	all	NAmer	1972	pr	136	n	bl	n	n	0	cig+/-ot	nev cigs	st
CHOI	3		m	0	0	all	-	all	As:oth	1985	CC	375	n	bl	n	n	0	cig+/-ot	nev cigs	st
CHOI	7		f	0	0	all	-	all	As:oth	1985	CC	375	n	bl	n	n	0	cig+/-ot	nev cigs	st
CHOW	25		m	0	0	wh	0	all	NAmer	1966	pr	219	n	bl	n	n	0	all/unsp	nev any	st
CHYOU	4	x	m	0	0	jap	0	all	NAmer	1965	pr	227	n	bl	n	y	0	cig+/-ot	nev cigs	st
COMSTO	3		m	0	0	all	-	all	NAmer	1975	ot	258	n	bl	n	n	0	cig+/-ot	nev any	st
COMSTO	8		f	0	0	all	-	all	NAmer	1975	ot	258	n	bl	n	n	0	cig+/-ot	nev any	st
CORREA	41	x	c	0	0	all	-	all	NAmer	1979	CC	1359	n	bl	y	n	0	cig+/-ot	nev cigs	st
CPSI	220		m	35	84	all	6	all	NAmer	1959	pr	5138	n	bl	n	n	1	cig+/-ot	nev any	ot
CPSI	279		f	40	74	all	6	all	NAmer	1959	pr	5138	n	bl	n	n	1	cig+/-ot	nev cigs	ot
CPSII	36	x	m	0	0	all	6	all	NAmer	1982	pr	3229	n	bl	n	n	0	cig only	nev any	st
CPSII	71	x	f	0	0	all	6	all	NAmer	1982	pr	3229	n	bl	n	n	0	cig+/-ot	nev cigs	st
DAMBER	14		m	0	0	all	-	all	Eu:Sca	1972	CC	579	n	bl	y	n	1	all/unsp	nev any	ot
DARBY	4		m	0	0	wh	-	all	Eu:UK	1988	CC	982	n	V	n	n	0	cig+/-ot	nev any	st
DARBY	11		f	0	0	wh	-	all	Eu:UK	1988	CC	982	n	V	n	n	0	cig+/-ot	nev any	st
DEAN2	2		m	0	0	all	-	all	Eu:UK	1960	CC	954	n	V	y	n	0	all/unsp	nev any	st
DEAN2	6		f	0	0	all	-	all	Eu:UK	1960	CC	954	n	V	y	n	0	all/unsp	nev any	st
DEAN3	40	x	m	0	0	all	-	all	Eu:UK	1969	CC	766	n	V	y	n	0	all/unsp	nev any	st
DEAN3	117	x	f	0	0	all	-	all	Eu:UK	1969	CC	766	n	V	y	n	0	cig only	nev any	st
DEKLER	8		m	0	0	all	0	all	Auslia	1961	pr	138	m	V	n	n	2	all/unsp	nev any	ot
DESTE2	2	x	c	0	0	all	-	all	SCAmer	1993	CC	463	n	bl	n	n	0	all/unsp	nev any	st
DESTEF	40	x	m	0	0	all	-	all	SCAmer	1988	CC	497	n	bl	n	y	0	all/unsp	nev any	st
DOCKER	1		c	0	0	wh	0	all	NAmer	1974	pr	120	n	bl	n	n	4	cig+/-ot	nev cigs	or
DOLL	90		m	0	0	all	-	all	Eu:UK	1948	CC	1465	n	V	n	n	0	all/unsp	nev any	st
DOLL	93		f	0	0	all	-	all	Eu:UK	1948	CC	1465	n	V	n	n	0	all/unsp	nev any	st
DOLL2	54		m	0	0	all	0	all	Eu:UK	1951	pr	920	n	V	n	n	1	all/unsp	nev any	ot
DOLL2	63		f	0	0	all	22	all	Eu:UK	1951	pr	920	n	V	n	n	1	cig only	nev any	ot
DORANT	2		m	0	0	all	0	all	Eu:wst	1986	ot	550	n	bl	n	y	0	all/unsp	nev any	st
DORGAN	9		m	0	0	wh	-	all	NAmer	1980	CC	2026	n	bl	y	y	0	cig+/-ot	nev any	st
DORGAN	33		m	0	0	bl	-	all	NAmer	1980	CC	2026	n	bl	y	y	0	cig+/-ot	nev any	st
DORGAN	56		f	0	0	wh	-	all	NAmer	1980	CC	2026	n	bl	y	y	0	cig+/-ot	nev any	st
DORGAN	79		f	0	0	bl	-	all	NAmer	1980	CC	2026	n	bl	y	y	0	cig+/-ot	nev any	st
DORN	51		m	35	84	wh	8	all	NAmer	1954	pr	5097	n	bl	n	n	1	all/unsp	nev any	ot
DROSTE	2	x	m	0	0	all	-	all	Eu:wst	1995	CC	478	n	bl	n	y	0	all/unsp	nev any	st
ENGELA	154	x	m	0	0	all	12	all	Eu:Sca	1964	pr	435	n	bl	n	n	0	all/unsp	nev any	st
ENGELA	161	x	f	0	0	all	12	all	Eu:Sca	1964	pr	435	n	bl	n	n	0	all/unsp	nev any	st
ENSTRO	1		m	0	0	all	0	all	NAmer	1959	pr	2879	n	bl	n	n	1	cig only	nev any	or
ENSTRO	2		f	0	0	all	0	all	NAmer	1959	pr	2879	n	bl	n	n	1	cig only	nev any	or
GAO	29	x	m	0	0	all	-	all	As:Chi	1984	CC	1405	n	ot	n	n	0	cig+/-ot	nev cigs	st
GAO	30	x	f	0	0	all	-	all	As:Chi	1984	CC	1405	n	ot	n	n	0	cig+/-ot	nev cigs	st
GAO2	1	x	m	0	0	all	-	all	As:Jap	1988	CC	282	n	bl	n	n	0	cig+/-ot	nev cigs	st
GARCIA	2		c	0	0	all	-	all	NAmer	1992	CC	416	n	bl	n	y	0	cig+/-ot	nev cigs	st

Table 1B1 - 4

IESLC - Meta-analysis of Current Smoking (vs never smoking), Any product (or Cigarettes if Any not available)
All LC types
Least adjusted

REF	NRR	X	SEX	AGEL	AGEH	RACE	YF	LC TYPE	LOC	START	ST	NLC	R	VB	P	H	AD	PRODUCT	DENOM	De
GARDIN	2		c	0	0	all	-	all	Eu:UK	1988	CC	143	n	V	y	n	0	all/unsp	nev any	st
GARSHI	23	x	m	0	0	all	-	all	NAmer	1981	CC	1081	o	bl	y	n	0	all/unsp	nev any	st
GOODMA	2		m	0	0	w+o	-	all	NAmer	1983	CC	326	n	bl	y	y	0	cig+/-ot	nev any	st
GOODMA	6		f	0	0	w+o	-	all	NAmer	1983	CC	326	n	bl	y	y	0	cig+/-ot	nev any	st
GRAHAM	20	x	m	0	0	wh	-	all	NAmer	1956	CC	685	n	bl	n	n	0	all/unsp	nev any	st
GREGOR	2		m	0	0	all	-	all	Eu:UK	1976	CC	104	n	V	n	y	0	cig+/-ot	nev cigs	st
GREGOR	6		f	0	0	all	-	all	Eu:UK	1976	CC	104	n	V	n	y	0	cig+/-ot	nev cigs	st
HAENSZ	54		f	0	0	all	-	not alv	NAmer	1955	CC	158	n	bl	n	y	0	cig+/-ot	nev any	st
HAMMO2	22	x	m	0	0	all	0	all	NAmer	1967	pr	450	o	bl	n	n	0	cig+/-ot	nev any	st
HAMMON	139		m	0	0	wh	0	all	NAmer	1952	pr	448	n	bl	n	n	1	cig only	nev any	ot
HEIN	5		m	0	0	all	0	all	Eu:Sca	1970	pr	144	n	bl	n	n	0	all/unsp	nev any	st
HENNEK	2		m	0	0	all	0	all	NAmer	1982	pr	169	n	bl	n	n	0	all/unsp	nev any	st
HIRAYA	1		m	0	0	all	0	all	As:Jap	1965	pr	1917	n	bl	n	n	1	cig+/-ot	nev any	st
HIRAYA	3		f	0	0	all	0	all	As:Jap	1965	pr	1917	n	bl	n	n	1	cig+/-ot	nev any	st
HITOSU	2	x	m	0	0	all	-	all	As:Jap	1960	CC	216	n	bl	y	n	0	all/unsp	nev any	st
HITOSU	9	x	f	0	0	all	-	all	As:Jap	1960	CC	216	n	bl	y	n	0	all/unsp	nev any	st
HOLE	47	x	m	0	0	all	0	all	Eu:UK	1972	pr	225	n	V	n	n	0	all/unsp	nev any	st
HOLE	29	x	f	0	0	all	11	all	Eu:UK	1972	pr	225	n	V	n	n	0	all/unsp	nev any	st
HUMBLE	13		m	0	0	w-hi	-	all	NAmer	1980	CC	521	n	bl	y	n	1	cig+/-ot	nev cigs	ot
HUMBLE	15		m	0	0	hi	-	all	NAmer	1980	CC	521	n	bl	y	n	1	cig+/-ot	nev cigs	ot
HUMBLE	17		f	0	0	w-hi	-	all	NAmer	1980	CC	521	n	bl	y	n	1	cig+/-ot	nev cigs	ot
HUMBLE	19		f	0	0	hi	-	all	NAmer	1980	CC	521	n	bl	y	n	1	cig+/-ot	nev cigs	ot
JAHN	5		m	0	0	all	-	all	Eu:Ger	1988	CC	1004	n	bl	n	n	0	cig+/-ot	nev any	st
JAIN	16	x	m	0	0	all	-	all	NAmer	1981	CC	845	n	V	y	n	0	cig+/-ot	nev cigs	st
JAIN	11	x	f	0	0	all	-	all	NAmer	1981	CC	845	n	V	y	n	0	cig+/-ot	nev cigs	st
JARVHO	2		m	0	0	all	-	all	Eu:Sca	1983	CC	147	n	bl	n	n	0	all/unsp	nev any	st
JARVHO	6		f	0	0	all	-	all	Eu:Sca	1983	CC	147	n	bl	n	n	0	all/unsp	nev any	st
JOLY	18		m	0	0	all	-	all	SCAmer	1978	CC	826	n	bl	n	n	0	all/unsp	nev any	st
JOLY	15		f	0	0	all	-	all	SCAmer	1978	CC	826	n	bl	n	n	0	cig+/-ot	nev any	st
KAISE2	68		m	35	99	all	9	all	NAmer	1979	pr	318	n	bl	n	n	1	cig only	nev any	st
KAISE2	60		f	35	99	all	9	all	NAmer	1979	pr	318	n	bl	n	n	1	cig only	nev any	st
KAISER	12		m	0	0	all	0	all	NAmer	1964	pr	714	n	bl	n	n	2	cig+/-ot	nev cigs	ot
KAISER	9		f	0	0	all	0	all	NAmer	1964	pr	714	n	bl	n	n	2	cig+/-ot	nev cigs	ot
KANELL	5	x	m	0	0	all	-	all	Eu:bal	1950	CC	862	n	bl	n	n	0	all/unsp	nev any	st
KATSOU	6	x	f	0	0	all	-	all	Eu:bal	1987	CC	101	n	bl	n	n	0	all/unsp	nev any	st
KAUFMA	7	x	c	0	0	all	-	all	NAmer	1981	CC	881	n	bl	n	n	0	cig+/-ot	nev cigs	st
KELLER	1		m	0	0	wh	-	all	NAmer	1985	CC	15038	n	bl	n	n	0	all/unsp	nev any	st
KELLER	9		m	0	0	nonw	-	all	NAmer	1985	CC	15038	n	bl	n	n	0	all/unsp	nev any	st
KELLER	5		f	0	0	wh	-	all	NAmer	1985	CC	15038	n	bl	n	n	0	all/unsp	nev any	st
KELLER	13		f	0	0	nonw	-	all	NAmer	1985	CC	15038	n	bl	n	n	0	all/unsp	nev any	st
KHUDER	19		m	0	0	all	-	all	NAmer	1985	CC	482	n	bl	n	y	0	cig+/-ot	nev cigs	or
KIHARA	7		c	0	0	jap	-	all	As:Jap	1991	CC	440	n	bl	n	n	0	all/unsp	nev any	st
KINLEN	9	x	m	0	0	all	0	all	Eu:UK	1967	pr	718	n	V	n	n	0	all/unsp	nev any	st
KJUUS	1		m	0	0	all	-	all	Eu:Sca	1979	CC	176	n	bl	n	n	0	all/unsp	nev any	st
KNEKT	15	x	m	20	69	all	21	all	Eu:Sca	1966	pr	515	n	bl	n	n	0	all/unsp	nev any	st
KOO	9		f	0	0	all	-	all	As:HK	1981	CC	200	n	bl	n	n	0	all/unsp	nev any	st
KREUZE	39		m	1	45	all	-	all	Eu:Ger	1990	CC	2260	n	bl	n	n	0	all/unsp	nev any	st
KREUZE	41		m	55	69	all	-	all	Eu:Ger	1990	CC	2260	n	bl	n	n	0	all/unsp	nev any	st
KREUZE	40		f	1	45	all	-	all	Eu:Ger	1990	CC	2260	n	bl	n	n	0	all/unsp	nev any	st
KREUZE	42		f	55	69	all	-	all	Eu:Ger	1990	CC	2260	n	bl	n	n	0	all/unsp	nev any	st
KUBIK	12		m	0	0	all	0	all	Eu:est	1965	pr	108	n	bl	n	n	0	cig+/-ot	nev any	st
LANGE	32	x	m	0	0	all	0	all	Eu:Sca	1976	pr	268	n	bl	n	n	0	all/unsp	nev any	st
LANGE	29	x	f	0	0	all	0	all	Eu:Sca	1976	pr	268	n	bl	n	n	0	all/unsp	nev any	st
LEMARC	2		c	0	0	w+o	-	all	NAmer	1992	CC	341	n	bl	n	y	0	all/unsp	nev any	st
LIAW	1		m	0	0	all	0	all	As:oth	1982	pr	127	n	ot	n	n	1	all/unsp	nev any	or
LIAW	2		f	0	0	all	0	all	As:oth	1982	pr	127	n	ot	n	n	1	all/unsp	nev any	or
LIDDEL	4		m	0	0	all	18	all	NAmer	1970	pr	304	m	V	n	n	1	cig+/-ot	nev cigs	ot
LOMBAR	9		m	0	0	all	-	all	NAmer	1951	CC	1040	n	bl	n	n	0	cig+/-ot	nev any	st
LUBIN	40		m	0	0	all	-	all	As:Chi	1984	CC	427	m	ot	y	n	0	cig+/-ot	nev any	st
LUBIN2	25	x	m	0	0	all	-	all	Eu:mul	1976	CC	7804	n	bl	n	y	0	all/unsp	nev any	st
LUBIN2	317		f	0	0	all	-	all	Eu:mul	1976	CC	7804	n	bl	n	y	0	cig+/-ot	nev any	st
MACLEN	19		m	0	0	ch	-	all	As:oth	1972	CC	233	n	bl	n	n	0	cig+/-ot	nev cigs	st
MACLEN	32		f	0	0	ch	-	all	As:oth	1972	CC	233	n	bl	n	n	0	cig+/-ot	nev cigs	st
MATOS	2	x	m	0	0	all	-	all	SCAmer	1994	CC	200	n	bl	n	n	0	cig+/-ot	nev any	st
MIGRAN	19	x	m	0	0	all	0	all	Eu:UK	1964	pr	259	n	V	n	n	0	all/unsp	nev any	st
MIGRAN	135	x	f	0	0	all	0	all	Eu:UK	1964	pr	259	n	V	n	n	0	all/unsp	nev any	st
MRFITR	2		m	0	0	all	0	all	NAmer	1973	pr	119	n	bl	n	n	0	cig+/-ot	nev cigs	ot
NAM	68	x	m	0	0	all	-	all	NAmer	1986	CC	1199	n	bl	y	n	0	cig+/-ot	nev cigs	ot
NAM	84	x	f	0	0	all	-	all	NAmer	1986	CC	1199	n	bl	y	n	0	cig+/-ot	nev cigs	ot
ODRISC	1		c	0	0	all	-	all	Eu:UK	1992	CC	446	n	V	n	n	0	all/unsp	nev any	st
OSANN	9	x	m	0	0	all	-	all	NAmer	1984	CC	1986	n	bl	n	n	0	cig+/-ot	nev cigs	st
OSANN	13	x	f	0	0	all	-	all	NAmer	1984	CC	1986	n	bl	n	n	0	cig+/-ot	nev cigs	st

Table 1B1 - 4

IESLC - Meta-analysis of Current Smoking (vs never smoking), Any product (or Cigarettes if Any not available)
All LC types
Least adjusted

REF	NRR	X	SEX	AGEL	AGEH	RACE	YF	LC TYPE	LOC	START	ST	NLC	R	VB	P	H	AD	PRODUCT	DENOM	De
PARKIN	27	x	m	0	0	bl	-	all	Africa	1963	CC	877	n	V	y	n	0	all/unsp	nev any	st
PERSH2	4	x	c	0	0	all	-	all	Eu:Sca	1980	CC	1022	n	bl	y	n	0	all/unsp	nev any	st
PETO	4		m	0	0	all	0	all	Eu:UK	1954	pr	103	n	V	n	n	0	all/unsp	nev any	st
PEZZO2	2		m	0	0	all	-	all	SCAmer	1992	CC	367	n	bl	n	y	0	cig+/-ot	nev cigs	st
PEZZOT	5		m	0	0	all	-	all	SCAmer	1987	CC	215	n	bl	n	y	0	cig only	nev cigs	st
QIAO2	2	x	m	0	0	all	0	all	As:Chi	1992	pr	241	m	ot	n	n	0	all/unsp	nev any	st
RACHTA	2	x	f	0	0	all	-	all	Eu:est	1991	CC	118	n	bl	n	y	0	cig+/-ot	nev cigs	st
SCHWAR	25		m	0	0	wh	-	all	NAmer	1984	CC	5588	n	bl	y	y	0	cig+/-ot	nev cigs	st
SCHWAR	26		m	0	0	bl	-	all	NAmer	1984	CC	5588	n	bl	y	y	0	cig+/-ot	nev cigs	st
SCHWAR	27		f	0	0	wh	-	all	NAmer	1984	CC	5588	n	bl	y	y	0	cig+/-ot	nev cigs	st
SCHWAR	28		f	0	0	bl	-	all	NAmer	1984	CC	5588	n	bl	y	y	0	cig+/-ot	nev cigs	st
SEGI2	19	x	m	0	0	all	-	all	As:Jap	1962	CC	378	n	bl	n	n	0	cig+/-ot	nev any	st
SEGI2	27	x	f	0	0	all	-	all	As:Jap	1962	CC	378	n	bl	n	n	0	cig+/-ot	nev any	st
SHAW	6		c	0	0	wh	-	all	NAmer	1988	CC	335	n	V	n	y	0	all/unsp	nev any	st
SOBUE	90	x	m	0	0	all	-	q+s+l+a	As:Jap	1986	CC	1376	n	bl	n	y	0	cig+/-ot	nev cigs	st
SOBUE	94	x	f	0	0	all	-	q+s+l+a	As:Jap	1986	CC	1376	n	bl	n	y	0	cig+/-ot	nev cigs	st
SOBUE2	10		m	0	0	all	-	q+s+l+a	As:Jap	1965	CC	2083	n	bl	n	n	2	cig+/-ot	nev any	ot
SOBUE2	12		f	0	0	all	-	q+s+l+a	As:Jap	1965	CC	2083	n	bl	n	n	2	cig+/-ot	nev any	ot
SPEIZE	6	x	f	0	0	all	0	all	NAmer	1976	pr	593	n	bl	n	y	0	cig+/-ot	nev cigs	st
SPITZ	2		c	0	0	b+hi	-	all	NAmer	1992	CC	177	n	bl	n	y	0	cig+/-ot	nev cigs	st
STOCKW	7		c	0	0	all	-	all	NAmer	1981	CC	22161	n	bl	n	n	0	cig+/-ot	nev any	st
STUCKE	2		m	0	0	all	-	all	Eu:wst	1989	CC	247	n	bl	n	y	0	all/unsp	nev any	ot
SUZUK2	2	x	c	0	0	all	-	all	SCAmer	1991	CC	123	n	bl	n	y	0	all/unsp	nev any	st
SVENSS	61	x	f	0	0	all	-	all	Eu:Sca	1983	CC	210	n	bl	n	n	0	all/unsp	nev any	st
TANG	1		c	0	0	all	-	not s	NAmer	1992	CC	119	n	bl	n	y	0	cig+/-ot	nev cigs	st
TENKAN	24		m	0	0	all	17	all	Eu:Sca	1962	pr	242	n	bl	n	n	1	all/unsp	nev any	ot
TIZZAN	5		m	0	0	all	-	all	Eu:wst	1959	CC	1358	n	bl	n	n	0	all/unsp	nev any	st
TIZZAN	13		f	0	0	all	-	all	Eu:wst	1959	CC	1358	n	bl	n	n	0	all/unsp	nev any	st
TOKARS	1		m	0	0	all	-	all	Eu:est	1966	ot	162	o	bl	n	y	0	all/unsp	nev any	st
TOUSEY	4	x	m	0	0	all	-	all	NAmer	1993	CC	507	n	bl	y	y	0	cig+/-ot	nev any	st
TOUSEY	8	x	f	0	0	all	-	all	NAmer	1993	CC	507	n	bl	y	y	0	cig+/-ot	nev any	st
TSUGAN	28		m	0	0	all	-	q+a	As:Jap	1976	CC	134	n	bl	n	y	0	all/unsp	nev any	st
TULINI	14	x	m	0	0	all	0	all	Eu:Sca	1967	pr	472	n	bl	n	n	1	all/unsp	nev any	ot
TULINI	20	x	f	0	0	all	0	all	Eu:Sca	1967	pr	472	n	bl	n	n	1	all/unsp	nev any	ot
TVERDA	5		m	0	0	all	0	all	Eu:Sca	1972	pr	238	n	bl	n	n	2	cig+/-ot	nev cigs	ot
TVERDA	15		f	0	0	all	0	all	Eu:Sca	1972	pr	238	n	bl	n	n	2	cig only	nev cigs	ot
WAKAI	2	x	m	0	0	all	-	all	As:Jap	1988	CC	333	n	bl	n	y	0	all/unsp	nev any	st
WAKAI	20	x	f	0	0	all	-	all	As:Jap	1988	CC	333	n	bl	n	y	0	all/unsp	nev any	st
WALD	2	x	m	0	0	all	0	all	Eu:UK	1975	pr	102	n	V	n	n	0	cig only	nev any	st
WANG2	17	x	c	0	0	all	-	all	As:Chi	1980	CC	103	n	ot	n	n	0	cig+/-ot	nev cigs	st
WIGLE	3	x	m	0	0	all	-	all	NAmer	1971	CC	728	n	V	n	n	0	all/unsp	nev any	st
WIGLE	6	x	f	0	0	all	-	all	NAmer	1971	CC	728	n	V	n	n	0	all/unsp	nev any	st
WU	34	x	f	0	0	wh	-	q+a	NAmer	1981	CC	220	n	bl	n	y	0	all/unsp	nev any	st
WUNSCH	2	x	m	0	0	all	-	all	SCAmer	1990	CC	398	n	bl	y	n	0	cig+/-ot	nev any	st
WUNSCH	8	x	f	0	0	all	-	all	SCAmer	1990	CC	398	n	bl	y	n	0	cig+/-ot	nev any	st
WYNDE3	50		m	0	0	all	-	all	NAmer	1966	CC	350	n	bl	n	y	0	all/unsp	nev any	st
WYNDE6	18		m	0	0	all	-	all	NAmer	1969	CC	4423	n	bl	n	y	0	cig+/-ot	nev any	st
WYNDE6	207		f	0	0	all	-	all	NAmer	1969	CC	4423	n	bl	n	y	0	cig+/-ot	nev cigs	st
YAMAGU	1	x	c	0	0	all	-	all	As:Jap	1989	CC	144	n	bl	n	y	0	all/unsp	nev any	st
YONG	12		m	0	0	all	0	all	NAmer	1971	pr	216	n	bl	n	n	1	cig+/-ot	nev cigs	or
YONG	15		f	0	0	all	0	all	NAmer	1971	pr	216	n	bl	n	n	1	cig+/-ot	nev cigs	or

Cigarette type is all/unspec for all RRs
except for the following:

REF	NRR	CIGTYPE
ALDERS	177	MC+-HR
ALDERS	176	MC only
DEAN3	117	MC only

Table 1B1 - 5

IESLC - Meta-analysis of Current Smoking (vs never smoking), Any product (or Cigarettes if Any not available)
All LC types
Least adjusted

REF	NRR	SEX	AD	Number Exposed Case	Number Exposed Cont	Non-exposed Case	Non-exposed Cont	RR	95.00%CI
AGUDO	10	f	0	20	17	80	183	2.69	(1.34- 5.41)
*AKIBA	2	m	0	345	171379	18	35833	4.01	(2.50- 6.44)
*AKIBA	6	f	0	74	51237	116	359850	4.48	(3.35- 6.00)
Subtotal AKIBA								4.35	(3.39- 5.57)
ALDERS	177	m	0	519	322	15	133	14.29	(8.23- 24.81)
ALDERS	176	f	0	410	229	75	243	5.80	(4.27- 7.87)
Subtotal ALDERS								7.17	(5.49- 9.36)
*AMANDU	1	m	0	115	96708	6	25350	5.02	(2.21- 11.41)
AMES	1	m	0	150	136	15	62	4.56	(2.48- 8.39)
*ANDERS	2	f	0	212	41262	46	195158	21.80	(15.85- 29.98)
*ARCHER	5	m	0	122	32529	6	9842	6.15	(2.71- 13.96)
ARMADA	27	m	0	188	122	4	64	24.66	(8.75- 69.44)
AUSTIN	2	c	0	111	125	5	88	15.63	(6.13- 39.87)
AXELSS	2	m	0	194	130	16	160	14.92	(8.53- 26.12)
AXELSS	10	f	0	96	69	18	154	11.90	(6.68- 21.22)
Subtotal AXELSS								13.38	(8.95- 20.00)
BARBON	3	m	0	562	362	22	188	13.27	(8.37- 21.04)
BECHER	13	m	0	101	122	3	54	14.90	(4.52- 49.09)
BECHER	14	f	0	33	26	10	52	6.60	(2.82- 15.44)
Subtotal BECHER								8.68	(4.35- 17.35)
*BENSHL	16	m	1	-	-	-	-	8.18	(3.62- 18.51)
*BEST	2	m	1	-	-	-	-	14.91	(7.05- 31.52)
BLOHMK	1	m	0	419	313	126	301	3.20	(2.48- 4.12)
*BOUCOT	2	m	0	85	22177	0	7551	58.23~	(3.61- 938.34)
*BRETT	4	m	0	135	37448	6	6530	3.92	(1.73- 8.88)
BROSS	11	m	0	690	638	38	170	4.84	(3.35- 6.99)
BROWN2	12	m	2	-	-	-	-	11.30	(10.20- 12.40)
BROWN2	11	f	2	-	-	-	-	13.60	(12.30- 15.10)
Subtotal BROWN2								12.34	(11.50- 13.25)
BUFFLE	3	m	0	257	219	5	47	11.03	(4.31- 28.22)
BUFFLE	7	f	0	313	183	41	198	8.26	(5.63- 12.11)
Subtotal BUFFLE								8.61	(6.04- 12.27)
CARPEN	9	c	0	219	162	15	241	21.72	(12.41- 38.01)
*CEDERL	106	m	2	-	-	-	-	7.72	(5.01- 11.89)
*CEDERL	75	f	2	-	-	-	-	4.82	(3.38- 6.88)
Subtotal CEDERL								5.83	(4.43- 7.67)
*CHANG	5	m	0	35	419	5	502	8.39	(3.32- 21.21)
*CHANG	11	f	0	30	603	11	1139	5.15	(2.60- 10.21)
Subtotal CHANG								6.12	(3.53- 10.60)
CHOI	3	m	0	232	329	13	95	5.15	(2.82- 9.42)
CHOI	7	f	0	13	23	76	164	1.22	(0.59- 2.54)
Subtotal CHOI								2.88	(1.81- 4.58)
*CHOW	25	m	0	167	124415	6	62913	14.07	(6.23- 31.78)
*CHYOU	4	m	0	181	3470	13	2406	9.65	(5.51- 16.91)
COMSTO	3	m	0	105	100	4	69	18.11	(6.37- 51.48)
COMSTO	8	f	0	77	52	13	115	13.10	(6.68- 25.67)
Subtotal COMSTO								14.40	(8.18- 25.36)
CORREA	41	c	0	943	571	51	388	12.56	(9.22- 17.13)
*CPSI	220	m	1	-	-	-	-	11.94	(9.52- 14.97)
*CPSI	279	f	1	-	-	-	-	3.20	(2.53- 4.04)
Subtotal CPSI								6.32	(5.37- 7.43)
*CPSII	36	m	0	1781	583646	124	742207	18.26	(15.23- 21.91)
*CPSII	71	f	0	1014	744217	310	2091302	9.19	(8.09- 10.44)
Subtotal CPSII								11.51	(10.37- 12.78)
DAMBER	14	m	1	-	-	-	-	9.60	(6.60- 14.20)
DARBY	4	m	0	322	453	3	384	90.98	(28.96- 285.90)
DARBY	11	f	0	195	217	23	529	20.67	(13.05- 32.74)
Subtotal DARBY								25.40	(16.57- 38.92)
DEAN2	2	m	0	671	600	33	112	3.80	(2.54- 5.68)
DEAN2	6	f	0	59	28	88	121	2.90	(1.71- 4.91)
Subtotal DEAN2								3.43	(2.49- 4.73)
DEAN3	40	m	0	502	1636	25	510	6.26	(4.14- 9.47)
DEAN3	117	f	0	102	1158	41	1538	3.30	(2.28- 4.79)
Subtotal DEAN3								4.39	(3.33- 5.79)
*DEKLER	8	m	2	-	-	-	-	23.03	(3.21- 164.97)
DESTE2	2	c	0	216	151	20	108	7.72	(4.59- 13.00)
DESTEF	40	m	0	362	226	27	163	9.67	(6.23- 15.01)
*DOCKER	1	c	4	-	-	-	-	8.00	(2.97- 21.60)
DOLL	90	m	0	1280	1172	7	61	9.52	(4.34- 20.89)
DOLL	93	f	0	58	41	40	59	2.09	(1.18- 3.68)
Subtotal DOLL								3.51	(2.21- 5.55)

Table 1B1 - 5

IESLC - Meta-analysis of Current Smoking (vs never smoking), Any product (or Cigarettes if Any not available)
All LC types
Least adjusted

REF	NRR	SEX	AD	Number Exposed Case	Cont	Non-exposed Case	Cont	RR	95.00%CI
*DOLL2	54	m	1	-	-	-	-	10.99	(6.97- 17.36)
*DOLL2	63	f	1	-	-	-	-	8.65	(2.93- 25.55)
Subtotal DOLL2								10.60	(6.96- 16.14)
DORANT	2	m	0	332	697	7	159	10.82	(5.02- 23.32)
DORGAN	9	m	0	464	170	15	93	16.92	(9.54- 30.01)
DORGAN	33	m	0	214	61	3	35	40.93	(12.17- 137.66)
DORGAN	56	f	0	611	119	103	244	12.16	(8.99- 16.46)
DORGAN	79	f	0	68	17	7	20	11.43	(4.16- 31.43)
Subtotal DORGAN								13.62	(10.58- 17.54)
*DORN	51	m	1	-	-	-	-	8.23	(6.55- 10.35)
DROSTE	2	m	0	379	267	7	93	18.86	(8.61- 41.30)
*ENGELA	154	m	0	100	6636	7	2683	5.78	(2.69- 12.41)
*ENGELA	161	f	0	13	2674	13	10708	4.00	(1.86- 8.63)
Subtotal ENGELA								4.81	(2.80- 8.27)
*ENSTRO	1	m	1	-	-	-	-	12.99	(10.46- 16.13)
*ENSTRO	2	f	1	-	-	-	-	6.95	(6.01- 8.04)
Subtotal ENSTRO								8.44	(7.48- 9.53)
GAO	29	m	0	529	438	62	202	3.93	(2.88- 5.37)
GAO	30	f	0	170	100	435	605	2.36	(1.79- 3.12)
Subtotal GAO								2.96	(2.41- 3.64)
GAO2	1	m	0	184	117	13	56	6.77	(3.55- 12.93)
GARCIA	2	c	0	169	74	21	139	15.12	(8.86- 25.79)
GARDIN	2	c	0	97	58	5	41	13.71	(5.13- 36.68)
GARSHI	23	m	0	657	782	41	363	7.44	(5.30- 10.44)
GOODMA	2	m	0	148	169	10	199	17.43	(8.90- 34.14)
GOODMA	6	f	0	58	56	19	177	9.65	(5.30- 17.56)
Subtotal GOODMA								12.53	(8.01- 19.60)
GRAHAM	20	m	0	517	1473	18	346	6.75	(4.16- 10.95)
GREGOR	2	m	0	49	53	10	14	1.29	(0.53- 3.18)
GREGOR	6	f	0	17	26	1	22	14.38	(1.77- 116.90)
Subtotal GREGOR								1.88	(0.82- 4.30)
HAENSZ	54	f	0	69	94	81	236	2.14	(1.43- 3.19)
*HAMMO2	22	m	0	209	4472	5	891	8.33	(3.44- 20.16)
*HAMMON	139	m	1	-	-	-	-	11.52	(6.83- 19.42)
*HEIN	5	m	0	132	3492	1	457	17.27	(2.42- 123.25)
*HENNEK	2	m	0	79	2438	23	10919	15.38	(9.69- 24.42)
*HIRAYA	1	m	1	-	-	-	-	4.45	(3.60- 5.50)
*HIRAYA	3	f	1	-	-	-	-	2.34	(1.87- 2.92)
Subtotal HIRAYA								3.28	(2.81- 3.82)
HITOSU	2	m	0	117	1597	7	242	2.53	(1.17- 5.50)
HITOSU	9	f	0	28	459	33	1893	3.50	(2.09- 5.85)
Subtotal HITOSU								3.17	(2.07- 4.86)
*HOLE	47	m	0	163	4130	7	1189	6.70	(3.15- 14.25)
*HOLE	29	f	0	13	2144	8	1917	1.45	(0.60- 3.50)
Subtotal HOLE								3.51	(1.98- 6.21)
HUMBLE	13	m	1	-	-	-	-	19.96	(8.27- 48.21)
HUMBLE	15	m	1	-	-	-	-	15.79	(3.43- 72.69)
HUMBLE	17	f	1	-	-	-	-	16.72	(7.44- 37.61)
HUMBLE	19	f	1	-	-	-	-	23.50	(6.79- 81.36)
Subtotal HUMBLE								18.65	(11.23- 30.97)
JAHN	5	m	0	352	269	18	138	10.03	(5.99- 16.81)
JAIN	16	m	0	265	118	12	85	15.91	(8.37- 30.23)
JAIN	11	f	0	305	99	52	214	12.68	(8.68- 18.51)
Subtotal JAIN								13.44	(9.70- 18.62)
JARVHO	2	m	0	73	29	1	16	40.28	(5.10- 317.77)
JARVHO	6	f	0	31	7	6	21	15.50	(4.56- 52.66)
Subtotal JARVHO								19.86	(6.93- 56.89)
JOLY	18	m	0	487	665	12	218	13.30	(7.35- 24.07)
JOLY	15	f	0	132	96	52	283	7.48	(5.04- 11.12)
Subtotal JOLY								8.94	(6.43- 12.42)
*KAISE2	68	m	1	-	-	-	-	8.04	(4.41- 14.66)
*KAISE2	60	f	1	-	-	-	-	14.48	(7.47- 28.04)
Subtotal KAISE2								10.49	(6.72- 16.36)
*KAISER	12	m	2	-	-	-	-	19.61	(13.32- 28.87)
*KAISER	9	f	2	-	-	-	-	6.53	(4.50- 9.48)
Subtotal KAISER								11.09	(8.48- 14.50)
KANELL	5	m	0	814	441	48	172	6.61	(4.71- 9.30)
KATSOU	6	f	0	45	18	48	67	3.49	(1.80- 6.75)
KAUFMA	7	c	0	621	886	35	925	18.52	(13.02- 26.36)
KELLER	1	m	0	5063	1210	323	1017	13.17	(11.45- 15.15)
KELLER	9	m	0	1053	212	38	117	15.29	(10.31- 22.69)

Table 1B1 - 5

IESLC - Meta-analysis of Current Smoking (vs never smoking), Any product (or Cigarettes if Any not available)
All LC types
Least adjusted

REF	NRR	SEX	AD	Number Exposed Case	Number Exposed Cont	Non-exposed Case	Non-exposed Cont	RR	95.00%CI
KELLER	5	f	0	2904	792	469	1860	14.54	(12.79- 16.53)
KELLER	13	f	0	454	135	67	232	11.64	(8.35- 16.24)
Subtotal KELLER								13.79	(12.62- 15.07)
KHUDER	19	m	0	275	-	23	-	8.10	(5.20- 12.70)
KIHARA	7	c	0	283	162	102	237	4.06	(3.00- 5.49)
*KINLEN	9	m	0	636	9879	7	1333	12.26	(5.83- 25.76)
KJUUS	1	m	0	135	77	2	24	21.04	(4.84- 91.45)
*KNEKT	15	m	0	93	36489	6	17814	7.57	(3.31- 17.27)
KOO	9	f	0	42	25	56	85	2.55	(1.40- 4.64)
KREUZE	39	m	0	168	99	6	54	15.27	(6.34- 36.79)
KREUZE	41	m	0	1252	524	23	403	41.86	(27.17- 64.51)
KREUZE	40	f	0	55	23	6	38	15.14	(5.63- 40.72)
KREUZE	42	f	0	170	54	95	177	5.87	(3.95- 8.70)
Subtotal KREUZE								14.48	(11.09- 18.90)
*KUBIK	12	m	0	98	6342	2	4271	33.00	(8.14- 133.74)
*LANGE	32	m	0	174	4537	5	721	5.53	(2.28- 13.41)
*LANGE	29	f	0	53	4455	7	2159	3.67	(1.67- 8.06)
Subtotal LANGE								4.40	(2.44- 7.92)
LEMARC	2	c	0	167	65	32	168	13.49	(8.39- 21.68)
*LIAW	1	m	1	-	-	-	-	3.70	(2.10- 6.60)
*LIAW	2	f	1	-	-	-	-	3.60	(1.00- 12.20)
Subtotal LIAW								3.68	(2.19- 6.20)
*LIDDEL	4	m	1	-	-	-	-	4.41	(2.77- 7.01)
LOMBAR	9	m	0	852	610	14	112	11.17	(6.35- 19.66)
LUBIN	40	m	0	296	650	9	72	3.64	(1.80- 7.38)
LUBIN2	25	m	0	5700	7744	190	2617	10.14	(8.72- 11.79)
LUBIN2	317	f	0	384	410	288	1180	3.84	(3.17- 4.64)
Subtotal LUBIN2								6.95	(6.18- 7.82)
MACLEN	19	m	0	137	108	5	15	3.81	(1.34- 10.80)
MACLEN	32	f	0	42	47	41	109	2.38	(1.37- 4.12)
Subtotal MACLEN								2.63	(1.62- 4.28)
MATOS	2	m	0	112	132	11	110	8.48	(4.35- 16.56)
*MIGRAN	19	m	0	182	5145	4	867	7.67	(2.85- 20.59)
*MIGRAN	135	f	0	30	3465	4	3814	8.26	(2.91- 23.41)
Subtotal MIGRAN								7.94	(3.88- 16.26)
*MRFITR	2	m	0	106	8194	0	1859	48.33~	(3.01- 777.42)
NAM	68	m	0	241	589	30	520	7.09	(4.77- 10.55)
NAM	84	f	0	133	234	52	885	9.67	(6.81- 13.75)
Subtotal NAM								8.44	(6.49- 10.98)
ODRISC	1	c	0	293	598	6	664	54.22	(23.98- 122.60)
OSANN	9	m	0	791	541	45	833	27.07	(19.67- 37.25)
OSANN	13	f	0	597	367	96	1093	18.52	(14.48- 23.68)
Subtotal OSANN								21.33	(17.55- 25.92)
PARKIN	27	m	0	375	946	107	1248	4.62	(3.67- 5.82)
PERSH2	4	c	0	736	631	178	1164	7.63	(6.31- 9.23)
*PETO	4	m	0	99	2036	2	295	7.17	(1.78- 28.92)
PEZZO2	2	m	0	233	198	6	117	22.95	(9.89- 53.26)
PEZZOT	5	m	0	145	129	4	116	32.60	(11.70- 90.81)
*QIAO2	2	m	0	198	6101	10	709	2.30	(1.22- 4.32)
RACHTA	2	f	0	72	33	33	98	6.48	(3.66- 11.46)
SCHWAR	25	m	0	1652	349	119	376	14.96	(11.81- 18.94)
SCHWAR	26	m	0	644	139	50	104	9.64	(6.56- 14.15)
SCHWAR	27	f	0	1029	309	182	855	15.64	(12.75- 19.19)
SCHWAR	28	f	0	256	90	40	247	17.56	(11.64- 26.50)
Subtotal SCHWAR								14.70	(12.84- 16.83)
SEGI2	19	m	0	267	485	8	53	3.65	(1.71- 7.79)
SEGI2	27	f	0	24	34	56	126	1.59	(0.86- 2.92)
Subtotal SEGI2								2.20	(1.37- 3.54)
SHAW	6	c	0	212	97	11	107	21.26	(10.93- 41.36)
SOBUE	90	m	0	736	650	34	128	4.26	(2.88- 6.31)
SOBUE	94	f	0	95	168	167	857	2.90	(2.15- 3.92)
Subtotal SOBUE								3.35	(2.64- 4.25)
SOBUE2	10	m	2	-	-	-	-	4.47	(3.89- 5.14)
SOBUE2	12	f	2	-	-	-	-	3.28	(2.79- 3.87)
Subtotal SOBUE2								3.92	(3.53- 4.36)
*SPEIZE	6	f	0	391	489993	58	776300	10.68	(8.11- 14.07)
SPITZ	2	c	0	103	89	7	128	21.16	(9.40- 47.66)
STOCKW	7	c	0	12470	3357	2791	10641	14.16	(13.38- 14.99)
STUCKE	2	m	0	69	68	0	51	104.50~	(6.32-1727.39)
SUZUK2	2	c	0	78	30	11	53	12.53	(5.78- 27.16)
SVENSS	61	f	0	142	53	38	120	8.46	(5.22- 13.71)

Table 1B1 - 5

IESLC - Meta-analysis of Current Smoking (vs never smoking), Any product (or Cigarettes if Any not available)
All LC types
Least adjusted

REF	NRR	SEX	AD	Number Exposed Case	Number Exposed Cont	Non-exposed Case	Non-exposed Cont	RR	95.00%CI
TANG	1	c	0	52	25	9	39	9.01	(3.78- 21.46)
*TENKAN	24	m	1	-	-	-	-	16.81	(7.22- 39.14)
TIZZAN	5	m	0	693	619	180	305	1.90	(1.53- 2.35)
TIZZAN	13	f	0	17	18	25	114	4.31	(1.95- 9.51)
Subtotal TIZZAN								2.01	(1.63- 2.47)
TOKARS	1	m	0	110	157	1	53	37.13	(5.06- 272.56)
TOUSEY	4	m	0	160	91	4	130	57.14	(20.45- 159.69)
TOUSEY	8	f	0	127	78	13	226	28.31	(15.13- 52.94)
Subtotal TOUSEY								34.23	(20.06- 58.43)
TSUGAN	28	m	0	63	63	18	22	1.22	(0.60- 2.50)
*TULINI	14	m	1	-	-	-	-	10.69	(5.80- 19.72)
*TULINI	20	f	1	-	-	-	-	18.99	(10.52- 34.26)
Subtotal TULINI								14.40	(9.41- 22.02)
*TVERDA	5	m	2	-	-	-	-	4.09	(2.65- 6.31)
*TVERDA	15	f	2	-	-	-	-	11.05	(3.33- 36.71)
Subtotal TVERDA								4.59	(3.05- 6.90)
WAKAI	2	m	0	181	284	10	65	4.14	(2.07- 8.27)
WAKAI	20	f	0	33	26	50	145	3.68	(2.01- 6.75)
Subtotal WAKAI								3.87	(2.46- 6.11)
*WALD	2	m	0	77	4182	7	6539	17.20	(7.94- 37.25)
WANG2	17	c	0	49	78	11	43	2.46	(1.16- 5.21)
WIGLE	3	m	0	454	522	15	204	11.83	(6.90- 20.28)
WIGLE	6	f	0	68	169	36	439	4.91	(3.16- 7.63)
Subtotal WIGLE								6.98	(4.96- 9.83)
WU	34	f	0	160	73	31	92	6.50	(3.98- 10.64)
WUNSCH	2	m	0	189	234	14	99	5.71	(3.16- 10.32)
WUNSCH	8	f	0	42	51	29	208	5.91	(3.36- 10.38)
Subtotal WUNSCH								5.81	(3.87- 8.74)
WYNDE3	50	m	0	227	207	9	88	10.72	(5.26- 21.84)
WYNDE6	18	m	0	1677	741	87	617	16.05	(12.62- 20.41)
WYNDE6	207	f	0	1022	376	159	856	14.63	(11.90- 17.99)
Subtotal WYNDE6								15.22	(13.01- 17.80)
YAMAGU	1	c	0	76	247	24	267	3.42	(2.10- 5.59)
*YONG	12	m	1	-	-	-	-	28.71	(6.98- 118.16)
*YONG	15	f	1	-	-	-	-	5.20	(2.38- 11.35)
Subtotal YONG								7.75	(3.91- 15.36)
Partial Totals				70900	2563023	9573	4433323		

*prospective study ~ With 0.5 adjustment for zero

REF	NRR	SEX	AD	Ys	Ws	Qs	Ps
AGUDO	10	f	0	0.99	7.89	11.75	0.0054
*AKIBA	2	m	0	1.39	17.12	11.58	0.0000
*AKIBA	6	f	0	1.50	45.22	22.87	0.0000
Subtotal AKIBA				1.47	62.34	34.45	
ALDERS	177	m	0	2.66	12.62	2.54	0.0000
ALDERS	176	f	0	1.76	41.23	8.45	0.0000
Subtotal ALDERS				1.97	53.85	10.99	
*AMANDU	1	m	0	1.61	5.70	2.03	0.0001
AMES	1	m	0	1.52	10.33	4.97	0.0000
*ANDERS	2	f	0	3.08	37.84	28.71	0.0000
*ARCHER	5	m	0	1.82	5.72	0.89	0.0000
ARMADA	27	m	0	3.21	3.58	3.54	0.0000
AUSTIN	2	c	0	2.75	4.38	1.27	0.0000
AXELSS	2	m	0	2.70	12.26	2.97	0.0000
AXELSS	10	f	0	2.48	11.50	0.81	0.0000
Subtotal AXELSS				2.59	23.76	3.78	
BARBON	3	m	0	2.59	18.08	2.54	0.0000
BECHER	13	m	0	2.70	2.70	0.65	0.0000
BECHER	14	f	0	1.89	5.32	0.56	0.0000
Subtotal BECHER				2.16	8.02	1.21	
*BENSHL	16	m	1	2.10	5.77	0.07	0.0000
*BEST	2	m	1	2.70	6.85	1.65	0.0000
BLOHMK	1	m	0	1.16	59.38	65.25	0.0000
*BOUCOT	2	m	0	4.06	0.50	1.71	0.0042
*BRETT	4	m	0	1.37	5.75	4.09	0.0010
BROSS	11	m	0	1.58	28.40	11.42	0.0000
BROWN2	12	m	2	2.42	402.82	18.46	0.0000
BROWN2	11	f	2	2.61	365.29	58.25	0.0000
Subtotal BROWN2				2.51	768.12	76.70	
BUFFLE	3	m	0	2.40	4.35	0.16	0.0000

Table 1B1 - 5

IESLC - Meta-analysis of Current Smoking (vs never smoking), Any product (or Cigarettes if Any not available)
All LC types
Least adjusted

REF	NRR	SEX	AD	Ys	Ws	Qs	Ps
BUFFLE	7	f	0	2.11	26.25	0.26	0.0000
Subtotal BUFFLE				2.15	30.60	0.42	
CARPEN	9	c	0	3.08	12.26	9.23	0.0000
*CEDERL	106	m	2	2.04	20.57	0.57	0.0000
*CEDERL	75	f	2	1.57	30.42	12.38	0.0000
Subtotal CEDERL				1.76	50.99	12.95	
*CHANG	5	m	0	2.13	4.46	0.03	0.0000
*CHANG	11	f	0	1.64	8.22	2.68	0.0000
Subtotal CHANG				1.81	12.68	2.71	
CHOI	3	m	0	1.64	10.55	3.44	0.0000
CHOI	7	f	0	0.20	7.16	28.99	0.5951
Subtotal CHOI				1.06	17.71	32.43	
*CHOW	25	m	0	2.64	5.79	1.09	0.0000
*CHYOU	4	m	0	2.27	12.23	0.04	0.0000
COMSTO	3	m	0	2.90	3.52	1.66	0.0000
COMSTO	8	f	0	2.57	8.49	1.11	0.0000
Subtotal COMSTO				2.67	12.01	2.77	
CORREA	41	c	0	2.53	40.00	4.10	0.0000
*CPSI	220	m	1	2.48	74.99	5.43	0.0000
*CPSI	279	f	1	1.16	70.15	76.99	0.0000
Subtotal CPSI				1.84	145.14	82.42	
*CPSII	36	m	0	2.90	115.97	55.89	0.0000
*CPSII	71	f	0	2.22	237.52	0.01	0.0000
Subtotal CPSII				2.44	353.49	55.91	
DAMBER	14	m	1	2.26	26.18	0.07	0.0000
DARBY	4	m	0	4.51	2.93	15.50	0.0000
DARBY	11	f	0	3.03	18.15	12.14	0.0000
Subtotal DARBY				3.23	21.08	27.64	
DEAN2	2	m	0	1.33	23.59	18.14	0.0000
DEAN2	6	f	0	1.06	13.83	18.20	0.0001
Subtotal DEAN2				1.23	37.42	36.34	
DEAN3	40	m	0	1.83	22.44	3.18	0.0000
DEAN3	117	f	0	1.20	28.00	28.88	0.0000
Subtotal DEAN3				1.48	50.44	32.07	
*DEKLER	8	m	2	3.14	0.99	0.85	0.0018
DESTE2	2	c	0	2.04	14.18	0.39	0.0000
DESTEF	40	m	0	2.27	19.86	0.07	0.0000
*DOCKER	1	c	4	2.08	3.90	0.07	0.0000
DOLL	90	m	0	2.25	6.22	0.01	0.0000
DOLL	93	f	0	0.74	11.96	26.04	0.0110
Subtotal DOLL				1.25	18.18	26.05	
*DOLL2	54	m	1	2.40	18.45	0.64	0.0000
*DOLL2	63	f	1	2.16	3.28	0.01	0.0001
Subtotal DOLL2				2.36	21.73	0.65	
DORANT	2	m	0	2.38	6.51	0.19	0.0000
DORGAN	9	m	0	2.83	11.70	4.47	0.0000
DORGAN	33	m	0	3.71	2.61	5.88	0.0000
DORGAN	56	f	0	2.50	41.93	3.47	0.0000
DORGAN	79	f	0	2.44	3.75	0.19	0.0000
Subtotal DORGAN				2.61	60.00	14.01	
*DORN	51	m	1	2.11	73.41	0.78	0.0000
DROSTE	2	m	0	2.94	6.25	3.30	0.0000
*ENGELA	154	m	0	1.75	6.56	1.37	0.0000
*ENGELA	161	f	0	1.39	6.52	4.42	0.0004
Subtotal ENGELA				1.57	13.08	5.79	
*ENSTRO	1	m	1	2.56	81.91	10.23	0.0000
*ENSTRO	2	f	1	1.94	181.45	13.43	0.0000
Subtotal ENSTRO				2.13	263.36	23.66	
GAO	29	m	0	1.37	39.60	28.00	0.0000
GAO	30	f	0	0.86	50.42	91.92	0.0000
Subtotal GAO				1.08	90.02	119.92	
GAO2	1	m	0	1.91	9.19	0.81	0.0000
GARCIA	2	c	0	2.72	13.47	3.44	0.0000
GARDIN	2	c	0	2.62	3.97	0.66	0.0000
GARSHI	23	m	0	2.01	33.39	1.39	0.0000
GOODMA	2	m	0	2.86	8.50	3.56	0.0000
GOODMA	6	f	0	2.27	10.71	0.03	0.0000
Subtotal GOODMA				2.53	19.21	3.59	
GRAHAM	20	m	0	1.91	16.38	1.49	0.0000
GREGOR	2	m	0	0.26	4.75	18.10	0.5741
GREGOR	6	f	0	2.67	0.88	0.18	0.0126

Table 1B1 - 5

IESLC - Meta-analysis of Current Smoking (vs never smoking), Any product (or Cigarettes if Any not available)
All LC types
Least adjusted

REF	NRR	SEX	AD	Ys	Ws	Qs	Ps
Subtotal GREGOR				0.63	5.62	18.28	
HAENSZ	54	f	0	0.76	23.97	50.44	0.0002
*HAMMO2	22	m	0	2.12	4.92	0.04	0.0000
*HAMMON	139	m	1	2.44	14.07	0.77	0.0000
*HEIN	5	m	0	2.85	0.99	0.41	0.0045
*HENNEK	2	m	0	2.73	17.97	4.91	0.0000
*HIRAYA	1	m	1	1.49	85.55	44.08	0.0000
*HIRAYA	3	f	1	0.85	77.37	143.23	0.0000
Subtotal HIRAYA				1.19	162.92	187.32	
HITOSU	2	m	0	0.93	6.40	10.52	0.0187
HITOSU	9	f	0	1.25	14.55	13.36	0.0000
Subtotal HITOSU				1.15	20.95	23.87	
*HOLE	47	m	0	1.90	6.76	0.64	0.0000
*HOLE	29	f	0	0.37	4.98	16.80	0.4046
Subtotal HOLE				1.25	11.74	17.44	
HUMBLE	13	m	1	2.99	4.94	3.03	0.0000
HUMBLE	15	m	1	2.76	1.65	0.50	0.0004
HUMBLE	17	f	1	2.82	5.85	2.15	0.0000
HUMBLE	19	f	1	3.16	2.49	2.23	0.0000
Subtotal HUMBLE				2.93	14.94	7.91	
JAHN	5	m	0	2.31	14.42	0.13	0.0000
JAIN	16	m	0	2.77	9.32	2.88	0.0000
JAIN	11	f	0	2.54	26.82	2.91	0.0000
Subtotal JAIN				2.60	36.14	5.79	
JARVHO	2	m	0	3.70	0.90	1.99	0.0005
JARVHO	6	f	0	2.74	2.57	0.72	0.0000
Subtotal JARVHO				2.99	3.47	2.71	
JOLY	18	m	0	2.59	10.93	1.56	0.0000
JOLY	15	f	0	2.01	24.54	0.96	0.0000
Subtotal JOLY				2.19	35.47	2.52	
*KAISE2	68	m	1	2.08	10.65	0.17	0.0000
*KAISE2	60	f	1	2.67	8.78	1.87	0.0000
Subtotal KAISE2				2.35	19.43	2.04	
*KAISER	12	m	2	2.98	25.68	15.04	0.0000
*KAISER	9	f	2	1.88	27.68	3.09	0.0000
Subtotal KAISER				2.41	53.36	18.13	
KANELL	5	m	0	1.89	33.17	3.43	0.0000
KATSOU	6	f	0	1.25	8.81	8.13	0.0002
KAUFMA	7	c	0	2.92	30.87	15.49	0.0000
KELLER	1	m	0	2.58	195.95	26.47	0.0000
KELLER	9	m	0	2.73	24.67	6.59	0.0000
KELLER	5	f	0	2.68	233.82	50.83	0.0000
KELLER	13	f	0	2.45	34.67	2.07	0.0000
Subtotal KELLER				2.62	489.11	85.95	
KHUDER	19	m	0	2.09	19.27	0.27	0.0000
KIHARA	7	c	0	1.40	42.14	27.64	0.0000
*KINLEN	9	m	0	2.51	6.96	0.61	0.0000
KJUUS	1	m	0	3.05	1.78	1.24	0.0000
*KNEKT	15	m	0	2.02	5.64	0.20	0.0000
KOO	9	f	0	0.94	10.70	17.39	0.0022
KREUZE	39	m	0	2.73	4.97	1.32	0.0000
KREUZE	41	m	0	3.73	20.55	47.70	0.0000
KREUZE	40	f	0	2.72	3.93	1.01	0.0000
KREUZE	42	f	0	1.77	24.64	4.81	0.0000
Subtotal KREUZE				2.67	54.09	54.84	
*KUBIK	12	m	0	3.50	1.96	3.24	0.0000
*LANGE	32	m	0	1.71	4.90	1.23	0.0002
*LANGE	29	f	0	1.30	6.21	5.15	0.0012
Subtotal LANGE				1.48	11.11	6.38	
LEMARC	2	c	0	2.60	17.07	2.61	0.0000
*LIAW	1	m	1	1.31	11.72	9.54	0.0000
*LIAW	2	f	1	1.28	2.46	2.12	0.0447
Subtotal LIAW				1.30	14.17	11.67	
*LIDDEL	4	m	1	1.48	17.82	9.42	0.0000
LOMBAR	9	m	0	2.41	12.02	0.49	0.0000
LUBIN	40	m	0	1.29	7.70	6.49	0.0003
LUBIN2	25	m	0	2.32	168.07	1.87	0.0000
LUBIN2	317	f	0	1.34	106.80	80.09	0.0000
Subtotal LUBIN2				1.94	274.88	81.96	
MACLEN	19	m	0	1.34	3.53	2.70	0.0120
MACLEN	32	f	0	0.87	12.71	23.02	0.0020

Table 1B1 - 5

IESLC - Meta-analysis of Current Smoking (vs never smoking), Any product (or Cigarettes if Any not available)
All LC types
Least adjusted

REF	NRR	SEX	AD	Ys	Ws	Qs	Ps
Subtotal MACLEN				0.97	16.25	25.72	
MATOS	2	m	0	2.14	8.58	0.05	0.0000
*MIGRAN	19	m	0	2.04	3.93	0.12	0.0001
*MIGRAN	135	f	0	2.11	3.54	0.04	0.0001
Subtotal MIGRAN				2.07	7.47	0.15	
*MRFITR	2	m	0	3.88	0.50	1.38	0.0062
NAM	68	m	0	1.96	24.33	1.54	0.0000
NAM	84	f	0	2.27	31.10	0.11	0.0000
Subtotal NAM				2.13	55.43	1.65	
ODRISC	1	c	0	3.99	5.77	18.34	0.0000
OSANN	9	m	0	3.30	37.69	44.57	0.0000
OSANN	13	f	0	2.92	63.57	31.88	0.0000
Subtotal OSANN				3.06	101.25	76.44	
PARKIN	27	m	0	1.53	72.09	33.30	0.0000
PERSH2	4	c	0	2.03	106.15	3.40	0.0000
*PETO	4	m	0	1.97	1.98	0.11	0.0056
PEZZO2	2	m	0	3.13	5.42	4.61	0.0000
PEZZOT	5	m	0	3.48	3.66	5.93	0.0000
*QIAO2	2	m	0	0.83	9.66	18.34	0.0096
RACHTA	2	f	0	1.87	11.81	1.38	0.0000
SCHWAR	25	m	0	2.71	68.81	16.82	0.0000
SCHWAR	26	m	0	2.27	26.07	0.08	0.0000
SCHWAR	27	f	0	2.75	91.98	26.76	0.0000
SCHWAR	28	f	0	2.87	22.69	9.74	0.0000
Subtotal SCHWAR				2.69	209.54	53.39	
SEGI2	19	m	0	1.29	6.68	5.62	0.0008
SEGI2	27	f	0	0.46	10.32	31.55	0.1372
Subtotal SEGI2				0.79	17.00	37.16	
SHAW	6	c	0	3.06	8.67	6.21	0.0000
SOBUE	90	m	0	1.45	24.92	14.43	0.0000
SOBUE	94	f	0	1.07	42.31	55.51	0.0000
Subtotal SOBUE				1.21	67.24	69.94	
SOBUE2	10	m	2	1.50	197.90	100.71	0.0000
SOBUE2	12	f	2	1.19	143.51	150.17	0.0000
Subtotal SOBUE2				1.37	341.42	250.88	
*SPEIZE	6	f	0	2.37	50.52	1.26	0.0000
SPITZ	2	c	0	3.05	5.83	4.13	0.0000
STOCKW	7	c	0	2.65	1204.31	232.98	0.0000
STUCKE	2	m	0	4.65	0.49	2.90	0.0012
SUZUK2	2	c	0	2.53	6.41	0.65	0.0000
SVENSS	61	f	0	2.14	16.51	0.09	0.0000
TANG	1	c	0	2.20	5.10	0.00	0.0000
*TENKAN	24	m	1	2.82	5.38	2.01	0.0000
TIZZAN	5	m	0	0.64	84.08	207.39	0.0000
TIZZAN	13	f	0	1.46	6.13	3.45	0.0003
Subtotal TIZZAN				0.70	90.21	210.84	
TOKARS	1	m	0	3.61	0.97	1.91	0.0004
TOUSEY	4	m	0	4.05	3.64	12.24	0.0000
TOUSEY	8	f	0	3.34	9.80	12.56	0.0000
Subtotal TOUSEY				3.53	13.44	24.81	
TSUGAN	28	m	0	0.20	7.53	30.44	0.5818
*TULINI	14	m	1	2.37	10.26	0.26	0.0000
*TULINI	20	f	1	2.94	11.02	5.92	0.0000
Subtotal TULINI				2.67	21.28	6.18	
*TVERDA	5	m	2	1.41	20.41	13.14	0.0000
*TVERDA	15	f	2	2.40	2.67	0.10	0.0001
Subtotal TVERDA				1.52	23.08	13.24	
WAKAI	2	m	0	1.42	8.04	5.01	0.0001
WAKAI	20	f	0	1.30	10.45	8.61	0.0000
Subtotal WAKAI				1.35	18.49	13.62	
*WALD	2	m	0	2.84	6.43	2.59	0.0000
WANG2	17	c	0	0.90	6.78	11.68	0.0193
WIGLE	3	m	0	2.47	13.21	0.89	0.0000
WIGLE	6	f	0	1.59	19.73	7.59	0.0000
Subtotal WIGLE				1.94	32.94	8.48	
WU	34	f	0	1.87	15.85	1.81	0.0000
WUNSCH	2	m	0	1.74	10.98	2.41	0.0000
WUNSCH	8	f	0	1.78	12.09	2.28	0.0000
Subtotal WUNSCH				1.76	23.07	4.69	
WYNDE3	50	m	0	2.37	7.59	0.20	0.0000
WYNDE6	18	m	0	2.78	66.40	21.19	0.0000

Table 1B1 - 5

IESLC - Meta-analysis of Current Smoking (vs never smoking), Any product (or Cigarettes if Any not available)
All LC types
Least adjusted

REF	NRR	SEX	AD	Ys	Ws	Qs	Ps
WYNDE6	207	f	0	2.68	90.13	20.12	0.0000
Subtotal WYNDE6				2.72	156.52	41.32	
YAMAGU	1	c	0	1.23	15.97	15.34	0.0000
*YONG	12	m	1	3.36	1.92	2.52	0.0000
*YONG	15	f	1	1.65	6.30	1.99	0.0000
Subtotal YONG				2.05	8.22	4.51	

	N	195
	NS	131
	Wt	6923.88
Het	Chi	2698.51
Het	df	194
Het	P	***
Fixed	RR	9.12
	RRl	8.91
	RRu	9.34
	P	+++
Random	RR	8.26
	RRl	7.49
	RRu	9.12
	P	+++
Asymm	P	*

Table 1B1 - 6

IESLC - Meta-analysis of Current Smoking (vs never smoking), Any product (or Cigarettes if Any not available)
All LC types
Least adjusted

	Sex			
	combined	male	female	Total
N	19	108	68	195
NS	19	103	63	185
Wt	1547.26	2766.85	2609.76	6923.88
Het Chi	171.65	1077.28	1192.93	2698.51
Het df	18	107	67	194
Het P	***	***	***	***
Fixed RR	12.90	8.72	7.79	9.12
RRl	12.28	8.40	7.50	8.91
RRu	13.56	9.05	8.09	9.34
P	+++	+++	+++	+++
Random RR	11.19	9.14	6.55	8.26
RRl	8.57	8.00	5.50	7.49
RRu	14.61	10.44	7.81	9.12
P	+++	+++	+++	+++
Between Chi				256.65
Between df				2
Between P				***
Btwn(F) P				***
Btwn(R) P				**

	Lung cancer type		
	all	other	Total
N	187	8	195
NS	125	6	131
Wt	6462.76	461.12	6923.88
Het Chi	2268.90	37.63	2698.51
Het df	186	7	194
Het P	***	***	***
Fixed RR	9.72	3.74	9.12
RRl	9.49	3.42	8.91
RRu	9.96	4.10	9.34
P	+++	+++	+++
Random RR	8.58	3.54	8.26
RRl	7.80	2.73	7.49
RRu	9.45	4.58	9.12
P	+++	+++	+++
Between Chi			391.99
Between df			1
Between P			***
Btwn(F) P			***
Btwn(R) P			***

	Location								
	NAmer	UK	Scand	othEur	China	Japan	othAs	other	Total
N	84	25	21	23	5	18	7	12	195
NS	55	16	14	17	4	11	4	10	131
Wt	4621.86	264.17	309.40	600.51	114.16	765.20	58.83	189.73	6923.88
Het Chi	610.22	166.74	50.93	344.46	6.90	54.52	10.61	40.22	2698.51
Het df	83	24	20	22	4	17	6	11	194
Het P	***	***	***	***	N.S.	***	N.S.	***	***
Fixed RR	12.19	6.54	7.81	5.92	2.90	3.66	2.91	7.03	9.12
RRl	11.85	5.79	6.98	5.47	2.42	3.41	2.26	6.10	8.91
RRu	12.55	7.37	8.73	6.42	3.49	3.93	3.76	8.11	9.34
P	+++	+++	+++	+++	+++	+++	+++	+++	+++
Random RR	11.41	7.56	8.38	8.69	2.91	3.49	2.90	9.16	8.26
RRl	10.38	5.38	6.78	6.04	2.21	3.01	2.04	6.71	7.49
RRu	12.55	10.61	10.36	12.51	3.82	4.05	4.13	12.50	9.12
P	+++	+++	+++	+++	+++	+++	+++	+++	+++
Between Chi									1413.93
Between df									7
Between P									***
Btwn(F) P									***
Btwn(R) P									***

Table 1B1 - 6

IESLC - Meta-analysis of Current Smoking (vs never smoking), Any product (or Cigarettes if Any not available)
All LC types
Least adjusted

Detailed Country in "other Europe"

	multi	Germany	othWest	East	Balkans	Total
N	2	8	8	3	2	23
NS	1	4	7	3	2	17
Wt	274.88	135.91	133.01	14.73	41.98	600.51
Het Chi	61.64	111.74	109.74	6.53	2.85	344.46
Het df	1	7	7	2	1	22
Het P	***	***	***	*	(*)	***
Fixed RR	6.95	6.98	3.46	9.02	5.78	5.92
RRl	6.18	5.90	2.92	5.42	4.27	5.47
RRu	7.82	8.26	4.10	15.04	7.83	6.42
P	+++	+++	+++	+++	+++	+++
Random RR	6.25	10.53	8.62	16.26	5.13	8.69
RRl	2.41	4.96	3.59	4.36	2.78	6.04
RRu	16.19	22.37	20.69	60.67	9.47	12.51
P	+++	+++	+++	+++	+++	+++
Between Chi						51.97
Between df						4
Between P						***
Btwn(F) P						N.S.
Btwn(R) P						N.S.

Detailed Country in "other Asia"

	India	HongKong	other	Total
N		1	6	7
NS		1	3	4
Wt		10.70	48.13	58.83
Het Chi		0.00	10.38	10.61
Het df		0	5	6
Het P		N.S.	(*)	N.S.
Fixed RR		2.55	3.00	2.91
RRl		1.40	2.26	2.26
RRu		4.64	3.98	3.76
P		++	+++	+++
Random RR		2.55	2.98	2.90
RRl		1.40	1.94	2.04
RRu		4.64	4.57	4.13
P		++	+++	+++
Between Chi				0.23
Between df				1
Between P				N.S.
Btwn(F) P				N.S.
Btwn(R) P				N.S.

Detailed other continent

	SCAmer	Auslia	Africa	Total
N	10	1	1	12
NS	8	1	1	10
Wt	116.65	0.99	72.09	189.73
Het Chi	18.89	0.00	0.00	40.22
Het df	9	0	0	11
Het P	*	N.S.	N.S.	***
Fixed RR	9.02	23.03	4.62	7.03
RRl	7.53	3.21	3.67	6.10
RRu	10.82	165.10	5.82	8.11
P	+++	++	+++	+++
Random RR	9.62	23.03	4.62	9.16
RRl	7.31	3.21	3.67	6.71
RRu	12.65	165.10	5.82	12.50
P	+++	++	+++	+++
Between Chi				21.33
Between df				2
Between P				***
Btwn(F) P				*
Btwn(R) P				***

Table 1B1 - 6

IESLC - Meta-analysis of Current Smoking (vs never smoking), Any product (or Cigarettes if Any not available)
All LC types
Least adjusted

		Start year of study				
	<1960	1960-69	1970-79	1980-89	1990+	Total
N	22	40	41	70	22	195
NS	17	27	27	43	17	131
Wt	742.73	1159.66	785.37	3981.56	254.56	6923.88
Het Chi	302.47	422.57	259.09	749.83	175.18	2698.51
Het df	21	39	40	69	21	194
Het P	***	***	***	***	***	***
Fixed RR	6.21	5.38	6.95	11.98	10.21	9.12
RRl	5.78	5.08	6.48	11.61	9.03	8.91
RRu	6.67	5.70	7.45	12.35	11.55	9.34
P	+++	+++	+++	+++	+++	+++
Random RR	6.45	6.32	7.15	9.82	12.57	8.26
RRl	4.75	5.12	5.83	8.67	8.71	7.49
RRu	8.76	7.81	8.78	11.12	18.14	9.12
P	+++	+++	+++	+++	+++	+++
Between Chi						789.37
Between df						4
Between P						***
Btwn(F) P						***
Btwn(R) P						***

	Study type (1)		
	CC	other	Total
N	128	67	195
NS	83	48	131
Wt	5329.20	1594.68	6923.88
Het Chi	2105.36	547.69	2698.51
Het df	127	66	194
Het P	***	***	***
Fixed RR	9.54	7.87	9.12
RRl	9.28	7.49	8.91
RRu	9.80	8.26	9.34
P	+++	+++	+++
Random RR	8.39	7.98	8.26
RRl	7.43	6.79	7.49
RRu	9.48	9.39	9.12
P	+++	+++	+++
Between Chi			45.46
Between df			1
Between P			***
Btwn(F) P			(*)
Btwn(R) P			N.S.

	Study type (2)			
	CC	prosp	other	Total
N	128	62	5	195
NS	83	44	4	131
Wt	5329.20	1564.86	29.81	6923.88
Het Chi	2105.36	536.96	9.81	2698.51
Het df	127	61	4	194
Het P	***	***	*	***
Fixed RR	9.54	7.84	9.37	9.12
RRl	9.28	7.46	6.54	8.91
RRu	9.80	8.24	13.41	9.34
P	+++	+++	+++	+++
Random RR	8.39	7.82	10.82	8.26
RRl	7.43	6.60	5.88	7.49
RRu	9.48	9.25	19.89	9.12
P	+++	+++	+++	+++
Between Chi				46.38
Between df				2
Between P				***
Btwn(F) P				N.S.
Btwn(R) P				N.S.

Table 1B1 - 6

IESLC - Meta-analysis of Current Smoking (vs never smoking), Any product (or Cigarettes if Any not available)
All LC types
Least adjusted

	Study size (number of LC cases)				
	100-249	250-499	500-999	1000+	Total
N	56	48	38	53	195
NS	46	35	24	26	131
Wt	376.67	544.64	764.08	5238.49	6923.88
Het Chi	234.22	259.53	301.71	1736.72	2698.51
Het df	55	47	37	52	194
Het P	***	***	***	***	***
Fixed RR	5.48	8.31	7.42	9.85	9.12
RRl	4.95	7.64	6.91	9.59	8.91
RRu	6.06	9.03	7.96	10.12	9.34
P	+++	+++	+++	+++	+++
Random RR	6.70	8.49	9.32	8.83	8.26
RRl	5.35	6.93	7.53	7.47	7.49
RRu	8.38	10.41	11.55	10.44	9.12
P	+++	+++	+++	+++	+++
Between Chi					166.34
Between df					3
Between P					***
Btwn(F) P					**
Btwn(R) P					N.S.

	Risky occupational population			
	no	mining	othRisky	Total
N	184	7	4	195
NS	120	7	4	131
Wt	6822.29	57.93	43.65	6923.88
Het Chi	2650.58	7.65	4.32	2698.51
Het df	183	6	3	194
Het P	***	N.S.	N.S.	***
Fixed RR	9.19	4.18	8.41	9.12
RRl	8.97	3.23	6.25	8.91
RRu	9.41	5.41	11.31	9.34
P	+++	+++	+++	+++
Random RR	8.39	4.23	9.56	8.26
RRl	7.58	3.13	5.95	7.49
RRu	9.28	5.71	15.35	9.12
P	+++	+++	+++	+++
Between Chi				35.96
Between df				2
Between P				***
Btwn(F) P				N.S.
Btwn(R) P				***

	National cigarette tobacco type			
	Virginia	blended	other	Total
N	34	154	7	195
NS	23	103	5	131
Wt	439.69	6355.85	128.34	6923.88
Het Chi	221.44	2256.61	7.61	2698.51
Het df	33	153	6	194
Het P	***	***	N.S.	***
Fixed RR	6.74	9.53	2.98	9.12
RRl	6.14	9.30	2.51	8.91
RRu	7.40	9.77	3.54	9.34
P	+++	+++	+++	+++
Random RR	8.07	8.68	3.01	8.26
RRl	6.21	7.80	2.43	7.49
RRu	10.48	9.65	3.73	9.12
P	+++	+++	+++	+++
Between Chi				212.86
Between df				2
Between P				***
Btwn(F) P				***
Btwn(R) P				***

Table 1B1 - 6

IESLC - Meta-analysis of Current Smoking (vs never smoking), Any product (or Cigarettes if Any not available)
All LC types
Least adjusted

	Any proxy use No/nk	Yes	Total
N	156	39	195
NS	110	21	131
Wt	6040.75	883.12	6923.88
Het Chi	2436.77	261.73	2698.51
Het df	155	38	194
Het P	***	***	***
Fixed RR	9.12	9.16	9.12
RRl	8.89	8.57	8.91
RRu	9.35	9.78	9.34
P	+++	+++	+++
Random RR	7.92	9.63	8.26
RRl	7.08	7.98	7.49
RRu	8.87	11.63	9.12
P	+++	+++	+++
Between Chi			0.02
Between df			1
Between P			N.S.
Btwn(F) P			N.S.
Btwn(R) P			(*)

	Full histological confirmation No	Yes	Total
N	144	51	195
NS	95	36	131
Wt	4977.30	1946.57	6923.88
Het Chi	2081.50	582.37	2698.51
Het df	143	50	194
Het P	***	***	***
Fixed RR	8.73	10.22	9.12
RRl	8.49	9.77	8.91
RRu	8.97	10.68	9.34
P	+++	+++	+++
Random RR	7.75	9.86	8.26
RRl	6.88	8.27	7.49
RRu	8.74	11.76	9.12
P	+++	+++	+++
Between Chi			34.64
Between df			1
Between P			***
Btwn(F) P			N.S.
Btwn(R) P			*

	Number of adjustment variables (1) 0	1	2+/+nk	Total
N	156	27	12	195
NS	108	16	7	131
Wt	4861.36	820.66	1241.86	6923.88
Het Chi	1963.27	272.03	367.17	2698.51
Het df	155	26	11	194
Het P	***	***	***	***
Fixed RR	9.75	6.83	8.53	9.12
RRl	9.48	6.37	8.07	8.91
RRu	10.02	7.31	9.02	9.34
P	+++	+++	+++	+++
Random RR	8.25	8.69	7.53	8.26
RRl	7.37	6.76	5.14	7.49
RRu	9.24	11.19	11.03	9.12
P	+++	+++	+++	+++
Between Chi				96.05
Between df				2
Between P				***
Btwn(F) P				*
Btwn(R) P				N.S.

Table 1B1 - 6

IESLC - Meta-analysis of Current Smoking (vs never smoking), Any product (or Cigarettes if Any not available)
All LC types
Least adjusted

	Number of adjustment variables (2) 0	1	2	3-5	6+/+nk	Total
N	156	27	11	1		195
NS	108	16	6	1		131
Wt	4861.36	820.66	1237.95	3.90		6923.88
Het Chi	1963.27	272.03	367.15	0.00		2698.51
Het df	155	26	10	0		194
Het P	***	***	***	N.S.		***
Fixed RR	9.75	6.83	8.53	8.00		9.12
RRl	9.48	6.37	8.07	2.97		8.91
RRu	10.02	7.31	9.02	21.57		9.34
P	+++	+++	+++	+++		+++
Random RR	8.25	8.69	7.50	8.00		8.26
RRl	7.37	6.76	5.05	2.97		7.49
RRu	9.24	11.19	11.14	21.57		9.12
P	+++	+++	+++	+++		+++
Between Chi						96.07
Between df						3
Between P						***
Btwn(F) P						(*)
Btwn(R) P						N.S.

	Product all/unsp	cig+/-ot	cig only	Total
N	85	95	15	195
NS	63	62	13	138
Wt	1901.55	4508.99	513.34	6923.88
Het Chi	794.26	1710.25	142.43	2698.51
Het df	84	94	14	194
Het P	***	***	***	***
Fixed RR	7.93	9.63	9.54	9.12
RRl	7.58	9.35	8.75	8.91
RRu	8.29	9.91	10.40	9.34
P	+++	+++	+++	+++
Random RR	7.55	8.71	9.77	8.26
RRl	6.48	7.55	6.98	7.49
RRu	8.80	10.03	13.67	9.12
P	+++	+++	+++	+++
Between Chi				51.56
Between df				2
Between P				***
Btwn(F) P				N.S.
Btwn(R) P				N.S.

	Denominator nev any	nev cigs	Total
N	134	61	195
NS	93	41	134
Wt	4676.12	2247.76	6923.88
Het Chi	1921.20	750.35	2698.51
Het df	133	60	194
Het P	***	***	***
Fixed RR	8.74	9.98	9.12
RRl	8.49	9.58	8.91
RRu	8.99	10.40	9.34
P	+++	+++	+++
Random RR	8.14	8.58	8.26
RRl	7.19	7.27	7.49
RRu	9.21	10.13	9.12
P	+++	+++	+++
Between Chi			26.97
Between df			1
Between P			***
Btwn(F) P			N.S.
Btwn(R) P			N.S.

Table 1B1 - 6

IESLC - Meta-analysis of Current Smoking (vs never smoking), Any product (or Cigarettes if Any not available)
All LC types
Least adjusted

	Derivation of RR/CI			
	Orig	StdCalc	Other	Total
N	14	151	30	195
NS	9	103	20	132
Wt	1180.13	4894.85	848.90	6923.88
Het Chi	156.09	2117.16	269.56	2698.51
Het df	13	150	29	194
Het P	***	***	***	***
Fixed RR	10.12	9.53	6.13	9.12
RRl	9.55	9.27	5.74	8.91
RRu	10.71	9.80	6.56	9.34
P	+++	+++	+++	+++
Random RR	7.04	8.21	9.36	8.26
RRl	5.47	7.30	7.34	7.49
RRu	9.05	9.24	11.93	9.12
P	+++	+++	+++	+++
Between Chi				155.70
Between df				2
Between P				***
Btwn(F) P				**
Btwn(R) P				N.S.

Table 1B1 - 7

IESLC - Meta-analysis of Current Smoking (vs never smoking), Any product (or Cigarettes if Any not available)
All LC types
Excluded studies (and stage at which they were excluded)

1	BOUCHA BUELL LAURIL MZILEN
2	ABELIN ABRAHA AUVINE AXELSO BAND BERRIN BLOT1 BLOT2 BLOT3 BLOT4 BRESLO BROCKM BROWN1 BYERS1 CASCO2 CASCOR CHAN CHATZI CHEN CHEN2 CHEN3 CHIAZZ COOKSO DAVEYS DEAN DOSEME DU DUNN EBELIN ESAKI FAN GENG GER GODLEY GOLLED GSELL HANSEN HEGMAN HINDS HIRAOK HOROWI HORWIT HU HU2 HUANG ISHIMA JARUP JIANG JONES JUSSAW KO KOHLME KOULUM KREYBE LAMTH LAMWK LAMWK2 LAUSSM LEI LETOUR LEVIN LIU LIU2 LIU3 LIU4 LIU5 LOMBA2 LUO MAGNUS MARSH MARSH2 MARTIS MASTRA MATSUD MCCONN MCDUFF MCLAUG MILLER MILLS MOLLO NOTAN2 NOU ORMOS PASTOR PAWLEG PERNU PERSHA PIKE POFFIJ POLEDN PRESCO QIAO RADZIK RANDIG REN RONCO ROOTS ROTHSC SADOWS SANKAR SCHWA2 SEGI SEOW SIEMIA SIMARA STASZE STOCKS SUN TAO ULMER VANDER WANG WANG3 WANG4 WICKLU WILKIN WUWILL WYNDE2 WYNDE4 XIANGZ XU XU2 XU3 XU4 YUAN ZHANG ZHENG ZHOU
3	RESTRE
4	BOFFET
5	RIMING TANG2 WYNDE5 WYNDE8
6	BYERS2 HIRAY2 LICKIN MRFIT MURATA NOTANI SAARIK SHIMIZ SITAS STAYNE WARSIN WATSON WYNDER
8	JEDRYC SUZUKI WU2
10	BENHAM GILLIS OSANN2 PISANI SIMONA VEIERO VUTUC WYNDE7

Table 1B1 - 8

Potentially overlapping studies

REF	REFGP	PRINC	.	OVERLAP/LINK
LUBIN2	LUBIN2	1		Lubin-combined
KOO	KOO	1		KOO/LAMTH/LAMWK
TVERDA	TVERDA	1		VEIERO/TVERDAL
AKIBA	AKIBA	1		AKIBA/ISHIMA
HEIN	PRESCO	1		Subset of PRESCO
LANGE	PRESCO	1		Subset of PRESCO
BROSS	BYERS1	1		GRAHAM/BROSS/BYERS1
GRAHAM	BYERS1	1		GRAHAM/BROSS/BYERS1
CHYOU	CHYOU	1		GOODMA/CHYOU
GOODMA	GOODMA	1		GOODMA/CHYOU
HOLE	TANG2	1		Subset of TANG2
BENSHL	TANG2	1		Subset of TANG2
WALD	TANG2	1		Subset of TANG2
KAISER	KAISER	1		KAISER/OSANN2
MRFITR	MRFIT	2		Subset of MRFIT
WYNDE6	WYNDE6	1		WYNDE5/6/7/8
CPSI	CPSI	1		CPSI overall
ENSTRO	ENSTRO	1		Subset of CPSI
SOBUE2	SOBUE2	1		SOBUE2/MATSUD
JAHN	BOFFET	2		Subset of BOFFET
LUBIN	XIANGZ	2		LUBIN/XIANGZ/QIAO

Table 1B1 - 9

Most adjusted - insufficient data for metaanalysis

REF	NRR	SEX	AGEL	AGEH	RACE	YF	LC TYPE	LOC	START	ST	NLC	R	VB	P	H	AD	PRODUCT	DENOM	De
DOLL2	45	f	0	0	all	11	all	Eu:UK	1951	pr	920	n	V	n	n	1	all/unsp	nev any	st
HIRAYA	152	m	0	0	all	0	all	As:Jap	1965	pr	1917	n	bl	n	n	4	cig+/-ot	nev any	or
RESTRE	2	c	0	0	all	-	all	SCAmer	1978	CC	102	n	bl	n	n	2	cig+/-ot	nev cigs	ot

REF	NRR	RR	SIG	RRDATA comment
DOLL2	45	5.00		0
HIRAYA	152	4.26		0
RESTRE	2	10.28	y	0

Table 1B2 -

IESLC - Meta-analysis of Current Smoking (vs never smoking), Cigarettes (or Any Product if Cigarettes not available)
All LC types

This analysis is restricted to results for:
1) Non-dose-response data
2) Current smokers
3) Results complete enough for use in metaanalysis

Within each study, results are then selected (in the following order of preference, within each sex) for:
4) PRODUCT: cigarettes regardless of other products, cigarettes only, all/unspec
5) CIGTYPE: all/unspecified, MC regardless of HR, MC only
6) DENOM: never smoked anything, never smoked cigarettes, (never +1 = +long term ex, +2 = +amount unknown, +3 = never cigs+long term ex)
7) Followup period (YF, prospective studies): whole study (coded as 0) or longest available
8) LCtype: all or nearest available, at least Squamous and Adeno. (q = squamous, s = small, l = large, a = adeno, mix = mixed, alv = alveolar)
9) Race: all or nearest available, otherwise by race (wh or w = white, bl or b = black, hi = hispanic ch = chinese, jap = japanese, haw = hawaiian, w+o = white + oriental, sca = scandinavian, as = asian)
10) For overlapping studies: principal rather than subsidiary studies
Finally by Age: whole study (coded as 0) if available, otherwise by widest available age group and then for single sex results (m, f) in preference to combined sex results (c).

Results adjusted (AD) for the most potential confounders are then chosen in Sections -1 to -3 (and those which actually differ from the adjusted results in Table 1B1 - 1 are marked 'x' in Section -1) and results adjusted for the least confounders in Sections -4 to -6. (Those least adjusted results which actually differ from the most adjusted as marked 'x' in column X in Section -4) (Results adjusted for an unknown number of confounder(s) are coded as 20.)

Section -7 shows excluded studies, together with the stage (as above) at which no qualifying results were found.

Section -8 lists the potentially overlapping studies which have been included (1=principal, 2=subsidiary).

Section -9 lists any results which would have been included in preference except that they had data not complete enough for use in meta-analysis, with their significance (yes/no), if known, and any further comment as entered on the database.

In addition to those mentioned above, the following fields, levels and abbreviations are used:
* or nk = not known, n = no, y = yes, ot = other
nev = never
all/unspec = all or unspecified, cig+/-ot = cigarettes irrespective of other products (cigar, pipe etc)
MC = manufactured cigarettes, HR = hand-rolled cigarettes
REF: 6-character study reference
NRR: number of the RR on the database within the study
ST : study type (CC = case control, pr or prosp = prospective)
NLC: number of lung cancer cases in whole study
R : risky occupational population (n = no, m = mining, o = other risky)
VB : national cigarette type (V = at least 75% Virginia, bl = at least 75% blended, ot = other)
P : any proxy use
H : full histological confirmation
De : derivation of RR/CI (or = original, st = standard method, ot = other method of estimation)

Table 1B2 - 1

IESLC - Meta-analysis of Current Smoking (vs never smoking), Cigarettes (or Any Product if Cigarettes not available)
All LC types
Most adjusted

REF	NRR	1B1	SEX	AGEL	AGEH	RACE	YF	LC TYPE	LOC	START	ST	NLC	R	VB	P	H	AD	PRODUCT	DENOM	De
AGUDO	3		f	0	0	all	-	all	Eu:wst	1989	CC	103	n	bl	n	n	3	cig only	nev any	or
AKIBA	10		m	0	0	all	0	all	As:Jap	1963	pr	610	n	bl	n	n	5	cig+/-ot	nev cigs	ot
AKIBA	14		f	0	0	all	0	all	As:Jap	1963	pr	610	n	bl	n	n	5	cig+/-ot	nev cigs	or
ALDERS	177		m	0	0	all	-	all	Eu:UK	1977	CC	1448	n	V	n	n	0	cig+/-ot	nev any	st
ALDERS	176		f	0	0	all	-	all	Eu:UK	1977	CC	1448	n	V	n	n	0	cig only	nev any	st
AMANDU	5		m	0	0	wh	0	all	NAmer	1959	pr	132	m	bl	n	n	2	cig+/-ot	nev cigs	ot
AMES	1		m	0	0	wh	-	all	NAmer	1959	ot	317	m	bl	n	n	0	all/unsp	nev any	or
ANDERS	6		f	0	0	all	0	all	NAmer	1986	pr	343	n	bl	n	n	1	cig+/-ot	nev cigs	or
ARCHER	5		m	0	0	wh	0	all	NAmer	1950	pr	146	m	bl	n	n	0	cig+/-ot	nev cigs	st
ARMADA	27		m	0	0	all	-	all	Eu:wst	1986	CC	325	n	bl	n	y	0	cig+/-ot	nev any	st
AUSTIN	6		c	0	0	all	-	all	NAmer	1970	CC	166	o	bl	y	n	3	cig+/-ot	nev cigs	or
AXELSS	2		m	0	0	sca	-	all	Eu:Sca	1989	CC	436	n	bl	n	n	0	all/unsp	nev any	st
AXELSS	10		f	0	0	sca	-	all	Eu:Sca	1989	CC	436	n	bl	n	n	0	all/unsp	nev any	st
BARBON	4		m	0	0	all	-	all	Eu:wst	1979	CC	755	n	bl	y	y	1	all/unsp	nev any	or
BECHER	13		m	0	0	all	-	all	Eu:Ger	1985	CC	194	n	bl	n	y	0	all/unsp	nev any	st
BECHER	14		f	0	0	all	-	all	Eu:Ger	1985	CC	194	n	bl	n	y	0	all/unsp	nev any	st
BENSHL	4	x	m	0	0	all	0	all	Eu:UK	1967	pr	486	n	V	n	n	1	cig+/-ot	nev any	ot
BEST	2		m	0	0	all	0	all	NAmer	1955	pr	381	n	V	n	n	1	cig only	nev any	ot
BLOHMK	1		m	0	0	all	-	all	Eu:Ger	1978	CC	888	n	bl	n	y	0	all/unsp	nev any	st
BOUCOT	114		m	0	0	all	0	all	NAmer	1951	pr	121	n	bl	n	n	2	cig only	nev any	ot
BRETT	4		m	0	0	all	0	all	Eu:UK	1960	pr	150	n	V	n	n	0	cig+/-ot	nev cigs	st
BROSS	4	x	m	0	0	wh	-	all	NAmer	1960	CC	974	n	bl	n	n	0	cig+/-ot	nev any	st
BROWN2	12		m	0	0	wh	-	all	NAmer	1984	CC	14596	n	bl	n	y	2	cig+/-ot	nev cigs	or
BROWN2	11		f	0	0	wh	-	all	NAmer	1984	CC	14596	n	bl	n	y	2	cig+/-ot	nev cigs	or
BUFFLE	3		m	0	0	wh	-	all	NAmer	1976	CC	943	n	bl	y	n	0	cig+/-ot	nev any	st
BUFFLE	7		f	0	0	wh	-	all	NAmer	1976	CC	943	n	bl	y	n	0	cig+/-ot	nev any	st
CARPEN	11		c	0	0	w+b	-	all	NAmer	1991	CC	356	n	bl	n	n	3	cig+/-ot	nev cigs	or
CEDERL	26	x	m	0	0	all	10	all	Eu:Sca	1963	pr	491	n	bl	n	n	1	cig+/-ot	nev any	ot
CEDERL	119	x	f	0	0	all	10	all	Eu:Sca	1963	pr	491	n	bl	n	n	1	cig+/-ot	nev any	ot
CHANG	5		m	0	0	all	0	all	NAmer	1972	pr	136	n	bl	n	n	0	cig+/-ot	nev cigs	st
CHANG	11		f	0	0	all	0	all	NAmer	1972	pr	136	n	bl	n	n	0	cig+/-ot	nev cigs	st
CHOI	3		m	0	0	all	-	all	As:oth	1985	CC	375	n	bl	n	n	0	cig+/-ot	nev cigs	st
CHOI	7		f	0	0	all	-	all	As:oth	1985	CC	375	n	bl	n	n	0	cig+/-ot	nev cigs	st
CHOW	56	x	m	0	0	wh	0	all	NAmer	1966	pr	219	n	bl	n	n	2	cig+/-ot	nev any	ot
CHYOU	2		m	0	0	jap	0	all	NAmer	1965	pr	227	n	bl	n	y	1	cig+/-ot	nev cigs	or
COMSTO	3		m	0	0	all	-	all	NAmer	1975	ot	258	n	bl	n	n	0	cig+/-ot	nev any	st
COMSTO	8		f	0	0	all	-	all	NAmer	1975	ot	258	n	bl	n	n	0	cig+/-ot	nev any	st
CORREA	42		c	0	0	all	-	all	NAmer	1979	CC	1359	n	bl	y	n	1	cig+/-ot	nev cigs	or
CPSI	220		m	35	84	all	6	all	NAmer	1959	pr	5138	n	bl	n	n	1	cig+/-ot	nev any	ot
CPSI	279		f	40	74	all	6	all	NAmer	1959	pr	5138	n	bl	n	n	1	cig+/-ot	nev cigs	ot
CPSII	126		m	0	0	all	6	all	NAmer	1982	pr	3229	n	bl	n	n	1	cig only	nev any	ot
CPSII	133		f	0	0	all	6	all	NAmer	1982	pr	3229	n	bl	n	n	1	cig+/-ot	nev cigs	ot
DAMBER	16	x	m	0	0	all	-	all	Eu:Sca	1972	CC	579	n	bl	y	n	1	cig only	nev any	ot
DARBY	4		m	0	0	wh	-	all	Eu:UK	1988	CC	982	n	V	n	n	0	cig+/-ot	nev any	st
DARBY	11		f	0	0	wh	-	all	Eu:UK	1988	CC	982	n	V	n	n	0	cig+/-ot	nev any	st
DEAN2	2		m	0	0	all	-	all	Eu:UK	1960	CC	954	n	V	y	n	0	all/unsp	nev any	st
DEAN2	6		f	0	0	all	-	all	Eu:UK	1960	CC	954	n	V	y	n	0	all/unsp	nev any	st
DEAN3	239	x	m	0	0	all	-	all	Eu:UK	1969	CC	766	n	V	y	n	1	cig+/-ot	nev any	ot
DEAN3	119		f	0	0	all	-	all	Eu:UK	1969	CC	766	n	V	y	n	3	cig only	nev any	ot
DEKLER	7	x	m	0	0	all	0	all	Auslia	1961	pr	138	m	V	n	n	2	cig+/-ot	nev any	ot
DESTE2	4		c	0	0	all	-	all	SCAmer	1993	CC	463	n	bl	n	n	7	all/unsp	nev any	or
DESTEF	41		m	0	0	all	-	all	SCAmer	1988	CC	497	n	bl	n	y	4	all/unsp	nev any	or
DOCKER	1		c	0	0	wh	0	all	NAmer	1974	pr	120	n	bl	n	n	4	cig+/-ot	nev cigs	or
DOLL	90		m	0	0	all	-	all	Eu:UK	1948	CC	1465	n	V	n	n	0	all/unsp	nev any	st
DOLL	93		f	0	0	all	-	all	Eu:UK	1948	CC	1465	n	V	n	n	0	all/unsp	nev any	st
DOLL2	68	x	m	0	0	all	20	all	Eu:UK	1951	pr	920	n	V	n	n	1	cig+/-ot	nev any	ot
DOLL2	63		f	0	0	all	22	all	Eu:UK	1951	pr	920	n	V	n	n	1	cig only	nev any	ot
DORANT	9	x	c	0	0	all	0	all	Eu:wst	1986	ot	550	n	bl	n	y	0	cig+/-ot	nev any	st
DORGAN	9		m	0	0	wh	-	all	NAmer	1980	CC	2026	n	bl	y	y	0	cig+/-ot	nev any	st
DORGAN	33		m	0	0	bl	-	all	NAmer	1980	CC	2026	n	bl	y	y	0	cig+/-ot	nev any	st
DORGAN	56		f	0	0	wh	-	all	NAmer	1980	CC	2026	n	bl	y	y	0	cig+/-ot	nev any	st
DORGAN	79		f	0	0	bl	-	all	NAmer	1980	CC	2026	n	bl	y	y	0	cig+/-ot	nev any	st
DORN	391	x	m	0	0	wh	25	all	NAmer	1954	pr	5097	n	bl	n	n	1	cig+/-ot	nev any	or
DROSTE	6		m	0	0	all	-	all	Eu:wst	1995	CC	478	n	bl	n	y	4	all/unsp	nev any	or
ENGELA	168	x	m	0	0	all	12	all	Eu:Sca	1964	pr	435	n	bl	n	n	1	cig+/-ot	nev any	ot
ENGELA	177	x	f	0	0	all	12	all	Eu:Sca	1964	pr	435	n	bl	n	n	1	cig+/-ot	nev any	ot
ENSTRO	1		m	0	0	all	0	all	NAmer	1959	pr	2879	n	bl	n	n	1	cig only	nev any	or
ENSTRO	2		f	0	0	all	0	all	NAmer	1959	pr	2879	n	bl	n	n	1	cig only	nev any	or
GAO	33		m	0	0	all	-	all	As:Chi	1984	CC	1405	n	ot	n	n	2	cig+/-ot	nev cigs	or
GAO	34		f	0	0	all	-	all	As:Chi	1984	CC	1405	n	ot	n	n	2	cig+/-ot	nev cigs	or
GAO2	8		m	0	0	all	-	all	As:Jap	1988	CC	282	n	bl	n	n	1	cig+/-ot	nev cigs	or
GARCIA	2		c	0	0	all	-	all	NAmer	1992	CC	416	n	bl	n	y	0	cig+/-ot	nev cigs	st

Table 1B2 - 1

IESLC - Meta-analysis of Current Smoking (vs never smoking), Cigarettes (or Any Product if Cigarettes not available)
All LC types
Most adjusted

REF	NRR	1B1	SEX	AGEL	AGEH	RACE	YF	LC TYPE	LOC	START	ST	NLC	R	VB	P	H	AD	PRODUCT	DENOM	De
GARDIN	6	x	c	0	0	all	-	all	Eu:UK	1988	CC	143	n	V	y	n	0	cig only	nev any	st
GARSHI	31		m	0	0	all	-	all	NAmer	1981	CC	1081	o	bl	y	n	1	all/unsp	nev any	st
GOODMA	2		m	0	0	w+o	-	all	NAmer	1983	CC	326	n	bl	y	y	0	cig+/-ot	nev any	st
GOODMA	6		f	0	0	w+o	-	all	NAmer	1983	CC	326	n	bl	y	y	0	cig+/-ot	nev any	st
GRAHAM	10	x	m	0	0	wh	-	all	NAmer	1956	CC	685	n	bl	n	n	1	cig+/-ot	nev any	ot
GREGOR	2		m	0	0	all	-	all	Eu:UK	1976	CC	104	n	V	n	y	0	cig+/-ot	nev cigs	st
GREGOR	6		f	0	0	all	-	all	Eu:UK	1976	CC	104	n	V	n	y	0	cig+/-ot	nev cigs	st
HAENSZ	54		f	0	0	all	-	not alv	NAmer	1955	CC	158	n	bl	n	y	0	cig+/-ot	nev any	st
HAMMO2	8		m	0	0	all	0	all	NAmer	1967	pr	450	o	bl	n	n	1	cig+/-ot	nev any	ot
HAMMON	139		m	0	0	wh	0	all	NAmer	1952	pr	448	n	bl	n	n	1	cig only	nev any	ot
HEIN	1	x	m	0	0	all	0	all	Eu:Sca	1970	pr	144	n	bl	n	n	0	cig only	nev any	st
HENNEK	2		m	0	0	all	0	all	NAmer	1982	pr	169	n	bl	n	n	0	all/unsp	nev any	st
HIRAYA	1		m	0	0	all	0	all	As:Jap	1965	pr	1917	n	bl	n	n	1	cig+/-ot	nev any	st
HIRAYA	3		f	0	0	all	0	all	As:Jap	1965	pr	1917	n	bl	n	n	1	cig+/-ot	nev any	st
HITOSU	34		m	0	0	all	-	all	As:Jap	1960	CC	216	n	bl	y	n	1	all/unsp	nev any	st
HITOSU	59		f	0	0	all	-	all	As:Jap	1960	CC	216	n	bl	y	n	1	all/unsp	nev any	st
HOLE	32	x	m	0	0	all	0	all	Eu:UK	1972	pr	225	n	V	n	n	1	cig+/-ot	nev any	ot
HOLE	31		f	0	0	all	11	all	Eu:UK	1972	pr	225	n	V	n	n	1	all/unsp	nev any	ot
HUMBLE	13		m	0	0	w-hi	-	all	NAmer	1980	CC	521	n	bl	y	n	1	cig+/-ot	nev cigs	ot
HUMBLE	15		m	0	0	hi	-	all	NAmer	1980	CC	521	n	bl	y	n	1	cig+/-ot	nev cigs	ot
HUMBLE	17		f	0	0	w-hi	-	all	NAmer	1980	CC	521	n	bl	y	n	1	cig+/-ot	nev cigs	ot
HUMBLE	19		f	0	0	hi	-	all	NAmer	1980	CC	521	n	bl	y	n	1	cig+/-ot	nev cigs	ot
JAHN	5		m	0	0	all	-	all	Eu:Ger	1988	CC	1004	n	bl	n	n	0	cig+/-ot	nev any	st
JAIN	52		m	0	0	all	-	all	NAmer	1981	CC	845	n	V	y	n	2	cig+/-ot	nev cigs	or
JAIN	51		f	0	0	all	-	all	NAmer	1981	CC	845	n	V	y	n	2	cig+/-ot	nev cigs	or
JARVHO	2		m	0	0	all	-	all	Eu:Sca	1983	CC	147	n	bl	n	n	0	all/unsp	nev any	st
JARVHO	6		f	0	0	all	-	all	Eu:Sca	1983	CC	147	n	bl	n	n	0	all/unsp	nev any	st
JOLY	16	x	m	0	0	all	-	all	SCAmer	1978	CC	826	n	bl	n	n	0	cig+/-ot	nev any	st
JOLY	15		f	0	0	all	-	all	SCAmer	1978	CC	826	n	bl	n	n	0	cig+/-ot	nev any	st
KAISE2	68		m	35	99	all	9	all	NAmer	1979	pr	318	n	bl	n	n	1	cig only	nev any	st
KAISE2	60		f	35	99	all	9	all	NAmer	1979	pr	318	n	bl	n	n	1	cig only	nev any	st
KAISER	12		m	0	0	all	0	all	NAmer	1964	pr	714	n	bl	n	n	2	cig+/-ot	nev cigs	ot
KAISER	9		f	0	0	all	0	all	NAmer	1964	pr	714	n	bl	n	n	2	cig+/-ot	nev cigs	ot
KANELL	30		m	0	0	all	-	all	Eu:bal	1950	CC	862	n	bl	n	n	1	all/unsp	nev any	st
KATSOU	2		f	0	0	all	-	all	Eu:bal	1987	CC	101	n	bl	n	n	1	all/unsp	nev any	or
KAUFMA	16		c	0	0	all	-	all	NAmer	1981	CC	881	n	bl	n	n	6	cig+/-ot	nev cigs	ot
KELLER	1		m	0	0	wh	-	all	NAmer	1985	CC	15038	n	bl	n	n	0	all/unsp	nev any	st
KELLER	9		m	0	0	nonw	-	all	NAmer	1985	CC	15038	n	bl	n	n	0	all/unsp	nev any	st
KELLER	5		f	0	0	wh	-	all	NAmer	1985	CC	15038	n	bl	n	n	0	all/unsp	nev any	st
KELLER	13		f	0	0	nonw	-	all	NAmer	1985	CC	15038	n	bl	n	n	0	all/unsp	nev any	st
KHUDER	19		m	0	0	all	-	all	NAmer	1985	CC	482	n	bl	n	y	0	cig+/-ot	nev cigs	or
KIHARA	7		c	0	0	jap	-	all	As:Jap	1991	CC	440	n	bl	n	n	0	all/unsp	nev any	st
KINLEN	19	x	m	0	0	all	0	all	Eu:UK	1967	pr	718	n	V	n	n	2	cig+/-ot	nev any	ot
KJUUS	1		m	0	0	all	-	all	Eu:Sca	1979	CC	176	n	bl	n	n	0	all/unsp	nev any	st
KNEKT	85	x	m	20	69	all	21	all	Eu:Sca	1966	pr	515	n	bl	n	n	1	cig+/-ot	nev any	ot
KOO	9		f	0	0	all	-	all	As:HK	1981	CC	200	n	bl	n	n	0	all/unsp	nev any	st
KREUZE	18	x	m	1	45	all	-	all	Eu:Ger	1990	CC	2260	n	bl	n	n	3	cig+/-ot	nev any	or
KREUZE	29	x	m	55	69	all	-	all	Eu:Ger	1990	CC	2260	n	bl	n	n	3	cig+/-ot	nev any	or
KREUZE	24	x	f	1	45	all	-	all	Eu:Ger	1990	CC	2260	n	bl	n	n	3	cig+/-ot	nev any	or
KREUZE	35	x	f	55	69	all	-	all	Eu:Ger	1990	CC	2260	n	bl	n	n	3	cig+/-ot	nev any	or
KUBIK	12		m	0	0	all	0	all	Eu:est	1965	pr	108	n	bl	n	n	0	cig+/-ot	nev any	st
LANGE	38		m	0	0	all	0	all	Eu:Sca	1976	pr	268	n	bl	n	n	1	all/unsp	nev any	ot
LANGE	35		f	0	0	all	0	all	Eu:Sca	1976	pr	268	n	bl	n	n	1	all/unsp	nev any	ot
LEMARC	2		c	0	0	w+o	-	all	NAmer	1992	CC	341	n	bl	n	y	0	all/unsp	nev any	st
LIAW	1		m	0	0	all	0	all	As:oth	1982	pr	127	n	ot	n	n	1	all/unsp	nev any	or
LIAW	2		f	0	0	all	0	all	As:oth	1982	pr	127	n	ot	n	n	1	all/unsp	nev any	or
LIDDEL	4		m	0	0	all	18	all	NAmer	1970	pr	304	m	V	n	n	1	cig+/-ot	nev cigs	ot
LOMBAR	9		m	0	0	all	-	all	NAmer	1951	CC	1040	n	bl	n	n	0	cig+/-ot	nev any	st
LUBIN	40		m	0	0	all	-	all	As:Chi	1984	CC	427	m	ot	y	n	0	cig+/-ot	nev any	st
LUBIN2	28	x	m	0	0	all	-	all	Eu:mul	1976	CC	7804	n	bl	n	y	2	cig+/-ot	nev any	ot
LUBIN2	317		f	0	0	all	-	all	Eu:mul	1976	CC	7804	n	bl	n	y	0	cig+/-ot	nev any	st
MACLEN	19		m	0	0	ch	-	all	As:oth	1972	CC	233	n	bl	n	n	0	cig+/-ot	nev cigs	st
MACLEN	32		f	0	0	ch	-	all	As:oth	1972	CC	233	n	bl	n	n	0	cig+/-ot	nev cigs	st
MATOS	3		m	0	0	all	-	all	SCAmer	1994	CC	200	n	bl	n	n	2	cig+/-ot	nev any	or
MIGRAN	12	x	m	0	0	all	0	all	Eu:UK	1964	pr	259	n	V	n	n	2	cig+/-ot	nev any	ot
MIGRAN	38	x	f	0	0	all	0	all	Eu:UK	1964	pr	259	n	V	n	n	2	cig+/-ot	nev any	ot
MRFITR	2		m	0	0	all	0	all	NAmer	1973	pr	119	n	bl	n	n	0	cig+/-ot	nev cigs	ot
NAM	76		m	0	0	all	-	all	NAmer	1986	CC	1199	n	bl	y	n	1	cig+/-ot	nev cigs	ot
NAM	92		f	0	0	all	-	all	NAmer	1986	CC	1199	n	bl	y	n	1	cig+/-ot	nev cigs	ot
ODRISC	1		c	0	0	all	-	all	Eu:UK	1992	CC	446	n	V	n	n	0	all/unsp	nev any	st
OSANN	33		m	0	0	all	-	all	NAmer	1984	CC	1986	n	bl	n	n	2	cig+/-ot	nev cigs	or
OSANN	34		f	0	0	all	-	all	NAmer	1984	CC	1986	n	bl	n	n	2	cig+/-ot	nev cigs	or

Table 1B2 - 1

IESLC - Meta-analysis of Current Smoking (vs never smoking), Cigarettes (or Any Product if Cigarettes not available)
All LC types
Most adjusted

REF	NRR	1B1	SEX	AGEL	AGEH	RACE	YF	LC TYPE	LOC	START	ST	NLC	R	VB	P	H	AD	PRODUCT	DENOM	De
PARKIN	30	x	m	0	0	bl	-	all	Africa	1963	CC	877	n	V	y	n	0	cig+/-ot	nev any	st
PERSH2	10		c	0	0	all	-	all	Eu:Sca	1980	CC	1022	n	bl	y	n	4	all/unsp	nev any	ot
PETO	4		m	0	0	all	0	all	Eu:UK	1954	pr	103	n	V	n	n	0	all/unsp	nev any	st
PEZZO2	2		m	0	0	all	-	all	SCAmer	1992	CC	367	n	bl	n	y	0	cig+/-ot	nev cigs	st
PEZZOT	5		m	0	0	all	-	all	SCAmer	1987	CC	215	n	bl	n	y	0	cig only	nev cigs	st
QIAO2	8	x	m	0	0	all	0	all	As:Chi	1992	pr	241	m	ot	n	n	0	cig+/-ot	nev any	st
RACHTA	9		f	0	0	all	-	all	Eu:est	1991	CC	118	n	bl	n	y	1	cig+/-ot	nev cigs	or
SCHWAR	25		m	0	0	wh	-	all	NAmer	1984	CC	5588	n	bl	y	y	0	cig+/-ot	nev cigs	st
SCHWAR	26		m	0	0	bl	-	all	NAmer	1984	CC	5588	n	bl	y	y	0	cig+/-ot	nev cigs	st
SCHWAR	27		f	0	0	wh	-	all	NAmer	1984	CC	5588	n	bl	y	y	0	cig+/-ot	nev cigs	st
SCHWAR	28		f	0	0	bl	-	all	NAmer	1984	CC	5588	n	bl	y	y	0	cig+/-ot	nev cigs	st
SEGI2	20		m	0	0	all	-	all	As:Jap	1962	CC	378	n	bl	n	n	1	cig+/-ot	nev any	ot
SEGI2	28		f	0	0	all	-	all	As:Jap	1962	CC	378	n	bl	n	n	1	cig+/-ot	nev any	ot
SHAW	6		c	0	0	wh	-	all	NAmer	1988	CC	335	n	V	n	y	0	all/unsp	nev any	st
SOBUE	42		m	0	0	all	-	q+s+l+a	As:Jap	1986	CC	1376	n	bl	n	y	1	cig+/-ot	nev cigs	or
SOBUE	52		f	0	0	all	-	q+s+l+a	As:Jap	1986	CC	1376	n	bl	n	y	1	cig+/-ot	nev cigs	or
SOBUE2	10		m	0	0	all	-	q+s+l+a	As:Jap	1965	CC	2083	n	bl	n	n	2	cig+/-ot	nev any	ot
SOBUE2	12		f	0	0	all	-	q+s+l+a	As:Jap	1965	CC	2083	n	bl	n	n	2	cig+/-ot	nev any	ot
SPEIZE	10		f	0	0	all	0	all	NAmer	1976	pr	593	n	bl	n	y	1	cig+/-ot	nev cigs	ot
SPITZ	2		c	0	0	b+hi	-	all	NAmer	1992	CC	177	n	bl	n	y	0	cig+/-ot	nev cigs	st
STOCKW	7		c	0	0	all	-	all	NAmer	1981	CC	22161	n	bl	n	n	0	cig+/-ot	nev any	st
STUCKE	2		m	0	0	all	-	all	Eu:wst	1989	CC	247	n	bl	n	y	0	all/unsp	nev any	ot
SUZUK2	6		c	0	0	all	-	all	SCAmer	1991	CC	123	n	bl	n	y	3	all/unsp	nev any	or
SVENSS	96		f	0	0	all	-	all	Eu:Sca	1983	CC	210	n	bl	n	n	1	all/unsp	nev any	ot
TANG	1		c	0	0	all	-	not s	NAmer	1992	CC	119	n	bl	n	y	0	cig+/-ot	nev cigs	st
TENKAN	24		m	0	0	all	17	all	Eu:Sca	1962	pr	242	n	bl	n	n	1	all/unsp	nev any	ot
TIZZAN	5		m	0	0	all	-	all	Eu:wst	1959	CC	1358	n	bl	n	n	0	all/unsp	nev any	st
TIZZAN	13		f	0	0	all	-	all	Eu:wst	1959	CC	1358	n	bl	n	n	0	all/unsp	nev any	st
TOKARS	1		m	0	0	all	-	all	Eu:est	1966	ot	162	o	bl	n	y	0	all/unsp	nev any	st
TOUSEY	12		m	0	0	all	-	all	NAmer	1993	CC	507	n	bl	y	y	3	cig+/-ot	nev any	or
TOUSEY	15		f	0	0	all	-	all	NAmer	1993	CC	507	n	bl	y	y	3	cig+/-ot	nev any	or
TSUGAN	28		m	0	0	all	-	q+a	As:Jap	1976	CC	134	n	bl	n	y	0	all/unsp	nev any	st
TULINI	36	x	m	0	0	all	0	all	Eu:Sca	1967	pr	472	n	bl	n	n	3	cig+/-ot	nev any	ot
TULINI	42	x	f	0	0	all	0	all	Eu:Sca	1967	pr	472	n	bl	n	n	3	cig+/-ot	nev any	ot
TVERDA	5		m	0	0	all	0	all	Eu:Sca	1972	pr	238	n	bl	n	n	2	cig+/-ot	nev cigs	ot
TVERDA	15		f	0	0	all	0	all	Eu:Sca	1972	pr	238	n	bl	n	n	2	cig only	nev cigs	ot
WAKAI	8		m	0	0	all	-	all	As:Jap	1988	CC	333	n	bl	n	y	2	all/unsp	nev any	or
WAKAI	26		f	0	0	all	-	all	As:Jap	1988	CC	333	n	bl	n	y	2	all/unsp	nev any	or
WALD	4		m	0	0	all	0	all	Eu:UK	1975	pr	102	n	V	n	n	1	cig only	nev any	or
WANG2	18		c	0	0	all	-	all	As:Chi	1980	CC	103	n	ot	n	n	4	cig+/-ot	nev cigs	ot
WIGLE	28	x	m	0	0	all	-	all	NAmer	1971	CC	728	n	V	n	n	1	cig only	nev any	ot
WIGLE	33	x	f	0	0	all	-	all	NAmer	1971	CC	728	n	V	n	n	1	cig+/-ot	nev any	ot
WU	42		f	0	0	wh	-	q+a	NAmer	1981	CC	220	n	bl	n	y	2	all/unsp	nev any	st
WUNSCH	5		m	0	0	all	-	all	SCAmer	1990	CC	398	n	bl	y	n	1	cig+/-ot	nev any	or
WUNSCH	11		f	0	0	all	-	all	SCAmer	1990	CC	398	n	bl	y	n	1	cig+/-ot	nev any	or
WYNDE3	50		m	0	0	all	-	all	NAmer	1966	CC	350	n	bl	n	y	0	all/unsp	nev any	st
WYNDE6	18		m	0	0	all	-	all	NAmer	1969	CC	4423	n	bl	n	y	0	cig+/-ot	nev any	st
WYNDE6	207		f	0	0	all	-	all	NAmer	1969	CC	4423	n	bl	n	y	0	cig+/-ot	nev cigs	st
YAMAGU	10		c	0	0	all	-	all	As:Jap	1989	CC	144	n	bl	n	y	1	all/unsp	nev any	ot
YONG	12		m	0	0	all	0	all	NAmer	1971	pr	216	n	bl	n	n	1	cig+/-ot	nev cigs	or
YONG	15		f	0	0	all	0	all	NAmer	1971	pr	216	n	bl	n	n	1	cig+/-ot	nev cigs	or

Cigarette type is all/unspec for all RRs
except for the following:

REF	NRR	CIGTYPE
ALDERS	177	MC+-HR
ALDERS	176	MC only
DEAN3	239	MC only
DEAN3	119	MC only
GARDIN	6	MC only

Table 1B2 - 2

IESLC - Meta-analysis of Current Smoking (vs never smoking), Cigarettes (or Any Product if Cigarettes not available)
All LC types
Most adjusted

REF	NRR	SEX	AD	Number Exposed Case	Number Exposed Cont	Non-exposed Case	Non-exposed Cont	RR	95.00%CI
AGUDO	3	f	3	-	-	-	-	3.61	(1.57- 8.32)
*AKIBA	10	m	5	-	-	-	-	5.10	(3.30- 7.90)
*AKIBA	14	f	5	-	-	-	-	3.90	(2.90- 5.30)
Subtotal AKIBA								4.25	(3.32- 5.45)
ALDERS	177	m	0	519	322	15	133	14.29	(8.23- 24.81)
ALDERS	176	f	0	410	229	75	243	5.80	(4.27- 7.87)
Subtotal ALDERS								7.17	(5.49- 9.36)
*AMANDU	5	m	2	-	-	-	-	6.54	(2.52- 16.98)
AMES	1	m	0	150	136	15	62	4.56	(2.48- 8.39)
*ANDERS	6	f	1	-	-	-	-	23.43	(17.02- 32.27)
*ARCHER	5	m	0	122	32529	6	9842	6.15	(2.71- 13.96)
ARMADA	27	m	0	188	122	4	64	24.66	(8.75- 69.44)
AUSTIN	6	c	3	-	-	-	-	19.60	(6.70- 57.00)
AXELSS	2	m	0	194	130	16	160	14.92	(8.53- 26.12)
AXELSS	10	f	0	96	69	18	154	11.90	(6.68- 21.22)
Subtotal AXELSS								13.38	(8.95- 20.00)
BARBON	4	m	1	-	-	-	-	13.40	(8.50- 21.40)
BECHER	13	m	0	101	122	3	54	14.90	(4.52- 49.09)
BECHER	14	f	0	33	26	10	52	6.60	(2.82- 15.44)
Subtotal BECHER								8.68	(4.35- 17.35)
*BENSHL	4	m	1	-	-	-	-	11.92	(6.36- 22.34)
*BEST	2	m	1	-	-	-	-	14.91	(7.05- 31.52)
BLOHMK	1	m	0	419	313	126	301	3.20	(2.48- 4.12)
*BOUCOT	114	m	2	-	-	-	-	62.29	(3.86-1004.01)
*BRETT	4	m	0	135	37448	6	6530	3.92	(1.73- 8.88)
BROSS	4	m	0	565	427	38	170	5.92	(4.07- 8.60)
BROWN2	12	m	2	-	-	-	-	11.30	(10.20- 12.40)
BROWN2	11	f	2	-	-	-	-	13.60	(12.30- 15.10)
Subtotal BROWN2								12.34	(11.50- 13.25)
BUFFLE	3	m	0	257	219	5	47	11.03	(4.31- 28.22)
BUFFLE	7	f	0	313	183	41	198	8.26	(5.63- 12.11)
Subtotal BUFFLE								8.61	(6.04- 12.27)
CARPEN	11	c	3	-	-	-	-	23.03	(12.96- 40.85)
*CEDERL	26	m	1	-	-	-	-	8.16	(3.70- 17.99)
*CEDERL	119	f	1	-	-	-	-	4.54	(1.85- 11.12)
Subtotal CEDERL								6.31	(3.49- 11.43)
*CHANG	5	m	0	35	419	5	502	8.39	(3.32- 21.21)
*CHANG	11	f	0	30	603	11	1139	5.15	(2.60- 10.21)
Subtotal CHANG								6.12	(3.53- 10.60)
CHOI	3	m	0	232	329	13	95	5.15	(2.82- 9.42)
CHOI	7	f	0	13	23	76	164	1.22	(0.59- 2.54)
Subtotal CHOI								2.88	(1.81- 4.58)
*CHOW	56	m	2	-	-	-	-	21.46	(9.38- 49.10)
*CHYOU	2	m	1	-	-	-	-	11.40	(6.50- 20.10)
COMSTO	3	m	0	105	100	4	69	18.11	(6.37- 51.48)
COMSTO	8	f	0	77	52	13	115	13.10	(6.68- 25.67)
Subtotal COMSTO								14.40	(8.18- 25.36)
CORREA	42	c	1	-	-	-	-	14.20	(10.80- 18.70)
*CPSI	220	m	1	-	-	-	-	11.94	(9.52- 14.97)
*CPSI	279	f	1	-	-	-	-	3.20	(2.53- 4.04)
Subtotal CPSI								6.32	(5.37- 7.43)
*CPSII	126	m	1	-	-	-	-	20.25	(16.37- 25.05)
*CPSII	133	f	1	-	-	-	-	11.78	(10.14- 13.68)
Subtotal CPSII								14.10	(12.47- 15.93)
DAMBER	16	m	1	-	-	-	-	9.80	(6.30- 15.30)
DARBY	4	m	0	322	453	3	384	90.98	(28.96- 285.90)
DARBY	11	f	0	195	217	23	529	20.67	(13.05- 32.74)
Subtotal DARBY								25.40	(16.57- 38.92)
DEAN2	2	m	0	671	600	33	112	3.80	(2.54- 5.68)
DEAN2	6	f	0	59	28	88	121	2.90	(1.71- 4.91)
Subtotal DEAN2								3.43	(2.49- 4.73)
DEAN3	239	m	1	-	-	-	-	7.04	(4.60- 10.77)
DEAN3	119	f	3	-	-	-	-	5.77	(3.75- 8.86)
Subtotal DEAN3								6.38	(4.72- 8.63)
*DEKLER	7	m	2	-	-	-	-	23.79	(3.32- 170.45)
DESTE2	4	c	7	-	-	-	-	9.10	(5.20- 15.90)
DESTEF	41	m	4	-	-	-	-	10.90	(6.90- 17.10)
*DOCKER	1	c	4	-	-	-	-	8.00	(2.97- 21.60)
DOLL	90	m	0	1280	1172	7	61	9.52	(4.34- 20.89)
DOLL	93	f	0	58	41	40	59	2.09	(1.18- 3.68)
Subtotal DOLL								3.51	(2.21- 5.55)

Table 1B2 - 2

IESLC - Meta-analysis of Current Smoking (vs never smoking), Cigarettes (or Any Product if Cigarettes not available)
All LC types
Most adjusted

REF	NRR	SEX	AD	Number Exposed Case	Number Exposed Cont	Non-exposed Case	Non-exposed Cont	RR	95.00%CI
*DOLL2	68	m	1	-	-	-	-	12.20	(5.77- 25.82)
*DOLL2	63	f	1	-	-	-	-	8.65	(2.93- 25.55)
Subtotal DOLL2								10.91	(5.89- 20.21)
DORANT	9	c	0	292	876	14	1090	25.95	(15.07- 44.69)
DORGAN	9	m	0	464	170	15	93	16.92	(9.54- 30.01)
DORGAN	33	m	0	214	61	3	35	40.93	(12.17- 137.66)
DORGAN	56	f	0	611	119	103	244	12.16	(8.99- 16.46)
DORGAN	79	f	0	68	17	7	20	11.43	(4.16- 31.43)
Subtotal DORGAN								13.62	(10.58- 17.54)
*DORN	391	m	1	-	-	-	-	10.86	(9.73- 12.13)
DROSTE	6	m	4	-	-	-	-	14.50	(6.30- 33.40)
*ENGELA	168	m	1	-	-	-	-	9.70	(4.49- 20.94)
*ENGELA	177	f	1	-	-	-	-	5.80	(2.69- 12.51)
Subtotal ENGELA								7.50	(4.35- 12.92)
*ENSTRO	1	m	1	-	-	-	-	12.99	(10.46- 16.13)
*ENSTRO	2	f	1	-	-	-	-	6.95	(6.01- 8.04)
Subtotal ENSTRO								8.44	(7.48- 9.53)
GAO	33	m	2	-	-	-	-	3.90	(2.90- 5.40)
GAO	34	f	2	-	-	-	-	2.90	(2.20- 3.80)
Subtotal GAO								3.30	(2.69- 4.05)
GAO2	8	m	1	-	-	-	-	6.61	(3.47- 12.58)
GARCIA	2	c	0	169	74	21	139	15.12	(8.86- 25.79)
GARDIN	6	c	0	72	39	5	41	15.14	(5.53- 41.44)
GARSHI	31	m	1	-	-	-	-	7.70	(5.48- 10.83)
GOODMA	2	m	0	148	169	10	199	17.43	(8.90- 34.14)
GOODMA	6	f	0	58	56	19	177	9.65	(5.30- 17.56)
Subtotal GOODMA								12.53	(8.01- 19.60)
GRAHAM	10	m	1	-	-	-	-	7.26	(4.52- 11.65)
GREGOR	2	m	0	49	53	10	14	1.29	(0.53- 3.18)
GREGOR	6	f	0	17	26	1	22	14.38	(1.77- 116.90)
Subtotal GREGOR								1.88	(0.82- 4.30)
HAENSZ	54	f	0	69	94	81	236	2.14	(1.43- 3.19)
*HAMMO2	8	m	1	-	-	-	-	10.14	(4.19- 24.55)
*HAMMON	139	m	1	-	-	-	-	11.52	(6.83- 19.42)
*HEIN	1	m	0	45	912	1	457	22.55	(3.12- 163.06)
*HENNEK	2	m	0	79	2438	23	10919	15.38	(9.69- 24.42)
*HIRAYA	1	m	1	-	-	-	-	4.45	(3.60- 5.50)
*HIRAYA	3	f	1	-	-	-	-	2.34	(1.87- 2.92)
Subtotal HIRAYA								3.28	(2.81- 3.82)
HITOSU	34	m	1	-	-	-	-	2.79	(1.27- 6.09)
HITOSU	59	f	1	-	-	-	-	3.09	(1.82- 5.27)
Subtotal HITOSU								2.99	(1.93- 4.65)
*HOLE	32	m	1	-	-	-	-	8.36	(3.92- 17.83)
*HOLE	31	f	1	-	-	-	-	1.53	(0.64- 3.70)
Subtotal HOLE								4.05	(2.28- 7.18)
HUMBLE	13	m	1	-	-	-	-	19.96	(8.27- 48.21)
HUMBLE	15	m	1	-	-	-	-	15.79	(3.43- 72.69)
HUMBLE	17	f	1	-	-	-	-	16.72	(7.44- 37.61)
HUMBLE	19	f	1	-	-	-	-	23.50	(6.79- 81.36)
Subtotal HUMBLE								18.65	(11.23- 30.97)
JAHN	5	m	0	352	269	18	138	10.03	(5.99- 16.81)
JAIN	52	m	2	-	-	-	-	12.40	(6.45- 26.60)
JAIN	51	f	2	-	-	-	-	16.80	(9.93- 30.60)
Subtotal JAIN								14.94	(9.61- 23.21)
JARVHO	2	m	0	73	29	1	16	40.28	(5.10- 317.77)
JARVHO	6	f	0	31	7	6	21	15.50	(4.56- 52.66)
Subtotal JARVHO								19.86	(6.93- 56.89)
JOLY	16	m	0	451	524	12	218	15.64	(8.63- 28.34)
JOLY	15	f	0	132	96	52	283	7.48	(5.04- 11.12)
Subtotal JOLY								9.38	(6.75- 13.04)
*KAISE2	68	m	1	-	-	-	-	8.04	(4.41- 14.66)
*KAISE2	60	f	1	-	-	-	-	14.48	(7.47- 28.04)
Subtotal KAISE2								10.49	(6.72- 16.36)
*KAISER	12	m	2	-	-	-	-	19.61	(13.32- 28.87)
*KAISER	9	f	2	-	-	-	-	6.53	(4.50- 9.48)
Subtotal KAISER								11.09	(8.48- 14.50)
KANELL	30	m	1	-	-	-	-	4.94	(3.47- 7.03)
KATSOU	2	f	1	-	-	-	-	3.40	(1.75- 6.61)
KAUFMA	16	c	6	-	-	-	-	20.63	(14.18- 30.01)
KELLER	1	m	0	5063	1210	323	1017	13.17	(11.45- 15.15)
KELLER	9	m	0	1053	212	38	117	15.29	(10.31- 22.69)

Table 1B2 - 2

IESLC - Meta-analysis of Current Smoking (vs never smoking), Cigarettes (or Any Product if Cigarettes not available)
All LC types
Most adjusted

REF	NRR	SEX	AD	Number Exposed Case	Number Exposed Cont	Non-exposed Case	Non-exposed Cont	RR	95.00%CI
KELLER	5	f	0	2904	792	469	1860	14.54	(12.79- 16.53)
KELLER	13	f	0	454	135	67	232	11.64	(8.35- 16.24)
Subtotal KELLER								13.79	(12.62- 15.07)
KHUDER	19	m	0	275	-	23	-	8.10	(5.20- 12.70)
KIHARA	7	c	0	283	162	102	237	4.06	(3.00- 5.49)
*KINLEN	19	m	2	-	-	-	-	13.97	(6.65- 29.34)
KJUUS	1	m	0	135	77	2	24	21.04	(4.84- 91.45)
*KNEKT	85	m	1	-	-	-	-	9.22	(4.02- 21.13)
KOO	9	f	0	42	25	56	85	2.55	(1.40- 4.64)
KREUZE	18	m	3	-	-	-	-	15.90	(6.50- 38.50)
KREUZE	29	m	3	-	-	-	-	41.90	(27.10- 64.60)
KREUZE	24	f	3	-	-	-	-	29.90	(9.50- 94.60)
KREUZE	35	f	3	-	-	-	-	6.40	(4.20- 9.60)
Subtotal KREUZE								16.25	(12.34- 21.41)
*KUBIK	12	m	0	98	6342	2	4271	33.00	(8.14- 133.74)
*LANGE	38	m	1	-	-	-	-	5.70	(2.13- 15.27)
*LANGE	35	f	1	-	-	-	-	5.02	(2.52- 10.01)
Subtotal LANGE								5.23	(2.98- 9.21)
LEMARC	2	c	0	167	65	32	168	13.49	(8.39- 21.68)
*LIAW	1	m	1	-	-	-	-	3.70	(2.10- 6.60)
*LIAW	2	f	1	-	-	-	-	3.60	(1.00- 12.20)
Subtotal LIAW								3.68	(2.19- 6.20)
*LIDDEL	4	m	1	-	-	-	-	4.41	(2.77- 7.01)
LOMBAR	9	m	0	852	610	14	112	11.17	(6.35- 19.66)
LUBIN	40	m	0	296	650	9	72	3.64	(1.80- 7.38)
LUBIN2	28	m	2	-	-	-	-	11.18	(9.57- 13.05)
LUBIN2	317	f	0	384	410	288	1180	3.84	(3.17- 4.64)
Subtotal LUBIN2								7.28	(6.46- 8.21)
MACLEN	19	m	0	137	108	5	15	3.81	(1.34- 10.80)
MACLEN	32	f	0	42	47	41	109	2.38	(1.37- 4.12)
Subtotal MACLEN								2.63	(1.62- 4.28)
MATOS	3	m	2	-	-	-	-	8.50	(4.30- 16.70)
*MIGRAN	12	m	2	-	-	-	-	4.04	(1.50- 10.90)
*MIGRAN	38	f	2	-	-	-	-	5.00	(1.76- 14.18)
Subtotal MIGRAN								4.47	(2.18- 9.17)
*MRFITR	2	m	0	106	8194	0	1859	48.33~	(3.01- 777.42)
NAM	76	m	1	-	-	-	-	8.67	(5.66- 13.26)
NAM	92	f	1	-	-	-	-	10.84	(7.26- 16.18)
Subtotal NAM								9.76	(7.29- 13.07)
ODRISC	1	c	0	293	598	6	664	54.22	(23.98- 122.60)
OSANN	33	m	2	-	-	-	-	26.50	(19.20- 36.50)
OSANN	34	f	2	-	-	-	-	19.60	(15.20- 25.20)
Subtotal OSANN								22.00	(18.03- 26.83)
PARKIN	30	m	0	346	874	107	1248	4.62	(3.66- 5.83)
PERSH2	10	c	4	-	-	-	-	8.29	(6.86- 10.02)
*PETO	4	m	0	99	2036	2	295	7.17	(1.78- 28.92)
PEZZO2	2	m	0	233	198	6	117	22.95	(9.89- 53.26)
PEZZOT	5	m	0	145	129	4	116	32.60	(11.70- 90.81)
*QIAO2	8	m	0	156	5399	10	709	2.05	(1.09- 3.86)
RACHTA	9	f	1	-	-	-	-	6.77	(3.71- 12.35)
SCHWAR	25	m	0	1652	349	119	376	14.96	(11.81- 18.94)
SCHWAR	26	m	0	644	139	50	104	9.64	(6.56- 14.15)
SCHWAR	27	f	0	1029	309	182	855	15.64	(12.75- 19.19)
SCHWAR	28	f	0	256	90	40	247	17.56	(11.64- 26.50)
Subtotal SCHWAR								14.70	(12.84- 16.83)
SEGI2	20	m	1	-	-	-	-	3.74	(1.75- 8.00)
SEGI2	28	f	1	-	-	-	-	1.65	(0.90- 3.02)
Subtotal SEGI2								2.27	(1.41- 3.64)
SHAW	6	c	0	212	97	11	107	21.26	(10.93- 41.36)
SOBUE	42	m	1	-	-	-	-	4.10	(2.80- 5.90)
SOBUE	52	f	1	-	-	-	-	2.80	(2.00- 3.90)
Subtotal SOBUE								3.32	(2.59- 4.26)
SOBUE2	10	m	2	-	-	-	-	4.47	(3.89- 5.14)
SOBUE2	12	f	2	-	-	-	-	3.28	(2.79- 3.87)
Subtotal SOBUE2								3.92	(3.53- 4.36)
*SPEIZE	10	f	1	-	-	-	-	12.69	(9.97- 16.16)
SPITZ	2	c	0	103	89	7	128	21.16	(9.40- 47.66)
STOCKW	7	c	0	12470	3357	2791	10641	14.16	(13.38- 14.99)
STUCKE	2	m	0	69	68	0	51	104.50~	(6.32-1727.39)
SUZUK2	6	c	3	-	-	-	-	22.00	(6.50- 76.00)
SVENSS	96	f	1	-	-	-	-	9.06	(5.31- 15.48)

Table 1B2 - 2

IESLC - Meta-analysis of Current Smoking (vs never smoking), Cigarettes (or Any Product if Cigarettes not available)
All LC types
Most adjusted

REF	NRR	SEX	AD	Number Exposed Case	Cont	Non-exposed Case	Cont	RR	95.00%CI
TANG	1	c	0	52	25	9	39	9.01	(3.78- 21.46)
*TENKAN	24	m	1	-	-	-	-	16.81	(7.22- 39.14)
TIZZAN	5	m	0	693	619	180	305	1.90	(1.53- 2.35)
TIZZAN	13	f	0	17	18	25	114	4.31	(1.95- 9.51)
Subtotal TIZZAN								2.01	(1.63- 2.47)
TOKARS	1	m	0	110	157	1	53	37.13	(5.06- 272.56)
TOUSEY	12	m	3	-	-	-	-	59.20	(21.00- 167.30)
TOUSEY	15	f	3	-	-	-	-	30.20	(16.00- 57.40)
Subtotal TOUSEY								36.34	(21.09- 62.60)
TSUGAN	28	m	0	63	63	18	22	1.22	(0.60- 2.50)
*TULINI	36	m	3	-	-	-	-	12.17	(6.56- 22.59)
*TULINI	42	f	3	-	-	-	-	16.34	(9.07- 29.47)
Subtotal TULINI								14.20	(9.27- 21.76)
*TVERDA	5	m	2	-	-	-	-	4.09	(2.65- 6.31)
*TVERDA	15	f	2	-	-	-	-	11.05	(3.33- 36.71)
Subtotal TVERDA								4.59	(3.05- 6.90)
WAKAI	8	m	2	-	-	-	-	4.40	(2.19- 8.85)
WAKAI	26	f	2	-	-	-	-	4.37	(2.21- 8.62)
Subtotal WAKAI								4.38	(2.69- 7.14)
*WALD	4	m	1	-	-	-	-	16.40	(7.55- 44.20)
WANG2	18	c	4	-	-	-	-	2.40	(1.14- 5.05)
WIGLE	28	m	1	-	-	-	-	12.40	(7.21- 21.31)
WIGLE	33	f	1	-	-	-	-	5.20	(3.34- 8.09)
Subtotal WIGLE								7.36	(5.23- 10.37)
WU	42	f	2	-	-	-	-	4.86	(2.76- 8.57)
WUNSCH	5	m	1	-	-	-	-	6.59	(3.59- 12.10)
WUNSCH	11	f	1	-	-	-	-	5.98	(3.25- 11.00)
Subtotal WUNSCH								6.28	(4.08- 9.66)
WYNDE3	50	m	0	227	207	9	88	10.72	(5.26- 21.84)
WYNDE6	18	m	0	1677	741	87	617	16.05	(12.62- 20.41)
WYNDE6	207	f	0	1022	376	159	856	14.63	(11.90- 17.99)
Subtotal WYNDE6								15.22	(13.01- 17.80)
YAMAGU	10	c	1	-	-	-	-	4.90	(2.55- 9.44)
*YONG	12	m	1	-	-	-	-	28.71	(6.98- 118.16)
*YONG	15	f	1	-	-	-	-	5.20	(2.38- 11.35)
Subtotal YONG								7.75	(3.91- 15.36)
Partial Totals				43832	117318	6390	64832		

*prospective study ~ With 0.5 adjustment for zero

REF	NRR	SEX	AD	Ys	Ws	Qs	Ps
AGUDO	3	f	3	1.28	5.53	5.13	0.0025
*AKIBA	10	m	5	1.63	20.16	7.71	0.0000
*AKIBA	14	f	5	1.36	42.26	33.23	0.0000
Subtotal AKIBA				1.45	62.42	40.94	
ALDERS	177	m	0	2.66	12.62	2.14	0.0000
ALDERS	176	f	0	1.76	41.23	9.89	0.0000
Subtotal ALDERS				1.97	53.85	12.03	
*AMANDU	5	m	2	1.88	4.22	0.58	0.0001
AMES	1	m	0	1.52	10.33	5.51	0.0000
*ANDERS	6	f	1	3.15	37.54	30.84	0.0000
*ARCHER	5	m	0	1.82	5.72	1.06	0.0000
ARMADA	27	m	0	3.21	3.58	3.28	0.0000
AUSTIN	6	c	3	2.98	3.35	1.78	0.0000
AXELSS	2	m	0	2.70	12.26	2.54	0.0000
AXELSS	10	f	0	2.48	11.50	0.60	0.0000
Subtotal AXELSS				2.59	23.76	3.14	
BARBON	4	m	1	2.60	18.02	2.18	0.0000
BECHER	13	m	0	2.70	2.70	0.56	0.0000
BECHER	14	f	0	1.89	5.32	0.69	0.0000
Subtotal BECHER				2.16	8.02	1.25	
*BENSHL	4	m	1	2.48	9.73	0.52	0.0000
*BEST	2	m	1	2.70	6.85	1.41	0.0000
BLOHMK	1	m	0	1.16	59.38	69.93	0.0000
*BOUCOT	114	m	2	4.13	0.50	1.76	0.0036
*BRETT	4	m	0	1.37	5.75	4.46	0.0010
BROSS	4	m	0	1.78	27.54	6.07	0.0000
BROWN2	12	m	2	2.42	402.82	12.64	0.0000
BROWN2	11	f	2	2.61	365.29	47.98	0.0000
Subtotal BROWN2				2.51	768.12	60.62	
BUFFLE	3	m	0	2.40	4.35	0.10	0.0000

Table 1B2 - 2

IESLC - Meta-analysis of Current Smoking (vs never smoking), Cigarettes (or Any Product if Cigarettes not available)
All LC types
Most adjusted

REF	NRR	SEX	AD	Ys	Ws	Qs	Ps
BUFFLE	7	f	0	2.11	26.25	0.49	0.0000
Subtotal BUFFLE				2.15	30.60	0.59	
CARPEN	11	c	3	3.14	11.66	9.22	0.0000
*CEDERL	26	m	1	2.10	6.14	0.14	0.0000
*CEDERL	119	f	1	1.51	4.78	2.58	0.0009
Subtotal CEDERL				1.84	10.92	2.71	
*CHANG	5	m	0	2.13	4.46	0.07	0.0000
*CHANG	11	f	0	1.64	8.22	3.04	0.0000
Subtotal CHANG				1.81	12.68	3.11	
CHOI	3	m	0	1.64	10.55	3.90	0.0000
CHOI	7	f	0	0.20	7.16	30.06	0.5951
Subtotal CHOI				1.06	17.71	33.96	
*CHOW	56	m	2	3.07	5.61	3.76	0.0000
*CHYOU	2	m	1	2.43	12.06	0.42	0.0000
COMSTO	3	m	0	2.90	3.52	1.48	0.0000
COMSTO	8	f	0	2.57	8.49	0.90	0.0000
Subtotal COMSTO				2.67	12.01	2.38	
CORREA	42	c	1	2.65	50.99	8.39	0.0000
*CPSI	220	m	1	2.48	74.99	4.04	0.0000
*CPSI	279	f	1	1.16	70.15	82.51	0.0000
Subtotal CPSI				1.84	145.14	86.55	
*CPSII	126	m	1	3.01	84.90	49.10	0.0000
*CPSII	133	f	1	2.47	171.36	8.20	0.0000
Subtotal CPSII				2.65	256.26	57.30	
DAMBER	16	m	1	2.28	19.52	0.02	0.0000
DARBY	4	m	0	4.51	2.93	15.01	0.0000
DARBY	11	f	0	3.03	18.15	11.07	0.0000
Subtotal DARBY				3.23	21.08	26.07	
DEAN2	2	m	0	1.33	23.59	19.70	0.0000
DEAN2	6	f	0	1.06	13.83	19.39	0.0001
Subtotal DEAN2				1.23	37.42	39.09	
DEAN3	239	m	1	1.95	21.23	1.86	0.0000
DEAN3	119	f	3	1.75	20.79	5.09	0.0000
Subtotal DEAN3				1.85	42.02	6.95	
*DEKLER	7	m	2	3.17	0.99	0.84	0.0016
DESTE2	4	c	7	2.21	12.30	0.02	0.0000
DESTEF	41	m	4	2.39	18.66	0.37	0.0000
*DOCKER	1	c	4	2.08	3.90	0.11	0.0000
DOLL	90	m	0	2.25	6.22	0.00	0.0000
DOLL	93	f	0	0.74	11.96	27.36	0.0110
Subtotal DOLL				1.25	18.18	27.36	
*DOLL2	68	m	1	2.50	6.84	0.44	0.0000
*DOLL2	63	f	1	2.16	3.28	0.03	0.0001
Subtotal DOLL2				2.39	10.12	0.47	
DORANT	9	c	0	3.26	13.00	13.23	0.0000
DORGAN	9	m	0	2.83	11.70	3.95	0.0000
DORGAN	33	m	0	3.71	2.61	5.60	0.0000
DORGAN	56	f	0	2.50	41.93	2.64	0.0000
DORGAN	79	f	0	2.44	3.75	0.13	0.0000
Subtotal DORGAN				2.61	60.00	12.32	
*DORN	391	m	1	2.39	316.13	5.97	0.0000
DROSTE	6	m	4	2.67	5.52	1.00	0.0000
*ENGELA	168	m	1	2.27	6.48	0.00	0.0000
*ENGELA	177	f	1	1.76	6.50	1.56	0.0000
Subtotal ENGELA				2.01	12.99	1.56	
*ENSTRO	1	m	1	2.56	81.91	8.21	0.0000
*ENSTRO	2	f	1	1.94	181.45	17.32	0.0000
Subtotal ENSTRO				2.13	263.36	25.52	
GAO	33	m	2	1.36	39.76	31.26	0.0000
GAO	34	f	2	1.06	51.44	71.99	0.0000
Subtotal GAO				1.19	91.20	103.24	
GAO2	8	m	1	1.89	9.26	1.19	0.0000
GARCIA	2	c	0	2.72	13.47	2.95	0.0000
GARDIN	6	c	0	2.72	3.79	0.84	0.0000
GARSHI	31	m	1	2.04	33.11	1.41	0.0000
GOODMA	2	m	0	2.86	8.50	3.17	0.0000
GOODMA	6	f	0	2.27	10.71	0.00	0.0000
Subtotal GOODMA				2.53	19.21	3.17	
GRAHAM	10	m	1	1.98	17.14	1.21	0.0000
GREGOR	2	m	0	0.26	4.75	18.79	0.5741
GREGOR	6	f	0	2.67	0.88	0.15	0.0126

Table 1B2 - 2

IESLC - Meta-analysis of Current Smoking (vs never smoking), Cigarettes (or Any Product if Cigarettes not available)
All LC types
Most adjusted

REF	NRR	SEX	AD	Ys	Ws	Qs	Ps
Subtotal GREGOR				0.63	5.62	18.94	
HAENSZ	54	f	0	0.76	23.97	53.04	0.0002
*HAMMO2	8	m	1	2.32	4.92	0.02	0.0000
*HAMMON	139	m	1	2.44	14.07	0.54	0.0000
*HEIN	1	m	0	3.12	0.98	0.74	0.0020
*HENNEK	2	m	0	2.73	17.97	4.24	0.0000
*HIRAYA	1	m	1	1.49	85.55	48.73	0.0000
*HIRAYA	3	f	1	0.85	77.37	151.11	0.0000
Subtotal HIRAYA				1.19	162.92	199.84	
HITOSU	34	m	1	1.03	6.25	9.33	0.0103
HITOSU	59	f	1	1.13	13.59	17.04	0.0000
Subtotal HITOSU				1.10	19.85	26.37	
*HOLE	32	m	1	2.12	6.70	0.10	0.0000
*HOLE	31	f	1	0.43	4.99	16.58	0.3421
Subtotal HOLE				1.40	11.69	16.68	
HUMBLE	13	m	1	2.99	4.94	2.75	0.0000
HUMBLE	15	m	1	2.76	1.65	0.43	0.0004
HUMBLE	17	f	1	2.82	5.85	1.89	0.0000
HUMBLE	19	f	1	3.16	2.49	2.06	0.0000
Subtotal HUMBLE				2.93	14.94	7.14	
JAHN	5	m	0	2.31	14.42	0.05	0.0000
JAIN	52	m	2	2.52	7.65	0.56	0.0000
JAIN	51	f	2	2.82	12.13	3.99	0.0000
Subtotal JAIN				2.70	19.79	4.55	
JARVHO	2	m	0	3.70	0.90	1.89	0.0005
JARVHO	6	f	0	2.74	2.57	0.62	0.0000
Subtotal JARVHO				2.99	3.47	2.51	
JOLY	16	m	0	2.75	10.86	2.74	0.0000
JOLY	15	f	0	2.01	24.54	1.36	0.0000
Subtotal JOLY				2.24	35.40	4.09	
*KAISE2	68	m	1	2.08	10.65	0.28	0.0000
*KAISE2	60	f	1	2.67	8.78	1.59	0.0000
Subtotal KAISE2				2.35	19.43	1.87	
*KAISER	12	m	2	2.98	25.68	13.62	0.0000
*KAISER	9	f	2	1.88	27.68	3.81	0.0000
Subtotal KAISER				2.41	53.36	17.44	
KANELL	30	m	1	1.60	30.83	13.04	0.0000
KATSOU	2	f	1	1.22	8.70	9.12	0.0003
KAUFMA	16	c	6	3.03	27.34	16.59	0.0000
KELLER	1	m	0	2.58	195.95	21.42	0.0000
KELLER	9	m	0	2.73	24.67	5.68	0.0000
KELLER	5	f	0	2.68	233.82	43.10	0.0000
KELLER	13	f	0	2.45	34.67	1.49	0.0000
Subtotal KELLER				2.62	489.11	71.69	
KHUDER	19	m	0	2.09	19.27	0.47	0.0000
KIHARA	7	c	0	1.40	42.14	30.21	0.0000
*KINLEN	19	m	2	2.64	6.97	1.06	0.0000
KJUUS	1	m	0	3.05	1.78	1.13	0.0000
*KNEKT	85	m	1	2.22	5.58	0.00	0.0000
KOO	9	f	0	0.94	10.70	18.41	0.0022
KREUZE	18	m	3	2.77	4.86	1.31	0.0000
KREUZE	29	m	3	3.74	20.36	45.06	0.0000
KREUZE	24	f	3	3.40	2.91	3.85	0.0000
KREUZE	35	f	3	1.86	22.48	3.44	0.0000
Subtotal KREUZE				2.79	50.61	53.66	
*KUBIK	12	m	0	3.50	1.96	3.06	0.0000
*LANGE	38	m	1	1.74	3.96	1.02	0.0005
*LANGE	35	f	1	1.61	8.08	3.25	0.0000
Subtotal LANGE				1.66	12.04	4.27	
LEMARC	2	c	0	2.60	17.07	2.14	0.0000
*LIAW	1	m	1	1.31	11.72	10.34	0.0000
*LIAW	2	f	1	1.28	2.46	2.30	0.0447
Subtotal LIAW				1.30	14.17	12.63	
*LIDDEL	4	m	1	1.48	17.82	10.40	0.0000
LOMBAR	9	m	0	2.41	12.02	0.33	0.0000
LUBIN	40	m	0	1.29	7.70	7.02	0.0003
LUBIN2	28	m	2	2.41	159.73	4.43	0.0000
LUBIN2	317	f	0	1.34	106.80	87.07	0.0000
Subtotal LUBIN2				1.99	266.54	91.49	
MACLEN	19	m	0	1.34	3.53	2.93	0.0120
MACLEN	32	f	0	0.87	12.71	24.30	0.0020

Table 1B2 - 2

IESLC - Meta-analysis of Current Smoking (vs never smoking), Cigarettes (or Any Product if Cigarettes not available)
All LC types
Most adjusted

REF	NRR	SEX	AD	Ys	Ws	Qs	Ps
Subtotal MACLEN				0.97	16.25	27.23	
MATOS	3	m	2	2.14	8.35	0.10	0.0000
*MIGRAN	12	m	2	1.40	3.91	2.83	0.0058
*MIGRAN	38	f	2	1.61	3.53	1.44	0.0025
Subtotal MIGRAN				1.50	7.44	4.27	
*MRFITR	2	m	0	3.88	0.50	1.32	0.0062
NAM	76	m	1	2.16	21.20	0.16	0.0000
NAM	92	f	1	2.38	23.93	0.44	0.0000
Subtotal NAM				2.28	45.13	0.60	
ODRISC	1	c	0	3.99	5.77	17.58	0.0000
OSANN	33	m	2	3.28	37.23	39.46	0.0000
OSANN	34	f	2	2.98	60.12	31.85	0.0000
Subtotal OSANN				3.09	97.36	71.31	
PARKIN	30	m	0	1.53	70.51	36.34	0.0000
PERSH2	10	c	4	2.12	107.04	1.88	0.0000
*PETO	4	m	0	1.97	1.98	0.15	0.0056
PEZZO2	2	m	0	3.13	5.42	4.25	0.0000
PEZZOT	5	m	0	3.48	3.66	5.60	0.0000
*QIAO2	8	m	0	0.72	9.54	22.35	0.0267
RACHTA	9	f	1	1.91	10.62	1.19	0.0000
SCHWAR	25	m	0	2.71	68.81	14.40	0.0000
SCHWAR	26	m	0	2.27	26.07	0.01	0.0000
SCHWAR	27	f	0	2.75	91.98	23.22	0.0000
SCHWAR	28	f	0	2.87	22.69	8.67	0.0000
Subtotal SCHWAR				2.69	209.54	46.30	
SEGI2	20	m	1	1.32	6.65	5.74	0.0007
SEGI2	28	f	1	0.50	10.48	31.99	0.1049
Subtotal SEGI2				0.82	17.14	37.73	
SHAW	6	c	0	3.06	8.67	5.68	0.0000
SOBUE	42	m	1	1.41	27.66	19.36	0.0000
SOBUE	52	f	1	1.03	34.45	51.12	0.0000
Subtotal SOBUE				1.20	62.11	70.48	
SOBUE2	10	m	2	1.50	197.90	111.40	0.0000
SOBUE2	12	f	2	1.19	143.51	161.20	0.0000
Subtotal SOBUE2				1.37	341.42	272.60	
*SPEIZE	10	f	1	2.54	65.88	5.66	0.0000
SPITZ	2	c	0	3.05	5.83	3.77	0.0000
STOCKW	7	c	0	2.65	1204.31	195.52	0.0000
STUCKE	2	m	0	4.65	0.49	2.82	0.0012
SUZUK2	6	c	3	3.09	2.54	1.81	0.0000
SVENSS	96	f	1	2.20	13.42	0.03	0.0000
TANG	1	c	0	2.20	5.10	0.01	0.0000
*TENKAN	24	m	1	2.82	5.38	1.77	0.0000
TIZZAN	5	m	0	0.64	84.08	217.25	0.0000
TIZZAN	13	f	0	1.46	6.13	3.80	0.0003
Subtotal TIZZAN				0.70	90.21	221.05	
TOKARS	1	m	0	3.61	0.97	1.81	0.0004
TOUSEY	12	m	3	4.08	3.57	11.99	0.0000
TOUSEY	15	f	3	3.41	9.42	12.67	0.0000
Subtotal TOUSEY				3.59	12.98	24.66	
TSUGAN	28	m	0	0.20	7.53	31.56	0.5818
*TULINI	36	m	3	2.50	10.05	0.63	0.0000
*TULINI	42	f	3	2.79	11.07	3.30	0.0000
Subtotal TULINI				2.65	21.12	3.93	
*TVERDA	5	m	2	1.41	20.41	14.37	0.0000
*TVERDA	15	f	2	2.40	2.67	0.06	0.0001
Subtotal TVERDA				1.52	23.08	14.44	
WAKAI	8	m	2	1.48	7.88	4.62	0.0000
WAKAI	26	f	2	1.47	8.29	4.95	0.0000
Subtotal WAKAI				1.48	16.17	9.58	
*WALD	4	m	1	2.80	4.92	1.49	0.0000
WANG2	18	c	4	0.88	6.94	13.06	0.0211
WIGLE	28	m	1	2.52	13.08	0.95	0.0000
WIGLE	33	f	1	1.65	19.63	7.04	0.0000
Subtotal WIGLE				2.00	32.72	8.00	
WU	42	f	2	1.58	11.97	5.32	0.0000
WUNSCH	5	m	1	1.89	10.41	1.36	0.0000
WUNSCH	11	f	1	1.79	10.34	2.18	0.0000
Subtotal WUNSCH				1.84	20.74	3.54	
WYNDE3	50	m	0	2.37	7.59	0.12	0.0000
WYNDE6	18	m	0	2.78	66.40	18.51	0.0000

Table 1B2 - 2

IESLC - Meta-analysis of Current Smoking (vs never smoking), Cigarettes (or Any Product if Cigarettes not available)
All LC types
Most adjusted

REF	NRR	SEX	AD	Ys	Ws	Qs	Ps
WYNDE6	207	f	0	2.68	90.13	17.10	0.0000
Subtotal WYNDE6				2.72	156.52	35.62	
YAMAGU	10	c	1	1.59	8.97	3.89	0.0000
*YONG	12	m	1	3.36	1.92	2.36	0.0000
*YONG	15	f	1	1.65	6.30	2.26	0.0000
Subtotal YONG				2.05	8.22	4.62	

	N	195
	NS	131
	Wt	6949.42
Het	Chi	2661.64
Het	df	194
Het	P	***
Fixed	RR	9.47
	RRl	9.25
	RRu	9.69
	P	+++
Random	RR	8.64
	RRl	7.83
	RRu	9.54
	P	+++
Asymm	P	*

Table 1B2 - 3

IESLC - Meta-analysis of Current Smoking (vs never smoking), Cigarettes (or Any Product if Cigarettes not available)
All LC types
Most adjusted

	Sex: combined	male	female	Total
N	20	107	68	195
NS	20	102	63	185
Wt	1554.19	2921.63	2473.60	6949.42
Het Chi	151.67	1088.20	1178.89	2661.64
Het df	19	106	67	194
Het P	***	***	***	***
Fixed RR	13.27	9.01	8.11	9.47
RRl	12.62	8.69	7.80	9.25
RRu	13.94	9.35	8.44	9.69
P	+++	+++	+++	+++
Random RR	12.65	9.43	6.81	8.64
RRl	9.87	8.26	5.69	7.83
RRu	16.21	10.76	8.16	9.54
P	+++	+++	+++	+++
Between Chi				242.88
Between df				2
Between P				***
Btwn(F) P				***
Btwn(R) P				***

	Lung cancer type: all	other	Total
N	187	8	195
NS	125	6	131
Wt	6497.31	452.11	6949.42
Het Chi	2200.82	33.50	2661.64
Het df	186	7	194
Het P	***	***	***
Fixed RR	10.11	3.70	9.47
RRl	9.86	3.37	9.25
RRu	10.35	4.05	9.69
P	+++	+++	+++
Random RR	9.02	3.38	8.64
RRl	8.20	2.63	7.83
RRu	9.93	4.35	9.54
P	+++	+++	+++
Between Chi			427.31
Between df			1
Between P			***
Btwn(F) P			***
Btwn(R) P			***

	Location: NAmer	UK	Scand	othEur	China	Japan	othAs	other	Total
N	84	25	21	23	5	18	7	12	195
NS	55	16	14	17	4	11	4	10	131
Wt	4750.90	246.33	261.07	588.41	115.37	749.94	58.83	178.57	6949.42
Het Chi	575.91	154.51	36.49	383.21	4.60	55.13	10.61	45.20	2661.64
Het df	83	24	20	22	4	17	6	11	194
Het P	***	***	*	***	N.S.	***	N.S.	***	***
Fixed RR	12.45	6.95	8.74	6.14	3.13	3.68	2.91	7.32	9.47
RRl	12.10	6.14	7.74	5.67	2.61	3.43	2.26	6.32	9.25
RRu	12.81	7.88	9.87	6.66	3.76	3.95	3.76	8.48	9.69
P	+++	+++	+++	+++	+++	+++	+++	+++	+++
Random RR	11.85	7.77	9.24	9.40	3.11	3.55	2.90	10.08	8.64
RRl	10.80	5.54	7.57	6.39	2.53	3.05	2.04	7.14	7.83
RRu	13.00	10.89	11.27	13.82	3.83	4.14	4.13	14.23	9.54
P	+++	+++	+++	+++	+++	+++	+++	+++	+++
Between Chi									1395.97
Between df									7
Between P									***
Btwn(F) P									***
Btwn(R) P									***

Table 1B2 - 3

IESLC - Meta-analysis of Current Smoking (vs never smoking), Cigarettes (or Any Product if Cigarettes not available)
All LC types
Most adjusted

	Detailed Country in "other Europe"					
	multi	Germany	othWest	East	Balkans	Total
N	2	8	8	3	2	23
NS	1	4	7	3	2	17
Wt	266.54	132.43	136.36	13.55	39.53	588.41
Het Chi	73.19	114.58	144.89	6.06	0.95	383.21
Het df	1	7	7	2	1	22
Het P	***	***	***	*	N.S.	***
Fixed RR	7.28	7.16	3.96	9.61	4.55	6.14
RRl	6.46	6.04	3.34	5.65	3.33	5.67
RRu	8.21	8.49	4.68	16.37	6.21	6.66
P	+++	+++	+++	+++	+++	+++
Random RR	6.56	11.51	10.06	16.43	4.55	9.40
RRl	2.30	5.29	3.79	4.59	3.33	6.39
RRu	18.71	25.06	26.66	58.84	6.21	13.82
P	+++	+++	+++	+++	+++	+++
Between Chi						43.55
Between df						4
Between P						***
Btwn(F) P						N.S.
Btwn(R) P						(*)

	Detailed Country in "other Asia"			
	India	HongKong	other	Total
N		1	6	7
NS		1	3	4
Wt		10.70	48.13	58.83
Het Chi		0.00	10.38	10.61
Het df		0	5	6
Het P		N.S.	(*)	N.S.
Fixed RR		2.55	3.00	2.91
RRl		1.40	2.26	2.26
RRu		4.64	3.98	3.76
P		++	+++	+++
Random RR		2.55	2.98	2.90
RRl		1.40	1.94	2.04
RRu		4.64	4.57	4.13
P		++	+++	+++
Between Chi				0.23
Between df				1
Between P				N.S.
Btwn(F) P				N.S.
Btwn(R) P				N.S.

	Detailed other continent			
	SCAmer	Auslia	Africa	Total
N	10	1	1	12
NS	8	1	1	10
Wt	107.07	0.99	70.51	178.57
Het Chi	19.63	0.00	0.00	45.20
Het df	9	0	0	11
Het P	*	N.S.	N.S.	***
Fixed RR	9.82	23.79	4.62	7.32
RRl	8.12	3.32	3.66	6.32
RRu	11.86	170.46	5.83	8.48
P	+++	++	+++	+++
Random RR	10.61	23.79	4.62	10.08
RRl	7.89	3.32	3.66	7.14
RRu	14.26	170.46	5.83	14.23
P	+++	++	+++	+++
Between Chi				25.56
Between df				2
Between P				***
Btwn(F) P				*
Btwn(R) P				***

Table 1B2 - 3

IESLC - Meta-analysis of Current Smoking (vs never smoking), Cigarettes (or Any Product if Cigarettes not available)
All LC types
Most adjusted

	Start year of study <1960	1960-69	1970-79	1980-89	1990+	Total
N	22	40	41	70	22	195
NS	17	27	27	43	17	131
Wt	970.78	1111.09	794.69	3833.19	239.68	6949.42
Het Chi	372.94	431.00	294.59	666.21	175.30	2661.64
Het df	21	39	40	69	21	194
Het P	***	***	***	***	***	***
Fixed RR	7.20	5.54	7.37	12.39	10.62	9.47
RRl	6.76	5.23	6.87	12.00	9.36	9.25
RRu	7.67	5.88	7.90	12.79	12.05	9.69
P	+++	+++	+++	+++	+++	+++
Random RR	6.55	6.76	7.45	10.33	13.35	8.64
RRl	4.81	5.44	6.01	9.15	9.12	7.83
RRu	8.93	8.41	9.25	11.65	19.53	9.54
P	+++	+++	+++	+++	+++	+++
Between Chi						721.60
Between df						4
Between P						***
Btwn(F) P						***
Btwn(R) P						***

	Study type (1) CC	other	Total
N	128	67	195
NS	83	48	131
Wt	5238.29	1711.13	6949.42
Het Chi	2031.85	612.86	2661.64
Het df	127	66	194
Het P	***	***	***
Fixed RR	9.74	8.68	9.47
RRl	9.48	8.28	9.25
RRu	10.00	9.10	9.69
P	+++	+++	+++
Random RR	8.67	8.59	8.64
RRl	7.68	7.26	7.83
RRu	9.80	10.17	9.54
P	+++	+++	+++
Between Chi			16.93
Between df			1
Between P			***
Btwn(F) P			N.S.
Btwn(R) P			N.S.

	Study type (2) CC	prosp	other	Total
N	128	62	5	195
NS	83	44	4	131
Wt	5238.29	1674.83	36.30	6949.42
Het Chi	2031.85	587.47	19.00	2661.64
Het df	127	61	4	194
Het P	***	***	***	***
Fixed RR	9.74	8.60	13.15	9.47
RRl	9.48	8.20	9.50	9.25
RRu	10.00	9.03	18.20	9.69
P	+++	+++	+++	+++
Random RR	8.67	8.30	14.19	8.64
RRl	7.68	6.98	6.50	7.83
RRu	9.80	9.87	30.98	9.54
P	+++	+++	+++	+++
Between Chi				23.32
Between df				2
Between P				***
Btwn(F) P				N.S.
Btwn(R) P				N.S.

Table 1B2 - 3

IESLC - Meta-analysis of Current Smoking (vs never smoking), Cigarettes (or Any Product if Cigarettes not available)
All LC types
Most adjusted

	Study size (number of LC cases) 100-249	250-499	500-999	1000+	Total
N	56	48	38	53	195
NS	46	35	24	26	131
Wt	349.23	500.02	734.56	5365.62	6949.42
Het Chi	239.15	244.84	319.40	1709.75	2661.64
Het df	55	47	37	52	194
Het P	***	***	***	***	***
Fixed RR	5.59	8.88	7.84	10.11	9.47
RRl	5.03	8.13	7.30	9.84	9.25
RRu	6.20	9.69	8.43	10.39	9.69
P	+++	+++	+++	+++	+++
Random RR	7.04	8.80	10.10	9.16	8.64
RRl	5.57	7.16	8.07	7.77	7.83
RRu	8.90	10.81	12.64	10.80	9.54
P	+++	+++	+++	+++	+++
Between Chi					148.49
Between df					3
Between P					***
Btwn(F) P					*
Btwn(R) P					N.S.

	Risky occupational population no	mining	othRisky	Total
N	184	7	4	195
NS	120	7	4	131
Wt	6850.75	56.33	42.35	6949.42
Het Chi	2608.51	9.82	4.84	2661.64
Het df	183	6	3	194
Het P	***	N.S.	N.S.	***
Fixed RR	9.53	4.17	8.87	9.47
RRl	9.31	3.21	6.57	9.25
RRu	9.76	5.41	11.99	9.69
P	+++	+++	+++	+++
Random RR	8.78	4.31	10.82	8.64
RRl	7.94	3.02	6.30	7.83
RRu	9.70	6.15	18.58	9.54
P	+++	+++	+++	+++
Between Chi				38.46
Between df				2
Between P				***
Btwn(F) P				N.S.
Btwn(R) P				***

	National cigarette tobacco type Virginia	blended	other	Total
N	34	154	7	195
NS	23	103	5	131
Wt	403.69	6416.19	129.55	6949.42
Het Chi	204.35	2246.86	4.94	2661.64
Het df	33	153	6	194
Het P	***	***	N.S.	***
Fixed RR	6.89	9.87	3.19	9.47
RRl	6.25	9.63	2.68	9.25
RRu	7.59	10.12	3.79	9.69
P	+++	+++	+++	+++
Random RR	8.27	9.13	3.19	8.64
RRl	6.36	8.21	2.68	7.83
RRu	10.75	10.16	3.79	9.54
P	+++	+++	+++	+++
Between Chi				205.50
Between df				2
Between P				***
Btwn(F) P				***
Btwn(R) P				***

Table 1B2 - 3

IESLC - Meta-analysis of Current Smoking (vs never smoking), Cigarettes (or Any Product if Cigarettes not available)
All LC types
Most adjusted

	Any proxy use No/nk	Yes	Total
N	156	39	195
NS	110	21	131
Wt	6103.17	846.25	6949.42
Het Chi	2426.43	234.59	2661.64
Het df	155	38	194
Het P	***	***	***
Fixed RR	9.43	9.71	9.47
RRl	9.20	9.08	9.25
RRu	9.67	10.39	9.69
P	+++	+++	+++
Random RR	8.28	10.09	8.64
RRl	7.40	8.40	7.83
RRu	9.28	12.13	9.54
P	+++	+++	+++
Between Chi			0.62
Between df			1
Between P			N.S.
Btwn(F) P			N.S.
Btwn(R) P			(*)

	Full histological confirmation No	Yes	Total
N	144	51	195
NS	95	36	131
Wt	5015.33	1934.10	6949.42
Het Chi	2055.94	574.14	2661.64
Het df	143	50	194
Het P	***	***	***
Fixed RR	9.08	10.55	9.47
RRl	8.83	10.09	9.25
RRu	9.33	11.03	9.69
P	+++	+++	+++
Random RR	8.12	10.29	8.64
RRl	7.21	8.63	7.83
RRu	9.15	12.28	9.54
P	+++	+++	+++
Between Chi			31.56
Between df			1
Between P			***
Btwn(F) P			N.S.
Btwn(R) P			*

	Number of adjustment variables (1) 0	1	2+/+nk	Total
N	83	64	48	195
NS	57	43	33	133
Wt	3077.89	1882.57	1988.96	6949.42
Het Chi	1197.40	638.30	750.27	2661.64
Het df	82	63	47	194
Het P	***	***	***	***
Fixed RR	10.59	8.31	9.01	9.47
RRl	10.22	7.94	8.62	9.25
RRu	10.97	8.69	9.41	9.69
P	+++	+++	+++	+++
Random RR	9.04	7.58	9.71	8.64
RRl	7.68	6.47	7.93	7.83
RRu	10.64	8.90	11.88	9.54
P	+++	+++	+++	+++
Between Chi				75.67
Between df				2
Between P				***
Btwn(F) P				(*)
Btwn(R) P				N.S.

Table 1B2 - 3

IESLC - Meta-analysis of Current Smoking (vs never smoking), Cigarettes (or Any Product if Cigarettes not available)
All LC types
Most adjusted

	Number of adjustment variables (2) 0	1	2	3-5	6+/+nk	Total
N	83	64	26	20	2	195
NS	57	43	17	14	2	133
Wt	3077.89	1882.57	1616.26	333.06	39.64	6949.42
Het Chi	1197.40	638.30	569.35	161.83	5.68	2661.64
Het df	82	63	25	19	1	194
Het P	***	***	***	***	*	***
Fixed RR	10.59	8.31	8.88	9.01	16.00	9.47
RRl	10.22	7.94	8.46	8.09	11.72	9.25
RRu	10.97	8.69	9.33	10.03	21.85	9.69
P	+++	+++	+++	+++	+++	+++
Random RR	9.04	7.58	8.35	11.51	14.08	8.64
RRl	7.68	6.47	6.35	8.12	6.33	7.83
RRu	10.64	8.90	10.98	16.30	31.35	9.54
P	+++	+++	+++	+++	+++	+++
Between Chi						89.08
Between df						4
Between P						***
Btwn(F) P						N.S.
Btwn(R) P						N.S.

	Product all/unsp	cig+/-ot	cig only	Total
N	52	124	19	195
NS	39	82	17	138
Wt	1206.25	5234.63	508.55	6949.42
Het Chi	629.97	1875.72	109.13	2661.64
Het df	51	123	18	194
Het P	***	***	***	***
Fixed RR	7.91	9.82	9.88	9.47
RRl	7.48	9.56	9.05	9.25
RRu	8.37	10.09	10.77	9.69
P	+++	+++	+++	+++
Random RR	7.09	9.07	10.80	8.64
RRl	5.68	8.04	8.27	7.83
RRu	8.86	10.23	14.12	9.54
P	+++	+++	+++	+++
Between Chi				46.82
Between df				2
Between P				***
Btwn(F) P				N.S.
Btwn(R) P				*

	Denominator nev any	nev cigs	Total
N	134	61	195
NS	93	41	134
Wt	4783.97	2165.45	6949.42
Het Chi	1896.36	735.66	2661.64
Het df	133	60	194
Het P	***	***	***
Fixed RR	9.06	10.43	9.47
RRl	8.81	10.00	9.25
RRu	9.32	10.88	9.69
P	+++	+++	+++
Random RR	8.55	8.89	8.64
RRl	7.56	7.52	7.83
RRu	9.67	10.51	9.54
P	+++	+++	+++
Between Chi			29.62
Between df			1
Between P			***
Btwn(F) P			N.S.
Btwn(R) P			N.S.

Table 1B2 - 3

IESLC - Meta-analysis of Current Smoking (vs never smoking), Cigarettes (or Any Product if Cigarettes not available)
All LC types
Most adjusted

	Derivation of RR/CI Orig	StdCalc	Other	Total
N	47	88	60	195
NS	33	59	41	133
Wt	2036.75	3325.40	1587.28	6949.42
Het Chi	549.69	1469.05	568.99	2661.64
Het df	46	87	59	194
Het P	***	***	***	***
Fixed RR	10.29	9.83	7.86	9.47
RRl	9.85	9.50	7.48	9.25
RRu	10.75	10.17	8.25	9.69
P	+++	+++	+++	+++
Random RR	9.49	8.46	8.32	8.64
RRl	7.97	7.18	7.00	7.83
RRu	11.29	9.96	9.91	9.54
P	+++	+++	+++	+++
Between Chi				73.91
Between df				2
Between P				***
Btwn(F) P				(*)
Btwn(R) P				N.S.

Table 1B2 - 4

IESLC - Meta-analysis of Current Smoking (vs never smoking), Cigarettes (or Any Product if Cigarettes not available)
All LC types
Least adjusted

REF	NRR	X	SEX	AGEL	AGEH	RACE	YF	LC TYPE	LOC	START	ST	NLC	R	VB	P	H	AD	PRODUCT	DENOM	De
AGUDO	10	x	f	0	0	all	-	all	Eu:wst	1989	CC	103	n	bl	n	n	0	cig only	nev any	st
AKIBA	2	x	m	0	0	all	0	all	As:Jap	1963	pr	610	n	bl	n	n	0	cig+/-ot	nev cigs	or
AKIBA	6	x	f	0	0	all	0	all	As:Jap	1963	pr	610	n	bl	n	n	0	cig+/-ot	nev cigs	or
ALDERS	177		m	0	0	all	-	all	Eu:UK	1977	CC	1448	n	V	n	n	0	cig+/-ot	nev any	st
ALDERS	176		f	0	0	all	-	all	Eu:UK	1977	CC	1448	n	V	n	n	0	cig only	nev any	st
AMANDU	1	x	m	0	0	wh	0	all	NAmer	1959	pr	132	m	bl	n	n	0	cig+/-ot	nev cigs	st
AMES	1		m	0	0	wh	-	all	NAmer	1959	ot	317	m	bl	n	n	0	all/unsp	nev any	or
ANDERS	2	x	f	0	0	all	0	all	NAmer	1986	pr	343	n	bl	n	n	0	cig+/-ot	nev cigs	st
ARCHER	5		m	0	0	wh	0	all	NAmer	1950	pr	146	m	bl	n	n	0	cig+/-ot	nev cigs	st
ARMADA	27		m	0	0	all	-	all	Eu:wst	1986	CC	325	n	bl	n	y	0	cig+/-ot	nev any	st
AUSTIN	2	x	c	0	0	all	-	all	NAmer	1970	CC	166	o	bl	y	n	0	cig+/-ot	nev cigs	st
AXELSS	2		m	0	0	sca	-	all	Eu:Sca	1989	CC	436	n	bl	n	n	0	all/unsp	nev any	st
AXELSS	10		f	0	0	sca	-	all	Eu:Sca	1989	CC	436	n	bl	n	n	0	all/unsp	nev any	st
BARBON	3	x	m	0	0	all	-	all	Eu:wst	1979	CC	755	n	bl	y	y	0	all/unsp	nev any	st
BECHER	13		m	0	0	all	-	all	Eu:Ger	1985	CC	194	n	bl	n	y	0	all/unsp	nev any	st
BECHER	14		f	0	0	all	-	all	Eu:Ger	1985	CC	194	n	bl	n	y	0	all/unsp	nev any	st
BENSHL	4		m	0	0	all	0	all	Eu:UK	1967	pr	486	n	V	n	n	1	cig+/-ot	nev any	ot
BEST	2		m	0	0	all	0	all	NAmer	1955	pr	381	n	V	n	n	1	cig only	nev any	ot
BLOHMK	1		m	0	0	all	-	all	Eu:Ger	1978	CC	888	n	bl	n	y	0	all/unsp	nev any	st
BOUCOT	2	x	m	0	0	all	0	all	NAmer	1951	pr	121	n	bl	n	n	0	cig only	nev any	ot
BRETT	4		m	0	0	all	0	all	Eu:UK	1960	pr	150	n	V	n	n	0	cig+/-ot	nev cigs	st
BROSS	4		m	0	0	wh	-	all	NAmer	1960	CC	974	n	bl	n	n	0	cig+/-ot	nev any	st
BROWN2	12		m	0	0	wh	-	all	NAmer	1984	CC	14596	n	bl	n	y	2	cig+/-ot	nev cigs	or
BROWN2	11		f	0	0	wh	-	all	NAmer	1984	CC	14596	n	bl	n	y	2	cig+/-ot	nev cigs	or
BUFFLE	3		m	0	0	wh	-	all	NAmer	1976	CC	943	n	bl	y	n	0	cig+/-ot	nev any	st
BUFFLE	7		f	0	0	wh	-	all	NAmer	1976	CC	943	n	bl	y	n	0	cig+/-ot	nev any	st
CARPEN	9	x	c	0	0	w+b	-	all	NAmer	1991	CC	356	n	bl	n	n	0	cig+/-ot	nev cigs	st
CEDERL	2	x	m	0	0	all	10	all	Eu:Sca	1963	pr	491	n	bl	n	n	0	cig+/-ot	nev any	st
CEDERL	119		f	0	0	all	10	all	Eu:Sca	1963	pr	491	n	bl	n	n	1	cig+/-ot	nev any	ot
CHANG	5		m	0	0	all	0	all	NAmer	1972	pr	136	n	bl	n	n	0	cig+/-ot	nev cigs	st
CHANG	11		f	0	0	all	0	all	NAmer	1972	pr	136	n	bl	n	n	0	cig+/-ot	nev cigs	st
CHOI	3		m	0	0	all	-	all	As:oth	1985	CC	375	n	bl	n	n	0	cig+/-ot	nev cigs	st
CHOI	7		f	0	0	all	-	all	As:oth	1985	CC	375	n	bl	n	n	0	cig+/-ot	nev cigs	st
CHOW	18	x	m	0	0	wh	0	all	NAmer	1966	pr	219	n	bl	n	n	0	cig+/-ot	nev any	st
CHYOU	4	x	m	0	0	jap	0	all	NAmer	1965	pr	227	n	bl	n	y	0	cig+/-ot	nev cigs	st
COMSTO	3		m	0	0	all	-	all	NAmer	1975	ot	258	n	bl	n	n	0	cig+/-ot	nev any	st
COMSTO	8		f	0	0	all	-	all	NAmer	1975	ot	258	n	bl	n	n	0	cig+/-ot	nev any	st
CORREA	41	x	c	0	0	all	-	all	NAmer	1979	CC	1359	n	bl	y	n	0	cig+/-ot	nev cigs	st
CPSI	220		m	35	84	all	6	all	NAmer	1959	pr	5138	n	bl	n	n	1	cig+/-ot	nev any	ot
CPSI	279		f	40	74	all	6	all	NAmer	1959	pr	5138	n	bl	n	n	1	cig+/-ot	nev cigs	ot
CPSII	36	x	m	0	0	all	6	all	NAmer	1982	pr	3229	n	bl	n	n	0	cig only	nev any	st
CPSII	71	x	f	0	0	all	6	all	NAmer	1982	pr	3229	n	bl	n	n	0	cig+/-ot	nev cigs	st
DAMBER	16		m	0	0	all	-	all	Eu:Sca	1972	CC	579	n	bl	y	n	1	cig only	nev any	ot
DARBY	4		m	0	0	wh	-	all	Eu:UK	1988	CC	982	n	V	n	n	0	cig+/-ot	nev any	st
DARBY	11		f	0	0	wh	-	all	Eu:UK	1988	CC	982	n	V	n	n	0	cig+/-ot	nev any	st
DEAN2	2		m	0	0	all	-	all	Eu:UK	1960	CC	954	n	V	y	n	0	all/unsp	nev any	st
DEAN2	6		f	0	0	all	-	all	Eu:UK	1960	CC	954	n	V	y	n	0	all/unsp	nev any	st
DEAN3	238	x	m	0	0	all	-	all	Eu:UK	1969	CC	766	n	V	y	n	0	cig+/-ot	nev any	st
DEAN3	117	x	f	0	0	all	-	all	Eu:UK	1969	CC	766	n	V	y	n	0	cig only	nev any	st
DEKLER	7		m	0	0	all	0	all	Auslia	1961	pr	138	m	V	n	n	2	cig+/-ot	nev any	ot
DESTE2	2	x	c	0	0	all	-	all	SCAmer	1993	CC	463	n	bl	n	n	0	all/unsp	nev any	st
DESTEF	40	x	m	0	0	all	-	all	SCAmer	1988	CC	497	n	bl	n	y	0	all/unsp	nev any	st
DOCKER	1		c	0	0	wh	0	all	NAmer	1974	pr	120	n	bl	n	n	4	cig+/-ot	nev cigs	or
DOLL	90		m	0	0	all	-	all	Eu:UK	1948	CC	1465	n	V	n	n	0	all/unsp	nev any	st
DOLL	93		f	0	0	all	-	all	Eu:UK	1948	CC	1465	n	V	n	n	0	all/unsp	nev any	st
DOLL2	68		m	0	0	all	20	all	Eu:UK	1951	pr	920	n	V	n	n	1	cig+/-ot	nev any	ot
DOLL2	63		f	0	0	all	22	all	Eu:UK	1951	pr	920	n	V	n	n	1	cig only	nev any	ot
DORANT	9		c	0	0	all	0	all	Eu:wst	1986	ot	550	n	bl	n	y	0	cig+/-ot	nev any	st
DORGAN	9		m	0	0	wh	-	all	NAmer	1980	CC	2026	n	bl	y	y	0	cig+/-ot	nev any	st
DORGAN	33		m	0	0	bl	-	all	NAmer	1980	CC	2026	n	bl	y	y	0	cig+/-ot	nev any	st
DORGAN	56		f	0	0	wh	-	all	NAmer	1980	CC	2026	n	bl	y	y	0	cig+/-ot	nev any	st
DORGAN	79		f	0	0	bl	-	all	NAmer	1980	CC	2026	n	bl	y	y	0	cig+/-ot	nev any	st
DORN	391		m	0	0	wh	25	all	NAmer	1954	pr	5097	n	bl	n	n	1	cig+/-ot	nev any	or
DROSTE	2	x	m	0	0	all	-	all	Eu:wst	1995	CC	478	n	bl	n	y	0	all/unsp	nev any	st
ENGELA	168		m	0	0	all	12	all	Eu:Sca	1964	pr	435	n	bl	n	n	1	cig+/-ot	nev any	ot
ENGELA	177		f	0	0	all	12	all	Eu:Sca	1964	pr	435	n	bl	n	n	1	cig+/-ot	nev any	ot
ENSTRO	1		m	0	0	all	0	all	NAmer	1959	pr	2879	n	bl	n	n	1	cig only	nev any	or
ENSTRO	2		f	0	0	all	0	all	NAmer	1959	pr	2879	n	bl	n	n	1	cig only	nev any	or
GAO	29	x	m	0	0	all	-	all	As:Chi	1984	CC	1405	n	ot	n	n	0	cig+/-ot	nev cigs	st
GAO	30	x	f	0	0	all	-	all	As:Chi	1984	CC	1405	n	ot	n	n	0	cig+/-ot	nev cigs	st
GAO2	1	x	m	0	0	all	-	all	As:Jap	1988	CC	282	n	bl	n	n	0	cig+/-ot	nev cigs	st
GARCIA	2		c	0	0	all	-	all	NAmer	1992	CC	416	n	bl	n	y	0	cig+/-ot	nev cigs	st

Table 1B2 - 4

IESLC - Meta-analysis of Current Smoking (vs never smoking), Cigarettes (or Any Product if Cigarettes not available)
All LC types
Least adjusted

REF	NRR	X	SEX	AGEL	AGEH	RACE	YF	LC TYPE	LOC	START	ST	NLC	R	VB	P	H	AD	PRODUCT	DENOM	De
GARDIN	6		c	0	0	all	-	all	Eu:UK	1988	CC	143	n	V	y	n	0	cig only	nev any	st
GARSHI	23	x	m	0	0	all	-	all	NAmer	1981	CC	1081	o	bl	y	n	0	all/unsp	nev any	st
GOODMA	2		m	0	0	w+o	-	all	NAmer	1983	CC	326	n	bl	y	y	0	cig+/-ot	nev any	st
GOODMA	6		f	0	0	w+o	-	all	NAmer	1983	CC	326	n	bl	y	y	0	cig+/-ot	nev any	st
GRAHAM	9	x	m	0	0	wh	-	all	NAmer	1956	CC	685	n	bl	n	n	0	cig+/-ot	nev any	st
GREGOR	2		m	0	0	all	-	all	Eu:UK	1976	CC	104	n	V	n	y	0	cig+/-ot	nev cigs	st
GREGOR	6		f	0	0	all	-	all	Eu:UK	1976	CC	104	n	V	n	y	0	cig+/-ot	nev cigs	st
HAENSZ	54		f	0	0	all	-	not alv	NAmer	1955	CC	158	n	bl	n	y	0	cig+/-ot	nev any	st
HAMMO2	22	x	m	0	0	all	0	all	NAmer	1967	pr	450	o	bl	n	n	0	cig+/-ot	nev any	st
HAMMON	139		m	0	0	wh	0	all	NAmer	1952	pr	448	n	bl	n	n	1	cig only	nev any	ot
HEIN	1		m	0	0	all	0	all	Eu:Sca	1970	pr	144	n	bl	n	n	0	cig only	nev any	st
HENNEK	2		m	0	0	all	0	all	NAmer	1982	pr	169	n	bl	n	n	0	all/unsp	nev any	st
HIRAYA	1		m	0	0	all	0	all	As:Jap	1965	pr	1917	n	bl	n	n	1	cig+/-ot	nev any	st
HIRAYA	3		f	0	0	all	0	all	As:Jap	1965	pr	1917	n	bl	n	n	1	cig+/-ot	nev any	st
HITOSU	2	x	m	0	0	all	-	all	As:Jap	1960	CC	216	n	bl	y	n	0	all/unsp	nev any	st
HITOSU	9	x	f	0	0	all	-	all	As:Jap	1960	CC	216	n	bl	y	n	0	all/unsp	nev any	st
HOLE	46	x	m	0	0	all	0	all	Eu:UK	1972	pr	225	n	V	n	n	0	cig+/-ot	nev any	st
HOLE	29	x	f	0	0	all	11	all	Eu:UK	1972	pr	225	n	V	n	n	0	all/unsp	nev any	st
HUMBLE	13		m	0	0	w-hi	-	all	NAmer	1980	CC	521	n	bl	y	n	1	cig+/-ot	nev cigs	ot
HUMBLE	15		m	0	0	hi	-	all	NAmer	1980	CC	521	n	bl	y	n	1	cig+/-ot	nev cigs	ot
HUMBLE	17		f	0	0	w-hi	-	all	NAmer	1980	CC	521	n	bl	y	n	1	cig+/-ot	nev cigs	ot
HUMBLE	19		f	0	0	hi	-	all	NAmer	1980	CC	521	n	bl	y	n	1	cig+/-ot	nev cigs	ot
JAHN	5		m	0	0	all	-	all	Eu:Ger	1988	CC	1004	n	bl	n	n	0	cig+/-ot	nev any	st
JAIN	16	x	m	0	0	all	-	all	NAmer	1981	CC	845	n	V	y	n	0	cig+/-ot	nev cigs	st
JAIN	11	x	f	0	0	all	-	all	NAmer	1981	CC	845	n	V	y	n	0	cig+/-ot	nev cigs	st
JARVHO	2		m	0	0	all	-	all	Eu:Sca	1983	CC	147	n	bl	n	n	0	all/unsp	nev any	st
JARVHO	6		f	0	0	all	-	all	Eu:Sca	1983	CC	147	n	bl	n	n	0	all/unsp	nev any	st
JOLY	16		m	0	0	all	-	all	SCAmer	1978	CC	826	n	bl	n	n	0	cig+/-ot	nev any	st
JOLY	15		f	0	0	all	-	all	SCAmer	1978	CC	826	n	bl	n	n	0	cig+/-ot	nev any	st
KAISE2	68		m	35	99	all	9	all	NAmer	1979	pr	318	n	bl	n	n	1	cig only	nev any	st
KAISE2	60		f	35	99	all	9	all	NAmer	1979	pr	318	n	bl	n	n	1	cig only	nev any	st
KAISER	12		m	0	0	all	0	all	NAmer	1964	pr	714	n	bl	n	n	2	cig+/-ot	nev cigs	ot
KAISER	9		f	0	0	all	0	all	NAmer	1964	pr	714	n	bl	n	n	2	cig+/-ot	nev cigs	ot
KANELL	5	x	m	0	0	all	-	all	Eu:bal	1950	CC	862	n	bl	n	n	0	all/unsp	nev any	st
KATSOU	6	x	f	0	0	all	-	all	Eu:bal	1987	CC	101	n	bl	n	n	0	all/unsp	nev any	st
KAUFMA	7	x	c	0	0	all	-	all	NAmer	1981	CC	881	n	bl	n	n	0	cig+/-ot	nev cigs	st
KELLER	1		m	0	0	wh	-	all	NAmer	1985	CC	15038	n	bl	n	n	0	all/unsp	nev any	st
KELLER	9		m	0	0	nonw	-	all	NAmer	1985	CC	15038	n	bl	n	n	0	all/unsp	nev any	st
KELLER	5		f	0	0	wh	-	all	NAmer	1985	CC	15038	n	bl	n	n	0	all/unsp	nev any	st
KELLER	13		f	0	0	nonw	-	all	NAmer	1985	CC	15038	n	bl	n	n	0	all/unsp	nev any	st
KHUDER	19		m	0	0	all	-	all	NAmer	1985	CC	482	n	bl	n	y	0	cig+/-ot	nev cigs	or
KIHARA	7		c	0	0	jap	-	all	As:Jap	1991	CC	440	n	bl	n	n	0	all/unsp	nev any	st
KINLEN	8	x	m	0	0	all	0	all	Eu:UK	1967	pr	718	n	V	n	n	0	cig+/-ot	nev any	st
KJUUS	1		m	0	0	all	-	all	Eu:Sca	1979	CC	176	n	bl	n	n	0	all/unsp	nev any	st
KNEKT	75	x	m	20	69	all	21	all	Eu:Sca	1966	pr	515	n	bl	n	n	0	cig+/-ot	nev any	st
KOO	9		f	0	0	all	-	all	As:HK	1981	CC	200	n	bl	n	n	0	all/unsp	nev any	st
KREUZE	5	x	m	1	45	all	-	all	Eu:Ger	1990	CC	2260	n	bl	n	n	0	cig+/-ot	nev any	st
KREUZE	7	x	m	55	69	all	-	all	Eu:Ger	1990	CC	2260	n	bl	n	n	0	cig+/-ot	nev any	st
KREUZE	6	x	f	1	45	all	-	all	Eu:Ger	1990	CC	2260	n	bl	n	n	0	cig+/-ot	nev any	st
KREUZE	8	x	f	55	69	all	-	all	Eu:Ger	1990	CC	2260	n	bl	n	n	0	cig+/-ot	nev any	st
KUBIK	12		m	0	0	all	0	all	Eu:est	1965	pr	108	n	bl	n	n	0	cig+/-ot	nev any	st
LANGE	32	x	m	0	0	all	0	all	Eu:Sca	1976	pr	268	n	bl	n	n	0	all/unsp	nev any	st
LANGE	29	x	f	0	0	all	0	all	Eu:Sca	1976	pr	268	n	bl	n	n	0	all/unsp	nev any	st
LEMARC	2		c	0	0	w+o	-	all	NAmer	1992	CC	341	n	bl	n	y	0	all/unsp	nev any	st
LIAW	1		m	0	0	all	0	all	As:oth	1982	pr	127	n	ot	n	n	1	all/unsp	nev any	or
LIAW	2		f	0	0	all	0	all	As:oth	1982	pr	127	n	ot	n	n	1	all/unsp	nev any	or
LIDDEL	4		m	0	0	all	18	all	NAmer	1970	pr	304	m	V	n	n	1	cig+/-ot	nev cigs	ot
LOMBAR	9		m	0	0	all	-	all	NAmer	1951	CC	1040	n	bl	n	n	0	cig+/-ot	nev any	st
LUBIN	40		m	0	0	all	-	all	As:Chi	1984	CC	427	m	ot	y	n	0	cig+/-ot	nev any	st
LUBIN2	27	x	m	0	0	all	-	all	Eu:mul	1976	CC	7804	n	bl	n	y	0	cig+/-ot	nev any	st
LUBIN2	317		f	0	0	all	-	all	Eu:mul	1976	CC	7804	n	bl	n	y	0	cig+/-ot	nev any	st
MACLEN	19		m	0	0	ch	-	all	As:oth	1972	CC	233	n	bl	n	n	0	cig+/-ot	nev cigs	st
MACLEN	32		f	0	0	ch	-	all	As:oth	1972	CC	233	n	bl	n	n	0	cig+/-ot	nev cigs	st
MATOS	2	x	m	0	0	all	-	all	SCAmer	1994	CC	200	n	bl	n	n	0	cig+/-ot	nev any	st
MIGRAN	11	x	m	0	0	all	0	all	Eu:UK	1964	pr	259	n	V	n	n	0	cig+/-ot	nev any	st
MIGRAN	37	x	f	0	0	all	0	all	Eu:UK	1964	pr	259	n	V	n	n	0	cig+/-ot	nev any	st
MRFITR	2		m	0	0	all	0	all	NAmer	1973	pr	119	n	bl	n	n	0	cig+/-ot	nev cigs	ot
NAM	68	x	m	0	0	all	-	all	NAmer	1986	CC	1199	n	bl	y	n	0	cig+/-ot	nev cigs	ot
NAM	84	x	f	0	0	all	-	all	NAmer	1986	CC	1199	n	bl	y	n	0	cig+/-ot	nev cigs	ot
ODRISC	1		c	0	0	all	-	all	Eu:UK	1992	CC	446	n	V	n	n	0	all/unsp	nev any	st
OSANN	9	x	m	0	0	all	-	all	NAmer	1984	CC	1986	n	bl	n	n	0	cig+/-ot	nev cigs	st
OSANN	13	x	f	0	0	all	-	all	NAmer	1984	CC	1986	n	bl	n	n	0	cig+/-ot	nev cigs	st

Table 1B2 - 4

IESLC - Meta-analysis of Current Smoking (vs never smoking), Cigarettes (or Any Product if Cigarettes not available)
All LC types
Least adjusted

REF	NRR	X	SEX	AGEL	AGEH	RACE	YF	LC TYPE	LOC	START	ST	NLC	R	VB	P	H	AD	PRODUCT	DENOM	De
PARKIN	30		m	0	0	bl	-	all	Africa	1963	CC	877	n	V	y	n	0	cig+/-ot	nev any	st
PERSH2	4	x	c	0	0	all	-	all	Eu:Sca	1980	CC	1022	n	bl	y	n	0	all/unsp	nev any	st
PETO	4		m	0	0	all	0	all	Eu:UK	1954	pr	103	n	V	n	n	0	all/unsp	nev any	st
PEZZO2	2		m	0	0	all	-	all	SCAmer	1992	CC	367	n	bl	n	y	0	cig+/-ot	nev cigs	st
PEZZOT	5		m	0	0	all	-	all	SCAmer	1987	CC	215	n	bl	n	y	0	cig only	nev cigs	st
QIAO2	8		m	0	0	all	0	all	As:Chi	1992	pr	241	m	ot	n	n	0	cig+/-ot	nev any	st
RACHTA	2	x	f	0	0	all	-	all	Eu:est	1991	CC	118	n	bl	n	y	0	cig+/-ot	nev cigs	st
SCHWAR	25		m	0	0	wh	-	all	NAmer	1984	CC	5588	n	bl	y	y	0	cig+/-ot	nev cigs	st
SCHWAR	26		m	0	0	bl	-	all	NAmer	1984	CC	5588	n	bl	y	y	0	cig+/-ot	nev cigs	st
SCHWAR	27		f	0	0	wh	-	all	NAmer	1984	CC	5588	n	bl	y	y	0	cig+/-ot	nev cigs	st
SCHWAR	28		f	0	0	bl	-	all	NAmer	1984	CC	5588	n	bl	y	y	0	cig+/-ot	nev cigs	st
SEGI2	19	x	m	0	0	all	-	all	As:Jap	1962	CC	378	n	bl	n	n	0	cig+/-ot	nev any	st
SEGI2	27	x	f	0	0	all	-	all	As:Jap	1962	CC	378	n	bl	n	n	0	cig+/-ot	nev any	st
SHAW	6		c	0	0	wh	-	all	NAmer	1988	CC	335	n	V	n	y	0	all/unsp	nev any	st
SOBUE	90	x	m	0	0	all	-	q+s+l+a	As:Jap	1986	CC	1376	n	bl	n	y	0	cig+/-ot	nev cigs	st
SOBUE	94	x	f	0	0	all	-	q+s+l+a	As:Jap	1986	CC	1376	n	bl	n	y	0	cig+/-ot	nev cigs	st
SOBUE2	10		m	0	0	all	-	q+s+l+a	As:Jap	1965	CC	2083	n	bl	n	n	2	cig+/-ot	nev any	ot
SOBUE2	12		f	0	0	all	-	q+s+l+a	As:Jap	1965	CC	2083	n	bl	n	n	2	cig+/-ot	nev any	ot
SPEIZE	6	x	f	0	0	all	0	all	NAmer	1976	pr	593	n	bl	n	y	0	cig+/-ot	nev cigs	st
SPITZ	2		c	0	0	b+hi	-	all	NAmer	1992	CC	177	n	bl	n	y	0	cig+/-ot	nev cigs	st
STOCKW	7		c	0	0	all	-	all	NAmer	1981	CC	22161	n	bl	n	n	0	cig+/-ot	nev any	st
STUCKE	2		m	0	0	all	-	all	Eu:wst	1989	CC	247	n	bl	n	y	0	all/unsp	nev any	ot
SUZUK2	2	x	c	0	0	all	-	all	SCAmer	1991	CC	123	n	bl	n	y	0	all/unsp	nev any	st
SVENSS	61	x	f	0	0	all	-	all	Eu:Sca	1983	CC	210	n	bl	n	n	0	all/unsp	nev any	st
TANG	1		c	0	0	all	-	not s	NAmer	1992	CC	119	n	bl	n	y	0	cig+/-ot	nev cigs	st
TENKAN	24		m	0	0	all	17	all	Eu:Sca	1962	pr	242	n	bl	n	n	1	all/unsp	nev any	ot
TIZZAN	5		m	0	0	all	-	all	Eu:wst	1959	CC	1358	n	bl	n	n	0	all/unsp	nev any	st
TIZZAN	13		f	0	0	all	-	all	Eu:wst	1959	CC	1358	n	bl	n	n	0	all/unsp	nev any	st
TOKARS	1		m	0	0	all	-	all	Eu:est	1966	ot	162	o	bl	n	y	0	all/unsp	nev any	st
TOUSEY	4	x	m	0	0	all	-	all	NAmer	1993	CC	507	n	bl	y	y	0	cig+/-ot	nev any	st
TOUSEY	8	x	f	0	0	all	-	all	NAmer	1993	CC	507	n	bl	y	y	0	cig+/-ot	nev any	st
TSUGAN	28		m	0	0	all	-	q+a	As:Jap	1976	CC	134	n	bl	n	y	0	all/unsp	nev any	st
TULINI	13	x	m	0	0	all	0	all	Eu:Sca	1967	pr	472	n	bl	n	n	1	cig+/-ot	nev any	ot
TULINI	19	x	f	0	0	all	0	all	Eu:Sca	1967	pr	472	n	bl	n	n	1	cig+/-ot	nev any	ot
TVERDA	5		m	0	0	all	0	all	Eu:Sca	1972	pr	238	n	bl	n	n	2	cig+/-ot	nev cigs	ot
TVERDA	15		f	0	0	all	0	all	Eu:Sca	1972	pr	238	n	bl	n	n	2	cig only	nev cigs	ot
WAKAI	2	x	m	0	0	all	-	all	As:Jap	1988	CC	333	n	bl	n	y	0	all/unsp	nev any	st
WAKAI	20	x	f	0	0	all	-	all	As:Jap	1988	CC	333	n	bl	n	y	0	all/unsp	nev any	st
WALD	2	x	m	0	0	all	0	all	Eu:UK	1975	pr	102	n	V	n	n	0	cig only	nev any	st
WANG2	17	x	c	0	0	all	-	all	As:Chi	1980	CC	103	n	ot	n	n	0	cig+/-ot	nev cigs	st
WIGLE	1	x	m	0	0	all	-	all	NAmer	1971	CC	728	n	V	n	n	0	cig only	nev any	st
WIGLE	33		f	0	0	all	-	all	NAmer	1971	CC	728	n	V	n	n	1	cig+/-ot	nev any	ot
WU	34	x	f	0	0	wh	-	q+a	NAmer	1981	CC	220	n	bl	n	y	0	all/unsp	nev any	st
WUNSCH	2	x	m	0	0	all	-	all	SCAmer	1990	CC	398	n	bl	y	n	0	cig+/-ot	nev any	st
WUNSCH	8	x	f	0	0	all	-	all	SCAmer	1990	CC	398	n	bl	y	n	0	cig+/-ot	nev any	st
WYNDE3	50		m	0	0	all	-	all	NAmer	1966	CC	350	n	bl	n	y	0	all/unsp	nev any	st
WYNDE6	18		m	0	0	all	-	all	NAmer	1969	CC	4423	n	bl	n	y	0	cig+/-ot	nev any	st
WYNDE6	207		f	0	0	all	-	all	NAmer	1969	CC	4423	n	bl	n	y	0	cig+/-ot	nev cigs	st
YAMAGU	1	x	c	0	0	all	-	all	As:Jap	1989	CC	144	n	bl	n	y	0	all/unsp	nev any	st
YONG	12		m	0	0	all	0	all	NAmer	1971	pr	216	n	bl	n	n	1	cig+/-ot	nev cigs	or
YONG	15		f	0	0	all	0	all	NAmer	1971	pr	216	n	bl	n	n	1	cig+/-ot	nev cigs	or

Cigarette type is all/unspec for all RRs
except for the following:

REF	NRR	CIGTYPE
ALDERS	177	MC+-HR
ALDERS	176	MC only
DEAN3	238	MC only
DEAN3	117	MC only
GARDIN	6	MC only

Table 1B2 - 5

IESLC - Meta-analysis of Current Smoking (vs never smoking), Cigarettes (or Any Product if Cigarettes not available)
All LC types
Least adjusted

REF	NRR	SEX	AD	Number Exposed Case	Number Exposed Cont	Non-exposed Case	Non-exposed Cont	RR	95.00%CI
AGUDO	10	f	0	20	17	80	183	2.69	(1.34- 5.41)
*AKIBA	2	m	0	345	171379	18	35833	4.01	(2.50- 6.44)
*AKIBA	6	f	0	74	51237	116	359850	4.48	(3.35- 6.00)
Subtotal AKIBA								4.35	(3.39- 5.57)
ALDERS	177	m	0	519	322	15	133	14.29	(8.23- 24.81)
ALDERS	176	f	0	410	229	75	243	5.80	(4.27- 7.87)
Subtotal ALDERS								7.17	(5.49- 9.36)
*AMANDU	1	m	0	115	96708	6	25350	5.02	(2.21- 11.41)
AMES	1	m	0	150	136	15	62	4.56	(2.48- 8.39)
*ANDERS	2	f	0	212	41262	46	195158	21.80	(15.85- 29.98)
*ARCHER	5	m	0	122	32529	6	9842	6.15	(2.71- 13.96)
ARMADA	27	m	0	188	122	4	64	24.66	(8.75- 69.44)
AUSTIN	2	c	0	111	125	5	88	15.63	(6.13- 39.87)
AXELSS	2	m	0	194	130	16	160	14.92	(8.53- 26.12)
AXELSS	10	f	0	96	69	18	154	11.90	(6.68- 21.22)
Subtotal AXELSS								13.38	(8.95- 20.00)
BARBON	3	m	0	562	362	22	188	13.27	(8.37- 21.04)
BECHER	13	m	0	101	122	3	54	14.90	(4.52- 49.09)
BECHER	14	f	0	33	26	10	52	6.60	(2.82- 15.44)
Subtotal BECHER								8.68	(4.35- 17.35)
*BENSHL	4	m	1	-	-	-	-	11.92	(6.36- 22.34)
*BEST	2	m	1	-	-	-	-	14.91	(7.05- 31.52)
BLOHMK	1	m	0	419	313	126	301	3.20	(2.48- 4.12)
*BOUCOT	2	m	0	85	22177	0	7551	58.23~	(3.61- 938.34)
*BRETT	4	m	0	135	37448	6	6530	3.92	(1.73- 8.88)
BROSS	4	m	0	565	427	38	170	5.92	(4.07- 8.60)
BROWN2	12	m	2	-	-	-	-	11.30	(10.20- 12.40)
BROWN2	11	f	2	-	-	-	-	13.60	(12.30- 15.10)
Subtotal BROWN2								12.34	(11.50- 13.25)
BUFFLE	3	m	0	257	219	5	47	11.03	(4.31- 28.22)
BUFFLE	7	f	0	313	183	41	198	8.26	(5.63- 12.11)
Subtotal BUFFLE								8.61	(6.04- 12.27)
CARPEN	9	c	0	219	162	15	241	21.72	(12.41- 38.01)
*CEDERL	2	m	0	55	8030	7	6352	6.22	(2.83- 13.64)
*CEDERL	119	f	1	-	-	-	-	4.54	(1.85- 11.12)
Subtotal CEDERL								5.42	(3.00- 9.79)
*CHANG	5	m	0	35	419	5	502	8.39	(3.32- 21.21)
*CHANG	11	f	0	30	603	11	1139	5.15	(2.60- 10.21)
Subtotal CHANG								6.12	(3.53- 10.60)
CHOI	3	m	0	232	329	13	95	5.15	(2.82- 9.42)
CHOI	7	f	0	13	23	76	164	1.22	(0.59- 2.54)
Subtotal CHOI								2.88	(1.81- 4.58)
*CHOW	18	m	0	138	81725	6	62913	17.71	(7.82- 40.09)
*CHYOU	4	m	0	181	3470	13	2406	9.65	(5.51- 16.91)
COMSTO	3	m	0	105	100	4	69	18.11	(6.37- 51.48)
COMSTO	8	f	0	77	52	13	115	13.10	(6.68- 25.67)
Subtotal COMSTO								14.40	(8.18- 25.36)
CORREA	41	c	0	943	571	51	388	12.56	(9.22- 17.13)
*CPSI	220	m	1	-	-	-	-	11.94	(9.52- 14.97)
*CPSI	279	f	1	-	-	-	-	3.20	(2.53- 4.04)
Subtotal CPSI								6.32	(5.37- 7.43)
*CPSII	36	m	0	1781	583646	124	742207	18.26	(15.23- 21.91)
*CPSII	71	f	0	1014	744217	310	2091302	9.19	(8.09- 10.44)
Subtotal CPSII								11.51	(10.37- 12.78)
DAMBER	16	m	1	-	-	-	-	9.80	(6.30- 15.30)
DARBY	4	m	0	322	453	3	384	90.98	(28.96- 285.90)
DARBY	11	f	0	195	217	23	529	20.67	(13.05- 32.74)
Subtotal DARBY								25.40	(16.57- 38.92)
DEAN2	2	m	0	671	600	33	112	3.80	(2.54- 5.68)
DEAN2	6	f	0	59	28	88	121	2.90	(1.71- 4.91)
Subtotal DEAN2								3.43	(2.49- 4.73)
DEAN3	238	m	0	408	1192	24	510	7.27	(4.76- 11.12)
DEAN3	117	f	0	102	1158	41	1538	3.30	(2.28- 4.79)
Subtotal DEAN3								4.65	(3.52- 6.14)
*DEKLER	7	m	2	-	-	-	-	23.79	(3.32- 170.45)
DESTE2	2	c	0	216	151	20	108	7.72	(4.59- 13.00)
DESTEF	40	m	0	362	226	27	163	9.67	(6.23- 15.01)
*DOCKER	1	c	4	-	-	-	-	8.00	(2.97- 21.60)
DOLL	90	m	0	1280	1172	7	61	9.52	(4.34- 20.89)
DOLL	93	f	0	58	41	40	59	2.09	(1.18- 3.68)
Subtotal DOLL								3.51	(2.21- 5.55)

Table 1B2 - 5

IESLC - Meta-analysis of Current Smoking (vs never smoking), Cigarettes (or Any Product if Cigarettes not available)
All LC types
Least adjusted

REF	NRR	SEX	AD	Number Exposed Case	Number Exposed Cont	Non-exposed Case	Non-exposed Cont	RR	95.00%CI
*DOLL2	68	m	1	-	-	-	-	12.20	(5.77- 25.82)
*DOLL2	63	f	1	-	-	-	-	8.65	(2.93- 25.55)
Subtotal DOLL2								10.91	(5.89- 20.21)
DORANT	9	c	0	292	876	14	1090	25.95	(15.07- 44.69)
DORGAN	9	m	0	464	170	15	93	16.92	(9.54- 30.01)
DORGAN	33	m	0	214	61	3	35	40.93	(12.17- 137.66)
DORGAN	56	f	0	611	119	103	244	12.16	(8.99- 16.46)
DORGAN	79	f	0	68	17	7	20	11.43	(4.16- 31.43)
Subtotal DORGAN								13.62	(10.58- 17.54)
*DORN	391	m	1	-	-	-	-	10.86	(9.73- 12.13)
DROSTE	2	m	0	379	267	7	93	18.86	(8.61- 41.30)
*ENGELA	168	m	1	-	-	-	-	9.70	(4.49- 20.94)
*ENGELA	177	f	1	-	-	-	-	5.80	(2.69- 12.51)
Subtotal ENGELA								7.50	(4.35- 12.92)
*ENSTRO	1	m	1	-	-	-	-	12.99	(10.46- 16.13)
*ENSTRO	2	f	1	-	-	-	-	6.95	(6.01- 8.04)
Subtotal ENSTRO								8.44	(7.48- 9.53)
GAO	29	m	0	529	438	62	202	3.93	(2.88- 5.37)
GAO	30	f	0	170	100	435	605	2.36	(1.79- 3.12)
Subtotal GAO								2.96	(2.41- 3.64)
GAO2	1	m	0	184	117	13	56	6.77	(3.55- 12.93)
GARCIA	2	c	0	169	74	21	139	15.12	(8.86- 25.79)
GARDIN	6	c	0	72	39	5	41	15.14	(5.53- 41.44)
GARSHI	23	m	0	657	782	41	363	7.44	(5.30- 10.44)
GOODMA	2	m	0	148	169	10	199	17.43	(8.90- 34.14)
GOODMA	6	f	0	58	56	19	177	9.65	(5.30- 17.56)
Subtotal GOODMA								12.53	(8.01- 19.60)
GRAHAM	9	m	0	453	1075	18	346	8.10	(4.98- 13.17)
GREGOR	2	m	0	49	53	10	14	1.29	(0.53- 3.18)
GREGOR	6	f	0	17	26	1	22	14.38	(1.77- 116.90)
Subtotal GREGOR								1.88	(0.82- 4.30)
HAENSZ	54	f	0	69	94	81	236	2.14	(1.43- 3.19)
*HAMMO2	22	m	0	209	4472	5	891	8.33	(3.44- 20.16)
*HAMMON	139	m	1	-	-	-	-	11.52	(6.83- 19.42)
*HEIN	1	m	0	45	912	1	457	22.55	(3.12- 163.06)
*HENNEK	2	m	0	79	2438	23	10919	15.38	(9.69- 24.42)
*HIRAYA	1	m	1	-	-	-	-	4.45	(3.60- 5.50)
*HIRAYA	3	f	1	-	-	-	-	2.34	(1.87- 2.92)
Subtotal HIRAYA								3.28	(2.81- 3.82)
HITOSU	2	m	0	117	1597	7	242	2.53	(1.17- 5.50)
HITOSU	9	f	0	28	459	33	1893	3.50	(2.09- 5.85)
Subtotal HITOSU								3.17	(2.07- 4.86)
*HOLE	46	m	0	161	3989	7	1189	6.86	(3.23- 14.57)
*HOLE	29	f	0	13	2144	8	1917	1.45	(0.60- 3.50)
Subtotal HOLE								3.55	(2.00- 6.29)
HUMBLE	13	m	1	-	-	-	-	19.96	(8.27- 48.21)
HUMBLE	15	m	1	-	-	-	-	15.79	(3.43- 72.69)
HUMBLE	17	f	1	-	-	-	-	16.72	(7.44- 37.61)
HUMBLE	19	f	1	-	-	-	-	23.50	(6.79- 81.36)
Subtotal HUMBLE								18.65	(11.23- 30.97)
JAHN	5	m	0	352	269	18	138	10.03	(5.99- 16.81)
JAIN	16	m	0	265	118	12	85	15.91	(8.37- 30.23)
JAIN	11	f	0	305	99	52	214	12.68	(8.68- 18.51)
Subtotal JAIN								13.44	(9.70- 18.62)
JARVHO	2	m	0	73	29	1	16	40.28	(5.10- 317.77)
JARVHO	6	f	0	31	7	6	21	15.50	(4.56- 52.66)
Subtotal JARVHO								19.86	(6.93- 56.89)
JOLY	16	m	0	451	524	12	218	15.64	(8.63- 28.34)
JOLY	15	f	0	132	96	52	283	7.48	(5.04- 11.12)
Subtotal JOLY								9.38	(6.75- 13.04)
*KAISE2	68	m	1	-	-	-	-	8.04	(4.41- 14.66)
*KAISE2	60	f	1	-	-	-	-	14.48	(7.47- 28.04)
Subtotal KAISE2								10.49	(6.72- 16.36)
*KAISER	12	m	2	-	-	-	-	19.61	(13.32- 28.87)
*KAISER	9	f	2	-	-	-	-	6.53	(4.50- 9.48)
Subtotal KAISER								11.09	(8.48- 14.50)
KANELL	5	m	0	814	441	48	172	6.61	(4.71- 9.30)
KATSOU	6	f	0	45	18	48	67	3.49	(1.80- 6.75)
KAUFMA	7	c	0	621	886	35	925	18.52	(13.02- 26.36)
KELLER	1	m	0	5063	1210	323	1017	13.17	(11.45- 15.15)
KELLER	9	m	0	1053	212	38	117	15.29	(10.31- 22.69)

Table 1B2 - 5

IESLC - Meta-analysis of Current Smoking (vs never smoking), Cigarettes (or Any Product if Cigarettes not available)
All LC types
Least adjusted

REF	NRR	SEX	AD	Number Exposed Case	Number Exposed Cont	Non-exposed Case	Non-exposed Cont	RR	95.00%CI
KELLER	5	f	0	2904	792	469	1860	14.54	(12.79- 16.53)
KELLER	13	f	0	454	135	67	232	11.64	(8.35- 16.24)
Subtotal KELLER								13.79	(12.62- 15.07)
KHUDER	19	m	0	275	-	23	-	8.10	(5.20- 12.70)
KIHARA	7	c	0	283	162	102	237	4.06	(3.00- 5.49)
*KINLEN	8	m	0	589	8512	7	1333	13.18	(6.27- 27.70)
KJUUS	1	m	0	135	77	2	24	21.04	(4.84- 91.45)
*KNEKT	75	m	0	86	33667	6	17814	7.58	(3.32- 17.35)
KOO	9	f	0	42	25	56	85	2.55	(1.40- 4.64)
KREUZE	5	m	0	168	93	6	54	16.26	(6.74- 39.22)
KREUZE	7	m	0	1226	509	23	403	42.20	(27.38- 65.05)
KREUZE	6	f	0	55	22	6	38	15.83	(5.87- 42.73)
KREUZE	8	f	0	167	54	95	177	5.76	(3.88- 8.56)
Subtotal KREUZE								14.54	(11.13- 18.99)
*KUBIK	12	m	0	98	6342	2	4271	33.00	(8.14- 133.74)
*LANGE	32	m	0	174	4537	5	721	5.53	(2.28- 13.41)
*LANGE	29	f	0	53	4455	7	2159	3.67	(1.67- 8.06)
Subtotal LANGE								4.40	(2.44- 7.92)
LEMARC	2	c	0	167	65	32	168	13.49	(8.39- 21.68)
*LIAW	1	m	1	-	-	-	-	3.70	(2.10- 6.60)
*LIAW	2	f	1	-	-	-	-	3.60	(1.00- 12.20)
Subtotal LIAW								3.68	(2.19- 6.20)
*LIDDEL	4	m	1	-	-	-	-	4.41	(2.77- 7.01)
LOMBAR	9	m	0	852	610	14	112	11.17	(6.35- 19.66)
LUBIN	40	m	0	296	650	9	72	3.64	(1.80- 7.38)
LUBIN2	27	m	0	5557	7279	190	2617	10.52	(9.04- 12.23)
LUBIN2	317	f	0	384	410	288	1180	3.84	(3.17- 4.64)
Subtotal LUBIN2								7.10	(6.31- 8.00)
MACLEN	19	m	0	137	108	5	15	3.81	(1.34- 10.80)
MACLEN	32	f	0	42	47	41	109	2.38	(1.37- 4.12)
Subtotal MACLEN								2.63	(1.62- 4.28)
MATOS	2	m	0	112	132	11	110	8.48	(4.35- 16.56)
*MIGRAN	11	m	0	166	4570	4	867	7.87	(2.93- 21.17)
*MIGRAN	37	f	0	30	3459	4	3814	8.27	(2.92- 23.45)
Subtotal MIGRAN								8.06	(3.93- 16.51)
*MRFITR	2	m	0	106	8194	0	1859	48.33~	(3.01- 777.42)
NAM	68	m	0	241	589	30	520	7.09	(4.77- 10.55)
NAM	84	f	0	133	234	52	885	9.67	(6.81- 13.75)
Subtotal NAM								8.44	(6.49- 10.98)
ODRISC	1	c	0	293	598	6	664	54.22	(23.98- 122.60)
OSANN	9	m	0	791	541	45	833	27.07	(19.67- 37.25)
OSANN	13	f	0	597	367	96	1093	18.52	(14.48- 23.68)
Subtotal OSANN								21.33	(17.55- 25.92)
PARKIN	30	m	0	346	874	107	1248	4.62	(3.66- 5.83)
PERSH2	4	c	0	736	631	178	1164	7.63	(6.31- 9.23)
*PETO	4	m	0	99	2036	2	295	7.17	(1.78- 28.92)
PEZZO2	2	m	0	233	198	6	117	22.95	(9.89- 53.26)
PEZZOT	5	m	0	145	129	4	116	32.60	(11.70- 90.81)
*QIAO2	8	m	0	156	5399	10	709	2.05	(1.09- 3.86)
RACHTA	2	f	0	72	33	33	98	6.48	(3.66- 11.46)
SCHWAR	25	m	0	1652	349	119	376	14.96	(11.81- 18.94)
SCHWAR	26	m	0	644	139	50	104	9.64	(6.56- 14.15)
SCHWAR	27	f	0	1029	309	182	855	15.64	(12.75- 19.19)
SCHWAR	28	f	0	256	90	40	247	17.56	(11.64- 26.50)
Subtotal SCHWAR								14.70	(12.84- 16.83)
SEGI2	19	m	0	267	485	8	53	3.65	(1.71- 7.79)
SEGI2	27	f	0	24	34	56	126	1.59	(0.86- 2.92)
Subtotal SEGI2								2.20	(1.37- 3.54)
SHAW	6	c	0	212	97	11	107	21.26	(10.93- 41.36)
SOBUE	90	m	0	736	650	34	128	4.26	(2.88- 6.31)
SOBUE	94	f	0	95	168	167	857	2.90	(2.15- 3.92)
Subtotal SOBUE								3.35	(2.64- 4.25)
SOBUE2	10	m	2	-	-	-	-	4.47	(3.89- 5.14)
SOBUE2	12	f	2	-	-	-	-	3.28	(2.79- 3.87)
Subtotal SOBUE2								3.92	(3.53- 4.36)
*SPEIZE	6	f	0	391	489993	58	776300	10.68	(8.11- 14.07)
SPITZ	2	c	0	103	89	7	128	21.16	(9.40- 47.66)
STOCKW	7	c	0	12470	3357	2791	10641	14.16	(13.38- 14.99)
STUCKE	2	m	0	69	68	0	51	104.50~	(6.32-1727.39)
SUZUK2	2	c	0	78	30	11	53	12.53	(5.78- 27.16)
SVENSS	61	f	0	142	53	38	120	8.46	(5.22- 13.71)

Table 1B2 - 5

IESLC - Meta-analysis of Current Smoking (vs never smoking), Cigarettes (or Any Product if Cigarettes not available)
All LC types
Least adjusted

REF	NRR	SEX	AD	Number Exposed Case	Number Exposed Cont	Non-exposed Case	Non-exposed Cont	RR	95.00%CI
TANG	1	c	0	52	25	9	39	9.01	(3.78- 21.46)
*TENKAN	24	m	1	-	-	-	-	16.81	(7.22- 39.14)
TIZZAN	5	m	0	693	619	180	305	1.90	(1.53- 2.35)
TIZZAN	13	f	0	17	18	25	114	4.31	(1.95- 9.51)
Subtotal TIZZAN								2.01	(1.63- 2.47)
TOKARS	1	m	0	110	157	1	53	37.13	(5.06- 272.56)
TOUSEY	4	m	0	160	91	4	130	57.14	(20.45- 159.69)
TOUSEY	8	f	0	127	78	13	226	28.31	(15.13- 52.94)
Subtotal TOUSEY								34.23	(20.06- 58.43)
TSUGAN	28	m	0	63	63	18	22	1.22	(0.60- 2.50)
*TULINI	13	m	1	-	-	-	-	13.35	(7.18- 24.79)
*TULINI	19	f	1	-	-	-	-	19.20	(10.63- 34.68)
Subtotal TULINI								16.15	(10.53- 24.77)
*TVERDA	5	m	2	-	-	-	-	4.09	(2.65- 6.31)
*TVERDA	15	f	2	-	-	-	-	11.05	(3.33- 36.71)
Subtotal TVERDA								4.59	(3.05- 6.90)
WAKAI	2	m	0	181	284	10	65	4.14	(2.07- 8.27)
WAKAI	20	f	0	33	26	50	145	3.68	(2.01- 6.75)
Subtotal WAKAI								3.87	(2.46- 6.11)
*WALD	2	m	0	77	4182	7	6539	17.20	(7.94- 37.25)
WANG2	17	c	0	49	78	11	43	2.46	(1.16- 5.21)
WIGLE	1	m	0	415	415	15	204	13.60	(7.91- 23.38)
WIGLE	33	f	1	-	-	-	-	5.20	(3.34- 8.09)
Subtotal WIGLE								7.64	(5.42- 10.76)
WU	34	f	0	160	73	31	92	6.50	(3.98- 10.64)
WUNSCH	2	m	0	189	234	14	99	5.71	(3.16- 10.32)
WUNSCH	8	f	0	42	51	29	208	5.91	(3.36- 10.38)
Subtotal WUNSCH								5.81	(3.87- 8.74)
WYNDE3	50	m	0	227	207	9	88	10.72	(5.26- 21.84)
WYNDE6	18	m	0	1677	741	87	617	16.05	(12.62- 20.41)
WYNDE6	207	f	0	1022	376	159	856	14.63	(11.90- 17.99)
Subtotal WYNDE6								15.22	(13.01- 17.80)
YAMAGU	1	c	0	76	247	24	267	3.42	(2.10- 5.59)
*YONG	12	m	1	-	-	-	-	28.71	(6.98- 118.16)
*YONG	15	f	1	-	-	-	-	5.20	(2.38- 11.35)
Subtotal YONG								7.75	(3.91- 15.36)
Partial Totals				69920	2508991	9530	4426776		

*prospective study ~ With 0.5 adjustment for zero

REF	NRR	SEX	AD	Ys	Ws	Qs	Ps
AGUDO	10	f	0	0.99	7.89	12.11	0.0054
*AKIBA	2	m	0	1.39	17.12	12.10	0.0000
*AKIBA	6	f	0	1.50	45.22	24.04	0.0000
Subtotal AKIBA				1.47	62.34	36.14	
ALDERS	177	m	0	2.66	12.62	2.34	0.0000
ALDERS	176	f	0	1.76	41.23	9.14	0.0000
Subtotal ALDERS				1.97	53.85	11.48	
*AMANDU	1	m	0	1.61	5.70	2.15	0.0001
AMES	1	m	0	1.52	10.33	5.23	0.0000
*ANDERS	2	f	0	3.08	37.84	27.53	0.0000
*ARCHER	5	m	0	1.82	5.72	0.97	0.0000
ARMADA	27	m	0	3.21	3.58	3.41	0.0000
AUSTIN	2	c	0	2.75	4.38	1.19	0.0000
AXELSS	2	m	0	2.70	12.26	2.75	0.0000
AXELSS	10	f	0	2.48	11.50	0.71	0.0000
Subtotal AXELSS				2.59	23.76	3.46	
BARBON	3	m	0	2.59	18.08	2.30	0.0000
BECHER	13	m	0	2.70	2.70	0.60	0.0000
BECHER	14	f	0	1.89	5.32	0.62	0.0000
Subtotal BECHER				2.16	8.02	1.23	
*BENSHL	4	m	1	2.48	9.73	0.61	0.0000
*BEST	2	m	1	2.70	6.85	1.53	0.0000
BLOHMK	1	m	0	1.16	59.38	67.52	0.0000
*BOUCOT	2	m	0	4.06	0.50	1.67	0.0042
*BRETT	4	m	0	1.37	5.75	4.27	0.0010
BROSS	4	m	0	1.78	27.54	5.59	0.0000
BROWN2	12	m	2	2.42	402.82	15.47	0.0000
BROWN2	11	f	2	2.61	365.29	53.09	0.0000
Subtotal BROWN2				2.51	768.12	68.56	
BUFFLE	3	m	0	2.40	4.35	0.13	0.0000

Table 1B2 - 5

IESLC - Meta-analysis of Current Smoking (vs never smoking), Cigarettes (or Any Product if Cigarettes not available)
All LC types
Least adjusted

REF	NRR	SEX	AD	Ys	Ws	Qs	Ps
BUFFLE	7	f	0	2.11	26.25	0.36	0.0000
Subtotal BUFFLE				2.15	30.60	0.49	
CARPEN	9	c	0	3.08	12.26	8.85	0.0000
*CEDERL	2	m	0	1.83	6.22	1.00	0.0000
*CEDERL	119	f	1	1.51	4.78	2.45	0.0009
Subtotal CEDERL				1.69	11.00	3.45	
*CHANG	5	m	0	2.13	4.46	0.05	0.0000
*CHANG	11	f	0	1.64	8.22	2.86	0.0000
Subtotal CHANG				1.81	12.68	2.90	
CHOI	3	m	0	1.64	10.55	3.66	0.0000
CHOI	7	f	0	0.20	7.16	29.51	0.5951
Subtotal CHOI				1.06	17.71	33.18	
*CHOW	18	m	0	2.87	5.75	2.39	0.0000
*CHYOU	4	m	0	2.27	12.23	0.02	0.0000
COMSTO	3	m	0	2.90	3.52	1.57	0.0000
COMSTO	8	f	0	2.57	8.49	1.00	0.0000
Subtotal COMSTO				2.67	12.01	2.57	
CORREA	41	c	0	2.53	40.00	3.65	0.0000
*CPSI	220	m	1	2.48	74.99	4.73	0.0000
*CPSI	279	f	1	1.16	70.15	79.67	0.0000
Subtotal CPSI				1.84	145.14	84.39	
*CPSII	36	m	0	2.90	115.97	53.02	0.0000
*CPSII	71	f	0	2.22	237.52	0.03	0.0000
Subtotal CPSII				2.44	353.49	53.04	
DAMBER	16	m	1	2.28	19.52	0.06	0.0000
DARBY	4	m	0	4.51	2.93	15.26	0.0000
DARBY	11	f	0	3.03	18.15	11.61	0.0000
Subtotal DARBY				3.23	21.08	26.87	
DEAN2	2	m	0	1.33	23.59	18.90	0.0000
DEAN2	6	f	0	1.06	13.83	18.78	0.0001
Subtotal DEAN2				1.23	37.42	37.67	
DEAN3	238	m	0	1.98	21.31	1.28	0.0000
DEAN3	117	f	0	1.20	28.00	29.92	0.0000
Subtotal DEAN3				1.54	49.32	31.20	
*DEKLER	7	m	2	3.17	0.99	0.88	0.0016
DESTE2	2	c	0	2.04	14.18	0.48	0.0000
DESTEF	40	m	0	2.27	19.86	0.03	0.0000
*DOCKER	1	c	4	2.08	3.90	0.09	0.0000
DOLL	90	m	0	2.25	6.22	0.00	0.0000
DOLL	93	f	0	0.74	11.96	26.68	0.0110
Subtotal DOLL				1.25	18.18	26.68	
*DOLL2	68	m	1	2.50	6.84	0.51	0.0000
*DOLL2	63	f	1	2.16	3.28	0.02	0.0001
Subtotal DOLL2				2.39	10.12	0.53	
DORANT	9	c	0	3.26	13.00	13.72	0.0000
DORGAN	9	m	0	2.83	11.70	4.21	0.0000
DORGAN	33	m	0	3.71	2.61	5.74	0.0000
DORGAN	56	f	0	2.50	41.93	3.05	0.0000
DORGAN	79	f	0	2.44	3.75	0.16	0.0000
Subtotal DORGAN				2.61	60.00	13.16	
*DORN	391	m	1	2.39	316.13	7.72	0.0000
DROSTE	2	m	0	2.94	6.25	3.13	0.0000
*ENGELA	168	m	1	2.27	6.48	0.01	0.0000
*ENGELA	177	f	1	1.76	6.50	1.44	0.0000
Subtotal ENGELA				2.01	12.99	1.46	
*ENSTRO	1	m	1	2.56	81.91	9.21	0.0000
*ENSTRO	2	f	1	1.94	181.45	15.27	0.0000
Subtotal ENSTRO				2.13	263.36	24.48	
GAO	29	m	0	1.37	39.60	29.22	0.0000
GAO	30	f	0	0.86	50.42	94.40	0.0000
Subtotal GAO				1.08	90.02	123.61	
GAO2	1	m	0	1.91	9.19	0.92	0.0000
GARCIA	2	c	0	2.72	13.47	3.19	0.0000
GARDIN	6	c	0	2.72	3.79	0.90	0.0000
GARSHI	23	m	0	2.01	33.39	1.65	0.0000
GOODMA	2	m	0	2.86	8.50	3.36	0.0000
GOODMA	6	f	0	2.27	10.71	0.02	0.0000
Subtotal GOODMA				2.53	19.21	3.38	
GRAHAM	9	m	0	2.09	16.24	0.30	0.0000
GREGOR	2	m	0	0.26	4.75	18.43	0.5741
GREGOR	6	f	0	2.67	0.88	0.17	0.0126

Table 1B2 - 5

IESLC - Meta-analysis of Current Smoking (vs never smoking), Cigarettes (or Any Product if Cigarettes not available)
All LC types
Least adjusted

REF	NRR	SEX	AD	Ys	Ws	Qs	Ps
Subtotal GREGOR				0.63	5.62	18.60	
HAENSZ	54	f	0	0.76	23.97	51.71	0.0002
*HAMMO2	22	m	0	2.12	4.92	0.06	0.0000
*HAMMON	139	m	1	2.44	14.07	0.65	0.0000
*HEIN	1	m	0	3.12	0.98	0.77	0.0020
*HENNEK	2	m	0	2.73	17.97	4.57	0.0000
*HIRAYA	1	m	1	1.49	85.55	46.33	0.0000
*HIRAYA	3	f	1	0.85	77.37	147.07	0.0000
Subtotal HIRAYA				1.19	162.92	193.40	
HITOSU	2	m	0	0.93	6.40	10.81	0.0187
HITOSU	9	f	0	1.25	14.55	13.87	0.0000
Subtotal HITOSU				1.15	20.95	24.68	
*HOLE	46	m	0	1.93	6.76	0.62	0.0000
*HOLE	29	f	0	0.37	4.98	17.13	0.4046
Subtotal HOLE				1.27	11.73	17.75	
HUMBLE	13	m	1	2.99	4.94	2.89	0.0000
HUMBLE	15	m	1	2.76	1.65	0.46	0.0004
HUMBLE	17	f	1	2.82	5.85	2.02	0.0000
HUMBLE	19	f	1	3.16	2.49	2.15	0.0000
Subtotal HUMBLE				2.93	14.94	7.52	
JAHN	5	m	0	2.31	14.42	0.09	0.0000
JAIN	16	m	0	2.77	9.32	2.70	0.0000
JAIN	11	f	0	2.54	26.82	2.60	0.0000
Subtotal JAIN				2.60	36.14	5.29	
JARVHO	2	m	0	3.70	0.90	1.94	0.0005
JARVHO	6	f	0	2.74	2.57	0.67	0.0000
Subtotal JARVHO				2.99	3.47	2.61	
JOLY	16	m	0	2.75	10.86	2.95	0.0000
JOLY	15	f	0	2.01	24.54	1.15	0.0000
Subtotal JOLY				2.24	35.40	4.09	
*KAISE2	68	m	1	2.08	10.65	0.22	0.0000
*KAISE2	60	f	1	2.67	8.78	1.73	0.0000
Subtotal KAISE2				2.35	19.43	1.95	
*KAISER	12	m	2	2.98	25.68	14.34	0.0000
*KAISER	9	f	2	1.88	27.68	3.44	0.0000
Subtotal KAISER				2.41	53.36	17.78	
KANELL	5	m	0	1.89	33.17	3.83	0.0000
KATSOU	6	f	0	1.25	8.81	8.44	0.0002
KAUFMA	7	c	0	2.92	30.87	14.71	0.0000
KELLER	1	m	0	2.58	195.95	23.93	0.0000
KELLER	9	m	0	2.73	24.67	6.13	0.0000
KELLER	5	f	0	2.68	233.82	46.96	0.0000
KELLER	13	f	0	2.45	34.67	1.77	0.0000
Subtotal KELLER				2.62	489.11	78.80	
KHUDER	19	m	0	2.09	19.27	0.36	0.0000
KIHARA	7	c	0	1.40	42.14	28.88	0.0000
*KINLEN	8	m	0	2.58	6.96	0.85	0.0000
KJUUS	1	m	0	3.05	1.78	1.19	0.0000
*KNEKT	75	m	0	2.03	5.61	0.23	0.0000
KOO	9	f	0	0.94	10.70	17.89	0.0022
KREUZE	5	m	0	2.79	4.95	1.55	0.0000
KREUZE	7	m	0	3.74	20.52	47.01	0.0000
KREUZE	6	f	0	2.76	3.90	1.11	0.0000
KREUZE	8	f	0	1.75	24.58	5.61	0.0000
Subtotal KREUZE				2.68	53.95	55.27	
*KUBIK	12	m	0	3.50	1.96	3.15	0.0000
*LANGE	32	m	0	1.71	4.90	1.32	0.0002
*LANGE	29	f	0	1.30	6.21	5.36	0.0012
Subtotal LANGE				1.48	11.11	6.67	
LEMARC	2	c	0	2.60	17.07	2.38	0.0000
*LIAW	1	m	1	1.31	11.72	9.93	0.0000
*LIAW	2	f	1	1.28	2.46	2.21	0.0447
Subtotal LIAW				1.30	14.17	12.14	
*LIDDEL	4	m	1	1.48	17.82	9.89	0.0000
LOMBAR	9	m	0	2.41	12.02	0.41	0.0000
LUBIN	40	m	0	1.29	7.70	6.74	0.0003
LUBIN2	27	m	0	2.35	167.71	2.58	0.0000
LUBIN2	317	f	0	1.34	106.80	83.47	0.0000
Subtotal LUBIN2				1.96	274.52	86.05	
MACLEN	19	m	0	1.34	3.53	2.81	0.0120
MACLEN	32	f	0	0.87	12.71	23.64	0.0020

Table 1B2 - 5

IESLC - Meta-analysis of Current Smoking (vs never smoking), Cigarettes (or Any Product if Cigarettes not available)
All LC types
Least adjusted

REF	NRR	SEX	AD	Ys	Ws	Qs	Ps
Subtotal MACLEN				0.97	16.25	26.45	
MATOS	2	m	0	2.14	8.58	0.07	0.0000
*MIGRAN	11	m	0	2.06	3.93	0.11	0.0000
*MIGRAN	37	f	0	2.11	3.54	0.05	0.0001
Subtotal MIGRAN				2.09	7.46	0.16	
*MRFITR	2	m	0	3.88	0.50	1.35	0.0062
NAM	68	m	0	1.96	24.33	1.77	0.0000
NAM	84	f	0	2.27	31.10	0.05	0.0000
Subtotal NAM				2.13	55.43	1.82	
ODRISC	1	c	0	3.99	5.77	17.97	0.0000
OSANN	9	m	0	3.30	37.69	43.10	0.0000
OSANN	13	f	0	2.92	63.57	30.27	0.0000
Subtotal OSANN				3.06	101.25	73.37	
PARKIN	30	m	0	1.53	70.51	34.45	0.0000
PERSH2	4	c	0	2.03	106.15	4.12	0.0000
*PETO	4	m	0	1.97	1.98	0.13	0.0056
PEZZO2	2	m	0	3.13	5.42	4.43	0.0000
PEZZOT	5	m	0	3.48	3.66	5.77	0.0000
*QIAO2	8	m	0	0.72	9.54	21.80	0.0267
RACHTA	2	f	0	1.87	11.81	1.53	0.0000
SCHWAR	25	m	0	2.71	68.81	15.61	0.0000
SCHWAR	26	m	0	2.27	26.07	0.04	0.0000
SCHWAR	27	f	0	2.75	91.98	24.99	0.0000
SCHWAR	28	f	0	2.87	22.69	9.21	0.0000
Subtotal SCHWAR				2.69	209.54	49.84	
SEGI2	19	m	0	1.29	6.68	5.84	0.0008
SEGI2	27	f	0	0.46	10.32	32.20	0.1372
Subtotal SEGI2				0.79	17.00	38.04	
SHAW	6	c	0	3.06	8.67	5.95	0.0000
SOBUE	90	m	0	1.45	24.92	15.12	0.0000
SOBUE	94	f	0	1.07	42.31	57.28	0.0000
Subtotal SOBUE				1.21	67.24	72.40	
SOBUE2	10	m	2	1.50	197.90	105.88	0.0000
SOBUE2	12	f	2	1.19	143.51	155.52	0.0000
Subtotal SOBUE2				1.37	341.42	261.41	
*SPEIZE	6	f	0	2.37	50.52	0.98	0.0000
SPITZ	2	c	0	3.05	5.83	3.95	0.0000
STOCKW	7	c	0	2.65	1204.31	214.22	0.0000
STUCKE	2	m	0	4.65	0.49	2.86	0.0012
SUZUK2	2	c	0	2.53	6.41	0.57	0.0000
SVENSS	61	f	0	2.14	16.51	0.14	0.0000
TANG	1	c	0	2.20	5.10	0.00	0.0000
*TENKAN	24	m	1	2.82	5.38	1.89	0.0000
TIZZAN	5	m	0	0.64	84.08	212.19	0.0000
TIZZAN	13	f	0	1.46	6.13	3.62	0.0003
Subtotal TIZZAN				0.70	90.21	215.81	
TOKARS	1	m	0	3.61	0.97	1.86	0.0004
TOUSEY	4	m	0	4.05	3.64	12.00	0.0000
TOUSEY	8	f	0	3.34	9.80	12.17	0.0000
Subtotal TOUSEY				3.53	13.44	24.17	
TSUGAN	28	m	0	0.20	7.53	30.99	0.5818
*TULINI	13	m	1	2.59	10.01	1.32	0.0000
*TULINI	19	f	1	2.95	10.99	5.79	0.0000
Subtotal TULINI				2.78	21.00	7.11	
*TVERDA	5	m	2	1.41	20.41	13.74	0.0000
*TVERDA	15	f	2	2.40	2.67	0.08	0.0001
Subtotal TVERDA				1.52	23.08	13.82	
WAKAI	2	m	0	1.42	8.04	5.24	0.0001
WAKAI	20	f	0	1.30	10.45	8.96	0.0000
Subtotal WAKAI				1.35	18.49	14.20	
*WALD	2	m	0	2.84	6.43	2.44	0.0000
WANG2	17	c	0	0.90	6.78	12.01	0.0193
WIGLE	1	m	0	2.61	13.09	1.90	0.0000
WIGLE	33	f	1	1.65	19.63	6.61	0.0000
Subtotal WIGLE				2.03	32.72	8.51	
WU	34	f	0	1.87	15.85	2.01	0.0000
WUNSCH	2	m	0	1.74	10.98	2.60	0.0000
WUNSCH	8	f	0	1.78	12.09	2.48	0.0000
Subtotal WUNSCH				1.76	23.07	5.08	
WYNDE3	50	m	0	2.37	7.59	0.16	0.0000
WYNDE6	18	m	0	2.78	66.40	19.86	0.0000

Table 1B2 - 5

IESLC - Meta-analysis of Current Smoking (vs never smoking), Cigarettes (or Any Product if Cigarettes not available)
All LC types
Least adjusted

REF	NRR	SEX	AD	Ys	Ws	Qs	Ps
WYNDE6	207	f	0	2.68	90.13	18.61	0.0000
Subtotal WYNDE6				2.72	156.52	38.47	
YAMAGU	1	c	0	1.23	15.97	15.92	0.0000
*YONG	12	m	1	3.36	1.92	2.44	0.0000
*YONG	15	f	1	1.65	6.30	2.12	0.0000
Subtotal YONG				2.05	8.22	4.56	

	N	195
	NS	131
	Wt	7113.52
Het	Chi	2706.23
Het	df	194
Het	P	***
Fixed	RR	9.29
	RRl	9.08
	RRu	9.51
	P	+++
Random	RR	8.44
	RRl	7.66
	RRu	9.31
	P	+++
Asymm	P	*

Table 1B2 - 6

IESLC - Meta-analysis of Current Smoking (vs never smoking), Cigarettes (or Any Product if Cigarettes not available)
All LC types
Least adjusted

	combined	Sex: male	female	Total
N	20	107	68	195
NS	20	102	63	185
Wt	1560.08	2969.56	2583.88	7113.52
Het Chi	178.02	1091.90	1184.55	2706.23
Het df	19	106	67	194
Het P	***	***	***	***
Fixed RR	12.98	9.03	7.84	9.29
RRl	12.35	8.71	7.54	9.08
RRu	13.64	9.36	8.14	9.51
P	+++	+++	+++	+++
Random RR	11.73	9.35	6.60	8.44
RRl	9.05	8.20	5.53	7.66
RRu	15.21	10.67	7.87	9.31
P	+++	+++	+++	+++
Between Chi				251.75
Between df				2
Between P				***
Btwn(F) P				***
Btwn(R) P				***

Table 1B2 - 7

IESLC - Meta-analysis of Current Smoking (vs never smoking), Cigarettes (or Any Product if Cigarettes not available)
All LC types
Excluded studies (and stage at which they were excluded)

1	BOUCHA BUELL LAURIL MZILEN
2	ABELIN ABRAHA AUVINE AXELSO BAND BERRIN BLOT1 BLOT2 BLOT3 BLOT4 BRESLO BROCKM BROWN1 BYERS1 CASCO2 CASCOR CHAN CHATZI CHEN CHEN2 CHEN3 CHIAZZ COOKSO DAVEYS DEAN DOSEME DU DUNN EBELIN ESAKI FAN GENG GER GODLEY GOLLED GSELL HANSEN HEGMAN HINDS HIRAOK HOROWI HORWIT HU HU2 HUANG ISHIMA JARUP JIANG JONES JUSSAW KO KOHLME KOULUM KREYBE LAMTH LAMWK LAMWK2 LAUSSM LEI LETOUR LEVIN LIU LIU2 LIU3 LIU4 LIU5 LOMBA2 LUO MAGNUS MARSH MARSH2 MARTIS MASTRA MATSUD MCCONN MCDUFF MCLAUG MILLER MILLS MOLLO NOTAN2 NOU ORMOS PASTOR PAWLEG PERNU PERSHA PIKE POFFIJ POLEDN PRESCO QIAO RADZIK RANDIG REN RONCO ROOTS ROTHSC SADOWS SANKAR SCHWA2 SEGI SEOW SIEMIA SIMARA STASZE STOCKS SUN TAO ULMER VANDER WANG WANG3 WANG4 WICKLU WILKIN WUWILL WYNDE2 WYNDE4 XIANGZ XU XU2 XU3 XU4 YUAN ZHANG ZHENG ZHOU
3	RESTRE
4	BOFFET
5	RIMING TANG2 WYNDE5 WYNDE8
6	BYERS2 HIRAY2 LICKIN MRFIT MURATA NOTANI SAARIK SHIMIZ SITAS STAYNE WARSIN WATSON WYNDER
8	JEDRYC SUZUKI WU2
10	BENHAM GILLIS OSANN2 PISANI SIMONA VEIERO VUTUC WYNDE7

Table 1B2 - 8

Potentially overlapping studies

REF	REFGP	PRINC	. OVERLAP/LINK
LUBIN2	LUBIN2	1	Lubin-combined
KOO	KOO	1	KOO/LAMTH/LAMWK
TVERDA	TVERDA	1	VEIERO/TVERDAL
AKIBA	AKIBA	1	AKIBA/ISHIMA
HEIN	PRESCO	1	Subset of PRESCO
LANGE	PRESCO	1	Subset of PRESCO
BROSS	BYERS1	1	GRAHAM/BROSS/BYERS1
GRAHAM	BYERS1	1	GRAHAM/BROSS/BYERS1
CHYOU	CHYOU	1	GOODMA/CHYOU
GOODMA	GOODMA	1	GOODMA/CHYOU
HOLE	TANG2	1	Subset of TANG2
BENSHL	TANG2	1	Subset of TANG2
WALD	TANG2	1	Subset of TANG2
KAISER	KAISER	1	KAISER/OSANN2
MRFITR	MRFIT	2	Subset of MRFIT
WYNDE6	WYNDE6	1	WYNDE5/6/7/8
CPSI	CPSI	1	CPSI overall
ENSTRO	ENSTRO	1	Subset of CPSI
SOBUE2	SOBUE2	1	SOBUE2/MATSUD
JAHN	BOFFET	2	Subset of BOFFET
LUBIN	XIANGZ	2	LUBIN/XIANGZ/QIAO

Table 1B2 - 9

Most adjusted - insufficient data for metaanalysis

REF	NRR	SEX	AGEL	AGEH	RACE	YF	LC TYPE	LOC	START	ST	NLC	R	VB	P	H	AD	PRODUCT	DENOM	De
HIRAYA	152	m	0	0	all	0	all	As:Jap	1965	pr	1917	n	bl	n	n	4	cig+/-ot	nev any	or
RESTRE	2	c	0	0	all	-	all	SCAmer	1978	CC	102	n	bl	n	n	2	cig+/-ot	nev cigs	ot

REF	NRR	RR	SIG	RRDATA comment
HIRAYA	152	4.26		0
RESTRE	2	10.28	y	0

Table 1B3 -

IESLC - Meta-analysis of Current Smoking (vs never smoking), Cigarettes only
All LC types

This analysis is restricted to results for:
1) Non-dose-response data
2) Current smokers
3) Results complete enough for use in metaanalysis

Within each study, results are then selected (in the following order of preference, within each sex) for:
4) PRODUCT: cigarettes only
5) CIGTYPE: all/unspecified, MC regardless of HR, MC only
6) DENOM: never smoked anything, never smoked cigarettes, (never +1 = +long term ex, +2 = +amount unknown,
+3 = never cigs+long term ex)
7) Followup period (YF, prospective studies): whole study (coded as 0) or longest available
8) LCtype: all or nearest available, at least Squamous and Adeno. (q = squamous, s = small,
l = large, a = adeno, mix = mixed, alv = alveolar)
9) Race: all or nearest available, otherwise by race (wh or w = white, bl or b = black, hi = hispanic
ch = chinese, jap = japanese, haw = hawaiian, w+o = white + oriental, sca = scandinavian, as = asian)
10) For overlapping studies: principal rather than subsidiary studies
Finally by Age: whole study (coded as 0) if available, otherwise by widest available age group
and then for single sex results (m, f) in preference to combined sex results (c).

Results adjusted (AD) for the most potential confounders are then chosen in Sections -1 to -3
(and those which actually differ from the adjusted results in Table 1B1 - 1 are marked 'x' in Section -1)
and results adjusted for the least confounders in Sections -4 to -6. (Those least adjusted results which
actually differ from the most adjusted as marked 'x' in column X in Section -4)
(Results adjusted for an unknown number of confounder(s) are coded as 20.)

Section -7 shows excluded studies, together with the stage (as above) at which no qualifying
results were found.

Section -8 lists the potentially overlapping studies which have been included (1=principal, 2=subsidiary).

Section -9 lists any results which would have been included in preference except that they had data not complete
enough for use in meta-analysis, with their significance (yes/no), if known, and any further comment as entered
on the database.

In addition to those mentioned above, the following fields, levels and abbreviations are used:
* or nk = not known, n = no, y = yes, ot = other
nev = never
all/unspec = all or unspecified, MC = manufactured cigarettes, HR = hand-rolled cigarettes
REF: 6-character study reference
NRR: number of the RR on the database within the study
ST : study type (CC = case control, pr or prosp = prospective)
NLC: number of lung cancer cases in whole study
R : risky occupational population (n = no, m = mining, o = other risky)
VB : national cigarette type (V = at least 75% Virginia, bl = at least 75% blended, ot = other)
P : any proxy use
H : full histological confirmation
De : derivation of RR/CI (or = original, st = standard method, ot = other method of estimation)

Table 1B3 - 1

IESLC - Meta-analysis of Current Smoking (vs never smoking), Cigarettes only
All LC types
Most adjusted

REF	NRR	1B1	SEX	AGEL	AGEH	RACE	YF	LC TYPE	LOC	START	ST	NLC	R	VB	P	H	AD	PRODUCT	DENOM	De
AGUDO	3		f	0	0	all	-	all	Eu:wst	1989	CC	103	n	bl	n	n	3	cig only	nev any	or
ALDERS	174	x	m	0	0	all	-	all	Eu:UK	1977	CC	1448	n	V	n	n	0	cig only	nev any	st
ALDERS	176		f	0	0	all	-	all	Eu:UK	1977	CC	1448	n	V	n	n	0	cig only	nev any	st
BEST	2		m	0	0	all	0	all	NAmer	1955	pr	381	n	V	n	n	1	cig only	nev any	ot
BOUCOT	114		m	0	0	all	0	all	NAmer	1951	pr	121	n	bl	n	n	2	cig only	nev any	ot
CEDERL	116	x	m	0	0	all	0	all	Eu:Sca	1963	pr	491	n	bl	n	n	2	cig only	nev any	or
CEDERL	41	x	f	0	0	all	10	all	Eu:Sca	1963	pr	491	n	bl	n	n	1	cig only	nev any	ot
CHOW	16	x	m	0	0	wh	0	all	NAmer	1966	pr	219	n	bl	n	n	0	cig only	nev any	st
CPSI	76	x	m	0	0	all	0	all	NAmer	1959	pr	5138	n	bl	n	n	1	cig only	nev any	st
CPSI	152	x	f	0	0	all	0	all	NAmer	1959	pr	5138	n	bl	n	n	1	cig only	nev any	st
CPSII	126		m	0	0	all	6	all	NAmer	1982	pr	3229	n	bl	n	n	1	cig only	nev any	ot
DAMBER	16	x	m	0	0	all	-	all	Eu:Sca	1972	CC	579	n	bl	y	n	1	cig only	nev any	ot
DEAN3	35	x	m	0	0	all	-	all	Eu:UK	1969	CC	766	n	V	y	n	3	cig only	nev any	ot
DEAN3	119		f	0	0	all	-	all	Eu:UK	1969	CC	766	n	V	y	n	3	cig only	nev any	ot
DOLL2	2	x	m	0	0	all	0	all	Eu:UK	1951	pr	920	n	V	n	n	1	cig only	nev any	ot
DOLL2	63		f	0	0	all	22	all	Eu:UK	1951	pr	920	n	V	n	n	1	cig only	nev any	ot
DORN	3	x	m	0	0	wh	0	all	NAmer	1954	pr	5097	n	bl	n	n	2	cig only	nev any	or
ENGELA	210	x	m	0	0	all	12	all	Eu:Sca	1964	pr	435	n	bl	n	n	1	cig only	nev any	ot
ENGELA	225	x	f	0	0	all	12	all	Eu:Sca	1964	pr	435	n	bl	n	n	1	cig only	nev any	ot
ENSTRO	1		m	0	0	all	0	all	NAmer	1959	pr	2879	n	bl	n	n	1	cig only	nev any	or
ENSTRO	2		f	0	0	all	0	all	NAmer	1959	pr	2879	n	bl	n	n	1	cig only	nev any	or
GARDIN	6	x	c	0	0	all	-	all	Eu:UK	1988	CC	143	n	V	y	n	0	cig only	nev any	st
GRAHAM	2	x	m	0	0	wh	-	all	NAmer	1956	CC	685	n	bl	n	n	0	cig only	nev any	st
HAMMON	139		m	0	0	wh	0	all	NAmer	1952	pr	448	n	bl	n	n	1	cig only	nev any	ot
HEIN	1	x	m	0	0	all	0	all	Eu:Sca	1970	pr	144	n	bl	n	n	0	cig only	nev any	st
KAISE2	68		m	35	99	all	9	all	NAmer	1979	pr	318	n	bl	n	n	1	cig only	nev any	st
KAISE2	60		f	35	99	all	9	all	NAmer	1979	pr	318	n	bl	n	n	1	cig only	nev any	st
LOMBAR	5	x	m	0	0	all	-	all	NAmer	1951	CC	1040	n	bl	n	n	0	cig only	nev any	st
LUBIN2	2	x	m	0	0	all	-	all	Eu:mul	1976	CC	7804	n	bl	n	y	2	cig only	nev any	ot
MIGRAN	10	x	m	0	0	all	0	all	Eu:UK	1964	pr	259	n	V	n	n	2	cig only	nev any	ot
MIGRAN	36	x	f	0	0	all	0	all	Eu:UK	1964	pr	259	n	V	n	n	2	cig only	nev any	ot
PEZZOT	5		m	0	0	all	-	all	SCAmer	1987	CC	215	n	bl	n	y	0	cig only	nev cigs	st
TVERDA	3	x	m	0	0	all	0	all	Eu:Sca	1972	pr	238	n	bl	n	n	2	cig only	nev cigs	ot
TVERDA	15		f	0	0	all	0	all	Eu:Sca	1972	pr	238	n	bl	n	n	2	cig only	nev cigs	ot
WALD	4		m	0	0	all	0	all	Eu:UK	1975	pr	102	n	V	n	n	1	cig only	nev any	or
WIGLE	28	x	m	0	0	all	-	all	NAmer	1971	CC	728	n	V	n	n	1	cig only	nev any	ot
WIGLE	4	x	f	0	0	all	-	all	NAmer	1971	CC	728	n	V	n	n	0	cig only	nev any	st
WYNDE7	1	x	m	0	0	all	-	all	NAmer	1977	CC	2085	n	bl	n	y	0	cig only	nev any	st

Cigarette type is all/unspec for all RRs
except for the following:

REF	NRR	CIGTYPE
ALDERS	174	MC only
ALDERS	176	MC only
DEAN3	35	MC only
DEAN3	119	MC only
GARDIN	6	MC only

Table 1B3 - 2

IESLC - Meta-analysis of Current Smoking (vs never smoking), Cigarettes only
All LC types
Most adjusted

REF	NRR	SEX	AD	Number Exposed Case	Exposed Cont	Non-exposed Case	Non-exposed Cont	RR	95.00%CI
AGUDO	3	f	3	-	-	-	-	3.61	(1.57- 8.32)
ALDERS	174	m	0	312	213	15	133	12.99	(7.41- 22.77)
ALDERS	176	f	0	410	229	75	243	5.80	(4.27- 7.87)
Subtotal ALDERS								6.97	(5.33- 9.12)
*BEST	2	m	1	-	-	-	-	14.91	(7.05- 31.52)
*BOUCOT	114	m	2	-	-	-	-	62.29	(3.86-1004.01)
*CEDERL	116	m	2	-	-	-	-	8.43	(5.49- 12.94)
*CEDERL	41	f	1	-	-	-	-	4.50	(1.78- 11.36)
Subtotal CEDERL								7.55	(5.11- 11.14)
*CHOW	16	m	0	71	40726	6	62913	18.28	(7.95- 42.06)
*CPSI	76	m	1	-	-	-	-	12.30	(10.71- 14.13)
*CPSI	152	f	1	-	-	-	-	3.58	(3.12- 4.10)
Subtotal CPSI								6.58	(5.97- 7.25)
*CPSII	126	m	1	-	-	-	-	20.25	(16.37- 25.05)
DAMBER	16	m	1	-	-	-	-	9.80	(6.30- 15.30)
DEAN3	35	m	3	-	-	-	-	8.48	(5.35- 13.42)
DEAN3	119	f	3	-	-	-	-	5.77	(3.75- 8.86)
Subtotal DEAN3								6.91	(5.04- 9.45)
*DOLL2	2	m	1	-	-	-	-	14.93	(9.41- 23.68)
*DOLL2	63	f	1	-	-	-	-	8.65	(2.93- 25.55)
Subtotal DOLL2								13.73	(8.98- 20.99)
*DORN	3	m	2	-	-	-	-	11.60	(10.40- 13.00)
*ENGELA	210	m	1	-	-	-	-	9.50	(4.37- 20.66)
*ENGELA	225	f	1	-	-	-	-	5.80	(2.69- 12.51)
Subtotal ENGELA								7.40	(4.29- 12.78)
*ENSTRO	1	m	1	-	-	-	-	12.99	(10.46- 16.13)
*ENSTRO	2	f	1	-	-	-	-	6.95	(6.01- 8.04)
Subtotal ENSTRO								8.44	(7.48- 9.53)
GARDIN	6	c	0	72	39	5	41	15.14	(5.53- 41.44)
GRAHAM	2	m	0	371	821	18	346	8.69	(5.32- 14.17)
*HAMMON	139	m	1	-	-	-	-	11.52	(6.83- 19.42)
*HEIN	1	m	0	45	912	1	457	22.55	(3.12- 163.06)
*KAISE2	68	m	1	-	-	-	-	8.04	(4.41- 14.66)
*KAISE2	60	f	1	-	-	-	-	14.48	(7.47- 28.04)
Subtotal KAISE2								10.49	(6.72- 16.36)
LOMBAR	5	m	0	432	249	14	112	13.88	(7.79- 24.72)
LUBIN2	2	m	2	-	-	-	-	11.21	(9.60- 13.09)
*MIGRAN	10	m	2	-	-	-	-	4.20	(1.55- 11.37)
*MIGRAN	36	f	2	-	-	-	-	5.11	(1.77- 14.79)
Subtotal MIGRAN								4.60	(2.23- 9.52)
PEZZOT	5	m	0	145	129	4	116	32.60	(11.70- 90.81)
*TVERDA	3	m	2	-	-	-	-	3.83	(2.47- 5.95)
*TVERDA	15	f	2	-	-	-	-	11.05	(3.33- 36.71)
Subtotal TVERDA								4.34	(2.87- 6.56)
*WALD	4	m	1	-	-	-	-	16.40	(7.55- 44.20)
WIGLE	28	m	1	-	-	-	-	12.40	(7.21- 21.31)
WIGLE	4	f	0	67	169	36	439	4.83	(3.11- 7.52)
Subtotal WIGLE								7.04	(5.00- 9.92)
WYNDE7	1	m	0	1107	993	64	918	15.99	(12.24- 20.89)
Partial Totals				3032	44480	238	65718		

*prospective study

REF	NRR	SEX	AD	Ys	Ws	Qs	Ps
AGUDO	3	f	3	1.28	5.53	4.89	0.0025
ALDERS	174	m	0	2.56	12.18	1.40	0.0000
ALDERS	176	f	0	1.76	41.23	8.98	0.0000
Subtotal ALDERS				1.94	53.41	10.38	
*BEST	2	m	1	2.70	6.85	1.56	0.0000
*BOUCOT	114	m	2	4.13	0.50	1.81	0.0036
*CEDERL	116	m	2	2.13	20.90	0.18	0.0000
*CEDERL	41	f	1	1.50	4.47	2.32	0.0015
Subtotal CEDERL				2.02	25.38	2.50	
*CHOW	16	m	0	2.91	5.53	2.57	0.0000
*CPSI	76	m	1	2.51	200.08	16.24	0.0000
*CPSI	152	f	1	1.28	205.94	185.59	0.0000
Subtotal CPSI				1.88	406.02	201.84	
*CPSII	126	m	1	3.01	84.90	52.12	0.0000
DAMBER	16	m	1	2.28	19.52	0.06	0.0000
DEAN3	35	m	3	2.14	18.17	0.14	0.0000
DEAN3	119	f	3	1.75	20.79	4.63	0.0000

Table 1B3 - 2

IESLC - Meta-analysis of Current Smoking (vs never smoking), Cigarettes only
All LC types
Most adjusted

REF	NRR	SEX	AD	Ys	Ws	Qs	Ps
Subtotal DEAN3				1.93	38.95	4.77	
*DOLL2	2	m	1	2.70	18.04	4.13	0.0000
*DOLL2	63	f	1	2.16	3.28	0.01	0.0001
Subtotal DOLL2				2.62	21.32	4.15	
*DORN	3	m	2	2.45	308.59	15.81	0.0000
*ENGELA	210	m	1	2.25	6.37	0.00	0.0000
*ENGELA	225	f	1	1.76	6.50	1.42	0.0000
Subtotal ENGELA				2.00	12.87	1.42	
*ENSTRO	1	m	1	2.56	81.91	9.44	0.0000
*ENSTRO	2	f	1	1.94	181.45	14.84	0.0000
Subtotal ENSTRO				2.13	263.36	24.28	
GARDIN	6	c	0	2.72	3.79	0.92	0.0000
GRAHAM	2	m	0	2.16	16.04	0.06	0.0000
*HAMMON	139	m	1	2.44	14.07	0.68	0.0000
*HEIN	1	m	0	3.12	0.98	0.78	0.0020
*KAISE2	68	m	1	2.08	10.65	0.21	0.0000
*KAISE2	60	f	1	2.67	8.78	1.76	0.0000
Subtotal KAISE2				2.35	19.43	1.97	
LOMBAR	5	m	0	2.63	11.54	1.90	0.0000
LUBIN2	2	m	2	2.42	159.81	5.90	0.0000
*MIGRAN	10	m	2	1.44	3.87	2.41	0.0048
*MIGRAN	36	f	2	1.63	3.41	1.20	0.0026
Subtotal MIGRAN				1.53	7.28	3.61	
PEZZOT	5	m	0	3.48	3.66	5.81	0.0000
*TVERDA	3	m	2	1.34	19.88	15.46	0.0000
*TVERDA	15	f	2	2.40	2.67	0.08	0.0001
Subtotal TVERDA				1.47	22.55	15.54	
*WALD	4	m	1	2.80	4.92	1.61	0.0000
WIGLE	28	m	1	2.52	13.08	1.12	0.0000
WIGLE	4	f	0	1.58	19.65	8.27	0.0000
Subtotal WIGLE				1.95	32.73	9.40	
WYNDE7	1	m	0	2.77	53.69	16.08	0.0000

	N	38
	NS	27
	Wt	1603.21
Het	Chi	392.42
Het	df	37
Het	P	***
Fixed	RR	9.25
	RRl	8.81
	RRu	9.71
	P	+++
Random	RR	9.52
	RRl	7.89
	RRu	11.49
	P	+++
Asymm	P	N.S.

Table 1B3 - 3

IESLC - Meta-analysis of Current Smoking (vs never smoking), Cigarettes only
All LC types
Most adjusted

	Sex			
	combined	male	female	Total
N	1	25	12	38
NS	1	25	12	38
Wt	3.79	1095.73	503.69	1603.21
Het Chi	0.00	77.20	57.13	392.42
Het df	0	24	11	37
Het P	N.S.	***	***	***
Fixed RR	15.14	12.13	5.11	9.25
RRl	5.53	11.43	4.69	8.81
RRu	41.44	12.87	5.58	9.71
P	+++	+++	+++	+++
Random RR	15.14	11.68	5.82	9.52
RRl	5.53	10.19	4.43	7.89
RRu	41.44	13.39	7.63	11.49
P	+++	+++	+++	+++
Between Chi				258.09
Between df				2
Between P				***
Btwn(F) P				***
Btwn(R) P				***

	Lung cancer type		
	all	other	Total
N	38		38
NS	27		27
Wt	1603.21		1603.21
Het Chi	392.42		392.42
Het df	37		37
Het P	***		***
Fixed RR	9.25		9.25
RRl	8.81		8.81
RRu	9.71		9.71
P	+++		+++
Random RR	9.52		9.52
RRl	7.89		7.89
RRu	11.49		11.49
P	+++		+++
Between Chi			
Between df			
Between P			N.S.
Btwn(F) P			N.S.
Btwn(R) P			N.S.

	Location								
	NAmer	UK	Scand	othEur	China	Japan	othAs	other	Total
N	17	10	8	2				1	38
NS	13	6	5	2				1	27
Wt	1223.26	129.67	81.29	165.33				3.66	1603.21
Het Chi	329.96	22.81	13.76	6.86				0.00	392.42
Het df	16	9	7	1				0	37
Het P	***	**	(*)	**				N.S.	***
Fixed RR	9.34	8.02	6.96	10.79				32.60	9.25
RRl	8.83	6.75	5.60	9.27				11.70	8.81
RRu	9.88	9.53	8.65	12.57				90.81	9.71
P	+++	+++	+++	+++				+++	+++
Random RR	10.87	8.68	7.12	6.87				32.60	9.52
RRl	8.13	6.38	5.07	2.29				11.70	7.89
RRu	14.53	11.81	10.00	20.64				90.81	11.49
P	+++	+++	+++	+++				+++	+++
Between Chi									19.04
Between df									4
Between P									***
Btwn(F) P									N.S.
Btwn(R) P									*

Table 1B3 - 3

IESLC - Meta-analysis of Current Smoking (vs never smoking), Cigarettes only
All LC types
Most adjusted

Detailed Country in "other Europe"

	multi	Germany	othWest	East	Balkans	Total
N	1		1			2
NS	1		1			2
Wt	159.81		5.53			165.33
Het Chi	0.00		0.00			6.86
Het df	0		0			1
Het P	N.S.		N.S.			**
Fixed RR	11.21		3.61			10.79
RRl	9.60		1.57			9.27
RRu	13.09		8.31			12.57
P	+++		++			+++
Random RR	11.21		3.61			6.87
RRl	9.60		1.57			2.29
RRu	13.09		8.31			20.64
P	+++		++			+++
Between Chi						6.86
Between df						1
Between P						**
Btwn(F) P						N.S.
Btwn(R) P						**

Detailed Country in "other Asia"

	India	HongKong	other	Total
N				
NS				
Wt				
Het Chi				
Het df				
Het P				
Fixed RR				
RRl				
RRu				
P				
Random RR				
RRl				
RRu				
P				
Between Chi				
Between df				
Between P				N.S.
Btwn(F) P				N.S.
Btwn(R) P				N.S.

Detailed other continent

	SCAmer	Auslia	Africa	Total
N	1			1
NS	1			1
Wt	3.66			3.66
Het Chi	0.00			0.00
Het df	0			0
Het P	N.S.			N.S.
Fixed RR	32.60			32.60
RRl	11.70			11.70
RRu	90.81			90.81
P	+++			+++
Random RR	32.60			32.60
RRl	11.70			11.70
RRu	90.81			90.81
P	+++			+++
Between Chi				
Between df				
Between P				N.S.
Btwn(F) P				N.S.
Btwn(R) P				N.S.

Table 1B3 - 3

IESLC - Meta-analysis of Current Smoking (vs never smoking), Cigarettes only
All LC types
Most adjusted

	Start year of study					
	<1960	1960-69	1970-79	1980-89	1990+	Total
N	12	9	13	4		38
NS	9	5	9	4		27
Wt	1048.29	90.01	367.04	97.88		1603.21
Het Chi	246.95	10.10	59.85	16.77		392.42
Het df	11	8	12	3		37
Het P	***	N.S.	***	***		***
Fixed RR	8.63	7.35	9.94	18.49		9.25
RRl	8.12	5.98	8.97	15.17		8.81
RRu	9.16	9.03	11.01	22.54		9.71
P	+++	+++	+++	+++		+++
Random RR	10.25	7.32	9.46	13.81		9.52
RRl	7.30	5.74	7.17	5.91		7.89
RRu	14.40	9.34	12.47	32.24		11.49
P	+++	+++	+++	+++		+++
Between Chi						58.74
Between df						3
Between P						***
Btwn(F) P						N.S.
Btwn(R) P						N.S.

	Study type (1)		
	CC	other	Total
N	14	24	38
NS	11	16	27
Wt	398.66	1204.56	1603.21
Het Chi	57.80	331.46	392.42
Het df	13	23	37
Het P	***	***	***
Fixed RR	9.99	9.02	9.25
RRl	9.06	8.52	8.81
RRu	11.02	9.54	9.71
P	+++	+++	+++
Random RR	9.47	9.52	9.52
RRl	7.44	7.32	7.89
RRu	12.05	12.39	11.49
P	+++	+++	+++
Between Chi			3.16
Between df			1
Between P			(*)
Btwn(F) P			N.S.
Btwn(R) P			N.S.

	Study type (2)			
	CC	prosp	other	Total
N	14	24		38
NS	11	16		27
Wt	398.66	1204.56		1603.21
Het Chi	57.80	331.46		392.42
Het df	13	23		37
Het P	***	***		***
Fixed RR	9.99	9.02		9.25
RRl	9.06	8.52		8.81
RRu	11.02	9.54		9.71
P	+++	+++		+++
Random RR	9.47	9.52		9.52
RRl	7.44	7.32		7.89
RRu	12.05	12.39		11.49
P	+++	+++		+++
Between Chi				3.16
Between df				1
Between P				(*)
Btwn(F) P				N.S.
Btwn(R) P				N.S.

Table 1B3 - 3

IESLC - Meta-analysis of Current Smoking (vs never smoking), Cigarettes only
All LC types
Most adjusted

	Study size (number of LC cases)				
	100-249	250-499	500-999	1000+	Total
N	9	10	8	11	38
NS	8	6	5	8	27
Wt	47.45	85.88	128.56	1341.32	1603.21
Het Chi	32.78	11.53	17.36	327.89	392.42
Het df	8	9	7	10	37
Het P	***	N.S.	*	***	***
Fixed RR	7.92	8.80	8.44	9.41	9.25
RRl	5.96	7.12	7.10	8.92	8.81
RRu	10.53	10.87	10.03	9.93	9.71
P	+++	+++	+++	+++	+++
Random RR	12.11	8.66	8.59	10.41	9.52
RRl	6.27	6.76	6.49	7.54	7.89
RRu	23.39	11.10	11.36	14.37	11.49
P	+++	+++	+++	+++	+++
Between Chi					2.85
Between df					3
Between P					N.S.
Btwn(F) P					N.S.
Btwn(R) P					N.S.

	Risky occupational population			
	no	mining	othRisky	Total
N	38			38
NS	27			27
Wt	1603.21			1603.21
Het Chi	392.42			392.42
Het df	37			37
Het P	***			***
Fixed RR	9.25			9.25
RRl	8.81			8.81
RRu	9.71			9.71
P	+++			+++
Random RR	9.52			9.52
RRl	7.89			7.89
RRu	11.49			11.49
P	+++			+++
Between Chi				
Between df				
Between P				N.S.
Btwn(F) P				N.S.
Btwn(R) P				N.S.

	National cigarette tobacco type			
	Virginia	blended	other	Total
N	13	25		38
NS	8	19		27
Wt	169.25	1433.96		1603.21
Het Chi	32.96	355.61		392.42
Het df	12	24		37
Het P	***	***		***
Fixed RR	8.02	9.41		9.25
RRl	6.90	8.93		8.81
RRu	9.33	9.91		9.71
P	+++	+++		+++
Random RR	8.77	9.88		9.52
RRl	6.67	7.81		7.89
RRu	11.52	12.51		11.49
P	+++	+++		+++
Between Chi				3.84
Between df				1
Between P				*
Btwn(F) P				N.S.
Btwn(R) P				N.S.

Table 1B3 - 3

IESLC - Meta-analysis of Current Smoking (vs never smoking), Cigarettes only
All LC types
Most adjusted

	Any proxy use No/nk	Yes	Total
N	34	4	38
NS	24	3	27
Wt	1540.95	62.26	1603.21
Het Chi	386.62	4.62	392.42
Het df	33	3	37
Het P	***	N.S.	***
Fixed RR	9.30	8.08	9.25
RRl	8.85	6.31	8.81
RRu	9.78	10.36	9.71
P	+++	+++	+++
Random RR	9.63	8.27	9.52
RRl	7.87	5.99	7.89
RRu	11.80	11.40	11.49
P	+++	+++	+++
Between Chi			1.18
Between df			1
Between P			N.S.
Btwn(F) P			N.S.
Btwn(R) P			N.S.

	Full histological confirmation No	Yes	Total
N	35	3	38
NS	24	3	27
Wt	1386.06	217.16	1603.21
Het Chi	361.61	8.51	392.42
Het df	34	2	37
Het P	***	*	***
Fixed RR	8.83	12.46	9.25
RRl	8.38	10.91	8.81
RRu	9.31	14.23	9.71
P	+++	+++	+++
Random RR	9.06	14.62	9.52
RRl	7.40	9.93	7.89
RRu	11.11	21.52	11.49
P	+++	+++	+++
Between Chi			22.30
Between df			1
Between P			***
Btwn(F) P			N.S.
Btwn(R) P			*

	Number of adjustment variables (1) 0	1	2+/+nk	Total
N	10	17	11	38
NS	9	12	8	29
Wt	168.29	870.82	564.10	1603.21
Het Chi	45.19	286.30	45.95	392.42
Het df	9	16	10	37
Het P	***	***	***	***
Fixed RR	10.19	8.47	10.30	9.25
RRl	8.76	7.92	9.49	8.81
RRu	11.85	9.05	11.19	9.71
P	+++	+++	+++	+++
Random RR	11.55	10.08	7.48	9.52
RRl	7.84	7.30	5.76	7.89
RRu	17.03	13.92	9.73	11.49
P	+++	+++	+++	+++
Between Chi				14.98
Between df				2
Between P				***
Btwn(F) P				N.S.
Btwn(R) P				N.S.

Table 1B3 - 3

IESLC - Meta-analysis of Current Smoking (vs never smoking), Cigarettes only
All LC types
Most adjusted

	Number of adjustment variables (2) 0	1	2	3-5	6+/+nk	Total
N	10	17	8	3		38
NS	9	12	6	2		29
Wt	168.29	870.82	519.62	44.48		1603.21
Het Chi	45.19	286.30	31.32	3.47		392.42
Het df	9	16	7	2		37
Het P	***	***	***	N.S.		***
Fixed RR	10.19	8.47	10.74	6.37		9.25
RRl	8.76	7.92	9.85	4.75		8.81
RRu	11.85	9.05	11.70	8.55		9.71
P	+++	+++	+++	+++		+++
Random RR	11.55	10.08	8.26	6.16		9.52
RRl	7.84	7.30	6.18	4.09		7.89
RRu	17.03	13.92	11.06	9.27		11.49
P	+++	+++	+++	+++		+++
Between Chi						26.14
Between df						3
Between P						***
Btwn(F) P						N.S.
Btwn(R) P						N.S.

	Product all/unsp	cig+/-ot	cig only	Total
N			38	38
NS			27	27
Wt			1603.21	1603.21
Het Chi			392.42	392.42
Het df			37	37
Het P			***	***
Fixed RR			9.25	9.25
RRl			8.81	8.81
RRu			9.71	9.71
P			+++	+++
Random RR			9.52	9.52
RRl			7.89	7.89
RRu			11.49	11.49
P			+++	+++
Between Chi				
Between df				
Between P				N.S.
Btwn(F) P				N.S.
Btwn(R) P				N.S.

	Denominator nev any	nev cigs	Total
N	35	3	38
NS	25	2	27
Wt	1577.01	26.21	1603.21
Het Chi	370.97	15.44	392.42
Het df	34	2	37
Het P	***	***	***
Fixed RR	9.32	5.75	9.25
RRl	8.87	3.92	8.81
RRu	9.80	8.44	9.71
P	+++	+++	+++
Random RR	9.57	10.52	9.52
RRl	7.90	2.61	7.89
RRu	11.59	42.44	11.49
P	+++	+++	+++
Between Chi			6.01
Between df			1
Between P			*
Btwn(F) P			N.S.
Btwn(R) P			N.S.

Table 1B3 - 3

IESLC - Meta-analysis of Current Smoking (vs never smoking), Cigarettes only
All LC types
Most adjusted

		Derivation of RR/CI			
		Orig	StdCalc	Other	Total
	N	6	14	18	38
	NS	5	11	13	29
	Wt	603.30	593.74	406.17	1603.21
Het	Chi	43.92	226.44	79.98	392.42
Het	df	5	13	17	37
Het	P	***	***	***	***
Fixed	RR	9.91	7.56	11.22	9.25
	RRl	9.15	6.98	10.18	8.81
	RRu	10.73	8.19	12.36	9.71
	P	+++	+++	+++	+++
Random	RR	9.34	10.61	9.15	9.52
	RRl	6.87	7.09	7.05	7.89
	RRu	12.69	15.88	11.88	11.49
	P	+++	+++	+++	+++
Between	Chi				42.08
Between	df				2
Between	P				***
Btwn(F)	P				N.S.
Btwn(R)	P				N.S.

Table 1B3 - 4

IESLC - Meta-analysis of Current Smoking (vs never smoking), Cigarettes only
All LC types
Least adjusted

REF	NRR	X	SEX	AGEL	AGEH	RACE	YF	LC TYPE	LOC	START	ST	NLC	R	VB	P	H	AD	PRODUCT	DENOM	De
AGUDO	10	x	f	0	0	all	-	all	Eu:wst	1989	CC	103	n	bl	n	n	0	cig only	nev any	st
ALDERS	174		m	0	0	all	-	all	Eu:UK	1977	CC	1448	n	V	n	n	0	cig only	nev any	st
ALDERS	176		f	0	0	all	-	all	Eu:UK	1977	CC	1448	n	V	n	n	0	cig only	nev any	st
BEST	2		m	0	0	all	0	all	NAmer	1955	pr	381	n	V	n	n	1	cig only	nev any	ot
BOUCOT	2	x	m	0	0	all	0	all	NAmer	1951	pr	121	n	bl	n	n	0	cig only	nev any	ot
CEDERL	116		m	0	0	all	0	all	Eu:Sca	1963	pr	491	n	bl	n	n	2	cig only	nev any	or
CEDERL	31	x	f	0	0	all	10	all	Eu:Sca	1963	pr	491	n	bl	n	n	0	cig only	nev any	st
CHOW	16		m	0	0	wh	0	all	NAmer	1966	pr	219	n	bl	n	n	0	cig only	nev any	st
CPSI	76		m	0	0	all	0	all	NAmer	1959	pr	5138	n	bl	n	n	1	cig only	nev any	st
CPSI	152		f	0	0	all	0	all	NAmer	1959	pr	5138	n	bl	n	n	1	cig only	nev any	st
CPSII	36	x	m	0	0	all	6	all	NAmer	1982	pr	3229	n	bl	n	n	0	cig only	nev any	st
DAMBER	16		m	0	0	all	-	all	Eu:Sca	1972	CC	579	n	bl	y	n	1	cig only	nev any	ot
DEAN3	33	x	m	0	0	all	-	all	Eu:UK	1969	CC	766	n	V	y	n	0	cig only	nev any	st
DEAN3	117	x	f	0	0	all	-	all	Eu:UK	1969	CC	766	n	V	y	n	0	cig only	nev any	st
DOLL2	2		m	0	0	all	0	all	Eu:UK	1951	pr	920	n	V	n	n	1	cig only	nev any	ot
DOLL2	63		f	0	0	all	22	all	Eu:UK	1951	pr	920	n	V	n	n	1	cig only	nev any	ot
DORN	3		m	0	0	wh	0	all	NAmer	1954	pr	5097	n	bl	n	n	2	cig only	nev any	or
ENGELA	210		m	0	0	all	12	all	Eu:Sca	1964	pr	435	n	bl	n	n	1	cig only	nev any	ot
ENGELA	225		f	0	0	all	12	all	Eu:Sca	1964	pr	435	n	bl	n	n	1	cig only	nev any	ot
ENSTRO	1		m	0	0	all	0	all	NAmer	1959	pr	2879	n	bl	n	n	1	cig only	nev any	or
ENSTRO	2		f	0	0	all	0	all	NAmer	1959	pr	2879	n	bl	n	n	1	cig only	nev any	or
GARDIN	6		c	0	0	all	-	all	Eu:UK	1988	CC	143	n	V	y	n	0	cig only	nev any	st
GRAHAM	2		m	0	0	wh	-	all	NAmer	1956	CC	685	n	bl	n	n	0	cig only	nev any	st
HAMMON	139		m	0	0	wh	0	all	NAmer	1952	pr	448	n	bl	n	n	1	cig only	nev any	ot
HEIN	1		m	0	0	all	0	all	Eu:Sca	1970	pr	144	n	bl	n	n	0	cig only	nev any	st
KAISE2	68		m	35	99	all	9	all	NAmer	1979	pr	318	n	bl	n	n	1	cig only	nev any	st
KAISE2	60		f	35	99	all	9	all	NAmer	1979	pr	318	n	bl	n	n	1	cig only	nev any	st
LOMBAR	5		m	0	0	all	-	all	NAmer	1951	CC	1040	n	bl	n	n	0	cig only	nev any	st
LUBIN2	1	x	m	0	0	all	-	all	Eu:mul	1976	CC	7804	n	bl	n	y	0	cig only	nev any	st
MIGRAN	9	x	m	0	0	all	0	all	Eu:UK	1964	pr	259	n	V	n	n	0	cig only	nev any	st
MIGRAN	35	x	f	0	0	all	0	all	Eu:UK	1964	pr	259	n	V	n	n	0	cig only	nev any	st
PEZZOT	5		m	0	0	all	-	all	SCAmer	1987	CC	215	n	bl	n	y	0	cig only	nev cigs	st
TVERDA	3		m	0	0	all	0	all	Eu:Sca	1972	pr	238	n	bl	n	n	2	cig only	nev cigs	ot
TVERDA	15		f	0	0	all	0	all	Eu:Sca	1972	pr	238	n	bl	n	n	2	cig only	nev cigs	ot
WALD	2	x	m	0	0	all	0	all	Eu:UK	1975	pr	102	n	V	n	n	0	cig only	nev any	st
WIGLE	1	x	m	0	0	all	-	all	NAmer	1971	CC	728	n	V	n	n	0	cig only	nev any	st
WIGLE	4		f	0	0	all	-	all	NAmer	1971	CC	728	n	V	n	n	0	cig only	nev any	st
WYNDE7	1		m	0	0	all	-	all	NAmer	1977	CC	2085	n	bl	n	y	0	cig only	nev any	st

Cigarette type is all/unspec for all RRs
except for the following:

REF	NRR	CIGTYPE
ALDERS	174	MC only
ALDERS	176	MC only
DEAN3	33	MC only
DEAN3	117	MC only
GARDIN	6	MC only

Table 1B3 - 5

IESLC - Meta-analysis of Current Smoking (vs never smoking), Cigarettes only
All LC types
Least adjusted

REF	NRR	SEX	AD	Number Exposed Case	Cont	Non-exposed Case	Cont	RR	95.00%CI
AGUDO	10	f	0	20	17	80	183	2.69	(1.34- 5.41)
ALDERS	174	m	0	312	213	15	133	12.99	(7.41- 22.77)
ALDERS	176	f	0	410	229	75	243	5.80	(4.27- 7.87)
Subtotal ALDERS								6.97	(5.33- 9.12)
*BEST	2	m	1	-	-	-	-	14.91	(7.05- 31.52)
*BOUCOT	2	m	0	85	22177	0	7551	58.23~	(3.61- 938.34)
*CEDERL	116	m	2	-	-	-	-	8.43	(5.49- 12.94)
*CEDERL	31	f	0	8	4709	19	17679	1.58	(0.69- 3.61)
Subtotal CEDERL								5.91	(4.04- 8.64)
*CHOW	16	m	0	71	40726	6	62913	18.28	(7.95- 42.06)
*CPSI	76	m	1	-	-	-	-	12.30	(10.71- 14.13)
*CPSI	152	f	1	-	-	-	-	3.58	(3.12- 4.10)
Subtotal CPSI								6.58	(5.97- 7.25)
*CPSII	36	m	0	1781	583646	124	742207	18.26	(15.23- 21.91)
DAMBER	16	m	1	-	-	-	-	9.80	(6.30- 15.30)
DEAN3	33	m	0	337	930	25	510	7.39	(4.86- 11.26)
DEAN3	117	f	0	102	1158	41	1538	3.30	(2.28- 4.79)
Subtotal DEAN3								4.70	(3.56- 6.20)
*DOLL2	2	m	1	-	-	-	-	14.93	(9.41- 23.68)
*DOLL2	63	f	1	-	-	-	-	8.65	(2.93- 25.55)
Subtotal DOLL2								13.73	(8.98- 20.99)
*DORN	3	m	2	-	-	-	-	11.60	(10.40- 13.00)
*ENGELA	210	m	1	-	-	-	-	9.50	(4.37- 20.66)
*ENGELA	225	f	1	-	-	-	-	5.80	(2.69- 12.51)
Subtotal ENGELA								7.40	(4.29- 12.78)
*ENSTRO	1	m	1	-	-	-	-	12.99	(10.46- 16.13)
*ENSTRO	2	f	1	-	-	-	-	6.95	(6.01- 8.04)
Subtotal ENSTRO								8.44	(7.48- 9.53)
GARDIN	6	c	0	72	39	5	41	15.14	(5.53- 41.44)
GRAHAM	2	m	0	371	821	18	346	8.69	(5.32- 14.17)
*HAMMON	139	m	1	-	-	-	-	11.52	(6.83- 19.42)
*HEIN	1	m	0	45	912	1	457	22.55	(3.12- 163.06)
*KAISE2	68	m	1	-	-	-	-	8.04	(4.41- 14.66)
*KAISE2	60	f	1	-	-	-	-	14.48	(7.47- 28.04)
Subtotal KAISE2								10.49	(6.72- 16.36)
LOMBAR	5	m	0	432	249	14	112	13.88	(7.79- 24.72)
LUBIN2	1	m	0	5243	6835	190	2617	10.57	(9.08- 12.29)
*MIGRAN	9	m	0	137	3707	4	867	8.01	(2.97- 21.59)
*MIGRAN	35	f	0	23	2749	4	3814	7.98	(2.76- 23.04)
Subtotal MIGRAN								8.00	(3.88- 16.50)
PEZZOT	5	m	0	145	129	4	116	32.60	(11.70- 90.81)
*TVERDA	3	m	2	-	-	-	-	3.83	(2.47- 5.95)
*TVERDA	15	f	2	-	-	-	-	11.05	(3.33- 36.71)
Subtotal TVERDA								4.34	(2.87- 6.56)
*WALD	2	m	0	77	4182	7	6539	17.20	(7.94- 37.25)
WIGLE	1	m	0	415	415	15	204	13.60	(7.91- 23.38)
WIGLE	4	f	0	67	169	36	439	4.83	(3.11- 7.52)
Subtotal WIGLE								7.31	(5.19- 10.30)
WYNDE7	1	m	0	1107	993	64	918	15.99	(12.24- 20.89)
Partial Totals				11260	675005	747	849427		

*prospective study ~ With 0.5 adjustment for zero

REF	NRR	SEX	AD	Ys	Ws	Qs	Ps
AGUDO	10	f	0	0.99	7.89	11.75	0.0054
ALDERS	174	m	0	2.56	12.18	1.52	0.0000
ALDERS	176	f	0	1.76	41.23	8.45	0.0000
Subtotal ALDERS				1.94	53.41	9.97	
*BEST	2	m	1	2.70	6.85	1.65	0.0000
*BOUCOT	2	m	0	4.06	0.50	1.71	0.0042
*CEDERL	116	m	2	2.13	20.90	0.13	0.0000
*CEDERL	31	f	0	0.46	5.64	17.32	0.2769
Subtotal CEDERL				1.78	26.54	17.45	
*CHOW	16	m	0	2.91	5.53	2.67	0.0000
*CPSI	76	m	1	2.51	200.08	17.89	0.0000
*CPSI	152	f	1	1.28	205.94	180.13	0.0000
Subtotal CPSI				1.88	406.02	198.02	
*CPSII	36	m	0	2.90	115.97	55.92	0.0000
DAMBER	16	m	1	2.28	19.52	0.10	0.0000
DEAN3	33	m	0	2.00	21.74	0.96	0.0000
DEAN3	117	f	0	1.20	28.00	28.87	0.0000

Table 1B3 - 5

IESLC - Meta-analysis of Current Smoking (vs never smoking), Cigarettes only
All LC types
Least adjusted

REF	NRR	SEX	AD	Ys	Ws	Qs	Ps
Subtotal DEAN3				1.55	49.74	29.83	
*DOLL2	2	m	1	2.70	18.04	4.38	0.0000
*DOLL2	63	f	1	2.16	3.28	0.01	0.0001
Subtotal DOLL2				2.62	21.32	4.39	
*DORN	3	m	2	2.45	308.59	17.84	0.0000
*ENGELA	210	m	1	2.25	6.37	0.01	0.0000
*ENGELA	225	f	1	1.76	6.50	1.33	0.0000
Subtotal ENGELA				2.00	12.87	1.34	
*ENSTRO	1	m	1	2.56	81.91	10.24	0.0000
*ENSTRO	2	f	1	1.94	181.45	13.41	0.0000
Subtotal ENSTRO				2.13	263.36	23.65	
GARDIN	6	c	0	2.72	3.79	0.97	0.0000
GRAHAM	2	m	0	2.16	16.04	0.04	0.0000
*HAMMON	139	m	1	2.44	14.07	0.77	0.0000
*HEIN	1	m	0	3.12	0.98	0.80	0.0020
*KAISE2	68	m	1	2.08	10.65	0.17	0.0000
*KAISE2	60	f	1	2.67	8.78	1.88	0.0000
Subtotal KAISE2				2.35	19.43	2.05	
LOMBAR	5	m	0	2.63	11.54	2.03	0.0000
LUBIN2	1	m	0	2.36	167.16	3.61	0.0000
*MIGRAN	9	m	0	2.08	3.91	0.07	0.0000
*MIGRAN	35	f	0	2.08	3.41	0.06	0.0001
Subtotal MIGRAN				2.08	7.32	0.13	
PEZZOT	5	m	0	3.48	3.66	5.94	0.0000
*TVERDA	3	m	2	1.34	19.88	14.97	0.0000
*TVERDA	15	f	2	2.40	2.67	0.10	0.0001
Subtotal TVERDA				1.47	22.55	15.07	
*WALD	2	m	0	2.84	6.43	2.59	0.0000
WIGLE	1	m	0	2.61	13.09	2.09	0.0000
WIGLE	4	f	0	1.58	19.65	7.92	0.0000
Subtotal WIGLE				1.99	32.74	10.01	
WYNDE7	1	m	0	2.77	53.69	16.92	0.0000

	N	38
	NS	27
	Wt	1657.51
Het	Chi	437.21
Het	df	37
Het	P	***
Fixed	RR	9.12
	RRl	8.69
	RRu	9.57
	P	+++
Random	RR	9.17
	RRl	7.56
	RRu	11.13
	P	+++
Asymm	P	N.S.

Table 1B3 - 6

IESLC - Meta-analysis of Current Smoking (vs never smoking), Cigarettes only
All LC types
Least adjusted

		Sex			
		combined	male	female	Total
	N	1	25	12	38
	NS	1	25	12	38
	Wt	3.79	1139.28	514.44	1657.51
Het	Chi	0.00	76.20	72.16	437.21
Het	df	0	24	11	37
Het	P	N.S.	***	***	***
Fixed	RR	15.14	12.06	4.90	9.12
	RRl	5.53	11.38	4.49	8.69
	RRu	41.44	12.78	5.34	9.57
	P	+++	+++	+++	+++
Random	RR	15.14	11.66	5.11	9.17
	RRl	5.53	10.20	3.82	7.56
	RRu	41.44	13.32	6.84	11.13
	P	+++	+++	+++	+++
Between	Chi				288.86
Between	df				2
Between	P				***
Btwn(F)	P				***
Btwn(R)	P				***

Table 1B3 - 7

IESLC - Meta-analysis of Current Smoking (vs never smoking), Cigarettes only
All LC types
Excluded studies (and stage at which they were excluded)

Stage	Studies
1	BOUCHA BUELL LAURIL MZILEN
2	ABELIN ABRAHA AUVINE AXELSO BAND BERRIN BLOT1 BLOT2 BLOT3 BLOT4 BRESLO BROCKM BROWN1 BYERS1 CASCO2 CASCOR CHAN CHATZI CHEN CHEN2 CHEN3 CHIAZZ COOKSO DAVEYS DEAN DOSEME DU DUNN EBELIN ESAKI FAN GENG GER GODLEY GOLLED GSELL HANSEN HEGMAN HINDS HIRAOK HOROWI HORWIT HU HU2 HUANG ISHIMA JARUP JIANG JONES JUSSAW KO KOHLME KOULUM KREYBE LAMTH LAMWK LAMWK2 LAUSSM LEI LETOUR LEVIN LIU LIU2 LIU3 LIU4 LIU5 LOMBA2 LUO MAGNUS MARSH MARSH2 MARTIS MASTRA MATSUD MCCONN MCDUFF MCLAUG MILLER MILLS MOLLO NOTAN2 NOU ORMOS PASTOR PAWLEG PERNU PERSHA PIKE POFFIJ POLEDN PRESCO QIAO RADZIK RANDIG REN RONCO ROOTS ROTHSC SADOWS SANKAR SCHWA2 SEGI SEOW SIEMIA SIMARA STASZE STOCKS SUN TAO ULMER VANDER WANG WANG3 WANG4 WICKLU WILKIN WUWILL WYNDE2 WYNDE4 XIANGZ XU XU2 XU3 XU4 YUAN ZHANG ZHENG ZHOU
3	RESTRE
4	AKIBA AMANDU AMES ANDERS ARCHER ARMADA AUSTIN AXELSS BARBON BECHER BENSHL BLOHMK BOFFET BRETT BROSS BROWN2 BUFFLE BYERS2 CARPEN CHANG CHOI CHYOU COMSTO CORREA DARBY DEAN2 DEKLER DESTE2 DESTEF DOCKER DOLL DORANT DORGAN DROSTE GAO GAO2 GARCIA GARSHI GILLIS GOODMA GREGOR HAENSZ HAMMO2 HENNEK HIRAYA HITOSU HOLE HUMBLE JAHN JAIN JARVHO JEDRYC JOLY KAISER KANELL KATSOU KAUFMA KELLER KHUDER KIHARA KINLEN KJUUS KNEKT KOO KREUZE KUBIK LEMARC LIAW LICKIN LIDDEL LUBIN MACLEN MATOS MRFIT MRFITR MURATA NAM NOTANI ODRISC OSANN OSANN2 PARKIN PERSH2 PETO PEZZO2 PISANI QIAO2 RACHTA SAARIK SCHWAR SEGI2 SHAW SHIMIZ SIMONA SITAS SOBUE SOBUE2 SPEIZE SPITZ STAYNE STOCKW STUCKE SUZUK2 SUZUKI SVENSS TANG TENKAN TIZZAN TOKARS TOUSEY TSUGAN TULINI VUTUC WAKAI WANG2 WARSIN WATSON WU WU2 WUNSCH WYNDE3 WYNDE5 WYNDE6 WYNDE8 YAMAGU YONG
5	LANGE RIMING TANG2
6	HIRAY2 WYNDER
10	BENHAM VEIERO

Table 1B3 - 8
Potentially overlapping studies

REF	REFGP	PRINC	OVERLAP/LINK
LUBIN2	LUBIN2	1	Lubin-combined
TVERDA	TVERDA	1	VEIERO/TVERDAL
HEIN	PRESCO	1	Subset of PRESCO
GRAHAM	BYERS1	1	GRAHAM/BROSS/BYERS1
WALD	TANG2	1	Subset of TANG2
WYNDE7	WYNDE6	2	WYNDE5/6/7/8
CPSI	CPSI	1	CPSI overall
ENSTRO	ENSTRO	1	Subset of CPSI

Table 1B4 -

IESLC - Meta-analysis of Current Smoking, Any product (or Cigarettes if Any not available), Age <56
All LC types

This analysis is restricted to results for:
1) Non-dose-response data
2) Current smokers
3) Age <56
4) Results complete enough for use in metaanalysis

Within each study, results are then selected (in the following order of preference, within each sex) for:
5) PRODUCT: all/unspec, cigarettes regardless of other products, cigarettes only
6) CIGTYPE: all/unspecified, MC regardless of HR, MC only
7) DENOM: never smoked anything, never smoked cigarettes, (never +1 = +long term ex, +2 = +amount unknown,
+3 = never cigs+long term ex)
8) Followup period (YF, prospective studies): whole study (coded as 0) or longest available
9) LCtype: all or nearest available, at least Squamous and Adeno. (q = squamous, s = small,
l = large, a = adeno, mix = mixed, alv = alveolar)
10) Race: all or nearest available, otherwise by race (wh or w = white, bl or b = black, hi = hispanic
ch = chinese, jap = japanese, haw = hawaiian, w+o = white + oriental, sca = scandinavian, as = asian)
11) For overlapping studies: principal rather than subsidiary studies
Finally by Age: whole study (actual age shown) if available, otherwise by widest available age group
and then for single sex results (m, f) in preference to combined sex results (c).

Results adjusted (AD) for the most potential confounders are then chosen in Sections -1 to -3
(and those which actually differ from the adjusted results in Table 1B1 - 1 are marked 'x' in Section -1)
and results adjusted for the least confounders in Sections -4 to -6. (Those least adjusted results which
actually differ from the most adjusted as marked 'x' in column X in Section -4)
(Results adjusted for an unknown number of confounder(s) are coded as 20.)

Section -7 shows excluded studies, together with the stage (as above) at which no qualifying
results were found.

Section -8 lists the potentially overlapping studies which have been included (1=principal, 2=subsidiary).

Section -9 lists any results which would have been included in preference except that they had data not complete
enough for use in meta-analysis, with their significance (yes/no), if known, and any further comment as entered
on the database.

In addition to those mentioned above, the following fields, levels and abbreviations are used:
* or nk = not known, n = no, y = yes, ot = other
nev = never
all/unspec = all or unspecified, cig+/-ot = cigarettes irrespective of other products (cigar, pipe etc)
MC = manufactured cigarettes, HR = hand-rolled cigarettes
REF: 6-character study reference
NRR: number of the RR on the database within the study
ST : study type (CC = case control, pr or prosp = prospective)
NLC: number of lung cancer cases in whole study
R : risky occupational population (n = no, m = mining, o = other risky)
VB : national cigarette type (V = at least 75% Virginia, bl = at least 75% blended, ot = other)
P : any proxy use
H : full histological confirmation
De : derivation of RR/CI (or = original, st = standard method, ot = other method of estimation)

Table 1B4 - 1

IESLC - Meta-analysis of Current Smoking, Any product (or Cigarettes if Any not available), Age <56
All LC types
Most adjusted

REF	NRR	1B1	SEX	AGEL	AGEH	RACE	YF	LC TYPE	LOC	START	ST	NLC	R	VB	P	H	AD	PRODUCT	DENOM	De
BEST	31	x	m	30	49	all	0	all	NAmer	1955	pr	381	n	V	n	n	1	cig only	nev any	ot
COMSTO	15	x	c	25	44	all	-	all	NAmer	1975	ot	258	n	bl	n	n	0	cig+/-ot	nev cigs	st
CPSI	205	x	m	35	54	all	6	all	NAmer	1959	pr	5138	n	bl	n	n	1	cig+/-ot	nev any	ot
CPSI	255	x	f	40	54	all	6	all	NAmer	1959	pr	5138	n	bl	n	n	1	cig+/-ot	nev cigs	ot
CPSII	8	x	m	30	54	all	6	all	NAmer	1982	pr	3229	n	bl	n	n	0	cig only	nev any	st
CPSII	43	x	f	30	54	all	6	all	NAmer	1982	pr	3229	n	bl	n	n	0	cig+/-ot	nev cigs	st
DEAN3	36	x	m	35	44	all	-	all	Eu:UK	1969	CC	766	n	V	y	n	0	all/unsp	nev any	st
DEAN3	37	x	m	45	54	all	-	all	Eu:UK	1969	CC	766	n	V	y	n	0	all/unsp	nev any	st
DEAN3	113	x	f	35	44	all	-	all	Eu:UK	1969	CC	766	n	V	y	n	0	cig only	nev any	st
DEAN3	114	x	f	45	54	all	-	all	Eu:UK	1969	CC	766	n	V	y	n	0	cig only	nev any	st
HITOSU	20	x	m	1	49	all	-	all	As:Jap	1960	CC	216	n	bl	y	n	0	all/unsp	nev any	st
HITOSU	41	x	f	1	49	all	-	all	As:Jap	1960	CC	216	n	bl	y	n	0	all/unsp	nev any	st
KANELL	10	x	m	1	49	all	-	all	Eu:bal	1950	CC	862	n	bl	n	n	0	all/unsp	nev any	st
KREUZE	39		m	1	45	all	-	all	Eu:Ger	1990	CC	2260	n	bl	n	n	0	all/unsp	nev any	st
KREUZE	40		f	1	45	all	-	all	Eu:Ger	1990	CC	2260	n	bl	n	n	0	all/unsp	nev any	st
KUBIK	1	x	m	40	54	all	4	all	Eu:est	1965	pr	108	n	bl	n	n	0	cig+/-ot	nev any	ot
NAM	4	x	m	25	54	all	-	all	NAmer	1986	CC	1199	n	bl	y	n	0	cig+/-ot	nev cigs	st
NAM	36	x	f	25	54	all	-	all	NAmer	1986	CC	1199	n	bl	y	n	0	cig+/-ot	nev cigs	st
SEGI2	53	x	m	1	54	all	-	all	As:Jap	1962	CC	378	n	bl	n	n	0	cig+/-ot	nev any	st
SPEIZE	10		f	30	55	all	0	all	NAmer	1976	pr	593	n	bl	n	y	1	cig+/-ot	nev cigs	ot
TSUGAN	28		m	30	49	all	-	q+a	As:Jap	1976	CC	134	n	bl	n	y	0	all/unsp	nev any	st
TVERDA	5		m	35	49	all	0	all	Eu:Sca	1972	pr	238	n	bl	n	n	2	cig+/-ot	nev cigs	ot
TVERDA	15		f	35	49	all	0	all	Eu:Sca	1972	pr	238	n	bl	n	n	2	cig only	nev cigs	ot
VUTUC	12	x	m	1	39	all	-	all	Eu:wst	1976	CC	1877	n	bl	n	n	0	cig+/-ot	nev cigs	st
VUTUC	35	x	m	41	50	all	-	all	Eu:wst	1976	CC	1877	n	bl	n	n	0	cig+/-ot	nev cigs	st

Cigarette type is all/unspec for all RRs
except for the following:

REF	NRR	CIGTYPE
DEAN3	113	MC only
DEAN3	114	MC only

Table 1B4 - 2

IESLC - Meta-analysis of Current Smoking, Any product (or Cigarettes if Any not available), Age <56
All LC types
Most adjusted

REF	NRR	SEX	AD	Number Exposed Case	Number Exposed Cont	Non-exposed Case	Non-exposed Cont	RR	95.00%CI
*BEST	31	m	1	-	-	-	-	3.93	(0.53- 29.13)
COMSTO	15	c	0	24	27	1	17	15.11	(1.87- 122.22)
*CPSI	205	m	1	-	-	-	-	9.48	(6.11- 14.71)
*CPSI	255	f	1	-	-	-	-	3.85	(2.68- 5.52)
Subtotal CPSI								5.54	(4.19- 7.32)
*CPSII	8	m	0	167	225147	12	248565	15.36	(8.55- 27.60)
*CPSII	43	f	0	141	323063	23	670327	12.72	(8.19- 19.77)
Subtotal CPSII								13.62	(9.58- 19.37)
DEAN3	36	m	0	12	462	1	187	4.86	(0.63- 37.62)
DEAN3	37	m	0	80	541	1	145	21.44	(2.96- 155.40)
DEAN3	113	f	0	6	409	1	328	4.81	(0.58- 40.17)
DEAN3	114	f	0	22	404	4	326	4.44	(1.51- 13.01)
Subtotal DEAN3								5.87	(2.65- 13.00)
HITOSU	20	m	0	7	916	1	118	0.90	(0.11- 7.39)
HITOSU	41	f	0	3	225	7	1108	2.11	(0.54- 8.22)
Subtotal HITOSU								1.64	(0.52- 5.15)
KANELL	10	m	0	65	136	9	60	3.19	(1.49- 6.82)
KREUZE	39	m	0	168	99	6	54	15.27	(6.34- 36.79)
KREUZE	40	f	0	55	23	6	38	15.14	(5.63- 40.72)
Subtotal KREUZE								15.22	(7.89- 29.36)
*KUBIK	1	m	0	10	3688	0	2420	13.78~	(0.81- 235.06)
NAM	4	m	0	31	353	1	176	15.46	(2.09- 114.15)
NAM	36	f	0	24	99	2	93	11.27	(2.59- 49.03)
Subtotal NAM								12.59	(3.85- 41.16)
SEGI2	53	m	0	92	169	2	18	4.90	(1.11- 21.58)
*SPEIZE	10	f	1	-	-	-	-	12.69	(9.97- 16.16)
TSUGAN	28	m	0	63	63	18	22	1.22	(0.60- 2.50)
*TVERDA	5	m	2	-	-	-	-	4.09	(2.65- 6.31)
*TVERDA	15	f	2	-	-	-	-	11.05	(3.33- 36.71)
Subtotal TVERDA								4.59	(3.05- 6.90)
VUTUC	12	m	0	12	15	1	9	7.20	(0.80- 65.05)
VUTUC	35	m	0	74	104	11	64	4.14	(2.04- 8.38)
Subtotal VUTUC								4.36	(2.23- 8.54)
Partial Totals				1056	555943	107	924075		

*prospective study ~ With 0.5 adjustment for zero

REF	NRR	SEX	AD	Ys	Ws	Qs	Ps
*BEST	31	m	1	1.37	0.96	0.42	0.1806
COMSTO	15	c	0	2.72	0.88	0.41	0.0109
*CPSI	205	m	1	2.25	19.91	0.92	0.0000
*CPSI	255	f	1	1.35	29.43	13.85	0.0000
Subtotal CPSI				1.71	49.34	14.77	
*CPSII	8	m	0	2.73	11.20	5.46	0.0000
*CPSII	43	f	0	2.54	19.78	5.13	0.0000
Subtotal CPSII				2.61	30.97	10.58	
DEAN3	36	m	0	1.58	0.92	0.19	0.1302
DEAN3	37	m	0	3.07	0.98	1.04	0.0024
DEAN3	113	f	0	1.57	0.85	0.18	0.1468
DEAN3	114	f	0	1.49	3.32	0.98	0.0066
Subtotal DEAN3				1.77	6.07	2.40	
HITOSU	20	m	0	-0.10	0.87	3.96	0.9233
HITOSU	41	f	0	0.75	2.08	3.44	0.2818
Subtotal HITOSU				0.50	2.94	7.40	
KANELL	10	m	0	1.16	6.64	5.09	0.0028
KREUZE	39	m	0	2.73	4.97	2.38	0.0000
KREUZE	40	f	0	2.72	3.93	1.84	0.0000
Subtotal KREUZE				2.72	8.90	4.22	
*KUBIK	1	m	0	2.62	0.48	0.17	0.0699
NAM	4	m	0	2.74	0.96	0.48	0.0073
NAM	36	f	0	2.42	1.78	0.27	0.0012
Subtotal NAM				2.53	2.74	0.74	
SEGI2	53	m	0	1.59	1.75	0.35	0.0357
*SPEIZE	10	f	1	2.54	65.88	16.92	0.0000
TSUGAN	28	m	0	0.20	7.53	25.32	0.5818
*TVERDA	5	m	2	1.41	20.41	7.99	0.0000
*TVERDA	15	f	2	2.40	2.67	0.36	0.0001
Subtotal TVERDA				1.52	23.08	8.35	
VUTUC	12	m	0	1.97	0.79	0.00	0.0788
VUTUC	35	m	0	1.42	7.71	2.90	0.0001
Subtotal VUTUC				1.47	8.51	2.90	

Table 1B4 - 2

IESLC - Meta-analysis of Current Smoking, Any product (or Cigarettes if Any not available), Age <56
All LC types
Most adjusted

	N	25
	NS	15
	Wt	216.66
Het	Chi	100.03
Het	df	24
Het	P	***
Fixed	RR	7.64
	RRl	6.69
	RRu	8.73
	P	+++
Random	RR	6.57
	RRl	4.68
	RRu	9.23
	P	+++
Asymm	P	N.S.

Table 1B4 - 3

IESLC - Meta-analysis of Current Smoking, Any product (or Cigarettes if Any not available), Age <56
All LC types
Most adjusted

	combined	Sex male	female	Total
N	1	15	9	25
NS	1	13	8	22
Wt	0.88	86.07	129.71	216.66
Het Chi	0.00	50.33	38.98	100.03
Het df	0	14	8	24
Het P	N.S.	***	***	***
Fixed RR	15.11	5.83	9.11	7.64
RRl	1.87	4.72	7.67	6.69
RRu	122.22	7.20	10.82	8.73
P	+	+++	+++	+++
Random RR	15.11	5.69	7.76	6.57
RRl	1.87	3.52	4.69	4.68
RRu	122.22	9.20	12.86	9.23
P	+	+++	+++	+++
Between Chi				10.73
Between df				2
Between P				**
Btwn(F) P				N.S.
Btwn(R) P				N.S.

Table 1B4 - 4

IESLC - Meta-analysis of Current Smoking, Any product (or Cigarettes if Any not available), Age <56
All LC types
Least adjusted

REF	NRR	X	SEX	AGEL	AGEH	RACE	YF	LC TYPE	LOC	START	ST	NLC	R	VB	P	H	AD	PRODUCT	DENOM	De
BEST	31		m	30	49	all	0	all	NAmer	1955	pr	381	n	V	n	n	1	cig only	nev any	ot
COMSTO	15		c	25	44	all	-	all	NAmer	1975	ot	258	n	bl	n	n	0	cig+/-ot	nev cigs	st
CPSI	205		m	35	54	all	6	all	NAmer	1959	pr	5138	n	bl	n	n	1	cig+/-ot	nev any	ot
CPSI	255		f	40	54	all	6	all	NAmer	1959	pr	5138	n	bl	n	n	1	cig+/-ot	nev cigs	ot
CPSII	8		m	30	54	all	6	all	NAmer	1982	pr	3229	n	bl	n	n	0	cig only	nev any	st
CPSII	43		f	30	54	all	6	all	NAmer	1982	pr	3229	n	bl	n	n	0	cig+/-ot	nev cigs	st
DEAN3	36		m	35	44	all	-	all	Eu:UK	1969	CC	766	n	V	y	n	0	all/unsp	nev any	st
DEAN3	37		m	45	54	all	-	all	Eu:UK	1969	CC	766	n	V	y	n	0	all/unsp	nev any	st
DEAN3	113		f	35	44	all	-	all	Eu:UK	1969	CC	766	n	V	y	n	0	cig only	nev any	st
DEAN3	114		f	45	54	all	-	all	Eu:UK	1969	CC	766	n	V	y	n	0	cig only	nev any	st
HITOSU	20		m	1	49	all	-	all	As:Jap	1960	CC	216	n	bl	y	n	0	all/unsp	nev any	st
HITOSU	41		f	1	49	all	-	all	As:Jap	1960	CC	216	n	bl	y	n	0	all/unsp	nev any	st
KANELL	10		m	1	49	all	-	all	Eu:bal	1950	CC	862	n	bl	n	n	0	all/unsp	nev any	st
KREUZE	39		m	1	45	all	-	all	Eu:Ger	1990	CC	2260	n	bl	n	n	0	all/unsp	nev any	st
KREUZE	40		f	1	45	all	-	all	Eu:Ger	1990	CC	2260	n	bl	n	n	0	all/unsp	nev any	st
KUBIK	1		m	40	54	all	4	all	Eu:est	1965	pr	108	n	bl	n	n	0	cig+/-ot	nev any	ot
NAM	4		m	25	54	all	-	all	NAmer	1986	CC	1199	n	bl	y	n	0	cig+/-ot	nev cigs	st
NAM	36		f	25	54	all	-	all	NAmer	1986	CC	1199	n	bl	y	n	0	cig+/-ot	nev cigs	st
SEGI2	53		m	1	54	all	-	all	As:Jap	1962	CC	378	n	bl	n	n	0	cig+/-ot	nev any	st
SPEIZE	6	x	f	30	55	all	0	all	NAmer	1976	pr	593	n	bl	n	y	0	cig+/-ot	nev cigs	st
TSUGAN	28		m	30	49	all	-	q+a	As:Jap	1976	CC	134	n	bl	n	y	0	all/unsp	nev any	st
TVERDA	5		m	35	49	all	0	all	Eu:Sca	1972	pr	238	n	bl	n	n	2	cig+/-ot	nev cigs	ot
TVERDA	15		f	35	49	all	0	all	Eu:Sca	1972	pr	238	n	bl	n	n	2	cig only	nev cigs	ot
VUTUC	12		m	1	39	all	-	all	Eu:wst	1976	CC	1877	n	bl	n	n	0	cig+/-ot	nev cigs	st
VUTUC	35		m	41	50	all	-	all	Eu:wst	1976	CC	1877	n	bl	n	n	0	cig+/-ot	nev cigs	st

Cigarette type is all/unspec for all RRs
except for the following:

REF	NRR	CIGTYPE
DEAN3	113	MC only
DEAN3	114	MC only

Table 1B4 - 5

IESLC - Meta-analysis of Current Smoking, Any product (or Cigarettes if Any not available), Age <56
All LC types
Least adjusted

REF	NRR	SEX	AD	Number Exposed Case	Number Exposed Cont	Non-exposed Case	Non-exposed Cont	RR	95.00%CI
*BEST	31	m	1	-	-	-	-	3.93	(0.53- 29.13)
COMSTO	15	c	0	24	27	1	17	15.11	(1.87- 122.22)
*CPSI	205	m	1	-	-	-	-	9.48	(6.11- 14.71)
*CPSI	255	f	1	-	-	-	-	3.85	(2.68- 5.52)
Subtotal CPSI								5.54	(4.19- 7.32)
*CPSII	8	m	0	167	225147	12	248565	15.36	(8.55- 27.60)
*CPSII	43	f	0	141	323063	23	670327	12.72	(8.19- 19.77)
Subtotal CPSII								13.62	(9.58- 19.37)
DEAN3	36	m	0	12	462	1	187	4.86	(0.63- 37.62)
DEAN3	37	m	0	80	541	1	145	21.44	(2.96- 155.40)
DEAN3	113	f	0	6	409	1	328	4.81	(0.58- 40.17)
DEAN3	114	f	0	22	404	4	326	4.44	(1.51- 13.01)
Subtotal DEAN3								5.87	(2.65- 13.00)
HITOSU	20	m	0	7	916	1	118	0.90	(0.11- 7.39)
HITOSU	41	f	0	3	225	7	1108	2.11	(0.54- 8.22)
Subtotal HITOSU								1.64	(0.52- 5.15)
KANELL	10	m	0	65	136	9	60	3.19	(1.49- 6.82)
KREUZE	39	m	0	168	99	6	54	15.27	(6.34- 36.79)
KREUZE	40	f	0	55	23	6	38	15.14	(5.63- 40.72)
Subtotal KREUZE								15.22	(7.89- 29.36)
*KUBIK	1	m	0	10	3688	0	2420	13.78~	(0.81- 235.06)
NAM	4	m	0	31	353	1	176	15.46	(2.09- 114.15)
NAM	36	f	0	24	99	2	93	11.27	(2.59- 49.03)
Subtotal NAM								12.59	(3.85- 41.16)
SEGI2	53	m	0	92	169	2	18	4.90	(1.11- 21.58)
*SPEIZE	6	f	0	391	489993	58	776300	10.68	(8.11- 14.07)
TSUGAN	28	m	0	63	63	18	22	1.22	(0.60- 2.50)
*TVERDA	5	m	2	-	-	-	-	4.09	(2.65- 6.31)
*TVERDA	15	f	2	-	-	-	-	11.05	(3.33- 36.71)
Subtotal TVERDA								4.59	(3.05- 6.90)
VUTUC	12	m	0	12	15	1	9	7.20	(0.80- 65.05)
VUTUC	35	m	0	74	104	11	64	4.14	(2.04- 8.38)
Subtotal VUTUC								4.36	(2.23- 8.54)
Partial Totals				1447	1045936	165	1700375		

*prospective study ~ With 0.5 adjustment for zero

REF	NRR	SEX	AD	Ys	Ws	Qs	Ps
*BEST	31	m	1	1.37	0.96	0.33	0.1806
COMSTO	15	c	0	2.72	0.88	0.51	0.0109
*CPSI	205	m	1	2.25	19.91	1.76	0.0000
*CPSI	255	f	1	1.35	29.43	10.74	0.0000
Subtotal CPSI				1.71	49.34	12.49	
*CPSII	8	m	0	2.73	11.20	6.81	0.0000
*CPSII	43	f	0	2.54	19.78	6.91	0.0000
Subtotal CPSII				2.61	30.97	13.72	
DEAN3	36	m	0	1.58	0.92	0.13	0.1302
DEAN3	37	m	0	3.07	0.98	1.21	0.0024
DEAN3	113	f	0	1.57	0.85	0.12	0.1468
DEAN3	114	f	0	1.49	3.32	0.71	0.0066
Subtotal DEAN3				1.77	6.07	2.17	
HITOSU	20	m	0	-0.10	0.87	3.67	0.9233
HITOSU	41	f	0	0.75	2.08	3.02	0.2818
Subtotal HITOSU				0.50	2.94	6.68	
KANELL	10	m	0	1.16	6.64	4.18	0.0028
KREUZE	39	m	0	2.73	4.97	2.98	0.0000
KREUZE	40	f	0	2.72	3.93	2.30	0.0000
Subtotal KREUZE				2.72	8.90	5.28	
*KUBIK	1	m	0	2.62	0.48	0.22	0.0699
NAM	4	m	0	2.74	0.96	0.59	0.0073
NAM	36	f	0	2.42	1.78	0.39	0.0012
Subtotal NAM				2.53	2.74	0.99	
SEGI2	53	m	0	1.59	1.75	0.23	0.0357
*SPEIZE	6	f	0	2.37	50.52	8.76	0.0000
TSUGAN	28	m	0	0.20	7.53	23.11	0.5818
*TVERDA	5	m	2	1.41	20.41	6.03	0.0000
*TVERDA	15	f	2	2.40	2.67	0.54	0.0001
Subtotal TVERDA				1.52	23.08	6.57	
VUTUC	12	m	0	1.97	0.79	0.00	0.0788
VUTUC	35	m	0	1.42	7.71	2.18	0.0001
Subtotal VUTUC				1.47	8.51	2.18	

Table 1B4 - 5

IESLC - Meta-analysis of Current Smoking, Any product (or Cigarettes if Any not available), Age <56
All LC types
Least adjusted

	N	25
	NS	15
	Wt	201.30
Het	Chi	87.41
Het	df	24
Het	P	***
Fixed	RR	7.04
	RRl	6.13
	RRu	8.09
	P	+++
Random	RR	6.49
	RRl	4.69
	RRu	8.99
	P	+++
Asymm	P	N.S.

Table 1B4 - 6

IESLC - Meta-analysis of Current Smoking, Any product (or Cigarettes if Any not available), Age <56
All LC types
Least adjusted

	combined	Sex male	female	Total
N	1	15	9	25
NS	1	13	8	22
Wt	0.88	86.07	114.35	201.30
Het Chi	0.00	50.33	31.35	87.41
Het df	0	14	8	24
Het P	N.S.	***	***	***
Fixed RR	15.11	5.83	8.07	7.04
RRl	1.87	4.72	6.72	6.13
RRu	122.22	7.20	9.70	8.09
P	+	+++	+++	+++
Random RR	15.11	5.69	7.57	6.49
RRl	1.87	3.52	4.73	4.69
RRu	122.22	9.20	12.09	8.99
P	+	+++	+++	+++
Between Chi				5.73
Between df				2
Between P				(*)
Btwn(F) P				N.S.
Btwn(R) P				N.S.

Table 1B4 - 7

IESLC - Meta-analysis of Current Smoking, Any product (or Cigarettes if Any not available), Age <56
All LC types
Excluded studies (and stage at which they were excluded)

1	BOUCHA	BUELL	LAURIL	MZILEN											
2	ABELIN	ABRAHA	AUVINE	AXELSO	BAND	BERRIN	BLOT1	BLOT2	BLOT3	BLOT4	BRESLO	BROCKM	BROWN1	BYERS1	CASCO2	CASCOR
	CHAN	CHATZI	CHEN	CHEN2	CHEN3	CHIAZZ	COOKSO	DAVEYS	DEAN	DOSEME	DU	DUNN	EBELIN	ESAKI	FAN	GENG
	GER	GODLEY	GOLLED	GSELL	HANSEN	HEGMAN	HINDS	HIRAOK	HOROWI	HORWIT	HU	HU2	HUANG	ISHIMA	JARUP	JIANG
	JONES	JUSSAW	KO	KOHLME	KOULUM	KREYBE	LAMTH	LAMWK	LAMWK2	LAUSSM	LEI	LETOUR	LEVIN	LIU	LIU2	LIU3
	LIU4	LIU5	LOMBA2	LUO	MAGNUS	MARSH	MARSH2	MARTIS	MASTRA	MATSUD	MCCONN	MCDUFF	MCLAUG	MILLER	MILLS	MOLLO
	NOTAN2	NOU	ORMOS	PASTOR	PAWLEG	PERNU	PERSHA	PIKE	POFFIJ	POLEDN	PRESCO	QIAO	RADZIK	RANDIG	REN	RONCO
	ROOTS	ROTHSC	SADOWS	SANKAR	SCHWA2	SEGI	SEOW	SIEMIA	SIMARA	STASZE	STOCKS	SUN	TAO	ULMER	VANDER	WANG
	WANG3	WANG4	WICKLU	WILKIN	WUWILL	WYNDE2	WYNDE4	XIANGZ	XU	XU2	XU3	XU4	YUAN	ZHANG	ZHENG	ZHOU
3	AGUDO	AKIBA	ALDERS	AMANDU	AMES	ANDERS	ARCHER	ARMADA	AUSTIN	AXELSS	BARBON	BECHER	BENHAM	BENSHL	BLOHMK	BOFFET
	BOUCOT	BRETT	BROSS	BROWN2	BUFFLE	BYERS2	CARPEN	CEDERL	CHANG	CHOI	CHOW	CHYOU	CORREA	DAMBER	DARBY	DEAN2
	DEKLER	DESTE2	DESTEF	DOCKER	DOLL	DOLL2	DORANT	DORGAN	DORN	DROSTE	ENGELA	ENSTRO	GAO	GAO2	GARCIA	GARDIN
	GARSHI	GILLIS	GOODMA	GRAHAM	GREGOR	HAENSZ	HAMMO2	HAMMON	HEIN	HENNEK	HIRAY2	HUMBLE	JAHN	JAIN	JARVHO	JEDRYC
	JOLY	KAISER	KATSOU	KAUFMA	KELLER	KHUDER	KIHARA	KINLEN	KJUUS	KNEKT	KOO	LANGE	LEMARC	LIAW	LICKIN	LIDDEL
	LOMBAR	LUBIN	LUBIN2	MACLEN	MATOS	MIGRAN	MRFIT	MRFITR	MURATA	NOTANI	ODRISC	OSANN	OSANN2	PARKIN	PERSH2	PETO
	PEZZO2	PEZZOT	PISANI	QIAO2	RACHTA	RESTRE	RIMING	SAARIK	SCHWAR	SHAW	SHIMIZ	SIMONA	SITAS	SOBUE	SOBUE2	SPITZ
	STAYNE	STOCKW	STUCKE	SUZUK2	SUZUKI	SVENSS	TANG	TANG2	TENKAN	TIZZAN	TOKARS	TOUSEY	TULINI	VEIERO	WAKAI	WALD
	WANG2	WARSIN	WATSON	WIGLE	WU	WU2	WUNSCH	WYNDE3	WYNDE5	WYNDE6	WYNDE7	WYNDE8	WYNDER	YAMAGU	YONG	
4	HIRAYA															
6	KAISE2															
7	HOLE															

Table 1B4 - 8
Potentially overlapping studies

REF	REFGP	PRINC	OVERLAP/LINK
VUTUC	LUBIN2	2	Subset of Lubin2
TVERDA	TVERDA	1	VEIERO/TVERDAL
CPSI	CPSI	1	CPSI overall

Table 1B4 - 9
Most adjusted - insufficient data for metaanalysis

REF	NRR	SEX	AGEL	AGEH	RACE	YF	LC TYPE	LOC	START	ST	NLC	R	VB	P	H	AD	PRODUCT	DENOM	De
HIRAYA	136	m	40	49	all	8	all	As:Jap	1965	pr	1917	n	bl	n	n	1	cig+/-ot	nev any	or

REF	NRR	RR	SIG	RRDATA comment
HIRAYA	136	2.08		0

Table 1B5 -

IESLC - Meta-analysis of Current Smoking, Any product (or Cigarettes if Any not available), Age 50-70
All LC types

This analysis is restricted to results for:
1) Non-dose-response data
2) Current smokers
3) Maximum age range 50-70
4) Results complete enough for use in metaanalysis

Within each study, results are then selected (in the following order of preference, within each sex) for:
5) PRODUCT: all/unspec, cigarettes regardless of other products, cigarettes only
6) CIGTYPE: all/unspecified, MC regardless of HR, MC only
7) DENOM: never smoked anything, never smoked cigarettes, (never +1 = +long term ex, +2 = +amount unknown,
+3 = never cigs+long term ex)
8) Followup period (YF, prospective studies): whole study (coded as 0) or longest available
9) LCtype: all or nearest available, at least Squamous and Adeno. (q = squamous, s = small,
l = large, a = adeno, mix = mixed, alv = alveolar)
10) Race: all or nearest available, otherwise by race (wh or w = white, bl or b = black, hi = hispanic
ch = chinese, jap = japanese, haw = hawaiian, w+o = white + oriental, sca = scandinavian, as = asian)
11) For overlapping studies: principal rather than subsidiary studies
Finally by Age: whole study (actual age shown) if available, otherwise by widest available age group
and then for single sex results (m, f) in preference to combined sex results (c).

Results adjusted (AD) for the most potential confounders are then chosen in Sections -1 to -3
(and those which actually differ from the adjusted results in Table 1B1 - 1 are marked 'x' in Section -1)
and results adjusted for the least confounders in Sections -4 to -6. (Those least adjusted results which
actually differ from the most adjusted as marked 'x' in column X in Section -4)
(Results adjusted for an unknown number of confounder(s) are coded as 20.)

Section -7 shows excluded studies, together with the stage (as above) at which no qualifying
results were found.

Section -8 lists the potentially overlapping studies which have been included (1=principal, 2=subsidiary).

Section -9 lists any results which would have been included in preference except that they had data not complete
enough for use in meta-analysis, with their significance (yes/no), if known, and any further comment as entered
on the database.

In addition to those mentioned above, the following fields, levels and abbreviations are used:
* or nk = not known, n = no, y = yes, ot = other
nev = never
all/unspec = all or unspecified, cig+/-ot = cigarettes irrespective of other products (cigar, pipe etc)
MC = manufactured cigarettes, HR = hand-rolled cigarettes
REF: 6-character study reference
NRR: number of the RR on the database within the study
ST : study type (CC = case control, pr or prosp = prospective)
NLC: number of lung cancer cases in whole study
R : risky occupational population (n = no, m = mining, o = other risky)
VB : national cigarette type (V = at least 75% Virginia, bl = at least 75% blended, ot = other)
P : any proxy use
H : full histological confirmation
De : derivation of RR/CI (or = original, st = standard method, ot = other method of estimation)

Table 1B5 - 1

IESLC - Meta-analysis of Current Smoking, Any product (or Cigarettes if Any not available), Age 50-70
All LC types
Most adjusted

REF	NRR	1B1	SEX	AGEL	AGEH	RACE	YF	LC TYPE	LOC	START	ST	NLC	R	VB	P	H	AD	PRODUCT	DENOM	De
ANDERS	6		f	55	69	all	0	all	NAmer	1986	pr	343	n	bl	n	n	1	cig+/-ot	nev cigs	or
BEST	32	x	m	50	69	all	0	all	NAmer	1955	pr	381	n	V	n	n	1	cig only	nev any	ot
CEDERL	9	x	m	50	59	all	10	all	Eu:Sca	1963	pr	491	n	bl	n	n	0	all/unsp	nev any	st
CEDERL	17	x	m	60	69	all	10	all	Eu:Sca	1963	pr	491	n	bl	n	n	0	all/unsp	nev any	st
CEDERL	35	x	f	50	59	all	10	all	Eu:Sca	1963	pr	491	n	bl	n	n	0	cig only	nev any	st
CEDERL	38	x	f	60	69	all	10	all	Eu:Sca	1963	pr	491	n	bl	n	n	0	cig only	nev any	st
CPSI	210	x	m	55	69	all	6	all	NAmer	1959	pr	5138	n	bl	n	n	1	cig+/-ot	nev any	ot
CPSI	103	x	f	55	64	wh	0	all	NAmer	1959	pr	5138	n	bl	n	n	0	cig only	nev any	st
CPSII	15	x	m	55	64	all	6	all	NAmer	1982	pr	3229	n	bl	n	n	0	cig only	nev any	st
CPSII	50	x	f	55	64	all	6	all	NAmer	1982	pr	3229	n	bl	n	n	0	cig+/-ot	nev cigs	st
DEAN3	38	x	m	55	64	all	-	all	Eu:UK	1969	CC	766	n	V	y	n	0	all/unsp	nev any	st
DEAN3	115	x	f	55	64	all	-	all	Eu:UK	1969	CC	766	n	V	y	n	0	cig only	nev any	st
DORANT	2		m	55	69	all	0	all	Eu:wst	1986	ot	550	n	bl	n	y	0	all/unsp	nev any	st
DORN	366	x	m	55	64	wh	25	all	NAmer	1954	pr	5097	n	bl	n	n	1	cig+/-ot	nev any	or
HAMMON	139		m	50	69	wh	0	all	NAmer	1952	pr	448	n	bl	n	n	1	cig only	nev any	ot
HITOSU	47	x	f	50	59	all	-	all	As:Jap	1960	CC	216	n	bl	y	n	0	all/unsp	nev any	st
KANELL	15	x	m	50	59	all	-	all	Eu:bal	1950	CC	862	n	bl	n	n	0	all/unsp	nev any	st
KANELL	20	x	m	60	69	all	-	all	Eu:bal	1950	CC	862	n	bl	n	n	0	all/unsp	nev any	st
KREUZE	41		m	55	69	all	-	all	Eu:Ger	1990	CC	2260	n	bl	n	n	0	all/unsp	nev any	st
KREUZE	42		f	55	69	all	-	all	Eu:Ger	1990	CC	2260	n	bl	n	n	0	all/unsp	nev any	st
KUBIK	2	x	m	55	64	all	4	all	Eu:est	1965	pr	108	n	bl	n	n	0	cig+/-ot	nev any	st
NAM	12	x	m	55	64	all	-	all	NAmer	1986	CC	1199	n	bl	y	n	0	cig+/-ot	nev cigs	st
NAM	44	x	f	55	64	all	-	all	NAmer	1986	CC	1199	n	bl	y	n	0	cig+/-ot	nev cigs	st
SEGI2	54	x	m	55	64	all	-	all	As:Jap	1962	CC	378	n	bl	n	n	0	cig+/-ot	nev any	st

Cigarette type is all/unspec for all RRs
except for the following:

REF	NRR	CIGTYPE
DEAN3	115	MC only

Table 1B5 - 2

IESLC - Meta-analysis of Current Smoking, Any product (or Cigarettes if Any not available), Age 50-70
All LC types
Most adjusted

REF	NRR	SEX	AD	Number Exposed Case	Cont	Non-exposed Case	Cont	RR	95.00%CI
*ANDERS	6	f	1	-	-	-	-	23.43	(17.02- 32.27)
*BEST	32	m	1	-	-	-	-	23.30	(7.45- 72.88)
*CEDERL	9	m	0	37	4232	4	1954	4.27	(1.52- 11.97)
*CEDERL	17	m	0	55	2628	3	1645	11.48	(3.60- 36.62)
*CEDERL	35	f	0	4	1159	9	6407	2.46	(0.76- 7.96)
*CEDERL	38	f	0	4	407	10	5877	5.78	(1.82- 18.34)
Subtotal CEDERL								5.10	(2.91- 8.95)
*CPSI	210	m	1	-	-	-	-	14.22	(10.37- 19.52)
*CPSI	103	f	0	249	591967	144	1366561	3.99	(3.25- 4.90)
Subtotal CPSI								5.82	(4.90- 6.91)
*CPSII	15	m	0	641	231659	21	253604	33.42	(21.64- 51.61)
*CPSII	50	f	0	390	267703	67	686189	14.92	(11.51- 19.33)
Subtotal CPSII								18.43	(14.76- 23.03)
DEAN3	38	m	0	183	358	7	96	7.01	(3.19- 15.41)
DEAN3	115	f	0	36	217	9	310	5.71	(2.70- 12.11)
Subtotal DEAN3								6.30	(3.66- 10.85)
DORANT	2	m	0	332	697	7	159	10.82	(5.02- 23.32)
*DORN	366	m	1	-	-	-	-	11.10	(9.78- 12.61)
*HAMMON	139	m	1	-	-	-	-	11.52	(6.83- 19.42)
HITOSU	47	f	0	6	148	8	478	2.42	(0.83- 7.09)
KANELL	15	m	0	354	112	9	38	13.35	(6.26- 28.45)
KANELL	20	m	0	336	127	16	35	5.79	(3.10- 10.82)
Subtotal KANELL								8.12	(5.02- 13.16)
KREUZE	41	m	0	1252	524	23	403	41.86	(27.17- 64.51)
KREUZE	42	f	0	170	54	95	177	5.87	(3.95- 8.70)
Subtotal KREUZE								14.33	(10.71- 19.19)
*KUBIK	2	m	0	45	2654	1	1851	31.38	(4.33- 227.48)
NAM	12	m	0	83	88	5	42	7.92	(2.99- 20.99)
NAM	44	f	0	28	48	10	69	4.03	(1.79- 9.05)
Subtotal NAM								5.31	(2.85- 9.90)
SEGI2	54	m	0	111	193	2	20	5.75	(1.32- 25.07)
Partial Totals				4316	1104975	450	2325915		

*prospective study

REF	NRR	SEX	AD	Ys	Ws	Qs	Ps
*ANDERS	6	f	1	3.15	37.54	24.48	0.0000
*BEST	32	m	1	3.15	2.95	1.90	0.0000
*CEDERL	9	m	0	1.45	3.62	2.90	0.0057
*CEDERL	17	m	0	2.44	2.85	0.03	0.0000
*CEDERL	35	f	0	0.90	2.78	5.82	0.1341
*CEDERL	38	f	0	1.75	2.88	1.01	0.0029
Subtotal CEDERL				1.63	12.13	9.75	
*CPSI	210	m	1	2.65	38.41	3.65	0.0000
*CPSI	103	f	0	1.38	91.26	84.50	0.0000
Subtotal CPSI				1.76	129.66	88.15	
*CPSII	15	m	0	3.51	20.34	27.48	0.0000
*CPSII	50	f	0	2.70	57.19	7.26	0.0000
Subtotal CPSII				2.91	77.53	34.74	
DEAN3	38	m	0	1.95	6.19	0.99	0.0000
DEAN3	115	f	0	1.74	6.82	2.48	0.0000
Subtotal DEAN3				1.84	13.01	3.47	
DORANT	2	m	0	2.38	6.51	0.01	0.0000
*DORN	366	m	1	2.41	237.89	0.87	0.0000
*HAMMON	139	m	1	2.44	14.07	0.13	0.0000
HITOSU	47	f	0	0.88	3.33	7.11	0.1066
KANELL	15	m	0	2.59	6.70	0.40	0.0000
KANELL	20	m	0	1.76	9.81	3.43	0.0000
Subtotal KANELL				2.09	16.51	3.83	
KREUZE	41	m	0	3.73	20.55	39.58	0.0000
KREUZE	42	f	0	1.77	24.64	8.22	0.0000
Subtotal KREUZE				2.66	45.19	47.80	
*KUBIK	2	m	0	3.45	0.98	1.18	0.0006
NAM	12	m	0	2.07	4.04	0.31	0.0000
NAM	44	f	0	1.39	5.85	5.32	0.0008
Subtotal NAM				1.67	9.89	5.63	
SEGI2	54	m	0	1.75	1.77	0.63	0.0199

Table 1B5 - 2

IESLC - Meta-analysis of Current Smoking, Any product (or Cigarettes if Any not available), Age 50-70
All LC types
Most adjusted

	N	24
	NS	15
	Wt	608.98
Het	Chi	229.69
Het	df	23
Het	P	***
Fixed	RR	10.45
	RRl	9.65
	RRu	11.31
	P	+++
Random	RR	9.62
	RRl	7.10
	RRu	13.05
	P	+++
Asymm	P	N.S.

Table 1B5 - 3

IESLC - Meta-analysis of Current Smoking, Any product (or Cigarettes if Any not available), Age 50-70
All LC types
Most adjusted

	Sex combined	male	female	Total
N		15	9	24
NS		13	8	21
Wt		376.69	232.28	608.98
Het Chi		69.86	124.11	229.69
Het df		14	8	23
Het P		***	***	***
Fixed RR		12.64	7.68	10.45
RRl		11.42	6.75	9.65
RRu		13.98	8.73	11.31
P		+++	+++	+++
Random RR		12.84	6.15	9.62
RRl		9.28	3.42	7.10
RRu		17.77	11.06	13.05
P		+++	+++	+++
Between Chi				35.72
Between df				1
Between P				***
Btwn(F) P				(*)
Btwn(R) P				*

Table 1B5 - 4

IESLC - Meta-analysis of Current Smoking, Any product (or Cigarettes if Any not available), Age 50-70
All LC types
Least adjusted

REF	NRR	X	SEX	AGEL	AGEH	RACE	YF	LC TYPE	LOC	START	ST	NLC	R	VB	P	H	AD	PRODUCT	DENOM	De
ANDERS	2	x	f	55	69	all	0	all	NAmer	1986	pr	343	n	bl	n	n	0	cig+/-ot	nev cigs	st
BEST	32		m	50	69	all	0	all	NAmer	1955	pr	381	n	V	n	n	1	cig only	nev any	ot
CEDERL	9		m	50	59	all	10	all	Eu:Sca	1963	pr	491	n	bl	n	n	0	all/unsp	nev any	st
CEDERL	17		m	60	69	all	10	all	Eu:Sca	1963	pr	491	n	bl	n	n	0	all/unsp	nev any	st
CEDERL	35		f	50	59	all	10	all	Eu:Sca	1963	pr	491	n	bl	n	n	0	cig only	nev any	st
CEDERL	38		f	60	69	all	10	all	Eu:Sca	1963	pr	491	n	bl	n	n	0	cig only	nev any	st
CPSI	210		m	55	69	all	6	all	NAmer	1959	pr	5138	n	bl	n	n	1	cig+/-ot	nev any	ot
CPSI	103		f	55	64	wh	0	all	NAmer	1959	pr	5138	n	bl	n	n	0	cig only	nev any	st
CPSII	15		m	55	64	all	6	all	NAmer	1982	pr	3229	n	bl	n	n	0	cig only	nev any	st
CPSII	50		f	55	64	all	6	all	NAmer	1982	pr	3229	n	bl	n	n	0	cig+/-ot	nev cigs	st
DEAN3	38		m	55	64	all	-	all	Eu:UK	1969	CC	766	n	V	y	n	0	all/unsp	nev any	st
DEAN3	115		f	55	64	all	-	all	Eu:UK	1969	CC	766	n	V	y	n	0	cig only	nev any	st
DORANT	2		m	55	69	all	0	all	Eu:wst	1986	ot	550	n	bl	n	y	0	all/unsp	nev any	st
DORN	366		m	55	64	wh	25	all	NAmer	1954	pr	5097	n	bl	n	n	1	cig+/-ot	nev any	or
HAMMON	139		m	50	69	wh	0	all	NAmer	1952	pr	448	n	bl	n	n	1	cig only	nev any	ot
HITOSU	47		f	50	59	all	-	all	As:Jap	1960	CC	216	n	bl	y	n	0	all/unsp	nev any	st
KANELL	15		m	50	59	all	-	all	Eu:bal	1950	CC	862	n	bl	n	n	0	all/unsp	nev any	st
KANELL	20		m	60	69	all	-	all	Eu:bal	1950	CC	862	n	bl	n	n	0	all/unsp	nev any	st
KREUZE	41		m	55	69	all	-	all	Eu:Ger	1990	CC	2260	n	bl	n	n	0	all/unsp	nev any	st
KREUZE	42		f	55	69	all	-	all	Eu:Ger	1990	CC	2260	n	bl	n	n	0	all/unsp	nev any	st
KUBIK	2		m	55	64	all	4	all	Eu:est	1965	pr	108	n	bl	n	n	0	cig+/-ot	nev any	st
NAM	12		m	55	64	all	-	all	NAmer	1986	CC	1199	n	bl	y	n	0	cig+/-ot	nev cigs	st
NAM	44		f	55	64	all	-	all	NAmer	1986	CC	1199	n	bl	y	n	0	cig+/-ot	nev cigs	st
SEGI2	54		m	55	64	all	-	all	As:Jap	1962	CC	378	n	bl	n	n	0	cig+/-ot	nev any	st

Cigarette type is all/unspec for all RRs
except for the following:

REF	NRR	CIGTYPE
DEAN3	115	MC only

Table 1B5 - 5

IESLC - Meta-analysis of Current Smoking, Any product (or Cigarettes if Any not available), Age 50-70
All LC types
Least adjusted

REF	NRR	SEX	AD	Number Exposed Case	Number Exposed Cont	Non-exposed Case	Non-exposed Cont	RR	95.00%CI
*ANDERS	2	f	0	212	41262	46	195158	21.80	(15.85- 29.98)
*BEST	32	m	1	-	-	-	-	23.30	(7.45- 72.88)
*CEDERL	9	m	0	37	4232	4	1954	4.27	(1.52- 11.97)
*CEDERL	17	m	0	55	2628	3	1645	11.48	(3.60- 36.62)
*CEDERL	35	f	0	4	1159	9	6407	2.46	(0.76- 7.96)
*CEDERL	38	f	0	4	407	10	5877	5.78	(1.82- 18.34)
Subtotal CEDERL								5.10	(2.91- 8.95)
*CPSI	210	m	1	-	-	-	-	14.22	(10.37- 19.52)
*CPSI	103	f	0	249	591967	144	1366561	3.99	(3.25- 4.90)
Subtotal CPSI								5.82	(4.90- 6.91)
*CPSII	15	m	0	641	231659	21	253604	33.42	(21.64- 51.61)
*CPSII	50	f	0	390	267703	67	686189	14.92	(11.51- 19.33)
Subtotal CPSII								18.43	(14.76- 23.03)
DEAN3	38	m	0	183	358	7	96	7.01	(3.19- 15.41)
DEAN3	115	f	0	36	217	9	310	5.71	(2.70- 12.11)
Subtotal DEAN3								6.30	(3.66- 10.85)
DORANT	2	m	0	332	697	7	159	10.82	(5.02- 23.32)
*DORN	366	m	1	-	-	-	-	11.10	(9.78- 12.61)
*HAMMON	139	m	1	-	-	-	-	11.52	(6.83- 19.42)
HITOSU	47	f	0	6	148	8	478	2.42	(0.83- 7.09)
KANELL	15	m	0	354	112	9	38	13.35	(6.26- 28.45)
KANELL	20	m	0	336	127	16	35	5.79	(3.10- 10.82)
Subtotal KANELL								8.12	(5.02- 13.16)
KREUZE	41	m	0	1252	524	23	403	41.86	(27.17- 64.51)
KREUZE	42	f	0	170	54	95	177	5.87	(3.95- 8.70)
Subtotal KREUZE								14.33	(10.71- 19.19)
*KUBIK	2	m	0	45	2654	1	1851	31.38	(4.33- 227.48)
NAM	12	m	0	83	88	5	42	7.92	(2.99- 20.99)
NAM	44	f	0	28	48	10	69	4.03	(1.79- 9.05)
Subtotal NAM								5.31	(2.85- 9.90)
SEGI2	54	m	0	111	193	2	20	5.75	(1.32- 25.07)
Partial Totals				4528	1146237	496	2521073		

*prospective study

REF	NRR	SEX	AD	Ys	Ws	Qs	Ps
*ANDERS	2	f	0	3.08	37.84	20.69	0.0000
*BEST	32	m	1	3.15	2.95	1.92	0.0000
*CEDERL	9	m	0	1.45	3.62	2.87	0.0057
*CEDERL	17	m	0	2.44	2.85	0.03	0.0000
*CEDERL	35	f	0	0.90	2.78	5.79	0.1341
*CEDERL	38	f	0	1.75	2.88	1.00	0.0029
Subtotal CEDERL				1.63	12.13	9.68	
*CPSI	210	m	1	2.65	38.41	3.74	0.0000
*CPSI	103	f	0	1.38	91.26	83.79	0.0000
Subtotal CPSI				1.76	129.66	87.53	
*CPSII	15	m	0	3.51	20.34	27.68	0.0000
*CPSII	50	f	0	2.70	57.19	7.42	0.0000
Subtotal CPSII				2.91	77.53	35.10	
DEAN3	38	m	0	1.95	6.19	0.97	0.0000
DEAN3	115	f	0	1.74	6.82	2.45	0.0000
Subtotal DEAN3				1.84	13.01	3.42	
DORANT	2	m	0	2.38	6.51	0.01	0.0000
*DORN	366	m	1	2.41	237.89	0.99	0.0000
*HAMMON	139	m	1	2.44	14.07	0.15	0.0000
HITOSU	47	f	0	0.88	3.33	7.07	0.1066
KANELL	15	m	0	2.59	6.70	0.41	0.0000
KANELL	20	m	0	1.76	9.81	3.38	0.0000
Subtotal KANELL				2.09	16.51	3.79	
KREUZE	41	m	0	3.73	20.55	39.82	0.0000
KREUZE	42	f	0	1.77	24.64	8.10	0.0000
Subtotal KREUZE				2.66	45.19	47.92	
*KUBIK	2	m	0	3.45	0.98	1.19	0.0006
NAM	12	m	0	2.07	4.04	0.30	0.0000
NAM	44	f	0	1.39	5.85	5.28	0.0008
Subtotal NAM				1.67	9.89	5.58	
SEGI2	54	m	0	1.75	1.77	0.62	0.0199

Table 1B5 - 5

IESLC - Meta-analysis of Current Smoking, Any product (or Cigarettes if Any not available), Age 50-70
All LC types
Least adjusted

	N	24
	NS	15
	Wt	609.27
Het	Chi	225.65
Het	df	23
Het	P	***
Fixed	RR	10.41
	RRl	9.61
	RRu	11.27
	P	+++
Random	RR	9.59
	RRl	7.10
	RRu	12.97
	P	+++
Asymm	P	N.S.

Table 1B5 - 6

IESLC - Meta-analysis of Current Smoking, Any product (or Cigarettes if Any not available), Age 50-70
All LC types
Least adjusted

	Sex combined	male	female	Total
N		15	9	24
NS		13	8	21
Wt		376.69	232.58	609.27
Het Chi		69.86	118.55	225.65
Het df		14	8	23
Het P		***	***	***
Fixed RR		12.64	7.60	10.41
RRl		11.42	6.68	9.61
RRu		13.98	8.64	11.27
P		+++	+++	+++
Random RR		12.84	6.11	9.59
RRl		9.28	3.44	7.10
RRu		17.77	10.85	12.97
P		+++	+++	+++
Between Chi				37.24
Between df				1
Between P				***
Btwn(F) P				*
Btwn(R) P				*

Table 1B5 - 7

IESLC - Meta-analysis of Current Smoking, Any product (or Cigarettes if Any not available), Age 50-70
All LC types
Excluded studies (and stage at which they were excluded)

1	BOUCHA BUELL LAURIL MZILEN
2	ABELIN ABRAHA AUVINE AXELSO BAND BERRIN BLOT1 BLOT2 BLOT3 BLOT4 BRESLO BROCKM BROWN1 BYERS1 CASCO2 CASCOR CHAN CHATZI CHEN CHEN2 CHEN3 CHIAZZ COOKSO DAVEYS DEAN DOSEME DU DUNN EBELIN ESAKI FAN GENG GER GODLEY GOLLED GSELL HANSEN HEGMAN HINDS HIRAOK HOROWI HORWIT HU HU2 HUANG ISHIMA JARUP JIANG JONES JUSSAW KO KOHLME KOULUM KREYBE LAMTH LAMWK LAMWK2 LAUSSM LEI LETOUR LEVIN LIU LIU2 LIU3 LIU4 LIU5 LOMBA2 LUO MAGNUS MARSH MARSH2 MARTIS MASTRA MATSUD MCCONN MCDUFF MCLAUG MILLER MILLS MOLLO NOTAN2 NOU ORMOS PASTOR PAWLEG PERNU PERSHA PIKE POFFIJ POLEDN PRESCO QIAO RADZIK RANDIG REN RONCO ROOTS ROTHSC SADOWS SANKAR SCHWA2 SEGI SEOW SIEMIA SIMARA STASZE STOCKS SUN TAO ULMER VANDER WANG WANG3 WANG4 WICKLU WILKIN WUWILL WYNDE2 WYNDE4 XIANGZ XU XU2 XU3 XU4 YUAN ZHANG ZHENG ZHOU
3	AGUDO AKIBA ALDERS AMANDU AMES ARCHER ARMADA AUSTIN AXELSS BARBON BECHER BENHAM BENSHL BLOHMK BOFFET BOUCOT BRETT BROSS BROWN2 BUFFLE BYERS2 CARPEN CHANG CHOI CHOW CHYOU COMSTO CORREA DAMBER DARBY DEAN2 DEKLER DESTE2 DESTEF DOCKER DOLL DOLL2 DORGAN DROSTE ENGELA ENSTRO GAO GAO2 GARCIA GARDIN GARSHI GILLIS GOODMA GRAHAM GREGOR HAENSZ HAMMO2 HEIN HENNEK HIRAY2 HUMBLE JAHN JAIN JARVHO JEDRYC JOLY KAISER KATSOU KAUFMA KELLER KHUDER KIHARA KINLEN KJUUS KNEKT KOO LANGE LEMARC LIAW LICKIN LIDDEL LOMBAR LUBIN LUBIN2 MACLEN MATOS MIGRAN MRFIT MRFITR MURATA NOTANI ODRISC OSANN OSANN2 PARKIN PERSH2 PETO PEZZO2 PEZZOT PISANI QIAO2 RACHTA RESTRE RIMING SAARIK SCHWAR SHAW SHIMIZ SIMONA SITAS SOBUE SOBUE2 SPEIZE SPITZ STAYNE STOCKW STUCKE SUZUK2 SUZUKI SVENSS TANG TANG2 TENKAN TIZZAN TOKARS TOUSEY TSUGAN TULINI TVERDA VEIERO VUTUC WAKAI WALD WANG2 WARSIN WATSON WIGLE WU WU2 WUNSCH WYNDE3 WYNDE5 WYNDE6 WYNDE7 WYNDE8 WYNDER YAMAGU YONG
4	HIRAYA
6	KAISE2
7	HOLE

Table 1B5 - 8
Potentially overlapping studies

REF	REFGP	PRINC	.	OVERLAP/LINK
CPSI	CPSI	1		CPSI overall

Table 1B5 - 9
Most adjusted - insufficient data for metaanalysis

REF	NRR	SEX	AGEL	AGEH	RACE	YF	LC TYPE	LOC	START	ST	NLC	R	VB	P	H	AD	PRODUCT	DENOM	De
HIRAYA	5	m	60	69	all	0	all	As:Jap	1965	pr	1917	n	bl	n	n	0	cig+/-ot	nev	any or

REF	NRR	RR	SIG	RRDATA	comment
HIRAYA	5	4.22		0	

Table 1B6 -

IESLC - Meta-analysis of Current Smoking, Any product (or Cigarettes if Any not available), Age 65+
All LC types

This analysis is restricted to results for:
1) Non-dose-response data
2) Current smokers
3) Age 65+
4) Results complete enough for use in metaanalysis

Within each study, results are then selected (in the following order of preference, within each sex) for:
5) PRODUCT: all/unspec, cigarettes regardless of other products, cigarettes only
6) CIGTYPE: all/unspecified, MC regardless of HR, MC only
7) DENOM: never smoked anything, never smoked cigarettes, (never +1 = +long term ex, +2 = +amount unknown,
+3 = never cigs+long term ex)
8) Followup period (YF, prospective studies): whole study (coded as 0) or longest available
9) LCtype: all or nearest available, at least Squamous and Adeno. (q = squamous, s = small,
l = large, a = adeno, mix = mixed, alv = alveolar)
10) Race: all or nearest available, otherwise by race (wh or w = white, bl or b = black, hi = hispanic
ch = chinese, jap = japanese, haw = hawaiian, w+o = white + oriental, sca = scandinavian, as = asian)
11) For overlapping studies: principal rather than subsidiary studies
Finally by Age: whole study (actual age shown) if available, otherwise by widest available age group
and then for single sex results (m, f) in preference to combined sex results (c).

Results adjusted (AD) for the most potential confounders are then chosen in Sections -1 to -3
(and those which actually differ from the adjusted results in Table 1B1 - 1 are marked 'x' in Section -1)
and results adjusted for the least confounders in Sections -4 to -6. (Those least adjusted results which
actually differ from the most adjusted as marked 'x' in column X in Section -4)
(Results adjusted for an unknown number of confounder(s) are coded as 20.)

Section -7 shows excluded studies, together with the stage (as above) at which no qualifying
results were found.

Section -8 lists the potentially overlapping studies which have been included (1=principal, 2=subsidiary).

Section -9 lists any results which would have been included in preference except that they had data not complete
enough for use in meta-analysis, with their significance (yes/no), if known, and any further comment as entered
on the database.

In addition to those mentioned above, the following fields, levels and abbreviations are used:
* or nk = not known, n = no, y = yes, ot = other
nev = never
all/unspec = all or unspecified, cig+/-ot = cigarettes irrespective of other products (cigar, pipe etc)
MC = manufactured cigarettes, HR = hand-rolled cigarettes
REF: 6-character study reference
NRR: number of the RR on the database within the study
ST : study type (CC = case control, pr or prosp = prospective)
NLC: number of lung cancer cases in whole study
R : risky occupational population (n = no, m = mining, o = other risky)
VB : national cigarette type (V = at least 75% Virginia, bl = at least 75% blended, ot = other)
P : any proxy use
H : full histological confirmation
De : derivation of RR/CI (or = original, st = standard method, ot = other method of estimation)

Table 1B6 - 1

IESLC - Meta-analysis of Current Smoking, Any product (or Cigarettes if Any not available), Age 65+
All LC types
Most adjusted

REF	NRR	1B1	SEX	AGEL	AGEH	RACE	YF	LC TYPE	LOC	START	ST	NLC	R	VB	P	H	AD	PRODUCT	DENOM	De
BEST	33	x	m	70	99	all	0	all	NAmer	1955	pr	381	n	V	n	n	1	cig only	nev any	ot
COMSTO	17	x	c	65	99	all	-	all	NAmer	1975	ot	258	n	bl	n	n	0	cig+/-ot	nev cigs	st
CPSI	215	x	m	70	84	all	6	all	NAmer	1959	pr	5138	n	bl	n	n	1	cig+/-ot	nev any	ot
CPSI	104	x	f	65	74	wh	0	all	NAmer	1959	pr	5138	n	bl	n	n	0	cig only	nev any	st
CPSI	105	x	f	75	84	wh	0	all	NAmer	1959	pr	5138	n	bl	n	n	0	cig only	nev any	st
CPSII	22	x	m	65	74	all	6	all	NAmer	1982	pr	3229	n	bl	n	n	0	cig only	nev any	st
CPSII	29	x	m	75	99	all	6	all	NAmer	1982	pr	3229	n	bl	n	n	0	cig only	nev any	st
CPSII	57	x	f	65	74	all	6	all	NAmer	1982	pr	3229	n	bl	n	n	0	cig+/-ot	nev cigs	st
CPSII	64	x	f	75	99	all	6	all	NAmer	1982	pr	3229	n	bl	n	n	0	cig+/-ot	nev cigs	st
DEAN3	39	x	m	65	99	all	-	all	Eu:UK	1969	CC	766	n	V	y	n	0	all/unsp	nev any	st
DEAN3	116	x	f	65	99	all	-	all	Eu:UK	1969	CC	766	n	V	y	n	0	cig only	nev any	st
DORN	50	x	m	65	84	wh	8	all	NAmer	1954	pr	5097	n	bl	n	n	1	all/unsp	nev any	ot
GARSHI	15	x	m	65	82	all	-	all	NAmer	1981	CC	1081	o	bl	y	n	0	all/unsp	nev any	st
HUMBLE	22	x	m	65	84	w-hi	-	not alv	NAmer	1980	CC	521	n	bl	y	n	0	cig+/-ot	nev cigs	st
HUMBLE	30	x	m	65	84	hi	-	not alv	NAmer	1980	CC	521	n	bl	y	n	0	cig+/-ot	nev cigs	st
HUMBLE	24	x	f	65	84	w-hi	-	not alv	NAmer	1980	CC	521	n	bl	y	n	0	cig+/-ot	nev cigs	st
HUMBLE	32	x	f	65	84	hi	-	not alv	NAmer	1980	CC	521	n	bl	y	n	0	cig+/-ot	nev cigs	st
KAISE2	36	x	m	65	74	all	9	all	NAmer	1979	pr	318	n	bl	n	n	0	cig only	nev any	st
KAISE2	44	x	m	75	99	all	9	all	NAmer	1979	pr	318	n	bl	n	n	0	cig only	nev any	st
KAISE2	12	x	f	65	74	all	9	all	NAmer	1979	pr	318	n	bl	n	n	0	cig only	nev any	st
KAISE2	20	x	f	75	99	all	9	all	NAmer	1979	pr	318	n	bl	n	n	0	cig only	nev any	st
KANELL	25	x	m	70	99	all	-	all	Eu:bal	1950	CC	862	n	bl	n	n	0	all/unsp	nev any	st
NAM	20	x	m	65	79	all	-	all	NAmer	1986	CC	1199	n	bl	y	n	0	cig+/-ot	nev cigs	st
NAM	28	x	m	80	99	all	-	all	NAmer	1986	CC	1199	n	bl	y	n	0	cig+/-ot	nev cigs	st
NAM	52	x	f	65	79	all	-	all	NAmer	1986	CC	1199	n	bl	y	n	0	cig+/-ot	nev cigs	st
NAM	60	x	f	80	99	all	-	all	NAmer	1986	CC	1199	n	bl	y	n	0	cig+/-ot	nev cigs	st
SEGI2	55	x	m	65	99	all	-	all	As:Jap	1962	CC	378	n	bl	n	n	0	cig+/-ot	nev any	st

Cigarette type is all/unspec for all RRs
except for the following:

REF	NRR	CIGTYPE
DEAN3	116	MC only

Table 1B6 - 2

IESLC - Meta-analysis of Current Smoking, Any product (or Cigarettes if Any not available), Age 65+
All LC types
Most adjusted

REF	NRR	SEX	AD	Number Exposed Case	Number Exposed Cont	Non-exposed Case	Non-exposed Cont	RR	95.00%CI
*BEST	33	m	1	-	-	-	-	12.43	(3.92- 39.43)
COMSTO	17	c	0	24	16	6	44	11.00	(3.80- 31.81)
*CPSI	215	m	1	-	-	-	-	10.48	(6.48- 16.96)
*CPSI	104	f	0	145	177746	178	957682	4.39	(3.53- 5.46)
*CPSI	105	f	0	28	31384	163	392909	2.15	(1.44- 3.21)
Subtotal CPSI								4.29	(3.59- 5.13)
*CPSII	22	m	0	743	106505	47	173660	25.78	(19.20- 34.61)
*CPSII	29	m	0	230	20335	44	66378	17.06	(12.36- 23.55)
*CPSII	57	f	0	374	126848	110	487073	13.06	(10.56- 16.15)
*CPSII	64	f	0	109	26603	110	247713	9.23	(7.08- 12.02)
Subtotal CPSII								14.35	(12.58- 16.37)
DEAN3	39	m	0	227	285	16	82	4.08	(2.32- 7.17)
DEAN3	116	f	0	38	128	27	574	6.31	(3.72- 10.71)
Subtotal DEAN3								5.15	(3.50- 7.57)
*DORN	50	m	1	-	-	-	-	6.80	(5.13- 9.00)
GARSHI	15	m	0	455	484	32	294	8.64	(5.87- 12.71)
HUMBLE	22	m	0	74	58	6	60	12.76	(5.15- 31.60)
HUMBLE	30	m	0	38	30	2	21	13.30	(2.89- 61.26)
HUMBLE	24	f	0	34	14	11	52	11.48	(4.67- 28.24)
HUMBLE	32	f	0	15	6	4	34	21.25	(5.22- 86.47)
Subtotal HUMBLE								13.32	(7.74- 22.94)
*KAISE2	36	m	0	16	4735	5	11466	7.75	(2.84- 21.14)
*KAISE2	44	m	0	7	1035	4	4486	7.59	(2.22- 25.86)
*KAISE2	12	f	0	19	7165	2	24159	32.03	(7.46- 137.49)
*KAISE2	20	f	0	6	1394	3	12285	17.63	(4.41- 70.40)
Subtotal KAISE2								11.66	(6.31- 21.55)
KANELL	25	m	0	59	66	14	39	2.49	(1.23- 5.04)
NAM	20	m	0	106	122	18	130	6.28	(3.59- 10.96)
NAM	28	m	0	21	26	6	172	23.15	(8.55- 62.73)
NAM	52	f	0	67	75	18	251	12.46	(6.97- 22.27)
NAM	60	f	0	14	12	22	472	25.03	(10.37- 60.44)
Subtotal NAM								11.49	(8.15- 16.20)
SEGI2	55	m	0	62	120	4	15	1.94	(0.62- 6.09)
Partial Totals				2911	505192	852	2380051		

*prospective study

REF	NRR	SEX	AD	Ys	Ws	Qs	Ps
*BEST	33	m	1	2.52	2.88	0.35	0.0000
COMSTO	17	c	0	2.40	3.41	0.17	0.0000
*CPSI	215	m	1	2.35	16.60	0.51	0.0000
*CPSI	104	f	0	1.48	79.95	38.56	0.0000
*CPSI	105	f	0	0.77	23.91	47.40	0.0002
Subtotal CPSI				1.46	120.46	86.48	
*CPSII	22	m	0	3.25	44.23	51.20	0.0000
*CPSII	29	m	0	2.84	37.02	16.29	0.0000
*CPSII	57	f	0	2.57	85.07	13.31	0.0000
*CPSII	64	f	0	2.22	54.87	0.13	0.0000
Subtotal CPSII				2.66	221.20	80.93	
DEAN3	39	m	0	1.41	12.11	7.12	0.0000
DEAN3	116	f	0	1.84	13.72	1.51	0.0000
Subtotal DEAN3				1.64	25.82	8.63	
*DORN	50	m	1	1.92	48.63	3.20	0.0000
GARSHI	15	m	0	2.16	25.70	0.01	0.0000
HUMBLE	22	m	0	2.55	4.67	0.65	0.0000
HUMBLE	30	m	0	2.59	1.65	0.28	0.0009
HUMBLE	24	f	0	2.44	4.74	0.34	0.0000
HUMBLE	32	f	0	3.06	1.95	1.52	0.0000
Subtotal HUMBLE				2.59	13.01	2.79	
*KAISE2	36	m	0	2.05	3.81	0.06	0.0001
*KAISE2	44	m	0	2.03	2.55	0.06	0.0012
*KAISE2	12	f	0	3.47	1.81	3.03	0.0000
*KAISE2	20	f	0	2.87	2.00	0.97	0.0000
Subtotal KAISE2				2.46	10.18	4.11	
KANELL	25	m	0	0.91	7.74	12.31	0.0111
NAM	20	m	0	1.84	12.36	1.40	0.0000
NAM	28	m	0	3.14	3.87	3.63	0.0000
NAM	52	f	0	2.52	11.39	1.38	0.0000
NAM	60	f	0	3.22	4.94	5.41	0.0000
Subtotal NAM				2.44	32.56	11.83	
SEGI2	55	m	0	0.66	2.93	6.70	0.2575

Table 1B6 - 2

IESLC - Meta-analysis of Current Smoking, Any product (or Cigarettes if Any not available), Age 65+
All LC types
Most adjusted

	N	27
	NS	12
	Wt	514.53
Het	Chi	217.51
Het	df	26
Het	P	***
Fixed	RR	8.79
	RRl	8.06
	RRu	9.58
	P	+++
Random	RR	9.07
	RRl	6.83
	RRu	12.04
	P	+++
Asymm	P	N.S.

Table 1B6 - 3

IESLC - Meta-analysis of Current Smoking, Any product (or Cigarettes if Any not available), Age 65+
All LC types
Most adjusted

	Sex combined	male	female	Total
N	1	15	11	27
NS	1	11	6	18
Wt	3.41	226.76	284.36	514.53
Het Chi	0.00	96.44	107.49	217.51
Het df	0	14	10	26
Het P	N.S.	***	***	***
Fixed RR	11.00	10.52	7.59	8.79
RRl	3.80	9.24	6.76	8.06
RRu	31.81	11.99	8.53	9.58
P	+++	+++	+++	+++
Random RR	11.00	8.68	9.54	9.07
RRl	3.80	5.92	6.07	6.83
RRu	31.81	12.74	15.00	12.04
P	+++	+++	+++	+++
Between Chi				13.58
Between df				2
Between P				**
Btwn(F) P				N.S.
Btwn(R) P				N.S.

Table 1B6 - 4

IESLC - Meta-analysis of Current Smoking, Any product (or Cigarettes if Any not available), Age 65+
All LC types
Least adjusted

REF	NRR	X	SEX	AGEL	AGEH	RACE	YF	LC TYPE	LOC	START	ST	NLC	R	VB	P	H	AD	PRODUCT	DENOM	De
BEST	33		m	70	99	all	0	all	NAmer	1955	pr	381	n	V	n	n	1	cig only	nev any	ot
COMSTO	17		c	65	99	all	-	all	NAmer	1975	ot	258	n	bl	n	n	0	cig+/-ot	nev cigs	st
CPSI	215		m	70	84	all	6	all	NAmer	1959	pr	5138	n	bl	n	n	1	cig+/-ot	nev any	ot
CPSI	104		f	65	74	wh	0	all	NAmer	1959	pr	5138	n	bl	n	n	0	cig only	nev any	st
CPSI	105		f	75	84	wh	0	all	NAmer	1959	pr	5138	n	bl	n	n	0	cig only	nev any	st
CPSII	22		m	65	74	all	6	all	NAmer	1982	pr	3229	n	bl	n	n	0	cig only	nev any	st
CPSII	29		m	75	99	all	6	all	NAmer	1982	pr	3229	n	bl	n	n	0	cig only	nev any	st
CPSII	57		f	65	74	all	6	all	NAmer	1982	pr	3229	n	bl	n	n	0	cig+/-ot	nev cigs	st
CPSII	64		f	75	99	all	6	all	NAmer	1982	pr	3229	n	bl	n	n	0	cig+/-ot	nev cigs	st
DEAN3	39		m	65	99	all	-	all	Eu:UK	1969	CC	766	n	V	y	n	0	all/unsp	nev any	st
DEAN3	116		f	65	99	all	-	all	Eu:UK	1969	CC	766	n	V	y	n	0	cig only	nev any	st
DORN	50		m	65	84	wh	8	all	NAmer	1954	pr	5097	n	bl	n	n	1	all/unsp	nev any	ot
GARSHI	15		m	65	82	all	-	all	NAmer	1981	CC	1081	o	bl	y	n	0	all/unsp	nev any	st
HUMBLE	22		m	65	84	w-hi	-	not alv	NAmer	1980	CC	521	n	bl	y	n	0	cig+/-ot	nev cigs	st
HUMBLE	30		m	65	84	hi	-	not alv	NAmer	1980	CC	521	n	bl	y	n	0	cig+/-ot	nev cigs	st
HUMBLE	24		f	65	84	w-hi	-	not alv	NAmer	1980	CC	521	n	bl	y	n	0	cig+/-ot	nev cigs	st
HUMBLE	32		f	65	84	hi	-	not alv	NAmer	1980	CC	521	n	bl	y	n	0	cig+/-ot	nev cigs	st
KAISE2	36		m	65	74	all	9	all	NAmer	1979	pr	318	n	bl	n	n	0	cig only	nev any	st
KAISE2	44		m	75	99	all	9	all	NAmer	1979	pr	318	n	bl	n	n	0	cig only	nev any	st
KAISE2	12		f	65	74	all	9	all	NAmer	1979	pr	318	n	bl	n	n	0	cig only	nev any	st
KAISE2	20		f	75	99	all	9	all	NAmer	1979	pr	318	n	bl	n	n	0	cig only	nev any	st
KANELL	25		m	70	99	all	-	all	Eu:bal	1950	CC	862	n	bl	n	n	0	all/unsp	nev any	st
NAM	20		m	65	79	all	-	all	NAmer	1986	CC	1199	n	bl	y	n	0	cig+/-ot	nev cigs	st
NAM	28		m	80	99	all	-	all	NAmer	1986	CC	1199	n	bl	y	n	0	cig+/-ot	nev cigs	st
NAM	52		f	65	79	all	-	all	NAmer	1986	CC	1199	n	bl	y	n	0	cig+/-ot	nev cigs	st
NAM	60		f	80	99	all	-	all	NAmer	1986	CC	1199	n	bl	y	n	0	cig+/-ot	nev cigs	st
SEGI2	55		m	65	99	all	-	all	As:Jap	1962	CC	378	n	bl	n	n	0	cig+/-ot	nev any	st

Cigarette type is all/unspec for all RRs
except for the following:

REF	NRR	CIGTYPE
DEAN3	116	MC only

Table 1B6 - 5

IESLC - Meta-analysis of Current Smoking, Any product (or Cigarettes if Any not available), Age 65+
All LC types
Least adjusted

REF	NRR	SEX	AD	Number Exposed Case	Number Exposed Cont	Non-exposed Case	Non-exposed Cont	RR	95.00%CI
*BEST	33	m	1	-	-	-	-	12.43	(3.92- 39.43)
COMSTO	17	c	0	24	16	6	44	11.00	(3.80- 31.81)
*CPSI	215	m	1	-	-	-	-	10.48	(6.48- 16.96)
*CPSI	104	f	0	145	177746	178	957682	4.39	(3.53- 5.46)
*CPSI	105	f	0	28	31384	163	392909	2.15	(1.44- 3.21)
Subtotal CPSI								4.29	(3.59- 5.13)
*CPSII	22	m	0	743	106505	47	173660	25.78	(19.20- 34.61)
*CPSII	29	m	0	230	20335	44	66378	17.06	(12.36- 23.55)
*CPSII	57	f	0	374	126848	110	487073	13.06	(10.56- 16.15)
*CPSII	64	f	0	109	26603	110	247713	9.23	(7.08- 12.02)
Subtotal CPSII								14.35	(12.58- 16.37)
DEAN3	39	m	0	227	285	16	82	4.08	(2.32- 7.17)
DEAN3	116	f	0	38	128	27	574	6.31	(3.72- 10.71)
Subtotal DEAN3								5.15	(3.50- 7.57)
*DORN	50	m	1	-	-	-	-	6.80	(5.13- 9.00)
GARSHI	15	m	0	455	484	32	294	8.64	(5.87- 12.71)
HUMBLE	22	m	0	74	58	6	60	12.76	(5.15- 31.60)
HUMBLE	30	m	0	38	30	2	21	13.30	(2.89- 61.26)
HUMBLE	24	f	0	34	14	11	52	11.48	(4.67- 28.24)
HUMBLE	32	f	0	15	6	4	34	21.25	(5.22- 86.47)
Subtotal HUMBLE								13.32	(7.74- 22.94)
*KAISE2	36	m	0	16	4735	5	11466	7.75	(2.84- 21.14)
*KAISE2	44	m	0	7	1035	4	4486	7.59	(2.22- 25.86)
*KAISE2	12	f	0	19	7165	2	24159	32.03	(7.46- 137.49)
*KAISE2	20	f	0	6	1394	3	12285	17.63	(4.41- 70.40)
Subtotal KAISE2								11.66	(6.31- 21.55)
KANELL	25	m	0	59	66	14	39	2.49	(1.23- 5.04)
NAM	20	m	0	106	122	18	130	6.28	(3.59- 10.96)
NAM	28	m	0	21	26	6	172	23.15	(8.55- 62.73)
NAM	52	f	0	67	75	18	251	12.46	(6.97- 22.27)
NAM	60	f	0	14	12	22	472	25.03	(10.37- 60.44)
Subtotal NAM								11.49	(8.15- 16.20)
SEGI2	55	m	0	62	120	4	15	1.94	(0.62- 6.09)
Partial Totals				2911	505192	852	2380051		

*prospective study

REF	NRR	SEX	AD	Ys	Ws	Qs	Ps
*BEST	33	m	1	2.52	2.88	0.35	0.0000
COMSTO	17	c	0	2.40	3.41	0.17	0.0000
*CPSI	215	m	1	2.35	16.60	0.51	0.0000
*CPSI	104	f	0	1.48	79.95	38.56	0.0000
*CPSI	105	f	0	0.77	23.91	47.40	0.0002
Subtotal CPSI				1.46	120.46	86.48	
*CPSII	22	m	0	3.25	44.23	51.20	0.0000
*CPSII	29	m	0	2.84	37.02	16.29	0.0000
*CPSII	57	f	0	2.57	85.07	13.31	0.0000
*CPSII	64	f	0	2.22	54.87	0.13	0.0000
Subtotal CPSII				2.66	221.20	80.93	
DEAN3	39	m	0	1.41	12.11	7.12	0.0000
DEAN3	116	f	0	1.84	13.72	1.51	0.0000
Subtotal DEAN3				1.64	25.82	8.63	
*DORN	50	m	1	1.92	48.63	3.20	0.0000
GARSHI	15	m	0	2.16	25.70	0.01	0.0000
HUMBLE	22	m	0	2.55	4.67	0.65	0.0000
HUMBLE	30	m	0	2.59	1.65	0.28	0.0009
HUMBLE	24	f	0	2.44	4.74	0.34	0.0000
HUMBLE	32	f	0	3.06	1.95	1.52	0.0000
Subtotal HUMBLE				2.59	13.01	2.79	
*KAISE2	36	m	0	2.05	3.81	0.06	0.0001
*KAISE2	44	m	0	2.03	2.55	0.06	0.0012
*KAISE2	12	f	0	3.47	1.81	3.03	0.0000
*KAISE2	20	f	0	2.87	2.00	0.97	0.0000
Subtotal KAISE2				2.46	10.18	4.11	
KANELL	25	m	0	0.91	7.74	12.31	0.0111
NAM	20	m	0	1.84	12.36	1.40	0.0000
NAM	28	m	0	3.14	3.87	3.63	0.0000
NAM	52	f	0	2.52	11.39	1.38	0.0000
NAM	60	f	0	3.22	4.94	5.41	0.0000
Subtotal NAM				2.44	32.56	11.83	
SEGI2	55	m	0	0.66	2.93	6.70	0.2575

Table 1B6 - 5

IESLC - Meta-analysis of Current Smoking, Any product (or Cigarettes if Any not available), Age 65+
All LC types
Least adjusted

	N	27
	NS	12
	Wt	514.53
Het	Chi	217.51
Het	df	26
Het	P	***
Fixed	RR	8.79
	RRl	8.06
	RRu	9.58
	P	+++
Random	RR	9.07
	RRl	6.83
	RRu	12.04
	P	+++
Asymm	P	N.S.

Table 1B6 - 6

IESLC - Meta-analysis of Current Smoking, Any product (or Cigarettes if Any not available), Age 65+
All LC types
Least adjusted

	combined	Sex male	female	Total
N	1	15	11	27
NS	1	11	6	18
Wt	3.41	226.76	284.36	514.53
Het Chi	0.00	96.44	107.49	217.51
Het df	0	14	10	26
Het P	N.S.	***	***	***
Fixed RR	11.00	10.52	7.59	8.79
RRl	3.80	9.24	6.76	8.06
RRu	31.81	11.99	8.53	9.58
P	+++	+++	+++	+++
Random RR	11.00	8.68	9.54	9.07
RRl	3.80	5.92	6.07	6.83
RRu	31.81	12.74	15.00	12.04
P	+++	+++	+++	+++
Between Chi				13.58
Between df				2
Between P				**
Btwn(F) P				N.S.
Btwn(R) P				N.S.

Table 1B6 - 7

IESLC - Meta-analysis of Current Smoking, Any product (or Cigarettes if Any not available), Age 65+
All LC types
Excluded studies (and stage at which they were excluded)

1 BOUCHA BUELL LAURIL MZILEN

2 ABELIN ABRAHA AUVINE AXELSO BAND BERRIN BLOT1 BLOT2 BLOT3 BLOT4 BRESLO BROCKM BROWN1 BYERS1 CASCO2 CASCOR
CHAN CHATZI CHEN CHEN2 CHEN3 CHIAZZ COOKSO DAVEYS DEAN DOSEME DU DUNN EBELIN ESAKI FAN GENG
GER GODLEY GOLLED GSELL HANSEN HEGMAN HINDS HIRAOK HOROWI HORWIT HU HU2 HUANG ISHIMA JARUP JIANG
JONES JUSSAW KO KOHLME KOULUM KREYBE LAMTH LAMWK LAMWK2 LAUSSM LEI LETOUR LEVIN LIU LIU2 LIU3
LIU4 LIU5 LOMBA2 LUO MAGNUS MARSH MARSH2 MARTIS MASTRA MATSUD MCCONN MCDUFF MCLAUG MILLER MILLS MOLLO
NOTAN2 NOU ORMOS PASTOR PAWLEG PERNU PERSHA PIKE POFFIJ POLEDN PRESCO QIAO RADZIK RANDIG REN RONCO
ROOTS ROTHSC SADOWS SANKAR SCHWA2 SEGI SEOW SIEMIA SIMARA STASZE STOCKS SUN TAO ULMER VANDER WANG
WANG3 WANG4 WICKLU WILKIN WUWILL WYNDE2 WYNDE4 XIANGZ XU XU2 XU3 XU4 YUAN ZHANG ZHENG ZHOU

3 AGUDO AKIBA ALDERS AMANDU AMES ANDERS ARCHER ARMADA AUSTIN AXELSS BARBON BECHER BENHAM BENSHL BLOHMK BOFFET
BOUCOT BRETT BROSS BROWN2 BUFFLE BYERS2 CARPEN CEDERL CHANG CHOI CHOW CHYOU CORREA DAMBER DARBY DEAN2
DEKLER DESTE2 DESTEF DOCKER DOLL DOLL2 DORANT DORGAN DROSTE ENGELA ENSTRO GAO GAO2 GARCIA GARDIN GILLIS
GOODMA GRAHAM GREGOR HAENSZ HAMMO2 HAMMON HEIN HENNEK HIRAY2 HITOSU HOLE JAHN JAIN JARVHO JEDRYC JOLY
KAISER KATSOU KAUFMA KELLER KHUDER KIHARA KINLEN KJUUS KNEKT KOO KREUZE KUBIK LANGE LEMARC LIAW LICKIN
LIDDEL LOMBAR LUBIN LUBIN2 MACLEN MATOS MIGRAN MRFIT MRFITR MURATA NOTANI ODRISC OSANN OSANN2 PARKIN PERSH2
PETO PEZZO2 PEZZOT PISANI QIAO2 RACHTA RESTRE RIMING SAARIK SCHWAR SHAW SHIMIZ SIMONA SITAS SOBUE SOBUE2
SPEIZE SPITZ STAYNE STOCKW STUCKE SUZUK2 SUZUKI SVENSS TANG TANG2 TIZZAN TOKARS TOUSEY TSUGAN TULINI TVERDA
VEIERO VUTUC WAKAI WALD WANG2 WARSIN WATSON WIGLE WU WU2 WUNSCH WYNDE3 WYNDE5 WYNDE6 WYNDE7 WYNDE8
WYNDER YAMAGU YONG

4 HIRAYA

7 TENKAN

Table 1B6 - 8
Potentially overlapping studies

REF	REFGP	PRINC	.	OVERLAP/LINK
CPSI	CPSI	1		CPSI overall

Table 1B6 - 9
Most adjusted - insufficient data for metaanalysis

REF	NRR	SEX	AGEL	AGEH	RACE	YF	LC TYPE	LOC	START	ST	NLC	R	VB	P	H	AD	PRODUCT	DENOM	De
HIRAYA	6	m	70	99	all	0	all	As:Jap	1965	pr	1917	n	bl	n	n	0	cig+/-ot	nev	any or

REF	NRR	RR	SIG	RRDATA	comment
HIRAYA	6	5.10		0	

Table 1B7 -

IESLC - Meta-analysis of Current Smoking, Cigarettes (or Any Product if Cigarettes not available), Age <56
All LC types

This analysis is restricted to results for:
1) Non-dose-response data
2) Current smokers
3) Age <56
4) Results complete enough for use in metaanalysis

Within each study, results are then selected (in the following order of preference, within each sex) for:
5) PRODUCT: cigarettes regardless of other products, cigarettes only, all/unspec
6) CIGTYPE: all/unspecified, MC regardless of HR, MC only
7) DENOM: never smoked anything, never smoked cigarettes, (never +1 = +long term ex, +2 = +amount unknown,
 +3 = never cigs+long term ex)
8) Followup period (YF, prospective studies): whole study (coded as 0) or longest available
9) LCtype: all or nearest available, at least Squamous and Adeno. (q = squamous, s = small,
 l = large, a = adeno, mix = mixed, alv = alveolar)
10) Race: all or nearest available, otherwise by race (wh or w = white, bl or b = black, hi = hispanic
 ch = chinese, jap = japanese, haw = hawaiian, w+o = white + oriental, sca = scandinavian, as = asian)
11) For overlapping studies: principal rather than subsidiary studies
Finally by Age: whole study (actual age shown) if available, otherwise by widest available age group
and then for single sex results (m, f) in preference to combined sex results (c).

Results adjusted (AD) for the most potential confounders are then chosen in Sections -1 to -3
(and those which actually differ from the adjusted results in Table 1B2 - 1 are marked 'x' in Section -1)
and results adjusted for the least confounders in Sections -4 to -6. (Those least adjusted results which
actually differ from the most adjusted as marked 'x' in column X in Section -4)
(Results adjusted for an unknown number of confounder(s) are coded as 20.)

Section -7 shows excluded studies, together with the stage (as above) at which no qualifying
results were found.

Section -8 lists the potentially overlapping studies which have been included (1=principal, 2=subsidiary).

Section -9 lists any results which would have been included in preference except that they had data not complete
enough for use in meta-analysis, with their significance (yes/no), if known, and any further comment as entered
on the database.

In addition to those mentioned above, the following fields, levels and abbreviations are used:
* or nk = not known, n = no, y = yes, ot = other
nev = never
all/unspec = all or unspecified, cig+/-ot = cigarettes irrespective of other products (cigar, pipe etc)
MC = manufactured cigarettes, HR = hand-rolled cigarettes
REF: 6-character study reference
NRR: number of the RR on the database within the study
ST : study type (CC = case control, pr or prosp = prospective)
NLC: number of lung cancer cases in whole study
R : risky occupational population (n = no, m = mining, o = other risky)
VB : national cigarette type (V = at least 75% Virginia, bl = at least 75% blended, ot = other)
P : any proxy use
H : full histological confirmation
De : derivation of RR/CI (or = original, st = standard method, ot = other method of estimation)

Table 1B7 - 1

IESLC - Meta-analysis of Current Smoking, Cigarettes (or Any Product if Cigarettes not available), Age <56
All LC types
Most adjusted

REF	NRR	1B2	SEX	AGEL	AGEH	RACE	YF	LC TYPE	LOC	START	ST	NLC	R	VB	P	H	AD	PRODUCT	DENOM	De
BEST	31	x	m	30	49	all	0	all	NAmer	1955	pr	381	n	V	n	n	1	cig only	nev any	ot
COMSTO	15	x	c	25	44	all	-	all	NAmer	1975	ot	258	n	bl	n	n	0	cig+/-ot	nev cigs	st
CPSI	205	x	m	35	54	all	6	all	NAmer	1959	pr	5138	n	bl	n	n	1	cig+/-ot	nev any	ot
CPSI	255	x	f	40	54	all	6	all	NAmer	1959	pr	5138	n	bl	n	n	1	cig+/-ot	nev cigs	ot
CPSII	8	x	m	30	54	all	6	all	NAmer	1982	pr	3229	n	bl	n	n	0	cig only	nev any	st
CPSII	43	x	f	30	54	all	6	all	NAmer	1982	pr	3229	n	bl	n	n	0	cig+/-ot	nev cigs	st
DEAN3	29	x	m	35	44	all	-	all	Eu:UK	1969	CC	766	n	V	y	n	0	cig only	nev any	st
DEAN3	30	x	m	45	54	all	-	all	Eu:UK	1969	CC	766	n	V	y	n	0	cig only	nev any	st
DEAN3	113	x	f	35	44	all	-	all	Eu:UK	1969	CC	766	n	V	y	n	0	cig only	nev any	st
DEAN3	114	x	f	45	54	all	-	all	Eu:UK	1969	CC	766	n	V	y	n	0	cig only	nev any	st
HITOSU	20	x	m	1	49	all	-	all	As:Jap	1960	CC	216	n	bl	y	n	0	all/unsp	nev any	st
HITOSU	41	x	f	1	49	all	-	all	As:Jap	1960	CC	216	n	bl	y	n	0	all/unsp	nev any	st
KANELL	10	x	m	1	49	all	-	all	Eu:bal	1950	CC	862	n	bl	n	n	0	all/unsp	nev any	st
KREUZE	18		m	1	45	all	-	all	Eu:Ger	1990	CC	2260	n	bl	n	n	3	cig+/-ot	nev any	or
KREUZE	24		f	1	45	all	-	all	Eu:Ger	1990	CC	2260	n	bl	n	n	3	cig+/-ot	nev any	or
KUBIK	1	x	m	40	54	all	4	all	Eu:est	1965	pr	108	n	bl	n	n	0	cig+/-ot	nev any	ot
NAM	4	x	m	25	54	all	-	all	NAmer	1986	CC	1199	n	bl	y	n	0	cig+/-ot	nev cigs	st
NAM	36	x	f	25	54	all	-	all	NAmer	1986	CC	1199	n	bl	y	n	0	cig+/-ot	nev cigs	st
SEGI2	53	x	m	1	54	all	-	all	As:Jap	1962	CC	378	n	bl	n	n	0	cig+/-ot	nev any	st
SPEIZE	10		f	30	55	all	0	all	NAmer	1976	pr	593	n	bl	n	y	1	cig+/-ot	nev cigs	ot
TSUGAN	28		m	30	49	all	-	q+a	As:Jap	1976	CC	134	n	bl	n	y	0	all/unsp	nev any	st
TVERDA	5		m	35	49	all	0	all	Eu:Sca	1972	pr	238	n	bl	n	n	2	cig+/-ot	nev cigs	ot
TVERDA	15		f	35	49	all	0	all	Eu:Sca	1972	pr	238	n	bl	n	n	2	cig only	nev cigs	ot
VUTUC	12	x	m	1	39	all	-	all	Eu:wst	1976	CC	1877	n	bl	n	n	0	cig+/-ot	nev cigs	st
VUTUC	35	x	m	41	50	all	-	all	Eu:wst	1976	CC	1877	n	bl	n	n	0	cig+/-ot	nev cigs	st

Cigarette type is all/unspec for all RRs
except for the following:

REF	NRR	CIGTYPE
DEAN3	29	MC only
DEAN3	30	MC only
DEAN3	113	MC only
DEAN3	114	MC only

Table 1B7 - 2

IESLC - Meta-analysis of Current Smoking, Cigarettes (or Any Product if Cigarettes not available), Age <56
All LC types
Most adjusted

REF	NRR	SEX	AD	Number Exposed Case	Number Exposed Cont	Non-exposed Case	Non-exposed Cont	RR	95.00%CI
*BEST	31	m	1	-	-	-	-	3.93	(0.53- 29.13)
COMSTO	15	c	0	24	27	1	17	15.11	(1.87- 122.22)
*CPSI	205	m	1	-	-	-	-	9.48	(6.11- 14.71)
*CPSI	255	f	1	-	-	-	-	3.85	(2.68- 5.52)
Subtotal CPSI								5.54	(4.19- 7.32)
*CPSII	8	m	0	167	225147	12	248565	15.36	(8.55- 27.60)
*CPSII	43	f	0	141	323063	23	670327	12.72	(8.19- 19.77)
Subtotal CPSII								13.62	(9.58- 19.37)
DEAN3	29	m	0	7	263	1	187	4.98	(0.61- 40.79)
DEAN3	30	m	0	44	311	1	145	20.51	(2.80- 150.36)
DEAN3	113	f	0	6	409	1	328	4.81	(0.58- 40.17)
DEAN3	114	f	0	22	404	4	326	4.44	(1.51- 13.01)
Subtotal DEAN3								5.84	(2.63- 12.99)
HITOSU	20	m	0	7	916	1	118	0.90	(0.11- 7.39)
HITOSU	41	f	0	3	225	7	1108	2.11	(0.54- 8.22)
Subtotal HITOSU								1.64	(0.52- 5.15)
KANELL	10	m	0	65	136	9	60	3.19	(1.49- 6.82)
KREUZE	18	m	3	-	-	-	-	15.90	(6.50- 38.50)
KREUZE	24	f	3	-	-	-	-	29.90	(9.50- 94.60)
Subtotal KREUZE								20.14	(9.97- 40.70)
*KUBIK	1	m	0	10	3688	0	2420	13.78~	(0.81- 235.06)
NAM	4	m	0	31	353	1	176	15.46	(2.09- 114.15)
NAM	36	f	0	24	99	2	93	11.27	(2.59- 49.03)
Subtotal NAM								12.59	(3.85- 41.16)
SEGI2	53	m	0	92	169	2	18	4.90	(1.11- 21.58)
*SPEIZE	10	f	1	-	-	-	-	12.69	(9.97- 16.16)
TSUGAN	28	m	0	63	63	18	22	1.22	(0.60- 2.50)
*TVERDA	5	m	2	-	-	-	-	4.09	(2.65- 6.31)
*TVERDA	15	f	2	-	-	-	-	11.05	(3.33- 36.71)
Subtotal TVERDA								4.59	(3.05- 6.90)
VUTUC	12	m	0	12	15	1	9	7.20	(0.80- 65.05)
VUTUC	35	m	0	74	104	11	64	4.14	(2.04- 8.38)
Subtotal VUTUC								4.36	(2.23- 8.54)
Partial Totals				792	555392	95	923983		

*prospective study ~ With 0.5 adjustment for zero

REF	NRR	SEX	AD	Ys	Ws	Qs	Ps
*BEST	31	m	1	1.37	0.96	0.43	0.1806
COMSTO	15	c	0	2.72	0.88	0.40	0.0109
*CPSI	205	m	1	2.25	19.91	0.87	0.0000
*CPSI	255	f	1	1.35	29.43	14.11	0.0000
Subtotal CPSI				1.71	49.34	14.98	
*CPSII	8	m	0	2.73	11.20	5.36	0.0000
*CPSII	43	f	0	2.54	19.78	5.00	0.0000
Subtotal CPSII				2.61	30.97	10.35	
DEAN3	29	m	0	1.60	0.87	0.16	0.1349
DEAN3	30	m	0	3.02	0.97	0.93	0.0030
DEAN3	113	f	0	1.57	0.85	0.19	0.1468
DEAN3	114	f	0	1.49	3.32	1.01	0.0066
Subtotal DEAN3				1.76	6.01	2.29	
HITOSU	20	m	0	-0.10	0.87	3.99	0.9233
HITOSU	41	f	0	0.75	2.08	3.47	0.2818
Subtotal HITOSU				0.50	2.94	7.46	
KANELL	10	m	0	1.16	6.64	5.16	0.0028
KREUZE	18	m	3	2.77	4.86	2.56	0.0000
KREUZE	24	f	3	3.40	2.91	5.36	0.0000
Subtotal KREUZE				3.00	7.76	7.92	
*KUBIK	1	m	0	2.62	0.48	0.16	0.0699
NAM	4	m	0	2.74	0.96	0.47	0.0073
NAM	36	f	0	2.42	1.78	0.26	0.0012
Subtotal NAM				2.53	2.74	0.73	
SEGI2	53	m	0	1.59	1.75	0.36	0.0357
*SPEIZE	10	f	1	2.54	65.88	16.49	0.0000
TSUGAN	28	m	0	0.20	7.53	25.50	0.5818
*TVERDA	5	m	2	1.41	20.41	8.15	0.0000
*TVERDA	15	f	2	2.40	2.67	0.35	0.0001
Subtotal TVERDA				1.52	23.08	8.50	
VUTUC	12	m	0	1.97	0.79	0.00	0.0788
VUTUC	35	m	0	1.42	7.71	2.96	0.0001
Subtotal VUTUC				1.47	8.51	2.97	

Table 1B7 - 2

IESLC - Meta-analysis of Current Smoking, Cigarettes (or Any Product if Cigarettes not available), Age <56
All LC types
Most adjusted

	N	25
	NS	15
	Wt	215.47
Het	Chi	103.70
Het	df	24
Het	P	***
Fixed	RR	7.69
	RRl	6.73
	RRu	8.79
	P	+++
Random	RR	6.73
	RRl	4.76
	RRu	9.53
	P	+++
Asymm	P	N.S.

Table 1B7 - 3

IESLC - Meta-analysis of Current Smoking, Cigarettes (or Any Product if Cigarettes not available), Age <56
All LC types
Most adjusted

		Sex		
	combined	male	female	Total
N	1	15	9	25
NS	1	13	8	22
Wt	0.88	85.90	128.69	215.47
Het Chi	0.00	50.47	42.05	103.70
Het df	0	14	8	24
Het P	N.S.	***	***	***
Fixed RR	15.11	5.83	9.21	7.69
RRl	1.87	4.72	7.75	6.73
RRu	122.22	7.21	10.95	8.79
P	+	+++	+++	+++
Random RR	15.11	5.70	8.19	6.73
RRl	1.87	3.52	4.83	4.76
RRu	122.22	9.24	13.89	9.53
P	+	+++	+++	+++
Between Chi				11.18
Between df				2
Between P				**
Btwn(F) P				N.S.
Btwn(R) P				N.S.

Table 1B7 - 4

IESLC - Meta-analysis of Current Smoking, Cigarettes (or Any Product if Cigarettes not available), Age <56
All LC types
Least adjusted

REF	NRR	X	SEX	AGEL	AGEH	RACE	YF	LC TYPE	LOC	START	ST	NLC	R	VB	P	H	AD	PRODUCT	DENOM	De
BEST	31		m	30	49	all	0	all	NAmer	1955	pr	381	n	V	n	n	1	cig only	nev any	ot
COMSTO	15		c	25	44	all	-	all	NAmer	1975	ot	258	n	bl	n	n	0	cig+/-ot	nev cigs	st
CPSI	205		m	35	54	all	6	all	NAmer	1959	pr	5138	n	bl	n	n	1	cig+/-ot	nev any	ot
CPSI	255		f	40	54	all	6	all	NAmer	1959	pr	5138	n	bl	n	n	1	cig+/-ot	nev cigs	ot
CPSII	8		m	30	54	all	6	all	NAmer	1982	pr	3229	n	bl	n	n	0	cig only	nev any	st
CPSII	43		f	30	54	all	6	all	NAmer	1982	pr	3229	n	bl	n	n	0	cig+/-ot	nev cigs	st
DEAN3	29		m	35	44	all	-	all	Eu:UK	1969	CC	766	n	V	y	n	0	cig only	nev any	st
DEAN3	30		m	45	54	all	-	all	Eu:UK	1969	CC	766	n	V	y	n	0	cig only	nev any	st
DEAN3	113		f	35	44	all	-	all	Eu:UK	1969	CC	766	n	V	y	n	0	cig only	nev any	st
DEAN3	114		f	45	54	all	-	all	Eu:UK	1969	CC	766	n	V	y	n	0	cig only	nev any	st
HITOSU	20		m	1	49	all	-	all	As:Jap	1960	CC	216	n	bl	y	n	0	all/unsp	nev any	st
HITOSU	41		f	1	49	all	-	all	As:Jap	1960	CC	216	n	bl	y	n	0	all/unsp	nev any	st
KANELL	10		m	1	49	all	-	all	Eu:bal	1950	CC	862	n	bl	n	n	0	all/unsp	nev any	st
KREUZE	5	x	m	1	45	all	-	all	Eu:Ger	1990	CC	2260	n	bl	n	n	0	cig+/-ot	nev any	st
KREUZE	6	x	f	1	45	all	-	all	Eu:Ger	1990	CC	2260	n	bl	n	n	0	cig+/-ot	nev any	st
KUBIK	1		m	40	54	all	4	all	Eu:est	1965	pr	108	n	bl	n	n	0	cig+/-ot	nev any	ot
NAM	4		m	25	54	all	-	all	NAmer	1986	CC	1199	n	bl	y	n	0	cig+/-ot	nev cigs	st
NAM	36		f	25	54	all	-	all	NAmer	1986	CC	1199	n	bl	y	n	0	cig+/-ot	nev cigs	st
SEGI2	53		m	1	54	all	-	all	As:Jap	1962	CC	378	n	bl	n	n	0	cig+/-ot	nev any	st
SPEIZE	6	x	f	30	55	all	0	all	NAmer	1976	pr	593	n	bl	n	y	0	cig+/-ot	nev cigs	st
TSUGAN	28		m	30	49	all	-	q+a	As:Jap	1976	CC	134	n	bl	n	y	0	all/unsp	nev any	st
TVERDA	5		m	35	49	all	0	all	Eu:Sca	1972	pr	238	n	bl	n	n	2	cig+/-ot	nev cigs	ot
TVERDA	15		f	35	49	all	0	all	Eu:Sca	1972	pr	238	n	bl	n	n	2	cig only	nev cigs	ot
VUTUC	12		m	1	39	all	-	all	Eu:wst	1976	CC	1877	n	bl	n	n	0	cig+/-ot	nev cigs	st
VUTUC	35		m	41	50	all	-	all	Eu:wst	1976	CC	1877	n	bl	n	n	0	cig+/-ot	nev cigs	st

Cigarette type is all/unspec for all RRs
except for the following:

REF	NRR	CIGTYPE
DEAN3	29	MC only
DEAN3	30	MC only
DEAN3	113	MC only
DEAN3	114	MC only

Table 1B7 - 5

IESLC - Meta-analysis of Current Smoking, Cigarettes (or Any Product if Cigarettes not available), Age <56
All LC types
Least adjusted

REF	NRR	SEX	AD	Number Exposed Case	Number Exposed Cont	Non-exposed Case	Non-exposed Cont	RR	95.00%CI
*BEST	31	m	1	-	-	-	-	3.93	(0.53- 29.13)
COMSTO	15	c	0	24	27	1	17	15.11	(1.87- 122.22)
*CPSI	205	m	1	-	-	-	-	9.48	(6.11- 14.71)
*CPSI	255	f	1	-	-	-	-	3.85	(2.68- 5.52)
Subtotal CPSI								5.54	(4.19- 7.32)
*CPSII	8	m	0	167	225147	12	248565	15.36	(8.55- 27.60)
*CPSII	43	f	0	141	323063	23	670327	12.72	(8.19- 19.77)
Subtotal CPSII								13.62	(9.58- 19.37)
DEAN3	29	m	0	7	263	1	187	4.98	(0.61- 40.79)
DEAN3	30	m	0	44	311	1	145	20.51	(2.80- 150.36)
DEAN3	113	f	0	6	409	1	328	4.81	(0.58- 40.17)
DEAN3	114	f	0	22	404	4	326	4.44	(1.51- 13.01)
Subtotal DEAN3								5.84	(2.63- 12.99)
HITOSU	20	m	0	7	916	1	118	0.90	(0.11- 7.39)
HITOSU	41	f	0	3	225	7	1108	2.11	(0.54- 8.22)
Subtotal HITOSU								1.64	(0.52- 5.15)
KANELL	10	m	0	65	136	9	60	3.19	(1.49- 6.82)
KREUZE	5	m	0	168	93	6	54	16.26	(6.74- 39.22)
KREUZE	6	f	0	55	22	6	38	15.83	(5.87- 42.73)
Subtotal KREUZE								16.07	(8.32- 31.06)
*KUBIK	1	m	0	10	3688	0	2420	13.78~	(0.81- 235.06)
NAM	4	m	0	31	353	1	176	15.46	(2.09- 114.15)
NAM	36	f	0	24	99	2	93	11.27	(2.59- 49.03)
Subtotal NAM								12.59	(3.85- 41.16)
SEGI2	53	m	0	92	169	2	18	4.90	(1.11- 21.58)
*SPEIZE	6	f	0	391	489993	58	776300	10.68	(8.11- 14.07)
TSUGAN	28	m	0	63	63	18	22	1.22	(0.60- 2.50)
*TVERDA	5	m	2	-	-	-	-	4.09	(2.65- 6.31)
*TVERDA	15	f	2	-	-	-	-	11.05	(3.33- 36.71)
Subtotal TVERDA								4.59	(3.05- 6.90)
VUTUC	12	m	0	12	15	1	9	7.20	(0.80- 65.05)
VUTUC	35	m	0	74	104	11	64	4.14	(2.04- 8.38)
Subtotal VUTUC								4.36	(2.23- 8.54)
Partial Totals				1406	1045500	165	1700375		

*prospective study ~ With 0.5 adjustment for zero

REF	NRR	SEX	AD	Ys	Ws	Qs	Ps
*BEST	31	m	1	1.37	0.96	0.33	0.1806
COMSTO	15	c	0	2.72	0.88	0.51	0.0109
*CPSI	205	m	1	2.25	19.91	1.73	0.0000
*CPSI	255	f	1	1.35	29.43	10.81	0.0000
Subtotal CPSI				1.71	49.34	12.55	
*CPSII	8	m	0	2.73	11.20	6.77	0.0000
*CPSII	43	f	0	2.54	19.78	6.86	0.0000
Subtotal CPSII				2.61	30.97	13.63	
DEAN3	29	m	0	1.60	0.87	0.11	0.1349
DEAN3	30	m	0	3.02	0.97	1.10	0.0030
DEAN3	113	f	0	1.57	0.85	0.13	0.1468
DEAN3	114	f	0	1.49	3.32	0.72	0.0066
Subtotal DEAN3				1.76	6.01	2.05	
HITOSU	20	m	0	-0.10	0.87	3.67	0.9233
HITOSU	41	f	0	0.75	2.08	3.03	0.2818
Subtotal HITOSU				0.50	2.94	6.70	
KANELL	10	m	0	1.16	6.64	4.20	0.0028
KREUZE	5	m	0	2.79	4.95	3.45	0.0000
KREUZE	6	f	0	2.76	3.90	2.54	0.0000
Subtotal KREUZE				2.78	8.85	5.99	
*KUBIK	1	m	0	2.62	0.48	0.21	0.0699
NAM	4	m	0	2.74	0.96	0.59	0.0073
NAM	36	f	0	2.42	1.78	0.39	0.0012
Subtotal NAM				2.53	2.74	0.98	
SEGI2	53	m	0	1.59	1.75	0.23	0.0357
*SPEIZE	6	f	0	2.37	50.52	8.67	0.0000
TSUGAN	28	m	0	0.20	7.53	23.16	0.5818
*TVERDA	5	m	2	1.41	20.41	6.08	0.0000
*TVERDA	15	f	2	2.40	2.67	0.54	0.0001
Subtotal TVERDA				1.52	23.08	6.61	
VUTUC	12	m	0	1.97	0.79	0.00	0.0788
VUTUC	35	m	0	1.42	7.71	2.20	0.0001
Subtotal VUTUC				1.47	8.51	2.20	

Table 1B7 - 5

IESLC - Meta-analysis of Current Smoking, Cigarettes (or Any Product if Cigarettes not available), Age <56
All LC types
Least adjusted

	N	25
	NS	15
	Wt	201.19
Het	Chi	88.03
Het	df	24
Het	P	***
Fixed	RR	7.06
	RRl	6.15
	RRu	8.10
	P	+++
Random	RR	6.52
	RRl	4.70
	RRu	9.04
	P	+++
Asymm	P	N.S.

Table 1B7 - 6

IESLC - Meta-analysis of Current Smoking, Cigarettes (or Any Product if Cigarettes not available), Age <56
All LC types
Least adjusted

		Sex		
	combined	male	female	Total
N	1	15	9	25
NS	1	13	8	22
Wt	0.88	86.00	114.32	201.19
Het Chi	0.00	50.79	31.57	88.03
Het df	0	14	8	24
Het P	N.S.	***	***	***
Fixed RR	15.11	5.85	8.09	7.06
RRl	1.87	4.73	6.73	6.15
RRu	122.22	7.22	9.71	8.10
P	+	+++	+++	+++
Random RR	15.11	5.71	7.60	6.52
RRl	1.87	3.52	4.74	4.70
RRu	122.22	9.27	12.16	9.04
P	+	+++	+++	+++
Between Chi				5.67
Between df				2
Between P				(*)
Btwn(F) P				N.S.
Btwn(R) P				N.S.

Table 1B7 - 7

IESLC - Meta-analysis of Current Smoking, Cigarettes (or Any Product if Cigarettes not available), Age <56

All LC types

Excluded studies (and stage at which they were excluded)

1 BOUCHA BUELL LAURIL MZILEN

2 ABELIN ABRAHA AUVINE AXELSO BAND BERRIN BLOT1 BLOT2 BLOT3 BLOT4 BRESLO BROCKM BROWN1 BYERS1 CASCO2 CASCOR CHAN CHATZI CHEN CHEN2 CHEN3 CHIAZZ COOKSO DAVEYS DEAN DOSEME DU DUNN EBELIN ESAKI FAN GENG GER GODLEY GOLLED GSELL HANSEN HEGMAN HINDS HIRAOK HOROWI HORWIT HU HU2 HUANG ISHIMA JARUP JIANG JONES JUSSAW KO KOHLME KOULUM KREYBE LAMTH LAMWK LAMWK2 LAUSSM LEI LETOUR LEVIN LIU LIU2 LIU3 LIU4 LIU5 LOMBA2 LUO MAGNUS MARSH MARSH2 MARTIS MASTRA MATSUD MCCONN MCDUFF MCLAUG MILLER MILLS MOLLO NOTAN2 NOU ORMOS PASTOR PAWLEG PERNU PERSHA PIKE POFFIJ POLEDN PRESCO QIAO RADZIK RANDIG REN RONCO ROOTS ROTHSC SADOWS SANKAR SCHWA2 SEGI SEOW SIEMIA SIMARA STASZE STOCKS SUN TAO ULMER VANDER WANG WANG3 WANG4 WICKLU WILKIN WUWILL WYNDE2 WYNDE4 XIANGZ XU XU2 XU3 XU4 YUAN ZHANG ZHENG ZHOU

3 AGUDO AKIBA ALDERS AMANDU AMES ANDERS ARCHER ARMADA AUSTIN AXELSS BARBON BECHER BENHAM BENSHL BLOHMK BOFFET BOUCOT BRETT BROSS BROWN2 BUFFLE BYERS2 CARPEN CEDERL CHANG CHOI CHOW CHYOU CORREA DAMBER DARBY DEAN2 DEKLER DESTE2 DESTEF DOCKER DOLL DOLL2 DORANT DORGAN DORN DROSTE ENGELA ENSTRO GAO GAO2 GARCIA GARDIN GARSHI GILLIS GOODMA GRAHAM GREGOR HAENSZ HAMMO2 HAMMON HEIN HENNEK HIRAY2 HUMBLE JAHN JAIN JARVHO JEDRYC JOLY KAISER KATSOU KAUFMA KELLER KHUDER KIHARA KINLEN KJUUS KNEKT KOO LANGE LEMARC LIAW LICKIN LIDDEL LOMBAR LUBIN LUBIN2 MACLEN MATOS MIGRAN MRFIT MRFITR MURATA NOTANI ODRISC OSANN OSANN2 PARKIN PERSH2 PETO PEZZO2 PEZZOT PISANI QIAO2 RACHTA RESTRE RIMING SAARIK SCHWAR SHAW SHIMIZ SIMONA SITAS SOBUE SOBUE2 SPITZ STAYNE STOCKW STUCKE SUZUK2 SUZUKI SVENSS TANG TANG2 TENKAN TIZZAN TOKARS TOUSEY TULINI VEIERO WAKAI WALD WANG2 WARSIN WATSON WIGLE WU WU2 WUNSCH WYNDE3 WYNDE5 WYNDE6 WYNDE7 WYNDE8 WYNDER YAMAGU YONG

4 HIRAYA

6 KAISE2

7 HOLE

Table 1B7 - 8

Potentially overlapping studies

REF	REFGP	PRINC	.	OVERLAP/LINK
VUTUC	LUBIN2	2		Subset of Lubin2
TVERDA	TVERDA	1		VEIERO/TVERDAL
CPSI	CPSI	1		CPSI overall

Table 1B7 - 9

Most adjusted - insufficient data for metaanalysis

REF	NRR	SEX	AGEL	AGEH	RACE	YF	LC TYPE	LOC	START	ST	NLC	R	VB	P	H	AD	PRODUCT	DENOM	De
HIRAYA	136	m	40	49	all	8	all	As:Jap	1965	pr	1917	n	bl	n	n	1	cig+/-ot	nev any	or

REF	NRR	RR	SIG	RRDATA comment
HIRAYA	136	2.08		0

Table 1B8 -

IESLC - Meta-analysis of Current Smoking, Cigarettes (or Any Product if Cigarettes not available), Age 50-70
All LC types

This analysis is restricted to results for:
1) Non-dose-response data
2) Current smokers
3) Maximum age range 50-70
4) Results complete enough for use in metaanalysis

Within each study, results are then selected (in the following order of preference, within each sex) for:
5) PRODUCT: cigarettes regardless of other products, cigarettes only, all/unspec
6) CIGTYPE: all/unspecified, MC regardless of HR, MC only
7) DENOM: never smoked anything, never smoked cigarettes, (never +1 = +long term ex, +2 = +amount unknown,
+3 = never cigs+long term ex)
8) Followup period (YF, prospective studies): whole study (coded as 0) or longest available
9) LCtype: all or nearest available, at least Squamous and Adeno. (q = squamous, s = small,
l = large, a = adeno, mix = mixed, alv = alveolar)
10) Race: all or nearest available, otherwise by race (wh or w = white, bl or b = black, hi = hispanic
ch = chinese, jap = japanese, haw = hawaiian, w+o = white + oriental, sca = scandinavian, as = asian)
11) For overlapping studies: principal rather than subsidiary studies
Finally by Age: whole study (actual age shown) if available, otherwise by widest available age group
and then for single sex results (m, f) in preference to combined sex results (c).

Results adjusted (AD) for the most potential confounders are then chosen in Sections -1 to -3
(and those which actually differ from the adjusted results in Table 1B2 - 1 are marked 'x' in Section -1)
and results adjusted for the least confounders in Sections -4 to -6. (Those least adjusted results which
actually differ from the most adjusted as marked 'x' in column X in Section -4)
(Results adjusted for an unknown number of confounder(s) are coded as 20.)

Section -7 shows excluded studies, together with the stage (as above) at which no qualifying
results were found.

Section -8 lists the potentially overlapping studies which have been included (1=principal, 2=subsidiary).

Section -9 lists any results which would have been included in preference except that they had data not complete
enough for use in meta-analysis, with their significance (yes/no), if known, and any further comment as entered
on the database.

In addition to those mentioned above, the following fields, levels and abbreviations are used:
* or nk = not known, n = no, y = yes, ot = other
nev = never
all/unspec = all or unspecified, cig+/-ot = cigarettes irrespective of other products (cigar, pipe etc)
MC = manufactured cigarettes, HR = hand-rolled cigarettes
REF: 6-character study reference
NRR: number of the RR on the database within the study
ST : study type (CC = case control, pr or prosp = prospective)
NLC: number of lung cancer cases in whole study
R : risky occupational population (n = no, m = mining, o = other risky)
VB : national cigarette type (V = at least 75% Virginia, bl = at least 75% blended, ot = other)
P : any proxy use
H : full histological confirmation
De : derivation of RR/CI (or = original, st = standard method, ot = other method of estimation)

Table 1B8 - 1

IESLC - Meta-analysis of Current Smoking, Cigarettes (or Any Product if Cigarettes not available), Age 50-70
All LC types
Most adjusted

REF	NRR	1B2	SEX	AGEL	AGEH	RACE	YF	LC TYPE	LOC	START	ST	NLC	R	VB	P	H	AD	PRODUCT	DENOM	De
ANDERS	6		f	55	69	all	0	all	NAmer	1986	pr	343	n	bl	n	n	1	cig+/-ot	nev cigs	or
BEST	32	x	m	50	69	all	0	all	NAmer	1955	pr	381	n	V	n	n	1	cig only	nev any	ot
CEDERL	10	x	m	50	59	all	10	all	Eu:Sca	1963	pr	491	n	bl	n	n	0	cig+/-ot	nev any	st
CEDERL	18	x	m	60	69	all	10	all	Eu:Sca	1963	pr	491	n	bl	n	n	0	cig+/-ot	nev any	st
CEDERL	35	x	f	50	59	all	10	all	Eu:Sca	1963	pr	491	n	bl	n	n	0	cig only	nev any	st
CEDERL	38	x	f	60	69	all	10	all	Eu:Sca	1963	pr	491	n	bl	n	n	0	cig only	nev any	st
CPSI	210	x	m	55	69	all	6	all	NAmer	1959	pr	5138	n	bl	n	n	1	cig+/-ot	nev any	ot
CPSI	103	x	f	55	64	wh	0	all	NAmer	1959	pr	5138	n	bl	n	n	0	cig only	nev any	st
CPSII	15	x	m	55	64	all	6	all	NAmer	1982	pr	3229	n	bl	n	n	0	cig only	nev any	st
CPSII	50	x	f	55	64	all	6	all	NAmer	1982	pr	3229	n	bl	n	n	0	cig+/-ot	nev cigs	st
DEAN3	31	x	m	55	64	all	-	all	Eu:UK	1969	CC	766	n	V	y	n	0	cig only	nev any	st
DEAN3	115	x	f	55	64	all	-	all	Eu:UK	1969	CC	766	n	V	y	n	0	cig only	nev any	st
DORANT	9		c	55	69	all	0	all	Eu:wst	1986	ot	550	n	bl	n	y	0	cig+/-ot	nev any	st
DORN	366	x	m	55	64	wh	25	all	NAmer	1954	pr	5097	n	bl	n	n	1	cig+/-ot	nev any	or
HAMMON	139		m	50	69	wh	0	all	NAmer	1952	pr	448	n	bl	n	n	1	cig only	nev any	ot
HITOSU	47	x	f	50	59	all	-	all	As:Jap	1960	CC	216	n	bl	y	n	0	all/unsp	nev any	st
KANELL	15	x	m	50	59	all	-	all	Eu:bal	1950	CC	862	n	bl	n	n	0	all/unsp	nev any	st
KANELL	20	x	m	60	69	all	-	all	Eu:bal	1950	CC	862	n	bl	n	n	0	all/unsp	nev any	st
KREUZE	29		m	55	69	all	-	all	Eu:Ger	1990	CC	2260	n	bl	n	n	3	cig+/-ot	nev any	or
KREUZE	35		f	55	69	all	-	all	Eu:Ger	1990	CC	2260	n	bl	n	n	3	cig+/-ot	nev any	or
KUBIK	2	x	m	55	64	all	4	all	Eu:est	1965	pr	108	n	bl	n	n	0	cig+/-ot	nev any	st
NAM	12	x	m	55	64	all	-	all	NAmer	1986	CC	1199	n	bl	y	n	0	cig+/-ot	nev cigs	st
NAM	44	x	f	55	64	all	-	all	NAmer	1986	CC	1199	n	bl	y	n	0	cig+/-ot	nev cigs	st
SEGI2	54	x	m	55	64	all	-	all	As:Jap	1962	CC	378	n	bl	n	n	0	cig+/-ot	nev any	st

Cigarette type is all/unspec for all RRs
except for the following:

REF	NRR	CIGTYPE
DEAN3	31	MC only
DEAN3	115	MC only

Table 1B8 - 2

IESLC - Meta-analysis of Current Smoking, Cigarettes (or Any Product if Cigarettes not available), Age 50-70
All LC types
Most adjusted

REF	NRR	SEX	AD	Number Exposed Case	Number Exposed Cont	Non-exposed Case	Non-exposed Cont	RR	95.00%CI
*ANDERS	6	f	1	-	-	-	-	23.43	(17.02- 32.27)
*BEST	32	m	1	-	-	-	-	23.30	(7.45- 72.88)
*CEDERL	10	m	0	21	2441	4	1954	4.20	(1.44- 12.22)
*CEDERL	18	m	0	30	1338	3	1645	12.29	(3.76- 40.20)
*CEDERL	35	f	0	4	1159	9	6407	2.46	(0.76- 7.96)
*CEDERL	38	f	0	4	407	10	5877	5.78	(1.82- 18.34)
Subtotal CEDERL								5.14	(2.90- 9.10)
*CPSI	210	m	1	-	-	-	-	14.22	(10.37- 19.52)
*CPSI	103	f	0	249	591967	144	1366561	3.99	(3.25- 4.90)
Subtotal CPSI								5.82	(4.90- 6.91)
*CPSII	15	m	0	641	231659	21	253604	33.42	(21.64- 51.61)
*CPSII	50	f	0	390	267703	67	686189	14.92	(11.51- 19.33)
Subtotal CPSII								18.43	(14.76- 23.03)
DEAN3	31	m	0	129	211	7	96	8.38	(3.78- 18.62)
DEAN3	115	f	0	36	217	9	310	5.71	(2.70- 12.11)
Subtotal DEAN3								6.84	(3.96- 11.82)
DORANT	9	c	0	292	876	14	1090	25.95	(15.07- 44.69)
*DORN	366	m	1	-	-	-	-	11.10	(9.78- 12.61)
*HAMMON	139	m	1	-	-	-	-	11.52	(6.83- 19.42)
HITOSU	47	f	0	6	148	8	478	2.42	(0.83- 7.09)
KANELL	15	m	0	354	112	9	38	13.35	(6.26- 28.45)
KANELL	20	m	0	336	127	16	35	5.79	(3.10- 10.82)
Subtotal KANELL								8.12	(5.02- 13.16)
KREUZE	29	m	3	-	-	-	-	41.90	(27.10- 64.60)
KREUZE	35	f	3	-	-	-	-	6.40	(4.20- 9.60)
Subtotal KREUZE								15.63	(11.59- 21.09)
*KUBIK	2	m	0	45	2654	1	1851	31.38	(4.33- 227.48)
NAM	12	m	0	83	88	5	42	7.92	(2.99- 20.99)
NAM	44	f	0	28	48	10	69	4.03	(1.79- 9.05)
Subtotal NAM								5.31	(2.85- 9.90)
SEGI2	54	m	0	111	193	2	20	5.75	(1.32- 25.07)
Partial Totals				2759	1101348	339	2326266		

*prospective study

REF	NRR	SEX	AD	Ys	Ws	Qs	Ps
*ANDERS	6	f	1	3.15	37.54	22.92	0.0000
*BEST	32	m	1	3.15	2.95	1.78	0.0000
*CEDERL	10	m	0	1.44	3.37	2.96	0.0084
*CEDERL	18	m	0	2.51	2.74	0.05	0.0000
*CEDERL	35	f	0	0.90	2.78	6.03	0.1341
*CEDERL	38	f	0	1.75	2.88	1.10	0.0029
Subtotal CEDERL				1.64	11.76	10.15	
*CPSI	210	m	1	2.65	38.41	3.05	0.0000
*CPSI	103	f	0	1.38	91.26	89.17	0.0000
Subtotal CPSI				1.76	129.66	92.22	
*CPSII	15	m	0	3.51	20.34	26.26	0.0000
*CPSII	50	f	0	2.70	57.19	6.23	0.0000
Subtotal CPSII				2.91	77.53	32.49	
DEAN3	31	m	0	2.13	6.03	0.37	0.0000
DEAN3	115	f	0	1.74	6.82	2.70	0.0000
Subtotal DEAN3				1.92	12.85	3.07	
DORANT	9	c	0	3.26	13.00	10.15	0.0000
*DORN	366	m	1	2.41	237.89	0.28	0.0000
*HAMMON	139	m	1	2.44	14.07	0.07	0.0000
HITOSU	47	f	0	0.88	3.33	7.37	0.1066
KANELL	15	m	0	2.59	6.70	0.32	0.0000
KANELL	20	m	0	1.76	9.81	3.74	0.0000
Subtotal KANELL				2.09	16.51	4.06	
KREUZE	29	m	3	3.74	20.36	37.80	0.0000
KREUZE	35	f	3	1.86	22.48	6.00	0.0000
Subtotal KREUZE				2.75	42.85	43.80	
*KUBIK	2	m	0	3.45	0.98	1.13	0.0006
NAM	12	m	0	2.07	4.04	0.37	0.0000
NAM	44	f	0	1.39	5.85	5.62	0.0008
Subtotal NAM				1.67	9.89	5.99	
SEGI2	54	m	0	1.75	1.77	0.69	0.0199

Table 1B8 - 2

IESLC - Meta-analysis of Current Smoking, Cigarettes (or Any Product if Cigarettes not available), Age 50-70
All LC types
Most adjusted

	N	24
	NS	15
	Wt	612.60
Het	Chi	236.15
Het	df	23
Het	P	***
Fixed	RR	10.73
	RRl	9.91
	RRu	11.61
	P	+++
Random	RR	10.18
	RRl	7.50
	RRu	13.83
	P	+++
Asymm	P	N.S.

Table 1B8 - 3

IESLC - Meta-analysis of Current Smoking, Cigarettes (or Any Product if Cigarettes not available), Age 50-70
All LC types
Most adjusted

	Sex: combined	male	female	Total
N	1	14	9	24
NS	1	12	8	21
Wt	13.00	369.47	230.12	612.60
Het Chi	0.00	68.13	123.04	236.15
Het df	0	13	8	23
Het P	N.S.	***	***	***
Fixed RR	25.95	12.72	7.76	10.73
RRl	15.07	11.49	6.82	9.91
RRu	44.69	14.09	8.83	11.61
P	+++	+++	+++	+++
Random RR	25.95	13.22	6.22	10.18
RRl	15.07	9.40	3.45	7.50
RRu	44.69	18.59	11.19	13.83
P	+++	+++	+++	+++
Between Chi				44.98
Between df				2
Between P				***
Btwn(F) P				N.S.
Btwn(R) P				**

Table 1B8 - 4

IESLC - Meta-analysis of Current Smoking, Cigarettes (or Any Product if Cigarettes not available), Age 50-70
All LC types
Least adjusted

REF	NRR	X	SEX	AGEL	AGEH	RACE	YF	LC TYPE	LOC	START	ST	NLC	R	VB	P	H	AD	PRODUCT	DENOM	De
ANDERS	2	x	f	55	69	all	0	all	NAmer	1986	pr	343	n	bl	n	n	0	cig+/-ot	nev cigs	st
BEST	32		m	50	69	all	0	all	NAmer	1955	pr	381	n	V	n	n	1	cig only	nev any	ot
CEDERL	10		m	50	59	all	10	all	Eu:Sca	1963	pr	491	n	bl	n	n	0	cig+/-ot	nev any	st
CEDERL	18		m	60	69	all	10	all	Eu:Sca	1963	pr	491	n	bl	n	n	0	cig+/-ot	nev any	st
CEDERL	35		f	50	59	all	10	all	Eu:Sca	1963	pr	491	n	bl	n	n	0	cig only	nev any	st
CEDERL	38		f	60	69	all	10	all	Eu:Sca	1963	pr	491	n	bl	n	n	0	cig only	nev any	st
CPSI	210		m	55	69	all	6	all	NAmer	1959	pr	5138	n	bl	n	n	1	cig+/-ot	nev any	ot
CPSI	103		f	55	64	wh	0	all	NAmer	1959	pr	5138	n	bl	n	n	0	cig only	nev any	st
CPSII	15		m	55	64	all	6	all	NAmer	1982	pr	3229	n	bl	n	n	0	cig only	nev any	st
CPSII	50		f	55	64	all	6	all	NAmer	1982	pr	3229	n	bl	n	n	0	cig+/-ot	nev cigs	st
DEAN3	31		m	55	64	all	-	all	Eu:UK	1969	CC	766	n	V	y	n	0	cig only	nev any	st
DEAN3	115		f	55	64	all	-	all	Eu:UK	1969	CC	766	n	V	y	n	0	cig only	nev any	st
DORANT	9		c	55	69	all	0	all	Eu:wst	1986	ot	550	n	bl	n	y	0	cig+/-ot	nev any	st
DORN	366		m	55	64	wh	25	all	NAmer	1954	pr	5097	n	bl	n	n	1	cig+/-ot	nev any	or
HAMMON	139		m	50	69	wh	0	all	NAmer	1952	pr	448	n	bl	n	n	1	cig only	nev any	ot
HITOSU	47		f	50	59	all	-	all	As:Jap	1960	CC	216	n	bl	y	n	0	all/unsp	nev any	st
KANELL	15		m	50	59	all	-	all	Eu:bal	1950	CC	862	n	bl	n	n	0	all/unsp	nev any	st
KANELL	20		m	60	69	all	-	all	Eu:bal	1950	CC	862	n	bl	n	n	0	all/unsp	nev any	st
KREUZE	7	x	m	55	69	all	-	all	Eu:Ger	1990	CC	2260	n	bl	n	n	0	cig+/-ot	nev any	st
KREUZE	8	x	f	55	69	all	-	all	Eu:Ger	1990	CC	2260	n	bl	n	n	0	cig+/-ot	nev any	st
KUBIK	2		m	55	64	all	4	all	Eu:est	1965	pr	108	n	bl	n	n	0	cig+/-ot	nev any	st
NAM	12		m	55	64	all	-	all	NAmer	1986	CC	1199	n	bl	y	n	0	cig+/-ot	nev cigs	st
NAM	44		f	55	64	all	-	all	NAmer	1986	CC	1199	n	bl	y	n	0	cig+/-ot	nev cigs	st
SEGI2	54		m	55	64	all	-	all	As:Jap	1962	CC	378	n	bl	n	n	0	cig+/-ot	nev any	st

Cigarette type is all/unspec for all RRs
except for the following:

REF	NRR	CIGTYPE
DEAN3	31	MC only
DEAN3	115	MC only

Table 1B8 - 5

IESLC - Meta-analysis of Current Smoking, Cigarettes (or Any Product if Cigarettes not available), Age 50-70
All LC types
Least adjusted

REF	NRR	SEX	AD	Number Exposed Case	Number Exposed Cont	Non-exposed Case	Non-exposed Cont	RR	95.00%CI
*ANDERS	2	f	0	212	41262	46	195158	21.80	(15.85- 29.98)
*BEST	32	m	1	-	-	-	-	23.30	(7.45- 72.88)
*CEDERL	10	m	0	21	2441	4	1954	4.20	(1.44- 12.22)
*CEDERL	18	m	0	30	1338	3	1645	12.29	(3.76- 40.20)
*CEDERL	35	f	0	4	1159	9	6407	2.46	(0.76- 7.96)
*CEDERL	38	f	0	4	407	10	5877	5.78	(1.82- 18.34)
Subtotal CEDERL								5.14	(2.90- 9.10)
*CPSI	210	m	1	-	-	-	-	14.22	(10.37- 19.52)
*CPSI	103	f	0	249	591967	144	1366561	3.99	(3.25- 4.90)
Subtotal CPSI								5.82	(4.90- 6.91)
*CPSII	15	m	0	641	231659	21	253604	33.42	(21.64- 51.61)
*CPSII	50	f	0	390	267703	67	686189	14.92	(11.51- 19.33)
Subtotal CPSII								18.43	(14.76- 23.03)
DEAN3	31	m	0	129	211	7	96	8.38	(3.78- 18.62)
DEAN3	115	f	0	36	217	9	310	5.71	(2.70- 12.11)
Subtotal DEAN3								6.84	(3.96- 11.82)
DORANT	9	c	0	292	876	14	1090	25.95	(15.07- 44.69)
*DORN	366	m	1	-	-	-	-	11.10	(9.78- 12.61)
*HAMMON	139	m	1	-	-	-	-	11.52	(6.83- 19.42)
HITOSU	47	f	0	6	148	8	478	2.42	(0.83- 7.09)
KANELL	15	m	0	354	112	9	38	13.35	(6.26- 28.45)
KANELL	20	m	0	336	127	16	35	5.79	(3.10- 10.82)
Subtotal KANELL								8.12	(5.02- 13.16)
KREUZE	7	m	0	1226	509	23	403	42.20	(27.38- 65.05)
KREUZE	8	f	0	167	54	95	177	5.76	(3.88- 8.56)
Subtotal KREUZE								14.26	(10.65- 19.09)
*KUBIK	2	m	0	45	2654	1	1851	31.38	(4.33- 227.48)
NAM	12	m	0	83	88	5	42	7.92	(2.99- 20.99)
NAM	44	f	0	28	48	10	69	4.03	(1.79- 9.05)
Subtotal NAM								5.31	(2.85- 9.90)
SEGI2	54	m	0	111	193	2	20	5.75	(1.32- 25.07)
Partial Totals				4364	1143173	503	2522004		

*prospective study

REF	NRR	SEX	AD	Ys	Ws	Qs	Ps
*ANDERS	2	f	0	3.08	37.84	19.54	0.0000
*BEST	32	m	1	3.15	2.95	1.82	0.0000
*CEDERL	10	m	0	1.44	3.37	2.90	0.0084
*CEDERL	18	m	0	2.51	2.74	0.06	0.0000
*CEDERL	35	f	0	0.90	2.78	5.96	0.1341
*CEDERL	38	f	0	1.75	2.88	1.07	0.0029
Subtotal CEDERL				1.64	11.76	9.98	
*CPSI	210	m	1	2.65	38.41	3.26	0.0000
*CPSI	103	f	0	1.38	91.26	87.47	0.0000
Subtotal CPSI				1.76	129.66	90.73	
*CPSII	15	m	0	3.51	20.34	26.70	0.0000
*CPSII	50	f	0	2.70	57.19	6.59	0.0000
Subtotal CPSII				2.91	77.53	33.29	
DEAN3	31	m	0	2.13	6.03	0.34	0.0000
DEAN3	115	f	0	1.74	6.82	2.62	0.0000
Subtotal DEAN3				1.92	12.85	2.96	
DORANT	9	c	0	3.26	13.00	10.37	0.0000
*DORN	366	m	1	2.41	237.89	0.45	0.0000
*HAMMON	139	m	1	2.44	14.07	0.09	0.0000
HITOSU	47	f	0	0.88	3.33	7.27	0.1066
KANELL	15	m	0	2.59	6.70	0.35	0.0000
KANELL	20	m	0	1.76	9.81	3.62	0.0000
Subtotal KANELL				2.09	16.51	3.97	
KREUZE	7	m	0	3.74	20.52	39.03	0.0000
KREUZE	8	f	0	1.75	24.58	9.21	0.0000
Subtotal KREUZE				2.66	45.10	48.23	
*KUBIK	2	m	0	3.45	0.98	1.15	0.0006
NAM	12	m	0	2.07	4.04	0.35	0.0000
NAM	44	f	0	1.39	5.85	5.51	0.0008
Subtotal NAM				1.67	9.89	5.86	
SEGI2	54	m	0	1.75	1.77	0.67	0.0199

Table 1B8 - 5

IESLC - Meta-analysis of Current Smoking, Cigarettes (or Any Product if Cigarettes not available), Age 50-70
All LC types
Least adjusted

	N	24
	NS	15
	Wt	615.15
Het	Chi	236.39
Het	df	23
Het	P	***
Fixed	RR	10.63
	RRl	9.82
	RRu	11.50
	P	+++
Random	RR	10.09
	RRl	7.43
	RRu	13.70
	P	+++
Asymm	P	N.S.

Table 1B8 - 6

IESLC - Meta-analysis of Current Smoking, Cigarettes (or Any Product if Cigarettes not available), Age 50-70
All LC types
Least adjusted

	Sex combined	male	female	Total
N	1	14	9	24
NS	1	12	8	21
Wt	13.00	369.63	232.52	615.15
Het Chi	0.00	68.70	118.78	236.39
Het df	0	13	8	23
Het P	N.S.	***	***	***
Fixed RR	25.95	12.73	7.58	10.63
RRl	15.07	11.50	6.67	9.82
RRu	44.69	14.10	8.62	11.50
P	+++	+++	+++	+++
Random RR	25.95	13.22	6.10	10.09
RRl	15.07	9.39	3.43	7.43
RRu	44.69	18.62	10.83	13.70
P	+++	+++	+++	+++
Between Chi				48.90
Between df				2
Between P				***
Btwn(F) P				(*)
Btwn(R) P				**

Table 1B8 - 7

IESLC - Meta-analysis of Current Smoking, Cigarettes (or Any Product if Cigarettes not available), Age 50-70
All LC types
Excluded studies (and stage at which they were excluded)

1	BOUCHA BUELL LAURIL MZILEN
2	ABELIN ABRAHA AUVINE AXELSO BAND BERRIN BLOT1 BLOT2 BLOT3 BLOT4 BRESLO BROCKM BROWN1 BYERS1 CASCO2 CASCOR CHAN CHATZI CHEN CHEN2 CHEN3 CHIAZZ COOKSO DAVEYS DEAN DOSEME DU DUNN EBELIN ESAKI FAN GENG GER GODLEY GOLLED GSELL HANSEN HEGMAN HINDS HIRAOK HOROWI HORWIT HU HU2 HUANG ISHIMA JARUP JIANG JONES JUSSAW KO KOHLME KOULUM KREYBE LAMTH LAMWK LAMWK2 LAUSSM LEI LETOUR LEVIN LIU LIU2 LIU3 LIU4 LIU5 LOMBA2 LUO MAGNUS MARSH MARSH2 MARTIS MASTRA MATSUD MCCONN MCDUFF MCLAUG MILLER MILLS MOLLO NOTAN2 NOU ORMOS PASTOR PAWLEG PERNU PERSHA PIKE POFFIJ POLEDN PRESCO QIAO RADZIK RANDIG REN RONCO ROOTS ROTHSC SADOWS SANKAR SCHWA2 SEGI SEOW SIEMIA SIMARA STASZE STOCKS SUN TAO ULMER VANDER WANG WANG3 WANG4 WICKLU WILKIN WUWILL WYNDE2 WYNDE4 XIANGZ XU XU2 XU3 XU4 YUAN ZHANG ZHENG ZHOU
3	AGUDO AKIBA ALDERS AMANDU AMES ARCHER ARMADA AUSTIN AXELSS BARBON BECHER BENHAM BENSHL BLOHMK BOFFET BOUCOT BRETT BROSS BROWN2 BUFFLE BYERS2 CARPEN CHANG CHOI CHOW CHYOU COMSTO CORREA DAMBER DARBY DEAN2 DEKLER DESTE2 DESTEF DOCKER DOLL DOLL2 DORGAN DROSTE ENGELA ENSTRO GAO GAO2 GARCIA GARDIN GARSHI GILLIS GOODMA GRAHAM GREGOR HAENSZ HAMMO2 HEIN HENNEK HIRAY2 HUMBLE JAHN JAIN JARVHO JEDRYC JOLY KAISER KATSOU KAUFMA KELLER KHUDER KIHARA KINLEN KJUUS KNEKT KOO LANGE LEMARC LIAW LICKIN LIDDEL LOMBAR LUBIN LUBIN2 MACLEN MATOS MIGRAN MRFIT MRFITR MURATA NOTANI ODRISC OSANN OSANN2 PARKIN PERSH2 PETO PEZZO2 PEZZOT PISANI QIAO2 RACHTA RESTRE RIMING SAARIK SCHWAR SHAW SHIMIZ SIMONA SITAS SOBUE SOBUE2 SPEIZE SPITZ STAYNE STOCKW STUCKE SUZUK2 SUZUKI SVENSS TANG TANG2 TENKAN TIZZAN TOKARS TOUSEY TSUGAN TULINI TVERDA VEIERO VUTUC WAKAI WALD WANG2 WARSIN WATSON WIGLE WU WU2 WUNSCH WYNDE3 WYNDE5 WYNDE6 WYNDE7 WYNDE8 WYNDER YAMAGU YONG
4	HIRAYA
6	KAISE2
7	HOLE

Table 1B8 - 8
Potentially overlapping studies

REF	REFGP	PRINC	.	OVERLAP/LINK
CPSI	CPSI	1		CPSI overall

Table 1B8 - 9
Most adjusted - insufficient data for metaanalysis

REF	NRR	SEX	AGEL	AGEH	RACE	YF	LC TYPE	LOC	START	ST	NLC	R	VB	P	H	AD	PRODUCT	DENOM	De
HIRAYA	5	m	60	69	all	0	all	As:Jap	1965	pr	1917	n	bl	n	n	0	cig+/-ot	nev any	or

REF	NRR	RR	SIG	RRDATA comment
HIRAYA	5	4.22		0

Table 1B9 -

IESLC - Meta-analysis of Current Smoking, Cigarettes (or Any Product if Cigarettes not available), Age 65+
All LC types

This analysis is restricted to results for:
1) Non-dose-response data
2) Current smokers
3) Age 65+
4) Results complete enough for use in metaanalysis

Within each study, results are then selected (in the following order of preference, within each sex) for:
5) PRODUCT: cigarettes regardless of other products, cigarettes only, all/unspec
6) CIGTYPE: all/unspecified, MC regardless of HR, MC only
7) DENOM: never smoked anything, never smoked cigarettes, (never +1 = +long term ex, +2 = +amount unknown,
+3 = never cigs+long term ex)
8) Followup period (YF, prospective studies): whole study (coded as 0) or longest available
9) LCtype: all or nearest available, at least Squamous and Adeno. (q = squamous, s = small,
l = large, a = adeno, mix = mixed, alv = alveolar)
10) Race: all or nearest available, otherwise by race (wh or w = white, bl or b = black, hi = hispanic
ch = chinese, jap = japanese, haw = hawaiian, w+o = white + oriental, sca = scandinavian, as = asian)
11) For overlapping studies: principal rather than subsidiary studies
Finally by Age: whole study (actual age shown) if available, otherwise by widest available age group
and then for single sex results (m, f) in preference to combined sex results (c).

Results adjusted (AD) for the most potential confounders are then chosen in Sections -1 to -3
(and those which actually differ from the adjusted results in Table 1B2 - 1 are marked 'x' in Section -1)
and results adjusted for the least confounders in Sections -4 to -6. (Those least adjusted results which
actually differ from the most adjusted as marked 'x' in column X in Section -4)
(Results adjusted for an unknown number of confounder(s) are coded as 20.)

Section -7 shows excluded studies, together with the stage (as above) at which no qualifying
results were found.

Section -8 lists the potentially overlapping studies which have been included (1=principal, 2=subsidiary).

Section -9 lists any results which would have been included in preference except that they had data not complete
enough for use in meta-analysis, with their significance (yes/no), if known, and any further comment as entered
on the database.

In addition to those mentioned above, the following fields, levels and abbreviations are used:
* or nk = not known, n = no, y = yes, ot = other
nev = never
all/unspec = all or unspecified, cig+/-ot = cigarettes irrespective of other products (cigar, pipe etc)
MC = manufactured cigarettes, HR = hand-rolled cigarettes
REF: 6-character study reference
NRR: number of the RR on the database within the study
ST : study type (CC = case control, pr or prosp = prospective)
NLC: number of lung cancer cases in whole study
R : risky occupational population (n = no, m = mining, o = other risky)
VB : national cigarette type (V = at least 75% Virginia, bl = at least 75% blended, ot = other)
P : any proxy use
H : full histological confirmation
De : derivation of RR/CI (or = original, st = standard method, ot = other method of estimation)

Table 1B9 - 1

IESLC - Meta-analysis of Current Smoking, Cigarettes (or Any Product if Cigarettes not available), Age 65+
All LC types
Most adjusted

REF	NRR	1B2	SEX	AGEL	AGEH	RACE	YF	LC TYPE	LOC	START	ST	NLC	R	VB	P	H	AD	PRODUCT	DENOM	De
BEST	33	x	m	70	99	all	0	all	NAmer	1955	pr	381	n	V	n	n	1	cig only	nev any	ot
COMSTO	17	x	c	65	99	all	-	all	NAmer	1975	ot	258	n	bl	n	n	0	cig+/-ot	nev cigs	st
CPSI	215	x	m	70	84	all	6	all	NAmer	1959	pr	5138	n	bl	n	n	1	cig+/-ot	nev any	ot
CPSI	104	x	f	65	74	wh	0	all	NAmer	1959	pr	5138	n	bl	n	n	0	cig only	nev any	st
CPSI	105	x	f	75	84	wh	0	all	NAmer	1959	pr	5138	n	bl	n	n	0	cig only	nev any	st
CPSII	22	x	m	65	74	all	6	all	NAmer	1982	pr	3229	n	bl	n	n	0	cig only	nev any	st
CPSII	29	x	m	75	99	all	6	all	NAmer	1982	pr	3229	n	bl	n	n	0	cig only	nev any	st
CPSII	57	x	f	65	74	all	6	all	NAmer	1982	pr	3229	n	bl	n	n	0	cig+/-ot	nev cigs	st
CPSII	64	x	f	75	99	all	6	all	NAmer	1982	pr	3229	n	bl	n	n	0	cig+/-ot	nev cigs	st
DEAN3	32	x	m	65	99	all	-	all	Eu:UK	1969	CC	766	n	V	y	n	0	cig only	nev any	st
DEAN3	116	x	f	65	99	all	-	all	Eu:UK	1969	CC	766	n	V	y	n	0	cig only	nev any	st
DORN	53	x	m	65	84	wh	8	all	NAmer	1954	pr	5097	n	bl	n	n	1	cig+/-ot	nev any	ot
GARSHI	15	x	m	65	82	all	-	all	NAmer	1981	CC	1081	o	bl	y	n	0	all/unsp	nev any	st
HUMBLE	22	x	m	65	84	w-hi	-	not alv	NAmer	1980	CC	521	n	bl	y	n	0	cig+/-ot	nev cigs	st
HUMBLE	30	x	m	65	84	hi	-	not alv	NAmer	1980	CC	521	n	bl	y	n	0	cig+/-ot	nev cigs	st
HUMBLE	24	x	f	65	84	w-hi	-	not alv	NAmer	1980	CC	521	n	bl	y	n	0	cig+/-ot	nev cigs	st
HUMBLE	32	x	f	65	84	hi	-	not alv	NAmer	1980	CC	521	n	bl	y	n	0	cig+/-ot	nev cigs	st
KAISE2	36	x	m	65	74	all	9	all	NAmer	1979	pr	318	n	bl	n	n	0	cig only	nev any	st
KAISE2	44	x	m	75	99	all	9	all	NAmer	1979	pr	318	n	bl	n	n	0	cig only	nev any	st
KAISE2	12	x	f	65	74	all	9	all	NAmer	1979	pr	318	n	bl	n	n	0	cig only	nev any	st
KAISE2	20	x	f	75	99	all	9	all	NAmer	1979	pr	318	n	bl	n	n	0	cig only	nev any	st
KANELL	25	x	m	70	99	all	-	all	Eu:bal	1950	CC	862	n	bl	n	n	0	all/unsp	nev any	st
NAM	20	x	m	65	79	all	-	all	NAmer	1986	CC	1199	n	bl	y	n	0	cig+/-ot	nev cigs	st
NAM	28	x	m	80	99	all	-	all	NAmer	1986	CC	1199	n	bl	y	n	0	cig+/-ot	nev cigs	st
NAM	52	x	f	65	79	all	-	all	NAmer	1986	CC	1199	n	bl	y	n	0	cig+/-ot	nev cigs	st
NAM	60	x	f	80	99	all	-	all	NAmer	1986	CC	1199	n	bl	y	n	0	cig+/-ot	nev cigs	st
SEGI2	55	x	m	65	99	all	-	all	As:Jap	1962	CC	378	n	bl	n	n	0	cig+/-ot	nev any	st

Cigarette type is all/unspec for all RRs
except for the following:

REF	NRR	CIGTYPE
DEAN3	32	MC only
DEAN3	116	MC only

Table 1B9 - 2

IESLC - Meta-analysis of Current Smoking, Cigarettes (or Any Product if Cigarettes not available), Age 65+
All LC types
Most adjusted

REF	NRR	SEX	AD	Number Exposed Case	Number Exposed Cont	Non-exposed Case	Non-exposed Cont	RR	95.00%CI
*BEST	33	m	1	-	-	-	-	12.43	(3.92- 39.43)
COMSTO	17	c	0	24	16	6	44	11.00	(3.80- 31.81)
*CPSI	215	m	1	-	-	-	-	10.48	(6.48- 16.96)
*CPSI	104	f	0	145	177746	178	957682	4.39	(3.53- 5.46)
*CPSI	105	f	0	28	31384	163	392909	2.15	(1.44- 3.21)
Subtotal CPSI								4.29	(3.59- 5.13)
*CPSII	22	m	0	743	106505	47	173660	25.78	(19.20- 34.61)
*CPSII	29	m	0	230	20335	44	66378	17.06	(12.36- 23.55)
*CPSII	57	f	0	374	126848	110	487073	13.06	(10.56- 16.15)
*CPSII	64	f	0	109	26603	110	247713	9.23	(7.08- 12.02)
Subtotal CPSII								14.35	(12.58- 16.37)
DEAN3	32	m	0	157	145	16	82	5.55	(3.10- 9.92)
DEAN3	116	f	0	38	128	27	574	6.31	(3.72- 10.71)
Subtotal DEAN3								5.95	(4.03- 8.81)
*DORN	53	m	1	-	-	-	-	8.98	(6.78- 11.90)
GARSHI	15	m	0	455	484	32	294	8.64	(5.87- 12.71)
HUMBLE	22	m	0	74	58	6	60	12.76	(5.15- 31.60)
HUMBLE	30	m	0	38	30	2	21	13.30	(2.89- 61.26)
HUMBLE	24	f	0	34	14	11	52	11.48	(4.67- 28.24)
HUMBLE	32	f	0	15	6	4	34	21.25	(5.22- 86.47)
Subtotal HUMBLE								13.32	(7.74- 22.94)
*KAISE2	36	m	0	16	4735	5	11466	7.75	(2.84- 21.14)
*KAISE2	44	m	0	7	1035	4	4486	7.59	(2.22- 25.86)
*KAISE2	12	f	0	19	7165	2	24159	32.03	(7.46- 137.49)
*KAISE2	20	f	0	6	1394	3	12285	17.63	(4.41- 70.40)
Subtotal KAISE2								11.66	(6.31- 21.55)
KANELL	25	m	0	59	66	14	39	2.49	(1.23- 5.04)
NAM	20	m	0	106	122	18	130	6.28	(3.59- 10.96)
NAM	28	m	0	21	26	6	172	23.15	(8.55- 62.73)
NAM	52	f	0	67	75	18	251	12.46	(6.97- 22.27)
NAM	60	f	0	14	12	22	472	25.03	(10.37- 60.44)
Subtotal NAM								11.49	(8.15- 16.20)
SEGI2	55	m	0	62	120	4	15	1.94	(0.62- 6.09)
Partial Totals				2841	505052	852	2380051		

*prospective study

REF	NRR	SEX	AD	Ys	Ws	Qs	Ps
*BEST	33	m	1	2.52	2.88	0.28	0.0000
COMSTO	17	c	0	2.40	3.41	0.12	0.0000
*CPSI	215	m	1	2.35	16.60	0.33	0.0000
*CPSI	104	f	0	1.48	79.95	42.45	0.0000
*CPSI	105	f	0	0.77	23.91	49.73	0.0002
Subtotal CPSI				1.46	120.46	92.52	
*CPSII	22	m	0	3.25	44.23	47.99	0.0000
*CPSII	29	m	0	2.84	37.02	14.65	0.0000
*CPSII	57	f	0	2.57	85.07	11.11	0.0000
*CPSII	64	f	0	2.22	54.87	0.01	0.0000
Subtotal CPSII				2.66	221.20	73.77	
DEAN3	32	m	0	1.71	11.37	2.78	0.0000
DEAN3	116	f	0	1.84	13.72	1.83	0.0000
Subtotal DEAN3				1.78	25.08	4.61	
*DORN	53	m	1	2.19	48.55	0.01	0.0000
GARSHI	15	m	0	2.16	25.70	0.07	0.0000
HUMBLE	22	m	0	2.55	4.67	0.53	0.0000
HUMBLE	30	m	0	2.59	1.65	0.24	0.0009
HUMBLE	24	f	0	2.44	4.74	0.26	0.0000
HUMBLE	32	f	0	3.06	1.95	1.40	0.0000
Subtotal HUMBLE				2.59	13.01	2.43	
*KAISE2	36	m	0	2.05	3.81	0.10	0.0001
*KAISE2	44	m	0	2.03	2.55	0.08	0.0012
*KAISE2	12	f	0	3.47	1.81	2.87	0.0000
*KAISE2	20	f	0	2.87	2.00	0.88	0.0000
Subtotal KAISE2				2.46	10.18	3.93	
KANELL	25	m	0	0.91	7.74	12.99	0.0111
NAM	20	m	0	1.84	12.36	1.70	0.0000
NAM	28	m	0	3.14	3.87	3.38	0.0000
NAM	52	f	0	2.52	11.39	1.13	0.0000
NAM	60	f	0	3.22	4.94	5.06	0.0000
Subtotal NAM				2.44	32.56	11.27	
SEGI2	55	m	0	0.66	2.93	7.01	0.2575

Table 1B9 - 2

IESLC - Meta-analysis of Current Smoking, Cigarettes (or Any Product if Cigarettes not available), Age 65+
All LC types
Most adjusted

	N	27
	NS	12
	Wt	513.71
Het	Chi	209.01
Het	df	26
Het	P	***
Fixed	RR	9.10
	RRl	8.34
	RRu	9.92
	P	+++
Random	RR	9.31
	RRl	7.04
	RRu	12.30
	P	+++
Asymm	P	N.S.

Table 1B9 - 3

IESLC - Meta-analysis of Current Smoking, Cigarettes (or Any Product if Cigarettes not available), Age 65+
All LC types
Most adjusted

	Sex			
	combined	male	female	Total
N	1	15	11	27
NS	1	11	6	18
Wt	3.41	225.95	284.36	513.71
Het Chi	0.00	80.80	107.49	209.01
Het df	0	14	10	26
Het P	N.S.	***	***	***
Fixed RR	11.00	11.38	7.59	9.10
RRl	3.80	9.99	6.76	8.34
RRu	31.81	12.97	8.53	9.92
P	+++	+++	+++	+++
Random RR	11.00	9.16	9.54	9.31
RRl	3.80	6.42	6.07	7.04
RRu	31.81	13.06	15.00	12.30
P	+++	+++	+++	+++
Between Chi				20.72
Between df				2
Between P				***
Btwn(F) P				N.S.
Btwn(R) P				N.S.

Table 1B9 - 4

IESLC - Meta-analysis of Current Smoking, Cigarettes (or Any Product if Cigarettes not available), Age 65+
All LC types
Least adjusted

REF	NRR	X	SEX	AGEL	AGEH	RACE	YF	LC TYPE	LOC	START	ST	NLC	R	VB	P	H	AD	PRODUCT	DENOM	De
BEST	33		m	70	99	all	0	all	NAmer	1955	pr	381	n	V	n	n	1	cig only	nev any	ot
COMSTO	17		c	65	99	all	-	all	NAmer	1975	ot	258	n	bl	n	n	0	cig+/-ot	nev cigs	st
CPSI	215		m	70	84	all	6	all	NAmer	1959	pr	5138	n	bl	n	n	1	cig+/-ot	nev any	ot
CPSI	104		f	65	74	wh	0	all	NAmer	1959	pr	5138	n	bl	n	n	0	cig only	nev any	st
CPSI	105		f	75	84	wh	0	all	NAmer	1959	pr	5138	n	bl	n	n	0	cig only	nev any	st
CPSII	22		m	65	74	all	6	all	NAmer	1982	pr	3229	n	bl	n	n	0	cig only	nev any	st
CPSII	29		m	75	99	all	6	all	NAmer	1982	pr	3229	n	bl	n	n	0	cig only	nev any	st
CPSII	57		f	65	74	all	6	all	NAmer	1982	pr	3229	n	bl	n	n	0	cig+/-ot	nev cigs	st
CPSII	64		f	75	99	all	6	all	NAmer	1982	pr	3229	n	bl	n	n	0	cig+/-ot	nev cigs	st
DEAN3	32		m	65	99	all	-	all	Eu:UK	1969	CC	766	n	V	y	n	0	cig only	nev any	st
DEAN3	116		f	65	99	all	-	all	Eu:UK	1969	CC	766	n	V	y	n	0	cig only	nev any	st
DORN	53		m	65	84	wh	8	all	NAmer	1954	pr	5097	n	bl	n	n	1	cig+/-ot	nev any	ot
GARSHI	15		m	65	82	all	-	all	NAmer	1981	CC	1081	o	bl	y	n	0	all/unsp	nev any	st
HUMBLE	22		m	65	84	w-hi	-	not alv	NAmer	1980	CC	521	n	bl	y	n	0	cig+/-ot	nev cigs	st
HUMBLE	30		m	65	84	hi	-	not alv	NAmer	1980	CC	521	n	bl	y	n	0	cig+/-ot	nev cigs	st
HUMBLE	24		f	65	84	w-hi	-	not alv	NAmer	1980	CC	521	n	bl	y	n	0	cig+/-ot	nev cigs	st
HUMBLE	32		f	65	84	hi	-	not alv	NAmer	1980	CC	521	n	bl	y	n	0	cig+/-ot	nev cigs	st
KAISE2	36		m	65	74	all	9	all	NAmer	1979	pr	318	n	bl	n	n	0	cig only	nev any	st
KAISE2	44		m	75	99	all	9	all	NAmer	1979	pr	318	n	bl	n	n	0	cig only	nev any	st
KAISE2	12		f	65	74	all	9	all	NAmer	1979	pr	318	n	bl	n	n	0	cig only	nev any	st
KAISE2	20		f	75	99	all	9	all	NAmer	1979	pr	318	n	bl	n	n	0	cig only	nev any	st
KANELL	25		m	70	99	all	-	all	Eu:bal	1950	CC	862	n	bl	n	n	0	all/unsp	nev any	st
NAM	20		m	65	79	all	-	all	NAmer	1986	CC	1199	n	bl	y	n	0	cig+/-ot	nev cigs	st
NAM	28		m	80	99	all	-	all	NAmer	1986	CC	1199	n	bl	y	n	0	cig+/-ot	nev cigs	st
NAM	52		f	65	79	all	-	all	NAmer	1986	CC	1199	n	bl	y	n	0	cig+/-ot	nev cigs	st
NAM	60		f	80	99	all	-	all	NAmer	1986	CC	1199	n	bl	y	n	0	cig+/-ot	nev cigs	st
SEGI2	55		m	65	99	all	-	all	As:Jap	1962	CC	378	n	bl	n	n	0	cig+/-ot	nev any	st

Cigarette type is all/unspec for all RRs
except for the following:

REF	NRR	CIGTYPE
DEAN3	32	MC only
DEAN3	116	MC only

Table 1B9 - 5

IESLC - Meta-analysis of Current Smoking, Cigarettes (or Any Product if Cigarettes not available), Age 65+
All LC types
Least adjusted

REF	NRR	SEX	AD	Number Exposed Case	Number Exposed Cont	Non-exposed Case	Non-exposed Cont	RR	95.00%CI
*BEST	33	m	1	-	-	-	-	12.43	(3.92- 39.43)
COMSTO	17	c	0	24	16	6	44	11.00	(3.80- 31.81)
*CPSI	215	m	1	-	-	-	-	10.48	(6.48- 16.96)
*CPSI	104	f	0	145	177746	178	957682	4.39	(3.53- 5.46)
*CPSI	105	f	0	28	31384	163	392909	2.15	(1.44- 3.21)
Subtotal CPSI								4.29	(3.59- 5.13)
*CPSII	22	m	0	743	106505	47	173660	25.78	(19.20- 34.61)
*CPSII	29	m	0	230	20335	44	66378	17.06	(12.36- 23.55)
*CPSII	57	f	0	374	126848	110	487073	13.06	(10.56- 16.15)
*CPSII	64	f	0	109	26603	110	247713	9.23	(7.08- 12.02)
Subtotal CPSII								14.35	(12.58- 16.37)
DEAN3	32	m	0	157	145	16	82	5.55	(3.10- 9.92)
DEAN3	116	f	0	38	128	27	574	6.31	(3.72- 10.71)
Subtotal DEAN3								5.95	(4.03- 8.81)
*DORN	53	m	1	-	-	-	-	8.98	(6.78- 11.90)
GARSHI	15	m	0	455	484	32	294	8.64	(5.87- 12.71)
HUMBLE	22	m	0	74	58	6	60	12.76	(5.15- 31.60)
HUMBLE	30	m	0	38	30	2	21	13.30	(2.89- 61.26)
HUMBLE	24	f	0	34	14	11	52	11.48	(4.67- 28.24)
HUMBLE	32	f	0	15	6	4	34	21.25	(5.22- 86.47)
Subtotal HUMBLE								13.32	(7.74- 22.94)
*KAISE2	36	m	0	16	4735	5	11466	7.75	(2.84- 21.14)
*KAISE2	44	m	0	7	1035	4	4486	7.59	(2.22- 25.86)
*KAISE2	12	f	0	19	7165	2	24159	32.03	(7.46- 137.49)
*KAISE2	20	f	0	6	1394	3	12285	17.63	(4.41- 70.40)
Subtotal KAISE2								11.66	(6.31- 21.55)
KANELL	25	m	0	59	66	14	39	2.49	(1.23- 5.04)
NAM	20	m	0	106	122	18	130	6.28	(3.59- 10.96)
NAM	28	m	0	21	26	6	172	23.15	(8.55- 62.73)
NAM	52	f	0	67	75	18	251	12.46	(6.97- 22.27)
NAM	60	f	0	14	12	22	472	25.03	(10.37- 60.44)
Subtotal NAM								11.49	(8.15- 16.20)
SEGI2	55	m	0	62	120	4	15	1.94	(0.62- 6.09)
Partial Totals				2841	505052	852	2380051		

*prospective study

REF	NRR	SEX	AD	Ys	Ws	Qs	Ps
*BEST	33	m	1	2.52	2.88	0.28	0.0000
COMSTO	17	c	0	2.40	3.41	0.12	0.0000
*CPSI	215	m	1	2.35	16.60	0.33	0.0000
*CPSI	104	f	0	1.48	79.95	42.45	0.0000
*CPSI	105	f	0	0.77	23.91	49.73	0.0002
Subtotal CPSI				1.46	120.46	92.52	
*CPSII	22	m	0	3.25	44.23	47.99	0.0000
*CPSII	29	m	0	2.84	37.02	14.65	0.0000
*CPSII	57	f	0	2.57	85.07	11.11	0.0000
*CPSII	64	f	0	2.22	54.87	0.01	0.0000
Subtotal CPSII				2.66	221.20	73.77	
DEAN3	32	m	0	1.71	11.37	2.78	0.0000
DEAN3	116	f	0	1.84	13.72	1.83	0.0000
Subtotal DEAN3				1.78	25.08	4.61	
*DORN	53	m	1	2.19	48.55	0.01	0.0000
GARSHI	15	m	0	2.16	25.70	0.07	0.0000
HUMBLE	22	m	0	2.55	4.67	0.53	0.0000
HUMBLE	30	m	0	2.59	1.65	0.24	0.0009
HUMBLE	24	f	0	2.44	4.74	0.26	0.0000
HUMBLE	32	f	0	3.06	1.95	1.40	0.0000
Subtotal HUMBLE				2.59	13.01	2.43	
*KAISE2	36	m	0	2.05	3.81	0.10	0.0001
*KAISE2	44	m	0	2.03	2.55	0.08	0.0012
*KAISE2	12	f	0	3.47	1.81	2.87	0.0000
*KAISE2	20	f	0	2.87	2.00	0.88	0.0000
Subtotal KAISE2				2.46	10.18	3.93	
KANELL	25	m	0	0.91	7.74	12.99	0.0111
NAM	20	m	0	1.84	12.36	1.70	0.0000
NAM	28	m	0	3.14	3.87	3.38	0.0000
NAM	52	f	0	2.52	11.39	1.13	0.0000
NAM	60	f	0	3.22	4.94	5.06	0.0000
Subtotal NAM				2.44	32.56	11.27	
SEGI2	55	m	0	0.66	2.93	7.01	0.2575

Table 1B9 - 5

IESLC - Meta-analysis of Current Smoking, Cigarettes (or Any Product if Cigarettes not available), Age 65+
All LC types
Least adjusted

	N	27
	NS	12
	Wt	513.71
Het	Chi	209.01
Het	df	26
Het	P	***
Fixed	RR	9.10
	RRl	8.34
	RRu	9.92
	P	+++
Random	RR	9.31
	RRl	7.04
	RRu	12.30
	P	+++
Asymm	P	N.S.

Table 1B9 - 6

IESLC - Meta-analysis of Current Smoking, Cigarettes (or Any Product if Cigarettes not available), Age 65+
All LC types
Least adjusted

	combined	Sex male	female	Total
N	1	15	11	27
NS	1	11	6	18
Wt	3.41	225.95	284.36	513.71
Het Chi	0.00	80.80	107.49	209.01
Het df	0	14	10	26
Het P	N.S.	***	***	***
Fixed RR	11.00	11.38	7.59	9.10
RRl	3.80	9.99	6.76	8.34
RRu	31.81	12.97	8.53	9.92
P	+++	+++	+++	+++
Random RR	11.00	9.16	9.54	9.31
RRl	3.80	6.42	6.07	7.04
RRu	31.81	13.06	15.00	12.30
P	+++	+++	+++	+++
Between Chi				20.72
Between df				2
Between P				***
Btwn(F) P				N.S.
Btwn(R) P				N.S.

Table 1B9 - 7

IESLC - Meta-analysis of Current Smoking, Cigarettes (or Any Product if Cigarettes not available), Age 65+
All LC types
Excluded studies (and stage at which they were excluded)

```
1      BOUCHA  BUELL LAURIL MZILEN

2      ABELIN ABRAHA AUVINE AXELSO   BAND BERRIN  BLOT1  BLOT2  BLOT3  BLOT4 BRESLO BROCKM BROWN1 BYERS1 CASCO2 CASCOR
         CHAN CHATZI   CHEN  CHEN2  CHEN3 CHIAZZ COOKSO DAVEYS   DEAN DOSEME     DU   DUNN EBELIN  ESAKI    FAN   GENG
          GER GODLEY GOLLED  GSELL HANSEN HEGMAN  HINDS HIRAOK HOROWI HORWIT     HU    HU2  HUANG ISHIMA  JARUP  JIANG
        JONES JUSSAW     KO KOHLME KOULUM KREYBE  LAMTH  LAMWK LAMWK2 LAUSSM    LEI LETOUR  LEVIN    LIU   LIU2   LIU3
         LIU4   LIU5 LOMBA2    LUO MAGNUS  MARSH MARSH2 MARTIS MASTRA MATSUD MCCONN MCDUFF MCLAUG MILLER  MILLS  MOLLO
       NOTAN2    NOU  ORMOS PASTOR PAWLEG  PERNU PERSHA   PIKE POFFIJ POLEDN PRESCO   QIAO RADZIK RANDIG    REN  RONCO
        ROOTS ROTHSC SADOWS SANKAR SCHWA2   SEGI   SEOW SIEMIA SIMARA STASZE STOCKS    SUN    TAO  ULMER VANDER   WANG
        WANG3  WANG4 WICKLU WILKIN WUWILL WYNDE2 WYNDE4 XIANGZ     XU    XU2    XU3    XU4   YUAN  ZHANG  ZHENG   ZHOU

3       AGUDO  AKIBA ALDERS AMANDU   AMES ANDERS ARCHER ARMADA AUSTIN AXELSS BARBON BECHER BENHAM BENSHL BLOHMK BOFFET
       BOUCOT  BRETT  BROSS BROWN2 BUFFLE BYERS2 CARPEN CEDERL  CHANG   CHOI   CHOW  CHYOU CORREA DAMBER  DARBY  DEAN2
       DEKLER DESTE2 DESTEF DOCKER   DOLL  DOLL2 DORANT DORGAN DROSTE ENGELA ENSTRO    GAO   GAO2 GARCIA GARDIN GILLIS
       GOODMA GRAHAM GREGOR HAENSZ HAMMO2 HAMMON   HEIN HENNEK HIRAY2 HITOSU   HOLE   JAHN   JAIN JARVHO JEDRYC   JOLY
       KAISER KATSOU KAUFMA KELLER KHUDER KIHARA KINLEN  KJUUS  KNEKT    KOO KREUZE  KUBIK  LANGE LEMARC   LIAW LICKIN
       LIDDEL LOMBAR  LUBIN LUBIN2 MACLEN  MATOS MIGRAN  MRFIT MRFITR MURATA NOTANI ODRISC  OSANN OSANN2 PARKIN PERSH2
         PETO PEZZO2 PEZZOT PISANI  QIAO2 RACHTA RESTRE RIMING SAARIK SCHWAR   SHAW SHIMIZ SIMONA  SITAS  SOBUE SOBUE2
       SPEIZE  SPITZ STAYNE STOCKW STUCKE SUZUK2 SUZUKI SVENSS   TANG  TANG2 TIZZAN TOKARS TOUSEY TSUGAN TULINI TVERDA
       VEIERO  VUTUC  WAKAI   WALD  WANG2 WARSIN WATSON  WIGLE     WU    WU2 WUNSCH WYNDE3 WYNDE5 WYNDE6 WYNDE7 WYNDE8
       WYNDER YAMAGU   YONG

4      HIRAYA

7      TENKAN
```

Table 1B9 - 8
Potentially overlapping studies

REF	REFGP	PRINC	.	OVERLAP/LINK
CPSI	CPSI	1		CPSI overall

Table 1B9 - 9
Most adjusted - insufficient data for metaanalysis

REF	NRR	SEX	AGEL	AGEH	RACE	YF	LC TYPE	LOC	START	ST	NLC	R	VB	P	H	AD	PRODUCT	DENOM	De
HIRAYA	6	m	70	99	all	0	all	As:Jap	1965	pr	1917	n	bl	n	n	0	cig+/-ot	nev any	or

REF	NRR	RR	SIG	RRDATA comment
HIRAYA	6	5.10		0

Table 1B10 -

IESLC - Meta-analysis of Current Smoking, Cigarettes only, Age <56
All LC types

This analysis is restricted to results for:
1) Non-dose-response data
2) Current smokers
3) Age <56
4) Results complete enough for use in metaanalysis

Within each study, results are then selected (in the following order of preference, within each sex) for:
5) PRODUCT: cigarettes only
6) CIGTYPE: all/unspecified, MC regardless of HR, MC only
7) DENOM: never smoked anything, never smoked cigarettes, (never +1 = +long term ex, +2 = +amount unknown, +3 = never cigs+long term ex)
8) Followup period (YF, prospective studies): whole study (coded as 0) or longest available
9) LCtype: all or nearest available, at least Squamous and Adeno. (q = squamous, s = small, l = large, a = adeno, mix = mixed, alv = alveolar)
10) Race: all or nearest available, otherwise by race (wh or w = white, bl or b = black, hi = hispanic ch = chinese, jap = japanese, haw = hawaiian, w+o = white + oriental, sca = scandinavian, as = asian)
11) For overlapping studies: principal rather than subsidiary studies
Finally by Age: whole study (actual age shown) if available, otherwise by widest available age group and then for single sex results (m, f) in preference to combined sex results (c).

Results adjusted (AD) for the most potential confounders are then chosen in Sections -1 to -3 (and those which actually differ from the adjusted results in Table 1B3 - 1 are marked 'x' in Section -1) and results adjusted for the least confounders in Sections -4 to -6. (Those least adjusted results which actually differ from the most adjusted as marked 'x' in column X in Section -4) (Results adjusted for an unknown number of confounder(s) are coded as 20.)

Section -7 shows excluded studies, together with the stage (as above) at which no qualifying results were found.

Section -8 lists the potentially overlapping studies which have been included (1=principal, 2=subsidiary).

Section -9 lists any results which would have been included in preference except that they had data not complete enough for use in meta-analysis, with their significance (yes/no), if known, and any further comment as entered on the database.

In addition to those mentioned above, the following fields, levels and abbreviations are used:
* or nk = not known, n = no, y = yes, ot = other
nev = never
all/unspec = all or unspecified, MC = manufactured cigarettes, HR = hand-rolled cigarettes
REF: 6-character study reference
NRR: number of the RR on the database within the study
ST : study type (CC = case control, pr or prosp = prospective)
NLC: number of lung cancer cases in whole study
R : risky occupational population (n = no, m = mining, o = other risky)
VB : national cigarette type (V = at least 75% Virginia, bl = at least 75% blended, ot = other)
P : any proxy use
H : full histological confirmation
De : derivation of RR/CI (or = original, st = standard method, ot = other method of estimation)

Table 1B10 - 1

IESLC - Meta-analysis of Current Smoking, Cigarettes only, Age <56
All LC types
Most adjusted

REF	NRR	1B3	SEX	AGEL	AGEH	RACE	YF	LC TYPE	LOC	START	ST	NLC	R	VB	P	H	AD	PRODUCT	DENOM	De
BEST	31	x	m	30	49	all	0	all	NAmer	1955	pr	381	n	V	n	n	1	cig only	nev any	ot
CPSI	26	x	m	40	54	wh	0	all	NAmer	1959	pr	5138	n	bl	n	n	0	cig only	nev any	st
CPSI	102	x	f	40	54	wh	0	all	NAmer	1959	pr	5138	n	bl	n	n	0	cig only	nev any	st
CPSII	8	x	m	30	54	all	6	all	NAmer	1982	pr	3229	n	bl	n	n	0	cig only	nev any	st
DEAN3	29	x	m	35	44	all	-	all	Eu:UK	1969	CC	766	n	V	y	n	0	cig only	nev any	st
DEAN3	30	x	m	45	54	all	-	all	Eu:UK	1969	CC	766	n	V	y	n	0	cig only	nev any	st
DEAN3	113	x	f	35	44	all	-	all	Eu:UK	1969	CC	766	n	V	y	n	0	cig only	nev any	st
DEAN3	114	x	f	45	54	all	-	all	Eu:UK	1969	CC	766	n	V	y	n	0	cig only	nev any	st
TVERDA	3		m	35	49	all	0	all	Eu:Sca	1972	pr	238	n	bl	n	n	2	cig only	nev cigs	ot
TVERDA	15		f	35	49	all	0	all	Eu:Sca	1972	pr	238	n	bl	n	n	2	cig only	nev cigs	ot

Cigarette type is all/unspec for all RRs
except for the following:

REF	NRR	CIGTYPE
DEAN3	29	MC only
DEAN3	30	MC only
DEAN3	113	MC only
DEAN3	114	MC only

Table 1B10 - 2

IESLC - Meta-analysis of Current Smoking, Cigarettes only, Age <56
All LC types
Most adjusted

REF	NRR	SEX	AD	Number Exposed Case	Cont	Non-exposed Case	Cont	RR	95.00%CI
*BEST	31	m	1	-	-	-	-	3.93	(0.53- 29.13)
*CPSI	26	m	0	411	630755	14	255642	11.90	(6.98- 20.27)
*CPSI	102	f	0	178	827881	47	1160026	5.31	(3.85- 7.32)
Subtotal CPSI								6.58	(5.00- 8.67)
*CPSII	8	m	0	167	225147	12	248565	15.36	(8.55- 27.60)
DEAN3	29	m	0	7	263	1	187	4.98	(0.61- 40.79)
DEAN3	30	m	0	44	311	1	145	20.51	(2.80- 150.36)
DEAN3	113	f	0	6	409	1	328	4.81	(0.58- 40.17)
DEAN3	114	f	0	22	404	4	326	4.44	(1.51- 13.01)
Subtotal DEAN3								5.84	(2.63- 12.99)
*TVERDA	3	m	2	-	-	-	-	3.83	(2.47- 5.95)
*TVERDA	15	f	2	-	-	-	-	11.05	(3.33- 36.71)
Subtotal TVERDA								4.34	(2.87- 6.56)
Partial Totals				835	1685170	80	1665219		

*prospective study

REF	NRR	SEX	AD	Ys	Ws	Qs	Ps
*BEST	31	m	1	1.37	0.96	0.24	0.1806
*CPSI	26	m	0	2.48	13.54	4.94	0.0000
*CPSI	102	f	0	1.67	37.19	1.54	0.0000
Subtotal CPSI				1.88	50.72	6.48	
*CPSII	8	m	0	2.73	11.20	8.27	0.0000
DEAN3	29	m	0	1.60	0.87	0.06	0.1349
DEAN3	30	m	0	3.02	0.97	1.28	0.0030
DEAN3	113	f	0	1.57	0.85	0.08	0.1468
DEAN3	114	f	0	1.49	3.32	0.49	0.0066
Subtotal DEAN3				1.76	6.01	1.90	
*TVERDA	3	m	2	1.34	19.88	5.57	0.0000
*TVERDA	15	f	2	2.40	2.67	0.75	0.0001
Subtotal TVERDA				1.47	22.55	6.32	

	N	10
	NS	5
	Wt	91.44
Het	Chi	23.22
Het	df	9
Het	P	**
Fixed	RR	6.50
	RRl	5.30
	RRu	7.98
	P	+++
Random	RR	7.26
	RRl	4.77
	RRu	11.04
	P	+++
Asymm	P	N.S.

Table 1B10 - 3

IESLC - Meta-analysis of Current Smoking, Cigarettes only, Age <56
All LC types
Most adjusted

	combined	Sex male	female	Total
N		6	4	10
NS		5	3	8
Wt		47.41	44.03	91.44
Het Chi		19.13	1.51	23.22
Het df		5	3	9
Het P		**	N.S.	**
Fixed RR		7.65	5.46	6.50
RRl		5.75	4.07	5.30
RRu		10.16	7.34	7.98
P		+++	+++	+++
Random RR		8.38	5.46	7.26
RRl		4.18	4.07	4.77
RRu		16.81	7.34	11.04
P		+++	+++	+++
Between Chi				2.58
Between df				1
Between P				N.S.
Btwn(F) P				N.S.
Btwn(R) P				N.S.

Table 1B10 - 4

IESLC - Meta-analysis of Current Smoking, Cigarettes only, Age <56
All LC types
Least adjusted

REF	NRR	X	SEX	AGEL	AGEH	RACE	YF	LC TYPE	LOC	START	ST	NLC	R	VB	P	H	AD	PRODUCT	DENOM	De
BEST	31		m	30	49	all	0	all	NAmer	1955	pr	381	n	V	n	n	1	cig only	nev any	ot
CPSI	26		m	40	54	wh	0	all	NAmer	1959	pr	5138	n	bl	n	n	0	cig only	nev any	st
CPSI	102		f	40	54	wh	0	all	NAmer	1959	pr	5138	n	bl	n	n	0	cig only	nev any	st
CPSII	8		m	30	54	all	6	all	NAmer	1982	pr	3229	n	bl	n	n	0	cig only	nev any	st
DEAN3	29		m	35	44	all	-	all	Eu:UK	1969	CC	766	n	V	y	n	0	cig only	nev any	st
DEAN3	30		m	45	54	all	-	all	Eu:UK	1969	CC	766	n	V	y	n	0	cig only	nev any	st
DEAN3	113		f	35	44	all	-	all	Eu:UK	1969	CC	766	n	V	y	n	0	cig only	nev any	st
DEAN3	114		f	45	54	all	-	all	Eu:UK	1969	CC	766	n	V	y	n	0	cig only	nev any	st
TVERDA	3		m	35	49	all	0	all	Eu:Sca	1972	pr	238	n	bl	n	n	2	cig only	nev cigs	ot
TVERDA	15		f	35	49	all	0	all	Eu:Sca	1972	pr	238	n	bl	n	n	2	cig only	nev cigs	ot

Cigarette type is all/unspec for all RRs
except for the following:

REF	NRR	CIGTYPE
DEAN3	29	MC only
DEAN3	30	MC only
DEAN3	113	MC only
DEAN3	114	MC only

Table 1B10 - 5

IESLC - Meta-analysis of Current Smoking, Cigarettes only, Age <56
All LC types
Least adjusted

REF	NRR	SEX	AD	Number Exposed Case	Cont	Non-exposed Case	Cont	RR	95.00%CI
*BEST	31	m	1	-	-	-	-	3.93	(0.53- 29.13)
*CPSI	26	m	0	411	630755	14	255642	11.90	(6.98- 20.27)
*CPSI	102	f	0	178	827881	47	1160026	5.31	(3.85- 7.32)
Subtotal CPSI								6.58	(5.00- 8.67)
*CPSII	8	m	0	167	225147	12	248565	15.36	(8.55- 27.60)
DEAN3	29	m	0	7	263	1	187	4.98	(0.61- 40.79)
DEAN3	30	m	0	44	311	1	145	20.51	(2.80- 150.36)
DEAN3	113	f	0	6	409	1	328	4.81	(0.58- 40.17)
DEAN3	114	f	0	22	404	4	326	4.44	(1.51- 13.01)
Subtotal DEAN3								5.84	(2.63- 12.99)
*TVERDA	3	m	2	-	-	-	-	3.83	(2.47- 5.95)
*TVERDA	15	f	2	-	-	-	-	11.05	(3.33- 36.71)
Subtotal TVERDA								4.34	(2.87- 6.56)
Partial Totals				835	1685170	80	1665219		

*prospective study

REF	NRR	SEX	AD	Ys	Ws	Qs	Ps
*BEST	31	m	1	1.37	0.96	0.24	0.1806
*CPSI	26	m	0	2.48	13.54	4.94	0.0000
*CPSI	102	f	0	1.67	37.19	1.54	0.0000
Subtotal CPSI				1.88	50.72	6.48	
*CPSII	8	m	0	2.73	11.20	8.27	0.0000
DEAN3	29	m	0	1.60	0.87	0.06	0.1349
DEAN3	30	m	0	3.02	0.97	1.28	0.0030
DEAN3	113	f	0	1.57	0.85	0.08	0.1468
DEAN3	114	f	0	1.49	3.32	0.49	0.0066
Subtotal DEAN3				1.76	6.01	1.90	
*TVERDA	3	m	2	1.34	19.88	5.57	0.0000
*TVERDA	15	f	2	2.40	2.67	0.75	0.0001
Subtotal TVERDA				1.47	22.55	6.32	

	N	10
	NS	5
	Wt	91.44
Het	Chi	23.22
Het	df	9
Het	P	**
Fixed	RR	6.50
	RRl	5.30
	RRu	7.98
	P	+++
Random	RR	7.26
	RRl	4.77
	RRu	11.04
	P	+++
Asymm	P	N.S.

Table 1B10 - 6

IESLC - Meta-analysis of Current Smoking, Cigarettes only, Age <56
All LC types
Least adjusted

	Sex combined	male	female	Total
N		6	4	10
NS		5	3	8
Wt		47.41	44.03	91.44
Het Chi		19.13	1.51	23.22
Het df		5	3	9
Het P		**	N.S.	**
Fixed RR		7.65	5.46	6.50
RRl		5.75	4.07	5.30
RRu		10.16	7.34	7.98
P		+++	+++	+++
Random RR		8.38	5.46	7.26
RRl		4.18	4.07	4.77
RRu		16.81	7.34	11.04
P		+++	+++	+++
Between Chi				2.58
Between df				1
Between P				N.S.
Btwn(F) P				N.S.
Btwn(R) P				N.S.

Table 1B10 - 7

IESLC - Meta-analysis of Current Smoking, Cigarettes only, Age <56
All LC types
Excluded studies (and stage at which they were excluded)

1 BOUCHA BUELL LAURIL MZILEN

2 ABELIN ABRAHA AUVINE AXELSO BAND BERRIN BLOT1 BLOT2 BLOT3 BLOT4 BRESLO BROCKM BROWN1 BYERS1 CASCO2 CASCOR
CHAN CHATZI CHEN CHEN2 CHEN3 CHIAZZ COOKSO DAVEYS DEAN DOSEME DU DUNN EBELIN ESAKI FAN GENG
GER GODLEY GOLLED GSELL HANSEN HEGMAN HINDS HIRAOK HOROWI HORWIT HU HU2 HUANG ISHIMA JARUP JIANG
JONES JUSSAW KO KOHLME KOULUM KREYBE LAMTH LAMWK LAMWK2 LAUSSM LEI LETOUR LEVIN LIU LIU2 LIU3
LIU4 LIU5 LOMBA2 LUO MAGNUS MARSH MARSH2 MARTIS MASTRA MATSUD MCCONN MCDUFF MCLAUG MILLER MILLS MOLLO
NOTAN2 NOU ORMOS PASTOR PAWLEG PERNU PERSHA PIKE POFFIJ POLEDN PRESCO QIAO RADZIK RANDIG REN RONCO
ROOTS ROTHSC SADOWS SANKAR SCHWA2 SEGI SEOW SIEMIA SIMARA STASZE STOCKS SUN TAO ULMER VANDER WANG
WANG3 WANG4 WICKLU WILKIN WUWILL WYNDE2 WYNDE4 XIANGZ XU XU2 XU3 XU4 YUAN ZHANG ZHENG ZHOU

3 AGUDO AKIBA ALDERS AMANDU AMES ANDERS ARCHER ARMADA AUSTIN AXELSS BARBON BECHER BENHAM BENSHL BLOHMK BOFFET
BOUCOT BRETT BROSS BROWN2 BUFFLE BYERS2 CARPEN CEDERL CHANG CHOI CHOW CHYOU CORREA DAMBER DARBY DEAN2
DEKLER DESTE2 DESTEF DOCKER DOLL DOLL2 DORANT DORGAN DORN DROSTE ENGELA ENSTRO GAO GAO2 GARCIA GARDIN
GARSHI GILLIS GOODMA GRAHAM GREGOR HAENSZ HAMMO2 HAMMON HEIN HENNEK HIRAY2 HUMBLE JAHN JAIN JARVHO JEDRYC
JOLY KAISER KATSOU KAUFMA KELLER KHUDER KIHARA KINLEN KJUUS KNEKT KOO LANGE LEMARC LIAW LICKIN LIDDEL
LOMBAR LUBIN LUBIN2 MACLEN MATOS MIGRAN MRFIT MRFITR MURATA NOTANI ODRISC OSANN OSANN2 PARKIN PERSH2 PETO
PEZZO2 PEZZOT PISANI QIAO2 RACHTA RESTRE RIMING SAARIK SCHWAR SHAW SHIMIZ SIMONA SITAS SOBUE SOBUE2 SPITZ
STAYNE STOCKW STUCKE SUZUK2 SUZUKI SVENSS TANG TANG2 TENKAN TIZZAN TOKARS TOUSEY TULINI VEIERO WAKAI WALD
WANG2 WARSIN WATSON WIGLE WU WU2 WUNSCH WYNDE3 WYNDE5 WYNDE6 WYNDE7 WYNDE8 WYNDER YAMAGU YONG

4 HIRAYA

5 COMSTO HITOSU HOLE KANELL KREUZE KUBIK NAM SEGI2 SPEIZE TSUGAN VUTUC

6 KAISE2

Table 1B10 - 8
Potentially overlapping studies

REF	REFGP	PRINC	.	OVERLAP/LINK
TVERDA	TVERDA	1		VEIERO/TVERDAL
CPSI	CPSI	1		CPSI overall

Table 1B11 -

IESLC - Meta-analysis of Current Smoking, Cigarettes only, Age 50-70
All LC types

This analysis is restricted to results for:
1) Non-dose-response data
2) Current smokers
3) Maximum age range 50-70
4) Results complete enough for use in metaanalysis

Within each study, results are then selected (in the following order of preference, within each sex) for:
5) PRODUCT: cigarettes only
6) CIGTYPE: all/unspecified, MC regardless of HR, MC only
7) DENOM: never smoked anything, never smoked cigarettes, (never +1 = +long term ex, +2 = +amount unknown,
+3 = never cigs+long term ex)
8) Followup period (YF, prospective studies): whole study (coded as 0) or longest available
9) LCtype: all or nearest available, at least Squamous and Adeno. (q = squamous, s = small,
l = large, a = adeno, mix = mixed, alv = alveolar)
10) Race: all or nearest available, otherwise by race (wh or w = white, bl or b = black, hi = hispanic
ch = chinese, jap = japanese, haw = hawaiian, w+o = white + oriental, sca = scandinavian, as = asian)
11) For overlapping studies: principal rather than subsidiary studies
Finally by Age: whole study (actual age shown) if available, otherwise by widest available age group
and then for single sex results (m, f) in preference to combined sex results (c).

Results adjusted (AD) for the most potential confounders are then chosen in Sections -1 to -3
(and those which actually differ from the adjusted results in Table 1B3 - 1 are marked 'x' in Section -1)
and results adjusted for the least confounders in Sections -4 to -6. (Those least adjusted results which
actually differ from the most adjusted as marked 'x' in column X in Section -4)
(Results adjusted for an unknown number of confounder(s) are coded as 20.)

Section -7 shows excluded studies, together with the stage (as above) at which no qualifying
results were found.

Section -8 lists the potentially overlapping studies which have been included (1=principal, 2=subsidiary).

Section -9 lists any results which would have been included in preference except that they had data not complete
enough for use in meta-analysis, with their significance (yes/no), if known, and any further comment as entered
on the database.

In addition to those mentioned above, the following fields, levels and abbreviations are used:
* or nk = not known, n = no, y = yes, ot = other
nev = never
all/unspec = all or unspecified, MC = manufactured cigarettes, HR = hand-rolled cigarettes
REF: 6-character study reference
NRR: number of the RR on the database within the study
ST : study type (CC = case control, pr or prosp = prospective)
NLC: number of lung cancer cases in whole study
R : risky occupational population (n = no, m = mining, o = other risky)
VB : national cigarette type (V = at least 75% Virginia, bl = at least 75% blended, ot = other)
P : any proxy use
H : full histological confirmation
De : derivation of RR/CI (or = original, st = standard method, ot = other method of estimation)

Table 1B11 - 1

IESLC - Meta-analysis of Current Smoking, Cigarettes only, Age 50-70
All LC types
Most adjusted

REF	NRR	1B3	SEX	AGEL	AGEH	RACE	YF	LC TYPE	LOC	START	ST	NLC	R	VB	P	H	AD	PRODUCT	DENOM	De
BEST	32	x	m	50	69	all	0	all	NAmer	1955	pr	381	n	V	n	n	1	cig only	nev any	ot
CEDERL	11	x	m	50	59	all	10	all	Eu:Sca	1963	pr	491	n	bl	n	n	0	cig only	nev any	st
CEDERL	19	x	m	60	69	all	10	all	Eu:Sca	1963	pr	491	n	bl	n	n	0	cig only	nev any	st
CEDERL	35	x	f	50	59	all	10	all	Eu:Sca	1963	pr	491	n	bl	n	n	0	cig only	nev any	st
CEDERL	38	x	f	60	69	all	10	all	Eu:Sca	1963	pr	491	n	bl	n	n	0	cig only	nev any	st
CPSI	27	x	m	55	64	wh	0	all	NAmer	1959	pr	5138	n	bl	n	n	0	cig only	nev any	st
CPSI	103	x	f	55	64	wh	0	all	NAmer	1959	pr	5138	n	bl	n	n	0	cig only	nev any	st
CPSII	15	x	m	55	64	all	6	all	NAmer	1982	pr	3229	n	bl	n	n	0	cig only	nev any	st
DEAN3	31	x	m	55	64	all	-	all	Eu:UK	1969	CC	766	n	V	y	n	0	cig only	nev any	st
DEAN3	115	x	f	55	64	all	-	all	Eu:UK	1969	CC	766	n	V	y	n	0	cig only	nev any	st
HAMMON	139		m	50	69	wh	0	all	NAmer	1952	pr	448	n	bl	n	n	1	cig only	nev any	ot

Cigarette type is all/unspec for all RRs
except for the following:

REF	NRR	CIGTYPE
DEAN3	31	MC only
DEAN3	115	MC only

Table 1B11 - 2

IESLC - Meta-analysis of Current Smoking, Cigarettes only, Age 50-70
All LC types
Most adjusted

REF	NRR	SEX	AD	Number Exposed Case	Number Exposed Cont	Non-exposed Case	Non-exposed Cont	RR	95.00%CI
*BEST	32	m	1	-	-	-	-	23.30	(7.45- 72.88)
*CEDERL	11	m	0	10	1418	4	1954	3.44	(1.08- 10.96)
*CEDERL	19	m	0	16	818	3	1645	10.73	(3.13- 36.70)
*CEDERL	35	f	0	4	1159	9	6407	2.46	(0.76- 7.96)
*CEDERL	38	f	0	4	407	10	5877	5.78	(1.82- 18.34)
Subtotal CEDERL								4.70	(2.61- 8.47)
*CPSI	27	m	0	1494	721686	51	352725	14.32	(10.83- 18.93)
*CPSI	103	f	0	249	591967	144	1366561	3.99	(3.25- 4.90)
Subtotal CPSI								6.25	(5.30- 7.37)
*CPSII	15	m	0	641	231659	21	253604	33.42	(21.64- 51.61)
DEAN3	31	m	0	129	211	7	96	8.38	(3.78- 18.62)
DEAN3	115	f	0	36	217	9	310	5.71	(2.70- 12.11)
Subtotal DEAN3								6.84	(3.96- 11.82)
*HAMMON	139	m	1	-	-	-	-	11.52	(6.83- 19.42)
Partial Totals				2583	1549542	258	1989179		

*prospective study

REF	NRR	SEX	AD	Ys	Ws	Qs	Ps
*BEST	32	m	1	3.15	2.95	3.54	0.0000
*CEDERL	11	m	0	1.24	2.87	1.91	0.0362
*CEDERL	19	m	0	2.37	2.54	0.26	0.0002
*CEDERL	35	f	0	0.90	2.78	3.70	0.1341
*CEDERL	38	f	0	1.75	2.88	0.26	0.0029
Subtotal CEDERL				1.55	11.06	6.13	
*CPSI	27	m	0	2.66	49.33	18.24	0.0000
*CPSI	103	f	0	1.38	91.26	40.86	0.0000
Subtotal CPSI				1.83	140.58	59.10	
*CPSII	15	m	0	3.51	20.34	43.09	0.0000
DEAN3	31	m	0	2.13	6.03	0.03	0.0000
DEAN3	115	f	0	1.74	6.82	0.66	0.0000
Subtotal DEAN3				1.92	12.85	0.69	
*HAMMON	139	m	1	2.44	14.07	2.15	0.0000

	N	11
	NS	6
	Wt	201.86
Het	Chi	114.70
Het	df	10
Het	P	***
Fixed	RR	7.79
	RRl	6.79
	RRu	8.95
	P	+++
Random	RR	8.62
	RRl	4.91
	RRu	15.11
	P	+++
Asymm	P	N.S.

Table 1B11 - 3

IESLC - Meta-analysis of Current Smoking, Cigarettes only, Age 50-70
All LC types
Most adjusted

	Sex combined	male	female	Total
N		7	4	11
NS		6	3	9
Wt		98.13	103.73	201.86
Het Chi		23.15	1.88	114.70
Het df		6	3	10
Het P		***	N.S.	***
Fixed RR		15.47	4.08	7.79
RRl		12.69	3.36	6.79
RRu		18.85	4.94	8.95
P		+++	+++	+++
Random RR		13.40	4.08	8.62
RRl		8.34	3.36	4.91
RRu		21.52	4.94	15.11
P		+++	+++	+++
Between Chi				89.68
Between df				1
Between P				***
Btwn(F) P				***
Btwn(R) P				***

Table 1B11 - 4

IESLC - Meta-analysis of Current Smoking, Cigarettes only, Age 50-70
All LC types
Least adjusted

REF	NRR	X	SEX	AGEL	AGEH	RACE	YF	LC TYPE	LOC	START	ST	NLC	R	VB	P	H	AD	PRODUCT	DENOM	De
BEST	32		m	50	69	all	0	all	NAmer	1955	pr	381	n	V	n	n	1	cig only	nev any	ot
CEDERL	11		m	50	59	all	10	all	Eu:Sca	1963	pr	491	n	bl	n	n	0	cig only	nev any	st
CEDERL	19		m	60	69	all	10	all	Eu:Sca	1963	pr	491	n	bl	n	n	0	cig only	nev any	st
CEDERL	35		f	50	59	all	10	all	Eu:Sca	1963	pr	491	n	bl	n	n	0	cig only	nev any	st
CEDERL	38		f	60	69	all	10	all	Eu:Sca	1963	pr	491	n	bl	n	n	0	cig only	nev any	st
CPSI	27		m	55	64	wh	0	all	NAmer	1959	pr	5138	n	bl	n	n	0	cig only	nev any	st
CPSI	103		f	55	64	wh	0	all	NAmer	1959	pr	5138	n	bl	n	n	0	cig only	nev any	st
CPSII	15		m	55	64	all	6	all	NAmer	1982	pr	3229	n	bl	n	n	0	cig only	nev any	st
DEAN3	31		m	55	64	all	-	all	Eu:UK	1969	CC	766	n	V	y	n	0	cig only	nev any	st
DEAN3	115		f	55	64	all	-	all	Eu:UK	1969	CC	766	n	V	y	n	0	cig only	nev any	st
HAMMON	139		m	50	69	wh	0	all	NAmer	1952	pr	448	n	bl	n	n	1	cig only	nev any	ot

Cigarette type is all/unspec for all RRs
except for the following:

REF	NRR	CIGTYPE
DEAN3	31	MC only
DEAN3	115	MC only

Table 1B11 - 5

IESLC - Meta-analysis of Current Smoking, Cigarettes only, Age 50-70
All LC types
Least adjusted

REF	NRR	SEX	AD	Number Exposed Case	Number Exposed Cont	Non-exposed Case	Non-exposed Cont	RR	95.00%CI
*BEST	32	m	1	-	-	-	-	23.30	(7.45- 72.88)
*CEDERL	11	m	0	10	1418	4	1954	3.44	(1.08- 10.96)
*CEDERL	19	m	0	16	818	3	1645	10.73	(3.13- 36.70)
*CEDERL	35	f	0	4	1159	9	6407	2.46	(0.76- 7.96)
*CEDERL	38	f	0	4	407	10	5877	5.78	(1.82- 18.34)
Subtotal CEDERL								4.70	(2.61- 8.47)
*CPSI	27	m	0	1494	721686	51	352725	14.32	(10.83- 18.93)
*CPSI	103	f	0	249	591967	144	1366561	3.99	(3.25- 4.90)
Subtotal CPSI								6.25	(5.30- 7.37)
*CPSII	15	m	0	641	231659	21	253604	33.42	(21.64- 51.61)
DEAN3	31	m	0	129	211	7	96	8.38	(3.78- 18.62)
DEAN3	115	f	0	36	217	9	310	5.71	(2.70- 12.11)
Subtotal DEAN3								6.84	(3.96- 11.82)
*HAMMON	139	m	1	-	-	-	-	11.52	(6.83- 19.42)
Partial Totals				2583	1549542	258	1989179		

*prospective study

REF	NRR	SEX	AD	Ys	Ws	Qs	Ps
*BEST	32	m	1	3.15	2.95	3.54	0.0000
*CEDERL	11	m	0	1.24	2.87	1.91	0.0362
*CEDERL	19	m	0	2.37	2.54	0.26	0.0002
*CEDERL	35	f	0	0.90	2.78	3.70	0.1341
*CEDERL	38	f	0	1.75	2.88	0.26	0.0029
Subtotal CEDERL				1.55	11.06	6.13	
*CPSI	27	m	0	2.66	49.33	18.24	0.0000
*CPSI	103	f	0	1.38	91.26	40.86	0.0000
Subtotal CPSI				1.83	140.58	59.10	
*CPSII	15	m	0	3.51	20.34	43.09	0.0000
DEAN3	31	m	0	2.13	6.03	0.03	0.0000
DEAN3	115	f	0	1.74	6.82	0.66	0.0000
Subtotal DEAN3				1.92	12.85	0.69	
*HAMMON	139	m	1	2.44	14.07	2.15	0.0000

	N	11
	NS	6
	Wt	201.86
Het	Chi	114.70
Het	df	10
Het	P	***
Fixed	RR	7.79
	RRl	6.79
	RRu	8.95
	P	+++
Random	RR	8.62
	RRl	4.91
	RRu	15.11
	P	+++
Asymm	P	N.S.

Table 1B11 - 6

IESLC - Meta-analysis of Current Smoking, Cigarettes only, Age 50-70
All LC types
Least adjusted

	Sex		
combined	male	female	Total
N	7	4	11
NS	6	3	9
Wt	98.13	103.73	201.86
Het Chi	23.15	1.88	114.70
Het df	6	3	10
Het P	***	N.S.	***
Fixed RR	15.47	4.08	7.79
RRl	12.69	3.36	6.79
RRu	18.85	4.94	8.95
P	+++	+++	+++
Random RR	13.40	4.08	8.62
RRl	8.34	3.36	4.91
RRu	21.52	4.94	15.11
P	+++	+++	+++
Between Chi			89.68
Between df			1
Between P			***
Btwn(F) P			***
Btwn(R) P			***

Table 1B11 - 7

IESLC - Meta-analysis of Current Smoking, Cigarettes only, Age 50-70
All LC types
Excluded studies (and stage at which they were excluded)

1	BOUCHA BUELL LAURIL MZILEN
2	ABELIN ABRAHA AUVINE AXELSO BAND BERRIN BLOT1 BLOT2 BLOT3 BLOT4 BRESLO BROCKM BROWN1 BYERS1 CASCO2 CASCOR CHAN CHATZI CHEN CHEN2 CHEN3 CHIAZZ COOKSO DAVEYS DEAN DOSEME DU DUNN EBELIN ESAKI FAN GENG GER GODLEY GOLLED GSELL HANSEN HEGMAN HINDS HIRAOK HOROWI HORWIT HU HU2 HUANG ISHIMA JARUP JIANG JONES JUSSAW KO KOHLME KOULUM KREYBE LAMTH LAMWK LAMWK2 LAUSSM LEI LETOUR LEVIN LIU LIU2 LIU3 LIU4 LIU5 LOMBA2 LUO MAGNUS MARSH MARSH2 MARTIS MASTRA MATSUD MCCONN MCDUFF MCLAUG MILLER MILLS MOLLO NOTAN2 NOU ORMOS PASTOR PAWLEG PERNU PERSHA PIKE POFFIJ POLEDN PRESCO QIAO RADZIK RANDIG REN RONCO ROOTS ROTHSC SADOWS SANKAR SCHWA2 SEGI SEOW SIEMIA SIMARA STASZE STOCKS SUN TAO ULMER VANDER WANG WANG3 WANG4 WICKLU WILKIN WUWILL WYNDE2 WYNDE4 XIANGZ XU XU2 XU3 XU4 YUAN ZHANG ZHENG ZHOU
3	AGUDO AKIBA ALDERS AMANDU AMES ARCHER ARMADA AUSTIN AXELSS BARBON BECHER BENHAM BENSHL BLOHMK BOFFET BOUCOT BRETT BROSS BROWN2 BUFFLE BYERS2 CARPEN CHANG CHOI CHOW CHYOU COMSTO CORREA DAMBER DARBY DEAN2 DEKLER DESTE2 DESTEF DOCKER DOLL DOLL2 DORGAN DROSTE ENGELA ENSTRO GAO GAO2 GARCIA GARDIN GARSHI GILLIS GOODMA GRAHAM GREGOR HAENSZ HAMMO2 HEIN HENNEK HIRAY2 HUMBLE JAHN JAIN JARVHO JEDRYC JOLY KAISER KATSOU KAUFMA KELLER KHUDER KIHARA KINLEN KJUUS KNEKT KOO LANGE LEMARC LIAW LICKIN LIDDEL LOMBAR LUBIN LUBIN2 MACLEN MATOS MIGRAN MRFIT MRFITR MURATA NOTANI ODRISC OSANN OSANN2 PARKIN PERSH2 PETO PEZZO2 PEZZOT PISANI QIAO2 RACHTA RESTRE RIMING SAARIK SCHWAR SHAW SHIMIZ SIMONA SITAS SOBUE SOBUE2 SPEIZE SPITZ STAYNE STOCKW STUCKE SUZUK2 SUZUKI SVENSS TANG TANG2 TENKAN TIZZAN TOKARS TOUSEY TSUGAN TULINI TVERDA VEIERO VUTUC WAKAI WALD WANG2 WARSIN WATSON WIGLE WU WU2 WUNSCH WYNDE3 WYNDE5 WYNDE6 WYNDE7 WYNDE8 WYNDER YAMAGU YONG
4	HIRAYA
5	ANDERS DORANT DORN HITOSU HOLE KANELL KREUZE KUBIK NAM SEGI2
6	KAISE2

Table 1B11 - 8
Potentially overlapping studies

REF	REFGP	PRINC	.	OVERLAP/LINK
CPSI	CPSI	1		CPSI overall

Table 1B12 -

IESLC - Meta-analysis of Current Smoking, Cigarettes only, Age 65+
All LC types

This analysis is restricted to results for:
1) Non-dose-response data
2) Current smokers
3) Age 65+
4) Results complete enough for use in metaanalysis

Within each study, results are then selected (in the following order of preference, within each sex) for:
5) PRODUCT: cigarettes only
6) CIGTYPE: all/unspecified, MC regardless of HR, MC only
7) DENOM: never smoked anything, never smoked cigarettes, (never +1 = +long term ex, +2 = +amount unknown,
+3 = never cigs+long term ex)
8) Followup period (YF, prospective studies): whole study (coded as 0) or longest available
9) LCtype: all or nearest available, at least Squamous and Adeno. (q = squamous, s = small,
l = large, a = adeno, mix = mixed, alv = alveolar)
10) Race: all or nearest available, otherwise by race (wh or w = white, bl or b = black, hi = hispanic
ch = chinese, jap = japanese, haw = hawaiian, w+o = white + oriental, sca = scandinavian, as = asian)
11) For overlapping studies: principal rather than subsidiary studies
Finally by Age: whole study (actual age shown) if available, otherwise by widest available age group
and then for single sex results (m, f) in preference to combined sex results (c).

Results adjusted (AD) for the most potential confounders are then chosen in Sections -1 to -3
(and those which actually differ from the adjusted results in Table 1B3 - 1 are marked 'x' in Section -1)
and results adjusted for the least confounders in Sections -4 to -6. (Those least adjusted results which
actually differ from the most adjusted as marked 'x' in column X in Section -4)
(Results adjusted for an unknown number of confounder(s) are coded as 20.)

Section -7 shows excluded studies, together with the stage (as above) at which no qualifying
results were found.

Section -8 lists the potentially overlapping studies which have been included (1=principal, 2=subsidiary).

Section -9 lists any results which would have been included in preference except that they had data not complete
enough for use in meta-analysis, with their significance (yes/no), if known, and any further comment as entered
on the database.

In addition to those mentioned above, the following fields, levels and abbreviations are used:
* or nk = not known, n = no, y = yes, ot = other
nev = never
all/unspec = all or unspecified, MC = manufactured cigarettes, HR = hand-rolled cigarettes
REF: 6-character study reference
NRR: number of the RR on the database within the study
ST : study type (CC = case control, pr or prosp = prospective)
NLC: number of lung cancer cases in whole study
R : risky occupational population (n = no, m = mining, o = other risky)
VB : national cigarette type (V = at least 75% Virginia, bl = at least 75% blended, ot = other)
P : any proxy use
H : full histological confirmation
De : derivation of RR/CI (or = original, st = standard method, ot = other method of estimation)

Table 1B12 - 1

IESLC - Meta-analysis of Current Smoking, Cigarettes only, Age 65+
All LC types
Most adjusted

REF	NRR	1B3	SEX	AGEL	AGEH	RACE	YF	LC TYPE	LOC	START	ST	NLC	R	VB	P	H	AD	PRODUCT	DENOM	De
BEST	33	x	m	70	99	all	0	all	NAmer	1955	pr	381	n	V	n	n	1	cig only	nev any	ot
CPSI	28	x	m	65	74	wh	0	all	NAmer	1959	pr	5138	n	bl	n	n	0	cig only	nev any	st
CPSI	29	x	m	75	84	wh	0	all	NAmer	1959	pr	5138	n	bl	n	n	0	cig only	nev any	st
CPSI	104	x	f	65	74	wh	0	all	NAmer	1959	pr	5138	n	bl	n	n	0	cig only	nev any	st
CPSI	105	x	f	75	84	wh	0	all	NAmer	1959	pr	5138	n	bl	n	n	0	cig only	nev any	st
CPSII	22	x	m	65	74	all	6	all	NAmer	1982	pr	3229	n	bl	n	n	0	cig only	nev any	st
CPSII	29	x	m	75	99	all	6	all	NAmer	1982	pr	3229	n	bl	n	n	0	cig only	nev any	st
DEAN3	32	x	m	65	99	all	-	all	Eu:UK	1969	CC	766	n	V	y	n	0	cig only	nev any	st
DEAN3	116	x	f	65	99	all	-	all	Eu:UK	1969	CC	766	n	V	y	n	0	cig only	nev any	st
DORN	71	x	m	65	84	wh	8	all	NAmer	1954	pr	5097	n	bl	n	n	1	cig only	nev any	ot
KAISE2	36	x	m	65	74	all	9	all	NAmer	1979	pr	318	n	bl	n	n	0	cig only	nev any	st
KAISE2	44	x	m	75	99	all	9	all	NAmer	1979	pr	318	n	bl	n	n	0	cig only	nev any	st
KAISE2	12	x	f	65	74	all	9	all	NAmer	1979	pr	318	n	bl	n	n	0	cig only	nev any	st
KAISE2	20	x	f	75	99	all	9	all	NAmer	1979	pr	318	n	bl	n	n	0	cig only	nev any	st

Cigarette type is all/unspec for all RRs
except for the following:

REF	NRR	CIGTYPE
DEAN3	32	MC only
DEAN3	116	MC only

Table 1B12 - 2

IESLC - Meta-analysis of Current Smoking, Cigarettes only, Age 65+
All LC types
Most adjusted

REF	NRR	SEX	AD	Number Exposed Case	Number Exposed Cont	Non-exposed Case	Non-exposed Cont	RR	95.00%CI
*BEST	33	m	1	-	-	-	-	12.43	(3.92- 39.43)
*CPSI	28	m	0	1210	275308	73	226284	13.62	(10.76- 17.25)
*CPSI	29	m	0	339	50531	58	91417	10.57	(8.01- 13.97)
*CPSI	104	f	0	145	177746	178	957682	4.39	(3.53- 5.46)
*CPSI	105	f	0	28	31384	163	392909	2.15	(1.44- 3.21)
Subtotal CPSI								7.02	(6.16- 8.01)
*CPSII	22	m	0	743	106505	47	173660	25.78	(19.20- 34.61)
*CPSII	29	m	0	230	20335	44	66378	17.06	(12.36- 23.55)
Subtotal CPSII								21.36	(17.19- 26.55)
DEAN3	32	m	0	157	145	16	82	5.55	(3.10- 9.92)
DEAN3	116	f	0	38	128	27	574	6.31	(3.72- 10.71)
Subtotal DEAN3								5.95	(4.03- 8.81)
*DORN	71	m	1	-	-	-	-	9.58	(7.18- 12.79)
*KAISE2	36	m	0	16	4735	5	11466	7.75	(2.84- 21.14)
*KAISE2	44	m	0	7	1035	4	4486	7.59	(2.22- 25.86)
*KAISE2	12	f	0	19	7165	2	24159	32.03	(7.46- 137.49)
*KAISE2	20	f	0	6	1394	3	12285	17.63	(4.41- 70.40)
Subtotal KAISE2								11.66	(6.31- 21.55)
Partial Totals				2938	676411	620	1961382		

*prospective study

REF	NRR	SEX	AD	Ys	Ws	Qs	Ps
*BEST	33	m	1	2.52	2.88	0.25	0.0000
*CPSI	28	m	0	2.61	68.88	10.25	0.0000
*CPSI	29	m	0	2.36	49.60	0.87	0.0000
*CPSI	104	f	0	1.48	79.95	44.62	0.0000
*CPSI	105	f	0	0.77	23.91	51.01	0.0002
Subtotal CPSI				1.95	222.35	106.74	
*CPSII	22	m	0	3.25	44.23	46.32	0.0000
*CPSII	29	m	0	2.84	37.02	13.81	0.0000
Subtotal CPSII				3.06	81.26	60.13	
DEAN3	32	m	0	1.71	11.37	2.99	0.0000
DEAN3	116	f	0	1.84	13.72	2.02	0.0000
Subtotal DEAN3				1.78	25.08	5.01	
*DORN	71	m	1	2.26	46.10	0.05	0.0000
*KAISE2	36	m	0	2.05	3.81	0.12	0.0001
*KAISE2	44	m	0	2.03	2.55	0.10	0.0012
*KAISE2	12	f	0	3.47	1.81	2.79	0.0000
*KAISE2	20	f	0	2.87	2.00	0.83	0.0000
Subtotal KAISE2				2.46	10.18	3.84	

	N	14
	NS	6
	Wt	387.85
Het	Chi	176.01
Het	df	13
Het	P	***
Fixed	RR	9.26
	RRl	8.39
	RRu	10.23
	P	+++
Random	RR	9.34
	RRl	6.21
	RRu	14.06
	P	+++
Asymm	P	N.S.

Table 1B12 - 3

IESLC - Meta-analysis of Current Smoking, Cigarettes only, Age 65+
All LC types
Most adjusted

	Sex combined	male	female	Total
N		9	5	14
NS		6	3	9
Wt		266.46	121.39	387.85
Het Chi		39.89	24.74	176.01
Het df		8	4	13
Het P		***	***	***
Fixed RR		13.30	4.19	9.26
RRl		11.80	3.51	8.39
RRu		15.00	5.00	10.23
P		+++	+++	+++
Random RR		12.03	5.81	9.34
RRl		8.84	3.16	6.21
RRu		16.37	10.67	14.06
P		+++	+++	+++
Between Chi				111.38
Between df				1
Between P				***
Btwn(F) P				***
Btwn(R) P				*

Table 1B12 - 4

IESLC - Meta-analysis of Current Smoking, Cigarettes only, Age 65+
All LC types
Least adjusted

REF	NRR	X	SEX	AGEL	AGEH	RACE	YF	LC TYPE	LOC	START	ST	NLC	R	VB	P	H	AD	PRODUCT	DENOM	De
BEST	33		m	70	99	all	0	all	NAmer	1955	pr	381	n	V	n	n	1	cig only	nev any	ot
CPSI	28		m	65	74	wh	0	all	NAmer	1959	pr	5138	n	bl	n	n	0	cig only	nev any	st
CPSI	29		m	75	84	wh	0	all	NAmer	1959	pr	5138	n	bl	n	n	0	cig only	nev any	st
CPSI	104		f	65	74	wh	0	all	NAmer	1959	pr	5138	n	bl	n	n	0	cig only	nev any	st
CPSI	105		f	75	84	wh	0	all	NAmer	1959	pr	5138	n	bl	n	n	0	cig only	nev any	st
CPSII	22		m	65	74	all	6	all	NAmer	1982	pr	3229	n	bl	n	n	0	cig only	nev any	st
CPSII	29		m	75	99	all	6	all	NAmer	1982	pr	3229	n	bl	n	n	0	cig only	nev any	st
DEAN3	32		m	65	99	all	-	all	Eu:UK	1969	CC	766	n	V	y	n	0	cig only	nev any	st
DEAN3	116		f	65	99	all	-	all	Eu:UK	1969	CC	766	n	V	y	n	0	cig only	nev any	st
DORN	71		m	65	84	wh	8	all	NAmer	1954	pr	5097	n	bl	n	n	1	cig only	nev any	ot
KAISE2	36		m	65	74	all	9	all	NAmer	1979	pr	318	n	bl	n	n	0	cig only	nev any	st
KAISE2	44		m	75	99	all	9	all	NAmer	1979	pr	318	n	bl	n	n	0	cig only	nev any	st
KAISE2	12		f	65	74	all	9	all	NAmer	1979	pr	318	n	bl	n	n	0	cig only	nev any	st
KAISE2	20		f	75	99	all	9	all	NAmer	1979	pr	318	n	bl	n	n	0	cig only	nev any	st

Cigarette type is all/unspec for all RRs
except for the following:

REF	NRR	CIGTYPE
DEAN3	32	MC only
DEAN3	116	MC only

Table 1B12 - 5

IESLC - Meta-analysis of Current Smoking, Cigarettes only, Age 65+
All LC types
Least adjusted

REF	NRR	SEX	AD	Number Exposed Case	Number Exposed Cont	Non-exposed Case	Non-exposed Cont	RR	95.00%CI
*BEST	33	m	1	-	-	-	-	12.43	(3.92- 39.43)
*CPSI	28	m	0	1210	275308	73	226284	13.62	(10.76- 17.25)
*CPSI	29	m	0	339	50531	58	91417	10.57	(8.01- 13.97)
*CPSI	104	f	0	145	177746	178	957682	4.39	(3.53- 5.46)
*CPSI	105	f	0	28	31384	163	392909	2.15	(1.44- 3.21)
Subtotal CPSI								7.02	(6.16- 8.01)
*CPSII	22	m	0	743	106505	47	173660	25.78	(19.20- 34.61)
*CPSII	29	m	0	230	20335	44	66378	17.06	(12.36- 23.55)
Subtotal CPSII								21.36	(17.19- 26.55)
DEAN3	32	m	0	157	145	16	82	5.55	(3.10- 9.92)
DEAN3	116	f	0	38	128	27	574	6.31	(3.72- 10.71)
Subtotal DEAN3								5.95	(4.03- 8.81)
*DORN	71	m	1	-	-	-	-	9.58	(7.18- 12.79)
*KAISE2	36	m	0	16	4735	5	11466	7.75	(2.84- 21.14)
*KAISE2	44	m	0	7	1035	4	4486	7.59	(2.22- 25.86)
*KAISE2	12	f	0	19	7165	2	24159	32.03	(7.46- 137.49)
*KAISE2	20	f	0	6	1394	3	12285	17.63	(4.41- 70.40)
Subtotal KAISE2								11.66	(6.31- 21.55)
Partial Totals				2938	676411	620	1961382		

*prospective study

REF	NRR	SEX	AD	Ys	Ws	Qs	Ps
*BEST	33	m	1	2.52	2.88	0.25	0.0000
*CPSI	28	m	0	2.61	68.88	10.25	0.0000
*CPSI	29	m	0	2.36	49.60	0.87	0.0000
*CPSI	104	f	0	1.48	79.95	44.62	0.0000
*CPSI	105	f	0	0.77	23.91	51.01	0.0002
Subtotal CPSI				1.95	222.35	106.74	
*CPSII	22	m	0	3.25	44.23	46.32	0.0000
*CPSII	29	m	0	2.84	37.02	13.81	0.0000
Subtotal CPSII				3.06	81.26	60.13	
DEAN3	32	m	0	1.71	11.37	2.99	0.0000
DEAN3	116	f	0	1.84	13.72	2.02	0.0000
Subtotal DEAN3				1.78	25.08	5.01	
*DORN	71	m	1	2.26	46.10	0.05	0.0000
*KAISE2	36	m	0	2.05	3.81	0.12	0.0001
*KAISE2	44	m	0	2.03	2.55	0.10	0.0012
*KAISE2	12	f	0	3.47	1.81	2.79	0.0000
*KAISE2	20	f	0	2.87	2.00	0.83	0.0000
Subtotal KAISE2				2.46	10.18	3.84	

	N	14
	NS	6
	Wt	387.85
Het	Chi	176.01
Het	df	13
Het	P	***
Fixed	RR	9.26
	RRl	8.39
	RRu	10.23
	P	+++
Random	RR	9.34
	RRl	6.21
	RRu	14.06
	P	+++
Asymm	P	N.S.

Table 1B12 - 6

IESLC - Meta-analysis of Current Smoking, Cigarettes only, Age 65+
All LC types
Least adjusted

	Sex combined	male	female	Total
N		9	5	14
NS		6	3	9
Wt		266.46	121.39	387.85
Het Chi		39.89	24.74	176.01
Het df		8	4	13
Het P		***	***	***
Fixed RR		13.30	4.19	9.26
RRl		11.80	3.51	8.39
RRu		15.00	5.00	10.23
P		+++	+++	+++
Random RR		12.03	5.81	9.34
RRl		8.84	3.16	6.21
RRu		16.37	10.67	14.06
P		+++	+++	+++
Between Chi				111.38
Between df				1
Between P				***
Btwn(F) P				***
Btwn(R) P				*

Table 1B12 - 7

IESLC - Meta-analysis of Current Smoking, Cigarettes only, Age 65+
All LC types
Excluded studies (and stage at which they were excluded)

1 BOUCHA BUELL LAURIL MZILEN

2 ABELIN ABRAHA AUVINE AXELSO BAND BERRIN BLOT1 BLOT2 BLOT3 BLOT4 BRESLO BROCKM BROWN1 BYERS1 CASCO2 CASCOR
CHAN CHATZI CHEN CHEN2 CHEN3 CHIAZZ COOKSO DAVEYS DEAN DOSEME DU DUNN EBELIN ESAKI FAN GENG
GER GODLEY GOLLED GSELL HANSEN HEGMAN HINDS HIRAOK HOROWI HORWIT HU HU2 HUANG ISHIMA JARUP JIANG
JONES JUSSAW KO KOHLME KOULUM KREYBE LAMTH LAMWK LAMWK2 LAUSSM LEI LETOUR LEVIN LIU LIU2 LIU3
LIU4 LIU5 LOMBA2 LUO MAGNUS MARSH MARSH2 MARTIS MASTRA MATSUD MCCONN MCDUFF MCLAUG MILLER MILLS MOLLO
NOTAN2 NOU ORMOS PASTOR PAWLEG PERNU PERSHA PIKE POFFIJ POLEDN PRESCO QIAO RADZIK RANDIG REN RONCO
ROOTS ROTHSC SADOWS SANKAR SCHWA2 SEGI SEOW SIEMIA SIMARA STASZE STOCKS SUN TAO ULMER VANDER WANG
WANG3 WANG4 WICKLU WILKIN WUWILL WYNDE2 WYNDE4 XIANGZ XU XU2 XU3 XU4 YUAN ZHANG ZHENG ZHOU

3 AGUDO AKIBA ALDERS AMANDU AMES ANDERS ARCHER ARMADA AUSTIN AXELSS BARBON BECHER BENHAM BENSHL BLOHMK BOFFET
BOUCOT BRETT BROSS BROWN2 BUFFLE BYERS2 CARPEN CEDERL CHANG CHOI CHOW CHYOU CORREA DAMBER DARBY DEAN2
DEKLER DESTE2 DESTEF DOCKER DOLL DOLL2 DORANT DORGAN DROSTE ENGELA ENSTRO GAO GAO2 GARCIA GARDIN GILLIS
GOODMA GRAHAM GREGOR HAENSZ HAMMO2 HAMMON HEIN HENNEK HIRAY2 HITOSU HOLE JAHN JAIN JARVHO JEDRYC JOLY
KAISER KATSOU KAUFMA KELLER KHUDER KIHARA KINLEN KJUUS KNEKT KOO KREUZE KUBIK LANGE LEMARC LIAW LICKIN
LIDDEL LOMBAR LUBIN LUBIN2 MACLEN MATOS MIGRAN MRFIT MRFITR MURATA NOTANI ODRISC OSANN OSANN2 PARKIN PERSH2
PETO PEZZO2 PEZZOT PISANI QIAO2 RACHTA RESTRE RIMING SAARIK SCHWAR SHAW SHIMIZ SIMONA SITAS SOBUE SOBUE2
SPEIZE SPITZ STAYNE STOCKW STUCKE SUZUK2 SUZUKI SVENSS TANG TANG2 TIZZAN TOKARS TOUSEY TSUGAN TULINI TVERDA
VEIERO VUTUC WAKAI WALD WANG2 WARSIN WATSON WIGLE WU WU2 WUNSCH WYNDE3 WYNDE5 WYNDE6 WYNDE7 WYNDE8
WYNDER YAMAGU YONG

4 HIRAYA

5 COMSTO GARSHI HUMBLE KANELL NAM SEGI2 TENKAN

Table 1B12 - 8

Potentially overlapping studies

REF	REFGP	PRINC	.	OVERLAP/LINK
CPSI	CPSI	1		CPSI overall

Table 1B13 -

IESLC - Meta-analysis of Current Smoking (vs non-current), Any product (or Cigarettes if Any not available)
All LC types

This analysis is restricted to results for:
1) Non-dose-response data
2) Current smokers
3) Results complete enough for use in metaanalysis

Within each study, results are then selected (in the following order of preference, within each sex) for:
4) PRODUCT: all/unspec, cigarettes regardless of other products, cigarettes only
5) CIGTYPE: all/unspecified, MC regardless of HR, MC only
6) DENOM: non smoker of anything, non smoker of cigarettes
7) Followup period (YF, prospective studies): whole study (coded as 0) or longest available
8) LCtype: all or nearest available, at least Squamous and Adeno. (q = squamous, s = small,
l = large, a = adeno, mix = mixed, alv = alveolar)
9) Race: all or nearest available, otherwise by race (wh or w = white, bl or b = black, hi = hispanic
ch = chinese, jap = japanese, haw = hawaiian, w+o = white + oriental, sca = scandinavian, as = asian)
10) For overlapping studies: principal rather than subsidiary studies
Finally by Age: whole study (coded as 0) if available, otherwise by widest available age group
and then for single sex results (m, f) in preference to combined sex results (c).

Results adjusted (AD) for the most potential confounders are then chosen in Sections -1 to -3
and results adjusted for the least confounders in Sections -4 to -6. (Those least adjusted results which
actually differ from the most adjusted as marked 'x' in column X in Section -4)
(Results adjusted for an unknown number of confounder(s) are coded as 20.)

Section -7 shows excluded studies, together with the stage (as above) at which no qualifying
results were found.

Section -8 lists the potentially overlapping studies which have been included (1=principal, 2=subsidiary).

Section -9 lists any results which would have been included in preference except that they had data not complete
enough for use in meta-analysis, with their significance (yes/no), if known, and any further comment as entered
on the database.

In addition to those mentioned above, the following fields, levels and abbreviations are used:
* or nk = not known, n = no, y = yes, ot = other
non = not current
all/unspec = all or unspecified, cig+/-ot = cigarettes irrespective of other products (cigar, pipe etc)
MC = manufactured cigarettes, HR = hand-rolled cigarettes
REF: 6-character study reference
NRR: number of the RR on the database within the study
ST : study type (CC = case control, pr or prosp = prospective)
NLC: number of lung cancer cases in whole study
R : risky occupational population (n = no, m = mining, o = other risky)
VB : national cigarette type (V = at least 75% Virginia, bl = at least 75% blended, ot = other)
P : any proxy use
H : full histological confirmation
De : derivation of RR/CI (or = original, st = standard method, ot = other method of estimation)

Table 1B13 - 1

IESLC - Meta-analysis of Current Smoking (vs non-current), Any product (or Cigarettes if Any not available)
All LC types
Most adjusted

REF	NRR	SEX	AGEL	AGEH	RACE	YF	LC TYPE	LOC	START	ST	NLC	R	VB	P	H	AD	PRODUCT	DENOM	De
AGUDO	15	f	0	0	all	-	all	Eu:wst	1989	CC	103	n	bl	n	n	3	cig only	non any	ot
AKIBA	12	m	0	0	all	0	all	As:Jap	1963	pr	610	n	bl	n	n	5	cig+/-ot	non cigs	ot
AKIBA	16	f	0	0	all	0	all	As:Jap	1963	pr	610	n	bl	n	n	5	cig+/-ot	non cigs	ot
AMANDU	8	m	0	0	wh	0	all	NAmer	1959	pr	132	m	bl	n	n	2	cig+/-ot	non cigs	ot
AMES	5	m	0	0	wh	-	all	NAmer	1959	ot	317	m	bl	n	n	0	all/unsp	non any	st
ANDERS	4	f	0	0	all	0	all	NAmer	1986	pr	343	n	bl	n	n	0	cig+/-ot	non cigs	st
ARCHER	10	m	0	0	wh	0	all	NAmer	1950	pr	146	m	bl	n	n	0	cig+/-ot	non cigs	st
ARMADA	24	m	0	0	all	-	all	Eu:wst	1986	CC	325	n	bl	n	y	1	cig+/-ot	non cigs	ot
AUSTIN	8	c	0	0	all	-	all	NAmer	1970	CC	166	o	bl	y	n	3	cig+/-ot	non cigs	ot
AXELSS	3	m	0	0	sca	-	all	Eu:Sca	1989	CC	436	n	bl	n	n	0	all/unsp	non any	st
AXELSS	12	f	0	0	sca	-	all	Eu:Sca	1989	CC	436	n	bl	n	n	0	all/unsp	non any	st
BARBON	109	m	0	0	all	-	all	Eu:wst	1979	CC	755	n	bl	y	y	1	all/unsp	non any	ot
BECHER	19	m	0	0	all	-	all	Eu:Ger	1985	CC	194	n	bl	n	y	0	all/unsp	non any	st
BECHER	20	f	0	0	all	-	all	Eu:Ger	1985	CC	194	n	bl	n	y	0	all/unsp	non any	st
BENSHL	17	m	40	64	all	10	all	Eu:UK	1967	pr	486	n	V	n	n	1	all/unsp	non any	ot
BLOHMK	4	m	0	0	all	-	all	Eu:Ger	1978	CC	888	n	bl	n	y	0	all/unsp	non any	st
BRETT	8	m	0	0	all	0	all	Eu:UK	1960	pr	150	n	V	n	n	0	cig+/-ot	non cigs	st
BROSS	17	m	0	0	wh	-	all	NAmer	1960	CC	974	n	bl	n	n	0	cig+/-ot	non cigs	st
BUFFLE	41	m	0	0	wh	-	all	NAmer	1976	CC	943	n	bl	y	n	0	cig+/-ot	non cigs	st
BUFFLE	42	f	0	0	wh	-	all	NAmer	1976	CC	943	n	bl	y	n	0	cig+/-ot	non cigs	st
BYERS2	1	m	0	0	wh	-	all	NAmer	1980	CC	448	n	bl	n	y	0	cig+/-ot	non cigs	st
BYERS2	2	f	0	0	wh	-	all	NAmer	1980	CC	448	n	bl	n	y	0	cig+/-ot	non cigs	st
CARPEN	13	c	0	0	w+b	-	all	NAmer	1991	CC	356	n	bl	n	n	3	cig+/-ot	non cigs	ot
CEDERL	108	m	0	0	all	16	all	Eu:Sca	1963	pr	491	n	bl	n	n	2	all/unsp	non any	ot
CEDERL	113	f	0	0	all	0	all	Eu:Sca	1963	pr	491	n	bl	n	n	2	all/unsp	non any	ot
CHANG	13	m	0	0	all	0	all	NAmer	1972	pr	136	n	bl	n	n	0	cig+/-ot	non cigs	st
CHANG	14	f	0	0	all	0	all	NAmer	1972	pr	136	n	bl	n	n	0	cig+/-ot	non cigs	st
CHOI	4	m	0	0	all	-	all	As:oth	1985	CC	375	n	bl	n	n	0	cig+/-ot	non cigs	st
CHOI	8	f	0	0	all	-	all	As:oth	1985	CC	375	n	bl	n	n	0	cig+/-ot	non cigs	st
CHOW	47	m	0	0	wh	0	all	NAmer	1966	pr	219	n	bl	n	n	0	all/unsp	non any	st
CHYOU	8	m	0	0	jap	0	all	NAmer	1965	pr	227	n	bl	n	y	1	cig+/-ot	non cigs	ot
COMSTO	90	c	0	0	all	-	all	NAmer	1975	ot	258	n	bl	n	n	1	cig+/-ot	non cigs	st
CORREA	54	c	0	0	all	-	all	NAmer	1979	CC	1359	n	bl	y	n	1	cig+/-ot	non cigs	ot
CPSI	74	m	0	0	wh	0	all	NAmer	1959	pr	5138	n	bl	n	n	1	cig only	non any	st
CPSI	285	f	40	74	all	6	all	NAmer	1959	pr	5138	n	bl	n	n	1	cig+/-ot	non cigs	ot
CPSII	110	m	35	99	all	4	all	NAmer	1982	pr	3229	n	bl	n	n	1	cig only	non cigs	ot
CPSII	85	f	0	0	all	4	all	NAmer	1982	pr	3229	n	bl	n	n	1	cig+/-ot	non cigs	ot
DARBY	21	m	0	0	wh	-	all	Eu:UK	1988	CC	982	n	V	n	n	0	all/unsp	non any	st
DARBY	30	f	0	0	wh	-	all	Eu:UK	1988	CC	982	n	V	n	n	0	all/unsp	non any	st
DEAN2	4	m	0	0	all	-	all	Eu:UK	1960	CC	954	n	V	y	n	0	all/unsp	non any	st
DEAN2	8	f	0	0	all	-	all	Eu:UK	1960	CC	954	n	V	y	n	0	all/unsp	non any	st
DEAN3	84	m	0	0	all	-	all	Eu:UK	1969	CC	766	n	V	y	n	3	all/unsp	non any	ot
DEAN3	154	f	0	0	all	-	all	Eu:UK	1969	CC	766	n	V	y	n	3	cig only	non any	ot
DEKLER	9	m	0	0	all	0	all	Auslia	1961	pr	138	m	V	n	n	2	all/unsp	non any	ot
DESTE2	19	c	0	0	all	-	all	SCAmer	1993	CC	463	n	bl	n	n	7	all/unsp	non any	ot
DESTEF	55	m	0	0	all	-	all	SCAmer	1988	CC	497	n	bl	n	y	4	all/unsp	non any	ot
DOCKER	4	c	0	0	wh	0	all	NAmer	1974	pr	120	n	bl	n	n	4	cig+/-ot	non cigs	ot
DOLL	92	m	0	0	all	-	all	Eu:UK	1948	CC	1465	n	V	n	n	0	all/unsp	non any	st
DOLL	95	f	0	0	all	-	all	Eu:UK	1948	CC	1465	n	V	n	n	0	all/unsp	non any	st
DOLL2	57	m	0	0	all	0	all	Eu:UK	1951	pr	920	n	V	n	n	1	all/unsp	non any	ot
DORANT	4	m	0	0	all	0	all	Eu:wst	1986	ot	550	n	bl	n	y	0	all/unsp	non any	st
DORGAN	14	m	0	0	wh	-	all	NAmer	1980	CC	2026	n	bl	y	y	0	cig+/-ot	non cigs	st
DORGAN	38	m	0	0	bl	-	all	NAmer	1980	CC	2026	n	bl	y	y	0	cig+/-ot	non cigs	st
DORGAN	61	f	0	0	wh	-	all	NAmer	1980	CC	2026	n	bl	y	y	0	cig+/-ot	non cigs	st
DORGAN	84	f	0	0	bl	-	all	NAmer	1980	CC	2026	n	bl	y	y	0	cig+/-ot	non cigs	st
DORN	199	m	35	84	wh	8	all	NAmer	1954	pr	5097	n	bl	n	n	1	all/unsp	non any	ot
DROSTE	8	m	0	0	all	-	all	Eu:wst	1995	CC	478	n	bl	n	y	4	all/unsp	non any	ot
ENGELA	160	m	0	0	all	12	all	Eu:Sca	1964	pr	435	n	bl	n	n	1	all/unsp	non any	ot
ENGELA	166	f	0	0	all	12	all	Eu:Sca	1964	pr	435	n	bl	n	n	1	all/unsp	non any	ot
GAO	37	m	0	0	all	-	all	As:Chi	1984	CC	1405	n	ot	n	n	2	cig+/-ot	non cigs	ot
GAO	38	f	0	0	all	-	all	As:Chi	1984	CC	1405	n	ot	n	n	2	cig+/-ot	non cigs	ot
GAO2	11	m	0	0	all	-	all	As:Jap	1988	CC	282	n	bl	n	n	1	cig+/-ot	non cigs	ot
GARCIA	4	c	0	0	all	-	all	NAmer	1992	CC	416	n	bl	n	y	0	cig+/-ot	non cigs	st
GARDIN	8	c	0	0	all	-	all	Eu:UK	1988	CC	143	n	V	y	n	0	all/unsp	non any	st
GARSHI	32	m	0	0	all	-	all	NAmer	1981	CC	1081	o	bl	y	n	1	all/unsp	non any	st
GRAHAM	36	m	0	0	wh	-	all	NAmer	1956	CC	685	n	bl	n	n	1	all/unsp	non any	ot
GREGOR	4	m	0	0	all	-	all	Eu:UK	1976	CC	104	n	V	n	y	0	cig+/-ot	non cigs	st
GREGOR	8	f	0	0	all	-	all	Eu:UK	1976	CC	104	n	V	n	y	0	cig+/-ot	non cigs	st
HAENSZ	66	f	0	0	all	-	not alv	NAmer	1955	CC	158	n	bl	n	y	0	cig+/-ot	non cigs	st
HAMMO2	29	m	0	0	all	0	all	NAmer	1967	pr	450	o	bl	n	n	1	cig+/-ot	non cigs	ot
HEIN	12	m	0	0	all	0	all	Eu:Sca	1970	pr	144	n	bl	n	n	0	all/unsp	non any	st
HENNEK	4	m	0	0	all	0	all	NAmer	1982	pr	169	n	bl	n	n	0	all/unsp	non any	st

Table 1B13 - 1

IESLC - Meta-analysis of Current Smoking (vs non-current), Any product (or Cigarettes if Any not available)
All LC types
Most adjusted

REF	NRR	SEX	AGEL	AGEH	RACE	YF	LC TYPE	LOC	START	ST	NLC	R	VB	P	H	AD	PRODUCT	DENOM	De
HIRAY2	10	m	0	0	all	-	all	As:Jap	1950	CC	145	n	bl	*	n	1	all/unsp	non any	ot
HIRAYA	148	m	0	0	all	0	all	As:Jap	1965	pr	1917	n	bl	n	n	1	cig+/-ot	non any	ot
HIRAYA	151	f	0	0	all	0	all	As:Jap	1965	pr	1917	n	bl	n	n	1	cig+/-ot	non any	ot
HITOSU	39	m	0	0	all	-	all	As:Jap	1960	CC	216	n	bl	y	n	1	all/unsp	non any	st
HITOSU	63	f	0	0	all	-	all	As:Jap	1960	CC	216	n	bl	y	n	1	all/unsp	non any	st
HOLE	40	m	0	0	all	0	all	Eu:UK	1972	pr	225	n	V	n	n	1	all/unsp	non any	ot
HOLE	71	f	0	0	all	9	all	Eu:UK	1972	pr	225	n	V	n	n	1	cig+/-ot	non any	st
HUMBLE	63	m	0	0	w-hi	-	all	NAmer	1980	CC	521	n	bl	y	n	1	cig+/-ot	non cigs	ot
HUMBLE	64	m	0	0	hi	-	all	NAmer	1980	CC	521	n	bl	y	n	1	cig+/-ot	non cigs	ot
HUMBLE	65	f	0	0	w-hi	-	all	NAmer	1980	CC	521	n	bl	y	n	1	cig+/-ot	non cigs	ot
HUMBLE	66	f	0	0	hi	-	all	NAmer	1980	CC	521	n	bl	y	n	1	cig+/-ot	non cigs	ot
JAHN	32	m	0	0	all	-	all	Eu:Ger	1988	CC	1004	n	bl	n	n	0	cig+/-ot	non cigs	st
JAIN	56	m	0	0	all	-	all	NAmer	1981	CC	845	n	V	y	n	2	cig+/-ot	non cigs	ot
JAIN	54	f	0	0	all	-	all	NAmer	1981	CC	845	n	V	y	n	2	cig+/-ot	non cigs	ot
JARVHO	4	m	0	0	all	-	all	Eu:Sca	1983	CC	147	n	bl	n	n	0	all/unsp	non any	st
JARVHO	8	f	0	0	all	-	all	Eu:Sca	1983	CC	147	n	bl	n	n	0	all/unsp	non any	st
JOLY	32	m	0	0	all	-	all	SCAmer	1978	CC	826	n	bl	n	n	0	all/unsp	non any	st
JOLY	29	f	0	0	all	-	all	SCAmer	1978	CC	826	n	bl	n	n	0	cig+/-ot	non any	st
KAISE2	71	m	35	99	all	9	all	NAmer	1979	pr	318	n	bl	n	n	1	cig only	non any	st
KAISE2	63	f	35	99	all	9	all	NAmer	1979	pr	318	n	bl	n	n	1	cig only	non any	st
KAISER	14	m	0	0	all	0	all	NAmer	1964	pr	714	n	bl	n	n	2	cig+/-ot	non cigs	ot
KAISER	11	f	0	0	all	0	all	NAmer	1964	pr	714	n	bl	n	n	2	cig+/-ot	non cigs	ot
KATSOU	30	f	0	0	all	-	all	Eu:bal	1987	CC	101	n	bl	n	n	1	all/unsp	non any	ot
KAUFMA	18	c	0	0	all	-	all	NAmer	1981	CC	881	n	bl	n	n	6	cig+/-ot	non cigs	ot
KELLER	4	m	0	0	wh	-	all	NAmer	1985	CC	15038	n	bl	n	n	0	all/unsp	non any	st
KELLER	12	m	0	0	nonw	-	all	NAmer	1985	CC	15038	n	bl	n	n	0	all/unsp	non any	st
KELLER	8	f	0	0	wh	-	all	NAmer	1985	CC	15038	n	bl	n	n	0	all/unsp	non any	st
KELLER	16	f	0	0	nonw	-	all	NAmer	1985	CC	15038	n	bl	n	n	0	all/unsp	non any	st
KHUDER	34	m	0	0	all	-	all	NAmer	1985	CC	482	n	bl	n	y	0	cig+/-ot	non cigs	ot
KIHARA	23	c	0	0	jap	-	all	As:Jap	1991	CC	440	n	bl	n	n	0	all/unsp	non any	st
KINLEN	22	m	0	0	all	0	all	Eu:UK	1967	pr	718	n	V	n	n	2	all/unsp	non any	ot
KJUUS	13	m	0	0	all	-	all	Eu:Sca	1979	CC	176	n	bl	n	n	0	all/unsp	non any	st
KNEKT	1	c	0	0	all	0	all	Eu:Sca	1966	pr	515	n	bl	n	n	2	all/unsp	non any	ot
KOO	10	f	0	0	all	-	all	As:HK	1981	CC	200	n	bl	n	n	0	all/unsp	non any	st
KREUZE	51	m	1	45	all	-	all	Eu:Ger	1990	CC	2260	n	bl	n	n	0	all/unsp	non any	st
KREUZE	53	m	55	69	all	-	all	Eu:Ger	1990	CC	2260	n	bl	n	n	0	all/unsp	non any	st
KREUZE	52	f	1	45	all	-	all	Eu:Ger	1990	CC	2260	n	bl	n	n	0	all/unsp	non any	st
KREUZE	54	f	55	69	all	-	all	Eu:Ger	1990	CC	2260	n	bl	n	n	0	all/unsp	non any	st
KUBIK	32	m	0	0	all	0	all	Eu:est	1965	pr	108	n	bl	n	n	0	cig+/-ot	non cigs	st
LANGE	39	m	0	0	all	0	all	Eu:Sca	1976	pr	268	n	bl	n	n	1	all/unsp	non any	ot
LANGE	36	f	0	0	all	0	all	Eu:Sca	1976	pr	268	n	bl	n	n	1	all/unsp	non any	ot
LEMARC	4	c	0	0	w+o	-	all	NAmer	1992	CC	341	n	bl	n	y	0	all/unsp	non any	st
LICKIN	1	m	0	0	all	-	all	Eu:Ger	1950	CC	224	n	bl	*	n	0	all/unsp	non any	st
LIDDEL	8	m	0	0	all	18	all	NAmer	1970	pr	304	m	V	n	n	1	cig+/-ot	non cigs	ot
LOMBAR	24	m	0	0	all	-	all	NAmer	1951	CC	1040	n	bl	n	n	0	cig+/-ot	non cigs	st
LUBIN2	82	m	0	0	all	-	all	Eu:mul	1976	CC	7804	n	bl	n	y	2	all/unsp	non any	ot
LUBIN2	318	f	0	0	all	-	all	Eu:mul	1976	CC	7804	n	bl	n	y	0	cig+/-ot	non any	st
MACLEN	50	m	0	0	ch	-	all	As:oth	1972	CC	233	n	bl	n	n	0	cig+/-ot	non cigs	st
MACLEN	63	f	0	0	ch	-	all	As:oth	1972	CC	233	n	bl	n	n	0	cig+/-ot	non cigs	st
MATOS	82	m	0	0	all	-	all	SCAmer	1994	CC	200	n	bl	n	n	2	cig+/-ot	non any	ot
MIGRAN	62	m	0	0	all	0	all	Eu:UK	1964	pr	259	n	V	n	n	2	all/unsp	non any	ot
MIGRAN	138	f	0	0	all	0	all	Eu:UK	1964	pr	259	n	V	n	n	2	all/unsp	non any	ot
MRFIT	6	m	0	0	all	0	all	NAmer	1973	pr	2004	n	bl	n	n	4	cig+/-ot	non cigs	ot
MURATA	4	m	0	0	all	-	all	As:Jap	1984	ot	107	n	bl	n	n	0	cig+/-ot	non cigs	st
NAM	80	m	0	0	all	-	all	NAmer	1986	CC	1199	n	bl	y	n	1	cig+/-ot	non cigs	ot
NAM	96	f	0	0	all	-	all	NAmer	1986	CC	1199	n	bl	y	n	1	cig+/-ot	non cigs	ot
NOTANI	1	m	0	0	all	-	all	As:Ind	1986	CC	246	n	V	n	n	1	all/unsp	non any	or
ODRISC	4	c	0	0	all	-	all	Eu:UK	1992	CC	446	n	V	n	n	0	all/unsp	non any	st
OSANN	73	m	0	0	all	-	all	NAmer	1984	CC	1986	n	bl	n	n	2	cig+/-ot	non cigs	ot
OSANN	74	f	0	0	all	-	all	NAmer	1984	CC	1986	n	bl	n	n	2	cig+/-ot	non cigs	ot
PARKIN	45	m	0	0	bl	-	all	Africa	1963	CC	877	n	V	y	n	6	all/unsp	non any	ot
PERSH2	12	c	0	0	all	-	all	Eu:Sca	1980	CC	1022	n	bl	y	n	4	all/unsp	non any	ot
PETO	6	m	0	0	all	0	all	Eu:UK	1954	pr	103	n	V	n	n	0	all/unsp	non any	st
PEZZO2	6	m	0	0	all	-	all	SCAmer	1992	CC	367	n	bl	n	y	0	cig+/-ot	non cigs	st
PEZZOT	26	m	0	0	all	-	all	SCAmer	1987	CC	215	n	bl	n	y	0	cig only	non cigs	st
QIAO2	23	m	0	0	all	0	all	As:Chi	1992	pr	241	m	ot	n	n	1	all/unsp	non any	ot
RACHTA	14	f	0	0	all	-	all	Eu:est	1991	CC	118	n	bl	n	y	1	cig+/-ot	non cigs	ot
SAARIK	1	c	0	0	wh	-	all	Eu:Sca	1988	CC	205	n	bl	n	y	0	all/unsp	non any	st
SCHWAR	29	m	0	0	wh	-	all	NAmer	1984	CC	5588	n	bl	y	y	0	cig+/-ot	non cigs	st
SCHWAR	30	m	0	0	bl	-	all	NAmer	1984	CC	5588	n	bl	y	y	0	cig+/-ot	non cigs	st
SCHWAR	31	f	0	0	wh	-	all	NAmer	1984	CC	5588	n	bl	y	y	0	cig+/-ot	non cigs	st
SCHWAR	32	f	0	0	bl	-	all	NAmer	1984	CC	5588	n	bl	y	y	0	cig+/-ot	non cigs	st

Table 1B13 - 1

IESLC - Meta-analysis of Current Smoking (vs non-current), Any product (or Cigarettes if Any not available)
All LC types
Most adjusted

REF	NRR	SEX	AGEL	AGEH	RACE	YF	LC TYPE	LOC	START	ST	NLC	R	VB	P	H	AD	PRODUCT	DENOM	De
SHAW	9	c	0	0	wh	-	all	NAmer	1988	CC	335	n	V	n	y	0	all/unsp	non any	st
SHIMIZ	5	m	0	0	all	-	all	As:Jap	1977	CC	751	n	bl	y	n	2	all/unsp	non any	or
SHIMIZ	10	f	0	0	all	-	all	As:Jap	1977	CC	751	n	bl	y	n	2	all/unsp	non any	or
SITAS	1	m	0	0	bl	-	all	Africa	1994	CC	*	n	V	n	n	1	all/unsp	non any	or
SOBUE	106	m	0	0	all	-	q+s+l+a	As:Jap	1986	CC	1376	n	bl	n	y	1	cig+/-ot	non cigs	ot
SOBUE	116	f	0	0	all	-	q+s+l+a	As:Jap	1986	CC	1376	n	bl	n	y	1	cig+/-ot	non cigs	ot
SPEIZE	9	f	0	0	all	0	all	NAmer	1976	pr	593	n	bl	n	y	0	cig+/-ot	non cigs	st
SPITZ	4	c	0	0	b+hi	-	all	NAmer	1992	CC	177	n	bl	n	y	0	cig+/-ot	non cigs	st
STAYNE	9	m	0	0	all	-	all	NAmer	1969	CC	420	n	bl	n	n	1	cig+/-ot	non cigs	st
STOCKW	12	c	0	0	all	-	all	NAmer	1981	CC	22161	n	bl	n	n	0	cig+/-ot	non cigs	st
STUCKE	4	m	0	0	all	-	all	Eu:wst	1989	CC	247	n	bl	n	y	0	all/unsp	non any	st
SUZUK2	21	c	0	0	all	-	all	SCAmer	1991	CC	123	n	bl	n	y	3	all/unsp	non any	ot
SVENSS	91	f	0	0	all	-	all	Eu:Sca	1983	CC	210	n	bl	n	n	1	all/unsp	non any	ot
TANG	4	c	0	0	all	-	not s	NAmer	1992	CC	119	n	bl	n	y	0	cig+/-ot	non cigs	st
TENKAN	21	m	0	0	all	18	all	Eu:Sca	1962	pr	242	n	bl	n	n	1	all/unsp	non any	st
TIZZAN	11	m	0	0	all	-	all	Eu:wst	1959	CC	1358	n	bl	n	n	0	all/unsp	non any	st
TIZZAN	17	f	0	0	all	-	all	Eu:wst	1959	CC	1358	n	bl	n	n	0	all/unsp	non any	st
TOKARS	4	m	0	0	all	-	all	Eu:est	1966	ot	162	o	bl	n	y	0	all/unsp	non any	st
TOUSEY	25	m	0	0	all	-	all	NAmer	1993	CC	507	n	bl	y	y	3	cig+/-ot	non cigs	ot
TOUSEY	30	f	0	0	all	-	all	NAmer	1993	CC	507	n	bl	y	y	0	cig+/-ot	non cigs	st
TSUGAN	32	m	0	0	all	-	q+a	As:Jap	1976	CC	134	n	bl	n	y	0	all/unsp	non any	st
TSUGAN	25	f	0	0	all	-	q+a	As:Jap	1976	CC	134	n	bl	n	y	0	all/unsp	non any	or
TULINI	41	m	0	0	all	0	all	Eu:Sca	1967	pr	472	n	bl	n	n	3	all/unsp	non any	ot
TULINI	46	f	0	0	all	0	all	Eu:Sca	1967	pr	472	n	bl	n	n	3	all/unsp	non any	ot
TVERDA	26	m	0	0	all	0	all	Eu:Sca	1972	pr	238	n	bl	n	n	2	cig+/-ot	non any	ot
TVERDA	19	f	0	0	all	0	all	Eu:Sca	1972	pr	238	n	bl	n	n	0	cig only	non any	st
WAKAI	73	m	0	0	all	-	all	As:Jap	1988	CC	333	n	bl	n	y	2	all/unsp	non any	ot
WAKAI	79	f	0	0	all	-	all	As:Jap	1988	CC	333	n	bl	n	y	2	all/unsp	non any	ot
WANG2	22	c	0	0	all	-	all	As:Chi	1980	CC	103	n	ot	n	n	4	cig+/-ot	non cigs	ot
WARSIN	1	m	0	0	all	-	all	Eu:wst	1945	CC	134	n	bl	*	n	0	all/unsp	non any	st
WATSON	5	m	0	0	all	-	all	NAmer	1950	CC	301	n	bl	n	y	0	all/unsp	non any	st
WATSON	6	f	0	0	all	-	all	NAmer	1950	CC	301	n	bl	n	y	0	all/unsp	non any	st
WIGLE	34	m	0	0	all	-	all	NAmer	1971	CC	728	n	V	n	n	1	all/unsp	non any	ot
WIGLE	35	f	0	0	all	-	all	NAmer	1971	CC	728	n	V	n	n	1	all/unsp	non any	ot
WU	46	f	0	0	wh	-	q+a	NAmer	1981	CC	220	n	bl	n	y	2	all/unsp	non any	ot
WUNSCH	14	m	0	0	all	-	all	SCAmer	1990	CC	398	n	bl	y	n	1	cig+/-ot	non any	ot
WUNSCH	16	f	0	0	all	-	all	SCAmer	1990	CC	398	n	bl	y	n	1	cig+/-ot	non any	ot
WYNDE3	53	m	0	0	all	-	all	NAmer	1966	CC	350	n	bl	n	y	0	all/unsp	non any	st
WYNDE6	153	m	0	0	all	-	all	NAmer	1969	CC	4423	n	bl	n	y	0	cig+/-ot	non cigs	st
WYNDE6	261	f	0	0	all	-	all	NAmer	1969	CC	4423	n	bl	n	y	0	cig+/-ot	non cigs	st
WYNDER	13	m	0	0	all	-	all	SCAmer	1956	CC	120	n	bl	n	n	0	all/unsp	non any	ot
WYNDER	20	f	0	0	all	-	all	SCAmer	1956	CC	120	n	bl	n	n	0	all/unsp	non any	st
YAMAGU	12	c	0	0	all	-	all	As:Jap	1989	CC	144	n	bl	n	y	1	all/unsp	non any	ot
YONG	3	c	0	0	all	0	all	NAmer	1971	pr	216	n	bl	n	n	1	cig+/-ot	non cigs	ot

Cigarette type is all/unspec for all RRs
except for the following:

REF	NRR	CIGTYPE
DEAN3	154	MC only

Table 1B13 - 2

IESLC - Meta-analysis of Current Smoking (vs non-current), Any product (or Cigarettes if Any not available)
All LC types
Most adjusted

REF	NRR	SEX	AD	Number Exposed Case	Number Exposed Cont	Non-exposed Case	Non-exposed Cont	RR	95.00%CI
AGUDO	15	f	3	-	-	-	-	3.53	(1.54- 8.10)
*AKIBA	12	m	5	-	-	-	-	3.08	(2.36- 4.00)
*AKIBA	16	f	5	-	-	-	-	3.82	(2.84- 5.15)
Subtotal AKIBA								3.39	(2.78- 4.13)
*AMANDU	8	m	2	-	-	-	-	4.53	(2.40- 8.55)
AMES	5	m	0	150	136	162	177	1.21	(0.88- 1.65)
*ANDERS	4	f	0	212	41262	131	250060	9.81	(7.89- 12.19)
*ARCHER	10	m	0	122	32529	24	13582	2.12	(1.37- 3.29)
ARMADA	24	m	1	-	-	-	-	2.40	(1.75- 3.29)
AUSTIN	8	c	3	-	-	-	-	3.79	(2.38- 6.03)
AXELSS	3	m	0	194	130	114	374	4.90	(3.61- 6.64)
AXELSS	12	f	0	96	69	32	194	8.43	(5.19- 13.70)
Subtotal AXELSS								5.71	(4.41- 7.40)
BARBON	109	m	1	-	-	-	-	3.16	(2.54- 3.92)
BECHER	19	m	0	101	122	45	170	3.13	(2.05- 4.77)
BECHER	20	f	0	33	26	15	70	5.92	(2.77- 12.64)
Subtotal BECHER								3.64	(2.52- 5.25)
*BENSHL	17	m	1	-	-	-	-	3.46	(2.49- 4.81)
BLOHMK	4	m	0	419	313	469	575	1.64	(1.36- 1.99)
*BRETT	8	m	0	135	37448	15	17012	4.09	(2.40- 6.97)
BROSS	17	m	0	565	427	304	355	1.55	(1.27- 1.88)
BUFFLE	41	m	0	257	219	218	247	1.33	(1.03- 1.72)
BUFFLE	42	f	0	313	183	147	299	3.48	(2.66- 4.55)
Subtotal BUFFLE								2.10	(1.75- 2.53)
BYERS2	1	m	0	212	197	83	387	5.02	(3.69- 6.82)
BYERS2	2	f	0	121	91	32	222	9.22	(5.83- 14.61)
Subtotal BYERS2								6.05	(4.69- 7.81)
CARPEN	13	c	3	-	-	-	-	5.78	(4.43- 7.53)
*CEDERL	108	m	2	-	-	-	-	7.43	(5.29- 10.44)
*CEDERL	113	f	2	-	-	-	-	4.80	(3.38- 6.82)
Subtotal CEDERL								6.01	(4.71- 7.68)
*CHANG	13	m	0	35	419	48	1589	2.77	(1.81- 4.22)
*CHANG	14	f	0	30	603	23	1719	3.72	(2.18- 6.35)
Subtotal CHANG								3.10	(2.22- 4.32)
CHOI	4	m	0	232	329	48	231	3.39	(2.38- 4.83)
CHOI	8	f	0	13	23	82	167	1.15	(0.55- 2.39)
Subtotal CHOI								2.76	(2.01- 3.80)
*CHOW	47	m	0	167	124415	33	134936	5.49	(3.78- 7.97)
*CHYOU	8	m	1	-	-	-	-	5.80	(4.22- 7.98)
COMSTO	90	c	1	-	-	-	-	6.34	(4.49- 8.94)
CORREA	54	c	1	-	-	-	-	5.45	(4.62- 6.44)
*CPSI	74	m	1	-	-	-	-	6.81	(6.20- 7.47)
*CPSI	285	f	1	-	-	-	-	3.12	(2.48- 3.92)
Subtotal CPSI								6.09	(5.59- 6.64)
*CPSII	110	m	1	-	-	-	-	3.85	(3.55- 4.18)
*CPSII	85	f	1	-	-	-	-	6.47	(5.70- 7.34)
Subtotal CPSII								4.49	(4.19- 4.80)
DARBY	21	m	0	379	618	288	1490	3.17	(2.65- 3.80)
DARBY	30	f	0	198	231	117	846	6.20	(4.73- 8.13)
Subtotal DARBY								3.89	(3.35- 4.52)
DEAN2	4	m	0	671	600	131	200	1.71	(1.33- 2.18)
DEAN2	8	f	0	59	28	93	123	2.79	(1.65- 4.71)
Subtotal DEAN2								1.87	(1.49- 2.33)
DEAN3	84	m	3	-	-	-	-	2.41	(1.94- 3.01)
DEAN3	154	f	3	-	-	-	-	5.59	(3.78- 8.28)
Subtotal DEAN3								2.95	(2.43- 3.57)
*DEKLER	9	m	2	-	-	-	-	3.64	(2.07- 6.41)
DESTE2	19	c	7	-	-	-	-	2.27	(1.65- 3.14)
DESTEF	55	m	4	-	-	-	-	3.42	(2.62- 4.45)
*DOCKER	4	c	4	-	-	-	-	4.52	(2.34- 8.70)
DOLL	92	m	0	1280	1172	77	185	2.62	(1.99- 3.46)
DOLL	95	f	0	58	41	50	67	1.90	(1.10- 3.26)
Subtotal DOLL								2.45	(1.92- 3.14)
*DOLL2	57	m	1	-	-	-	-	3.29	(2.84- 3.82)
DORANT	4	m	0	332	697	153	930	2.90	(2.33- 3.59)
DORGAN	14	m	0	464	170	272	378	3.79	(3.00- 4.80)
DORGAN	38	m	0	214	61	55	109	6.95	(4.52- 10.70)
DORGAN	61	f	0	611	119	249	354	7.30	(5.66- 9.42)
DORGAN	84	f	0	68	17	18	30	6.67	(3.03- 14.69)
Subtotal DORGAN								5.39	(4.61- 6.31)
*DORN	199	m	1	-	-	-	-	3.52	(3.11- 3.98)

Table 1B13 - 2

IESLC - Meta-analysis of Current Smoking (vs non-current), Any product (or Cigarettes if Any not available)
All LC types
Most adjusted

REF	NRR	SEX	AD	Number Exposed Case	Number Exposed Cont	Non-exposed Case	Non-exposed Cont	RR	95.00%CI
DROSTE	8	m	4	-	-	-	-	4.34	(3.08- 6.13)
*ENGELA	160	m	1	-	-	-	-	1.24	(0.75- 2.05)
*ENGELA	166	f	1	-	-	-	-	6.10	(2.83- 13.15)
Subtotal ENGELA								2.00	(1.31- 3.05)
GAO	37	m	2	-	-	-	-	1.87	(1.50- 2.33)
GAO	38	f	2	-	-	-	-	2.55	(1.94- 3.34)
Subtotal GAO								2.11	(1.78- 2.51)
GAO2	11	m	1	-	-	-	-	2.47	(1.76- 3.46)
GARCIA	4	c	0	169	74	247	372	3.44	(2.50- 4.72)
GARDIN	8	c	0	97	58	46	85	3.09	(1.90- 5.02)
GARSHI	32	m	1	-	-	-	-	2.64	(2.24- 3.11)
GRAHAM	36	m	1	-	-	-	-	1.04	(0.85- 1.27)
GREGOR	4	m	0	49	53	33	59	1.65	(0.93- 2.94)
GREGOR	8	f	0	17	26	5	38	4.97	(1.63- 15.15)
Subtotal GREGOR								2.09	(1.25- 3.48)
HAENSZ	66	f	0	69	94	88	245	2.04	(1.38- 3.03)
*HAMMO2	29	m	1	-	-	-	-	2.31	(1.83- 2.93)
*HEIN	12	m	0	132	3492	12	1436	4.52	(2.51- 8.14)
*HENNEK	4	m	0	79	2438	90	19593	7.05	(5.23- 9.51)
HIRAY2	10	m	1	-	-	-	-	3.22	(1.60- 6.50)
*HIRAYA	148	m	1	-	-	-	-	4.08	(3.37- 4.94)
*HIRAYA	151	f	1	-	-	-	-	2.32	(1.86- 2.90)
Subtotal HIRAYA								3.21	(2.78- 3.71)
HITOSU	39	m	1	-	-	-	-	1.23	(0.81- 1.86)
HITOSU	63	f	1	-	-	-	-	2.69	(1.61- 4.51)
Subtotal HITOSU								1.67	(1.21- 2.31)
*HOLE	40	m	1	-	-	-	-	4.16	(2.84- 6.11)
*HOLE	71	f	1	-	-	-	-	1.88	(0.92- 3.80)
Subtotal HOLE								3.48	(2.48- 4.87)
HUMBLE	63	m	1	-	-	-	-	4.02	(2.77- 5.83)
HUMBLE	64	m	1	-	-	-	-	2.94	(1.59- 5.45)
HUMBLE	65	f	1	-	-	-	-	6.14	(3.47- 10.85)
HUMBLE	66	f	1	-	-	-	-	12.47	(4.53- 34.34)
Subtotal HUMBLE								4.50	(3.45- 5.89)
JAHN	32	m	0	352	269	487	570	1.53	(1.25- 1.87)
JAIN	56	m	2	-	-	-	-	4.00	(2.77- 5.76)
JAIN	54	f	2	-	-	-	-	8.58	(5.46- 13.48)
Subtotal JAIN								5.41	(4.07- 7.19)
JARVHO	4	m	0	73	29	27	44	4.10	(2.15- 7.81)
JARVHO	8	f	0	31	7	16	29	8.03	(2.89- 22.31)
Subtotal JARVHO								4.96	(2.88- 8.56)
JOLY	32	m	0	487	665	120	441	2.69	(2.13- 3.40)
JOLY	29	f	0	132	96	86	310	4.96	(3.47- 7.07)
Subtotal JOLY								3.23	(2.66- 3.93)
*KAISE2	71	m	1	-	-	-	-	4.35	(2.86- 6.62)
*KAISE2	63	f	1	-	-	-	-	8.61	(5.32- 13.93)
Subtotal KAISE2								5.84	(4.26- 8.02)
*KAISER	14	m	2	-	-	-	-	10.41	(8.11- 13.37)
*KAISER	11	f	2	-	-	-	-	4.49	(3.36- 6.00)
Subtotal KAISER								7.27	(6.02- 8.79)
KATSOU	30	f	1	-	-	-	-	3.08	(1.60- 5.92)
KAUFMA	18	c	6	-	-	-	-	5.18	(4.33- 6.19)
KELLER	4	m	0	5063	1210	3326	2324	2.92	(2.69- 3.17)
KELLER	12	m	0	1053	212	478	245	2.55	(2.06- 3.15)
KELLER	8	f	0	2904	792	1563	2337	5.48	(4.95- 6.07)
KELLER	16	f	0	454	135	197	311	5.31	(4.08- 6.90)
Subtotal KELLER								3.70	(3.49- 3.93)
KHUDER	34	m	0	275	-	207	-	1.82	(1.46- 2.25)
KIHARA	23	c	0	283	162	157	307	3.42	(2.60- 4.49)
*KINLEN	22	m	2	-	-	-	-	3.49	(2.78- 4.39)
KJUUS	13	m	0	135	77	41	99	4.23	(2.67- 6.70)
*KNEKT	1	c	2	-	-	-	-	3.76	(2.65- 5.32)
KOO	10	f	0	42	25	78	95	2.05	(1.15- 3.65)
KREUZE	51	m	0	168	99	15	101	11.43	(6.29- 20.75)
KREUZE	53	m	0	1252	524	457	1237	6.47	(5.58- 7.50)
KREUZE	52	f	0	55	23	13	57	10.48	(4.83- 22.75)
KREUZE	54	f	0	170	54	130	224	5.42	(3.73- 7.89)
Subtotal KREUZE								6.60	(5.78- 7.53)
*KUBIK	32	m	0	98	6342	10	5980	9.24	(4.83- 17.70)
*LANGE	39	m	1	-	-	-	-	3.26	(2.08- 5.10)
*LANGE	36	f	1	-	-	-	-	3.85	(2.17- 6.83)

Table 1B13 - 2

IESLC - Meta-analysis of Current Smoking (vs non-current), Any product (or Cigarettes if Any not available)
All LC types
Most adjusted

REF	NRR	SEX	AD	Number Exposed Case	Number Exposed Cont	Non-exposed Case	Non-exposed Cont	RR	95.00%CI
Subtotal LANGE								3.47	(2.44- 4.94)
LEMARC	4	c	0	167	65	174	391	5.77	(4.12- 8.09)
LICKIN	1	m	0	220	840	4	160	10.48	(3.84- 28.57)
*LIDDEL	8	m	1	-	-	-	-	3.65	(2.68- 4.98)
LOMBAR	24	m	0	852	610	188	430	3.19	(2.61- 3.90)
LUBIN2	82	m	2	-	-	-	-	3.57	(3.33- 3.83)
LUBIN2	318	f	0	384	410	388	1337	3.23	(2.70- 3.86)
Subtotal LUBIN2								3.52	(3.30- 3.76)
MACLEN	50	m	0	137	108	10	26	3.30	(1.52- 7.14)
MACLEN	63	f	0	42	47	44	119	2.42	(1.41- 4.15)
Subtotal MACLEN								2.68	(1.72- 4.17)
MATOS	82	m	2	-	-	-	-	2.60	(1.83- 3.70)
*MIGRAN	62	m	2	-	-	-	-	1.36	(0.91- 2.02)
*MIGRAN	138	f	2	-	-	-	-	4.02	(1.55- 10.41)
Subtotal MIGRAN								1.60	(1.11- 2.31)
*MRFIT	6	m	4	-	-	-	-	6.70	(6.04- 7.44)
MURATA	4	m	0	76	93	31	121	3.19	(1.94- 5.25)
NAM	80	m	1	-	-	-	-	1.34	(1.09- 1.66)
NAM	96	f	1	-	-	-	-	2.77	(2.10- 3.65)
Subtotal NAM								1.75	(1.48- 2.07)
NOTANI	1	m	1	-	-	-	-	5.79	(3.50- 9.70)
ODRISC	4	c	0	293	598	153	1062	3.40	(2.73- 4.24)
OSANN	73	m	2	-	-	-	-	4.98	(4.23- 5.85)
OSANN	74	f	2	-	-	-	-	9.13	(7.52- 11.08)
Subtotal OSANN								6.39	(5.64- 7.24)
PARKIN	45	m	6	-	-	-	-	3.60	(2.85- 4.55)
PERSH2	12	c	4	-	-	-	-	6.44	(5.45- 7.60)
*PETO	6	m	0	99	2036	4	682	8.29	(3.06- 22.44)
PEZZO2	6	m	0	233	198	134	388	3.41	(2.59- 4.48)
PEZZOT	26	m	0	145	129	70	304	4.88	(3.43- 6.94)
*QIAO2	23	m	1	-	-	-	-	1.30	(0.91- 1.86)
RACHTA	14	f	1	-	-	-	-	5.30	(3.01- 9.32)
SAARIK	1	c	0	102	66	103	224	3.36	(2.28- 4.95)
SCHWAR	29	m	0	1652	349	1115	1046	4.44	(3.85- 5.12)
SCHWAR	30	m	0	644	139	269	240	4.13	(3.21- 5.32)
SCHWAR	31	f	0	1029	309	504	1183	7.82	(6.63- 9.21)
SCHWAR	32	f	0	256	90	119	336	8.03	(5.84- 11.04)
Subtotal SCHWAR								5.59	(5.08- 6.15)
SHAW	9	c	0	212	97	123	276	4.90	(3.56- 6.76)
SHIMIZ	5	m	2	-	-	-	-	3.70	(2.70- 5.10)
SHIMIZ	10	f	2	-	-	-	-	3.40	(2.10- 5.30)
Subtotal SHIMIZ								3.60	(2.77- 4.68)
SITAS	1	m	1	-	-	-	-	8.40	(3.20- 22.20)
SOBUE	106	m	1	-	-	-	-	1.82	(1.52- 2.17)
SOBUE	116	f	1	-	-	-	-	2.52	(1.82- 3.49)
Subtotal SOBUE								1.96	(1.68- 2.29)
*SPEIZE	9	f	0	391	489993	202	1298381	5.13	(4.33- 6.08)
SPITZ	4	c	0	103	89	74	208	3.25	(2.21- 4.80)
STAYNE	9	m	1	-	-	-	-	3.30	(2.45- 4.45)
STOCKW	12	c	0	12470	3357	9691	14353	5.50	(5.25- 5.76)
STUCKE	4	m	0	69	68	178	186	1.06	(0.72- 1.57)
SUZUK2	21	c	3	-	-	-	-	5.73	(2.75- 11.95)
SVENSS	91	f	1	-	-	-	-	6.48	(4.05- 10.37)
TANG	4	c	0	52	25	67	73	2.27	(1.27- 4.05)
*TENKAN	21	m	1	-	-	-	-	6.32	(4.35- 9.19)
TIZZAN	11	m	0	693	619	526	597	1.27	(1.08- 1.49)
TIZZAN	17	f	0	17	18	33	124	3.55	(1.65- 7.63)
Subtotal TIZZAN								1.33	(1.13- 1.55)
TOKARS	4	m	0	110	157	38	139	2.56	(1.66- 3.95)
TOUSEY	25	m	3	-	-	-	-	6.61	(4.64- 9.42)
TOUSEY	30	f	0	127	78	79	362	7.46	(5.14- 10.83)
Subtotal TOUSEY								7.00	(5.42- 9.05)
TSUGAN	32	m	0	63	63	28	30	1.07	(0.58- 2.00)
TSUGAN	25	f	0	6	10	33	30	0.55	(0.18- 1.68)
Subtotal TSUGAN								0.91	(0.53- 1.58)
*TULINI	41	m	3	-	-	-	-	4.78	(3.51- 6.50)
*TULINI	46	f	3	-	-	-	-	9.76	(6.47- 14.73)
Subtotal TULINI								6.18	(4.83- 7.91)
*TVERDA	26	m	2	-	-	-	-	10.64	(6.28- 18.03)
*TVERDA	19	f	0	24	113761	3	196384	13.81	(4.16- 45.86)
Subtotal TVERDA								11.10	(6.85- 17.99)

Table 1B13 - 2

IESLC - Meta-analysis of Current Smoking (vs non-current), Any product (or Cigarettes if Any not available)
All LC types
Most adjusted

REF	NRR	SEX	AD	Number Exposed Case	Cont	Non-exposed Case	Cont	RR	95.00%CI
WAKAI	73	m	2	-	-	-	-	2.19	(1.57- 3.05)
WAKAI	79	f	2	-	-	-	-	3.93	(2.01- 7.67)
Subtotal WAKAI								2.46	(1.83- 3.31)
WANG2	22	c	4	-	-	-	-	1.78	(0.98- 3.23)
WARSIN	1	m	0	129	81	5	19	6.05	(2.17- 16.84)
WATSON	5	m	0	260	250	5	27	5.62	(2.13- 14.81)
WATSON	6	f	0	15	33	21	148	3.20	(1.49- 6.87)
Subtotal WATSON								3.97	(2.18- 7.23)
WIGLE	34	m	1	-	-	-	-	2.47	(1.97- 3.09)
WIGLE	35	f	1	-	-	-	-	4.56	(3.02- 6.87)
Subtotal WIGLE								2.85	(2.34- 3.47)
WU	46	f	2	-	-	-	-	4.42	(2.79- 7.01)
WUNSCH	14	m	1	-	-	-	-	2.52	(1.88- 3.39)
WUNSCH	16	f	1	-	-	-	-	4.25	(2.44- 7.41)
Subtotal WUNSCH								2.83	(2.18- 3.67)
WYNDE3	53	m	0	227	207	57	213	4.10	(2.89- 5.80)
WYNDE6	153	m	0	1677	741	1233	1872	3.44	(3.07- 3.84)
WYNDE6	261	f	0	1022	376	491	1181	6.54	(5.58- 7.66)
Subtotal WYNDE6								4.26	(3.89- 4.67)
WYNDER	13	m	0	80	185	0	35	30.81~	(1.87- 508.45)
WYNDER	20	f	0	39	71	2	141	38.73	(9.09- 164.98)
Subtotal WYNDER								36.90	(10.18- 133.72)
YAMAGU	12	c	1	-	-	-	-	2.73	(1.72- 4.35)
*YONG	3	c	1	-	-	-	-	5.08	(3.74- 6.90)
Partial Totals				45497	876017	27656	1986445		

*prospective study ~ With 0.5 adjustment for zero

REF	NRR	SEX	AD	Ys	Ws	Qs	Ps
AGUDO	15	f	3	1.26	5.58	0.08	0.0029
*AKIBA	12	m	5	1.12	55.19	3.67	0.0000
*AKIBA	16	f	5	1.34	43.38	0.08	0.0000
Subtotal AKIBA				1.22	98.57	3.75	
*AMANDU	8	m	2	1.51	9.52	0.16	0.0000
AMES	5	m	0	0.19	38.70	55.39	0.2459
*ANDERS	4	f	0	2.28	81.15	65.77	0.0000
*ARCHER	10	m	0	0.75	20.10	7.99	0.0007
ARMADA	24	m	1	0.88	38.56	9.93	0.0000
AUSTIN	8	c	3	1.33	17.78	0.05	0.0000
AXELSS	3	m	0	1.59	41.16	1.74	0.0000
AXELSS	12	f	0	2.13	16.31	9.16	0.0000
Subtotal AXELSS				1.74	57.47	10.90	
BARBON	109	m	1	1.15	81.61	4.41	0.0000
BECHER	19	m	0	1.14	21.64	1.27	0.0000
BECHER	20	f	0	1.78	6.68	1.05	0.0000
Subtotal BECHER				1.29	28.32	2.32	
*BENSHL	17	m	1	1.24	35.45	0.71	0.0000
BLOHMK	4	m	0	0.50	105.79	83.32	0.0000
*BRETT	8	m	0	1.41	13.52	0.01	0.0000
BROSS	17	m	0	0.44	97.86	87.91	0.0000
BUFFLE	41	m	0	0.28	58.50	70.54	0.0293
BUFFLE	42	f	0	1.25	53.17	0.99	0.0000
Subtotal BUFFLE				0.74	111.68	71.52	
BYERS2	1	m	0	1.61	40.94	2.17	0.0000
BYERS2	2	f	0	2.22	18.18	12.79	0.0000
Subtotal BYERS2				1.80	59.12	14.96	
CARPEN	13	c	3	1.75	54.60	7.53	0.0000
*CEDERL	108	m	2	2.01	33.25	12.89	0.0000
*CEDERL	113	f	2	1.57	31.18	1.08	0.0000
Subtotal CEDERL				1.79	64.43	13.96	
*CHANG	13	m	0	1.02	21.56	2.88	0.0000
*CHANG	14	f	0	1.31	13.41	0.07	0.0000
Subtotal CHANG				1.13	34.97	2.95	
CHOI	4	m	0	1.22	30.76	0.80	0.0000
CHOI	8	f	0	0.14	7.22	11.13	0.7054
Subtotal CHOI				1.02	37.97	11.93	
*CHOW	47	m	0	1.70	27.57	2.82	0.0000
*CHYOU	8	m	1	1.76	37.86	5.32	0.0000
COMSTO	90	c	1	1.85	32.40	6.97	0.0000
CORREA	54	c	1	1.70	139.29	13.62	0.0000
*CPSI	74	m	1	1.92	442.50	126.87	0.0000

Table 1B13 - 2

IESLC - Meta-analysis of Current Smoking (vs non-current), Any product (or Cigarettes if Any not available)
All LC types
Most adjusted

REF	NRR	SEX	AD	Ys	Ws	Qs	Ps
*CPSI	285	f	1	1.14	73.31	4.40	0.0000
Subtotal CPSI				1.81	515.81	131.28	
*CPSII	110	m	1	1.35	575.76	0.70	0.0000
*CPSII	85	f	1	1.87	240.30	56.35	0.0000
Subtotal CPSII				1.50	816.06	57.05	
DARBY	21	m	0	1.15	119.05	6.21	0.0000
DARBY	30	f	0	1.82	52.33	10.19	0.0000
Subtotal DARBY				1.36	171.38	16.40	
DEAN2	4	m	0	0.53	63.33	45.54	0.0000
DEAN2	8	f	0	1.02	13.98	1.79	0.0001
Subtotal DEAN2				0.62	77.31	47.33	
DEAN3	84	m	3	0.88	79.64	20.17	0.0000
DEAN3	154	f	3	1.72	24.99	2.86	0.0000
Subtotal DEAN3				1.08	104.63	23.03	
*DEKLER	9	m	2	1.29	12.03	0.10	0.0000
DESTE2	19	c	7	0.82	37.11	11.77	0.0000
DESTEF	55	m	4	1.23	54.76	1.29	0.0000
*DOCKER	4	c	4	1.51	8.91	0.14	0.0000
DOLL	92	m	0	0.96	49.93	8.73	0.0000
DOLL	95	f	0	0.64	13.06	7.22	0.0208
Subtotal DOLL				0.90	62.99	15.95	
*DOLL2	57	m	1	1.19	174.85	6.45	0.0000
DORANT	4	m	0	1.06	82.93	8.48	0.0000
DORGAN	14	m	0	1.33	69.64	0.17	0.0000
DORGAN	38	m	0	1.94	20.65	6.39	0.0000
DORGAN	61	f	0	1.99	59.24	21.67	0.0000
DORGAN	84	f	0	1.90	6.16	1.63	0.0000
Subtotal DORGAN				1.68	155.69	29.86	
*DORN	199	m	1	1.26	252.56	3.91	0.0000
DROSTE	8	m	4	1.47	32.44	0.23	0.0000
*ENGELA	160	m	1	0.22	15.20	20.73	0.4017
*ENGELA	166	f	1	1.81	6.51	1.18	0.0000
Subtotal ENGELA				0.69	21.71	21.90	
GAO	37	m	2	0.63	79.22	45.40	0.0000
GAO	38	f	2	0.94	52.06	10.39	0.0000
Subtotal GAO				0.75	131.28	55.79	
GAO2	11	m	1	0.90	33.63	7.71	0.0000
GARCIA	4	c	0	1.24	38.22	0.83	0.0000
GARDIN	8	c	0	1.13	16.38	1.06	0.0000
GARSHI	32	m	1	0.97	142.70	24.24	0.0000
GRAHAM	36	m	1	0.04	95.30	172.08	0.7018
GREGOR	4	m	0	0.50	11.56	8.96	0.0875
GREGOR	8	f	0	1.60	3.09	0.15	0.0048
Subtotal GREGOR				0.73	14.65	9.11	
HAENSZ	66	f	0	0.71	24.64	11.00	0.0004
*HAMMO2	29	m	1	0.84	69.36	20.65	0.0000
*HEIN	12	m	0	1.51	11.12	0.18	0.0000
*HENNEK	4	m	0	1.95	42.90	13.97	0.0000
HIRAY2	10	m	1	1.17	7.82	0.36	0.0011
*HIRAYA	148	m	1	1.41	105.05	0.06	0.0000
*HIRAYA	151	f	1	0.84	77.90	22.83	0.0000
Subtotal HIRAYA				1.17	182.95	22.89	
HITOSU	39	m	1	0.21	22.24	30.75	0.3290
HITOSU	63	f	1	0.99	14.48	2.24	0.0002
Subtotal HITOSU				0.52	36.72	32.99	
*HOLE	40	m	1	1.43	26.18	0.05	0.0000
*HOLE	71	f	1	0.63	7.64	4.32	0.0811
Subtotal HOLE				1.25	33.82	4.36	
HUMBLE	63	m	1	1.39	27.75	0.00	0.0000
HUMBLE	64	m	1	1.08	10.13	0.94	0.0006
HUMBLE	65	f	1	1.81	11.82	2.21	0.0000
HUMBLE	66	f	1	2.52	3.75	4.87	0.0000
Subtotal HUMBLE				1.51	53.44	8.02	
JAHN	32	m	0	0.43	96.47	88.28	0.0000
JAIN	56	m	2	1.39	28.67	0.00	0.0000
JAIN	54	f	2	2.15	18.81	11.05	0.0000
Subtotal JAIN				1.69	47.48	11.05	
JARVHO	4	m	0	1.41	9.26	0.01	0.0000
JARVHO	8	f	0	2.08	3.68	1.80	0.0001
Subtotal JARVHO				1.60	12.94	1.81	
JOLY	32	m	0	0.99	70.63	10.90	0.0000

Table 1B13 - 2

IESLC - Meta-analysis of Current Smoking (vs non-current), Any product (or Cigarettes if Any not available)
All LC types
Most adjusted

REF	NRR	SEX	AD	Ys	Ws	Qs	Ps
JOLY	29	f	0	1.60	30.44	1.44	0.0000
Subtotal JOLY				1.17	101.08	12.35	
*KAISE2	71	m	1	1.47	21.81	0.17	0.0000
*KAISE2	63	f	1	2.15	16.58	9.83	0.0000
Subtotal KAISE2				1.77	38.40	10.00	
*KAISER	14	m	2	2.34	61.48	56.64	0.0000
*KAISER	11	f	2	1.50	45.71	0.65	0.0000
Subtotal KAISER				1.98	107.19	57.29	
KATSOU	30	f	1	1.12	8.98	0.60	0.0008
KAUFMA	18	c	6	1.64	120.32	8.25	0.0000
KELLER	4	m	0	1.07	569.83	54.79	0.0000
KELLER	12	m	0	0.93	84.46	16.99	0.0000
KELLER	8	f	0	1.70	373.88	37.95	0.0000
KELLER	16	f	0	1.67	55.86	4.58	0.0000
Subtotal KELLER				1.31	1084.02	114.31	
KHUDER	34	m	0	0.60	82.15	50.51	0.0000
KIHARA	23	c	0	1.23	51.72	1.23	0.0000
*KINLEN	22	m	2	1.25	73.61	1.30	0.0000
KJUUS	13	m	0	1.44	18.22	0.07	0.0000
*KNEKT	1	c	2	1.32	31.64	0.11	0.0000
KOO	10	f	0	0.72	11.47	5.10	0.0153
KREUZE	51	m	0	2.44	10.80	11.97	0.0000
KREUZE	53	m	0	1.87	175.32	41.04	0.0000
KREUZE	52	f	0	2.35	6.41	5.99	0.0000
KREUZE	54	f	0	1.69	27.35	2.59	0.0000
Subtotal KREUZE				1.89	219.88	61.60	
*KUBIK	32	m	0	2.22	9.10	6.43	0.0000
*LANGE	39	m	1	1.18	19.10	0.77	0.0000
*LANGE	36	f	1	1.35	11.69	0.01	0.0000
Subtotal LANGE				1.24	30.79	0.79	
LEMARC	4	c	0	1.75	33.70	4.62	0.0000
LICKIN	1	m	0	2.35	3.82	3.56	0.0000
*LIDDEL	8	m	1	1.29	40.02	0.31	0.0000
LOMBAR	24	m	0	1.16	95.62	4.69	0.0000
LUBIN2	82	m	2	1.27	785.18	9.56	0.0000
LUBIN2	318	f	0	1.17	119.50	5.33	0.0000
Subtotal LUBIN2				1.26	904.67	14.90	
MACLEN	50	m	0	1.19	6.45	0.23	0.0024
MACLEN	63	f	0	0.88	13.12	3.29	0.0014
Subtotal MACLEN				0.98	19.57	3.52	
MATOS	82	m	2	0.96	31.00	5.66	0.0000
*MIGRAN	62	m	2	0.31	24.17	27.95	0.1307
*MIGRAN	138	f	2	1.39	4.24	0.00	0.0042
Subtotal MIGRAN				0.47	28.40	27.95	
*MRFIT	6	m	4	1.90	353.58	95.30	0.0000
MURATA	4	m	0	1.16	15.52	0.77	0.0000
NAM	80	m	1	0.29	86.84	103.23	0.0064
NAM	96	f	1	1.02	50.28	6.67	0.0000
Subtotal NAM				0.56	137.13	109.89	
NOTANI	1	m	1	1.76	14.79	2.06	0.0000
ODRISC	4	c	0	1.22	79.60	2.01	0.0000
OSANN	73	m	2	1.61	146.16	7.24	0.0000
OSANN	74	f	2	2.21	102.29	70.24	0.0000
Subtotal OSANN				1.85	248.45	77.47	
PARKIN	45	m	6	1.28	70.21	0.73	0.0000
PERSH2	12	c	4	1.86	138.96	31.96	0.0000
*PETO	6	m	0	2.12	3.87	2.08	0.0000
PEZZO2	6	m	0	1.23	51.59	1.27	0.0000
PEZZOT	26	m	0	1.59	31.03	1.27	0.0000
*QIAO2	23	m	1	0.26	30.07	37.75	0.1503
RACHTA	14	f	1	1.67	12.03	0.98	0.0000
SAARIK	1	c	0	1.21	25.56	0.74	0.0000
SCHWAR	29	m	0	1.49	187.84	2.19	0.0000
SCHWAR	30	m	0	1.42	60.13	0.08	0.0000
SCHWAR	31	f	0	2.06	142.10	64.42	0.0000
SCHWAR	32	f	0	2.08	37.88	18.59	0.0000
Subtotal SCHWAR				1.72	427.95	85.27	
SHAW	9	c	0	1.59	37.34	1.60	0.0000
SHIMIZ	5	m	2	1.31	37.99	0.21	0.0000
SHIMIZ	10	f	2	1.22	17.93	0.45	0.0000
Subtotal SHIMIZ				1.28	55.92	0.67	

Table 1B13 - 2

IESLC - Meta-analysis of Current Smoking (vs non-current), Any product (or Cigarettes if Any not available)
All LC types
Most adjusted

REF	NRR	SEX	AD	Ys	Ws	Qs	Ps
SITAS	1	m	1	2.13	4.10	2.28	0.0000
SOBUE	106	m	1	0.60	121.23	74.53	0.0000
SOBUE	116	f	1	0.92	36.25	7.63	0.0000
Subtotal SOBUE				0.67	157.48	82.16	
*SPEIZE	9	f	0	1.63	133.24	8.46	0.0000
SPITZ	4	c	0	1.18	25.47	1.05	0.0000
STAYNE	9	m	1	1.19	43.14	1.54	0.0000
STOCKW	12	c	0	1.71	1815.09	188.32	0.0000
STUCKE	4	m	0	0.06	24.88	43.64	0.7702
SUZUK2	21	c	3	1.75	7.12	0.94	0.0000
SVENSS	91	f	1	1.87	17.38	4.10	0.0000
TANG	4	c	0	0.82	11.38	3.63	0.0058
*TENKAN	21	m	1	1.84	27.47	5.83	0.0000
TIZZAN	11	m	0	0.24	150.72	197.05	0.0033
TIZZAN	17	f	0	1.27	6.55	0.09	0.0012
Subtotal TIZZAN				0.28	157.27	197.14	
TOKARS	4	m	0	0.94	20.42	3.99	0.0000
TOUSEY	25	m	3	1.89	30.64	7.84	0.0000
TOUSEY	30	f	0	2.01	27.69	10.88	0.0000
Subtotal TOUSEY				1.95	58.33	18.71	
TSUGAN	32	m	0	0.07	9.92	17.13	0.8280
TSUGAN	25	f	0	-0.61	3.03	11.98	0.2916
Subtotal TSUGAN				-0.09	12.95	29.11	
*TULINI	41	m	3	1.56	40.47	1.33	0.0000
*TULINI	46	f	3	2.28	22.70	18.20	0.0000
Subtotal TULINI				1.82	63.17	19.53	
*TVERDA	26	m	2	2.36	13.81	13.31	0.0000
*TVERDA	19	f	0	2.63	2.67	4.12	0.0000
Subtotal TVERDA				2.41	16.48	17.43	
WAKAI	73	m	2	0.78	34.84	12.50	0.0000
WAKAI	79	f	2	1.37	8.57	0.00	0.0001
Subtotal WAKAI				0.90	43.41	12.51	
WANG2	22	c	4	0.58	10.80	7.02	0.0581
WARSIN	1	m	0	1.80	3.67	0.64	0.0006
WATSON	5	m	0	1.73	4.08	0.48	0.0005
WATSON	6	f	0	1.16	6.61	0.32	0.0028
Subtotal WATSON				1.38	10.69	0.80	
WIGLE	34	m	1	0.90	75.83	17.38	0.0000
WIGLE	35	f	1	1.52	22.75	0.41	0.0000
Subtotal WIGLE				1.05	98.58	17.79	
WU	46	f	2	1.49	18.10	0.19	0.0000
WUNSCH	14	m	1	0.92	44.21	9.30	0.0000
WUNSCH	16	f	1	1.45	12.45	0.05	0.0000
Subtotal WUNSCH				1.04	56.66	9.35	
WYNDE3	53	m	0	1.41	31.77	0.02	0.0000
WYNDE6	153	m	0	1.23	303.85	6.71	0.0000
WYNDE6	261	f	0	1.88	153.34	37.52	0.0000
Subtotal WYNDE6				1.45	457.19	44.23	
WYNDER	13	m	0	3.43	0.49	2.04	0.0166
WYNDER	20	f	0	3.66	1.83	9.45	0.0000
Subtotal WYNDER				3.61	2.32	11.50	
YAMAGU	12	c	1	1.00	17.85	2.56	0.0000
*YONG	3	c	1	1.63	40.97	2.41	0.0000

Table 1B13 - 2

IESLC - Meta-analysis of Current Smoking (vs non-current), Any product (or Cigarettes if Any not available)
All LC types
Most adjusted

	N	188
	NS	129
	Wt	13269.19
Het	Chi	2929.69
Het	df	187
Het	P	***
Fixed	RR	3.99
	RRl	3.92
	RRu	4.05
	P	+++
Random	RR	3.75
	RRl	3.48
	RRu	4.03
	P	+++
Asymm	P	*

Table 1B13 - 3

IESLC - Meta-analysis of Current Smoking (vs non-current), Any product (or Cigarettes if Any not available)
All LC types
Most adjusted

		Sex		
	combined	male	female	Total
N	23	102	63	188
NS	23	97	58	178
Wt	2792.20	7932.11	2544.89	13269.19
Het Chi	117.93	1767.21	468.76	2929.69
Het df	22	101	62	187
Het P	***	***	***	***
Fixed RR	5.14	3.36	5.14	3.99
RRl	4.95	3.29	4.94	3.92
RRu	5.33	3.44	5.34	4.05
P	+++	+++	+++	+++
Random RR	4.17	3.26	4.67	3.75
RRl	3.68	2.96	4.14	3.48
RRu	4.72	3.60	5.26	4.03
P	+++	+++	+++	+++
Between Chi				575.78
Between df				2
Between P				***
Btwn(F) P				***
Btwn(R) P				***

Table 1B13 - 4

IESLC - Meta-analysis of Current Smoking (vs non-current), Any product (or Cigarettes if Any not available)
All LC types
Least adjusted

REF	NRR	X	SEX	AGEL	AGEH	RACE	YF	LC TYPE	LOC	START	ST	NLC	R	VB	P	H	AD	PRODUCT	DENOM	De
AGUDO	16	x	f	0	0	all	-	all	Eu:wst	1989	CC	103	n	bl	n	n	0	cig only	non any	st
AKIBA	4	x	m	0	0	all	0	all	As:Jap	1963	pr	610	n	bl	n	n	0	cig+/-ot	non cigs	st
AKIBA	8	x	f	0	0	all	0	all	As:Jap	1963	pr	610	n	bl	n	n	0	cig+/-ot	non cigs	st
AMANDU	4	x	m	0	0	wh	0	all	NAmer	1959	pr	132	m	bl	n	n	0	cig+/-ot	non cigs	st
AMES	5		m	0	0	wh	-	all	NAmer	1959	ot	317	m	bl	n	n	0	all/unsp	non any	st
ANDERS	4		f	0	0	all	0	all	NAmer	1986	pr	343	n	bl	n	n	0	cig+/-ot	non cigs	st
ARCHER	10		m	0	0	wh	0	all	NAmer	1950	pr	146	m	bl	n	n	0	cig+/-ot	non cigs	st
ARMADA	10	x	m	0	0	all	-	all	Eu:wst	1986	CC	325	n	bl	n	y	0	cig+/-ot	non cigs	st
AUSTIN	4	x	c	0	0	all	-	all	NAmer	1970	CC	166	o	bl	y	n	0	cig+/-ot	non cigs	st
AXELSS	3		m	0	0	sca	-	all	Eu:Sca	1989	CC	436	n	bl	n	n	0	all/unsp	non any	st
AXELSS	12		f	0	0	sca	-	all	Eu:Sca	1989	CC	436	n	bl	n	n	0	all/unsp	non any	st
BARBON	108	x	m	0	0	all	-	all	Eu:wst	1979	CC	755	n	bl	y	y	0	all/unsp	non any	st
BECHER	19		m	0	0	all	-	all	Eu:Ger	1985	CC	194	n	bl	n	y	0	all/unsp	non any	st
BECHER	20		f	0	0	all	-	all	Eu:Ger	1985	CC	194	n	bl	n	y	0	all/unsp	non any	st
BENSHL	17		m	40	64	all	10	all	Eu:UK	1967	pr	486	n	V	n	n	1	all/unsp	non any	ot
BLOHMK	4		m	0	0	all	-	all	Eu:Ger	1978	CC	888	n	bl	n	y	0	all/unsp	non any	st
BRETT	8		m	0	0	all	0	all	Eu:UK	1960	pr	150	n	V	n	n	0	cig+/-ot	non cigs	st
BROSS	17		m	0	0	wh	-	all	NAmer	1960	CC	974	n	bl	n	n	0	cig+/-ot	non cigs	st
BUFFLE	41		m	0	0	wh	-	all	NAmer	1976	CC	943	n	bl	y	n	0	cig+/-ot	non cigs	st
BUFFLE	42		f	0	0	wh	-	all	NAmer	1976	CC	943	n	bl	y	n	0	cig+/-ot	non cigs	st
BYERS2	1		m	0	0	wh	-	all	NAmer	1980	CC	448	n	bl	n	y	0	cig+/-ot	non cigs	st
BYERS2	2		f	0	0	wh	-	all	NAmer	1980	CC	448	n	bl	n	y	0	cig+/-ot	non cigs	st
CARPEN	17	x	c	0	0	w+b	-	all	NAmer	1991	CC	356	n	bl	n	n	0	cig+/-ot	non cigs	st
CEDERL	108		m	0	0	all	16	all	Eu:Sca	1963	pr	491	n	bl	n	n	2	all/unsp	non any	ot
CEDERL	113		f	0	0	all	0	all	Eu:Sca	1963	pr	491	n	bl	n	n	2	all/unsp	non any	ot
CHANG	13		m	0	0	all	0	all	NAmer	1972	pr	136	n	bl	n	n	0	cig+/-ot	non cigs	st
CHANG	14		f	0	0	all	0	all	NAmer	1972	pr	136	n	bl	n	n	0	cig+/-ot	non cigs	st
CHOI	4		m	0	0	all	-	all	As:oth	1985	CC	375	n	bl	n	n	0	cig+/-ot	non cigs	st
CHOI	8		f	0	0	all	-	all	As:oth	1985	CC	375	n	bl	n	n	0	cig+/-ot	non cigs	st
CHOW	47		m	0	0	wh	0	all	NAmer	1966	pr	219	n	bl	n	n	0	all/unsp	non any	st
CHYOU	10	x	m	0	0	jap	0	all	NAmer	1965	pr	227	n	bl	n	y	0	cig+/-ot	non cigs	st
COMSTO	41	x	m	0	0	all	-	all	NAmer	1975	ot	258	n	bl	n	n	0	cig+/-ot	non cigs	st
COMSTO	53	x	f	0	0	all	-	all	NAmer	1975	ot	258	n	bl	n	n	0	cig+/-ot	non cigs	st
CORREA	53	x	c	0	0	all	-	all	NAmer	1979	CC	1359	n	bl	y	n	0	cig+/-ot	non cigs	st
CPSI	74		m	0	0	wh	0	all	NAmer	1959	pr	5138	n	bl	n	n	1	cig only	non any	st
CPSI	285		f	40	74	all	6	all	NAmer	1959	pr	5138	n	bl	n	n	1	cig+/-ot	non cigs	ot
CPSII	110		m	35	99	all	4	all	NAmer	1982	pr	3229	n	bl	n	n	1	cig only	non cigs	ot
CPSII	85		f	0	0	all	4	all	NAmer	1982	pr	3229	n	bl	n	n	1	cig+/-ot	non cigs	ot
DARBY	21		m	0	0	wh	-	all	Eu:UK	1988	CC	982	n	V	n	n	0	all/unsp	non any	st
DARBY	30		f	0	0	wh	-	all	Eu:UK	1988	CC	982	n	V	n	n	0	all/unsp	non any	st
DEAN2	4		m	0	0	all	-	all	Eu:UK	1960	CC	954	n	V	y	n	0	all/unsp	non any	st
DEAN2	8		f	0	0	all	-	all	Eu:UK	1960	CC	954	n	V	y	n	0	all/unsp	non any	st
DEAN3	82	x	m	0	0	all	-	all	Eu:UK	1969	CC	766	n	V	y	n	0	all/unsp	non any	st
DEAN3	152	x	f	0	0	all	-	all	Eu:UK	1969	CC	766	n	V	y	n	0	cig only	non any	st
DEKLER	9		m	0	0	all	0	all	Auslia	1961	pr	138	m	V	n	n	2	all/unsp	non any	ot
DESTE2	18	x	c	0	0	all	-	all	SCAmer	1993	CC	463	n	bl	n	n	0	all/unsp	non any	st
DESTEF	54	x	m	0	0	all	-	all	SCAmer	1988	CC	497	n	bl	n	y	0	all/unsp	non any	st
DOCKER	4		c	0	0	wh	0	all	NAmer	1974	pr	120	n	bl	n	n	4	cig+/-ot	non cigs	ot
DOLL	92		m	0	0	all	-	all	Eu:UK	1948	CC	1465	n	V	n	n	0	all/unsp	non any	st
DOLL	95		f	0	0	all	-	all	Eu:UK	1948	CC	1465	n	V	n	n	0	all/unsp	non any	st
DOLL2	57		m	0	0	all	0	all	Eu:UK	1951	pr	920	n	V	n	n	1	all/unsp	non any	ot
DORANT	4		m	0	0	all	0	all	Eu:wst	1986	ot	550	n	bl	n	y	0	all/unsp	non any	st
DORGAN	14		m	0	0	wh	-	all	NAmer	1980	CC	2026	n	bl	y	y	0	cig+/-ot	non cigs	st
DORGAN	38		m	0	0	bl	-	all	NAmer	1980	CC	2026	n	bl	y	y	0	cig+/-ot	non cigs	st
DORGAN	61		f	0	0	wh	-	all	NAmer	1980	CC	2026	n	bl	y	y	0	cig+/-ot	non cigs	st
DORGAN	84		f	0	0	bl	-	all	NAmer	1980	CC	2026	n	bl	y	y	0	cig+/-ot	non cigs	st
DORN	199		m	35	84	wh	8	all	NAmer	1954	pr	5097	n	bl	n	n	1	all/unsp	non any	ot
DROSTE	4	x	m	0	0	all	-	all	Eu:wst	1995	CC	478	n	bl	n	y	0	all/unsp	non any	st
ENGELA	156	x	m	0	0	all	12	all	Eu:Sca	1964	pr	435	n	bl	n	n	0	all/unsp	non any	st
ENGELA	163	x	f	0	0	all	12	all	Eu:Sca	1964	pr	435	n	bl	n	n	0	all/unsp	non any	st
GAO	35	x	m	0	0	all	-	all	As:Chi	1984	CC	1405	n	ot	n	n	0	cig+/-ot	non cigs	st
GAO	36	x	f	0	0	all	-	all	As:Chi	1984	CC	1405	n	ot	n	n	0	cig+/-ot	non cigs	st
GAO2	7	x	m	0	0	all	-	all	As:Jap	1988	CC	282	n	bl	n	n	0	cig+/-ot	non cigs	st
GARCIA	4		c	0	0	all	-	all	NAmer	1992	CC	416	n	bl	n	y	0	cig+/-ot	non cigs	st
GARDIN	8		c	0	0	all	-	all	Eu:UK	1988	CC	143	n	V	y	n	0	all/unsp	non any	st
GARSHI	24	x	m	0	0	all	-	all	NAmer	1981	CC	1081	o	bl	y	n	0	all/unsp	non any	st
GRAHAM	33	x	m	0	0	wh	-	all	NAmer	1956	CC	685	n	bl	n	n	0	all/unsp	non any	st
GREGOR	4		m	0	0	all	-	all	Eu:UK	1976	CC	104	n	V	n	y	0	cig+/-ot	non cigs	st
GREGOR	8		f	0	0	all	-	all	Eu:UK	1976	CC	104	n	V	n	y	0	cig+/-ot	non cigs	st
HAENSZ	66		f	0	0	all	-	not alv	NAmer	1955	CC	158	n	bl	n	y	0	cig+/-ot	non cigs	st
HAMMO2	34	x	m	0	0	all	0	all	NAmer	1967	pr	450	o	bl	n	n	0	cig+/-ot	non cigs	st
HEIN	12		m	0	0	all	0	all	Eu:Sca	1970	pr	144	n	bl	n	n	0	all/unsp	non any	st

Table 1B13 - 4

IESLC - Meta-analysis of Current Smoking (vs non-current), Any product (or Cigarettes if Any not available)
All LC types
Least adjusted

REF	NRR	X	SEX	AGEL	AGEH	RACE	YF	LC TYPE	LOC	START	ST	NLC	R	VB	P	H	AD	PRODUCT	DENOM	De
HENNEK	4		m	0	0	all	0	all	NAmer	1982	pr	169	n	bl	n	n	0	all/unsp	non any	st
HIRAY2	10		m	0	0	all	-	all	As:Jap	1950	CC	145	n	bl	*	n	1	all/unsp	non any	ot
HIRAYA	148		m	0	0	all	0	all	As:Jap	1965	pr	1917	n	bl	n	n	1	cig+/-ot	non any	ot
HIRAYA	151		f	0	0	all	0	all	As:Jap	1965	pr	1917	n	bl	n	n	1	cig+/-ot	non any	ot
HITOSU	7	x	m	0	0	all	-	all	As:Jap	1960	CC	216	n	bl	y	n	0	all/unsp	non any	st
HITOSU	13	x	f	0	0	all	-	all	As:Jap	1960	CC	216	n	bl	y	n	0	all/unsp	non any	st
HOLE	54	x	m	0	0	all	0	all	Eu:UK	1972	pr	225	n	V	n	n	0	all/unsp	non any	st
HOLE	68	x	f	0	0	all	9	all	Eu:UK	1972	pr	225	n	V	n	n	0	cig+/-ot	non any	st
HUMBLE	63		m	0	0	w-hi	-	all	NAmer	1980	CC	521	n	bl	y	n	1	cig+/-ot	non cigs	ot
HUMBLE	64		m	0	0	hi	-	all	NAmer	1980	CC	521	n	bl	y	n	1	cig+/-ot	non cigs	ot
HUMBLE	65		f	0	0	w-hi	-	all	NAmer	1980	CC	521	n	bl	y	n	1	cig+/-ot	non cigs	ot
HUMBLE	66		f	0	0	hi	-	all	NAmer	1980	CC	521	n	bl	y	n	1	cig+/-ot	non cigs	ot
JAHN	32		m	0	0	all	-	all	Eu:Ger	1988	CC	1004	n	bl	n	n	0	cig+/-ot	non cigs	st
JAIN	36	x	m	0	0	all	-	all	NAmer	1981	CC	845	n	V	y	n	0	cig+/-ot	non cigs	st
JAIN	31	x	f	0	0	all	-	all	NAmer	1981	CC	845	n	V	y	n	0	cig+/-ot	non cigs	st
JARVHO	4		m	0	0	all	-	all	Eu:Sca	1983	CC	147	n	bl	n	n	0	all/unsp	non any	st
JARVHO	8		f	0	0	all	-	all	Eu:Sca	1983	CC	147	n	bl	n	n	0	all/unsp	non any	st
JOLY	32		m	0	0	all	-	all	SCAmer	1978	CC	826	n	bl	n	n	0	all/unsp	non any	st
JOLY	29		f	0	0	all	-	all	SCAmer	1978	CC	826	n	bl	n	n	0	cig+/-ot	non any	st
KAISE2	71		m	35	99	all	9	all	NAmer	1979	pr	318	n	bl	n	n	1	cig only	non any	st
KAISE2	63		f	35	99	all	9	all	NAmer	1979	pr	318	n	bl	n	n	1	cig only	non any	st
KAISER	14		m	0	0	all	0	all	NAmer	1964	pr	714	n	bl	n	n	2	cig+/-ot	non cigs	ot
KAISER	11		f	0	0	all	0	all	NAmer	1964	pr	714	n	bl	n	n	2	cig+/-ot	non cigs	ot
KATSOU	28	x	f	0	0	all	-	all	Eu:bal	1987	CC	101	n	bl	n	n	0	all/unsp	non any	st
KAUFMA	9	x	c	0	0	all	-	all	NAmer	1981	CC	881	n	bl	n	n	0	cig+/-ot	non cigs	st
KELLER	4		m	0	0	wh	-	all	NAmer	1985	CC	15038	n	bl	n	n	0	all/unsp	non any	st
KELLER	12		m	0	0	nonw	-	all	NAmer	1985	CC	15038	n	bl	n	n	0	all/unsp	non any	st
KELLER	8		f	0	0	wh	-	all	NAmer	1985	CC	15038	n	bl	n	n	0	all/unsp	non any	st
KELLER	16		f	0	0	nonw	-	all	NAmer	1985	CC	15038	n	bl	n	n	0	all/unsp	non any	st
KHUDER	34		m	0	0	all	-	all	NAmer	1985	CC	482	n	bl	n	y	0	cig+/-ot	non cigs	ot
KIHARA	23		c	0	0	jap	-	all	As:Jap	1991	CC	440	n	bl	n	n	0	all/unsp	non any	st
KINLEN	11	x	m	0	0	all	0	all	Eu:UK	1967	pr	718	n	V	n	n	0	all/unsp	non any	st
KJUUS	13		m	0	0	all	-	all	Eu:Sca	1979	CC	176	n	bl	n	n	0	all/unsp	non any	st
KNEKT	81	x	m	20	69	all	21	all	Eu:Sca	1966	pr	515	n	bl	n	n	0	all/unsp	non any	st
KOO	10		f	0	0	all	-	all	As:HK	1981	CC	200	n	bl	n	n	0	all/unsp	non any	st
KREUZE	51		m	1	45	all	-	all	Eu:Ger	1990	CC	2260	n	bl	n	n	0	all/unsp	non any	st
KREUZE	53		m	55	69	all	-	all	Eu:Ger	1990	CC	2260	n	bl	n	n	0	all/unsp	non any	st
KREUZE	52		f	1	45	all	-	all	Eu:Ger	1990	CC	2260	n	bl	n	n	0	all/unsp	non any	st
KREUZE	54		f	55	69	all	-	all	Eu:Ger	1990	CC	2260	n	bl	n	n	0	all/unsp	non any	st
KUBIK	32		m	0	0	all	0	all	Eu:est	1965	pr	108	n	bl	n	n	0	cig+/-ot	non cigs	st
LANGE	33	x	m	0	0	all	0	all	Eu:Sca	1976	pr	268	n	bl	n	n	0	all/unsp	non any	st
LANGE	30	x	f	0	0	all	0	all	Eu:Sca	1976	pr	268	n	bl	n	n	0	all/unsp	non any	st
LEMARC	4		c	0	0	w+o	-	all	NAmer	1992	CC	341	n	bl	n	y	0	all/unsp	non any	st
LICKIN	1		m	0	0	all	-	all	Eu:Ger	1950	CC	224	n	bl	*	n	0	all/unsp	non any	st
LIDDEL	8		m	0	0	all	18	all	NAmer	1970	pr	304	m	V	n	n	1	cig+/-ot	non cigs	ot
LOMBAR	24		m	0	0	all	-	all	NAmer	1951	CC	1040	n	bl	n	n	0	cig+/-ot	non cigs	st
LUBIN2	81	x	m	0	0	all	-	all	Eu:mul	1976	CC	7804	n	bl	n	y	0	all/unsp	non any	st
LUBIN2	318		f	0	0	all	-	all	Eu:mul	1976	CC	7804	n	bl	n	y	0	cig+/-ot	non any	st
MACLEN	50		m	0	0	ch	-	all	As:oth	1972	CC	233	n	bl	n	n	0	cig+/-ot	non cigs	st
MACLEN	63		f	0	0	ch	-	all	As:oth	1972	CC	233	n	bl	n	n	0	cig+/-ot	non cigs	st
MATOS	75	x	m	0	0	all	-	all	SCAmer	1994	CC	200	n	bl	n	n	0	cig+/-ot	non any	st
MIGRAN	61	x	m	0	0	all	0	all	Eu:UK	1964	pr	259	n	V	n	n	0	all/unsp	non any	st
MIGRAN	137	x	f	0	0	all	0	all	Eu:UK	1964	pr	259	n	V	n	n	0	all/unsp	non any	st
MRFIT	23	x	m	0	0	all	0	all	NAmer	1973	pr	2004	n	bl	n	n	0	cig+/-ot	non cigs	st
MURATA	4		m	0	0	all	-	all	As:Jap	1984	ot	107	n	bl	n	n	0	cig+/-ot	non cigs	st
NAM	72	x	m	0	0	all	-	all	NAmer	1986	CC	1199	n	bl	y	n	0	cig+/-ot	non cigs	ot
NAM	88	x	f	0	0	all	-	all	NAmer	1986	CC	1199	n	bl	y	n	0	cig+/-ot	non cigs	ot
NOTANI	1		m	0	0	all	-	all	As:Ind	1986	CC	246	n	V	n	n	1	all/unsp	non any	or
ODRISC	4		c	0	0	all	-	all	Eu:UK	1992	CC	446	n	V	n	n	0	all/unsp	non any	st
OSANN	65	x	m	0	0	all	-	all	NAmer	1984	CC	1986	n	bl	n	n	0	cig+/-ot	non cigs	st
OSANN	69	x	f	0	0	all	-	all	NAmer	1984	CC	1986	n	bl	n	n	0	cig+/-ot	non cigs	st
PARKIN	46	x	m	0	0	bl	-	all	Africa	1963	CC	877	n	V	y	n	0	all/unsp	non any	st
PERSH2	6	x	c	0	0	all	-	all	Eu:Sca	1980	CC	1022	n	bl	y	n	0	all/unsp	non any	st
PETO	6		m	0	0	all	0	all	Eu:UK	1954	pr	103	n	V	n	n	0	all/unsp	non any	st
PEZZO2	6		m	0	0	all	-	all	SCAmer	1992	CC	367	n	bl	n	y	0	cig+/-ot	non cigs	st
PEZZOT	26		m	0	0	all	-	all	SCAmer	1987	CC	215	n	bl	n	y	0	cig only	non cigs	st
QIAO2	19	x	m	0	0	all	0	all	As:Chi	1992	pr	241	m	ot	n	n	0	all/unsp	non any	st
RACHTA	4	x	f	0	0	all	-	all	Eu:est	1991	CC	118	n	bl	n	y	0	cig+/-ot	non cigs	st
SAARIK	1		c	0	0	wh	-	all	Eu:Sca	1988	CC	205	n	bl	n	y	0	all/unsp	non any	st
SCHWAR	29		m	0	0	wh	-	all	NAmer	1984	CC	5588	n	bl	y	y	0	cig+/-ot	non cigs	st
SCHWAR	30		m	0	0	bl	-	all	NAmer	1984	CC	5588	n	bl	y	y	0	cig+/-ot	non cigs	st
SCHWAR	31		f	0	0	wh	-	all	NAmer	1984	CC	5588	n	bl	y	y	0	cig+/-ot	non cigs	st

Table 1B13 - 4

IESLC - Meta-analysis of Current Smoking (vs non-current), Any product (or Cigarettes if Any not available)
All LC types
Least adjusted

REF	NRR	X	SEX	AGEL	AGEH	RACE	YF	LC TYPE	LOC	START	ST	NLC	R	VB	P	H	AD	PRODUCT	DENOM	De
SCHWAR	32		f	0	0	bl	-	all	NAmer	1984	CC	5588	n	bl	y	y	0	cig+/-ot	non cigs	st
SHAW	9		c	0	0	wh	-	all	NAmer	1988	CC	335	n	V	n	y	0	all/unsp	non any	st
SHIMIZ	5		m	0	0	all	-	all	As:Jap	1977	CC	751	n	bl	y	n	2	all/unsp	non any	or
SHIMIZ	10		f	0	0	all	-	all	As:Jap	1977	CC	751	n	bl	y	n	2	all/unsp	non any	or
SITAS	1		m	0	0	bl	-	all	Africa	1994	CC	*	n	V	n	n	1	all/unsp	non any	or
SOBUE	92	x	m	0	0	all	-	q+s+l+a	As:Jap	1986	CC	1376	n	bl	n	y	0	cig+/-ot	non cigs	st
SOBUE	96	x	f	0	0	all	-	q+s+l+a	As:Jap	1986	CC	1376	n	bl	n	y	0	cig+/-ot	non cigs	st
SPEIZE	9		f	0	0	all	0	all	NAmer	1976	pr	593	n	bl	n	y	0	cig+/-ot	non cigs	st
SPITZ	4		c	0	0	b+hi	-	all	NAmer	1992	CC	177	n	bl	n	y	0	cig+/-ot	non cigs	st
STAYNE	9		m	0	0	all	-	all	NAmer	1969	CC	420	n	bl	n	n	1	cig+/-ot	non cigs	st
STOCKW	12		c	0	0	all	-	all	NAmer	1981	CC	22161	n	bl	n	n	0	cig+/-ot	non cigs	st
STUCKE	4		m	0	0	all	-	all	Eu:wst	1989	CC	247	n	bl	n	y	0	all/unsp	non any	st
SUZUK2	19	x	c	0	0	all	-	all	SCAmer	1991	CC	123	n	bl	n	y	0	all/unsp	non any	st
SVENSS	66	x	f	0	0	all	-	all	Eu:Sca	1983	CC	210	n	bl	n	n	0	all/unsp	non any	st
TANG	4		c	0	0	all	-	not s	NAmer	1992	CC	119	n	bl	n	y	0	cig+/-ot	non cigs	st
TENKAN	21		m	0	0	all	18	all	Eu:Sca	1962	pr	242	n	bl	n	n	1	all/unsp	non any	st
TIZZAN	11		m	0	0	all	-	all	Eu:wst	1959	CC	1358	n	bl	n	n	0	all/unsp	non any	st
TIZZAN	17		f	0	0	all	-	all	Eu:wst	1959	CC	1358	n	bl	n	n	0	all/unsp	non any	st
TOKARS	4		m	0	0	all	-	all	Eu:est	1966	ot	162	o	bl	n	y	0	all/unsp	non any	st
TOUSEY	20	x	m	0	0	all	-	all	NAmer	1993	CC	507	n	bl	y	y	0	cig+/-ot	non cigs	st
TOUSEY	30		f	0	0	all	-	all	NAmer	1993	CC	507	n	bl	y	y	0	cig+/-ot	non cigs	st
TSUGAN	32		m	0	0	all	-	q+a	As:Jap	1976	CC	134	n	bl	n	y	0	all/unsp	non any	st
TSUGAN	25		f	0	0	all	-	q+a	As:Jap	1976	CC	134	n	bl	n	y	0	all/unsp	non any	or
TULINI	18	x	m	0	0	all	0	all	Eu:Sca	1967	pr	472	n	bl	n	n	1	all/unsp	non any	ot
TULINI	23	x	f	0	0	all	0	all	Eu:Sca	1967	pr	472	n	bl	n	n	1	all/unsp	non any	ot
TVERDA	26		m	0	0	all	0	all	Eu:Sca	1972	pr	238	n	bl	n	n	2	cig+/-ot	non any	ot
TVERDA	19		f	0	0	all	0	all	Eu:Sca	1972	pr	238	n	bl	n	n	0	cig only	non any	st
WAKAI	14	x	m	0	0	all	-	all	As:Jap	1988	CC	333	n	bl	n	y	0	all/unsp	non any	st
WAKAI	32	x	f	0	0	all	-	all	As:Jap	1988	CC	333	n	bl	n	y	0	all/unsp	non any	st
WANG2	21	x	c	0	0	all	-	all	As:Chi	1980	CC	103	n	ot	n	n	0	cig+/-ot	non cigs	st
WARSIN	1		m	0	0	all	-	all	Eu:wst	1945	CC	134	n	bl	*	n	0	all/unsp	non any	st
WATSON	5		m	0	0	all	-	all	NAmer	1950	CC	301	n	bl	n	y	0	all/unsp	non any	st
WATSON	6		f	0	0	all	-	all	NAmer	1950	CC	301	n	bl	n	y	0	all/unsp	non any	st
WIGLE	21	x	m	0	0	all	-	all	NAmer	1971	CC	728	n	V	n	n	0	all/unsp	non any	st
WIGLE	24	x	f	0	0	all	-	all	NAmer	1971	CC	728	n	V	n	n	0	all/unsp	non any	st
WU	38	x	f	0	0	wh	-	q+a	NAmer	1981	CC	220	n	bl	n	y	0	all/unsp	non any	st
WUNSCH	13	x	m	0	0	all	-	all	SCAmer	1990	CC	398	n	bl	y	n	0	cig+/-ot	non any	st
WUNSCH	15	x	f	0	0	all	-	all	SCAmer	1990	CC	398	n	bl	y	n	0	cig+/-ot	non any	st
WYNDE3	53		m	0	0	all	-	all	NAmer	1966	CC	350	n	bl	n	y	0	all/unsp	non any	st
WYNDE6	153		m	0	0	all	-	all	NAmer	1969	CC	4423	n	bl	n	y	0	cig+/-ot	non cigs	st
WYNDE6	261		f	0	0	all	-	all	NAmer	1969	CC	4423	n	bl	n	y	0	cig+/-ot	non cigs	st
WYNDER	13		m	0	0	all	-	all	SCAmer	1956	CC	120	n	bl	n	n	0	all/unsp	non any	ot
WYNDER	20		f	0	0	all	-	all	SCAmer	1956	CC	120	n	bl	n	n	0	all/unsp	non any	st
YAMAGU	6	x	c	0	0	all	-	all	As:Jap	1989	CC	144	n	bl	n	y	0	all/unsp	non any	st
YONG	3		c	0	0	all	0	all	NAmer	1971	pr	216	n	bl	n	n	1	cig+/-ot	non cigs	ot

Cigarette type is all/unspec for all RRs
except for the following:

REF	NRR	CIGTYPE
DEAN3	152	MC only

Table 1B13 - 5

IESLC - Meta-analysis of Current Smoking (vs non-current), Any product (or Cigarettes if Any not available)
All LC types
Least adjusted

REF	NRR	SEX	AD	Number Exposed Case	Number Exposed Cont	Non-exposed Case	Non-exposed Cont	RR	95.00%CI
AGUDO	16	f	0	20	17	83	189	2.68	(1.34- 5.37)
*AKIBA	4	m	0	345	171379	66	72136	2.20	(1.69- 2.86)
*AKIBA	8	f	0	74	51237	125	373792	4.32	(3.24- 5.76)
Subtotal AKIBA								2.99	(2.46- 3.63)
*AMANDU	4	m	0	115	96708	17	40037	2.80	(1.68- 4.66)
AMES	5	m	0	150	136	162	177	1.21	(0.88- 1.65)
*ANDERS	4	f	0	212	41262	131	250060	9.81	(7.89- 12.19)
*ARCHER	10	m	0	122	32529	24	13582	2.12	(1.37- 3.29)
ARMADA	10	m	0	188	122	137	203	2.28	(1.67- 3.13)
AUSTIN	4	c	0	111	125	55	200	3.23	(2.18- 4.78)
AXELSS	3	m	0	194	130	114	374	4.90	(3.61- 6.64)
AXELSS	12	f	0	96	69	32	194	8.43	(5.19- 13.70)
Subtotal AXELSS								5.71	(4.41- 7.40)
BARBON	108	m	0	562	362	193	393	3.16	(2.54- 3.93)
BECHER	19	m	0	101	122	45	170	3.13	(2.05- 4.77)
BECHER	20	f	0	33	26	15	70	5.92	(2.77- 12.64)
Subtotal BECHER								3.64	(2.52- 5.25)
*BENSHL	17	m	1	-	-	-	-	3.46	(2.49- 4.81)
BLOHMK	4	m	0	419	313	469	575	1.64	(1.36- 1.99)
*BRETT	8	m	0	135	37448	15	17012	4.09	(2.40- 6.97)
BROSS	17	m	0	565	427	304	355	1.55	(1.27- 1.88)
BUFFLE	41	m	0	257	219	218	247	1.33	(1.03- 1.72)
BUFFLE	42	f	0	313	183	147	299	3.48	(2.66- 4.55)
Subtotal BUFFLE								2.10	(1.75- 2.53)
BYERS2	1	m	0	212	197	83	387	5.02	(3.69- 6.82)
BYERS2	2	f	0	121	91	32	222	9.22	(5.83- 14.61)
Subtotal BYERS2								6.05	(4.69- 7.81)
CARPEN	17	c	0	219	162	124	548	5.97	(4.51- 7.92)
*CEDERL	108	m	2	-	-	-	-	7.43	(5.29- 10.44)
*CEDERL	113	f	2	-	-	-	-	4.80	(3.38- 6.82)
Subtotal CEDERL								6.01	(4.71- 7.68)
*CHANG	13	m	0	35	419	48	1589	2.77	(1.81- 4.22)
*CHANG	14	f	0	30	603	23	1719	3.72	(2.18- 6.35)
Subtotal CHANG								3.10	(2.22- 4.32)
CHOI	4	m	0	232	329	48	231	3.39	(2.38- 4.83)
CHOI	8	f	0	13	23	82	167	1.15	(0.55- 2.39)
Subtotal CHOI								2.76	(2.01- 3.80)
*CHOW	47	m	0	167	124415	33	134936	5.49	(3.78- 7.97)
*CHYOU	10	m	0	181	3470	46	4490	5.09	(3.69- 7.02)
COMSTO	41	m	0	105	100	52	213	4.30	(2.86- 6.47)
COMSTO	53	f	0	77	52	24	150	9.25	(5.31- 16.14)
Subtotal COMSTO								5.63	(4.05- 7.82)
CORREA	53	c	0	943	571	309	703	3.76	(3.17- 4.45)
*CPSI	74	m	1	-	-	-	-	6.81	(6.20- 7.47)
*CPSI	285	f	1	-	-	-	-	3.12	(2.48- 3.92)
Subtotal CPSI								6.09	(5.59- 6.64)
*CPSII	110	m	1	-	-	-	-	3.85	(3.55- 4.18)
*CPSII	85	f	1	-	-	-	-	6.47	(5.70- 7.34)
Subtotal CPSII								4.49	(4.19- 4.80)
DARBY	21	m	0	379	618	288	1490	3.17	(2.65- 3.80)
DARBY	30	f	0	198	231	117	846	6.20	(4.73- 8.13)
Subtotal DARBY								3.89	(3.35- 4.52)
DEAN2	4	m	0	671	600	131	200	1.71	(1.33- 2.18)
DEAN2	8	f	0	59	28	93	123	2.79	(1.65- 4.71)
Subtotal DEAN2								1.87	(1.49- 2.33)
DEAN3	82	m	0	502	1636	114	927	2.50	(2.00- 3.11)
DEAN3	152	f	0	102	1158	48	1800	3.30	(2.33- 4.69)
Subtotal DEAN3								2.70	(2.24- 3.25)
*DEKLER	9	m	2	-	-	-	-	3.64	(2.07- 6.41)
DESTE2	18	c	0	216	151	104	169	2.32	(1.69- 3.20)
DESTEF	54	m	0	362	226	135	271	3.22	(2.47- 4.19)
*DOCKER	4	c	4	-	-	-	-	4.52	(2.34- 8.70)
DOLL	92	m	0	1280	1172	77	185	2.62	(1.99- 3.46)
DOLL	95	f	0	58	41	50	67	1.90	(1.10- 3.26)
Subtotal DOLL								2.45	(1.92- 3.14)
*DOLL2	57	m	1	-	-	-	-	3.29	(2.84- 3.82)
DORANT	4	m	0	332	697	153	930	2.90	(2.33- 3.59)
DORGAN	14	m	0	464	170	272	378	3.79	(3.00- 4.80)
DORGAN	38	m	0	214	61	55	109	6.95	(4.52- 10.70)
DORGAN	61	f	0	611	119	249	354	7.30	(5.66- 9.42)
DORGAN	84	f	0	68	17	18	30	6.67	(3.03- 14.69)

Table 1B13 - 5

IESLC - Meta-analysis of Current Smoking (vs non-current), Any product (or Cigarettes if Any not available)
All LC types
Least adjusted

REF	NRR	SEX	AD	Number Exposed Case	Cont	Non-exposed Case	Cont	RR	95.00%CI
Subtotal DORGAN								5.39	(4.61- 6.31)
*DORN	199	m	1	-	-	-	-	3.52	(3.11- 3.98)
DROSTE	4	m	0	379	267	99	269	3.86	(2.92- 5.10)
*ENGELA	156	m	0	100	6636	18	5282	4.42	(2.68- 7.29)
*ENGELA	163	f	0	13	2674	13	11296	4.22	(1.96- 9.10)
Subtotal ENGELA								4.36	(2.87- 6.63)
GAO	35	m	0	529	438	204	322	1.91	(1.54- 2.37)
GAO	36	f	0	170	100	502	635	2.15	(1.64- 2.83)
Subtotal GAO								2.00	(1.69- 2.37)
GAO2	7	m	0	184	117	98	165	2.65	(1.88- 3.72)
GARCIA	4	c	0	169	74	247	372	3.44	(2.50- 4.72)
GARDIN	8	c	0	97	58	46	85	3.09	(1.90- 5.02)
GARSHI	24	m	0	657	782	332	996	2.52	(2.14- 2.96)
GRAHAM	33	m	0	517	1473	168	524	1.09	(0.90- 1.34)
GREGOR	4	m	0	49	53	33	59	1.65	(0.93- 2.94)
GREGOR	8	f	0	17	26	5	38	4.97	(1.63- 15.15)
Subtotal GREGOR								2.09	(1.25- 3.48)
HAENSZ	66	f	0	69	94	88	245	2.04	(1.38- 3.03)
*HAMMO2	34	m	0	209	4472	98	3580	1.71	(1.35- 2.16)
*HEIN	12	m	0	132	3492	12	1436	4.52	(2.51- 8.14)
*HENNEK	4	m	0	79	2438	90	19593	7.05	(5.23- 9.51)
HIRAY2	10	m	1	-	-	-	-	3.22	(1.60- 6.50)
*HIRAYA	148	m	1	-	-	-	-	4.08	(3.37- 4.94)
*HIRAYA	151	f	1	-	-	-	-	2.32	(1.86- 2.90)
Subtotal HIRAYA								3.21	(2.78- 3.71)
HITOSU	7	m	0	117	1597	32	432	0.99	(0.66- 1.48)
HITOSU	13	f	0	28	459	39	1934	3.03	(1.84- 4.97)
Subtotal HITOSU								1.55	(1.13- 2.12)
*HOLE	54	m	0	163	4130	31	2925	3.72	(2.54- 5.45)
*HOLE	68	f	0	17	3437	14	4958	1.75	(0.86- 3.55)
Subtotal HOLE								3.14	(2.25- 4.39)
HUMBLE	63	m	1	-	-	-	-	4.02	(2.77- 5.83)
HUMBLE	64	m	1	-	-	-	-	2.94	(1.59- 5.45)
HUMBLE	65	f	1	-	-	-	-	6.14	(3.47- 10.85)
HUMBLE	66	f	1	-	-	-	-	12.47	(4.53- 34.34)
Subtotal HUMBLE								4.50	(3.45- 5.89)
JAHN	32	m	0	352	269	487	570	1.53	(1.25- 1.87)
JAIN	36	m	0	265	118	138	244	3.97	(2.94- 5.37)
JAIN	31	f	0	305	99	137	311	6.99	(5.17- 9.47)
Subtotal JAIN								5.26	(4.25- 6.51)
JARVHO	4	m	0	73	29	27	44	4.10	(2.15- 7.81)
JARVHO	8	f	0	31	7	16	29	8.03	(2.89- 22.31)
Subtotal JARVHO								4.96	(2.88- 8.56)
JOLY	32	m	0	487	665	120	441	2.69	(2.13- 3.40)
JOLY	29	f	0	132	96	86	310	4.96	(3.47- 7.07)
Subtotal JOLY								3.23	(2.66- 3.93)
*KAISE2	71	m	1	-	-	-	-	4.35	(2.86- 6.62)
*KAISE2	63	f	1	-	-	-	-	8.61	(5.32- 13.93)
Subtotal KAISE2								5.84	(4.26- 8.02)
*KAISER	14	m	2	-	-	-	-	10.41	(8.11- 13.37)
*KAISER	11	f	2	-	-	-	-	4.49	(3.36- 6.00)
Subtotal KAISER								7.27	(6.02- 8.79)
KATSOU	28	f	0	45	18	56	71	3.17	(1.66- 6.07)
KAUFMA	9	c	0	621	886	260	1684	4.54	(3.85- 5.36)
KELLER	4	m	0	5063	1210	3326	2324	2.92	(2.69- 3.17)
KELLER	12	m	0	1053	212	478	245	2.55	(2.06- 3.15)
KELLER	8	f	0	2904	792	1563	2337	5.48	(4.95- 6.07)
KELLER	16	f	0	454	135	197	311	5.31	(4.08- 6.90)
Subtotal KELLER								3.70	(3.49- 3.93)
KHUDER	34	m	0	275	-	207	-	1.82	(1.46- 2.25)
KIHARA	23	c	0	283	162	157	307	3.42	(2.60- 4.49)
*KINLEN	11	m	0	636	9879	82	4176	3.28	(2.61- 4.11)
KJUUS	13	m	0	135	77	41	99	4.23	(2.67- 6.70)
*KNEKT	81	m	0	93	36489	24	33123	3.52	(2.25- 5.51)
KOO	10	f	0	42	25	78	95	2.05	(1.15- 3.65)
KREUZE	51	m	0	168	99	15	101	11.43	(6.29- 20.75)
KREUZE	53	m	0	1252	524	457	1237	6.47	(5.58- 7.50)
KREUZE	52	f	0	55	23	13	57	10.48	(4.83- 22.75)
KREUZE	54	f	0	170	54	130	224	5.42	(3.73- 7.89)
Subtotal KREUZE								6.60	(5.78- 7.53)
*KUBIK	32	m	0	98	6342	10	5980	9.24	(4.83- 17.70)

Table 1B13 - 5

IESLC - Meta-analysis of Current Smoking (vs non-current), Any product (or Cigarettes if Any not available)
All LC types
Least adjusted

REF	NRR	SEX	AD	Number Exposed Case	Number Exposed Cont	Non-exposed Case	Non-exposed Cont	RR	95.00%CI
*LANGE	33	m	0	174	4537	26	1974	2.91	(1.93- 4.38)
*LANGE	30	f	0	53	4455	15	3248	2.58	(1.45- 4.56)
Subtotal LANGE								2.79	(2.00- 3.90)
LEMARC	4	c	0	167	65	174	391	5.77	(4.12- 8.09)
LICKIN	1	m	0	220	840	4	160	10.48	(3.84- 28.57)
*LIDDEL	8	m	1	-	-	-	-	3.65	(2.68- 4.98)
LOMBAR	24	m	0	852	610	188	430	3.19	(2.61- 3.90)
LUBIN2	81	m	0	5700	7744	1219	5714	3.45	(3.21- 3.70)
LUBIN2	318	f	0	384	410	388	1337	3.23	(2.70- 3.86)
Subtotal LUBIN2								3.42	(3.20- 3.65)
MACLEN	50	m	0	137	108	10	26	3.30	(1.52- 7.14)
MACLEN	63	f	0	42	47	44	119	2.42	(1.41- 4.15)
Subtotal MACLEN								2.68	(1.72- 4.17)
MATOS	75	m	0	112	132	87	261	2.55	(1.79- 3.61)
*MIGRAN	61	m	0	182	5145	28	2441	3.08	(2.08- 4.58)
*MIGRAN	137	f	0	30	3465	5	4435	7.68	(2.98- 19.77)
Subtotal MIGRAN								3.53	(2.45- 5.08)
*MRFIT	23	m	0	1548	133117	456	228545	5.83	(5.25- 6.47)
MURATA	4	m	0	76	93	31	121	3.19	(1.94- 5.25)
NAM	72	m	0	241	589	399	1006	1.03	(0.85- 1.25)
NAM	88	f	0	133	234	211	1147	3.09	(2.39- 4.00)
Subtotal NAM								1.51	(1.30- 1.76)
NOTANI	1	m	1	-	-	-	-	5.79	(3.50- 9.70)
ODRISC	4	c	0	293	598	153	1062	3.40	(2.73- 4.24)
OSANN	65	m	0	791	541	362	1310	5.29	(4.51- 6.21)
OSANN	69	f	0	597	367	236	1289	8.88	(7.35- 10.75)
Subtotal OSANN								6.56	(5.80- 7.41)
PARKIN	46	m	0	375	946	133	1309	3.90	(3.15- 4.84)
PERSH2	6	c	0	736	631	286	1457	5.94	(5.04- 7.01)
*PETO	6	m	0	99	2036	4	682	8.29	(3.06- 22.44)
PEZZO2	6	m	0	233	198	134	388	3.41	(2.59- 4.48)
PEZZOT	26	m	0	145	129	70	304	4.88	(3.43- 6.94)
*QIAO2	19	m	0	198	6101	43	1525	1.15	(0.83- 1.59)
RACHTA	4	f	0	72	33	46	108	5.12	(2.99- 8.77)
SAARIK	1	c	0	102	66	103	224	3.36	(2.28- 4.95)
SCHWAR	29	m	0	1652	349	1115	1046	4.44	(3.85- 5.12)
SCHWAR	30	m	0	644	139	269	240	4.13	(3.21- 5.32)
SCHWAR	31	f	0	1029	309	504	1183	7.82	(6.63- 9.21)
SCHWAR	32	f	0	256	90	119	336	8.03	(5.84- 11.04)
Subtotal SCHWAR								5.59	(5.08- 6.15)
SHAW	9	c	0	212	97	123	276	4.90	(3.56- 6.76)
SHIMIZ	5	m	2	-	-	-	-	3.70	(2.70- 5.10)
SHIMIZ	10	f	2	-	-	-	-	3.40	(2.10- 5.30)
Subtotal SHIMIZ								3.60	(2.77- 4.68)
SITAS	1	m	1	-	-	-	-	8.40	(3.20- 22.20)
SOBUE	92	m	0	736	650	321	491	1.73	(1.45- 2.06)
SOBUE	96	f	0	95	168	199	921	2.62	(1.95- 3.51)
Subtotal SOBUE								1.93	(1.66- 2.24)
*SPEIZE	9	f	0	391	489993	202	1298381	5.13	(4.33- 6.08)
SPITZ	4	c	0	103	89	74	208	3.25	(2.21- 4.80)
STAYNE	9	m	1	-	-	-	-	3.30	(2.45- 4.45)
STOCKW	12	c	0	12470	3357	9691	14353	5.50	(5.25- 5.76)
STUCKE	4	m	0	69	68	178	186	1.06	(0.72- 1.57)
SUZUK2	19	c	0	78	30	45	93	5.37	(3.10- 9.33)
SVENSS	66	f	0	142	53	68	156	6.15	(4.02- 9.40)
TANG	4	c	0	52	25	67	73	2.27	(1.27- 4.05)
*TENKAN	21	m	1	-	-	-	-	6.32	(4.35- 9.19)
TIZZAN	11	m	0	693	619	526	597	1.27	(1.08- 1.49)
TIZZAN	17	f	0	17	18	33	124	3.55	(1.65- 7.63)
Subtotal TIZZAN								1.33	(1.13- 1.55)
TOKARS	4	m	0	110	157	38	139	2.56	(1.66- 3.95)
TOUSEY	20	m	0	160	91	141	476	5.94	(4.32- 8.16)
TOUSEY	30	f	0	127	78	79	362	7.46	(5.14- 10.83)
Subtotal TOUSEY								6.54	(5.13- 8.33)
TSUGAN	32	m	0	63	63	28	30	1.07	(0.58- 2.00)
TSUGAN	25	f	0	6	10	33	30	0.55	(0.18- 1.68)
Subtotal TSUGAN								0.91	(0.53- 1.58)
*TULINI	18	m	1	-	-	-	-	5.24	(3.85- 7.13)
*TULINI	23	f	1	-	-	-	-	11.25	(7.49- 16.90)
Subtotal TULINI								6.92	(5.41- 8.85)
*TVERDA	26	m	2	-	-	-	-	10.64	(6.28- 18.03)

Table 1B13 - 5

IESLC - Meta-analysis of Current Smoking (vs non-current), Any product (or Cigarettes if Any not available)
All LC types
Least adjusted

REF	NRR	SEX	AD	Number Exposed Case	Cont	Non-exposed Case	Cont	RR	95.00%CI
*TVERDA	19	f	0	24	113761	3	196384	13.81	(4.16- 45.86)
Subtotal TVERDA								11.10	(6.85- 17.99)
WAKAI	14	m	0	181	284	64	205	2.04	(1.46- 2.86)
WAKAI	32	f	0	33	26	55	150	3.46	(1.90- 6.31)
Subtotal WAKAI								2.32	(1.73- 3.11)
WANG2	21	c	0	49	78	22	64	1.83	(1.00- 3.34)
WARSIN	1	m	0	129	81	5	19	6.05	(2.17- 16.84)
WATSON	5	m	0	260	250	5	27	5.62	(2.13- 14.81)
WATSON	6	f	0	15	33	21	148	3.20	(1.49- 6.87)
Subtotal WATSON								3.97	(2.18- 7.23)
WIGLE	21	m	0	454	522	159	480	2.63	(2.11- 3.27)
WIGLE	24	f	0	68	169	47	505	4.32	(2.87- 6.52)
Subtotal WIGLE								2.93	(2.42- 3.56)
WU	38	f	0	160	73	60	147	5.37	(3.57- 8.08)
WUNSCH	13	m	0	189	234	114	299	2.12	(1.59- 2.83)
WUNSCH	15	f	0	42	51	46	244	4.37	(2.61- 7.32)
Subtotal WUNSCH								2.52	(1.96- 3.24)
WYNDE3	53	m	0	227	207	57	213	4.10	(2.89- 5.80)
WYNDE6	153	m	0	1677	741	1233	1872	3.44	(3.07- 3.84)
WYNDE6	261	f	0	1022	376	491	1181	6.54	(5.58- 7.66)
Subtotal WYNDE6								4.26	(3.89- 4.67)
WYNDER	13	m	0	80	185	0	35	30.81~	(1.87- 508.45)
WYNDER	20	f	0	39	71	2	141	38.73	(9.09- 164.98)
Subtotal WYNDER								36.90	(10.18- 133.72)
YAMAGU	6	c	0	76	247	68	429	1.94	(1.35- 2.79)
*YONG	3	c	1	-	-	-	-	5.08	(3.74- 6.90)
Partial Totals				68072	1448277	36594	2816022		

*prospective study ~ With 0.5 adjustment for zero

REF	NRR	SEX	AD	Ys	Ws	Qs	Ps
AGUDO	16	f	0	0.99	7.93	1.13	0.0055
*AKIBA	4	m	0	0.79	55.46	18.28	0.0000
*AKIBA	8	f	0	1.46	46.53	0.47	0.0000
Subtotal AKIBA				1.10	101.99	18.75	
*AMANDU	4	m	0	1.03	14.82	1.64	0.0001
AMES	5	m	0	0.19	38.70	53.53	0.2459
*ANDERS	4	f	0	2.28	81.15	68.76	0.0000
*ARCHER	10	m	0	0.75	20.10	7.48	0.0007
ARMADA	10	m	0	0.83	38.85	11.20	0.0000
AUSTIN	4	c	0	1.17	24.88	0.90	0.0000
AXELSS	3	m	0	1.59	41.16	2.10	0.0000
AXELSS	12	f	0	2.13	16.31	9.66	0.0000
Subtotal AXELSS				1.74	57.47	11.76	
BARBON	108	m	0	1.15	81.52	3.65	0.0000
BECHER	19	m	0	1.14	21.64	1.07	0.0000
BECHER	20	f	0	1.78	6.68	1.16	0.0000
Subtotal BECHER				1.29	28.32	2.23	
*BENSHL	17	m	1	1.24	35.45	0.52	0.0000
BLOHMK	4	m	0	0.50	105.79	79.56	0.0000
*BRETT	8	m	0	1.41	13.52	0.03	0.0000
BROSS	17	m	0	0.44	97.86	84.20	0.0000
BUFFLE	41	m	0	0.28	58.50	67.96	0.0293
BUFFLE	42	f	0	1.25	53.17	0.71	0.0000
Subtotal BUFFLE				0.74	111.68	68.67	
BYERS2	1	m	0	1.61	40.94	2.56	0.0000
BYERS2	2	f	0	2.22	18.18	13.42	0.0000
Subtotal BYERS2				1.80	59.12	15.99	
CARPEN	17	c	0	1.79	48.48	8.75	0.0000
*CEDERL	108	m	2	2.01	33.25	13.74	0.0000
*CEDERL	113	f	2	1.57	31.18	1.32	0.0000
Subtotal CEDERL				1.79	64.43	15.06	
*CHANG	13	m	0	1.02	21.56	2.57	0.0000
*CHANG	14	f	0	1.31	13.41	0.03	0.0000
Subtotal CHANG				1.13	34.97	2.61	
CHOI	4	m	0	1.22	30.76	0.61	0.0000
CHOI	8	f	0	0.14	7.22	10.77	0.7054
Subtotal CHOI				1.02	37.97	11.38	
*CHOW	47	m	0	1.70	27.57	3.19	0.0000
*CHYOU	10	m	0	1.63	37.38	2.62	0.0000
COMSTO	41	m	0	1.46	23.02	0.21	0.0000

Table 1B13 - 5

IESLC - Meta-analysis of Current Smoking (vs non-current), Any product (or Cigarettes if Any not available)
All LC types
Least adjusted

REF	NRR	SEX	AD	Ys	Ws	Qs	Ps
COMSTO	53	f	0	2.23	12.41	9.23	0.0000
Subtotal COMSTO				1.73	35.43	9.45	
CORREA	53	c	0	1.32	133.86	0.20	0.0000
*CPSI	74	m	1	1.92	442.50	136.66	0.0000
*CPSI	285	f	1	1.14	73.31	3.71	0.0000
Subtotal CPSI				1.81	515.81	140.36	
*CPSII	110	m	1	1.35	575.76	0.12	0.0000
*CPSII	85	f	1	1.87	240.30	61.16	0.0000
Subtotal CPSII				1.50	816.06	61.29	
DARBY	21	m	0	1.15	119.05	5.15	0.0000
DARBY	30	f	0	1.82	52.33	11.15	0.0000
Subtotal DARBY				1.36	171.38	16.30	
DEAN2	4	m	0	0.53	63.33	43.39	0.0000
DEAN2	8	f	0	1.02	13.98	1.59	0.0001
Subtotal DEAN2				0.62	77.31	44.98	
DEAN3	82	m	0	0.91	80.30	16.14	0.0000
DEAN3	152	f	0	1.19	31.20	0.88	0.0000
Subtotal DEAN3				0.99	111.49	17.02	
*DEKLER	9	m	2	1.29	12.03	0.06	0.0000
DESTE2	18	c	0	0.84	37.33	10.06	0.0000
DESTEF	54	m	0	1.17	54.69	2.07	0.0000
*DOCKER	4	c	4	1.51	8.91	0.19	0.0000
DOLL	92	m	0	0.96	49.93	7.91	0.0000
DOLL	95	f	0	0.64	13.06	6.83	0.0208
Subtotal DOLL				0.90	62.99	14.74	
*DOLL2	57	m	1	1.19	174.85	5.16	0.0000
DORANT	4	m	0	1.06	82.93	7.44	0.0000
DORGAN	14	m	0	1.33	69.64	0.06	0.0000
DORGAN	38	m	0	1.94	20.65	6.86	0.0000
DORGAN	61	f	0	1.99	59.24	23.15	0.0000
DORGAN	84	f	0	1.90	6.16	1.76	0.0000
Subtotal DORGAN				1.68	155.69	31.83	
*DORN	199	m	1	1.26	252.56	2.74	0.0000
DROSTE	4	m	0	1.35	49.50	0.01	0.0000
*ENGELA	156	m	0	1.49	15.33	0.24	0.0000
*ENGELA	163	f	0	1.44	6.52	0.04	0.0002
Subtotal ENGELA				1.47	21.85	0.28	
GAO	35	m	0	0.65	82.09	42.26	0.0000
GAO	36	f	0	0.77	51.42	18.33	0.0000
Subtotal GAO				0.69	133.51	60.59	
GAO2	7	m	0	0.97	33.06	5.00	0.0000
GARCIA	4	c	0	1.24	38.22	0.62	0.0000
GARDIN	8	c	0	1.13	16.38	0.90	0.0000
GARSHI	24	m	0	0.92	146.69	28.17	0.0000
GRAHAM	33	m	0	0.09	95.48	154.52	0.3765
GREGOR	4	m	0	0.50	11.56	8.55	0.0875
GREGOR	8	f	0	1.60	3.09	0.18	0.0048
Subtotal GREGOR				0.73	14.65	8.73	
HAENSZ	66	f	0	0.71	24.64	10.35	0.0004
*HAMMO2	34	m	0	0.53	69.03	47.30	0.0000
*HEIN	12	m	0	1.51	11.12	0.24	0.0000
*HENNEK	4	m	0	1.95	42.90	14.98	0.0000
HIRAY2	10	m	1	1.17	7.82	0.29	0.0011
*HIRAYA	148	m	1	1.41	105.05	0.20	0.0000
*HIRAYA	151	f	1	0.84	77.90	21.15	0.0000
Subtotal HIRAYA				1.17	182.95	21.35	
HITOSU	7	m	0	-0.01	23.40	44.15	0.9575
HITOSU	13	f	0	1.11	15.61	1.02	0.0000
Subtotal HITOSU				0.44	39.01	45.17	
*HOLE	54	m	0	1.31	26.45	0.06	0.0000
*HOLE	68	f	0	0.56	7.71	4.96	0.1197
Subtotal HOLE				1.14	34.16	5.02	
HUMBLE	63	m	1	1.39	27.75	0.02	0.0000
HUMBLE	64	m	1	1.08	10.13	0.82	0.0006
HUMBLE	65	f	1	1.81	11.82	2.42	0.0000
HUMBLE	66	f	1	2.52	3.75	5.04	0.0000
Subtotal HUMBLE				1.51	53.44	8.30	
JAHN	32	m	0	0.43	96.47	84.58	0.0000
JAIN	36	m	0	1.38	42.39	0.01	0.0000
JAIN	31	f	0	1.95	41.85	14.19	0.0000
Subtotal JAIN				1.66	84.24	14.20	

Table 1B13 - 5

IESLC - Meta-analysis of Current Smoking (vs non-current), Any product (or Cigarettes if Any not available)
All LC types
Least adjusted

REF	NRR	SEX	AD	Ys	Ws	Qs	Ps
JARVHO	4	m	0	1.41	9.26	0.02	0.0000
JARVHO	8	f	0	2.08	3.68	1.91	0.0001
Subtotal JARVHO				1.60	12.94	1.93	
JOLY	32	m	0	0.99	70.63	9.81	0.0000
JOLY	29	f	0	1.60	30.44	1.72	0.0000
Subtotal JOLY				1.17	101.08	11.53	
*KAISE2	71	m	1	1.47	21.81	0.25	0.0000
*KAISE2	63	f	1	2.15	16.58	10.36	0.0000
Subtotal KAISE2				1.77	38.40	10.61	
*KAISER	14	m	2	2.34	61.48	59.06	0.0000
*KAISER	11	f	2	1.50	45.71	0.89	0.0000
Subtotal KAISER				1.98	107.19	59.95	
KATSOU	28	f	0	1.15	9.11	0.40	0.0005
KAUFMA	9	c	0	1.51	139.30	3.14	0.0000
KELLER	4	m	0	1.07	569.83	47.86	0.0000
KELLER	12	m	0	0.93	84.46	15.49	0.0000
KELLER	8	f	0	1.70	373.88	42.93	0.0000
KELLER	16	f	0	1.67	55.86	5.26	0.0000
Subtotal KELLER				1.31	1084.02	111.54	
KHUDER	34	m	0	0.60	82.15	47.93	0.0000
KIHARA	23	c	0	1.23	51.72	0.93	0.0000
*KINLEN	11	m	0	1.19	74.48	2.29	0.0000
KJUUS	13	m	0	1.44	18.22	0.12	0.0000
*KNEKT	81	m	0	1.26	19.10	0.21	0.0000
KOO	10	f	0	0.72	11.47	4.80	0.0153
KREUZE	51	m	0	2.44	10.80	12.44	0.0000
KREUZE	53	m	0	1.87	175.32	44.55	0.0000
KREUZE	52	f	0	2.35	6.41	6.24	0.0000
KREUZE	54	f	0	1.69	27.35	2.95	0.0000
Subtotal KREUZE				1.89	219.88	66.18	
*KUBIK	32	m	0	2.22	9.10	6.75	0.0000
*LANGE	33	m	0	1.07	23.00	1.99	0.0000
*LANGE	30	f	0	0.95	11.76	2.04	0.0012
Subtotal LANGE				1.03	34.76	4.03	
LEMARC	4	c	0	1.75	33.70	5.14	0.0000
LICKIN	1	m	0	2.35	3.82	3.71	0.0000
*LIDDEL	8	m	1	1.29	40.02	0.18	0.0000
LOMBAR	24	m	0	1.16	95.62	3.87	0.0000
LUBIN2	81	m	0	1.24	769.28	11.87	0.0000
LUBIN2	318	f	0	1.17	119.50	4.36	0.0000
Subtotal LUBIN2				1.23	888.77	16.23	
MACLEN	50	m	0	1.19	6.45	0.18	0.0024
MACLEN	63	f	0	0.88	13.12	3.03	0.0014
Subtotal MACLEN				0.98	19.57	3.21	
MATOS	75	m	0	0.93	31.42	5.76	0.0000
*MIGRAN	61	m	0	1.13	24.63	1.38	0.0000
*MIGRAN	137	f	0	2.04	4.30	1.96	0.0000
Subtotal MIGRAN				1.26	28.92	3.34	
*MRFIT	23	m	0	1.76	353.72	56.61	0.0000
MURATA	4	m	0	1.16	15.52	0.64	0.0000
NAM	72	m	0	0.03	106.98	189.67	0.7473
NAM	88	f	0	1.13	57.46	3.16	0.0000
Subtotal NAM				0.41	164.44	192.83	
NOTANI	1	m	1	1.76	14.79	2.29	0.0000
ODRISC	4	c	0	1.22	79.60	1.53	0.0000
OSANN	65	m	0	1.67	150.64	13.86	0.0000
OSANN	69	f	0	2.18	106.24	71.73	0.0000
Subtotal OSANN				1.88	256.87	85.59	
PARKIN	46	m	0	1.36	83.29	0.00	0.0000
PERSH2	6	c	0	1.78	140.32	24.68	0.0000
*PETO	6	m	0	2.12	3.87	2.19	0.0000
PEZZO2	6	m	0	1.23	51.59	0.96	0.0000
PEZZOT	26	m	0	1.59	31.03	1.54	0.0000
*QIAO2	19	m	0	0.14	36.38	54.33	0.3964
RACHTA	4	f	0	1.63	13.30	0.98	0.0000
SAARIK	1	c	0	1.21	25.56	0.58	0.0000
SCHWAR	29	m	0	1.49	187.84	3.08	0.0000
SCHWAR	30	m	0	1.42	60.13	0.19	0.0000
SCHWAR	31	f	0	2.06	142.10	68.35	0.0000
SCHWAR	32	f	0	2.08	37.88	19.68	0.0000
Subtotal SCHWAR				1.72	427.95	91.30	

Table 1B13 - 5

IESLC - Meta-analysis of Current Smoking (vs non-current), Any product (or Cigarettes if Any not available)
All LC types
Least adjusted

REF	NRR	SEX	AD	Ys	Ws	Qs	Ps
SHAW	9	c	0	1.59	37.34	1.93	0.0000
SHIMIZ	5	m	2	1.31	37.99	0.11	0.0000
SHIMIZ	10	f	2	1.22	17.93	0.35	0.0000
Subtotal SHIMIZ				1.28	55.92	0.46	
SITAS	1	m	1	2.13	4.10	2.40	0.0000
SOBUE	92	m	0	0.55	124.24	82.20	0.0000
SOBUE	96	f	0	0.96	44.27	7.10	0.0000
Subtotal SOBUE				0.66	168.51	89.30	
*SPEIZE	9	f	0	1.63	133.24	9.88	0.0000
SPITZ	4	c	0	1.18	25.47	0.85	0.0000
STAYNE	9	m	1	1.19	43.14	1.23	0.0000
STOCKW	12	c	0	1.71	1815.09	212.76	0.0000
STUCKE	4	m	0	0.06	24.88	42.31	0.7702
SUZUK2	19	c	0	1.68	12.64	1.28	0.0000
SVENSS	66	f	0	1.82	21.26	4.37	0.0000
TANG	4	c	0	0.82	11.38	3.38	0.0058
*TENKAN	21	m	1	1.84	27.47	6.36	0.0000
TIZZAN	11	m	0	0.24	150.72	190.12	0.0033
TIZZAN	17	f	0	1.27	6.55	0.06	0.0012
Subtotal TIZZAN				0.28	157.27	190.19	
TOKARS	4	m	0	0.94	20.42	3.63	0.0000
TOUSEY	20	m	0	1.78	37.83	6.62	0.0000
TOUSEY	30	f	0	2.01	27.69	11.59	0.0000
Subtotal TOUSEY				1.88	65.52	18.21	
TSUGAN	32	m	0	0.07	9.92	16.60	0.8280
TSUGAN	25	f	0	-0.61	3.03	11.74	0.2916
Subtotal TSUGAN				-0.09	12.95	28.34	
*TULINI	18	m	1	1.66	40.46	3.49	0.0000
*TULINI	23	f	1	2.42	23.20	25.96	0.0000
Subtotal TULINI				1.93	63.67	29.45	
*TVERDA	26	m	2	2.36	13.81	13.87	0.0000
*TVERDA	19	f	0	2.63	2.67	4.25	0.0000
Subtotal TVERDA				2.41	16.48	18.12	
WAKAI	14	m	0	0.71	33.84	14.26	0.0000
WAKAI	32	f	0	1.24	10.68	0.16	0.0000
Subtotal WAKAI				0.84	44.52	14.41	
WANG2	21	c	0	0.60	10.60	6.12	0.0496
WARSIN	1	m	0	1.80	3.67	0.70	0.0006
WATSON	5	m	0	1.73	4.08	0.54	0.0005
WATSON	6	f	0	1.16	6.61	0.26	0.0028
Subtotal WATSON				1.38	10.69	0.80	
WIGLE	21	m	0	0.97	80.06	12.64	0.0000
WIGLE	24	f	0	1.46	22.79	0.23	0.0000
Subtotal WIGLE				1.08	102.85	12.87	
WU	38	f	0	1.68	23.03	2.33	0.0000
WUNSCH	13	m	0	0.75	46.12	17.28	0.0000
WUNSCH	15	f	0	1.47	14.44	0.18	0.0000
Subtotal WUNSCH				0.92	60.56	17.46	
WYNDE3	53	m	0	1.41	31.77	0.07	0.0000
WYNDE6	153	m	0	1.23	303.85	5.01	0.0000
WYNDE6	261	f	0	1.88	153.34	40.66	0.0000
Subtotal WYNDE6				1.45	457.19	45.66	
WYNDER	13	m	0	3.43	0.49	2.08	0.0166
WYNDER	20	f	0	3.66	1.83	9.62	0.0000
Subtotal WYNDER				3.61	2.32	11.71	
YAMAGU	6	c	0	0.66	29.20	14.28	0.0003
*YONG	3	c	1	1.63	40.97	2.83	0.0000

Table 1B13 - 5

IESLC - Meta-analysis of Current Smoking (vs non-current), Any product (or Cigarettes if Any not available)
All LC types
Least adjusted

	N	189
	NS	129
	Wt	13455.50
Het	Chi	3031.64
Het	df	188
Het	P	***
Fixed	RR	3.91
	RRl	3.84
	RRu	3.97
	P	+++
Random	RR	3.70
	RRl	3.44
	RRu	3.98
	P	+++
Asymm	P	*

Table 1B13 - 6

IESLC - Meta-analysis of Current Smoking (vs non-current), Any product (or Cigarettes if Any not available)
All LC types
Least adjusted

		Sex		
	combined	male	female	Total
N	21	104	64	189
NS	21	99	59	179
Wt	2760.95	8067.81	2626.75	13455.50
Het Chi	143.13	1813.62	497.06	3031.64
Het df	20	103	63	188
Het P	***	***	***	***
Fixed RR	4.96	3.30	5.11	3.91
RRl	4.78	3.23	4.92	3.84
RRu	5.15	3.37	5.31	3.97
P	+++	+++	+++	+++
Random RR	3.84	3.25	4.64	3.70
RRl	3.33	2.95	4.12	3.44
RRu	4.43	3.59	5.23	3.98
P	+++	+++	+++	+++
Between Chi				577.83
Between df				2
Between P				***
Btwn(F) P				***
Btwn(R) P				***

Table 1B13 - 7

IESLC - Meta-analysis of Current Smoking (vs non-current), Any product (or Cigarettes if Any not available)
All LC types
Excluded studies (and stage at which they were excluded)

1	BOUCHA BUELL LAURIL MZILEN
2	ABELIN ABRAHA AUVINE AXELSO BAND BERRIN BLOT1 BLOT2 BLOT3 BLOT4 BRESLO BROCKM BROWN1 BYERS1 CASCO2 CASCOR CHAN CHATZI CHEN CHEN2 CHEN3 CHIAZZ COOKSO DAVEYS DEAN DOSEME DU DUNN EBELIN ESAKI FAN GENG GER GODLEY GOLLED GSELL HANSEN HEGMAN HINDS HIRAOK HOROWI HORWIT HU HU2 HUANG ISHIMA JARUP JIANG JONES JUSSAW KO KOHLME KOULUM KREYBE LAMTH LAMWK LAMWK2 LAUSSM LEI LETOUR LEVIN LIU LIU2 LIU3 LIU4 LIU5 LOMBA2 LUO MAGNUS MARSH MARSH2 MARTIS MASTRA MATSUD MCCONN MCDUFF MCLAUG MILLER MILLS MOLLO NOTAN2 NOU ORMOS PASTOR PAWLEG PERNU PERSHA PIKE POFFIJ POLEDN PRESCO QIAO RADZIK RANDIG REN RONCO ROOTS ROTHSC SADOWS SANKAR SCHWA2 SEGI SEOW SIEMIA SIMARA STASZE STOCKS SUN TAO ULMER VANDER WANG WANG3 WANG4 WICKLU WILKIN WUWILL WYNDE2 WYNDE4 XIANGZ XU XU2 XU3 XU4 YUAN ZHANG ZHENG ZHOU
3	RESTRE
4	BOFFET
5	RIMING TANG2 WYNDE5 WYNDE8
6	ALDERS BENHAM BEST BOUCOT BROWN2 DAMBER ENSTRO GILLIS GOODMA HAMMON KANELL LIAW LUBIN SOBUE2 WALD WU2 WYNDE7
8	JEDRYC SEGI2 SUZUKI
10	MRFITR OSANN2 PISANI SIMONA VEIERO VUTUC

Table 1B13 - 8
Potentially overlapping studies

REF	REFGP	PRINC	OVERLAP/LINK
LUBIN2	LUBIN2	1	Lubin-combined
KOO	KOO	1	KOO/LAMTH/LAMWK
TVERDA	TVERDA	1	VEIERO/TVERDAL
AKIBA	AKIBA	1	AKIBA/ISHIMA
HEIN	PRESCO	1	Subset of PRESCO
LANGE	PRESCO	1	Subset of PRESCO
BROSS	BYERS1	1	GRAHAM/BROSS/BYERS1
GRAHAM	BYERS1	1	GRAHAM/BROSS/BYERS1
CHYOU	CHYOU	1	GOODMA/CHYOU
HOLE	TANG2	1	Subset of TANG2
BENSHL	TANG2	1	Subset of TANG2
KAISER	KAISER	1	KAISER/OSANN2
MRFIT	MRFIT	1	MRFIT-overall
WYNDE6	WYNDE6	1	WYNDE5/6/7/8
CPSI	CPSI	1	CPSI overall
JAHN	BOFFET	2	Subset of BOFFET

Table 1B14 -

IESLC - Meta-analysis of Current Smoking (vs non-current), Cigarettes (or Any Product if Cigarettes not available)
All LC types

This analysis is restricted to results for:
1) Non-dose-response data
2) Current smokers
3) Results complete enough for use in metaanalysis

Within each study, results are then selected (in the following order of preference, within each sex) for:
4) PRODUCT: cigarettes regardless of other products, cigarettes only, all/unspec
5) CIGTYPE: all/unspecified, MC regardless of HR, MC only
6) DENOM: non smoker of anything, non smoker of cigarettes
7) Followup period (YF, prospective studies): whole study (coded as 0) or longest available
8) LCtype: all or nearest available, at least Squamous and Adeno. (q = squamous, s = small,
l = large, a = adeno, mix = mixed, alv = alveolar)
9) Race: all or nearest available, otherwise by race (wh or w = white, bl or b = black, hi = hispanic
ch = chinese, jap = japanese, haw = hawaiian, w+o = white + oriental, sca = scandinavian, as = asian)
10) For overlapping studies: principal rather than subsidiary studies
Finally by Age: whole study (coded as 0) if available, otherwise by widest available age group
and then for single sex results (m, f) in preference to combined sex results (c).

Results adjusted (AD) for the most potential confounders are then chosen in Sections -1 to -3
and results adjusted for the least confounders in Sections -4 to -6. (Those least adjusted results which
actually differ from the most adjusted as marked 'x' in column X in Section -4)
(Results adjusted for an unknown number of confounder(s) are coded as 20.)

Section -7 shows excluded studies, together with the stage (as above) at which no qualifying
results were found.

Section -8 lists the potentially overlapping studies which have been included (1=principal, 2=subsidiary).

Section -9 lists any results which would have been included in preference except that they had data not complete
enough for use in meta-analysis, with their significance (yes/no), if known, and any further comment as entered
on the database.

In addition to those mentioned above, the following fields, levels and abbreviations are used:
* or nk = not known, n = no, y = yes, ot = other
non = not current
all/unspec = all or unspecified, cig+/-ot = cigarettes irrespective of other products (cigar, pipe etc)
MC = manufactured cigarettes, HR = hand-rolled cigarettes
REF: 6-character study reference
NRR: number of the RR on the database within the study
ST : study type (CC = case control, pr or prosp = prospective)
NLC: number of lung cancer cases in whole study
R : risky occupational population (n = no, m = mining, o = other risky)
VB : national cigarette type (V = at least 75% Virginia, bl = at least 75% blended, ot = other)
P : any proxy use
H : full histological confirmation
De : derivation of RR/CI (or = original, st = standard method, ot = other method of estimation)

Table 1B14 - 1

IESLC - Meta-analysis of Current Smoking (vs non-current), Cigarettes (or Any Product if Cigarettes not available)
All LC types
Most adjusted

REF	NRR	SEX	AGEL	AGEH	RACE	YF	LC TYPE	LOC	START	ST	NLC	R	VB	P	H	AD	PRODUCT	DENOM	De
AGUDO	15	f	0	0	all	-	all	Eu:wst	1989	CC	103	n	bl	n	n	3	cig only	non any	ot
AKIBA	12	m	0	0	all	0	all	As:Jap	1963	pr	610	n	bl	n	n	5	cig+/-ot	non cigs	ot
AKIBA	16	f	0	0	all	0	all	As:Jap	1963	pr	610	n	bl	n	n	5	cig+/-ot	non cigs	ot
AMANDU	8	m	0	0	wh	0	all	NAmer	1959	pr	132	m	bl	n	n	2	cig+/-ot	non cigs	ot
AMES	5	m	0	0	wh	-	all	NAmer	1959	ot	317	m	bl	n	n	0	all/unsp	non any	st
ANDERS	4	f	0	0	all	0	all	NAmer	1986	pr	343	n	bl	n	n	0	cig+/-ot	non cigs	st
ARCHER	10	m	0	0	wh	0	all	NAmer	1950	pr	146	m	bl	n	n	0	cig+/-ot	non cigs	st
ARMADA	24	m	0	0	all	-	all	Eu:wst	1986	CC	325	n	bl	n	y	1	cig+/-ot	non cigs	ot
AUSTIN	8	c	0	0	all	-	all	NAmer	1970	CC	166	o	bl	y	n	3	cig+/-ot	non cigs	ot
AXELSS	3	m	0	0	sca	-	all	Eu:Sca	1989	CC	436	n	bl	n	n	0	all/unsp	non any	st
AXELSS	12	f	0	0	sca	-	all	Eu:Sca	1989	CC	436	n	bl	n	n	0	all/unsp	non any	st
BARBON	109	m	0	0	all	-	all	Eu:wst	1979	CC	755	n	bl	y	y	1	all/unsp	non any	ot
BECHER	19	m	0	0	all	-	all	Eu:Ger	1985	CC	194	n	bl	n	y	0	all/unsp	non any	st
BECHER	20	f	0	0	all	-	all	Eu:Ger	1985	CC	194	n	bl	n	y	0	all/unsp	non any	st
BENSHL	26	m	40	64	all	10	all	Eu:UK	1967	pr	486	n	V	n	n	1	cig+/-ot	non any	ot
BLOHMK	4	m	0	0	all	-	all	Eu:Ger	1978	CC	888	n	bl	n	y	0	all/unsp	non any	st
BRETT	8	m	0	0	all	0	all	Eu:UK	1960	pr	150	n	V	n	n	0	cig+/-ot	non cigs	st
BROSS	17	m	0	0	wh	-	all	NAmer	1960	CC	974	n	bl	n	n	0	cig+/-ot	non cigs	st
BUFFLE	41	m	0	0	wh	-	all	NAmer	1976	CC	943	n	bl	y	n	0	cig+/-ot	non cigs	st
BUFFLE	42	f	0	0	wh	-	all	NAmer	1976	CC	943	n	bl	y	n	0	cig+/-ot	non cigs	st
BYERS2	1	m	0	0	wh	-	all	NAmer	1980	CC	448	n	bl	n	y	0	cig+/-ot	non cigs	st
BYERS2	2	f	0	0	wh	-	all	NAmer	1980	CC	448	n	bl	n	y	0	cig+/-ot	non cigs	st
CARPEN	13	c	0	0	w+b	-	all	NAmer	1991	CC	356	n	bl	n	n	3	cig+/-ot	non cigs	ot
CEDERL	90	m	0	0	all	10	all	Eu:Sca	1963	pr	491	n	bl	n	n	1	cig+/-ot	non any	ot
CEDERL	102	f	0	0	all	10	all	Eu:Sca	1963	pr	491	n	bl	n	n	1	cig+/-ot	non any	ot
CHANG	13	m	0	0	all	0	all	NAmer	1972	pr	136	n	bl	n	n	0	cig+/-ot	non cigs	st
CHANG	14	f	0	0	all	0	all	NAmer	1972	pr	136	n	bl	n	n	0	cig+/-ot	non cigs	st
CHOI	4	m	0	0	all	-	all	As:oth	1985	CC	375	n	bl	n	n	0	cig+/-ot	non cigs	st
CHOI	8	f	0	0	all	-	all	As:oth	1985	CC	375	n	bl	n	n	0	cig+/-ot	non cigs	st
CHOW	41	m	0	0	wh	0	all	NAmer	1966	pr	219	n	bl	n	n	0	cig+/-ot	non any	st
CHYOU	8	m	0	0	jap	0	all	NAmer	1965	pr	227	n	bl	n	y	1	cig+/-ot	non cigs	ot
COMSTO	90	c	0	0	all	-	all	NAmer	1975	ot	258	n	bl	n	n	1	cig+/-ot	non cigs	st
CORREA	54	c	0	0	all	-	all	NAmer	1979	CC	1359	n	bl	y	n	1	cig+/-ot	non cigs	ot
CPSI	74	m	0	0	wh	0	all	NAmer	1959	pr	5138	n	bl	n	n	1	cig only	non any	st
CPSI	285	f	40	74	all	6	all	NAmer	1959	pr	5138	n	bl	n	n	1	cig+/-ot	non cigs	ot
CPSII	110	m	35	99	all	4	all	NAmer	1982	pr	3229	n	bl	n	n	1	cig only	non cigs	ot
CPSII	85	f	0	0	all	4	all	NAmer	1982	pr	3229	n	bl	n	n	1	cig+/-ot	non cigs	ot
DARBY	7	m	0	0	wh	-	all	Eu:UK	1988	CC	982	n	V	n	n	0	cig+/-ot	non any	st
DARBY	14	f	0	0	wh	-	all	Eu:UK	1988	CC	982	n	V	n	n	0	cig+/-ot	non any	st
DEAN2	4	m	0	0	all	-	all	Eu:UK	1960	CC	954	n	V	y	n	0	all/unsp	non any	st
DEAN2	8	f	0	0	all	-	all	Eu:UK	1960	CC	954	n	V	y	n	0	all/unsp	non any	st
DEAN3	247	m	0	0	all	-	all	Eu:UK	1969	CC	766	n	V	y	n	1	cig+/-ot	non any	ot
DEAN3	154	f	0	0	all	-	all	Eu:UK	1969	CC	766	n	V	y	n	3	cig only	non any	ot
DEKLER	10	m	0	0	all	0	all	Auslia	1961	pr	138	m	V	n	n	2	cig+/-ot	non any	ot
DESTE2	19	c	0	0	all	-	all	SCAmer	1993	CC	463	n	bl	n	n	7	all/unsp	non any	ot
DESTEF	55	m	0	0	all	-	all	SCAmer	1988	CC	497	n	bl	n	y	4	all/unsp	non any	ot
DOCKER	4	c	0	0	wh	0	all	NAmer	1974	pr	120	n	bl	n	n	4	cig+/-ot	non cigs	ot
DOLL	92	m	0	0	all	-	all	Eu:UK	1948	CC	1465	n	V	n	n	0	all/unsp	non any	st
DOLL	95	f	0	0	all	-	all	Eu:UK	1948	CC	1465	n	V	n	n	0	all/unsp	non any	st
DOLL2	76	m	0	0	all	20	all	Eu:UK	1951	pr	920	n	V	n	n	1	cig+/-ot	non any	ot
DORANT	4	m	0	0	all	0	all	Eu:wst	1986	ot	550	n	bl	n	y	0	all/unsp	non any	st
DORGAN	14	m	0	0	wh	-	all	NAmer	1980	CC	2026	n	bl	y	y	0	cig+/-ot	non cigs	st
DORGAN	38	m	0	0	bl	-	all	NAmer	1980	CC	2026	n	bl	y	y	0	cig+/-ot	non cigs	st
DORGAN	61	f	0	0	wh	-	all	NAmer	1980	CC	2026	n	bl	y	y	0	cig+/-ot	non cigs	st
DORGAN	84	f	0	0	bl	-	all	NAmer	1980	CC	2026	n	bl	y	y	0	cig+/-ot	non cigs	st
DORN	202	m	35	84	wh	8	all	NAmer	1954	pr	5097	n	bl	n	n	1	cig+/-ot	non any	ot
DROSTE	8	m	0	0	all	-	all	Eu:wst	1995	CC	478	n	bl	n	y	4	all/unsp	non any	ot
ENGELA	173	m	0	0	all	12	all	Eu:Sca	1964	pr	435	n	bl	n	n	1	cig+/-ot	non any	ot
ENGELA	181	f	0	0	all	12	all	Eu:Sca	1964	pr	435	n	bl	n	n	1	cig+/-ot	non any	ot
GAO	37	m	0	0	all	-	all	As:Chi	1984	CC	1405	n	ot	n	n	2	cig+/-ot	non cigs	ot
GAO	38	f	0	0	all	-	all	As:Chi	1984	CC	1405	n	ot	n	n	2	cig+/-ot	non cigs	ot
GAO2	11	m	0	0	all	-	all	As:Jap	1988	CC	282	n	bl	n	n	1	cig+/-ot	non cigs	ot
GARCIA	4	c	0	0	all	-	all	NAmer	1992	CC	416	n	bl	n	y	0	cig+/-ot	non cigs	st
GARDIN	12	c	0	0	all	-	all	Eu:UK	1988	CC	143	n	V	y	n	0	cig only	non any	st
GARSHI	32	m	0	0	all	-	all	NAmer	1981	CC	1081	o	bl	y	n	1	all/unsp	non any	st
GRAHAM	34	m	0	0	wh	-	all	NAmer	1956	CC	685	n	bl	n	n	1	cig+/-ot	non any	ot
GREGOR	4	m	0	0	all	-	all	Eu:UK	1976	CC	104	n	V	n	y	0	cig+/-ot	non cigs	st
GREGOR	8	f	0	0	all	-	all	Eu:UK	1976	CC	104	n	V	n	y	0	cig+/-ot	non cigs	st
HAENSZ	66	f	0	0	all	-	not alv	NAmer	1955	CC	158	n	bl	n	y	0	cig+/-ot	non cigs	st
HAMMO2	29	m	0	0	all	0	all	NAmer	1967	pr	450	o	bl	n	n	1	cig+/-ot	non cigs	ot
HEIN	8	m	0	0	all	0	all	Eu:Sca	1970	pr	144	n	bl	n	n	0	cig only	non any	st
HENNEK	4	m	0	0	all	0	all	NAmer	1982	pr	169	n	bl	n	n	0	all/unsp	non any	st

Table 1B14 - 1

IESLC - Meta-analysis of Current Smoking (vs non-current), Cigarettes (or Any Product if Cigarettes not available)
All LC types
Most adjusted

REF	NRR	SEX	AGEL	AGEH	RACE	YF	LC TYPE	LOC	START	ST	NLC	R	VB	P	H	AD	PRODUCT	DENOM	De
HIRAY2	15	m	0	0	all	-	all	As:Jap	1950	CC	145	n	bl	*	n	1	cig+/-ot	non any	ot
HIRAYA	148	m	0	0	all	0	all	As:Jap	1965	pr	1917	n	bl	n	n	1	cig+/-ot	non any	ot
HIRAYA	151	f	0	0	all	0	all	As:Jap	1965	pr	1917	n	bl	n	n	1	cig+/-ot	non any	ot
HITOSU	39	m	0	0	all	-	all	As:Jap	1960	CC	216	n	bl	y	n	1	all/unsp	non any	st
HITOSU	63	f	0	0	all	-	all	As:Jap	1960	CC	216	n	bl	y	n	1	all/unsp	non any	st
HOLE	38	m	0	0	all	0	all	Eu:UK	1972	pr	225	n	V	n	n	1	cig+/-ot	non any	ot
HOLE	71	f	0	0	all	9	all	Eu:UK	1972	pr	225	n	V	n	n	1	cig+/-ot	non any	st
HUMBLE	63	m	0	0	w-hi	-	all	NAmer	1980	CC	521	n	bl	y	n	1	cig+/-ot	non cigs	ot
HUMBLE	64	m	0	0	hi	-	all	NAmer	1980	CC	521	n	bl	y	n	1	cig+/-ot	non cigs	ot
HUMBLE	65	f	0	0	w-hi	-	all	NAmer	1980	CC	521	n	bl	y	n	1	cig+/-ot	non cigs	ot
HUMBLE	66	f	0	0	hi	-	all	NAmer	1980	CC	521	n	bl	y	n	1	cig+/-ot	non cigs	ot
JAHN	32	m	0	0	all	-	all	Eu:Ger	1988	CC	1004	n	bl	n	n	0	cig+/-ot	non cigs	st
JAIN	56	m	0	0	all	-	all	NAmer	1981	CC	845	n	V	y	n	2	cig+/-ot	non cigs	ot
JAIN	54	f	0	0	all	-	all	NAmer	1981	CC	845	n	V	y	n	2	cig+/-ot	non cigs	ot
JARVHO	4	m	0	0	all	-	all	Eu:Sca	1983	CC	147	n	bl	n	n	0	all/unsp	non any	st
JARVHO	8	f	0	0	all	-	all	Eu:Sca	1983	CC	147	n	bl	n	n	0	all/unsp	non any	st
JOLY	30	m	0	0	all	-	all	SCAmer	1978	CC	826	n	bl	n	n	0	cig+/-ot	non any	st
JOLY	29	f	0	0	all	-	all	SCAmer	1978	CC	826	n	bl	n	n	0	cig+/-ot	non any	st
KAISE2	71	m	35	99	all	9	all	NAmer	1979	pr	318	n	bl	n	n	1	cig only	non any	st
KAISE2	63	f	35	99	all	9	all	NAmer	1979	pr	318	n	bl	n	n	1	cig only	non any	st
KAISER	14	m	0	0	all	0	all	NAmer	1964	pr	714	n	bl	n	n	2	cig+/-ot	non cigs	ot
KAISER	11	f	0	0	all	0	all	NAmer	1964	pr	714	n	bl	n	n	2	cig+/-ot	non cigs	ot
KATSOU	30	f	0	0	all	-	all	Eu:bal	1987	CC	101	n	bl	n	n	1	all/unsp	non any	ot
KAUFMA	18	c	0	0	all	-	all	NAmer	1981	CC	881	n	bl	n	n	6	cig+/-ot	non cigs	ot
KELLER	4	m	0	0	wh	-	all	NAmer	1985	CC	15038	n	bl	n	n	0	all/unsp	non any	st
KELLER	12	m	0	0	nonw	-	all	NAmer	1985	CC	15038	n	bl	n	n	0	all/unsp	non any	st
KELLER	8	f	0	0	wh	-	all	NAmer	1985	CC	15038	n	bl	n	n	0	all/unsp	non any	st
KELLER	16	f	0	0	nonw	-	all	NAmer	1985	CC	15038	n	bl	n	n	0	all/unsp	non any	st
KHUDER	34	m	0	0	all	-	all	NAmer	1985	CC	482	n	bl	n	y	0	cig+/-ot	non cigs	ot
KIHARA	23	c	0	0	jap	-	all	As:Jap	1991	CC	440	n	bl	n	n	0	all/unsp	non any	st
KINLEN	21	m	0	0	all	0	all	Eu:UK	1967	pr	718	n	V	n	n	2	cig+/-ot	non any	ot
KJUUS	13	m	0	0	all	-	all	Eu:Sca	1979	CC	176	n	bl	n	n	0	all/unsp	non any	st
KNEKT	91	m	20	69	all	21	all	Eu:Sca	1966	pr	515	n	bl	n	n	1	cig+/-ot	non any	ot
KOO	10	f	0	0	all	-	all	As:HK	1981	CC	200	n	bl	n	n	0	all/unsp	non any	st
KREUZE	43	m	1	45	all	-	all	Eu:Ger	1990	CC	2260	n	bl	n	n	0	cig+/-ot	non any	st
KREUZE	45	m	55	69	all	-	all	Eu:Ger	1990	CC	2260	n	bl	n	n	0	cig+/-ot	non any	st
KREUZE	44	f	1	45	all	-	all	Eu:Ger	1990	CC	2260	n	bl	n	n	0	cig+/-ot	non any	st
KREUZE	46	f	55	69	all	-	all	Eu:Ger	1990	CC	2260	n	bl	n	n	0	cig+/-ot	non any	st
KUBIK	32	m	0	0	all	0	all	Eu:est	1965	pr	108	n	bl	n	n	0	cig+/-ot	non cigs	st
LANGE	39	m	0	0	all	0	all	Eu:Sca	1976	pr	268	n	bl	n	n	1	all/unsp	non any	ot
LANGE	36	f	0	0	all	0	all	Eu:Sca	1976	pr	268	n	bl	n	n	1	all/unsp	non any	ot
LEMARC	4	c	0	0	w+o	-	all	NAmer	1992	CC	341	n	bl	n	y	0	all/unsp	non any	st
LICKIN	1	m	0	0	all	-	all	Eu:Ger	1950	CC	224	n	bl	*	n	0	all/unsp	non any	st
LIDDEL	8	m	0	0	all	18	all	NAmer	1970	pr	304	m	V	n	n	1	cig+/-ot	non cigs	ot
LOMBAR	24	m	0	0	all	-	all	NAmer	1951	CC	1040	n	bl	n	n	0	cig+/-ot	non cigs	st
LUBIN2	84	m	0	0	all	-	all	Eu:mul	1976	CC	7804	n	bl	n	y	2	cig+/-ot	non any	ot
LUBIN2	318	f	0	0	all	-	all	Eu:mul	1976	CC	7804	n	bl	n	y	0	cig+/-ot	non any	st
MACLEN	50	m	0	0	ch	-	all	As:oth	1972	CC	233	n	bl	n	n	0	cig+/-ot	non cigs	st
MACLEN	63	f	0	0	ch	-	all	As:oth	1972	CC	233	n	bl	n	n	0	cig+/-ot	non cigs	st
MATOS	82	m	0	0	all	-	all	SCAmer	1994	CC	200	n	bl	n	n	2	cig+/-ot	non any	ot
MIGRAN	54	m	0	0	all	0	all	Eu:UK	1964	pr	259	n	V	n	n	2	cig+/-ot	non any	ot
MIGRAN	73	f	0	0	all	0	all	Eu:UK	1964	pr	259	n	V	n	n	2	cig+/-ot	non any	ot
MRFIT	6	m	0	0	all	0	all	NAmer	1973	pr	2004	n	bl	n	n	4	cig+/-ot	non cigs	ot
MURATA	4	m	0	0	all	-	all	As:Jap	1984	ot	107	n	bl	n	n	0	cig+/-ot	non cigs	st
NAM	80	m	0	0	all	-	all	NAmer	1986	CC	1199	n	bl	y	n	1	cig+/-ot	non cigs	ot
NAM	96	f	0	0	all	-	all	NAmer	1986	CC	1199	n	bl	y	n	1	cig+/-ot	non cigs	ot
NOTANI	1	m	0	0	all	-	all	As:Ind	1986	CC	246	n	V	n	n	1	all/unsp	non any	or
ODRISC	4	c	0	0	all	-	all	Eu:UK	1992	CC	446	n	V	n	n	0	all/unsp	non any	st
OSANN	73	m	0	0	all	-	all	NAmer	1984	CC	1986	n	bl	n	n	2	cig+/-ot	non cigs	ot
OSANN	74	f	0	0	all	-	all	NAmer	1984	CC	1986	n	bl	n	n	2	cig+/-ot	non cigs	ot
PARKIN	40	m	0	0	bl	-	all	Africa	1963	CC	877	n	V	y	n	0	cig+/-ot	non any	st
PERSH2	12	c	0	0	all	-	all	Eu:Sca	1980	CC	1022	n	bl	y	n	4	all/unsp	non any	ot
PETO	6	m	0	0	all	0	all	Eu:UK	1954	pr	103	n	V	n	n	0	all/unsp	non any	st
PEZZO2	6	m	0	0	all	-	all	SCAmer	1992	CC	367	n	bl	n	y	0	cig+/-ot	non cigs	st
PEZZOT	26	m	0	0	all	-	all	SCAmer	1987	CC	215	n	bl	n	y	0	cig only	non cigs	st
QIAO2	21	m	0	0	all	0	all	As:Chi	1992	pr	241	m	ot	n	n	0	cig+/-ot	non any	st
RACHTA	14	f	0	0	all	-	all	Eu:est	1991	CC	118	n	bl	n	y	1	cig+/-ot	non cigs	ot
SAARIK	1	c	0	0	wh	-	all	Eu:Sca	1988	CC	205	n	bl	n	y	0	all/unsp	non any	st
SCHWAR	29	m	0	0	wh	-	all	NAmer	1984	CC	5588	n	bl	y	y	0	cig+/-ot	non cigs	st
SCHWAR	30	m	0	0	bl	-	all	NAmer	1984	CC	5588	n	bl	y	y	0	cig+/-ot	non cigs	st
SCHWAR	31	f	0	0	wh	-	all	NAmer	1984	CC	5588	n	bl	y	y	0	cig+/-ot	non cigs	st
SCHWAR	32	f	0	0	bl	-	all	NAmer	1984	CC	5588	n	bl	y	y	0	cig+/-ot	non cigs	st

Table 1B14 - 1

IESLC - Meta-analysis of Current Smoking (vs non-current), Cigarettes (or Any Product if Cigarettes not available)
All LC types
Most adjusted

REF	NRR	SEX	AGEL	AGEH	RACE	YF	LC TYPE	LOC	START	ST	NLC	R	VB	P	H	AD	PRODUCT	DENOM	De
SHAW	9	c	0	0	wh	-	all	NAmer	1988	CC	335	n	V	n	y	0	all/unsp	non any	st
SHIMIZ	5	m	0	0	all	-	all	As:Jap	1977	CC	751	n	bl	y	n	2	all/unsp	non any	or
SHIMIZ	10	f	0	0	all	-	all	As:Jap	1977	CC	751	n	bl	y	n	2	all/unsp	non any	or
SITAS	1	m	0	0	bl	-	all	Africa	1994	CC	*	n	V	n	n	1	all/unsp	non any	or
SOBUE	106	m	0	0	all	-	q+s+l+a	As:Jap	1986	CC	1376	n	bl	n	y	1	cig+/-ot	non cigs	ot
SOBUE	116	f	0	0	all	-	q+s+l+a	As:Jap	1986	CC	1376	n	bl	n	y	1	cig+/-ot	non cigs	ot
SPEIZE	9	f	0	0	all	0	all	NAmer	1976	pr	593	n	bl	n	y	0	cig+/-ot	non cigs	st
SPITZ	4	c	0	0	b+hi	-	all	NAmer	1992	CC	177	n	bl	n	y	0	cig+/-ot	non cigs	st
STAYNE	9	m	0	0	all	-	all	NAmer	1969	CC	420	n	bl	n	n	1	cig+/-ot	non cigs	st
STOCKW	12	c	0	0	all	-	all	NAmer	1981	CC	22161	n	bl	n	n	0	cig+/-ot	non cigs	st
STUCKE	4	m	0	0	all	-	all	Eu:wst	1989	CC	247	n	bl	n	y	0	all/unsp	non any	st
SUZUK2	21	c	0	0	all	-	all	SCAmer	1991	CC	123	n	bl	n	y	3	all/unsp	non any	ot
SVENSS	91	f	0	0	all	-	all	Eu:Sca	1983	CC	210	n	bl	n	n	1	all/unsp	non any	ot
TANG	4	c	0	0	all	-	not s	NAmer	1992	CC	119	n	bl	n	y	0	cig+/-ot	non cigs	st
TENKAN	21	m	0	0	all	18	all	Eu:Sca	1962	pr	242	n	bl	n	n	1	all/unsp	non any	st
TIZZAN	11	m	0	0	all	-	all	Eu:wst	1959	CC	1358	n	bl	n	n	0	all/unsp	non any	st
TIZZAN	17	f	0	0	all	-	all	Eu:wst	1959	CC	1358	n	bl	n	n	0	all/unsp	non any	st
TOKARS	4	m	0	0	all	-	all	Eu:est	1966	ot	162	o	bl	n	y	0	all/unsp	non any	st
TOUSEY	25	m	0	0	all	-	all	NAmer	1993	CC	507	n	bl	y	y	3	cig+/-ot	non cigs	ot
TOUSEY	30	f	0	0	all	-	all	NAmer	1993	CC	507	n	bl	y	y	0	cig+/-ot	non cigs	st
TSUGAN	32	m	0	0	all	-	q+a	As:Jap	1976	CC	134	n	bl	n	y	0	all/unsp	non any	st
TSUGAN	25	f	0	0	all	-	q+a	As:Jap	1976	CC	134	n	bl	n	y	0	all/unsp	non any	or
TULINI	40	m	0	0	all	0	all	Eu:Sca	1967	pr	472	n	bl	n	n	3	cig+/-ot	non any	ot
TULINI	45	f	0	0	all	0	all	Eu:Sca	1967	pr	472	n	bl	n	n	3	cig+/-ot	non any	ot
TVERDA	26	m	0	0	all	0	all	Eu:Sca	1972	pr	238	n	bl	n	n	2	cig+/-ot	non any	ot
TVERDA	19	f	0	0	all	0	all	Eu:Sca	1972	pr	238	n	bl	n	n	0	cig only	non any	st
WAKAI	73	m	0	0	all	-	all	As:Jap	1988	CC	333	n	bl	n	y	2	all/unsp	non any	ot
WAKAI	79	f	0	0	all	-	all	As:Jap	1988	CC	333	n	bl	n	y	2	all/unsp	non any	ot
WANG2	22	c	0	0	all	-	all	As:Chi	1980	CC	103	n	ot	n	n	4	cig+/-ot	non cigs	ot
WARSIN	1	m	0	0	all	-	all	Eu:wst	1945	CC	134	n	bl	*	n	0	all/unsp	non any	st
WATSON	5	m	0	0	all	-	all	NAmer	1950	CC	301	n	bl	n	y	0	all/unsp	non any	st
WATSON	6	f	0	0	all	-	all	NAmer	1950	CC	301	n	bl	n	y	0	all/unsp	non any	st
WIGLE	19	m	0	0	all	-	all	NAmer	1971	CC	728	n	V	n	n	0	cig only	non any	st
WIGLE	22	f	0	0	all	-	all	NAmer	1971	CC	728	n	V	n	n	0	cig only	non any	st
WU	46	f	0	0	wh	-	q+a	NAmer	1981	CC	220	n	bl	n	y	2	all/unsp	non any	ot
WUNSCH	14	m	0	0	all	-	all	SCAmer	1990	CC	398	n	bl	y	n	1	cig+/-ot	non any	ot
WUNSCH	16	f	0	0	all	-	all	SCAmer	1990	CC	398	n	bl	y	n	1	cig+/-ot	non any	ot
WYNDE3	53	m	0	0	all	-	all	NAmer	1966	CC	350	n	bl	n	y	0	all/unsp	non any	st
WYNDE6	153	m	0	0	all	-	all	NAmer	1969	CC	4423	n	bl	n	y	0	cig+/-ot	non cigs	st
WYNDE6	261	f	0	0	all	-	all	NAmer	1969	CC	4423	n	bl	n	y	0	cig+/-ot	non cigs	st
WYNDER	14	m	0	0	all	-	all	SCAmer	1956	CC	120	n	bl	n	n	0	cig+/-ot	non any	ot
WYNDER	21	f	0	0	all	-	all	SCAmer	1956	CC	120	n	bl	n	n	0	cig+/-ot	non any	st
YAMAGU	12	c	0	0	all	-	all	As:Jap	1989	CC	144	n	bl	n	y	1	all/unsp	non any	ot
YONG	3	c	0	0	all	0	all	NAmer	1971	pr	216	n	bl	n	n	1	cig+/-ot	non cigs	ot

Cigarette type is all/unspec for all RRs
except for the following:

REF	NRR	CIGTYPE
DEAN3	247	MC only
DEAN3	154	MC only
GARDIN	12	MC only

Table 1B14 - 2

IESLC - Meta-analysis of Current Smoking (vs non-current), Cigarettes (or Any Product if Cigarettes not available)
All LC types
Most adjusted

REF	NRR	SEX	AD	Number Exposed Case	Cont	Non-exposed Case	Cont	RR	95.00%CI
AGUDO	15	f	3	-	-	-	-	3.53	(1.54- 8.10)
*AKIBA	12	m	5	-	-	-	-	3.08	(2.36- 4.00)
*AKIBA	16	f	5	-	-	-	-	3.82	(2.84- 5.15)
Subtotal AKIBA								3.39	(2.78- 4.13)
*AMANDU	8	m	2	-	-	-	-	4.53	(2.40- 8.55)
AMES	5	m	0	150	136	162	177	1.21	(0.88- 1.65)
*ANDERS	4	f	0	212	41262	131	250060	9.81	(7.89- 12.19)
*ARCHER	10	m	0	122	32529	24	13582	2.12	(1.37- 3.29)
ARMADA	24	m	1	-	-	-	-	2.40	(1.75- 3.29)
AUSTIN	8	c	3	-	-	-	-	3.79	(2.38- 6.03)
AXELSS	3	m	0	194	130	114	374	4.90	(3.61- 6.64)
AXELSS	12	f	0	96	69	32	194	8.43	(5.19- 13.70)
Subtotal AXELSS								5.71	(4.41- 7.40)
BARBON	109	m	1	-	-	-	-	3.16	(2.54- 3.92)
BECHER	19	m	0	101	122	45	170	3.13	(2.05- 4.77)
BECHER	20	f	0	33	26	15	70	5.92	(2.77- 12.64)
Subtotal BECHER								3.64	(2.52- 5.25)
*BENSHL	26	m	1	-	-	-	-	3.65	(2.63- 5.08)
BLOHMK	4	m	0	419	313	469	575	1.64	(1.36- 1.99)
*BRETT	8	m	0	135	37448	15	17012	4.09	(2.40- 6.97)
BROSS	17	m	0	565	427	304	355	1.55	(1.27- 1.88)
BUFFLE	41	m	0	257	219	218	247	1.33	(1.03- 1.72)
BUFFLE	42	f	0	313	183	147	299	3.48	(2.66- 4.55)
Subtotal BUFFLE								2.10	(1.75- 2.53)
BYERS2	1	m	0	212	197	83	387	5.02	(3.69- 6.82)
BYERS2	2	f	0	121	91	32	222	9.22	(5.83- 14.61)
Subtotal BYERS2								6.05	(4.69- 7.81)
CARPEN	13	c	3	-	-	-	-	5.78	(4.43- 7.53)
*CEDERL	90	m	1	-	-	-	-	3.88	(2.29- 6.57)
*CEDERL	102	f	1	-	-	-	-	4.39	(1.80- 10.71)
Subtotal CEDERL								4.01	(2.54- 6.31)
*CHANG	13	m	0	35	419	48	1589	2.77	(1.81- 4.22)
*CHANG	14	f	0	30	603	23	1719	3.72	(2.18- 6.35)
Subtotal CHANG								3.10	(2.22- 4.32)
CHOI	4	m	0	232	329	48	231	3.39	(2.38- 4.83)
CHOI	8	f	0	13	23	82	167	1.15	(0.55- 2.39)
Subtotal CHOI								2.76	(2.01- 3.80)
*CHOW	41	m	0	138	81725	33	134936	6.90	(4.72- 10.09)
*CHYOU	8	m	1	-	-	-	-	5.80	(4.22- 7.98)
COMSTO	90	c	1	-	-	-	-	6.34	(4.49- 8.94)
CORREA	54	c	1	-	-	-	-	5.45	(4.62- 6.44)
*CPSI	74	m	1	-	-	-	-	6.81	(6.20- 7.47)
*CPSI	285	f	1	-	-	-	-	3.12	(2.48- 3.92)
Subtotal CPSI								6.09	(5.59- 6.64)
*CPSII	110	m	1	-	-	-	-	3.85	(3.55- 4.18)
*CPSII	85	f	1	-	-	-	-	6.47	(5.70- 7.34)
Subtotal CPSII								4.49	(4.19- 4.80)
DARBY	7	m	0	322	453	288	1490	3.68	(3.04- 4.45)
DARBY	14	f	0	195	217	117	846	6.50	(4.94- 8.54)
Subtotal DARBY								4.43	(3.79- 5.18)
DEAN2	4	m	0	671	600	131	200	1.71	(1.33- 2.18)
DEAN2	8	f	0	59	28	93	123	2.79	(1.65- 4.71)
Subtotal DEAN2								1.87	(1.49- 2.33)
DEAN3	247	m	1	-	-	-	-	3.32	(2.64- 4.17)
DEAN3	154	f	3	-	-	-	-	5.59	(3.78- 8.28)
Subtotal DEAN3								3.79	(3.11- 4.62)
*DEKLER	10	m	2	-	-	-	-	3.76	(2.13- 6.63)
DESTE2	19	c	7	-	-	-	-	2.27	(1.65- 3.14)
DESTEF	55	m	4	-	-	-	-	3.42	(2.62- 4.45)
*DOCKER	4	c	4	-	-	-	-	4.52	(2.34- 8.70)
DOLL	92	m	0	1280	1172	77	185	2.62	(1.99- 3.46)
DOLL	95	f	0	58	41	50	67	1.90	(1.10- 3.26)
Subtotal DOLL								2.45	(1.92- 3.14)
*DOLL2	76	m	1	-	-	-	-	3.60	(2.83- 4.58)
DORANT	4	m	0	332	697	153	930	2.90	(2.33- 3.59)
DORGAN	14	m	0	464	170	272	378	3.79	(3.00- 4.80)
DORGAN	38	m	0	214	61	55	109	6.95	(4.52- 10.70)
DORGAN	61	f	0	611	119	249	354	7.30	(5.66- 9.42)
DORGAN	84	f	0	68	17	18	30	6.67	(3.03- 14.69)
Subtotal DORGAN								5.39	(4.61- 6.31)
*DORN	202	m	1	-	-	-	-	4.50	(3.98- 5.01)

Table 1B14 - 2

IESLC - Meta-analysis of Current Smoking (vs non-current), Cigarettes (or Any Product if Cigarettes not available)
All LC types
Most adjusted

REF	NRR	SEX	AD	Number Exposed Case	Number Exposed Cont	Non-exposed Case	Non-exposed Cont	RR	95.00%CI
DROSTE	8	m	4	-	-	-	-	4.34	(3.08- 6.13)
*ENGELA	173	m	1	-	-	-	-	5.89	(3.55- 9.78)
*ENGELA	181	f	1	-	-	-	-	6.12	(2.84- 13.20)
Subtotal ENGELA								5.96	(3.90- 9.10)
GAO	37	m	2	-	-	-	-	1.87	(1.50- 2.33)
GAO	38	f	2	-	-	-	-	2.55	(1.94- 3.34)
Subtotal GAO								2.11	(1.78- 2.51)
GAO2	11	m	1	-	-	-	-	2.47	(1.76- 3.46)
GARCIA	4	c	0	169	74	247	372	3.44	(2.50- 4.72)
GARDIN	12	c	0	72	39	46	85	3.41	(2.01- 5.79)
GARSHI	32	m	1	-	-	-	-	2.64	(2.24- 3.11)
GRAHAM	34	m	1	-	-	-	-	1.24	(1.01- 1.53)
GREGOR	4	m	0	49	53	33	59	1.65	(0.93- 2.94)
GREGOR	8	f	0	17	26	5	38	4.97	(1.63- 15.15)
Subtotal GREGOR								2.09	(1.25- 3.48)
HAENSZ	66	f	0	69	94	88	245	2.04	(1.38- 3.03)
*HAMMO2	29	m	1	-	-	-	-	2.31	(1.83- 2.93)
*HEIN	8	m	0	45	912	12	1436	5.90	(3.14- 11.10)
*HENNEK	4	m	0	79	2438	90	19593	7.05	(5.23- 9.51)
HIRAY2	15	m	1	-	-	-	-	3.26	(1.61- 6.62)
*HIRAYA	148	m	1	-	-	-	-	4.08	(3.37- 4.94)
*HIRAYA	151	f	1	-	-	-	-	2.32	(1.86- 2.90)
Subtotal HIRAYA								3.21	(2.78- 3.71)
HITOSU	39	m	1	-	-	-	-	1.23	(0.81- 1.86)
HITOSU	63	f	1	-	-	-	-	2.69	(1.61- 4.51)
Subtotal HITOSU								1.67	(1.21- 2.31)
*HOLE	38	m	1	-	-	-	-	4.30	(2.93- 6.31)
*HOLE	71	f	1	-	-	-	-	1.88	(0.92- 3.80)
Subtotal HOLE								3.57	(2.54- 5.00)
HUMBLE	63	m	1	-	-	-	-	4.02	(2.77- 5.83)
HUMBLE	64	m	1	-	-	-	-	2.94	(1.59- 5.45)
HUMBLE	65	f	1	-	-	-	-	6.14	(3.47- 10.85)
HUMBLE	66	f	1	-	-	-	-	12.47	(4.53- 34.34)
Subtotal HUMBLE								4.50	(3.45- 5.89)
JAHN	32	m	0	352	269	487	570	1.53	(1.25- 1.87)
JAIN	56	m	2	-	-	-	-	4.00	(2.77- 5.76)
JAIN	54	f	2	-	-	-	-	8.58	(5.46- 13.48)
Subtotal JAIN								5.41	(4.07- 7.19)
JARVHO	4	m	0	73	29	27	44	4.10	(2.15- 7.81)
JARVHO	8	f	0	31	7	16	29	8.03	(2.89- 22.31)
Subtotal JARVHO								4.96	(2.88- 8.56)
JOLY	30	m	0	451	524	120	441	3.16	(2.49- 4.01)
JOLY	29	f	0	132	96	86	310	4.96	(3.47- 7.07)
Subtotal JOLY								3.63	(2.98- 4.43)
*KAISE2	71	m	1	-	-	-	-	4.35	(2.86- 6.62)
*KAISE2	63	f	1	-	-	-	-	8.61	(5.32- 13.93)
Subtotal KAISE2								5.84	(4.26- 8.02)
*KAISER	14	m	2	-	-	-	-	10.41	(8.11- 13.37)
*KAISER	11	f	2	-	-	-	-	4.49	(3.36- 6.00)
Subtotal KAISER								7.27	(6.02- 8.79)
KATSOU	30	f	1	-	-	-	-	3.08	(1.60- 5.92)
KAUFMA	18	c	6	-	-	-	-	5.18	(4.33- 6.19)
KELLER	4	m	0	5063	1210	3326	2324	2.92	(2.69- 3.17)
KELLER	12	m	0	1053	212	478	245	2.55	(2.06- 3.15)
KELLER	8	f	0	2904	792	1563	2337	5.48	(4.95- 6.07)
KELLER	16	f	0	454	135	197	311	5.31	(4.08- 6.90)
Subtotal KELLER								3.70	(3.49- 3.93)
KHUDER	34	m	0	275	-	207	-	1.82	(1.46- 2.25)
KIHARA	23	c	0	283	162	157	307	3.42	(2.60- 4.49)
*KINLEN	21	m	2	-	-	-	-	3.78	(3.01- 4.76)
KJUUS	13	m	0	135	77	41	99	4.23	(2.67- 6.70)
*KNEKT	91	m	1	-	-	-	-	4.98	(3.16- 7.86)
KOO	10	f	0	42	25	78	95	2.05	(1.15- 3.65)
KREUZE	43	m	0	168	93	15	101	12.16	(6.68- 22.13)
KREUZE	45	m	0	1226	509	457	1237	6.52	(5.62- 7.57)
KREUZE	44	f	0	55	22	13	57	10.96	(5.03- 23.90)
KREUZE	46	f	0	167	54	130	224	5.33	(3.66- 7.76)
Subtotal KREUZE								6.66	(5.83- 7.60)
*KUBIK	32	m	0	98	6342	10	5980	9.24	(4.83- 17.70)
*LANGE	39	m	1	-	-	-	-	3.26	(2.08- 5.10)
*LANGE	36	f	1	-	-	-	-	3.85	(2.17- 6.83)

Table 1B14 - 2

IESLC - Meta-analysis of Current Smoking (vs non-current), Cigarettes (or Any Product if Cigarettes not available)
All LC types
Most adjusted

REF	NRR	SEX	AD	Number Exposed Case	Cont	Non-exposed Case	Cont	RR	95.00%CI
Subtotal LANGE								3.47	(2.44- 4.94)
LEMARC	4	c	0	167	65	174	391	5.77	(4.12- 8.09)
LICKIN	1	m	0	220	840	4	160	10.48	(3.84- 28.57)
*LIDDEL	8	m	1	-	-	-	-	3.65	(2.68- 4.98)
LOMBAR	24	m	0	852	610	188	430	3.19	(2.61- 3.90)
LUBIN2	84	m	2	-	-	-	-	3.74	(3.49- 4.01)
LUBIN2	318	f	0	384	410	388	1337	3.23	(2.70- 3.86)
Subtotal LUBIN2								3.67	(3.44- 3.91)
MACLEN	50	m	0	137	108	10	26	3.30	(1.52- 7.14)
MACLEN	63	f	0	42	47	44	119	2.42	(1.41- 4.15)
Subtotal MACLEN								2.68	(1.72- 4.17)
MATOS	82	m	2	-	-	-	-	2.60	(1.83- 3.70)
*MIGRAN	54	m	2	-	-	-	-	1.37	(0.92- 2.03)
*MIGRAN	73	f	2	-	-	-	-	4.02	(1.55- 10.42)
Subtotal MIGRAN								1.61	(1.11- 2.31)
*MRFIT	6	m	4	-	-	-	-	6.70	(6.04- 7.44)
MURATA	4	m	0	76	93	31	121	3.19	(1.94- 5.25)
NAM	80	m	1	-	-	-	-	1.34	(1.09- 1.66)
NAM	96	f	1	-	-	-	-	2.77	(2.10- 3.65)
Subtotal NAM								1.75	(1.48- 2.07)
NOTANI	1	m	1	-	-	-	-	5.79	(3.50- 9.70)
ODRISC	4	c	0	293	598	153	1062	3.40	(2.73- 4.24)
OSANN	73	m	2	-	-	-	-	4.98	(4.23- 5.85)
OSANN	74	f	2	-	-	-	-	9.13	(7.52- 11.08)
Subtotal OSANN								6.39	(5.64- 7.24)
PARKIN	40	m	0	346	874	133	1309	3.90	(3.13- 4.84)
PERSH2	12	c	4	-	-	-	-	6.44	(5.45- 7.60)
*PETO	6	m	0	99	2036	4	682	8.29	(3.06- 22.44)
PEZZO2	6	m	0	233	198	134	388	3.41	(2.59- 4.48)
PEZZOT	26	m	0	145	129	70	304	4.88	(3.43- 6.94)
*QIAO2	21	m	0	156	5399	43	1525	1.02	(0.73- 1.43)
RACHTA	14	f	1	-	-	-	-	5.30	(3.01- 9.32)
SAARIK	1	c	0	102	66	103	224	3.36	(2.28- 4.95)
SCHWAR	29	m	0	1652	349	1115	1046	4.44	(3.85- 5.12)
SCHWAR	30	m	0	644	139	269	240	4.13	(3.21- 5.32)
SCHWAR	31	f	0	1029	309	504	1183	7.82	(6.63- 9.21)
SCHWAR	32	f	0	256	90	119	336	8.03	(5.84- 11.04)
Subtotal SCHWAR								5.59	(5.08- 6.15)
SHAW	9	c	0	212	97	123	276	4.90	(3.56- 6.76)
SHIMIZ	5	m	2	-	-	-	-	3.70	(2.70- 5.10)
SHIMIZ	10	f	2	-	-	-	-	3.40	(2.10- 5.30)
Subtotal SHIMIZ								3.60	(2.77- 4.68)
SITAS	1	m	1	-	-	-	-	8.40	(3.20- 22.20)
SOBUE	106	m	1	-	-	-	-	1.82	(1.52- 2.17)
SOBUE	116	f	1	-	-	-	-	2.52	(1.82- 3.49)
Subtotal SOBUE								1.96	(1.68- 2.29)
*SPEIZE	9	f	0	391	489993	202	1298381	5.13	(4.33- 6.08)
SPITZ	4	c	0	103	89	74	208	3.25	(2.21- 4.80)
STAYNE	9	m	1	-	-	-	-	3.30	(2.45- 4.45)
STOCKW	12	c	0	12470	3357	9691	14353	5.50	(5.25- 5.76)
STUCKE	4	m	0	69	68	178	186	1.06	(0.72- 1.57)
SUZUK2	21	c	3	-	-	-	-	5.73	(2.75- 11.95)
SVENSS	91	f	1	-	-	-	-	6.48	(4.05- 10.37)
TANG	4	c	0	52	25	67	73	2.27	(1.27- 4.05)
*TENKAN	21	m	1	-	-	-	-	6.32	(4.35- 9.19)
TIZZAN	11	m	0	693	619	526	597	1.27	(1.08- 1.49)
TIZZAN	17	f	0	17	18	33	124	3.55	(1.65- 7.63)
Subtotal TIZZAN								1.33	(1.13- 1.55)
TOKARS	4	m	0	110	157	38	139	2.56	(1.66- 3.95)
TOUSEY	25	m	3	-	-	-	-	6.61	(4.64- 9.42)
TOUSEY	30	f	0	127	78	79	362	7.46	(5.14- 10.83)
Subtotal TOUSEY								7.00	(5.42- 9.05)
TSUGAN	32	m	0	63	63	28	30	1.07	(0.58- 2.00)
TSUGAN	25	f	0	6	10	33	30	0.55	(0.18- 1.68)
Subtotal TSUGAN								0.91	(0.53- 1.58)
*TULINI	40	m	3	-	-	-	-	5.82	(4.22- 8.03)
*TULINI	45	f	3	-	-	-	-	9.83	(6.50- 14.85)
Subtotal TULINI								7.09	(5.50- 9.14)
*TVERDA	26	m	2	-	-	-	-	10.64	(6.28- 18.03)
*TVERDA	19	f	0	24	113761	3	196384	13.81	(4.16- 45.86)
Subtotal TVERDA								11.10	(6.85- 17.99)

Table 1B14 - 2

IESLC - Meta-analysis of Current Smoking (vs non-current), Cigarettes (or Any Product if Cigarettes not available)
All LC types
Most adjusted

REF	NRR	SEX	AD	Number Exposed Case	Number Exposed Cont	Non-exposed Case	Non-exposed Cont	RR	95.00%CI
WAKAI	73	m	2	-	-	-	-	2.19	(1.57- 3.05)
WAKAI	79	f	2	-	-	-	-	3.93	(2.01- 7.67)
Subtotal WAKAI								2.46	(1.83- 3.31)
WANG2	22	c	4	-	-	-	-	1.78	(0.98- 3.23)
WARSIN	1	m	0	129	81	5	19	6.05	(2.17- 16.84)
WATSON	5	m	0	260	250	5	27	5.62	(2.13- 14.81)
WATSON	6	f	0	15	33	21	148	3.20	(1.49- 6.87)
Subtotal WATSON								3.97	(2.18- 7.23)
WIGLE	19	m	0	415	415	159	480	3.02	(2.41- 3.78)
WIGLE	22	f	0	67	169	47	505	4.26	(2.82- 6.43)
Subtotal WIGLE								3.27	(2.68- 3.98)
WU	46	f	2	-	-	-	-	4.42	(2.79- 7.01)
WUNSCH	14	m	1	-	-	-	-	2.52	(1.88- 3.39)
WUNSCH	16	f	1	-	-	-	-	4.25	(2.44- 7.41)
Subtotal WUNSCH								2.83	(2.18- 3.67)
WYNDE3	53	m	0	227	207	57	213	4.10	(2.89- 5.80)
WYNDE6	153	m	0	1677	741	1233	1872	3.44	(3.07- 3.84)
WYNDE6	261	f	0	1022	376	491	1181	6.54	(5.58- 7.66)
Subtotal WYNDE6								4.26	(3.89- 4.67)
WYNDER	14	m	0	73	154	0	35	33.78~	(2.04- 558.25)
WYNDER	21	f	0	38	60	2	141	44.65	(10.44- 191.04)
Subtotal WYNDER								42.09	(11.58- 153.00)
YAMAGU	12	c	1	-	-	-	-	2.73	(1.72- 4.35)
*YONG	3	c	1	-	-	-	-	5.08	(3.74- 6.90)
Partial Totals				46207	837201	28038	1990264		

*prospective study ~ With 0.5 adjustment for zero

REF	NRR	SEX	AD	Ys	Ws	Qs	Ps
AGUDO	15	f	3	1.26	5.58	0.11	0.0029
*AKIBA	12	m	5	1.12	55.19	4.23	0.0000
*AKIBA	16	f	5	1.34	43.38	0.16	0.0000
Subtotal AKIBA				1.22	98.57	4.40	
*AMANDU	8	m	2	1.51	9.52	0.11	0.0000
AMES	5	m	0	0.19	38.70	57.15	0.2459
*ANDERS	4	f	0	2.28	81.15	63.03	0.0000
*ARCHER	10	m	0	0.75	20.10	8.47	0.0007
ARMADA	24	m	1	0.88	38.56	10.68	0.0000
AUSTIN	8	c	3	1.33	17.78	0.09	0.0000
AXELSS	3	m	0	1.59	41.16	1.43	0.0000
AXELSS	12	f	0	2.13	16.31	8.70	0.0000
Subtotal AXELSS				1.74	57.47	10.14	
BARBON	109	m	1	1.15	81.61	5.15	0.0000
BECHER	19	m	0	1.14	21.64	1.48	0.0000
BECHER	20	f	0	1.78	6.68	0.95	0.0000
Subtotal BECHER				1.29	28.32	2.43	
*BENSHL	26	m	1	1.29	35.45	0.41	0.0000
BLOHMK	4	m	0	0.50	105.79	86.91	0.0000
*BRETT	8	m	0	1.41	13.52	0.00	0.0000
BROSS	17	m	0	0.44	97.86	91.46	0.0000
BUFFLE	41	m	0	0.28	58.50	72.99	0.0293
BUFFLE	42	f	0	1.25	53.17	1.28	0.0000
Subtotal BUFFLE				0.74	111.68	74.27	
BYERS2	1	m	0	1.61	40.94	1.83	0.0000
BYERS2	2	f	0	2.22	18.18	12.22	0.0000
Subtotal BYERS2				1.80	59.12	14.05	
CARPEN	13	c	3	1.75	54.60	6.79	0.0000
*CEDERL	90	m	1	1.36	13.83	0.03	0.0000
*CEDERL	102	f	1	1.48	4.83	0.03	0.0011
Subtotal CEDERL				1.39	18.66	0.06	
*CHANG	13	m	0	1.02	21.56	3.19	0.0000
*CHANG	14	f	0	1.31	13.41	0.11	0.0000
Subtotal CHANG				1.13	34.97	3.30	
CHOI	4	m	0	1.22	30.76	1.00	0.0000
CHOI	8	f	0	0.14	7.22	11.48	0.7054
Subtotal CHOI				1.02	37.97	12.47	
*CHOW	41	m	0	1.93	26.65	7.49	0.0000
*CHYOU	8	m	1	1.76	37.86	4.80	0.0000
COMSTO	90	c	1	1.85	32.40	6.42	0.0000
CORREA	54	c	1	1.70	139.29	12.02	0.0000
*CPSI	74	m	1	1.92	442.50	118.07	0.0000

Table 1B14 - 2

IESLC - Meta-analysis of Current Smoking (vs non-current), Cigarettes (or Any Product if Cigarettes not available)
All LC types
Most adjusted

REF	NRR	SEX	AD	Ys	Ws	Qs	Ps
*CPSI	285	f	1	1.14	73.31	5.11	0.0000
Subtotal CPSI				1.81	515.81	123.18	
*CPSII	110	m	1	1.35	575.76	1.66	0.0000
*CPSII	85	f	1	1.87	240.30	52.04	0.0000
Subtotal CPSII				1.50	816.06	53.70	
DARBY	7	m	0	1.30	105.75	1.05	0.0000
DARBY	14	f	0	1.87	51.37	11.33	0.0000
Subtotal DARBY				1.49	157.12	12.38	
DEAN2	4	m	0	0.53	63.33	47.59	0.0000
DEAN2	8	f	0	1.02	13.98	1.99	0.0001
Subtotal DEAN2				0.62	77.31	49.58	
DEAN3	247	m	1	1.20	73.53	3.00	0.0000
DEAN3	154	f	3	1.72	24.99	2.55	0.0000
Subtotal DEAN3				1.33	98.52	5.54	
*DEKLER	10	m	2	1.32	11.92	0.07	0.0000
DESTE2	19	c	7	0.82	37.11	12.57	0.0000
DESTEF	55	m	4	1.23	54.76	1.62	0.0000
*DOCKER	4	c	4	1.51	8.91	0.10	0.0000
DOLL	92	m	0	0.96	49.93	9.54	0.0000
DOLL	95	f	0	0.64	13.06	7.59	0.0208
Subtotal DOLL				0.90	62.99	17.13	
*DOLL2	76	m	1	1.28	66.30	0.97	0.0000
DORANT	4	m	0	1.06	82.93	9.52	0.0000
DORGAN	14	m	0	1.33	69.64	0.33	0.0000
DORGAN	38	m	0	1.94	20.65	5.96	0.0000
DORGAN	61	f	0	1.99	59.24	20.34	0.0000
DORGAN	84	f	0	1.90	6.16	1.51	0.0000
Subtotal DORGAN				1.68	155.69	28.14	
*DORN	202	m	1	1.50	290.08	3.03	0.0000
DROSTE	8	m	4	1.47	32.44	0.14	0.0000
*ENGELA	173	m	1	1.77	14.96	2.06	0.0000
*ENGELA	181	f	1	1.81	6.51	1.09	0.0000
Subtotal ENGELA				1.78	21.47	3.16	
GAO	37	m	2	0.63	79.22	47.69	0.0000
GAO	38	f	2	0.94	52.06	11.29	0.0000
Subtotal GAO				0.75	131.28	58.99	
GAO2	11	m	1	0.90	33.63	8.33	0.0000
GARCIA	4	c	0	1.24	38.22	1.06	0.0000
GARDIN	12	c	0	1.23	13.69	0.42	0.0000
GARSHI	32	m	1	0.97	142.70	26.51	0.0000
GRAHAM	34	m	1	0.22	89.08	125.46	0.0423
GREGOR	4	m	0	0.50	11.56	9.35	0.0875
GREGOR	8	f	0	1.60	3.09	0.13	0.0048
Subtotal GREGOR				0.73	14.65	9.47	
HAENSZ	66	f	0	0.71	24.64	11.64	0.0004
*HAMMO2	29	m	1	0.84	69.36	22.11	0.0000
*HEIN	8	m	0	1.78	9.64	1.35	0.0000
*HENNEK	4	m	0	1.95	42.90	13.06	0.0000
HIRAY2	15	m	1	1.18	7.69	0.37	0.0011
*HIRAYA	148	m	1	1.41	105.05	0.00	0.0000
*HIRAYA	151	f	1	0.84	77.90	24.45	0.0000
Subtotal HIRAYA				1.17	182.95	24.45	
HITOSU	39	m	1	0.21	22.24	31.74	0.3290
HITOSU	63	f	1	0.99	14.48	2.46	0.0002
Subtotal HITOSU				0.52	36.72	34.20	
*HOLE	38	m	1	1.46	26.11	0.08	0.0000
*HOLE	71	f	1	0.63	7.64	4.54	0.0811
Subtotal HOLE				1.27	33.75	4.62	
HUMBLE	63	m	1	1.39	27.75	0.00	0.0000
HUMBLE	64	m	1	1.08	10.13	1.06	0.0006
HUMBLE	65	f	1	1.81	11.82	2.02	0.0000
HUMBLE	66	f	1	2.52	3.75	4.71	0.0000
Subtotal HUMBLE				1.51	53.44	7.79	
JAHN	32	m	0	0.43	96.47	91.81	0.0000
JAIN	56	m	2	1.39	28.67	0.01	0.0000
JAIN	54	f	2	2.15	18.81	10.51	0.0000
Subtotal JAIN				1.69	47.48	10.52	
JARVHO	4	m	0	1.41	9.26	0.00	0.0000
JARVHO	8	f	0	2.08	3.68	1.70	0.0001
Subtotal JARVHO				1.60	12.94	1.71	
JOLY	30	m	0	1.15	67.90	4.25	0.0000

Table 1B14 - 2

IESLC - Meta-analysis of Current Smoking (vs non-current), Cigarettes (or Any Product if Cigarettes not available)
All LC types
Most adjusted

REF	NRR	SEX	AD	Ys	Ws	Qs	Ps
JOLY	29	f	0	1.60	30.44	1.20	0.0000
Subtotal JOLY				1.29	98.35	5.46	
*KAISE2	71	m	1	1.47	21.81	0.10	0.0000
*KAISE2	63	f	1	2.15	16.58	9.36	0.0000
Subtotal KAISE2				1.77	38.40	9.46	
*KAISER	14	m	2	2.34	61.48	54.44	0.0000
*KAISER	11	f	2	1.50	45.71	0.46	0.0000
Subtotal KAISER				1.98	107.19	54.89	
KATSOU	30	f	1	1.12	8.98	0.69	0.0008
KAUFMA	18	c	6	1.64	120.32	7.10	0.0000
KELLER	4	m	0	1.07	569.83	61.67	0.0000
KELLER	12	m	0	0.93	84.46	18.45	0.0000
KELLER	8	f	0	1.70	373.88	33.58	0.0000
KELLER	16	f	0	1.67	55.86	4.00	0.0000
Subtotal KELLER				1.31	1084.02	117.70	
KHUDER	34	m	0	0.60	82.15	52.97	0.0000
KIHARA	23	c	0	1.23	51.72	1.55	0.0000
*KINLEN	21	m	2	1.33	73.15	0.38	0.0000
KJUUS	13	m	0	1.44	18.22	0.03	0.0000
*KNEKT	91	m	1	1.61	18.51	0.77	0.0000
KOO	10	f	0	0.72	11.47	5.40	0.0153
KREUZE	43	m	0	2.50	10.72	12.89	0.0000
KREUZE	45	m	0	1.87	173.10	38.73	0.0000
KREUZE	44	f	0	2.39	6.32	6.23	0.0000
KREUZE	46	f	0	1.67	27.28	2.01	0.0000
Subtotal KREUZE				1.90	217.43	59.86	
*KUBIK	32	m	0	2.22	9.10	6.15	0.0000
*LANGE	39	m	1	1.18	19.10	0.93	0.0000
*LANGE	36	f	1	1.35	11.69	0.03	0.0000
Subtotal LANGE				1.24	30.79	0.96	
LEMARC	4	c	0	1.75	33.70	4.16	0.0000
LICKIN	1	m	0	2.35	3.82	3.43	0.0000
*LIDDEL	8	m	1	1.29	40.02	0.46	0.0000
LOMBAR	24	m	0	1.16	95.62	5.52	0.0000
LUBIN2	84	m	2	1.32	796.56	5.45	0.0000
LUBIN2	318	f	0	1.17	119.50	6.33	0.0000
Subtotal LUBIN2				1.30	916.05	11.79	
MACLEN	50	m	0	1.19	6.45	0.28	0.0024
MACLEN	63	f	0	0.88	13.12	3.54	0.0014
Subtotal MACLEN				0.98	19.57	3.82	
MATOS	82	m	2	0.96	31.00	6.18	0.0000
*MIGRAN	54	m	2	0.31	24.53	28.99	0.1189
*MIGRAN	73	f	2	1.39	4.23	0.00	0.0042
Subtotal MIGRAN				0.47	28.76	28.99	
*MRFIT	6	m	4	1.90	353.58	88.49	0.0000
MURATA	4	m	0	1.16	15.52	0.91	0.0000
NAM	80	m	1	0.29	86.84	106.84	0.0064
NAM	96	f	1	1.02	50.28	7.38	0.0000
Subtotal NAM				0.56	137.13	114.21	
NOTANI	1	m	1	1.76	14.79	1.86	0.0000
ODRISC	4	c	0	1.22	79.60	2.52	0.0000
OSANN	73	m	2	1.61	146.16	6.06	0.0000
OSANN	74	f	2	2.21	102.29	67.07	0.0000
Subtotal OSANN				1.85	248.45	73.13	
PARKIN	40	m	0	1.36	81.19	0.14	0.0000
PERSH2	12	c	4	1.86	138.96	29.49	0.0000
*PETO	6	m	0	2.12	3.87	1.97	0.0000
PEZZO2	6	m	0	1.23	51.59	1.60	0.0000
PEZZOT	26	m	0	1.59	31.03	1.05	0.0000
*QIAO2	21	m	0	0.02	34.69	65.82	0.8856
RACHTA	14	f	1	1.67	12.03	0.85	0.0000
SAARIK	1	c	0	1.21	25.56	0.92	0.0000
SCHWAR	29	m	0	1.49	187.84	1.49	0.0000
SCHWAR	30	m	0	1.42	60.13	0.02	0.0000
SCHWAR	31	f	0	2.06	142.10	60.85	0.0000
SCHWAR	32	f	0	2.08	37.88	17.60	0.0000
Subtotal SCHWAR				1.72	427.95	79.95	
SHAW	9	c	0	1.59	37.34	1.32	0.0000
SHIMIZ	5	m	2	1.31	37.99	0.33	0.0000
SHIMIZ	10	f	2	1.22	17.93	0.57	0.0000
Subtotal SHIMIZ				1.28	55.92	0.90	

Table 1B14 - 2

IESLC - Meta-analysis of Current Smoking (vs non-current), Cigarettes (or Any Product if Cigarettes not available)
All LC types
Most adjusted

REF	NRR	SEX	AD	Ys	Ws	Qs	Ps
SITAS	1	m	1	2.13	4.10	2.16	0.0000
SOBUE	106	m	1	0.60	121.23	78.17	0.0000
SOBUE	116	f	1	0.92	36.25	8.27	0.0000
Subtotal SOBUE				0.67	157.48	86.44	
*SPEIZE	9	f	0	1.63	133.24	7.24	0.0000
SPITZ	4	c	0	1.18	25.47	1.26	0.0000
STAYNE	9	m	1	1.19	43.14	1.86	0.0000
STOCKW	12	c	0	1.71	1815.09	166.87	0.0000
STUCKE	4	m	0	0.06	24.88	44.89	0.7702
SUZUK2	21	c	3	1.75	7.12	0.84	0.0000
SVENSS	91	f	1	1.87	17.38	3.79	0.0000
TANG	4	c	0	0.82	11.38	3.88	0.0058
*TENKAN	21	m	1	1.84	27.47	5.36	0.0000
TIZZAN	11	m	0	0.24	150.72	203.62	0.0033
TIZZAN	17	f	0	1.27	6.55	0.12	0.0012
Subtotal TIZZAN				0.28	157.27	203.74	
TOKARS	4	m	0	0.94	20.42	4.33	0.0000
TOUSEY	25	m	3	1.89	30.64	7.26	0.0000
TOUSEY	30	f	0	2.01	27.69	10.23	0.0000
Subtotal TOUSEY				1.95	58.33	17.49	
TSUGAN	32	m	0	0.07	9.92	17.62	0.8280
TSUGAN	25	f	0	-0.61	3.03	12.21	0.2916
Subtotal TSUGAN				-0.09	12.95	29.83	
*TULINI	40	m	3	1.76	37.12	4.80	0.0000
*TULINI	45	f	3	2.29	22.51	17.58	0.0000
Subtotal TULINI				1.96	59.64	22.37	
*TVERDA	26	m	2	2.36	13.81	12.81	0.0000
*TVERDA	19	f	0	2.63	2.67	3.99	0.0000
Subtotal TVERDA				2.41	16.48	16.80	
WAKAI	73	m	2	0.78	34.84	13.31	0.0000
WAKAI	79	f	2	1.37	8.57	0.01	0.0001
Subtotal WAKAI				0.90	43.41	13.31	
WANG2	22	c	4	0.58	10.80	7.36	0.0581
WARSIN	1	m	0	1.80	3.67	0.58	0.0006
WATSON	5	m	0	1.73	4.08	0.43	0.0005
WATSON	6	f	0	1.16	6.61	0.37	0.0028
Subtotal WATSON				1.38	10.69	0.80	
WIGLE	19	m	0	1.10	75.80	6.68	0.0000
WIGLE	22	f	0	1.45	22.68	0.05	0.0000
Subtotal WIGLE				1.18	98.48	6.74	
WU	46	f	2	1.49	18.10	0.13	0.0000
WUNSCH	14	m	1	0.92	44.21	10.08	0.0000
WUNSCH	16	f	1	1.45	12.45	0.03	0.0000
Subtotal WUNSCH				1.04	56.66	10.11	
WYNDE3	53	m	0	1.41	31.77	0.00	0.0000
WYNDE6	153	m	0	1.23	303.85	8.53	0.0000
WYNDE6	261	f	0	1.88	153.34	34.71	0.0000
Subtotal WYNDE6				1.45	457.19	43.24	
WYNDER	14	m	0	3.52	0.49	2.19	0.0139
WYNDER	21	f	0	3.80	1.82	10.45	0.0000
Subtotal WYNDER				3.74	2.31	12.64	
YAMAGU	12	c	1	1.00	17.85	2.82	0.0000
*YONG	3	c	1	1.63	40.97	2.05	0.0000

Table 1B14 - 2

IESLC - Meta-analysis of Current Smoking (vs non-current), Cigarettes (or Any Product if Cigarettes not available)
All LC types
Most adjusted

	N	188
	NS	129
	Wt	13125.10
Het	Chi	2828.65
Het	df	187
Het	P	***
Fixed	RR	4.06
	RRl	3.99
	RRu	4.13
	P	+++
Random	RR	3.81
	RRl	3.55
	RRu	4.10
	P	+++
Asymm	P	*

Table 1B14 - 3

IESLC - Meta-analysis of Current Smoking (vs non-current), Cigarettes (or Any Product if Cigarettes not available)
All LC types
Most adjusted

	Sex combined	male	female	Total
N	22	103	63	188
NS	22	98	58	178
Wt	2757.87	7850.09	2517.14	13125.10
Het Chi	112.82	1745.16	471.71	2828.65
Het df	21	102	62	187
Het P	***	***	***	***
Fixed RR	5.16	3.46	5.14	4.06
RRl	4.98	3.39	4.94	3.99
RRu	5.36	3.54	5.35	4.13
P	+++	+++	+++	+++
Random RR	4.20	3.37	4.66	3.81
RRl	3.70	3.06	4.13	3.55
RRu	4.77	3.72	5.26	4.10
P	+++	+++	+++	+++
Between Chi				498.97
Between df				2
Between P				***
Btwn(F) P				***
Btwn(R) P				***

Table 1B14 - 4

IESLC - Meta-analysis of Current Smoking (vs non-current), Cigarettes (or Any Product if Cigarettes not available)
All LC types
Least adjusted

REF	NRR	X	SEX	AGEL	AGEH	RACE	YF	LC TYPE	LOC	START	ST	NLC	R	VB	P	H	AD	PRODUCT	DENOM	De
AGUDO	16	x	f	0	0	all	-	all	Eu:wst	1989	CC	103	n	bl	n	n	0	cig only	non any	st
AKIBA	4	x	m	0	0	all	0	all	As:Jap	1963	pr	610	n	bl	n	n	0	cig+/-ot	non cigs	st
AKIBA	8	x	f	0	0	all	0	all	As:Jap	1963	pr	610	n	bl	n	n	0	cig+/-ot	non cigs	st
AMANDU	4	x	m	0	0	wh	0	all	NAmer	1959	pr	132	m	bl	n	n	0	cig+/-ot	non cigs	st
AMES	5		m	0	0	wh	-	all	NAmer	1959	ot	317	m	bl	n	n	0	all/unsp	non any	st
ANDERS	4		f	0	0	all	0	all	NAmer	1986	pr	343	n	bl	n	n	0	cig+/-ot	non cigs	st
ARCHER	10		m	0	0	wh	0	all	NAmer	1950	pr	146	m	bl	n	n	0	cig+/-ot	non cigs	st
ARMADA	10	x	m	0	0	all	-	all	Eu:wst	1986	CC	325	n	bl	n	y	0	cig+/-ot	non cigs	st
AUSTIN	4	x	c	0	0	all	-	all	NAmer	1970	CC	166	o	bl	y	n	0	cig+/-ot	non cigs	st
AXELSS	3		m	0	0	sca	-	all	Eu:Sca	1989	CC	436	n	bl	n	n	0	all/unsp	non any	st
AXELSS	12		f	0	0	sca	-	all	Eu:Sca	1989	CC	436	n	bl	n	n	0	all/unsp	non any	st
BARBON	108	x	m	0	0	all	-	all	Eu:wst	1979	CC	755	n	bl	y	y	0	all/unsp	non any	st
BECHER	19		m	0	0	all	-	all	Eu:Ger	1985	CC	194	n	bl	n	y	0	all/unsp	non any	st
BECHER	20		f	0	0	all	-	all	Eu:Ger	1985	CC	194	n	bl	n	y	0	all/unsp	non any	st
BENSHL	26		m	40	64	all	10	all	Eu:UK	1967	pr	486	n	V	n	n	1	cig+/-ot	non any	ot
BLOHMK	4		m	0	0	all	-	all	Eu:Ger	1978	CC	888	n	bl	n	y	0	all/unsp	non any	st
BRETT	8		m	0	0	all	0	all	Eu:UK	1960	pr	150	n	V	n	n	0	cig+/-ot	non cigs	st
BROSS	17		m	0	0	wh	-	all	NAmer	1960	CC	974	n	bl	n	n	0	cig+/-ot	non cigs	st
BUFFLE	41		m	0	0	wh	-	all	NAmer	1976	CC	943	n	bl	y	n	0	cig+/-ot	non cigs	st
BUFFLE	42		f	0	0	wh	-	all	NAmer	1976	CC	943	n	bl	y	n	0	cig+/-ot	non cigs	st
BYERS2	1		m	0	0	wh	-	all	NAmer	1980	CC	448	n	bl	n	y	0	cig+/-ot	non cigs	st
BYERS2	2		f	0	0	wh	-	all	NAmer	1980	CC	448	n	bl	n	y	0	cig+/-ot	non cigs	st
CARPEN	17	x	c	0	0	w+b	-	all	NAmer	1991	CC	356	n	bl	n	n	0	cig+/-ot	non cigs	st
CEDERL	90		m	0	0	all	10	all	Eu:Sca	1963	pr	491	n	bl	n	n	1	cig+/-ot	non any	ot
CEDERL	102		f	0	0	all	10	all	Eu:Sca	1963	pr	491	n	bl	n	n	1	cig+/-ot	non any	ot
CHANG	13		m	0	0	all	0	all	NAmer	1972	pr	136	n	bl	n	n	0	cig+/-ot	non cigs	st
CHANG	14		f	0	0	all	0	all	NAmer	1972	pr	136	n	bl	n	n	0	cig+/-ot	non cigs	st
CHOI	4		m	0	0	all	-	all	As:oth	1985	CC	375	n	bl	n	n	0	cig+/-ot	non cigs	st
CHOI	8		f	0	0	all	-	all	As:oth	1985	CC	375	n	bl	n	n	0	cig+/-ot	non cigs	st
CHOW	41		m	0	0	wh	0	all	NAmer	1966	pr	219	n	bl	n	n	0	cig+/-ot	non any	st
CHYOU	10	x	m	0	0	jap	0	all	NAmer	1965	pr	227	n	bl	n	y	0	cig+/-ot	non cigs	st
COMSTO	41	x	m	0	0	all	-	all	NAmer	1975	ot	258	n	bl	n	n	0	cig+/-ot	non cigs	st
COMSTO	53	x	f	0	0	all	-	all	NAmer	1975	ot	258	n	bl	n	n	0	cig+/-ot	non cigs	st
CORREA	53	x	c	0	0	all	-	all	NAmer	1979	CC	1359	n	bl	y	n	0	cig+/-ot	non cigs	st
CPSI	74		m	0	0	wh	0	all	NAmer	1959	pr	5138	n	bl	n	n	1	cig only	non any	st
CPSI	285		f	40	74	all	6	all	NAmer	1959	pr	5138	n	bl	n	n	1	cig+/-ot	non cigs	ot
CPSII	110		m	35	99	all	4	all	NAmer	1982	pr	3229	n	bl	n	n	1	cig only	non cigs	ot
CPSII	85		f	0	0	all	4	all	NAmer	1982	pr	3229	n	bl	n	n	1	cig+/-ot	non cigs	ot
DARBY	7		m	0	0	wh	-	all	Eu:UK	1988	CC	982	n	V	n	n	0	cig+/-ot	non any	st
DARBY	14		f	0	0	wh	-	all	Eu:UK	1988	CC	982	n	V	n	n	0	cig+/-ot	non any	st
DEAN2	4		m	0	0	all	-	all	Eu:UK	1960	CC	954	n	V	y	n	0	all/unsp	non any	st
DEAN2	8		f	0	0	all	-	all	Eu:UK	1960	CC	954	n	V	y	n	0	all/unsp	non any	st
DEAN3	246	x	m	0	0	all	-	all	Eu:UK	1969	CC	766	n	V	y	n	0	cig+/-ot	non any	st
DEAN3	152	x	f	0	0	all	-	all	Eu:UK	1969	CC	766	n	V	y	n	0	cig only	non any	st
DEKLER	10		m	0	0	all	0	all	Auslia	1961	pr	138	m	V	n	n	2	cig+/-ot	non any	ot
DESTE2	18	x	c	0	0	all	-	all	SCAmer	1993	CC	463	n	bl	n	n	0	all/unsp	non any	st
DESTEF	54	x	m	0	0	all	-	all	SCAmer	1988	CC	497	n	bl	n	y	0	all/unsp	non any	st
DOCKER	4		c	0	0	wh	0	all	NAmer	1974	pr	120	n	bl	n	n	4	cig+/-ot	non cigs	ot
DOLL	92		m	0	0	all	-	all	Eu:UK	1948	CC	1465	n	V	n	n	0	all/unsp	non any	st
DOLL	95		f	0	0	all	-	all	Eu:UK	1948	CC	1465	n	V	n	n	0	all/unsp	non any	st
DOLL2	76		m	0	0	all	20	all	Eu:UK	1951	pr	920	n	V	n	n	1	cig+/-ot	non any	ot
DORANT	4		m	0	0	all	0	all	Eu:wst	1986	ot	550	n	bl	n	y	0	all/unsp	non any	st
DORGAN	14		m	0	0	wh	-	all	NAmer	1980	CC	2026	n	bl	y	y	0	cig+/-ot	non cigs	st
DORGAN	38		m	0	0	bl	-	all	NAmer	1980	CC	2026	n	bl	y	y	0	cig+/-ot	non cigs	st
DORGAN	61		f	0	0	wh	-	all	NAmer	1980	CC	2026	n	bl	y	y	0	cig+/-ot	non cigs	st
DORGAN	84		f	0	0	bl	-	all	NAmer	1980	CC	2026	n	bl	y	y	0	cig+/-ot	non cigs	st
DORN	202		m	35	84	wh	8	all	NAmer	1954	pr	5097	n	bl	n	n	1	cig+/-ot	non any	ot
DROSTE	4	x	m	0	0	all	-	all	Eu:wst	1995	CC	478	n	bl	n	y	0	all/unsp	non any	st
ENGELA	173		m	0	0	all	12	all	Eu:Sca	1964	pr	435	n	bl	n	n	1	cig+/-ot	non any	ot
ENGELA	181		f	0	0	all	12	all	Eu:Sca	1964	pr	435	n	bl	n	n	1	cig+/-ot	non any	ot
GAO	35	x	m	0	0	all	-	all	As:Chi	1984	CC	1405	n	ot	n	n	0	cig+/-ot	non cigs	st
GAO	36	x	f	0	0	all	-	all	As:Chi	1984	CC	1405	n	ot	n	n	0	cig+/-ot	non cigs	st
GAO2	7	x	m	0	0	all	-	all	As:Jap	1988	CC	282	n	bl	n	n	0	cig+/-ot	non cigs	st
GARCIA	4		c	0	0	all	-	all	NAmer	1992	CC	416	n	bl	n	y	0	cig+/-ot	non cigs	st
GARDIN	12		c	0	0	all	-	all	Eu:UK	1988	CC	143	n	V	y	n	0	cig only	non any	st
GARSHI	24	x	m	0	0	all	-	all	NAmer	1981	CC	1081	o	bl	y	n	0	all/unsp	non any	st
GRAHAM	29	x	m	0	0	wh	-	all	NAmer	1956	CC	685	n	bl	n	n	0	cig+/-ot	non any	st
GREGOR	4		m	0	0	all	-	all	Eu:UK	1976	CC	104	n	V	n	y	0	cig+/-ot	non cigs	st
GREGOR	8		f	0	0	all	-	all	Eu:UK	1976	CC	104	n	V	n	y	0	cig+/-ot	non cigs	st
HAENSZ	66		f	0	0	all	-	not alv	NAmer	1955	CC	158	n	bl	n	y	0	cig+/-ot	non cigs	st
HAMMO2	34	x	m	0	0	all	0	all	NAmer	1967	pr	450	o	bl	n	n	0	cig+/-ot	non cigs	st
HEIN	8		m	0	0	all	0	all	Eu:Sca	1970	pr	144	n	bl	n	n	0	cig only	non any	st

Table 1B14 - 4

IESLC - Meta-analysis of Current Smoking (vs non-current), Cigarettes (or Any Product if Cigarettes not available)
All LC types
Least adjusted

REF	NRR	X	SEX	AGEL	AGEH	RACE	YF	LC TYPE	LOC	START	ST	NLC	R	VB	P	H	AD	PRODUCT	DENOM	De
HENNEK	4		m	0	0	all	0	all	NAmer	1982	pr	169	n	bl	n	n	0	all/unsp	non any	st
HIRAY2	15		m	0	0	all	-	all	As:Jap	1950	CC	145	n	bl	*	n	1	cig+/-ot	non any	ot
HIRAYA	148		m	0	0	all	0	all	As:Jap	1965	pr	1917	n	bl	n	n	1	cig+/-ot	non any	ot
HIRAYA	151		f	0	0	all	0	all	As:Jap	1965	pr	1917	n	bl	n	n	1	cig+/-ot	non any	ot
HITOSU	7	x	m	0	0	all	-	all	As:Jap	1960	CC	216	n	bl	y	n	0	all/unsp	non any	st
HITOSU	13	x	f	0	0	all	-	all	As:Jap	1960	CC	216	n	bl	y	n	0	all/unsp	non any	st
HOLE	52	x	m	0	0	all	0	all	Eu:UK	1972	pr	225	n	V	n	n	0	cig+/-ot	non any	st
HOLE	68	x	f	0	0	all	9	all	Eu:UK	1972	pr	225	n	V	n	n	0	cig+/-ot	non any	st
HUMBLE	63		m	0	0	w-hi	-	all	NAmer	1980	CC	521	n	bl	y	n	1	cig+/-ot	non cigs	ot
HUMBLE	64		m	0	0	hi	-	all	NAmer	1980	CC	521	n	bl	y	n	1	cig+/-ot	non cigs	ot
HUMBLE	65		f	0	0	w-hi	-	all	NAmer	1980	CC	521	n	bl	y	n	1	cig+/-ot	non cigs	ot
HUMBLE	66		f	0	0	hi	-	all	NAmer	1980	CC	521	n	bl	y	n	1	cig+/-ot	non cigs	ot
JAHN	32		m	0	0	all	-	all	Eu:Ger	1988	CC	1004	n	bl	n	n	0	cig+/-ot	non cigs	st
JAIN	36	x	m	0	0	all	-	all	NAmer	1981	CC	845	n	V	y	n	0	cig+/-ot	non cigs	st
JAIN	31	x	f	0	0	all	-	all	NAmer	1981	CC	845	n	V	y	n	0	cig+/-ot	non cigs	st
JARVHO	4		m	0	0	all	-	all	Eu:Sca	1983	CC	147	n	bl	n	n	0	all/unsp	non any	st
JARVHO	8		f	0	0	all	-	all	Eu:Sca	1983	CC	147	n	bl	n	n	0	all/unsp	non any	st
JOLY	30		m	0	0	all	-	all	SCAmer	1978	CC	826	n	bl	n	n	0	cig+/-ot	non any	st
JOLY	29		f	0	0	all	-	all	SCAmer	1978	CC	826	n	bl	n	n	0	cig+/-ot	non any	st
KAISE2	71		m	35	99	all	9	all	NAmer	1979	pr	318	n	bl	n	n	1	cig only	non any	st
KAISE2	63		f	35	99	all	9	all	NAmer	1979	pr	318	n	bl	n	n	1	cig only	non any	st
KAISER	14		m	0	0	all	0	all	NAmer	1964	pr	714	n	bl	n	n	2	cig+/-ot	non cigs	ot
KAISER	11		f	0	0	all	0	all	NAmer	1964	pr	714	n	bl	n	n	2	cig+/-ot	non cigs	ot
KATSOU	28	x	f	0	0	all	-	all	Eu:bal	1987	CC	101	n	bl	n	n	0	all/unsp	non any	st
KAUFMA	9	x	c	0	0	all	-	all	NAmer	1981	CC	881	n	bl	n	n	0	cig+/-ot	non cigs	st
KELLER	4		m	0	0	wh	-	all	NAmer	1985	CC	15038	n	bl	n	n	0	all/unsp	non any	st
KELLER	12		m	0	0	nonw	-	all	NAmer	1985	CC	15038	n	bl	n	n	0	all/unsp	non any	st
KELLER	8		f	0	0	wh	-	all	NAmer	1985	CC	15038	n	bl	n	n	0	all/unsp	non any	st
KELLER	16		f	0	0	nonw	-	all	NAmer	1985	CC	15038	n	bl	n	n	0	all/unsp	non any	st
KHUDER	34		m	0	0	all	-	all	NAmer	1985	CC	482	n	bl	n	y	0	cig+/-ot	non cigs	ot
KIHARA	23		c	0	0	jap	-	all	As:Jap	1991	CC	440	n	bl	n	n	0	all/unsp	non any	st
KINLEN	10	x	m	0	0	all	0	all	Eu:UK	1967	pr	718	n	V	n	n	0	cig+/-ot	non any	st
KJUUS	13		m	0	0	all	-	all	Eu:Sca	1979	CC	176	n	bl	n	n	0	all/unsp	non any	st
KNEKT	80	x	m	20	69	all	21	all	Eu:Sca	1966	pr	515	n	bl	n	n	0	cig+/-ot	non any	st
KOO	10		f	0	0	all	-	all	As:HK	1981	CC	200	n	bl	n	n	0	all/unsp	non any	st
KREUZE	43		m	1	45	all	-	all	Eu:Ger	1990	CC	2260	n	bl	n	n	0	cig+/-ot	non any	st
KREUZE	45		m	55	69	all	-	all	Eu:Ger	1990	CC	2260	n	bl	n	n	0	cig+/-ot	non any	st
KREUZE	44		f	1	45	all	-	all	Eu:Ger	1990	CC	2260	n	bl	n	n	0	cig+/-ot	non any	st
KREUZE	46		f	55	69	all	-	all	Eu:Ger	1990	CC	2260	n	bl	n	n	0	cig+/-ot	non any	st
KUBIK	32		m	0	0	all	0	all	Eu:est	1965	pr	108	n	bl	n	n	0	cig+/-ot	non cigs	st
LANGE	33	x	m	0	0	all	0	all	Eu:Sca	1976	pr	268	n	bl	n	n	0	all/unsp	non any	st
LANGE	30	x	f	0	0	all	0	all	Eu:Sca	1976	pr	268	n	bl	n	n	0	all/unsp	non any	st
LEMARC	4		c	0	0	w+o	-	all	NAmer	1992	CC	341	n	bl	n	y	0	all/unsp	non any	st
LICKIN	1		m	0	0	all	-	all	Eu:Ger	1950	CC	224	n	bl	*	n	0	all/unsp	non any	st
LIDDEL	8		m	0	0	all	18	all	NAmer	1970	pr	304	m	V	n	n	1	cig+/-ot	non cigs	ot
LOMBAR	24		m	0	0	all	-	all	NAmer	1951	CC	1040	n	bl	n	n	0	cig+/-ot	non cigs	st
LUBIN2	83	x	m	0	0	all	-	all	Eu:mul	1976	CC	7804	n	bl	n	y	0	cig+/-ot	non any	st
LUBIN2	318		f	0	0	all	-	all	Eu:mul	1976	CC	7804	n	bl	n	y	0	cig+/-ot	non any	st
MACLEN	50		m	0	0	ch	-	all	As:oth	1972	CC	233	n	bl	n	n	0	cig+/-ot	non cigs	st
MACLEN	63		f	0	0	ch	-	all	As:oth	1972	CC	233	n	bl	n	n	0	cig+/-ot	non cigs	st
MATOS	75	x	m	0	0	all	-	all	SCAmer	1994	CC	200	n	bl	n	n	0	cig+/-ot	non any	st
MIGRAN	53	x	m	0	0	all	0	all	Eu:UK	1964	pr	259	n	V	n	n	0	cig+/-ot	non any	st
MIGRAN	72	x	f	0	0	all	0	all	Eu:UK	1964	pr	259	n	V	n	n	0	cig+/-ot	non any	st
MRFIT	23	x	m	0	0	all	0	all	NAmer	1973	pr	2004	n	bl	n	n	0	cig+/-ot	non cigs	st
MURATA	4		m	0	0	all	-	all	As:Jap	1984	ot	107	n	bl	n	n	0	cig+/-ot	non cigs	st
NAM	72	x	m	0	0	all	-	all	NAmer	1986	CC	1199	n	bl	y	n	0	cig+/-ot	non cigs	ot
NAM	88	x	f	0	0	all	-	all	NAmer	1986	CC	1199	n	bl	y	n	0	cig+/-ot	non cigs	ot
NOTANI	1		m	0	0	all	-	all	As:Ind	1986	CC	246	n	V	n	n	1	all/unsp	non any	or
ODRISC	4		c	0	0	all	-	all	Eu:UK	1992	CC	446	n	V	n	n	0	all/unsp	non any	st
OSANN	65	x	m	0	0	all	-	all	NAmer	1984	CC	1986	n	bl	n	n	0	cig+/-ot	non cigs	st
OSANN	69	x	f	0	0	all	-	all	NAmer	1984	CC	1986	n	bl	n	n	0	cig+/-ot	non cigs	st
PARKIN	40		m	0	0	bl	-	all	Africa	1963	CC	877	n	V	y	n	0	cig+/-ot	non any	st
PERSH2	6	x	c	0	0	all	-	all	Eu:Sca	1980	CC	1022	n	bl	y	n	0	all/unsp	non any	st
PETO	6		m	0	0	all	0	all	Eu:UK	1954	pr	103	n	V	n	n	0	all/unsp	non any	st
PEZZO2	6		m	0	0	all	-	all	SCAmer	1992	CC	367	n	bl	n	y	0	cig+/-ot	non cigs	st
PEZZOT	26		m	0	0	all	-	all	SCAmer	1987	CC	215	n	bl	n	y	0	cig only	non cigs	st
QIAO2	21		m	0	0	all	0	all	As:Chi	1992	pr	241	m	ot	n	n	0	cig+/-ot	non any	st
RACHTA	4	x	f	0	0	all	-	all	Eu:est	1991	CC	118	n	bl	n	y	0	cig+/-ot	non cigs	st
SAARIK	1		c	0	0	wh	-	all	Eu:Sca	1988	CC	205	n	bl	n	y	0	all/unsp	non any	st
SCHWAR	29		m	0	0	wh	-	all	NAmer	1984	CC	5588	n	bl	y	y	0	cig+/-ot	non cigs	st
SCHWAR	30		m	0	0	bl	-	all	NAmer	1984	CC	5588	n	bl	y	y	0	cig+/-ot	non cigs	st
SCHWAR	31		f	0	0	wh	-	all	NAmer	1984	CC	5588	n	bl	y	y	0	cig+/-ot	non cigs	st

Table 1B14 - 4

IESLC - Meta-analysis of Current Smoking (vs non-current), Cigarettes (or Any Product if Cigarettes not available)
All LC types
Least adjusted

REF	NRR	X	SEX	AGEL	AGEH	RACE	YF	LC TYPE	LOC	START	ST	NLC	R	VB	P	H	AD	PRODUCT	DENOM	De
SCHWAR	32		f	0	0	bl	-	all	NAmer	1984	CC	5588	n	bl	y	y	0	cig+/-ot	non cigs	st
SHAW	9		c	0	0	wh	-	all	NAmer	1988	CC	335	n	V	n	y	0	all/unsp	non any	st
SHIMIZ	5		m	0	0	all	-	all	As:Jap	1977	CC	751	n	bl	y	n	2	all/unsp	non any	or
SHIMIZ	10		f	0	0	all	-	all	As:Jap	1977	CC	751	n	bl	y	n	2	all/unsp	non any	or
SITAS	1		m	0	0	bl	-	all	Africa	1994	CC	*	n	V	n	n	1	all/unsp	non any	or
SOBUE	92	x	m	0	0	all	-	q+s+l+a	As:Jap	1986	CC	1376	n	bl	n	y	0	cig+/-ot	non cigs	st
SOBUE	96	x	f	0	0	all	-	q+s+l+a	As:Jap	1986	CC	1376	n	bl	n	y	0	cig+/-ot	non cigs	st
SPEIZE	9		f	0	0	all	0	all	NAmer	1976	pr	593	n	bl	n	y	0	cig+/-ot	non cigs	st
SPITZ	4		c	0	0	b+hi	-	all	NAmer	1992	CC	177	n	bl	n	y	0	cig+/-ot	non cigs	st
STAYNE	9		m	0	0	all	-	all	NAmer	1969	CC	420	n	bl	n	n	1	cig+/-ot	non cigs	st
STOCKW	12		c	0	0	all	-	all	NAmer	1981	CC	22161	n	bl	n	n	0	cig+/-ot	non cigs	st
STUCKE	4		m	0	0	all	-	all	Eu:wst	1989	CC	247	n	bl	n	y	0	all/unsp	non any	st
SUZUK2	19	x	c	0	0	all	-	all	SCAmer	1991	CC	123	n	bl	n	y	0	all/unsp	non any	st
SVENSS	66	x	f	0	0	all	-	all	Eu:Sca	1983	CC	210	n	bl	n	n	0	all/unsp	non any	st
TANG	4		c	0	0	all	-	not s	NAmer	1992	CC	119	n	bl	n	y	0	cig+/-ot	non cigs	st
TENKAN	21		m	0	0	all	18	all	Eu:Sca	1962	pr	242	n	bl	n	n	1	all/unsp	non any	st
TIZZAN	11		m	0	0	all	-	all	Eu:wst	1959	CC	1358	n	bl	n	n	0	all/unsp	non any	st
TIZZAN	17		f	0	0	all	-	all	Eu:wst	1959	CC	1358	n	bl	n	n	0	all/unsp	non any	st
TOKARS	4		m	0	0	all	-	all	Eu:est	1966	ot	162	o	bl	n	y	0	all/unsp	non any	st
TOUSEY	20	x	m	0	0	all	-	all	NAmer	1993	CC	507	n	bl	y	y	0	cig+/-ot	non cigs	st
TOUSEY	30		f	0	0	all	-	all	NAmer	1993	CC	507	n	bl	y	y	0	cig+/-ot	non cigs	st
TSUGAN	32		m	0	0	all	-	q+a	As:Jap	1976	CC	134	n	bl	n	y	0	all/unsp	non any	st
TSUGAN	25		f	0	0	all	-	q+a	As:Jap	1976	CC	134	n	bl	n	y	0	all/unsp	non any	or
TULINI	17	x	m	0	0	all	0	all	Eu:Sca	1967	pr	472	n	bl	n	n	1	cig+/-ot	non any	ot
TULINI	22	x	f	0	0	all	0	all	Eu:Sca	1967	pr	472	n	bl	n	n	1	cig+/-ot	non any	ot
TVERDA	26		m	0	0	all	0	all	Eu:Sca	1972	pr	238	n	bl	n	n	2	cig+/-ot	non any	ot
TVERDA	19		f	0	0	all	0	all	Eu:Sca	1972	pr	238	n	bl	n	n	0	cig only	non any	st
WAKAI	14	x	m	0	0	all	-	all	As:Jap	1988	CC	333	n	bl	n	y	0	all/unsp	non any	st
WAKAI	32	x	f	0	0	all	-	all	As:Jap	1988	CC	333	n	bl	n	y	0	all/unsp	non any	st
WANG2	21	x	c	0	0	all	-	all	As:Chi	1980	CC	103	n	ot	n	n	0	cig+/-ot	non cigs	st
WARSIN	1		m	0	0	all	-	all	Eu:wst	1945	CC	134	n	bl	*	n	0	all/unsp	non any	st
WATSON	5		m	0	0	all	-	all	NAmer	1950	CC	301	n	bl	n	y	0	all/unsp	non any	st
WATSON	6		f	0	0	all	-	all	NAmer	1950	CC	301	n	bl	n	y	0	all/unsp	non any	st
WIGLE	19		m	0	0	all	-	all	NAmer	1971	CC	728	n	V	n	n	0	cig only	non any	st
WIGLE	22		f	0	0	all	-	all	NAmer	1971	CC	728	n	V	n	n	0	cig only	non any	st
WU	38	x	f	0	0	wh	-	q+a	NAmer	1981	CC	220	n	bl	n	y	0	all/unsp	non any	st
WUNSCH	13	x	m	0	0	all	-	all	SCAmer	1990	CC	398	n	bl	y	n	0	cig+/-ot	non any	st
WUNSCH	15	x	f	0	0	all	-	all	SCAmer	1990	CC	398	n	bl	y	n	0	cig+/-ot	non any	st
WYNDE3	53		m	0	0	all	-	all	NAmer	1966	CC	350	n	bl	n	y	0	all/unsp	non any	st
WYNDE6	153		m	0	0	all	-	all	NAmer	1969	CC	4423	n	bl	n	y	0	cig+/-ot	non cigs	st
WYNDE6	261		f	0	0	all	-	all	NAmer	1969	CC	4423	n	bl	n	y	0	cig+/-ot	non cigs	st
WYNDER	14		m	0	0	all	-	all	SCAmer	1956	CC	120	n	bl	n	n	0	cig+/-ot	non any	ot
WYNDER	21		f	0	0	all	-	all	SCAmer	1956	CC	120	n	bl	n	n	0	cig+/-ot	non any	st
YAMAGU	6	x	c	0	0	all	-	all	As:Jap	1989	CC	144	n	bl	n	y	0	all/unsp	non any	st
YONG	3		c	0	0	all	0	all	NAmer	1971	pr	216	n	bl	n	n	1	cig+/-ot	non cigs	ot

Cigarette type is all/unspec for all RRs
except for the following:

REF	NRR	CIGTYPE
DEAN3	246	MC only
DEAN3	152	MC only
GARDIN	12	MC only

Table 1B14 - 5

IESLC - Meta-analysis of Current Smoking (vs non-current), Cigarettes (or Any Product if Cigarettes not available)
All LC types
Least adjusted

REF	NRR	SEX	AD	Number Exposed Case	Number Exposed Cont	Non-exposed Case	Non-exposed Cont	RR	95.00%CI
AGUDO	16	f	0	20	17	83	189	2.68	(1.34- 5.37)
*AKIBA	4	m	0	345	171379	66	72136	2.20	(1.69- 2.86)
*AKIBA	8	f	0	74	51237	125	373792	4.32	(3.24- 5.76)
Subtotal AKIBA								2.99	(2.46- 3.63)
*AMANDU	4	m	0	115	96708	17	40037	2.80	(1.68- 4.66)
AMES	5	m	0	150	136	162	177	1.21	(0.88- 1.65)
*ANDERS	4	f	0	212	41262	131	250060	9.81	(7.89- 12.19)
*ARCHER	10	m	0	122	32529	24	13582	2.12	(1.37- 3.29)
ARMADA	10	m	0	188	122	137	203	2.28	(1.67- 3.13)
AUSTIN	4	c	0	111	125	55	200	3.23	(2.18- 4.78)
AXELSS	3	m	0	194	130	114	374	4.90	(3.61- 6.64)
AXELSS	12	f	0	96	69	32	194	8.43	(5.19- 13.70)
Subtotal AXELSS								5.71	(4.41- 7.40)
BARBON	108	m	0	562	362	193	393	3.16	(2.54- 3.93)
BECHER	19	m	0	101	122	45	170	3.13	(2.05- 4.77)
BECHER	20	f	0	33	26	15	70	5.92	(2.77- 12.64)
Subtotal BECHER								3.64	(2.52- 5.25)
*BENSHL	26	m	1	-	-	-	-	3.65	(2.63- 5.08)
BLOHMK	4	m	0	419	313	469	575	1.64	(1.36- 1.99)
*BRETT	8	m	0	135	37448	15	17012	4.09	(2.40- 6.97)
BROSS	17	m	0	565	427	304	355	1.55	(1.27- 1.88)
BUFFLE	41	m	0	257	219	218	247	1.33	(1.03- 1.72)
BUFFLE	42	f	0	313	183	147	299	3.48	(2.66- 4.55)
Subtotal BUFFLE								2.10	(1.75- 2.53)
BYERS2	1	m	0	212	197	83	387	5.02	(3.69- 6.82)
BYERS2	2	f	0	121	91	32	222	9.22	(5.83- 14.61)
Subtotal BYERS2								6.05	(4.69- 7.81)
CARPEN	17	c	0	219	162	124	548	5.97	(4.51- 7.92)
*CEDERL	90	m	1	-	-	-	-	3.88	(2.29- 6.57)
*CEDERL	102	f	1	-	-	-	-	4.39	(1.80- 10.71)
Subtotal CEDERL								4.01	(2.54- 6.31)
*CHANG	13	m	0	35	419	48	1589	2.77	(1.81- 4.22)
*CHANG	14	f	0	30	603	23	1719	3.72	(2.18- 6.35)
Subtotal CHANG								3.10	(2.22- 4.32)
CHOI	4	m	0	232	329	48	231	3.39	(2.38- 4.83)
CHOI	8	f	0	13	23	82	167	1.15	(0.55- 2.39)
Subtotal CHOI								2.76	(2.01- 3.80)
*CHOW	41	m	0	138	81725	33	134936	6.90	(4.72- 10.09)
*CHYOU	10	m	0	181	3470	46	4490	5.09	(3.69- 7.02)
COMSTO	41	m	0	105	100	52	213	4.30	(2.86- 6.47)
COMSTO	53	f	0	77	52	24	150	9.25	(5.31- 16.14)
Subtotal COMSTO								5.63	(4.05- 7.82)
CORREA	53	c	0	943	571	309	703	3.76	(3.17- 4.45)
*CPSI	74	m	1	-	-	-	-	6.81	(6.20- 7.47)
*CPSI	285	f	1	-	-	-	-	3.12	(2.48- 3.92)
Subtotal CPSI								6.09	(5.59- 6.64)
*CPSII	110	m	1	-	-	-	-	3.85	(3.55- 4.18)
*CPSII	85	f	1	-	-	-	-	6.47	(5.70- 7.34)
Subtotal CPSII								4.49	(4.19- 4.80)
DARBY	7	m	0	322	453	288	1490	3.68	(3.04- 4.45)
DARBY	14	f	0	195	217	117	846	6.50	(4.94- 8.54)
Subtotal DARBY								4.43	(3.79- 5.18)
DEAN2	4	m	0	671	600	131	200	1.71	(1.33- 2.18)
DEAN2	8	f	0	59	28	93	123	2.79	(1.65- 4.71)
Subtotal DEAN2								1.87	(1.49- 2.33)
DEAN3	246	m	0	408	1192	113	928	2.81	(2.24- 3.52)
DEAN3	152	f	0	102	1158	48	1800	3.30	(2.33- 4.69)
Subtotal DEAN3								2.95	(2.44- 3.56)
*DEKLER	10	m	2	-	-	-	-	3.76	(2.13- 6.63)
DESTE2	18	c	0	216	151	104	169	2.32	(1.69- 3.20)
DESTEF	54	m	0	362	226	135	271	3.22	(2.47- 4.19)
*DOCKER	4	c	4	-	-	-	-	4.52	(2.34- 8.70)
DOLL	92	m	0	1280	1172	77	185	2.62	(1.99- 3.46)
DOLL	95	f	0	58	41	50	67	1.90	(1.10- 3.26)
Subtotal DOLL								2.45	(1.92- 3.14)
*DOLL2	76	m	1	-	-	-	-	3.60	(2.83- 4.58)
DORANT	4	m	0	332	697	153	930	2.90	(2.33- 3.59)
DORGAN	14	m	0	464	170	272	378	3.79	(3.00- 4.80)
DORGAN	38	m	0	214	61	55	109	6.95	(4.52- 10.70)
DORGAN	61	f	0	611	119	249	354	7.30	(5.66- 9.42)
DORGAN	84	f	0	68	17	18	30	6.67	(3.03- 14.69)

Table 1B14 - 5

IESLC - Meta-analysis of Current Smoking (vs non-current), Cigarettes (or Any Product if Cigarettes not available)
All LC types
Least adjusted

REF	NRR	SEX	AD	Number Exposed Case	Number Exposed Cont	Non-exposed Case	Non-exposed Cont	RR	95.00%CI
Subtotal DORGAN								5.39	(4.61- 6.31)
*DORN	202	m	1	-	-	-	-	4.50	(3.98- 5.01)
DROSTE	4	m	0	379	267	99	269	3.86	(2.92- 5.10)
*ENGELA	173	m	1	-	-	-	-	5.89	(3.55- 9.78)
*ENGELA	181	f	1	-	-	-	-	6.12	(2.84- 13.20)
Subtotal ENGELA								5.96	(3.90- 9.10)
GAO	35	m	0	529	438	204	322	1.91	(1.54- 2.37)
GAO	36	f	0	170	100	502	635	2.15	(1.64- 2.83)
Subtotal GAO								2.00	(1.69- 2.37)
GAO2	7	m	0	184	117	98	165	2.65	(1.88- 3.72)
GARCIA	4	c	0	169	74	247	372	3.44	(2.50- 4.72)
GARDIN	12	c	0	72	39	46	85	3.41	(2.01- 5.79)
GARSHI	24	m	0	657	782	332	996	2.52	(2.14- 2.96)
GRAHAM	29	m	0	453	1075	168	524	1.31	(1.07- 1.61)
GREGOR	4	m	0	49	53	33	59	1.65	(0.93- 2.94)
GREGOR	8	f	0	17	26	5	38	4.97	(1.63- 15.15)
Subtotal GREGOR								2.09	(1.25- 3.48)
HAENSZ	66	f	0	69	94	88	245	2.04	(1.38- 3.03)
*HAMMO2	34	m	0	209	4472	98	3580	1.71	(1.35- 2.16)
*HEIN	8	m	0	45	912	12	1436	5.90	(3.14- 11.10)
*HENNEK	4	m	0	79	2438	90	19593	7.05	(5.23- 9.51)
HIRAY2	15	m	1	-	-	-	-	3.26	(1.61- 6.62)
*HIRAYA	148	m	1	-	-	-	-	4.08	(3.37- 4.94)
*HIRAYA	151	f	1	-	-	-	-	2.32	(1.86- 2.90)
Subtotal HIRAYA								3.21	(2.78- 3.71)
HITOSU	7	m	0	117	1597	32	432	0.99	(0.66- 1.48)
HITOSU	13	f	0	28	459	39	1934	3.03	(1.84- 4.97)
Subtotal HITOSU								1.55	(1.13- 2.12)
*HOLE	52	m	0	161	3989	31	2925	3.81	(2.60- 5.58)
*HOLE	68	f	0	17	3437	14	4958	1.75	(0.86- 3.55)
Subtotal HOLE								3.20	(2.28- 4.47)
HUMBLE	63	m	1	-	-	-	-	4.02	(2.77- 5.83)
HUMBLE	64	m	1	-	-	-	-	2.94	(1.59- 5.45)
HUMBLE	65	f	1	-	-	-	-	6.14	(3.47- 10.85)
HUMBLE	66	f	1	-	-	-	-	12.47	(4.53- 34.34)
Subtotal HUMBLE								4.50	(3.45- 5.89)
JAHN	32	m	0	352	269	487	570	1.53	(1.25- 1.87)
JAIN	36	m	0	265	118	138	244	3.97	(2.94- 5.37)
JAIN	31	f	0	305	99	137	311	6.99	(5.17- 9.47)
Subtotal JAIN								5.26	(4.25- 6.51)
JARVHO	4	m	0	73	29	27	44	4.10	(2.15- 7.81)
JARVHO	8	f	0	31	7	16	29	8.03	(2.89- 22.31)
Subtotal JARVHO								4.96	(2.88- 8.56)
JOLY	30	m	0	451	524	120	441	3.16	(2.49- 4.01)
JOLY	29	f	0	132	96	86	310	4.96	(3.47- 7.07)
Subtotal JOLY								3.63	(2.98- 4.43)
*KAISE2	71	m	1	-	-	-	-	4.35	(2.86- 6.62)
*KAISE2	63	f	1	-	-	-	-	8.61	(5.32- 13.93)
Subtotal KAISE2								5.84	(4.26- 8.02)
*KAISER	14	m	2	-	-	-	-	10.41	(8.11- 13.37)
*KAISER	11	f	2	-	-	-	-	4.49	(3.36- 6.00)
Subtotal KAISER								7.27	(6.02- 8.79)
KATSOU	28	f	0	45	18	56	71	3.17	(1.66- 6.07)
KAUFMA	9	c	0	621	886	260	1684	4.54	(3.85- 5.36)
KELLER	4	m	0	5063	1210	3326	2324	2.92	(2.69- 3.17)
KELLER	12	m	0	1053	212	478	245	2.55	(2.06- 3.15)
KELLER	8	f	0	2904	792	1563	2337	5.48	(4.95- 6.07)
KELLER	16	f	0	454	135	197	311	5.31	(4.08- 6.90)
Subtotal KELLER								3.70	(3.49- 3.93)
KHUDER	34	m	0	275	-	207	-	1.82	(1.46- 2.25)
KIHARA	23	c	0	283	162	157	307	3.42	(2.60- 4.49)
*KINLEN	10	m	0	589	8512	82	4176	3.52	(2.81- 4.43)
KJUUS	13	m	0	135	77	41	99	4.23	(2.67- 6.70)
*KNEKT	80	m	0	86	33667	24	33123	3.53	(2.24- 5.54)
KOO	10	f	0	42	25	78	95	2.05	(1.15- 3.65)
KREUZE	43	m	0	168	93	15	101	12.16	(6.68- 22.13)
KREUZE	45	m	0	1226	509	457	1237	6.52	(5.62- 7.57)
KREUZE	44	f	0	55	22	13	57	10.96	(5.03- 23.90)
KREUZE	46	f	0	167	54	130	224	5.33	(3.66- 7.76)
Subtotal KREUZE								6.66	(5.83- 7.60)
*KUBIK	32	m	0	98	6342	10	5980	9.24	(4.83- 17.70)

Table 1B14 - 5

IESLC - Meta-analysis of Current Smoking (vs non-current), Cigarettes (or Any Product if Cigarettes not available)
All LC types
Least adjusted

REF	NRR	SEX	AD	Number Exposed Case	Number Exposed Cont	Non-exposed Case	Non-exposed Cont	RR	95.00%CI
*LANGE	33	m	0	174	4537	26	1974	2.91	(1.93- 4.38)
*LANGE	30	f	0	53	4455	15	3248	2.58	(1.45- 4.56)
Subtotal LANGE								2.79	(2.00- 3.90)
LEMARC	4	c	0	167	65	174	391	5.77	(4.12- 8.09)
LICKIN	1	m	0	220	840	4	160	10.48	(3.84- 28.57)
*LIDDEL	8	m	1	-	-	-	-	3.65	(2.68- 4.98)
LOMBAR	24	m	0	852	610	188	430	3.19	(2.61- 3.90)
LUBIN2	83	m	0	5557	7279	1219	5714	3.58	(3.33- 3.84)
LUBIN2	318	f	0	384	410	388	1337	3.23	(2.70- 3.86)
Subtotal LUBIN2								3.53	(3.30- 3.77)
MACLEN	50	m	0	137	108	10	26	3.30	(1.52- 7.14)
MACLEN	63	f	0	42	47	44	119	2.42	(1.41- 4.15)
Subtotal MACLEN								2.68	(1.72- 4.17)
MATOS	75	m	0	112	132	87	261	2.55	(1.79- 3.61)
*MIGRAN	53	m	0	166	4570	28	2441	3.17	(2.13- 4.71)
*MIGRAN	72	f	0	30	3459	5	4435	7.69	(2.99- 19.81)
Subtotal MIGRAN								3.62	(2.51- 5.22)
*MRFIT	23	m	0	1548	133117	456	228545	5.83	(5.25- 6.47)
MURATA	4	m	0	76	93	31	121	3.19	(1.94- 5.25)
NAM	72	m	0	241	589	399	1006	1.03	(0.85- 1.25)
NAM	88	f	0	133	234	211	1147	3.09	(2.39- 4.00)
Subtotal NAM								1.51	(1.30- 1.76)
NOTANI	1	m	1	-	-	-	-	5.79	(3.50- 9.70)
ODRISC	4	c	0	293	598	153	1062	3.40	(2.73- 4.24)
OSANN	65	m	0	791	541	362	1310	5.29	(4.51- 6.21)
OSANN	69	f	0	597	367	236	1289	8.88	(7.35- 10.75)
Subtotal OSANN								6.56	(5.80- 7.41)
PARKIN	40	m	0	346	874	133	1309	3.90	(3.13- 4.84)
PERSH2	6	c	0	736	631	286	1457	5.94	(5.04- 7.01)
*PETO	6	m	0	99	2036	4	682	8.29	(3.06- 22.44)
PEZZO2	6	m	0	233	198	134	388	3.41	(2.59- 4.48)
PEZZOT	26	m	0	145	129	70	304	4.88	(3.43- 6.94)
*QIAO2	21	m	0	156	5399	43	1525	1.02	(0.73- 1.43)
RACHTA	4	f	0	72	33	46	108	5.12	(2.99- 8.77)
SAARIK	1	c	0	102	66	103	224	3.36	(2.28- 4.95)
SCHWAR	29	m	0	1652	349	1115	1046	4.44	(3.85- 5.12)
SCHWAR	30	m	0	644	139	269	240	4.13	(3.21- 5.32)
SCHWAR	31	f	0	1029	309	504	1183	7.82	(6.63- 9.21)
SCHWAR	32	f	0	256	90	119	336	8.03	(5.84- 11.04)
Subtotal SCHWAR								5.59	(5.08- 6.15)
SHAW	9	c	0	212	97	123	276	4.90	(3.56- 6.76)
SHIMIZ	5	m	2	-	-	-	-	3.70	(2.70- 5.10)
SHIMIZ	10	f	2	-	-	-	-	3.40	(2.10- 5.30)
Subtotal SHIMIZ								3.60	(2.77- 4.68)
SITAS	1	m	1	-	-	-	-	8.40	(3.20- 22.20)
SOBUE	92	m	0	736	650	321	491	1.73	(1.45- 2.06)
SOBUE	96	f	0	95	168	199	921	2.62	(1.95- 3.51)
Subtotal SOBUE								1.93	(1.66- 2.24)
*SPEIZE	9	f	0	391	489993	202	1298381	5.13	(4.33- 6.08)
SPITZ	4	c	0	103	89	74	208	3.25	(2.21- 4.80)
STAYNE	9	m	1	-	-	-	-	3.30	(2.45- 4.45)
STOCKW	12	c	0	12470	3357	9691	14353	5.50	(5.25- 5.76)
STUCKE	4	m	0	69	68	178	186	1.06	(0.72- 1.57)
SUZUK2	19	c	0	78	30	45	93	5.37	(3.10- 9.33)
SVENSS	66	f	0	142	53	68	156	6.15	(4.02- 9.40)
TANG	4	c	0	52	25	67	73	2.27	(1.27- 4.05)
*TENKAN	21	m	1	-	-	-	-	6.32	(4.35- 9.19)
TIZZAN	11	m	0	693	619	526	597	1.27	(1.08- 1.49)
TIZZAN	17	f	0	17	18	33	124	3.55	(1.65- 7.63)
Subtotal TIZZAN								1.33	(1.13- 1.55)
TOKARS	4	m	0	110	157	38	139	2.56	(1.66- 3.95)
TOUSEY	20	m	0	160	91	141	476	5.94	(4.32- 8.16)
TOUSEY	30	f	0	127	78	79	362	7.46	(5.14- 10.83)
Subtotal TOUSEY								6.54	(5.13- 8.33)
TSUGAN	32	m	0	63	63	28	30	1.07	(0.58- 2.00)
TSUGAN	25	f	0	6	10	33	30	0.55	(0.18- 1.68)
Subtotal TSUGAN								0.91	(0.53- 1.58)
*TULINI	17	m	1	-	-	-	-	6.54	(4.74- 9.03)
*TULINI	22	f	1	-	-	-	-	11.38	(7.57- 17.11)
Subtotal TULINI								8.09	(6.28- 10.42)
*TVERDA	26	m	2	-	-	-	-	10.64	(6.28- 18.03)

Table 1B14 - 5

IESLC - Meta-analysis of Current Smoking (vs non-current), Cigarettes (or Any Product if Cigarettes not available)
All LC types
Least adjusted

REF	NRR	SEX	AD	Number Exposed Case	Number Exposed Cont	Non-exposed Case	Non-exposed Cont	RR	95.00%CI
*TVERDA	19	f	0	24	113761	3	196384	13.81	(4.16- 45.86)
Subtotal TVERDA								11.10	(6.85- 17.99)
WAKAI	14	m	0	181	284	64	205	2.04	(1.46- 2.86)
WAKAI	32	f	0	33	26	55	150	3.46	(1.90- 6.31)
Subtotal WAKAI								2.32	(1.73- 3.11)
WANG2	21	c	0	49	78	22	64	1.83	(1.00- 3.34)
WARSIN	1	m	0	129	81	5	19	6.05	(2.17- 16.84)
WATSON	5	m	0	260	250	5	27	5.62	(2.13- 14.81)
WATSON	6	f	0	15	33	21	148	3.20	(1.49- 6.87)
Subtotal WATSON								3.97	(2.18- 7.23)
WIGLE	19	m	0	415	415	159	480	3.02	(2.41- 3.78)
WIGLE	22	f	0	67	169	47	505	4.26	(2.82- 6.43)
Subtotal WIGLE								3.27	(2.68- 3.98)
WU	38	f	0	160	73	60	147	5.37	(3.57- 8.08)
WUNSCH	13	m	0	189	234	114	299	2.12	(1.59- 2.83)
WUNSCH	15	f	0	42	51	46	244	4.37	(2.61- 7.32)
Subtotal WUNSCH								2.52	(1.96- 3.24)
WYNDE3	53	m	0	227	207	57	213	4.10	(2.89- 5.80)
WYNDE6	153	m	0	1677	741	1233	1872	3.44	(3.07- 3.84)
WYNDE6	261	f	0	1022	376	491	1181	6.54	(5.58- 7.66)
Subtotal WYNDE6								4.26	(3.89- 4.67)
WYNDER	14	m	0	73	154	0	35	33.78~	(2.04- 558.25)
WYNDER	21	f	0	38	60	2	141	44.65	(10.44- 191.04)
Subtotal WYNDER								42.09	(11.58- 153.00)
YAMAGU	6	c	0	76	247	68	429	1.94	(1.35- 2.79)
*YONG	3	c	1	-	-	-	-	5.08	(3.74- 6.90)
Partial Totals				67201	1386195	36562	2799445		

*prospective study ~ With 0.5 adjustment for zero

REF	NRR	SEX	AD	Ys	Ws	Qs	Ps
AGUDO	16	f	0	0.99	7.93	1.22	0.0055
*AKIBA	4	m	0	0.79	55.46	19.27	0.0000
*AKIBA	8	f	0	1.46	46.53	0.34	0.0000
Subtotal AKIBA				1.10	101.99	19.60	
*AMANDU	4	m	0	1.03	14.82	1.80	0.0001
AMES	5	m	0	0.19	38.70	54.93	0.2459
*ANDERS	4	f	0	2.28	81.15	66.49	0.0000
*ARCHER	10	m	0	0.75	20.10	7.86	0.0007
ARMADA	10	m	0	0.83	38.85	11.85	0.0000
AUSTIN	4	c	0	1.17	24.88	1.05	0.0000
AXELSS	3	m	0	1.59	41.16	1.82	0.0000
AXELSS	12	f	0	2.13	16.31	9.28	0.0000
Subtotal AXELSS				1.74	57.47	11.10	
BARBON	108	m	0	1.15	81.52	4.20	0.0000
BECHER	19	m	0	1.14	21.64	1.22	0.0000
BECHER	20	f	0	1.78	6.68	1.07	0.0000
Subtotal BECHER				1.29	28.32	2.30	
*BENSHL	26	m	1	1.29	35.45	0.25	0.0000
BLOHMK	4	m	0	0.50	105.79	82.40	0.0000
*BRETT	8	m	0	1.41	13.52	0.01	0.0000
BROSS	17	m	0	0.44	97.86	87.00	0.0000
BUFFLE	41	m	0	0.28	58.50	69.90	0.0293
BUFFLE	42	f	0	1.25	53.17	0.92	0.0000
Subtotal BUFFLE				0.74	111.68	70.82	
BYERS2	1	m	0	1.61	40.94	2.26	0.0000
BYERS2	2	f	0	2.22	18.18	12.95	0.0000
Subtotal BYERS2				1.80	59.12	15.21	
CARPEN	17	c	0	1.79	48.48	8.13	0.0000
*CEDERL	90	m	1	1.36	13.83	0.01	0.0000
*CEDERL	102	f	1	1.48	4.83	0.05	0.0011
Subtotal CEDERL				1.39	18.66	0.06	
*CHANG	13	m	0	1.02	21.56	2.81	0.0000
*CHANG	14	f	0	1.31	13.41	0.06	0.0000
Subtotal CHANG				1.13	34.97	2.86	
CHOI	4	m	0	1.22	30.76	0.75	0.0000
CHOI	8	f	0	0.14	7.22	11.05	0.7054
Subtotal CHOI				1.02	37.97	11.80	
*CHOW	41	m	0	1.93	26.65	8.18	0.0000
*CHYOU	10	m	0	1.63	37.38	2.33	0.0000
COMSTO	41	m	0	1.46	23.02	0.15	0.0000

Table 1B14 - 5

IESLC - Meta-analysis of Current Smoking (vs non-current), Cigarettes (or Any Product if Cigarettes not available)
All LC types
Least adjusted

REF	NRR	SEX	AD	Ys	Ws	Qs	Ps
COMSTO	53	f	0	2.23	12.41	8.91	0.0000
Subtotal COMSTO				1.73	35.43	9.06	
CORREA	53	c	0	1.32	133.86	0.39	0.0000
*CPSI	74	m	1	1.92	442.50	129.23	0.0000
*CPSI	285	f	1	1.14	73.31	4.23	0.0000
Subtotal CPSI				1.81	515.81	133.46	
*CPSII	110	m	1	1.35	575.76	0.51	0.0000
*CPSII	85	f	1	1.87	240.30	57.51	0.0000
Subtotal CPSII				1.50	816.06	58.02	
DARBY	7	m	0	1.30	105.75	0.61	0.0000
DARBY	14	f	0	1.87	51.37	12.51	0.0000
Subtotal DARBY				1.49	157.12	13.12	
DEAN2	4	m	0	0.53	63.33	45.01	0.0000
DEAN2	8	f	0	1.02	13.98	1.74	0.0001
Subtotal DEAN2				0.62	77.31	46.75	
DEAN3	246	m	0	1.03	75.66	8.98	0.0000
DEAN3	152	f	0	1.19	31.20	1.05	0.0000
Subtotal DEAN3				1.08	106.85	10.02	
*DEKLER	10	m	2	1.32	11.92	0.03	0.0000
DESTE2	18	c	0	0.84	37.33	10.66	0.0000
DESTEF	54	m	0	1.17	54.69	2.41	0.0000
*DOCKER	4	c	4	1.51	8.91	0.15	0.0000
DOLL	92	m	0	0.96	49.93	8.53	0.0000
DOLL	95	f	0	0.64	13.06	7.12	0.0208
Subtotal DOLL				0.90	62.99	15.65	
*DOLL2	76	m	1	1.28	66.30	0.62	0.0000
DORANT	4	m	0	1.06	82.93	8.22	0.0000
DORGAN	14	m	0	1.33	69.64	0.14	0.0000
DORGAN	38	m	0	1.94	20.65	6.50	0.0000
DORGAN	61	f	0	1.99	59.24	22.03	0.0000
DORGAN	84	f	0	1.90	6.16	1.66	0.0000
Subtotal DORGAN				1.68	155.69	30.33	
*DORN	202	m	1	1.50	290.08	4.61	0.0000
DROSTE	4	m	0	1.35	49.50	0.04	0.0000
*ENGELA	173	m	1	1.77	14.96	2.34	0.0000
*ENGELA	181	f	1	1.81	6.51	1.22	0.0000
Subtotal ENGELA				1.78	21.47	3.56	
GAO	35	m	0	0.65	82.09	44.08	0.0000
GAO	36	f	0	0.77	51.42	19.28	0.0000
Subtotal GAO				0.69	133.51	63.36	
GAO2	7	m	0	0.97	33.06	5.40	0.0000
GARCIA	4	c	0	1.24	38.22	0.78	0.0000
GARDIN	12	c	0	1.23	13.69	0.31	0.0000
GARSHI	24	m	0	0.92	146.69	30.17	0.0000
GRAHAM	29	m	0	0.27	90.92	110.94	0.0091
GREGOR	4	m	0	0.50	11.56	8.86	0.0875
GREGOR	8	f	0	1.60	3.09	0.16	0.0048
Subtotal GREGOR				0.73	14.65	9.01	
HAENSZ	66	f	0	0.71	24.64	10.84	0.0004
*HAMMO2	34	m	0	0.53	69.03	49.07	0.0000
*HEIN	8	m	0	1.78	9.64	1.52	0.0000
*HENNEK	4	m	0	1.95	42.90	14.22	0.0000
HIRAY2	15	m	1	1.18	7.69	0.30	0.0011
*HIRAYA	148	m	1	1.41	105.05	0.08	0.0000
*HIRAYA	151	f	1	0.84	77.90	22.41	0.0000
Subtotal HIRAYA				1.17	182.95	22.50	
HITOSU	7	m	0	-0.01	23.40	45.14	0.9575
HITOSU	13	f	0	1.11	15.61	1.15	0.0000
Subtotal HITOSU				0.44	39.01	46.29	
*HOLE	52	m	0	1.34	26.40	0.04	0.0000
*HOLE	68	f	0	0.56	7.71	5.15	0.1197
Subtotal HOLE				1.16	34.11	5.19	
HUMBLE	63	m	1	1.39	27.75	0.00	0.0000
HUMBLE	64	m	1	1.08	10.13	0.91	0.0006
HUMBLE	65	f	1	1.81	11.82	2.26	0.0000
HUMBLE	66	f	1	2.52	3.75	4.91	0.0000
Subtotal HUMBLE				1.51	53.44	8.08	
JAHN	32	m	0	0.43	96.47	87.37	0.0000
JAIN	36	m	0	1.38	42.39	0.00	0.0000
JAIN	31	f	0	1.95	41.85	13.46	0.0000
Subtotal JAIN				1.66	84.24	13.46	

Table 1B14 - 5

IESLC - Meta-analysis of Current Smoking (vs non-current), Cigarettes (or Any Product if Cigarettes not available)
All LC types
Least adjusted

REF	NRR	SEX	AD	Ys	Ws	Qs	Ps
JARVHO	4	m	0	1.41	9.26	0.01	0.0000
JARVHO	8	f	0	2.08	3.68	1.83	0.0001
Subtotal JARVHO				1.60	12.94	1.84	
JOLY	30	m	0	1.15	67.90	3.48	0.0000
JOLY	29	f	0	1.60	30.44	1.51	0.0000
Subtotal JOLY				1.29	98.35	4.99	
*KAISE2	71	m	1	1.47	21.81	0.19	0.0000
*KAISE2	63	f	1	2.15	16.58	9.96	0.0000
Subtotal KAISE2				1.77	38.40	10.14	
*KAISER	14	m	2	2.34	61.48	57.23	0.0000
*KAISER	11	f	2	1.50	45.71	0.70	0.0000
Subtotal KAISER				1.98	107.19	57.93	
KATSOU	28	f	0	1.15	9.11	0.46	0.0005
KAUFMA	9	c	0	1.51	139.30	2.53	0.0000
KELLER	4	m	0	1.07	569.83	53.05	0.0000
KELLER	12	m	0	0.93	84.46	16.61	0.0000
KELLER	8	f	0	1.70	373.88	39.14	0.0000
KELLER	16	f	0	1.67	55.86	4.74	0.0000
Subtotal KELLER				1.31	1084.02	113.55	
KHUDER	34	m	0	0.60	82.15	49.87	0.0000
KIHARA	23	c	0	1.23	51.72	1.16	0.0000
*KINLEN	10	m	0	1.26	73.88	1.04	0.0000
KJUUS	13	m	0	1.44	18.22	0.08	0.0000
*KNEKT	80	m	0	1.26	18.78	0.26	0.0000
KOO	10	f	0	0.72	11.47	5.03	0.0153
KREUZE	43	m	0	2.50	10.72	13.46	0.0000
KREUZE	45	m	0	1.87	173.10	42.73	0.0000
KREUZE	44	f	0	2.39	6.32	6.53	0.0000
KREUZE	46	f	0	1.67	27.28	2.38	0.0000
Subtotal KREUZE				1.90	217.43	65.10	
*KUBIK	32	m	0	2.22	9.10	6.51	0.0000
*LANGE	33	m	0	1.07	23.00	2.20	0.0000
*LANGE	30	f	0	0.95	11.76	2.19	0.0012
Subtotal LANGE				1.03	34.76	4.39	
LEMARC	4	c	0	1.75	33.70	4.75	0.0000
LICKIN	1	m	0	2.35	3.82	3.60	0.0000
*LIDDEL	8	m	1	1.29	40.02	0.28	0.0000
LOMBAR	24	m	0	1.16	95.62	4.48	0.0000
LUBIN2	83	m	0	1.27	761.80	8.08	0.0000
LUBIN2	318	f	0	1.17	119.50	5.09	0.0000
Subtotal LUBIN2				1.26	881.29	13.17	
MACLEN	50	m	0	1.19	6.45	0.22	0.0024
MACLEN	63	f	0	0.88	13.12	3.22	0.0014
Subtotal MACLEN				0.98	19.57	3.44	
MATOS	75	m	0	0.93	31.42	6.18	0.0000
*MIGRAN	53	m	0	1.15	24.33	1.23	0.0000
*MIGRAN	72	f	0	2.04	4.30	1.88	0.0000
Subtotal MIGRAN				1.29	28.62	3.12	
*MRFIT	23	m	0	1.76	353.72	52.36	0.0000
MURATA	4	m	0	1.16	15.52	0.74	0.0000
NAM	72	m	0	0.03	106.98	194.06	0.7473
NAM	88	f	0	1.13	57.46	3.59	0.0000
Subtotal NAM				0.41	164.44	197.65	
NOTANI	1	m	1	1.76	14.79	2.11	0.0000
ODRISC	4	c	0	1.22	79.60	1.89	0.0000
OSANN	65	m	0	1.67	150.64	12.50	0.0000
OSANN	69	f	0	2.18	106.24	69.08	0.0000
Subtotal OSANN				1.88	256.87	81.58	
PARKIN	40	m	0	1.36	81.19	0.03	0.0000
PERSH2	6	c	0	1.78	140.32	22.91	0.0000
*PETO	6	m	0	2.12	3.87	2.10	0.0000
PEZZO2	6	m	0	1.23	51.59	1.19	0.0000
PEZZOT	26	m	0	1.59	31.03	1.34	0.0000
*QIAO2	21	m	0	0.02	34.69	63.56	0.8856
RACHTA	4	f	0	1.63	13.30	0.87	0.0000
SAARIK	1	c	0	1.21	25.56	0.70	0.0000
SCHWAR	29	m	0	1.49	187.84	2.39	0.0000
SCHWAR	30	m	0	1.42	60.13	0.10	0.0000
SCHWAR	31	f	0	2.06	142.10	65.37	0.0000
SCHWAR	32	f	0	2.08	37.88	18.85	0.0000
Subtotal SCHWAR				1.72	427.95	86.71	

Table 1B14 - 5

IESLC - Meta-analysis of Current Smoking (vs non-current), Cigarettes (or Any Product if Cigarettes not available)
All LC types
Least adjusted

REF	NRR	SEX	AD	Ys	Ws	Qs	Ps
SHAW	9	c	0	1.59	37.34	1.68	0.0000
SHIMIZ	5	m	2	1.31	37.99	0.18	0.0000
SHIMIZ	10	f	2	1.22	17.93	0.43	0.0000
Subtotal SHIMIZ				1.28	55.92	0.61	
SITAS	1	m	1	2.13	4.10	2.31	0.0000
SOBUE	92	m	0	0.55	124.24	85.32	0.0000
SOBUE	96	f	0	0.96	44.27	7.66	0.0000
Subtotal SOBUE				0.66	168.51	92.98	
*SPEIZE	9	f	0	1.63	133.24	8.80	0.0000
SPITZ	4	c	0	1.18	25.47	1.00	0.0000
STAYNE	9	m	1	1.19	43.14	1.46	0.0000
STOCKW	12	c	0	1.71	1815.09	194.16	0.0000
STUCKE	4	m	0	0.06	24.88	43.31	0.7702
SUZUK2	19	c	0	1.68	12.64	1.16	0.0000
SVENSS	66	f	0	1.82	21.26	4.08	0.0000
TANG	4	c	0	0.82	11.38	3.57	0.0058
*TENKAN	21	m	1	1.84	27.47	5.96	0.0000
TIZZAN	11	m	0	0.24	150.72	195.34	0.0033
TIZZAN	17	f	0	1.27	6.55	0.08	0.0012
Subtotal TIZZAN				0.28	157.27	195.42	
TOKARS	4	m	0	0.94	20.42	3.90	0.0000
TOUSEY	20	m	0	1.78	37.83	6.14	0.0000
TOUSEY	30	f	0	2.01	27.69	11.05	0.0000
Subtotal TOUSEY				1.88	65.52	17.19	
TSUGAN	32	m	0	0.07	9.92	17.00	0.8280
TSUGAN	25	f	0	-0.61	3.03	11.92	0.2916
Subtotal TSUGAN				-0.09	12.95	28.92	
*TULINI	17	m	1	1.88	36.99	9.25	0.0000
*TULINI	22	f	1	2.43	23.11	25.66	0.0000
Subtotal TULINI				2.09	60.10	34.91	
*TVERDA	26	m	2	2.36	13.81	13.45	0.0000
*TVERDA	19	f	0	2.63	2.67	4.15	0.0000
Subtotal TVERDA				2.41	16.48	17.60	
WAKAI	14	m	0	0.71	33.84	14.94	0.0000
WAKAI	32	f	0	1.24	10.68	0.20	0.0000
Subtotal WAKAI				0.84	44.52	15.13	
WANG2	21	c	0	0.60	10.60	6.37	0.0496
WARSIN	1	m	0	1.80	3.67	0.65	0.0006
WATSON	5	m	0	1.73	4.08	0.49	0.0005
WATSON	6	f	0	1.16	6.61	0.30	0.0028
Subtotal WATSON				1.38	10.69	0.80	
WIGLE	19	m	0	1.10	75.80	5.65	0.0000
WIGLE	22	f	0	1.45	22.68	0.12	0.0000
Subtotal WIGLE				1.18	98.48	5.77	
WU	38	f	0	1.68	23.03	2.11	0.0000
WUNSCH	13	m	0	0.75	46.12	18.15	0.0000
WUNSCH	15	f	0	1.47	14.44	0.13	0.0000
Subtotal WUNSCH				0.92	60.56	18.28	
WYNDE3	53	m	0	1.41	31.77	0.03	0.0000
WYNDE6	153	m	0	1.23	303.85	6.27	0.0000
WYNDE6	261	f	0	1.88	153.34	38.28	0.0000
Subtotal WYNDE6				1.45	457.19	44.55	
WYNDER	14	m	0	3.52	0.49	2.24	0.0139
WYNDER	21	f	0	3.80	1.82	10.65	0.0000
Subtotal WYNDER				3.74	2.31	12.89	
YAMAGU	6	c	0	0.66	29.20	14.92	0.0003
*YONG	3	c	1	1.63	40.97	2.51	0.0000

Table 1B14 - 5

IESLC - Meta-analysis of Current Smoking (vs non-current), Cigarettes (or Any Product if Cigarettes not available)
All LC types
Least adjusted

	N	189
	NS	129
	Wt	13283.89
Het	Chi	2960.06
Het	df	188
Het	P	***
Fixed	RR	3.97
	RRl	3.90
	RRu	4.03
	P	+++
Random	RR	3.74
	RRl	3.48
	RRu	4.03
	P	+++
Asymm	P	*

Table 1B14 - 6

IESLC - Meta-analysis of Current Smoking (vs non-current), Cigarettes (or Any Product if Cigarettes not available)
All LC types
Least adjusted

	combined	Sex: male	female	Total
N	21	104	64	189
NS	21	99	59	179
Wt	2758.26	7926.58	2599.05	13283.89
Het Chi	141.38	1802.50	499.88	2960.06
Het df	20	103	63	188
Het P	***	***	***	***
Fixed RR	4.97	3.37	5.12	3.97
RRl	4.78	3.30	4.93	3.90
RRu	5.16	3.45	5.32	4.03
P	+++	+++	+++	+++
Random RR	3.86	3.31	4.66	3.74
RRl	3.35	3.00	4.13	3.48
RRu	4.45	3.65	5.26	4.03
P	+++	+++	+++	+++
Between Chi				516.31
Between df				2
Between P				***
Btwn(F) P				***
Btwn(R) P				***

Table 1B14 - 7

IESLC - Meta-analysis of Current Smoking (vs non-current), Cigarettes (or Any Product if Cigarettes not available)
All LC types
Excluded studies (and stage at which they were excluded)

1 BOUCHA BUELL LAURIL MZILEN

2 ABELIN ABRAHA AUVINE AXELSO BAND BERRIN BLOT1 BLOT2 BLOT3 BLOT4 BRESLO BROCKM BROWN1 BYERS1 CASCO2 CASCOR
CHAN CHATZI CHEN CHEN2 CHEN3 CHIAZZ COOKSO DAVEYS DEAN DOSEME DU DUNN EBELIN ESAKI FAN GENG
GER GODLEY GOLLED GSELL HANSEN HEGMAN HINDS HIRAOK HOROWI HORWIT HU HU2 HUANG ISHIMA JARUP JIANG
JONES JUSSAW KO KOHLME KOULUM KREYBE LAMTH LAMWK LAMWK2 LAUSSM LEI LETOUR LEVIN LIU LIU2 LIU3
LIU4 LIU5 LOMBA2 LUO MAGNUS MARSH MARSH2 MARTIS MASTRA MATSUD MCCONN MCDUFF MCLAUG MILLER MILLS MOLLO
NOTAN2 NOU ORMOS PASTOR PAWLEG PERNU PERSHA PIKE POFFIJ POLEDN PRESCO QIAO RADZIK RANDIG REN RONCO
ROOTS ROTHSC SADOWS SANKAR SCHWA2 SEGI SEOW SIEMIA SIMARA STASZE STOCKS SUN TAO ULMER VANDER WANG
WANG3 WANG4 WICKLU WILKIN WUWILL WYNDE2 WYNDE4 XIANGZ XU XU2 XU3 XU4 YUAN ZHANG ZHENG ZHOU

3 RESTRE

4 BOFFET

5 RIMING TANG2 WYNDE5 WYNDE8

6 ALDERS BENHAM BEST BOUCOT BROWN2 DAMBER ENSTRO GILLIS GOODMA HAMMON KANELL LIAW LUBIN SOBUE2 WALD WU2
WYNDE7

8 JEDRYC SEGI2 SUZUKI

10 MRFITR OSANN2 PISANI SIMONA VEIERO VUTUC

Table 1B14 - 8

Potentially overlapping studies

REF	REFGP	PRINC	OVERLAP/LINK
LUBIN2	LUBIN2	1	Lubin-combined
KOO	KOO	1	KOO/LAMTH/LAMWK
TVERDA	TVERDA	1	VEIERO/TVERDAL
AKIBA	AKIBA	1	AKIBA/ISHIMA
HEIN	PRESCO	1	Subset of PRESCO
LANGE	PRESCO	1	Subset of PRESCO
BROSS	BYERS1	1	GRAHAM/BROSS/BYERS1
GRAHAM	BYERS1	1	GRAHAM/BROSS/BYERS1
CHYOU	CHYOU	1	GOODMA/CHYOU
HOLE	TANG2	1	Subset of TANG2
BENSHL	TANG2	1	Subset of TANG2
KAISER	KAISER	1	KAISER/OSANN2
MRFIT	MRFIT	1	MRFIT-overall
WYNDE6	WYNDE6	1	WYNDE5/6/7/8
CPSI	CPSI	1	CPSI overall
JAHN	BOFFET	2	Subset of BOFFET

Table 1B15 -

IESLC - Meta-analysis of Current Smoking (vs non-current), Cigarettes only
All LC types

This analysis is restricted to results for:
1) Non-dose-response data
2) Current smokers
3) Results complete enough for use in metaanalysis

Within each study, results are then selected (in the following order of preference, within each sex) for:
4) PRODUCT: cigarettes only
5) CIGTYPE: all/unspecified, MC regardless of HR, MC only
6) DENOM: non smoker of anything, non smoker of cigarettes
7) Followup period (YF, prospective studies): whole study (coded as 0) or longest available
8) LCtype: all or nearest available, at least Squamous and Adeno. (q = squamous, s = small,
l = large, a = adeno, mix = mixed, alv = alveolar)
9) Race: all or nearest available, otherwise by race (wh or w = white, bl or b = black, hi = hispanic
ch = chinese, jap = japanese, haw = hawaiian, w+o = white + oriental, sca = scandinavian, as = asian)
10) For overlapping studies: principal rather than subsidiary studies
Finally by Age: whole study (coded as 0) if available, otherwise by widest available age group
and then for single sex results (m, f) in preference to combined sex results (c).

Results adjusted (AD) for the most potential confounders are then chosen in Sections -1 to -3
and results adjusted for the least confounders in Sections -4 to -6. (Those least adjusted results which
actually differ from the most adjusted as marked 'x' in column X in Section -4)
(Results adjusted for an unknown number of confounder(s) are coded as 20.)

Section -7 shows excluded studies, together with the stage (as above) at which no qualifying
results were found.

Section -8 lists the potentially overlapping studies which have been included (1=principal, 2=subsidiary).

Section -9 lists any results which would have been included in preference except that they had data not complete
enough for use in meta-analysis, with their significance (yes/no), if known, and any further comment as entered
on the database.

In addition to those mentioned above, the following fields, levels and abbreviations are used:
* or nk = not known, n = no, y = yes, ot = other
non = not current
all/unspec = all or unspecified, MC = manufactured cigarettes, HR = hand-rolled cigarettes
REF: 6-character study reference
NRR: number of the RR on the database within the study
ST : study type (CC = case control, pr or prosp = prospective)
NLC: number of lung cancer cases in whole study
R : risky occupational population (n = no, m = mining, o = other risky)
VB : national cigarette type (V = at least 75% Virginia, bl = at least 75% blended, ot = other)
P : any proxy use
H : full histological confirmation
De : derivation of RR/CI (or = original, st = standard method, ot = other method of estimation)

Table 1B15 - 1

IESLC - Meta-analysis of Current Smoking (vs non-current), Cigarettes only
All LC types
Most adjusted

REF	NRR	SEX	AGEL	AGEH	RACE	YF	LC TYPE	LOC	START	ST	NLC	R	VB	P	H	AD	PRODUCT	DENOM	De
AGUDO	15	f	0	0	all	-	all	Eu:wst	1989	CC	103	n	bl	n	n	3	cig only	non any	ot
CEDERL	117	m	0	0	all	0	all	Eu:Sca	1963	pr	491	n	bl	n	n	2	cig only	non any	ot
CEDERL	103	f	0	0	all	10	all	Eu:Sca	1963	pr	491	n	bl	n	n	1	cig only	non any	ot
CPSI	74	m	0	0	wh	0	all	NAmer	1959	pr	5138	n	bl	n	n	1	cig only	non any	st
CPSI	150	f	0	0	wh	0	all	NAmer	1959	pr	5138	n	bl	n	n	1	cig only	non any	st
CPSII	110	m	35	99	all	4	all	NAmer	1982	pr	3229	n	bl	n	n	1	cig only	non cigs	ot
DEAN3	77	m	0	0	all	-	all	Eu:UK	1969	CC	766	n	V	y	n	3	cig only	non any	ot
DEAN3	154	f	0	0	all	-	all	Eu:UK	1969	CC	766	n	V	y	n	3	cig only	non any	ot
DOLL2	58	m	0	0	all	0	all	Eu:UK	1951	pr	920	n	V	n	n	1	cig only	non any	ot
DORN	220	m	35	84	wh	8	all	NAmer	1954	pr	5097	n	bl	n	n	1	cig only	non any	ot
ENGELA	216	m	0	0	all	12	all	Eu:Sca	1964	pr	435	n	bl	n	n	1	cig only	non any	ot
ENGELA	226	f	0	0	all	12	all	Eu:Sca	1964	pr	435	n	bl	n	n	1	cig only	non any	ot
GARDIN	12	c	0	0	all	-	all	Eu:UK	1988	CC	143	n	V	y	n	0	cig only	non any	st
GRAHAM	28	m	0	0	wh	-	all	NAmer	1956	CC	685	n	bl	n	n	0	cig only	non any	st
HEIN	8	m	0	0	all	0	all	Eu:Sca	1970	pr	144	n	bl	n	n	0	cig only	non any	st
HIRAY2	8	m	0	0	all	-	all	As:Jap	1950	CC	145	n	bl	*	n	1	cig only	non any	ot
KAISE2	71	m	35	99	all	9	all	NAmer	1979	pr	318	n	bl	n	n	1	cig only	non any	st
KAISE2	63	f	35	99	all	9	all	NAmer	1979	pr	318	n	bl	n	n	1	cig only	non any	st
LOMBAR	22	m	0	0	all	-	all	NAmer	1951	CC	1040	n	bl	n	n	0	cig only	non cigs	st
LUBIN2	74	m	0	0	all	-	all	Eu:mul	1976	CC	7804	n	bl	n	y	2	cig only	non any	ot
MIGRAN	52	m	0	0	all	0	all	Eu:UK	1964	pr	259	n	V	n	n	2	cig only	non any	ot
MIGRAN	71	f	0	0	all	0	all	Eu:UK	1964	pr	259	n	V	n	n	2	cig only	non any	ot
PEZZOT	26	m	0	0	all	-	all	SCAmer	1987	CC	215	n	bl	n	y	0	cig only	non cigs	st
TVERDA	24	m	0	0	all	0	all	Eu:Sca	1972	pr	238	n	bl	n	n	2	cig only	non any	ot
TVERDA	19	f	0	0	all	0	all	Eu:Sca	1972	pr	238	n	bl	n	n	0	cig only	non any	st
WIGLE	19	m	0	0	all	-	all	NAmer	1971	CC	728	n	V	n	n	0	cig only	non any	st
WIGLE	22	f	0	0	all	-	all	NAmer	1971	CC	728	n	V	n	n	0	cig only	non any	st
WYNDER	9	m	0	0	all	-	all	SCAmer	1956	CC	120	n	bl	n	n	0	cig only	non any	ot
WYNDER	11	f	0	0	all	-	all	SCAmer	1956	CC	120	n	bl	n	n	0	cig only	non any	st

Cigarette type is all/unspec for all RRs
except for the following:

REF	NRR	CIGTYPE
DEAN3	77	MC only
DEAN3	154	MC only
GARDIN	12	MC only

Table 1B15 - 2

IESLC - Meta-analysis of Current Smoking (vs non-current), Cigarettes only
All LC types
Most adjusted

REF	NRR	SEX	AD	Number Exposed Case	Number Exposed Cont	Non-exposed Case	Non-exposed Cont	RR	95.00%CI
AGUDO	15	f	3	-	-	-	-	3.53	(1.54- 8.10)
*CEDERL	117	m	2	-	-	-	-	7.80	(5.45- 11.17)
*CEDERL	103	f	1	-	-	-	-	4.36	(1.74- 10.94)
Subtotal CEDERL								7.22	(5.17- 10.09)
*CPSI	74	m	1	-	-	-	-	6.81	(6.20- 7.47)
*CPSI	150	f	1	-	-	-	-	3.57	(3.12- 4.09)
Subtotal CPSI								5.53	(5.12- 5.97)
*CPSII	110	m	1	-	-	-	-	3.85	(3.55- 4.18)
DEAN3	77	m	3	-	-	-	-	3.04	(2.40- 3.86)
DEAN3	154	f	3	-	-	-	-	5.59	(3.78- 8.28)
Subtotal DEAN3								3.58	(2.92- 4.39)
*DOLL2	58	m	1	-	-	-	-	4.47	(3.80- 5.25)
*DORN	220	m	1	-	-	-	-	4.97	(4.35- 5.67)
*ENGELA	216	m	1	-	-	-	-	5.77	(3.44- 9.68)
*ENGELA	226	f	1	-	-	-	-	6.12	(2.84- 13.20)
Subtotal ENGELA								5.88	(3.83- 9.03)
GARDIN	12	c	0	72	39	46	85	3.41	(2.01- 5.79)
GRAHAM	28	m	0	371	821	168	524	1.41	(1.14- 1.74)
*HEIN	8	m	0	45	912	12	1436	5.90	(3.14- 11.10)
HIRAY2	8	m	1	-	-	-	-	3.42	(1.68- 6.96)
*KAISE2	71	m	1	-	-	-	-	4.35	(2.86- 6.62)
*KAISE2	63	f	1	-	-	-	-	8.61	(5.32- 13.93)
Subtotal KAISE2								5.84	(4.26- 8.02)
LOMBAR	22	m	0	432	249	188	430	3.97	(3.15- 5.00)
LUBIN2	74	m	2	-	-	-	-	3.75	(3.49- 4.02)
*MIGRAN	52	m	2	-	-	-	-	1.38	(0.92- 2.07)
*MIGRAN	71	f	2	-	-	-	-	4.15	(1.57- 10.98)
Subtotal MIGRAN								1.62	(1.12- 2.36)
PEZZOT	26	m	0	145	129	70	304	4.88	(3.43- 6.94)
*TVERDA	24	m	2	-	-	-	-	9.98	(5.87- 16.99)
*TVERDA	19	f	0	24	113761	3	196384	13.81	(4.16- 45.86)
Subtotal TVERDA								10.53	(6.47- 17.11)
WIGLE	19	m	0	415	415	159	480	3.02	(2.41- 3.78)
WIGLE	22	f	0	67	169	47	505	4.26	(2.82- 6.43)
Subtotal WIGLE								3.27	(2.68- 3.98)
WYNDER	9	m	0	46	77	0	35	42.60~	(2.55- 711.01)
WYNDER	11	f	0	36	58	2	141	43.76	(10.20- 187.73)
Subtotal WYNDER								43.51	(11.94- 158.62)
Partial Totals				1653	116630	695	200324		

*prospective study ~ With 0.5 adjustment for zero

REF	NRR	SEX	AD	Ys	Ws	Qs	Ps
AGUDO	15	f	3	1.26	5.58	0.16	0.0029
*CEDERL	117	m	2	2.05	29.84	11.59	0.0000
*CEDERL	103	f	1	1.47	4.55	0.01	0.0017
Subtotal CEDERL				1.98	34.38	11.60	
*CPSI	74	m	1	1.92	442.50	105.22	0.0000
*CPSI	150	f	1	1.27	209.67	5.25	0.0000
Subtotal CPSI				1.71	652.18	110.47	
*CPSII	110	m	1	1.35	575.76	3.94	0.0000
DEAN3	77	m	3	1.11	68.05	6.92	0.0000
DEAN3	154	f	3	1.72	24.99	2.11	0.0000
Subtotal DEAN3				1.28	93.04	9.02	
*DOLL2	58	m	1	1.50	147.08	0.65	0.0000
*DORN	220	m	1	1.60	218.79	6.52	0.0000
*ENGELA	216	m	1	1.75	14.36	1.49	0.0000
*ENGELA	226	f	1	1.81	6.51	0.94	0.0000
Subtotal ENGELA				1.77	20.86	2.43	
GARDIN	12	c	0	1.23	13.69	0.57	0.0000
GRAHAM	28	m	0	0.34	84.93	100.45	0.0016
*HEIN	8	m	0	1.78	9.64	1.15	0.0000
HIRAY2	8	m	1	1.23	7.61	0.31	0.0007
*KAISE2	71	m	1	1.47	21.81	0.03	0.0000
*KAISE2	63	f	1	2.15	16.58	8.65	0.0000
Subtotal KAISE2				1.77	38.40	8.68	
LOMBAR	22	m	0	1.38	71.55	0.20	0.0000
LUBIN2	74	m	2	1.32	768.74	9.13	0.0000
*MIGRAN	52	m	2	0.32	23.37	28.72	0.1195
*MIGRAN	71	f	2	1.42	4.06	0.00	0.0041
Subtotal MIGRAN				0.49	27.43	28.72	

Table 1B15 - 2

IESLC - Meta-analysis of Current Smoking (vs non-current), Cigarettes only
All LC types
Most adjusted

REF	NRR	SEX	AD	Ys	Ws	Qs	Ps
PEZZOT	26	m	0	1.59	31.03	0.74	0.0000
*TVERDA	24	m	2	2.30	13.60	10.29	0.0000
*TVERDA	19	f	0	2.63	2.67	3.81	0.0000
Subtotal TVERDA				2.35	16.27	14.10	
WIGLE	19	m	0	1.10	75.80	8.05	0.0000
WIGLE	22	f	0	1.45	22.68	0.01	0.0000
Subtotal WIGLE				1.18	98.48	8.06	
WYNDER	9	m	0	3.75	0.48	2.61	0.0090
WYNDER	11	f	0	3.78	1.81	9.98	0.0000
Subtotal WYNDER				3.77	2.30	12.60	

	N	29
	NS	20
	Wt	2917.73
Het	Chi	329.51
Het	df	28
Het	P	***
Fixed	RR	4.18
	RRl	4.03
	RRu	4.34
	P	+++
Random	RR	4.39
	RRl	3.76
	RRu	5.12
	P	+++
Asymm	P	N.S.

Table 1B15 - 3

IESLC - Meta-analysis of Current Smoking (vs non-current), Cigarettes only
All LC types
Most adjusted

	Sex combined	male	female	Total
N	1	18	10	29
NS	1	18	10	29
Wt	13.69	2604.94	299.09	2917.73
Het Chi	0.00	298.01	30.85	329.51
Het df	0	17	9	28
Het P	N.S.	***	***	***
Fixed RR	3.41	4.19	4.12	4.18
RRl	2.01	4.04	3.68	4.03
RRu	5.79	4.36	4.62	4.34
P	+++	+++	+++	+++
Random RR	3.41	4.09	5.57	4.39
RRl	2.01	3.39	4.03	3.76
RRu	5.79	4.95	7.70	5.12
P	+++	+++	+++	+++
Between Chi				0.65
Between df				2
Between P				N.S.
Btwn(F) P				N.S.
Btwn(R) P				N.S.

Table 1B15 - 4

IESLC - Meta-analysis of Current Smoking (vs non-current), Cigarettes only
All LC types
Least adjusted

REF	NRR	X	SEX	AGEL	AGEH	RACE	YF	LC TYPE	LOC	START	ST	NLC	R	VB	P	H	AD	PRODUCT	DENOM	De
AGUDO	16	x	f	0	0	all	-	all	Eu:wst	1989	CC	103	n	bl	n	n	0	cig only	non any	st
CEDERL	117		m	0	0	all	0	all	Eu:Sca	1963	pr	491	n	bl	n	n	2	cig only	non any	ot
CEDERL	34	x	f	0	0	all	10	all	Eu:Sca	1963	pr	491	n	bl	n	n	0	cig only	non any	st
CPSI	74		m	0	0	wh	0	all	NAmer	1959	pr	5138	n	bl	n	n	1	cig only	non any	st
CPSI	150		f	0	0	wh	0	all	NAmer	1959	pr	5138	n	bl	n	n	1	cig only	non any	st
CPSII	110		m	35	99	all	4	all	NAmer	1982	pr	3229	n	bl	n	n	1	cig only	non cigs	ot
DEAN3	75	x	m	0	0	all	-	all	Eu:UK	1969	CC	766	n	V	y	n	0	cig only	non any	st
DEAN3	152	x	f	0	0	all	-	all	Eu:UK	1969	CC	766	n	V	y	n	0	cig only	non any	st
DOLL2	58		m	0	0	all	0	all	Eu:UK	1951	pr	920	n	V	n	n	1	cig only	non any	ot
DORN	220		m	35	84	wh	8	all	NAmer	1954	pr	5097	n	bl	n	n	1	cig only	non any	ot
ENGELA	216		m	0	0	all	12	all	Eu:Sca	1964	pr	435	n	bl	n	n	1	cig only	non any	ot
ENGELA	226		f	0	0	all	12	all	Eu:Sca	1964	pr	435	n	bl	n	n	1	cig only	non any	ot
GARDIN	12		c	0	0	all	-	all	Eu:UK	1988	CC	143	n	V	y	n	0	cig only	non any	st
GRAHAM	28		m	0	0	wh	-	all	NAmer	1956	CC	685	n	bl	n	n	0	cig only	non any	st
HEIN	8		m	0	0	all	0	all	Eu:Sca	1970	pr	144	n	bl	n	n	0	cig only	non any	st
HIRAY2	8		m	0	0	all	-	all	As:Jap	1950	CC	145	n	bl	*	n	1	cig only	non any	ot
KAISE2	71		m	35	99	all	9	all	NAmer	1979	pr	318	n	bl	n	n	1	cig only	non any	st
KAISE2	63		f	35	99	all	9	all	NAmer	1979	pr	318	n	bl	n	n	1	cig only	non any	st
LOMBAR	22		m	0	0	all	-	all	NAmer	1951	CC	1040	n	bl	n	n	0	cig only	non cigs	st
LUBIN2	73	x	m	0	0	all	-	all	Eu:mul	1976	CC	7804	n	bl	n	y	0	cig only	non any	st
MIGRAN	51	x	m	0	0	all	0	all	Eu:UK	1964	pr	259	n	V	n	n	0	cig only	non any	st
MIGRAN	70	x	f	0	0	all	0	all	Eu:UK	1964	pr	259	n	V	n	n	0	cig only	non any	st
PEZZOT	26		m	0	0	all	-	all	SCAmer	1987	CC	215	n	bl	n	y	0	cig only	non cigs	st
TVERDA	24		m	0	0	all	0	all	Eu:Sca	1972	pr	238	n	bl	n	n	2	cig only	non any	ot
TVERDA	19		f	0	0	all	0	all	Eu:Sca	1972	pr	238	n	bl	n	n	0	cig only	non any	st
WIGLE	19		m	0	0	all	-	all	NAmer	1971	CC	728	n	V	n	n	0	cig only	non any	st
WIGLE	22		f	0	0	all	-	all	NAmer	1971	CC	728	n	V	n	n	0	cig only	non any	st
WYNDER	9		m	0	0	all	-	all	SCAmer	1956	CC	120	n	bl	n	n	0	cig only	non any	ot
WYNDER	11		f	0	0	all	-	all	SCAmer	1956	CC	120	n	bl	n	n	0	cig only	non any	st

Cigarette type is all/unspec for all RRs
except for the following:

REF	NRR	CIGTYPE
DEAN3	75	MC only
DEAN3	152	MC only
GARDIN	12	MC only

Table 1B15 - 5

IESLC - Meta-analysis of Current Smoking (vs non-current), Cigarettes only
All LC types
Least adjusted

REF	NRR	SEX	AD	Number Exposed Case	Cont	Non-exposed Case	Cont	RR	95.00%CI
AGUDO	16	f	0	20	17	83	189	2.68	(1.34- 5.37)
*CEDERL	117	m	2	-	-	-	-	7.80	(5.45- 11.17)
*CEDERL	34	f	0	8	4709	20	18586	1.58	(0.70- 3.58)
Subtotal CEDERL								6.03	(4.34- 8.38)
*CPSI	74	m	1	-	-	-	-	6.81	(6.20- 7.47)
*CPSI	150	f	1	-	-	-	-	3.57	(3.12- 4.09)
Subtotal CPSI								5.53	(5.12- 5.97)
*CPSII	110	m	1	-	-	-	-	3.85	(3.55- 4.18)
DEAN3	75	m	0	337	930	114	927	2.95	(2.34- 3.71)
DEAN3	152	f	0	102	1158	48	1800	3.30	(2.33- 4.69)
Subtotal DEAN3								3.05	(2.51- 3.70)
*DOLL2	58	m	1	-	-	-	-	4.47	(3.80- 5.25)
*DORN	220	m	1	-	-	-	-	4.97	(4.35- 5.67)
*ENGELA	216	m	1	-	-	-	-	5.77	(3.44- 9.68)
*ENGELA	226	f	1	-	-	-	-	6.12	(2.84- 13.20)
Subtotal ENGELA								5.88	(3.83- 9.03)
GARDIN	12	c	0	72	39	46	85	3.41	(2.01- 5.79)
GRAHAM	28	m	0	371	821	168	524	1.41	(1.14- 1.74)
*HEIN	8	m	0	45	912	12	1436	5.90	(3.14- 11.10)
HIRAY2	8	m	1	-	-	-	-	3.42	(1.68- 6.96)
*KAISE2	71	m	1	-	-	-	-	4.35	(2.86- 6.62)
*KAISE2	63	f	1	-	-	-	-	8.61	(5.32- 13.93)
Subtotal KAISE2								5.84	(4.26- 8.02)
LOMBAR	22	m	0	432	249	188	430	3.97	(3.15- 5.00)
LUBIN2	73	m	0	5243	6835	1219	5714	3.60	(3.35- 3.86)
*MIGRAN	51	m	0	137	3707	28	2441	3.22	(2.15- 4.82)
*MIGRAN	70	f	0	23	2749	5	4435	7.42	(2.82- 19.50)
Subtotal MIGRAN								3.65	(2.51- 5.29)
PEZZOT	26	m	0	145	129	70	304	4.88	(3.43- 6.94)
*TVERDA	24	m	2	-	-	-	-	9.98	(5.87- 16.99)
*TVERDA	19	f	0	24	113761	3	196384	13.81	(4.16- 45.86)
Subtotal TVERDA								10.53	(6.47- 17.11)
WIGLE	19	m	0	415	415	159	480	3.02	(2.41- 3.78)
WIGLE	22	f	0	67	169	47	505	4.26	(2.82- 6.43)
Subtotal WIGLE								3.27	(2.68- 3.98)
WYNDER	9	m	0	46	77	0	35	42.60~	(2.55- 711.01)
WYNDER	11	f	0	36	58	2	141	43.76	(10.20- 187.73)
Subtotal WYNDER								43.51	(11.94- 158.62)
Partial Totals				7523	136735	2212	234416		

*prospective study ~ With 0.5 adjustment for zero

REF	NRR	SEX	AD	Ys	Ws	Qs	Ps
AGUDO	16	f	0	0.99	7.93	1.49	0.0055
*CEDERL	117	m	2	2.05	29.84	12.03	0.0000
*CEDERL	34	f	0	0.46	5.72	5.30	0.2747
Subtotal CEDERL				1.80	35.56	17.33	
*CPSI	74	m	1	1.92	442.50	110.30	0.0000
*CPSI	150	f	1	1.27	209.67	4.50	0.0000
Subtotal CPSI				1.71	652.18	114.81	
*CPSII	110	m	1	1.35	575.76	2.91	0.0000
DEAN3	75	m	0	1.08	71.98	8.25	0.0000
DEAN3	152	f	0	1.19	31.20	1.57	0.0000
Subtotal DEAN3				1.12	103.17	9.81	
*DOLL2	58	m	1	1.50	147.08	0.90	0.0000
*DORN	220	m	1	1.60	218.79	7.43	0.0000
*ENGELA	216	m	1	1.75	14.36	1.60	0.0000
*ENGELA	226	f	1	1.81	6.51	1.00	0.0000
Subtotal ENGELA				1.77	20.86	2.60	
GARDIN	12	c	0	1.23	13.69	0.50	0.0000
GRAHAM	28	m	0	0.34	84.93	98.32	0.0016
*HEIN	8	m	0	1.78	9.64	1.23	0.0000
HIRAY2	8	m	1	1.23	7.61	0.27	0.0007
*KAISE2	71	m	1	1.47	21.81	0.06	0.0000
*KAISE2	63	f	1	2.15	16.58	8.93	0.0000
Subtotal KAISE2				1.77	38.40	8.99	
LOMBAR	22	m	0	1.38	71.55	0.12	0.0000
LUBIN2	73	m	0	1.28	750.53	14.58	0.0000
*MIGRAN	51	m	0	1.17	23.62	1.47	0.0000
*MIGRAN	70	f	0	2.00	4.12	1.41	0.0000
Subtotal MIGRAN				1.29	27.74	2.88	

Table 1B15 - 5

IESLC - Meta-analysis of Current Smoking (vs non-current), Cigarettes only
All LC types
Least adjusted

REF	NRR	SEX	AD	Ys	Ws	Qs	Ps
PEZZOT	26	m	0	1.59	31.03	0.86	0.0000
*TVERDA	24	m	2	2.30	13.60	10.57	0.0000
*TVERDA	19	f	0	2.63	2.67	3.88	0.0000
Subtotal TVERDA				2.35	16.27	14.45	
WIGLE	19	m	0	1.10	75.80	7.49	0.0000
WIGLE	22	f	0	1.45	22.68	0.02	0.0000
Subtotal WIGLE				1.18	98.48	7.51	
WYNDER	9	m	0	3.75	0.48	2.64	0.0090
WYNDER	11	f	0	3.78	1.81	10.08	0.0000
Subtotal WYNDER				3.77	2.30	12.72	

	N	29
	NS	20
	Wt	2913.50
Het	Chi	319.71
Het	df	28
Het	P	***
Fixed	RR	4.13
	RRl	3.99
	RRu	4.29
	P	+++
Random	RR	4.33
	RRl	3.72
	RRu	5.04
	P	+++
Asymm	P	N.S.

Table 1B15 - 6

IESLC - Meta-analysis of Current Smoking (vs non-current), Cigarettes only
All LC types
Least adjusted

	combined	Sex male	female	Total
N	1	18	10	29
NS	1	18	10	29
Wt	13.69	2590.92	308.88	2913.50
Het Chi	0.00	280.79	36.71	319.71
Het df	0	17	9	28
Het P	N.S.	***	***	***
Fixed RR	3.41	4.17	3.86	4.13
RRl	2.01	4.01	3.45	3.99
RRu	5.79	4.34	4.31	4.29
P	+++	+++	+++	+++
Random RR	3.41	4.26	4.81	4.33
RRl	2.01	3.54	3.44	3.72
RRu	5.79	5.12	6.72	5.04
P	+++	+++	+++	+++
Between Chi				2.20
Between df				2
Between P				N.S.
Btwn(F) P				N.S.
Btwn(R) P				N.S.

Table 1B15 - 7

IESLC - Meta-analysis of Current Smoking (vs non-current), Cigarettes only
All LC types
Excluded studies (and stage at which they were excluded)

1 BOUCHA BUELL LAURIL MZILEN

2 ABELIN ABRAHA AUVINE AXELSO BAND BERRIN BLOT1 BLOT2 BLOT3 BLOT4 BRESLO BROCKM BROWN1 BYERS1 CASCO2 CASCOR
CHAN CHATZI CHEN CHEN2 CHEN3 CHIAZZ COOKSO DAVEYS DEAN DOSEME DU DUNN EBELIN ESAKI FAN GENG
GER GODLEY GOLLED GSELL HANSEN HEGMAN HINDS HIRAOK HOROWI HORWIT HU HU2 HUANG ISHIMA JARUP JIANG
JONES JUSSAW KO KOHLME KOULUM KREYBE LAMTH LAMWK LAMWK2 LAUSSM LEI LETOUR LEVIN LIU LIU2 LIU3
LIU4 LIU5 LOMBA2 LUO MAGNUS MARSH MARSH2 MARTIS MASTRA MATSUD MCCONN MCDUFF MCLAUG MILLER MILLS MOLLO
NOTAN2 NOU ORMOS PASTOR PAWLEG PERNU PERSHA PIKE POFFIJ POLEDN PRESCO QIAO RADZIK RANDIG REN RONCO
ROOTS ROTHSC SADOWS SANKAR SCHWA2 SEGI SEOW SIEMIA SIMARA STASZE STOCKS SUN TAO ULMER VANDER WANG
WANG3 WANG4 WICKLU WILKIN WUWILL WYNDE2 WYNDE4 XIANGZ XU XU2 XU3 XU4 YUAN ZHANG ZHENG ZHOU

3 RESTRE

4 AKIBA AMANDU AMES ANDERS ARCHER ARMADA AUSTIN AXELSS BARBON BECHER BENSHL BLOHMK BOFFET BRETT BROSS BROWN2
BUFFLE BYERS2 CARPEN CHANG CHOI CHYOU COMSTO CORREA DARBY DEAN2 DEKLER DESTE2 DESTEF DOCKER DOLL DORANT
DORGAN DROSTE GAO GAO2 GARCIA GARSHI GILLIS GOODMA GREGOR HAENSZ HAMMO2 HENNEK HIRAYA HITOSU HOLE HUMBLE
JAHN JAIN JARVHO JEDRYC JOLY KAISER KANELL KATSOU KAUFMA KELLER KHUDER KIHARA KINLEN KJUUS KNEKT KOO
KREUZE KUBIK LEMARC LIAW LICKIN LIDDEL LUBIN MACLEN MATOS MRFIT MRFITR MURATA NAM NOTANI ODRISC OSANN
OSANN2 PARKIN PERSH2 PETO PEZZO2 PISANI QIAO2 RACHTA SAARIK SCHWAR SEGI2 SHAW SHIMIZ SIMONA SITAS SOBUE
SOBUE2 SPEIZE SPITZ STAYNE STOCKW STUCKE SUZUK2 SUZUKI SVENSS TANG TENKAN TIZZAN TOKARS TOUSEY TSUGAN TULINI
VUTUC WAKAI WANG2 WARSIN WATSON WU WU2 WUNSCH WYNDE3 WYNDE5 WYNDE6 WYNDE8 YAMAGU YONG

5 LANGE RIMING TANG2

6 ALDERS BENHAM BEST BOUCOT CHOW DAMBER ENSTRO HAMMON WALD WYNDE7

10 VEIERO

Table 1B15 - 8
Potentially overlapping studies

REF	REFGP	PRINC	OVERLAP/LINK
LUBIN2	LUBIN2	1	Lubin-combined
TVERDA	TVERDA	1	VEIERO/TVERDAL
HEIN	PRESCO	1	Subset of PRESCO
GRAHAM	BYERS1	1	GRAHAM/BROSS/BYERS1
CPSI	CPSI	1	CPSI overall
