# Supplementary material for: Systematic review with meta-analysis of the epidemiological evidence in the 1900s relating smoking to lung cancer
Source: BMC Cancer. 2012 Sep 3;12:385. doi: 10.1186/1471-2407-12-385 (PMC3505152; doi:10.1186/1471-2407-12-385)
Supplement: Additional file 5 — Detailed Analysis Tables (Individual file names as described in Additional file 1: Methods, Table1). [file 1471-2407-12-385-S5.zip › PDF/1C.pdf]

Table 1C1 -

IESLC - Meta-anal of Ever Smoking (or Current if Ever not available), Any prod (or Cigs if Any not avail)
All LC types

This analysis is restricted to results for:
1) Non-dose-response data
2) Results complete enough for use in metaanalysis

Within each study, results are then selected (in the following order of preference, within each sex) for:
3) SMKSTA: ever smokers, current smokers
4) PRODUCT: all/unspec, cigarettes regardless of other products, cigarettes only
5) CIGTYPE: all/unspecified, MC regardless of HR, MC only
6) DENOM: never smoked anything, never smoked cigarettes, (never +1 = +long term ex, +2 = +amount unknown,
+3 = never cigs+long term ex)
7) Followup period (YF, prospective studies): whole study (coded as 0) or longest available
8) LCtype: all or nearest available, at least Squamous and Adeno. (q = squamous, s = small,
l = large, a = adeno, mix = mixed, alv = alveolar)
9) Race: all or nearest available, otherwise by race (wh or w = white, bl or b = black, hi = hispanic
ch = chinese, jap = japanese, haw = hawaiian, w+o = white + oriental, sca = scandinavian, as = asian)
10) For overlapping studies: principal rather than subsidiary studies
Finally by Age: whole study (coded as 0) if available, otherwise by widest available age group
and then for single sex results (m, f) in preference to combined sex results (c).

Results adjusted (AD) for the most potential confounders are then chosen in Sections -1 to -3
and results adjusted for the least confounders in Sections -4 to -6. (Those least adjusted results which
actually differ from the most adjusted as marked 'x' in column X in Section -4)
(Results adjusted for an unknown number of confounder(s) are coded as 20.)

Section -7 shows excluded studies, together with the stage (as above) at which no qualifying
results were found.

Section -8 lists the potentially overlapping studies which have been included (1=principal, 2=subsidiary).

Section -9 lists any results which would have been included in preference except that they had data not complete
enough for use in meta-analysis, with their significance (yes/no), if known, and any further comment as entered
on the database.

In addition to those mentioned above, the following fields, levels and abbreviations are used:
* or nk = not known, n = no, y = yes, ot = other
ev = ever, cu = current, nev = never
all/unspec = all or unspecified, cig+/-ot = cigarettes irrespective of other products (cigar, pipe etc)
MC = manufactured cigarettes, HR = hand-rolled cigarettes
REF: 6-character study reference
NRR: number of the RR on the database within the study
ST : study type (CC = case control, pr or prosp = prospective)
NLC: number of lung cancer cases in whole study
R : risky occupational population (n = no, m = mining, o = other risky)
VB : national cigarette type (V = at least 75% Virginia, bl = at least 75% blended, ot = other)
P : any proxy use
H : full histological confirmation
De : derivation of RR/CI (or = original, st = standard method, ot = other method of estimation)

Table 1C1 - 1

IESLC - Meta-anal of Ever Smoking (or Current if Ever not available), Any prod (or Cigs if Any not avail)
All LC types
Most adjusted

REF	NRR	SEX	AGEL	AGEH	RACE	YF	LC TYPE	LOC	START	ST	NLC	R	VB	P	H	AD	SM	PRODUCT	DENOM	De
ABELIN	44	m	0	0	all	-	all	Eu:wst	1941	CC	118	n	bl	y	n	1	ev	all/unsp	nev any	st
ABRAHA	7	m	0	0	all	0	q+s+a	Eu:est	1975	pr	571	n	bl	n	n	0	ev	all/unsp	nev any	ot
ABRAHA	8	f	0	0	all	0	q+s+a	Eu:est	1975	pr	571	n	bl	n	n	0	ev	all/unsp	nev any	ot
AGUDO	1	f	0	0	all	-	all	Eu:wst	1989	CC	103	n	bl	n	n	3	ev	cig only	nev any	or
AKIBA	11	m	0	0	all	0	all	As:Jap	1963	pr	610	n	bl	n	n	5	ev	cig+/-ot	nev cigs	ot
AKIBA	15	f	0	0	all	0	all	As:Jap	1963	pr	610	n	bl	n	n	5	ev	cig+/-ot	nev cigs	ot
ALDERS	69	m	0	0	all	-	all	Eu:UK	1977	CC	1448	n	V	n	n	1	ev	all/unsp	nev any	ot
ALDERS	6	f	0	0	all	-	all	Eu:UK	1977	CC	1448	n	V	n	n	1	ev	cig only	nev any	ot
AMANDU	7	m	0	0	wh	0	all	NAmer	1959	pr	132	m	bl	n	n	2	ev	cig+/-ot	nev cigs	ot
AMES	4	m	0	0	wh	-	all	NAmer	1959	ot	317	m	bl	n	n	0	ev	all/unsp	nev any	st
ANDERS	3	f	0	0	all	0	all	NAmer	1986	pr	343	n	bl	n	n	0	ev	cig+/-ot	nev cigs	ot
ARCHER	6	m	0	0	wh	0	all	NAmer	1950	pr	146	m	bl	n	n	0	ev	cig+/-ot	nev cigs	st
ARMADA	29	m	0	0	all	-	all	Eu:wst	1986	CC	325	n	bl	n	y	0	ev	all/unsp	nev any	st
AUSTIN	7	c	0	0	all	-	all	NAmer	1970	CC	166	o	bl	y	n	3	ev	cig+/-ot	nev cigs	ot
AUVINE	19	c	0	0	all	-	all	Eu:Sca	1986	CC	517	n	bl	y	n	2	ev	cig+/-ot	nev cigs	ot
AXELSO	1	c	0	0	all	-	all	Eu:Sca	1960	CC	152	n	bl	y	n	0	ev	all/unsp	nev any	st
AXELSS	8	m	0	0	sca	-	all	Eu:Sca	1989	CC	436	n	bl	n	n	6	ev	all/unsp	nev any	ot
AXELSS	11	f	0	0	sca	-	all	Eu:Sca	1989	CC	436	n	bl	n	n	0	ev	all/unsp	nev any	st
BAND	1	m	0	0	all	-	all	NAmer	1983	CC	2831	n	V	y	y	2	ev	cig only	nev any	ot
BARBON	131	m	0	0	all	-	all	Eu:wst	1979	CC	755	n	bl	y	y	3	ev	all/unsp	nev any	ot
BECHER	1	m	0	0	all	-	all	Eu:Ger	1985	CC	194	n	bl	n	y	0	ev	all/unsp	nev any	st
BECHER	24	f	0	0	all	-	all	Eu:Ger	1985	CC	194	n	bl	n	y	2	ev	all/unsp	nev any	ot
BENSHL	18	m	40	64	all	10	all	Eu:UK	1967	pr	486	n	V	n	n	1	ev	all/unsp	nev any	ot
BEST	22	m	55	79	all	3	all	NAmer	1955	pr	381	n	V	n	n	0	ev	all/unsp	nev any	st
BEST	18	f	0	0	all	0	all	NAmer	1955	pr	381	n	V	n	n	1	ev	cig only	nev any	ot
BLOHMK	3	m	0	0	all	-	all	Eu:Ger	1978	CC	888	n	bl	n	y	0	ev	all/unsp	nev any	st
BLOT4	1	m	0	0	wh	-	all	NAmer	1974	CC	335	n	bl	y	n	0	ev	cig+/-ot	nev cigs	st
BOFFET	33	m	0	0	all	-	all	Eu:mul	1988	CC	5621	n	bl	y	n	2	ev	all/unsp	nev any	or
BOUCOT	121	m	0	0	all	0	all	NAmer	1951	pr	121	n	bl	n	n	2	ev	all/unsp	nev any	ot
BRESLO	37	m	0	0	all	-	all	NAmer	1949	CC	518	n	bl	n	y	0	ev	all/unsp	nev+1	st
BRESLO	38	f	0	0	all	-	all	NAmer	1949	CC	518	n	bl	n	y	0	ev	all/unsp	nev+1	st
BRETT	10	m	0	0	all	0	all	Eu:UK	1960	pr	150	n	V	n	n	0	ev	cig+/-ot	nev cigs	st
BROCKM	1	m	0	0	wh	-	all	Eu:Ger	1990	CC	117	n	bl	n	y	0	ev	cig+/-ot	nev cigs	st
BROCKM	2	f	0	0	wh	-	all	Eu:Ger	1990	CC	117	n	bl	n	y	0	ev	cig+/-ot	nev cigs	st
BROSS	12	m	0	0	wh	-	all	NAmer	1960	CC	974	n	bl	n	n	0	ev	all/unsp	nev any	st
BROWN2	2	m	0	0	wh	-	all	NAmer	1984	CC	14596	n	bl	n	y	2	ev	cig+/-ot	nev cigs	or
BROWN2	1	f	0	0	wh	-	all	NAmer	1984	CC	14596	n	bl	n	y	2	ev	cig+/-ot	nev cigs	or
BUFFLE	1	m	0	0	wh	-	all	NAmer	1976	CC	943	n	bl	y	n	0	ev	all/unsp	nev any	st
BUFFLE	5	f	0	0	wh	-	all	NAmer	1976	CC	943	n	bl	y	n	0	ev	all/unsp	nev any	st
CARPEN	12	c	0	0	w+b	-	all	NAmer	1991	CC	356	n	bl	n	n	3	ev	cig+/-ot	nev cigs	ot
CASCO2	1	c	0	0	wh	-	all	Eu:Ger	1991	CC	155	n	bl	n	n	0	ev	all/unsp	nev any	st
CASCOR	1	c	0	0	wh	-	all	Eu:Ger	1985	CC	389	n	bl	n	y	0	ev	all/unsp	nev any	st
CEDERL	107	m	0	0	all	16	all	Eu:Sca	1963	pr	491	n	bl	n	n	2	ev	all/unsp	nev any	ot
CEDERL	112	f	0	0	all	0	all	Eu:Sca	1963	pr	491	n	bl	n	n	2	ev	all/unsp	nev any	ot
CHAN	9	m	0	0	all	-	all	As:HK	1976	CC	397	n	bl	n	n	0	ev	all/unsp	nev any	st
CHAN	10	f	0	0	all	-	all	As:HK	1976	CC	397	n	bl	n	n	0	ev	all/unsp	nev any	st
CHANG	6	m	0	0	all	0	all	NAmer	1972	pr	136	n	bl	n	n	0	ev	cig+/-ot	nev cigs	st
CHANG	12	f	0	0	all	0	all	NAmer	1972	pr	136	n	bl	n	n	0	ev	cig+/-ot	nev cigs	st
CHATZI	4	c	0	0	all	-	all	Eu:bal	1987	CC	282	n	bl	n	y	0	ev	all/unsp	nev any	st
CHEN2	1	m	0	0	all	-	all	As:Chi	1983	CC	193	n	ot	y	n	0	ev	all/unsp	nev any	st
CHEN2	2	f	0	0	all	-	all	As:Chi	1983	CC	193	n	ot	y	n	0	ev	all/unsp	nev any	st
CHEN3	1	c	0	0	all	-	all	As:Chi	1981	CC	254	n	ot	y	n	0	ev	all/unsp	nev any	st
CHIAZZ	3	m	0	0	all	-	all	NAmer	1940	CC	144	o	bl	y	n	11	ev	cig+/-ot	nev cigs	or
CHOI	1	m	0	0	all	-	all	As:oth	1985	CC	375	n	bl	n	n	0	ev	cig+/-ot	nev cigs	st
CHOI	5	f	0	0	all	-	all	As:oth	1985	CC	375	n	bl	n	n	0	ev	cig+/-ot	nev cigs	st
CHOW	55	m	0	0	wh	0	all	NAmer	1966	pr	219	n	bl	n	n	2	ev	all/unsp	nev any	ot
CHYOU	7	m	0	0	jap	0	all	NAmer	1965	pr	227	n	bl	n	y	1	ev	cig+/-ot	nev cigs	ot
COMSTO	34	m	0	0	all	-	all	NAmer	1975	ot	258	n	bl	n	n	0	ev	all/unsp	nev any	st
COMSTO	46	f	0	0	all	-	all	NAmer	1975	ot	258	n	bl	n	n	0	ev	all/unsp	nev any	st
COOKSO	5	c	0	0	bl	-	all	Africa	1961	CC	234	n	V	n	y	0	ev	all/unsp	nev any	st
CORREA	34	c	0	0	all	-	all	NAmer	1979	CC	1359	n	bl	y	n	1	ev	cig+/-ot	nev cigs	or
CPSI	187	m	35	84	all	6	all	NAmer	1959	pr	5138	n	bl	n	n	1	ev	cig+/-ot	nev any	ot
CPSI	274	f	40	74	all	6	all	NAmer	1959	pr	5138	n	bl	n	n	1	ev	cig+/-ot	nev cigs	ot
CPSII	104	m	35	99	all	4	all	NAmer	1982	pr	3229	n	bl	n	n	1	ev	cig only	nev any	ot
CPSII	79	f	0	0	all	4	all	NAmer	1982	pr	3229	n	bl	n	n	1	ev	cig+/-ot	nev cigs	ot
DAMBER	25	m	0	0	all	-	all	Eu:Sca	1972	CC	579	n	bl	y	n	1	ev	all/unsp	nev any	st
DARBY	15	m	0	0	wh	-	all	Eu:UK	1988	CC	982	n	V	n	n	0	ev	all/unsp	nev any	st
DARBY	16	f	0	0	wh	-	all	Eu:UK	1988	CC	982	n	V	n	n	0	ev	all/unsp	nev any	st
DAVEYS	5	m	0	0	all	-	all	Eu:Ger	1930	CC	109	n	bl	y	n	0	ev	all/unsp	nev any	st
DAVEYS	6	f	0	0	all	-	all	Eu:Ger	1930	CC	109	n	bl	y	n	0	ev	all/unsp	nev any	ot
DEAN	7	m	0	0	wh	-	all	Africa	1947	CC	603	n	V	y	n	0	ev	all/unsp	nev any	st
DEAN2	3	m	0	0	all	-	all	Eu:UK	1960	CC	954	n	V	y	n	0	ev	all/unsp	nev any	st

Table 1C1 - 1

IESLC - Meta-anal of Ever Smoking (or Current if Ever not available), Any prod (or Cigs if Any not avail)
All LC types
Most adjusted

REF	NRR	SEX	AGEL	AGEH	RACE	YF	LC TYPE	LOC	START	ST	NLC	R	VB	P	H	AD	SM	PRODUCT	DENOM	De
DEAN2	7	f	0	0	all	-	all	Eu:UK	1960	CC	954	n	V	y	n	0	ev	all/unsp	nev any	st
DEAN3	49	m	0	0	all	-	all	Eu:UK	1969	CC	766	n	V	y	n	3	ev	all/unsp	nev any	ot
DEAN3	126	f	0	0	all	-	all	Eu:UK	1969	CC	766	n	V	y	n	3	ev	cig only	nev any	ot
DEKLER	6	m	0	0	all	0	all	Auslia	1961	pr	138	m	V	n	n	2	ev	all/unsp	nev any	ot
DESTE2	14	c	0	0	all	-	all	SCAmer	1993	CC	463	n	bl	n	n	7	ev	all/unsp	nev any	st
DESTEF	48	m	0	0	all	-	all	SCAmer	1988	CC	497	n	bl	n	y	4	ev	all/unsp	nev any	ot
DOCKER	3	c	0	0	wh	0	all	NAmer	1974	pr	120	n	bl	n	n	4	ev	cig+/-ot	nev cigs	ot
DOLL	6	m	0	0	all	-	all	Eu:UK	1948	CC	1465	n	V	n	n	0	ev	all/unsp	nev any	st
DOLL	12	f	0	0	all	-	all	Eu:UK	1948	CC	1465	n	V	n	n	0	ev	all/unsp	nev any	st
DOLL2	56	m	0	0	all	0	all	Eu:UK	1951	pr	920	n	V	n	n	1	ev	all/unsp	nev any	ot
DOLL2	63	f	0	0	all	22	all	Eu:UK	1951	pr	920	n	V	n	n	1	cu	cig only	nev any	ot
DORANT	10	c	0	0	all	0	all	Eu:wst	1986	ot	550	n	bl	n	y	0	ev	all/unsp	nev any	st
DORGAN	6	m	0	0	wh	-	all	NAmer	1980	CC	2026	n	bl	y	y	0	ev	all/unsp	nev any	st
DORGAN	30	m	0	0	bl	-	all	NAmer	1980	CC	2026	n	bl	y	y	0	ev	all/unsp	nev any	st
DORGAN	53	f	0	0	wh	-	all	NAmer	1980	CC	2026	n	bl	y	y	0	ev	all/unsp	nev any	st
DORGAN	76	f	0	0	bl	-	all	NAmer	1980	CC	2026	n	bl	y	y	0	ev	all/unsp	nev any	st
DORN	196	m	35	84	wh	8	all	NAmer	1954	pr	5097	n	bl	n	n	1	ev	all/unsp	nev any	ot
DOSEME	1	m	0	0	all	-	all	Eu:bal	1979	CC	1210	n	bl	n	n	2	ev	cig+/-ot	nev cigs	or
DROSTE	7	m	0	0	all	-	all	Eu:wst	1995	CC	478	n	bl	n	y	4	ev	all/unsp	nev any	ot
DU	1	m	0	0	all	-	all	As:Chi	1985	CC	849	n	ot	y	n	0	ev	all/unsp	nev any	or
DU	2	f	0	0	all	-	all	As:Chi	1985	CC	849	n	ot	y	n	0	ev	all/unsp	nev any	or
DUNN	6	m	0	0	all	0	all	NAmer	1954	pr	139	o	bl	n	n	0	ev	cig+/-ot	nev cigs	st
EBELIN	1	m	0	0	all	-	all	Eu:Ger	1980	CC	130	n	bl	n	n	0	ev	all/unsp	nev any	st
ENGELA	159	m	0	0	all	12	all	Eu:Sca	1964	pr	435	n	bl	n	n	1	ev	all/unsp	nev any	ot
ENGELA	165	f	0	0	all	12	all	Eu:Sca	1964	pr	435	n	bl	n	n	1	ev	all/unsp	nev any	ot
ENSTRO	1	m	0	0	all	0	all	NAmer	1959	pr	2879	n	bl	n	n	1	cu	cig only	nev any	or
ENSTRO	2	f	0	0	all	0	all	NAmer	1959	pr	2879	n	bl	n	n	1	cu	cig only	nev any	or
ESAKI	4	m	0	0	all	-	all	As:Jap	1961	CC	245	n	bl	y	n	0	ev	cig+/-ot	nev cigs	st
ESAKI	5	f	0	0	all	-	all	As:Jap	1961	CC	245	n	bl	y	n	0	ev	cig+/-ot	nev cigs	st
FAN	1	m	0	0	all	-	all	As:Chi	1990	CC	403	n	ot	y	n	0	ev	cig+/-ot	nev cigs	st
FAN	2	f	0	0	all	-	all	As:Chi	1990	CC	403	n	ot	y	n	0	ev	cig+/-ot	nev cigs	st
GAO	1	m	0	0	all	-	all	As:Chi	1984	CC	1405	n	ot	n	n	2	ev	cig+/-ot	nev cigs	or
GAO	11	f	0	0	all	-	all	As:Chi	1984	CC	1405	n	ot	n	n	2	ev	cig+/-ot	nev cigs	or
GAO2	10	m	0	0	all	-	all	As:Jap	1988	CC	282	n	bl	n	n	1	ev	cig+/-ot	nev cigs	ot
GARCIA	3	c	0	0	all	-	all	NAmer	1992	CC	416	n	bl	n	y	0	ev	cig+/-ot	nev cigs	st
GARDIN	7	c	0	0	all	-	all	Eu:UK	1988	CC	143	n	V	y	n	0	ev	all/unsp	nev any	st
GARSHI	25	m	0	0	all	-	all	NAmer	1981	CC	1081	o	bl	y	n	1	ev	all/unsp	nev any	st
GENG	1	m	0	0	all	-	all	As:Chi	1985	CC	292	n	ot	*	n	0	ev	cig+/-ot	nev any	st
GENG	2	f	0	0	all	-	all	As:Chi	1985	CC	292	n	ot	*	n	0	ev	cig+/-ot	nev any	st
GER	21	c	0	0	all	-	all	As:oth	1990	CC	141	n	ot	y	n	14	ev	all/unsp	nev any	ot
GODLEY	5	m	0	0	all	-	all	NAmer	1966	CC	1986	n	bl	y	n	1	ev	cig+/-ot	nev cigs	ot
GODLEY	6	f	0	0	all	-	all	NAmer	1966	CC	1986	n	bl	y	n	1	ev	cig+/-ot	nev cigs	ot
GOLLED	7	m	35	99	all	-	all	Eu:UK	1952	CC	443	n	V	y	n	1	ev	cig+/-ot	nev any	ot
GOODMA	3	m	0	0	w+o	-	all	NAmer	1983	CC	326	n	bl	y	y	0	ev	cig+/-ot	nev any	st
GOODMA	7	f	0	0	w+o	-	all	NAmer	1983	CC	326	n	bl	y	y	0	ev	cig+/-ot	nev any	st
GRAHAM	27	m	0	0	wh	-	all	NAmer	1956	CC	685	n	bl	n	n	1	ev	all/unsp	nev any	ot
GREGOR	3	m	0	0	all	-	all	Eu:UK	1976	CC	104	n	V	n	y	0	ev	cig+/-ot	nev cigs	st
GREGOR	7	f	0	0	all	-	all	Eu:UK	1976	CC	104	n	V	n	y	0	ev	cig+/-ot	nev cigs	st
GSELL	8	m	0	0	all	-	all	Eu:wst	1937	CC	150	n	bl	n	y	0	ev	all/unsp	nev any	st
HAENSZ	11	f	0	0	all	-	not alv	NAmer	1955	CC	158	n	bl	n	y	2	ev	all/unsp	nev any	ot
HAMMO2	4	m	0	0	all	6	all	NAmer	1967	pr	450	o	bl	n	n	1	ev	all/unsp	nev any	ot
HAMMON	117	m	0	0	wh	0	all	NAmer	1952	pr	448	n	bl	n	n	1	ev	all/unsp	nev any	ot
HANSEN	3	m	0	0	all	0	all	Eu:Sca	1968	pr	105	o	bl	y	n	2	ev	all/unsp	nev any	ot
HEGMAN	1	c	0	0	all	-	all	NAmer	1989	CC	282	n	bl	y	y	0	ev	all/unsp	nev any	st
HEIN	7	m	0	0	all	0	all	Eu:Sca	1970	pr	144	n	bl	n	n	0	ev	all/unsp	nev any	st
HENNEK	3	m	0	0	all	0	all	NAmer	1982	pr	169	n	bl	n	n	0	ev	all/unsp	nev any	st
HINDS	22	f	0	0	o	-	all	NAmer	1968	CC	292	n	bl	n	n	3	ev	all/unsp	nev any	st
HIRAYA	147	m	0	0	all	0	all	As:Jap	1965	pr	1917	n	bl	n	n	1	ev	cig+/-ot	nev any	ot
HIRAYA	150	f	0	0	all	0	all	As:Jap	1965	pr	1917	n	bl	n	n	1	ev	cig+/-ot	nev any	ot
HITOSU	38	m	0	0	all	-	all	As:Jap	1960	CC	216	n	bl	y	n	1	ev	all/unsp	nev any	st
HITOSU	62	f	0	0	all	-	all	As:Jap	1960	CC	216	n	bl	y	n	1	ev	all/unsp	nev any	st
HOLE	8	m	0	0	all	0	all	Eu:UK	1972	pr	225	n	V	n	n	1	ev	all/unsp	nev any	ot
HOLE	31	f	0	0	all	11	all	Eu:UK	1972	pr	225	n	V	n	n	1	cu	all/unsp	nev any	ot
HOROWI	1	m	0	0	all	-	all	NAmer	1956	CC	236	n	V	n	n	0	ev	cig+/-ot	nev any	st
HOROWI	2	f	0	0	all	-	all	NAmer	1956	CC	236	n	V	n	n	0	ev	cig+/-ot	nev any	st
HORWIT	1	f	0	0	all	-	all	NAmer	1977	CC	112	n	bl	n	n	0	ev	cig+/-ot	nev cigs	st
HU	15	m	0	0	all	-	all	As:Chi	1985	CC	227	n	ot	n	y	0	ev	cig+/-ot	nev any	st
HU	16	f	0	0	all	-	all	As:Chi	1985	CC	227	n	ot	n	y	0	ev	cig+/-ot	nev any	st
HU2	9	m	0	0	all	-	all	As:Chi	1977	CC	523	n	ot	y	n	0	ev	cig+/-ot	nev cigs	st
HU2	10	f	0	0	all	-	all	As:Chi	1977	CC	523	n	ot	y	n	0	ev	cig+/-ot	nev cigs	st
HUANG	1	c	0	0	all	-	all	As:Chi	1990	CC	135	n	ot	y	n	0	ev	all/unsp	nev any	st
HUMBLE	14	m	0	0	w-hi	-	all	NAmer	1980	CC	521	n	bl	y	n	1	ev	cig+/-ot	nev cigs	ot

Table 1C1 - 1

IESLC - Meta-anal of Ever Smoking (or Current if Ever not available), Any prod (or Cigs if Any not avail)
All LC types
Most adjusted

REF	NRR	SEX	AGEL	AGEH	RACE	YF	LC TYPE	LOC	START	ST	NLC	R	VB	P	H	AD	SM	PRODUCT	DENOM	De
HUMBLE	16	m	0	0	hi	-	all	NAmer	1980	CC	521	n	bl	y	n	1	ev	cig+/-ot	nev cigs	ot
HUMBLE	18	f	0	0	w-hi	-	all	NAmer	1980	CC	521	n	bl	y	n	1	ev	cig+/-ot	nev cigs	ot
HUMBLE	20	f	0	0	hi	-	all	NAmer	1980	CC	521	n	bl	y	n	1	ev	cig+/-ot	nev cigs	ot
JAHN	22	f	0	0	all	-	all	Eu:Ger	1988	CC	1004	n	bl	n	n	2	ev	cig+/-ot	nev any	ot
JAIN	46	m	0	0	all	-	all	NAmer	1981	CC	845	n	V	y	n	2	ev	cig+/-ot	nev cigs	or
JAIN	41	f	0	0	all	-	all	NAmer	1981	CC	845	n	V	y	n	2	ev	cig+/-ot	nev cigs	or
JARUP	6	m	0	0	all	-	all	Eu:Sca	1928	CC	102	o	bl	y	n	2	ev	all/unsp	nev any	ot
JARVHO	3	m	0	0	all	-	all	Eu:Sca	1983	CC	147	n	bl	n	n	0	ev	all/unsp	nev any	st
JARVHO	7	f	0	0	all	-	all	Eu:Sca	1983	CC	147	n	bl	n	n	0	ev	all/unsp	nev any	st
JEDRYC	58	m	0	0	all	-	all	Eu:est	1980	CC	1630	n	bl	y	n	4	ev	cig+/-ot	nev any	ot
JEDRYC	59	f	0	0	all	-	all	Eu:est	1980	CC	1630	n	bl	y	n	4	ev	cig+/-ot	nev any	ot
JIANG	1	m	0	0	all	-	all	As:Chi	1984	CC	125	n	ot	n	n	0	ev	all/unsp	nev any	st
JIANG	2	f	0	0	all	-	all	As:Chi	1984	CC	125	n	ot	n	n	0	ev	all/unsp	nev any	st
JOLY	14	m	0	0	all	-	all	SCAmer	1978	CC	826	n	bl	n	n	0	ev	all/unsp	nev any	st
JOLY	1	f	0	0	all	-	all	SCAmer	1978	CC	826	n	bl	n	n	0	ev	cig+/-ot	nev any	st
JUSSAW	29	m	0	0	all	-	all	As:Ind	1964	CC	792	n	V	n	n	2	ev	all/unsp	nev any	st
KAISE2	72	m	35	99	all	9	all	NAmer	1979	pr	318	n	bl	n	n	1	ev	cig only	nev any	st
KAISE2	64	f	35	99	all	9	all	NAmer	1979	pr	318	n	bl	n	n	1	ev	cig only	nev any	st
KAISER	13	m	0	0	all	0	all	NAmer	1964	pr	714	n	bl	n	n	2	ev	cig+/-ot	nev cigs	ot
KAISER	10	f	0	0	all	0	all	NAmer	1964	pr	714	n	bl	n	n	2	ev	cig+/-ot	nev cigs	ot
KANELL	30	m	0	0	all	-	all	Eu:bal	1950	CC	862	n	bl	n	n	1	cu	all/unsp	nev any	st
KATSOU	29	f	0	0	all	-	all	Eu:bal	1987	CC	101	n	bl	n	n	1	ev	all/unsp	nev any	ot
KAUFMA	17	c	0	0	all	-	all	NAmer	1981	CC	881	n	bl	n	n	6	ev	cig+/-ot	nev cigs	ot
KELLER	3	m	0	0	wh	-	all	NAmer	1985	CC	15038	n	bl	n	n	0	ev	all/unsp	nev any	st
KELLER	11	m	0	0	nonw	-	all	NAmer	1985	CC	15038	n	bl	n	n	0	ev	all/unsp	nev any	st
KELLER	7	f	0	0	wh	-	all	NAmer	1985	CC	15038	n	bl	n	n	0	ev	all/unsp	nev any	st
KELLER	15	f	0	0	nonw	-	all	NAmer	1985	CC	15038	n	bl	n	n	0	ev	all/unsp	nev any	st
KHUDER	4	m	0	0	all	-	all	NAmer	1985	CC	482	n	bl	n	y	0	ev	cig+/-ot	nev cigs	st
KIHARA	31	c	0	0	jap	-	all	As:Jap	1991	CC	440	n	bl	n	n	0	ev	all/unsp	nev any	st
KINLEN	17	m	0	0	all	0	all	Eu:UK	1967	pr	718	n	V	n	n	2	ev	all/unsp	nev any	ot
KJUUS	10	m	0	0	all	-	all	Eu:Sca	1979	CC	176	n	bl	n	n	0	ev	all/unsp	nev any	st
KNEKT	87	m	20	69	all	21	all	Eu:Sca	1966	pr	515	n	bl	n	n	1	ev	all/unsp	nev any	ot
KO	1	f	0	0	all	-	all	As:oth	1992	CC	117	n	ot	n	y	3	ev	cig+/-ot	nev cigs	or
KOHLME	2	c	0	0	all	-	all	Eu:Ger	1990	CC	239	n	bl	n	n	4	ev	all/unsp	nev any	or
KOO	1	f	0	0	all	-	all	As:HK	1981	CC	200	n	bl	n	n	0	ev	all/unsp	nev any	st
KOULUM	1	m	0	0	all	-	all	Eu:Sca	1936	CC	812	n	bl	n	n	0	ev	all/unsp	nev any	st
KREUZE	14	f	1	45	all	-	all	Eu:Ger	1990	CC	2260	n	bl	n	n	0	ev	all/unsp	nev any	st
KREUZE	16	f	55	69	all	-	all	Eu:Ger	1990	CC	2260	n	bl	n	n	0	ev	all/unsp	nev any	st
KREYBE	12	m	0	0	all	-	all	Eu:Sca	1948	CC	300	n	bl	n	y	1	ev	all/unsp	nev any	ot
KREYBE	30	f	0	0	all	-	all	Eu:Sca	1948	CC	300	n	bl	n	y	1	ev	all/unsp	nev any	ot
KUBIK	28	m	0	0	all	0	all	Eu:est	1965	pr	108	n	bl	n	n	0	ev	all/unsp	nev any	st
LAMTH	6	f	0	0	ch	-	all	As:HK	1983	CC	445	n	bl	n	n	0	ev	all/unsp	nev any	or
LAMWK	1	f	0	0	ch	-	all	As:HK	1981	CC	163	n	bl	n	n	0	ev	all/unsp	nev any	st
LAMWK2	9	m	0	0	all	-	q+s+l+a	As:HK	1976	CC	480	n	bl	n	n	0	ev	all/unsp	nev any	st
LAMWK2	10	f	0	0	all	-	q+s+l+a	As:HK	1976	CC	480	n	bl	n	n	0	ev	all/unsp	nev any	st
LANGE	40	m	0	0	all	0	all	Eu:Sca	1976	pr	268	n	bl	n	n	1	ev	all/unsp	nev any	ot
LANGE	37	f	0	0	all	0	all	Eu:Sca	1976	pr	268	n	bl	n	n	1	ev	all/unsp	nev any	ot
LAUSSM	11	m	0	0	all	-	all	Eu:Ger	1982	CC	432	n	bl	n	n	3	ev	all/unsp	nev any	or
LEI	1	m	0	0	all	-	all	As:Chi	1986	CC	792	n	ot	y	n	0	ev	all/unsp	nev any	st
LEI	2	f	0	0	all	-	all	As:Chi	1986	CC	792	n	ot	y	n	0	ev	all/unsp	nev any	st
LEMARC	3	c	0	0	w+o	-	all	NAmer	1992	CC	341	n	bl	n	y	0	ev	all/unsp	nev any	st
LETOUR	1	c	0	0	all	-	all	NAmer	1983	CC	738	n	V	y	y	0	ev	cig+/-ot	nev cigs	st
LEVIN	32	m	0	0	all	-	all	NAmer	1938	CC	475	n	bl	n	n	1	ev	all/unsp	nev any	st
LIAW	1	m	0	0	all	0	all	As:oth	1982	pr	127	n	ot	n	n	1	cu	all/unsp	nev any	or
LIAW	2	f	0	0	all	0	all	As:oth	1982	pr	127	n	ot	n	n	1	cu	all/unsp	nev any	or
LIDDEL	5	m	0	0	all	18	all	NAmer	1970	pr	304	m	V	n	n	1	ev	cig+/-ot	nev cigs	ot
LIU	2	c	0	0	all	-	all	As:Chi	1980	CC	229	n	ot	*	n	2	ev	all/unsp	nev any	or
LIU2	2	m	0	0	all	-	all	As:Chi	1983	CC	316	n	ot	n	n	3	ev	all/unsp	nev any	ot
LIU2	4	f	0	0	all	-	all	As:Chi	1983	CC	316	n	ot	n	n	3	ev	all/unsp	nev any	ot
LIU3	2	m	0	0	all	-	all	As:Chi	1985	CC	110	n	ot	n	n	2	ev	all/unsp	nev any	or
LIU4	11	m	0	0	all	-	all	As:Chi	1986	CC	1000-00	n	ot	y	n	2	ev	all/unsp	nev any	ot
LIU4	12	f	0	0	all	-	all	As:Chi	1986	CC	1000-00	n	ot	y	n	2	ev	all/unsp	nev any	ot
LIU5	1	c	0	0	all	-	all	As:Chi	1978	CC	111	n	ot	y	n	0	ev	all/unsp	nev any	st
LOMBA2	1	f	0	0	all	-	all	NAmer	1960	CC	225	n	bl	n	n	0	ev	cig+/-ot	nev cigs	st
LOMBAR	12	m	0	0	all	-	all	NAmer	1951	CC	1040	n	bl	n	n	0	ev	all/unsp	nev any	st
LUBIN2	46	m	0	0	all	-	all	Eu:mul	1976	CC	7804	n	bl	n	y	2	ev	all/unsp	nev any	ot
LUBIN2	102	f	0	0	all	-	all	Eu:mul	1976	CC	7804	n	bl	n	y	1	ev	all/unsp	nev any	ot
LUO	7	c	0	0	all	-	all	As:Chi	1990	CC	102	n	ot	n	y	20	ev	cig+/-ot	nev cigs	or
MACLEN	73	c	0	0	ch	-	all	As:oth	1972	CC	233	n	bl	n	n	2	ev	cig+/-ot	nev cigs	ot
MAGNUS	5	m	0	0	all	0	all	Eu:Sca	1953	pr	203	o	bl	y	n	3	ev	all/unsp	nev any	ot

Table 1C1 - 1

IESLC - Meta-anal of Ever Smoking (or Current if Ever not available), Any prod (or Cigs if Any not avail)
All LC types
Most adjusted

REF	NRR	SEX	AGEL	AGEH	RACE	YF	LC TYPE	LOC	START	ST	NLC	R	VB	P	H	AD	SM	PRODUCT	DENOM	De
MARSH	7	c	0	0	all	-	all	NAmer	1979	CC	150	n	bl	y	n	2	ev	all/unsp	nev any	or
MARSH2	5	m	0	0	all	-	all	NAmer	1979	CC	114	n	bl	y	n	1	ev	all/unsp	nev any	or
MARSH2	6	f	0	0	all	-	all	NAmer	1979	CC	114	n	bl	y	n	1	ev	all/unsp	nev any	ot
MARTIS	4	m	0	0	all	-	all	Eu:UK	1972	CC	201	n	V	n	n	0	ev	cig+/-ot	nev cigs	st
MASTRA	2	m	0	0	all	-	all	Eu:wst	1973	CC	309	n	bl	n	n	2	ev	all/unsp	nev any	st
MATOS	27	m	0	0	all	-	all	SCAmer	1994	CC	200	n	bl	n	n	2	ev	cig+/-ot	nev any	or
MATSUD	10	m	0	0	all	-	all	As:Jap	1965	CC	179	n	bl	n	n	0	ev	cig+/-ot	nev cigs	st
MCCONN	1	m	0	0	all	-	all	Eu:UK	1946	CC	100	n	V	n	y	0	ev	all/unsp	nev any	st
MCCONN	2	f	0	0	all	-	all	Eu:UK	1946	CC	100	n	V	n	y	0	ev	all/unsp	nev any	st
MCDUFF	1	m	0	0	all	-	all	NAmer	1979	CC	165	n	V	y	n	0	ev	cig+/-ot	nev cigs	st
MCLAUG	1	m	0	0	all	-	all	As:Chi	1972	CC	316	o	ot	y	n	0	ev	all/unsp	nev any	st
MIGRAN	27	m	0	0	all	0	all	Eu:UK	1964	pr	259	n	V	n	n	2	ev	all/unsp	nev any	ot
MIGRAN	42	f	0	0	all	0	all	Eu:UK	1964	pr	259	n	V	n	n	2	ev	all/unsp	nev any	ot
MILLER	2	f	0	0	all	-	all	NAmer	1972	CC	168	n	bl	y	n	1	ev	cig+/-ot	nev any	ot
MILLS	3	m	0	0	wh	-	all	NAmer	1940	CC	444	n	bl	y	n	1	ev	all/unsp	nev any	ot
MRFITR	6	m	0	0	all	0	all	NAmer	1973	pr	119	n	bl	n	n	0	ev	cig+/-ot	nev cigs	ot
NAM	77	m	0	0	all	-	all	NAmer	1986	CC	1199	n	bl	y	n	1	ev	cig+/-ot	nev cigs	ot
NAM	93	f	0	0	all	-	all	NAmer	1986	CC	1199	n	bl	y	n	1	ev	cig+/-ot	nev cigs	ot
NOTAN2	15	m	0	0	all	-	all	As:Ind	1963	CC	683	n	V	n	n	2	ev	all/unsp	nev any	ot
NOU	11	m	30	64	all	-	all	Eu:Sca	1971	CC	273	n	bl	y	n	0	ev	all/unsp	nev any	st
NOU	12	f	30	64	all	-	all	Eu:Sca	1971	CC	273	n	bl	y	n	0	ev	all/unsp	nev any	st
ODRISC	3	c	0	0	all	-	all	Eu:UK	1992	CC	446	n	V	n	n	0	ev	all/unsp	nev any	st
ORMOS	4	m	0	0	all	-	all	Eu:est	1947	CC	119	n	bl	y	y	0	ev	cig+/-ot	nev any	st
ORMOS	26	f	0	0	all	-	all	Eu:est	1947	CC	119	n	bl	y	y	0	ev	cig+/-ot	nev any	st
OSANN	41	m	0	0	all	-	all	NAmer	1984	CC	1986	n	bl	n	n	2	ev	cig+/-ot	nev cigs	or
OSANN	42	f	0	0	all	-	all	NAmer	1984	CC	1986	n	bl	n	n	2	ev	cig+/-ot	nev cigs	or
PARKIN	28	m	0	0	bl	-	all	Africa	1963	CC	877	n	V	y	n	6	ev	all/unsp	nev any	ot
PASTOR	10	m	0	0	all	-	all	Eu:wst	1976	CC	204	n	bl	y	n	1	ev	all/unsp	nev any	or
PAWLEG	2	m	0	0	all	-	all	Eu:est	1992	CC	176	n	bl	n	y	6	ev	all/unsp	nev any	ot
PERNU	2	m	0	0	all	-	all	Eu:Sca	1944	CC	1606	n	bl	n	n	0	ev	all/unsp	nev any	st
PERNU	1	f	0	0	all	-	all	Eu:Sca	1944	CC	1606	n	bl	n	n	0	ev	all/unsp	nev any	st
PERSH2	11	c	0	0	all	-	all	Eu:Sca	1980	CC	1022	n	bl	y	n	4	ev	all/unsp	nev any	ot
PETO	5	m	0	0	all	0	all	Eu:UK	1954	pr	103	n	V	n	n	0	ev	all/unsp	nev any	st
PEZZO2	10	m	0	0	all	-	all	SCAmer	1992	CC	367	n	bl	n	y	0	ev	cig+/-ot	nev cigs	st
PEZZOT	25	m	0	0	all	-	all	SCAmer	1987	CC	215	n	bl	n	y	0	ev	cig only	nev cigs	st
PIKE	4	m	0	0	w-hi	-	all	NAmer	1972	CC	731	n	bl	y	n	0	ev	all/unsp	nev any	st
PIKE	8	f	0	0	w-hi	-	all	NAmer	1972	CC	731	n	bl	y	n	0	ev	all/unsp	nev any	st
POFFIJ	1	c	0	0	all	-	all	Eu:mul	1990	CC	971	n	bl	n	n	0	ev	all/unsp	nev any	st
POLEDN	1	c	0	0	all	-	all	NAmer	1978	CC	209	n	bl	y	n	1	ev	cig+/-ot	nev cigs	or
QIAO2	15	m	0	0	all	0	all	As:Chi	1992	pr	241	m	ot	n	n	1	ev	all/unsp	nev any	ot
RACHTA	15	f	0	0	all	-	all	Eu:est	1991	CC	118	n	bl	n	y	4	ev	cig+/-ot	nev cigs	ot
RADZIK	1	c	0	0	all	-	all	Eu:est	1986	CC	189	n	bl	n	n	0	ev	all/unsp	nev any	st
RANDIG	23	m	0	0	all	-	all	Eu:Ger	1951	CC	448	n	bl	n	n	0	ev	all/unsp	nev any	st
RANDIG	24	f	0	0	all	-	all	Eu:Ger	1951	CC	448	n	bl	n	n	0	ev	all/unsp	nev any	st
REN	1	m	0	0	all	-	all	As:Chi	1980	CC	244	n	ot	*	n	0	ev	all/unsp	nev any	st
REN	2	f	0	0	all	-	all	As:Chi	1980	CC	244	n	ot	*	n	0	ev	all/unsp	nev any	st
RONCO	1	m	0	0	all	-	all	Eu:wst	1976	CC	126	n	bl	y	n	0	ev	all/unsp	nev any	st
ROTHSC	2	c	0	0	all	-	all	NAmer	1971	CC	284	n	bl	y	n	1	ev	all/unsp	nev any	st
SADOWS	31	m	0	0	wh	-	all	NAmer	1938	CC	477	n	bl	n	n	1	ev	all/unsp	nev any	ot
SANKAR	2	m	0	0	all	-	all	As:Ind	1990	CC	281	n	V	n	n	3	ev	all/unsp	nev any	ot
SCHWAR	1	m	0	0	wh	-	all	NAmer	1984	CC	5588	n	bl	y	y	0	ev	cig+/-ot	nev cigs	st
SCHWAR	2	m	0	0	bl	-	all	NAmer	1984	CC	5588	n	bl	y	y	0	ev	cig+/-ot	nev cigs	st
SCHWAR	3	f	0	0	wh	-	all	NAmer	1984	CC	5588	n	bl	y	y	0	ev	cig+/-ot	nev cigs	st
SCHWAR	4	f	0	0	bl	-	all	NAmer	1984	CC	5588	n	bl	y	y	0	ev	cig+/-ot	nev cigs	st
SEGI	1	m	0	0	all	-	all	As:Jap	1948	CC	159	n	bl	n	n	0	ev	all/unsp	nev any	ot
SEGI2	20	m	0	0	all	-	all	As:Jap	1962	CC	378	n	bl	n	n	1	cu	cig+/-ot	nev any	ot
SEGI2	28	f	0	0	all	-	all	As:Jap	1962	CC	378	n	bl	n	n	1	cu	cig+/-ot	nev any	ot
SEOW	6	f	0	0	ch	-	q+s+l+a	As:oth	1997	CC	153	n	bl	n	y	1	ev	cig+/-ot	nev cigs	st
SHAW	12	c	0	0	wh	-	all	NAmer	1988	CC	335	n	V	n	y	0	ev	all/unsp	nev any	st
SIEMIA	5	m	0	0	all	-	all	NAmer	1979	CC	857	n	V	y	y	7	ev	cig+/-ot	nev cigs	or
SIMARA	3	m	0	0	all	-	all	As:oth	1971	CC	115	n	bl	n	n	6	ev	cig+/-ot	nev cigs	ot
SIMARA	4	f	0	0	all	-	all	As:oth	1971	CC	115	n	bl	n	n	6	ev	cig+/-ot	nev cigs	ot
SOBUE	105	m	0	0	all	-	q+s+l+a	As:Jap	1986	CC	1376	n	bl	n	y	1	ev	cig+/-ot	nev cigs	ot
SOBUE	115	f	0	0	all	-	q+s+l+a	As:Jap	1986	CC	1376	n	bl	n	y	1	ev	cig+/-ot	nev cigs	ot
SOBUE2	10	m	0	0	all	-	q+s+l+a	As:Jap	1965	CC	2083	n	bl	n	n	2	cu	cig+/-ot	nev any	ot
SOBUE2	12	f	0	0	all	-	q+s+l+a	As:Jap	1965	CC	2083	n	bl	n	n	2	cu	cig+/-ot	nev any	ot
SPEIZE	8	f	0	0	all	0	all	NAmer	1976	pr	593	n	bl	n	y	0	ev	cig+/-ot	nev cigs	st
SPITZ	3	c	0	0	b+hi	-	all	NAmer	1992	CC	177	n	bl	n	y	0	ev	cig+/-ot	nev cigs	st
STASZE	1	m	0	0	all	-	all	Eu:est	1954	CC	281	n	bl	n	y	0	ev	all/unsp	nev any	st
STASZE	5	f	0	0	all	-	all	Eu:est	1954	CC	281	n	bl	n	y	0	ev	all/unsp	nev any	st
STAYNE	1	m	0	0	all	-	all	NAmer	1969	CC	420	n	bl	n	n	0	ev	all/unsp	nev any	st
STOCKS	47	m	0	0	all	-	all	Eu:UK	1952	CC	2932	n	V	y	n	2	ev	all/unsp	nev any	st

Table 1C1 - 1

IESLC - Meta-anal of Ever Smoking (or Current if Ever not available), Any prod (or Cigs if Any not avail)
All LC types
Most adjusted

REF	NRR	SEX	AGEL	AGEH	RACE	YF	LC TYPE	LOC	START	ST	NLC	R	VB	P	H	AD	SM	PRODUCT	DENOM	De
STOCKS	50	f	0	0	all	-	all	Eu:UK	1952	CC	2932	n	V	y	n	1	ev	cig+/-ot	nev any	ot
STOCKW	6	c	0	0	all	-	all	NAmer	1981	CC	22161	n	bl	n	n	0	ev	all/unsp	nev any	st
STUCKE	3	m	0	0	all	-	all	Eu:wst	1989	CC	247	n	bl	n	y	0	ev	all/unsp	nev any	ot
SUN	1	c	0	0	all	-	all	As:Chi	1992	CC	207	n	ot	n	y	0	ev	all/unsp	nev any	st
SUZUK2	20	c	0	0	all	-	all	SCAmer	1991	CC	123	n	bl	n	y	3	ev	all/unsp	nev any	ot
SVENSS	71	f	0	0	all	-	all	Eu:Sca	1983	CC	210	n	bl	n	n	1	ev	all/unsp	nev any	ot
TANG	3	c	0	0	all	-	not s	NAmer	1992	CC	119	n	bl	n	y	0	ev	cig+/-ot	nev cigs	st
TENKAN	22	m	0	0	all	17	all	Eu:Sca	1962	pr	242	n	bl	n	n	1	ev	all/unsp	nev any	ot
TIZZAN	1	m	0	0	all	-	all	Eu:wst	1959	CC	1358	n	bl	n	n	0	ev	all/unsp	nev any	st
TIZZAN	12	f	0	0	all	-	all	Eu:wst	1959	CC	1358	n	bl	n	n	0	ev	all/unsp	nev any	st
TOKARS	6	c	0	0	all	-	all	Eu:est	1966	ot	162	o	bl	n	y	3	ev	all/unsp	nev any	or
TOUSEY	21	m	0	0	all	-	all	NAmer	1993	CC	507	n	bl	y	y	3	ev	all/unsp	nev any	ot
TOUSEY	26	f	0	0	all	-	all	NAmer	1993	CC	507	n	bl	y	y	0	ev	all/unsp	nev any	st
TSUGAN	27	m	0	0	all	-	q+a	As:Jap	1976	CC	134	n	bl	n	y	0	ev	all/unsp	nev any	st
TULINI	38	m	0	0	all	0	all	Eu:Sca	1967	pr	472	n	bl	n	n	3	ev	all/unsp	nev any	ot
TULINI	44	f	0	0	all	0	all	Eu:Sca	1967	pr	472	n	bl	n	n	3	ev	all/unsp	nev any	ot
TVERDA	22	m	0	0	all	0	all	Eu:Sca	1972	pr	238	n	bl	n	n	2	ev	cig+/-ot	nev cigs	ot
TVERDA	15	f	0	0	all	0	all	Eu:Sca	1972	pr	238	n	bl	n	n	2	cu	cig only	nev cigs	ot
WAKAI	72	m	0	0	all	-	all	As:Jap	1988	CC	333	n	bl	n	y	2	ev	all/unsp	nev any	ot
WAKAI	78	f	0	0	all	-	all	As:Jap	1988	CC	333	n	bl	n	y	2	ev	all/unsp	nev any	ot
WALD	4	m	0	0	all	0	all	Eu:UK	1975	pr	102	n	V	n	n	1	cu	cig only	nev any	or
WANG	5	c	0	0	all	-	all	As:Chi	1990	CC	390	n	ot	*	y	6	ev	all/unsp	nev any	or
WANG2	16	c	0	0	all	-	all	As:Chi	1980	CC	103	n	ot	n	n	4	ev	cig+/-ot	nev cigs	ot
WANG3	1	c	0	0	all	-	all	As:Chi	1981	CC	293	n	ot	*	n	0	ev	all/unsp	nev any	st
WANG4	2	m	0	0	all	-	all	As:Chi	1976	CC	1170	n	ot	y	n	2	ev	all/unsp	nev any	st
WICKLU	1	m	0	0	wh	-	all	NAmer	1968	CC	155	n	bl	y	n	0	ev	cig+/-ot	nev+3	or
WIGLE	27	m	0	0	all	-	all	NAmer	1971	CC	728	n	V	n	n	1	ev	all/unsp	nev any	ot
WIGLE	32	f	0	0	all	-	all	NAmer	1971	CC	728	n	V	n	n	1	ev	all/unsp	nev any	ot
WILKIN	3	c	0	0	all	-	all	Eu:UK	1992	CC	271	n	V	n	n	4	ev	cig+/-ot	nev cigs	ot
WU	45	f	0	0	wh	-	q+a	NAmer	1981	CC	220	n	bl	n	y	2	ev	all/unsp	nev any	ot
WUNSCH	4	m	0	0	all	-	all	SCAmer	1990	CC	398	n	bl	y	n	1	ev	cig+/-ot	nev any	or
WUNSCH	10	f	0	0	all	-	all	SCAmer	1990	CC	398	n	bl	y	n	1	ev	cig+/-ot	nev any	or
WUWILL	8	f	0	0	all	-	all	As:Chi	1985	CC	965	n	ot	n	n	3	ev	cig+/-ot	nev cigs	or
WYNDE2	21	m	0	0	all	-	all	NAmer	1962	CC	404	n	bl	n	y	0	ev	all/unsp	nev any	st
WYNDE3	49	m	0	0	all	-	all	NAmer	1966	CC	350	n	bl	n	y	0	ev	all/unsp	nev any	st
WYNDE3	138	f	0	0	all	-	all	NAmer	1966	CC	350	n	bl	n	y	0	ev	all/unsp	nev any	st
WYNDE4	48	m	0	0	all	-	all	NAmer	1948	CC	684	n	bl	y	n	0	ev	all/unsp	nev any	st
WYNDE4	62	f	0	0	all	-	all	NAmer	1948	CC	684	n	bl	y	n	2	ev	all/unsp	nev any	ot
WYNDE6	72	m	0	0	all	-	all	NAmer	1969	CC	4423	n	bl	n	y	0	ev	all/unsp	nev any	st
WYNDE6	252	f	0	0	all	-	all	NAmer	1969	CC	4423	n	bl	n	y	0	ev	cig+/-ot	nev cigs	st
XIANGZ	13	m	0	0	all	0	all	As:Chi	1976	pr	983	m	ot	n	n	2	ev	all/unsp	nev any	ot
XU	2	m	0	0	all	-	all	As:Chi	1985	CC	729	n	ot	n	n	2	ev	all/unsp	nev any	or
XU2	2	c	0	0	all	-	all	As:Chi	1987	CC	610	o	ot	y	n	7	ev	all/unsp	nev any	ot
XU3	2	m	0	0	all	-	all	As:Chi	1981	CC	135	n	ot	n	n	1	ev	all/unsp	nev any	or
XU3	4	f	0	0	all	-	all	As:Chi	1981	CC	135	n	ot	n	n	1	ev	all/unsp	nev any	or
XU4	1	c	0	0	all	-	all	As:Chi	1981	CC	206	n	ot	*	n	0	ev	all/unsp	nev any	st
YAMAGU	11	c	0	0	all	-	all	As:Jap	1989	CC	144	n	bl	n	y	1	ev	all/unsp	nev any	ot
YONG	12	m	0	0	all	0	all	NAmer	1971	pr	216	n	bl	n	n	1	cu	cig+/-ot	nev cigs	or
YONG	15	f	0	0	all	0	all	NAmer	1971	pr	216	n	bl	n	n	1	cu	cig+/-ot	nev cigs	or
YUAN	1	m	0	0	all	0	all	As:Chi	1986	pr	142	n	ot	n	n	2	ev	cig+/-ot	nev cigs	ot
ZHANG	2	m	0	0	all	-	all	As:Chi	1988	CC	100	n	ot	n	n	7	ev	all/unsp	nev any	or
ZHANG	3	f	0	0	all	-	all	As:Chi	1988	CC	100	n	ot	n	n	7	ev	all/unsp	nev any	or
ZHENG	15	m	0	0	all	-	all	As:Chi	1982	CC	540	n	ot	*	y	0	ev	cig+/-ot	nev cigs	st
ZHENG	24	f	0	0	all	-	all	As:Chi	1982	CC	540	n	ot	*	y	0	ev	cig+/-ot	nev cigs	st
ZHOU	2	m	0	0	all	-	all	As:Chi	1978	CC	1360	n	ot	n	n	0	ev	all/unsp	nev any	st
ZHOU	3	f	0	0	all	-	all	As:Chi	1978	CC	1360	n	ot	n	n	0	ev	all/unsp	nev any	st

Cigarette type is all/unspec for all RRs
except for the following:

REF	NRR	CIGTYPE
ALDERS	6	MC only
DEAN3	126	MC only

Table 1C1 - 2

IESLC - Meta-anal of Ever Smoking (or Current if Ever not available), Any prod (or Cigs if Any not avail)
All LC types
Most adjusted

REF	NRR	SEX	AD	Number Exposed Case	Number Exposed Cont	Non-exposed Case	Non-exposed Cont	RR	95.00%CI
ABELIN	44	m	1	-	-	-	-	35.38	(8.62- 145.24)
*ABRAHA	7	m	0	269	10351	10	3365	8.74	(4.66- 16.42)
*ABRAHA	8	f	0	62	5256	28	11589	4.88	(3.13- 7.62)
Subtotal ABRAHA								5.93	(4.12- 8.53)
AGUDO	1	f	3	-	-	-	-	3.10	(1.42- 6.75)
*AKIBA	11	m	5	-	-	-	-	4.75	(3.07- 7.34)
*AKIBA	15	f	5	-	-	-	-	3.16	(2.37- 4.21)
Subtotal AKIBA								3.58	(2.81- 4.54)
ALDERS	69	m	1	-	-	-	-	9.65	(5.61- 16.61)
ALDERS	6	f	1	-	-	-	-	4.75	(3.55- 6.35)
Subtotal ALDERS								5.56	(4.31- 7.19)
*AMANDU	7	m	2	-	-	-	-	5.89	(2.27- 15.28)
AMES	4	m	0	297	251	15	62	4.89	(2.72- 8.81)
*ANDERS	3	f	0	297	96164	46	195158	13.10	(9.61- 17.87)
*ARCHER	6	m	0	140	36269	6	9842	6.33	(2.80- 14.33)
ARMADA	29	m	0	321	261	4	64	19.68	(7.07- 54.75)
AUSTIN	7	c	3	-	-	-	-	11.12	(3.95- 31.28)
AUVINE	19	c	2	-	-	-	-	13.84	(7.90- 24.25)
AXELSO	1	c	0	90	86	62	371	6.26	(4.20- 9.34)
AXELSS	8	m	6	-	-	-	-	8.02	(4.62- 13.94)
AXELSS	11	f	0	110	109	18	154	8.63	(4.95- 15.05)
Subtotal AXELSS								8.32	(5.62- 12.31)
BAND	1	m	2	-	-	-	-	9.96	(7.38- 13.44)
BARBON	131	m	3	-	-	-	-	11.13	(7.02- 17.64)
BECHER	1	m	0	143	238	3	54	10.82	(3.32- 35.23)
BECHER	24	f	2	-	-	-	-	11.81	(3.20- 43.56)
Subtotal BECHER								11.25	(4.69- 27.01)
*BENSHL	18	m	1	-	-	-	-	5.90	(2.62- 13.31)
*BEST	22	m	0	221	24776	1	2854	25.46	(3.57- 181.46)
*BEST	18	f	1	-	-	-	-	2.24	(0.59- 8.44)
Subtotal BEST								4.81	(1.60- 14.47)
BLOHMK	3	m	0	762	587	126	301	3.10	(2.45- 3.92)
BLOT4	1	m	0	327	245	8	87	14.51	(6.91- 30.51)
BOFFET	33	m	2	-	-	-	-	14.20	(11.70- 17.20)
*BOUCOT	121	m	2	-	-	-	-	40.95	(2.55- 658.45)
BRESLO	37	m	0	486	451	7	42	6.47	(2.88- 14.54)
BRESLO	38	f	0	13	11	12	14	1.38	(0.45- 4.20)
Subtotal BRESLO								3.79	(1.97- 7.29)
*BRETT	10	m	0	144	47930	6	6530	3.27	(1.45- 7.40)
BROCKM	1	m	0	87	81	2	2	1.07	(0.15- 7.80)
BROCKM	2	f	0	24	54	4	18	2.00	(0.61- 6.54)
Subtotal BROCKM								1.70	(0.61- 4.70)
BROSS	12	m	0	902	784	38	170	5.15	(3.57- 7.41)
BROWN2	2	m	2	-	-	-	-	9.10	(8.30- 10.00)
BROWN2	1	f	2	-	-	-	-	12.70	(11.50- 13.90)
Subtotal BROWN2								10.72	(10.03- 11.46)
BUFFLE	1	m	0	470	419	5	47	10.54	(4.15- 26.76)
BUFFLE	5	f	0	419	284	41	198	7.12	(4.93- 10.30)
Subtotal BUFFLE								7.51	(5.33- 10.58)
CARPEN	12	c	3	-	-	-	-	14.88	(8.46- 26.18)
CASCO2	1	c	0	149	212	6	98	11.48	(4.90- 26.87)
CASCOR	1	c	0	365	362	22	295	13.52	(8.56- 21.35)
*CEDERL	107	m	2	-	-	-	-	5.92	(3.85- 9.12)
*CEDERL	112	f	2	-	-	-	-	4.18	(2.94- 5.93)
Subtotal CEDERL								4.80	(3.66- 6.30)
CHAN	9	m	0	206	161	2	43	27.51	(6.57- 115.26)
CHAN	10	f	0	105	50	84	139	3.48	(2.26- 5.35)
Subtotal CHAN								4.13	(2.73- 6.25)
*CHANG	6	m	0	78	1506	5	502	5.20	(2.12- 12.77)
*CHANG	12	f	0	42	1183	11	1139	3.68	(1.90- 7.10)
Subtotal CHANG								4.15	(2.44- 7.06)
CHATZI	4	c	0	255	365	27	129	3.34	(2.14- 5.21)
CHEN2	1	m	0	121	97	9	33	4.57	(2.09- 10.02)
CHEN2	2	f	0	38	30	25	33	1.67	(0.82- 3.39)
Subtotal CHEN2								2.62	(1.55- 4.44)
CHEN3	1	c	0	182	156	72	98	1.59	(1.09- 2.30)
CHIAZZ	3	m	11	-	-	-	-	26.17	(3.32- 206.50)
CHOI	1	m	0	267	465	13	95	4.20	(2.31- 7.64)
CHOI	5	f	0	19	26	76	164	1.58	(0.82- 3.02)
Subtotal CHOI								2.68	(1.72- 4.16)
*CHOW	55	m	2	-	-	-	-	11.08	(4.87- 25.21)

Table 1C1 - 2

IESLC - Meta-anal of Ever Smoking (or Current if Ever not available), Any prod (or Cigs if Any not avail)
All LC types
Most adjusted

REF	NRR	SEX	AD	Number Exposed Case	Number Exposed Cont	Non-exposed Case	Non-exposed Cont	RR	95.00%CI
*CHYOU	7	m	1	-	-	-	-	8.35	(4.76- 14.64)
COMSTO	34	m	0	153	244	4	69	10.82	(3.87- 30.24)
COMSTO	46	f	0	88	87	13	115	8.95	(4.69- 17.06)
Subtotal COMSTO								9.44	(5.46- 16.31)
COOKSO	5	c	0	189	39	45	61	6.57	(3.92- 11.02)
CORREA	34	c	1	-	-	-	-	11.40	(8.70- 15.00)
*CPSI	187	m	1	-	-	-	-	9.18	(7.36- 11.45)
*CPSI	274	f	1	-	-	-	-	2.79	(2.22- 3.51)
Subtotal CPSI								5.17	(4.41- 6.06)
*CPSII	104	m	1	-	-	-	-	12.83	(10.28- 16.01)
*CPSII	79	f	1	-	-	-	-	8.16	(6.93- 9.62)
Subtotal CPSII								9.58	(8.40- 10.93)
DAMBER	25	m	1	-	-	-	-	7.15	(4.98- 10.26)
DARBY	15	m	0	664	1724	3	384	49.30	(15.77- 154.07)
DARBY	16	f	0	292	548	23	529	12.26	(7.89- 19.05)
Subtotal DARBY								14.69	(9.74- 22.16)
DAVEYS	5	m	0	90	144	3	23	4.79	(1.40- 16.42)
DAVEYS	6	f	0	0	3	16	83	0.72~	(0.04- 14.66)
Subtotal DAVEYS								3.65	(1.17- 11.42)
DEAN	7	m	0	591	574	12	61	5.23	(2.79- 9.82)
DEAN2	3	m	0	769	688	33	112	3.79	(2.54- 5.67)
DEAN2	7	f	0	64	30	88	121	2.93	(1.76- 4.90)
Subtotal DEAN2								3.44	(2.51- 4.72)
DEAN3	49	m	3	-	-	-	-	6.14	(3.92- 9.61)
DEAN3	126	f	3	-	-	-	-	4.63	(3.03- 7.09)
Subtotal DEAN3								5.29	(3.89- 7.20)
*DEKLER	6	m	2	-	-	-	-	20.29	(2.84- 145.18)
DESTE2	14	c	7	-	-	-	-	8.70	(5.10- 14.90)
DESTEF	48	m	4	-	-	-	-	9.11	(5.86- 14.15)
*DOCKER	3	c	4	-	-	-	-	4.29	(1.66- 11.06)
DOLL	6	m	0	1350	1296	7	61	9.08	(4.14- 19.92)
DOLL	12	f	0	68	49	40	59	2.05	(1.19- 3.53)
Subtotal DOLL								3.32	(2.12- 5.19)
*DOLL2	56	m	1	-	-	-	-	7.66	(4.86- 12.07)
*DOLL2	63	f	1	-	-	-	-	8.65	(2.93- 25.55)
Subtotal DOLL2								7.80	(5.13- 11.86)
DORANT	10	c	0	470	2033	14	1090	18.00	(10.52- 30.78)
DORGAN	6	m	0	721	455	15	93	9.82	(5.62- 17.16)
DORGAN	30	m	0	266	135	3	35	22.99	(6.94- 76.10)
DORGAN	53	f	0	757	229	103	244	7.83	(5.96- 10.30)
DORGAN	76	f	0	79	27	7	20	8.36	(3.18- 21.95)
Subtotal DORGAN								8.52	(6.75- 10.76)
*DORN	196	m	1	-	-	-	-	7.04	(5.60- 8.84)
DOSEME	1	m	2	-	-	-	-	3.30	(2.60- 4.40)
DROSTE	7	m	4	-	-	-	-	8.62	(3.80- 19.56)
DU	1	m	0	538	-	28	-	3.53	(2.44- 5.11)
DU	2	f	0	191	-	92	-	1.93	(1.30- 2.87)
Subtotal DU								2.66	(2.03- 3.49)
*DUNN	6	m	0	137	52634	2	14160	18.43	(4.56- 74.42)
EBELIN	1	m	0	101	142	12	117	6.93	(3.63- 13.24)
*ENGELA	159	m	1	-	-	-	-	6.34	(2.95- 13.60)
*ENGELA	165	f	1	-	-	-	-	4.74	(2.20- 10.22)
Subtotal ENGELA								5.49	(3.19- 9.43)
*ENSTRO	1	m	1	-	-	-	-	12.99	(10.46- 16.13)
*ENSTRO	2	f	1	-	-	-	-	6.95	(6.01- 8.04)
Subtotal ENSTRO								8.44	(7.48- 9.53)
ESAKI	4	m	0	155	143	16	28	1.90	(0.99- 3.65)
ESAKI	5	f	0	34	19	40	55	2.46	(1.23- 4.92)
Subtotal ESAKI								2.14	(1.33- 3.45)
FAN	1	m	0	216	498	36	236	2.84	(1.93- 4.18)
FAN	2	f	0	82	97	69	320	3.92	(2.65- 5.81)
Subtotal FAN								3.33	(2.53- 4.38)
GAO	1	m	2	-	-	-	-	3.90	(2.90- 5.40)
GAO	11	f	2	-	-	-	-	3.30	(2.50- 4.20)
Subtotal GAO								3.53	(2.90- 4.31)
GAO2	10	m	1	-	-	-	-	5.17	(2.76- 9.69)
GARCIA	3	c	0	395	307	21	139	8.52	(5.26- 13.80)
GARDIN	7	c	0	138	102	5	41	11.09	(4.23- 29.06)
GARSHI	25	m	1	-	-	-	-	5.81	(4.17- 8.10)
GENG	1	m	0	92	68	7	31	5.99	(2.49- 14.42)
GENG	2	f	0	126	75	67	118	2.96	(1.96- 4.48)

Table 1C1 - 2

IESLC - Meta-anal of Ever Smoking (or Current if Ever not available), Any prod (or Cigs if Any not avail)
All LC types
Most adjusted

REF	NRR	SEX	AD	Number Exposed Case	Number Exposed Cont	Non-exposed Case	Non-exposed Cont	RR	95.00%CI
Subtotal GENG								3.36	(2.31- 4.89)
GER	21	c	14	-	-	-	-	1.84	(1.06- 3.20)
GODLEY	5	m	1	-	-	-	-	6.84	(5.60- 8.35)
GODLEY	6	f	1	-	-	-	-	5.54	(4.29- 7.15)
Subtotal GODLEY								6.31	(5.39- 7.39)
GOLLED	7	m	1	-	-	-	-	7.51	(4.44- 12.71)
GOODMA	3	m	0	216	398	10	199	10.80	(5.60- 20.82)
GOODMA	7	f	0	81	91	19	177	8.29	(4.74- 14.52)
Subtotal GOODMA								9.27	(6.05- 14.19)
GRAHAM	27	m	1	-	-	-	-	7.01	(4.39- 11.20)
GREGOR	3	m	0	72	98	10	14	1.03	(0.43- 2.45)
GREGOR	7	f	0	21	42	1	22	11.00	(1.39- 87.29)
Subtotal GREGOR								1.46	(0.66- 3.26)
GSELL	8	m	0	148	121	2	29	17.74	(4.15- 75.83)
HAENSZ	11	f	2	-	-	-	-	2.19	(1.48- 3.24)
*HAMMO2	4	m	1	-	-	-	-	22.82	(3.20- 162.75)
*HAMMON	117	m	1	-	-	-	-	6.80	(4.06- 11.37)
*HANSEN	3	m	2	-	-	-	-	1.53	(0.71- 3.91)
HEGMAN	1	c	0	255	1202	27	2080	16.34	(10.92- 24.45)
*HEIN	7	m	0	143	4471	1	457	14.62	(2.05- 104.23)
*HENNEK	3	m	0	146	11112	23	10919	6.24	(4.02- 9.67)
HINDS	22	f	3	-	-	-	-	5.65	(4.14- 7.72)
*HIRAYA	147	m	1	-	-	-	-	4.36	(3.53- 5.39)
*HIRAYA	150	f	1	-	-	-	-	2.36	(1.90- 2.94)
Subtotal HIRAYA								3.24	(2.78- 3.77)
HITOSU	38	m	1	-	-	-	-	2.91	(1.34- 6.34)
HITOSU	62	f	1	-	-	-	-	3.40	(2.05- 5.64)
Subtotal HITOSU								3.25	(2.12- 4.96)
*HOLE	8	m	1	-	-	-	-	6.44	(3.03- 13.69)
*HOLE	31	f	1	-	-	-	-	1.53	(0.64- 3.70)
Subtotal HOLE								3.50	(1.97- 6.19)
HOROWI	1	m	0	182	525	19	196	3.58	(2.17- 5.90)
HOROWI	2	f	0	21	382	14	463	1.82	(0.91- 3.62)
Subtotal HOROWI								2.83	(1.89- 4.25)
HORWIT	1	f	0	97	92	11	118	11.31	(5.73- 22.34)
HU	15	m	0	120	94	41	67	2.09	(1.30- 3.35)
HU	16	f	0	26	18	40	48	1.73	(0.83- 3.61)
Subtotal HU								1.98	(1.33- 2.94)
HU2	9	m	0	294	228	49	115	3.03	(2.08- 4.41)
HU2	10	f	0	108	80	72	100	1.88	(1.23- 2.85)
Subtotal HU2								2.44	(1.85- 3.23)
HUANG	1	c	0	98	77	37	58	2.00	(1.20- 3.32)
HUMBLE	14	m	1	-	-	-	-	12.10	(5.12- 28.60)
HUMBLE	16	m	1	-	-	-	-	11.88	(2.65- 53.30)
HUMBLE	18	f	1	-	-	-	-	11.36	(5.32- 24.23)
HUMBLE	20	f	1	-	-	-	-	15.40	(4.87- 48.74)
Subtotal HUMBLE								12.28	(7.58- 19.90)
JAHN	22	f	2	-	-	-	-	3.30	(1.99- 5.49)
JAIN	46	m	2	-	-	-	-	8.30	(4.53- 17.00)
JAIN	41	f	2	-	-	-	-	9.20	(5.95- 15.10)
Subtotal JAIN								8.89	(6.08- 13.01)
JARUP	6	m	2	-	-	-	-	7.54	(2.80- 20.33)
JARVHO	3	m	0	99	57	1	16	27.79	(3.59- 215.09)
JARVHO	7	f	0	41	15	6	21	9.57	(3.24- 28.26)
Subtotal JARVHO								12.08	(4.64- 31.46)
JEDRYC	58	m	4	-	-	-	-	5.46	(3.85- 7.73)
JEDRYC	59	f	4	-	-	-	-	4.54	(2.56- 8.05)
Subtotal JEDRYC								5.19	(3.86- 7.00)
JIANG	1	m	0	93	83	7	17	2.72	(1.08- 6.89)
JIANG	2	f	0	11	6	14	19	2.49	(0.74- 8.35)
Subtotal JIANG								2.63	(1.26- 5.50)
JOLY	14	m	0	595	888	12	218	12.17	(6.75- 21.97)
JOLY	1	f	0	166	123	52	283	7.34	(5.04- 10.70)
Subtotal JOLY								8.50	(6.19- 11.68)
JUSSAW	29	m	2	-	-	-	-	16.83	(11.65- 25.21)
*KAISE2	72	m	1	-	-	-	-	5.40	(3.05- 9.57)
*KAISE2	64	f	1	-	-	-	-	10.09	(5.29- 19.27)
Subtotal KAISE2								7.11	(4.63- 10.90)
*KAISER	13	m	2	-	-	-	-	17.63	(11.98- 25.96)
*KAISER	10	f	2	-	-	-	-	5.63	(3.89- 8.14)
Subtotal KAISER								9.70	(7.43- 12.67)

Table 1C1 - 2

IESLC - Meta-anal of Ever Smoking (or Current if Ever not available), Any prod (or Cigs if Any not avail)
All LC types
Most adjusted

REF	NRR	SEX	AD	Number Exposed Case	Number Exposed Cont	Non-exposed Case	Non-exposed Cont	RR	95.00%CI
KANELL	30	m	1	-	-	-	-	4.94	(3.47- 7.03)
KATSOU	29	f	1	-	-	-	-	3.30	(1.77- 6.15)
KAUFMA	17	c	6	-	-	-	-	12.38	(8.59- 17.85)
KELLER	3	m	0	8066	2517	323	1017	10.09	(8.83- 11.52)
KELLER	11	m	0	1493	340	38	117	13.52	(9.20- 19.86)
KELLER	7	f	0	3998	1269	469	1860	12.49	(11.09- 14.08)
KELLER	15	f	0	584	214	67	232	9.45	(6.91- 12.93)
Subtotal KELLER								11.30	(10.40- 12.29)
KHUDER	4	m	0	459	785	23	309	7.86	(5.06- 12.19)
KIHARA	31	c	0	338	232	102	237	3.39	(2.54- 4.51)
*KINLEN	17	m	2	-	-	-	-	10.99	(5.24- 23.06)
KJUUS	10	m	0	174	152	2	24	13.74	(3.19- 59.08)
*KNEKT	87	m	1	-	-	-	-	6.42	(2.82- 14.62)
KO	1	f	3	-	-	-	-	4.20	(1.10- 15.60)
KOHLME	2	c	4	-	-	-	-	16.40	(6.90- 38.42)
KOO	1	f	0	112	63	88	137	2.77	(1.84- 4.16)
KOULUM	1	m	0	807	246	5	54	35.43	(14.02- 89.55)
KREUZE	14	f	0	62	42	6	38	9.35	(3.63- 24.08)
KREUZE	16	f	0	205	101	95	177	3.78	(2.68- 5.34)
Subtotal KREUZE								4.21	(3.04- 5.81)
KREYBE	12	m	1	-	-	-	-	6.61	(2.93- 14.92)
KREYBE	30	f	1	-	-	-	-	1.43	(0.71- 2.86)
Subtotal KREYBE								2.73	(1.61- 4.64)
*KUBIK	28	m	0	106	8051	2	4271	28.12	(6.94- 113.84)
LAMTH	6	f	0	242	106	202	337	3.81	(2.86- 5.08)
LAMWK	1	f	0	88	41	75	144	4.12	(2.59- 6.55)
LAMWK2	9	m	0	244	161	23	43	2.83	(1.64- 4.88)
LAMWK2	10	f	0	75	50	65	139	3.21	(2.02- 5.10)
Subtotal LAMWK2								3.04	(2.14- 4.33)
*LANGE	40	m	1	-	-	-	-	4.74	(1.77- 12.67)
*LANGE	37	f	1	-	-	-	-	4.93	(2.48- 9.81)
Subtotal LANGE								4.87	(2.77- 8.55)
LAUSSM	11	m	3	-	-	-	-	5.70	(4.10- 7.80)
LEI	1	m	0	443	361	41	123	3.68	(2.52- 5.38)
LEI	2	f	0	123	61	85	147	3.49	(2.32- 5.24)
Subtotal LEI								3.59	(2.72- 4.74)
LEMARC	3	c	0	309	288	32	168	5.63	(3.74- 8.49)
LETOUR	1	c	0	714	514	24	224	12.96	(8.38- 20.05)
LEVIN	32	m	1	-	-	-	-	4.86	(3.41- 6.92)
*LIAW	1	m	1	-	-	-	-	3.70	(2.10- 6.60)
*LIAW	2	f	1	-	-	-	-	3.60	(1.00- 12.20)
Subtotal LIAW								3.68	(2.19- 6.20)
*LIDDEL	5	m	1	-	-	-	-	3.61	(2.27- 5.73)
LIU	2	c	2	-	-	-	-	1.92	(1.40- 2.64)
LIU2	2	m	3	-	-	-	-	5.19	(2.03- 13.25)
LIU2	4	f	3	-	-	-	-	4.65	(2.18- 9.93)
Subtotal LIU2								4.86	(2.69- 8.76)
LIU3	2	m	2	-	-	-	-	1.26	(0.30- 5.26)
LIU4	11	m	2	-	-	-	-	2.76	(2.69- 2.83)
LIU4	12	f	2	-	-	-	-	2.86	(2.77- 2.95)
Subtotal LIU4								2.80	(2.74- 2.85)
LIU5	1	c	0	85	70	26	41	1.91	(1.07- 3.44)
LOMBA2	1	f	0	149	353	76	239	1.33	(0.96- 1.83)
LOMBAR	12	m	0	1026	928	14	112	8.84	(5.04- 15.53)
LUBIN2	46	m	2	-	-	-	-	8.50	(7.29- 9.91)
LUBIN2	102	f	1	-	-	-	-	3.90	(3.29- 4.62)
Subtotal LUBIN2								5.99	(5.34- 6.71)
LUO	7	c	20	-	-	-	-	2.70	(1.50- 5.00)
MACLEN	73	c	2	-	-	-	-	2.67	(1.66- 4.29)
*MAGNUS	5	m	3	-	-	-	-	4.13	(1.94- 8.77)
MARSH	7	c	2	-	-	-	-	6.80	(3.30- 13.99)
MARSH2	5	m	1	-	-	-	-	1.89	(0.70- 5.14)
MARSH2	6	f	1	-	-	-	-	5.28	(1.89- 14.72)
Subtotal MARSH2								3.11	(1.52- 6.36)
MARTIS	4	m	0	197	176	4	25	7.00	(2.39- 20.49)
MASTRA	2	m	2	-	-	-	-	8.14	(3.32- 20.00)
MATOS	27	m	2	-	-	-	-	6.80	(3.50- 13.10)
MATSUD	10	m	0	170	3314	3	1255	21.46	(6.84- 67.33)
MCCONN	1	m	0	88	174	5	12	1.21	(0.41- 3.55)
MCCONN	2	f	0	3	3	4	11	2.75	(0.38- 19.67)
Subtotal MCCONN								1.46	(0.57- 3.76)

Table 1C1 - 2

IESLC - Meta-anal of Ever Smoking (or Current if Ever not available), Any prod (or Cigs if Any not avail)
All LC types
Most adjusted

REF	NRR	SEX	AD	Number Exposed Case	Cont	Non-exposed Case	Cont	RR	95.00%CI
MCDUFF	1	m	0	159	134	6	31	6.13	(2.48- 15.14)
MCLAUG	1	m	0	294	1082	22	270	3.33	(2.12- 5.25)
*MIGRAN	27	m	2	-	-	-	-	3.61	(1.34- 9.72)
*MIGRAN	42	f	2	-	-	-	-	4.62	(1.63- 13.09)
Subtotal MIGRAN								4.06	(1.98- 8.32)
MILLER	2	f	1	-	-	-	-	4.99	(2.06- 12.10)
MILLS	3	m	1	-	-	-	-	1.33	(1.09- 1.63)
*MRFITR	6	m	0	119	11007	0	1859	40.37~	(2.51- 648.95)
NAM	77	m	1	-	-	-	-	8.71	(5.87- 12.93)
NAM	93	f	1	-	-	-	-	8.88	(6.35- 12.40)
Subtotal NAM								8.81	(6.82- 11.37)
NOTAN2	15	m	2	-	-	-	-	2.99	(2.40- 3.72)
NOU	11	m	0	74	247	6	122	6.09	(2.58- 14.39)
NOU	12	f	0	10	92	4	261	7.09	(2.17- 23.17)
Subtotal NOU								6.42	(3.20- 12.87)
ODRISC	3	c	0	440	996	6	664	48.89	(21.71- 110.07)
ORMOS	4	m	0	87	1034	7	777	9.34	(4.30- 20.28)
ORMOS	26	f	0	1	234	23	1044	0.19	(0.03- 1.44)
Subtotal ORMOS								5.65	(2.74- 11.64)
OSANN	41	m	2	-	-	-	-	19.70	(14.40- 26.80)
OSANN	42	f	2	-	-	-	-	15.00	(11.80- 19.10)
Subtotal OSANN								16.62	(13.74- 20.10)
PARKIN	28	m	6	-	-	-	-	4.03	(3.15- 5.15)
PASTOR	10	m	1	-	-	-	-	6.81	(3.38- 13.70)
PAWLEG	2	m	6	-	-	-	-	12.26	(4.07- 36.95)
PERNU	2	m	0	1380	438	97	275	8.93	(6.92- 11.53)
PERNU	1	f	0	19	89	110	971	1.88	(1.11- 3.21)
Subtotal PERNU								6.68	(5.31- 8.41)
PERSH2	11	c	4	-	-	-	-	6.55	(5.46- 7.86)
*PETO	5	m	0	101	2423	2	295	6.15	(1.52- 24.79)
PEZZO2	10	m	0	361	469	6	117	15.01	(6.53- 34.48)
PEZZOT	25	m	0	211	317	4	116	19.30	(7.02- 53.10)
PIKE	4	m	0	514	375	18	69	5.25	(3.08- 8.98)
PIKE	8	f	0	163	90	36	96	4.83	(3.04- 7.66)
Subtotal PIKE								5.01	(3.53- 7.10)
POFFIJ	1	c	0	913	918	58	452	7.75	(5.81- 10.34)
POLEDN	1	c	1	-	-	-	-	9.24	(5.23- 16.33)
*QIAO2	15	m	1	-	-	-	-	1.53	(0.81- 2.89)
RACHTA	15	f	4	-	-	-	-	8.21	(3.96- 17.05)
RADZIK	1	c	0	180	198	9	13	1.31	(0.55- 3.15)
RANDIG	23	m	0	410	359	5	22	5.03	(1.88- 13.41)
RANDIG	24	f	0	16	39	17	92	2.22	(1.02- 4.84)
Subtotal RANDIG								3.04	(1.65- 5.60)
REN	1	m	0	106	84	12	34	3.58	(1.74- 7.33)
REN	2	f	0	78	20	48	50	4.06	(2.16- 7.64)
Subtotal REN								3.84	(2.39- 6.17)
RONCO	1	m	0	120	306	6	78	5.10	(2.16- 12.01)
ROTHSC	2	c	1	-	-	-	-	5.55	(2.97- 10.37)
SADOWS	31	m	1	-	-	-	-	3.63	(1.81- 7.30)
SANKAR	2	m	3	-	-	-	-	13.62	(9.00- 20.62)
SCHWAR	1	m	0	2648	1019	119	376	8.21	(6.60- 10.22)
SCHWAR	2	m	0	863	275	50	104	6.53	(4.54- 9.39)
SCHWAR	3	f	0	1351	637	182	855	9.96	(8.28- 12.00)
SCHWAR	4	f	0	335	179	40	247	11.56	(7.90- 16.90)
Subtotal SCHWAR								9.05	(7.99- 10.25)
SEGI	1	m	0	140	1742	18	382	1.71	(1.03- 2.82)
SEGI2	20	m	1	-	-	-	-	3.74	(1.75- 8.00)
SEGI2	28	f	1	-	-	-	-	1.65	(0.90- 3.02)
Subtotal SEGI2								2.27	(1.41- 3.64)
SEOW	6	f	1	-	-	-	-	5.25	(2.80- 9.84)
SHAW	12	c	0	324	266	11	107	11.85	(6.24- 22.50)
SIEMIA	5	m	7	-	-	-	-	12.10	(6.60- 22.30)
SIMARA	3	m	6	-	-	-	-	1.65	(0.97- 2.81)
SIMARA	4	f	6	-	-	-	-	1.63	(0.87- 3.06)
Subtotal SIMARA								1.64	(1.09- 2.46)
SOBUE	105	m	1	-	-	-	-	3.72	(2.57- 5.38)
SOBUE	115	f	1	-	-	-	-	2.51	(1.89- 3.33)
Subtotal SOBUE								2.90	(2.32- 3.64)
SOBUE2	10	m	2	-	-	-	-	4.47	(3.89- 5.14)
SOBUE2	12	f	2	-	-	-	-	3.28	(2.79- 3.87)
Subtotal SOBUE2								3.92	(3.53- 4.36)

Table 1C1 - 2

IESLC - Meta-anal of Ever Smoking (or Current if Ever not available), Any prod (or Cigs if Any not avail)
All LC types
Most adjusted

REF	NRR	SEX	AD	Number Exposed Case	Number Exposed Cont	Non-exposed Case	Non-exposed Cont	RR	95.00%CI
*SPEIZE	8	f	0	535	1012074	58	776300	7.08	(5.40- 9.28)
SPITZ	3	c	0	170	169	7	128	18.39	(8.35- 40.53)
STASZE	1	m	0	255	754	5	158	10.69	(4.34- 26.33)
STASZE	5	f	0	6	153	15	1660	4.34	(1.66- 11.35)
Subtotal STASZE								7.01	(3.63- 13.53)
STAYNE	1	m	0	362	567	58	333	3.67	(2.69- 4.99)
STOCKS	47	m	2	-	-	-	-	5.95	(4.23- 8.36)
STOCKS	50	f	1	-	-	-	-	3.04	(2.35- 3.93)
Subtotal STOCKS								3.88	(3.16- 4.76)
STOCKW	6	c	0	19370	7069	2791	10641	10.45	(9.94- 10.98)
STUCKE	3	m	0	247	203	0	51	125.27~	(7.68-2042.38)
SUN	1	c	0	140	173	67	191	2.31	(1.62- 3.30)
SUZUK2	20	c	3	-	-	-	-	14.02	(4.36- 45.04)
SVENSS	71	f	1	-	-	-	-	6.18	(3.79- 10.07)
TANG	3	c	0	110	59	9	39	8.08	(3.66- 17.82)
*TENKAN	22	m	1	-	-	-	-	14.64	(6.29- 34.07)
TIZZAN	1	m	0	1036	911	180	305	1.93	(1.57- 2.36)
TIZZAN	12	f	0	25	28	25	114	4.07	(2.04- 8.13)
Subtotal TIZZAN								2.05	(1.68- 2.49)
TOKARS	6	c	3	-	-	-	-	6.60	(3.20- 13.70)
TOUSEY	21	m	3	-	-	-	-	19.63	(7.18- 53.68)
TOUSEY	26	f	0	193	214	13	226	15.68	(8.67- 28.34)
Subtotal TOUSEY								16.61	(9.97- 27.67)
TSUGAN	27	m	0	73	71	18	22	1.26	(0.62- 2.54)
*TULINI	38	m	3	-	-	-	-	7.71	(4.19- 14.18)
*TULINI	44	f	3	-	-	-	-	13.01	(7.24- 23.40)
Subtotal TULINI								10.12	(6.63- 15.44)
*TVERDA	22	m	2	-	-	-	-	4.58	(2.97- 7.06)
*TVERDA	15	f	2	-	-	-	-	11.05	(3.33- 36.71)
Subtotal TVERDA								5.07	(3.37- 7.62)
WAKAI	72	m	2	-	-	-	-	3.67	(1.84- 7.32)
WAKAI	78	f	2	-	-	-	-	4.49	(2.35- 8.59)
Subtotal WAKAI								4.09	(2.55- 6.55)
*WALD	4	m	1	-	-	-	-	16.40	(7.55- 44.20)
WANG	5	c	6	-	-	-	-	2.88	(1.74- 4.77)
WANG2	16	c	4	-	-	-	-	2.29	(1.12- 4.70)
WANG3	1	c	0	235	172	58	121	2.85	(1.97- 4.13)
WANG4	2	m	2	-	-	-	-	1.16	(0.96- 1.42)
WICKLU	1	m	0	-	-	-	-	4.60	(2.80- 7.60)
WIGLE	27	m	1	-	-	-	-	8.80	(5.15- 15.02)
WIGLE	32	f	1	-	-	-	-	4.40	(2.88- 6.73)
Subtotal WIGLE								5.75	(4.12- 8.02)
WILKIN	3	c	4	-	-	-	-	7.83	(4.45- 13.78)
WU	45	f	2	-	-	-	-	3.03	(1.81- 5.07)
WUNSCH	4	m	1	-	-	-	-	4.75	(2.66- 8.50)
WUNSCH	10	f	1	-	-	-	-	4.43	(2.62- 7.47)
Subtotal WUNSCH								4.57	(3.10- 6.74)
WUWILL	8	f	3	-	-	-	-	2.30	(1.90- 2.80)
WYNDE2	21	m	0	396	616	8	105	8.44	(4.07- 17.51)
WYNDE3	49	m	0	275	332	9	88	8.10	(4.00- 16.38)
WYNDE3	138	f	0	46	56	20	76	3.12	(1.67- 5.85)
Subtotal WYNDE3								4.76	(2.98- 7.61)
WYNDE4	48	m	0	632	665	12	115	9.11	(4.98- 16.67)
WYNDE4	62	f	2	-	-	-	-	2.87	(1.48- 5.55)
Subtotal WYNDE4								5.38	(3.45- 8.41)
WYNDE6	72	m	0	2823	1996	87	617	10.03	(7.96- 12.65)
WYNDE6	252	f	0	1354	701	159	856	10.40	(8.58- 12.60)
Subtotal WYNDE6								10.25	(8.84- 11.88)
*XIANGZ	13	m	2	-	-	-	-	2.16	(1.46- 3.18)
XU	2	m	2	-	-	-	-	2.70	(2.10- 3.50)
XU2	2	c	7	-	-	-	-	3.80	(2.84- 5.07)
XU3	2	m	1	-	-	-	-	5.99	(2.65- 13.50)
XU3	4	f	1	-	-	-	-	3.86	(1.39- 10.70)
Subtotal XU3								5.05	(2.67- 9.54)
XU4	1	c	0	161	113	45	93	2.94	(1.92- 4.52)
YAMAGU	11	c	1	-	-	-	-	3.97	(2.12- 7.40)
*YONG	12	m	1	-	-	-	-	28.71	(6.98- 118.16)
*YONG	15	f	1	-	-	-	-	5.20	(2.38- 11.35)
Subtotal YONG								7.75	(3.91- 15.36)
*YUAN	1	m	2	-	-	-	-	6.50	(3.64- 11.60)
ZHANG	2	m	7	-	-	-	-	4.00	(1.61- 9.91)

Table 1C1 - 2

IESLC - Meta-anal of Ever Smoking (or Current if Ever not available), Any prod (or Cigs if Any not avail)
All LC types
Most adjusted

REF	NRR	SEX	AD	Number Exposed Case	Number Exposed Cont	Non-exposed Case	Non-exposed Cont	RR	95.00%CI
ZHANG	3	f	7	-	-	-	-	3.75	(1.80- 10.76)
Subtotal ZHANG								3.87	(2.05- 7.32)
ZHENG	15	m	0	279	218	33	94	3.65	(2.36- 5.63)
ZHENG	24	f	0	76	44	152	184	2.09	(1.36- 3.21)
Subtotal ZHENG								2.75	(2.03- 3.73)
ZHOU	2	m	0	740	41	275	36	2.36	(1.48- 3.77)
ZHOU	3	f	0	112	7	231	32	2.22	(0.95- 5.18)
Subtotal ZHOU								2.33	(1.54- 3.51)
Partial Totals				80688	1384900	9242	1081588		

*prospective study ~ With 0.5 adjustment for zero

REF	NRR	SEX	AD	Ys	Ws	Qs	Ps
ABELIN	44	m	1	3.57	1.93	8.64	0.0000
*ABRAHA	7	m	0	2.17	9.68	5.02	0.0000
*ABRAHA	8	f	0	1.59	19.39	0.37	0.0000
Subtotal ABRAHA				1.78	29.07	5.39	
AGUDO	1	f	3	1.13	6.32	0.63	0.0044
*AKIBA	11	m	5	1.56	20.22	0.25	0.0000
*AKIBA	15	f	5	1.15	46.54	4.12	0.0000
Subtotal AKIBA				1.27	66.77	4.36	
ALDERS	69	m	1	2.27	13.04	8.75	0.0000
ALDERS	6	f	1	1.56	45.44	0.55	0.0000
Subtotal ALDERS				1.72	58.48	9.30	
*AMANDU	7	m	2	1.77	4.23	0.45	0.0003
AMES	4	m	0	1.59	11.09	0.22	0.0000
*ANDERS	3	f	0	2.57	39.86	50.43	0.0000
*ARCHER	6	m	0	1.85	5.76	0.91	0.0000
ARMADA	29	m	0	2.98	3.67	8.60	0.0000
AUSTIN	7	c	3	2.41	3.59	3.31	0.0000
AUVINE	19	c	2	2.63	12.22	17.00	0.0000
AXELSO	1	c	0	1.83	24.06	3.59	0.0000
AXELSS	8	m	6	2.08	12.60	5.06	0.0000
AXELSS	11	f	0	2.16	12.45	6.24	0.0000
Subtotal AXELSS				2.12	25.05	11.30	
BAND	1	m	2	2.30	42.76	30.93	0.0000
BARBON	131	m	3	2.41	18.10	16.74	0.0000
BECHER	1	m	0	2.38	2.75	2.40	0.0001
BECHER	24	f	2	2.47	2.25	2.35	0.0002
Subtotal BECHER				2.42	5.01	4.75	
*BENSHL	18	m	1	1.77	5.82	0.62	0.0000
*BEST	22	m	0	3.24	1.00	3.19	0.0012
*BEST	18	f	1	0.81	2.17	0.89	0.2348
Subtotal BEST				1.57	3.17	4.08	
BLOHMK	3	m	0	1.13	70.05	7.01	0.0000
BLOT4	1	m	0	2.68	6.96	10.48	0.0000
BOFFET	33	m	2	2.65	103.49	150.33	0.0000
*BOUCOT	121	m	2	3.71	0.50	2.55	0.0088
BRESLO	37	m	0	1.87	5.85	1.02	0.0000
BRESLO	38	f	0	0.32	3.10	3.94	0.5717
Subtotal BRESLO				1.33	8.95	4.96	
*BRETT	10	m	0	1.18	5.77	0.40	0.0044
BROCKM	1	m	0	0.07	0.98	1.85	0.9437
BROCKM	2	f	0	0.69	2.73	1.56	0.2517
Subtotal BROCKM				0.53	3.71	3.41	
BROSS	12	m	0	1.64	28.92	1.05	0.0000
BROWN2	2	m	2	2.21	442.58	255.81	0.0000
BROWN2	1	f	2	2.54	427.71	511.50	0.0000
Subtotal BROWN2				2.37	870.29	767.31	
BUFFLE	1	m	0	2.36	4.43	3.65	0.0000
BUFFLE	5	f	0	1.96	28.29	7.52	0.0000
Subtotal BUFFLE				2.02	32.72	11.17	
CARPEN	12	c	3	2.70	12.04	18.87	0.0000
CASCO2	1	c	0	2.44	5.31	5.23	0.0000
CASCOR	1	c	0	2.60	18.40	24.60	0.0000
*CEDERL	107	m	2	1.78	20.66	2.25	0.0000
*CEDERL	112	f	2	1.43	31.21	0.01	0.0000
Subtotal CEDERL				1.57	51.88	2.26	
CHAN	9	m	0	3.31	1.87	6.52	0.0000
CHAN	10	f	0	1.25	20.57	0.84	0.0000
Subtotal CHAN				1.42	22.44	7.36	

Table 1C1 - 2

IESLC - Meta-anal of Ever Smoking (or Current if Ever not available), Any prod (or Cigs if Any not avail)
All LC types
Most adjusted

REF	NRR	SEX	AD	Ys	Ws	Qs	Ps
*CHANG	6	m	0	1.65	4.76	0.19	0.0003
*CHANG	12	f	0	1.30	8.85	0.19	0.0001
Subtotal CHANG				1.42	13.61	0.38	
CHATZI	4	c	0	1.21	19.44	1.14	0.0000
CHEN2	1	m	0	1.52	6.25	0.03	0.0001
CHEN2	2	f	0	0.51	7.70	6.71	0.1539
Subtotal CHEN2				0.97	13.95	6.75	
CHEN3	1	c	0	0.46	27.78	26.98	0.0148
CHIAZZ	3	m	11	3.26	0.90	2.97	0.0019
CHOI	1	m	0	1.43	10.71	0.00	0.0000
CHOI	5	f	0	0.46	9.06	8.93	0.1703
Subtotal CHOI				0.99	19.78	8.93	
*CHOW	55	m	2	2.41	5.68	5.21	0.0000
*CHYOU	7	m	1	2.12	12.17	5.53	0.0000
COMSTO	34	m	0	2.38	3.63	3.16	0.0000
COMSTO	46	f	0	2.19	9.22	5.09	0.0000
Subtotal COMSTO				2.25	12.85	8.26	
COOKSO	5	c	0	1.88	14.38	2.71	0.0000
CORREA	34	c	1	2.43	51.78	50.30	0.0000
*CPSI	187	m	1	2.22	78.68	46.53	0.0000
*CPSI	274	f	1	1.03	73.22	13.04	0.0000
Subtotal CPSI				1.64	151.90	59.57	
*CPSII	104	m	1	2.55	78.29	95.38	0.0000
*CPSII	79	f	1	2.10	142.84	60.58	0.0000
Subtotal CPSII				2.26	221.13	155.96	
DAMBER	25	m	1	1.97	29.41	7.92	0.0000
DARBY	15	m	0	3.90	2.96	17.76	0.0000
DARBY	16	f	0	2.51	19.76	22.11	0.0000
Subtotal DARBY				2.69	22.71	39.87	
DAVEYS	5	m	0	1.57	2.53	0.04	0.0126
DAVEYS	6	f	0	-0.32	0.42	1.33	0.8327
Subtotal DAVEYS				1.30	2.96	1.37	
DEAN	7	m	0	1.66	9.69	0.42	0.0000
DEAN2	3	m	0	1.33	23.82	0.31	0.0000
DEAN2	7	f	0	1.08	14.58	2.02	0.0000
Subtotal DEAN2				1.24	38.40	2.33	
DEAN3	49	m	3	1.81	19.11	2.57	0.0000
DEAN3	126	f	3	1.53	21.26	0.15	0.0000
Subtotal DEAN3				1.67	40.37	2.72	
*DEKLER	6	m	2	3.01	0.99	2.42	0.0027
DESTE2	14	c	7	2.16	13.37	6.84	0.0000
DESTEF	48	m	4	2.21	19.77	11.46	0.0000
*DOCKER	3	c	4	1.46	4.27	0.00	0.0026
DOLL	6	m	0	2.21	6.22	3.57	0.0000
DOLL	12	f	0	0.72	12.98	6.95	0.0099
Subtotal DOLL				1.20	19.20	10.52	
*DOLL2	56	m	1	2.04	18.57	6.42	0.0000
*DOLL2	63	f	1	2.16	3.28	1.65	0.0001
Subtotal DOLL2				2.05	21.84	8.07	
DORANT	10	c	0	2.89	13.34	27.75	0.0000
DORGAN	6	m	0	2.28	12.35	8.65	0.0000
DORGAN	30	m	0	3.13	2.68	7.63	0.0000
DORGAN	53	f	0	2.06	51.30	19.09	0.0000
DORGAN	76	f	0	2.12	4.12	1.88	0.0000
Subtotal DORGAN				2.14	70.44	37.25	
*DORN	196	m	1	1.95	73.73	18.70	0.0000
DOSEME	1	m	2	1.19	55.52	3.58	0.0000
DROSTE	7	m	4	2.15	5.72	2.85	0.0000
DU	1	m	0	1.26	28.12	0.98	0.0000
DU	2	f	0	0.66	24.50	15.31	0.0011
Subtotal DU				0.98	52.62	16.29	
*DUNN	6	m	0	2.91	1.97	4.24	0.0000
EBELIN	1	m	0	1.94	9.19	2.19	0.0000
*ENGELA	159	m	1	1.85	6.58	1.05	0.0000
*ENGELA	165	f	1	1.56	6.51	0.08	0.0001
Subtotal ENGELA				1.70	13.09	1.12	
*ENSTRO	1	m	1	2.56	81.91	102.04	0.0000
*ENSTRO	2	f	1	1.94	181.45	43.69	0.0000
Subtotal ENSTRO				2.13	263.36	145.74	
ESAKI	4	m	0	0.64	8.96	5.84	0.0554
ESAKI	5	f	0	0.90	7.99	2.40	0.0109

Table 1C1 - 2

IESLC - Meta-anal of Ever Smoking (or Current if Ever not available), Any prod (or Cigs if Any not avail)
All LC types
Most adjusted

REF	NRR	SEX	AD	Ys	Ws	Qs	Ps
Subtotal ESAKI				0.76	16.94	8.24	
FAN	1	m	0	1.04	25.87	4.20	0.0000
FAN	2	f	0	1.37	24.92	0.17	0.0000
Subtotal FAN				1.20	50.80	4.37	
GAO	1	m	2	1.36	39.76	0.30	0.0000
GAO	11	f	2	1.19	57.09	3.69	0.0000
Subtotal GAO				1.26	96.85	3.99	
GAO2	10	m	1	1.64	9.74	0.37	0.0000
GARCIA	3	c	0	2.14	16.50	7.95	0.0000
GARDIN	7	c	0	2.41	4.14	3.80	0.0000
GARSHI	25	m	1	1.76	34.86	3.38	0.0000
GENG	1	m	0	1.79	4.98	0.58	0.0001
GENG	2	f	0	1.08	22.39	2.95	0.0000
Subtotal GENG				1.21	27.37	3.54	
GER	21	c	14	0.61	12.59	8.84	0.0305
GODLEY	5	m	1	1.92	96.28	21.70	0.0000
GODLEY	6	f	1	1.71	58.89	4.10	0.0000
Subtotal GODLEY				1.84	155.17	25.81	
GOLLED	7	m	1	2.02	13.89	4.49	0.0000
GOODMA	3	m	0	2.38	8.92	7.74	0.0000
GOODMA	7	f	0	2.12	12.25	5.46	0.0000
Subtotal GOODMA				2.23	21.17	13.19	
GRAHAM	27	m	1	1.95	17.52	4.37	0.0000
GREGOR	3	m	0	0.03	5.11	10.31	0.9492
GREGOR	7	f	0	2.40	0.90	0.81	0.0233
Subtotal GREGOR				0.38	6.01	11.12	
GSELL	8	m	0	2.88	1.82	3.71	0.0001
HAENSZ	11	f	2	0.78	25.03	11.04	0.0001
*HAMMO2	4	m	1	3.13	1.00	2.81	0.0018
*HAMMON	117	m	1	1.92	14.49	3.19	0.0000
*HANSEN	3	m	2	0.43	5.28	5.52	0.3285
HEGMAN	1	c	0	2.79	23.66	42.85	0.0000
*HEIN	7	m	0	2.68	1.00	1.52	0.0074
*HENNEK	3	m	0	1.83	19.94	2.92	0.0000
HINDS	22	f	3	1.73	39.57	3.18	0.0000
*HIRAYA	147	m	1	1.47	85.78	0.05	0.0000
*HIRAYA	150	f	1	0.86	80.63	28.00	0.0000
Subtotal HIRAYA				1.18	166.40	28.06	
HITOSU	38	m	1	1.07	6.36	0.92	0.0071
HITOSU	62	f	1	1.22	15.00	0.75	0.0000
Subtotal HITOSU				1.18	21.36	1.67	
*HOLE	8	m	1	1.86	6.76	1.16	0.0000
*HOLE	31	f	1	0.43	4.99	5.22	0.3421
Subtotal HOLE				1.25	11.75	6.38	
HOROWI	1	m	0	1.27	15.35	0.46	0.0000
HOROWI	2	f	0	0.60	8.08	5.84	0.0894
Subtotal HOROWI				1.04	23.43	6.30	
HORWIT	1	f	0	2.43	8.29	7.93	0.0000
HU	15	m	0	0.74	17.16	8.71	0.0023
HU	16	f	0	0.55	7.15	5.77	0.1413
Subtotal HU				0.68	24.31	14.48	
HU2	9	m	0	1.11	27.11	3.15	0.0000
HU2	10	f	0	0.63	21.91	14.71	0.0033
Subtotal HU2				0.89	49.01	17.85	
HUANG	1	c	0	0.69	14.82	8.50	0.0078
HUMBLE	14	m	1	2.49	5.19	5.67	0.0000
HUMBLE	16	m	1	2.47	1.71	1.80	0.0012
HUMBLE	18	f	1	2.43	6.68	6.45	0.0000
HUMBLE	20	f	1	2.73	2.90	4.79	0.0000
Subtotal HUMBLE				2.51	16.48	18.71	
JAHN	22	f	2	1.19	14.92	0.96	0.0000
JAIN	46	m	2	2.12	8.79	3.92	0.0000
JAIN	41	f	2	2.22	17.72	10.54	0.0000
Subtotal JAIN				2.19	26.50	14.46	
JARUP	6	m	2	2.02	3.91	1.28	0.0001
JARVHO	3	m	0	3.32	0.92	3.23	0.0015
JARVHO	7	f	0	2.26	3.28	2.15	0.0000
Subtotal JARVHO				2.49	4.19	5.38	
JEDRYC	58	m	4	1.70	31.63	1.97	0.0000
JEDRYC	59	f	4	1.51	11.71	0.05	0.0000
Subtotal JEDRYC				1.65	43.33	2.02	

Table 1C1 - 2

IESLC - Meta-anal of Ever Smoking (or Current if Ever not available), Any prod (or Cigs if Any not avail)
All LC types
Most adjusted

REF	NRR	SEX	AD	Ys	Ws	Qs	Ps
JIANG	1	m	0	1.00	4.45	0.89	0.0346
JIANG	2	f	0	0.91	2.62	0.75	0.1401
Subtotal JIANG				0.97	7.08	1.64	
JOLY	14	m	0	2.50	11.02	12.18	0.0000
JOLY	1	f	0	1.99	27.09	8.07	0.0000
Subtotal JOLY				2.14	38.11	20.25	
JUSSAW	29	m	2	2.82	25.79	48.76	0.0000
*KAISE2	72	m	1	1.69	11.75	0.67	0.0000
*KAISE2	64	f	1	2.31	9.19	6.86	0.0000
Subtotal KAISE2				1.96	20.95	7.52	
*KAISER	13	m	2	2.87	25.69	51.93	0.0000
*KAISER	10	f	2	1.73	28.18	2.21	0.0000
Subtotal KAISER				2.27	53.88	54.14	
KANELL	30	m	1	1.60	30.83	0.69	0.0000
KATSOU	29	f	1	1.19	9.91	0.64	0.0002
KAUFMA	17	c	6	2.52	28.72	32.77	0.0000
KELLER	3	m	0	2.31	217.37	162.09	0.0000
KELLER	11	m	0	2.60	25.99	34.74	0.0000
KELLER	7	f	0	2.53	269.69	312.98	0.0000
KELLER	15	f	0	2.25	39.03	24.85	0.0000
Subtotal KELLER				2.43	552.08	534.66	
KHUDER	4	m	0	2.06	19.93	7.50	0.0000
KIHARA	31	c	0	1.22	46.97	2.45	0.0000
*KINLEN	17	m	2	2.40	7.00	6.30	0.0000
KJUUS	10	m	0	2.62	1.81	2.48	0.0004
*KNEKT	87	m	1	1.86	5.67	0.96	0.0000
KO	1	f	3	1.44	2.18	0.00	0.0339
KOHLME	2	c	4	2.80	5.21	9.49	0.0000
KOO	1	f	0	1.02	23.01	4.25	0.0000
KOULUM	1	m	0	3.57	4.47	20.07	0.0000
KREUZE	14	f	0	2.24	4.29	2.66	0.0000
KREUZE	16	f	0	1.33	32.30	0.45	0.0000
Subtotal KREUZE				1.44	36.60	3.11	
KREYBE	12	m	1	1.89	5.80	1.13	0.0000
KREYBE	30	f	1	0.36	7.92	9.41	0.3143
Subtotal KREYBE				1.01	13.71	10.54	
*KUBIK	28	m	0	3.34	1.96	7.00	0.0000
LAMTH	6	f	0	1.34	46.55	0.57	0.0000
LAMWK	1	f	0	1.42	17.85	0.02	0.0000
LAMWK2	9	m	0	1.04	12.98	2.15	0.0002
LAMWK2	10	f	0	1.17	17.89	1.43	0.0000
Subtotal LAMWK2				1.11	30.86	3.57	
*LANGE	40	m	1	1.56	3.97	0.05	0.0019
*LANGE	37	f	1	1.60	8.13	0.18	0.0000
Subtotal LANGE				1.58	12.09	0.22	
LAUSSM	11	m	3	1.74	37.15	3.18	0.0000
LEI	1	m	0	1.30	26.63	0.56	0.0000
LEI	2	f	0	1.25	23.21	0.92	0.0000
Subtotal LEI				1.28	49.84	1.48	
LEMARC	3	c	0	1.73	22.77	1.79	0.0000
LETOUR	1	c	0	2.56	20.21	25.09	0.0000
LEVIN	32	m	1	1.58	30.68	0.54	0.0000
*LIAW	1	m	1	1.31	11.72	0.23	0.0000
*LIAW	2	f	1	1.28	2.46	0.07	0.0447
Subtotal LIAW				1.30	14.17	0.30	
*LIDDEL	5	m	1	1.28	17.92	0.48	0.0000
LIU	2	c	2	0.65	38.19	24.18	0.0001
LIU2	2	m	3	1.65	4.37	0.17	0.0006
LIU2	4	f	3	1.54	6.68	0.05	0.0001
Subtotal LIU2				1.58	11.05	0.23	
LIU3	2	m	2	0.23	1.87	2.77	0.7518
LIU4	11	m	2	1.02	5969.41	1118.11	0.0000
LIU4	12	f	2	1.05	3876.64	611.60	0.0000
Subtotal LIU4				1.03	9846.05	1729.71	
LIU5	1	c	0	0.65	11.25	7.17	0.0293
LOMBA2	1	f	0	0.28	37.19	50.46	0.0841
LOMBAR	12	m	0	2.18	12.13	6.50	0.0000
LUBIN2	46	m	2	2.14	162.99	78.06	0.0000
LUBIN2	102	f	1	1.36	133.31	1.01	0.0000
Subtotal LUBIN2				1.79	296.30	79.07	
LUO	7	c	20	0.99	10.60	2.19	0.0012

Table 1C1 - 2

IESLC - Meta-anal of Ever Smoking (or Current if Ever not available), Any prod (or Cigs if Any not avail)
All LC types
Most adjusted

REF	NRR	SEX	AD	Ys	Ws	Qs	Ps
MACLEN	73	c	2	0.98	17.04	3.70	0.0001
*MAGNUS	5	m	3	1.42	6.75	0.01	0.0002
MARSH	7	c	2	1.92	7.36	1.62	0.0000
MARSH2	5	m	1	0.64	3.87	2.55	0.2107
MARSH2	6	f	1	1.66	3.65	0.17	0.0015
Subtotal MARSH2				1.14	7.51	2.72	
MARTIS	4	m	0	1.95	3.32	0.82	0.0004
MASTRA	2	m	2	2.10	4.76	2.01	0.0000
MATOS	27	m	2	1.92	8.82	1.94	0.0000
MATSUD	10	m	0	3.07	2.94	7.69	0.0000
MCCONN	1	m	0	0.19	3.33	5.24	0.7237
MCCONN	2	f	0	1.01	0.99	0.19	0.3136
Subtotal MCCONN				0.38	4.32	5.43	
MCDUFF	1	m	0	1.81	4.70	0.63	0.0001
MCLAUG	1	m	0	1.20	18.70	1.11	0.0000
*MIGRAN	27	m	2	1.28	3.91	0.11	0.0111
*MIGRAN	42	f	2	1.53	3.54	0.02	0.0040
Subtotal MIGRAN				1.40	7.45	0.13	
MILLER	2	f	1	1.61	4.90	0.12	0.0004
MILLS	3	m	1	0.29	96.62	129.90	0.0046
*MRFITR	6	m	0	3.70	0.50	2.52	0.0091
NAM	77	m	1	2.16	24.64	12.65	0.0000
NAM	93	f	1	2.18	34.31	18.57	0.0000
Subtotal NAM				2.18	58.95	31.22	
NOTAN2	15	m	2	1.10	80.00	9.95	0.0000
NOU	11	m	0	1.81	5.20	0.67	0.0000
NOU	12	f	0	1.96	2.74	0.72	0.0012
Subtotal NOU				1.86	7.94	1.39	
ODRISC	3	c	0	3.89	5.83	34.77	0.0000
ORMOS	4	m	0	2.23	6.39	3.95	0.0000
ORMOS	26	f	0	-1.64	0.95	9.09	0.1093
Subtotal ORMOS				1.73	7.34	13.04	
OSANN	41	m	2	2.98	39.82	93.54	0.0000
OSANN	42	f	2	2.71	66.25	105.19	0.0000
Subtotal OSANN				2.81	106.08	198.73	
PARKIN	28	m	6	1.39	63.58	0.19	0.0000
PASTOR	10	m	1	1.92	7.85	1.74	0.0000
PAWLEG	2	m	6	2.51	3.16	3.54	0.0000
PERNU	2	m	0	2.19	58.99	32.45	0.0000
PERNU	1	f	0	0.63	13.52	8.96	0.0198
Subtotal PERNU				1.90	72.50	41.41	
PERSH2	11	c	4	1.88	115.76	21.55	0.0000
*PETO	5	m	0	1.82	1.98	0.27	0.0107
PEZZO2	10	m	0	2.71	5.55	8.82	0.0000
PEZZOT	25	m	0	2.96	3.75	8.58	0.0000
PIKE	4	m	0	1.66	13.39	0.60	0.0000
PIKE	8	f	0	1.57	18.04	0.29	0.0000
Subtotal PIKE				1.61	31.43	0.89	
POFFIJ	1	c	0	2.05	46.21	16.62	0.0000
POLEDN	1	c	1	2.22	11.85	7.13	0.0000
*QIAO2	15	m	1	0.43	9.50	9.93	0.1900
RACHTA	15	f	4	2.11	7.21	3.12	0.0000
RADZIK	1	c	0	0.27	5.03	6.96	0.5411
RANDIG	23	m	0	1.61	3.99	0.11	0.0013
RANDIG	24	f	0	0.80	6.34	2.68	0.0447
Subtotal RANDIG				1.11	10.32	2.79	
REN	1	m	0	1.27	7.46	0.23	0.0005
REN	2	f	0	1.40	9.65	0.02	0.0000
Subtotal REN				1.35	17.11	0.25	
RONCO	1	m	0	1.63	5.23	0.17	0.0002
ROTHSC	2	c	1	1.71	9.83	0.69	0.0000
SADOWS	31	m	1	1.29	7.90	0.20	0.0003
SANKAR	2	m	3	2.61	22.36	30.27	0.0000
SCHWAR	1	m	0	2.11	80.50	34.79	0.0000
SCHWAR	2	m	0	1.88	29.06	5.32	0.0000
SCHWAR	3	f	0	2.30	111.43	80.68	0.0000
SCHWAR	4	f	0	2.45	26.58	26.54	0.0000
Subtotal SCHWAR				2.20	247.58	147.34	
SEGI	1	m	0	0.53	15.18	12.68	0.0375
SEGI2	20	m	1	1.32	6.65	0.11	0.0007
SEGI2	28	f	1	0.50	10.48	9.41	0.1049

Table 1C1 - 2

IESLC - Meta-anal of Ever Smoking (or Current if Ever not available), Any prod (or Cigs if Any not avail)
All LC types
Most adjusted

REF	NRR	SEX	AD	Ys	Ws	Qs	Ps
Subtotal SEGI2				0.82	17.14	9.52	
SEOW	6	f	1	1.66	9.73	0.43	0.0000
SHAW	12	c	0	2.47	9.34	9.79	0.0000
SIEMIA	5	m	7	2.49	10.37	11.32	0.0000
SIMARA	3	m	6	0.50	13.58	12.19	0.0650
SIMARA	4	f	6	0.49	9.71	8.94	0.1278
Subtotal SIMARA				0.50	23.30	21.13	
SOBUE	105	m	1	1.31	28.15	0.51	0.0000
SOBUE	115	f	1	0.92	47.90	13.34	0.0000
Subtotal SOBUE				1.07	76.05	13.85	
SOBUE2	10	m	2	1.50	197.90	0.48	0.0000
SOBUE2	12	f	2	1.19	143.51	9.71	0.0000
Subtotal SOBUE2				1.37	341.42	10.20	
*SPEIZE	8	f	0	1.96	52.33	13.54	0.0000
SPITZ	3	c	0	2.91	6.16	13.19	0.0000
STASZE	1	m	0	2.37	4.73	4.01	0.0000
STASZE	5	f	0	1.47	4.16	0.00	0.0028
Subtotal STASZE				1.95	8.88	4.01	
STAYNE	1	m	0	1.30	40.37	0.90	0.0000
STOCKS	47	m	2	1.78	33.11	3.72	0.0000
STOCKS	50	f	1	1.11	58.11	6.57	0.0000
Subtotal STOCKS				1.36	91.22	10.29	
STOCKW	6	c	0	2.35	1549.52	1250.38	0.0000
STUCKE	3	m	0	4.83	0.49	5.64	0.0007
SUN	1	c	0	0.84	30.23	11.32	0.0000
SUZUK2	20	c	3	2.64	2.82	4.01	0.0000
SVENSS	71	f	1	1.82	16.09	2.24	0.0000
TANG	3	c	0	2.09	6.14	2.53	0.0000
*TENKAN	22	m	1	2.68	5.38	8.22	0.0000
TIZZAN	1	m	0	0.66	91.77	57.57	0.0000
TIZZAN	12	f	0	1.40	8.03	0.02	0.0001
Subtotal TIZZAN				0.72	99.80	57.59	
TOKARS	6	c	3	1.89	7.27	1.40	0.0000
TOUSEY	21	m	3	2.98	3.80	8.88	0.0000
TOUSEY	26	f	0	2.75	10.96	18.65	0.0000
Subtotal TOUSEY				2.81	14.76	27.53	
TSUGAN	27	m	0	0.23	7.76	11.55	0.5244
*TULINI	38	m	3	2.04	10.34	3.65	0.0000
*TULINI	44	f	3	2.57	11.17	13.95	0.0000
Subtotal TULINI				2.31	21.50	17.60	
*TVERDA	22	m	2	1.52	20.49	0.11	0.0000
*TVERDA	15	f	2	2.40	2.67	2.43	0.0001
Subtotal TVERDA				1.62	23.16	2.54	
WAKAI	72	m	2	1.30	8.06	0.18	0.0002
WAKAI	78	f	2	1.50	9.15	0.03	0.0000
Subtotal WAKAI				1.41	17.20	0.20	
*WALD	4	m	1	2.80	4.92	8.96	0.0000
WANG	5	c	6	1.06	15.11	2.30	0.0000
WANG2	16	c	4	0.83	7.47	2.87	0.0235
WANG3	1	c	0	1.05	28.11	4.51	0.0000
WANG4	2	m	2	0.15	100.26	169.34	0.1372
WICKLU	1	m	0	1.53	15.41	0.09	0.0000
WIGLE	27	m	1	2.17	13.41	7.08	0.0000
WIGLE	32	f	1	1.48	21.33	0.02	0.0000
Subtotal WIGLE				1.75	34.74	7.11	
WILKIN	3	c	4	2.06	12.03	4.47	0.0000
WU	45	f	2	1.11	14.48	1.67	0.0000
WUNSCH	4	m	1	1.56	11.39	0.14	0.0000
WUNSCH	10	f	1	1.49	14.00	0.02	0.0000
Subtotal WUNSCH				1.52	25.38	0.16	
WUWILL	8	f	3	0.83	102.19	38.67	0.0000
WYNDE2	21	m	0	2.13	7.21	3.38	0.0000
WYNDE3	49	m	0	2.09	7.74	3.21	0.0000
WYNDE3	138	f	0	1.14	9.73	0.93	0.0004
Subtotal WYNDE3				1.56	17.48	4.14	
WYNDE4	48	m	0	2.21	10.51	6.09	0.0000
WYNDE4	62	f	2	1.05	8.80	1.36	0.0018
Subtotal WYNDE4				1.68	19.31	7.45	
WYNDE6	72	m	0	2.31	71.58	52.65	0.0000
WYNDE6	252	f	0	2.34	103.92	82.99	0.0000
Subtotal WYNDE6				2.33	175.50	135.64	

Table 1C1 - 2

IESLC - Meta-anal of Ever Smoking (or Current if Ever not available), Any prod (or Cigs if Any not avail)
All LC types
Most adjusted

REF	NRR	SEX	AD	Ys	Ws	Qs	Ps
*XIANGZ	13	m	2	0.77	25.36	11.65	0.0001
XU	2	m	2	0.99	58.89	12.18	0.0000
XU2	2	c	7	1.34	45.75	0.58	0.0000
XU3	2	m	1	1.79	5.80	0.68	0.0000
XU3	4	f	1	1.35	3.69	0.03	0.0095
Subtotal XU3				1.62	9.49	0.71	
XU4	1	c	0	1.08	20.82	2.82	0.0000
YAMAGU	11	c	1	1.38	9.83	0.05	0.0000
*YONG	12	m	1	3.36	1.92	7.00	0.0000
*YONG	15	f	1	1.65	6.30	0.25	0.0000
Subtotal YONG				2.05	8.22	7.25	
*YUAN	1	m	2	1.87	11.44	2.05	0.0000
ZHANG	2	m	7	1.39	4.65	0.02	0.0028
ZHANG	3	f	7	1.32	4.81	0.08	0.0038
Subtotal ZHANG				1.35	9.46	0.09	
ZHENG	15	m	0	1.29	20.36	0.49	0.0000
ZHENG	24	f	0	0.74	20.88	10.54	0.0008
Subtotal ZHENG				1.01	41.24	11.02	
ZHOU	2	m	0	0.86	17.50	6.05	0.0003
ZHOU	3	f	0	0.80	5.34	2.27	0.0660
Subtotal ZHOU				0.84	22.83	8.32	

	N	342
	NS	242
	Wt	20014.39
Het	Chi	7658.52
Het	df	341
Het	P	***
Fixed	RR	4.25
	RRl	4.20
	RRu	4.31
	P	+++
Random	RR	5.48
	RRl	5.07
	RRu	5.93
	P	+++
Asymm	P	***

Table 1C1 - 3

IESLC - Meta-anal of Ever Smoking (or Current if Ever not available), Any prod (or Cigs if Any not avail)
All LC types
Most adjusted

	Sex			
	combined	male	female	Total
N	48	178	116	342
NS	48	174	111	333
Wt	2445.12	10193.04	7376.23	20014.39
Het Chi	684.99	3298.81	2430.04	7658.52
Het df	47	177	115	341
Het P	***	***	***	***
Fixed RR	8.28	3.80	3.99	4.25
RRl	7.96	3.73	3.90	4.20
RRu	8.61	3.87	4.08	4.31
P	+++	+++	+++	+++
Random RR	6.07	6.21	4.41	5.48
RRl	4.95	5.54	3.85	5.07
RRu	7.46	6.96	5.04	5.93
P	+++	+++	+++	+++
Between Chi				1244.68
Between df				2
Between P				***
Btwn(F) P				***
Btwn(R) P				***

	Lung cancer type		
	all	other	Total
N	329	13	342
NS	233	9	242
Wt	19473.83	540.55	20014.39
Het Chi	7597.95	47.68	7658.52
Het df	328	12	341
Het P	***	***	***
Fixed RR	4.27	3.65	4.25
RRl	4.21	3.36	4.20
RRu	4.33	3.97	4.31
P	+++	+++	+++
Random RR	5.59	3.53	5.48
RRl	5.16	2.88	5.07
RRu	6.06	4.32	5.93
P	+++	+++	+++
Between Chi			12.89
Between df			1
Between P			***
Btwn(F) P			N.S.
Btwn(R) P			***

	Location								
	NAmer	UK	Scand	othEur	China	Japan	othAs	other	Total
N	119	32	33	51	51	22	20	14	342
NS	82	21	23	40	35	14	15	12	242
Wt	5791.26	386.45	476.92	1062.82	10907.37	815.71	367.64	206.22	20014.39
Het Chi	1201.52	154.36	103.61	429.88	166.47	75.19	143.01	37.28	7658.52
Het df	118	31	32	50	50	21	19	13	341
Het P	***	***	***	***	***	***	***	***	***
Fixed RR	8.85	5.24	6.45	5.53	2.77	3.45	3.78	6.25	4.25
RRl	8.63	4.74	5.90	5.21	2.72	3.22	3.42	5.45	4.20
RRu	9.08	5.79	7.05	5.87	2.83	3.70	4.19	7.16	4.31
P	+++	+++	+++	+++	+++	+++	+++	+++	+++
Random RR	7.55	5.84	6.44	6.06	2.69	3.23	3.75	7.41	5.48
RRl	6.85	4.57	5.35	4.94	2.50	2.77	2.78	5.72	5.07
RRu	8.32	7.46	7.76	7.43	2.88	3.77	5.06	9.60	5.93
P	+++	+++	+++	+++	+++	+++	+++	+++	+++
Between Chi									5347.20
Between df									7
Between P									***
Btwn(F) P									***
Btwn(R) P									***

Table 1C1 - 3

IESLC - Meta-anal of Ever Smoking (or Current if Ever not available), Any prod (or Cigs if Any not avail)
All LC types
Most adjusted

	Detailed Country in "other Europe"					
	multi	Germany	othWest	East	Balkans	Total
N	4	17	13	13	4	51
NS	3	12	12	9	4	40
Wt	446.01	218.84	169.04	113.26	115.68	1062.82
Het Chi	101.78	66.23	135.12	36.35	3.63	429.88
Het df	3	16	12	12	3	50
Het P	***	***	***	***	N.S.	***
Fixed RR	7.51	4.66	3.95	5.74	3.68	5.53
RRl	6.85	4.09	3.40	4.77	3.07	5.21
RRu	8.24	5.32	4.59	6.90	4.42	5.87
P	+++	+++	+++	+++	+++	+++
Random RR	7.76	5.47	8.78	5.85	3.70	6.06
RRl	4.45	3.94	4.70	4.10	3.00	4.94
RRu	13.54	7.57	16.40	8.34	4.56	7.43
P	+++	+++	+++	+++	+++	+++
Between Chi						86.76
Between df						4
Between P						***
Btwn(F) P						*
Btwn(R) P						**

	Detailed Country in "other Asia"			
	India	HongKong	other	Total
N	3	7	10	20
NS	3	5	7	15
Wt	128.15	140.70	98.79	367.64
Het Chi	80.36	10.74	18.17	143.01
Het df	2	6	9	19
Het P	***	(*)	*	***
Fixed RR	5.52	3.52	2.57	3.78
RRl	4.64	2.99	2.11	3.42
RRu	6.56	4.15	3.13	4.19
P	+++	+++	+++	+++
Random RR	8.74	3.56	2.62	3.75
RRl	2.58	2.81	1.96	2.78
RRu	29.65	4.50	3.50	5.06
P	+++	+++	+++	+++
Between Chi				33.74
Between df				2
Between P				***
Btwn(F) P				N.S.
Btwn(R) P				(*)

	Detailed other continent			
	SCAmer	Auslia	Africa	Total
N	10	1	3	14
NS	8	1	3	12
Wt	117.57	0.99	87.66	206.22
Het Chi	16.77	0.00	3.05	37.28
Het df	9	0	2	13
Het P	(*)	N.S.	N.S.	***
Fixed RR	7.91	20.29	4.49	6.25
RRl	6.61	2.84	3.65	5.45
RRu	9.48	145.07	5.54	7.16
P	+++	++	+++	+++
Random RR	8.23	20.29	4.78	7.41
RRl	6.34	2.84	3.52	5.72
RRu	10.69	145.07	6.50	9.60
P	+++	++	+++	+++
Between Chi				17.46
Between df				2
Between P				***
Btwn(F) P				*
Btwn(R) P				*

Table 1C1 - 3

IESLC - Meta-anal of Ever Smoking (or Current if Ever not available), Any prod (or Cigs if Any not avail)
All LC types
Most adjusted

	Start year of study <1960	1960-69	1970-79	1980-89	1990+	Total
N	58	56	75	116	37	342
NS	41	40	53	76	32	242
Wt	1215.20	1648.17	1370.40	15290.44	490.19	20014.39
Het Chi	618.87	459.65	599.70	5654.65	238.00	7658.52
Het df	57	55	74	115	36	341
Het P	***	***	***	***	***	***
Fixed RR	4.70	4.83	4.57	4.11	5.28	4.25
RRl	4.45	4.60	4.34	4.04	4.83	4.20
RRu	4.98	5.07	4.82	4.17	5.77	4.31
P	+++	+++	+++	+++	+++	+++
Random RR	4.88	5.21	5.13	5.91	6.22	5.48
RRl	3.95	4.46	4.35	5.15	4.89	5.07
RRu	6.02	6.07	6.05	6.77	7.92	5.93
P	+++	+++	+++	+++	+++	+++
Between Chi						87.65
Between df						4
Between P						***
Btwn(F) P						N.S.
Btwn(R) P						N.S.

	Study type (1) CC	other	Total
N	267	75	342
NS	188	54	242
Wt	18441.29	1573.10	20014.39
Het Chi	6909.30	479.43	7658.52
Het df	266	74	341
Het P	***	***	***
Fixed RR	4.11	6.33	4.25
RRl	4.05	6.03	4.20
RRu	4.17	6.65	4.31
P	+++	+++	+++
Random RR	5.27	6.32	5.48
RRl	4.83	5.47	5.07
RRu	5.76	7.30	5.93
P	+++	+++	+++
Between Chi			269.79
Between df			1
Between P			***
Btwn(F) P			***
Btwn(R) P			*

	Study type (2) CC	prosp	other	Total
N	267	70	5	342
NS	188	50	4	242
Wt	18441.29	1528.55	44.55	20014.39
Het Chi	6909.30	461.79	11.34	7658.52
Het df	266	69	4	341
Het P	***	***	*	***
Fixed RR	4.11	6.26	9.17	4.25
RRl	4.05	5.96	6.84	4.20
RRu	4.17	6.59	12.30	4.31
P	+++	+++	+++	+++
Random RR	5.27	6.16	8.92	5.48
RRl	4.83	5.30	5.36	5.07
RRu	5.76	7.16	14.86	5.93
P	+++	+++	+++	+++
Between Chi				276.09
Between df				2
Between P				***
Btwn(F) P				**
Btwn(R) P				*

Table 1C1 - 3

IESLC - Meta-anal of Ever Smoking (or Current if Ever not available), Any prod (or Cigs if Any not avail)
All LC types
Most adjusted

	Study size (number of LC cases)				
	100-249	250-499	500-999	1000+	Total
N	121	88	66	67	342
NS	98	64	44	36	242
Wt	946.76	1278.02	1495.37	16294.24	20014.39
Het Chi	478.12	585.53	574.98	5948.73	7658.52
Het df	120	87	65	66	341
Het P	***	***	***	***	***
Fixed RR	3.73	4.80	4.93	4.19	4.25
RRl	3.50	4.54	4.69	4.13	4.20
RRu	3.98	5.07	5.19	4.25	4.31
P	+++	+++	+++	+++	+++
Random RR	4.49	5.65	6.16	6.13	5.48
RRl	3.91	4.86	5.26	5.19	5.07
RRu	5.15	6.56	7.21	7.25	5.93
P	+++	+++	+++	+++	+++
Between Chi					71.16
Between df					3
Between P					***
Btwn(F) P					N.S.
Btwn(R) P					**

	Risky occupational population			
	no	mining	othRisky	Total
N	324	7	11	342
NS	224	7	11	242
Wt	19809.57	74.85	129.97	20014.39
Het Chi	7605.83	18.94	25.75	7658.52
Het df	323	6	10	341
Het P	***	**	**	***
Fixed RR	4.26	3.13	4.62	4.25
RRl	4.20	2.49	3.89	4.20
RRu	4.32	3.92	5.48	4.31
P	+++	+++	+++	+++
Random RR	5.52	3.66	5.25	5.48
RRl	5.09	2.34	3.74	5.07
RRu	5.98	5.73	7.38	5.93
P	+++	+++	+++	+++
Between Chi				8.00
Between df				2
Between P				*
Btwn(F) P				N.S.
Btwn(R) P				N.S.

	National cigarette tobacco type			
	Virginia	blended	other	Total
N	53	234	55	342
NS	38	166	38	242
Wt	796.38	8281.69	10936.31	20014.39
Het Chi	315.85	2710.70	170.10	7658.52
Het df	52	233	54	341
Het P	***	***	***	***
Fixed RR	5.60	7.29	2.77	4.25
RRl	5.22	7.13	2.72	4.20
RRu	6.00	7.45	2.83	4.31
P	+++	+++	+++	+++
Random RR	6.22	6.26	2.69	5.48
RRl	5.16	5.76	2.51	5.07
RRu	7.50	6.81	2.88	5.93
P	+++	+++	+++	+++
Between Chi				4461.87
Between df				2
Between P				***
Btwn(F) P				***
Btwn(R) P				***

Table 1C1 - 3

IESLC - Meta-anal of Ever Smoking (or Current if Ever not available), Any prod (or Cigs if Any not avail)
All LC types
Most adjusted

	Any proxy use		
	No/nk	Yes	Total
N	241	101	342
NS	175	67	242
Wt	7952.65	12061.73	20014.39
Het Chi	3047.78	1845.32	7658.52
Het df	240	100	341
Het P	***	***	***
Fixed RR	6.72	3.15	4.25
RRl	6.58	3.09	4.20
RRu	6.87	3.20	4.31
P	+++	+++	+++
Random RR	5.49	5.39	5.48
RRl	5.02	4.84	5.07
RRu	5.99	6.02	5.93
P	+++	+++	+++
Between Chi			2765.42
Between df			1
Between P			***
Btwn(F) P			***
Btwn(R) P			N.S.

	Full histological confirmation		
	No	Yes	Total
N	259	83	342
NS	183	59	242
Wt	17494.68	2519.71	20014.39
Het Chi	5866.42	789.61	7658.52
Het df	258	82	341
Het P	***	***	***
Fixed RR	3.91	7.67	4.25
RRl	3.85	7.38	4.20
RRu	3.97	7.98	4.31
P	+++	+++	+++
Random RR	5.25	6.30	5.48
RRl	4.81	5.47	5.07
RRu	5.72	7.25	5.93
P	+++	+++	+++
Between Chi			1002.49
Between df			1
Between P			***
Btwn(F) P			***
Btwn(R) P			*

	Number of adjustment variables (1)			
	0	1	2+/+nk	Total
N	164	80	98	342
NS	114	55	80	249
Wt	4667.87	2148.70	13197.82	20014.39
Het Chi	1870.84	720.64	3064.29	7658.52
Het df	163	79	97	341
Het P	***	***	***	***
Fixed RR	7.12	5.40	3.41	4.25
RRl	6.92	5.18	3.35	4.20
RRu	7.33	5.64	3.47	4.31
P	+++	+++	+++	+++
Random RR	5.44	5.47	5.52	5.48
RRl	4.86	4.75	4.86	5.07
RRu	6.09	6.29	6.27	5.93
P	+++	+++	+++	+++
Between Chi				2002.75
Between df				2
Between P				***
Btwn(F) P				***
Btwn(R) P				N.S.

Table 1C1 - 3

IESLC - Meta-anal of Ever Smoking (or Current if Ever not available), Any prod (or Cigs if Any not avail)
All LC types
Most adjusted

	Number of adjustment variables (2) 0	1	2	3-5	6+/+nk	Total
N	164	80	52	31	15	342
NS	114	55	41	26	13	249
Wt	4667.87	2148.70	12323.71	624.61	249.50	20014.39
Het Chi	1870.84	720.64	2635.19	180.15	97.34	7658.52
Het df	163	79	51	30	14	341
Het P	***	***	***	***	***	***
Fixed RR	7.12	5.40	3.32	5.33	4.45	4.25
RRl	6.92	5.18	3.26	4.93	3.93	4.20
RRu	7.33	5.64	3.38	5.77	5.04	4.31
P	+++	+++	+++	+++	+++	+++
Random RR	5.44	5.47	5.34	6.41	4.55	5.48
RRl	4.86	4.75	4.50	5.18	3.18	5.07
RRu	6.09	6.29	6.35	7.93	6.51	5.93
P	+++	+++	+++	+++	+++	+++
Between Chi						2154.36
Between df						4
Between P						***
Btwn(F) P						***
Btwn(R) P						N.S.

	Product all/unsp	cig+/-ot	cig only	Total
N	209	119	14	342
NS	156	83	12	251
Wt	15593.14	3926.08	495.17	20014.39
Het Chi	5016.90	1566.15	77.35	7658.52
Het df	208	118	13	341
Het P	***	***	***	***
Fixed RR	3.79	6.21	8.36	4.25
RRl	3.73	6.02	7.66	4.20
RRu	3.85	6.40	9.13	4.31
P	+++	+++	+++	+++
Random RR	5.36	5.45	7.92	5.48
RRl	4.86	4.81	6.07	5.07
RRu	5.91	6.18	10.33	5.93
P	+++	+++	+++	+++
Between Chi				998.12
Between df				2
Between P				***
Btwn(F) P				***
Btwn(R) P				*

	Denominator nev any	nev cigs	Total
N	248	94	342
NS	177	68	245
Wt	16985.59	3028.80	20014.39
Het Chi	5555.09	1209.54	7658.52
Het df	247	93	341
Het P	***	***	***
Fixed RR	3.89	7.02	4.25
RRl	3.83	6.77	4.20
RRu	3.95	7.27	4.31
P	+++	+++	+++
Random RR	5.28	6.03	5.48
RRl	4.83	5.22	5.07
RRu	5.76	6.95	5.93
P	+++	+++	+++
Between Chi			893.89
Between df			1
Between P			***
Btwn(F) P			***
Btwn(R) P			N.S.

Table 1C1 - 3

IESLC - Meta-anal of Ever Smoking (or Current if Ever not available), Any prod (or Cigs if Any not avail)
All LC types
Most adjusted

	Derivation of RR/CI			
	Orig	StdCalc	Other	Total
N	46	171	125	342
NS	35	122	93	250
Wt	2096.08	4914.06	13004.24	20014.39
Het Chi	817.54	2134.55	1831.56	7658.52
Het df	45	170	124	341
Het P	***	***	***	***
Fixed RR	7.51	6.96	3.22	4.25
RRl	7.20	6.77	3.17	4.20
RRu	7.84	7.16	3.28	4.31
P	+++	+++	+++	+++
Random RR	5.67	5.56	5.21	5.48
RRl	4.61	4.97	4.75	5.07
RRu	6.98	6.23	5.72	5.93
P	+++	+++	+++	+++
Between Chi				2874.87
Between df				2
Between P				***
Btwn(F) P				***
Btwn(R) P				N.S.

	Smoking status		
	ever	current	Total
N	327	15	342
NS	235	10	245
Wt	19323.40	690.99	20014.39
Het Chi	7465.31	156.73	7658.52
Het df	326	14	341
Het P	***	***	***
Fixed RR	4.22	5.33	4.25
RRl	4.16	4.95	4.20
RRu	4.28	5.75	4.31
P	+++	+++	+++
Random RR	5.49	5.27	5.48
RRl	5.07	3.85	5.07
RRu	5.95	7.21	5.93
P	+++	+++	+++
Between Chi			36.48
Between df			1
Between P			***
Btwn(F) P			N.S.
Btwn(R) P			N.S.

	Study LIU4		
	LIU4	others	Total
N	2	340	342
NS	1	241	242
Wt	9846.05	10168.33	20014.39
Het Chi	2.98	4256.80	7658.52
Het df	1	339	341
Het P	(*)	***	***
Fixed RR	2.80	6.38	4.25
RRl	2.74	6.26	4.20
RRu	2.85	6.51	4.31
P	+++	+++	+++
Random RR	2.81	5.51	5.48
RRl	2.71	5.10	5.07
RRu	2.91	5.94	5.93
P	+++	+++	+++
Between Chi			3398.74
Between df			1
Between P			***
Btwn(F) P			***
Btwn(R) P			***

Table 1C1 - 4

IESLC - Meta-anal of Ever Smoking (or Current if Ever not available), Any prod (or Cigs if Any not avail)
All LC types
Least adjusted

REF	NRR	X	SEX	AGEL	AGEH	RACE	YF	LC TYPE	LOC	START	ST	NLC	R	VB	P	H	AD	SM	PRODUCT	DENOM	De
ABELIN	1	x	m	0	0	all	-	all	Eu:wst	1941	CC	118	n	bl	y	n	0	ev	all/unsp	nev any	st
ABRAHA	7		m	0	0	all	0	q+s+a	Eu:est	1975	pr	571	n	bl	n	n	0	ev	all/unsp	nev any	ot
ABRAHA	8		f	0	0	all	0	q+s+a	Eu:est	1975	pr	571	n	bl	n	n	0	ev	all/unsp	nev any	ot
AGUDO	8	x	f	0	0	all	-	all	Eu:wst	1989	CC	103	n	bl	n	n	0	ev	cig only	nev any	st
AKIBA	3	x	m	0	0	all	0	all	As:Jap	1963	pr	610	n	bl	n	n	0	ev	cig+/-ot	nev cigs	st
AKIBA	7	x	f	0	0	all	0	all	As:Jap	1963	pr	610	n	bl	n	n	0	ev	cig+/-ot	nev cigs	st
ALDERS	62	x	m	0	0	all	-	all	Eu:UK	1977	CC	1448	n	V	n	n	0	ev	all/unsp	nev any	st
ALDERS	12	x	f	0	0	all	-	all	Eu:UK	1977	CC	1448	n	V	n	n	0	ev	cig only	nev any	st
AMANDU	3	x	m	0	0	wh	0	all	NAmer	1959	pr	132	m	bl	n	n	0	ev	cig+/-ot	nev cigs	st
AMES	4		m	0	0	wh	-	all	NAmer	1959	ot	317	m	bl	n	n	0	ev	all/unsp	nev any	st
ANDERS	3		f	0	0	all	0	all	NAmer	1986	pr	343	n	bl	n	n	0	ev	cig+/-ot	nev cigs	st
ARCHER	6		m	0	0	wh	0	all	NAmer	1950	pr	146	m	bl	n	n	0	ev	cig+/-ot	nev cigs	st
ARMADA	29		m	0	0	all	-	all	Eu:wst	1986	CC	325	n	bl	n	y	0	ev	all/unsp	nev any	st
AUSTIN	3	x	c	0	0	all	-	all	NAmer	1970	CC	166	o	bl	y	n	0	ev	cig+/-ot	nev cigs	st
AUVINE	1	x	c	0	0	all	-	all	Eu:Sca	1986	CC	517	n	bl	y	n	0	ev	cig+/-ot	nev cigs	st
AXELSO	1		c	0	0	all	-	all	Eu:Sca	1960	CC	152	n	bl	y	n	0	ev	all/unsp	nev any	st
AXELSS	1	x	m	0	0	sca	-	all	Eu:Sca	1989	CC	436	n	bl	n	n	0	ev	all/unsp	nev any	st
AXELSS	11		f	0	0	sca	-	all	Eu:Sca	1989	CC	436	n	bl	n	n	0	ev	all/unsp	nev any	st
BAND	1		m	0	0	all	-	all	NAmer	1983	CC	2831	n	V	y	y	2	ev	cig only	nev any	ot
BARBON	106	x	m	0	0	all	-	all	Eu:wst	1979	CC	755	n	bl	y	y	0	ev	all/unsp	nev any	st
BECHER	1		m	0	0	all	-	all	Eu:Ger	1985	CC	194	n	bl	n	y	0	ev	all/unsp	nev any	st
BECHER	2	x	f	0	0	all	-	all	Eu:Ger	1985	CC	194	n	bl	n	y	0	ev	all/unsp	nev any	st
BENSHL	18		m	40	64	all	10	all	Eu:UK	1967	pr	486	n	V	n	n	1	ev	all/unsp	nev any	ot
BEST	22		m	55	79	all	3	all	NAmer	1955	pr	381	n	V	n	n	0	ev	all/unsp	nev any	st
BEST	18		f	0	0	all	0	all	NAmer	1955	pr	381	n	V	n	n	1	ev	cig only	nev any	ot
BLOHMK	3		m	0	0	all	-	all	Eu:Ger	1978	CC	888	n	bl	n	y	0	ev	all/unsp	nev any	st
BLOT4	1		m	0	0	wh	-	all	NAmer	1974	CC	335	n	bl	y	n	0	ev	cig+/-ot	nev cigs	st
BOFFET	32	x	m	0	0	all	-	all	Eu:mul	1988	CC	5621	n	bl	y	n	0	ev	all/unsp	nev any	st
BOUCOT	9	x	m	0	0	all	0	all	NAmer	1951	pr	121	n	bl	n	n	0	ev	all/unsp	nev any	ot
BRESLO	37		m	0	0	all	-	all	NAmer	1949	CC	518	n	bl	n	y	0	ev	all/unsp	nev+1	st
BRESLO	38		f	0	0	all	-	all	NAmer	1949	CC	518	n	bl	n	y	0	ev	all/unsp	nev+1	st
BRETT	10		m	0	0	all	0	all	Eu:UK	1960	pr	150	n	V	n	n	0	ev	cig+/-ot	nev cigs	st
BROCKM	1		m	0	0	wh	-	all	Eu:Ger	1990	CC	117	n	bl	n	y	0	ev	cig+/-ot	nev cigs	st
BROCKM	2		f	0	0	wh	-	all	Eu:Ger	1990	CC	117	n	bl	n	y	0	ev	cig+/-ot	nev cigs	st
BROSS	12		m	0	0	wh	-	all	NAmer	1960	CC	974	n	bl	n	n	0	ev	all/unsp	nev any	st
BROWN2	2		m	0	0	wh	-	all	NAmer	1984	CC	14596	n	bl	n	y	2	ev	cig+/-ot	nev cigs	or
BROWN2	1		f	0	0	wh	-	all	NAmer	1984	CC	14596	n	bl	n	y	2	ev	cig+/-ot	nev cigs	or
BUFFLE	1		m	0	0	wh	-	all	NAmer	1976	CC	943	n	bl	y	n	0	ev	all/unsp	nev any	st
BUFFLE	5		f	0	0	wh	-	all	NAmer	1976	CC	943	n	bl	y	n	0	ev	all/unsp	nev any	st
CARPEN	7	x	c	0	0	w+b	-	all	NAmer	1991	CC	356	n	bl	n	n	0	ev	cig+/-ot	nev cigs	st
CASCO2	1		c	0	0	wh	-	all	Eu:Ger	1991	CC	155	n	bl	n	n	0	ev	all/unsp	nev any	st
CASCOR	1		c	0	0	wh	-	all	Eu:Ger	1985	CC	389	n	bl	n	y	0	ev	all/unsp	nev any	st
CEDERL	107		m	0	0	all	16	all	Eu:Sca	1963	pr	491	n	bl	n	n	2	ev	all/unsp	nev any	ot
CEDERL	112		f	0	0	all	0	all	Eu:Sca	1963	pr	491	n	bl	n	n	2	ev	all/unsp	nev any	ot
CHAN	9		m	0	0	all	-	all	As:HK	1976	CC	397	n	bl	n	n	0	ev	all/unsp	nev any	st
CHAN	10		f	0	0	all	-	all	As:HK	1976	CC	397	n	bl	n	n	0	ev	all/unsp	nev any	st
CHANG	6		m	0	0	all	0	all	NAmer	1972	pr	136	n	bl	n	n	0	ev	cig+/-ot	nev cigs	st
CHANG	12		f	0	0	all	0	all	NAmer	1972	pr	136	n	bl	n	n	0	ev	cig+/-ot	nev cigs	st
CHATZI	4		c	0	0	all	-	all	Eu:bal	1987	CC	282	n	bl	n	y	0	ev	all/unsp	nev any	st
CHEN2	1		m	0	0	all	-	all	As:Chi	1983	CC	193	n	ot	y	n	0	ev	all/unsp	nev any	st
CHEN2	2		f	0	0	all	-	all	As:Chi	1983	CC	193	n	ot	y	n	0	ev	all/unsp	nev any	st
CHEN3	1		c	0	0	all	-	all	As:Chi	1981	CC	254	n	ot	y	n	0	ev	all/unsp	nev any	st
CHIAZZ	2	x	m	0	0	all	-	all	NAmer	1940	CC	144	o	bl	y	n	0	ev	cig+/-ot	nev cigs	st
CHOI	1		m	0	0	all	-	all	As:oth	1985	CC	375	n	bl	n	n	0	ev	cig+/-ot	nev cigs	st
CHOI	5		f	0	0	all	-	all	As:oth	1985	CC	375	n	bl	n	n	0	ev	cig+/-ot	nev cigs	st
CHOW	7	x	m	0	0	wh	0	all	NAmer	1966	pr	219	n	bl	n	n	0	ev	all/unsp	nev any	st
CHYOU	9	x	m	0	0	jap	0	all	NAmer	1965	pr	227	n	bl	n	y	0	ev	cig+/-ot	nev cigs	st
COMSTO	34		m	0	0	all	-	all	NAmer	1975	ot	258	n	bl	n	n	0	ev	all/unsp	nev any	st
COMSTO	46		f	0	0	all	-	all	NAmer	1975	ot	258	n	bl	n	n	0	ev	all/unsp	nev any	st
COOKSO	5		c	0	0	bl	-	all	Africa	1961	CC	234	n	V	n	y	0	ev	all/unsp	nev any	st
CORREA	33	x	c	0	0	all	-	all	NAmer	1979	CC	1359	n	bl	y	n	0	ev	cig+/-ot	nev cigs	st
CPSI	187		m	35	84	all	6	all	NAmer	1959	pr	5138	n	bl	n	n	1	ev	cig+/-ot	nev any	ot
CPSI	274		f	40	74	all	6	all	NAmer	1959	pr	5138	n	bl	n	n	1	ev	cig+/-ot	nev cigs	ot
CPSII	104		m	35	99	all	4	all	NAmer	1982	pr	3229	n	bl	n	n	1	ev	cig only	nev any	ot
CPSII	79		f	0	0	all	4	all	NAmer	1982	pr	3229	n	bl	n	n	1	ev	cig+/-ot	nev cigs	ot
DAMBER	5	x	m	0	0	all	-	all	Eu:Sca	1972	CC	579	n	bl	y	n	0	ev	all/unsp	nev any	st
DARBY	15		m	0	0	wh	-	all	Eu:UK	1988	CC	982	n	V	n	n	0	ev	all/unsp	nev any	st
DARBY	16		f	0	0	wh	-	all	Eu:UK	1988	CC	982	n	V	n	n	0	ev	all/unsp	nev any	st
DAVEYS	5		m	0	0	all	-	all	Eu:Ger	1930	CC	109	n	bl	y	n	0	ev	all/unsp	nev any	st
DAVEYS	6		f	0	0	all	-	all	Eu:Ger	1930	CC	109	n	bl	y	n	0	ev	all/unsp	nev any	ot
DEAN	7		m	0	0	wh	-	all	Africa	1947	CC	603	n	V	y	n	0	ev	all/unsp	nev any	st
DEAN2	3		m	0	0	all	-	all	Eu:UK	1960	CC	954	n	V	y	n	0	ev	all/unsp	nev any	st

Table 1C1 - 4

IESLC - Meta-anal of Ever Smoking (or Current if Ever not available), Any prod (or Cigs if Any not avail)
All LC types
Least adjusted

REF	NRR	X	SEX	AGEL	AGEH	RACE	YF	LC TYPE	LOC	START	ST	NLC	R	VB	P	H	AD	SM	PRODUCT	DENOM	De
DEAN2	7		f	0	0	all	-	all	Eu:UK	1960	CC	954	n	V	y	n	0	ev	all/unsp	nev any	st
DEAN3	47	x	m	0	0	all	-	all	Eu:UK	1969	CC	766	n	V	y	n	0	ev	all/unsp	nev any	st
DEAN3	124	x	f	0	0	all	-	all	Eu:UK	1969	CC	766	n	V	y	n	0	ev	cig only	nev any	st
DEKLER	6		m	0	0	all	0	all	Auslia	1961	pr	138	m	V	n	n	2	ev	all/unsp	nev any	ot
DESTE2	13	x	c	0	0	all	-	all	SCAmer	1993	CC	463	n	bl	n	n	0	ev	all/unsp	nev any	st
DESTEF	5	x	m	0	0	all	-	all	SCAmer	1988	CC	497	n	bl	n	y	0	ev	all/unsp	nev any	st
DOCKER	3		c	0	0	wh	0	all	NAmer	1974	pr	120	n	bl	n	n	4	ev	cig+/-ot	nev cigs	ot
DOLL	6		m	0	0	all	-	all	Eu:UK	1948	CC	1465	n	V	n	n	0	ev	all/unsp	nev any	st
DOLL	12		f	0	0	all	-	all	Eu:UK	1948	CC	1465	n	V	n	n	0	ev	all/unsp	nev any	st
DOLL2	56		m	0	0	all	0	all	Eu:UK	1951	pr	920	n	V	n	n	1	ev	all/unsp	nev any	ot
DOLL2	63		f	0	0	all	22	all	Eu:UK	1951	pr	920	n	V	n	n	1	cu	cig only	nev any	ot
DORANT	10		c	0	0	all	0	all	Eu:wst	1986	ot	550	n	bl	n	y	0	ev	all/unsp	nev any	st
DORGAN	6		m	0	0	wh	-	all	NAmer	1980	CC	2026	n	bl	y	y	0	ev	all/unsp	nev any	st
DORGAN	30		m	0	0	bl	-	all	NAmer	1980	CC	2026	n	bl	y	y	0	ev	all/unsp	nev any	st
DORGAN	53		f	0	0	wh	-	all	NAmer	1980	CC	2026	n	bl	y	y	0	ev	all/unsp	nev any	st
DORGAN	76		f	0	0	bl	-	all	NAmer	1980	CC	2026	n	bl	y	y	0	ev	all/unsp	nev any	st
DORN	196		m	35	84	wh	8	all	NAmer	1954	pr	5097	n	bl	n	n	1	ev	all/unsp	nev any	ot
DOSEME	17	x	m	0	0	all	-	all	Eu:bal	1979	CC	1210	n	bl	n	n	0	ev	cig+/-ot	nev cigs	st
DROSTE	3	x	m	0	0	all	-	all	Eu:wst	1995	CC	478	n	bl	n	y	0	ev	all/unsp	nev any	st
DU	1		m	0	0	all	-	all	As:Chi	1985	CC	849	n	ot	y	n	0	ev	all/unsp	nev any	or
DU	2		f	0	0	all	-	all	As:Chi	1985	CC	849	n	ot	y	n	0	ev	all/unsp	nev any	or
DUNN	6		m	0	0	all	0	all	NAmer	1954	pr	139	o	bl	n	n	0	ev	cig+/-ot	nev cigs	st
EBELIN	1		m	0	0	all	-	all	Eu:Ger	1980	CC	130	n	bl	n	n	0	ev	all/unsp	nev any	st
ENGELA	155	x	m	0	0	all	12	all	Eu:Sca	1964	pr	435	n	bl	n	n	0	ev	all/unsp	nev any	st
ENGELA	162	x	f	0	0	all	12	all	Eu:Sca	1964	pr	435	n	bl	n	n	0	ev	all/unsp	nev any	st
ENSTRO	1		m	0	0	all	0	all	NAmer	1959	pr	2879	n	bl	n	n	1	cu	cig only	nev any	or
ENSTRO	2		f	0	0	all	0	all	NAmer	1959	pr	2879	n	bl	n	n	1	cu	cig only	nev any	or
ESAKI	4		m	0	0	all	-	all	As:Jap	1961	CC	245	n	bl	y	n	0	ev	cig+/-ot	nev cigs	st
ESAKI	5		f	0	0	all	-	all	As:Jap	1961	CC	245	n	bl	y	n	0	ev	cig+/-ot	nev cigs	st
FAN	1		m	0	0	all	-	all	As:Chi	1990	CC	403	n	ot	y	n	0	ev	cig+/-ot	nev cigs	st
FAN	2		f	0	0	all	-	all	As:Chi	1990	CC	403	n	ot	y	n	0	ev	cig+/-ot	nev cigs	st
GAO	6	x	m	0	0	all	-	all	As:Chi	1984	CC	1405	n	ot	n	n	0	ev	cig+/-ot	nev cigs	st
GAO	16	x	f	0	0	all	-	all	As:Chi	1984	CC	1405	n	ot	n	n	0	ev	cig+/-ot	nev cigs	st
GAO2	6	x	m	0	0	all	-	all	As:Jap	1988	CC	282	n	bl	n	n	0	ev	cig+/-ot	nev cigs	st
GARCIA	3		c	0	0	all	-	all	NAmer	1992	CC	416	n	bl	n	y	0	ev	cig+/-ot	nev cigs	st
GARDIN	7		c	0	0	all	-	all	Eu:UK	1988	CC	143	n	V	y	n	0	ev	all/unsp	nev any	st
GARSHI	17	x	m	0	0	all	-	all	NAmer	1981	CC	1081	o	bl	y	n	0	ev	all/unsp	nev any	st
GENG	1		m	0	0	all	-	all	As:Chi	1985	CC	292	n	ot	*	n	0	ev	cig+/-ot	nev any	st
GENG	2		f	0	0	all	-	all	As:Chi	1985	CC	292	n	ot	*	n	0	ev	cig+/-ot	nev any	st
GER	17	x	c	0	0	all	-	all	As:oth	1990	CC	141	n	ot	y	n	0	ev	all/unsp	nev any	st
GODLEY	5		m	0	0	all	-	all	NAmer	1966	CC	1986	n	bl	y	n	1	ev	cig+/-ot	nev cigs	ot
GODLEY	6		f	0	0	all	-	all	NAmer	1966	CC	1986	n	bl	y	n	1	ev	cig+/-ot	nev cigs	ot
GOLLED	21	x	m	35	99	all	-	all	Eu:UK	1952	CC	443	n	V	y	n	0	ev	cig+/-ot	nev any	st
GOODMA	3		m	0	0	w+o	-	all	NAmer	1983	CC	326	n	bl	y	y	0	ev	cig+/-ot	nev any	st
GOODMA	7		f	0	0	w+o	-	all	NAmer	1983	CC	326	n	bl	y	y	0	ev	cig+/-ot	nev any	st
GRAHAM	22	x	m	0	0	wh	-	all	NAmer	1956	CC	685	n	bl	n	n	0	ev	all/unsp	nev any	st
GREGOR	3		m	0	0	all	-	all	Eu:UK	1976	CC	104	n	V	n	y	0	ev	cig+/-ot	nev cigs	st
GREGOR	7		f	0	0	all	-	all	Eu:UK	1976	CC	104	n	V	n	y	0	ev	cig+/-ot	nev cigs	st
GSELL	8		m	0	0	all	-	all	Eu:wst	1937	CC	150	n	bl	n	y	0	ev	all/unsp	nev any	st
HAENSZ	50	x	f	0	0	all	-	not alv	NAmer	1955	CC	158	n	bl	n	y	0	ev	all/unsp	nev any	st
HAMMO2	18	x	m	0	0	all	6	all	NAmer	1967	pr	450	o	bl	n	n	0	ev	all/unsp	nev any	st
HAMMON	129	x	m	0	0	wh	0	all	NAmer	1952	pr	448	n	bl	n	n	0	ev	all/unsp	nev any	st
HANSEN	3		m	0	0	all	0	all	Eu:Sca	1968	pr	105	o	bl	y	n	2	ev	all/unsp	nev any	ot
HEGMAN	1		c	0	0	all	-	all	NAmer	1989	CC	282	n	bl	y	y	0	ev	all/unsp	nev any	st
HEIN	7		m	0	0	all	0	all	Eu:Sca	1970	pr	144	n	bl	n	n	0	ev	all/unsp	nev any	st
HENNEK	3		m	0	0	all	0	all	NAmer	1982	pr	169	n	bl	n	n	0	ev	all/unsp	nev any	st
HINDS	26	x	f	0	0	o	-	all	NAmer	1968	CC	292	n	bl	n	n	0	ev	all/unsp	nev any	st
HIRAYA	147		m	0	0	all	0	all	As:Jap	1965	pr	1917	n	bl	n	n	1	ev	cig+/-ot	nev any	ot
HIRAYA	150		f	0	0	all	0	all	As:Jap	1965	pr	1917	n	bl	n	n	1	ev	cig+/-ot	nev any	ot
HITOSU	6	x	m	0	0	all	-	all	As:Jap	1960	CC	216	n	bl	y	n	0	ev	all/unsp	nev any	st
HITOSU	12	x	f	0	0	all	-	all	As:Jap	1960	CC	216	n	bl	y	n	0	ev	all/unsp	nev any	st
HOLE	15	x	m	0	0	all	0	all	Eu:UK	1972	pr	225	n	V	n	n	0	ev	all/unsp	nev any	st
HOLE	29	x	f	0	0	all	11	all	Eu:UK	1972	pr	225	n	V	n	n	0	cu	all/unsp	nev any	st
HOROWI	1		m	0	0	all	-	all	NAmer	1956	CC	236	n	V	n	n	0	ev	cig+/-ot	nev any	st
HOROWI	2		f	0	0	all	-	all	NAmer	1956	CC	236	n	V	n	n	0	ev	cig+/-ot	nev any	st
HORWIT	1		f	0	0	all	-	all	NAmer	1977	CC	112	n	bl	n	n	0	ev	cig+/-ot	nev cigs	st
HU	15		m	0	0	all	-	all	As:Chi	1985	CC	227	n	ot	n	y	0	ev	cig+/-ot	nev any	st
HU	16		f	0	0	all	-	all	As:Chi	1985	CC	227	n	ot	n	y	0	ev	cig+/-ot	nev any	st
HU2	9		m	0	0	all	-	all	As:Chi	1977	CC	523	n	ot	y	n	0	ev	cig+/-ot	nev cigs	st
HU2	10		f	0	0	all	-	all	As:Chi	1977	CC	523	n	ot	y	n	0	ev	cig+/-ot	nev cigs	st
HUANG	1		c	0	0	all	-	all	As:Chi	1990	CC	135	n	ot	y	n	0	ev	all/unsp	nev any	st
HUMBLE	14		m	0	0	w-hi	-	all	NAmer	1980	CC	521	n	bl	y	n	1	ev	cig+/-ot	nev cigs	ot

Table 1C1 - 4

IESLC - Meta-anal of Ever Smoking (or Current if Ever not available), Any prod (or Cigs if Any not avail)
All LC types
Least adjusted

REF	NRR	X	SEX	AGEL	AGEH	RACE	YF	LC TYPE	LOC	START	ST	NLC	R	VB	P	H	AD	SM	PRODUCT	DENOM	De
HUMBLE	16		m	0	0	hi	-	all	NAmer	1980	CC	521	n	bl	y	n	1	ev	cig+/-ot	nev cigs	ot
HUMBLE	18		f	0	0	w-hi	-	all	NAmer	1980	CC	521	n	bl	y	n	1	ev	cig+/-ot	nev cigs	ot
HUMBLE	20		f	0	0	hi	-	all	NAmer	1980	CC	521	n	bl	y	n	1	ev	cig+/-ot	nev cigs	ot
JAHN	3	x	f	0	0	all	-	all	Eu:Ger	1988	CC	1004	n	bl	n	n	0	ev	cig+/-ot	nev any	st
JAIN	6	x	m	0	0	all	-	all	NAmer	1981	CC	845	n	V	y	n	0	ev	cig+/-ot	nev cigs	st
JAIN	1	x	f	0	0	all	-	all	NAmer	1981	CC	845	n	V	y	n	0	ev	cig+/-ot	nev cigs	st
JARUP	3	x	m	0	0	all	-	all	Eu:Sca	1928	CC	102	o	bl	y	n	0	ev	all/unsp	nev any	st
JARVHO	3		m	0	0	all	-	all	Eu:Sca	1983	CC	147	n	bl	n	n	0	ev	all/unsp	nev any	st
JARVHO	7		f	0	0	all	-	all	Eu:Sca	1983	CC	147	n	bl	n	n	0	ev	all/unsp	nev any	st
JEDRYC	63	x	m	0	0	all	-	all	Eu:est	1980	CC	1630	n	bl	y	n	0	ev	cig+/-ot	nev any	st
JEDRYC	68	x	f	0	0	all	-	all	Eu:est	1980	CC	1630	n	bl	y	n	0	ev	cig+/-ot	nev any	st
JIANG	1		m	0	0	all	-	all	As:Chi	1984	CC	125	n	ot	n	n	0	ev	all/unsp	nev any	st
JIANG	2		f	0	0	all	-	all	As:Chi	1984	CC	125	n	ot	n	n	0	ev	all/unsp	nev any	st
JOLY	14		m	0	0	all	-	all	SCAmer	1978	CC	826	n	bl	n	n	0	ev	all/unsp	nev any	st
JOLY	1		f	0	0	all	-	all	SCAmer	1978	CC	826	n	bl	n	n	0	ev	cig+/-ot	nev any	st
JUSSAW	3	x	m	0	0	all	-	all	As:Ind	1964	CC	792	n	V	n	n	0	ev	all/unsp	nev any	st
KAISE2	72		m	35	99	all	9	all	NAmer	1979	pr	318	n	bl	n	n	1	ev	cig only	nev any	st
KAISE2	64		f	35	99	all	9	all	NAmer	1979	pr	318	n	bl	n	n	1	ev	cig only	nev any	st
KAISER	13		m	0	0	all	0	all	NAmer	1964	pr	714	n	bl	n	n	2	ev	cig+/-ot	nev cigs	ot
KAISER	10		f	0	0	all	0	all	NAmer	1964	pr	714	n	bl	n	n	2	ev	cig+/-ot	nev cigs	ot
KANELL	5	x	m	0	0	all	-	all	Eu:bal	1950	CC	862	n	bl	n	n	0	cu	all/unsp	nev any	st
KATSOU	27	x	f	0	0	all	-	all	Eu:bal	1987	CC	101	n	bl	n	n	0	ev	all/unsp	nev any	st
KAUFMA	8	x	c	0	0	all	-	all	NAmer	1981	CC	881	n	bl	n	n	0	ev	cig+/-ot	nev cigs	st
KELLER	3		m	0	0	wh	-	all	NAmer	1985	CC	15038	n	bl	n	n	0	ev	all/unsp	nev any	st
KELLER	11		m	0	0	nonw	-	all	NAmer	1985	CC	15038	n	bl	n	n	0	ev	all/unsp	nev any	st
KELLER	7		f	0	0	wh	-	all	NAmer	1985	CC	15038	n	bl	n	n	0	ev	all/unsp	nev any	st
KELLER	15		f	0	0	nonw	-	all	NAmer	1985	CC	15038	n	bl	n	n	0	ev	all/unsp	nev any	st
KHUDER	4		m	0	0	all	-	all	NAmer	1985	CC	482	n	bl	n	y	0	ev	cig+/-ot	nev cigs	st
KIHARA	31		c	0	0	jap	-	all	As:Jap	1991	CC	440	n	bl	n	n	0	ev	all/unsp	nev any	st
KINLEN	6	x	m	0	0	all	0	all	Eu:UK	1967	pr	718	n	V	n	n	0	ev	all/unsp	nev any	st
KJUUS	10		m	0	0	all	-	all	Eu:Sca	1979	CC	176	n	bl	n	n	0	ev	all/unsp	nev any	st
KNEKT	76	x	m	20	69	all	21	all	Eu:Sca	1966	pr	515	n	bl	n	n	0	ev	all/unsp	nev any	st
KO	1		f	0	0	all	-	all	As:oth	1992	CC	117	n	ot	n	y	3	ev	cig+/-ot	nev cigs	or
KOHLME	1	x	c	0	0	all	-	all	Eu:Ger	1990	CC	239	n	bl	n	n	0	ev	all/unsp	nev any	st
KOO	1		f	0	0	all	-	all	As:HK	1981	CC	200	n	bl	n	n	0	ev	all/unsp	nev any	st
KOULUM	1		m	0	0	all	-	all	Eu:Sca	1936	CC	812	n	bl	n	n	0	ev	all/unsp	nev any	st
KREUZE	14		f	1	45	all	-	all	Eu:Ger	1990	CC	2260	n	bl	n	n	0	ev	all/unsp	nev any	st
KREUZE	16		f	55	69	all	-	all	Eu:Ger	1990	CC	2260	n	bl	n	n	0	ev	all/unsp	nev any	st
KREYBE	24	x	m	0	0	all	-	all	Eu:Sca	1948	CC	300	n	bl	n	y	0	ev	all/unsp	nev any	st
KREYBE	39	x	f	0	0	all	-	all	Eu:Sca	1948	CC	300	n	bl	n	y	0	ev	all/unsp	nev any	st
KUBIK	28		m	0	0	all	0	all	Eu:est	1965	pr	108	n	bl	n	n	0	ev	all/unsp	nev any	st
LAMTH	6		f	0	0	ch	-	all	As:HK	1983	CC	445	n	bl	n	n	0	ev	all/unsp	nev any	or
LAMWK	1		f	0	0	ch	-	all	As:HK	1981	CC	163	n	bl	n	n	0	ev	all/unsp	nev any	st
LAMWK2	9		m	0	0	all	-	q+s+l+a	As:HK	1976	CC	480	n	bl	n	n	0	ev	all/unsp	nev any	st
LAMWK2	10		f	0	0	all	-	q+s+l+a	As:HK	1976	CC	480	n	bl	n	n	0	ev	all/unsp	nev any	st
LANGE	34	x	m	0	0	all	0	all	Eu:Sca	1976	pr	268	n	bl	n	n	0	ev	all/unsp	nev any	st
LANGE	31	x	f	0	0	all	0	all	Eu:Sca	1976	pr	268	n	bl	n	n	0	ev	all/unsp	nev any	st
LAUSSM	10	x	m	0	0	all	-	all	Eu:Ger	1982	CC	432	n	bl	n	n	0	ev	all/unsp	nev any	st
LEI	1		m	0	0	all	-	all	As:Chi	1986	CC	792	n	ot	y	n	0	ev	all/unsp	nev any	st
LEI	2		f	0	0	all	-	all	As:Chi	1986	CC	792	n	ot	y	n	0	ev	all/unsp	nev any	st
LEMARC	3		c	0	0	w+o	-	all	NAmer	1992	CC	341	n	bl	n	y	0	ev	all/unsp	nev any	st
LETOUR	1		c	0	0	all	-	all	NAmer	1983	CC	738	n	V	y	y	0	ev	cig+/-ot	nev cigs	st
LEVIN	32		m	0	0	all	-	all	NAmer	1938	CC	475	n	bl	n	n	1	ev	all/unsp	nev any	st
LIAW	1		m	0	0	all	0	all	As:oth	1982	pr	127	n	ot	n	n	1	cu	all/unsp	nev any	or
LIAW	2		f	0	0	all	0	all	As:oth	1982	pr	127	n	ot	n	n	1	cu	all/unsp	nev any	or
LIDDEL	5		m	0	0	all	18	all	NAmer	1970	pr	304	m	V	n	n	1	ev	cig+/-ot	nev cigs	ot
LIU	2		c	0	0	all	-	all	As:Chi	1980	CC	229	n	ot	*	n	2	ev	all/unsp	nev any	or
LIU2	1	x	m	0	0	all	-	all	As:Chi	1983	CC	316	n	ot	n	n	0	ev	all/unsp	nev any	st
LIU2	3	x	f	0	0	all	-	all	As:Chi	1983	CC	316	n	ot	n	n	0	ev	all/unsp	nev any	st
LIU3	1	x	m	0	0	all	-	all	As:Chi	1985	CC	110	n	ot	n	n	0	ev	all/unsp	nev any	st
LIU4	11		m	0	0	all	-	all	As:Chi	1986	CC	1000-00	n	ot	y	n	2	ev	all/unsp	nev any	ot
LIU4	12		f	0	0	all	-	all	As:Chi	1986	CC	1000-00	n	ot	y	n	2	ev	all/unsp	nev any	ot
LIU5	1		c	0	0	all	-	all	As:Chi	1978	CC	111	n	ot	y	n	0	ev	all/unsp	nev any	st
LOMBA2	1		f	0	0	all	-	all	NAmer	1960	CC	225	n	bl	n	n	0	ev	cig+/-ot	nev cigs	st
LOMBAR	12		m	0	0	all	-	all	NAmer	1951	CC	1040	n	bl	n	n	0	ev	all/unsp	nev any	st
LUBIN2	45	x	m	0	0	all	-	all	Eu:mul	1976	CC	7804	n	bl	n	y	0	ev	all/unsp	nev any	st
LUBIN2	101	x	f	0	0	all	-	all	Eu:mul	1976	CC	7804	n	bl	n	y	0	ev	all/unsp	nev any	st
LUO	1	x	c	0	0	all	-	all	As:Chi	1990	CC	102	n	ot	n	y	0	ev	cig+/-ot	nev cigs	st
MACLEN	71	x	m	0	0	ch	-	all	As:oth	1972	CC	233	n	bl	n	n	0	ev	cig+/-ot	nev cigs	st
MACLEN	72	x	f	0	0	ch	-	all	As:oth	1972	CC	233	n	bl	n	n	0	ev	cig+/-ot	nev cigs	st

Table 1C1 - 4

IESLC - Meta-anal of Ever Smoking (or Current if Ever not available), Any prod (or Cigs if Any not avail)
All LC types
Least adjusted

REF	NRR	X	SEX	AGEL	AGEH	RACE	YF	LC TYPE	LOC	START	ST	NLC	R	VB	P	H	AD	SM	PRODUCT	DENOM	De
MAGNUS	1	x	m	0	0	all	0	all	Eu:Sca	1953	pr	203	o	bl	y	n	0	ev	all/unsp	nev any	st
MARSH	2	x	m	0	0	all	-	all	NAmer	1979	CC	150	n	bl	y	n	0	ev	all/unsp	nev any	st
MARSH	4	x	f	0	0	all	-	all	NAmer	1979	CC	150	n	bl	y	n	0	ev	all/unsp	nev any	st
MARSH2	2	x	c	0	0	all	-	all	NAmer	1979	CC	114	n	bl	y	n	0	ev	all/unsp	nev any	st
MARTIS	4		m	0	0	all	-	all	Eu:UK	1972	CC	201	n	V	n	n	0	ev	cig+/-ot	nev cigs	st
MASTRA	1	x	m	0	0	all	-	all	Eu:wst	1973	CC	309	n	bl	n	n	0	ev	all/unsp	nev any	st
MATOS	26	x	m	0	0	all	-	all	SCAmer	1994	CC	200	n	bl	n	n	0	ev	cig+/-ot	nev any	st
MATSUD	10		m	0	0	all	-	all	As:Jap	1965	CC	179	n	bl	n	n	0	ev	cig+/-ot	nev cigs	st
MCCONN	1		m	0	0	all	-	all	Eu:UK	1946	CC	100	n	V	n	y	0	ev	all/unsp	nev any	st
MCCONN	2		f	0	0	all	-	all	Eu:UK	1946	CC	100	n	V	n	y	0	ev	all/unsp	nev any	st
MCDUFF	1		m	0	0	all	-	all	NAmer	1979	CC	165	n	V	y	n	0	ev	cig+/-ot	nev cigs	st
MCLAUG	1		m	0	0	all	-	all	As:Chi	1972	CC	316	o	ot	y	n	0	ev	all/unsp	nev any	st
MIGRAN	26	x	m	0	0	all	0	all	Eu:UK	1964	pr	259	n	V	n	n	0	ev	all/unsp	nev any	st
MIGRAN	41	x	f	0	0	all	0	all	Eu:UK	1964	pr	259	n	V	n	n	0	ev	all/unsp	nev any	st
MILLER	1	x	f	0	0	all	-	all	NAmer	1972	CC	168	n	bl	y	n	0	ev	cig+/-ot	nev any	st
MILLS	3		m	0	0	wh	-	all	NAmer	1940	CC	444	n	bl	y	n	1	ev	all/unsp	nev any	ot
MRFITR	6		m	0	0	all	0	all	NAmer	1973	pr	119	n	bl	n	n	0	ev	cig+/-ot	nev cigs	ot
NAM	69	x	m	0	0	all	-	all	NAmer	1986	CC	1199	n	bl	y	n	0	ev	cig+/-ot	nev cigs	ot
NAM	85	x	f	0	0	all	-	all	NAmer	1986	CC	1199	n	bl	y	n	0	ev	cig+/-ot	nev cigs	ot
NOTAN2	1	x	m	0	0	all	-	all	As:Ind	1963	CC	683	n	V	n	n	0	ev	all/unsp	nev any	st
NOU	11		m	30	64	all	-	all	Eu:Sca	1971	CC	273	n	bl	y	n	0	ev	all/unsp	nev any	st
NOU	12		f	30	64	all	-	all	Eu:Sca	1971	CC	273	n	bl	y	n	0	ev	all/unsp	nev any	st
ODRISC	3		c	0	0	all	-	all	Eu:UK	1992	CC	446	n	V	n	n	0	ev	all/unsp	nev any	st
ORMOS	4		m	0	0	all	-	all	Eu:est	1947	CC	119	n	bl	y	y	0	ev	cig+/-ot	nev any	st
ORMOS	26		f	0	0	all	-	all	Eu:est	1947	CC	119	n	bl	y	y	0	ev	cig+/-ot	nev any	st
OSANN	17	x	m	0	0	all	-	all	NAmer	1984	CC	1986	n	bl	n	n	0	ev	cig+/-ot	nev cigs	st
OSANN	21	x	f	0	0	all	-	all	NAmer	1984	CC	1986	n	bl	n	n	0	ev	cig+/-ot	nev cigs	st
PARKIN	29	x	m	0	0	bl	-	all	Africa	1963	CC	877	n	V	y	n	0	ev	all/unsp	nev any	st
PASTOR	5	x	m	0	0	all	-	all	Eu:wst	1976	CC	204	n	bl	y	n	0	ev	all/unsp	nev any	st
PAWLEG	1	x	m	0	0	all	-	all	Eu:est	1992	CC	176	n	bl	n	y	0	ev	all/unsp	nev any	st
PERNU	2		m	0	0	all	-	all	Eu:Sca	1944	CC	1606	n	bl	n	n	0	ev	all/unsp	nev any	st
PERNU	1		f	0	0	all	-	all	Eu:Sca	1944	CC	1606	n	bl	n	n	0	ev	all/unsp	nev any	st
PERSH2	5	x	c	0	0	all	-	all	Eu:Sca	1980	CC	1022	n	bl	y	n	0	ev	all/unsp	nev any	st
PETO	5		m	0	0	all	0	all	Eu:UK	1954	pr	103	n	V	n	n	0	ev	all/unsp	nev any	st
PEZZO2	10		m	0	0	all	-	all	SCAmer	1992	CC	367	n	bl	n	y	0	ev	cig+/-ot	nev cigs	st
PEZZOT	25		m	0	0	all	-	all	SCAmer	1987	CC	215	n	bl	n	y	0	ev	cig only	nev cigs	st
PIKE	4		m	0	0	w-hi	-	all	NAmer	1972	CC	731	n	bl	y	n	0	ev	all/unsp	nev any	st
PIKE	8		f	0	0	w-hi	-	all	NAmer	1972	CC	731	n	bl	y	n	0	ev	all/unsp	nev any	st
POFFIJ	1		c	0	0	all	-	all	Eu:mul	1990	CC	971	n	bl	n	n	0	ev	all/unsp	nev any	st
POLEDN	3	x	c	0	0	all	-	all	NAmer	1978	CC	209	n	bl	y	n	0	ev	cig+/-ot	nev cigs	st
QIAO2	3	x	m	0	0	all	0	all	As:Chi	1992	pr	241	m	ot	n	n	0	ev	all/unsp	nev any	st
RACHTA	3	x	f	0	0	all	-	all	Eu:est	1991	CC	118	n	bl	n	y	0	ev	cig+/-ot	nev cigs	st
RADZIK	1		c	0	0	all	-	all	Eu:est	1986	CC	189	n	bl	n	n	0	ev	all/unsp	nev any	st
RANDIG	23		m	0	0	all	-	all	Eu:Ger	1951	CC	448	n	bl	n	n	0	ev	all/unsp	nev any	st
RANDIG	24		f	0	0	all	-	all	Eu:Ger	1951	CC	448	n	bl	n	n	0	ev	all/unsp	nev any	st
REN	1		m	0	0	all	-	all	As:Chi	1980	CC	244	n	ot	*	n	0	ev	all/unsp	nev any	st
REN	2		f	0	0	all	-	all	As:Chi	1980	CC	244	n	ot	*	n	0	ev	all/unsp	nev any	st
RONCO	1		m	0	0	all	-	all	Eu:wst	1976	CC	126	n	bl	y	n	0	ev	all/unsp	nev any	st
ROTHSC	1	x	c	0	0	all	-	all	NAmer	1971	CC	284	n	bl	y	n	0	ev	all/unsp	nev any	st
SADOWS	7	x	m	0	0	wh	-	all	NAmer	1938	CC	477	n	bl	n	n	0	ev	all/unsp	nev any	st
SANKAR	1	x	m	0	0	all	-	all	As:Ind	1990	CC	281	n	V	n	n	0	ev	all/unsp	nev any	st
SCHWAR	1		m	0	0	wh	-	all	NAmer	1984	CC	5588	n	bl	y	y	0	ev	cig+/-ot	nev cigs	st
SCHWAR	2		m	0	0	bl	-	all	NAmer	1984	CC	5588	n	bl	y	y	0	ev	cig+/-ot	nev cigs	st
SCHWAR	3		f	0	0	wh	-	all	NAmer	1984	CC	5588	n	bl	y	y	0	ev	cig+/-ot	nev cigs	st
SCHWAR	4		f	0	0	bl	-	all	NAmer	1984	CC	5588	n	bl	y	y	0	ev	cig+/-ot	nev cigs	st
SEGI	1		m	0	0	all	-	all	As:Jap	1948	CC	159	n	bl	n	n	0	ev	all/unsp	nev any	ot
SEGI2	19	x	m	0	0	all	-	all	As:Jap	1962	CC	378	n	bl	n	n	0	cu	cig+/-ot	nev any	st
SEGI2	27	x	f	0	0	all	-	all	As:Jap	1962	CC	378	n	bl	n	n	0	cu	cig+/-ot	nev any	st
SEOW	1	x	f	0	0	ch	-	q+s+l+a	As:oth	1997	CC	153	n	bl	n	y	0	ev	cig+/-ot	nev cigs	st
SHAW	12		c	0	0	wh	-	all	NAmer	1988	CC	335	n	V	n	y	0	ev	all/unsp	nev any	st
SIEMIA	9	x	m	0	0	all	-	all	NAmer	1979	CC	857	n	V	y	y	0	ev	cig+/-ot	nev cigs	st
SIMARA	5	x	m	0	0	all	-	all	As:oth	1971	CC	115	n	bl	n	n	0	ev	cig+/-ot	nev cigs	st
SIMARA	6	x	f	0	0	all	-	all	As:oth	1971	CC	115	n	bl	n	n	0	ev	cig+/-ot	nev cigs	st
SOBUE	91	x	m	0	0	all	-	q+s+l+a	As:Jap	1986	CC	1376	n	bl	n	y	0	ev	cig+/-ot	nev cigs	st
SOBUE	95	x	f	0	0	all	-	q+s+l+a	As:Jap	1986	CC	1376	n	bl	n	y	0	ev	cig+/-ot	nev cigs	st
SOBUE2	10		m	0	0	all	-	q+s+l+a	As:Jap	1965	CC	2083	n	bl	n	n	2	cu	cig+/-ot	nev any	ot
SOBUE2	12		f	0	0	all	-	q+s+l+a	As:Jap	1965	CC	2083	n	bl	n	n	2	cu	cig+/-ot	nev any	ot
SPEIZE	8		f	0	0	all	0	all	NAmer	1976	pr	593	n	bl	n	y	0	ev	cig+/-ot	nev cigs	st
SPITZ	3		c	0	0	b+hi	-	all	NAmer	1992	CC	177	n	bl	n	y	0	ev	cig+/-ot	nev cigs	st
STASZE	1		m	0	0	all	-	all	Eu:est	1954	CC	281	n	bl	n	y	0	ev	all/unsp	nev any	st
STASZE	5		f	0	0	all	-	all	Eu:est	1954	CC	281	n	bl	n	y	0	ev	all/unsp	nev any	st
STAYNE	1		m	0	0	all	-	all	NAmer	1969	CC	420	n	bl	n	n	0	ev	all/unsp	nev any	st

Table 1C1 - 4

IESLC - Meta-anal of Ever Smoking (or Current if Ever not available), Any prod (or Cigs if Any not avail)
All LC types
Least adjusted

REF	NRR	X	SEX	AGEL	AGEH	RACE	YF	LC TYPE	LOC	START	ST	NLC	R	VB	P	H	AD	SM	PRODUCT	DENOM	De
STOCKS	31	x	m	0	0	all	-	all	Eu:UK	1952	CC	2932	n	V	y	n	0	ev	all/unsp	nev any	st
STOCKS	50		f	0	0	all	-	all	Eu:UK	1952	CC	2932	n	V	y	n	1	ev	cig+/-ot	nev any	ot
STOCKW	6		c	0	0	all	-	all	NAmer	1981	CC	22161	n	bl	n	n	0	ev	all/unsp	nev any	st
STUCKE	3		m	0	0	all	-	all	Eu:wst	1989	CC	247	n	bl	n	y	0	ev	all/unsp	nev any	ot
SUN	1		c	0	0	all	-	all	As:Chi	1992	CC	207	n	ot	n	y	0	ev	all/unsp	nev any	st
SUZUK2	18	x	c	0	0	all	-	all	SCAmer	1991	CC	123	n	bl	n	y	0	ev	all/unsp	nev any	st
SVENSS	56	x	f	0	0	all	-	all	Eu:Sca	1983	CC	210	n	bl	n	n	0	ev	all/unsp	nev any	st
TANG	3		c	0	0	all	-	not s	NAmer	1992	CC	119	n	bl	n	y	0	ev	cig+/-ot	nev cigs	st
TENKAN	22		m	0	0	all	17	all	Eu:Sca	1962	pr	242	n	bl	n	n	1	ev	all/unsp	nev any	ot
TIZZAN	1		m	0	0	all	-	all	Eu:wst	1959	CC	1358	n	bl	n	n	0	ev	all/unsp	nev any	st
TIZZAN	12		f	0	0	all	-	all	Eu:wst	1959	CC	1358	n	bl	n	n	0	ev	all/unsp	nev any	st
TOKARS	3	x	m	0	0	all	-	all	Eu:est	1966	ot	162	o	bl	n	y	0	ev	all/unsp	nev any	st
TOKARS	5	x	f	0	0	all	-	all	Eu:est	1966	ot	162	o	bl	n	y	0	ev	all/unsp	nev any	st
TOUSEY	16	x	m	0	0	all	-	all	NAmer	1993	CC	507	n	bl	y	y	0	ev	all/unsp	nev any	st
TOUSEY	26		f	0	0	all	-	all	NAmer	1993	CC	507	n	bl	y	y	0	ev	all/unsp	nev any	st
TSUGAN	27		m	0	0	all	-	q+a	As:Jap	1976	CC	134	n	bl	n	y	0	ev	all/unsp	nev any	st
TULINI	15	x	m	0	0	all	0	all	Eu:Sca	1967	pr	472	n	bl	n	n	1	ev	all/unsp	nev any	ot
TULINI	21	x	f	0	0	all	0	all	Eu:Sca	1967	pr	472	n	bl	n	n	1	ev	all/unsp	nev any	ot
TVERDA	22		m	0	0	all	0	all	Eu:Sca	1972	pr	238	n	bl	n	n	2	ev	cig+/-ot	nev cigs	ot
TVERDA	15		f	0	0	all	0	all	Eu:Sca	1972	pr	238	n	bl	n	n	2	cu	cig only	nev cigs	ot
WAKAI	13	x	m	0	0	all	-	all	As:Jap	1988	CC	333	n	bl	n	y	0	ev	all/unsp	nev any	st
WAKAI	31	x	f	0	0	all	-	all	As:Jap	1988	CC	333	n	bl	n	y	0	ev	all/unsp	nev any	st
WALD	2	x	m	0	0	all	0	all	Eu:UK	1975	pr	102	n	V	n	n	0	cu	cig only	nev any	st
WANG	1	x	m	0	0	all	-	all	As:Chi	1990	CC	390	n	ot	*	y	0	ev	all/unsp	nev any	or
WANG	2	x	f	0	0	all	-	all	As:Chi	1990	CC	390	n	ot	*	y	0	ev	all/unsp	nev any	or
WANG2	8	x	c	0	0	all	-	all	As:Chi	1980	CC	103	n	ot	n	n	0	ev	cig+/-ot	nev cigs	st
WANG3	1		c	0	0	all	-	all	As:Chi	1981	CC	293	n	ot	*	n	0	ev	all/unsp	nev any	st
WANG4	1	x	m	0	0	all	-	all	As:Chi	1976	CC	1170	n	ot	y	n	0	ev	all/unsp	nev any	st
WICKLU	1		m	0	0	wh	-	all	NAmer	1968	CC	155	n	bl	y	n	0	ev	cig+/-ot	nev+3	or
WIGLE	15	x	m	0	0	all	-	all	NAmer	1971	CC	728	n	V	n	n	0	ev	all/unsp	nev any	st
WIGLE	18	x	f	0	0	all	-	all	NAmer	1971	CC	728	n	V	n	n	0	ev	all/unsp	nev any	st
WILKIN	1	x	m	0	0	all	-	all	Eu:UK	1992	CC	271	n	V	n	n	0	ev	cig+/-ot	nev cigs	st
WILKIN	2	x	f	0	0	all	-	all	Eu:UK	1992	CC	271	n	V	n	n	0	ev	cig+/-ot	nev cigs	st
WU	37	x	f	0	0	wh	-	q+a	NAmer	1981	CC	220	n	bl	n	y	0	ev	all/unsp	nev any	st
WUNSCH	1	x	m	0	0	all	-	all	SCAmer	1990	CC	398	n	bl	y	n	0	ev	cig+/-ot	nev any	st
WUNSCH	7	x	f	0	0	all	-	all	SCAmer	1990	CC	398	n	bl	y	n	0	ev	cig+/-ot	nev any	st
WUWILL	6	x	f	0	0	all	-	all	As:Chi	1985	CC	965	n	ot	n	n	0	ev	cig+/-ot	nev cigs	st
WYNDE2	21		m	0	0	all	-	all	NAmer	1962	CC	404	n	bl	n	y	0	ev	all/unsp	nev any	st
WYNDE3	49		m	0	0	all	-	all	NAmer	1966	CC	350	n	bl	n	y	0	ev	all/unsp	nev any	st
WYNDE3	138		f	0	0	all	-	all	NAmer	1966	CC	350	n	bl	n	y	0	ev	all/unsp	nev any	st
WYNDE4	48		m	0	0	all	-	all	NAmer	1948	CC	684	n	bl	y	n	0	ev	all/unsp	nev any	st
WYNDE4	62		f	0	0	all	-	all	NAmer	1948	CC	684	n	bl	y	n	2	ev	all/unsp	nev any	ot
WYNDE6	72		m	0	0	all	-	all	NAmer	1969	CC	4423	n	bl	n	y	0	ev	all/unsp	nev any	st
WYNDE6	252		f	0	0	all	-	all	NAmer	1969	CC	4423	n	bl	n	y	0	ev	cig+/-ot	nev cigs	st
XIANGZ	8	x	m	0	0	all	0	all	As:Chi	1976	pr	983	m	ot	n	n	0	ev	all/unsp	nev any	st
XU	1	x	m	0	0	all	-	all	As:Chi	1985	CC	729	n	ot	n	n	0	ev	all/unsp	nev any	st
XU2	1	x	c	0	0	all	-	all	As:Chi	1987	CC	610	o	ot	y	n	0	ev	all/unsp	nev any	st
XU3	1	x	m	0	0	all	-	all	As:Chi	1981	CC	135	n	ot	n	n	0	ev	all/unsp	nev any	st
XU3	3	x	f	0	0	all	-	all	As:Chi	1981	CC	135	n	ot	n	n	0	ev	all/unsp	nev any	st
XU4	1		c	0	0	all	-	all	As:Chi	1981	CC	206	n	ot	*	n	0	ev	all/unsp	nev any	st
YAMAGU	5	x	c	0	0	all	-	all	As:Jap	1989	CC	144	n	bl	n	y	0	ev	all/unsp	nev any	st
YONG	12		m	0	0	all	0	all	NAmer	1971	pr	216	n	bl	n	n	1	cu	cig+/-ot	nev cigs	or
YONG	15		f	0	0	all	0	all	NAmer	1971	pr	216	n	bl	n	n	1	cu	cig+/-ot	nev cigs	or
YUAN	1		m	0	0	all	0	all	As:Chi	1986	pr	142	n	ot	n	n	2	ev	cig+/-ot	nev cigs	ot
ZHANG	1	x	c	0	0	all	-	all	As:Chi	1988	CC	100	n	ot	n	n	0	ev	all/unsp	nev any	st
ZHENG	15		m	0	0	all	-	all	As:Chi	1982	CC	540	n	ot	*	y	0	ev	cig+/-ot	nev cigs	st
ZHENG	24		f	0	0	all	-	all	As:Chi	1982	CC	540	n	ot	*	y	0	ev	cig+/-ot	nev cigs	st
ZHOU	2		m	0	0	all	-	all	As:Chi	1978	CC	1360	n	ot	n	n	0	ev	all/unsp	nev any	st
ZHOU	3		f	0	0	all	-	all	As:Chi	1978	CC	1360	n	ot	n	n	0	ev	all/unsp	nev any	st

Cigarette type is all/unspec for all RRs
except for the following:

REF	NRR	CIGTYPE
ALDERS	12	MC only
DEAN3	124	MC only

Table 1C1 - 5

IESLC - Meta-anal of Ever Smoking (or Current if Ever not available), Any prod (or Cigs if Any not avail)
All LC types
Least adjusted

REF	NRR	SEX	AD	Number Exposed Case	Number Exposed Cont	Non-exposed Case	Non-exposed Cont	RR	95.00%CI
ABELIN	1	m	0	116	341	2	183	31.13	(7.60- 127.40)
*ABRAHA	7	m	0	269	10351	10	3365	8.74	(4.66- 16.42)
*ABRAHA	8	f	0	62	5256	28	11589	4.88	(3.13- 7.62)
Subtotal ABRAHA								5.93	(4.12- 8.53)
AGUDO	8	f	0	23	23	80	183	2.29	(1.21- 4.32)
*AKIBA	3	m	0	393	207682	18	35833	3.77	(2.35- 6.04)
*AKIBA	7	f	0	83	65179	116	359850	3.95	(2.98- 5.24)
Subtotal AKIBA								3.90	(3.06- 4.97)
ALDERS	62	m	0	799	676	15	133	10.48	(6.09- 18.05)
ALDERS	12	f	0	530	371	75	243	4.63	(3.46- 6.19)
Subtotal ALDERS								5.55	(4.30- 7.18)
*AMANDU	3	m	0	126	111395	6	25350	4.78	(2.11- 10.84)
AMES	4	m	0	297	251	15	62	4.89	(2.72- 8.81)
*ANDERS	3	f	0	297	96164	46	195158	13.10	(9.61- 17.87)
*ARCHER	6	m	0	140	36269	6	9842	6.33	(2.80- 14.33)
ARMADA	29	m	0	321	261	4	64	19.68	(7.07- 54.75)
AUSTIN	3	c	0	161	237	5	88	11.96	(4.75- 30.09)
AUVINE	1	c	0	473	288	44	229	8.55	(6.00- 12.18)
AXELSO	1	c	0	90	86	62	371	6.26	(4.20- 9.34)
AXELSS	1	m	0	292	344	16	160	8.49	(4.96- 14.52)
AXELSS	11	f	0	110	109	18	154	8.63	(4.95- 15.05)
Subtotal AXELSS								8.56	(5.82- 12.59)
BAND	1	m	2	-	-	-	-	9.96	(7.38- 13.44)
BARBON	106	m	0	733	567	22	188	11.05	(7.01- 17.41)
BECHER	1	m	0	143	238	3	54	10.82	(3.32- 35.23)
BECHER	2	f	0	38	44	10	52	4.49	(2.01- 10.03)
Subtotal BECHER								5.93	(3.05- 11.53)
*BENSHL	18	m	1	-	-	-	-	5.90	(2.62- 13.31)
*BEST	22	m	0	221	24776	1	2854	25.46	(3.57- 181.46)
*BEST	18	f	1	-	-	-	-	2.24	(0.59- 8.44)
Subtotal BEST								4.81	(1.60- 14.47)
BLOHMK	3	m	0	762	587	126	301	3.10	(2.45- 3.92)
BLOT4	1	m	0	327	245	8	87	14.51	(6.91- 30.51)
BOFFET	32	m	0	5504	5505	117	1750	14.95	(12.36- 18.10)
*BOUCOT	9	m	0	121	44937	0	7551	40.83~	(2.54- 656.51)
BRESLO	37	m	0	486	451	7	42	6.47	(2.88- 14.54)
BRESLO	38	f	0	13	11	12	14	1.38	(0.45- 4.20)
Subtotal BRESLO								3.79	(1.97- 7.29)
*BRETT	10	m	0	144	47930	6	6530	3.27	(1.45- 7.40)
BROCKM	1	m	0	87	81	2	2	1.07	(0.15- 7.80)
BROCKM	2	f	0	24	54	4	18	2.00	(0.61- 6.54)
Subtotal BROCKM								1.70	(0.61- 4.70)
BROSS	12	m	0	902	784	38	170	5.15	(3.57- 7.41)
BROWN2	2	m	2	-	-	-	-	9.10	(8.30- 10.00)
BROWN2	1	f	2	-	-	-	-	12.70	(11.50- 13.90)
Subtotal BROWN2								10.72	(10.03- 11.46)
BUFFLE	1	m	0	470	419	5	47	10.54	(4.15- 26.76)
BUFFLE	5	f	0	419	284	41	198	7.12	(4.93- 10.30)
Subtotal BUFFLE								7.51	(5.33- 10.58)
CARPEN	7	c	0	328	469	15	241	11.24	(6.55- 19.29)
CASCO2	1	c	0	149	212	6	98	11.48	(4.90- 26.87)
CASCOR	1	c	0	365	362	22	295	13.52	(8.56- 21.35)
*CEDERL	107	m	2	-	-	-	-	5.92	(3.85- 9.12)
*CEDERL	112	f	2	-	-	-	-	4.18	(2.94- 5.93)
Subtotal CEDERL								4.80	(3.66- 6.30)
CHAN	9	m	0	206	161	2	43	27.51	(6.57- 115.26)
CHAN	10	f	0	105	50	84	139	3.48	(2.26- 5.35)
Subtotal CHAN								4.13	(2.73- 6.25)
*CHANG	6	m	0	78	1506	5	502	5.20	(2.12- 12.77)
*CHANG	12	f	0	42	1183	11	1139	3.68	(1.90- 7.10)
Subtotal CHANG								4.15	(2.44- 7.06)
CHATZI	4	c	0	255	365	27	129	3.34	(2.14- 5.21)
CHEN2	1	m	0	121	97	9	33	4.57	(2.09- 10.02)
CHEN2	2	f	0	38	30	25	33	1.67	(0.82- 3.39)
Subtotal CHEN2								2.62	(1.55- 4.44)
CHEN3	1	c	0	182	156	72	98	1.59	(1.09- 2.30)
CHIAZZ	2	m	0	139	209	4	47	7.81	(2.75- 22.18)
CHOI	1	m	0	267	465	13	95	4.20	(2.31- 7.64)
CHOI	5	f	0	19	26	76	164	1.58	(0.82- 3.02)
Subtotal CHOI								2.68	(1.72- 4.16)
*CHOW	7	m	0	206	202852	6	62913	10.65	(4.73- 23.98)

Table 1C1 - 5

IESLC - Meta-anal of Ever Smoking (or Current if Ever not available), Any prod (or Cigs if Any not avail)
All LC types
Least adjusted

REF	NRR	SEX	AD	Number Exposed Case	Cont	Non-exposed Case	Cont	RR	95.00%CI
*CHYOU	9	m	0	214	5554	13	2406	7.13	(4.08- 12.46)
COMSTO	34	m	0	153	244	4	69	10.82	(3.87- 30.24)
COMSTO	46	f	0	88	87	13	115	8.95	(4.69- 17.06)
Subtotal COMSTO								9.44	(5.46- 16.31)
COOKSO	5	c	0	189	39	45	61	6.57	(3.92- 11.02)
CORREA	33	c	0	1202	886	51	388	10.32	(7.61- 14.00)
*CPSI	187	m	1	-	-	-	-	9.18	(7.36- 11.45)
*CPSI	274	f	1	-	-	-	-	2.79	(2.22- 3.51)
Subtotal CPSI								5.17	(4.41- 6.06)
*CPSII	104	m	1	-	-	-	-	12.83	(10.28- 16.01)
*CPSII	79	f	1	-	-	-	-	8.16	(6.93- 9.62)
Subtotal CPSII								9.58	(8.40- 10.93)
DAMBER	5	m	0	537	364	42	208	7.31	(5.11- 10.44)
DARBY	15	m	0	664	1724	3	384	49.30	(15.77- 154.07)
DARBY	16	f	0	292	548	23	529	12.26	(7.89- 19.05)
Subtotal DARBY								14.69	(9.74- 22.16)
DAVEYS	5	m	0	90	144	3	23	4.79	(1.40- 16.42)
DAVEYS	6	f	0	0	3	16	83	0.72~	(0.04- 14.66)
Subtotal DAVEYS								3.65	(1.17- 11.42)
DEAN	7	m	0	591	574	12	61	5.23	(2.79- 9.82)
DEAN2	3	m	0	769	688	33	112	3.79	(2.54- 5.67)
DEAN2	7	f	0	64	30	88	121	2.93	(1.76- 4.90)
Subtotal DEAN2								3.44	(2.51- 4.72)
DEAN3	47	m	0	591	2053	25	510	5.87	(3.89- 8.86)
DEAN3	124	f	0	109	1420	41	1538	2.88	(2.00- 4.15)
Subtotal DEAN3								3.94	(3.00- 5.19)
*DEKLER	6	m	2	-	-	-	-	20.29	(2.84- 145.18)
DESTE2	13	c	0	300	212	20	108	7.64	(4.60- 12.71)
DESTEF	5	m	0	470	334	27	163	8.50	(5.52- 13.07)
*DOCKER	3	c	4	-	-	-	-	4.29	(1.66- 11.06)
DOLL	6	m	0	1350	1296	7	61	9.08	(4.14- 19.92)
DOLL	12	f	0	68	49	40	59	2.05	(1.19- 3.53)
Subtotal DOLL								3.32	(2.12- 5.19)
*DOLL2	56	m	1	-	-	-	-	7.66	(4.86- 12.07)
*DOLL2	63	f	1	-	-	-	-	8.65	(2.93- 25.55)
Subtotal DOLL2								7.80	(5.13- 11.86)
DORANT	10	c	0	470	2033	14	1090	18.00	(10.52- 30.78)
DORGAN	6	m	0	721	455	15	93	9.82	(5.62- 17.16)
DORGAN	30	m	0	266	135	3	35	22.99	(6.94- 76.10)
DORGAN	53	f	0	757	229	103	244	7.83	(5.96- 10.30)
DORGAN	76	f	0	79	27	7	20	8.36	(3.18- 21.95)
Subtotal DORGAN								8.52	(6.75- 10.76)
*DORN	196	m	1	-	-	-	-	7.04	(5.60- 8.84)
DOSEME	17	m	0	1068	536	142	293	4.11	(3.28- 5.15)
DROSTE	3	m	0	471	443	7	93	14.13	(6.48- 30.78)
DU	1	m	0	538	-	28	-	3.53	(2.44- 5.11)
DU	2	f	0	191	-	92	-	1.93	(1.30- 2.87)
Subtotal DU								2.66	(2.03- 3.49)
*DUNN	6	m	0	137	52634	2	14160	18.43	(4.56- 74.42)
EBELIN	1	m	0	101	142	12	117	6.93	(3.63- 13.24)
*ENGELA	155	m	0	111	9235	7	2683	4.61	(2.15- 9.88)
*ENGELA	162	f	0	13	3262	13	10708	3.28	(1.52- 7.07)
Subtotal ENGELA								3.89	(2.27- 6.69)
*ENSTRO	1	m	1	-	-	-	-	12.99	(10.46- 16.13)
*ENSTRO	2	f	1	-	-	-	-	6.95	(6.01- 8.04)
Subtotal ENSTRO								8.44	(7.48- 9.53)
ESAKI	4	m	0	155	143	16	28	1.90	(0.99- 3.65)
ESAKI	5	f	0	34	19	40	55	2.46	(1.23- 4.92)
Subtotal ESAKI								2.14	(1.33- 3.45)
FAN	1	m	0	216	498	36	236	2.84	(1.93- 4.18)
FAN	2	f	0	82	97	69	320	3.92	(2.65- 5.81)
Subtotal FAN								3.33	(2.53- 4.38)
GAO	6	m	0	671	558	62	202	3.92	(2.89- 5.32)
GAO	16	f	0	237	130	435	605	2.54	(1.98- 3.25)
Subtotal GAO								3.01	(2.48- 3.65)
GAO2	6	m	0	269	226	13	56	5.13	(2.73- 9.62)
GARCIA	3	c	0	395	307	21	139	8.52	(5.26- 13.80)
GARDIN	7	c	0	138	102	5	41	11.09	(4.23- 29.06)
GARSHI	17	m	0	1040	1596	41	363	5.77	(4.14- 8.04)
GENG	1	m	0	92	68	7	31	5.99	(2.49- 14.42)
GENG	2	f	0	126	75	67	118	2.96	(1.96- 4.48)

Table 1C1 - 5

IESLC - Meta-anal of Ever Smoking (or Current if Ever not available), Any prod (or Cigs if Any not avail)
All LC types
Least adjusted

REF	NRR	SEX	AD	Number Exposed Case	Number Exposed Cont	Non-exposed Case	Non-exposed Cont	RR	95.00%CI
Subtotal GENG								3.36	(2.31- 4.89)
GER	17	c	0	90	318	51	246	1.37	(0.93- 2.00)
GODLEY	5	m	1	-	-	-	-	6.84	(5.60- 8.35)
GODLEY	6	f	1	-	-	-	-	5.54	(4.29- 7.15)
Subtotal GODLEY								6.31	(5.39- 7.39)
GOLLED	21	m	0	380	1966	15	490	6.31	(3.73- 10.68)
GOODMA	3	m	0	216	398	10	199	10.80	(5.60- 20.82)
GOODMA	7	f	0	81	91	19	177	8.29	(4.74- 14.52)
Subtotal GOODMA								9.27	(6.05- 14.19)
GRAHAM	22	m	0	667	1651	18	346	7.77	(4.79- 12.58)
GREGOR	3	m	0	72	98	10	14	1.03	(0.43- 2.45)
GREGOR	7	f	0	21	42	1	22	11.00	(1.39- 87.29)
Subtotal GREGOR								1.46	(0.66- 3.26)
GSELL	8	m	0	148	121	2	29	17.74	(4.15- 75.83)
HAENSZ	50	f	0	76	103	81	236	2.15	(1.46- 3.17)
*HAMMO2	18	m	0	180	10199	1	1457	25.71	(3.61- 183.40)
*HAMMON	129	m	0	425	510108	15	115884	6.44	(3.85- 10.77)
*HANSEN	3	m	2	-	-	-	-	1.53	(0.71- 3.91)
HEGMAN	1	c	0	255	1202	27	2080	16.34	(10.92- 24.45)
*HEIN	7	m	0	143	4471	1	457	14.62	(2.05- 104.23)
*HENNEK	3	m	0	146	11112	23	10919	6.24	(4.02- 9.67)
HINDS	26	f	0	167	592	124	1812	4.12	(3.21- 5.29)
*HIRAYA	147	m	1	-	-	-	-	4.36	(3.53- 5.39)
*HIRAYA	150	f	1	-	-	-	-	2.36	(1.90- 2.94)
Subtotal HIRAYA								3.24	(2.78- 3.77)
HITOSU	6	m	0	142	1787	7	242	2.75	(1.27- 5.94)
HITOSU	12	f	0	34	500	33	1893	3.90	(2.39- 6.36)
Subtotal HITOSU								3.53	(2.33- 5.33)
*HOLE	15	m	0	187	5866	7	1189	5.41	(2.55- 11.49)
*HOLE	29	f	0	13	2144	8	1917	1.45	(0.60- 3.50)
Subtotal HOLE								3.10	(1.75- 5.50)
HOROWI	1	m	0	182	525	19	196	3.58	(2.17- 5.90)
HOROWI	2	f	0	21	382	14	463	1.82	(0.91- 3.62)
Subtotal HOROWI								2.83	(1.89- 4.25)
HORWIT	1	f	0	97	92	11	118	11.31	(5.73- 22.34)
HU	15	m	0	120	94	41	67	2.09	(1.30- 3.35)
HU	16	f	0	26	18	40	48	1.73	(0.83- 3.61)
Subtotal HU								1.98	(1.33- 2.94)
HU2	9	m	0	294	228	49	115	3.03	(2.08- 4.41)
HU2	10	f	0	108	80	72	100	1.88	(1.23- 2.85)
Subtotal HU2								2.44	(1.85- 3.23)
HUANG	1	c	0	98	77	37	58	2.00	(1.20- 3.32)
HUMBLE	14	m	1	-	-	-	-	12.10	(5.12- 28.60)
HUMBLE	16	m	1	-	-	-	-	11.88	(2.65- 53.30)
HUMBLE	18	f	1	-	-	-	-	11.36	(5.32- 24.23)
HUMBLE	20	f	1	-	-	-	-	15.40	(4.87- 48.74)
Subtotal HUMBLE								12.28	(7.58- 19.90)
JAHN	3	f	0	112	67	53	98	3.09	(1.97- 4.85)
JAIN	6	m	0	391	277	12	85	10.00	(5.36- 18.66)
JAIN	1	f	0	390	196	52	214	8.19	(5.78- 11.60)
Subtotal JAIN								8.59	(6.34- 11.64)
JARUP	3	m	0	91	52	11	42	6.68	(3.17- 14.09)
JARVHO	3	m	0	99	57	1	16	27.79	(3.59- 215.09)
JARVHO	7	f	0	41	15	6	21	9.57	(3.24- 28.26)
Subtotal JARVHO								12.08	(4.64- 31.46)
JEDRYC	63	m	0	852	656	49	219	5.80	(4.19- 8.04)
JEDRYC	68	f	0	120	32	78	166	7.98	(4.97- 12.82)
Subtotal JEDRYC								6.43	(4.92- 8.41)
JIANG	1	m	0	93	83	7	17	2.72	(1.08- 6.89)
JIANG	2	f	0	11	6	14	19	2.49	(0.74- 8.35)
Subtotal JIANG								2.63	(1.26- 5.50)
JOLY	14	m	0	595	888	12	218	12.17	(6.75- 21.97)
JOLY	1	f	0	166	123	52	283	7.34	(5.04- 10.70)
Subtotal JOLY								8.50	(6.19- 11.68)
JUSSAW	3	m	0	643	168	149	624	16.03	(12.53- 20.51)
*KAISE2	72	m	1	-	-	-	-	5.40	(3.05- 9.57)
*KAISE2	64	f	1	-	-	-	-	10.09	(5.29- 19.27)
Subtotal KAISE2								7.11	(4.63- 10.90)
*KAISER	13	m	2	-	-	-	-	17.63	(11.98- 25.96)
*KAISER	10	f	2	-	-	-	-	5.63	(3.89- 8.14)
Subtotal KAISER								9.70	(7.43- 12.67)

Table 1C1 - 5

IESLC - Meta-anal of Ever Smoking (or Current if Ever not available), Any prod (or Cigs if Any not avail)
All LC types
Least adjusted

REF	NRR	SEX	AD	Number Exposed Case	Number Exposed Cont	Non-exposed Case	Non-exposed Cont	RR	95.00%CI
KANELL	5	m	0	814	441	48	172	6.61	(4.71- 9.30)
KATSOU	27	f	0	53	22	48	67	3.36	(1.81- 6.25)
KAUFMA	8	c	0	846	1645	35	925	13.59	(9.60- 19.24)
KELLER	3	m	0	8066	2517	323	1017	10.09	(8.83- 11.52)
KELLER	11	m	0	1493	340	38	117	13.52	(9.20- 19.86)
KELLER	7	f	0	3998	1269	469	1860	12.49	(11.09- 14.08)
KELLER	15	f	0	584	214	67	232	9.45	(6.91- 12.93)
Subtotal KELLER								11.30	(10.40- 12.29)
KHUDER	4	m	0	459	785	23	309	7.86	(5.06- 12.19)
KIHARA	31	c	0	338	232	102	237	3.39	(2.54- 4.51)
*KINLEN	6	m	0	711	12722	7	1333	10.64	(5.07- 22.36)
KJUUS	10	m	0	174	152	2	24	13.74	(3.19- 59.08)
*KNEKT	76	m	0	111	51798	6	17814	6.36	(2.80- 14.46)
KO	1	f	3	-	-	-	-	4.20	(1.10- 15.60)
KOHLME	1	c	0	228	236	11	193	16.95	(8.99- 31.96)
KOO	1	f	0	112	63	88	137	2.77	(1.84- 4.16)
KOULUM	1	m	0	807	246	5	54	35.43	(14.02- 89.55)
KREUZE	14	f	0	62	42	6	38	9.35	(3.63- 24.08)
KREUZE	16	f	0	205	101	95	177	3.78	(2.68- 5.34)
Subtotal KREUZE								4.21	(3.04- 5.81)
KREYBE	24	m	0	252	3514	6	644	7.70	(3.41- 17.37)
KREYBE	39	f	0	12	328	30	657	0.80	(0.40- 1.59)
Subtotal KREYBE								2.04	(1.21- 3.44)
*KUBIK	28	m	0	106	8051	2	4271	28.12	(6.94- 113.84)
LAMTH	6	f	0	242	106	202	337	3.81	(2.86- 5.08)
LAMWK	1	f	0	88	41	75	144	4.12	(2.59- 6.55)
LAMWK2	9	m	0	244	161	23	43	2.83	(1.64- 4.88)
LAMWK2	10	f	0	75	50	65	139	3.21	(2.02- 5.10)
Subtotal LAMWK2								3.04	(2.14- 4.33)
*LANGE	34	m	0	195	5790	5	721	4.86	(2.01- 11.76)
*LANGE	31	f	0	61	5544	7	2159	3.39	(1.55- 7.41)
Subtotal LANGE								3.97	(2.21- 7.13)
LAUSSM	10	m	0	347	188	85	226	4.91	(3.61- 6.66)
LEI	1	m	0	443	361	41	123	3.68	(2.52- 5.38)
LEI	2	f	0	123	61	85	147	3.49	(2.32- 5.24)
Subtotal LEI								3.59	(2.72- 4.74)
LEMARC	3	c	0	309	288	32	168	5.63	(3.74- 8.49)
LETOUR	1	c	0	714	514	24	224	12.96	(8.38- 20.05)
LEVIN	32	m	1	-	-	-	-	4.86	(3.41- 6.92)
*LIAW	1	m	1	-	-	-	-	3.70	(2.10- 6.60)
*LIAW	2	f	1	-	-	-	-	3.60	(1.00- 12.20)
Subtotal LIAW								3.68	(2.19- 6.20)
*LIDDEL	5	m	1	-	-	-	-	3.61	(2.27- 5.73)
LIU	2	c	2	-	-	-	-	1.92	(1.40- 2.64)
LIU2	1	m	0	212	180	12	44	4.32	(2.21- 8.43)
LIU2	3	f	0	54	23	38	69	4.26	(2.27- 7.99)
Subtotal LIU2								4.29	(2.71- 6.78)
LIU3	1	m	0	52	205	4	19	1.20	(0.39- 3.69)
LIU4	11	m	2	-	-	-	-	2.76	(2.69- 2.83)
LIU4	12	f	2	-	-	-	-	2.86	(2.77- 2.95)
Subtotal LIU4								2.80	(2.74- 2.85)
LIU5	1	c	0	85	70	26	41	1.91	(1.07- 3.44)
LOMBA2	1	f	0	149	353	76	239	1.33	(0.96- 1.83)
LOMBAR	12	m	0	1026	928	14	112	8.84	(5.04- 15.53)
LUBIN2	45	m	0	6729	10841	190	2617	8.55	(7.36- 9.94)
LUBIN2	101	f	0	549	561	336	1188	3.46	(2.92- 4.10)
Subtotal LUBIN2								5.73	(5.12- 6.41)
LUO	1	c	0	65	146	37	160	1.93	(1.21- 3.06)
MACLEN	71	m	0	142	119	5	15	3.58	(1.26- 10.14)
MACLEN	72	f	0	45	57	41	109	2.10	(1.23- 3.57)
Subtotal MACLEN								2.34	(1.46- 3.76)
*MAGNUS	1	m	0	189	3439	11	1086	5.43	(2.97- 9.93)
MARSH	2	m	0	98	155	2	31	9.80	(2.29- 41.87)
MARSH	4	f	0	42	64	8	71	5.82	(2.54- 13.33)
Subtotal MARSH								6.62	(3.22- 13.59)
MARSH2	2	c	0	102	149	12	56	3.19	(1.63- 6.26)
MARTIS	4	m	0	197	176	4	25	7.00	(2.39- 20.49)
MASTRA	1	m	0	303	265	6	44	8.38	(3.52- 19.99)
MATOS	26	m	0	188	283	11	110	6.64	(3.48- 12.68)
MATSUD	10	m	0	170	3314	3	1255	21.46	(6.84- 67.33)
MCCONN	1	m	0	88	174	5	12	1.21	(0.41- 3.55)

Table 1C1 - 5

IESLC - Meta-anal of Ever Smoking (or Current if Ever not available), Any prod (or Cigs if Any not avail)
All LC types
Least adjusted

REF	NRR	SEX	AD	Number Exposed Case	Exposed Cont	Non-exposed Case	Non-exposed Cont	RR	95.00%CI
MCCONN	2	f	0	3	3	4	11	2.75 (	0.38- 19.67)
Subtotal MCCONN								1.46 (	0.57- 3.76)
MCDUFF	1	m	0	159	134	6	31	6.13 (	2.48- 15.14)
MCLAUG	1	m	0	294	1082	22	270	3.33 (	2.12- 5.25)
*MIGRAN	26	m	0	206	6719	4	867	6.65 (	2.48- 17.83)
*MIGRAN	41	f	0	31	4086	4	3814	7.23 (	2.56- 20.47)
Subtotal MIGRAN								6.92 (	3.38- 14.16)
MILLER	1	f	0	140	1607	28	3638	11.32 (	7.51- 17.06)
MILLS	3	m	1	-	-	-	-	1.33 (	1.09- 1.63)
*MRFITR	6	m	0	119	11007	0	1859	40.37~(	2.51- 648.95)
NAM	69	m	0	610	1075	30	520	9.84 (	6.72- 14.40)
NAM	85	f	0	292	496	52	885	10.02 (	7.31- 13.73)
Subtotal NAM								9.94 (	7.80- 12.68)
NOTAN2	1	m	0	549	735	134	544	3.03 (	2.44- 3.77)
NOU	11	m	0	74	247	6	122	6.09 (	2.58- 14.39)
NOU	12	f	0	10	92	4	261	7.09 (	2.17- 23.17)
Subtotal NOU								6.42 (	3.20- 12.87)
ODRISC	3	c	0	440	996	6	664	48.89 (	21.71- 110.07)
ORMOS	4	m	0	87	1034	7	777	9.34 (	4.30- 20.28)
ORMOS	26	f	0	1	234	23	1044	0.19 (	0.03- 1.44)
Subtotal ORMOS								5.65 (	2.74- 11.64)
OSANN	17	m	0	1108	1018	45	833	20.15 (	14.75- 27.52)
OSANN	21	f	0	737	563	96	1093	14.90 (	11.77- 18.87)
Subtotal OSANN								16.63 (	13.78- 20.07)
PARKIN	29	m	0	401	1007	107	1248	4.64 (	3.69- 5.84)
PASTOR	5	m	0	194	262	10	89	6.59 (	3.34- 13.00)
PAWLEG	1	m	0	172	249	4	92	15.89 (	5.73- 44.05)
PERNU	2	m	0	1380	438	97	275	8.93 (	6.92- 11.53)
PERNU	1	f	0	19	89	110	971	1.88 (	1.11- 3.21)
Subtotal PERNU								6.68 (	5.31- 8.41)
PERSH2	5	c	0	844	924	178	1164	5.97 (	4.97- 7.17)
*PETO	5	m	0	101	2423	2	295	6.15 (	1.52- 24.79)
PEZZO2	10	m	0	361	469	6	117	15.01 (	6.53- 34.48)
PEZZOT	25	m	0	211	317	4	116	19.30 (	7.02- 53.10)
PIKE	4	m	0	514	375	18	69	5.25 (	3.08- 8.98)
PIKE	8	f	0	163	90	36	96	4.83 (	3.04- 7.66)
Subtotal PIKE								5.01 (	3.53- 7.10)
POFFIJ	1	c	0	913	918	58	452	7.75 (	5.81- 10.34)
POLEDN	3	c	0	196	271	12	139	8.38 (	4.52- 15.54)
*QIAO2	3	m	0	231	6917	10	709	2.37 (	1.26- 4.44)
RACHTA	3	f	0	85	43	33	98	5.87 (	3.43- 10.06)
RADZIK	1	c	0	180	198	9	13	1.31 (	0.55- 3.15)
RANDIG	23	m	0	410	359	5	22	5.03 (	1.88- 13.41)
RANDIG	24	f	0	16	39	17	92	2.22 (	1.02- 4.84)
Subtotal RANDIG								3.04 (	1.65- 5.60)
REN	1	m	0	106	84	12	34	3.58 (	1.74- 7.33)
REN	2	f	0	78	20	48	50	4.06 (	2.16- 7.64)
Subtotal REN								3.84 (	2.39- 6.17)
RONCO	1	m	0	120	306	6	78	5.10 (	2.16- 12.01)
ROTHSC	1	c	0	271	222	13	62	5.82 (	3.12- 10.86)
SADOWS	7	m	0	459	534	18	81	3.87 (	2.29- 6.54)
SANKAR	1	m	0	251	439	28	767	15.66 (	10.42- 23.55)
SCHWAR	1	m	0	2648	1019	119	376	8.21 (	6.60- 10.22)
SCHWAR	2	m	0	863	275	50	104	6.53 (	4.54- 9.39)
SCHWAR	3	f	0	1351	637	182	855	9.96 (	8.28- 12.00)
SCHWAR	4	f	0	335	179	40	247	11.56 (	7.90- 16.90)
Subtotal SCHWAR								9.05 (	7.99- 10.25)
SEGI	1	m	0	140	1742	18	382	1.71 (	1.03- 2.82)
SEGI2	19	m	0	267	485	8	53	3.65 (	1.71- 7.79)
SEGI2	27	f	0	24	34	56	126	1.59 (	0.86- 2.92)
Subtotal SEGI2								2.20 (	1.37- 3.54)
SEOW	1	f	0	61	15	92	125	5.53 (	2.96- 10.33)
SHAW	12	c	0	324	266	11	107	11.85 (	6.24- 22.50)
SIEMIA	9	m	0	844	428	13	105	15.93 (	8.85- 28.67)
SIMARA	5	m	0	33	264	27	433	2.00 (	1.18- 3.41)
SIMARA	6	f	0	17	67	38	349	2.33 (	1.24- 4.37)
Subtotal SIMARA								2.13 (	1.42- 3.20)
SOBUE	91	m	0	1023	1013	34	128	3.80 (	2.58- 5.60)
SOBUE	95	f	0	127	232	167	857	2.81 (	2.14- 3.69)
Subtotal SOBUE								3.10 (	2.48- 3.88)
SOBUE2	10	m	2	-	-	-	-	4.47 (	3.89- 5.14)

Table 1C1 - 5

IESLC - Meta-anal of Ever Smoking (or Current if Ever not available), Any prod (or Cigs if Any not avail)
All LC types
Least adjusted

REF	NRR	SEX	AD	Number Exposed Case	Number Exposed Cont	Non-exposed Case	Non-exposed Cont	RR	95.00%CI
SOBUE2	12	f	2	-	-	-	-	3.28	(2.79- 3.87)
Subtotal SOBUE2								3.92	(3.53- 4.36)
*SPEIZE	8	f	0	535	1012074	58	776300	7.08	(5.40- 9.28)
SPITZ	3	c	0	170	169	7	128	18.39	(8.35- 40.53)
STASZE	1	m	0	255	754	5	158	10.69	(4.34- 26.33)
STASZE	5	f	0	6	153	15	1660	4.34	(1.66- 11.35)
Subtotal STASZE								7.01	(3.63- 13.53)
STAYNE	1	m	0	362	567	58	333	3.67	(2.69- 4.99)
STOCKS	31	m	0	2632	6477	45	638	5.76	(4.24- 7.82)
STOCKS	50	f	1	-	-	-	-	3.04	(2.35- 3.93)
Subtotal STOCKS								3.96	(3.25- 4.82)
STOCKW	6	c	0	19370	7069	2791	10641	10.45	(9.94- 10.98)
STUCKE	3	m	0	247	203	0	51	125.27~	(7.68-2042.38)
SUN	1	c	0	140	173	67	191	2.31	(1.62- 3.30)
SUZUK2	18	c	0	112	70	11	53	7.71	(3.77- 15.76)
SVENSS	56	f	0	172	89	38	120	6.10	(3.91- 9.53)
TANG	3	c	0	110	59	9	39	8.08	(3.66- 17.82)
*TENKAN	22	m	1	-	-	-	-	14.64	(6.29- 34.07)
TIZZAN	1	m	0	1036	911	180	305	1.93	(1.57- 2.36)
TIZZAN	12	f	0	25	28	25	114	4.07	(2.04- 8.13)
Subtotal TIZZAN								2.05	(1.68- 2.49)
TOKARS	3	m	0	147	243	1	53	32.06	(4.39- 234.30)
TOKARS	5	f	0	1	2	13	40	1.54	(0.13- 18.38)
Subtotal TOKARS								9.77	(2.07- 46.11)
TOUSEY	16	m	0	297	437	4	130	22.09	(8.08- 60.39)
TOUSEY	26	f	0	193	214	13	226	15.68	(8.67- 28.34)
Subtotal TOUSEY								17.12	(10.28- 28.52)
TSUGAN	27	m	0	73	71	18	22	1.26	(0.62- 2.54)
*TULINI	15	m	1	-	-	-	-	8.06	(4.38- 14.84)
*TULINI	21	f	1	-	-	-	-	14.95	(8.30- 26.95)
Subtotal TULINI								11.10	(7.26- 16.95)
*TVERDA	22	m	2	-	-	-	-	4.58	(2.97- 7.06)
*TVERDA	15	f	2	-	-	-	-	11.05	(3.33- 36.71)
Subtotal TVERDA								5.07	(3.37- 7.62)
WAKAI	13	m	0	235	424	10	65	3.60	(1.82- 7.14)
WAKAI	31	f	0	38	31	50	145	3.55	(2.00- 6.30)
Subtotal WAKAI								3.57	(2.30- 5.55)
*WALD	2	m	0	77	4182	7	6539	17.20	(7.94- 37.25)
WANG	1	m	0	262	-	29	-	3.47	(2.10- 5.80)
WANG	2	f	0	17	-	82	-	4.00	(1.30- 12.00)
Subtotal WANG								3.56	(2.24- 5.64)
WANG2	8	c	0	60	99	11	43	2.37	(1.14- 4.94)
WANG3	1	c	0	235	172	58	121	2.85	(1.97- 4.13)
WANG4	1	m	0	1043	18164	127	2374	1.07	(0.89- 1.30)
WICKLU	1	m	0	-	-	-	-	4.60	(2.80- 7.60)
WIGLE	15	m	0	598	798	15	204	10.19	(5.97- 17.40)
WIGLE	18	f	0	79	235	36	439	4.10	(2.68- 6.27)
Subtotal WIGLE								5.83	(4.18- 8.13)
WILKIN	1	m	0	173	372	2	108	25.11	(6.13- 102.89)
WILKIN	2	f	0	84	109	12	89	5.72	(2.93- 11.13)
Subtotal WILKIN								7.49	(4.10- 13.68)
WU	37	f	0	189	128	31	92	4.38	(2.75- 6.97)
WUNSCH	1	m	0	290	441	14	99	4.65	(2.61- 8.30)
WUNSCH	7	f	0	60	98	29	208	4.39	(2.65- 7.27)
Subtotal WUNSCH								4.50	(3.08- 6.58)
WUWILL	6	f	0	539	351	417	601	2.21	(1.84- 2.66)
WYNDE2	21	m	0	396	616	8	105	8.44	(4.07- 17.51)
WYNDE3	49	m	0	275	332	9	88	8.10	(4.00- 16.38)
WYNDE3	138	f	0	46	56	20	76	3.12	(1.67- 5.85)
Subtotal WYNDE3								4.76	(2.98- 7.61)
WYNDE4	48	m	0	632	665	12	115	9.11	(4.98- 16.67)
WYNDE4	62	f	2	-	-	-	-	2.87	(1.48- 5.55)
Subtotal WYNDE4								5.38	(3.45- 8.41)
WYNDE6	72	m	0	2823	1996	87	617	10.03	(7.96- 12.65)
WYNDE6	252	f	0	1354	701	159	856	10.40	(8.58- 12.60)
Subtotal WYNDE6								10.25	(8.84- 11.88)
*XIANGZ	8	m	0	907	13037	25	974	2.71	(1.83- 4.01)
XU	1	m	0	627	552	102	236	2.63	(2.03- 3.40)
XU2	1	c	0	501	582	82	377	3.96	(3.03- 5.17)
XU3	1	m	0	92	68	7	31	5.99	(2.49- 14.42)
XU3	3	f	0	23	11	13	25	4.02	(1.51- 10.74)

Table 1C1 - 5

IESLC - Meta-anal of Ever Smoking (or Current if Ever not available), Any prod (or Cigs if Any not avail)
All LC types
Least adjusted

REF	NRR	SEX	AD	Number Case	Exposed Cont	Non-exposed Case	Cont	RR	95.00%CI
Subtotal XU3								5.02	(2.61- 9.66)
XU4	1	c	0	161	113	45	93	2.94	(1.92- 4.52)
YAMAGU	5	c	0	120	409	24	267	3.26	(2.05- 5.19)
*YONG	12	m	1	-	-	-	-	28.71	(6.98- 118.16)
*YONG	15	f	1	-	-	-	-	5.20	(2.38- 11.35)
Subtotal YONG								7.75	(3.91- 15.36)
*YUAN	1	m	2	-	-	-	-	6.50	(3.64- 11.60)
ZHANG	1	c	0	72	102	28	98	2.47	(1.47- 4.14)
ZHENG	15	m	0	279	218	33	94	3.65	(2.36- 5.63)
ZHENG	24	f	0	76	44	152	184	2.09	(1.36- 3.21)
Subtotal ZHENG								2.75	(2.03- 3.73)
ZHOU	2	m	0	740	41	275	36	2.36	(1.48- 3.77)
ZHOU	3	f	0	112	7	231	32	2.22	(0.95- 5.18)
Subtotal ZHOU								2.33	(1.54- 3.51)
Partial Totals				134036	2765441	15003	1787463		

*prospective study ~ With 0.5 adjustment for zero

REF	NRR	SEX	AD	Ys	Ws	Qs	Ps
ABELIN	1	m	0	3.44	1.93	7.64	0.0000
*ABRAHA	7	m	0	2.17	9.68	5.00	0.0000
*ABRAHA	8	f	0	1.59	19.39	0.36	0.0000
Subtotal ABRAHA				1.78	29.07	5.35	
AGUDO	8	f	0	0.83	9.53	3.69	0.0106
*AKIBA	3	m	0	1.33	17.22	0.26	0.0000
*AKIBA	7	f	0	1.37	48.42	0.28	0.0000
Subtotal AKIBA				1.36	65.65	0.54	
ALDERS	62	m	0	2.35	13.00	10.52	0.0000
ALDERS	12	f	0	1.53	45.39	0.31	0.0000
Subtotal ALDERS				1.71	58.39	10.82	
*AMANDU	3	m	0	1.56	5.73	0.07	0.0002
AMES	4	m	0	1.59	11.09	0.21	0.0000
*ANDERS	3	f	0	2.57	39.86	50.25	0.0000
*ARCHER	6	m	0	1.85	5.76	0.90	0.0000
ARMADA	29	m	0	2.98	3.67	8.58	0.0000
AUSTIN	3	c	0	2.48	4.51	4.79	0.0000
AUVINE	1	c	0	2.15	30.60	14.81	0.0000
AXELSO	1	c	0	1.83	24.06	3.56	0.0000
AXELSS	1	m	0	2.14	13.32	6.32	0.0000
AXELSS	11	f	0	2.16	12.45	6.20	0.0000
Subtotal AXELSS				2.15	25.77	12.52	
BAND	1	m	2	2.30	42.76	30.79	0.0000
BARBON	106	m	0	2.40	18.55	16.82	0.0000
BECHER	1	m	0	2.38	2.75	2.39	0.0001
BECHER	2	f	0	1.50	5.94	0.02	0.0003
Subtotal BECHER				1.78	8.70	2.40	
*BENSHL	18	m	1	1.77	5.82	0.61	0.0000
*BEST	22	m	0	3.24	1.00	3.18	0.0012
*BEST	18	f	1	0.81	2.17	0.90	0.2348
Subtotal BEST				1.57	3.17	4.08	
BLOHMK	3	m	0	1.13	70.05	7.10	0.0000
BLOT4	1	m	0	2.68	6.96	10.45	0.0000
BOFFET	32	m	0	2.71	105.47	166.11	0.0000
*BOUCOT	9	m	0	3.71	0.50	2.54	0.0089
BRESLO	37	m	0	1.87	5.85	1.01	0.0000
BRESLO	38	f	0	0.32	3.10	3.95	0.5717
Subtotal BRESLO				1.33	8.95	4.96	
*BRETT	10	m	0	1.18	5.77	0.41	0.0044
BROCKM	1	m	0	0.07	0.98	1.86	0.9437
BROCKM	2	f	0	0.69	2.73	1.57	0.2517
Subtotal BROCKM				0.53	3.71	3.42	
BROSS	12	m	0	1.64	28.92	1.03	0.0000
BROWN2	2	m	2	2.21	442.58	254.46	0.0000
BROWN2	1	f	2	2.54	427.71	509.63	0.0000
Subtotal BROWN2				2.37	870.29	764.09	
BUFFLE	1	m	0	2.36	4.43	3.63	0.0000
BUFFLE	5	f	0	1.96	28.29	7.46	0.0000
Subtotal BUFFLE				2.02	32.72	11.09	
CARPEN	7	c	0	2.42	13.16	12.36	0.0000
CASCO2	1	c	0	2.44	5.31	5.21	0.0000
CASCOR	1	c	0	2.60	18.40	24.51	0.0000

Table 1C1 - 5

IESLC - Meta-anal of Ever Smoking (or Current if Ever not available), Any prod (or Cigs if Any not avail)
All LC types
Least adjusted

REF	NRR	SEX	AD	Ys	Ws	Qs	Ps
*CEDERL	107	m	2	1.78	20.66	2.23	0.0000
*CEDERL	112	f	2	1.43	31.21	0.01	0.0000
Subtotal CEDERL				1.57	51.88	2.24	
CHAN	9	m	0	3.31	1.87	6.51	0.0000
CHAN	10	f	0	1.25	20.57	0.86	0.0000
Subtotal CHAN				1.42	22.44	7.37	
*CHANG	6	m	0	1.65	4.76	0.19	0.0003
*CHANG	12	f	0	1.30	8.85	0.19	0.0001
Subtotal CHANG				1.42	13.61	0.38	
CHATZI	4	c	0	1.21	19.44	1.16	0.0000
CHEN2	1	m	0	1.52	6.25	0.03	0.0001
CHEN2	2	f	0	0.51	7.70	6.74	0.1539
Subtotal CHEN2				0.97	13.95	6.77	
CHEN3	1	c	0	0.46	27.78	27.09	0.0148
CHIAZZ	2	m	0	2.06	3.53	1.30	0.0001
CHOI	1	m	0	1.43	10.71	0.00	0.0000
CHOI	5	f	0	0.46	9.06	8.96	0.1703
Subtotal CHOI				0.99	19.78	8.97	
*CHOW	7	m	0	2.37	5.83	4.89	0.0000
*CHYOU	9	m	0	1.96	12.35	3.27	0.0000
COMSTO	34	m	0	2.38	3.63	3.15	0.0000
COMSTO	46	f	0	2.19	9.22	5.07	0.0000
Subtotal COMSTO				2.25	12.85	8.22	
COOKSO	5	c	0	1.88	14.38	2.69	0.0000
CORREA	33	c	0	2.33	41.42	32.38	0.0000
*CPSI	187	m	1	2.22	78.68	46.29	0.0000
*CPSI	274	f	1	1.03	73.22	13.16	0.0000
Subtotal CPSI				1.64	151.90	59.45	
*CPSII	104	m	1	2.55	78.29	95.04	0.0000
*CPSII	79	f	1	2.10	142.84	60.20	0.0000
Subtotal CPSII				2.26	221.13	155.24	
DAMBER	5	m	0	1.99	30.10	8.73	0.0000
DARBY	15	m	0	3.90	2.96	17.73	0.0000
DARBY	16	f	0	2.51	19.76	22.03	0.0000
Subtotal DARBY				2.69	22.71	39.76	
DAVEYS	5	m	0	1.57	2.53	0.03	0.0126
DAVEYS	6	f	0	-0.32	0.42	1.34	0.8327
Subtotal DAVEYS				1.30	2.96	1.37	
DEAN	7	m	0	1.66	9.69	0.41	0.0000
DEAN2	3	m	0	1.33	23.82	0.32	0.0000
DEAN2	7	f	0	1.08	14.58	2.04	0.0000
Subtotal DEAN2				1.24	38.40	2.36	
DEAN3	47	m	0	1.77	22.66	2.32	0.0000
DEAN3	124	f	0	1.06	28.64	4.41	0.0000
Subtotal DEAN3				1.37	51.29	6.73	
*DEKLER	6	m	2	3.01	0.99	2.42	0.0027
DESTE2	13	c	0	2.03	14.86	5.06	0.0000
DESTEF	5	m	0	2.14	20.71	9.84	0.0000
*DOCKER	3	c	4	1.46	4.27	0.00	0.0026
DOLL	6	m	0	2.21	6.22	3.55	0.0000
DOLL	12	f	0	0.72	12.98	6.98	0.0099
Subtotal DOLL				1.20	19.20	10.54	
*DOLL2	56	m	1	2.04	18.57	6.38	0.0000
*DOLL2	63	f	1	2.16	3.28	1.64	0.0001
Subtotal DOLL2				2.05	21.84	8.02	
DORANT	10	c	0	2.89	13.34	27.67	0.0000
DORGAN	6	m	0	2.28	12.35	8.60	0.0000
DORGAN	30	m	0	3.13	2.68	7.61	0.0000
DORGAN	53	f	0	2.06	51.30	18.97	0.0000
DORGAN	76	f	0	2.12	4.12	1.87	0.0000
Subtotal DORGAN				2.14	70.44	37.05	
*DORN	196	m	1	1.95	73.73	18.55	0.0000
DOSEME	17	m	0	1.41	75.43	0.10	0.0000
DROSTE	3	m	0	2.65	6.33	9.08	0.0000
DU	1	m	0	1.26	28.12	1.00	0.0000
DU	2	f	0	0.66	24.50	15.39	0.0011
Subtotal DU				0.98	52.62	16.39	
*DUNN	6	m	0	2.91	1.97	4.22	0.0000
EBELIN	1	m	0	1.94	9.19	2.18	0.0000
*ENGELA	155	m	0	1.53	6.61	0.04	0.0001
*ENGELA	162	f	0	1.19	6.52	0.45	0.0024

Table 1C1 - 5

IESLC - Meta-anal of Ever Smoking (or Current if Ever not available), Any prod (or Cigs if Any not avail)
All LC types
Least adjusted

REF	NRR	SEX	AD	Ys	Ws	Qs	Ps
Subtotal ENGELA				1.36	13.12	0.48	
*ENSTRO	1	m	1	2.56	81.91	101.68	0.0000
*ENSTRO	2	f	1	1.94	181.45	43.34	0.0000
Subtotal ENSTRO				2.13	263.36	145.02	
ESAKI	4	m	0	0.64	8.96	5.87	0.0554
ESAKI	5	f	0	0.90	7.99	2.41	0.0109
Subtotal ESAKI				0.76	16.94	8.29	
FAN	1	m	0	1.04	25.87	4.24	0.0000
FAN	2	f	0	1.37	24.92	0.18	0.0000
Subtotal FAN				1.20	50.80	4.42	
GAO	6	m	0	1.37	41.05	0.29	0.0000
GAO	16	f	0	0.93	63.04	17.02	0.0000
Subtotal GAO				1.10	104.09	17.31	
GAO2	6	m	0	1.63	9.72	0.33	0.0000
GARCIA	3	c	0	2.14	16.50	7.90	0.0000
GARDIN	7	c	0	2.41	4.14	3.79	0.0000
GARSHI	17	m	0	1.75	34.80	3.19	0.0000
GENG	1	m	0	1.79	4.98	0.58	0.0001
GENG	2	f	0	1.08	22.39	2.99	0.0000
Subtotal GENG				1.21	27.37	3.56	
GER	17	c	0	0.31	26.37	34.19	0.1100
GODLEY	5	m	1	1.92	96.28	21.52	0.0000
GODLEY	6	f	1	1.71	58.89	4.04	0.0000
Subtotal GODLEY				1.84	155.17	25.56	
GOLLED	21	m	0	1.84	13.92	2.15	0.0000
GOODMA	3	m	0	2.38	8.92	7.70	0.0000
GOODMA	7	f	0	2.12	12.25	5.42	0.0000
Subtotal GOODMA				2.23	21.17	13.13	
GRAHAM	22	m	0	2.05	16.52	5.94	0.0000
GREGOR	3	m	0	0.03	5.11	10.34	0.9492
GREGOR	7	f	0	2.40	0.90	0.80	0.0233
Subtotal GREGOR				0.38	6.01	11.14	
GSELL	8	m	0	2.88	1.82	3.70	0.0001
HAENSZ	50	f	0	0.77	25.35	11.88	0.0001
*HAMMO2	18	m	0	3.25	1.00	3.21	0.0012
*HAMMON	129	m	0	1.86	14.49	2.46	0.0000
*HANSEN	3	m	2	0.43	5.28	5.54	0.3285
HEGMAN	1	c	0	2.79	23.66	42.72	0.0000
*HEIN	7	m	0	2.68	1.00	1.51	0.0074
*HENNEK	3	m	0	1.83	19.94	2.89	0.0000
HINDS	26	f	0	1.42	61.37	0.07	0.0000
*HIRAYA	147	m	1	1.47	85.78	0.04	0.0000
*HIRAYA	150	f	1	0.86	80.63	28.20	0.0000
Subtotal HIRAYA				1.18	166.40	28.24	
HITOSU	6	m	0	1.01	6.47	1.25	0.0102
HITOSU	12	f	0	1.36	16.07	0.13	0.0000
Subtotal HITOSU				1.26	22.53	1.38	
*HOLE	15	m	0	1.69	6.79	0.39	0.0000
*HOLE	29	f	0	0.37	4.98	5.77	0.4046
Subtotal HOLE				1.13	11.77	6.15	
HOROWI	1	m	0	1.27	15.35	0.47	0.0000
HOROWI	2	f	0	0.60	8.08	5.87	0.0894
Subtotal HOROWI				1.04	23.43	6.34	
HORWIT	1	f	0	2.43	8.29	7.90	0.0000
HU	15	m	0	0.74	17.16	8.76	0.0023
HU	16	f	0	0.55	7.15	5.79	0.1413
Subtotal HU				0.68	24.31	14.56	
HU2	9	m	0	1.11	27.11	3.18	0.0000
HU2	10	f	0	0.63	21.91	14.78	0.0033
Subtotal HU2				0.89	49.01	17.96	
HUANG	1	c	0	0.69	14.82	8.55	0.0078
HUMBLE	14	m	1	2.49	5.19	5.65	0.0000
HUMBLE	16	m	1	2.47	1.71	1.79	0.0012
HUMBLE	18	f	1	2.43	6.68	6.42	0.0000
HUMBLE	20	f	1	2.73	2.90	4.78	0.0000
Subtotal HUMBLE				2.51	16.48	18.64	
JAHN	3	f	0	1.13	18.89	1.95	0.0000
JAIN	6	m	0	2.30	9.88	7.18	0.0000
JAIN	1	f	0	2.10	31.68	13.50	0.0000
Subtotal JAIN				2.15	41.55	20.67	
JARUP	3	m	0	1.90	6.90	1.39	0.0000

Table 1C1 - 5

IESLC - Meta-anal of Ever Smoking (or Current if Ever not available), Any prod (or Cigs if Any not avail)
All LC types
Least adjusted

REF	NRR	SEX	AD	Ys	Ws	Qs	Ps
JARVHO	3	m	0	3.32	0.92	3.22	0.0015
JARVHO	7	f	0	2.26	3.28	2.14	0.0000
Subtotal JARVHO				2.49	4.19	5.36	
JEDRYC	63	m	0	1.76	36.14	3.44	0.0000
JEDRYC	68	f	0	2.08	17.12	6.73	0.0000
Subtotal JEDRYC				1.86	53.25	10.17	
JIANG	1	m	0	1.00	4.45	0.90	0.0346
JIANG	2	f	0	0.91	2.62	0.76	0.1401
Subtotal JIANG				0.97	7.08	1.66	
JOLY	14	m	0	2.50	11.02	12.13	0.0000
JOLY	1	f	0	1.99	27.09	8.02	0.0000
Subtotal JOLY				2.14	38.11	20.15	
JUSSAW	3	m	0	2.77	63.20	110.86	0.0000
*KAISE2	72	m	1	1.69	11.75	0.66	0.0000
*KAISE2	64	f	1	2.31	9.19	6.82	0.0000
Subtotal KAISE2				1.96	20.95	7.48	
*KAISER	13	m	2	2.87	25.69	51.78	0.0000
*KAISER	10	f	2	1.73	28.18	2.18	0.0000
Subtotal KAISER				2.27	53.88	53.96	
KANELL	5	m	0	1.89	33.17	6.40	0.0000
KATSOU	27	f	0	1.21	9.99	0.56	0.0001
KAUFMA	8	c	0	2.61	31.80	42.75	0.0000
KELLER	3	m	0	2.31	217.37	161.33	0.0000
KELLER	11	m	0	2.60	25.99	34.62	0.0000
KELLER	7	f	0	2.53	269.69	311.81	0.0000
KELLER	15	f	0	2.25	39.03	24.73	0.0000
Subtotal KELLER				2.43	552.08	532.50	
KHUDER	4	m	0	2.06	19.93	7.45	0.0000
KIHARA	31	c	0	1.22	46.97	2.50	0.0000
*KINLEN	6	m	0	2.36	6.97	5.83	0.0000
KJUUS	10	m	0	2.62	1.81	2.47	0.0004
*KNEKT	76	m	0	1.85	5.69	0.91	0.0000
KO	1	f	3	1.44	2.18	0.00	0.0339
KOHLME	1	c	0	2.83	9.55	18.19	0.0000
KOO	1	f	0	1.02	23.01	4.29	0.0000
KOULUM	1	m	0	3.57	4.47	20.03	0.0000
KREUZE	14	f	0	2.24	4.29	2.65	0.0000
KREUZE	16	f	0	1.33	32.30	0.46	0.0000
Subtotal KREUZE				1.44	36.60	3.11	
KREYBE	24	m	0	2.04	5.80	2.02	0.0000
KREYBE	39	f	0	-0.22	8.25	23.05	0.5245
Subtotal KREYBE				0.71	14.05	25.07	
*KUBIK	28	m	0	3.34	1.96	6.99	0.0000
LAMTH	6	f	0	1.34	46.55	0.59	0.0000
LAMWK	1	f	0	1.42	17.85	0.02	0.0000
LAMWK2	9	m	0	1.04	12.98	2.17	0.0002
LAMWK2	10	f	0	1.17	17.89	1.45	0.0000
Subtotal LAMWK2				1.11	30.86	3.61	
*LANGE	34	m	0	1.58	4.91	0.08	0.0005
*LANGE	31	f	0	1.22	6.30	0.33	0.0022
Subtotal LANGE				1.38	11.22	0.41	
LAUSSM	10	m	0	1.59	41.00	0.81	0.0000
LEI	1	m	0	1.30	26.63	0.57	0.0000
LEI	2	f	0	1.25	23.21	0.94	0.0000
Subtotal LEI				1.28	49.84	1.51	
LEMARC	3	c	0	1.73	22.77	1.77	0.0000
LETOUR	1	c	0	2.56	20.21	25.00	0.0000
LEVIN	32	m	1	1.58	30.68	0.53	0.0000
*LIAW	1	m	1	1.31	11.72	0.24	0.0000
*LIAW	2	f	1	1.28	2.46	0.07	0.0447
Subtotal LIAW				1.30	14.17	0.31	
*LIDDEL	5	m	1	1.28	17.92	0.50	0.0000
LIU	2	c	2	0.65	38.19	24.30	0.0001
LIU2	1	m	0	1.46	8.60	0.00	0.0000
LIU2	3	f	0	1.45	9.73	0.00	0.0000
Subtotal LIU2				1.46	18.32	0.00	
LIU3	1	m	0	0.19	3.06	4.89	0.7444
LIU4	11	m	2	1.02	5969.41	1128.50	0.0000
LIU4	12	f	2	1.05	3876.64	617.80	0.0000
Subtotal LIU4				1.03	9846.05	1746.29	
LIU5	1	c	0	0.65	11.25	7.21	0.0293

Table 1C1 - 5

IESLC - Meta-anal of Ever Smoking (or Current if Ever not available), Any prod (or Cigs if Any not avail)
All LC types
Least adjusted

REF	NRR	SEX	AD	Ys	Ws	Qs	Ps
LOMBA2	1	f	0	0.28	37.19	50.64	0.0841
LOMBAR	12	m	0	2.18	12.13	6.46	0.0000
LUBIN2	45	m	0	2.15	169.89	82.26	0.0000
LUBIN2	101	f	0	1.24	134.74	5.87	0.0000
Subtotal LUBIN2				1.75	304.63	88.13	
LUO	1	c	0	0.66	18.01	11.39	0.0054
MACLEN	71	m	0	1.28	3.54	0.11	0.0163
MACLEN	72	f	0	0.74	13.64	6.85	0.0062
Subtotal MACLEN				0.85	17.18	6.96	
*MAGNUS	1	m	0	1.69	10.53	0.61	0.0000
MARSH	2	m	0	2.28	1.82	1.26	0.0021
MARSH	4	f	0	1.76	5.60	0.55	0.0000
Subtotal MARSH				1.89	7.42	1.81	
MARSH2	2	c	0	1.16	8.50	0.71	0.0007
MARTIS	4	m	0	1.95	3.32	0.82	0.0004
MASTRA	1	m	0	2.13	5.09	2.33	0.0000
MATOS	26	m	0	1.89	9.19	1.81	0.0000
MATSUD	10	m	0	3.07	2.94	7.67	0.0000
MCCONN	1	m	0	0.19	3.33	5.25	0.7237
MCCONN	2	f	0	1.01	0.99	0.19	0.3136
Subtotal MCCONN				0.38	4.32	5.44	
MCDUFF	1	m	0	1.81	4.70	0.62	0.0001
MCLAUG	1	m	0	1.20	18.70	1.13	0.0000
*MIGRAN	26	m	0	1.89	3.94	0.78	0.0002
*MIGRAN	41	f	0	1.98	3.55	0.99	0.0002
Subtotal MIGRAN				1.93	7.49	1.77	
MILLER	1	f	0	2.43	22.85	21.79	0.0000
MILLS	3	m	1	0.29	96.62	130.35	0.0046
*MRFITR	6	m	0	3.70	0.50	2.52	0.0091
NAM	69	m	0	2.29	26.44	18.48	0.0000
NAM	85	f	0	2.30	38.76	28.30	0.0000
Subtotal NAM				2.30	65.19	46.78	
NOTAN2	1	m	0	1.11	80.11	9.30	0.0000
NOU	11	m	0	1.81	5.20	0.66	0.0000
NOU	12	f	0	1.96	2.74	0.71	0.0012
Subtotal NOU				1.86	7.94	1.37	
ODRISC	3	c	0	3.89	5.83	34.71	0.0000
ORMOS	4	m	0	2.23	6.39	3.93	0.0000
ORMOS	26	f	0	-1.64	0.95	9.10	0.1093
Subtotal ORMOS				1.73	7.34	13.03	
OSANN	17	m	0	3.00	39.51	95.31	0.0000
OSANN	21	f	0	2.70	69.13	108.30	0.0000
Subtotal OSANN				2.81	108.65	203.61	
PARKIN	29	m	0	1.54	73.35	0.54	0.0000
PASTOR	5	m	0	1.89	8.32	1.58	0.0000
PAWLEG	1	m	0	2.77	3.69	6.39	0.0000
PERNU	2	m	0	2.19	58.99	32.27	0.0000
PERNU	1	f	0	0.63	13.52	9.01	0.0198
Subtotal PERNU				1.90	72.50	41.28	
PERSH2	5	c	0	1.79	114.36	13.01	0.0000
*PETO	5	m	0	1.82	1.98	0.26	0.0107
PEZZO2	10	m	0	2.71	5.55	8.80	0.0000
PEZZOT	25	m	0	2.96	3.75	8.56	0.0000
PIKE	4	m	0	1.66	13.39	0.59	0.0000
PIKE	8	f	0	1.57	18.04	0.28	0.0000
Subtotal PIKE				1.61	31.43	0.87	
POFFIJ	1	c	0	2.05	46.21	16.51	0.0000
POLEDN	3	c	0	2.13	10.07	4.59	0.0000
*QIAO2	3	m	0	0.86	9.73	3.36	0.0072
RACHTA	3	f	0	1.77	13.24	1.35	0.0000
RADZIK	1	c	0	0.27	5.03	6.98	0.5411
RANDIG	23	m	0	1.61	3.99	0.11	0.0013
RANDIG	24	f	0	0.80	6.34	2.70	0.0447
Subtotal RANDIG				1.11	10.32	2.80	
REN	1	m	0	1.27	7.46	0.23	0.0005
REN	2	f	0	1.40	9.65	0.02	0.0000
Subtotal REN				1.35	17.11	0.25	
RONCO	1	m	0	1.63	5.23	0.17	0.0002
ROTHSC	1	c	0	1.76	9.88	0.96	0.0000
SADOWS	7	m	0	1.35	13.90	0.13	0.0000
SANKAR	1	m	0	2.75	23.11	39.12	0.0000

Table 1C1 - 5

IESLC - Meta-anal of Ever Smoking (or Current if Ever not available), Any prod (or Cigs if Any not avail)
All LC types
Least adjusted

REF	NRR	SEX	AD	Ys	Ws	Qs	Ps
SCHWAR	1	m	0	2.11	80.50	34.58	0.0000
SCHWAR	2	m	0	1.88	29.06	5.27	0.0000
SCHWAR	3	f	0	2.30	111.43	80.30	0.0000
SCHWAR	4	f	0	2.45	26.58	26.43	0.0000
Subtotal SCHWAR				2.20	247.58	146.59	
SEGI	1	m	0	0.53	15.18	12.74	0.0375
SEGI2	19	m	0	1.29	6.68	0.16	0.0008
SEGI2	27	f	0	0.46	10.32	10.06	0.1372
Subtotal SEGI2				0.79	17.00	10.23	
SEOW	1	f	0	1.71	9.81	0.66	0.0000
SHAW	12	c	0	2.47	9.34	9.76	0.0000
SIEMIA	9	m	0	2.77	11.12	19.31	0.0000
SIMARA	5	m	0	0.70	13.62	7.75	0.0103
SIMARA	6	f	0	0.85	9.72	3.54	0.0084
Subtotal SIMARA				0.76	23.33	11.30	
SOBUE	91	m	0	1.34	25.52	0.33	0.0000
SOBUE	95	f	0	1.03	51.71	9.00	0.0000
Subtotal SOBUE				1.13	77.23	9.33	
SOBUE2	10	m	2	1.50	197.90	0.44	0.0000
SOBUE2	12	f	2	1.19	143.51	9.87	0.0000
Subtotal SOBUE2				1.37	341.42	10.31	
*SPEIZE	8	f	0	1.96	52.33	13.43	0.0000
SPITZ	3	c	0	2.91	6.16	13.16	0.0000
STASZE	1	m	0	2.37	4.73	3.99	0.0000
STASZE	5	f	0	1.47	4.16	0.00	0.0028
Subtotal STASZE				1.95	8.88	3.99	
STAYNE	1	m	0	1.30	40.37	0.92	0.0000
STOCKS	31	m	0	1.75	41.11	3.73	0.0000
STOCKS	50	f	1	1.11	58.11	6.65	0.0000
Subtotal STOCKS				1.38	99.22	10.37	
STOCKW	6	c	0	2.35	1549.52	1244.80	0.0000
STUCKE	3	m	0	4.83	0.49	5.63	0.0007
SUN	1	c	0	0.84	30.23	11.40	0.0000
SUZUK2	18	c	0	2.04	7.52	2.64	0.0000
SVENSS	56	f	0	1.81	19.34	2.49	0.0000
TANG	3	c	0	2.09	6.14	2.51	0.0000
*TENKAN	22	m	1	2.68	5.38	8.19	0.0000
TIZZAN	1	m	0	0.66	91.77	57.87	0.0000
TIZZAN	12	f	0	1.40	8.03	0.02	0.0001
Subtotal TIZZAN				0.72	99.80	57.88	
TOKARS	3	m	0	3.47	0.97	3.95	0.0006
TOKARS	5	f	0	0.43	0.62	0.65	0.7336
Subtotal TOKARS				2.28	1.60	4.60	
TOUSEY	16	m	0	3.10	3.80	10.28	0.0000
TOUSEY	26	f	0	2.75	10.96	18.60	0.0000
Subtotal TOUSEY				2.84	14.76	28.87	
TSUGAN	27	m	0	0.23	7.76	11.59	0.5244
*TULINI	15	m	1	2.09	10.32	4.19	0.0000
*TULINI	21	f	1	2.70	11.08	17.44	0.0000
Subtotal TULINI				2.41	21.40	21.63	
*TVERDA	22	m	2	1.52	20.49	0.11	0.0000
*TVERDA	15	f	2	2.40	2.67	2.42	0.0001
Subtotal TVERDA				1.62	23.16	2.52	
WAKAI	13	m	0	1.28	8.20	0.23	0.0002
WAKAI	31	f	0	1.27	11.70	0.39	0.0000
Subtotal WAKAI				1.27	19.90	0.62	
*WALD	2	m	0	2.84	6.43	12.52	0.0000
WANG	1	m	0	1.24	14.89	0.63	0.0000
WANG	2	f	0	1.39	3.11	0.01	0.0145
Subtotal WANG				1.27	18.00	0.64	
WANG2	8	c	0	0.86	7.10	2.45	0.0216
WANG3	1	c	0	1.05	28.11	4.56	0.0000
WANG4	1	m	0	0.07	107.42	204.34	0.4630
WICKLU	1	m	0	1.53	15.41	0.09	0.0000
WIGLE	15	m	0	2.32	13.42	10.20	0.0000
WIGLE	18	f	0	1.41	21.29	0.03	0.0000
Subtotal WIGLE				1.76	34.71	10.23	
WILKIN	1	m	0	3.22	1.93	6.07	0.0000
WILKIN	2	f	0	1.74	8.65	0.74	0.0000
Subtotal WILKIN				2.01	10.58	6.82	
WU	37	f	0	1.48	17.78	0.01	0.0000

Table 1C1 - 5

IESLC - Meta-anal of Ever Smoking (or Current if Ever not available), Any prod (or Cigs if Any not avail)
All LC types
Least adjusted

REF	NRR	SEX	AD	Ys	Ws	Qs	Ps
WUNSCH	1	m	0	1.54	11.46	0.09	0.0000
WUNSCH	7	f	0	1.48	15.11	0.01	0.0000
Subtotal WUNSCH				1.50	26.58	0.10	
WUWILL	6	f	0	0.79	114.07	49.03	0.0000
WYNDE2	21	m	0	2.13	7.21	3.36	0.0000
WYNDE3	49	m	0	2.09	7.74	3.19	0.0000
WYNDE3	138	f	0	1.14	9.73	0.95	0.0004
Subtotal WYNDE3				1.56	17.48	4.14	
WYNDE4	48	m	0	2.21	10.51	6.06	0.0000
WYNDE4	62	f	2	1.05	8.80	1.38	0.0018
Subtotal WYNDE4				1.68	19.31	7.44	
WYNDE6	72	m	0	2.31	71.58	52.40	0.0000
WYNDE6	252	f	0	2.34	103.92	82.62	0.0000
Subtotal WYNDE6				2.33	175.50	135.02	
*XIANGZ	8	m	0	1.00	25.00	5.13	0.0000
XU	1	m	0	0.97	57.31	13.41	0.0000
XU2	1	c	0	1.38	53.87	0.30	0.0000
XU3	1	m	0	1.79	4.98	0.58	0.0001
XU3	3	f	0	1.39	3.98	0.01	0.0055
Subtotal XU3				1.61	8.96	0.59	
XU4	1	c	0	1.08	20.82	2.85	0.0000
YAMAGU	5	c	0	1.18	17.80	1.27	0.0000
*YONG	12	m	1	3.36	1.92	6.98	0.0000
*YONG	15	f	1	1.65	6.30	0.25	0.0000
Subtotal YONG				2.05	8.22	7.23	
*YUAN	1	m	2	1.87	11.44	2.03	0.0000
ZHANG	1	c	0	0.90	14.37	4.28	0.0006
ZHENG	15	m	0	1.29	20.36	0.50	0.0000
ZHENG	24	f	0	0.74	20.88	10.60	0.0008
Subtotal ZHENG				1.01	41.24	11.10	
ZHOU	2	m	0	0.86	17.50	6.09	0.0003
ZHOU	3	f	0	0.80	5.34	2.28	0.0660
Subtotal ZHOU				0.84	22.83	8.38	

	N	345
	NS	242
	Wt	20334.92
Het	Chi	7908.22
Het	df	344
Het	P	***
Fixed	RR	4.26
	RRl	4.21
	RRu	4.32
	P	+++
Random	RR	5.47
	RRl	5.06
	RRu	5.91
	P	+++
Asymm	P	***

Table 1C1 - 6

IESLC - Meta-anal of Ever Smoking (or Current if Ever not available), Any prod (or Cigs if Any not avail)
All LC types
Least adjusted

	combined	Sex male	female	Total
N	45	181	119	345
NS	45	177	114	336
Wt	2466.61	10339.96	7528.35	20334.92
Het Chi	766.53	3481.00	2514.85	7908.22
Het df	44	180	118	344
Het P	***	***	***	***
Fixed RR	8.06	3.85	3.98	4.26
RRl	7.75	3.78	3.89	4.21
RRu	8.39	3.92	4.07	4.32
P	+++	+++	+++	+++
Random RR	5.77	6.36	4.34	5.47
RRl	4.63	5.67	3.81	5.06
RRu	7.18	7.13	4.96	5.91
P	+++	+++	+++	+++
Between Chi				1145.84
Between df				2
Between P				***
Btwn(F) P				***
Btwn(R) P				***

	Lung cancer type all	other	Total
N	332	13	345
NS	233	9	242
Wt	19789.49	545.43	20334.92
Het Chi	7852.69	45.37	7908.22
Het df	331	12	344
Het P	***	***	***
Fixed RR	4.28	3.73	4.26
RRl	4.22	3.43	4.21
RRu	4.34	4.05	4.32
P	+++	+++	+++
Random RR	5.57	3.67	5.47
RRl	5.14	3.02	5.06
RRu	6.03	4.47	5.91
P	+++	+++	+++
Between Chi			10.16
Between df			1
Between P			**
Btwn(F) P			N.S.
Btwn(R) P			***

	NAmer	UK	Scand	Location othEur	China	Japan	othAs	other	Total
N	119	33	33	52	51	22	21	14	345
NS	82	21	23	40	35	14	15	12	242
Wt	5862.67	405.41	504.74	1127.21	10962.85	827.43	419.95	224.67	20334.92
Het Chi	1233.07	169.30	124.12	473.44	189.20	71.16	237.46	28.60	7908.22
Het df	118	32	32	51	50	21	20	13	344
Het P	***	***	***	***	***	***	***	**	***
Fixed RR	8.82	5.05	6.22	5.59	2.77	3.48	4.21	6.30	4.26
RRl	8.60	4.59	5.70	5.27	2.72	3.25	3.82	5.53	4.21
RRu	9.05	5.57	6.79	5.92	2.82	3.73	4.63	7.19	4.32
P	+++	+++	+++	+++	+++	+++	+++	+++	+++
Random RR	7.61	5.91	6.15	6.13	2.66	3.23	3.79	7.07	5.47
RRl	6.90	4.62	5.06	4.99	2.48	2.79	2.67	5.69	5.06
RRu	8.39	7.57	7.48	7.54	2.86	3.75	5.39	8.79	5.91
P	+++	+++	+++	+++	+++	+++	+++	+++	+++
Between Chi									5381.87
Between df									7
Between P									***
Btwn(F) P									***
Btwn(R) P									***

Table 1C1 - 6

IESLC - Meta-anal of Ever Smoking (or Current if Ever not available), Any prod (or Cigs if Any not avail)
All LC types
Least adjusted

	Detailed Country in "other Europe"					
	multi	Germany	othWest	East	Balkans	Total
N	4	17	13	14	4	52
NS	3	12	12	9	4	40
Wt	456.31	234.69	174.11	124.07	138.03	1127.21
Het Chi	133.70	71.89	143.82	41.71	8.06	473.44
Het df	3	16	12	13	3	51
Het P	***	***	***	***	*	***
Fixed RR	7.37	4.58	3.96	6.18	4.41	5.59
RRl	6.73	4.03	3.41	5.19	3.73	5.27
RRu	8.08	5.20	4.59	7.37	5.21	5.92
P	+++	+++	+++	+++	+++	+++
Random RR	7.65	5.23	8.80	6.25	4.34	6.13
RRl	4.07	3.79	4.70	4.33	3.19	4.99
RRu	14.36	7.22	16.49	9.01	5.91	7.54
P	+++	+++	+++	+++	+++	+++
Between Chi						74.25
Between df						4
Between P						***
Btwn(F) P						(*)
Btwn(R) P						N.S.

	Detailed Country in "other Asia"			
	India	HongKong	other	Total
N	3	7	11	21
NS	3	5	7	15
Wt	166.42	140.70	112.82	419.95
Het Chi	114.34	10.74	24.70	237.46
Het df	2	6	10	20
Het P	***	(*)	**	***
Fixed RR	7.17	3.52	2.39	4.21
RRl	6.16	2.99	1.99	3.82
RRu	8.34	4.15	2.88	4.63
P	+++	+++	+++	+++
Random RR	9.08	3.56	2.64	3.79
RRl	2.71	2.81	1.94	2.67
RRu	30.40	4.50	3.60	5.39
P	+++	+++	+++	+++
Between Chi				87.68
Between df				2
Between P				***
Btwn(F) P				*
Btwn(R) P				(*)

	Detailed other continent			
	SCAmer	Auslia	Africa	Total
N	10	1	3	14
NS	8	1	3	12
Wt	126.26	0.99	97.42	224.67
Het Chi	16.04	0.00	1.48	28.60
Het df	9	0	2	13
Het P	(*)	N.S.	N.S.	**
Fixed RR	7.53	20.29	4.95	6.30
RRl	6.33	2.84	4.06	5.53
RRu	8.97	145.07	6.03	7.19
P	+++	++	+++	+++
Random RR	7.74	20.29	4.95	7.07
RRl	6.07	2.84	4.06	5.69
RRu	9.86	145.07	6.03	8.79
P	+++	++	+++	+++
Between Chi				11.09
Between df				2
Between P				**
Btwn(F) P				(*)
Btwn(R) P				**

Table 1C1 - 6

IESLC - Meta-anal of Ever Smoking (or Current if Ever not available), Any prod (or Cigs if Any not avail)
All LC types
Least adjusted

	Start year of study <1960	1960-69	1970-79	1980-89	1990+	Total
N	58	57	76	115	39	345
NS	41	40	53	76	32	242
Wt	1242.13	1722.70	1416.66	15419.17	534.26	20334.92
Het Chi	634.93	515.86	645.48	5730.64	289.24	7908.22
Het df	57	56	75	114	38	344
Het P	***	***	***	***	***	***
Fixed RR	4.72	4.91	4.57	4.11	5.09	4.26
RRl	4.47	4.69	4.34	4.05	4.68	4.21
RRu	4.99	5.15	4.82	4.18	5.54	4.32
P	+++	+++	+++	+++	+++	+++
Random RR	4.83	5.17	5.26	5.82	6.23	5.47
RRl	3.92	4.41	4.45	5.08	4.87	5.06
RRu	5.95	6.06	6.22	6.67	7.97	5.91
P	+++	+++	+++	+++	+++	+++
Between Chi						92.09
Between df						4
Between P						***
Btwn(F) P						N.S.
Btwn(R) P						N.S.

	Study type (1) CC	other	Total
N	269	76	345
NS	188	54	242
Wt	18762.52	1572.40	20334.92
Het Chi	7169.47	460.59	7908.22
Het df	268	75	344
Het P	***	***	***
Fixed RR	4.12	6.39	4.26
RRl	4.06	6.08	4.21
RRu	4.18	6.71	4.32
P	+++	+++	+++
Random RR	5.25	6.37	5.47
RRl	4.81	5.52	5.06
RRu	5.73	7.34	5.91
P	+++	+++	+++
Between Chi			278.17
Between df			1
Between P			***
Btwn(F) P			***
Btwn(R) P			*

	Study type (2) CC	prosp	other	Total
N	269	70	6	345
NS	188	50	4	242
Wt	18762.52	1533.52	38.88	20334.92
Het Chi	7169.47	439.45	13.90	7908.22
Het df	268	69	5	344
Het P	***	***	*	***
Fixed RR	4.12	6.32	9.78	4.26
RRl	4.06	6.01	7.14	4.21
RRu	4.18	6.64	13.39	4.32
P	+++	+++	+++	+++
Random RR	5.25	6.20	9.53	5.47
RRl	4.81	5.35	5.20	5.06
RRu	5.73	7.17	17.49	5.91
P	+++	+++	+++	+++
Between Chi				285.41
Between df				2
Between P				***
Btwn(F) P				**
Btwn(R) P				*

Table 1C1 - 6

IESLC - Meta-anal of Ever Smoking (or Current if Ever not available), Any prod (or Cigs if Any not avail)
All LC types
Least adjusted

	Study size (number of LC cases)				
	100-249	250-499	500-999	1000+	Total
N	122	90	66	67	345
NS	98	64	44	36	242
Wt	1038.30	1327.54	1610.26	16358.83	20334.92
Het Chi	535.83	611.89	650.13	6019.12	7908.22
Het df	121	89	65	66	344
Het P	***	***	***	***	***
Fixed RR	3.77	4.69	5.15	4.18	4.26
RRl	3.55	4.44	4.91	4.12	4.21
RRu	4.00	4.95	5.41	4.25	4.32
P	+++	+++	+++	+++	+++
Random RR	4.48	5.58	6.21	6.18	5.47
RRl	3.90	4.81	5.29	5.23	5.06
RRu	5.14	6.47	7.30	7.31	5.91
P	+++	+++	+++	+++	+++
Between Chi					91.25
Between df					3
Between P					***
Btwn(F) P					N.S.
Btwn(R) P					**

	Risky occupational population			
	no	mining	othRisky	Total
N	326	7	12	345
NS	224	7	11	242
Wt	20116.01	76.23	142.68	20334.92
Het Chi	7865.34	10.01	28.73	7908.22
Het df	325	6	11	344
Het P	***	N.S.	**	***
Fixed RR	4.26	3.55	4.73	4.26
RRl	4.20	2.83	4.02	4.21
RRu	4.32	4.44	5.58	4.32
P	+++	+++	+++	+++
Random RR	5.49	3.79	5.46	5.47
RRl	5.07	2.77	3.90	5.06
RRu	5.95	5.18	7.63	5.91
P	+++	+++	+++	+++
Between Chi				4.14
Between df				2
Between P				N.S.
Btwn(F) P				N.S.
Btwn(R) P				(*)

	National cigarette tobacco type			
	Virginia	blended	other	Total
N	54	236	55	345
NS	38	166	38	242
Wt	879.15	8450.20	11005.57	20334.92
Het Chi	381.95	2776.59	203.88	7908.22
Het df	53	235	54	344
Het P	***	***	***	***
Fixed RR	5.89	7.25	2.76	4.26
RRl	5.51	7.10	2.71	4.21
RRu	6.29	7.41	2.82	4.32
P	+++	+++	+++	+++
Random RR	6.37	6.23	2.64	5.47
RRl	5.24	5.74	2.46	5.06
RRu	7.74	6.77	2.84	5.91
P	+++	+++	+++	+++
Between Chi				4545.81
Between df				2
Between P				***
Btwn(F) P				***
Btwn(R) P				***

Table 1C1 - 6

IESLC - Meta-anal of Ever Smoking (or Current if Ever not available), Any prod (or Cigs if Any not avail)
All LC types
Least adjusted

	Any proxy use No/nk	Yes	Total
N	244	101	345
NS	175	67	242
Wt	8143.35	12191.57	20334.92
Het Chi	3207.55	1973.63	7908.22
Het df	243	100	344
Het P	***	***	***
Fixed RR	6.67	3.16	4.26
RRl	6.53	3.10	4.21
RRu	6.82	3.22	4.32
P	+++	+++	+++
Random RR	5.44	5.45	5.47
RRl	4.98	4.88	5.06
RRu	5.95	6.08	5.91
P	+++	+++	+++
Between Chi			2727.05
Between df			1
Between P			***
Btwn(F) P			***
Btwn(R) P			N.S.

	Full histological confirmation No	Yes	Total
N	260	85	345
NS	183	59	242
Wt	17768.52	2566.40	20334.92
Het Chi	6086.75	862.70	7908.22
Het df	259	84	344
Het P	***	***	***
Fixed RR	3.93	7.55	4.26
RRl	3.87	7.26	4.21
RRu	3.98	7.85	4.32
P	+++	+++	+++
Random RR	5.26	6.18	5.47
RRl	4.82	5.36	5.06
RRu	5.73	7.13	5.91
P	+++	+++	+++
Between Chi			958.78
Between df			1
Between P			***
Btwn(F) P			***
Btwn(R) P			(*)

	Number of adjustment variables (1) 0	1	2+/+nk	Total
N	293	32	20	345
NS	212	19	14	245
Wt	7682.89	1351.45	11300.59	20334.92
Het Chi	3502.09	535.47	1675.74	7908.22
Het df	292	31	19	344
Het P	***	***	***	***
Fixed RR	6.24	5.65	3.18	4.26
RRl	6.10	5.36	3.12	4.21
RRu	6.38	5.96	3.24	4.32
P	+++	+++	+++	+++
Random RR	5.41	6.32	5.10	5.47
RRl	4.97	4.95	3.99	5.06
RRu	5.89	8.07	6.52	5.91
P	+++	+++	+++	+++
Between Chi				2194.91
Between df				2
Between P				***
Btwn(F) P				***
Btwn(R) P				N.S.

Table 1C1 - 6

IESLC - Meta-anal of Ever Smoking (or Current if Ever not available), Any prod (or Cigs if Any not avail)
All LC types
Least adjusted

	Number of adjustment variables (2) 0	1	2	3-5	6+/+nk	Total
N	293	32	18	2		345
NS	212	19	12	2		245
Wt	7682.89	1351.45	11294.13	6.46		20334.92
Het Chi	3502.09	535.47	1675.19	0.00		7908.22
Het df	292	31	17	1		344
Het P	***	***	***	N.S.		***
Fixed RR	6.24	5.65	3.18	4.26		4.26
RRl	6.10	5.36	3.12	1.97		4.21
RRu	6.38	5.96	3.24	9.21		4.32
P	+++	+++	+++	+++		+++
Random RR	5.41	6.32	5.15	4.26		5.47
RRl	4.97	4.95	4.00	1.97		5.06
RRu	5.89	8.07	6.64	9.21		5.91
P	+++	+++	+++	+++		+++
Between Chi						2195.47
Between df						3
Between P						***
Btwn(F) P						***
Btwn(R) P						N.S.

	Product all/unsp	cig+/-ot	cig only	Total
N	210	121	14	345
NS	156	83	12	251
Wt	15777.12	4050.58	507.22	20334.92
Het Chi	5205.31	1612.43	113.59	7908.22
Het df	209	120	13	344
Het P	***	***	***	***
Fixed RR	3.80	6.19	7.98	4.26
RRl	3.74	6.01	7.31	4.21
RRu	3.86	6.39	8.70	4.32
P	+++	+++	+++	+++
Random RR	5.35	5.48	7.37	5.47
RRl	4.85	4.84	5.42	5.06
RRu	5.90	6.19	10.03	5.91
P	+++	+++	+++	+++
Between Chi				976.89
Between df				2
Between P				***
Btwn(F) P				***
Btwn(R) P				N.S.

	Denominator nev any	nev cigs	Total
N	249	96	345
NS	177	68	245
Wt	17214.91	3120.01	20334.92
Het Chi	5783.99	1251.49	7908.22
Het df	248	95	344
Het P	***	***	***
Fixed RR	3.90	6.94	4.26
RRl	3.85	6.70	4.21
RRu	3.96	7.18	4.32
P	+++	+++	+++
Random RR	5.28	5.97	5.47
RRl	4.83	5.18	5.06
RRu	5.76	6.87	5.91
P	+++	+++	+++
Between Chi			872.75
Between df			1
Between P			***
Btwn(F) P			***
Btwn(R) P			N.S.

Table 1C1 - 6

IESLC - Meta-anal of Ever Smoking (or Current if Ever not available), Any prod (or Cigs if Any not avail)
All LC types
Least adjusted

	Derivation of RR/CI			
	Orig	StdCalc	Other	Total
N	16	281	48	345
NS	10	204	32	246
Wt	1328.99	7490.58	11515.35	20334.92
Het Chi	318.07	3392.50	1132.97	7908.22
Het df	15	280	47	344
Het P	***	***	***	***
Fixed RR	8.54	6.30	3.05	4.26
RRl	8.09	6.16	3.00	4.21
RRu	9.01	6.44	3.11	4.32
P	+++	+++	+++	+++
Random RR	5.09	5.44	5.55	5.47
RRl	3.75	4.99	4.83	5.06
RRu	6.92	5.94	6.36	5.91
P	+++	+++	+++	+++
Between Chi				3064.68
Between df				2
Between P				***
Btwn(F) P				***
Btwn(R) P				N.S.

	Smoking status		
	ever	current	Total
N	330	15	345
NS	235	10	245
Wt	19640.22	694.70	20334.92
Het Chi	7704.99	162.01	7908.22
Het df	329	14	344
Het P	***	***	***
Fixed RR	4.23	5.42	4.26
RRl	4.17	5.03	4.21
RRu	4.29	5.83	4.32
P	+++	+++	+++
Random RR	5.47	5.42	5.47
RRl	5.05	3.96	5.06
RRu	5.93	7.44	5.91
P	+++	+++	+++
Between Chi			41.23
Between df			1
Between P			***
Btwn(F) P			N.S.
Btwn(R) P			N.S.

	Study LIU4		
	LIU4	others	Total
N	2	343	345
NS	1	241	242
Wt	9846.05	10488.87	20334.92
Het Chi	2.98	4525.46	7908.22
Het df	1	342	344
Het P	(*)	***	***
Fixed RR	2.80	6.33	4.26
RRl	2.74	6.21	4.21
RRu	2.85	6.45	4.32
P	+++	+++	+++
Random RR	2.81	5.49	5.47
RRl	2.71	5.09	5.06
RRu	2.91	5.93	5.91
P	+++	+++	+++
Between Chi			3379.79
Between df			1
Between P			***
Btwn(F) P			***
Btwn(R) P			***

Table 1C1 - 7

IESLC - Meta-anal of Ever Smoking (or Current if Ever not available), Any prod (or Cigs if Any not avail)
All LC types
Excluded studies (and stage at which they were excluded)

1	BOUCHA BUELL LAURIL MZILEN
2	BERRIN CHEN RESTRE TAO ULMER
5	RIMING TANG2 WYNDE5 WYNDE8
6	BLOT1 BLOT2 BLOT3 BYERS2 HIRAOK HIRAY2 JONES LICKIN MOLLO MRFIT MURATA NOTANI PERSHA SAARIK SCHWA2 SHIMIZ SITAS VANDER WARSIN WATSON WYNDER
8	BROWN1 BYERS1 SUZUKI WU2
10	BENHAM GILLIS ISHIMA LUBIN OSANN2 PISANI PRESCO QIAO ROOTS SIMONA VEIERO VUTUC WYNDE7

Table 1C1 - 8

Potentially overlapping studies

REF	REFGP	PRINC	.	OVERLAP/LINK
LUBIN2	LUBIN2	1		Lubin-combined
CHAN	CHAN	1		Chan/LamWK2
LAMWK2	LAMWK2	1		Chan/LamWK2
LAMTH	LAMTH	1		KOO/LAMTH/LAMWK
KOO	KOO	1		KOO/LAMTH/LAMWK
LAMWK	LAMWK	1		KOO/LAMTH/LAMWK
CASCOR	CASCOR	1		Roots/Cascor 1
TVERDA	TVERDA	1		VEIERO/TVERDAL
AKIBA	AKIBA	1		AKIBA/ISHIMA
HEIN	PRESCO	1		Subset of PRESCO
LANGE	PRESCO	1		Subset of PRESCO
BROSS	BYERS1	1		GRAHAM/BROSS/BYERS1
GRAHAM	BYERS1	1		GRAHAM/BROSS/BYERS1
CHYOU	CHYOU	1		GOODMA/CHYOU
GOODMA	GOODMA	1		GOODMA/CHYOU
HOLE	TANG2	1		Subset of TANG2
BENSHL	TANG2	1		Subset of TANG2
WALD	TANG2	1		Subset of TANG2
KAISER	KAISER	1		KAISER/OSANN2
MRFITR	MRFIT	2		Subset of MRFIT
WYNDE6	WYNDE6	1		WYNDE5/6/7/8
CPSI	CPSI	1		CPSI overall
ENSTRO	ENSTRO	1		Subset of CPSI
SOBUE2	SOBUE2	1		SOBUE2/MATSUD
MATSUD	MATSUD	1		SOBUE2/MATSUD
BOFFET	BOFFET	1		BOFFET-combined
JAHN	BOFFET	2		Subset of BOFFET
XIANGZ	XIANGZ	1		LUBIN/XIANGZ/QIAO

Table 1C1 - 9

Most adjusted - insufficient data for metaanalysis

REF	NRR	SEX	AGEL	AGEH	RACE	YF	LC TYPE	LOC	START	ST	NLC	R	VB	P	H	AD	SM	PRODUCT	DENOM	De
DOLL2	43	f	0	0	all	11	all	Eu:UK	1951	pr	920	n	V	n	n	1	ev	all/unsp	nev any	st
RESTRE	2	c	0	0	all	-	all	SCAmer	1978	CC	102	n	bl	n	n	2	cu	cig+/-ot	nev cigs	ot
TAO	1	m	0	0	all	-	all	As:Chi	1988	CC	723	n	ot	n	n	5	ev	all/unsp	nev any	or
TAO	2	f	0	0	all	-	all	As:Chi	1988	CC	723	n	ot	n	n	5	ev	all/unsp	nev any	or
ULMER	1	c	0	0	all	-	all	Eu:Ger	1971	CC	726	n	bl	y	n	0	ev	all/unsp	nev any	or

REF	NRR	RR	SIG	RRDATA comment
DOLL2	43	4.33		0
RESTRE	2	10.28	y	0
TAO	1	3.95		0
TAO	2	4.93		0
ULMER	1	12.72		0

Least adjusted - insufficient data for meta-analysis: as for adjusted plus the following

REF	NRR	SEX	AGEL	AGEH	RACE	YF	LC TYPE	LOC	START	ST	NLC	R	VB	P	H	AD	SM	PRODUCT	DENOM	De
CORREA	59	m	0	0	wh	-	all	NAmer	1979	CC	1359	n	bl	y	n	0	ev	cig+/-ot	nev cigs	or
CORREA	61	m	0	0	bl	-	all	NAmer	1979	CC	1359	n	bl	y	n	0	ev	cig+/-ot	nev cigs	or
CORREA	60	f	0	0	wh	-	all	NAmer	1979	CC	1359	n	bl	y	n	0	ev	cig+/-ot	nev cigs	or
CORREA	62	f	0	0	bl	-	all	NAmer	1979	CC	1359	n	bl	y	n	0	ev	cig+/-ot	nev cigs	or
LIU	1	c	0	0	all	-	all	As:Chi	1980	CC	229	n	ot	*	n	0	ev	all/unsp	nev any	or

Table 1C1 - 9

IESLC - Meta-anal of Ever Smoking (or Current if Ever not available), Any prod (or Cigs if Any not avail)
All LC types
Least adjusted - insufficient data for meta-analysis: as for adjusted plus the following

REF	NRR	RR	SIG	RRDATA comment
CORREA	59	17.20		0
CORREA	61	13.10		0
CORREA	60	8.60		0
CORREA	62	22.00		0
LIU	1	2.45		0

Table 1C2 -

IESLC - Meta-anal of Current Smoking (or Ever if Current not available), Any prod (or Cigs if Any not avail)
All LC types

This analysis is restricted to results for:
1) Non-dose-response data
2) Results complete enough for use in metaanalysis

Within each study, results are then selected (in the following order of preference, within each sex) for:
3) SMKSTA: current smokers, ever smokers
4) PRODUCT: all/unspec, cigarettes regardless of other products, cigarettes only
5) CIGTYPE: all/unspecified, MC regardless of HR, MC only
6) DENOM: never smoked anything, never smoked cigarettes, (never +1 = +long term ex, +2 = +amount unknown, +3 = never cigs+long term ex)
7) Followup period (YF, prospective studies): whole study (coded as 0) or longest available
8) LCtype: all or nearest available, at least Squamous and Adeno. (q = squamous, s = small, l = large, a = adeno, mix = mixed, alv = alveolar)
9) Race: all or nearest available, otherwise by race (wh or w = white, bl or b = black, hi = hispanic ch = chinese, jap = japanese, haw = hawaiian, w+o = white + oriental, sca = scandinavian, as = asian)
10) For overlapping studies: principal rather than subsidiary studies
Finally by Age: whole study (coded as 0) if available, otherwise by widest available age group and then for single sex results (m, f) in preference to combined sex results (c).

Results adjusted (AD) for the most potential confounders are then chosen in Sections -1 to -3 (and those which actually differ from the adjusted results in Table 1C1 - 1 are marked 'x' in Section -1) and results adjusted for the least confounders in Sections -4 to -6. (Those least adjusted results which actually differ from the most adjusted as marked 'x' in column X in Section -4) (Results adjusted for an unknown number of confounder(s) are coded as 20.)

Section -7 shows excluded studies, together with the stage (as above) at which no qualifying results were found.

Section -8 lists the potentially overlapping studies which have been included (1=principal, 2=subsidiary).

Section -9 lists any results which would have been included in preference except that they had data not complete enough for use in meta-analysis, with their significance (yes/no), if known, and any further comment as entered on the database.

In addition to those mentioned above, the following fields, levels and abbreviations are used:
* or nk = not known, n = no, y = yes, ot = other
ev = ever, cu = current, nev = never
all/unspec = all or unspecified, cig+/-ot = cigarettes irrespective of other products (cigar, pipe etc)
MC = manufactured cigarettes, HR = hand-rolled cigarettes
REF: 6-character study reference
NRR: number of the RR on the database within the study
ST : study type (CC = case control, pr or prosp = prospective)
NLC: number of lung cancer cases in whole study
R : risky occupational population (n = no, m = mining, o = other risky)
VB : national cigarette type (V = at least 75% Virginia, bl = at least 75% blended, ot = other)
P : any proxy use
H : full histological confirmation
De : derivation of RR/CI (or = original, st = standard method, ot = other method of estimation)

Table 1C2 - 1

IESLC - Meta-anal of Current Smoking (or Ever if Current not available), Any prod (or Cigs if Any not avail)
All LC types
Most adjusted

REF	NRR	1C1	SEX	AGEL	AGEH	RACE	YF	LC TYPE	LOC	START	ST	NLC	R	VB	P	H	AD	SM	PRODUCT	DENOM	De
ABELIN	44		m	0	0	all	-	all	Eu:wst	1941	CC	118	n	bl	y	n	1	ev	all/unsp	nev any	st
ABRAHA	7		m	0	0	all	0	q+s+a	Eu:est	1975	pr	571	n	bl	n	n	0	ev	all/unsp	nev any	ot
ABRAHA	8		f	0	0	all	0	q+s+a	Eu:est	1975	pr	571	n	bl	n	n	0	ev	all/unsp	nev any	ot
AGUDO	3	x	f	0	0	all	-	all	Eu:wst	1989	CC	103	n	bl	n	n	3	cu	cig only	nev any	or
AKIBA	10	x	m	0	0	all	0	all	As:Jap	1963	pr	610	n	bl	n	n	5	cu	cig+/-ot	nev cigs	ot
AKIBA	14	x	f	0	0	all	0	all	As:Jap	1963	pr	610	n	bl	n	n	5	cu	cig+/-ot	nev cigs	or
ALDERS	177	x	m	0	0	all	-	all	Eu:UK	1977	CC	1448	n	V	n	n	0	cu	cig+/-ot	nev any	st
ALDERS	176	x	f	0	0	all	-	all	Eu:UK	1977	CC	1448	n	V	n	n	0	cu	cig only	nev any	st
AMANDU	5	x	m	0	0	wh	0	all	NAmer	1959	pr	132	m	bl	n	n	2	cu	cig+/-ot	nev cigs	ot
AMES	1	x	m	0	0	wh	-	all	NAmer	1959	ot	317	m	bl	n	n	0	cu	all/unsp	nev any	or
ANDERS	6	x	f	0	0	all	0	all	NAmer	1986	pr	343	n	bl	n	n	1	cu	cig+/-ot	nev cigs	or
ARCHER	5	x	m	0	0	wh	0	all	NAmer	1950	pr	146	m	bl	n	n	0	cu	cig+/-ot	nev cigs	st
ARMADA	27	x	m	0	0	all	-	all	Eu:wst	1986	CC	325	n	bl	n	y	0	cu	cig+/-ot	nev any	st
AUSTIN	6	x	c	0	0	all	-	all	NAmer	1970	CC	166	o	bl	y	n	3	cu	cig+/-ot	nev cigs	or
AUVINE	19		c	0	0	all	-	all	Eu:Sca	1986	CC	517	n	bl	y	n	2	ev	cig+/-ot	nev cigs	ot
AXELSO	1		c	0	0	all	-	all	Eu:Sca	1960	CC	152	n	bl	y	n	0	ev	all/unsp	nev any	st
AXELSS	2	x	m	0	0	sca	-	all	Eu:Sca	1989	CC	436	n	bl	n	n	0	cu	all/unsp	nev any	st
AXELSS	10	x	f	0	0	sca	-	all	Eu:Sca	1989	CC	436	n	bl	n	n	0	cu	all/unsp	nev any	st
BAND	1		m	0	0	all	-	all	NAmer	1983	CC	2831	n	V	y	y	2	ev	cig only	nev any	ot
BARBON	4	x	m	0	0	all	-	all	Eu:wst	1979	CC	755	n	bl	y	y	1	cu	all/unsp	nev any	or
BECHER	13	x	m	0	0	all	-	all	Eu:Ger	1985	CC	194	n	bl	n	y	0	cu	all/unsp	nev any	st
BECHER	14	x	f	0	0	all	-	all	Eu:Ger	1985	CC	194	n	bl	n	y	0	cu	all/unsp	nev any	st
BENSHL	16	x	m	40	64	all	10	all	Eu:UK	1967	pr	486	n	V	n	n	1	cu	all/unsp	nev any	ot
BEST	2	x	m	0	0	all	0	all	NAmer	1955	pr	381	n	V	n	n	1	cu	cig only	nev any	ot
BEST	18		f	0	0	all	0	all	NAmer	1955	pr	381	n	V	n	n	1	ev	cig only	nev any	ot
BLOHMK	1	x	m	0	0	all	-	all	Eu:Ger	1978	CC	888	n	bl	n	y	0	cu	all/unsp	nev any	st
BLOT4	1		m	0	0	wh	-	all	NAmer	1974	CC	335	n	bl	y	n	0	ev	cig+/-ot	nev cigs	st
BOFFET	33		m	0	0	all	-	all	Eu:mul	1988	CC	5621	n	bl	y	n	2	ev	all/unsp	nev any	or
BOUCOT	114	x	m	0	0	all	0	all	NAmer	1951	pr	121	n	bl	n	n	2	cu	cig only	nev any	ot
BRESLO	37		m	0	0	all	-	all	NAmer	1949	CC	518	n	bl	n	y	0	ev	all/unsp	nev+1	st
BRESLO	38		f	0	0	all	-	all	NAmer	1949	CC	518	n	bl	n	y	0	ev	all/unsp	nev+1	st
BRETT	4	x	m	0	0	all	0	all	Eu:UK	1960	pr	150	n	V	n	n	0	cu	cig+/-ot	nev cigs	st
BROCKM	1		m	0	0	wh	-	all	Eu:Ger	1990	CC	117	n	bl	n	y	0	ev	cig+/-ot	nev cigs	st
BROCKM	2		f	0	0	wh	-	all	Eu:Ger	1990	CC	117	n	bl	n	y	0	ev	cig+/-ot	nev cigs	st
BROSS	11	x	m	0	0	wh	-	all	NAmer	1960	CC	974	n	bl	n	n	0	cu	all/unsp	nev any	st
BROWN2	12	x	m	0	0	wh	-	all	NAmer	1984	CC	14596	n	bl	n	y	2	cu	cig+/-ot	nev cigs	or
BROWN2	11	x	f	0	0	wh	-	all	NAmer	1984	CC	14596	n	bl	n	y	2	cu	cig+/-ot	nev cigs	or
BUFFLE	3	x	m	0	0	wh	-	all	NAmer	1976	CC	943	n	bl	y	n	0	cu	cig+/-ot	nev any	st
BUFFLE	7	x	f	0	0	wh	-	all	NAmer	1976	CC	943	n	bl	y	n	0	cu	cig+/-ot	nev any	st
CARPEN	11	x	c	0	0	w+b	-	all	NAmer	1991	CC	356	n	bl	n	n	3	cu	cig+/-ot	nev cigs	or
CASCO2	1		c	0	0	wh	-	all	Eu:Ger	1991	CC	155	n	bl	n	n	0	ev	all/unsp	nev any	st
CASCOR	1		c	0	0	wh	-	all	Eu:Ger	1985	CC	389	n	bl	n	y	0	ev	all/unsp	nev any	st
CEDERL	106	x	m	0	0	all	16	all	Eu:Sca	1963	pr	491	n	bl	n	n	2	cu	all/unsp	nev any	ot
CEDERL	75	x	f	0	0	all	0	all	Eu:Sca	1963	pr	491	n	bl	n	n	2	cu	all/unsp	nev any	or
CHAN	9		m	0	0	all	-	all	As:HK	1976	CC	397	n	bl	n	n	0	ev	all/unsp	nev any	st
CHAN	10		f	0	0	all	-	all	As:HK	1976	CC	397	n	bl	n	n	0	ev	all/unsp	nev any	st
CHANG	5	x	m	0	0	all	0	all	NAmer	1972	pr	136	n	bl	n	n	0	cu	cig+/-ot	nev cigs	st
CHANG	11	x	f	0	0	all	0	all	NAmer	1972	pr	136	n	bl	n	n	0	cu	cig+/-ot	nev cigs	st
CHATZI	4		c	0	0	all	-	all	Eu:bal	1987	CC	282	n	bl	n	y	0	ev	all/unsp	nev any	st
CHEN2	1		m	0	0	all	-	all	As:Chi	1983	CC	193	n	ot	y	n	0	ev	all/unsp	nev any	st
CHEN2	2		f	0	0	all	-	all	As:Chi	1983	CC	193	n	ot	y	n	0	ev	all/unsp	nev any	st
CHEN3	1		c	0	0	all	-	all	As:Chi	1981	CC	254	n	ot	y	n	0	ev	all/unsp	nev any	st
CHIAZZ	3		m	0	0	all	-	all	NAmer	1940	CC	144	o	bl	y	n	11	ev	cig+/-ot	nev cigs	or
CHOI	3	x	m	0	0	all	-	all	As:oth	1985	CC	375	n	bl	n	n	0	cu	cig+/-ot	nev cigs	st
CHOI	7	x	f	0	0	all	-	all	As:oth	1985	CC	375	n	bl	n	n	0	cu	cig+/-ot	nev cigs	st
CHOW	25	x	m	0	0	wh	0	all	NAmer	1966	pr	219	n	bl	n	n	0	cu	all/unsp	nev any	st
CHYOU	2	x	m	0	0	jap	0	all	NAmer	1965	pr	227	n	bl	n	y	1	cu	cig+/-ot	nev cigs	or
COMSTO	3	x	m	0	0	all	-	all	NAmer	1975	ot	258	n	bl	n	n	0	cu	cig+/-ot	nev any	st
COMSTO	8	x	f	0	0	all	-	all	NAmer	1975	ot	258	n	bl	n	n	0	cu	cig+/-ot	nev any	st
COOKSO	5		c	0	0	bl	-	all	Africa	1961	CC	234	n	V	n	y	0	ev	all/unsp	nev any	st
CORREA	42	x	c	0	0	all	-	all	NAmer	1979	CC	1359	n	bl	y	n	1	cu	cig+/-ot	nev cigs	or
CPSI	220	x	m	35	84	all	6	all	NAmer	1959	pr	5138	n	bl	n	n	1	cu	cig+/-ot	nev any	ot
CPSI	279	x	f	40	74	all	6	all	NAmer	1959	pr	5138	n	bl	n	n	1	cu	cig+/-ot	nev cigs	ot
CPSII	126	x	m	0	0	all	6	all	NAmer	1982	pr	3229	n	bl	n	n	1	cu	cig only	nev any	ot
CPSII	133	x	f	0	0	all	6	all	NAmer	1982	pr	3229	n	bl	n	n	1	cu	cig+/-ot	nev cigs	ot
DAMBER	14	x	m	0	0	all	-	all	Eu:Sca	1972	CC	579	n	bl	y	n	1	cu	all/unsp	nev any	ot
DARBY	4	x	m	0	0	wh	-	all	Eu:UK	1988	CC	982	n	V	n	n	0	cu	cig+/-ot	nev any	st
DARBY	11	x	f	0	0	wh	-	all	Eu:UK	1988	CC	982	n	V	n	n	0	cu	cig+/-ot	nev any	st
DAVEYS	5		m	0	0	all	-	all	Eu:Ger	1930	CC	109	n	bl	y	n	0	ev	all/unsp	nev any	st
DAVEYS	6		f	0	0	all	-	all	Eu:Ger	1930	CC	109	n	bl	y	n	0	ev	all/unsp	nev any	ot
DEAN	7		m	0	0	wh	-	all	Africa	1947	CC	603	n	V	y	n	0	ev	all/unsp	nev any	st
DEAN2	2	x	m	0	0	all	-	all	Eu:UK	1960	CC	954	n	V	y	n	0	cu	all/unsp	nev any	st

Table 1C2 - 1

IESLC - Meta-anal of Current Smoking (or Ever if Current not available), Any prod (or Cigs if Any not avail)
All LC types
Most adjusted

REF	NRR	1C1	SEX	AGEL	AGEH	RACE	YF	LC TYPE	LOC	START	ST	NLC	R	VB	P	H	AD	SM	PRODUCT	DENOM	De
DEAN2	6	x	f	0	0	all	-	all	Eu:UK	1960	CC	954	n	V	y	n	0	cu	all/unsp	nev any	st
DEAN3	42	x	m	0	0	all	-	all	Eu:UK	1969	CC	766	n	V	y	n	3	cu	all/unsp	nev any	ot
DEAN3	119	x	f	0	0	all	-	all	Eu:UK	1969	CC	766	n	V	y	n	3	cu	cig only	nev any	ot
DEKLER	8	x	m	0	0	all	0	all	Auslia	1961	pr	138	m	V	n	n	2	cu	all/unsp	nev any	ot
DESTE2	4	x	c	0	0	all	-	all	SCAmer	1993	CC	463	n	bl	n	n	7	cu	all/unsp	nev any	or
DESTEF	41	x	m	0	0	all	-	all	SCAmer	1988	CC	497	n	bl	n	y	4	cu	all/unsp	nev any	or
DOCKER	1	x	c	0	0	wh	0	all	NAmer	1974	pr	120	n	bl	n	n	4	cu	cig+/-ot	nev cigs	or
DOLL	90	x	m	0	0	all	-	all	Eu:UK	1948	CC	1465	n	V	n	n	0	cu	all/unsp	nev any	st
DOLL	93	x	f	0	0	all	-	all	Eu:UK	1948	CC	1465	n	V	n	n	0	cu	all/unsp	nev any	st
DOLL2	54	x	m	0	0	all	0	all	Eu:UK	1951	pr	920	n	V	n	n	1	cu	all/unsp	nev any	ot
DOLL2	63		f	0	0	all	22	all	Eu:UK	1951	pr	920	n	V	n	n	1	cu	cig only	nev any	ot
DORANT	2	x	m	0	0	all	0	all	Eu:wst	1986	ot	550	n	bl	n	y	0	cu	all/unsp	nev any	st
DORGAN	9	x	m	0	0	wh	-	all	NAmer	1980	CC	2026	n	bl	y	y	0	cu	cig+/-ot	nev any	st
DORGAN	33	x	m	0	0	bl	-	all	NAmer	1980	CC	2026	n	bl	y	y	0	cu	cig+/-ot	nev any	st
DORGAN	56	x	f	0	0	wh	-	all	NAmer	1980	CC	2026	n	bl	y	y	0	cu	cig+/-ot	nev any	st
DORGAN	79	x	f	0	0	bl	-	all	NAmer	1980	CC	2026	n	bl	y	y	0	cu	cig+/-ot	nev any	st
DORN	51	x	m	35	84	wh	8	all	NAmer	1954	pr	5097	n	bl	n	n	1	cu	all/unsp	nev any	ot
DOSEME	1		m	0	0	all	-	all	Eu:bal	1979	CC	1210	n	bl	n	n	2	ev	cig+/-ot	nev cigs	or
DROSTE	6	x	m	0	0	all	-	all	Eu:wst	1995	CC	478	n	bl	n	y	4	cu	all/unsp	nev any	or
DU	1		m	0	0	all	-	all	As:Chi	1985	CC	849	n	ot	y	n	0	ev	all/unsp	nev any	or
DU	2		f	0	0	all	-	all	As:Chi	1985	CC	849	n	ot	y	n	0	ev	all/unsp	nev any	or
DUNN	6		m	0	0	all	0	all	NAmer	1954	pr	139	o	bl	n	n	0	ev	cig+/-ot	nev cigs	st
EBELIN	1		m	0	0	all	-	all	Eu:Ger	1980	CC	130	n	bl	n	n	0	ev	all/unsp	nev any	st
ENGELA	158	x	m	0	0	all	12	all	Eu:Sca	1964	pr	435	n	bl	n	n	1	cu	all/unsp	nev any	ot
ENGELA	164	x	f	0	0	all	12	all	Eu:Sca	1964	pr	435	n	bl	n	n	1	cu	all/unsp	nev any	ot
ENSTRO	1		m	0	0	all	0	all	NAmer	1959	pr	2879	n	bl	n	n	1	cu	cig only	nev any	or
ENSTRO	2		f	0	0	all	0	all	NAmer	1959	pr	2879	n	bl	n	n	1	cu	cig only	nev any	or
ESAKI	4		m	0	0	all	-	all	As:Jap	1961	CC	245	n	bl	y	n	0	ev	cig+/-ot	nev cigs	st
ESAKI	5		f	0	0	all	-	all	As:Jap	1961	CC	245	n	bl	y	n	0	ev	cig+/-ot	nev cigs	st
FAN	1		m	0	0	all	-	all	As:Chi	1990	CC	403	n	ot	y	n	0	ev	cig+/-ot	nev cigs	st
FAN	2		f	0	0	all	-	all	As:Chi	1990	CC	403	n	ot	y	n	0	ev	cig+/-ot	nev cigs	st
GAO	33	x	m	0	0	all	-	all	As:Chi	1984	CC	1405	n	ot	n	n	2	cu	cig+/-ot	nev cigs	or
GAO	34	x	f	0	0	all	-	all	As:Chi	1984	CC	1405	n	ot	n	n	2	cu	cig+/-ot	nev cigs	or
GAO2	8	x	m	0	0	all	-	all	As:Jap	1988	CC	282	n	bl	n	n	1	cu	cig+/-ot	nev cigs	or
GARCIA	2	x	c	0	0	all	-	all	NAmer	1992	CC	416	n	bl	n	y	0	cu	cig+/-ot	nev cigs	st
GARDIN	2	x	c	0	0	all	-	all	Eu:UK	1988	CC	143	n	V	y	n	0	cu	all/unsp	nev any	st
GARSHI	31	x	m	0	0	all	-	all	NAmer	1981	CC	1081	o	bl	y	n	1	cu	all/unsp	nev any	st
GENG	1		m	0	0	all	-	all	As:Chi	1985	CC	292	n	ot	*	n	0	ev	cig+/-ot	nev any	st
GENG	2		f	0	0	all	-	all	As:Chi	1985	CC	292	n	ot	*	n	0	ev	cig+/-ot	nev any	st
GER	21		c	0	0	all	-	all	As:oth	1990	CC	141	n	ot	y	n	14	ev	all/unsp	nev any	ot
GODLEY	5		m	0	0	all	-	all	NAmer	1966	CC	1986	n	bl	y	n	1	ev	cig+/-ot	nev cigs	ot
GODLEY	6		f	0	0	all	-	all	NAmer	1966	CC	1986	n	bl	y	n	1	ev	cig+/-ot	nev cigs	ot
GOLLED	7		m	35	99	all	-	all	Eu:UK	1952	CC	443	n	V	y	n	1	ev	cig+/-ot	nev any	ot
GOODMA	2	x	m	0	0	w+o	-	all	NAmer	1983	CC	326	n	bl	y	y	0	cu	cig+/-ot	nev any	st
GOODMA	6	x	f	0	0	w+o	-	all	NAmer	1983	CC	326	n	bl	y	y	0	cu	cig+/-ot	nev any	st
GRAHAM	25	x	m	0	0	wh	-	all	NAmer	1956	CC	685	n	bl	n	n	1	cu	all/unsp	nev any	ot
GREGOR	2	x	m	0	0	all	-	all	Eu:UK	1976	CC	104	n	V	n	y	0	cu	cig+/-ot	nev cigs	st
GREGOR	6	x	f	0	0	all	-	all	Eu:UK	1976	CC	104	n	V	n	y	0	cu	cig+/-ot	nev cigs	st
GSELL	8		m	0	0	all	-	all	Eu:wst	1937	CC	150	n	bl	n	y	0	ev	all/unsp	nev any	st
HAENSZ	54	x	f	0	0	all	-	not alv	NAmer	1955	CC	158	n	bl	n	y	0	cu	cig+/-ot	nev any	st
HAMMO2	8	x	m	0	0	all	0	all	NAmer	1967	pr	450	o	bl	n	n	1	cu	cig+/-ot	nev any	ot
HAMMON	139	x	m	0	0	wh	0	all	NAmer	1952	pr	448	n	bl	n	n	1	cu	cig only	nev any	ot
HANSEN	3		m	0	0	all	0	all	Eu:Sca	1968	pr	105	o	bl	y	n	2	ev	all/unsp	nev any	ot
HEGMAN	1		c	0	0	all	-	all	NAmer	1989	CC	282	n	bl	y	y	0	ev	all/unsp	nev any	st
HEIN	5	x	m	0	0	all	0	all	Eu:Sca	1970	pr	144	n	bl	n	n	0	cu	all/unsp	nev any	st
HENNEK	2	x	m	0	0	all	0	all	NAmer	1982	pr	169	n	bl	n	n	0	cu	all/unsp	nev any	st
HINDS	22		f	0	0	o	-	all	NAmer	1968	CC	292	n	bl	n	n	3	ev	all/unsp	nev any	st
HIRAYA	1	x	m	0	0	all	0	all	As:Jap	1965	pr	1917	n	bl	n	n	1	cu	cig+/-ot	nev any	st
HIRAYA	3	x	f	0	0	all	0	all	As:Jap	1965	pr	1917	n	bl	n	n	1	cu	cig+/-ot	nev any	st
HITOSU	34	x	m	0	0	all	-	all	As:Jap	1960	CC	216	n	bl	y	n	1	cu	all/unsp	nev any	st
HITOSU	59	x	f	0	0	all	-	all	As:Jap	1960	CC	216	n	bl	y	n	1	cu	all/unsp	nev any	st
HOLE	33	x	m	0	0	all	0	all	Eu:UK	1972	pr	225	n	V	n	n	1	cu	all/unsp	nev any	ot
HOLE	31		f	0	0	all	11	all	Eu:UK	1972	pr	225	n	V	n	n	1	cu	all/unsp	nev any	ot
HOROWI	1		m	0	0	all	-	all	NAmer	1956	CC	236	n	V	n	n	0	ev	cig+/-ot	nev any	st
HOROWI	2		f	0	0	all	-	all	NAmer	1956	CC	236	n	V	n	n	0	ev	cig+/-ot	nev any	st
HORWIT	1		f	0	0	all	-	all	NAmer	1977	CC	112	n	bl	n	n	0	ev	cig+/-ot	nev cigs	st
HU	15		m	0	0	all	-	all	As:Chi	1985	CC	227	n	ot	n	y	0	ev	cig+/-ot	nev any	st
HU	16		f	0	0	all	-	all	As:Chi	1985	CC	227	n	ot	n	y	0	ev	cig+/-ot	nev any	st
HU2	9		m	0	0	all	-	all	As:Chi	1977	CC	523	n	ot	y	n	0	ev	cig+/-ot	nev cigs	st
HU2	10		f	0	0	all	-	all	As:Chi	1977	CC	523	n	ot	y	n	0	ev	cig+/-ot	nev cigs	st
HUANG	1		c	0	0	all	-	all	As:Chi	1990	CC	135	n	ot	y	n	0	ev	all/unsp	nev any	st
HUMBLE	13	x	m	0	0	w-hi	-	all	NAmer	1980	CC	521	n	bl	y	n	1	cu	cig+/-ot	nev cigs	ot

Table 1C2 - 1

IESLC - Meta-anal of Current Smoking (or Ever if Current not available), Any prod (or Cigs if Any not avail)
All LC types
Most adjusted

REF	NRR	1C1	SEX	AGEL	AGEH	RACE	YF	LC TYPE	LOC	START	ST	NLC	R	VB	P	H	AD	SM	PRODUCT	DENOM	De
HUMBLE	15	x	m	0	0	hi	-	all	NAmer	1980	CC	521	n	bl	y	n	1	cu	cig+/-ot	nev cigs	ot
HUMBLE	17	x	f	0	0	w-hi	-	all	NAmer	1980	CC	521	n	bl	y	n	1	cu	cig+/-ot	nev cigs	ot
HUMBLE	19	x	f	0	0	hi	-	all	NAmer	1980	CC	521	n	bl	y	n	1	cu	cig+/-ot	nev cigs	ot
JAHN	22		f	0	0	all	-	all	Eu:Ger	1988	CC	1004	n	bl	n	n	2	ev	cig+/-ot	nev any	ot
JAIN	52	x	m	0	0	all	-	all	NAmer	1981	CC	845	n	V	y	n	2	cu	cig+/-ot	nev cigs	or
JAIN	51	x	f	0	0	all	-	all	NAmer	1981	CC	845	n	V	y	n	2	cu	cig+/-ot	nev cigs	or
JARUP	6		m	0	0	all	-	all	Eu:Sca	1928	CC	102	o	bl	y	n	2	ev	all/unsp	nev any	ot
JARVHO	2	x	m	0	0	all	-	all	Eu:Sca	1983	CC	147	n	bl	n	n	0	cu	all/unsp	nev any	st
JARVHO	6	x	f	0	0	all	-	all	Eu:Sca	1983	CC	147	n	bl	n	n	0	cu	all/unsp	nev any	st
JEDRYC	58		m	0	0	all	-	all	Eu:est	1980	CC	1630	n	bl	y	n	4	ev	cig+/-ot	nev any	ot
JEDRYC	59		f	0	0	all	-	all	Eu:est	1980	CC	1630	n	bl	y	n	4	ev	cig+/-ot	nev any	ot
JIANG	1		m	0	0	all	-	all	As:Chi	1984	CC	125	n	ot	n	n	0	ev	all/unsp	nev any	st
JIANG	2		f	0	0	all	-	all	As:Chi	1984	CC	125	n	ot	n	n	0	ev	all/unsp	nev any	st
JOLY	18	x	m	0	0	all	-	all	SCAmer	1978	CC	826	n	bl	n	n	0	cu	all/unsp	nev any	st
JOLY	15	x	f	0	0	all	-	all	SCAmer	1978	CC	826	n	bl	n	n	0	cu	cig+/-ot	nev any	st
JUSSAW	29		m	0	0	all	-	all	As:Ind	1964	CC	792	n	V	n	n	2	ev	all/unsp	nev any	st
KAISE2	68	x	m	35	99	all	9	all	NAmer	1979	pr	318	n	bl	n	n	1	cu	cig only	nev any	st
KAISE2	60	x	f	35	99	all	9	all	NAmer	1979	pr	318	n	bl	n	n	1	cu	cig only	nev any	st
KAISER	12	x	m	0	0	all	0	all	NAmer	1964	pr	714	n	bl	n	n	2	cu	cig+/-ot	nev cigs	ot
KAISER	9	x	f	0	0	all	0	all	NAmer	1964	pr	714	n	bl	n	n	2	cu	cig+/-ot	nev cigs	ot
KANELL	30		m	0	0	all	-	all	Eu:bal	1950	CC	862	n	bl	n	n	1	cu	all/unsp	nev any	st
KATSOU	2	x	f	0	0	all	-	all	Eu:bal	1987	CC	101	n	bl	n	n	1	cu	all/unsp	nev any	or
KAUFMA	16	x	c	0	0	all	-	all	NAmer	1981	CC	881	n	bl	n	n	6	cu	cig+/-ot	nev cigs	ot
KELLER	1	x	m	0	0	wh	-	all	NAmer	1985	CC	15038	n	bl	n	n	0	cu	all/unsp	nev any	st
KELLER	9	x	m	0	0	nonw	-	all	NAmer	1985	CC	15038	n	bl	n	n	0	cu	all/unsp	nev any	st
KELLER	5	x	f	0	0	wh	-	all	NAmer	1985	CC	15038	n	bl	n	n	0	cu	all/unsp	nev any	st
KELLER	13	x	f	0	0	nonw	-	all	NAmer	1985	CC	15038	n	bl	n	n	0	cu	all/unsp	nev any	st
KHUDER	19	x	m	0	0	all	-	all	NAmer	1985	CC	482	n	bl	n	y	0	cu	cig+/-ot	nev cigs	or
KIHARA	7	x	c	0	0	jap	-	all	As:Jap	1991	CC	440	n	bl	n	n	0	cu	all/unsp	nev any	st
KINLEN	20	x	m	0	0	all	0	all	Eu:UK	1967	pr	718	n	V	n	n	2	cu	all/unsp	nev any	ot
KJUUS	1	x	m	0	0	all	-	all	Eu:Sca	1979	CC	176	n	bl	n	n	0	cu	all/unsp	nev any	st
KNEKT	86	x	m	20	69	all	21	all	Eu:Sca	1966	pr	515	n	bl	n	n	1	cu	all/unsp	nev any	ot
KO	1		f	0	0	all	-	all	As:oth	1992	CC	117	n	ot	n	y	3	ev	cig+/-ot	nev cigs	or
KOHLME	2		c	0	0	all	-	all	Eu:Ger	1990	CC	239	n	bl	n	n	4	ev	all/unsp	nev any	or
KOO	9	x	f	0	0	all	-	all	As:HK	1981	CC	200	n	bl	n	n	0	cu	all/unsp	nev any	st
KOULUM	1		m	0	0	all	-	all	Eu:Sca	1936	CC	812	n	bl	n	n	0	ev	all/unsp	nev any	st
KREUZE	40	x	f	1	45	all	-	all	Eu:Ger	1990	CC	2260	n	bl	n	n	0	cu	all/unsp	nev any	st
KREUZE	42	x	f	55	69	all	-	all	Eu:Ger	1990	CC	2260	n	bl	n	n	0	cu	all/unsp	nev any	st
KREYBE	12		m	0	0	all	-	all	Eu:Sca	1948	CC	300	n	bl	n	y	1	ev	all/unsp	nev any	ot
KREYBE	30		f	0	0	all	-	all	Eu:Sca	1948	CC	300	n	bl	n	y	1	ev	all/unsp	nev any	ot
KUBIK	12	x	m	0	0	all	0	all	Eu:est	1965	pr	108	n	bl	n	n	0	cu	cig+/-ot	nev any	st
LAMTH	6		f	0	0	ch	-	all	As:HK	1983	CC	445	n	bl	n	n	0	ev	all/unsp	nev any	or
LAMWK	1		f	0	0	ch	-	all	As:HK	1981	CC	163	n	bl	n	n	0	ev	all/unsp	nev any	st
LAMWK2	9		m	0	0	all	-	q+s+l+a	As:HK	1976	CC	480	n	bl	n	n	0	ev	all/unsp	nev any	st
LAMWK2	10		f	0	0	all	-	q+s+l+a	As:HK	1976	CC	480	n	bl	n	n	0	ev	all/unsp	nev any	st
LANGE	38	x	m	0	0	all	0	all	Eu:Sca	1976	pr	268	n	bl	n	n	1	cu	all/unsp	nev any	ot
LANGE	35	x	f	0	0	all	0	all	Eu:Sca	1976	pr	268	n	bl	n	n	1	cu	all/unsp	nev any	ot
LAUSSM	11		m	0	0	all	-	all	Eu:Ger	1982	CC	432	n	bl	n	n	3	ev	all/unsp	nev any	or
LEI	1		m	0	0	all	-	all	As:Chi	1986	CC	792	n	ot	y	n	0	ev	all/unsp	nev any	st
LEI	2		f	0	0	all	-	all	As:Chi	1986	CC	792	n	ot	y	n	0	ev	all/unsp	nev any	st
LEMARC	2	x	c	0	0	w+o	-	all	NAmer	1992	CC	341	n	bl	n	y	0	cu	all/unsp	nev any	st
LETOUR	1		c	0	0	all	-	all	NAmer	1983	CC	738	n	V	y	y	0	ev	cig+/-ot	nev cigs	st
LEVIN	32		m	0	0	all	-	all	NAmer	1938	CC	475	n	bl	n	n	1	ev	all/unsp	nev any	st
LIAW	1		m	0	0	all	0	all	As:oth	1982	pr	127	n	ot	n	n	1	cu	all/unsp	nev any	or
LIAW	2		f	0	0	all	0	all	As:oth	1982	pr	127	n	ot	n	n	1	cu	all/unsp	nev any	or
LIDDEL	4	x	m	0	0	all	18	all	NAmer	1970	pr	304	m	V	n	n	1	cu	cig+/-ot	nev cigs	ot
LIU	2		c	0	0	all	-	all	As:Chi	1980	CC	229	n	ot	*	n	2	ev	all/unsp	nev any	or
LIU2	2		m	0	0	all	-	all	As:Chi	1983	CC	316	n	ot	n	n	3	ev	all/unsp	nev any	ot
LIU2	4		f	0	0	all	-	all	As:Chi	1983	CC	316	n	ot	n	n	3	ev	all/unsp	nev any	ot
LIU3	2		m	0	0	all	-	all	As:Chi	1985	CC	110	n	ot	n	n	2	ev	all/unsp	nev any	or
LIU4	11		m	0	0	all	-	all	As:Chi	1986	CC	1000-00	n	ot	y	n	2	ev	all/unsp	nev any	ot
LIU4	12		f	0	0	all	-	all	As:Chi	1986	CC	1000-00	n	ot	y	n	2	ev	all/unsp	nev any	ot
LIU5	1		c	0	0	all	-	all	As:Chi	1978	CC	111	n	ot	y	n	0	ev	all/unsp	nev any	st
LOMBA2	1		f	0	0	all	-	all	NAmer	1960	CC	225	n	bl	n	n	0	ev	cig+/-ot	nev cigs	st
LOMBAR	9	x	m	0	0	all	-	all	NAmer	1951	CC	1040	n	bl	n	n	0	cu	cig+/-ot	nev any	st
LUBIN2	26	x	m	0	0	all	-	all	Eu:mul	1976	CC	7804	n	bl	n	y	2	cu	all/unsp	nev any	ot
LUBIN2	317	x	f	0	0	all	-	all	Eu:mul	1976	CC	7804	n	bl	n	y	0	cu	cig+/-ot	nev any	st
LUO	7		c	0	0	all	-	all	As:Chi	1990	CC	102	n	ot	n	y	20	ev	cig+/-ot	nev cigs	or
MACLEN	19	x	m	0	0	ch	-	all	As:oth	1972	CC	233	n	bl	n	n	0	cu	cig+/-ot	nev cigs	st
MACLEN	32	x	f	0	0	ch	-	all	As:oth	1972	CC	233	n	bl	n	n	0	cu	cig+/-ot	nev cigs	st

Table 1C2 - 1

IESLC - Meta-anal of Current Smoking (or Ever if Current not available), Any prod (or Cigs if Any not avail)
All LC types
Most adjusted

REF	NRR	1C1	SEX	AGEL	AGEH	RACE	YF	LC TYPE	LOC	START	ST	NLC	R	VB	P	H	AD	SM	PRODUCT	DENOM	De
MAGNUS	5		m	0	0	all	0	all	Eu:Sca	1953	pr	203	o	bl	y	n	3	ev	all/unsp	nev any	ot
MARSH	7		c	0	0	all	-	all	NAmer	1979	CC	150	n	bl	y	n	2	ev	all/unsp	nev any	or
MARSH2	5		m	0	0	all	-	all	NAmer	1979	CC	114	n	bl	y	n	1	ev	all/unsp	nev any	or
MARSH2	6		f	0	0	all	-	all	NAmer	1979	CC	114	n	bl	y	n	1	ev	all/unsp	nev any	ot
MARTIS	4		m	0	0	all	-	all	Eu:UK	1972	CC	201	n	V	n	n	0	ev	cig+/-ot	nev cigs	st
MASTRA	2		m	0	0	all	-	all	Eu:wst	1973	CC	309	n	bl	n	n	2	ev	all/unsp	nev any	st
MATOS	3	x	m	0	0	all	-	all	SCAmer	1994	CC	200	n	bl	n	n	2	cu	cig+/-ot	nev any	or
MATSUD	10		m	0	0	all	-	all	As:Jap	1965	CC	179	n	bl	n	n	0	ev	cig+/-ot	nev cigs	st
MCCONN	1		m	0	0	all	-	all	Eu:UK	1946	CC	100	n	V	n	y	0	ev	all/unsp	nev any	st
MCCONN	2		f	0	0	all	-	all	Eu:UK	1946	CC	100	n	V	n	y	0	ev	all/unsp	nev any	st
MCDUFF	1		m	0	0	all	-	all	NAmer	1979	CC	165	n	V	y	n	0	ev	cig+/-ot	nev cigs	st
MCLAUG	1		m	0	0	all	-	all	As:Chi	1972	CC	316	o	ot	y	n	0	ev	all/unsp	nev any	st
MIGRAN	20	x	m	0	0	all	0	all	Eu:UK	1964	pr	259	n	V	n	n	2	cu	all/unsp	nev any	ot
MIGRAN	136	x	f	0	0	all	0	all	Eu:UK	1964	pr	259	n	V	n	n	2	cu	all/unsp	nev any	ot
MILLER	2		f	0	0	all	-	all	NAmer	1972	CC	168	n	bl	y	n	1	ev	cig+/-ot	nev any	ot
MILLS	3		m	0	0	wh	-	all	NAmer	1940	CC	444	n	bl	y	n	1	ev	all/unsp	nev any	ot
MRFITR	2	x	m	0	0	all	0	all	NAmer	1973	pr	119	n	bl	n	n	0	cu	cig+/-ot	nev cigs	ot
NAM	76	x	m	0	0	all	-	all	NAmer	1986	CC	1199	n	bl	y	n	1	cu	cig+/-ot	nev cigs	ot
NAM	92	x	f	0	0	all	-	all	NAmer	1986	CC	1199	n	bl	y	n	1	cu	cig+/-ot	nev cigs	ot
NOTAN2	15		m	0	0	all	-	all	As:Ind	1963	CC	683	n	V	n	n	2	ev	all/unsp	nev any	ot
NOU	11		m	30	64	all	-	all	Eu:Sca	1971	CC	273	n	bl	y	n	0	ev	all/unsp	nev any	st
NOU	12		f	30	64	all	-	all	Eu:Sca	1971	CC	273	n	bl	y	n	0	ev	all/unsp	nev any	st
ODRISC	1	x	c	0	0	all	-	all	Eu:UK	1992	CC	446	n	V	n	n	0	cu	all/unsp	nev any	st
ORMOS	4		m	0	0	all	-	all	Eu:est	1947	CC	119	n	bl	y	y	0	ev	cig+/-ot	nev any	st
ORMOS	26		f	0	0	all	-	all	Eu:est	1947	CC	119	n	bl	y	y	0	ev	cig+/-ot	nev any	st
OSANN	33	x	m	0	0	all	-	all	NAmer	1984	CC	1986	n	bl	n	n	2	cu	cig+/-ot	nev cigs	or
OSANN	34	x	f	0	0	all	-	all	NAmer	1984	CC	1986	n	bl	n	n	2	cu	cig+/-ot	nev cigs	or
PARKIN	26	x	m	0	0	bl	-	all	Africa	1963	CC	877	n	V	y	n	6	cu	all/unsp	nev any	ot
PASTOR	10		m	0	0	all	-	all	Eu:wst	1976	CC	204	n	bl	y	n	1	ev	all/unsp	nev any	or
PAWLEG	2		m	0	0	all	-	all	Eu:est	1992	CC	176	n	bl	n	y	6	ev	all/unsp	nev any	ot
PERNU	2		m	0	0	all	-	all	Eu:Sca	1944	CC	1606	n	bl	n	n	0	ev	all/unsp	nev any	st
PERNU	1		f	0	0	all	-	all	Eu:Sca	1944	CC	1606	n	bl	n	n	0	ev	all/unsp	nev any	st
PERSH2	10	x	c	0	0	all	-	all	Eu:Sca	1980	CC	1022	n	bl	y	n	4	cu	all/unsp	nev any	ot
PETO	4	x	m	0	0	all	0	all	Eu:UK	1954	pr	103	n	V	n	n	0	cu	all/unsp	nev any	st
PEZZO2	2	x	m	0	0	all	-	all	SCAmer	1992	CC	367	n	bl	n	y	0	cu	cig+/-ot	nev cigs	st
PEZZOT	5	x	m	0	0	all	-	all	SCAmer	1987	CC	215	n	bl	n	y	0	cu	cig only	nev cigs	st
PIKE	4		m	0	0	w-hi	-	all	NAmer	1972	CC	731	n	bl	y	n	0	ev	all/unsp	nev any	st
PIKE	8		f	0	0	w-hi	-	all	NAmer	1972	CC	731	n	bl	y	n	0	ev	all/unsp	nev any	st
POFFIJ	1		c	0	0	all	-	all	Eu:mul	1990	CC	971	n	bl	n	n	0	ev	all/unsp	nev any	st
POLEDN	1		c	0	0	all	-	all	NAmer	1978	CC	209	n	bl	y	n	1	ev	cig+/-ot	nev cigs	or
QIAO2	14	x	m	0	0	all	0	all	As:Chi	1992	pr	241	m	ot	n	n	1	cu	all/unsp	nev any	or
RACHTA	9	x	f	0	0	all	-	all	Eu:est	1991	CC	118	n	bl	n	y	1	cu	cig+/-ot	nev cigs	or
RADZIK	1		c	0	0	all	-	all	Eu:est	1986	CC	189	n	bl	n	n	0	ev	all/unsp	nev any	st
RANDIG	23		m	0	0	all	-	all	Eu:Ger	1951	CC	448	n	bl	n	n	0	ev	all/unsp	nev any	st
RANDIG	24		f	0	0	all	-	all	Eu:Ger	1951	CC	448	n	bl	n	n	0	ev	all/unsp	nev any	st
REN	1		m	0	0	all	-	all	As:Chi	1980	CC	244	n	ot	*	n	0	ev	all/unsp	nev any	st
REN	2		f	0	0	all	-	all	As:Chi	1980	CC	244	n	ot	*	n	0	ev	all/unsp	nev any	st
RONCO	1		m	0	0	all	-	all	Eu:wst	1976	CC	126	n	bl	y	n	0	ev	all/unsp	nev any	st
ROTHSC	2		c	0	0	all	-	all	NAmer	1971	CC	284	n	bl	y	n	1	ev	all/unsp	nev any	st
SADOWS	31		m	0	0	wh	-	all	NAmer	1938	CC	477	n	bl	n	n	1	ev	all/unsp	nev any	ot
SANKAR	2		m	0	0	all	-	all	As:Ind	1990	CC	281	n	V	n	n	3	ev	all/unsp	nev any	ot
SCHWAR	25	x	m	0	0	wh	-	all	NAmer	1984	CC	5588	n	bl	y	y	0	cu	cig+/-ot	nev cigs	st
SCHWAR	26	x	m	0	0	bl	-	all	NAmer	1984	CC	5588	n	bl	y	y	0	cu	cig+/-ot	nev cigs	st
SCHWAR	27	x	f	0	0	wh	-	all	NAmer	1984	CC	5588	n	bl	y	y	0	cu	cig+/-ot	nev cigs	st
SCHWAR	28	x	f	0	0	bl	-	all	NAmer	1984	CC	5588	n	bl	y	y	0	cu	cig+/-ot	nev cigs	st
SEGI	1		m	0	0	all	-	all	As:Jap	1948	CC	159	n	bl	n	n	0	ev	all/unsp	nev any	ot
SEGI2	20		m	0	0	all	-	all	As:Jap	1962	CC	378	n	bl	n	n	1	cu	cig+/-ot	nev any	ot
SEGI2	28		f	0	0	all	-	all	As:Jap	1962	CC	378	n	bl	n	n	1	cu	cig+/-ot	nev any	ot
SEOW	6		f	0	0	ch	-	q+s+l+a	As:oth	1997	CC	153	n	bl	n	y	1	ev	cig+/-ot	nev cigs	st
SHAW	6	x	c	0	0	wh	-	all	NAmer	1988	CC	335	n	V	n	y	0	cu	all/unsp	nev any	st
SIEMIA	5		m	0	0	all	-	all	NAmer	1979	CC	857	n	V	y	y	7	ev	cig+/-ot	nev cigs	or
SIMARA	3		m	0	0	all	-	all	As:oth	1971	CC	115	n	bl	n	n	6	ev	cig+/-ot	nev cigs	ot
SIMARA	4		f	0	0	all	-	all	As:oth	1971	CC	115	n	bl	n	n	6	ev	cig+/-ot	nev cigs	ot
SOBUE	42	x	m	0	0	all	-	q+s+l+a	As:Jap	1986	CC	1376	n	bl	n	y	1	cu	cig+/-ot	nev cigs	or
SOBUE	52	x	f	0	0	all	-	q+s+l+a	As:Jap	1986	CC	1376	n	bl	n	y	1	cu	cig+/-ot	nev cigs	or
SOBUE2	10		m	0	0	all	-	q+s+l+a	As:Jap	1965	CC	2083	n	bl	n	n	2	cu	cig+/-ot	nev any	ot
SOBUE2	12		f	0	0	all	-	q+s+l+a	As:Jap	1965	CC	2083	n	bl	n	n	2	cu	cig+/-ot	nev any	ot
SPEIZE	10	x	f	0	0	all	0	all	NAmer	1976	pr	593	n	bl	n	y	1	cu	cig+/-ot	nev cigs	ot
SPITZ	2	x	c	0	0	b+hi	-	all	NAmer	1992	CC	177	n	bl	n	y	0	cu	cig+/-ot	nev cigs	st
STASZE	1		m	0	0	all	-	all	Eu:est	1954	CC	281	n	bl	n	y	0	ev	all/unsp	nev any	st
STASZE	5		f	0	0	all	-	all	Eu:est	1954	CC	281	n	bl	n	y	0	ev	all/unsp	nev any	st
STAYNE	1		m	0	0	all	-	all	NAmer	1969	CC	420	n	bl	n	n	0	ev	all/unsp	nev any	st

Table 1C2 - 1

IESLC - Meta-anal of Current Smoking (or Ever if Current not available), Any prod (or Cigs if Any not avail)
All LC types
Most adjusted

REF	NRR	1C1	SEX	AGEL	AGEH	RACE	YF	LC TYPE	LOC	START	ST	NLC	R	VB	P	H	AD	SM	PRODUCT	DENOM	De
STOCKS	47		m	0	0	all	-	all	Eu:UK	1952	CC	2932	n	V	y	n	2	ev	all/unsp	nev any	st
STOCKS	50		f	0	0	all	-	all	Eu:UK	1952	CC	2932	n	V	y	n	1	ev	cig+/-ot	nev any	ot
STOCKW	7	x	c	0	0	all	-	all	NAmer	1981	CC	22161	n	bl	n	n	0	cu	cig+/-ot	nev any	st
STUCKE	2	x	m	0	0	all	-	all	Eu:wst	1989	CC	247	n	bl	n	y	0	cu	all/unsp	nev any	ot
SUN	1		c	0	0	all	-	all	As:Chi	1992	CC	207	n	ot	n	y	0	ev	all/unsp	nev any	st
SUZUK2	6	x	c	0	0	all	-	all	SCAmer	1991	CC	123	n	bl	n	y	3	cu	all/unsp	nev any	or
SVENSS	96	x	f	0	0	all	-	all	Eu:Sca	1983	CC	210	n	bl	n	n	1	cu	all/unsp	nev any	ot
TANG	1	x	c	0	0	all	-	not s	NAmer	1992	CC	119	n	bl	n	y	0	cu	cig+/-ot	nev cigs	st
TENKAN	24	x	m	0	0	all	17	all	Eu:Sca	1962	pr	242	n	bl	n	n	1	cu	all/unsp	nev any	ot
TIZZAN	5	x	m	0	0	all	-	all	Eu:wst	1959	CC	1358	n	bl	n	n	0	cu	all/unsp	nev any	st
TIZZAN	13	x	f	0	0	all	-	all	Eu:wst	1959	CC	1358	n	bl	n	n	0	cu	all/unsp	nev any	st
TOKARS	1	x	m	0	0	all	-	all	Eu:est	1966	ot	162	o	bl	n	y	0	cu	all/unsp	nev any	st
TOKARS	5	x	f	0	0	all	-	all	Eu:est	1966	ot	162	o	bl	n	y	0	ev	all/unsp	nev any	st
TOUSEY	12	x	m	0	0	all	-	all	NAmer	1993	CC	507	n	bl	y	y	3	cu	cig+/-ot	nev any	or
TOUSEY	15	x	f	0	0	all	-	all	NAmer	1993	CC	507	n	bl	y	y	3	cu	cig+/-ot	nev any	or
TSUGAN	28	x	m	0	0	all	-	q+a	As:Jap	1976	CC	134	n	bl	n	y	0	cu	all/unsp	nev any	st
TULINI	37	x	m	0	0	all	0	all	Eu:Sca	1967	pr	472	n	bl	n	n	3	cu	all/unsp	nev any	ot
TULINI	43	x	f	0	0	all	0	all	Eu:Sca	1967	pr	472	n	bl	n	n	3	cu	all/unsp	nev any	ot
TVERDA	5	x	m	0	0	all	0	all	Eu:Sca	1972	pr	238	n	bl	n	n	2	cu	cig+/-ot	nev cigs	ot
TVERDA	15		f	0	0	all	0	all	Eu:Sca	1972	pr	238	n	bl	n	n	2	cu	cig only	nev cigs	ot
WAKAI	8	x	m	0	0	all	-	all	As:Jap	1988	CC	333	n	bl	n	y	2	cu	all/unsp	nev any	or
WAKAI	26	x	f	0	0	all	-	all	As:Jap	1988	CC	333	n	bl	n	y	2	cu	all/unsp	nev any	or
WALD	4		m	0	0	all	0	all	Eu:UK	1975	pr	102	n	V	n	n	1	cu	cig only	nev any	or
WANG	5		c	0	0	all	-	all	As:Chi	1990	CC	390	n	ot	*	y	6	ev	all/unsp	nev any	or
WANG2	18	x	c	0	0	all	-	all	As:Chi	1980	CC	103	n	ot	n	n	4	cu	cig+/-ot	nev cigs	ot
WANG3	1		c	0	0	all	-	all	As:Chi	1981	CC	293	n	ot	*	n	0	ev	all/unsp	nev any	st
WANG4	2		m	0	0	all	-	all	As:Chi	1976	CC	1170	n	ot	y	n	2	ev	all/unsp	nev any	st
WICKLU	1		m	0	0	wh	-	all	NAmer	1968	CC	155	n	bl	y	n	0	ev	cig+/-ot	nev+3	or
WIGLE	25	x	m	0	0	all	-	all	NAmer	1971	CC	728	n	V	n	n	1	cu	all/unsp	nev any	ot
WIGLE	30	x	f	0	0	all	-	all	NAmer	1971	CC	728	n	V	n	n	1	cu	all/unsp	nev any	ot
WILKIN	3		c	0	0	all	-	all	Eu:UK	1992	CC	271	n	V	n	n	4	ev	cig+/-ot	nev cigs	ot
WU	42	x	f	0	0	wh	-	q+a	NAmer	1981	CC	220	n	bl	n	y	2	cu	all/unsp	nev any	st
WUNSCH	5	x	m	0	0	all	-	all	SCAmer	1990	CC	398	n	bl	y	n	1	cu	cig+/-ot	nev any	or
WUNSCH	11	x	f	0	0	all	-	all	SCAmer	1990	CC	398	n	bl	y	n	1	cu	cig+/-ot	nev any	or
WUWILL	8		f	0	0	all	-	all	As:Chi	1985	CC	965	n	ot	n	n	3	ev	cig+/-ot	nev cigs	or
WYNDE2	21		m	0	0	all	-	all	NAmer	1962	CC	404	n	bl	n	y	0	ev	all/unsp	nev any	st
WYNDE3	50	x	m	0	0	all	-	all	NAmer	1966	CC	350	n	bl	n	y	0	cu	all/unsp	nev any	st
WYNDE3	138		f	0	0	all	-	all	NAmer	1966	CC	350	n	bl	n	y	0	ev	all/unsp	nev any	st
WYNDE4	48		m	0	0	all	-	all	NAmer	1948	CC	684	n	bl	y	n	0	ev	all/unsp	nev any	st
WYNDE4	62		f	0	0	all	-	all	NAmer	1948	CC	684	n	bl	y	n	2	ev	all/unsp	nev any	ot
WYNDE6	18	x	m	0	0	all	-	all	NAmer	1969	CC	4423	n	bl	n	y	0	cu	cig+/-ot	nev any	st
WYNDE6	207	x	f	0	0	all	-	all	NAmer	1969	CC	4423	n	bl	n	y	0	cu	cig+/-ot	nev cigs	st
XIANGZ	13		m	0	0	all	0	all	As:Chi	1976	pr	983	m	ot	n	n	2	ev	all/unsp	nev any	ot
XU	2		m	0	0	all	-	all	As:Chi	1985	CC	729	n	ot	n	n	2	ev	all/unsp	nev any	or
XU2	2		c	0	0	all	-	all	As:Chi	1987	CC	610	o	ot	y	n	7	ev	all/unsp	nev any	ot
XU3	2		m	0	0	all	-	all	As:Chi	1981	CC	135	n	ot	n	n	1	ev	all/unsp	nev any	or
XU3	4		f	0	0	all	-	all	As:Chi	1981	CC	135	n	ot	n	n	1	ev	all/unsp	nev any	or
XU4	1		c	0	0	all	-	all	As:Chi	1981	CC	206	n	ot	*	n	0	ev	all/unsp	nev any	st
YAMAGU	10	x	c	0	0	all	-	all	As:Jap	1989	CC	144	n	bl	n	y	1	cu	all/unsp	nev any	ot
YONG	12		m	0	0	all	0	all	NAmer	1971	pr	216	n	bl	n	n	1	cu	cig+/-ot	nev cigs	or
YONG	15		f	0	0	all	0	all	NAmer	1971	pr	216	n	bl	n	n	1	cu	cig+/-ot	nev cigs	or
YUAN	1		m	0	0	all	0	all	As:Chi	1986	pr	142	n	ot	n	n	2	ev	cig+/-ot	nev cigs	ot
ZHANG	2		m	0	0	all	-	all	As:Chi	1988	CC	100	n	ot	n	n	7	ev	all/unsp	nev any	or
ZHANG	3		f	0	0	all	-	all	As:Chi	1988	CC	100	n	ot	n	n	7	ev	all/unsp	nev any	or
ZHENG	15		m	0	0	all	-	all	As:Chi	1982	CC	540	n	ot	*	y	0	ev	cig+/-ot	nev cigs	st
ZHENG	24		f	0	0	all	-	all	As:Chi	1982	CC	540	n	ot	*	y	0	ev	cig+/-ot	nev cigs	st
ZHOU	2		m	0	0	all	-	all	As:Chi	1978	CC	1360	n	ot	n	n	0	ev	all/unsp	nev any	st
ZHOU	3		f	0	0	all	-	all	As:Chi	1978	CC	1360	n	ot	n	n	0	ev	all/unsp	nev any	st

Cigarette type is all/unspec for all RRs
except for the following:

REF	NRR	CIGTYPE
ALDERS	177	MC+-HR
ALDERS	176	MC only
DEAN3	119	MC only

Table 1C2 - 2

IESLC - Meta-anal of Current Smoking (or Ever if Current not available), Any prod (or Cigs if Any not avail)
All LC types
Most adjusted

REF	NRR	SEX	AD	Number Exposed Case	Number Exposed Cont	Non-exposed Case	Non-exposed Cont	RR	95.00%CI
ABELIN	44	m	1	-	-	-	-	35.38	(8.62- 145.24)
*ABRAHA	7	m	0	269	10351	10	3365	8.74	(4.66- 16.42)
*ABRAHA	8	f	0	62	5256	28	11589	4.88	(3.13- 7.62)
Subtotal ABRAHA								5.93	(4.12- 8.53)
AGUDO	3	f	3	-	-	-	-	3.61	(1.57- 8.32)
*AKIBA	10	m	5	-	-	-	-	5.10	(3.30- 7.90)
*AKIBA	14	f	5	-	-	-	-	3.90	(2.90- 5.30)
Subtotal AKIBA								4.25	(3.32- 5.45)
ALDERS	177	m	0	519	322	15	133	14.29	(8.23- 24.81)
ALDERS	176	f	0	410	229	75	243	5.80	(4.27- 7.87)
Subtotal ALDERS								7.17	(5.49- 9.36)
*AMANDU	5	m	2	-	-	-	-	6.54	(2.52- 16.98)
AMES	1	m	0	150	136	15	62	4.56	(2.48- 8.39)
*ANDERS	6	f	1	-	-	-	-	23.43	(17.02- 32.27)
*ARCHER	5	m	0	122	32529	6	9842	6.15	(2.71- 13.96)
ARMADA	27	m	0	188	122	4	64	24.66	(8.75- 69.44)
AUSTIN	6	c	3	-	-	-	-	19.60	(6.70- 57.00)
AUVINE	19	c	2	-	-	-	-	13.84	(7.90- 24.25)
AXELSO	1	c	0	90	86	62	371	6.26	(4.20- 9.34)
AXELSS	2	m	0	194	130	16	160	14.92	(8.53- 26.12)
AXELSS	10	f	0	96	69	18	154	11.90	(6.68- 21.22)
Subtotal AXELSS								13.38	(8.95- 20.00)
BAND	1	m	2	-	-	-	-	9.96	(7.38- 13.44)
BARBON	4	m	1	-	-	-	-	13.40	(8.50- 21.40)
BECHER	13	m	0	101	122	3	54	14.90	(4.52- 49.09)
BECHER	14	f	0	33	26	10	52	6.60	(2.82- 15.44)
Subtotal BECHER								8.68	(4.35- 17.35)
*BENSHL	16	m	1	-	-	-	-	8.18	(3.62- 18.51)
*BEST	2	m	1	-	-	-	-	14.91	(7.05- 31.52)
*BEST	18	f	1	-	-	-	-	2.24	(0.59- 8.44)
Subtotal BEST								9.45	(4.92- 18.15)
BLOHMK	1	m	0	419	313	126	301	3.20	(2.48- 4.12)
BLOT4	1	m	0	327	245	8	87	14.51	(6.91- 30.51)
BOFFET	33	m	2	-	-	-	-	14.20	(11.70- 17.20)
*BOUCOT	114	m	2	-	-	-	-	62.29	(3.86-1004.01)
BRESLO	37	m	0	486	451	7	42	6.47	(2.88- 14.54)
BRESLO	38	f	0	13	11	12	14	1.38	(0.45- 4.20)
Subtotal BRESLO								3.79	(1.97- 7.29)
*BRETT	4	m	0	135	37448	6	6530	3.92	(1.73- 8.88)
BROCKM	1	m	0	87	81	2	2	1.07	(0.15- 7.80)
BROCKM	2	f	0	24	54	4	18	2.00	(0.61- 6.54)
Subtotal BROCKM								1.70	(0.61- 4.70)
BROSS	11	m	0	690	638	38	170	4.84	(3.35- 6.99)
BROWN2	12	m	2	-	-	-	-	11.30	(10.20- 12.40)
BROWN2	11	f	2	-	-	-	-	13.60	(12.30- 15.10)
Subtotal BROWN2								12.34	(11.50- 13.25)
BUFFLE	3	m	0	257	219	5	47	11.03	(4.31- 28.22)
BUFFLE	7	f	0	313	183	41	198	8.26	(5.63- 12.11)
Subtotal BUFFLE								8.61	(6.04- 12.27)
CARPEN	11	c	3	-	-	-	-	23.03	(12.96- 40.85)
CASCO2	1	c	0	149	212	6	98	11.48	(4.90- 26.87)
CASCOR	1	c	0	365	362	22	295	13.52	(8.56- 21.35)
*CEDERL	106	m	2	-	-	-	-	7.72	(5.01- 11.89)
*CEDERL	75	f	2	-	-	-	-	4.82	(3.38- 6.88)
Subtotal CEDERL								5.83	(4.43- 7.67)
CHAN	9	m	0	206	161	2	43	27.51	(6.57- 115.26)
CHAN	10	f	0	105	50	84	139	3.48	(2.26- 5.35)
Subtotal CHAN								4.13	(2.73- 6.25)
*CHANG	5	m	0	35	419	5	502	8.39	(3.32- 21.21)
*CHANG	11	f	0	30	603	11	1139	5.15	(2.60- 10.21)
Subtotal CHANG								6.12	(3.53- 10.60)
CHATZI	4	c	0	255	365	27	129	3.34	(2.14- 5.21)
CHEN2	1	m	0	121	97	9	33	4.57	(2.09- 10.02)
CHEN2	2	f	0	38	30	25	33	1.67	(0.82- 3.39)
Subtotal CHEN2								2.62	(1.55- 4.44)
CHEN3	1	c	0	182	156	72	98	1.59	(1.09- 2.30)
CHIAZZ	3	m	11	-	-	-	-	26.17	(3.32- 206.50)
CHOI	3	m	0	232	329	13	95	5.15	(2.82- 9.42)
CHOI	7	f	0	13	23	76	164	1.22	(0.59- 2.54)
Subtotal CHOI								2.88	(1.81- 4.58)
*CHOW	25	m	0	167	124415	6	62913	14.07	(6.23- 31.78)

Table 1C2 - 2

IESLC - Meta-anal of Current Smoking (or Ever if Current not available), Any prod (or Cigs if Any not avail)
All LC types
Most adjusted

REF	NRR	SEX	AD	Number Exposed Case	Cont	Non-exposed Case	Cont	RR	95.00%CI
*CHYOU	2	m	1	-	-	-	-	11.40	(6.50- 20.10)
COMSTO	3	m	0	105	100	4	69	18.11	(6.37- 51.48)
COMSTO	8	f	0	77	52	13	115	13.10	(6.68- 25.67)
Subtotal COMSTO								14.40	(8.18- 25.36)
COOKSO	5	c	0	189	39	45	61	6.57	(3.92- 11.02)
CORREA	42	c	1	-	-	-	-	14.20	(10.80- 18.70)
*CPSI	220	m	1	-	-	-	-	11.94	(9.52- 14.97)
*CPSI	279	f	1	-	-	-	-	3.20	(2.53- 4.04)
Subtotal CPSI								6.32	(5.37- 7.43)
*CPSII	126	m	1	-	-	-	-	20.25	(16.37- 25.05)
*CPSII	133	f	1	-	-	-	-	11.78	(10.14- 13.68)
Subtotal CPSII								14.10	(12.47- 15.93)
DAMBER	14	m	1	-	-	-	-	9.60	(6.60- 14.20)
DARBY	4	m	0	322	453	3	384	90.98	(28.96- 285.90)
DARBY	11	f	0	195	217	23	529	20.67	(13.05- 32.74)
Subtotal DARBY								25.40	(16.57- 38.92)
DAVEYS	5	m	0	90	144	3	23	4.79	(1.40- 16.42)
DAVEYS	6	f	0	0	3	16	83	0.72~	(0.04- 14.66)
Subtotal DAVEYS								3.65	(1.17- 11.42)
DEAN	7	m	0	591	574	12	61	5.23	(2.79- 9.82)
DEAN2	2	m	0	671	600	33	112	3.80	(2.54- 5.68)
DEAN2	6	f	0	59	28	88	121	2.90	(1.71- 4.91)
Subtotal DEAN2								3.43	(2.49- 4.73)
DEAN3	42	m	3	-	-	-	-	6.72	(4.28- 10.55)
DEAN3	119	f	3	-	-	-	-	5.77	(3.75- 8.86)
Subtotal DEAN3								6.20	(4.54- 8.47)
*DEKLER	8	m	2	-	-	-	-	23.03	(3.21- 164.97)
DESTE2	4	c	7	-	-	-	-	9.10	(5.20- 15.90)
DESTEF	41	m	4	-	-	-	-	10.90	(6.90- 17.10)
*DOCKER	1	c	4	-	-	-	-	8.00	(2.97- 21.60)
DOLL	90	m	0	1280	1172	7	61	9.52	(4.34- 20.89)
DOLL	93	f	0	58	41	40	59	2.09	(1.18- 3.68)
Subtotal DOLL								3.51	(2.21- 5.55)
*DOLL2	54	m	1	-	-	-	-	10.99	(6.97- 17.36)
*DOLL2	63	f	1	-	-	-	-	8.65	(2.93- 25.55)
Subtotal DOLL2								10.60	(6.96- 16.14)
DORANT	2	m	0	332	697	7	159	10.82	(5.02- 23.32)
DORGAN	9	m	0	464	170	15	93	16.92	(9.54- 30.01)
DORGAN	33	m	0	214	61	3	35	40.93	(12.17- 137.66)
DORGAN	56	f	0	611	119	103	244	12.16	(8.99- 16.46)
DORGAN	79	f	0	68	17	7	20	11.43	(4.16- 31.43)
Subtotal DORGAN								13.62	(10.58- 17.54)
*DORN	51	m	1	-	-	-	-	8.23	(6.55- 10.35)
DOSEME	1	m	2	-	-	-	-	3.30	(2.60- 4.40)
DROSTE	6	m	4	-	-	-	-	14.50	(6.30- 33.40)
DU	1	m	0	538	-	28	-	3.53	(2.44- 5.11)
DU	2	f	0	191	-	92	-	1.93	(1.30- 2.87)
Subtotal DU								2.66	(2.03- 3.49)
*DUNN	6	m	0	137	52634	2	14160	18.43	(4.56- 74.42)
EBELIN	1	m	0	101	142	12	117	6.93	(3.63- 13.24)
*ENGELA	158	m	1	-	-	-	-	7.37	(3.42- 15.86)
*ENGELA	164	f	1	-	-	-	-	5.78	(2.68- 12.46)
Subtotal ENGELA								6.53	(3.79- 11.23)
*ENSTRO	1	m	1	-	-	-	-	12.99	(10.46- 16.13)
*ENSTRO	2	f	1	-	-	-	-	6.95	(6.01- 8.04)
Subtotal ENSTRO								8.44	(7.48- 9.53)
ESAKI	4	m	0	155	143	16	28	1.90	(0.99- 3.65)
ESAKI	5	f	0	34	19	40	55	2.46	(1.23- 4.92)
Subtotal ESAKI								2.14	(1.33- 3.45)
FAN	1	m	0	216	498	36	236	2.84	(1.93- 4.18)
FAN	2	f	0	82	97	69	320	3.92	(2.65- 5.81)
Subtotal FAN								3.33	(2.53- 4.38)
GAO	33	m	2	-	-	-	-	3.90	(2.90- 5.40)
GAO	34	f	2	-	-	-	-	2.90	(2.20- 3.80)
Subtotal GAO								3.30	(2.69- 4.05)
GAO2	8	m	1	-	-	-	-	6.61	(3.47- 12.58)
GARCIA	2	c	0	169	74	21	139	15.12	(8.86- 25.79)
GARDIN	2	c	0	97	58	5	41	13.71	(5.13- 36.68)
GARSHI	31	m	1	-	-	-	-	7.70	(5.48- 10.83)
GENG	1	m	0	92	68	7	31	5.99	(2.49- 14.42)
GENG	2	f	0	126	75	67	118	2.96	(1.96- 4.48)

Table 1C2 - 2

IESLC - Meta-anal of Current Smoking (or Ever if Current not available), Any prod (or Cigs if Any not avail)
All LC types
Most adjusted

REF	NRR	SEX	AD	Number Exposed Case	Number Exposed Cont	Non-exposed Case	Non-exposed Cont	RR	95.00%CI
Subtotal GENG								3.36	(2.31- 4.89)
GER	21	c	14	-	-	-	-	1.84	(1.06- 3.20)
GODLEY	5	m	1	-	-	-	-	6.84	(5.60- 8.35)
GODLEY	6	f	1	-	-	-	-	5.54	(4.29- 7.15)
Subtotal GODLEY								6.31	(5.39- 7.39)
GOLLED	7	m	1	-	-	-	-	7.51	(4.44- 12.71)
GOODMA	2	m	0	148	169	10	199	17.43	(8.90- 34.14)
GOODMA	6	f	0	58	56	19	177	9.65	(5.30- 17.56)
Subtotal GOODMA								12.53	(8.01- 19.60)
GRAHAM	25	m	1	-	-	-	-	6.06	(3.78- 9.70)
GREGOR	2	m	0	49	53	10	14	1.29	(0.53- 3.18)
GREGOR	6	f	0	17	26	1	22	14.38	(1.77- 116.90)
Subtotal GREGOR								1.88	(0.82- 4.30)
GSELL	8	m	0	148	121	2	29	17.74	(4.15- 75.83)
HAENSZ	54	f	0	69	94	81	236	2.14	(1.43- 3.19)
*HAMMO2	8	m	1	-	-	-	-	10.14	(4.19- 24.55)
*HAMMON	139	m	1	-	-	-	-	11.52	(6.83- 19.42)
*HANSEN	3	m	2	-	-	-	-	1.53	(0.71- 3.91)
HEGMAN	1	c	0	255	1202	27	2080	16.34	(10.92- 24.45)
*HEIN	5	m	0	132	3492	1	457	17.27	(2.42- 123.25)
*HENNEK	2	m	0	79	2438	23	10919	15.38	(9.69- 24.42)
HINDS	22	f	3	-	-	-	-	5.65	(4.14- 7.72)
*HIRAYA	1	m	1	-	-	-	-	4.45	(3.60- 5.50)
*HIRAYA	3	f	1	-	-	-	-	2.34	(1.87- 2.92)
Subtotal HIRAYA								3.28	(2.81- 3.82)
HITOSU	34	m	1	-	-	-	-	2.79	(1.27- 6.09)
HITOSU	59	f	1	-	-	-	-	3.09	(1.82- 5.27)
Subtotal HITOSU								2.99	(1.93- 4.65)
*HOLE	33	m	1	-	-	-	-	8.10	(3.80- 17.26)
*HOLE	31	f	1	-	-	-	-	1.53	(0.64- 3.70)
Subtotal HOLE								3.98	(2.24- 7.06)
HOROWI	1	m	0	182	525	19	196	3.58	(2.17- 5.90)
HOROWI	2	f	0	21	382	14	463	1.82	(0.91- 3.62)
Subtotal HOROWI								2.83	(1.89- 4.25)
HORWIT	1	f	0	97	92	11	118	11.31	(5.73- 22.34)
HU	15	m	0	120	94	41	67	2.09	(1.30- 3.35)
HU	16	f	0	26	18	40	48	1.73	(0.83- 3.61)
Subtotal HU								1.98	(1.33- 2.94)
HU2	9	m	0	294	228	49	115	3.03	(2.08- 4.41)
HU2	10	f	0	108	80	72	100	1.88	(1.23- 2.85)
Subtotal HU2								2.44	(1.85- 3.23)
HUANG	1	c	0	98	77	37	58	2.00	(1.20- 3.32)
HUMBLE	13	m	1	-	-	-	-	19.96	(8.27- 48.21)
HUMBLE	15	m	1	-	-	-	-	15.79	(3.43- 72.69)
HUMBLE	17	f	1	-	-	-	-	16.72	(7.44- 37.61)
HUMBLE	19	f	1	-	-	-	-	23.50	(6.79- 81.36)
Subtotal HUMBLE								18.65	(11.23- 30.97)
JAHN	22	f	2	-	-	-	-	3.30	(1.99- 5.49)
JAIN	52	m	2	-	-	-	-	12.40	(6.45- 26.60)
JAIN	51	f	2	-	-	-	-	16.80	(9.93- 30.60)
Subtotal JAIN								14.94	(9.61- 23.21)
JARUP	6	m	2	-	-	-	-	7.54	(2.80- 20.33)
JARVHO	2	m	0	73	29	1	16	40.28	(5.10- 317.77)
JARVHO	6	f	0	31	7	6	21	15.50	(4.56- 52.66)
Subtotal JARVHO								19.86	(6.93- 56.89)
JEDRYC	58	m	4	-	-	-	-	5.46	(3.85- 7.73)
JEDRYC	59	f	4	-	-	-	-	4.54	(2.56- 8.05)
Subtotal JEDRYC								5.19	(3.86- 7.00)
JIANG	1	m	0	93	83	7	17	2.72	(1.08- 6.89)
JIANG	2	f	0	11	6	14	19	2.49	(0.74- 8.35)
Subtotal JIANG								2.63	(1.26- 5.50)
JOLY	18	m	0	487	665	12	218	13.30	(7.35- 24.07)
JOLY	15	f	0	132	96	52	283	7.48	(5.04- 11.12)
Subtotal JOLY								8.94	(6.43- 12.42)
JUSSAW	29	m	2	-	-	-	-	16.83	(11.65- 25.21)
*KAISE2	68	m	1	-	-	-	-	8.04	(4.41- 14.66)
*KAISE2	60	f	1	-	-	-	-	14.48	(7.47- 28.04)
Subtotal KAISE2								10.49	(6.72- 16.36)
*KAISER	12	m	2	-	-	-	-	19.61	(13.32- 28.87)
*KAISER	9	f	2	-	-	-	-	6.53	(4.50- 9.48)
Subtotal KAISER								11.09	(8.48- 14.50)

Table 1C2 - 2

IESLC - Meta-anal of Current Smoking (or Ever if Current not available), Any prod (or Cigs if Any not avail)
All LC types
Most adjusted

REF	NRR	SEX	AD	Number Exposed Case	Number Exposed Cont	Non-exposed Case	Non-exposed Cont	RR	95.00%CI
KANELL	30	m	1	-	-	-	-	4.94	(3.47- 7.03)
KATSOU	2	f	1	-	-	-	-	3.40	(1.75- 6.61)
KAUFMA	16	c	6	-	-	-	-	20.63	(14.18- 30.01)
KELLER	1	m	0	5063	1210	323	1017	13.17	(11.45- 15.15)
KELLER	9	m	0	1053	212	38	117	15.29	(10.31- 22.69)
KELLER	5	f	0	2904	792	469	1860	14.54	(12.79- 16.53)
KELLER	13	f	0	454	135	67	232	11.64	(8.35- 16.24)
Subtotal KELLER								13.79	(12.62- 15.07)
KHUDER	19	m	0	275	-	23	-	8.10	(5.20- 12.70)
KIHARA	7	c	0	283	162	102	237	4.06	(3.00- 5.49)
*KINLEN	20	m	2	-	-	-	-	12.89	(6.14- 27.06)
KJUUS	1	m	0	135	77	2	24	21.04	(4.84- 91.45)
*KNEKT	86	m	1	-	-	-	-	8.86	(3.87- 20.27)
KO	1	f	3	-	-	-	-	4.20	(1.10- 15.60)
KOHLME	2	c	4	-	-	-	-	16.40	(6.90- 38.42)
KOO	9	f	0	42	25	56	85	2.55	(1.40- 4.64)
KOULUM	1	m	0	807	246	5	54	35.43	(14.02- 89.55)
KREUZE	40	f	0	55	23	6	38	15.14	(5.63- 40.72)
KREUZE	42	f	0	170	54	95	177	5.87	(3.95- 8.70)
Subtotal KREUZE								6.68	(4.63- 9.64)
KREYBE	12	m	1	-	-	-	-	6.61	(2.93- 14.92)
KREYBE	30	f	1	-	-	-	-	1.43	(0.71- 2.86)
Subtotal KREYBE								2.73	(1.61- 4.64)
*KUBIK	12	m	0	98	6342	2	4271	33.00	(8.14- 133.74)
LAMTH	6	f	0	242	106	202	337	3.81	(2.86- 5.08)
LAMWK	1	f	0	88	41	75	144	4.12	(2.59- 6.55)
LAMWK2	9	m	0	244	161	23	43	2.83	(1.64- 4.88)
LAMWK2	10	f	0	75	50	65	139	3.21	(2.02- 5.10)
Subtotal LAMWK2								3.04	(2.14- 4.33)
*LANGE	38	m	1	-	-	-	-	5.70	(2.13- 15.27)
*LANGE	35	f	1	-	-	-	-	5.02	(2.52- 10.01)
Subtotal LANGE								5.23	(2.98- 9.21)
LAUSSM	11	m	3	-	-	-	-	5.70	(4.10- 7.80)
LEI	1	m	0	443	361	41	123	3.68	(2.52- 5.38)
LEI	2	f	0	123	61	85	147	3.49	(2.32- 5.24)
Subtotal LEI								3.59	(2.72- 4.74)
LEMARC	2	c	0	167	65	32	168	13.49	(8.39- 21.68)
LETOUR	1	c	0	714	514	24	224	12.96	(8.38- 20.05)
LEVIN	32	m	1	-	-	-	-	4.86	(3.41- 6.92)
*LIAW	1	m	1	-	-	-	-	3.70	(2.10- 6.60)
*LIAW	2	f	1	-	-	-	-	3.60	(1.00- 12.20)
Subtotal LIAW								3.68	(2.19- 6.20)
*LIDDEL	4	m	1	-	-	-	-	4.41	(2.77- 7.01)
LIU	2	c	2	-	-	-	-	1.92	(1.40- 2.64)
LIU2	2	m	3	-	-	-	-	5.19	(2.03- 13.25)
LIU2	4	f	3	-	-	-	-	4.65	(2.18- 9.93)
Subtotal LIU2								4.86	(2.69- 8.76)
LIU3	2	m	2	-	-	-	-	1.26	(0.30- 5.26)
LIU4	11	m	2	-	-	-	-	2.76	(2.69- 2.83)
LIU4	12	f	2	-	-	-	-	2.86	(2.77- 2.95)
Subtotal LIU4								2.80	(2.74- 2.85)
LIU5	1	c	0	85	70	26	41	1.91	(1.07- 3.44)
LOMBA2	1	f	0	149	353	76	239	1.33	(0.96- 1.83)
LOMBAR	9	m	0	852	610	14	112	11.17	(6.35- 19.66)
LUBIN2	26	m	2	-	-	-	-	10.67	(9.14- 12.46)
LUBIN2	317	f	0	384	410	288	1180	3.84	(3.17- 4.64)
Subtotal LUBIN2								7.09	(6.28- 7.99)
LUO	7	c	20	-	-	-	-	2.70	(1.50- 5.00)
MACLEN	19	m	0	137	108	5	15	3.81	(1.34- 10.80)
MACLEN	32	f	0	42	47	41	109	2.38	(1.37- 4.12)
Subtotal MACLEN								2.63	(1.62- 4.28)
*MAGNUS	5	m	3	-	-	-	-	4.13	(1.94- 8.77)
MARSH	7	c	2	-	-	-	-	6.80	(3.30- 13.99)
MARSH2	5	m	1	-	-	-	-	1.89	(0.70- 5.14)
MARSH2	6	f	1	-	-	-	-	5.28	(1.89- 14.72)
Subtotal MARSH2								3.11	(1.52- 6.36)
MARTIS	4	m	0	197	176	4	25	7.00	(2.39- 20.49)
MASTRA	2	m	2	-	-	-	-	8.14	(3.32- 20.00)
MATOS	3	m	2	-	-	-	-	8.50	(4.30- 16.70)
MATSUD	10	m	0	170	3314	3	1255	21.46	(6.84- 67.33)
MCCONN	1	m	0	88	174	5	12	1.21	(0.41- 3.55)

Table 1C2 - 2

IESLC - Meta-anal of Current Smoking (or Ever if Current not available), Any prod (or Cigs if Any not avail)
All LC types
Most adjusted

REF	NRR	SEX	AD	Number Exposed Case	Cont	Non-exposed Case	Cont	RR	95.00%CI
MCCONN	2	f	0	3	3	4	11	2.75	(0.38- 19.67)
Subtotal MCCONN								1.46	(0.57- 3.76)
MCDUFF	1	m	0	159	134	6	31	6.13	(2.48- 15.14)
MCLAUG	1	m	0	294	1082	22	270	3.33	(2.12- 5.25)
*MIGRAN	20	m	2	-	-	-	-	3.93	(1.46- 10.60)
*MIGRAN	136	f	2	-	-	-	-	4.99	(1.76- 14.16)
Subtotal MIGRAN								4.40	(2.15- 9.03)
MILLER	2	f	1	-	-	-	-	4.99	(2.06- 12.10)
MILLS	3	m	1	-	-	-	-	1.33	(1.09- 1.63)
*MRFITR	2	m	0	106	8194	0	1859	48.33~	(3.01- 777.42)
NAM	76	m	1	-	-	-	-	8.67	(5.66- 13.26)
NAM	92	f	1	-	-	-	-	10.84	(7.26- 16.18)
Subtotal NAM								9.76	(7.29- 13.07)
NOTAN2	15	m	2	-	-	-	-	2.99	(2.40- 3.72)
NOU	11	m	0	74	247	6	122	6.09	(2.58- 14.39)
NOU	12	f	0	10	92	4	261	7.09	(2.17- 23.17)
Subtotal NOU								6.42	(3.20- 12.87)
ODRISC	1	c	0	293	598	6	664	54.22	(23.98- 122.60)
ORMOS	4	m	0	87	1034	7	777	9.34	(4.30- 20.28)
ORMOS	26	f	0	1	234	23	1044	0.19	(0.03- 1.44)
Subtotal ORMOS								5.65	(2.74- 11.64)
OSANN	33	m	2	-	-	-	-	26.50	(19.20- 36.50)
OSANN	34	f	2	-	-	-	-	19.60	(15.20- 25.20)
Subtotal OSANN								22.00	(18.03- 26.83)
PARKIN	26	m	6	-	-	-	-	4.08	(3.18- 5.23)
PASTOR	10	m	1	-	-	-	-	6.81	(3.38- 13.70)
PAWLEG	2	m	6	-	-	-	-	12.26	(4.07- 36.95)
PERNU	2	m	0	1380	438	97	275	8.93	(6.92- 11.53)
PERNU	1	f	0	19	89	110	971	1.88	(1.11- 3.21)
Subtotal PERNU								6.68	(5.31- 8.41)
PERSH2	10	c	4	-	-	-	-	8.29	(6.86- 10.02)
*PETO	4	m	0	99	2036	2	295	7.17	(1.78- 28.92)
PEZZO2	2	m	0	233	198	6	117	22.95	(9.89- 53.26)
PEZZOT	5	m	0	145	129	4	116	32.60	(11.70- 90.81)
PIKE	4	m	0	514	375	18	69	5.25	(3.08- 8.98)
PIKE	8	f	0	163	90	36	96	4.83	(3.04- 7.66)
Subtotal PIKE								5.01	(3.53- 7.10)
POFFIJ	1	c	0	913	918	58	452	7.75	(5.81- 10.34)
POLEDN	1	c	1	-	-	-	-	9.24	(5.23- 16.33)
*QIAO2	14	m	1	-	-	-	-	1.59	(0.84- 3.01)
RACHTA	9	f	1	-	-	-	-	6.77	(3.71- 12.35)
RADZIK	1	c	0	180	198	9	13	1.31	(0.55- 3.15)
RANDIG	23	m	0	410	359	5	22	5.03	(1.88- 13.41)
RANDIG	24	f	0	16	39	17	92	2.22	(1.02- 4.84)
Subtotal RANDIG								3.04	(1.65- 5.60)
REN	1	m	0	106	84	12	34	3.58	(1.74- 7.33)
REN	2	f	0	78	20	48	50	4.06	(2.16- 7.64)
Subtotal REN								3.84	(2.39- 6.17)
RONCO	1	m	0	120	306	6	78	5.10	(2.16- 12.01)
ROTHSC	2	c	1	-	-	-	-	5.55	(2.97- 10.37)
SADOWS	31	m	1	-	-	-	-	3.63	(1.81- 7.30)
SANKAR	2	m	3	-	-	-	-	13.62	(9.00- 20.62)
SCHWAR	25	m	0	1652	349	119	376	14.96	(11.81- 18.94)
SCHWAR	26	m	0	644	139	50	104	9.64	(6.56- 14.15)
SCHWAR	27	f	0	1029	309	182	855	15.64	(12.75- 19.19)
SCHWAR	28	f	0	256	90	40	247	17.56	(11.64- 26.50)
Subtotal SCHWAR								14.70	(12.84- 16.83)
SEGI	1	m	0	140	1742	18	382	1.71	(1.03- 2.82)
SEGI2	20	m	1	-	-	-	-	3.74	(1.75- 8.00)
SEGI2	28	f	1	-	-	-	-	1.65	(0.90- 3.02)
Subtotal SEGI2								2.27	(1.41- 3.64)
SEOW	6	f	1	-	-	-	-	5.25	(2.80- 9.84)
SHAW	6	c	0	212	97	11	107	21.26	(10.93- 41.36)
SIEMIA	5	m	7	-	-	-	-	12.10	(6.60- 22.30)
SIMARA	3	m	6	-	-	-	-	1.65	(0.97- 2.81)
SIMARA	4	f	6	-	-	-	-	1.63	(0.87- 3.06)
Subtotal SIMARA								1.64	(1.09- 2.46)
SOBUE	42	m	1	-	-	-	-	4.10	(2.80- 5.90)
SOBUE	52	f	1	-	-	-	-	2.80	(2.00- 3.90)
Subtotal SOBUE								3.32	(2.59- 4.26)
SOBUE2	10	m	2	-	-	-	-	4.47	(3.89- 5.14)

Table 1C2 - 2

IESLC - Meta-anal of Current Smoking (or Ever if Current not available), Any prod (or Cigs if Any not avail)
All LC types
Most adjusted

REF	NRR	SEX	AD	Number Exposed Case	Cont	Non-exposed Case	Cont	RR	95.00%CI
SOBUE2	12	f	2	-	-	-	-	3.28	(2.79- 3.87)
Subtotal SOBUE2								3.92	(3.53- 4.36)
*SPEIZE	10	f	1	-	-	-	-	12.69	(9.97- 16.16)
SPITZ	2	c	0	103	89	7	128	21.16	(9.40- 47.66)
STASZE	1	m	0	255	754	5	158	10.69	(4.34- 26.33)
STASZE	5	f	0	6	153	15	1660	4.34	(1.66- 11.35)
Subtotal STASZE								7.01	(3.63- 13.53)
STAYNE	1	m	0	362	567	58	333	3.67	(2.69- 4.99)
STOCKS	47	m	2	-	-	-	-	5.95	(4.23- 8.36)
STOCKS	50	f	1	-	-	-	-	3.04	(2.35- 3.93)
Subtotal STOCKS								3.88	(3.16- 4.76)
STOCKW	7	c	0	12470	3357	2791	10641	14.16	(13.38- 14.99)
STUCKE	2	m	0	69	68	0	51	104.50~	(6.32-1727.39)
SUN	1	c	0	140	173	67	191	2.31	(1.62- 3.30)
SUZUK2	6	c	3	-	-	-	-	22.00	(6.50- 76.00)
SVENSS	96	f	1	-	-	-	-	9.06	(5.31- 15.48)
TANG	1	c	0	52	25	9	39	9.01	(3.78- 21.46)
*TENKAN	24	m	1	-	-	-	-	16.81	(7.22- 39.14)
TIZZAN	5	m	0	693	619	180	305	1.90	(1.53- 2.35)
TIZZAN	13	f	0	17	18	25	114	4.31	(1.95- 9.51)
Subtotal TIZZAN								2.01	(1.63- 2.47)
TOKARS	1	m	0	110	157	1	53	37.13	(5.06- 272.56)
TOKARS	5	f	0	1	2	13	40	1.54	(0.13- 18.38)
Subtotal TOKARS								10.65	(2.25- 50.36)
TOUSEY	12	m	3	-	-	-	-	59.20	(21.00- 167.30)
TOUSEY	15	f	3	-	-	-	-	30.20	(16.00- 57.40)
Subtotal TOUSEY								36.34	(21.09- 62.60)
TSUGAN	28	m	0	63	63	18	22	1.22	(0.60- 2.50)
*TULINI	37	m	3	-	-	-	-	9.99	(5.42- 18.40)
*TULINI	43	f	3	-	-	-	-	16.24	(9.02- 29.25)
Subtotal TULINI								12.86	(8.41- 19.64)
*TVERDA	5	m	2	-	-	-	-	4.09	(2.65- 6.31)
*TVERDA	15	f	2	-	-	-	-	11.05	(3.33- 36.71)
Subtotal TVERDA								4.59	(3.05- 6.90)
WAKAI	8	m	2	-	-	-	-	4.40	(2.19- 8.85)
WAKAI	26	f	2	-	-	-	-	4.37	(2.21- 8.62)
Subtotal WAKAI								4.38	(2.69- 7.14)
*WALD	4	m	1	-	-	-	-	16.40	(7.55- 44.20)
WANG	5	c	6	-	-	-	-	2.88	(1.74- 4.77)
WANG2	18	c	4	-	-	-	-	2.40	(1.14- 5.05)
WANG3	1	c	0	235	172	58	121	2.85	(1.97- 4.13)
WANG4	2	m	2	-	-	-	-	1.16	(0.96- 1.42)
WICKLU	1	m	0	-	-	-	-	4.60	(2.80- 7.60)
WIGLE	25	m	1	-	-	-	-	10.40	(6.07- 17.83)
WIGLE	30	f	1	-	-	-	-	5.20	(3.34- 8.08)
Subtotal WIGLE								6.87	(4.88- 9.67)
WILKIN	3	c	4	-	-	-	-	7.83	(4.45- 13.78)
WU	42	f	2	-	-	-	-	4.86	(2.76- 8.57)
WUNSCH	5	m	1	-	-	-	-	6.59	(3.59- 12.10)
WUNSCH	11	f	1	-	-	-	-	5.98	(3.25- 11.00)
Subtotal WUNSCH								6.28	(4.08- 9.66)
WUWILL	8	f	3	-	-	-	-	2.30	(1.90- 2.80)
WYNDE2	21	m	0	396	616	8	105	8.44	(4.07- 17.51)
WYNDE3	50	m	0	227	207	9	88	10.72	(5.26- 21.84)
WYNDE3	138	f	0	46	56	20	76	3.12	(1.67- 5.85)
Subtotal WYNDE3								5.36	(3.35- 8.58)
WYNDE4	48	m	0	632	665	12	115	9.11	(4.98- 16.67)
WYNDE4	62	f	2	-	-	-	-	2.87	(1.48- 5.55)
Subtotal WYNDE4								5.38	(3.45- 8.41)
WYNDE6	18	m	0	1677	741	87	617	16.05	(12.62- 20.41)
WYNDE6	207	f	0	1022	376	159	856	14.63	(11.90- 17.99)
Subtotal WYNDE6								15.22	(13.01- 17.80)
*XIANGZ	13	m	2	-	-	-	-	2.16	(1.46- 3.18)
XU	2	m	2	-	-	-	-	2.70	(2.10- 3.50)
XU2	2	c	7	-	-	-	-	3.80	(2.84- 5.07)
XU3	2	m	1	-	-	-	-	5.99	(2.65- 13.50)
XU3	4	f	1	-	-	-	-	3.86	(1.39- 10.70)
Subtotal XU3								5.05	(2.67- 9.54)
XU4	1	c	0	161	113	45	93	2.94	(1.92- 4.52)
YAMAGU	10	c	1	-	-	-	-	4.90	(2.55- 9.44)
*YONG	12	m	1	-	-	-	-	28.71	(6.98- 118.16)

Table 1C2 - 2

IESLC - Meta-anal of Current Smoking (or Ever if Current not available), Any prod (or Cigs if Any not avail)
All LC types
Most adjusted

REF	NRR	SEX	AD	Number Exposed Case	Number Exposed Cont	Non-exposed Case	Non-exposed Cont	RR	95.00%CI
*YONG	15	f	1	-	-	-	-	5.20	(2.38- 11.35)
Subtotal YONG								7.75	(3.91- 15.36)
*YUAN	1	m	2	-	-	-	-	6.50	(3.64- 11.60)
ZHANG	2	m	7	-	-	-	-	4.00	(1.61- 9.91)
ZHANG	3	f	7	-	-	-	-	3.75	(1.80- 10.76)
Subtotal ZHANG								3.87	(2.05- 7.32)
ZHENG	15	m	0	279	218	33	94	3.65	(2.36- 5.63)
ZHENG	24	f	0	76	44	152	184	2.09	(1.36- 3.21)
Subtotal ZHENG								2.75	(2.03- 3.73)
ZHOU	2	m	0	740	41	275	36	2.36	(1.48- 3.77)
ZHOU	3	f	0	112	7	231	32	2.22	(0.95- 5.18)
Subtotal ZHOU								2.33	(1.54- 3.51)
Partial Totals				61694	329394	9636	170892		

*prospective study ~ With 0.5 adjustment for zero

REF	NRR	SEX	AD	Ys	Ws	Qs	Ps
ABELIN	44	m	1	3.57	1.93	8.28	0.0000
*ABRAHA	7	m	0	2.17	9.68	4.41	0.0000
*ABRAHA	8	f	0	1.59	19.39	0.17	0.0000
Subtotal ABRAHA				1.78	29.07	4.58	
AGUDO	3	f	3	1.28	5.53	0.24	0.0025
*AKIBA	10	m	5	1.63	20.16	0.37	0.0000
*AKIBA	14	f	5	1.36	42.26	0.74	0.0000
Subtotal AKIBA				1.45	62.42	1.11	
ALDERS	177	m	0	2.66	12.62	17.17	0.0000
ALDERS	176	f	0	1.76	41.23	2.89	0.0000
Subtotal ALDERS				1.97	53.85	20.06	
*AMANDU	5	m	2	1.88	4.22	0.62	0.0001
AMES	1	m	0	1.52	10.33	0.01	0.0000
*ANDERS	6	f	1	3.15	37.54	103.55	0.0000
*ARCHER	5	m	0	1.82	5.72	0.60	0.0000
ARMADA	27	m	0	3.21	3.58	10.50	0.0000
AUSTIN	6	c	3	2.98	3.35	7.37	0.0000
AUVINE	19	c	2	2.63	12.22	15.72	0.0000
AXELSO	1	c	0	1.83	24.06	2.80	0.0000
AXELSS	2	m	0	2.70	12.26	17.93	0.0000
AXELSS	10	f	0	2.48	11.50	11.12	0.0000
Subtotal AXELSS				2.59	23.76	29.06	
BAND	1	m	2	2.30	42.76	27.73	0.0000
BARBON	4	m	1	2.60	18.02	21.89	0.0000
BECHER	13	m	0	2.70	2.70	3.95	0.0000
BECHER	14	f	0	1.89	5.32	0.82	0.0000
Subtotal BECHER				2.16	8.02	4.77	
*BENSHL	16	m	1	2.10	5.77	2.14	0.0000
*BEST	2	m	1	2.70	6.85	10.01	0.0000
*BEST	18	f	1	0.81	2.17	1.02	0.2348
Subtotal BEST				2.25	9.02	11.03	
BLOHMK	1	m	0	1.16	59.38	6.50	0.0000
BLOT4	1	m	0	2.68	6.96	9.73	0.0000
BOFFET	33	m	2	2.65	103.49	139.26	0.0000
*BOUCOT	114	m	2	4.13	0.50	3.46	0.0036
BRESLO	37	m	0	1.87	5.85	0.81	0.0000
BRESLO	38	f	0	0.32	3.10	4.26	0.5717
Subtotal BRESLO				1.33	8.95	5.07	
*BRETT	4	m	0	1.37	5.75	0.09	0.0010
BROCKM	1	m	0	0.07	0.98	1.97	0.9437
BROCKM	2	f	0	0.69	2.73	1.75	0.2517
Subtotal BROCKM				0.53	3.71	3.72	
BROSS	11	m	0	1.58	28.40	0.20	0.0000
BROWN2	12	m	2	2.42	402.82	349.56	0.0000
BROWN2	11	f	2	2.61	365.29	455.62	0.0000
Subtotal BROWN2				2.51	768.12	805.17	
BUFFLE	3	m	0	2.40	4.35	3.58	0.0000
BUFFLE	7	f	0	2.11	26.25	10.03	0.0000
Subtotal BUFFLE				2.15	30.60	13.61	
CARPEN	11	c	3	3.14	11.66	31.49	0.0000
CASCO2	1	c	0	2.44	5.31	4.77	0.0000
CASCOR	1	c	0	2.60	18.40	22.71	0.0000
*CEDERL	106	m	2	2.04	20.57	6.24	0.0000
*CEDERL	75	f	2	1.57	30.42	0.19	0.0000

Table 1C2 - 2

IESLC - Meta-anal of Current Smoking (or Ever if Current not available), Any prod (or Cigs if Any not avail)
All LC types
Most adjusted

REF	NRR	SEX	AD	Ys	Ws	Qs	Ps
Subtotal CEDERL				1.76	50.99	6.43	
CHAN	9	m	0	3.31	1.87	6.21	0.0000
CHAN	10	f	0	1.25	20.57	1.26	0.0000
Subtotal CHAN				1.42	22.44	7.47	
*CHANG	5	m	0	2.13	4.46	1.79	0.0000
*CHANG	11	f	0	1.64	8.22	0.18	0.0000
Subtotal CHANG				1.81	12.68	1.96	
CHATZI	4	c	0	1.21	19.44	1.61	0.0000
CHEN2	1	m	0	1.52	6.25	0.00	0.0001
CHEN2	2	f	0	0.51	7.70	7.38	0.1539
Subtotal CHEN2				0.97	13.95	7.38	
CHEN3	1	c	0	0.46	27.78	29.52	0.0148
CHIAZZ	3	m	11	3.26	0.90	2.83	0.0019
CHOI	3	m	0	1.64	10.55	0.23	0.0000
CHOI	7	f	0	0.20	7.16	12.00	0.5951
Subtotal CHOI				1.06	17.71	12.23	
*CHOW	25	m	0	2.64	5.79	7.68	0.0000
*CHYOU	2	m	1	2.43	12.06	10.66	0.0000
COMSTO	3	m	0	2.90	3.52	6.93	0.0000
COMSTO	8	f	0	2.57	8.49	9.89	0.0000
Subtotal COMSTO				2.67	12.01	16.82	
COOKSO	5	c	0	1.88	14.38	2.18	0.0000
CORREA	42	c	1	2.65	50.99	68.60	0.0000
*CPSI	220	m	1	2.48	74.99	73.00	0.0000
*CPSI	279	f	1	1.16	70.15	7.64	0.0000
Subtotal CPSI				1.84	145.14	80.65	
*CPSII	126	m	1	3.01	84.90	194.84	0.0000
*CPSII	133	f	1	2.47	171.36	162.28	0.0000
Subtotal CPSII				2.65	256.26	357.12	
DAMBER	14	m	1	2.26	26.18	15.46	0.0000
DARBY	4	m	0	4.51	2.93	26.68	0.0000
DARBY	11	f	0	3.03	18.15	42.78	0.0000
Subtotal DARBY				3.23	21.08	69.46	
DAVEYS	5	m	0	1.57	2.53	0.01	0.0126
DAVEYS	6	f	0	-0.32	0.42	1.40	0.8327
Subtotal DAVEYS				1.30	2.96	1.41	
DEAN	7	m	0	1.66	9.69	0.25	0.0000
DEAN2	2	m	0	1.33	23.59	0.60	0.0000
DEAN2	6	f	0	1.06	13.83	2.55	0.0001
Subtotal DEAN2				1.23	37.42	3.15	
DEAN3	42	m	3	1.91	18.88	3.20	0.0000
DEAN3	119	f	3	1.75	20.79	1.40	0.0000
Subtotal DEAN3				1.83	39.66	4.60	
*DEKLER	8	m	2	3.14	0.99	2.67	0.0018
DESTE2	4	c	7	2.21	12.30	6.29	0.0000
DESTEF	41	m	4	2.39	18.66	14.96	0.0000
*DOCKER	1	c	4	2.08	3.90	1.34	0.0000
DOLL	90	m	0	2.25	6.22	3.59	0.0000
DOLL	93	f	0	0.74	11.96	6.87	0.0110
Subtotal DOLL				1.25	18.18	10.46	
*DOLL2	54	m	1	2.40	18.45	15.07	0.0000
*DOLL2	63	f	1	2.16	3.28	1.45	0.0001
Subtotal DOLL2				2.36	21.73	16.52	
DORANT	2	m	0	2.38	6.51	5.13	0.0000
DORGAN	9	m	0	2.83	11.70	20.87	0.0000
DORGAN	33	m	0	3.71	2.61	12.85	0.0000
DORGAN	56	f	0	2.50	41.93	42.37	0.0000
DORGAN	79	f	0	2.44	3.75	3.34	0.0000
Subtotal DORGAN				2.61	60.00	79.42	
*DORN	51	m	1	2.11	73.41	27.72	0.0000
DOSEME	1	m	2	1.19	55.52	4.97	0.0000
DROSTE	6	m	4	2.67	5.52	7.70	0.0000
DU	1	m	0	1.26	28.12	1.51	0.0000
DU	2	f	0	0.66	24.50	17.11	0.0011
Subtotal DU				0.98	52.62	18.63	
*DUNN	6	m	0	2.91	1.97	3.98	0.0000
EBELIN	1	m	0	1.94	9.19	1.81	0.0000
*ENGELA	158	m	1	2.00	6.53	1.66	0.0000
*ENGELA	164	f	1	1.75	6.51	0.44	0.0000
Subtotal ENGELA				1.88	13.04	2.10	
*ENSTRO	1	m	1	2.56	81.91	93.94	0.0000

Table 1C2 - 2

IESLC - Meta-anal of Current Smoking (or Ever if Current not available), Any prod (or Cigs if Any not avail)
All LC types
Most adjusted

REF	NRR	SEX	AD	Ys	Ws	Qs	Ps
*ENSTRO	2	f	1	1.94	181.45	36.01	0.0000
Subtotal ENSTRO				2.13	263.36	129.95	
ESAKI	4	m	0	0.64	8.96	6.52	0.0554
ESAKI	5	f	0	0.90	7.99	2.81	0.0109
Subtotal ESAKI				0.76	16.94	9.32	
FAN	1	m	0	1.04	25.87	5.20	0.0000
FAN	2	f	0	1.37	24.92	0.40	0.0000
Subtotal FAN				1.20	50.80	5.60	
GAO	33	m	2	1.36	39.76	0.70	0.0000
GAO	34	f	2	1.06	51.44	9.45	0.0000
Subtotal GAO				1.19	91.20	10.14	
GAO2	8	m	1	1.89	9.26	1.45	0.0000
GARCIA	2	c	0	2.72	13.47	20.13	0.0000
GARDIN	2	c	0	2.62	3.97	5.02	0.0000
GARSHI	31	m	1	2.04	33.11	9.94	0.0000
GENG	1	m	0	1.79	4.98	0.44	0.0001
GENG	2	f	0	1.08	22.39	3.74	0.0000
Subtotal GENG				1.21	27.37	4.18	
GER	21	c	14	0.61	12.59	9.82	0.0305
GODLEY	5	m	1	1.92	96.28	17.76	0.0000
GODLEY	6	f	1	1.71	58.89	2.82	0.0000
Subtotal GODLEY				1.84	155.17	20.58	
GOLLED	7	m	1	2.02	13.89	3.80	0.0000
GOODMA	2	m	0	2.86	8.50	15.83	0.0000
GOODMA	6	f	0	2.27	10.71	6.41	0.0000
Subtotal GOODMA				2.53	19.21	22.23	
GRAHAM	25	m	1	1.80	17.30	1.65	0.0000
GREGOR	2	m	0	0.26	4.75	7.24	0.5741
GREGOR	6	f	0	2.67	0.88	1.20	0.0126
Subtotal GREGOR				0.63	5.62	8.45	
GSELL	8	m	0	2.88	1.82	3.48	0.0001
HAENSZ	54	f	0	0.76	23.97	12.88	0.0002
*HAMMO2	8	m	1	2.32	4.92	3.33	0.0000
*HAMMON	139	m	1	2.44	14.07	12.72	0.0000
*HANSEN	3	m	2	0.43	5.28	6.02	0.3285
HEGMAN	1	c	0	2.79	23.66	40.01	0.0000
*HEIN	5	m	0	2.85	0.99	1.83	0.0045
*HENNEK	2	m	0	2.73	17.97	27.64	0.0000
HINDS	22	f	3	1.73	39.57	2.25	0.0000
*HIRAYA	1	m	1	1.49	85.55	0.00	0.0000
*HIRAYA	3	f	1	0.85	77.37	32.00	0.0000
Subtotal HIRAYA				1.19	162.92	32.00	
HITOSU	34	m	1	1.03	6.25	1.36	0.0103
HITOSU	59	f	1	1.13	13.59	1.81	0.0000
Subtotal HITOSU				1.10	19.85	3.18	
*HOLE	33	m	1	2.09	6.71	2.40	0.0000
*HOLE	31	f	1	0.43	4.99	5.69	0.3421
Subtotal HOLE				1.38	11.70	8.10	
HOROWI	1	m	0	1.27	15.35	0.74	0.0000
HOROWI	2	f	0	0.60	8.08	6.48	0.0894
Subtotal HOROWI				1.04	23.43	7.21	
HORWIT	1	f	0	2.43	8.29	7.21	0.0000
HU	15	m	0	0.74	17.16	9.86	0.0023
HU	16	f	0	0.55	7.15	6.36	0.1413
Subtotal HU				0.68	24.31	16.22	
HU2	9	m	0	1.11	27.11	4.04	0.0000
HU2	10	f	0	0.63	21.91	16.38	0.0033
Subtotal HU2				0.89	49.01	20.41	
HUANG	1	c	0	0.69	14.82	9.55	0.0078
HUMBLE	13	m	1	2.99	4.94	11.13	0.0000
HUMBLE	15	m	1	2.76	1.65	2.64	0.0004
HUMBLE	17	f	1	2.82	5.85	10.25	0.0000
HUMBLE	19	f	1	3.16	2.49	6.90	0.0000
Subtotal HUMBLE				2.93	14.94	30.92	
JAHN	22	f	2	1.19	14.92	1.34	0.0000
JAIN	52	m	2	2.52	7.65	8.03	0.0000
JAIN	51	f	2	2.82	12.13	21.40	0.0000
Subtotal JAIN				2.70	19.79	29.43	
JARUP	6	m	2	2.02	3.91	1.09	0.0001
JARVHO	2	m	0	3.70	0.90	4.37	0.0005
JARVHO	6	f	0	2.74	2.57	4.00	0.0000

Table 1C2 - 2

IESLC - Meta-anal of Current Smoking (or Ever if Current not available), Any prod (or Cigs if Any not avail)
All LC types
Most adjusted

REF	NRR	SEX	AD	Ys	Ws	Qs	Ps
Subtotal JARVHO				2.99	3.47	8.36	
JEDRYC	58	m	4	1.70	31.63	1.32	0.0000
JEDRYC	59	f	4	1.51	11.71	0.00	0.0000
Subtotal JEDRYC				1.65	43.33	1.32	
JIANG	1	m	0	1.00	4.45	1.08	0.0346
JIANG	2	f	0	0.91	2.62	0.89	0.1401
Subtotal JIANG				0.97	7.08	1.97	
JOLY	18	m	0	2.59	10.93	13.10	0.0000
JOLY	15	f	0	2.01	24.54	6.62	0.0000
Subtotal JOLY				2.19	35.47	19.72	
JUSSAW	29	m	2	2.82	25.79	45.61	0.0000
*KAISE2	68	m	1	2.08	10.65	3.72	0.0000
*KAISE2	60	f	1	2.67	8.78	12.22	0.0000
Subtotal KAISE2				2.35	19.43	15.94	
*KAISER	12	m	2	2.98	25.68	56.46	0.0000
*KAISER	9	f	2	1.88	27.68	4.06	0.0000
Subtotal KAISER				2.41	53.36	60.52	
KANELL	30	m	1	1.60	30.83	0.33	0.0000
KATSOU	2	f	1	1.22	8.70	0.63	0.0003
KAUFMA	16	c	6	3.03	27.34	64.29	0.0000
KELLER	1	m	0	2.58	195.95	230.70	0.0000
KELLER	9	m	0	2.73	24.67	37.58	0.0000
KELLER	5	f	0	2.68	233.82	327.64	0.0000
KELLER	13	f	0	2.45	34.67	32.06	0.0000
Subtotal KELLER				2.62	489.11	627.98	
KHUDER	19	m	0	2.09	19.27	6.91	0.0000
KIHARA	7	c	0	1.40	42.14	0.36	0.0000
*KINLEN	20	m	2	2.56	6.98	7.90	0.0000
KJUUS	1	m	0	3.05	1.78	4.29	0.0000
*KNEKT	86	m	1	2.18	5.60	2.65	0.0000
KO	1	f	3	1.44	2.18	0.01	0.0339
KOHLME	2	c	4	2.80	5.21	8.86	0.0000
KOO	9	f	0	0.94	10.70	3.32	0.0022
KOULUM	1	m	0	3.57	4.47	19.22	0.0000
KREUZE	40	f	0	2.72	3.93	5.89	0.0000
KREUZE	42	f	0	1.77	24.64	1.87	0.0000
Subtotal KREUZE				1.90	28.57	7.76	
KREYBE	12	m	1	1.89	5.80	0.91	0.0000
KREYBE	30	f	1	0.36	7.92	10.21	0.3143
Subtotal KREYBE				1.01	13.71	11.11	
*KUBIK	12	m	0	3.50	1.96	7.87	0.0000
LAMTH	6	f	0	1.34	46.55	1.13	0.0000
LAMWK	1	f	0	1.42	17.85	0.11	0.0000
LAMWK2	9	m	0	1.04	12.98	2.65	0.0002
LAMWK2	10	f	0	1.17	17.89	1.92	0.0000
Subtotal LAMWK2				1.11	30.86	4.57	
*LANGE	38	m	1	1.74	3.96	0.24	0.0005
*LANGE	35	f	1	1.61	8.08	0.12	0.0000
Subtotal LANGE				1.66	12.04	0.36	
LAUSSM	11	m	3	1.74	37.15	2.27	0.0000
LEI	1	m	0	1.30	26.63	0.96	0.0000
LEI	2	f	0	1.25	23.21	1.38	0.0000
Subtotal LEI				1.28	49.84	2.34	
LEMARC	2	c	0	2.60	17.07	20.98	0.0000
LETOUR	1	c	0	2.56	20.21	23.10	0.0000
LEVIN	32	m	1	1.58	30.68	0.24	0.0000
*LIAW	1	m	1	1.31	11.72	0.40	0.0000
*LIAW	2	f	1	1.28	2.46	0.11	0.0447
Subtotal LIAW				1.30	14.17	0.51	
*LIDDEL	4	m	1	1.48	17.82	0.00	0.0000
LIU	2	c	2	0.65	38.19	27.01	0.0001
LIU2	2	m	3	1.65	4.37	0.10	0.0006
LIU2	4	f	3	1.54	6.68	0.01	0.0001
Subtotal LIU2				1.58	11.05	0.12	
LIU3	2	m	2	0.23	1.87	2.98	0.7518
LIU4	11	m	2	1.02	5969.41	1364.09	0.0000
LIU4	12	f	2	1.05	3876.64	758.86	0.0000
Subtotal LIU4				1.03	9846.05	2122.95	
LIU5	1	c	0	0.65	11.25	8.01	0.0293
LOMBA2	1	f	0	0.28	37.19	54.46	0.0841
LOMBAR	9	m	0	2.41	12.02	10.18	0.0000

Table 1C2 - 2

IESLC - Meta-anal of Current Smoking (or Ever if Current not available), Any prod (or Cigs if Any not avail)
All LC types
Most adjusted

REF	NRR	SEX	AD	Ys	Ws	Qs	Ps
LUBIN2	26	m	2	2.37	160.04	122.30	0.0000
LUBIN2	317	f	0	1.34	106.80	2.35	0.0000
Subtotal LUBIN2				1.96	266.84	124.65	
LUO	7	c	20	0.99	10.60	2.65	0.0012
MACLEN	19	m	0	1.34	3.53	0.09	0.0120
MACLEN	32	f	0	0.87	12.71	5.01	0.0020
Subtotal MACLEN				0.97	16.25	5.10	
*MAGNUS	5	m	3	1.42	6.75	0.04	0.0002
MARSH	7	c	2	1.92	7.36	1.32	0.0000
MARSH2	5	m	1	0.64	3.87	2.84	0.2107
MARSH2	6	f	1	1.66	3.65	0.11	0.0015
Subtotal MARSH2				1.14	7.51	2.94	
MARTIS	4	m	0	1.95	3.32	0.68	0.0004
MASTRA	2	m	2	2.10	4.76	1.74	0.0000
MATOS	3	m	2	2.14	8.35	3.49	0.0000
MATSUD	10	m	0	3.07	2.94	7.27	0.0000
MCCONN	1	m	0	0.19	3.33	5.62	0.7237
MCCONN	2	f	0	1.01	0.99	0.23	0.3136
Subtotal MCCONN				0.38	4.32	5.85	
MCDUFF	1	m	0	1.81	4.70	0.48	0.0001
MCLAUG	1	m	0	1.20	18.70	1.56	0.0000
*MIGRAN	20	m	2	1.37	3.91	0.06	0.0068
*MIGRAN	136	f	2	1.61	3.53	0.05	0.0025
Subtotal MIGRAN				1.48	7.44	0.11	
MILLER	2	f	1	1.61	4.90	0.06	0.0004
MILLS	3	m	1	0.29	96.62	140.23	0.0046
*MRFITR	2	m	0	3.88	0.50	2.83	0.0062
NAM	76	m	1	2.16	21.20	9.42	0.0000
NAM	92	f	1	2.38	23.93	18.95	0.0000
Subtotal NAM				2.28	45.13	28.37	
NOTAN2	15	m	2	1.10	80.00	12.67	0.0000
NOU	11	m	0	1.81	5.20	0.51	0.0000
NOU	12	f	0	1.96	2.74	0.59	0.0012
Subtotal NOU				1.86	7.94	1.11	
ODRISC	1	c	0	3.99	5.77	36.07	0.0000
ORMOS	4	m	0	2.23	6.39	3.51	0.0000
ORMOS	26	f	0	-1.64	0.95	9.36	0.1093
Subtotal ORMOS				1.73	7.34	12.87	
OSANN	33	m	2	3.28	37.23	118.49	0.0000
OSANN	34	f	2	2.98	60.12	132.09	0.0000
Subtotal OSANN				3.09	97.36	250.58	
PARKIN	26	m	6	1.41	62.08	0.47	0.0000
PASTOR	10	m	1	1.92	7.85	1.42	0.0000
PAWLEG	2	m	6	2.51	3.16	3.24	0.0000
PERNU	2	m	0	2.19	58.99	28.61	0.0000
PERNU	1	f	0	0.63	13.52	9.99	0.0198
Subtotal PERNU				1.90	72.50	38.60	
PERSH2	10	c	4	2.12	107.04	41.39	0.0000
*PETO	4	m	0	1.97	1.98	0.45	0.0056
PEZZO2	2	m	0	3.13	5.42	14.57	0.0000
PEZZOT	5	m	0	3.48	3.66	14.51	0.0000
PIKE	4	m	0	1.66	13.39	0.37	0.0000
PIKE	8	f	0	1.57	18.04	0.12	0.0000
Subtotal PIKE				1.61	31.43	0.49	
POFFIJ	1	c	0	2.05	46.21	14.21	0.0000
POLEDN	1	c	1	2.22	11.85	6.32	0.0000
*QIAO2	14	m	1	0.46	9.43	10.00	0.1544
RACHTA	9	f	1	1.91	10.62	1.87	0.0000
RADZIK	1	c	0	0.27	5.03	7.50	0.5411
RANDIG	23	m	0	1.61	3.99	0.06	0.0013
RANDIG	24	f	0	0.80	6.34	3.07	0.0447
Subtotal RANDIG				1.11	10.32	3.12	
REN	1	m	0	1.27	7.46	0.36	0.0005
REN	2	f	0	1.40	9.65	0.08	0.0000
Subtotal REN				1.35	17.11	0.44	
RONCO	1	m	0	1.63	5.23	0.10	0.0002
ROTHSC	2	c	1	1.71	9.83	0.48	0.0000
SADOWS	31	m	1	1.29	7.90	0.33	0.0003
SANKAR	2	m	3	2.61	22.36	27.96	0.0000
SCHWAR	25	m	0	2.71	68.81	101.05	0.0000
SCHWAR	26	m	0	2.27	26.07	15.55	0.0000

Table 1C2 - 2

IESLC - Meta-anal of Current Smoking (or Ever if Current not available), Any prod (or Cigs if Any not avail)
All LC types
Most adjusted

REF	NRR	SEX	AD	Ys	Ws	Qs	Ps
SCHWAR	27	f	0	2.75	91.98	145.29	0.0000
SCHWAR	28	f	0	2.87	22.69	42.76	0.0000
Subtotal SCHWAR				2.69	209.54	304.65	
SEGI	1	m	0	0.53	15.18	13.97	0.0375
SEGI2	20	m	1	1.32	6.65	0.20	0.0007
SEGI2	28	f	1	0.50	10.48	10.33	0.1049
Subtotal SEGI2				0.82	17.14	10.53	
SEOW	6	f	1	1.66	9.73	0.26	0.0000
SHAW	6	c	0	3.06	8.67	21.21	0.0000
SIEMIA	5	m	7	2.49	10.37	10.36	0.0000
SIMARA	3	m	6	0.50	13.58	13.38	0.0650
SIMARA	4	f	6	0.49	9.71	9.81	0.1278
Subtotal SIMARA				0.50	23.30	23.18	
SOBUE	42	m	1	1.41	27.66	0.19	0.0000
SOBUE	52	f	1	1.03	34.45	7.41	0.0000
Subtotal SOBUE				1.20	62.11	7.59	
SOBUE2	10	m	2	1.50	197.90	0.00	0.0000
SOBUE2	12	f	2	1.19	143.51	13.39	0.0000
Subtotal SOBUE2				1.37	341.42	13.39	
*SPEIZE	10	f	1	2.54	65.88	72.29	0.0000
SPITZ	2	c	0	3.05	5.83	14.16	0.0000
STASZE	1	m	0	2.37	4.73	3.63	0.0000
STASZE	5	f	0	1.47	4.16	0.00	0.0028
Subtotal STASZE				1.95	8.88	3.63	
STAYNE	1	m	0	1.30	40.37	1.52	0.0000
STOCKS	47	m	2	1.78	33.11	2.79	0.0000
STOCKS	50	f	1	1.11	58.11	8.45	0.0000
Subtotal STOCKS				1.36	91.22	11.24	
STOCKW	7	c	0	2.65	1204.31	1613.08	0.0000
STUCKE	2	m	0	4.65	0.49	4.86	0.0012
SUN	1	c	0	0.84	30.23	13.06	0.0000
SUZUK2	6	c	3	3.09	2.54	6.49	0.0000
SVENSS	96	f	1	2.20	13.42	6.78	0.0000
TANG	1	c	0	2.20	5.10	2.54	0.0000
*TENKAN	24	m	1	2.82	5.38	9.50	0.0000
TIZZAN	5	m	0	0.64	84.08	61.18	0.0000
TIZZAN	13	f	0	1.46	6.13	0.01	0.0003
Subtotal TIZZAN				0.70	90.21	61.18	
TOKARS	1	m	0	3.61	0.97	4.35	0.0004
TOKARS	5	f	0	0.43	0.62	0.70	0.7336
Subtotal TOKARS				2.37	1.59	5.06	
TOUSEY	12	m	3	4.08	3.57	23.89	0.0000
TOUSEY	15	f	3	3.41	9.42	34.52	0.0000
Subtotal TOUSEY				3.59	12.98	58.41	
TSUGAN	28	m	0	0.20	7.53	12.59	0.5818
*TULINI	37	m	3	2.30	10.29	6.72	0.0000
*TULINI	43	f	3	2.79	11.10	18.60	0.0000
Subtotal TULINI				2.55	21.39	25.32	
*TVERDA	5	m	2	1.41	20.41	0.15	0.0000
*TVERDA	15	f	2	2.40	2.67	2.20	0.0001
Subtotal TVERDA				1.52	23.08	2.35	
WAKAI	8	m	2	1.48	7.88	0.00	0.0000
WAKAI	26	f	2	1.47	8.29	0.00	0.0000
Subtotal WAKAI				1.48	16.17	0.00	
*WALD	4	m	1	2.80	4.92	8.37	0.0000
WANG	5	c	6	1.06	15.11	2.87	0.0000
WANG2	18	c	4	0.88	6.94	2.65	0.0211
WANG3	1	c	0	1.05	28.11	5.59	0.0000
WANG4	2	m	2	0.15	100.26	181.33	0.1372
WICKLU	1	m	0	1.53	15.41	0.02	0.0000
WIGLE	25	m	1	2.34	13.23	9.53	0.0000
WIGLE	30	f	1	1.65	19.69	0.48	0.0000
Subtotal WIGLE				1.93	32.92	10.00	
WILKIN	3	c	4	2.06	12.03	3.84	0.0000
WU	42	f	2	1.58	11.97	0.09	0.0000
WUNSCH	5	m	1	1.89	10.41	1.60	0.0000
WUNSCH	11	f	1	1.79	10.34	0.90	0.0000
Subtotal WUNSCH				1.84	20.74	2.50	
WUWILL	8	f	3	0.83	102.19	44.56	0.0000
WYNDE2	21	m	0	2.13	7.21	2.95	0.0000
WYNDE3	50	m	0	2.37	7.59	5.87	0.0000

Table 1C2 - 2

IESLC - Meta-anal of Current Smoking (or Ever if Current not available), Any prod (or Cigs if Any not avail)
All LC types
Most adjusted

REF	NRR	SEX	AD	Ys	Ws	Qs	Ps
WYNDE3	138	f	0	1.14	9.73	1.23	0.0004
Subtotal WYNDE3				1.68	17.32	7.09	
WYNDE4	48	m	0	2.21	10.51	5.39	0.0000
WYNDE4	62	f	2	1.05	8.80	1.69	0.0018
Subtotal WYNDE4				1.68	19.31	7.08	
WYNDE6	18	m	0	2.78	66.40	109.20	0.0000
WYNDE6	207	f	0	2.68	90.13	127.63	0.0000
Subtotal WYNDE6				2.72	156.52	236.84	
*XIANGZ	13	m	2	0.77	25.36	13.26	0.0001
XU	2	m	2	0.99	58.89	14.72	0.0000
XU2	2	c	7	1.34	45.75	1.15	0.0000
XU3	2	m	1	1.79	5.80	0.51	0.0000
XU3	4	f	1	1.35	3.69	0.08	0.0095
Subtotal XU3				1.62	9.49	0.59	
XU4	1	c	0	1.08	20.82	3.56	0.0000
YAMAGU	10	c	1	1.59	8.97	0.08	0.0000
*YONG	12	m	1	3.36	1.92	6.67	0.0000
*YONG	15	f	1	1.65	6.30	0.15	0.0000
Subtotal YONG				2.05	8.22	6.82	
*YUAN	1	m	2	1.87	11.44	1.64	0.0000
ZHANG	2	m	7	1.39	4.65	0.05	0.0028
ZHANG	3	f	7	1.32	4.81	0.14	0.0038
Subtotal ZHANG				1.35	9.46	0.19	
ZHENG	15	m	0	1.29	20.36	0.81	0.0000
ZHENG	24	f	0	0.74	20.88	11.92	0.0008
Subtotal ZHENG				1.01	41.24	12.73	
ZHOU	2	m	0	0.86	17.50	7.02	0.0003
ZHOU	3	f	0	0.80	5.34	2.60	0.0660
Subtotal ZHOU				0.84	22.83	9.62	

	N	344
	NS	242
	Wt	19261.12
Het	Chi	9715.82
Het	df	343
Het	P	***
Fixed	RR	4.45
	RRl	4.39
	RRu	4.51
	P	+++
Random	RR	6.20
	RRl	5.68
	RRu	6.77
	P	+++
Asymm	P	***

Table 1C2 - 3

IESLC - Meta-anal of Current Smoking (or Ever if Current not available), Any prod (or Cigs if Any not avail)
All LC types
Most adjusted

	Sex combined	male	female	Total
N	45	181	118	344
NS	45	177	113	335
Wt	2031.82	10083.12	7146.18	19261.12
Het Chi	863.31	4269.62	3045.45	9715.82
Het df	44	180	117	343
Het P	***	***	***	***
Fixed RR	10.11	3.97	4.14	4.45
RRl	9.68	3.89	4.05	4.39
RRu	10.56	4.05	4.24	4.51
P	+++	+++	+++	+++
Random RR	6.95	7.12	4.85	6.20
RRl	5.42	6.26	4.17	5.68
RRu	8.90	8.09	5.65	6.77
P	+++	+++	+++	+++
Between Chi				1537.45
Between df				2
Between P				***
Btwn(F) P				***
Btwn(R) P				***

	Smoking status ever	current	Total
N	153	191	344
NS	116	129	245
Wt	12559.25	6701.87	19261.12
Het Chi	1598.66	2614.15	9715.82
Het df	152	190	343
Het P	***	***	***
Fixed RR	3.01	9.25	4.45
RRl	2.96	9.03	4.39
RRu	3.07	9.48	4.51
P	+++	+++	+++
Random RR	4.14	8.35	6.20
RRl	3.81	7.56	5.68
RRu	4.50	9.23	6.77
P	+++	+++	+++
Between Chi			5503.01
Between df			1
Between P			***
Btwn(F) P			***
Btwn(R) P			***

	Study LIU4 LIU4	others	Total
N	2	342	344
NS	1	241	242
Wt	9846.05	9415.06	19261.12
Het Chi	2.98	5375.86	9715.82
Het df	1	341	343
Het P	(*)	***	***
Fixed RR	2.80	7.23	4.45
RRl	2.74	7.09	4.39
RRu	2.85	7.38	4.51
P	+++	+++	+++
Random RR	2.81	6.23	6.20
RRl	2.71	5.71	5.68
RRu	2.91	6.80	6.77
P	+++	+++	+++
Between Chi			4336.99
Between df			1
Between P			***
Btwn(F) P			***
Btwn(R) P			***

Table 1C2 - 4

IESLC - Meta-anal of Current Smoking (or Ever if Current not available), Any prod (or Cigs if Any not avail)
All LC types
Least adjusted

REF	NRR	X	SEX	AGEL	AGEH	RACE	YF	LC TYPE	LOC	START	ST	NLC	R	VB	P	H	AD	SM	PRODUCT	DENOM	De
ABELIN	1	x	m	0	0	all	-	all	Eu:wst	1941	CC	118	n	bl	y	n	0	ev	all/unsp	nev any	st
ABRAHA	7		m	0	0	all	0	q+s+a	Eu:est	1975	pr	571	n	bl	n	n	0	ev	all/unsp	nev any	ot
ABRAHA	8		f	0	0	all	0	q+s+a	Eu:est	1975	pr	571	n	bl	n	n	0	ev	all/unsp	nev any	ot
AGUDO	10	x	f	0	0	all	-	all	Eu:wst	1989	CC	103	n	bl	n	n	0	cu	cig only	nev any	st
AKIBA	2	x	m	0	0	all	0	all	As:Jap	1963	pr	610	n	bl	n	n	0	cu	cig+/-ot	nev cigs	or
AKIBA	6	x	f	0	0	all	0	all	As:Jap	1963	pr	610	n	bl	n	n	0	cu	cig+/-ot	nev cigs	or
ALDERS	177		m	0	0	all	-	all	Eu:UK	1977	CC	1448	n	V	n	n	0	cu	cig+/-ot	nev any	st
ALDERS	176		f	0	0	all	-	all	Eu:UK	1977	CC	1448	n	V	n	n	0	cu	cig only	nev any	st
AMANDU	1	x	m	0	0	wh	0	all	NAmer	1959	pr	132	m	bl	n	n	0	cu	cig+/-ot	nev cigs	st
AMES	1		m	0	0	wh	-	all	NAmer	1959	ot	317	m	bl	n	n	0	cu	all/unsp	nev any	or
ANDERS	2	x	f	0	0	all	0	all	NAmer	1986	pr	343	n	bl	n	n	0	cu	cig+/-ot	nev cigs	st
ARCHER	5		m	0	0	wh	0	all	NAmer	1950	pr	146	m	bl	n	n	0	cu	cig+/-ot	nev cigs	st
ARMADA	27		m	0	0	all	-	all	Eu:wst	1986	CC	325	n	bl	n	y	0	cu	cig+/-ot	nev any	st
AUSTIN	2	x	c	0	0	all	-	all	NAmer	1970	CC	166	o	bl	y	n	0	cu	cig+/-ot	nev cigs	st
AUVINE	1	x	c	0	0	all	-	all	Eu:Sca	1986	CC	517	n	bl	y	n	0	ev	cig+/-ot	nev cigs	st
AXELSO	1		c	0	0	all	-	all	Eu:Sca	1960	CC	152	n	bl	y	n	0	ev	all/unsp	nev any	st
AXELSS	2		m	0	0	sca	-	all	Eu:Sca	1989	CC	436	n	bl	n	n	0	cu	all/unsp	nev any	st
AXELSS	10		f	0	0	sca	-	all	Eu:Sca	1989	CC	436	n	bl	n	n	0	cu	all/unsp	nev any	st
BAND	1		m	0	0	all	-	all	NAmer	1983	CC	2831	n	V	y	y	2	ev	cig only	nev any	ot
BARBON	3	x	m	0	0	all	-	all	Eu:wst	1979	CC	755	n	bl	y	y	0	cu	all/unsp	nev any	st
BECHER	13		m	0	0	all	-	all	Eu:Ger	1985	CC	194	n	bl	n	y	0	cu	all/unsp	nev any	st
BECHER	14		f	0	0	all	-	all	Eu:Ger	1985	CC	194	n	bl	n	y	0	cu	all/unsp	nev any	st
BENSHL	16		m	40	64	all	10	all	Eu:UK	1967	pr	486	n	V	n	n	1	cu	all/unsp	nev any	ot
BEST	2		m	0	0	all	0	all	NAmer	1955	pr	381	n	V	n	n	1	cu	cig only	nev any	ot
BEST	18		f	0	0	all	0	all	NAmer	1955	pr	381	n	V	n	n	1	ev	cig only	nev any	ot
BLOHMK	1		m	0	0	all	-	all	Eu:Ger	1978	CC	888	n	bl	n	y	0	cu	all/unsp	nev any	st
BLOT4	1		m	0	0	wh	-	all	NAmer	1974	CC	335	n	bl	y	n	0	ev	cig+/-ot	nev cigs	st
BOFFET	32	x	m	0	0	all	-	all	Eu:mul	1988	CC	5621	n	bl	y	n	0	ev	all/unsp	nev any	st
BOUCOT	2	x	m	0	0	all	0	all	NAmer	1951	pr	121	n	bl	n	n	0	cu	cig only	nev any	ot
BRESLO	37		m	0	0	all	-	all	NAmer	1949	CC	518	n	bl	n	y	0	ev	all/unsp	nev+1	st
BRESLO	38		f	0	0	all	-	all	NAmer	1949	CC	518	n	bl	n	y	0	ev	all/unsp	nev+1	st
BRETT	4		m	0	0	all	0	all	Eu:UK	1960	pr	150	n	V	n	n	0	cu	cig+/-ot	nev cigs	st
BROCKM	1		m	0	0	wh	-	all	Eu:Ger	1990	CC	117	n	bl	n	y	0	ev	cig+/-ot	nev cigs	st
BROCKM	2		f	0	0	wh	-	all	Eu:Ger	1990	CC	117	n	bl	n	y	0	ev	cig+/-ot	nev cigs	st
BROSS	11		m	0	0	wh	-	all	NAmer	1960	CC	974	n	bl	n	n	0	cu	all/unsp	nev any	st
BROWN2	12		m	0	0	wh	-	all	NAmer	1984	CC	14596	n	bl	n	y	2	cu	cig+/-ot	nev cigs	or
BROWN2	11		f	0	0	wh	-	all	NAmer	1984	CC	14596	n	bl	n	y	2	cu	cig+/-ot	nev cigs	or
BUFFLE	3		m	0	0	wh	-	all	NAmer	1976	CC	943	n	bl	y	n	0	cu	cig+/-ot	nev any	st
BUFFLE	7		f	0	0	wh	-	all	NAmer	1976	CC	943	n	bl	y	n	0	cu	cig+/-ot	nev any	st
CARPEN	9	x	c	0	0	w+b	-	all	NAmer	1991	CC	356	n	bl	n	n	0	cu	cig+/-ot	nev cigs	st
CASCO2	1		c	0	0	wh	-	all	Eu:Ger	1991	CC	155	n	bl	n	n	0	ev	all/unsp	nev any	st
CASCOR	1		c	0	0	wh	-	all	Eu:Ger	1985	CC	389	n	bl	n	y	0	ev	all/unsp	nev any	st
CEDERL	106		m	0	0	all	16	all	Eu:Sca	1963	pr	491	n	bl	n	n	2	cu	all/unsp	nev any	ot
CEDERL	75		f	0	0	all	0	all	Eu:Sca	1963	pr	491	n	bl	n	n	2	cu	all/unsp	nev any	or
CHAN	9		m	0	0	all	-	all	As:HK	1976	CC	397	n	bl	n	n	0	ev	all/unsp	nev any	st
CHAN	10		f	0	0	all	-	all	As:HK	1976	CC	397	n	bl	n	n	0	ev	all/unsp	nev any	st
CHANG	5		m	0	0	all	0	all	NAmer	1972	pr	136	n	bl	n	n	0	cu	cig+/-ot	nev cigs	st
CHANG	11		f	0	0	all	0	all	NAmer	1972	pr	136	n	bl	n	n	0	cu	cig+/-ot	nev cigs	st
CHATZI	4		c	0	0	all	-	all	Eu:bal	1987	CC	282	n	bl	n	y	0	ev	all/unsp	nev any	st
CHEN2	1		m	0	0	all	-	all	As:Chi	1983	CC	193	n	ot	y	n	0	ev	all/unsp	nev any	st
CHEN2	2		f	0	0	all	-	all	As:Chi	1983	CC	193	n	ot	y	n	0	ev	all/unsp	nev any	st
CHEN3	1		c	0	0	all	-	all	As:Chi	1981	CC	254	n	ot	y	n	0	ev	all/unsp	nev any	st
CHIAZZ	2	x	m	0	0	all	-	all	NAmer	1940	CC	144	o	bl	y	n	0	ev	cig+/-ot	nev cigs	st
CHOI	3		m	0	0	all	-	all	As:oth	1985	CC	375	n	bl	n	n	0	cu	cig+/-ot	nev cigs	st
CHOI	7		f	0	0	all	-	all	As:oth	1985	CC	375	n	bl	n	n	0	cu	cig+/-ot	nev cigs	st
CHOW	25		m	0	0	wh	0	all	NAmer	1966	pr	219	n	bl	n	n	0	cu	all/unsp	nev any	st
CHYOU	4	x	m	0	0	jap	0	all	NAmer	1965	pr	227	n	bl	n	y	0	cu	cig+/-ot	nev cigs	st
COMSTO	3		m	0	0	all	-	all	NAmer	1975	ot	258	n	bl	n	n	0	cu	cig+/-ot	nev any	st
COMSTO	8		f	0	0	all	-	all	NAmer	1975	ot	258	n	bl	n	n	0	cu	cig+/-ot	nev any	st
COOKSO	5		c	0	0	bl	-	all	Africa	1961	CC	234	n	V	n	y	0	ev	all/unsp	nev any	st
CORREA	41	x	c	0	0	all	-	all	NAmer	1979	CC	1359	n	bl	y	n	0	cu	cig+/-ot	nev cigs	st
CPSI	220		m	35	84	all	6	all	NAmer	1959	pr	5138	n	bl	n	n	1	cu	cig+/-ot	nev any	ot
CPSI	279		f	40	74	all	6	all	NAmer	1959	pr	5138	n	bl	n	n	1	cu	cig+/-ot	nev cigs	ot
CPSII	36	x	m	0	0	all	6	all	NAmer	1982	pr	3229	n	bl	n	n	0	cu	cig only	nev any	st
CPSII	71	x	f	0	0	all	6	all	NAmer	1982	pr	3229	n	bl	n	n	0	cu	cig+/-ot	nev cigs	st
DAMBER	14		m	0	0	all	-	all	Eu:Sca	1972	CC	579	n	bl	y	n	1	cu	all/unsp	nev any	ot
DARBY	4		m	0	0	wh	-	all	Eu:UK	1988	CC	982	n	V	n	n	0	cu	cig+/-ot	nev any	st
DARBY	11		f	0	0	wh	-	all	Eu:UK	1988	CC	982	n	V	n	n	0	cu	cig+/-ot	nev any	st
DAVEYS	5		m	0	0	all	-	all	Eu:Ger	1930	CC	109	n	bl	y	n	0	ev	all/unsp	nev any	st
DAVEYS	6		f	0	0	all	-	all	Eu:Ger	1930	CC	109	n	bl	y	n	0	ev	all/unsp	nev any	ot
DEAN	7		m	0	0	wh	-	all	Africa	1947	CC	603	n	V	y	n	0	ev	all/unsp	nev any	st
DEAN2	2		m	0	0	all	-	all	Eu:UK	1960	CC	954	n	V	y	n	0	cu	all/unsp	nev any	st

Table 1C2 - 4

IESLC - Meta-anal of Current Smoking (or Ever if Current not available), Any prod (or Cigs if Any not avail)
All LC types
Least adjusted

REF	NRR	X	SEX	AGEL	AGEH	RACE	YF	LC TYPE	LOC	START	ST	NLC	R	VB	P	H	AD	SM	PRODUCT	DENOM	De
DEAN2	6		f	0	0	all	-	all	Eu:UK	1960	CC	954	n	V	y	n	0	cu	all/unsp	nev any	st
DEAN3	40	x	m	0	0	all	-	all	Eu:UK	1969	CC	766	n	V	y	n	0	cu	all/unsp	nev any	st
DEAN3	117	x	f	0	0	all	-	all	Eu:UK	1969	CC	766	n	V	y	n	0	cu	cig only	nev any	st
DEKLER	8		m	0	0	all	0	all	Auslia	1961	pr	138	m	V	n	n	2	cu	all/unsp	nev any	ot
DESTE2	2	x	c	0	0	all	-	all	SCAmer	1993	CC	463	n	bl	n	n	0	cu	all/unsp	nev any	st
DESTEF	40	x	m	0	0	all	-	all	SCAmer	1988	CC	497	n	bl	n	y	0	cu	all/unsp	nev any	st
DOCKER	1		c	0	0	wh	0	all	NAmer	1974	pr	120	n	bl	n	n	4	cu	cig+/-ot	nev cigs	or
DOLL	90		m	0	0	all	-	all	Eu:UK	1948	CC	1465	n	V	n	n	0	cu	all/unsp	nev any	st
DOLL	93		f	0	0	all	-	all	Eu:UK	1948	CC	1465	n	V	n	n	0	cu	all/unsp	nev any	st
DOLL2	54		m	0	0	all	0	all	Eu:UK	1951	pr	920	n	V	n	n	1	cu	all/unsp	nev any	ot
DOLL2	63		f	0	0	all	22	all	Eu:UK	1951	pr	920	n	V	n	n	1	cu	cig only	nev any	ot
DORANT	2		m	0	0	all	0	all	Eu:wst	1986	ot	550	n	bl	n	y	0	cu	all/unsp	nev any	st
DORGAN	9		m	0	0	wh	-	all	NAmer	1980	CC	2026	n	bl	y	y	0	cu	cig+/-ot	nev any	st
DORGAN	33		m	0	0	bl	-	all	NAmer	1980	CC	2026	n	bl	y	y	0	cu	cig+/-ot	nev any	st
DORGAN	56		f	0	0	wh	-	all	NAmer	1980	CC	2026	n	bl	y	y	0	cu	cig+/-ot	nev any	st
DORGAN	79		f	0	0	bl	-	all	NAmer	1980	CC	2026	n	bl	y	y	0	cu	cig+/-ot	nev any	st
DORN	51		m	35	84	wh	8	all	NAmer	1954	pr	5097	n	bl	n	n	1	cu	all/unsp	nev any	ot
DOSEME	17	x	m	0	0	all	-	all	Eu:bal	1979	CC	1210	n	bl	n	n	0	ev	cig+/-ot	nev cigs	st
DROSTE	2	x	m	0	0	all	-	all	Eu:wst	1995	CC	478	n	bl	n	y	0	cu	all/unsp	nev any	st
DU	1		m	0	0	all	-	all	As:Chi	1985	CC	849	n	ot	y	n	0	ev	all/unsp	nev any	or
DU	2		f	0	0	all	-	all	As:Chi	1985	CC	849	n	ot	y	n	0	ev	all/unsp	nev any	or
DUNN	6		m	0	0	all	0	all	NAmer	1954	pr	139	o	bl	n	n	0	ev	cig+/-ot	nev cigs	st
EBELIN	1		m	0	0	all	-	all	Eu:Ger	1980	CC	130	n	bl	n	n	0	ev	all/unsp	nev any	st
ENGELA	154	x	m	0	0	all	12	all	Eu:Sca	1964	pr	435	n	bl	n	n	0	cu	all/unsp	nev any	st
ENGELA	161	x	f	0	0	all	12	all	Eu:Sca	1964	pr	435	n	bl	n	n	0	cu	all/unsp	nev any	st
ENSTRO	1		m	0	0	all	0	all	NAmer	1959	pr	2879	n	bl	n	n	1	cu	cig only	nev any	or
ENSTRO	2		f	0	0	all	0	all	NAmer	1959	pr	2879	n	bl	n	n	1	cu	cig only	nev any	or
ESAKI	4		m	0	0	all	-	all	As:Jap	1961	CC	245	n	bl	y	n	0	ev	cig+/-ot	nev cigs	st
ESAKI	5		f	0	0	all	-	all	As:Jap	1961	CC	245	n	bl	y	n	0	ev	cig+/-ot	nev cigs	st
FAN	1		m	0	0	all	-	all	As:Chi	1990	CC	403	n	ot	y	n	0	ev	cig+/-ot	nev cigs	st
FAN	2		f	0	0	all	-	all	As:Chi	1990	CC	403	n	ot	y	n	0	ev	cig+/-ot	nev cigs	st
GAO	29	x	m	0	0	all	-	all	As:Chi	1984	CC	1405	n	ot	n	n	0	cu	cig+/-ot	nev cigs	st
GAO	30	x	f	0	0	all	-	all	As:Chi	1984	CC	1405	n	ot	n	n	0	cu	cig+/-ot	nev cigs	st
GAO2	1	x	m	0	0	all	-	all	As:Jap	1988	CC	282	n	bl	n	n	0	cu	cig+/-ot	nev cigs	st
GARCIA	2		c	0	0	all	-	all	NAmer	1992	CC	416	n	bl	n	y	0	cu	cig+/-ot	nev cigs	st
GARDIN	2		c	0	0	all	-	all	Eu:UK	1988	CC	143	n	V	y	n	0	cu	all/unsp	nev any	st
GARSHI	23	x	m	0	0	all	-	all	NAmer	1981	CC	1081	o	bl	y	n	0	cu	all/unsp	nev any	st
GENG	1		m	0	0	all	-	all	As:Chi	1985	CC	292	n	ot	*	n	0	ev	cig+/-ot	nev any	st
GENG	2		f	0	0	all	-	all	As:Chi	1985	CC	292	n	ot	*	n	0	ev	cig+/-ot	nev any	st
GER	17	x	c	0	0	all	-	all	As:oth	1990	CC	141	n	ot	y	n	0	ev	all/unsp	nev any	st
GODLEY	5		m	0	0	all	-	all	NAmer	1966	CC	1986	n	bl	y	n	1	ev	cig+/-ot	nev cigs	ot
GODLEY	6		f	0	0	all	-	all	NAmer	1966	CC	1986	n	bl	y	n	1	ev	cig+/-ot	nev cigs	ot
GOLLED	21	x	m	35	99	all	-	all	Eu:UK	1952	CC	443	n	V	y	n	0	ev	cig+/-ot	nev any	st
GOODMA	2		m	0	0	w+o	-	all	NAmer	1983	CC	326	n	bl	y	y	0	cu	cig+/-ot	nev any	st
GOODMA	6		f	0	0	w+o	-	all	NAmer	1983	CC	326	n	bl	y	y	0	cu	cig+/-ot	nev any	st
GRAHAM	20	x	m	0	0	wh	-	all	NAmer	1956	CC	685	n	bl	n	n	0	cu	all/unsp	nev any	st
GREGOR	2		m	0	0	all	-	all	Eu:UK	1976	CC	104	n	V	n	y	0	cu	cig+/-ot	nev cigs	st
GREGOR	6		f	0	0	all	-	all	Eu:UK	1976	CC	104	n	V	n	y	0	cu	cig+/-ot	nev cigs	st
GSELL	8		m	0	0	all	-	all	Eu:wst	1937	CC	150	n	bl	n	y	0	ev	all/unsp	nev any	st
HAENSZ	54		f	0	0	all	-	not alv	NAmer	1955	CC	158	n	bl	n	y	0	cu	cig+/-ot	nev any	st
HAMMO2	22	x	m	0	0	all	0	all	NAmer	1967	pr	450	o	bl	n	n	0	cu	cig+/-ot	nev any	st
HAMMON	139		m	0	0	wh	0	all	NAmer	1952	pr	448	n	bl	n	n	1	cu	cig only	nev any	ot
HANSEN	3		m	0	0	all	0	all	Eu:Sca	1968	pr	105	o	bl	y	n	2	ev	all/unsp	nev any	ot
HEGMAN	1		c	0	0	all	-	all	NAmer	1989	CC	282	n	bl	y	y	0	ev	all/unsp	nev any	st
HEIN	5		m	0	0	all	0	all	Eu:Sca	1970	pr	144	n	bl	n	n	0	cu	all/unsp	nev any	st
HENNEK	2		m	0	0	all	0	all	NAmer	1982	pr	169	n	bl	n	n	0	cu	all/unsp	nev any	st
HINDS	26	x	f	0	0	o	-	all	NAmer	1968	CC	292	n	bl	n	n	0	ev	all/unsp	nev any	st
HIRAYA	1		m	0	0	all	0	all	As:Jap	1965	pr	1917	n	bl	n	n	1	cu	cig+/-ot	nev any	st
HIRAYA	3		f	0	0	all	0	all	As:Jap	1965	pr	1917	n	bl	n	n	1	cu	cig+/-ot	nev any	st
HITOSU	2	x	m	0	0	all	-	all	As:Jap	1960	CC	216	n	bl	y	n	0	cu	all/unsp	nev any	st
HITOSU	9	x	f	0	0	all	-	all	As:Jap	1960	CC	216	n	bl	y	n	0	cu	all/unsp	nev any	st
HOLE	47	x	m	0	0	all	0	all	Eu:UK	1972	pr	225	n	V	n	n	0	cu	all/unsp	nev any	st
HOLE	29	x	f	0	0	all	11	all	Eu:UK	1972	pr	225	n	V	n	n	0	cu	all/unsp	nev any	st
HOROWI	1		m	0	0	all	-	all	NAmer	1956	CC	236	n	V	n	n	0	ev	cig+/-ot	nev any	st
HOROWI	2		f	0	0	all	-	all	NAmer	1956	CC	236	n	V	n	n	0	ev	cig+/-ot	nev any	st
HORWIT	1		f	0	0	all	-	all	NAmer	1977	CC	112	n	bl	n	n	0	ev	cig+/-ot	nev cigs	st
HU	15		m	0	0	all	-	all	As:Chi	1985	CC	227	n	ot	n	y	0	ev	cig+/-ot	nev any	st
HU	16		f	0	0	all	-	all	As:Chi	1985	CC	227	n	ot	n	y	0	ev	cig+/-ot	nev any	st
HU2	9		m	0	0	all	-	all	As:Chi	1977	CC	523	n	ot	y	n	0	ev	cig+/-ot	nev cigs	st
HU2	10		f	0	0	all	-	all	As:Chi	1977	CC	523	n	ot	y	n	0	ev	cig+/-ot	nev cigs	st
HUANG	1		c	0	0	all	-	all	As:Chi	1990	CC	135	n	ot	y	n	0	ev	all/unsp	nev any	st
HUMBLE	13		m	0	0	w-hi	-	all	NAmer	1980	CC	521	n	bl	y	n	1	cu	cig+/-ot	nev cigs	ot

Table 1C2 - 4

IESLC - Meta-anal of Current Smoking (or Ever if Current not available), Any prod (or Cigs if Any not avail)
All LC types
Least adjusted

REF	NRR	X	SEX	AGEL	AGEH	RACE	YF	LC TYPE	LOC	START	ST	NLC	R	VB	P	H	AD	SM	PRODUCT	DENOM	De
HUMBLE	15		m	0	0	hi	-	all	NAmer	1980	CC	521	n	bl	y	n	1	cu	cig+/-ot	nev cigs	ot
HUMBLE	17		f	0	0	w-hi	-	all	NAmer	1980	CC	521	n	bl	y	n	1	cu	cig+/-ot	nev cigs	ot
HUMBLE	19		f	0	0	hi	-	all	NAmer	1980	CC	521	n	bl	y	n	1	cu	cig+/-ot	nev cigs	ot
JAHN	3	x	f	0	0	all	-	all	Eu:Ger	1988	CC	1004	n	bl	n	n	0	ev	cig+/-ot	nev any	st
JAIN	16	x	m	0	0	all	-	all	NAmer	1981	CC	845	n	V	y	n	0	cu	cig+/-ot	nev cigs	st
JAIN	11	x	f	0	0	all	-	all	NAmer	1981	CC	845	n	V	y	n	0	cu	cig+/-ot	nev cigs	st
JARUP	3	x	m	0	0	all	-	all	Eu:Sca	1928	CC	102	o	bl	y	n	0	ev	all/unsp	nev any	st
JARVHO	2		m	0	0	all	-	all	Eu:Sca	1983	CC	147	n	bl	n	n	0	cu	all/unsp	nev any	st
JARVHO	6		f	0	0	all	-	all	Eu:Sca	1983	CC	147	n	bl	n	n	0	cu	all/unsp	nev any	st
JEDRYC	63	x	m	0	0	all	-	all	Eu:est	1980	CC	1630	n	bl	y	n	0	ev	cig+/-ot	nev any	st
JEDRYC	68	x	f	0	0	all	-	all	Eu:est	1980	CC	1630	n	bl	y	n	0	ev	cig+/-ot	nev any	st
JIANG	1		m	0	0	all	-	all	As:Chi	1984	CC	125	n	ot	n	n	0	ev	all/unsp	nev any	st
JIANG	2		f	0	0	all	-	all	As:Chi	1984	CC	125	n	ot	n	n	0	ev	all/unsp	nev any	st
JOLY	18		m	0	0	all	-	all	SCAmer	1978	CC	826	n	bl	n	n	0	cu	all/unsp	nev any	st
JOLY	15		f	0	0	all	-	all	SCAmer	1978	CC	826	n	bl	n	n	0	cu	cig+/-ot	nev any	st
JUSSAW	3	x	m	0	0	all	-	all	As:Ind	1964	CC	792	n	V	n	n	0	ev	all/unsp	nev any	st
KAISE2	68		m	35	99	all	9	all	NAmer	1979	pr	318	n	bl	n	n	1	cu	cig only	nev any	st
KAISE2	60		f	35	99	all	9	all	NAmer	1979	pr	318	n	bl	n	n	1	cu	cig only	nev any	st
KAISER	12		m	0	0	all	0	all	NAmer	1964	pr	714	n	bl	n	n	2	cu	cig+/-ot	nev cigs	ot
KAISER	9		f	0	0	all	0	all	NAmer	1964	pr	714	n	bl	n	n	2	cu	cig+/-ot	nev cigs	ot
KANELL	5	x	m	0	0	all	-	all	Eu:bal	1950	CC	862	n	bl	n	n	0	cu	all/unsp	nev any	st
KATSOU	6	x	f	0	0	all	-	all	Eu:bal	1987	CC	101	n	bl	n	n	0	cu	all/unsp	nev any	st
KAUFMA	7	x	c	0	0	all	-	all	NAmer	1981	CC	881	n	bl	n	n	0	cu	cig+/-ot	nev cigs	st
KELLER	1		m	0	0	wh	-	all	NAmer	1985	CC	15038	n	bl	n	n	0	cu	all/unsp	nev any	st
KELLER	9		m	0	0	nonw	-	all	NAmer	1985	CC	15038	n	bl	n	n	0	cu	all/unsp	nev any	st
KELLER	5		f	0	0	wh	-	all	NAmer	1985	CC	15038	n	bl	n	n	0	cu	all/unsp	nev any	st
KELLER	13		f	0	0	nonw	-	all	NAmer	1985	CC	15038	n	bl	n	n	0	cu	all/unsp	nev any	st
KHUDER	19		m	0	0	all	-	all	NAmer	1985	CC	482	n	bl	n	y	0	cu	cig+/-ot	nev cigs	or
KIHARA	7		c	0	0	jap	-	all	As:Jap	1991	CC	440	n	bl	n	n	0	cu	all/unsp	nev any	st
KINLEN	9	x	m	0	0	all	0	all	Eu:UK	1967	pr	718	n	V	n	n	0	cu	all/unsp	nev any	st
KJUUS	1		m	0	0	all	-	all	Eu:Sca	1979	CC	176	n	bl	n	n	0	cu	all/unsp	nev any	st
KNEKT	15	x	m	20	69	all	21	all	Eu:Sca	1966	pr	515	n	bl	n	n	0	cu	all/unsp	nev any	st
KO	1		f	0	0	all	-	all	As:oth	1992	CC	117	n	ot	n	y	3	ev	cig+/-ot	nev cigs	or
KOHLME	1	x	c	0	0	all	-	all	Eu:Ger	1990	CC	239	n	bl	n	n	0	ev	all/unsp	nev any	st
KOO	9		f	0	0	all	-	all	As:HK	1981	CC	200	n	bl	n	n	0	cu	all/unsp	nev any	st
KOULUM	1		m	0	0	all	-	all	Eu:Sca	1936	CC	812	n	bl	n	n	0	ev	all/unsp	nev any	st
KREUZE	40		f	1	45	all	-	all	Eu:Ger	1990	CC	2260	n	bl	n	n	0	cu	all/unsp	nev any	st
KREUZE	42		f	55	69	all	-	all	Eu:Ger	1990	CC	2260	n	bl	n	n	0	cu	all/unsp	nev any	st
KREYBE	24	x	m	0	0	all	-	all	Eu:Sca	1948	CC	300	n	bl	n	y	0	ev	all/unsp	nev any	st
KREYBE	39	x	f	0	0	all	-	all	Eu:Sca	1948	CC	300	n	bl	n	y	0	ev	all/unsp	nev any	st
KUBIK	12		m	0	0	all	0	all	Eu:est	1965	pr	108	n	bl	n	n	0	cu	cig+/-ot	nev any	st
LAMTH	6		f	0	0	ch	-	all	As:HK	1983	CC	445	n	bl	n	n	0	ev	all/unsp	nev any	or
LAMWK	1		f	0	0	ch	-	all	As:HK	1981	CC	163	n	bl	n	n	0	ev	all/unsp	nev any	st
LAMWK2	9		m	0	0	all	-	q+s+l+a	As:HK	1976	CC	480	n	bl	n	n	0	ev	all/unsp	nev any	st
LAMWK2	10		f	0	0	all	-	q+s+l+a	As:HK	1976	CC	480	n	bl	n	n	0	ev	all/unsp	nev any	st
LANGE	32	x	m	0	0	all	0	all	Eu:Sca	1976	pr	268	n	bl	n	n	0	cu	all/unsp	nev any	st
LANGE	29	x	f	0	0	all	0	all	Eu:Sca	1976	pr	268	n	bl	n	n	0	cu	all/unsp	nev any	st
LAUSSM	10	x	m	0	0	all	-	all	Eu:Ger	1982	CC	432	n	bl	n	n	0	ev	all/unsp	nev any	st
LEI	1		m	0	0	all	-	all	As:Chi	1986	CC	792	n	ot	y	n	0	ev	all/unsp	nev any	st
LEI	2		f	0	0	all	-	all	As:Chi	1986	CC	792	n	ot	y	n	0	ev	all/unsp	nev any	st
LEMARC	2		c	0	0	w+o	-	all	NAmer	1992	CC	341	n	bl	n	y	0	cu	all/unsp	nev any	st
LETOUR	1		c	0	0	all	-	all	NAmer	1983	CC	738	n	V	y	y	0	ev	cig+/-ot	nev cigs	st
LEVIN	32		m	0	0	all	-	all	NAmer	1938	CC	475	n	bl	n	n	1	ev	all/unsp	nev any	st
LIAW	1		m	0	0	all	0	all	As:oth	1982	pr	127	n	ot	n	n	1	cu	all/unsp	nev any	or
LIAW	2		f	0	0	all	0	all	As:oth	1982	pr	127	n	ot	n	n	1	cu	all/unsp	nev any	or
LIDDEL	4		m	0	0	all	18	all	NAmer	1970	pr	304	m	V	n	n	1	cu	cig+/-ot	nev cigs	ot
LIU	2		c	0	0	all	-	all	As:Chi	1980	CC	229	n	ot	*	n	2	ev	all/unsp	nev any	or
LIU2	1	x	m	0	0	all	-	all	As:Chi	1983	CC	316	n	ot	n	n	0	ev	all/unsp	nev any	st
LIU2	3	x	f	0	0	all	-	all	As:Chi	1983	CC	316	n	ot	n	n	0	ev	all/unsp	nev any	st
LIU3	1	x	m	0	0	all	-	all	As:Chi	1985	CC	110	n	ot	n	n	0	ev	all/unsp	nev any	st
LIU4	11		m	0	0	all	-	all	As:Chi	1986	CC	1000-00	n	ot	y	n	2	ev	all/unsp	nev any	ot
LIU4	12		f	0	0	all	-	all	As:Chi	1986	CC	1000-00	n	ot	y	n	2	ev	all/unsp	nev any	ot
LIU5	1		c	0	0	all	-	all	As:Chi	1978	CC	111	n	ot	y	n	0	ev	all/unsp	nev any	st
LOMBA2	1		f	0	0	all	-	all	NAmer	1960	CC	225	n	bl	n	n	0	ev	cig+/-ot	nev cigs	st
LOMBAR	9		m	0	0	all	-	all	NAmer	1951	CC	1040	n	bl	n	n	0	cu	cig+/-ot	nev any	st
LUBIN2	25	x	m	0	0	all	-	all	Eu:mul	1976	CC	7804	n	bl	n	y	0	cu	all/unsp	nev any	st
LUBIN2	317		f	0	0	all	-	all	Eu:mul	1976	CC	7804	n	bl	n	y	0	cu	cig+/-ot	nev any	st
LUO	1	x	c	0	0	all	-	all	As:Chi	1990	CC	102	n	ot	n	y	0	ev	cig+/-ot	nev cigs	st
MACLEN	19		m	0	0	ch	-	all	As:oth	1972	CC	233	n	bl	n	n	0	cu	cig+/-ot	nev cigs	st
MACLEN	32		f	0	0	ch	-	all	As:oth	1972	CC	233	n	bl	n	n	0	cu	cig+/-ot	nev cigs	st

Table 1C2 - 4

IESLC - Meta-anal of Current Smoking (or Ever if Current not available), Any prod (or Cigs if Any not avail)
All LC types
Least adjusted

REF	NRR	X	SEX	AGEL	AGEH	RACE	YF	LC TYPE	LOC	START	ST	NLC	R	VB	P	H	AD	SM	PRODUCT	DENOM	De
MAGNUS	1	x	m	0	0	all	0	all	Eu:Sca	1953	pr	203	o	bl	y	n	0	ev	all/unsp	nev any	st
MARSH	2	x	m	0	0	all	-	all	NAmer	1979	CC	150	n	bl	y	n	0	ev	all/unsp	nev any	st
MARSH	4	x	f	0	0	all	-	all	NAmer	1979	CC	150	n	bl	y	n	0	ev	all/unsp	nev any	st
MARSH2	2	x	c	0	0	all	-	all	NAmer	1979	CC	114	n	bl	y	n	0	ev	all/unsp	nev any	st
MARTIS	4		m	0	0	all	-	all	Eu:UK	1972	CC	201	n	V	n	n	0	ev	cig+/-ot	nev cigs	st
MASTRA	1	x	m	0	0	all	-	all	Eu:wst	1973	CC	309	n	bl	n	n	0	ev	all/unsp	nev any	st
MATOS	2	x	m	0	0	all	-	all	SCAmer	1994	CC	200	n	bl	n	n	0	cu	cig+/-ot	nev any	st
MATSUD	10		m	0	0	all	-	all	As:Jap	1965	CC	179	n	bl	n	n	0	ev	cig+/-ot	nev cigs	st
MCCONN	1		m	0	0	all	-	all	Eu:UK	1946	CC	100	n	V	n	y	0	ev	all/unsp	nev any	st
MCCONN	2		f	0	0	all	-	all	Eu:UK	1946	CC	100	n	V	n	y	0	ev	all/unsp	nev any	st
MCDUFF	1		m	0	0	all	-	all	NAmer	1979	CC	165	n	V	y	n	0	ev	cig+/-ot	nev cigs	st
MCLAUG	1		m	0	0	all	-	all	As:Chi	1972	CC	316	o	ot	y	n	0	ev	all/unsp	nev any	st
MIGRAN	19	x	m	0	0	all	0	all	Eu:UK	1964	pr	259	n	V	n	n	0	cu	all/unsp	nev any	st
MIGRAN	135	x	f	0	0	all	0	all	Eu:UK	1964	pr	259	n	V	n	n	0	cu	all/unsp	nev any	st
MILLER	1	x	f	0	0	all	-	all	NAmer	1972	CC	168	n	bl	y	n	0	ev	cig+/-ot	nev any	st
MILLS	3		m	0	0	wh	-	all	NAmer	1940	CC	444	n	bl	y	n	1	ev	all/unsp	nev any	ot
MRFITR	2		m	0	0	all	0	all	NAmer	1973	pr	119	n	bl	n	n	0	cu	cig+/-ot	nev cigs	ot
NAM	68	x	m	0	0	all	-	all	NAmer	1986	CC	1199	n	bl	y	n	0	cu	cig+/-ot	nev cigs	ot
NAM	84	x	f	0	0	all	-	all	NAmer	1986	CC	1199	n	bl	y	n	0	cu	cig+/-ot	nev cigs	ot
NOTAN2	1	x	m	0	0	all	-	all	As:Ind	1963	CC	683	n	V	n	n	0	ev	all/unsp	nev any	st
NOU	11		m	30	64	all	-	all	Eu:Sca	1971	CC	273	n	bl	y	n	0	ev	all/unsp	nev any	st
NOU	12		f	30	64	all	-	all	Eu:Sca	1971	CC	273	n	bl	y	n	0	ev	all/unsp	nev any	st
ODRISC	1		c	0	0	all	-	all	Eu:UK	1992	CC	446	n	V	n	n	0	cu	all/unsp	nev any	st
ORMOS	4		m	0	0	all	-	all	Eu:est	1947	CC	119	n	bl	y	y	0	ev	cig+/-ot	nev any	st
ORMOS	26		f	0	0	all	-	all	Eu:est	1947	CC	119	n	bl	y	y	0	ev	cig+/-ot	nev any	st
OSANN	9	x	m	0	0	all	-	all	NAmer	1984	CC	1986	n	bl	n	n	0	cu	cig+/-ot	nev cigs	st
OSANN	13	x	f	0	0	all	-	all	NAmer	1984	CC	1986	n	bl	n	n	0	cu	cig+/-ot	nev cigs	st
PARKIN	27	x	m	0	0	bl	-	all	Africa	1963	CC	877	n	V	y	n	0	cu	all/unsp	nev any	st
PASTOR	5	x	m	0	0	all	-	all	Eu:wst	1976	CC	204	n	bl	y	n	0	ev	all/unsp	nev any	st
PAWLEG	1	x	m	0	0	all	-	all	Eu:est	1992	CC	176	n	bl	n	y	0	ev	all/unsp	nev any	st
PERNU	2		m	0	0	all	-	all	Eu:Sca	1944	CC	1606	n	bl	n	n	0	ev	all/unsp	nev any	st
PERNU	1		f	0	0	all	-	all	Eu:Sca	1944	CC	1606	n	bl	n	n	0	ev	all/unsp	nev any	st
PERSH2	4	x	c	0	0	all	-	all	Eu:Sca	1980	CC	1022	n	bl	y	n	0	cu	all/unsp	nev any	st
PETO	4		m	0	0	all	0	all	Eu:UK	1954	pr	103	n	V	n	n	0	cu	all/unsp	nev any	st
PEZZO2	2		m	0	0	all	-	all	SCAmer	1992	CC	367	n	bl	n	y	0	cu	cig+/-ot	nev cigs	st
PEZZOT	5		m	0	0	all	-	all	SCAmer	1987	CC	215	n	bl	n	y	0	cu	cig only	nev cigs	st
PIKE	4		m	0	0	w-hi	-	all	NAmer	1972	CC	731	n	bl	y	n	0	ev	all/unsp	nev any	st
PIKE	8		f	0	0	w-hi	-	all	NAmer	1972	CC	731	n	bl	y	n	0	ev	all/unsp	nev any	st
POFFIJ	1		c	0	0	all	-	all	Eu:mul	1990	CC	971	n	bl	n	n	0	ev	all/unsp	nev any	st
POLEDN	3	x	c	0	0	all	-	all	NAmer	1978	CC	209	n	bl	y	n	0	ev	cig+/-ot	nev cigs	st
QIAO2	2	x	m	0	0	all	0	all	As:Chi	1992	pr	241	m	ot	n	n	0	cu	all/unsp	nev any	st
RACHTA	2	x	f	0	0	all	-	all	Eu:est	1991	CC	118	n	bl	n	y	0	cu	cig+/-ot	nev cigs	st
RADZIK	1		c	0	0	all	-	all	Eu:est	1986	CC	189	n	bl	n	n	0	ev	all/unsp	nev any	st
RANDIG	23		m	0	0	all	-	all	Eu:Ger	1951	CC	448	n	bl	n	n	0	ev	all/unsp	nev any	st
RANDIG	24		f	0	0	all	-	all	Eu:Ger	1951	CC	448	n	bl	n	n	0	ev	all/unsp	nev any	st
REN	1		m	0	0	all	-	all	As:Chi	1980	CC	244	n	ot	*	n	0	ev	all/unsp	nev any	st
REN	2		f	0	0	all	-	all	As:Chi	1980	CC	244	n	ot	*	n	0	ev	all/unsp	nev any	st
RONCO	1		m	0	0	all	-	all	Eu:wst	1976	CC	126	n	bl	y	n	0	ev	all/unsp	nev any	st
ROTHSC	1	x	c	0	0	all	-	all	NAmer	1971	CC	284	n	bl	y	n	0	ev	all/unsp	nev any	st
SADOWS	7	x	m	0	0	wh	-	all	NAmer	1938	CC	477	n	bl	n	n	0	ev	all/unsp	nev any	st
SANKAR	1	x	m	0	0	all	-	all	As:Ind	1990	CC	281	n	V	n	n	0	ev	all/unsp	nev any	st
SCHWAR	25		m	0	0	wh	-	all	NAmer	1984	CC	5588	n	bl	y	y	0	cu	cig+/-ot	nev cigs	st
SCHWAR	26		m	0	0	bl	-	all	NAmer	1984	CC	5588	n	bl	y	y	0	cu	cig+/-ot	nev cigs	st
SCHWAR	27		f	0	0	wh	-	all	NAmer	1984	CC	5588	n	bl	y	y	0	cu	cig+/-ot	nev cigs	st
SCHWAR	28		f	0	0	bl	-	all	NAmer	1984	CC	5588	n	bl	y	y	0	cu	cig+/-ot	nev cigs	st
SEGI	1		m	0	0	all	-	all	As:Jap	1948	CC	159	n	bl	n	n	0	ev	all/unsp	nev any	ot
SEGI2	19	x	m	0	0	all	-	all	As:Jap	1962	CC	378	n	bl	n	n	0	cu	cig+/-ot	nev any	st
SEGI2	27	x	f	0	0	all	-	all	As:Jap	1962	CC	378	n	bl	n	n	0	cu	cig+/-ot	nev any	st
SEOW	1	x	f	0	0	ch	-	q+s+l+a	As:oth	1997	CC	153	n	bl	n	y	0	ev	cig+/-ot	nev cigs	st
SHAW	6		c	0	0	wh	-	all	NAmer	1988	CC	335	n	V	n	y	0	cu	all/unsp	nev any	st
SIEMIA	9	x	m	0	0	all	-	all	NAmer	1979	CC	857	n	V	y	y	0	ev	cig+/-ot	nev cigs	st
SIMARA	5	x	m	0	0	all	-	all	As:oth	1971	CC	115	n	bl	n	n	0	ev	cig+/-ot	nev cigs	st
SIMARA	6	x	f	0	0	all	-	all	As:oth	1971	CC	115	n	bl	n	n	0	ev	cig+/-ot	nev cigs	st
SOBUE	90	x	m	0	0	all	-	q+s+l+a	As:Jap	1986	CC	1376	n	bl	n	y	0	cu	cig+/-ot	nev cigs	st
SOBUE	94	x	f	0	0	all	-	q+s+l+a	As:Jap	1986	CC	1376	n	bl	n	y	0	cu	cig+/-ot	nev cigs	st
SOBUE2	10		m	0	0	all	-	q+s+l+a	As:Jap	1965	CC	2083	n	bl	n	n	2	cu	cig+/-ot	nev any	ot
SOBUE2	12		f	0	0	all	-	q+s+l+a	As:Jap	1965	CC	2083	n	bl	n	n	2	cu	cig+/-ot	nev any	ot
SPEIZE	6	x	f	0	0	all	0	all	NAmer	1976	pr	593	n	bl	n	y	0	cu	cig+/-ot	nev cigs	st
SPITZ	2		c	0	0	b+hi	-	all	NAmer	1992	CC	177	n	bl	n	y	0	cu	cig+/-ot	nev cigs	st
STASZE	1		m	0	0	all	-	all	Eu:est	1954	CC	281	n	bl	n	y	0	ev	all/unsp	nev any	st
STASZE	5		f	0	0	all	-	all	Eu:est	1954	CC	281	n	bl	n	y	0	ev	all/unsp	nev any	st
STAYNE	1		m	0	0	all	-	all	NAmer	1969	CC	420	n	bl	n	n	0	ev	all/unsp	nev any	st

Table 1C2 - 4

IESLC - Meta-anal of Current Smoking (or Ever if Current not available), Any prod (or Cigs if Any not avail)
All LC types
Least adjusted

REF	NRR	X	SEX	AGEL	AGEH	RACE	YF	LC TYPE	LOC	START	ST	NLC	R	VB	P	H	AD	SM	PRODUCT	DENOM	De
STOCKS	31	x	m	0	0	all	-	all	Eu:UK	1952	CC	2932	n	V	y	n	0	ev	all/unsp	nev any	st
STOCKS	50		f	0	0	all	-	all	Eu:UK	1952	CC	2932	n	V	y	n	1	ev	cig+/-ot	nev any	ot
STOCKW	7		c	0	0	all	-	all	NAmer	1981	CC	22161	n	bl	n	n	0	cu	cig+/-ot	nev any	st
STUCKE	2		m	0	0	all	-	all	Eu:wst	1989	CC	247	n	bl	n	y	0	cu	all/unsp	nev any	ot
SUN	1		c	0	0	all	-	all	As:Chi	1992	CC	207	n	ot	n	y	0	ev	all/unsp	nev any	st
SUZUK2	2	x	c	0	0	all	-	all	SCAmer	1991	CC	123	n	bl	n	y	0	cu	all/unsp	nev any	st
SVENSS	61	x	f	0	0	all	-	all	Eu:Sca	1983	CC	210	n	bl	n	n	0	cu	all/unsp	nev any	st
TANG	1		c	0	0	all	-	not s	NAmer	1992	CC	119	n	bl	n	y	0	cu	cig+/-ot	nev cigs	st
TENKAN	24		m	0	0	all	17	all	Eu:Sca	1962	pr	242	n	bl	n	n	1	cu	all/unsp	nev any	ot
TIZZAN	5		m	0	0	all	-	all	Eu:wst	1959	CC	1358	n	bl	n	n	0	cu	all/unsp	nev any	st
TIZZAN	13		f	0	0	all	-	all	Eu:wst	1959	CC	1358	n	bl	n	n	0	cu	all/unsp	nev any	st
TOKARS	1		m	0	0	all	-	all	Eu:est	1966	ot	162	o	bl	n	y	0	cu	all/unsp	nev any	st
TOKARS	5		f	0	0	all	-	all	Eu:est	1966	ot	162	o	bl	n	y	0	ev	all/unsp	nev any	st
TOUSEY	4	x	m	0	0	all	-	all	NAmer	1993	CC	507	n	bl	y	y	0	cu	cig+/-ot	nev any	st
TOUSEY	8	x	f	0	0	all	-	all	NAmer	1993	CC	507	n	bl	y	y	0	cu	cig+/-ot	nev any	st
TSUGAN	28		m	0	0	all	-	q+a	As:Jap	1976	CC	134	n	bl	n	y	0	cu	all/unsp	nev any	st
TULINI	14	x	m	0	0	all	0	all	Eu:Sca	1967	pr	472	n	bl	n	n	1	cu	all/unsp	nev any	ot
TULINI	20	x	f	0	0	all	0	all	Eu:Sca	1967	pr	472	n	bl	n	n	1	cu	all/unsp	nev any	ot
TVERDA	5		m	0	0	all	0	all	Eu:Sca	1972	pr	238	n	bl	n	n	2	cu	cig+/-ot	nev cigs	ot
TVERDA	15		f	0	0	all	0	all	Eu:Sca	1972	pr	238	n	bl	n	n	2	cu	cig only	nev cigs	ot
WAKAI	2	x	m	0	0	all	-	all	As:Jap	1988	CC	333	n	bl	n	y	0	cu	all/unsp	nev any	st
WAKAI	20	x	f	0	0	all	-	all	As:Jap	1988	CC	333	n	bl	n	y	0	cu	all/unsp	nev any	st
WALD	2	x	m	0	0	all	0	all	Eu:UK	1975	pr	102	n	V	n	n	0	cu	cig only	nev any	st
WANG	1	x	m	0	0	all	-	all	As:Chi	1990	CC	390	n	ot	*	y	0	ev	all/unsp	nev any	or
WANG	2	x	f	0	0	all	-	all	As:Chi	1990	CC	390	n	ot	*	y	0	ev	all/unsp	nev any	or
WANG2	17	x	c	0	0	all	-	all	As:Chi	1980	CC	103	n	ot	n	n	0	cu	cig+/-ot	nev cigs	st
WANG3	1		c	0	0	all	-	all	As:Chi	1981	CC	293	n	ot	*	n	0	ev	all/unsp	nev any	st
WANG4	1	x	m	0	0	all	-	all	As:Chi	1976	CC	1170	n	ot	y	n	0	ev	all/unsp	nev any	st
WICKLU	1		m	0	0	wh	-	all	NAmer	1968	CC	155	n	bl	y	n	0	ev	cig+/-ot	nev+3	or
WIGLE	3	x	m	0	0	all	-	all	NAmer	1971	CC	728	n	V	n	n	0	cu	all/unsp	nev any	st
WIGLE	6	x	f	0	0	all	-	all	NAmer	1971	CC	728	n	V	n	n	0	cu	all/unsp	nev any	st
WILKIN	1	x	m	0	0	all	-	all	Eu:UK	1992	CC	271	n	V	n	n	0	ev	cig+/-ot	nev cigs	st
WILKIN	2	x	f	0	0	all	-	all	Eu:UK	1992	CC	271	n	V	n	n	0	ev	cig+/-ot	nev cigs	st
WU	34	x	f	0	0	wh	-	q+a	NAmer	1981	CC	220	n	bl	n	y	0	cu	all/unsp	nev any	st
WUNSCH	2	x	m	0	0	all	-	all	SCAmer	1990	CC	398	n	bl	y	n	0	cu	cig+/-ot	nev any	st
WUNSCH	8	x	f	0	0	all	-	all	SCAmer	1990	CC	398	n	bl	y	n	0	cu	cig+/-ot	nev any	st
WUWILL	6	x	f	0	0	all	-	all	As:Chi	1985	CC	965	n	ot	n	n	0	ev	cig+/-ot	nev cigs	st
WYNDE2	21		m	0	0	all	-	all	NAmer	1962	CC	404	n	bl	n	y	0	ev	all/unsp	nev any	st
WYNDE3	50		m	0	0	all	-	all	NAmer	1966	CC	350	n	bl	n	y	0	cu	all/unsp	nev any	st
WYNDE3	138		f	0	0	all	-	all	NAmer	1966	CC	350	n	bl	n	y	0	ev	all/unsp	nev any	st
WYNDE4	48		m	0	0	all	-	all	NAmer	1948	CC	684	n	bl	y	n	0	ev	all/unsp	nev any	st
WYNDE4	62		f	0	0	all	-	all	NAmer	1948	CC	684	n	bl	y	n	2	ev	all/unsp	nev any	ot
WYNDE6	18		m	0	0	all	-	all	NAmer	1969	CC	4423	n	bl	n	y	0	cu	cig+/-ot	nev any	st
WYNDE6	207		f	0	0	all	-	all	NAmer	1969	CC	4423	n	bl	n	y	0	cu	cig+/-ot	nev cigs	st
XIANGZ	8	x	m	0	0	all	0	all	As:Chi	1976	pr	983	m	ot	n	n	0	ev	all/unsp	nev any	st
XU	1	x	m	0	0	all	-	all	As:Chi	1985	CC	729	n	ot	n	n	0	ev	all/unsp	nev any	st
XU2	1	x	c	0	0	all	-	all	As:Chi	1987	CC	610	o	ot	y	n	0	ev	all/unsp	nev any	st
XU3	1	x	m	0	0	all	-	all	As:Chi	1981	CC	135	n	ot	n	n	0	ev	all/unsp	nev any	st
XU3	3	x	f	0	0	all	-	all	As:Chi	1981	CC	135	n	ot	n	n	0	ev	all/unsp	nev any	st
XU4	1		c	0	0	all	-	all	As:Chi	1981	CC	206	n	ot	*	n	0	ev	all/unsp	nev any	st
YAMAGU	1	x	c	0	0	all	-	all	As:Jap	1989	CC	144	n	bl	n	y	0	cu	all/unsp	nev any	st
YONG	12		m	0	0	all	0	all	NAmer	1971	pr	216	n	bl	n	n	1	cu	cig+/-ot	nev cigs	or
YONG	15		f	0	0	all	0	all	NAmer	1971	pr	216	n	bl	n	n	1	cu	cig+/-ot	nev cigs	or
YUAN	1		m	0	0	all	0	all	As:Chi	1986	pr	142	n	ot	n	n	2	ev	cig+/-ot	nev cigs	ot
ZHANG	1	x	c	0	0	all	-	all	As:Chi	1988	CC	100	n	ot	n	n	0	ev	all/unsp	nev any	st
ZHENG	15		m	0	0	all	-	all	As:Chi	1982	CC	540	n	ot	*	y	0	ev	cig+/-ot	nev cigs	st
ZHENG	24		f	0	0	all	-	all	As:Chi	1982	CC	540	n	ot	*	y	0	ev	cig+/-ot	nev cigs	st
ZHOU	2		m	0	0	all	-	all	As:Chi	1978	CC	1360	n	ot	n	n	0	ev	all/unsp	nev any	st
ZHOU	3		f	0	0	all	-	all	As:Chi	1978	CC	1360	n	ot	n	n	0	ev	all/unsp	nev any	st

Cigarette type is all/unspec for all RRs
except for the following:

REF	NRR	CIGTYPE
ALDERS	177	MC+-HR
ALDERS	176	MC only
DEAN3	117	MC only

Table 1C2 - 5

IESLC - Meta-anal of Current Smoking (or Ever if Current not available), Any prod (or Cigs if Any not avail)
All LC types
Least adjusted

REF	NRR	SEX	AD	Number Exposed Case	Number Exposed Cont	Non-exposed Case	Non-exposed Cont	RR	95.00%CI
ABELIN	1	m	0	116	341	2	183	31.13	(7.60- 127.40)
*ABRAHA	7	m	0	269	10351	10	3365	8.74	(4.66- 16.42)
*ABRAHA	8	f	0	62	5256	28	11589	4.88	(3.13- 7.62)
Subtotal ABRAHA								5.93	(4.12- 8.53)
AGUDO	10	f	0	20	17	80	183	2.69	(1.34- 5.41)
*AKIBA	2	m	0	345	171379	18	35833	4.01	(2.50- 6.44)
*AKIBA	6	f	0	74	51237	116	359850	4.48	(3.35- 6.00)
Subtotal AKIBA								4.35	(3.39- 5.57)
ALDERS	177	m	0	519	322	15	133	14.29	(8.23- 24.81)
ALDERS	176	f	0	410	229	75	243	5.80	(4.27- 7.87)
Subtotal ALDERS								7.17	(5.49- 9.36)
*AMANDU	1	m	0	115	96708	6	25350	5.02	(2.21- 11.41)
AMES	1	m	0	150	136	15	62	4.56	(2.48- 8.39)
*ANDERS	2	f	0	212	41262	46	195158	21.80	(15.85- 29.98)
*ARCHER	5	m	0	122	32529	6	9842	6.15	(2.71- 13.96)
ARMADA	27	m	0	188	122	4	64	24.66	(8.75- 69.44)
AUSTIN	2	c	0	111	125	5	88	15.63	(6.13- 39.87)
AUVINE	1	c	0	473	288	44	229	8.55	(6.00- 12.18)
AXELSO	1	c	0	90	86	62	371	6.26	(4.20- 9.34)
AXELSS	2	m	0	194	130	16	160	14.92	(8.53- 26.12)
AXELSS	10	f	0	96	69	18	154	11.90	(6.68- 21.22)
Subtotal AXELSS								13.38	(8.95- 20.00)
BAND	1	m	2	-	-	-	-	9.96	(7.38- 13.44)
BARBON	3	m	0	562	362	22	188	13.27	(8.37- 21.04)
BECHER	13	m	0	101	122	3	54	14.90	(4.52- 49.09)
BECHER	14	f	0	33	26	10	52	6.60	(2.82- 15.44)
Subtotal BECHER								8.68	(4.35- 17.35)
*BENSHL	16	m	1	-	-	-	-	8.18	(3.62- 18.51)
*BEST	2	m	1	-	-	-	-	14.91	(7.05- 31.52)
*BEST	18	f	1	-	-	-	-	2.24	(0.59- 8.44)
Subtotal BEST								9.45	(4.92- 18.15)
BLOHMK	1	m	0	419	313	126	301	3.20	(2.48- 4.12)
BLOT4	1	m	0	327	245	8	87	14.51	(6.91- 30.51)
BOFFET	32	m	0	5504	5505	117	1750	14.95	(12.36- 18.10)
*BOUCOT	2	m	0	85	22177	0	7551	58.23~	(3.61- 938.34)
BRESLO	37	m	0	486	451	7	42	6.47	(2.88- 14.54)
BRESLO	38	f	0	13	11	12	14	1.38	(0.45- 4.20)
Subtotal BRESLO								3.79	(1.97- 7.29)
*BRETT	4	m	0	135	37448	6	6530	3.92	(1.73- 8.88)
BROCKM	1	m	0	87	81	2	2	1.07	(0.15- 7.80)
BROCKM	2	f	0	24	54	4	18	2.00	(0.61- 6.54)
Subtotal BROCKM								1.70	(0.61- 4.70)
BROSS	11	m	0	690	638	38	170	4.84	(3.35- 6.99)
BROWN2	12	m	2	-	-	-	-	11.30	(10.20- 12.40)
BROWN2	11	f	2	-	-	-	-	13.60	(12.30- 15.10)
Subtotal BROWN2								12.34	(11.50- 13.25)
BUFFLE	3	m	0	257	219	5	47	11.03	(4.31- 28.22)
BUFFLE	7	f	0	313	183	41	198	8.26	(5.63- 12.11)
Subtotal BUFFLE								8.61	(6.04- 12.27)
CARPEN	9	c	0	219	162	15	241	21.72	(12.41- 38.01)
CASCO2	1	c	0	149	212	6	98	11.48	(4.90- 26.87)
CASCOR	1	c	0	365	362	22	295	13.52	(8.56- 21.35)
*CEDERL	106	m	2	-	-	-	-	7.72	(5.01- 11.89)
*CEDERL	75	f	2	-	-	-	-	4.82	(3.38- 6.88)
Subtotal CEDERL								5.83	(4.43- 7.67)
CHAN	9	m	0	206	161	2	43	27.51	(6.57- 115.26)
CHAN	10	f	0	105	50	84	139	3.48	(2.26- 5.35)
Subtotal CHAN								4.13	(2.73- 6.25)
*CHANG	5	m	0	35	419	5	502	8.39	(3.32- 21.21)
*CHANG	11	f	0	30	603	11	1139	5.15	(2.60- 10.21)
Subtotal CHANG								6.12	(3.53- 10.60)
CHATZI	4	c	0	255	365	27	129	3.34	(2.14- 5.21)
CHEN2	1	m	0	121	97	9	33	4.57	(2.09- 10.02)
CHEN2	2	f	0	38	30	25	33	1.67	(0.82- 3.39)
Subtotal CHEN2								2.62	(1.55- 4.44)
CHEN3	1	c	0	182	156	72	98	1.59	(1.09- 2.30)
CHIAZZ	2	m	0	139	209	4	47	7.81	(2.75- 22.18)
CHOI	3	m	0	232	329	13	95	5.15	(2.82- 9.42)
CHOI	7	f	0	13	23	76	164	1.22	(0.59- 2.54)
Subtotal CHOI								2.88	(1.81- 4.58)
*CHOW	25	m	0	167	124415	6	62913	14.07	(6.23- 31.78)

Table 1C2 - 5

IESLC - Meta-anal of Current Smoking (or Ever if Current not available), Any prod (or Cigs if Any not avail)
All LC types
Least adjusted

REF	NRR	SEX	AD	Number Exposed Case	Number Exposed Cont	Non-exposed Case	Non-exposed Cont	RR	95.00%CI
*CHYOU	4	m	0	181	3470	13	2406	9.65	(5.51- 16.91)
COMSTO	3	m	0	105	100	4	69	18.11	(6.37- 51.48)
COMSTO	8	f	0	77	52	13	115	13.10	(6.68- 25.67)
Subtotal COMSTO								14.40	(8.18- 25.36)
COOKSO	5	c	0	189	39	45	61	6.57	(3.92- 11.02)
CORREA	41	c	0	943	571	51	388	12.56	(9.22- 17.13)
*CPSI	220	m	1	-	-	-	-	11.94	(9.52- 14.97)
*CPSI	279	f	1	-	-	-	-	3.20	(2.53- 4.04)
Subtotal CPSI								6.32	(5.37- 7.43)
*CPSII	36	m	0	1781	583646	124	742207	18.26	(15.23- 21.91)
*CPSII	71	f	0	1014	744217	310	2091302	9.19	(8.09- 10.44)
Subtotal CPSII								11.51	(10.37- 12.78)
DAMBER	14	m	1	-	-	-	-	9.60	(6.60- 14.20)
DARBY	4	m	0	322	453	3	384	90.98	(28.96- 285.90)
DARBY	11	f	0	195	217	23	529	20.67	(13.05- 32.74)
Subtotal DARBY								25.40	(16.57- 38.92)
DAVEYS	5	m	0	90	144	3	23	4.79	(1.40- 16.42)
DAVEYS	6	f	0	0	3	16	83	0.72~	(0.04- 14.66)
Subtotal DAVEYS								3.65	(1.17- 11.42)
DEAN	7	m	0	591	574	12	61	5.23	(2.79- 9.82)
DEAN2	2	m	0	671	600	33	112	3.80	(2.54- 5.68)
DEAN2	6	f	0	59	28	88	121	2.90	(1.71- 4.91)
Subtotal DEAN2								3.43	(2.49- 4.73)
DEAN3	40	m	0	502	1636	25	510	6.26	(4.14- 9.47)
DEAN3	117	f	0	102	1158	41	1538	3.30	(2.28- 4.79)
Subtotal DEAN3								4.39	(3.33- 5.79)
*DEKLER	8	m	2	-	-	-	-	23.03	(3.21- 164.97)
DESTE2	2	c	0	216	151	20	108	7.72	(4.59- 13.00)
DESTEF	40	m	0	362	226	27	163	9.67	(6.23- 15.01)
*DOCKER	1	c	4	-	-	-	-	8.00	(2.97- 21.60)
DOLL	90	m	0	1280	1172	7	61	9.52	(4.34- 20.89)
DOLL	93	f	0	58	41	40	59	2.09	(1.18- 3.68)
Subtotal DOLL								3.51	(2.21- 5.55)
*DOLL2	54	m	1	-	-	-	-	10.99	(6.97- 17.36)
*DOLL2	63	f	1	-	-	-	-	8.65	(2.93- 25.55)
Subtotal DOLL2								10.60	(6.96- 16.14)
DORANT	2	m	0	332	697	7	159	10.82	(5.02- 23.32)
DORGAN	9	m	0	464	170	15	93	16.92	(9.54- 30.01)
DORGAN	33	m	0	214	61	3	35	40.93	(12.17- 137.66)
DORGAN	56	f	0	611	119	103	244	12.16	(8.99- 16.46)
DORGAN	79	f	0	68	17	7	20	11.43	(4.16- 31.43)
Subtotal DORGAN								13.62	(10.58- 17.54)
*DORN	51	m	1	-	-	-	-	8.23	(6.55- 10.35)
DOSEME	17	m	0	1068	536	142	293	4.11	(3.28- 5.15)
DROSTE	2	m	0	379	267	7	93	18.86	(8.61- 41.30)
DU	1	m	0	538	-	28	-	3.53	(2.44- 5.11)
DU	2	f	0	191	-	92	-	1.93	(1.30- 2.87)
Subtotal DU								2.66	(2.03- 3.49)
*DUNN	6	m	0	137	52634	2	14160	18.43	(4.56- 74.42)
EBELIN	1	m	0	101	142	12	117	6.93	(3.63- 13.24)
*ENGELA	154	m	0	100	6636	7	2683	5.78	(2.69- 12.41)
*ENGELA	161	f	0	13	2674	13	10708	4.00	(1.86- 8.63)
Subtotal ENGELA								4.81	(2.80- 8.27)
*ENSTRO	1	m	1	-	-	-	-	12.99	(10.46- 16.13)
*ENSTRO	2	f	1	-	-	-	-	6.95	(6.01- 8.04)
Subtotal ENSTRO								8.44	(7.48- 9.53)
ESAKI	4	m	0	155	143	16	28	1.90	(0.99- 3.65)
ESAKI	5	f	0	34	19	40	55	2.46	(1.23- 4.92)
Subtotal ESAKI								2.14	(1.33- 3.45)
FAN	1	m	0	216	498	36	236	2.84	(1.93- 4.18)
FAN	2	f	0	82	97	69	320	3.92	(2.65- 5.81)
Subtotal FAN								3.33	(2.53- 4.38)
GAO	29	m	0	529	438	62	202	3.93	(2.88- 5.37)
GAO	30	f	0	170	100	435	605	2.36	(1.79- 3.12)
Subtotal GAO								2.96	(2.41- 3.64)
GAO2	1	m	0	184	117	13	56	6.77	(3.55- 12.93)
GARCIA	2	c	0	169	74	21	139	15.12	(8.86- 25.79)
GARDIN	2	c	0	97	58	5	41	13.71	(5.13- 36.68)
GARSHI	23	m	0	657	782	41	363	7.44	(5.30- 10.44)
GENG	1	m	0	92	68	7	31	5.99	(2.49- 14.42)
GENG	2	f	0	126	75	67	118	2.96	(1.96- 4.48)

Table 1C2 - 5

IESLC - Meta-anal of Current Smoking (or Ever if Current not available), Any prod (or Cigs if Any not avail)
All LC types
Least adjusted

REF	NRR	SEX	AD	Number Exposed Case	Number Exposed Cont	Non-exposed Case	Non-exposed Cont	RR	95.00%CI
Subtotal GENG								3.36	(2.31- 4.89)
GER	17	c	0	90	318	51	246	1.37	(0.93- 2.00)
GODLEY	5	m	1	-	-	-	-	6.84	(5.60- 8.35)
GODLEY	6	f	1	-	-	-	-	5.54	(4.29- 7.15)
Subtotal GODLEY								6.31	(5.39- 7.39)
GOLLED	21	m	0	380	1966	15	490	6.31	(3.73- 10.68)
GOODMA	2	m	0	148	169	10	199	17.43	(8.90- 34.14)
GOODMA	6	f	0	58	56	19	177	9.65	(5.30- 17.56)
Subtotal GOODMA								12.53	(8.01- 19.60)
GRAHAM	20	m	0	517	1473	18	346	6.75	(4.16- 10.95)
GREGOR	2	m	0	49	53	10	14	1.29	(0.53- 3.18)
GREGOR	6	f	0	17	26	1	22	14.38	(1.77- 116.90)
Subtotal GREGOR								1.88	(0.82- 4.30)
GSELL	8	m	0	148	121	2	29	17.74	(4.15- 75.83)
HAENSZ	54	f	0	69	94	81	236	2.14	(1.43- 3.19)
*HAMMO2	22	m	0	209	4472	5	891	8.33	(3.44- 20.16)
*HAMMON	139	m	1	-	-	-	-	11.52	(6.83- 19.42)
*HANSEN	3	m	2	-	-	-	-	1.53	(0.71- 3.91)
HEGMAN	1	c	0	255	1202	27	2080	16.34	(10.92- 24.45)
*HEIN	5	m	0	132	3492	1	457	17.27	(2.42- 123.25)
*HENNEK	2	m	0	79	2438	23	10919	15.38	(9.69- 24.42)
HINDS	26	f	0	167	592	124	1812	4.12	(3.21- 5.29)
*HIRAYA	1	m	1	-	-	-	-	4.45	(3.60- 5.50)
*HIRAYA	3	f	1	-	-	-	-	2.34	(1.87- 2.92)
Subtotal HIRAYA								3.28	(2.81- 3.82)
HITOSU	2	m	0	117	1597	7	242	2.53	(1.17- 5.50)
HITOSU	9	f	0	28	459	33	1893	3.50	(2.09- 5.85)
Subtotal HITOSU								3.17	(2.07- 4.86)
*HOLE	47	m	0	163	4130	7	1189	6.70	(3.15- 14.25)
*HOLE	29	f	0	13	2144	8	1917	1.45	(0.60- 3.50)
Subtotal HOLE								3.51	(1.98- 6.21)
HOROWI	1	m	0	182	525	19	196	3.58	(2.17- 5.90)
HOROWI	2	f	0	21	382	14	463	1.82	(0.91- 3.62)
Subtotal HOROWI								2.83	(1.89- 4.25)
HORWIT	1	f	0	97	92	11	118	11.31	(5.73- 22.34)
HU	15	m	0	120	94	41	67	2.09	(1.30- 3.35)
HU	16	f	0	26	18	40	48	1.73	(0.83- 3.61)
Subtotal HU								1.98	(1.33- 2.94)
HU2	9	m	0	294	228	49	115	3.03	(2.08- 4.41)
HU2	10	f	0	108	80	72	100	1.88	(1.23- 2.85)
Subtotal HU2								2.44	(1.85- 3.23)
HUANG	1	c	0	98	77	37	58	2.00	(1.20- 3.32)
HUMBLE	13	m	1	-	-	-	-	19.96	(8.27- 48.21)
HUMBLE	15	m	1	-	-	-	-	15.79	(3.43- 72.69)
HUMBLE	17	f	1	-	-	-	-	16.72	(7.44- 37.61)
HUMBLE	19	f	1	-	-	-	-	23.50	(6.79- 81.36)
Subtotal HUMBLE								18.65	(11.23- 30.97)
JAHN	3	f	0	112	67	53	98	3.09	(1.97- 4.85)
JAIN	16	m	0	265	118	12	85	15.91	(8.37- 30.23)
JAIN	11	f	0	305	99	52	214	12.68	(8.68- 18.51)
Subtotal JAIN								13.44	(9.70- 18.62)
JARUP	3	m	0	91	52	11	42	6.68	(3.17- 14.09)
JARVHO	2	m	0	73	29	1	16	40.28	(5.10- 317.77)
JARVHO	6	f	0	31	7	6	21	15.50	(4.56- 52.66)
Subtotal JARVHO								19.86	(6.93- 56.89)
JEDRYC	63	m	0	852	656	49	219	5.80	(4.19- 8.04)
JEDRYC	68	f	0	120	32	78	166	7.98	(4.97- 12.82)
Subtotal JEDRYC								6.43	(4.92- 8.41)
JIANG	1	m	0	93	83	7	17	2.72	(1.08- 6.89)
JIANG	2	f	0	11	6	14	19	2.49	(0.74- 8.35)
Subtotal JIANG								2.63	(1.26- 5.50)
JOLY	18	m	0	487	665	12	218	13.30	(7.35- 24.07)
JOLY	15	f	0	132	96	52	283	7.48	(5.04- 11.12)
Subtotal JOLY								8.94	(6.43- 12.42)
JUSSAW	3	m	0	643	168	149	624	16.03	(12.53- 20.51)
*KAISE2	68	m	1	-	-	-	-	8.04	(4.41- 14.66)
*KAISE2	60	f	1	-	-	-	-	14.48	(7.47- 28.04)
Subtotal KAISE2								10.49	(6.72- 16.36)
*KAISER	12	m	2	-	-	-	-	19.61	(13.32- 28.87)
*KAISER	9	f	2	-	-	-	-	6.53	(4.50- 9.48)
Subtotal KAISER								11.09	(8.48- 14.50)

Table 1C2 - 5

IESLC - Meta-anal of Current Smoking (or Ever if Current not available), Any prod (or Cigs if Any not avail)
All LC types
Least adjusted

REF	NRR	SEX	AD	Number Exposed Case	Number Exposed Cont	Non-exposed Case	Non-exposed Cont	RR	95.00%CI
KANELL	5	m	0	814	441	48	172	6.61	(4.71- 9.30)
KATSOU	6	f	0	45	18	48	67	3.49	(1.80- 6.75)
KAUFMA	7	c	0	621	886	35	925	18.52	(13.02- 26.36)
KELLER	1	m	0	5063	1210	323	1017	13.17	(11.45- 15.15)
KELLER	9	m	0	1053	212	38	117	15.29	(10.31- 22.69)
KELLER	5	f	0	2904	792	469	1860	14.54	(12.79- 16.53)
KELLER	13	f	0	454	135	67	232	11.64	(8.35- 16.24)
Subtotal KELLER								13.79	(12.62- 15.07)
KHUDER	19	m	0	275	-	23	-	8.10	(5.20- 12.70)
KIHARA	7	c	0	283	162	102	237	4.06	(3.00- 5.49)
*KINLEN	9	m	0	636	9879	7	1333	12.26	(5.83- 25.76)
KJUUS	1	m	0	135	77	2	24	21.04	(4.84- 91.45)
*KNEKT	15	m	0	93	36489	6	17814	7.57	(3.31- 17.27)
KO	1	f	3	-	-	-	-	4.20	(1.10- 15.60)
KOHLME	1	c	0	228	236	11	193	16.95	(8.99- 31.96)
KOO	9	f	0	42	25	56	85	2.55	(1.40- 4.64)
KOULUM	1	m	0	807	246	5	54	35.43	(14.02- 89.55)
KREUZE	40	f	0	55	23	6	38	15.14	(5.63- 40.72)
KREUZE	42	f	0	170	54	95	177	5.87	(3.95- 8.70)
Subtotal KREUZE								6.68	(4.63- 9.64)
KREYBE	24	m	0	252	3514	6	644	7.70	(3.41- 17.37)
KREYBE	39	f	0	12	328	30	657	0.80	(0.40- 1.59)
Subtotal KREYBE								2.04	(1.21- 3.44)
*KUBIK	12	m	0	98	6342	2	4271	33.00	(8.14- 133.74)
LAMTH	6	f	0	242	106	202	337	3.81	(2.86- 5.08)
LAMWK	1	f	0	88	41	75	144	4.12	(2.59- 6.55)
LAMWK2	9	m	0	244	161	23	43	2.83	(1.64- 4.88)
LAMWK2	10	f	0	75	50	65	139	3.21	(2.02- 5.10)
Subtotal LAMWK2								3.04	(2.14- 4.33)
*LANGE	32	m	0	174	4537	5	721	5.53	(2.28- 13.41)
*LANGE	29	f	0	53	4455	7	2159	3.67	(1.67- 8.06)
Subtotal LANGE								4.40	(2.44- 7.92)
LAUSSM	10	m	0	347	188	85	226	4.91	(3.61- 6.66)
LEI	1	m	0	443	361	41	123	3.68	(2.52- 5.38)
LEI	2	f	0	123	61	85	147	3.49	(2.32- 5.24)
Subtotal LEI								3.59	(2.72- 4.74)
LEMARC	2	c	0	167	65	32	168	13.49	(8.39- 21.68)
LETOUR	1	c	0	714	514	24	224	12.96	(8.38- 20.05)
LEVIN	32	m	1	-	-	-	-	4.86	(3.41- 6.92)
*LIAW	1	m	1	-	-	-	-	3.70	(2.10- 6.60)
*LIAW	2	f	1	-	-	-	-	3.60	(1.00- 12.20)
Subtotal LIAW								3.68	(2.19- 6.20)
*LIDDEL	4	m	1	-	-	-	-	4.41	(2.77- 7.01)
LIU	2	c	2	-	-	-	-	1.92	(1.40- 2.64)
LIU2	1	m	0	212	180	12	44	4.32	(2.21- 8.43)
LIU2	3	f	0	54	23	38	69	4.26	(2.27- 7.99)
Subtotal LIU2								4.29	(2.71- 6.78)
LIU3	1	m	0	52	205	4	19	1.20	(0.39- 3.69)
LIU4	11	m	2	-	-	-	-	2.76	(2.69- 2.83)
LIU4	12	f	2	-	-	-	-	2.86	(2.77- 2.95)
Subtotal LIU4								2.80	(2.74- 2.85)
LIU5	1	c	0	85	70	26	41	1.91	(1.07- 3.44)
LOMBA2	1	f	0	149	353	76	239	1.33	(0.96- 1.83)
LOMBAR	9	m	0	852	610	14	112	11.17	(6.35- 19.66)
LUBIN2	25	m	0	5700	7744	190	2617	10.14	(8.72- 11.79)
LUBIN2	317	f	0	384	410	288	1180	3.84	(3.17- 4.64)
Subtotal LUBIN2								6.95	(6.18- 7.82)
LUO	1	c	0	65	146	37	160	1.93	(1.21- 3.06)
MACLEN	19	m	0	137	108	5	15	3.81	(1.34- 10.80)
MACLEN	32	f	0	42	47	41	109	2.38	(1.37- 4.12)
Subtotal MACLEN								2.63	(1.62- 4.28)
*MAGNUS	1	m	0	189	3439	11	1086	5.43	(2.97- 9.93)
MARSH	2	m	0	98	155	2	31	9.80	(2.29- 41.87)
MARSH	4	f	0	42	64	8	71	5.82	(2.54- 13.33)
Subtotal MARSH								6.62	(3.22- 13.59)
MARSH2	2	c	0	102	149	12	56	3.19	(1.63- 6.26)
MARTIS	4	m	0	197	176	4	25	7.00	(2.39- 20.49)
MASTRA	1	m	0	303	265	6	44	8.38	(3.52- 19.99)
MATOS	2	m	0	112	132	11	110	8.48	(4.35- 16.56)
MATSUD	10	m	0	170	3314	3	1255	21.46	(6.84- 67.33)
MCCONN	1	m	0	88	174	5	12	1.21	(0.41- 3.55)

Table 1C2 - 5

IESLC - Meta-anal of Current Smoking (or Ever if Current not available), Any prod (or Cigs if Any not avail)
All LC types
Least adjusted

REF	NRR	SEX	AD	Number Exposed Case	Cont	Non-exposed Case	Cont	RR	95.00%CI
MCCONN	2	f	0	3	3	4	11	2.75	(0.38- 19.67)
Subtotal MCCONN								1.46	(0.57- 3.76)
MCDUFF	1	m	0	159	134	6	31	6.13	(2.48- 15.14)
MCLAUG	1	m	0	294	1082	22	270	3.33	(2.12- 5.25)
*MIGRAN	19	m	0	182	5145	4	867	7.67	(2.85- 20.59)
*MIGRAN	135	f	0	30	3465	4	3814	8.26	(2.91- 23.41)
Subtotal MIGRAN								7.94	(3.88- 16.26)
MILLER	1	f	0	140	1607	28	3638	11.32	(7.51- 17.06)
MILLS	3	m	1	-	-	-	-	1.33	(1.09- 1.63)
*MRFITR	2	m	0	106	8194	0	1859	48.33~	(3.01- 777.42)
NAM	68	m	0	241	589	30	520	7.09	(4.77- 10.55)
NAM	84	f	0	133	234	52	885	9.67	(6.81- 13.75)
Subtotal NAM								8.44	(6.49- 10.98)
NOTAN2	1	m	0	549	735	134	544	3.03	(2.44- 3.77)
NOU	11	m	0	74	247	6	122	6.09	(2.58- 14.39)
NOU	12	f	0	10	92	4	261	7.09	(2.17- 23.17)
Subtotal NOU								6.42	(3.20- 12.87)
ODRISC	1	c	0	293	598	6	664	54.22	(23.98- 122.60)
ORMOS	4	m	0	87	1034	7	777	9.34	(4.30- 20.28)
ORMOS	26	f	0	1	234	23	1044	0.19	(0.03- 1.44)
Subtotal ORMOS								5.65	(2.74- 11.64)
OSANN	9	m	0	791	541	45	833	27.07	(19.67- 37.25)
OSANN	13	f	0	597	367	96	1093	18.52	(14.48- 23.68)
Subtotal OSANN								21.33	(17.55- 25.92)
PARKIN	27	m	0	375	946	107	1248	4.62	(3.67- 5.82)
PASTOR	5	m	0	194	262	10	89	6.59	(3.34- 13.00)
PAWLEG	1	m	0	172	249	4	92	15.89	(5.73- 44.05)
PERNU	2	m	0	1380	438	97	275	8.93	(6.92- 11.53)
PERNU	1	f	0	19	89	110	971	1.88	(1.11- 3.21)
Subtotal PERNU								6.68	(5.31- 8.41)
PERSH2	4	c	0	736	631	178	1164	7.63	(6.31- 9.23)
*PETO	4	m	0	99	2036	2	295	7.17	(1.78- 28.92)
PEZZO2	2	m	0	233	198	6	117	22.95	(9.89- 53.26)
PEZZOT	5	m	0	145	129	4	116	32.60	(11.70- 90.81)
PIKE	4	m	0	514	375	18	69	5.25	(3.08- 8.98)
PIKE	8	f	0	163	90	36	96	4.83	(3.04- 7.66)
Subtotal PIKE								5.01	(3.53- 7.10)
POFFIJ	1	c	0	913	918	58	452	7.75	(5.81- 10.34)
POLEDN	3	c	0	196	271	12	139	8.38	(4.52- 15.54)
*QIAO2	2	m	0	198	6101	10	709	2.30	(1.22- 4.32)
RACHTA	2	f	0	72	33	33	98	6.48	(3.66- 11.46)
RADZIK	1	c	0	180	198	9	13	1.31	(0.55- 3.15)
RANDIG	23	m	0	410	359	5	22	5.03	(1.88- 13.41)
RANDIG	24	f	0	16	39	17	92	2.22	(1.02- 4.84)
Subtotal RANDIG								3.04	(1.65- 5.60)
REN	1	m	0	106	84	12	34	3.58	(1.74- 7.33)
REN	2	f	0	78	20	48	50	4.06	(2.16- 7.64)
Subtotal REN								3.84	(2.39- 6.17)
RONCO	1	m	0	120	306	6	78	5.10	(2.16- 12.01)
ROTHSC	1	c	0	271	222	13	62	5.82	(3.12- 10.86)
SADOWS	7	m	0	459	534	18	81	3.87	(2.29- 6.54)
SANKAR	1	m	0	251	439	28	767	15.66	(10.42- 23.55)
SCHWAR	25	m	0	1652	349	119	376	14.96	(11.81- 18.94)
SCHWAR	26	m	0	644	139	50	104	9.64	(6.56- 14.15)
SCHWAR	27	f	0	1029	309	182	855	15.64	(12.75- 19.19)
SCHWAR	28	f	0	256	90	40	247	17.56	(11.64- 26.50)
Subtotal SCHWAR								14.70	(12.84- 16.83)
SEGI	1	m	0	140	1742	18	382	1.71	(1.03- 2.82)
SEGI2	19	m	0	267	485	8	53	3.65	(1.71- 7.79)
SEGI2	27	f	0	24	34	56	126	1.59	(0.86- 2.92)
Subtotal SEGI2								2.20	(1.37- 3.54)
SEOW	1	f	0	61	15	92	125	5.53	(2.96- 10.33)
SHAW	6	c	0	212	97	11	107	21.26	(10.93- 41.36)
SIEMIA	9	m	0	844	428	13	105	15.93	(8.85- 28.67)
SIMARA	5	m	0	33	264	27	433	2.00	(1.18- 3.41)
SIMARA	6	f	0	17	67	38	349	2.33	(1.24- 4.37)
Subtotal SIMARA								2.13	(1.42- 3.20)
SOBUE	90	m	0	736	650	34	128	4.26	(2.88- 6.31)
SOBUE	94	f	0	95	168	167	857	2.90	(2.15- 3.92)
Subtotal SOBUE								3.35	(2.64- 4.25)
SOBUE2	10	m	2	-	-	-	-	4.47	(3.89- 5.14)

Table 1C2 - 5

IESLC - Meta-anal of Current Smoking (or Ever if Current not available), Any prod (or Cigs if Any not avail)
All LC types
Least adjusted

REF	NRR	SEX	AD	Number Exposed Case	Number Exposed Cont	Non-exposed Case	Non-exposed Cont	RR	95.00%CI
SOBUE2	12	f	2	-	-	-	-	3.28	(2.79- 3.87)
Subtotal SOBUE2								3.92	(3.53- 4.36)
*SPEIZE	6	f	0	391	489993	58	776300	10.68	(8.11- 14.07)
SPITZ	2	c	0	103	89	7	128	21.16	(9.40- 47.66)
STASZE	1	m	0	255	754	5	158	10.69	(4.34- 26.33)
STASZE	5	f	0	6	153	15	1660	4.34	(1.66- 11.35)
Subtotal STASZE								7.01	(3.63- 13.53)
STAYNE	1	m	0	362	567	58	333	3.67	(2.69- 4.99)
STOCKS	31	m	0	2632	6477	45	638	5.76	(4.24- 7.82)
STOCKS	50	f	1	-	-	-	-	3.04	(2.35- 3.93)
Subtotal STOCKS								3.96	(3.25- 4.82)
STOCKW	7	c	0	12470	3357	2791	10641	14.16	(13.38- 14.99)
STUCKE	2	m	0	69	68	0	51	104.50~	(6.32-1727.39)
SUN	1	c	0	140	173	67	191	2.31	(1.62- 3.30)
SUZUK2	2	c	0	78	30	11	53	12.53	(5.78- 27.16)
SVENSS	61	f	0	142	53	38	120	8.46	(5.22- 13.71)
TANG	1	c	0	52	25	9	39	9.01	(3.78- 21.46)
*TENKAN	24	m	1	-	-	-	-	16.81	(7.22- 39.14)
TIZZAN	5	m	0	693	619	180	305	1.90	(1.53- 2.35)
TIZZAN	13	f	0	17	18	25	114	4.31	(1.95- 9.51)
Subtotal TIZZAN								2.01	(1.63- 2.47)
TOKARS	1	m	0	110	157	1	53	37.13	(5.06- 272.56)
TOKARS	5	f	0	1	2	13	40	1.54	(0.13- 18.38)
Subtotal TOKARS								10.65	(2.25- 50.36)
TOUSEY	4	m	0	160	91	4	130	57.14	(20.45- 159.69)
TOUSEY	8	f	0	127	78	13	226	28.31	(15.13- 52.94)
Subtotal TOUSEY								34.23	(20.06- 58.43)
TSUGAN	28	m	0	63	63	18	22	1.22	(0.60- 2.50)
*TULINI	14	m	1	-	-	-	-	10.69	(5.80- 19.72)
*TULINI	20	f	1	-	-	-	-	18.99	(10.52- 34.26)
Subtotal TULINI								14.40	(9.41- 22.02)
*TVERDA	5	m	2	-	-	-	-	4.09	(2.65- 6.31)
*TVERDA	15	f	2	-	-	-	-	11.05	(3.33- 36.71)
Subtotal TVERDA								4.59	(3.05- 6.90)
WAKAI	2	m	0	181	284	10	65	4.14	(2.07- 8.27)
WAKAI	20	f	0	33	26	50	145	3.68	(2.01- 6.75)
Subtotal WAKAI								3.87	(2.46- 6.11)
*WALD	2	m	0	77	4182	7	6539	17.20	(7.94- 37.25)
WANG	1	m	0	262	-	29	-	3.47	(2.10- 5.80)
WANG	2	f	0	17	-	82	-	4.00	(1.30- 12.00)
Subtotal WANG								3.56	(2.24- 5.64)
WANG2	17	c	0	49	78	11	43	2.46	(1.16- 5.21)
WANG3	1	c	0	235	172	58	121	2.85	(1.97- 4.13)
WANG4	1	m	0	1043	18164	127	2374	1.07	(0.89- 1.30)
WICKLU	1	m	0	-	-	-	-	4.60	(2.80- 7.60)
WIGLE	3	m	0	454	522	15	204	11.83	(6.90- 20.28)
WIGLE	6	f	0	68	169	36	439	4.91	(3.16- 7.63)
Subtotal WIGLE								6.98	(4.96- 9.83)
WILKIN	1	m	0	173	372	2	108	25.11	(6.13- 102.89)
WILKIN	2	f	0	84	109	12	89	5.72	(2.93- 11.13)
Subtotal WILKIN								7.49	(4.10- 13.68)
WU	34	f	0	160	73	31	92	6.50	(3.98- 10.64)
WUNSCH	2	m	0	189	234	14	99	5.71	(3.16- 10.32)
WUNSCH	8	f	0	42	51	29	208	5.91	(3.36- 10.38)
Subtotal WUNSCH								5.81	(3.87- 8.74)
WUWILL	6	f	0	539	351	417	601	2.21	(1.84- 2.66)
WYNDE2	21	m	0	396	616	8	105	8.44	(4.07- 17.51)
WYNDE3	50	m	0	227	207	9	88	10.72	(5.26- 21.84)
WYNDE3	138	f	0	46	56	20	76	3.12	(1.67- 5.85)
Subtotal WYNDE3								5.36	(3.35- 8.58)
WYNDE4	48	m	0	632	665	12	115	9.11	(4.98- 16.67)
WYNDE4	62	f	2	-	-	-	-	2.87	(1.48- 5.55)
Subtotal WYNDE4								5.38	(3.45- 8.41)
WYNDE6	18	m	0	1677	741	87	617	16.05	(12.62- 20.41)
WYNDE6	207	f	0	1022	376	159	856	14.63	(11.90- 17.99)
Subtotal WYNDE6								15.22	(13.01- 17.80)
*XIANGZ	8	m	0	907	13037	25	974	2.71	(1.83- 4.01)
XU	1	m	0	627	552	102	236	2.63	(2.03- 3.40)
XU2	1	c	0	501	582	82	377	3.96	(3.03- 5.17)
XU3	1	m	0	92	68	7	31	5.99	(2.49- 14.42)
XU3	3	f	0	23	11	13	25	4.02	(1.51- 10.74)

Table 1C2 - 5

IESLC - Meta-anal of Current Smoking (or Ever if Current not available), Any prod (or Cigs if Any not avail)
All LC types
Least adjusted

REF	NRR	SEX	AD	Number Exposed Case	Number Exposed Cont	Non-exposed Case	Non-exposed Cont	RR	95.00%CI
Subtotal XU3								5.02	(2.61- 9.66)
XU4	1	c	0	161	113	45	93	2.94	(1.92- 4.52)
YAMAGU	1	c	0	76	247	24	267	3.42	(2.10- 5.59)
*YONG	12	m	1	-	-	-	-	28.71	(6.98- 118.16)
*YONG	15	f	1	-	-	-	-	5.20	(2.38- 11.35)
Subtotal YONG								7.75	(3.91- 15.36)
*YUAN	1	m	2	-	-	-	-	6.50	(3.64- 11.60)
ZHANG	1	c	0	72	102	28	98	2.47	(1.47- 4.14)
ZHENG	15	m	0	279	218	33	94	3.65	(2.36- 5.63)
ZHENG	24	f	0	76	44	152	184	2.09	(1.36- 3.21)
Subtotal ZHENG								2.75	(2.03- 3.73)
ZHOU	2	m	0	740	41	275	36	2.36	(1.48- 3.77)
ZHOU	3	f	0	112	7	231	32	2.22	(0.95- 5.18)
Subtotal ZHOU								2.33	(1.54- 3.51)
Partial Totals				109009	2718025	15296	4500160		

*prospective study ~ With 0.5 adjustment for zero

REF	NRR	SEX	AD	Ys	Ws	Qs	Ps
ABELIN	1	m	0	3.44	1.93	7.30	0.0000
*ABRAHA	7	m	0	2.17	9.68	4.39	0.0000
*ABRAHA	8	f	0	1.59	19.39	0.16	0.0000
Subtotal ABRAHA				1.78	29.07	4.54	
AGUDO	10	f	0	0.99	7.89	2.01	0.0054
*AKIBA	2	m	0	1.39	17.12	0.20	0.0000
*AKIBA	6	f	0	1.50	45.22	0.00	0.0000
Subtotal AKIBA				1.47	62.34	0.20	
ALDERS	177	m	0	2.66	12.62	17.11	0.0000
ALDERS	176	f	0	1.76	41.23	2.85	0.0000
Subtotal ALDERS				1.97	53.85	19.96	
*AMANDU	1	m	0	1.61	5.70	0.08	0.0001
AMES	1	m	0	1.52	10.33	0.00	0.0000
*ANDERS	2	f	0	3.08	37.84	95.25	0.0000
*ARCHER	5	m	0	1.82	5.72	0.59	0.0000
ARMADA	27	m	0	3.21	3.58	10.47	0.0000
AUSTIN	2	c	0	2.75	4.38	6.88	0.0000
AUVINE	1	c	0	2.15	30.60	12.94	0.0000
AXELSO	1	c	0	1.83	24.06	2.77	0.0000
AXELSS	2	m	0	2.70	12.26	17.87	0.0000
AXELSS	10	f	0	2.48	11.50	11.08	0.0000
Subtotal AXELSS				2.59	23.76	28.95	
BAND	1	m	2	2.30	42.76	27.59	0.0000
BARBON	3	m	0	2.59	18.08	21.48	0.0000
BECHER	13	m	0	2.70	2.70	3.93	0.0000
BECHER	14	f	0	1.89	5.32	0.82	0.0000
Subtotal BECHER				2.16	8.02	4.75	
*BENSHL	16	m	1	2.10	5.77	2.12	0.0000
*BEST	2	m	1	2.70	6.85	9.98	0.0000
*BEST	18	f	1	0.81	2.17	1.03	0.2348
Subtotal BEST				2.25	9.02	11.01	
BLOHMK	1	m	0	1.16	59.38	6.58	0.0000
BLOT4	1	m	0	2.68	6.96	9.69	0.0000
BOFFET	32	m	0	2.71	105.47	154.34	0.0000
*BOUCOT	2	m	0	4.06	0.50	3.28	0.0042
BRESLO	37	m	0	1.87	5.85	0.81	0.0000
BRESLO	38	f	0	0.32	3.10	4.27	0.5717
Subtotal BRESLO				1.33	8.95	5.08	
*BRETT	4	m	0	1.37	5.75	0.09	0.0010
BROCKM	1	m	0	0.07	0.98	1.98	0.9437
BROCKM	2	f	0	0.69	2.73	1.76	0.2517
Subtotal BROCKM				0.53	3.71	3.74	
BROSS	11	m	0	1.58	28.40	0.19	0.0000
BROWN2	12	m	2	2.42	402.82	348.03	0.0000
BROWN2	11	f	2	2.61	365.29	453.96	0.0000
Subtotal BROWN2				2.51	768.12	801.98	
BUFFLE	3	m	0	2.40	4.35	3.57	0.0000
BUFFLE	7	f	0	2.11	26.25	9.96	0.0000
Subtotal BUFFLE				2.15	30.60	13.53	
CARPEN	9	c	0	3.08	12.26	30.72	0.0000
CASCO2	1	c	0	2.44	5.31	4.75	0.0000
CASCOR	1	c	0	2.60	18.40	22.63	0.0000

Table 1C2 - 5

IESLC - Meta-anal of Current Smoking (or Ever if Current not available), Any prod (or Cigs if Any not avail)
All LC types
Least adjusted

REF	NRR	SEX	AD	Ys	Ws	Qs	Ps
*CEDERL	106	m	2	2.04	20.57	6.19	0.0000
*CEDERL	75	f	2	1.57	30.42	0.18	0.0000
Subtotal CEDERL				1.76	50.99	6.37	
CHAN	9	m	0	3.31	1.87	6.19	0.0000
CHAN	10	f	0	1.25	20.57	1.28	0.0000
Subtotal CHAN				1.42	22.44	7.48	
*CHANG	5	m	0	2.13	4.46	1.78	0.0000
*CHANG	11	f	0	1.64	8.22	0.17	0.0000
Subtotal CHANG				1.81	12.68	1.95	
CHATZI	4	c	0	1.21	19.44	1.63	0.0000
CHEN2	1	m	0	1.52	6.25	0.00	0.0001
CHEN2	2	f	0	0.51	7.70	7.41	0.1539
Subtotal CHEN2				0.97	13.95	7.41	
CHEN3	1	c	0	0.46	27.78	29.63	0.0148
CHIAZZ	2	m	0	2.06	3.53	1.11	0.0001
CHOI	3	m	0	1.64	10.55	0.22	0.0000
CHOI	7	f	0	0.20	7.16	12.04	0.5951
Subtotal CHOI				1.06	17.71	12.26	
*CHOW	25	m	0	2.64	5.79	7.65	0.0000
*CHYOU	4	m	0	2.27	12.23	7.29	0.0000
COMSTO	3	m	0	2.90	3.52	6.91	0.0000
COMSTO	8	f	0	2.57	8.49	9.85	0.0000
Subtotal COMSTO				2.67	12.01	16.76	
COOKSO	5	c	0	1.88	14.38	2.15	0.0000
CORREA	41	c	0	2.53	40.00	42.90	0.0000
*CPSI	220	m	1	2.48	74.99	72.70	0.0000
*CPSI	279	f	1	1.16	70.15	7.74	0.0000
Subtotal CPSI				1.84	145.14	80.44	
*CPSII	36	m	0	2.90	115.97	230.46	0.0000
*CPSII	71	f	0	2.22	237.52	124.16	0.0000
Subtotal CPSII				2.44	353.49	354.61	
DAMBER	14	m	1	2.26	26.18	15.38	0.0000
DARBY	4	m	0	4.51	2.93	26.64	0.0000
DARBY	11	f	0	3.03	18.15	42.66	0.0000
Subtotal DARBY				3.23	21.08	69.31	
DAVEYS	5	m	0	1.57	2.53	0.01	0.0126
DAVEYS	6	f	0	-0.32	0.42	1.40	0.8327
Subtotal DAVEYS				1.30	2.96	1.42	
DEAN	7	m	0	1.66	9.69	0.25	0.0000
DEAN2	2	m	0	1.33	23.59	0.62	0.0000
DEAN2	6	f	0	1.06	13.83	2.58	0.0001
Subtotal DEAN2				1.23	37.42	3.19	
DEAN3	40	m	0	1.83	22.44	2.58	0.0000
DEAN3	117	f	0	1.20	28.00	2.52	0.0000
Subtotal DEAN3				1.48	50.44	5.10	
*DEKLER	8	m	2	3.14	0.99	2.67	0.0018
DESTE2	2	c	0	2.04	14.18	4.28	0.0000
DESTEF	40	m	0	2.27	19.86	11.89	0.0000
*DOCKER	1	c	4	2.08	3.90	1.33	0.0000
DOLL	90	m	0	2.25	6.22	3.57	0.0000
DOLL	93	f	0	0.74	11.96	6.91	0.0110
Subtotal DOLL				1.25	18.18	10.48	
*DOLL2	54	m	1	2.40	18.45	15.00	0.0000
*DOLL2	63	f	1	2.16	3.28	1.44	0.0001
Subtotal DOLL2				2.36	21.73	16.44	
DORANT	2	m	0	2.38	6.51	5.11	0.0000
DORGAN	9	m	0	2.83	11.70	20.80	0.0000
DORGAN	33	m	0	3.71	2.61	12.83	0.0000
DORGAN	56	f	0	2.50	41.93	42.20	0.0000
DORGAN	79	f	0	2.44	3.75	3.32	0.0000
Subtotal DORGAN				2.61	60.00	79.15	
*DORN	51	m	1	2.11	73.41	27.54	0.0000
DOSEME	17	m	0	1.41	75.43	0.50	0.0000
DROSTE	2	m	0	2.94	6.25	12.99	0.0000
DU	1	m	0	1.26	28.12	1.54	0.0000
DU	2	f	0	0.66	24.50	17.20	0.0011
Subtotal DU				0.98	52.62	18.74	
*DUNN	6	m	0	2.91	1.97	3.97	0.0000
EBELIN	1	m	0	1.94	9.19	1.79	0.0000
*ENGELA	154	m	0	1.75	6.56	0.44	0.0000
*ENGELA	161	f	0	1.39	6.52	0.08	0.0004

Table 1C2 - 5

IESLC - Meta-anal of Current Smoking (or Ever if Current not available), Any prod (or Cigs if Any not avail)
All LC types
Least adjusted

REF	NRR	SEX	AD	Ys	Ws	Qs	Ps
Subtotal ENGELA				1.57	13.08	0.51	
*ENSTRO	1	m	1	2.56	81.91	93.58	0.0000
*ENSTRO	2	f	1	1.94	181.45	35.68	0.0000
Subtotal ENSTRO				2.13	263.36	129.26	
ESAKI	4	m	0	0.64	8.96	6.55	0.0554
ESAKI	5	f	0	0.90	7.99	2.83	0.0109
Subtotal ESAKI				0.76	16.94	9.37	
FAN	1	m	0	1.04	25.87	5.25	0.0000
FAN	2	f	0	1.37	24.92	0.42	0.0000
Subtotal FAN				1.20	50.80	5.66	
GAO	29	m	0	1.37	39.60	0.62	0.0000
GAO	30	f	0	0.86	50.42	20.32	0.0000
Subtotal GAO				1.08	90.02	20.94	
GAO2	1	m	0	1.91	9.19	1.61	0.0000
GARCIA	2	c	0	2.72	13.47	20.06	0.0000
GARDIN	2	c	0	2.62	3.97	5.01	0.0000
GARSHI	23	m	0	2.01	33.39	8.73	0.0000
GENG	1	m	0	1.79	4.98	0.43	0.0001
GENG	2	f	0	1.08	22.39	3.77	0.0000
Subtotal GENG				1.21	27.37	4.21	
GER	17	c	0	0.31	26.37	36.96	0.1100
GODLEY	5	m	1	1.92	96.28	17.59	0.0000
GODLEY	6	f	1	1.71	58.89	2.77	0.0000
Subtotal GODLEY				1.84	155.17	20.36	
GOLLED	21	m	0	1.84	13.92	1.68	0.0000
GOODMA	2	m	0	2.86	8.50	15.78	0.0000
GOODMA	6	f	0	2.27	10.71	6.37	0.0000
Subtotal GOODMA				2.53	19.21	22.15	
GRAHAM	20	m	0	1.91	16.38	2.80	0.0000
GREGOR	2	m	0	0.26	4.75	7.27	0.5741
GREGOR	6	f	0	2.67	0.88	1.20	0.0126
Subtotal GREGOR				0.63	5.62	8.47	
GSELL	8	m	0	2.88	1.82	3.47	0.0001
HAENSZ	54	f	0	0.76	23.97	12.95	0.0002
*HAMMO2	22	m	0	2.12	4.92	1.92	0.0000
*HAMMON	139	m	1	2.44	14.07	12.67	0.0000
*HANSEN	3	m	2	0.43	5.28	6.04	0.3285
HEGMAN	1	c	0	2.79	23.66	39.89	0.0000
*HEIN	5	m	0	2.85	0.99	1.82	0.0045
*HENNEK	2	m	0	2.73	17.97	27.55	0.0000
HINDS	26	f	0	1.42	61.37	0.38	0.0000
*HIRAYA	1	m	1	1.49	85.55	0.00	0.0000
*HIRAYA	3	f	1	0.85	77.37	32.20	0.0000
Subtotal HIRAYA				1.19	162.92	32.20	
HITOSU	2	m	0	0.93	6.40	2.05	0.0187
HITOSU	9	f	0	1.25	14.55	0.86	0.0000
Subtotal HITOSU				1.15	20.95	2.91	
*HOLE	47	m	0	1.90	6.76	1.12	0.0000
*HOLE	29	f	0	0.37	4.98	6.26	0.4046
Subtotal HOLE				1.25	11.74	7.38	
HOROWI	1	m	0	1.27	15.35	0.75	0.0000
HOROWI	2	f	0	0.60	8.08	6.51	0.0894
Subtotal HOROWI				1.04	23.43	7.26	
HORWIT	1	f	0	2.43	8.29	7.18	0.0000
HU	15	m	0	0.74	17.16	9.91	0.0023
HU	16	f	0	0.55	7.15	6.39	0.1413
Subtotal HU				0.68	24.31	16.30	
HU2	9	m	0	1.11	27.11	4.08	0.0000
HU2	10	f	0	0.63	21.91	16.46	0.0033
Subtotal HU2				0.89	49.01	20.54	
HUANG	1	c	0	0.69	14.82	9.60	0.0078
HUMBLE	13	m	1	2.99	4.94	11.10	0.0000
HUMBLE	15	m	1	2.76	1.65	2.63	0.0004
HUMBLE	17	f	1	2.82	5.85	10.22	0.0000
HUMBLE	19	f	1	3.16	2.49	6.88	0.0000
Subtotal HUMBLE				2.93	14.94	30.83	
JAHN	3	f	0	1.13	18.89	2.54	0.0000
JAIN	16	m	0	2.77	9.32	15.06	0.0000
JAIN	11	f	0	2.54	26.82	29.27	0.0000
Subtotal JAIN				2.60	36.14	44.33	
JARUP	3	m	0	1.90	6.90	1.13	0.0000

Table 1C2 - 5

IESLC - Meta-anal of Current Smoking (or Ever if Current not available), Any prod (or Cigs if Any not avail)
All LC types
Least adjusted

REF	NRR	SEX	AD	Ys	Ws	Qs	Ps
JARVHO	2	m	0	3.70	0.90	4.36	0.0005
JARVHO	6	f	0	2.74	2.57	3.98	0.0000
Subtotal JARVHO				2.99	3.47	8.34	
JEDRYC	63	m	0	1.76	36.14	2.51	0.0000
JEDRYC	68	f	0	2.08	17.12	5.79	0.0000
Subtotal JEDRYC				1.86	53.25	8.30	
JIANG	1	m	0	1.00	4.45	1.09	0.0346
JIANG	2	f	0	0.91	2.62	0.89	0.1401
Subtotal JIANG				0.97	7.08	1.98	
JOLY	18	m	0	2.59	10.93	13.05	0.0000
JOLY	15	f	0	2.01	24.54	6.57	0.0000
Subtotal JOLY				2.19	35.47	19.62	
JUSSAW	3	m	0	2.77	63.20	103.41	0.0000
*KAISE2	68	m	1	2.08	10.65	3.70	0.0000
*KAISE2	60	f	1	2.67	8.78	12.18	0.0000
Subtotal KAISE2				2.35	19.43	15.87	
*KAISER	12	m	2	2.98	25.68	56.31	0.0000
*KAISER	9	f	2	1.88	27.68	4.02	0.0000
Subtotal KAISER				2.41	53.36	60.33	
KANELL	5	m	0	1.89	33.17	5.15	0.0000
KATSOU	6	f	0	1.25	8.81	0.53	0.0002
KAUFMA	7	c	0	2.92	30.87	62.58	0.0000
KELLER	1	m	0	2.58	195.95	229.83	0.0000
KELLER	9	m	0	2.73	24.67	37.46	0.0000
KELLER	5	f	0	2.68	233.82	326.52	0.0000
KELLER	13	f	0	2.45	34.67	31.92	0.0000
Subtotal KELLER				2.62	489.11	625.72	
KHUDER	19	m	0	2.09	19.27	6.86	0.0000
KIHARA	7	c	0	1.40	42.14	0.38	0.0000
*KINLEN	9	m	0	2.51	6.96	7.12	0.0000
KJUUS	1	m	0	3.05	1.78	4.28	0.0000
*KNEKT	15	m	0	2.02	5.64	1.58	0.0000
KO	1	f	3	1.44	2.18	0.01	0.0339
KOHLME	1	c	0	2.83	9.55	17.02	0.0000
KOO	9	f	0	0.94	10.70	3.35	0.0022
KOULUM	1	m	0	3.57	4.47	19.19	0.0000
KREUZE	40	f	0	2.72	3.93	5.87	0.0000
KREUZE	42	f	0	1.77	24.64	1.85	0.0000
Subtotal KREUZE				1.90	28.57	7.72	
KREYBE	24	m	0	2.04	5.80	1.73	0.0000
KREYBE	39	f	0	-0.22	8.25	24.31	0.5245
Subtotal KREYBE				0.71	14.05	26.04	
*KUBIK	12	m	0	3.50	1.96	7.86	0.0000
LAMTH	6	f	0	1.34	46.55	1.16	0.0000
LAMWK	1	f	0	1.42	17.85	0.11	0.0000
LAMWK2	9	m	0	1.04	12.98	2.67	0.0002
LAMWK2	10	f	0	1.17	17.89	1.94	0.0000
Subtotal LAMWK2				1.11	30.86	4.62	
*LANGE	32	m	0	1.71	4.90	0.23	0.0002
*LANGE	29	f	0	1.30	6.21	0.24	0.0012
Subtotal LANGE				1.48	11.11	0.46	
LAUSSM	10	m	0	1.59	41.00	0.37	0.0000
LEI	1	m	0	1.30	26.63	0.98	0.0000
LEI	2	f	0	1.25	23.21	1.41	0.0000
Subtotal LEI				1.28	49.84	2.39	
LEMARC	2	c	0	2.60	17.07	20.90	0.0000
LETOUR	1	c	0	2.56	20.21	23.01	0.0000
LEVIN	32	m	1	1.58	30.68	0.23	0.0000
*LIAW	1	m	1	1.31	11.72	0.41	0.0000
*LIAW	2	f	1	1.28	2.46	0.11	0.0447
Subtotal LIAW				1.30	14.17	0.52	
*LIDDEL	4	m	1	1.48	17.82	0.00	0.0000
LIU	2	c	2	0.65	38.19	27.14	0.0001
LIU2	1	m	0	1.46	8.60	0.01	0.0000
LIU2	3	f	0	1.45	9.73	0.02	0.0000
Subtotal LIU2				1.46	18.32	0.03	
LIU3	1	m	0	0.19	3.06	5.24	0.7444
LIU4	11	m	2	1.02	5969.41	1375.75	0.0000
LIU4	12	f	2	1.05	3876.64	765.87	0.0000
Subtotal LIU4				1.03	9846.05	2141.62	
LIU5	1	c	0	0.65	11.25	8.04	0.0293

Table 1C2 - 5

IESLC - Meta-anal of Current Smoking (or Ever if Current not available), Any prod (or Cigs if Any not avail)
All LC types
Least adjusted

REF	NRR	SEX	AD	Ys	Ws	Qs	Ps
LOMBA2	1	f	0	0.28	37.19	54.64	0.0841
LOMBAR	9	m	0	2.41	12.02	10.14	0.0000
LUBIN2	25	m	0	2.32	168.07	113.29	0.0000
LUBIN2	317	f	0	1.34	106.80	2.42	0.0000
Subtotal LUBIN2				1.94	274.88	115.71	
LUO	1	c	0	0.66	18.01	12.72	0.0054
MACLEN	19	m	0	1.34	3.53	0.09	0.0120
MACLEN	32	f	0	0.87	12.71	5.05	0.0020
Subtotal MACLEN				0.97	16.25	5.14	
*MAGNUS	1	m	0	1.69	10.53	0.40	0.0000
MARSH	2	m	0	2.28	1.82	1.13	0.0021
MARSH	4	f	0	1.76	5.60	0.40	0.0000
Subtotal MARSH				1.89	7.42	1.53	
MARSH2	2	c	0	1.16	8.50	0.95	0.0007
MARTIS	4	m	0	1.95	3.32	0.67	0.0004
MASTRA	1	m	0	2.13	5.09	2.03	0.0000
MATOS	2	m	0	2.14	8.58	3.55	0.0000
MATSUD	10	m	0	3.07	2.94	7.25	0.0000
MCCONN	1	m	0	0.19	3.33	5.64	0.7237
MCCONN	2	f	0	1.01	0.99	0.23	0.3136
Subtotal MCCONN				0.38	4.32	5.87	
MCDUFF	1	m	0	1.81	4.70	0.48	0.0001
MCLAUG	1	m	0	1.20	18.70	1.58	0.0000
*MIGRAN	19	m	0	2.04	3.93	1.15	0.0001
*MIGRAN	135	f	0	2.11	3.54	1.34	0.0001
Subtotal MIGRAN				2.07	7.47	2.49	
MILLER	1	f	0	2.43	22.85	19.82	0.0000
MILLS	3	m	1	0.29	96.62	140.71	0.0046
*MRFITR	2	m	0	3.88	0.50	2.83	0.0062
NAM	68	m	0	1.96	24.33	5.23	0.0000
NAM	84	f	0	2.27	31.10	18.64	0.0000
Subtotal NAM				2.13	55.43	23.87	
NOTAN2	1	m	0	1.11	80.11	11.93	0.0000
NOU	11	m	0	1.81	5.20	0.50	0.0000
NOU	12	f	0	1.96	2.74	0.59	0.0012
Subtotal NOU				1.86	7.94	1.09	
ODRISC	1	c	0	3.99	5.77	36.01	0.0000
ORMOS	4	m	0	2.23	6.39	3.49	0.0000
ORMOS	26	f	0	-1.64	0.95	9.37	0.1093
Subtotal ORMOS				1.73	7.34	12.86	
OSANN	9	m	0	3.30	37.69	122.50	0.0000
OSANN	13	f	0	2.92	63.57	128.83	0.0000
Subtotal OSANN				3.06	101.25	251.33	
PARKIN	27	m	0	1.53	72.09	0.09	0.0000
PASTOR	5	m	0	1.89	8.32	1.27	0.0000
PAWLEG	1	m	0	2.77	3.69	5.96	0.0000
PERNU	2	m	0	2.19	58.99	28.44	0.0000
PERNU	1	f	0	0.63	13.52	10.03	0.0198
Subtotal PERNU				1.90	72.50	38.48	
PERSH2	4	c	0	2.03	106.15	30.55	0.0000
*PETO	4	m	0	1.97	1.98	0.45	0.0056
PEZZO2	2	m	0	3.13	5.42	14.54	0.0000
PEZZOT	5	m	0	3.48	3.66	14.48	0.0000
PIKE	4	m	0	1.66	13.39	0.36	0.0000
PIKE	8	f	0	1.57	18.04	0.11	0.0000
Subtotal PIKE				1.61	31.43	0.47	
POFFIJ	1	c	0	2.05	46.21	14.11	0.0000
POLEDN	3	c	0	2.13	10.07	4.00	0.0000
*QIAO2	2	m	0	0.83	9.66	4.23	0.0096
RACHTA	2	f	0	1.87	11.81	1.65	0.0000
RADZIK	1	c	0	0.27	5.03	7.53	0.5411
RANDIG	23	m	0	1.61	3.99	0.06	0.0013
RANDIG	24	f	0	0.80	6.34	3.08	0.0447
Subtotal RANDIG				1.11	10.32	3.14	
REN	1	m	0	1.27	7.46	0.36	0.0005
REN	2	f	0	1.40	9.65	0.08	0.0000
Subtotal REN				1.35	17.11	0.45	
RONCO	1	m	0	1.63	5.23	0.09	0.0002
ROTHSC	1	c	0	1.76	9.88	0.70	0.0000
SADOWS	7	m	0	1.35	13.90	0.28	0.0000
SANKAR	1	m	0	2.75	23.11	36.45	0.0000

Table 1C2 - 5

IESLC - Meta-anal of Current Smoking (or Ever if Current not available), Any prod (or Cigs if Any not avail)
All LC types
Least adjusted

REF	NRR	SEX	AD	Ys	Ws	Qs	Ps
SCHWAR	25	m	0	2.71	68.81	100.71	0.0000
SCHWAR	26	m	0	2.27	26.07	15.47	0.0000
SCHWAR	27	f	0	2.75	91.98	144.82	0.0000
SCHWAR	28	f	0	2.87	22.69	42.63	0.0000
Subtotal SCHWAR				2.69	209.54	303.63	
SEGI	1	m	0	0.53	15.18	14.03	0.0375
SEGI2	19	m	0	1.29	6.68	0.27	0.0008
SEGI2	27	f	0	0.46	10.32	11.01	0.1372
Subtotal SEGI2				0.79	17.00	11.28	
SEOW	1	f	0	1.71	9.81	0.45	0.0000
SHAW	6	c	0	3.06	8.67	21.15	0.0000
SIEMIA	9	m	0	2.77	11.12	18.00	0.0000
SIMARA	5	m	0	0.70	13.62	8.71	0.0103
SIMARA	6	f	0	0.85	9.72	4.10	0.0084
Subtotal SIMARA				0.76	23.33	12.81	
SOBUE	90	m	0	1.45	24.92	0.05	0.0000
SOBUE	94	f	0	1.07	42.31	7.82	0.0000
Subtotal SOBUE				1.21	67.24	7.87	
SOBUE2	10	m	2	1.50	197.90	0.00	0.0000
SOBUE2	12	f	2	1.19	143.51	13.57	0.0000
Subtotal SOBUE2				1.37	341.42	13.57	
*SPEIZE	6	f	0	2.37	50.52	38.51	0.0000
SPITZ	2	c	0	3.05	5.83	14.12	0.0000
STASZE	1	m	0	2.37	4.73	3.61	0.0000
STASZE	5	f	0	1.47	4.16	0.00	0.0028
Subtotal STASZE				1.95	8.88	3.61	
STAYNE	1	m	0	1.30	40.37	1.56	0.0000
STOCKS	31	m	0	1.75	41.11	2.69	0.0000
STOCKS	50	f	1	1.11	58.11	8.54	0.0000
Subtotal STOCKS				1.38	99.22	11.24	
STOCKW	7	c	0	2.65	1204.31	1607.40	0.0000
STUCKE	2	m	0	4.65	0.49	4.86	0.0012
SUN	1	c	0	0.84	30.23	13.14	0.0000
SUZUK2	2	c	0	2.53	6.41	6.84	0.0000
SVENSS	61	f	0	2.14	16.51	6.77	0.0000
TANG	1	c	0	2.20	5.10	2.52	0.0000
*TENKAN	24	m	1	2.82	5.38	9.47	0.0000
TIZZAN	5	m	0	0.64	84.08	61.47	0.0000
TIZZAN	13	f	0	1.46	6.13	0.01	0.0003
Subtotal TIZZAN				0.70	90.21	61.48	
TOKARS	1	m	0	3.61	0.97	4.34	0.0004
TOKARS	5	f	0	0.43	0.62	0.71	0.7336
Subtotal TOKARS				2.37	1.59	5.05	
TOUSEY	4	m	0	4.05	3.64	23.66	0.0000
TOUSEY	8	f	0	3.34	9.80	33.46	0.0000
Subtotal TOUSEY				3.53	13.44	57.12	
TSUGAN	28	m	0	0.20	7.53	12.63	0.5818
*TULINI	14	m	1	2.37	10.26	7.84	0.0000
*TULINI	20	f	1	2.94	11.02	23.13	0.0000
Subtotal TULINI				2.67	21.28	30.97	
*TVERDA	5	m	2	1.41	20.41	0.15	0.0000
*TVERDA	15	f	2	2.40	2.67	2.20	0.0001
Subtotal TVERDA				1.52	23.08	2.35	
WAKAI	2	m	0	1.42	8.04	0.04	0.0001
WAKAI	20	f	0	1.30	10.45	0.39	0.0000
Subtotal WAKAI				1.35	18.49	0.43	
*WALD	2	m	0	2.84	6.43	11.72	0.0000
WANG	1	m	0	1.24	14.89	0.94	0.0000
WANG	2	f	0	1.39	3.11	0.04	0.0145
Subtotal WANG				1.27	18.00	0.98	
WANG2	17	c	0	0.90	6.78	2.42	0.0193
WANG3	1	c	0	1.05	28.11	5.64	0.0000
WANG4	1	m	0	0.07	107.42	217.98	0.4630
WICKLU	1	m	0	1.53	15.41	0.01	0.0000
WIGLE	3	m	0	2.47	13.21	12.57	0.0000
WIGLE	6	f	0	1.59	19.73	0.18	0.0000
Subtotal WIGLE				1.94	32.94	12.74	
WILKIN	1	m	0	3.22	1.93	5.77	0.0000
WILKIN	2	f	0	1.74	8.65	0.53	0.0000
Subtotal WILKIN				2.01	10.58	6.30	
WU	34	f	0	1.87	15.85	2.26	0.0000

Table 1C2 - 5

IESLC - Meta-anal of Current Smoking (or Ever if Current not available), Any prod (or Cigs if Any not avail)
All LC types
Least adjusted

REF	NRR	SEX	AD	Ys	Ws	Qs	Ps
WUNSCH	2	m	0	1.74	10.98	0.67	0.0000
WUNSCH	8	f	0	1.78	12.09	0.95	0.0000
Subtotal WUNSCH				1.76	23.07	1.62	
WUWILL	6	f	0	0.79	114.07	56.03	0.0000
WYNDE2	21	m	0	2.13	7.21	2.93	0.0000
WYNDE3	50	m	0	2.37	7.59	5.84	0.0000
WYNDE3	138	f	0	1.14	9.73	1.24	0.0004
Subtotal WYNDE3				1.68	17.32	7.08	
WYNDE4	48	m	0	2.21	10.51	5.36	0.0000
WYNDE4	62	f	2	1.05	8.80	1.71	0.0018
Subtotal WYNDE4				1.68	19.31	7.07	
WYNDE6	18	m	0	2.78	66.40	108.86	0.0000
WYNDE6	207	f	0	2.68	90.13	127.20	0.0000
Subtotal WYNDE6				2.72	156.52	236.05	
*XIANGZ	8	m	0	1.00	25.00	6.20	0.0000
XU	1	m	0	0.97	57.31	16.04	0.0000
XU2	1	c	0	1.38	53.87	0.77	0.0000
XU3	1	m	0	1.79	4.98	0.43	0.0001
XU3	3	f	0	1.39	3.98	0.04	0.0055
Subtotal XU3				1.61	8.96	0.48	
XU4	1	c	0	1.08	20.82	3.59	0.0000
YAMAGU	1	c	0	1.23	15.97	1.12	0.0000
*YONG	12	m	1	3.36	1.92	6.66	0.0000
*YONG	15	f	1	1.65	6.30	0.15	0.0000
Subtotal YONG				2.05	8.22	6.80	
*YUAN	1	m	2	1.87	11.44	1.62	0.0000
ZHANG	1	c	0	0.90	14.37	5.01	0.0006
ZHENG	15	m	0	1.29	20.36	0.83	0.0000
ZHENG	24	f	0	0.74	20.88	11.99	0.0008
Subtotal ZHENG				1.01	41.24	12.82	
ZHOU	2	m	0	0.86	17.50	7.07	0.0003
ZHOU	3	f	0	0.80	5.34	2.61	0.0660
Subtotal ZHOU				0.84	22.83	9.68	

	N	345
	NS	242
	Wt	19661.85
Het	Chi	9854.69
Het	df	344
Het	P	***
Fixed	RR	4.46
	RRl	4.40
	RRu	4.52
	P	+++
Random	RR	6.15
	RRl	5.63
	RRu	6.71
	P	+++
Asymm	P	***

Table 1C2 - 6

IESLC - Meta-anal of Current Smoking (or Ever if Current not available), Any prod (or Cigs if Any not avail)
All LC types
Least adjusted

	Sex combined	male	female	Total
N	44	182	119	345
NS	44	178	114	336
Wt	2076.37	10258.39	7327.09	19661.85
Het Chi	986.58	4447.23	3034.60	9854.69
Het df	43	181	118	344
Het P	***	***	***	***
Fixed RR	9.65	4.03	4.14	4.46
RRl	9.25	3.95	4.05	4.40
RRu	10.08	4.10	4.23	4.52
P	+++	+++	+++	+++
Random RR	6.43	7.20	4.82	6.15
RRl	4.96	6.34	4.16	5.63
RRu	8.34	8.18	5.59	6.71
P	+++	+++	+++	+++
Between Chi				1386.29
Between df				2
Between P				***
Btwn(F) P				***
Btwn(R) P				***

	Smoking status ever	current	Total
N	154	191	345
NS	116	129	245
Wt	12785.61	6876.24	19661.85
Het Chi	1860.97	2642.76	9854.69
Het df	153	190	344
Het P	***	***	***
Fixed RR	3.04	9.09	4.46
RRl	2.99	8.87	4.40
RRu	3.10	9.30	4.52
P	+++	+++	+++
Random RR	4.19	8.18	6.15
RRl	3.85	7.41	5.63
RRu	4.57	9.03	6.71
P	+++	+++	+++
Between Chi			5350.96
Between df			1
Between P			***
Btwn(F) P			***
Btwn(R) P			***

	Study LIU4 LIU4	others	Total
N	2	343	345
NS	1	241	242
Wt	9846.05	9815.80	19661.85
Het Chi	2.98	5567.83	9854.69
Het df	1	342	344
Het P	(*)	***	***
Fixed RR	2.80	7.12	4.46
RRl	2.74	6.98	4.40
RRu	2.85	7.26	4.52
P	+++	+++	+++
Random RR	2.81	6.18	6.15
RRl	2.71	5.67	5.63
RRu	2.91	6.74	6.71
P	+++	+++	+++
Between Chi			4283.88
Between df			1
Between P			***
Btwn(F) P			***
Btwn(R) P			***

Table 1C2 - 7

IESLC - Meta-anal of Current Smoking (or Ever if Current not available), Any prod (or Cigs if Any not avail)
All LC types
Excluded studies (and stage at which they were excluded)

1	BOUCHA BUELL LAURIL MZILEN
2	BERRIN CHEN RESTRE TAO ULMER
5	RIMING TANG2 WYNDE5 WYNDE8
6	BLOT1 BLOT2 BLOT3 BYERS2 HIRAOK HIRAY2 JONES LICKIN MOLLO MRFIT MURATA NOTANI PERSHA SAARIK SCHWA2 SHIMIZ SITAS VANDER WARSIN WATSON WYNDER
8	BROWN1 BYERS1 SUZUKI WU2
10	BENHAM GILLIS ISHIMA LUBIN OSANN2 PISANI PRESCO QIAO ROOTS SIMONA VEIERO VUTUC WYNDE7

Table 1C2 - 8
Potentially overlapping studies

REF	REFGP	PRINC	. OVERLAP/LINK
LUBIN2	LUBIN2	1	Lubin-combined
CHAN	CHAN	1	Chan/LamWK2
LAMWK2	LAMWK2	1	Chan/LamWK2
LAMTH	LAMTH	1	KOO/LAMTH/LAMWK
KOO	KOO	1	KOO/LAMTH/LAMWK
LAMWK	LAMWK	1	KOO/LAMTH/LAMWK
CASCOR	CASCOR	1	Roots/Cascor 1
TVERDA	TVERDA	1	VEIERO/TVERDAL
AKIBA	AKIBA	1	AKIBA/ISHIMA
HEIN	PRESCO	1	Subset of PRESCO
LANGE	PRESCO	1	Subset of PRESCO
BROSS	BYERS1	1	GRAHAM/BROSS/BYERS1
GRAHAM	BYERS1	1	GRAHAM/BROSS/BYERS1
CHYOU	CHYOU	1	GOODMA/CHYOU
GOODMA	GOODMA	1	GOODMA/CHYOU
HOLE	TANG2	1	Subset of TANG2
BENSHL	TANG2	1	Subset of TANG2
WALD	TANG2	1	Subset of TANG2
KAISER	KAISER	1	KAISER/OSANN2
MRFITR	MRFIT	2	Subset of MRFIT
WYNDE6	WYNDE6	1	WYNDE5/6/7/8
CPSI	CPSI	1	CPSI overall
ENSTRO	ENSTRO	1	Subset of CPSI
SOBUE2	SOBUE2	1	SOBUE2/MATSUD
MATSUD	MATSUD	1	SOBUE2/MATSUD
BOFFET	BOFFET	1	BOFFET-combined
JAHN	BOFFET	2	Subset of BOFFET
XIANGZ	XIANGZ	1	LUBIN/XIANGZ/QIAO

Table 1C2 - 9
Most adjusted - insufficient data for metaanalysis

REF	NRR	SEX	AGEL	AGEH	RACE	YF	LC TYPE	LOC	START	ST	NLC	R	VB	P	H	AD	SM	PRODUCT	DENOM	De
DOLL2	45	f	0	0	all	11	all	Eu:UK	1951	pr	920	n	V	n	n	1	cu	all/unsp	nev any	st
HIRAYA	152	m	0	0	all	0	all	As:Jap	1965	pr	1917	n	bl	n	n	4	cu	cig+/-ot	nev any	or
RESTRE	2	c	0	0	all	-	all	SCAmer	1978	CC	102	n	bl	n	n	2	cu	cig+/-ot	nev cigs	ot
TAO	1	m	0	0	all	-	all	As:Chi	1988	CC	723	n	ot	n	n	5	ev	all/unsp	nev any	or
TAO	2	f	0	0	all	-	all	As:Chi	1988	CC	723	n	ot	n	n	5	ev	all/unsp	nev any	or
ULMER	1	c	0	0	all	-	all	Eu:Ger	1971	CC	726	n	bl	y	n	0	ev	all/unsp	nev any	or

REF	NRR	RR	SIG	RRDATA comment
DOLL2	45	5.00		0
HIRAYA	152	4.26		0
RESTRE	2	10.28	y	0
TAO	1	3.95		0
TAO	2	4.93		0
ULMER	1	12.72		0

Least adjusted - insufficient data for meta-analysis: as for adjusted plus the following

REF	NRR	SEX	AGEL	AGEH	RACE	YF	LC TYPE	LOC	START	ST	NLC	R	VB	P	H	AD	SM	PRODUCT	DENOM	De
CORREA	59	m	0	0	wh	-	all	NAmer	1979	CC	1359	n	bl	y	n	0	ev	cig+/-ot	nev cigs	or
CORREA	61	m	0	0	bl	-	all	NAmer	1979	CC	1359	n	bl	y	n	0	ev	cig+/-ot	nev cigs	or
CORREA	60	f	0	0	wh	-	all	NAmer	1979	CC	1359	n	bl	y	n	0	ev	cig+/-ot	nev cigs	or

Table 1C2 - 9

IESLC - Meta-anal of Current Smoking (or Ever if Current not available), Any prod (or Cigs if Any not avail)
All LC types
Least adjusted - insufficient data for meta-analysis: as for adjusted plus the following

REF	NRR	SEX	AGEL	AGEH	RACE	YF	LC TYPE	LOC	START	ST	NLC	R	VB	P	H	AD	SM	PRODUCT	DENOM	De
CORREA	62	f	0	0	bl	-	all	NAmer	1979	CC	1359	n	bl	y	n	0	ev	cig+/-ot	nev cigs	or
LIU	1	c	0	0	all	-	all	As:Chi	1980	CC	229	n	ot	*	n	0	ev	all/unsp	nev any	or

REF	NRR	RR	SIG	RRDATA comment
CORREA	59	17.20		0
CORREA	61	13.10		0
CORREA	60	8.60		0
CORREA	62	22.00		0
LIU	1	2.45		0

Table 1C3 -

IESLC - Meta-anal of Ever Smoking (or Current if Ever not available), Cigs (or Any Prod if Cigs not avail)
All LC types

This analysis is restricted to results for:
1) Non-dose-response data
2) Results complete enough for use in metaanalysis

Within each study, results are then selected (in the following order of preference, within each sex) for:
3) SMKSTA: ever smokers, current smokers
4) PRODUCT: cigarettes regardless of other products, cigarettes only, all/unspec
5) CIGTYPE: all/unspecified, MC regardless of HR, MC only
6) DENOM: never smoked anything, never smoked cigarettes, (never +1 = +long term ex, +2 = +amount unknown,
+3 = never cigs+long term ex)
7) Followup period (YF, prospective studies): whole study (coded as 0) or longest available
8) LCtype: all or nearest available, at least Squamous and Adeno. (q = squamous, s = small,
l = large, a = adeno, mix = mixed, alv = alveolar)
9) Race: all or nearest available, otherwise by race (wh or w = white, bl or b = black, hi = hispanic
ch = chinese, jap = japanese, haw = hawaiian, w+o = white + oriental, sca = scandinavian, as = asian)
10) For overlapping studies: principal rather than subsidiary studies
Finally by Age: whole study (coded as 0) if available, otherwise by widest available age group
and then for single sex results (m, f) in preference to combined sex results (c).

Results adjusted (AD) for the most potential confounders are then chosen in Sections -1 to -3
(and those which actually differ from the adjusted results in Table 1C1 - 1 are marked 'x' in Section -1)
and results adjusted for the least confounders in Sections -4 to -6. (Those least adjusted results which
actually differ from the most adjusted as marked 'x' in column X in Section -4)
(Results adjusted for an unknown number of confounder(s) are coded as 20.)

Section -7 shows excluded studies, together with the stage (as above) at which no qualifying
results were found.

Section -8 lists the potentially overlapping studies which have been included (1=principal, 2=subsidiary).

Section -9 lists any results which would have been included in preference except that they had data not complete
enough for use in meta-analysis, with their significance (yes/no), if known, and any further comment as entered
on the database.

In addition to those mentioned above, the following fields, levels and abbreviations are used:
* or nk = not known, n = no, y = yes, ot = other
ev = ever, cu = current, nev = never
all/unspec = all or unspecified, cig+/-ot = cigarettes irrespective of other products (cigar, pipe etc)
MC = manufactured cigarettes, HR = hand-rolled cigarettes
REF: 6-character study reference
NRR: number of the RR on the database within the study
ST : study type (CC = case control, pr or prosp = prospective)
NLC: number of lung cancer cases in whole study
R : risky occupational population (n = no, m = mining, o = other risky)
VB : national cigarette type (V = at least 75% Virginia, bl = at least 75% blended, ot = other)
P : any proxy use
H : full histological confirmation
De : derivation of RR/CI (or = original, st = standard method, ot = other method of estimation)

Table 1C3 - 1

IESLC - Meta-anal of Ever Smoking (or Current if Ever not available), Cigs (or Any Prod if Cigs not avail)
All LC types
Most adjusted

REF	NRR	1C1	SEX	AGEL	AGEH	RACE	YF	LC TYPE	LOC	START	ST	NLC	R	VB	P	H	AD	SM	PRODUCT	DENOM	De
ABELIN	45	x	m	0	0	all	-	all	Eu:wst	1941	CC	118	n	bl	y	n	1	ev	cig+/-ot	nev any	st
ABRAHA	7		m	0	0	all	0	q+s+a	Eu:est	1975	pr	571	n	bl	n	n	0	ev	all/unsp	nev any	ot
ABRAHA	8		f	0	0	all	0	q+s+a	Eu:est	1975	pr	571	n	bl	n	n	0	ev	all/unsp	nev any	ot
AGUDO	1		f	0	0	all	-	all	Eu:wst	1989	CC	103	n	bl	n	n	3	ev	cig only	nev any	or
AKIBA	11		m	0	0	all	0	all	As:Jap	1963	pr	610	n	bl	n	n	5	ev	cig+/-ot	nev cigs	ot
AKIBA	15		f	0	0	all	0	all	As:Jap	1963	pr	610	n	bl	n	n	5	ev	cig+/-ot	nev cigs	ot
ALDERS	68	x	m	0	0	all	-	all	Eu:UK	1977	CC	1448	n	V	n	n	1	ev	cig+/-ot	nev any	ot
ALDERS	6		f	0	0	all	-	all	Eu:UK	1977	CC	1448	n	V	n	n	1	ev	cig only	nev any	ot
AMANDU	7		m	0	0	wh	0	all	NAmer	1959	pr	132	m	bl	n	n	2	ev	cig+/-ot	nev cigs	ot
AMES	4		m	0	0	wh	-	all	NAmer	1959	ot	317	m	bl	n	n	0	ev	all/unsp	nev any	st
ANDERS	3		f	0	0	all	0	all	NAmer	1986	pr	343	n	bl	n	n	0	ev	cig+/-ot	nev cigs	st
ARCHER	6		m	0	0	wh	0	all	NAmer	1950	pr	146	m	bl	n	n	0	ev	cig+/-ot	nev cigs	st
ARMADA	4	x	m	0	0	all	-	all	Eu:wst	1986	CC	325	n	bl	n	y	0	ev	cig+/-ot	nev any	st
AUSTIN	7		c	0	0	all	-	all	NAmer	1970	CC	166	o	bl	y	n	3	ev	cig+/-ot	nev cigs	ot
AUVINE	19		c	0	0	all	-	all	Eu:Sca	1986	CC	517	n	bl	y	n	2	ev	cig+/-ot	nev cigs	ot
AXELSO	1		c	0	0	all	-	all	Eu:Sca	1960	CC	152	n	bl	y	n	0	ev	all/unsp	nev any	st
AXELSS	8		m	0	0	sca	-	all	Eu:Sca	1989	CC	436	n	bl	n	n	6	ev	all/unsp	nev any	ot
AXELSS	11		f	0	0	sca	-	all	Eu:Sca	1989	CC	436	n	bl	n	n	0	ev	all/unsp	nev any	st
BAND	1		m	0	0	all	-	all	NAmer	1983	CC	2831	n	V	y	y	2	ev	cig only	nev any	ot
BARBON	131		m	0	0	all	-	all	Eu:wst	1979	CC	755	n	bl	y	y	3	ev	all/unsp	nev any	ot
BECHER	21	x	m	0	0	all	-	all	Eu:Ger	1985	CC	194	n	bl	n	y	2	ev	cig+/-ot	nev any	ot
BECHER	16	x	f	0	0	all	-	all	Eu:Ger	1985	CC	194	n	bl	n	y	0	ev	cig+/-ot	nev any	st
BENSHL	15	x	m	0	0	all	0	all	Eu:UK	1967	pr	486	n	V	n	n	1	ev	cig+/-ot	nev any	ot
BEST	23	x	m	55	79	all	3	all	NAmer	1955	pr	381	n	V	n	n	0	ev	cig+/-ot	nev any	st
BEST	18		f	0	0	all	0	all	NAmer	1955	pr	381	n	V	n	n	1	ev	cig only	nev any	ot
BLOHMK	3		m	0	0	all	-	all	Eu:Ger	1978	CC	888	n	bl	n	y	0	ev	all/unsp	nev any	st
BLOT4	1		m	0	0	wh	-	all	NAmer	1974	CC	335	n	bl	y	n	0	ev	cig+/-ot	nev cigs	st
BOFFET	27	x	m	0	0	all	-	all	Eu:mul	1988	CC	5621	n	bl	y	n	2	ev	cig+/-ot	nev any	ot
BOUCOT	122	x	m	0	0	all	0	all	NAmer	1951	pr	121	n	bl	n	n	2	ev	cig+/-ot	nev any	ot
BRESLO	17	x	m	0	0	all	-	all	NAmer	1949	CC	518	n	bl	n	y	0	ev	cig+/-ot	nev+1	st
BRESLO	23	x	f	0	0	all	-	all	NAmer	1949	CC	518	n	bl	n	y	0	ev	cig+/-ot	nev+1	st
BRETT	10		m	0	0	all	0	all	Eu:UK	1960	pr	150	n	V	n	n	0	ev	cig+/-ot	nev cigs	st
BROCKM	1		m	0	0	wh	-	all	Eu:Ger	1990	CC	117	n	bl	n	y	0	ev	cig+/-ot	nev cigs	st
BROCKM	2		f	0	0	wh	-	all	Eu:Ger	1990	CC	117	n	bl	n	y	0	ev	cig+/-ot	nev cigs	st
BROSS	13	x	m	0	0	wh	-	all	NAmer	1960	CC	974	n	bl	n	n	0	ev	cig+/-ot	nev any	st
BROWN2	2		m	0	0	wh	-	all	NAmer	1984	CC	14596	n	bl	n	y	2	ev	cig+/-ot	nev cigs	or
BROWN2	1		f	0	0	wh	-	all	NAmer	1984	CC	14596	n	bl	n	y	2	ev	cig+/-ot	nev cigs	or
BUFFLE	2	x	m	0	0	wh	-	all	NAmer	1976	CC	943	n	bl	y	n	0	ev	cig+/-ot	nev any	st
BUFFLE	6	x	f	0	0	wh	-	all	NAmer	1976	CC	943	n	bl	y	n	0	ev	cig+/-ot	nev any	st
CARPEN	12		c	0	0	w+b	-	all	NAmer	1991	CC	356	n	bl	n	n	3	ev	cig+/-ot	nev cigs	ot
CASCO2	1		c	0	0	wh	-	all	Eu:Ger	1991	CC	155	n	bl	n	n	0	ev	all/unsp	nev any	st
CASCOR	1		c	0	0	wh	-	all	Eu:Ger	1985	CC	389	n	bl	n	y	0	ev	all/unsp	nev any	st
CEDERL	107		m	0	0	all	16	all	Eu:Sca	1963	pr	491	n	bl	n	n	2	ev	all/unsp	nev any	ot
CEDERL	112		f	0	0	all	0	all	Eu:Sca	1963	pr	491	n	bl	n	n	2	ev	all/unsp	nev any	ot
CHAN	5	x	m	0	0	all	-	all	As:HK	1976	CC	397	n	bl	n	n	0	ev	cig+/-ot	nev any	st
CHAN	6	x	f	0	0	all	-	all	As:HK	1976	CC	397	n	bl	n	n	0	ev	cig+/-ot	nev any	st
CHANG	6		m	0	0	all	0	all	NAmer	1972	pr	136	n	bl	n	n	0	ev	cig+/-ot	nev cigs	st
CHANG	12		f	0	0	all	0	all	NAmer	1972	pr	136	n	bl	n	n	0	ev	cig+/-ot	nev cigs	st
CHATZI	4		c	0	0	all	-	all	Eu:bal	1987	CC	282	n	bl	n	y	0	ev	all/unsp	nev any	st
CHEN2	1		m	0	0	all	-	all	As:Chi	1983	CC	193	n	ot	y	n	0	ev	all/unsp	nev any	st
CHEN2	2		f	0	0	all	-	all	As:Chi	1983	CC	193	n	ot	y	n	0	ev	all/unsp	nev any	st
CHEN3	1		c	0	0	all	-	all	As:Chi	1981	CC	254	n	ot	y	n	0	ev	all/unsp	nev any	st
CHIAZZ	3		m	0	0	all	-	all	NAmer	1940	CC	144	o	bl	y	n	11	ev	cig+/-ot	nev cigs	or
CHOI	1		m	0	0	all	-	all	As:oth	1985	CC	375	n	bl	n	n	0	ev	cig+/-ot	nev cigs	st
CHOI	5		f	0	0	all	-	all	As:oth	1985	CC	375	n	bl	n	n	0	ev	cig+/-ot	nev cigs	st
CHOW	54	x	m	0	0	wh	0	all	NAmer	1966	pr	219	n	bl	n	n	2	ev	cig+/-ot	nev any	ot
CHYOU	7		m	0	0	jap	0	all	NAmer	1965	pr	227	n	bl	n	y	1	ev	cig+/-ot	nev cigs	ot
COMSTO	33	x	m	0	0	all	-	all	NAmer	1975	ot	258	n	bl	n	n	0	ev	cig+/-ot	nev any	st
COMSTO	45	x	f	0	0	all	-	all	NAmer	1975	ot	258	n	bl	n	n	0	ev	cig+/-ot	nev any	st
COOKSO	4	x	c	0	0	bl	-	all	Africa	1961	CC	234	n	V	n	y	0	ev	cig+/-ot	nev any	st
CORREA	34		c	0	0	all	-	all	NAmer	1979	CC	1359	n	bl	y	n	1	ev	cig+/-ot	nev cigs	or
CPSI	187		m	35	84	all	6	all	NAmer	1959	pr	5138	n	bl	n	n	1	ev	cig+/-ot	nev any	ot
CPSI	274		f	40	74	all	6	all	NAmer	1959	pr	5138	n	bl	n	n	1	ev	cig+/-ot	nev cigs	ot
CPSII	104		m	35	99	all	4	all	NAmer	1982	pr	3229	n	bl	n	n	1	ev	cig only	nev any	ot
CPSII	79		f	0	0	all	4	all	NAmer	1982	pr	3229	n	bl	n	n	1	ev	cig+/-ot	nev cigs	ot
DAMBER	37	x	m	0	0	all	-	all	Eu:Sca	1972	CC	579	n	bl	y	n	1	ev	cig+/-ot	nev any	ot
DARBY	15		m	0	0	wh	-	all	Eu:UK	1988	CC	982	n	V	n	n	0	ev	all/unsp	nev any	st
DARBY	16		f	0	0	wh	-	all	Eu:UK	1988	CC	982	n	V	n	n	0	ev	all/unsp	nev any	st
DAVEYS	5		m	0	0	all	-	all	Eu:Ger	1930	CC	109	n	bl	y	n	0	ev	all/unsp	nev any	st
DAVEYS	6		f	0	0	all	-	all	Eu:Ger	1930	CC	109	n	bl	y	n	0	ev	all/unsp	nev any	ot
DEAN	8	x	m	0	0	wh	-	all	Africa	1947	CC	603	n	V	y	n	0	ev	cig+/-ot	nev any	st
DEAN2	12	x	m	0	0	all	-	all	Eu:UK	1960	CC	954	n	V	y	n	0	ev	cig+/-ot	nev any	st

Table 1C3 - 1

IESLC - Meta-anal of Ever Smoking (or Current if Ever not available), Cigs (or Any Prod if Cigs not avail)
All LC types
Most adjusted

REF	NRR	1C1	SEX	AGEL	AGEH	RACE	YF	LC TYPE	LOC	START	ST	NLC	R	VB	P	H	AD	SM	PRODUCT	DENOM	De
DEAN2	20	x	f	0	0	all	-	all	Eu:UK	1960	CC	954	n	V	y	n	0	ev	cig+/-ot	nev any	st
DEAN3	241	x	m	0	0	all	-	all	Eu:UK	1969	CC	766	n	V	y	n	1	ev	cig only	nev any	ot
DEAN3	126		f	0	0	all	-	all	Eu:UK	1969	CC	766	n	V	y	n	3	ev	cig only	nev any	ot
DEKLER	6		m	0	0	all	0	all	Auslia	1961	pr	138	m	V	n	n	2	ev	all/unsp	nev any	ot
DESTE2	15	x	m	0	0	all	-	all	SCAmer	1993	CC	463	n	bl	n	n	0	ev	all/unsp	nev any	st
DESTEF	13	x	m	0	0	all	-	all	SCAmer	1988	CC	497	n	bl	n	y	4	ev	cig+/-ot	nev any	or
DOCKER	3		c	0	0	wh	0	all	NAmer	1974	pr	120	n	bl	n	n	4	ev	cig+/-ot	nev cigs	ot
DOLL	20	x	m	0	0	all	-	all	Eu:UK	1948	CC	1465	n	V	n	n	0	ev	cig+/-ot	nev any	st
DOLL	12		f	0	0	all	-	all	Eu:UK	1948	CC	1465	n	V	n	n	0	ev	all/unsp	nev any	st
DOLL2	88	x	m	0	0	all	10	all	Eu:UK	1951	pr	920	n	V	n	n	1	ev	cig+/-ot	nev any	ot
DOLL2	63		f	0	0	all	22	all	Eu:UK	1951	pr	920	n	V	n	n	1	cu	cig only	nev any	ot
DORANT	10		c	0	0	all	0	all	Eu:wst	1986	ot	550	n	bl	n	y	0	ev	all/unsp	nev any	st
DORGAN	107	x	m	0	0	wh	-	all	NAmer	1980	CC	2026	n	bl	y	y	2	ev	cig+/-ot	nev any	or
DORGAN	95	x	f	0	0	all	-	all	NAmer	1980	CC	2026	n	bl	y	y	3	ev	cig+/-ot	nev any	or
DORN	413	x	m	0	0	wh	5	all	NAmer	1954	pr	5097	n	bl	n	n	1	ev	cig+/-ot	nev any	ot
DOSEME	1		m	0	0	all	-	all	Eu:bal	1979	CC	1210	n	bl	n	n	2	ev	cig+/-ot	nev cigs	or
DROSTE	7		m	0	0	all	-	all	Eu:wst	1995	CC	478	n	bl	n	y	4	ev	all/unsp	nev any	ot
DU	1		m	0	0	all	-	all	As:Chi	1985	CC	849	n	ot	y	n	0	ev	all/unsp	nev any	or
DU	2		f	0	0	all	-	all	As:Chi	1985	CC	849	n	ot	y	n	0	ev	all/unsp	nev any	or
DUNN	6		m	0	0	all	0	all	NAmer	1954	pr	139	o	bl	n	n	0	ev	cig+/-ot	nev cigs	st
EBELIN	1		m	0	0	all	-	all	Eu:Ger	1980	CC	130	n	bl	n	n	0	ev	all/unsp	nev any	st
ENGELA	36	x	m	0	0	all	0	all	Eu:Sca	1964	pr	435	n	bl	n	n	7	ev	cig+/-ot	nev cigs	ot
ENGELA	49	x	f	0	0	all	0	all	Eu:Sca	1964	pr	435	n	bl	n	n	5	ev	cig+/-ot	nev cigs	ot
ENSTRO	1		m	0	0	all	0	all	NAmer	1959	pr	2879	n	bl	n	n	1	cu	cig only	nev any	or
ENSTRO	2		f	0	0	all	0	all	NAmer	1959	pr	2879	n	bl	n	n	1	cu	cig only	nev any	or
ESAKI	4		m	0	0	all	-	all	As:Jap	1961	CC	245	n	bl	y	n	0	ev	cig+/-ot	nev cigs	st
ESAKI	5		f	0	0	all	-	all	As:Jap	1961	CC	245	n	bl	y	n	0	ev	cig+/-ot	nev cigs	st
FAN	1		m	0	0	all	-	all	As:Chi	1990	CC	403	n	ot	y	n	0	ev	cig+/-ot	nev cigs	st
FAN	2		f	0	0	all	-	all	As:Chi	1990	CC	403	n	ot	y	n	0	ev	cig+/-ot	nev cigs	st
GAO	1		m	0	0	all	-	all	As:Chi	1984	CC	1405	n	ot	n	n	2	ev	cig+/-ot	nev cigs	or
GAO	11		f	0	0	all	-	all	As:Chi	1984	CC	1405	n	ot	n	n	2	ev	cig+/-ot	nev cigs	or
GAO2	10		m	0	0	all	-	all	As:Jap	1988	CC	282	n	bl	n	n	1	ev	cig+/-ot	nev cigs	ot
GARCIA	3		c	0	0	all	-	all	NAmer	1992	CC	416	n	bl	n	y	0	ev	cig+/-ot	nev cigs	st
GARDIN	7		c	0	0	all	-	all	Eu:UK	1988	CC	143	n	V	y	n	0	ev	all/unsp	nev any	st
GARSHI	25		m	0	0	all	-	all	NAmer	1981	CC	1081	o	bl	y	n	1	ev	all/unsp	nev any	st
GENG	1		m	0	0	all	-	all	As:Chi	1985	CC	292	n	ot	*	n	0	ev	cig+/-ot	nev any	st
GENG	2		f	0	0	all	-	all	As:Chi	1985	CC	292	n	ot	*	n	0	ev	cig+/-ot	nev any	st
GER	21		c	0	0	all	-	all	As:oth	1990	CC	141	n	ot	y	n	14	ev	all/unsp	nev any	ot
GODLEY	5		m	0	0	all	-	all	NAmer	1966	CC	1986	n	bl	y	n	1	ev	cig+/-ot	nev cigs	ot
GODLEY	6		f	0	0	all	-	all	NAmer	1966	CC	1986	n	bl	y	n	1	ev	cig+/-ot	nev cigs	ot
GOLLED	7		m	35	99	all	-	all	Eu:UK	1952	CC	443	n	V	y	n	1	ev	cig+/-ot	nev any	ot
GOODMA	3		m	0	0	w+o	-	all	NAmer	1983	CC	326	n	bl	y	y	0	ev	cig+/-ot	nev any	st
GOODMA	7		f	0	0	w+o	-	all	NAmer	1983	CC	326	n	bl	y	y	0	ev	cig+/-ot	nev any	st
GRAHAM	23	x	m	0	0	wh	-	all	NAmer	1956	CC	685	n	bl	n	n	1	ev	cig+/-ot	nev any	ot
GREGOR	3		m	0	0	all	-	all	Eu:UK	1976	CC	104	n	V	n	y	0	ev	cig+/-ot	nev cigs	st
GREGOR	7		f	0	0	all	-	all	Eu:UK	1976	CC	104	n	V	n	y	0	ev	cig+/-ot	nev cigs	st
GSELL	6	x	m	0	0	all	-	all	Eu:wst	1937	CC	150	n	bl	n	y	0	ev	cig+/-ot	nev any	st
HAENSZ	56	x	f	0	0	all	-	not alv	NAmer	1955	CC	158	n	bl	n	y	0	ev	cig+/-ot	nev any	st
HAMMO2	2	x	m	0	0	all	6	all	NAmer	1967	pr	450	o	bl	n	n	1	ev	cig+/-ot	nev any	ot
HAMMON	116	x	m	0	0	wh	0	all	NAmer	1952	pr	448	n	bl	n	n	1	ev	cig+/-ot	nev any	ot
HANSEN	3		m	0	0	all	0	all	Eu:Sca	1968	pr	105	o	bl	y	n	2	ev	all/unsp	nev any	ot
HEGMAN	1		c	0	0	all	-	all	NAmer	1989	CC	282	n	bl	y	y	0	ev	all/unsp	nev any	st
HEIN	7		m	0	0	all	0	all	Eu:Sca	1970	pr	144	n	bl	n	n	0	ev	all/unsp	nev any	st
HENNEK	3		m	0	0	all	0	all	NAmer	1982	pr	169	n	bl	n	n	0	ev	all/unsp	nev any	st
HINDS	22		f	0	0	o	-	all	NAmer	1968	CC	292	n	bl	n	n	3	ev	all/unsp	nev any	st
HIRAYA	147		m	0	0	all	0	all	As:Jap	1965	pr	1917	n	bl	n	n	1	ev	cig+/-ot	nev any	ot
HIRAYA	150		f	0	0	all	0	all	As:Jap	1965	pr	1917	n	bl	n	n	1	ev	cig+/-ot	nev any	ot
HITOSU	38		m	0	0	all	-	all	As:Jap	1960	CC	216	n	bl	y	n	1	ev	all/unsp	nev any	st
HITOSU	62		f	0	0	all	-	all	As:Jap	1960	CC	216	n	bl	y	n	1	ev	all/unsp	nev any	st
HOLE	8		m	0	0	all	0	all	Eu:UK	1972	pr	225	n	V	n	n	1	ev	all/unsp	nev any	ot
HOLE	31		f	0	0	all	11	all	Eu:UK	1972	pr	225	n	V	n	n	1	cu	all/unsp	nev any	ot
HOROWI	1		m	0	0	all	-	all	NAmer	1956	CC	236	n	V	n	n	0	ev	cig+/-ot	nev any	st
HOROWI	2		f	0	0	all	-	all	NAmer	1956	CC	236	n	V	n	n	0	ev	cig+/-ot	nev any	st
HORWIT	1		f	0	0	all	-	all	NAmer	1977	CC	112	n	bl	n	n	0	ev	cig+/-ot	nev cigs	st
HU	15		m	0	0	all	-	all	As:Chi	1985	CC	227	n	ot	n	y	0	ev	cig+/-ot	nev any	st
HU	16		f	0	0	all	-	all	As:Chi	1985	CC	227	n	ot	n	y	0	ev	cig+/-ot	nev any	st
HU2	9		m	0	0	all	-	all	As:Chi	1977	CC	523	n	ot	y	n	0	ev	cig+/-ot	nev cigs	st
HU2	10		f	0	0	all	-	all	As:Chi	1977	CC	523	n	ot	y	n	0	ev	cig+/-ot	nev cigs	st
HUANG	1		c	0	0	all	-	all	As:Chi	1990	CC	135	n	ot	y	n	0	ev	all/unsp	nev any	st
HUMBLE	14		m	0	0	w-hi	-	all	NAmer	1980	CC	521	n	bl	y	n	1	ev	cig+/-ot	nev cigs	ot
HUMBLE	16		m	0	0	hi	-	all	NAmer	1980	CC	521	n	bl	y	n	1	ev	cig+/-ot	nev cigs	ot
HUMBLE	18		f	0	0	w-hi	-	all	NAmer	1980	CC	521	n	bl	y	n	1	ev	cig+/-ot	nev cigs	ot

Table 1C3 - 1

IESLC - Meta-anal of Ever Smoking (or Current if Ever not available), Cigs (or Any Prod if Cigs not avail)
All LC types
Most adjusted

REF	NRR	1C1	SEX	AGEL	AGEH	RACE	YF	LC TYPE	LOC	START	ST	NLC	R	VB	P	H	AD	SM	PRODUCT	DENOM	De
HUMBLE	20		f	0	0	hi	-	all	NAmer	1980	CC	521	n	bl	y	n	1	ev	cig+/-ot	nev cigs	ot
JAHN	22		f	0	0	all	-	all	Eu:Ger	1988	CC	1004	n	bl	n	n	2	ev	cig+/-ot	nev any	ot
JAIN	46		m	0	0	all	-	all	NAmer	1981	CC	845	n	V	y	n	2	ev	cig+/-ot	nev cigs	or
JAIN	41		f	0	0	all	-	all	NAmer	1981	CC	845	n	V	y	n	2	ev	cig+/-ot	nev cigs	or
JARUP	6		m	0	0	all	-	all	Eu:Sca	1928	CC	102	o	bl	y	n	2	ev	all/unsp	nev any	ot
JARVHO	3		m	0	0	all	-	all	Eu:Sca	1983	CC	147	n	bl	n	n	0	ev	all/unsp	nev any	st
JARVHO	7		f	0	0	all	-	all	Eu:Sca	1983	CC	147	n	bl	n	n	0	ev	all/unsp	nev any	st
JEDRYC	58		m	0	0	all	-	all	Eu:est	1980	CC	1630	n	bl	y	n	4	ev	cig+/-ot	nev any	ot
JEDRYC	59		f	0	0	all	-	all	Eu:est	1980	CC	1630	n	bl	y	n	4	ev	cig+/-ot	nev any	ot
JIANG	1		m	0	0	all	-	all	As:Chi	1984	CC	125	n	ot	n	n	0	ev	all/unsp	nev any	st
JIANG	2		f	0	0	all	-	all	As:Chi	1984	CC	125	n	ot	n	n	0	ev	all/unsp	nev any	st
JOLY	2	x	m	0	0	all	-	all	SCAmer	1978	CC	826	n	bl	n	n	0	ev	cig+/-ot	nev any	st
JOLY	1		f	0	0	all	-	all	SCAmer	1978	CC	826	n	bl	n	n	0	ev	cig+/-ot	nev any	st
JUSSAW	31	x	m	0	0	all	-	all	As:Ind	1964	CC	792	n	V	n	n	2	ev	cig only	nev any	st
KAISE2	72		m	35	99	all	9	all	NAmer	1979	pr	318	n	bl	n	n	1	ev	cig only	nev any	st
KAISE2	64		f	35	99	all	9	all	NAmer	1979	pr	318	n	bl	n	n	1	ev	cig only	nev any	st
KAISER	13		m	0	0	all	0	all	NAmer	1964	pr	714	n	bl	n	n	2	ev	cig+/-ot	nev cigs	ot
KAISER	10		f	0	0	all	0	all	NAmer	1964	pr	714	n	bl	n	n	2	ev	cig+/-ot	nev cigs	ot
KANELL	30		m	0	0	all	-	all	Eu:bal	1950	CC	862	n	bl	n	n	1	cu	all/unsp	nev any	st
KATSOU	29		f	0	0	all	-	all	Eu:bal	1987	CC	101	n	bl	n	n	1	ev	all/unsp	nev any	ot
KAUFMA	17		c	0	0	all	-	all	NAmer	1981	CC	881	n	bl	n	n	6	ev	cig+/-ot	nev cigs	ot
KELLER	3		m	0	0	wh	-	all	NAmer	1985	CC	15038	n	bl	n	n	0	ev	all/unsp	nev any	st
KELLER	11		m	0	0	nonw	-	all	NAmer	1985	CC	15038	n	bl	n	n	0	ev	all/unsp	nev any	st
KELLER	7		f	0	0	wh	-	all	NAmer	1985	CC	15038	n	bl	n	n	0	ev	all/unsp	nev any	st
KELLER	15		f	0	0	nonw	-	all	NAmer	1985	CC	15038	n	bl	n	n	0	ev	all/unsp	nev any	st
KHUDER	4		m	0	0	all	-	all	NAmer	1985	CC	482	n	bl	n	y	0	ev	cig+/-ot	nev cigs	st
KIHARA	31		c	0	0	jap	-	all	As:Jap	1991	CC	440	n	bl	n	n	0	ev	all/unsp	nev any	st
KINLEN	17		m	0	0	all	0	all	Eu:UK	1967	pr	718	n	V	n	n	2	ev	all/unsp	nev any	ot
KJUUS	3	x	m	0	0	all	-	all	Eu:Sca	1979	CC	176	n	bl	n	n	0	ev	cig only	nev any	st
KNEKT	87		m	20	69	all	21	all	Eu:Sca	1966	pr	515	n	bl	n	n	1	ev	all/unsp	nev any	ot
KO	1		f	0	0	all	-	all	As:oth	1992	CC	117	n	ot	n	y	3	ev	cig+/-ot	nev cigs	or
KOHLME	2		c	0	0	all	-	all	Eu:Ger	1990	CC	239	n	bl	n	n	4	ev	all/unsp	nev any	or
KOO	1		f	0	0	all	-	all	As:HK	1981	CC	200	n	bl	n	n	0	ev	all/unsp	nev any	st
KOULUM	2	x	m	0	0	all	-	all	Eu:Sca	1936	CC	812	n	bl	n	n	0	ev	cig only	nev any	st
KREUZE	60	x	f	1	45	all	-	all	Eu:Ger	1990	CC	2260	n	bl	n	n	3	ev	cig+/-ot	nev any	ot
KREUZE	62	x	f	55	69	all	-	all	Eu:Ger	1990	CC	2260	n	bl	n	n	3	ev	cig+/-ot	nev any	ot
KREYBE	12		m	0	0	all	-	all	Eu:Sca	1948	CC	300	n	bl	n	y	1	ev	all/unsp	nev any	ot
KREYBE	30		f	0	0	all	-	all	Eu:Sca	1948	CC	300	n	bl	n	y	1	ev	all/unsp	nev any	ot
KUBIK	27	x	m	0	0	all	0	all	Eu:est	1965	pr	108	n	bl	n	n	0	ev	cig+/-ot	nev any	st
LAMTH	6		f	0	0	ch	-	all	As:HK	1983	CC	445	n	bl	n	n	0	ev	all/unsp	nev any	or
LAMWK	1		f	0	0	ch	-	all	As:HK	1981	CC	163	n	bl	n	n	0	ev	all/unsp	nev any	st
LAMWK2	9		m	0	0	all	-	q+s+l+a	As:HK	1976	CC	480	n	bl	n	n	0	ev	all/unsp	nev any	st
LAMWK2	10		f	0	0	all	-	q+s+l+a	As:HK	1976	CC	480	n	bl	n	n	0	ev	all/unsp	nev any	st
LANGE	40		m	0	0	all	0	all	Eu:Sca	1976	pr	268	n	bl	n	n	1	ev	all/unsp	nev any	ot
LANGE	37		f	0	0	all	0	all	Eu:Sca	1976	pr	268	n	bl	n	n	1	ev	all/unsp	nev any	ot
LAUSSM	11		m	0	0	all	-	all	Eu:Ger	1982	CC	432	n	bl	n	n	3	ev	all/unsp	nev any	or
LEI	1		m	0	0	all	-	all	As:Chi	1986	CC	792	n	ot	y	n	0	ev	all/unsp	nev any	st
LEI	2		f	0	0	all	-	all	As:Chi	1986	CC	792	n	ot	y	n	0	ev	all/unsp	nev any	st
LEMARC	3		c	0	0	w+o	-	all	NAmer	1992	CC	341	n	bl	n	y	0	ev	all/unsp	nev any	st
LETOUR	1		c	0	0	all	-	all	NAmer	1983	CC	738	n	V	y	y	0	ev	cig+/-ot	nev cigs	st
LEVIN	30	x	m	0	0	all	-	all	NAmer	1938	CC	475	n	bl	n	n	1	ev	cig+/-ot	nev any	st
LIAW	1		m	0	0	all	0	all	As:oth	1982	pr	127	n	ot	n	n	1	cu	all/unsp	nev any	or
LIAW	2		f	0	0	all	0	all	As:oth	1982	pr	127	n	ot	n	n	1	cu	all/unsp	nev any	or
LIDDEL	5		m	0	0	all	18	all	NAmer	1970	pr	304	m	V	n	n	1	ev	cig+/-ot	nev cigs	ot
LIU	2		c	0	0	all	-	all	As:Chi	1980	CC	229	n	ot	*	n	2	ev	all/unsp	nev any	or
LIU2	2		m	0	0	all	-	all	As:Chi	1983	CC	316	n	ot	n	n	3	ev	all/unsp	nev any	ot
LIU2	4		f	0	0	all	-	all	As:Chi	1983	CC	316	n	ot	n	n	3	ev	all/unsp	nev any	ot
LIU3	2		m	0	0	all	-	all	As:Chi	1985	CC	110	n	ot	n	n	2	ev	all/unsp	nev any	or
LIU4	10	x	m	35	69	all	-	all	As:Chi	1986	CC	1000-00	n	ot	y	n	2	ev	cig only	nev any	ot
LIU4	12		f	0	0	all	-	all	As:Chi	1986	CC	1000-00	n	ot	y	n	2	ev	all/unsp	nev any	ot
LIU5	1		c	0	0	all	-	all	As:Chi	1978	CC	111	n	ot	y	n	0	ev	all/unsp	nev any	st
LOMBA2	1		f	0	0	all	-	all	NAmer	1960	CC	225	n	bl	n	n	0	ev	cig+/-ot	nev cigs	st
LOMBAR	2	x	m	0	0	all	-	all	NAmer	1951	CC	1040	n	bl	n	n	0	ev	cig+/-ot	nev any	st
LUBIN2	48	x	m	0	0	all	-	all	Eu:mul	1976	CC	7804	n	bl	n	y	2	ev	cig+/-ot	nev any	ot
LUBIN2	98	x	f	0	0	all	-	all	Eu:mul	1976	CC	7804	n	bl	n	y	1	ev	cig only	nev any	ot
LUO	7		c	0	0	all	-	all	As:Chi	1990	CC	102	n	ot	n	y	20	ev	cig+/-ot	nev cigs	or
MACLEN	73		c	0	0	ch	-	all	As:oth	1972	CC	233	n	bl	n	n	2	ev	cig+/-ot	nev cigs	ot
MAGNUS	5		m	0	0	all	0	all	Eu:Sca	1953	pr	203	o	bl	y	n	3	ev	all/unsp	nev any	ot
MARSH	1	x	m	0	0	all	-	all	NAmer	1979	CC	150	n	bl	y	n	0	ev	cig+/-ot	nev any	st
MARSH	3	x	f	0	0	all	-	all	NAmer	1979	CC	150	n	bl	y	n	0	ev	cig+/-ot	nev any	st

Table 1C3 - 1

IESLC - Meta-anal of Ever Smoking (or Current if Ever not available), Cigs (or Any Prod if Cigs not avail)
All LC types
Most adjusted

REF	NRR	1C1	SEX	AGEL	AGEH	RACE	YF	LC TYPE	LOC	START	ST	NLC	R	VB	P	H	AD	SM	PRODUCT	DENOM	De
MARSH2	5		m	0	0	all	-	all	NAmer	1979	CC	114	n	bl	y	n	1	ev	all/unsp	nev any	or
MARSH2	6		f	0	0	all	-	all	NAmer	1979	CC	114	n	bl	y	n	1	ev	all/unsp	nev any	ot
MARTIS	4		m	0	0	all	-	all	Eu:UK	1972	CC	201	n	V	n	n	0	ev	cig+/-ot	nev cigs	st
MASTRA	2		m	0	0	all	-	all	Eu:wst	1973	CC	309	n	bl	n	n	2	ev	all/unsp	nev any	st
MATOS	27		m	0	0	all	-	all	SCAmer	1994	CC	200	n	bl	n	n	2	ev	cig+/-ot	nev any	or
MATSUD	10		m	0	0	all	-	all	As:Jap	1965	CC	179	n	bl	n	n	0	ev	cig+/-ot	nev cigs	st
MCCONN	1		m	0	0	all	-	all	Eu:UK	1946	CC	100	n	V	n	y	0	ev	all/unsp	nev any	st
MCCONN	2		f	0	0	all	-	all	Eu:UK	1946	CC	100	n	V	n	y	0	ev	all/unsp	nev any	st
MCDUFF	1		m	0	0	all	-	all	NAmer	1979	CC	165	n	V	y	n	0	ev	cig+/-ot	nev cigs	st
MCLAUG	1		m	0	0	all	-	all	As:Chi	1972	CC	316	o	ot	y	n	0	ev	all/unsp	nev any	st
MIGRAN	27		m	0	0	all	0	all	Eu:UK	1964	pr	259	n	V	n	n	2	ev	all/unsp	nev any	ot
MIGRAN	42		f	0	0	all	0	all	Eu:UK	1964	pr	259	n	V	n	n	2	ev	all/unsp	nev any	ot
MILLER	2		f	0	0	all	-	all	NAmer	1972	CC	168	n	bl	y	n	1	ev	cig+/-ot	nev any	ot
MILLS	1	x	m	0	0	wh	-	all	NAmer	1940	CC	444	n	bl	y	n	1	ev	cig only	nev any	ot
MRFITR	6		m	0	0	all	0	all	NAmer	1973	pr	119	n	bl	n	n	0	ev	cig+/-ot	nev cigs	ot
NAM	77		m	0	0	all	-	all	NAmer	1986	CC	1199	n	bl	y	n	1	ev	cig+/-ot	nev cigs	ot
NAM	93		f	0	0	all	-	all	NAmer	1986	CC	1199	n	bl	y	n	1	ev	cig+/-ot	nev cigs	ot
NOTAN2	19	x	m	0	0	all	-	all	As:Ind	1963	CC	683	n	V	n	n	2	ev	cig only	nev any	ot
NOU	11		m	30	64	all	-	all	Eu:Sca	1971	CC	273	n	bl	y	n	0	ev	all/unsp	nev any	st
NOU	12		f	30	64	all	-	all	Eu:Sca	1971	CC	273	n	bl	y	n	0	ev	all/unsp	nev any	st
ODRISC	3		c	0	0	all	-	all	Eu:UK	1992	CC	446	n	V	n	n	0	ev	all/unsp	nev any	st
ORMOS	4		m	0	0	all	-	all	Eu:est	1947	CC	119	n	bl	y	y	0	ev	cig+/-ot	nev any	st
ORMOS	26		f	0	0	all	-	all	Eu:est	1947	CC	119	n	bl	y	y	0	ev	cig+/-ot	nev any	st
OSANN	41		m	0	0	all	-	all	NAmer	1984	CC	1986	n	bl	n	n	2	ev	cig+/-ot	nev cigs	or
OSANN	42		f	0	0	all	-	all	NAmer	1984	CC	1986	n	bl	n	n	2	ev	cig+/-ot	nev cigs	or
PARKIN	31	x	m	0	0	bl	-	all	Africa	1963	CC	877	n	V	y	n	0	ev	cig+/-ot	nev any	st
PASTOR	10		m	0	0	all	-	all	Eu:wst	1976	CC	204	n	bl	y	n	1	ev	all/unsp	nev any	or
PAWLEG	2		m	0	0	all	-	all	Eu:est	1992	CC	176	n	bl	n	y	6	ev	all/unsp	nev any	ot
PERNU	8	x	m	0	0	all	-	all	Eu:Sca	1944	CC	1606	n	bl	n	n	0	ev	cig only	nev any	st
PERNU	4	x	f	0	0	all	-	all	Eu:Sca	1944	CC	1606	n	bl	n	n	0	ev	cig only	nev any	st
PERSH2	11		c	0	0	all	-	all	Eu:Sca	1980	CC	1022	n	bl	y	n	4	ev	all/unsp	nev any	ot
PETO	5		m	0	0	all	0	all	Eu:UK	1954	pr	103	n	V	n	n	0	ev	all/unsp	nev any	st
PEZZO2	10		m	0	0	all	-	all	SCAmer	1992	CC	367	n	bl	n	y	0	ev	cig+/-ot	nev cigs	st
PEZZOT	25		m	0	0	all	-	all	SCAmer	1987	CC	215	n	bl	n	y	0	ev	cig only	nev cigs	st
PIKE	4		m	0	0	w-hi	-	all	NAmer	1972	CC	731	n	bl	y	n	0	ev	all/unsp	nev any	st
PIKE	8		f	0	0	w-hi	-	all	NAmer	1972	CC	731	n	bl	y	n	0	ev	all/unsp	nev any	st
POFFIJ	1		c	0	0	all	-	all	Eu:mul	1990	CC	971	n	bl	n	n	0	ev	all/unsp	nev any	st
POLEDN	1		c	0	0	all	-	all	NAmer	1978	CC	209	n	bl	y	n	1	ev	cig+/-ot	nev cigs	or
QIAO2	9	x	m	0	0	all	0	all	As:Chi	1992	pr	241	m	ot	n	n	0	ev	cig+/-ot	nev any	st
RACHTA	15		f	0	0	all	-	all	Eu:est	1991	CC	118	n	bl	n	y	4	ev	cig+/-ot	nev cigs	ot
RADZIK	1		c	0	0	all	-	all	Eu:est	1986	CC	189	n	bl	n	n	0	ev	all/unsp	nev any	st
RANDIG	9	x	m	0	0	all	-	all	Eu:Ger	1951	CC	448	n	bl	n	n	0	ev	cig+/-ot	nev any	st
RANDIG	10	x	f	0	0	all	-	all	Eu:Ger	1951	CC	448	n	bl	n	n	0	ev	cig+/-ot	nev any	st
REN	1		m	0	0	all	-	all	As:Chi	1980	CC	244	n	ot	*	n	0	ev	all/unsp	nev any	st
REN	2		f	0	0	all	-	all	As:Chi	1980	CC	244	n	ot	*	n	0	ev	all/unsp	nev any	st
RONCO	3	x	m	0	0	all	-	all	Eu:wst	1976	CC	126	n	bl	y	n	2	ev	cig only	nev any	ot
ROTHSC	2		c	0	0	all	-	all	NAmer	1971	CC	284	n	bl	y	n	1	ev	all/unsp	nev any	st
SADOWS	4	x	m	0	0	wh	-	all	NAmer	1938	CC	477	n	bl	n	n	0	ev	cig+/-ot	nev any	st
SANKAR	2		m	0	0	all	-	all	As:Ind	1990	CC	281	n	V	n	n	3	ev	all/unsp	nev any	ot
SCHWAR	1		m	0	0	wh	-	all	NAmer	1984	CC	5588	n	bl	y	y	0	ev	cig+/-ot	nev cigs	st
SCHWAR	2		m	0	0	bl	-	all	NAmer	1984	CC	5588	n	bl	y	y	0	ev	cig+/-ot	nev cigs	st
SCHWAR	3		f	0	0	wh	-	all	NAmer	1984	CC	5588	n	bl	y	y	0	ev	cig+/-ot	nev cigs	st
SCHWAR	4		f	0	0	bl	-	all	NAmer	1984	CC	5588	n	bl	y	y	0	ev	cig+/-ot	nev cigs	st
SEGI	1		m	0	0	all	-	all	As:Jap	1948	CC	159	n	bl	n	n	0	ev	all/unsp	nev any	ot
SEGI2	20		m	0	0	all	-	all	As:Jap	1962	CC	378	n	bl	n	n	1	cu	cig+/-ot	nev any	ot
SEGI2	28		f	0	0	all	-	all	As:Jap	1962	CC	378	n	bl	n	n	1	cu	cig+/-ot	nev any	ot
SEOW	6		f	0	0	ch	-	q+s+l+a	As:oth	1997	CC	153	n	bl	n	y	1	ev	cig+/-ot	nev cigs	st
SHAW	12		c	0	0	wh	-	all	NAmer	1988	CC	335	n	V	n	y	0	ev	all/unsp	nev any	st
SIEMIA	5		m	0	0	all	-	all	NAmer	1979	CC	857	n	V	y	y	7	ev	cig+/-ot	nev cigs	or
SIMARA	3		m	0	0	all	-	all	As:oth	1971	CC	115	n	bl	n	n	6	ev	cig+/-ot	nev cigs	ot
SIMARA	4		f	0	0	all	-	all	As:oth	1971	CC	115	n	bl	n	n	6	ev	cig+/-ot	nev cigs	ot
SOBUE	105		m	0	0	all	-	q+s+l+a	As:Jap	1986	CC	1376	n	bl	n	y	1	ev	cig+/-ot	nev cigs	ot
SOBUE	115		f	0	0	all	-	q+s+l+a	As:Jap	1986	CC	1376	n	bl	n	y	1	ev	cig+/-ot	nev cigs	ot
SOBUE2	10		m	0	0	all	-	q+s+l+a	As:Jap	1965	CC	2083	n	bl	n	n	2	cu	cig+/-ot	nev any	ot
SOBUE2	12		f	0	0	all	-	q+s+l+a	As:Jap	1965	CC	2083	n	bl	n	n	2	cu	cig+/-ot	nev any	ot
SPEIZE	8		f	0	0	all	0	all	NAmer	1976	pr	593	n	bl	n	y	0	ev	cig+/-ot	nev cigs	st
SPITZ	3		c	0	0	b+hi	-	all	NAmer	1992	CC	177	n	bl	n	y	0	ev	cig+/-ot	nev cigs	st
STASZE	7	x	m	0	0	all	-	all	Eu:est	1954	CC	281	n	bl	n	y	0	ev	cig+/-ot	nev any	st
STASZE	5		f	0	0	all	-	all	Eu:est	1954	CC	281	n	bl	n	y	0	ev	all/unsp	nev any	st
STAYNE	1		m	0	0	all	-	all	NAmer	1969	CC	420	n	bl	n	n	0	ev	all/unsp	nev any	st
STOCKS	46	x	m	0	0	all	-	all	Eu:UK	1952	CC	2932	n	V	y	n	2	ev	cig+/-ot	nev any	st
STOCKS	50		f	0	0	all	-	all	Eu:UK	1952	CC	2932	n	V	y	n	1	ev	cig+/-ot	nev any	ot

Table 1C3 - 1

IESLC - Meta-anal of Ever Smoking (or Current if Ever not available), Cigs (or Any Prod if Cigs not avail)
All LC types
Most adjusted

REF	NRR	1C1	SEX	AGEL	AGEH	RACE	YF	LC TYPE	LOC	START	ST	NLC	R	VB	P	H	AD	SM	PRODUCT	DENOM	De
STOCKW	8	x	c	0	0	all	-	all	NAmer	1981	CC	22161	n	bl	n	n	0	ev	cig+/-ot	nev any	st
STUCKE	3		m	0	0	all	-	all	Eu:wst	1989	CC	247	n	bl	n	y	0	ev	all/unsp	nev any	ot
SUN	1		c	0	0	all	-	all	As:Chi	1992	CC	207	n	ot	n	y	0	ev	all/unsp	nev any	st
SUZUK2	7	x	c	0	0	all	-	all	SCAmer	1991	CC	123	n	bl	n	y	3	ev	cig only	nev any	or
SVENSS	71		f	0	0	all	-	all	Eu:Sca	1983	CC	210	n	bl	n	n	1	ev	all/unsp	nev any	ot
TANG	3		c	0	0	all	-	not s	NAmer	1992	CC	119	n	bl	n	y	0	ev	cig+/-ot	nev cigs	st
TENKAN	22		m	0	0	all	17	all	Eu:Sca	1962	pr	242	n	bl	n	n	1	ev	all/unsp	nev any	ot
TIZZAN	2	x	m	0	0	all	-	all	Eu:wst	1959	CC	1358	n	bl	n	n	0	ev	cig only	nev any	st
TIZZAN	22	x	f	0	0	all	-	all	Eu:wst	1959	CC	1358	n	bl	n	n	0	ev	cig only	nev any	st
TOKARS	6		c	0	0	all	-	all	Eu:est	1966	ot	162	o	bl	n	y	3	ev	all/unsp	nev any	or
TOUSEY	10	x	m	0	0	all	-	all	NAmer	1993	CC	507	n	bl	y	y	3	ev	cig+/-ot	nev any	or
TOUSEY	13	x	f	0	0	all	-	all	NAmer	1993	CC	507	n	bl	y	y	3	ev	cig+/-ot	nev any	or
TSUGAN	27		m	0	0	all	-	q+a	As:Jap	1976	CC	134	n	bl	n	y	0	ev	all/unsp	nev any	st
TULINI	38		m	0	0	all	0	all	Eu:Sca	1967	pr	472	n	bl	n	n	3	ev	all/unsp	nev any	ot
TULINI	44		f	0	0	all	0	all	Eu:Sca	1967	pr	472	n	bl	n	n	3	ev	all/unsp	nev any	ot
TVERDA	22		m	0	0	all	0	all	Eu:Sca	1972	pr	238	n	bl	n	n	2	ev	cig+/-ot	nev cigs	ot
TVERDA	15		f	0	0	all	0	all	Eu:Sca	1972	pr	238	n	bl	n	n	2	cu	cig only	nev cigs	ot
WAKAI	72		m	0	0	all	-	all	As:Jap	1988	CC	333	n	bl	n	y	2	ev	all/unsp	nev any	ot
WAKAI	78		f	0	0	all	-	all	As:Jap	1988	CC	333	n	bl	n	y	2	ev	all/unsp	nev any	ot
WALD	4		m	0	0	all	0	all	Eu:UK	1975	pr	102	n	V	n	n	1	cu	cig only	nev any	or
WANG	5		c	0	0	all	-	all	As:Chi	1990	CC	390	n	ot	*	y	6	ev	all/unsp	nev any	or
WANG2	16		c	0	0	all	-	all	As:Chi	1980	CC	103	n	ot	n	n	4	ev	cig+/-ot	nev cigs	ot
WANG3	1		c	0	0	all	-	all	As:Chi	1981	CC	293	n	ot	*	n	0	ev	all/unsp	nev any	st
WANG4	2		m	0	0	all	-	all	As:Chi	1976	CC	1170	n	ot	y	n	2	ev	all/unsp	nev any	st
WICKLU	1		m	0	0	wh	-	all	NAmer	1968	CC	155	n	bl	y	n	0	ev	cig+/-ot	nev+3	or
WIGLE	13	x	m	0	0	all	-	all	NAmer	1971	CC	728	n	V	n	n	0	ev	cig only	nev any	st
WIGLE	16	x	f	0	0	all	-	all	NAmer	1971	CC	728	n	V	n	n	0	ev	cig only	nev any	st
WILKIN	3		c	0	0	all	-	all	Eu:UK	1992	CC	271	n	V	n	n	4	ev	cig+/-ot	nev cigs	ot
WU	45		f	0	0	wh	-	q+a	NAmer	1981	CC	220	n	bl	n	y	2	ev	all/unsp	nev any	ot
WUNSCH	4		m	0	0	all	-	all	SCAmer	1990	CC	398	n	bl	y	n	1	ev	cig+/-ot	nev any	or
WUNSCH	10		f	0	0	all	-	all	SCAmer	1990	CC	398	n	bl	y	n	1	ev	cig+/-ot	nev any	or
WUWILL	8		f	0	0	all	-	all	As:Chi	1985	CC	965	n	ot	n	n	3	ev	cig+/-ot	nev cigs	or
WYNDE2	16	x	m	0	0	all	-	all	NAmer	1962	CC	404	n	bl	n	y	0	ev	cig+/-ot	nev any	st
WYNDE3	48	x	m	0	0	all	-	all	NAmer	1966	CC	350	n	bl	n	y	0	ev	cig+/-ot	nev any	st
WYNDE3	83	x	f	0	0	all	-	all	NAmer	1966	CC	350	n	bl	n	y	0	ev	cig+/-ot	nev any	st
WYNDE4	48		m	0	0	all	-	all	NAmer	1948	CC	684	n	bl	y	n	0	ev	all/unsp	nev any	st
WYNDE4	62		f	0	0	all	-	all	NAmer	1948	CC	684	n	bl	y	n	2	ev	all/unsp	nev any	ot
WYNDE6	81	x	m	0	0	all	-	all	NAmer	1969	CC	4423	n	bl	n	y	0	ev	cig+/-ot	nev any	st
WYNDE6	252		f	0	0	all	-	all	NAmer	1969	CC	4423	n	bl	n	y	0	ev	cig+/-ot	nev cigs	st
XIANGZ	14	x	m	0	0	all	0	all	As:Chi	1976	pr	983	m	ot	n	n	2	ev	cig+/-ot	nev any	ot
XU	2		m	0	0	all	-	all	As:Chi	1985	CC	729	n	ot	n	n	2	ev	all/unsp	nev any	or
XU2	2		c	0	0	all	-	all	As:Chi	1987	CC	610	o	ot	y	n	7	ev	all/unsp	nev any	ot
XU3	2		m	0	0	all	-	all	As:Chi	1981	CC	135	n	ot	n	n	1	ev	all/unsp	nev any	or
XU3	4		f	0	0	all	-	all	As:Chi	1981	CC	135	n	ot	n	n	1	ev	all/unsp	nev any	or
XU4	1		c	0	0	all	-	all	As:Chi	1981	CC	206	n	ot	*	n	0	ev	all/unsp	nev any	st
YAMAGU	11		c	0	0	all	-	all	As:Jap	1989	CC	144	n	bl	n	y	1	ev	all/unsp	nev any	ot
YONG	12		m	0	0	all	0	all	NAmer	1971	pr	216	n	bl	n	n	1	cu	cig+/-ot	nev cigs	or
YONG	15		f	0	0	all	0	all	NAmer	1971	pr	216	n	bl	n	n	1	cu	cig+/-ot	nev cigs	or
YUAN	1		m	0	0	all	0	all	As:Chi	1986	pr	142	n	ot	n	n	2	ev	cig+/-ot	nev cigs	ot
ZHANG	2		m	0	0	all	-	all	As:Chi	1988	CC	100	n	ot	n	n	7	ev	all/unsp	nev any	or
ZHANG	3		f	0	0	all	-	all	As:Chi	1988	CC	100	n	ot	n	n	7	ev	all/unsp	nev any	or
ZHENG	15		m	0	0	all	-	all	As:Chi	1982	CC	540	n	ot	*	y	0	ev	cig+/-ot	nev cigs	st
ZHENG	24		f	0	0	all	-	all	As:Chi	1982	CC	540	n	ot	*	y	0	ev	cig+/-ot	nev cigs	st
ZHOU	2		m	0	0	all	-	all	As:Chi	1978	CC	1360	n	ot	n	n	0	ev	all/unsp	nev any	st
ZHOU	3		f	0	0	all	-	all	As:Chi	1978	CC	1360	n	ot	n	n	0	ev	all/unsp	nev any	st

Cigarette type is all/unspec for all RRs
except for the following:

REF	NRR	CIGTYPE
ALDERS	6	MC only
DEAN3	241	MC only
DEAN3	126	MC only
DESTEF	13	MC only
JUSSAW	31	MC only
NOTAN2	19	MC only
PERNU	8	MC only
PERNU	4	MC only
SUZUK2	7	MC only

Table 1C3 - 2

IESLC - Meta-anal of Ever Smoking (or Current if Ever not available), Cigs (or Any Prod if Cigs not avail)
All LC types
Most adjusted

REF	NRR	SEX	AD	Number Exposed Case	Number Exposed Cont	Non-exposed Case	Non-exposed Cont	RR	95.00%CI
ABELIN	45	m	1	-	-	-	-	39.29	(9.04- 170.68)
*ABRAHA	7	m	0	269	10351	10	3365	8.74	(4.66- 16.42)
*ABRAHA	8	f	0	62	5256	28	11589	4.88	(3.13- 7.62)
Subtotal ABRAHA								5.93	(4.12- 8.53)
AGUDO	1	f	3	-	-	-	-	3.10	(1.42- 6.75)
*AKIBA	11	m	5	-	-	-	-	4.75	(3.07- 7.34)
*AKIBA	15	f	5	-	-	-	-	3.16	(2.37- 4.21)
Subtotal AKIBA								3.58	(2.81- 4.54)
ALDERS	68	m	1	-	-	-	-	10.00	(5.81- 17.22)
ALDERS	6	f	1	-	-	-	-	4.75	(3.55- 6.35)
Subtotal ALDERS								5.61	(4.34- 7.24)
*AMANDU	7	m	2	-	-	-	-	5.89	(2.27- 15.28)
AMES	4	m	0	297	251	15	62	4.89	(2.72- 8.81)
*ANDERS	3	f	0	297	96164	46	195158	13.10	(9.61- 17.87)
*ARCHER	6	m	0	140	36269	6	9842	6.33	(2.80- 14.33)
ARMADA	4	m	0	317	254	4	64	19.97	(7.18- 55.57)
AUSTIN	7	c	3	-	-	-	-	11.12	(3.95- 31.28)
AUVINE	19	c	2	-	-	-	-	13.84	(7.90- 24.25)
AXELSO	1	c	0	90	86	62	371	6.26	(4.20- 9.34)
AXELSS	8	m	6	-	-	-	-	8.02	(4.62- 13.94)
AXELSS	11	f	0	110	109	18	154	8.63	(4.95- 15.05)
Subtotal AXELSS								8.32	(5.62- 12.31)
BAND	1	m	2	-	-	-	-	9.96	(7.38- 13.44)
BARBON	131	m	3	-	-	-	-	11.13	(7.02- 17.64)
BECHER	21	m	2	-	-	-	-	9.03	(3.09- 26.33)
BECHER	16	f	0	38	44	10	52	4.49	(2.01- 10.03)
Subtotal BECHER								5.78	(3.04- 10.99)
*BENSHL	15	m	1	-	-	-	-	8.02	(4.29- 15.01)
*BEST	23	m	0	212	21711	1	2854	27.87	(3.91- 198.68)
*BEST	18	f	1	-	-	-	-	2.24	(0.59- 8.44)
Subtotal BEST								4.95	(1.65- 14.89)
BLOHMK	3	m	0	762	587	126	301	3.10	(2.45- 3.92)
BLOT4	1	m	0	327	245	8	87	14.51	(6.91- 30.51)
BOFFET	27	m	2	-	-	-	-	14.43	(11.91- 17.49)
*BOUCOT	122	m	2	-	-	-	-	51.72	(3.22- 831.81)
BRESLO	17	m	0	471	383	7	42	7.38	(3.28- 16.61)
BRESLO	23	f	0	13	11	12	14	1.38	(0.45- 4.20)
Subtotal BRESLO								4.12	(2.14- 7.94)
*BRETT	10	m	0	144	47930	6	6530	3.27	(1.45- 7.40)
BROCKM	1	m	0	87	81	2	2	1.07	(0.15- 7.80)
BROCKM	2	f	0	24	54	4	18	2.00	(0.61- 6.54)
Subtotal BROCKM								1.70	(0.61- 4.70)
BROSS	13	m	0	831	612	38	170	6.07	(4.21- 8.77)
BROWN2	2	m	2	-	-	-	-	9.10	(8.30- 10.00)
BROWN2	1	f	2	-	-	-	-	12.70	(11.50- 13.90)
Subtotal BROWN2								10.72	(10.03- 11.46)
BUFFLE	2	m	0	461	373	5	47	11.62	(4.57- 29.50)
BUFFLE	6	f	0	419	284	41	198	7.12	(4.93- 10.30)
Subtotal BUFFLE								7.61	(5.40- 10.72)
CARPEN	12	c	3	-	-	-	-	14.88	(8.46- 26.18)
CASCO2	1	c	0	149	212	6	98	11.48	(4.90- 26.87)
CASCOR	1	c	0	365	362	22	295	13.52	(8.56- 21.35)
*CEDERL	107	m	2	-	-	-	-	5.92	(3.85- 9.12)
*CEDERL	112	f	2	-	-	-	-	4.18	(2.94- 5.93)
Subtotal CEDERL								4.80	(3.66- 6.30)
CHAN	5	m	0	206	161	2	43	27.51	(6.57- 115.26)
CHAN	6	f	0	105	50	84	139	3.48	(2.26- 5.35)
Subtotal CHAN								4.13	(2.73- 6.25)
*CHANG	6	m	0	78	1506	5	502	5.20	(2.12- 12.77)
*CHANG	12	f	0	42	1183	11	1139	3.68	(1.90- 7.10)
Subtotal CHANG								4.15	(2.44- 7.06)
CHATZI	4	c	0	255	365	27	129	3.34	(2.14- 5.21)
CHEN2	1	m	0	121	97	9	33	4.57	(2.09- 10.02)
CHEN2	2	f	0	38	30	25	33	1.67	(0.82- 3.39)
Subtotal CHEN2								2.62	(1.55- 4.44)
CHEN3	1	c	0	182	156	72	98	1.59	(1.09- 2.30)
CHIAZZ	3	m	11	-	-	-	-	26.17	(3.32- 206.50)
CHOI	1	m	0	267	465	13	95	4.20	(2.31- 7.64)
CHOI	5	f	0	19	26	76	164	1.58	(0.82- 3.02)
Subtotal CHOI								2.68	(1.72- 4.16)
*CHOW	54	m	2	-	-	-	-	11.58	(5.09- 26.34)

Table 1C3 - 2

IESLC - Meta-anal of Ever Smoking (or Current if Ever not available), Cigs (or Any Prod if Cigs not avail)
All LC types
Most adjusted

REF	NRR	SEX	AD	Number Exposed Case	Number Exposed Cont	Non-exposed Case	Non-exposed Cont	RR	95.00%CI
*CHYOU	7	m	1	-	-	-	-	8.35	(4.76- 14.64)
COMSTO	33	m	0	151	229	4	69	11.37	(4.07- 31.82)
COMSTO	45	f	0	88	87	13	115	8.95	(4.69- 17.06)
Subtotal COMSTO								9.58	(5.54- 16.54)
COOKSO	4	c	0	184	38	45	61	6.56	(3.90- 11.04)
CORREA	34	c	1	-	-	-	-	11.40	(8.70- 15.00)
*CPSI	187	m	1	-	-	-	-	9.18	(7.36- 11.45)
*CPSI	274	f	1	-	-	-	-	2.79	(2.22- 3.51)
Subtotal CPSI								5.17	(4.41- 6.06)
*CPSII	104	m	1	-	-	-	-	12.83	(10.28- 16.01)
*CPSII	79	f	1	-	-	-	-	8.16	(6.93- 9.62)
Subtotal CPSII								9.58	(8.40- 10.93)
DAMBER	37	m	1	-	-	-	-	7.78	(5.29- 11.44)
DARBY	15	m	0	664	1724	3	384	49.30	(15.77- 154.07)
DARBY	16	f	0	292	548	23	529	12.26	(7.89- 19.05)
Subtotal DARBY								14.69	(9.74- 22.16)
DAVEYS	5	m	0	90	144	3	23	4.79	(1.40- 16.42)
DAVEYS	6	f	0	0	3	16	83	0.72~	(0.04- 14.66)
Subtotal DAVEYS								3.65	(1.17- 11.42)
DEAN	8	m	0	540	500	12	61	5.49	(2.92- 10.32)
DEAN2	12	m	0	686	556	33	112	4.19	(2.80- 6.27)
DEAN2	20	f	0	63	29	88	121	2.99	(1.78- 5.02)
Subtotal DEAN2								3.69	(2.68- 5.07)
DEAN3	241	m	1	-	-	-	-	6.11	(3.99- 9.34)
DEAN3	126	f	3	-	-	-	-	4.63	(3.03- 7.09)
Subtotal DEAN3								5.32	(3.94- 7.18)
*DEKLER	6	m	2	-	-	-	-	20.29	(2.84- 145.18)
DESTE2	15	m	0	432	314	31	151	6.70	(4.43- 10.13)
DESTEF	13	m	4	-	-	-	-	6.10	(3.70- 10.00)
*DOCKER	3	c	4	-	-	-	-	4.29	(1.66- 11.06)
DOLL	20	m	0	504	467	7	61	9.40	(4.26- 20.77)
DOLL	12	f	0	68	49	40	59	2.05	(1.19- 3.53)
Subtotal DOLL								3.34	(2.13- 5.23)
*DOLL2	88	m	1	-	-	-	-	11.58	(3.70- 36.24)
*DOLL2	63	f	1	-	-	-	-	8.65	(2.93- 25.55)
Subtotal DOLL2								9.93	(4.53- 21.78)
DORANT	10	c	0	470	2033	14	1090	18.00	(10.52- 30.78)
DORGAN	107	m	2	-	-	-	-	11.60	(6.50- 20.70)
DORGAN	95	f	3	-	-	-	-	8.50	(6.70- 10.80)
Subtotal DORGAN								8.89	(7.13- 11.09)
*DORN	413	m	1	-	-	-	-	8.83	(6.64- 11.75)
DOSEME	1	m	2	-	-	-	-	3.30	(2.60- 4.40)
DROSTE	7	m	4	-	-	-	-	8.62	(3.80- 19.56)
DU	1	m	0	538	-	28	-	3.53	(2.44- 5.11)
DU	2	f	0	191	-	92	-	1.93	(1.30- 2.87)
Subtotal DU								2.66	(2.03- 3.49)
*DUNN	6	m	0	137	52634	2	14160	18.43	(4.56- 74.42)
EBELIN	1	m	0	101	142	12	117	6.93	(3.63- 13.24)
*ENGELA	36	m	7	-	-	-	-	1.67	(1.03- 2.71)
*ENGELA	49	f	5	-	-	-	-	6.05	(3.08- 11.90)
Subtotal ENGELA								2.58	(1.74- 3.83)
*ENSTRO	1	m	1	-	-	-	-	12.99	(10.46- 16.13)
*ENSTRO	2	f	1	-	-	-	-	6.95	(6.01- 8.04)
Subtotal ENSTRO								8.44	(7.48- 9.53)
ESAKI	4	m	0	155	143	16	28	1.90	(0.99- 3.65)
ESAKI	5	f	0	34	19	40	55	2.46	(1.23- 4.92)
Subtotal ESAKI								2.14	(1.33- 3.45)
FAN	1	m	0	216	498	36	236	2.84	(1.93- 4.18)
FAN	2	f	0	82	97	69	320	3.92	(2.65- 5.81)
Subtotal FAN								3.33	(2.53- 4.38)
GAO	1	m	2	-	-	-	-	3.90	(2.90- 5.40)
GAO	11	f	2	-	-	-	-	3.30	(2.50- 4.20)
Subtotal GAO								3.53	(2.90- 4.31)
GAO2	10	m	1	-	-	-	-	5.17	(2.76- 9.69)
GARCIA	3	c	0	395	307	21	139	8.52	(5.26- 13.80)
GARDIN	7	c	0	138	102	5	41	11.09	(4.23- 29.06)
GARSHI	25	m	1	-	-	-	-	5.81	(4.17- 8.10)
GENG	1	m	0	92	68	7	31	5.99	(2.49- 14.42)
GENG	2	f	0	126	75	67	118	2.96	(1.96- 4.48)
Subtotal GENG								3.36	(2.31- 4.89)
GER	21	c	14	-	-	-	-	1.84	(1.06- 3.20)

Table 1C3 - 2

IESLC - Meta-anal of Ever Smoking (or Current if Ever not available), Cigs (or Any Prod if Cigs not avail)
All LC types
Most adjusted

REF	NRR	SEX	AD	Number Exposed Case	Number Exposed Cont	Non-exposed Case	Non-exposed Cont	RR	95.00%CI
GODLEY	5	m	1	-	-	-	-	6.84	(5.60- 8.35)
GODLEY	6	f	1	-	-	-	-	5.54	(4.29- 7.15)
Subtotal GODLEY								6.31	(5.39- 7.39)
GOLLED	7	m	1	-	-	-	-	7.51	(4.44- 12.71)
GOODMA	3	m	0	216	398	10	199	10.80	(5.60- 20.82)
GOODMA	7	f	0	81	91	19	177	8.29	(4.74- 14.52)
Subtotal GOODMA								9.27	(6.05- 14.19)
GRAHAM	23	m	1	-	-	-	-	8.31	(5.19- 13.30)
GREGOR	3	m	0	72	98	10	14	1.03	(0.43- 2.45)
GREGOR	7	f	0	21	42	1	22	11.00	(1.39- 87.29)
Subtotal GREGOR								1.46	(0.66- 3.26)
GSELL	6	m	0	60	42	2	29	20.71	(4.69- 91.56)
HAENSZ	56	f	0	74	103	81	236	2.09	(1.42- 3.09)
*HAMMO2	2	m	1	-	-	-	-	24.92	(3.49- 177.78)
*HAMMON	116	m	1	-	-	-	-	8.93	(5.33- 14.96)
*HANSEN	3	m	2	-	-	-	-	1.53	(0.71- 3.91)
HEGMAN	1	c	0	255	1202	27	2080	16.34	(10.92- 24.45)
*HEIN	7	m	0	143	4471	1	457	14.62	(2.05- 104.23)
*HENNEK	3	m	0	146	11112	23	10919	6.24	(4.02- 9.67)
HINDS	22	f	3	-	-	-	-	5.65	(4.14- 7.72)
*HIRAYA	147	m	1	-	-	-	-	4.36	(3.53- 5.39)
*HIRAYA	150	f	1	-	-	-	-	2.36	(1.90- 2.94)
Subtotal HIRAYA								3.24	(2.78- 3.77)
HITOSU	38	m	1	-	-	-	-	2.91	(1.34- 6.34)
HITOSU	62	f	1	-	-	-	-	3.40	(2.05- 5.64)
Subtotal HITOSU								3.25	(2.12- 4.96)
*HOLE	8	m	1	-	-	-	-	6.44	(3.03- 13.69)
*HOLE	31	f	1	-	-	-	-	1.53	(0.64- 3.70)
Subtotal HOLE								3.50	(1.97- 6.19)
HOROWI	1	m	0	182	525	19	196	3.58	(2.17- 5.90)
HOROWI	2	f	0	21	382	14	463	1.82	(0.91- 3.62)
Subtotal HOROWI								2.83	(1.89- 4.25)
HORWIT	1	f	0	97	92	11	118	11.31	(5.73- 22.34)
HU	15	m	0	120	94	41	67	2.09	(1.30- 3.35)
HU	16	f	0	26	18	40	48	1.73	(0.83- 3.61)
Subtotal HU								1.98	(1.33- 2.94)
HU2	9	m	0	294	228	49	115	3.03	(2.08- 4.41)
HU2	10	f	0	108	80	72	100	1.88	(1.23- 2.85)
Subtotal HU2								2.44	(1.85- 3.23)
HUANG	1	c	0	98	77	37	58	2.00	(1.20- 3.32)
HUMBLE	14	m	1	-	-	-	-	12.10	(5.12- 28.60)
HUMBLE	16	m	1	-	-	-	-	11.88	(2.65- 53.30)
HUMBLE	18	f	1	-	-	-	-	11.36	(5.32- 24.23)
HUMBLE	20	f	1	-	-	-	-	15.40	(4.87- 48.74)
Subtotal HUMBLE								12.28	(7.58- 19.90)
JAHN	22	f	2	-	-	-	-	3.30	(1.99- 5.49)
JAIN	46	m	2	-	-	-	-	8.30	(4.53- 17.00)
JAIN	41	f	2	-	-	-	-	9.20	(5.95- 15.10)
Subtotal JAIN								8.89	(6.08- 13.01)
JARUP	6	m	2	-	-	-	-	7.54	(2.80- 20.33)
JARVHO	3	m	0	99	57	1	16	27.79	(3.59- 215.09)
JARVHO	7	f	0	41	15	6	21	9.57	(3.24- 28.26)
Subtotal JARVHO								12.08	(4.64- 31.46)
JEDRYC	58	m	4	-	-	-	-	5.46	(3.85- 7.73)
JEDRYC	59	f	4	-	-	-	-	4.54	(2.56- 8.05)
Subtotal JEDRYC								5.19	(3.86- 7.00)
JIANG	1	m	0	93	83	7	17	2.72	(1.08- 6.89)
JIANG	2	f	0	11	6	14	19	2.49	(0.74- 8.35)
Subtotal JIANG								2.63	(1.26- 5.50)
JOLY	2	m	0	552	709	12	218	14.14	(7.83- 25.56)
JOLY	1	f	0	166	123	52	283	7.34	(5.04- 10.70)
Subtotal JOLY								8.87	(6.46- 12.19)
JUSSAW	31	m	2	-	-	-	-	8.64	(4.61- 17.88)
*KAISE2	72	m	1	-	-	-	-	5.40	(3.05- 9.57)
*KAISE2	64	f	1	-	-	-	-	10.09	(5.29- 19.27)
Subtotal KAISE2								7.11	(4.63- 10.90)
*KAISER	13	m	2	-	-	-	-	17.63	(11.98- 25.96)
*KAISER	10	f	2	-	-	-	-	5.63	(3.89- 8.14)
Subtotal KAISER								9.70	(7.43- 12.67)
KANELL	30	m	1	-	-	-	-	4.94	(3.47- 7.03)
KATSOU	29	f	1	-	-	-	-	3.30	(1.77- 6.15)

Table 1C3 - 2

IESLC - Meta-anal of Ever Smoking (or Current if Ever not available), Cigs (or Any Prod if Cigs not avail)
All LC types
Most adjusted

REF	NRR	SEX	AD	Number Exposed Case	Number Exposed Cont	Non-exposed Case	Non-exposed Cont	RR	95.00%CI
KAUFMA	17	c	6	-	-	-	-	12.38	(8.59- 17.85)
KELLER	3	m	0	8066	2517	323	1017	10.09	(8.83- 11.52)
KELLER	11	m	0	1493	340	38	117	13.52	(9.20- 19.86)
KELLER	7	f	0	3998	1269	469	1860	12.49	(11.09- 14.08)
KELLER	15	f	0	584	214	67	232	9.45	(6.91- 12.93)
Subtotal KELLER								11.30	(10.40- 12.29)
KHUDER	4	m	0	459	785	23	309	7.86	(5.06- 12.19)
KIHARA	31	c	0	338	232	102	237	3.39	(2.54- 4.51)
*KINLEN	17	m	2	-	-	-	-	10.99	(5.24- 23.06)
KJUUS	3	m	0	151	127	2	24	14.27	(3.31- 61.54)
*KNEKT	87	m	1	-	-	-	-	6.42	(2.82- 14.62)
KO	1	f	3	-	-	-	-	4.20	(1.10- 15.60)
KOHLME	2	c	4	-	-	-	-	16.40	(6.90- 38.42)
KOO	1	f	0	112	63	88	137	2.77	(1.84- 4.16)
KOULUM	2	m	0	625	229	5	54	29.48	(11.65- 74.60)
KREUZE	60	f	3	-	-	-	-	9.21	(3.45- 24.53)
KREUZE	62	f	3	-	-	-	-	4.05	(2.81- 5.86)
Subtotal KREUZE								4.48	(3.18- 6.32)
KREYBE	12	m	1	-	-	-	-	6.61	(2.93- 14.92)
KREYBE	30	f	1	-	-	-	-	1.43	(0.71- 2.86)
Subtotal KREYBE								2.73	(1.61- 4.64)
*KUBIK	27	m	0	106	7829	2	4271	28.91	(7.14- 117.06)
LAMTH	6	f	0	242	106	202	337	3.81	(2.86- 5.08)
LAMWK	1	f	0	88	41	75	144	4.12	(2.59- 6.55)
LAMWK2	9	m	0	244	161	23	43	2.83	(1.64- 4.88)
LAMWK2	10	f	0	75	50	65	139	3.21	(2.02- 5.10)
Subtotal LAMWK2								3.04	(2.14- 4.33)
*LANGE	40	m	1	-	-	-	-	4.74	(1.77- 12.67)
*LANGE	37	f	1	-	-	-	-	4.93	(2.48- 9.81)
Subtotal LANGE								4.87	(2.77- 8.55)
LAUSSM	11	m	3	-	-	-	-	5.70	(4.10- 7.80)
LEI	1	m	0	443	361	41	123	3.68	(2.52- 5.38)
LEI	2	f	0	123	61	85	147	3.49	(2.32- 5.24)
Subtotal LEI								3.59	(2.72- 4.74)
LEMARC	3	c	0	309	288	32	168	5.63	(3.74- 8.49)
LETOUR	1	c	0	714	514	24	224	12.96	(8.38- 20.05)
LEVIN	30	m	1	-	-	-	-	6.97	(4.87- 9.97)
*LIAW	1	m	1	-	-	-	-	3.70	(2.10- 6.60)
*LIAW	2	f	1	-	-	-	-	3.60	(1.00- 12.20)
Subtotal LIAW								3.68	(2.19- 6.20)
*LIDDEL	5	m	1	-	-	-	-	3.61	(2.27- 5.73)
LIU	2	c	2	-	-	-	-	1.92	(1.40- 2.64)
LIU2	2	m	3	-	-	-	-	5.19	(2.03- 13.25)
LIU2	4	f	3	-	-	-	-	4.65	(2.18- 9.93)
Subtotal LIU2								4.86	(2.69- 8.76)
LIU3	2	m	2	-	-	-	-	1.26	(0.30- 5.26)
LIU4	10	m	2	-	-	-	-	3.88	(3.78- 3.98)
LIU4	12	f	2	-	-	-	-	2.86	(2.77- 2.95)
Subtotal LIU4								3.43	(3.37- 3.50)
LIU5	1	c	0	85	70	26	41	1.91	(1.07- 3.44)
LOMBA2	1	f	0	149	353	76	239	1.33	(0.96- 1.83)
LOMBAR	2	m	0	978	782	14	112	10.01	(5.70- 17.58)
LUBIN2	48	m	2	-	-	-	-	8.74	(7.49- 10.19)
LUBIN2	98	f	1	-	-	-	-	3.90	(3.29- 4.62)
Subtotal LUBIN2								6.07	(5.42- 6.81)
LUO	7	c	20	-	-	-	-	2.70	(1.50- 5.00)
MACLEN	73	c	2	-	-	-	-	2.67	(1.66- 4.29)
*MAGNUS	5	m	3	-	-	-	-	4.13	(1.94- 8.77)
MARSH	1	m	0	98	150	2	31	10.13	(2.37- 43.27)
MARSH	3	f	0	42	64	8	71	5.82	(2.54- 13.33)
Subtotal MARSH								6.67	(3.25- 13.70)
MARSH2	5	m	1	-	-	-	-	1.89	(0.70- 5.14)
MARSH2	6	f	1	-	-	-	-	5.28	(1.89- 14.72)
Subtotal MARSH2								3.11	(1.52- 6.36)
MARTIS	4	m	0	197	176	4	25	7.00	(2.39- 20.49)
MASTRA	2	m	2	-	-	-	-	8.14	(3.32- 20.00)
MATOS	27	m	2	-	-	-	-	6.80	(3.50- 13.10)
MATSUD	10	m	0	170	3314	3	1255	21.46	(6.84- 67.33)
MCCONN	1	m	0	88	174	5	12	1.21	(0.41- 3.55)
MCCONN	2	f	0	3	3	4	11	2.75	(0.38- 19.67)
Subtotal MCCONN								1.46	(0.57- 3.76)

Table 1C3 - 2

IESLC - Meta-anal of Ever Smoking (or Current if Ever not available), Cigs (or Any Prod if Cigs not avail)
All LC types
Most adjusted

REF	NRR	SEX	AD	Number Exposed Case	Number Exposed Cont	Non-exposed Case	Non-exposed Cont	RR	95.00%CI
MCDUFF	1	m	0	159	134	6	31	6.13	(2.48- 15.14)
MCLAUG	1	m	0	294	1082	22	270	3.33	(2.12- 5.25)
*MIGRAN	27	m	2	-	-	-	-	3.61	(1.34- 9.72)
*MIGRAN	42	f	2	-	-	-	-	4.62	(1.63- 13.09)
Subtotal MIGRAN								4.06	(1.98- 8.32)
MILLER	2	f	1	-	-	-	-	4.99	(2.06- 12.10)
MILLS	1	m	1	-	-	-	-	1.27	(1.01- 1.61)
*MRFITR	6	m	0	119	11007	0	1859	40.37~	(2.51- 648.95)
NAM	77	m	1	-	-	-	-	8.71	(5.87- 12.93)
NAM	93	f	1	-	-	-	-	8.88	(6.35- 12.40)
Subtotal NAM								8.81	(6.82- 11.37)
NOTAN2	19	m	2	-	-	-	-	2.36	(1.68- 3.31)
NOU	11	m	0	74	247	6	122	6.09	(2.58- 14.39)
NOU	12	f	0	10	92	4	261	7.09	(2.17- 23.17)
Subtotal NOU								6.42	(3.20- 12.87)
ODRISC	3	c	0	440	996	6	664	48.89	(21.71- 110.07)
ORMOS	4	m	0	87	1034	7	777	9.34	(4.30- 20.28)
ORMOS	26	f	0	1	234	23	1044	0.19	(0.03- 1.44)
Subtotal ORMOS								5.65	(2.74- 11.64)
OSANN	41	m	2	-	-	-	-	19.70	(14.40- 26.80)
OSANN	42	f	2	-	-	-	-	15.00	(11.80- 19.10)
Subtotal OSANN								16.62	(13.74- 20.10)
PARKIN	31	m	0	372	933	107	1248	4.65	(3.69- 5.86)
PASTOR	10	m	1	-	-	-	-	6.81	(3.38- 13.70)
PAWLEG	2	m	6	-	-	-	-	12.26	(4.07- 36.95)
PERNU	8	m	0	706	216	97	275	9.27	(7.02- 12.23)
PERNU	4	f	0	7	24	110	971	2.57	(1.08- 6.11)
Subtotal PERNU								8.22	(6.32- 10.71)
PERSH2	11	c	4	-	-	-	-	6.55	(5.46- 7.86)
*PETO	5	m	0	101	2423	2	295	6.15	(1.52- 24.79)
PEZZO2	10	m	0	361	469	6	117	15.01	(6.53- 34.48)
PEZZOT	25	m	0	211	317	4	116	19.30	(7.02- 53.10)
PIKE	4	m	0	514	375	18	69	5.25	(3.08- 8.98)
PIKE	8	f	0	163	90	36	96	4.83	(3.04- 7.66)
Subtotal PIKE								5.01	(3.53- 7.10)
POFFIJ	1	c	0	913	918	58	452	7.75	(5.81- 10.34)
POLEDN	1	c	1	-	-	-	-	9.24	(5.23- 16.33)
*QIAO2	9	m	0	197	6360	10	709	2.20	(1.17- 4.13)
RACHTA	15	f	4	-	-	-	-	8.21	(3.96- 17.05)
RADZIK	1	c	0	180	198	9	13	1.31	(0.55- 3.15)
RANDIG	9	m	0	277	245	5	22	4.97	(1.86- 13.34)
RANDIG	10	f	0	16	39	17	92	2.22	(1.02- 4.84)
Subtotal RANDIG								3.03	(1.64- 5.58)
REN	1	m	0	106	84	12	34	3.58	(1.74- 7.33)
REN	2	f	0	78	20	48	50	4.06	(2.16- 7.64)
Subtotal REN								3.84	(2.39- 6.17)
RONCO	3	m	2	-	-	-	-	5.43	(2.27- 12.96)
ROTHSC	2	c	1	-	-	-	-	5.55	(2.97- 10.37)
SADOWS	4	m	0	421	446	18	81	4.25	(2.51- 7.20)
SANKAR	2	m	3	-	-	-	-	13.62	(9.00- 20.62)
SCHWAR	1	m	0	2648	1019	119	376	8.21	(6.60- 10.22)
SCHWAR	2	m	0	863	275	50	104	6.53	(4.54- 9.39)
SCHWAR	3	f	0	1351	637	182	855	9.96	(8.28- 12.00)
SCHWAR	4	f	0	335	179	40	247	11.56	(7.90- 16.90)
Subtotal SCHWAR								9.05	(7.99- 10.25)
SEGI	1	m	0	140	1742	18	382	1.71	(1.03- 2.82)
SEGI2	20	m	1	-	-	-	-	3.74	(1.75- 8.00)
SEGI2	28	f	1	-	-	-	-	1.65	(0.90- 3.02)
Subtotal SEGI2								2.27	(1.41- 3.64)
SEOW	6	f	1	-	-	-	-	5.25	(2.80- 9.84)
SHAW	12	c	0	324	266	11	107	11.85	(6.24- 22.50)
SIEMIA	5	m	7	-	-	-	-	12.10	(6.60- 22.30)
SIMARA	3	m	6	-	-	-	-	1.65	(0.97- 2.81)
SIMARA	4	f	6	-	-	-	-	1.63	(0.87- 3.06)
Subtotal SIMARA								1.64	(1.09- 2.46)
SOBUE	105	m	1	-	-	-	-	3.72	(2.57- 5.38)
SOBUE	115	f	1	-	-	-	-	2.51	(1.89- 3.33)
Subtotal SOBUE								2.90	(2.32- 3.64)
SOBUE2	10	m	2	-	-	-	-	4.47	(3.89- 5.14)
SOBUE2	12	f	2	-	-	-	-	3.28	(2.79- 3.87)
Subtotal SOBUE2								3.92	(3.53- 4.36)

Table 1C3 - 2

IESLC - Meta-anal of Ever Smoking (or Current if Ever not available), Cigs (or Any Prod if Cigs not avail)
All LC types
Most adjusted

REF	NRR	SEX	AD	Number Exposed Case	Number Exposed Cont	Non-exposed Case	Non-exposed Cont	RR	95.00%CI
*SPEIZE	8	f	0	535	1012074	58	776300	7.08	(5.40- 9.28)
SPITZ	3	c	0	170	169	7	128	18.39	(8.35- 40.53)
STASZE	7	m	0	251	653	5	158	12.15	(4.93- 29.94)
STASZE	5	f	0	6	153	15	1660	4.34	(1.66- 11.35)
Subtotal STASZE								7.50	(3.89- 14.48)
STAYNE	1	m	0	362	567	58	333	3.67	(2.69- 4.99)
STOCKS	46	m	2	-	-	-	-	6.94	(4.93- 9.77)
STOCKS	50	f	1	-	-	-	-	3.04	(2.35- 3.93)
Subtotal STOCKS								4.10	(3.33- 5.03)
STOCKW	8	c	0	18655	6414	2791	10641	11.09	(10.54- 11.66)
STUCKE	3	m	0	247	203	0	51	125.27~	(7.68-2042.38)
SUN	1	c	0	140	173	67	191	2.31	(1.62- 3.30)
SUZUK2	7	c	3	-	-	-	-	11.00	(3.40- 36.00)
SVENSS	71	f	1	-	-	-	-	6.18	(3.79- 10.07)
TANG	3	c	0	110	59	9	39	8.08	(3.66- 17.82)
*TENKAN	22	m	1	-	-	-	-	14.64	(6.29- 34.07)
TIZZAN	2	m	0	994	836	180	305	2.01	(1.64- 2.48)
TIZZAN	22	f	0	25	28	25	114	4.07	(2.04- 8.13)
Subtotal TIZZAN								2.13	(1.75- 2.60)
TOKARS	6	c	3	-	-	-	-	6.60	(3.20- 13.70)
TOUSEY	10	m	3	-	-	-	-	21.40	(7.80- 59.00)
TOUSEY	13	f	3	-	-	-	-	16.80	(9.20- 30.70)
Subtotal TOUSEY								17.90	(10.67- 30.04)
TSUGAN	27	m	0	73	71	18	22	1.26	(0.62- 2.54)
*TULINI	38	m	3	-	-	-	-	7.71	(4.19- 14.18)
*TULINI	44	f	3	-	-	-	-	13.01	(7.24- 23.40)
Subtotal TULINI								10.12	(6.63- 15.44)
*TVERDA	22	m	2	-	-	-	-	4.58	(2.97- 7.06)
*TVERDA	15	f	2	-	-	-	-	11.05	(3.33- 36.71)
Subtotal TVERDA								5.07	(3.37- 7.62)
WAKAI	72	m	2	-	-	-	-	3.67	(1.84- 7.32)
WAKAI	78	f	2	-	-	-	-	4.49	(2.35- 8.59)
Subtotal WAKAI								4.09	(2.55- 6.55)
*WALD	4	m	1	-	-	-	-	16.40	(7.55- 44.20)
WANG	5	c	6	-	-	-	-	2.88	(1.74- 4.77)
WANG2	16	c	4	-	-	-	-	2.29	(1.12- 4.70)
WANG3	1	c	0	235	172	58	121	2.85	(1.97- 4.13)
WANG4	2	m	2	-	-	-	-	1.16	(0.96- 1.42)
WICKLU	1	m	0	-	-	-	-	4.60	(2.80- 7.60)
WIGLE	13	m	0	543	632	15	204	11.68	(6.83- 19.99)
WIGLE	16	f	0	78	235	36	439	4.05	(2.64- 6.19)
Subtotal WIGLE								6.09	(4.37- 8.51)
WILKIN	3	c	4	-	-	-	-	7.83	(4.45- 13.78)
WU	45	f	2	-	-	-	-	3.03	(1.81- 5.07)
WUNSCH	4	m	1	-	-	-	-	4.75	(2.66- 8.50)
WUNSCH	10	f	1	-	-	-	-	4.43	(2.62- 7.47)
Subtotal WUNSCH								4.57	(3.10- 6.74)
WUWILL	8	f	3	-	-	-	-	2.30	(1.90- 2.80)
WYNDE2	16	m	0	382	512	8	105	9.79	(4.71- 20.34)
WYNDE3	48	m	0	261	264	9	88	9.67	(4.77- 19.60)
WYNDE3	83	f	0	46	56	20	76	3.12	(1.67- 5.85)
Subtotal WYNDE3								5.14	(3.21- 8.22)
WYNDE4	48	m	0	632	665	12	115	9.11	(4.98- 16.67)
WYNDE4	62	f	2	-	-	-	-	2.87	(1.48- 5.55)
Subtotal WYNDE4								5.38	(3.45- 8.41)
WYNDE6	81	m	0	2765	1797	87	617	10.91	(8.65- 13.76)
WYNDE6	252	f	0	1354	701	159	856	10.40	(8.58- 12.60)
Subtotal WYNDE6								10.60	(9.14- 12.30)
*XIANGZ	14	m	2	-	-	-	-	1.79	(1.21- 2.65)
XU	2	m	2	-	-	-	-	2.70	(2.10- 3.50)
XU2	2	c	7	-	-	-	-	3.80	(2.84- 5.07)
XU3	2	m	1	-	-	-	-	5.99	(2.65- 13.50)
XU3	4	f	1	-	-	-	-	3.86	(1.39- 10.70)
Subtotal XU3								5.05	(2.67- 9.54)
XU4	1	c	0	161	113	45	93	2.94	(1.92- 4.52)
YAMAGU	11	c	1	-	-	-	-	3.97	(2.12- 7.40)
*YONG	12	m	1	-	-	-	-	28.71	(6.98- 118.16)
*YONG	15	f	1	-	-	-	-	5.20	(2.38- 11.35)
Subtotal YONG								7.75	(3.91- 15.36)
*YUAN	1	m	2	-	-	-	-	6.50	(3.64- 11.60)
ZHANG	2	m	7	-	-	-	-	4.00	(1.61- 9.91)

Table 1C3 - 2

IESLC - Meta-anal of Ever Smoking (or Current if Ever not available), Cigs (or Any Prod if Cigs not avail)
All LC types
Most adjusted

REF	NRR	SEX	AD	Number Exposed Case	Number Exposed Cont	Non-exposed Case	Non-exposed Cont	RR	95.00%CI
ZHANG	3	f	7	-	-	-	-	3.75	(1.80- 10.76)
Subtotal ZHANG								3.87	(2.05- 7.32)
ZHENG	15	m	0	279	218	33	94	3.65	(2.36- 5.63)
ZHENG	24	f	0	76	44	152	184	2.09	(1.36- 3.21)
Subtotal ZHENG								2.75	(2.03- 3.73)
ZHOU	2	m	0	740	41	275	36	2.36	(1.48- 3.77)
ZHOU	3	f	0	112	7	231	32	2.22	(0.95- 5.18)
Subtotal ZHOU								2.33	(1.54- 3.51)
Partial Totals				77291	1385753	9309	1083845		

*prospective study ~ With 0.5 adjustment for zero

REF	NRR	SEX	AD	Ys	Ws	Qs	Ps
ABELIN	45	m	1	3.67	1.78	7.95	0.0000
*ABRAHA	7	m	0	2.17	9.68	3.61	0.0000
*ABRAHA	8	f	0	1.59	19.39	0.02	0.0000
Subtotal ABRAHA				1.78	29.07	3.62	
AGUDO	1	f	3	1.13	6.32	1.15	0.0044
*AKIBA	11	m	5	1.56	20.22	0.00	0.0000
*AKIBA	15	f	5	1.15	46.54	7.72	0.0000
Subtotal AKIBA				1.27	66.77	7.72	
ALDERS	68	m	1	2.30	13.02	7.22	0.0000
ALDERS	6	f	1	1.56	45.44	0.00	0.0000
Subtotal ALDERS				1.72	58.46	7.22	
*AMANDU	7	m	2	1.77	4.23	0.20	0.0003
AMES	4	m	0	1.59	11.09	0.01	0.0000
*ANDERS	3	f	0	2.57	39.86	41.06	0.0000
*ARCHER	6	m	0	1.85	5.76	0.48	0.0000
ARMADA	4	m	0	2.99	3.67	7.57	0.0000
AUSTIN	7	c	3	2.41	3.59	2.60	0.0000
AUVINE	19	c	2	2.63	12.22	13.98	0.0000
AXELSO	1	c	0	1.83	24.06	1.84	0.0000
AXELSS	8	m	6	2.08	12.60	3.46	0.0000
AXELSS	11	f	0	2.16	12.45	4.45	0.0000
Subtotal AXELSS				2.12	25.05	7.91	
BAND	1	m	2	2.30	42.76	23.47	0.0000
BARBON	131	m	3	2.41	18.10	13.13	0.0000
BECHER	21	m	2	2.20	3.35	1.38	0.0001
BECHER	16	f	0	1.50	5.94	0.02	0.0003
Subtotal BECHER				1.75	9.29	1.40	
*BENSHL	15	m	1	2.08	9.80	2.69	0.0000
*BEST	23	m	0	3.33	1.00	3.12	0.0009
*BEST	18	f	1	0.81	2.17	1.23	0.2348
Subtotal BEST				1.60	3.17	4.34	
BLOHMK	3	m	0	1.13	70.05	12.72	0.0000
BLOT4	1	m	0	2.68	6.96	8.69	0.0000
BOFFET	27	m	2	2.67	104.07	128.58	0.0000
*BOUCOT	122	m	2	3.95	0.50	2.84	0.0054
BRESLO	17	m	0	2.00	5.83	1.13	0.0000
BRESLO	23	f	0	0.32	3.10	4.74	0.5717
Subtotal BRESLO				1.42	8.93	5.87	
*BRETT	10	m	0	1.18	5.77	0.80	0.0044
BROCKM	1	m	0	0.07	0.98	2.16	0.9437
BROCKM	2	f	0	0.69	2.73	2.04	0.2517
Subtotal BROCKM				0.53	3.71	4.20	
BROSS	13	m	0	1.80	28.54	1.73	0.0000
BROWN2	2	m	2	2.21	442.58	187.27	0.0000
BROWN2	1	f	2	2.54	427.71	413.97	0.0000
Subtotal BROWN2				2.37	870.29	601.24	
BUFFLE	2	m	0	2.45	4.42	3.54	0.0000
BUFFLE	6	f	0	1.96	28.29	4.66	0.0000
Subtotal BUFFLE				2.03	32.71	8.20	
CARPEN	12	c	3	2.70	12.04	15.71	0.0000
CASCO2	1	c	0	2.44	5.31	4.14	0.0000
CASCOR	1	c	0	2.60	18.40	20.15	0.0000
*CEDERL	107	m	2	1.78	20.66	1.00	0.0000
*CEDERL	112	f	2	1.43	31.21	0.51	0.0000
Subtotal CEDERL				1.57	51.88	1.51	
CHAN	5	m	0	3.31	1.87	5.78	0.0000
CHAN	6	f	0	1.25	20.57	2.00	0.0000
Subtotal CHAN				1.42	22.44	7.78	

Table 1C3 - 2

IESLC - Meta-anal of Ever Smoking (or Current if Ever not available), Cigs (or Any Prod if Cigs not avail)
All LC types
Most adjusted

REF	NRR	SEX	AD	Ys	Ws	Qs	Ps
*CHANG	6	m	0	1.65	4.76	0.04	0.0003
*CHANG	12	f	0	1.30	8.85	0.58	0.0001
Subtotal CHANG				1.42	13.61	0.62	
CHATZI	4	c	0	1.21	19.44	2.41	0.0000
CHEN2	1	m	0	1.52	6.25	0.01	0.0001
CHEN2	2	f	0	0.51	7.70	8.38	0.1539
Subtotal CHEN2				0.97	13.95	8.39	
CHEN3	1	c	0	0.46	27.78	33.33	0.0148
CHIAZZ	3	m	11	3.26	0.90	2.62	0.0019
CHOI	1	m	0	1.43	10.71	0.16	0.0000
CHOI	5	f	0	0.46	9.06	11.01	0.1703
Subtotal CHOI				0.99	19.78	11.18	
*CHOW	54	m	2	2.45	5.69	4.52	0.0000
*CHYOU	7	m	1	2.12	12.17	3.88	0.0000
COMSTO	33	m	0	2.43	3.63	2.77	0.0000
COMSTO	45	f	0	2.19	9.22	3.70	0.0000
Subtotal COMSTO				2.26	12.85	6.47	
COOKSO	4	c	0	1.88	14.21	1.49	0.0000
CORREA	34	c	1	2.43	51.78	39.72	0.0000
*CPSI	187	m	1	2.22	78.68	34.19	0.0000
*CPSI	274	f	1	1.03	73.22	20.70	0.0000
Subtotal CPSI				1.64	151.90	54.90	
*CPSII	104	m	1	2.55	78.29	77.36	0.0000
*CPSII	79	f	1	2.10	142.84	41.88	0.0000
Subtotal CPSII				2.26	221.13	119.23	
DAMBER	37	m	1	2.05	25.83	6.30	0.0000
DARBY	15	m	0	3.90	2.96	16.20	0.0000
DARBY	16	f	0	2.51	19.76	17.76	0.0000
Subtotal DARBY				2.69	22.71	33.96	
DAVEYS	5	m	0	1.57	2.53	0.00	0.0126
DAVEYS	6	f	0	-0.32	0.42	1.50	0.8327
Subtotal DAVEYS				1.30	2.96	1.50	
DEAN	8	m	0	1.70	9.65	0.20	0.0000
DEAN2	12	m	0	1.43	23.54	0.37	0.0000
DEAN2	20	f	0	1.09	14.29	3.07	0.0000
Subtotal DEAN2				1.30	37.83	3.44	
DEAN3	241	m	1	1.81	21.24	1.35	0.0000
DEAN3	126	f	3	1.53	21.26	0.01	0.0000
Subtotal DEAN3				1.67	42.50	1.36	
*DEKLER	6	m	2	3.01	0.99	2.09	0.0027
DESTE2	15	m	0	1.90	22.53	2.67	0.0000
DESTEF	13	m	4	1.81	15.54	0.98	0.0000
*DOCKER	3	c	4	1.46	4.27	0.04	0.0026
DOLL	20	m	0	2.24	6.12	2.86	0.0000
DOLL	12	f	0	0.72	12.98	9.19	0.0099
Subtotal DOLL				1.21	19.10	12.05	
*DOLL2	88	m	1	2.45	2.95	2.35	0.0000
*DOLL2	63	f	1	2.16	3.28	1.18	0.0001
Subtotal DOLL2				2.30	6.23	3.52	
DORANT	10	c	0	2.89	13.34	23.69	0.0000
DORGAN	107	m	2	2.45	11.45	9.14	0.0000
DORGAN	95	f	3	2.14	67.41	22.85	0.0000
Subtotal DORGAN				2.19	78.86	31.99	
*DORN	413	m	1	2.18	47.17	18.15	0.0000
DOSEME	1	m	2	1.19	55.52	7.35	0.0000
DROSTE	7	m	4	2.15	5.72	2.04	0.0000
DU	1	m	0	1.26	28.12	2.47	0.0000
DU	2	f	0	0.66	24.50	19.86	0.0011
Subtotal DU				0.98	52.62	22.33	
*DUNN	6	m	0	2.91	1.97	3.63	0.0000
EBELIN	1	m	0	1.94	9.19	1.32	0.0000
*ENGELA	36	m	7	0.51	16.42	17.93	0.0377
*ENGELA	49	f	5	1.80	8.41	0.49	0.0000
Subtotal ENGELA				0.95	24.83	18.42	
*ENSTRO	1	m	1	2.56	81.91	82.96	0.0000
*ENSTRO	2	f	1	1.94	181.45	26.33	0.0000
Subtotal ENSTRO				2.13	263.36	109.29	
ESAKI	4	m	0	0.64	8.96	7.54	0.0554
ESAKI	5	f	0	0.90	7.99	3.45	0.0109
Subtotal ESAKI				0.76	16.94	10.99	
FAN	1	m	0	1.04	25.87	6.80	0.0000

Table 1C3 - 2

IESLC - Meta-anal of Ever Smoking (or Current if Ever not available), Cigs (or Any Prod if Cigs not avail)
All LC types
Most adjusted

REF	NRR	SEX	AD	Ys	Ws	Qs	Ps
FAN	2	f	0	1.37	24.92	0.91	0.0000
Subtotal FAN				1.20	50.80	7.72	
GAO	1	m	2	1.36	39.76	1.54	0.0000
GAO	11	f	2	1.19	57.09	7.56	0.0000
Subtotal GAO				1.26	96.85	9.10	
GAO2	10	m	1	1.64	9.74	0.07	0.0000
GARCIA	3	c	0	2.14	16.50	5.63	0.0000
GARDIN	7	c	0	2.41	4.14	2.98	0.0000
GARSHI	25	m	1	1.76	34.86	1.42	0.0000
GENG	1	m	0	1.79	4.98	0.27	0.0001
GENG	2	f	0	1.08	22.39	5.01	0.0000
Subtotal GENG				1.21	27.37	5.28	
GER	21	c	14	0.61	12.59	11.31	0.0305
GODLEY	5	m	1	1.92	96.28	12.83	0.0000
GODLEY	6	f	1	1.71	58.89	1.40	0.0000
Subtotal GODLEY				1.84	155.17	14.23	
GOLLED	7	m	1	2.02	13.89	2.92	0.0000
GOODMA	3	m	0	2.38	8.92	6.02	0.0000
GOODMA	7	f	0	2.12	12.25	3.81	0.0000
Subtotal GOODMA				2.23	21.17	9.83	
GRAHAM	23	m	1	2.12	17.35	5.44	0.0000
GREGOR	3	m	0	0.03	5.11	11.97	0.9492
GREGOR	7	f	0	2.40	0.90	0.63	0.0233
Subtotal GREGOR				0.38	6.01	12.60	
GSELL	6	m	0	3.03	1.74	3.77	0.0001
HAENSZ	56	f	0	0.74	25.12	16.85	0.0002
*HAMMO2	2	m	1	3.22	0.99	2.73	0.0013
*HAMMON	116	m	1	2.19	14.43	5.76	0.0000
*HANSEN	3	m	2	0.43	5.28	6.77	0.3285
HEGMAN	1	c	0	2.79	23.66	36.14	0.0000
*HEIN	7	m	0	2.68	1.00	1.26	0.0074
*HENNEK	3	m	0	1.83	19.94	1.48	0.0000
HINDS	22	f	3	1.73	39.57	1.20	0.0000
*HIRAYA	147	m	1	1.47	85.78	0.62	0.0000
*HIRAYA	150	f	1	0.86	80.63	39.41	0.0000
Subtotal HIRAYA				1.18	166.40	40.03	
HITOSU	38	m	1	1.07	6.36	1.53	0.0071
HITOSU	62	f	1	1.22	15.00	1.67	0.0000
Subtotal HITOSU				1.18	21.36	3.20	
*HOLE	8	m	1	1.86	6.76	0.63	0.0000
*HOLE	31	f	1	0.43	4.99	6.40	0.3421
Subtotal HOLE				1.25	11.75	7.03	
HOROWI	1	m	0	1.27	15.35	1.23	0.0000
HOROWI	2	f	0	0.60	8.08	7.44	0.0894
Subtotal HOROWI				1.04	23.43	8.68	
HORWIT	1	f	0	2.43	8.29	6.25	0.0000
HU	15	m	0	0.74	17.16	11.61	0.0023
HU	16	f	0	0.55	7.15	7.26	0.1413
Subtotal HU				0.68	24.31	18.87	
HU2	9	m	0	1.11	27.11	5.50	0.0000
HU2	10	f	0	0.63	21.91	18.91	0.0033
Subtotal HU2				0.89	49.01	24.41	
HUANG	1	c	0	0.69	14.82	11.15	0.0078
HUMBLE	14	m	1	2.49	5.19	4.54	0.0000
HUMBLE	16	m	1	2.47	1.71	1.43	0.0012
HUMBLE	18	f	1	2.43	6.68	5.09	0.0000
HUMBLE	20	f	1	2.73	2.90	4.01	0.0000
Subtotal HUMBLE				2.51	16.48	15.07	
JAHN	22	f	2	1.19	14.92	1.98	0.0000
JAIN	46	m	2	2.12	8.79	2.74	0.0000
JAIN	41	f	2	2.22	17.72	7.75	0.0000
Subtotal JAIN				2.19	26.50	10.49	
JARUP	6	m	2	2.02	3.91	0.84	0.0001
JARVHO	3	m	0	3.32	0.92	2.86	0.0015
JARVHO	7	f	0	2.26	3.28	1.61	0.0000
Subtotal JARVHO				2.49	4.19	4.47	
JEDRYC	58	m	4	1.70	31.63	0.62	0.0000
JEDRYC	59	f	4	1.51	11.71	0.02	0.0000
Subtotal JEDRYC				1.65	43.33	0.64	
JIANG	1	m	0	1.00	4.45	1.38	0.0346
JIANG	2	f	0	0.91	2.62	1.09	0.1401

Table 1C3 - 2

IESLC - Meta-anal of Ever Smoking (or Current if Ever not available), Cigs (or Any Prod if Cigs not avail)
All LC types
Most adjusted

REF	NRR	SEX	AD	Ys	Ws	Qs	Ps
Subtotal JIANG				0.97	7.08	2.48	
JOLY	2	m	0	2.65	10.97	13.07	0.0000
JOLY	1	f	0	1.99	27.09	5.15	0.0000
Subtotal JOLY				2.18	38.06	18.23	
JUSSAW	31	m	2	2.16	8.36	3.00	0.0000
*KAISE2	72	m	1	1.69	11.75	0.19	0.0000
*KAISE2	64	f	1	2.31	9.19	5.22	0.0000
Subtotal KAISE2				1.96	20.95	5.42	
*KAISER	13	m	2	2.87	25.69	44.22	0.0000
*KAISER	10	f	2	1.73	28.18	0.82	0.0000
Subtotal KAISER				2.27	53.88	45.03	
KANELL	30	m	1	1.60	30.83	0.05	0.0000
KATSOU	29	f	1	1.19	9.91	1.31	0.0002
KAUFMA	17	c	6	2.52	28.72	26.38	0.0000
KELLER	3	m	0	2.31	217.37	123.50	0.0000
KELLER	11	m	0	2.60	25.99	28.46	0.0000
KELLER	7	f	0	2.53	269.69	252.45	0.0000
KELLER	15	f	0	2.25	39.03	18.48	0.0000
Subtotal KELLER				2.43	552.08	422.89	
KHUDER	4	m	0	2.06	19.93	5.05	0.0000
KIHARA	31	c	0	1.22	46.97	5.38	0.0000
*KINLEN	17	m	2	2.40	7.00	4.93	0.0000
KJUUS	3	m	0	2.66	1.80	2.18	0.0004
*KNEKT	87	m	1	1.86	5.67	0.52	0.0000
KO	1	f	3	1.44	2.18	0.03	0.0339
KOHLME	2	c	4	2.80	5.21	8.01	0.0000
KOO	1	f	0	1.02	23.01	6.70	0.0000
KOULUM	2	m	0	3.38	4.45	14.85	0.0000
KREUZE	60	f	3	2.22	3.99	1.75	0.0000
KREUZE	62	f	3	1.40	28.45	0.72	0.0000
Subtotal KREUZE				1.50	32.44	2.47	
KREYBE	12	m	1	1.89	5.80	0.63	0.0000
KREYBE	30	f	1	0.36	7.92	11.40	0.3143
Subtotal KREYBE				1.01	13.71	12.03	
*KUBIK	27	m	0	3.36	1.96	6.41	0.0000
LAMTH	6	f	0	1.34	46.55	2.26	0.0000
LAMWK	1	f	0	1.42	17.85	0.36	0.0000
LAMWK2	9	m	0	1.04	12.98	3.46	0.0002
LAMWK2	10	f	0	1.17	17.89	2.75	0.0000
Subtotal LAMWK2				1.11	30.86	6.21	
*LANGE	40	m	1	1.56	3.97	0.00	0.0019
*LANGE	37	f	1	1.60	8.13	0.01	0.0000
Subtotal LANGE				1.58	12.09	0.01	
LAUSSM	11	m	3	1.74	37.15	1.24	0.0000
LEI	1	m	0	1.30	26.63	1.72	0.0000
LEI	2	f	0	1.25	23.21	2.21	0.0000
Subtotal LEI				1.28	49.84	3.94	
LEMARC	3	c	0	1.73	22.77	0.66	0.0000
LETOUR	1	c	0	2.56	20.21	20.39	0.0000
LEVIN	30	m	1	1.94	29.93	4.41	0.0000
*LIAW	1	m	1	1.31	11.72	0.73	0.0000
*LIAW	2	f	1	1.28	2.46	0.19	0.0447
Subtotal LIAW				1.30	14.17	0.92	
*LIDDEL	5	m	1	1.28	17.92	1.35	0.0000
LIU	2	c	2	0.65	38.19	31.31	0.0001
LIU2	2	m	3	1.65	4.37	0.03	0.0006
LIU2	4	f	3	1.54	6.68	0.00	0.0001
Subtotal LIU2				1.58	11.05	0.04	
LIU3	2	m	2	0.23	1.87	3.30	0.7518
LIU4	10	m	2	1.36	5780.52	235.76	0.0000
LIU4	12	f	2	1.05	3876.64	996.36	0.0000
Subtotal LIU4				1.23	9657.16	1232.12	
LIU5	1	c	0	0.65	11.25	9.28	0.0293
LOMBA2	1	f	0	0.28	37.19	60.42	0.0841
LOMBAR	2	m	0	2.30	12.10	6.72	0.0000
LUBIN2	48	m	2	2.17	162.15	60.36	0.0000
LUBIN2	98	f	1	1.36	133.31	5.16	0.0000
Subtotal LUBIN2				1.80	295.46	65.52	
LUO	7	c	20	0.99	10.60	3.38	0.0012
MACLEN	73	c	2	0.98	17.04	5.65	0.0001
*MAGNUS	5	m	3	1.42	6.75	0.13	0.0002

Table 1C3 - 2

IESLC - Meta-anal of Ever Smoking (or Current if Ever not available), Cigs (or Any Prod if Cigs not avail)
All LC types
Most adjusted

REF	NRR	SEX	AD	Ys	Ws	Qs	Ps
MARSH	1	m	0	2.32	1.82	1.04	0.0018
MARSH	3	f	0	1.76	5.60	0.23	0.0000
Subtotal MARSH				1.90	7.42	1.28	
MARSH2	5	m	1	0.64	3.87	3.28	0.2107
MARSH2	6	f	1	1.66	3.65	0.04	0.0015
Subtotal MARSH2				1.14	7.51	3.32	
MARTIS	4	m	0	1.95	3.32	0.50	0.0004
MASTRA	2	m	2	2.10	4.76	1.38	0.0000
MATOS	27	m	2	1.92	8.82	1.14	0.0000
MATSUD	10	m	0	3.07	2.94	6.69	0.0000
MCCONN	1	m	0	0.19	3.33	6.19	0.7237
MCCONN	2	f	0	1.01	0.99	0.30	0.3136
Subtotal MCCONN				0.38	4.32	6.49	
MCDUFF	1	m	0	1.81	4.70	0.31	0.0001
MCLAUG	1	m	0	1.20	18.70	2.34	0.0000
*MIGRAN	27	m	2	1.28	3.91	0.29	0.0111
*MIGRAN	42	f	2	1.53	3.54	0.00	0.0040
Subtotal MIGRAN				1.40	7.45	0.30	
MILLER	2	f	1	1.61	4.90	0.01	0.0004
MILLS	1	m	1	0.24	71.48	123.71	0.0406
*MRFITR	6	m	0	3.70	0.50	2.28	0.0091
NAM	77	m	1	2.16	24.64	9.07	0.0000
NAM	93	f	1	2.18	34.31	13.44	0.0000
Subtotal NAM				2.18	58.95	22.51	
NOTAN2	19	m	2	0.86	33.41	16.33	0.0000
NOU	11	m	0	1.81	5.20	0.32	0.0000
NOU	12	f	0	1.96	2.74	0.44	0.0012
Subtotal NOU				1.86	7.94	0.76	
ODRISC	3	c	0	3.89	5.83	31.71	0.0000
ORMOS	4	m	0	2.23	6.39	2.92	0.0000
ORMOS	26	f	0	-1.64	0.95	9.75	0.1093
Subtotal ORMOS				1.73	7.34	12.67	
OSANN	41	m	2	2.98	39.82	80.62	0.0000
OSANN	42	f	2	2.71	66.25	87.66	0.0000
Subtotal OSANN				2.81	106.08	168.28	
PARKIN	31	m	0	1.54	71.91	0.03	0.0000
PASTOR	10	m	1	1.92	7.85	1.02	0.0000
PAWLEG	2	m	6	2.51	3.16	2.84	0.0000
PERNU	8	m	0	2.23	50.02	22.36	0.0000
PERNU	4	f	0	0.95	5.14	1.92	0.0321
Subtotal PERNU				2.11	55.16	24.29	
PERSH2	11	c	4	1.88	115.76	11.98	0.0000
*PETO	5	m	0	1.82	1.98	0.13	0.0107
PEZZO2	10	m	0	2.71	5.55	7.35	0.0000
PEZZOT	25	m	0	2.96	3.75	7.38	0.0000
PIKE	4	m	0	1.66	13.39	0.14	0.0000
PIKE	8	f	0	1.57	18.04	0.01	0.0000
Subtotal PIKE				1.61	31.43	0.14	
POFFIJ	1	c	0	2.05	46.21	11.10	0.0000
POLEDN	1	c	1	2.22	11.85	5.25	0.0000
*QIAO2	9	m	0	0.79	9.66	5.74	0.0145
RACHTA	15	f	4	2.11	7.21	2.16	0.0000
RADZIK	1	c	0	0.27	5.03	8.32	0.5411
RANDIG	9	m	0	1.60	3.95	0.01	0.0014
RANDIG	10	f	0	0.80	6.34	3.66	0.0447
Subtotal RANDIG				1.11	10.29	3.67	
REN	1	m	0	1.27	7.46	0.60	0.0005
REN	2	f	0	1.40	9.65	0.23	0.0000
Subtotal REN				1.35	17.11	0.84	
RONCO	3	m	2	1.69	5.06	0.09	0.0001
ROTHSC	2	c	1	1.71	9.83	0.24	0.0000
SADOWS	4	m	0	1.45	13.79	0.17	0.0000
SANKAR	2	m	3	2.61	22.36	24.82	0.0000
SCHWAR	1	m	0	2.11	80.50	24.15	0.0000
SCHWAR	2	m	0	1.88	29.06	2.94	0.0000
SCHWAR	3	f	0	2.30	111.43	61.21	0.0000
SCHWAR	4	f	0	2.45	26.58	21.03	0.0000
Subtotal SCHWAR				2.20	247.58	109.33	
SEGI	1	m	0	0.53	15.18	15.91	0.0375
SEGI2	20	m	1	1.32	6.65	0.38	0.0007
SEGI2	28	f	1	0.50	10.48	11.71	0.1049

Table 1C3 - 2

IESLC - Meta-anal of Ever Smoking (or Current if Ever not available), Cigs (or Any Prod if Cigs not avail)
All LC types
Most adjusted

REF	NRR	SEX	AD	Ys	Ws	Qs	Ps
Subtotal SEGI2				0.82	17.14	12.09	
SEOW	6	f	1	1.66	9.73	0.10	0.0000
SHAW	12	c	0	2.47	9.34	7.81	0.0000
SIEMIA	5	m	7	2.49	10.37	9.07	0.0000
SIMARA	3	m	6	0.50	13.58	15.17	0.0650
SIMARA	4	f	6	0.49	9.71	11.11	0.1278
Subtotal SIMARA				0.50	23.30	26.28	
SOBUE	105	m	1	1.31	28.15	1.68	0.0000
SOBUE	115	f	1	0.92	47.90	19.47	0.0000
Subtotal SOBUE				1.07	76.05	21.14	
SOBUE2	10	m	2	1.50	197.90	0.72	0.0000
SOBUE2	12	f	2	1.19	143.51	19.64	0.0000
Subtotal SOBUE2				1.37	341.42	20.36	
*SPEIZE	8	f	0	1.96	52.33	8.32	0.0000
SPITZ	3	c	0	2.91	6.16	11.29	0.0000
STASZE	7	m	0	2.50	4.72	4.16	0.0000
STASZE	5	f	0	1.47	4.16	0.03	0.0028
Subtotal STASZE				2.02	8.88	4.20	
STAYNE	1	m	0	1.30	40.37	2.70	0.0000
STOCKS	46	m	2	1.94	32.85	4.73	0.0000
STOCKS	50	f	1	1.11	58.11	11.56	0.0000
Subtotal STOCKS				1.41	90.96	16.29	
STOCKW	8	c	0	2.41	1511.07	1087.01	0.0000
STUCKE	3	m	0	4.83	0.49	5.28	0.0007
SUN	1	c	0	0.84	30.23	15.75	0.0000
SUZUK2	7	c	3	2.40	2.76	1.95	0.0001
SVENSS	71	f	1	1.82	16.09	1.12	0.0000
TANG	3	c	0	2.09	6.14	1.74	0.0000
*TENKAN	22	m	1	2.68	5.38	6.83	0.0000
TIZZAN	2	m	0	0.70	90.61	66.60	0.0000
TIZZAN	22	f	0	1.40	8.03	0.19	0.0001
Subtotal TIZZAN				0.76	98.64	66.79	
TOKARS	6	c	3	1.89	7.27	0.79	0.0000
TOUSEY	10	m	3	3.06	3.75	8.51	0.0000
TOUSEY	13	f	3	2.82	10.58	16.89	0.0000
Subtotal TOUSEY				2.88	14.33	25.40	
TSUGAN	27	m	0	0.23	7.76	13.72	0.5244
*TULINI	38	m	3	2.04	10.34	2.43	0.0000
*TULINI	44	f	3	2.57	11.17	11.34	0.0000
Subtotal TULINI				2.31	21.50	13.77	
*TVERDA	22	m	2	1.52	20.49	0.03	0.0000
*TVERDA	15	f	2	2.40	2.67	1.90	0.0001
Subtotal TVERDA				1.62	23.16	1.93	
WAKAI	72	m	2	1.30	8.06	0.53	0.0002
WAKAI	78	f	2	1.50	9.15	0.03	0.0000
Subtotal WAKAI				1.41	17.20	0.56	
*WALD	4	m	1	2.80	4.92	7.56	0.0000
WANG	5	c	6	1.06	15.11	3.78	0.0000
WANG2	16	c	4	0.83	7.47	3.97	0.0235
WANG3	1	c	0	1.05	28.11	7.32	0.0000
WANG4	2	m	2	0.15	100.26	199.15	0.1372
WICKLU	1	m	0	1.53	15.41	0.02	0.0000
WIGLE	13	m	0	2.46	13.33	10.81	0.0000
WIGLE	16	f	0	1.40	21.22	0.54	0.0000
Subtotal WIGLE				1.81	34.55	11.35	
WILKIN	3	c	4	2.06	12.03	3.01	0.0000
WU	45	f	2	1.11	14.48	2.92	0.0000
WUNSCH	4	m	1	1.56	11.39	0.00	0.0000
WUNSCH	10	f	1	1.49	14.00	0.07	0.0000
Subtotal WUNSCH				1.52	25.38	0.07	
WUWILL	8	f	3	0.83	102.19	53.70	0.0000
WYNDE2	16	m	0	2.28	7.19	3.77	0.0000
WYNDE3	48	m	0	2.27	7.69	3.88	0.0000
WYNDE3	83	f	0	1.14	9.73	1.71	0.0004
Subtotal WYNDE3				1.64	17.42	5.60	
WYNDE4	48	m	0	2.21	10.51	4.46	0.0000
WYNDE4	62	f	2	1.05	8.80	2.23	0.0018
Subtotal WYNDE4				1.68	19.31	6.69	
WYNDE6	81	m	0	2.39	71.26	49.34	0.0000
WYNDE6	252	f	0	2.34	103.92	63.86	0.0000
Subtotal WYNDE6				2.36	175.18	113.20	

Table 1C3 - 2

IESLC - Meta-anal of Ever Smoking (or Current if Ever not available), Cigs (or Any Prod if Cigs not avail)
All LC types
Most adjusted

REF	NRR	SEX	AD	Ys	Ws	Qs	Ps
*XIANGZ	14	m	2	0.58	25.00	23.80	0.0036
XU	2	m	2	0.99	58.89	18.77	0.0000
XU2	2	c	7	1.34	45.75	2.27	0.0000
XU3	2	m	1	1.79	5.80	0.31	0.0000
XU3	4	f	1	1.35	3.69	0.16	0.0095
Subtotal XU3				1.62	9.49	0.47	
XU4	1	c	0	1.08	20.82	4.75	0.0000
YAMAGU	11	c	1	1.38	9.83	0.32	0.0000
*YONG	12	m	1	3.36	1.92	6.22	0.0000
*YONG	15	f	1	1.65	6.30	0.05	0.0000
Subtotal YONG				2.05	8.22	6.27	
*YUAN	1	m	2	1.87	11.44	1.13	0.0000
ZHANG	2	m	7	1.39	4.65	0.14	0.0028
ZHANG	3	f	7	1.32	4.81	0.27	0.0038
Subtotal ZHANG				1.35	9.46	0.40	
ZHENG	15	m	0	1.29	20.36	1.42	0.0000
ZHENG	24	f	0	0.74	20.88	14.04	0.0008
Subtotal ZHENG				1.01	41.24	15.47	
ZHOU	2	m	0	0.86	17.50	8.52	0.0003
ZHOU	3	f	0	0.80	5.34	3.10	0.0660
Subtotal ZHOU				0.84	22.83	11.62	

	N	341
	NS	242
	Wt	19674.69
Het	Chi	6483.61
Het	df	340
Het	P	***
Fixed	RR	4.75
	RRl	4.68
	RRu	4.82
	P	+++
Random	RR	5.48
	RRl	5.09
	RRu	5.90
	P	+++
Asymm	P	**

Table 1C3 - 3

IESLC - Meta-anal of Ever Smoking (or Current if Ever not available), Cigs (or Any Prod if Cigs not avail)
All LC types
Most adjusted

	Sex			
	combined	male	female	Total
N	46	179	116	341
NS	46	176	112	334
Wt	2385.71	9902.80	7386.18	19674.69
Het Chi	726.54	2270.65	2437.82	6483.61
Het df	45	178	115	340
Het P	***	***	***	***
Fixed RR	8.57	4.68	4.00	4.75
RRl	8.23	4.59	3.91	4.68
RRu	8.92	4.77	4.09	4.82
P	+++	+++	+++	+++
Random RR	6.01	6.18	4.40	5.48
RRl	4.83	5.60	3.85	5.09
RRu	7.47	6.82	5.04	5.90
P	+++	+++	+++	+++
Between Chi				1048.61
Between df				2
Between P				***
Btwn(F) P				***
Btwn(R) P				***

	Lung cancer type		
	all	other	Total
N	328	13	341
NS	233	9	242
Wt	19134.04	540.65	19674.69
Het Chi	6395.87	48.91	6483.61
Het df	327	12	340
Het P	***	***	***
Fixed RR	4.78	3.65	4.75
RRl	4.72	3.35	4.68
RRu	4.85	3.97	4.82
P	+++	+++	+++
Random RR	5.59	3.52	5.48
RRl	5.18	2.87	5.09
RRu	6.03	4.31	5.90
P	+++	+++	+++
Between Chi			38.83
Between df			1
Between P			***
Btwn(F) P			N.S.
Btwn(R) P			***

	Location								
	NAmer	UK	Scand	othEur	China	Japan	othAs	other	Total
N	118	32	33	51	51	22	20	14	341
NS	82	21	23	40	35	14	15	12	242
Wt	5713.12	375.99	467.72	1061.08	10718.29	815.71	303.62	219.17	19674.69
Het Chi	1143.61	156.26	115.04	426.07	455.89	75.19	91.59	28.46	6483.61
Het df	117	31	32	50	50	21	19	13	340
Het P	***	***	***	***	***	***	***	**	***
Fixed RR	9.14	5.34	6.33	5.59	3.33	3.45	3.44	6.17	4.75
RRl	8.90	4.83	5.78	5.27	3.27	3.22	3.08	5.41	4.68
RRu	9.38	5.91	6.93	5.94	3.40	3.70	3.85	7.05	4.82
P	+++	+++	+++	+++	+++	+++	+++	+++	+++
Random RR	7.69	6.02	6.32	6.01	2.77	3.23	3.50	6.97	5.48
RRl	6.99	4.69	5.19	4.91	2.51	2.77	2.70	5.58	5.09
RRu	8.47	7.73	7.70	7.36	3.06	3.77	4.55	8.70	5.90
P	+++	+++	+++	+++	+++	+++	+++	+++	+++
Between Chi									3991.49
Between df									7
Between P									***
Btwn(F) P									***
Btwn(R) P									***

Table 1C3 - 3

IESLC - Meta-anal of Ever Smoking (or Current if Ever not available), Cigs (or Any Prod if Cigs not avail)
All LC types
Most adjusted

	Detailed Country in "other Europe"					
	multi	Germany	othWest	East	Balkans	Total
N	4	17	13	13	4	51
NS	3	12	12	9	4	40
Wt	445.74	218.92	167.48	113.25	115.68	1061.08
Het Chi	105.30	62.72	129.90	37.35	3.63	426.07
Het df	3	16	12	12	3	50
Het P	***	***	***	***	N.S.	***
Fixed RR	7.62	4.66	4.07	5.77	3.68	5.59
RRl	6.95	4.09	3.50	4.80	3.07	5.27
RRu	8.36	5.33	4.74	6.94	4.42	5.94
P	+++	+++	+++	+++	+++	+++
Random RR	7.85	5.25	8.96	5.91	3.70	6.01
RRl	4.46	3.83	4.84	4.12	3.00	4.91
RRu	13.82	7.18	16.60	8.46	4.56	7.36
P	+++	+++	+++	+++	+++	+++
Between Chi						87.16
Between df						4
Between P						***
Btwn(F) P						*
Btwn(R) P						**

	Detailed Country in "other Asia"			
	India	HongKong	other	Total
N	3	7	10	20
NS	3	5	7	15
Wt	64.13	140.70	98.79	303.62
Het Chi	43.73	10.74	18.17	91.59
Het df	2	6	9	19
Het P	***	(*)	*	***
Fixed RR	5.15	3.52	2.57	3.44
RRl	4.03	2.99	2.11	3.08
RRu	6.58	4.15	3.13	3.85
P	+++	+++	+++	+++
Random RR	6.46	3.56	2.62	3.50
RRl	1.90	2.81	1.96	2.70
RRu	21.92	4.50	3.50	4.55
P	++	+++	+++	+++
Between Chi				18.95
Between df				2
Between P				***
Btwn(F) P				N.S.
Btwn(R) P				N.S.

	Detailed other continent			
	SCAmer	Auslia	Africa	Total
N	10	1	3	14
NS	8	1	3	12
Wt	122.40	0.99	95.77	219.17
Het Chi	17.99	0.00	1.51	28.46
Het df	9	0	2	13
Het P	*	N.S.	N.S.	**
Fixed RR	7.24	20.29	4.98	6.17
RRl	6.06	2.84	4.07	5.41
RRu	8.64	145.07	6.08	7.05
P	+++	++	+++	+++
Random RR	7.62	20.29	4.98	6.97
RRl	5.84	2.84	4.07	5.58
RRu	9.94	145.07	6.08	8.70
P	+++	++	+++	+++
Between Chi				8.95
Between df				2
Between P				*
Btwn(F) P				N.S.
Btwn(R) P				*

Table 1C3 - 3

IESLC - Meta-anal of Ever Smoking (or Current if Ever not available), Cigs (or Any Prod if Cigs not avail)
All LC types
Most adjusted

	Start year of study <1960	1960-69	1970-79	1980-89	1990+	Total
N	58	56	76	114	37	341
NS	41	40	53	76	32	242
Wt	1133.64	1608.81	1365.23	15072.13	494.87	19674.69
Het Chi	589.08	452.98	626.25	4576.39	227.79	6483.61
Het df	57	55	75	113	36	340
Het P	***	***	***	***	***	***
Fixed RR	4.95	4.83	4.59	4.72	5.33	4.75
RRl	4.67	4.60	4.35	4.65	4.88	4.68
RRu	5.24	5.07	4.84	4.80	5.82	4.82
P	+++	+++	+++	+++	+++	+++
Random RR	5.12	5.11	5.19	5.82	6.23	5.48
RRl	4.14	4.38	4.39	5.13	4.93	5.09
RRu	6.33	5.96	6.14	6.60	7.88	5.90
P	+++	+++	+++	+++	+++	+++
Between Chi						11.12
Between df						4
Between P						*
Btwn(F) P						N.S.
Btwn(R) P						N.S.

	Study type (1) CC	other	Total
N	266	75	341
NS	188	54	242
Wt	18128.30	1546.38	19674.69
Het Chi	5830.71	518.83	6483.61
Het df	265	74	340
Het P	***	***	***
Fixed RR	4.64	6.30	4.75
RRl	4.57	5.99	4.68
RRu	4.70	6.62	4.82
P	+++	+++	+++
Random RR	5.27	6.34	5.48
RRl	4.85	5.45	5.09
RRu	5.72	7.37	5.90
P	+++	+++	+++
Between Chi			134.08
Between df			1
Between P			***
Btwn(F) P			**
Btwn(R) P			*

	Study type (2) CC	prosp	other	Total
N	266	70	5	341
NS	188	50	4	242
Wt	18128.30	1501.84	44.55	19674.69
Het Chi	5830.71	500.80	11.41	6483.61
Het df	265	69	4	340
Het P	***	***	*	***
Fixed RR	4.64	6.23	9.21	4.75
RRl	4.57	5.92	6.87	4.68
RRu	4.70	6.55	12.35	4.82
P	+++	+++	+++	+++
Random RR	5.27	6.18	8.98	5.48
RRl	4.85	5.29	5.39	5.09
RRu	5.72	7.23	14.98	5.90
P	+++	+++	+++	+++
Between Chi				140.69
Between df				2
Between P				***
Btwn(F) P				*
Btwn(R) P				*

Table 1C3 - 3

IESLC - Meta-anal of Ever Smoking (or Current if Ever not available), Cigs (or Any Prod if Cigs not avail)
All LC types
Most adjusted

	Study size (number of LC cases)				
	100-249	250-499	500-999	1000+	Total
N	122	88	66	65	341
NS	98	64	44	36	242
Wt	950.73	1278.47	1420.41	16025.08	19674.69
Het Chi	472.31	576.27	554.60	4821.16	6483.61
Het df	121	87	65	64	340
Het P	***	***	***	***	***
Fixed RR	3.74	4.89	4.94	4.79	4.75
RRl	3.51	4.63	4.69	4.71	4.68
RRu	3.98	5.17	5.21	4.86	4.82
P	+++	+++	+++	+++	+++
Random RR	4.48	5.62	6.18	6.23	5.48
RRl	3.92	4.85	5.27	5.34	5.09
RRu	5.13	6.52	7.25	7.27	5.90
P	+++	+++	+++	+++	+++
Between Chi					59.26
Between df					3
Between P					***
Btwn(F) P					N.S.
Btwn(R) P					**

	Risky occupational population			
	no	mining	othRisky	Total
N	323	7	11	341
NS	224	7	11	242
Wt	19470.07	74.66	129.97	19674.69
Het Chi	6423.75	19.59	26.04	6483.61
Het df	322	6	10	340
Het P	***	**	**	***
Fixed RR	4.76	3.08	4.62	4.75
RRl	4.69	2.45	3.89	4.68
RRu	4.82	3.86	5.49	4.82
P	+++	+++	+++	+++
Random RR	5.51	3.75	5.27	5.48
RRl	5.11	2.38	3.75	5.09
RRu	5.94	5.91	7.42	5.90
P	+++	+++	+++	+++
Between Chi				14.24
Between df				2
Between P				***
Btwn(F) P				N.S.
Btwn(R) P				N.S.

	National cigarette tobacco type			
	Virginia	blended	other	Total
N	53	233	55	341
NS	38	166	38	242
Wt	729.83	8197.63	10747.23	19674.69
Het Chi	281.64	2721.41	460.59	6483.61
Het df	52	232	54	340
Het P	***	***	***	***
Fixed RR	5.72	7.43	3.33	4.75
RRl	5.32	7.27	3.27	4.68
RRu	6.15	7.59	3.40	4.82
P	+++	+++	+++	+++
Random RR	6.25	6.27	2.78	5.48
RRl	5.19	5.76	2.52	5.09
RRu	7.51	6.82	3.06	5.90
P	+++	+++	+++	+++
Between Chi				3019.97
Between df				2
Between P				***
Btwn(F) P				***
Btwn(R) P				***

Table 1C3 - 3

IESLC - Meta-anal of Ever Smoking (or Current if Ever not available), Cigs (or Any Prod if Cigs not avail)
All LC types
Most adjusted

	Any proxy use No/nk	Yes	Total
N	241	100	341
NS	175	67	242
Wt	7812.69	11862.00	19674.69
Het Chi	3105.55	1672.15	6483.61
Het df	240	99	340
Het P	***	***	***
Fixed RR	6.83	3.74	4.75
RRl	6.68	3.67	4.68
RRu	6.98	3.81	4.82
P	+++	+++	+++
Random RR	5.49	5.42	5.48
RRl	5.02	4.88	5.09
RRu	6.01	6.03	5.90
P	+++	+++	+++
Between Chi			1705.91
Between df			1
Between P			***
Btwn(F) P			***
Btwn(R) P			N.S.

	Full histological confirmation No	Yes	Total
N	260	81	341
NS	183	59	242
Wt	17148.42	2526.27	19674.69
Het Chi	5009.67	799.74	6483.61
Het df	259	80	340
Het P	***	***	***
Fixed RR	4.42	7.69	4.75
RRl	4.36	7.40	4.68
RRu	4.49	8.00	4.82
P	+++	+++	+++
Random RR	5.27	6.19	5.48
RRl	4.86	5.37	5.09
RRu	5.72	7.14	5.90
P	+++	+++	+++
Between Chi			674.21
Between df			1
Between P			***
Btwn(F) P			***
Btwn(R) P			(*)

	Number of adjustment variables (1) 0	1	2+/+nk	Total
N	165	75	101	341
NS	117	52	80	249
Wt	4673.94	2036.64	12964.11	19674.69
Het Chi	1964.14	673.91	2616.19	6483.61
Het df	164	74	100	340
Het P	***	***	***	***
Fixed RR	7.15	5.59	3.99	4.75
RRl	6.95	5.35	3.92	4.68
RRu	7.36	5.84	4.06	4.82
P	+++	+++	+++	+++
Random RR	5.33	5.71	5.54	5.48
RRl	4.75	4.94	4.92	5.09
RRu	5.98	6.60	6.24	5.90
P	+++	+++	+++	+++
Between Chi				1229.38
Between df				2
Between P				***
Btwn(F) P				***
Btwn(R) P				N.S.

Table 1C3 - 3

IESLC - Meta-anal of Ever Smoking (or Current if Ever not available), Cigs (or Any Prod if Cigs not avail)
All LC types
Most adjusted

	Number of adjustment variables (2) 0	1	2	3-5	6+/+nk	Total
N	165	75	52	35	14	341
NS	117	52	41	29	12	251
Wt	4673.94	2036.64	12055.13	720.01	188.97	19674.69
Het Chi	1964.14	673.91	2224.86	205.04	104.60	6483.61
Het df	164	74	51	34	13	340
Het P	***	***	***	***	***	***
Fixed RR	7.15	5.59	3.91	5.54	4.03	4.75
RRl	6.95	5.35	3.85	5.15	3.49	4.68
RRu	7.36	5.84	3.99	5.96	4.64	4.82
P	+++	+++	+++	+++	+++	+++
Random RR	5.33	5.71	5.43	6.50	4.08	5.48
RRl	4.75	4.94	4.62	5.33	2.64	5.09
RRu	5.98	6.60	6.39	7.92	6.29	5.90
P	+++	+++	+++	+++	+++	+++
Between Chi						1311.07
Between df						4
Between P						***
Btwn(F) P						***
Btwn(R) P						N.S.

	Product all/unsp	cig+/-ot	cig only	Total
N	131	180	30	341
NS	99	128	24	251
Wt	6379.33	6549.44	6745.92	19674.69
Het Chi	1789.75	2280.96	580.15	6483.61
Het df	130	179	29	340
Het P	***	***	***	***
Fixed RR	3.61	7.27	4.07	4.75
RRl	3.52	7.10	3.97	4.68
RRu	3.70	7.45	4.16	4.82
P	+++	+++	+++	+++
Random RR	4.76	5.96	6.03	5.48
RRl	4.19	5.40	4.79	5.09
RRu	5.39	6.58	7.58	5.90
P	+++	+++	+++	+++
Between Chi				1832.75
Between df				2
Between P				***
Btwn(F) P				***
Btwn(R) P				*

	Denominator nev any	nev cigs	Total
N	245	96	341
NS	176	69	245
Wt	16621.06	3053.63	19674.69
Het Chi	4711.21	1243.37	6483.61
Het df	244	95	340
Het P	***	***	***
Fixed RR	4.43	6.96	4.75
RRl	4.36	6.72	4.68
RRu	4.49	7.21	4.82
P	+++	+++	+++
Random RR	5.30	5.94	5.48
RRl	4.88	5.15	5.09
RRu	5.76	6.85	5.90
P	+++	+++	+++
Between Chi			529.04
Between df			1
Between P			***
Btwn(F) P			***
Btwn(R) P			N.S.

Table 1C3 - 3

IESLC - Meta-anal of Ever Smoking (or Current if Ever not available), Cigs (or Any Prod if Cigs not avail)
All LC types
Most adjusted

	Derivation of RR/CI			
	Orig	StdCalc	Other	Total
N	50	170	121	341
NS	37	123	89	249
Wt	2096.72	4858.77	12719.20	19674.69
Het Chi	789.92	2204.04	1746.29	6483.61
Het df	49	169	120	340
Het P	***	***	***	***
Fixed RR	7.37	6.99	3.81	4.75
RRl	7.06	6.79	3.75	4.68
RRu	7.69	7.19	3.88	4.82
P	+++	+++	+++	+++
Random RR	5.97	5.42	5.32	5.48
RRl	4.91	4.83	4.84	5.09
RRu	7.27	6.09	5.85	5.90
P	+++	+++	+++	+++
Between Chi				1743.36
Between df				2
Between P				***
Btwn(F) P				***
Btwn(R) P				N.S.

	Smoking status		
	ever	current	Total
N	326	15	341
NS	235	10	245
Wt	18983.70	690.99	19674.69
Het Chi	6317.25	156.73	6483.61
Het df	325	14	340
Het P	***	***	***
Fixed RR	4.73	5.33	4.75
RRl	4.66	4.95	4.68
RRu	4.80	5.75	4.82
P	+++	+++	+++
Random RR	5.49	5.27	5.48
RRl	5.09	3.85	5.09
RRu	5.92	7.21	5.90
P	+++	+++	+++
Between Chi			9.64
Between df			1
Between P			**
Btwn(F) P			N.S.
Btwn(R) P			N.S.

	Study LIU4		
	LIU4	others	Total
N	2	339	341
NS	1	241	242
Wt	9657.16	10017.53	19674.69
Het Chi	215.88	4271.81	6483.61
Het df	1	338	340
Het P	***	***	***
Fixed RR	3.43	6.49	4.75
RRl	3.37	6.37	4.68
RRu	3.50	6.62	4.82
P	+++	+++	+++
Random RR	3.33	5.51	5.48
RRl	2.47	5.10	5.09
RRu	4.49	5.95	5.90
P	+++	+++	+++
Between Chi			1995.92
Between df			1
Between P			***
Btwn(F) P			***
Btwn(R) P			**

Table 1C3 - 4

IESLC - Meta-anal of Ever Smoking (or Current if Ever not available), Cigs (or Any Prod if Cigs not avail)
All LC types
Least adjusted

REF	NRR	X	SEX	AGEL	AGEH	RACE	YF	LC TYPE	LOC	START	ST	NLC	R	VB	P	H	AD	SM	PRODUCT	DENOM	De
ABELIN	2	x	m	0	0	all	-	all	Eu:wst	1941	CC	118	n	bl	y	n	0	ev	cig+/-ot	nev any	st
ABRAHA	7		m	0	0	all	0	q+s+a	Eu:est	1975	pr	571	n	bl	n	n	0	ev	all/unsp	nev any	ot
ABRAHA	8		f	0	0	all	0	q+s+a	Eu:est	1975	pr	571	n	bl	n	n	0	ev	all/unsp	nev any	ot
AGUDO	8	x	f	0	0	all	-	all	Eu:wst	1989	CC	103	n	bl	n	n	0	ev	cig only	nev any	st
AKIBA	3	x	m	0	0	all	0	all	As:Jap	1963	pr	610	n	bl	n	n	0	ev	cig+/-ot	nev cigs	st
AKIBA	7	x	f	0	0	all	0	all	As:Jap	1963	pr	610	n	bl	n	n	0	ev	cig+/-ot	nev cigs	st
ALDERS	61	x	m	0	0	all	-	all	Eu:UK	1977	CC	1448	n	V	n	n	0	ev	cig+/-ot	nev any	st
ALDERS	12	x	f	0	0	all	-	all	Eu:UK	1977	CC	1448	n	V	n	n	0	ev	cig only	nev any	st
AMANDU	3	x	m	0	0	wh	0	all	NAmer	1959	pr	132	m	bl	n	n	0	ev	cig+/-ot	nev cigs	st
AMES	4		m	0	0	wh	-	all	NAmer	1959	ot	317	m	bl	n	n	0	ev	all/unsp	nev any	st
ANDERS	3		f	0	0	all	0	all	NAmer	1986	pr	343	n	bl	n	n	0	ev	cig+/-ot	nev cigs	st
ARCHER	6		m	0	0	wh	0	all	NAmer	1950	pr	146	m	bl	n	n	0	ev	cig+/-ot	nev cigs	st
ARMADA	4		m	0	0	all	-	all	Eu:wst	1986	CC	325	n	bl	n	y	0	ev	cig+/-ot	nev any	st
AUSTIN	3	x	c	0	0	all	-	all	NAmer	1970	CC	166	o	bl	y	n	0	ev	cig+/-ot	nev cigs	st
AUVINE	1	x	c	0	0	all	-	all	Eu:Sca	1986	CC	517	n	bl	y	n	0	ev	cig+/-ot	nev cigs	st
AXELSO	1		c	0	0	all	-	all	Eu:Sca	1960	CC	152	n	bl	y	n	0	ev	all/unsp	nev any	st
AXELSS	1	x	m	0	0	sca	-	all	Eu:Sca	1989	CC	436	n	bl	n	n	0	ev	all/unsp	nev any	st
AXELSS	11		f	0	0	sca	-	all	Eu:Sca	1989	CC	436	n	bl	n	n	0	ev	all/unsp	nev any	st
BAND	1		m	0	0	all	-	all	NAmer	1983	CC	2831	n	V	y	y	2	ev	cig only	nev any	ot
BARBON	106	x	m	0	0	all	-	all	Eu:wst	1979	CC	755	n	bl	y	y	0	ev	all/unsp	nev any	st
BECHER	15	x	m	0	0	all	-	all	Eu:Ger	1985	CC	194	n	bl	n	y	0	ev	cig+/-ot	nev any	st
BECHER	16		f	0	0	all	-	all	Eu:Ger	1985	CC	194	n	bl	n	y	0	ev	cig+/-ot	nev any	st
BENSHL	15		m	0	0	all	0	all	Eu:UK	1967	pr	486	n	V	n	n	1	ev	cig+/-ot	nev any	ot
BEST	23		m	55	79	all	3	all	NAmer	1955	pr	381	n	V	n	n	0	ev	cig+/-ot	nev any	st
BEST	18		f	0	0	all	0	all	NAmer	1955	pr	381	n	V	n	n	1	ev	cig only	nev any	ot
BLOHMK	3		m	0	0	all	-	all	Eu:Ger	1978	CC	888	n	bl	n	y	0	ev	all/unsp	nev any	st
BLOT4	1		m	0	0	wh	-	all	NAmer	1974	CC	335	n	bl	y	n	0	ev	cig+/-ot	nev cigs	st
BOFFET	7	x	m	0	0	all	-	all	Eu:mul	1988	CC	5621	n	bl	y	n	0	ev	cig+/-ot	nev any	st
BOUCOT	58	x	m	0	0	all	0	all	NAmer	1951	pr	121	n	bl	n	n	0	ev	cig+/-ot	nev any	ot
BRESLO	17		m	0	0	all	-	all	NAmer	1949	CC	518	n	bl	n	y	0	ev	cig+/-ot	nev+1	st
BRESLO	23		f	0	0	all	-	all	NAmer	1949	CC	518	n	bl	n	y	0	ev	cig+/-ot	nev+1	st
BRETT	10		m	0	0	all	0	all	Eu:UK	1960	pr	150	n	V	n	n	0	ev	cig+/-ot	nev cigs	st
BROCKM	1		m	0	0	wh	-	all	Eu:Ger	1990	CC	117	n	bl	n	y	0	ev	cig+/-ot	nev cigs	st
BROCKM	2		f	0	0	wh	-	all	Eu:Ger	1990	CC	117	n	bl	n	y	0	ev	cig+/-ot	nev cigs	st
BROSS	13		m	0	0	wh	-	all	NAmer	1960	CC	974	n	bl	n	n	0	ev	cig+/-ot	nev any	st
BROWN2	2		m	0	0	wh	-	all	NAmer	1984	CC	14596	n	bl	n	y	2	ev	cig+/-ot	nev cigs	or
BROWN2	1		f	0	0	wh	-	all	NAmer	1984	CC	14596	n	bl	n	y	2	ev	cig+/-ot	nev cigs	or
BUFFLE	2		m	0	0	wh	-	all	NAmer	1976	CC	943	n	bl	y	n	0	ev	cig+/-ot	nev any	st
BUFFLE	6		f	0	0	wh	-	all	NAmer	1976	CC	943	n	bl	y	n	0	ev	cig+/-ot	nev any	st
CARPEN	7	x	c	0	0	w+b	-	all	NAmer	1991	CC	356	n	bl	n	n	0	ev	cig+/-ot	nev cigs	st
CASCO2	1		c	0	0	wh	-	all	Eu:Ger	1991	CC	155	n	bl	n	n	0	ev	all/unsp	nev any	st
CASCOR	1		c	0	0	wh	-	all	Eu:Ger	1985	CC	389	n	bl	n	y	0	ev	all/unsp	nev any	st
CEDERL	107		m	0	0	all	16	all	Eu:Sca	1963	pr	491	n	bl	n	n	2	ev	all/unsp	nev any	ot
CEDERL	112		f	0	0	all	0	all	Eu:Sca	1963	pr	491	n	bl	n	n	2	ev	all/unsp	nev any	ot
CHAN	5		m	0	0	all	-	all	As:HK	1976	CC	397	n	bl	n	n	0	ev	cig+/-ot	nev any	st
CHAN	6		f	0	0	all	-	all	As:HK	1976	CC	397	n	bl	n	n	0	ev	cig+/-ot	nev any	st
CHANG	6		m	0	0	all	0	all	NAmer	1972	pr	136	n	bl	n	n	0	ev	cig+/-ot	nev cigs	st
CHANG	12		f	0	0	all	0	all	NAmer	1972	pr	136	n	bl	n	n	0	ev	cig+/-ot	nev cigs	st
CHATZI	4		c	0	0	all	-	all	Eu:bal	1987	CC	282	n	bl	n	y	0	ev	all/unsp	nev any	st
CHEN2	1		m	0	0	all	-	all	As:Chi	1983	CC	193	n	ot	y	n	0	ev	all/unsp	nev any	st
CHEN2	2		f	0	0	all	-	all	As:Chi	1983	CC	193	n	ot	y	n	0	ev	all/unsp	nev any	st
CHEN3	1		c	0	0	all	-	all	As:Chi	1981	CC	254	n	ot	y	n	0	ev	all/unsp	nev any	st
CHIAZZ	2	x	m	0	0	all	-	all	NAmer	1940	CC	144	o	bl	y	n	0	ev	cig+/-ot	nev cigs	st
CHOI	1		m	0	0	all	-	all	As:oth	1985	CC	375	n	bl	n	n	0	ev	cig+/-ot	nev cigs	st
CHOI	5		f	0	0	all	-	all	As:oth	1985	CC	375	n	bl	n	n	0	ev	cig+/-ot	nev cigs	st
CHOW	6	x	m	0	0	wh	0	all	NAmer	1966	pr	219	n	bl	n	n	0	ev	cig+/-ot	nev any	st
CHYOU	9	x	m	0	0	jap	0	all	NAmer	1965	pr	227	n	bl	n	y	0	ev	cig+/-ot	nev cigs	st
COMSTO	33		m	0	0	all	-	all	NAmer	1975	ot	258	n	bl	n	n	0	ev	cig+/-ot	nev any	st
COMSTO	45		f	0	0	all	-	all	NAmer	1975	ot	258	n	bl	n	n	0	ev	cig+/-ot	nev any	st
COOKSO	4		c	0	0	bl	-	all	Africa	1961	CC	234	n	V	n	y	0	ev	cig+/-ot	nev any	st
CORREA	33	x	c	0	0	all	-	all	NAmer	1979	CC	1359	n	bl	y	n	0	ev	cig+/-ot	nev cigs	st
CPSI	187		m	35	84	all	6	all	NAmer	1959	pr	5138	n	bl	n	n	1	ev	cig+/-ot	nev any	ot
CPSI	274		f	40	74	all	6	all	NAmer	1959	pr	5138	n	bl	n	n	1	ev	cig+/-ot	nev cigs	ot
CPSII	104		m	35	99	all	4	all	NAmer	1982	pr	3229	n	bl	n	n	1	ev	cig only	nev any	ot
CPSII	79		f	0	0	all	4	all	NAmer	1982	pr	3229	n	bl	n	n	1	ev	cig+/-ot	nev cigs	ot
DAMBER	26	x	m	0	0	all	-	all	Eu:Sca	1972	CC	579	n	bl	y	n	0	ev	cig+/-ot	nev any	st
DARBY	15		m	0	0	wh	-	all	Eu:UK	1988	CC	982	n	V	n	n	0	ev	all/unsp	nev any	st
DARBY	16		f	0	0	wh	-	all	Eu:UK	1988	CC	982	n	V	n	n	0	ev	all/unsp	nev any	st
DAVEYS	5		m	0	0	all	-	all	Eu:Ger	1930	CC	109	n	bl	y	n	0	ev	all/unsp	nev any	st
DAVEYS	6		f	0	0	all	-	all	Eu:Ger	1930	CC	109	n	bl	y	n	0	ev	all/unsp	nev any	ot
DEAN	8		m	0	0	wh	-	all	Africa	1947	CC	603	n	V	y	n	0	ev	cig+/-ot	nev any	st
DEAN2	12		m	0	0	all	-	all	Eu:UK	1960	CC	954	n	V	y	n	0	ev	cig+/-ot	nev any	st

Table 1C3 - 4

IESLC - Meta-anal of Ever Smoking (or Current if Ever not available), Cigs (or Any Prod if Cigs not avail)
All LC types
Least adjusted

REF	NRR	X	SEX	AGEL	AGEH	RACE	YF	LC TYPE	LOC	START	ST	NLC	R	VB	P	H	AD	SM	PRODUCT	DENOM	De
DEAN2	20		f	0	0	all	-	all	Eu:UK	1960	CC	954	n	V	y	n	0	ev	cig+/-ot	nev any	st
DEAN3	240	x	m	0	0	all	-	all	Eu:UK	1969	CC	766	n	V	y	n	0	ev	cig only	nev any	st
DEAN3	124	x	f	0	0	all	-	all	Eu:UK	1969	CC	766	n	V	y	n	0	ev	cig only	nev any	st
DEKLER	6		m	0	0	all	0	all	Auslia	1961	pr	138	m	V	n	n	2	ev	all/unsp	nev any	ot
DESTE2	15		m	0	0	all	-	all	SCAmer	1993	CC	463	n	bl	n	n	0	ev	all/unsp	nev any	st
DESTEF	11	x	m	0	0	all	-	all	SCAmer	1988	CC	497	n	bl	n	y	0	ev	cig+/-ot	nev any	st
DOCKER	3		c	0	0	wh	0	all	NAmer	1974	pr	120	n	bl	n	n	4	ev	cig+/-ot	nev cigs	ot
DOLL	20		m	0	0	all	-	all	Eu:UK	1948	CC	1465	n	V	n	n	0	ev	cig+/-ot	nev any	st
DOLL	12		f	0	0	all	-	all	Eu:UK	1948	CC	1465	n	V	n	n	0	ev	all/unsp	nev any	st
DOLL2	88		m	0	0	all	10	all	Eu:UK	1951	pr	920	n	V	n	n	1	ev	cig+/-ot	nev any	ot
DOLL2	63		f	0	0	all	22	all	Eu:UK	1951	pr	920	n	V	n	n	1	cu	cig only	nev any	ot
DORANT	10		c	0	0	all	0	all	Eu:wst	1986	ot	550	n	bl	n	y	0	ev	all/unsp	nev any	st
DORGAN	7	x	m	0	0	wh	-	all	NAmer	1980	CC	2026	n	bl	y	y	0	ev	cig+/-ot	nev any	st
DORGAN	31	x	m	0	0	bl	-	all	NAmer	1980	CC	2026	n	bl	y	y	0	ev	cig+/-ot	nev any	st
DORGAN	95		f	0	0	all	-	all	NAmer	1980	CC	2026	n	bl	y	y	3	ev	cig+/-ot	nev any	or
DORN	413		m	0	0	wh	5	all	NAmer	1954	pr	5097	n	bl	n	n	1	ev	cig+/-ot	nev any	ot
DOSEME	17	x	m	0	0	all	-	all	Eu:bal	1979	CC	1210	n	bl	n	n	0	ev	cig+/-ot	nev cigs	st
DROSTE	3	x	m	0	0	all	-	all	Eu:wst	1995	CC	478	n	bl	n	y	0	ev	all/unsp	nev any	st
DU	1		m	0	0	all	-	all	As:Chi	1985	CC	849	n	ot	y	n	0	ev	all/unsp	nev any	or
DU	2		f	0	0	all	-	all	As:Chi	1985	CC	849	n	ot	y	n	0	ev	all/unsp	nev any	or
DUNN	6		m	0	0	all	0	all	NAmer	1954	pr	139	o	bl	n	n	0	ev	cig+/-ot	nev cigs	st
EBELIN	1		m	0	0	all	-	all	Eu:Ger	1980	CC	130	n	bl	n	n	0	ev	all/unsp	nev any	st
ENGELA	8	x	m	0	0	all	0	all	Eu:Sca	1964	pr	435	n	bl	n	n	0	ev	cig+/-ot	nev cigs	st
ENGELA	22	x	f	0	0	all	0	all	Eu:Sca	1964	pr	435	n	bl	n	n	0	ev	cig+/-ot	nev cigs	st
ENSTRO	1		m	0	0	all	0	all	NAmer	1959	pr	2879	n	bl	n	n	1	cu	cig only	nev any	or
ENSTRO	2		f	0	0	all	0	all	NAmer	1959	pr	2879	n	bl	n	n	1	cu	cig only	nev any	or
ESAKI	4		m	0	0	all	-	all	As:Jap	1961	CC	245	n	bl	y	n	0	ev	cig+/-ot	nev cigs	st
ESAKI	5		f	0	0	all	-	all	As:Jap	1961	CC	245	n	bl	y	n	0	ev	cig+/-ot	nev cigs	st
FAN	1		m	0	0	all	-	all	As:Chi	1990	CC	403	n	ot	y	n	0	ev	cig+/-ot	nev cigs	st
FAN	2		f	0	0	all	-	all	As:Chi	1990	CC	403	n	ot	y	n	0	ev	cig+/-ot	nev cigs	st
GAO	6	x	m	0	0	all	-	all	As:Chi	1984	CC	1405	n	ot	n	n	0	ev	cig+/-ot	nev cigs	st
GAO	16	x	f	0	0	all	-	all	As:Chi	1984	CC	1405	n	ot	n	n	0	ev	cig+/-ot	nev cigs	st
GAO2	6	x	m	0	0	all	-	all	As:Jap	1988	CC	282	n	bl	n	n	0	ev	cig+/-ot	nev cigs	st
GARCIA	3		c	0	0	all	-	all	NAmer	1992	CC	416	n	bl	n	y	0	ev	cig+/-ot	nev cigs	st
GARDIN	7		c	0	0	all	-	all	Eu:UK	1988	CC	143	n	V	y	n	0	ev	all/unsp	nev any	st
GARSHI	17	x	m	0	0	all	-	all	NAmer	1981	CC	1081	o	bl	y	n	0	ev	all/unsp	nev any	st
GENG	1		m	0	0	all	-	all	As:Chi	1985	CC	292	n	ot	*	n	0	ev	cig+/-ot	nev any	st
GENG	2		f	0	0	all	-	all	As:Chi	1985	CC	292	n	ot	*	n	0	ev	cig+/-ot	nev any	st
GER	17	x	c	0	0	all	-	all	As:oth	1990	CC	141	n	ot	y	n	0	ev	all/unsp	nev any	st
GODLEY	5		m	0	0	all	-	all	NAmer	1966	CC	1986	n	bl	y	n	1	ev	cig+/-ot	nev cigs	ot
GODLEY	6		f	0	0	all	-	all	NAmer	1966	CC	1986	n	bl	y	n	1	ev	cig+/-ot	nev cigs	ot
GOLLED	21	x	m	35	99	all	-	all	Eu:UK	1952	CC	443	n	V	y	n	0	ev	cig+/-ot	nev any	st
GOODMA	3		m	0	0	w+o	-	all	NAmer	1983	CC	326	n	bl	y	y	0	ev	cig+/-ot	nev any	st
GOODMA	7		f	0	0	w+o	-	all	NAmer	1983	CC	326	n	bl	y	y	0	ev	cig+/-ot	nev any	st
GRAHAM	12	x	m	0	0	wh	-	all	NAmer	1956	CC	685	n	bl	n	n	0	ev	cig+/-ot	nev any	st
GREGOR	3		m	0	0	all	-	all	Eu:UK	1976	CC	104	n	V	n	y	0	ev	cig+/-ot	nev cigs	st
GREGOR	7		f	0	0	all	-	all	Eu:UK	1976	CC	104	n	V	n	y	0	ev	cig+/-ot	nev cigs	st
GSELL	6		m	0	0	all	-	all	Eu:wst	1937	CC	150	n	bl	n	y	0	ev	cig+/-ot	nev any	st
HAENSZ	56		f	0	0	all	-	not alv	NAmer	1955	CC	158	n	bl	n	y	0	ev	cig+/-ot	nev any	st
HAMMO2	16	x	m	0	0	all	6	all	NAmer	1967	pr	450	o	bl	n	n	0	ev	cig+/-ot	nev any	st
HAMMON	128	x	m	0	0	wh	0	all	NAmer	1952	pr	448	n	bl	n	n	0	ev	cig+/-ot	nev any	st
HANSEN	3		m	0	0	all	0	all	Eu:Sca	1968	pr	105	o	bl	y	n	2	ev	all/unsp	nev any	ot
HEGMAN	1		c	0	0	all	-	all	NAmer	1989	CC	282	n	bl	y	y	0	ev	all/unsp	nev any	st
HEIN	7		m	0	0	all	0	all	Eu:Sca	1970	pr	144	n	bl	n	n	0	ev	all/unsp	nev any	st
HENNEK	3		m	0	0	all	0	all	NAmer	1982	pr	169	n	bl	n	n	0	ev	all/unsp	nev any	st
HINDS	26	x	f	0	0	o	-	all	NAmer	1968	CC	292	n	bl	n	n	0	ev	all/unsp	nev any	st
HIRAYA	147		m	0	0	all	0	all	As:Jap	1965	pr	1917	n	bl	n	n	1	ev	cig+/-ot	nev any	ot
HIRAYA	150		f	0	0	all	0	all	As:Jap	1965	pr	1917	n	bl	n	n	1	ev	cig+/-ot	nev any	ot
HITOSU	6	x	m	0	0	all	-	all	As:Jap	1960	CC	216	n	bl	y	n	0	ev	all/unsp	nev any	st
HITOSU	12	x	f	0	0	all	-	all	As:Jap	1960	CC	216	n	bl	y	n	0	ev	all/unsp	nev any	st
HOLE	15	x	m	0	0	all	0	all	Eu:UK	1972	pr	225	n	V	n	n	0	ev	all/unsp	nev any	st
HOLE	29	x	f	0	0	all	11	all	Eu:UK	1972	pr	225	n	V	n	n	0	cu	all/unsp	nev any	st
HOROWI	1		m	0	0	all	-	all	NAmer	1956	CC	236	n	V	n	n	0	ev	cig+/-ot	nev any	st
HOROWI	2		f	0	0	all	-	all	NAmer	1956	CC	236	n	V	n	n	0	ev	cig+/-ot	nev any	st
HORWIT	1		f	0	0	all	-	all	NAmer	1977	CC	112	n	bl	n	n	0	ev	cig+/-ot	nev cigs	st
HU	15		m	0	0	all	-	all	As:Chi	1985	CC	227	n	ot	n	y	0	ev	cig+/-ot	nev any	st
HU	16		f	0	0	all	-	all	As:Chi	1985	CC	227	n	ot	n	y	0	ev	cig+/-ot	nev any	st
HU2	9		m	0	0	all	-	all	As:Chi	1977	CC	523	n	ot	y	n	0	ev	cig+/-ot	nev cigs	st
HU2	10		f	0	0	all	-	all	As:Chi	1977	CC	523	n	ot	y	n	0	ev	cig+/-ot	nev cigs	st
HUANG	1		c	0	0	all	-	all	As:Chi	1990	CC	135	n	ot	y	n	0	ev	all/unsp	nev any	st
HUMBLE	14		m	0	0	w-hi	-	all	NAmer	1980	CC	521	n	bl	y	n	1	ev	cig+/-ot	nev cigs	ot
HUMBLE	16		m	0	0	hi	-	all	NAmer	1980	CC	521	n	bl	y	n	1	ev	cig+/-ot	nev cigs	ot

Table 1C3 - 4

IESLC - Meta-anal of Ever Smoking (or Current if Ever not available), Cigs (or Any Prod if Cigs not avail)
All LC types
Least adjusted

REF	NRR	X	SEX	AGEL	AGEH	RACE	YF	LC TYPE	LOC	START	ST	NLC	R	VB	P	H	AD	SM	PRODUCT	DENOM	De
HUMBLE	18		f	0	0	w-hi	-	all	NAmer	1980	CC	521	n	bl	y	n	1	ev	cig+/-ot	nev cigs	ot
HUMBLE	20		f	0	0	hi	-	all	NAmer	1980	CC	521	n	bl	y	n	1	ev	cig+/-ot	nev cigs	ot
JAHN	3	x	f	0	0	all	-	all	Eu:Ger	1988	CC	1004	n	bl	n	n	0	ev	cig+/-ot	nev any	st
JAIN	6	x	m	0	0	all	-	all	NAmer	1981	CC	845	n	V	y	n	0	ev	cig+/-ot	nev cigs	st
JAIN	1	x	f	0	0	all	-	all	NAmer	1981	CC	845	n	V	y	n	0	ev	cig+/-ot	nev cigs	st
JARUP	3	x	m	0	0	all	-	all	Eu:Sca	1928	CC	102	o	bl	y	n	0	ev	all/unsp	nev any	st
JARVHO	3		m	0	0	all	-	all	Eu:Sca	1983	CC	147	n	bl	n	n	0	ev	all/unsp	nev any	st
JARVHO	7		f	0	0	all	-	all	Eu:Sca	1983	CC	147	n	bl	n	n	0	ev	all/unsp	nev any	st
JEDRYC	63	x	m	0	0	all	-	all	Eu:est	1980	CC	1630	n	bl	y	n	0	ev	cig+/-ot	nev any	st
JEDRYC	68	x	f	0	0	all	-	all	Eu:est	1980	CC	1630	n	bl	y	n	0	ev	cig+/-ot	nev any	st
JIANG	1		m	0	0	all	-	all	As:Chi	1984	CC	125	n	ot	n	n	0	ev	all/unsp	nev any	st
JIANG	2		f	0	0	all	-	all	As:Chi	1984	CC	125	n	ot	n	n	0	ev	all/unsp	nev any	st
JOLY	2		m	0	0	all	-	all	SCAmer	1978	CC	826	n	bl	n	n	0	ev	cig+/-ot	nev any	st
JOLY	1		f	0	0	all	-	all	SCAmer	1978	CC	826	n	bl	n	n	0	ev	cig+/-ot	nev any	st
JUSSAW	2	x	m	0	0	all	-	all	As:Ind	1964	CC	792	n	V	n	n	0	ev	cig only	nev any	st
KAISE2	72		m	35	99	all	9	all	NAmer	1979	pr	318	n	bl	n	n	1	ev	cig only	nev any	st
KAISE2	64		f	35	99	all	9	all	NAmer	1979	pr	318	n	bl	n	n	1	ev	cig only	nev any	st
KAISER	13		m	0	0	all	0	all	NAmer	1964	pr	714	n	bl	n	n	2	ev	cig+/-ot	nev cigs	ot
KAISER	10		f	0	0	all	0	all	NAmer	1964	pr	714	n	bl	n	n	2	ev	cig+/-ot	nev cigs	ot
KANELL	5	x	m	0	0	all	-	all	Eu:bal	1950	CC	862	n	bl	n	n	0	cu	all/unsp	nev any	st
KATSOU	27	x	f	0	0	all	-	all	Eu:bal	1987	CC	101	n	bl	n	n	0	ev	all/unsp	nev any	st
KAUFMA	8	x	c	0	0	all	-	all	NAmer	1981	CC	881	n	bl	n	n	0	ev	cig+/-ot	nev cigs	st
KELLER	3		m	0	0	wh	-	all	NAmer	1985	CC	15038	n	bl	n	n	0	ev	all/unsp	nev any	st
KELLER	11		m	0	0	nonw	-	all	NAmer	1985	CC	15038	n	bl	n	n	0	ev	all/unsp	nev any	st
KELLER	7		f	0	0	wh	-	all	NAmer	1985	CC	15038	n	bl	n	n	0	ev	all/unsp	nev any	st
KELLER	15		f	0	0	nonw	-	all	NAmer	1985	CC	15038	n	bl	n	n	0	ev	all/unsp	nev any	st
KHUDER	4		m	0	0	all	-	all	NAmer	1985	CC	482	n	bl	n	y	0	ev	cig+/-ot	nev cigs	st
KIHARA	31		c	0	0	jap	-	all	As:Jap	1991	CC	440	n	bl	n	n	0	ev	all/unsp	nev any	st
KINLEN	6	x	m	0	0	all	0	all	Eu:UK	1967	pr	718	n	V	n	n	0	ev	all/unsp	nev any	st
KJUUS	3		m	0	0	all	-	all	Eu:Sca	1979	CC	176	n	bl	n	n	0	ev	cig only	nev any	st
KNEKT	76	x	m	20	69	all	21	all	Eu:Sca	1966	pr	515	n	bl	n	n	0	ev	all/unsp	nev any	st
KO	1		f	0	0	all	-	all	As:oth	1992	CC	117	n	ot	n	y	3	ev	cig+/-ot	nev cigs	or
KOHLME	1	x	c	0	0	all	-	all	Eu:Ger	1990	CC	239	n	bl	n	n	0	ev	all/unsp	nev any	st
KOO	1		f	0	0	all	-	all	As:HK	1981	CC	200	n	bl	n	n	0	ev	all/unsp	nev any	st
KOULUM	2		m	0	0	all	-	all	Eu:Sca	1936	CC	812	n	bl	n	n	0	ev	cig only	nev any	st
KREUZE	60		f	1	45	all	-	all	Eu:Ger	1990	CC	2260	n	bl	n	n	3	ev	cig+/-ot	nev any	ot
KREUZE	62		f	55	69	all	-	all	Eu:Ger	1990	CC	2260	n	bl	n	n	3	ev	cig+/-ot	nev any	ot
KREYBE	24	x	m	0	0	all	-	all	Eu:Sca	1948	CC	300	n	bl	n	y	0	ev	all/unsp	nev any	st
KREYBE	39	x	f	0	0	all	-	all	Eu:Sca	1948	CC	300	n	bl	n	y	0	ev	all/unsp	nev any	st
KUBIK	27		m	0	0	all	0	all	Eu:est	1965	pr	108	n	bl	n	n	0	ev	cig+/-ot	nev any	st
LAMTH	6		f	0	0	ch	-	all	As:HK	1983	CC	445	n	bl	n	n	0	ev	all/unsp	nev any	or
LAMWK	1		f	0	0	ch	-	all	As:HK	1981	CC	163	n	bl	n	n	0	ev	all/unsp	nev any	st
LAMWK2	9		m	0	0	all	-	q+s+l+a	As:HK	1976	CC	480	n	bl	n	n	0	ev	all/unsp	nev any	st
LAMWK2	10		f	0	0	all	-	q+s+l+a	As:HK	1976	CC	480	n	bl	n	n	0	ev	all/unsp	nev any	st
LANGE	34	x	m	0	0	all	0	all	Eu:Sca	1976	pr	268	n	bl	n	n	0	ev	all/unsp	nev any	st
LANGE	31	x	f	0	0	all	0	all	Eu:Sca	1976	pr	268	n	bl	n	n	0	ev	all/unsp	nev any	st
LAUSSM	10	x	m	0	0	all	-	all	Eu:Ger	1982	CC	432	n	bl	n	n	0	ev	all/unsp	nev any	st
LEI	1		m	0	0	all	-	all	As:Chi	1986	CC	792	n	ot	y	n	0	ev	all/unsp	nev any	st
LEI	2		f	0	0	all	-	all	As:Chi	1986	CC	792	n	ot	y	n	0	ev	all/unsp	nev any	st
LEMARC	3		c	0	0	w+o	-	all	NAmer	1992	CC	341	n	bl	n	y	0	ev	all/unsp	nev any	st
LETOUR	1		c	0	0	all	-	all	NAmer	1983	CC	738	n	V	y	y	0	ev	cig+/-ot	nev cigs	st
LEVIN	30		m	0	0	all	-	all	NAmer	1938	CC	475	n	bl	n	n	1	ev	cig+/-ot	nev any	st
LIAW	1		m	0	0	all	0	all	As:oth	1982	pr	127	n	ot	n	n	1	cu	all/unsp	nev any	or
LIAW	2		f	0	0	all	0	all	As:oth	1982	pr	127	n	ot	n	n	1	cu	all/unsp	nev any	or
LIDDEL	5		m	0	0	all	18	all	NAmer	1970	pr	304	m	V	n	n	1	ev	cig+/-ot	nev cigs	ot
LIU	2		c	0	0	all	-	all	As:Chi	1980	CC	229	n	ot	*	n	2	ev	all/unsp	nev any	or
LIU2	1	x	m	0	0	all	-	all	As:Chi	1983	CC	316	n	ot	n	n	0	ev	all/unsp	nev any	st
LIU2	3	x	f	0	0	all	-	all	As:Chi	1983	CC	316	n	ot	n	n	0	ev	all/unsp	nev any	st
LIU3	1	x	m	0	0	all	-	all	As:Chi	1985	CC	110	n	ot	n	n	0	ev	all/unsp	nev any	st
LIU4	10		m	35	69	all	-	all	As:Chi	1986	CC	1000-00	n	ot	y	n	2	ev	cig only	nev any	ot
LIU4	12		f	0	0	all	-	all	As:Chi	1986	CC	1000-00	n	ot	y	n	2	ev	all/unsp	nev any	ot
LIU5	1		c	0	0	all	-	all	As:Chi	1978	CC	111	n	ot	y	n	0	ev	all/unsp	nev any	st
LOMBA2	1		f	0	0	all	-	all	NAmer	1960	CC	225	n	bl	n	n	0	ev	cig+/-ot	nev cigs	st
LOMBAR	2		m	0	0	all	-	all	NAmer	1951	CC	1040	n	bl	n	n	0	ev	cig+/-ot	nev any	st
LUBIN2	47	x	m	0	0	all	-	all	Eu:mul	1976	CC	7804	n	bl	n	y	0	ev	cig+/-ot	nev any	st
LUBIN2	97	x	f	0	0	all	-	all	Eu:mul	1976	CC	7804	n	bl	n	y	0	ev	cig only	nev any	st
LUO	1	x	c	0	0	all	-	all	As:Chi	1990	CC	102	n	ot	n	y	0	ev	cig+/-ot	nev cigs	st
MACLEN	71	x	m	0	0	ch	-	all	As:oth	1972	CC	233	n	bl	n	n	0	ev	cig+/-ot	nev cigs	st
MACLEN	72	x	f	0	0	ch	-	all	As:oth	1972	CC	233	n	bl	n	n	0	ev	cig+/-ot	nev cigs	st
MAGNUS	1	x	m	0	0	all	0	all	Eu:Sca	1953	pr	203	o	bl	y	n	0	ev	all/unsp	nev any	st

Table 1C3 - 4

IESLC - Meta-anal of Ever Smoking (or Current if Ever not available), Cigs (or Any Prod if Cigs not avail)
All LC types
Least adjusted

REF	NRR	X	SEX	AGEL	AGEH	RACE	YF	LC TYPE	LOC	START	ST	NLC	R	VB	P	H	AD	SM	PRODUCT	DENOM	De
MARSH	1		m	0	0	all	-	all	NAmer	1979	CC	150	n	bl	y	n	0	ev	cig+/-ot	nev any	st
MARSH	3		f	0	0	all	-	all	NAmer	1979	CC	150	n	bl	y	n	0	ev	cig+/-ot	nev any	st
MARSH2	1	x	c	0	0	all	-	all	NAmer	1979	CC	114	n	bl	y	n	0	ev	cig+/-ot	nev any	st
MARTIS	4		m	0	0	all	-	all	Eu:UK	1972	CC	201	n	V	n	n	0	ev	cig+/-ot	nev cigs	st
MASTRA	1	x	m	0	0	all	-	all	Eu:wst	1973	CC	309	n	bl	n	n	0	ev	all/unsp	nev any	st
MATOS	26	x	m	0	0	all	-	all	SCAmer	1994	CC	200	n	bl	n	n	0	ev	cig+/-ot	nev any	st
MATSUD	10		m	0	0	all	-	all	As:Jap	1965	CC	179	n	bl	n	n	0	ev	cig+/-ot	nev cigs	st
MCCONN	1		m	0	0	all	-	all	Eu:UK	1946	CC	100	n	V	n	y	0	ev	all/unsp	nev any	st
MCCONN	2		f	0	0	all	-	all	Eu:UK	1946	CC	100	n	V	n	y	0	ev	all/unsp	nev any	st
MCDUFF	1		m	0	0	all	-	all	NAmer	1979	CC	165	n	V	y	n	0	ev	cig+/-ot	nev cigs	st
MCLAUG	1		m	0	0	all	-	all	As:Chi	1972	CC	316	o	ot	y	n	0	ev	all/unsp	nev any	st
MIGRAN	26	x	m	0	0	all	0	all	Eu:UK	1964	pr	259	n	V	n	n	0	ev	all/unsp	nev any	st
MIGRAN	41	x	f	0	0	all	0	all	Eu:UK	1964	pr	259	n	V	n	n	0	ev	all/unsp	nev any	st
MILLER	1	x	f	0	0	all	-	all	NAmer	1972	CC	168	n	bl	y	n	0	ev	cig+/-ot	nev any	st
MILLS	1		m	0	0	wh	-	all	NAmer	1940	CC	444	n	bl	y	n	1	ev	cig only	nev any	ot
MRFITR	6		m	0	0	all	0	all	NAmer	1973	pr	119	n	bl	n	n	0	ev	cig+/-ot	nev cigs	ot
NAM	69	x	m	0	0	all	-	all	NAmer	1986	CC	1199	n	bl	y	n	0	ev	cig+/-ot	nev cigs	ot
NAM	85	x	f	0	0	all	-	all	NAmer	1986	CC	1199	n	bl	y	n	0	ev	cig+/-ot	nev cigs	ot
NOTAN2	7	x	m	0	0	all	-	all	As:Ind	1963	CC	683	n	V	n	n	0	ev	cig only	nev any	st
NOU	11		m	30	64	all	-	all	Eu:Sca	1971	CC	273	n	bl	y	n	0	ev	all/unsp	nev any	st
NOU	12		f	30	64	all	-	all	Eu:Sca	1971	CC	273	n	bl	y	n	0	ev	all/unsp	nev any	st
ODRISC	3		c	0	0	all	-	all	Eu:UK	1992	CC	446	n	V	n	n	0	ev	all/unsp	nev any	st
ORMOS	4		m	0	0	all	-	all	Eu:est	1947	CC	119	n	bl	y	y	0	ev	cig+/-ot	nev any	st
ORMOS	26		f	0	0	all	-	all	Eu:est	1947	CC	119	n	bl	y	y	0	ev	cig+/-ot	nev any	st
OSANN	17	x	m	0	0	all	-	all	NAmer	1984	CC	1986	n	bl	n	n	0	ev	cig+/-ot	nev cigs	st
OSANN	21	x	f	0	0	all	-	all	NAmer	1984	CC	1986	n	bl	n	n	0	ev	cig+/-ot	nev cigs	st
PARKIN	31		m	0	0	bl	-	all	Africa	1963	CC	877	n	V	y	n	0	ev	cig+/-ot	nev any	st
PASTOR	5	x	m	0	0	all	-	all	Eu:wst	1976	CC	204	n	bl	y	n	0	ev	all/unsp	nev any	st
PAWLEG	1	x	m	0	0	all	-	all	Eu:est	1992	CC	176	n	bl	n	y	0	ev	all/unsp	nev any	st
PERNU	8		m	0	0	all	-	all	Eu:Sca	1944	CC	1606	n	bl	n	n	0	ev	cig only	nev any	st
PERNU	4		f	0	0	all	-	all	Eu:Sca	1944	CC	1606	n	bl	n	n	0	ev	cig only	nev any	st
PERSH2	5	x	c	0	0	all	-	all	Eu:Sca	1980	CC	1022	n	bl	y	n	0	ev	all/unsp	nev any	st
PETO	5		m	0	0	all	0	all	Eu:UK	1954	pr	103	n	V	n	n	0	ev	all/unsp	nev any	st
PEZZO2	10		m	0	0	all	-	all	SCAmer	1992	CC	367	n	bl	n	y	0	ev	cig+/-ot	nev cigs	st
PEZZOT	25		m	0	0	all	-	all	SCAmer	1987	CC	215	n	bl	n	y	0	ev	cig only	nev cigs	st
PIKE	4		m	0	0	w-hi	-	all	NAmer	1972	CC	731	n	bl	y	n	0	ev	all/unsp	nev any	st
PIKE	8		f	0	0	w-hi	-	all	NAmer	1972	CC	731	n	bl	y	n	0	ev	all/unsp	nev any	st
POFFIJ	1		c	0	0	all	-	all	Eu:mul	1990	CC	971	n	bl	n	n	0	ev	all/unsp	nev any	st
POLEDN	3	x	c	0	0	all	-	all	NAmer	1978	CC	209	n	bl	y	n	0	ev	cig+/-ot	nev cigs	st
QIAO2	9		m	0	0	all	0	all	As:Chi	1992	pr	241	m	ot	n	n	0	ev	cig+/-ot	nev any	st
RACHTA	3	x	f	0	0	all	-	all	Eu:est	1991	CC	118	n	bl	n	y	0	ev	cig+/-ot	nev cigs	st
RADZIK	1		c	0	0	all	-	all	Eu:est	1986	CC	189	n	bl	n	n	0	ev	all/unsp	nev any	st
RANDIG	9		m	0	0	all	-	all	Eu:Ger	1951	CC	448	n	bl	n	n	0	ev	cig+/-ot	nev any	st
RANDIG	10		f	0	0	all	-	all	Eu:Ger	1951	CC	448	n	bl	n	n	0	ev	cig+/-ot	nev any	st
REN	1		m	0	0	all	-	all	As:Chi	1980	CC	244	n	ot	*	n	0	ev	all/unsp	nev any	st
REN	2		f	0	0	all	-	all	As:Chi	1980	CC	244	n	ot	*	n	0	ev	all/unsp	nev any	st
RONCO	2	x	m	0	0	all	-	all	Eu:wst	1976	CC	126	n	bl	y	n	0	ev	cig only	nev any	st
ROTHSC	1	x	c	0	0	all	-	all	NAmer	1971	CC	284	n	bl	y	n	0	ev	all/unsp	nev any	st
SADOWS	4		m	0	0	wh	-	all	NAmer	1938	CC	477	n	bl	n	n	0	ev	cig+/-ot	nev any	st
SANKAR	1	x	m	0	0	all	-	all	As:Ind	1990	CC	281	n	V	n	n	0	ev	all/unsp	nev any	st
SCHWAR	1		m	0	0	wh	-	all	NAmer	1984	CC	5588	n	bl	y	y	0	ev	cig+/-ot	nev cigs	st
SCHWAR	2		m	0	0	bl	-	all	NAmer	1984	CC	5588	n	bl	y	y	0	ev	cig+/-ot	nev cigs	st
SCHWAR	3		f	0	0	wh	-	all	NAmer	1984	CC	5588	n	bl	y	y	0	ev	cig+/-ot	nev cigs	st
SCHWAR	4		f	0	0	bl	-	all	NAmer	1984	CC	5588	n	bl	y	y	0	ev	cig+/-ot	nev cigs	st
SEGI	1		m	0	0	all	-	all	As:Jap	1948	CC	159	n	bl	n	n	0	ev	all/unsp	nev any	ot
SEGI2	19	x	m	0	0	all	-	all	As:Jap	1962	CC	378	n	bl	n	n	0	cu	cig+/-ot	nev any	st
SEGI2	27	x	f	0	0	all	-	all	As:Jap	1962	CC	378	n	bl	n	n	0	cu	cig+/-ot	nev any	st
SEOW	1	x	f	0	0	ch	-	q+s+l+a	As:oth	1997	CC	153	n	bl	n	y	0	ev	cig+/-ot	nev cigs	st
SHAW	12		c	0	0	wh	-	all	NAmer	1988	CC	335	n	V	n	y	0	ev	all/unsp	nev any	st
SIEMIA	9	x	m	0	0	all	-	all	NAmer	1979	CC	857	n	V	y	y	0	ev	cig+/-ot	nev cigs	st
SIMARA	5	x	m	0	0	all	-	all	As:oth	1971	CC	115	n	bl	n	n	0	ev	cig+/-ot	nev cigs	st
SIMARA	6	x	f	0	0	all	-	all	As:oth	1971	CC	115	n	bl	n	n	0	ev	cig+/-ot	nev cigs	st
SOBUE	91	x	m	0	0	all	-	q+s+l+a	As:Jap	1986	CC	1376	n	bl	n	y	0	ev	cig+/-ot	nev cigs	st
SOBUE	95	x	f	0	0	all	-	q+s+l+a	As:Jap	1986	CC	1376	n	bl	n	y	0	ev	cig+/-ot	nev cigs	st
SOBUE2	10		m	0	0	all	-	q+s+l+a	As:Jap	1965	CC	2083	n	bl	n	n	2	cu	cig+/-ot	nev any	ot
SOBUE2	12		f	0	0	all	-	q+s+l+a	As:Jap	1965	CC	2083	n	bl	n	n	2	cu	cig+/-ot	nev any	ot
SPEIZE	8		f	0	0	all	0	all	NAmer	1976	pr	593	n	bl	n	y	0	ev	cig+/-ot	nev cigs	st
SPITZ	3		c	0	0	b+hi	-	all	NAmer	1992	CC	177	n	bl	n	y	0	ev	cig+/-ot	nev cigs	st
STASZE	7		m	0	0	all	-	all	Eu:est	1954	CC	281	n	bl	n	y	0	ev	cig+/-ot	nev any	st
STASZE	5		f	0	0	all	-	all	Eu:est	1954	CC	281	n	bl	n	y	0	ev	all/unsp	nev any	st
STAYNE	1		m	0	0	all	-	all	NAmer	1969	CC	420	n	bl	n	n	0	ev	all/unsp	nev any	st
STOCKS	30	x	m	0	0	all	-	all	Eu:UK	1952	CC	2932	n	V	y	n	0	ev	cig+/-ot	nev any	st

Table 1C3 - 4

IESLC - Meta-anal of Ever Smoking (or Current if Ever not available), Cigs (or Any Prod if Cigs not avail)
All LC types
Least adjusted

REF	NRR	X	SEX	AGEL	AGEH	RACE	YF	LC TYPE	LOC	START	ST	NLC	R	VB	P	H	AD	SM	PRODUCT	DENOM	De
STOCKS	50		f	0	0	all	-	all	Eu:UK	1952	CC	2932	n	V	y	n	1	ev	cig+/-ot	nev any	ot
STOCKW	8		c	0	0	all	-	all	NAmer	1981	CC	22161	n	bl	n	n	0	ev	cig+/-ot	nev any	st
STUCKE	3		m	0	0	all	-	all	Eu:wst	1989	CC	247	n	bl	n	y	0	ev	all/unsp	nev any	ot
SUN	1		c	0	0	all	-	all	As:Chi	1992	CC	207	n	ot	n	y	0	ev	all/unsp	nev any	st
SUZUK2	3	x	c	0	0	all	-	all	SCAmer	1991	CC	123	n	bl	n	y	0	ev	cig only	nev any	st
SVENSS	56	x	f	0	0	all	-	all	Eu:Sca	1983	CC	210	n	bl	n	n	0	ev	all/unsp	nev any	st
TANG	3		c	0	0	all	-	not s	NAmer	1992	CC	119	n	bl	n	y	0	ev	cig+/-ot	nev cigs	st
TENKAN	22		m	0	0	all	17	all	Eu:Sca	1962	pr	242	n	bl	n	n	1	ev	all/unsp	nev any	ot
TIZZAN	2		m	0	0	all	-	all	Eu:wst	1959	CC	1358	n	bl	n	n	0	ev	cig only	nev any	st
TIZZAN	22		f	0	0	all	-	all	Eu:wst	1959	CC	1358	n	bl	n	n	0	ev	cig only	nev any	st
TOKARS	3	x	m	0	0	all	-	all	Eu:est	1966	ot	162	o	bl	n	y	0	ev	all/unsp	nev any	st
TOKARS	5	x	f	0	0	all	-	all	Eu:est	1966	ot	162	o	bl	n	y	0	ev	all/unsp	nev any	st
TOUSEY	2	x	m	0	0	all	-	all	NAmer	1993	CC	507	n	bl	y	y	0	ev	cig+/-ot	nev any	st
TOUSEY	6	x	f	0	0	all	-	all	NAmer	1993	CC	507	n	bl	y	y	0	ev	cig+/-ot	nev any	st
TSUGAN	27		m	0	0	all	-	q+a	As:Jap	1976	CC	134	n	bl	n	y	0	ev	all/unsp	nev any	st
TULINI	15	x	m	0	0	all	0	all	Eu:Sca	1967	pr	472	n	bl	n	n	1	ev	all/unsp	nev any	ot
TULINI	21	x	f	0	0	all	0	all	Eu:Sca	1967	pr	472	n	bl	n	n	1	ev	all/unsp	nev any	ot
TVERDA	22		m	0	0	all	0	all	Eu:Sca	1972	pr	238	n	bl	n	n	2	ev	cig+/-ot	nev cigs	ot
TVERDA	15		f	0	0	all	0	all	Eu:Sca	1972	pr	238	n	bl	n	n	2	cu	cig only	nev cigs	ot
WAKAI	13	x	m	0	0	all	-	all	As:Jap	1988	CC	333	n	bl	n	y	0	ev	all/unsp	nev any	st
WAKAI	31	x	f	0	0	all	-	all	As:Jap	1988	CC	333	n	bl	n	y	0	ev	all/unsp	nev any	st
WALD	2	x	m	0	0	all	0	all	Eu:UK	1975	pr	102	n	V	n	n	0	cu	cig only	nev any	st
WANG	1	x	m	0	0	all	-	all	As:Chi	1990	CC	390	n	ot	*	y	0	ev	all/unsp	nev any	or
WANG	2	x	f	0	0	all	-	all	As:Chi	1990	CC	390	n	ot	*	y	0	ev	all/unsp	nev any	or
WANG2	8	x	c	0	0	all	-	all	As:Chi	1980	CC	103	n	ot	n	n	0	ev	cig+/-ot	nev cigs	st
WANG3	1		c	0	0	all	-	all	As:Chi	1981	CC	293	n	ot	*	n	0	ev	all/unsp	nev any	st
WANG4	1	x	m	0	0	all	-	all	As:Chi	1976	CC	1170	n	ot	y	n	0	ev	all/unsp	nev any	st
WICKLU	1		m	0	0	wh	-	all	NAmer	1968	CC	155	n	bl	y	n	0	ev	cig+/-ot	nev+3	or
WIGLE	13		m	0	0	all	-	all	NAmer	1971	CC	728	n	V	n	n	0	ev	cig only	nev any	st
WIGLE	16		f	0	0	all	-	all	NAmer	1971	CC	728	n	V	n	n	0	ev	cig only	nev any	st
WILKIN	1	x	m	0	0	all	-	all	Eu:UK	1992	CC	271	n	V	n	n	0	ev	cig+/-ot	nev cigs	st
WILKIN	2	x	f	0	0	all	-	all	Eu:UK	1992	CC	271	n	V	n	n	0	ev	cig+/-ot	nev cigs	st
WU	37	x	f	0	0	wh	-	q+a	NAmer	1981	CC	220	n	bl	n	y	0	ev	all/unsp	nev any	st
WUNSCH	1	x	m	0	0	all	-	all	SCAmer	1990	CC	398	n	bl	y	n	0	ev	cig+/-ot	nev any	st
WUNSCH	7	x	f	0	0	all	-	all	SCAmer	1990	CC	398	n	bl	y	n	0	ev	cig+/-ot	nev any	st
WUWILL	6	x	f	0	0	all	-	all	As:Chi	1985	CC	965	n	ot	n	n	0	ev	cig+/-ot	nev cigs	st
WYNDE2	16		m	0	0	all	-	all	NAmer	1962	CC	404	n	bl	n	y	0	ev	cig+/-ot	nev any	st
WYNDE3	48		m	0	0	all	-	all	NAmer	1966	CC	350	n	bl	n	y	0	ev	cig+/-ot	nev any	st
WYNDE3	83		f	0	0	all	-	all	NAmer	1966	CC	350	n	bl	n	y	0	ev	cig+/-ot	nev any	st
WYNDE4	48		m	0	0	all	-	all	NAmer	1948	CC	684	n	bl	y	n	0	ev	all/unsp	nev any	st
WYNDE4	62		f	0	0	all	-	all	NAmer	1948	CC	684	n	bl	y	n	2	ev	all/unsp	nev any	ot
WYNDE6	81		m	0	0	all	-	all	NAmer	1969	CC	4423	n	bl	n	y	0	ev	cig+/-ot	nev any	st
WYNDE6	252		f	0	0	all	-	all	NAmer	1969	CC	4423	n	bl	n	y	0	ev	cig+/-ot	nev cigs	st
XIANGZ	6	x	m	0	0	all	0	all	As:Chi	1976	pr	983	m	ot	n	n	0	ev	cig+/-ot	nev any	st
XU	1	x	m	0	0	all	-	all	As:Chi	1985	CC	729	n	ot	n	n	0	ev	all/unsp	nev any	st
XU2	1	x	c	0	0	all	-	all	As:Chi	1987	CC	610	o	ot	y	n	0	ev	all/unsp	nev any	st
XU3	1	x	m	0	0	all	-	all	As:Chi	1981	CC	135	n	ot	n	n	0	ev	all/unsp	nev any	st
XU3	3	x	f	0	0	all	-	all	As:Chi	1981	CC	135	n	ot	n	n	0	ev	all/unsp	nev any	st
XU4	1		c	0	0	all	-	all	As:Chi	1981	CC	206	n	ot	*	n	0	ev	all/unsp	nev any	st
YAMAGU	5	x	c	0	0	all	-	all	As:Jap	1989	CC	144	n	bl	n	y	0	ev	all/unsp	nev any	st
YONG	12		m	0	0	all	0	all	NAmer	1971	pr	216	n	bl	n	n	1	cu	cig+/-ot	nev cigs	or
YONG	15		f	0	0	all	0	all	NAmer	1971	pr	216	n	bl	n	n	1	cu	cig+/-ot	nev cigs	or
YUAN	1		m	0	0	all	0	all	As:Chi	1986	pr	142	n	ot	n	n	2	ev	cig+/-ot	nev cigs	ot
ZHANG	1	x	c	0	0	all	-	all	As:Chi	1988	CC	100	n	ot	n	n	0	ev	all/unsp	nev any	st
ZHENG	15		m	0	0	all	-	all	As:Chi	1982	CC	540	n	ot	*	y	0	ev	cig+/-ot	nev cigs	st
ZHENG	24		f	0	0	all	-	all	As:Chi	1982	CC	540	n	ot	*	y	0	ev	cig+/-ot	nev cigs	st
ZHOU	2		m	0	0	all	-	all	As:Chi	1978	CC	1360	n	ot	n	n	0	ev	all/unsp	nev any	st
ZHOU	3		f	0	0	all	-	all	As:Chi	1978	CC	1360	n	ot	n	n	0	ev	all/unsp	nev any	st

Cigarette type is all/unspec for all RRs
except for the following:

REF	NRR	CIGTYPE
ALDERS	12	MC only
DEAN3	240	MC only
DEAN3	124	MC only
DESTEF	11	MC only
JUSSAW	2	MC only
NOTAN2	7	MC only

Table 1C3 - 4

IESLC - Meta-anal of Ever Smoking (or Current if Ever not available), Cigs (or Any Prod if Cigs not avail)
All LC types
Least adjusted

REF	NRR	CIGTYPE
PERNU	8	MC only
PERNU	4	MC only
SUZUK2	3	MC only

Table 1C3 - 5

IESLC - Meta-anal of Ever Smoking (or Current if Ever not available), Cigs (or Any Prod if Cigs not avail)
All LC types
Least adjusted

REF	NRR	SEX	AD	Number Exposed Case	Cont	Non-exposed Case	Cont	RR	95.00%CI
ABELIN	2	m	0	47	154	2	183	27.93	(6.67- 116.83)
*ABRAHA	7	m	0	269	10351	10	3365	8.74	(4.66- 16.42)
*ABRAHA	8	f	0	62	5256	28	11589	4.88	(3.13- 7.62)
Subtotal ABRAHA								5.93	(4.12- 8.53)
AGUDO	8	f	0	23	23	80	183	2.29	(1.21- 4.32)
*AKIBA	3	m	0	393	207682	18	35833	3.77	(2.35- 6.04)
*AKIBA	7	f	0	83	65179	116	359850	3.95	(2.98- 5.24)
Subtotal AKIBA								3.90	(3.06- 4.97)
ALDERS	61	m	0	782	641	15	133	10.82	(6.28- 18.64)
ALDERS	12	f	0	530	371	75	243	4.63	(3.46- 6.19)
Subtotal ALDERS								5.59	(4.33- 7.23)
*AMANDU	3	m	0	126	111395	6	25350	4.78	(2.11- 10.84)
AMES	4	m	0	297	251	15	62	4.89	(2.72- 8.81)
*ANDERS	3	f	0	297	96164	46	195158	13.10	(9.61- 17.87)
*ARCHER	6	m	0	140	36269	6	9842	6.33	(2.80- 14.33)
ARMADA	4	m	0	317	254	4	64	19.97	(7.18- 55.57)
AUSTIN	3	c	0	161	237	5	88	11.96	(4.75- 30.09)
AUVINE	1	c	0	473	288	44	229	8.55	(6.00- 12.18)
AXELSO	1	c	0	90	86	62	371	6.26	(4.20- 9.34)
AXELSS	1	m	0	292	344	16	160	8.49	(4.96- 14.52)
AXELSS	11	f	0	110	109	18	154	8.63	(4.95- 15.05)
Subtotal AXELSS								8.56	(5.82- 12.59)
BAND	1	m	2	-	-	-	-	9.96	(7.38- 13.44)
BARBON	106	m	0	733	567	22	188	11.05	(7.01- 17.41)
BECHER	15	m	0	137	217	3	54	11.36	(3.48- 37.06)
BECHER	16	f	0	38	44	10	52	4.49	(2.01- 10.03)
Subtotal BECHER								6.02	(3.10- 11.71)
*BENSHL	15	m	1	-	-	-	-	8.02	(4.29- 15.01)
*BEST	23	m	0	212	21711	1	2854	27.87	(3.91- 198.68)
*BEST	18	f	1	-	-	-	-	2.24	(0.59- 8.44)
Subtotal BEST								4.95	(1.65- 14.89)
BLOHMK	3	m	0	762	587	126	301	3.10	(2.45- 3.92)
BLOT4	1	m	0	327	245	8	87	14.51	(6.91- 30.51)
BOFFET	7	m	0	5386	5239	117	1750	15.38	(12.70- 18.61)
*BOUCOT	58	m	0	117	35761	0	7551	49.62~	(3.09- 797.95)
BRESLO	17	m	0	471	383	7	42	7.38	(3.28- 16.61)
BRESLO	23	f	0	13	11	12	14	1.38	(0.45- 4.20)
Subtotal BRESLO								4.12	(2.14- 7.94)
*BRETT	10	m	0	144	47930	6	6530	3.27	(1.45- 7.40)
BROCKM	1	m	0	87	81	2	2	1.07	(0.15- 7.80)
BROCKM	2	f	0	24	54	4	18	2.00	(0.61- 6.54)
Subtotal BROCKM								1.70	(0.61- 4.70)
BROSS	13	m	0	831	612	38	170	6.07	(4.21- 8.77)
BROWN2	2	m	2	-	-	-	-	9.10	(8.30- 10.00)
BROWN2	1	f	2	-	-	-	-	12.70	(11.50- 13.90)
Subtotal BROWN2								10.72	(10.03- 11.46)
BUFFLE	2	m	0	461	373	5	47	11.62	(4.57- 29.50)
BUFFLE	6	f	0	419	284	41	198	7.12	(4.93- 10.30)
Subtotal BUFFLE								7.61	(5.40- 10.72)
CARPEN	7	c	0	328	469	15	241	11.24	(6.55- 19.29)
CASCO2	1	c	0	149	212	6	98	11.48	(4.90- 26.87)
CASCOR	1	c	0	365	362	22	295	13.52	(8.56- 21.35)
*CEDERL	107	m	2	-	-	-	-	5.92	(3.85- 9.12)
*CEDERL	112	f	2	-	-	-	-	4.18	(2.94- 5.93)
Subtotal CEDERL								4.80	(3.66- 6.30)
CHAN	5	m	0	206	161	2	43	27.51	(6.57- 115.26)
CHAN	6	f	0	105	50	84	139	3.48	(2.26- 5.35)
Subtotal CHAN								4.13	(2.73- 6.25)
*CHANG	6	m	0	78	1506	5	502	5.20	(2.12- 12.77)
*CHANG	12	f	0	42	1183	11	1139	3.68	(1.90- 7.10)
Subtotal CHANG								4.15	(2.44- 7.06)
CHATZI	4	c	0	255	365	27	129	3.34	(2.14- 5.21)
CHEN2	1	m	0	121	97	9	33	4.57	(2.09- 10.02)
CHEN2	2	f	0	38	30	25	33	1.67	(0.82- 3.39)
Subtotal CHEN2								2.62	(1.55- 4.44)
CHEN3	1	c	0	182	156	72	98	1.59	(1.09- 2.30)
CHIAZZ	2	m	0	139	209	4	47	7.81	(2.75- 22.18)
CHOI	1	m	0	267	465	13	95	4.20	(2.31- 7.64)
CHOI	5	f	0	19	26	76	164	1.58	(0.82- 3.02)
Subtotal CHOI								2.68	(1.72- 4.16)
*CHOW	6	m	0	201	189175	6	62913	11.14	(4.95- 25.09)

Table 1C3 - 5

IESLC - Meta-anal of Ever Smoking (or Current if Ever not available), Cigs (or Any Prod if Cigs not avail)
All LC types
Least adjusted

REF	NRR	SEX	AD	Number Exposed Case	Number Exposed Cont	Non-exposed Case	Non-exposed Cont	RR	95.00%CI
*CHYOU	9	m	0	214	5554	13	2406	7.13	(4.08- 12.46)
COMSTO	33	m	0	151	229	4	69	11.37	(4.07- 31.82)
COMSTO	45	f	0	88	87	13	115	8.95	(4.69- 17.06)
Subtotal COMSTO								9.58	(5.54- 16.54)
COOKSO	4	c	0	184	38	45	61	6.56	(3.90- 11.04)
CORREA	33	c	0	1202	886	51	388	10.32	(7.61- 14.00)
*CPSI	187	m	1	-	-	-	-	9.18	(7.36- 11.45)
*CPSI	274	f	1	-	-	-	-	2.79	(2.22- 3.51)
Subtotal CPSI								5.17	(4.41- 6.06)
*CPSII	104	m	1	-	-	-	-	12.83	(10.28- 16.01)
*CPSII	79	f	1	-	-	-	-	8.16	(6.93- 9.62)
Subtotal CPSII								9.58	(8.40- 10.93)
DAMBER	26	m	0	332	215	42	208	7.65	(5.26- 11.11)
DARBY	15	m	0	664	1724	3	384	49.30	(15.77- 154.07)
DARBY	16	f	0	292	548	23	529	12.26	(7.89- 19.05)
Subtotal DARBY								14.69	(9.74- 22.16)
DAVEYS	5	m	0	90	144	3	23	4.79	(1.40- 16.42)
DAVEYS	6	f	0	0	3	16	83	0.72~	(0.04- 14.66)
Subtotal DAVEYS								3.65	(1.17- 11.42)
DEAN	8	m	0	540	500	12	61	5.49	(2.92- 10.32)
DEAN2	12	m	0	686	556	33	112	4.19	(2.80- 6.27)
DEAN2	20	f	0	63	29	88	121	2.99	(1.78- 5.02)
Subtotal DEAN2								3.69	(2.68- 5.07)
DEAN3	240	m	0	399	1227	24	510	6.91	(4.52- 10.57)
DEAN3	124	f	0	109	1420	41	1538	2.88	(2.00- 4.15)
Subtotal DEAN3								4.18	(3.17- 5.52)
*DEKLER	6	m	2	-	-	-	-	20.29	(2.84- 145.18)
DESTE2	15	m	0	432	314	31	151	6.70	(4.43- 10.13)
DESTEF	11	m	0	108	113	27	163	5.77	(3.55- 9.37)
*DOCKER	3	c	4	-	-	-	-	4.29	(1.66- 11.06)
DOLL	20	m	0	504	467	7	61	9.40	(4.26- 20.77)
DOLL	12	f	0	68	49	40	59	2.05	(1.19- 3.53)
Subtotal DOLL								3.34	(2.13- 5.23)
*DOLL2	88	m	1	-	-	-	-	11.58	(3.70- 36.24)
*DOLL2	63	f	1	-	-	-	-	8.65	(2.93- 25.55)
Subtotal DOLL2								9.93	(4.53- 21.78)
DORANT	10	c	0	470	2033	14	1090	18.00	(10.52- 30.78)
DORGAN	7	m	0	699	400	15	93	10.83	(6.19- 18.95)
DORGAN	31	m	0	264	116	3	35	26.55	(8.00- 88.08)
DORGAN	95	f	3	-	-	-	-	8.50	(6.70- 10.80)
Subtotal DORGAN								9.15	(7.37- 11.35)
*DORN	413	m	1	-	-	-	-	8.83	(6.64- 11.75)
DOSEME	17	m	0	1068	536	142	293	4.11	(3.28- 5.15)
DROSTE	3	m	0	471	443	7	93	14.13	(6.48- 30.78)
DU	1	m	0	538	-	28	-	3.53	(2.44- 5.11)
DU	2	f	0	191	-	92	-	1.93	(1.30- 2.87)
Subtotal DU								2.66	(2.03- 3.49)
*DUNN	6	m	0	137	52634	2	14160	18.43	(4.56- 74.42)
EBELIN	1	m	0	101	142	12	117	6.93	(3.63- 13.24)
*ENGELA	8	m	0	306	168817	27	58716	3.94	(2.66- 5.84)
*ENGELA	22	f	0	71	104832	31	207789	4.54	(2.98- 6.92)
Subtotal ENGELA								4.21	(3.16- 5.61)
*ENSTRO	1	m	1	-	-	-	-	12.99	(10.46- 16.13)
*ENSTRO	2	f	1	-	-	-	-	6.95	(6.01- 8.04)
Subtotal ENSTRO								8.44	(7.48- 9.53)
ESAKI	4	m	0	155	143	16	28	1.90	(0.99- 3.65)
ESAKI	5	f	0	34	19	40	55	2.46	(1.23- 4.92)
Subtotal ESAKI								2.14	(1.33- 3.45)
FAN	1	m	0	216	498	36	236	2.84	(1.93- 4.18)
FAN	2	f	0	82	97	69	320	3.92	(2.65- 5.81)
Subtotal FAN								3.33	(2.53- 4.38)
GAO	6	m	0	671	558	62	202	3.92	(2.89- 5.32)
GAO	16	f	0	237	130	435	605	2.54	(1.98- 3.25)
Subtotal GAO								3.01	(2.48- 3.65)
GAO2	6	m	0	269	226	13	56	5.13	(2.73- 9.62)
GARCIA	3	c	0	395	307	21	139	8.52	(5.26- 13.80)
GARDIN	7	c	0	138	102	5	41	11.09	(4.23- 29.06)
GARSHI	17	m	0	1040	1596	41	363	5.77	(4.14- 8.04)
GENG	1	m	0	92	68	7	31	5.99	(2.49- 14.42)
GENG	2	f	0	126	75	67	118	2.96	(1.96- 4.48)
Subtotal GENG								3.36	(2.31- 4.89)

Table 1C3 - 5

IESLC - Meta-anal of Ever Smoking (or Current if Ever not available), Cigs (or Any Prod if Cigs not avail)
All LC types
Least adjusted

REF	NRR	SEX	AD	Number Exposed Case	Cont	Non-exposed Case	Cont	RR	95.00%CI
GER	17	c	0	90	318	51	246	1.37	(0.93- 2.00)
GODLEY	5	m	1	-	-	-	-	6.84	(5.60- 8.35)
GODLEY	6	f	1	-	-	-	-	5.54	(4.29- 7.15)
Subtotal GODLEY								6.31	(5.39- 7.39)
GOLLED	21	m	0	380	1966	15	490	6.31	(3.73- 10.68)
GOODMA	3	m	0	216	398	10	199	10.80	(5.60- 20.82)
GOODMA	7	f	0	81	91	19	177	8.29	(4.74- 14.52)
Subtotal GOODMA								9.27	(6.05- 14.19)
GRAHAM	12	m	0	618	1284	18	346	9.25	(5.71- 15.00)
GREGOR	3	m	0	72	98	10	14	1.03	(0.43- 2.45)
GREGOR	7	f	0	21	42	1	22	11.00	(1.39- 87.29)
Subtotal GREGOR								1.46	(0.66- 3.26)
GSELL	6	m	0	60	42	2	29	20.71	(4.69- 91.56)
HAENSZ	56	f	0	74	103	81	236	2.09	(1.42- 3.09)
*HAMMO2	16	m	0	179	9590	1	1457	27.20	(3.81- 193.97)
*HAMMON	128	m	0	397	382338	15	115884	8.02	(4.79- 13.43)
*HANSEN	3	m	2	-	-	-	-	1.53	(0.71- 3.91)
HEGMAN	1	c	0	255	1202	27	2080	16.34	(10.92- 24.45)
*HEIN	7	m	0	143	4471	1	457	14.62	(2.05- 104.23)
*HENNEK	3	m	0	146	11112	23	10919	6.24	(4.02- 9.67)
HINDS	26	f	0	167	592	124	1812	4.12	(3.21- 5.29)
*HIRAYA	147	m	1	-	-	-	-	4.36	(3.53- 5.39)
*HIRAYA	150	f	1	-	-	-	-	2.36	(1.90- 2.94)
Subtotal HIRAYA								3.24	(2.78- 3.77)
HITOSU	6	m	0	142	1787	7	242	2.75	(1.27- 5.94)
HITOSU	12	f	0	34	500	33	1893	3.90	(2.39- 6.36)
Subtotal HITOSU								3.53	(2.33- 5.33)
*HOLE	15	m	0	187	5866	7	1189	5.41	(2.55- 11.49)
*HOLE	29	f	0	13	2144	8	1917	1.45	(0.60- 3.50)
Subtotal HOLE								3.10	(1.75- 5.50)
HOROWI	1	m	0	182	525	19	196	3.58	(2.17- 5.90)
HOROWI	2	f	0	21	382	14	463	1.82	(0.91- 3.62)
Subtotal HOROWI								2.83	(1.89- 4.25)
HORWIT	1	f	0	97	92	11	118	11.31	(5.73- 22.34)
HU	15	m	0	120	94	41	67	2.09	(1.30- 3.35)
HU	16	f	0	26	18	40	48	1.73	(0.83- 3.61)
Subtotal HU								1.98	(1.33- 2.94)
HU2	9	m	0	294	228	49	115	3.03	(2.08- 4.41)
HU2	10	f	0	108	80	72	100	1.88	(1.23- 2.85)
Subtotal HU2								2.44	(1.85- 3.23)
HUANG	1	c	0	98	77	37	58	2.00	(1.20- 3.32)
HUMBLE	14	m	1	-	-	-	-	12.10	(5.12- 28.60)
HUMBLE	16	m	1	-	-	-	-	11.88	(2.65- 53.30)
HUMBLE	18	f	1	-	-	-	-	11.36	(5.32- 24.23)
HUMBLE	20	f	1	-	-	-	-	15.40	(4.87- 48.74)
Subtotal HUMBLE								12.28	(7.58- 19.90)
JAHN	3	f	0	112	67	53	98	3.09	(1.97- 4.85)
JAIN	6	m	0	391	277	12	85	10.00	(5.36- 18.66)
JAIN	1	f	0	390	196	52	214	8.19	(5.78- 11.60)
Subtotal JAIN								8.59	(6.34- 11.64)
JARUP	3	m	0	91	52	11	42	6.68	(3.17- 14.09)
JARVHO	3	m	0	99	57	1	16	27.79	(3.59- 215.09)
JARVHO	7	f	0	41	15	6	21	9.57	(3.24- 28.26)
Subtotal JARVHO								12.08	(4.64- 31.46)
JEDRYC	63	m	0	852	656	49	219	5.80	(4.19- 8.04)
JEDRYC	68	f	0	120	32	78	166	7.98	(4.97- 12.82)
Subtotal JEDRYC								6.43	(4.92- 8.41)
JIANG	1	m	0	93	83	7	17	2.72	(1.08- 6.89)
JIANG	2	f	0	11	6	14	19	2.49	(0.74- 8.35)
Subtotal JIANG								2.63	(1.26- 5.50)
JOLY	2	m	0	552	709	12	218	14.14	(7.83- 25.56)
JOLY	1	f	0	166	123	52	283	7.34	(5.04- 10.70)
Subtotal JOLY								8.87	(6.46- 12.19)
JUSSAW	2	m	0	126	77	149	624	6.85	(4.90- 9.58)
*KAISE2	72	m	1	-	-	-	-	5.40	(3.05- 9.57)
*KAISE2	64	f	1	-	-	-	-	10.09	(5.29- 19.27)
Subtotal KAISE2								7.11	(4.63- 10.90)
*KAISER	13	m	2	-	-	-	-	17.63	(11.98- 25.96)
*KAISER	10	f	2	-	-	-	-	5.63	(3.89- 8.14)
Subtotal KAISER								9.70	(7.43- 12.67)
KANELL	5	m	0	814	441	48	172	6.61	(4.71- 9.30)

Table 1C3 - 5

IESLC - Meta-anal of Ever Smoking (or Current if Ever not available), Cigs (or Any Prod if Cigs not avail)
All LC types
Least adjusted

REF	NRR	SEX	AD	Number Exposed Case	Number Exposed Cont	Non-exposed Case	Non-exposed Cont	RR	95.00%CI
KATSOU	27	f	0	53	22	48	67	3.36	(1.81- 6.25)
KAUFMA	8	c	0	846	1645	35	925	13.59	(9.60- 19.24)
KELLER	3	m	0	8066	2517	323	1017	10.09	(8.83- 11.52)
KELLER	11	m	0	1493	340	38	117	13.52	(9.20- 19.86)
KELLER	7	f	0	3998	1269	469	1860	12.49	(11.09- 14.08)
KELLER	15	f	0	584	214	67	232	9.45	(6.91- 12.93)
Subtotal KELLER								11.30	(10.40- 12.29)
KHUDER	4	m	0	459	785	23	309	7.86	(5.06- 12.19)
KIHARA	31	c	0	338	232	102	237	3.39	(2.54- 4.51)
*KINLEN	6	m	0	711	12722	7	1333	10.64	(5.07- 22.36)
KJUUS	3	m	0	151	127	2	24	14.27	(3.31- 61.54)
*KNEKT	76	m	0	111	51798	6	17814	6.36	(2.80- 14.46)
KO	1	f	3	-	-	-	-	4.20	(1.10- 15.60)
KOHLME	1	c	0	228	236	11	193	16.95	(8.99- 31.96)
KOO	1	f	0	112	63	88	137	2.77	(1.84- 4.16)
KOULUM	2	m	0	625	229	5	54	29.48	(11.65- 74.60)
KREUZE	60	f	3	-	-	-	-	9.21	(3.45- 24.53)
KREUZE	62	f	3	-	-	-	-	4.05	(2.81- 5.86)
Subtotal KREUZE								4.48	(3.18- 6.32)
KREYBE	24	m	0	252	3514	6	644	7.70	(3.41- 17.37)
KREYBE	39	f	0	12	328	30	657	0.80	(0.40- 1.59)
Subtotal KREYBE								2.04	(1.21- 3.44)
*KUBIK	27	m	0	106	7829	2	4271	28.91	(7.14- 117.06)
LAMTH	6	f	0	242	106	202	337	3.81	(2.86- 5.08)
LAMWK	1	f	0	88	41	75	144	4.12	(2.59- 6.55)
LAMWK2	9	m	0	244	161	23	43	2.83	(1.64- 4.88)
LAMWK2	10	f	0	75	50	65	139	3.21	(2.02- 5.10)
Subtotal LAMWK2								3.04	(2.14- 4.33)
*LANGE	34	m	0	195	5790	5	721	4.86	(2.01- 11.76)
*LANGE	31	f	0	61	5544	7	2159	3.39	(1.55- 7.41)
Subtotal LANGE								3.97	(2.21- 7.13)
LAUSSM	10	m	0	347	188	85	226	4.91	(3.61- 6.66)
LEI	1	m	0	443	361	41	123	3.68	(2.52- 5.38)
LEI	2	f	0	123	61	85	147	3.49	(2.32- 5.24)
Subtotal LEI								3.59	(2.72- 4.74)
LEMARC	3	c	0	309	288	32	168	5.63	(3.74- 8.49)
LETOUR	1	c	0	714	514	24	224	12.96	(8.38- 20.05)
LEVIN	30	m	1	-	-	-	-	6.97	(4.87- 9.97)
*LIAW	1	m	1	-	-	-	-	3.70	(2.10- 6.60)
*LIAW	2	f	1	-	-	-	-	3.60	(1.00- 12.20)
Subtotal LIAW								3.68	(2.19- 6.20)
*LIDDEL	5	m	1	-	-	-	-	3.61	(2.27- 5.73)
LIU	2	c	2	-	-	-	-	1.92	(1.40- 2.64)
LIU2	1	m	0	212	180	12	44	4.32	(2.21- 8.43)
LIU2	3	f	0	54	23	38	69	4.26	(2.27- 7.99)
Subtotal LIU2								4.29	(2.71- 6.78)
LIU3	1	m	0	52	205	4	19	1.20	(0.39- 3.69)
LIU4	10	m	2	-	-	-	-	3.88	(3.78- 3.98)
LIU4	12	f	2	-	-	-	-	2.86	(2.77- 2.95)
Subtotal LIU4								3.43	(3.37- 3.50)
LIU5	1	c	0	85	70	26	41	1.91	(1.07- 3.44)
LOMBA2	1	f	0	149	353	76	239	1.33	(0.96- 1.83)
LOMBAR	2	m	0	978	782	14	112	10.01	(5.70- 17.58)
LUBIN2	47	m	0	6630	10435	190	2617	8.75	(7.53- 10.17)
LUBIN2	97	f	0	548	559	336	1188	3.47	(2.93- 4.10)
Subtotal LUBIN2								5.81	(5.19- 6.50)
LUO	1	c	0	65	146	37	160	1.93	(1.21- 3.06)
MACLEN	71	m	0	142	119	5	15	3.58	(1.26- 10.14)
MACLEN	72	f	0	45	57	41	109	2.10	(1.23- 3.57)
Subtotal MACLEN								2.34	(1.46- 3.76)
*MAGNUS	1	m	0	189	3439	11	1086	5.43	(2.97- 9.93)
MARSH	1	m	0	98	150	2	31	10.13	(2.37- 43.27)
MARSH	3	f	0	42	64	8	71	5.82	(2.54- 13.33)
Subtotal MARSH								6.67	(3.25- 13.70)
MARSH2	1	c	0	102	145	12	56	3.28	(1.67- 6.43)
MARTIS	4	m	0	197	176	4	25	7.00	(2.39- 20.49)
MASTRA	1	m	0	303	265	6	44	8.38	(3.52- 19.99)
MATOS	26	m	0	188	283	11	110	6.64	(3.48- 12.68)
MATSUD	10	m	0	170	3314	3	1255	21.46	(6.84- 67.33)
MCCONN	1	m	0	88	174	5	12	1.21	(0.41- 3.55)
MCCONN	2	f	0	3	3	4	11	2.75	(0.38- 19.67)

Table 1C3 - 5

IESLC - Meta-anal of Ever Smoking (or Current if Ever not available), Cigs (or Any Prod if Cigs not avail)
All LC types
Least adjusted

REF	NRR	SEX	AD	Number Exposed Case	Number Exposed Cont	Non-exposed Case	Non-exposed Cont	RR	95.00%CI
Subtotal MCCONN								1.46	(0.57- 3.76)
MCDUFF	1	m	0	159	134	6	31	6.13	(2.48- 15.14)
MCLAUG	1	m	0	294	1082	22	270	3.33	(2.12- 5.25)
*MIGRAN	26	m	0	206	6719	4	867	6.65	(2.48- 17.83)
*MIGRAN	41	f	0	31	4086	4	3814	7.23	(2.56- 20.47)
Subtotal MIGRAN								6.92	(3.38- 14.16)
MILLER	1	f	0	140	1607	28	3638	11.32	(7.51- 17.06)
MILLS	1	m	1	-	-	-	-	1.27	(1.01- 1.61)
*MRFITR	6	m	0	119	11007	0	1859	40.37~	(2.51- 648.95)
NAM	69	m	0	610	1075	30	520	9.84	(6.72- 14.40)
NAM	85	f	0	292	496	52	885	10.02	(7.31- 13.73)
Subtotal NAM								9.94	(7.80- 12.68)
NOTAN2	7	m	0	78	129	134	544	2.45	(1.75- 3.44)
NOU	11	m	0	74	247	6	122	6.09	(2.58- 14.39)
NOU	12	f	0	10	92	4	261	7.09	(2.17- 23.17)
Subtotal NOU								6.42	(3.20- 12.87)
ODRISC	3	c	0	440	996	6	664	48.89	(21.71- 110.07)
ORMOS	4	m	0	87	1034	7	777	9.34	(4.30- 20.28)
ORMOS	26	f	0	1	234	23	1044	0.19	(0.03- 1.44)
Subtotal ORMOS								5.65	(2.74- 11.64)
OSANN	17	m	0	1108	1018	45	833	20.15	(14.75- 27.52)
OSANN	21	f	0	737	563	96	1093	14.90	(11.77- 18.87)
Subtotal OSANN								16.63	(13.78- 20.07)
PARKIN	31	m	0	372	933	107	1248	4.65	(3.69- 5.86)
PASTOR	5	m	0	194	262	10	89	6.59	(3.34- 13.00)
PAWLEG	1	m	0	172	249	4	92	15.89	(5.73- 44.05)
PERNU	8	m	0	706	216	97	275	9.27	(7.02- 12.23)
PERNU	4	f	0	7	24	110	971	2.57	(1.08- 6.11)
Subtotal PERNU								8.22	(6.32- 10.71)
PERSH2	5	c	0	844	924	178	1164	5.97	(4.97- 7.17)
*PETO	5	m	0	101	2423	2	295	6.15	(1.52- 24.79)
PEZZO2	10	m	0	361	469	6	117	15.01	(6.53- 34.48)
PEZZOT	25	m	0	211	317	4	116	19.30	(7.02- 53.10)
PIKE	4	m	0	514	375	18	69	5.25	(3.08- 8.98)
PIKE	8	f	0	163	90	36	96	4.83	(3.04- 7.66)
Subtotal PIKE								5.01	(3.53- 7.10)
POFFIJ	1	c	0	913	918	58	452	7.75	(5.81- 10.34)
POLEDN	3	c	0	196	271	12	139	8.38	(4.52- 15.54)
*QIAO2	9	m	0	197	6360	10	709	2.20	(1.17- 4.13)
RACHTA	3	f	0	85	43	33	98	5.87	(3.43- 10.06)
RADZIK	1	c	0	180	198	9	13	1.31	(0.55- 3.15)
RANDIG	9	m	0	277	245	5	22	4.97	(1.86- 13.34)
RANDIG	10	f	0	16	39	17	92	2.22	(1.02- 4.84)
Subtotal RANDIG								3.03	(1.64- 5.58)
REN	1	m	0	106	84	12	34	3.58	(1.74- 7.33)
REN	2	f	0	78	20	48	50	4.06	(2.16- 7.64)
Subtotal REN								3.84	(2.39- 6.17)
RONCO	2	m	0	116	274	6	78	5.50	(2.33- 12.98)
ROTHSC	1	c	0	271	222	13	62	5.82	(3.12- 10.86)
SADOWS	4	m	0	421	446	18	81	4.25	(2.51- 7.20)
SANKAR	1	m	0	251	439	28	767	15.66	(10.42- 23.55)
SCHWAR	1	m	0	2648	1019	119	376	8.21	(6.60- 10.22)
SCHWAR	2	m	0	863	275	50	104	6.53	(4.54- 9.39)
SCHWAR	3	f	0	1351	637	182	855	9.96	(8.28- 12.00)
SCHWAR	4	f	0	335	179	40	247	11.56	(7.90- 16.90)
Subtotal SCHWAR								9.05	(7.99- 10.25)
SEGI	1	m	0	140	1742	18	382	1.71	(1.03- 2.82)
SEGI2	19	m	0	267	485	8	53	3.65	(1.71- 7.79)
SEGI2	27	f	0	24	34	56	126	1.59	(0.86- 2.92)
Subtotal SEGI2								2.20	(1.37- 3.54)
SEOW	1	f	0	61	15	92	125	5.53	(2.96- 10.33)
SHAW	12	c	0	324	266	11	107	11.85	(6.24- 22.50)
SIEMIA	9	m	0	844	428	13	105	15.93	(8.85- 28.67)
SIMARA	5	m	0	33	264	27	433	2.00	(1.18- 3.41)
SIMARA	6	f	0	17	67	38	349	2.33	(1.24- 4.37)
Subtotal SIMARA								2.13	(1.42- 3.20)
SOBUE	91	m	0	1023	1013	34	128	3.80	(2.58- 5.60)
SOBUE	95	f	0	127	232	167	857	2.81	(2.14- 3.69)
Subtotal SOBUE								3.10	(2.48- 3.88)
SOBUE2	10	m	2	-	-	-	-	4.47	(3.89- 5.14)
SOBUE2	12	f	2	-	-	-	-	3.28	(2.79- 3.87)

Table 1C3 - 5

IESLC - Meta-anal of Ever Smoking (or Current if Ever not available), Cigs (or Any Prod if Cigs not avail)
All LC types
Least adjusted

REF	NRR	SEX	AD	Number Exposed Case	Number Exposed Cont	Non-exposed Case	Non-exposed Cont	RR	95.00%CI
Subtotal SOBUE2								3.92	(3.53- 4.36)
*SPEIZE	8	f	0	535	1012074	58	776300	7.08	(5.40- 9.28)
SPITZ	3	c	0	170	169	7	128	18.39	(8.35- 40.53)
STASZE	7	m	0	251	653	5	158	12.15	(4.93- 29.94)
STASZE	5	f	0	6	153	15	1660	4.34	(1.66- 11.35)
Subtotal STASZE								7.50	(3.89- 14.48)
STAYNE	1	m	0	362	567	58	333	3.67	(2.69- 4.99)
STOCKS	30	m	0	2421	5483	45	638	6.26	(4.61- 8.50)
STOCKS	50	f	1	-	-	-	-	3.04	(2.35- 3.93)
Subtotal STOCKS								4.10	(3.37- 4.99)
STOCKW	8	c	0	18655	6414	2791	10641	11.09	(10.54- 11.66)
STUCKE	3	m	0	247	203	0	51	125.27~	(7.68-2042.38)
SUN	1	c	0	140	173	67	191	2.31	(1.62- 3.30)
SUZUK2	3	c	0	82	63	11	53	6.27	(3.03- 12.98)
SVENSS	56	f	0	172	89	38	120	6.10	(3.91- 9.53)
TANG	3	c	0	110	59	9	39	8.08	(3.66- 17.82)
*TENKAN	22	m	1	-	-	-	-	14.64	(6.29- 34.07)
TIZZAN	2	m	0	994	836	180	305	2.01	(1.64- 2.48)
TIZZAN	22	f	0	25	28	25	114	4.07	(2.04- 8.13)
Subtotal TIZZAN								2.13	(1.75- 2.60)
TOKARS	3	m	0	147	243	1	53	32.06	(4.39- 234.30)
TOKARS	5	f	0	1	2	13	40	1.54	(0.13- 18.38)
Subtotal TOKARS								9.77	(2.07- 46.11)
TOUSEY	2	m	0	293	389	4	130	24.48	(8.95- 66.97)
TOUSEY	6	f	0	192	212	13	226	15.74	(8.71- 28.46)
Subtotal TOUSEY								17.64	(10.59- 29.38)
TSUGAN	27	m	0	73	71	18	22	1.26	(0.62- 2.54)
*TULINI	15	m	1	-	-	-	-	8.06	(4.38- 14.84)
*TULINI	21	f	1	-	-	-	-	14.95	(8.30- 26.95)
Subtotal TULINI								11.10	(7.26- 16.95)
*TVERDA	22	m	2	-	-	-	-	4.58	(2.97- 7.06)
*TVERDA	15	f	2	-	-	-	-	11.05	(3.33- 36.71)
Subtotal TVERDA								5.07	(3.37- 7.62)
WAKAI	13	m	0	235	424	10	65	3.60	(1.82- 7.14)
WAKAI	31	f	0	38	31	50	145	3.55	(2.00- 6.30)
Subtotal WAKAI								3.57	(2.30- 5.55)
*WALD	2	m	0	77	4182	7	6539	17.20	(7.94- 37.25)
WANG	1	m	0	262	-	29	-	3.47	(2.10- 5.80)
WANG	2	f	0	17	-	82	-	4.00	(1.30- 12.00)
Subtotal WANG								3.56	(2.24- 5.64)
WANG2	8	c	0	60	99	11	43	2.37	(1.14- 4.94)
WANG3	1	c	0	235	172	58	121	2.85	(1.97- 4.13)
WANG4	1	m	0	1043	18164	127	2374	1.07	(0.89- 1.30)
WICKLU	1	m	0	-	-	-	-	4.60	(2.80- 7.60)
WIGLE	13	m	0	543	632	15	204	11.68	(6.83- 19.99)
WIGLE	16	f	0	78	235	36	439	4.05	(2.64- 6.19)
Subtotal WIGLE								6.09	(4.37- 8.51)
WILKIN	1	m	0	173	372	2	108	25.11	(6.13- 102.89)
WILKIN	2	f	0	84	109	12	89	5.72	(2.93- 11.13)
Subtotal WILKIN								7.49	(4.10- 13.68)
WU	37	f	0	189	128	31	92	4.38	(2.75- 6.97)
WUNSCH	1	m	0	290	441	14	99	4.65	(2.61- 8.30)
WUNSCH	7	f	0	60	98	29	208	4.39	(2.65- 7.27)
Subtotal WUNSCH								4.50	(3.08- 6.58)
WUWILL	6	f	0	539	351	417	601	2.21	(1.84- 2.66)
WYNDE2	16	m	0	382	512	8	105	9.79	(4.71- 20.34)
WYNDE3	48	m	0	261	264	9	88	9.67	(4.77- 19.60)
WYNDE3	83	f	0	46	56	20	76	3.12	(1.67- 5.85)
Subtotal WYNDE3								5.14	(3.21- 8.22)
WYNDE4	48	m	0	632	665	12	115	9.11	(4.98- 16.67)
WYNDE4	62	f	2	-	-	-	-	2.87	(1.48- 5.55)
Subtotal WYNDE4								5.38	(3.45- 8.41)
WYNDE6	81	m	0	2765	1797	87	617	10.91	(8.65- 13.76)
WYNDE6	252	f	0	1354	701	159	856	10.40	(8.58- 12.60)
Subtotal WYNDE6								10.60	(9.14- 12.30)
*XIANGZ	6	m	0	526	10580	25	974	1.94	(1.30- 2.88)
XU	1	m	0	627	552	102	236	2.63	(2.03- 3.40)
XU2	1	c	0	501	582	82	377	3.96	(3.03- 5.17)
XU3	1	m	0	92	68	7	31	5.99	(2.49- 14.42)
XU3	3	f	0	23	11	13	25	4.02	(1.51- 10.74)
Subtotal XU3								5.02	(2.61- 9.66)

Table 1C3 - 5

IESLC - Meta-anal of Ever Smoking (or Current if Ever not available), Cigs (or Any Prod if Cigs not avail)
All LC types
Least adjusted

REF	NRR	SEX	AD	Number Exposed Case	Number Exposed Cont	Non-exposed Case	Non-exposed Cont	RR	95.00%CI
XU4	1	c	0	161	113	45	93	2.94	(1.92- 4.52)
YAMAGU	5	c	0	120	409	24	267	3.26	(2.05- 5.19)
*YONG	12	m	1	-	-	-	-	28.71	(6.98- 118.16)
*YONG	15	f	1	-	-	-	-	5.20	(2.38- 11.35)
Subtotal YONG								7.75	(3.91- 15.36)
*YUAN	1	m	2	-	-	-	-	6.50	(3.64- 11.60)
ZHANG	1	c	0	72	102	28	98	2.47	(1.47- 4.14)
ZHENG	15	m	0	279	218	33	94	3.65	(2.36- 5.63)
ZHENG	24	f	0	76	44	152	184	2.09	(1.36- 3.21)
Subtotal ZHENG								2.75	(2.03- 3.73)
ZHOU	2	m	0	740	41	275	36	2.36	(1.48- 3.77)
ZHOU	3	f	0	112	7	231	32	2.22	(0.95- 5.18)
Subtotal ZHOU								2.33	(1.54- 3.51)
Partial Totals				127214	2860698	14840	2040141		

*prospective study ~ With 0.5 adjustment for zero

REF	NRR	SEX	AD	Ys	Ws	Qs	Ps
ABELIN	2	m	0	3.33	1.88	5.89	0.0000
*ABRAHA	7	m	0	2.17	9.68	3.62	0.0000
*ABRAHA	8	f	0	1.59	19.39	0.02	0.0000
Subtotal ABRAHA				1.78	29.07	3.64	
AGUDO	8	f	0	0.83	9.53	5.07	0.0106
*AKIBA	3	m	0	1.33	17.22	0.91	0.0000
*AKIBA	7	f	0	1.37	48.42	1.62	0.0000
Subtotal AKIBA				1.36	65.65	2.53	
ALDERS	61	m	0	2.38	12.98	8.83	0.0000
ALDERS	12	f	0	1.53	45.39	0.03	0.0000
Subtotal ALDERS				1.72	58.37	8.85	
*AMANDU	3	m	0	1.56	5.73	0.00	0.0002
AMES	4	m	0	1.59	11.09	0.01	0.0000
*ANDERS	3	f	0	2.57	39.86	41.16	0.0000
*ARCHER	6	m	0	1.85	5.76	0.48	0.0000
ARMADA	4	m	0	2.99	3.67	7.58	0.0000
AUSTIN	3	c	0	2.48	4.51	3.86	0.0000
AUVINE	1	c	0	2.15	30.60	10.62	0.0000
AXELSO	1	c	0	1.83	24.06	1.86	0.0000
AXELSS	1	m	0	2.14	13.32	4.51	0.0000
AXELSS	11	f	0	2.16	12.45	4.47	0.0000
Subtotal AXELSS				2.15	25.77	8.98	
BAND	1	m	2	2.30	42.76	23.54	0.0000
BARBON	106	m	0	2.40	18.55	13.27	0.0000
BECHER	15	m	0	2.43	2.75	2.10	0.0001
BECHER	16	f	0	1.50	5.94	0.02	0.0003
Subtotal BECHER				1.80	8.69	2.12	
*BENSHL	15	m	1	2.08	9.80	2.70	0.0000
*BEST	23	m	0	3.33	1.00	3.12	0.0009
*BEST	18	f	1	0.81	2.17	1.22	0.2348
Subtotal BEST				1.60	3.17	4.34	
BLOHMK	3	m	0	1.13	70.05	12.64	0.0000
BLOT4	1	m	0	2.68	6.96	8.71	0.0000
BOFFET	7	m	0	2.73	105.32	145.73	0.0000
*BOUCOT	58	m	0	3.90	0.50	2.74	0.0059
BRESLO	17	m	0	2.00	5.83	1.14	0.0000
BRESLO	23	f	0	0.32	3.10	4.73	0.5717
Subtotal BRESLO				1.42	8.93	5.87	
*BRETT	10	m	0	1.18	5.77	0.80	0.0044
BROCKM	1	m	0	0.07	0.98	2.15	0.9437
BROCKM	2	f	0	0.69	2.73	2.04	0.2517
Subtotal BROCKM				0.53	3.71	4.19	
BROSS	13	m	0	1.80	28.54	1.75	0.0000
BROWN2	2	m	2	2.21	442.58	187.97	0.0000
BROWN2	1	f	2	2.54	427.71	415.00	0.0000
Subtotal BROWN2				2.37	870.29	602.97	
BUFFLE	2	m	0	2.45	4.42	3.55	0.0000
BUFFLE	6	f	0	1.96	28.29	4.69	0.0000
Subtotal BUFFLE				2.03	32.71	8.24	
CARPEN	7	c	0	2.42	13.16	9.79	0.0000
CASCO2	1	c	0	2.44	5.31	4.15	0.0000
CASCOR	1	c	0	2.60	18.40	20.19	0.0000
*CEDERL	107	m	2	1.78	20.66	1.02	0.0000

Table 1C3 - 5

IESLC - Meta-anal of Ever Smoking (or Current if Ever not available), Cigs (or Any Prod if Cigs not avail)
All LC types
Least adjusted

REF	NRR	SEX	AD	Ys	Ws	Qs	Ps
*CEDERL	112	f	2	1.43	31.21	0.50	0.0000
Subtotal CEDERL				1.57	51.88	1.51	
CHAN	5	m	0	3.31	1.87	5.78	0.0000
CHAN	6	f	0	1.25	20.57	1.99	0.0000
Subtotal CHAN				1.42	22.44	7.77	
*CHANG	6	m	0	1.65	4.76	0.04	0.0003
*CHANG	12	f	0	1.30	8.85	0.57	0.0001
Subtotal CHANG				1.42	13.61	0.61	
CHATZI	4	c	0	1.21	19.44	2.40	0.0000
CHEN2	1	m	0	1.52	6.25	0.01	0.0001
CHEN2	2	f	0	0.51	7.70	8.36	0.1539
Subtotal CHEN2				0.97	13.95	8.37	
CHEN3	1	c	0	0.46	27.78	33.25	0.0148
CHIAZZ	2	m	0	2.06	3.53	0.88	0.0001
CHOI	1	m	0	1.43	10.71	0.16	0.0000
CHOI	5	f	0	0.46	9.06	10.99	0.1703
Subtotal CHOI				0.99	19.78	11.15	
*CHOW	6	m	0	2.41	5.83	4.25	0.0000
*CHYOU	9	m	0	1.96	12.35	2.05	0.0000
COMSTO	33	m	0	2.43	3.63	2.78	0.0000
COMSTO	45	f	0	2.19	9.22	3.72	0.0000
Subtotal COMSTO				2.26	12.85	6.49	
COOKSO	4	c	0	1.88	14.21	1.50	0.0000
CORREA	33	c	0	2.33	41.42	25.04	0.0000
*CPSI	187	m	1	2.22	78.68	34.32	0.0000
*CPSI	274	f	1	1.03	73.22	20.61	0.0000
Subtotal CPSI				1.64	151.90	54.93	
*CPSII	104	m	1	2.55	78.29	77.55	0.0000
*CPSII	79	f	1	2.10	142.84	42.07	0.0000
Subtotal CPSII				2.26	221.13	119.61	
DAMBER	26	m	0	2.03	27.56	6.29	0.0000
DARBY	15	m	0	3.90	2.96	16.22	0.0000
DARBY	16	f	0	2.51	19.76	17.81	0.0000
Subtotal DARBY				2.69	22.71	34.02	
DAVEYS	5	m	0	1.57	2.53	0.00	0.0126
DAVEYS	6	f	0	-0.32	0.42	1.50	0.8327
Subtotal DAVEYS				1.30	2.96	1.50	
DEAN	8	m	0	1.70	9.65	0.21	0.0000
DEAN2	12	m	0	1.43	23.54	0.36	0.0000
DEAN2	20	f	0	1.09	14.29	3.05	0.0000
Subtotal DEAN2				1.30	37.83	3.42	
DEAN3	240	m	0	1.93	21.30	3.02	0.0000
DEAN3	124	f	0	1.06	28.64	7.13	0.0000
Subtotal DEAN3				1.43	49.94	10.15	
*DEKLER	6	m	2	3.01	0.99	2.10	0.0027
DESTE2	15	m	0	1.90	22.53	2.69	0.0000
DESTEF	11	m	0	1.75	16.32	0.63	0.0000
*DOCKER	3	c	4	1.46	4.27	0.04	0.0026
DOLL	20	m	0	2.24	6.12	2.87	0.0000
DOLL	12	f	0	0.72	12.98	9.16	0.0099
Subtotal DOLL				1.21	19.10	12.03	
*DOLL2	88	m	1	2.45	2.95	2.35	0.0000
*DOLL2	63	f	1	2.16	3.28	1.18	0.0001
Subtotal DOLL2				2.30	6.23	3.54	
DORANT	10	c	0	2.89	13.34	23.73	0.0000
DORGAN	7	m	0	2.38	12.29	8.39	0.0000
DORGAN	31	m	0	3.28	2.67	7.93	0.0000
DORGAN	95	f	3	2.14	67.41	22.95	0.0000
Subtotal DORGAN				2.21	82.37	39.27	
*DORN	413	m	1	2.18	47.17	18.23	0.0000
DOSEME	17	m	0	1.41	75.43	1.54	0.0000
DROSTE	3	m	0	2.65	6.33	7.54	0.0000
DU	1	m	0	1.26	28.12	2.45	0.0000
DU	2	f	0	0.66	24.50	19.80	0.0011
Subtotal DU				0.98	52.62	22.25	
*DUNN	6	m	0	2.91	1.97	3.63	0.0000
EBELIN	1	m	0	1.94	9.19	1.33	0.0000
*ENGELA	8	m	0	1.37	24.82	0.85	0.0000
*ENGELA	22	f	0	1.51	21.59	0.04	0.0000
Subtotal ENGELA				1.44	46.41	0.89	
*ENSTRO	1	m	1	2.56	81.91	83.16	0.0000

Table 1C3 - 5

IESLC - Meta-anal of Ever Smoking (or Current if Ever not available), Cigs (or Any Prod if Cigs not avail)
All LC types
Least adjusted

REF	NRR	SEX	AD	Ys	Ws	Qs	Ps
*ENSTRO	2	f	1	1.94	181.45	26.50	0.0000
Subtotal ENSTRO				2.13	263.36	109.66	
ESAKI	4	m	0	0.64	8.96	7.52	0.0554
ESAKI	5	f	0	0.90	7.99	3.44	0.0109
Subtotal ESAKI				0.76	16.94	10.96	
FAN	1	m	0	1.04	25.87	6.77	0.0000
FAN	2	f	0	1.37	24.92	0.90	0.0000
Subtotal FAN				1.20	50.80	7.67	
GAO	6	m	0	1.37	41.05	1.50	0.0000
GAO	16	f	0	0.93	63.04	24.72	0.0000
Subtotal GAO				1.10	104.09	26.21	
GAO2	6	m	0	1.63	9.72	0.06	0.0000
GARCIA	3	c	0	2.14	16.50	5.66	0.0000
GARDIN	7	c	0	2.41	4.14	2.99	0.0000
GARSHI	17	m	0	1.75	34.80	1.34	0.0000
GENG	1	m	0	1.79	4.98	0.27	0.0001
GENG	2	f	0	1.08	22.39	4.98	0.0000
Subtotal GENG				1.21	27.37	5.26	
GER	17	c	0	0.31	26.37	40.89	0.1100
GODLEY	5	m	1	1.92	96.28	12.91	0.0000
GODLEY	6	f	1	1.71	58.89	1.42	0.0000
Subtotal GODLEY				1.84	155.17	14.34	
GOLLED	21	m	0	1.84	13.92	1.14	0.0000
GOODMA	3	m	0	2.38	8.92	6.04	0.0000
GOODMA	7	f	0	2.12	12.25	3.83	0.0000
Subtotal GOODMA				2.23	21.17	9.86	
GRAHAM	12	m	0	2.22	16.44	7.34	0.0000
GREGOR	3	m	0	0.03	5.11	11.95	0.9492
GREGOR	7	f	0	2.40	0.90	0.63	0.0233
Subtotal GREGOR				0.38	6.01	12.58	
GSELL	6	m	0	3.03	1.74	3.78	0.0001
HAENSZ	56	f	0	0.74	25.12	16.80	0.0002
*HAMMO2	16	m	0	3.30	1.00	3.04	0.0010
*HAMMON	128	m	0	2.08	14.46	3.99	0.0000
*HANSEN	3	m	2	0.43	5.28	6.76	0.3285
HEGMAN	1	c	0	2.79	23.66	36.21	0.0000
*HEIN	7	m	0	2.68	1.00	1.26	0.0074
*HENNEK	3	m	0	1.83	19.94	1.50	0.0000
HINDS	26	f	0	1.42	61.37	1.21	0.0000
*HIRAYA	147	m	1	1.47	85.78	0.61	0.0000
*HIRAYA	150	f	1	0.86	80.63	39.27	0.0000
Subtotal HIRAYA				1.18	166.40	39.88	
HITOSU	6	m	0	1.01	6.47	1.93	0.0102
HITOSU	12	f	0	1.36	16.07	0.61	0.0000
Subtotal HITOSU				1.26	22.53	2.54	
*HOLE	15	m	0	1.69	6.79	0.12	0.0000
*HOLE	29	f	0	0.37	4.98	6.96	0.4046
Subtotal HOLE				1.13	11.77	7.08	
HOROWI	1	m	0	1.27	15.35	1.22	0.0000
HOROWI	2	f	0	0.60	8.08	7.42	0.0894
Subtotal HOROWI				1.04	23.43	8.65	
HORWIT	1	f	0	2.43	8.29	6.27	0.0000
HU	15	m	0	0.74	17.16	11.57	0.0023
HU	16	f	0	0.55	7.15	7.24	0.1413
Subtotal HU				0.68	24.31	18.82	
HU2	9	m	0	1.11	27.11	5.47	0.0000
HU2	10	f	0	0.63	21.91	18.86	0.0033
Subtotal HU2				0.89	49.01	24.33	
HUANG	1	c	0	0.69	14.82	11.11	0.0078
HUMBLE	14	m	1	2.49	5.19	4.56	0.0000
HUMBLE	16	m	1	2.47	1.71	1.44	0.0012
HUMBLE	18	f	1	2.43	6.68	5.10	0.0000
HUMBLE	20	f	1	2.73	2.90	4.02	0.0000
Subtotal HUMBLE				2.51	16.48	15.11	
JAHN	3	f	0	1.13	18.89	3.46	0.0000
JAIN	6	m	0	2.30	9.88	5.49	0.0000
JAIN	1	f	0	2.10	31.68	9.45	0.0000
Subtotal JAIN				2.15	41.55	14.94	
JARUP	3	m	0	1.90	6.90	0.81	0.0000
JARVHO	3	m	0	3.32	0.92	2.87	0.0015
JARVHO	7	f	0	2.26	3.28	1.61	0.0000

Table 1C3 - 5

IESLC - Meta-anal of Ever Smoking (or Current if Ever not available), Cigs (or Any Prod if Cigs not avail)
All LC types
Least adjusted

REF	NRR	SEX	AD	Ys	Ws	Qs	Ps
Subtotal JARVHO				2.49	4.19	4.48	
JEDRYC	63	m	0	1.76	36.14	1.48	0.0000
JEDRYC	68	f	0	2.08	17.12	4.64	0.0000
Subtotal JEDRYC				1.86	53.25	6.11	
JIANG	1	m	0	1.00	4.45	1.37	0.0346
JIANG	2	f	0	0.91	2.62	1.09	0.1401
Subtotal JIANG				0.97	7.08	2.46	
JOLY	2	m	0	2.65	10.97	13.10	0.0000
JOLY	1	f	0	1.99	27.09	5.18	0.0000
Subtotal JOLY				2.18	38.06	18.28	
JUSSAW	2	m	0	1.92	34.20	4.63	0.0000
*KAISE2	72	m	1	1.69	11.75	0.20	0.0000
*KAISE2	64	f	1	2.31	9.19	5.24	0.0000
Subtotal KAISE2				1.96	20.95	5.44	
*KAISER	13	m	2	2.87	25.69	44.30	0.0000
*KAISER	10	f	2	1.73	28.18	0.83	0.0000
Subtotal KAISER				2.27	53.88	45.13	
KANELL	5	m	0	1.89	33.17	3.67	0.0000
KATSOU	27	f	0	1.21	9.99	1.18	0.0001
KAUFMA	8	c	0	2.61	31.80	35.26	0.0000
KELLER	3	m	0	2.31	217.37	123.90	0.0000
KELLER	11	m	0	2.60	25.99	28.53	0.0000
KELLER	7	f	0	2.53	269.69	253.08	0.0000
KELLER	15	f	0	2.25	39.03	18.55	0.0000
Subtotal KELLER				2.43	552.08	424.06	
KHUDER	4	m	0	2.06	19.93	5.08	0.0000
KIHARA	31	c	0	1.22	46.97	5.34	0.0000
*KINLEN	6	m	0	2.36	6.97	4.55	0.0000
KJUUS	3	m	0	2.66	1.80	2.18	0.0004
*KNEKT	76	m	0	1.85	5.69	0.49	0.0000
KO	1	f	3	1.44	2.18	0.03	0.0339
KOHLME	1	c	0	2.83	9.55	15.49	0.0000
KOO	1	f	0	1.02	23.01	6.67	0.0000
KOULUM	2	m	0	3.38	4.45	14.87	0.0000
KREUZE	60	f	3	2.22	3.99	1.76	0.0000
KREUZE	62	f	3	1.40	28.45	0.71	0.0000
Subtotal KREUZE				1.50	32.44	2.47	
KREYBE	24	m	0	2.04	5.80	1.36	0.0000
KREYBE	39	f	0	-0.22	8.25	26.08	0.5245
Subtotal KREYBE				0.71	14.05	27.44	
*KUBIK	27	m	0	3.36	1.96	6.42	0.0000
LAMTH	6	f	0	1.34	46.55	2.24	0.0000
LAMWK	1	f	0	1.42	17.85	0.35	0.0000
LAMWK2	9	m	0	1.04	12.98	3.44	0.0002
LAMWK2	10	f	0	1.17	17.89	2.73	0.0000
Subtotal LAMWK2				1.11	30.86	6.18	
*LANGE	34	m	0	1.58	4.91	0.00	0.0005
*LANGE	31	f	0	1.22	6.30	0.71	0.0022
Subtotal LANGE				1.38	11.22	0.71	
LAUSSM	10	m	0	1.59	41.00	0.05	0.0000
LEI	1	m	0	1.30	26.63	1.71	0.0000
LEI	2	f	0	1.25	23.21	2.19	0.0000
Subtotal LEI				1.28	49.84	3.90	
LEMARC	3	c	0	1.73	22.77	0.67	0.0000
LETOUR	1	c	0	2.56	20.21	20.44	0.0000
LEVIN	30	m	1	1.94	29.93	4.44	0.0000
*LIAW	1	m	1	1.31	11.72	0.72	0.0000
*LIAW	2	f	1	1.28	2.46	0.19	0.0447
Subtotal LIAW				1.30	14.17	0.91	
*LIDDEL	5	m	1	1.28	17.92	1.33	0.0000
LIU	2	c	2	0.65	38.19	31.23	0.0001
LIU2	1	m	0	1.46	8.60	0.08	0.0000
LIU2	3	f	0	1.45	9.73	0.11	0.0000
Subtotal LIU2				1.46	18.32	0.19	
LIU3	1	m	0	0.19	3.06	5.75	0.7444
LIU4	10	m	2	1.36	5780.52	232.92	0.0000
LIU4	12	f	2	1.05	3876.64	991.57	0.0000
Subtotal LIU4				1.23	9657.16	1224.48	
LIU5	1	c	0	0.65	11.25	9.25	0.0293
LOMBA2	1	f	0	0.28	37.19	60.31	0.0841
LOMBAR	2	m	0	2.30	12.10	6.74	0.0000

Table 1C3 - 5

IESLC - Meta-anal of Ever Smoking (or Current if Ever not available), Cigs (or Any Prod if Cigs not avail)
All LC types
Least adjusted

REF	NRR	SEX	AD	Ys	Ws	Qs	Ps
LUBIN2	47	m	0	2.17	169.72	63.70	0.0000
LUBIN2	97	f	0	1.24	134.56	13.23	0.0000
Subtotal LUBIN2				1.76	304.28	76.93	
LUO	1	c	0	0.66	18.01	14.64	0.0054
MACLEN	71	m	0	1.28	3.54	0.28	0.0163
MACLEN	72	f	0	0.74	13.64	9.06	0.0062
Subtotal MACLEN				0.85	17.18	9.34	
*MAGNUS	1	m	0	1.69	10.53	0.19	0.0000
MARSH	1	m	0	2.32	1.82	1.05	0.0018
MARSH	3	f	0	1.76	5.60	0.24	0.0000
Subtotal MARSH				1.90	7.42	1.28	
MARSH2	1	c	0	1.19	8.48	1.15	0.0005
MARTIS	4	m	0	1.95	3.32	0.50	0.0004
MASTRA	1	m	0	2.13	5.09	1.65	0.0000
MATOS	26	m	0	1.89	9.19	1.04	0.0000
MATSUD	10	m	0	3.07	2.94	6.70	0.0000
MCCONN	1	m	0	0.19	3.33	6.18	0.7237
MCCONN	2	f	0	1.01	0.99	0.29	0.3136
Subtotal MCCONN				0.38	4.32	6.48	
MCDUFF	1	m	0	1.81	4.70	0.31	0.0001
MCLAUG	1	m	0	1.20	18.70	2.32	0.0000
*MIGRAN	26	m	0	1.89	3.94	0.45	0.0002
*MIGRAN	41	f	0	1.98	3.55	0.63	0.0002
Subtotal MIGRAN				1.93	7.49	1.08	
MILLER	1	f	0	2.43	22.85	17.30	0.0000
MILLS	1	m	1	0.24	71.48	123.48	0.0406
*MRFITR	6	m	0	3.70	0.50	2.28	0.0091
NAM	69	m	0	2.29	26.44	14.07	0.0000
NAM	85	f	0	2.30	38.76	21.68	0.0000
Subtotal NAM				2.30	65.19	35.75	
NOTAN2	7	m	0	0.90	33.47	14.52	0.0000
NOU	11	m	0	1.81	5.20	0.33	0.0000
NOU	12	f	0	1.96	2.74	0.44	0.0012
Subtotal NOU				1.86	7.94	0.77	
ODRISC	3	c	0	3.89	5.83	31.75	0.0000
ORMOS	4	m	0	2.23	6.39	2.93	0.0000
ORMOS	26	f	0	-1.64	0.95	9.74	0.1093
Subtotal ORMOS				1.73	7.34	12.68	
OSANN	17	m	0	3.00	39.51	82.68	0.0000
OSANN	21	f	0	2.70	69.13	90.65	0.0000
Subtotal OSANN				2.81	108.65	173.33	
PARKIN	31	m	0	1.54	71.91	0.03	0.0000
PASTOR	5	m	0	1.89	8.32	0.90	0.0000
PAWLEG	1	m	0	2.77	3.69	5.40	0.0000
PERNU	8	m	0	2.23	50.02	22.44	0.0000
PERNU	4	f	0	0.95	5.14	1.92	0.0321
Subtotal PERNU				2.11	55.16	24.36	
PERSH2	5	c	0	1.79	114.36	6.09	0.0000
*PETO	5	m	0	1.82	1.98	0.13	0.0107
PEZZO2	10	m	0	2.71	5.55	7.37	0.0000
PEZZOT	25	m	0	2.96	3.75	7.39	0.0000
PIKE	4	m	0	1.66	13.39	0.14	0.0000
PIKE	8	f	0	1.57	18.04	0.01	0.0000
Subtotal PIKE				1.61	31.43	0.15	
POFFIJ	1	c	0	2.05	46.21	11.15	0.0000
POLEDN	3	c	0	2.13	10.07	3.26	0.0000
*QIAO2	9	m	0	0.79	9.66	5.73	0.0145
RACHTA	3	f	0	1.77	13.24	0.60	0.0000
RADZIK	1	c	0	0.27	5.03	8.30	0.5411
RANDIG	9	m	0	1.60	3.95	0.01	0.0014
RANDIG	10	f	0	0.80	6.34	3.65	0.0447
Subtotal RANDIG				1.11	10.29	3.66	
REN	1	m	0	1.27	7.46	0.60	0.0005
REN	2	f	0	1.40	9.65	0.23	0.0000
Subtotal REN				1.35	17.11	0.83	
RONCO	2	m	0	1.71	5.21	0.12	0.0001
ROTHSC	1	c	0	1.76	9.88	0.42	0.0000
SADOWS	4	m	0	1.45	13.79	0.17	0.0000
SANKAR	1	m	0	2.75	23.11	32.98	0.0000
SCHWAR	1	m	0	2.11	80.50	24.25	0.0000
SCHWAR	2	m	0	1.88	29.06	2.97	0.0000

Table 1C3 - 5

IESLC - Meta-anal of Ever Smoking (or Current if Ever not available), Cigs (or Any Prod if Cigs not avail)
All LC types
Least adjusted

REF	NRR	SEX	AD	Ys	Ws	Qs	Ps
SCHWAR	3	f	0	2.30	111.43	61.41	0.0000
SCHWAR	4	f	0	2.45	26.58	21.09	0.0000
Subtotal SCHWAR				2.20	247.58	109.72	
SEGI	1	m	0	0.53	15.18	15.87	0.0375
SEGI2	19	m	0	1.29	6.68	0.46	0.0008
SEGI2	27	f	0	0.46	10.32	12.35	0.1372
Subtotal SEGI2				0.79	17.00	12.81	
SEOW	1	f	0	1.71	9.81	0.23	0.0000
SHAW	12	c	0	2.47	9.34	7.83	0.0000
SIEMIA	9	m	0	2.77	11.12	16.31	0.0000
SIMARA	5	m	0	0.70	13.62	10.10	0.0103
SIMARA	6	f	0	0.85	9.72	4.91	0.0084
Subtotal SIMARA				0.76	23.33	15.00	
SOBUE	91	m	0	1.34	25.52	1.25	0.0000
SOBUE	95	f	0	1.03	51.71	14.18	0.0000
Subtotal SOBUE				1.13	77.23	15.43	
SOBUE2	10	m	2	1.50	197.90	0.69	0.0000
SOBUE2	12	f	2	1.19	143.51	19.51	0.0000
Subtotal SOBUE2				1.37	341.42	20.20	
*SPEIZE	8	f	0	1.96	52.33	8.37	0.0000
SPITZ	3	c	0	2.91	6.16	11.31	0.0000
STASZE	7	m	0	2.50	4.72	4.18	0.0000
STASZE	5	f	0	1.47	4.16	0.03	0.0028
Subtotal STASZE				2.02	8.88	4.21	
STAYNE	1	m	0	1.30	40.37	2.68	0.0000
STOCKS	30	m	0	1.83	41.01	3.16	0.0000
STOCKS	50	f	1	1.11	58.11	11.49	0.0000
Subtotal STOCKS				1.41	99.12	14.65	
STOCKW	8	c	0	2.41	1511.07	1090.15	0.0000
STUCKE	3	m	0	4.83	0.49	5.28	0.0007
SUN	1	c	0	0.84	30.23	15.70	0.0000
SUZUK2	3	c	0	1.84	7.25	0.57	0.0000
SVENSS	56	f	0	1.81	19.34	1.23	0.0000
TANG	3	c	0	2.09	6.14	1.74	0.0000
*TENKAN	22	m	1	2.68	5.38	6.84	0.0000
TIZZAN	2	m	0	0.70	90.61	66.41	0.0000
TIZZAN	22	f	0	1.40	8.03	0.19	0.0001
Subtotal TIZZAN				0.76	98.64	66.60	
TOKARS	3	m	0	3.47	0.97	3.55	0.0006
TOKARS	5	f	0	0.43	0.62	0.79	0.7336
Subtotal TOKARS				2.28	1.60	4.34	
TOUSEY	2	m	0	3.20	3.79	10.22	0.0000
TOUSEY	6	f	0	2.76	10.96	15.77	0.0000
Subtotal TOUSEY				2.87	14.75	25.99	
TSUGAN	27	m	0	0.23	7.76	13.70	0.5244
*TULINI	15	m	1	2.09	10.32	2.90	0.0000
*TULINI	21	f	1	2.70	11.08	14.60	0.0000
Subtotal TULINI				2.41	21.40	17.51	
*TVERDA	22	m	2	1.52	20.49	0.02	0.0000
*TVERDA	15	f	2	2.40	2.67	1.91	0.0001
Subtotal TVERDA				1.62	23.16	1.93	
WAKAI	13	m	0	1.28	8.20	0.62	0.0002
WAKAI	31	f	0	1.27	11.70	0.97	0.0000
Subtotal WAKAI				1.27	19.90	1.59	
*WALD	2	m	0	2.84	6.43	10.68	0.0000
WANG	1	m	0	1.24	14.89	1.45	0.0000
WANG	2	f	0	1.39	3.11	0.09	0.0145
Subtotal WANG				1.27	18.00	1.54	
WANG2	8	c	0	0.86	7.10	3.42	0.0216
WANG3	1	c	0	1.05	28.11	7.29	0.0000
WANG4	1	m	0	0.07	107.42	237.13	0.4630
WICKLU	1	m	0	1.53	15.41	0.01	0.0000
WIGLE	13	m	0	2.46	13.33	10.84	0.0000
WIGLE	16	f	0	1.40	21.22	0.53	0.0000
Subtotal WIGLE				1.81	34.55	11.38	
WILKIN	1	m	0	3.22	1.93	5.37	0.0000
WILKIN	2	f	0	1.74	8.65	0.30	0.0000
Subtotal WILKIN				2.01	10.58	5.67	
WU	37	f	0	1.48	17.78	0.11	0.0000
WUNSCH	1	m	0	1.54	11.46	0.00	0.0000
WUNSCH	7	f	0	1.48	15.11	0.09	0.0000

Table 1C3 - 5

IESLC - Meta-anal of Ever Smoking (or Current if Ever not available), Cigs (or Any Prod if Cigs not avail)
All LC types
Least adjusted

REF	NRR	SEX	AD	Ys	Ws	Qs	Ps
Subtotal WUNSCH				1.50	26.58	0.09	
WUWILL	6	f	0	0.79	114.07	66.26	0.0000
WYNDE2	16	m	0	2.28	7.19	3.78	0.0000
WYNDE3	48	m	0	2.27	7.69	3.90	0.0000
WYNDE3	83	f	0	1.14	9.73	1.70	0.0004
Subtotal WYNDE3				1.64	17.42	5.60	
WYNDE4	48	m	0	2.21	10.51	4.48	0.0000
WYNDE4	62	f	2	1.05	8.80	2.22	0.0018
Subtotal WYNDE4				1.68	19.31	6.70	
WYNDE6	81	m	0	2.39	71.26	49.48	0.0000
WYNDE6	252	f	0	2.34	103.92	64.06	0.0000
Subtotal WYNDE6				2.36	175.18	113.54	
*XIANGZ	6	m	0	0.66	24.52	19.66	0.0011
XU	1	m	0	0.97	57.31	19.97	0.0000
XU2	1	c	0	1.38	53.87	1.76	0.0000
XU3	1	m	0	1.79	4.98	0.27	0.0001
XU3	3	f	0	1.39	3.98	0.11	0.0055
Subtotal XU3				1.61	8.96	0.38	
XU4	1	c	0	1.08	20.82	4.73	0.0000
YAMAGU	5	c	0	1.18	17.80	2.48	0.0000
*YONG	12	m	1	3.36	1.92	6.23	0.0000
*YONG	15	f	1	1.65	6.30	0.05	0.0000
Subtotal YONG				2.05	8.22	6.28	
*YUAN	1	m	2	1.87	11.44	1.14	0.0000
ZHANG	1	c	0	0.90	14.37	6.11	0.0006
ZHENG	15	m	0	1.29	20.36	1.41	0.0000
ZHENG	24	f	0	0.74	20.88	14.00	0.0008
Subtotal ZHENG				1.01	41.24	15.41	
ZHOU	2	m	0	0.86	17.50	8.49	0.0003
ZHOU	3	f	0	0.80	5.34	3.09	0.0660
Subtotal ZHOU				0.84	22.83	11.58	

	N	344
	NS	242
	Wt	19984.30
Het	Chi	6678.56
Het	df	343
Het	P	***
Fixed	RR	4.74
	RRl	4.68
	RRu	4.81
	P	+++
Random	RR	5.50
	RRl	5.11
	RRu	5.91
	P	+++
Asymm	P	**

Table 1C3 - 6

IESLC - Meta-anal of Ever Smoking (or Current if Ever not available), Cigs (or Any Prod if Cigs not avail)
All LC types
Least adjusted

	combined	Sex male	female	Total
N	44	182	118	344
NS	44	178	114	336
Wt	2412.85	10029.35	7542.10	19984.30
Het Chi	813.29	2354.29	2521.54	6678.56
Het df	43	181	117	343
Het P	***	***	***	***
Fixed RR	8.34	4.71	4.00	4.74
RRl	8.01	4.62	3.91	4.68
RRu	8.68	4.80	4.09	4.81
P	+++	+++	+++	+++
Random RR	5.72	6.34	4.36	5.50
RRl	4.55	5.75	3.82	5.11
RRu	7.21	7.00	4.98	5.91
P	+++	+++	+++	+++
Between Chi				989.43
Between df				2
Between P				***
Btwn(F) P				***
Btwn(R) P				***

Table 1C3 - 7

IESLC - Meta-anal of Ever Smoking (or Current if Ever not available), Cigs (or Any Prod if Cigs not avail)
All LC types
Excluded studies (and stage at which they were excluded)

1	BOUCHA BUELL LAURIL MZILEN
2	BERRIN CHEN RESTRE TAO ULMER
5	RIMING TANG2 WYNDE5 WYNDE8
6	BLOT1 BLOT2 BLOT3 BYERS2 HIRAOK HIRAY2 JONES LICKIN MOLLO MRFIT MURATA NOTANI PERSHA SAARIK SCHWA2 SHIMIZ SITAS VANDER WARSIN WATSON WYNDER
8	BROWN1 BYERS1 SUZUKI WU2
10	BENHAM GILLIS ISHIMA LUBIN OSANN2 PISANI PRESCO QIAO ROOTS SIMONA VEIERO VUTUC WYNDE7

Table 1C3 - 8
Potentially overlapping studies

REF	REFGP	PRINC	.	OVERLAP/LINK
LUBIN2	LUBIN2	1		Lubin-combined
CHAN	CHAN	1		Chan/LamWK2
LAMWK2	LAMWK2	1		Chan/LamWK2
LAMTH	LAMTH	1		KOO/LAMTH/LAMWK
KOO	KOO	1		KOO/LAMTH/LAMWK
LAMWK	LAMWK	1		KOO/LAMTH/LAMWK
CASCOR	CASCOR	1		Roots/Cascor 1
TVERDA	TVERDA	1		VEIERO/TVERDAL
AKIBA	AKIBA	1		AKIBA/ISHIMA
HEIN	PRESCO	1		Subset of PRESCO
LANGE	PRESCO	1		Subset of PRESCO
BROSS	BYERS1	1		GRAHAM/BROSS/BYERS1
GRAHAM	BYERS1	1		GRAHAM/BROSS/BYERS1
CHYOU	CHYOU	1		GOODMA/CHYOU
GOODMA	GOODMA	1		GOODMA/CHYOU
HOLE	TANG2	1		Subset of TANG2
BENSHL	TANG2	1		Subset of TANG2
WALD	TANG2	1		Subset of TANG2
KAISER	KAISER	1		KAISER/OSANN2
MRFITR	MRFIT	2		Subset of MRFIT
WYNDE6	WYNDE6	1		WYNDE5/6/7/8
CPSI	CPSI	1		CPSI overall
ENSTRO	ENSTRO	1		Subset of CPSI
SOBUE2	SOBUE2	1		SOBUE2/MATSUD
MATSUD	MATSUD	1		SOBUE2/MATSUD
BOFFET	BOFFET	1		BOFFET-combined
JAHN	BOFFET	2		Subset of BOFFET
XIANGZ	XIANGZ	1		LUBIN/XIANGZ/QIAO

Table 1C3 - 9
Most adjusted - insufficient data for metaanalysis

REF	NRR	SEX	AGEL	AGEH	RACE	YF	LC TYPE	LOC	START	ST	NLC	R	VB	P	H	AD	SM	PRODUCT	DENOM	De
DOLL2	43	f	0	0	all	11	all	Eu:UK	1951	pr	920	n	V	n	n	1	ev	all/unsp	nev any	st
RESTRE	2	c	0	0	all	-	all	SCAmer	1978	CC	102	n	bl	n	n	2	cu	cig+/-ot	nev cigs	ot
TAO	1	m	0	0	all	-	all	As:Chi	1988	CC	723	n	ot	n	n	5	ev	all/unsp	nev any	or
TAO	2	f	0	0	all	-	all	As:Chi	1988	CC	723	n	ot	n	n	5	ev	all/unsp	nev any	or
ULMER	1	c	0	0	all	-	all	Eu:Ger	1971	CC	726	n	bl	y	n	0	ev	all/unsp	nev any	or

REF	NRR	RR	SIG	RRDATA comment
DOLL2	43	4.33		0
RESTRE	2	10.28	y	0
TAO	1	3.95		0
TAO	2	4.93		0
ULMER	1	12.72		0

Least adjusted - insufficient data for meta-analysis: as for adjusted plus the following

REF	NRR	SEX	AGEL	AGEH	RACE	YF	LC TYPE	LOC	START	ST	NLC	R	VB	P	H	AD	SM	PRODUCT	DENOM	De
CORREA	59	m	0	0	wh	-	all	NAmer	1979	CC	1359	n	bl	y	n	0	ev	cig+/-ot	nev cigs	or
CORREA	61	m	0	0	bl	-	all	NAmer	1979	CC	1359	n	bl	y	n	0	ev	cig+/-ot	nev cigs	or
CORREA	60	f	0	0	wh	-	all	NAmer	1979	CC	1359	n	bl	y	n	0	ev	cig+/-ot	nev cigs	or
CORREA	62	f	0	0	bl	-	all	NAmer	1979	CC	1359	n	bl	y	n	0	ev	cig+/-ot	nev cigs	or
LIU	1	c	0	0	all	-	all	As:Chi	1980	CC	229	n	ot	*	n	0	ev	all/unsp	nev any	or

Table 1C3 - 9

IESLC - Meta-anal of Ever Smoking (or Current if Ever not available), Cigs (or Any Prod if Cigs not avail)
All LC types
Least adjusted - insufficient data for meta-analysis: as for adjusted plus the following

REF	NRR	RR	SIG	RRDATA comment
CORREA	59	17.20		0
CORREA	61	13.10		0
CORREA	60	8.60		0
CORREA	62	22.00		0
LIU	1	2.45		0

Table 1C4 -

IESLC - Meta-anal of Current Smoking (or Ever if Current not available), Cigs (or Any Prod if Cigs not avail)
All LC types

This analysis is restricted to results for:
1) Non-dose-response data
2) Results complete enough for use in metaanalysis

Within each study, results are then selected (in the following order of preference, within each sex) for:
3) SMKSTA: current smokers, ever smokers
4) PRODUCT: cigarettes regardless of other products, cigarettes only, all/unspec
5) CIGTYPE: all/unspecified, MC regardless of HR, MC only
6) DENOM: never smoked anything, never smoked cigarettes, (never +1 = +long term ex, +2 = +amount unknown, +3 = never cigs+long term ex)
7) Followup period (YF, prospective studies): whole study (coded as 0) or longest available
8) LCtype: all or nearest available, at least Squamous and Adeno. (q = squamous, s = small, l = large, a = adeno, mix = mixed, alv = alveolar)
9) Race: all or nearest available, otherwise by race (wh or w = white, bl or b = black, hi = hispanic ch = chinese, jap = japanese, haw = hawaiian, w+o = white + oriental, sca = scandinavian, as = asian)
10) For overlapping studies: principal rather than subsidiary studies
Finally by Age: whole study (coded as 0) if available, otherwise by widest available age group and then for single sex results (m, f) in preference to combined sex results (c).

Results adjusted (AD) for the most potential confounders are then chosen in Sections -1 to -3 (and those which actually differ from the adjusted results in Table 1C3 - 1 are marked 'x' in Section -1) and results adjusted for the least confounders in Sections -4 to -6. (Those least adjusted results which actually differ from the most adjusted as marked 'x' in column X in Section -4)
(Results adjusted for an unknown number of confounder(s) are coded as 20.)

Section -7 shows excluded studies, together with the stage (as above) at which no qualifying results were found.

Section -8 lists the potentially overlapping studies which have been included (1=principal, 2=subsidiary).

Section -9 lists any results which would have been included in preference except that they had data not complete enough for use in meta-analysis, with their significance (yes/no), if known, and any further comment as entered on the database.

In addition to those mentioned above, the following fields, levels and abbreviations are used:
* or nk = not known, n = no, y = yes, ot = other
ev = ever, cu = current, nev = never
all/unspec = all or unspecified, cig+/-ot = cigarettes irrespective of other products (cigar, pipe etc)
MC = manufactured cigarettes, HR = hand-rolled cigarettes
REF: 6-character study reference
NRR: number of the RR on the database within the study
ST : study type (CC = case control, pr or prosp = prospective)
NLC: number of lung cancer cases in whole study
R : risky occupational population (n = no, m = mining, o = other risky)
VB : national cigarette type (V = at least 75% Virginia, bl = at least 75% blended, ot = other)
P : any proxy use
H : full histological confirmation
De : derivation of RR/CI (or = original, st = standard method, ot = other method of estimation)

Table 1C4 - 1

IESLC - Meta-anal of Current Smoking (or Ever if Current not available), Cigs (or Any Prod if Cigs not avail)
All LC types
Most adjusted

REF	NRR	1C3	SEX	AGEL	AGEH	RACE	YF	LC TYPE	LOC	START	ST	NLC	R	VB	P	H	AD	SM	PRODUCT	DENOM	De
ABELIN	45		m	0	0	all	-	all	Eu:wst	1941	CC	118	n	bl	y	n	1	ev	cig+/-ot	nev any	st
ABRAHA	7		m	0	0	all	0	q+s+a	Eu:est	1975	pr	571	n	bl	n	n	0	ev	all/unsp	nev any	ot
ABRAHA	8		f	0	0	all	0	q+s+a	Eu:est	1975	pr	571	n	bl	n	n	0	ev	all/unsp	nev any	ot
AGUDO	3	x	f	0	0	all	-	all	Eu:wst	1989	CC	103	n	bl	n	n	3	cu	cig only	nev any	or
AKIBA	10	x	m	0	0	all	0	all	As:Jap	1963	pr	610	n	bl	n	n	5	cu	cig+/-ot	nev cigs	ot
AKIBA	14	x	f	0	0	all	0	all	As:Jap	1963	pr	610	n	bl	n	n	5	cu	cig+/-ot	nev cigs	or
ALDERS	177	x	m	0	0	all	-	all	Eu:UK	1977	CC	1448	n	V	n	n	0	cu	cig+/-ot	nev any	st
ALDERS	176	x	f	0	0	all	-	all	Eu:UK	1977	CC	1448	n	V	n	n	0	cu	cig only	nev any	st
AMANDU	5	x	m	0	0	wh	0	all	NAmer	1959	pr	132	m	bl	n	n	2	cu	cig+/-ot	nev cigs	ot
AMES	1	x	m	0	0	wh	-	all	NAmer	1959	ot	317	m	bl	n	n	0	cu	all/unsp	nev any	or
ANDERS	6	x	f	0	0	all	0	all	NAmer	1986	pr	343	n	bl	n	n	1	cu	cig+/-ot	nev cigs	or
ARCHER	5	x	m	0	0	wh	0	all	NAmer	1950	pr	146	m	bl	n	n	0	cu	cig+/-ot	nev cigs	st
ARMADA	27	x	m	0	0	all	-	all	Eu:wst	1986	CC	325	n	bl	n	y	0	cu	cig+/-ot	nev any	st
AUSTIN	6	x	c	0	0	all	-	all	NAmer	1970	CC	166	o	bl	y	n	3	cu	cig+/-ot	nev cigs	or
AUVINE	19		c	0	0	all	-	all	Eu:Sca	1986	CC	517	n	bl	y	n	2	ev	cig+/-ot	nev cigs	ot
AXELSO	1		c	0	0	all	-	all	Eu:Sca	1960	CC	152	n	bl	y	n	0	ev	all/unsp	nev any	st
AXELSS	2	x	m	0	0	sca	-	all	Eu:Sca	1989	CC	436	n	bl	n	n	0	cu	all/unsp	nev any	st
AXELSS	10	x	f	0	0	sca	-	all	Eu:Sca	1989	CC	436	n	bl	n	n	0	cu	all/unsp	nev any	st
BAND	1		m	0	0	all	-	all	NAmer	1983	CC	2831	n	V	y	y	2	ev	cig only	nev any	ot
BARBON	4	x	m	0	0	all	-	all	Eu:wst	1979	CC	755	n	bl	y	y	1	cu	all/unsp	nev any	or
BECHER	13	x	m	0	0	all	-	all	Eu:Ger	1985	CC	194	n	bl	n	y	0	cu	all/unsp	nev any	st
BECHER	14	x	f	0	0	all	-	all	Eu:Ger	1985	CC	194	n	bl	n	y	0	cu	all/unsp	nev any	st
BENSHL	4	x	m	0	0	all	0	all	Eu:UK	1967	pr	486	n	V	n	n	1	cu	cig+/-ot	nev any	ot
BEST	2	x	m	0	0	all	0	all	NAmer	1955	pr	381	n	V	n	n	1	cu	cig only	nev any	ot
BEST	18		f	0	0	all	0	all	NAmer	1955	pr	381	n	V	n	n	1	ev	cig only	nev any	ot
BLOHMK	1	x	m	0	0	all	-	all	Eu:Ger	1978	CC	888	n	bl	n	y	0	cu	all/unsp	nev any	st
BLOT4	1		m	0	0	wh	-	all	NAmer	1974	CC	335	n	bl	y	n	0	ev	cig+/-ot	nev cigs	st
BOFFET	27		m	0	0	all	-	all	Eu:mul	1988	CC	5621	n	bl	y	n	2	ev	cig+/-ot	nev any	ot
BOUCOT	114	x	m	0	0	all	0	all	NAmer	1951	pr	121	n	bl	n	n	2	cu	cig only	nev any	ot
BRESLO	17		m	0	0	all	-	all	NAmer	1949	CC	518	n	bl	n	y	0	ev	cig+/-ot	nev+1	st
BRESLO	23		f	0	0	all	-	all	NAmer	1949	CC	518	n	bl	n	y	0	ev	cig+/-ot	nev+1	st
BRETT	4	x	m	0	0	all	0	all	Eu:UK	1960	pr	150	n	V	n	n	0	cu	cig+/-ot	nev cigs	st
BROCKM	1		m	0	0	wh	-	all	Eu:Ger	1990	CC	117	n	bl	n	y	0	ev	cig+/-ot	nev cigs	st
BROCKM	2		f	0	0	wh	-	all	Eu:Ger	1990	CC	117	n	bl	n	y	0	ev	cig+/-ot	nev cigs	st
BROSS	4	x	m	0	0	wh	-	all	NAmer	1960	CC	974	n	bl	n	n	0	cu	cig+/-ot	nev any	st
BROWN2	12	x	m	0	0	wh	-	all	NAmer	1984	CC	14596	n	bl	n	y	2	cu	cig+/-ot	nev cigs	or
BROWN2	11	x	f	0	0	wh	-	all	NAmer	1984	CC	14596	n	bl	n	y	2	cu	cig+/-ot	nev cigs	or
BUFFLE	3	x	m	0	0	wh	-	all	NAmer	1976	CC	943	n	bl	y	n	0	cu	cig+/-ot	nev any	st
BUFFLE	7	x	f	0	0	wh	-	all	NAmer	1976	CC	943	n	bl	y	n	0	cu	cig+/-ot	nev any	st
CARPEN	11	x	c	0	0	w+b	-	all	NAmer	1991	CC	356	n	bl	n	n	3	cu	cig+/-ot	nev cigs	or
CASCO2	1		c	0	0	wh	-	all	Eu:Ger	1991	CC	155	n	bl	n	n	0	ev	all/unsp	nev any	st
CASCOR	1		c	0	0	wh	-	all	Eu:Ger	1985	CC	389	n	bl	n	y	0	ev	all/unsp	nev any	st
CEDERL	26	x	m	0	0	all	10	all	Eu:Sca	1963	pr	491	n	bl	n	n	1	cu	cig+/-ot	nev any	ot
CEDERL	119	x	f	0	0	all	10	all	Eu:Sca	1963	pr	491	n	bl	n	n	1	cu	cig+/-ot	nev any	ot
CHAN	5		m	0	0	all	-	all	As:HK	1976	CC	397	n	bl	n	n	0	ev	cig+/-ot	nev any	st
CHAN	6		f	0	0	all	-	all	As:HK	1976	CC	397	n	bl	n	n	0	ev	cig+/-ot	nev any	st
CHANG	5	x	m	0	0	all	0	all	NAmer	1972	pr	136	n	bl	n	n	0	cu	cig+/-ot	nev cigs	st
CHANG	11	x	f	0	0	all	0	all	NAmer	1972	pr	136	n	bl	n	n	0	cu	cig+/-ot	nev cigs	st
CHATZI	4		c	0	0	all	-	all	Eu:bal	1987	CC	282	n	bl	n	y	0	ev	all/unsp	nev any	st
CHEN2	1		m	0	0	all	-	all	As:Chi	1983	CC	193	n	ot	y	n	0	ev	all/unsp	nev any	st
CHEN2	2		f	0	0	all	-	all	As:Chi	1983	CC	193	n	ot	y	n	0	ev	all/unsp	nev any	st
CHEN3	1		c	0	0	all	-	all	As:Chi	1981	CC	254	n	ot	y	n	0	ev	all/unsp	nev any	st
CHIAZZ	3		m	0	0	all	-	all	NAmer	1940	CC	144	o	bl	y	n	11	ev	cig+/-ot	nev cigs	or
CHOI	3	x	m	0	0	all	-	all	As:oth	1985	CC	375	n	bl	n	n	0	cu	cig+/-ot	nev cigs	st
CHOI	7	x	f	0	0	all	-	all	As:oth	1985	CC	375	n	bl	n	n	0	cu	cig+/-ot	nev cigs	st
CHOW	56	x	m	0	0	wh	0	all	NAmer	1966	pr	219	n	bl	n	n	2	cu	cig+/-ot	nev any	ot
CHYOU	2	x	m	0	0	jap	0	all	NAmer	1965	pr	227	n	bl	n	y	1	cu	cig+/-ot	nev cigs	or
COMSTO	3	x	m	0	0	all	-	all	NAmer	1975	ot	258	n	bl	n	n	0	cu	cig+/-ot	nev any	st
COMSTO	8	x	f	0	0	all	-	all	NAmer	1975	ot	258	n	bl	n	n	0	cu	cig+/-ot	nev any	st
COOKSO	4		c	0	0	bl	-	all	Africa	1961	CC	234	n	V	n	y	0	ev	cig+/-ot	nev any	st
CORREA	42	x	c	0	0	all	-	all	NAmer	1979	CC	1359	n	bl	y	n	1	cu	cig+/-ot	nev cigs	or
CPSI	220	x	m	35	84	all	6	all	NAmer	1959	pr	5138	n	bl	n	n	1	cu	cig+/-ot	nev any	ot
CPSI	279	x	f	40	74	all	6	all	NAmer	1959	pr	5138	n	bl	n	n	1	cu	cig+/-ot	nev cigs	ot
CPSII	126	x	m	0	0	all	6	all	NAmer	1982	pr	3229	n	bl	n	n	1	cu	cig only	nev any	ot
CPSII	133	x	f	0	0	all	6	all	NAmer	1982	pr	3229	n	bl	n	n	1	cu	cig+/-ot	nev cigs	ot
DAMBER	16	x	m	0	0	all	-	all	Eu:Sca	1972	CC	579	n	bl	y	n	1	cu	cig only	nev any	ot
DARBY	4	x	m	0	0	wh	-	all	Eu:UK	1988	CC	982	n	V	n	n	0	cu	cig+/-ot	nev any	st
DARBY	11	x	f	0	0	wh	-	all	Eu:UK	1988	CC	982	n	V	n	n	0	cu	cig+/-ot	nev any	st
DAVEYS	5		m	0	0	all	-	all	Eu:Ger	1930	CC	109	n	bl	y	n	0	ev	all/unsp	nev any	st
DAVEYS	6		f	0	0	all	-	all	Eu:Ger	1930	CC	109	n	bl	y	n	0	ev	all/unsp	nev any	ot
DEAN	8		m	0	0	wh	-	all	Africa	1947	CC	603	n	V	y	n	0	ev	cig+/-ot	nev any	st
DEAN2	2	x	m	0	0	all	-	all	Eu:UK	1960	CC	954	n	V	y	n	0	cu	all/unsp	nev any	st

Table 1C4 - 1

IESLC - Meta-anal of Current Smoking (or Ever if Current not available), Cigs (or Any Prod if Cigs not avail)
All LC types
Most adjusted

REF	NRR	1C3	SEX	AGEL	AGEH	RACE	YF	LC TYPE	LOC	START	ST	NLC	R	VB	P	H	AD	SM	PRODUCT	DENOM	De
DEAN2	6	x	f	0	0	all	-	all	Eu:UK	1960	CC	954	n	V	y	n	0	cu	all/unsp	nev any	st
DEAN3	239	x	m	0	0	all	-	all	Eu:UK	1969	CC	766	n	V	y	n	1	cu	cig+/-ot	nev any	ot
DEAN3	119	x	f	0	0	all	-	all	Eu:UK	1969	CC	766	n	V	y	n	3	cu	cig only	nev any	ot
DEKLER	7	x	m	0	0	all	0	all	Auslia	1961	pr	138	m	V	n	n	2	cu	cig+/-ot	nev any	ot
DESTE2	4	x	c	0	0	all	-	all	SCAmer	1993	CC	463	n	bl	n	n	7	cu	all/unsp	nev any	or
DESTEF	41	x	m	0	0	all	-	all	SCAmer	1988	CC	497	n	bl	n	y	4	cu	all/unsp	nev any	or
DOCKER	1	x	c	0	0	wh	0	all	NAmer	1974	pr	120	n	bl	n	n	4	cu	cig+/-ot	nev cigs	or
DOLL	90	x	m	0	0	all	-	all	Eu:UK	1948	CC	1465	n	V	n	n	0	cu	all/unsp	nev any	st
DOLL	93	x	f	0	0	all	-	all	Eu:UK	1948	CC	1465	n	V	n	n	0	cu	all/unsp	nev any	st
DOLL2	68	x	m	0	0	all	20	all	Eu:UK	1951	pr	920	n	V	n	n	1	cu	cig+/-ot	nev any	ot
DOLL2	63		f	0	0	all	22	all	Eu:UK	1951	pr	920	n	V	n	n	1	cu	cig only	nev any	ot
DORANT	9	x	c	0	0	all	0	all	Eu:wst	1986	ot	550	n	bl	n	y	0	cu	cig+/-ot	nev any	st
DORGAN	9	x	m	0	0	wh	-	all	NAmer	1980	CC	2026	n	bl	y	y	0	cu	cig+/-ot	nev any	st
DORGAN	33	x	m	0	0	bl	-	all	NAmer	1980	CC	2026	n	bl	y	y	0	cu	cig+/-ot	nev any	st
DORGAN	56	x	f	0	0	wh	-	all	NAmer	1980	CC	2026	n	bl	y	y	0	cu	cig+/-ot	nev any	st
DORGAN	79	x	f	0	0	bl	-	all	NAmer	1980	CC	2026	n	bl	y	y	0	cu	cig+/-ot	nev any	st
DORN	391	x	m	0	0	wh	25	all	NAmer	1954	pr	5097	n	bl	n	n	1	cu	cig+/-ot	nev any	or
DOSEME	1		m	0	0	all	-	all	Eu:bal	1979	CC	1210	n	bl	n	n	2	ev	cig+/-ot	nev cigs	or
DROSTE	6	x	m	0	0	all	-	all	Eu:wst	1995	CC	478	n	bl	n	y	4	cu	all/unsp	nev any	or
DU	1		m	0	0	all	-	all	As:Chi	1985	CC	849	n	ot	y	n	0	ev	all/unsp	nev any	or
DU	2		f	0	0	all	-	all	As:Chi	1985	CC	849	n	ot	y	n	0	ev	all/unsp	nev any	or
DUNN	6		m	0	0	all	0	all	NAmer	1954	pr	139	o	bl	n	n	0	ev	cig+/-ot	nev cigs	st
EBELIN	1		m	0	0	all	-	all	Eu:Ger	1980	CC	130	n	bl	n	n	0	ev	all/unsp	nev any	st
ENGELA	168	x	m	0	0	all	12	all	Eu:Sca	1964	pr	435	n	bl	n	n	1	cu	cig+/-ot	nev any	ot
ENGELA	177	x	f	0	0	all	12	all	Eu:Sca	1964	pr	435	n	bl	n	n	1	cu	cig+/-ot	nev any	ot
ENSTRO	1		m	0	0	all	0	all	NAmer	1959	pr	2879	n	bl	n	n	1	cu	cig only	nev any	or
ENSTRO	2		f	0	0	all	0	all	NAmer	1959	pr	2879	n	bl	n	n	1	cu	cig only	nev any	or
ESAKI	4		m	0	0	all	-	all	As:Jap	1961	CC	245	n	bl	y	n	0	ev	cig+/-ot	nev cigs	st
ESAKI	5		f	0	0	all	-	all	As:Jap	1961	CC	245	n	bl	y	n	0	ev	cig+/-ot	nev cigs	st
FAN	1		m	0	0	all	-	all	As:Chi	1990	CC	403	n	ot	y	n	0	ev	cig+/-ot	nev cigs	st
FAN	2		f	0	0	all	-	all	As:Chi	1990	CC	403	n	ot	y	n	0	ev	cig+/-ot	nev cigs	st
GAO	33	x	m	0	0	all	-	all	As:Chi	1984	CC	1405	n	ot	n	n	2	cu	cig+/-ot	nev cigs	or
GAO	34	x	f	0	0	all	-	all	As:Chi	1984	CC	1405	n	ot	n	n	2	cu	cig+/-ot	nev cigs	or
GAO2	8	x	m	0	0	all	-	all	As:Jap	1988	CC	282	n	bl	n	n	1	cu	cig+/-ot	nev cigs	or
GARCIA	2	x	c	0	0	all	-	all	NAmer	1992	CC	416	n	bl	n	y	0	cu	cig+/-ot	nev cigs	st
GARDIN	6	x	c	0	0	all	-	all	Eu:UK	1988	CC	143	n	V	y	n	0	cu	cig only	nev any	st
GARSHI	31	x	m	0	0	all	-	all	NAmer	1981	CC	1081	o	bl	y	n	1	cu	all/unsp	nev any	st
GENG	1		m	0	0	all	-	all	As:Chi	1985	CC	292	n	ot	*	n	0	ev	cig+/-ot	nev any	st
GENG	2		f	0	0	all	-	all	As:Chi	1985	CC	292	n	ot	*	n	0	ev	cig+/-ot	nev any	st
GER	21		c	0	0	all	-	all	As:oth	1990	CC	141	n	ot	y	n	14	ev	all/unsp	nev any	ot
GODLEY	5		m	0	0	all	-	all	NAmer	1966	CC	1986	n	bl	y	n	1	ev	cig+/-ot	nev cigs	ot
GODLEY	6		f	0	0	all	-	all	NAmer	1966	CC	1986	n	bl	y	n	1	ev	cig+/-ot	nev cigs	ot
GOLLED	7		m	35	99	all	-	all	Eu:UK	1952	CC	443	n	V	y	n	1	ev	cig+/-ot	nev any	ot
GOODMA	2	x	m	0	0	w+o	-	all	NAmer	1983	CC	326	n	bl	y	y	0	cu	cig+/-ot	nev any	st
GOODMA	6	x	f	0	0	w+o	-	all	NAmer	1983	CC	326	n	bl	y	y	0	cu	cig+/-ot	nev any	st
GRAHAM	10	x	m	0	0	wh	-	all	NAmer	1956	CC	685	n	bl	n	n	1	cu	cig+/-ot	nev any	ot
GREGOR	2	x	m	0	0	all	-	all	Eu:UK	1976	CC	104	n	V	n	y	0	cu	cig+/-ot	nev cigs	st
GREGOR	6	x	f	0	0	all	-	all	Eu:UK	1976	CC	104	n	V	n	y	0	cu	cig+/-ot	nev cigs	st
GSELL	6		m	0	0	all	-	all	Eu:wst	1937	CC	150	n	bl	n	y	0	ev	cig+/-ot	nev any	st
HAENSZ	54	x	f	0	0	all	-	not alv	NAmer	1955	CC	158	n	bl	n	y	0	cu	cig+/-ot	nev any	st
HAMMO2	8	x	m	0	0	all	0	all	NAmer	1967	pr	450	o	bl	n	n	1	cu	cig+/-ot	nev any	ot
HAMMON	139	x	m	0	0	wh	0	all	NAmer	1952	pr	448	n	bl	n	n	1	cu	cig only	nev any	ot
HANSEN	3		m	0	0	all	0	all	Eu:Sca	1968	pr	105	o	bl	y	n	2	ev	all/unsp	nev any	ot
HEGMAN	1		c	0	0	all	-	all	NAmer	1989	CC	282	n	bl	y	y	0	ev	all/unsp	nev any	st
HEIN	1	x	m	0	0	all	0	all	Eu:Sca	1970	pr	144	n	bl	n	n	0	cu	cig only	nev any	st
HENNEK	2	x	m	0	0	all	0	all	NAmer	1982	pr	169	n	bl	n	n	0	cu	all/unsp	nev any	st
HINDS	22		f	0	0	o	-	all	NAmer	1968	CC	292	n	bl	n	n	3	ev	all/unsp	nev any	st
HIRAYA	1	x	m	0	0	all	0	all	As:Jap	1965	pr	1917	n	bl	n	n	1	cu	cig+/-ot	nev any	st
HIRAYA	3	x	f	0	0	all	0	all	As:Jap	1965	pr	1917	n	bl	n	n	1	cu	cig+/-ot	nev any	st
HITOSU	34	x	m	0	0	all	-	all	As:Jap	1960	CC	216	n	bl	y	n	1	cu	all/unsp	nev any	st
HITOSU	59	x	f	0	0	all	-	all	As:Jap	1960	CC	216	n	bl	y	n	1	cu	all/unsp	nev any	st
HOLE	32	x	m	0	0	all	0	all	Eu:UK	1972	pr	225	n	V	n	n	1	cu	cig+/-ot	nev any	ot
HOLE	31		f	0	0	all	11	all	Eu:UK	1972	pr	225	n	V	n	n	1	cu	all/unsp	nev any	ot
HOROWI	1		m	0	0	all	-	all	NAmer	1956	CC	236	n	V	n	n	0	ev	cig+/-ot	nev any	st
HOROWI	2		f	0	0	all	-	all	NAmer	1956	CC	236	n	V	n	n	0	ev	cig+/-ot	nev any	st
HORWIT	1		f	0	0	all	-	all	NAmer	1977	CC	112	n	bl	n	n	0	ev	cig+/-ot	nev cigs	st
HU	15		m	0	0	all	-	all	As:Chi	1985	CC	227	n	ot	n	y	0	ev	cig+/-ot	nev any	st
HU	16		f	0	0	all	-	all	As:Chi	1985	CC	227	n	ot	n	y	0	ev	cig+/-ot	nev any	st
HU2	9		m	0	0	all	-	all	As:Chi	1977	CC	523	n	ot	y	n	0	ev	cig+/-ot	nev cigs	st
HU2	10		f	0	0	all	-	all	As:Chi	1977	CC	523	n	ot	y	n	0	ev	cig+/-ot	nev cigs	st
HUANG	1		c	0	0	all	-	all	As:Chi	1990	CC	135	n	ot	y	n	0	ev	all/unsp	nev any	st
HUMBLE	13	x	m	0	0	w-hi	-	all	NAmer	1980	CC	521	n	bl	y	n	1	cu	cig+/-ot	nev cigs	ot

Table 1C4 - 1

IESLC - Meta-anal of Current Smoking (or Ever if Current not available), Cigs (or Any Prod if Cigs not avail)
All LC types
Most adjusted

REF	NRR	1C3	SEX	AGEL	AGEH	RACE	YF	LC TYPE	LOC	START	ST	NLC	R	VB	P	H	AD	SM	PRODUCT	DENOM	De
HUMBLE	15	x	m	0	0	hi	-	all	NAmer	1980	CC	521	n	bl	y	n	1	cu	cig+/-ot	nev cigs	ot
HUMBLE	17	x	f	0	0	w-hi	-	all	NAmer	1980	CC	521	n	bl	y	n	1	cu	cig+/-ot	nev cigs	ot
HUMBLE	19	x	f	0	0	hi	-	all	NAmer	1980	CC	521	n	bl	y	n	1	cu	cig+/-ot	nev cigs	ot
JAHN	22		f	0	0	all	-	all	Eu:Ger	1988	CC	1004	n	bl	n	n	2	ev	cig+/-ot	nev any	ot
JAIN	52	x	m	0	0	all	-	all	NAmer	1981	CC	845	n	V	y	n	2	cu	cig+/-ot	nev cigs	or
JAIN	51	x	f	0	0	all	-	all	NAmer	1981	CC	845	n	V	y	n	2	cu	cig+/-ot	nev cigs	or
JARUP	6		m	0	0	all	-	all	Eu:Sca	1928	CC	102	o	bl	y	n	2	ev	all/unsp	nev any	ot
JARVHO	2	x	m	0	0	all	-	all	Eu:Sca	1983	CC	147	n	bl	n	n	0	cu	all/unsp	nev any	st
JARVHO	6	x	f	0	0	all	-	all	Eu:Sca	1983	CC	147	n	bl	n	n	0	cu	all/unsp	nev any	st
JEDRYC	58		m	0	0	all	-	all	Eu:est	1980	CC	1630	n	bl	y	n	4	ev	cig+/-ot	nev any	ot
JEDRYC	59		f	0	0	all	-	all	Eu:est	1980	CC	1630	n	bl	y	n	4	ev	cig+/-ot	nev any	ot
JIANG	1		m	0	0	all	-	all	As:Chi	1984	CC	125	n	ot	n	n	0	ev	all/unsp	nev any	st
JIANG	2		f	0	0	all	-	all	As:Chi	1984	CC	125	n	ot	n	n	0	ev	all/unsp	nev any	st
JOLY	16	x	m	0	0	all	-	all	SCAmer	1978	CC	826	n	bl	n	n	0	cu	cig+/-ot	nev any	st
JOLY	15	x	f	0	0	all	-	all	SCAmer	1978	CC	826	n	bl	n	n	0	cu	cig+/-ot	nev any	st
JUSSAW	31		m	0	0	all	-	all	As:Ind	1964	CC	792	n	V	n	n	2	ev	cig only	nev any	st
KAISE2	68	x	m	35	99	all	9	all	NAmer	1979	pr	318	n	bl	n	n	1	cu	cig only	nev any	st
KAISE2	60	x	f	35	99	all	9	all	NAmer	1979	pr	318	n	bl	n	n	1	cu	cig only	nev any	st
KAISER	12	x	m	0	0	all	0	all	NAmer	1964	pr	714	n	bl	n	n	2	cu	cig+/-ot	nev cigs	ot
KAISER	9	x	f	0	0	all	0	all	NAmer	1964	pr	714	n	bl	n	n	2	cu	cig+/-ot	nev cigs	ot
KANELL	30		m	0	0	all	-	all	Eu:bal	1950	CC	862	n	bl	n	n	1	cu	all/unsp	nev any	st
KATSOU	2	x	f	0	0	all	-	all	Eu:bal	1987	CC	101	n	bl	n	n	1	cu	all/unsp	nev any	or
KAUFMA	16	x	c	0	0	all	-	all	NAmer	1981	CC	881	n	bl	n	n	6	cu	cig+/-ot	nev cigs	ot
KELLER	1	x	m	0	0	wh	-	all	NAmer	1985	CC	15038	n	bl	n	n	0	cu	all/unsp	nev any	st
KELLER	9	x	m	0	0	nonw	-	all	NAmer	1985	CC	15038	n	bl	n	n	0	cu	all/unsp	nev any	st
KELLER	5	x	f	0	0	wh	-	all	NAmer	1985	CC	15038	n	bl	n	n	0	cu	all/unsp	nev any	st
KELLER	13	x	f	0	0	nonw	-	all	NAmer	1985	CC	15038	n	bl	n	n	0	cu	all/unsp	nev any	st
KHUDER	19	x	m	0	0	all	-	all	NAmer	1985	CC	482	n	bl	n	y	0	cu	cig+/-ot	nev cigs	or
KIHARA	7	x	c	0	0	jap	-	all	As:Jap	1991	CC	440	n	bl	n	n	0	cu	all/unsp	nev any	st
KINLEN	19	x	m	0	0	all	0	all	Eu:UK	1967	pr	718	n	V	n	n	2	cu	cig+/-ot	nev any	ot
KJUUS	1	x	m	0	0	all	-	all	Eu:Sca	1979	CC	176	n	bl	n	n	0	cu	all/unsp	nev any	st
KNEKT	85	x	m	20	69	all	21	all	Eu:Sca	1966	pr	515	n	bl	n	n	1	cu	cig+/-ot	nev any	ot
KO	1		f	0	0	all	-	all	As:oth	1992	CC	117	n	ot	n	y	3	ev	cig+/-ot	nev cigs	or
KOHLME	2		c	0	0	all	-	all	Eu:Ger	1990	CC	239	n	bl	n	n	4	ev	all/unsp	nev any	or
KOO	9	x	f	0	0	all	-	all	As:HK	1981	CC	200	n	bl	n	n	0	cu	all/unsp	nev any	st
KOULUM	2		m	0	0	all	-	all	Eu:Sca	1936	CC	812	n	bl	n	n	0	ev	cig only	nev any	st
KREUZE	24	x	f	1	45	all	-	all	Eu:Ger	1990	CC	2260	n	bl	n	n	3	cu	cig+/-ot	nev any	or
KREUZE	35	x	f	55	69	all	-	all	Eu:Ger	1990	CC	2260	n	bl	n	n	3	cu	cig+/-ot	nev any	or
KREYBE	12		m	0	0	all	-	all	Eu:Sca	1948	CC	300	n	bl	n	y	1	ev	all/unsp	nev any	ot
KREYBE	30		f	0	0	all	-	all	Eu:Sca	1948	CC	300	n	bl	n	y	1	ev	all/unsp	nev any	ot
KUBIK	12	x	m	0	0	all	0	all	Eu:est	1965	pr	108	n	bl	n	n	0	cu	cig+/-ot	nev any	st
LAMTH	6		f	0	0	ch	-	all	As:HK	1983	CC	445	n	bl	n	n	0	ev	all/unsp	nev any	or
LAMWK	1		f	0	0	ch	-	all	As:HK	1981	CC	163	n	bl	n	n	0	ev	all/unsp	nev any	st
LAMWK2	9		m	0	0	all	-	q+s+l+a	As:HK	1976	CC	480	n	bl	n	n	0	ev	all/unsp	nev any	st
LAMWK2	10		f	0	0	all	-	q+s+l+a	As:HK	1976	CC	480	n	bl	n	n	0	ev	all/unsp	nev any	st
LANGE	38	x	m	0	0	all	0	all	Eu:Sca	1976	pr	268	n	bl	n	n	1	cu	all/unsp	nev any	ot
LANGE	35	x	f	0	0	all	0	all	Eu:Sca	1976	pr	268	n	bl	n	n	1	cu	all/unsp	nev any	ot
LAUSSM	11		m	0	0	all	-	all	Eu:Ger	1982	CC	432	n	bl	n	n	3	ev	all/unsp	nev any	or
LEI	1		m	0	0	all	-	all	As:Chi	1986	CC	792	n	ot	y	n	0	ev	all/unsp	nev any	st
LEI	2		f	0	0	all	-	all	As:Chi	1986	CC	792	n	ot	y	n	0	ev	all/unsp	nev any	st
LEMARC	2	x	c	0	0	w+o	-	all	NAmer	1992	CC	341	n	bl	n	y	0	cu	all/unsp	nev any	st
LETOUR	1		c	0	0	all	-	all	NAmer	1983	CC	738	n	V	y	y	0	ev	cig+/-ot	nev cigs	st
LEVIN	30		m	0	0	all	-	all	NAmer	1938	CC	475	n	bl	n	n	1	ev	cig+/-ot	nev any	st
LIAW	1		m	0	0	all	0	all	As:oth	1982	pr	127	n	ot	n	n	1	cu	all/unsp	nev any	or
LIAW	2		f	0	0	all	0	all	As:oth	1982	pr	127	n	ot	n	n	1	cu	all/unsp	nev any	or
LIDDEL	4	x	m	0	0	all	18	all	NAmer	1970	pr	304	m	V	n	n	1	cu	cig+/-ot	nev cigs	ot
LIU	2		c	0	0	all	-	all	As:Chi	1980	CC	229	n	ot	*	n	2	ev	all/unsp	nev any	or
LIU2	2		m	0	0	all	-	all	As:Chi	1983	CC	316	n	ot	n	n	3	ev	all/unsp	nev any	ot
LIU2	4		f	0	0	all	-	all	As:Chi	1983	CC	316	n	ot	n	n	3	ev	all/unsp	nev any	ot
LIU3	2		m	0	0	all	-	all	As:Chi	1985	CC	110	n	ot	n	n	2	ev	all/unsp	nev any	or
LIU4	10		m	35	69	all	-	all	As:Chi	1986	CC	1000-00	n	ot	y	n	2	ev	cig only	nev any	ot
LIU4	12		f	0	0	all	-	all	As:Chi	1986	CC	1000-00	n	ot	y	n	2	ev	all/unsp	nev any	ot
LIU5	1		c	0	0	all	-	all	As:Chi	1978	CC	111	n	ot	y	n	0	ev	all/unsp	nev any	st
LOMBA2	1		f	0	0	all	-	all	NAmer	1960	CC	225	n	bl	n	n	0	ev	cig+/-ot	nev cigs	st
LOMBAR	9	x	m	0	0	all	-	all	NAmer	1951	CC	1040	n	bl	n	n	0	cu	cig+/-ot	nev any	st
LUBIN2	28	x	m	0	0	all	-	all	Eu:mul	1976	CC	7804	n	bl	n	y	2	cu	cig+/-ot	nev any	ot
LUBIN2	317	x	f	0	0	all	-	all	Eu:mul	1976	CC	7804	n	bl	n	y	0	cu	cig+/-ot	nev any	st
LUO	7		c	0	0	all	-	all	As:Chi	1990	CC	102	n	ot	n	y	20	ev	cig+/-ot	nev cigs	or
MACLEN	19	x	m	0	0	ch	-	all	As:oth	1972	CC	233	n	bl	n	n	0	cu	cig+/-ot	nev cigs	st
MACLEN	32	x	f	0	0	ch	-	all	As:oth	1972	CC	233	n	bl	n	n	0	cu	cig+/-ot	nev cigs	st

Table 1C4 - 1

IESLC - Meta-anal of Current Smoking (or Ever if Current not available), Cigs (or Any Prod if Cigs not avail)
All LC types
Most adjusted

REF	NRR	1C3	SEX	AGEL	AGEH	RACE	YF	LC TYPE	LOC	START	ST	NLC	R	VB	P	H	AD	SM	PRODUCT	DENOM	De
MAGNUS	5		m	0	0	all	0	all	Eu:Sca	1953	pr	203	o	bl	y	n	3	ev	all/unsp	nev any	ot
MARSH	1		m	0	0	all	-	all	NAmer	1979	CC	150	n	bl	y	n	0	ev	cig+/-ot	nev any	st
MARSH	3		f	0	0	all	-	all	NAmer	1979	CC	150	n	bl	y	n	0	ev	cig+/-ot	nev any	st
MARSH2	5		m	0	0	all	-	all	NAmer	1979	CC	114	n	bl	y	n	1	ev	all/unsp	nev any	or
MARSH2	6		f	0	0	all	-	all	NAmer	1979	CC	114	n	bl	y	n	1	ev	all/unsp	nev any	ot
MARTIS	4		m	0	0	all	-	all	Eu:UK	1972	CC	201	n	V	n	n	0	ev	cig+/-ot	nev cigs	st
MASTRA	2		m	0	0	all	-	all	Eu:wst	1973	CC	309	n	bl	n	n	2	ev	all/unsp	nev any	st
MATOS	3	x	m	0	0	all	-	all	SCAmer	1994	CC	200	n	bl	n	n	2	cu	cig+/-ot	nev any	or
MATSUD	10		m	0	0	all	-	all	As:Jap	1965	CC	179	n	bl	n	n	0	ev	cig+/-ot	nev cigs	st
MCCONN	1		m	0	0	all	-	all	Eu:UK	1946	CC	100	n	V	n	y	0	ev	all/unsp	nev any	st
MCCONN	2		f	0	0	all	-	all	Eu:UK	1946	CC	100	n	V	n	y	0	ev	all/unsp	nev any	st
MCDUFF	1		m	0	0	all	-	all	NAmer	1979	CC	165	n	V	y	n	0	ev	cig+/-ot	nev cigs	st
MCLAUG	1		m	0	0	all	-	all	As:Chi	1972	CC	316	o	ot	y	n	0	ev	all/unsp	nev any	st
MIGRAN	12	x	m	0	0	all	0	all	Eu:UK	1964	pr	259	n	V	n	n	2	cu	cig+/-ot	nev any	ot
MIGRAN	38	x	f	0	0	all	0	all	Eu:UK	1964	pr	259	n	V	n	n	2	cu	cig+/-ot	nev any	ot
MILLER	2		f	0	0	all	-	all	NAmer	1972	CC	168	n	bl	y	n	1	ev	cig+/-ot	nev any	ot
MILLS	1		m	0	0	wh	-	all	NAmer	1940	CC	444	n	bl	y	n	1	ev	cig only	nev any	ot
MRFITR	2	x	m	0	0	all	0	all	NAmer	1973	pr	119	n	bl	n	n	0	cu	cig+/-ot	nev cigs	ot
NAM	76	x	m	0	0	all	-	all	NAmer	1986	CC	1199	n	bl	y	n	1	cu	cig+/-ot	nev cigs	ot
NAM	92	x	f	0	0	all	-	all	NAmer	1986	CC	1199	n	bl	y	n	1	cu	cig+/-ot	nev cigs	ot
NOTAN2	19		m	0	0	all	-	all	As:Ind	1963	CC	683	n	V	n	n	2	ev	cig only	nev any	ot
NOU	11		m	30	64	all	-	all	Eu:Sca	1971	CC	273	n	bl	y	n	0	ev	all/unsp	nev any	st
NOU	12		f	30	64	all	-	all	Eu:Sca	1971	CC	273	n	bl	y	n	0	ev	all/unsp	nev any	st
ODRISC	1	x	c	0	0	all	-	all	Eu:UK	1992	CC	446	n	V	n	n	0	cu	all/unsp	nev any	st
ORMOS	4		m	0	0	all	-	all	Eu:est	1947	CC	119	n	bl	y	y	0	ev	cig+/-ot	nev any	st
ORMOS	26		f	0	0	all	-	all	Eu:est	1947	CC	119	n	bl	y	y	0	ev	cig+/-ot	nev any	st
OSANN	33	x	m	0	0	all	-	all	NAmer	1984	CC	1986	n	bl	n	n	2	cu	cig+/-ot	nev cigs	or
OSANN	34	x	f	0	0	all	-	all	NAmer	1984	CC	1986	n	bl	n	n	2	cu	cig+/-ot	nev cigs	or
PARKIN	30	x	m	0	0	bl	-	all	Africa	1963	CC	877	n	V	y	n	0	cu	cig+/-ot	nev any	st
PASTOR	10		m	0	0	all	-	all	Eu:wst	1976	CC	204	n	bl	y	n	1	ev	all/unsp	nev any	or
PAWLEG	2		m	0	0	all	-	all	Eu:est	1992	CC	176	n	bl	n	y	6	ev	all/unsp	nev any	ot
PERNU	8		m	0	0	all	-	all	Eu:Sca	1944	CC	1606	n	bl	n	n	0	ev	cig only	nev any	st
PERNU	4		f	0	0	all	-	all	Eu:Sca	1944	CC	1606	n	bl	n	n	0	ev	cig only	nev any	st
PERSH2	10	x	c	0	0	all	-	all	Eu:Sca	1980	CC	1022	n	bl	y	n	4	cu	all/unsp	nev any	ot
PETO	4	x	m	0	0	all	0	all	Eu:UK	1954	pr	103	n	V	n	n	0	cu	all/unsp	nev any	st
PEZZO2	2	x	m	0	0	all	-	all	SCAmer	1992	CC	367	n	bl	n	y	0	cu	cig+/-ot	nev cigs	st
PEZZOT	5	x	m	0	0	all	-	all	SCAmer	1987	CC	215	n	bl	n	y	0	cu	cig only	nev cigs	st
PIKE	4		m	0	0	w-hi	-	all	NAmer	1972	CC	731	n	bl	y	n	0	ev	all/unsp	nev any	st
PIKE	8		f	0	0	w-hi	-	all	NAmer	1972	CC	731	n	bl	y	n	0	ev	all/unsp	nev any	st
POFFIJ	1		c	0	0	all	-	all	Eu:mul	1990	CC	971	n	bl	n	n	0	ev	all/unsp	nev any	st
POLEDN	1		c	0	0	all	-	all	NAmer	1978	CC	209	n	bl	y	n	1	ev	cig+/-ot	nev cigs	or
QIAO2	8	x	m	0	0	all	0	all	As:Chi	1992	pr	241	m	ot	n	n	0	cu	cig+/-ot	nev any	st
RACHTA	9	x	f	0	0	all	-	all	Eu:est	1991	CC	118	n	bl	n	y	1	cu	cig+/-ot	nev cigs	or
RADZIK	1		c	0	0	all	-	all	Eu:est	1986	CC	189	n	bl	n	n	0	ev	all/unsp	nev any	st
RANDIG	9		m	0	0	all	-	all	Eu:Ger	1951	CC	448	n	bl	n	n	0	ev	cig+/-ot	nev any	st
RANDIG	10		f	0	0	all	-	all	Eu:Ger	1951	CC	448	n	bl	n	n	0	ev	cig+/-ot	nev any	st
REN	1		m	0	0	all	-	all	As:Chi	1980	CC	244	n	ot	*	n	0	ev	all/unsp	nev any	st
REN	2		f	0	0	all	-	all	As:Chi	1980	CC	244	n	ot	*	n	0	ev	all/unsp	nev any	st
RONCO	3		m	0	0	all	-	all	Eu:wst	1976	CC	126	n	bl	y	n	2	ev	cig only	nev any	ot
ROTHSC	2		c	0	0	all	-	all	NAmer	1971	CC	284	n	bl	y	n	1	ev	all/unsp	nev any	st
SADOWS	4		m	0	0	wh	-	all	NAmer	1938	CC	477	n	bl	n	n	0	ev	cig+/-ot	nev any	st
SANKAR	2		m	0	0	all	-	all	As:Ind	1990	CC	281	n	V	n	n	3	ev	all/unsp	nev any	ot
SCHWAR	25	x	m	0	0	wh	-	all	NAmer	1984	CC	5588	n	bl	y	y	0	cu	cig+/-ot	nev cigs	st
SCHWAR	26	x	m	0	0	bl	-	all	NAmer	1984	CC	5588	n	bl	y	y	0	cu	cig+/-ot	nev cigs	st
SCHWAR	27	x	f	0	0	wh	-	all	NAmer	1984	CC	5588	n	bl	y	y	0	cu	cig+/-ot	nev cigs	st
SCHWAR	28	x	f	0	0	bl	-	all	NAmer	1984	CC	5588	n	bl	y	y	0	cu	cig+/-ot	nev cigs	st
SEGI	1		m	0	0	all	-	all	As:Jap	1948	CC	159	n	bl	n	n	0	ev	all/unsp	nev any	ot
SEGI2	20		m	0	0	all	-	all	As:Jap	1962	CC	378	n	bl	n	n	1	cu	cig+/-ot	nev any	ot
SEGI2	28		f	0	0	all	-	all	As:Jap	1962	CC	378	n	bl	n	n	1	cu	cig+/-ot	nev any	ot
SEOW	6		f	0	0	ch	-	q+s+l+a	As:oth	1997	CC	153	n	bl	n	y	1	ev	cig+/-ot	nev cigs	st
SHAW	6	x	c	0	0	wh	-	all	NAmer	1988	CC	335	n	V	n	y	0	cu	all/unsp	nev any	st
SIEMIA	5		m	0	0	all	-	all	NAmer	1979	CC	857	n	V	y	y	7	ev	cig+/-ot	nev cigs	or
SIMARA	3		m	0	0	all	-	all	As:oth	1971	CC	115	n	bl	n	n	6	ev	cig+/-ot	nev cigs	ot
SIMARA	4		f	0	0	all	-	all	As:oth	1971	CC	115	n	bl	n	n	6	ev	cig+/-ot	nev cigs	ot
SOBUE	42	x	m	0	0	all	-	q+s+l+a	As:Jap	1986	CC	1376	n	bl	n	y	1	cu	cig+/-ot	nev cigs	or
SOBUE	52	x	f	0	0	all	-	q+s+l+a	As:Jap	1986	CC	1376	n	bl	n	y	1	cu	cig+/-ot	nev cigs	or
SOBUE2	10		m	0	0	all	-	q+s+l+a	As:Jap	1965	CC	2083	n	bl	n	n	2	cu	cig+/-ot	nev any	ot
SOBUE2	12		f	0	0	all	-	q+s+l+a	As:Jap	1965	CC	2083	n	bl	n	n	2	cu	cig+/-ot	nev any	ot
SPEIZE	10	x	f	0	0	all	0	all	NAmer	1976	pr	593	n	bl	n	y	1	cu	cig+/-ot	nev cigs	ot
SPITZ	2	x	c	0	0	b+hi	-	all	NAmer	1992	CC	177	n	bl	n	y	0	cu	cig+/-ot	nev cigs	st
STASZE	7		m	0	0	all	-	all	Eu:est	1954	CC	281	n	bl	n	y	0	ev	cig+/-ot	nev any	st
STASZE	5		f	0	0	all	-	all	Eu:est	1954	CC	281	n	bl	n	y	0	ev	all/unsp	nev any	st

Table 1C4 - 1

IESLC - Meta-anal of Current Smoking (or Ever if Current not available), Cigs (or Any Prod if Cigs not avail)
All LC types
Most adjusted

REF	NRR	1C3	SEX	AGEL	AGEH	RACE	YF	LC TYPE	LOC	START	ST	NLC	R	VB	P	H	AD	SM	PRODUCT	DENOM	De
STAYNE	1		m	0	0	all	-	all	NAmer	1969	CC	420	n	bl	n	n	0	ev	all/unsp	nev any	st
STOCKS	46		m	0	0	all	-	all	Eu:UK	1952	CC	2932	n	V	y	n	2	ev	cig+/-ot	nev any	st
STOCKS	50		f	0	0	all	-	all	Eu:UK	1952	CC	2932	n	V	y	n	1	ev	cig+/-ot	nev any	ot
STOCKW	7	x	c	0	0	all	-	all	NAmer	1981	CC	22161	n	bl	n	n	0	cu	cig+/-ot	nev any	st
STUCKE	2	x	m	0	0	all	-	all	Eu:wst	1989	CC	247	n	bl	n	y	0	cu	all/unsp	nev any	ot
SUN	1		c	0	0	all	-	all	As:Chi	1992	CC	207	n	ot	n	y	0	ev	all/unsp	nev any	st
SUZUK2	6	x	c	0	0	all	-	all	SCAmer	1991	CC	123	n	bl	n	y	3	cu	all/unsp	nev any	or
SVENSS	96	x	f	0	0	all	-	all	Eu:Sca	1983	CC	210	n	bl	n	n	1	cu	all/unsp	nev any	ot
TANG	1	x	c	0	0	all	-	not s	NAmer	1992	CC	119	n	bl	n	y	0	cu	cig+/-ot	nev cigs	st
TENKAN	24	x	m	0	0	all	17	all	Eu:Sca	1962	pr	242	n	bl	n	n	1	cu	all/unsp	nev any	ot
TIZZAN	5	x	m	0	0	all	-	all	Eu:wst	1959	CC	1358	n	bl	n	n	0	cu	all/unsp	nev any	st
TIZZAN	13	x	f	0	0	all	-	all	Eu:wst	1959	CC	1358	n	bl	n	n	0	cu	all/unsp	nev any	st
TOKARS	1	x	m	0	0	all	-	all	Eu:est	1966	ot	162	o	bl	n	y	0	cu	all/unsp	nev any	st
TOKARS	5	x	f	0	0	all	-	all	Eu:est	1966	ot	162	o	bl	n	y	0	ev	all/unsp	nev any	st
TOUSEY	12	x	m	0	0	all	-	all	NAmer	1993	CC	507	n	bl	y	y	3	cu	cig+/-ot	nev any	or
TOUSEY	15	x	f	0	0	all	-	all	NAmer	1993	CC	507	n	bl	y	y	3	cu	cig+/-ot	nev any	or
TSUGAN	28	x	m	0	0	all	-	q+a	As:Jap	1976	CC	134	n	bl	n	y	0	cu	all/unsp	nev any	st
TULINI	36	x	m	0	0	all	0	all	Eu:Sca	1967	pr	472	n	bl	n	n	3	cu	cig+/-ot	nev any	ot
TULINI	42	x	f	0	0	all	0	all	Eu:Sca	1967	pr	472	n	bl	n	n	3	cu	cig+/-ot	nev any	ot
TVERDA	5	x	m	0	0	all	0	all	Eu:Sca	1972	pr	238	n	bl	n	n	2	cu	cig+/-ot	nev cigs	ot
TVERDA	15		f	0	0	all	0	all	Eu:Sca	1972	pr	238	n	bl	n	n	2	cu	cig only	nev cigs	ot
WAKAI	8	x	m	0	0	all	-	all	As:Jap	1988	CC	333	n	bl	n	y	2	cu	all/unsp	nev any	or
WAKAI	26	x	f	0	0	all	-	all	As:Jap	1988	CC	333	n	bl	n	y	2	cu	all/unsp	nev any	or
WALD	4		m	0	0	all	0	all	Eu:UK	1975	pr	102	n	V	n	n	1	cu	cig only	nev any	or
WANG	5		c	0	0	all	-	all	As:Chi	1990	CC	390	n	ot	*	y	6	ev	all/unsp	nev any	or
WANG2	18	x	c	0	0	all	-	all	As:Chi	1980	CC	103	n	ot	n	n	4	cu	cig+/-ot	nev cigs	ot
WANG3	1		c	0	0	all	-	all	As:Chi	1981	CC	293	n	ot	*	n	0	ev	all/unsp	nev any	st
WANG4	2		m	0	0	all	-	all	As:Chi	1976	CC	1170	n	ot	y	n	2	ev	all/unsp	nev any	st
WICKLU	1		m	0	0	wh	-	all	NAmer	1968	CC	155	n	bl	y	n	0	ev	cig+/-ot	nev+3	or
WIGLE	28	x	m	0	0	all	-	all	NAmer	1971	CC	728	n	V	n	n	1	cu	cig only	nev any	ot
WIGLE	33	x	f	0	0	all	-	all	NAmer	1971	CC	728	n	V	n	n	1	cu	cig+/-ot	nev any	ot
WILKIN	3		c	0	0	all	-	all	Eu:UK	1992	CC	271	n	V	n	n	4	ev	cig+/-ot	nev cigs	ot
WU	42	x	f	0	0	wh	-	q+a	NAmer	1981	CC	220	n	bl	n	y	2	cu	all/unsp	nev any	st
WUNSCH	5	x	m	0	0	all	-	all	SCAmer	1990	CC	398	n	bl	y	n	1	cu	cig+/-ot	nev any	or
WUNSCH	11	x	f	0	0	all	-	all	SCAmer	1990	CC	398	n	bl	y	n	1	cu	cig+/-ot	nev any	or
WUWILL	8		f	0	0	all	-	all	As:Chi	1985	CC	965	n	ot	n	n	3	ev	cig+/-ot	nev cigs	or
WYNDE2	16		m	0	0	all	-	all	NAmer	1962	CC	404	n	bl	n	y	0	ev	cig+/-ot	nev any	st
WYNDE3	50	x	m	0	0	all	-	all	NAmer	1966	CC	350	n	bl	n	y	0	cu	all/unsp	nev any	st
WYNDE3	83		f	0	0	all	-	all	NAmer	1966	CC	350	n	bl	n	y	0	ev	cig+/-ot	nev any	st
WYNDE4	48		m	0	0	all	-	all	NAmer	1948	CC	684	n	bl	y	n	0	ev	all/unsp	nev any	st
WYNDE4	62		f	0	0	all	-	all	NAmer	1948	CC	684	n	bl	y	n	2	ev	all/unsp	nev any	ot
WYNDE6	18	x	m	0	0	all	-	all	NAmer	1969	CC	4423	n	bl	n	y	0	cu	cig+/-ot	nev any	st
WYNDE6	207	x	f	0	0	all	-	all	NAmer	1969	CC	4423	n	bl	n	y	0	cu	cig+/-ot	nev cigs	st
XIANGZ	14		m	0	0	all	0	all	As:Chi	1976	pr	983	m	ot	n	n	2	ev	cig+/-ot	nev any	ot
XU	2		m	0	0	all	-	all	As:Chi	1985	CC	729	n	ot	n	n	2	ev	all/unsp	nev any	or
XU2	2		c	0	0	all	-	all	As:Chi	1987	CC	610	o	ot	y	n	7	ev	all/unsp	nev any	ot
XU3	2		m	0	0	all	-	all	As:Chi	1981	CC	135	n	ot	n	n	1	ev	all/unsp	nev any	or
XU3	4		f	0	0	all	-	all	As:Chi	1981	CC	135	n	ot	n	n	1	ev	all/unsp	nev any	or
XU4	1		c	0	0	all	-	all	As:Chi	1981	CC	206	n	ot	*	n	0	ev	all/unsp	nev any	st
YAMAGU	10	x	c	0	0	all	-	all	As:Jap	1989	CC	144	n	bl	n	y	1	cu	all/unsp	nev any	ot
YONG	12		m	0	0	all	0	all	NAmer	1971	pr	216	n	bl	n	n	1	cu	cig+/-ot	nev cigs	or
YONG	15		f	0	0	all	0	all	NAmer	1971	pr	216	n	bl	n	n	1	cu	cig+/-ot	nev cigs	or
YUAN	1		m	0	0	all	0	all	As:Chi	1986	pr	142	n	ot	n	n	2	ev	cig+/-ot	nev cigs	ot
ZHANG	2		m	0	0	all	-	all	As:Chi	1988	CC	100	n	ot	n	n	7	ev	all/unsp	nev any	or
ZHANG	3		f	0	0	all	-	all	As:Chi	1988	CC	100	n	ot	n	n	7	ev	all/unsp	nev any	or
ZHENG	15		m	0	0	all	-	all	As:Chi	1982	CC	540	n	ot	*	y	0	ev	cig+/-ot	nev cigs	st
ZHENG	24		f	0	0	all	-	all	As:Chi	1982	CC	540	n	ot	*	y	0	ev	cig+/-ot	nev cigs	st
ZHOU	2		m	0	0	all	-	all	As:Chi	1978	CC	1360	n	ot	n	n	0	ev	all/unsp	nev any	st
ZHOU	3		f	0	0	all	-	all	As:Chi	1978	CC	1360	n	ot	n	n	0	ev	all/unsp	nev any	st

Cigarette type is all/unspec for all RRs
except for the following:

REF	NRR	CIGTYPE
ALDERS	177	MC+-HR
ALDERS	176	MC only
DEAN3	239	MC only
DEAN3	119	MC only
GARDIN	6	MC only

Table 1C4 - 1

IESLC - Meta-anal of Current Smoking (or Ever if Current not available), Cigs (or Any Prod if Cigs not avail)
All LC types
Most adjusted

REF	NRR	CIGTYPE
JUSSAW	31	MC only
NOTAN2	19	MC only
PERNU	8	MC only
PERNU	4	MC only

Table 1C4 - 2

IESLC - Meta-anal of Current Smoking (or Ever if Current not available), Cigs (or Any Prod if Cigs not avail)
All LC types
Most adjusted

REF	NRR	SEX	AD	Number Exposed Case	Number Exposed Cont	Non-exposed Case	Non-exposed Cont	RR	95.00%CI
ABELIN	45	m	1	-	-	-	-	39.29	(9.04- 170.68)
*ABRAHA	7	m	0	269	10351	10	3365	8.74	(4.66- 16.42)
*ABRAHA	8	f	0	62	5256	28	11589	4.88	(3.13- 7.62)
Subtotal ABRAHA								5.93	(4.12- 8.53)
AGUDO	3	f	3	-	-	-	-	3.61	(1.57- 8.32)
*AKIBA	10	m	5	-	-	-	-	5.10	(3.30- 7.90)
*AKIBA	14	f	5	-	-	-	-	3.90	(2.90- 5.30)
Subtotal AKIBA								4.25	(3.32- 5.45)
ALDERS	177	m	0	519	322	15	133	14.29	(8.23- 24.81)
ALDERS	176	f	0	410	229	75	243	5.80	(4.27- 7.87)
Subtotal ALDERS								7.17	(5.49- 9.36)
*AMANDU	5	m	2	-	-	-	-	6.54	(2.52- 16.98)
AMES	1	m	0	150	136	15	62	4.56	(2.48- 8.39)
*ANDERS	6	f	1	-	-	-	-	23.43	(17.02- 32.27)
*ARCHER	5	m	0	122	32529	6	9842	6.15	(2.71- 13.96)
ARMADA	27	m	0	188	122	4	64	24.66	(8.75- 69.44)
AUSTIN	6	c	3	-	-	-	-	19.60	(6.70- 57.00)
AUVINE	19	c	2	-	-	-	-	13.84	(7.90- 24.25)
AXELSO	1	c	0	90	86	62	371	6.26	(4.20- 9.34)
AXELSS	2	m	0	194	130	16	160	14.92	(8.53- 26.12)
AXELSS	10	f	0	96	69	18	154	11.90	(6.68- 21.22)
Subtotal AXELSS								13.38	(8.95- 20.00)
BAND	1	m	2	-	-	-	-	9.96	(7.38- 13.44)
BARBON	4	m	1	-	-	-	-	13.40	(8.50- 21.40)
BECHER	13	m	0	101	122	3	54	14.90	(4.52- 49.09)
BECHER	14	f	0	33	26	10	52	6.60	(2.82- 15.44)
Subtotal BECHER								8.68	(4.35- 17.35)
*BENSHL	4	m	1	-	-	-	-	11.92	(6.36- 22.34)
*BEST	2	m	1	-	-	-	-	14.91	(7.05- 31.52)
*BEST	18	f	1	-	-	-	-	2.24	(0.59- 8.44)
Subtotal BEST								9.45	(4.92- 18.15)
BLOHMK	1	m	0	419	313	126	301	3.20	(2.48- 4.12)
BLOT4	1	m	0	327	245	8	87	14.51	(6.91- 30.51)
BOFFET	27	m	2	-	-	-	-	14.43	(11.91- 17.49)
*BOUCOT	114	m	2	-	-	-	-	62.29	(3.86-1004.01)
BRESLO	17	m	0	471	383	7	42	7.38	(3.28- 16.61)
BRESLO	23	f	0	13	11	12	14	1.38	(0.45- 4.20)
Subtotal BRESLO								4.12	(2.14- 7.94)
*BRETT	4	m	0	135	37448	6	6530	3.92	(1.73- 8.88)
BROCKM	1	m	0	87	81	2	2	1.07	(0.15- 7.80)
BROCKM	2	f	0	24	54	4	18	2.00	(0.61- 6.54)
Subtotal BROCKM								1.70	(0.61- 4.70)
BROSS	4	m	0	565	427	38	170	5.92	(4.07- 8.60)
BROWN2	12	m	2	-	-	-	-	11.30	(10.20- 12.40)
BROWN2	11	f	2	-	-	-	-	13.60	(12.30- 15.10)
Subtotal BROWN2								12.34	(11.50- 13.25)
BUFFLE	3	m	0	257	219	5	47	11.03	(4.31- 28.22)
BUFFLE	7	f	0	313	183	41	198	8.26	(5.63- 12.11)
Subtotal BUFFLE								8.61	(6.04- 12.27)
CARPEN	11	c	3	-	-	-	-	23.03	(12.96- 40.85)
CASCO2	1	c	0	149	212	6	98	11.48	(4.90- 26.87)
CASCOR	1	c	0	365	362	22	295	13.52	(8.56- 21.35)
*CEDERL	26	m	1	-	-	-	-	8.16	(3.70- 17.99)
*CEDERL	119	f	1	-	-	-	-	4.54	(1.85- 11.12)
Subtotal CEDERL								6.31	(3.49- 11.43)
CHAN	5	m	0	206	161	2	43	27.51	(6.57- 115.26)
CHAN	6	f	0	105	50	84	139	3.48	(2.26- 5.35)
Subtotal CHAN								4.13	(2.73- 6.25)
*CHANG	5	m	0	35	419	5	502	8.39	(3.32- 21.21)
*CHANG	11	f	0	30	603	11	1139	5.15	(2.60- 10.21)
Subtotal CHANG								6.12	(3.53- 10.60)
CHATZI	4	c	0	255	365	27	129	3.34	(2.14- 5.21)
CHEN2	1	m	0	121	97	9	33	4.57	(2.09- 10.02)
CHEN2	2	f	0	38	30	25	33	1.67	(0.82- 3.39)
Subtotal CHEN2								2.62	(1.55- 4.44)
CHEN3	1	c	0	182	156	72	98	1.59	(1.09- 2.30)
CHIAZZ	3	m	11	-	-	-	-	26.17	(3.32- 206.50)
CHOI	3	m	0	232	329	13	95	5.15	(2.82- 9.42)
CHOI	7	f	0	13	23	76	164	1.22	(0.59- 2.54)
Subtotal CHOI								2.88	(1.81- 4.58)
*CHOW	56	m	2	-	-	-	-	21.46	(9.38- 49.10)

Table 1C4 - 2

IESLC - Meta-anal of Current Smoking (or Ever if Current not available), Cigs (or Any Prod if Cigs not avail)
All LC types
Most adjusted

REF	NRR	SEX	AD	Number Exposed Case	Cont	Non-exposed Case	Cont	RR	95.00%CI
*CHYOU	2	m	1	-	-	-	-	11.40	(6.50- 20.10)
COMSTO	3	m	0	105	100	4	69	18.11	(6.37- 51.48)
COMSTO	8	f	0	77	52	13	115	13.10	(6.68- 25.67)
Subtotal COMSTO								14.40	(8.18- 25.36)
COOKSO	4	c	0	184	38	45	61	6.56	(3.90- 11.04)
CORREA	42	c	1	-	-	-	-	14.20	(10.80- 18.70)
*CPSI	220	m	1	-	-	-	-	11.94	(9.52- 14.97)
*CPSI	279	f	1	-	-	-	-	3.20	(2.53- 4.04)
Subtotal CPSI								6.32	(5.37- 7.43)
*CPSII	126	m	1	-	-	-	-	20.25	(16.37- 25.05)
*CPSII	133	f	1	-	-	-	-	11.78	(10.14- 13.68)
Subtotal CPSII								14.10	(12.47- 15.93)
DAMBER	16	m	1	-	-	-	-	9.80	(6.30- 15.30)
DARBY	4	m	0	322	453	3	384	90.98	(28.96- 285.90)
DARBY	11	f	0	195	217	23	529	20.67	(13.05- 32.74)
Subtotal DARBY								25.40	(16.57- 38.92)
DAVEYS	5	m	0	90	144	3	23	4.79	(1.40- 16.42)
DAVEYS	6	f	0	0	3	16	83	0.72~	(0.04- 14.66)
Subtotal DAVEYS								3.65	(1.17- 11.42)
DEAN	8	m	0	540	500	12	61	5.49	(2.92- 10.32)
DEAN2	2	m	0	671	600	33	112	3.80	(2.54- 5.68)
DEAN2	6	f	0	59	28	88	121	2.90	(1.71- 4.91)
Subtotal DEAN2								3.43	(2.49- 4.73)
DEAN3	239	m	1	-	-	-	-	7.04	(4.60- 10.77)
DEAN3	119	f	3	-	-	-	-	5.77	(3.75- 8.86)
Subtotal DEAN3								6.38	(4.72- 8.63)
*DEKLER	7	m	2	-	-	-	-	23.79	(3.32- 170.45)
DESTE2	4	c	7	-	-	-	-	9.10	(5.20- 15.90)
DESTEF	41	m	4	-	-	-	-	10.90	(6.90- 17.10)
*DOCKER	1	c	4	-	-	-	-	8.00	(2.97- 21.60)
DOLL	90	m	0	1280	1172	7	61	9.52	(4.34- 20.89)
DOLL	93	f	0	58	41	40	59	2.09	(1.18- 3.68)
Subtotal DOLL								3.51	(2.21- 5.55)
*DOLL2	68	m	1	-	-	-	-	12.20	(5.77- 25.82)
*DOLL2	63	f	1	-	-	-	-	8.65	(2.93- 25.55)
Subtotal DOLL2								10.91	(5.89- 20.21)
DORANT	9	c	0	292	876	14	1090	25.95	(15.07- 44.69)
DORGAN	9	m	0	464	170	15	93	16.92	(9.54- 30.01)
DORGAN	33	m	0	214	61	3	35	40.93	(12.17- 137.66)
DORGAN	56	f	0	611	119	103	244	12.16	(8.99- 16.46)
DORGAN	79	f	0	68	17	7	20	11.43	(4.16- 31.43)
Subtotal DORGAN								13.62	(10.58- 17.54)
*DORN	391	m	1	-	-	-	-	10.86	(9.73- 12.13)
DOSEME	1	m	2	-	-	-	-	3.30	(2.60- 4.40)
DROSTE	6	m	4	-	-	-	-	14.50	(6.30- 33.40)
DU	1	m	0	538	-	28	-	3.53	(2.44- 5.11)
DU	2	f	0	191	-	92	-	1.93	(1.30- 2.87)
Subtotal DU								2.66	(2.03- 3.49)
*DUNN	6	m	0	137	52634	2	14160	18.43	(4.56- 74.42)
EBELIN	1	m	0	101	142	12	117	6.93	(3.63- 13.24)
*ENGELA	168	m	1	-	-	-	-	9.70	(4.49- 20.94)
*ENGELA	177	f	1	-	-	-	-	5.80	(2.69- 12.51)
Subtotal ENGELA								7.50	(4.35- 12.92)
*ENSTRO	1	m	1	-	-	-	-	12.99	(10.46- 16.13)
*ENSTRO	2	f	1	-	-	-	-	6.95	(6.01- 8.04)
Subtotal ENSTRO								8.44	(7.48- 9.53)
ESAKI	4	m	0	155	143	16	28	1.90	(0.99- 3.65)
ESAKI	5	f	0	34	19	40	55	2.46	(1.23- 4.92)
Subtotal ESAKI								2.14	(1.33- 3.45)
FAN	1	m	0	216	498	36	236	2.84	(1.93- 4.18)
FAN	2	f	0	82	97	69	320	3.92	(2.65- 5.81)
Subtotal FAN								3.33	(2.53- 4.38)
GAO	33	m	2	-	-	-	-	3.90	(2.90- 5.40)
GAO	34	f	2	-	-	-	-	2.90	(2.20- 3.80)
Subtotal GAO								3.30	(2.69- 4.05)
GAO2	8	m	1	-	-	-	-	6.61	(3.47- 12.58)
GARCIA	2	c	0	169	74	21	139	15.12	(8.86- 25.79)
GARDIN	6	c	0	72	39	5	41	15.14	(5.53- 41.44)
GARSHI	31	m	1	-	-	-	-	7.70	(5.48- 10.83)
GENG	1	m	0	92	68	7	31	5.99	(2.49- 14.42)
GENG	2	f	0	126	75	67	118	2.96	(1.96- 4.48)

Table 1C4 - 2

IESLC - Meta-anal of Current Smoking (or Ever if Current not available), Cigs (or Any Prod if Cigs not avail)
All LC types
Most adjusted

REF	NRR	SEX	AD	Number Exposed Case	Number Exposed Cont	Non-exposed Case	Non-exposed Cont	RR	95.00%CI
Subtotal GENG								3.36	(2.31- 4.89)
GER	21	c	14	-	-	-	-	1.84	(1.06- 3.20)
GODLEY	5	m	1	-	-	-	-	6.84	(5.60- 8.35)
GODLEY	6	f	1	-	-	-	-	5.54	(4.29- 7.15)
Subtotal GODLEY								6.31	(5.39- 7.39)
GOLLED	7	m	1	-	-	-	-	7.51	(4.44- 12.71)
GOODMA	2	m	0	148	169	10	199	17.43	(8.90- 34.14)
GOODMA	6	f	0	58	56	19	177	9.65	(5.30- 17.56)
Subtotal GOODMA								12.53	(8.01- 19.60)
GRAHAM	10	m	1	-	-	-	-	7.26	(4.52- 11.65)
GREGOR	2	m	0	49	53	10	14	1.29	(0.53- 3.18)
GREGOR	6	f	0	17	26	1	22	14.38	(1.77- 116.90)
Subtotal GREGOR								1.88	(0.82- 4.30)
GSELL	6	m	0	60	42	2	29	20.71	(4.69- 91.56)
HAENSZ	54	f	0	69	94	81	236	2.14	(1.43- 3.19)
*HAMMO2	8	m	1	-	-	-	-	10.14	(4.19- 24.55)
*HAMMON	139	m	1	-	-	-	-	11.52	(6.83- 19.42)
*HANSEN	3	m	2	-	-	-	-	1.53	(0.71- 3.91)
HEGMAN	1	c	0	255	1202	27	2080	16.34	(10.92- 24.45)
*HEIN	1	m	0	45	912	1	457	22.55	(3.12- 163.06)
*HENNEK	2	m	0	79	2438	23	10919	15.38	(9.69- 24.42)
HINDS	22	f	3	-	-	-	-	5.65	(4.14- 7.72)
*HIRAYA	1	m	1	-	-	-	-	4.45	(3.60- 5.50)
*HIRAYA	3	f	1	-	-	-	-	2.34	(1.87- 2.92)
Subtotal HIRAYA								3.28	(2.81- 3.82)
HITOSU	34	m	1	-	-	-	-	2.79	(1.27- 6.09)
HITOSU	59	f	1	-	-	-	-	3.09	(1.82- 5.27)
Subtotal HITOSU								2.99	(1.93- 4.65)
*HOLE	32	m	1	-	-	-	-	8.36	(3.92- 17.83)
*HOLE	31	f	1	-	-	-	-	1.53	(0.64- 3.70)
Subtotal HOLE								4.05	(2.28- 7.18)
HOROWI	1	m	0	182	525	19	196	3.58	(2.17- 5.90)
HOROWI	2	f	0	21	382	14	463	1.82	(0.91- 3.62)
Subtotal HOROWI								2.83	(1.89- 4.25)
HORWIT	1	f	0	97	92	11	118	11.31	(5.73- 22.34)
HU	15	m	0	120	94	41	67	2.09	(1.30- 3.35)
HU	16	f	0	26	18	40	48	1.73	(0.83- 3.61)
Subtotal HU								1.98	(1.33- 2.94)
HU2	9	m	0	294	228	49	115	3.03	(2.08- 4.41)
HU2	10	f	0	108	80	72	100	1.88	(1.23- 2.85)
Subtotal HU2								2.44	(1.85- 3.23)
HUANG	1	c	0	98	77	37	58	2.00	(1.20- 3.32)
HUMBLE	13	m	1	-	-	-	-	19.96	(8.27- 48.21)
HUMBLE	15	m	1	-	-	-	-	15.79	(3.43- 72.69)
HUMBLE	17	f	1	-	-	-	-	16.72	(7.44- 37.61)
HUMBLE	19	f	1	-	-	-	-	23.50	(6.79- 81.36)
Subtotal HUMBLE								18.65	(11.23- 30.97)
JAHN	22	f	2	-	-	-	-	3.30	(1.99- 5.49)
JAIN	52	m	2	-	-	-	-	12.40	(6.45- 26.60)
JAIN	51	f	2	-	-	-	-	16.80	(9.93- 30.60)
Subtotal JAIN								14.94	(9.61- 23.21)
JARUP	6	m	2	-	-	-	-	7.54	(2.80- 20.33)
JARVHO	2	m	0	73	29	1	16	40.28	(5.10- 317.77)
JARVHO	6	f	0	31	7	6	21	15.50	(4.56- 52.66)
Subtotal JARVHO								19.86	(6.93- 56.89)
JEDRYC	58	m	4	-	-	-	-	5.46	(3.85- 7.73)
JEDRYC	59	f	4	-	-	-	-	4.54	(2.56- 8.05)
Subtotal JEDRYC								5.19	(3.86- 7.00)
JIANG	1	m	0	93	83	7	17	2.72	(1.08- 6.89)
JIANG	2	f	0	11	6	14	19	2.49	(0.74- 8.35)
Subtotal JIANG								2.63	(1.26- 5.50)
JOLY	16	m	0	451	524	12	218	15.64	(8.63- 28.34)
JOLY	15	f	0	132	96	52	283	7.48	(5.04- 11.12)
Subtotal JOLY								9.38	(6.75- 13.04)
JUSSAW	31	m	2	-	-	-	-	8.64	(4.61- 17.88)
*KAISE2	68	m	1	-	-	-	-	8.04	(4.41- 14.66)
*KAISE2	60	f	1	-	-	-	-	14.48	(7.47- 28.04)
Subtotal KAISE2								10.49	(6.72- 16.36)
*KAISER	12	m	2	-	-	-	-	19.61	(13.32- 28.87)
*KAISER	9	f	2	-	-	-	-	6.53	(4.50- 9.48)
Subtotal KAISER								11.09	(8.48- 14.50)

Table 1C4 - 2

IESLC - Meta-anal of Current Smoking (or Ever if Current not available), Cigs (or Any Prod if Cigs not avail)
All LC types
Most adjusted

REF	NRR	SEX	AD	Number Exposed Case	Number Exposed Cont	Non-exposed Case	Non-exposed Cont	RR	95.00%CI
KANELL	30	m	1	-	-	-	-	4.94	(3.47- 7.03)
KATSOU	2	f	1	-	-	-	-	3.40	(1.75- 6.61)
KAUFMA	16	c	6	-	-	-	-	20.63	(14.18- 30.01)
KELLER	1	m	0	5063	1210	323	1017	13.17	(11.45- 15.15)
KELLER	9	m	0	1053	212	38	117	15.29	(10.31- 22.69)
KELLER	5	f	0	2904	792	469	1860	14.54	(12.79- 16.53)
KELLER	13	f	0	454	135	67	232	11.64	(8.35- 16.24)
Subtotal KELLER								13.79	(12.62- 15.07)
KHUDER	19	m	0	275	-	23	-	8.10	(5.20- 12.70)
KIHARA	7	c	0	283	162	102	237	4.06	(3.00- 5.49)
*KINLEN	19	m	2	-	-	-	-	13.97	(6.65- 29.34)
KJUUS	1	m	0	135	77	2	24	21.04	(4.84- 91.45)
*KNEKT	85	m	1	-	-	-	-	9.22	(4.02- 21.13)
KO	1	f	3	-	-	-	-	4.20	(1.10- 15.60)
KOHLME	2	c	4	-	-	-	-	16.40	(6.90- 38.42)
KOO	9	f	0	42	25	56	85	2.55	(1.40- 4.64)
KOULUM	2	m	0	625	229	5	54	29.48	(11.65- 74.60)
KREUZE	24	f	3	-	-	-	-	29.90	(9.50- 94.60)
KREUZE	35	f	3	-	-	-	-	6.40	(4.20- 9.60)
Subtotal KREUZE								7.64	(5.18- 11.27)
KREYBE	12	m	1	-	-	-	-	6.61	(2.93- 14.92)
KREYBE	30	f	1	-	-	-	-	1.43	(0.71- 2.86)
Subtotal KREYBE								2.73	(1.61- 4.64)
*KUBIK	12	m	0	98	6342	2	4271	33.00	(8.14- 133.74)
LAMTH	6	f	0	242	106	202	337	3.81	(2.86- 5.08)
LAMWK	1	f	0	88	41	75	144	4.12	(2.59- 6.55)
LAMWK2	9	m	0	244	161	23	43	2.83	(1.64- 4.88)
LAMWK2	10	f	0	75	50	65	139	3.21	(2.02- 5.10)
Subtotal LAMWK2								3.04	(2.14- 4.33)
*LANGE	38	m	1	-	-	-	-	5.70	(2.13- 15.27)
*LANGE	35	f	1	-	-	-	-	5.02	(2.52- 10.01)
Subtotal LANGE								5.23	(2.98- 9.21)
LAUSSM	11	m	3	-	-	-	-	5.70	(4.10- 7.80)
LEI	1	m	0	443	361	41	123	3.68	(2.52- 5.38)
LEI	2	f	0	123	61	85	147	3.49	(2.32- 5.24)
Subtotal LEI								3.59	(2.72- 4.74)
LEMARC	2	c	0	167	65	32	168	13.49	(8.39- 21.68)
LETOUR	1	c	0	714	514	24	224	12.96	(8.38- 20.05)
LEVIN	30	m	1	-	-	-	-	6.97	(4.87- 9.97)
*LIAW	1	m	1	-	-	-	-	3.70	(2.10- 6.60)
*LIAW	2	f	1	-	-	-	-	3.60	(1.00- 12.20)
Subtotal LIAW								3.68	(2.19- 6.20)
*LIDDEL	4	m	1	-	-	-	-	4.41	(2.77- 7.01)
LIU	2	c	2	-	-	-	-	1.92	(1.40- 2.64)
LIU2	2	m	3	-	-	-	-	5.19	(2.03- 13.25)
LIU2	4	f	3	-	-	-	-	4.65	(2.18- 9.93)
Subtotal LIU2								4.86	(2.69- 8.76)
LIU3	2	m	2	-	-	-	-	1.26	(0.30- 5.26)
LIU4	10	m	2	-	-	-	-	3.88	(3.78- 3.98)
LIU4	12	f	2	-	-	-	-	2.86	(2.77- 2.95)
Subtotal LIU4								3.43	(3.37- 3.50)
LIU5	1	c	0	85	70	26	41	1.91	(1.07- 3.44)
LOMBA2	1	f	0	149	353	76	239	1.33	(0.96- 1.83)
LOMBAR	9	m	0	852	610	14	112	11.17	(6.35- 19.66)
LUBIN2	28	m	2	-	-	-	-	11.18	(9.57- 13.05)
LUBIN2	317	f	0	384	410	288	1180	3.84	(3.17- 4.64)
Subtotal LUBIN2								7.28	(6.46- 8.21)
LUO	7	c	20	-	-	-	-	2.70	(1.50- 5.00)
MACLEN	19	m	0	137	108	5	15	3.81	(1.34- 10.80)
MACLEN	32	f	0	42	47	41	109	2.38	(1.37- 4.12)
Subtotal MACLEN								2.63	(1.62- 4.28)
*MAGNUS	5	m	3	-	-	-	-	4.13	(1.94- 8.77)
MARSH	1	m	0	98	150	2	31	10.13	(2.37- 43.27)
MARSH	3	f	0	42	64	8	71	5.82	(2.54- 13.33)
Subtotal MARSH								6.67	(3.25- 13.70)
MARSH2	5	m	1	-	-	-	-	1.89	(0.70- 5.14)
MARSH2	6	f	1	-	-	-	-	5.28	(1.89- 14.72)
Subtotal MARSH2								3.11	(1.52- 6.36)
MARTIS	4	m	0	197	176	4	25	7.00	(2.39- 20.49)
MASTRA	2	m	2	-	-	-	-	8.14	(3.32- 20.00)
MATOS	3	m	2	-	-	-	-	8.50	(4.30- 16.70)

Table 1C4 - 2

IESLC - Meta-anal of Current Smoking (or Ever if Current not available), Cigs (or Any Prod if Cigs not avail)
All LC types
Most adjusted

REF	NRR	SEX	AD	Number Exposed Case	Number Exposed Cont	Non-exposed Case	Non-exposed Cont	RR	95.00%CI
MATSUD	10	m	0	170	3314	3	1255	21.46	(6.84- 67.33)
MCCONN	1	m	0	88	174	5	12	1.21	(0.41- 3.55)
MCCONN	2	f	0	3	3	4	11	2.75	(0.38- 19.67)
Subtotal MCCONN								1.46	(0.57- 3.76)
MCDUFF	1	m	0	159	134	6	31	6.13	(2.48- 15.14)
MCLAUG	1	m	0	294	1082	22	270	3.33	(2.12- 5.25)
*MIGRAN	12	m	2	-	-	-	-	4.04	(1.50- 10.90)
*MIGRAN	38	f	2	-	-	-	-	5.00	(1.76- 14.18)
Subtotal MIGRAN								4.47	(2.18- 9.17)
MILLER	2	f	1	-	-	-	-	4.99	(2.06- 12.10)
MILLS	1	m	1	-	-	-	-	1.27	(1.01- 1.61)
*MRFITR	2	m	0	106	8194	0	1859	48.33~	(3.01- 777.42)
NAM	76	m	1	-	-	-	-	8.67	(5.66- 13.26)
NAM	92	f	1	-	-	-	-	10.84	(7.26- 16.18)
Subtotal NAM								9.76	(7.29- 13.07)
NOTAN2	19	m	2	-	-	-	-	2.36	(1.68- 3.31)
NOU	11	m	0	74	247	6	122	6.09	(2.58- 14.39)
NOU	12	f	0	10	92	4	261	7.09	(2.17- 23.17)
Subtotal NOU								6.42	(3.20- 12.87)
ODRISC	1	c	0	293	598	6	664	54.22	(23.98- 122.60)
ORMOS	4	m	0	87	1034	7	777	9.34	(4.30- 20.28)
ORMOS	26	f	0	1	234	23	1044	0.19	(0.03- 1.44)
Subtotal ORMOS								5.65	(2.74- 11.64)
OSANN	33	m	2	-	-	-	-	26.50	(19.20- 36.50)
OSANN	34	f	2	-	-	-	-	19.60	(15.20- 25.20)
Subtotal OSANN								22.00	(18.03- 26.83)
PARKIN	30	m	0	346	874	107	1248	4.62	(3.66- 5.83)
PASTOR	10	m	1	-	-	-	-	6.81	(3.38- 13.70)
PAWLEG	2	m	6	-	-	-	-	12.26	(4.07- 36.95)
PERNU	8	m	0	706	216	97	275	9.27	(7.02- 12.23)
PERNU	4	f	0	7	24	110	971	2.57	(1.08- 6.11)
Subtotal PERNU								8.22	(6.32- 10.71)
PERSH2	10	c	4	-	-	-	-	8.29	(6.86- 10.02)
*PETO	4	m	0	99	2036	2	295	7.17	(1.78- 28.92)
PEZZO2	2	m	0	233	198	6	117	22.95	(9.89- 53.26)
PEZZOT	5	m	0	145	129	4	116	32.60	(11.70- 90.81)
PIKE	4	m	0	514	375	18	69	5.25	(3.08- 8.98)
PIKE	8	f	0	163	90	36	96	4.83	(3.04- 7.66)
Subtotal PIKE								5.01	(3.53- 7.10)
POFFIJ	1	c	0	913	918	58	452	7.75	(5.81- 10.34)
POLEDN	1	c	1	-	-	-	-	9.24	(5.23- 16.33)
*QIAO2	8	m	0	156	5399	10	709	2.05	(1.09- 3.86)
RACHTA	9	f	1	-	-	-	-	6.77	(3.71- 12.35)
RADZIK	1	c	0	180	198	9	13	1.31	(0.55- 3.15)
RANDIG	9	m	0	277	245	5	22	4.97	(1.86- 13.34)
RANDIG	10	f	0	16	39	17	92	2.22	(1.02- 4.84)
Subtotal RANDIG								3.03	(1.64- 5.58)
REN	1	m	0	106	84	12	34	3.58	(1.74- 7.33)
REN	2	f	0	78	20	48	50	4.06	(2.16- 7.64)
Subtotal REN								3.84	(2.39- 6.17)
RONCO	3	m	2	-	-	-	-	5.43	(2.27- 12.96)
ROTHSC	2	c	1	-	-	-	-	5.55	(2.97- 10.37)
SADOWS	4	m	0	421	446	18	81	4.25	(2.51- 7.20)
SANKAR	2	m	3	-	-	-	-	13.62	(9.00- 20.62)
SCHWAR	25	m	0	1652	349	119	376	14.96	(11.81- 18.94)
SCHWAR	26	m	0	644	139	50	104	9.64	(6.56- 14.15)
SCHWAR	27	f	0	1029	309	182	855	15.64	(12.75- 19.19)
SCHWAR	28	f	0	256	90	40	247	17.56	(11.64- 26.50)
Subtotal SCHWAR								14.70	(12.84- 16.83)
SEGI	1	m	0	140	1742	18	382	1.71	(1.03- 2.82)
SEGI2	20	m	1	-	-	-	-	3.74	(1.75- 8.00)
SEGI2	28	f	1	-	-	-	-	1.65	(0.90- 3.02)
Subtotal SEGI2								2.27	(1.41- 3.64)
SEOW	6	f	1	-	-	-	-	5.25	(2.80- 9.84)
SHAW	6	c	0	212	97	11	107	21.26	(10.93- 41.36)
SIEMIA	5	m	7	-	-	-	-	12.10	(6.60- 22.30)
SIMARA	3	m	6	-	-	-	-	1.65	(0.97- 2.81)
SIMARA	4	f	6	-	-	-	-	1.63	(0.87- 3.06)
Subtotal SIMARA								1.64	(1.09- 2.46)
SOBUE	42	m	1	-	-	-	-	4.10	(2.80- 5.90)
SOBUE	52	f	1	-	-	-	-	2.80	(2.00- 3.90)

Table 1C4 - 2

IESLC - Meta-anal of Current Smoking (or Ever if Current not available), Cigs (or Any Prod if Cigs not avail)
All LC types
Most adjusted

REF	NRR	SEX	AD	Number Exposed Case	Number Exposed Cont	Non-exposed Case	Non-exposed Cont	RR	95.00%CI
Subtotal SOBUE								3.32	(2.59- 4.26)
SOBUE2	10	m	2	-	-	-	-	4.47	(3.89- 5.14)
SOBUE2	12	f	2	-	-	-	-	3.28	(2.79- 3.87)
Subtotal SOBUE2								3.92	(3.53- 4.36)
*SPEIZE	10	f	1	-	-	-	-	12.69	(9.97- 16.16)
SPITZ	2	c	0	103	89	7	128	21.16	(9.40- 47.66)
STASZE	7	m	0	251	653	5	158	12.15	(4.93- 29.94)
STASZE	5	f	0	6	153	15	1660	4.34	(1.66- 11.35)
Subtotal STASZE								7.50	(3.89- 14.48)
STAYNE	1	m	0	362	567	58	333	3.67	(2.69- 4.99)
STOCKS	46	m	2	-	-	-	-	6.94	(4.93- 9.77)
STOCKS	50	f	1	-	-	-	-	3.04	(2.35- 3.93)
Subtotal STOCKS								4.10	(3.33- 5.03)
STOCKW	7	c	0	12470	3357	2791	10641	14.16	(13.38- 14.99)
STUCKE	2	m	0	69	68	0	51	104.50~	(6.32-1727.39)
SUN	1	c	0	140	173	67	191	2.31	(1.62- 3.30)
SUZUK2	6	c	3	-	-	-	-	22.00	(6.50- 76.00)
SVENSS	96	f	1	-	-	-	-	9.06	(5.31- 15.48)
TANG	1	c	0	52	25	9	39	9.01	(3.78- 21.46)
*TENKAN	24	m	1	-	-	-	-	16.81	(7.22- 39.14)
TIZZAN	5	m	0	693	619	180	305	1.90	(1.53- 2.35)
TIZZAN	13	f	0	17	18	25	114	4.31	(1.95- 9.51)
Subtotal TIZZAN								2.01	(1.63- 2.47)
TOKARS	1	m	0	110	157	1	53	37.13	(5.06- 272.56)
TOKARS	5	f	0	1	2	13	40	1.54	(0.13- 18.38)
Subtotal TOKARS								10.65	(2.25- 50.36)
TOUSEY	12	m	3	-	-	-	-	59.20	(21.00- 167.30)
TOUSEY	15	f	3	-	-	-	-	30.20	(16.00- 57.40)
Subtotal TOUSEY								36.34	(21.09- 62.60)
TSUGAN	28	m	0	63	63	18	22	1.22	(0.60- 2.50)
*TULINI	36	m	3	-	-	-	-	12.17	(6.56- 22.59)
*TULINI	42	f	3	-	-	-	-	16.34	(9.07- 29.47)
Subtotal TULINI								14.20	(9.27- 21.76)
*TVERDA	5	m	2	-	-	-	-	4.09	(2.65- 6.31)
*TVERDA	15	f	2	-	-	-	-	11.05	(3.33- 36.71)
Subtotal TVERDA								4.59	(3.05- 6.90)
WAKAI	8	m	2	-	-	-	-	4.40	(2.19- 8.85)
WAKAI	26	f	2	-	-	-	-	4.37	(2.21- 8.62)
Subtotal WAKAI								4.38	(2.69- 7.14)
*WALD	4	m	1	-	-	-	-	16.40	(7.55- 44.20)
WANG	5	c	6	-	-	-	-	2.88	(1.74- 4.77)
WANG2	18	c	4	-	-	-	-	2.40	(1.14- 5.05)
WANG3	1	c	0	235	172	58	121	2.85	(1.97- 4.13)
WANG4	2	m	2	-	-	-	-	1.16	(0.96- 1.42)
WICKLU	1	m	0	-	-	-	-	4.60	(2.80- 7.60)
WIGLE	28	m	1	-	-	-	-	12.40	(7.21- 21.31)
WIGLE	33	f	1	-	-	-	-	5.20	(3.34- 8.09)
Subtotal WIGLE								7.36	(5.23- 10.37)
WILKIN	3	c	4	-	-	-	-	7.83	(4.45- 13.78)
WU	42	f	2	-	-	-	-	4.86	(2.76- 8.57)
WUNSCH	5	m	1	-	-	-	-	6.59	(3.59- 12.10)
WUNSCH	11	f	1	-	-	-	-	5.98	(3.25- 11.00)
Subtotal WUNSCH								6.28	(4.08- 9.66)
WUWILL	8	f	3	-	-	-	-	2.30	(1.90- 2.80)
WYNDE2	16	m	0	382	512	8	105	9.79	(4.71- 20.34)
WYNDE3	50	m	0	227	207	9	88	10.72	(5.26- 21.84)
WYNDE3	83	f	0	46	56	20	76	3.12	(1.67- 5.85)
Subtotal WYNDE3								5.36	(3.35- 8.58)
WYNDE4	48	m	0	632	665	12	115	9.11	(4.98- 16.67)
WYNDE4	62	f	2	-	-	-	-	2.87	(1.48- 5.55)
Subtotal WYNDE4								5.38	(3.45- 8.41)
WYNDE6	18	m	0	1677	741	87	617	16.05	(12.62- 20.41)
WYNDE6	207	f	0	1022	376	159	856	14.63	(11.90- 17.99)
Subtotal WYNDE6								15.22	(13.01- 17.80)
*XIANGZ	14	m	2	-	-	-	-	1.79	(1.21- 2.65)
XU	2	m	2	-	-	-	-	2.70	(2.10- 3.50)
XU2	2	c	7	-	-	-	-	3.80	(2.84- 5.07)
XU3	2	m	1	-	-	-	-	5.99	(2.65- 13.50)
XU3	4	f	1	-	-	-	-	3.86	(1.39- 10.70)
Subtotal XU3								5.05	(2.67- 9.54)
XU4	1	c	0	161	113	45	93	2.94	(1.92- 4.52)

Table 1C4 - 2

IESLC - Meta-anal of Current Smoking (or Ever if Current not available), Cigs (or Any Prod if Cigs not avail)
All LC types
Most adjusted

REF	NRR	SEX	AD	Number Exposed Case	Cont	Non-exposed Case	Cont	RR	95.00%CI
YAMAGU	10	c	1	-	-	-	-	4.90	(2.55- 9.44)
*YONG	12	m	1	-	-	-	-	28.71	(6.98- 118.16)
*YONG	15	f	1	-	-	-	-	5.20	(2.38- 11.35)
Subtotal YONG								7.75	(3.91- 15.36)
*YUAN	1	m	2	-	-	-	-	6.50	(3.64- 11.60)
ZHANG	2	m	7	-	-	-	-	4.00	(1.61- 9.91)
ZHANG	3	f	7	-	-	-	-	3.75	(1.80- 10.76)
Subtotal ZHANG								3.87	(2.05- 7.32)
ZHENG	15	m	0	279	218	33	94	3.65	(2.36- 5.63)
ZHENG	24	f	0	76	44	152	184	2.09	(1.36- 3.21)
Subtotal ZHENG								2.75	(2.03- 3.73)
ZHOU	2	m	0	740	41	275	36	2.36	(1.48- 3.77)
ZHOU	3	f	0	112	7	231	32	2.22	(0.95- 5.18)
Subtotal ZHOU								2.33	(1.54- 3.51)
Partial Totals				60754	207912	9675	110757		

*prospective study ~ With 0.5 adjustment for zero

REF	NRR	SEX	AD	Ys	Ws	Qs	Ps
ABELIN	45	m	1	3.67	1.78	7.51	0.0000
*ABRAHA	7	m	0	2.17	9.68	2.95	0.0000
*ABRAHA	8	f	0	1.59	19.39	0.02	0.0000
Subtotal ABRAHA				1.78	29.07	2.97	
AGUDO	3	f	3	1.28	5.53	0.61	0.0025
*AKIBA	10	m	5	1.63	20.16	0.00	0.0000
*AKIBA	14	f	5	1.36	42.26	2.76	0.0000
Subtotal AKIBA				1.45	62.42	2.77	
ALDERS	177	m	0	2.66	12.62	13.73	0.0000
ALDERS	176	f	0	1.76	41.23	0.82	0.0000
Subtotal ALDERS				1.97	53.85	14.56	
*AMANDU	5	m	2	1.88	4.22	0.29	0.0001
AMES	1	m	0	1.52	10.33	0.10	0.0000
*ANDERS	6	f	1	3.15	37.54	88.73	0.0000
*ARCHER	5	m	0	1.82	5.72	0.23	0.0000
ARMADA	27	m	0	3.21	3.58	9.04	0.0000
AUSTIN	6	c	3	2.98	3.35	6.19	0.0000
AUVINE	19	c	2	2.63	12.22	12.48	0.0000
AXELSO	1	c	0	1.83	24.06	1.14	0.0000
AXELSS	2	m	0	2.70	12.26	14.46	0.0000
AXELSS	10	f	0	2.48	11.50	8.51	0.0000
Subtotal AXELSS				2.59	23.76	22.97	
BAND	1	m	2	2.30	42.76	19.88	0.0000
BARBON	4	m	1	2.60	18.02	17.26	0.0000
BECHER	13	m	0	2.70	2.70	3.18	0.0000
BECHER	14	f	0	1.89	5.32	0.39	0.0000
Subtotal BECHER				2.16	8.02	3.57	
*BENSHL	4	m	1	2.48	9.73	7.23	0.0000
*BEST	2	m	1	2.70	6.85	8.07	0.0000
*BEST	18	f	1	0.81	2.17	1.42	0.2348
Subtotal BEST				2.25	9.02	9.50	
BLOHMK	1	m	0	1.16	59.38	12.25	0.0000
BLOT4	1	m	0	2.68	6.96	7.80	0.0000
BOFFET	27	m	2	2.67	104.07	115.31	0.0000
*BOUCOT	114	m	2	4.13	0.50	3.14	0.0036
BRESLO	17	m	0	2.00	5.83	0.85	0.0000
BRESLO	23	f	0	0.32	3.10	5.20	0.5717
Subtotal BRESLO				1.42	8.93	6.05	
*BRETT	4	m	0	1.37	5.75	0.36	0.0010
BROCKM	1	m	0	0.07	0.98	2.33	0.9437
BROCKM	2	f	0	0.69	2.73	2.33	0.2517
Subtotal BROCKM				0.53	3.71	4.66	
BROSS	4	m	0	1.78	27.54	0.72	0.0000
BROWN2	12	m	2	2.42	402.82	263.07	0.0000
BROWN2	11	f	2	2.61	365.29	360.48	0.0000
Subtotal BROWN2				2.51	768.12	623.55	
BUFFLE	3	m	0	2.40	4.35	2.68	0.0000
BUFFLE	7	f	0	2.11	26.25	6.42	0.0000
Subtotal BUFFLE				2.15	30.60	9.10	
CARPEN	11	c	3	3.14	11.66	26.94	0.0000
CASCO2	1	c	0	2.44	5.31	3.60	0.0000
CASCOR	1	c	0	2.60	18.40	17.94	0.0000

Table 1C4 - 2

IESLC - Meta-anal of Current Smoking (or Ever if Current not available), Cigs (or Any Prod if Cigs not avail)
All LC types
Most adjusted

REF	NRR	SEX	AD	Ys	Ws	Qs	Ps
*CEDERL	26	m	1	2.10	6.14	1.43	0.0000
*CEDERL	119	f	1	1.51	4.78	0.05	0.0009
Subtotal CEDERL				1.84	10.92	1.48	
CHAN	5	m	0	3.31	1.87	5.40	0.0000
CHAN	6	f	0	1.25	20.57	2.83	0.0000
Subtotal CHAN				1.42	22.44	8.23	
*CHANG	5	m	0	2.13	4.46	1.16	0.0000
*CHANG	11	f	0	1.64	8.22	0.00	0.0000
Subtotal CHANG				1.81	12.68	1.16	
CHATZI	4	c	0	1.21	19.44	3.29	0.0000
CHEN2	1	m	0	1.52	6.25	0.06	0.0001
CHEN2	2	f	0	0.51	7.70	9.36	0.1539
Subtotal CHEN2				0.97	13.95	9.41	
CHEN3	1	c	0	0.46	27.78	37.01	0.0148
CHIAZZ	3	m	11	3.26	0.90	2.45	0.0019
CHOI	3	m	0	1.64	10.55	0.01	0.0000
CHOI	7	f	0	0.20	7.16	14.40	0.5951
Subtotal CHOI				1.06	17.71	14.40	
*CHOW	56	m	2	3.07	5.61	11.78	0.0000
*CHYOU	2	m	1	2.43	12.06	8.05	0.0000
COMSTO	3	m	0	2.90	3.52	5.77	0.0000
COMSTO	8	f	0	2.57	8.49	7.75	0.0000
Subtotal COMSTO				2.67	12.01	13.52	
COOKSO	4	c	0	1.88	14.21	1.00	0.0000
CORREA	42	c	1	2.65	50.99	54.78	0.0000
*CPSI	220	m	1	2.48	74.99	55.88	0.0000
*CPSI	279	f	1	1.16	70.15	14.43	0.0000
Subtotal CPSI				1.84	145.14	70.31	
*CPSII	126	m	1	3.01	84.90	164.39	0.0000
*CPSII	133	f	1	2.47	171.36	123.73	0.0000
Subtotal CPSII				2.65	256.26	288.12	
DAMBER	16	m	1	2.28	19.52	8.65	0.0000
DARBY	4	m	0	4.51	2.93	24.54	0.0000
DARBY	11	f	0	3.03	18.15	36.18	0.0000
Subtotal DARBY				3.23	21.08	60.72	
DAVEYS	5	m	0	1.57	2.53	0.01	0.0126
DAVEYS	6	f	0	-0.32	0.42	1.60	0.8327
Subtotal DAVEYS				1.30	2.96	1.60	
DEAN	8	m	0	1.70	9.65	0.07	0.0000
DEAN2	2	m	0	1.33	23.59	1.89	0.0000
DEAN2	6	f	0	1.06	13.83	4.23	0.0001
Subtotal DEAN2				1.23	37.42	6.12	
DEAN3	239	m	1	1.95	21.23	2.38	0.0000
DEAN3	119	f	3	1.75	20.79	0.38	0.0000
Subtotal DEAN3				1.85	42.02	2.77	
*DEKLER	7	m	2	3.17	0.99	2.39	0.0016
DESTE2	4	c	7	2.21	12.30	4.31	0.0000
DESTEF	41	m	4	2.39	18.66	11.12	0.0000
*DOCKER	1	c	4	2.08	3.90	0.84	0.0000
DOLL	90	m	0	2.25	6.22	2.52	0.0000
DOLL	93	f	0	0.74	11.96	9.29	0.0110
Subtotal DOLL				1.25	18.18	11.81	
*DOLL2	68	m	1	2.50	6.84	5.36	0.0000
*DOLL2	63	f	1	2.16	3.28	0.96	0.0001
Subtotal DOLL2				2.39	10.12	6.32	
DORANT	9	c	0	3.26	13.00	34.95	0.0000
DORGAN	9	m	0	2.83	11.70	17.19	0.0000
DORGAN	33	m	0	3.71	2.61	11.46	0.0000
DORGAN	56	f	0	2.50	41.93	32.60	0.0000
DORGAN	79	f	0	2.44	3.75	2.52	0.0000
Subtotal DORGAN				2.61	60.00	63.77	
*DORN	391	m	1	2.39	316.13	186.66	0.0000
DOSEME	1	m	2	1.19	55.52	9.92	0.0000
DROSTE	6	m	4	2.67	5.52	6.18	0.0000
DU	1	m	0	1.26	28.12	3.55	0.0000
DU	2	f	0	0.66	24.50	22.54	0.0011
Subtotal DU				0.98	52.62	26.09	
*DUNN	6	m	0	2.91	1.97	3.32	0.0000
EBELIN	1	m	0	1.94	9.19	0.94	0.0000
*ENGELA	168	m	1	2.27	6.48	2.78	0.0000
*ENGELA	177	f	1	1.76	6.50	0.13	0.0000

Table 1C4 - 2

IESLC - Meta-anal of Current Smoking (or Ever if Current not available), Cigs (or Any Prod if Cigs not avail)
All LC types
Most adjusted

REF	NRR	SEX	AD	Ys	Ws	Qs	Ps
Subtotal ENGELA				2.01	12.99	2.91	
*ENSTRO	1	m	1	2.56	81.91	73.54	0.0000
*ENSTRO	2	f	1	1.94	181.45	18.82	0.0000
Subtotal ENSTRO				2.13	263.36	92.36	
ESAKI	4	m	0	0.64	8.96	8.54	0.0554
ESAKI	5	f	0	0.90	7.99	4.10	0.0109
Subtotal ESAKI				0.76	16.94	12.64	
FAN	1	m	0	1.04	25.87	8.46	0.0000
FAN	2	f	0	1.37	24.92	1.56	0.0000
Subtotal FAN				1.20	50.80	10.02	
GAO	33	m	2	1.36	39.76	2.60	0.0000
GAO	34	f	2	1.06	51.44	15.67	0.0000
Subtotal GAO				1.19	91.20	18.27	
GAO2	8	m	1	1.89	9.26	0.68	0.0000
GARCIA	2	c	0	2.72	13.47	16.27	0.0000
GARDIN	6	c	0	2.72	3.79	4.59	0.0000
GARSHI	31	m	1	2.04	33.11	5.97	0.0000
GENG	1	m	0	1.79	4.98	0.15	0.0001
GENG	2	f	0	1.08	22.39	6.33	0.0000
Subtotal GENG				1.21	27.37	6.48	
GER	21	c	14	0.61	12.59	12.76	0.0305
GODLEY	5	m	1	1.92	96.28	9.02	0.0000
GODLEY	6	f	1	1.71	58.89	0.54	0.0000
Subtotal GODLEY				1.84	155.17	9.56	
GOLLED	7	m	1	2.02	13.89	2.22	0.0000
GOODMA	2	m	0	2.86	8.50	13.09	0.0000
GOODMA	6	f	0	2.27	10.71	4.53	0.0000
Subtotal GOODMA				2.53	19.21	17.62	
GRAHAM	10	m	1	1.98	17.14	2.29	0.0000
GREGOR	2	m	0	0.26	4.75	8.76	0.5741
GREGOR	6	f	0	2.67	0.88	0.96	0.0126
Subtotal GREGOR				0.63	5.62	9.72	
GSELL	6	m	0	3.03	1.74	3.48	0.0001
HAENSZ	54	f	0	0.76	23.97	17.59	0.0002
*HAMMO2	8	m	1	2.32	4.92	2.41	0.0000
*HAMMON	139	m	1	2.44	14.07	9.63	0.0000
*HANSEN	3	m	2	0.43	5.28	7.49	0.3285
HEGMAN	1	c	0	2.79	23.66	32.78	0.0000
*HEIN	1	m	0	3.12	0.98	2.21	0.0020
*HENNEK	2	m	0	2.73	17.97	22.41	0.0000
HINDS	22	f	3	1.73	39.57	0.52	0.0000
*HIRAYA	1	m	1	1.49	85.55	1.31	0.0000
*HIRAYA	3	f	1	0.85	77.37	45.46	0.0000
Subtotal HIRAYA				1.19	162.92	46.77	
HITOSU	34	m	1	1.03	6.25	2.18	0.0103
HITOSU	59	f	1	1.13	13.59	3.24	0.0000
Subtotal HITOSU				1.10	19.85	5.43	
*HOLE	32	m	1	2.12	6.70	1.72	0.0000
*HOLE	31	f	1	0.43	4.99	7.08	0.3421
Subtotal HOLE				1.40	11.69	8.80	
HOROWI	1	m	0	1.27	15.35	1.80	0.0000
HOROWI	2	f	0	0.60	8.08	8.38	0.0894
Subtotal HOROWI				1.04	23.43	10.18	
HORWIT	1	f	0	2.43	8.29	5.43	0.0000
HU	15	m	0	0.74	17.16	13.33	0.0023
HU	16	f	0	0.55	7.15	8.14	0.1413
Subtotal HU				0.68	24.31	21.46	
HU2	9	m	0	1.11	27.11	7.03	0.0000
HU2	10	f	0	0.63	21.91	21.39	0.0033
Subtotal HU2				0.89	49.01	28.42	
HUANG	1	c	0	0.69	14.82	12.71	0.0078
HUMBLE	13	m	1	2.99	4.94	9.38	0.0000
HUMBLE	15	m	1	2.76	1.65	2.15	0.0004
HUMBLE	17	f	1	2.82	5.85	8.43	0.0000
HUMBLE	19	f	1	3.16	2.49	5.91	0.0000
Subtotal HUMBLE				2.93	14.94	25.86	
JAHN	22	f	2	1.19	14.92	2.67	0.0000
JAIN	52	m	2	2.52	7.65	6.21	0.0000
JAIN	51	f	2	2.82	12.13	17.61	0.0000
Subtotal JAIN				2.70	19.79	23.82	
JARUP	6	m	2	2.02	3.91	0.64	0.0001

Table 1C4 - 2

IESLC - Meta-anal of Current Smoking (or Ever if Current not available), Cigs (or Any Prod if Cigs not avail)
All LC types
Most adjusted

REF	NRR	SEX	AD	Ys	Ws	Qs	Ps
JARVHO	2	m	0	3.70	0.90	3.89	0.0005
JARVHO	6	f	0	2.74	2.57	3.25	0.0000
Subtotal JARVHO				2.99	3.47	7.14	
JEDRYC	58	m	4	1.70	31.63	0.21	0.0000
JEDRYC	59	f	4	1.51	11.71	0.13	0.0000
Subtotal JEDRYC				1.65	43.33	0.33	
JIANG	1	m	0	1.00	4.45	1.69	0.0346
JIANG	2	f	0	0.91	2.62	1.30	0.1401
Subtotal JIANG				0.97	7.08	2.99	
JOLY	16	m	0	2.75	10.86	13.94	0.0000
JOLY	15	f	0	2.01	24.54	3.85	0.0000
Subtotal JOLY				2.24	35.40	17.79	
JUSSAW	31	m	2	2.16	8.36	2.44	0.0000
*KAISE2	68	m	1	2.08	10.65	2.33	0.0000
*KAISE2	60	f	1	2.67	8.78	9.80	0.0000
Subtotal KAISE2				2.35	19.43	12.12	
*KAISER	12	m	2	2.98	25.68	47.45	0.0000
*KAISER	9	f	2	1.88	27.68	1.87	0.0000
Subtotal KAISER				2.41	53.36	49.32	
KANELL	30	m	1	1.60	30.83	0.01	0.0000
KATSOU	2	f	1	1.22	8.70	1.34	0.0003
KAUFMA	16	c	6	3.03	27.34	54.36	0.0000
KELLER	1	m	0	2.58	195.95	181.20	0.0000
KELLER	9	m	0	2.73	24.67	30.44	0.0000
KELLER	5	f	0	2.68	233.82	262.89	0.0000
KELLER	13	f	0	2.45	34.67	24.36	0.0000
Subtotal KELLER				2.62	489.11	498.88	
KHUDER	19	m	0	2.09	19.27	4.35	0.0000
KIHARA	7	c	0	1.40	42.14	1.96	0.0000
*KINLEN	19	m	2	2.64	6.97	7.26	0.0000
KJUUS	1	m	0	3.05	1.78	3.64	0.0000
*KNEKT	85	m	1	2.22	5.58	2.04	0.0000
KO	1	f	3	1.44	2.18	0.07	0.0339
KOHLME	2	c	4	2.80	5.21	7.26	0.0000
KOO	9	f	0	0.94	10.70	4.96	0.0022
KOULUM	2	m	0	3.38	4.45	13.91	0.0000
KREUZE	24	f	3	3.40	2.91	9.23	0.0000
KREUZE	35	f	3	1.86	22.48	1.29	0.0000
Subtotal KREUZE				2.03	25.39	10.52	
KREYBE	12	m	1	1.89	5.80	0.43	0.0000
KREYBE	30	f	1	0.36	7.92	12.55	0.3143
Subtotal KREYBE				1.01	13.71	12.97	
*KUBIK	12	m	0	3.50	1.96	6.93	0.0000
LAMTH	6	f	0	1.34	46.55	3.63	0.0000
LAMWK	1	f	0	1.42	17.85	0.72	0.0000
LAMWK2	9	m	0	1.04	12.98	4.29	0.0002
LAMWK2	10	f	0	1.17	17.89	3.64	0.0000
Subtotal LAMWK2				1.11	30.86	7.93	
*LANGE	38	m	1	1.74	3.96	0.06	0.0005
*LANGE	35	f	1	1.61	8.08	0.00	0.0000
Subtotal LANGE				1.66	12.04	0.06	
LAUSSM	11	m	3	1.74	37.15	0.57	0.0000
LEI	1	m	0	1.30	26.63	2.62	0.0000
LEI	2	f	0	1.25	23.21	3.14	0.0000
Subtotal LEI				1.28	49.84	5.75	
LEMARC	2	c	0	2.60	17.07	16.57	0.0000
LETOUR	1	c	0	2.56	20.21	18.07	0.0000
LEVIN	30	m	1	1.94	29.93	3.16	0.0000
*LIAW	1	m	1	1.31	11.72	1.11	0.0000
*LIAW	2	f	1	1.28	2.46	0.28	0.0447
Subtotal LIAW				1.30	14.17	1.39	
*LIDDEL	4	m	1	1.48	17.82	0.31	0.0000
LIU	2	c	2	0.65	38.19	35.52	0.0001
LIU2	2	m	3	1.65	4.37	0.00	0.0006
LIU2	4	f	3	1.54	6.68	0.04	0.0001
Subtotal LIU2				1.58	11.05	0.05	
LIU3	2	m	2	0.23	1.87	3.60	0.7518
LIU4	10	m	2	1.36	5780.52	393.30	0.0000
LIU4	12	f	2	1.05	3876.64	1241.27	0.0000
Subtotal LIU4				1.23	9657.16	1634.57	
LIU5	1	c	0	0.65	11.25	10.52	0.0293

Table 1C4 - 2

IESLC - Meta-anal of Current Smoking (or Ever if Current not available), Cigs (or Any Prod if Cigs not avail)
All LC types
Most adjusted

REF	NRR	SEX	AD	Ys	Ws	Qs	Ps
LOMBA2	1	f	0	0.28	37.19	66.14	0.0841
LOMBAR	9	m	0	2.41	12.02	7.64	0.0000
LUBIN2	28	m	2	2.41	159.73	101.58	0.0000
LUBIN2	317	f	0	1.34	106.80	7.90	0.0000
Subtotal LUBIN2				1.99	266.54	109.47	
LUO	7	c	20	0.99	10.60	4.12	0.0012
MACLEN	19	m	0	1.34	3.53	0.28	0.0120
MACLEN	32	f	0	0.87	12.71	7.18	0.0020
Subtotal MACLEN				0.97	16.25	7.46	
*MAGNUS	5	m	3	1.42	6.75	0.27	0.0002
MARSH	1	m	0	2.32	1.82	0.89	0.0018
MARSH	3	f	0	1.76	5.60	0.12	0.0000
Subtotal MARSH				1.90	7.42	1.01	
MARSH2	5	m	1	0.64	3.87	3.71	0.2107
MARSH2	6	f	1	1.66	3.65	0.01	0.0015
Subtotal MARSH2				1.14	7.51	3.72	
MARTIS	4	m	0	1.95	3.32	0.36	0.0004
MASTRA	2	m	2	2.10	4.76	1.10	0.0000
MATOS	3	m	2	2.14	8.35	2.29	0.0000
MATSUD	10	m	0	3.07	2.94	6.17	0.0000
MCCONN	1	m	0	0.19	3.33	6.74	0.7237
MCCONN	2	f	0	1.01	0.99	0.36	0.3136
Subtotal MCCONN				0.38	4.32	7.10	
MCDUFF	1	m	0	1.81	4.70	0.18	0.0001
MCLAUG	1	m	0	1.20	18.70	3.18	0.0000
*MIGRAN	12	m	2	1.40	3.91	0.19	0.0058
*MIGRAN	38	f	2	1.61	3.53	0.00	0.0025
Subtotal MIGRAN				1.50	7.44	0.19	
MILLER	2	f	1	1.61	4.90	0.00	0.0004
MILLS	1	m	1	0.24	71.48	135.04	0.0406
*MRFITR	2	m	0	3.88	0.50	2.55	0.0062
NAM	76	m	1	2.16	21.20	6.26	0.0000
NAM	92	f	1	2.38	23.93	14.06	0.0000
Subtotal NAM				2.28	45.13	20.31	
NOTAN2	19	m	2	0.86	33.41	19.20	0.0000
NOU	11	m	0	1.81	5.20	0.19	0.0000
NOU	12	f	0	1.96	2.74	0.32	0.0012
Subtotal NOU				1.86	7.94	0.51	
ODRISC	1	c	0	3.99	5.77	32.60	0.0000
ORMOS	4	m	0	2.23	6.39	2.44	0.0000
ORMOS	26	f	0	-1.64	0.95	10.11	0.1093
Subtotal ORMOS				1.73	7.34	12.55	
OSANN	33	m	2	3.28	37.23	102.66	0.0000
OSANN	34	f	2	2.98	60.12	111.01	0.0000
Subtotal OSANN				3.09	97.36	213.67	
PARKIN	30	m	0	1.53	70.51	0.53	0.0000
PASTOR	10	m	1	1.92	7.85	0.71	0.0000
PAWLEG	2	m	6	2.51	3.16	2.50	0.0000
PERNU	8	m	0	2.23	50.02	18.60	0.0000
PERNU	4	f	0	0.95	5.14	2.31	0.0321
Subtotal PERNU				2.11	55.16	20.91	
PERSH2	10	c	4	2.12	107.04	26.59	0.0000
*PETO	4	m	0	1.97	1.98	0.25	0.0056
PEZZO2	2	m	0	3.13	5.42	12.46	0.0000
PEZZOT	5	m	0	3.48	3.66	12.76	0.0000
PIKE	4	m	0	1.66	13.39	0.02	0.0000
PIKE	8	f	0	1.57	18.04	0.03	0.0000
Subtotal PIKE				1.61	31.43	0.06	
POFFIJ	1	c	0	2.05	46.21	8.59	0.0000
POLEDN	1	c	1	2.22	11.85	4.37	0.0000
*QIAO2	8	m	0	0.72	9.54	7.72	0.0267
RACHTA	9	f	1	1.91	10.62	0.93	0.0000
RADZIK	1	c	0	0.27	5.03	9.10	0.5411
RANDIG	9	m	0	1.60	3.95	0.00	0.0014
RANDIG	10	f	0	0.80	6.34	4.25	0.0447
Subtotal RANDIG				1.11	10.29	4.25	
REN	1	m	0	1.27	7.46	0.88	0.0005
REN	2	f	0	1.40	9.65	0.45	0.0000
Subtotal REN				1.35	17.11	1.32	
RONCO	3	m	2	1.69	5.06	0.03	0.0001
ROTHSC	2	c	1	1.71	9.83	0.09	0.0000

Table 1C4 - 2

IESLC - Meta-anal of Current Smoking (or Ever if Current not available), Cigs (or Any Prod if Cigs not avail)
All LC types
Most adjusted

REF	NRR	SEX	AD	Ys	Ws	Qs	Ps
SADOWS	4	m	0	1.45	13.79	0.40	0.0000
SANKAR	2	m	3	2.61	22.36	22.13	0.0000
SCHWAR	25	m	0	2.71	68.81	81.52	0.0000
SCHWAR	26	m	0	2.27	26.07	10.98	0.0000
SCHWAR	27	f	0	2.75	91.98	118.16	0.0000
SCHWAR	28	f	0	2.87	22.69	35.41	0.0000
Subtotal SCHWAR				2.69	209.54	246.07	
SEGI	1	m	0	0.53	15.18	17.79	0.0375
SEGI2	20	m	1	1.32	6.65	0.59	0.0007
SEGI2	28	f	1	0.50	10.48	13.06	0.1049
Subtotal SEGI2				0.82	17.14	13.64	
SEOW	6	f	1	1.66	9.73	0.02	0.0000
SHAW	6	c	0	3.06	8.67	17.99	0.0000
SIEMIA	5	m	7	2.49	10.37	7.96	0.0000
SIMARA	3	m	6	0.50	13.58	16.91	0.0650
SIMARA	4	f	6	0.49	9.71	12.36	0.1278
Subtotal SIMARA				0.50	23.30	29.28	
SOBUE	42	m	1	1.41	27.66	1.17	0.0000
SOBUE	52	f	1	1.03	34.45	11.87	0.0000
Subtotal SOBUE				1.20	62.11	13.04	
SOBUE2	10	m	2	1.50	197.90	2.82	0.0000
SOBUE2	12	f	2	1.19	143.51	26.39	0.0000
Subtotal SOBUE2				1.37	341.42	29.21	
*SPEIZE	10	f	1	2.54	65.88	56.26	0.0000
SPITZ	2	c	0	3.05	5.83	12.01	0.0000
STASZE	7	m	0	2.50	4.72	3.66	0.0000
STASZE	5	f	0	1.47	4.16	0.09	0.0028
Subtotal STASZE				2.02	8.88	3.75	
STAYNE	1	m	0	1.30	40.37	4.07	0.0000
STOCKS	46	m	2	1.94	32.85	3.38	0.0000
STOCKS	50	f	1	1.11	58.11	14.81	0.0000
Subtotal STOCKS				1.41	90.96	18.19	
STOCKW	7	c	0	2.65	1204.31	1287.39	0.0000
STUCKE	2	m	0	4.65	0.49	4.49	0.0012
SUN	1	c	0	0.84	30.23	18.42	0.0000
SUZUK2	6	c	3	3.09	2.54	5.52	0.0000
SVENSS	96	f	1	2.20	13.42	4.63	0.0000
TANG	1	c	0	2.20	5.10	1.73	0.0000
*TENKAN	24	m	1	2.82	5.38	7.81	0.0000
TIZZAN	5	m	0	0.64	84.08	80.16	0.0000
TIZZAN	13	f	0	1.46	6.13	0.15	0.0003
Subtotal TIZZAN				0.70	90.21	80.31	
TOKARS	1	m	0	3.61	0.97	3.86	0.0004
TOKARS	5	f	0	0.43	0.62	0.88	0.7336
Subtotal TOKARS				2.37	1.59	4.74	
TOUSEY	12	m	3	4.08	3.57	21.67	0.0000
TOUSEY	15	f	3	3.41	9.42	30.21	0.0000
Subtotal TOUSEY				3.59	12.98	51.87	
TSUGAN	28	m	0	0.20	7.53	15.10	0.5818
*TULINI	36	m	3	2.50	10.05	7.82	0.0000
*TULINI	42	f	3	2.79	11.07	15.33	0.0000
Subtotal TULINI				2.65	21.12	23.15	
*TVERDA	5	m	2	1.41	20.41	0.88	0.0000
*TVERDA	15	f	2	2.40	2.67	1.65	0.0001
Subtotal TVERDA				1.52	23.08	2.53	
WAKAI	8	m	2	1.48	7.88	0.14	0.0000
WAKAI	26	f	2	1.47	8.29	0.17	0.0000
Subtotal WAKAI				1.48	16.17	0.31	
*WALD	4	m	1	2.80	4.92	6.86	0.0000
WANG	5	c	6	1.06	15.11	4.72	0.0000
WANG2	18	c	4	0.88	6.94	3.81	0.0211
WANG3	1	c	0	1.05	28.11	9.11	0.0000
WANG4	2	m	2	0.15	100.26	216.14	0.1372
WICKLU	1	m	0	1.53	15.41	0.13	0.0000
WIGLE	28	m	1	2.52	13.08	10.62	0.0000
WIGLE	33	f	1	1.65	19.63	0.02	0.0000
Subtotal WIGLE				2.00	32.72	10.64	
WILKIN	3	c	4	2.06	12.03	2.34	0.0000
WU	42	f	2	1.58	11.97	0.02	0.0000
WUNSCH	5	m	1	1.89	10.41	0.75	0.0000
WUNSCH	11	f	1	1.79	10.34	0.30	0.0000

Table 1C4 - 2

IESLC - Meta-anal of Current Smoking (or Ever if Current not available), Cigs (or Any Prod if Cigs not avail)
All LC types
Most adjusted

REF	NRR	SEX	AD	Ys	Ws	Qs	Ps
Subtotal WUNSCH				1.84	20.74	1.06	
WUWILL	8	f	3	0.83	102.19	62.78	0.0000
WYNDE2	16	m	0	2.28	7.19	3.18	0.0000
WYNDE3	50	m	0	2.37	7.59	4.34	0.0000
WYNDE3	83	f	0	1.14	9.73	2.23	0.0004
Subtotal WYNDE3				1.68	17.32	6.56	
WYNDE4	48	m	0	2.21	10.51	3.69	0.0000
WYNDE4	62	f	2	1.05	8.80	2.78	0.0018
Subtotal WYNDE4				1.68	19.31	6.47	
WYNDE6	18	m	0	2.78	66.40	89.20	0.0000
WYNDE6	207	f	0	2.68	90.13	102.53	0.0000
Subtotal WYNDE6				2.72	156.52	191.73	
*XIANGZ	14	m	2	0.58	25.00	26.76	0.0036
XU	2	m	2	0.99	58.89	22.89	0.0000
XU2	2	c	7	1.34	45.75	3.63	0.0000
XU3	2	m	1	1.79	5.80	0.17	0.0000
XU3	4	f	1	1.35	3.69	0.26	0.0095
Subtotal XU3				1.62	9.49	0.44	
XU4	1	c	0	1.08	20.82	6.00	0.0000
YAMAGU	10	c	1	1.59	8.97	0.01	0.0000
*YONG	12	m	1	3.36	1.92	5.82	0.0000
*YONG	15	f	1	1.65	6.30	0.01	0.0000
Subtotal YONG				2.05	8.22	5.82	
*YUAN	1	m	2	1.87	11.44	0.74	0.0000
ZHANG	2	m	7	1.39	4.65	0.25	0.0028
ZHANG	3	f	7	1.32	4.81	0.42	0.0038
Subtotal ZHANG				1.35	9.46	0.66	
ZHENG	15	m	0	1.29	20.36	2.13	0.0000
ZHENG	24	f	0	0.74	20.88	16.13	0.0008
Subtotal ZHENG				1.01	41.24	18.26	
ZHOU	2	m	0	0.86	17.50	10.02	0.0003
ZHOU	3	f	0	0.80	5.34	3.60	0.0660
Subtotal ZHOU				0.84	22.83	13.62	

	N	345
	NS	242
	Wt	19170.40
Het	Chi	8406.25
Het	df	344
Het	P	***
Fixed	RR	5.04
	RRl	4.97
	RRu	5.11
	P	+++
Random	RR	6.29
	RRl	5.79
	RRu	6.83
	P	+++
Asymm	P	***

Table 1C4 - 3

IESLC - Meta-anal of Current Smoking (or Ever if Current not available), Cigs (or Any Prod if Cigs not avail)
All LC types
Most adjusted

	Sex combined	male	female	Total
N	45	181	119	345
NS	45	177	114	336
Wt	2037.11	10018.81	7114.48	19170.40
Het Chi	873.80	3207.65	3044.38	8406.25
Het df	44	180	118	344
Het P	***	***	***	***
Fixed RR	10.19	5.01	4.15	5.04
RRl	9.76	4.91	4.05	4.97
RRu	10.64	5.11	4.25	5.11
P	+++	+++	+++	+++
Random RR	7.17	7.17	4.90	6.29
RRl	5.60	6.41	4.21	5.79
RRu	9.20	8.03	5.70	6.83
P	+++	+++	+++	+++
Between Chi				1280.42
Between df				2
Between P				***
Btwn(F) P				***
Btwn(R) P				***

	Smoking status ever	current	Total
N	154	191	345
NS	116	129	245
Wt	12268.31	6902.09	19170.40
Het Chi	1555.75	2608.10	8406.25
Het df	153	190	344
Het P	***	***	***
Fixed RR	3.54	9.43	5.04
RRl	3.48	9.21	4.97
RRu	3.60	9.65	5.11
P	+++	+++	+++
Random RR	4.16	8.57	6.29
RRl	3.83	7.76	5.79
RRu	4.52	9.45	6.83
P	+++	+++	+++
Between Chi			4242.40
Between df			1
Between P			***
Btwn(F) P			***
Btwn(R) P			***

	Study LIU4 LIU4	others	Total
N	2	343	345
NS	1	241	242
Wt	9657.16	9513.24	19170.40
Het Chi	215.88	5331.52	8406.25
Het df	1	342	344
Het P	***	***	***
Fixed RR	3.43	7.43	5.04
RRl	3.37	7.28	4.97
RRu	3.50	7.58	5.11
P	+++	+++	+++
Random RR	3.33	6.33	6.29
RRl	2.47	5.80	5.79
RRu	4.49	6.90	6.83
P	+++	+++	+++
Between Chi			2858.84
Between df			1
Between P			***
Btwn(F) P			***
Btwn(R) P			***

Table 1C4 - 4

IESLC - Meta-anal of Current Smoking (or Ever if Current not available), Cigs (or Any Prod if Cigs not avail)
All LC types
Least adjusted

REF	NRR	X	SEX	AGEL	AGEH	RACE	YF	LC TYPE	LOC	START	ST	NLC	R	VB	P	H	AD	SM	PRODUCT	DENOM	De
ABELIN	2	x	m	0	0	all	-	all	Eu:wst	1941	CC	118	n	bl	y	n	0	ev	cig+/-ot	nev any	st
ABRAHA	7		m	0	0	all	0	q+s+a	Eu:est	1975	pr	571	n	bl	n	n	0	ev	all/unsp	nev any	ot
ABRAHA	8		f	0	0	all	0	q+s+a	Eu:est	1975	pr	571	n	bl	n	n	0	ev	all/unsp	nev any	ot
AGUDO	10	x	f	0	0	all	-	all	Eu:wst	1989	CC	103	n	bl	n	n	0	cu	cig only	nev any	st
AKIBA	2	x	m	0	0	all	0	all	As:Jap	1963	pr	610	n	bl	n	n	0	cu	cig+/-ot	nev cigs	or
AKIBA	6	x	f	0	0	all	0	all	As:Jap	1963	pr	610	n	bl	n	n	0	cu	cig+/-ot	nev cigs	or
ALDERS	177		m	0	0	all	-	all	Eu:UK	1977	CC	1448	n	V	n	n	0	cu	cig+/-ot	nev any	st
ALDERS	176		f	0	0	all	-	all	Eu:UK	1977	CC	1448	n	V	n	n	0	cu	cig only	nev any	st
AMANDU	1	x	m	0	0	wh	0	all	NAmer	1959	pr	132	m	bl	n	n	0	cu	cig+/-ot	nev cigs	st
AMES	1		m	0	0	wh	-	all	NAmer	1959	ot	317	m	bl	n	n	0	cu	all/unsp	nev any	or
ANDERS	2	x	f	0	0	all	0	all	NAmer	1986	pr	343	n	bl	n	n	0	cu	cig+/-ot	nev cigs	st
ARCHER	5		m	0	0	wh	0	all	NAmer	1950	pr	146	m	bl	n	n	0	cu	cig+/-ot	nev cigs	st
ARMADA	27		m	0	0	all	-	all	Eu:wst	1986	CC	325	n	bl	n	y	0	cu	cig+/-ot	nev any	st
AUSTIN	2	x	c	0	0	all	-	all	NAmer	1970	CC	166	o	bl	y	n	0	cu	cig+/-ot	nev cigs	st
AUVINE	1	x	c	0	0	all	-	all	Eu:Sca	1986	CC	517	n	bl	y	n	0	ev	cig+/-ot	nev cigs	st
AXELSO	1		c	0	0	all	-	all	Eu:Sca	1960	CC	152	n	bl	y	n	0	ev	all/unsp	nev any	st
AXELSS	2		m	0	0	sca	-	all	Eu:Sca	1989	CC	436	n	bl	n	n	0	cu	all/unsp	nev any	st
AXELSS	10		f	0	0	sca	-	all	Eu:Sca	1989	CC	436	n	bl	n	n	0	cu	all/unsp	nev any	st
BAND	1		m	0	0	all	-	all	NAmer	1983	CC	2831	n	V	y	y	2	ev	cig only	nev any	ot
BARBON	3	x	m	0	0	all	-	all	Eu:wst	1979	CC	755	n	bl	y	y	0	cu	all/unsp	nev any	st
BECHER	13		m	0	0	all	-	all	Eu:Ger	1985	CC	194	n	bl	n	y	0	cu	all/unsp	nev any	st
BECHER	14		f	0	0	all	-	all	Eu:Ger	1985	CC	194	n	bl	n	y	0	cu	all/unsp	nev any	st
BENSHL	4		m	0	0	all	0	all	Eu:UK	1967	pr	486	n	V	n	n	1	cu	cig+/-ot	nev any	ot
BEST	2		m	0	0	all	0	all	NAmer	1955	pr	381	n	V	n	n	1	cu	cig only	nev any	ot
BEST	18		f	0	0	all	0	all	NAmer	1955	pr	381	n	V	n	n	1	ev	cig only	nev any	ot
BLOHMK	1		m	0	0	all	-	all	Eu:Ger	1978	CC	888	n	bl	n	y	0	cu	all/unsp	nev any	st
BLOT4	1		m	0	0	wh	-	all	NAmer	1974	CC	335	n	bl	y	n	0	ev	cig+/-ot	nev cigs	st
BOFFET	7	x	m	0	0	all	-	all	Eu:mul	1988	CC	5621	n	bl	y	n	0	ev	cig+/-ot	nev any	st
BOUCOT	2	x	m	0	0	all	0	all	NAmer	1951	pr	121	n	bl	n	n	0	cu	cig only	nev any	ot
BRESLO	17		m	0	0	all	-	all	NAmer	1949	CC	518	n	bl	n	y	0	ev	cig+/-ot	nev+1	st
BRESLO	23		f	0	0	all	-	all	NAmer	1949	CC	518	n	bl	n	y	0	ev	cig+/-ot	nev+1	st
BRETT	4		m	0	0	all	0	all	Eu:UK	1960	pr	150	n	V	n	n	0	cu	cig+/-ot	nev cigs	st
BROCKM	1		m	0	0	wh	-	all	Eu:Ger	1990	CC	117	n	bl	n	y	0	ev	cig+/-ot	nev cigs	st
BROCKM	2		f	0	0	wh	-	all	Eu:Ger	1990	CC	117	n	bl	n	y	0	ev	cig+/-ot	nev cigs	st
BROSS	4		m	0	0	wh	-	all	NAmer	1960	CC	974	n	bl	n	n	0	cu	cig+/-ot	nev any	st
BROWN2	12		m	0	0	wh	-	all	NAmer	1984	CC	14596	n	bl	n	y	2	cu	cig+/-ot	nev cigs	or
BROWN2	11		f	0	0	wh	-	all	NAmer	1984	CC	14596	n	bl	n	y	2	cu	cig+/-ot	nev cigs	or
BUFFLE	3		m	0	0	wh	-	all	NAmer	1976	CC	943	n	bl	y	n	0	cu	cig+/-ot	nev any	st
BUFFLE	7		f	0	0	wh	-	all	NAmer	1976	CC	943	n	bl	y	n	0	cu	cig+/-ot	nev any	st
CARPEN	9	x	c	0	0	w+b	-	all	NAmer	1991	CC	356	n	bl	n	n	0	cu	cig+/-ot	nev cigs	st
CASCO2	1		c	0	0	wh	-	all	Eu:Ger	1991	CC	155	n	bl	n	n	0	ev	all/unsp	nev any	st
CASCOR	1		c	0	0	wh	-	all	Eu:Ger	1985	CC	389	n	bl	n	y	0	ev	all/unsp	nev any	st
CEDERL	2	x	m	0	0	all	10	all	Eu:Sca	1963	pr	491	n	bl	n	n	0	cu	cig+/-ot	nev any	st
CEDERL	119		f	0	0	all	10	all	Eu:Sca	1963	pr	491	n	bl	n	n	1	cu	cig+/-ot	nev any	ot
CHAN	5		m	0	0	all	-	all	As:HK	1976	CC	397	n	bl	n	n	0	ev	cig+/-ot	nev any	st
CHAN	6		f	0	0	all	-	all	As:HK	1976	CC	397	n	bl	n	n	0	ev	cig+/-ot	nev any	st
CHANG	5		m	0	0	all	0	all	NAmer	1972	pr	136	n	bl	n	n	0	cu	cig+/-ot	nev cigs	st
CHANG	11		f	0	0	all	0	all	NAmer	1972	pr	136	n	bl	n	n	0	cu	cig+/-ot	nev cigs	st
CHATZI	4		c	0	0	all	-	all	Eu:bal	1987	CC	282	n	bl	n	y	0	ev	all/unsp	nev any	st
CHEN2	1		m	0	0	all	-	all	As:Chi	1983	CC	193	n	ot	y	n	0	ev	all/unsp	nev any	st
CHEN2	2		f	0	0	all	-	all	As:Chi	1983	CC	193	n	ot	y	n	0	ev	all/unsp	nev any	st
CHEN3	1		c	0	0	all	-	all	As:Chi	1981	CC	254	n	ot	y	n	0	ev	all/unsp	nev any	st
CHIAZZ	2	x	m	0	0	all	-	all	NAmer	1940	CC	144	o	bl	y	n	0	ev	cig+/-ot	nev cigs	st
CHOI	3		m	0	0	all	-	all	As:oth	1985	CC	375	n	bl	n	n	0	cu	cig+/-ot	nev cigs	st
CHOI	7		f	0	0	all	-	all	As:oth	1985	CC	375	n	bl	n	n	0	cu	cig+/-ot	nev cigs	st
CHOW	18	x	m	0	0	wh	0	all	NAmer	1966	pr	219	n	bl	n	n	0	cu	cig+/-ot	nev any	st
CHYOU	4	x	m	0	0	jap	0	all	NAmer	1965	pr	227	n	bl	n	y	0	cu	cig+/-ot	nev cigs	st
COMSTO	3		m	0	0	all	-	all	NAmer	1975	ot	258	n	bl	n	n	0	cu	cig+/-ot	nev any	st
COMSTO	8		f	0	0	all	-	all	NAmer	1975	ot	258	n	bl	n	n	0	cu	cig+/-ot	nev any	st
COOKSO	4		c	0	0	bl	-	all	Africa	1961	CC	234	n	V	n	y	0	ev	cig+/-ot	nev any	st
CORREA	41	x	c	0	0	all	-	all	NAmer	1979	CC	1359	n	bl	y	n	0	cu	cig+/-ot	nev cigs	st
CPSI	220		m	35	84	all	6	all	NAmer	1959	pr	5138	n	bl	n	n	1	cu	cig+/-ot	nev any	ot
CPSI	279		f	40	74	all	6	all	NAmer	1959	pr	5138	n	bl	n	n	1	cu	cig+/-ot	nev cigs	ot
CPSII	36	x	m	0	0	all	6	all	NAmer	1982	pr	3229	n	bl	n	n	0	cu	cig only	nev any	st
CPSII	71	x	f	0	0	all	6	all	NAmer	1982	pr	3229	n	bl	n	n	0	cu	cig+/-ot	nev cigs	st
DAMBER	16		m	0	0	all	-	all	Eu:Sca	1972	CC	579	n	bl	y	n	1	cu	cig only	nev any	ot
DARBY	4		m	0	0	wh	-	all	Eu:UK	1988	CC	982	n	V	n	n	0	cu	cig+/-ot	nev any	st
DARBY	11		f	0	0	wh	-	all	Eu:UK	1988	CC	982	n	V	n	n	0	cu	cig+/-ot	nev any	st
DAVEYS	5		m	0	0	all	-	all	Eu:Ger	1930	CC	109	n	bl	y	n	0	ev	all/unsp	nev any	st
DAVEYS	6		f	0	0	all	-	all	Eu:Ger	1930	CC	109	n	bl	y	n	0	ev	all/unsp	nev any	ot
DEAN	8		m	0	0	wh	-	all	Africa	1947	CC	603	n	V	y	n	0	ev	cig+/-ot	nev any	st
DEAN2	2		m	0	0	all	-	all	Eu:UK	1960	CC	954	n	V	y	n	0	cu	all/unsp	nev any	st

Table 1C4 - 4

IESLC - Meta-anal of Current Smoking (or Ever if Current not available), Cigs (or Any Prod if Cigs not avail)
All LC types
Least adjusted

REF	NRR	X	SEX	AGEL	AGEH	RACE	YF	LC TYPE	LOC	START	ST	NLC	R	VB	P	H	AD	SM	PRODUCT	DENOM	De
DEAN2	6		f	0	0	all	-	all	Eu:UK	1960	CC	954	n	V	y	n	0	cu	all/unsp	nev any	st
DEAN3	238	x	m	0	0	all	-	all	Eu:UK	1969	CC	766	n	V	y	n	0	cu	cig+/-ot	nev any	st
DEAN3	117	x	f	0	0	all	-	all	Eu:UK	1969	CC	766	n	V	y	n	0	cu	cig only	nev any	st
DEKLER	7		m	0	0	all	0	all	Auslia	1961	pr	138	m	V	n	n	2	cu	cig+/-ot	nev any	ot
DESTE2	2	x	c	0	0	all	-	all	SCAmer	1993	CC	463	n	bl	n	n	0	cu	all/unsp	nev any	st
DESTEF	40	x	m	0	0	all	-	all	SCAmer	1988	CC	497	n	bl	n	y	0	cu	all/unsp	nev any	st
DOCKER	1		c	0	0	wh	0	all	NAmer	1974	pr	120	n	bl	n	n	4	cu	cig+/-ot	nev cigs	or
DOLL	90		m	0	0	all	-	all	Eu:UK	1948	CC	1465	n	V	n	n	0	cu	all/unsp	nev any	st
DOLL	93		f	0	0	all	-	all	Eu:UK	1948	CC	1465	n	V	n	n	0	cu	all/unsp	nev any	st
DOLL2	68		m	0	0	all	20	all	Eu:UK	1951	pr	920	n	V	n	n	1	cu	cig+/-ot	nev any	ot
DOLL2	63		f	0	0	all	22	all	Eu:UK	1951	pr	920	n	V	n	n	1	cu	cig only	nev any	ot
DORANT	9		c	0	0	all	0	all	Eu:wst	1986	ot	550	n	bl	n	y	0	cu	cig+/-ot	nev any	st
DORGAN	9		m	0	0	wh	-	all	NAmer	1980	CC	2026	n	bl	y	y	0	cu	cig+/-ot	nev any	st
DORGAN	33		m	0	0	bl	-	all	NAmer	1980	CC	2026	n	bl	y	y	0	cu	cig+/-ot	nev any	st
DORGAN	56		f	0	0	wh	-	all	NAmer	1980	CC	2026	n	bl	y	y	0	cu	cig+/-ot	nev any	st
DORGAN	79		f	0	0	bl	-	all	NAmer	1980	CC	2026	n	bl	y	y	0	cu	cig+/-ot	nev any	st
DORN	391		m	0	0	wh	25	all	NAmer	1954	pr	5097	n	bl	n	n	1	cu	cig+/-ot	nev any	or
DOSEME	17	x	m	0	0	all	-	all	Eu:bal	1979	CC	1210	n	bl	n	n	0	ev	cig+/-ot	nev cigs	st
DROSTE	2	x	m	0	0	all	-	all	Eu:wst	1995	CC	478	n	bl	n	y	0	cu	all/unsp	nev any	st
DU	1		m	0	0	all	-	all	As:Chi	1985	CC	849	n	ot	y	n	0	ev	all/unsp	nev any	or
DU	2		f	0	0	all	-	all	As:Chi	1985	CC	849	n	ot	y	n	0	ev	all/unsp	nev any	or
DUNN	6		m	0	0	all	0	all	NAmer	1954	pr	139	o	bl	n	n	0	ev	cig+/-ot	nev cigs	st
EBELIN	1		m	0	0	all	-	all	Eu:Ger	1980	CC	130	n	bl	n	n	0	ev	all/unsp	nev any	st
ENGELA	168		m	0	0	all	12	all	Eu:Sca	1964	pr	435	n	bl	n	n	1	cu	cig+/-ot	nev any	ot
ENGELA	177		f	0	0	all	12	all	Eu:Sca	1964	pr	435	n	bl	n	n	1	cu	cig+/-ot	nev any	ot
ENSTRO	1		m	0	0	all	0	all	NAmer	1959	pr	2879	n	bl	n	n	1	cu	cig only	nev any	or
ENSTRO	2		f	0	0	all	0	all	NAmer	1959	pr	2879	n	bl	n	n	1	cu	cig only	nev any	or
ESAKI	4		m	0	0	all	-	all	As:Jap	1961	CC	245	n	bl	y	n	0	ev	cig+/-ot	nev cigs	st
ESAKI	5		f	0	0	all	-	all	As:Jap	1961	CC	245	n	bl	y	n	0	ev	cig+/-ot	nev cigs	st
FAN	1		m	0	0	all	-	all	As:Chi	1990	CC	403	n	ot	y	n	0	ev	cig+/-ot	nev cigs	st
FAN	2		f	0	0	all	-	all	As:Chi	1990	CC	403	n	ot	y	n	0	ev	cig+/-ot	nev cigs	st
GAO	29	x	m	0	0	all	-	all	As:Chi	1984	CC	1405	n	ot	n	n	0	cu	cig+/-ot	nev cigs	st
GAO	30	x	f	0	0	all	-	all	As:Chi	1984	CC	1405	n	ot	n	n	0	cu	cig+/-ot	nev cigs	st
GAO2	1	x	m	0	0	all	-	all	As:Jap	1988	CC	282	n	bl	n	n	0	cu	cig+/-ot	nev cigs	st
GARCIA	2		c	0	0	all	-	all	NAmer	1992	CC	416	n	bl	n	y	0	cu	cig+/-ot	nev cigs	st
GARDIN	6		c	0	0	all	-	all	Eu:UK	1988	CC	143	n	V	y	n	0	cu	cig only	nev any	st
GARSHI	23	x	m	0	0	all	-	all	NAmer	1981	CC	1081	o	bl	y	n	0	cu	all/unsp	nev any	st
GENG	1		m	0	0	all	-	all	As:Chi	1985	CC	292	n	ot	*	n	0	ev	cig+/-ot	nev any	st
GENG	2		f	0	0	all	-	all	As:Chi	1985	CC	292	n	ot	*	n	0	ev	cig+/-ot	nev any	st
GER	17	x	c	0	0	all	-	all	As:oth	1990	CC	141	n	ot	y	n	0	ev	all/unsp	nev any	st
GODLEY	5		m	0	0	all	-	all	NAmer	1966	CC	1986	n	bl	y	n	1	ev	cig+/-ot	nev cigs	ot
GODLEY	6		f	0	0	all	-	all	NAmer	1966	CC	1986	n	bl	y	n	1	ev	cig+/-ot	nev cigs	ot
GOLLED	21	x	m	35	99	all	-	all	Eu:UK	1952	CC	443	n	V	y	n	0	ev	cig+/-ot	nev any	st
GOODMA	2		m	0	0	w+o	-	all	NAmer	1983	CC	326	n	bl	y	y	0	cu	cig+/-ot	nev any	st
GOODMA	6		f	0	0	w+o	-	all	NAmer	1983	CC	326	n	bl	y	y	0	cu	cig+/-ot	nev any	st
GRAHAM	9	x	m	0	0	wh	-	all	NAmer	1956	CC	685	n	bl	n	n	0	cu	cig+/-ot	nev any	st
GREGOR	2		m	0	0	all	-	all	Eu:UK	1976	CC	104	n	V	n	y	0	cu	cig+/-ot	nev cigs	st
GREGOR	6		f	0	0	all	-	all	Eu:UK	1976	CC	104	n	V	n	y	0	cu	cig+/-ot	nev cigs	st
GSELL	6		m	0	0	all	-	all	Eu:wst	1937	CC	150	n	bl	n	y	0	ev	cig+/-ot	nev any	st
HAENSZ	54		f	0	0	all	-	not alv	NAmer	1955	CC	158	n	bl	n	y	0	cu	cig+/-ot	nev any	st
HAMMO2	22	x	m	0	0	all	0	all	NAmer	1967	pr	450	o	bl	n	n	0	cu	cig+/-ot	nev any	st
HAMMON	139		m	0	0	wh	0	all	NAmer	1952	pr	448	n	bl	n	n	1	cu	cig only	nev any	ot
HANSEN	3		m	0	0	all	0	all	Eu:Sca	1968	pr	105	o	bl	y	n	2	ev	all/unsp	nev any	ot
HEGMAN	1		c	0	0	all	-	all	NAmer	1989	CC	282	n	bl	y	y	0	ev	all/unsp	nev any	st
HEIN	1		m	0	0	all	0	all	Eu:Sca	1970	pr	144	n	bl	n	n	0	cu	cig only	nev any	st
HENNEK	2		m	0	0	all	0	all	NAmer	1982	pr	169	n	bl	n	n	0	cu	all/unsp	nev any	st
HINDS	26	x	f	0	0	o	-	all	NAmer	1968	CC	292	n	bl	n	n	0	ev	all/unsp	nev any	st
HIRAYA	1		m	0	0	all	0	all	As:Jap	1965	pr	1917	n	bl	n	n	1	cu	cig+/-ot	nev any	st
HIRAYA	3		f	0	0	all	0	all	As:Jap	1965	pr	1917	n	bl	n	n	1	cu	cig+/-ot	nev any	st
HITOSU	2	x	m	0	0	all	-	all	As:Jap	1960	CC	216	n	bl	y	n	0	cu	all/unsp	nev any	st
HITOSU	9	x	f	0	0	all	-	all	As:Jap	1960	CC	216	n	bl	y	n	0	cu	all/unsp	nev any	st
HOLE	46	x	m	0	0	all	0	all	Eu:UK	1972	pr	225	n	V	n	n	0	cu	cig+/-ot	nev any	st
HOLE	29	x	f	0	0	all	11	all	Eu:UK	1972	pr	225	n	V	n	n	0	cu	all/unsp	nev any	st
HOROWI	1		m	0	0	all	-	all	NAmer	1956	CC	236	n	V	n	n	0	ev	cig+/-ot	nev any	st
HOROWI	2		f	0	0	all	-	all	NAmer	1956	CC	236	n	V	n	n	0	ev	cig+/-ot	nev any	st
HORWIT	1		f	0	0	all	-	all	NAmer	1977	CC	112	n	bl	n	n	0	ev	cig+/-ot	nev cigs	st
HU	15		m	0	0	all	-	all	As:Chi	1985	CC	227	n	ot	n	y	0	ev	cig+/-ot	nev any	st
HU	16		f	0	0	all	-	all	As:Chi	1985	CC	227	n	ot	n	y	0	ev	cig+/-ot	nev any	st
HU2	9		m	0	0	all	-	all	As:Chi	1977	CC	523	n	ot	y	n	0	ev	cig+/-ot	nev cigs	st
HU2	10		f	0	0	all	-	all	As:Chi	1977	CC	523	n	ot	y	n	0	ev	cig+/-ot	nev cigs	st
HUANG	1		c	0	0	all	-	all	As:Chi	1990	CC	135	n	ot	y	n	0	ev	all/unsp	nev any	st
HUMBLE	13		m	0	0	w-hi	-	all	NAmer	1980	CC	521	n	bl	y	n	1	cu	cig+/-ot	nev cigs	ot

Table 1C4 - 4

IESLC - Meta-anal of Current Smoking (or Ever if Current not available), Cigs (or Any Prod if Cigs not avail)
All LC types
Least adjusted

REF	NRR	X	SEX	AGEL	AGEH	RACE	YF	LC TYPE	LOC	START	ST	NLC	R	VB	P	H	AD	SM	PRODUCT	DENOM	De
HUMBLE	15		m	0	0	hi	-	all	NAmer	1980	CC	521	n	bl	y	n	1	cu	cig+/-ot	nev cigs	ot
HUMBLE	17		f	0	0	w-hi	-	all	NAmer	1980	CC	521	n	bl	y	n	1	cu	cig+/-ot	nev cigs	ot
HUMBLE	19		f	0	0	hi	-	all	NAmer	1980	CC	521	n	bl	y	n	1	cu	cig+/-ot	nev cigs	ot
JAHN	3	x	f	0	0	all	-	all	Eu:Ger	1988	CC	1004	n	bl	n	n	0	ev	cig+/-ot	nev any	st
JAIN	16	x	m	0	0	all	-	all	NAmer	1981	CC	845	n	V	y	n	0	cu	cig+/-ot	nev cigs	st
JAIN	11	x	f	0	0	all	-	all	NAmer	1981	CC	845	n	V	y	n	0	cu	cig+/-ot	nev cigs	st
JARUP	3	x	m	0	0	all	-	all	Eu:Sca	1928	CC	102	o	bl	y	n	0	ev	all/unsp	nev any	st
JARVHO	2		m	0	0	all	-	all	Eu:Sca	1983	CC	147	n	bl	n	n	0	cu	all/unsp	nev any	st
JARVHO	6		f	0	0	all	-	all	Eu:Sca	1983	CC	147	n	bl	n	n	0	cu	all/unsp	nev any	st
JEDRYC	63	x	m	0	0	all	-	all	Eu:est	1980	CC	1630	n	bl	y	n	0	ev	cig+/-ot	nev any	st
JEDRYC	68	x	f	0	0	all	-	all	Eu:est	1980	CC	1630	n	bl	y	n	0	ev	cig+/-ot	nev any	st
JIANG	1		m	0	0	all	-	all	As:Chi	1984	CC	125	n	ot	n	n	0	ev	all/unsp	nev any	st
JIANG	2		f	0	0	all	-	all	As:Chi	1984	CC	125	n	ot	n	n	0	ev	all/unsp	nev any	st
JOLY	16		m	0	0	all	-	all	SCAmer	1978	CC	826	n	bl	n	n	0	cu	cig+/-ot	nev any	st
JOLY	15		f	0	0	all	-	all	SCAmer	1978	CC	826	n	bl	n	n	0	cu	cig+/-ot	nev any	st
JUSSAW	2	x	m	0	0	all	-	all	As:Ind	1964	CC	792	n	V	n	n	0	ev	cig only	nev any	st
KAISE2	68		m	35	99	all	9	all	NAmer	1979	pr	318	n	bl	n	n	1	cu	cig only	nev any	st
KAISE2	60		f	35	99	all	9	all	NAmer	1979	pr	318	n	bl	n	n	1	cu	cig only	nev any	st
KAISER	12		m	0	0	all	0	all	NAmer	1964	pr	714	n	bl	n	n	2	cu	cig+/-ot	nev cigs	ot
KAISER	9		f	0	0	all	0	all	NAmer	1964	pr	714	n	bl	n	n	2	cu	cig+/-ot	nev cigs	ot
KANELL	5	x	m	0	0	all	-	all	Eu:bal	1950	CC	862	n	bl	n	n	0	cu	all/unsp	nev any	st
KATSOU	6	x	f	0	0	all	-	all	Eu:bal	1987	CC	101	n	bl	n	n	0	cu	all/unsp	nev any	st
KAUFMA	7	x	c	0	0	all	-	all	NAmer	1981	CC	881	n	bl	n	n	0	cu	cig+/-ot	nev cigs	st
KELLER	1		m	0	0	wh	-	all	NAmer	1985	CC	15038	n	bl	n	n	0	cu	all/unsp	nev any	st
KELLER	9		m	0	0	nonw	-	all	NAmer	1985	CC	15038	n	bl	n	n	0	cu	all/unsp	nev any	st
KELLER	5		f	0	0	wh	-	all	NAmer	1985	CC	15038	n	bl	n	n	0	cu	all/unsp	nev any	st
KELLER	13		f	0	0	nonw	-	all	NAmer	1985	CC	15038	n	bl	n	n	0	cu	all/unsp	nev any	st
KHUDER	19		m	0	0	all	-	all	NAmer	1985	CC	482	n	bl	n	y	0	cu	cig+/-ot	nev cigs	or
KIHARA	7		c	0	0	jap	-	all	As:Jap	1991	CC	440	n	bl	n	n	0	cu	all/unsp	nev any	st
KINLEN	8	x	m	0	0	all	0	all	Eu:UK	1967	pr	718	n	V	n	n	0	cu	cig+/-ot	nev any	st
KJUUS	1		m	0	0	all	-	all	Eu:Sca	1979	CC	176	n	bl	n	n	0	cu	all/unsp	nev any	st
KNEKT	75	x	m	20	69	all	21	all	Eu:Sca	1966	pr	515	n	bl	n	n	0	cu	cig+/-ot	nev any	st
KO	1		f	0	0	all	-	all	As:oth	1992	CC	117	n	ot	n	y	3	ev	cig+/-ot	nev cigs	or
KOHLME	1	x	c	0	0	all	-	all	Eu:Ger	1990	CC	239	n	bl	n	n	0	ev	all/unsp	nev any	st
KOO	9		f	0	0	all	-	all	As:HK	1981	CC	200	n	bl	n	n	0	cu	all/unsp	nev any	st
KOULUM	2		m	0	0	all	-	all	Eu:Sca	1936	CC	812	n	bl	n	n	0	ev	cig only	nev any	st
KREUZE	6	x	f	1	45	all	-	all	Eu:Ger	1990	CC	2260	n	bl	n	n	0	cu	cig+/-ot	nev any	st
KREUZE	8	x	f	55	69	all	-	all	Eu:Ger	1990	CC	2260	n	bl	n	n	0	cu	cig+/-ot	nev any	st
KREYBE	24	x	m	0	0	all	-	all	Eu:Sca	1948	CC	300	n	bl	n	y	0	ev	all/unsp	nev any	st
KREYBE	39	x	f	0	0	all	-	all	Eu:Sca	1948	CC	300	n	bl	n	y	0	ev	all/unsp	nev any	st
KUBIK	12		m	0	0	all	0	all	Eu:est	1965	pr	108	n	bl	n	n	0	cu	cig+/-ot	nev any	st
LAMTH	6		f	0	0	ch	-	all	As:HK	1983	CC	445	n	bl	n	n	0	ev	all/unsp	nev any	or
LAMWK	1		f	0	0	ch	-	all	As:HK	1981	CC	163	n	bl	n	n	0	ev	all/unsp	nev any	st
LAMWK2	9		m	0	0	all	-	q+s+l+a	As:HK	1976	CC	480	n	bl	n	n	0	ev	all/unsp	nev any	st
LAMWK2	10		f	0	0	all	-	q+s+l+a	As:HK	1976	CC	480	n	bl	n	n	0	ev	all/unsp	nev any	st
LANGE	32	x	m	0	0	all	0	all	Eu:Sca	1976	pr	268	n	bl	n	n	0	cu	all/unsp	nev any	st
LANGE	29	x	f	0	0	all	0	all	Eu:Sca	1976	pr	268	n	bl	n	n	0	cu	all/unsp	nev any	st
LAUSSM	10	x	m	0	0	all	-	all	Eu:Ger	1982	CC	432	n	bl	n	n	0	ev	all/unsp	nev any	st
LEI	1		m	0	0	all	-	all	As:Chi	1986	CC	792	n	ot	y	n	0	ev	all/unsp	nev any	st
LEI	2		f	0	0	all	-	all	As:Chi	1986	CC	792	n	ot	y	n	0	ev	all/unsp	nev any	st
LEMARC	2		c	0	0	w+o	-	all	NAmer	1992	CC	341	n	bl	n	y	0	cu	all/unsp	nev any	st
LETOUR	1		c	0	0	all	-	all	NAmer	1983	CC	738	n	V	y	y	0	ev	cig+/-ot	nev cigs	st
LEVIN	30		m	0	0	all	-	all	NAmer	1938	CC	475	n	bl	n	n	1	ev	cig+/-ot	nev any	st
LIAW	1		m	0	0	all	0	all	As:oth	1982	pr	127	n	ot	n	n	1	cu	all/unsp	nev any	or
LIAW	2		f	0	0	all	0	all	As:oth	1982	pr	127	n	ot	n	n	1	cu	all/unsp	nev any	or
LIDDEL	4		m	0	0	all	18	all	NAmer	1970	pr	304	m	V	n	n	1	cu	cig+/-ot	nev cigs	ot
LIU	2		c	0	0	all	-	all	As:Chi	1980	CC	229	n	ot	*	n	2	ev	all/unsp	nev any	or
LIU2	1	x	m	0	0	all	-	all	As:Chi	1983	CC	316	n	ot	n	n	0	ev	all/unsp	nev any	st
LIU2	3	x	f	0	0	all	-	all	As:Chi	1983	CC	316	n	ot	n	n	0	ev	all/unsp	nev any	st
LIU3	1	x	m	0	0	all	-	all	As:Chi	1985	CC	110	n	ot	n	n	0	ev	all/unsp	nev any	st
LIU4	10		m	35	69	all	-	all	As:Chi	1986	CC	1000-00	n	ot	y	n	2	ev	cig only	nev any	ot
LIU4	12		f	0	0	all	-	all	As:Chi	1986	CC	1000-00	n	ot	y	n	2	ev	all/unsp	nev any	ot
LIU5	1		c	0	0	all	-	all	As:Chi	1978	CC	111	n	ot	y	n	0	ev	all/unsp	nev any	st
LOMBA2	1		f	0	0	all	-	all	NAmer	1960	CC	225	n	bl	n	n	0	ev	cig+/-ot	nev cigs	st
LOMBAR	9		m	0	0	all	-	all	NAmer	1951	CC	1040	n	bl	n	n	0	cu	cig+/-ot	nev any	st
LUBIN2	27	x	m	0	0	all	-	all	Eu:mul	1976	CC	7804	n	bl	n	y	0	cu	cig+/-ot	nev any	st
LUBIN2	317		f	0	0	all	-	all	Eu:mul	1976	CC	7804	n	bl	n	y	0	cu	cig+/-ot	nev any	st
LUO	1	x	c	0	0	all	-	all	As:Chi	1990	CC	102	n	ot	n	y	0	ev	cig+/-ot	nev cigs	st
MACLEN	19		m	0	0	ch	-	all	As:oth	1972	CC	233	n	bl	n	n	0	cu	cig+/-ot	nev cigs	st
MACLEN	32		f	0	0	ch	-	all	As:oth	1972	CC	233	n	bl	n	n	0	cu	cig+/-ot	nev cigs	st

Table 1C4 - 4

IESLC - Meta-anal of Current Smoking (or Ever if Current not available), Cigs (or Any Prod if Cigs not avail)
All LC types
Least adjusted

REF	NRR	X	SEX	AGEL	AGEH	RACE	YF	LC TYPE	LOC	START	ST	NLC	R	VB	P	H	AD	SM	PRODUCT	DENOM	De
MAGNUS	1	x	m	0	0	all	0	all	Eu:Sca	1953	pr	203	o	bl	y	n	0	ev	all/unsp	nev any	st
MARSH	1		m	0	0	all	-	all	NAmer	1979	CC	150	n	bl	y	n	0	ev	cig+/-ot	nev any	st
MARSH	3		f	0	0	all	-	all	NAmer	1979	CC	150	n	bl	y	n	0	ev	cig+/-ot	nev any	st
MARSH2	1	x	c	0	0	all	-	all	NAmer	1979	CC	114	n	bl	y	n	0	ev	cig+/-ot	nev any	st
MARTIS	4		m	0	0	all	-	all	Eu:UK	1972	CC	201	n	V	n	n	0	ev	cig+/-ot	nev cigs	st
MASTRA	1	x	m	0	0	all	-	all	Eu:wst	1973	CC	309	n	bl	n	n	0	ev	all/unsp	nev any	st
MATOS	2	x	m	0	0	all	-	all	SCAmer	1994	CC	200	n	bl	n	n	0	cu	cig+/-ot	nev any	st
MATSUD	10		m	0	0	all	-	all	As:Jap	1965	CC	179	n	bl	n	n	0	ev	cig+/-ot	nev cigs	st
MCCONN	1		m	0	0	all	-	all	Eu:UK	1946	CC	100	n	V	n	y	0	ev	all/unsp	nev any	st
MCCONN	2		f	0	0	all	-	all	Eu:UK	1946	CC	100	n	V	n	y	0	ev	all/unsp	nev any	st
MCDUFF	1		m	0	0	all	-	all	NAmer	1979	CC	165	n	V	y	n	0	ev	cig+/-ot	nev cigs	st
MCLAUG	1		m	0	0	all	-	all	As:Chi	1972	CC	316	o	ot	y	n	0	ev	all/unsp	nev any	st
MIGRAN	11	x	m	0	0	all	0	all	Eu:UK	1964	pr	259	n	V	n	n	0	cu	cig+/-ot	nev any	st
MIGRAN	37	x	f	0	0	all	0	all	Eu:UK	1964	pr	259	n	V	n	n	0	cu	cig+/-ot	nev any	st
MILLER	1	x	f	0	0	all	-	all	NAmer	1972	CC	168	n	bl	y	n	0	ev	cig+/-ot	nev any	st
MILLS	1		m	0	0	wh	-	all	NAmer	1940	CC	444	n	bl	y	n	1	ev	cig only	nev any	ot
MRFITR	2		m	0	0	all	0	all	NAmer	1973	pr	119	n	bl	n	n	0	cu	cig+/-ot	nev cigs	ot
NAM	68	x	m	0	0	all	-	all	NAmer	1986	CC	1199	n	bl	y	n	0	cu	cig+/-ot	nev cigs	ot
NAM	84	x	f	0	0	all	-	all	NAmer	1986	CC	1199	n	bl	y	n	0	cu	cig+/-ot	nev cigs	ot
NOTAN2	7	x	m	0	0	all	-	all	As:Ind	1963	CC	683	n	V	n	n	0	ev	cig only	nev any	st
NOU	11		m	30	64	all	-	all	Eu:Sca	1971	CC	273	n	bl	y	n	0	ev	all/unsp	nev any	st
NOU	12		f	30	64	all	-	all	Eu:Sca	1971	CC	273	n	bl	y	n	0	ev	all/unsp	nev any	st
ODRISC	1		c	0	0	all	-	all	Eu:UK	1992	CC	446	n	V	n	n	0	cu	all/unsp	nev any	st
ORMOS	4		m	0	0	all	-	all	Eu:est	1947	CC	119	n	bl	y	y	0	ev	cig+/-ot	nev any	st
ORMOS	26		f	0	0	all	-	all	Eu:est	1947	CC	119	n	bl	y	y	0	ev	cig+/-ot	nev any	st
OSANN	9	x	m	0	0	all	-	all	NAmer	1984	CC	1986	n	bl	n	n	0	cu	cig+/-ot	nev cigs	st
OSANN	13	x	f	0	0	all	-	all	NAmer	1984	CC	1986	n	bl	n	n	0	cu	cig+/-ot	nev cigs	st
PARKIN	30		m	0	0	bl	-	all	Africa	1963	CC	877	n	V	y	n	0	cu	cig+/-ot	nev any	st
PASTOR	5	x	m	0	0	all	-	all	Eu:wst	1976	CC	204	n	bl	y	n	0	ev	all/unsp	nev any	st
PAWLEG	1	x	m	0	0	all	-	all	Eu:est	1992	CC	176	n	bl	n	y	0	ev	all/unsp	nev any	st
PERNU	8		m	0	0	all	-	all	Eu:Sca	1944	CC	1606	n	bl	n	n	0	ev	cig only	nev any	st
PERNU	4		f	0	0	all	-	all	Eu:Sca	1944	CC	1606	n	bl	n	n	0	ev	cig only	nev any	st
PERSH2	4	x	c	0	0	all	-	all	Eu:Sca	1980	CC	1022	n	bl	y	n	0	cu	all/unsp	nev any	st
PETO	4		m	0	0	all	0	all	Eu:UK	1954	pr	103	n	V	n	n	0	cu	all/unsp	nev any	st
PEZZO2	2		m	0	0	all	-	all	SCAmer	1992	CC	367	n	bl	n	y	0	cu	cig+/-ot	nev cigs	st
PEZZOT	5		m	0	0	all	-	all	SCAmer	1987	CC	215	n	bl	n	y	0	cu	cig only	nev cigs	st
PIKE	4		m	0	0	w-hi	-	all	NAmer	1972	CC	731	n	bl	y	n	0	ev	all/unsp	nev any	st
PIKE	8		f	0	0	w-hi	-	all	NAmer	1972	CC	731	n	bl	y	n	0	ev	all/unsp	nev any	st
POFFIJ	1		c	0	0	all	-	all	Eu:mul	1990	CC	971	n	bl	n	n	0	ev	all/unsp	nev any	st
POLEDN	3	x	c	0	0	all	-	all	NAmer	1978	CC	209	n	bl	y	n	0	ev	cig+/-ot	nev cigs	st
QIAO2	8		m	0	0	all	0	all	As:Chi	1992	pr	241	m	ot	n	n	0	cu	cig+/-ot	nev any	st
RACHTA	2	x	f	0	0	all	-	all	Eu:est	1991	CC	118	n	bl	n	y	0	cu	cig+/-ot	nev cigs	st
RADZIK	1		c	0	0	all	-	all	Eu:est	1986	CC	189	n	bl	n	n	0	ev	all/unsp	nev any	st
RANDIG	9		m	0	0	all	-	all	Eu:Ger	1951	CC	448	n	bl	n	n	0	ev	cig+/-ot	nev any	st
RANDIG	10		f	0	0	all	-	all	Eu:Ger	1951	CC	448	n	bl	n	n	0	ev	cig+/-ot	nev any	st
REN	1		m	0	0	all	-	all	As:Chi	1980	CC	244	n	ot	*	n	0	ev	all/unsp	nev any	st
REN	2		f	0	0	all	-	all	As:Chi	1980	CC	244	n	ot	*	n	0	ev	all/unsp	nev any	st
RONCO	2	x	m	0	0	all	-	all	Eu:wst	1976	CC	126	n	bl	y	n	0	ev	cig only	nev any	st
ROTHSC	1	x	c	0	0	all	-	all	NAmer	1971	CC	284	n	bl	y	n	0	ev	all/unsp	nev any	st
SADOWS	4		m	0	0	wh	-	all	NAmer	1938	CC	477	n	bl	n	n	0	ev	cig+/-ot	nev any	st
SANKAR	1	x	m	0	0	all	-	all	As:Ind	1990	CC	281	n	V	n	n	0	ev	all/unsp	nev any	st
SCHWAR	25		m	0	0	wh	-	all	NAmer	1984	CC	5588	n	bl	y	y	0	cu	cig+/-ot	nev cigs	st
SCHWAR	26		m	0	0	bl	-	all	NAmer	1984	CC	5588	n	bl	y	y	0	cu	cig+/-ot	nev cigs	st
SCHWAR	27		f	0	0	wh	-	all	NAmer	1984	CC	5588	n	bl	y	y	0	cu	cig+/-ot	nev cigs	st
SCHWAR	28		f	0	0	bl	-	all	NAmer	1984	CC	5588	n	bl	y	y	0	cu	cig+/-ot	nev cigs	st
SEGI	1		m	0	0	all	-	all	As:Jap	1948	CC	159	n	bl	n	n	0	ev	all/unsp	nev any	ot
SEGI2	19	x	m	0	0	all	-	all	As:Jap	1962	CC	378	n	bl	n	n	0	cu	cig+/-ot	nev any	st
SEGI2	27	x	f	0	0	all	-	all	As:Jap	1962	CC	378	n	bl	n	n	0	cu	cig+/-ot	nev any	st
SEOW	1	x	f	0	0	ch	-	q+s+l+a	As:oth	1997	CC	153	n	bl	n	y	0	ev	cig+/-ot	nev cigs	st
SHAW	6		c	0	0	wh	-	all	NAmer	1988	CC	335	n	V	n	y	0	cu	all/unsp	nev any	st
SIEMIA	9	x	m	0	0	all	-	all	NAmer	1979	CC	857	n	V	y	y	0	ev	cig+/-ot	nev cigs	st
SIMARA	5	x	m	0	0	all	-	all	As:oth	1971	CC	115	n	bl	n	n	0	ev	cig+/-ot	nev cigs	st
SIMARA	6	x	f	0	0	all	-	all	As:oth	1971	CC	115	n	bl	n	n	0	ev	cig+/-ot	nev cigs	st
SOBUE	90	x	m	0	0	all	-	q+s+l+a	As:Jap	1986	CC	1376	n	bl	n	y	0	cu	cig+/-ot	nev cigs	st
SOBUE	94	x	f	0	0	all	-	q+s+l+a	As:Jap	1986	CC	1376	n	bl	n	y	0	cu	cig+/-ot	nev cigs	st
SOBUE2	10		m	0	0	all	-	q+s+l+a	As:Jap	1965	CC	2083	n	bl	n	n	2	cu	cig+/-ot	nev any	ot
SOBUE2	12		f	0	0	all	-	q+s+l+a	As:Jap	1965	CC	2083	n	bl	n	n	2	cu	cig+/-ot	nev any	ot
SPEIZE	6	x	f	0	0	all	0	all	NAmer	1976	pr	593	n	bl	n	y	0	cu	cig+/-ot	nev cigs	st
SPITZ	2		c	0	0	b+hi	-	all	NAmer	1992	CC	177	n	bl	n	y	0	cu	cig+/-ot	nev cigs	st
STASZE	7		m	0	0	all	-	all	Eu:est	1954	CC	281	n	bl	n	y	0	ev	cig+/-ot	nev any	st
STASZE	5		f	0	0	all	-	all	Eu:est	1954	CC	281	n	bl	n	y	0	ev	all/unsp	nev any	st
STAYNE	1		m	0	0	all	-	all	NAmer	1969	CC	420	n	bl	n	n	0	ev	all/unsp	nev any	st

Table 1C4 - 4

IESLC - Meta-anal of Current Smoking (or Ever if Current not available), Cigs (or Any Prod if Cigs not avail)
All LC types
Least adjusted

REF	NRR	X	SEX	AGEL	AGEH	RACE	YF	LC TYPE	LOC	START	ST	NLC	R	VB	P	H	AD	SM	PRODUCT	DENOM	De
STOCKS	30	x	m	0	0	all	-	all	Eu:UK	1952	CC	2932	n	V	y	n	0	ev	cig+/-ot	nev any	st
STOCKS	50		f	0	0	all	-	all	Eu:UK	1952	CC	2932	n	V	y	n	1	ev	cig+/-ot	nev any	ot
STOCKW	7		c	0	0	all	-	all	NAmer	1981	CC	22161	n	bl	n	n	0	cu	cig+/-ot	nev any	st
STUCKE	2		m	0	0	all	-	all	Eu:wst	1989	CC	247	n	bl	n	y	0	cu	all/unsp	nev any	ot
SUN	1		c	0	0	all	-	all	As:Chi	1992	CC	207	n	ot	n	y	0	ev	all/unsp	nev any	st
SUZUK2	2	x	c	0	0	all	-	all	SCAmer	1991	CC	123	n	bl	n	y	0	cu	all/unsp	nev any	st
SVENSS	61	x	f	0	0	all	-	all	Eu:Sca	1983	CC	210	n	bl	n	n	0	cu	all/unsp	nev any	st
TANG	1		c	0	0	all	-	not s	NAmer	1992	CC	119	n	bl	n	y	0	cu	cig+/-ot	nev cigs	st
TENKAN	24		m	0	0	all	17	all	Eu:Sca	1962	pr	242	n	bl	n	n	1	cu	all/unsp	nev any	ot
TIZZAN	5		m	0	0	all	-	all	Eu:wst	1959	CC	1358	n	bl	n	n	0	cu	all/unsp	nev any	st
TIZZAN	13		f	0	0	all	-	all	Eu:wst	1959	CC	1358	n	bl	n	n	0	cu	all/unsp	nev any	st
TOKARS	1		m	0	0	all	-	all	Eu:est	1966	ot	162	o	bl	n	y	0	cu	all/unsp	nev any	st
TOKARS	5		f	0	0	all	-	all	Eu:est	1966	ot	162	o	bl	n	y	0	ev	all/unsp	nev any	st
TOUSEY	4	x	m	0	0	all	-	all	NAmer	1993	CC	507	n	bl	y	y	0	cu	cig+/-ot	nev any	st
TOUSEY	8	x	f	0	0	all	-	all	NAmer	1993	CC	507	n	bl	y	y	0	cu	cig+/-ot	nev any	st
TSUGAN	28		m	0	0	all	-	q+a	As:Jap	1976	CC	134	n	bl	n	y	0	cu	all/unsp	nev any	st
TULINI	13	x	m	0	0	all	0	all	Eu:Sca	1967	pr	472	n	bl	n	n	1	cu	cig+/-ot	nev any	ot
TULINI	19	x	f	0	0	all	0	all	Eu:Sca	1967	pr	472	n	bl	n	n	1	cu	cig+/-ot	nev any	ot
TVERDA	5		m	0	0	all	0	all	Eu:Sca	1972	pr	238	n	bl	n	n	2	cu	cig+/-ot	nev cigs	ot
TVERDA	15		f	0	0	all	0	all	Eu:Sca	1972	pr	238	n	bl	n	n	2	cu	cig only	nev cigs	ot
WAKAI	2	x	m	0	0	all	-	all	As:Jap	1988	CC	333	n	bl	n	y	0	cu	all/unsp	nev any	st
WAKAI	20	x	f	0	0	all	-	all	As:Jap	1988	CC	333	n	bl	n	y	0	cu	all/unsp	nev any	st
WALD	2	x	m	0	0	all	0	all	Eu:UK	1975	pr	102	n	V	n	n	0	cu	cig only	nev any	st
WANG	1	x	m	0	0	all	-	all	As:Chi	1990	CC	390	n	ot	*	y	0	ev	all/unsp	nev any	or
WANG	2	x	f	0	0	all	-	all	As:Chi	1990	CC	390	n	ot	*	y	0	ev	all/unsp	nev any	or
WANG2	17	x	c	0	0	all	-	all	As:Chi	1980	CC	103	n	ot	n	n	0	cu	cig+/-ot	nev cigs	st
WANG3	1		c	0	0	all	-	all	As:Chi	1981	CC	293	n	ot	*	n	0	ev	all/unsp	nev any	st
WANG4	1	x	m	0	0	all	-	all	As:Chi	1976	CC	1170	n	ot	y	n	0	ev	all/unsp	nev any	st
WICKLU	1		m	0	0	wh	-	all	NAmer	1968	CC	155	n	bl	y	n	0	ev	cig+/-ot	nev+3	or
WIGLE	1	x	m	0	0	all	-	all	NAmer	1971	CC	728	n	V	n	n	0	cu	cig only	nev any	st
WIGLE	33		f	0	0	all	-	all	NAmer	1971	CC	728	n	V	n	n	1	cu	cig+/-ot	nev any	ot
WILKIN	1	x	m	0	0	all	-	all	Eu:UK	1992	CC	271	n	V	n	n	0	ev	cig+/-ot	nev cigs	st
WILKIN	2	x	f	0	0	all	-	all	Eu:UK	1992	CC	271	n	V	n	n	0	ev	cig+/-ot	nev cigs	st
WU	34	x	f	0	0	wh	-	q+a	NAmer	1981	CC	220	n	bl	n	y	0	cu	all/unsp	nev any	st
WUNSCH	2	x	m	0	0	all	-	all	SCAmer	1990	CC	398	n	bl	y	n	0	cu	cig+/-ot	nev any	st
WUNSCH	8	x	f	0	0	all	-	all	SCAmer	1990	CC	398	n	bl	y	n	0	cu	cig+/-ot	nev any	st
WUWILL	6	x	f	0	0	all	-	all	As:Chi	1985	CC	965	n	ot	n	n	0	ev	cig+/-ot	nev cigs	st
WYNDE2	16		m	0	0	all	-	all	NAmer	1962	CC	404	n	bl	n	y	0	ev	cig+/-ot	nev any	st
WYNDE3	50		m	0	0	all	-	all	NAmer	1966	CC	350	n	bl	n	y	0	cu	all/unsp	nev any	st
WYNDE3	83		f	0	0	all	-	all	NAmer	1966	CC	350	n	bl	n	y	0	ev	cig+/-ot	nev any	st
WYNDE4	48		m	0	0	all	-	all	NAmer	1948	CC	684	n	bl	y	n	0	ev	all/unsp	nev any	st
WYNDE4	62		f	0	0	all	-	all	NAmer	1948	CC	684	n	bl	y	n	2	ev	all/unsp	nev any	ot
WYNDE6	18		m	0	0	all	-	all	NAmer	1969	CC	4423	n	bl	n	y	0	cu	cig+/-ot	nev any	st
WYNDE6	207		f	0	0	all	-	all	NAmer	1969	CC	4423	n	bl	n	y	0	cu	cig+/-ot	nev cigs	st
XIANGZ	6	x	m	0	0	all	0	all	As:Chi	1976	pr	983	m	ot	n	n	0	ev	cig+/-ot	nev any	st
XU	1	x	m	0	0	all	-	all	As:Chi	1985	CC	729	n	ot	n	n	0	ev	all/unsp	nev any	st
XU2	1	x	c	0	0	all	-	all	As:Chi	1987	CC	610	o	ot	y	n	0	ev	all/unsp	nev any	st
XU3	1	x	m	0	0	all	-	all	As:Chi	1981	CC	135	n	ot	n	n	0	ev	all/unsp	nev any	st
XU3	3	x	f	0	0	all	-	all	As:Chi	1981	CC	135	n	ot	n	n	0	ev	all/unsp	nev any	st
XU4	1		c	0	0	all	-	all	As:Chi	1981	CC	206	n	ot	*	n	0	ev	all/unsp	nev any	st
YAMAGU	1	x	c	0	0	all	-	all	As:Jap	1989	CC	144	n	bl	n	y	0	cu	all/unsp	nev any	st
YONG	12		m	0	0	all	0	all	NAmer	1971	pr	216	n	bl	n	n	1	cu	cig+/-ot	nev cigs	or
YONG	15		f	0	0	all	0	all	NAmer	1971	pr	216	n	bl	n	n	1	cu	cig+/-ot	nev cigs	or
YUAN	1		m	0	0	all	0	all	As:Chi	1986	pr	142	n	ot	n	n	2	ev	cig+/-ot	nev cigs	ot
ZHANG	1	x	c	0	0	all	-	all	As:Chi	1988	CC	100	n	ot	n	n	0	ev	all/unsp	nev any	st
ZHENG	15		m	0	0	all	-	all	As:Chi	1982	CC	540	n	ot	*	y	0	ev	cig+/-ot	nev cigs	st
ZHENG	24		f	0	0	all	-	all	As:Chi	1982	CC	540	n	ot	*	y	0	ev	cig+/-ot	nev cigs	st
ZHOU	2		m	0	0	all	-	all	As:Chi	1978	CC	1360	n	ot	n	n	0	ev	all/unsp	nev any	st
ZHOU	3		f	0	0	all	-	all	As:Chi	1978	CC	1360	n	ot	n	n	0	ev	all/unsp	nev any	st

Cigarette type is all/unspec for all RRs
except for the following:

REF	NRR	CIGTYPE
ALDERS	177	MC+-HR
ALDERS	176	MC only
DEAN3	238	MC only
DEAN3	117	MC only
GARDIN	6	MC only

Table 1C4 - 4

IESLC - Meta-anal of Current Smoking (or Ever if Current not available), Cigs (or Any Prod if Cigs not avail)
All LC types
Least adjusted

REF	NRR	CIGTYPE
JUSSAW	2	MC only
NOTAN2	7	MC only
PERNU	8	MC only
PERNU	4	MC only

Table 1C4 - 5

IESLC - Meta-anal of Current Smoking (or Ever if Current not available), Cigs (or Any Prod if Cigs not avail)
All LC types
Least adjusted

REF	NRR	SEX	AD	Number Exposed Case	Number Exposed Cont	Non-exposed Case	Non-exposed Cont	RR	95.00%CI
ABELIN	2	m	0	47	154	2	183	27.93	(6.67- 116.83)
*ABRAHA	7	m	0	269	10351	10	3365	8.74	(4.66- 16.42)
*ABRAHA	8	f	0	62	5256	28	11589	4.88	(3.13- 7.62)
Subtotal ABRAHA								5.93	(4.12- 8.53)
AGUDO	10	f	0	20	17	80	183	2.69	(1.34- 5.41)
*AKIBA	2	m	0	345	171379	18	35833	4.01	(2.50- 6.44)
*AKIBA	6	f	0	74	51237	116	359850	4.48	(3.35- 6.00)
Subtotal AKIBA								4.35	(3.39- 5.57)
ALDERS	177	m	0	519	322	15	133	14.29	(8.23- 24.81)
ALDERS	176	f	0	410	229	75	243	5.80	(4.27- 7.87)
Subtotal ALDERS								7.17	(5.49- 9.36)
*AMANDU	1	m	0	115	96708	6	25350	5.02	(2.21- 11.41)
AMES	1	m	0	150	136	15	62	4.56	(2.48- 8.39)
*ANDERS	2	f	0	212	41262	46	195158	21.80	(15.85- 29.98)
*ARCHER	5	m	0	122	32529	6	9842	6.15	(2.71- 13.96)
ARMADA	27	m	0	188	122	4	64	24.66	(8.75- 69.44)
AUSTIN	2	c	0	111	125	5	88	15.63	(6.13- 39.87)
AUVINE	1	c	0	473	288	44	229	8.55	(6.00- 12.18)
AXELSO	1	c	0	90	86	62	371	6.26	(4.20- 9.34)
AXELSS	2	m	0	194	130	16	160	14.92	(8.53- 26.12)
AXELSS	10	f	0	96	69	18	154	11.90	(6.68- 21.22)
Subtotal AXELSS								13.38	(8.95- 20.00)
BAND	1	m	2	-	-	-	-	9.96	(7.38- 13.44)
BARBON	3	m	0	562	362	22	188	13.27	(8.37- 21.04)
BECHER	13	m	0	101	122	3	54	14.90	(4.52- 49.09)
BECHER	14	f	0	33	26	10	52	6.60	(2.82- 15.44)
Subtotal BECHER								8.68	(4.35- 17.35)
*BENSHL	4	m	1	-	-	-	-	11.92	(6.36- 22.34)
*BEST	2	m	1	-	-	-	-	14.91	(7.05- 31.52)
*BEST	18	f	1	-	-	-	-	2.24	(0.59- 8.44)
Subtotal BEST								9.45	(4.92- 18.15)
BLOHMK	1	m	0	419	313	126	301	3.20	(2.48- 4.12)
BLOT4	1	m	0	327	245	8	87	14.51	(6.91- 30.51)
BOFFET	7	m	0	5386	5239	117	1750	15.38	(12.70- 18.61)
*BOUCOT	2	m	0	85	22177	0	7551	58.23~	(3.61- 938.34)
BRESLO	17	m	0	471	383	7	42	7.38	(3.28- 16.61)
BRESLO	23	f	0	13	11	12	14	1.38	(0.45- 4.20)
Subtotal BRESLO								4.12	(2.14- 7.94)
*BRETT	4	m	0	135	37448	6	6530	3.92	(1.73- 8.88)
BROCKM	1	m	0	87	81	2	2	1.07	(0.15- 7.80)
BROCKM	2	f	0	24	54	4	18	2.00	(0.61- 6.54)
Subtotal BROCKM								1.70	(0.61- 4.70)
BROSS	4	m	0	565	427	38	170	5.92	(4.07- 8.60)
BROWN2	12	m	2	-	-	-	-	11.30	(10.20- 12.40)
BROWN2	11	f	2	-	-	-	-	13.60	(12.30- 15.10)
Subtotal BROWN2								12.34	(11.50- 13.25)
BUFFLE	3	m	0	257	219	5	47	11.03	(4.31- 28.22)
BUFFLE	7	f	0	313	183	41	198	8.26	(5.63- 12.11)
Subtotal BUFFLE								8.61	(6.04- 12.27)
CARPEN	9	c	0	219	162	15	241	21.72	(12.41- 38.01)
CASCO2	1	c	0	149	212	6	98	11.48	(4.90- 26.87)
CASCOR	1	c	0	365	362	22	295	13.52	(8.56- 21.35)
*CEDERL	2	m	0	55	8030	7	6352	6.22	(2.83- 13.64)
*CEDERL	119	f	1	-	-	-	-	4.54	(1.85- 11.12)
Subtotal CEDERL								5.42	(3.00- 9.79)
CHAN	5	m	0	206	161	2	43	27.51	(6.57- 115.26)
CHAN	6	f	0	105	50	84	139	3.48	(2.26- 5.35)
Subtotal CHAN								4.13	(2.73- 6.25)
*CHANG	5	m	0	35	419	5	502	8.39	(3.32- 21.21)
*CHANG	11	f	0	30	603	11	1139	5.15	(2.60- 10.21)
Subtotal CHANG								6.12	(3.53- 10.60)
CHATZI	4	c	0	255	365	27	129	3.34	(2.14- 5.21)
CHEN2	1	m	0	121	97	9	33	4.57	(2.09- 10.02)
CHEN2	2	f	0	38	30	25	33	1.67	(0.82- 3.39)
Subtotal CHEN2								2.62	(1.55- 4.44)
CHEN3	1	c	0	182	156	72	98	1.59	(1.09- 2.30)
CHIAZZ	2	m	0	139	209	4	47	7.81	(2.75- 22.18)
CHOI	3	m	0	232	329	13	95	5.15	(2.82- 9.42)
CHOI	7	f	0	13	23	76	164	1.22	(0.59- 2.54)
Subtotal CHOI								2.88	(1.81- 4.58)
*CHOW	18	m	0	138	81725	6	62913	17.71	(7.82- 40.09)

Table 1C4 - 5

IESLC - Meta-anal of Current Smoking (or Ever if Current not available), Cigs (or Any Prod if Cigs not avail)
All LC types
Least adjusted

REF	NRR	SEX	AD	Number Exposed Case	Number Exposed Cont	Non-exposed Case	Non-exposed Cont	RR	95.00%CI
*CHYOU	4	m	0	181	3470	13	2406	9.65	(5.51- 16.91)
COMSTO	3	m	0	105	100	4	69	18.11	(6.37- 51.48)
COMSTO	8	f	0	77	52	13	115	13.10	(6.68- 25.67)
Subtotal COMSTO								14.40	(8.18- 25.36)
COOKSO	4	c	0	184	38	45	61	6.56	(3.90- 11.04)
CORREA	41	c	0	943	571	51	388	12.56	(9.22- 17.13)
*CPSI	220	m	1	-	-	-	-	11.94	(9.52- 14.97)
*CPSI	279	f	1	-	-	-	-	3.20	(2.53- 4.04)
Subtotal CPSI								6.32	(5.37- 7.43)
*CPSII	36	m	0	1781	583646	124	742207	18.26	(15.23- 21.91)
*CPSII	71	f	0	1014	744217	310	2091302	9.19	(8.09- 10.44)
Subtotal CPSII								11.51	(10.37- 12.78)
DAMBER	16	m	1	-	-	-	-	9.80	(6.30- 15.30)
DARBY	4	m	0	322	453	3	384	90.98	(28.96- 285.90)
DARBY	11	f	0	195	217	23	529	20.67	(13.05- 32.74)
Subtotal DARBY								25.40	(16.57- 38.92)
DAVEYS	5	m	0	90	144	3	23	4.79	(1.40- 16.42)
DAVEYS	6	f	0	0	3	16	83	0.72~	(0.04- 14.66)
Subtotal DAVEYS								3.65	(1.17- 11.42)
DEAN	8	m	0	540	500	12	61	5.49	(2.92- 10.32)
DEAN2	2	m	0	671	600	33	112	3.80	(2.54- 5.68)
DEAN2	6	f	0	59	28	88	121	2.90	(1.71- 4.91)
Subtotal DEAN2								3.43	(2.49- 4.73)
DEAN3	238	m	0	408	1192	24	510	7.27	(4.76- 11.12)
DEAN3	117	f	0	102	1158	41	1538	3.30	(2.28- 4.79)
Subtotal DEAN3								4.65	(3.52- 6.14)
*DEKLER	7	m	2	-	-	-	-	23.79	(3.32- 170.45)
DESTE2	2	c	0	216	151	20	108	7.72	(4.59- 13.00)
DESTEF	40	m	0	362	226	27	163	9.67	(6.23- 15.01)
*DOCKER	1	c	4	-	-	-	-	8.00	(2.97- 21.60)
DOLL	90	m	0	1280	1172	7	61	9.52	(4.34- 20.89)
DOLL	93	f	0	58	41	40	59	2.09	(1.18- 3.68)
Subtotal DOLL								3.51	(2.21- 5.55)
*DOLL2	68	m	1	-	-	-	-	12.20	(5.77- 25.82)
*DOLL2	63	f	1	-	-	-	-	8.65	(2.93- 25.55)
Subtotal DOLL2								10.91	(5.89- 20.21)
DORANT	9	c	0	292	876	14	1090	25.95	(15.07- 44.69)
DORGAN	9	m	0	464	170	15	93	16.92	(9.54- 30.01)
DORGAN	33	m	0	214	61	3	35	40.93	(12.17- 137.66)
DORGAN	56	f	0	611	119	103	244	12.16	(8.99- 16.46)
DORGAN	79	f	0	68	17	7	20	11.43	(4.16- 31.43)
Subtotal DORGAN								13.62	(10.58- 17.54)
*DORN	391	m	1	-	-	-	-	10.86	(9.73- 12.13)
DOSEME	17	m	0	1068	536	142	293	4.11	(3.28- 5.15)
DROSTE	2	m	0	379	267	7	93	18.86	(8.61- 41.30)
DU	1	m	0	538	-	28	-	3.53	(2.44- 5.11)
DU	2	f	0	191	-	92	-	1.93	(1.30- 2.87)
Subtotal DU								2.66	(2.03- 3.49)
*DUNN	6	m	0	137	52634	2	14160	18.43	(4.56- 74.42)
EBELIN	1	m	0	101	142	12	117	6.93	(3.63- 13.24)
*ENGELA	168	m	1	-	-	-	-	9.70	(4.49- 20.94)
*ENGELA	177	f	1	-	-	-	-	5.80	(2.69- 12.51)
Subtotal ENGELA								7.50	(4.35- 12.92)
*ENSTRO	1	m	1	-	-	-	-	12.99	(10.46- 16.13)
*ENSTRO	2	f	1	-	-	-	-	6.95	(6.01- 8.04)
Subtotal ENSTRO								8.44	(7.48- 9.53)
ESAKI	4	m	0	155	143	16	28	1.90	(0.99- 3.65)
ESAKI	5	f	0	34	19	40	55	2.46	(1.23- 4.92)
Subtotal ESAKI								2.14	(1.33- 3.45)
FAN	1	m	0	216	498	36	236	2.84	(1.93- 4.18)
FAN	2	f	0	82	97	69	320	3.92	(2.65- 5.81)
Subtotal FAN								3.33	(2.53- 4.38)
GAO	29	m	0	529	438	62	202	3.93	(2.88- 5.37)
GAO	30	f	0	170	100	435	605	2.36	(1.79- 3.12)
Subtotal GAO								2.96	(2.41- 3.64)
GAO2	1	m	0	184	117	13	56	6.77	(3.55- 12.93)
GARCIA	2	c	0	169	74	21	139	15.12	(8.86- 25.79)
GARDIN	6	c	0	72	39	5	41	15.14	(5.53- 41.44)
GARSHI	23	m	0	657	782	41	363	7.44	(5.30- 10.44)
GENG	1	m	0	92	68	7	31	5.99	(2.49- 14.42)
GENG	2	f	0	126	75	67	118	2.96	(1.96- 4.48)

Table 1C4 - 5

IESLC - Meta-anal of Current Smoking (or Ever if Current not available), Cigs (or Any Prod if Cigs not avail)
All LC types
Least adjusted

REF	NRR	SEX	AD	Number Exposed Case	Number Exposed Cont	Non-exposed Case	Non-exposed Cont	RR	95.00%CI
Subtotal GENG								3.36	(2.31- 4.89)
GER	17	c	0	90	318	51	246	1.37	(0.93- 2.00)
GODLEY	5	m	1	-	-	-	-	6.84	(5.60- 8.35)
GODLEY	6	f	1	-	-	-	-	5.54	(4.29- 7.15)
Subtotal GODLEY								6.31	(5.39- 7.39)
GOLLED	21	m	0	380	1966	15	490	6.31	(3.73- 10.68)
GOODMA	2	m	0	148	169	10	199	17.43	(8.90- 34.14)
GOODMA	6	f	0	58	56	19	177	9.65	(5.30- 17.56)
Subtotal GOODMA								12.53	(8.01- 19.60)
GRAHAM	9	m	0	453	1075	18	346	8.10	(4.98- 13.17)
GREGOR	2	m	0	49	53	10	14	1.29	(0.53- 3.18)
GREGOR	6	f	0	17	26	1	22	14.38	(1.77- 116.90)
Subtotal GREGOR								1.88	(0.82- 4.30)
GSELL	6	m	0	60	42	2	29	20.71	(4.69- 91.56)
HAENSZ	54	f	0	69	94	81	236	2.14	(1.43- 3.19)
*HAMMO2	22	m	0	209	4472	5	891	8.33	(3.44- 20.16)
*HAMMON	139	m	1	-	-	-	-	11.52	(6.83- 19.42)
*HANSEN	3	m	2	-	-	-	-	1.53	(0.71- 3.91)
HEGMAN	1	c	0	255	1202	27	2080	16.34	(10.92- 24.45)
*HEIN	1	m	0	45	912	1	457	22.55	(3.12- 163.06)
*HENNEK	2	m	0	79	2438	23	10919	15.38	(9.69- 24.42)
HINDS	26	f	0	167	592	124	1812	4.12	(3.21- 5.29)
*HIRAYA	1	m	1	-	-	-	-	4.45	(3.60- 5.50)
*HIRAYA	3	f	1	-	-	-	-	2.34	(1.87- 2.92)
Subtotal HIRAYA								3.28	(2.81- 3.82)
HITOSU	2	m	0	117	1597	7	242	2.53	(1.17- 5.50)
HITOSU	9	f	0	28	459	33	1893	3.50	(2.09- 5.85)
Subtotal HITOSU								3.17	(2.07- 4.86)
*HOLE	46	m	0	161	3989	7	1189	6.86	(3.23- 14.57)
*HOLE	29	f	0	13	2144	8	1917	1.45	(0.60- 3.50)
Subtotal HOLE								3.55	(2.00- 6.29)
HOROWI	1	m	0	182	525	19	196	3.58	(2.17- 5.90)
HOROWI	2	f	0	21	382	14	463	1.82	(0.91- 3.62)
Subtotal HOROWI								2.83	(1.89- 4.25)
HORWIT	1	f	0	97	92	11	118	11.31	(5.73- 22.34)
HU	15	m	0	120	94	41	67	2.09	(1.30- 3.35)
HU	16	f	0	26	18	40	48	1.73	(0.83- 3.61)
Subtotal HU								1.98	(1.33- 2.94)
HU2	9	m	0	294	228	49	115	3.03	(2.08- 4.41)
HU2	10	f	0	108	80	72	100	1.88	(1.23- 2.85)
Subtotal HU2								2.44	(1.85- 3.23)
HUANG	1	c	0	98	77	37	58	2.00	(1.20- 3.32)
HUMBLE	13	m	1	-	-	-	-	19.96	(8.27- 48.21)
HUMBLE	15	m	1	-	-	-	-	15.79	(3.43- 72.69)
HUMBLE	17	f	1	-	-	-	-	16.72	(7.44- 37.61)
HUMBLE	19	f	1	-	-	-	-	23.50	(6.79- 81.36)
Subtotal HUMBLE								18.65	(11.23- 30.97)
JAHN	3	f	0	112	67	53	98	3.09	(1.97- 4.85)
JAIN	16	m	0	265	118	12	85	15.91	(8.37- 30.23)
JAIN	11	f	0	305	99	52	214	12.68	(8.68- 18.51)
Subtotal JAIN								13.44	(9.70- 18.62)
JARUP	3	m	0	91	52	11	42	6.68	(3.17- 14.09)
JARVHO	2	m	0	73	29	1	16	40.28	(5.10- 317.77)
JARVHO	6	f	0	31	7	6	21	15.50	(4.56- 52.66)
Subtotal JARVHO								19.86	(6.93- 56.89)
JEDRYC	63	m	0	852	656	49	219	5.80	(4.19- 8.04)
JEDRYC	68	f	0	120	32	78	166	7.98	(4.97- 12.82)
Subtotal JEDRYC								6.43	(4.92- 8.41)
JIANG	1	m	0	93	83	7	17	2.72	(1.08- 6.89)
JIANG	2	f	0	11	6	14	19	2.49	(0.74- 8.35)
Subtotal JIANG								2.63	(1.26- 5.50)
JOLY	16	m	0	451	524	12	218	15.64	(8.63- 28.34)
JOLY	15	f	0	132	96	52	283	7.48	(5.04- 11.12)
Subtotal JOLY								9.38	(6.75- 13.04)
JUSSAW	2	m	0	126	77	149	624	6.85	(4.90- 9.58)
*KAISE2	68	m	1	-	-	-	-	8.04	(4.41- 14.66)
*KAISE2	60	f	1	-	-	-	-	14.48	(7.47- 28.04)
Subtotal KAISE2								10.49	(6.72- 16.36)
*KAISER	12	m	2	-	-	-	-	19.61	(13.32- 28.87)
*KAISER	9	f	2	-	-	-	-	6.53	(4.50- 9.48)
Subtotal KAISER								11.09	(8.48- 14.50)

Table 1C4 - 5

IESLC - Meta-anal of Current Smoking (or Ever if Current not available), Cigs (or Any Prod if Cigs not avail)
All LC types
Least adjusted

REF	NRR	SEX	AD	Number Exposed Case	Number Exposed Cont	Non-exposed Case	Non-exposed Cont	RR	95.00%CI
KANELL	5	m	0	814	441	48	172	6.61	(4.71- 9.30)
KATSOU	6	f	0	45	18	48	67	3.49	(1.80- 6.75)
KAUFMA	7	c	0	621	886	35	925	18.52	(13.02- 26.36)
KELLER	1	m	0	5063	1210	323	1017	13.17	(11.45- 15.15)
KELLER	9	m	0	1053	212	38	117	15.29	(10.31- 22.69)
KELLER	5	f	0	2904	792	469	1860	14.54	(12.79- 16.53)
KELLER	13	f	0	454	135	67	232	11.64	(8.35- 16.24)
Subtotal KELLER								13.79	(12.62- 15.07)
KHUDER	19	m	0	275	-	23	-	8.10	(5.20- 12.70)
KIHARA	7	c	0	283	162	102	237	4.06	(3.00- 5.49)
*KINLEN	8	m	0	589	8512	7	1333	13.18	(6.27- 27.70)
KJUUS	1	m	0	135	77	2	24	21.04	(4.84- 91.45)
*KNEKT	75	m	0	86	33667	6	17814	7.58	(3.32- 17.35)
KO	1	f	3	-	-	-	-	4.20	(1.10- 15.60)
KOHLME	1	c	0	228	236	11	193	16.95	(8.99- 31.96)
KOO	9	f	0	42	25	56	85	2.55	(1.40- 4.64)
KOULUM	2	m	0	625	229	5	54	29.48	(11.65- 74.60)
KREUZE	6	f	0	55	22	6	38	15.83	(5.87- 42.73)
KREUZE	8	f	0	167	54	95	177	5.76	(3.88- 8.56)
Subtotal KREUZE								6.62	(4.58- 9.55)
KREYBE	24	m	0	252	3514	6	644	7.70	(3.41- 17.37)
KREYBE	39	f	0	12	328	30	657	0.80	(0.40- 1.59)
Subtotal KREYBE								2.04	(1.21- 3.44)
*KUBIK	12	m	0	98	6342	2	4271	33.00	(8.14- 133.74)
LAMTH	6	f	0	242	106	202	337	3.81	(2.86- 5.08)
LAMWK	1	f	0	88	41	75	144	4.12	(2.59- 6.55)
LAMWK2	9	m	0	244	161	23	43	2.83	(1.64- 4.88)
LAMWK2	10	f	0	75	50	65	139	3.21	(2.02- 5.10)
Subtotal LAMWK2								3.04	(2.14- 4.33)
*LANGE	32	m	0	174	4537	5	721	5.53	(2.28- 13.41)
*LANGE	29	f	0	53	4455	7	2159	3.67	(1.67- 8.06)
Subtotal LANGE								4.40	(2.44- 7.92)
LAUSSM	10	m	0	347	188	85	226	4.91	(3.61- 6.66)
LEI	1	m	0	443	361	41	123	3.68	(2.52- 5.38)
LEI	2	f	0	123	61	85	147	3.49	(2.32- 5.24)
Subtotal LEI								3.59	(2.72- 4.74)
LEMARC	2	c	0	167	65	32	168	13.49	(8.39- 21.68)
LETOUR	1	c	0	714	514	24	224	12.96	(8.38- 20.05)
LEVIN	30	m	1	-	-	-	-	6.97	(4.87- 9.97)
*LIAW	1	m	1	-	-	-	-	3.70	(2.10- 6.60)
*LIAW	2	f	1	-	-	-	-	3.60	(1.00- 12.20)
Subtotal LIAW								3.68	(2.19- 6.20)
*LIDDEL	4	m	1	-	-	-	-	4.41	(2.77- 7.01)
LIU	2	c	2	-	-	-	-	1.92	(1.40- 2.64)
LIU2	1	m	0	212	180	12	44	4.32	(2.21- 8.43)
LIU2	3	f	0	54	23	38	69	4.26	(2.27- 7.99)
Subtotal LIU2								4.29	(2.71- 6.78)
LIU3	1	m	0	52	205	4	19	1.20	(0.39- 3.69)
LIU4	10	m	2	-	-	-	-	3.88	(3.78- 3.98)
LIU4	12	f	2	-	-	-	-	2.86	(2.77- 2.95)
Subtotal LIU4								3.43	(3.37- 3.50)
LIU5	1	c	0	85	70	26	41	1.91	(1.07- 3.44)
LOMBA2	1	f	0	149	353	76	239	1.33	(0.96- 1.83)
LOMBAR	9	m	0	852	610	14	112	11.17	(6.35- 19.66)
LUBIN2	27	m	0	5557	7279	190	2617	10.52	(9.04- 12.23)
LUBIN2	317	f	0	384	410	288	1180	3.84	(3.17- 4.64)
Subtotal LUBIN2								7.10	(6.31- 8.00)
LUO	1	c	0	65	146	37	160	1.93	(1.21- 3.06)
MACLEN	19	m	0	137	108	5	15	3.81	(1.34- 10.80)
MACLEN	32	f	0	42	47	41	109	2.38	(1.37- 4.12)
Subtotal MACLEN								2.63	(1.62- 4.28)
*MAGNUS	1	m	0	189	3439	11	1086	5.43	(2.97- 9.93)
MARSH	1	m	0	98	150	2	31	10.13	(2.37- 43.27)
MARSH	3	f	0	42	64	8	71	5.82	(2.54- 13.33)
Subtotal MARSH								6.67	(3.25- 13.70)
MARSH2	1	c	0	102	145	12	56	3.28	(1.67- 6.43)
MARTIS	4	m	0	197	176	4	25	7.00	(2.39- 20.49)
MASTRA	1	m	0	303	265	6	44	8.38	(3.52- 19.99)
MATOS	2	m	0	112	132	11	110	8.48	(4.35- 16.56)
MATSUD	10	m	0	170	3314	3	1255	21.46	(6.84- 67.33)
MCCONN	1	m	0	88	174	5	12	1.21	(0.41- 3.55)

Table 1C4 - 5

IESLC - Meta-anal of Current Smoking (or Ever if Current not available), Cigs (or Any Prod if Cigs not avail)
All LC types
Least adjusted

REF	NRR	SEX	AD	Number Exposed Case	Number Exposed Cont	Non-exposed Case	Non-exposed Cont	RR	95.00%CI
MCCONN	2	f	0	3	3	4	11	2.75	(0.38- 19.67)
Subtotal MCCONN								1.46	(0.57- 3.76)
MCDUFF	1	m	0	159	134	6	31	6.13	(2.48- 15.14)
MCLAUG	1	m	0	294	1082	22	270	3.33	(2.12- 5.25)
*MIGRAN	11	m	0	166	4570	4	867	7.87	(2.93- 21.17)
*MIGRAN	37	f	0	30	3459	4	3814	8.27	(2.92- 23.45)
Subtotal MIGRAN								8.06	(3.93- 16.51)
MILLER	1	f	0	140	1607	28	3638	11.32	(7.51- 17.06)
MILLS	1	m	1	-	-	-	-	1.27	(1.01- 1.61)
*MRFITR	2	m	0	106	8194	0	1859	48.33~	(3.01- 777.42)
NAM	68	m	0	241	589	30	520	7.09	(4.77- 10.55)
NAM	84	f	0	133	234	52	885	9.67	(6.81- 13.75)
Subtotal NAM								8.44	(6.49- 10.98)
NOTAN2	7	m	0	78	129	134	544	2.45	(1.75- 3.44)
NOU	11	m	0	74	247	6	122	6.09	(2.58- 14.39)
NOU	12	f	0	10	92	4	261	7.09	(2.17- 23.17)
Subtotal NOU								6.42	(3.20- 12.87)
ODRISC	1	c	0	293	598	6	664	54.22	(23.98- 122.60)
ORMOS	4	m	0	87	1034	7	777	9.34	(4.30- 20.28)
ORMOS	26	f	0	1	234	23	1044	0.19	(0.03- 1.44)
Subtotal ORMOS								5.65	(2.74- 11.64)
OSANN	9	m	0	791	541	45	833	27.07	(19.67- 37.25)
OSANN	13	f	0	597	367	96	1093	18.52	(14.48- 23.68)
Subtotal OSANN								21.33	(17.55- 25.92)
PARKIN	30	m	0	346	874	107	1248	4.62	(3.66- 5.83)
PASTOR	5	m	0	194	262	10	89	6.59	(3.34- 13.00)
PAWLEG	1	m	0	172	249	4	92	15.89	(5.73- 44.05)
PERNU	8	m	0	706	216	97	275	9.27	(7.02- 12.23)
PERNU	4	f	0	7	24	110	971	2.57	(1.08- 6.11)
Subtotal PERNU								8.22	(6.32- 10.71)
PERSH2	4	c	0	736	631	178	1164	7.63	(6.31- 9.23)
*PETO	4	m	0	99	2036	2	295	7.17	(1.78- 28.92)
PEZZO2	2	m	0	233	198	6	117	22.95	(9.89- 53.26)
PEZZOT	5	m	0	145	129	4	116	32.60	(11.70- 90.81)
PIKE	4	m	0	514	375	18	69	5.25	(3.08- 8.98)
PIKE	8	f	0	163	90	36	96	4.83	(3.04- 7.66)
Subtotal PIKE								5.01	(3.53- 7.10)
POFFIJ	1	c	0	913	918	58	452	7.75	(5.81- 10.34)
POLEDN	3	c	0	196	271	12	139	8.38	(4.52- 15.54)
*QIAO2	8	m	0	156	5399	10	709	2.05	(1.09- 3.86)
RACHTA	2	f	0	72	33	33	98	6.48	(3.66- 11.46)
RADZIK	1	c	0	180	198	9	13	1.31	(0.55- 3.15)
RANDIG	9	m	0	277	245	5	22	4.97	(1.86- 13.34)
RANDIG	10	f	0	16	39	17	92	2.22	(1.02- 4.84)
Subtotal RANDIG								3.03	(1.64- 5.58)
REN	1	m	0	106	84	12	34	3.58	(1.74- 7.33)
REN	2	f	0	78	20	48	50	4.06	(2.16- 7.64)
Subtotal REN								3.84	(2.39- 6.17)
RONCO	2	m	0	116	274	6	78	5.50	(2.33- 12.98)
ROTHSC	1	c	0	271	222	13	62	5.82	(3.12- 10.86)
SADOWS	4	m	0	421	446	18	81	4.25	(2.51- 7.20)
SANKAR	1	m	0	251	439	28	767	15.66	(10.42- 23.55)
SCHWAR	25	m	0	1652	349	119	376	14.96	(11.81- 18.94)
SCHWAR	26	m	0	644	139	50	104	9.64	(6.56- 14.15)
SCHWAR	27	f	0	1029	309	182	855	15.64	(12.75- 19.19)
SCHWAR	28	f	0	256	90	40	247	17.56	(11.64- 26.50)
Subtotal SCHWAR								14.70	(12.84- 16.83)
SEGI	1	m	0	140	1742	18	382	1.71	(1.03- 2.82)
SEGI2	19	m	0	267	485	8	53	3.65	(1.71- 7.79)
SEGI2	27	f	0	24	34	56	126	1.59	(0.86- 2.92)
Subtotal SEGI2								2.20	(1.37- 3.54)
SEOW	1	f	0	61	15	92	125	5.53	(2.96- 10.33)
SHAW	6	c	0	212	97	11	107	21.26	(10.93- 41.36)
SIEMIA	9	m	0	844	428	13	105	15.93	(8.85- 28.67)
SIMARA	5	m	0	33	264	27	433	2.00	(1.18- 3.41)
SIMARA	6	f	0	17	67	38	349	2.33	(1.24- 4.37)
Subtotal SIMARA								2.13	(1.42- 3.20)
SOBUE	90	m	0	736	650	34	128	4.26	(2.88- 6.31)
SOBUE	94	f	0	95	168	167	857	2.90	(2.15- 3.92)
Subtotal SOBUE								3.35	(2.64- 4.25)
SOBUE2	10	m	2	-	-	-	-	4.47	(3.89- 5.14)

Table 1C4 - 5

IESLC - Meta-anal of Current Smoking (or Ever if Current not available), Cigs (or Any Prod if Cigs not avail)
All LC types
Least adjusted

REF	NRR	SEX	AD	Number Exposed Case	Number Exposed Cont	Non-exposed Case	Non-exposed Cont	RR	95.00%CI
SOBUE2	12	f	2	-	-	-	-	3.28	(2.79- 3.87)
Subtotal SOBUE2								3.92	(3.53- 4.36)
*SPEIZE	6	f	0	391	489993	58	776300	10.68	(8.11- 14.07)
SPITZ	2	c	0	103	89	7	128	21.16	(9.40- 47.66)
STASZE	7	m	0	251	653	5	158	12.15	(4.93- 29.94)
STASZE	5	f	0	6	153	15	1660	4.34	(1.66- 11.35)
Subtotal STASZE								7.50	(3.89- 14.48)
STAYNE	1	m	0	362	567	58	333	3.67	(2.69- 4.99)
STOCKS	30	m	0	2421	5483	45	638	6.26	(4.61- 8.50)
STOCKS	50	f	1	-	-	-	-	3.04	(2.35- 3.93)
Subtotal STOCKS								4.10	(3.37- 4.99)
STOCKW	7	c	0	12470	3357	2791	10641	14.16	(13.38- 14.99)
STUCKE	2	m	0	69	68	0	51	104.50~	(6.32-1727.39)
SUN	1	c	0	140	173	67	191	2.31	(1.62- 3.30)
SUZUK2	2	c	0	78	30	11	53	12.53	(5.78- 27.16)
SVENSS	61	f	0	142	53	38	120	8.46	(5.22- 13.71)
TANG	1	c	0	52	25	9	39	9.01	(3.78- 21.46)
*TENKAN	24	m	1	-	-	-	-	16.81	(7.22- 39.14)
TIZZAN	5	m	0	693	619	180	305	1.90	(1.53- 2.35)
TIZZAN	13	f	0	17	18	25	114	4.31	(1.95- 9.51)
Subtotal TIZZAN								2.01	(1.63- 2.47)
TOKARS	1	m	0	110	157	1	53	37.13	(5.06- 272.56)
TOKARS	5	f	0	1	2	13	40	1.54	(0.13- 18.38)
Subtotal TOKARS								10.65	(2.25- 50.36)
TOUSEY	4	m	0	160	91	4	130	57.14	(20.45- 159.69)
TOUSEY	8	f	0	127	78	13	226	28.31	(15.13- 52.94)
Subtotal TOUSEY								34.23	(20.06- 58.43)
TSUGAN	28	m	0	63	63	18	22	1.22	(0.60- 2.50)
*TULINI	13	m	1	-	-	-	-	13.35	(7.18- 24.79)
*TULINI	19	f	1	-	-	-	-	19.20	(10.63- 34.68)
Subtotal TULINI								16.15	(10.53- 24.77)
*TVERDA	5	m	2	-	-	-	-	4.09	(2.65- 6.31)
*TVERDA	15	f	2	-	-	-	-	11.05	(3.33- 36.71)
Subtotal TVERDA								4.59	(3.05- 6.90)
WAKAI	2	m	0	181	284	10	65	4.14	(2.07- 8.27)
WAKAI	20	f	0	33	26	50	145	3.68	(2.01- 6.75)
Subtotal WAKAI								3.87	(2.46- 6.11)
*WALD	2	m	0	77	4182	7	6539	17.20	(7.94- 37.25)
WANG	1	m	0	262	-	29	-	3.47	(2.10- 5.80)
WANG	2	f	0	17	-	82	-	4.00	(1.30- 12.00)
Subtotal WANG								3.56	(2.24- 5.64)
WANG2	17	c	0	49	78	11	43	2.46	(1.16- 5.21)
WANG3	1	c	0	235	172	58	121	2.85	(1.97- 4.13)
WANG4	1	m	0	1043	18164	127	2374	1.07	(0.89- 1.30)
WICKLU	1	m	0	-	-	-	-	4.60	(2.80- 7.60)
WIGLE	1	m	0	415	415	15	204	13.60	(7.91- 23.38)
WIGLE	33	f	1	-	-	-	-	5.20	(3.34- 8.09)
Subtotal WIGLE								7.64	(5.42- 10.76)
WILKIN	1	m	0	173	372	2	108	25.11	(6.13- 102.89)
WILKIN	2	f	0	84	109	12	89	5.72	(2.93- 11.13)
Subtotal WILKIN								7.49	(4.10- 13.68)
WU	34	f	0	160	73	31	92	6.50	(3.98- 10.64)
WUNSCH	2	m	0	189	234	14	99	5.71	(3.16- 10.32)
WUNSCH	8	f	0	42	51	29	208	5.91	(3.36- 10.38)
Subtotal WUNSCH								5.81	(3.87- 8.74)
WUWILL	6	f	0	539	351	417	601	2.21	(1.84- 2.66)
WYNDE2	16	m	0	382	512	8	105	9.79	(4.71- 20.34)
WYNDE3	50	m	0	227	207	9	88	10.72	(5.26- 21.84)
WYNDE3	83	f	0	46	56	20	76	3.12	(1.67- 5.85)
Subtotal WYNDE3								5.36	(3.35- 8.58)
WYNDE4	48	m	0	632	665	12	115	9.11	(4.98- 16.67)
WYNDE4	62	f	2	-	-	-	-	2.87	(1.48- 5.55)
Subtotal WYNDE4								5.38	(3.45- 8.41)
WYNDE6	18	m	0	1677	741	87	617	16.05	(12.62- 20.41)
WYNDE6	207	f	0	1022	376	159	856	14.63	(11.90- 17.99)
Subtotal WYNDE6								15.22	(13.01- 17.80)
*XIANGZ	6	m	0	526	10580	25	974	1.94	(1.30- 2.88)
XU	1	m	0	627	552	102	236	2.63	(2.03- 3.40)
XU2	1	c	0	501	582	82	377	3.96	(3.03- 5.17)
XU3	1	m	0	92	68	7	31	5.99	(2.49- 14.42)
XU3	3	f	0	23	11	13	25	4.02	(1.51- 10.74)

Table 1C4 - 5

IESLC - Meta-anal of Current Smoking (or Ever if Current not available), Cigs (or Any Prod if Cigs not avail)
All LC types
Least adjusted

REF	NRR	SEX	AD	Number Exposed Case	Number Exposed Cont	Non-exposed Case	Non-exposed Cont	RR	95.00%CI
Subtotal XU3								5.02	(2.61- 9.66)
XU4	1	c	0	161	113	45	93	2.94	(1.92- 4.52)
YAMAGU	1	c	0	76	247	24	267	3.42	(2.10- 5.59)
*YONG	12	m	1	-	-	-	-	28.71	(6.98- 118.16)
*YONG	15	f	1	-	-	-	-	5.20	(2.38- 11.35)
Subtotal YONG								7.75	(3.91- 15.36)
*YUAN	1	m	2	-	-	-	-	6.50	(3.64- 11.60)
ZHANG	1	c	0	72	102	28	98	2.47	(1.47- 4.14)
ZHENG	15	m	0	279	218	33	94	3.65	(2.36- 5.63)
ZHENG	24	f	0	76	44	152	184	2.09	(1.36- 3.21)
Subtotal ZHENG								2.75	(2.03- 3.73)
ZHOU	2	m	0	740	41	275	36	2.36	(1.48- 3.77)
ZHOU	3	f	0	112	7	231	32	2.22	(0.95- 5.18)
Subtotal ZHOU								2.33	(1.54- 3.51)
Partial Totals				105068	2658439	15253	4493613		

*prospective study ~ With 0.5 adjustment for zero

REF	NRR	SEX	AD	Ys	Ws	Qs	Ps
ABELIN	2	m	0	3.33	1.88	5.52	0.0000
*ABRAHA	7	m	0	2.17	9.68	2.98	0.0000
*ABRAHA	8	f	0	1.59	19.39	0.02	0.0000
Subtotal ABRAHA				1.78	29.07	2.99	
AGUDO	10	f	0	0.99	7.89	3.07	0.0054
*AKIBA	2	m	0	1.39	17.12	0.87	0.0000
*AKIBA	6	f	0	1.50	45.22	0.59	0.0000
Subtotal AKIBA				1.47	62.34	1.46	
ALDERS	177	m	0	2.66	12.62	13.81	0.0000
ALDERS	176	f	0	1.76	41.23	0.86	0.0000
Subtotal ALDERS				1.97	53.85	14.67	
*AMANDU	1	m	0	1.61	5.70	0.00	0.0001
AMES	1	m	0	1.52	10.33	0.10	0.0000
*ANDERS	2	f	0	3.08	37.84	81.57	0.0000
*ARCHER	5	m	0	1.82	5.72	0.24	0.0000
ARMADA	27	m	0	3.21	3.58	9.07	0.0000
AUSTIN	2	c	0	2.75	4.38	5.65	0.0000
AUVINE	1	c	0	2.15	30.60	8.66	0.0000
AXELSO	1	c	0	1.83	24.06	1.17	0.0000
AXELSS	2	m	0	2.70	12.26	14.54	0.0000
AXELSS	10	f	0	2.48	11.50	8.57	0.0000
Subtotal AXELSS				2.59	23.76	23.11	
BAND	1	m	2	2.30	42.76	20.06	0.0000
BARBON	3	m	0	2.59	18.08	17.07	0.0000
BECHER	13	m	0	2.70	2.70	3.20	0.0000
BECHER	14	f	0	1.89	5.32	0.40	0.0000
Subtotal BECHER				2.16	8.02	3.60	
*BENSHL	4	m	1	2.48	9.73	7.28	0.0000
*BEST	2	m	1	2.70	6.85	8.12	0.0000
*BEST	18	f	1	0.81	2.17	1.41	0.2348
Subtotal BEST				2.25	9.02	9.53	
BLOHMK	1	m	0	1.16	59.38	12.09	0.0000
BLOT4	1	m	0	2.68	6.96	7.85	0.0000
BOFFET	7	m	0	2.73	105.32	131.93	0.0000
*BOUCOT	2	m	0	4.06	0.50	2.99	0.0042
BRESLO	17	m	0	2.00	5.83	0.86	0.0000
BRESLO	23	f	0	0.32	3.10	5.18	0.5717
Subtotal BRESLO				1.42	8.93	6.04	
*BRETT	4	m	0	1.37	5.75	0.35	0.0010
BROCKM	1	m	0	0.07	0.98	2.32	0.9437
BROCKM	2	f	0	0.69	2.73	2.32	0.2517
Subtotal BROCKM				0.53	3.71	4.64	
BROSS	4	m	0	1.78	27.54	0.75	0.0000
BROWN2	12	m	2	2.42	402.82	265.06	0.0000
BROWN2	11	f	2	2.61	365.29	362.70	0.0000
Subtotal BROWN2				2.51	768.12	627.76	
BUFFLE	3	m	0	2.40	4.35	2.70	0.0000
BUFFLE	7	f	0	2.11	26.25	6.50	0.0000
Subtotal BUFFLE				2.15	30.60	9.20	
CARPEN	9	c	0	3.08	12.26	26.30	0.0000
CASCO2	1	c	0	2.44	5.31	3.63	0.0000
CASCOR	1	c	0	2.60	18.40	18.05	0.0000

Table 1C4 - 5

IESLC - Meta-anal of Current Smoking (or Ever if Current not available), Cigs (or Any Prod if Cigs not avail)
All LC types
Least adjusted

REF	NRR	SEX	AD	Ys	Ws	Qs	Ps
*CEDERL	2	m	0	1.83	6.22	0.28	0.0000
*CEDERL	119	f	1	1.51	4.78	0.05	0.0009
Subtotal CEDERL				1.69	11.00	0.33	
CHAN	5	m	0	3.31	1.87	5.41	0.0000
CHAN	6	f	0	1.25	20.57	2.79	0.0000
Subtotal CHAN				1.42	22.44	8.20	
*CHANG	5	m	0	2.13	4.46	1.17	0.0000
*CHANG	11	f	0	1.64	8.22	0.01	0.0000
Subtotal CHANG				1.81	12.68	1.18	
CHATZI	4	c	0	1.21	19.44	3.24	0.0000
CHEN2	1	m	0	1.52	6.25	0.05	0.0001
CHEN2	2	f	0	0.51	7.70	9.30	0.1539
Subtotal CHEN2				0.97	13.95	9.36	
CHEN3	1	c	0	0.46	27.78	36.81	0.0148
CHIAZZ	2	m	0	2.06	3.53	0.69	0.0001
CHOI	3	m	0	1.64	10.55	0.01	0.0000
CHOI	7	f	0	0.20	7.16	14.34	0.5951
Subtotal CHOI				1.06	17.71	14.34	
*CHOW	18	m	0	2.87	5.75	9.13	0.0000
*CHYOU	4	m	0	2.27	12.23	5.23	0.0000
COMSTO	3	m	0	2.90	3.52	5.80	0.0000
COMSTO	8	f	0	2.57	8.49	7.80	0.0000
Subtotal COMSTO				2.67	12.01	13.60	
COOKSO	4	c	0	1.88	14.21	1.02	0.0000
CORREA	41	c	0	2.53	40.00	33.66	0.0000
*CPSI	220	m	1	2.48	74.99	56.28	0.0000
*CPSI	279	f	1	1.16	70.15	14.23	0.0000
Subtotal CPSI				1.84	145.14	70.51	
*CPSII	36	m	0	2.90	115.97	193.39	0.0000
*CPSII	71	f	0	2.22	237.52	86.84	0.0000
Subtotal CPSII				2.44	353.49	280.24	
DAMBER	16	m	1	2.28	19.52	8.73	0.0000
DARBY	4	m	0	4.51	2.93	24.59	0.0000
DARBY	11	f	0	3.03	18.15	36.33	0.0000
Subtotal DARBY				3.23	21.08	60.93	
DAVEYS	5	m	0	1.57	2.53	0.01	0.0126
DAVEYS	6	f	0	-0.32	0.42	1.59	0.8327
Subtotal DAVEYS				1.30	2.96	1.60	
DEAN	8	m	0	1.70	9.65	0.08	0.0000
DEAN2	2	m	0	1.33	23.59	1.85	0.0000
DEAN2	6	f	0	1.06	13.83	4.18	0.0001
Subtotal DEAN2				1.23	37.42	6.03	
DEAN3	238	m	0	1.98	21.31	2.93	0.0000
DEAN3	117	f	0	1.20	28.00	4.90	0.0000
Subtotal DEAN3				1.54	49.32	7.83	
*DEKLER	7	m	2	3.17	0.99	2.40	0.0016
DESTE2	2	c	0	2.04	14.18	2.63	0.0000
DESTEF	40	m	0	2.27	19.86	8.53	0.0000
*DOCKER	1	c	4	2.08	3.90	0.85	0.0000
DOLL	90	m	0	2.25	6.22	2.54	0.0000
DOLL	93	f	0	0.74	11.96	9.23	0.0110
Subtotal DOLL				1.25	18.18	11.77	
*DOLL2	68	m	1	2.50	6.84	5.39	0.0000
*DOLL2	63	f	1	2.16	3.28	0.97	0.0001
Subtotal DOLL2				2.39	10.12	6.36	
DORANT	9	c	0	3.26	13.00	35.08	0.0000
DORGAN	9	m	0	2.83	11.70	17.27	0.0000
DORGAN	33	m	0	3.71	2.61	11.50	0.0000
DORGAN	56	f	0	2.50	41.93	32.83	0.0000
DORGAN	79	f	0	2.44	3.75	2.54	0.0000
Subtotal DORGAN				2.61	60.00	64.14	
*DORN	391	m	1	2.39	316.13	188.15	0.0000
DOSEME	17	m	0	1.41	75.43	3.01	0.0000
DROSTE	2	m	0	2.94	6.25	10.95	0.0000
DU	1	m	0	1.26	28.12	3.49	0.0000
DU	2	f	0	0.66	24.50	22.40	0.0011
Subtotal DU				0.98	52.62	25.89	
*DUNN	6	m	0	2.91	1.97	3.33	0.0000
EBELIN	1	m	0	1.94	9.19	0.96	0.0000
*ENGELA	168	m	1	2.27	6.48	2.81	0.0000
*ENGELA	177	f	1	1.76	6.50	0.14	0.0000

Table 1C4 - 5

IESLC - Meta-anal of Current Smoking (or Ever if Current not available), Cigs (or Any Prod if Cigs not avail)
All LC types
Least adjusted

REF	NRR	SEX	AD	Ys	Ws	Qs	Ps
Subtotal ENGELA				2.01	12.99	2.95	
*ENSTRO	1	m	1	2.56	81.91	74.01	0.0000
*ENSTRO	2	f	1	1.94	181.45	19.18	0.0000
Subtotal ENSTRO				2.13	263.36	93.19	
ESAKI	4	m	0	0.64	8.96	8.49	0.0554
ESAKI	5	f	0	0.90	7.99	4.06	0.0109
Subtotal ESAKI				0.76	16.94	12.55	
FAN	1	m	0	1.04	25.87	8.37	0.0000
FAN	2	f	0	1.37	24.92	1.53	0.0000
Subtotal FAN				1.20	50.80	9.89	
GAO	29	m	0	1.37	39.60	2.35	0.0000
GAO	30	f	0	0.86	50.42	28.60	0.0000
Subtotal GAO				1.08	90.02	30.95	
GAO2	1	m	0	1.91	9.19	0.82	0.0000
GARCIA	2	c	0	2.72	13.47	16.36	0.0000
GARDIN	6	c	0	2.72	3.79	4.61	0.0000
GARSHI	23	m	0	2.01	33.39	5.16	0.0000
GENG	1	m	0	1.79	4.98	0.16	0.0001
GENG	2	f	0	1.08	22.39	6.26	0.0000
Subtotal GENG				1.21	27.37	6.42	
GER	17	c	0	0.31	26.37	44.72	0.1100
GODLEY	5	m	1	1.92	96.28	9.20	0.0000
GODLEY	6	f	1	1.71	58.89	0.57	0.0000
Subtotal GODLEY				1.84	155.17	9.77	
GOLLED	21	m	0	1.84	13.92	0.73	0.0000
GOODMA	2	m	0	2.86	8.50	13.16	0.0000
GOODMA	6	f	0	2.27	10.71	4.57	0.0000
Subtotal GOODMA				2.53	19.21	17.73	
GRAHAM	9	m	0	2.09	16.24	3.71	0.0000
GREGOR	2	m	0	0.26	4.75	8.72	0.5741
GREGOR	6	f	0	2.67	0.88	0.97	0.0126
Subtotal GREGOR				0.63	5.62	9.69	
GSELL	6	m	0	3.03	1.74	3.49	0.0001
HAENSZ	54	f	0	0.76	23.97	17.46	0.0002
*HAMMO2	22	m	0	2.12	4.92	1.26	0.0000
*HAMMON	139	m	1	2.44	14.07	9.70	0.0000
*HANSEN	3	m	2	0.43	5.28	7.46	0.3285
HEGMAN	1	c	0	2.79	23.66	32.95	0.0000
*HEIN	1	m	0	3.12	0.98	2.21	0.0020
*HENNEK	2	m	0	2.73	17.97	22.53	0.0000
HINDS	26	f	0	1.42	61.37	2.39	0.0000
*HIRAYA	1	m	1	1.49	85.55	1.25	0.0000
*HIRAYA	3	f	1	0.85	77.37	45.10	0.0000
Subtotal HIRAYA				1.19	162.92	46.35	
HITOSU	2	m	0	0.93	6.40	3.00	0.0187
HITOSU	9	f	0	1.25	14.55	1.90	0.0000
Subtotal HITOSU				1.15	20.95	4.90	
*HOLE	46	m	0	1.93	6.76	0.66	0.0000
*HOLE	29	f	0	0.37	4.98	7.65	0.4046
Subtotal HOLE				1.27	11.73	8.31	
HOROWI	1	m	0	1.27	15.35	1.77	0.0000
HOROWI	2	f	0	0.60	8.08	8.33	0.0894
Subtotal HOROWI				1.04	23.43	10.10	
HORWIT	1	f	0	2.43	8.29	5.47	0.0000
HU	15	m	0	0.74	17.16	13.23	0.0023
HU	16	f	0	0.55	7.15	8.09	0.1413
Subtotal HU				0.68	24.31	21.32	
HU2	9	m	0	1.11	27.11	6.95	0.0000
HU2	10	f	0	0.63	21.91	21.26	0.0033
Subtotal HU2				0.89	49.01	28.20	
HUANG	1	c	0	0.69	14.82	12.63	0.0078
HUMBLE	13	m	1	2.99	4.94	9.42	0.0000
HUMBLE	15	m	1	2.76	1.65	2.16	0.0004
HUMBLE	17	f	1	2.82	5.85	8.47	0.0000
HUMBLE	19	f	1	3.16	2.49	5.93	0.0000
Subtotal HUMBLE				2.93	14.94	25.98	
JAHN	3	f	0	1.13	18.89	4.45	0.0000
JAIN	16	m	0	2.77	9.32	12.39	0.0000
JAIN	11	f	0	2.54	26.82	23.01	0.0000
Subtotal JAIN				2.60	36.14	35.40	
JARUP	3	m	0	1.90	6.90	0.56	0.0000

Table 1C4 - 5

IESLC - Meta-anal of Current Smoking (or Ever if Current not available), Cigs (or Any Prod if Cigs not avail)
All LC types
Least adjusted

REF	NRR	SEX	AD	Ys	Ws	Qs	Ps
JARVHO	2	m	0	3.70	0.90	3.90	0.0005
JARVHO	6	f	0	2.74	2.57	3.26	0.0000
Subtotal JARVHO				2.99	3.47	7.17	
JEDRYC	63	m	0	1.76	36.14	0.76	0.0000
JEDRYC	68	f	0	2.08	17.12	3.68	0.0000
Subtotal JEDRYC				1.86	53.25	4.44	
JIANG	1	m	0	1.00	4.45	1.67	0.0346
JIANG	2	f	0	0.91	2.62	1.29	0.1401
Subtotal JIANG				0.97	7.08	2.96	
JOLY	16	m	0	2.75	10.86	14.02	0.0000
JOLY	15	f	0	2.01	24.54	3.91	0.0000
Subtotal JOLY				2.24	35.40	17.93	
JUSSAW	2	m	0	1.92	34.20	3.31	0.0000
*KAISE2	68	m	1	2.08	10.65	2.36	0.0000
*KAISE2	60	f	1	2.67	8.78	9.85	0.0000
Subtotal KAISE2				2.35	19.43	12.21	
*KAISER	12	m	2	2.98	25.68	47.67	0.0000
*KAISER	9	f	2	1.88	27.68	1.91	0.0000
Subtotal KAISER				2.41	53.36	49.58	
KANELL	5	m	0	1.89	33.17	2.52	0.0000
KATSOU	6	f	0	1.25	8.81	1.17	0.0002
KAUFMA	7	c	0	2.92	30.87	52.61	0.0000
KELLER	1	m	0	2.58	195.95	182.36	0.0000
KELLER	9	m	0	2.73	24.67	30.61	0.0000
KELLER	5	f	0	2.68	233.82	264.40	0.0000
KELLER	13	f	0	2.45	34.67	24.53	0.0000
Subtotal KELLER				2.62	489.11	501.90	
KHUDER	19	m	0	2.09	19.27	4.41	0.0000
KIHARA	7	c	0	1.40	42.14	1.91	0.0000
*KINLEN	8	m	0	2.58	6.96	6.48	0.0000
KJUUS	1	m	0	3.05	1.78	3.65	0.0000
*KNEKT	75	m	0	2.03	5.61	0.95	0.0000
KO	1	f	3	1.44	2.18	0.07	0.0339
KOHLME	1	c	0	2.83	9.55	14.14	0.0000
KOO	9	f	0	0.94	10.70	4.91	0.0022
KOULUM	2	m	0	3.38	4.45	13.96	0.0000
KREUZE	6	f	0	2.76	3.90	5.14	0.0000
KREUZE	8	f	0	1.75	24.58	0.47	0.0000
Subtotal KREUZE				1.89	28.48	5.61	
KREYBE	24	m	0	2.04	5.80	1.06	0.0000
KREYBE	39	f	0	-0.22	8.25	27.78	0.5245
Subtotal KREYBE				0.71	14.05	28.84	
*KUBIK	12	m	0	3.50	1.96	6.95	0.0000
LAMTH	6	f	0	1.34	46.55	3.55	0.0000
LAMWK	1	f	0	1.42	17.85	0.70	0.0000
LAMWK2	9	m	0	1.04	12.98	4.25	0.0002
LAMWK2	10	f	0	1.17	17.89	3.59	0.0000
Subtotal LAMWK2				1.11	30.86	7.84	
*LANGE	32	m	0	1.71	4.90	0.05	0.0002
*LANGE	29	f	0	1.30	6.21	0.61	0.0012
Subtotal LANGE				1.48	11.11	0.66	
LAUSSM	10	m	0	1.59	41.00	0.02	0.0000
LEI	1	m	0	1.30	26.63	2.56	0.0000
LEI	2	f	0	1.25	23.21	3.08	0.0000
Subtotal LEI				1.28	49.84	5.65	
LEMARC	2	c	0	2.60	17.07	16.67	0.0000
LETOUR	1	c	0	2.56	20.21	18.19	0.0000
LEVIN	30	m	1	1.94	29.93	3.22	0.0000
*LIAW	1	m	1	1.31	11.72	1.09	0.0000
*LIAW	2	f	1	1.28	2.46	0.27	0.0447
Subtotal LIAW				1.30	14.17	1.36	
*LIDDEL	4	m	1	1.48	17.82	0.30	0.0000
LIU	2	c	2	0.65	38.19	35.29	0.0001
LIU2	1	m	0	1.46	8.60	0.20	0.0000
LIU2	3	f	0	1.45	9.73	0.26	0.0000
Subtotal LIU2				1.46	18.32	0.46	
LIU3	1	m	0	0.19	3.06	6.23	0.7444
LIU4	10	m	2	1.36	5780.52	384.14	0.0000
LIU4	12	f	2	1.05	3876.64	1227.91	0.0000
Subtotal LIU4				1.23	9657.16	1612.06	
LIU5	1	c	0	0.65	11.25	10.45	0.0293

Table 1C4 - 5

IESLC - Meta-anal of Current Smoking (or Ever if Current not available), Cigs (or Any Prod if Cigs not avail)
All LC types
Least adjusted

REF	NRR	SEX	AD	Ys	Ws	Qs	Ps
LOMBA2	1	f	0	0.28	37.19	65.83	0.0841
LOMBAR	9	m	0	2.41	12.02	7.69	0.0000
LUBIN2	27	m	0	2.35	167.71	91.64	0.0000
LUBIN2	317	f	0	1.34	106.80	7.72	0.0000
Subtotal LUBIN2				1.96	274.52	99.36	
LUO	1	c	0	0.66	18.01	16.55	0.0054
MACLEN	19	m	0	1.34	3.53	0.27	0.0120
MACLEN	32	f	0	0.87	12.71	7.12	0.0020
Subtotal MACLEN				0.97	16.25	7.39	
*MAGNUS	1	m	0	1.69	10.53	0.06	0.0000
MARSH	1	m	0	2.32	1.82	0.90	0.0018
MARSH	3	f	0	1.76	5.60	0.12	0.0000
Subtotal MARSH				1.90	7.42	1.02	
MARSH2	1	c	0	1.19	8.48	1.53	0.0005
MARTIS	4	m	0	1.95	3.32	0.37	0.0004
MASTRA	1	m	0	2.13	5.09	1.34	0.0000
MATOS	2	m	0	2.14	8.58	2.36	0.0000
MATSUD	10	m	0	3.07	2.94	6.20	0.0000
MCCONN	1	m	0	0.19	3.33	6.71	0.7237
MCCONN	2	f	0	1.01	0.99	0.36	0.3136
Subtotal MCCONN				0.38	4.32	7.07	
MCDUFF	1	m	0	1.81	4.70	0.19	0.0001
MCLAUG	1	m	0	1.20	18.70	3.13	0.0000
*MIGRAN	11	m	0	2.06	3.93	0.79	0.0000
*MIGRAN	37	f	0	2.11	3.54	0.88	0.0001
Subtotal MIGRAN				2.09	7.46	1.68	
MILLER	1	f	0	2.43	22.85	15.10	0.0000
MILLS	1	m	1	0.24	71.48	134.44	0.0406
*MRFITR	2	m	0	3.88	0.50	2.55	0.0062
NAM	68	m	0	1.96	24.33	2.90	0.0000
NAM	84	f	0	2.27	31.10	13.37	0.0000
Subtotal NAM				2.13	55.43	16.28	
NOTAN2	7	m	0	0.90	33.47	17.14	0.0000
NOU	11	m	0	1.81	5.20	0.19	0.0000
NOU	12	f	0	1.96	2.74	0.33	0.0012
Subtotal NOU				1.86	7.94	0.52	
ODRISC	1	c	0	3.99	5.77	32.68	0.0000
ORMOS	4	m	0	2.23	6.39	2.46	0.0000
ORMOS	26	f	0	-1.64	0.95	10.09	0.1093
Subtotal ORMOS				1.73	7.34	12.55	
OSANN	9	m	0	3.30	37.69	106.95	0.0000
OSANN	13	f	0	2.92	63.57	108.30	0.0000
Subtotal OSANN				3.06	101.25	215.25	
PARKIN	30	m	0	1.53	70.51	0.50	0.0000
PASTOR	5	m	0	1.89	8.32	0.62	0.0000
PAWLEG	1	m	0	2.77	3.69	4.90	0.0000
PERNU	8	m	0	2.23	50.02	18.78	0.0000
PERNU	4	f	0	0.95	5.14	2.29	0.0321
Subtotal PERNU				2.11	55.16	21.07	
PERSH2	4	c	0	2.03	106.15	18.56	0.0000
*PETO	4	m	0	1.97	1.98	0.25	0.0056
PEZZO2	2	m	0	3.13	5.42	12.51	0.0000
PEZZOT	5	m	0	3.48	3.66	12.80	0.0000
PIKE	4	m	0	1.66	13.39	0.03	0.0000
PIKE	8	f	0	1.57	18.04	0.03	0.0000
Subtotal PIKE				1.61	31.43	0.05	
POFFIJ	1	c	0	2.05	46.21	8.71	0.0000
POLEDN	3	c	0	2.13	10.07	2.64	0.0000
*QIAO2	8	m	0	0.72	9.54	7.67	0.0267
RACHTA	2	f	0	1.87	11.81	0.77	0.0000
RADZIK	1	c	0	0.27	5.03	9.06	0.5411
RANDIG	9	m	0	1.60	3.95	0.00	0.0014
RANDIG	10	f	0	0.80	6.34	4.22	0.0447
Subtotal RANDIG				1.11	10.29	4.22	
REN	1	m	0	1.27	7.46	0.86	0.0005
REN	2	f	0	1.40	9.65	0.43	0.0000
Subtotal REN				1.35	17.11	1.29	
RONCO	2	m	0	1.71	5.21	0.04	0.0001
ROTHSC	1	c	0	1.76	9.88	0.22	0.0000
SADOWS	4	m	0	1.45	13.79	0.39	0.0000
SANKAR	1	m	0	2.75	23.11	29.90	0.0000

Table 1C4 - 5

IESLC - Meta-anal of Current Smoking (or Ever if Current not available), Cigs (or Any Prod if Cigs not avail)
All LC types
Least adjusted

REF	NRR	SEX	AD	Ys	Ws	Qs	Ps
SCHWAR	25	m	0	2.71	68.81	81.98	0.0000
SCHWAR	26	m	0	2.27	26.07	11.08	0.0000
SCHWAR	27	f	0	2.75	91.98	118.80	0.0000
SCHWAR	28	f	0	2.87	22.69	35.59	0.0000
Subtotal SCHWAR				2.69	209.54	247.44	
SEGI	1	m	0	0.53	15.18	17.69	0.0375
SEGI2	19	m	0	1.29	6.68	0.68	0.0008
SEGI2	27	f	0	0.46	10.32	13.68	0.1372
Subtotal SEGI2				0.79	17.00	14.36	
SEOW	1	f	0	1.71	9.81	0.09	0.0000
SHAW	6	c	0	3.06	8.67	18.07	0.0000
SIEMIA	9	m	0	2.77	11.12	14.81	0.0000
SIMARA	5	m	0	0.70	13.62	11.48	0.0103
SIMARA	6	f	0	0.85	9.72	5.72	0.0084
Subtotal SIMARA				0.76	23.33	17.20	
SOBUE	90	m	0	1.45	24.92	0.67	0.0000
SOBUE	94	f	0	1.07	42.31	12.72	0.0000
Subtotal SOBUE				1.21	67.24	13.39	
SOBUE2	10	m	2	1.50	197.90	2.67	0.0000
SOBUE2	12	f	2	1.19	143.51	26.02	0.0000
Subtotal SOBUE2				1.37	341.42	28.69	
*SPEIZE	6	f	0	2.37	50.52	28.78	0.0000
SPITZ	2	c	0	3.05	5.83	12.06	0.0000
STASZE	7	m	0	2.50	4.72	3.68	0.0000
STASZE	5	f	0	1.47	4.16	0.09	0.0028
Subtotal STASZE				2.02	8.88	3.77	
STAYNE	1	m	0	1.30	40.37	4.00	0.0000
STOCKS	30	m	0	1.83	41.01	2.00	0.0000
STOCKS	50	f	1	1.11	58.11	14.63	0.0000
Subtotal STOCKS				1.41	99.12	16.63	
STOCKW	7	c	0	2.65	1204.31	1295.00	0.0000
STUCKE	2	m	0	4.65	0.49	4.50	0.0012
SUN	1	c	0	0.84	30.23	18.28	0.0000
SUZUK2	2	c	0	2.53	6.41	5.36	0.0000
SVENSS	61	f	0	2.14	16.51	4.50	0.0000
TANG	1	c	0	2.20	5.10	1.75	0.0000
*TENKAN	24	m	1	2.82	5.38	7.85	0.0000
TIZZAN	5	m	0	0.64	84.08	79.66	0.0000
TIZZAN	13	f	0	1.46	6.13	0.14	0.0003
Subtotal TIZZAN				0.70	90.21	79.81	
TOKARS	1	m	0	3.61	0.97	3.87	0.0004
TOKARS	5	f	0	0.43	0.62	0.87	0.7336
Subtotal TOKARS				2.37	1.59	4.74	
TOUSEY	4	m	0	4.05	3.64	21.51	0.0000
TOUSEY	8	f	0	3.34	9.80	29.31	0.0000
Subtotal TOUSEY				3.53	13.44	50.82	
TSUGAN	28	m	0	0.20	7.53	15.04	0.5818
*TULINI	13	m	1	2.59	10.01	9.57	0.0000
*TULINI	19	f	1	2.95	10.99	19.77	0.0000
Subtotal TULINI				2.78	21.00	29.34	
*TVERDA	5	m	2	1.41	20.41	0.86	0.0000
*TVERDA	15	f	2	2.40	2.67	1.66	0.0001
Subtotal TVERDA				1.52	23.08	2.52	
WAKAI	2	m	0	1.42	8.04	0.30	0.0001
WAKAI	20	f	0	1.30	10.45	1.01	0.0000
Subtotal WAKAI				1.35	18.49	1.31	
*WALD	2	m	0	2.84	6.43	9.75	0.0000
WANG	1	m	0	1.24	14.89	2.03	0.0000
WANG	2	f	0	1.39	3.11	0.16	0.0145
Subtotal WANG				1.27	18.00	2.19	
WANG2	17	c	0	0.90	6.78	3.47	0.0193
WANG3	1	c	0	1.05	28.11	9.01	0.0000
WANG4	1	m	0	0.07	107.42	255.70	0.4630
WICKLU	1	m	0	1.53	15.41	0.12	0.0000
WIGLE	1	m	0	2.61	13.09	13.00	0.0000
WIGLE	33	f	1	1.65	19.63	0.02	0.0000
Subtotal WIGLE				2.03	32.72	13.02	
WILKIN	1	m	0	3.22	1.93	5.01	0.0000
WILKIN	2	f	0	1.74	8.65	0.15	0.0000
Subtotal WILKIN				2.01	10.58	5.15	
WU	34	f	0	1.87	15.85	1.06	0.0000

Table 1C4 - 5

IESLC - Meta-anal of Current Smoking (or Ever if Current not available), Cigs (or Any Prod if Cigs not avail)
All LC types
Least adjusted

REF	NRR	SEX	AD	Ys	Ws	Qs	Ps
WUNSCH	2	m	0	1.74	10.98	0.18	0.0000
WUNSCH	8	f	0	1.78	12.09	0.32	0.0000
Subtotal WUNSCH				1.76	23.07	0.50	
WUWILL	6	f	0	0.79	114.07	76.55	0.0000
WYNDE2	16	m	0	2.28	7.19	3.21	0.0000
WYNDE3	50	m	0	2.37	7.59	4.37	0.0000
WYNDE3	83	f	0	1.14	9.73	2.20	0.0004
Subtotal WYNDE3				1.68	17.32	6.57	
WYNDE4	48	m	0	2.21	10.51	3.73	0.0000
WYNDE4	62	f	2	1.05	8.80	2.75	0.0018
Subtotal WYNDE4				1.68	19.31	6.48	
WYNDE6	18	m	0	2.78	66.40	89.67	0.0000
WYNDE6	207	f	0	2.68	90.13	103.12	0.0000
Subtotal WYNDE6				2.72	156.52	192.79	
*XIANGZ	6	m	0	0.66	24.52	22.25	0.0011
XU	1	m	0	0.97	57.31	24.02	0.0000
XU2	1	c	0	1.38	53.87	3.05	0.0000
XU3	1	m	0	1.79	4.98	0.16	0.0001
XU3	3	f	0	1.39	3.98	0.20	0.0055
Subtotal XU3				1.61	8.96	0.35	
XU4	1	c	0	1.08	20.82	5.93	0.0000
YAMAGU	1	c	0	1.23	15.97	2.34	0.0000
*YONG	12	m	1	3.36	1.92	5.84	0.0000
*YONG	15	f	1	1.65	6.30	0.01	0.0000
Subtotal YONG				2.05	8.22	5.84	
*YUAN	1	m	2	1.87	11.44	0.76	0.0000
ZHANG	1	c	0	0.90	14.37	7.22	0.0006
ZHENG	15	m	0	1.29	20.36	2.09	0.0000
ZHENG	24	f	0	0.74	20.88	16.02	0.0008
Subtotal ZHENG				1.01	41.24	18.11	
ZHOU	2	m	0	0.86	17.50	9.94	0.0003
ZHOU	3	f	0	0.80	5.34	3.57	0.0660
Subtotal ZHOU				0.84	22.83	13.51	

	N	345
	NS	242
	Wt	19542.47
Het	Chi	8497.07
Het	df	344
Het	P	***
Fixed	RR	5.02
	RRl	4.95
	RRu	5.09
	P	+++
Random	RR	6.21
	RRl	5.72
	RRu	6.74
	P	+++
Asymm	P	***

Table 1C4 - 6

IESLC - Meta-anal of Current Smoking (or Ever if Current not available), Cigs (or Any Prod if Cigs not avail)
All LC types
Least adjusted

		combined	Sex male	female	Total
	N	45	181	119	345
	NS	45	177	114	336
	Wt	2089.01	10160.62	7292.83	19542.47
Het	Chi	998.95	3290.53	3028.31	8497.07
Het	df	44	180	118	344
Het	P	***	***	***	***
Fixed	RR	9.71	5.03	4.14	5.02
	RRl	9.31	4.94	4.05	4.95
	RRu	10.14	5.13	4.24	5.09
	P	+++	+++	+++	+++
Random	RR	6.65	7.19	4.85	6.21
	RRl	5.14	6.43	4.19	5.72
	RRu	8.60	8.05	5.63	6.74
	P	+++	+++	+++	+++
Between	Chi				1179.29
Between	df				2
Between	P				***
Btwn(F)	P				***
Btwn(R)	P				***

Table 1C4 - 7

IESLC - Meta-anal of Current Smoking (or Ever if Current not available), Cigs (or Any Prod if Cigs not avail)
All LC types
Excluded studies (and stage at which they were excluded)

1	BOUCHA BUELL LAURIL MZILEN
2	BERRIN CHEN RESTRE TAO ULMER
5	RIMING TANG2 WYNDE5 WYNDE8
6	BLOT1 BLOT2 BLOT3 BYERS2 HIRAOK HIRAY2 JONES LICKIN MOLLO MRFIT MURATA NOTANI PERSHA SAARIK SCHWA2 SHIMIZ SITAS VANDER WARSIN WATSON WYNDER
8	BROWN1 BYERS1 SUZUKI WU2
10	BENHAM GILLIS ISHIMA LUBIN OSANN2 PISANI PRESCO QIAO ROOTS SIMONA VEIERO VUTUC WYNDE7

Table 1C4 - 8

Potentially overlapping studies

REF	REFGP	PRINC	.	OVERLAP/LINK
LUBIN2	LUBIN2	1		Lubin-combined
CHAN	CHAN	1		Chan/LamWK2
LAMWK2	LAMWK2	1		Chan/LamWK2
LAMTH	LAMTH	1		KOO/LAMTH/LAMWK
KOO	KOO	1		KOO/LAMTH/LAMWK
LAMWK	LAMWK	1		KOO/LAMTH/LAMWK
CASCOR	CASCOR	1		Roots/Cascor 1
TVERDA	TVERDA	1		VEIERO/TVERDAL
AKIBA	AKIBA	1		AKIBA/ISHIMA
HEIN	PRESCO	1		Subset of PRESCO
LANGE	PRESCO	1		Subset of PRESCO
BROSS	BYERS1	1		GRAHAM/BROSS/BYERS1
GRAHAM	BYERS1	1		GRAHAM/BROSS/BYERS1
CHYOU	CHYOU	1		GOODMA/CHYOU
GOODMA	GOODMA	1		GOODMA/CHYOU
HOLE	TANG2	1		Subset of TANG2
BENSHL	TANG2	1		Subset of TANG2
WALD	TANG2	1		Subset of TANG2
KAISER	KAISER	1		KAISER/OSANN2
MRFITR	MRFIT	2		Subset of MRFIT
WYNDE6	WYNDE6	1		WYNDE5/6/7/8
CPSI	CPSI	1		CPSI overall
ENSTRO	ENSTRO	1		Subset of CPSI
SOBUE2	SOBUE2	1		SOBUE2/MATSUD
MATSUD	MATSUD	1		SOBUE2/MATSUD
BOFFET	BOFFET	1		BOFFET-combined
JAHN	BOFFET	2		Subset of BOFFET
XIANGZ	XIANGZ	1		LUBIN/XIANGZ/QIAO

Table 1C4 - 9

Most adjusted - insufficient data for metaanalysis

REF	NRR	SEX	AGEL	AGEH	RACE	YF	LC TYPE	LOC	START	ST	NLC	R	VB	P	H	AD	SM	PRODUCT	DENOM	De
HIRAYA	152	m	0	0	all	0	all	As:Jap	1965	pr	1917	n	bl	n	n	4	cu	cig+/-ot	nev any	or
RESTRE	2	c	0	0	all	-	all	SCAmer	1978	CC	102	n	bl	n	n	2	cu	cig+/-ot	nev cigs	ot
TAO	1	m	0	0	all	-	all	As:Chi	1988	CC	723	n	ot	n	n	5	ev	all/unsp	nev any	or
TAO	2	f	0	0	all	-	all	As:Chi	1988	CC	723	n	ot	n	n	5	ev	all/unsp	nev any	or
ULMER	1	c	0	0	all	-	all	Eu:Ger	1971	CC	726	n	bl	y	n	0	ev	all/unsp	nev any	or

REF	NRR	RR	SIG	RRDATA comment
HIRAYA	152	4.26		0
RESTRE	2	10.28	y	0
TAO	1	3.95		0
TAO	2	4.93		0
ULMER	1	12.72		0

Least adjusted - insufficient data for meta-analysis: as for adjusted plus the following

REF	NRR	SEX	AGEL	AGEH	RACE	YF	LC TYPE	LOC	START	ST	NLC	R	VB	P	H	AD	SM	PRODUCT	DENOM	De
CORREA	59	m	0	0	wh	-	all	NAmer	1979	CC	1359	n	bl	y	n	0	ev	cig+/-ot	nev cigs	or
CORREA	61	m	0	0	bl	-	all	NAmer	1979	CC	1359	n	bl	y	n	0	ev	cig+/-ot	nev cigs	or
CORREA	60	f	0	0	wh	-	all	NAmer	1979	CC	1359	n	bl	y	n	0	ev	cig+/-ot	nev cigs	or
CORREA	62	f	0	0	bl	-	all	NAmer	1979	CC	1359	n	bl	y	n	0	ev	cig+/-ot	nev cigs	or
LIU	1	c	0	0	all	-	all	As:Chi	1980	CC	229	n	ot	*	n	0	ev	all/unsp	nev any	or

Table 1C4 - 9

IESLC - Meta-anal of Current Smoking (or Ever if Current not available), Cigs (or Any Prod if Cigs not avail)
All LC types
Least adjusted - insufficient data for meta-analysis: as for adjusted plus the following

REF	NRR	RR	SIG	RRDATA comment
CORREA	59	17.20		0
CORREA	61	13.10		0
CORREA	60	8.60		0
CORREA	62	22.00		0
LIU	1	2.45		0

Table 1C5 -

IESLC - Meta-anal of Ever Smoking (or Current if Ever not available), Cigarettes only
All LC types

This analysis is restricted to results for:
1) Non-dose-response data
2) Results complete enough for use in metaanalysis

Within each study, results are then selected (in the following order of preference, within each sex) for:
3) SMKSTA: ever smokers, current smokers
4) PRODUCT: cigarettes only
5) CIGTYPE: all/unspecified, MC regardless of HR, MC only
6) DENOM: never smoked anything, never smoked cigarettes, (never +1 = +long term ex, +2 = +amount unknown, +3 = never cigs+long term ex)
7) Followup period (YF, prospective studies): whole study (coded as 0) or longest available
8) LCtype: all or nearest available, at least Squamous and Adeno. (q = squamous, s = small, l = large, a = adeno, mix = mixed, alv = alveolar)
9) Race: all or nearest available, otherwise by race (wh or w = white, bl or b = black, hi = hispanic ch = chinese, jap = japanese, haw = hawaiian, w+o = white + oriental, sca = scandinavian, as = asian)
10) For overlapping studies: principal rather than subsidiary studies
Finally by Age: whole study (coded as 0) if available, otherwise by widest available age group and then for single sex results (m, f) in preference to combined sex results (c).

Results adjusted (AD) for the most potential confounders are then chosen in Sections -1 to -3 (and those which actually differ from the adjusted results in Table 1C1 - 1 are marked 'x' in Section -1) and results adjusted for the least confounders in Sections -4 to -6. (Those least adjusted results which actually differ from the most adjusted as marked 'x' in column X in Section -4) (Results adjusted for an unknown number of confounder(s) are coded as 20.)

Section -7 shows excluded studies, together with the stage (as above) at which no qualifying results were found.

Section -8 lists the potentially overlapping studies which have been included (1=principal, 2=subsidiary).

Section -9 lists any results which would have been included in preference except that they had data not complete enough for use in meta-analysis, with their significance (yes/no), if known, and any further comment as entered on the database.

In addition to those mentioned above, the following fields, levels and abbreviations are used:
* or nk = not known, n = no, y = yes, ot = other
ev = ever, cu = current, nev = never
all/unspec = all or unspecified, MC = manufactured cigarettes, HR = hand-rolled cigarettes
REF: 6-character study reference
NRR: number of the RR on the database within the study
ST : study type (CC = case control, pr or prosp = prospective)
NLC: number of lung cancer cases in whole study
R : risky occupational population (n = no, m = mining, o = other risky)
VB : national cigarette type (V = at least 75% Virginia, bl = at least 75% blended, ot = other)
P : any proxy use
H : full histological confirmation
De : derivation of RR/CI (or = original, st = standard method, ot = other method of estimation)

Table 1C5 - 1

IESLC - Meta-anal of Ever Smoking (or Current if Ever not available), Cigarettes only
All LC types
Most adjusted

REF	NRR	1C1	SEX	AGEL	AGEH	RACE	YF	LC TYPE	LOC	START	ST	NLC	R	VB	P	H	AD	SM	PRODUCT	DENOM	De
ABELIN	47	x	m	0	0	all	-	all	Eu:wst	1941	CC	118	n	bl	y	n	1	ev	cig only	nev any	st
AGUDO	1		f	0	0	all	-	all	Eu:wst	1989	CC	103	n	bl	n	n	3	ev	cig only	nev any	or
ALDERS	67	x	m	0	0	all	-	all	Eu:UK	1977	CC	1448	n	V	n	n	1	ev	cig only	nev any	ot
ALDERS	6		f	0	0	all	-	all	Eu:UK	1977	CC	1448	n	V	n	n	1	ev	cig only	nev any	ot
ARMADA	1	x	m	0	0	all	-	all	Eu:wst	1986	CC	325	n	bl	n	y	0	ev	cig only	nev any	st
BAND	1		m	0	0	all	-	all	NAmer	1983	CC	2831	n	V	y	y	2	ev	cig only	nev any	ot
BEST	1	x	m	0	0	all	0	all	NAmer	1955	pr	381	n	V	n	n	1	ev	cig only	nev any	ot
BEST	18		f	0	0	all	0	all	NAmer	1955	pr	381	n	V	n	n	1	ev	cig only	nev any	ot
BOFFET	3	x	m	0	0	all	-	all	Eu:mul	1988	CC	5621	n	bl	y	n	2	ev	cig only	nev any	or
BOUCOT	113	x	m	0	0	all	0	all	NAmer	1951	pr	121	n	bl	n	n	2	ev	cig only	nev any	ot
BRESLO	18	x	m	0	0	all	-	all	NAmer	1949	CC	518	n	bl	n	y	0	ev	cig only	nev+1	st
BRESLO	24	x	f	0	0	all	-	all	NAmer	1949	CC	518	n	bl	n	y	0	ev	cig only	nev+1	st
CEDERL	116	x	m	0	0	all	0	all	Eu:Sca	1963	pr	491	n	bl	n	n	2	cu	cig only	nev any	or
CEDERL	41	x	f	0	0	all	10	all	Eu:Sca	1963	pr	491	n	bl	n	n	1	cu	cig only	nev any	ot
CHOW	15	x	m	0	0	wh	0	all	NAmer	1966	pr	219	n	bl	n	n	0	ev	cig only	nev any	st
CPSI	73	x	m	0	0	wh	0	all	NAmer	1959	pr	5138	n	bl	n	n	1	ev	cig only	nev any	st
CPSI	149	x	f	0	0	wh	0	all	NAmer	1959	pr	5138	n	bl	n	n	1	ev	cig only	nev any	st
CPSII	104		m	35	99	all	4	all	NAmer	1982	pr	3229	n	bl	n	n	1	ev	cig only	nev any	ot
DAMBER	35	x	m	0	0	all	-	all	Eu:Sca	1972	CC	579	n	bl	y	n	1	ev	cig only	nev any	ot
DEAN	4	x	m	0	0	wh	-	all	Africa	1947	CC	603	n	V	y	n	0	ev	cig only	nev any	st
DEAN2	9	x	m	0	0	all	-	all	Eu:UK	1960	CC	954	n	V	y	n	0	ev	cig only	nev any	st
DEAN2	17	x	f	0	0	all	-	all	Eu:UK	1960	CC	954	n	V	y	n	0	ev	cig only	nev any	st
DEAN3	241	x	m	0	0	all	-	all	Eu:UK	1969	CC	766	n	V	y	n	1	ev	cig only	nev any	ot
DEAN3	126		f	0	0	all	-	all	Eu:UK	1969	CC	766	n	V	y	n	3	ev	cig only	nev any	ot
DOLL	13	x	m	0	0	all	-	all	Eu:UK	1948	CC	1465	n	V	n	n	0	ev	cig only	nev any	st
DOLL2	62	x	m	0	0	all	0	all	Eu:UK	1951	pr	920	n	V	n	n	1	ev	cig only	nev any	ot
DOLL2	63		f	0	0	all	22	all	Eu:UK	1951	pr	920	n	V	n	n	1	cu	cig only	nev any	ot
DORN	1	x	m	0	0	wh	0	all	NAmer	1954	pr	5097	n	bl	n	n	2	ev	cig only	nev any	or
ENGELA	210	x	m	0	0	all	12	all	Eu:Sca	1964	pr	435	n	bl	n	n	1	cu	cig only	nev any	ot
ENGELA	225	x	f	0	0	all	12	all	Eu:Sca	1964	pr	435	n	bl	n	n	1	cu	cig only	nev any	ot
ENSTRO	1		m	0	0	all	0	all	NAmer	1959	pr	2879	n	bl	n	n	1	cu	cig only	nev any	or
ENSTRO	2		f	0	0	all	0	all	NAmer	1959	pr	2879	n	bl	n	n	1	cu	cig only	nev any	or
GARDIN	6	x	c	0	0	all	-	all	Eu:UK	1988	CC	143	n	V	y	n	0	cu	cig only	nev any	st
GOLLED	6	x	m	35	99	all	-	all	Eu:UK	1952	CC	443	n	V	y	n	1	ev	cig only	nev any	ot
GRAHAM	3	x	m	0	0	wh	-	all	NAmer	1956	CC	685	n	bl	n	n	0	ev	cig only	nev any	st
HAMMON	115	x	m	0	0	wh	0	all	NAmer	1952	pr	448	n	bl	n	n	1	ev	cig only	nev any	ot
HEIN	1	x	m	0	0	all	0	all	Eu:Sca	1970	pr	144	n	bl	n	n	0	cu	cig only	nev any	st
JOLY	48	x	m	0	0	all	-	all	SCAmer	1978	CC	826	n	bl	n	n	0	ev	cig only	nev any	st
JUSSAW	31	x	m	0	0	all	-	all	As:Ind	1964	CC	792	n	V	n	n	2	ev	cig only	nev any	st
KAISE2	72		m	35	99	all	9	all	NAmer	1979	pr	318	n	bl	n	n	1	ev	cig only	nev any	st
KAISE2	64		f	35	99	all	9	all	NAmer	1979	pr	318	n	bl	n	n	1	ev	cig only	nev any	st
KJUUS	3	x	m	0	0	all	-	all	Eu:Sca	1979	CC	176	n	bl	n	n	0	ev	cig only	nev any	st
KOULUM	2	x	m	0	0	all	-	all	Eu:Sca	1936	CC	812	n	bl	n	n	0	ev	cig only	nev any	st
LIU4	10	x	m	35	69	all	-	all	As:Chi	1986	CC	1000-00	n	ot	y	n	2	ev	cig only	nev any	ot
LOMBAR	10	x	m	0	0	all	-	all	NAmer	1951	CC	1040	n	bl	n	n	0	ev	cig only	nev any	st
LUBIN2	18	x	m	0	0	all	-	all	Eu:mul	1976	CC	7804	n	bl	n	y	2	ev	cig only	nev any	ot
LUBIN2	98	x	f	0	0	all	-	all	Eu:mul	1976	CC	7804	n	bl	n	y	1	ev	cig only	nev any	ot
MCCONN	16	x	c	0	0	all	-	all	Eu:UK	1946	CC	100	n	V	n	y	0	ev	cig only	nev any	st
MIGRAN	10	x	m	0	0	all	0	all	Eu:UK	1964	pr	259	n	V	n	n	2	cu	cig only	nev any	ot
MIGRAN	36	x	f	0	0	all	0	all	Eu:UK	1964	pr	259	n	V	n	n	2	cu	cig only	nev any	ot
MILLS	1	x	m	0	0	wh	-	all	NAmer	1940	CC	444	n	bl	y	n	1	ev	cig only	nev any	ot
NOTAN2	19	x	m	0	0	all	-	all	As:Ind	1963	CC	683	n	V	n	n	2	ev	cig only	nev any	ot
PERNU	8	x	m	0	0	all	-	all	Eu:Sca	1944	CC	1606	n	bl	n	n	0	ev	cig only	nev any	st
PERNU	4	x	f	0	0	all	-	all	Eu:Sca	1944	CC	1606	n	bl	n	n	0	ev	cig only	nev any	st
PEZZOT	25		m	0	0	all	-	all	SCAmer	1987	CC	215	n	bl	n	y	0	ev	cig only	nev cigs	st
RONCO	3	x	m	0	0	all	-	all	Eu:wst	1976	CC	126	n	bl	y	n	2	ev	cig only	nev any	ot
SADOWS	28	x	m	0	0	wh	-	all	NAmer	1938	CC	477	n	bl	n	n	1	ev	cig only	nev any	ot
STASZE	2	x	m	0	0	all	-	all	Eu:est	1954	CC	281	n	bl	n	y	0	ev	cig only	nev any	st
SUZUK2	7	x	c	0	0	all	-	all	SCAmer	1991	CC	123	n	bl	n	y	3	ev	cig only	nev any	or
TIZZAN	2	x	m	0	0	all	-	all	Eu:wst	1959	CC	1358	n	bl	n	n	0	ev	cig only	nev any	st
TIZZAN	22	x	f	0	0	all	-	all	Eu:wst	1959	CC	1358	n	bl	n	n	0	ev	cig only	nev any	st
TVERDA	3	x	m	0	0	all	0	all	Eu:Sca	1972	pr	238	n	bl	n	n	2	cu	cig only	nev cigs	ot
TVERDA	15		f	0	0	all	0	all	Eu:Sca	1972	pr	238	n	bl	n	n	2	cu	cig only	nev cigs	ot
WALD	4		m	0	0	all	0	all	Eu:UK	1975	pr	102	n	V	n	n	1	cu	cig only	nev any	or
WIGLE	13	x	m	0	0	all	-	all	NAmer	1971	CC	728	n	V	n	n	0	ev	cig only	nev any	st
WIGLE	16	x	f	0	0	all	-	all	NAmer	1971	CC	728	n	V	n	n	0	ev	cig only	nev any	st
WYNDE7	39	x	m	0	0	all	-	all	NAmer	1977	CC	2085	n	bl	n	y	0	ev	cig only	nev any	st
XIANGZ	9	x	m	0	0	all	0	all	As:Chi	1976	pr	983	m	ot	n	n	2	ev	cig only	nev any	ot

Table 1C5 - 1

IESLC - Meta-anal of Ever Smoking (or Current if Ever not available), Cigarettes only
All LC types
Most adjusted

Cigarette type is all/unspec for all RRs
except for the following:

REF	NRR	CIGTYPE
ALDERS	6	MC only
DEAN3	241	MC only
DEAN3	126	MC only
GARDIN	6	MC only
JUSSAW	31	MC only
NOTAN2	19	MC only
PERNU	8	MC only
PERNU	4	MC only
SUZUK2	7	MC only

Table 1C5 - 2

IESLC - Meta-anal of Ever Smoking (or Current if Ever not available), Cigarettes only
All LC types
Most adjusted

REF	NRR	SEX	AD	Number Case	Exposed Cont	Non-exposed Case	Cont	RR	95.00%CI
ABELIN	47	m	1	-	-	-	-	53.81	(11.36- 254.76)
AGUDO	1	f	3	-	-	-	-	3.10	(1.42- 6.75)
ALDERS	67	m	1	-	-	-	-	10.38	(6.00- 17.93)
ALDERS	6	f	1	-	-	-	-	4.75	(3.55- 6.35)
Subtotal ALDERS								5.64	(4.36- 7.29)
ARMADA	1	m	0	245	197	4	64	19.90	(7.12- 55.59)
BAND	1	m	2	-	-	-	-	9.96	(7.38- 13.44)
*BEST	1	m	1	-	-	-	-	14.20	(6.70- 30.10)
*BEST	18	f	1	-	-	-	-	2.24	(0.59- 8.44)
Subtotal BEST								9.09	(4.72- 17.48)
BOFFET	3	m	2	-	-	-	-	14.90	(12.30- 18.10)
*BOUCOT	113	m	2	-	-	-	-	53.14	(3.30- 856.06)
BRESLO	18	m	0	316	229	7	42	8.28	(3.65- 18.76)
BRESLO	24	f	0	13	11	12	14	1.38	(0.45- 4.20)
Subtotal BRESLO								4.42	(2.28- 8.54)
*CEDERL	116	m	2	-	-	-	-	8.43	(5.49- 12.94)
*CEDERL	41	f	1	-	-	-	-	4.50	(1.78- 11.36)
Subtotal CEDERL								7.55	(5.11- 11.14)
*CHOW	15	m	0	57	57162	6	62913	10.46	(4.51- 24.25)
*CPSI	73	m	1	-	-	-	-	10.07	(8.77- 11.56)
*CPSI	149	f	1	-	-	-	-	3.20	(2.81- 3.65)
Subtotal CPSI								5.50	(5.00- 6.05)
*CPSII	104	m	1	-	-	-	-	12.83	(10.28- 16.01)
DAMBER	35	m	1	-	-	-	-	7.07	(4.67- 10.68)
DEAN	4	m	0	403	385	12	61	5.32	(2.82- 10.04)
DEAN2	9	m	0	629	508	33	112	4.20	(2.80- 6.30)
DEAN2	17	f	0	62	29	88	121	2.94	(1.75- 4.94)
Subtotal DEAN2								3.67	(2.67- 5.05)
DEAN3	241	m	1	-	-	-	-	6.11	(3.99- 9.34)
DEAN3	126	f	3	-	-	-	-	4.63	(3.03- 7.09)
Subtotal DEAN3								5.32	(3.94- 7.18)
DOLL	13	m	0	1004	899	7	61	9.73	(4.43- 21.39)
*DOLL2	62	m	1	-	-	-	-	8.78	(5.55- 13.88)
*DOLL2	63	f	1	-	-	-	-	8.65	(2.93- 25.55)
Subtotal DOLL2								8.76	(5.74- 13.36)
*DORN	1	m	2	-	-	-	-	8.40	(7.50- 9.40)
*ENGELA	210	m	1	-	-	-	-	9.50	(4.37- 20.66)
*ENGELA	225	f	1	-	-	-	-	5.80	(2.69- 12.51)
Subtotal ENGELA								7.40	(4.29- 12.78)
*ENSTRO	1	m	1	-	-	-	-	12.99	(10.46- 16.13)
*ENSTRO	2	f	1	-	-	-	-	6.95	(6.01- 8.04)
Subtotal ENSTRO								8.44	(7.48- 9.53)
GARDIN	6	c	0	72	39	5	41	15.14	(5.53- 41.44)
GOLLED	6	m	1	-	-	-	-	7.65	(4.52- 12.94)
GRAHAM	3	m	0	474	951	18	346	9.58	(5.89- 15.58)
*HAMMON	115	m	1	-	-	-	-	9.94	(5.90- 16.73)
*HEIN	1	m	0	45	912	1	457	22.55	(3.12- 163.06)
JOLY	48	m	0	379	499	12	218	13.80	(7.60- 25.05)
JUSSAW	31	m	2	-	-	-	-	8.64	(4.61- 17.88)
*KAISE2	72	m	1	-	-	-	-	5.40	(3.05- 9.57)
*KAISE2	64	f	1	-	-	-	-	10.09	(5.29- 19.27)
Subtotal KAISE2								7.11	(4.63- 10.90)
KJUUS	3	m	0	151	127	2	24	14.27	(3.31- 61.54)
KOULUM	2	m	0	625	229	5	54	29.48	(11.65- 74.60)
LIU4	10	m	2	-	-	-	-	3.88	(3.78- 3.98)
LOMBAR	10	m	0	486	302	14	112	12.87	(7.25- 22.85)
LUBIN2	18	m	2	-	-	-	-	8.87	(7.60- 10.35)
LUBIN2	98	f	1	-	-	-	-	3.90	(3.29- 4.62)
Subtotal LUBIN2								6.11	(5.45- 6.85)
MCCONN	16	c	0	68	138	9	23	1.26	(0.55- 2.87)
*MIGRAN	10	m	2	-	-	-	-	4.20	(1.55- 11.37)
*MIGRAN	36	f	2	-	-	-	-	5.11	(1.77- 14.79)
Subtotal MIGRAN								4.60	(2.23- 9.52)
MILLS	1	m	1	-	-	-	-	1.27	(1.01- 1.61)
NOTAN2	19	m	2	-	-	-	-	2.36	(1.68- 3.31)
PERNU	8	m	0	706	216	97	275	9.27	(7.02- 12.23)
PERNU	4	f	0	7	24	110	971	2.57	(1.08- 6.11)
Subtotal PERNU								8.22	(6.32- 10.71)
PEZZOT	25	m	0	211	317	4	116	19.30	(7.02- 53.10)
RONCO	3	m	2	-	-	-	-	5.43	(2.27- 12.96)
SADOWS	28	m	1	-	-	-	-	4.46	(2.28- 8.72)

Table 1C5 - 2

IESLC - Meta-anal of Ever Smoking (or Current if Ever not available), Cigarettes only
All LC types
Most adjusted

REF	NRR	SEX	AD	Number Exposed Case	Cont	Non-exposed Case	Cont	RR	95.00%CI
STASZE	2	m	0	218	552	5	158	12.48	(5.05- 30.82)
SUZUK2	7	c	3	-	-	-	-	11.00	(3.40- 36.00)
TIZZAN	2	m	0	994	836	180	305	2.01	(1.64- 2.48)
TIZZAN	22	f	0	25	28	25	114	4.07	(2.04- 8.13)
Subtotal TIZZAN								2.13	(1.75- 2.60)
*TVERDA	3	m	2	-	-	-	-	3.83	(2.47- 5.95)
*TVERDA	15	f	2	-	-	-	-	11.05	(3.33- 36.71)
Subtotal TVERDA								4.34	(2.87- 6.56)
*WALD	4	m	1	-	-	-	-	16.40	(7.55- 44.20)
WIGLE	13	m	0	543	632	15	204	11.68	(6.83- 19.99)
WIGLE	16	f	0	78	235	36	439	4.05	(2.64- 6.19)
Subtotal WIGLE								6.09	(4.37- 8.51)
WYNDE7	39	m	0	1645	2108	64	918	11.19	(8.62- 14.54)
*XIANGZ	9	m	2	-	-	-	-	1.70	(1.14- 2.54)
Partial Totals				9456	67565	771	68163		

*prospective study

REF	NRR	SEX	AD	Ys	Ws	Qs	Ps
ABELIN	47	m	1	3.99	1.59	9.80	0.0000
AGUDO	1	f	3	1.13	6.32	0.87	0.0044
ALDERS	67	m	1	2.34	12.82	9.01	0.0000
ALDERS	6	f	1	1.56	45.44	0.15	0.0000
Subtotal ALDERS				1.73	58.26	9.16	
ARMADA	1	m	0	2.99	3.64	8.07	0.0000
BAND	1	m	2	2.30	42.76	27.17	0.0000
*BEST	1	m	1	2.65	6.81	9.03	0.0000
*BEST	18	f	1	0.81	2.17	1.05	0.2348
Subtotal BEST				2.21	8.98	10.08	
BOFFET	3	m	2	2.70	102.96	148.23	0.0000
*BOUCOT	113	m	2	3.97	0.50	3.04	0.0051
BRESLO	18	m	0	2.11	5.74	2.15	0.0000
BRESLO	24	f	0	0.32	3.10	4.32	0.5717
Subtotal BRESLO				1.49	8.84	6.47	
*CEDERL	116	m	2	2.13	20.90	8.30	0.0000
*CEDERL	41	f	1	1.50	4.47	0.00	0.0015
Subtotal CEDERL				2.02	25.38	8.30	
*CHOW	15	m	0	2.35	5.43	3.88	0.0000
*CPSI	73	m	1	2.31	201.40	131.50	0.0000
*CPSI	149	f	1	1.16	224.63	25.72	0.0000
Subtotal CPSI				1.71	426.03	157.22	
*CPSII	104	m	1	2.55	78.29	86.36	0.0000
DAMBER	35	m	1	1.96	22.46	4.64	0.0000
DEAN	4	m	0	1.67	9.54	0.28	0.0000
DEAN2	9	m	0	1.44	23.37	0.10	0.0000
DEAN2	17	f	0	1.08	14.24	2.55	0.0000
Subtotal DEAN2				1.30	37.61	2.65	
DEAN3	241	m	1	1.81	21.24	2.02	0.0000
DEAN3	126	f	3	1.53	21.26	0.02	0.0000
Subtotal DEAN3				1.67	42.50	2.04	
DOLL	13	m	0	2.28	6.20	3.71	0.0000
*DOLL2	62	m	1	2.17	18.29	8.23	0.0000
*DOLL2	63	f	1	2.16	3.28	1.41	0.0001
Subtotal DOLL2				2.17	21.56	9.64	
*DORN	1	m	2	2.13	301.36	118.36	0.0000
*ENGELA	210	m	1	2.25	6.37	3.58	0.0000
*ENGELA	225	f	1	1.76	6.50	0.43	0.0000
Subtotal ENGELA				2.00	12.87	4.01	
*ENSTRO	1	m	1	2.56	81.91	92.50	0.0000
*ENSTRO	2	f	1	1.94	181.45	34.69	0.0000
Subtotal ENSTRO				2.13	263.36	127.18	
GARDIN	6	c	0	2.72	3.79	5.60	0.0000
GOLLED	6	m	1	2.03	13.89	3.95	0.0000
GRAHAM	3	m	0	2.26	16.23	9.33	0.0000
*HAMMON	115	m	1	2.30	14.15	8.94	0.0000
*HEIN	1	m	0	3.12	0.98	2.56	0.0020
JOLY	48	m	0	2.62	10.80	13.62	0.0000
JUSSAW	31	m	2	2.16	8.36	3.59	0.0000
*KAISE2	72	m	1	1.69	11.75	0.40	0.0000
*KAISE2	64	f	1	2.31	9.19	6.03	0.0000
Subtotal KAISE2				1.96	20.95	6.43	

Table 1C5 - 2

IESLC - Meta-anal of Ever Smoking (or Current if Ever not available), Cigarettes only
All LC types
Most adjusted

REF	NRR	SEX	AD	Ys	Ws	Qs	Ps
KJUUS	3	m	0	2.66	1.80	2.40	0.0004
KOULUM	2	m	0	3.38	4.45	15.78	0.0000
LIU4	10	m	2	1.36	5780.52	122.68	0.0000
LOMBAR	10	m	0	2.56	11.67	12.95	0.0000
LUBIN2	18	m	2	2.18	161.10	74.75	0.0000
LUBIN2	98	f	1	1.36	133.31	2.63	0.0000
Subtotal LUBIN2				1.81	294.41	77.38	
MCCONN	16	c	0	0.23	5.66	9.15	0.5832
*MIGRAN	10	m	2	1.44	3.87	0.02	0.0048
*MIGRAN	36	f	2	1.63	3.41	0.06	0.0026
Subtotal MIGRAN				1.53	7.28	0.07	
MILLS	1	m	1	0.24	71.48	113.36	0.0406
NOTAN2	19	m	2	0.86	33.41	13.81	0.0000
PERNU	8	m	0	2.23	50.02	26.28	0.0000
PERNU	4	f	0	0.95	5.14	1.59	0.0321
Subtotal PERNU				2.11	55.16	27.87	
PEZZOT	25	m	0	2.96	3.75	7.98	0.0000
RONCO	3	m	2	1.69	5.06	0.18	0.0001
SADOWS	28	m	1	1.50	8.54	0.00	0.0000
STASZE	2	m	0	2.52	4.70	4.92	0.0000
SUZUK2	7	c	3	2.40	2.76	2.22	0.0001
TIZZAN	2	m	0	0.70	90.61	58.14	0.0000
TIZZAN	22	f	0	1.40	8.03	0.08	0.0001
Subtotal TIZZAN				0.76	98.64	58.22	
*TVERDA	3	m	2	1.34	19.88	0.50	0.0000
*TVERDA	15	f	2	2.40	2.67	2.17	0.0001
Subtotal TVERDA				1.47	22.55	2.67	
*WALD	4	m	1	2.80	4.92	8.26	0.0000
WIGLE	13	m	0	2.46	13.33	12.21	0.0000
WIGLE	16	f	0	1.40	21.22	0.23	0.0000
Subtotal WIGLE				1.81	34.55	12.43	
WYNDE7	39	m	0	2.42	56.19	46.92	0.0000
*XIANGZ	9	m	2	0.53	23.94	22.57	0.0094

	N	68
	NS	51
	Wt	8107.03
Het	Chi	1367.01
Het	df	67
Het	P	***
Fixed	RR	4.49
	RRl	4.39
	RRu	4.59
	P	+++
Random	RR	6.70
	RRl	5.72
	RRu	7.84
	P	+++
Asymm	P	***

Table 1C5 - 3

IESLC - Meta-anal of Ever Smoking (or Current if Ever not available), Cigarettes only
All LC types
Most adjusted

	Sex			
	combined	male	female	Total
N	3	47	18	68
NS	3	47	18	68
Wt	12.21	7398.98	695.84	8107.03
Het Chi	16.97	1266.03	83.54	1367.01
Het df	2	46	17	67
Het P	***	***	***	***
Fixed RR	4.44	4.50	4.38	4.49
RRl	2.54	4.40	4.06	4.39
RRu	7.79	4.60	4.72	4.59
P	+++	+++	+++	+++
Random RR	5.76	8.00	4.39	6.70
RRl	1.08	6.49	3.52	5.72
RRu	30.72	9.86	5.47	7.84
P	+	+++	+++	+++
Between Chi				0.48
Between df				2
Between P				N.S.
Btwn(F) P				N.S.
Btwn(R) P				***

	Lung cancer type		
	all	other	Total
N	68		68
NS	51		51
Wt	8107.03		8107.03
Het Chi	1367.01		1367.01
Het df	67		67
Het P	***		***
Fixed RR	4.49		4.49
RRl	4.39		4.39
RRu	4.59		4.59
P	+++		+++
Random RR	6.70		6.70
RRl	5.72		5.72
RRu	7.84		7.84
P	+++		+++
Between Chi			
Between df			
Between P			N.S.
Btwn(F) P			N.S.
Btwn(R) P			N.S.

	Location								
	NAmer	UK	Scand	othEur	China	Japan	othAs	other	Total
N	23	15	12	10	2		2	4	68
NS	17	10	8	8	2		2	4	51
Wt	1369.30	201.68	145.64	517.33	5804.46		41.78	26.86	8107.03
Het Chi	497.98	44.83	30.08	261.84	16.24		11.27	6.56	1367.01
Het df	22	14	11	9	1		1	3	67
Het P	***	***	**	***	***		***	(*)	***
Fixed RR	6.89	5.57	7.49	6.04	3.87		3.06	10.07	4.49
RRl	6.54	4.85	6.37	5.55	3.77		2.26	6.90	4.39
RRu	7.27	6.39	8.81	6.59	3.97		4.14	14.70	4.59
P	+++	+++	+++	+++	+++		+++	+++	+++
Random RR	7.24	5.92	7.49	7.06	2.63		4.36	10.58	6.70
RRl	5.44	4.53	5.46	4.10	1.17		1.23	5.83	5.72
RRu	9.64	7.76	10.28	12.15	5.90		15.53	19.22	7.84
P	+++	+++	+++	+++	+		+	+++	+++
Between Chi									498.21
Between df									6
Between P									***
Btwn(F) P									***
Btwn(R) P									N.S.

Table 1C5 - 3

IESLC - Meta-anal of Ever Smoking (or Current if Ever not available), Cigarettes only
All LC types
Most adjusted

	Detailed Country in "other Europe"					
	multi	Germany	othWest	East	Balkans	Total
N	3		6	1		10
NS	2		5	1		8
Wt	397.37		115.26	4.70		517.33
Het Chi	109.78		40.06	0.00		261.84
Het df	2		5	0		9
Het P	***		***	N.S.		***
Fixed RR	7.70		2.55	12.48		6.04
RRl	6.98		2.12	5.05		5.55
RRu	8.50		3.06	30.82		6.59
P	+++		+++	+++		+++
Random RR	8.01		6.11	12.48		7.06
RRl	3.84		2.72	5.05		4.10
RRu	16.70		13.73	30.82		12.15
P	+++		+++	+++		+++
Between Chi						112.00
Between df						2
Between P						***
Btwn(F) P						N.S.
Btwn(R) P						N.S.

	Detailed Country in "other Asia"			
	India	HongKong	other	Total
N	2			2
NS	2			2
Wt	41.78			41.78
Het Chi	11.27			11.27
Het df	1			1
Het P	***			***
Fixed RR	3.06			3.06
RRl	2.26			2.26
RRu	4.14			4.14
P	+++			+++
Random RR	4.36			4.36
RRl	1.23			1.23
RRu	15.53			15.53
P	+			+
Between Chi				
Between df				
Between P				N.S.
Btwn(F) P				N.S.
Btwn(R) P				N.S.

	Detailed other continent			
	SCAmer	Auslia	Africa	Total
N	3		1	4
NS	3		1	4
Wt	17.32		9.54	26.86
Het Chi	0.54		0.00	6.56
Het df	2		0	3
Het P	N.S.		N.S.	(*)
Fixed RR	14.31		5.32	10.07
RRl	8.94		2.82	6.90
RRu	22.92		10.04	14.70
P	+++		+++	+++
Random RR	14.31		5.32	10.58
RRl	8.94		2.82	5.83
RRu	22.92		10.04	19.22
P	+++		+++	+++
Between Chi				6.02
Between df				1
Between P				*
Btwn(F) P				*
Btwn(R) P				*

Table 1C5 - 3

IESLC - Meta-anal of Ever Smoking (or Current if Ever not available), Cigarettes only
All LC types
Most adjusted

	Start year of study <1960	1960-69	1970-79	1980-89	1990+	Total
N	28	13	18	8	1	68
NS	21	8	13	8	1	51
Wt	1352.53	172.84	556.87	6022.04	2.76	8107.03
Het Chi	595.83	37.98	147.64	349.33	0.00	1367.01
Het df	27	12	17	7	0	67
Het P	***	***	***	***	N.S.	***
Fixed RR	6.00	4.70	6.26	4.07	11.00	4.49
RRl	5.69	4.05	5.76	3.97	3.38	4.39
RRu	6.33	5.46	6.80	4.17	35.79	4.59
P	+++	+++	+++	+++	+++	+++
Random RR	6.48	5.21	6.93	10.02	11.00	6.70
RRl	4.85	3.92	5.20	5.31	3.38	5.72
RRu	8.66	6.93	9.22	18.89	35.79	7.84
P	+++	+++	+++	+++	+++	+++
Between Chi						236.23
Between df						4
Between P						***
Btwn(F) P						*
Btwn(R) P						N.S.

	Study type (1) CC	other	Total
N	42	26	68
NS	34	17	51
Wt	6868.51	1238.52	8107.03
Het Chi	739.21	312.24	1367.01
Het df	41	25	67
Het P	***	***	***
Fixed RR	4.13	7.14	4.49
RRl	4.03	6.76	4.39
RRu	4.23	7.55	4.59
P	+++	+++	+++
Random RR	6.36	7.34	6.70
RRl	5.18	5.76	5.72
RRu	7.79	9.36	7.84
P	+++	+++	+++
Between Chi			315.55
Between df			1
Between P			***
Btwn(F) P			***
Btwn(R) P			N.S.

	Study type (2) CC	prosp	other	Total
N	42	26		68
NS	34	17		51
Wt	6868.51	1238.52		8107.03
Het Chi	739.21	312.24		1367.01
Het df	41	25		67
Het P	***	***		***
Fixed RR	4.13	7.14		4.49
RRl	4.03	6.76		4.39
RRu	4.23	7.55		4.59
P	+++	+++		+++
Random RR	6.36	7.34		6.70
RRl	5.18	5.76		5.72
RRu	7.79	9.36		7.84
P	+++	+++		+++
Between Chi				315.55
Between df				1
Between P				***
Btwn(F) P				***
Btwn(R) P				N.S.

Table 1C5 - 3

IESLC - Meta-anal of Ever Smoking (or Current if Ever not available), Cigarettes only
All LC types
Most adjusted

	Study size (number of LC cases)				
	100-249	250-499	500-999	1000+	Total
N	14	16	18	20	68
NS	13	11	13	14	51
Wt	65.11	191.84	274.27	7575.81	8107.03
Het Chi	50.46	164.76	113.59	1023.32	1367.01
Het df	13	15	17	19	67
Het P	***	***	***	***	***
Fixed RR	6.39	3.93	5.01	4.47	4.49
RRl	5.01	3.41	4.45	4.37	4.39
RRu	8.15	4.53	5.63	4.57	4.59
P	+++	+++	+++	+++	+++
Random RR	9.13	6.42	5.77	7.08	6.70
RRl	5.40	3.84	4.20	5.46	5.72
RRu	15.46	10.75	7.93	9.20	7.84
P	+++	+++	+++	+++	+++
Between Chi					14.87
Between df					3
Between P					**
Btwn(F) P					N.S.
Btwn(R) P					N.S.

	Risky occupational population			
	no	mining	othRisky	Total
N	67	1		68
NS	50	1		51
Wt	8083.09	23.94		8107.03
Het Chi	1344.37	0.00		1367.01
Het df	66	0		67
Het P	***	N.S.		***
Fixed RR	4.50	1.70		4.49
RRl	4.40	1.14		4.39
RRu	4.60	2.54		4.59
P	+++	++		+++
Random RR	6.86	1.70		6.70
RRl	5.85	1.14		5.72
RRu	8.05	2.54		7.84
P	+++	++		+++
Between Chi				22.63
Between df				1
Between P				***
Btwn(F) P				N.S.
Btwn(R) P				***

	National cigarette tobacco type			
	Virginia	blended	other	Total
N	23	43	2	68
NS	16	33	2	51
Wt	339.28	1963.29	5804.46	8107.03
Het Chi	102.65	786.13	16.24	1367.01
Het df	22	42	1	67
Het P	***	***	***	***
Fixed RR	5.68	6.70	3.87	4.49
RRl	5.11	6.41	3.77	4.39
RRu	6.32	7.00	3.97	4.59
P	+++	+++	+++	+++
Random RR	6.04	7.54	2.63	6.70
RRl	4.72	6.06	1.17	5.72
RRu	7.74	9.39	5.90	7.84
P	+++	+++	+	+++
Between Chi				461.99
Between df				2
Between P				***
Btwn(F) P				***
Btwn(R) P				*

Table 1C5 - 3

IESLC - Meta-anal of Ever Smoking (or Current if Ever not available), Cigarettes only
All LC types
Most adjusted

	Any proxy use No/nk	Yes	Total
N	54	14	68
NS	39	12	51
Wt	1972.88	6134.15	8107.03
Het Chi	644.27	349.82	1367.01
Het df	53	13	67
Het P	***	***	***
Fixed RR	6.55	3.97	4.49
RRl	6.27	3.88	4.39
RRu	6.85	4.08	4.59
P	+++	+++	+++
Random RR	6.95	6.01	6.70
RRl	5.81	4.04	5.72
RRu	8.31	8.95	7.84
P	+++	+++	+++
Between Chi			372.92
Between df			1
Between P			***
Btwn(F) P			***
Btwn(R) P			N.S.

	Full histological confirmation No	Yes	Total
N	57	11	68
NS	42	9	51
Wt	7684.32	422.71	8107.03
Het Chi	1172.17	107.32	1367.01
Het df	56	10	67
Het P	***	***	***
Fixed RR	4.38	6.99	4.49
RRl	4.28	6.35	4.39
RRu	4.48	7.69	4.59
P	+++	+++	+++
Random RR	6.60	7.28	6.70
RRl	5.55	4.90	5.72
RRu	7.86	10.81	7.84
P	+++	+++	+++
Between Chi			87.52
Between df			1
Between P			***
Btwn(F) P			*
Btwn(R) P			N.S.

	Number of adjustment variables (1) 0	1	2+/+nk	Total
N	25	25	18	68
NS	20	18	16	54
Wt	379.64	1186.35	6541.05	8107.03
Het Chi	234.81	476.06	522.87	1367.01
Het df	24	24	17	67
Het P	***	***	***	***
Fixed RR	5.65	5.89	4.22	4.49
RRl	5.11	5.56	4.12	4.39
RRu	6.24	6.24	4.32	4.59
P	+++	+++	+++	+++
Random RR	7.27	7.00	5.88	6.70
RRl	5.14	5.27	4.28	5.72
RRu	10.28	9.30	8.07	7.84
P	+++	+++	+++	+++
Between Chi				133.26
Between df				2
Between P				***
Btwn(F) P				*
Btwn(R) P				N.S.

Table 1C5 - 3

IESLC - Meta-anal of Ever Smoking (or Current if Ever not available), Cigarettes only
All LC types
Most adjusted

	Number of adjustment variables (2) 0	1	2	3-5	6+/+nk	Total
N	25	25	15	3		68
NS	20	18	13	3		54
Wt	379.64	1186.35	6510.70	30.34		8107.03
Het Chi	234.81	476.06	519.55	3.08		1367.01
Het df	24	24	14	2		67
Het P	***	***	***	N.S.		***
Fixed RR	5.65	5.89	4.21	4.61		4.49
RRl	5.11	5.56	4.11	3.23		4.39
RRu	6.24	6.24	4.32	6.58		4.59
P	+++	+++	+++	+++		+++
Random RR	7.27	7.00	6.06	4.72		6.70
RRl	5.14	5.27	4.27	2.82		5.72
RRu	10.28	9.30	8.59	7.89		7.84
P	+++	+++	+++	+++		+++
Between Chi						133.50
Between df						3
Between P						***
Btwn(F) P						(*)
Btwn(R) P						N.S.

	Product all/unsp	cig+/-ot	cig only	Total
N			68	68
NS			51	51
Wt			8107.03	8107.03
Het Chi			1367.01	1367.01
Het df			67	67
Het P			***	***
Fixed RR			4.49	4.49
RRl			4.39	4.39
RRu			4.59	4.59
P			+++	+++
Random RR			6.70	6.70
RRl			5.72	5.72
RRu			7.84	7.84
P			+++	+++
Between Chi				
Between df				
Between P				N.S.
Btwn(F) P				N.S.
Btwn(R) P				N.S.

	Denominator nev any	nev cigs	Total
N	65	3	68
NS	49	2	51
Wt	8080.73	26.30	8107.03
Het Chi	1356.35	9.80	1367.01
Het df	64	2	67
Het P	***	**	***
Fixed RR	4.49	5.37	4.49
RRl	4.39	3.67	4.39
RRu	4.58	7.87	4.59
P	+++	+++	+++
Random RR	6.65	8.62	6.70
RRl	5.66	2.85	5.72
RRu	7.82	26.06	7.84
P	+++	+++	+++
Between Chi			0.85
Between df			1
Between P			N.S.
Btwn(F) P			N.S.
Btwn(R) P			N.S.

Table 1C5 - 3

IESLC - Meta-anal of Ever Smoking (or Current if Ever not available), Cigarettes only
All LC types
Most adjusted

	Derivation of RR/CI			
	Orig	StdCalc	Other	Total
N	8	31	29	68
NS	7	24	21	52
Wt	702.58	836.57	6567.88	8107.03
Het Chi	59.78	387.36	448.95	1367.01
Het df	7	30	28	67
Het P	***	***	***	***
Fixed RR	9.12	5.65	4.04	4.49
RRl	8.47	5.28	3.94	4.39
RRu	9.83	6.05	4.14	4.59
P	+++	+++	+++	+++
Random RR	9.44	7.40	5.59	6.70
RRl	7.18	5.57	4.45	5.72
RRu	12.42	9.82	7.01	7.84
P	+++	+++	+++	+++
Between Chi				470.91
Between df				2
Between P				***
Btwn(F) P				***
Btwn(R) P				*

	Smoking status		
	ever	current	Total
N	54	14	68
NS	43	9	52
Wt	7762.63	344.40	8107.03
Het Chi	1201.78	43.74	1367.01
Het df	53	13	67
Het P	***	***	***
Fixed RR	4.37	8.03	4.49
RRl	4.28	7.22	4.39
RRu	4.47	8.92	4.59
P	+++	+++	+++
Random RR	6.45	7.88	6.70
RRl	5.41	5.96	5.72
RRu	7.70	10.40	7.84
P	+++	+++	+++
Between Chi			121.48
Between df			1
Between P			***
Btwn(F) P			*
Btwn(R) P			N.S.

	Study LIU4		
	LIU4	others	Total
N	1	67	68
NS	1	50	51
Wt	5780.52	2326.51	8107.03
Het Chi	0.00	939.51	1367.01
Het df	0	66	67
Het P	N.S.	***	***
Fixed RR	3.88	6.45	4.49
RRl	3.78	6.19	4.39
RRu	3.98	6.71	4.59
P	+++	+++	+++
Random RR	3.88	6.81	6.70
RRl	3.78	5.73	5.72
RRu	3.98	8.09	7.84
P	+++	+++	+++
Between Chi			427.50
Between df			1
Between P			***
Btwn(F) P			***
Btwn(R) P			***

Table 1C5 - 4

IESLC - Meta-anal of Ever Smoking (or Current if Ever not available), Cigarettes only
All LC types
Least adjusted

REF	NRR	X	SEX	AGEL	AGEH	RACE	YF	LC TYPE	LOC	START	ST	NLC	R	VB	P	H	AD	SM	PRODUCT	DENOM	De
ABELIN	4	x	m	0	0	all	-	all	Eu:wst	1941	CC	118	n	bl	y	n	0	ev	cig only	nev any	st
AGUDO	8	x	f	0	0	all	-	all	Eu:wst	1989	CC	103	n	bl	n	n	0	ev	cig only	nev any	st
ALDERS	60	x	m	0	0	all	-	all	Eu:UK	1977	CC	1448	n	V	n	n	0	ev	cig only	nev any	st
ALDERS	12	x	f	0	0	all	-	all	Eu:UK	1977	CC	1448	n	V	n	n	0	ev	cig only	nev any	st
ARMADA	1		m	0	0	all	-	all	Eu:wst	1986	CC	325	n	bl	n	y	0	ev	cig only	nev any	st
BAND	1		m	0	0	all	-	all	NAmer	1983	CC	2831	n	V	y	y	2	ev	cig only	nev any	ot
BEST	1		m	0	0	all	0	all	NAmer	1955	pr	381	n	V	n	n	1	ev	cig only	nev any	ot
BEST	18		f	0	0	all	0	all	NAmer	1955	pr	381	n	V	n	n	1	ev	cig only	nev any	ot
BOFFET	6	x	m	0	0	all	-	all	Eu:mul	1988	CC	5621	n	bl	y	n	0	ev	cig only	nev any	st
BOUCOT	1	x	m	0	0	all	0	all	NAmer	1951	pr	121	n	bl	n	n	0	ev	cig only	nev any	ot
BRESLO	18		m	0	0	all	-	all	NAmer	1949	CC	518	n	bl	n	y	0	ev	cig only	nev+1	st
BRESLO	24		f	0	0	all	-	all	NAmer	1949	CC	518	n	bl	n	y	0	ev	cig only	nev+1	st
CEDERL	116		m	0	0	all	0	all	Eu:Sca	1963	pr	491	n	bl	n	n	2	cu	cig only	nev any	or
CEDERL	31	x	f	0	0	all	10	all	Eu:Sca	1963	pr	491	n	bl	n	n	0	cu	cig only	nev any	st
CHOW	15		m	0	0	wh	0	all	NAmer	1966	pr	219	n	bl	n	n	0	ev	cig only	nev any	st
CPSI	73		m	0	0	wh	0	all	NAmer	1959	pr	5138	n	bl	n	n	1	ev	cig only	nev any	st
CPSI	149		f	0	0	wh	0	all	NAmer	1959	pr	5138	n	bl	n	n	1	ev	cig only	nev any	st
CPSII	104		m	35	99	all	4	all	NAmer	1982	pr	3229	n	bl	n	n	1	ev	cig only	nev any	ot
DAMBER	1	x	m	0	0	all	-	all	Eu:Sca	1972	CC	579	n	bl	y	n	0	ev	cig only	nev any	st
DEAN	4		m	0	0	wh	-	all	Africa	1947	CC	603	n	V	y	n	0	ev	cig only	nev any	st
DEAN2	9		m	0	0	all	-	all	Eu:UK	1960	CC	954	n	V	y	n	0	ev	cig only	nev any	st
DEAN2	17		f	0	0	all	-	all	Eu:UK	1960	CC	954	n	V	y	n	0	ev	cig only	nev any	st
DEAN3	240	x	m	0	0	all	-	all	Eu:UK	1969	CC	766	n	V	y	n	0	ev	cig only	nev any	st
DEAN3	124	x	f	0	0	all	-	all	Eu:UK	1969	CC	766	n	V	y	n	0	ev	cig only	nev any	st
DOLL	13		m	0	0	all	-	all	Eu:UK	1948	CC	1465	n	V	n	n	0	ev	cig only	nev any	st
DOLL2	62		m	0	0	all	0	all	Eu:UK	1951	pr	920	n	V	n	n	1	ev	cig only	nev any	ot
DOLL2	63		f	0	0	all	22	all	Eu:UK	1951	pr	920	n	V	n	n	1	cu	cig only	nev any	ot
DORN	1		m	0	0	wh	0	all	NAmer	1954	pr	5097	n	bl	n	n	2	ev	cig only	nev any	or
ENGELA	210		m	0	0	all	12	all	Eu:Sca	1964	pr	435	n	bl	n	n	1	cu	cig only	nev any	ot
ENGELA	225		f	0	0	all	12	all	Eu:Sca	1964	pr	435	n	bl	n	n	1	cu	cig only	nev any	ot
ENSTRO	1		m	0	0	all	0	all	NAmer	1959	pr	2879	n	bl	n	n	1	cu	cig only	nev any	or
ENSTRO	2		f	0	0	all	0	all	NAmer	1959	pr	2879	n	bl	n	n	1	cu	cig only	nev any	or
GARDIN	6		c	0	0	all	-	all	Eu:UK	1988	CC	143	n	V	y	n	0	cu	cig only	nev any	st
GOLLED	20	x	m	35	99	all	-	all	Eu:UK	1952	CC	443	n	V	y	n	0	ev	cig only	nev any	st
GRAHAM	3		m	0	0	wh	-	all	NAmer	1956	CC	685	n	bl	n	n	0	ev	cig only	nev any	st
HAMMON	127	x	m	0	0	wh	0	all	NAmer	1952	pr	448	n	bl	n	n	0	ev	cig only	nev any	st
HEIN	1		m	0	0	all	0	all	Eu:Sca	1970	pr	144	n	bl	n	n	0	cu	cig only	nev any	st
JOLY	48		m	0	0	all	-	all	SCAmer	1978	CC	826	n	bl	n	n	0	ev	cig only	nev any	st
JUSSAW	2	x	m	0	0	all	-	all	As:Ind	1964	CC	792	n	V	n	n	0	ev	cig only	nev any	st
KAISE2	72		m	35	99	all	9	all	NAmer	1979	pr	318	n	bl	n	n	1	ev	cig only	nev any	st
KAISE2	64		f	35	99	all	9	all	NAmer	1979	pr	318	n	bl	n	n	1	ev	cig only	nev any	st
KJUUS	3		m	0	0	all	-	all	Eu:Sca	1979	CC	176	n	bl	n	n	0	ev	cig only	nev any	st
KOULUM	2		m	0	0	all	-	all	Eu:Sca	1936	CC	812	n	bl	n	n	0	ev	cig only	nev any	st
LIU4	10		m	35	69	all	-	all	As:Chi	1986	CC	1000-00	n	ot	y	n	2	ev	cig only	nev any	ot
LOMBAR	10		m	0	0	all	-	all	NAmer	1951	CC	1040	n	bl	n	n	0	ev	cig only	nev any	st
LUBIN2	17	x	m	0	0	all	-	all	Eu:mul	1976	CC	7804	n	bl	n	y	0	ev	cig only	nev any	st
LUBIN2	97	x	f	0	0	all	-	all	Eu:mul	1976	CC	7804	n	bl	n	y	0	ev	cig only	nev any	st
MCCONN	16		c	0	0	all	-	all	Eu:UK	1946	CC	100	n	V	n	y	0	ev	cig only	nev any	st
MIGRAN	9	x	m	0	0	all	0	all	Eu:UK	1964	pr	259	n	V	n	n	0	cu	cig only	nev any	st
MIGRAN	35	x	f	0	0	all	0	all	Eu:UK	1964	pr	259	n	V	n	n	0	cu	cig only	nev any	st
MILLS	1		m	0	0	wh	-	all	NAmer	1940	CC	444	n	bl	y	n	1	ev	cig only	nev any	ot
NOTAN2	7	x	m	0	0	all	-	all	As:Ind	1963	CC	683	n	V	n	n	0	ev	cig only	nev any	st
PERNU	8		m	0	0	all	-	all	Eu:Sca	1944	CC	1606	n	bl	n	n	0	ev	cig only	nev any	st
PERNU	4		f	0	0	all	-	all	Eu:Sca	1944	CC	1606	n	bl	n	n	0	ev	cig only	nev any	st
PEZZOT	25		m	0	0	all	-	all	SCAmer	1987	CC	215	n	bl	n	y	0	ev	cig only	nev cigs	st
RONCO	2	x	m	0	0	all	-	all	Eu:wst	1976	CC	126	n	bl	y	n	0	ev	cig only	nev any	st
SADOWS	1	x	m	0	0	wh	-	all	NAmer	1938	CC	477	n	bl	n	n	0	ev	cig only	nev any	st
STASZE	2		m	0	0	all	-	all	Eu:est	1954	CC	281	n	bl	n	y	0	ev	cig only	nev any	st
SUZUK2	3	x	c	0	0	all	-	all	SCAmer	1991	CC	123	n	bl	n	y	0	ev	cig only	nev any	st
TIZZAN	2		m	0	0	all	-	all	Eu:wst	1959	CC	1358	n	bl	n	n	0	ev	cig only	nev any	st
TIZZAN	22		f	0	0	all	-	all	Eu:wst	1959	CC	1358	n	bl	n	n	0	ev	cig only	nev any	st
TVERDA	3		m	0	0	all	0	all	Eu:Sca	1972	pr	238	n	bl	n	n	2	cu	cig only	nev cigs	ot
TVERDA	15		f	0	0	all	0	all	Eu:Sca	1972	pr	238	n	bl	n	n	2	cu	cig only	nev cigs	ot
WALD	2	x	m	0	0	all	0	all	Eu:UK	1975	pr	102	n	V	n	n	0	cu	cig only	nev any	st
WIGLE	13		m	0	0	all	-	all	NAmer	1971	CC	728	n	V	n	n	0	ev	cig only	nev any	st
WIGLE	16		f	0	0	all	-	all	NAmer	1971	CC	728	n	V	n	n	0	ev	cig only	nev any	st
WYNDE7	39		m	0	0	all	-	all	NAmer	1977	CC	2085	n	bl	n	y	0	ev	cig only	nev any	st
XIANGZ	1	x	m	0	0	all	0	all	As:Chi	1976	pr	983	m	ot	n	n	0	ev	cig only	nev any	st

Table 1C5 - 4

IESLC - Meta-anal of Ever Smoking (or Current if Ever not available), Cigarettes only
All LC types
Least adjusted

Cigarette type is all/unspec for all RRs
except for the following:

REF	NRR	CIGTYPE
ALDERS	12	MC only
DEAN3	240	MC only
DEAN3	124	MC only
GARDIN	6	MC only
JUSSAW	2	MC only
NOTAN2	7	MC only
PERNU	8	MC only
PERNU	4	MC only
SUZUK2	3	MC only

Table 1C5 - 5

IESLC - Meta-anal of Ever Smoking (or Current if Ever not available), Cigarettes only
All LC types
Least adjusted

REF	NRR	SEX	AD	Number Exposed Case	Number Exposed Cont	Non-exposed Case	Non-exposed Cont	RR	95.00%CI
ABELIN	4	m	0	30	90	2	183	30.50	(7.13- 130.47)
AGUDO	8	f	0	23	23	80	183	2.29	(1.21- 4.32)
ALDERS	60	m	0	576	462	15	133	11.05	(6.39- 19.12)
ALDERS	12	f	0	530	371	75	243	4.63	(3.46- 6.19)
Subtotal ALDERS								5.61	(4.34- 7.25)
ARMADA	1	m	0	245	197	4	64	19.90	(7.12- 55.59)
BAND	1	m	2	-	-	-	-	9.96	(7.38- 13.44)
*BEST	1	m	1	-	-	-	-	14.20	(6.70- 30.10)
*BEST	18	f	1	-	-	-	-	2.24	(0.59- 8.44)
Subtotal BEST								9.09	(4.72- 17.48)
BOFFET	6	m	0	4204	3930	117	1750	16.00	(13.20- 19.39)
*BOUCOT	1	m	0	93	28154	0	7551	50.16~	(3.11- 807.74)
BRESLO	18	m	0	316	229	7	42	8.28	(3.65- 18.76)
BRESLO	24	f	0	13	11	12	14	1.38	(0.45- 4.20)
Subtotal BRESLO								4.42	(2.28- 8.54)
*CEDERL	116	m	2	-	-	-	-	8.43	(5.49- 12.94)
*CEDERL	31	f	0	8	4709	19	17679	1.58	(0.69- 3.61)
Subtotal CEDERL								5.91	(4.04- 8.64)
*CHOW	15	m	0	57	57162	6	62913	10.46	(4.51- 24.25)
*CPSI	73	m	1	-	-	-	-	10.07	(8.77- 11.56)
*CPSI	149	f	1	-	-	-	-	3.20	(2.81- 3.65)
Subtotal CPSI								5.50	(5.00- 6.05)
*CPSII	104	m	1	-	-	-	-	12.83	(10.28- 16.01)
DAMBER	1	m	0	198	140	42	208	7.00	(4.71- 10.41)
DEAN	4	m	0	403	385	12	61	5.32	(2.82- 10.04)
DEAN2	9	m	0	629	508	33	112	4.20	(2.80- 6.30)
DEAN2	17	f	0	62	29	88	121	2.94	(1.75- 4.94)
Subtotal DEAN2								3.67	(2.67- 5.05)
DEAN3	240	m	0	399	1227	24	510	6.91	(4.52- 10.57)
DEAN3	124	f	0	109	1420	41	1538	2.88	(2.00- 4.15)
Subtotal DEAN3								4.18	(3.17- 5.52)
DOLL	13	m	0	1004	899	7	61	9.73	(4.43- 21.39)
*DOLL2	62	m	1	-	-	-	-	8.78	(5.55- 13.88)
*DOLL2	63	f	1	-	-	-	-	8.65	(2.93- 25.55)
Subtotal DOLL2								8.76	(5.74- 13.36)
*DORN	1	m	2	-	-	-	-	8.40	(7.50- 9.40)
*ENGELA	210	m	1	-	-	-	-	9.50	(4.37- 20.66)
*ENGELA	225	f	1	-	-	-	-	5.80	(2.69- 12.51)
Subtotal ENGELA								7.40	(4.29- 12.78)
*ENSTRO	1	m	1	-	-	-	-	12.99	(10.46- 16.13)
*ENSTRO	2	f	1	-	-	-	-	6.95	(6.01- 8.04)
Subtotal ENSTRO								8.44	(7.48- 9.53)
GARDIN	6	c	0	72	39	5	41	15.14	(5.53- 41.44)
GOLLED	20	m	0	344	1791	15	490	6.27	(3.71- 10.62)
GRAHAM	3	m	0	474	951	18	346	9.58	(5.89- 15.58)
*HAMMON	127	m	0	249	225565	15	115884	8.53	(5.06- 14.36)
*HEIN	1	m	0	45	912	1	457	22.55	(3.12- 163.06)
JOLY	48	m	0	379	499	12	218	13.80	(7.60- 25.05)
JUSSAW	2	m	0	126	77	149	624	6.85	(4.90- 9.58)
*KAISE2	72	m	1	-	-	-	-	5.40	(3.05- 9.57)
*KAISE2	64	f	1	-	-	-	-	10.09	(5.29- 19.27)
Subtotal KAISE2								7.11	(4.63- 10.90)
KJUUS	3	m	0	151	127	2	24	14.27	(3.31- 61.54)
KOULUM	2	m	0	625	229	5	54	29.48	(11.65- 74.60)
LIU4	10	m	2	-	-	-	-	3.88	(3.78- 3.98)
LOMBAR	10	m	0	486	302	14	112	12.87	(7.25- 22.85)
LUBIN2	17	m	0	6042	9343	190	2617	8.91	(7.66- 10.36)
LUBIN2	97	f	0	548	559	336	1188	3.47	(2.93- 4.10)
Subtotal LUBIN2								5.86	(5.24- 6.56)
MCCONN	16	c	0	68	138	9	23	1.26	(0.55- 2.87)
*MIGRAN	9	m	0	137	3707	4	867	8.01	(2.97- 21.59)
*MIGRAN	35	f	0	23	2749	4	3814	7.98	(2.76- 23.04)
Subtotal MIGRAN								8.00	(3.88- 16.50)
MILLS	1	m	1	-	-	-	-	1.27	(1.01- 1.61)
NOTAN2	7	m	0	78	129	134	544	2.45	(1.75- 3.44)
PERNU	8	m	0	706	216	97	275	9.27	(7.02- 12.23)
PERNU	4	f	0	7	24	110	971	2.57	(1.08- 6.11)
Subtotal PERNU								8.22	(6.32- 10.71)
PEZZOT	25	m	0	211	317	4	116	19.30	(7.02- 53.10)
RONCO	2	m	0	116	274	6	78	5.50	(2.33- 12.98)
SADOWS	1	m	0	273	328	18	81	3.75	(2.19- 6.40)

Table 1C5 - 5

IESLC - Meta-anal of Ever Smoking (or Current if Ever not available), Cigarettes only
All LC types
Least adjusted

REF	NRR	SEX	AD	Number Exposed Case	Cont	Non-exposed Case	Cont	RR	95.00%CI
STASZE	2	m	0	218	552	5	158	12.48	(5.05- 30.82)
SUZUK2	3	c	0	82	63	11	53	6.27	(3.03- 12.98)
TIZZAN	2	m	0	994	836	180	305	2.01	(1.64- 2.48)
TIZZAN	22	f	0	25	28	25	114	4.07	(2.04- 8.13)
Subtotal TIZZAN								2.13	(1.75- 2.60)
*TVERDA	3	m	2	-	-	-	-	3.83	(2.47- 5.95)
*TVERDA	15	f	2	-	-	-	-	11.05	(3.33- 36.71)
Subtotal TVERDA								4.34	(2.87- 6.56)
*WALD	2	m	0	77	4182	7	6539	17.20	(7.94- 37.25)
WIGLE	13	m	0	543	632	15	204	11.68	(6.83- 19.99)
WIGLE	16	f	0	78	235	36	439	4.05	(2.64- 6.19)
Subtotal WIGLE								6.09	(4.37- 8.51)
WYNDE7	39	m	0	1645	2108	64	918	11.19	(8.62- 14.54)
*XIANGZ	1	m	0	289	8011	25	974	1.41	(0.94- 2.10)
Partial Totals				24010	364869	2100	231894		

*prospective study ~ With 0.5 adjustment for zero

REF	NRR	SEX	AD	Ys	Ws	Qs	Ps
ABELIN	4	m	0	3.42	1.82	6.69	0.0000
AGUDO	8	f	0	0.83	9.53	4.30	0.0106
ALDERS	60	m	0	2.40	12.81	10.45	0.0000
ALDERS	12	f	0	1.53	45.39	0.05	0.0000
Subtotal ALDERS				1.72	58.20	10.50	
ARMADA	1	m	0	2.99	3.64	8.09	0.0000
BAND	1	m	2	2.30	42.76	27.31	0.0000
*BEST	1	m	1	2.65	6.81	9.06	0.0000
*BEST	18	f	1	0.81	2.17	1.04	0.2348
Subtotal BEST				2.21	8.98	10.10	
BOFFET	6	m	0	2.77	104.05	168.66	0.0000
*BOUCOT	1	m	0	3.92	0.50	2.90	0.0058
BRESLO	18	m	0	2.11	5.74	2.17	0.0000
BRESLO	24	f	0	0.32	3.10	4.30	0.5717
Subtotal BRESLO				1.49	8.84	6.47	
*CEDERL	116	m	2	2.13	20.90	8.36	0.0000
*CEDERL	31	f	0	0.46	5.64	6.12	0.2769
Subtotal CEDERL				1.78	26.54	14.47	
*CHOW	15	m	0	2.35	5.43	3.90	0.0000
*CPSI	73	m	1	2.31	201.40	132.18	0.0000
*CPSI	149	f	1	1.16	224.63	25.40	0.0000
Subtotal CPSI				1.71	426.03	157.58	
*CPSII	104	m	1	2.55	78.29	86.70	0.0000
DAMBER	1	m	0	1.95	24.50	4.90	0.0000
DEAN	4	m	0	1.67	9.54	0.28	0.0000
DEAN2	9	m	0	1.44	23.37	0.10	0.0000
DEAN2	17	f	0	1.08	14.24	2.53	0.0000
Subtotal DEAN2				1.30	37.61	2.62	
DEAN3	240	m	0	1.93	21.30	4.00	0.0000
DEAN3	124	f	0	1.06	28.64	5.59	0.0000
Subtotal DEAN3				1.43	49.94	9.59	
DOLL	13	m	0	2.28	6.20	3.73	0.0000
*DOLL2	62	m	1	2.17	18.29	8.28	0.0000
*DOLL2	63	f	1	2.16	3.28	1.42	0.0001
Subtotal DOLL2				2.17	21.56	9.70	
*DORN	1	m	2	2.13	301.36	119.15	0.0000
*ENGELA	210	m	1	2.25	6.37	3.60	0.0000
*ENGELA	225	f	1	1.76	6.50	0.43	0.0000
Subtotal ENGELA				2.00	12.87	4.03	
*ENSTRO	1	m	1	2.56	81.91	92.86	0.0000
*ENSTRO	2	f	1	1.94	181.45	35.02	0.0000
Subtotal ENSTRO				2.13	263.36	127.88	
GARDIN	6	c	0	2.72	3.79	5.62	0.0000
GOLLED	20	m	0	1.84	13.86	1.57	0.0000
GRAHAM	3	m	0	2.26	16.23	9.38	0.0000
*HAMMON	127	m	0	2.14	14.15	5.87	0.0000
*HEIN	1	m	0	3.12	0.98	2.56	0.0020
JOLY	48	m	0	2.62	10.80	13.68	0.0000
JUSSAW	2	m	0	1.92	34.20	6.18	0.0000
*KAISE2	72	m	1	1.69	11.75	0.41	0.0000
*KAISE2	64	f	1	2.31	9.19	6.06	0.0000
Subtotal KAISE2				1.96	20.95	6.47	

Table 1C5 - 5

IESLC - Meta-anal of Ever Smoking (or Current if Ever not available), Cigarettes only
All LC types
Least adjusted

REF	NRR	SEX	AD	Ys	Ws	Qs	Ps
KJUUS	3	m	0	2.66	1.80	2.41	0.0004
KOULUM	2	m	0	3.38	4.45	15.81	0.0000
LIU4	10	m	2	1.36	5780.52	119.20	0.0000
LOMBAR	10	m	0	2.56	11.67	13.00	0.0000
LUBIN2	17	m	0	2.19	168.98	79.85	0.0000
LUBIN2	97	f	0	1.24	134.56	8.85	0.0000
Subtotal LUBIN2				1.77	303.54	88.70	
MCCONN	16	c	0	0.23	5.66	9.12	0.5832
*MIGRAN	9	m	0	2.08	3.91	1.32	0.0000
*MIGRAN	35	f	0	2.08	3.41	1.14	0.0001
Subtotal MIGRAN				2.08	7.32	2.46	
MILLS	1	m	1	0.24	71.48	112.98	0.0406
NOTAN2	7	m	0	0.90	33.47	12.11	0.0000
PERNU	8	m	0	2.23	50.02	26.43	0.0000
PERNU	4	f	0	0.95	5.14	1.58	0.0321
Subtotal PERNU				2.11	55.16	28.01	
PEZZOT	25	m	0	2.96	3.75	8.01	0.0000
RONCO	2	m	0	1.71	5.21	0.22	0.0001
SADOWS	1	m	0	1.32	13.40	0.43	0.0000
STASZE	2	m	0	2.52	4.70	4.94	0.0000
SUZUK2	3	c	0	1.84	7.25	0.82	0.0000
TIZZAN	2	m	0	0.70	90.61	57.84	0.0000
TIZZAN	22	f	0	1.40	8.03	0.07	0.0001
Subtotal TIZZAN				0.76	98.64	57.91	
*TVERDA	3	m	2	1.34	19.88	0.49	0.0000
*TVERDA	15	f	2	2.40	2.67	2.18	0.0001
Subtotal TVERDA				1.47	22.55	2.66	
*WALD	2	m	0	2.84	6.43	11.65	0.0000
WIGLE	13	m	0	2.46	13.33	12.26	0.0000
WIGLE	16	f	0	1.40	21.22	0.22	0.0000
Subtotal WIGLE				1.81	34.55	12.48	
WYNDE7	39	m	0	2.42	56.19	47.13	0.0000
*XIANGZ	1	m	0	0.34	23.64	31.75	0.0980

	N	68
	NS	51
	Wt	8167.91
Het	Chi	1422.74
Het	df	67
Het	P	***
Fixed	RR	4.48
	RRl	4.38
	RRu	4.58
	P	+++
Random	RR	6.46
	RRl	5.52
	RRu	7.57
	P	+++
Asymm	P	**

Table 1C5 - 6

IESLC - Meta-anal of Ever Smoking (or Current if Ever not available), Cigarettes only
All LC types
Least adjusted

	Sex combined	male	female	Total
N	3	47	18	68
NS	3	47	18	68
Wt	16.71	7442.41	708.79	8167.91
Het Chi	15.56	1300.48	102.03	1422.74
Het df	2	46	17	67
Het P	***	***	***	***
Fixed RR	4.44	4.51	4.14	4.48
RRl	2.75	4.41	3.85	4.38
RRu	7.18	4.62	4.46	4.58
P	+++	+++	+++	+++
Random RR	4.83	7.93	3.97	6.46
RRl	1.24	6.44	3.14	5.52
RRu	18.86	9.77	5.02	7.57
P	+	+++	+++	+++
Between Chi				4.68
Between df				2
Between P				(*)
Btwn(F) P				N.S.
Btwn(R) P				***

Table 1C5 - 7

IESLC - Meta-anal of Ever Smoking (or Current if Ever not available), Cigarettes only
All LC types
Excluded studies (and stage at which they were excluded)

1	BOUCHA BUELL LAURIL MZILEN
2	BERRIN CHEN RESTRE TAO ULMER
4	ABRAHA AKIBA AMANDU AMES ANDERS ARCHER AUSTIN AUVINE AXELSO AXELSS BARBON BECHER BENSHL BLOHMK BLOT1 BLOT2 BLOT3 BLOT4 BRETT BROCKM BROSS BROWN1 BROWN2 BUFFLE BYERS1 BYERS2 CARPEN CASCO2 CASCOR CHAN CHANG CHATZI CHEN2 CHEN3 CHIAZZ CHOI CHYOU COMSTO COOKSO CORREA DARBY DAVEYS DEKLER DESTE2 DESTEF DOCKER DORANT DORGAN DOSEME DROSTE DU DUNN EBELIN ESAKI FAN GAO GAO2 GARCIA GARSHI GENG GER GILLIS GODLEY GOODMA GREGOR GSELL HAENSZ HAMMO2 HANSEN HEGMAN HENNEK HINDS HIRAOK HIRAYA HITOSU HOLE HOROWI HORWIT HU HU2 HUANG HUMBLE ISHIMA JAHN JAIN JARUP JARVHO JEDRYC JIANG JONES KAISER KANELL KATSOU KAUFMA KELLER KHUDER KIHARA KINLEN KNEKT KO KOHLME KOO KREUZE KREYBE KUBIK LAMTH LAMWK LAMWK2 LAUSSM LEI LEMARC LETOUR LEVIN LIAW LICKIN LIDDEL LIU LIU2 LIU3 LIU5 LOMBA2 LUO MACLEN MAGNUS MARSH MARSH2 MARTIS MASTRA MATOS MATSUD MCDUFF MCLAUG MILLER MOLLO MRFIT MRFITR MURATA NAM NOTANI NOU ODRISC ORMOS OSANN OSANN2 PARKIN PASTOR PAWLEG PERSH2 PERSHA PETO PEZZO2 PIKE PISANI POFFIJ POLEDN PRESCO QIAO2 RACHTA RADZIK RANDIG REN ROOTS ROTHSC SAARIK SANKAR SCHWAR SEGI SEGI2 SEOW SHAW SHIMIZ SIEMIA SIMARA SIMONA SITAS SOBUE SOBUE2 SPEIZE SPITZ STAYNE STOCKS STOCKW STUCKE SUN SUZUKI SVENSS TANG TENKAN TOKARS TOUSEY TSUGAN TULINI VANDER VUTUC WAKAI WANG WANG2 WANG3 WANG4 WARSIN WATSON WICKLU WILKIN WU WU2 WUNSCH WUWILL WYNDE2 WYNDE3 WYNDE4 WYNDE5 WYNDE6 WYNDE8 XU XU2 XU3 XU4 YAMAGU YONG YUAN ZHANG ZHENG ZHOU
5	LANGE RIMING TANG2
6	HIRAY2 SCHWA2 WYNDER
10	BENHAM LUBIN QIAO VEIERO

Table 1C5 - 8
Potentially overlapping studies

REF	REFGP	PRINC	OVERLAP/LINK
LUBIN2	LUBIN2	1	Lubin-combined
TVERDA	TVERDA	1	VEIERO/TVERDAL
HEIN	PRESCO	1	Subset of PRESCO
GRAHAM	BYERS1	1	GRAHAM/BROSS/BYERS1
WALD	TANG2	1	Subset of TANG2
WYNDE7	WYNDE6	2	WYNDE5/6/7/8
CPSI	CPSI	1	CPSI overall
ENSTRO	ENSTRO	1	Subset of CPSI
BOFFET	BOFFET	1	BOFFET-combined
XIANGZ	XIANGZ	1	LUBIN/XIANGZ/QIAO

Table 1C6 -

IESLC - Meta-anal of Current Smoking (or Ever if Current not available), Cigarettes only
All LC types

This analysis is restricted to results for:
1) Non-dose-response data
2) Results complete enough for use in metaanalysis

Within each study, results are then selected (in the following order of preference, within each sex) for:
3) SMKSTA: current smokers, ever smokers
4) PRODUCT: cigarettes only
5) CIGTYPE: all/unspecified, MC regardless of HR, MC only
6) DENOM: never smoked anything, never smoked cigarettes, (never +1 = +long term ex, +2 = +amount unknown,
+3 = never cigs+long term ex)
7) Followup period (YF, prospective studies): whole study (coded as 0) or longest available
8) LCtype: all or nearest available, at least Squamous and Adeno. (q = squamous, s = small,
l = large, a = adeno, mix = mixed, alv = alveolar)
9) Race: all or nearest available, otherwise by race (wh or w = white, bl or b = black, hi = hispanic
ch = chinese, jap = japanese, haw = hawaiian, w+o = white + oriental, sca = scandinavian, as = asian)
10) For overlapping studies: principal rather than subsidiary studies
Finally by Age: whole study (coded as 0) if available, otherwise by widest available age group
and then for single sex results (m, f) in preference to combined sex results (c).

Results adjusted (AD) for the most potential confounders are then chosen in Sections -1 to -3
(and those which actually differ from the adjusted results in Table 1C5 - 1 are marked 'x' in Section -1)
and results adjusted for the least confounders in Sections -4 to -6. (Those least adjusted results which
actually differ from the most adjusted as marked 'x' in column X in Section -4)
(Results adjusted for an unknown number of confounder(s) are coded as 20.)

Section -7 shows excluded studies, together with the stage (as above) at which no qualifying
results were found.

Section -8 lists the potentially overlapping studies which have been included (1=principal, 2=subsidiary).

Section -9 lists any results which would have been included in preference except that they had data not complete
enough for use in meta-analysis, with their significance (yes/no), if known, and any further comment as entered
on the database.

In addition to those mentioned above, the following fields, levels and abbreviations are used:
* or nk = not known, n = no, y = yes, ot = other
ev = ever, cu = current, nev = never
all/unspec = all or unspecified, MC = manufactured cigarettes, HR = hand-rolled cigarettes
REF: 6-character study reference
NRR: number of the RR on the database within the study
ST : study type (CC = case control, pr or prosp = prospective)
NLC: number of lung cancer cases in whole study
R : risky occupational population (n = no, m = mining, o = other risky)
VB : national cigarette type (V = at least 75% Virginia, bl = at least 75% blended, ot = other)
P : any proxy use
H : full histological confirmation
De : derivation of RR/CI (or = original, st = standard method, ot = other method of estimation)

Table 1C6 - 1

IESLC - Meta-anal of Current Smoking (or Ever if Current not available), Cigarettes only
All LC types
Most adjusted

REF	NRR	1C5	SEX	AGEL	AGEH	RACE	YF	LC TYPE	LOC	START	ST	NLC	R	VB	P	H	AD	SM	PRODUCT	DENOM	De
ABELIN	47		m	0	0	all	-	all	Eu:wst	1941	CC	118	n	bl	y	n	1	ev	cig only	nev any	st
AGUDO	3	x	f	0	0	all	-	all	Eu:wst	1989	CC	103	n	bl	n	n	3	cu	cig only	nev any	or
ALDERS	174	x	m	0	0	all	-	all	Eu:UK	1977	CC	1448	n	V	n	n	0	cu	cig only	nev any	st
ALDERS	176	x	f	0	0	all	-	all	Eu:UK	1977	CC	1448	n	V	n	n	0	cu	cig only	nev any	st
ARMADA	1		m	0	0	all	-	all	Eu:wst	1986	CC	325	n	bl	n	y	0	ev	cig only	nev any	st
BAND	1		m	0	0	all	-	all	NAmer	1983	CC	2831	n	V	y	y	2	ev	cig only	nev any	ot
BEST	2	x	m	0	0	all	0	all	NAmer	1955	pr	381	n	V	n	n	1	cu	cig only	nev any	ot
BEST	18		f	0	0	all	0	all	NAmer	1955	pr	381	n	V	n	n	1	ev	cig only	nev any	ot
BOFFET	3		m	0	0	all	-	all	Eu:mul	1988	CC	5621	n	bl	y	n	2	ev	cig only	nev any	or
BOUCOT	114	x	m	0	0	all	0	all	NAmer	1951	pr	121	n	bl	n	n	2	cu	cig only	nev any	ot
BRESLO	18		m	0	0	all	-	all	NAmer	1949	CC	518	n	bl	n	y	0	ev	cig only	nev+1	st
BRESLO	24		f	0	0	all	-	all	NAmer	1949	CC	518	n	bl	n	y	0	ev	cig only	nev+1	st
CEDERL	116		m	0	0	all	0	all	Eu:Sca	1963	pr	491	n	bl	n	n	2	cu	cig only	nev any	or
CEDERL	41		f	0	0	all	10	all	Eu:Sca	1963	pr	491	n	bl	n	n	1	cu	cig only	nev any	ot
CHOW	16	x	m	0	0	wh	0	all	NAmer	1966	pr	219	n	bl	n	n	0	cu	cig only	nev any	st
CPSI	76	x	m	0	0	all	0	all	NAmer	1959	pr	5138	n	bl	n	n	1	cu	cig only	nev any	st
CPSI	152	x	f	0	0	all	0	all	NAmer	1959	pr	5138	n	bl	n	n	1	cu	cig only	nev any	st
CPSII	126	x	m	0	0	all	6	all	NAmer	1982	pr	3229	n	bl	n	n	1	cu	cig only	nev any	ot
DAMBER	16	x	m	0	0	all	-	all	Eu:Sca	1972	CC	579	n	bl	y	n	1	cu	cig only	nev any	ot
DEAN	4		m	0	0	wh	-	all	Africa	1947	CC	603	n	V	y	n	0	ev	cig only	nev any	st
DEAN2	9		m	0	0	all	-	all	Eu:UK	1960	CC	954	n	V	y	n	0	ev	cig only	nev any	st
DEAN2	17		f	0	0	all	-	all	Eu:UK	1960	CC	954	n	V	y	n	0	ev	cig only	nev any	st
DEAN3	35	x	m	0	0	all	-	all	Eu:UK	1969	CC	766	n	V	y	n	3	cu	cig only	nev any	ot
DEAN3	119	x	f	0	0	all	-	all	Eu:UK	1969	CC	766	n	V	y	n	3	cu	cig only	nev any	ot
DOLL	13		m	0	0	all	-	all	Eu:UK	1948	CC	1465	n	V	n	n	0	ev	cig only	nev any	st
DOLL2	2	x	m	0	0	all	0	all	Eu:UK	1951	pr	920	n	V	n	n	1	cu	cig only	nev any	ot
DOLL2	63		f	0	0	all	22	all	Eu:UK	1951	pr	920	n	V	n	n	1	cu	cig only	nev any	ot
DORN	3	x	m	0	0	wh	0	all	NAmer	1954	pr	5097	n	bl	n	n	2	cu	cig only	nev any	or
ENGELA	210		m	0	0	all	12	all	Eu:Sca	1964	pr	435	n	bl	n	n	1	cu	cig only	nev any	ot
ENGELA	225		f	0	0	all	12	all	Eu:Sca	1964	pr	435	n	bl	n	n	1	cu	cig only	nev any	ot
ENSTRO	1		m	0	0	all	0	all	NAmer	1959	pr	2879	n	bl	n	n	1	cu	cig only	nev any	or
ENSTRO	2		f	0	0	all	0	all	NAmer	1959	pr	2879	n	bl	n	n	1	cu	cig only	nev any	or
GARDIN	6		c	0	0	all	-	all	Eu:UK	1988	CC	143	n	V	y	n	0	cu	cig only	nev any	st
GOLLED	6		m	35	99	all	-	all	Eu:UK	1952	CC	443	n	V	y	n	1	ev	cig only	nev any	ot
GRAHAM	2	x	m	0	0	wh	-	all	NAmer	1956	CC	685	n	bl	n	n	0	cu	cig only	nev any	st
HAMMON	139	x	m	0	0	wh	0	all	NAmer	1952	pr	448	n	bl	n	n	1	cu	cig only	nev any	ot
HEIN	1		m	0	0	all	0	all	Eu:Sca	1970	pr	144	n	bl	n	n	0	cu	cig only	nev any	st
JOLY	48		m	0	0	all	-	all	SCAmer	1978	CC	826	n	bl	n	n	0	ev	cig only	nev any	st
JUSSAW	31		m	0	0	all	-	all	As:Ind	1964	CC	792	n	V	n	n	2	ev	cig only	nev any	st
KAISE2	68	x	m	35	99	all	9	all	NAmer	1979	pr	318	n	bl	n	n	1	cu	cig only	nev any	st
KAISE2	60	x	f	35	99	all	9	all	NAmer	1979	pr	318	n	bl	n	n	1	cu	cig only	nev any	st
KJUUS	3		m	0	0	all	-	all	Eu:Sca	1979	CC	176	n	bl	n	n	0	ev	cig only	nev any	st
KOULUM	2		m	0	0	all	-	all	Eu:Sca	1936	CC	812	n	bl	n	n	0	ev	cig only	nev any	st
LIU4	10		m	35	69	all	-	all	As:Chi	1986	CC	1000-00	n	ot	y	n	2	ev	cig only	nev any	ot
LOMBAR	5	x	m	0	0	all	-	all	NAmer	1951	CC	1040	n	bl	n	n	0	cu	cig only	nev any	st
LUBIN2	2	x	m	0	0	all	-	all	Eu:mul	1976	CC	7804	n	bl	n	y	2	cu	cig only	nev any	ot
LUBIN2	98		f	0	0	all	-	all	Eu:mul	1976	CC	7804	n	bl	n	y	1	ev	cig only	nev any	ot
MCCONN	16		c	0	0	all	-	all	Eu:UK	1946	CC	100	n	V	n	y	0	ev	cig only	nev any	st
MIGRAN	10		m	0	0	all	0	all	Eu:UK	1964	pr	259	n	V	n	n	2	cu	cig only	nev any	ot
MIGRAN	36		f	0	0	all	0	all	Eu:UK	1964	pr	259	n	V	n	n	2	cu	cig only	nev any	ot
MILLS	1		m	0	0	wh	-	all	NAmer	1940	CC	444	n	bl	y	n	1	ev	cig only	nev any	ot
NOTAN2	19		m	0	0	all	-	all	As:Ind	1963	CC	683	n	V	n	n	2	ev	cig only	nev any	ot
PERNU	8		m	0	0	all	-	all	Eu:Sca	1944	CC	1606	n	bl	n	n	0	ev	cig only	nev any	st
PERNU	4		f	0	0	all	-	all	Eu:Sca	1944	CC	1606	n	bl	n	n	0	ev	cig only	nev any	st
PEZZOT	5	x	m	0	0	all	-	all	SCAmer	1987	CC	215	n	bl	n	y	0	cu	cig only	nev cigs	st
RONCO	3		m	0	0	all	-	all	Eu:wst	1976	CC	126	n	bl	y	n	2	ev	cig only	nev any	ot
SADOWS	28		m	0	0	wh	-	all	NAmer	1938	CC	477	n	bl	n	n	1	ev	cig only	nev any	ot
STASZE	2		m	0	0	all	-	all	Eu:est	1954	CC	281	n	bl	n	y	0	ev	cig only	nev any	st
SUZUK2	7		c	0	0	all	-	all	SCAmer	1991	CC	123	n	bl	n	y	3	ev	cig only	nev any	or
TIZZAN	2		m	0	0	all	-	all	Eu:wst	1959	CC	1358	n	bl	n	n	0	ev	cig only	nev any	st
TIZZAN	22		f	0	0	all	-	all	Eu:wst	1959	CC	1358	n	bl	n	n	0	ev	cig only	nev any	st
TVERDA	3		m	0	0	all	0	all	Eu:Sca	1972	pr	238	n	bl	n	n	2	cu	cig only	nev cigs	ot
TVERDA	15		f	0	0	all	0	all	Eu:Sca	1972	pr	238	n	bl	n	n	2	cu	cig only	nev cigs	ot
WALD	4		m	0	0	all	0	all	Eu:UK	1975	pr	102	n	V	n	n	1	cu	cig only	nev any	or
WIGLE	28	x	m	0	0	all	-	all	NAmer	1971	CC	728	n	V	n	n	1	cu	cig only	nev any	ot
WIGLE	4	x	f	0	0	all	-	all	NAmer	1971	CC	728	n	V	n	n	0	cu	cig only	nev any	st
WYNDE7	1	x	m	0	0	all	-	all	NAmer	1977	CC	2085	n	bl	n	y	0	cu	cig only	nev any	st
XIANGZ	9		m	0	0	all	0	all	As:Chi	1976	pr	983	m	ot	n	n	2	ev	cig only	nev any	ot

Table 1C6 - 1

IESLC - Meta-anal of Current Smoking (or Ever if Current not available), Cigarettes only
All LC types
Most adjusted

Cigarette type is all/unspec for all RRs
except for the following:

REF	NRR	CIGTYPE
ALDERS	174	MC only
ALDERS	176	MC only
DEAN3	35	MC only
DEAN3	119	MC only
GARDIN	6	MC only
JUSSAW	31	MC only
NOTAN2	19	MC only
PERNU	8	MC only
PERNU	4	MC only
SUZUK2	7	MC only

Table 1C6 - 2

IESLC - Meta-anal of Current Smoking (or Ever if Current not available), Cigarettes only
All LC types
Most adjusted

REF	NRR	SEX	AD	Number Exposed Case	Number Exposed Cont	Non-exposed Case	Non-exposed Cont	RR	95.00%CI
ABELIN	47	m	1	-	-	-	-	53.81	(11.36- 254.76)
AGUDO	3	f	3	-	-	-	-	3.61	(1.57- 8.32)
ALDERS	174	m	0	312	213	15	133	12.99	(7.41- 22.77)
ALDERS	176	f	0	410	229	75	243	5.80	(4.27- 7.87)
Subtotal ALDERS								6.97	(5.33- 9.12)
ARMADA	1	m	0	245	197	4	64	19.90	(7.12- 55.59)
BAND	1	m	2	-	-	-	-	9.96	(7.38- 13.44)
*BEST	2	m	1	-	-	-	-	14.91	(7.05- 31.52)
*BEST	18	f	1	-	-	-	-	2.24	(0.59- 8.44)
Subtotal BEST								9.45	(4.92- 18.15)
BOFFET	3	m	2	-	-	-	-	14.90	(12.30- 18.10)
*BOUCOT	114	m	2	-	-	-	-	62.29	(3.86-1004.01)
BRESLO	18	m	0	316	229	7	42	8.28	(3.65- 18.76)
BRESLO	24	f	0	13	11	12	14	1.38	(0.45- 4.20)
Subtotal BRESLO								4.42	(2.28- 8.54)
*CEDERL	116	m	2	-	-	-	-	8.43	(5.49- 12.94)
*CEDERL	41	f	1	-	-	-	-	4.50	(1.78- 11.36)
Subtotal CEDERL								7.55	(5.11- 11.14)
*CHOW	16	m	0	71	40726	6	62913	18.28	(7.95- 42.06)
*CPSI	76	m	1	-	-	-	-	12.30	(10.71- 14.13)
*CPSI	152	f	1	-	-	-	-	3.58	(3.12- 4.10)
Subtotal CPSI								6.58	(5.97- 7.25)
*CPSII	126	m	1	-	-	-	-	20.25	(16.37- 25.05)
DAMBER	16	m	1	-	-	-	-	9.80	(6.30- 15.30)
DEAN	4	m	0	403	385	12	61	5.32	(2.82- 10.04)
DEAN2	9	m	0	629	508	33	112	4.20	(2.80- 6.30)
DEAN2	17	f	0	62	29	88	121	2.94	(1.75- 4.94)
Subtotal DEAN2								3.67	(2.67- 5.05)
DEAN3	35	m	3	-	-	-	-	8.48	(5.35- 13.42)
DEAN3	119	f	3	-	-	-	-	5.77	(3.75- 8.86)
Subtotal DEAN3								6.91	(5.04- 9.45)
DOLL	13	m	0	1004	899	7	61	9.73	(4.43- 21.39)
*DOLL2	2	m	1	-	-	-	-	14.93	(9.41- 23.68)
*DOLL2	63	f	1	-	-	-	-	8.65	(2.93- 25.55)
Subtotal DOLL2								13.73	(8.98- 20.99)
*DORN	3	m	2	-	-	-	-	11.60	(10.40- 13.00)
*ENGELA	210	m	1	-	-	-	-	9.50	(4.37- 20.66)
*ENGELA	225	f	1	-	-	-	-	5.80	(2.69- 12.51)
Subtotal ENGELA								7.40	(4.29- 12.78)
*ENSTRO	1	m	1	-	-	-	-	12.99	(10.46- 16.13)
*ENSTRO	2	f	1	-	-	-	-	6.95	(6.01- 8.04)
Subtotal ENSTRO								8.44	(7.48- 9.53)
GARDIN	6	c	0	72	39	5	41	15.14	(5.53- 41.44)
GOLLED	6	m	1	-	-	-	-	7.65	(4.52- 12.94)
GRAHAM	2	m	0	371	821	18	346	8.69	(5.32- 14.17)
*HAMMON	139	m	1	-	-	-	-	11.52	(6.83- 19.42)
*HEIN	1	m	0	45	912	1	457	22.55	(3.12- 163.06)
JOLY	48	m	0	379	499	12	218	13.80	(7.60- 25.05)
JUSSAW	31	m	2	-	-	-	-	8.64	(4.61- 17.88)
*KAISE2	68	m	1	-	-	-	-	8.04	(4.41- 14.66)
*KAISE2	60	f	1	-	-	-	-	14.48	(7.47- 28.04)
Subtotal KAISE2								10.49	(6.72- 16.36)
KJUUS	3	m	0	151	127	2	24	14.27	(3.31- 61.54)
KOULUM	2	m	0	625	229	5	54	29.48	(11.65- 74.60)
LIU4	10	m	2	-	-	-	-	3.88	(3.78- 3.98)
LOMBAR	5	m	0	432	249	14	112	13.88	(7.79- 24.72)
LUBIN2	2	m	2	-	-	-	-	11.21	(9.60- 13.09)
LUBIN2	98	f	1	-	-	-	-	3.90	(3.29- 4.62)
Subtotal LUBIN2								6.94	(6.19- 7.78)
MCCONN	16	c	0	68	138	9	23	1.26	(0.55- 2.87)
*MIGRAN	10	m	2	-	-	-	-	4.20	(1.55- 11.37)
*MIGRAN	36	f	2	-	-	-	-	5.11	(1.77- 14.79)
Subtotal MIGRAN								4.60	(2.23- 9.52)
MILLS	1	m	1	-	-	-	-	1.27	(1.01- 1.61)
NOTAN2	19	m	2	-	-	-	-	2.36	(1.68- 3.31)
PERNU	8	m	0	706	216	97	275	9.27	(7.02- 12.23)
PERNU	4	f	0	7	24	110	971	2.57	(1.08- 6.11)
Subtotal PERNU								8.22	(6.32- 10.71)
PEZZOT	5	m	0	145	129	4	116	32.60	(11.70- 90.81)
RONCO	3	m	2	-	-	-	-	5.43	(2.27- 12.96)
SADOWS	28	m	1	-	-	-	-	4.46	(2.28- 8.72)

Table 1C6 - 2

IESLC - Meta-anal of Current Smoking (or Ever if Current not available), Cigarettes only
All LC types
Most adjusted

REF	NRR	SEX	AD	Number Exposed Case	Number Exposed Cont	Non-exposed Case	Non-exposed Cont	RR	95.00%CI
STASZE	2	m	0	218	552	5	158	12.48	(5.05- 30.82)
SUZUK2	7	c	3	-	-	-	-	11.00	(3.40- 36.00)
TIZZAN	2	m	0	994	836	180	305	2.01	(1.64- 2.48)
TIZZAN	22	f	0	25	28	25	114	4.07	(2.04- 8.13)
Subtotal TIZZAN								2.13	(1.75- 2.60)
*TVERDA	3	m	2	-	-	-	-	3.83	(2.47- 5.95)
*TVERDA	15	f	2	-	-	-	-	11.05	(3.33- 36.71)
Subtotal TVERDA								4.34	(2.87- 6.56)
*WALD	4	m	1	-	-	-	-	16.40	(7.55- 44.20)
WIGLE	28	m	1	-	-	-	-	12.40	(7.21- 21.31)
WIGLE	4	f	0	67	169	36	439	4.83	(3.11- 7.52)
Subtotal WIGLE								7.04	(5.00- 9.92)
WYNDE7	1	m	0	1107	993	64	918	15.99	(12.24- 20.89)
*XIANGZ	9	m	2	-	-	-	-	1.70	(1.14- 2.54)
Partial Totals				8877	49387	846	68335		

*prospective study

REF	NRR	SEX	AD	Ys	Ws	Qs	Ps
ABELIN	47	m	1	3.99	1.59	9.49	0.0000
AGUDO	3	f	3	1.28	5.53	0.37	0.0025
ALDERS	174	m	0	2.56	12.18	12.73	0.0000
ALDERS	176	f	0	1.76	41.23	1.93	0.0000
Subtotal ALDERS				1.94	53.41	14.66	
ARMADA	1	m	0	2.99	3.64	7.64	0.0000
BAND	1	m	2	2.30	42.76	24.50	0.0000
*BEST	2	m	1	2.70	6.85	9.22	0.0000
*BEST	18	f	1	0.81	2.17	1.17	0.2348
Subtotal BEST				2.25	9.02	10.40	
BOFFET	3	m	2	2.70	102.96	138.47	0.0000
*BOUCOT	114	m	2	4.13	0.50	3.33	0.0036
BRESLO	18	m	0	2.11	5.74	1.88	0.0000
BRESLO	24	f	0	0.32	3.10	4.62	0.5717
Subtotal BRESLO				1.49	8.84	6.50	
*CEDERL	116	m	2	2.13	20.90	7.28	0.0000
*CEDERL	41	f	1	1.50	4.47	0.01	0.0015
Subtotal CEDERL				2.02	25.38	7.29	
*CHOW	16	m	0	2.91	5.53	10.30	0.0000
*CPSI	76	m	1	2.51	200.08	187.46	0.0000
*CPSI	152	f	1	1.28	205.94	14.61	0.0000
Subtotal CPSI				1.88	406.02	202.06	
*CPSII	126	m	1	3.01	84.90	182.59	0.0000
DAMBER	16	m	1	2.28	19.52	10.71	0.0000
DEAN	4	m	0	1.67	9.54	0.16	0.0000
DEAN2	9	m	0	1.44	23.37	0.26	0.0000
DEAN2	17	f	0	1.08	14.24	3.06	0.0000
Subtotal DEAN2				1.30	37.61	3.32	
DEAN3	35	m	3	2.14	18.17	6.45	0.0000
DEAN3	119	f	3	1.75	20.79	0.93	0.0000
Subtotal DEAN3				1.93	38.95	7.38	
DOLL	13	m	0	2.28	6.20	3.34	0.0000
*DOLL2	2	m	1	2.70	18.04	24.35	0.0000
*DOLL2	63	f	1	2.16	3.28	1.24	0.0001
Subtotal DOLL2				2.62	21.32	25.59	
*DORN	3	m	2	2.45	308.59	255.17	0.0000
*ENGELA	210	m	1	2.25	6.37	3.21	0.0000
*ENGELA	225	f	1	1.76	6.50	0.30	0.0000
Subtotal ENGELA				2.00	12.87	3.51	
*ENSTRO	1	m	1	2.56	81.91	85.64	0.0000
*ENSTRO	2	f	1	1.94	181.45	28.61	0.0000
Subtotal ENSTRO				2.13	263.36	114.25	
GARDIN	6	c	0	2.72	3.79	5.24	0.0000
GOLLED	6	m	1	2.03	13.89	3.38	0.0000
GRAHAM	2	m	0	2.16	16.04	6.17	0.0000
*HAMMON	139	m	1	2.44	14.07	11.46	0.0000
*HEIN	1	m	0	3.12	0.98	2.43	0.0020
JOLY	48	m	0	2.62	10.80	12.67	0.0000
JUSSAW	31	m	2	2.16	8.36	3.16	0.0000
*KAISE2	68	m	1	2.08	10.65	3.14	0.0000
*KAISE2	60	f	1	2.67	8.78	11.24	0.0000
Subtotal KAISE2				2.35	19.43	14.37	

Table 1C6 - 2

IESLC - Meta-anal of Current Smoking (or Ever if Current not available), Cigarettes only
All LC types
Most adjusted

REF	NRR	SEX	AD	Ys	Ws	Qs	Ps
KJUUS	3	m	0	2.66	1.80	2.24	0.0004
KOULUM	2	m	0	3.38	4.45	15.11	0.0000
LIU4	10	m	2	1.36	5780.52	199.63	0.0000
LOMBAR	5	m	0	2.63	11.54	13.67	0.0000
LUBIN2	2	m	2	2.42	159.81	122.39	0.0000
LUBIN2	98	f	1	1.36	133.31	4.35	0.0000
Subtotal LUBIN2				1.94	293.11	126.74	
MCCONN	16	c	0	0.23	5.66	9.74	0.5832
*MIGRAN	10	m	2	1.44	3.87	0.04	0.0048
*MIGRAN	36	f	2	1.63	3.41	0.03	0.0026
Subtotal MIGRAN				1.53	7.28	0.07	
MILLS	1	m	1	0.24	71.48	120.70	0.0406
NOTAN2	19	m	2	0.86	33.41	15.59	0.0000
PERNU	8	m	0	2.23	50.02	23.45	0.0000
PERNU	4	f	0	0.95	5.14	1.82	0.0321
Subtotal PERNU				2.11	55.16	25.28	
PEZZOT	5	m	0	3.48	3.66	13.81	0.0000
RONCO	3	m	2	1.69	5.06	0.11	0.0001
SADOWS	28	m	1	1.50	8.54	0.02	0.0000
STASZE	2	m	0	2.52	4.70	4.54	0.0000
SUZUK2	7	c	3	2.40	2.76	2.02	0.0001
TIZZAN	2	m	0	0.70	90.61	64.12	0.0000
TIZZAN	22	f	0	1.40	8.03	0.15	0.0001
Subtotal TIZZAN				0.76	98.64	64.27	
*TVERDA	3	m	2	1.34	19.88	0.79	0.0000
*TVERDA	15	f	2	2.40	2.67	1.98	0.0001
Subtotal TVERDA				1.47	22.55	2.76	
*WALD	4	m	1	2.80	4.92	7.76	0.0000
WIGLE	28	m	1	2.52	13.08	12.46	0.0000
WIGLE	4	f	0	1.58	19.65	0.02	0.0000
Subtotal WIGLE				1.95	32.73	12.49	
WYNDE7	1	m	0	2.77	53.69	81.27	0.0000
*XIANGZ	9	m	2	0.53	23.94	24.47	0.0094

	N	68
	NS	51
	Wt	8081.01
Het	Chi	1842.18
Het	df	67
Het	P	***
Fixed	RR	4.67
	RRl	4.57
	RRu	4.78
	P	+++
Random	RR	7.44
	RRl	6.21
	RRu	8.91
	P	+++
Asymm	P	***

Table 1C6 - 3

IESLC - Meta-anal of Current Smoking (or Ever if Current not available), Cigarettes only
All LC types
Most adjusted

	Sex			
	combined	male	female	Total
N	3	47	18	68
NS	3	47	18	68
Wt	12.21	7399.12	669.68	8081.01
Het Chi	16.97	1748.75	76.39	1842.18
Het df	2	46	17	67
Het P	***	***	***	***
Fixed RR	4.44	4.67	4.71	4.67
RRl	2.54	4.56	4.37	4.57
RRu	7.79	4.78	5.08	4.78
P	+++	+++	+++	+++
Random RR	5.76	9.04	4.78	7.44
RRl	1.08	7.10	3.85	6.21
RRu	30.72	11.51	5.92	8.91
P	+	+++	+++	+++
Between Chi				0.08
Between df				2
Between P				N.S.
Btwn(F) P				N.S.
Btwn(R) P				***

	Smoking status		
	ever	current	Total
N	30	38	68
NS	26	27	53
Wt	6477.80	1603.21	8081.01
Het Chi	516.76	392.42	1842.18
Het df	29	37	67
Het P	***	***	***
Fixed RR	3.95	9.25	4.67
RRl	3.85	8.81	4.57
RRu	4.04	9.71	4.78
P	+++	+++	+++
Random RR	5.31	9.52	7.44
RRl	4.13	7.89	6.21
RRu	6.83	11.49	8.91
P	+++	+++	+++
Between Chi			933.00
Between df			1
Between P			***
Btwn(F) P			***
Btwn(R) P			***

	Study LIU4		
	LIU4	others	Total
N	1	67	68
NS	1	50	51
Wt	5780.52	2300.49	8081.01
Het Chi	0.00	1140.93	1842.18
Het df	0	66	67
Het P	N.S.	***	***
Fixed RR	3.88	7.45	4.67
RRl	3.78	7.15	4.57
RRu	3.98	7.76	4.78
P	+++	+++	+++
Random RR	3.88	7.55	7.44
RRl	3.78	6.25	6.21
RRu	3.98	9.12	8.91
P	+++	+++	+++
Between Chi			701.25
Between df			1
Between P			***
Btwn(F) P			***
Btwn(R) P			***

Table 1C6 - 4

IESLC - Meta-anal of Current Smoking (or Ever if Current not available), Cigarettes only
All LC types
Least adjusted

REF	NRR	X	SEX	AGEL	AGEH	RACE	YF	LC TYPE	LOC	START	ST	NLC	R	VB	P	H	AD	SM	PRODUCT	DENOM	De
ABELIN	4	x	m	0	0	all	-	all	Eu:wst	1941	CC	118	n	bl	y	n	0	ev	cig only	nev any	st
AGUDO	10	x	f	0	0	all	-	all	Eu:wst	1989	CC	103	n	bl	n	n	0	cu	cig only	nev any	st
ALDERS	174		m	0	0	all	-	all	Eu:UK	1977	CC	1448	n	V	n	n	0	cu	cig only	nev any	st
ALDERS	176		f	0	0	all	-	all	Eu:UK	1977	CC	1448	n	V	n	n	0	cu	cig only	nev any	st
ARMADA	1		m	0	0	all	-	all	Eu:wst	1986	CC	325	n	bl	n	y	0	ev	cig only	nev any	st
BAND	1		m	0	0	all	-	all	NAmer	1983	CC	2831	n	V	y	y	2	ev	cig only	nev any	ot
BEST	2		m	0	0	all	0	all	NAmer	1955	pr	381	n	V	n	n	1	cu	cig only	nev any	ot
BEST	18		f	0	0	all	0	all	NAmer	1955	pr	381	n	V	n	n	1	ev	cig only	nev any	ot
BOFFET	6	x	m	0	0	all	-	all	Eu:mul	1988	CC	5621	n	bl	y	n	0	ev	cig only	nev any	st
BOUCOT	2	x	m	0	0	all	0	all	NAmer	1951	pr	121	n	bl	n	n	0	cu	cig only	nev any	ot
BRESLO	18		m	0	0	all	-	all	NAmer	1949	CC	518	n	bl	n	y	0	ev	cig only	nev+1	st
BRESLO	24		f	0	0	all	-	all	NAmer	1949	CC	518	n	bl	n	y	0	ev	cig only	nev+1	st
CEDERL	116		m	0	0	all	0	all	Eu:Sca	1963	pr	491	n	bl	n	n	2	cu	cig only	nev any	or
CEDERL	31	x	f	0	0	all	10	all	Eu:Sca	1963	pr	491	n	bl	n	n	0	cu	cig only	nev any	st
CHOW	16		m	0	0	wh	0	all	NAmer	1966	pr	219	n	bl	n	n	0	cu	cig only	nev any	st
CPSI	76		m	0	0	all	0	all	NAmer	1959	pr	5138	n	bl	n	n	1	cu	cig only	nev any	st
CPSI	152		f	0	0	all	0	all	NAmer	1959	pr	5138	n	bl	n	n	1	cu	cig only	nev any	st
CPSII	36	x	m	0	0	all	6	all	NAmer	1982	pr	3229	n	bl	n	n	0	cu	cig only	nev any	st
DAMBER	16		m	0	0	all	-	all	Eu:Sca	1972	CC	579	n	bl	y	n	1	cu	cig only	nev any	ot
DEAN	4		m	0	0	wh	-	all	Africa	1947	CC	603	n	V	y	n	0	ev	cig only	nev any	st
DEAN2	9		m	0	0	all	-	all	Eu:UK	1960	CC	954	n	V	y	n	0	ev	cig only	nev any	st
DEAN2	17		f	0	0	all	-	all	Eu:UK	1960	CC	954	n	V	y	n	0	ev	cig only	nev any	st
DEAN3	33	x	m	0	0	all	-	all	Eu:UK	1969	CC	766	n	V	y	n	0	cu	cig only	nev any	st
DEAN3	117	x	f	0	0	all	-	all	Eu:UK	1969	CC	766	n	V	y	n	0	cu	cig only	nev any	st
DOLL	13		m	0	0	all	-	all	Eu:UK	1948	CC	1465	n	V	n	n	0	ev	cig only	nev any	st
DOLL2	2		m	0	0	all	0	all	Eu:UK	1951	pr	920	n	V	n	n	1	cu	cig only	nev any	ot
DOLL2	63		f	0	0	all	22	all	Eu:UK	1951	pr	920	n	V	n	n	1	cu	cig only	nev any	ot
DORN	3		m	0	0	wh	0	all	NAmer	1954	pr	5097	n	bl	n	n	2	cu	cig only	nev any	or
ENGELA	210		m	0	0	all	12	all	Eu:Sca	1964	pr	435	n	bl	n	n	1	cu	cig only	nev any	ot
ENGELA	225		f	0	0	all	12	all	Eu:Sca	1964	pr	435	n	bl	n	n	1	cu	cig only	nev any	ot
ENSTRO	1		m	0	0	all	0	all	NAmer	1959	pr	2879	n	bl	n	n	1	cu	cig only	nev any	or
ENSTRO	2		f	0	0	all	0	all	NAmer	1959	pr	2879	n	bl	n	n	1	cu	cig only	nev any	or
GARDIN	6		c	0	0	all	-	all	Eu:UK	1988	CC	143	n	V	y	n	0	cu	cig only	nev any	st
GOLLED	20	x	m	35	99	all	-	all	Eu:UK	1952	CC	443	n	V	y	n	0	ev	cig only	nev any	st
GRAHAM	2		m	0	0	wh	-	all	NAmer	1956	CC	685	n	bl	n	n	0	cu	cig only	nev any	st
HAMMON	139		m	0	0	wh	0	all	NAmer	1952	pr	448	n	bl	n	n	1	cu	cig only	nev any	ot
HEIN	1		m	0	0	all	0	all	Eu:Sca	1970	pr	144	n	bl	n	n	0	cu	cig only	nev any	st
JOLY	48		m	0	0	all	-	all	SCAmer	1978	CC	826	n	bl	n	n	0	ev	cig only	nev any	st
JUSSAW	2	x	m	0	0	all	-	all	As:Ind	1964	CC	792	n	V	n	n	0	ev	cig only	nev any	st
KAISE2	68		m	35	99	all	9	all	NAmer	1979	pr	318	n	bl	n	n	1	cu	cig only	nev any	st
KAISE2	60		f	35	99	all	9	all	NAmer	1979	pr	318	n	bl	n	n	1	cu	cig only	nev any	st
KJUUS	3		m	0	0	all	-	all	Eu:Sca	1979	CC	176	n	bl	n	n	0	ev	cig only	nev any	st
KOULUM	2		m	0	0	all	-	all	Eu:Sca	1936	CC	812	n	bl	n	n	0	ev	cig only	nev any	st
LIU4	10		m	35	69	all	-	all	As:Chi	1986	CC	1000-00	n	ot	y	n	2	ev	cig only	nev any	ot
LOMBAR	5		m	0	0	all	-	all	NAmer	1951	CC	1040	n	bl	n	n	0	cu	cig only	nev any	st
LUBIN2	1	x	m	0	0	all	-	all	Eu:mul	1976	CC	7804	n	bl	n	y	0	cu	cig only	nev any	st
LUBIN2	97	x	f	0	0	all	-	all	Eu:mul	1976	CC	7804	n	bl	n	y	0	ev	cig only	nev any	st
MCCONN	16		c	0	0	all	-	all	Eu:UK	1946	CC	100	n	V	n	y	0	ev	cig only	nev any	st
MIGRAN	9	x	m	0	0	all	0	all	Eu:UK	1964	pr	259	n	V	n	n	0	cu	cig only	nev any	st
MIGRAN	35	x	f	0	0	all	0	all	Eu:UK	1964	pr	259	n	V	n	n	0	cu	cig only	nev any	st
MILLS	1		m	0	0	wh	-	all	NAmer	1940	CC	444	n	bl	y	n	1	ev	cig only	nev any	ot
NOTAN2	7	x	m	0	0	all	-	all	As:Ind	1963	CC	683	n	V	n	n	0	ev	cig only	nev any	st
PERNU	8		m	0	0	all	-	all	Eu:Sca	1944	CC	1606	n	bl	n	n	0	ev	cig only	nev any	st
PERNU	4		f	0	0	all	-	all	Eu:Sca	1944	CC	1606	n	bl	n	n	0	ev	cig only	nev any	st
PEZZOT	5		m	0	0	all	-	all	SCAmer	1987	CC	215	n	bl	n	y	0	cu	cig only	nev cigs	st
RONCO	2	x	m	0	0	all	-	all	Eu:wst	1976	CC	126	n	bl	y	n	0	ev	cig only	nev any	st
SADOWS	1	x	m	0	0	wh	-	all	NAmer	1938	CC	477	n	bl	n	n	0	ev	cig only	nev any	st
STASZE	2		m	0	0	all	-	all	Eu:est	1954	CC	281	n	bl	n	y	0	ev	cig only	nev any	st
SUZUK2	3	x	c	0	0	all	-	all	SCAmer	1991	CC	123	n	bl	n	y	0	ev	cig only	nev any	st
TIZZAN	2		m	0	0	all	-	all	Eu:wst	1959	CC	1358	n	bl	n	n	0	ev	cig only	nev any	st
TIZZAN	22		f	0	0	all	-	all	Eu:wst	1959	CC	1358	n	bl	n	n	0	ev	cig only	nev any	st
TVERDA	3		m	0	0	all	0	all	Eu:Sca	1972	pr	238	n	bl	n	n	2	cu	cig only	nev cigs	ot
TVERDA	15		f	0	0	all	0	all	Eu:Sca	1972	pr	238	n	bl	n	n	2	cu	cig only	nev cigs	ot
WALD	2	x	m	0	0	all	0	all	Eu:UK	1975	pr	102	n	V	n	n	0	cu	cig only	nev any	st
WIGLE	1	x	m	0	0	all	-	all	NAmer	1971	CC	728	n	V	n	n	0	cu	cig only	nev any	st
WIGLE	4		f	0	0	all	-	all	NAmer	1971	CC	728	n	V	n	n	0	cu	cig only	nev any	st
WYNDE7	1		m	0	0	all	-	all	NAmer	1977	CC	2085	n	bl	n	y	0	cu	cig only	nev any	st
XIANGZ	1	x	m	0	0	all	0	all	As:Chi	1976	pr	983	m	ot	n	n	0	ev	cig only	nev any	st

Table 1C6 - 4

IESLC - Meta-anal of Current Smoking (or Ever if Current not available), Cigarettes only
All LC types
Least adjusted

Cigarette type is all/unspec for all RRs
except for the following:

REF	NRR	CIGTYPE
ALDERS	174	MC only
ALDERS	176	MC only
DEAN3	33	MC only
DEAN3	117	MC only
GARDIN	6	MC only
JUSSAW	2	MC only
NOTAN2	7	MC only
PERNU	8	MC only
PERNU	4	MC only
SUZUK2	3	MC only

Table 1C6 - 5

IESLC - Meta-anal of Current Smoking (or Ever if Current not available), Cigarettes only
All LC types
Least adjusted

REF	NRR	SEX	AD	Number Exposed Case	Number Exposed Cont	Non-exposed Case	Non-exposed Cont	RR	95.00%CI
ABELIN	4	m	0	30	90	2	183	30.50	(7.13- 130.47)
AGUDO	10	f	0	20	17	80	183	2.69	(1.34- 5.41)
ALDERS	174	m	0	312	213	15	133	12.99	(7.41- 22.77)
ALDERS	176	f	0	410	229	75	243	5.80	(4.27- 7.87)
Subtotal ALDERS								6.97	(5.33- 9.12)
ARMADA	1	m	0	245	197	4	64	19.90	(7.12- 55.59)
BAND	1	m	2	-	-	-	-	9.96	(7.38- 13.44)
*BEST	2	m	1	-	-	-	-	14.91	(7.05- 31.52)
*BEST	18	f	1	-	-	-	-	2.24	(0.59- 8.44)
Subtotal BEST								9.45	(4.92- 18.15)
BOFFET	6	m	0	4204	3930	117	1750	16.00	(13.20- 19.39)
*BOUCOT	2	m	0	85	22177	0	7551	58.23~	(3.61- 938.34)
BRESLO	18	m	0	316	229	7	42	8.28	(3.65- 18.76)
BRESLO	24	f	0	13	11	12	14	1.38	(0.45- 4.20)
Subtotal BRESLO								4.42	(2.28- 8.54)
*CEDERL	116	m	2	-	-	-	-	8.43	(5.49- 12.94)
*CEDERL	31	f	0	8	4709	19	17679	1.58	(0.69- 3.61)
Subtotal CEDERL								5.91	(4.04- 8.64)
*CHOW	16	m	0	71	40726	6	62913	18.28	(7.95- 42.06)
*CPSI	76	m	1	-	-	-	-	12.30	(10.71- 14.13)
*CPSI	152	f	1	-	-	-	-	3.58	(3.12- 4.10)
Subtotal CPSI								6.58	(5.97- 7.25)
*CPSII	36	m	0	1781	583646	124	742207	18.26	(15.23- 21.91)
DAMBER	16	m	1	-	-	-	-	9.80	(6.30- 15.30)
DEAN	4	m	0	403	385	12	61	5.32	(2.82- 10.04)
DEAN2	9	m	0	629	508	33	112	4.20	(2.80- 6.30)
DEAN2	17	f	0	62	29	88	121	2.94	(1.75- 4.94)
Subtotal DEAN2								3.67	(2.67- 5.05)
DEAN3	33	m	0	337	930	25	510	7.39	(4.86- 11.26)
DEAN3	117	f	0	102	1158	41	1538	3.30	(2.28- 4.79)
Subtotal DEAN3								4.70	(3.56- 6.20)
DOLL	13	m	0	1004	899	7	61	9.73	(4.43- 21.39)
*DOLL2	2	m	1	-	-	-	-	14.93	(9.41- 23.68)
*DOLL2	63	f	1	-	-	-	-	8.65	(2.93- 25.55)
Subtotal DOLL2								13.73	(8.98- 20.99)
*DORN	3	m	2	-	-	-	-	11.60	(10.40- 13.00)
*ENGELA	210	m	1	-	-	-	-	9.50	(4.37- 20.66)
*ENGELA	225	f	1	-	-	-	-	5.80	(2.69- 12.51)
Subtotal ENGELA								7.40	(4.29- 12.78)
*ENSTRO	1	m	1	-	-	-	-	12.99	(10.46- 16.13)
*ENSTRO	2	f	1	-	-	-	-	6.95	(6.01- 8.04)
Subtotal ENSTRO								8.44	(7.48- 9.53)
GARDIN	6	c	0	72	39	5	41	15.14	(5.53- 41.44)
GOLLED	20	m	0	344	1791	15	490	6.27	(3.71- 10.62)
GRAHAM	2	m	0	371	821	18	346	8.69	(5.32- 14.17)
*HAMMON	139	m	1	-	-	-	-	11.52	(6.83- 19.42)
*HEIN	1	m	0	45	912	1	457	22.55	(3.12- 163.06)
JOLY	48	m	0	379	499	12	218	13.80	(7.60- 25.05)
JUSSAW	2	m	0	126	77	149	624	6.85	(4.90- 9.58)
*KAISE2	68	m	1	-	-	-	-	8.04	(4.41- 14.66)
*KAISE2	60	f	1	-	-	-	-	14.48	(7.47- 28.04)
Subtotal KAISE2								10.49	(6.72- 16.36)
KJUUS	3	m	0	151	127	2	24	14.27	(3.31- 61.54)
KOULUM	2	m	0	625	229	5	54	29.48	(11.65- 74.60)
LIU4	10	m	2	-	-	-	-	3.88	(3.78- 3.98)
LOMBAR	5	m	0	432	249	14	112	13.88	(7.79- 24.72)
LUBIN2	1	m	0	5243	6835	190	2617	10.57	(9.08- 12.29)
LUBIN2	97	f	0	548	559	336	1188	3.47	(2.93- 4.10)
Subtotal LUBIN2								6.43	(5.74- 7.19)
MCCONN	16	c	0	68	138	9	23	1.26	(0.55- 2.87)
*MIGRAN	9	m	0	137	3707	4	867	8.01	(2.97- 21.59)
*MIGRAN	35	f	0	23	2749	4	3814	7.98	(2.76- 23.04)
Subtotal MIGRAN								8.00	(3.88- 16.50)
MILLS	1	m	1	-	-	-	-	1.27	(1.01- 1.61)
NOTAN2	7	m	0	78	129	134	544	2.45	(1.75- 3.44)
PERNU	8	m	0	706	216	97	275	9.27	(7.02- 12.23)
PERNU	4	f	0	7	24	110	971	2.57	(1.08- 6.11)
Subtotal PERNU								8.22	(6.32- 10.71)
PEZZOT	5	m	0	145	129	4	116	32.60	(11.70- 90.81)
RONCO	2	m	0	116	274	6	78	5.50	(2.33- 12.98)
SADOWS	1	m	0	273	328	18	81	3.75	(2.19- 6.40)

Table 1C6 - 5

IESLC - Meta-anal of Current Smoking (or Ever if Current not available), Cigarettes only
All LC types
Least adjusted

REF	NRR	SEX	AD	Number Exposed Case	Cont	Non-exposed Case	Cont	RR	95.00%CI
STASZE	2	m	0	218	552	5	158	12.48	(5.05- 30.82)
SUZUK2	3	c	0	82	63	11	53	6.27	(3.03- 12.98)
TIZZAN	2	m	0	994	836	180	305	2.01	(1.64- 2.48)
TIZZAN	22	f	0	25	28	25	114	4.07	(2.04- 8.13)
Subtotal TIZZAN								2.13	(1.75- 2.60)
*TVERDA	3	m	2	-	-	-	-	3.83	(2.47- 5.95)
*TVERDA	15	f	2	-	-	-	-	11.05	(3.33- 36.71)
Subtotal TVERDA								4.34	(2.87- 6.56)
*WALD	2	m	0	77	4182	7	6539	17.20	(7.94- 37.25)
WIGLE	1	m	0	415	415	15	204	13.60	(7.91- 23.38)
WIGLE	4	f	0	67	169	36	439	4.83	(3.11- 7.52)
Subtotal WIGLE								7.31	(5.19- 10.30)
WYNDE7	1	m	0	1107	993	64	918	15.99	(12.24- 20.89)
*XIANGZ	1	m	0	289	8011	25	974	1.41	(0.94- 2.10)
Partial Totals				23195	695164	2168	858009		

*prospective study ~ With 0.5 adjustment for zero

REF	NRR	SEX	AD	Ys	Ws	Qs	Ps
ABELIN	4	m	0	3.42	1.82	6.40	0.0000
AGUDO	10	f	0	0.99	7.89	2.41	0.0054
ALDERS	174	m	0	2.56	12.18	12.72	0.0000
ALDERS	176	f	0	1.76	41.23	1.92	0.0000
Subtotal ALDERS				1.94	53.41	14.63	
ARMADA	1	m	0	2.99	3.64	7.63	0.0000
BAND	1	m	2	2.30	42.76	24.45	0.0000
*BEST	2	m	1	2.70	6.85	9.21	0.0000
*BEST	18	f	1	0.81	2.17	1.18	0.2348
Subtotal BEST				2.25	9.02	10.39	
BOFFET	6	m	0	2.77	104.05	157.48	0.0000
*BOUCOT	2	m	0	4.06	0.50	3.16	0.0042
BRESLO	18	m	0	2.11	5.74	1.87	0.0000
BRESLO	24	f	0	0.32	3.10	4.62	0.5717
Subtotal BRESLO				1.49	8.84	6.50	
*CEDERL	116	m	2	2.13	20.90	7.26	0.0000
*CEDERL	31	f	0	0.46	5.64	6.63	0.2769
Subtotal CEDERL				1.78	26.54	13.89	
*CHOW	16	m	0	2.91	5.53	10.29	0.0000
*CPSI	76	m	1	2.51	200.08	187.20	0.0000
*CPSI	152	f	1	1.28	205.94	14.68	0.0000
Subtotal CPSI				1.88	406.02	201.88	
*CPSII	36	m	0	2.90	115.97	215.33	0.0000
DAMBER	16	m	1	2.28	19.52	10.69	0.0000
DEAN	4	m	0	1.67	9.54	0.16	0.0000
DEAN2	9	m	0	1.44	23.37	0.27	0.0000
DEAN2	17	f	0	1.08	14.24	3.07	0.0000
Subtotal DEAN2				1.30	37.61	3.33	
DEAN3	33	m	0	2.00	21.74	4.56	0.0000
DEAN3	117	f	0	1.20	28.00	3.37	0.0000
Subtotal DEAN3				1.55	49.74	7.94	
DOLL	13	m	0	2.28	6.20	3.33	0.0000
*DOLL2	2	m	1	2.70	18.04	24.32	0.0000
*DOLL2	63	f	1	2.16	3.28	1.24	0.0001
Subtotal DOLL2				2.62	21.32	25.56	
*DORN	3	m	2	2.45	308.59	254.80	0.0000
*ENGELA	210	m	1	2.25	6.37	3.20	0.0000
*ENGELA	225	f	1	1.76	6.50	0.30	0.0000
Subtotal ENGELA				2.00	12.87	3.50	
*ENSTRO	1	m	1	2.56	81.91	85.53	0.0000
*ENSTRO	2	f	1	1.94	181.45	28.51	0.0000
Subtotal ENSTRO				2.13	263.36	114.04	
GARDIN	6	c	0	2.72	3.79	5.23	0.0000
GOLLED	20	m	0	1.84	13.86	1.20	0.0000
GRAHAM	2	m	0	2.16	16.04	6.15	0.0000
*HAMMON	139	m	1	2.44	14.07	11.44	0.0000
*HEIN	1	m	0	3.12	0.98	2.43	0.0020
JOLY	48	m	0	2.62	10.80	12.65	0.0000
JUSSAW	2	m	0	1.92	34.20	5.00	0.0000
*KAISE2	68	m	1	2.08	10.65	3.13	0.0000
*KAISE2	60	f	1	2.67	8.78	11.22	0.0000
Subtotal KAISE2				2.35	19.43	14.35	

Table 1C6 - 5

IESLC - Meta-anal of Current Smoking (or Ever if Current not available), Cigarettes only
All LC types
Least adjusted

REF	NRR	SEX	AD	Ys	Ws	Qs	Ps
KJUUS	3	m	0	2.66	1.80	2.24	0.0004
KOULUM	2	m	0	3.38	4.45	15.10	0.0000
LIU4	10	m	2	1.36	5780.52	201.06	0.0000
LOMBAR	5	m	0	2.63	11.54	13.66	0.0000
LUBIN2	1	m	0	2.36	167.16	111.10	0.0000
LUBIN2	97	f	0	1.24	134.56	12.05	0.0000
Subtotal LUBIN2				1.86	301.72	123.16	
MCCONN	16	c	0	0.23	5.66	9.75	0.5832
*MIGRAN	9	m	0	2.08	3.91	1.13	0.0000
*MIGRAN	35	f	0	2.08	3.41	0.97	0.0001
Subtotal MIGRAN				2.08	7.32	2.11	
MILLS	1	m	1	0.24	71.48	120.83	0.0406
NOTAN2	7	m	0	0.90	33.47	13.90	0.0000
PERNU	8	m	0	2.23	50.02	23.41	0.0000
PERNU	4	f	0	0.95	5.14	1.83	0.0321
Subtotal PERNU				2.11	55.16	25.24	
PEZZOT	5	m	0	3.48	3.66	13.80	0.0000
RONCO	2	m	0	1.71	5.21	0.14	0.0001
SADOWS	1	m	0	1.32	13.40	0.66	0.0000
STASZE	2	m	0	2.52	4.70	4.53	0.0000
SUZUK2	3	c	0	1.84	7.25	0.63	0.0000
TIZZAN	2	m	0	0.70	90.61	64.22	0.0000
TIZZAN	22	f	0	1.40	8.03	0.15	0.0001
Subtotal TIZZAN				0.76	98.64	64.37	
*TVERDA	3	m	2	1.34	19.88	0.79	0.0000
*TVERDA	15	f	2	2.40	2.67	1.97	0.0001
Subtotal TVERDA				1.47	22.55	2.76	
*WALD	2	m	0	2.84	6.43	10.91	0.0000
WIGLE	1	m	0	2.61	13.09	14.92	0.0000
WIGLE	4	f	0	1.58	19.65	0.02	0.0000
Subtotal WIGLE				1.99	32.74	14.95	
WYNDE7	1	m	0	2.77	53.69	81.19	0.0000
*XIANGZ	1	m	0	0.34	23.64	34.15	0.0980

	N	68
	NS	51
	Wt	8172.96
Het	Chi	1911.36
Het	df	67
Het	P	***
Fixed	RR	4.68
	RRl	4.58
	RRu	4.78
	P	+++
Random	RR	7.13
	RRl	5.96
	RRu	8.54
	P	+++
Asymm	P	**

Table 1C6 - 6

IESLC - Meta-anal of Current Smoking (or Ever if Current not available), Cigarettes only
All LC types
Least adjusted

		Sex		
	combined	male	female	Total
N	3	47	18	68
NS	3	47	18	68
Wt	16.71	7474.57	681.68	8172.96
Het Chi	15.56	1799.46	94.59	1911.36
Het df	2	46	17	67
Het P	***	***	***	***
Fixed RR	4.44	4.70	4.46	4.68
RRl	2.75	4.59	4.13	4.58
RRu	7.18	4.80	4.80	4.78
P	+++	+++	+++	+++
Random RR	4.83	8.90	4.31	7.13
RRl	1.24	7.01	3.42	5.96
RRu	18.86	11.30	5.42	8.54
P	+	+++	+++	+++
Between Chi				1.76
Between df				2
Between P				N.S.
Btwn(F) P				N.S.
Btwn(R) P				***

Table 1C6 - 7

IESLC - Meta-anal of Current Smoking (or Ever if Current not available), Cigarettes only
All LC types
Excluded studies (and stage at which they were excluded)

1	BOUCHA BUELL LAURIL MZILEN
2	BERRIN CHEN RESTRE TAO ULMER
4	ABRAHA AKIBA AMANDU AMES ANDERS ARCHER AUSTIN AUVINE AXELSO AXELSS BARBON BECHER BENSHL BLOHMK BLOT1 BLOT2 BLOT3 BLOT4 BRETT BROCKM BROSS BROWN1 BROWN2 BUFFLE BYERS1 BYERS2 CARPEN CASCO2 CASCOR CHAN CHANG CHATZI CHEN2 CHEN3 CHIAZZ CHOI CHYOU COMSTO COOKSO CORREA DARBY DAVEYS DEKLER DESTE2 DESTEF DOCKER DORANT DORGAN DOSEME DROSTE DU DUNN EBELIN ESAKI FAN GAO GAO2 GARCIA GARSHI GENG GER GILLIS GODLEY GOODMA GREGOR GSELL HAENSZ HAMMO2 HANSEN HEGMAN HENNEK HINDS HIRAOK HIRAYA HITOSU HOLE HOROWI HORWIT HU HU2 HUANG HUMBLE ISHIMA JAHN JAIN JARUP JARVHO JEDRYC JIANG JONES KAISER KANELL KATSOU KAUFMA KELLER KHUDER KIHARA KINLEN KNEKT KO KOHLME KOO KREUZE KREYBE KUBIK LAMTH LAMWK LAMWK2 LAUSSM LEI LEMARC LETOUR LEVIN LIAW LICKIN LIDDEL LIU LIU2 LIU3 LIU5 LOMBA2 LUO MACLEN MAGNUS MARSH MARSH2 MARTIS MASTRA MATOS MATSUD MCDUFF MCLAUG MILLER MOLLO MRFIT MRFITR MURATA NAM NOTANI NOU ODRISC ORMOS OSANN OSANN2 PARKIN PASTOR PAWLEG PERSH2 PERSHA PETO PEZZO2 PIKE PISANI POFFIJ POLEDN PRESCO QIAO2 RACHTA RADZIK RANDIG REN ROOTS ROTHSC SAARIK SANKAR SCHWAR SEGI SEGI2 SEOW SHAW SHIMIZ SIEMIA SIMARA SIMONA SITAS SOBUE SOBUE2 SPEIZE SPITZ STAYNE STOCKS STOCKW STUCKE SUN SUZUKI SVENSS TANG TENKAN TOKARS TOUSEY TSUGAN TULINI VANDER VUTUC WAKAI WANG WANG2 WANG3 WANG4 WARSIN WATSON WICKLU WILKIN WU WU2 WUNSCH WUWILL WYNDE2 WYNDE3 WYNDE4 WYNDE5 WYNDE6 WYNDE8 XU XU2 XU3 XU4 YAMAGU YONG YUAN ZHANG ZHENG ZHOU
5	LANGE RIMING TANG2
6	HIRAY2 SCHWA2 WYNDER
10	BENHAM LUBIN QIAO VEIERO

Table 1C6 - 8
Potentially overlapping studies

REF	REFGP	PRINC	OVERLAP/LINK
LUBIN2	LUBIN2	1	Lubin-combined
TVERDA	TVERDA	1	VEIERO/TVERDAL
HEIN	PRESCO	1	Subset of PRESCO
GRAHAM	BYERS1	1	GRAHAM/BROSS/BYERS1
WALD	TANG2	1	Subset of TANG2
WYNDE7	WYNDE6	2	WYNDE5/6/7/8
CPSI	CPSI	1	CPSI overall
ENSTRO	ENSTRO	1	Subset of CPSI
BOFFET	BOFFET	1	BOFFET-combined
XIANGZ	XIANGZ	1	LUBIN/XIANGZ/QIAO
