# Supplementary material for: Systematic review with meta-analysis of the epidemiological evidence in the 1900s relating smoking to lung cancer
Source: BMC Cancer. 2012 Sep 3;12:385. doi: 10.1186/1471-2407-12-385 (PMC3505152; doi:10.1186/1471-2407-12-385)
Supplement: Additional file 5 — Detailed Analysis Tables (Individual file names as described in Additional file 1: Methods, Table1). [file 1471-2407-12-385-S5.zip › PDF/3C.pdf]

Table 3C1 -

IESLC - Meta-anal of Ever Smoking (or Current if Ever not available), Any prod (or Cigs if Any not avail)
Adenocarcinoma

This analysis is restricted to results for:
1) Non-dose-response data
2) Results complete enough for use in metaanalysis

Within each study, results are then selected (in the following order of preference, within each sex) for:
3) SMKSTA: ever smokers, current smokers
4) PRODUCT: all/unspec, cigarettes regardless of other products, cigarettes only
5) CIGTYPE: all/unspecified, MC regardless of HR, MC only
6) DENOM: never smoked anything, never smoked cigarettes, (never +1 = +long term ex, +2 = +amount unknown, +3 = never cigs+long term ex)
7) Followup period (YF, prospective studies): whole study (coded as 0) or longest available
8) LCtype: adeno or nearest available, but not squamous. (q = squamous, s = small, a = adeno, l = large, KII = Kreyberg II, al = alveolar, br = bronchiolar, u = undifferentiated)
9) Race: all or nearest available, otherwise by race (wh or w = white, bl or b = black, hi = hispanic ch = chinese, jap = japanese, haw = hawaiian, w+o = white + oriental, sca = scandinavian, as = asian)
10) For overlapping studies: principal rather than subsidiary studies
Finally by Age: whole study (coded as 0) if available, otherwise by widest available age group
and then for single sex results (m, f) in preference to combined sex results (c).

Results adjusted (AD) for the most potential confounders are then chosen in Sections -1 to -3
and results adjusted for the least confounders in Sections -4 to -6. (Those least adjusted results which
actually differ from the most adjusted as marked 'x' in column X in Section -4)
(Results adjusted for an unknown number of confounder(s) are coded as 20.)

Section -7 shows excluded studies, together with the stage (as above) at which no qualifying
results were found.

Section -8 lists the potentially overlapping studies which have been included (1=principal, 2=subsidiary).

Section -9 lists any results which would have been included in preference except that they had data not complete
enough for use in meta-analysis, with their significance (yes/no), if known, and any further comment as entered
on the database.

In addition to those mentioned above, the following fields, levels and abbreviations are used:
* or nk = not known, n = no, y = yes, ot = other
ev = ever, cu = current, nev = never
all/unspec = all or unspecified, cig+/-ot = cigarettes irrespective of other products (cigar, pipe etc)
MC = manufactured cigarettes, HR = hand-rolled cigarettes
REF: 6-character study reference
NRR: number of the RR on the database within the study
ST : study type (CC = case control, pr or prosp = prospective)
NLC: number of lung cancer cases in whole study
R : risky occupational population (n = no, m = mining, o = other risky)
VB : national cigarette type (V = at least 75% Virginia, bl = at least 75% blended, ot = other)
P : any proxy use
H : full histological confirmation
De : derivation of RR/CI (or = original, st = standard method, ot = other method of estimation)

Table 3C1 - 1

IESLC - Meta-anal of Ever Smoking (or Current if Ever not available), Any prod (or Cigs if Any not avail)
Adenocarcinoma
Most adjusted

REF	NRR	SEX	AGEL	AGEH	RACE	YF	LC TYPE	LOC	START	ST	NLC	R	VB	P	H	AD	SM	PRODUCT	DENOM	De
ABRAHA	2	m	0	0	all	0	a	Eu:est	1975	pr	571	n	bl	n	n	0	ev	all/unsp	nev any	ot
ABRAHA	5	f	0	0	all	0	a	Eu:est	1975	pr	571	n	bl	n	n	0	ev	all/unsp	nev any	ot
ALDERS	54	m	0	0	all	-	a	Eu:UK	1977	CC	1448	n	V	n	n	2	ev	all/unsp	nev any	or
ALDERS	57	f	0	0	all	-	a	Eu:UK	1977	CC	1448	n	V	n	n	1	ev	all/unsp	nev any	or
ANDERS	12	f	0	0	all	0	a	NAmer	1986	pr	343	n	bl	n	n	0	ev	cig+/-ot	nev cigs	st
BAND	2	m	0	0	all	-	a	NAmer	1983	CC	2831	n	V	y	y	2	ev	cig only	nev any	ot
BARBON	130	m	0	0	all	-	a	Eu:wst	1979	CC	755	n	bl	y	y	3	ev	all/unsp	nev any	ot
BECHER	12	f	0	0	all	-	not q+s	Eu:Ger	1985	CC	194	n	bl	n	y	1	ev	all/unsp	nev any	or
BOUCOT	147	m	0	0	all	0	a	NAmer	1951	pr	121	n	bl	n	n	2	cu	cig only	nev any	ot
BRESLO	35	c	0	0	all	-	a	NAmer	1949	CC	518	n	bl	n	y	0	ev	all/unsp	nev+1	st
BROWN1	3	m	0	0	wh	-	a	NAmer	1979	CC	102	n	bl	y	y	1	ev	cig+/-ot	nev cigs	or
BROWN1	4	f	0	0	wh	-	a	NAmer	1979	CC	102	n	bl	y	y	1	ev	cig+/-ot	nev cigs	or
BROWN2	4	m	0	0	wh	-	a	NAmer	1984	CC	14596	n	bl	n	y	2	ev	cig+/-ot	nev cigs	or
BROWN2	3	f	0	0	wh	-	a	NAmer	1984	CC	14596	n	bl	n	y	2	ev	cig+/-ot	nev cigs	or
BUFFLE	50	m	0	0	wh	-	a	NAmer	1976	CC	943	n	bl	y	n	0	ev	cig+/-ot	nev cigs	ot
BUFFLE	45	f	0	0	wh	-	a	NAmer	1976	CC	943	n	bl	y	n	0	ev	cig+/-ot	nev cigs	ot
BYERS1	3	m	0	0	wh	-	a	NAmer	1957	CC	1002	n	bl	n	n	0	ev	cig+/-ot	nev cigs	st
CHAN	12	m	0	0	all	-	a+l	As:HK	1976	CC	397	n	bl	n	n	0	ev	all/unsp	nev any	ot
CHAN	16	f	0	0	all	-	a+l	As:HK	1976	CC	397	n	bl	n	n	0	ev	all/unsp	nev any	st
CHOI	63	m	0	0	all	-	a	As:oth	1985	CC	375	n	bl	n	n	0	ev	cig+/-ot	nev cigs	st
CHOI	65	f	0	0	all	-	a	As:oth	1985	CC	375	n	bl	n	n	0	ev	cig+/-ot	nev cigs	st
COMSTO	67	m	0	0	all	-	a	NAmer	1975	ot	258	n	bl	n	n	0	ev	cig+/-ot	nev cigs	st
COMSTO	79	f	0	0	all	-	a	NAmer	1975	ot	258	n	bl	n	n	0	ev	cig+/-ot	nev cigs	st
CORREA	36	c	0	0	all	-	a	NAmer	1979	CC	1359	n	bl	y	n	1	ev	cig+/-ot	nev cigs	or
CPSI	404	m	0	0	all	2	a	NAmer	1959	pr	5138	n	bl	n	n	1	cu	cig only	nev any	ot
CPSI	406	f	0	0	all	2	a	NAmer	1959	pr	5138	n	bl	n	n	1	cu	cig only	nev any	ot
CPSII	115	m	0	0	all	2	a	NAmer	1982	pr	3229	n	bl	n	n	1	cu	cig only	nev any	st
CPSII	118	f	0	0	all	2	a	NAmer	1982	pr	3229	n	bl	n	n	1	cu	cig+/-ot	nev cigs	st
DAMBER	32	m	0	0	all	-	a+al+br	Eu:Sca	1972	CC	579	n	bl	y	n	1	ev	all/unsp	nev any	or
DESTE2	17	m	0	0	all	-	a	SCAmer	1993	CC	463	n	bl	n	n	2	ev	all/unsp	nev any	or
DOLL	87	m	0	0	all	-	KII	Eu:UK	1948	CC	1465	n	V	n	n	1	ev	all/unsp	nev any	ot
DOLL	89	f	0	0	all	-	KII	Eu:UK	1948	CC	1465	n	V	n	n	1	ev	all/unsp	nev any	ot
DORGAN	125	m	0	0	wh	-	a	NAmer	1980	CC	2026	n	bl	y	y	2	ev	cig+/-ot	nev any	or
DORGAN	104	f	0	0	all	-	a	NAmer	1980	CC	2026	n	bl	y	y	3	ev	cig+/-ot	nev any	or
DORN	340	m	0	0	wh	8	a	NAmer	1954	pr	5097	n	bl	n	n	1	cu	cig only	nev any	ot
DOSEME	4	m	0	0	all	-	not q+s	Eu:bal	1979	CC	1210	n	bl	n	n	2	ev	cig+/-ot	nev cigs	or
ENGELA	76	m	0	0	all	0	a	Eu:Sca	1964	pr	435	n	bl	n	n	7	ev	cig+/-ot	nev cigs	ot
FAN	4	c	0	0	all	-	a	As:Chi	1990	CC	403	n	ot	y	n	0	ev	cig+/-ot	nev cigs	ot
GAO	3	m	0	0	all	-	a	As:Chi	1984	CC	1405	n	ot	n	n	2	ev	cig+/-ot	nev cigs	or
GAO	13	f	0	0	all	-	a	As:Chi	1984	CC	1405	n	ot	n	n	2	ev	cig+/-ot	nev cigs	or
GER	9	c	0	0	all	-	a	As:oth	1990	CC	141	n	ot	y	n	8	ev	all/unsp	nev any	ot
HAENSZ	5	f	0	0	all	-	a	NAmer	1955	CC	158	n	bl	n	y	2	ev	all/unsp	nev any	ot
HAMMON	92	m	0	0	wh	0	a	NAmer	1952	pr	448	n	bl	n	n	0	ev	all/unsp	nev any	st
HEGMAN	4	c	0	0	all	-	a	NAmer	1989	CC	282	n	bl	y	y	0	ev	all/unsp	nev any	st
HINDS	24	f	0	0	o	-	a	NAmer	1968	CC	292	n	bl	n	n	3	ev	all/unsp	nev any	st
ISHIMA	8	c	0	0	all	-	a	As:Jap	1961	CC	180	n	bl	y	y	5	ev	all/unsp	nev any	st
JAHN	47	m	0	0	all	-	a	Eu:Ger	1988	CC	1004	n	bl	n	n	0	ev	all/unsp	nev any	st
JAIN	47	m	0	0	all	-	a	NAmer	1981	CC	845	n	V	y	n	2	ev	cig+/-ot	nev cigs	or
JAIN	42	f	0	0	all	-	a	NAmer	1981	CC	845	n	V	y	n	2	ev	cig+/-ot	nev cigs	or
JEDRYC	56	m	0	0	all	-	a	Eu:est	1980	CC	1630	n	bl	y	n	3	ev	cig+/-ot	nev any	ot
JOLY	51	m	0	0	all	-	a	SCAmer	1978	CC	826	n	bl	n	n	0	ev	cig+/-ot	nev any	st
JOLY	50	f	0	0	all	-	a	SCAmer	1978	CC	826	n	bl	n	n	0	ev	cig+/-ot	nev any	st
JUSSAW	26	m	0	0	all	-	KII	As:Ind	1964	CC	792	n	V	n	n	0	ev	all/unsp	nev any	st
KATSOU	33	f	0	0	all	-	a	Eu:bal	1987	CC	101	n	bl	n	n	1	ev	all/unsp	nev any	ot
KHUDER	27	m	0	0	all	-	a	NAmer	1985	CC	482	n	bl	n	y	0	ev	cig+/-ot	nev cigs	ot
KIHARA	29	c	0	0	jap	-	a	As:Jap	1991	CC	440	n	bl	n	n	0	ev	all/unsp	nev any	st
KOO	7	f	0	0	all	-	a+l	As:HK	1981	CC	200	n	bl	n	n	0	ev	all/unsp	nev any	st
KREYBE	8	m	0	0	all	-	KII	Eu:Sca	1948	CC	300	n	bl	n	y	1	ev	all/unsp	nev any	ot
KREYBE	27	f	0	0	all	-	KII	Eu:Sca	1948	CC	300	n	bl	n	y	1	ev	all/unsp	nev any	ot
LAMTH	3	f	0	0	ch	-	a	As:HK	1983	CC	445	n	bl	n	n	0	ev	all/unsp	nev any	or
LAMWK	4	f	0	0	ch	-	a	As:HK	1981	CC	163	n	bl	n	n	0	ev	all/unsp	nev any	st
LAMWK2	3	m	0	0	all	-	a	As:HK	1976	CC	480	n	bl	n	n	0	ev	all/unsp	nev any	st
LAMWK2	7	f	0	0	all	-	a	As:HK	1976	CC	480	n	bl	n	n	0	ev	all/unsp	nev any	st
LOMBA2	3	f	0	0	all	-	not q+u	NAmer	1960	CC	225	n	bl	n	n	0	ev	cig+/-ot	nev cigs	st
LUBIN	36	m	0	0	all	-	KII	As:Chi	1984	CC	427	m	ot	y	n	0	ev	all/unsp	nev any	st
LUBIN2	148	m	0	0	all	-	a	Eu:mul	1976	CC	7804	n	bl	n	y	0	ev	cig+/-ot	nev any	st
LUBIN2	168	f	0	0	all	-	a	Eu:mul	1976	CC	7804	n	bl	n	y	0	ev	cig+/-ot	nev any	st
LUO	9	c	0	0	all	-	a	As:Chi	1990	CC	102	n	ot	n	y	20	ev	cig+/-ot	nev cigs	or
MATOS	69	m	0	0	all	-	a	SCAmer	1994	CC	200	n	bl	n	n	2	ev	cig+/-ot	nev any	ot
MATSUD	12	m	0	0	all	-	a	As:Jap	1965	CC	179	n	bl	n	n	0	ev	cig+/-ot	nev cigs	ot
NOU	3	m	0	0	all	-	a	Eu:Sca	1971	CC	273	n	bl	y	n	0	ev	all/unsp	nev any	st
NOU	8	f	0	0	all	-	a	Eu:Sca	1971	CC	273	n	bl	y	n	0	ev	all/unsp	nev any	st

Table 3C1 - 1

IESLC - Meta-anal of Ever Smoking (or Current if Ever not available), Any prod (or Cigs if Any not avail)
Adenocarcinoma
Most adjusted

REF	NRR	SEX	AGEL	AGEH	RACE	YF	LC TYPE	LOC	START	ST	NLC	R	VB	P	H	AD	SM	PRODUCT	DENOM	De
ORMOS	21	m	0	0	all	-	a	Eu:est	1947	CC	119	n	bl	y	y	0	ev	cig+/-ot	nev any	ot
OSANN	47	m	0	0	all	-	a	NAmer	1984	CC	1986	n	bl	n	n	2	ev	cig+/-ot	nev cigs	or
OSANN	48	f	0	0	all	-	a	NAmer	1984	CC	1986	n	bl	n	n	2	ev	cig+/-ot	nev cigs	or
OSANN2	31	f	0	0	all	-	KII	NAmer	1964	ot	217	n	bl	n	y	1	ev	cig+/-ot	nev cigs	or
PEZZOT	7	m	0	0	all	-	a	SCAmer	1987	CC	215	n	bl	n	y	0	ev	cig only	nev cigs	st
SCHWAR	8	m	40	54	wh	-	a	NAmer	1984	CC	5588	n	bl	y	y	0	ev	cig+/-ot	nev cigs	st
SCHWAR	7	m	40	54	bl	-	a	NAmer	1984	CC	5588	n	bl	y	y	0	ev	cig+/-ot	nev cigs	st
SCHWAR	16	f	40	54	wh	-	a	NAmer	1984	CC	5588	n	bl	y	y	0	ev	cig+/-ot	nev cigs	st
SCHWAR	15	f	40	54	bl	-	a	NAmer	1984	CC	5588	n	bl	y	y	0	ev	cig+/-ot	nev cigs	st
SEOW	2	f	0	0	ch	-	a	As:oth	1997	CC	153	n	bl	n	y	0	ev	cig+/-ot	nev cigs	st
SIEMIA	8	m	0	0	all	-	a	NAmer	1979	CC	857	n	V	y	y	7	ev	cig+/-ot	nev cigs	or
SOBUE	99	m	0	0	all	-	a	As:Jap	1986	CC	1376	n	bl	n	y	1	ev	cig+/-ot	nev cigs	ot
SOBUE	109	f	0	0	all	-	a	As:Jap	1986	CC	1376	n	bl	n	y	1	ev	cig+/-ot	nev cigs	ot
SOBUE2	2	m	0	0	all	-	a	As:Jap	1965	CC	2083	n	bl	n	n	2	cu	cig+/-ot	nev any	or
SOBUE2	6	f	0	0	all	-	a	As:Jap	1965	CC	2083	n	bl	n	n	2	cu	cig+/-ot	nev any	or
STASZE	21	m	0	0	all	-	a	Eu:est	1954	CC	281	n	bl	n	y	0	ev	all/unsp	nev any	ot
STASZE	4	f	0	0	all	-	a	Eu:est	1954	CC	281	n	bl	n	y	0	ev	all/unsp	nev any	st
STAYNE	4	m	0	0	all	-	a	NAmer	1969	CC	420	n	bl	n	n	0	ev	all/unsp	nev any	st
SUZUK2	16	c	0	0	all	-	a	SCAmer	1991	CC	123	n	bl	n	y	3	ev	all/unsp	nev any	or
SUZUKI	11	m	0	0	all	-	a	As:Jap	1978	CC	238	n	bl	n	y	2	ev	cig+/-ot	nev any	ot
SUZUKI	15	f	0	0	all	-	a	As:Jap	1978	CC	238	n	bl	n	y	2	ev	cig+/-ot	nev any	ot
SVENSS	74	f	0	0	all	-	a	Eu:Sca	1983	CC	210	n	bl	n	n	1	ev	all/unsp	nev any	ot
TIZZAN	19	c	0	0	all	-	a	Eu:wst	1959	CC	1358	n	bl	n	n	0	ev	all/unsp	nev any	st
TOKARS	8	c	0	0	all	-	a	Eu:est	1966	ot	162	o	bl	n	y	3	ev	all/unsp	nev any	or
TSUGAN	10	m	0	0	all	-	a	As:Jap	1976	CC	134	n	bl	n	y	3	ev	all/unsp	nev any	ot
TSUGAN	11	f	0	0	all	-	a	As:Jap	1976	CC	134	n	bl	n	y	3	ev	all/unsp	nev any	ot
WAKAI	76	m	0	0	all	-	a	As:Jap	1988	CC	333	n	bl	n	y	1	ev	all/unsp	nev any	ot
WAKAI	82	f	0	0	all	-	a	As:Jap	1988	CC	333	n	bl	n	y	1	ev	all/unsp	nev any	ot
WU	31	f	0	0	wh	-	a	NAmer	1981	CC	220	n	bl	n	y	2	ev	all/unsp	nev any	ot
WU2	1	f	0	0	all	-	a	NAmer	1983	CC	336	n	bl	n	y	2	cu	all/unsp	nev any	or
WUWILL	11	f	0	0	all	-	a	As:Chi	1985	CC	965	n	ot	n	n	3	ev	cig+/-ot	nev cigs	or
WYNDE2	14	m	0	0	all	-	KII	NAmer	1962	CC	404	n	bl	n	y	0	ev	all/unsp	nev any	st
WYNDE3	29	m	0	0	all	-	KII	NAmer	1966	CC	350	n	bl	n	y	0	ev	all/unsp	nev any	st
WYNDE3	135	f	0	0	all	-	KII	NAmer	1966	CC	350	n	bl	n	y	0	ev	all/unsp	nev any	st
WYNDE4	42	m	0	0	all	-	a	NAmer	1948	CC	684	n	bl	y	n	0	ev	all/unsp	nev any	st
WYNDE4	56	f	0	0	all	-	a	NAmer	1948	CC	684	n	bl	y	n	2	ev	all/unsp	nev any	ot
WYNDE6	69	m	0	0	all	-	KII	NAmer	1969	CC	4423	n	bl	n	y	0	ev	all/unsp	nev any	st
WYNDE6	414	f	0	0	wh	-	a	NAmer	1969	CC	4423	n	bl	n	y	1	ev	cig+/-ot	nev cigs	ot
XU3	22	m	0	0	all	-	KII	As:Chi	1981	CC	135	n	ot	n	n	1	ev	all/unsp	nev any	ot
XU3	26	f	0	0	all	-	KII	As:Chi	1981	CC	135	n	ot	n	n	1	ev	all/unsp	nev any	ot
ZHENG	10	m	0	0	all	-	a	As:Chi	1982	CC	540	n	ot	*	y	0	ev	cig+/-ot	nev cigs	st
ZHENG	21	f	0	0	all	-	a	As:Chi	1982	CC	540	n	ot	*	y	0	ev	cig+/-ot	nev cigs	st
ZHOU	26	m	0	0	all	-	a	As:Chi	1978	CC	1360	n	ot	n	n	0	ev	all/unsp	nev any	st
ZHOU	27	f	0	0	all	-	a	As:Chi	1978	CC	1360	n	ot	n	n	0	ev	all/unsp	nev any	st

Cigarette type is all/unspec for all RRs

Table 3C1 - 2

IESLC - Meta-anal of Ever Smoking (or Current if Ever not available), Any prod (or Cigs if Any not avail)
Adenocarcinoma
Most adjusted

REF	NRR	SEX	AD	Number Exposed Case	Number Exposed Cont	Non-exposed Case	Non-exposed Cont	RR	95.00%CI
*ABRAHA	2	m	0	59	10351	8	3365	2.40	(1.15- 5.01)
*ABRAHA	5	f	0	19	5256	16	11589	2.62	(1.35- 5.09)
Subtotal ABRAHA								2.52	(1.54- 4.12)
ALDERS	54	m	2	-	-	-	-	7.11	(1.49- 33.85)
ALDERS	57	f	1	-	-	-	-	3.58	(1.48- 8.65)
Subtotal ALDERS								4.23	(1.96- 9.12)
*ANDERS	12	f	0	99	96164	33	195158	6.09	(4.11- 9.03)
BAND	2	m	2	-	-	-	-	4.10	(3.01- 5.59)
BARBON	130	m	3	-	-	-	-	7.02	(3.24- 15.22)
BECHER	12	f	1	-	-	-	-	10.83	(1.32- 88.70)
*BOUCOT	147	m	2	-	-	-	-	10.95	(0.65- 183.57)
BRESLO	35	c	0	42	462	4	56	1.27	(0.44- 3.68)
BROWN1	3	m	1	-	-	-	-	4.49	(1.44- 13.98)
BROWN1	4	f	1	-	-	-	-	3.95	(1.76- 8.80)
Subtotal BROWN1								4.12	(2.14- 7.95)
BROWN2	4	m	2	-	-	-	-	8.20	(6.90- 9.70)
BROWN2	3	f	2	-	-	-	-	6.90	(6.10- 7.90)
Subtotal BROWN2								7.35	(6.63- 8.15)
BUFFLE	50	m	0	-	-	-	-	4.50	(1.85- 10.95)
BUFFLE	45	f	0	-	-	-	-	4.02	(2.42- 6.67)
Subtotal BUFFLE								4.13	(2.66- 6.42)
BYERS1	3	m	0	47	695	7	424	4.10	(1.83- 9.15)
CHAN	12	m	0	56	161	0	43	30.44~	(1.84- 502.58)
CHAN	16	f	0	28	50	40	139	1.95	(1.09- 3.48)
Subtotal CHAN								2.18	(1.23- 3.85)
CHOI	63	m	0	46	465	7	95	1.34	(0.59- 3.06)
CHOI	65	f	0	5	26	49	164	0.64	(0.23- 1.77)
Subtotal CHOI								1.00	(0.53- 1.89)
COMSTO	67	m	0	43	229	2	84	7.89	(1.87- 33.27)
COMSTO	79	f	0	29	87	8	115	4.79	(2.09- 11.00)
Subtotal COMSTO								5.43	(2.64- 11.14)
CORREA	36	c	1	-	-	-	-	5.60	(3.60- 8.80)
*CPSI	404	m	1	-	-	-	-	4.58	(1.74- 12.05)
*CPSI	406	f	1	-	-	-	-	1.43	(0.47- 4.39)
Subtotal CPSI								2.78	(1.34- 5.78)
*CPSII	115	m	1	-	-	-	-	19.22	(6.46- 57.16)
*CPSII	118	f	1	-	-	-	-	8.23	(4.36- 15.54)
Subtotal CPSII								10.21	(5.89- 17.67)
DAMBER	32	m	1	-	-	-	-	2.40	(1.10- 5.30)
DESTE2	17	m	2	-	-	-	-	4.30	(1.60- 11.40)
DOLL	87	m	1	-	-	-	-	0.95	(0.22- 4.02)
DOLL	89	f	1	-	-	-	-	1.97	(0.60- 6.46)
Subtotal DOLL								1.47	(0.59- 3.69)
DORGAN	125	m	2	-	-	-	-	4.80	(1.90- 12.00)
DORGAN	104	f	3	-	-	-	-	3.90	(2.80- 5.40)
Subtotal DORGAN								3.99	(2.93- 5.44)
*DORN	340	m	1	-	-	-	-	5.95	(3.85- 9.22)
DOSEME	4	m	2	-	-	-	-	2.60	(1.70- 4.20)
*ENGELA	76	m	7	-	-	-	-	2.33	(0.92- 5.89)
FAN	4	c	0	67	595	45	556	1.39	(0.94- 2.07)
GAO	3	m	2	-	-	-	-	1.60	(1.10- 2.40)
GAO	13	f	2	-	-	-	-	1.50	(1.00- 2.10)
Subtotal GAO								1.55	(1.18- 2.02)
GER	9	c	8	-	-	-	-	1.10	(0.55- 2.19)
HAENSZ	5	f	2	-	-	-	-	1.19	(0.65- 2.19)
*HAMMON	92	m	0	29	510108	2	115884	3.29	(0.79- 13.80)
HEGMAN	4	c	0	83	1202	15	2080	9.58	(5.50- 16.67)
HINDS	24	f	3	-	-	-	-	3.89	(2.49- 6.07)
ISHIMA	8	c	5	-	-	-	-	15.00	(2.31- 631.48)
JAHN	47	m	0	204	701	8	138	5.02	(2.42- 10.41)
JAIN	47	m	2	-	-	-	-	8.00	(2.28- 50.60)
JAIN	42	f	2	-	-	-	-	3.45	(1.83- 7.10)
Subtotal JAIN								3.95	(2.12- 7.35)
JEDRYC	56	m	3	-	-	-	-	3.44	(1.52- 7.78)
JOLY	51	m	0	72	709	5	218	4.43	(1.77- 11.10)
JOLY	50	f	0	33	122	25	283	3.06	(1.75- 5.37)
Subtotal JOLY								3.38	(2.10- 5.46)
JUSSAW	26	m	0	34	168	13	624	9.71	(5.01- 18.82)
KATSOU	33	f	1	-	-	-	-	1.72	(0.80- 3.71)
KHUDER	27	m	0	155	-	7	-	8.11	(3.67- 17.93)
KIHARA	29	c	0	130	232	78	237	1.70	(1.22- 2.38)

Table 3C1 - 2

IESLC - Meta-anal of Ever Smoking (or Current if Ever not available), Any prod (or Cigs if Any not avail)
Adenocarcinoma
Most adjusted

REF	NRR	SEX	AD	Number Exposed Case	Number Exposed Cont	Non-exposed Case	Non-exposed Cont	RR	95.00%CI
KOO	7	f	0	34	63	46	137	1.61	(0.94- 2.74)
KREYBE	8	m	1	-	-	-	-	2.44	(0.76- 7.86)
KREYBE	27	f	1	-	-	-	-	1.28	(0.60- 2.74)
Subtotal KREYBE								1.55	(0.82- 2.93)
LAMTH	3	f	0	79	51	131	158	1.87	(1.23- 2.85)
LAMWK	4	f	0	36	41	60	144	2.11	(1.23- 3.61)
LAMWK2	3	m	0	52	161	15	43	0.93	(0.48- 1.80)
LAMWK2	7	f	0	26	50	41	139	1.76	(0.98- 3.17)
Subtotal LAMWK2								1.33	(0.86- 2.07)
LOMBA2	3	f	0	42	353	54	239	0.53	(0.34- 0.81)
LUBIN	36	m	0	37	939	4	72	0.71	(0.25- 2.05)
LUBIN2	148	m	0	655	10433	57	2616	2.88	(2.19- 3.79)
LUBIN2	168	f	0	85	567	138	1180	1.28	(0.96- 1.71)
Subtotal LUBIN2								1.96	(1.61- 2.39)
LUO	9	c	20	-	-	-	-	1.50	(0.70- 3.00)
MATOS	69	m	2	-	-	-	-	6.21	(2.42- 15.96)
MATSUD	12	m	0	23	3314	0	1255	17.80~	(1.08- 293.32)
NOU	3	m	0	36	247	4	122	4.45	(1.55- 12.77)
NOU	8	f	0	9	92	29	261	0.88	(0.40- 1.93)
Subtotal NOU								1.57	(0.83- 2.94)
ORMOS	21	m	0	4	1034	0	777	6.76~	(0.36- 125.82)
OSANN	47	m	2	-	-	-	-	17.90	(10.40- 31.00)
OSANN	48	f	2	-	-	-	-	9.50	(6.80- 13.80)
Subtotal OSANN								11.46	(8.51- 15.42)
OSANN2	31	f	1	-	-	-	-	2.50	(1.30- 5.10)
PEZZOT	7	m	0	60	317	3	116	7.32	(2.25- 23.79)
SCHWAR	8	m	0	84	178	1	73	34.45	(4.71- 252.10)
SCHWAR	7	m	0	45	39	1	7	8.08	(0.95- 68.56)
SCHWAR	16	f	0	92	108	10	79	6.73	(3.29- 13.75)
SCHWAR	15	f	0	20	28	3	41	9.76	(2.65- 36.00)
Subtotal SCHWAR								8.40	(4.73- 14.94)
SEOW	2	f	0	19	15	67	125	2.36	(1.13- 4.95)
SIEMIA	8	m	7	-	-	-	-	6.30	(2.50- 16.20)
SOBUE	99	m	1	-	-	-	-	1.83	(1.21- 2.77)
SOBUE	109	f	1	-	-	-	-	1.41	(1.00- 1.99)
Subtotal SOBUE								1.57	(1.20- 2.04)
SOBUE2	2	m	2	-	-	-	-	3.10	(2.40- 3.70)
SOBUE2	6	f	2	-	-	-	-	1.80	(1.40- 2.20)
Subtotal SOBUE2								2.39	(2.04- 2.79)
STASZE	21	m	0	20	754	0	158	8.61~	(0.52- 143.15)
STASZE	4	f	0	1	153	10	1660	1.08	(0.14- 8.53)
Subtotal STASZE								2.24	(0.42- 11.81)
STAYNE	4	m	0	43	567	7	333	3.61	(1.60- 8.11)
SUZUK2	16	c	3	-	-	-	-	6.00	(0.70- 50.00)
SUZUKI	11	m	2	-	-	-	-	4.53	(2.48- 8.29)
SUZUKI	15	f	2	-	-	-	-	2.19	(1.16- 4.14)
Subtotal SUZUKI								3.21	(2.07- 4.97)
SVENSS	74	f	1	-	-	-	-	2.91	(1.56- 5.42)
TIZZAN	19	c	0	88	939	25	419	1.57	(0.99- 2.49)
TOKARS	8	c	3	-	-	-	-	4.30	(1.90- 9.90)
TSUGAN	10	m	3	-	-	-	-	0.93	(0.43- 1.99)
TSUGAN	11	f	3	-	-	-	-	0.67	(0.22- 2.07)
Subtotal TSUGAN								0.84	(0.45- 1.58)
WAKAI	76	m	1	-	-	-	-	1.93	(0.90- 4.18)
WAKAI	82	f	1	-	-	-	-	1.39	(0.66- 2.90)
Subtotal WAKAI								1.63	(0.96- 2.77)
WU	31	f	2	-	-	-	-	2.60	(1.53- 4.44)
WU2	1	f	2	-	-	-	-	4.50	(3.00- 6.90)
WUWILL	11	f	3	-	-	-	-	1.50	(1.10- 1.90)
WYNDE2	14	m	0	49	616	5	105	1.67	(0.65- 4.29)
WYNDE3	29	m	0	68	332	6	88	3.00	(1.26- 7.15)
WYNDE3	135	f	0	21	56	15	76	1.90	(0.90- 4.01)
Subtotal WYNDE3								2.31	(1.31- 4.07)
WYNDE4	42	m	0	35	665	4	115	1.51	(0.53- 4.34)
WYNDE4	56	f	2	-	-	-	-	0.60	(0.13- 2.69)
Subtotal WYNDE4								1.12	(0.47- 2.66)
WYNDE6	69	m	0	1079	1996	58	617	5.75	(4.35- 7.60)
WYNDE6	414	f	1	-	-	-	-	13.99	(10.18- 19.23)
Subtotal WYNDE6								8.47	(6.87- 10.44)
XU3	22	m	1	-	-	-	-	4.84	(1.37- 17.10)
XU3	26	f	1	-	-	-	-	1.09	(0.26- 4.50)

Table 3C1 - 2

IESLC - Meta-anal of Ever Smoking (or Current if Ever not available), Any prod (or Cigs if Any not avail)
Adenocarcinoma
Most adjusted

REF	NRR	SEX	AD	Number Exposed Case	Cont	Non-exposed Case	Cont	RR	95.00%CI
Subtotal XU3								2.51	(0.98- 6.47)
ZHENG	10	m	0	123	218	29	94	1.83	(1.14- 2.93)
ZHENG	21	f	0	33	44	119	184	1.16	(0.70- 1.93)
Subtotal ZHENG								1.48	(1.05- 2.09)
ZHOU	26	m	0	131	41	88	36	1.31	(0.77- 2.20)
ZHOU	27	f	0	30	7	96	32	1.43	(0.57- 3.57)
Subtotal ZHOU								1.34	(0.85- 2.10)
Partial Totals				4439	652201	1498	342753		

*prospective study ~ With 0.5 adjustment for zero

REF	NRR	SEX	AD	Ys	Ws	Qs	Ps
*ABRAHA	2	m	0	0.87	7.06	0.84	0.0201
*ABRAHA	5	f	0	0.96	8.71	0.57	0.0045
Subtotal ABRAHA				0.92	15.77	1.41	
ALDERS	54	m	2	1.96	1.58	0.87	0.0138
ALDERS	57	f	1	1.28	4.93	0.02	0.0046
Subtotal ALDERS				1.44	6.50	0.89	
*ANDERS	12	f	0	1.81	24.76	8.55	0.0000
BAND	2	m	2	1.41	40.10	1.48	0.0000
BARBON	130	m	3	1.95	6.42	3.42	0.0000
BECHER	12	f	1	2.38	0.87	1.18	0.0265
*BOUCOT	147	m	2	2.39	0.48	0.67	0.0964
BRESLO	35	c	0	0.24	3.40	3.25	0.6564
BROWN1	3	m	1	1.50	2.97	0.24	0.0096
BROWN1	4	f	1	1.37	5.93	0.14	0.0008
Subtotal BROWN1				1.42	8.91	0.38	
BROWN2	4	m	2	2.10	132.45	103.85	0.0000
BROWN2	3	f	2	1.93	229.82	116.78	0.0000
Subtotal BROWN2				1.99	362.27	220.63	
BUFFLE	50	m	0	1.50	4.86	0.40	0.0009
BUFFLE	45	f	0	1.39	14.95	0.45	0.0000
Subtotal BUFFLE				1.42	19.81	0.84	
BYERS1	3	m	0	1.41	5.95	0.22	0.0006
CHAN	12	m	0	3.42	0.49	2.36	0.0170
CHAN	16	f	0	0.67	11.38	3.48	0.0247
Subtotal CHAN				0.78	11.86	5.84	
CHOI	63	m	0	0.29	5.64	4.82	0.4842
CHOI	65	f	0	-0.44	3.77	10.39	0.3920
Subtotal CHOI				-0.00	9.42	15.21	
COMSTO	67	m	0	2.07	1.85	1.33	0.0049
COMSTO	79	f	0	1.57	5.57	0.67	0.0002
Subtotal COMSTO				1.69	7.42	2.00	
CORREA	36	c	1	1.72	19.23	4.89	0.0000
*CPSI	404	m	1	1.52	4.10	0.38	0.0021
*CPSI	406	f	1	0.36	3.08	2.28	0.5303
Subtotal CPSI				1.02	7.18	2.66	
*CPSII	115	m	1	2.96	3.23	9.76	0.0000
*CPSII	118	f	1	2.11	9.51	7.52	0.0000
Subtotal CPSII				2.32	12.75	17.28	
DAMBER	32	m	1	0.88	6.21	0.73	0.0291
DESTE2	17	m	2	1.46	3.99	0.23	0.0036
DOLL	87	m	1	-0.05	1.82	2.94	0.9448
DOLL	89	f	1	0.68	2.72	0.80	0.2634
Subtotal DOLL				0.39	4.54	3.73	
DORGAN	125	m	2	1.57	4.52	0.55	0.0008
DORGAN	104	f	3	1.36	35.62	0.72	0.0000
Subtotal DORGAN				1.38	40.15	1.28	
*DORN	340	m	1	1.78	20.15	6.43	0.0000
DOSEME	4	m	2	0.96	18.78	1.30	0.0000
*ENGELA	76	m	7	0.85	4.46	0.62	0.0741
FAN	4	c	0	0.33	24.61	19.43	0.1013
GAO	3	m	2	0.47	25.25	14.15	0.0182
GAO	13	f	2	0.41	27.91	18.46	0.0322
Subtotal GAO				0.44	53.16	32.61	
GER	9	c	8	0.10	8.05	10.16	0.7869
HAENSZ	5	f	2	0.17	10.41	11.37	0.5745
*HAMMON	92	m	0	1.19	1.87	0.00	0.1030
HEGMAN	4	c	0	2.26	12.50	13.53	0.0000
HINDS	24	f	3	1.36	19.35	0.38	0.0000
ISHIMA	8	c	5	2.71	0.49	1.08	0.0585

Table 3C1 - 2

IESLC - Meta-anal of Ever Smoking (or Current if Ever not available), Any prod (or Cigs if Any not avail)
Adenocarcinoma
Most adjusted

REF	NRR	SEX	AD	Ys	Ws	Qs	Ps
JAHN	47	m	0	1.61	7.22	1.12	0.0000
JAIN	47	m	2	2.08	1.60	1.18	0.0085
JAIN	42	f	2	1.24	8.36	0.00	0.0003
Subtotal JAIN				1.37	9.96	1.19	
JEDRYC	56	m	3	1.24	5.76	0.00	0.0030
JOLY	51	m	0	1.49	4.55	0.33	0.0015
JOLY	50	f	0	1.12	12.19	0.12	0.0001
Subtotal JOLY				1.22	16.74	0.45	
JUSSAW	26	m	0	2.27	8.78	9.77	0.0000
KATSOU	33	f	1	0.54	6.53	2.99	0.1658
KHUDER	27	m	0	2.09	6.11	4.67	0.0000
KIHARA	29	c	0	0.53	34.43	16.23	0.0018
KOO	7	f	0	0.47	13.45	7.45	0.0817
KREYBE	8	m	1	0.89	2.82	0.30	0.1345
KREYBE	27	f	1	0.25	6.66	6.29	0.5240
Subtotal KREYBE				0.44	9.48	6.59	
LAMTH	3	f	0	0.63	21.63	7.62	0.0036
LAMWK	4	f	0	0.75	13.20	2.96	0.0068
LAMWK2	3	m	0	-0.08	8.67	14.55	0.8206
LAMWK2	7	f	0	0.57	11.11	4.72	0.0588
Subtotal LAMWK2				0.28	19.77	19.27	
LOMBA2	3	f	0	-0.64	20.27	70.11	0.0039
LUBIN	36	m	0	-0.34	3.42	8.36	0.5249
LUBIN2	148	m	0	1.06	51.15	1.32	0.0000
LUBIN2	168	f	0	0.25	46.25	43.55	0.0913
Subtotal LUBIN2				0.67	97.40	44.86	
LUO	9	c	20	0.41	7.26	4.80	0.2748
MATOS	69	m	2	1.83	4.32	1.59	0.0001
MATSUD	12	m	0	2.88	0.49	1.35	0.0440
NOU	3	m	0	1.49	3.45	0.26	0.0056
NOU	8	f	0	-0.13	6.24	11.30	0.7504
Subtotal NOU				0.45	9.69	11.56	
ORMOS	21	m	0	1.91	0.45	0.22	0.1999
OSANN	47	m	2	2.88	12.88	35.76	0.0000
OSANN	48	f	2	2.25	30.68	32.71	0.0000
Subtotal OSANN				2.44	43.56	68.47	
OSANN2	31	f	1	0.92	8.22	0.75	0.0086
PEZZOT	7	m	0	1.99	2.76	1.65	0.0009
SCHWAR	8	m	0	3.54	0.97	5.22	0.0005
SCHWAR	7	m	0	2.09	0.84	0.64	0.0556
SCHWAR	16	f	0	1.91	7.53	3.56	0.0000
SCHWAR	15	f	0	2.28	2.26	2.53	0.0006
Subtotal SCHWAR				2.13	11.60	11.96	
SEOW	2	f	0	0.86	7.03	0.90	0.0226
SIEMIA	8	m	7	1.84	4.40	1.70	0.0001
SOBUE	99	m	1	0.60	22.40	8.45	0.0042
SOBUE	109	f	1	0.34	32.45	24.85	0.0503
Subtotal SOBUE				0.45	54.85	33.30	
SOBUE2	2	m	2	1.13	82.01	0.62	0.0000
SOBUE2	6	f	2	0.59	75.22	29.94	0.0000
Subtotal SOBUE2				0.87	157.22	30.56	
STASZE	21	m	0	2.15	0.49	0.42	0.1332
STASZE	4	f	0	0.08	0.90	1.17	0.9382
Subtotal STASZE				0.81	1.39	1.59	
STAYNE	4	m	0	1.28	5.85	0.02	0.0019
SUZUK2	16	c	3	1.79	0.84	0.28	0.0999
SUZUKI	11	m	2	1.51	10.55	0.90	0.0000
SUZUKI	15	f	2	0.78	9.49	1.79	0.0157
Subtotal SUZUKI				1.17	20.04	2.69	
SVENSS	74	f	1	1.07	9.91	0.22	0.0008
TIZZAN	19	c	0	0.45	18.24	10.74	0.0538
TOKARS	8	c	3	1.46	5.64	0.32	0.0005
TSUGAN	10	m	3	-0.07	6.55	10.91	0.8527
TSUGAN	11	f	3	-0.40	3.06	8.02	0.4837
Subtotal TSUGAN				-0.18	9.60	18.93	
WAKAI	76	m	1	0.66	6.52	2.05	0.0933
WAKAI	82	f	1	0.33	7.01	5.55	0.3832
Subtotal WAKAI				0.49	13.53	7.60	
WU	31	f	2	0.96	13.54	0.94	0.0004
WU2	1	f	2	1.50	22.15	1.80	0.0000
WUWILL	11	f	3	0.41	51.44	34.02	0.0036

Table 3C1 - 2

IESLC - Meta-anal of Ever Smoking (or Current if Ever not available), Any prod (or Cigs if Any not avail)
Adenocarcinoma
Most adjusted

REF	NRR	SEX	AD	Ys	Ws	Qs	Ps
WYNDE2	14	m	0	0.51	4.32	2.15	0.2863
WYNDE3	29	m	0	1.10	5.11	0.07	0.0129
WYNDE3	135	f	0	0.64	6.88	2.29	0.0922
Subtotal WYNDE3				0.84	11.99	2.36	
WYNDE4	42	m	0	0.41	3.46	2.24	0.4408
WYNDE4	56	f	2	-0.51	1.67	5.01	0.5087
Subtotal WYNDE4				0.11	5.14	7.25	
WYNDE6	69	m	0	1.75	49.29	13.88	0.0000
WYNDE6	414	f	1	2.64	37.98	76.55	0.0000
Subtotal WYNDE6				2.14	87.27	90.43	
XU3	22	m	1	1.58	2.41	0.31	0.0143
XU3	26	f	1	0.09	1.89	2.42	0.9057
Subtotal XU3				0.92	4.30	2.73	
ZHENG	10	m	0	0.60	17.29	6.54	0.0121
ZHENG	21	f	0	0.15	14.95	17.14	0.5668
Subtotal ZHENG				0.39	32.24	23.68	
ZHOU	26	m	0	0.27	14.05	12.70	0.3154
ZHOU	27	f	0	0.36	4.59	3.41	0.4448
Subtotal ZHOU				0.29	18.64	16.12	

	N	116
	NS	81
	Wt	1733.90
Het	Chi	989.98
Het	df	115
Het	P	***
Fixed	RR	3.38
	RRl	3.23
	RRu	3.55
	P	+++
Random	RR	2.94
	RRl	2.52
	RRu	3.43
	P	+++
Asymm	P	*

Table 3C1 - 3

IESLC - Meta-anal of Ever Smoking (or Current if Ever not available), Any prod (or Cigs if Any not avail)
Adenocarcinoma
Most adjusted

	Sex			
	combined	male	female	Total
N	11	56	49	116
NS	11	55	48	114
Wt	134.70	665.17	934.03	1733.90
Het Chi	64.23	288.45	592.06	989.98
Het df	10	55	48	115
Het P	***	***	***	***
Fixed RR	2.29	4.03	3.16	3.38
RRl	1.93	3.74	2.96	3.23
RRu	2.71	4.35	3.37	3.55
P	+++	+++	+++	+++
Random RR	2.52	3.70	2.39	2.94
RRl	1.56	3.02	1.86	2.52
RRu	4.08	4.55	3.05	3.43
P	+++	+++	+++	+++
Between Chi				45.24
Between df				2
Between P				***
Btwn(F) P				(*)
Btwn(R) P				*

	Lung cancer type						
	a	a+l	a+al+br	KII	not q+u	not q+s	Total
N	96	3	1	13	1	2	116
NS	67	2	1	9	1	2	82
Wt	1558.10	25.32	6.21	104.34	20.27	19.65	1733.90
Het Chi	851.35	4.13	0.00	49.79	0.00	1.69	989.98
Het df	95	2	0	12	0	1	115
Het P	***	N.S.	N.S.	***	N.S.	N.S.	***
Fixed RR	3.50	1.85	2.40	3.63	0.53	2.77	3.38
RRl	3.33	1.26	1.09	3.00	0.34	1.78	3.23
RRu	3.67	2.74	5.27	4.40	0.81	4.31	3.55
P	+++	++	+	+++	--	+++	+++
Random RR	3.10	2.05	2.40	2.39	0.53	3.61	2.94
RRl	2.62	1.05	1.09	1.51	0.34	1.11	2.52
RRu	3.65	3.99	5.27	3.79	0.81	11.71	3.43
P	+++	+	+	+++	--	+	+++
Between Chi							83.03
Between df							5
Between P							***
Btwn(F) P							(*)
Btwn(R) P							***

	Location								
	NAmer	UK	Scand	othEur	China	Japan	othAs	other	Total
N	47	4	7	15	12	13	12	6	116
NS	32	2	5	12	8	8	9	5	81
Wt	871.05	11.05	39.74	184.48	195.08	290.66	113.20	28.65	1733.90
Het Chi	317.56	4.12	9.53	41.55	7.56	41.19	40.04	2.85	989.98
Het df	46	3	6	14	11	12	11	5	115
Het P	***	N.S.	N.S.	***	N.S.	***	***	N.S.	***
Fixed RR	5.73	2.74	2.04	2.30	1.48	2.07	1.88	4.20	3.38
RRl	5.36	1.52	1.49	1.99	1.28	1.84	1.56	2.91	3.23
RRu	6.12	4.94	2.78	2.65	1.70	2.32	2.26	6.06	3.55
P	+++	+++	+++	+++	+++	+++	+++	+++	+++
Random RR	4.53	2.68	2.05	2.72	1.48	1.93	1.87	4.20	2.94
RRl	3.69	1.31	1.37	2.01	1.28	1.49	1.29	2.91	2.52
RRu	5.57	5.47	3.06	3.68	1.70	2.50	2.71	6.06	3.43
P	+++	++	+++	+++	+++	+++	+++	+++	+++
Between Chi									525.59
Between df									7
Between P									***
Btwn(F) P									***
Btwn(R) P									***

Table 3C1 - 3

IESLC - Meta-anal of Ever Smoking (or Current if Ever not available), Any prod (or Cigs if Any not avail)
Adenocarcinoma
Most adjusted

	Detailed Country in "other Europe"					
	multi	Germany	othWest	East	Balkans	Total
N	2	2	2	7	2	15
NS	1	2	2	5	2	12
Wt	97.40	8.08	24.66	29.01	25.31	184.48
Het Chi	15.93	0.46	10.65	3.13	0.83	41.55
Het df	1	1	1	6	1	14
Het P	***	N.S.	**	N.S.	N.S.	***
Fixed RR	1.96	5.45	2.32	3.00	2.34	2.30
RRl	1.61	2.74	1.56	2.09	1.58	1.99
RRu	2.39	10.86	3.44	4.32	3.45	2.65
P	+++	+++	+++	+++	+++	+++
Random RR	1.92	5.45	3.21	3.00	2.34	2.72
RRl	0.87	2.74	0.74	2.09	1.58	2.01
RRu	4.26	10.86	13.90	4.32	3.45	3.68
P	N.S.	+++	N.S.	+++	+++	+++
Between Chi						10.55
Between df						4
Between P						*
Btwn(F) P						N.S.
Btwn(R) P						N.S.

	Detailed Country in "other Asia"			
	India	HongKong	other	Total
N	1	7	4	12
NS	1	5	3	9
Wt	8.78	79.92	24.49	113.20
Het Chi	0.00	8.27	4.60	40.04
Het df	0	6	3	11
Het P	N.S.	N.S.	N.S.	***
Fixed RR	9.71	1.75	1.32	1.88
RRl	5.01	1.40	0.89	1.56
RRu	18.82	2.18	1.96	2.26
P	+++	+++	N.S.	+++
Random RR	9.71	1.74	1.29	1.87
RRl	5.01	1.33	0.79	1.29
RRu	18.82	2.28	2.13	2.71
P	+++	+++	N.S.	+++
Between Chi				27.16
Between df				2
Between P				***
Btwn(F) P				**
Btwn(R) P				***

	Detailed other continent			
	SCAmer	Auslia	Africa	Total
N	6			6
NS	5			5
Wt	28.65			28.65
Het Chi	2.85			2.85
Het df	5			5
Het P	N.S.			N.S.
Fixed RR	4.20			4.20
RRl	2.91			2.91
RRu	6.06			6.06
P	+++			+++
Random RR	4.20			4.20
RRl	2.91			2.91
RRu	6.06			6.06
P	+++			+++
Between Chi				
Between df				
Between P				N.S.
Btwn(F) P				N.S.
Btwn(R) P				N.S.

Table 3C1 - 3

IESLC - Meta-anal of Ever Smoking (or Current if Ever not available), Any prod (or Cigs if Any not avail)
Adenocarcinoma
Most adjusted

	Start year of study <1960	1960-69	1970-79	1980-89	1990+	Total
N	18	16	31	43	8	116
NS	13	13	18	29	8	81
Wt	88.69	334.35	317.22	903.11	90.53	1733.90
Het Chi	41.90	209.68	105.81	489.60	15.29	989.98
Het df	17	15	30	42	7	115
Het P	***	***	***	***	*	***
Fixed RR	2.31	3.30	2.47	4.23	1.76	3.38
RRl	1.88	2.96	2.21	3.96	1.44	3.23
RRu	2.85	3.67	2.76	4.51	2.17	3.55
P	+++	+++	+++	+++	+++	+++
Random RR	2.11	3.35	2.69	3.45	1.99	2.94
RRl	1.44	2.12	2.14	2.68	1.40	2.52
RRu	3.08	5.29	3.37	4.43	2.83	3.43
P	+++	+++	+++	+++	+++	+++
Between Chi						127.69
Between df						4
Between P						***
Btwn(F) P						**
Btwn(R) P						(*)

	Study type (1) CC	other	Total
N	101	15	116
NS	70	11	81
Wt	1625.20	108.70	1733.90
Het Chi	948.53	29.31	989.98
Het df	100	14	115
Het P	***	**	***
Fixed RR	3.31	4.67	3.38
RRl	3.15	3.87	3.23
RRu	3.48	5.64	3.55
P	+++	+++	+++
Random RR	2.78	4.38	2.94
RRl	2.36	3.24	2.52
RRu	3.29	5.91	3.43
P	+++	+++	+++
Between Chi			12.14
Between df			1
Between P			***
Btwn(F) P			N.S.
Btwn(R) P			**

	Study type (2) CC	prosp	other	Total
N	101	11	4	116
NS	70	8	3	81
Wt	1625.20	87.42	21.28	1733.90
Het Chi	948.53	25.31	2.81	989.98
Het df	100	10	3	115
Het P	***	**	N.S.	***
Fixed RR	3.31	4.92	3.78	3.38
RRl	3.15	3.99	2.47	3.23
RRu	3.48	6.07	5.78	3.55
P	+++	+++	+++	+++
Random RR	2.78	4.48	3.78	2.94
RRl	2.36	3.07	2.47	2.52
RRu	3.29	6.54	5.78	3.43
P	+++	+++	+++	+++
Between Chi				13.32
Between df				2
Between P				**
Btwn(F) P				N.S.
Btwn(R) P				*

Table 3C1 - 3

IESLC - Meta-anal of Ever Smoking (or Current if Ever not available), Any prod (or Cigs if Any not avail)
Adenocarcinoma
Most adjusted

	Study size (number of LC cases)				
	100-249	250-499	500-999	1000+	Total
N	28	32	18	38	116
NS	24	23	12	22	81
Wt	177.06	283.99	180.32	1092.53	1733.90
Het Chi	91.26	129.12	66.34	545.09	989.98
Het df	27	31	17	37	115
Het P	***	***	***	***	***
Fixed RR	1.94	2.49	2.40	4.24	3.38
RRl	1.68	2.22	2.07	4.00	3.23
RRu	2.25	2.80	2.77	4.50	3.55
P	+++	+++	+++	+++	+++
Random RR	2.35	2.43	2.86	3.85	2.94
RRl	1.74	1.88	2.08	2.99	2.52
RRu	3.16	3.15	3.92	4.97	3.43
P	+++	+++	+++	+++	+++
Between Chi					158.17
Between df					3
Between P					***
Btwn(F) P					***
Btwn(R) P					*

	Risky occupational population			
	no	mining	othRisky	Total
N	114	1	1	116
NS	79	1	1	81
Wt	1724.83	3.42	5.64	1733.90
Het Chi	981.29	0.00	0.00	989.98
Het df	113	0	0	115
Het P	***	N.S.	N.S.	***
Fixed RR	3.39	0.71	4.30	3.38
RRl	3.23	0.25	1.88	3.23
RRu	3.55	2.05	9.82	3.55
P	+++	N.S.	+++	+++
Random RR	2.96	0.71	4.30	2.94
RRl	2.54	0.25	1.88	2.52
RRu	3.46	2.05	9.82	3.43
P	+++	N.S.	+++	+++
Between Chi				8.69
Between df				2
Between P				*
Btwn(F) P				N.S.
Btwn(R) P				*

	National cigarette tobacco type			
	Virginia	blended	other	Total
N	9	94	13	116
NS	6	66	9	81
Wt	74.28	1456.48	203.13	1733.90
Het Chi	13.94	803.48	8.23	989.98
Het df	8	93	12	115
Het P	(*)	***	N.S.	***
Fixed RR	4.36	3.75	1.46	3.38
RRl	3.48	3.57	1.27	3.23
RRu	5.48	3.95	1.67	3.55
P	+++	+++	+++	+++
Random RR	4.37	3.17	1.46	2.94
RRl	3.01	2.68	1.27	2.52
RRu	6.34	3.76	1.67	3.43
P	+++	+++	+++	+++
Between Chi				164.33
Between df				2
Between P				***
Btwn(F) P				***
Btwn(R) P				***

Table 3C1 - 3

IESLC - Meta-anal of Ever Smoking (or Current if Ever not available), Any prod (or Cigs if Any not avail)
Adenocarcinoma
Most adjusted

	Any proxy use No/nk	Yes	Total
N	88	28	116
NS	62	19	81
Wt	1497.01	236.89	1733.90
Het Chi	890.44	98.85	989.98
Het df	87	27	115
Het P	***	***	***
Fixed RR	3.36	3.56	3.38
RRl	3.19	3.13	3.23
RRu	3.53	4.04	3.55
P	+++	+++	+++
Random RR	2.77	3.61	2.94
RRl	2.31	2.73	2.52
RRu	3.31	4.76	3.43
P	+++	+++	+++
Between Chi			0.69
Between df			1
Between P			N.S.
Btwn(F) P			N.S.
Btwn(R) P			N.S.

	Full histological confirmation No	Yes	Total
N	67	49	116
NS	48	33	81
Wt	816.28	917.62	1733.90
Het Chi	400.28	461.36	989.98
Het df	66	48	115
Het P	***	***	***
Fixed RR	2.54	4.37	3.38
RRl	2.37	4.10	3.23
RRu	2.72	4.66	3.55
P	+++	+++	+++
Random RR	2.73	3.25	2.94
RRl	2.26	2.57	2.52
RRu	3.28	4.11	3.43
P	+++	+++	+++
Between Chi			128.34
Between df			1
Between P			***
Btwn(F) P			***
Btwn(R) P			N.S.

	Number of adjustment variables (1) 0	1	2+/+nk	Total
N	54	25	37	116
NS	38	17	29	84
Wt	577.24	229.57	927.09	1733.90
Het Chi	295.04	166.95	422.19	989.98
Het df	53	24	36	115
Het P	***	***	***	***
Fixed RR	2.40	3.62	4.12	3.38
RRl	2.21	3.18	3.86	3.23
RRu	2.60	4.12	4.40	3.55
P	+++	+++	+++	+++
Random RR	2.61	3.12	3.28	2.94
RRl	2.11	2.16	2.54	2.52
RRu	3.23	4.51	4.23	3.43
P	+++	+++	+++	+++
Between Chi				105.79
Between df				2
Between P				***
Btwn(F) P				**
Btwn(R) P				N.S.

Table 3C1 - 3

IESLC - Meta-anal of Ever Smoking (or Current if Ever not available), Any prod (or Cigs if Any not avail)
Adenocarcinoma
Most adjusted

	Number of adjustment variables (2) 0	1	2	3-5	6+/+nk	Total
N	54	25	23	10	4	116
NS	38	17	17	9	4	85
Wt	577.24	229.57	767.75	135.17	24.16	1733.90
Het Chi	295.04	166.95	308.69	47.25	9.32	989.98
Het df	53	24	22	9	3	115
Het P	***	***	***	***	*	***
Fixed RR	2.40	3.62	4.61	2.51	1.91	3.38
RRl	2.21	3.18	4.29	2.12	1.28	3.23
RRu	2.60	4.12	4.94	2.98	2.84	3.55
P	+++	+++	+++	+++	++	+++
Random RR	2.61	3.12	3.74	2.79	2.11	2.94
RRl	2.11	2.16	2.74	1.74	1.03	2.52
RRu	3.23	4.51	5.08	4.47	4.31	3.43
P	+++	+++	+++	+++	+	+++
Between Chi						162.73
Between df						4
Between P						***
Btwn(F) P						***
Btwn(R) P						N.S.

	Product all/unsp	cig+/-ot	cig only	Total
N	56	53	7	116
NS	42	35	6	83
Wt	454.21	1205.78	73.91	1733.90
Het Chi	195.87	718.83	13.49	989.98
Het df	55	52	6	115
Het P	***	***	*	***
Fixed RR	2.49	3.71	4.81	3.38
RRl	2.28	3.51	3.83	3.23
RRu	2.73	3.93	6.04	3.55
P	+++	+++	+++	+++
Random RR	2.31	3.45	5.24	2.94
RRl	1.91	2.73	3.35	2.52
RRu	2.80	4.35	8.22	3.43
P	+++	+++	+++	+++
Between Chi				61.79
Between df				2
Between P				***
Btwn(F) P				*
Btwn(R) P				***

	Denominator nev any	nev cigs	Total
N	75	41	116
NS	55	28	83
Wt	867.44	866.46	1733.90
Het Chi	288.10	596.65	989.98
Het df	74	40	115
Het P	***	***	***
Fixed RR	2.64	4.33	3.38
RRl	2.47	4.05	3.23
RRu	2.83	4.63	3.55
P	+++	+++	+++
Random RR	2.59	3.64	2.94
RRl	2.23	2.73	2.52
RRu	3.01	4.86	3.43
P	+++	+++	+++
Between Chi			105.23
Between df			1
Between P			***
Btwn(F) P			***
Btwn(R) P			*

Table 3C1 - 3

IESLC - Meta-anal of Ever Smoking (or Current if Ever not available), Any prod (or Cigs if Any not avail)
Adenocarcinoma
Most adjusted

	Derivation of RR/CI			
	Orig	StdCalc	Other	Total
N	29	47	40	116
NS	21	34	30	85
Wt	852.39	519.98	361.52	1733.90
Het Chi	367.82	298.39	224.03	989.98
Het df	28	46	39	115
Het P	***	***	***	***
Fixed RR	4.29	2.52	2.96	3.38
RRl	4.01	2.31	2.67	3.23
RRu	4.58	2.74	3.28	3.55
P	+++	+++	+++	+++
Random RR	3.78	2.71	2.64	2.94
RRl	2.86	2.13	2.01	2.52
RRu	5.00	3.43	3.47	3.43
P	+++	+++	+++	+++
Between Chi				99.73
Between df				2
Between P				***
Btwn(F) P				**
Btwn(R) P				N.S.

	Smoking status		
	ever	current	Total
N	107	9	116
NS	75	6	81
Wt	1513.97	219.93	1733.90
Het Chi	930.22	56.84	989.98
Het df	106	8	115
Het P	***	***	***
Fixed RR	3.44	3.04	3.38
RRl	3.27	2.66	3.23
RRu	3.61	3.47	3.55
P	+++	+++	+++
Random RR	2.84	4.31	2.94
RRl	2.41	2.78	2.52
RRu	3.35	6.71	3.43
P	+++	+++	+++
Between Chi			2.92
Between df			1
Between P			(*)
Btwn(F) P			N.S.
Btwn(R) P			(*)

Table 3C1 - 4

IESLC - Meta-anal of Ever Smoking (or Current if Ever not available), Any prod (or Cigs if Any not avail)
Adenocarcinoma
Least adjusted

REF	NRR	X	SEX	AGEL	AGEH	RACE	YF	LC TYPE	LOC	START	ST	NLC	R	VB	P	H	AD	SM	PRODUCT	DENOM	De
ABRAHA	2		m	0	0	all	0	a	Eu:est	1975	pr	571	n	bl	n	n	0	ev	all/unsp	nev any	ot
ABRAHA	5		f	0	0	all	0	a	Eu:est	1975	pr	571	n	bl	n	n	0	ev	all/unsp	nev any	ot
ALDERS	54		m	0	0	all	-	a	Eu:UK	1977	CC	1448	n	V	n	n	2	ev	all/unsp	nev any	or
ALDERS	57		f	0	0	all	-	a	Eu:UK	1977	CC	1448	n	V	n	n	1	ev	all/unsp	nev any	or
ANDERS	12		f	0	0	all	0	a	NAmer	1986	pr	343	n	bl	n	n	0	ev	cig+/-ot	nev cigs	st
BAND	2		m	0	0	all	-	a	NAmer	1983	CC	2831	n	V	y	y	2	ev	cig only	nev any	ot
BARBON	122	x	m	0	0	all	-	a	Eu:wst	1979	CC	755	n	bl	y	y	0	ev	all/unsp	nev any	st
BECHER	12		f	0	0	all	-	not q+s	Eu:Ger	1985	CC	194	n	bl	n	y	1	ev	all/unsp	nev any	or
BOUCOT	72	x	m	0	0	all	0	a	NAmer	1951	pr	121	n	bl	n	n	0	cu	cig only	nev any	ot
BRESLO	35		c	0	0	all	-	a	NAmer	1949	CC	518	n	bl	n	y	0	ev	all/unsp	nev+1	st
BROWN1	1	x	m	0	0	wh	-	a	NAmer	1979	CC	102	n	bl	y	y	0	ev	cig+/-ot	nev cigs	st
BROWN1	2	x	f	0	0	wh	-	a	NAmer	1979	CC	102	n	bl	y	y	0	ev	cig+/-ot	nev cigs	st
BROWN2	4		m	0	0	wh	-	a	NAmer	1984	CC	14596	n	bl	n	y	2	ev	cig+/-ot	nev cigs	or
BROWN2	3		f	0	0	wh	-	a	NAmer	1984	CC	14596	n	bl	n	y	2	ev	cig+/-ot	nev cigs	or
BUFFLE	50		m	0	0	wh	-	a	NAmer	1976	CC	943	n	bl	y	n	0	ev	cig+/-ot	nev cigs	ot
BUFFLE	45		f	0	0	wh	-	a	NAmer	1976	CC	943	n	bl	y	n	0	ev	cig+/-ot	nev cigs	ot
BYERS1	3		m	0	0	wh	-	a	NAmer	1957	CC	1002	n	bl	n	n	0	ev	cig+/-ot	nev cigs	st
CHAN	12		m	0	0	all	-	a+l	As:HK	1976	CC	397	n	bl	n	n	0	ev	all/unsp	nev any	ot
CHAN	16		f	0	0	all	-	a+l	As:HK	1976	CC	397	n	bl	n	n	0	ev	all/unsp	nev any	st
CHOI	63		m	0	0	all	-	a	As:oth	1985	CC	375	n	bl	n	n	0	ev	cig+/-ot	nev cigs	st
CHOI	65		f	0	0	all	-	a	As:oth	1985	CC	375	n	bl	n	n	0	ev	cig+/-ot	nev cigs	st
COMSTO	67		m	0	0	all	-	a	NAmer	1975	ot	258	n	bl	n	n	0	ev	cig+/-ot	nev cigs	st
COMSTO	79		f	0	0	all	-	a	NAmer	1975	ot	258	n	bl	n	n	0	ev	cig+/-ot	nev cigs	st
CORREA	36		c	0	0	all	-	a	NAmer	1979	CC	1359	n	bl	y	n	1	ev	cig+/-ot	nev cigs	or
CPSI	404		m	0	0	all	2	a	NAmer	1959	pr	5138	n	bl	n	n	1	cu	cig only	nev any	ot
CPSI	406		f	0	0	all	2	a	NAmer	1959	pr	5138	n	bl	n	n	1	cu	cig only	nev any	ot
CPSII	115		m	0	0	all	2	a	NAmer	1982	pr	3229	n	bl	n	n	1	cu	cig only	nev any	st
CPSII	118		f	0	0	all	2	a	NAmer	1982	pr	3229	n	bl	n	n	1	cu	cig+/-ot	nev cigs	st
DAMBER	11	x	m	0	0	all	-	a+al+br	Eu:Sca	1972	CC	579	n	bl	y	n	0	ev	all/unsp	nev any	st
DESTE2	17		m	0	0	all	-	a	SCAmer	1993	CC	463	n	bl	n	n	2	ev	all/unsp	nev any	or
DOLL	83	x	m	0	0	all	-	KII	Eu:UK	1948	CC	1465	n	V	n	n	0	ev	all/unsp	nev any	st
DOLL	85	x	f	0	0	all	-	KII	Eu:UK	1948	CC	1465	n	V	n	n	0	ev	all/unsp	nev any	st
DORGAN	125		m	0	0	wh	-	a	NAmer	1980	CC	2026	n	bl	y	y	2	ev	cig+/-ot	nev any	or
DORGAN	104		f	0	0	all	-	a	NAmer	1980	CC	2026	n	bl	y	y	3	ev	cig+/-ot	nev any	or
DORN	340		m	0	0	wh	8	a	NAmer	1954	pr	5097	n	bl	n	n	1	cu	cig only	nev any	ot
DOSEME	20	x	m	0	0	all	-	not q+s	Eu:bal	1979	CC	1210	n	bl	n	n	0	ev	cig+/-ot	nev cigs	st
ENGELA	76		m	0	0	all	0	a	Eu:Sca	1964	pr	435	n	bl	n	n	7	ev	cig+/-ot	nev cigs	ot
FAN	4		c	0	0	all	-	a	As:Chi	1990	CC	403	n	ot	y	n	0	ev	cig+/-ot	nev cigs	ot
GAO	8	x	m	0	0	all	-	a	As:Chi	1984	CC	1405	n	ot	n	n	0	ev	cig+/-ot	nev cigs	st
GAO	18	x	f	0	0	all	-	a	As:Chi	1984	CC	1405	n	ot	n	n	0	ev	cig+/-ot	nev cigs	st
GER	1	x	c	0	0	all	-	a	As:oth	1990	CC	141	n	ot	y	n	0	ev	all/unsp	nev any	st
HAENSZ	33	x	f	0	0	all	-	a	NAmer	1955	CC	158	n	bl	n	y	0	ev	all/unsp	nev any	or
HAMMON	92		m	0	0	wh	0	a	NAmer	1952	pr	448	n	bl	n	n	0	ev	all/unsp	nev any	st
HEGMAN	4		c	0	0	all	-	a	NAmer	1989	CC	282	n	bl	y	y	0	ev	all/unsp	nev any	st
HINDS	24		f	0	0	o	-	a	NAmer	1968	CC	292	n	bl	n	n	3	ev	all/unsp	nev any	st
ISHIMA	3	x	c	0	0	all	-	a	As:Jap	1961	CC	180	n	bl	y	y	0	ev	all/unsp	nev any	st
JAHN	47		m	0	0	all	-	a	Eu:Ger	1988	CC	1004	n	bl	n	n	0	ev	all/unsp	nev any	st
JAIN	7	x	m	0	0	all	-	a	NAmer	1981	CC	845	n	V	y	n	0	ev	cig+/-ot	nev cigs	st
JAIN	2	x	f	0	0	all	-	a	NAmer	1981	CC	845	n	V	y	n	0	ev	cig+/-ot	nev cigs	st
JEDRYC	21	x	m	0	0	all	-	a	Eu:est	1980	CC	1630	n	bl	y	n	0	ev	cig+/-ot	nev any	st
JOLY	51		m	0	0	all	-	a	SCAmer	1978	CC	826	n	bl	n	n	0	ev	cig+/-ot	nev any	st
JOLY	50		f	0	0	all	-	a	SCAmer	1978	CC	826	n	bl	n	n	0	ev	cig+/-ot	nev any	st
JUSSAW	26		m	0	0	all	-	KII	As:Ind	1964	CC	792	n	V	n	n	0	ev	all/unsp	nev any	st
KATSOU	31	x	f	0	0	all	-	a	Eu:bal	1987	CC	101	n	bl	n	n	0	ev	all/unsp	nev any	st
KHUDER	27		m	0	0	all	-	a	NAmer	1985	CC	482	n	bl	n	y	0	ev	cig+/-ot	nev cigs	ot
KIHARA	29		c	0	0	jap	-	a	As:Jap	1991	CC	440	n	bl	n	n	0	ev	all/unsp	nev any	st
KOO	7		f	0	0	all	-	a+l	As:HK	1981	CC	200	n	bl	n	n	0	ev	all/unsp	nev any	st
KREYBE	20	x	m	0	0	all	-	KII	Eu:Sca	1948	CC	300	n	bl	n	y	0	ev	all/unsp	nev any	st
KREYBE	36	x	f	0	0	all	-	KII	Eu:Sca	1948	CC	300	n	bl	n	y	0	ev	all/unsp	nev any	st
LAMTH	3		f	0	0	ch	-	a	As:HK	1983	CC	445	n	bl	n	n	0	ev	all/unsp	nev any	or
LAMWK	4		f	0	0	ch	-	a	As:HK	1981	CC	163	n	bl	n	n	0	ev	all/unsp	nev any	st
LAMWK2	3		m	0	0	all	-	a	As:HK	1976	CC	480	n	bl	n	n	0	ev	all/unsp	nev any	st
LAMWK2	7		f	0	0	all	-	a	As:HK	1976	CC	480	n	bl	n	n	0	ev	all/unsp	nev any	st
LOMBA2	3		f	0	0	all	-	not q+u	NAmer	1960	CC	225	n	bl	n	n	0	ev	cig+/-ot	nev cigs	st
LUBIN	36		m	0	0	all	-	KII	As:Chi	1984	CC	427	m	ot	y	n	0	ev	all/unsp	nev any	st
LUBIN2	148		m	0	0	all	-	a	Eu:mul	1976	CC	7804	n	bl	n	y	0	ev	cig+/-ot	nev any	st
LUBIN2	168		f	0	0	all	-	a	Eu:mul	1976	CC	7804	n	bl	n	y	0	ev	cig+/-ot	nev any	st
LUO	3	x	c	0	0	all	-	a	As:Chi	1990	CC	102	n	ot	n	y	0	ev	cig+/-ot	nev cigs	st
MATOS	68	x	m	0	0	all	-	a	SCAmer	1994	CC	200	n	bl	n	n	0	ev	cig+/-ot	nev any	st
MATSUD	12		m	0	0	all	-	a	As:Jap	1965	CC	179	n	bl	n	n	0	ev	cig+/-ot	nev cigs	ot
NOU	3		m	0	0	all	-	a	Eu:Sca	1971	CC	273	n	bl	y	n	0	ev	all/unsp	nev any	st
NOU	8		f	0	0	all	-	a	Eu:Sca	1971	CC	273	n	bl	y	n	0	ev	all/unsp	nev any	st

Table 3C1 - 4

IESLC - Meta-anal of Ever Smoking (or Current if Ever not available), Any prod (or Cigs if Any not avail)
Adenocarcinoma
Least adjusted

REF	NRR	X	SEX	AGEL	AGEH	RACE	YF	LC TYPE	LOC	START	ST	NLC	R	VB	P	H	AD	SM	PRODUCT	DENOM	De
ORMOS	21		m	0	0	all	-	a	Eu:est	1947	CC	119	n	bl	y	y	0	ev	cig+/-ot	nev any	ot
OSANN	19	x	m	0	0	all	-	a	NAmer	1984	CC	1986	n	bl	n	n	0	ev	cig+/-ot	nev cigs	st
OSANN	23	x	f	0	0	all	-	a	NAmer	1984	CC	1986	n	bl	n	n	0	ev	cig+/-ot	nev cigs	st
OSANN2	13	x	f	0	0	all	-	KII	NAmer	1964	ot	217	n	bl	n	y	0	ev	cig+/-ot	nev cigs	st
PEZZOT	7		m	0	0	all	-	a	SCAmer	1987	CC	215	n	bl	n	y	0	ev	cig only	nev cigs	st
SCHWAR	8		m	40	54	wh	-	a	NAmer	1984	CC	5588	n	bl	y	y	0	ev	cig+/-ot	nev cigs	st
SCHWAR	7		m	40	54	bl	-	a	NAmer	1984	CC	5588	n	bl	y	y	0	ev	cig+/-ot	nev cigs	st
SCHWAR	16		f	40	54	wh	-	a	NAmer	1984	CC	5588	n	bl	y	y	0	ev	cig+/-ot	nev cigs	st
SCHWAR	15		f	40	54	bl	-	a	NAmer	1984	CC	5588	n	bl	y	y	0	ev	cig+/-ot	nev cigs	st
SEOW	2		f	0	0	ch	-	a	As:oth	1997	CC	153	n	bl	n	y	0	ev	cig+/-ot	nev cigs	st
SIEMIA	12	x	m	0	0	all	-	a	NAmer	1979	CC	857	n	V	y	y	0	ev	cig+/-ot	nev cigs	st
SOBUE	7	x	m	0	0	all	-	a	As:Jap	1986	CC	1376	n	bl	n	y	0	ev	cig+/-ot	nev cigs	st
SOBUE	23	x	f	0	0	all	-	a	As:Jap	1986	CC	1376	n	bl	n	y	0	ev	cig+/-ot	nev cigs	st
SOBUE2	2		m	0	0	all	-	a	As:Jap	1965	CC	2083	n	bl	n	n	2	cu	cig+/-ot	nev any	or
SOBUE2	6		f	0	0	all	-	a	As:Jap	1965	CC	2083	n	bl	n	n	2	cu	cig+/-ot	nev any	or
STASZE	21		m	0	0	all	-	a	Eu:est	1954	CC	281	n	bl	n	y	0	ev	all/unsp	nev any	ot
STASZE	4		f	0	0	all	-	a	Eu:est	1954	CC	281	n	bl	n	y	0	ev	all/unsp	nev any	st
STAYNE	4		m	0	0	all	-	a	NAmer	1969	CC	420	n	bl	n	n	0	ev	all/unsp	nev any	st
SUZUK2	13	x	c	0	0	all	-	a	SCAmer	1991	CC	123	n	bl	n	y	0	ev	all/unsp	nev any	st
SUZUKI	3	x	m	0	0	all	-	a	As:Jap	1978	CC	238	n	bl	n	y	0	ev	cig+/-ot	nev any	st
SUZUKI	7	x	f	0	0	all	-	a	As:Jap	1978	CC	238	n	bl	n	y	0	ev	cig+/-ot	nev any	st
SVENSS	59	x	f	0	0	all	-	a	Eu:Sca	1983	CC	210	n	bl	n	n	0	ev	all/unsp	nev any	st
TIZZAN	19		c	0	0	all	-	a	Eu:wst	1959	CC	1358	n	bl	n	n	0	ev	all/unsp	nev any	st
TOKARS	7	x	c	0	0	all	-	a	Eu:est	1966	ot	162	o	bl	n	y	0	ev	all/unsp	nev any	st
TSUGAN	2	x	m	0	0	all	-	a	As:Jap	1976	CC	134	n	bl	n	y	0	ev	all/unsp	nev any	st
TSUGAN	8	x	f	0	0	all	-	a	As:Jap	1976	CC	134	n	bl	n	y	0	ev	all/unsp	nev any	or
WAKAI	17	x	m	0	0	all	-	a	As:Jap	1988	CC	333	n	bl	n	y	0	ev	all/unsp	nev any	st
WAKAI	35	x	f	0	0	all	-	a	As:Jap	1988	CC	333	n	bl	n	y	0	ev	all/unsp	nev any	st
WU	5	x	f	0	0	wh	-	a	NAmer	1981	CC	220	n	bl	n	y	0	ev	all/unsp	nev any	st
WU2	1		f	0	0	all	-	a	NAmer	1983	CC	336	n	bl	n	y	2	cu	all/unsp	nev any	or
WUWILL	25	x	f	0	0	all	-	a	As:Chi	1985	CC	965	n	ot	n	n	0	ev	cig+/-ot	nev cigs	st
WYNDE2	14		m	0	0	all	-	KII	NAmer	1962	CC	404	n	bl	n	y	0	ev	all/unsp	nev any	st
WYNDE3	29		m	0	0	all	-	KII	NAmer	1966	CC	350	n	bl	n	y	0	ev	all/unsp	nev any	st
WYNDE3	135		f	0	0	all	-	KII	NAmer	1966	CC	350	n	bl	n	y	0	ev	all/unsp	nev any	st
WYNDE4	42		m	0	0	all	-	a	NAmer	1948	CC	684	n	bl	y	n	0	ev	all/unsp	nev any	st
WYNDE4	56		f	0	0	all	-	a	NAmer	1948	CC	684	n	bl	y	n	2	ev	all/unsp	nev any	ot
WYNDE6	69		m	0	0	all	-	KII	NAmer	1969	CC	4423	n	bl	n	y	0	ev	all/unsp	nev any	st
WYNDE6	413	x	f	0	0	wh	-	a	NAmer	1969	CC	4423	n	bl	n	y	0	ev	cig+/-ot	nev cigs	st
XU3	21	x	m	0	0	all	-	KII	As:Chi	1981	CC	135	n	ot	n	n	0	ev	all/unsp	nev any	st
XU3	25	x	f	0	0	all	-	KII	As:Chi	1981	CC	135	n	ot	n	n	0	ev	all/unsp	nev any	st
ZHENG	10		m	0	0	all	-	a	As:Chi	1982	CC	540	n	ot	*	y	0	ev	cig+/-ot	nev cigs	st
ZHENG	21		f	0	0	all	-	a	As:Chi	1982	CC	540	n	ot	*	y	0	ev	cig+/-ot	nev cigs	st
ZHOU	26		m	0	0	all	-	a	As:Chi	1978	CC	1360	n	ot	n	n	0	ev	all/unsp	nev any	st
ZHOU	27		f	0	0	all	-	a	As:Chi	1978	CC	1360	n	ot	n	n	0	ev	all/unsp	nev any	st

Cigarette type is all/unspec for all RRs

Table 3C1 - 5

IESLC - Meta-anal of Ever Smoking (or Current if Ever not available), Any prod (or Cigs if Any not avail)
Adenocarcinoma
Least adjusted

REF	NRR	SEX	AD	Number Exposed Case	Number Exposed Cont	Non-exposed Case	Non-exposed Cont	RR	95.00%CI
*ABRAHA	2	m	0	59	10351	8	3365	2.40	(1.15- 5.01)
*ABRAHA	5	f	0	19	5256	16	11589	2.62	(1.35- 5.09)
Subtotal ABRAHA								2.52	(1.54- 4.12)
ALDERS	54	m	2	-	-	-	-	7.11	(1.49- 33.85)
ALDERS	57	f	1	-	-	-	-	3.58	(1.48- 8.65)
Subtotal ALDERS								4.23	(1.96- 9.12)
*ANDERS	12	f	0	99	96164	33	195158	6.09	(4.11- 9.03)
BAND	2	m	2	-	-	-	-	4.10	(3.01- 5.59)
BARBON	122	m	0	151	567	7	188	7.15	(3.29- 15.53)
BECHER	12	f	1	-	-	-	-	10.83	(1.32- 88.70)
*BOUCOT	72	m	0	14	22177	0	7551	9.87~	(0.59- 165.51)
BRESLO	35	c	0	42	462	4	56	1.27	(0.44- 3.68)
BROWN1	1	m	0	46	46	4	19	4.75	(1.50- 15.05)
BROWN1	2	f	0	33	19	19	47	4.30	(1.98- 9.34)
Subtotal BROWN1								4.43	(2.33- 8.44)
BROWN2	4	m	2	-	-	-	-	8.20	(6.90- 9.70)
BROWN2	3	f	2	-	-	-	-	6.90	(6.10- 7.90)
Subtotal BROWN2								7.35	(6.63- 8.15)
BUFFLE	50	m	0	-	-	-	-	4.50	(1.85- 10.95)
BUFFLE	45	f	0	-	-	-	-	4.02	(2.42- 6.67)
Subtotal BUFFLE								4.13	(2.66- 6.42)
BYERS1	3	m	0	47	695	7	424	4.10	(1.83- 9.15)
CHAN	12	m	0	56	161	0	43	30.44~	(1.84- 502.58)
CHAN	16	f	0	28	50	40	139	1.95	(1.09- 3.48)
Subtotal CHAN								2.18	(1.23- 3.85)
CHOI	63	m	0	46	465	7	95	1.34	(0.59- 3.06)
CHOI	65	f	0	5	26	49	164	0.64	(0.23- 1.77)
Subtotal CHOI								1.00	(0.53- 1.89)
COMSTO	67	m	0	43	229	2	84	7.89	(1.87- 33.27)
COMSTO	79	f	0	29	87	8	115	4.79	(2.09- 11.00)
Subtotal COMSTO								5.43	(2.64- 11.14)
CORREA	36	c	1	-	-	-	-	5.60	(3.60- 8.80)
*CPSI	404	m	1	-	-	-	-	4.58	(1.74- 12.05)
*CPSI	406	f	1	-	-	-	-	1.43	(0.47- 4.39)
Subtotal CPSI								2.78	(1.34- 5.78)
*CPSII	115	m	1	-	-	-	-	19.22	(6.46- 57.16)
*CPSII	118	f	1	-	-	-	-	8.23	(4.36- 15.54)
Subtotal CPSII								10.21	(5.89- 17.67)
DAMBER	11	m	0	65	49	16	29	2.40	(1.18- 4.91)
DESTE2	17	m	2	-	-	-	-	4.30	(1.60- 11.40)
DOLL	83	m	0	38	1296	2	61	0.89	(0.21- 3.79)
DOLL	85	f	0	8	49	5	59	1.93	(0.59- 6.27)
Subtotal DOLL								1.42	(0.57- 3.53)
DORGAN	125	m	2	-	-	-	-	4.80	(1.90- 12.00)
DORGAN	104	f	3	-	-	-	-	3.90	(2.80- 5.40)
Subtotal DORGAN								3.99	(2.93- 5.44)
*DORN	340	m	1	-	-	-	-	5.95	(3.85- 9.22)
DOSEME	20	m	0	142	536	24	293	3.23	(2.05- 5.10)
*ENGELA	76	m	7	-	-	-	-	2.33	(0.92- 5.89)
FAN	4	c	0	67	595	45	556	1.39	(0.94- 2.07)
GAO	8	m	0	180	558	42	202	1.55	(1.07- 2.25)
GAO	18	f	0	62	130	266	605	1.08	(0.78- 1.52)
Subtotal GAO								1.27	(0.99- 1.63)
GER	1	c	0	35	139	37	149	1.01	(0.60- 1.70)
HAENSZ	33	f	0	18	103	37	236	1.11	(0.61- 2.05)
*HAMMON	92	m	0	29	510108	2	115884	3.29	(0.79- 13.80)
HEGMAN	4	c	0	83	1202	15	2080	9.58	(5.50- 16.67)
HINDS	24	f	3	-	-	-	-	3.89	(2.49- 6.07)
ISHIMA	3	c	0	39	25	13	27	3.24	(1.41- 7.43)
JAHN	47	m	0	204	701	8	138	5.02	(2.42- 10.41)
JAIN	7	m	0	90	277	4	85	6.90	(2.46- 19.35)
JAIN	2	f	0	86	196	24	214	3.91	(2.39- 6.40)
Subtotal JAIN								4.35	(2.79- 6.78)
JEDRYC	21	m	0	99	1054	7	289	3.88	(1.78- 8.44)
JOLY	51	m	0	72	709	5	218	4.43	(1.77- 11.10)
JOLY	50	f	0	33	122	25	283	3.06	(1.75- 5.37)
Subtotal JOLY								3.38	(2.10- 5.46)
JUSSAW	26	m	0	34	168	13	624	9.71	(5.01- 18.82)
KATSOU	31	f	0	18	22	30	67	1.83	(0.86- 3.90)
KHUDER	27	m	0	155	-	7	-	8.11	(3.67- 17.93)
KIHARA	29	c	0	130	232	78	237	1.70	(1.22- 2.38)

Table 3C1 - 5

IESLC - Meta-anal of Ever Smoking (or Current if Ever not available), Any prod (or Cigs if Any not avail)
Adenocarcinoma
Least adjusted

REF	NRR	SEX	AD	Number Exposed Case	Number Exposed Cont	Non-exposed Case	Non-exposed Cont	RR	95.00%CI
KOO	7	f	0	34	63	46	137	1.61	(0.94- 2.74)
KREYBE	20	m	0	42	3514	3	644	2.57	(0.79- 8.30)
KREYBE	36	f	0	10	328	27	657	0.74	(0.35- 1.55)
Subtotal KREYBE								1.05	(0.56- 1.97)
LAMTH	3	f	0	79	51	131	158	1.87	(1.23- 2.85)
LAMWK	4	f	0	36	41	60	144	2.11	(1.23- 3.61)
LAMWK2	3	m	0	52	161	15	43	0.93	(0.48- 1.80)
LAMWK2	7	f	0	26	50	41	139	1.76	(0.98- 3.17)
Subtotal LAMWK2								1.33	(0.86- 2.07)
LOMBA2	3	f	0	42	353	54	239	0.53	(0.34- 0.81)
LUBIN	36	m	0	37	939	4	72	0.71	(0.25- 2.05)
LUBIN2	148	m	0	655	10433	57	2616	2.88	(2.19- 3.79)
LUBIN2	168	f	0	85	567	138	1180	1.28	(0.96- 1.71)
Subtotal LUBIN2								1.96	(1.61- 2.39)
LUO	3	c	0	28	146	29	160	1.06	(0.60- 1.86)
MATOS	68	m	0	79	283	5	110	6.14	(2.42- 15.57)
MATSUD	12	m	0	23	3314	0	1255	17.80~	(1.08- 293.32)
NOU	3	m	0	36	247	4	122	4.45	(1.55- 12.77)
NOU	8	f	0	9	92	29	261	0.88	(0.40- 1.93)
Subtotal NOU								1.57	(0.83- 2.94)
ORMOS	21	m	0	4	1034	0	777	6.76~	(0.36- 125.82)
OSANN	19	m	0	319	1018	14	833	18.64	(10.83- 32.09)
OSANN	23	f	0	243	563	47	1093	10.04	(7.23- 13.94)
Subtotal OSANN								11.85	(8.95- 15.69)
OSANN2	13	f	0	61	40	22	43	2.98	(1.56- 5.71)
PEZZOT	7	m	0	60	317	3	116	7.32	(2.25- 23.79)
SCHWAR	8	m	0	84	178	1	73	34.45	(4.71- 252.10)
SCHWAR	7	m	0	45	39	1	7	8.08	(0.95- 68.56)
SCHWAR	16	f	0	92	108	10	79	6.73	(3.29- 13.75)
SCHWAR	15	f	0	20	28	3	41	9.76	(2.65- 36.00)
Subtotal SCHWAR								8.40	(4.73- 14.94)
SEOW	2	f	0	19	15	67	125	2.36	(1.13- 4.95)
SIEMIA	12	m	0	162	428	5	105	7.95	(3.18- 19.85)
SOBUE	7	m	0	393	1013	27	128	1.84	(1.20- 2.83)
SOBUE	23	f	0	58	232	137	857	1.56	(1.11- 2.20)
Subtotal SOBUE								1.66	(1.27- 2.17)
SOBUE2	2	m	2	-	-	-	-	3.10	(2.40- 3.70)
SOBUE2	6	f	2	-	-	-	-	1.80	(1.40- 2.20)
Subtotal SOBUE2								2.39	(2.04- 2.79)
STASZE	21	m	0	20	754	0	158	8.61~	(0.52- 143.15)
STASZE	4	f	0	1	153	10	1660	1.08	(0.14- 8.53)
Subtotal STASZE								2.24	(0.42- 11.81)
STAYNE	4	m	0	43	567	7	333	3.61	(1.60- 8.11)
SUZUK2	13	c	0	20	10	5	15	6.00	(1.69- 21.26)
SUZUKI	3	m	0	144	217	14	99	4.69	(2.58- 8.53)
SUZUKI	7	f	0	25	27	55	133	2.24	(1.19- 4.20)
Subtotal SUZUKI								3.30	(2.14- 5.09)
SVENSS	59	f	0	50	89	22	120	3.06	(1.73- 5.43)
TIZZAN	19	c	0	88	939	25	419	1.57	(0.99- 2.49)
TOKARS	7	c	0	68	112	10	54	3.28	(1.57- 6.86)
TSUGAN	2	m	0	53	56	18	17	0.89	(0.42- 1.91)
TSUGAN	8	f	0	6	10	33	30	0.55	(0.18- 1.68)
Subtotal TSUGAN								0.77	(0.41- 1.44)
WAKAI	17	m	0	98	424	8	65	1.88	(0.87- 4.04)
WAKAI	35	f	0	13	31	46	145	1.32	(0.64- 2.74)
Subtotal WAKAI								1.56	(0.92- 2.65)
WU	5	f	0	120	87	29	62	2.95	(1.75- 4.96)
WU2	1	f	2	-	-	-	-	4.50	(3.00- 6.90)
WUWILL	25	f	0	138	351	172	601	1.37	(1.06- 1.78)
WYNDE2	14	m	0	49	616	5	105	1.67	(0.65- 4.29)
WYNDE3	29	m	0	68	332	6	88	3.00	(1.26- 7.15)
WYNDE3	135	f	0	21	56	15	76	1.90	(0.90- 4.01)
Subtotal WYNDE3								2.31	(1.31- 4.07)
WYNDE4	42	m	0	35	665	4	115	1.51	(0.53- 4.34)
WYNDE4	56	f	2	-	-	-	-	0.60	(0.13- 2.69)
Subtotal WYNDE4								1.12	(0.47- 2.66)
WYNDE6	69	m	0	1079	1996	58	617	5.75	(4.35- 7.60)
WYNDE6	413	f	0	326	275	58	673	13.76	(10.06- 18.80)
Subtotal WYNDE6								8.47	(6.88- 10.43)
XU3	21	m	0	29	68	3	31	4.41	(1.25- 15.57)
XU3	25	f	0	4	11	7	25	1.30	(0.31- 5.36)

Table 3C1 - 5

IESLC - Meta-anal of Ever Smoking (or Current if Ever not available), Any prod (or Cigs if Any not avail)
Adenocarcinoma
Least adjusted

REF	NRR	SEX	AD	Number Exposed Case	Cont	Non-exposed Case	Cont	RR	95.00%CI
Subtotal XU3								2.57	(1.00- 6.59)
ZHENG	10	m	0	123	218	29	94	1.83	(1.14- 2.93)
ZHENG	21	f	0	33	44	119	184	1.16	(0.70- 1.93)
Subtotal ZHENG								1.48	(1.05- 2.09)
ZHOU	26	m	0	131	41	88	36	1.31	(0.77- 2.20)
ZHOU	27	f	0	30	7	96	32	1.43	(0.57- 3.57)
Subtotal ZHOU								1.34	(0.85- 2.10)
Partial Totals				8052	688777	2831	359574		

*prospective study ~ With 0.5 adjustment for zero

REF	NRR	SEX	AD	Ys	Ws	Qs	Ps
*ABRAHA	2	m	0	0.87	7.06	0.77	0.0201
*ABRAHA	5	f	0	0.96	8.71	0.51	0.0045
Subtotal ABRAHA				0.92	15.77	1.28	
ALDERS	54	m	2	1.96	1.58	0.90	0.0138
ALDERS	57	f	1	1.28	4.93	0.03	0.0046
Subtotal ALDERS				1.44	6.50	0.93	
*ANDERS	12	f	0	1.81	24.76	8.98	0.0000
BAND	2	m	2	1.41	40.10	1.72	0.0000
BARBON	122	m	0	1.97	6.39	3.72	0.0000
BECHER	12	f	1	2.38	0.87	1.20	0.0265
*BOUCOT	72	m	0	2.29	0.48	0.57	0.1114
BRESLO	35	c	0	0.24	3.40	3.16	0.6564
BROWN1	1	m	0	1.56	2.89	0.36	0.0081
BROWN1	2	f	0	1.46	6.38	0.41	0.0002
Subtotal BROWN1				1.49	9.27	0.77	
BROWN2	4	m	2	2.10	132.45	107.29	0.0000
BROWN2	3	f	2	1.93	229.82	121.60	0.0000
Subtotal BROWN2				1.99	362.27	228.89	
BUFFLE	50	m	0	1.50	4.86	0.44	0.0009
BUFFLE	45	f	0	1.39	14.95	0.52	0.0000
Subtotal BUFFLE				1.42	19.81	0.96	
BYERS1	3	m	0	1.41	5.95	0.25	0.0006
CHAN	12	m	0	3.42	0.49	2.39	0.0170
CHAN	16	f	0	0.67	11.38	3.30	0.0247
Subtotal CHAN				0.78	11.86	5.69	
CHOI	63	m	0	0.29	5.64	4.67	0.4842
CHOI	65	f	0	-0.44	3.77	10.21	0.3920
Subtotal CHOI				-0.00	9.42	14.88	
COMSTO	67	m	0	2.07	1.85	1.37	0.0049
COMSTO	79	f	0	1.57	5.57	0.73	0.0002
Subtotal COMSTO				1.69	7.42	2.11	
CORREA	36	c	1	1.72	19.23	5.17	0.0000
*CPSI	404	m	1	1.52	4.10	0.41	0.0021
*CPSI	406	f	1	0.36	3.08	2.21	0.5303
Subtotal CPSI				1.02	7.18	2.62	
*CPSII	115	m	1	2.96	3.23	9.92	0.0000
*CPSII	118	f	1	2.11	9.51	7.77	0.0000
Subtotal CPSII				2.32	12.75	17.69	
DAMBER	11	m	0	0.88	7.53	0.80	0.0161
DESTE2	17	m	2	1.46	3.99	0.26	0.0036
DOLL	83	m	0	-0.11	1.84	3.19	0.8795
DOLL	85	f	0	0.66	2.76	0.83	0.2760
Subtotal DOLL				0.35	4.60	4.02	
DORGAN	125	m	2	1.57	4.52	0.60	0.0008
DORGAN	104	f	3	1.36	35.62	0.88	0.0000
Subtotal DORGAN				1.38	40.15	1.48	
*DORN	340	m	1	1.78	20.15	6.76	0.0000
DOSEME	20	m	0	1.17	18.52	0.02	0.0000
*ENGELA	76	m	7	0.85	4.46	0.57	0.0741
FAN	4	c	0	0.33	24.61	18.80	0.1013
GAO	8	m	0	0.44	27.69	16.20	0.0208
GAO	18	f	0	0.08	34.21	43.12	0.6343
Subtotal GAO				0.24	61.90	59.33	
GER	1	c	0	0.01	14.39	20.38	0.9579
HAENSZ	33	f	0	0.11	10.36	12.43	0.7268
*HAMMON	92	m	0	1.19	1.87	0.00	0.1030
HEGMAN	4	c	0	2.26	12.50	13.91	0.0000
HINDS	24	f	3	1.36	19.35	0.46	0.0000
ISHIMA	3	c	0	1.18	5.57	0.00	0.0055

Table 3C1 - 5

IESLC - Meta-anal of Ever Smoking (or Current if Ever not available), Any prod (or Cigs if Any not avail)
Adenocarcinoma
Least adjusted

REF	NRR	SEX	AD	Ys	Ws	Qs	Ps
JAHN	47	m	0	1.61	7.22	1.21	0.0000
JAIN	7	m	0	1.93	3.62	1.92	0.0002
JAIN	2	f	0	1.36	15.86	0.41	0.0000
Subtotal JAIN				1.47	19.47	2.32	
JEDRYC	21	m	0	1.36	6.35	0.15	0.0006
JOLY	51	m	0	1.49	4.55	0.37	0.0015
JOLY	50	f	0	1.12	12.19	0.09	0.0001
Subtotal JOLY				1.22	16.74	0.45	
JUSSAW	26	m	0	2.27	8.78	10.04	0.0000
KATSOU	31	f	0	0.60	6.70	2.42	0.1187
KHUDER	27	m	0	2.09	6.11	4.83	0.0000
KIHARA	29	c	0	0.53	34.43	15.55	0.0018
KOO	7	f	0	0.47	13.45	7.16	0.0817
KREYBE	20	m	0	0.94	2.79	0.19	0.1158
KREYBE	36	f	0	-0.30	7.06	15.95	0.4275
Subtotal KREYBE				0.05	9.85	16.14	
LAMTH	3	f	0	0.63	21.63	7.25	0.0036
LAMWK	4	f	0	0.75	13.20	2.78	0.0068
LAMWK2	3	m	0	-0.08	8.67	14.23	0.8206
LAMWK2	7	f	0	0.57	11.11	4.51	0.0588
Subtotal LAMWK2				0.28	19.77	18.74	
LOMBA2	3	f	0	-0.64	20.27	69.02	0.0039
LUBIN	36	m	0	-0.34	3.42	8.20	0.5249
LUBIN2	148	m	0	1.06	51.15	1.09	0.0000
LUBIN2	168	f	0	0.25	46.25	42.25	0.0913
Subtotal LUBIN2				0.67	97.40	43.34	
LUO	3	c	0	0.06	12.01	15.81	0.8449
MATOS	68	m	0	1.82	4.44	1.66	0.0001
MATSUD	12	m	0	2.88	0.49	1.37	0.0440
NOU	3	m	0	1.49	3.45	0.29	0.0056
NOU	8	f	0	-0.13	6.24	11.06	0.7504
Subtotal NOU				0.45	9.69	11.34	
ORMOS	21	m	0	1.91	0.45	0.23	0.1999
OSANN	19	m	0	2.93	13.03	38.61	0.0000
OSANN	23	f	0	2.31	35.61	43.26	0.0000
Subtotal OSANN				2.47	48.64	81.87	
OSANN2	13	f	0	1.09	9.08	0.11	0.0010
PEZZOT	7	m	0	1.99	2.76	1.71	0.0009
SCHWAR	8	m	0	3.54	0.97	5.29	0.0005
SCHWAR	7	m	0	2.09	0.84	0.66	0.0556
SCHWAR	16	f	0	1.91	7.53	3.72	0.0000
SCHWAR	15	f	0	2.28	2.26	2.60	0.0006
Subtotal SCHWAR				2.13	11.60	12.26	
SEOW	2	f	0	0.86	7.03	0.83	0.0226
SIEMIA	12	m	0	2.07	4.59	3.46	0.0000
SOBUE	7	m	0	0.61	20.67	7.31	0.0056
SOBUE	23	f	0	0.45	33.31	19.09	0.0099
Subtotal SOBUE				0.51	53.98	26.40	
SOBUE2	2	m	2	1.13	82.01	0.43	0.0000
SOBUE2	6	f	2	0.59	75.22	28.57	0.0000
Subtotal SOBUE2				0.87	157.22	29.01	
STASZE	21	m	0	2.15	0.49	0.44	0.1332
STASZE	4	f	0	0.08	0.90	1.14	0.9382
Subtotal STASZE				0.81	1.39	1.58	
STAYNE	4	m	0	1.28	5.85	0.04	0.0019
SUZUK2	13	c	0	1.79	2.40	0.83	0.0055
SUZUKI	3	m	0	1.55	10.74	1.26	0.0000
SUZUKI	7	f	0	0.81	9.73	1.54	0.0119
Subtotal SUZUKI				1.19	20.48	2.80	
SVENSS	59	f	0	1.12	11.76	0.08	0.0001
TIZZAN	19	c	0	0.45	18.24	10.33	0.0538
TOKARS	7	c	0	1.19	7.03	0.00	0.0016
TSUGAN	2	m	0	-0.11	6.62	11.47	0.7728
TSUGAN	8	f	0	-0.61	3.03	9.92	0.2916
Subtotal TSUGAN				-0.27	9.65	21.39	
WAKAI	17	m	0	0.63	6.54	2.15	0.1071
WAKAI	35	f	0	0.28	7.26	6.21	0.4522
Subtotal WAKAI				0.45	13.79	8.36	
WU	5	f	0	1.08	14.20	0.21	0.0000
WU2	1	f	2	1.50	22.15	1.99	0.0000
WUWILL	25	f	0	0.32	56.90	44.73	0.0166

Table 3C1 - 5

IESLC - Meta-anal of Ever Smoking (or Current if Ever not available), Any prod (or Cigs if Any not avail)
Adenocarcinoma
Least adjusted

REF	NRR	SEX	AD	Ys	Ws	Qs	Ps
WYNDE2	14	m	0	0.51	4.32	2.06	0.2863
WYNDE3	29	m	0	1.10	5.11	0.06	0.0129
WYNDE3	135	f	0	0.64	6.88	2.18	0.0922
Subtotal WYNDE3				0.84	11.99	2.23	
WYNDE4	42	m	0	0.41	3.46	2.16	0.4408
WYNDE4	56	f	2	-0.51	1.67	4.92	0.5087
Subtotal WYNDE4				0.11	5.14	7.08	
WYNDE6	69	m	0	1.75	49.29	14.65	0.0000
WYNDE6	413	f	0	2.62	39.32	78.99	0.0000
Subtotal WYNDE6				2.14	88.61	93.64	
XU3	21	m	0	1.48	2.41	0.19	0.0213
XU3	25	f	0	0.26	1.91	1.70	0.7180
Subtotal XU3				0.94	4.32	1.88	
ZHENG	10	m	0	0.60	17.29	6.23	0.0121
ZHENG	21	f	0	0.15	14.95	16.68	0.5668
Subtotal ZHENG				0.39	32.24	22.91	
ZHOU	26	m	0	0.27	14.05	12.32	0.3154
ZHOU	27	f	0	0.36	4.59	3.30	0.4448
Subtotal ZHOU				0.29	18.64	15.62	

	N	116
	NS	81
	Wt	1789.25
Het	Chi	1073.23
Het	df	115
Het	P	***
Fixed	RR	3.33
	RRl	3.18
	RRu	3.49
	P	+++
Random	RR	2.92
	RRl	2.50
	RRu	3.42
	P	+++
Asymm	P	*

Table 3C1 - 6

IESLC - Meta-anal of Ever Smoking (or Current if Ever not available), Any prod (or Cigs if Any not avail)
Adenocarcinoma
Least adjusted

	combined	Sex male	female	Total
N	11	56	49	116
NS	11	55	48	114
Wt	153.82	670.17	965.26	1789.25
Het Chi	73.65	293.36	645.61	1073.23
Het df	10	55	48	115
Het P	***	***	***	***
Fixed RR	2.14	4.06	3.12	3.33
RRl	1.83	3.76	2.93	3.18
RRu	2.51	4.38	3.32	3.49
P	+++	+++	+++	+++
Random RR	2.36	3.75	2.37	2.92
RRl	1.50	3.06	1.84	2.50
RRu	3.71	4.61	3.05	3.42
P	+++	+++	+++	+++
Between Chi				60.61
Between df				2
Between P				***
Btwn(F) P				*
Btwn(R) P				*

	Lung cancer type a	a+l	a+al+br	KII	not q+u	not q+s	Total
N	96	3	1	13	1	2	116
NS	67	2	1	9	1	2	82
Wt	1611.09	25.32	7.53	105.65	20.27	19.39	1789.25
Het Chi	928.57	4.13	0.00	58.98	0.00	1.21	1073.23
Het df	95	2	0	12	0	1	115
Het P	***	N.S.	N.S.	***	N.S.	N.S.	***
Fixed RR	3.43	1.85	2.40	3.53	0.53	3.41	3.33
RRl	3.27	1.26	1.18	2.92	0.34	2.19	3.18
RRu	3.61	2.74	4.91	4.28	0.81	5.33	3.49
P	+++	++	+	+++	--	+++	+++
Random RR	3.08	2.05	2.40	2.29	0.53	3.76	2.92
RRl	2.60	1.05	1.18	1.40	0.34	1.72	2.50
RRu	3.64	3.99	4.91	3.76	0.81	8.20	3.42
P	+++	+	+	++	--	+++	+++
Between Chi							80.35
Between df							5
Between P							***
Btwn(F) P							N.S.
Btwn(R) P							***

	Location NAmer	UK	Scand	othEur	China	Japan	othAs	other	Total
N	47	4	7	15	12	13	12	6	116
NS	32	2	5	12	8	8	9	5	81
Wt	888.99	11.10	43.28	186.34	214.06	295.61	119.53	30.33	1789.25
Het Chi	320.36	4.43	15.96	42.83	9.68	41.23	43.03	3.02	1073.23
Het df	46	3	6	14	11	12	11	5	115
Het P	***	N.S.	*	***	N.S.	***	***	N.S.	***
Fixed RR	5.78	2.69	1.93	2.35	1.34	2.10	1.81	4.28	3.33
RRl	5.41	1.49	1.43	2.04	1.17	1.87	1.51	3.00	3.18
RRu	6.17	4.84	2.60	2.72	1.53	2.35	2.16	6.11	3.49
P	+++	+++	+++	+++	+++	+++	+++	+++	+++
Random RR	4.62	2.61	1.93	2.78	1.34	1.96	1.85	4.28	2.92
RRl	3.77	1.25	1.17	2.05	1.17	1.52	1.27	3.00	2.50
RRu	5.65	5.49	3.20	3.77	1.53	2.52	2.68	6.11	3.42
P	+++	+	+	+++	+++	+++	++	+++	+++
Between Chi									592.69
Between df									7
Between P									***
Btwn(F) P									***
Btwn(R) P									***

Table 3C1 - 6

IESLC - Meta-anal of Ever Smoking (or Current if Ever not available), Any prod (or Cigs if Any not avail)
Adenocarcinoma
Least adjusted

	Detailed Country in "other Europe"					
	multi	Germany	othWest	East	Balkans	Total
N	2	2	2	7	2	15
NS	1	2	2	5	2	12
Wt	97.40	8.08	24.63	31.00	25.22	186.34
Het Chi	15.93	0.46	10.87	2.75	1.60	42.83
Het df	1	1	1	6	1	14
Het P	***	N.S.	***	N.S.	N.S.	***
Fixed RR	1.96	5.45	2.33	2.95	2.78	2.35
RRl	1.61	2.74	1.57	2.07	1.88	2.04
RRu	2.39	10.86	3.45	4.19	4.11	2.72
P	+++	+++	+++	+++	+++	+++
Random RR	1.92	5.45	3.24	2.95	2.64	2.78
RRl	0.87	2.74	0.73	2.07	1.55	2.05
RRu	4.26	10.86	14.30	4.19	4.51	3.77
P	N.S.	+++	N.S.	+++	+++	+++
Between Chi						11.21
Between df						4
Between P						*
Btwn(F) P						N.S.
Btwn(R) P						N.S.

	Detailed Country in "other Asia"			
	India	HongKong	other	Total
N	1	7	4	12
NS	1	5	3	9
Wt	8.78	79.92	30.83	119.53
Het Chi	0.00	8.27	5.16	43.03
Het df	0	6	3	11
Het P	N.S.	N.S.	N.S.	***
Fixed RR	9.71	1.75	1.22	1.81
RRl	5.01	1.40	0.86	1.51
RRu	18.82	2.18	1.74	2.16
P	+++	+++	N.S.	+++
Random RR	9.71	1.74	1.24	1.85
RRl	5.01	1.33	0.76	1.27
RRu	18.82	2.28	2.01	2.68
P	+++	+++	N.S.	++
Between Chi				29.60
Between df				2
Between P				***
Btwn(F) P				**
Btwn(R) P				***

	Detailed other continent			
	SCAmer	Auslia	Africa	Total
N	6			6
NS	5			5
Wt	30.33			30.33
Het Chi	3.02			3.02
Het df	5			5
Het P	N.S.			N.S.
Fixed RR	4.28			4.28
RRl	3.00			3.00
RRu	6.11			6.11
P	+++			+++
Random RR	4.28			4.28
RRl	3.00			3.00
RRu	6.11			6.11
P	+++			+++
Between Chi				
Between df				
Between P				N.S.
Btwn(F) P				N.S.
Btwn(R) P				N.S.

Table 3C1 - 6

IESLC - Meta-anal of Ever Smoking (or Current if Ever not available), Any prod (or Cigs if Any not avail)
Adenocarcinoma
Least adjusted

	Start year of study <1960	1960-69	1970-79	1980-89	1990+	Total
N	18	16	31	43	8	116
NS	13	13	18	29	8	81
Wt	89.07	343.02	319.26	934.61	103.29	1789.25
Het Chi	49.48	208.52	113.24	542.32	22.76	1073.23
Het df	17	15	30	42	7	115
Het P	***	***	***	***	**	***
Fixed RR	2.19	3.30	2.52	4.15	1.65	3.33
RRl	1.78	2.97	2.25	3.89	1.36	3.18
RRu	2.70	3.67	2.81	4.42	2.00	3.49
P	+++	+++	+++	+++	+++	+++
Random RR	2.00	3.23	2.74	3.47	1.96	2.92
RRl	1.33	2.08	2.17	2.69	1.33	2.50
RRu	3.01	5.01	3.46	4.49	2.90	3.42
P	+++	+++	+++	+++	+++	+++
Between Chi						136.91
Between df						4
Between P						***
Btwn(F) P						**
Btwn(R) P						(*)

	Study type (1) CC	other	Total
N	101	15	116
NS	70	11	81
Wt	1678.30	110.95	1789.25
Het Chi	1031.75	28.69	1073.23
Het df	100	14	115
Het P	***	*	***
Fixed RR	3.26	4.63	3.33
RRl	3.11	3.85	3.18
RRu	3.42	5.58	3.49
P	+++	+++	+++
Random RR	2.77	4.34	2.92
RRl	2.34	3.24	2.50
RRu	3.28	5.83	3.42
P	+++	+++	+++
Between Chi			12.80
Between df			1
Between P			***
Btwn(F) P			N.S.
Btwn(R) P			**

	Study type (2) CC	prosp	other	Total
N	101	11	4	116
NS	70	8	3	81
Wt	1678.30	87.42	23.54	1789.25
Het Chi	1031.75	25.23	1.96	1073.23
Het df	100	10	3	115
Het P	***	**	N.S.	***
Fixed RR	3.26	4.92	3.70	3.33
RRl	3.11	3.99	2.47	3.18
RRu	3.42	6.07	5.55	3.49
P	+++	+++	+++	+++
Random RR	2.77	4.47	3.70	2.92
RRl	2.34	3.07	2.47	2.50
RRu	3.28	6.52	5.55	3.42
P	+++	+++	+++	+++
Between Chi				14.29
Between df				2
Between P				***
Btwn(F) P				N.S.
Btwn(R) P				*

Table 3C1 - 6

IESLC - Meta-anal of Ever Smoking (or Current if Ever not available), Any prod (or Cigs if Any not avail)
Adenocarcinoma
Least adjusted

	Study size (number of LC cases)				
	100-249	250-499	500-999	1000+	Total
N	28	32	18	38	116
NS	24	23	12	22	81
Wt	200.64	284.63	196.76	1107.21	1789.25
Het Chi	105.48	137.08	79.80	580.77	1073.23
Het df	27	31	17	37	115
Het P	***	***	***	***	***
Fixed RR	1.93	2.45	2.40	4.22	3.33
RRl	1.68	2.18	2.09	3.98	3.18
RRu	2.21	2.75	2.76	4.48	3.49
P	+++	+++	+++	+++	+++
Random RR	2.33	2.38	2.93	3.87	2.92
RRl	1.73	1.82	2.11	2.98	2.50
RRu	3.14	3.11	4.08	5.01	3.42
P	+++	+++	+++	+++	+++
Between Chi					170.11
Between df					3
Between P					***
Btwn(F) P					***
Btwn(R) P					*

	Risky occupational population			
	no	mining	othRisky	Total
N	114	1	1	116
NS	79	1	1	81
Wt	1778.79	3.42	7.03	1789.25
Het Chi	1065.01	0.00	0.00	1073.23
Het df	113	0	0	115
Het P	***	N.S.	N.S.	***
Fixed RR	3.34	0.71	3.28	3.33
RRl	3.19	0.25	1.57	3.18
RRu	3.50	2.05	6.86	3.49
P	+++	N.S.	++	+++
Random RR	2.95	0.71	3.28	2.92
RRl	2.52	0.25	1.57	2.50
RRu	3.46	2.05	6.86	3.42
P	+++	N.S.	++	+++
Between Chi				8.22
Between df				2
Between P				*
Btwn(F) P				N.S.
Btwn(R) P				*

	National cigarette tobacco type			
	Virginia	blended	other	Total
N	9	94	13	116
NS	6	66	9	81
Wt	84.04	1476.76	228.45	1789.25
Het Chi	15.38	819.24	10.73	1073.23
Het df	8	93	12	115
Het P	(*)	***	N.S.	***
Fixed RR	4.46	3.79	1.32	3.33
RRl	3.60	3.60	1.16	3.18
RRu	5.52	3.98	1.50	3.49
P	+++	+++	+++	+++
Random RR	4.55	3.17	1.32	2.92
RRl	3.18	2.68	1.16	2.50
RRu	6.52	3.76	1.50	3.42
P	+++	+++	+++	+++
Between Chi				227.88
Between df				2
Between P				***
Btwn(F) P				***
Btwn(R) P				***

Table 3C1 - 6

IESLC - Meta-anal of Ever Smoking (or Current if Ever not available), Any prod (or Cigs if Any not avail)
Adenocarcinoma
Least adjusted

	Any proxy use No/nk	Yes	Total
N	88	28	116
NS	62	19	81
Wt	1529.01	260.24	1789.25
Het Chi	960.37	112.21	1073.23
Het df	87	27	115
Het P	***	***	***
Fixed RR	3.31	3.49	3.33
RRl	3.15	3.09	3.18
RRu	3.48	3.94	3.49
P	+++	+++	+++
Random RR	2.74	3.62	2.92
RRl	2.28	2.75	2.50
RRu	3.29	4.77	3.42
P	+++	+++	+++
Between Chi			0.66
Between df			1
Between P			N.S.
Btwn(F) P			N.S.
Btwn(R) P			N.S.

	Full histological confirmation No	Yes	Total
N	67	49	116
NS	48	33	81
Wt	855.29	933.96	1789.25
Het Chi	453.46	486.89	1073.23
Het df	66	48	115
Het P	***	***	***
Fixed RR	2.51	4.33	3.33
RRl	2.35	4.06	3.18
RRu	2.68	4.61	3.49
P	+++	+++	+++
Random RR	2.74	3.19	2.92
RRl	2.26	2.52	2.50
RRu	3.32	4.03	3.42
P	+++	+++	+++
Between Chi			132.88
Between df			1
Between P			***
Btwn(F) P			***
Btwn(R) P			N.S.

	Number of adjustment variables (1) 0	1	2+/+nk	Total
N	95	8	13	116
NS	67	6	10	83
Wt	1071.22	65.11	652.93	1789.25
Het Chi	689.46	13.59	169.07	1073.23
Het df	94	7	12	115
Het P	***	(*)	***	***
Fixed RR	2.54	5.79	4.94	3.33
RRl	2.39	4.54	4.58	3.18
RRu	2.69	7.39	5.34	3.49
P	+++	+++	+++	+++
Random RR	2.67	5.69	3.88	2.92
RRl	2.24	3.88	2.75	2.50
RRu	3.18	8.34	5.47	3.42
P	+++	+++	+++	+++
Between Chi				201.11
Between df				2
Between P				***
Btwn(F) P				***
Btwn(R) P				***

Table 3C1 - 6

IESLC - Meta-anal of Ever Smoking (or Current if Ever not available), Any prod (or Cigs if Any not avail)
Adenocarcinoma
Least adjusted

	Number of adjustment variables (2) 0	1	2	3-5	6+/+nk	Total
N	95	8	10	2	1	116
NS	67	6	8	2	1	84
Wt	1071.22	65.11	593.50	54.97	4.46	1789.25
Het Chi	689.46	13.59	163.00	0.00	0.00	1073.23
Het df	94	7	9	1	0	115
Het P	***	(*)	***	N.S.	N.S.	***
Fixed RR	2.54	5.79	5.08	3.90	2.33	3.33
RRl	2.39	4.54	4.69	2.99	0.92	3.18
RRu	2.69	7.39	5.51	5.08	5.90	3.49
P	+++	+++	+++	+++	(+)	+++
Random RR	2.67	5.69	4.01	3.90	2.33	2.92
RRl	2.24	3.88	2.65	2.99	0.92	2.50
RRu	3.18	8.34	6.07	5.08	5.90	3.42
P	+++	+++	+++	+++	(+)	+++
Between Chi						207.19
Between df						4
Between P						***
Btwn(F) P						***
Btwn(R) P						**

	Product all/unsp	cig+/-ot	cig only	Total
N	56	53	7	116
NS	42	35	6	83
Wt	473.25	1242.09	73.91	1789.25
Het Chi	212.32	781.10	13.41	1073.23
Het df	55	52	6	115
Het P	***	***	*	***
Fixed RR	2.45	3.67	4.81	3.33
RRl	2.24	3.47	3.83	3.18
RRu	2.68	3.88	6.04	3.49
P	+++	+++	+++	+++
Random RR	2.28	3.48	5.23	2.92
RRl	1.88	2.75	3.34	2.50
RRu	2.76	4.41	8.19	3.42
P	+++	+++	+++	+++
Between Chi				66.40
Between df				2
Between P				***
Btwn(F) P				*
Btwn(R) P				***

	Denominator nev any	nev cigs	Total
N	75	41	116
NS	55	28	83
Wt	887.62	901.63	1789.25
Het Chi	306.54	664.35	1073.23
Het df	74	40	115
Het P	***	***	***
Fixed RR	2.62	4.23	3.33
RRl	2.45	3.96	3.18
RRu	2.80	4.51	3.49
P	+++	+++	+++
Random RR	2.57	3.67	2.92
RRl	2.21	2.73	2.50
RRu	2.99	4.93	3.42
P	+++	+++	+++
Between Chi			102.35
Between df			1
Between P			***
Btwn(F) P			***
Btwn(R) P			*

Table 3C1 - 6

IESLC - Meta-anal of Ever Smoking (or Current if Ever not available), Any prod (or Cigs if Any not avail)
Adenocarcinoma
Least adjusted

	Derivation of RR/CI: Orig	StdCalc	Other	Total
N	15	84	17	116
NS	11	60	14	85
Wt	647.40	999.60	142.26	1789.25
Het Chi	215.30	672.81	47.44	1073.23
Het df	14	83	16	115
Het P	***	***	***	***
Fixed RR	4.77	2.64	3.36	3.33
RRl	4.42	2.48	2.85	3.18
RRu	5.15	2.81	3.96	3.49
P	+++	+++	+++	+++
Random RR	3.47	2.76	3.42	2.92
RRl	2.41	2.29	2.42	2.50
RRu	4.99	3.32	4.84	3.42
P	+++	+++	+++	+++
Between Chi				137.69
Between df				2
Between P				***
Btwn(F) P				***
Btwn(R) P				N.S.

	Smoking status: ever	current	Total
N	107	9	116
NS	75	6	81
Wt	1569.32	219.93	1789.25
Het Chi	1014.33	56.72	1073.23
Het df	106	8	115
Het P	***	***	***
Fixed RR	3.38	3.04	3.33
RRl	3.21	2.66	3.18
RRu	3.55	3.47	3.49
P	+++	+++	+++
Random RR	2.83	4.30	2.92
RRl	2.39	2.77	2.50
RRu	3.34	6.69	3.42
P	+++	+++	+++
Between Chi			2.18
Between df			1
Between P			N.S.
Btwn(F) P			N.S.
Btwn(R) P			(*)

Table 3C1 - 7

IESLC - Meta-anal of Ever Smoking (or Current if Ever not available), Any prod (or Cigs if Any not avail)
Adenocarcinoma
Excluded studies (and stage at which they were excluded)

1	BOUCHA BUELL LAURIL MZILEN
2	BERRIN CHEN RESTRE TAO ULMER
5	RIMING TANG2 WYNDE5 WYNDE8
6	BLOT1 BLOT2 BLOT3 BYERS2 HIRAOK HIRAY2 JONES LICKIN MOLLO MRFIT MURATA NOTANI PERSHA SAARIK SCHWA2 SHIMIZ SITAS VANDER WARSIN WATSON WYNDER
8	ABELIN AGUDO AKIBA AMANDU AMES ARCHER ARMADA AUSTIN AUVINE AXELSO AXELSS BENSHL BEST BLOHMK BLOT4 BOFFET BRETT BROCKM BROSS CARPEN CASCO2 CASCOR CEDERL CHANG CHATZI CHEN2 CHEN3 CHIAZZ CHOW CHYOU COOKSO DARBY DAVEYS DEAN DEAN2 DEAN3 DEKLER DESTEF DOCKER DOLL2 DORANT DROSTE DU DUNN EBELIN ENSTRO ESAKI GAO2 GARCIA GARDIN GARSHI GENG GILLIS GODLEY GOLLED GOODMA GRAHAM GREGOR GSELL HAMMO2 HANSEN HEIN HENNEK HIRAYA HITOSU HOLE HOROWI HORWIT HU HU2 HUANG HUMBLE JARUP JARVHO JIANG KAISE2 KAISER KANELL KAUFMA KELLER KINLEN KJUUS KNEKT KO KOHLME KOULUM KREUZE KUBIK LANGE LAUSSM LEI LEMARC LETOUR LEVIN LIAW LIDDEL LIU LIU2 LIU3 LIU4 LIU5 LOMBAR MACLEN MAGNUS MARSH MARSH2 MARTIS MASTRA MCCONN MCDUFF MCLAUG MIGRAN MILLER MILLS MRFITR NAM NOTAN2 ODRISC PARKIN PASTOR PAWLEG PERNU PERSH2 PETO PEZZO2 PIKE PISANI POFFIJ POLEDN PRESCO QIAO QIAO2 RACHTA RADZIK RANDIG REN RONCO ROOTS ROTHSC SADOWS SANKAR SEGI SEGI2 SHAW SIMARA SIMONA SPEIZE SPITZ STOCKS STOCKW STUCKE SUN TANG TENKAN TOUSEY TULINI TVERDA VEIERO WALD WANG WANG2 WANG3 WANG4 WICKLU WIGLE WILKIN WUNSCH XIANGZ XU XU2 XU4 YAMAGU YONG YUAN ZHANG
10	BENHAM VUTUC WYNDE7

Table 3C1 - 8
Potentially overlapping studies

REF	REFGP	PRINC	. OVERLAP/LINK
LUBIN2	LUBIN2	1	Lubin-combined
CHAN	CHAN	1	Chan/LamWK2
LAMWK2	LAMWK2	1	Chan/LamWK2
LAMTH	LAMTH	1	KOO/LAMTH/LAMWK
KOO	KOO	1	KOO/LAMTH/LAMWK
LAMWK	LAMWK	1	KOO/LAMTH/LAMWK
ISHIMA	AKIBA	2	AKIBA/ISHIMA
BYERS1	BYERS1	2	GRAHAM/BROSS/BYERS1
OSANN2	KAISER	2	KAISER/OSANN2
WYNDE6	WYNDE6	1	WYNDE5/6/7/8
CPSI	CPSI	1	CPSI overall
SOBUE2	SOBUE2	1	SOBUE2/MATSUD
MATSUD	MATSUD	1	SOBUE2/MATSUD
JAHN	BOFFET	2	Subset of BOFFET
LUBIN	XIANGZ	2	LUBIN/XIANGZ/QIAO

Table 3C1 - 9
Most adjusted - insufficient data for metaanalysis

REF	NRR	SEX	AGEL	AGEH	RACE	YF	LC TYPE	LOC	START	ST	NLC	R	VB	P	H	AD	SM	PRODUCT	DENOM	De
CHEN	11	c	0	0	all	-	a	As:oth	1987	CC	323	n	ot	n	y	2	ev	cig+/-ot	nev cigs	ot
CHYOU	6	m	0	0	jap	0	a	NAmer	1965	pr	227	n	bl	n	y	1	cu	cig+/-ot	nev cigs	or
JIANG	5	m	0	0	all	-	a	As:Chi	1984	CC	125	n	ot	n	n	0	ev	all/unsp	nev any	ot
JIANG	6	f	0	0	all	-	a	As:Chi	1984	CC	125	n	ot	n	n	0	ev	all/unsp	nev any	ot
KOO	4	f	0	0	all	-	a	As:HK	1981	CC	200	n	bl	n	n	0	ev	all/unsp	nev any	or
WANG	4	c	0	0	all	-	a	As:Chi	1990	CC	390	n	ot	*	y	0	ev	all/unsp	nev any	ot

REF	NRR	RR	SIG	RRDATA comment
CHEN	11	2.08	y	P < 0.001
CHYOU	6	6.80		0
JIANG	5	*	n	0
JIANG	6	*	n	0
KOO	4	1.88		0
WANG	4	2.22	n	P>0.05

Table 3C2 -

IESLC - Meta-anal of Current Smoking (or Ever if Current not available), Any prod (or Cigs if Any not avail)
Adenocarcinoma

This analysis is restricted to results for:
1) Non-dose-response data
2) Results complete enough for use in metaanalysis

Within each study, results are then selected (in the following order of preference, within each sex) for:
3) SMKSTA: current smokers, ever smokers
4) PRODUCT: all/unspec, cigarettes regardless of other products, cigarettes only
5) CIGTYPE: all/unspecified, MC regardless of HR, MC only
6) DENOM: never smoked anything, never smoked cigarettes, (never +1 = +long term ex, +2 = +amount unknown,
+3 = never cigs+long term ex)
7) Followup period (YF, prospective studies): whole study (coded as 0) or longest available
8) LCtype: adeno or nearest available, but not squamous. (q = squamous, s = small,
a = adeno, l = large, KII = Kreyberg II, al = alveolar, br = bronchiolar, u = undifferentiated)
9) Race: all or nearest available, otherwise by race (wh or w = white, bl or b = black, hi = hispanic
ch = chinese, jap = japanese, haw = hawaiian, w+o = white + oriental, sca = scandinavian, as = asian)
10) For overlapping studies: principal rather than subsidiary studies
Finally by Age: whole study (coded as 0) if available, otherwise by widest available age group
and then for single sex results (m, f) in preference to combined sex results (c).

Results adjusted (AD) for the most potential confounders are then chosen in Sections -1 to -3
(and those which actually differ from the adjusted results in Table 3C1 - 1 are marked 'x' in Section -1)
and results adjusted for the least confounders in Sections -4 to -6. (Those least adjusted results which
actually differ from the most adjusted as marked 'x' in column X in Section -4)
(Results adjusted for an unknown number of confounder(s) are coded as 20.)

Section -7 shows excluded studies, together with the stage (as above) at which no qualifying
results were found.

Section -8 lists the potentially overlapping studies which have been included (1=principal, 2=subsidiary).

Section -9 lists any results which would have been included in preference except that they had data not complete
enough for use in meta-analysis, with their significance (yes/no), if known, and any further comment as entered
on the database.

In addition to those mentioned above, the following fields, levels and abbreviations are used:
* or nk = not known, n = no, y = yes, ot = other
ev = ever, cu = current, nev = never
all/unspec = all or unspecified, cig+/-ot = cigarettes irrespective of other products (cigar, pipe etc)
MC = manufactured cigarettes, HR = hand-rolled cigarettes
REF: 6-character study reference
NRR: number of the RR on the database within the study
ST : study type (CC = case control, pr or prosp = prospective)
NLC: number of lung cancer cases in whole study
R : risky occupational population (n = no, m = mining, o = other risky)
VB : national cigarette type (V = at least 75% Virginia, bl = at least 75% blended, ot = other)
P : any proxy use
H : full histological confirmation
De : derivation of RR/CI (or = original, st = standard method, ot = other method of estimation)

Table 3C2 - 1

IESLC - Meta-anal of Current Smoking (or Ever if Current not available), Any prod (or Cigs if Any not avail)
Adenocarcinoma
Most adjusted

REF	NRR	3C1	SEX	AGEL	AGEH	RACE	YF	LC TYPE	LOC	START	ST	NLC	R	VB	P	H	AD	SM	PRODUCT	DENOM	De
ABRAHA	2		m	0	0	all	0	a	Eu:est	1975	pr	571	n	bl	n	n	0	ev	all/unsp	nev any	ot
ABRAHA	5		f	0	0	all	0	a	Eu:est	1975	pr	571	n	bl	n	n	0	ev	all/unsp	nev any	ot
ALDERS	54		m	0	0	all	-	a	Eu:UK	1977	CC	1448	n	V	n	n	2	ev	all/unsp	nev any	or
ALDERS	57		f	0	0	all	-	a	Eu:UK	1977	CC	1448	n	V	n	n	1	ev	all/unsp	nev any	or
ANDERS	12		f	0	0	all	0	a	NAmer	1986	pr	343	n	bl	n	n	0	ev	cig+/-ot	nev cigs	st
BAND	2		m	0	0	all	-	a	NAmer	1983	CC	2831	n	V	y	y	2	ev	cig only	nev any	ot
BARBON	100	x	m	0	0	all	-	a	Eu:wst	1979	CC	755	n	bl	y	y	1	cu	all/unsp	nev any	or
BECHER	12		f	0	0	all	-	not q+s	Eu:Ger	1985	CC	194	n	bl	n	y	1	ev	all/unsp	nev any	or
BOUCOT	147		m	0	0	all	0	a	NAmer	1951	pr	121	n	bl	n	n	2	cu	cig only	nev any	ot
BRESLO	35		c	0	0	all	-	a	NAmer	1949	CC	518	n	bl	n	y	0	ev	all/unsp	nev+1	st
BROWN1	3		m	0	0	wh	-	a	NAmer	1979	CC	102	n	bl	y	y	1	ev	cig+/-ot	nev cigs	or
BROWN1	4		f	0	0	wh	-	a	NAmer	1979	CC	102	n	bl	y	y	1	ev	cig+/-ot	nev cigs	or
BROWN2	14	x	m	0	0	wh	-	a	NAmer	1984	CC	14596	n	bl	n	y	2	cu	cig+/-ot	nev cigs	or
BROWN2	13	x	f	0	0	wh	-	a	NAmer	1984	CC	14596	n	bl	n	y	2	cu	cig+/-ot	nev cigs	or
BUFFLE	50		m	0	0	wh	-	a	NAmer	1976	CC	943	n	bl	y	n	0	ev	cig+/-ot	nev cigs	ot
BUFFLE	71	x	f	0	0	w-hi	-	a	NAmer	1976	CC	943	n	bl	y	n	0	cu	cig+/-ot	nev cigs	st
BYERS1	3		m	0	0	wh	-	a	NAmer	1957	CC	1002	n	bl	n	n	0	ev	cig+/-ot	nev cigs	st
CHAN	12		m	0	0	all	-	a+l	As:HK	1976	CC	397	n	bl	n	n	0	ev	all/unsp	nev any	ot
CHAN	16		f	0	0	all	-	a+l	As:HK	1976	CC	397	n	bl	n	n	0	ev	all/unsp	nev any	st
CHOI	63		m	0	0	all	-	a	As:oth	1985	CC	375	n	bl	n	n	0	ev	cig+/-ot	nev cigs	st
CHOI	65		f	0	0	all	-	a	As:oth	1985	CC	375	n	bl	n	n	0	ev	cig+/-ot	nev cigs	st
COMSTO	24	x	m	0	0	all	-	a	NAmer	1975	ot	258	n	bl	n	n	0	cu	cig+/-ot	nev cigs	st
COMSTO	31	x	f	0	0	all	-	a	NAmer	1975	ot	258	n	bl	n	n	0	cu	cig+/-ot	nev cigs	st
CORREA	44	x	c	0	0	all	-	a	NAmer	1979	CC	1359	n	bl	y	n	1	cu	cig+/-ot	nev cigs	or
CPSI	404		m	0	0	all	2	a	NAmer	1959	pr	5138	n	bl	n	n	1	cu	cig only	nev any	ot
CPSI	406		f	0	0	all	2	a	NAmer	1959	pr	5138	n	bl	n	n	1	cu	cig only	nev any	ot
CPSII	115		m	0	0	all	2	a	NAmer	1982	pr	3229	n	bl	n	n	1	cu	cig only	nev any	st
CPSII	118		f	0	0	all	2	a	NAmer	1982	pr	3229	n	bl	n	n	1	cu	cig+/-ot	nev cigs	st
DAMBER	32		m	0	0	all	-	a+al+br	Eu:Sca	1972	CC	579	n	bl	y	n	1	ev	all/unsp	nev any	or
DESTE2	17		m	0	0	all	-	a	SCAmer	1993	CC	463	n	bl	n	n	2	ev	all/unsp	nev any	or
DOLL	87		m	0	0	all	-	KII	Eu:UK	1948	CC	1465	n	V	n	n	1	ev	all/unsp	nev any	ot
DOLL	89		f	0	0	all	-	KII	Eu:UK	1948	CC	1465	n	V	n	n	1	ev	all/unsp	nev any	ot
DORGAN	125		m	0	0	wh	-	a	NAmer	1980	CC	2026	n	bl	y	y	2	ev	cig+/-ot	nev any	or
DORGAN	104		f	0	0	all	-	a	NAmer	1980	CC	2026	n	bl	y	y	3	ev	cig+/-ot	nev any	or
DORN	340		m	0	0	wh	8	a	NAmer	1954	pr	5097	n	bl	n	n	1	cu	cig only	nev any	ot
DOSEME	4		m	0	0	all	-	not q+s	Eu:bal	1979	CC	1210	n	bl	n	n	2	ev	cig+/-ot	nev cigs	or
ENGELA	70	x	m	0	0	all	0	a	Eu:Sca	1964	pr	435	n	bl	n	n	7	cu	cig+/-ot	nev cigs	ot
FAN	4		c	0	0	all	-	a	As:Chi	1990	CC	403	n	ot	y	n	0	ev	cig+/-ot	nev cigs	ot
GAO	3		m	0	0	all	-	a	As:Chi	1984	CC	1405	n	ot	n	n	2	ev	cig+/-ot	nev cigs	or
GAO	13		f	0	0	all	-	a	As:Chi	1984	CC	1405	n	ot	n	n	2	ev	cig+/-ot	nev cigs	or
GER	9		c	0	0	all	-	a	As:oth	1990	CC	141	n	ot	y	n	8	ev	all/unsp	nev any	ot
HAENSZ	37	x	f	0	0	all	-	a	NAmer	1955	CC	158	n	bl	n	y	0	cu	cig+/-ot	nev any	st
HAMMON	92		m	0	0	wh	0	a	NAmer	1952	pr	448	n	bl	n	n	0	ev	all/unsp	nev any	st
HEGMAN	4		c	0	0	all	-	a	NAmer	1989	CC	282	n	bl	y	y	0	ev	all/unsp	nev any	st
HINDS	24		f	0	0	o	-	a	NAmer	1968	CC	292	n	bl	n	n	3	ev	all/unsp	nev any	st
ISHIMA	8		c	0	0	all	-	a	As:Jap	1961	CC	180	n	bl	y	y	5	ev	all/unsp	nev any	st
JAHN	8	x	m	0	0	all	-	a	Eu:Ger	1988	CC	1004	n	bl	n	n	0	cu	cig+/-ot	nev any	st
JAIN	17	x	m	0	0	all	-	a	NAmer	1981	CC	845	n	V	y	n	0	cu	cig+/-ot	nev cigs	st
JAIN	12	x	f	0	0	all	-	a	NAmer	1981	CC	845	n	V	y	n	0	cu	cig+/-ot	nev cigs	st
JEDRYC	26	x	m	0	0	all	-	a	Eu:est	1980	CC	1630	n	bl	y	n	0	cu	cig+/-ot	nev any	st
JOLY	51		m	0	0	all	-	a	SCAmer	1978	CC	826	n	bl	n	n	0	ev	cig+/-ot	nev any	st
JOLY	50		f	0	0	all	-	a	SCAmer	1978	CC	826	n	bl	n	n	0	ev	cig+/-ot	nev any	st
JUSSAW	26		m	0	0	all	-	KII	As:Ind	1964	CC	792	n	V	n	n	0	ev	all/unsp	nev any	st
KATSOU	12	x	f	0	0	all	-	a	Eu:bal	1987	CC	101	n	bl	n	n	1	cu	all/unsp	nev any	or
KHUDER	17	x	m	0	0	all	-	a	NAmer	1985	CC	482	n	bl	n	y	0	cu	cig+/-ot	nev cigs	or
KIHARA	5	x	c	0	0	jap	-	a	As:Jap	1991	CC	440	n	bl	n	n	0	cu	all/unsp	nev any	st
KOO	7		f	0	0	all	-	a+l	As:HK	1981	CC	200	n	bl	n	n	0	ev	all/unsp	nev any	st
KREYBE	8		m	0	0	all	-	KII	Eu:Sca	1948	CC	300	n	bl	n	y	1	ev	all/unsp	nev any	ot
KREYBE	27		f	0	0	all	-	KII	Eu:Sca	1948	CC	300	n	bl	n	y	1	ev	all/unsp	nev any	ot
LAMTH	3		f	0	0	ch	-	a	As:HK	1983	CC	445	n	bl	n	n	0	ev	all/unsp	nev any	or
LAMWK	4		f	0	0	ch	-	a	As:HK	1981	CC	163	n	bl	n	n	0	ev	all/unsp	nev any	st
LAMWK2	3		m	0	0	all	-	a	As:HK	1976	CC	480	n	bl	n	n	0	ev	all/unsp	nev any	st
LAMWK2	7		f	0	0	all	-	a	As:HK	1976	CC	480	n	bl	n	n	0	ev	all/unsp	nev any	st
LOMBA2	3		f	0	0	all	-	not q+u	NAmer	1960	CC	225	n	bl	n	n	0	ev	cig+/-ot	nev cigs	st
LUBIN	36		m	0	0	all	-	KII	As:Chi	1984	CC	427	m	ot	y	n	0	ev	all/unsp	nev any	st
LUBIN2	252	x	m	0	0	all	-	a	Eu:mul	1976	CC	7804	n	bl	n	y	0	cu	cig+/-ot	nev any	st
LUBIN2	264	x	f	0	0	all	-	a	Eu:mul	1976	CC	7804	n	bl	n	y	0	cu	cig+/-ot	nev any	st
LUO	9		c	0	0	all	-	a	As:Chi	1990	CC	102	n	ot	n	y	20	ev	cig+/-ot	nev cigs	or
MATOS	53	x	m	0	0	all	-	a	SCAmer	1994	CC	200	n	bl	n	n	2	cu	cig+/-ot	nev any	or
MATSUD	12		m	0	0	all	-	a	As:Jap	1965	CC	179	n	bl	n	n	0	ev	cig+/-ot	nev cigs	ot
NOU	3		m	0	0	all	-	a	Eu:Sca	1971	CC	273	n	bl	y	n	0	ev	all/unsp	nev any	st
NOU	8		f	0	0	all	-	a	Eu:Sca	1971	CC	273	n	bl	y	n	0	ev	all/unsp	nev any	st

Table 3C2 - 1

IESLC - Meta-anal of Current Smoking (or Ever if Current not available), Any prod (or Cigs if Any not avail)
Adenocarcinoma
Most adjusted

REF	NRR	3C1	SEX	AGEL	AGEH	RACE	YF	LC TYPE	LOC	START	ST	NLC	R	VB	P	H	AD	SM	PRODUCT	DENOM	De
ORMOS	21		m	0	0	all	-	a	Eu:est	1947	CC	119	n	bl	y	y	0	ev	cig+/-ot	nev any	ot
OSANN	39	x	m	0	0	all	-	a	NAmer	1984	CC	1986	n	bl	n	n	2	cu	cig+/-ot	nev cigs	or
OSANN	40	x	f	0	0	all	-	a	NAmer	1984	CC	1986	n	bl	n	n	2	cu	cig+/-ot	nev cigs	or
OSANN2	32	x	f	0	0	all	-	KII	NAmer	1964	ot	217	n	bl	n	y	1	cu	cig+/-ot	nev cigs	or
PEZZOT	7		m	0	0	all	-	a	SCAmer	1987	CC	215	n	bl	n	y	0	ev	cig only	nev cigs	st
SCHWAR	8		m	40	54	wh	-	a	NAmer	1984	CC	5588	n	bl	y	y	0	ev	cig+/-ot	nev cigs	st
SCHWAR	7		m	40	54	bl	-	a	NAmer	1984	CC	5588	n	bl	y	y	0	ev	cig+/-ot	nev cigs	st
SCHWAR	16		f	40	54	wh	-	a	NAmer	1984	CC	5588	n	bl	y	y	0	ev	cig+/-ot	nev cigs	st
SCHWAR	15		f	40	54	bl	-	a	NAmer	1984	CC	5588	n	bl	y	y	0	ev	cig+/-ot	nev cigs	st
SEOW	2		f	0	0	ch	-	a	As:oth	1997	CC	153	n	bl	n	y	0	ev	cig+/-ot	nev cigs	st
SIEMIA	8		m	0	0	all	-	a	NAmer	1979	CC	857	n	V	y	y	7	ev	cig+/-ot	nev cigs	or
SOBUE	36	x	m	0	0	all	-	a	As:Jap	1986	CC	1376	n	bl	n	y	1	cu	cig+/-ot	nev cigs	or
SOBUE	46	x	f	0	0	all	-	a	As:Jap	1986	CC	1376	n	bl	n	y	1	cu	cig+/-ot	nev cigs	or
SOBUE2	2		m	0	0	all	-	a	As:Jap	1965	CC	2083	n	bl	n	n	2	cu	cig+/-ot	nev any	or
SOBUE2	6		f	0	0	all	-	a	As:Jap	1965	CC	2083	n	bl	n	n	2	cu	cig+/-ot	nev any	or
STASZE	21		m	0	0	all	-	a	Eu:est	1954	CC	281	n	bl	n	y	0	ev	all/unsp	nev any	ot
STASZE	4		f	0	0	all	-	a	Eu:est	1954	CC	281	n	bl	n	y	0	ev	all/unsp	nev any	st
STAYNE	4		m	0	0	all	-	a	NAmer	1969	CC	420	n	bl	n	n	0	ev	all/unsp	nev any	st
SUZUK2	16		c	0	0	all	-	a	SCAmer	1991	CC	123	n	bl	n	y	3	ev	all/unsp	nev any	or
SUZUKI	10	x	m	0	0	all	-	a	As:Jap	1978	CC	238	n	bl	n	y	2	cu	cig+/-ot	nev any	or
SUZUKI	14	x	f	0	0	all	-	a	As:Jap	1978	CC	238	n	bl	n	y	2	cu	cig+/-ot	nev any	or
SVENSS	99	x	f	0	0	all	-	a	Eu:Sca	1983	CC	210	n	bl	n	n	1	cu	all/unsp	nev any	ot
TIZZAN	19		c	0	0	all	-	a	Eu:wst	1959	CC	1358	n	bl	n	n	0	ev	all/unsp	nev any	st
TOKARS	8		c	0	0	all	-	a	Eu:est	1966	ot	162	o	bl	n	y	3	ev	all/unsp	nev any	or
TSUGAN	3	x	m	0	0	all	-	a	As:Jap	1976	CC	134	n	bl	n	y	0	cu	all/unsp	nev any	st
TSUGAN	9	x	f	0	0	all	-	a	As:Jap	1976	CC	134	n	bl	n	y	0	cu	all/unsp	nev any	or
WAKAI	12	x	m	0	0	all	-	a	As:Jap	1988	CC	333	n	bl	n	y	1	cu	all/unsp	nev any	or
WAKAI	30	x	f	0	0	all	-	a	As:Jap	1988	CC	333	n	bl	n	y	1	cu	all/unsp	nev any	or
WU	7	x	f	0	0	wh	-	a	NAmer	1981	CC	220	n	bl	n	y	2	cu	all/unsp	nev any	or
WU2	1		f	0	0	all	-	a	NAmer	1983	CC	336	n	bl	n	y	2	cu	all/unsp	nev any	or
WUWILL	11		f	0	0	all	-	a	As:Chi	1985	CC	965	n	ot	n	n	3	ev	cig+/-ot	nev cigs	or
WYNDE2	14		m	0	0	all	-	KII	NAmer	1962	CC	404	n	bl	n	y	0	ev	all/unsp	nev any	st
WYNDE3	30	x	m	0	0	all	-	KII	NAmer	1966	CC	350	n	bl	n	y	0	cu	all/unsp	nev any	st
WYNDE3	135		f	0	0	all	-	KII	NAmer	1966	CC	350	n	bl	n	y	0	ev	all/unsp	nev any	st
WYNDE4	42		m	0	0	all	-	a	NAmer	1948	CC	684	n	bl	y	n	0	ev	all/unsp	nev any	st
WYNDE4	56		f	0	0	all	-	a	NAmer	1948	CC	684	n	bl	y	n	2	ev	all/unsp	nev any	ot
WYNDE6	15	x	m	0	0	all	-	KII	NAmer	1969	CC	4423	n	bl	n	y	0	cu	cig+/-ot	nev any	st
WYNDE6	204	x	f	0	0	all	-	KII	NAmer	1969	CC	4423	n	bl	n	y	0	cu	cig+/-ot	nev cigs	st
XU3	22		m	0	0	all	-	KII	As:Chi	1981	CC	135	n	ot	n	n	1	ev	all/unsp	nev any	ot
XU3	26		f	0	0	all	-	KII	As:Chi	1981	CC	135	n	ot	n	n	1	ev	all/unsp	nev any	ot
ZHENG	10		m	0	0	all	-	a	As:Chi	1982	CC	540	n	ot	*	y	0	ev	cig+/-ot	nev cigs	st
ZHENG	21		f	0	0	all	-	a	As:Chi	1982	CC	540	n	ot	*	y	0	ev	cig+/-ot	nev cigs	st
ZHOU	26		m	0	0	all	-	a	As:Chi	1978	CC	1360	n	ot	n	n	0	ev	all/unsp	nev any	st
ZHOU	27		f	0	0	all	-	a	As:Chi	1978	CC	1360	n	ot	n	n	0	ev	all/unsp	nev any	st

Cigarette type is all/unspec for all RRs

Table 3C2 - 2

IESLC - Meta-anal of Current Smoking (or Ever if Current not available), Any prod (or Cigs if Any not avail)
Adenocarcinoma
Most adjusted

REF	NRR	SEX	AD	Number Exposed Case	Number Exposed Cont	Non-exposed Case	Non-exposed Cont	RR	95.00%CI
*ABRAHA	2	m	0	59	10351	8	3365	2.40	(1.15- 5.01)
*ABRAHA	5	f	0	19	5256	16	11589	2.62	(1.35- 5.09)
Subtotal ABRAHA								2.52	(1.54- 4.12)
ALDERS	54	m	2	-	-	-	-	7.11	(1.49- 33.85)
ALDERS	57	f	1	-	-	-	-	3.58	(1.48- 8.65)
Subtotal ALDERS								4.23	(1.96- 9.12)
*ANDERS	12	f	0	99	96164	33	195158	6.09	(4.11- 9.03)
BAND	2	m	2	-	-	-	-	4.10	(3.01- 5.59)
BARBON	100	m	1	-	-	-	-	7.90	(3.60- 17.40)
BECHER	12	f	1	-	-	-	-	10.83	(1.32- 88.70)
*BOUCOT	147	m	2	-	-	-	-	10.95	(0.65- 183.57)
BRESLO	35	c	0	42	462	4	56	1.27	(0.44- 3.68)
BROWN1	3	m	1	-	-	-	-	4.49	(1.44- 13.98)
BROWN1	4	f	1	-	-	-	-	3.95	(1.76- 8.80)
Subtotal BROWN1								4.12	(2.14- 7.95)
BROWN2	14	m	2	-	-	-	-	9.10	(7.60- 10.80)
BROWN2	13	f	2	-	-	-	-	7.20	(6.20- 8.30)
Subtotal BROWN2								7.92	(7.08- 8.86)
BUFFLE	50	m	0	-	-	-	-	4.50	(1.85- 10.95)
BUFFLE	71	f	0	56	110	7	112	8.15	(3.56- 18.65)
Subtotal BUFFLE								6.18	(3.37- 11.33)
BYERS1	3	m	0	47	695	7	424	4.10	(1.83- 9.15)
CHAN	12	m	0	56	161	0	43	30.44~	(1.84- 502.58)
CHAN	16	f	0	28	50	40	139	1.95	(1.09- 3.48)
Subtotal CHAN								2.18	(1.23- 3.85)
CHOI	63	m	0	46	465	7	95	1.34	(0.59- 3.06)
CHOI	65	f	0	5	26	49	164	0.64	(0.23- 1.77)
Subtotal CHOI								1.00	(0.53- 1.89)
COMSTO	24	m	0	30	100	2	84	12.60	(2.92- 54.28)
COMSTO	31	f	0	23	52	8	115	6.36	(2.67- 15.16)
Subtotal COMSTO								7.60	(3.60- 16.04)
CORREA	44	c	1	-	-	-	-	6.70	(4.30- 10.60)
*CPSI	404	m	1	-	-	-	-	4.58	(1.74- 12.05)
*CPSI	406	f	1	-	-	-	-	1.43	(0.47- 4.39)
Subtotal CPSI								2.78	(1.34- 5.78)
*CPSII	115	m	1	-	-	-	-	19.22	(6.46- 57.16)
*CPSII	118	f	1	-	-	-	-	8.23	(4.36- 15.54)
Subtotal CPSII								10.21	(5.89- 17.67)
DAMBER	32	m	1	-	-	-	-	2.40	(1.10- 5.30)
DESTE2	17	m	2	-	-	-	-	4.30	(1.60- 11.40)
DOLL	87	m	1	-	-	-	-	0.95	(0.22- 4.02)
DOLL	89	f	1	-	-	-	-	1.97	(0.60- 6.46)
Subtotal DOLL								1.47	(0.59- 3.69)
DORGAN	125	m	2	-	-	-	-	4.80	(1.90- 12.00)
DORGAN	104	f	3	-	-	-	-	3.90	(2.80- 5.40)
Subtotal DORGAN								3.99	(2.93- 5.44)
*DORN	340	m	1	-	-	-	-	5.95	(3.85- 9.22)
DOSEME	4	m	2	-	-	-	-	2.60	(1.70- 4.20)
*ENGELA	70	m	7	-	-	-	-	7.06	(2.69- 18.50)
FAN	4	c	0	67	595	45	556	1.39	(0.94- 2.07)
GAO	3	m	2	-	-	-	-	1.60	(1.10- 2.40)
GAO	13	f	2	-	-	-	-	1.50	(1.00- 2.10)
Subtotal GAO								1.55	(1.18- 2.02)
GER	9	c	8	-	-	-	-	1.10	(0.55- 2.19)
HAENSZ	37	f	0	16	94	37	236	1.09	(0.58- 2.05)
*HAMMON	92	m	0	29	510108	2	115884	3.29	(0.79- 13.80)
HEGMAN	4	c	0	83	1202	15	2080	9.58	(5.50- 16.67)
HINDS	24	f	3	-	-	-	-	3.89	(2.49- 6.07)
ISHIMA	8	c	5	-	-	-	-	15.00	(2.31- 631.48)
JAHN	8	m	0	75	269	8	138	4.81	(2.26- 10.26)
JAIN	17	m	0	60	118	4	85	10.81	(3.78- 30.87)
JAIN	12	f	0	69	99	24	214	6.21	(3.69- 10.47)
Subtotal JAIN								6.93	(4.35- 11.07)
JEDRYC	26	m	0	68	516	7	289	5.44	(2.47- 12.00)
JOLY	51	m	0	72	709	5	218	4.43	(1.77- 11.10)
JOLY	50	f	0	33	122	25	283	3.06	(1.75- 5.37)
Subtotal JOLY								3.38	(2.10- 5.46)
JUSSAW	26	m	0	34	168	13	624	9.71	(5.01- 18.82)
KATSOU	12	f	1	-	-	-	-	1.70	(0.75- 3.89)
KHUDER	17	m	0	92	-	7	-	8.20	(3.60- 18.40)
KIHARA	5	c	0	103	162	78	237	1.93	(1.35- 2.76)

Table 3C2 - 2

IESLC - Meta-anal of Current Smoking (or Ever if Current not available), Any prod (or Cigs if Any not avail)
Adenocarcinoma
Most adjusted

REF	NRR	SEX	AD	Number Exposed Case	Number Exposed Cont	Non-exposed Case	Non-exposed Cont	RR	95.00%CI
KOO	7	f	0	34	63	46	137	1.61	(0.94- 2.74)
KREYBE	8	m	1	-	-	-	-	2.44	(0.76- 7.86)
KREYBE	27	f	1	-	-	-	-	1.28	(0.60- 2.74)
Subtotal KREYBE								1.55	(0.82- 2.93)
LAMTH	3	f	0	79	51	131	158	1.87	(1.23- 2.85)
LAMWK	4	f	0	36	41	60	144	2.11	(1.23- 3.61)
LAMWK2	3	m	0	52	161	15	43	0.93	(0.48- 1.80)
LAMWK2	7	f	0	26	50	41	139	1.76	(0.98- 3.17)
Subtotal LAMWK2								1.33	(0.86- 2.07)
LOMBA2	3	f	0	42	353	54	239	0.53	(0.34- 0.81)
LUBIN	36	m	0	37	939	4	72	0.71	(0.25- 2.05)
LUBIN2	252	m	0	454	6209	57	2616	3.36	(2.54- 4.44)
LUBIN2	264	f	0	69	410	138	1180	1.44	(1.06- 1.96)
Subtotal LUBIN2								2.30	(1.87- 2.83)
LUO	9	c	20	-	-	-	-	1.50	(0.70- 3.00)
MATOS	53	m	2	-	-	-	-	7.90	(3.00- 20.90)
MATSUD	12	m	0	23	3314	0	1255	17.80~	(1.08- 293.32)
NOU	3	m	0	36	247	4	122	4.45	(1.55- 12.77)
NOU	8	f	0	9	92	29	261	0.88	(0.40- 1.93)
Subtotal NOU								1.57	(0.83- 2.94)
ORMOS	21	m	0	4	1034	0	777	6.76~	(0.36- 125.82)
OSANN	39	m	2	-	-	-	-	21.70	(12.50- 39.70)
OSANN	40	f	2	-	-	-	-	11.60	(8.20- 16.40)
Subtotal OSANN								13.69	(10.17- 18.43)
OSANN2	32	f	1	-	-	-	-	3.20	(1.50- 6.60)
PEZZOT	7	m	0	60	317	3	116	7.32	(2.25- 23.79)
SCHWAR	8	m	0	84	178	1	73	34.45	(4.71- 252.10)
SCHWAR	7	m	0	45	39	1	7	8.08	(0.95- 68.56)
SCHWAR	16	f	0	92	108	10	79	6.73	(3.29- 13.75)
SCHWAR	15	f	0	20	28	3	41	9.76	(2.65- 36.00)
Subtotal SCHWAR								8.40	(4.73- 14.94)
SEOW	2	f	0	19	15	67	125	2.36	(1.13- 4.95)
SIEMIA	8	m	7	-	-	-	-	6.30	(2.50- 16.20)
SOBUE	36	m	1	-	-	-	-	1.90	(1.30- 3.00)
SOBUE	46	f	1	-	-	-	-	1.30	(0.90- 2.00)
Subtotal SOBUE								1.56	(1.17- 2.08)
SOBUE2	2	m	2	-	-	-	-	3.10	(2.40- 3.70)
SOBUE2	6	f	2	-	-	-	-	1.80	(1.40- 2.20)
Subtotal SOBUE2								2.39	(2.04- 2.79)
STASZE	21	m	0	20	754	0	158	8.61~	(0.52- 143.15)
STASZE	4	f	0	1	153	10	1660	1.08	(0.14- 8.53)
Subtotal STASZE								2.24	(0.42- 11.81)
STAYNE	4	m	0	43	567	7	333	3.61	(1.60- 8.11)
SUZUK2	16	c	3	-	-	-	-	6.00	(0.70- 50.00)
SUZUKI	10	m	2	-	-	-	-	5.00	(2.71- 9.27)
SUZUKI	14	f	2	-	-	-	-	2.40	(1.19- 4.86)
Subtotal SUZUKI								3.64	(2.29- 5.78)
SVENSS	99	f	1	-	-	-	-	3.78	(1.91- 7.48)
TIZZAN	19	c	0	88	939	25	419	1.57	(0.99- 2.49)
TOKARS	8	c	3	-	-	-	-	4.30	(1.90- 9.90)
TSUGAN	3	m	0	45	50	18	17	0.85	(0.39- 1.85)
TSUGAN	9	f	0	6	10	33	30	0.55	(0.18- 1.68)
Subtotal TSUGAN								0.74	(0.39- 1.40)
WAKAI	12	m	1	-	-	-	-	2.18	(1.00- 4.76)
WAKAI	30	f	1	-	-	-	-	1.14	(0.49- 2.61)
Subtotal WAKAI								1.61	(0.91- 2.85)
WU	7	f	2	-	-	-	-	4.10	(2.30- 7.50)
WU2	1	f	2	-	-	-	-	4.50	(3.00- 6.90)
WUWILL	11	f	3	-	-	-	-	1.50	(1.10- 1.90)
WYNDE2	14	m	0	49	616	5	105	1.67	(0.65- 4.29)
WYNDE3	30	m	0	56	207	6	88	3.97	(1.65- 9.55)
WYNDE3	135	f	0	21	56	15	76	1.90	(0.90- 4.01)
Subtotal WYNDE3								2.59	(1.47- 4.57)
WYNDE4	42	m	0	35	665	4	115	1.51	(0.53- 4.34)
WYNDE4	56	f	2	-	-	-	-	0.60	(0.13- 2.69)
Subtotal WYNDE4								1.12	(0.47- 2.66)
WYNDE6	15	m	0	651	741	58	617	9.35	(7.00- 12.48)
WYNDE6	204	f	0	472	376	119	856	9.03	(7.14- 11.42)
Subtotal WYNDE6								9.15	(7.63- 10.98)
XU3	22	m	1	-	-	-	-	4.84	(1.37- 17.10)
XU3	26	f	1	-	-	-	-	1.09	(0.26- 4.50)

Table 3C2 - 2

IESLC - Meta-anal of Current Smoking (or Ever if Current not available), Any prod (or Cigs if Any not avail)
Adenocarcinoma
Most adjusted

REF	NRR	SEX	AD	Number Exposed Case	Number Exposed Cont	Non-exposed Case	Non-exposed Cont	RR	95.00%CI
Subtotal XU3								2.51	(0.98- 6.47)
ZHENG	10	m	0	123	218	29	94	1.83	(1.14- 2.93)
ZHENG	21	f	0	33	44	119	184	1.16	(0.70- 1.93)
Subtotal ZHENG								1.48	(1.05- 2.09)
ZHOU	26	m	0	131	41	88	36	1.31	(0.77- 2.20)
ZHOU	27	f	0	30	7	96	32	1.43	(0.57- 3.57)
Subtotal ZHOU								1.34	(0.85- 2.10)
Partial Totals				4336	647147	1747	344592		

*prospective study ~ With 0.5 adjustment for zero

REF	NRR	SEX	AD	Ys	Ws	Qs	Ps
*ABRAHA	2	m	0	0.87	7.06	1.13	0.0201
*ABRAHA	5	f	0	0.96	8.71	0.85	0.0045
Subtotal ABRAHA				0.92	15.77	1.99	
ALDERS	54	m	2	1.96	1.58	0.74	0.0138
ALDERS	57	f	1	1.28	4.93	0.00	0.0046
Subtotal ALDERS				1.44	6.50	0.74	
*ANDERS	12	f	0	1.81	24.76	6.99	0.0000
BAND	2	m	2	1.41	40.10	0.74	0.0000
BARBON	100	m	1	2.07	6.19	3.88	0.0000
BECHER	12	f	1	2.38	0.87	1.06	0.0265
*BOUCOT	147	m	2	2.39	0.48	0.60	0.0964
BRESLO	35	c	0	0.24	3.40	3.64	0.6564
BROWN1	3	m	1	1.50	2.97	0.15	0.0096
BROWN1	4	f	1	1.37	5.93	0.06	0.0008
Subtotal BROWN1				1.42	8.91	0.21	
BROWN2	14	m	2	2.21	124.44	108.34	0.0000
BROWN2	13	f	2	1.97	180.58	88.20	0.0000
Subtotal BROWN2				2.07	305.02	196.54	
BUFFLE	50	m	0	1.50	4.86	0.25	0.0009
BUFFLE	71	f	0	2.10	5.59	3.78	0.0000
Subtotal BUFFLE				1.82	10.45	4.04	
BYERS1	3	m	0	1.41	5.95	0.11	0.0006
CHAN	12	m	0	3.42	0.49	2.24	0.0170
CHAN	16	f	0	0.67	11.38	4.22	0.0247
Subtotal CHAN				0.78	11.86	6.46	
CHOI	63	m	0	0.29	5.64	5.42	0.4842
CHOI	65	f	0	-0.44	3.77	11.11	0.3920
Subtotal CHOI				-0.00	9.42	16.54	
COMSTO	24	m	0	2.53	1.80	2.85	0.0007
COMSTO	31	f	0	1.85	5.09	1.68	0.0000
Subtotal COMSTO				2.03	6.89	4.53	
CORREA	44	c	1	1.90	18.88	7.42	0.0000
*CPSI	404	m	1	1.52	4.10	0.25	0.0021
*CPSI	406	f	1	0.36	3.08	2.59	0.5303
Subtotal CPSI				1.02	7.18	2.84	
*CPSII	115	m	1	2.96	3.23	9.13	0.0000
*CPSII	118	f	1	2.11	9.51	6.59	0.0000
Subtotal CPSII				2.32	12.75	15.73	
DAMBER	32	m	1	0.88	6.21	0.99	0.0291
DESTE2	17	m	2	1.46	3.99	0.13	0.0036
DOLL	87	m	1	-0.05	1.82	3.20	0.9448
DOLL	89	f	1	0.68	2.72	0.97	0.2634
Subtotal DOLL				0.39	4.54	4.17	
DORGAN	125	m	2	1.57	4.52	0.39	0.0008
DORGAN	104	f	3	1.36	35.62	0.26	0.0000
Subtotal DORGAN				1.38	40.15	0.65	
*DORN	340	m	1	1.78	20.15	5.20	0.0000
DOSEME	4	m	2	0.96	18.78	1.92	0.0000
*ENGELA	70	m	7	1.95	4.13	1.91	0.0001
FAN	4	c	0	0.33	24.61	21.98	0.1013
GAO	3	m	2	0.47	25.25	16.37	0.0182
GAO	13	f	2	0.41	27.91	21.12	0.0322
Subtotal GAO				0.44	53.16	37.48	
GER	9	c	8	0.10	8.05	11.20	0.7869
HAENSZ	37	f	0	0.08	9.58	13.63	0.7992
*HAMMON	92	m	0	1.19	1.87	0.01	0.1030
HEGMAN	4	c	0	2.26	12.50	12.10	0.0000
HINDS	24	f	3	1.36	19.35	0.13	0.0000
ISHIMA	8	c	5	2.71	0.49	1.00	0.0585

Table 3C2 - 2

IESLC - Meta-anal of Current Smoking (or Ever if Current not available), Any prod (or Cigs if Any not avail)
Adenocarcinoma
Most adjusted

REF	NRR	SEX	AD	Ys	Ws	Qs	Ps
JAHN	8	m	0	1.57	6.70	0.58	0.0000
JAIN	17	m	0	2.38	3.49	4.25	0.0000
JAIN	12	f	0	1.83	14.10	4.29	0.0000
Subtotal JAIN				1.94	17.58	8.55	
JEDRYC	26	m	0	1.69	6.14	1.08	0.0000
JOLY	51	m	0	1.49	4.55	0.21	0.0015
JOLY	50	f	0	1.12	12.19	0.30	0.0001
Subtotal JOLY				1.22	16.74	0.50	
JUSSAW	26	m	0	2.27	8.78	8.75	0.0000
KATSOU	12	f	1	0.53	5.67	3.14	0.2064
KHUDER	17	m	0	2.10	5.77	3.97	0.0000
KIHARA	5	c	0	0.66	30.38	11.55	0.0003
KOO	7	f	0	0.47	13.45	8.62	0.0817
KREYBE	8	m	1	0.89	2.82	0.41	0.1345
KREYBE	27	f	1	0.25	6.66	7.04	0.5240
Subtotal KREYBE				0.44	9.48	7.46	
LAMTH	3	f	0	0.63	21.63	9.14	0.0036
LAMWK	4	f	0	0.75	13.20	3.70	0.0068
LAMWK2	3	m	0	-0.08	8.67	15.85	0.8206
LAMWK2	7	f	0	0.57	11.11	5.57	0.0588
Subtotal LAMWK2				0.28	19.77	21.42	
LOMBA2	3	f	0	-0.64	20.27	74.44	0.0039
LUBIN	36	m	0	-0.34	3.42	8.97	0.5249
LUBIN2	252	m	0	1.21	49.29	0.21	0.0000
LUBIN2	264	f	0	0.36	39.96	33.18	0.0214
Subtotal LUBIN2				0.83	89.24	33.39	
LUO	9	c	20	0.41	7.26	5.49	0.2748
MATOS	53	m	2	2.07	4.08	2.56	0.0000
MATSUD	12	m	0	2.88	0.49	1.26	0.0440
NOU	3	m	0	1.49	3.45	0.16	0.0056
NOU	8	f	0	-0.13	6.24	12.27	0.7504
Subtotal NOU				0.45	9.69	12.43	
ORMOS	21	m	0	1.91	0.45	0.18	0.1999
OSANN	39	m	2	3.08	11.51	37.37	0.0000
OSANN	40	f	2	2.45	31.98	44.22	0.0000
Subtotal OSANN				2.62	43.49	81.58	
OSANN2	32	f	1	1.16	7.00	0.09	0.0021
PEZZOT	7	m	0	1.99	2.76	1.41	0.0009
SCHWAR	8	m	0	3.54	0.97	4.97	0.0005
SCHWAR	7	m	0	2.09	0.84	0.56	0.0556
SCHWAR	16	f	0	1.91	7.53	3.00	0.0000
SCHWAR	15	f	0	2.28	2.26	2.27	0.0006
Subtotal SCHWAR				2.13	11.60	10.80	
SEOW	2	f	0	0.86	7.03	1.21	0.0226
SIEMIA	8	m	7	1.84	4.40	1.41	0.0001
SOBUE	36	m	1	0.64	21.97	8.81	0.0026
SOBUE	46	f	1	0.26	24.10	24.72	0.1978
Subtotal SOBUE				0.44	46.07	33.54	
SOBUE2	2	m	2	1.13	82.01	1.70	0.0000
SOBUE2	6	f	2	0.59	75.22	35.54	0.0000
Subtotal SOBUE2				0.87	157.22	37.24	
STASZE	21	m	0	2.15	0.49	0.37	0.1332
STASZE	4	f	0	0.08	0.90	1.29	0.9382
Subtotal STASZE				0.81	1.39	1.66	
STAYNE	4	m	0	1.28	5.85	0.00	0.0019
SUZUK2	16	c	3	1.79	0.84	0.23	0.0999
SUZUKI	10	m	2	1.61	10.16	1.13	0.0000
SUZUKI	14	f	2	0.88	7.76	1.24	0.0147
Subtotal SUZUKI				1.29	17.92	2.38	
SVENSS	99	f	1	1.33	8.25	0.02	0.0001
TIZZAN	19	c	0	0.45	18.24	12.38	0.0538
TOKARS	8	c	3	1.46	5.64	0.19	0.0005
TSUGAN	3	m	0	-0.16	6.39	13.20	0.6813
TSUGAN	9	f	0	-0.61	3.03	10.72	0.2916
Subtotal TSUGAN				-0.31	9.41	23.92	
WAKAI	12	m	1	0.78	6.31	1.55	0.0502
WAKAI	30	f	1	0.13	5.49	7.19	0.7588
Subtotal WAKAI				0.48	11.80	8.74	
WU	7	f	2	1.41	11.00	0.20	0.0000
WU2	1	f	2	1.50	22.15	1.16	0.0000
WUWILL	11	f	3	0.41	51.44	38.91	0.0036

Table 3C2 - 2

IESLC - Meta-anal of Current Smoking (or Ever if Current not available), Any prod (or Cigs if Any not avail)
Adenocarcinoma
Most adjusted

REF	NRR	SEX	AD	Ys	Ws	Qs	Ps
WYNDE2	14	m	0	0.51	4.32	2.51	0.2863
WYNDE3	30	m	0	1.38	4.98	0.05	0.0021
WYNDE3	135	f	0	0.64	6.88	2.76	0.0922
Subtotal WYNDE3				0.95	11.86	2.81	
WYNDE4	42	m	0	0.41	3.46	2.57	0.4408
WYNDE4	56	f	2	-0.51	1.67	5.34	0.5087
Subtotal WYNDE4				0.11	5.14	7.91	
WYNDE6	15	m	0	2.23	45.98	42.35	0.0000
WYNDE6	204	f	0	2.20	69.69	59.67	0.0000
Subtotal WYNDE6				2.21	115.67	102.02	
XU3	22	m	1	1.58	2.41	0.22	0.0143
XU3	26	f	1	0.09	1.89	2.67	0.9057
Subtotal XU3				0.92	4.30	2.89	
ZHENG	10	m	0	0.60	17.29	7.80	0.0121
ZHENG	21	f	0	0.15	14.95	19.00	0.5668
Subtotal ZHENG				0.39	32.24	26.79	
ZHOU	26	m	0	0.27	14.05	14.26	0.3154
ZHOU	27	f	0	0.36	4.59	3.87	0.4448
Subtotal ZHOU				0.29	18.64	18.13	

	N	116
	NS	81
	Wt	1668.81
Het	Chi	1033.90
Het	df	115
Het	P	***
Fixed	RR	3.58
	RRl	3.41
	RRu	3.76
	P	+++
Random	RR	3.13
	RRl	2.67
	RRu	3.67
	P	+++
Asymm	P	*

Table 3C2 - 3

IESLC - Meta-anal of Current Smoking (or Ever if Current not available), Any prod (or Cigs if Any not avail)
Adenocarcinoma
Most adjusted

	combined	Sex: male	female	Total
N	11	56	49	116
NS	11	55	48	114
Wt	130.28	649.86	888.67	1668.81
Het Chi	67.95	330.87	582.96	1033.90
Het df	10	55	48	115
Het P	***	***	***	***
Fixed RR	2.44	4.37	3.27	3.58
RRl	2.05	4.05	3.07	3.41
RRu	2.89	4.72	3.50	3.76
P	+++	+++	+++	+++
Random RR	2.62	4.04	2.49	3.13
RRl	1.59	3.25	1.94	2.67
RRu	4.33	5.03	3.19	3.67
P	+++	+++	+++	+++
Between Chi				52.12
Between df				2
Between P				***
Btwn(F) P				(*)
Btwn(R) P				*

	Smoking status: ever	current	Total
N	72	44	116
NS	52	31	83
Wt	651.45	1017.36	1668.81
Het Chi	291.15	527.97	1033.90
Het df	71	43	115
Het P	***	***	***
Fixed RR	2.29	4.77	3.58
RRl	2.12	4.49	3.41
RRu	2.47	5.07	3.76
P	+++	+++	+++
Random RR	2.48	4.21	3.13
RRl	2.08	3.32	2.67
RRu	2.95	5.34	3.67
P	+++	+++	+++
Between Chi			214.78
Between df			1
Between P			***
Btwn(F) P			***
Btwn(R) P			***

Table 3C2 - 4

IESLC - Meta-anal of Current Smoking (or Ever if Current not available), Any prod (or Cigs if Any not avail)
Adenocarcinoma
Least adjusted

REF	NRR	X	SEX	AGEL	AGEH	RACE	YF	LC TYPE	LOC	START	ST	NLC	R	VB	P	H	AD	SM	PRODUCT	DENOM	De
ABRAHA	2		m	0	0	all	0	a	Eu:est	1975	pr	571	n	bl	n	n	0	ev	all/unsp	nev any	ot
ABRAHA	5		f	0	0	all	0	a	Eu:est	1975	pr	571	n	bl	n	n	0	ev	all/unsp	nev any	ot
ALDERS	54		m	0	0	all	-	a	Eu:UK	1977	CC	1448	n	V	n	n	2	ev	all/unsp	nev any	or
ALDERS	57		f	0	0	all	-	a	Eu:UK	1977	CC	1448	n	V	n	n	1	ev	all/unsp	nev any	or
ANDERS	12		f	0	0	all	0	a	NAmer	1986	pr	343	n	bl	n	n	0	ev	cig+/-ot	nev cigs	st
BAND	2		m	0	0	all	-	a	NAmer	1983	CC	2831	n	V	y	y	2	ev	cig only	nev any	ot
BARBON	44	x	m	0	0	all	-	a	Eu:wst	1979	CC	755	n	bl	y	y	0	cu	all/unsp	nev any	st
BECHER	12		f	0	0	all	-	not q+s	Eu:Ger	1985	CC	194	n	bl	n	y	1	ev	all/unsp	nev any	or
BOUCOT	72	x	m	0	0	all	0	a	NAmer	1951	pr	121	n	bl	n	n	0	cu	cig only	nev any	ot
BRESLO	35		c	0	0	all	-	a	NAmer	1949	CC	518	n	bl	n	y	0	ev	all/unsp	nev+1	st
BROWN1	1	x	m	0	0	wh	-	a	NAmer	1979	CC	102	n	bl	y	y	0	ev	cig+/-ot	nev cigs	st
BROWN1	2	x	f	0	0	wh	-	a	NAmer	1979	CC	102	n	bl	y	y	0	ev	cig+/-ot	nev cigs	st
BROWN2	14		m	0	0	wh	-	a	NAmer	1984	CC	14596	n	bl	n	y	2	cu	cig+/-ot	nev cigs	or
BROWN2	13		f	0	0	wh	-	a	NAmer	1984	CC	14596	n	bl	n	y	2	cu	cig+/-ot	nev cigs	or
BUFFLE	50		m	0	0	wh	-	a	NAmer	1976	CC	943	n	bl	y	n	0	ev	cig+/-ot	nev cigs	ot
BUFFLE	71		f	0	0	w-hi	-	a	NAmer	1976	CC	943	n	bl	y	n	0	cu	cig+/-ot	nev cigs	st
BYERS1	3		m	0	0	wh	-	a	NAmer	1957	CC	1002	n	bl	n	n	0	ev	cig+/-ot	nev cigs	st
CHAN	12		m	0	0	all	-	a+l	As:HK	1976	CC	397	n	bl	n	n	0	ev	all/unsp	nev any	ot
CHAN	16		f	0	0	all	-	a+l	As:HK	1976	CC	397	n	bl	n	n	0	ev	all/unsp	nev any	st
CHOI	63		m	0	0	all	-	a	As:oth	1985	CC	375	n	bl	n	n	0	ev	cig+/-ot	nev cigs	st
CHOI	65		f	0	0	all	-	a	As:oth	1985	CC	375	n	bl	n	n	0	ev	cig+/-ot	nev cigs	st
COMSTO	24		m	0	0	all	-	a	NAmer	1975	ot	258	n	bl	n	n	0	cu	cig+/-ot	nev cigs	st
COMSTO	31		f	0	0	all	-	a	NAmer	1975	ot	258	n	bl	n	n	0	cu	cig+/-ot	nev cigs	st
CORREA	44		c	0	0	all	-	a	NAmer	1979	CC	1359	n	bl	y	n	1	cu	cig+/-ot	nev cigs	or
CPSI	404		m	0	0	all	2	a	NAmer	1959	pr	5138	n	bl	n	n	1	cu	cig only	nev any	ot
CPSI	406		f	0	0	all	2	a	NAmer	1959	pr	5138	n	bl	n	n	1	cu	cig only	nev any	ot
CPSII	115		m	0	0	all	2	a	NAmer	1982	pr	3229	n	bl	n	n	1	cu	cig only	nev any	st
CPSII	118		f	0	0	all	2	a	NAmer	1982	pr	3229	n	bl	n	n	1	cu	cig+/-ot	nev cigs	st
DAMBER	11	x	m	0	0	all	-	a+al+br	Eu:Sca	1972	CC	579	n	bl	y	n	0	ev	all/unsp	nev any	st
DESTE2	17		m	0	0	all	-	a	SCAmer	1993	CC	463	n	bl	n	n	2	ev	all/unsp	nev any	or
DOLL	83	x	m	0	0	all	-	KII	Eu:UK	1948	CC	1465	n	V	n	n	0	ev	all/unsp	nev any	st
DOLL	85	x	f	0	0	all	-	KII	Eu:UK	1948	CC	1465	n	V	n	n	0	ev	all/unsp	nev any	st
DORGAN	125		m	0	0	wh	-	a	NAmer	1980	CC	2026	n	bl	y	y	2	ev	cig+/-ot	nev any	or
DORGAN	104		f	0	0	all	-	a	NAmer	1980	CC	2026	n	bl	y	y	3	ev	cig+/-ot	nev any	or
DORN	340		m	0	0	wh	8	a	NAmer	1954	pr	5097	n	bl	n	n	1	cu	cig only	nev any	ot
DOSEME	20	x	m	0	0	all	-	not q+s	Eu:bal	1979	CC	1210	n	bl	n	n	0	ev	cig+/-ot	nev cigs	st
ENGELA	70		m	0	0	all	0	a	Eu:Sca	1964	pr	435	n	bl	n	n	7	cu	cig+/-ot	nev cigs	ot
FAN	4		c	0	0	all	-	a	As:Chi	1990	CC	403	n	ot	y	n	0	ev	cig+/-ot	nev cigs	ot
GAO	8	x	m	0	0	all	-	a	As:Chi	1984	CC	1405	n	ot	n	n	0	ev	cig+/-ot	nev cigs	st
GAO	18	x	f	0	0	all	-	a	As:Chi	1984	CC	1405	n	ot	n	n	0	ev	cig+/-ot	nev cigs	st
GER	1	x	c	0	0	all	-	a	As:oth	1990	CC	141	n	ot	y	n	0	ev	all/unsp	nev any	st
HAENSZ	37		f	0	0	all	-	a	NAmer	1955	CC	158	n	bl	n	y	0	cu	cig+/-ot	nev any	st
HAMMON	92		m	0	0	wh	0	a	NAmer	1952	pr	448	n	bl	n	n	0	ev	all/unsp	nev any	st
HEGMAN	4		c	0	0	all	-	a	NAmer	1989	CC	282	n	bl	y	y	0	ev	all/unsp	nev any	st
HINDS	24		f	0	0	o	-	a	NAmer	1968	CC	292	n	bl	n	n	3	ev	all/unsp	nev any	st
ISHIMA	3	x	c	0	0	all	-	a	As:Jap	1961	CC	180	n	bl	y	y	0	ev	all/unsp	nev any	st
JAHN	8		m	0	0	all	-	a	Eu:Ger	1988	CC	1004	n	bl	n	n	0	cu	cig+/-ot	nev any	st
JAIN	17		m	0	0	all	-	a	NAmer	1981	CC	845	n	V	y	n	0	cu	cig+/-ot	nev cigs	st
JAIN	12		f	0	0	all	-	a	NAmer	1981	CC	845	n	V	y	n	0	cu	cig+/-ot	nev cigs	st
JEDRYC	26		m	0	0	all	-	a	Eu:est	1980	CC	1630	n	bl	y	n	0	cu	cig+/-ot	nev any	st
JOLY	51		m	0	0	all	-	a	SCAmer	1978	CC	826	n	bl	n	n	0	ev	cig+/-ot	nev any	st
JOLY	50		f	0	0	all	-	a	SCAmer	1978	CC	826	n	bl	n	n	0	ev	cig+/-ot	nev any	st
JUSSAW	26		m	0	0	all	-	KII	As:Ind	1964	CC	792	n	V	n	n	0	ev	all/unsp	nev any	st
KATSOU	16	x	f	0	0	all	-	a	Eu:bal	1987	CC	101	n	bl	n	n	0	cu	all/unsp	nev any	st
KHUDER	17		m	0	0	all	-	a	NAmer	1985	CC	482	n	bl	n	y	0	cu	cig+/-ot	nev cigs	or
KIHARA	5		c	0	0	jap	-	a	As:Jap	1991	CC	440	n	bl	n	n	0	cu	all/unsp	nev any	st
KOO	7		f	0	0	all	-	a+l	As:HK	1981	CC	200	n	bl	n	n	0	ev	all/unsp	nev any	st
KREYBE	20	x	m	0	0	all	-	KII	Eu:Sca	1948	CC	300	n	bl	n	y	0	ev	all/unsp	nev any	st
KREYBE	36	x	f	0	0	all	-	KII	Eu:Sca	1948	CC	300	n	bl	n	y	0	ev	all/unsp	nev any	st
LAMTH	3		f	0	0	ch	-	a	As:HK	1983	CC	445	n	bl	n	n	0	ev	all/unsp	nev any	or
LAMWK	4		f	0	0	ch	-	a	As:HK	1981	CC	163	n	bl	n	n	0	ev	all/unsp	nev any	st
LAMWK2	3		m	0	0	all	-	a	As:HK	1976	CC	480	n	bl	n	n	0	ev	all/unsp	nev any	st
LAMWK2	7		f	0	0	all	-	a	As:HK	1976	CC	480	n	bl	n	n	0	ev	all/unsp	nev any	st
LOMBA2	3		f	0	0	all	-	not q+u	NAmer	1960	CC	225	n	bl	n	n	0	ev	cig+/-ot	nev cigs	st
LUBIN	36		m	0	0	all	-	KII	As:Chi	1984	CC	427	m	ot	y	n	0	ev	all/unsp	nev any	st
LUBIN2	252		m	0	0	all	-	a	Eu:mul	1976	CC	7804	n	bl	n	y	0	cu	cig+/-ot	nev any	st
LUBIN2	264		f	0	0	all	-	a	Eu:mul	1976	CC	7804	n	bl	n	y	0	cu	cig+/-ot	nev any	st
LUO	3	x	c	0	0	all	-	a	As:Chi	1990	CC	102	n	ot	n	y	0	ev	cig+/-ot	nev cigs	st
MATOS	52	x	m	0	0	all	-	a	SCAmer	1994	CC	200	n	bl	n	n	0	cu	cig+/-ot	nev any	st
MATSUD	12		m	0	0	all	-	a	As:Jap	1965	CC	179	n	bl	n	n	0	ev	cig+/-ot	nev cigs	ot
NOU	3		m	0	0	all	-	a	Eu:Sca	1971	CC	273	n	bl	y	n	0	ev	all/unsp	nev any	st
NOU	8		f	0	0	all	-	a	Eu:Sca	1971	CC	273	n	bl	y	n	0	ev	all/unsp	nev any	st

Table 3C2 - 4

IESLC - Meta-anal of Current Smoking (or Ever if Current not available), Any prod (or Cigs if Any not avail)
Adenocarcinoma
Least adjusted

REF	NRR	X	SEX	AGEL	AGEH	RACE	YF	LC TYPE	LOC	START	ST	NLC	R	VB	P	H	AD	SM	PRODUCT	DENOM	De
ORMOS	21		m	0	0	all	-	a	Eu:est	1947	CC	119	n	bl	y	y	0	ev	cig+/-ot	nev any	ot
OSANN	11	x	m	0	0	all	-	a	NAmer	1984	CC	1986	n	bl	n	n	0	cu	cig+/-ot	nev cigs	st
OSANN	15	x	f	0	0	all	-	a	NAmer	1984	CC	1986	n	bl	n	n	0	cu	cig+/-ot	nev cigs	st
OSANN2	14	x	f	0	0	all	-	KII	NAmer	1964	ot	217	n	bl	n	y	0	cu	cig+/-ot	nev cigs	st
PEZZOT	7		m	0	0	all	-	a	SCAmer	1987	CC	215	n	bl	n	y	0	ev	cig only	nev cigs	st
SCHWAR	8		m	40	54	wh	-	a	NAmer	1984	CC	5588	n	bl	y	y	0	ev	cig+/-ot	nev cigs	st
SCHWAR	7		m	40	54	bl	-	a	NAmer	1984	CC	5588	n	bl	y	y	0	ev	cig+/-ot	nev cigs	st
SCHWAR	16		f	40	54	wh	-	a	NAmer	1984	CC	5588	n	bl	y	y	0	ev	cig+/-ot	nev cigs	st
SCHWAR	15		f	40	54	bl	-	a	NAmer	1984	CC	5588	n	bl	y	y	0	ev	cig+/-ot	nev cigs	st
SEOW	2		f	0	0	ch	-	a	As:oth	1997	CC	153	n	bl	n	y	0	ev	cig+/-ot	nev cigs	st
SIEMIA	12	x	m	0	0	all	-	a	NAmer	1979	CC	857	n	V	y	y	0	ev	cig+/-ot	nev cigs	st
SOBUE	6	x	m	0	0	all	-	a	As:Jap	1986	CC	1376	n	bl	n	y	0	cu	cig+/-ot	nev cigs	st
SOBUE	22	x	f	0	0	all	-	a	As:Jap	1986	CC	1376	n	bl	n	y	0	cu	cig+/-ot	nev cigs	st
SOBUE2	2		m	0	0	all	-	a	As:Jap	1965	CC	2083	n	bl	n	n	2	cu	cig+/-ot	nev any	or
SOBUE2	6		f	0	0	all	-	a	As:Jap	1965	CC	2083	n	bl	n	n	2	cu	cig+/-ot	nev any	or
STASZE	21		m	0	0	all	-	a	Eu:est	1954	CC	281	n	bl	n	y	0	ev	all/unsp	nev any	ot
STASZE	4		f	0	0	all	-	a	Eu:est	1954	CC	281	n	bl	n	y	0	ev	all/unsp	nev any	st
STAYNE	4		m	0	0	all	-	a	NAmer	1969	CC	420	n	bl	n	n	0	ev	all/unsp	nev any	st
SUZUK2	13	x	c	0	0	all	-	a	SCAmer	1991	CC	123	n	bl	n	y	0	ev	all/unsp	nev any	st
SUZUKI	2	x	m	0	0	all	-	a	As:Jap	1978	CC	238	n	bl	n	y	0	cu	cig+/-ot	nev any	st
SUZUKI	6	x	f	0	0	all	-	a	As:Jap	1978	CC	238	n	bl	n	y	0	cu	cig+/-ot	nev any	st
SVENSS	64	x	f	0	0	all	-	a	Eu:Sca	1983	CC	210	n	bl	n	n	0	cu	all/unsp	nev any	st
TIZZAN	19		c	0	0	all	-	a	Eu:wst	1959	CC	1358	n	bl	n	n	0	ev	all/unsp	nev any	st
TOKARS	7	x	c	0	0	all	-	a	Eu:est	1966	ot	162	o	bl	n	y	0	ev	all/unsp	nev any	st
TSUGAN	3		m	0	0	all	-	a	As:Jap	1976	CC	134	n	bl	n	y	0	cu	all/unsp	nev any	st
TSUGAN	9		f	0	0	all	-	a	As:Jap	1976	CC	134	n	bl	n	y	0	cu	all/unsp	nev any	or
WAKAI	6	x	m	0	0	all	-	a	As:Jap	1988	CC	333	n	bl	n	y	0	cu	all/unsp	nev any	st
WAKAI	24	x	f	0	0	all	-	a	As:Jap	1988	CC	333	n	bl	n	y	0	cu	all/unsp	nev any	st
WU	2	x	f	0	0	wh	-	a	NAmer	1981	CC	220	n	bl	n	y	0	cu	all/unsp	nev any	st
WU2	1		f	0	0	all	-	a	NAmer	1983	CC	336	n	bl	n	y	2	cu	all/unsp	nev any	or
WUWILL	25	x	f	0	0	all	-	a	As:Chi	1985	CC	965	n	ot	n	n	0	ev	cig+/-ot	nev cigs	st
WYNDE2	14		m	0	0	all	-	KII	NAmer	1962	CC	404	n	bl	n	y	0	ev	all/unsp	nev any	st
WYNDE3	30		m	0	0	all	-	KII	NAmer	1966	CC	350	n	bl	n	y	0	cu	all/unsp	nev any	st
WYNDE3	135		f	0	0	all	-	KII	NAmer	1966	CC	350	n	bl	n	y	0	ev	all/unsp	nev any	st
WYNDE4	42		m	0	0	all	-	a	NAmer	1948	CC	684	n	bl	y	n	0	ev	all/unsp	nev any	st
WYNDE4	56		f	0	0	all	-	a	NAmer	1948	CC	684	n	bl	y	n	2	ev	all/unsp	nev any	ot
WYNDE6	15		m	0	0	all	-	KII	NAmer	1969	CC	4423	n	bl	n	y	0	cu	cig+/-ot	nev any	st
WYNDE6	204		f	0	0	all	-	KII	NAmer	1969	CC	4423	n	bl	n	y	0	cu	cig+/-ot	nev cigs	st
XU3	21	x	m	0	0	all	-	KII	As:Chi	1981	CC	135	n	ot	n	n	0	ev	all/unsp	nev any	st
XU3	25	x	f	0	0	all	-	KII	As:Chi	1981	CC	135	n	ot	n	n	0	ev	all/unsp	nev any	st
ZHENG	10		m	0	0	all	-	a	As:Chi	1982	CC	540	n	ot	*	y	0	ev	cig+/-ot	nev cigs	st
ZHENG	21		f	0	0	all	-	a	As:Chi	1982	CC	540	n	ot	*	y	0	ev	cig+/-ot	nev cigs	st
ZHOU	26		m	0	0	all	-	a	As:Chi	1978	CC	1360	n	ot	n	n	0	ev	all/unsp	nev any	st
ZHOU	27		f	0	0	all	-	a	As:Chi	1978	CC	1360	n	ot	n	n	0	ev	all/unsp	nev any	st

Cigarette type is all/unspec for all RRs

Table 3C2 - 5

IESLC - Meta-anal of Current Smoking (or Ever if Current not available), Any prod (or Cigs if Any not avail)
Adenocarcinoma
Least adjusted

REF	NRR	SEX	AD	Number Exposed Case	Cont	Non-exposed Case	Cont	RR	95.00%CI
*ABRAHA	2	m	0	59	10351	8	3365	2.40	(1.15- 5.01)
*ABRAHA	5	f	0	19	5256	16	11589	2.62	(1.35- 5.09)
Subtotal ABRAHA								2.52	(1.54- 4.12)
ALDERS	54	m	2	-	-	-	-	7.11	(1.49- 33.85)
ALDERS	57	f	1	-	-	-	-	3.58	(1.48- 8.65)
Subtotal ALDERS								4.23	(1.96- 9.12)
*ANDERS	12	f	0	99	96164	33	195158	6.09	(4.11- 9.03)
BAND	2	m	2	-	-	-	-	4.10	(3.01- 5.59)
BARBON	44	m	0	109	362	7	188	8.09	(3.69- 17.72)
BECHER	12	f	1	-	-	-	-	10.83	(1.32- 88.70)
*BOUCOT	72	m	0	14	22177	0	7551	9.87~	(0.59- 165.51)
BRESLO	35	c	0	42	462	4	56	1.27	(0.44- 3.68)
BROWN1	1	m	0	46	46	4	19	4.75	(1.50- 15.05)
BROWN1	2	f	0	33	19	19	47	4.30	(1.98- 9.34)
Subtotal BROWN1								4.43	(2.33- 8.44)
BROWN2	14	m	2	-	-	-	-	9.10	(7.60- 10.80)
BROWN2	13	f	2	-	-	-	-	7.20	(6.20- 8.30)
Subtotal BROWN2								7.92	(7.08- 8.86)
BUFFLE	50	m	0	-	-	-	-	4.50	(1.85- 10.95)
BUFFLE	71	f	0	56	110	7	112	8.15	(3.56- 18.65)
Subtotal BUFFLE								6.18	(3.37- 11.33)
BYERS1	3	m	0	47	695	7	424	4.10	(1.83- 9.15)
CHAN	12	m	0	56	161	0	43	30.44~	(1.84- 502.58)
CHAN	16	f	0	28	50	40	139	1.95	(1.09- 3.48)
Subtotal CHAN								2.18	(1.23- 3.85)
CHOI	63	m	0	46	465	7	95	1.34	(0.59- 3.06)
CHOI	65	f	0	5	26	49	164	0.64	(0.23- 1.77)
Subtotal CHOI								1.00	(0.53- 1.89)
COMSTO	24	m	0	30	100	2	84	12.60	(2.92- 54.28)
COMSTO	31	f	0	23	52	8	115	6.36	(2.67- 15.16)
Subtotal COMSTO								7.60	(3.60- 16.04)
CORREA	44	c	1	-	-	-	-	6.70	(4.30- 10.60)
*CPSI	404	m	1	-	-	-	-	4.58	(1.74- 12.05)
*CPSI	406	f	1	-	-	-	-	1.43	(0.47- 4.39)
Subtotal CPSI								2.78	(1.34- 5.78)
*CPSII	115	m	1	-	-	-	-	19.22	(6.46- 57.16)
*CPSII	118	f	1	-	-	-	-	8.23	(4.36- 15.54)
Subtotal CPSII								10.21	(5.89- 17.67)
DAMBER	11	m	0	65	49	16	29	2.40	(1.18- 4.91)
DESTE2	17	m	2	-	-	-	-	4.30	(1.60- 11.40)
DOLL	83	m	0	38	1296	2	61	0.89	(0.21- 3.79)
DOLL	85	f	0	8	49	5	59	1.93	(0.59- 6.27)
Subtotal DOLL								1.42	(0.57- 3.53)
DORGAN	125	m	2	-	-	-	-	4.80	(1.90- 12.00)
DORGAN	104	f	3	-	-	-	-	3.90	(2.80- 5.40)
Subtotal DORGAN								3.99	(2.93- 5.44)
*DORN	340	m	1	-	-	-	-	5.95	(3.85- 9.22)
DOSEME	20	m	0	142	536	24	293	3.23	(2.05- 5.10)
*ENGELA	70	m	7	-	-	-	-	7.06	(2.69- 18.50)
FAN	4	c	0	67	595	45	556	1.39	(0.94- 2.07)
GAO	8	m	0	180	558	42	202	1.55	(1.07- 2.25)
GAO	18	f	0	62	130	266	605	1.08	(0.78- 1.52)
Subtotal GAO								1.27	(0.99- 1.63)
GER	1	c	0	35	139	37	149	1.01	(0.60- 1.70)
HAENSZ	37	f	0	16	94	37	236	1.09	(0.58- 2.05)
*HAMMON	92	m	0	29	510108	2	115884	3.29	(0.79- 13.80)
HEGMAN	4	c	0	83	1202	15	2080	9.58	(5.50- 16.67)
HINDS	24	f	3	-	-	-	-	3.89	(2.49- 6.07)
ISHIMA	3	c	0	39	25	13	27	3.24	(1.41- 7.43)
JAHN	8	m	0	75	269	8	138	4.81	(2.26- 10.26)
JAIN	17	m	0	60	118	4	85	10.81	(3.78- 30.87)
JAIN	12	f	0	69	99	24	214	6.21	(3.69- 10.47)
Subtotal JAIN								6.93	(4.35- 11.07)
JEDRYC	26	m	0	68	516	7	289	5.44	(2.47- 12.00)
JOLY	51	m	0	72	709	5	218	4.43	(1.77- 11.10)
JOLY	50	f	0	33	122	25	283	3.06	(1.75- 5.37)
Subtotal JOLY								3.38	(2.10- 5.46)
JUSSAW	26	m	0	34	168	13	624	9.71	(5.01- 18.82)
KATSOU	16	f	0	15	18	30	67	1.86	(0.83- 4.18)
KHUDER	17	m	0	92	-	7	-	8.20	(3.60- 18.40)
KIHARA	5	c	0	103	162	78	237	1.93	(1.35- 2.76)

Table 3C2 - 5

IESLC - Meta-anal of Current Smoking (or Ever if Current not available), Any prod (or Cigs if Any not avail)
Adenocarcinoma
Least adjusted

REF	NRR	SEX	AD	Number Exposed Case	Cont	Non-exposed Case	Cont	RR	95.00%CI
KOO	7	f	0	34	63	46	137	1.61 (	0.94- 2.74)
KREYBE	20	m	0	42	3514	3	644	2.57 (	0.79- 8.30)
KREYBE	36	f	0	10	328	27	657	0.74 (	0.35- 1.55)
Subtotal KREYBE								1.05 (	0.56- 1.97)
LAMTH	3	f	0	79	51	131	158	1.87 (	1.23- 2.85)
LAMWK	4	f	0	36	41	60	144	2.11 (	1.23- 3.61)
LAMWK2	3	m	0	52	161	15	43	0.93 (	0.48- 1.80)
LAMWK2	7	f	0	26	50	41	139	1.76 (	0.98- 3.17)
Subtotal LAMWK2								1.33 (	0.86- 2.07)
LOMBA2	3	f	0	42	353	54	239	0.53 (	0.34- 0.81)
LUBIN	36	m	0	37	939	4	72	0.71 (	0.25- 2.05)
LUBIN2	252	m	0	454	6209	57	2616	3.36 (	2.54- 4.44)
LUBIN2	264	f	0	69	410	138	1180	1.44 (	1.06- 1.96)
Subtotal LUBIN2								2.30 (	1.87- 2.83)
LUO	3	c	0	28	146	29	160	1.06 (	0.60- 1.86)
MATOS	52	m	0	46	132	5	110	7.67 (	2.94- 19.96)
MATSUD	12	m	0	23	3314	0	1255	17.80~(	1.08- 293.32)
NOU	3	m	0	36	247	4	122	4.45 (	1.55- 12.77)
NOU	8	f	0	9	92	29	261	0.88 (	0.40- 1.93)
Subtotal NOU								1.57 (	0.83- 2.94)
ORMOS	21	m	0	4	1034	0	777	6.76~(	0.36- 125.82)
OSANN	11	m	0	217	541	14	833	23.87 (	13.75- 41.41)
OSANN	15	f	0	193	367	47	1093	12.23 (	8.70- 17.18)
Subtotal OSANN								14.70 (	11.01- 19.64)
OSANN2	14	f	0	50	28	22	43	3.49 (	1.75- 6.97)
PEZZOT	7	m	0	60	317	3	116	7.32 (	2.25- 23.79)
SCHWAR	8	m	0	84	178	1	73	34.45 (	4.71- 252.10)
SCHWAR	7	m	0	45	39	1	7	8.08 (	0.95- 68.56)
SCHWAR	16	f	0	92	108	10	79	6.73 (	3.29- 13.75)
SCHWAR	15	f	0	20	28	3	41	9.76 (	2.65- 36.00)
Subtotal SCHWAR								8.40 (	4.73- 14.94)
SEOW	2	f	0	19	15	67	125	2.36 (	1.13- 4.95)
SIEMIA	12	m	0	162	428	5	105	7.95 (	3.18- 19.85)
SOBUE	6	m	0	276	650	27	128	2.01 (	1.30- 3.12)
SOBUE	22	f	0	38	168	137	857	1.41 (	0.95- 2.10)
Subtotal SOBUE								1.66 (	1.24- 2.22)
SOBUE2	2	m	2	-	-	-	-	3.10 (	2.40- 3.70)
SOBUE2	6	f	2	-	-	-	-	1.80 (	1.40- 2.20)
Subtotal SOBUE2								2.39 (	2.04- 2.79)
STASZE	21	m	0	20	754	0	158	8.61~(	0.52- 143.15)
STASZE	4	f	0	1	153	10	1660	1.08 (	0.14- 8.53)
Subtotal STASZE								2.24 (	0.42- 11.81)
STAYNE	4	m	0	43	567	7	333	3.61 (	1.60- 8.11)
SUZUK2	13	c	0	20	10	5	15	6.00 (	1.69- 21.26)
SUZUKI	2	m	0	119	162	14	99	5.19 (	2.83- 9.54)
SUZUKI	6	f	0	20	20	55	133	2.42 (	1.21- 4.84)
Subtotal SUZUKI								3.73 (	2.36- 5.89)
SVENSS	64	f	0	38	53	22	120	3.91 (	2.11- 7.25)
TIZZAN	19	c	0	88	939	25	419	1.57 (	0.99- 2.49)
TOKARS	7	c	0	68	112	10	54	3.28 (	1.57- 6.86)
TSUGAN	3	m	0	45	50	18	17	0.85 (	0.39- 1.85)
TSUGAN	9	f	0	6	10	33	30	0.55 (	0.18- 1.68)
Subtotal TSUGAN								0.74 (	0.39- 1.40)
WAKAI	6	m	0	75	284	8	65	2.15 (	0.99- 4.67)
WAKAI	24	f	0	9	26	46	145	1.09 (	0.48- 2.50)
Subtotal WAKAI								1.56 (	0.89- 2.75)
WU	2	f	0	99	50	29	62	4.23 (	2.43- 7.39)
WU2	1	f	2	-	-	-	-	4.50 (	3.00- 6.90)
WUWILL	25	f	0	138	351	172	601	1.37 (	1.06- 1.78)
WYNDE2	14	m	0	49	616	5	105	1.67 (	0.65- 4.29)
WYNDE3	30	m	0	56	207	6	88	3.97 (	1.65- 9.55)
WYNDE3	135	f	0	21	56	15	76	1.90 (	0.90- 4.01)
Subtotal WYNDE3								2.59 (	1.47- 4.57)
WYNDE4	42	m	0	35	665	4	115	1.51 (	0.53- 4.34)
WYNDE4	56	f	2	-	-	-	-	0.60 (	0.13- 2.69)
Subtotal WYNDE4								1.12 (	0.47- 2.66)
WYNDE6	15	m	0	651	741	58	617	9.35 (	7.00- 12.48)
WYNDE6	204	f	0	472	376	119	856	9.03 (	7.14- 11.42)
Subtotal WYNDE6								9.15 (	7.63- 10.98)
XU3	21	m	0	29	68	3	31	4.41 (	1.25- 15.57)
XU3	25	f	0	4	11	7	25	1.30 (	0.31- 5.36)

Table 3C2 - 5

IESLC - Meta-anal of Current Smoking (or Ever if Current not available), Any prod (or Cigs if Any not avail)
Adenocarcinoma
Least adjusted

REF	NRR	SEX	AD	Number Exposed Case	Cont	Non-exposed Case	Cont	RR	95.00%CI
Subtotal XU3								2.57	(1.00- 6.59)
ZHENG	10	m	0	123	218	29	94	1.83	(1.14- 2.93)
ZHENG	21	f	0	33	44	119	184	1.16	(0.70- 1.93)
Subtotal ZHENG								1.48	(1.05- 2.09)
ZHOU	26	m	0	131	41	88	36	1.31	(0.77- 2.20)
ZHOU	27	f	0	30	7	96	32	1.43	(0.57- 3.57)
Subtotal ZHOU								1.34	(0.85- 2.10)
Partial Totals				6803	680000	2899	359869		

*prospective study ~ With 0.5 adjustment for zero

REF	NRR	SEX	AD	Ys	Ws	Qs	Ps
*ABRAHA	2	m	0	0.87	7.06	1.02	0.0201
*ABRAHA	5	f	0	0.96	8.71	0.75	0.0045
Subtotal ABRAHA				0.92	15.77	1.77	
ALDERS	54	m	2	1.96	1.58	0.79	0.0138
ALDERS	57	f	1	1.28	4.93	0.00	0.0046
Subtotal ALDERS				1.44	6.50	0.79	
*ANDERS	12	f	0	1.81	24.76	7.52	0.0000
BAND	2	m	2	1.41	40.10	0.97	0.0000
BARBON	44	m	0	2.09	6.25	4.36	0.0000
BECHER	12	f	1	2.38	0.87	1.10	0.0265
*BOUCOT	72	m	0	2.29	0.48	0.52	0.1114
BRESLO	35	c	0	0.24	3.40	3.50	0.6564
BROWN1	1	m	0	1.56	2.89	0.27	0.0081
BROWN1	2	f	0	1.46	6.38	0.26	0.0002
Subtotal BROWN1				1.49	9.27	0.53	
BROWN2	14	m	2	2.21	124.44	113.06	0.0000
BROWN2	13	f	2	1.97	180.58	93.35	0.0000
Subtotal BROWN2				2.07	305.02	206.41	
BUFFLE	50	m	0	1.50	4.86	0.30	0.0009
BUFFLE	71	f	0	2.10	5.59	3.97	0.0000
Subtotal BUFFLE				1.82	10.45	4.27	
BYERS1	3	m	0	1.41	5.95	0.14	0.0006
CHAN	12	m	0	3.42	0.49	2.28	0.0170
CHAN	16	f	0	0.67	11.38	3.95	0.0247
Subtotal CHAN				0.78	11.86	6.23	
CHOI	63	m	0	0.29	5.64	5.20	0.4842
CHOI	65	f	0	-0.44	3.77	10.85	0.3920
Subtotal CHOI				-0.00	9.42	16.06	
COMSTO	24	m	0	2.53	1.80	2.94	0.0007
COMSTO	31	f	0	1.85	5.09	1.80	0.0000
Subtotal COMSTO				2.03	6.89	4.74	
CORREA	44	c	1	1.90	18.88	7.90	0.0000
*CPSI	404	m	1	1.52	4.10	0.29	0.0021
*CPSI	406	f	1	0.36	3.08	2.48	0.5303
Subtotal CPSI				1.02	7.18	2.77	
*CPSII	115	m	1	2.96	3.23	9.35	0.0000
*CPSII	118	f	1	2.11	9.51	6.92	0.0000
Subtotal CPSII				2.32	12.75	16.27	
DAMBER	11	m	0	0.88	7.53	1.08	0.0161
DESTE2	17	m	2	1.46	3.99	0.17	0.0036
DOLL	83	m	0	-0.11	1.84	3.44	0.8795
DOLL	85	f	0	0.66	2.76	0.99	0.2760
Subtotal DOLL				0.35	4.60	4.43	
DORGAN	125	m	2	1.57	4.52	0.44	0.0008
DORGAN	104	f	3	1.36	35.62	0.40	0.0000
Subtotal DORGAN				1.38	40.15	0.84	
*DORN	340	m	1	1.78	20.15	5.62	0.0000
DOSEME	20	m	0	1.17	18.52	0.12	0.0000
*ENGELA	70	m	7	1.95	4.13	2.02	0.0001
FAN	4	c	0	0.33	24.61	21.05	0.1013
GAO	8	m	0	0.44	27.69	18.44	0.0208
GAO	18	f	0	0.08	34.21	47.13	0.6343
Subtotal GAO				0.24	61.90	65.56	
GER	1	c	0	0.01	14.39	22.16	0.9579
HAENSZ	37	f	0	0.08	9.58	13.18	0.7992
*HAMMON	92	m	0	1.19	1.87	0.01	0.1030
HEGMAN	4	c	0	2.26	12.50	12.60	0.0000
HINDS	24	f	3	1.36	19.35	0.21	0.0000
ISHIMA	3	c	0	1.18	5.57	0.04	0.0055

Table 3C2 - 5

IESLC - Meta-anal of Current Smoking (or Ever if Current not available), Any prod (or Cigs if Any not avail)
Adenocarcinoma
Least adjusted

REF	NRR	SEX	AD	Ys	Ws	Qs	Ps
JAHN	8	m	0	1.57	6.70	0.67	0.0000
JAIN	17	m	0	2.38	3.49	4.41	0.0000
JAIN	12	f	0	1.83	14.10	4.61	0.0000
Subtotal JAIN				1.94	17.58	9.02	
JEDRYC	26	m	0	1.69	6.14	1.18	0.0000
JOLY	51	m	0	1.49	4.55	0.25	0.0015
JOLY	50	f	0	1.12	12.19	0.23	0.0001
Subtotal JOLY				1.22	16.74	0.47	
JUSSAW	26	m	0	2.27	8.78	9.11	0.0000
KATSOU	16	f	0	0.62	5.87	2.36	0.1325
KHUDER	17	m	0	2.10	5.77	4.16	0.0000
KIHARA	5	c	0	0.66	30.38	10.81	0.0003
KOO	7	f	0	0.47	13.45	8.20	0.0817
KREYBE	20	m	0	0.94	2.79	0.27	0.1158
KREYBE	36	f	0	-0.30	7.06	17.05	0.4275
Subtotal KREYBE				0.05	9.85	17.32	
LAMTH	3	f	0	0.63	21.63	8.59	0.0036
LAMWK	4	f	0	0.75	13.20	3.43	0.0068
LAMWK2	3	m	0	-0.08	8.67	15.38	0.8206
LAMWK2	7	f	0	0.57	11.11	5.26	0.0588
Subtotal LAMWK2				0.28	19.77	20.64	
LOMBA2	3	f	0	-0.64	20.27	72.88	0.0039
LUBIN	36	m	0	-0.34	3.42	8.75	0.5249
LUBIN2	252	m	0	1.21	49.29	0.10	0.0000
LUBIN2	264	f	0	0.36	39.96	31.73	0.0214
Subtotal LUBIN2				0.83	89.24	31.83	
LUO	3	c	0	0.06	12.01	17.25	0.8449
MATOS	52	m	0	2.04	4.19	2.56	0.0000
MATSUD	12	m	0	2.88	0.49	1.29	0.0440
NOU	3	m	0	1.49	3.45	0.19	0.0056
NOU	8	f	0	-0.13	6.24	11.92	0.7504
Subtotal NOU				0.45	9.69	12.12	
ORMOS	21	m	0	1.91	0.45	0.19	0.1999
OSANN	11	m	0	3.17	12.64	46.48	0.0000
OSANN	15	f	0	2.50	33.23	51.81	0.0000
Subtotal OSANN				2.69	45.87	98.30	
OSANN2	14	f	0	1.25	8.04	0.00	0.0004
PEZZOT	7	m	0	1.99	2.76	1.49	0.0009
SCHWAR	8	m	0	3.54	0.97	5.06	0.0005
SCHWAR	7	m	0	2.09	0.84	0.58	0.0556
SCHWAR	16	f	0	1.91	7.53	3.20	0.0000
SCHWAR	15	f	0	2.28	2.26	2.36	0.0006
Subtotal SCHWAR				2.13	11.60	11.20	
SEOW	2	f	0	0.86	7.03	1.10	0.0226
SIEMIA	12	m	0	2.07	4.59	3.07	0.0000
SOBUE	6	m	0	0.70	20.00	6.17	0.0018
SOBUE	22	f	0	0.35	24.55	20.24	0.0855
Subtotal SOBUE				0.51	44.54	26.41	
SOBUE2	2	m	2	1.13	82.01	1.25	0.0000
SOBUE2	6	f	2	0.59	75.22	33.49	0.0000
Subtotal SOBUE2				0.87	157.22	34.75	
STASZE	21	m	0	2.15	0.49	0.39	0.1332
STASZE	4	f	0	0.08	0.90	1.24	0.9382
Subtotal STASZE				0.81	1.39	1.64	
STAYNE	4	m	0	1.28	5.85	0.00	0.0019
SUZUK2	13	c	0	1.79	2.40	0.69	0.0055
SUZUKI	2	m	0	1.65	10.41	1.60	0.0000
SUZUKI	6	f	0	0.88	7.96	1.10	0.0128
Subtotal SUZUKI				1.32	18.36	2.70	
SVENSS	64	f	0	1.36	10.10	0.12	0.0000
TIZZAN	19	c	0	0.45	18.24	11.78	0.0538
TOKARS	7	c	0	1.19	7.03	0.03	0.0016
TSUGAN	3	m	0	-0.16	6.39	12.83	0.6813
TSUGAN	9	f	0	-0.61	3.03	10.49	0.2916
Subtotal TSUGAN				-0.31	9.41	23.32	
WAKAI	6	m	0	0.76	6.36	1.54	0.0542
WAKAI	24	f	0	0.09	5.61	7.65	0.8363
Subtotal WAKAI				0.45	11.97	9.19	
WU	2	f	0	1.44	12.39	0.44	0.0000
WU2	1	f	2	1.50	22.15	1.37	0.0000
WUWILL	25	f	0	0.32	56.90	50.02	0.0166

Table 3C2 - 5

IESLC - Meta-anal of Current Smoking (or Ever if Current not available), Any prod (or Cigs if Any not avail)
Adenocarcinoma
Least adjusted

REF	NRR	SEX	AD	Ys	Ws	Qs	Ps
WYNDE2	14	m	0	0.51	4.32	2.38	0.2863
WYNDE3	30	m	0	1.38	4.98	0.08	0.0021
WYNDE3	135	f	0	0.64	6.88	2.59	0.0922
Subtotal WYNDE3				0.95	11.86	2.66	
WYNDE4	42	m	0	0.41	3.46	2.45	0.4408
WYNDE4	56	f	2	-0.51	1.67	5.22	0.5087
Subtotal WYNDE4				0.11	5.14	7.67	
WYNDE6	15	m	0	2.23	45.98	44.15	0.0000
WYNDE6	204	f	0	2.20	69.69	62.29	0.0000
Subtotal WYNDE6				2.21	115.67	106.44	
XU3	21	m	0	1.48	2.41	0.13	0.0213
XU3	25	f	0	0.26	1.91	1.89	0.7180
Subtotal XU3				0.94	4.32	2.01	
ZHENG	10	m	0	0.60	17.29	7.34	0.0121
ZHENG	21	f	0	0.15	14.95	18.33	0.5668
Subtotal ZHENG				0.39	32.24	25.66	
ZHOU	26	m	0	0.27	14.05	13.70	0.3154
ZHOU	27	f	0	0.36	4.59	3.70	0.4448
Subtotal ZHOU				0.29	18.64	17.40	

	N	116
	NS	81
	Wt	1710.31
Het	Chi	1119.61
Het	df	115
Het	P	***
Fixed	RR	3.51
	RRl	3.35
	RRu	3.68
	P	+++
Random	RR	3.10
	RRl	2.64
	RRu	3.65
	P	+++
Asymm	P	(*)

Table 3C2 - 6

IESLC - Meta-anal of Current Smoking (or Ever if Current not available), Any prod (or Cigs if Any not avail)
Adenocarcinoma
Least adjusted

	combined	Sex: male	female	Total
N	11	56	49	116
NS	11	55	48	114
Wt	149.40	653.09	907.82	1710.31
Het Chi	78.56	337.31	631.88	1119.61
Het df	10	55	48	115
Het P	***	***	***	***
Fixed RR	2.25	4.42	3.20	3.51
RRl	1.92	4.09	3.00	3.35
RRu	2.65	4.77	3.41	3.68
P	+++	+++	+++	+++
Random RR	2.44	4.09	2.46	3.10
RRl	1.52	3.29	1.91	2.64
RRu	3.91	5.10	3.18	3.65
P	+++	+++	+++	+++
Between Chi				71.87
Between df				2
Between P				***
Btwn(F) P				*
Btwn(R) P				**

	Smoking status: ever	current	Total
N	72	44	116
NS	52	31	83
Wt	686.83	1023.48	1710.31
Het Chi	332.17	530.13	1119.61
Het df	71	43	115
Het P	***	***	***
Fixed RR	2.18	4.82	3.51
RRl	2.03	4.53	3.35
RRu	2.35	5.12	3.68
P	+++	+++	+++
Random RR	2.44	4.27	3.10
RRl	2.04	3.37	2.64
RRu	2.92	5.40	3.65
P	+++	+++	+++
Between Chi			257.31
Between df			1
Between P			***
Btwn(F) P			***
Btwn(R) P			***

Table 3C2 - 7

IESLC - Meta-anal of Current Smoking (or Ever if Current not available), Any prod (or Cigs if Any not avail)
Adenocarcinoma
Excluded studies (and stage at which they were excluded)

1 BOUCHA BUELL LAURIL MZILEN

2 BERRIN CHEN RESTRE TAO ULMER

5 RIMING TANG2 WYNDE5 WYNDE8

6 BLOT1 BLOT2 BLOT3 BYERS2 HIRAOK HIRAY2 JONES LICKIN MOLLO MRFIT MURATA NOTANI PERSHA SAARIK SCHWA2 SHIMIZ SITAS VANDER WARSIN WATSON WYNDER

8 ABELIN AGUDO AKIBA AMANDU AMES ARCHER ARMADA AUSTIN AUVINE AXELSO AXELSS BENSHL BEST BLOHMK BLOT4 BOFFET BRETT BROCKM BROSS CARPEN CASCO2 CASCOR CEDERL CHANG CHATZI CHEN2 CHEN3 CHIAZZ CHOW CHYOU COOKSO DARBY DAVEYS DEAN DEAN2 DEAN3 DEKLER DESTEF DOCKER DOLL2 DORANT DROSTE DU DUNN EBELIN ENSTRO ESAKI GAO2 GARCIA GARDIN GARSHI GENG GILLIS GODLEY GOLLED GOODMA GRAHAM GREGOR GSELL HAMMO2 HANSEN HEIN HENNEK HIRAYA HITOSU HOLE HOROWI HORWIT HU HU2 HUANG HUMBLE JARUP JARVHO JIANG KAISE2 KAISER KANELL KAUFMA KELLER KINLEN KJUUS KNEKT KO KOHLME KOULUM KREUZE KUBIK LANGE LAUSSM LEI LEMARC LETOUR LEVIN LIAW LIDDEL LIU LIU2 LIU3 LIU4 LIU5 LOMBAR MACLEN MAGNUS MARSH MARSH2 MARTIS MASTRA MCCONN MCDUFF MCLAUG MIGRAN MILLER MILLS MRFITR NAM NOTAN2 ODRISC PARKIN PASTOR PAWLEG PERNU PERSH2 PETO PEZZO2 PIKE PISANI POFFIJ POLEDN PRESCO QIAO QIAO2 RACHTA RADZIK RANDIG REN RONCO ROOTS ROTHSC SADOWS SANKAR SEGI SEGI2 SHAW SIMARA SIMONA SPEIZE SPITZ STOCKS STOCKW STUCKE SUN TANG TENKAN TOUSEY TULINI TVERDA VEIERO WALD WANG WANG2 WANG3 WANG4 WICKLU WIGLE WILKIN WUNSCH XIANGZ XU XU2 XU4 YAMAGU YONG YUAN ZHANG

10 BENHAM VUTUC WYNDE7

Table 3C2 - 8
Potentially overlapping studies

REF	REFGP	PRINC	.	OVERLAP/LINK
LUBIN2	LUBIN2	1		Lubin-combined
CHAN	CHAN	1		Chan/LamWK2
LAMWK2	LAMWK2	1		Chan/LamWK2
LAMTH	LAMTH	1		KOO/LAMTH/LAMWK
KOO	KOO	1		KOO/LAMTH/LAMWK
LAMWK	LAMWK	1		KOO/LAMTH/LAMWK
ISHIMA	AKIBA	2		AKIBA/ISHIMA
BYERS1	BYERS1	2		GRAHAM/BROSS/BYERS1
OSANN2	KAISER	2		KAISER/OSANN2
WYNDE6	WYNDE6	1		WYNDE5/6/7/8
CPSI	CPSI	1		CPSI overall
SOBUE2	SOBUE2	1		SOBUE2/MATSUD
MATSUD	MATSUD	1		SOBUE2/MATSUD
JAHN	BOFFET	2		Subset of BOFFET
LUBIN	XIANGZ	2		LUBIN/XIANGZ/QIAO

Table 3C2 - 9
Most adjusted - insufficient data for metaanalysis

REF	NRR	SEX	AGEL	AGEH	RACE	YF	LC TYPE	LOC	START	ST	NLC	R	VB	P	H	AD	SM	PRODUCT	DENOM	De
CHEN	11	c	0	0	all	-	a	As:oth	1987	CC	323	n	ot	n	y	2	ev	cig+/-ot	nev cigs	ot
CHYOU	6	m	0	0	jap	0	a	NAmer	1965	pr	227	n	bl	n	y	1	cu	cig+/-ot	nev cigs	or
JIANG	5	m	0	0	all	-	a	As:Chi	1984	CC	125	n	ot	n	n	0	ev	all/unsp	nev any	ot
JIANG	6	f	0	0	all	-	a	As:Chi	1984	CC	125	n	ot	n	n	0	ev	all/unsp	nev any	ot
KOO	4	f	0	0	all	-	a	As:HK	1981	CC	200	n	bl	n	n	0	ev	all/unsp	nev any	or
WANG	4	c	0	0	all	-	a	As:Chi	1990	CC	390	n	ot	*	y	0	ev	all/unsp	nev any	ot

REF	NRR	RR	SIG	RRDATA comment
CHEN	11	2.08	y	P < 0.001
CHYOU	6	6.80		0
JIANG	5	*	n	0
JIANG	6	*	n	0
KOO	4	1.88		0
WANG	4	2.22	n	P>0.05

Table 3C3 -

IESLC - Meta-anal of Ever Smoking (or Current if Ever not available), Cigs (or Any Prod if Cigs not avail)
Adenocarcinoma

This analysis is restricted to results for:
1) Non-dose-response data
2) Results complete enough for use in metaanalysis

Within each study, results are then selected (in the following order of preference, within each sex) for:
3) SMKSTA: ever smokers, current smokers
4) PRODUCT: cigarettes regardless of other products, cigarettes only, all/unspec
5) CIGTYPE: all/unspecified, MC regardless of HR, MC only
6) DENOM: never smoked anything, never smoked cigarettes, (never +1 = +long term ex, +2 = +amount unknown,
+3 = never cigs+long term ex)
7) Followup period (YF, prospective studies): whole study (coded as 0) or longest available
8) LCtype: adeno or nearest available, but not squamous. (q = squamous, s = small,
a = adeno, l = large, KII = Kreyberg II, al = alveolar, br = bronchiolar, u = undifferentiated)
9) Race: all or nearest available, otherwise by race (wh or w = white, bl or b = black, hi = hispanic
ch = chinese, jap = japanese, haw = hawaiian, w+o = white + oriental, sca = scandinavian, as = asian)
10) For overlapping studies: principal rather than subsidiary studies
Finally by Age: whole study (coded as 0) if available, otherwise by widest available age group
and then for single sex results (m, f) in preference to combined sex results (c).

Results adjusted (AD) for the most potential confounders are then chosen in Sections -1 to -3
(and those which actually differ from the adjusted results in Table 3C1 - 1 are marked 'x' in Section -1)
and results adjusted for the least confounders in Sections -4 to -6. (Those least adjusted results which
actually differ from the most adjusted as marked 'x' in column X in Section -4)
(Results adjusted for an unknown number of confounder(s) are coded as 20.)

Section -7 shows excluded studies, together with the stage (as above) at which no qualifying
results were found.

Section -8 lists the potentially overlapping studies which have been included (1=principal, 2=subsidiary).

Section -9 lists any results which would have been included in preference except that they had data not complete
enough for use in meta-analysis, with their significance (yes/no), if known, and any further comment as entered
on the database.

In addition to those mentioned above, the following fields, levels and abbreviations are used:
* or nk = not known, n = no, y = yes, ot = other
ev = ever, cu = current, nev = never
all/unspec = all or unspecified, cig+/-ot = cigarettes irrespective of other products (cigar, pipe etc)
MC = manufactured cigarettes, HR = hand-rolled cigarettes
REF: 6-character study reference
NRR: number of the RR on the database within the study
ST : study type (CC = case control, pr or prosp = prospective)
NLC: number of lung cancer cases in whole study
R : risky occupational population (n = no, m = mining, o = other risky)
VB : national cigarette type (V = at least 75% Virginia, bl = at least 75% blended, ot = other)
P : any proxy use
H : full histological confirmation
De : derivation of RR/CI (or = original, st = standard method, ot = other method of estimation)

Table 3C3 - 1

IESLC - Meta-anal of Ever Smoking (or Current if Ever not available), Cigs (or Any Prod if Cigs not avail)
Adenocarcinoma
Most adjusted

REF	NRR	3C1	SEX	AGEL	AGEH	RACE	YF	LC TYPE	LOC	START	ST	NLC	R	VB	P	H	AD	SM	PRODUCT	DENOM	De
ABRAHA	2		m	0	0	all	0	a	Eu:est	1975	pr	571	n	bl	n	n	0	ev	all/unsp	nev any	ot
ABRAHA	5		f	0	0	all	0	a	Eu:est	1975	pr	571	n	bl	n	n	0	ev	all/unsp	nev any	ot
ALDERS	95	x	m	0	0	all	-	not q+s	Eu:UK	1977	CC	1448	n	V	n	n	1	ev	cig+/-ot	nev any	ot
ALDERS	45	x	f	0	0	all	-	not q+s	Eu:UK	1977	CC	1448	n	V	n	n	1	ev	cig only	nev any	ot
ANDERS	12		f	0	0	all	0	a	NAmer	1986	pr	343	n	bl	n	n	0	ev	cig+/-ot	nev cigs	st
BAND	2		m	0	0	all	-	a	NAmer	1983	CC	2831	n	V	y	y	2	ev	cig only	nev any	ot
BARBON	130		m	0	0	all	-	a	Eu:wst	1979	CC	755	n	bl	y	y	3	ev	all/unsp	nev any	ot
BECHER	12		f	0	0	all	-	not q+s	Eu:Ger	1985	CC	194	n	bl	n	y	1	ev	all/unsp	nev any	or
BOUCOT	147		m	0	0	all	0	a	NAmer	1951	pr	121	n	bl	n	n	2	cu	cig only	nev any	ot
BRESLO	1	x	c	0	0	all	-	a	NAmer	1949	CC	518	n	bl	n	y	0	ev	cig+/-ot	nev+1	st
BROWN1	3		m	0	0	wh	-	a	NAmer	1979	CC	102	n	bl	y	y	1	ev	cig+/-ot	nev cigs	or
BROWN1	4		f	0	0	wh	-	a	NAmer	1979	CC	102	n	bl	y	y	1	ev	cig+/-ot	nev cigs	or
BROWN2	4		m	0	0	wh	-	a	NAmer	1984	CC	14596	n	bl	n	y	2	ev	cig+/-ot	nev cigs	or
BROWN2	3		f	0	0	wh	-	a	NAmer	1984	CC	14596	n	bl	n	y	2	ev	cig+/-ot	nev cigs	or
BUFFLE	50		m	0	0	wh	-	a	NAmer	1976	CC	943	n	bl	y	n	0	ev	cig+/-ot	nev cigs	ot
BUFFLE	45		f	0	0	wh	-	a	NAmer	1976	CC	943	n	bl	y	n	0	ev	cig+/-ot	nev cigs	ot
BYERS1	3		m	0	0	wh	-	a	NAmer	1957	CC	1002	n	bl	n	n	0	ev	cig+/-ot	nev cigs	st
CHAN	19	x	m	0	0	all	-	a+l	As:HK	1976	CC	397	n	bl	n	n	0	ev	cig+/-ot	nev any	ot
CHAN	23	x	f	0	0	all	-	a+l	As:HK	1976	CC	397	n	bl	n	n	0	ev	cig+/-ot	nev any	st
CHOI	63		m	0	0	all	-	a	As:oth	1985	CC	375	n	bl	n	n	0	ev	cig+/-ot	nev cigs	st
CHOI	65		f	0	0	all	-	a	As:oth	1985	CC	375	n	bl	n	n	0	ev	cig+/-ot	nev cigs	st
COMSTO	67		m	0	0	all	-	a	NAmer	1975	ot	258	n	bl	n	n	0	ev	cig+/-ot	nev cigs	st
COMSTO	79		f	0	0	all	-	a	NAmer	1975	ot	258	n	bl	n	n	0	ev	cig+/-ot	nev cigs	st
CORREA	36		c	0	0	all	-	a	NAmer	1979	CC	1359	n	bl	y	n	1	ev	cig+/-ot	nev cigs	or
CPSI	404		m	0	0	all	2	a	NAmer	1959	pr	5138	n	bl	n	n	1	cu	cig only	nev any	ot
CPSI	406		f	0	0	all	2	a	NAmer	1959	pr	5138	n	bl	n	n	1	cu	cig only	nev any	ot
CPSII	115		m	0	0	all	2	a	NAmer	1982	pr	3229	n	bl	n	n	1	cu	cig only	nev any	st
CPSII	118		f	0	0	all	2	a	NAmer	1982	pr	3229	n	bl	n	n	1	cu	cig+/-ot	nev cigs	st
DAMBER	32		m	0	0	all	-	a+al+br	Eu:Sca	1972	CC	579	n	bl	y	n	1	ev	all/unsp	nev any	or
DESTE2	17		m	0	0	all	-	a	SCAmer	1993	CC	463	n	bl	n	n	2	ev	all/unsp	nev any	or
DOLL	87		m	0	0	all	-	KII	Eu:UK	1948	CC	1465	n	V	n	n	1	ev	all/unsp	nev any	ot
DOLL	89		f	0	0	all	-	KII	Eu:UK	1948	CC	1465	n	V	n	n	1	ev	all/unsp	nev any	ot
DORGAN	125		m	0	0	wh	-	a	NAmer	1980	CC	2026	n	bl	y	y	2	ev	cig+/-ot	nev any	or
DORGAN	104		f	0	0	all	-	a	NAmer	1980	CC	2026	n	bl	y	y	3	ev	cig+/-ot	nev any	or
DORN	340		m	0	0	wh	8	a	NAmer	1954	pr	5097	n	bl	n	n	1	cu	cig only	nev any	ot
DOSEME	4		m	0	0	all	-	not q+s	Eu:bal	1979	CC	1210	n	bl	n	n	2	ev	cig+/-ot	nev cigs	or
ENGELA	76		m	0	0	all	0	a	Eu:Sca	1964	pr	435	n	bl	n	n	7	ev	cig+/-ot	nev cigs	ot
FAN	4		c	0	0	all	-	a	As:Chi	1990	CC	403	n	ot	y	n	0	ev	cig+/-ot	nev cigs	ot
GAO	3		m	0	0	all	-	a	As:Chi	1984	CC	1405	n	ot	n	n	2	ev	cig+/-ot	nev cigs	or
GAO	13		f	0	0	all	-	a	As:Chi	1984	CC	1405	n	ot	n	n	2	ev	cig+/-ot	nev cigs	or
GER	9		c	0	0	all	-	a	As:oth	1990	CC	141	n	ot	y	n	8	ev	all/unsp	nev any	ot
HAENSZ	39	x	f	0	0	all	-	a	NAmer	1955	CC	158	n	bl	n	y	0	ev	cig+/-ot	nev any	st
HAMMON	84	x	m	0	0	wh	0	a	NAmer	1952	pr	448	n	bl	n	n	1	ev	cig+/-ot	nev any	ot
HEGMAN	4		c	0	0	all	-	a	NAmer	1989	CC	282	n	bl	y	y	0	ev	all/unsp	nev any	st
HINDS	24		f	0	0	o	-	a	NAmer	1968	CC	292	n	bl	n	n	3	ev	all/unsp	nev any	st
ISHIMA	8		c	0	0	all	-	a	As:Jap	1961	CC	180	n	bl	y	y	5	ev	all/unsp	nev any	st
JAHN	43	x	m	0	0	all	-	a	Eu:Ger	1988	CC	1004	n	bl	n	n	0	ev	cig+/-ot	nev any	st
JAIN	47		m	0	0	all	-	a	NAmer	1981	CC	845	n	V	y	n	2	ev	cig+/-ot	nev cigs	or
JAIN	42		f	0	0	all	-	a	NAmer	1981	CC	845	n	V	y	n	2	ev	cig+/-ot	nev cigs	or
JEDRYC	56		m	0	0	all	-	a	Eu:est	1980	CC	1630	n	bl	y	n	3	ev	cig+/-ot	nev any	ot
JOLY	51		m	0	0	all	-	a	SCAmer	1978	CC	826	n	bl	n	n	0	ev	cig+/-ot	nev any	st
JOLY	50		f	0	0	all	-	a	SCAmer	1978	CC	826	n	bl	n	n	0	ev	cig+/-ot	nev any	st
JUSSAW	28	x	m	0	0	all	-	KII	As:Ind	1964	CC	792	n	V	n	n	0	ev	cig only	nev any	st
KATSOU	33		f	0	0	all	-	a	Eu:bal	1987	CC	101	n	bl	n	n	1	ev	all/unsp	nev any	ot
KHUDER	27		m	0	0	all	-	a	NAmer	1985	CC	482	n	bl	n	y	0	ev	cig+/-ot	nev cigs	ot
KIHARA	29		c	0	0	jap	-	a	As:Jap	1991	CC	440	n	bl	n	n	0	ev	all/unsp	nev any	st
KOO	7		f	0	0	all	-	a+l	As:HK	1981	CC	200	n	bl	n	n	0	ev	all/unsp	nev any	st
KREYBE	8		m	0	0	all	-	KII	Eu:Sca	1948	CC	300	n	bl	n	y	1	ev	all/unsp	nev any	ot
KREYBE	27		f	0	0	all	-	KII	Eu:Sca	1948	CC	300	n	bl	n	y	1	ev	all/unsp	nev any	ot
LAMTH	3		f	0	0	ch	-	a	As:HK	1983	CC	445	n	bl	n	n	0	ev	all/unsp	nev any	or
LAMWK	4		f	0	0	ch	-	a	As:HK	1981	CC	163	n	bl	n	n	0	ev	all/unsp	nev any	st
LAMWK2	3		m	0	0	all	-	a	As:HK	1976	CC	480	n	bl	n	n	0	ev	all/unsp	nev any	st
LAMWK2	7		f	0	0	all	-	a	As:HK	1976	CC	480	n	bl	n	n	0	ev	all/unsp	nev any	st
LOMBA2	3		f	0	0	all	-	not q+u	NAmer	1960	CC	225	n	bl	n	n	0	ev	cig+/-ot	nev cigs	st
LUBIN	37	x	m	0	0	all	-	KII	As:Chi	1984	CC	427	m	ot	y	n	0	ev	cig+/-ot	nev any	st
LUBIN2	148		m	0	0	all	-	a	Eu:mul	1976	CC	7804	n	bl	n	y	0	ev	cig+/-ot	nev any	st
LUBIN2	168		f	0	0	all	-	a	Eu:mul	1976	CC	7804	n	bl	n	y	0	ev	cig+/-ot	nev any	st
LUO	9		c	0	0	all	-	a	As:Chi	1990	CC	102	n	ot	n	y	20	ev	cig+/-ot	nev cigs	or
MATOS	69		m	0	0	all	-	a	SCAmer	1994	CC	200	n	bl	n	n	2	ev	cig+/-ot	nev any	ot
MATSUD	12		m	0	0	all	-	a	As:Jap	1965	CC	179	n	bl	n	n	0	ev	cig+/-ot	nev cigs	ot
NOU	3		m	0	0	all	-	a	Eu:Sca	1971	CC	273	n	bl	y	n	0	ev	all/unsp	nev any	st
NOU	8		f	0	0	all	-	a	Eu:Sca	1971	CC	273	n	bl	y	n	0	ev	all/unsp	nev any	st

Table 3C3 - 1

IESLC - Meta-anal of Ever Smoking (or Current if Ever not available), Cigs (or Any Prod if Cigs not avail)
Adenocarcinoma
Most adjusted

REF	NRR	3C1	SEX	AGEL	AGEH	RACE	YF	LC TYPE	LOC	START	ST	NLC	R	VB	P	H	AD	SM	PRODUCT	DENOM	De
ORMOS	21		m	0	0	all	-	a	Eu:est	1947	CC	119	n	bl	y	y	0	ev	cig+/-ot	nev any	ot
OSANN	47		m	0	0	all	-	a	NAmer	1984	CC	1986	n	bl	n	n	2	ev	cig+/-ot	nev cigs	or
OSANN	48		f	0	0	all	-	a	NAmer	1984	CC	1986	n	bl	n	n	2	ev	cig+/-ot	nev cigs	or
OSANN2	31		f	0	0	all	-	KII	NAmer	1964	ot	217	n	bl	n	y	1	ev	cig+/-ot	nev cigs	or
PEZZOT	7		m	0	0	all	-	a	SCAmer	1987	CC	215	n	bl	n	y	0	ev	cig only	nev cigs	st
SCHWAR	8		m	40	54	wh	-	a	NAmer	1984	CC	5588	n	bl	y	y	0	ev	cig+/-ot	nev cigs	st
SCHWAR	7		m	40	54	bl	-	a	NAmer	1984	CC	5588	n	bl	y	y	0	ev	cig+/-ot	nev cigs	st
SCHWAR	16		f	40	54	wh	-	a	NAmer	1984	CC	5588	n	bl	y	y	0	ev	cig+/-ot	nev cigs	st
SCHWAR	15		f	40	54	bl	-	a	NAmer	1984	CC	5588	n	bl	y	y	0	ev	cig+/-ot	nev cigs	st
SEOW	2		f	0	0	ch	-	a	As:oth	1997	CC	153	n	bl	n	y	0	ev	cig+/-ot	nev cigs	st
SIEMIA	8		m	0	0	all	-	a	NAmer	1979	CC	857	n	V	y	y	7	ev	cig+/-ot	nev cigs	or
SOBUE	99		m	0	0	all	-	a	As:Jap	1986	CC	1376	n	bl	n	y	1	ev	cig+/-ot	nev cigs	ot
SOBUE	109		f	0	0	all	-	a	As:Jap	1986	CC	1376	n	bl	n	y	1	ev	cig+/-ot	nev cigs	ot
SOBUE2	2		m	0	0	all	-	a	As:Jap	1965	CC	2083	n	bl	n	n	2	cu	cig+/-ot	nev any	or
SOBUE2	6		f	0	0	all	-	a	As:Jap	1965	CC	2083	n	bl	n	n	2	cu	cig+/-ot	nev any	or
STASZE	24	x	m	0	0	all	-	a	Eu:est	1954	CC	281	n	bl	n	y	0	ev	cig+/-ot	nev any	ot
STASZE	4		f	0	0	all	-	a	Eu:est	1954	CC	281	n	bl	n	y	0	ev	all/unsp	nev any	st
STAYNE	4		m	0	0	all	-	a	NAmer	1969	CC	420	n	bl	n	n	0	ev	all/unsp	nev any	st
SUZUK2	16		c	0	0	all	-	a	SCAmer	1991	CC	123	n	bl	n	y	3	ev	all/unsp	nev any	or
SUZUKI	11		m	0	0	all	-	a	As:Jap	1978	CC	238	n	bl	n	y	2	ev	cig+/-ot	nev any	ot
SUZUKI	15		f	0	0	all	-	a	As:Jap	1978	CC	238	n	bl	n	y	2	ev	cig+/-ot	nev any	ot
SVENSS	74		f	0	0	all	-	a	Eu:Sca	1983	CC	210	n	bl	n	n	1	ev	all/unsp	nev any	ot
TIZZAN	19		c	0	0	all	-	a	Eu:wst	1959	CC	1358	n	bl	n	n	0	ev	all/unsp	nev any	st
TOKARS	8		c	0	0	all	-	a	Eu:est	1966	ot	162	o	bl	n	y	3	ev	all/unsp	nev any	or
TSUGAN	10		m	0	0	all	-	a	As:Jap	1976	CC	134	n	bl	n	y	3	ev	all/unsp	nev any	ot
TSUGAN	11		f	0	0	all	-	a	As:Jap	1976	CC	134	n	bl	n	y	3	ev	all/unsp	nev any	ot
WAKAI	76		m	0	0	all	-	a	As:Jap	1988	CC	333	n	bl	n	y	1	ev	all/unsp	nev any	ot
WAKAI	82		f	0	0	all	-	a	As:Jap	1988	CC	333	n	bl	n	y	1	ev	all/unsp	nev any	ot
WU	31		f	0	0	wh	-	a	NAmer	1981	CC	220	n	bl	n	y	2	ev	all/unsp	nev any	ot
WU2	1		f	0	0	all	-	a	NAmer	1983	CC	336	n	bl	n	y	2	cu	all/unsp	nev any	or
WUWILL	11		f	0	0	all	-	a	As:Chi	1985	CC	965	n	ot	n	n	3	ev	cig+/-ot	nev cigs	or
WYNDE2	9	x	m	0	0	all	-	KII	NAmer	1962	CC	404	n	bl	n	y	0	ev	cig+/-ot	nev any	st
WYNDE3	28	x	m	0	0	all	-	KII	NAmer	1966	CC	350	n	bl	n	y	0	ev	cig+/-ot	nev any	st
WYNDE3	75	x	f	0	0	all	-	KII	NAmer	1966	CC	350	n	bl	n	y	0	ev	cig+/-ot	nev any	st
WYNDE4	42		m	0	0	all	-	a	NAmer	1948	CC	684	n	bl	y	n	0	ev	all/unsp	nev any	st
WYNDE4	56		f	0	0	all	-	a	NAmer	1948	CC	684	n	bl	y	n	2	ev	all/unsp	nev any	ot
WYNDE6	78	x	m	0	0	all	-	KII	NAmer	1969	CC	4423	n	bl	n	y	0	ev	cig+/-ot	nev any	st
WYNDE6	414		f	0	0	wh	-	a	NAmer	1969	CC	4423	n	bl	n	y	1	ev	cig+/-ot	nev cigs	ot
XU3	22		m	0	0	all	-	KII	As:Chi	1981	CC	135	n	ot	n	n	1	ev	all/unsp	nev any	ot
XU3	26		f	0	0	all	-	KII	As:Chi	1981	CC	135	n	ot	n	n	1	ev	all/unsp	nev any	ot
ZHENG	10		m	0	0	all	-	a	As:Chi	1982	CC	540	n	ot	*	y	0	ev	cig+/-ot	nev cigs	st
ZHENG	21		f	0	0	all	-	a	As:Chi	1982	CC	540	n	ot	*	y	0	ev	cig+/-ot	nev cigs	st
ZHOU	26		m	0	0	all	-	a	As:Chi	1978	CC	1360	n	ot	n	n	0	ev	all/unsp	nev any	st
ZHOU	27		f	0	0	all	-	a	As:Chi	1978	CC	1360	n	ot	n	n	0	ev	all/unsp	nev any	st

Cigarette type is all/unspec for all RRs

except for the following:

REF	NRR	CIGTYPE
ALDERS	45	MC only
CHAN	19	MC+-HR
CHAN	23	MC+-HR
JUSSAW	28	MC only

Table 3C3 - 2

IESLC - Meta-anal of Ever Smoking (or Current if Ever not available), Cigs (or Any Prod if Cigs not avail)
Adenocarcinoma
Most adjusted

REF	NRR	SEX	AD	Number Exposed Case	Number Exposed Cont	Non-exposed Case	Non-exposed Cont	RR	95.00%CI
*ABRAHA	2	m	0	59	10351	8	3365	2.40	(1.15- 5.01)
*ABRAHA	5	f	0	19	5256	16	11589	2.62	(1.35- 5.09)
Subtotal ABRAHA								2.52	(1.54- 4.12)
ALDERS	95	m	1	-	-	-	-	4.41	(1.92- 10.13)
ALDERS	45	f	1	-	-	-	-	3.69	(2.32- 5.88)
Subtotal ALDERS								3.85	(2.57- 5.78)
*ANDERS	12	f	0	99	96164	33	195158	6.09	(4.11- 9.03)
BAND	2	m	2	-	-	-	-	4.10	(3.01- 5.59)
BARBON	130	m	3	-	-	-	-	7.02	(3.24- 15.22)
BECHER	12	f	1	-	-	-	-	10.83	(1.32- 88.70)
*BOUCOT	147	m	2	-	-	-	-	10.95	(0.65- 183.57)
BRESLO	1	c	0	40	394	4	56	1.42	(0.49- 4.12)
BROWN1	3	m	1	-	-	-	-	4.49	(1.44- 13.98)
BROWN1	4	f	1	-	-	-	-	3.95	(1.76- 8.80)
Subtotal BROWN1								4.12	(2.14- 7.95)
BROWN2	4	m	2	-	-	-	-	8.20	(6.90- 9.70)
BROWN2	3	f	2	-	-	-	-	6.90	(6.10- 7.90)
Subtotal BROWN2								7.35	(6.63- 8.15)
BUFFLE	50	m	0	-	-	-	-	4.50	(1.85- 10.95)
BUFFLE	45	f	0	-	-	-	-	4.02	(2.42- 6.67)
Subtotal BUFFLE								4.13	(2.66- 6.42)
BYERS1	3	m	0	47	695	7	424	4.10	(1.83- 9.15)
CHAN	19	m	0	56	160	0	43	30.63~	(1.85- 505.72)
CHAN	23	f	0	24	38	40	139	2.19	(1.18- 4.08)
Subtotal CHAN								2.48	(1.35- 4.55)
CHOI	63	m	0	46	465	7	95	1.34	(0.59- 3.06)
CHOI	65	f	0	5	26	49	164	0.64	(0.23- 1.77)
Subtotal CHOI								1.00	(0.53- 1.89)
COMSTO	67	m	0	43	229	2	84	7.89	(1.87- 33.27)
COMSTO	79	f	0	29	87	8	115	4.79	(2.09- 11.00)
Subtotal COMSTO								5.43	(2.64- 11.14)
CORREA	36	c	1	-	-	-	-	5.60	(3.60- 8.80)
*CPSI	404	m	1	-	-	-	-	4.58	(1.74- 12.05)
*CPSI	406	f	1	-	-	-	-	1.43	(0.47- 4.39)
Subtotal CPSI								2.78	(1.34- 5.78)
*CPSII	115	m	1	-	-	-	-	19.22	(6.46- 57.16)
*CPSII	118	f	1	-	-	-	-	8.23	(4.36- 15.54)
Subtotal CPSII								10.21	(5.89- 17.67)
DAMBER	32	m	1	-	-	-	-	2.40	(1.10- 5.30)
DESTE2	17	m	2	-	-	-	-	4.30	(1.60- 11.40)
DOLL	87	m	1	-	-	-	-	0.95	(0.22- 4.02)
DOLL	89	f	1	-	-	-	-	1.97	(0.60- 6.46)
Subtotal DOLL								1.47	(0.59- 3.69)
DORGAN	125	m	2	-	-	-	-	4.80	(1.90- 12.00)
DORGAN	104	f	3	-	-	-	-	3.90	(2.80- 5.40)
Subtotal DORGAN								3.99	(2.93- 5.44)
*DORN	340	m	1	-	-	-	-	5.95	(3.85- 9.22)
DOSEME	4	m	2	-	-	-	-	2.60	(1.70- 4.20)
*ENGELA	76	m	7	-	-	-	-	2.33	(0.92- 5.89)
FAN	4	c	0	67	595	45	556	1.39	(0.94- 2.07)
GAO	3	m	2	-	-	-	-	1.60	(1.10- 2.40)
GAO	13	f	2	-	-	-	-	1.50	(1.00- 2.10)
Subtotal GAO								1.55	(1.18- 2.02)
GER	9	c	8	-	-	-	-	1.10	(0.55- 2.19)
HAENSZ	39	f	0	18	103	37	236	1.11	(0.61- 2.05)
*HAMMON	84	m	1	-	-	-	-	3.67	(0.87- 15.45)
HEGMAN	4	c	0	83	1202	15	2080	9.58	(5.50- 16.67)
HINDS	24	f	3	-	-	-	-	3.89	(2.49- 6.07)
ISHIMA	8	c	5	-	-	-	-	15.00	(2.31- 631.48)
JAHN	43	m	0	202	671	8	138	5.19	(2.50- 10.77)
JAIN	47	m	2	-	-	-	-	8.00	(2.28- 50.60)
JAIN	42	f	2	-	-	-	-	3.45	(1.83- 7.10)
Subtotal JAIN								3.95	(2.12- 7.35)
JEDRYC	56	m	3	-	-	-	-	3.44	(1.52- 7.78)
JOLY	51	m	0	72	709	5	218	4.43	(1.77- 11.10)
JOLY	50	f	0	33	122	25	283	3.06	(1.75- 5.37)
Subtotal JOLY								3.38	(2.10- 5.46)
JUSSAW	28	m	0	3	77	13	624	1.87	(0.52- 6.71)
KATSOU	33	f	1	-	-	-	-	1.72	(0.80- 3.71)
KHUDER	27	m	0	155	-	7	-	8.11	(3.67- 17.93)
KIHARA	29	c	0	130	232	78	237	1.70	(1.22- 2.38)

Table 3C3 - 2

IESLC - Meta-anal of Ever Smoking (or Current if Ever not available), Cigs (or Any Prod if Cigs not avail)
Adenocarcinoma
Most adjusted

REF	NRR	SEX	AD	Number Exposed Case	Cont	Non-exposed Case	Cont	RR	95.00%CI
KOO	7	f	0	34	63	46	137	1.61	(0.94- 2.74)
KREYBE	8	m	1	-	-	-	-	2.44	(0.76- 7.86)
KREYBE	27	f	1	-	-	-	-	1.28	(0.60- 2.74)
Subtotal KREYBE								1.55	(0.82- 2.93)
LAMTH	3	f	0	79	51	131	158	1.87	(1.23- 2.85)
LAMWK	4	f	0	36	41	60	144	2.11	(1.23- 3.61)
LAMWK2	3	m	0	52	161	15	43	0.93	(0.48- 1.80)
LAMWK2	7	f	0	26	50	41	139	1.76	(0.98- 3.17)
Subtotal LAMWK2								1.33	(0.86- 2.07)
LOMBA2	3	f	0	42	353	54	239	0.53	(0.34- 0.81)
LUBIN	37	m	0	32	788	4	72	0.73	(0.25- 2.12)
LUBIN2	148	m	0	655	10433	57	2616	2.88	(2.19- 3.79)
LUBIN2	168	f	0	85	567	138	1180	1.28	(0.96- 1.71)
Subtotal LUBIN2								1.96	(1.61- 2.39)
LUO	9	c	20	-	-	-	-	1.50	(0.70- 3.00)
MATOS	69	m	2	-	-	-	-	6.21	(2.42- 15.96)
MATSUD	12	m	0	23	3314	0	1255	17.80~	(1.08- 293.32)
NOU	3	m	0	36	247	4	122	4.45	(1.55- 12.77)
NOU	8	f	0	9	92	29	261	0.88	(0.40- 1.93)
Subtotal NOU								1.57	(0.83- 2.94)
ORMOS	21	m	0	4	1034	0	777	6.76~	(0.36- 125.82)
OSANN	47	m	2	-	-	-	-	17.90	(10.40- 31.00)
OSANN	48	f	2	-	-	-	-	9.50	(6.80- 13.80)
Subtotal OSANN								11.46	(8.51- 15.42)
OSANN2	31	f	1	-	-	-	-	2.50	(1.30- 5.10)
PEZZOT	7	m	0	60	317	3	116	7.32	(2.25- 23.79)
SCHWAR	8	m	0	84	178	1	73	34.45	(4.71- 252.10)
SCHWAR	7	m	0	45	39	1	7	8.08	(0.95- 68.56)
SCHWAR	16	f	0	92	108	10	79	6.73	(3.29- 13.75)
SCHWAR	15	f	0	20	28	3	41	9.76	(2.65- 36.00)
Subtotal SCHWAR								8.40	(4.73- 14.94)
SEOW	2	f	0	19	15	67	125	2.36	(1.13- 4.95)
SIEMIA	8	m	7	-	-	-	-	6.30	(2.50- 16.20)
SOBUE	99	m	1	-	-	-	-	1.83	(1.21- 2.77)
SOBUE	109	f	1	-	-	-	-	1.41	(1.00- 1.99)
Subtotal SOBUE								1.57	(1.20- 2.04)
SOBUE2	2	m	2	-	-	-	-	3.10	(2.40- 3.70)
SOBUE2	6	f	2	-	-	-	-	1.80	(1.40- 2.20)
Subtotal SOBUE2								2.39	(2.04- 2.79)
STASZE	24	m	0	20	653	0	158	9.94~	(0.60- 165.30)
STASZE	4	f	0	1	153	10	1660	1.08	(0.14- 8.53)
Subtotal STASZE								2.36	(0.45- 12.42)
STAYNE	4	m	0	43	567	7	333	3.61	(1.60- 8.11)
SUZUK2	16	c	3	-	-	-	-	6.00	(0.70- 50.00)
SUZUKI	11	m	2	-	-	-	-	4.53	(2.48- 8.29)
SUZUKI	15	f	2	-	-	-	-	2.19	(1.16- 4.14)
Subtotal SUZUKI								3.21	(2.07- 4.97)
SVENSS	74	f	1	-	-	-	-	2.91	(1.56- 5.42)
TIZZAN	19	c	0	88	939	25	419	1.57	(0.99- 2.49)
TOKARS	8	c	3	-	-	-	-	4.30	(1.90- 9.90)
TSUGAN	10	m	3	-	-	-	-	0.93	(0.43- 1.99)
TSUGAN	11	f	3	-	-	-	-	0.67	(0.22- 2.07)
Subtotal TSUGAN								0.84	(0.45- 1.58)
WAKAI	76	m	1	-	-	-	-	1.93	(0.90- 4.18)
WAKAI	82	f	1	-	-	-	-	1.39	(0.66- 2.90)
Subtotal WAKAI								1.63	(0.96- 2.77)
WU	31	f	2	-	-	-	-	2.60	(1.53- 4.44)
WU2	1	f	2	-	-	-	-	4.50	(3.00- 6.90)
WUWILL	11	f	3	-	-	-	-	1.50	(1.10- 1.90)
WYNDE2	9	m	0	46	512	5	105	1.89	(0.73- 4.86)
WYNDE3	28	m	0	64	264	6	88	3.56	(1.49- 8.49)
WYNDE3	75	f	0	21	56	15	76	1.90	(0.90- 4.01)
Subtotal WYNDE3								2.48	(1.41- 4.37)
WYNDE4	42	m	0	35	665	4	115	1.51	(0.53- 4.34)
WYNDE4	56	f	2	-	-	-	-	0.60	(0.13- 2.69)
Subtotal WYNDE4								1.12	(0.47- 2.66)
WYNDE6	78	m	0	1059	1797	58	617	6.27	(4.74- 8.29)
WYNDE6	414	f	1	-	-	-	-	13.99	(10.18- 19.23)
Subtotal WYNDE6								8.90	(7.21- 10.98)
XU3	22	m	1	-	-	-	-	4.84	(1.37- 17.10)
XU3	26	f	1	-	-	-	-	1.09	(0.26- 4.50)

Table 3C3 - 2

IESLC - Meta-anal of Ever Smoking (or Current if Ever not available), Cigs (or Any Prod if Cigs not avail)
Adenocarcinoma
Most adjusted

REF	NRR	SEX	AD	Number Exposed Case	Number Exposed Cont	Non-exposed Case	Non-exposed Cont	RR	95.00%CI
Subtotal XU3								2.51	(0.98- 6.47)
ZHENG	10	m	0	123	218	29	94	1.83	(1.14- 2.93)
ZHENG	21	f	0	33	44	119	184	1.16	(0.70- 1.93)
Subtotal ZHENG								1.48	(1.05- 2.09)
ZHOU	26	m	0	131	41	88	36	1.31	(0.77- 2.20)
ZHOU	27	f	0	30	7	96	32	1.43	(0.57- 3.57)
Subtotal ZHOU								1.34	(0.85- 2.10)
Partial Totals				4357	141371	1533	227105		

*prospective study ~ With 0.5 adjustment for zero

REF	NRR	SEX	AD	Ys	Ws	Qs	Ps
*ABRAHA	2	m	0	0.87	7.06	0.83	0.0201
*ABRAHA	5	f	0	0.96	8.71	0.57	0.0045
Subtotal ABRAHA				0.92	15.77	1.40	
ALDERS	95	m	1	1.48	5.55	0.39	0.0005
ALDERS	45	f	1	1.31	17.77	0.14	0.0000
Subtotal ALDERS				1.35	23.32	0.53	
*ANDERS	12	f	0	1.81	24.76	8.57	0.0000
BAND	2	m	2	1.41	40.10	1.49	0.0000
BARBON	130	m	3	1.95	6.42	3.43	0.0000
BECHER	12	f	1	2.38	0.87	1.18	0.0265
*BOUCOT	147	m	2	2.39	0.48	0.67	0.0964
BRESLO	1	c	0	0.35	3.39	2.54	0.5177
BROWN1	3	m	1	1.50	2.97	0.24	0.0096
BROWN1	4	f	1	1.37	5.93	0.14	0.0008
Subtotal BROWN1				1.42	8.91	0.38	
BROWN2	4	m	2	2.10	132.45	103.99	0.0000
BROWN2	3	f	2	1.93	229.82	116.99	0.0000
Subtotal BROWN2				1.99	362.27	220.98	
BUFFLE	50	m	0	1.50	4.86	0.40	0.0009
BUFFLE	45	f	0	1.39	14.95	0.45	0.0000
Subtotal BUFFLE				1.42	19.81	0.85	
BYERS1	3	m	0	1.41	5.95	0.22	0.0006
CHAN	19	m	0	3.42	0.49	2.37	0.0168
CHAN	23	f	0	0.79	9.98	1.86	0.0130
Subtotal CHAN				0.91	10.47	4.24	
CHOI	63	m	0	0.29	5.64	4.81	0.4842
CHOI	65	f	0	-0.44	3.77	10.38	0.3920
Subtotal CHOI				-0.00	9.42	15.19	
COMSTO	67	m	0	2.07	1.85	1.33	0.0049
COMSTO	79	f	0	1.57	5.57	0.68	0.0002
Subtotal COMSTO				1.69	7.42	2.01	
CORREA	36	c	1	1.72	19.23	4.90	0.0000
*CPSI	404	m	1	1.52	4.10	0.38	0.0021
*CPSI	406	f	1	0.36	3.08	2.28	0.5303
Subtotal CPSI				1.02	7.18	2.66	
*CPSII	115	m	1	2.96	3.23	9.76	0.0000
*CPSII	118	f	1	2.11	9.51	7.53	0.0000
Subtotal CPSII				2.32	12.75	17.29	
DAMBER	32	m	1	0.88	6.21	0.73	0.0291
DESTE2	17	m	2	1.46	3.99	0.23	0.0036
DOLL	87	m	1	-0.05	1.82	2.93	0.9448
DOLL	89	f	1	0.68	2.72	0.79	0.2634
Subtotal DOLL				0.39	4.54	3.73	
DORGAN	125	m	2	1.57	4.52	0.56	0.0008
DORGAN	104	f	3	1.36	35.62	0.73	0.0000
Subtotal DORGAN				1.38	40.15	1.28	
*DORN	340	m	1	1.78	20.15	6.44	0.0000
DOSEME	4	m	2	0.96	18.78	1.29	0.0000
*ENGELA	76	m	7	0.85	4.46	0.62	0.0741
FAN	4	c	0	0.33	24.61	19.40	0.1013
GAO	3	m	2	0.47	25.25	14.13	0.0182
GAO	13	f	2	0.41	27.91	18.43	0.0322
Subtotal GAO				0.44	53.16	32.56	
GER	9	c	8	0.10	8.05	10.15	0.7869
HAENSZ	39	f	0	0.11	10.36	12.75	0.7268
*HAMMON	84	m	1	1.30	1.86	0.01	0.0765
HEGMAN	4	c	0	2.26	12.50	13.54	0.0000
HINDS	24	f	3	1.36	19.35	0.38	0.0000
ISHIMA	8	c	5	2.71	0.49	1.08	0.0585

Table 3C3 - 2

IESLC - Meta-anal of Ever Smoking (or Current if Ever not available), Cigs (or Any Prod if Cigs not avail)
Adenocarcinoma
Most adjusted

REF	NRR	SEX	AD	Ys	Ws	Qs	Ps
JAHN	43	m	0	1.65	7.21	1.33	0.0000
JAIN	47	m	2	2.08	1.60	1.19	0.0085
JAIN	42	f	2	1.24	8.36	0.00	0.0003
Subtotal JAIN				1.37	9.96	1.19	
JEDRYC	56	m	3	1.24	5.76	0.00	0.0030
JOLY	51	m	0	1.49	4.55	0.33	0.0015
JOLY	50	f	0	1.12	12.19	0.12	0.0001
Subtotal JOLY				1.22	16.74	0.45	
JUSSAW	28	m	0	0.63	2.35	0.83	0.3368
KATSOU	33	f	1	0.54	6.53	2.98	0.1658
KHUDER	27	m	0	2.09	6.11	4.68	0.0000
KIHARA	29	c	0	0.53	34.43	16.20	0.0018
KOO	7	f	0	0.47	13.45	7.44	0.0817
KREYBE	8	m	1	0.89	2.82	0.30	0.1345
KREYBE	27	f	1	0.25	6.66	6.28	0.5240
Subtotal KREYBE				0.44	9.48	6.58	
LAMTH	3	f	0	0.63	21.63	7.61	0.0036
LAMWK	4	f	0	0.75	13.20	2.95	0.0068
LAMWK2	3	m	0	-0.08	8.67	14.54	0.8206
LAMWK2	7	f	0	0.57	11.11	4.71	0.0588
Subtotal LAMWK2				0.28	19.77	19.25	
LOMBA2	3	f	0	-0.64	20.27	70.06	0.0039
LUBIN	37	m	0	-0.31	3.37	7.91	0.5649
LUBIN2	148	m	0	1.06	51.15	1.31	0.0000
LUBIN2	168	f	0	0.25	46.25	43.49	0.0913
Subtotal LUBIN2				0.67	97.40	44.80	
LUO	9	c	20	0.41	7.26	4.79	0.2748
MATOS	69	m	2	1.83	4.32	1.60	0.0001
MATSUD	12	m	0	2.88	0.49	1.35	0.0440
NOU	3	m	0	1.49	3.45	0.26	0.0056
NOU	8	f	0	-0.13	6.24	11.29	0.7504
Subtotal NOU				0.45	9.69	11.55	
ORMOS	21	m	0	1.91	0.45	0.22	0.1999
OSANN	47	m	2	2.88	12.88	35.79	0.0000
OSANN	48	f	2	2.25	30.68	32.75	0.0000
Subtotal OSANN				2.44	43.56	68.54	
OSANN2	31	f	1	0.92	8.22	0.75	0.0086
PEZZOT	7	m	0	1.99	2.76	1.65	0.0009
SCHWAR	8	m	0	3.54	0.97	5.23	0.0005
SCHWAR	7	m	0	2.09	0.84	0.64	0.0556
SCHWAR	16	f	0	1.91	7.53	3.57	0.0000
SCHWAR	15	f	0	2.28	2.26	2.54	0.0006
Subtotal SCHWAR				2.13	11.60	11.97	
SEOW	2	f	0	0.86	7.03	0.90	0.0226
SIEMIA	8	m	7	1.84	4.40	1.71	0.0001
SOBUE	99	m	1	0.60	22.40	8.44	0.0042
SOBUE	109	f	1	0.34	32.45	24.81	0.0503
Subtotal SOBUE				0.45	54.85	33.25	
SOBUE2	2	m	2	1.13	82.01	0.62	0.0000
SOBUE2	6	f	2	0.59	75.22	29.88	0.0000
Subtotal SOBUE2				0.87	157.22	30.49	
STASZE	24	m	0	2.30	0.49	0.57	0.1092
STASZE	4	f	0	0.08	0.90	1.17	0.9382
Subtotal STASZE				0.86	1.39	1.73	
STAYNE	4	m	0	1.28	5.85	0.02	0.0019
SUZUK2	16	c	3	1.79	0.84	0.28	0.0999
SUZUKI	11	m	2	1.51	10.55	0.90	0.0000
SUZUKI	15	f	2	0.78	9.49	1.79	0.0157
Subtotal SUZUKI				1.17	20.04	2.69	
SVENSS	74	f	1	1.07	9.91	0.22	0.0008
TIZZAN	19	c	0	0.45	18.24	10.72	0.0538
TOKARS	8	c	3	1.46	5.64	0.33	0.0005
TSUGAN	10	m	3	-0.07	6.55	10.90	0.8527
TSUGAN	11	f	3	-0.40	3.06	8.01	0.4837
Subtotal TSUGAN				-0.18	9.60	18.91	
WAKAI	76	m	1	0.66	6.52	2.05	0.0933
WAKAI	82	f	1	0.33	7.01	5.54	0.3832
Subtotal WAKAI				0.49	13.53	7.59	
WU	31	f	2	0.96	13.54	0.93	0.0004
WU2	1	f	2	1.50	22.15	1.81	0.0000
WUWILL	11	f	3	0.41	51.44	33.97	0.0036

Table 3C3 - 2

IESLC - Meta-anal of Ever Smoking (or Current if Ever not available), Cigs (or Any Prod if Cigs not avail)
Adenocarcinoma
Most adjusted

REF	NRR	SEX	AD	Ys	Ws	Qs	Ps
WYNDE2	9	m	0	0.63	4.29	1.46	0.1887
WYNDE3	28	m	0	1.27	5.06	0.01	0.0043
WYNDE3	75	f	0	0.64	6.88	2.28	0.0922
Subtotal WYNDE3				0.91	11.95	2.30	
WYNDE4	42	m	0	0.41	3.46	2.24	0.4408
WYNDE4	56	f	2	-0.51	1.67	5.00	0.5087
Subtotal WYNDE4				0.11	5.14	7.24	
WYNDE6	78	m	0	1.84	49.11	18.73	0.0000
WYNDE6	414	f	1	2.64	37.98	76.62	0.0000
Subtotal WYNDE6				2.19	87.09	95.35	
XU3	22	m	1	1.58	2.41	0.31	0.0143
XU3	26	f	1	0.09	1.89	2.42	0.9057
Subtotal XU3				0.92	4.30	2.73	
ZHENG	10	m	0	0.60	17.29	6.53	0.0121
ZHENG	21	f	0	0.15	14.95	17.12	0.5668
Subtotal ZHENG				0.39	32.24	23.64	
ZHOU	26	m	0	0.27	14.05	12.69	0.3154
ZHOU	27	f	0	0.36	4.59	3.41	0.4448
Subtotal ZHOU				0.29	18.64	16.09	

	N	116
	NS	81
	Wt	1742.50
Het	Chi	983.75
Het	df	115
Het	P	***
Fixed	RR	3.38
	RRl	3.23
	RRu	3.54
	P	+++
Random	RR	2.91
	RRl	2.50
	RRu	3.40
	P	+++
Asymm	P	*

Table 3C3 - 3

IESLC - Meta-anal of Ever Smoking (or Current if Ever not available), Cigs (or Any Prod if Cigs not avail)
Adenocarcinoma
Most adjusted

		Sex		
	combined	male	female	Total
N	11	56	49	116
NS	11	55	48	114
Wt	134.68	662.40	945.42	1742.50
Het Chi	63.82	284.35	592.37	983.75
Het df	10	55	48	115
Het P	***	***	***	***
Fixed RR	2.30	4.01	3.17	3.38
RRl	1.94	3.71	2.97	3.23
RRu	2.72	4.33	3.38	3.54
P	+++	+++	+++	+++
Random RR	2.55	3.62	2.40	2.91
RRl	1.58	2.95	1.88	2.50
RRu	4.11	4.44	3.06	3.40
P	+++	+++	+++	+++
Between Chi				43.21
Between df				2
Between P				***
Btwn(F) P				(*)
Btwn(R) P				*

			Lung cancer type				
	a	a+l	a+al+br	KII	not q+u	not q+s	Total
N	94	3	1	13	1	4	116
NS	66	2	1	9	1	3	82
Wt	1551.51	23.93	6.21	97.62	20.27	42.97	1742.50
Het Chi	851.58	4.35	0.00	44.94	0.00	2.98	983.75
Het df	93	2	0	12	0	3	115
Het P	***	N.S.	N.S.	***	N.S.	N.S.	***
Fixed RR	3.49	1.94	2.40	3.47	0.53	3.31	3.38
RRl	3.32	1.30	1.09	2.85	0.34	2.46	3.23
RRu	3.67	2.90	5.27	4.24	0.81	4.47	3.54
P	+++	++	+	+++	--	+++	+++
Random RR	3.08	2.20	2.40	2.14	0.53	3.31	2.91
RRl	2.60	1.08	1.09	1.34	0.34	2.46	2.50
RRu	3.64	4.47	5.27	3.40	0.81	4.47	3.40
P	+++	+	+	++	--	+++	+++
Between Chi							79.90
Between df							5
Between P							***
Btwn(F) P							(*)
Btwn(R) P							***

					Location				
	NAmer	UK	Scand	othEur	China	Japan	othAs	other	Total
N	47	4	7	15	12	13	12	6	116
NS	32	2	5	12	8	8	9	5	81
Wt	870.71	27.86	39.74	184.47	195.03	290.66	105.38	28.65	1742.50
Het Chi	316.38	4.24	9.53	42.13	7.39	41.19	14.91	2.85	983.75
Het df	46	3	6	14	11	12	11	5	115
Het P	***	N.S.	N.S.	***	N.S.	***	N.S.	N.S.	***
Fixed RR	5.77	3.29	2.04	2.30	1.48	2.07	1.66	4.20	3.38
RRl	5.40	2.27	1.49	1.99	1.28	1.84	1.37	2.91	3.23
RRu	6.16	4.77	2.78	2.66	1.70	2.32	2.00	6.06	3.54
P	+++	+++	+++	+++	+++	+++	+++	+++	+++
Random RR	4.58	3.04	2.05	2.74	1.48	1.93	1.63	4.20	2.91
RRl	3.73	1.83	1.37	2.02	1.28	1.49	1.29	2.91	2.50
RRu	5.62	5.06	3.06	3.71	1.70	2.50	2.06	6.06	3.40
P	+++	+++	+++	+++	+++	+++	+++	+++	+++
Between Chi									545.15
Between df									7
Between P									***
Btwn(F) P									***
Btwn(R) P									***

Table 3C3 - 3

IESLC - Meta-anal of Ever Smoking (or Current if Ever not available), Cigs (or Any Prod if Cigs not avail)
Adenocarcinoma
Most adjusted

Detailed Country in "other Europe"

	multi	Germany	othWest	East	Balkans	Total
N	2	2	2	7	2	15
NS	1	2	2	5	2	12
Wt	97.40	8.08	24.66	29.01	25.31	184.47
Het Chi	15.93	0.42	10.65	3.28	0.83	42.13
Het df	1	1	1	6	1	14
Het P	***	N.S.	**	N.S.	N.S.	***
Fixed RR	1.96	5.62	2.32	3.01	2.34	2.30
RRl	1.61	2.82	1.56	2.09	1.58	1.99
RRu	2.39	11.20	3.44	4.33	3.45	2.66
P	+++	+++	+++	+++	+++	+++
Random RR	1.92	5.62	3.21	3.01	2.34	2.74
RRl	0.87	2.82	0.74	2.09	1.58	2.02
RRu	4.26	11.20	13.90	4.33	3.45	3.71
P	N.S.	+++	N.S.	+++	+++	+++
Between Chi						11.02
Between df						4
Between P						*
Btwn(F) P						N.S.
Btwn(R) P						N.S.

Detailed Country in "other Asia"

	India	HongKong	other	Total
N	1	7	4	12
NS	1	5	3	9
Wt	2.35	78.53	24.49	105.38
Het Chi	0.00	8.66	4.60	14.91
Het df	0	6	3	11
Het P	N.S.	N.S.	N.S.	N.S.
Fixed RR	1.87	1.77	1.32	1.66
RRl	0.52	1.42	0.89	1.37
RRu	6.71	2.21	1.96	2.00
P	N.S.	+++	N.S.	+++
Random RR	1.87	1.77	1.29	1.63
RRl	0.52	1.34	0.79	1.29
RRu	6.71	2.33	2.13	2.06
P	N.S.	+++	N.S.	+++
Between Chi				1.64
Between df				2
Between P				N.S.
Btwn(F) P				N.S.
Btwn(R) P				N.S.

Detailed other continent

	SCAmer	Auslia	Africa	Total
N	6			6
NS	5			5
Wt	28.65			28.65
Het Chi	2.85			2.85
Het df	5			5
Het P	N.S.			N.S.
Fixed RR	4.20			4.20
RRl	2.91			2.91
RRu	6.06			6.06
P	+++			+++
Random RR	4.20			4.20
RRl	2.91			2.91
RRu	6.06			6.06
P	+++			+++
Between Chi				
Between df				
Between P				N.S.
Btwn(F) P				N.S.
Btwn(R) P				N.S.

Table 3C3 - 3

IESLC - Meta-anal of Ever Smoking (or Current if Ever not available), Cigs (or Any Prod if Cigs not avail)
Adenocarcinoma
Most adjusted

	<1960	1960-69	1970-79	1980-89	1990+	Total
	Start year of study					
N	18	16	31	43	8	116
NS	13	13	18	29	8	81
Wt	88.60	327.67	332.64	903.06	90.53	1742.50
Het Chi	42.77	204.45	107.41	489.17	15.29	983.75
Het df	17	15	30	42	7	115
Het P	***	***	***	***	*	***
Fixed RR	2.31	3.24	2.53	4.23	1.76	3.38
RRl	1.88	2.91	2.27	3.96	1.44	3.23
RRu	2.85	3.61	2.82	4.51	2.17	3.54
P	+++	+++	+++	+++	+++	+++
Random RR	2.12	3.10	2.71	3.45	1.99	2.91
RRl	1.45	1.95	2.18	2.69	1.40	2.50
RRu	3.11	4.92	3.38	4.44	2.83	3.40
P	+++	+++	+++	+++	+++	+++
Between Chi						124.67
Between df						4
Between P						***
Btwn(F) P						**
Btwn(R) P						(*)

	CC	other	Total
	Study type (1)		
N	101	15	116
NS	70	11	81
Wt	1633.82	108.68	1742.50
Het Chi	942.24	29.19	983.75
Het df	100	14	115
Het P	***	**	***
Fixed RR	3.31	4.68	3.38
RRl	3.15	3.88	3.23
RRu	3.47	5.65	3.54
P	+++	+++	+++
Random RR	2.75	4.39	2.91
RRl	2.33	3.26	2.50
RRu	3.25	5.93	3.40
P	+++	+++	+++
Between Chi			12.32
Between df			1
Between P			***
Btwn(F) P			N.S.
Btwn(R) P			**

	CC	prosp	other	Total
	Study type (2)			
N	101	11	4	116
NS	70	8	3	81
Wt	1633.82	87.40	21.28	1742.50
Het Chi	942.24	25.17	2.81	983.75
Het df	100	10	3	115
Het P	***	**	N.S.	***
Fixed RR	3.31	4.93	3.78	3.38
RRl	3.15	4.00	2.47	3.23
RRu	3.47	6.08	5.78	3.54
P	+++	+++	+++	+++
Random RR	2.75	4.51	3.78	2.91
RRl	2.33	3.09	2.47	2.50
RRu	3.25	6.57	5.78	3.40
P	+++	+++	+++	+++
Between Chi				13.53
Between df				2
Between P				**
Btwn(F) P				N.S.
Btwn(R) P				*

Table 3C3 - 3

IESLC - Meta-anal of Ever Smoking (or Current if Ever not available), Cigs (or Any Prod if Cigs not avail)
Adenocarcinoma
Most adjusted

	Study size (number of LC cases) 100-249	250-499	500-999	1000+	Total
N	28	32	18	38	116
NS	24	23	12	22	81
Wt	177.01	282.46	173.87	1109.16	1742.50
Het Chi	91.95	128.64	47.96	547.90	983.75
Het df	27	31	17	37	115
Het P	***	***	***	***	***
Fixed RR	1.94	2.52	2.23	4.25	3.38
RRl	1.67	2.24	1.92	4.01	3.23
RRu	2.24	2.83	2.59	4.51	3.54
P	+++	+++	+++	+++	+++
Random RR	2.34	2.48	2.60	3.85	2.91
RRl	1.74	1.91	1.96	2.99	2.50
RRu	3.16	3.21	3.44	4.94	3.40
P	+++	+++	+++	+++	+++
Between Chi					167.30
Between df					3
Between P					***
Btwn(F) P					***
Btwn(R) P					*

	Risky occupational population no	mining	othRisky	Total
N	114	1	1	116
NS	79	1	1	81
Wt	1733.49	3.37	5.64	1742.50
Het Chi	975.51	0.00	0.00	983.75
Het df	113	0	0	115
Het P	***	N.S.	N.S.	***
Fixed RR	3.39	0.73	4.30	3.38
RRl	3.23	0.25	1.88	3.23
RRu	3.55	2.12	9.82	3.54
P	+++	N.S.	+++	+++
Random RR	2.94	0.73	4.30	2.91
RRl	2.52	0.25	1.88	2.50
RRu	3.42	2.12	9.82	3.40
P	+++	N.S.	+++	+++
Between Chi				8.25
Between df				2
Between P				*
Btwn(F) P				N.S.
Btwn(R) P				*

	National cigarette tobacco type Virginia	blended	other	Total
N	9	94	13	116
NS	6	66	9	81
Wt	84.67	1454.75	203.08	1742.50
Het Chi	8.31	805.30	8.06	983.75
Het df	8	93	12	115
Het P	N.S.	***	N.S.	***
Fixed RR	3.80	3.78	1.46	3.38
RRl	3.07	3.59	1.27	3.23
RRu	4.70	3.97	1.68	3.54
P	+++	+++	+++	+++
Random RR	3.78	3.20	1.46	2.91
RRl	3.01	2.70	1.27	2.50
RRu	4.73	3.79	1.68	3.40
P	+++	+++	+++	+++
Between Chi				162.07
Between df				2
Between P				***
Btwn(F) P				***
Btwn(R) P				***

Table 3C3 - 3

IESLC - Meta-anal of Ever Smoking (or Current if Ever not available), Cigs (or Any Prod if Cigs not avail)
Adenocarcinoma
Most adjusted

	Any proxy use No/nk	Yes	Total
N	88	28	116
NS	62	19	81
Wt	1505.66	236.84	1742.50
Het Chi	884.63	98.39	983.75
Het df	87	27	115
Het P	***	***	***
Fixed RR	3.35	3.56	3.38
RRl	3.19	3.13	3.23
RRu	3.53	4.04	3.54
P	+++	+++	+++
Random RR	2.74	3.61	2.91
RRl	2.29	2.74	2.50
RRu	3.27	4.76	3.40
P	+++	+++	+++
Between Chi			0.73
Between df			1
Between P			N.S.
Btwn(F) P			N.S.
Btwn(R) P			(*)

	Full histological confirmation No	Yes	Total
N	67	49	116
NS	48	33	81
Wt	825.20	917.30	1742.50
Het Chi	386.13	463.44	983.75
Het df	66	48	115
Het P	***	***	***
Fixed RR	2.52	4.40	3.38
RRl	2.36	4.12	3.23
RRu	2.70	4.69	3.54
P	+++	+++	+++
Random RR	2.67	3.28	2.91
RRl	2.23	2.59	2.50
RRu	3.20	4.15	3.40
P	+++	+++	+++
Between Chi			134.18
Between df			1
Between P			***
Btwn(F) P			***
Btwn(R) P			N.S.

	Number of adjustment variables (1) 0	1	2+/+nk	Total
N	54	27	35	116
NS	38	18	27	83
Wt	577.59	249.81	915.10	1742.50
Het Chi	290.81	167.17	405.49	983.75
Het df	53	26	34	115
Het P	***	***	***	***
Fixed RR	2.34	3.64	4.18	3.38
RRl	2.16	3.22	3.91	3.23
RRu	2.54	4.12	4.46	3.54
P	+++	+++	+++	+++
Random RR	2.51	3.19	3.35	2.91
RRl	2.03	2.27	2.58	2.50
RRu	3.10	4.48	4.33	3.40
P	+++	+++	+++	+++
Between Chi				120.29
Between df				2
Between P				***
Btwn(F) P				***
Btwn(R) P				N.S.

Table 3C3 - 3

IESLC - Meta-anal of Ever Smoking (or Current if Ever not available), Cigs (or Any Prod if Cigs not avail)
Adenocarcinoma
Most adjusted

	Number of adjustment variables (2) 0	1	2	3-5	6+/+nk	Total
N	54	27	21	10	4	116
NS	38	18	15	9	4	84
Wt	577.59	249.81	755.76	135.17	24.16	1742.50
Het Chi	290.81	167.17	289.07	47.25	9.32	983.75
Het df	53	26	20	9	3	115
Het P	***	***	***	***	*	***
Fixed RR	2.34	3.64	4.69	2.51	1.91	3.38
RRl	2.16	3.22	4.37	2.12	1.28	3.23
RRu	2.54	4.12	5.04	2.98	2.84	3.54
P	+++	+++	+++	+++	++	+++
Random RR	2.51	3.19	3.89	2.79	2.11	2.91
RRl	2.03	2.27	2.84	1.74	1.03	2.50
RRu	3.10	4.48	5.33	4.47	4.31	3.40
P	+++	+++	+++	+++	+	+++
Between Chi						180.14
Between df						4
Between P						***
Btwn(F) P						***
Btwn(R) P						N.S.

	Product all/unsp	cig+/-ot	cig only	Total
N	41	66	9	116
NS	31	45	8	84
Wt	334.65	1313.82	94.03	1742.50
Het Chi	114.83	770.77	16.32	983.75
Het df	40	65	8	115
Het P	***	***	*	***
Fixed RR	2.19	3.70	4.47	3.38
RRl	1.96	3.51	3.65	3.23
RRu	2.43	3.91	5.47	3.54
P	+++	+++	+++	+++
Random RR	2.14	3.31	4.58	2.91
RRl	1.76	2.69	3.20	2.50
RRu	2.61	4.08	6.55	3.40
P	+++	+++	+++	+++
Between Chi				81.82
Between df				2
Between P				***
Btwn(F) P				**
Btwn(R) P				***

	Denominator nev any	nev cigs	Total
N	75	41	116
NS	55	28	83
Wt	876.04	866.46	1742.50
Het Chi	281.85	596.65	983.75
Het df	74	40	115
Het P	***	***	***
Fixed RR	2.65	4.33	3.38
RRl	2.48	4.05	3.23
RRu	2.83	4.63	3.54
P	+++	+++	+++
Random RR	2.57	3.64	2.91
RRl	2.21	2.73	2.50
RRu	2.97	4.86	3.40
P	+++	+++	+++
Between Chi			105.25
Between df			1
Between P			***
Btwn(F) P			***
Btwn(R) P			*

Table 3C3 - 3

IESLC - Meta-anal of Ever Smoking (or Current if Ever not available), Cigs (or Any Prod if Cigs not avail)

Adenocarcinoma
Most adjusted

		Derivation of RR/CI			
		Orig	StdCalc	Other	Total
	N	27	47	42	116
	NS	20	34	31	85
	Wt	845.89	520.33	376.28	1742.50
Het	Chi	367.26	295.24	216.67	983.75
Het	df	26	46	41	115
Het	P	***	***	***	***
Fixed	RR	4.29	2.45	3.09	3.38
	RRl	4.01	2.25	2.80	3.23
	RRu	4.59	2.67	3.42	3.54
	P	+++	+++	+++	+++
Random	RR	3.74	2.58	2.78	2.91
	RRl	2.80	2.04	2.15	2.50
	RRu	5.00	3.27	3.60	3.40
	P	+++	+++	+++	+++
Between	Chi				104.58
Between	df				2
Between	P				***
Btwn(F)	P				**
Btwn(R)	P				N.S.

		Smoking status		
		ever	current	Total
	N	107	9	116
	NS	75	6	81
	Wt	1522.57	219.93	1742.50
Het	Chi	924.03	56.84	983.75
Het	df	106	8	115
Het	P	***	***	***
Fixed	RR	3.43	3.04	3.38
	RRl	3.27	2.66	3.23
	RRu	3.61	3.47	3.54
	P	+++	+++	+++
Random	RR	2.82	4.31	2.91
	RRl	2.39	2.78	2.50
	RRu	3.32	6.71	3.40
	P	+++	+++	+++
Between	Chi			2.88
Between	df			1
Between	P			(*)
Btwn(F)	P			N.S.
Btwn(R)	P			(*)

Table 3C3 - 4

IESLC - Meta-anal of Ever Smoking (or Current if Ever not available), Cigs (or Any Prod if Cigs not avail)
Adenocarcinoma
Least adjusted

REF	NRR	X	SEX	AGEL	AGEH	RACE	YF	LC TYPE	LOC	START	ST	NLC	R	VB	P	H	AD	SM	PRODUCT	DENOM	De
ABRAHA	2		m	0	0	all	0	a	Eu:est	1975	pr	571	n	bl	n	n	0	ev	all/unsp	nev any	ot
ABRAHA	5		f	0	0	all	0	a	Eu:est	1975	pr	571	n	bl	n	n	0	ev	all/unsp	nev any	ot
ALDERS	108	x	m	0	0	all	-	not q+s	Eu:UK	1977	CC	1448	n	V	n	n	0	ev	cig+/-ot	nev any	st
ALDERS	104	x	f	0	0	all	-	not q+s	Eu:UK	1977	CC	1448	n	V	n	n	0	ev	cig only	nev any	st
ANDERS	12		f	0	0	all	0	a	NAmer	1986	pr	343	n	bl	n	n	0	ev	cig+/-ot	nev cigs	st
BAND	2		m	0	0	all	-	a	NAmer	1983	CC	2831	n	V	y	y	2	ev	cig only	nev any	ot
BARBON	122	x	m	0	0	all	-	a	Eu:wst	1979	CC	755	n	bl	y	y	0	ev	all/unsp	nev any	st
BECHER	12		f	0	0	all	-	not q+s	Eu:Ger	1985	CC	194	n	bl	n	y	1	ev	all/unsp	nev any	or
BOUCOT	72	x	m	0	0	all	0	a	NAmer	1951	pr	121	n	bl	n	n	0	cu	cig only	nev any	ot
BRESLO	1		c	0	0	all	-	a	NAmer	1949	CC	518	n	bl	n	y	0	ev	cig+/-ot	nev+1	st
BROWN1	1	x	m	0	0	wh	-	a	NAmer	1979	CC	102	n	bl	y	y	0	ev	cig+/-ot	nev cigs	st
BROWN1	2	x	f	0	0	wh	-	a	NAmer	1979	CC	102	n	bl	y	y	0	ev	cig+/-ot	nev cigs	st
BROWN2	4		m	0	0	wh	-	a	NAmer	1984	CC	14596	n	bl	n	y	2	ev	cig+/-ot	nev cigs	or
BROWN2	3		f	0	0	wh	-	a	NAmer	1984	CC	14596	n	bl	n	y	2	ev	cig+/-ot	nev cigs	or
BUFFLE	50		m	0	0	wh	-	a	NAmer	1976	CC	943	n	bl	y	n	0	ev	cig+/-ot	nev cigs	ot
BUFFLE	45		f	0	0	wh	-	a	NAmer	1976	CC	943	n	bl	y	n	0	ev	cig+/-ot	nev cigs	ot
BYERS1	3		m	0	0	wh	-	a	NAmer	1957	CC	1002	n	bl	n	n	0	ev	cig+/-ot	nev cigs	st
CHAN	19		m	0	0	all	-	a+l	As:HK	1976	CC	397	n	bl	n	n	0	ev	cig+/-ot	nev any	ot
CHAN	23		f	0	0	all	-	a+l	As:HK	1976	CC	397	n	bl	n	n	0	ev	cig+/-ot	nev any	st
CHOI	63		m	0	0	all	-	a	As:oth	1985	CC	375	n	bl	n	n	0	ev	cig+/-ot	nev cigs	st
CHOI	65		f	0	0	all	-	a	As:oth	1985	CC	375	n	bl	n	n	0	ev	cig+/-ot	nev cigs	st
COMSTO	67		m	0	0	all	-	a	NAmer	1975	ot	258	n	bl	n	n	0	ev	cig+/-ot	nev cigs	st
COMSTO	79		f	0	0	all	-	a	NAmer	1975	ot	258	n	bl	n	n	0	ev	cig+/-ot	nev cigs	st
CORREA	36		c	0	0	all	-	a	NAmer	1979	CC	1359	n	bl	y	n	1	ev	cig+/-ot	nev cigs	or
CPSI	404		m	0	0	all	2	a	NAmer	1959	pr	5138	n	bl	n	n	1	cu	cig only	nev any	ot
CPSI	406		f	0	0	all	2	a	NAmer	1959	pr	5138	n	bl	n	n	1	cu	cig only	nev any	ot
CPSII	115		m	0	0	all	2	a	NAmer	1982	pr	3229	n	bl	n	n	1	cu	cig only	nev any	st
CPSII	118		f	0	0	all	2	a	NAmer	1982	pr	3229	n	bl	n	n	1	cu	cig+/-ot	nev cigs	st
DAMBER	11	x	m	0	0	all	-	a+al+br	Eu:Sca	1972	CC	579	n	bl	y	n	0	ev	all/unsp	nev any	st
DESTE2	17		m	0	0	all	-	a	SCAmer	1993	CC	463	n	bl	n	n	2	ev	all/unsp	nev any	or
DOLL	83	x	m	0	0	all	-	KII	Eu:UK	1948	CC	1465	n	V	n	n	0	ev	all/unsp	nev any	st
DOLL	85	x	f	0	0	all	-	KII	Eu:UK	1948	CC	1465	n	V	n	n	0	ev	all/unsp	nev any	st
DORGAN	125		m	0	0	wh	-	a	NAmer	1980	CC	2026	n	bl	y	y	2	ev	cig+/-ot	nev any	or
DORGAN	104		f	0	0	all	-	a	NAmer	1980	CC	2026	n	bl	y	y	3	ev	cig+/-ot	nev any	or
DORN	340		m	0	0	wh	8	a	NAmer	1954	pr	5097	n	bl	n	n	1	cu	cig only	nev any	ot
DOSEME	20	x	m	0	0	all	-	not q+s	Eu:bal	1979	CC	1210	n	bl	n	n	0	ev	cig+/-ot	nev cigs	st
ENGELA	76		m	0	0	all	0	a	Eu:Sca	1964	pr	435	n	bl	n	n	7	ev	cig+/-ot	nev cigs	ot
FAN	4		c	0	0	all	-	a	As:Chi	1990	CC	403	n	ot	y	n	0	ev	cig+/-ot	nev cigs	ot
GAO	8	x	m	0	0	all	-	a	As:Chi	1984	CC	1405	n	ot	n	n	0	ev	cig+/-ot	nev cigs	st
GAO	18	x	f	0	0	all	-	a	As:Chi	1984	CC	1405	n	ot	n	n	0	ev	cig+/-ot	nev cigs	st
GER	1	x	c	0	0	all	-	a	As:oth	1990	CC	141	n	ot	y	n	0	ev	all/unsp	nev any	st
HAENSZ	39		f	0	0	all	-	a	NAmer	1955	CC	158	n	bl	n	y	0	ev	cig+/-ot	nev any	st
HAMMON	91	x	m	0	0	wh	0	a	NAmer	1952	pr	448	n	bl	n	n	0	ev	cig+/-ot	nev any	st
HEGMAN	4		c	0	0	all	-	a	NAmer	1989	CC	282	n	bl	y	y	0	ev	all/unsp	nev any	st
HINDS	24		f	0	0	o	-	a	NAmer	1968	CC	292	n	bl	n	n	3	ev	all/unsp	nev any	st
ISHIMA	3	x	c	0	0	all	-	a	As:Jap	1961	CC	180	n	bl	y	y	0	ev	all/unsp	nev any	st
JAHN	43		m	0	0	all	-	a	Eu:Ger	1988	CC	1004	n	bl	n	n	0	ev	cig+/-ot	nev any	st
JAIN	7	x	m	0	0	all	-	a	NAmer	1981	CC	845	n	V	y	n	0	ev	cig+/-ot	nev cigs	st
JAIN	2	x	f	0	0	all	-	a	NAmer	1981	CC	845	n	V	y	n	0	ev	cig+/-ot	nev cigs	st
JEDRYC	21	x	m	0	0	all	-	a	Eu:est	1980	CC	1630	n	bl	y	n	0	ev	cig+/-ot	nev any	st
JOLY	51		m	0	0	all	-	a	SCAmer	1978	CC	826	n	bl	n	n	0	ev	cig+/-ot	nev any	st
JOLY	50		f	0	0	all	-	a	SCAmer	1978	CC	826	n	bl	n	n	0	ev	cig+/-ot	nev any	st
JUSSAW	28		m	0	0	all	-	KII	As:Ind	1964	CC	792	n	V	n	n	0	ev	cig only	nev any	st
KATSOU	31	x	f	0	0	all	-	a	Eu:bal	1987	CC	101	n	bl	n	n	0	ev	all/unsp	nev any	st
KHUDER	27		m	0	0	all	-	a	NAmer	1985	CC	482	n	bl	n	y	0	ev	cig+/-ot	nev cigs	ot
KIHARA	29		c	0	0	jap	-	a	As:Jap	1991	CC	440	n	bl	n	n	0	ev	all/unsp	nev any	st
KOO	7		f	0	0	all	-	a+l	As:HK	1981	CC	200	n	bl	n	n	0	ev	all/unsp	nev any	st
KREYBE	20	x	m	0	0	all	-	KII	Eu:Sca	1948	CC	300	n	bl	n	y	0	ev	all/unsp	nev any	st
KREYBE	36	x	f	0	0	all	-	KII	Eu:Sca	1948	CC	300	n	bl	n	y	0	ev	all/unsp	nev any	st
LAMTH	3		f	0	0	ch	-	a	As:HK	1983	CC	445	n	bl	n	n	0	ev	all/unsp	nev any	or
LAMWK	4		f	0	0	ch	-	a	As:HK	1981	CC	163	n	bl	n	n	0	ev	all/unsp	nev any	st
LAMWK2	3		m	0	0	all	-	a	As:HK	1976	CC	480	n	bl	n	n	0	ev	all/unsp	nev any	st
LAMWK2	7		f	0	0	all	-	a	As:HK	1976	CC	480	n	bl	n	n	0	ev	all/unsp	nev any	st
LOMBA2	3		f	0	0	all	-	not q+u	NAmer	1960	CC	225	n	bl	n	n	0	ev	cig+/-ot	nev cigs	st
LUBIN	37		m	0	0	all	-	KII	As:Chi	1984	CC	427	m	ot	y	n	0	ev	cig+/-ot	nev any	st
LUBIN2	148		m	0	0	all	-	a	Eu:mul	1976	CC	7804	n	bl	n	y	0	ev	cig+/-ot	nev any	st
LUBIN2	168		f	0	0	all	-	a	Eu:mul	1976	CC	7804	n	bl	n	y	0	ev	cig+/-ot	nev any	st
LUO	3	x	c	0	0	all	-	a	As:Chi	1990	CC	102	n	ot	n	y	0	ev	cig+/-ot	nev cigs	st
MATOS	68	x	m	0	0	all	-	a	SCAmer	1994	CC	200	n	bl	n	n	0	ev	cig+/-ot	nev any	st
MATSUD	12		m	0	0	all	-	a	As:Jap	1965	CC	179	n	bl	n	n	0	ev	cig+/-ot	nev cigs	ot
NOU	3		m	0	0	all	-	a	Eu:Sca	1971	CC	273	n	bl	y	n	0	ev	all/unsp	nev any	st
NOU	8		f	0	0	all	-	a	Eu:Sca	1971	CC	273	n	bl	y	n	0	ev	all/unsp	nev any	st

Table 3C3 - 4

IESLC - Meta-anal of Ever Smoking (or Current if Ever not available), Cigs (or Any Prod if Cigs not avail)
Adenocarcinoma
Least adjusted

REF	NRR	X	SEX	AGEL	AGEH	RACE	YF	LC TYPE	LOC	START	ST	NLC	R	VB	P	H	AD	SM	PRODUCT	DENOM	De
ORMOS	21		m	0	0	all	-	a	Eu:est	1947	CC	119	n	bl	y	y	0	ev	cig+/-ot	nev any	ot
OSANN	19	x	m	0	0	all	-	a	NAmer	1984	CC	1986	n	bl	n	n	0	ev	cig+/-ot	nev cigs	st
OSANN	23	x	f	0	0	all	-	a	NAmer	1984	CC	1986	n	bl	n	n	0	ev	cig+/-ot	nev cigs	st
OSANN2	13	x	f	0	0	all	-	KII	NAmer	1964	ot	217	n	bl	n	y	0	ev	cig+/-ot	nev cigs	st
PEZZOT	7		m	0	0	all	-	a	SCAmer	1987	CC	215	n	bl	n	y	0	ev	cig only	nev cigs	st
SCHWAR	8		m	40	54	wh	-	a	NAmer	1984	CC	5588	n	bl	y	y	0	ev	cig+/-ot	nev cigs	st
SCHWAR	7		m	40	54	bl	-	a	NAmer	1984	CC	5588	n	bl	y	y	0	ev	cig+/-ot	nev cigs	st
SCHWAR	16		f	40	54	wh	-	a	NAmer	1984	CC	5588	n	bl	y	y	0	ev	cig+/-ot	nev cigs	st
SCHWAR	15		f	40	54	bl	-	a	NAmer	1984	CC	5588	n	bl	y	y	0	ev	cig+/-ot	nev cigs	st
SEOW	2		f	0	0	ch	-	a	As:oth	1997	CC	153	n	bl	n	y	0	ev	cig+/-ot	nev cigs	st
SIEMIA	12	x	m	0	0	all	-	a	NAmer	1979	CC	857	n	V	y	y	0	ev	cig+/-ot	nev cigs	st
SOBUE	7	x	m	0	0	all	-	a	As:Jap	1986	CC	1376	n	bl	n	y	0	ev	cig+/-ot	nev cigs	st
SOBUE	23	x	f	0	0	all	-	a	As:Jap	1986	CC	1376	n	bl	n	y	0	ev	cig+/-ot	nev cigs	st
SOBUE2	2		m	0	0	all	-	a	As:Jap	1965	CC	2083	n	bl	n	n	2	cu	cig+/-ot	nev any	or
SOBUE2	6		f	0	0	all	-	a	As:Jap	1965	CC	2083	n	bl	n	n	2	cu	cig+/-ot	nev any	or
STASZE	24		m	0	0	all	-	a	Eu:est	1954	CC	281	n	bl	n	y	0	ev	cig+/-ot	nev any	ot
STASZE	4		f	0	0	all	-	a	Eu:est	1954	CC	281	n	bl	n	y	0	ev	all/unsp	nev any	st
STAYNE	4		m	0	0	all	-	a	NAmer	1969	CC	420	n	bl	n	n	0	ev	all/unsp	nev any	st
SUZUK2	13	x	c	0	0	all	-	a	SCAmer	1991	CC	123	n	bl	n	y	0	ev	all/unsp	nev any	st
SUZUKI	3	x	m	0	0	all	-	a	As:Jap	1978	CC	238	n	bl	n	y	0	ev	cig+/-ot	nev any	st
SUZUKI	7	x	f	0	0	all	-	a	As:Jap	1978	CC	238	n	bl	n	y	0	ev	cig+/-ot	nev any	st
SVENSS	59	x	f	0	0	all	-	a	Eu:Sca	1983	CC	210	n	bl	n	n	0	ev	all/unsp	nev any	st
TIZZAN	19		c	0	0	all	-	a	Eu:wst	1959	CC	1358	n	bl	n	n	0	ev	all/unsp	nev any	st
TOKARS	7	x	c	0	0	all	-	a	Eu:est	1966	ot	162	o	bl	n	y	0	ev	all/unsp	nev any	st
TSUGAN	2	x	m	0	0	all	-	a	As:Jap	1976	CC	134	n	bl	n	y	0	ev	all/unsp	nev any	st
TSUGAN	8	x	f	0	0	all	-	a	As:Jap	1976	CC	134	n	bl	n	y	0	ev	all/unsp	nev any	or
WAKAI	17	x	m	0	0	all	-	a	As:Jap	1988	CC	333	n	bl	n	y	0	ev	all/unsp	nev any	st
WAKAI	35	x	f	0	0	all	-	a	As:Jap	1988	CC	333	n	bl	n	y	0	ev	all/unsp	nev any	st
WU	5	x	f	0	0	wh	-	a	NAmer	1981	CC	220	n	bl	n	y	0	ev	all/unsp	nev any	st
WU2	1		f	0	0	all	-	a	NAmer	1983	CC	336	n	bl	n	y	2	cu	all/unsp	nev any	or
WUWILL	25	x	f	0	0	all	-	a	As:Chi	1985	CC	965	n	ot	n	n	0	ev	cig+/-ot	nev cigs	st
WYNDE2	9		m	0	0	all	-	KII	NAmer	1962	CC	404	n	bl	n	y	0	ev	cig+/-ot	nev any	st
WYNDE3	28		m	0	0	all	-	KII	NAmer	1966	CC	350	n	bl	n	y	0	ev	cig+/-ot	nev any	st
WYNDE3	75		f	0	0	all	-	KII	NAmer	1966	CC	350	n	bl	n	y	0	ev	cig+/-ot	nev any	st
WYNDE4	42		m	0	0	all	-	a	NAmer	1948	CC	684	n	bl	y	n	0	ev	all/unsp	nev any	st
WYNDE4	56		f	0	0	all	-	a	NAmer	1948	CC	684	n	bl	y	n	2	ev	all/unsp	nev any	ot
WYNDE6	78		m	0	0	all	-	KII	NAmer	1969	CC	4423	n	bl	n	y	0	ev	cig+/-ot	nev any	st
WYNDE6	413	x	f	0	0	wh	-	a	NAmer	1969	CC	4423	n	bl	n	y	0	ev	cig+/-ot	nev cigs	st
XU3	21	x	m	0	0	all	-	KII	As:Chi	1981	CC	135	n	ot	n	n	0	ev	all/unsp	nev any	st
XU3	25	x	f	0	0	all	-	KII	As:Chi	1981	CC	135	n	ot	n	n	0	ev	all/unsp	nev any	st
ZHENG	10		m	0	0	all	-	a	As:Chi	1982	CC	540	n	ot	*	y	0	ev	cig+/-ot	nev cigs	st
ZHENG	21		f	0	0	all	-	a	As:Chi	1982	CC	540	n	ot	*	y	0	ev	cig+/-ot	nev cigs	st
ZHOU	26		m	0	0	all	-	a	As:Chi	1978	CC	1360	n	ot	n	n	0	ev	all/unsp	nev any	st
ZHOU	27		f	0	0	all	-	a	As:Chi	1978	CC	1360	n	ot	n	n	0	ev	all/unsp	nev any	st

Cigarette type is all/unspec for all RRs

except for the following:

REF	NRR	CIGTYPE
ALDERS	104	MC only
CHAN	19	MC+-HR
CHAN	23	MC+-HR
JUSSAW	28	MC only

Table 3C3 - 5

IESLC - Meta-anal of Ever Smoking (or Current if Ever not available), Cigs (or Any Prod if Cigs not avail)
Adenocarcinoma
Least adjusted

REF	NRR	SEX	AD	Number Exposed Case	Number Exposed Cont	Non-exposed Case	Non-exposed Cont	RR	95.00%CI
*ABRAHA	2	m	0	59	10351	8	3365	2.40	(1.15- 5.01)
*ABRAHA	5	f	0	19	5256	16	11589	2.62	(1.35- 5.09)
Subtotal ABRAHA								2.52	(1.54- 4.12)
ALDERS	108	m	0	141	641	6	133	4.88	(2.11- 11.27)
ALDERS	104	f	0	104	371	25	243	2.72	(1.71- 4.34)
Subtotal ALDERS								3.13	(2.08- 4.70)
*ANDERS	12	f	0	99	96164	33	195158	6.09	(4.11- 9.03)
BAND	2	m	2	-	-	-	-	4.10	(3.01- 5.59)
BARBON	122	m	0	151	567	7	188	7.15	(3.29- 15.53)
BECHER	12	f	1	-	-	-	-	10.83	(1.32- 88.70)
*BOUCOT	72	m	0	14	22177	0	7551	9.87~	(0.59- 165.51)
BRESLO	1	c	0	40	394	4	56	1.42	(0.49- 4.12)
BROWN1	1	m	0	46	46	4	19	4.75	(1.50- 15.05)
BROWN1	2	f	0	33	19	19	47	4.30	(1.98- 9.34)
Subtotal BROWN1								4.43	(2.33- 8.44)
BROWN2	4	m	2	-	-	-	-	8.20	(6.90- 9.70)
BROWN2	3	f	2	-	-	-	-	6.90	(6.10- 7.90)
Subtotal BROWN2								7.35	(6.63- 8.15)
BUFFLE	50	m	0	-	-	-	-	4.50	(1.85- 10.95)
BUFFLE	45	f	0	-	-	-	-	4.02	(2.42- 6.67)
Subtotal BUFFLE								4.13	(2.66- 6.42)
BYERS1	3	m	0	47	695	7	424	4.10	(1.83- 9.15)
CHAN	19	m	0	56	160	0	43	30.63~	(1.85- 505.72)
CHAN	23	f	0	24	38	40	139	2.19	(1.18- 4.08)
Subtotal CHAN								2.48	(1.35- 4.55)
CHOI	63	m	0	46	465	7	95	1.34	(0.59- 3.06)
CHOI	65	f	0	5	26	49	164	0.64	(0.23- 1.77)
Subtotal CHOI								1.00	(0.53- 1.89)
COMSTO	67	m	0	43	229	2	84	7.89	(1.87- 33.27)
COMSTO	79	f	0	29	87	8	115	4.79	(2.09- 11.00)
Subtotal COMSTO								5.43	(2.64- 11.14)
CORREA	36	c	1	-	-	-	-	5.60	(3.60- 8.80)
*CPSI	404	m	1	-	-	-	-	4.58	(1.74- 12.05)
*CPSI	406	f	1	-	-	-	-	1.43	(0.47- 4.39)
Subtotal CPSI								2.78	(1.34- 5.78)
*CPSII	115	m	1	-	-	-	-	19.22	(6.46- 57.16)
*CPSII	118	f	1	-	-	-	-	8.23	(4.36- 15.54)
Subtotal CPSII								10.21	(5.89- 17.67)
DAMBER	11	m	0	65	49	16	29	2.40	(1.18- 4.91)
DESTE2	17	m	2	-	-	-	-	4.30	(1.60- 11.40)
DOLL	83	m	0	38	1296	2	61	0.89	(0.21- 3.79)
DOLL	85	f	0	8	49	5	59	1.93	(0.59- 6.27)
Subtotal DOLL								1.42	(0.57- 3.53)
DORGAN	125	m	2	-	-	-	-	4.80	(1.90- 12.00)
DORGAN	104	f	3	-	-	-	-	3.90	(2.80- 5.40)
Subtotal DORGAN								3.99	(2.93- 5.44)
*DORN	340	m	1	-	-	-	-	5.95	(3.85- 9.22)
DOSEME	20	m	0	142	536	24	293	3.23	(2.05- 5.10)
*ENGELA	76	m	7	-	-	-	-	2.33	(0.92- 5.89)
FAN	4	c	0	67	595	45	556	1.39	(0.94- 2.07)
GAO	8	m	0	180	558	42	202	1.55	(1.07- 2.25)
GAO	18	f	0	62	130	266	605	1.08	(0.78- 1.52)
Subtotal GAO								1.27	(0.99- 1.63)
GER	1	c	0	35	139	37	149	1.01	(0.60- 1.70)
HAENSZ	39	f	0	18	103	37	236	1.11	(0.61- 2.05)
*HAMMON	91	m	0	26	382338	2	115884	3.94	(0.94- 16.60)
HEGMAN	4	c	0	83	1202	15	2080	9.58	(5.50- 16.67)
HINDS	24	f	3	-	-	-	-	3.89	(2.49- 6.07)
ISHIMA	3	c	0	39	25	13	27	3.24	(1.41- 7.43)
JAHN	43	m	0	202	671	8	138	5.19	(2.50- 10.77)
JAIN	7	m	0	90	277	4	85	6.90	(2.46- 19.35)
JAIN	2	f	0	86	196	24	214	3.91	(2.39- 6.40)
Subtotal JAIN								4.35	(2.79- 6.78)
JEDRYC	21	m	0	99	1054	7	289	3.88	(1.78- 8.44)
JOLY	51	m	0	72	709	5	218	4.43	(1.77- 11.10)
JOLY	50	f	0	33	122	25	283	3.06	(1.75- 5.37)
Subtotal JOLY								3.38	(2.10- 5.46)
JUSSAW	28	m	0	3	77	13	624	1.87	(0.52- 6.71)
KATSOU	31	f	0	18	22	30	67	1.83	(0.86- 3.90)
KHUDER	27	m	0	155	-	7	-	8.11	(3.67- 17.93)
KIHARA	29	c	0	130	232	78	237	1.70	(1.22- 2.38)

Table 3C3 - 5

IESLC - Meta-anal of Ever Smoking (or Current if Ever not available), Cigs (or Any Prod if Cigs not avail)
Adenocarcinoma
Least adjusted

REF	NRR	SEX	AD	Number Exposed Case	Number Exposed Cont	Non-exposed Case	Non-exposed Cont	RR	95.00%CI
KOO	7	f	0	34	63	46	137	1.61	(0.94- 2.74)
KREYBE	20	m	0	42	3514	3	644	2.57	(0.79- 8.30)
KREYBE	36	f	0	10	328	27	657	0.74	(0.35- 1.55)
Subtotal KREYBE								1.05	(0.56- 1.97)
LAMTH	3	f	0	79	51	131	158	1.87	(1.23- 2.85)
LAMWK	4	f	0	36	41	60	144	2.11	(1.23- 3.61)
LAMWK2	3	m	0	52	161	15	43	0.93	(0.48- 1.80)
LAMWK2	7	f	0	26	50	41	139	1.76	(0.98- 3.17)
Subtotal LAMWK2								1.33	(0.86- 2.07)
LOMBA2	3	f	0	42	353	54	239	0.53	(0.34- 0.81)
LUBIN	37	m	0	32	788	4	72	0.73	(0.25- 2.12)
LUBIN2	148	m	0	655	10433	57	2616	2.88	(2.19- 3.79)
LUBIN2	168	f	0	85	567	138	1180	1.28	(0.96- 1.71)
Subtotal LUBIN2								1.96	(1.61- 2.39)
LUO	3	c	0	28	146	29	160	1.06	(0.60- 1.86)
MATOS	68	m	0	79	283	5	110	6.14	(2.42- 15.57)
MATSUD	12	m	0	23	3314	0	1255	17.80~	(1.08- 293.32)
NOU	3	m	0	36	247	4	122	4.45	(1.55- 12.77)
NOU	8	f	0	9	92	29	261	0.88	(0.40- 1.93)
Subtotal NOU								1.57	(0.83- 2.94)
ORMOS	21	m	0	4	1034	0	777	6.76~	(0.36- 125.82)
OSANN	19	m	0	319	1018	14	833	18.64	(10.83- 32.09)
OSANN	23	f	0	243	563	47	1093	10.04	(7.23- 13.94)
Subtotal OSANN								11.85	(8.95- 15.69)
OSANN2	13	f	0	61	40	22	43	2.98	(1.56- 5.71)
PEZZOT	7	m	0	60	317	3	116	7.32	(2.25- 23.79)
SCHWAR	8	m	0	84	178	1	73	34.45	(4.71- 252.10)
SCHWAR	7	m	0	45	39	1	7	8.08	(0.95- 68.56)
SCHWAR	16	f	0	92	108	10	79	6.73	(3.29- 13.75)
SCHWAR	15	f	0	20	28	3	41	9.76	(2.65- 36.00)
Subtotal SCHWAR								8.40	(4.73- 14.94)
SEOW	2	f	0	19	15	67	125	2.36	(1.13- 4.95)
SIEMIA	12	m	0	162	428	5	105	7.95	(3.18- 19.85)
SOBUE	7	m	0	393	1013	27	128	1.84	(1.20- 2.83)
SOBUE	23	f	0	58	232	137	857	1.56	(1.11- 2.20)
Subtotal SOBUE								1.66	(1.27- 2.17)
SOBUE2	2	m	2	-	-	-	-	3.10	(2.40- 3.70)
SOBUE2	6	f	2	-	-	-	-	1.80	(1.40- 2.20)
Subtotal SOBUE2								2.39	(2.04- 2.79)
STASZE	24	m	0	20	653	0	158	9.94~	(0.60- 165.30)
STASZE	4	f	0	1	153	10	1660	1.08	(0.14- 8.53)
Subtotal STASZE								2.36	(0.45- 12.42)
STAYNE	4	m	0	43	567	7	333	3.61	(1.60- 8.11)
SUZUK2	13	c	0	20	10	5	15	6.00	(1.69- 21.26)
SUZUKI	3	m	0	144	217	14	99	4.69	(2.58- 8.53)
SUZUKI	7	f	0	25	27	55	133	2.24	(1.19- 4.20)
Subtotal SUZUKI								3.30	(2.14- 5.09)
SVENSS	59	f	0	50	89	22	120	3.06	(1.73- 5.43)
TIZZAN	19	c	0	88	939	25	419	1.57	(0.99- 2.49)
TOKARS	7	c	0	68	112	10	54	3.28	(1.57- 6.86)
TSUGAN	2	m	0	53	56	18	17	0.89	(0.42- 1.91)
TSUGAN	8	f	0	6	10	33	30	0.55	(0.18- 1.68)
Subtotal TSUGAN								0.77	(0.41- 1.44)
WAKAI	17	m	0	98	424	8	65	1.88	(0.87- 4.04)
WAKAI	35	f	0	13	31	46	145	1.32	(0.64- 2.74)
Subtotal WAKAI								1.56	(0.92- 2.65)
WU	5	f	0	120	87	29	62	2.95	(1.75- 4.96)
WU2	1	f	2	-	-	-	-	4.50	(3.00- 6.90)
WUWILL	25	f	0	138	351	172	601	1.37	(1.06- 1.78)
WYNDE2	9	m	0	46	512	5	105	1.89	(0.73- 4.86)
WYNDE3	28	m	0	64	264	6	88	3.56	(1.49- 8.49)
WYNDE3	75	f	0	21	56	15	76	1.90	(0.90- 4.01)
Subtotal WYNDE3								2.48	(1.41- 4.37)
WYNDE4	42	m	0	35	665	4	115	1.51	(0.53- 4.34)
WYNDE4	56	f	2	-	-	-	-	0.60	(0.13- 2.69)
Subtotal WYNDE4								1.12	(0.47- 2.66)
WYNDE6	78	m	0	1059	1797	58	617	6.27	(4.74- 8.29)
WYNDE6	413	f	0	326	275	58	673	13.76	(10.06- 18.80)
Subtotal WYNDE6								8.89	(7.22- 10.95)
XU3	21	m	0	29	68	3	31	4.41	(1.25- 15.57)
XU3	25	f	0	4	11	7	25	1.30	(0.31- 5.36)

Table 3C3 - 5

IESLC - Meta-anal of Ever Smoking (or Current if Ever not available), Cigs (or Any Prod if Cigs not avail)
Adenocarcinoma
Least adjusted

REF	NRR	SEX	AD	Number Exposed Case	Exposed Cont	Non-exposed Case	Non-exposed Cont	RR	95.00%CI
Subtotal XU3								2.57	(1.00- 6.59)
ZHENG	10	m	0	123	218	29	94	1.83	(1.14- 2.93)
ZHENG	21	f	0	33	44	119	184	1.16	(0.70- 1.93)
Subtotal ZHENG								1.48	(1.05- 2.09)
ZHOU	26	m	0	131	41	88	36	1.31	(0.77- 2.20)
ZHOU	27	f	0	30	7	96	32	1.43	(0.57- 3.57)
Subtotal ZHOU								1.34	(0.85- 2.10)
Partial Totals				8223	561194	2862	359950		

*prospective study ~ With 0.5 adjustment for zero

REF	NRR	SEX	AD	Ys	Ws	Qs	Ps
*ABRAHA	2	m	0	0.87	7.06	0.75	0.0201
*ABRAHA	5	f	0	0.96	8.71	0.50	0.0045
Subtotal ABRAHA				0.92	15.77	1.25	
ALDERS	108	m	0	1.58	5.47	0.80	0.0002
ALDERS	104	f	0	1.00	17.72	0.70	0.0000
Subtotal ALDERS				1.14	23.19	1.50	
*ANDERS	12	f	0	1.81	24.76	9.06	0.0000
BAND	2	m	2	1.41	40.10	1.76	0.0000
BARBON	122	m	0	1.97	6.39	3.75	0.0000
BECHER	12	f	1	2.38	0.87	1.21	0.0265
*BOUCOT	72	m	0	2.29	0.48	0.57	0.1114
BRESLO	1	c	0	0.35	3.39	2.44	0.5177
BROWN1	1	m	0	1.56	2.89	0.37	0.0081
BROWN1	2	f	0	1.46	6.38	0.42	0.0002
Subtotal BROWN1				1.49	9.27	0.79	
BROWN2	4	m	2	2.10	132.45	107.96	0.0000
BROWN2	3	f	2	1.93	229.82	122.55	0.0000
Subtotal BROWN2				1.99	362.27	230.51	
BUFFLE	50	m	0	1.50	4.86	0.45	0.0009
BUFFLE	45	f	0	1.39	14.95	0.54	0.0000
Subtotal BUFFLE				1.42	19.81	0.99	
BYERS1	3	m	0	1.41	5.95	0.26	0.0006
CHAN	19	m	0	3.42	0.49	2.41	0.0168
CHAN	23	f	0	0.79	9.98	1.72	0.0130
Subtotal CHAN				0.91	10.47	4.13	
CHOI	63	m	0	0.29	5.64	4.64	0.4842
CHOI	65	f	0	-0.44	3.77	10.17	0.3920
Subtotal CHOI				-0.00	9.42	14.81	
COMSTO	67	m	0	2.07	1.85	1.38	0.0049
COMSTO	79	f	0	1.57	5.57	0.74	0.0002
Subtotal COMSTO				1.69	7.42	2.13	
CORREA	36	c	1	1.72	19.23	5.23	0.0000
*CPSI	404	m	1	1.52	4.10	0.42	0.0021
*CPSI	406	f	1	0.36	3.08	2.19	0.5303
Subtotal CPSI				1.02	7.18	2.61	
*CPSII	115	m	1	2.96	3.23	9.95	0.0000
*CPSII	118	f	1	2.11	9.51	7.82	0.0000
Subtotal CPSII				2.32	12.75	17.77	
DAMBER	11	m	0	0.88	7.53	0.79	0.0161
DESTE2	17	m	2	1.46	3.99	0.26	0.0036
DOLL	83	m	0	-0.11	1.84	3.17	0.8795
DOLL	85	f	0	0.66	2.76	0.82	0.2760
Subtotal DOLL				0.35	4.60	3.99	
DORGAN	125	m	2	1.57	4.52	0.61	0.0008
DORGAN	104	f	3	1.36	35.62	0.91	0.0000
Subtotal DORGAN				1.38	40.15	1.52	
*DORN	340	m	1	1.78	20.15	6.83	0.0000
DOSEME	20	m	0	1.17	18.52	0.01	0.0000
*ENGELA	76	m	7	0.85	4.46	0.56	0.0741
FAN	4	c	0	0.33	24.61	18.68	0.1013
GAO	8	m	0	0.44	27.69	16.09	0.0208
GAO	18	f	0	0.08	34.21	42.91	0.6343
Subtotal GAO				0.24	61.90	58.99	
GER	1	c	0	0.01	14.39	20.29	0.9579
HAENSZ	39	f	0	0.11	10.36	12.37	0.7268
*HAMMON	91	m	0	1.37	1.86	0.05	0.0617
HEGMAN	4	c	0	2.26	12.50	13.98	0.0000
HINDS	24	f	3	1.36	19.35	0.48	0.0000
ISHIMA	3	c	0	1.18	5.57	0.00	0.0055

Table 3C3 - 5

IESLC - Meta-anal of Ever Smoking (or Current if Ever not available), Cigs (or Any Prod if Cigs not avail)
Adenocarcinoma
Least adjusted

REF	NRR	SEX	AD	Ys	Ws	Qs	Ps
JAHN	43	m	0	1.65	7.21	1.43	0.0000
JAIN	7	m	0	1.93	3.62	1.93	0.0002
JAIN	2	f	0	1.36	15.86	0.42	0.0000
Subtotal JAIN				1.47	19.47	2.35	
JEDRYC	21	m	0	1.36	6.35	0.15	0.0006
JOLY	51	m	0	1.49	4.55	0.37	0.0015
JOLY	50	f	0	1.12	12.19	0.08	0.0001
Subtotal JOLY				1.22	16.74	0.46	
JUSSAW	28	m	0	0.63	2.35	0.78	0.3368
KATSOU	31	f	0	0.60	6.70	2.40	0.1187
KHUDER	27	m	0	2.09	6.11	4.86	0.0000
KIHARA	29	c	0	0.53	34.43	15.42	0.0018
KOO	7	f	0	0.47	13.45	7.11	0.0817
KREYBE	20	m	0	0.94	2.79	0.19	0.1158
KREYBE	36	f	0	-0.30	7.06	15.89	0.4275
Subtotal KREYBE				0.05	9.85	16.07	
LAMTH	3	f	0	0.63	21.63	7.18	0.0036
LAMWK	4	f	0	0.75	13.20	2.74	0.0068
LAMWK2	3	m	0	-0.08	8.67	14.16	0.8206
LAMWK2	7	f	0	0.57	11.11	4.47	0.0588
Subtotal LAMWK2				0.28	19.77	18.63	
LOMBA2	3	f	0	-0.64	20.27	68.81	0.0039
LUBIN	37	m	0	-0.31	3.37	7.74	0.5649
LUBIN2	148	m	0	1.06	51.15	1.05	0.0000
LUBIN2	168	f	0	0.25	46.25	42.00	0.0913
Subtotal LUBIN2				0.67	97.40	43.05	
LUO	3	c	0	0.06	12.01	15.73	0.8449
MATOS	68	m	0	1.82	4.44	1.67	0.0001
MATSUD	12	m	0	2.88	0.49	1.38	0.0440
NOU	3	m	0	1.49	3.45	0.29	0.0056
NOU	8	f	0	-0.13	6.24	11.01	0.7504
Subtotal NOU				0.45	9.69	11.30	
ORMOS	21	m	0	1.91	0.45	0.23	0.1999
OSANN	19	m	0	2.93	13.03	38.74	0.0000
OSANN	23	f	0	2.31	35.61	43.48	0.0000
Subtotal OSANN				2.47	48.64	82.22	
OSANN2	13	f	0	1.09	9.08	0.11	0.0010
PEZZOT	7	m	0	1.99	2.76	1.72	0.0009
SCHWAR	8	m	0	3.54	0.97	5.30	0.0005
SCHWAR	7	m	0	2.09	0.84	0.66	0.0556
SCHWAR	16	f	0	1.91	7.53	3.75	0.0000
SCHWAR	15	f	0	2.28	2.26	2.62	0.0006
Subtotal SCHWAR				2.13	11.60	12.33	
SEOW	2	f	0	0.86	7.03	0.82	0.0226
SIEMIA	12	m	0	2.07	4.59	3.49	0.0000
SOBUE	7	m	0	0.61	20.67	7.24	0.0056
SOBUE	23	f	0	0.45	33.31	18.95	0.0099
Subtotal SOBUE				0.51	53.98	26.19	
SOBUE2	2	m	2	1.13	82.01	0.40	0.0000
SOBUE2	6	f	2	0.59	75.22	28.31	0.0000
Subtotal SOBUE2				0.87	157.22	28.71	
STASZE	24	m	0	2.30	0.49	0.58	0.1092
STASZE	4	f	0	0.08	0.90	1.13	0.9382
Subtotal STASZE				0.86	1.39	1.72	
STAYNE	4	m	0	1.28	5.85	0.04	0.0019
SUZUK2	13	c	0	1.79	2.40	0.84	0.0055
SUZUKI	3	m	0	1.55	10.74	1.28	0.0000
SUZUKI	7	f	0	0.81	9.73	1.52	0.0119
Subtotal SUZUKI				1.19	20.48	2.80	
SVENSS	59	f	0	1.12	11.76	0.08	0.0001
TIZZAN	19	c	0	0.45	18.24	10.26	0.0538
TOKARS	7	c	0	1.19	7.03	0.00	0.0016
TSUGAN	2	m	0	-0.11	6.62	11.42	0.7728
TSUGAN	8	f	0	-0.61	3.03	9.89	0.2916
Subtotal TSUGAN				-0.27	9.65	21.31	
WAKAI	17	m	0	0.63	6.54	2.13	0.1071
WAKAI	35	f	0	0.28	7.26	6.17	0.4522
Subtotal WAKAI				0.45	13.79	8.30	
WU	5	f	0	1.08	14.20	0.20	0.0000
WU2	1	f	2	1.50	22.15	2.03	0.0000
WUWILL	25	f	0	0.32	56.90	44.44	0.0166

Table 3C3 - 5

IESLC - Meta-anal of Ever Smoking (or Current if Ever not available), Cigs (or Any Prod if Cigs not avail)
Adenocarcinoma
Least adjusted

REF	NRR	SEX	AD	Ys	Ws	Qs	Ps
WYNDE2	9	m	0	0.63	4.29	1.38	0.1887
WYNDE3	28	m	0	1.27	5.06	0.02	0.0043
WYNDE3	75	f	0	0.64	6.88	2.15	0.0922
Subtotal WYNDE3				0.91	11.95	2.18	
WYNDE4	42	m	0	0.41	3.46	2.15	0.4408
WYNDE4	56	f	2	-0.51	1.67	4.91	0.5087
Subtotal WYNDE4				0.11	5.14	7.05	
WYNDE6	78	m	0	1.84	49.11	19.76	0.0000
WYNDE6	413	f	0	2.62	39.32	79.30	0.0000
Subtotal WYNDE6				2.19	88.43	99.06	
XU3	21	m	0	1.48	2.41	0.19	0.0213
XU3	25	f	0	0.26	1.91	1.69	0.7180
Subtotal XU3				0.94	4.32	1.88	
ZHENG	10	m	0	0.60	17.29	6.17	0.0121
ZHENG	21	f	0	0.15	14.95	16.59	0.5668
Subtotal ZHENG				0.39	32.24	22.76	
ZHOU	26	m	0	0.27	14.05	12.24	0.3154
ZHOU	27	f	0	0.36	4.59	3.27	0.4448
Subtotal ZHOU				0.29	18.64	15.52	

	N	116
	NS	81
	Wt	1797.78
Het	Chi	1066.51
Het	df	115
Het	P	***
Fixed	RR	3.32
	RRl	3.17
	RRu	3.48
	P	+++
Random	RR	2.90
	RRl	2.48
	RRu	3.38
	P	+++
Asymm	P	*

Table 3C3 - 6

IESLC - Meta-anal of Ever Smoking (or Current if Ever not available), Cigs (or Any Prod if Cigs not avail)
Adenocarcinoma
Least adjusted

		Sex		
	combined	male	female	Total
N	11	56	49	116
NS	11	55	48	114
Wt	153.80	667.31	976.66	1797.78
Het Chi	73.30	289.46	644.54	1066.51
Het df	10	55	48	115
Het P	***	***	***	***
Fixed RR	2.14	4.04	3.12	3.32
RRl	1.83	3.75	2.93	3.17
RRu	2.51	4.36	3.32	3.48
P	+++	+++	+++	+++
Random RR	2.38	3.68	2.36	2.90
RRl	1.51	3.00	1.84	2.48
RRu	3.74	4.51	3.04	3.38
P	+++	+++	+++	+++
Between Chi				59.21
Between df				2
Between P				***
Btwn(F) P				*
Btwn(R) P				*

Table 3C3 - 7

IESLC - Meta-anal of Ever Smoking (or Current if Ever not available), Cigs (or Any Prod if Cigs not avail)
Adenocarcinoma
Excluded studies (and stage at which they were excluded)

1	BOUCHA BUELL LAURIL MZILEN
2	BERRIN CHEN RESTRE TAO ULMER
5	RIMING TANG2 WYNDE5 WYNDE8
6	BLOT1 BLOT2 BLOT3 BYERS2 HIRAOK HIRAY2 JONES LICKIN MOLLO MRFIT MURATA NOTANI PERSHA SAARIK SCHWA2 SHIMIZ SITAS VANDER WARSIN WATSON WYNDER
8	ABELIN AGUDO AKIBA AMANDU AMES ARCHER ARMADA AUSTIN AUVINE AXELSO AXELSS BENSHL BEST BLOHMK BLOT4 BOFFET BRETT BROCKM BROSS CARPEN CASCO2 CASCOR CEDERL CHANG CHATZI CHEN2 CHEN3 CHIAZZ CHOW CHYOU COOKSO DARBY DAVEYS DEAN DEAN2 DEAN3 DEKLER DESTEF DOCKER DOLL2 DORANT DROSTE DU DUNN EBELIN ENSTRO ESAKI GAO2 GARCIA GARDIN GARSHI GENG GILLIS GODLEY GOLLED GOODMA GRAHAM GREGOR GSELL HAMMO2 HANSEN HEIN HENNEK HIRAYA HITOSU HOLE HOROWI HORWIT HU HU2 HUANG HUMBLE JARUP JARVHO JIANG KAISE2 KAISER KANELL KAUFMA KELLER KINLEN KJUUS KNEKT KO KOHLME KOULUM KREUZE KUBIK LANGE LAUSSM LEI LEMARC LETOUR LEVIN LIAW LIDDEL LIU LIU2 LIU3 LIU4 LIU5 LOMBAR MACLEN MAGNUS MARSH MARSH2 MARTIS MASTRA MCCONN MCDUFF MCLAUG MIGRAN MILLER MILLS MRFITR NAM NOTAN2 ODRISC PARKIN PASTOR PAWLEG PERNU PERSH2 PETO PEZZO2 PIKE PISANI POFFIJ POLEDN PRESCO QIAO QIAO2 RACHTA RADZIK RANDIG REN RONCO ROOTS ROTHSC SADOWS SANKAR SEGI SEGI2 SHAW SIMARA SIMONA SPEIZE SPITZ STOCKS STOCKW STUCKE SUN TANG TENKAN TOUSEY TULINI TVERDA VEIERO WALD WANG WANG2 WANG3 WANG4 WICKLU WIGLE WILKIN WUNSCH XIANGZ XU XU2 XU4 YAMAGU YONG YUAN ZHANG
10	BENHAM VUTUC WYNDE7

Table 3C3 - 8
Potentially overlapping studies

REF	REFGP	PRINC	. OVERLAP/LINK
LUBIN2	LUBIN2	1	Lubin-combined
CHAN	CHAN	1	Chan/LamWK2
LAMWK2	LAMWK2	1	Chan/LamWK2
LAMTH	LAMTH	1	KOO/LAMTH/LAMWK
KOO	KOO	1	KOO/LAMTH/LAMWK
LAMWK	LAMWK	1	KOO/LAMTH/LAMWK
ISHIMA	AKIBA	2	AKIBA/ISHIMA
BYERS1	BYERS1	2	GRAHAM/BROSS/BYERS1
OSANN2	KAISER	2	KAISER/OSANN2
WYNDE6	WYNDE6	1	WYNDE5/6/7/8
CPSI	CPSI	1	CPSI overall
SOBUE2	SOBUE2	1	SOBUE2/MATSUD
MATSUD	MATSUD	1	SOBUE2/MATSUD
JAHN	BOFFET	2	Subset of BOFFET
LUBIN	XIANGZ	2	LUBIN/XIANGZ/QIAO

Table 3C3 - 9
Most adjusted - insufficient data for metaanalysis

REF	NRR	SEX	AGEL	AGEH	RACE	YF	LC TYPE	LOC	START	ST	NLC	R	VB	P	H	AD	SM	PRODUCT	DENOM	De
CHEN	11	c	0	0	all	-	a	As:oth	1987	CC	323	n	ot	n	y	2	ev	cig+/-ot	nev cigs	ot
CHYOU	6	m	0	0	jap	0	a	NAmer	1965	pr	227	n	bl	n	y	1	cu	cig+/-ot	nev cigs	or
JIANG	5	m	0	0	all	-	a	As:Chi	1984	CC	125	n	ot	n	n	0	ev	all/unsp	nev any	ot
JIANG	6	f	0	0	all	-	a	As:Chi	1984	CC	125	n	ot	n	n	0	ev	all/unsp	nev any	ot
KOO	4	f	0	0	all	-	a	As:HK	1981	CC	200	n	bl	n	n	0	ev	all/unsp	nev any	or
WANG	4	c	0	0	all	-	a	As:Chi	1990	CC	390	n	ot	*	y	0	ev	all/unsp	nev any	ot

REF	NRR	RR	SIG	RRDATA comment
CHEN	11	2.08	y	P < 0.001
CHYOU	6	6.80		0
JIANG	5	*	n	0
JIANG	6	*	n	0
KOO	4	1.88		0
WANG	4	2.22	n	P>0.05

Table 3C4 -

IESLC - Meta-anal of Current Smoking (or Ever if Current not available), Cigs (or Any Prod if Cigs not avail)
Adenocarcinoma

This analysis is restricted to results for:
1) Non-dose-response data
2) Results complete enough for use in metaanalysis

Within each study, results are then selected (in the following order of preference, within each sex) for:
3) SMKSTA: current smokers, ever smokers
4) PRODUCT: cigarettes regardless of other products, cigarettes only, all/unspec
5) CIGTYPE: all/unspecified, MC regardless of HR, MC only
6) DENOM: never smoked anything, never smoked cigarettes, (never +1 = +long term ex, +2 = +amount unknown, +3 = never cigs+long term ex)
7) Followup period (YF, prospective studies): whole study (coded as 0) or longest available
8) LCtype: adeno or nearest available, but not squamous. (q = squamous, s = small, a = adeno, l = large, KII = Kreyberg II, al = alveolar, br = bronchiolar, u = undifferentiated)
9) Race: all or nearest available, otherwise by race (wh or w = white, bl or b = black, hi = hispanic ch = chinese, jap = japanese, haw = hawaiian, w+o = white + oriental, sca = scandinavian, as = asian)
10) For overlapping studies: principal rather than subsidiary studies
Finally by Age: whole study (coded as 0) if available, otherwise by widest available age group
and then for single sex results (m, f) in preference to combined sex results (c).

Results adjusted (AD) for the most potential confounders are then chosen in Sections -1 to -3
(and those which actually differ from the adjusted results in Table 3C3 - 1 are marked 'x' in Section -1)
and results adjusted for the least confounders in Sections -4 to -6. (Those least adjusted results which
actually differ from the most adjusted as marked 'x' in column X in Section -4)
(Results adjusted for an unknown number of confounder(s) are coded as 20.)

Section -7 shows excluded studies, together with the stage (as above) at which no qualifying
results were found.

Section -8 lists the potentially overlapping studies which have been included (1=principal, 2=subsidiary).

Section -9 lists any results which would have been included in preference except that they had data not complete
enough for use in meta-analysis, with their significance (yes/no), if known, and any further comment as entered
on the database.

In addition to those mentioned above, the following fields, levels and abbreviations are used:
* or nk = not known, n = no, y = yes, ot = other
ev = ever, cu = current, nev = never
all/unspec = all or unspecified, cig+/-ot = cigarettes irrespective of other products (cigar, pipe etc)
MC = manufactured cigarettes, HR = hand-rolled cigarettes
REF: 6-character study reference
NRR: number of the RR on the database within the study
ST : study type (CC = case control, pr or prosp = prospective)
NLC: number of lung cancer cases in whole study
R : risky occupational population (n = no, m = mining, o = other risky)
VB : national cigarette type (V = at least 75% Virginia, bl = at least 75% blended, ot = other)
P : any proxy use
H : full histological confirmation
De : derivation of RR/CI (or = original, st = standard method, ot = other method of estimation)

Table 3C4 - 1

IESLC - Meta-anal of Current Smoking (or Ever if Current not available), Cigs (or Any Prod if Cigs not avail)
Adenocarcinoma
Most adjusted

REF	NRR	3C3	SEX	AGEL	AGEH	RACE	YF	LC TYPE	LOC	START	ST	NLC	R	VB	P	H	AD	SM	PRODUCT	DENOM	De
ABRAHA	2		m	0	0	all	0	a	Eu:est	1975	pr	571	n	bl	n	n	0	ev	all/unsp	nev any	ot
ABRAHA	5		f	0	0	all	0	a	Eu:est	1975	pr	571	n	bl	n	n	0	ev	all/unsp	nev any	ot
ALDERS	95		m	0	0	all	-	not q+s	Eu:UK	1977	CC	1448	n	V	n	n	1	ev	cig+/-ot	nev any	ot
ALDERS	45		f	0	0	all	-	not q+s	Eu:UK	1977	CC	1448	n	V	n	n	1	ev	cig only	nev any	ot
ANDERS	12		f	0	0	all	0	a	NAmer	1986	pr	343	n	bl	n	n	0	ev	cig+/-ot	nev cigs	st
BAND	2		m	0	0	all	-	a	NAmer	1983	CC	2831	n	V	y	y	2	ev	cig only	nev any	ot
BARBON	100	x	m	0	0	all	-	a	Eu:wst	1979	CC	755	n	bl	y	y	1	cu	all/unsp	nev any	or
BECHER	12		f	0	0	all	-	not q+s	Eu:Ger	1985	CC	194	n	bl	n	y	1	ev	all/unsp	nev any	or
BOUCOT	147		m	0	0	all	0	a	NAmer	1951	pr	121	n	bl	n	n	2	cu	cig only	nev any	ot
BRESLO	1		c	0	0	all	-	a	NAmer	1949	CC	518	n	bl	n	y	0	ev	cig+/-ot	nev+1	st
BROWN1	3		m	0	0	wh	-	a	NAmer	1979	CC	102	n	bl	y	y	1	ev	cig+/-ot	nev cigs	or
BROWN1	4		f	0	0	wh	-	a	NAmer	1979	CC	102	n	bl	y	y	1	ev	cig+/-ot	nev cigs	or
BROWN2	14	x	m	0	0	wh	-	a	NAmer	1984	CC	14596	n	bl	n	y	2	cu	cig+/-ot	nev cigs	or
BROWN2	13	x	f	0	0	wh	-	a	NAmer	1984	CC	14596	n	bl	n	y	2	cu	cig+/-ot	nev cigs	or
BUFFLE	50		m	0	0	wh	-	a	NAmer	1976	CC	943	n	bl	y	n	0	ev	cig+/-ot	nev cigs	ot
BUFFLE	71	x	f	0	0	w-hi	-	a	NAmer	1976	CC	943	n	bl	y	n	0	cu	cig+/-ot	nev cigs	st
BYERS1	3		m	0	0	wh	-	a	NAmer	1957	CC	1002	n	bl	n	n	0	ev	cig+/-ot	nev cigs	st
CHAN	19		m	0	0	all	-	a+l	As:HK	1976	CC	397	n	bl	n	n	0	ev	cig+/-ot	nev any	ot
CHAN	23		f	0	0	all	-	a+l	As:HK	1976	CC	397	n	bl	n	n	0	ev	cig+/-ot	nev any	st
CHOI	63		m	0	0	all	-	a	As:oth	1985	CC	375	n	bl	n	n	0	ev	cig+/-ot	nev cigs	st
CHOI	65		f	0	0	all	-	a	As:oth	1985	CC	375	n	bl	n	n	0	ev	cig+/-ot	nev cigs	st
COMSTO	24	x	m	0	0	all	-	a	NAmer	1975	ot	258	n	bl	n	n	0	cu	cig+/-ot	nev cigs	st
COMSTO	31	x	f	0	0	all	-	a	NAmer	1975	ot	258	n	bl	n	n	0	cu	cig+/-ot	nev cigs	st
CORREA	44	x	c	0	0	all	-	a	NAmer	1979	CC	1359	n	bl	y	n	1	cu	cig+/-ot	nev cigs	or
CPSI	404		m	0	0	all	2	a	NAmer	1959	pr	5138	n	bl	n	n	1	cu	cig only	nev any	ot
CPSI	406		f	0	0	all	2	a	NAmer	1959	pr	5138	n	bl	n	n	1	cu	cig only	nev any	ot
CPSII	115		m	0	0	all	2	a	NAmer	1982	pr	3229	n	bl	n	n	1	cu	cig only	nev any	st
CPSII	118		f	0	0	all	2	a	NAmer	1982	pr	3229	n	bl	n	n	1	cu	cig+/-ot	nev cigs	st
DAMBER	32		m	0	0	all	-	a+al+br	Eu:Sca	1972	CC	579	n	bl	y	n	1	ev	all/unsp	nev any	or
DESTE2	17		m	0	0	all	-	a	SCAmer	1993	CC	463	n	bl	n	n	2	ev	all/unsp	nev any	or
DOLL	87		m	0	0	all	-	KII	Eu:UK	1948	CC	1465	n	V	n	n	1	ev	all/unsp	nev any	ot
DOLL	89		f	0	0	all	-	KII	Eu:UK	1948	CC	1465	n	V	n	n	1	ev	all/unsp	nev any	ot
DORGAN	125		m	0	0	wh	-	a	NAmer	1980	CC	2026	n	bl	y	y	2	ev	cig+/-ot	nev any	or
DORGAN	104		f	0	0	all	-	a	NAmer	1980	CC	2026	n	bl	y	y	3	ev	cig+/-ot	nev any	or
DORN	340		m	0	0	wh	8	a	NAmer	1954	pr	5097	n	bl	n	n	1	cu	cig only	nev any	ot
DOSEME	4		m	0	0	all	-	not q+s	Eu:bal	1979	CC	1210	n	bl	n	n	2	ev	cig+/-ot	nev cigs	or
ENGELA	70	x	m	0	0	all	0	a	Eu:Sca	1964	pr	435	n	bl	n	n	7	cu	cig+/-ot	nev cigs	ot
FAN	4		c	0	0	all	-	a	As:Chi	1990	CC	403	n	ot	y	n	0	ev	cig+/-ot	nev cigs	ot
GAO	3		m	0	0	all	-	a	As:Chi	1984	CC	1405	n	ot	n	n	2	ev	cig+/-ot	nev cigs	or
GAO	13		f	0	0	all	-	a	As:Chi	1984	CC	1405	n	ot	n	n	2	ev	cig+/-ot	nev cigs	or
GER	9		c	0	0	all	-	a	As:oth	1990	CC	141	n	ot	y	n	8	ev	all/unsp	nev any	ot
HAENSZ	37	x	f	0	0	all	-	a	NAmer	1955	CC	158	n	bl	n	y	0	cu	cig+/-ot	nev any	st
HAMMON	84		m	0	0	wh	0	a	NAmer	1952	pr	448	n	bl	n	n	1	ev	cig+/-ot	nev any	ot
HEGMAN	4		c	0	0	all	-	a	NAmer	1989	CC	282	n	bl	y	y	0	ev	all/unsp	nev any	st
HINDS	24		f	0	0	o	-	a	NAmer	1968	CC	292	n	bl	n	n	3	ev	all/unsp	nev any	st
ISHIMA	8		c	0	0	all	-	a	As:Jap	1961	CC	180	n	bl	y	y	5	ev	all/unsp	nev any	st
JAHN	8	x	m	0	0	all	-	a	Eu:Ger	1988	CC	1004	n	bl	n	n	0	cu	cig+/-ot	nev any	st
JAIN	17	x	m	0	0	all	-	a	NAmer	1981	CC	845	n	V	y	n	0	cu	cig+/-ot	nev cigs	st
JAIN	12	x	f	0	0	all	-	a	NAmer	1981	CC	845	n	V	y	n	0	cu	cig+/-ot	nev cigs	st
JEDRYC	26	x	m	0	0	all	-	a	Eu:est	1980	CC	1630	n	bl	y	n	0	cu	cig+/-ot	nev any	st
JOLY	51		m	0	0	all	-	a	SCAmer	1978	CC	826	n	bl	n	n	0	ev	cig+/-ot	nev any	st
JOLY	50		f	0	0	all	-	a	SCAmer	1978	CC	826	n	bl	n	n	0	ev	cig+/-ot	nev any	st
JUSSAW	28		m	0	0	all	-	KII	As:Ind	1964	CC	792	n	V	n	n	0	ev	cig only	nev any	st
KATSOU	12	x	f	0	0	all	-	a	Eu:bal	1987	CC	101	n	bl	n	n	1	cu	all/unsp	nev any	or
KHUDER	17	x	m	0	0	all	-	a	NAmer	1985	CC	482	n	bl	n	y	0	cu	cig+/-ot	nev cigs	or
KIHARA	5	x	c	0	0	jap	-	a	As:Jap	1991	CC	440	n	bl	n	n	0	cu	all/unsp	nev any	st
KOO	7		f	0	0	all	-	a+l	As:HK	1981	CC	200	n	bl	n	n	0	ev	all/unsp	nev any	st
KREYBE	8		m	0	0	all	-	KII	Eu:Sca	1948	CC	300	n	bl	n	y	1	ev	all/unsp	nev any	ot
KREYBE	27		f	0	0	all	-	KII	Eu:Sca	1948	CC	300	n	bl	n	y	1	ev	all/unsp	nev any	ot
LAMTH	3		f	0	0	ch	-	a	As:HK	1983	CC	445	n	bl	n	n	0	ev	all/unsp	nev any	or
LAMWK	4		f	0	0	ch	-	a	As:HK	1981	CC	163	n	bl	n	n	0	ev	all/unsp	nev any	st
LAMWK2	3		m	0	0	all	-	a	As:HK	1976	CC	480	n	bl	n	n	0	ev	all/unsp	nev any	st
LAMWK2	7		f	0	0	all	-	a	As:HK	1976	CC	480	n	bl	n	n	0	ev	all/unsp	nev any	st
LOMBA2	3		f	0	0	all	-	not q+u	NAmer	1960	CC	225	n	bl	n	n	0	ev	cig+/-ot	nev cigs	st
LUBIN	37		m	0	0	all	-	KII	As:Chi	1984	CC	427	m	ot	y	n	0	ev	cig+/-ot	nev any	st
LUBIN2	252	x	m	0	0	all	-	a	Eu:mul	1976	CC	7804	n	bl	n	y	0	cu	cig+/-ot	nev any	st
LUBIN2	264	x	f	0	0	all	-	a	Eu:mul	1976	CC	7804	n	bl	n	y	0	cu	cig+/-ot	nev any	st
LUO	9		c	0	0	all	-	a	As:Chi	1990	CC	102	n	ot	n	y	20	ev	cig+/-ot	nev cigs	or
MATOS	53	x	m	0	0	all	-	a	SCAmer	1994	CC	200	n	bl	n	n	2	cu	cig+/-ot	nev any	or
MATSUD	12		m	0	0	all	-	a	As:Jap	1965	CC	179	n	bl	n	n	0	ev	cig+/-ot	nev cigs	ot
NOU	3		m	0	0	all	-	a	Eu:Sca	1971	CC	273	n	bl	y	n	0	ev	all/unsp	nev any	st
NOU	8		f	0	0	all	-	a	Eu:Sca	1971	CC	273	n	bl	y	n	0	ev	all/unsp	nev any	st

Table 3C4 - 1

IESLC - Meta-anal of Current Smoking (or Ever if Current not available), Cigs (or Any Prod if Cigs not avail)
Adenocarcinoma
Most adjusted

REF	NRR	3C3	SEX	AGEL	AGEH	RACE	YF	LC TYPE	LOC	START	ST	NLC	R	VB	P	H	AD	SM	PRODUCT	DENOM	De
ORMOS	21		m	0	0	all	-	a	Eu:est	1947	CC	119	n	bl	y	y	0	ev	cig+/-ot	nev any	ot
OSANN	39	x	m	0	0	all	-	a	NAmer	1984	CC	1986	n	bl	n	n	2	cu	cig+/-ot	nev cigs	or
OSANN	40	x	f	0	0	all	-	a	NAmer	1984	CC	1986	n	bl	n	n	2	cu	cig+/-ot	nev cigs	or
OSANN2	32	x	f	0	0	all	-	KII	NAmer	1964	ot	217	n	bl	n	y	1	cu	cig+/-ot	nev cigs	or
PEZZOT	7		m	0	0	all	-	a	SCAmer	1987	CC	215	n	bl	n	y	0	ev	cig only	nev cigs	st
SCHWAR	8		m	40	54	wh	-	a	NAmer	1984	CC	5588	n	bl	y	y	0	ev	cig+/-ot	nev cigs	st
SCHWAR	7		m	40	54	bl	-	a	NAmer	1984	CC	5588	n	bl	y	y	0	ev	cig+/-ot	nev cigs	st
SCHWAR	16		f	40	54	wh	-	a	NAmer	1984	CC	5588	n	bl	y	y	0	ev	cig+/-ot	nev cigs	st
SCHWAR	15		f	40	54	bl	-	a	NAmer	1984	CC	5588	n	bl	y	y	0	ev	cig+/-ot	nev cigs	st
SEOW	2		f	0	0	ch	-	a	As:oth	1997	CC	153	n	bl	n	y	0	ev	cig+/-ot	nev cigs	st
SIEMIA	8		m	0	0	all	-	a	NAmer	1979	CC	857	n	V	y	y	7	ev	cig+/-ot	nev cigs	or
SOBUE	36	x	m	0	0	all	-	a	As:Jap	1986	CC	1376	n	bl	n	y	1	cu	cig+/-ot	nev cigs	or
SOBUE	46	x	f	0	0	all	-	a	As:Jap	1986	CC	1376	n	bl	n	y	1	cu	cig+/-ot	nev cigs	or
SOBUE2	2		m	0	0	all	-	a	As:Jap	1965	CC	2083	n	bl	n	n	2	cu	cig+/-ot	nev any	or
SOBUE2	6		f	0	0	all	-	a	As:Jap	1965	CC	2083	n	bl	n	n	2	cu	cig+/-ot	nev any	or
STASZE	24		m	0	0	all	-	a	Eu:est	1954	CC	281	n	bl	n	y	0	ev	cig+/-ot	nev any	ot
STASZE	4		f	0	0	all	-	a	Eu:est	1954	CC	281	n	bl	n	y	0	ev	all/unsp	nev any	st
STAYNE	4		m	0	0	all	-	a	NAmer	1969	CC	420	n	bl	n	n	0	ev	all/unsp	nev any	st
SUZUK2	16		c	0	0	all	-	a	SCAmer	1991	CC	123	n	bl	n	y	3	ev	all/unsp	nev any	or
SUZUKI	10	x	m	0	0	all	-	a	As:Jap	1978	CC	238	n	bl	n	y	2	cu	cig+/-ot	nev any	or
SUZUKI	14	x	f	0	0	all	-	a	As:Jap	1978	CC	238	n	bl	n	y	2	cu	cig+/-ot	nev any	or
SVENSS	99	x	f	0	0	all	-	a	Eu:Sca	1983	CC	210	n	bl	n	n	1	cu	all/unsp	nev any	ot
TIZZAN	19		c	0	0	all	-	a	Eu:wst	1959	CC	1358	n	bl	n	n	0	ev	all/unsp	nev any	st
TOKARS	8		c	0	0	all	-	a	Eu:est	1966	ot	162	o	bl	n	y	3	ev	all/unsp	nev any	or
TSUGAN	3	x	m	0	0	all	-	a	As:Jap	1976	CC	134	n	bl	n	y	0	cu	all/unsp	nev any	st
TSUGAN	9	x	f	0	0	all	-	a	As:Jap	1976	CC	134	n	bl	n	y	0	cu	all/unsp	nev any	or
WAKAI	12	x	m	0	0	all	-	a	As:Jap	1988	CC	333	n	bl	n	y	1	cu	all/unsp	nev any	or
WAKAI	30	x	f	0	0	all	-	a	As:Jap	1988	CC	333	n	bl	n	y	1	cu	all/unsp	nev any	or
WU	7	x	f	0	0	wh	-	a	NAmer	1981	CC	220	n	bl	n	y	2	cu	all/unsp	nev any	or
WU2	1		f	0	0	all	-	a	NAmer	1983	CC	336	n	bl	n	y	2	cu	all/unsp	nev any	or
WUWILL	11		f	0	0	all	-	a	As:Chi	1985	CC	965	n	ot	n	n	3	ev	cig+/-ot	nev cigs	or
WYNDE2	9		m	0	0	all	-	KII	NAmer	1962	CC	404	n	bl	n	y	0	ev	cig+/-ot	nev any	st
WYNDE3	30	x	m	0	0	all	-	KII	NAmer	1966	CC	350	n	bl	n	y	0	cu	all/unsp	nev any	st
WYNDE3	75		f	0	0	all	-	KII	NAmer	1966	CC	350	n	bl	n	y	0	ev	cig+/-ot	nev any	st
WYNDE4	42		m	0	0	all	-	a	NAmer	1948	CC	684	n	bl	y	n	0	ev	all/unsp	nev any	st
WYNDE4	56		f	0	0	all	-	a	NAmer	1948	CC	684	n	bl	y	n	2	ev	all/unsp	nev any	ot
WYNDE6	15	x	m	0	0	all	-	KII	NAmer	1969	CC	4423	n	bl	n	y	0	cu	cig+/-ot	nev any	st
WYNDE6	204	x	f	0	0	all	-	KII	NAmer	1969	CC	4423	n	bl	n	y	0	cu	cig+/-ot	nev cigs	st
XU3	22		m	0	0	all	-	KII	As:Chi	1981	CC	135	n	ot	n	n	1	ev	all/unsp	nev any	ot
XU3	26		f	0	0	all	-	KII	As:Chi	1981	CC	135	n	ot	n	n	1	ev	all/unsp	nev any	ot
ZHENG	10		m	0	0	all	-	a	As:Chi	1982	CC	540	n	ot	*	y	0	ev	cig+/-ot	nev cigs	st
ZHENG	21		f	0	0	all	-	a	As:Chi	1982	CC	540	n	ot	*	y	0	ev	cig+/-ot	nev cigs	st
ZHOU	26		m	0	0	all	-	a	As:Chi	1978	CC	1360	n	ot	n	n	0	ev	all/unsp	nev any	st
ZHOU	27		f	0	0	all	-	a	As:Chi	1978	CC	1360	n	ot	n	n	0	ev	all/unsp	nev any	st

Cigarette type is all/unspec for all RRs

except for the following:

REF	NRR	CIGTYPE
ALDERS	45	MC only
CHAN	19	MC+-HR
CHAN	23	MC+-HR
JUSSAW	28	MC only

Table 3C4 - 2

IESLC - Meta-anal of Current Smoking (or Ever if Current not available), Cigs (or Any Prod if Cigs not avail)
Adenocarcinoma
Most adjusted

REF	NRR	SEX	AD	Number Exposed Case	Number Exposed Cont	Non-exposed Case	Non-exposed Cont	RR	95.00%CI
*ABRAHA	2	m	0	59	10351	8	3365	2.40	(1.15- 5.01)
*ABRAHA	5	f	0	19	5256	16	11589	2.62	(1.35- 5.09)
Subtotal ABRAHA								2.52	(1.54- 4.12)
ALDERS	95	m	1	-	-	-	-	4.41	(1.92- 10.13)
ALDERS	45	f	1	-	-	-	-	3.69	(2.32- 5.88)
Subtotal ALDERS								3.85	(2.57- 5.78)
*ANDERS	12	f	0	99	96164	33	195158	6.09	(4.11- 9.03)
BAND	2	m	2	-	-	-	-	4.10	(3.01- 5.59)
BARBON	100	m	1	-	-	-	-	7.90	(3.60- 17.40)
BECHER	12	f	1	-	-	-	-	10.83	(1.32- 88.70)
*BOUCOT	147	m	2	-	-	-	-	10.95	(0.65- 183.57)
BRESLO	1	c	0	40	394	4	56	1.42	(0.49- 4.12)
BROWN1	3	m	1	-	-	-	-	4.49	(1.44- 13.98)
BROWN1	4	f	1	-	-	-	-	3.95	(1.76- 8.80)
Subtotal BROWN1								4.12	(2.14- 7.95)
BROWN2	14	m	2	-	-	-	-	9.10	(7.60- 10.80)
BROWN2	13	f	2	-	-	-	-	7.20	(6.20- 8.30)
Subtotal BROWN2								7.92	(7.08- 8.86)
BUFFLE	50	m	0	-	-	-	-	4.50	(1.85- 10.95)
BUFFLE	71	f	0	56	110	7	112	8.15	(3.56- 18.65)
Subtotal BUFFLE								6.18	(3.37- 11.33)
BYERS1	3	m	0	47	695	7	424	4.10	(1.83- 9.15)
CHAN	19	m	0	56	160	0	43	30.63~	(1.85- 505.72)
CHAN	23	f	0	24	38	40	139	2.19	(1.18- 4.08)
Subtotal CHAN								2.48	(1.35- 4.55)
CHOI	63	m	0	46	465	7	95	1.34	(0.59- 3.06)
CHOI	65	f	0	5	26	49	164	0.64	(0.23- 1.77)
Subtotal CHOI								1.00	(0.53- 1.89)
COMSTO	24	m	0	30	100	2	84	12.60	(2.92- 54.28)
COMSTO	31	f	0	23	52	8	115	6.36	(2.67- 15.16)
Subtotal COMSTO								7.60	(3.60- 16.04)
CORREA	44	c	1	-	-	-	-	6.70	(4.30- 10.60)
*CPSI	404	m	1	-	-	-	-	4.58	(1.74- 12.05)
*CPSI	406	f	1	-	-	-	-	1.43	(0.47- 4.39)
Subtotal CPSI								2.78	(1.34- 5.78)
*CPSII	115	m	1	-	-	-	-	19.22	(6.46- 57.16)
*CPSII	118	f	1	-	-	-	-	8.23	(4.36- 15.54)
Subtotal CPSII								10.21	(5.89- 17.67)
DAMBER	32	m	1	-	-	-	-	2.40	(1.10- 5.30)
DESTE2	17	m	2	-	-	-	-	4.30	(1.60- 11.40)
DOLL	87	m	1	-	-	-	-	0.95	(0.22- 4.02)
DOLL	89	f	1	-	-	-	-	1.97	(0.60- 6.46)
Subtotal DOLL								1.47	(0.59- 3.69)
DORGAN	125	m	2	-	-	-	-	4.80	(1.90- 12.00)
DORGAN	104	f	3	-	-	-	-	3.90	(2.80- 5.40)
Subtotal DORGAN								3.99	(2.93- 5.44)
*DORN	340	m	1	-	-	-	-	5.95	(3.85- 9.22)
DOSEME	4	m	2	-	-	-	-	2.60	(1.70- 4.20)
*ENGELA	70	m	7	-	-	-	-	7.06	(2.69- 18.50)
FAN	4	c	0	67	595	45	556	1.39	(0.94- 2.07)
GAO	3	m	2	-	-	-	-	1.60	(1.10- 2.40)
GAO	13	f	2	-	-	-	-	1.50	(1.00- 2.10)
Subtotal GAO								1.55	(1.18- 2.02)
GER	9	c	8	-	-	-	-	1.10	(0.55- 2.19)
HAENSZ	37	f	0	16	94	37	236	1.09	(0.58- 2.05)
*HAMMON	84	m	1	-	-	-	-	3.67	(0.87- 15.45)
HEGMAN	4	c	0	83	1202	15	2080	9.58	(5.50- 16.67)
HINDS	24	f	3	-	-	-	-	3.89	(2.49- 6.07)
ISHIMA	8	c	5	-	-	-	-	15.00	(2.31- 631.48)
JAHN	8	m	0	75	269	8	138	4.81	(2.26- 10.26)
JAIN	17	m	0	60	118	4	85	10.81	(3.78- 30.87)
JAIN	12	f	0	69	99	24	214	6.21	(3.69- 10.47)
Subtotal JAIN								6.93	(4.35- 11.07)
JEDRYC	26	m	0	68	516	7	289	5.44	(2.47- 12.00)
JOLY	51	m	0	72	709	5	218	4.43	(1.77- 11.10)
JOLY	50	f	0	33	122	25	283	3.06	(1.75- 5.37)
Subtotal JOLY								3.38	(2.10- 5.46)
JUSSAW	28	m	0	3	77	13	624	1.87	(0.52- 6.71)
KATSOU	12	f	1	-	-	-	-	1.70	(0.75- 3.89)
KHUDER	17	m	0	92	-	7	-	8.20	(3.60- 18.40)
KIHARA	5	c	0	103	162	78	237	1.93	(1.35- 2.76)

Table 3C4 - 2

IESLC - Meta-anal of Current Smoking (or Ever if Current not available), Cigs (or Any Prod if Cigs not avail)
Adenocarcinoma
Most adjusted

REF	NRR	SEX	AD	Number Exposed Case	Cont	Non-exposed Case	Cont	RR	95.00%CI
KOO	7	f	0	34	63	46	137	1.61	(0.94- 2.74)
KREYBE	8	m	1	-	-	-	-	2.44	(0.76- 7.86)
KREYBE	27	f	1	-	-	-	-	1.28	(0.60- 2.74)
Subtotal KREYBE								1.55	(0.82- 2.93)
LAMTH	3	f	0	79	51	131	158	1.87	(1.23- 2.85)
LAMWK	4	f	0	36	41	60	144	2.11	(1.23- 3.61)
LAMWK2	3	m	0	52	161	15	43	0.93	(0.48- 1.80)
LAMWK2	7	f	0	26	50	41	139	1.76	(0.98- 3.17)
Subtotal LAMWK2								1.33	(0.86- 2.07)
LOMBA2	3	f	0	42	353	54	239	0.53	(0.34- 0.81)
LUBIN	37	m	0	32	788	4	72	0.73	(0.25- 2.12)
LUBIN2	252	m	0	454	6209	57	2616	3.36	(2.54- 4.44)
LUBIN2	264	f	0	69	410	138	1180	1.44	(1.06- 1.96)
Subtotal LUBIN2								2.30	(1.87- 2.83)
LUO	9	c	20	-	-	-	-	1.50	(0.70- 3.00)
MATOS	53	m	2	-	-	-	-	7.90	(3.00- 20.90)
MATSUD	12	m	0	23	3314	0	1255	17.80~	(1.08- 293.32)
NOU	3	m	0	36	247	4	122	4.45	(1.55- 12.77)
NOU	8	f	0	9	92	29	261	0.88	(0.40- 1.93)
Subtotal NOU								1.57	(0.83- 2.94)
ORMOS	21	m	0	4	1034	0	777	6.76~	(0.36- 125.82)
OSANN	39	m	2	-	-	-	-	21.70	(12.50- 39.70)
OSANN	40	f	2	-	-	-	-	11.60	(8.20- 16.40)
Subtotal OSANN								13.69	(10.17- 18.43)
OSANN2	32	f	1	-	-	-	-	3.20	(1.50- 6.60)
PEZZOT	7	m	0	60	317	3	116	7.32	(2.25- 23.79)
SCHWAR	8	m	0	84	178	1	73	34.45	(4.71- 252.10)
SCHWAR	7	m	0	45	39	1	7	8.08	(0.95- 68.56)
SCHWAR	16	f	0	92	108	10	79	6.73	(3.29- 13.75)
SCHWAR	15	f	0	20	28	3	41	9.76	(2.65- 36.00)
Subtotal SCHWAR								8.40	(4.73- 14.94)
SEOW	2	f	0	19	15	67	125	2.36	(1.13- 4.95)
SIEMIA	8	m	7	-	-	-	-	6.30	(2.50- 16.20)
SOBUE	36	m	1	-	-	-	-	1.90	(1.30- 3.00)
SOBUE	46	f	1	-	-	-	-	1.30	(0.90- 2.00)
Subtotal SOBUE								1.56	(1.17- 2.08)
SOBUE2	2	m	2	-	-	-	-	3.10	(2.40- 3.70)
SOBUE2	6	f	2	-	-	-	-	1.80	(1.40- 2.20)
Subtotal SOBUE2								2.39	(2.04- 2.79)
STASZE	24	m	0	20	653	0	158	9.94~	(0.60- 165.30)
STASZE	4	f	0	1	153	10	1660	1.08	(0.14- 8.53)
Subtotal STASZE								2.36	(0.45- 12.42)
STAYNE	4	m	0	43	567	7	333	3.61	(1.60- 8.11)
SUZUK2	16	c	3	-	-	-	-	6.00	(0.70- 50.00)
SUZUKI	10	m	2	-	-	-	-	5.00	(2.71- 9.27)
SUZUKI	14	f	2	-	-	-	-	2.40	(1.19- 4.86)
Subtotal SUZUKI								3.64	(2.29- 5.78)
SVENSS	99	f	1	-	-	-	-	3.78	(1.91- 7.48)
TIZZAN	19	c	0	88	939	25	419	1.57	(0.99- 2.49)
TOKARS	8	c	3	-	-	-	-	4.30	(1.90- 9.90)
TSUGAN	3	m	0	45	50	18	17	0.85	(0.39- 1.85)
TSUGAN	9	f	0	6	10	33	30	0.55	(0.18- 1.68)
Subtotal TSUGAN								0.74	(0.39- 1.40)
WAKAI	12	m	1	-	-	-	-	2.18	(1.00- 4.76)
WAKAI	30	f	1	-	-	-	-	1.14	(0.49- 2.61)
Subtotal WAKAI								1.61	(0.91- 2.85)
WU	7	f	2	-	-	-	-	4.10	(2.30- 7.50)
WU2	1	f	2	-	-	-	-	4.50	(3.00- 6.90)
WUWILL	11	f	3	-	-	-	-	1.50	(1.10- 1.90)
WYNDE2	9	m	0	46	512	5	105	1.89	(0.73- 4.86)
WYNDE3	30	m	0	56	207	6	88	3.97	(1.65- 9.55)
WYNDE3	75	f	0	21	56	15	76	1.90	(0.90- 4.01)
Subtotal WYNDE3								2.59	(1.47- 4.57)
WYNDE4	42	m	0	35	665	4	115	1.51	(0.53- 4.34)
WYNDE4	56	f	2	-	-	-	-	0.60	(0.13- 2.69)
Subtotal WYNDE4								1.12	(0.47- 2.66)
WYNDE6	15	m	0	651	741	58	617	9.35	(7.00- 12.48)
WYNDE6	204	f	0	472	376	119	856	9.03	(7.14- 11.42)
Subtotal WYNDE6								9.15	(7.63- 10.98)
XU3	22	m	1	-	-	-	-	4.84	(1.37- 17.10)
XU3	26	f	1	-	-	-	-	1.09	(0.26- 4.50)

Table 3C4 - 2

IESLC - Meta-anal of Current Smoking (or Ever if Current not available), Cigs (or Any Prod if Cigs not avail)
Adenocarcinoma
Most adjusted

REF	NRR	SEX	AD	Number Exposed Case	Cont	Non-exposed Case	Cont	RR	95.00%CI
Subtotal XU3								2.51	(0.98- 6.47)
ZHENG	10	m	0	123	218	29	94	1.83	(1.14- 2.93)
ZHENG	21	f	0	33	44	119	184	1.16	(0.70- 1.93)
Subtotal ZHENG								1.48	(1.05- 2.09)
ZHOU	26	m	0	131	41	88	36	1.31	(0.77- 2.20)
ZHOU	27	f	0	30	7	96	32	1.43	(0.57- 3.57)
Subtotal ZHOU								1.34	(0.85- 2.10)
Partial Totals				4262	136511	1745	228708		

*prospective study ~ With 0.5 adjustment for zero

REF	NRR	SEX	AD	Ys	Ws	Qs	Ps
*ABRAHA	2	m	0	0.87	7.06	1.11	0.0201
*ABRAHA	5	f	0	0.96	8.71	0.83	0.0045
Subtotal ABRAHA				0.92	15.77	1.94	
ALDERS	95	m	1	1.48	5.55	0.25	0.0005
ALDERS	45	f	1	1.31	17.77	0.02	0.0000
Subtotal ALDERS				1.35	23.32	0.27	
*ANDERS	12	f	0	1.81	24.76	7.08	0.0000
BAND	2	m	2	1.41	40.10	0.78	0.0000
BARBON	100	m	1	2.07	6.19	3.92	0.0000
BECHER	12	f	1	2.38	0.87	1.07	0.0265
*BOUCOT	147	m	2	2.39	0.48	0.61	0.0964
BRESLO	1	c	0	0.35	3.39	2.86	0.5177
BROWN1	3	m	1	1.50	2.97	0.16	0.0096
BROWN1	4	f	1	1.37	5.93	0.06	0.0008
Subtotal BROWN1				1.42	8.91	0.22	
BROWN2	14	m	2	2.21	124.44	109.20	0.0000
BROWN2	13	f	2	1.97	180.58	89.14	0.0000
Subtotal BROWN2				2.07	305.02	198.34	
BUFFLE	50	m	0	1.50	4.86	0.26	0.0009
BUFFLE	71	f	0	2.10	5.59	3.82	0.0000
Subtotal BUFFLE				1.82	10.45	4.08	
BYERS1	3	m	0	1.41	5.95	0.11	0.0006
CHAN	19	m	0	3.42	0.49	2.26	0.0168
CHAN	23	f	0	0.79	9.98	2.35	0.0130
Subtotal CHAN				0.91	10.47	4.61	
CHOI	63	m	0	0.29	5.64	5.38	0.4842
CHOI	65	f	0	-0.44	3.77	11.06	0.3920
Subtotal CHOI				-0.00	9.42	16.45	
COMSTO	24	m	0	2.53	1.80	2.87	0.0007
COMSTO	31	f	0	1.85	5.09	1.70	0.0000
Subtotal COMSTO				2.03	6.89	4.57	
CORREA	44	c	1	1.90	18.88	7.51	0.0000
*CPSI	404	m	1	1.52	4.10	0.26	0.0021
*CPSI	406	f	1	0.36	3.08	2.57	0.5303
Subtotal CPSI				1.02	7.18	2.83	
*CPSII	115	m	1	2.96	3.23	9.17	0.0000
*CPSII	118	f	1	2.11	9.51	6.65	0.0000
Subtotal CPSII				2.32	12.75	15.83	
DAMBER	32	m	1	0.88	6.21	0.97	0.0291
DESTE2	17	m	2	1.46	3.99	0.14	0.0036
DOLL	87	m	1	-0.05	1.82	3.19	0.9448
DOLL	89	f	1	0.68	2.72	0.96	0.2634
Subtotal DOLL				0.39	4.54	4.14	
DORGAN	125	m	2	1.57	4.52	0.40	0.0008
DORGAN	104	f	3	1.36	35.62	0.29	0.0000
Subtotal DORGAN				1.38	40.15	0.68	
*DORN	340	m	1	1.78	20.15	5.28	0.0000
DOSEME	4	m	2	0.96	18.78	1.88	0.0000
*ENGELA	70	m	7	1.95	4.13	1.93	0.0001
FAN	4	c	0	0.33	24.61	21.81	0.1013
GAO	3	m	2	0.47	25.25	16.22	0.0182
GAO	13	f	2	0.41	27.91	20.94	0.0322
Subtotal GAO				0.44	53.16	37.15	
GER	9	c	8	0.10	8.05	11.13	0.7869
HAENSZ	37	f	0	0.08	9.58	13.55	0.7992
*HAMMON	84	m	1	1.30	1.86	0.00	0.0765
HEGMAN	4	c	0	2.26	12.50	12.19	0.0000
HINDS	24	f	3	1.36	19.35	0.15	0.0000
ISHIMA	8	c	5	2.71	0.49	1.01	0.0585

Table 3C4 - 2

IESLC - Meta-anal of Current Smoking (or Ever if Current not available), Cigs (or Any Prod if Cigs not avail)
Adenocarcinoma
Most adjusted

REF	NRR	SEX	AD	Ys	Ws	Qs	Ps
JAHN	8	m	0	1.57	6.70	0.60	0.0000
JAIN	17	m	0	2.38	3.49	4.28	0.0000
JAIN	12	f	0	1.83	14.10	4.35	0.0000
Subtotal JAIN				1.94	17.58	8.63	
JEDRYC	26	m	0	1.69	6.14	1.10	0.0000
JOLY	51	m	0	1.49	4.55	0.21	0.0015
JOLY	50	f	0	1.12	12.19	0.28	0.0001
Subtotal JOLY				1.22	16.74	0.50	
JUSSAW	28	m	0	0.63	2.35	0.98	0.3368
KATSOU	12	f	1	0.53	5.67	3.11	0.2064
KHUDER	17	m	0	2.10	5.77	4.00	0.0000
KIHARA	5	c	0	0.66	30.38	11.41	0.0003
KOO	7	f	0	0.47	13.45	8.54	0.0817
KREYBE	8	m	1	0.89	2.82	0.41	0.1345
KREYBE	27	f	1	0.25	6.66	6.99	0.5240
Subtotal KREYBE				0.44	9.48	7.40	
LAMTH	3	f	0	0.63	21.63	9.04	0.0036
LAMWK	4	f	0	0.75	13.20	3.65	0.0068
LAMWK2	3	m	0	-0.08	8.67	15.76	0.8206
LAMWK2	7	f	0	0.57	11.11	5.51	0.0588
Subtotal LAMWK2				0.28	19.77	21.27	
LOMBA2	3	f	0	-0.64	20.27	74.15	0.0039
LUBIN	37	m	0	-0.31	3.37	8.47	0.5649
LUBIN2	252	m	0	1.21	49.29	0.18	0.0000
LUBIN2	264	f	0	0.36	39.96	32.91	0.0214
Subtotal LUBIN2				0.83	89.24	33.09	
LUO	9	c	20	0.41	7.26	5.44	0.2748
MATOS	53	m	2	2.07	4.08	2.58	0.0000
MATSUD	12	m	0	2.88	0.49	1.27	0.0440
NOU	3	m	0	1.49	3.45	0.17	0.0056
NOU	8	f	0	-0.13	6.24	12.21	0.7504
Subtotal NOU				0.45	9.69	12.37	
ORMOS	21	m	0	1.91	0.45	0.18	0.1999
OSANN	39	m	2	3.08	11.51	37.52	0.0000
OSANN	40	f	2	2.45	31.98	44.50	0.0000
Subtotal OSANN				2.62	43.49	82.02	
OSANN2	32	f	1	1.16	7.00	0.08	0.0021
PEZZOT	7	m	0	1.99	2.76	1.43	0.0009
SCHWAR	8	m	0	3.54	0.97	4.99	0.0005
SCHWAR	7	m	0	2.09	0.84	0.56	0.0556
SCHWAR	16	f	0	1.91	7.53	3.04	0.0000
SCHWAR	15	f	0	2.28	2.26	2.29	0.0006
Subtotal SCHWAR				2.13	11.60	10.87	
SEOW	2	f	0	0.86	7.03	1.19	0.0226
SIEMIA	8	m	7	1.84	4.40	1.42	0.0001
SOBUE	36	m	1	0.64	21.97	8.71	0.0026
SOBUE	46	f	1	0.26	24.10	24.54	0.1978
Subtotal SOBUE				0.44	46.07	33.25	
SOBUE2	2	m	2	1.13	82.01	1.61	0.0000
SOBUE2	6	f	2	0.59	75.22	35.16	0.0000
Subtotal SOBUE2				0.87	157.22	36.77	
STASZE	24	m	0	2.30	0.49	0.51	0.1092
STASZE	4	f	0	0.08	0.90	1.28	0.9382
Subtotal STASZE				0.86	1.39	1.79	
STAYNE	4	m	0	1.28	5.85	0.00	0.0019
SUZUK2	16	c	3	1.79	0.84	0.23	0.0999
SUZUKI	10	m	2	1.61	10.16	1.16	0.0000
SUZUKI	14	f	2	0.88	7.76	1.22	0.0147
Subtotal SUZUKI				1.29	17.92	2.38	
SVENSS	99	f	1	1.33	8.25	0.03	0.0001
TIZZAN	19	c	0	0.45	18.24	12.27	0.0538
TOKARS	8	c	3	1.46	5.64	0.20	0.0005
TSUGAN	3	m	0	-0.16	6.39	13.13	0.6813
TSUGAN	9	f	0	-0.61	3.03	10.67	0.2916
Subtotal TSUGAN				-0.31	9.41	23.80	
WAKAI	12	m	1	0.78	6.31	1.53	0.0502
WAKAI	30	f	1	0.13	5.49	7.14	0.7588
Subtotal WAKAI				0.48	11.80	8.67	
WU	7	f	2	1.41	11.00	0.21	0.0000
WU2	1	f	2	1.50	22.15	1.20	0.0000
WUWILL	11	f	3	0.41	51.44	38.58	0.0036

Table 3C4 - 2

IESLC - Meta-anal of Current Smoking (or Ever if Current not available), Cigs (or Any Prod if Cigs not avail)
Adenocarcinoma
Most adjusted

REF	NRR	SEX	AD	Ys	Ws	Qs	Ps
WYNDE2	9	m	0	0.63	4.29	1.74	0.1887
WYNDE3	30	m	0	1.38	4.98	0.06	0.0021
WYNDE3	75	f	0	0.64	6.88	2.73	0.0922
Subtotal WYNDE3				0.95	11.86	2.79	
WYNDE4	42	m	0	0.41	3.46	2.55	0.4408
WYNDE4	56	f	2	-0.51	1.67	5.32	0.5087
Subtotal WYNDE4				0.11	5.14	7.86	
WYNDE6	15	m	0	2.23	45.98	42.68	0.0000
WYNDE6	204	f	0	2.20	69.69	60.15	0.0000
Subtotal WYNDE6				2.21	115.67	102.83	
XU3	22	m	1	1.58	2.41	0.22	0.0143
XU3	26	f	1	0.09	1.89	2.66	0.9057
Subtotal XU3				0.92	4.30	2.88	
ZHENG	10	m	0	0.60	17.29	7.71	0.0121
ZHENG	21	f	0	0.15	14.95	18.87	0.5668
Subtotal ZHENG				0.39	32.24	26.58	
ZHOU	26	m	0	0.27	14.05	14.16	0.3154
ZHOU	27	f	0	0.36	4.59	3.84	0.4448
Subtotal ZHOU				0.29	18.64	18.00	

	N	116
	NS	81
	Wt	1677.69
Het	Chi	1021.97
Het	df	115
Het	P	***
Fixed	RR	3.57
	RRl	3.40
	RRu	3.74
	P	+++
Random	RR	3.09
	RRl	2.64
	RRu	3.62
	P	+++
Asymm	P	*

Table 3C4 - 3

IESLC - Meta-anal of Current Smoking (or Ever if Current not available), Cigs (or Any Prod if Cigs not avail)
Adenocarcinoma
Most adjusted

	Sex combined	male	female	Total
N	11	56	49	116
NS	11	55	48	114
Wt	130.26	647.32	900.11	1677.69
Het Chi	67.50	324.99	581.68	1021.97
Het df	10	55	48	115
Het P	***	***	***	***
Fixed RR	2.44	4.31	3.29	3.57
RRl	2.06	3.99	3.08	3.40
RRu	2.90	4.66	3.51	3.74
P	+++	+++	+++	+++
Random RR	2.65	3.92	2.50	3.09
RRl	1.61	3.16	1.95	2.64
RRu	4.36	4.88	3.20	3.62
P	+++	+++	+++	+++
Between Chi				47.81
Between df				2
Between P				***
Btwn(F) P				(*)
Btwn(R) P				*

	Smoking status ever	current	Total
N	72	44	116
NS	52	31	83
Wt	660.33	1017.36	1677.69
Het Chi	275.43	527.97	1021.97
Het df	71	43	115
Het P	***	***	***
Fixed RR	2.28	4.77	3.57
RRl	2.11	4.49	3.40
RRu	2.46	5.07	3.74
P	+++	+++	+++
Random RR	2.43	4.21	3.09
RRl	2.06	3.32	2.64
RRu	2.88	5.34	3.62
P	+++	+++	+++
Between Chi			218.56
Between df			1
Between P			***
Btwn(F) P			***
Btwn(R) P			***

Table 3C4 - 4

IESLC - Meta-anal of Current Smoking (or Ever if Current not available), Cigs (or Any Prod if Cigs not avail)
Adenocarcinoma
Least adjusted

REF	NRR	X	SEX	AGEL	AGEH	RACE	YF	LC TYPE	LOC	START	ST	NLC	R	VB	P	H	AD	SM	PRODUCT	DENOM	De
ABRAHA	2		m	0	0	all	0	a	Eu:est	1975	pr	571	n	bl	n	n	0	ev	all/unsp	nev any	ot
ABRAHA	5		f	0	0	all	0	a	Eu:est	1975	pr	571	n	bl	n	n	0	ev	all/unsp	nev any	ot
ALDERS	108	x	m	0	0	all	-	not q+s	Eu:UK	1977	CC	1448	n	V	n	n	0	ev	cig+/-ot	nev any	st
ALDERS	104	x	f	0	0	all	-	not q+s	Eu:UK	1977	CC	1448	n	V	n	n	0	ev	cig only	nev any	st
ANDERS	12		f	0	0	all	0	a	NAmer	1986	pr	343	n	bl	n	n	0	ev	cig+/-ot	nev cigs	st
BAND	2		m	0	0	all	-	a	NAmer	1983	CC	2831	n	V	y	y	2	ev	cig only	nev any	ot
BARBON	44	x	m	0	0	all	-	a	Eu:wst	1979	CC	755	n	bl	y	y	0	cu	all/unsp	nev any	st
BECHER	12		f	0	0	all	-	not q+s	Eu:Ger	1985	CC	194	n	bl	n	y	1	ev	all/unsp	nev any	or
BOUCOT	72	x	m	0	0	all	0	a	NAmer	1951	pr	121	n	bl	n	n	0	cu	cig only	nev any	ot
BRESLO	1		c	0	0	all	-	a	NAmer	1949	CC	518	n	bl	n	y	0	ev	cig+/-ot	nev+1	st
BROWN1	1	x	m	0	0	wh	-	a	NAmer	1979	CC	102	n	bl	y	y	0	ev	cig+/-ot	nev cigs	st
BROWN1	2	x	f	0	0	wh	-	a	NAmer	1979	CC	102	n	bl	y	y	0	ev	cig+/-ot	nev cigs	st
BROWN2	14		m	0	0	wh	-	a	NAmer	1984	CC	14596	n	bl	n	y	2	cu	cig+/-ot	nev cigs	or
BROWN2	13		f	0	0	wh	-	a	NAmer	1984	CC	14596	n	bl	n	y	2	cu	cig+/-ot	nev cigs	or
BUFFLE	50		m	0	0	wh	-	a	NAmer	1976	CC	943	n	bl	y	n	0	ev	cig+/-ot	nev cigs	ot
BUFFLE	71		f	0	0	w-hi	-	a	NAmer	1976	CC	943	n	bl	y	n	0	cu	cig+/-ot	nev cigs	st
BYERS1	3		m	0	0	wh	-	a	NAmer	1957	CC	1002	n	bl	n	n	0	ev	cig+/-ot	nev cigs	st
CHAN	19		m	0	0	all	-	a+l	As:HK	1976	CC	397	n	bl	n	n	0	ev	cig+/-ot	nev any	ot
CHAN	23		f	0	0	all	-	a+l	As:HK	1976	CC	397	n	bl	n	n	0	ev	cig+/-ot	nev any	st
CHOI	63		m	0	0	all	-	a	As:oth	1985	CC	375	n	bl	n	n	0	ev	cig+/-ot	nev cigs	st
CHOI	65		f	0	0	all	-	a	As:oth	1985	CC	375	n	bl	n	n	0	ev	cig+/-ot	nev cigs	st
COMSTO	24		m	0	0	all	-	a	NAmer	1975	ot	258	n	bl	n	n	0	cu	cig+/-ot	nev cigs	st
COMSTO	31		f	0	0	all	-	a	NAmer	1975	ot	258	n	bl	n	n	0	cu	cig+/-ot	nev cigs	st
CORREA	44		c	0	0	all	-	a	NAmer	1979	CC	1359	n	bl	y	n	1	cu	cig+/-ot	nev cigs	or
CPSI	404		m	0	0	all	2	a	NAmer	1959	pr	5138	n	bl	n	n	1	cu	cig only	nev any	ot
CPSI	406		f	0	0	all	2	a	NAmer	1959	pr	5138	n	bl	n	n	1	cu	cig only	nev any	ot
CPSII	115		m	0	0	all	2	a	NAmer	1982	pr	3229	n	bl	n	n	1	cu	cig only	nev any	st
CPSII	118		f	0	0	all	2	a	NAmer	1982	pr	3229	n	bl	n	n	1	cu	cig+/-ot	nev cigs	st
DAMBER	11	x	m	0	0	all	-	a+al+br	Eu:Sca	1972	CC	579	n	bl	y	n	0	ev	all/unsp	nev any	st
DESTE2	17		m	0	0	all	-	a	SCAmer	1993	CC	463	n	bl	n	n	2	ev	all/unsp	nev any	or
DOLL	83	x	m	0	0	all	-	KII	Eu:UK	1948	CC	1465	n	V	n	n	0	ev	all/unsp	nev any	st
DOLL	85	x	f	0	0	all	-	KII	Eu:UK	1948	CC	1465	n	V	n	n	0	ev	all/unsp	nev any	st
DORGAN	125		m	0	0	wh	-	a	NAmer	1980	CC	2026	n	bl	y	y	2	ev	cig+/-ot	nev any	or
DORGAN	104		f	0	0	all	-	a	NAmer	1980	CC	2026	n	bl	y	y	3	ev	cig+/-ot	nev any	or
DORN	340		m	0	0	wh	8	a	NAmer	1954	pr	5097	n	bl	n	n	1	cu	cig only	nev any	ot
DOSEME	20	x	m	0	0	all	-	not q+s	Eu:bal	1979	CC	1210	n	bl	n	n	0	ev	cig+/-ot	nev cigs	st
ENGELA	70		m	0	0	all	0	a	Eu:Sca	1964	pr	435	n	bl	n	n	7	cu	cig+/-ot	nev cigs	ot
FAN	4		c	0	0	all	-	a	As:Chi	1990	CC	403	n	ot	y	n	0	ev	cig+/-ot	nev cigs	ot
GAO	8	x	m	0	0	all	-	a	As:Chi	1984	CC	1405	n	ot	n	n	0	ev	cig+/-ot	nev cigs	st
GAO	18	x	f	0	0	all	-	a	As:Chi	1984	CC	1405	n	ot	n	n	0	ev	cig+/-ot	nev cigs	st
GER	1	x	c	0	0	all	-	a	As:oth	1990	CC	141	n	ot	y	n	0	ev	all/unsp	nev any	st
HAENSZ	37		f	0	0	all	-	a	NAmer	1955	CC	158	n	bl	n	y	0	cu	cig+/-ot	nev any	st
HAMMON	91	x	m	0	0	wh	0	a	NAmer	1952	pr	448	n	bl	n	n	0	ev	cig+/-ot	nev any	st
HEGMAN	4		c	0	0	all	-	a	NAmer	1989	CC	282	n	bl	y	y	0	ev	all/unsp	nev any	st
HINDS	24		f	0	0	o	-	a	NAmer	1968	CC	292	n	bl	n	n	3	ev	all/unsp	nev any	st
ISHIMA	3	x	c	0	0	all	-	a	As:Jap	1961	CC	180	n	bl	y	y	0	ev	all/unsp	nev any	st
JAHN	8		m	0	0	all	-	a	Eu:Ger	1988	CC	1004	n	bl	n	n	0	cu	cig+/-ot	nev any	st
JAIN	17		m	0	0	all	-	a	NAmer	1981	CC	845	n	V	y	n	0	cu	cig+/-ot	nev cigs	st
JAIN	12		f	0	0	all	-	a	NAmer	1981	CC	845	n	V	y	n	0	cu	cig+/-ot	nev cigs	st
JEDRYC	26		m	0	0	all	-	a	Eu:est	1980	CC	1630	n	bl	y	n	0	cu	cig+/-ot	nev any	st
JOLY	51		m	0	0	all	-	a	SCAmer	1978	CC	826	n	bl	n	n	0	ev	cig+/-ot	nev any	st
JOLY	50		f	0	0	all	-	a	SCAmer	1978	CC	826	n	bl	n	n	0	ev	cig+/-ot	nev any	st
JUSSAW	28		m	0	0	all	-	KII	As:Ind	1964	CC	792	n	V	n	n	0	ev	cig only	nev any	st
KATSOU	16	x	f	0	0	all	-	a	Eu:bal	1987	CC	101	n	bl	n	n	0	cu	all/unsp	nev any	st
KHUDER	17		m	0	0	all	-	a	NAmer	1985	CC	482	n	bl	n	y	0	cu	cig+/-ot	nev cigs	or
KIHARA	5		c	0	0	jap	-	a	As:Jap	1991	CC	440	n	bl	n	n	0	cu	all/unsp	nev any	st
KOO	7		f	0	0	all	-	a+l	As:HK	1981	CC	200	n	bl	n	n	0	ev	all/unsp	nev any	st
KREYBE	20	x	m	0	0	all	-	KII	Eu:Sca	1948	CC	300	n	bl	n	y	0	ev	all/unsp	nev any	st
KREYBE	36	x	f	0	0	all	-	KII	Eu:Sca	1948	CC	300	n	bl	n	y	0	ev	all/unsp	nev any	st
LAMTH	3		f	0	0	ch	-	a	As:HK	1983	CC	445	n	bl	n	n	0	ev	all/unsp	nev any	or
LAMWK	4		f	0	0	ch	-	a	As:HK	1981	CC	163	n	bl	n	n	0	ev	all/unsp	nev any	st
LAMWK2	3		m	0	0	all	-	a	As:HK	1976	CC	480	n	bl	n	n	0	ev	all/unsp	nev any	st
LAMWK2	7		f	0	0	all	-	a	As:HK	1976	CC	480	n	bl	n	n	0	ev	all/unsp	nev any	st
LOMBA2	3		f	0	0	all	-	not q+u	NAmer	1960	CC	225	n	bl	n	n	0	ev	cig+/-ot	nev cigs	st
LUBIN	37		m	0	0	all	-	KII	As:Chi	1984	CC	427	m	ot	y	n	0	ev	cig+/-ot	nev any	st
LUBIN2	252		m	0	0	all	-	a	Eu:mul	1976	CC	7804	n	bl	n	y	0	cu	cig+/-ot	nev any	st
LUBIN2	264		f	0	0	all	-	a	Eu:mul	1976	CC	7804	n	bl	n	y	0	cu	cig+/-ot	nev any	st
LUO	3	x	c	0	0	all	-	a	As:Chi	1990	CC	102	n	ot	n	y	0	ev	cig+/-ot	nev cigs	st
MATOS	52	x	m	0	0	all	-	a	SCAmer	1994	CC	200	n	bl	n	n	0	cu	cig+/-ot	nev any	st
MATSUD	12		m	0	0	all	-	a	As:Jap	1965	CC	179	n	bl	n	n	0	ev	cig+/-ot	nev cigs	ot
NOU	3		m	0	0	all	-	a	Eu:Sca	1971	CC	273	n	bl	y	n	0	ev	all/unsp	nev any	st
NOU	8		f	0	0	all	-	a	Eu:Sca	1971	CC	273	n	bl	y	n	0	ev	all/unsp	nev any	st

Table 3C4 - 4

IESLC - Meta-anal of Current Smoking (or Ever if Current not available), Cigs (or Any Prod if Cigs not avail)
Adenocarcinoma
Least adjusted

REF	NRR	X	SEX	AGEL	AGEH	RACE	YF	LC TYPE	LOC	START	ST	NLC	R	VB	P	H	AD	SM	PRODUCT	DENOM	De
ORMOS	21		m	0	0	all	-	a	Eu:est	1947	CC	119	n	bl	y	y	0	ev	cig+/-ot	nev any	ot
OSANN	11	x	m	0	0	all	-	a	NAmer	1984	CC	1986	n	bl	n	n	0	cu	cig+/-ot	nev cigs	st
OSANN	15	x	f	0	0	all	-	a	NAmer	1984	CC	1986	n	bl	n	n	0	cu	cig+/-ot	nev cigs	st
OSANN2	14	x	f	0	0	all	-	KII	NAmer	1964	ot	217	n	bl	n	y	0	cu	cig+/-ot	nev cigs	st
PEZZOT	7		m	0	0	all	-	a	SCAmer	1987	CC	215	n	bl	n	y	0	ev	cig only	nev cigs	st
SCHWAR	8		m	40	54	wh	-	a	NAmer	1984	CC	5588	n	bl	y	y	0	ev	cig+/-ot	nev cigs	st
SCHWAR	7		m	40	54	bl	-	a	NAmer	1984	CC	5588	n	bl	y	y	0	ev	cig+/-ot	nev cigs	st
SCHWAR	16		f	40	54	wh	-	a	NAmer	1984	CC	5588	n	bl	y	y	0	ev	cig+/-ot	nev cigs	st
SCHWAR	15		f	40	54	bl	-	a	NAmer	1984	CC	5588	n	bl	y	y	0	ev	cig+/-ot	nev cigs	st
SEOW	2		f	0	0	ch	-	a	As:oth	1997	CC	153	n	bl	n	y	0	ev	cig+/-ot	nev cigs	st
SIEMIA	12	x	m	0	0	all	-	a	NAmer	1979	CC	857	n	V	y	y	0	ev	cig+/-ot	nev cigs	st
SOBUE	6	x	m	0	0	all	-	a	As:Jap	1986	CC	1376	n	bl	n	y	0	cu	cig+/-ot	nev cigs	st
SOBUE	22	x	f	0	0	all	-	a	As:Jap	1986	CC	1376	n	bl	n	y	0	cu	cig+/-ot	nev cigs	st
SOBUE2	2		m	0	0	all	-	a	As:Jap	1965	CC	2083	n	bl	n	n	2	cu	cig+/-ot	nev any	or
SOBUE2	6		f	0	0	all	-	a	As:Jap	1965	CC	2083	n	bl	n	n	2	cu	cig+/-ot	nev any	or
STASZE	24		m	0	0	all	-	a	Eu:est	1954	CC	281	n	bl	n	y	0	ev	cig+/-ot	nev any	ot
STASZE	4		f	0	0	all	-	a	Eu:est	1954	CC	281	n	bl	n	y	0	ev	all/unsp	nev any	st
STAYNE	4		m	0	0	all	-	a	NAmer	1969	CC	420	n	bl	n	n	0	ev	all/unsp	nev any	st
SUZUK2	13	x	c	0	0	all	-	a	SCAmer	1991	CC	123	n	bl	n	y	0	ev	all/unsp	nev any	st
SUZUKI	2	x	m	0	0	all	-	a	As:Jap	1978	CC	238	n	bl	n	y	0	cu	cig+/-ot	nev any	st
SUZUKI	6	x	f	0	0	all	-	a	As:Jap	1978	CC	238	n	bl	n	y	0	cu	cig+/-ot	nev any	st
SVENSS	64	x	f	0	0	all	-	a	Eu:Sca	1983	CC	210	n	bl	n	n	0	cu	all/unsp	nev any	st
TIZZAN	19		c	0	0	all	-	a	Eu:wst	1959	CC	1358	n	bl	n	n	0	ev	all/unsp	nev any	st
TOKARS	7	x	c	0	0	all	-	a	Eu:est	1966	ot	162	o	bl	n	y	0	ev	all/unsp	nev any	st
TSUGAN	3		m	0	0	all	-	a	As:Jap	1976	CC	134	n	bl	n	y	0	cu	all/unsp	nev any	st
TSUGAN	9		f	0	0	all	-	a	As:Jap	1976	CC	134	n	bl	n	y	0	cu	all/unsp	nev any	or
WAKAI	6	x	m	0	0	all	-	a	As:Jap	1988	CC	333	n	bl	n	y	0	cu	all/unsp	nev any	st
WAKAI	24	x	f	0	0	all	-	a	As:Jap	1988	CC	333	n	bl	n	y	0	cu	all/unsp	nev any	st
WU	2	x	f	0	0	wh	-	a	NAmer	1981	CC	220	n	bl	n	y	0	cu	all/unsp	nev any	st
WU2	1		f	0	0	all	-	a	NAmer	1983	CC	336	n	bl	n	y	2	cu	all/unsp	nev any	or
WUWILL	25	x	f	0	0	all	-	a	As:Chi	1985	CC	965	n	ot	n	n	0	ev	cig+/-ot	nev cigs	st
WYNDE2	9		m	0	0	all	-	KII	NAmer	1962	CC	404	n	bl	n	y	0	ev	cig+/-ot	nev any	st
WYNDE3	30		m	0	0	all	-	KII	NAmer	1966	CC	350	n	bl	n	y	0	cu	all/unsp	nev any	st
WYNDE3	75		f	0	0	all	-	KII	NAmer	1966	CC	350	n	bl	n	y	0	ev	cig+/-ot	nev any	st
WYNDE4	42		m	0	0	all	-	a	NAmer	1948	CC	684	n	bl	y	n	0	ev	all/unsp	nev any	st
WYNDE4	56		f	0	0	all	-	a	NAmer	1948	CC	684	n	bl	y	n	2	ev	all/unsp	nev any	ot
WYNDE6	15		m	0	0	all	-	KII	NAmer	1969	CC	4423	n	bl	n	y	0	cu	cig+/-ot	nev any	st
WYNDE6	204		f	0	0	all	-	KII	NAmer	1969	CC	4423	n	bl	n	y	0	cu	cig+/-ot	nev cigs	st
XU3	21	x	m	0	0	all	-	KII	As:Chi	1981	CC	135	n	ot	n	n	0	ev	all/unsp	nev any	st
XU3	25	x	f	0	0	all	-	KII	As:Chi	1981	CC	135	n	ot	n	n	0	ev	all/unsp	nev any	st
ZHENG	10		m	0	0	all	-	a	As:Chi	1982	CC	540	n	ot	*	y	0	ev	cig+/-ot	nev cigs	st
ZHENG	21		f	0	0	all	-	a	As:Chi	1982	CC	540	n	ot	*	y	0	ev	cig+/-ot	nev cigs	st
ZHOU	26		m	0	0	all	-	a	As:Chi	1978	CC	1360	n	ot	n	n	0	ev	all/unsp	nev any	st
ZHOU	27		f	0	0	all	-	a	As:Chi	1978	CC	1360	n	ot	n	n	0	ev	all/unsp	nev any	st

Cigarette type is all/unspec for all RRs

except for the following:

REF	NRR	CIGTYPE
ALDERS	104	MC only
CHAN	19	MC+-HR
CHAN	23	MC+-HR
JUSSAW	28	MC only

Table 3C4 - 5

IESLC - Meta-anal of Current Smoking (or Ever if Current not available), Cigs (or Any Prod if Cigs not avail)
Adenocarcinoma
Least adjusted

REF	NRR	SEX	AD	Number Exposed Case	Number Exposed Cont	Non-exposed Case	Non-exposed Cont	RR	95.00%CI
*ABRAHA	2	m	0	59	10351	8	3365	2.40	(1.15- 5.01)
*ABRAHA	5	f	0	19	5256	16	11589	2.62	(1.35- 5.09)
Subtotal ABRAHA								2.52	(1.54- 4.12)
ALDERS	108	m	0	141	641	6	133	4.88	(2.11- 11.27)
ALDERS	104	f	0	104	371	25	243	2.72	(1.71- 4.34)
Subtotal ALDERS								3.13	(2.08- 4.70)
*ANDERS	12	f	0	99	96164	33	195158	6.09	(4.11- 9.03)
BAND	2	m	2	-	-	-	-	4.10	(3.01- 5.59)
BARBON	44	m	0	109	362	7	188	8.09	(3.69- 17.72)
BECHER	12	f	1	-	-	-	-	10.83	(1.32- 88.70)
*BOUCOT	72	m	0	14	22177	0	7551	9.87~	(0.59- 165.51)
BRESLO	1	c	0	40	394	4	56	1.42	(0.49- 4.12)
BROWN1	1	m	0	46	46	4	19	4.75	(1.50- 15.05)
BROWN1	2	f	0	33	19	19	47	4.30	(1.98- 9.34)
Subtotal BROWN1								4.43	(2.33- 8.44)
BROWN2	14	m	2	-	-	-	-	9.10	(7.60- 10.80)
BROWN2	13	f	2	-	-	-	-	7.20	(6.20- 8.30)
Subtotal BROWN2								7.92	(7.08- 8.86)
BUFFLE	50	m	0	-	-	-	-	4.50	(1.85- 10.95)
BUFFLE	71	f	0	56	110	7	112	8.15	(3.56- 18.65)
Subtotal BUFFLE								6.18	(3.37- 11.33)
BYERS1	3	m	0	47	695	7	424	4.10	(1.83- 9.15)
CHAN	19	m	0	56	160	0	43	30.63~	(1.85- 505.72)
CHAN	23	f	0	24	38	40	139	2.19	(1.18- 4.08)
Subtotal CHAN								2.48	(1.35- 4.55)
CHOI	63	m	0	46	465	7	95	1.34	(0.59- 3.06)
CHOI	65	f	0	5	26	49	164	0.64	(0.23- 1.77)
Subtotal CHOI								1.00	(0.53- 1.89)
COMSTO	24	m	0	30	100	2	84	12.60	(2.92- 54.28)
COMSTO	31	f	0	23	52	8	115	6.36	(2.67- 15.16)
Subtotal COMSTO								7.60	(3.60- 16.04)
CORREA	44	c	1	-	-	-	-	6.70	(4.30- 10.60)
*CPSI	404	m	1	-	-	-	-	4.58	(1.74- 12.05)
*CPSI	406	f	1	-	-	-	-	1.43	(0.47- 4.39)
Subtotal CPSI								2.78	(1.34- 5.78)
*CPSII	115	m	1	-	-	-	-	19.22	(6.46- 57.16)
*CPSII	118	f	1	-	-	-	-	8.23	(4.36- 15.54)
Subtotal CPSII								10.21	(5.89- 17.67)
DAMBER	11	m	0	65	49	16	29	2.40	(1.18- 4.91)
DESTE2	17	m	2	-	-	-	-	4.30	(1.60- 11.40)
DOLL	83	m	0	38	1296	2	61	0.89	(0.21- 3.79)
DOLL	85	f	0	8	49	5	59	1.93	(0.59- 6.27)
Subtotal DOLL								1.42	(0.57- 3.53)
DORGAN	125	m	2	-	-	-	-	4.80	(1.90- 12.00)
DORGAN	104	f	3	-	-	-	-	3.90	(2.80- 5.40)
Subtotal DORGAN								3.99	(2.93- 5.44)
*DORN	340	m	1	-	-	-	-	5.95	(3.85- 9.22)
DOSEME	20	m	0	142	536	24	293	3.23	(2.05- 5.10)
*ENGELA	70	m	7	-	-	-	-	7.06	(2.69- 18.50)
FAN	4	c	0	67	595	45	556	1.39	(0.94- 2.07)
GAO	8	m	0	180	558	42	202	1.55	(1.07- 2.25)
GAO	18	f	0	62	130	266	605	1.08	(0.78- 1.52)
Subtotal GAO								1.27	(0.99- 1.63)
GER	1	c	0	35	139	37	149	1.01	(0.60- 1.70)
HAENSZ	37	f	0	16	94	37	236	1.09	(0.58- 2.05)
*HAMMON	91	m	0	26	382338	2	115884	3.94	(0.94- 16.60)
HEGMAN	4	c	0	83	1202	15	2080	9.58	(5.50- 16.67)
HINDS	24	f	3	-	-	-	-	3.89	(2.49- 6.07)
ISHIMA	3	c	0	39	25	13	27	3.24	(1.41- 7.43)
JAHN	8	m	0	75	269	8	138	4.81	(2.26- 10.26)
JAIN	17	m	0	60	118	4	85	10.81	(3.78- 30.87)
JAIN	12	f	0	69	99	24	214	6.21	(3.69- 10.47)
Subtotal JAIN								6.93	(4.35- 11.07)
JEDRYC	26	m	0	68	516	7	289	5.44	(2.47- 12.00)
JOLY	51	m	0	72	709	5	218	4.43	(1.77- 11.10)
JOLY	50	f	0	33	122	25	283	3.06	(1.75- 5.37)
Subtotal JOLY								3.38	(2.10- 5.46)
JUSSAW	28	m	0	3	77	13	624	1.87	(0.52- 6.71)
KATSOU	16	f	0	15	18	30	67	1.86	(0.83- 4.18)
KHUDER	17	m	0	92	-	7	-	8.20	(3.60- 18.40)
KIHARA	5	c	0	103	162	78	237	1.93	(1.35- 2.76)

Table 3C4 - 5

IESLC - Meta-anal of Current Smoking (or Ever if Current not available), Cigs (or Any Prod if Cigs not avail)
Adenocarcinoma
Least adjusted

REF	NRR	SEX	AD	Number Exposed Case	Number Exposed Cont	Non-exposed Case	Non-exposed Cont	RR	95.00%CI
KOO	7	f	0	34	63	46	137	1.61	(0.94- 2.74)
KREYBE	20	m	0	42	3514	3	644	2.57	(0.79- 8.30)
KREYBE	36	f	0	10	328	27	657	0.74	(0.35- 1.55)
Subtotal KREYBE								1.05	(0.56- 1.97)
LAMTH	3	f	0	79	51	131	158	1.87	(1.23- 2.85)
LAMWK	4	f	0	36	41	60	144	2.11	(1.23- 3.61)
LAMWK2	3	m	0	52	161	15	43	0.93	(0.48- 1.80)
LAMWK2	7	f	0	26	50	41	139	1.76	(0.98- 3.17)
Subtotal LAMWK2								1.33	(0.86- 2.07)
LOMBA2	3	f	0	42	353	54	239	0.53	(0.34- 0.81)
LUBIN	37	m	0	32	788	4	72	0.73	(0.25- 2.12)
LUBIN2	252	m	0	454	6209	57	2616	3.36	(2.54- 4.44)
LUBIN2	264	f	0	69	410	138	1180	1.44	(1.06- 1.96)
Subtotal LUBIN2								2.30	(1.87- 2.83)
LUO	3	c	0	28	146	29	160	1.06	(0.60- 1.86)
MATOS	52	m	0	46	132	5	110	7.67	(2.94- 19.96)
MATSUD	12	m	0	23	3314	0	1255	17.80~	(1.08- 293.32)
NOU	3	m	0	36	247	4	122	4.45	(1.55- 12.77)
NOU	8	f	0	9	92	29	261	0.88	(0.40- 1.93)
Subtotal NOU								1.57	(0.83- 2.94)
ORMOS	21	m	0	4	1034	0	777	6.76~	(0.36- 125.82)
OSANN	11	m	0	217	541	14	833	23.87	(13.75- 41.41)
OSANN	15	f	0	193	367	47	1093	12.23	(8.70- 17.18)
Subtotal OSANN								14.70	(11.01- 19.64)
OSANN2	14	f	0	50	28	22	43	3.49	(1.75- 6.97)
PEZZOT	7	m	0	60	317	3	116	7.32	(2.25- 23.79)
SCHWAR	8	m	0	84	178	1	73	34.45	(4.71- 252.10)
SCHWAR	7	m	0	45	39	1	7	8.08	(0.95- 68.56)
SCHWAR	16	f	0	92	108	10	79	6.73	(3.29- 13.75)
SCHWAR	15	f	0	20	28	3	41	9.76	(2.65- 36.00)
Subtotal SCHWAR								8.40	(4.73- 14.94)
SEOW	2	f	0	19	15	67	125	2.36	(1.13- 4.95)
SIEMIA	12	m	0	162	428	5	105	7.95	(3.18- 19.85)
SOBUE	6	m	0	276	650	27	128	2.01	(1.30- 3.12)
SOBUE	22	f	0	38	168	137	857	1.41	(0.95- 2.10)
Subtotal SOBUE								1.66	(1.24- 2.22)
SOBUE2	2	m	2	-	-	-	-	3.10	(2.40- 3.70)
SOBUE2	6	f	2	-	-	-	-	1.80	(1.40- 2.20)
Subtotal SOBUE2								2.39	(2.04- 2.79)
STASZE	24	m	0	20	653	0	158	9.94~	(0.60- 165.30)
STASZE	4	f	0	1	153	10	1660	1.08	(0.14- 8.53)
Subtotal STASZE								2.36	(0.45- 12.42)
STAYNE	4	m	0	43	567	7	333	3.61	(1.60- 8.11)
SUZUK2	13	c	0	20	10	5	15	6.00	(1.69- 21.26)
SUZUKI	2	m	0	119	162	14	99	5.19	(2.83- 9.54)
SUZUKI	6	f	0	20	20	55	133	2.42	(1.21- 4.84)
Subtotal SUZUKI								3.73	(2.36- 5.89)
SVENSS	64	f	0	38	53	22	120	3.91	(2.11- 7.25)
TIZZAN	19	c	0	88	939	25	419	1.57	(0.99- 2.49)
TOKARS	7	c	0	68	112	10	54	3.28	(1.57- 6.86)
TSUGAN	3	m	0	45	50	18	17	0.85	(0.39- 1.85)
TSUGAN	9	f	0	6	10	33	30	0.55	(0.18- 1.68)
Subtotal TSUGAN								0.74	(0.39- 1.40)
WAKAI	6	m	0	75	284	8	65	2.15	(0.99- 4.67)
WAKAI	24	f	0	9	26	46	145	1.09	(0.48- 2.50)
Subtotal WAKAI								1.56	(0.89- 2.75)
WU	2	f	0	99	50	29	62	4.23	(2.43- 7.39)
WU2	1	f	2	-	-	-	-	4.50	(3.00- 6.90)
WUWILL	25	f	0	138	351	172	601	1.37	(1.06- 1.78)
WYNDE2	9	m	0	46	512	5	105	1.89	(0.73- 4.86)
WYNDE3	30	m	0	56	207	6	88	3.97	(1.65- 9.55)
WYNDE3	75	f	0	21	56	15	76	1.90	(0.90- 4.01)
Subtotal WYNDE3								2.59	(1.47- 4.57)
WYNDE4	42	m	0	35	665	4	115	1.51	(0.53- 4.34)
WYNDE4	56	f	2	-	-	-	-	0.60	(0.13- 2.69)
Subtotal WYNDE4								1.12	(0.47- 2.66)
WYNDE6	15	m	0	651	741	58	617	9.35	(7.00- 12.48)
WYNDE6	204	f	0	472	376	119	856	9.03	(7.14- 11.42)
Subtotal WYNDE6								9.15	(7.63- 10.98)
XU3	21	m	0	29	68	3	31	4.41	(1.25- 15.57)
XU3	25	f	0	4	11	7	25	1.30	(0.31- 5.36)

Table 3C4 - 5

IESLC - Meta-anal of Current Smoking (or Ever if Current not available), Cigs (or Any Prod if Cigs not avail)
Adenocarcinoma
Least adjusted

REF	NRR	SEX	AD	Number Exposed Case	Cont	Non-exposed Case	Cont	RR	95.00%CI
Subtotal XU3								2.57	(1.00- 6.59)
ZHENG	10	m	0	123	218	29	94	1.83	(1.14- 2.93)
ZHENG	21	f	0	33	44	119	184	1.16	(0.70- 1.93)
Subtotal ZHENG								1.48	(1.05- 2.09)
ZHOU	26	m	0	131	41	88	36	1.31	(0.77- 2.20)
ZHOU	27	f	0	30	7	96	32	1.43	(0.57- 3.57)
Subtotal ZHOU								1.34	(0.85- 2.10)
Partial Totals				7000	552714	2930	360245		

*prospective study ~ With 0.5 adjustment for zero

REF	NRR	SEX	AD	Ys	Ws	Qs	Ps
*ABRAHA	2	m	0	0.87	7.06	0.99	0.0201
*ABRAHA	5	f	0	0.96	8.71	0.71	0.0045
Subtotal ABRAHA				0.92	15.77	1.70	
ALDERS	108	m	0	1.58	5.47	0.62	0.0002
ALDERS	104	f	0	1.00	17.72	1.08	0.0000
Subtotal ALDERS				1.14	23.19	1.69	
*ANDERS	12	f	0	1.81	24.76	7.70	0.0000
BAND	2	m	2	1.41	40.10	1.05	0.0000
BARBON	44	m	0	2.09	6.25	4.42	0.0000
BECHER	12	f	1	2.38	0.87	1.12	0.0265
*BOUCOT	72	m	0	2.29	0.48	0.52	0.1114
BRESLO	1	c	0	0.35	3.39	2.73	0.5177
BROWN1	1	m	0	1.56	2.89	0.28	0.0081
BROWN1	2	f	0	1.46	6.38	0.28	0.0002
Subtotal BROWN1				1.49	9.27	0.55	
BROWN2	14	m	2	2.21	124.44	114.55	0.0000
BROWN2	13	f	2	1.97	180.58	94.98	0.0000
Subtotal BROWN2				2.07	305.02	209.53	
BUFFLE	50	m	0	1.50	4.86	0.32	0.0009
BUFFLE	71	f	0	2.10	5.59	4.03	0.0000
Subtotal BUFFLE				1.82	10.45	4.35	
BYERS1	3	m	0	1.41	5.95	0.15	0.0006
CHAN	19	m	0	3.42	0.49	2.31	0.0168
CHAN	23	f	0	0.79	9.98	2.14	0.0130
Subtotal CHAN				0.91	10.47	4.44	
CHOI	63	m	0	0.29	5.64	5.14	0.4842
CHOI	65	f	0	-0.44	3.77	10.77	0.3920
Subtotal CHOI				-0.00	9.42	15.91	
COMSTO	24	m	0	2.53	1.80	2.97	0.0007
COMSTO	31	f	0	1.85	5.09	1.84	0.0000
Subtotal COMSTO				2.03	6.89	4.81	
CORREA	44	c	1	1.90	18.88	8.06	0.0000
*CPSI	404	m	1	1.52	4.10	0.31	0.0021
*CPSI	406	f	1	0.36	3.08	2.44	0.5303
Subtotal CPSI				1.02	7.18	2.75	
*CPSII	115	m	1	2.96	3.23	9.42	0.0000
*CPSII	118	f	1	2.11	9.51	7.02	0.0000
Subtotal CPSII				2.32	12.75	16.44	
DAMBER	11	m	0	0.88	7.53	1.04	0.0161
DESTE2	17	m	2	1.46	3.99	0.18	0.0036
DOLL	83	m	0	-0.11	1.84	3.41	0.8795
DOLL	85	f	0	0.66	2.76	0.97	0.2760
Subtotal DOLL				0.35	4.60	4.38	
DORGAN	125	m	2	1.57	4.52	0.46	0.0008
DORGAN	104	f	3	1.36	35.62	0.45	0.0000
Subtotal DORGAN				1.38	40.15	0.91	
*DORN	340	m	1	1.78	20.15	5.76	0.0000
DOSEME	20	m	0	1.17	18.52	0.10	0.0000
*ENGELA	70	m	7	1.95	4.13	2.06	0.0001
FAN	4	c	0	0.33	24.61	20.77	0.1013
GAO	8	m	0	0.44	27.69	18.15	0.0208
GAO	18	f	0	0.08	34.21	46.63	0.6343
Subtotal GAO				0.24	61.90	64.78	
GER	1	c	0	0.01	14.39	21.94	0.9579
HAENSZ	37	f	0	0.08	9.58	13.04	0.7992
*HAMMON	91	m	0	1.37	1.86	0.03	0.0617
HEGMAN	4	c	0	2.26	12.50	12.76	0.0000
HINDS	24	f	3	1.36	19.35	0.23	0.0000
ISHIMA	3	c	0	1.18	5.57	0.03	0.0055

Table 3C4 - 5

IESLC - Meta-anal of Current Smoking (or Ever if Current not available), Cigs (or Any Prod if Cigs not avail)
Adenocarcinoma
Least adjusted

REF	NRR	SEX	AD	Ys	Ws	Qs	Ps
JAHN	8	m	0	1.57	6.70	0.69	0.0000
JAIN	17	m	0	2.38	3.49	4.46	0.0000
JAIN	12	f	0	1.83	14.10	4.71	0.0000
Subtotal JAIN				1.94	17.58	9.17	
JEDRYC	26	m	0	1.69	6.14	1.22	0.0000
JOLY	51	m	0	1.49	4.55	0.26	0.0015
JOLY	50	f	0	1.12	12.19	0.21	0.0001
Subtotal JOLY				1.22	16.74	0.47	
JUSSAW	28	m	0	0.63	2.35	0.91	0.3368
KATSOU	16	f	0	0.62	5.87	2.31	0.1325
KHUDER	17	m	0	2.10	5.77	4.22	0.0000
KIHARA	5	c	0	0.66	30.38	10.59	0.0003
KOO	7	f	0	0.47	13.45	8.07	0.0817
KREYBE	20	m	0	0.94	2.79	0.26	0.1158
KREYBE	36	f	0	-0.30	7.06	16.91	0.4275
Subtotal KREYBE				0.05	9.85	17.17	
LAMTH	3	f	0	0.63	21.63	8.42	0.0036
LAMWK	4	f	0	0.75	13.20	3.34	0.0068
LAMWK2	3	m	0	-0.08	8.67	15.24	0.8206
LAMWK2	7	f	0	0.57	11.11	5.16	0.0588
Subtotal LAMWK2				0.28	19.77	20.40	
LOMBA2	3	f	0	-0.64	20.27	72.40	0.0039
LUBIN	37	m	0	-0.31	3.37	8.23	0.5649
LUBIN2	252	m	0	1.21	49.29	0.07	0.0000
LUBIN2	264	f	0	0.36	39.96	31.29	0.0214
Subtotal LUBIN2				0.83	89.24	31.36	
LUO	3	c	0	0.06	12.01	17.07	0.8449
MATOS	52	m	0	2.04	4.19	2.60	0.0000
MATSUD	12	m	0	2.88	0.49	1.30	0.0440
NOU	3	m	0	1.49	3.45	0.20	0.0056
NOU	8	f	0	-0.13	6.24	11.81	0.7504
Subtotal NOU				0.45	9.69	12.02	
ORMOS	21	m	0	1.91	0.45	0.20	0.1999
OSANN	11	m	0	3.17	12.64	46.79	0.0000
OSANN	15	f	0	2.50	33.23	52.33	0.0000
Subtotal OSANN				2.69	45.87	99.12	
OSANN2	14	f	0	1.25	8.04	0.00	0.0004
PEZZOT	7	m	0	1.99	2.76	1.52	0.0009
SCHWAR	8	m	0	3.54	0.97	5.09	0.0005
SCHWAR	7	m	0	2.09	0.84	0.59	0.0556
SCHWAR	16	f	0	1.91	7.53	3.26	0.0000
SCHWAR	15	f	0	2.28	2.26	2.39	0.0006
Subtotal SCHWAR				2.13	11.60	11.33	
SEOW	2	f	0	0.86	7.03	1.06	0.0226
SIEMIA	12	m	0	2.07	4.59	3.12	0.0000
SOBUE	6	m	0	0.70	20.00	6.03	0.0018
SOBUE	22	f	0	0.35	24.55	19.96	0.0855
Subtotal SOBUE				0.51	44.54	25.99	
SOBUE2	2	m	2	1.13	82.01	1.13	0.0000
SOBUE2	6	f	2	0.59	75.22	32.87	0.0000
Subtotal SOBUE2				0.87	157.22	34.00	
STASZE	24	m	0	2.30	0.49	0.53	0.1092
STASZE	4	f	0	0.08	0.90	1.23	0.9382
Subtotal STASZE				0.86	1.39	1.76	
STAYNE	4	m	0	1.28	5.85	0.01	0.0019
SUZUK2	13	c	0	1.79	2.40	0.71	0.0055
SUZUKI	2	m	0	1.65	10.41	1.65	0.0000
SUZUKI	6	f	0	0.88	7.96	1.06	0.0128
Subtotal SUZUKI				1.32	18.36	2.72	
SVENSS	64	f	0	1.36	10.10	0.13	0.0000
TIZZAN	19	c	0	0.45	18.24	11.60	0.0538
TOKARS	7	c	0	1.19	7.03	0.03	0.0016
TSUGAN	3	m	0	-0.16	6.39	12.72	0.6813
TSUGAN	9	f	0	-0.61	3.03	10.42	0.2916
Subtotal TSUGAN				-0.31	9.41	23.14	
WAKAI	6	m	0	0.76	6.36	1.50	0.0542
WAKAI	24	f	0	0.09	5.61	7.57	0.8363
Subtotal WAKAI				0.45	11.97	9.07	
WU	2	f	0	1.44	12.39	0.47	0.0000
WU2	1	f	2	1.50	22.15	1.44	0.0000
WUWILL	25	f	0	0.32	56.90	49.35	0.0166

Table 3C4 - 5

IESLC - Meta-anal of Current Smoking (or Ever if Current not available), Cigs (or Any Prod if Cigs not avail)
Adenocarcinoma
Least adjusted

REF	NRR	SEX	AD	Ys	Ws	Qs	Ps
WYNDE2	9	m	0	0.63	4.29	1.62	0.1887
WYNDE3	30	m	0	1.38	4.98	0.08	0.0021
WYNDE3	75	f	0	0.64	6.88	2.54	0.0922
Subtotal WYNDE3				0.95	11.86	2.62	
WYNDE4	42	m	0	0.41	3.46	2.41	0.4408
WYNDE4	56	f	2	-0.51	1.67	5.18	0.5087
Subtotal WYNDE4				0.11	5.14	7.60	
WYNDE6	15	m	0	2.23	45.98	44.71	0.0000
WYNDE6	204	f	0	2.20	69.69	63.12	0.0000
Subtotal WYNDE6				2.21	115.67	107.83	
XU3	21	m	0	1.48	2.41	0.13	0.0213
XU3	25	f	0	0.26	1.91	1.86	0.7180
Subtotal XU3				0.94	4.32	1.99	
ZHENG	10	m	0	0.60	17.29	7.20	0.0121
ZHENG	21	f	0	0.15	14.95	18.12	0.5668
Subtotal ZHENG				0.39	32.24	25.31	
ZHOU	26	m	0	0.27	14.05	13.52	0.3154
ZHOU	27	f	0	0.36	4.59	3.65	0.4448
Subtotal ZHOU				0.29	18.64	17.18	

	N	116
	NS	81
	Wt	1719.06
Het	Chi	1108.80
Het	df	115
Het	P	***
Fixed	RR	3.49
	RRl	3.33
	RRu	3.66
	P	+++
Random	RR	3.06
	RRl	2.61
	RRu	3.60
	P	+++
Asymm	P	*

Table 3C4 - 6

IESLC - Meta-anal of Current Smoking (or Ever if Current not available), Cigs (or Any Prod if Cigs not avail)
Adenocarcinoma
Least adjusted

		Sex		
	combined	male	female	Total
N	11	56	49	116
NS	11	55	48	114
Wt	149.39	650.46	919.22	1719.06
Het Chi	78.16	331.65	630.88	1108.80
Het df	10	55	48	115
Het P	***	***	***	***
Fixed RR	2.26	4.36	3.19	3.49
RRl	1.92	4.04	2.99	3.33
RRu	2.65	4.71	3.40	3.66
P	+++	+++	+++	+++
Random RR	2.46	3.98	2.46	3.06
RRl	1.53	3.20	1.91	2.61
RRu	3.94	4.96	3.17	3.60
P	+++	+++	+++	+++
Between Chi				68.11
Between df				2
Between P				***
Btwn(F) P				*
Btwn(R) P				*

Table 3C4 - 7

IESLC - Meta-anal of Current Smoking (or Ever if Current not available), Cigs (or Any Prod if Cigs not avail)
Adenocarcinoma
Excluded studies (and stage at which they were excluded)

1	BOUCHA BUELL LAURIL MZILEN
2	BERRIN CHEN RESTRE TAO ULMER
5	RIMING TANG2 WYNDE5 WYNDE8
6	BLOT1 BLOT2 BLOT3 BYERS2 HIRAOK HIRAY2 JONES LICKIN MOLLO MRFIT MURATA NOTANI PERSHA SAARIK SCHWA2 SHIMIZ SITAS VANDER WARSIN WATSON WYNDER
8	ABELIN AGUDO AKIBA AMANDU AMES ARCHER ARMADA AUSTIN AUVINE AXELSO AXELSS BENSHL BEST BLOHMK BLOT4 BOFFET BRETT BROCKM BROSS CARPEN CASCO2 CASCOR CEDERL CHANG CHATZI CHEN2 CHEN3 CHIAZZ CHOW CHYOU COOKSO DARBY DAVEYS DEAN DEAN2 DEAN3 DEKLER DESTEF DOCKER DOLL2 DORANT DROSTE DU DUNN EBELIN ENSTRO ESAKI GAO2 GARCIA GARDIN GARSHI GENG GILLIS GODLEY GOLLED GOODMA GRAHAM GREGOR GSELL HAMMO2 HANSEN HEIN HENNEK HIRAYA HITOSU HOLE HOROWI HORWIT HU HU2 HUANG HUMBLE JARUP JARVHO JIANG KAISE2 KAISER KANELL KAUFMA KELLER KINLEN KJUUS KNEKT KO KOHLME KOULUM KREUZE KUBIK LANGE LAUSSM LEI LEMARC LETOUR LEVIN LIAW LIDDEL LIU LIU2 LIU3 LIU4 LIU5 LOMBAR MACLEN MAGNUS MARSH MARSH2 MARTIS MASTRA MCCONN MCDUFF MCLAUG MIGRAN MILLER MILLS MRFITR NAM NOTAN2 ODRISC PARKIN PASTOR PAWLEG PERNU PERSH2 PETO PEZZO2 PIKE PISANI POFFIJ POLEDN PRESCO QIAO QIAO2 RACHTA RADZIK RANDIG REN RONCO ROOTS ROTHSC SADOWS SANKAR SEGI SEGI2 SHAW SIMARA SIMONA SPEIZE SPITZ STOCKS STOCKW STUCKE SUN TANG TENKAN TOUSEY TULINI TVERDA VEIERO WALD WANG WANG2 WANG3 WANG4 WICKLU WIGLE WILKIN WUNSCH XIANGZ XU XU2 XU4 YAMAGU YONG YUAN ZHANG
10	BENHAM VUTUC WYNDE7

Table 3C4 - 8
Potentially overlapping studies

REF	REFGP	PRINC	OVERLAP/LINK
LUBIN2	LUBIN2	1	Lubin-combined
CHAN	CHAN	1	Chan/LamWK2
LAMWK2	LAMWK2	1	Chan/LamWK2
LAMTH	LAMTH	1	KOO/LAMTH/LAMWK
KOO	KOO	1	KOO/LAMTH/LAMWK
LAMWK	LAMWK	1	KOO/LAMTH/LAMWK
ISHIMA	AKIBA	2	AKIBA/ISHIMA
BYERS1	BYERS1	2	GRAHAM/BROSS/BYERS1
OSANN2	KAISER	2	KAISER/OSANN2
WYNDE6	WYNDE6	1	WYNDE5/6/7/8
CPSI	CPSI	1	CPSI overall
SOBUE2	SOBUE2	1	SOBUE2/MATSUD
MATSUD	MATSUD	1	SOBUE2/MATSUD
JAHN	BOFFET	2	Subset of BOFFET
LUBIN	XIANGZ	2	LUBIN/XIANGZ/QIAO

Table 3C4 - 9
Most adjusted - insufficient data for metaanalysis

REF	NRR	SEX	AGEL	AGEH	RACE	YF	LC TYPE	LOC	START	ST	NLC	R	VB	P	H	AD	SM	PRODUCT	DENOM	De
CHEN	11	c	0	0	all	-	a	As:oth	1987	CC	323	n	ot	n	y	2	ev	cig+/-ot	nev cigs	ot
CHYOU	6	m	0	0	jap	0	a	NAmer	1965	pr	227	n	bl	n	y	1	cu	cig+/-ot	nev cigs	or
JIANG	5	m	0	0	all	-	a	As:Chi	1984	CC	125	n	ot	n	n	0	ev	all/unsp	nev any	ot
JIANG	6	f	0	0	all	-	a	As:Chi	1984	CC	125	n	ot	n	n	0	ev	all/unsp	nev any	ot
KOO	4	f	0	0	all	-	a	As:HK	1981	CC	200	n	bl	n	n	0	ev	all/unsp	nev any	or
WANG	4	c	0	0	all	-	a	As:Chi	1990	CC	390	n	ot	*	y	0	ev	all/unsp	nev any	ot

REF	NRR	RR	SIG	RRDATA comment
CHEN	11	2.08	y	P < 0.001
CHYOU	6	6.80		0
JIANG	5	*	n	0
JIANG	6	*	n	0
KOO	4	1.88		0
WANG	4	2.22	n	P>0.05

Table 3C5 -

IESLC - Meta-anal of Ever Smoking (or Current if Ever not available), Cigarettes only
Adenocarcinoma

This analysis is restricted to results for:
1) Non-dose-response data
2) Results complete enough for use in metaanalysis

Within each study, results are then selected (in the following order of preference, within each sex) for:
3) SMKSTA: ever smokers, current smokers
4) PRODUCT: cigarettes only
5) CIGTYPE: all/unspecified, MC regardless of HR, MC only
6) DENOM: never smoked anything, never smoked cigarettes, (never +1 = +long term ex, +2 = +amount unknown, +3 = never cigs+long term ex)
7) Followup period (YF, prospective studies): whole study (coded as 0) or longest available
8) LCtype: adeno or nearest available, but not squamous. (q = squamous, s = small, a = adeno, l = large, KII = Kreyberg II, al = alveolar, br = bronchiolar, u = undifferentiated)
9) Race: all or nearest available, otherwise by race (wh or w = white, bl or b = black, hi = hispanic ch = chinese, jap = japanese, haw = hawaiian, w+o = white + oriental, sca = scandinavian, as = asian)
10) For overlapping studies: principal rather than subsidiary studies
Finally by Age: whole study (coded as 0) if available, otherwise by widest available age group and then for single sex results (m, f) in preference to combined sex results (c).

Results adjusted (AD) for the most potential confounders are then chosen in Sections -1 to -3 (and those which actually differ from the adjusted results in Table 3C1 - 1 are marked 'x' in Section -1) and results adjusted for the least confounders in Sections -4 to -6. (Those least adjusted results which actually differ from the most adjusted as marked 'x' in column X in Section -4) (Results adjusted for an unknown number of confounder(s) are coded as 20.)

Section -7 shows excluded studies, together with the stage (as above) at which no qualifying results were found.

Section -8 lists the potentially overlapping studies which have been included (1=principal, 2=subsidiary).

Section -9 lists any results which would have been included in preference except that they had data not complete enough for use in meta-analysis, with their significance (yes/no), if known, and any further comment as entered on the database.

In addition to those mentioned above, the following fields, levels and abbreviations are used:
* or nk = not known, n = no, y = yes, ot = other
ev = ever, cu = current, nev = never
all/unspec = all or unspecified, MC = manufactured cigarettes, HR = hand-rolled cigarettes
REF: 6-character study reference
NRR: number of the RR on the database within the study
ST : study type (CC = case control, pr or prosp = prospective)
NLC: number of lung cancer cases in whole study
R : risky occupational population (n = no, m = mining, o = other risky)
VB : national cigarette type (V = at least 75% Virginia, bl = at least 75% blended, ot = other)
P : any proxy use
H : full histological confirmation
De : derivation of RR/CI (or = original, st = standard method, ot = other method of estimation)

Table 3C5 - 1

IESLC - Meta-anal of Ever Smoking (or Current if Ever not available), Cigarettes only
Adenocarcinoma
Most adjusted

REF	NRR	3C1	SEX	AGEL	AGEH	RACE	YF	LC TYPE	LOC	START	ST	NLC	R	VB	P	H	AD	SM	PRODUCT	DENOM	De
ALDERS	94	x	m	0	0	all	-	not q+s	Eu:UK	1977	CC	1448	n	V	n	n	1	ev	cig only	nev any	ot
ALDERS	45	x	f	0	0	all	-	not q+s	Eu:UK	1977	CC	1448	n	V	n	n	1	ev	cig only	nev any	ot
BAND	2		m	0	0	all	-	a	NAmer	1983	CC	2831	n	V	y	y	2	ev	cig only	nev any	ot
BOUCOT	147		m	0	0	all	0	a	NAmer	1951	pr	121	n	bl	n	n	2	cu	cig only	nev any	ot
BRESLO	2	x	c	0	0	all	-	a	NAmer	1949	CC	518	n	bl	n	y	0	ev	cig only	nev+1	st
CPSI	404		m	0	0	all	2	a	NAmer	1959	pr	5138	n	bl	n	n	1	cu	cig only	nev any	ot
CPSI	406		f	0	0	all	2	a	NAmer	1959	pr	5138	n	bl	n	n	1	cu	cig only	nev any	ot
CPSII	115		m	0	0	all	2	a	NAmer	1982	pr	3229	n	bl	n	n	1	cu	cig only	nev any	st
DORN	340		m	0	0	wh	8	a	NAmer	1954	pr	5097	n	bl	n	n	1	cu	cig only	nev any	ot
HAMMON	83	x	m	0	0	wh	0	a	NAmer	1952	pr	448	n	bl	n	n	1	ev	cig only	nev any	ot
JUSSAW	28	x	m	0	0	all	-	KII	As:Ind	1964	CC	792	n	V	n	n	0	ev	cig only	nev any	st
LUBIN	18	x	m	0	0	all	-	KII	As:Chi	1984	CC	427	m	ot	y	n	0	ev	cig only	nev any	st
LUBIN2	144	x	m	0	0	all	-	a	Eu:mul	1976	CC	7804	n	bl	n	y	0	ev	cig only	nev any	st
PEZZOT	7		m	0	0	all	-	a	SCAmer	1987	CC	215	n	bl	n	y	0	ev	cig only	nev cigs	st
STASZE	22	x	m	0	0	all	-	a	Eu:est	1954	CC	281	n	bl	n	y	0	ev	cig only	nev any	ot
WYNDE7	64	x	m	0	0	all	-	KII	NAmer	1977	CC	2085	n	bl	n	y	0	ev	cig only	nev any	st

Cigarette type is all/unspec for all RRs

except for the following:

REF	NRR	CIGTYPE
ALDERS	45	MC only
JUSSAW	28	MC only

Table 3C5 - 2

IESLC - Meta-anal of Ever Smoking (or Current if Ever not available), Cigarettes only
Adenocarcinoma
Most adjusted

REF	NRR	SEX	AD	Number Exposed Case	Number Exposed Cont	Non-exposed Case	Non-exposed Cont	RR	95.00%CI
ALDERS	94	m	1	-	-	-	-	4.53	(1.96- 10.50)
ALDERS	45	f	1	-	-	-	-	3.69	(2.32- 5.88)
Subtotal ALDERS								3.87	(2.58- 5.82)
BAND	2	m	2	-	-	-	-	4.10	(3.01- 5.59)
*BOUCOT	147	m	2	-	-	-	-	10.95	(0.65- 183.57)
BRESLO	2	c	0	31	240	4	56	1.81	(0.61- 5.33)
*CPSI	404	m	1	-	-	-	-	4.58	(1.74- 12.05)
*CPSI	406	f	1	-	-	-	-	1.43	(0.47- 4.39)
Subtotal CPSI								2.78	(1.34- 5.78)
*CPSII	115	m	1	-	-	-	-	19.22	(6.46- 57.16)
*DORN	340	m	1	-	-	-	-	5.95	(3.85- 9.22)
*HAMMON	83	m	1	-	-	-	-	3.39	(0.77- 14.91)
JUSSAW	28	m	0	3	77	13	624	1.87	(0.52- 6.71)
LUBIN	18	m	0	5	191	4	72	0.47	(0.12- 1.80)
LUBIN2	144	m	0	686	9345	195	2616	0.98	(0.84- 1.16)
PEZZOT	7	m	0	60	317	3	116	7.32	(2.25- 23.79)
STASZE	22	m	0	15	552	0	158	8.89~	(0.53- 149.45)
WYNDE7	64	m	0	645	2108	42	918	6.69	(4.85- 9.22)
Partial Totals				1445	12830	261	4560		

*prospective study ~ With 0.5 adjustment for zero

REF	NRR	SEX	AD	Ys	Ws	Qs	Ps
ALDERS	94	m	1	1.51	5.45	2.96	0.0004
ALDERS	45	f	1	1.31	17.77	5.03	0.0000
Subtotal ALDERS				1.35	23.22	7.99	
BAND	2	m	2	1.41	40.10	16.29	0.0000
*BOUCOT	147	m	2	2.39	0.48	1.27	0.0964
BRESLO	2	c	0	0.59	3.29	0.11	0.2828
*CPSI	404	m	1	1.52	4.10	2.30	0.0021
*CPSI	406	f	1	0.36	3.08	0.53	0.5303
Subtotal CPSI				1.02	7.18	2.83	
*CPSII	115	m	1	2.96	3.23	15.40	0.0000
*DORN	340	m	1	1.78	20.15	20.55	0.0000
*HAMMON	83	m	1	1.22	1.75	0.35	0.1063
JUSSAW	28	m	0	0.63	2.35	0.05	0.3368
LUBIN	18	m	0	-0.75	2.13	4.96	0.2719
LUBIN2	144	m	0	-0.02	141.34	87.95	0.8555
PEZZOT	7	m	0	1.99	2.76	4.09	0.0009
STASZE	22	m	0	2.19	0.48	0.96	0.1290
WYNDE7	64	m	0	1.90	37.14	47.16	0.0000

	N	16
	NS	14
	Wt	285.61
Het	Chi	209.96
Het	df	15
Het	P	***
Fixed	RR	2.17
	RRl	1.93
	RRu	2.43
	P	+++
Random	RR	3.51
	RRl	2.02
	RRu	6.10
	P	+++
Asymm	P	(*)

Table 3C5 - 3

IESLC - Meta-anal of Ever Smoking (or Current if Ever not available), Cigarettes only
Adenocarcinoma
Most adjusted

		Sex		
	combined	male	female	Total
N	1	13	2	16
NS	1	13	2	16
Wt	3.29	261.48	20.84	285.61
Het Chi	0.00	204.07	2.36	209.96
Het df	0	12	1	15
Het P	N.S.	***	N.S.	***
Fixed RR	1.81	2.11	3.21	2.17
RRl	0.61	1.87	2.09	1.93
RRu	5.33	2.38	4.93	2.43
P	N.S.	+++	+++	+++
Random RR	1.81	3.95	2.65	3.51
RRl	0.61	2.05	1.09	2.02
RRu	5.33	7.63	6.42	6.10
P	N.S.	+++	+	+++
Between Chi				3.53
Between df				2
Between P				N.S.
Btwn(F) P				N.S.
Btwn(R) P				N.S.

			Lung cancer type				
	a	a+l	a+al+br	KII	not q+u	not q+s	Total
N	11			3		2	16
NS	10			3		1	14
Wt	220.76			41.63		23.22	285.61
Het Chi	137.68			17.02		0.18	209.96
Het df	10			2		1	15
Het P	***			***		N.S.	***
Fixed RR	1.71			5.43		3.87	2.17
RRl	1.50			4.01		2.58	1.93
RRu	1.96			7.36		5.82	2.43
P	+++			+++		+++	+++
Random RR	3.94			1.99		3.87	3.51
RRl	1.95			0.38		2.58	2.02
RRu	8.00			10.29		5.82	6.10
P	+++			N.S.		+++	+++
Between Chi							55.09
Between df							2
Between P							***
Btwn(F) P							N.S.
Btwn(R) P							N.S.

				Location					
	NAmer	UK	Scand	othEur	China	Japan	othAs	other	Total
N	9	2		2	1		1	1	16
NS	8	1		2	1		1	1	14
Wt	113.32	23.22		141.82	2.13		2.35	2.76	285.61
Het Chi	19.93	0.18		2.33	0.00		0.00	0.00	209.96
Het df	8	1		1	0		0	0	15
Het P	*	N.S.		N.S.	N.S.		N.S.	N.S.	***
Fixed RR	5.13	3.87		0.99	0.47		1.87	7.32	2.17
RRl	4.26	2.58		0.84	0.12		0.52	2.25	1.93
RRu	6.16	5.82		1.17	1.80		6.71	23.79	2.43
P	+++	+++		N.S.	N.S.		N.S.	+++	+++
Random RR	4.83	3.87		1.85	0.47		1.87	7.32	3.51
RRl	3.33	2.58		0.26	0.12		0.52	2.25	2.02
RRu	7.00	5.82		13.02	1.80		6.71	23.79	6.10
P	+++	+++		N.S.	N.S.		N.S.	+++	+++
Between Chi									187.52
Between df									5
Between P									***
Btwn(F) P									***
Btwn(R) P									*

Table 3C5 - 3

IESLC - Meta-anal of Ever Smoking (or Current if Ever not available), Cigarettes only
Adenocarcinoma
Most adjusted

		Detailed Country in "other Europe"					
		multi	Germany	othWest	East	Balkans	Total
	N	1			1		2
	NS	1			1		2
	Wt	141.34			0.48		141.82
Het	Chi	0.00			0.00		2.33
Het	df	0			0		1
Het	P	N.S.			N.S.		N.S.
Fixed	RR	0.98			8.89		0.99
	RRl	0.84			0.53		0.84
	RRu	1.16			149.45		1.17
	P	N.S.			N.S.		N.S.
Random	RR	0.98			8.89		1.85
	RRl	0.84			0.53		0.26
	RRu	1.16			149.45		13.02
	P	N.S.			N.S.		N.S.
Between	Chi						2.33
Between	df						1
Between	P						N.S.
Btwn(F)	P						N.S.
Btwn(R)	P						N.S.

		Detailed Country in "other Asia"			
		India	HongKong	other	Total
	N	1			1
	NS	1			1
	Wt	2.35			2.35
Het	Chi	0.00			0.00
Het	df	0			0
Het	P	N.S.			N.S.
Fixed	RR	1.87			1.87
	RRl	0.52			0.52
	RRu	6.71			6.71
	P	N.S.			N.S.
Random	RR	1.87			1.87
	RRl	0.52			0.52
	RRu	6.71			6.71
	P	N.S.			N.S.
Between	Chi				
Between	df				
Between	P				N.S.
Btwn(F)	P				N.S.
Btwn(R)	P				N.S.

		Detailed other continent			
		SCAmer	Auslia	Africa	Total
	N	1			1
	NS	1			1
	Wt	2.76			2.76
Het	Chi	0.00			0.00
Het	df	0			0
Het	P	N.S.			N.S.
Fixed	RR	7.32			7.32
	RRl	2.25			2.25
	RRu	23.79			23.79
	P	+++			+++
Random	RR	7.32			7.32
	RRl	2.25			2.25
	RRu	23.79			23.79
	P	+++			+++
Between	Chi				
Between	df				
Between	P				N.S.
Btwn(F)	P				N.S.
Btwn(R)	P				N.S.

Table 3C5 - 3

IESLC - Meta-anal of Ever Smoking (or Current if Ever not available), Cigarettes only
Adenocarcinoma
Most adjusted

	Start year of study <1960	1960-69	1970-79	1980-89	1990+	Total
N	7	1	4	4		16
NS	6	1	3	4		14
Wt	33.33	2.35	201.70	48.23		285.61
Het Chi	9.09	0.00	127.46	18.54		209.96
Het df	6	0	3	3		15
Het P	N.S.	N.S.	***	***		***
Fixed RR	4.42	1.87	1.64	4.27		2.17
RRl	3.15	0.52	1.43	3.22		1.93
RRu	6.21	6.71	1.88	5.67		2.43
P	+++	N.S.	+++	+++		+++
Random RR	3.72	1.87	3.19	4.27		3.51
RRl	2.20	0.52	1.01	1.37		2.02
RRu	6.29	6.71	10.13	13.31		6.10
P	+++	N.S.	+	+		+++
Between Chi						54.88
Between df						3
Between P						***
Btwn(F) P						N.S.
Btwn(R) P						N.S.

	Study type (1) CC	other	Total
N	10	6	16
NS	9	5	14
Wt	252.82	32.79	285.61
Het Chi	165.83	11.55	209.96
Het df	9	5	15
Het P	***	*	***
Fixed RR	1.92	5.54	2.17
RRl	1.70	3.93	1.93
RRu	2.17	7.80	2.43
P	+++	+++	+++
Random RR	2.84	5.22	3.51
RRl	1.42	2.70	2.02
RRu	5.65	10.10	6.10
P	++	+++	+++
Between Chi			32.59
Between df			1
Between P			***
Btwn(F) P			N.S.
Btwn(R) P			N.S.

	Study type (2) CC	prosp	other	Total
N	10	6		16
NS	9	5		14
Wt	252.82	32.79		285.61
Het Chi	165.83	11.55		209.96
Het df	9	5		15
Het P	***	*		***
Fixed RR	1.92	5.54		2.17
RRl	1.70	3.93		1.93
RRu	2.17	7.80		2.43
P	+++	+++		+++
Random RR	2.84	5.22		3.51
RRl	1.42	2.70		2.02
RRu	5.65	10.10		6.10
P	++	+++		+++
Between Chi				32.59
Between df				1
Between P				***
Btwn(F) P				N.S.
Btwn(R) P				N.S.

Table 3C5 - 3

IESLC - Meta-anal of Ever Smoking (or Current if Ever not available), Cigarettes only
Adenocarcinoma
Most adjusted

	Study size (number of LC cases)				
	100-249	250-499	500-999	1000+	Total
N	2	3	2	9	16
NS	2	3	2	7	14
Wt	3.25	4.36	5.64	272.36	285.61
Het Chi	0.07	5.54	0.00	198.16	209.96
Het df	1	2	1	8	15
Het P	N.S.	(*)	N.S.	***	***
Fixed RR	7.77	1.44	1.83	2.16	2.17
RRl	2.62	0.56	0.80	1.91	1.93
RRu	23.06	3.68	4.19	2.43	2.43
P	+++	N.S.	N.S.	+++	+++
Random RR	7.77	1.89	1.83	4.04	3.51
RRl	2.62	0.35	0.80	2.00	2.02
RRu	23.06	10.23	4.19	8.14	6.10
P	+++	N.S.	N.S.	+++	+++
Between Chi					6.19
Between df					3
Between P					N.S.
Btwn(F) P					N.S.
Btwn(R) P					N.S.

	Risky occupational population			
	no	mining	othRisky	Total
N	15	1		16
NS	13	1		14
Wt	283.48	2.13		285.61
Het Chi	204.96	0.00		209.96
Het df	14	0		15
Het P	***	N.S.		***
Fixed RR	2.19	0.47		2.17
RRl	1.95	0.12		1.93
RRu	2.46	1.80		2.43
P	+++	N.S.		+++
Random RR	3.94	0.47		3.51
RRl	2.24	0.12		2.02
RRu	6.96	1.80		6.10
P	+++	N.S.		+++
Between Chi				5.00
Between df				1
Between P				*
Btwn(F) P				N.S.
Btwn(R) P				**

	National cigarette tobacco type			
	Virginia	blended	other	Total
N	4	11	1	16
NS	3	10	1	14
Wt	65.67	217.81	2.13	285.61
Het Chi	1.55	174.89	0.00	209.96
Het df	3	10	0	15
Het P	N.S.	***	N.S.	***
Fixed RR	3.91	1.84	0.47	2.17
RRl	3.07	1.61	0.12	1.93
RRu	4.98	2.10	1.80	2.43
P	+++	+++	N.S.	+++
Random RR	3.91	4.24	0.47	3.51
RRl	3.07	1.92	0.12	2.02
RRu	4.98	9.37	1.80	6.10
P	+++	+++	N.S.	+++
Between Chi				33.52
Between df				2
Between P				***
Btwn(F) P				N.S.
Btwn(R) P				**

Table 3C5 - 3

IESLC - Meta-anal of Ever Smoking (or Current if Ever not available), Cigarettes only
Adenocarcinoma
Most adjusted

	Any proxy use		
	No/nk	Yes	Total
N	14	2	16
NS	12	2	14
Wt	243.38	42.23	285.61
Het Chi	186.66	9.47	209.96
Het df	13	1	15
Het P	***	**	***
Fixed RR	1.98	3.68	2.17
RRl	1.74	2.72	1.93
RRu	2.24	4.97	2.43
P	+++	+++	+++
Random RR	3.95	1.54	3.51
RRl	2.09	0.19	2.02
RRu	7.48	12.71	6.10
P	+++	N.S.	+++
Between Chi			13.83
Between df			1
Between P			***
Btwn(F) P			N.S.
Btwn(R) P			N.S.

	Full histological confirmation		
	No	Yes	Total
N	10	6	16
NS	8	6	14
Wt	60.50	225.11	285.61
Het Chi	26.11	149.23	209.96
Het df	9	5	15
Het P	**	***	***
Fixed RR	4.24	1.81	2.17
RRl	3.30	1.59	1.93
RRu	5.46	2.06	2.43
P	+++	+++	+++
Random RR	3.69	3.43	3.51
RRl	2.23	1.31	2.02
RRu	6.10	8.98	6.10
P	+++	+	+++
Between Chi			34.62
Between df			1
Between P			***
Btwn(F) P			N.S.
Btwn(R) P			N.S.

	Number of adjustment variables (1)			
	0	1	2+/+nk	Total
N	7	7	2	16
NS	7	5	2	14
Wt	189.50	55.53	40.58	285.61
Het Chi	119.58	13.12	0.46	209.96
Het df	6	6	1	15
Het P	***	*	N.S.	***
Fixed RR	1.50	4.74	4.15	2.17
RRl	1.30	3.64	3.05	1.93
RRu	1.73	6.17	5.64	2.43
P	+++	+++	+++	+++
Random RR	2.36	4.68	4.15	3.51
RRl	0.86	3.01	3.05	2.02
RRu	6.48	7.30	5.64	6.10
P	(+)	+++	+++	+++
Between Chi				76.80
Between df				2
Between P				***
Btwn(F) P				(*)
Btwn(R) P				N.S.

Table 3C5 - 3

IESLC - Meta-anal of Ever Smoking (or Current if Ever not available), Cigarettes only
Adenocarcinoma
Most adjusted

	Number of adjustment variables (2) 0	1	2	3-5	6+/+nk	Total
N	7	7	2			16
NS	7	5	2			14
Wt	189.50	55.53	40.58			285.61
Het Chi	119.58	13.12	0.46			209.96
Het df	6	6	1			15
Het P	***	*	N.S.			***
Fixed RR	1.50	4.74	4.15			2.17
RRl	1.30	3.64	3.05			1.93
RRu	1.73	6.17	5.64			2.43
P	+++	+++	+++			+++
Random RR	2.36	4.68	4.15			3.51
RRl	0.86	3.01	3.05			2.02
RRu	6.48	7.30	5.64			6.10
P	(+)	+++	+++			+++
Between Chi						76.80
Between df						2
Between P						***
Btwn(F) P						(*)
Btwn(R) P						N.S.

	Product all/unsp	cig+/-ot	cig only	Total
N			16	16
NS			14	14
Wt			285.61	285.61
Het Chi			209.96	209.96
Het df			15	15
Het P			***	***
Fixed RR			2.17	2.17
RRl			1.93	1.93
RRu			2.43	2.43
P			+++	+++
Random RR			3.51	3.51
RRl			2.02	2.02
RRu			6.10	6.10
P			+++	+++
Between Chi				
Between df				
Between P				N.S.
Btwn(F) P				N.S.
Btwn(R) P				N.S.

	Denominator nev any	nev cigs	Total
N	15	1	16
NS	13	1	14
Wt	282.85	2.76	285.61
Het Chi	205.83	0.00	209.96
Het df	14	0	15
Het P	***	N.S.	***
Fixed RR	2.14	7.32	2.17
RRl	1.91	2.25	1.93
RRu	2.41	23.79	2.43
P	+++	+++	+++
Random RR	3.34	7.32	3.51
RRl	1.89	2.25	2.02
RRu	5.92	23.79	6.10
P	+++	+++	+++
Between Chi			4.13
Between df			1
Between P			*
Btwn(F) P			N.S.
Btwn(R) P			N.S.

Table 3C5 - 3

IESLC - Meta-anal of Ever Smoking (or Current if Ever not available), Cigarettes only
Adenocarcinoma
Most adjusted

	Derivation of RR/CI Orig	StdCalc	Other	Total
N		7	9	16
NS		7	7	14
Wt		192.25	93.36	285.61
Het Chi		138.80	7.17	209.96
Het df		6	8	15
Het P		***	N.S.	***
Fixed RR		1.56	4.28	2.17
RRl		1.35	3.49	1.93
RRu		1.79	5.24	2.43
P		+++	+++	+++
Random RR		2.87	4.28	3.51
RRl		1.03	3.49	2.02
RRu		7.96	5.24	6.10
P		+	+++	+++
Between Chi				64.00
Between df				1
Between P				***
Btwn(F) P				*
Btwn(R) P				N.S.

	Smoking status ever	current	Total
N	11	5	16
NS	10	4	14
Wt	254.57	31.04	285.61
Het Chi	166.39	11.10	209.96
Het df	10	4	15
Het P	***	*	***
Fixed RR	1.93	5.69	2.17
RRl	1.70	4.00	1.93
RRu	2.18	8.09	2.43
P	+++	+++	+++
Random RR	2.87	5.56	3.51
RRl	1.49	2.61	2.02
RRu	5.55	11.85	6.10
P	++	+++	+++
Between Chi			32.47
Between df			1
Between P			***
Btwn(F) P			N.S.
Btwn(R) P			N.S.

Table 3C5 - 4

IESLC - Meta-anal of Ever Smoking (or Current if Ever not available), Cigarettes only
Adenocarcinoma
Least adjusted

REF	NRR	X	SEX	AGEL	AGEH	RACE	YF	LC TYPE	LOC	START	ST	NLC	R	VB	P	H	AD	SM	PRODUCT	DENOM	De
ALDERS	107	x	m	0	0	all	-	not q+s	Eu:UK	1977	CC	1448	n	V	n	n	0	ev	cig only	nev any	st
ALDERS	104	x	f	0	0	all	-	not q+s	Eu:UK	1977	CC	1448	n	V	n	n	0	ev	cig only	nev any	st
BAND	2		m	0	0	all	-	a	NAmer	1983	CC	2831	n	V	y	y	2	ev	cig only	nev any	ot
BOUCOT	72	x	m	0	0	all	0	a	NAmer	1951	pr	121	n	bl	n	n	0	cu	cig only	nev any	ot
BRESLO	2		c	0	0	all	-	a	NAmer	1949	CC	518	n	bl	n	y	0	ev	cig only	nev+1	st
CPSI	404		m	0	0	all	2	a	NAmer	1959	pr	5138	n	bl	n	n	1	cu	cig only	nev any	ot
CPSI	406		f	0	0	all	2	a	NAmer	1959	pr	5138	n	bl	n	n	1	cu	cig only	nev any	ot
CPSII	115		m	0	0	all	2	a	NAmer	1982	pr	3229	n	bl	n	n	1	cu	cig only	nev any	st
DORN	340		m	0	0	wh	8	a	NAmer	1954	pr	5097	n	bl	n	n	1	cu	cig only	nev any	ot
HAMMON	90	x	m	0	0	wh	0	a	NAmer	1952	pr	448	n	bl	n	n	0	ev	cig only	nev any	st
JUSSAW	28		m	0	0	all	-	KII	As:Ind	1964	CC	792	n	V	n	n	0	ev	cig only	nev any	st
LUBIN	18		m	0	0	all	-	KII	As:Chi	1984	CC	427	m	ot	y	n	0	ev	cig only	nev any	st
LUBIN2	144		m	0	0	all	-	a	Eu:mul	1976	CC	7804	n	bl	n	y	0	ev	cig only	nev any	st
PEZZOT	7		m	0	0	all	-	a	SCAmer	1987	CC	215	n	bl	n	y	0	ev	cig only	nev cigs	st
STASZE	22		m	0	0	all	-	a	Eu:est	1954	CC	281	n	bl	n	y	0	ev	cig only	nev any	ot
WYNDE7	64		m	0	0	all	-	KII	NAmer	1977	CC	2085	n	bl	n	y	0	ev	cig only	nev any	st

Cigarette type is all/unspec for all RRs

except for the following:

REF	NRR	CIGTYPE
ALDERS	104	MC only
JUSSAW	28	MC only

Table 3C5 - 5

IESLC - Meta-anal of Ever Smoking (or Current if Ever not available), Cigarettes only
Adenocarcinoma
Least adjusted

REF	NRR	SEX	AD	Number Exposed Case	Cont	Non-exposed Case	Cont	RR	95.00%CI
ALDERS	107	m	0	102	462	6	133	4.89	(2.10- 11.40)
ALDERS	104	f	0	104	371	25	243	2.72	(1.71- 4.34)
Subtotal ALDERS								3.12	(2.08- 4.69)
BAND	2	m	2	-	-	-	-	4.10	(3.01- 5.59)
*BOUCOT	72	m	0	14	22177	0	7551	9.87~	(0.59- 165.51)
BRESLO	2	c	0	31	240	4	56	1.81	(0.61- 5.33)
*CPSI	404	m	1	-	-	-	-	4.58	(1.74- 12.05)
*CPSI	406	f	1	-	-	-	-	1.43	(0.47- 4.39)
Subtotal CPSI								2.78	(1.34- 5.78)
*CPSII	115	m	1	-	-	-	-	19.22	(6.46- 57.16)
*DORN	340	m	1	-	-	-	-	5.95	(3.85- 9.22)
*HAMMON	90	m	0	14	225565	2	115884	3.60	(0.82- 15.82)
JUSSAW	28	m	0	3	77	13	624	1.87	(0.52- 6.71)
LUBIN	18	m	0	5	191	4	72	0.47	(0.12- 1.80)
LUBIN2	144	m	0	686	9345	195	2616	0.98	(0.84- 1.16)
PEZZOT	7	m	0	60	317	3	116	7.32	(2.25- 23.79)
STASZE	22	m	0	15	552	0	158	8.89~	(0.53- 149.45)
WYNDE7	64	m	0	645	2108	42	918	6.69	(4.85- 9.22)
Partial Totals				1679	261405	294	128371		

*prospective study ~ With 0.5 adjustment for zero

REF	NRR	SEX	AD	Ys	Ws	Qs	Ps
ALDERS	107	m	0	1.59	5.37	3.72	0.0002
ALDERS	104	f	0	1.00	17.72	1.08	0.0000
Subtotal ALDERS				1.14	23.09	4.79	
BAND	2	m	2	1.41	40.10	17.20	0.0000
*BOUCOT	72	m	0	2.29	0.48	1.14	0.1114
BRESLO	2	c	0	0.59	3.29	0.09	0.2828
*CPSI	404	m	1	1.52	4.10	2.41	0.0021
*CPSI	406	f	1	0.36	3.08	0.49	0.5303
Subtotal CPSI				1.02	7.18	2.89	
*CPSII	115	m	1	2.96	3.23	15.64	0.0000
*DORN	340	m	1	1.78	20.15	21.26	0.0000
*HAMMON	90	m	0	1.28	1.75	0.48	0.0904
JUSSAW	28	m	0	0.63	2.35	0.04	0.3368
LUBIN	18	m	0	-0.75	2.13	4.85	0.2719
LUBIN2	144	m	0	-0.02	141.34	84.10	0.8555
PEZZOT	7	m	0	1.99	2.76	4.21	0.0009
STASZE	22	m	0	2.19	0.48	0.99	0.1290
WYNDE7	64	m	0	1.90	37.14	48.63	0.0000

	N	16
	NS	14
	Wt	285.49
Het	Chi	206.32
Het	df	15
Het	P	***
Fixed	RR	2.13
	RRl	1.90
	RRu	2.39
	P	+++
Random	RR	3.44
	RRl	1.99
	RRu	5.96
	P	+++
Asymm	P	(*)

Table 3C5 - 6

IESLC - Meta-anal of Ever Smoking (or Current if Ever not available), Cigarettes only
Adenocarcinoma
Least adjusted

		Sex		
	combined	male	female	Total
N	1	13	2	16
NS	1	13	2	16
Wt	3.29	261.40	20.80	285.49
Het Chi	0.00	204.64	1.09	206.32
Het df	0	12	1	15
Het P	N.S.	***	N.S.	***
Fixed RR	1.81	2.11	2.48	2.13
RRl	0.61	1.87	1.61	1.90
RRu	5.33	2.38	3.81	2.39
P	N.S.	+++	+++	+++
Random RR	1.81	3.98	2.43	3.44
RRl	0.61	2.06	1.50	1.99
RRu	5.33	7.70	3.94	5.96
P	N.S.	+++	+++	+++
Between Chi				0.59
Between df				2
Between P				N.S.
Btwn(F) P				N.S.
Btwn(R) P				N.S.

Table 3C5 - 7

IESLC - Meta-anal of Ever Smoking (or Current if Ever not available), Cigarettes only
Adenocarcinoma
Excluded studies (and stage at which they were excluded)

1	BOUCHA BUELL LAURIL MZILEN
2	BERRIN CHEN RESTRE TAO ULMER
4	ABRAHA AKIBA AMANDU AMES ANDERS ARCHER AUSTIN AUVINE AXELSO AXELSS BARBON BECHER BENSHL BLOHMK BLOT1 BLOT2 BLOT3 BLOT4 BRETT BROCKM BROSS BROWN1 BROWN2 BUFFLE BYERS1 BYERS2 CARPEN CASCO2 CASCOR CHAN CHANG CHATZI CHEN2 CHEN3 CHIAZZ CHOI CHYOU COMSTO COOKSO CORREA DARBY DAVEYS DEKLER DESTE2 DESTEF DOCKER DORANT DORGAN DOSEME DROSTE DU DUNN EBELIN ESAKI FAN GAO GAO2 GARCIA GARSHI GENG GER GILLIS GODLEY GOODMA GREGOR GSELL HAENSZ HAMMO2 HANSEN HEGMAN HENNEK HINDS HIRAOK HIRAYA HITOSU HOLE HOROWI HORWIT HU HU2 HUANG HUMBLE ISHIMA JAHN JAIN JARUP JARVHO JEDRYC JIANG JONES KAISER KANELL KATSOU KAUFMA KELLER KHUDER KIHARA KINLEN KNEKT KO KOHLME KOO KREUZE KREYBE KUBIK LAMTH LAMWK LAMWK2 LAUSSM LEI LEMARC LETOUR LEVIN LIAW LICKIN LIDDEL LIU LIU2 LIU3 LIU5 LOMBA2 LUO MACLEN MAGNUS MARSH MARSH2 MARTIS MASTRA MATOS MATSUD MCDUFF MCLAUG MILLER MOLLO MRFIT MRFITR MURATA NAM NOTANI NOU ODRISC ORMOS OSANN OSANN2 PARKIN PASTOR PAWLEG PERSH2 PERSHA PETO PEZZO2 PIKE PISANI POFFIJ POLEDN PRESCO QIAO2 RACHTA RADZIK RANDIG REN ROOTS ROTHSC SAARIK SANKAR SCHWAR SEGI SEGI2 SEOW SHAW SHIMIZ SIEMIA SIMARA SIMONA SITAS SOBUE SOBUE2 SPEIZE SPITZ STAYNE STOCKS STOCKW STUCKE SUN SUZUKI SVENSS TANG TENKAN TOKARS TOUSEY TSUGAN TULINI VANDER VUTUC WAKAI WANG WANG2 WANG3 WANG4 WARSIN WATSON WICKLU WILKIN WU WU2 WUNSCH WUWILL WYNDE2 WYNDE3 WYNDE4 WYNDE5 WYNDE6 WYNDE8 XU XU2 XU3 XU4 YAMAGU YONG YUAN ZHANG ZHENG ZHOU
5	LANGE RIMING TANG2
6	HIRAY2 SCHWA2 WYNDER
8	ABELIN AGUDO ARMADA BEST BOFFET CEDERL CHOW DAMBER DEAN DEAN2 DEAN3 DOLL DOLL2 ENGELA ENSTRO GARDIN GOLLED GRAHAM HEIN JOLY KAISE2 KJUUS KOULUM LIU4 LOMBAR MCCONN MIGRAN MILLS NOTAN2 PERNU QIAO RONCO SADOWS SUZUK2 TIZZAN TVERDA VEIERO WALD WIGLE XIANGZ
10	BENHAM

Table 3C5 - 8

Potentially overlapping studies

REF	REFGP	PRINC	OVERLAP/LINK
LUBIN2	LUBIN2	1	Lubin-combined
WYNDE7	WYNDE6	2	WYNDE5/6/7/8
CPSI	CPSI	1	CPSI overall
LUBIN	XIANGZ	2	LUBIN/XIANGZ/QIAO

Table 3C6 -

IESLC - Meta-anal of Current Smoking (or Ever if Current not available), Cigarettes only
Adenocarcinoma

This analysis is restricted to results for:
1) Non-dose-response data
2) Results complete enough for use in metaanalysis

Within each study, results are then selected (in the following order of preference, within each sex) for:
3) SMKSTA: current smokers, ever smokers
4) PRODUCT: cigarettes only
5) CIGTYPE: all/unspecified, MC regardless of HR, MC only
6) DENOM: never smoked anything, never smoked cigarettes, (never +1 = +long term ex, +2 = +amount unknown, +3 = never cigs+long term ex)
7) Followup period (YF, prospective studies): whole study (coded as 0) or longest available
8) LCtype: adeno or nearest available, but not squamous. (q = squamous, s = small, a = adeno, l = large, KII = Kreyberg II, al = alveolar, br = bronchiolar, u = undifferentiated)
9) Race: all or nearest available, otherwise by race (wh or w = white, bl or b = black, hi = hispanic ch = chinese, jap = japanese, haw = hawaiian, w+o = white + oriental, sca = scandinavian, as = asian)
10) For overlapping studies: principal rather than subsidiary studies
Finally by Age: whole study (coded as 0) if available, otherwise by widest available age group
and then for single sex results (m, f) in preference to combined sex results (c).

Results adjusted (AD) for the most potential confounders are then chosen in Sections -1 to -3
(and those which actually differ from the adjusted results in Table 3C5 - 1 are marked 'x' in Section -1)
and results adjusted for the least confounders in Sections -4 to -6. (Those least adjusted results which
actually differ from the most adjusted as marked 'x' in column X in Section -4)
(Results adjusted for an unknown number of confounder(s) are coded as 20.)

Section -7 shows excluded studies, together with the stage (as above) at which no qualifying
results were found.

Section -8 lists the potentially overlapping studies which have been included (1=principal, 2=subsidiary).

Section -9 lists any results which would have been included in preference except that they had data not complete
enough for use in meta-analysis, with their significance (yes/no), if known, and any further comment as entered
on the database.

In addition to those mentioned above, the following fields, levels and abbreviations are used:
* or nk = not known, n = no, y = yes, ot = other
ev = ever, cu = current, nev = never
all/unspec = all or unspecified, MC = manufactured cigarettes, HR = hand-rolled cigarettes
REF: 6-character study reference
NRR: number of the RR on the database within the study
ST : study type (CC = case control, pr or prosp = prospective)
NLC: number of lung cancer cases in whole study
R : risky occupational population (n = no, m = mining, o = other risky)
VB : national cigarette type (V = at least 75% Virginia, bl = at least 75% blended, ot = other)
P : any proxy use
H : full histological confirmation
De : derivation of RR/CI (or = original, st = standard method, ot = other method of estimation)

Table 3C6 - 1

IESLC - Meta-anal of Current Smoking (or Ever if Current not available), Cigarettes only
Adenocarcinoma
Most adjusted

REF	NRR	3C5	SEX	AGEL	AGEH	RACE	YF	LC TYPE	LOC	START	ST	NLC	R	VB	P	H	AD	SM	PRODUCT	DENOM	De
ALDERS	94		m	0	0	all	-	not q+s	Eu:UK	1977	CC	1448	n	V	n	n	1	ev	cig only	nev any	ot
ALDERS	45		f	0	0	all	-	not q+s	Eu:UK	1977	CC	1448	n	V	n	n	1	ev	cig only	nev any	ot
BAND	2		m	0	0	all	-	a	NAmer	1983	CC	2831	n	V	y	y	2	ev	cig only	nev any	ot
BOUCOT	147		m	0	0	all	0	a	NAmer	1951	pr	121	n	bl	n	n	2	cu	cig only	nev any	ot
BRESLO	2		c	0	0	all	-	a	NAmer	1949	CC	518	n	bl	n	y	0	ev	cig only	nev+1	st
CPSI	404		m	0	0	all	2	a	NAmer	1959	pr	5138	n	bl	n	n	1	cu	cig only	nev any	ot
CPSI	406		f	0	0	all	2	a	NAmer	1959	pr	5138	n	bl	n	n	1	cu	cig only	nev any	ot
CPSII	115		m	0	0	all	2	a	NAmer	1982	pr	3229	n	bl	n	n	1	cu	cig only	nev any	st
DORN	340		m	0	0	wh	8	a	NAmer	1954	pr	5097	n	bl	n	n	1	cu	cig only	nev any	ot
HAMMON	83		m	0	0	wh	0	a	NAmer	1952	pr	448	n	bl	n	n	1	ev	cig only	nev any	ot
JUSSAW	28		m	0	0	all	-	KII	As:Ind	1964	CC	792	n	V	n	n	0	ev	cig only	nev any	st
LUBIN	18		m	0	0	all	-	KII	As:Chi	1984	CC	427	m	ot	y	n	0	ev	cig only	nev any	st
LUBIN2	144		m	0	0	all	-	a	Eu:mul	1976	CC	7804	n	bl	n	y	0	ev	cig only	nev any	st
PEZZOT	7		m	0	0	all	-	a	SCAmer	1987	CC	215	n	bl	n	y	0	ev	cig only	nev cigs	st
STASZE	22		m	0	0	all	-	a	Eu:est	1954	CC	281	n	bl	n	y	0	ev	cig only	nev any	ot
WYNDE7	32	x	m	0	0	all	-	KII	NAmer	1977	CC	2085	n	bl	n	y	0	cu	cig only	nev any	st

Cigarette type is all/unspec for all RRs

except for the following:

REF	NRR	CIGTYPE
ALDERS	45	MC only
JUSSAW	28	MC only

Table 3C6 - 2

IESLC - Meta-anal of Current Smoking (or Ever if Current not available), Cigarettes only
Adenocarcinoma
Most adjusted

REF	NRR	SEX	AD	Number Exposed Case	Number Exposed Cont	Non-exposed Case	Non-exposed Cont	RR	95.00%CI
ALDERS	94	m	1	-	-	-	-	4.53	(1.96- 10.50)
ALDERS	45	f	1	-	-	-	-	3.69	(2.32- 5.88)
Subtotal ALDERS								3.87	(2.58- 5.82)
BAND	2	m	2	-	-	-	-	4.10	(3.01- 5.59)
*BOUCOT	147	m	2	-	-	-	-	10.95	(0.65- 183.57)
BRESLO	2	c	0	31	240	4	56	1.81	(0.61- 5.33)
*CPSI	404	m	1	-	-	-	-	4.58	(1.74- 12.05)
*CPSI	406	f	1	-	-	-	-	1.43	(0.47- 4.39)
Subtotal CPSI								2.78	(1.34- 5.78)
*CPSII	115	m	1	-	-	-	-	19.22	(6.46- 57.16)
*DORN	340	m	1	-	-	-	-	5.95	(3.85- 9.22)
*HAMMON	83	m	1	-	-	-	-	3.39	(0.77- 14.91)
JUSSAW	28	m	0	3	77	13	624	1.87	(0.52- 6.71)
LUBIN	18	m	0	5	191	4	72	0.47	(0.12- 1.80)
LUBIN2	144	m	0	686	9345	195	2616	0.98	(0.84- 1.16)
PEZZOT	7	m	0	60	317	3	116	7.32	(2.25- 23.79)
STASZE	22	m	0	15	552	0	158	8.89~	(0.53- 149.45)
WYNDE7	32	m	0	441	993	42	918	9.71	(6.99- 13.49)
Partial Totals				1241	11715	261	4560		

*prospective study

~ With 0.5 adjustment for zero

REF	NRR	SEX	AD	Ys	Ws	Qs	Ps
ALDERS	94	m	1	1.51	5.45	2.65	0.0004
ALDERS	45	f	1	1.31	17.77	4.30	0.0000
Subtotal ALDERS				1.35	23.22	6.95	
BAND	2	m	2	1.41	40.10	14.31	0.0000
*BOUCOT	147	m	2	2.39	0.48	1.20	0.0964
BRESLO	2	c	0	0.59	3.29	0.16	0.2828
*CPSI	404	m	1	1.52	4.10	2.06	0.0021
*CPSI	406	f	1	0.36	3.08	0.64	0.5303
Subtotal CPSI				1.02	7.18	2.70	
*CPSII	115	m	1	2.96	3.23	14.84	0.0000
*DORN	340	m	1	1.78	20.15	18.95	0.0000
*HAMMON	83	m	1	1.22	1.75	0.29	0.1063
JUSSAW	28	m	0	0.63	2.35	0.08	0.3368
LUBIN	18	m	0	-0.75	2.13	5.23	0.2719
LUBIN2	144	m	0	-0.02	141.34	97.10	0.8555
PEZZOT	7	m	0	1.99	2.76	3.83	0.0009
STASZE	22	m	0	2.19	0.48	0.91	0.1290
WYNDE7	32	m	0	2.27	35.49	75.59	0.0000

	N	16
	NS	14
	Wt	283.96
Het	Chi	242.14
Het	df	15
Het	P	***
Fixed	RR	2.26
	RRl	2.01
	RRu	2.53
	P	+++
Random	RR	3.62
	RRl	2.00
	RRu	6.53
	P	+++
Asymm	P	N.S.

Table 3C6 - 3

IESLC - Meta-anal of Current Smoking (or Ever if Current not available), Cigarettes only
Adenocarcinoma
Most adjusted

		Sex		
	combined	male	female	Total
N	1	13	2	16
NS	1	13	2	16
Wt	3.29	259.83	20.84	283.96
Het Chi	0.00	236.87	2.36	242.14
Het df	0	12	1	15
Het P	N.S.	***	N.S.	***
Fixed RR	1.81	2.20	3.21	2.26
RRl	0.61	1.95	2.09	2.01
RRu	5.33	2.48	4.93	2.53
P	N.S.	+++	+++	+++
Random RR	1.81	4.11	2.65	3.62
RRl	0.61	2.03	1.09	2.00
RRu	5.33	8.33	6.42	6.53
P	N.S.	+++	+	+++
Between Chi				2.91
Between df				2
Between P				N.S.
Btwn(F) P				N.S.
Btwn(R) P				N.S.

	Smoking status		
	ever	current	Total
N	10	6	16
NS	9	5	14
Wt	217.43	66.54	283.96
Het Chi	99.04	15.82	242.14
Het df	9	5	15
Het P	***	**	***
Fixed RR	1.56	7.57	2.26
RRl	1.36	5.95	2.01
RRu	1.78	9.62	2.53
P	+++	+++	+++
Random RR	2.57	6.47	3.62
RRl	1.35	3.71	2.00
RRu	4.88	11.28	6.53
P	++	+++	+++
Between Chi			127.28
Between df			1
Between P			***
Btwn(F) P			**
Btwn(R) P			*

Table 3C6 - 4

IESLC - Meta-anal of Current Smoking (or Ever if Current not available), Cigarettes only
Adenocarcinoma
Least adjusted

REF	NRR	X	SEX	AGEL	AGEH	RACE	YF	LC TYPE	LOC	START	ST	NLC	R	VB	P	H	AD	SM	PRODUCT	DENOM	De
ALDERS	107	x	m	0	0	all	-	not q+s	Eu:UK	1977	CC	1448	n	V	n	n	0	ev	cig only	nev any	st
ALDERS	104	x	f	0	0	all	-	not q+s	Eu:UK	1977	CC	1448	n	V	n	n	0	ev	cig only	nev any	st
BAND	2		m	0	0	all	-	a	NAmer	1983	CC	2831	n	V	y	y	2	ev	cig only	nev any	ot
BOUCOT	72	x	m	0	0	all	0	a	NAmer	1951	pr	121	n	bl	n	n	0	cu	cig only	nev any	ot
BRESLO	2		c	0	0	all	-	a	NAmer	1949	CC	518	n	bl	n	y	0	ev	cig only	nev+1	st
CPSI	404		m	0	0	all	2	a	NAmer	1959	pr	5138	n	bl	n	n	1	cu	cig only	nev any	ot
CPSI	406		f	0	0	all	2	a	NAmer	1959	pr	5138	n	bl	n	n	1	cu	cig only	nev any	ot
CPSII	115		m	0	0	all	2	a	NAmer	1982	pr	3229	n	bl	n	n	1	cu	cig only	nev any	st
DORN	340		m	0	0	wh	8	a	NAmer	1954	pr	5097	n	bl	n	n	1	cu	cig only	nev any	ot
HAMMON	90	x	m	0	0	wh	0	a	NAmer	1952	pr	448	n	bl	n	n	0	ev	cig only	nev any	st
JUSSAW	28		m	0	0	all	-	KII	As:Ind	1964	CC	792	n	V	n	n	0	ev	cig only	nev any	st
LUBIN	18		m	0	0	all	-	KII	As:Chi	1984	CC	427	m	ot	y	n	0	ev	cig only	nev any	st
LUBIN2	144		m	0	0	all	-	a	Eu:mul	1976	CC	7804	n	bl	n	y	0	ev	cig only	nev any	st
PEZZOT	7		m	0	0	all	-	a	SCAmer	1987	CC	215	n	bl	n	y	0	ev	cig only	nev cigs	st
STASZE	22		m	0	0	all	-	a	Eu:est	1954	CC	281	n	bl	n	y	0	ev	cig only	nev any	ot
WYNDE7	32		m	0	0	all	-	KII	NAmer	1977	CC	2085	n	bl	n	y	0	cu	cig only	nev any	st

Cigarette type is all/unspec for all RRs

except for the following:

REF	NRR	CIGTYPE
ALDERS	104	MC only
JUSSAW	28	MC only

Table 3C6 - 5

IESLC - Meta-anal of Current Smoking (or Ever if Current not available), Cigarettes only
Adenocarcinoma
Least adjusted

REF	NRR	SEX	AD	Number Exposed Case	Cont	Non-exposed Case	Cont	RR	95.00%CI
ALDERS	107	m	0	102	462	6	133	4.89	(2.10- 11.40)
ALDERS	104	f	0	104	371	25	243	2.72	(1.71- 4.34)
Subtotal ALDERS								3.12	(2.08- 4.69)
BAND	2	m	2	-	-	-	-	4.10	(3.01- 5.59)
*BOUCOT	72	m	0	14	22177	0	7551	9.87~	(0.59- 165.51)
BRESLO	2	c	0	31	240	4	56	1.81	(0.61- 5.33)
*CPSI	404	m	1	-	-	-	-	4.58	(1.74- 12.05)
*CPSI	406	f	1	-	-	-	-	1.43	(0.47- 4.39)
Subtotal CPSI								2.78	(1.34- 5.78)
*CPSII	115	m	1	-	-	-	-	19.22	(6.46- 57.16)
*DORN	340	m	1	-	-	-	-	5.95	(3.85- 9.22)
*HAMMON	90	m	0	14	225565	2	115884	3.60	(0.82- 15.82)
JUSSAW	28	m	0	3	77	13	624	1.87	(0.52- 6.71)
LUBIN	18	m	0	5	191	4	72	0.47	(0.12- 1.80)
LUBIN2	144	m	0	686	9345	195	2616	0.98	(0.84- 1.16)
PEZZOT	7	m	0	60	317	3	116	7.32	(2.25- 23.79)
STASZE	22	m	0	15	552	0	158	8.89~	(0.53- 149.45)
WYNDE7	32	m	0	441	993	42	918	9.71	(6.99- 13.49)
Partial Totals				1475	260290	294	128371		

*prospective study ~ With 0.5 adjustment for zero

REF	NRR	SEX	AD	Ys	Ws	Qs	Ps
ALDERS	107	m	0	1.59	5.37	3.37	0.0002
ALDERS	104	f	0	1.00	17.72	0.75	0.0000
Subtotal ALDERS				1.14	23.09	4.12	
BAND	2	m	2	1.41	40.10	15.17	0.0000
*BOUCOT	72	m	0	2.29	0.48	1.08	0.1114
BRESLO	2	c	0	0.59	3.29	0.14	0.2828
*CPSI	404	m	1	1.52	4.10	2.16	0.0021
*CPSI	406	f	1	0.36	3.08	0.59	0.5303
Subtotal CPSI				1.02	7.18	2.75	
*CPSII	115	m	1	2.96	3.23	15.08	0.0000
*DORN	340	m	1	1.78	20.15	19.64	0.0000
*HAMMON	90	m	0	1.28	1.75	0.41	0.0904
JUSSAW	28	m	0	0.63	2.35	0.07	0.3368
LUBIN	18	m	0	-0.75	2.13	5.11	0.2719
LUBIN2	144	m	0	-0.02	141.34	93.03	0.8555
PEZZOT	7	m	0	1.99	2.76	3.94	0.0009
STASZE	22	m	0	2.19	0.48	0.93	0.1290
WYNDE7	32	m	0	2.27	35.49	77.42	0.0000

	N	16
	NS	14
	Wt	283.84
Het	Chi	238.89
Het	df	15
Het	P	***
Fixed	RR	2.22
	RRl	1.97
	RRu	2.49
	P	+++
Random	RR	3.55
	RRl	1.97
	RRu	6.40
	P	+++
Asymm	P	N.S.

Table 3C6 - 6

IESLC - Meta-anal of Current Smoking (or Ever if Current not available), Cigarettes only
Adenocarcinoma
Least adjusted

		Sex		
	combined	male	female	Total
N	1	13	2	16
NS	1	13	2	16
Wt	3.29	259.75	20.80	283.84
Het Chi	0.00	237.40	1.09	238.89
Het df	0	12	1	15
Het P	N.S.	***	N.S.	***
Fixed RR	1.81	2.20	2.48	2.22
RRl	0.61	1.95	1.61	1.97
RRu	5.33	2.49	3.81	2.49
P	N.S.	+++	+++	+++
Random RR	1.81	4.14	2.43	3.55
RRl	0.61	2.04	1.50	1.97
RRu	5.33	8.39	3.94	6.40
P	N.S.	+++	+++	+++
Between Chi				0.40
Between df				2
Between P				N.S.
Btwn(F) P				N.S.
Btwn(R) P				N.S.

Table 3C6 - 7

IESLC - Meta-anal of Current Smoking (or Ever if Current not available), Cigarettes only
Adenocarcinoma
Excluded studies (and stage at which they were excluded)

Stage	Excluded studies
1	BOUCHA BUELL LAURIL MZILEN
2	BERRIN CHEN RESTRE TAO ULMER
4	ABRAHA AKIBA AMANDU AMES ANDERS ARCHER AUSTIN AUVINE AXELSO AXELSS BARBON BECHER BENSHL BLOHMK BLOT1 BLOT2 BLOT3 BLOT4 BRETT BROCKM BROSS BROWN1 BROWN2 BUFFLE BYERS1 BYERS2 CARPEN CASCO2 CASCOR CHAN CHANG CHATZI CHEN2 CHEN3 CHIAZZ CHOI CHYOU COMSTO COOKSO CORREA DARBY DAVEYS DEKLER DESTE2 DESTEF DOCKER DORANT DORGAN DOSEME DROSTE DU DUNN EBELIN ESAKI FAN GAO GAO2 GARCIA GARSHI GENG GER GILLIS GODLEY GOODMA GREGOR GSELL HAENSZ HAMMO2 HANSEN HEGMAN HENNEK HINDS HIRAOK HIRAYA HITOSU HOLE HOROWI HORWIT HU HU2 HUANG HUMBLE ISHIMA JAHN JAIN JARUP JARVHO JEDRYC JIANG JONES KAISER KANELL KATSOU KAUFMA KELLER KHUDER KIHARA KINLEN KNEKT KO KOHLME KOO KREUZE KREYBE KUBIK LAMTH LAMWK LAMWK2 LAUSSM LEI LEMARC LETOUR LEVIN LIAW LICKIN LIDDEL LIU LIU2 LIU3 LIU5 LOMBA2 LUO MACLEN MAGNUS MARSH MARSH2 MARTIS MASTRA MATOS MATSUD MCDUFF MCLAUG MILLER MOLLO MRFIT MRFITR MURATA NAM NOTANI NOU ODRISC ORMOS OSANN OSANN2 PARKIN PASTOR PAWLEG PERSH2 PERSHA PETO PEZZO2 PIKE PISANI POFFIJ POLEDN PRESCO QIAO2 RACHTA RADZIK RANDIG REN ROOTS ROTHSC SAARIK SANKAR SCHWAR SEGI SEGI2 SEOW SHAW SHIMIZ SIEMIA SIMARA SIMONA SITAS SOBUE SOBUE2 SPEIZE SPITZ STAYNE STOCKS STOCKW STUCKE SUN SUZUKI SVENSS TANG TENKAN TOKARS TOUSEY TSUGAN TULINI VANDER VUTUC WAKAI WANG WANG2 WANG3 WANG4 WARSIN WATSON WICKLU WILKIN WU WU2 WUNSCH WUWILL WYNDE2 WYNDE3 WYNDE4 WYNDE5 WYNDE6 WYNDE8 XU XU2 XU3 XU4 YAMAGU YONG YUAN ZHANG ZHENG ZHOU
5	LANGE RIMING TANG2
6	HIRAY2 SCHWA2 WYNDER
8	ABELIN AGUDO ARMADA BEST BOFFET CEDERL CHOW DAMBER DEAN DEAN2 DEAN3 DOLL DOLL2 ENGELA ENSTRO GARDIN GOLLED GRAHAM HEIN JOLY KAISE2 KJUUS KOULUM LIU4 LOMBAR MCCONN MIGRAN MILLS NOTAN2 PERNU QIAO RONCO SADOWS SUZUK2 TIZZAN TVERDA VEIERO WALD WIGLE XIANGZ
10	BENHAM

Table 3C6 - 8
Potentially overlapping studies

REF	REFGP	PRINC	OVERLAP/LINK
LUBIN2	LUBIN2	1	Lubin-combined
WYNDE7	WYNDE6	2	WYNDE5/6/7/8
CPSI	CPSI	1	CPSI overall
LUBIN	XIANGZ	2	LUBIN/XIANGZ/QIAO
