# Supplementary material for: Systematic review with meta-analysis of the epidemiological evidence in the 1900s relating smoking to lung cancer
Source: BMC Cancer. 2012 Sep 3;12:385. doi: 10.1186/1471-2407-12-385 (PMC3505152; doi:10.1186/1471-2407-12-385)
Supplement: Additional file 5 — Detailed Analysis Tables (Individual file names as described in Additional file 1: Methods, Table1). [file 1471-2407-12-385-S5.zip › PDF/2BR.pdf]

Table 2B1R - 1

IESLC - Meta-regression of current smoking, any product (or cigs if any not available)
Multiple regression of data from Table 2B1
Squamous

Stepwise allowing only characteristics from the fixed model

Log Relative risk
WEIGHTED on Weight

		Deviance	(DF)				
Model 1		288.688	(40)				
		Estimate	S.E.	P	RR	95%CIl	95%CIu
Constant		2.623	0.039	+++	13.770	12.748	14.874

		Deviance	(DF)	Drop Dev	P		
Model 2		194.753	(36)	93.934	**		
		Estimate	S.E.	P	RR	95%CIl	95%CIu
Constant		2.890	0.050	+++	17.986	16.295	19.853
Location							
NAmer	24	Aliased			17.986	14.295	22.631
UK	0	Aliased			17.986	14.295	22.631
Scand	2	-0.148	0.437	N.S.	15.506	2.147	111.980
othEur	6	-0.387	0.107	---	12.218	7.956	18.765
China	0	Aliased			17.986	14.295	22.631
Japan	8	-0.966	0.101	---	6.848	4.598	10.199
othAs	0	Aliased			17.986	14.295	22.631
other	1	-0.548	0.586	N.S.	10.400	0.726	148.901

		Deviance	(DF)	Drop Dev	P		
Model 3		143.741	(32)	51.013	*		
		Estimate	S.E.	P	RR	95%CIl	95%CIu
Constant		1.927	0.170	+++	6.870	4.928	9.579
Location							
NAmer	24	Aliased			20.005	15.794	25.339
UK	0	Aliased			20.005	15.794	25.339
Scand	2	-0.275	0.439	N.S.	15.194	2.487	92.809
othEur	6	-0.666	0.170	---	10.278	5.571	18.962
China	0	Aliased			20.005	15.794	25.339
Japan	8	-1.204	0.129	---	6.000	3.762	9.571
othAs	0	Aliased			20.005	15.794	25.339
other	1	-2.343	0.760	--	1.921	0.084	43.954
Start year of study							
<1960	6	Aliased			4.729	2.291	9.763
1960-69	7	1.174	0.203	+++	15.292	10.197	22.935
1970-79	8	1.274	0.233	+++	16.912	9.522	30.040
1980-89	18	1.006	0.179	+++	12.936	9.923	16.865
1990+	2	2.757	0.515	+++	74.535	10.234	542.873

		Deviance	(DF)	Drop Dev	P		
Model 4		100.916	(30)	42.824	**		
		Estimate	S.E.	P	RR	95%CIl	95%CIu
Constant		1.578	0.191	+++	4.847	3.334	7.046
Location							
NAmer	24	Aliased			20.089	16.366	24.661
UK	0	Aliased			20.089	16.366	24.661
Scand	2	-0.713	0.448	N.S.	9.848	1.994	48.636
othEur	6	-0.478	0.196	-	12.450	6.543	23.687
China	0	Aliased			20.089	16.366	24.661
Japan	8	-1.370	0.160	---	5.105	3.034	8.589
othAs	0	Aliased			20.089	16.366	24.661
other	1	-2.291	0.795	--	2.033	0.120	34.529
Start year of study							
<1960	6	Aliased			3.219	1.651	6.273
1960-69	7	1.764	0.225	+++	18.791	12.265	28.787
1970-79	8	1.315	0.236	+++	11.994	7.038	20.440
1980-89	18	1.460	0.201	+++	13.856	10.624	18.070
1990+	2	3.272	0.545	+++	84.874	13.432	536.304
Number of adjustment variables (1)							
0	16	Aliased			14.214	9.753	20.715
1	15	0.739	0.181	+++	29.749	18.517	47.792
2+/+nk	10	-0.218	0.146	N.S.	11.432	9.000	14.521

Table 2B1R - 1

IESLC - Meta-regression of current smoking, any product (or cigs if any not available)
Multiple regression of data from Table 2B1
Squamous

Fixed model

Log Relative risk
WEIGHTED on Weight

		Deviance	(DF)				
Model 7		86.049	(24)				
		Estimate	S.E.	P	RR	95%CIl	95%CIu
Constant		1.193	0.239	+++	3.295	2.062	5.267
Sex(RR)							
Male	22	Aliased			13.653	11.046	16.875
Female	17	-0.002	0.088	N.S.	13.627	10.606	17.509
Combined	2	0.224	0.459	N.S.	17.086	3.374	86.528
Location							
NAmer	24	Aliased			18.999	14.356	25.144
UK	0	Aliased			18.999	14.356	25.144
Scand	2	-0.177	0.513	N.S.	15.911	2.425	104.407
othEur	6	-0.440	0.266	N.S.	12.238	5.315	28.177
China	0	Aliased			18.999	14.356	25.144
Japan	8	-1.174	0.193	---	5.875	3.209	10.755
othAs	0	Aliased			18.999	14.356	25.144
other	1	-1.221	1.045	N.S.	5.605	0.132	238.773
Start year of study							
<1960	6	Aliased			4.248	1.372	13.156
1960-69	7	1.374	0.321	+++	16.782	10.480	26.873
1970-79	8	0.896	0.418	+	10.407	4.653	23.276
1980-89	18	1.276	0.325	+++	15.212	10.355	22.347
1990+	2	2.817	0.823	++	71.073	4.598	1098.710
Study type (1)							
CC	30	Aliased			13.543	11.580	15.840
other	11	0.404	0.371	N.S.	20.284	5.382	76.452
Study size (number of LC cases)							
100-249	8	Aliased			6.514	2.256	18.807
250-499	9	0.420	0.384	N.S.	9.917	4.009	24.530
500-999	4	0.852	0.400	+	15.267	5.490	42.455
1000+	20	0.803	0.298	+	14.538	12.342	17.124
Number of adjustment variables (1)							
0	16	Aliased			16.784	10.386	27.124
1	15	0.428	0.277	N.S.	25.755	12.393	53.525
2+/+nk	10	-0.447	0.175	-	10.731	8.210	14.027

Table 2B1R - 2

IESLC - Meta-regression of current smoking, any product (or cigs if any not available)
Multiple regression of data from Table 2B1
Squamous
Effect of removing characteristics

Log Relative risk
WEIGHTED on Weight

		Deviance	(DF)				
Model 7		86.049	(24)				
		Estimate	S.E.	P	RR	95%CIl	95%CIu
Constant		1.193	0.239	+++	3.295	2.062	5.267
Sex(RR)							
Male	22	Aliased			13.653	11.046	16.875
Female	17	-0.002	0.088	N.S.	13.627	10.606	17.509
Combined	2	0.224	0.459	N.S.	17.086	3.374	86.528
Location							
NAmer	24	Aliased			18.999	14.356	25.144
UK	0	Aliased			18.999	14.356	25.144
Scand	2	-0.177	0.513	N.S.	15.911	2.425	104.407
othEur	6	-0.440	0.266	N.S.	12.238	5.315	28.177
China	0	Aliased			18.999	14.356	25.144
Japan	8	-1.174	0.193	---	5.875	3.209	10.755
othAs	0	Aliased			18.999	14.356	25.144
other	1	-1.221	1.045	N.S.	5.605	0.132	238.773
Start year of study							
<1960	6	Aliased			4.248	1.372	13.156
1960-69	7	1.374	0.321	+++	16.782	10.480	26.873
1970-79	8	0.896	0.418	+	10.407	4.653	23.276
1980-89	18	1.276	0.325	+++	15.212	10.355	22.347
1990+	2	2.817	0.823	++	71.073	4.598	1098.710
Study type (1)							
CC	30	Aliased			13.543	11.580	15.840
other	11	0.404	0.371	N.S.	20.284	5.382	76.452
Study size (number of LC cases)							
100-249	8	Aliased			6.514	2.256	18.807
250-499	9	0.420	0.384	N.S.	9.917	4.009	24.530
500-999	4	0.852	0.400	+	15.267	5.490	42.455
1000+	20	0.803	0.298	+	14.538	12.342	17.124
Number of adjustment variables (1)							
0	16	Aliased			16.784	10.386	27.124
1	15	0.428	0.277	N.S.	25.755	12.393	53.525
2+/+nk	10	-0.447	0.175	-	10.731	8.210	14.027

Omit Sex		Deviance	(DF)	Drop Dev	P		
Model 8		86.300	(26)	-0.251	N.S.		
		Estimate	S.E.	P	RR	95%CIl	95%CIu
Constant		1.185	0.223	+++	3.270	2.114	5.058
Number of adjustment variables (1)							
0	16	Aliased			16.371	10.721	25.001
1	15	0.517	0.213	+	27.465	16.197	46.571
2+/+nk	10	-0.422	0.166	-	10.733	8.327	13.835
Location							
NAmer	24	Aliased			19.457	15.838	23.904
UK	0	Aliased			19.457	15.838	23.904
Scand	2	-0.196	0.512	N.S.	16.002	2.628	97.430
othEur	6	-0.519	0.208	-	11.576	5.850	22.907
China	0	Aliased			19.457	15.838	23.904
Japan	8	-1.216	0.174	---	5.769	3.275	10.164
othAs	0	Aliased			19.457	15.838	23.904
other	1	-1.506	0.860	(-)	4.314	0.207	89.714
Start year of study							
<1960	6	Aliased			4.167	1.424	12.197
1960-69	7	1.392	0.319	+++	16.769	10.688	26.311
1970-79	8	0.989	0.371	+	11.206	6.413	19.582
1980-89	18	1.261	0.323	+++	14.705	11.120	19.445
1990+	2	3.085	0.616	+++	91.164	13.325	623.703
Study type (1)							
CC	30	Aliased			13.585	11.707	15.765
other	11	0.329	0.338	N.S.	18.875	5.890	60.488

Table 2B1R - 2

IESLC - Meta-regression of current smoking, any product (or cigs if any not available)
Multiple regression of data from Table 2B1
Squamous
Effect of removing characteristics

Log Relative risk
WEIGHTED on Weight

		Estimate	S.E.	P	RR	95%CIl	95%CIu
Study size (number of LC cases)							
100-249	8	Aliased			6.521	2.391	17.784
250-499	9	0.426	0.374	N.S.	9.983	4.228	23.573
500-999	4	0.812	0.387	+	14.681	5.729	37.618
1000+	20	0.802	0.294	+	14.548	12.431	17.025

Omit Location		Deviance	(DF)	Drop Dev	P		
Model 8		130.112	(28)	-44.063	*		
		Estimate	S.E.	P	RR	95%CIl	95%CIu
Constant		1.323	0.234	+++	3.753	2.371	5.940
Number of adjustment variables (1)							
0	16	Aliased			26.210	19.044	36.074
1	15	-0.716	0.189	---	12.805	7.499	21.867
2+/+nk	10	-0.994	0.128	---	9.697	7.765	12.110
Sex(RR)							
Male	22	Aliased			12.834	10.687	15.413
Female	17	0.006	0.087	N.S.	12.915	10.258	16.258
Combined	2	1.643	0.285	+++	66.386	25.819	170.691
Start year of study							
<1960	6	Aliased			3.826	1.554	9.422
1960-69	7	1.075	0.283	+++	11.206	8.528	14.724
1970-79	8	0.606	0.284	+	7.015	4.652	10.579
1980-89	18	1.817	0.286	+++	23.554	18.562	29.889
1990+	2	1.219	0.475	+	12.945	2.908	57.619
Study type (1)							
CC	30	Aliased			12.914	11.203	14.887
other	11	1.560	0.311	+++	61.442	21.895	172.419
Study size (number of LC cases)							
100-249	8	Aliased			8.119	3.505	18.809
250-499	9	-0.193	0.332	N.S.	6.696	3.112	14.408
500-999	4	0.879	0.355	+	19.545	7.837	48.742
1000+	20	0.583	0.255	+	14.544	12.534	16.876

Omit Start year		Deviance	(DF)	Drop Dev	P		
Model 8		117.898	(28)	-31.848	(*)		
		Estimate	S.E.	P	RR	95%CIl	95%CIu
Constant		1.660	0.220	+++	5.257	3.416	8.090
Number of adjustment variables (1)							
0	16	Aliased			16.306	12.021	22.118
1	15	0.328	0.178	(+)	22.640	13.371	38.335
2+/+nk	10	-0.374	0.124	--	11.221	8.974	14.031
Sex(RR)							
Male	22	Aliased			13.514	11.235	16.255
Female	17	0.020	0.088	N.S.	13.784	10.975	17.312
Combined	2	0.273	0.252	N.S.	17.755	7.758	40.634
Location							
NAmer	24	Aliased			18.740	15.656	22.432
UK	0	Aliased			18.740	15.656	22.432
Scand	2	0.581	0.467	N.S.	33.492	6.746	166.268
othEur	6	-0.642	0.139	---	9.858	6.495	14.964
China	0	Aliased			18.740	15.656	22.432
Japan	8	-1.013	0.108	---	6.803	4.906	9.433
othAs	0	Aliased			18.740	15.656	22.432
other	1	1.056	0.627	N.S.	53.874	6.267	463.152
Study type (1)							
CC	30	Aliased			14.003	12.174	16.107
other	11	-0.407	0.257	N.S.	9.319	3.955	21.957
Study size (number of LC cases)							
100-249	8	Aliased			3.269	1.608	6.644
250-499	9	1.438	0.293	+++	13.772	6.549	28.959
500-999	4	1.555	0.324	+++	15.479	6.208	38.596
1000+	20	1.514	0.215	+++	14.854	12.823	17.206

Table 2B1R - 2

IESLC - Meta-regression of current smoking, any product (or cigs if any not available)
Multiple regression of data from Table 2B1
Squamous
Effect of removing characteristics

Log Relative risk
WEIGHTED on Weight

Omit Study type		Deviance	(DF)	Drop Dev	P		
Model 8		87.237	(25)	-1.188	N.S.		
		Estimate	S.E.	P	RR	95%CIl	95%CIu
Constant		1.230	0.237	+++	3.423	2.152	5.443
Number of adjustment variables (1)							
0	16	Aliased			15.822	10.323	24.251
1	15	0.611	0.221	+	29.152	16.231	52.357
2+/+nk	10	-0.382	0.165	-	10.802	8.317	14.031
Sex(RR)							
Male	22	Aliased			13.791	11.236	16.926
Female	17	-0.006	0.088	N.S.	13.707	10.730	17.508
Combined	2	0.020	0.419	N.S.	14.071	3.296	60.078
Location							
NAmer	24	Aliased			19.424	14.910	25.305
UK	0	Aliased			19.424	14.910	25.305
Scand	2	-0.050	0.500	N.S.	18.473	3.132	108.963
othEur	6	-0.459	0.265	(-)	12.279	5.424	27.797
China	0	Aliased			19.424	14.910	25.305
Japan	8	-1.268	0.172	---	5.463	3.181	9.382
othAs	0	Aliased			19.424	14.910	25.305
other	1	-1.369	1.036	N.S.	4.941	0.128	190.439
Start year of study							
<1960	6	Aliased			5.097	2.020	12.865
1960-69	7	1.220	0.288	+++	17.259	10.985	27.114
1970-79	8	0.741	0.393	(+)	10.694	4.885	23.408
1980-89	18	1.041	0.243	+++	14.432	10.340	20.143
1990+	2	2.862	0.822	++	89.182	6.800	1169.652
Study size (number of LC cases)							
100-249	8	Aliased			5.697	2.230	14.550
250-499	9	0.637	0.328	(+)	10.766	4.630	25.037
500-999	4	0.948	0.390	+	14.706	5.441	39.746
1000+	20	0.941	0.269	++	14.602	12.446	17.131

Omit Study size		Deviance	(DF)	Drop Dev	P		
Model 8		95.505	(27)	-9.456	N.S.		
		Estimate	S.E.	P	RR	95%CIl	95%CIu
Constant		1.394	0.224	+++	4.032	2.599	6.255
Number of adjustment variables (1)							
0	16	Aliased			15.569	10.226	23.706
1	15	0.485	0.263	(+)	25.298	13.099	48.859
2+/+nk	10	-0.325	0.160	(-)	11.244	8.880	14.239
Sex(RR)							
Male	22	Aliased			13.831	11.364	16.833
Female	17	-0.024	0.086	N.S.	13.500	10.703	17.029
Combined	2	0.115	0.439	N.S.	15.522	3.596	67.001
Location							
NAmer	24	Aliased			19.627	15.191	25.360
UK	0	Aliased			19.627	15.191	25.360
Scand	2	-0.890	0.457	(-)	8.056	1.641	39.553
othEur	6	-0.501	0.250	(-)	11.892	5.681	24.896
China	0	Aliased			19.627	15.191	25.360
Japan	8	-1.237	0.185	---	5.699	3.313	9.803
othAs	0	Aliased			19.627	15.191	25.360
other	1	-1.935	0.979	(-)	2.836	0.103	78.101
Start year of study							
<1960	6	Aliased			2.559	1.194	5.483
1960-69	7	1.933	0.243	+++	17.678	11.549	27.058
1970-79	8	1.549	0.318	+++	12.042	5.884	24.646
1980-89	18	1.754	0.241	+++	14.785	10.399	21.022
1990+	2	3.208	0.757	+++	63.249	5.205	768.548
Study type (1)							
CC	30	Aliased			13.405	11.598	15.495
other	11	0.653	0.312	+	25.746	8.949	74.069

Table 2B1R - 2

IESLC - Meta-regression of current smoking, any product (or cigs if any not available)
Multiple regression of data from Table 2B1
Squamous
Effect of removing characteristics

Log Relative risk
WEIGHTED on Weight

Omit N adjustment vars		Deviance	(DF)	Drop Dev	P		
Model 8		111.118	(26)	-25.069	*		
		Estimate	S.E.	P	RR	95%CIl	95%CIu
Constant		1.182	0.235	+++	3.260	2.057	5.168
Study size (number of LC cases)							
100-249	8	Aliased			7.948	2.901	21.774
250-499	9	0.483	0.372	N.S.	12.886	5.792	28.667
500-999	4	1.024	0.398	+	22.123	8.630	56.716
1000+	20	0.570	0.294	(+)	14.059	12.038	16.420
Sex(RR)							
Male	22	Aliased			12.704	10.455	15.437
Female	17	0.085	0.087	N.S.	13.826	10.921	17.502
Combined	2	1.172	0.340	++	41.028	12.938	130.107
Location							
NAmer	24	Aliased			17.361	13.675	22.041
UK	0	Aliased			17.361	13.675	22.041
Scand	2	-0.353	0.507	N.S.	12.202	2.033	73.235
othEur	6	0.040	0.248	N.S.	18.071	8.634	37.820
China	0	Aliased			17.361	13.675	22.041
Japan	8	-1.149	0.130	---	5.503	3.630	8.344
othAs	0	Aliased			17.361	13.675	22.041
other	1	-0.632	0.939	N.S.	9.227	0.351	242.277
Start year of study							
<1960	6	Aliased			5.036	1.726	14.693
1960-69	7	1.275	0.319	+++	18.017	12.254	26.489
1970-79	8	0.619	0.414	N.S.	9.357	4.437	19.731
1980-89	18	1.112	0.305	++	15.310	11.691	20.049
1990+	2	1.792	0.722	+	30.230	3.420	267.195
Study type (1)							
CC	30	Aliased			13.278	11.465	15.377
other	11	0.885	0.294	++	32.165	11.669	88.661

Table 2B1R - 3

IESLC - Meta-regression of current smoking, any product (or cigs if any not available)
Multiple regression of data from Table 2B1
Squamous
Study outliers

Study Ref	NRR	LOGRR	FITVAL	SEFITV	STDRES
LUBIN2	261	1.817	2.450	0.289	-2.184
BROWN2	16	2.617	2.824	0.154	-1.341
CPSI	405	1.447	2.826	1.161	-1.188
KHUDER	14	2.152	2.888	0.672	-1.096
KATSOU	20	1.864	2.455	0.778	-0.759
SOBUE	44	2.272	2.523	0.528	-0.476
SOBUE2	1	1.649	1.748	0.210	-0.474
COMSTO	23	2.428	2.913	1.278	-0.379
ENGELA	56	2.388	2.766	1.021	-0.370
CPSII	114	3.670	4.103	1.222	-0.354
BUFFLE	63	2.583	2.938	1.098	-0.324
HAENSZ	20	1.107	1.191	0.449	-0.187
MATOS	39	2.342	2.342	0.898	0.000
CORREA	43	3.544	3.544	0.393	0.000
KIHARA	2	3.481	3.481	0.784	0.000
BARBON	18	2.934	2.929	0.753	0.007
DORN	338	2.838	2.827	0.606	0.018
WYNDE6	12	3.383	3.369	0.368	0.038
JAIN	13	3.374	3.318	0.791	0.070
WAKAI	10	2.284	2.143	1.323	0.107
OSANN2	26	3.664	3.397	1.659	0.161
CPSII	117	4.368	4.101	1.480	0.181
WYNDE3	10	3.188	2.987	1.088	0.185
WYNDE6	201	3.444	3.367	0.329	0.233
JAIN	18	3.652	3.320	1.308	0.254
CPSI	403	3.379	2.827	1.866	0.296
JEDRYC	22	3.088	2.831	0.764	0.337
SVENSS	97	3.087	2.717	1.012	0.366
JAHN	7	3.264	2.831	1.077	0.402
SOBUE	34	2.896	2.525	0.781	0.475
COMSTO	30	4.285	2.911	2.469	0.556
SOBUE2	5	1.974	1.746	0.389	0.586
TSUGAN	14	2.713	0.915	2.571	0.699
WU	16	3.564	2.019	1.871	0.826
BOUCOT	141	3.316	1.149	2.535	0.855
HAMMON	102	3.274	2.445	0.916	0.905
BROWN2	15	3.025	2.822	0.209	0.976
WAKAI	28	3.339	2.141	1.218	0.983
OSANN	36	3.561	2.822	0.590	1.252
OSANN	35	3.898	2.824	0.690	1.556
LUBIN2	249	2.978	2.451	0.263	2.004

Table 2B1R - 4

IESLC - Meta-regression of current smoking, any product (or cigs if any not available)
Multiple regression of data from Table 2B1
Squamous
Effect of additional characteristics

Log Relative risk
WEIGHTED on Weight

		Deviance	(DF)				
Model 7		86.049	(24)				
		Estimate	S.E.	P	RR	95%CIl	95%CIu
Constant		1.193	0.239	+++	3.295	2.062	5.267
Sex(RR)							
Male	22	Aliased			13.653	11.046	16.875
Female	17	-0.002	0.088	N.S.	13.627	10.606	17.509
Combined	2	0.224	0.459	N.S.	17.086	3.374	86.528
Location							
NAmer	24	Aliased			18.999	14.356	25.144
UK	0	Aliased			18.999	14.356	25.144
Scand	2	-0.177	0.513	N.S.	15.911	2.425	104.407
othEur	6	-0.440	0.266	N.S.	12.238	5.315	28.177
China	0	Aliased			18.999	14.356	25.144
Japan	8	-1.174	0.193	---	5.875	3.209	10.755
othAs	0	Aliased			18.999	14.356	25.144
other	1	-1.221	1.045	N.S.	5.605	0.132	238.773
Start year of study							
<1960	6	Aliased			4.248	1.372	13.156
1960-69	7	1.374	0.321	+++	16.782	10.480	26.873
1970-79	8	0.896	0.418	+	10.407	4.653	23.276
1980-89	18	1.276	0.325	+++	15.212	10.355	22.347
1990+	2	2.817	0.823	++	71.073	4.598	1098.710
Study type (1)							
CC	30	Aliased			13.543	11.580	15.840
other	11	0.404	0.371	N.S.	20.284	5.382	76.452
Study size (number of LC cases)							
100-249	8	Aliased			6.514	2.256	18.807
250-499	9	0.420	0.384	N.S.	9.917	4.009	24.530
500-999	4	0.852	0.400	+	15.267	5.490	42.455
1000+	20	0.803	0.298	+	14.538	12.342	17.124
Number of adjustment variables (1)							
0	16	Aliased			16.784	10.386	27.124
1	15	0.428	0.277	N.S.	25.755	12.393	53.525
2+/+nk	10	-0.447	0.175	-	10.731	8.210	14.027

		Deviance	(DF)	Drop Dev	P		
Model 8		77.537	(20)	8.512	N.S.		
		Estimate	S.E.	P	RR	95%CIl	95%CIu
Constant		1.179	0.242	+++	3.251	2.023	5.225
Sex(RR)							
Male	22	Aliased			14.094	11.038	17.995
Female	17	0.004	0.089	N.S.	14.145	10.683	18.729
Combined	2	-0.594	0.792	N.S.	7.784	0.419	144.508
Location							
NAmer	24	Aliased			20.235	14.739	27.779
UK	0	Aliased			20.235	14.739	27.779
Scand	2	-1.114	0.616	(-)	6.639	0.692	63.708
othEur	6	Aliased			20.235	14.739	27.779
China	0	Aliased			20.235	14.739	27.779
Japan	8	-1.771	0.319	---	3.442	1.308	9.057
othAs	0	Aliased			20.235	14.739	27.779
other	1	-3.646	1.501	-	0.528	0.002	140.720
Start year of study							
<1960	6	Aliased			2.851	0.772	10.528
1960-69	7	2.146	0.427	+++	24.385	11.006	54.026
1970-79	8	1.361	0.559	+	11.122	2.202	56.172
1980-89	18	1.452	0.381	++	12.185	5.822	25.505
1990+	2	4.636	1.189	+++	293.921	4.405	19613.463
Study type (1)							
CC	30	Aliased			13.792	11.631	16.355
other	11	-0.039	0.487	N.S.	13.268	2.172	81.059

Table 2B1R - 4

IESLC - Meta-regression of current smoking, any product (or cigs if any not available)
Multiple regression of data from Table 2B1
Squamous
Effect of additional characteristics

WEIGHTED on Weight

		Estimate	S.E.	P	RR	95%CIl	95%CIu
Study size (number of LC cases)							
100-249	8	Aliased			13.335	2.942	60.438
250-499	9	0.031	0.429	N.S.	13.753	4.698	40.263
500-999	4	0.602	0.489	N.S.	24.348	5.013	118.249
1000+	20	0.019	0.417	N.S.	13.588	11.150	16.558
Number of adjustment variables (1)							
0	16	Aliased			10.311	4.033	26.363
1	15	1.579	0.560	+	49.987	13.299	187.893
2+/+nk	10	0.173	0.315	N.S.	12.255	8.613	17.439
Detailed Country in othEur							
not o E	35	Aliased			14.376	10.155	20.352
multi	2	-0.106	0.536	N.S.	12.926	2.208	75.675
Germany	1	0.614	0.671	N.S.	26.568	1.984	355.752
othWest	1	-1.787	1.002	(-)	2.408	0.057	102.039
East	1	0.438	0.525	N.S.	22.275	2.917	170.100
Balkans	1	-2.350	0.720	--	1.372	0.082	22.947

		Deviance	(DF)	Drop Dev	P		
Model 8		86.049	(24)	0.000	N.S.		
		Estimate	S.E.	P	RR	95%CIl	95%CIu
Constant		1.193	0.239	+++	3.295	2.062	5.267
Sex(RR)							
Male	22	Aliased			13.653	11.046	16.875
Female	17	-0.002	0.088	N.S.	13.627	10.606	17.509
Combined	2	0.224	0.459	N.S.	17.086	3.374	86.528
Location							
NAmer	24	Aliased			18.999	14.356	25.144
UK	0	Aliased			18.999	14.356	25.144
Scand	2	-0.177	0.513	N.S.	15.911	2.425	104.407
othEur	6	-0.440	0.266	N.S.	12.238	5.315	28.177
China	0	Aliased			18.999	14.356	25.144
Japan	8	-1.174	0.193	---	5.875	3.209	10.755
othAs	0	Aliased			18.999	14.356	25.144
other	1	-1.221	1.045	N.S.	5.605	0.132	238.773
Start year of study							
<1960	6	Aliased			4.248	1.372	13.156
1960-69	7	1.374	0.321	+++	16.782	10.480	26.873
1970-79	8	0.896	0.418	+	10.407	4.653	23.276
1980-89	18	1.276	0.325	+++	15.212	10.355	22.347
1990+	2	2.817	0.823	++	71.073	4.598	1098.710
Study type (1)							
CC	30	Aliased			13.543	11.580	15.840
other	11	0.404	0.371	N.S.	20.284	5.382	76.452
Study size (number of LC cases)							
100-249	8	Aliased			6.514	2.256	18.807
250-499	9	0.420	0.384	N.S.	9.917	4.009	24.530
500-999	4	0.852	0.400	+	15.267	5.490	42.455
1000+	20	0.803	0.298	+	14.538	12.342	17.124
Number of adjustment variables (1)							
0	16	Aliased			16.784	10.386	27.124
1	15	0.428	0.277	N.S.	25.755	12.393	53.525
2+/+nk	10	-0.447	0.175	-	10.731	8.210	14.027
		Estimate					
Detailed Country in othAsia							
not o A	41	Aliased					

		Deviance	(DF)	Drop Dev	P		
Model 8		84.943	(23)	1.106	N.S.		
		Estimate	S.E.	P	RR	95%CIl	95%CIu
Constant		0.759	0.477	N.S.	2.135	0.838	5.438
Sex(RR)							
Male	22	Aliased			13.816	11.097	17.203
Female	17	-0.011	0.089	N.S.	13.671	10.598	17.635
Combined	2	0.016	0.500	N.S.	14.043	2.345	84.108

Table 2B1R - 4

IESLC - Meta-regression of current smoking, any product (or cigs if any not available)
Multiple regression of data from Table 2B1
Squamous
Effect of additional characteristics

WEIGHTED on Weight

		Estimate	S.E.	P	RR	95%CIl	95%CIu
Location							
NAmer	24	Aliased			17.393	11.367	26.614
UK	0	Aliased			17.393	11.367	26.614
Scand	2	0.159	0.605	N.S.	20.384	2.483	167.370
othEur	6	-0.344	0.281	N.S.	12.331	5.287	28.760
China	0	Aliased			17.393	11.367	26.614
Japan	8	-0.838	0.373	-	7.524	2.560	22.107
othAs	0	Aliased			17.393	11.367	26.614
other	1	-1.062	1.056	N.S.	6.014	0.132	273.193
Start year of study							
<1960	6	Aliased			4.192	1.330	13.216
1960-69	7	1.133	0.395	++	13.010	4.648	36.414
1970-79	8	1.013	0.433	+	11.549	4.705	28.345
1980-89	18	1.384	0.341	+++	16.722	9.972	28.041
1990+	2	2.984	0.838	++	82.884	4.876	1408.808
Study type (1)							
CC	30	Aliased			13.398	11.376	15.779
other	11	0.667	0.447	N.S.	26.100	5.159	132.051
Study size (number of LC cases)							
100-249	8	Aliased			5.267	1.410	19.677
250-499	9	0.560	0.406	N.S.	9.216	3.544	23.971
500-999	4	1.204	0.522	+	17.553	5.547	55.551
1000+	20	1.027	0.366	+	14.706	12.392	17.452
Number of adjustment variables (1)							
0	16	Aliased			16.128	9.707	26.795
1	15	0.259	0.321	N.S.	20.896	7.280	59.978
2+/+nk	10	-0.339	0.203	N.S.	11.491	7.970	16.565
Squamous (or nearest)							
q	33	Aliased			12.698	9.167	17.591
oth	8	0.470	0.447	N.S.	20.314	5.008	82.399

		Deviance	(DF)	Drop Dev	P		
Model 8		81.502	(21)	4.548	N.S.		
		Estimate	S.E.	P	RR	95%CIl	95%CIu
Constant		1.542	0.691	+	4.676	1.207	18.122
Sex(RR)							
Male	22	Aliased			13.810	11.306	16.868
Female	17	-0.008	0.089	N.S.	13.706	10.547	17.810
Combined	2	Aliased			13.810	11.306	16.868
Location							
NAmer	24	Aliased			18.748	10.535	33.363
UK	0	Aliased			18.748	10.535	33.363
Scand	2	-0.030	0.687	N.S.	18.203	1.771	187.131
othEur	6	-0.546	0.307	(-)	10.864	4.328	27.273
China	0	Aliased			18.748	10.535	33.363
Japan	8	-1.013	0.535	(-)	6.805	1.422	32.569
othAs	0	Aliased			18.748	10.535	33.363
other	1	-1.642	1.040	N.S.	3.630	0.088	150.335
Start year of study							
<1960	6	Aliased			4.436	0.556	35.385
1960-69	7	1.209	0.845	N.S.	14.863	3.011	73.358
1970-79	8	0.901	0.545	N.S.	10.922	3.836	31.098
1980-89	18	1.269	0.505	+	15.779	8.555	29.102
1990+	2	2.993	0.933	++	88.449	8.703	898.957
Study type (1)							
CC	30	Aliased			13.550	11.434	16.058
other	11	0.392	0.477	N.S.	20.046	3.403	118.087
Study size (number of LC cases)							
100-249	8	Aliased			8.238	1.596	42.516
250-499	9	-0.041	0.522	N.S.	7.906	2.781	22.472
500-999	4	0.633	0.625	N.S.	15.510	4.095	58.746
1000+	20	0.566	0.442	N.S.	14.505	12.127	17.349

Table 2B1R - 4

IESLC - Meta-regression of current smoking, any product (or cigs if any not available)
Multiple regression of data from Table 2B1
Squamous
Effect of additional characteristics

WEIGHTED on Weight

		Estimate	S.E.	P	RR	95%CIl	95%CIu
Number of adjustment variables (1)							
0	16	Aliased			18.332	10.152	33.105
1	15	0.183	0.417	N.S.	22.003	4.672	103.625
2+/+nk	10	-0.551	0.253	-	10.562	6.372	17.508
Squamous (or nearest)							
q	33	Aliased			13.566	9.105	20.213
q+s	1	0.352	0.615	N.S.	19.292	2.312	160.987
q+u	1	-0.428	0.730	N.S.	8.840	0.612	127.712
KI	5	0.070	0.692	N.S.	14.556	1.427	148.508
not a	1	1.199	0.640	(+)	44.978	3.789	533.873

		Deviance	(DF)	Drop Dev	P		
Model 8		86.048	(23)	0.002	N.S.		
		Estimate	S.E.	P	RR	95%CIl	95%CIu
Constant		1.193	0.239	+++	3.297	2.062	5.271
Sex(RR)							
Male	22	Aliased			13.652	10.993	16.953
Female	17	-0.002	0.088	N.S.	13.626	10.548	17.603
Combined	2	0.226	0.461	N.S.	17.117	3.235	90.555
Location							
NAmer	24	Aliased			18.987	14.172	25.439
UK	0	Aliased			18.987	14.172	25.439
Scand	2	-0.168	0.561	N.S.	16.043	2.006	128.311
othEur	6	-0.436	0.286	N.S.	12.281	4.907	30.739
China	0	Aliased			18.987	14.172	25.439
Japan	8	-1.175	0.195	---	5.865	3.100	11.097
othAs	0	Aliased			18.987	14.172	25.439
other	1	-1.214	1.059	N.S.	5.640	0.117	272.391
Start year of study							
<1960	6	Aliased			4.289	0.990	18.578
1960-69	7	1.365	0.388	++	16.796	10.314	27.351
1970-79	8	0.883	0.534	N.S.	10.369	4.233	25.395
1980-89	18	1.266	0.403	++	15.212	10.269	22.533
1990+	2	2.809	0.848	++	71.166	4.328	1170.152
		Estimate					
Study type (1)							
CC	30	Aliased					
other	11	Aliased					
		Estimate	S.E.	P	RR	95%CIl	95%CIu
Study size (number of LC cases)							
100-249	8	Aliased			6.464	1.739	24.030
250-499	9	0.427	0.421	N.S.	9.908	3.913	25.088
500-999	4	0.860	0.454	(+)	15.279	5.361	43.548
1000+	20	0.811	0.363	+	14.545	12.238	17.286
Number of adjustment variables (1)							
0	16	Aliased			16.770	10.201	27.568
1	15	0.431	0.285	N.S.	25.802	11.983	55.557
2+/+nk	10	-0.446	0.177	-	10.732	8.162	14.111
Study type (2)							
CC	30	Aliased			13.547	11.515	15.938
prosp	8	0.393	0.465	N.S.	20.065	3.639	110.649
other	3	0.425	0.645	N.S.	20.718	1.805	237.777

		Deviance	(DF)	Drop Dev	P		
Model 8		85.026	(23)	1.024	N.S.		
		Estimate	S.E.	P	RR	95%CIl	95%CIu
Constant		1.147	0.243	+++	3.148	1.953	5.073
Sex(RR)							
Male	22	Aliased			13.546	10.902	16.830
Female	17	0.004	0.089	N.S.	13.604	10.547	17.549
Combined	2	0.358	0.478	N.S.	19.372	3.496	107.353

Table 2B1R - 4

IESLC - Meta-regression of current smoking, any product (or cigs if any not available)
Multiple regression of data from Table 2B1
Squamous
Effect of additional characteristics

WEIGHTED on Weight

		Estimate	S.E.	P	RR	95%CIl	95%CIu
Location							
NAmer	24	Aliased			18.659	13.928	24.996
UK	0	Aliased			18.659	13.928	24.996
Scand	2	0.306	0.701	N.S.	25.344	1.921	334.437
othEur	6	-0.376	0.273	N.S.	12.817	5.401	30.413
China	0	Aliased			18.659	13.928	24.996
Japan	8	-1.165	0.193	---	5.822	3.148	10.769
othAs	0	Aliased			18.659	13.928	24.996
other	1	-0.996	1.069	N.S.	6.893	0.141	336.025
Start year of study							
<1960	6	Aliased			4.320	1.369	13.637
1960-69	7	1.373	0.321	+++	17.052	10.533	27.607
1970-79	8	0.840	0.422	(+)	10.011	4.365	22.958
1980-89	18	1.268	0.325	+++	15.359	10.376	22.734
1990+	2	2.623	0.845	++	59.545	3.419	1037.115
Study type (1)							
CC	30	Aliased			13.486	11.495	15.823
other	11	0.506	0.384	N.S.	22.375	5.540	90.374
Study size (number of LC cases)							
100-249	8	Aliased			6.281	2.122	18.590
250-499	9	0.517	0.395	N.S.	10.536	4.087	27.157
500-999	4	0.928	0.407	+	15.892	5.566	45.379
1000+	20	0.837	0.300	+	14.509	12.285	17.136
		Estimate					
Number of adjustment variables (1)							
0	16	Aliased					
1	15	Aliased					
2+/+nk	10	Aliased					
		Estimate	S.E.	P	RR	95%CIl	95%CIu
Number of adjustment variables (2)							
0	16	Aliased			16.852	10.349	27.443
1	15	0.362	0.285	N.S.	24.197	11.111	52.696
2	9	-0.433	0.176	-	10.935	8.258	14.480
3-5	0	Aliased			16.852	10.349	27.443
6+/+nk	1	-1.462	1.018	N.S.	3.907	0.090	170.032
		Deviance	(DF)	Drop Dev	P		
Model 8		86.049	(24)	0.000	N.S.		
		Estimate	S.E.	P	RR	95%CIl	95%CIu
Constant		1.193	0.239	+++	3.295	2.062	5.267
Sex(RR)							
Male	22	Aliased			13.653	11.046	16.875
Female	17	-0.002	0.088	N.S.	13.627	10.606	17.509
Combined	2	0.224	0.459	N.S.	17.086	3.374	86.528
Location							
NAmer	24	Aliased			18.894	14.393	24.803
UK	0	Aliased			18.894	14.393	24.803
Scand	2	-0.177	0.513	N.S.	15.823	2.408	103.987
othEur	6	-0.440	0.266	N.S.	12.170	5.264	28.138
China	0	Aliased			18.894	14.393	24.803
Japan	8	-1.174	0.193	---	5.842	3.176	10.747
othAs	0	Aliased			18.894	14.393	24.803
other	1	Aliased			18.894	14.393	24.803
Start year of study							
<1960	6	Aliased			4.309	1.406	13.202
1960-69	7	1.374	0.321	+++	17.021	10.556	27.446
1970-79	8	0.896	0.418	+	10.556	4.629	24.071
1980-89	18	1.276	0.325	+++	15.429	10.706	22.236
1990+	2	1.597	0.644	+	21.268	1.846	245.014
Study type (1)							
CC	30	Aliased			13.543	11.580	15.840
other	11	0.404	0.371	N.S.	20.284	5.382	76.452

Table 2B1R - 4

IESLC - Meta-regression of current smoking, any product (or cigs if any not available)
Multiple regression of data from Table 2B1
Squamous
Effect of additional characteristics

WEIGHTED on Weight

		Estimate	S.E.	P	RR	95%CIl	95%CIu
Study size (number of LC cases)							
100-249	8	Aliased			6.514	2.256	18.807
250-499	9	0.420	0.384	N.S.	9.917	4.009	24.530
500-999	4	0.852	0.400	+	15.267	5.490	42.455
1000+	20	0.803	0.298	+	14.538	12.342	17.124
Number of adjustment variables (1)							
0	16	Aliased			16.784	10.386	27.124
1	15	0.428	0.277	N.S.	25.755	12.393	53.525
2+/+nk	10	-0.447	0.175	-	10.731	8.210	14.027
RR adjusted for or study matched on age							
Yes	40	Aliased			13.652	11.768	15.839
No	1	1.221	1.045	N.S.	46.273	0.980	2185.437

		Deviance	(DF)	Drop Dev	P		
Model 8		85.906	(23)	0.144	N.S.		
		Estimate	S.E.	P	RR	95%CIl	95%CIu
Constant		1.316	0.405	++	3.730	1.687	8.248
Sex(RR)							
Male	22	Aliased			13.702	11.005	17.061
Female	17	-0.003	0.089	N.S.	13.665	10.565	17.675
Combined	2	0.145	0.505	N.S.	15.834	2.561	97.919
Location							
NAmer	24	Aliased			19.018	14.285	25.321
UK	0	Aliased			19.018	14.285	25.321
Scand	2	-0.107	0.546	N.S.	17.085	2.204	132.418
othEur	6	-0.489	0.296	N.S.	11.660	4.382	31.029
China	0	Aliased			19.018	14.285	25.321
Japan	8	-1.136	0.218	---	6.109	2.942	12.686
othAs	0	Aliased			19.018	14.285	25.321
other	1	-1.374	1.121	N.S.	4.812	0.078	296.748
Start year of study							
<1960	6	Aliased			4.632	1.096	19.581
1960-69	7	1.252	0.454	+	16.200	8.926	29.402
1970-79	8	0.816	0.469	(+)	10.470	4.594	23.863
1980-89	18	1.192	0.392	++	15.260	10.293	22.625
1990+	2	2.893	0.847	++	83.613	3.300	2118.677
Study type (1)							
CC	30	Aliased			13.548	11.546	15.897
other	11	0.396	0.371	N.S.	20.130	5.186	78.147
Study size (number of LC cases)							
100-249	8	Aliased			6.491	2.198	19.167
250-499	9	0.397	0.389	N.S.	9.652	3.684	25.289
500-999	4	0.817	0.410	(+)	14.694	4.835	44.658
1000+	20	0.809	0.298	+	14.575	12.308	17.259
Number of adjustment variables (1)							
0	16	Aliased			17.160	10.023	29.380
1	15	0.458	0.289	N.S.	27.141	10.903	67.563
2+/+nk	10	-0.494	0.214	-	10.476	7.279	15.078
RR adjusted for or matched on factor other than sex, age							
Yes	29	Aliased			13.961	11.399	17.099
No	12	-0.135	0.355	N.S.	12.202	3.610	41.241

		Deviance	(DF)	Drop Dev	P		
Model 8		82.939	(22)	3.110	N.S.		
		Estimate	S.E.	P	RR	95%CIl	95%CIu
Constant		2.010	0.544	++	7.467	2.569	21.700
Sex(RR)							
Male	22	Aliased			13.364	10.700	16.691
Female	17	0.005	0.089	N.S.	13.430	10.362	17.405
Combined	2	0.679	0.526	N.S.	26.344	3.909	177.523

Table 2B1R - 4

IESLC - Meta-regression of current smoking, any product (or cigs if any not available)
Multiple regression of data from Table 2B1
Squamous
Effect of additional characteristics

WEIGHTED on Weight

		Estimate	S.E.	P	RR	95%CIl	95%CIu
Location							
NAmer	24	Aliased			18.386	13.669	24.731
UK	0	Aliased			18.386	13.669	24.731
Scand	2	-0.110	0.520	N.S.	16.468	2.336	116.102
othEur	6	-0.596	0.282	-	10.136	3.904	26.315
China	0	Aliased			18.386	13.669	24.731
Japan	8	-0.921	0.241	---	7.323	3.345	16.030
othAs	0	Aliased			18.386	13.669	24.731
other	1	0.565	1.472	N.S.	32.365	0.138	7601.456
Start year of study							
<1960	6	Aliased			5.246	1.046	26.301
1960-69	7	1.006	0.472	+	14.345	7.951	25.882
1970-79	8	0.681	0.541	N.S.	10.369	4.438	24.226
1980-89	18	1.160	0.431	+	16.732	10.720	26.114
1990+	2	1.315	1.216	N.S.	19.541	0.374	1020.579
Study type (1)							
CC	30	Aliased			13.306	11.198	15.811
other	11	0.833	0.546	N.S.	30.599	4.145	225.894
Study size (number of LC cases)							
100-249	8	Aliased			4.570	0.969	21.563
250-499	9	0.397	0.407	N.S.	6.796	1.911	24.167
500-999	4	1.018	0.450	+	12.646	4.087	39.135
1000+	20	1.200	0.437	+	15.174	12.411	18.551
Number of adjustment variables (1)							
0	16	Aliased			20.745	10.595	40.617
1	15	-0.172	0.441	N.S.	17.459	5.610	54.338
2+/+nk	10	-0.695	0.225	--	10.354	7.776	13.786
Product							
all/unsp	9	Aliased			31.312	5.013	195.574
cig+/-ot	26	-0.854	0.494	(-)	13.325	11.171	15.894
cig only	6	-0.916	0.925	N.S.	12.523	1.040	150.763

		Deviance	(DF)	Drop Dev	P		
Model 8		85.538	(23)	0.512	N.S.		
		Estimate	S.E.	P	RR	95%CIl	95%CIu
Constant		1.164	0.242	+++	3.204	1.992	5.153
Sex(RR)							
Male	22	Aliased			13.542	10.867	16.875
Female	17	0.018	0.093	N.S.	13.783	10.603	17.915
Combined	2	0.243	0.460	N.S.	17.272	3.307	90.221
Location							
NAmer	24	Aliased			19.702	13.968	27.789
UK	0	Aliased			19.702	13.968	27.789
Scand	2	-0.181	0.513	N.S.	16.441	2.401	112.577
othEur	6	-0.582	0.332	(-)	11.006	3.978	30.452
China	0	Aliased			19.702	13.968	27.789
Japan	8	-1.230	0.208	---	5.758	3.082	10.757
othAs	0	Aliased			19.702	13.968	27.789
other	1	-1.214	1.045	N.S.	5.853	0.127	269.034
Start year of study							
<1960	6	Aliased			3.852	1.090	13.610
1960-69	7	1.421	0.328	+++	15.959	9.224	27.611
1970-79	8	1.010	0.448	+	10.579	4.639	24.123
1980-89	18	1.407	0.373	+++	15.730	10.234	24.177
1990+	2	2.851	0.824	++	66.635	4.014	1106.236
Study type (1)							
CC	30	Aliased			13.545	11.548	15.888
other	11	0.401	0.371	N.S.	20.221	5.235	78.112
Study size (number of LC cases)							
100-249	8	Aliased			6.223	2.058	18.818
250-499	9	0.452	0.386	N.S.	9.781	3.878	24.673
500-999	4	0.896	0.405	+	15.242	5.378	43.195
1000+	20	0.852	0.306	+	14.585	12.334	17.247

Table 2B1R - 4

IESLC - Meta-regression of current smoking, any product (or cigs if any not available)
Multiple regression of data from Table 2B1
Squamous
Effect of additional characteristics

WEIGHTED on Weight

		Estimate	S.E.	P	RR	95%CIl	95%CIu
Number of adjustment variables (1)							
0	16	Aliased			16.912	10.356	27.619
1	15	0.422	0.278	N.S.	25.795	12.245	54.339
2+/+nk	10	-0.459	0.176	-	10.682	8.123	14.047
Denominator							
nev any	24	Aliased			14.914	9.539	23.317
nev cigs	17	-0.148	0.207	N.S.	12.860	8.699	19.012

		Deviance	(DF)	Drop Dev	P		
Model 8		85.664	(23)	0.385	N.S.		
		Estimate	S.E.	P	RR	95%CIl	95%CIu
Constant		1.602	0.702	+	4.963	1.254	19.646
Sex(RR)							
Male	22	Aliased			13.770	11.027	17.196
Female	17	-0.006	0.089	N.S.	13.687	10.587	17.695
Combined	2	0.056	0.533	N.S.	14.560	2.135	99.279
Location							
NAmer	24	Aliased			19.379	14.212	26.425
UK	0	Aliased			19.379	14.212	26.425
Scand	2	-0.211	0.516	N.S.	15.691	2.302	106.957
othEur	6	-0.464	0.269	(-)	12.187	5.207	28.524
China	0	Aliased			19.379	14.212	26.425
Japan	8	-1.243	0.223	---	5.590	2.813	11.108
othAs	0	Aliased			19.379	14.212	26.425
other	1	-1.498	1.137	N.S.	4.333	0.069	270.399
Start year of study							
<1960	6	Aliased			4.279	1.351	13.554
1960-69	7	1.391	0.322	+++	17.199	10.404	28.432
1970-79	8	0.949	0.427	+	11.058	4.496	27.195
1980-89	18	1.224	0.336	++	14.547	9.023	23.452
1990+	2	3.036	0.895	++	89.086	3.965	2001.615
Study type (1)							
CC	30	Aliased			13.602	11.570	15.990
other	11	0.299	0.407	N.S.	18.351	4.161	80.921
Study size (number of LC cases)							
100-249	8	Aliased			6.569	2.227	19.380
250-499	9	0.452	0.387	N.S.	10.318	3.974	26.791
500-999	4	0.627	0.540	N.S.	12.296	2.288	66.077
1000+	20	0.798	0.298	+	14.587	12.330	17.258
Number of adjustment variables (1)							
0	16	Aliased			15.876	8.755	28.790
1	15	0.560	0.350	N.S.	27.801	11.540	66.976
2+/+nk	10	-0.377	0.209	(-)	10.893	8.169	14.527
National cigarette tobacco type							
Virginia	2	Aliased			20.891	1.639	266.283
blended	39	-0.421	0.679	N.S.	13.707	11.781	15.949

		Deviance	(DF)	Drop Dev	P		
Model 8		85.383	(23)	0.667	N.S.		
		Estimate	S.E.	P	RR	95%CIl	95%CIu
Constant		1.193	0.239	+++	3.297	2.063	5.269
Sex(RR)							
Male	22	Aliased			14.030	10.930	18.009
Female	17	0.002	0.089	N.S.	14.056	10.491	18.833
Combined	2	-0.468	0.964	N.S.	8.788	0.268	288.086
Location							
NAmer	24	Aliased			19.783	14.067	27.822
UK	0	Aliased			19.783	14.067	27.822
Scand	2	-0.220	0.516	N.S.	15.881	2.342	107.707
othEur	6	-0.590	0.323	(-)	10.965	4.079	29.478
China	0	Aliased			19.783	14.067	27.822
Japan	8	-1.224	0.202	---	5.818	3.139	10.783
othAs	0	Aliased			19.783	14.067	27.822
other	1	-2.030	1.441	N.S.	2.598	0.014	479.400

Table 2B1R - 4

IESLC - Meta-regression of current smoking, any product (or cigs if any not available)
Multiple regression of data from Table 2B1
Squamous
Effect of additional characteristics

WEIGHTED on Weight

		Estimate	S.E.	P	RR	95%CIl	95%CIu
Start year of study							
<1960	6	Aliased			4.059	1.260	13.069
1960-69	7	1.409	0.324	+++	16.605	10.259	26.877
1970-79	8	1.071	0.470	+	11.850	4.292	32.713
1980-89	18	1.261	0.326	+++	14.329	8.873	23.140
1990+	2	3.577	1.242	++	145.163	1.929	10925.870
Study type (1)							
CC	30	Aliased			13.581	11.575	15.936
other	11	0.336	0.380	N.S.	19.001	4.763	75.792
Study size (number of LC cases)							
100-249	8	Aliased			6.816	2.271	20.459
250-499	9	0.402	0.384	N.S.	10.190	4.020	25.830
500-999	4	0.324	0.760	N.S.	9.427	0.805	110.393
1000+	20	0.766	0.301	+	14.670	12.355	17.419
Number of adjustment variables (1)							
0	16	Aliased			16.140	9.587	27.172
1	15	0.523	0.301	(+)	27.239	12.386	59.905
2+/+nk	10	-0.398	0.186	-	10.840	8.222	14.292
Any proxy use							
No/nk	35	Aliased			13.357	10.882	16.394
Yes	6	0.457	0.560	N.S.	21.100	2.915	152.713

		Deviance	(DF)	Drop Dev	P		
Model 8		80.231	(23)	5.818	N.S.		
		Estimate	S.E.	P	RR	95%CIl	95%CIu
Constant		1.575	0.287	+++	4.832	2.753	8.481
Sex(RR)							
Male	22	Aliased			14.362	11.495	17.944
Female	17	-0.014	0.089	N.S.	14.168	10.988	18.269
Combined	2	-0.892	0.652	N.S.	5.887	0.605	57.264
Location							
NAmer	24	Aliased			22.156	15.431	31.812
UK	0	Aliased			22.156	15.431	31.812
Scand	2	-0.348	0.518	N.S.	15.648	2.446	100.098
othEur	6	-0.840	0.314	-	9.560	3.872	23.607
China	0	Aliased			22.156	15.431	31.812
Japan	8	-1.551	0.248	---	4.699	2.367	9.332
othAs	0	Aliased			22.156	15.431	31.812
other	1	-2.514	1.175	-	1.793	0.030	106.576
Start year of study							
<1960	6	Aliased			4.107	1.345	12.539
1960-69	7	1.308	0.322	+++	15.194	9.324	24.759
1970-79	8	1.235	0.441	+	14.117	5.632	35.386
1980-89	18	1.220	0.326	++	13.912	9.299	20.814
1990+	2	3.765	0.912	+++	177.222	8.511	3690.243
Study type (1)							
CC	30	Aliased			13.941	11.872	16.369
other	11	-0.298	0.471	N.S.	10.343	1.965	54.431
Study size (number of LC cases)							
100-249	8	Aliased			5.866	2.037	16.895
250-499	9	0.583	0.390	N.S.	10.510	4.283	25.790
500-999	4	0.524	0.423	N.S.	9.904	2.972	33.006
1000+	20	0.922	0.302	++	14.750	12.532	17.361
Number of adjustment variables (1)							
0	16	Aliased			16.563	10.312	26.604
1	15	0.704	0.300	+	33.480	14.686	76.326
2+/+nk	10	-0.484	0.176	-	10.211	7.758	13.439
Full histological confirmation							
No	24	Aliased			18.611	11.524	30.055
Yes	17	-0.439	0.182	-	11.994	9.301	15.467

	Deviance	(DF)	Drop Dev	P
Model 8	86.049	(24)	0.000	N.S.

Table 2B1R - 4

IESLC - Meta-regression of current smoking, any product (or cigs if any not available)
Multiple regression of data from Table 2B1
Squamous
Effect of additional characteristics

WEIGHTED on Weight

		Estimate	S.E.	P	RR	95%CIl	95%CIu
Constant		1.193	0.239	+++	3.295	2.062	5.267
Sex(RR)							
Male	22	Aliased			13.653	11.046	16.875
Female	17	-0.002	0.088	N.S.	13.627	10.606	17.509
Combined	2	0.224	0.459	N.S.	17.086	3.374	86.528
Location							
NAmer	24	Aliased			18.999	14.356	25.144
UK	0	Aliased			18.999	14.356	25.144
Scand	2	-0.177	0.513	N.S.	15.911	2.425	104.407
othEur	6	-0.440	0.266	N.S.	12.238	5.315	28.177
China	0	Aliased			18.999	14.356	25.144
Japan	8	-1.174	0.193	---	5.875	3.209	10.755
othAs	0	Aliased			18.999	14.356	25.144
other	1	-1.221	1.045	N.S.	5.605	0.132	238.773
Start year of study							
<1960	6	Aliased			4.248	1.372	13.156
1960-69	7	1.374	0.321	+++	16.782	10.480	26.873
1970-79	8	0.896	0.418	+	10.407	4.653	23.276
1980-89	18	1.276	0.325	+++	15.212	10.355	22.347
1990+	2	2.817	0.823	++	71.073	4.598	1098.710
Study type (1)							
CC	30	Aliased			13.543	11.580	15.840
other	11	0.404	0.371	N.S.	20.284	5.382	76.452
Study size (number of LC cases)							
100-249	8	Aliased			6.514	2.256	18.807
250-499	9	0.420	0.384	N.S.	9.917	4.009	24.530
500-999	4	0.852	0.400	+	15.267	5.490	42.455
1000+	20	0.803	0.298	+	14.538	12.342	17.124
Number of adjustment variables (1)							
0	16	Aliased			16.784	10.386	27.124
1	15	0.428	0.277	N.S.	25.755	12.393	53.525
2+/+nk	10	-0.447	0.175	-	10.731	8.210	14.027
		Estimate					
Risky occupational population							
No	41	Aliased					

		Deviance	(DF)	Drop Dev	P		
Model 8		73.921	(22)	12.128	N.S.		
		Estimate	S.E.	P	RR	95%CIl	95%CIu
Constant		1.174	0.240	+++	3.236	2.022	5.181
Sex(RR)							
Male	22	Aliased			13.567	11.033	16.682
Female	17	-0.018	0.089	N.S.	13.330	10.447	17.009
Combined	2	0.521	0.481	N.S.	22.841	4.405	118.431
Location							
NAmer	24	Aliased			18.788	14.210	24.842
UK	0	Aliased			18.788	14.210	24.842
Scand	2	0.212	0.545	N.S.	23.230	3.412	158.158
othEur	6	-0.526	0.267	(-)	11.102	4.899	25.161
China	0	Aliased			18.788	14.210	24.842
Japan	8	-1.057	0.212	---	6.530	3.449	12.365
othAs	0	Aliased			18.788	14.210	24.842
other	1	-1.375	1.088	N.S.	4.750	0.109	206.354
Start year of study							
<1960	6	Aliased			3.426	1.103	10.642
1960-69	7	1.523	0.338	+++	15.704	9.673	25.496
1970-79	8	1.078	0.434	+	10.072	4.533	22.378
1980-89	18	1.561	0.336	+++	16.323	10.871	24.508
1990+	2	3.186	0.862	++	82.891	5.395	1273.591
Study type (1)							
CC	30	Aliased			13.251	11.266	15.586
other	11	0.934	0.539	(+)	33.728	5.236	217.276

Table 2B1R - 4

IESLC - Meta-regression of current smoking, any product (or cigs if any not available)
Multiple regression of data from Table 2B1
Squamous
Effect of additional characteristics

WEIGHTED on Weight

		Estimate	S.E.	P	RR	95%CIl	95%CIu
Study size (number of LC cases)							
100-249	8	Aliased			7.073	2.437	20.533
250-499	9	-0.344	0.442	N.S.	5.014	1.596	15.751
500-999	4	1.038	0.530	(+)	19.979	4.902	81.427
1000+	20	0.742	0.308	+	14.853	12.656	17.431
Number of adjustment variables (1)							
0	16	Aliased			19.671	11.493	33.669
1	15	0.028	0.319	N.S.	20.234	9.356	43.762
2+/+nk	10	-0.644	0.202	--	10.334	7.848	13.607
Lowest age in RR							
<25/unlim	27	Aliased			13.705	11.671	16.095
25-39	10	-0.336	0.422	N.S.	9.794	2.293	41.826
40+	4	1.512	0.480	++	62.193	11.414	338.877

		Deviance	(DF)	Drop Dev	P		
Model 8		81.662	(21)	4.387	N.S.		
		Estimate	S.E.	P	RR	95%CIl	95%CIu
Constant		2.805	1.606	(+)	16.534	0.710	385.138
Sex(RR)							
Male	22	Aliased			13.777	11.013	17.235
Female	17	-0.000	0.089	N.S.	13.772	10.587	17.917
Combined	2	-0.009	0.496	N.S.	13.655	2.203	84.652
Location							
NAmer	24	Aliased			19.674	14.432	26.819
UK	0	Aliased			19.674	14.432	26.819
Scand	2	-0.539	0.577	N.S.	11.478	1.305	100.972
othEur	6	-0.532	0.289	(-)	11.555	4.516	29.567
China	0	Aliased			19.674	14.432	26.819
Japan	8	-1.240	0.207	---	5.695	2.892	11.212
othAs	0	Aliased			19.674	14.432	26.819
other	1	-1.750	1.115	N.S.	3.419	0.054	216.755
Start year of study							
<1960	6	Aliased			3.629	0.970	13.576
1960-69	7	1.546	0.351	+++	17.032	10.135	28.623
1970-79	8	1.147	0.489	+	11.429	4.520	28.902
1980-89	18	1.395	0.355	+++	14.637	9.501	22.551
1990+	2	3.372	0.910	++	105.720	5.233	2135.708
Study type (1)							
CC	30	Aliased			13.718	11.621	16.194
other	11	0.092	0.417	N.S.	15.039	3.187	70.970
Study size (number of LC cases)							
100-249	8	Aliased			7.195	2.140	24.195
250-499	9	0.228	0.456	N.S.	9.041	2.948	27.728
500-999	4	0.454	0.524	N.S.	11.334	2.779	46.231
1000+	20	0.710	0.325	+	14.640	12.335	17.375
Number of adjustment variables (1)							
0	16	Aliased			16.156	9.299	28.070
1	15	0.566	0.302	(+)	28.459	12.756	63.492
2+/+nk	10	-0.409	0.195	-	10.731	7.986	14.420
Highest age in RR							
<65	1	Aliased			71.879	0.138	37424.586
65-74	2	-0.788	1.805	N.S.	32.680	3.097	344.896
75-84	6	-1.279	1.667	N.S.	20.013	5.772	69.391
85+/unlim	32	-1.677	1.618	N.S.	13.443	11.432	15.807

		Deviance	(DF)	Drop Dev	P		
Model 8		83.793	(23)	2.256	N.S.		
		Estimate	S.E.	P	RR	95%CIl	95%CIu
Constant		3.795	1.749	+	44.477	1.443	1370.744
Sex(RR)							
Male	22	Aliased			13.766	11.108	17.060
Female	17	0.003	0.089	N.S.	13.814	10.705	17.825
Combined	2	-0.024	0.488	N.S.	13.441	2.357	76.656

Table 2B1R - 4

IESLC - Meta-regression of current smoking, any product (or cigs if any not available)
Multiple regression of data from Table 2B1
Squamous
Effect of additional characteristics

WEIGHTED on Weight

		Estimate	S.E.	P	RR	95%CIl	95%CIu
Location							
NAmer	24	Aliased			19.751	14.653	26.622
UK	0	Aliased			19.751	14.653	26.622
Scand	2	-0.380	0.531	N.S.	13.508	1.942	93.975
othEur	6	-0.522	0.272	(-)	11.714	5.018	27.343
China	0	Aliased			19.751	14.653	26.622
Japan	8	-1.278	0.205	---	5.504	2.929	10.343
othAs	0	Aliased			19.751	14.653	26.622
other	1	-1.579	1.072	N.S.	4.073	0.085	194.234
Start year of study							
<1960	6	Aliased			4.307	1.378	13.467
1960-69	7	1.347	0.322	+++	16.567	10.296	26.659
1970-79	8	0.946	0.420	+	11.089	4.852	25.346
1980-89	18	1.234	0.326	+++	14.793	9.977	21.934
1990+	2	3.105	0.845	++	96.055	5.499	1677.790
Study type (1)							
CC	30	Aliased			13.583	11.597	15.909
other	11	0.333	0.374	N.S.	18.944	4.920	72.949
Study size (number of LC cases)							
100-249	8	Aliased			6.195	2.112	18.171
250-499	9	0.516	0.389	N.S.	10.380	4.137	26.046
500-999	4	0.751	0.406	(+)	13.128	4.381	39.335
1000+	20	0.858	0.300	++	14.605	12.378	17.232
Number of adjustment variables (1)							
0	16	Aliased			15.520	9.211	26.149
1	15	0.603	0.301	(+)	28.368	13.061	61.614
2+/+nk	10	-0.345	0.188	(-)	10.987	8.335	14.483
Midpoint age in RR		-0.046	0.031	N.S.	189.337	0.276	129740.973

		Deviance	(DF)	Drop Dev	P		
Model 8		82.456	(23)	3.593	N.S.		
		Estimate	S.E.	P	RR	95%CIl	95%CIu
Constant		0.441	0.463	N.S.	1.554	0.627	3.852
Sex(RR)							
Male	22	Aliased			13.598	11.001	16.809
Female	17	-0.009	0.089	N.S.	13.475	10.477	17.329
Combined	2	0.387	0.467	N.S.	20.031	3.840	104.478
Location							
NAmer	24	Aliased			17.026	11.962	24.234
UK	0	Aliased			17.026	11.962	24.234
Scand	2	-0.730	0.590	N.S.	8.205	0.835	80.595
othEur	6	-0.117	0.316	N.S.	15.148	5.961	38.495
China	0	Aliased			17.026	11.962	24.234
Japan	8	-0.912	0.237	---	6.842	3.486	13.429
othAs	0	Aliased			17.026	11.962	24.234
other	1	-0.203	1.175	N.S.	13.896	0.219	882.601
Start year of study							
<1960	6	Aliased			3.272	0.947	11.313
1960-69	7	1.438	0.323	+++	13.779	7.496	25.328
1970-79	8	1.380	0.490	++	13.006	5.206	32.491
1980-89	18	1.607	0.369	+++	16.317	10.847	24.545
1990+	2	2.086	0.909	+	26.340	0.917	756.441
Study type (1)							
CC	30	Aliased			13.666	11.674	15.999
other	11	0.185	0.388	N.S.	16.438	4.098	65.939
Study size (number of LC cases)							
100-249	8	Aliased			6.708	2.320	19.398
250-499	9	0.709	0.413	(+)	13.626	4.542	40.881
500-999	4	0.667	0.412	N.S.	13.071	4.497	37.990
1000+	20	0.761	0.299	+	14.352	12.161	16.937

Table 2B1R - 4

IESLC - Meta-regression of current smoking, any product (or cigs if any not available)
Multiple regression of data from Table 2B1
Squamous
Effect of additional characteristics

WEIGHTED on Weight

		Estimate	S.E.	P	RR	95%CIl	95%CIu
Number of adjustment variables (1)							
0	16	Aliased			12.702	6.142	26.269
1	15	0.576	0.288	(+)	22.585	10.402	49.041
2+/+nk	10	0.019	0.302	N.S.	12.943	8.218	20.385
Derivation of RR/CI							
Orig/2x2	24	Aliased			12.007	8.847	16.296
Other	17	0.770	0.406	(+)	25.936	7.446	90.342

		Deviance	(DF)	Drop Dev	P		
Model 8		77.049	(22)	9.000	N.S.		
		Estimate	S.E.	P	RR	95%CIl	95%CIu
Constant		-0.147	0.543	N.S.	0.863	0.298	2.499
Sex(RR)							
Male	22	Aliased			13.581	11.010	16.753
Female	17	-0.014	0.089	N.S.	13.390	10.439	17.175
Combined	2	0.464	0.467	N.S.	21.600	4.230	110.289
Location							
NAmer	24	Aliased			19.016	14.412	25.091
UK	0	Aliased			19.016	14.412	25.091
Scand	2	-1.531	0.735	-	4.113	0.284	59.615
othEur	6	-0.536	0.268	(-)	11.124	4.831	25.612
China	0	Aliased			19.016	14.412	25.091
Japan	8	-1.057	0.197	---	6.609	3.571	12.230
othAs	0	Aliased			19.016	14.412	25.091
other	1	-0.475	1.079	N.S.	11.824	0.256	546.566
Start year of study							
<1960	6	Aliased			2.804	0.804	9.774
1960-69	7	1.741	0.353	+++	15.988	9.963	25.658
1970-79	8	1.367	0.453	++	11.006	4.948	24.482
1980-89	18	1.766	0.368	+++	16.397	11.079	24.267
1990+	2	2.226	0.854	+	25.963	1.321	510.450
Study type (1)							
CC	30	Aliased			14.070	11.911	16.620
other	11	-0.523	0.551	N.S.	8.338	1.195	58.154
Study size (number of LC cases)							
100-249	8	Aliased			8.684	2.844	26.514
250-499	9	0.681	0.402	N.S.	17.162	5.559	52.985
500-999	4	0.568	0.412	N.S.	15.321	5.564	42.184
1000+	20	0.473	0.320	N.S.	13.933	11.755	16.515
Number of adjustment variables (1)							
0	16	Aliased			7.691	2.325	25.439
1	15	1.387	0.491	++	30.784	13.297	71.267
2+/+nk	10	0.739	0.481	N.S.	16.099	8.772	29.546
Derivation of RR/CI							
Orig	17	Aliased			8.647	4.645	16.096
StcCalc	13	1.314	0.498	+	32.174	9.294	111.383
Other	11	1.529	0.652	+	39.896	4.691	339.321
