# Supplementary material for: Systematic review with meta-analysis of the epidemiological evidence in the 1900s relating smoking to lung cancer
Source: BMC Cancer. 2012 Sep 3;12:385. doi: 10.1186/1471-2407-12-385 (PMC3505152; doi:10.1186/1471-2407-12-385)
Supplement: Additional file 5 — Detailed Analysis Tables (Individual file names as described in Additional file 1: Methods, Table1). [file 1471-2407-12-385-S5.zip › PDF/3F.pdf]

Table 3F1 -

IESLC - Meta-analysis of Cigarette Smoking, only Filter vs only Plain (or nearest available)
Adenocarcinoma

This analysis is restricted to results for:
1) Non-dose-response data
2) Results complete enough for use in metaanalysis

Within each study, results are then selected (in the following order of preference, within each sex) for:
3) CIGTYP: filter only/NOS, always, mainly, both, equally, ever
4) DENOM: plain only/NOS, always, mainly, ever
5) PRODUCT: cigarettes regardless of other products, cigarettes only (Note only study ALDERS has both product definitions available)
6) SMKSTA: ever, current. (Note only study MATOS has both ever and current available)
7) LCtype: all or nearest available, at least Squamous and Adeno. (q = squamous, s = small, l = large, a = adeno, mix = mixed, alv = alveolar)
8) Race: all or nearest available, otherwise by race (wh or w = white, bl or b = black, hi = hispanic ch = chinese, jap = japanese, haw = hawaiian, w+o = white + oriental, sca = scandinavian, as = asian)
9) Followup period (YF, prospective studies): whole study (coded as 0) or longest available
10) For overlapping studies: principal rather than subsidiary studies
Finally by Age: whole study (coded as 0) if available, otherwise by widest available age group
and then for single sex results (m, f) in preference to combined sex results (c).

Results adjusted (AD) for the most potential confounders are then chosen in Sections -1 to -3 and results adjusted for the least confounders in Sections -4 to -6. (Those least adjusted results which actually differ from the most adjusted as marked 'x' in column X in Section -4)
(Results adjusted for an unknown number of confounder(s) are coded as 20.)

Section -7 shows excluded studies, together with the stage (as above) at which no qualifying results were found.

Section -8 lists the potentially overlapping studies which have been included (1=principal, 2=subsidiary).

Section -9 lists any results which would have been included in preference except that they had data not complete enough for use in meta-analysis, with their significance (yes/no), if known, and any further comment as entered on the database.

In addition to those mentioned above, the following fields, levels and abbreviations are used:
* or nk = not known, n = no, y = yes, ot = other
ev = ever, cu = current, cig+/-ot = cigarettes irrespective of other products (cigar, pipe etc)
f = filter, p = plain, NOS = not otherwise specified
REF: 6-character study reference
NRR: number of the RR on the database within the study
ST : study type (CC = case control, pr or prosp = prospective)
NLC: number of lung cancer cases in whole study
R : risky occupational population (n = no, m = mining, o = other risky)
VB : national cigarette type (V = at least 75% Virginia, bl = at least 75% blended, ot = other)
P : any proxy use
H : full histological confirmation
De : derivation of RR/CI (or = original, st = standard method, ot = other method of estimation)

Table 3F1 - 1

IESLC - Meta-analysis of Cigarette Smoking, only Filter vs only Plain (or nearest available)
Adenocarcinoma
Most adjusted

REF	NRR	SEX	AGEL	AGEH	RACE	YF	LC TYPE	LOC	START	ST	NLC	R	VB	P	H	AD	SM	PRODUCT	CIGTYP	DENOM	De
LUBIN2	204	m	0	0	all	-	a	Eu:mul	1976	CC	7804	n	bl	n	y	3	ev	cig+/-ot	only f	always p	ot
LUBIN2	236	f	0	0	all	-	a	Eu:mul	1976	CC	7804	n	bl	n	y	3	ev	cig+/-ot	only f	always p	ot
MATOS	65	m	0	0	all	-	a	SCAmer	1994	CC	200	n	bl	n	n	3	ev	cig+/-ot	mainly f	mainly p	st
PEZZOT	16	m	0	0	all	-	a	SCAmer	1987	CC	215	n	bl	n	y	4	ev	cig only	only f	ever p	ot
SOBUE	86	m	0	0	all	-	a	As:Jap	1986	CC	1376	n	bl	n	y	5	cu	cig+/-ot	only f	p NOS	st
WAKAI	71	m	0	0	all	-	a	As:Jap	1988	CC	333	n	bl	n	y	0	cu	cig+/-ot	only f	p NOS	ot
WYNDE5	2	m	0	0	all	-	KII	NAmer	1969	CC	1365	n	bl	n	y	0	cu	cig+/-ot	ever f	always p	st
WYNDE5	5	f	0	0	all	-	KII	NAmer	1969	CC	1365	n	bl	n	y	0	cu	cig+/-ot	ever f	always p	st
WYNDE6	313	m	0	0	all	-	a	NAmer	1969	CC	4423	n	bl	n	y	3	cu	cig+/-ot	only f	always p	or
WYNDE6	319	f	0	0	all	-	a	NAmer	1969	CC	4423	n	bl	n	y	3	cu	cig+/-ot	only f	always p	or

Table 3F1 - 2

IESLC - Meta-analysis of Cigarette Smoking, only Filter vs only Plain (or nearest available)
Adenocarcinoma
Most adjusted

REF	NRR	SEX	AD	Number Exposed Case	Number Exposed Cont	Non-exposed Case	Non-exposed Cont	RR	95.00%CI
LUBIN2	204	m	3	-	-	-	-	0.71	(0.52- 0.99)
LUBIN2	236	f	3	-	-	-	-	0.45	(0.20- 1.06)
Subtotal LUBIN2								0.67	(0.50- 0.90)
MATOS	65	m	3	-	-	-	-	1.43	(0.63- 3.33)
PEZZOT	16	m	4	-	-	-	-	0.38	(0.19- 0.76)
SOBUE	86	m	5	-	-	-	-	0.83	(0.40- 1.67)
WAKAI	71	m	0	73	271	0	9	5.14~	(0.30- 89.41)
WYNDE5	2	m	0	139	629	73	398	1.20	(0.88- 1.64)
WYNDE5	5	f	0	68	200	13	30	0.78	(0.39- 1.59)
Subtotal WYNDE5								1.12	(0.85- 1.49)
WYNDE6	313	m	3	-	-	-	-	1.00	(0.70- 1.50)
WYNDE6	319	f	3	-	-	-	-	0.90	(0.50- 1.70)
Subtotal WYNDE6								0.97	(0.70- 1.34)
Partial Totals				280	1100	86	437		

*prospective study

~ With 0.5 adjustment for zero

REF	NRR	SEX	AD	Ys	Ws	Qs	Ps
LUBIN2	204	m	3	-0.34	37.06	1.71	0.0371
LUBIN2	236	f	3	-0.80	5.52	2.49	0.0605
Subtotal LUBIN2				-0.40	42.59	4.19	
MATOS	65	m	3	0.36	5.54	1.31	0.3997
PEZZOT	16	m	4	-0.97	8.00	5.64	0.0062
SOBUE	86	m	5	-0.19	7.52	0.03	0.6093
WAKAI	71	m	0	1.64	0.47	1.47	0.2610
WYNDE5	2	m	0	0.19	40.01	3.95	0.2386
WYNDE5	5	f	0	-0.24	7.69	0.10	0.5010
Subtotal WYNDE5				0.12	47.70	4.05	
WYNDE6	313	m	3	0.00	26.45	0.43	1.0000
WYNDE6	319	f	3	-0.11	10.26	0.01	0.7358
Subtotal WYNDE6				-0.03	36.71	0.44	

	N	10
	NS	7
	Wt	148.54
Het	Chi	17.12
Het	df	9
Het	P	*
Fixed	RR	0.88
	RRl	0.75
	RRu	1.03
	P	N.S.
Random	RR	0.84
	RRl	0.66
	RRu	1.08
	P	N.S.
Asymm	P	N.S.

Table 3F1 - 3

IESLC - Meta-analysis of Cigarette Smoking, only Filter vs only Plain (or nearest available)
Adenocarcinoma
Most adjusted

	Sex			
	combined	male	female	Total
N		7	3	10
NS		7	3	10
Wt		125.06	23.48	148.54
Het Chi		14.38	1.78	17.12
Het df		6	2	9
Het P		*	N.S.	*
Fixed RR		0.91	0.73	0.88
RRl		0.76	0.49	0.75
RRu		1.09	1.10	1.03
P		N.S.	N.S.	N.S.
Random RR		0.89	0.73	0.84
RRl		0.65	0.49	0.66
RRu		1.22	1.10	1.08
P		N.S.	N.S.	N.S.
Between Chi				0.96
Between df				1
Between P				N.S.
Btwn(F) P				N.S.
Btwn(R) P				N.S.

	All LC (or nearest)						
	a	a+l	a+al+br	KII	not q+u	not q+s	Total
N	8			2			10
NS	6			1			7
Wt	100.84			47.70			148.54
Het Chi	11.72			1.19			17.12
Het df	7			1			9
Het P	N.S.			N.S.			*
Fixed RR	0.78			1.12			0.88
RRl	0.64			0.85			0.75
RRu	0.95			1.49			1.03
P	-			N.S.			N.S.
Random RR	0.78			1.10			0.84
RRl	0.59			0.78			0.66
RRu	1.04			1.55			1.08
P	(-)			N.S.			N.S.
Between Chi							4.22
Between df							1
Between P							*
Btwn(F) P							N.S.
Btwn(R) P							N.S.

	Location								
	NAmer	UK	Scand	othEur	China	Japan	othAs	other	Total
N	4			2		2		2	10
NS	2			1		2		2	7
Wt	84.42			42.59		7.99		13.54	148.54
Het Chi	1.72			1.00		1.48		5.75	17.12
Het df	3			1		1		1	9
Het P	N.S.			N.S.		N.S.		*	*
Fixed RR	1.05			0.67		0.92		0.65	0.88
RRl	0.85			0.50		0.46		0.38	0.75
RRu	1.31			0.90		1.85		1.11	1.03
P	N.S.			--		N.S.		N.S.	N.S.
Random RR	1.05			0.67		1.20		0.72	0.84
RRl	0.85			0.50		0.29		0.20	0.66
RRu	1.31			0.90		5.02		2.64	1.08
P	N.S.			--		N.S.		N.S.	N.S.
Between Chi									7.18
Between df									3
Between P									(*)
Btwn(F) P									N.S.
Btwn(R) P									N.S.

Table 3F1 - 3

IESLC - Meta-analysis of Cigarette Smoking, only Filter vs only Plain (or nearest available)
Adenocarcinoma
Most adjusted

	Detailed Country in "other Europe" multi	Germany	othWest	East	Balkans	Total
N	2					2
NS	1					1
Wt	42.59					42.59
Het Chi	1.00					1.00
Het df	1					1
Het P	N.S.					N.S.
Fixed RR	0.67					0.67
RRl	0.50					0.50
RRu	0.90					0.90
P	--					--
Random RR	0.67					0.67
RRl	0.50					0.50
RRu	0.90					0.90
P	--					--
Between Chi						
Between df						
Between P						N.S.
Btwn(F) P						N.S.
Btwn(R) P						N.S.

	Detailed Country in "other Asia" India	HongKong	other	Total
N				
NS				
Wt				
Het Chi				
Het df				
Het P				
Fixed RR				
RRl				
RRu				
P				
Random RR				
RRl				
RRu				
P				
Between Chi				
Between df				
Between P				N.S.
Btwn(F) P				N.S.
Btwn(R) P				N.S.

	Detailed other continent SCAmer	Auslia	Africa	Total
N	2			2
NS	2			2
Wt	13.54			13.54
Het Chi	5.75			5.75
Het df	1			1
Het P	*			*
Fixed RR	0.65			0.65
RRl	0.38			0.38
RRu	1.11			1.11
P	N.S.			N.S.
Random RR	0.72			0.72
RRl	0.20			0.20
RRu	2.64			2.64
P	N.S.			N.S.
Between Chi				
Between df				
Between P				N.S.
Btwn(F) P				N.S.
Btwn(R) P				N.S.

Table 3F1 - 3

IESLC - Meta-analysis of Cigarette Smoking, only Filter vs only Plain (or nearest available)
Adenocarcinoma
Most adjusted

		Start year of study				
	<1960	1960-69	1970-79	1980-89	1990+	Total
N		4	2	3	1	10
NS		2	1	3	1	7
Wt		84.42	42.59	15.99	5.54	148.54
Het Chi		1.72	1.00	4.63	0.00	17.12
Het df		3	1	2	0	9
Het P		N.S.	N.S.	(*)	N.S.	*
Fixed RR		1.05	0.67	0.59	1.43	0.88
RRl		0.85	0.50	0.36	0.62	0.75
RRu		1.31	0.90	0.97	3.29	1.03
P		N.S.	--	-	N.S.	N.S.
Random RR		1.05	0.67	0.67	1.43	0.84
RRl		0.85	0.50	0.28	0.62	0.66
RRu		1.31	0.90	1.62	3.29	1.08
P		N.S.	--	N.S.	N.S.	N.S.
Between Chi						9.77
Between df						3
Between P						*
Btwn(F) P						N.S.
Btwn(R) P						(*)

	Study type (1)		
	CC	other	Total
N	10		10
NS	7		7
Wt	148.54		148.54
Het Chi	17.12		17.12
Het df	9		9
Het P	*		*
Fixed RR	0.88		0.88
RRl	0.75		0.75
RRu	1.03		1.03
P	N.S.		N.S.
Random RR	0.84		0.84
RRl	0.66		0.66
RRu	1.08		1.08
P	N.S.		N.S.
Between Chi			
Between df			
Between P			N.S.
Btwn(F) P			N.S.
Btwn(R) P			N.S.

	Study type (2)			
	CC	prosp	other	Total
N	10			10
NS	7			7
Wt	148.54			148.54
Het Chi	17.12			17.12
Het df	9			9
Het P	*			*
Fixed RR	0.88			0.88
RRl	0.75			0.75
RRu	1.03			1.03
P	N.S.			N.S.
Random RR	0.84			0.84
RRl	0.66			0.66
RRu	1.08			1.08
P	N.S.			N.S.
Between Chi				
Between df				
Between P				N.S.
Btwn(F) P				N.S.
Btwn(R) P				N.S.

Table 3F1 - 3

IESLC - Meta-analysis of Cigarette Smoking, only Filter vs only Plain (or nearest available)
Adenocarcinoma
Most adjusted

	Study size (number of LC cases) 100-249	250-499	500-999	1000+	Total
N	2	1		7	10
NS	2	1		4	7
Wt	13.54	0.47		134.53	148.54
Het Chi	5.75	0.00		8.63	17.12
Het df	1	0		6	9
Het P	*	N.S.		N.S.	*
Fixed RR	0.65	5.14		0.90	0.88
RRl	0.38	0.30		0.76	0.75
RRu	1.11	89.41		1.07	1.03
P	N.S.	N.S.		N.S.	N.S.
Random RR	0.72	5.14		0.88	0.84
RRl	0.20	0.30		0.71	0.66
RRu	2.64	89.41		1.09	1.08
P	N.S.	N.S.		N.S.	N.S.
Between Chi					2.74
Between df					2
Between P					N.S.
Btwn(F) P					N.S.
Btwn(R) P					N.S.

	Risky occupational population no	mining	othRisky	Total
N	10			10
NS	7			7
Wt	148.54			148.54
Het Chi	17.12			17.12
Het df	9			9
Het P	*			*
Fixed RR	0.88			0.88
RRl	0.75			0.75
RRu	1.03			1.03
P	N.S.			N.S.
Random RR	0.84			0.84
RRl	0.66			0.66
RRu	1.08			1.08
P	N.S.			N.S.
Between Chi				
Between df				
Between P				N.S.
Btwn(F) P				N.S.
Btwn(R) P				N.S.

	National cigarette tobacco type Virginia	blended	other	Total
N		10		10
NS		7		7
Wt		148.54		148.54
Het Chi		17.12		17.12
Het df		9		9
Het P		*		*
Fixed RR		0.88		0.88
RRl		0.75		0.75
RRu		1.03		1.03
P		N.S.		N.S.
Random RR		0.84		0.84
RRl		0.66		0.66
RRu		1.08		1.08
P		N.S.		N.S.
Between Chi				
Between df				
Between P				N.S.
Btwn(F) P				N.S.
Btwn(R) P				N.S.

Table 3F1 - 3

IESLC - Meta-analysis of Cigarette Smoking, only Filter vs only Plain (or nearest available)
Adenocarcinoma
Most adjusted

	Any proxy use No/nk	Yes	Total
N	10		10
NS	7		7
Wt	148.54		148.54
Het Chi	17.12		17.12
Het df	9		9
Het P	*		*
Fixed RR	0.88		0.88
RRl	0.75		0.75
RRu	1.03		1.03
P	N.S.		N.S.
Random RR	0.84		0.84
RRl	0.66		0.66
RRu	1.08		1.08
P	N.S.		N.S.
Between Chi			
Between df			
Between P			N.S.
Btwn(F) P			N.S.
Btwn(R) P			N.S.

	Full histological confirmation No	Yes	Total
N	1	9	10
NS	1	6	7
Wt	5.54	142.99	148.54
Het Chi	0.00	15.76	17.12
Het df	0	8	9
Het P	N.S.	*	*
Fixed RR	1.43	0.86	0.88
RRl	0.62	0.73	0.75
RRu	3.29	1.02	1.03
P	N.S.	(-)	N.S.
Random RR	1.43	0.81	0.84
RRl	0.62	0.62	0.66
RRu	3.29	1.05	1.08
P	N.S.	N.S.	N.S.
Between Chi			1.36
Between df			1
Between P			N.S.
Btwn(F) P			N.S.
Btwn(R) P			N.S.

	Number of adjustment variables (1) 0	1	2+/+nk	Total
N	3		7	10
NS	2		5	7
Wt	48.17		100.36	148.54
Het Chi	2.27		10.04	17.12
Het df	2		6	9
Het P	N.S.		N.S.	*
Fixed RR	1.14		0.78	0.88
RRl	0.86		0.64	0.75
RRu	1.51		0.94	1.03
P	N.S.		-	N.S.
Random RR	1.12		0.77	0.84
RRl	0.78		0.58	0.66
RRu	1.60		1.01	1.08
P	N.S.		(-)	N.S.
Between Chi				4.81
Between df				1
Between P				*
Btwn(F) P				N.S.
Btwn(R) P				N.S.

Table 3F1 - 3

IESLC - Meta-analysis of Cigarette Smoking, only Filter vs only Plain (or nearest available)
Adenocarcinoma
Most adjusted

	Number of adjustment variables (2) 0	1	2	3-5	6+/+nk	Total
N	3			7		10
NS	2			5		7
Wt	48.17			100.36		148.54
Het Chi	2.27			10.04		17.12
Het df	2			6		9
Het P	N.S.			N.S.		*
Fixed RR	1.14			0.78		0.88
RRl	0.86			0.64		0.75
RRu	1.51			0.94		1.03
P	N.S.			-		N.S.
Random RR	1.12			0.77		0.84
RRl	0.78			0.58		0.66
RRu	1.60			1.01		1.08
P	N.S.			(-)		N.S.
Between Chi						4.81
Between df						1
Between P						*
Btwn(F) P						N.S.
Btwn(R) P						N.S.

	Smoking status ever	current	Total
N	4	6	10
NS	3	4	7
Wt	56.13	92.41	148.54
Het Chi	6.75	3.32	17.12
Het df	3	5	9
Het P	(*)	N.S.	*
Fixed RR	0.67	1.04	0.88
RRl	0.51	0.85	0.75
RRu	0.86	1.28	1.03
P	--	N.S.	N.S.
Random RR	0.64	1.04	0.84
RRl	0.40	0.85	0.66
RRu	1.04	1.28	1.08
P	(-)	N.S.	N.S.
Between Chi			7.05
Between df			1
Between P			**
Btwn(F) P			*
Btwn(R) P			(*)

	Product cig+/-ot	cig only	Total
N	9	1	10
NS	6	1	7
Wt	140.54	8.00	148.54
Het Chi	11.16	0.00	17.12
Het df	8	0	9
Het P	N.S.	N.S.	*
Fixed RR	0.92	0.38	0.88
RRl	0.78	0.19	0.75
RRu	1.09	0.76	1.03
P	N.S.	--	N.S.
Random RR	0.91	0.38	0.84
RRl	0.74	0.19	0.66
RRu	1.13	0.76	1.08
P	N.S.	--	N.S.
Between Chi			5.96
Between df			1
Between P			*
Btwn(F) P			(*)
Btwn(R) P			*

Table 3F1 - 3

IESLC - Meta-analysis of Cigarette Smoking, only Filter vs only Plain (or nearest available)
Adenocarcinoma
Most adjusted

	Cigarette type						
	only f	always f	mainly f	equl p&f	both p&f	ever f	Total
N	7		1			2	10
NS	5		1			1	7
Wt	95.29		5.54			47.70	148.54
Het Chi	9.60		0.00			1.19	17.12
Het df	6		0			1	9
Het P	N.S.		N.S.			N.S.	*
Fixed RR	0.76		1.43			1.12	0.88
RRl	0.62		0.62			0.85	0.75
RRu	0.93		3.29			1.49	1.03
P	--		N.S.			N.S.	N.S.
Random RR	0.74		1.43			1.10	0.84
RRl	0.55		0.62			0.78	0.66
RRu	0.98		3.29			1.55	1.08
P	-		N.S.			N.S.	N.S.
Between Chi							6.34
Between df							2
Between P							*
Btwn(F) P							N.S.
Btwn(R) P							N.S.

	Denominator				
	ever p	mainly p	p NOS	always p	Total
N	1	1	2	6	10
NS	1	1	2	3	7
Wt	8.00	5.54	7.99	127.00	148.54
Het Chi	0.00	0.00	1.48	8.58	17.12
Het df	0	0	1	5	9
Het P	N.S.	N.S.	N.S.	N.S.	*
Fixed RR	0.38	1.43	0.92	0.91	0.88
RRl	0.19	0.62	0.46	0.76	0.75
RRu	0.76	3.29	1.85	1.08	1.03
P	--	N.S.	N.S.	N.S.	N.S.
Random RR	0.38	1.43	1.20	0.88	0.84
RRl	0.19	0.62	0.29	0.69	0.66
RRu	0.76	3.29	5.02	1.12	1.08
P	--	N.S.	N.S.	N.S.	N.S.
Between Chi					7.07
Between df					3
Between P					(*)
Btwn(F) P					N.S.
Btwn(R) P					(*)

	Derivation of RR/CI			
	Orig	StdCalc	Other	Total
N	2	4	4	10
NS	1	3	3	7
Wt	36.71	60.77	51.06	148.54
Het Chi	0.08	2.19	5.27	17.12
Het df	1	3	3	9
Het P	N.S.	N.S.	N.S.	*
Fixed RR	0.97	1.11	0.62	0.88
RRl	0.70	0.86	0.47	0.75
RRu	1.34	1.42	0.82	1.03
P	N.S.	N.S.	---	N.S.
Random RR	0.97	1.11	0.57	0.84
RRl	0.70	0.86	0.35	0.66
RRu	1.34	1.42	0.93	1.08
P	N.S.	N.S.	-	N.S.
Between Chi				9.58
Between df				2
Between P				**
Btwn(F) P				(*)
Btwn(R) P				(*)

Table 3F1 - 4

IESLC - Meta-analysis of Cigarette Smoking, only Filter vs only Plain (or nearest available)
Adenocarcinoma
Least adjusted

REF	NRR	X	SEX	AGEL	AGEH	RACE	YF	LC TYPE	LOC	START	ST	NLC	R	VB	P	H	AD	SM	PRODUCT	CIGTYP	DENOM	De
LUBIN2	188	x	m	0	0	all	-	a	Eu:mul	1976	CC	7804	n	bl	n	y	0	ev	cig+/-ot	only f	always p	st
LUBIN2	220	x	f	0	0	all	-	a	Eu:mul	1976	CC	7804	n	bl	n	y	0	ev	cig+/-ot	only f	always p	st
MATOS	74	x	m	0	0	all	-	a	SCAmer	1994	CC	200	n	bl	n	n	0	ev	cig+/-ot	mainly f	mainly p	st
PEZZOT	15	x	m	0	0	all	-	a	SCAmer	1987	CC	215	n	bl	n	y	2	ev	cig only	only f	ever p	ot
SOBUE	74	x	m	0	0	all	-	a	As:Jap	1986	CC	1376	n	bl	n	y	0	cu	cig+/-ot	only f	p NOS	st
WAKAI	71		m	0	0	all	-	a	As:Jap	1988	CC	333	n	bl	n	y	0	cu	cig+/-ot	only f	p NOS	ot
WYNDE5	2		m	0	0	all	-	KII	NAmer	1969	CC	1365	n	bl	n	y	0	cu	cig+/-ot	ever f	always p	st
WYNDE5	5		f	0	0	all	-	KII	NAmer	1969	CC	1365	n	bl	n	y	0	cu	cig+/-ot	ever f	always p	st
WYNDE6	301	x	m	0	0	all	-	a	NAmer	1969	CC	4423	n	bl	n	y	0	cu	cig+/-ot	only f	always p	st
WYNDE6	307	x	f	0	0	all	-	a	NAmer	1969	CC	4423	n	bl	n	y	0	cu	cig+/-ot	only f	always p	st

Table 3F1 - 5

IESLC - Meta-analysis of Cigarette Smoking, only Filter vs only Plain (or nearest available)
Adenocarcinoma
Least adjusted

REF	NRR	SEX	AD	Number Exposed Case	Number Exposed Cont	Non-exposed Case	Non-exposed Cont	RR	95.00%CI
LUBIN2	188	m	0	47	1018	197	3810	0.89	(0.64- 1.24)
LUBIN2	220	f	0	19	213	9	62	0.61	(0.26- 1.43)
Subtotal LUBIN2								0.85	(0.63- 1.15)
MATOS	74	m	0	70	229	8	46	1.76	(0.79- 3.90)
PEZZOT	15	m	2	-	-	-	-	0.28	(0.14- 0.55)
SOBUE	74	m	0	218	540	16	26	0.66	(0.35- 1.25)
WAKAI	71	m	0	73	271	0	9	5.14~	(0.30- 89.41)
WYNDE5	2	m	0	139	629	73	398	1.20	(0.88- 1.64)
WYNDE5	5	f	0	68	200	13	30	0.78	(0.39- 1.59)
Subtotal WYNDE5								1.12	(0.85- 1.49)
WYNDE6	301	m	0	88	122	135	165	0.88	(0.62- 1.26)
WYNDE6	307	f	0	158	158	32	30	0.94	(0.54- 1.62)
Subtotal WYNDE6								0.90	(0.67- 1.21)
Partial Totals				880	3380	483	4576		

*prospective study

~ With 0.5 adjustment for zero

REF	NRR	SEX	AD	Ys	Ws	Qs	Ps
LUBIN2	188	m	0	-0.11	36.24	0.00	0.4954
LUBIN2	220	f	0	-0.49	5.42	0.80	0.2570
Subtotal LUBIN2				-0.16	41.65	0.81	
MATOS	74	m	0	0.56	6.05	2.68	0.1655
PEZZOT	15	m	2	-1.27	8.21	11.25	0.0003
SOBUE	74	m	0	-0.42	9.31	0.95	0.1983
WAKAI	71	m	0	1.64	0.47	1.43	0.2610
WYNDE5	2	m	0	0.19	40.01	3.33	0.2386
WYNDE5	5	f	0	-0.24	7.69	0.15	0.5010
Subtotal WYNDE5				0.12	47.70	3.48	
WYNDE6	301	m	0	-0.13	30.28	0.02	0.4881
WYNDE6	307	f	0	-0.06	12.95	0.02	0.8164
Subtotal WYNDE6				-0.11	43.22	0.04	

	N	10
	NS	7
	Wt	156.61
Het	Chi	20.63
Het	df	9
Het	P	*
Fixed	RR	0.90
	RRl	0.77
	RRu	1.06
	P	N.S.
Random	RR	0.85
	RRl	0.65
	RRu	1.11
	P	N.S.
Asymm	P	N.S.

Table 3F1 - 6

IESLC - Meta-analysis of Cigarette Smoking, only Filter vs only Plain (or nearest available)
Adenocarcinoma
Least adjusted

	Sex combined	male	female	Total
N		7	3	10
NS		7	3	10
Wt		130.56	26.06	156.61
Het Chi		19.61	0.70	20.63
Het df		6	2	9
Het P		**	N.S.	*
Fixed RR		0.92	0.81	0.90
RRl		0.78	0.55	0.77
RRu		1.09	1.20	1.06
P		N.S.	N.S.	N.S.
Random RR		0.86	0.81	0.85
RRl		0.60	0.55	0.65
RRu		1.24	1.20	1.11
P		N.S.	N.S.	N.S.
Between Chi				0.33
Between df				1
Between P				N.S.
Btwn(F) P				N.S.
Btwn(R) P				N.S.

Table 3F1 - 7

IESLC - Meta-analysis of Cigarette Smoking, only Filter vs only Plain (or nearest available)
Adenocarcinoma
Excluded studies (and stage at which they were excluded)

1	BOUCHA BUELL LAURIL MZILEN
2	BERRIN CHEN RESTRE TAO ULMER
3	ABELIN ABRAHA AKIBA AMANDU AMES ANDERS ARCHER AUSTIN AUVINE AXELSO AXELSS BAND BARBON BENSHL BEST BLOHMK BLOT1 BLOT2 BLOT3 BLOT4 BOFFET BOUCOT BRESLO BRETT BROCKM BROWN1 BROWN2 BYERS1 BYERS2 CARPEN CASCO2 CASCOR CEDERL CHAN CHANG CHATZI CHEN2 CHEN3 CHIAZZ CHOW CHYOU COMSTO COOKSO CPSI DAMBER DARBY DAVEYS DEAN DEKLER DOCKER DOLL2 DORANT DORGAN DORN DOSEME DROSTE DU DUNN EBELIN ENSTRO ESAKI FAN GAO GAO2 GARCIA GARDIN GARSHI GENG GER GILLIS GODLEY GOLLED GOODMA GRAHAM GREGOR GSELL HAENSZ HAMMO2 HAMMON HANSEN HEGMAN HEIN HENNEK HINDS HIRAOK HIRAY2 HIRAYA HITOSU HOLE HOROWI HORWIT HU HU2 HUANG HUMBLE ISHIMA JAHN JAIN JARUP JARVHO JEDRYC JIANG JOLY JONES JUSSAW KAISER KANELL KATSOU KAUFMA KELLER KIHARA KINLEN KJUUS KNEKT KO KOHLME KOO KOULUM KREUZE KREYBE KUBIK LAMTH LAMWK LAMWK2 LAUSSM LEI LEMARC LETOUR LEVIN LIAW LICKIN LIDDEL LIU LIU2 LIU3 LIU4 LIU5 LOMBA2 LOMBAR LUBIN LUO MAGNUS MARSH MARSH2 MARTIS MASTRA MATSUD MCCONN MCDUFF MCLAUG MILLER MILLS MOLLO MRFIT MURATA NAM NOTAN2 NOTANI NOU ODRISC ORMOS OSANN OSANN2 PARKIN PASTOR PAWLEG PERNU PERSH2 PERSHA PETO PEZZO2 PIKE PISANI POFFIJ POLEDN PRESCO QIAO QIAO2 RACHTA RADZIK RANDIG REN RONCO ROOTS ROTHSC SAARIK SADOWS SANKAR SCHWA2 SCHWAR SEGI SEOW SHAW SHIMIZ SIEMIA SIMARA SIMONA SITAS SOBUE2 SPEIZE SPITZ STASZE STAYNE STOCKS STOCKW STUCKE SUN SUZUK2 SUZUKI SVENSS TANG TENKAN TIZZAN TOKARS TOUSEY TSUGAN TULINI TVERDA VANDER VEIERO VUTUC WALD WANG WANG2 WANG3 WANG4 WARSIN WATSON WICKLU WIGLE WILKIN WU WU2 WUNSCH WUWILL WYNDE2 WYNDE4 WYNDE7 WYNDE8 WYNDER XIANGZ XU XU2 XU3 XU4 YAMAGU YONG YUAN ZHANG ZHENG ZHOU
7	AGUDO ALDERS ARMADA BECHER BENHAM BROSS BUFFLE CHOI CORREA CPSII DEAN2 DEAN3 DESTE2 DESTEF DOLL ENGELA KAISE2 KHUDER LANGE MACLEN MIGRAN MRFITR RIMING SEGI2 TANG2 WYNDE3

Table 3F1 - 8

Potentially overlapping studies

REF	REFGP	PRINC	.	OVERLAP/LINK
LUBIN2	LUBIN2	1		Lubin-combined
WYNDE5	WYNDE6	2		WYNDE5/6/7/8
WYNDE6	WYNDE6	1		WYNDE5/6/7/8

Table 3F2 -

IESLC - Meta-analysis of Cigarette Smoking, ever Filter vs only Plain (or nearest available)
Adenocarcinoma

This analysis is restricted to results for:
1) Non-dose-response data
2) Results complete enough for use in metaanalysis

Within each study, results are then selected (in the following order of preference, within each sex) for:
3) CIGTYP: filter ever, equally, both, mainly, always, only/NOS
4) DENOM: plain only/NOS, always, mainly, ever
5) PRODUCT: cigarettes regardless of other products, cigarettes only (Note only study ALDERS has both product definitions available)
6) SMKSTA: ever, current. (Note only study MATOS has both ever and current available)
7) LCtype: all or nearest available, at least Squamous and Adeno. (q = squamous, s = small,
l = large, a = adeno, mix = mixed, alv = alveolar)
8) Race: all or nearest available, otherwise by race (wh or w = white, bl or b = black, hi = hispanic
ch = chinese, jap = japanese, haw = hawaiian, w+o = white + oriental, sca = scandinavian, as = asian)
9) Followup period (YF, prospective studies): whole study (coded as 0) or longest available
10) For overlapping studies: principal rather than subsidiary studies
Finally by Age: whole study (coded as 0) if available, otherwise by widest available age group
and then for single sex results (m, f) in preference to combined sex results (c).

Results adjusted (AD) for the most potential confounders are then chosen in Sections -1 to -3
(and those which actually differ from the adjusted results in Table 3F1 - 1 are marked 'x' in Section -1)
and results adjusted for the least confounders in Sections -4 to -6. (Those least adjusted results which
actually differ from the most adjusted as marked 'x' in column X in Section -4)
(Results adjusted for an unknown number of confounder(s) are coded as 20.)

Section -7 shows excluded studies, together with the stage (as above) at which no qualifying
results were found.

Section -8 lists the potentially overlapping studies which have been included (1=principal, 2=subsidiary).

Section -9 lists any results which would have been included in preference except that they had data not complete
enough for use in meta-analysis, with their significance (yes/no), if known, and any further comment as entered
on the database.

In addition to those mentioned above, the following fields, levels and abbreviations are used:
* or nk = not known, n = no, y = yes, ot = other
ev = ever, cu = current, cig+/-ot = cigarettes irrespective of other products (cigar, pipe etc)
f = filter, p = plain, NOS = not otherwise specified
REF: 6-character study reference
NRR: number of the RR on the database within the study
ST : study type (CC = case control, pr or prosp = prospective)
NLC: number of lung cancer cases in whole study
R : risky occupational population (n = no, m = mining, o = other risky)
VB : national cigarette type (V = at least 75% Virginia, bl = at least 75% blended, ot = other)
P : any proxy use
H : full histological confirmation
De : derivation of RR/CI (or = original, st = standard method, ot = other method of estimation)

Table 3F2 - 1

IESLC - Meta-analysis of Cigarette Smoking, ever Filter vs only Plain (or nearest available)
Adenocarcinoma
Most adjusted

REF	NRR	3F1	SEX	AGEL	AGEH	RACE	YF	LC TYPE	LOC	START	ST	NLC	R	VB	P	H	AD	SM	PRODUCT	CIGTYP	DENOM	De
LUBIN2	212	x	m	0	0	all	-	a	Eu:mul	1976	CC	7804	n	bl	n	y	3	ev	cig+/-ot	ever f	always p	ot
LUBIN2	244	x	f	0	0	all	-	a	Eu:mul	1976	CC	7804	n	bl	n	y	3	ev	cig+/-ot	ever f	always p	ot
MATOS	65		m	0	0	all	-	a	SCAmer	1994	CC	200	n	bl	n	n	3	ev	cig+/-ot	mainly f	mainly p	st
PEZZOT	16		m	0	0	all	-	a	SCAmer	1987	CC	215	n	bl	n	y	4	ev	cig only	only f	ever p	ot
SOBUE	86		m	0	0	all	-	a	As:Jap	1986	CC	1376	n	bl	n	y	5	cu	cig+/-ot	only f	p NOS	st
WAKAI	71		m	0	0	all	-	a	As:Jap	1988	CC	333	n	bl	n	y	0	cu	cig+/-ot	only f	p NOS	ot
WYNDE5	2		m	0	0	all	-	KII	NAmer	1969	CC	1365	n	bl	n	y	0	cu	cig+/-ot	ever f	always p	st
WYNDE5	5		f	0	0	all	-	KII	NAmer	1969	CC	1365	n	bl	n	y	0	cu	cig+/-ot	ever f	always p	st
WYNDE6	315	x	m	0	0	all	-	a	NAmer	1969	CC	4423	n	bl	n	y	3	cu	cig+/-ot	ever f	always p	ot
WYNDE6	321	x	f	0	0	all	-	a	NAmer	1969	CC	4423	n	bl	n	y	3	cu	cig+/-ot	ever f	always p	ot

Table 3F2 - 2

IESLC - Meta-analysis of Cigarette Smoking, ever Filter vs only Plain (or nearest available)
Adenocarcinoma
Most adjusted

REF	NRR	SEX	AD	Number Exposed Case	Number Exposed Cont	Non-exposed Case	Non-exposed Cont	RR	95.00%CI
LUBIN2	212	m	3	-	-	-	-	1.01	(0.85- 1.19)
LUBIN2	244	f	3	-	-	-	-	0.84	(0.46- 1.53)
Subtotal LUBIN2								1.00	(0.85- 1.17)
MATOS	65	m	3	-	-	-	-	1.43	(0.63- 3.33)
PEZZOT	16	m	4	-	-	-	-	0.38	(0.19- 0.76)
SOBUE	86	m	5	-	-	-	-	0.83	(0.40- 1.67)
WAKAI	71	m	0	73	271	0	9	5.14~	(0.30- 89.41)
WYNDE5	2	m	0	139	629	73	398	1.20	(0.88- 1.64)
WYNDE5	5	f	0	68	200	13	30	0.78	(0.39- 1.59)
Subtotal WYNDE5								1.12	(0.85- 1.49)
WYNDE6	315	m	3	-	-	-	-	1.00	(0.79- 1.27)
WYNDE6	321	f	3	-	-	-	-	1.15	(0.68- 1.94)
Subtotal WYNDE6								1.02	(0.82- 1.27)
Partial Totals				280	1100	86	437		

*prospective study

~ With 0.5 adjustment for zero

REF	NRR	SEX	AD	Ys	Ws	Qs	Ps
LUBIN2	212	m	3	0.01	135.72	0.01	0.9077
LUBIN2	244	f	3	-0.17	10.64	0.33	0.5696
Subtotal LUBIN2				-0.00	146.36	0.34	
MATOS	65	m	3	0.36	5.54	0.70	0.3997
PEZZOT	16	m	4	-0.97	8.00	7.52	0.0062
SOBUE	86	m	5	-0.19	7.52	0.27	0.6093
WAKAI	71	m	0	1.64	0.47	1.26	0.2610
WYNDE5	2	m	0	0.19	40.01	1.36	0.2386
WYNDE5	5	f	0	-0.24	7.69	0.46	0.5010
Subtotal WYNDE5				0.12	47.70	1.82	
WYNDE6	315	m	3	0.00	68.18	0.00	1.0000
WYNDE6	321	f	3	0.14	13.98	0.26	0.6013
Subtotal WYNDE6				0.02	82.16	0.26	

	N	10
	NS	7
	Wt	297.76
Het	Chi	12.17
Het	df	9
Het	P	N.S.
Fixed	RR	1.00
	RRl	0.89
	RRu	1.12
	P	N.S.
Random	RR	0.99
	RRl	0.84
	RRu	1.16
	P	N.S.
Asymm	P	N.S.

Table 3F2 - 3

IESLC - Meta-analysis of Cigarette Smoking, ever Filter vs only Plain (or nearest available)
Adenocarcinoma
Most adjusted

	combined	Sex male	female	Total
N		7	3	10
NS		7	3	10
Wt		265.44	32.31	297.76
Het Chi		11.10	0.95	12.17
Het df		6	2	9
Het P		(*)	N.S.	N.S.
Fixed RR		1.01	0.95	1.00
RRl		0.89	0.67	0.89
RRu		1.14	1.34	1.12
P		N.S.	N.S.	N.S.
Random RR		0.99	0.95	0.99
RRl		0.80	0.67	0.84
RRu		1.22	1.34	1.16
P		N.S.	N.S.	N.S.
Between Chi				0.12
Between df				1
Between P				N.S.
Btwn(F) P				N.S.
Btwn(R) P				N.S.

	a	All LC (or nearest) a+l	a+al+br	KII	not q+u	not q+s	Total
N	8			2			10
NS	6			1			7
Wt	250.06			47.70			297.76
Het Chi	10.23			1.19			12.17
Het df	7			1			9
Het P	N.S.			N.S.			N.S.
Fixed RR	0.98			1.12			1.00
RRl	0.87			0.85			0.89
RRu	1.11			1.49			1.12
P	N.S.			N.S.			N.S.
Random RR	0.95			1.10			0.99
RRl	0.78			0.78			0.84
RRu	1.16			1.55			1.16
P	N.S.			N.S.			N.S.
Between Chi							0.75
Between df							1
Between P							N.S.
Btwn(F) P							N.S.
Btwn(R) P							N.S.

	NAmer	UK	Scand	Location othEur	China	Japan	othAs	other	Total
N	4			2		2		2	10
NS	2			1		2		2	7
Wt	129.86			146.36		7.99		13.54	297.76
Het Chi	1.68			0.34		1.48		5.75	12.17
Het df	3			1		1		1	9
Het P	N.S.			N.S.		N.S.		*	N.S.
Fixed RR	1.06			1.00		0.92		0.65	1.00
RRl	0.89			0.85		0.46		0.38	0.89
RRu	1.26			1.17		1.85		1.11	1.12
P	N.S.			N.S.		N.S.		N.S.	N.S.
Random RR	1.06			1.00		1.20		0.72	0.99
RRl	0.89			0.85		0.29		0.20	0.84
RRu	1.26			1.17		5.02		2.64	1.16
P	N.S.			N.S.		N.S.		N.S.	N.S.
Between Chi									2.93
Between df									3
Between P									N.S.
Btwn(F) P									N.S.
Btwn(R) P									N.S.

Table 3F2 - 3

IESLC - Meta-analysis of Cigarette Smoking, ever Filter vs only Plain (or nearest available)
Adenocarcinoma
Most adjusted

	Detailed Country in "other Europe" multi	Germany	othWest	East	Balkans	Total
N	2					2
NS	1					1
Wt	146.36					146.36
Het Chi	0.34					0.34
Het df	1					1
Het P	N.S.					N.S.
Fixed RR	1.00					1.00
RRl	0.85					0.85
RRu	1.17					1.17
P	N.S.					N.S.
Random RR	1.00					1.00
RRl	0.85					0.85
RRu	1.17					1.17
P	N.S.					N.S.
Between Chi						
Between df						
Between P						N.S.
Btwn(F) P						N.S.
Btwn(R) P						N.S.

	Detailed Country in "other Asia" India	HongKong	other	Total
N				
NS				
Wt				
Het Chi				
Het df				
Het P				
Fixed RR				
RRl				
RRu				
P				
Random RR				
RRl				
RRu				
P				
Between Chi				
Between df				
Between P				N.S.
Btwn(F) P				N.S.
Btwn(R) P				N.S.

	Detailed other continent SCAmer	Auslia	Africa	Total
N	2			2
NS	2			2
Wt	13.54			13.54
Het Chi	5.75			5.75
Het df	1			1
Het P	*			*
Fixed RR	0.65			0.65
RRl	0.38			0.38
RRu	1.11			1.11
P	N.S.			N.S.
Random RR	0.72			0.72
RRl	0.20			0.20
RRu	2.64			2.64
P	N.S.			N.S.
Between Chi				
Between df				
Between P				N.S.
Btwn(F) P				N.S.
Btwn(R) P				N.S.

Table 3F2 - 3

IESLC - Meta-analysis of Cigarette Smoking, ever Filter vs only Plain (or nearest available)
Adenocarcinoma
Most adjusted

	<1960	Start year of study 1960-69	1970-79	1980-89	1990+	Total
N		4	2	3	1	10
NS		2	1	3	1	7
Wt		129.86	146.36	15.99	5.54	297.76
Het Chi		1.68	0.34	4.63	0.00	12.17
Het df		3	1	2	0	9
Het P		N.S.	N.S.	(*)	N.S.	N.S.
Fixed RR		1.06	1.00	0.59	1.43	1.00
RRl		0.89	0.85	0.36	0.62	0.89
RRu		1.26	1.17	0.97	3.29	1.12
P		N.S.	N.S.	-	N.S.	N.S.
Random RR		1.06	1.00	0.67	1.43	0.99
RRl		0.89	0.85	0.28	0.62	0.84
RRu		1.26	1.17	1.62	3.29	1.16
P		N.S.	N.S.	N.S.	N.S.	N.S.
Between Chi						5.53
Between df						3
Between P						N.S.
Btwn(F) P						N.S.
Btwn(R) P						N.S.

	Study type (1) CC	other	Total
N	10		10
NS	7		7
Wt	297.76		297.76
Het Chi	12.17		12.17
Het df	9		9
Het P	N.S.		N.S.
Fixed RR	1.00		1.00
RRl	0.89		0.89
RRu	1.12		1.12
P	N.S.		N.S.
Random RR	0.99		0.99
RRl	0.84		0.84
RRu	1.16		1.16
P	N.S.		N.S.
Between Chi			
Between df			
Between P			N.S.
Btwn(F) P			N.S.
Btwn(R) P			N.S.

	Study type (2) CC	prosp	other	Total
N	10			10
NS	7			7
Wt	297.76			297.76
Het Chi	12.17			12.17
Het df	9			9
Het P	N.S.			N.S.
Fixed RR	1.00			1.00
RRl	0.89			0.89
RRu	1.12			1.12
P	N.S.			N.S.
Random RR	0.99			0.99
RRl	0.84			0.84
RRu	1.16			1.16
P	N.S.			N.S.
Between Chi				
Between df				
Between P				N.S.
Btwn(F) P				N.S.
Btwn(R) P				N.S.

Table 3F2 - 3

IESLC - Meta-analysis of Cigarette Smoking, ever Filter vs only Plain (or nearest available)
Adenocarcinoma
Most adjusted

	Study size (number of LC cases)				
	100-249	250-499	500-999	1000+	Total
N	2	1		7	10
NS	2	1		4	7
Wt	13.54	0.47		283.75	297.76
Het Chi	5.75	0.00		2.60	12.17
Het df	1	0		6	9
Het P	*	N.S.		N.S.	N.S.
Fixed RR	0.65	5.14		1.02	1.00
RRl	0.38	0.30		0.91	0.89
RRu	1.11	89.41		1.15	1.12
P	N.S.	N.S.		N.S.	N.S.
Random RR	0.72	5.14		1.02	0.99
RRl	0.20	0.30		0.91	0.84
RRu	2.64	89.41		1.15	1.16
P	N.S.	N.S.		N.S.	N.S.
Between Chi					3.82
Between df					2
Between P					N.S.
Btwn(F) P					N.S.
Btwn(R) P					N.S.

	Risky occupational population			
	no	mining	othRisky	Total
N	10			10
NS	7			7
Wt	297.76			297.76
Het Chi	12.17			12.17
Het df	9			9
Het P	N.S.			N.S.
Fixed RR	1.00			1.00
RRl	0.89			0.89
RRu	1.12			1.12
P	N.S.			N.S.
Random RR	0.99			0.99
RRl	0.84			0.84
RRu	1.16			1.16
P	N.S.			N.S.
Between Chi				
Between df				
Between P				N.S.
Btwn(F) P				N.S.
Btwn(R) P				N.S.

	National cigarette tobacco type			
	Virginia	blended	other	Total
N		10		10
NS		7		7
Wt		297.76		297.76
Het Chi		12.17		12.17
Het df		9		9
Het P		N.S.		N.S.
Fixed RR		1.00		1.00
RRl		0.89		0.89
RRu		1.12		1.12
P		N.S.		N.S.
Random RR		0.99		0.99
RRl		0.84		0.84
RRu		1.16		1.16
P		N.S.		N.S.
Between Chi				
Between df				
Between P				N.S.
Btwn(F) P				N.S.
Btwn(R) P				N.S.

Table 3F2 - 3

IESLC - Meta-analysis of Cigarette Smoking, ever Filter vs only Plain (or nearest available)
Adenocarcinoma
Most adjusted

	Any proxy use No/nk	Yes	Total
N	10		10
NS	7		7
Wt	297.76		297.76
Het Chi	12.17		12.17
Het df	9		9
Het P	N.S.		N.S.
Fixed RR	1.00		1.00
RRl	0.89		0.89
RRu	1.12		1.12
P	N.S.		N.S.
Random RR	0.99		0.99
RRl	0.84		0.84
RRu	1.16		1.16
P	N.S.		N.S.
Between Chi			
Between df			
Between P			N.S.
Btwn(F) P			N.S.
Btwn(R) P			N.S.

	Full histological confirmation No	Yes	Total
N	1	9	10
NS	1	6	7
Wt	5.54	292.21	297.76
Het Chi	0.00	11.46	12.17
Het df	0	8	9
Het P	N.S.	N.S.	N.S.
Fixed RR	1.43	1.00	1.00
RRl	0.62	0.89	0.89
RRu	3.29	1.12	1.12
P	N.S.	N.S.	N.S.
Random RR	1.43	0.97	0.99
RRl	0.62	0.82	0.84
RRu	3.29	1.15	1.16
P	N.S.	N.S.	N.S.
Between Chi			0.71
Between df			1
Between P			N.S.
Btwn(F) P			N.S.
Btwn(R) P			N.S.

	Number of adjustment variables (1) 0	1	2+/+nk	Total
N	3		7	10
NS	2		5	7
Wt	48.17		249.58	297.76
Het Chi	2.27		8.94	12.17
Het df	2		6	9
Het P	N.S.		N.S.	N.S.
Fixed RR	1.14		0.98	1.00
RRl	0.86		0.86	0.89
RRu	1.51		1.11	1.12
P	N.S.		N.S.	N.S.
Random RR	1.12		0.95	0.99
RRl	0.78		0.78	0.84
RRu	1.60		1.15	1.16
P	N.S.		N.S.	N.S.
Between Chi				0.97
Between df				1
Between P				N.S.
Btwn(F) P				N.S.
Btwn(R) P				N.S.

Table 3F2 - 3

IESLC - Meta-analysis of Cigarette Smoking, ever Filter vs only Plain (or nearest available)
Adenocarcinoma
Most adjusted

	Number of adjustment variables (2) 0	1	2	3-5	6+/+nk	Total
N	3			7		10
NS	2			5		7
Wt	48.17			249.58		297.76
Het Chi	2.27			8.94		12.17
Het df	2			6		9
Het P	N.S.			N.S.		N.S.
Fixed RR	1.14			0.98		1.00
RRl	0.86			0.86		0.89
RRu	1.51			1.11		1.12
P	N.S.			N.S.		N.S.
Random RR	1.12			0.95		0.99
RRl	0.78			0.78		0.84
RRu	1.60			1.15		1.16
P	N.S.			N.S.		N.S.
Between Chi						0.97
Between df						1
Between P						N.S.
Btwn(F) P						N.S.
Btwn(R) P						N.S.

	Smoking status ever	current	Total
N	4	6	10
NS	3	4	7
Wt	159.90	137.86	297.76
Het Chi	8.29	3.29	12.17
Het df	3	5	9
Het P	*	N.S.	N.S.
Fixed RR	0.96	1.05	1.00
RRl	0.82	0.89	0.89
RRu	1.12	1.24	1.12
P	N.S.	N.S.	N.S.
Random RR	0.84	1.05	0.99
RRl	0.54	0.89	0.84
RRu	1.30	1.24	1.16
P	N.S.	N.S.	N.S.
Between Chi			0.59
Between df			1
Between P			N.S.
Btwn(F) P			N.S.
Btwn(R) P			N.S.

	Product cig+/-ot	cig only	Total
N	9	1	10
NS	6	1	7
Wt	289.76	8.00	297.76
Het Chi	4.44	0.00	12.17
Het df	8	0	9
Het P	N.S.	N.S.	N.S.
Fixed RR	1.03	0.38	1.00
RRl	0.92	0.19	0.89
RRu	1.15	0.76	1.12
P	N.S.	--	N.S.
Random RR	1.03	0.38	0.99
RRl	0.92	0.19	0.84
RRu	1.15	0.76	1.16
P	N.S.	--	N.S.
Between Chi			7.73
Between df			1
Between P			**
Btwn(F) P			**
Btwn(R) P			**

Table 3F2 - 3

IESLC - Meta-analysis of Cigarette Smoking, ever Filter vs only Plain (or nearest available)
Adenocarcinoma
Most adjusted

	Cigarette type						
	only f	always f	mainly f	equl p&f	both p&f	ever f	Total
N	3		1			6	10
NS	3		1			3	7
Wt	15.99		5.54			276.22	297.76
Het Chi	4.63		0.00			2.27	12.17
Het df	2		0			5	9
Het P	(*)		N.S.			N.S.	N.S.
Fixed RR	0.59		1.43			1.03	1.00
RRl	0.36		0.62			0.91	0.89
RRu	0.97		3.29			1.15	1.12
P	-		N.S.			N.S.	N.S.
Random RR	0.67		1.43			1.03	0.99
RRl	0.28		0.62			0.91	0.84
RRu	1.62		3.29			1.15	1.16
P	N.S.		N.S.			N.S.	N.S.
Between Chi							5.26
Between df							2
Between P							(*)
Btwn(F) P							N.S.
Btwn(R) P							N.S.

	Denominator				
	ever p	mainly p	p NOS	always p	Total
N	1	1	2	6	10
NS	1	1	2	3	7
Wt	8.00	5.54	7.99	276.22	297.76
Het Chi	0.00	0.00	1.48	2.27	12.17
Het df	0	0	1	5	9
Het P	N.S.	N.S.	N.S.	N.S.	N.S.
Fixed RR	0.38	1.43	0.92	1.03	1.00
RRl	0.19	0.62	0.46	0.91	0.89
RRu	0.76	3.29	1.85	1.15	1.12
P	--	N.S.	N.S.	N.S.	N.S.
Random RR	0.38	1.43	1.20	1.03	0.99
RRl	0.19	0.62	0.29	0.91	0.84
RRu	0.76	3.29	5.02	1.15	1.16
P	--	N.S.	N.S.	N.S.	N.S.
Between Chi					8.42
Between df					3
Between P					*
Btwn(F) P					(*)
Btwn(R) P					*

	Derivation of RR/CI			
	Orig	StdCalc	Other	Total
N		4	6	10
NS		3	4	7
Wt		60.77	236.99	297.76
Het Chi		2.19	9.23	12.17
Het df		3	5	9
Het P		N.S.	N.S.	N.S.
Fixed RR		1.11	0.98	1.00
RRl		0.86	0.86	0.89
RRu		1.42	1.11	1.12
P		N.S.	N.S.	N.S.
Random RR		1.11	0.93	0.99
RRl		0.86	0.74	0.84
RRu		1.42	1.17	1.16
P		N.S.	N.S.	N.S.
Between Chi				0.75
Between df				1
Between P				N.S.
Btwn(F) P				N.S.
Btwn(R) P				N.S.

Table 3F2 - 4

IESLC - Meta-analysis of Cigarette Smoking, ever Filter vs only Plain (or nearest available)
Adenocarcinoma
Least adjusted

REF	NRR	X	SEX	AGEL	AGEH	RACE	YF	LC TYPE	LOC	START	ST	NLC	R	VB	P	H	AD	SM	PRODUCT	CIGTYP	DENOM	De
LUBIN2	196	x	m	0	0	all	-	a	Eu:mul	1976	CC	7804	n	bl	n	y	0	ev	cig+/-ot	ever f	always p	st
LUBIN2	228	x	f	0	0	all	-	a	Eu:mul	1976	CC	7804	n	bl	n	y	0	ev	cig+/-ot	ever f	always p	st
MATOS	74	x	m	0	0	all	-	a	SCAmer	1994	CC	200	n	bl	n	n	0	ev	cig+/-ot	mainly f	mainly p	st
PEZZOT	15	x	m	0	0	all	-	a	SCAmer	1987	CC	215	n	bl	n	y	2	ev	cig only	only f	ever p	ot
SOBUE	74	x	m	0	0	all	-	a	As:Jap	1986	CC	1376	n	bl	n	y	0	cu	cig+/-ot	only f	p NOS	st
WAKAI	71		m	0	0	all	-	a	As:Jap	1988	CC	333	n	bl	n	y	0	cu	cig+/-ot	only f	p NOS	ot
WYNDE5	2		m	0	0	all	-	KII	NAmer	1969	CC	1365	n	bl	n	y	0	cu	cig+/-ot	ever f	always p	st
WYNDE5	5		f	0	0	all	-	KII	NAmer	1969	CC	1365	n	bl	n	y	0	cu	cig+/-ot	ever f	always p	st
WYNDE6	303	x	m	0	0	all	-	a	NAmer	1969	CC	4423	n	bl	n	y	0	cu	cig+/-ot	ever f	always p	st
WYNDE6	309	x	f	0	0	all	-	a	NAmer	1969	CC	4423	n	bl	n	y	0	cu	cig+/-ot	ever f	always p	st

Table 3F2 - 5

IESLC - Meta-analysis of Cigarette Smoking, ever Filter vs only Plain (or nearest available)
Adenocarcinoma
Least adjusted

REF	NRR	SEX	AD	Number Exposed Case	Number Exposed Cont	Non-exposed Case	Non-exposed Cont	RR	95.00%CI
LUBIN2	196	m	0	475	6627	197	3810	1.39	(1.17- 1.64)
LUBIN2	228	f	0	75	505	9	62	1.02	(0.49- 2.14)
Subtotal LUBIN2								1.37	(1.16- 1.61)
MATOS	74	m	0	70	229	8	46	1.76	(0.79- 3.90)
PEZZOT	15	m	2	-	-	-	-	0.28	(0.14- 0.55)
SOBUE	74	m	0	218	540	16	26	0.66	(0.35- 1.25)
WAKAI	71	m	0	73	271	0	9	5.14~	(0.30- 89.41)
WYNDE5	2	m	0	139	629	73	398	1.20	(0.88- 1.64)
WYNDE5	5	f	0	68	200	13	30	0.78	(0.39- 1.59)
Subtotal WYNDE5								1.12	(0.85- 1.49)
WYNDE6	303	m	0	577	711	135	165	0.99	(0.77- 1.28)
WYNDE6	309	f	0	537	437	32	30	1.15	(0.69- 1.93)
Subtotal WYNDE6								1.02	(0.81- 1.28)
Partial Totals				2232	10149	483	4576		

*prospective study

~ With 0.5 adjustment for zero

REF	NRR	SEX	AD	Ys	Ws	Qs	Ps
LUBIN2	196	m	0	0.33	131.67	4.55	0.0002
LUBIN2	228	f	0	0.02	7.01	0.10	0.9518
Subtotal LUBIN2				0.31	138.68	4.65	
MATOS	74	m	0	0.56	6.05	1.08	0.1655
PEZZOT	15	m	2	-1.27	8.21	16.40	0.0003
SOBUE	74	m	0	-0.42	9.31	2.94	0.1983
WAKAI	71	m	0	1.64	0.47	1.06	0.2610
WYNDE5	2	m	0	0.19	40.01	0.08	0.2386
WYNDE5	5	f	0	-0.24	7.69	1.13	0.5010
Subtotal WYNDE5				0.12	47.70	1.21	
WYNDE6	303	m	0	-0.01	60.21	1.33	0.9495
WYNDE6	309	f	0	0.14	14.55	0.00	0.5893
Subtotal WYNDE6				0.02	74.76	1.33	

	N	10
	NS	7
	Wt	285.18
Het	Chi	28.68
Het	df	9
Het	P	***
Fixed	RR	1.15
	RRl	1.02
	RRu	1.29
	P	+
Random	RR	0.99
	RRl	0.76
	RRu	1.29
	P	N.S.
Asymm	P	N.S.

Table 3F2 - 6

IESLC - Meta-analysis of Cigarette Smoking, ever Filter vs only Plain (or nearest available)
Adenocarcinoma
Least adjusted

	combined	Sex male	female	Total
N		7	3	10
NS		7	3	10
Wt		255.93	29.26	285.18
Het Chi		27.40	0.74	28.68
Het df		6	2	9
Het P		***	N.S.	***
Fixed RR		1.17	1.01	1.15
RRl		1.03	0.70	1.02
RRu		1.32	1.45	1.29
P		+	N.S.	+
Random RR		0.98	1.01	0.99
RRl		0.70	0.70	0.76
RRu		1.38	1.45	1.29
P		N.S.	N.S.	N.S.
Between Chi				0.54
Between df				1
Between P				N.S.
Btwn(F) P				N.S.
Btwn(R) P				N.S.

Table 3F2 - 7

IESLC - Meta-analysis of Cigarette Smoking, ever Filter vs only Plain (or nearest available)
Adenocarcinoma
Excluded studies (and stage at which they were excluded)

1 BOUCHA BUELL LAURIL MZILEN

2 BERRIN CHEN RESTRE TAO ULMER

3 ABELIN ABRAHA AKIBA AMANDU AMES ANDERS ARCHER AUSTIN AUVINE AXELSO AXELSS BAND BARBON BENSHL BEST BLOHMK
BLOT1 BLOT2 BLOT3 BLOT4 BOFFET BOUCOT BRESLO BRETT BROCKM BROWN1 BROWN2 BYERS1 BYERS2 CARPEN CASCO2 CASCOR
CEDERL CHAN CHANG CHATZI CHEN2 CHEN3 CHIAZZ CHOW CHYOU COMSTO COOKSO CPSI DAMBER DARBY DAVEYS DEAN
DEKLER DOCKER DOLL2 DORANT DORGAN DORN DOSEME DROSTE DU DUNN EBELIN ENSTRO ESAKI FAN GAO GAO2
GARCIA GARDIN GARSHI GENG GER GILLIS GODLEY GOLLED GOODMA GRAHAM GREGOR GSELL HAENSZ HAMMO2 HAMMON HANSEN
HEGMAN HEIN HENNEK HINDS HIRAOK HIRAY2 HIRAYA HITOSU HOLE HOROWI HORWIT HU HU2 HUANG HUMBLE ISHIMA
JAHN JAIN JARUP JARVHO JEDRYC JIANG JOLY JONES JUSSAW KAISER KANELL KATSOU KAUFMA KELLER KIHARA KINLEN
KJUUS KNEKT KO KOHLME KOO KOULUM KREUZE KREYBE KUBIK LAMTH LAMWK LAMWK2 LAUSSM LEI LEMARC LETOUR
LEVIN LIAW LICKIN LIDDEL LIU LIU2 LIU3 LIU4 LIU5 LOMBA2 LOMBAR LUBIN LUO MAGNUS MARSH MARSH2
MARTIS MASTRA MATSUD MCCONN MCDUFF MCLAUG MILLER MILLS MOLLO MRFIT MURATA NAM NOTAN2 NOTANI NOU ODRISC
ORMOS OSANN OSANN2 PARKIN PASTOR PAWLEG PERNU PERSH2 PERSHA PETO PEZZO2 PIKE PISANI POFFIJ POLEDN PRESCO
QIAO QIAO2 RACHTA RADZIK RANDIG REN RONCO ROOTS ROTHSC SAARIK SADOWS SANKAR SCHWA2 SCHWAR SEGI SEOW
SHAW SHIMIZ SIEMIA SIMARA SIMONA SITAS SOBUE2 SPEIZE SPITZ STASZE STAYNE STOCKS STOCKW STUCKE SUN SUZUK2
SUZUKI SVENSS TANG TENKAN TIZZAN TOKARS TOUSEY TSUGAN TULINI TVERDA VANDER VEIERO VUTUC WALD WANG WANG2
WANG3 WANG4 WARSIN WATSON WICKLU WIGLE WILKIN WU WU2 WUNSCH WUWILL WYNDE2 WYNDE4 WYNDE7 WYNDE8 WYNDER
XIANGZ XU XU2 XU3 XU4 YAMAGU YONG YUAN ZHANG ZHENG ZHOU

7 AGUDO ALDERS ARMADA BECHER BENHAM BROSS BUFFLE CHOI CORREA CPSII DEAN2 DEAN3 DESTE2 DESTEF DOLL ENGELA
KAISE2 KHUDER LANGE MACLEN MIGRAN MRFITR RIMING SEGI2 TANG2 WYNDE3

Table 3F2 - 8

Potentially overlapping studies

REF	REFGP	PRINC	. OVERLAP/LINK
LUBIN2	LUBIN2	1	Lubin-combined
WYNDE5	WYNDE6	2	WYNDE5/6/7/8
WYNDE6	WYNDE6	1	WYNDE5/6/7/8

Table 3F3 -

IESLC - Meta-analysis of Cigarette Smoking, only Filter vs ever Plain (or nearest available)
Adenocarcinoma

This analysis is restricted to results for:
1) Non-dose-response data
2) Results complete enough for use in metaanalysis

Within each study, results are then selected (in the following order of preference, within each sex) for:
3) CIGTYP: filter only/NOS, always, mainly, both, equally, ever
4) DENOM: plain ever, mainly, always, only/NOS
5) PRODUCT: cigarettes regardless of other products, cigarettes only (Note only study ALDERS has both product definitions available)
6) SMKSTA: ever, current (Note only study MATOS has both ever and current available)
7) LCtype: all or nearest available, at least Squamous and Adeno. (q = squamous, s = small, l = large, a = adeno, mix = mixed, alv = alveolar)
8) Race: all or nearest available, otherwise by race (wh or w = white, bl or b = black, hi = hispanic ch = chinese, jap = japanese, haw = hawaiian, w+o = white + oriental, sca = scandinavian, as = asian)
9) Followup period (YF, prospective studies): whole study (coded as 0) or longest available
10) For overlapping studies: principal rather than subsidiary studies
Finally by Age: whole study (coded as 0) if available, otherwise by widest available age group and then for single sex results (m, f) in preference to combined sex results (c).

Results adjusted (AD) for the most potential confounders are then chosen in Sections -1 to -3 (and those which actually differ from the adjusted results in Table 3F1 - 1 are marked 'x' in Section -1) and results adjusted for the least confounders in Sections -4 to -6. (Those least adjusted results which actually differ from the most adjusted as marked 'x' in column X in Section -4) (Results adjusted for an unknown number of confounder(s) are coded as 20.)

Section -7 shows excluded studies, together with the stage (as above) at which no qualifying results were found.

Section -8 lists the potentially overlapping studies which have been included (1=principal, 2=subsidiary).

Section -9 lists any results which would have been included in preference except that they had data not complete enough for use in meta-analysis, with their significance (yes/no), if known, and any further comment as entered on the database.

In addition to those mentioned above, the following fields, levels and abbreviations are used:
* or nk = not known, n = no, y = yes, ot = other
ev = ever, cu = current, cig+/-ot = cigarettes irrespective of other products (cigar, pipe etc)
f = filter, p = plain, NOS = not otherwise specified
REF: 6-character study reference
NRR: number of the RR on the database within the study
ST : study type (CC = case control, pr or prosp = prospective)
NLC: number of lung cancer cases in whole study
R : risky occupational population (n = no, m = mining, o = other risky)
VB : national cigarette type (V = at least 75% Virginia, bl = at least 75% blended, ot = other)
P : any proxy use
H : full histological confirmation
De : derivation of RR/CI (or = original, st = standard method, ot = other method of estimation)

Table 3F3 - 1

IESLC - Meta-analysis of Cigarette Smoking, only Filter vs ever Plain (or nearest available)
Adenocarcinoma
Most adjusted

REF	NRR	3F1	SEX	AGEL	AGEH	RACE	YF	LC TYPE	LOC	START	ST	NLC	R	VB	P	H	AD	SM	PRODUCT	CIGTYP	DENOM	De
LUBIN2	216	x	m	0	0	all	-	a	Eu:mul	1976	CC	7804	n	bl	n	y	3	ev	cig+/-ot	only f	ever p	ot
LUBIN2	248	x	f	0	0	all	-	a	Eu:mul	1976	CC	7804	n	bl	n	y	3	ev	cig+/-ot	only f	ever p	ot
MATOS	65		m	0	0	all	-	a	SCAmer	1994	CC	200	n	bl	n	n	3	ev	cig+/-ot	mainly f	mainly p	st
PEZZOT	16		m	0	0	all	-	a	SCAmer	1987	CC	215	n	bl	n	y	4	ev	cig only	only f	ever p	ot
SOBUE	86		m	0	0	all	-	a	As:Jap	1986	CC	1376	n	bl	n	y	5	cu	cig+/-ot	only f	p NOS	st
WAKAI	71		m	0	0	all	-	a	As:Jap	1988	CC	333	n	bl	n	y	0	cu	cig+/-ot	only f	p NOS	ot
WYNDE5	2		m	0	0	all	-	KII	NAmer	1969	CC	1365	n	bl	n	y	0	cu	cig+/-ot	ever f	always p	st
WYNDE5	5		f	0	0	all	-	KII	NAmer	1969	CC	1365	n	bl	n	y	0	cu	cig+/-ot	ever f	always p	st
WYNDE6	314	x	m	0	0	all	-	a	NAmer	1969	CC	4423	n	bl	n	y	3	cu	cig+/-ot	only f	ever p	ot
WYNDE6	320	x	f	0	0	all	-	a	NAmer	1969	CC	4423	n	bl	n	y	3	cu	cig+/-ot	only f	ever p	ot

Table 3F3 - 2

IESLC - Meta-analysis of Cigarette Smoking, only Filter vs ever Plain (or nearest available)
Adenocarcinoma
Most adjusted

REF	NRR	SEX	AD	Number Exposed Case	Number Exposed Cont	Non-exposed Case	Non-exposed Cont	RR	95.00%CI
LUBIN2	216	m	3	-	-	-	-	1.14	(0.97- 1.35)
LUBIN2	248	f	3	-	-	-	-	1.56	(0.74- 3.27)
Subtotal LUBIN2								1.16	(0.98- 1.36)
MATOS	65	m	3	-	-	-	-	1.43	(0.63- 3.33)
PEZZOT	16	m	4	-	-	-	-	0.38	(0.19- 0.76)
SOBUE	86	m	5	-	-	-	-	0.83	(0.40- 1.67)
WAKAI	71	m	0	73	271	0	9	5.14~	(0.30- 89.41)
WYNDE5	2	m	0	139	629	73	398	1.20	(0.88- 1.64)
WYNDE5	5	f	0	68	200	13	30	0.78	(0.39- 1.59)
Subtotal WYNDE5								1.12	(0.85- 1.49)
WYNDE6	314	m	3	-	-	-	-	1.00	(0.72- 1.39)
WYNDE6	320	f	3	-	-	-	-	0.76	(0.53- 1.10)
Subtotal WYNDE6								0.88	(0.69- 1.13)
Partial Totals				280	1100	86	437		

*prospective study

~ With 0.5 adjustment for zero

REF	NRR	SEX	AD	Ys	Ws	Qs	Ps
LUBIN2	216	m	3	0.13	140.62	1.03	0.1202
LUBIN2	248	f	3	0.44	6.96	1.11	0.2407
Subtotal LUBIN2				0.15	147.58	2.14	
MATOS	65	m	3	0.36	5.54	0.54	0.3997
PEZZOT	16	m	4	-0.97	8.00	8.21	0.0062
SOBUE	86	m	5	-0.19	7.52	0.40	0.6093
WAKAI	71	m	0	1.64	0.47	1.19	0.2610
WYNDE5	2	m	0	0.19	40.01	0.79	0.2386
WYNDE5	5	f	0	-0.24	7.69	0.64	0.5010
Subtotal WYNDE5				0.12	47.70	1.43	
WYNDE6	314	m	3	0.00	35.51	0.07	1.0000
WYNDE6	320	f	3	-0.27	28.82	2.95	0.1407
Subtotal WYNDE6				-0.12	64.33	3.03	

	N	10
	NS	7
	Wt	281.14
Het	Chi	16.94
Het	df	9
Het	P	*
Fixed	RR	1.05
	RRl	0.93
	RRu	1.18
	P	N.S.
Random	RR	0.98
	RRl	0.80
	RRu	1.21
	P	N.S.
Asymm	P	N.S.

Table 3F3 - 3

IESLC - Meta-analysis of Cigarette Smoking, only Filter vs ever Plain (or nearest available)
Adenocarcinoma
Most adjusted

	Sex			
	combined	male	female	Total
N		7	3	10
NS		7	3	10
Wt		237.67	43.47	281.14
Het Chi		11.92	2.97	16.94
Het df		6	2	9
Het P		(*)	N.S.	*
Fixed RR		1.09	0.86	1.05
RRl		0.96	0.64	0.93
RRu		1.23	1.15	1.18
P		N.S.	N.S.	N.S.
Random RR		1.01	0.90	0.98
RRl		0.80	0.60	0.80
RRu		1.29	1.35	1.21
P		N.S.	N.S.	N.S.
Between Chi				2.04
Between df				1
Between P				N.S.
Btwn(F) P				N.S.
Btwn(R) P				N.S.

	All LC (or nearest)						
	a	a+l	a+al+br	KII	not q+u	not q+s	Total
N	8			2			10
NS	6			1			7
Wt	233.44			47.70			281.14
Het Chi	15.46			1.19			16.94
Het df	7			1			9
Het P	*			N.S.			*
Fixed RR	1.03			1.12			1.05
RRl	0.91			0.85			0.93
RRu	1.17			1.49			1.18
P	N.S.			N.S.			N.S.
Random RR	0.95			1.10			0.98
RRl	0.73			0.78			0.80
RRu	1.24			1.55			1.21
P	N.S.			N.S.			N.S.
Between Chi							0.29
Between df							1
Between P							N.S.
Btwn(F) P							N.S.
Btwn(R) P							N.S.

	Location								
	NAmer	UK	Scand	othEur	China	Japan	othAs	other	Total
N	4			2		2		2	10
NS	2			1		2		2	7
Wt	112.03			147.58		7.99		13.54	281.14
Het Chi	3.96			0.65		1.48		5.75	16.94
Het df	3			1		1		1	9
Het P	N.S.			N.S.		N.S.		*	*
Fixed RR	0.98			1.16		0.92		0.65	1.05
RRl	0.81			0.98		0.46		0.38	0.93
RRu	1.18			1.36		1.85		1.11	1.18
P	N.S.			(+)		N.S.		N.S.	N.S.
Random RR	0.97			1.16		1.20		0.72	0.98
RRl	0.78			0.98		0.29		0.20	0.80
RRu	1.21			1.36		5.02		2.64	1.21
P	N.S.			(+)		N.S.		N.S.	N.S.
Between Chi									5.10
Between df									3
Between P									N.S.
Btwn(F) P									N.S.
Btwn(R) P									N.S.

Table 3F3 - 3

IESLC - Meta-analysis of Cigarette Smoking, only Filter vs ever Plain (or nearest available)
Adenocarcinoma
Most adjusted

	Detailed Country in "other Europe" multi	Germany	othWest	East	Balkans	Total
N	2					2
NS	1					1
Wt	147.58					147.58
Het Chi	0.65					0.65
Het df	1					1
Het P	N.S.					N.S.
Fixed RR	1.16					1.16
RRl	0.98					0.98
RRu	1.36					1.36
P	(+)					(+)
Random RR	1.16					1.16
RRl	0.98					0.98
RRu	1.36					1.36
P	(+)					(+)
Between Chi						
Between df						
Between P						N.S.
Btwn(F) P						N.S.
Btwn(R) P						N.S.

	Detailed Country in "other Asia" India	HongKong	other	Total
N				
NS				
Wt				
Het Chi				
Het df				
Het P				
Fixed RR				
RRl				
RRu				
P				
Random RR				
RRl				
RRu				
P				
Between Chi				
Between df				
Between P				N.S.
Btwn(F) P				N.S.
Btwn(R) P				N.S.

	Detailed other continent SCAmer	Auslia	Africa	Total
N	2			2
NS	2			2
Wt	13.54			13.54
Het Chi	5.75			5.75
Het df	1			1
Het P	*			*
Fixed RR	0.65			0.65
RRl	0.38			0.38
RRu	1.11			1.11
P	N.S.			N.S.
Random RR	0.72			0.72
RRl	0.20			0.20
RRu	2.64			2.64
P	N.S.			N.S.
Between Chi				
Between df				
Between P				N.S.
Btwn(F) P				N.S.
Btwn(R) P				N.S.

Table 3F3 - 3

IESLC - Meta-analysis of Cigarette Smoking, only Filter vs ever Plain (or nearest available)
Adenocarcinoma
Most adjusted

	Start year of study					
	<1960	1960-69	1970-79	1980-89	1990+	Total
N		4	2	3	1	10
NS		2	1	3	1	7
Wt		112.03	147.58	15.99	5.54	281.14
Het Chi		3.96	0.65	4.63	0.00	16.94
Het df		3	1	2	0	9
Het P		N.S.	N.S.	(*)	N.S.	*
Fixed RR		0.98	1.16	0.59	1.43	1.05
RRl		0.81	0.98	0.36	0.62	0.93
RRu		1.18	1.36	0.97	3.29	1.18
P		N.S.	(+)	-	N.S.	N.S.
Random RR		0.97	1.16	0.67	1.43	0.98
RRl		0.78	0.98	0.28	0.62	0.80
RRu		1.21	1.36	1.62	3.29	1.21
P		N.S.	(+)	N.S.	N.S.	N.S.
Between Chi						7.69
Between df						3
Between P						(*)
Btwn(F) P						N.S.
Btwn(R) P						N.S.

	Study type (1)		
	CC	other	Total
N	10		10
NS	7		7
Wt	281.14		281.14
Het Chi	16.94		16.94
Het df	9		9
Het P	*		*
Fixed RR	1.05		1.05
RRl	0.93		0.93
RRu	1.18		1.18
P	N.S.		N.S.
Random RR	0.98		0.98
RRl	0.80		0.80
RRu	1.21		1.21
P	N.S.		N.S.
Between Chi			
Between df			
Between P			N.S.
Btwn(F) P			N.S.
Btwn(R) P			N.S.

	Study type (2)			
	CC	prosp	other	Total
N	10			10
NS	7			7
Wt	281.14			281.14
Het Chi	16.94			16.94
Het df	9			9
Het P	*			*
Fixed RR	1.05			1.05
RRl	0.93			0.93
RRu	1.18			1.18
P	N.S.			N.S.
Random RR	0.98			0.98
RRl	0.80			0.80
RRu	1.21			1.21
P	N.S.			N.S.
Between Chi				
Between df				
Between P				N.S.
Btwn(F) P				N.S.
Btwn(R) P				N.S.

Table 3F3 - 3

IESLC - Meta-analysis of Cigarette Smoking, only Filter vs ever Plain (or nearest available)
Adenocarcinoma
Most adjusted

	Study size (number of LC cases) 100-249	250-499	500-999	1000+	Total
N	2	1		7	10
NS	2	1		4	7
Wt	13.54	0.47		267.13	281.14
Het Chi	5.75	0.00		6.88	16.94
Het df	1	0		6	9
Het P	*	N.S.		N.S.	*
Fixed RR	0.65	5.14		1.07	1.05
RRl	0.38	0.30		0.95	0.93
RRu	1.11	89.41		1.21	1.18
P	N.S.	N.S.		N.S.	N.S.
Random RR	0.72	5.14		1.05	0.98
RRl	0.20	0.30		0.91	0.80
RRu	2.64	89.41		1.21	1.21
P	N.S.	N.S.		N.S.	N.S.
Between Chi					4.31
Between df					2
Between P					N.S.
Btwn(F) P					N.S.
Btwn(R) P					N.S.

	Risky occupational population no	mining	othRisky	Total
N	10			10
NS	7			7
Wt	281.14			281.14
Het Chi	16.94			16.94
Het df	9			9
Het P	*			*
Fixed RR	1.05			1.05
RRl	0.93			0.93
RRu	1.18			1.18
P	N.S.			N.S.
Random RR	0.98			0.98
RRl	0.80			0.80
RRu	1.21			1.21
P	N.S.			N.S.
Between Chi				
Between df				
Between P				N.S.
Btwn(F) P				N.S.
Btwn(R) P				N.S.

	National cigarette tobacco type Virginia	blended	other	Total
N		10		10
NS		7		7
Wt		281.14		281.14
Het Chi		16.94		16.94
Het df		9		9
Het P		*		*
Fixed RR		1.05		1.05
RRl		0.93		0.93
RRu		1.18		1.18
P		N.S.		N.S.
Random RR		0.98		0.98
RRl		0.80		0.80
RRu		1.21		1.21
P		N.S.		N.S.
Between Chi				
Between df				
Between P				N.S.
Btwn(F) P				N.S.
Btwn(R) P				N.S.

Table 3F3 - 3

IESLC - Meta-analysis of Cigarette Smoking, only Filter vs ever Plain (or nearest available)
Adenocarcinoma
Most adjusted

	Any proxy use No/nk	Yes	Total
N	10		10
NS	7		7
Wt	281.14		281.14
Het Chi	16.94		16.94
Het df	9		9
Het P	*		*
Fixed RR	1.05		1.05
RRl	0.93		0.93
RRu	1.18		1.18
P	N.S.		N.S.
Random RR	0.98		0.98
RRl	0.80		0.80
RRu	1.21		1.21
P	N.S.		N.S.
Between Chi			
Between df			
Between P			N.S.
Btwn(F) P			N.S.
Btwn(R) P			N.S.

	Full histological confirmation No	Yes	Total
N	1	9	10
NS	1	6	7
Wt	5.54	275.60	281.14
Het Chi	0.00	16.39	16.94
Het df	0	8	9
Het P	N.S.	*	*
Fixed RR	1.43	1.04	1.05
RRl	0.62	0.92	0.93
RRu	3.29	1.17	1.18
P	N.S.	N.S.	N.S.
Random RR	1.43	0.96	0.98
RRl	0.62	0.78	0.80
RRu	3.29	1.19	1.21
P	N.S.	N.S.	N.S.
Between Chi			0.55
Between df			1
Between P			N.S.
Btwn(F) P			N.S.
Btwn(R) P			N.S.

	Number of adjustment variables (1) 0	1	2+/+nk	Total
N	3		7	10
NS	2		5	7
Wt	48.17		232.97	281.14
Het Chi	2.27		14.24	16.94
Het df	2		6	9
Het P	N.S.		*	*
Fixed RR	1.14		1.03	1.05
RRl	0.86		0.90	0.93
RRu	1.51		1.17	1.18
P	N.S.		N.S.	N.S.
Random RR	1.12		0.94	0.98
RRl	0.78		0.72	0.80
RRu	1.60		1.22	1.21
P	N.S.		N.S.	N.S.
Between Chi				0.43
Between df				1
Between P				N.S.
Btwn(F) P				N.S.
Btwn(R) P				N.S.

Table 3F3 - 3

IESLC - Meta-analysis of Cigarette Smoking, only Filter vs ever Plain (or nearest available)
Adenocarcinoma
Most adjusted

	Number of adjustment variables (2) 0	1	2	3-5	6+/+nk	Total
N	3			7		10
NS	2			5		7
Wt	48.17			232.97		281.14
Het Chi	2.27			14.24		16.94
Het df	2			6		9
Het P	N.S.			*		*
Fixed RR	1.14			1.03		1.05
RRl	0.86			0.90		0.93
RRu	1.51			1.17		1.18
P	N.S.			N.S.		N.S.
Random RR	1.12			0.94		0.98
RRl	0.78			0.72		0.80
RRu	1.60			1.22		1.21
P	N.S.			N.S.		N.S.
Between Chi						0.43
Between df						1
Between P						N.S.
Btwn(F) P						N.S.
Btwn(R) P						N.S.

	Smoking status ever	current	Total
N	4	6	10
NS	3	4	7
Wt	161.12	120.03	281.14
Het Chi	10.44	5.46	16.94
Het df	3	5	9
Het P	*	N.S.	*
Fixed RR	1.10	0.98	1.05
RRl	0.95	0.82	0.93
RRu	1.29	1.17	1.18
P	N.S.	N.S.	N.S.
Random RR	0.99	0.97	0.98
RRl	0.58	0.80	0.80
RRu	1.69	1.18	1.21
P	N.S.	N.S.	N.S.
Between Chi			1.03
Between df			1
Between P			N.S.
Btwn(F) P			N.S.
Btwn(R) P			N.S.

	Product cig+/-ot	cig only	Total
N	9	1	10
NS	6	1	7
Wt	273.15	8.00	281.14
Het Chi	8.49	0.00	16.94
Het df	8	0	9
Het P	N.S.	N.S.	*
Fixed RR	1.08	0.38	1.05
RRl	0.96	0.19	0.93
RRu	1.21	0.76	1.18
P	N.S.	--	N.S.
Random RR	1.07	0.38	0.98
RRl	0.94	0.19	0.80
RRu	1.22	0.76	1.21
P	N.S.	--	N.S.
Between Chi			8.45
Between df			1
Between P			**
Btwn(F) P			*
Btwn(R) P			**

Table 3F3 - 3

IESLC - Meta-analysis of Cigarette Smoking, only Filter vs ever Plain (or nearest available)
Adenocarcinoma
Most adjusted

	Cigarette type						
	only f	always f	mainly f	equl p&f	both p&f	ever f	Total
N	7		1			2	10
NS	5		1			1	7
Wt	227.90		5.54			47.70	281.14
Het Chi	14.85		0.00			1.19	16.94
Het df	6		0			1	9
Het P	*		N.S.			N.S.	*
Fixed RR	1.02		1.43			1.12	1.05
RRl	0.90		0.62			0.85	0.93
RRu	1.17		3.29			1.49	1.18
P	N.S.		N.S.			N.S.	N.S.
Random RR	0.92		1.43			1.10	0.98
RRl	0.70		0.62			0.78	0.80
RRu	1.22		3.29			1.55	1.21
P	N.S.		N.S.			N.S.	N.S.
Between Chi							0.90
Between df							2
Between P							N.S.
Btwn(F) P							N.S.
Btwn(R) P							N.S.

	Denominator				
	ever p	mainly p	p NOS	always p	Total
N	5	1	2	2	10
NS	3	1	2	1	7
Wt	219.90	5.54	7.99	47.70	281.14
Het Chi	13.29	0.00	1.48	1.19	16.94
Het df	4	0	1	1	9
Het P	**	N.S.	N.S.	N.S.	*
Fixed RR	1.03	1.43	0.92	1.12	1.05
RRl	0.90	0.62	0.46	0.85	0.93
RRu	1.17	3.29	1.85	1.49	1.18
P	N.S.	N.S.	N.S.	N.S.	N.S.
Random RR	0.91	1.43	1.20	1.10	0.98
RRl	0.67	0.62	0.29	0.78	0.80
RRu	1.24	3.29	5.02	1.55	1.21
P	N.S.	N.S.	N.S.	N.S.	N.S.
Between Chi					0.99
Between df					3
Between P					N.S.
Btwn(F) P					N.S.
Btwn(R) P					N.S.

	Derivation of RR/CI			
	Orig	StdCalc	Other	Total
N		4	6	10
NS		3	4	7
Wt		60.77	220.38	281.14
Het Chi		2.19	14.51	16.94
Het df		3	5	9
Het P		N.S.	*	*
Fixed RR		1.11	1.03	1.05
RRl		0.86	0.90	0.93
RRu		1.42	1.18	1.18
P		N.S.	N.S.	N.S.
Random RR		1.11	0.93	0.98
RRl		0.86	0.68	0.80
RRu		1.42	1.27	1.21
P		N.S.	N.S.	N.S.
Between Chi				0.24
Between df				1
Between P				N.S.
Btwn(F) P				N.S.
Btwn(R) P				N.S.

Table 3F3 - 4

IESLC - Meta-analysis of Cigarette Smoking, only Filter vs ever Plain (or nearest available)
Adenocarcinoma
Least adjusted

REF	NRR	X	SEX	AGEL	AGEH	RACE	YF	LC TYPE	LOC	START	ST	NLC	R	VB	P	H	AD	SM	PRODUCT	CIGTYP	DENOM	De
LUBIN2	200	x	m	0	0	all	-	a	Eu:mul	1976	CC	7804	n	bl	n	y	0	ev	cig+/-ot	only f	ever p	st
LUBIN2	232	x	f	0	0	all	-	a	Eu:mul	1976	CC	7804	n	bl	n	y	0	ev	cig+/-ot	only f	ever p	st
MATOS	74	x	m	0	0	all	-	a	SCAmer	1994	CC	200	n	bl	n	n	0	ev	cig+/-ot	mainly f	mainly p	st
PEZZOT	15	x	m	0	0	all	-	a	SCAmer	1987	CC	215	n	bl	n	y	2	ev	cig only	only f	ever p	ot
SOBUE	74	x	m	0	0	all	-	a	As:Jap	1986	CC	1376	n	bl	n	y	0	cu	cig+/-ot	only f	p NOS	st
WAKAI	71		m	0	0	all	-	a	As:Jap	1988	CC	333	n	bl	n	y	0	cu	cig+/-ot	only f	p NOS	ot
WYNDE5	2		m	0	0	all	-	KII	NAmer	1969	CC	1365	n	bl	n	y	0	cu	cig+/-ot	ever f	always p	st
WYNDE5	5		f	0	0	all	-	KII	NAmer	1969	CC	1365	n	bl	n	y	0	cu	cig+/-ot	ever f	always p	st
WYNDE6	302	x	m	0	0	all	-	a	NAmer	1969	CC	4423	n	bl	n	y	0	cu	cig+/-ot	only f	ever p	st
WYNDE6	308	x	f	0	0	all	-	a	NAmer	1969	CC	4423	n	bl	n	y	0	cu	cig+/-ot	only f	ever p	st

Table 3F3 - 5

IESLC - Meta-analysis of Cigarette Smoking, only Filter vs ever Plain (or nearest available)
Adenocarcinoma
Least adjusted

REF	NRR	SEX	AD	Number Exposed Case	Number Exposed Cont	Non-exposed Case	Non-exposed Cont	RR	95.00%CI
LUBIN2	200	m	0	47	1018	624	9419	0.70	(0.51- 0.94)
LUBIN2	232	f	0	19	213	65	354	0.49	(0.28- 0.83)
Subtotal LUBIN2								0.64	(0.49- 0.83)
MATOS	74	m	0	70	229	8	46	1.76	(0.79- 3.90)
PEZZOT	15	m	2	-	-	-	-	0.28	(0.14- 0.55)
SOBUE	74	m	0	218	540	16	26	0.66	(0.35- 1.25)
WAKAI	71	m	0	73	271	0	9	5.14~	(0.30- 89.41)
WYNDE5	2	m	0	139	629	73	398	1.20	(0.88- 1.64)
WYNDE5	5	f	0	68	200	13	30	0.78	(0.39- 1.59)
Subtotal WYNDE5								1.12	(0.85- 1.49)
WYNDE6	302	m	0	88	122	624	754	0.87	(0.65- 1.17)
WYNDE6	308	f	0	158	158	411	309	0.75	(0.58- 0.98)
Subtotal WYNDE6								0.80	(0.66- 0.98)
Partial Totals				880	3380	1834	11345		

*prospective study

~ With 0.5 adjustment for zero

REF	NRR	SEX	AD	Ys	Ws	Qs	Ps
LUBIN2	200	m	0	-0.36	41.72	0.77	0.0197
LUBIN2	232	f	0	-0.72	13.24	3.26	0.0086
Subtotal LUBIN2				-0.45	54.96	4.03	
MATOS	74	m	0	0.56	6.05	3.77	0.1655
PEZZOT	15	m	2	-1.27	8.21	9.00	0.0003
SOBUE	74	m	0	-0.42	9.31	0.36	0.1983
WAKAI	71	m	0	1.64	0.47	1.64	0.2610
WYNDE5	2	m	0	0.19	40.01	6.79	0.2386
WYNDE5	5	f	0	-0.24	7.69	0.00	0.5010
Subtotal WYNDE5				0.12	47.70	6.79	
WYNDE6	302	m	0	-0.14	44.47	0.34	0.3594
WYNDE6	308	f	0	-0.29	54.56	0.19	0.0351
Subtotal WYNDE6				-0.22	99.03	0.54	

	N	10
	NS	7
	Wt	225.73
Het	Chi	26.12
Het	df	9
Het	P	**
Fixed	RR	0.80
	RRl	0.70
	RRu	0.91
	P	---
Random	RR	0.77
	RRl	0.59
	RRu	0.99
	P	-
Asymm	P	N.S.

Table 3F3 - 6

IESLC - Meta-analysis of Cigarette Smoking, only Filter vs ever Plain (or nearest available)
Adenocarcinoma
Least adjusted

	Sex combined	male	female	Total
N		7	3	10
NS		7	3	10
Wt		150.23	75.50	225.73
Het Chi		22.00	2.14	26.12
Het df		6	2	9
Het P		**	N.S.	**
Fixed RR		0.85	0.70	0.80
RRl		0.73	0.56	0.70
RRu		1.00	0.88	0.91
P		(-)	--	---
Random RR		0.82	0.69	0.77
RRl		0.57	0.54	0.59
RRu		1.18	0.89	0.99
P		N.S.	--	-
Between Chi				1.98
Between df				1
Between P				N.S.
Btwn(F) P				N.S.
Btwn(R) P				N.S.

Table 3F3 - 7

IESLC - Meta-analysis of Cigarette Smoking, only Filter vs ever Plain (or nearest available)
Adenocarcinoma
Excluded studies (and stage at which they were excluded)

1	BOUCHA	BUELL	LAURIL	MZILEN												
2	BERRIN	CHEN	RESTRE	TAO	ULMER											
3	ABELIN	ABRAHA	AKIBA	AMANDU	AMES	ANDERS	ARCHER	AUSTIN	AUVINE	AXELSO	AXELSS	BAND	BARBON	BENSHL	BEST	BLOHMK
	BLOT1	BLOT2	BLOT3	BLOT4	BOFFET	BOUCOT	BRESLO	BRETT	BROCKM	BROWN1	BROWN2	BYERS1	BYERS2	CARPEN	CASCO2	CASCOR
	CEDERL	CHAN	CHANG	CHATZI	CHEN2	CHEN3	CHIAZZ	CHOW	CHYOU	COMSTO	COOKSO	CPSI	DAMBER	DARBY	DAVEYS	DEAN
	DEKLER	DOCKER	DOLL2	DORANT	DORGAN	DORN	DOSEME	DROSTE	DU	DUNN	EBELIN	ENSTRO	ESAKI	FAN	GAO	GAO2
	GARCIA	GARDIN	GARSHI	GENG	GER	GILLIS	GODLEY	GOLLED	GOODMA	GRAHAM	GREGOR	GSELL	HAENSZ	HAMMO2	HAMMON	HANSEN
	HEGMAN	HEIN	HENNEK	HINDS	HIRAOK	HIRAY2	HIRAYA	HITOSU	HOLE	HOROWI	HORWIT	HU	HU2	HUANG	HUMBLE	ISHIMA
	JAHN	JAIN	JARUP	JARVHO	JEDRYC	JIANG	JOLY	JONES	JUSSAW	KAISER	KANELL	KATSOU	KAUFMA	KELLER	KIHARA	KINLEN
	KJUUS	KNEKT	KO	KOHLME	KOO	KOULUM	KREUZE	KREYBE	KUBIK	LAMTH	LAMWK	LAMWK2	LAUSSM	LEI	LEMARC	LETOUR
	LEVIN	LIAW	LICKIN	LIDDEL	LIU	LIU2	LIU3	LIU4	LIU5	LOMBA2	LOMBAR	LUBIN	LUO	MAGNUS	MARSH	MARSH2
	MARTIS	MASTRA	MATSUD	MCCONN	MCDUFF	MCLAUG	MILLER	MILLS	MOLLO	MRFIT	MURATA	NAM	NOTAN2	NOTANI	NOU	ODRISC
	ORMOS	OSANN	OSANN2	PARKIN	PASTOR	PAWLEG	PERNU	PERSH2	PERSHA	PETO	PEZZO2	PIKE	PISANI	POFFIJ	POLEDN	PRESCO
	QIAO	QIAO2	RACHTA	RADZIK	RANDIG	REN	RONCO	ROOTS	ROTHSC	SAARIK	SADOWS	SANKAR	SCHWA2	SCHWAR	SEGI	SEOW
	SHAW	SHIMIZ	SIEMIA	SIMARA	SIMONA	SITAS	SOBUE2	SPEIZE	SPITZ	STASZE	STAYNE	STOCKS	STOCKW	STUCKE	SUN	SUZUK2
	SUZUKI	SVENSS	TANG	TENKAN	TIZZAN	TOKARS	TOUSEY	TSUGAN	TULINI	TVERDA	VANDER	VEIERO	VUTUC	WALD	WANG	WANG2
	WANG3	WANG4	WARSIN	WATSON	WICKLU	WIGLE	WILKIN	WU	WU2	WUNSCH	WUWILL	WYNDE2	WYNDE4	WYNDE7	WYNDE8	WYNDER
	XIANGZ	XU	XU2	XU3	XU4	YAMAGU	YONG	YUAN	ZHANG	ZHENG	ZHOU					
7	AGUDO	ALDERS	ARMADA	BECHER	BENHAM	BROSS	BUFFLE	CHOI	CORREA	CPSII	DEAN2	DEAN3	DESTE2	DESTEF	DOLL	ENGELA
	KAISE2	KHUDER	LANGE	MACLEN	MIGRAN	MRFITR	RIMING	SEGI2	TANG2	WYNDE3						

Table 3F3 - 8

Potentially overlapping studies

REF	REFGP	PRINC	OVERLAP/LINK
LUBIN2	LUBIN2	1	Lubin-combined
WYNDE5	WYNDE6	2	WYNDE5/6/7/8
WYNDE6	WYNDE6	1	WYNDE5/6/7/8

Table 3F4 -

IESLC - Meta-analysis of Cigarette Smoking, Hand-rolled vs Manufactured
Adenocarcinoma

This analysis is restricted to results for:
1) Non-dose-response data
2) Results complete enough for use in metaanalysis

Within each study, results are then selected (in the following order of preference, within each sex) for:
3) CIGTYP: hand-rolled any, both, mainly, only
4) DENOM: manufactured only ever, only current, any, ever
5) PRODUCT: cigarettes regardless of other products, cigarettes only
6) SMKSTA: ever, current
7) LCtype: all or nearest available, at least Squamous and Adeno. (q = squamous, s = small, l = large, a = adeno, mix = mixed, alv = alveolar)
8) Race: all or nearest available, otherwise by race (wh or w = white, bl or b = black, hi = hispanic ch = chinese, jap = japanese, haw = hawaiian, w+o = white + oriental, sca = scandinavian, as = asian)
9) Followup period (YF, prospective studies): whole study (coded as 0) or longest available
10) For overlapping studies: principal rather than subsidiary studies
Finally by Age: whole study (coded as 0) if available, otherwise by widest available age group
and then for single sex results (m, f) in preference to combined sex results (c).

Results adjusted (AD) for the most potential confounders are then chosen in Sections -1 to -3
and results adjusted for the least confounders in Sections -4 to -6. (Those least adjusted results which
actually differ from the most adjusted as marked 'x' in column X in Section -4)
(Results adjusted for an unknown number of confounder(s) are coded as 20.)

Section -7 shows excluded studies, together with the stage (as above) at which no qualifying
results were found.

Section -8 lists the potentially overlapping studies which have been included (1=principal, 2=subsidiary).

Section -9 lists any results which would have been included in preference except that they had data not complete
enough for use in meta-analysis, with their significance (yes/no), if known, and any further comment as entered
on the database.

In addition to those mentioned above, the following fields, levels and abbreviations are used:
* or nk = not known, n = no, y = yes, ot = other
ev = ever, cu = current, cig+/-ot = cigarettes irrespective of other products (cigar, pipe etc)
m or mc = manufactured cigarettes, h or hr = hand-rolled cigarettes
REF: 6-character study reference
NRR: number of the RR on the database within the study
ST : study type (CC = case control, pr or prosp = prospective)
NLC: number of lung cancer cases in whole study
R : risky occupational population (n = no, m = mining, o = other risky)
VB : national cigarette type (V = at least 75% Virginia, bl = at least 75% blended, ot = other)
P : any proxy use
H : full histological confirmation
De : derivation of RR/CI (or = original, st = standard method, ot = other method of estimation)

Table 3F4 - 1

IESLC - Meta-analysis of Cigarette Smoking, Hand-rolled vs Manufactured
Adenocarcinoma
Most adjusted

REF	NRR	SEX	AGEL	AGEH	RACE	YF	LC TYPE	LOC	START	ST	NLC	R	VB	P	H	AD	SM	PRODUCT	CIGTYP	DENOM	De
ALDERS	151	m	0	0	all	-	not q+s	Eu:UK	1977	CC	1448	n	V	n	n	1	ev	cig only	both m&h	only mc	ot
DESTEF	37	m	0	0	all	-	a	SCAmer	1988	CC	497	n	bl	n	y	6	ev	cig+/-ot	both m&h	only mc	or
ENGELA	192	m	0	0	all	0	a	Eu:Sca	1964	pr	435	n	bl	n	n	7	cu	cig+/-ot	both m&h	only mc	ot
JUSSAW	40	m	0	0	all	-	KII	As:Ind	1964	CC	792	n	V	n	n	0	ev	cig only	only hr	only mc	st

Table 3F4 - 2

IESLC - Meta-analysis of Cigarette Smoking, Hand-rolled vs Manufactured
Adenocarcinoma
Most adjusted

REF	NRR	SEX	AD	Number Exposed Case	Number Exposed Cont	Non-exposed Case	Non-exposed Cont	RR	95.00%CI
ALDERS	151	m	1	-	-	-	-	2.70	(1.67- 4.37)
DESTEF	37	m	6	-	-	-	-	2.30	(1.30- 4.30)
*ENGELA	192	m	7	-	-	-	-	0.43	(0.18- 0.99)
JUSSAW	40	m	0	29	85	3	77	8.76	(2.56- 29.90)
Partial Totals				29	85	3	77		

*prospective study

REF	NRR	SEX	AD	Ys	Ws	Qs	Ps
ALDERS	151	m	1	0.99	16.61	0.96	0.0001
DESTEF	37	m	6	0.83	10.74	0.07	0.0063
*ENGELA	192	m	7	-0.84	5.29	13.49	0.0523
JUSSAW	40	m	0	2.17	2.55	5.11	0.0005

	N	4
	NS	4
	Wt	35.18
Het	Chi	19.62
Het	df	3
Het	P	***
Fixed	RR	2.12
	RRl	1.53
	RRu	2.96
	P	+++
Random	RR	2.09
	RRl	0.83
	RRu	5.25
	P	N.S.
Asymm	P	N.S.

Table 3F4 - 3

IESLC - Meta-analysis of Cigarette Smoking, Hand-rolled vs Manufactured
Adenocarcinoma
Most adjusted

	Sex: combined	male	female	Total
N		4		4
NS		4		4
Wt		35.18		35.18
Het Chi		19.62		19.62
Het df		3		3
Het P		***		***
Fixed RR		2.12		2.12
RRl		1.53		1.53
RRu		2.96		2.96
P		+++		+++
Random RR		2.09		2.09
RRl		0.83		0.83
RRu		5.25		5.25
P		N.S.		N.S.
Between Chi				
Between df				
Between P				N.S.
Btwn(F) P				N.S.
Btwn(R) P				N.S.

Too few RRs for analysis by factor

Table 3F4 - 4

IESLC - Meta-analysis of Cigarette Smoking, Hand-rolled vs Manufactured
Adenocarcinoma
Least adjusted

REF	NRR	X	SEX	AGEL	AGEH	RACE	YF	LC TYPE	LOC	START	ST	NLC	R	VB	P	H	AD	SM	PRODUCT	CIGTYP	DENOM	De
ALDERS	159	x	m	0	0	all	-	not q+s	Eu:UK	1977	CC	1448	n	V	n	n	0	ev	cig only	both m&h	only mc	st
DESTEF	37		m	0	0	all	-	a	SCAmer	1988	CC	497	n	bl	n	y	6	ev	cig+/-ot	both m&h	only mc	or
ENGELA	192		m	0	0	all	0	a	Eu:Sca	1964	pr	435	n	bl	n	n	7	cu	cig+/-ot	both m&h	only mc	ot
JUSSAW	40		m	0	0	all	-	KII	As:Ind	1964	CC	792	n	V	n	n	0	ev	cig only	only hr	only mc	st

Table 3F4 - 5

IESLC - Meta-analysis of Cigarette Smoking, Hand-rolled vs Manufactured
Adenocarcinoma
Least adjusted

REF	NRR	SEX	AD	Number Exposed Case	Number Exposed Cont	Non-exposed Case	Non-exposed Cont	RR	95.00%CI
ALDERS	159	m	0	40	113	62	349	1.99	(1.27- 3.13)
DESTEF	37	m	6	-	-	-	-	2.30	(1.30- 4.30)
*ENGELA	192	m	7	-	-	-	-	0.43	(0.18- 0.99)
JUSSAW	40	m	0	29	85	3	77	8.76	(2.56- 29.90)
Partial Totals				69	198	65	426		

*prospective study

REF	NRR	SEX	AD	Ys	Ws	Qs	Ps
ALDERS	159	m	0	0.69	18.92	0.11	0.0027
DESTEF	37	m	6	0.83	10.74	0.51	0.0063
*ENGELA	192	m	7	-0.84	5.29	11.25	0.0523
JUSSAW	40	m	0	2.17	2.55	6.16	0.0005

	N	4
	NS	4
	Wt	37.50
Het	Chi	18.03
Het	df	3
Het	P	***
Fixed	RR	1.85
	RRl	1.34
	RRu	2.55
	P	+++
Random	RR	1.91
	RRl	0.80
	RRu	4.55
	P	N.S.
Asymm	P	N.S.

Table 3F4 - 6

IESLC - Meta-analysis of Cigarette Smoking, Hand-rolled vs Manufactured
Adenocarcinoma
Least adjusted

	Sex combined	male	female	Total
N		4		4
NS		4		4
Wt		37.50		37.50
Het Chi		18.03		18.03
Het df		3		3
Het P		***		***
Fixed RR		1.85		1.85
RRl		1.34		1.34
RRu		2.55		2.55
P		+++		+++
Random RR		1.91		1.91
RRl		0.80		0.80
RRu		4.55		4.55
P		N.S.		N.S.
Between Chi				
Between df				
Between P				N.S.
Btwn(F) P				N.S.
Btwn(R) P				N.S.

Table 3F4 - 7

IESLC - Meta-analysis of Cigarette Smoking, Hand-rolled vs Manufactured
Adenocarcinoma
Excluded studies (and stage at which they were excluded)

1	BOUCHA	BUELL	LAURIL	MZILEN												
2	BERRIN	CHEN	RESTRE	TAO	ULMER											
3	ABELIN	ABRAHA	AGUDO	AKIBA	AMANDU	AMES	ANDERS	ARCHER	ARMADA	AUSTIN	AUVINE	AXELSO	AXELSS	BAND	BARBON	BECHER
	BENSHL	BEST	BLOHMK	BLOT1	BLOT2	BLOT3	BLOT4	BOFFET	BOUCOT	BRESLO	BRETT	BROCKM	BROSS	BROWN1	BROWN2	BYERS1
	BYERS2	CARPEN	CASCO2	CASCOR	CEDERL	CHANG	CHATZI	CHEN2	CHEN3	CHIAZZ	CHOI	CHOW	CHYOU	COMSTO	COOKSO	CORREA
	CPSI	CPSII	DAMBER	DARBY	DAVEYS	DEAN	DEAN2	DEKLER	DOCKER	DOLL	DOLL2	DORANT	DORGAN	DORN	DOSEME	DROSTE
	DU	DUNN	EBELIN	ENSTRO	ESAKI	FAN	GAO	GAO2	GARCIA	GARSHI	GENG	GER	GILLIS	GODLEY	GOLLED	GOODMA
	GRAHAM	GREGOR	GSELL	HAENSZ	HAMMO2	HAMMON	HANSEN	HEGMAN	HEIN	HENNEK	HINDS	HIRAOK	HIRAY2	HIRAYA	HITOSU	HOLE
	HOROWI	HORWIT	HUANG	HUMBLE	ISHIMA	JAHN	JAIN	JARUP	JARVHO	JEDRYC	JIANG	JOLY	JONES	KAISE2	KAISER	KANELL
	KATSOU	KAUFMA	KELLER	KHUDER	KIHARA	KINLEN	KJUUS	KNEKT	KO	KOHLME	KOO	KOULUM	KREUZE	KREYBE	KUBIK	LAMTH
	LAMWK	LAMWK2	LANGE	LAUSSM	LEI	LEMARC	LETOUR	LEVIN	LIAW	LICKIN	LIDDEL	LIU	LIU2	LIU3	LIU4	LIU5
	LOMBA2	LOMBAR	LUBIN	LUBIN2	LUO	MAGNUS	MARSH	MARSH2	MARTIS	MASTRA	MATOS	MATSUD	MCCONN	MCDUFF	MCLAUG	MILLER
	MILLS	MOLLO	MRFIT	MRFITR	MURATA	NAM	NOTANI	NOU	ODRISC	ORMOS	OSANN	OSANN2	PARKIN	PASTOR	PAWLEG	PERSH2
	PERSHA	PETO	PEZZO2	PEZZOT	PIKE	PISANI	POFFIJ	POLEDN	PRESCO	QIAO	QIAO2	RACHTA	RADZIK	RANDIG	REN	RIMING
	RONCO	ROOTS	ROTHSC	SAARIK	SADOWS	SANKAR	SCHWA2	SCHWAR	SEGI	SEGI2	SEOW	SHAW	SHIMIZ	SIEMIA	SIMARA	SIMONA
	SITAS	SOBUE	SOBUE2	SPEIZE	SPITZ	STASZE	STAYNE	STOCKS	STOCKW	STUCKE	SUN	SUZUKI	SVENSS	TANG	TANG2	TENKAN
	TIZZAN	TOKARS	TOUSEY	TSUGAN	TULINI	TVERDA	VANDER	VEIERO	VUTUC	WAKAI	WALD	WANG	WANG2	WANG3	WANG4	WARSIN
	WATSON	WICKLU	WIGLE	WILKIN	WU	WU2	WUNSCH	WUWILL	WYNDE2	WYNDE3	WYNDE4	WYNDE5	WYNDE6	WYNDE7	WYNDE8	WYNDER
	XIANGZ	XU	XU2	XU3	XU4	YAMAGU	YONG	YUAN	ZHANG	ZHENG	ZHOU					
4	BUFFLE	DEAN3														
6	BENHAM	CHAN	DESTE2	GARDIN	HU	HU2	MACLEN	MIGRAN	NOTAN2	PERNU	SUZUK2					

Table 3F5 -

IESLC - Meta-analysis of Cigarette Smoking, Menthol vs non-menthol
Adenocarcinoma

This analysis is restricted to results for:
1) Non-dose-response data
2) Results complete enough for use in metaanalysis

Within each study, results are then selected (in the following order of preference, within each sex) for:
3) Cigarette type: menthol
4) Denominator: non-menthol
5) PRODUCT: cigarettes regardless of other products, cigarettes only
6) SMKSTA: ever, current
7) LCtype: all or nearest available, at least Squamous and Adeno. (q = squamous, s = small,
l = large, a = adeno, mix = mixed, alv = alveolar)
8) Race: all or nearest available, otherwise by race (wh or w = white, bl or b = black, hi = hispanic
ch = chinese, jap = japanese, haw = hawaiian, w+o = white + oriental, sca = scandinavian, as = asian)
9) Followup period (YF, prospective studies): whole study (coded as 0) or longest available
10) For overlapping studies: principal rather than subsidiary studies
Finally by Age: whole study (coded as 0) if available, otherwise by widest available age group
and then for single sex results (m, f) in preference to combined sex results (c).

Results adjusted (AD) for the most potential confounders are then chosen in Sections -1 to -3
and results adjusted for the least confounders in Sections -4 to -6. (Those least adjusted results which
actually differ from the most adjusted as marked 'x' in column X in Section -4)
(Results adjusted for an unknown number of confounder(s) are coded as 20.)

Section -7 shows excluded studies, together with the stage (as above) at which no qualifying
results were found.

Section -8 lists the potentially overlapping studies which have been included (1=principal, 2=subsidiary).

Section -9 lists any results which would have been included in preference except that they had data not complete
enough for use in meta-analysis, with their significance (yes/no), if known, and any further comment as entered
on the database.

In addition to those mentioned above, the following fields, levels and abbreviations are used:
* or nk = not known, n = no, y = yes, ot = other
ev = ever, cu = current, cig+/-ot = cigarettes irrespective of other products (cigar, pipe etc)
REF: 6-character study reference
NRR: number of the RR on the database within the study
ST : study type (CC = case control, pr or prosp = prospective)
NLC: number of lung cancer cases in whole study
R : risky occupational population (n = no, m = mining, o = other risky)
VB : national cigarette type (V = at least 75% Virginia, bl = at least 75% blended, ot = other)
P : any proxy use
H : full histological confirmation
De : derivation of RR/CI (or = original, st = standard method, ot = other method of estimation)

Table 3F5 - 1

IESLC - Meta-analysis of Cigarette Smoking, Menthol vs non-menthol
Adenocarcinoma
Most adjusted

REF	NRR	SEX	AGEL	AGEH	RACE	YF	LC TYPE	LOC	START	ST	NLC	R	VB	P	H	AD	SM	PRODUCT	De
WYNDE8	8	c	0	0	all	-	a	NAmer	1985	CC	1044	n	bl	n	y	8	cu	cig+/-ot	ot

Table 3F5 - 2

IESLC - Meta-analysis of Cigarette Smoking, Menthol vs non-menthol
Adenocarcinoma
Most adjusted

REF	NRR	SEX	AD	Number Exposed Case	Cont	Non-exposed Case	Cont	RR	95.00%CI
WYNDE8	8	c	8	-	-	-	-	0.96	(0.73- 1.27)
Partial Totals				0	0	0	0		

*prospective study

REF	NRR	SEX	AD	Ys	Ws	Qs	Ps
WYNDE8	8	c	8	-0.04	50.11	0.00	0.7726

	N	1
	NS	1
	Wt	50.11
Het	Chi	0.00
Het	df	0
Het	P	N.S.
Fixed	RR	0.96
	RRl	0.73
	RRu	1.27
	P	N.S.
Random	RR	0.96
	RRl	0.73
	RRu	1.27
	P	N.S.
Asymm	P	

Table 3F5 - 3

IESLC - Meta-analysis of Cigarette Smoking, Menthol vs non-menthol
Adenocarcinoma
Most adjusted

	Sex combined	male	female	Total
N	1			1
NS	1			1
Wt	50.11			50.11
Het Chi	0.00			0.00
Het df	0			0
Het P	N.S.			N.S.
Fixed RR	0.96			0.96
RRl	0.73			0.73
RRu	1.27			1.27
P	N.S.			N.S.
Random RR	0.96			0.96
RRl	0.73			0.73
RRu	1.27			1.27
P	N.S.			N.S.
Between Chi				
Between df				
Between P				N.S.
Btwn(F) P				N.S.
Btwn(R) P				N.S.

Too few RRs for analysis by factor

Table 3F5 - 4

IESLC - Meta-analysis of Cigarette Smoking, Menthol vs non-menthol
Adenocarcinoma
Least adjusted

REF	NRR	X	SEX	AGEL	AGEH	RACE	YF	LC TYPE	LOC	START	ST	NLC	R	VB	P	H	AD	SM	PRODUCT	De
WYNDE8	8		c	0	0	all	-	a	NAmer	1985	CC	1044	n	bl	n	y	8	cu	cig+/-ot	ot

Table 3F5 - 5

IESLC - Meta-analysis of Cigarette Smoking, Menthol vs non-menthol
Adenocarcinoma
Least adjusted

REF	NRR	SEX	AD	Number Exposed Case	Cont	Non-exposed Case	Cont	RR	95.00%CI
WYNDE8	8	c	8	-	-	-	-	0.96	(0.73- 1.27)
Partial Totals				0	0	0	0		

*prospective study

REF	NRR	SEX	AD	Ys	Ws	Qs	Ps
WYNDE8	8	c	8	-0.04	50.11	0.00	0.7726

	N	1
	NS	1
	Wt	50.11
Het	Chi	0.00
Het	df	0
Het	P	N.S.
Fixed	RR	0.96
	RRl	0.73
	RRu	1.27
	P	N.S.
Random	RR	0.96
	RRl	0.73
	RRu	1.27
	P	N.S.
Asymm	P	

Table 3F5 - 6

IESLC - Meta-analysis of Cigarette Smoking, Menthol vs non-menthol
Adenocarcinoma
Least adjusted

		Sex		
	combined	male	female	Total
N	1			1
NS	1			1
Wt	50.11			50.11
Het Chi	0.00			0.00
Het df	0			0
Het P	N.S.			N.S.
Fixed RR	0.96			0.96
RRl	0.73			0.73
RRu	1.27			1.27
P	N.S.			N.S.
Random RR	0.96			0.96
RRl	0.73			0.73
RRu	1.27			1.27
P	N.S.			N.S.
Between Chi				
Between df				
Between P				N.S.
Btwn(F) P				N.S.
Btwn(R) P				N.S.

Table 3F5 - 7

IESLC - Meta-analysis of Cigarette Smoking, Menthol vs non-menthol
Adenocarcinoma
Excluded studies (and stage at which they were excluded)

1	BOUCHA BUELL LAURIL MZILEN
2	BERRIN CHEN RESTRE TAO ULMER
3	ABELIN ABRAHA AGUDO AKIBA ALDERS AMANDU AMES ANDERS ARCHER ARMADA AUSTIN AUVINE AXELSO AXELSS BAND BARBON BECHER BENHAM BENSHL BEST BLOHMK BLOT1 BLOT2 BLOT3 BLOT4 BOFFET BOUCOT BRESLO BRETT BROCKM BROWN1 BROWN2 BUFFLE BYERS1 BYERS2 CASCO2 CASCOR CEDERL CHAN CHANG CHATZI CHEN2 CHEN3 CHIAZZ CHOI CHOW CHYOU COMSTO COOKSO CORREA CPSI CPSII DAMBER DARBY DAVEYS DEAN DEAN2 DEAN3 DEKLER DESTE2 DESTEF DOCKER DOLL DOLL2 DORANT DORGAN DORN DOSEME DROSTE DU DUNN EBELIN ENGELA ENSTRO ESAKI FAN GAO GAO2 GARCIA GARDIN GARSHI GENG GER GILLIS GODLEY GOLLED GOODMA GRAHAM GREGOR GSELL HAENSZ HAMMO2 HAMMON HANSEN HEGMAN HEIN HENNEK HINDS HIRAOK HIRAY2 HIRAYA HITOSU HOLE HOROWI HORWIT HU HU2 HUANG HUMBLE ISHIMA JAHN JAIN JARUP JARVHO JEDRYC JIANG JOLY JONES JUSSAW KAISER KANELL KATSOU KAUFMA KELLER KHUDER KIHARA KINLEN KJUUS KNEKT KO KOHLME KOO KOULUM KREUZE KREYBE KUBIK LAMTH LAMWK LAMWK2 LANGE LAUSSM LEI LEMARC LETOUR LEVIN LIAW LICKIN LIDDEL LIU LIU2 LIU3 LIU4 LIU5 LOMBA2 LOMBAR LUBIN LUBIN2 LUO MACLEN MAGNUS MARSH MARSH2 MARTIS MASTRA MATOS MATSUD MCCONN MCDUFF MCLAUG MIGRAN MILLER MILLS MOLLO MRFIT MRFITR MURATA NAM NOTAN2 NOTANI NOU ODRISC ORMOS OSANN OSANN2 PARKIN PASTOR PAWLEG PERNU PERSH2 PERSHA PETO PEZZO2 PEZZOT PIKE PISANI POFFIJ POLEDN PRESCO QIAO QIAO2 RACHTA RADZIK RANDIG REN RIMING RONCO ROOTS ROTHSC SAARIK SADOWS SANKAR SCHWA2 SCHWAR SEGI SEGI2 SEOW SHAW SHIMIZ SIEMIA SIMARA SIMONA SITAS SOBUE SOBUE2 SPEIZE SPITZ STASZE STAYNE STOCKS STOCKW STUCKE SUN SUZUK2 SUZUKI SVENSS TANG TANG2 TENKAN TIZZAN TOKARS TOUSEY TSUGAN TULINI TVERDA VANDER VEIERO VUTUC WAKAI WALD WANG WANG2 WANG3 WANG4 WARSIN WATSON WICKLU WIGLE WILKIN WU WU2 WUNSCH WUWILL WYNDE2 WYNDE3 WYNDE4 WYNDE5 WYNDE6 WYNDE7 WYNDER XIANGZ XU XU2 XU3 XU4 YAMAGU YONG YUAN ZHANG ZHENG ZHOU
4	BROSS
7	CARPEN KAISE2

Table 3F5 - 8
Potentially overlapping studies

REF	REFGP	PRINC	.	OVERLAP/LINK
WYNDE8	WYNDE6	2		WYNDE5/6/7/8
