# Supplementary material for: Systematic review with meta-analysis of the epidemiological evidence in the 1900s relating smoking to lung cancer
Source: BMC Cancer. 2012 Sep 3;12:385. doi: 10.1186/1471-2407-12-385 (PMC3505152; doi:10.1186/1471-2407-12-385)
Supplement: Additional file 5 — Detailed Analysis Tables (Individual file names as described in Additional file 1: Methods, Table1). [file 1471-2407-12-385-S5.zip › PDF/1G.pdf]

Table 1G1 -

IESLC - Meta-analysis of Ever Smoking by Amount, Overview, Any product (or Cigarettes if Any not available)
All LC types

This analysis is restricted to results for:
1) Results by Amount smoked
Results by Amount smoked (in numbers of cigarettes or cigarette equivalents) are grouped under 2 schemes (S1, S2).
Each scheme has a set of "key values". An interval is allocated to the category whose key value
it includes and intervals which include none or more than one of the key values are excluded.
(Open-ended intervals are coded as 99.)

S1	key value	maximum range
1	5	1-19
2	20	6-44
3	45	21+

S2	key value	maximum range
1	1	1-9
2	10	2-19
3	20	11-29
4	30	21-39
5	40	31-98
6	99	41+

For all/unspec product, the definition of cigarette equivalents is shown at the end of Sections -1 and -4.
2) Ever smokers
3) Results complete enough for use in metaanalysis

Within each study, results are then selected (in the following order of preference, within each sex) for:
4) PRODUCT: all/unspec, cigarettes regardless of other products, cigarettes only
5) CIGTYPE: all/unspecified, MC regardless of HR, MC only
6) DENOM: never smoked anything, never smoked cigarettes, (never +1 = +long term ex, +2 = +amount unknown,
+3 = never cigs+long term ex)
7) Followup period (YF, prospective studies): whole study (coded as 0) or longest available
8) LCtype: all or nearest available, at least Squamous and Adeno. (q = squamous, s = small,
l = large, a = adeno, mix = mixed, alv = alveolar)
9) Race: all or nearest available, otherwise by race (wh or w = white, bl or b = black, hi = hispanic
ch = chinese, jap = japanese, haw = hawaiian, w+o = white + oriental, sca = scandinavian, as = asian)
10) For overlapping studies: principal rather than subsidiary studies
Finally by Age: whole study (coded as 0) if available, otherwise by widest available age group
and then for single sex results (m, f) in preference to combined sex results (c).

Results adjusted (AD) for the most potential confounders are then chosen in Sections -1 to -3
and results adjusted for the least confounders in Sections -4 to -6. (Those least adjusted results which
actually differ from the most adjusted as marked 'x' in column X in Section -4)
(Results adjusted for an unknown number of confounder(s) are coded as 20.)

Section -7 shows excluded studies, together with the stage (as above) at which no qualifying
results were found.

Section -8 lists the potentially overlapping studies which have been included (1=principal, 2=subsidiary).

Section -9 lists any results which would have been included in preference except that they had data not complete
enough for use in meta-analysis, with their significance (yes/no), if known, and any further comment as entered
on the database.

In addition to those mentioned above, the following fields, levels and abbreviations are used:
* or nk = not known, n = no, y = yes, ot = other
nev = never
all/unspec = all or unspecified, cig+/-ot = cigarettes irrespective of other products (cigar, pipe etc)
MC = manufactured cigarettes, HR = hand-rolled cigarettes
exL, exH = range of exposure (low and high) in the smoking group, in terms of Amount smoked,
cigarettes or cigarette equivalents
REF: 6-character study reference
NRR: number of the RR on the database within the study
ST : study type (CC = case control, pr or prosp = prospective)
NLC: number of lung cancer cases in whole study
R : risky occupational population (n = no, m = mining, o = other risky)
VB : national cigarette type (V = at least 75% Virginia, bl = at least 75% blended, ot = other)
P : any proxy use
H : full histological confirmation
De : derivation of RR/CI (or = original, st = standard method, ot = other method of estimation)

Table 1G1 - 1

IESLC - Meta-analysis of Ever Smoking by Amount, Overview, Any product (or Cigarettes if Any not available)
All LC types
Most adjusted

REF	NRR	SEX	AGEL	AGEH	RACE	YF	LC TYPE	LOC	START	ST	NLC	R	VB	P	H	AD	PRODUCT	exL	exH	S1	S2	DENOM	De
AGUDO	4	f	0	0	all	-	all	Eu:wst	1989	CC	103	n	bl	n	n	3	cig only	1	10	1	0	nev any	or
AGUDO	5	f	0	0	all	-	all	Eu:wst	1989	CC	103	n	bl	n	n	3	cig only	11	99	0	0	nev any	or
ALDERS	18	m	0	0	all	-	all	Eu:UK	1977	CC	1448	n	V	n	n	1	cig only	1	17	1	0	nev+2	ot
ALDERS	19	m	0	0	all	-	all	Eu:UK	1977	CC	1448	n	V	n	n	1	cig only	18	27	2	3	nev+2	ot
ALDERS	20	m	0	0	all	-	all	Eu:UK	1977	CC	1448	n	V	n	n	1	cig only	28	99	3	0	nev+2	ot
ALDERS	21	f	0	0	all	-	all	Eu:UK	1977	CC	1448	n	V	n	n	1	cig only	1	17	1	0	nev+2	ot
ALDERS	22	f	0	0	all	-	all	Eu:UK	1977	CC	1448	n	V	n	n	1	cig only	18	27	2	3	nev+2	ot
ALDERS	23	f	0	0	all	-	all	Eu:UK	1977	CC	1448	n	V	n	n	1	cig only	28	99	3	0	nev+2	ot
ARMADA	46	m	0	0	all	-	all	Eu:wst	1986	CC	325	n	bl	n	y	0	cig+/-ot	1	14	1	0	nev any	st
ARMADA	47	m	0	0	all	-	all	Eu:wst	1986	CC	325	n	bl	n	y	0	cig+/-ot	15	24	2	3	nev any	st
ARMADA	48	m	0	0	all	-	all	Eu:wst	1986	CC	325	n	bl	n	y	0	cig+/-ot	25	99	3	0	nev any	st
AUVINE	13	c	0	0	all	-	all	Eu:Sca	1986	CC	517	n	bl	y	n	2	cig+/-ot	1	10	1	0	nev cigs	or
AUVINE	14	c	0	0	all	-	all	Eu:Sca	1986	CC	517	n	bl	y	n	2	cig+/-ot	11	20	2	3	nev cigs	or
AUVINE	15	c	0	0	all	-	all	Eu:Sca	1986	CC	517	n	bl	y	n	2	cig+/-ot	21	99	3	0	nev cigs	or
AXELSS	5	m	0	0	sca	-	all	Eu:Sca	1989	CC	436	n	bl	n	n	6	all/unsp	1	9	1	1	nev any	ot
AXELSS	6	m	0	0	sca	-	all	Eu:Sca	1989	CC	436	n	bl	n	n	6	all/unsp	10	19	0	2	nev any	ot
AXELSS	7	m	0	0	sca	-	all	Eu:Sca	1989	CC	436	n	bl	n	n	6	all/unsp	20	99	0	0	nev any	ot
AXELSS	13	f	0	0	sca	-	all	Eu:Sca	1989	CC	436	n	bl	n	n	0	all/unsp	1	9	1	1	nev any	st
AXELSS	14	f	0	0	sca	-	all	Eu:Sca	1989	CC	436	n	bl	n	n	0	all/unsp	10	19	0	2	nev any	st
AXELSS	15	f	0	0	sca	-	all	Eu:Sca	1989	CC	436	n	bl	n	n	0	all/unsp	20	29	2	3	nev any	st
AXELSS	16	f	0	0	sca	-	all	Eu:Sca	1989	CC	436	n	bl	n	n	0	all/unsp	30	99	3	0	nev any	st
BARBON	82	m	0	0	all	-	all	Eu:wst	1979	CC	755	n	bl	y	y	3	all/unsp	1	19	1	0	nev any	or
BARBON	83	m	0	0	all	-	all	Eu:wst	1979	CC	755	n	bl	y	y	3	all/unsp	20	39	2	0	nev any	or
BARBON	84	m	0	0	all	-	all	Eu:wst	1979	CC	755	n	bl	y	y	3	all/unsp	40	99	3	0	nev any	or
BOUCOT	99	m	0	0	all	9	all	NAmer	1951	pr	121	n	bl	n	n	0	cig+/-ot	1	20	0	0	nev any	ot
BOUCOT	100	m	0	0	all	9	all	NAmer	1951	pr	121	n	bl	n	n	0	cig+/-ot	21	99	3	0	nev any	ot
BRESLO	13	m	0	0	all	-	all	NAmer	1949	CC	518	n	bl	n	y	0	cig+/-ot	1	9	1	1	nev+3	st
BRESLO	14	m	0	0	all	-	all	NAmer	1949	CC	518	n	bl	n	y	0	cig+/-ot	10	19	0	2	nev+3	st
BRESLO	15	m	0	0	all	-	all	NAmer	1949	CC	518	n	bl	n	y	0	cig+/-ot	20	39	2	0	nev+3	st
BRESLO	16	m	0	0	all	-	all	NAmer	1949	CC	518	n	bl	n	y	0	cig+/-ot	40	99	3	0	nev+3	st
BRESLO	29	f	0	0	all	-	all	NAmer	1949	CC	518	n	bl	n	y	0	cig+/-ot	1	19	1	0	nev+3	st
BRESLO	30	f	0	0	all	-	all	NAmer	1949	CC	518	n	bl	n	y	0	cig+/-ot	20	99	0	0	nev+3	st
BROWN2	32	m	0	0	wh	-	all	NAmer	1984	CC	14596	n	bl	n	y	2	cig+/-ot	1	19	1	0	nev cigs	or
BROWN2	42	m	0	0	wh	-	all	NAmer	1984	CC	14596	n	bl	n	y	2	cig+/-ot	20	99	0	0	nev cigs	or
BROWN2	31	f	0	0	wh	-	all	NAmer	1984	CC	14596	n	bl	n	y	2	cig+/-ot	1	19	1	0	nev cigs	or
BROWN2	41	f	0	0	wh	-	all	NAmer	1984	CC	14596	n	bl	n	y	2	cig+/-ot	20	99	0	0	nev cigs	or
BUFFLE	28	f	0	0	w-hi	-	all	NAmer	1976	CC	943	n	bl	y	n	0	cig+/-ot	1	19	1	0	nev cigs	or
BUFFLE	29	f	0	0	w-hi	-	all	NAmer	1976	CC	943	n	bl	y	n	0	cig+/-ot	20	20	2	3	nev cigs	or
BUFFLE	35	f	0	0	w-hi	-	all	NAmer	1976	CC	943	n	bl	y	n	0	cig+/-ot	21	99	3	0	nev cigs	or
CHATZI	1	c	0	0	all	-	all	Eu:bal	1987	CC	282	n	bl	n	y	0	all/unsp	1	45	0	0	nev any	st
CHATZI	2	c	0	0	all	-	all	Eu:bal	1987	CC	282	n	bl	n	y	0	all/unsp	46	74	0	0	nev any	st
CHATZI	3	c	0	0	all	-	all	Eu:bal	1987	CC	282	n	bl	n	y	0	all/unsp	75	99	0	6	nev any	st
CHEN2	3	m	0	0	all	-	all	As:Chi	1983	CC	193	n	ot	y	n	0	all/unsp	1	9	1	1	nev any	st
CHEN2	4	m	0	0	all	-	all	As:Chi	1983	CC	193	n	ot	y	n	0	all/unsp	10	20	2	0	nev any	st
CHEN2	5	m	0	0	all	-	all	As:Chi	1983	CC	193	n	ot	y	n	0	all/unsp	21	30	0	4	nev any	st
CHEN2	6	m	0	0	all	-	all	As:Chi	1983	CC	193	n	ot	y	n	0	all/unsp	31	99	3	0	nev any	st
CHEN2	7	f	0	0	all	-	all	As:Chi	1983	CC	193	n	ot	y	n	0	all/unsp	1	9	1	1	nev any	st
CHEN2	8	f	0	0	all	-	all	As:Chi	1983	CC	193	n	ot	y	n	0	all/unsp	10	20	2	0	nev any	st
CHEN2	9	f	0	0	all	-	all	As:Chi	1983	CC	193	n	ot	y	n	0	all/unsp	21	30	0	4	nev any	st
CHEN2	10	f	0	0	all	-	all	As:Chi	1983	CC	193	n	ot	y	n	0	all/unsp	31	99	3	0	nev any	st
CHOI	12	m	0	0	all	-	all	As:oth	1985	CC	375	n	bl	n	n	0	cig+/-ot	1	10	1	0	nev cigs	st
CHOI	13	m	0	0	all	-	all	As:oth	1985	CC	375	n	bl	n	n	0	cig+/-ot	11	20	2	3	nev cigs	st
CHOI	14	m	0	0	all	-	all	As:oth	1985	CC	375	n	bl	n	n	0	cig+/-ot	21	30	0	4	nev cigs	st
CHOI	15	m	0	0	all	-	all	As:oth	1985	CC	375	n	bl	n	n	0	cig+/-ot	31	40	0	5	nev cigs	st
CHOI	16	m	0	0	all	-	all	As:oth	1985	CC	375	n	bl	n	n	0	cig+/-ot	41	99	3	6	nev cigs	st
CHOI	17	f	0	0	all	-	all	As:oth	1985	CC	375	n	bl	n	n	0	cig+/-ot	1	10	1	0	nev cigs	st
CHOI	18	f	0	0	all	-	all	As:oth	1985	CC	375	n	bl	n	n	0	cig+/-ot	11	30	2	0	nev cigs	st
CHOI	20	f	0	0	all	-	all	As:oth	1985	CC	375	n	bl	n	n	0	cig+/-ot	31	99	3	0	nev cigs	st
COOKSO	1	c	0	0	bl	-	all	Africa	1961	CC	234	n	V	n	y	0	cig+/-ot	1	9	1	1	nev any	st
COOKSO	2	c	0	0	bl	-	all	Africa	1961	CC	234	n	V	n	y	0	cig+/-ot	10	99	0	0	nev any	st
CPSI	243	m	50	74	all	6	all	NAmer	1959	pr	5138	n	bl	n	n	1	cig only	1	19	1	0	nev any	ot
CPSI	246	m	50	74	all	6	all	NAmer	1959	pr	5138	n	bl	n	n	1	cig only	20	99	0	0	nev any	ot
CPSII	102	m	35	99	all	4	all	NAmer	1982	pr	3229	n	bl	n	n	1	cig only	1	20	0	0	nev any	ot
CPSII	103	m	35	99	all	4	all	NAmer	1982	pr	3229	n	bl	n	n	1	cig only	21	99	3	0	nev any	ot
CPSII	105	f	35	99	all	4	all	NAmer	1982	pr	3229	n	bl	n	n	1	cig+/-ot	1	19	1	0	nev cigs	ot
CPSII	106	f	35	99	all	4	all	NAmer	1982	pr	3229	n	bl	n	n	1	cig+/-ot	20	99	0	0	nev cigs	ot
DAMBER	6	m	0	0	all	-	all	Eu:Sca	1972	CC	579	n	bl	y	n	1	cig only	1	7	1	1	nev any	ot
DAMBER	7	m	0	0	all	-	all	Eu:Sca	1972	CC	579	n	bl	y	n	1	cig only	8	15	0	2	nev any	ot
DAMBER	8	m	0	0	all	-	all	Eu:Sca	1972	CC	579	n	bl	y	n	1	cig only	16	25	2	3	nev any	ot
DAMBER	9	m	0	0	all	-	all	Eu:Sca	1972	CC	579	n	bl	y	n	1	cig only	26	99	3	0	nev any	ot
DAVEYS	1	m	0	0	all	-	all	Eu:Ger	1930	CC	109	n	bl	y	n	0	all/unsp	1	5	1	1	nev any	st
DAVEYS	2	m	0	0	all	-	all	Eu:Ger	1930	CC	109	n	bl	y	n	0	all/unsp	6	10	0	2	nev any	st

Table 1G1 - 1

IESLC - Meta-analysis of Ever Smoking by Amount, Overview, Any product (or Cigarettes if Any not available)
All LC types
Most adjusted

REF	NRR	SEX	AGEL	AGEH	RACE	YF	LC TYPE	LOC	START	ST	NLC	R	VB	P	H	AD	PRODUCT	exL	exH	S1	S2	DENOM	De
DAVEYS	3	m	0	0	all	-	all	Eu:Ger	1930	CC	109	n	bl	y	n	0	all/unsp	11	20	2	3	nev any	st
DAVEYS	4	m	0	0	all	-	all	Eu:Ger	1930	CC	109	n	bl	y	n	0	all/unsp	21	99	3	0	nev any	st
DEAN	1	m	0	0	wh	-	all	Africa	1947	CC	603	n	V	y	n	0	cig only	1	20	0	0	nev any	st
DEAN	2	m	0	0	wh	-	all	Africa	1947	CC	603	n	V	y	n	0	cig only	25	45	3	0	nev any	st
DEAN	3	m	0	0	wh	-	all	Africa	1947	CC	603	n	V	y	n	0	cig only	50	99	0	6	nev any	st
DEAN2	25	m	0	0	all	-	all	Eu:UK	1960	CC	954	n	V	y	n	0	cig only	1	22	0	0	nev any	st
DEAN2	26	m	0	0	all	-	all	Eu:UK	1960	CC	954	n	V	y	n	0	cig only	23	99	3	0	nev any	st
DEAN2	29	f	0	0	all	-	all	Eu:UK	1960	CC	954	n	V	y	n	0	cig only	1	22	0	0	nev any	st
DEAN2	30	f	0	0	all	-	all	Eu:UK	1960	CC	954	n	V	y	n	0	cig only	23	99	3	0	nev any	st
DESTEF	6	m	0	0	all	-	all	SCAmer	1988	CC	497	n	bl	n	y	4	all/unsp	1	10	1	0	nev any	or
DESTEF	7	m	0	0	all	-	all	SCAmer	1988	CC	497	n	bl	n	y	4	all/unsp	11	20	2	3	nev any	or
DESTEF	8	m	0	0	all	-	all	SCAmer	1988	CC	497	n	bl	n	y	4	all/unsp	21	40	0	0	nev any	or
DESTEF	9	m	0	0	all	-	all	SCAmer	1988	CC	497	n	bl	n	y	4	all/unsp	41	99	3	6	nev any	or
DOLL	1	m	0	0	all	-	all	Eu:UK	1948	CC	1465	n	V	n	n	0	all/unsp	1	4	0	1	nev any	st
DOLL	2	m	0	0	all	-	all	Eu:UK	1948	CC	1465	n	V	n	n	0	all/unsp	5	14	1	2	nev any	st
DOLL	3	m	0	0	all	-	all	Eu:UK	1948	CC	1465	n	V	n	n	0	all/unsp	15	24	2	3	nev any	st
DOLL	4	m	0	0	all	-	all	Eu:UK	1948	CC	1465	n	V	n	n	0	all/unsp	25	49	3	0	nev any	st
DOLL	5	m	0	0	all	-	all	Eu:UK	1948	CC	1465	n	V	n	n	0	all/unsp	50	99	0	6	nev any	st
DOLL	7	f	0	0	all	-	all	Eu:UK	1948	CC	1465	n	V	n	n	0	all/unsp	1	4	0	1	nev any	st
DOLL	8	f	0	0	all	-	all	Eu:UK	1948	CC	1465	n	V	n	n	0	all/unsp	5	14	1	2	nev any	st
DOLL	9	f	0	0	all	-	all	Eu:UK	1948	CC	1465	n	V	n	n	0	all/unsp	15	24	2	3	nev any	st
DOLL	10	f	0	0	all	-	all	Eu:UK	1948	CC	1465	n	V	n	n	0	all/unsp	25	49	3	0	nev any	st
DOLL2	46	m	35	99	all	5	all	Eu:UK	1951	pr	920	n	V	n	n	1	all/unsp	1	14	1	0	nev any	ot
DOLL2	47	m	35	99	all	5	all	Eu:UK	1951	pr	920	n	V	n	n	1	all/unsp	15	24	2	3	nev any	ot
DOLL2	48	m	35	99	all	5	all	Eu:UK	1951	pr	920	n	V	n	n	1	all/unsp	25	99	3	0	nev any	ot
DORGAN	108	m	0	0	wh	-	all	NAmer	1980	CC	2026	n	bl	y	y	2	cig+/-ot	1	19	1	0	nev any	ot
DORGAN	109	m	0	0	wh	-	all	NAmer	1980	CC	2026	n	bl	y	y	2	cig+/-ot	20	99	0	0	nev any	ot
DORGAN	96	f	0	0	all	-	all	NAmer	1980	CC	2026	n	bl	y	y	3	cig+/-ot	1	19	1	0	nev any	ot
DORGAN	97	f	0	0	all	-	all	NAmer	1980	CC	2026	n	bl	y	y	3	cig+/-ot	20	99	0	0	nev any	ot
DOSEME	5	m	0	0	all	-	all	Eu:bal	1979	CC	1210	n	bl	n	n	2	cig+/-ot	1	10	1	0	nev cigs	or
DOSEME	9	m	0	0	all	-	all	Eu:bal	1979	CC	1210	n	bl	n	n	2	cig+/-ot	11	20	2	3	nev cigs	or
DOSEME	13	m	0	0	all	-	all	Eu:bal	1979	CC	1210	n	bl	n	n	2	cig+/-ot	21	99	3	0	nev cigs	or
DUNN	1	m	0	0	all	0	all	NAmer	1954	pr	139	o	bl	n	n	0	cig+/-ot	1	4	0	1	nev cigs	st
DUNN	2	m	0	0	all	0	all	NAmer	1954	pr	139	o	bl	n	n	0	cig+/-ot	5	14	1	2	nev cigs	st
DUNN	3	m	0	0	all	0	all	NAmer	1954	pr	139	o	bl	n	n	0	cig+/-ot	15	24	2	3	nev cigs	st
DUNN	4	m	0	0	all	0	all	NAmer	1954	pr	139	o	bl	n	n	0	cig+/-ot	25	34	0	4	nev cigs	st
DUNN	5	m	0	0	all	0	all	NAmer	1954	pr	139	o	bl	n	n	0	cig+/-ot	35	99	3	0	nev cigs	st
EBELIN	2	m	0	0	all	-	all	Eu:Ger	1980	CC	130	n	bl	n	n	0	all/unsp	1	9	1	1	nev any	st
EBELIN	3	m	0	0	all	-	all	Eu:Ger	1980	CC	130	n	bl	n	n	0	all/unsp	10	19	0	2	nev any	st
EBELIN	4	m	0	0	all	-	all	Eu:Ger	1980	CC	130	n	bl	n	n	0	all/unsp	20	29	2	3	nev any	st
EBELIN	5	m	0	0	all	-	all	Eu:Ger	1980	CC	130	n	bl	n	n	0	all/unsp	30	39	0	4	nev any	st
EBELIN	6	m	0	0	all	-	all	Eu:Ger	1980	CC	130	n	bl	n	n	0	all/unsp	40	99	3	0	nev any	st
ESAKI	1	m	0	0	all	-	all	As:Jap	1961	CC	245	n	bl	y	n	0	cig+/-ot	1	14	1	0	nev cigs	st
ESAKI	2	m	0	0	all	-	all	As:Jap	1961	CC	245	n	bl	y	n	0	cig+/-ot	15	29	2	3	nev cigs	st
ESAKI	3	m	0	0	all	-	all	As:Jap	1961	CC	245	n	bl	y	n	0	cig+/-ot	30	99	3	0	nev cigs	st
FAN	6	m	0	0	all	-	all	As:Chi	1990	CC	403	n	ot	y	n	0	cig+/-ot	1	9	1	1	nev cigs	st
FAN	7	m	0	0	all	-	all	As:Chi	1990	CC	403	n	ot	y	n	0	cig+/-ot	10	19	0	2	nev cigs	st
FAN	8	m	0	0	all	-	all	As:Chi	1990	CC	403	n	ot	y	n	0	cig+/-ot	20	29	2	3	nev cigs	st
FAN	9	m	0	0	all	-	all	As:Chi	1990	CC	403	n	ot	y	n	0	cig+/-ot	30	99	3	0	nev cigs	st
FAN	10	f	0	0	all	-	all	As:Chi	1990	CC	403	n	ot	y	n	0	cig+/-ot	1	9	1	1	nev cigs	st
FAN	11	f	0	0	all	-	all	As:Chi	1990	CC	403	n	ot	y	n	0	cig+/-ot	10	19	0	2	nev cigs	st
FAN	12	f	0	0	all	-	all	As:Chi	1990	CC	403	n	ot	y	n	0	cig+/-ot	20	29	2	3	nev cigs	st
FAN	13	f	0	0	all	-	all	As:Chi	1990	CC	403	n	ot	y	n	0	cig+/-ot	30	99	3	0	nev cigs	st
GAO	21	f	0	0	all	-	all	As:Chi	1984	CC	1405	n	ot	n	n	2	cig+/-ot	1	9	1	1	nev cigs	ot
GAO	22	f	0	0	all	-	all	As:Chi	1984	CC	1405	n	ot	n	n	2	cig+/-ot	10	19	0	2	nev cigs	ot
GAO	23	f	0	0	all	-	all	As:Chi	1984	CC	1405	n	ot	n	n	2	cig+/-ot	20	99	0	0	nev cigs	ot
GARSHI	26	m	0	0	all	-	all	NAmer	1981	CC	1081	o	bl	y	n	1	all/unsp	1	15	1	0	nev any	st
GARSHI	27	m	0	0	all	-	all	NAmer	1981	CC	1081	o	bl	y	n	1	all/unsp	16	25	2	3	nev any	st
GARSHI	28	m	0	0	all	-	all	NAmer	1981	CC	1081	o	bl	y	n	1	all/unsp	26	35	0	4	nev any	st
GARSHI	29	m	0	0	all	-	all	NAmer	1981	CC	1081	o	bl	y	n	1	all/unsp	36	99	3	0	nev any	st
GER	22	c	0	0	all	-	all	As:oth	1990	CC	141	n	ot	y	n	14	all/unsp	1	10	1	0	nev any	ot
GER	23	c	0	0	all	-	all	As:oth	1990	CC	141	n	ot	y	n	14	all/unsp	11	20	2	3	nev any	ot
GER	24	c	0	0	all	-	all	As:oth	1990	CC	141	n	ot	y	n	14	all/unsp	21	99	3	0	nev any	ot
GOLLED	1	m	35	99	all	-	all	Eu:UK	1952	CC	443	n	V	y	n	1	cig only	1	10	1	0	nev any	ot
GOLLED	2	m	35	99	all	-	all	Eu:UK	1952	CC	443	n	V	y	n	1	cig only	11	22	2	3	nev any	ot
GOLLED	3	m	35	99	all	-	all	Eu:UK	1952	CC	443	n	V	y	n	1	cig only	23	99	3	0	nev any	ot
GSELL	1	m	0	0	all	-	all	Eu:wst	1937	CC	150	n	bl	n	y	0	all/unsp	1	9	1	1	nev any	st
GSELL	2	m	0	0	all	-	all	Eu:wst	1937	CC	150	n	bl	n	y	0	all/unsp	10	14	0	2	nev any	st
GSELL	3	m	0	0	all	-	all	Eu:wst	1937	CC	150	n	bl	n	y	0	all/unsp	15	20	2	3	nev any	st
GSELL	4	m	0	0	all	-	all	Eu:wst	1937	CC	150	n	bl	n	y	0	all/unsp	21	35	0	4	nev any	st
GSELL	5	m	0	0	all	-	all	Eu:wst	1937	CC	150	n	bl	n	y	0	all/unsp	36	99	3	0	nev any	st
HAMMON	153	m	0	0	wh	0	all	NAmer	1952	pr	448	n	bl	n	n	1	cig only	1	9	1	1	nev any	ot

Table 1G1 - 1

IESLC - Meta-analysis of Ever Smoking by Amount, Overview, Any product (or Cigarettes if Any not available)
All LC types
Most adjusted

REF	NRR	SEX	AGEL	AGEH	RACE	YF	LC TYPE	LOC	START	ST	NLC	R	VB	P	H	AD	PRODUCT	exL	exH	S1	S2	DENOM	De
HAMMON	154	m	0	0	wh	0	all	NAmer	1952	pr	448	n	bl	n	n	1	cig only	10	20	2	0	nev any	ot
HAMMON	155	m	0	0	wh	0	all	NAmer	1952	pr	448	n	bl	n	n	1	cig only	21	99	3	0	nev any	ot
HANSEN	1	m	0	0	all	0	all	Eu:Sca	1968	pr	105	o	bl	y	n	2	all/unsp	1	19	1	0	nev any	ot
HANSEN	2	m	0	0	all	0	all	Eu:Sca	1968	pr	105	o	bl	y	n	2	all/unsp	20	99	0	0	nev any	ot
HU	1	m	0	0	all	-	all	As:Chi	1985	CC	227	n	ot	n	y	0	cig+/-ot	1	14	1	0	nev any	st
HU	2	m	0	0	all	-	all	As:Chi	1985	CC	227	n	ot	n	y	0	cig+/-ot	14	24	2	3	nev any	st
HU	3	m	0	0	all	-	all	As:Chi	1985	CC	227	n	ot	n	y	0	cig+/-ot	25	99	3	0	nev any	st
HU	4	f	0	0	all	-	all	As:Chi	1985	CC	227	n	ot	n	y	0	cig+/-ot	1	14	1	0	nev any	st
HU	5	f	0	0	all	-	all	As:Chi	1985	CC	227	n	ot	n	y	0	cig+/-ot	14	24	2	3	nev any	st
HU	6	f	0	0	all	-	all	As:Chi	1985	CC	227	n	ot	n	y	0	cig+/-ot	25	99	3	0	nev any	st
HU2	2	c	0	0	all	-	all	As:Chi	1977	CC	523	n	ot	y	n	0	cig+/-ot	1	4	0	1	nev cigs	st
HU2	3	c	0	0	all	-	all	As:Chi	1977	CC	523	n	ot	y	n	0	cig+/-ot	5	9	1	0	nev cigs	st
HU2	4	c	0	0	all	-	all	As:Chi	1977	CC	523	n	ot	y	n	0	cig+/-ot	10	14	0	2	nev cigs	st
HU2	5	c	0	0	all	-	all	As:Chi	1977	CC	523	n	ot	y	n	0	cig+/-ot	15	19	0	0	nev cigs	or
HU2	6	c	0	0	all	-	all	As:Chi	1977	CC	523	n	ot	y	n	0	cig+/-ot	20	29	2	3	nev cigs	st
HU2	7	c	0	0	all	-	all	As:Chi	1977	CC	523	n	ot	y	n	0	cig+/-ot	30	99	3	0	nev cigs	st
JARUP	4	m	0	0	all	-	all	Eu:Sca	1928	CC	102	o	bl	y	n	2	all/unsp	1	10	1	0	nev any	or
JARUP	5	m	0	0	all	-	all	Eu:Sca	1928	CC	102	o	bl	y	n	2	all/unsp	11	99	0	0	nev any	or
JEDRYC	45	m	0	0	all	-	all	Eu:est	1980	CC	1630	n	bl	y	n	4	cig+/-ot	1	19	1	0	nev any	or
JEDRYC	46	m	0	0	all	-	all	Eu:est	1980	CC	1630	n	bl	y	n	4	cig+/-ot	20	29	2	3	nev any	or
JEDRYC	47	m	0	0	all	-	all	Eu:est	1980	CC	1630	n	bl	y	n	4	cig+/-ot	30	99	3	0	nev any	or
JEDRYC	48	f	0	0	all	-	all	Eu:est	1980	CC	1630	n	bl	y	n	4	cig+/-ot	1	19	1	0	nev any	or
JEDRYC	49	f	0	0	all	-	all	Eu:est	1980	CC	1630	n	bl	y	n	4	cig+/-ot	20	29	2	3	nev any	or
JEDRYC	50	f	0	0	all	-	all	Eu:est	1980	CC	1630	n	bl	y	n	4	cig+/-ot	30	99	3	0	nev any	or
JOLY	7	m	0	0	all	-	all	SCAmer	1978	CC	826	n	bl	n	n	0	cig+/-ot	1	9	1	1	nev any	st
JOLY	8	m	0	0	all	-	all	SCAmer	1978	CC	826	n	bl	n	n	0	cig+/-ot	10	19	0	2	nev any	st
JOLY	9	m	0	0	all	-	all	SCAmer	1978	CC	826	n	bl	n	n	0	cig+/-ot	20	29	2	3	nev any	st
JOLY	10	m	0	0	all	-	all	SCAmer	1978	CC	826	n	bl	n	n	0	cig+/-ot	30	99	3	0	nev any	st
JOLY	3	f	0	0	all	-	all	SCAmer	1978	CC	826	n	bl	n	n	0	cig+/-ot	1	9	1	1	nev any	st
JOLY	4	f	0	0	all	-	all	SCAmer	1978	CC	826	n	bl	n	n	0	cig+/-ot	10	19	0	2	nev any	st
JOLY	5	f	0	0	all	-	all	SCAmer	1978	CC	826	n	bl	n	n	0	cig+/-ot	20	29	2	3	nev any	st
JOLY	6	f	0	0	all	-	all	SCAmer	1978	CC	826	n	bl	n	n	0	cig+/-ot	30	99	3	0	nev any	st
JUSSAW	34	m	0	0	all	-	all	As:Ind	1964	CC	792	n	V	n	n	2	cig only	1	19	1	0	nev any	st
JUSSAW	35	m	0	0	all	-	all	As:Ind	1964	CC	792	n	V	n	n	2	cig only	20	99	0	0	nev any	st
KHUDER	1	m	0	0	all	-	all	NAmer	1985	CC	482	n	bl	n	y	0	cig+/-ot	1	19	1	0	nev cigs	st
KHUDER	2	m	0	0	all	-	all	NAmer	1985	CC	482	n	bl	n	y	0	cig+/-ot	20	39	2	0	nev cigs	st
KHUDER	3	m	0	0	all	-	all	NAmer	1985	CC	482	n	bl	n	y	0	cig+/-ot	40	99	3	0	nev cigs	st
KOULUM	6	m	0	0	all	-	all	Eu:Sca	1936	CC	812	n	bl	n	n	0	all/unsp	1	9	1	1	nev any	st
KOULUM	5	m	0	0	all	-	all	Eu:Sca	1936	CC	812	n	bl	n	n	0	all/unsp	10	19	0	2	nev any	st
KOULUM	4	m	0	0	all	-	all	Eu:Sca	1936	CC	812	n	bl	n	n	0	all/unsp	20	99	0	0	nev any	st
KREUZE	19	m	1	45	all	-	all	Eu:Ger	1990	CC	2260	n	bl	n	n	3	cig+/-ot	1	9	1	1	nev any	or
KREUZE	20	m	1	45	all	-	all	Eu:Ger	1990	CC	2260	n	bl	n	n	3	cig+/-ot	10	19	0	2	nev any	or
KREUZE	21	m	1	45	all	-	all	Eu:Ger	1990	CC	2260	n	bl	n	n	3	cig+/-ot	20	29	2	3	nev any	or
KREUZE	22	m	1	45	all	-	all	Eu:Ger	1990	CC	2260	n	bl	n	n	3	cig+/-ot	30	99	3	0	nev any	or
KREUZE	30	m	55	69	all	-	all	Eu:Ger	1990	CC	2260	n	bl	n	n	3	cig+/-ot	1	9	1	1	nev any	or
KREUZE	31	m	55	69	all	-	all	Eu:Ger	1990	CC	2260	n	bl	n	n	3	cig+/-ot	10	19	0	2	nev any	or
KREUZE	32	m	55	69	all	-	all	Eu:Ger	1990	CC	2260	n	bl	n	n	3	cig+/-ot	20	29	2	3	nev any	or
KREUZE	33	m	55	69	all	-	all	Eu:Ger	1990	CC	2260	n	bl	n	n	3	cig+/-ot	30	99	3	0	nev any	or
KREUZE	25	f	1	45	all	-	all	Eu:Ger	1990	CC	2260	n	bl	n	n	3	cig+/-ot	1	9	1	1	nev any	or
KREUZE	26	f	1	45	all	-	all	Eu:Ger	1990	CC	2260	n	bl	n	n	3	cig+/-ot	10	19	0	2	nev any	or
KREUZE	27	f	1	45	all	-	all	Eu:Ger	1990	CC	2260	n	bl	n	n	3	cig+/-ot	20	29	2	3	nev any	or
KREUZE	36	f	55	69	all	-	all	Eu:Ger	1990	CC	2260	n	bl	n	n	3	cig+/-ot	1	9	1	1	nev any	or
KREUZE	37	f	55	69	all	-	all	Eu:Ger	1990	CC	2260	n	bl	n	n	3	cig+/-ot	10	19	0	2	nev any	or
KREUZE	38	f	55	69	all	-	all	Eu:Ger	1990	CC	2260	n	bl	n	n	3	cig+/-ot	20	29	2	3	nev any	or
KREYBE	9	m	0	0	all	-	all	Eu:Sca	1948	CC	300	n	bl	n	y	1	all/unsp	1	14	1	0	nev any	ot
KREYBE	10	m	0	0	all	-	all	Eu:Sca	1948	CC	300	n	bl	n	y	1	all/unsp	15	24	2	3	nev any	ot
KREYBE	11	m	0	0	all	-	all	Eu:Sca	1948	CC	300	n	bl	n	y	1	all/unsp	25	99	3	0	nev any	ot
KREYBE	28	f	0	0	all	-	all	Eu:Sca	1948	CC	300	n	bl	n	y	1	all/unsp	1	14	1	0	nev any	ot
KREYBE	29	f	0	0	all	-	all	Eu:Sca	1948	CC	300	n	bl	n	y	1	all/unsp	15	99	0	0	nev any	ot
LAMTH	7	f	0	0	ch	-	all	As:HK	1983	CC	445	n	bl	n	n	0	all/unsp	1	10	1	0	nev any	or
LAMTH	2	f	0	0	ch	-	all	As:HK	1983	CC	445	n	bl	n	n	0	all/unsp	11	20	2	3	nev any	or
LAMTH	9	f	0	0	ch	-	all	As:HK	1983	CC	445	n	bl	n	n	0	all/unsp	21	99	3	0	nev any	or
LAUSSM	18	m	0	0	all	-	all	Eu:Ger	1982	CC	432	n	bl	n	n	3	all/unsp	1	9	1	1	nev any	ot
LAUSSM	19	m	0	0	all	-	all	Eu:Ger	1982	CC	432	n	bl	n	n	3	all/unsp	10	19	0	2	nev any	ot
LAUSSM	20	m	0	0	all	-	all	Eu:Ger	1982	CC	432	n	bl	n	n	3	all/unsp	20	99	0	0	nev any	ot
LETOUR	2	c	0	0	all	-	all	NAmer	1983	CC	738	n	V	y	y	0	cig+/-ot	1	19	1	0	nev cigs	st
LETOUR	3	c	0	0	all	-	all	NAmer	1983	CC	738	n	V	y	y	0	cig+/-ot	20	40	2	0	nev cigs	st
LETOUR	4	c	0	0	all	-	all	NAmer	1983	CC	738	n	V	y	y	0	cig+/-ot	41	99	3	6	nev cigs	st
LIU2	8	m	0	0	all	-	all	As:Chi	1983	CC	316	n	ot	n	n	3	all/unsp	1	19	1	0	nev any	or
LIU2	9	m	0	0	all	-	all	As:Chi	1983	CC	316	n	ot	n	n	3	all/unsp	20	29	2	3	nev any	or
LIU2	10	m	0	0	all	-	all	As:Chi	1983	CC	316	n	ot	n	n	3	all/unsp	30	99	3	0	nev any	or
LIU2	14	f	0	0	all	-	all	As:Chi	1983	CC	316	n	ot	n	n	3	all/unsp	1	9	1	1	nev any	or

Table 1G1 - 1

IESLC - Meta-analysis of Ever Smoking by Amount, Overview, Any product (or Cigarettes if Any not available)
All LC types
Most adjusted

REF	NRR	SEX	AGEL	AGEH	RACE	YF	LC TYPE	LOC	START	ST	NLC	R	VB	P	H	AD	PRODUCT	exL	exH	S1	S2	DENOM	De
LIU2	15	f	0	0	all	-	all	As:Chi	1983	CC	316	n	ot	n	n	3	all/unsp	10	19	0	2	nev any	or
LIU2	16	f	0	0	all	-	all	As:Chi	1983	CC	316	n	ot	n	n	3	all/unsp	20	99	0	0	nev any	or
LIU3	6	m	0	0	all	-	all	As:Chi	1985	CC	110	n	ot	n	n	2	all/unsp	1	15	1	0	nev any	or
LIU3	7	m	0	0	all	-	all	As:Chi	1985	CC	110	n	ot	n	n	2	all/unsp	16	30	2	0	nev any	or
LIU3	8	m	0	0	all	-	all	As:Chi	1985	CC	110	n	ot	n	n	2	all/unsp	31	99	3	0	nev any	or
LIU4	7	m	35	69	all	-	all	As:Chi	1986	CC	1000-00	n	ot	y	n	2	cig only	1	19	1	0	nev any	ot
LIU4	8	m	35	69	all	-	all	As:Chi	1986	CC	1000-00	n	ot	y	n	2	cig only	20	20	2	3	nev any	ot
LIU4	9	m	35	69	all	-	all	As:Chi	1986	CC	1000-00	n	ot	y	n	2	cig only	21	99	3	0	nev any	ot
LIU5	2	c	0	0	all	-	all	As:Chi	1978	CC	111	n	ot	y	n	0	all/unsp	1	9	1	1	nev any	st
LIU5	3	c	0	0	all	-	all	As:Chi	1978	CC	111	n	ot	y	n	0	all/unsp	10	19	0	2	nev any	st
LIU5	4	c	0	0	all	-	all	As:Chi	1978	CC	111	n	ot	y	n	0	all/unsp	20	99	0	0	nev any	st
LUBIN	11	m	0	0	all	-	all	As:Chi	1984	CC	427	m	ot	y	n	4	cig only	1	6	1	1	nev any	ot
LUBIN	12	m	0	0	all	-	all	As:Chi	1984	CC	427	m	ot	y	n	4	cig only	7	14	0	2	nev any	ot
LUBIN	13	m	0	0	all	-	all	As:Chi	1984	CC	427	m	ot	y	n	4	cig only	15	19	0	0	nev any	ot
LUBIN	14	m	0	0	all	-	all	As:Chi	1984	CC	427	m	ot	y	n	4	cig only	20	99	0	0	nev any	ot
LUBIN2	273	m	0	0	all	-	all	Eu:mul	1976	CC	7804	n	bl	n	y	0	cig+/-ot	1	9	1	1	nev any	st
LUBIN2	274	m	0	0	all	-	all	Eu:mul	1976	CC	7804	n	bl	n	y	0	cig+/-ot	10	19	0	2	nev any	st
LUBIN2	275	m	0	0	all	-	all	Eu:mul	1976	CC	7804	n	bl	n	y	0	cig+/-ot	20	29	2	3	nev any	st
LUBIN2	276	m	0	0	all	-	all	Eu:mul	1976	CC	7804	n	bl	n	y	0	cig+/-ot	30	99	3	0	nev any	st
LUBIN2	281	f	0	0	all	-	all	Eu:mul	1976	CC	7804	n	bl	n	y	0	cig+/-ot	1	9	1	1	nev any	st
LUBIN2	282	f	0	0	all	-	all	Eu:mul	1976	CC	7804	n	bl	n	y	0	cig+/-ot	10	19	0	2	nev any	st
LUBIN2	283	f	0	0	all	-	all	Eu:mul	1976	CC	7804	n	bl	n	y	0	cig+/-ot	20	29	2	3	nev any	st
LUBIN2	284	f	0	0	all	-	all	Eu:mul	1976	CC	7804	n	bl	n	y	0	cig+/-ot	30	99	3	0	nev any	st
MACLEN	36	c	0	0	ch	-	all	As:oth	1972	CC	233	n	bl	n	n	2	cig+/-ot	1	9	1	1	nev cigs	or
MACLEN	37	c	0	0	ch	-	all	As:oth	1972	CC	233	n	bl	n	n	2	cig+/-ot	10	19	0	2	nev cigs	or
MACLEN	38	c	0	0	ch	-	all	As:oth	1972	CC	233	n	bl	n	n	2	cig+/-ot	20	29	2	3	nev cigs	or
MACLEN	39	c	0	0	ch	-	all	As:oth	1972	CC	233	n	bl	n	n	2	cig+/-ot	30	99	3	0	nev cigs	or
MARTIS	1	m	0	0	all	-	all	Eu:UK	1972	CC	201	n	V	n	n	0	cig+/-ot	1	14	1	0	nev cigs	st
MARTIS	2	m	0	0	all	-	all	Eu:UK	1972	CC	201	n	V	n	n	0	cig+/-ot	15	24	2	3	nev cigs	st
MARTIS	3	m	0	0	all	-	all	Eu:UK	1972	CC	201	n	V	n	n	0	cig+/-ot	25	99	3	0	nev cigs	st
MATOS	29	m	0	0	all	-	all	SCAmer	1994	CC	200	n	bl	n	n	2	cig+/-ot	1	14	1	0	nev any	or
MATOS	31	m	0	0	all	-	all	SCAmer	1994	CC	200	n	bl	n	n	2	cig+/-ot	15	24	2	3	nev any	or
MATOS	33	m	0	0	all	-	all	SCAmer	1994	CC	200	n	bl	n	n	2	cig+/-ot	25	99	3	0	nev any	or
MATSUD	1	m	0	0	all	-	all	As:Jap	1965	CC	179	n	bl	n	n	0	cig+/-ot	1	10	1	0	nev cigs	st
MATSUD	2	m	0	0	all	-	all	As:Jap	1965	CC	179	n	bl	n	n	0	cig+/-ot	11	20	2	3	nev cigs	st
MATSUD	3	m	0	0	all	-	all	As:Jap	1965	CC	179	n	bl	n	n	0	cig+/-ot	21	99	3	0	nev cigs	st
MCCONN	26	c	0	0	all	-	all	Eu:UK	1946	CC	100	n	V	n	y	0	all/unsp	1	10	1	0	nev any	st
MCCONN	25	c	0	0	all	-	all	Eu:UK	1946	CC	100	n	V	n	y	0	all/unsp	10	20	2	0	nev any	st
MCCONN	24	c	0	0	all	-	all	Eu:UK	1946	CC	100	n	V	n	y	0	all/unsp	21	99	3	0	nev any	st
NOTAN2	8	m	0	0	all	-	all	As:Ind	1963	CC	683	n	V	n	n	0	cig only	1	9	1	1	nev any	st
NOTAN2	9	m	0	0	all	-	all	As:Ind	1963	CC	683	n	V	n	n	0	cig only	10	19	0	2	nev any	st
NOTAN2	10	m	0	0	all	-	all	As:Ind	1963	CC	683	n	V	n	n	0	cig only	20	99	0	0	nev any	st
ORMOS	1	m	0	0	all	-	all	Eu:est	1947	CC	119	n	bl	y	y	0	cig+/-ot	1	15	1	0	nev any	st
ORMOS	2	m	0	0	all	-	all	Eu:est	1947	CC	119	n	bl	y	y	0	cig+/-ot	16	30	2	0	nev any	st
ORMOS	3	m	0	0	all	-	all	Eu:est	1947	CC	119	n	bl	y	y	0	cig+/-ot	31	99	3	0	nev any	st
OSANN	49	m	0	0	all	-	all	NAmer	1984	CC	1986	n	bl	n	n	2	cig+/-ot	1	39	0	0	nev cigs	or
OSANN	57	m	0	0	all	-	all	NAmer	1984	CC	1986	n	bl	n	n	2	cig+/-ot	40	99	3	0	nev cigs	or
OSANN	50	f	0	0	all	-	all	NAmer	1984	CC	1986	n	bl	n	n	2	cig+/-ot	1	39	0	0	nev cigs	or
OSANN	58	f	0	0	all	-	all	NAmer	1984	CC	1986	n	bl	n	n	2	cig+/-ot	40	99	3	0	nev cigs	or
OSANN2	22	f	0	0	all	-	all	NAmer	1964	ot	217	n	bl	n	y	1	cig+/-ot	1	19	1	0	nev cigs	or
OSANN2	23	f	0	0	all	-	all	NAmer	1964	ot	217	n	bl	n	y	1	cig+/-ot	20	99	0	0	nev cigs	or
PASTOR	6	m	0	0	all	-	all	Eu:wst	1976	CC	204	n	bl	y	n	1	all/unsp	1	9	1	1	nev any	st
PASTOR	7	m	0	0	all	-	all	Eu:wst	1976	CC	204	n	bl	y	n	1	all/unsp	10	19	0	2	nev any	st
PASTOR	8	m	0	0	all	-	all	Eu:wst	1976	CC	204	n	bl	y	n	1	all/unsp	20	29	2	3	nev any	st
PASTOR	9	m	0	0	all	-	all	Eu:wst	1976	CC	204	n	bl	y	n	1	all/unsp	30	99	3	0	nev any	st
PERNU	17	m	0	0	all	-	all	Eu:Sca	1944	CC	1606	n	bl	n	n	0	all/unsp	1	4	0	1	nev any	st
PERNU	18	m	0	0	all	-	all	Eu:Sca	1944	CC	1606	n	bl	n	n	0	all/unsp	5	9	1	0	nev any	st
PERNU	19	m	0	0	all	-	all	Eu:Sca	1944	CC	1606	n	bl	n	n	0	all/unsp	10	14	0	2	nev any	st
PERNU	20	m	0	0	all	-	all	Eu:Sca	1944	CC	1606	n	bl	n	n	0	all/unsp	15	19	0	0	nev any	st
PERNU	21	m	0	0	all	-	all	Eu:Sca	1944	CC	1606	n	bl	n	n	0	all/unsp	20	24	2	3	nev any	st
PERNU	22	m	0	0	all	-	all	Eu:Sca	1944	CC	1606	n	bl	n	n	0	all/unsp	25	29	0	0	nev any	st
PERNU	23	m	0	0	all	-	all	Eu:Sca	1944	CC	1606	n	bl	n	n	0	all/unsp	30	49	3	0	nev any	st
PERNU	24	m	0	0	all	-	all	Eu:Sca	1944	CC	1606	n	bl	n	n	0	all/unsp	50	99	0	6	nev any	st
PERNU	11	f	0	0	all	-	all	Eu:Sca	1944	CC	1606	n	bl	n	n	0	all/unsp	1	4	0	1	nev any	st
PERNU	12	f	0	0	all	-	all	Eu:Sca	1944	CC	1606	n	bl	n	n	0	all/unsp	5	9	1	0	nev any	st
PERNU	13	f	0	0	all	-	all	Eu:Sca	1944	CC	1606	n	bl	n	n	0	all/unsp	10	14	0	2	nev any	st
PERNU	14	f	0	0	all	-	all	Eu:Sca	1944	CC	1606	n	bl	n	n	0	all/unsp	15	19	0	0	nev any	st
PERNU	15	f	0	0	all	-	all	Eu:Sca	1944	CC	1606	n	bl	n	n	0	all/unsp	20	24	2	3	nev any	st
PERNU	16	f	0	0	all	-	all	Eu:Sca	1944	CC	1606	n	bl	n	n	0	all/unsp	25	99	3	0	nev any	st

Table 1G1 - 1

IESLC - Meta-analysis of Ever Smoking by Amount, Overview, Any product (or Cigarettes if Any not available)
All LC types
Most adjusted

REF	NRR	SEX	AGEL	AGEH	RACE	YF	LC TYPE	LOC	START	ST	NLC	R	VB	P	H	AD	PRODUCT	exL	exH	S1	S2	DENOM	De
PIKE	1	m	0	0	w-hi	-	all	NAmer	1972	CC	731	n	bl	y	n	0	all/unsp	1	20	0	0	nev any	st
PIKE	2	m	0	0	w-hi	-	all	NAmer	1972	CC	731	n	bl	y	n	0	all/unsp	21	40	0	0	nev any	st
PIKE	3	m	0	0	w-hi	-	all	NAmer	1972	CC	731	n	bl	y	n	0	all/unsp	41	99	3	6	nev any	st
PIKE	5	f	0	0	w-hi	-	all	NAmer	1972	CC	731	n	bl	y	n	0	all/unsp	1	20	0	0	nev any	st
PIKE	6	f	0	0	w-hi	-	all	NAmer	1972	CC	731	n	bl	y	n	0	all/unsp	21	40	0	0	nev any	st
PIKE	7	f	0	0	w-hi	-	all	NAmer	1972	CC	731	n	bl	y	n	0	all/unsp	41	99	3	6	nev any	st
POLEDN	2	c	0	0	all	-	all	NAmer	1978	CC	209	n	bl	y	n	0	cig+/-ot	1	19	1	0	nev cigs	st
POLEDN	4	c	0	0	all	-	all	NAmer	1978	CC	209	n	bl	y	n	0	cig+/-ot	20	99	0	0	nev cigs	st
RACHTA	10	f	0	0	all	-	all	Eu:est	1991	CC	118	n	bl	n	y	1	cig+/-ot	1	9	1	1	nev cigs	or
RACHTA	11	f	0	0	all	-	all	Eu:est	1991	CC	118	n	bl	n	y	1	cig+/-ot	10	19	0	2	nev cigs	or
RACHTA	12	f	0	0	all	-	all	Eu:est	1991	CC	118	n	bl	n	y	1	cig+/-ot	20	99	0	0	nev cigs	or
RANDIG	1	m	0	0	all	-	all	Eu:Ger	1951	CC	448	n	bl	n	n	0	all/unsp	1	4	0	1	nev any	st
RANDIG	2	m	0	0	all	-	all	Eu:Ger	1951	CC	448	n	bl	n	n	0	all/unsp	5	9	1	0	nev any	st
RANDIG	3	m	0	0	all	-	all	Eu:Ger	1951	CC	448	n	bl	n	n	0	all/unsp	10	19	0	2	nev any	st
RANDIG	4	m	0	0	all	-	all	Eu:Ger	1951	CC	448	n	bl	n	n	0	all/unsp	20	99	0	0	nev any	st
RANDIG	5	f	0	0	all	-	all	Eu:Ger	1951	CC	448	n	bl	n	n	0	all/unsp	1	4	0	1	nev any	st
RANDIG	6	f	0	0	all	-	all	Eu:Ger	1951	CC	448	n	bl	n	n	0	all/unsp	5	9	1	0	nev any	st
RANDIG	7	f	0	0	all	-	all	Eu:Ger	1951	CC	448	n	bl	n	n	0	all/unsp	10	99	0	0	nev any	st
SHAW	10	c	0	0	wh	-	all	NAmer	1988	CC	335	n	V	n	y	0	all/unsp	1	19	1	0	nev any	st
SHAW	11	c	0	0	wh	-	all	NAmer	1988	CC	335	n	V	n	y	0	all/unsp	20	99	0	0	nev any	st
SIEMIA	13	m	0	0	all	-	all	NAmer	1979	CC	857	n	V	y	y	0	cig+/-ot	1	19	1	0	nev cigs	or
SIEMIA	14	m	0	0	all	-	all	NAmer	1979	CC	857	n	V	y	y	0	cig+/-ot	20	39	2	0	nev cigs	or
SIEMIA	15	m	0	0	all	-	all	NAmer	1979	CC	857	n	V	y	y	0	cig+/-ot	40	99	3	0	nev cigs	or
SPITZ	5	c	0	0	b+hi	-	all	NAmer	1992	CC	177	n	bl	n	y	0	cig+/-ot	1	19	1	0	nev cigs	st
SPITZ	6	c	0	0	b+hi	-	all	NAmer	1992	CC	177	n	bl	n	y	0	cig+/-ot	20	99	0	0	nev cigs	st
STOCKS	41	m	0	0	all	-	all	Eu:UK	1952	CC	2932	n	V	y	n	2	cig+/-ot	1	14	1	0	nev any	st
STOCKS	42	m	0	0	all	-	all	Eu:UK	1952	CC	2932	n	V	y	n	2	cig+/-ot	15	21	2	3	nev any	st
STOCKS	43	m	0	0	all	-	all	Eu:UK	1952	CC	2932	n	V	y	n	2	cig+/-ot	22	28	0	0	nev any	st
STOCKS	44	m	0	0	all	-	all	Eu:UK	1952	CC	2932	n	V	y	n	2	cig+/-ot	29	36	0	4	nev any	st
STOCKS	45	m	0	0	all	-	all	Eu:UK	1952	CC	2932	n	V	y	n	2	cig+/-ot	37	99	3	0	nev any	st
STOCKS	48	f	0	0	all	-	all	Eu:UK	1952	CC	2932	n	V	y	n	1	cig+/-ot	1	14	1	0	nev any	ot
STOCKS	49	f	0	0	all	-	all	Eu:UK	1952	CC	2932	n	V	y	n	1	cig+/-ot	15	99	0	0	nev any	ot
TIZZAN	7	m	0	0	all	-	all	Eu:wst	1959	CC	1358	n	bl	n	n	0	cig only	1	9	1	1	nev any	st
TIZZAN	8	m	0	0	all	-	all	Eu:wst	1959	CC	1358	n	bl	n	n	0	cig only	10	20	2	0	nev any	st
TIZZAN	9	m	0	0	all	-	all	Eu:wst	1959	CC	1358	n	bl	n	n	0	cig only	21	40	0	0	nev any	st
TIZZAN	10	m	0	0	all	-	all	Eu:wst	1959	CC	1358	n	bl	n	n	0	cig only	41	99	3	6	nev any	st
TIZZAN	15	f	0	0	all	-	all	Eu:wst	1959	CC	1358	n	bl	n	n	0	cig only	1	9	1	1	nev any	st
TIZZAN	16	f	0	0	all	-	all	Eu:wst	1959	CC	1358	n	bl	n	n	0	cig only	10	99	0	0	nev any	st
WANG2	9	c	0	0	all	-	all	As:Chi	1980	CC	103	n	ot	n	n	4	cig+/-ot	1	4	0	1	nev cigs	ot
WANG2	10	c	0	0	all	-	all	As:Chi	1980	CC	103	n	ot	n	n	4	cig+/-ot	5	9	1	0	nev cigs	ot
WANG2	11	c	0	0	all	-	all	As:Chi	1980	CC	103	n	ot	n	n	4	cig+/-ot	10	14	0	2	nev cigs	ot
WANG2	12	c	0	0	all	-	all	As:Chi	1980	CC	103	n	ot	n	n	4	cig+/-ot	15	19	0	0	nev cigs	ot
WANG2	13	c	0	0	all	-	all	As:Chi	1980	CC	103	n	ot	n	n	4	cig+/-ot	20	29	2	3	nev cigs	ot
WANG2	14	c	0	0	all	-	all	As:Chi	1980	CC	103	n	ot	n	n	4	cig+/-ot	30	39	0	4	nev cigs	ot
WANG2	15	c	0	0	all	-	all	As:Chi	1980	CC	103	n	ot	n	n	4	cig+/-ot	40	99	3	0	nev cigs	ot
WUWILL	12	f	0	0	all	-	all	As:Chi	1985	CC	965	n	ot	n	n	3	cig+/-ot	1	19	1	0	nev cigs	ot
WUWILL	13	f	0	0	all	-	all	As:Chi	1985	CC	965	n	ot	n	n	3	cig+/-ot	20	99	0	0	nev cigs	ot
WYNDE2	17	m	0	0	all	-	all	NAmer	1962	CC	404	n	bl	n	y	0	cig+/-ot	1	10	1	0	nev any	st
WYNDE2	18	m	0	0	all	-	all	NAmer	1962	CC	404	n	bl	n	y	0	cig+/-ot	11	20	2	3	nev any	st
WYNDE2	19	m	0	0	all	-	all	NAmer	1962	CC	404	n	bl	n	y	0	cig+/-ot	21	34	0	4	nev any	st
WYNDE2	20	m	0	0	all	-	all	NAmer	1962	CC	404	n	bl	n	y	0	cig+/-ot	35	99	3	0	nev any	st
WYNDE3	44	m	0	0	all	-	all	NAmer	1966	CC	350	n	bl	n	y	0	cig+/-ot	1	9	1	1	nev any	st
WYNDE3	45	m	0	0	all	-	all	NAmer	1966	CC	350	n	bl	n	y	0	cig+/-ot	10	20	2	0	nev any	st
WYNDE3	46	m	0	0	all	-	all	NAmer	1966	CC	350	n	bl	n	y	0	cig+/-ot	21	40	0	0	nev any	st
WYNDE3	47	m	0	0	all	-	all	NAmer	1966	CC	350	n	bl	n	y	0	cig+/-ot	41	99	3	6	nev any	st
WYNDE3	79	f	0	0	all	-	all	NAmer	1966	CC	350	n	bl	n	y	0	cig+/-ot	1	9	1	1	nev any	st
WYNDE3	80	f	0	0	all	-	all	NAmer	1966	CC	350	n	bl	n	y	0	cig+/-ot	10	20	2	0	nev any	st
WYNDE3	81	f	0	0	all	-	all	NAmer	1966	CC	350	n	bl	n	y	0	cig+/-ot	21	40	0	0	nev any	st
WYNDE3	82	f	0	0	all	-	all	NAmer	1966	CC	350	n	bl	n	y	0	cig+/-ot	41	99	3	6	nev any	st
WYNDE4	43	m	0	0	all	-	all	NAmer	1948	CC	684	n	bl	y	n	0	all/unsp	1	9	1	1	nev any	st
WYNDE4	44	m	0	0	all	-	all	NAmer	1948	CC	684	n	bl	y	n	0	all/unsp	10	15	0	2	nev any	st
WYNDE4	45	m	0	0	all	-	all	NAmer	1948	CC	684	n	bl	y	n	0	all/unsp	16	20	2	3	nev any	st
WYNDE4	46	m	0	0	all	-	all	NAmer	1948	CC	684	n	bl	y	n	0	all/unsp	21	34	0	4	nev any	st
WYNDE4	47	m	0	0	all	-	all	NAmer	1948	CC	684	n	bl	y	n	0	all/unsp	35	99	3	0	nev any	st
WYNDE4	57	f	0	0	all	-	all	NAmer	1948	CC	684	n	bl	y	n	2	all/unsp	1	9	1	1	nev any	ot
WYNDE4	58	f	0	0	all	-	all	NAmer	1948	CC	684	n	bl	y	n	2	all/unsp	10	15	0	2	nev any	ot
WYNDE4	59	f	0	0	all	-	all	NAmer	1948	CC	684	n	bl	y	n	2	all/unsp	16	20	2	3	nev any	ot
WYNDE4	60	f	0	0	all	-	all	NAmer	1948	CC	684	n	bl	y	n	2	all/unsp	21	34	0	4	nev any	ot
WYNDE4	61	f	0	0	all	-	all	NAmer	1948	CC	684	n	bl	y	n	2	all/unsp	35	99	3	0	nev any	ot
XU3	9	m	0	0	all	-	all	As:Chi	1981	CC	135	n	ot	n	n	1	all/unsp	1	9	1	1	nev any	ot
XU3	10	m	0	0	all	-	all	As:Chi	1981	CC	135	n	ot	n	n	1	all/unsp	10	19	0	2	nev any	ot
XU3	11	m	0	0	all	-	all	As:Chi	1981	CC	135	n	ot	n	n	1	all/unsp	20	29	2	3	nev any	ot

Table 1G1 - 1

IESLC - Meta-analysis of Ever Smoking by Amount, Overview, Any product (or Cigarettes if Any not available)
All LC types
Most adjusted

REF	NRR	SEX	AGEL	AGEH	RACE	YF	LC TYPE	LOC	START	ST	NLC	R	VB	P	H	AD	PRODUCT	exL	exH	S1	S2	DENOM	De
XU3	12	m	0	0	all	-	all	As:Chi	1981	CC	135	n	ot	n	n	1	all/unsp	30	99	3	0	nev any	ot
XU3	16	f	0	0	all	-	all	As:Chi	1981	CC	135	n	ot	n	n	1	all/unsp	1	9	1	1	nev any	ot
XU3	17	f	0	0	all	-	all	As:Chi	1981	CC	135	n	ot	n	n	1	all/unsp	10	19	0	2	nev any	ot
XU3	18	f	0	0	all	-	all	As:Chi	1981	CC	135	n	ot	n	n	1	all/unsp	20	99	0	0	nev any	ot
YUAN	2	m	0	0	all	0	all	As:Chi	1986	pr	142	n	ot	n	n	2	cig+/-ot	1	19	1	0	nev cigs	ot
YUAN	3	m	0	0	all	0	all	As:Chi	1986	pr	142	n	ot	n	n	2	cig+/-ot	20	99	0	0	nev cigs	ot
ZHENG	11	m	0	0	all	-	all	As:Chi	1982	CC	540	n	ot	*	y	0	cig+/-ot	1	9	1	1	nev cigs	st
ZHENG	12	m	0	0	all	-	all	As:Chi	1982	CC	540	n	ot	*	y	0	cig+/-ot	10	19	0	2	nev cigs	st
ZHENG	13	m	0	0	all	-	all	As:Chi	1982	CC	540	n	ot	*	y	0	cig+/-ot	20	29	2	3	nev cigs	st
ZHENG	14	m	0	0	all	-	all	As:Chi	1982	CC	540	n	ot	*	y	0	cig+/-ot	30	99	3	0	nev cigs	st
ZHENG	22	f	0	0	all	-	all	As:Chi	1982	CC	540	n	ot	*	y	0	cig+/-ot	1	9	1	1	nev cigs	st
ZHENG	23	f	0	0	all	-	all	As:Chi	1982	CC	540	n	ot	*	y	0	cig+/-ot	10	99	0	0	nev cigs	st
ZHOU	4	c	0	0	all	-	all	As:Chi	1978	CC	1360	n	ot	n	n	0	all/unsp	1	9	1	1	nev any	st
ZHOU	5	c	0	0	all	-	all	As:Chi	1978	CC	1360	n	ot	n	n	0	all/unsp	10	19	0	2	nev any	st
ZHOU	6	c	0	0	all	-	all	As:Chi	1978	CC	1360	n	ot	n	n	0	all/unsp	20	99	0	0	nev any	st

Cigarette type is all/unspec for all RRs
except for the following:

REF	NRR	CIGTYPE
ALDERS	18	MC only
ALDERS	19	MC only
ALDERS	20	MC only
ALDERS	21	MC only
ALDERS	22	MC only
ALDERS	23	MC only
JUSSAW	34	MC only
JUSSAW	35	MC only
NOTAN2	8	MC only
NOTAN2	9	MC only
NOTAN2	10	MC only

REF	NRR	Cigarette equivalent
AGUDO	4	-
AGUDO	5	-
ALDERS	18	-
ALDERS	19	-
ALDERS	20	-
ALDERS	21	-
ALDERS	22	-
ALDERS	23	-
ARMADA	46	*
ARMADA	47	*
ARMADA	48	*
AUVINE	13	*
AUVINE	14	*
AUVINE	15	*
AXELSS	5	includes 1 g pipe tob = 1 cig
AXELSS	6	includes 1 g pipe tob = 1 cig
AXELSS	7	includes 1 g pipe tob = 1 cig
AXELSS	13	includes 1 g pipe tob = 1 cig
AXELSS	14	includes 1 g pipe tob = 1 cig
AXELSS	15	includes 1 g pipe tob = 1 cig
AXELSS	16	includes 1 g pipe tob = 1 cig
BARBON	82	*
BARBON	83	*
BARBON	84	*
BOUCOT	99	up to 1 pk cigs, 4 cigars or 10 pipes
BOUCOT	100	> 1 pk cigs, 4 cigars or 10 pipes
BRESLO	13	*
BRESLO	14	*
BRESLO	15	*
BRESLO	16	*
BRESLO	29	*
BRESLO	30	*
BROWN2	32	*
BROWN2	42	*
BROWN2	31	*
BROWN2	41	*

Table 1G1 - 1

IESLC - Meta-analysis of Ever Smoking by Amount, Overview, Any product (or Cigarettes if Any not available)
All LC types
Most adjusted

REF	NRR	Cigarette equivalent
BUFFLE	28	*
BUFFLE	29	*
BUFFLE	35	*
CHATZI	1	*
CHATZI	2	*
CHATZI	3	*
CHEN2	3	*
CHEN2	4	*
CHEN2	5	*
CHEN2	6	*
CHEN2	7	*
CHEN2	8	*
CHEN2	9	*
CHEN2	10	*
CHOI	12	*
CHOI	13	*
CHOI	14	*
CHOI	15	*
CHOI	16	*
CHOI	17	*
CHOI	18	*
CHOI	20	*
COOKSO	1	*
COOKSO	2	*
CPSI	243	-
CPSI	246	-
CPSII	102	-
CPSII	103	-
CPSII	105	*
CPSII	106	*
DAMBER	6	-
DAMBER	7	-
DAMBER	8	-
DAMBER	9	-
DAVEYS	1	1 cigar or up to 5 cigs
DAVEYS	2	2 cigars or 6-10 cigs
DAVEYS	3	3-4 cigars or 11-20 cigs
DAVEYS	4	>4 cigars or >20 cigarettes
DEAN	1	-
DEAN	2	-
DEAN	3	-
DEAN2	25	-
DEAN2	26	-
DEAN2	29	-
DEAN2	30	-
DESTEF	6	*
DESTEF	7	*
DESTEF	8	*
DESTEF	9	*
DOLL	1	*
DOLL	2	*
DOLL	3	*
DOLL	4	*
DOLL	5	*
DOLL	7	*
DOLL	8	*
DOLL	9	*
DOLL	10	*
DOLL2	46	grams
DOLL2	47	grams
DOLL2	48	grams
DORGAN	108	*
DORGAN	109	*
DORGAN	96	*
DORGAN	97	*
DOSEME	5	*
DOSEME	9	*
DOSEME	13	*
DUNN	1	*
DUNN	2	*
DUNN	3	*
DUNN	4	*

Table 1G1 - 1

IESLC - Meta-analysis of Ever Smoking by Amount, Overview, Any product (or Cigarettes if Any not available)
All LC types
Most adjusted

REF	NRR	Cigarette equivalent
DUNN	5	*
EBELIN	2	*
EBELIN	3	*
EBELIN	4	*
EBELIN	5	*
EBELIN	6	*
ESAKI	1	*
ESAKI	2	*
ESAKI	3	*
FAN	6	*
FAN	7	*
FAN	8	*
FAN	9	*
FAN	10	*
FAN	11	*
FAN	12	*
FAN	13	*
GAO	21	*
GAO	22	*
GAO	23	*
GARSHI	26	*
GARSHI	27	*
GARSHI	28	*
GARSHI	29	*
GER	22	*
GER	23	*
GER	24	*
GOLLED	1	-
GOLLED	2	-
GOLLED	3	-
GSELL	1	inc cigar = 5, cheroot = 4, pipe = 2.5
GSELL	2	inc cigar = 5, cheroot = 4, pipe = 2.5
GSELL	3	inc cigar = 5, cheroot = 4, pipe = 2.5
GSELL	4	inc cigar = 5, cheroot = 4, pipe = 2.5
GSELL	5	inc cigar = 5, cheroot = 4, pipe = 2.5
HAMMON	153	-
HAMMON	154	-
HAMMON	155	-
HANSEN	1	cig equivalents (not defined)
HANSEN	2	cig equivalents (not defined)
HU	1	*
HU	2	*
HU	3	*
HU	4	*
HU	5	*
HU	6	*
HU2	2	*
HU2	3	*
HU2	4	*
HU2	5	*
HU2	6	*
HU2	7	*
JARUP	4	gms, inc 1 pk pipe/wk = 7/d, 1 cigar = 4
JARUP	5	gms, inc 1 pk pipe/wk = 7/d, 1 cigar = 4
JEDRYC	45	*
JEDRYC	46	*
JEDRYC	47	*
JEDRYC	48	*
JEDRYC	49	*
JEDRYC	50	*
JOLY	7	*
JOLY	8	*
JOLY	9	*
JOLY	10	*
JOLY	3	*
JOLY	4	*
JOLY	5	*
JOLY	6	*
JUSSAW	34	-
JUSSAW	35	-
KHUDER	1	*
KHUDER	2	*

Table 1G1 - 1

IESLC - Meta-analysis of Ever Smoking by Amount, Overview, Any product (or Cigarettes if Any not available)
All LC types
Most adjusted

REF	NRR	Cigarette equivalent
KHUDER	3	*
KOULUM	6	*
KOULUM	5	*
KOULUM	4	*
KREUZE	19	*
KREUZE	20	*
KREUZE	21	*
KREUZE	22	*
KREUZE	30	*
KREUZE	31	*
KREUZE	32	*
KREUZE	33	*
KREUZE	25	*
KREUZE	26	*
KREUZE	27	*
KREUZE	36	*
KREUZE	37	*
KREUZE	38	*
KREYBE	9	grams inc 1 cig=1
KREYBE	10	grams inc 1 cig=1
KREYBE	11	grams inc 1 cig=1
KREYBE	28	grams inc 1 cig=1
KREYBE	29	grams inc 1 cig=1
LAMTH	7	*
LAMTH	2	*
LAMTH	9	*
LAUSSM	18	inc cigars and pipes in grams
LAUSSM	19	inc cigars and pipes in grams
LAUSSM	20	inc cigars and pipes in grams
LETOUR	2	*
LETOUR	3	*
LETOUR	4	*
LIU2	8	*
LIU2	9	*
LIU2	10	*
LIU2	14	*
LIU2	15	*
LIU2	16	*
LIU3	6	Converted from kg/month
LIU3	7	Converted from kg/month
LIU3	8	Converted from kg/month
LIU4	7	-
LIU4	8	-
LIU4	9	-
LIU5	2	*
LIU5	3	*
LIU5	4	*
LUBIN	11	-
LUBIN	12	-
LUBIN	13	-
LUBIN	14	-
LUBIN2	273	*
LUBIN2	274	*
LUBIN2	275	*
LUBIN2	276	*
LUBIN2	281	*
LUBIN2	282	*
LUBIN2	283	*
LUBIN2	284	*
MACLEN	36	*
MACLEN	37	*
MACLEN	38	*
MACLEN	39	*
MARTIS	1	*
MARTIS	2	*
MARTIS	3	*
MATOS	29	*
MATOS	31	*
MATOS	33	*
MATSUD	1	*
MATSUD	2	*
MATSUD	3	*

Table 1G1 - 1

IESLC - Meta-analysis of Ever Smoking by Amount, Overview, Any product (or Cigarettes if Any not available)
All LC types
Most adjusted

REF	NRR	Cigarette equivalent
MCCONN	26	N cigs exc mixed pipe, or <2oz pure pipe
MCCONN	25	N cigs exc mixed pipe, or 2-4oz pure pip
MCCONN	24	N cigs exc mixed pipe, or >4oz pure pipe
NOTAN2	8	-
NOTAN2	9	-
NOTAN2	10	-
ORMOS	1	*
ORMOS	2	*
ORMOS	3	*
OSANN	49	*
OSANN	57	*
OSANN	50	*
OSANN	58	*
OSANN2	22	*
OSANN2	23	*
PASTOR	6	*
PASTOR	7	*
PASTOR	8	*
PASTOR	9	*
PERNU	17	grams
PERNU	18	grams
PERNU	19	grams
PERNU	20	grams
PERNU	21	grams
PERNU	22	grams
PERNU	23	grams
PERNU	24	grams
PERNU	11	grams
PERNU	12	grams
PERNU	13	grams
PERNU	14	grams
PERNU	15	grams
PERNU	16	grams
PIKE	1	*
PIKE	2	*
PIKE	3	*
PIKE	5	*
PIKE	6	*
PIKE	7	*
POLEDN	2	*
POLEDN	4	*
RACHTA	10	*
RACHTA	11	*
RACHTA	12	*
RANDIG	1	inc 1g pip=1, cgr=5, chrt=4, cigarillo=3
RANDIG	2	inc 1g pip=1, cgr=5, chrt=4, cigarillo=3
RANDIG	3	inc 1g pip=1, cgr=5, chrt=4, cigarillo=3
RANDIG	4	inc 1g pip=1, cgr=5, chrt=4, cigarillo=3
RANDIG	5	inc 1g pip=1, cgr=5, chrt=4, cigarillo=3
RANDIG	6	inc 1g pip=1, cgr=5, chrt=4, cigarillo=3
RANDIG	7	inc 1g pip=1, cgr=5, chrt=4, cigarillo=3
SHAW	10	*
SHAW	11	*
SIEMIA	13	*
SIEMIA	14	*
SIEMIA	15	*
SPITZ	5	*
SPITZ	6	*
STOCKS	41	*
STOCKS	42	*
STOCKS	43	*
STOCKS	44	*
STOCKS	45	*
STOCKS	48	*
STOCKS	49	*
TIZZAN	7	-
TIZZAN	8	-
TIZZAN	9	-
TIZZAN	10	-
TIZZAN	15	-
TIZZAN	16	-
WANG2	9	*

Table 1G1 - 1

IESLC - Meta-analysis of Ever Smoking by Amount, Overview, Any product (or Cigarettes if Any not available)
All LC types
Most adjusted

REF	NRR	Cigarette equivalent
WANG2	10	*
WANG2	11	*
WANG2	12	*
WANG2	13	*
WANG2	14	*
WANG2	15	*
WUWILL	12	*
WUWILL	13	*
WYNDE2	17	*
WYNDE2	18	*
WYNDE2	19	*
WYNDE2	20	*
WYNDE3	44	*
WYNDE3	45	*
WYNDE3	46	*
WYNDE3	47	*
WYNDE3	79	*
WYNDE3	80	*
WYNDE3	81	*
WYNDE3	82	*
WYNDE4	43	inc 1 cigar = 5 cigs, 1 pipe = 2.5 cigs
WYNDE4	44	inc 1 cigar = 5 cigs, 1 pipe = 2.5 cigs
WYNDE4	45	inc 1 cigar = 5 cigs, 1 pipe = 2.5 cigs
WYNDE4	46	inc 1 cigar = 5 cigs, 1 pipe = 2.5 cigs
WYNDE4	47	inc 1 cigar = 5 cigs, 1 pipe = 2.5 cigs
WYNDE4	57	inc 1 cigar = 5 cigs, 1 pipe = 2.5 cigs
WYNDE4	58	inc 1 cigar = 5 cigs, 1 pipe = 2.5 cigs
WYNDE4	59	inc 1 cigar = 5 cigs, 1 pipe = 2.5 cigs
WYNDE4	60	inc 1 cigar = 5 cigs, 1 pipe = 2.5 cigs
WYNDE4	61	inc 1 cigar = 5 cigs, 1 pipe = 2.5 cigs
XU3	9	*
XU3	10	*
XU3	11	*
XU3	12	*
XU3	16	*
XU3	17	*
XU3	18	*
YUAN	2	*
YUAN	3	*
ZHENG	11	*
ZHENG	12	*
ZHENG	13	*
ZHENG	14	*
ZHENG	22	*
ZHENG	23	*
ZHOU	4	*
ZHOU	5	*
ZHOU	6	*

In this overview table, subtotals and Qs values may be invalid and should be ignored

Table 1G1 - 2

IESLC - Meta-analysis of Ever Smoking by Amount, Overview, Any product (or Cigarettes if Any not available)
All LC types
Most adjusted

REF	NRR	SEX	AD	Number Exposed Case	Number Exposed Cont	Non-exposed Case	Non-exposed Cont	RR	95.00%CI
AGUDO	4	f	3	-	-	-	-	1.57	(0.52- 4.70)
AGUDO	5	f	3	-	-	-	-	4.94	(1.86- 13.09)
Subtotal AGUDO								2.98	(1.44- 6.19)
ALDERS	18	m	1	-	-	-	-	3.55	(1.94- 6.49)
ALDERS	19	m	1	-	-	-	-	7.96	(4.63- 13.69)
ALDERS	20	m	1	-	-	-	-	8.52	(5.07- 14.33)
ALDERS	21	f	1	-	-	-	-	2.62	(1.88- 3.65)
ALDERS	22	f	1	-	-	-	-	5.28	(3.79- 7.36)
ALDERS	23	f	1	-	-	-	-	6.90	(4.69- 10.15)
Subtotal ALDERS								4.91	(4.15- 5.82)
ARMADA	46	m	0	44	117	4	64	6.02	(2.07- 17.51)
ARMADA	47	m	0	134	105	4	64	20.42	(7.20- 57.88)
ARMADA	48	m	0	139	32	4	64	69.50	(23.58- 204.81)
Subtotal ARMADA								20.25	(10.96- 37.40)
AUVINE	13	c	2	-	-	-	-	20.00	(9.72- 41.20)
AUVINE	14	c	2	-	-	-	-	33.90	(17.10- 67.00)
AUVINE	15	c	2	-	-	-	-	66.50	(25.80- 172.00)
Subtotal AUVINE								32.22	(20.76- 50.01)
AXELSS	5	m	6	-	-	-	-	3.82	(1.98- 7.36)
AXELSS	6	m	6	-	-	-	-	8.90	(4.91- 16.12)
AXELSS	7	m	6	-	-	-	-	10.40	(5.80- 18.66)
AXELSS	13	f	0	13	37	18	154	3.01	(1.35- 6.68)
AXELSS	14	f	0	63	50	18	154	10.78	(5.84- 19.91)
AXELSS	15	f	0	28	15	18	154	15.97	(7.21- 35.36)
AXELSS	16	f	0	6	7	18	154	7.33	(2.22- 24.22)
Subtotal AXELSS								7.81	(6.01- 10.15)
BARBON	82	m	3	-	-	-	-	6.70	(4.20- 11.00)
BARBON	83	m	3	-	-	-	-	12.80	(7.90- 21.00)
BARBON	84	m	3	-	-	-	-	21.30	(13.00- 36.00)
Subtotal BARBON								11.97	(9.01- 15.91)
*BOUCOT	99	m	0	38	2670	0	805	23.23~	(1.43- 377.62)
*BOUCOT	100	m	0	43	1519	0	805	46.12~	(2.84- 748.16)
Subtotal BOUCOT								32.74	(4.56- 235.00)
BRESLO	13	m	0	16	45	22	110	1.78	(0.86- 3.69)
BRESLO	14	m	0	69	105	22	110	3.29	(1.90- 5.69)
BRESLO	15	m	0	296	193	22	110	7.67	(4.69- 12.55)
BRESLO	16	m	0	80	22	22	110	18.18	(9.42- 35.09)
BRESLO	29	f	0	5	5	12	14	1.17	(0.27- 5.02)
BRESLO	30	f	0	8	6	12	14	1.56	(0.42- 5.76)
Subtotal BRESLO								5.03	(3.79- 6.65)
BROWN2	32	m	2	-	-	-	-	6.10	(5.30- 6.90)
BROWN2	42	m	2	-	-	-	-	14.10	(12.70- 15.50)
BROWN2	31	f	2	-	-	-	-	8.40	(7.20- 9.70)
BROWN2	41	f	2	-	-	-	-	17.10	(15.30- 19.10)
Subtotal BROWN2								11.59	(10.92- 12.30)
BUFFLE	28	f	0	21	42	12	112	4.67	(2.11- 10.31)
BUFFLE	29	f	0	76	60	12	112	11.82	(5.96- 23.45)
BUFFLE	35	f	0	141	62	12	112	21.23	(10.90- 41.32)
Subtotal BUFFLE								11.51	(7.65- 17.33)
CHATZI	1	c	0	68	127	27	129	2.56	(1.54- 4.25)
CHATZI	2	c	0	73	123	27	129	2.84	(1.71- 4.70)
CHATZI	3	c	0	114	115	27	129	4.74	(2.90- 7.72)
Subtotal CHATZI								3.28	(2.46- 4.38)
CHEN2	3	m	0	17	26	9	33	2.40	(0.92- 6.25)
CHEN2	4	m	0	44	50	9	33	3.23	(1.39- 7.48)
CHEN2	5	m	0	34	9	9	33	13.85	(4.89- 39.22)
CHEN2	6	m	0	26	12	9	33	7.94	(2.91- 21.72)
CHEN2	7	f	0	5	17	25	33	0.39	(0.13- 1.20)
CHEN2	8	f	0	22	10	25	33	2.90	(1.17- 7.22)
CHEN2	9	f	0	7	1	25	33	9.24	(1.07- 80.02)
CHEN2	10	f	0	4	2	25	33	2.64	(0.45- 15.58)
Subtotal CHEN2								3.36	(2.30- 4.90)
CHOI	12	m	0	20	90	13	95	1.62	(0.76- 3.46)
CHOI	13	m	0	144	281	13	95	3.74	(2.03- 6.92)
CHOI	14	m	0	50	49	13	95	7.46	(3.70- 15.03)
CHOI	15	m	0	37	39	13	95	6.93	(3.33- 14.44)
CHOI	16	m	0	16	6	13	95	19.49	(6.47- 58.71)
CHOI	17	f	0	9	16	76	164	1.21	(0.51- 2.87)
CHOI	18	f	0	7	9	76	164	1.68	(0.60- 4.68)
CHOI	20	f	0	3	1	76	164	6.47	(0.66- 63.26)
Subtotal CHOI								3.83	(2.86- 5.13)

Table 1G1 - 2

IESLC - Meta-analysis of Ever Smoking by Amount, Overview, Any product (or Cigarettes if Any not available)
All LC types
Most adjusted

REF	NRR	SEX	AD	Number Exposed Case	Number Exposed Cont	Non-exposed Case	Non-exposed Cont	RR	95.00%CI
COOKSO	1	c	0	102	27	45	61	5.12	(2.89- 9.08)
COOKSO	2	c	0	82	11	45	61	10.11	(4.83- 21.13)
Subtotal COOKSO								6.61	(4.21- 10.40)
*CPSI	243	m	1	-	-	-	-	5.81	(4.33- 7.80)
*CPSI	246	m	1	-	-	-	-	13.60	(10.46- 17.67)
Subtotal CPSI								9.33	(7.68- 11.35)
*CPSII	102	m	1	-	-	-	-	9.99	(7.97- 12.51)
*CPSII	103	m	1	-	-	-	-	17.60	(14.05- 22.05)
*CPSII	105	f	1	-	-	-	-	4.16	(3.41- 5.09)
*CPSII	106	f	1	-	-	-	-	13.34	(11.31- 15.75)
Subtotal CPSII								9.98	(9.03- 11.02)
DAMBER	6	m	1	-	-	-	-	2.30	(1.30- 4.40)
DAMBER	7	m	1	-	-	-	-	7.30	(4.40- 12.70)
DAMBER	8	m	1	-	-	-	-	9.10	(5.50- 15.30)
DAMBER	9	m	1	-	-	-	-	14.90	(6.70- 33.50)
Subtotal DAMBER								6.60	(4.92- 8.86)
DAVEYS	1	m	0	11	69	3	23	1.22	(0.31- 4.77)
DAVEYS	2	m	0	31	32	3	23	7.43	(2.02- 27.27)
DAVEYS	3	m	0	19	22	3	23	6.62	(1.72- 25.56)
DAVEYS	4	m	0	29	21	3	23	10.59	(2.81- 39.94)
Subtotal DAVEYS								5.12	(2.63- 9.98)
DEAN	1	m	0	73	168	12	61	2.21	(1.12- 4.35)
DEAN	2	m	0	228	172	12	61	6.74	(3.52- 12.91)
DEAN	3	m	0	102	45	12	61	11.52	(5.66- 23.47)
Subtotal DEAN								5.46	(3.69- 8.08)
DEAN2	25	m	0	377	396	33	112	3.23	(2.14- 4.88)
DEAN2	26	m	0	252	112	33	112	7.64	(4.88- 11.95)
DEAN2	29	f	0	44	24	88	121	2.52	(1.43- 4.45)
DEAN2	30	f	0	18	5	88	121	4.95	(1.77- 13.84)
Subtotal DEAN2								4.21	(3.25- 5.45)
DESTEF	6	m	4	-	-	-	-	2.90	(1.60- 5.00)
DESTEF	7	m	4	-	-	-	-	8.40	(5.20- 13.60)
DESTEF	8	m	4	-	-	-	-	10.40	(6.40- 16.90)
DESTEF	9	m	4	-	-	-	-	23.70	(13.40- 42.10)
Subtotal DESTEF								8.87	(6.83- 11.51)
DOLL	1	m	0	55	129	7	61	3.72	(1.60- 8.64)
DOLL	2	m	0	489	570	7	61	7.48	(3.39- 16.50)
DOLL	3	m	0	475	431	7	61	9.60	(4.35- 21.22)
DOLL	4	m	0	293	154	7	61	16.58	(7.40- 37.13)
DOLL	5	m	0	38	12	7	61	27.60	(9.99- 76.25)
DOLL	7	f	0	16	25	40	59	0.94	(0.45- 1.99)
DOLL	8	f	0	24	18	40	59	1.97	(0.95- 4.09)
DOLL	9	f	0	14	6	40	59	3.44	(1.22- 9.71)
DOLL	10	f	0	14	0	40	59	42.60~	(2.47- 734.58)
Subtotal DOLL								4.97	(3.72- 6.65)
*DOLL2	46	m	1	-	-	-	-	6.71	(0.91- 49.81)
*DOLL2	47	m	1	-	-	-	-	12.29	(1.67- 90.41)
*DOLL2	48	m	1	-	-	-	-	23.71	(3.25- 173.24)
Subtotal DOLL2								12.54	(3.96- 39.67)
DORGAN	108	m	2	-	-	-	-	6.88	(3.74- 12.66)
DORGAN	109	m	2	-	-	-	-	14.00	(7.81- 25.11)
DORGAN	96	f	3	-	-	-	-	5.67	(4.36- 7.36)
DORGAN	97	f	3	-	-	-	-	12.22	(9.31- 16.04)
Subtotal DORGAN								8.47	(7.13- 10.07)
DOSEME	5	m	2	-	-	-	-	2.20	(1.40- 3.30)
DOSEME	9	m	2	-	-	-	-	3.10	(2.30- 4.10)
DOSEME	13	m	2	-	-	-	-	6.60	(4.40- 10.20)
Subtotal DOSEME								3.44	(2.79- 4.24)
*DUNN	1	m	0	3	2538	2	14160	8.37	(1.40- 50.06)
*DUNN	2	m	0	12	9418	2	14160	9.02	(2.02- 40.30)
*DUNN	3	m	0	75	27720	2	14160	19.16	(4.70- 78.00)
*DUNN	4	m	0	32	9017	2	14160	25.13	(6.02- 104.82)
*DUNN	5	m	0	13	3206	2	14160	28.71	(6.48- 127.15)
Subtotal DUNN								16.88	(8.61- 33.09)
EBELIN	2	m	0	20	72	12	117	2.71	(1.25- 5.87)
EBELIN	3	m	0	19	26	12	117	7.13	(3.08- 16.48)
EBELIN	4	m	0	47	37	12	117	12.39	(5.95- 25.80)
EBELIN	5	m	0	5	3	12	117	16.25	(3.45- 76.54)
EBELIN	6	m	0	4	4	12	117	9.75	(2.16- 44.04)
Subtotal EBELIN								6.99	(4.62- 10.59)
ESAKI	1	m	0	47	75	16	28	1.10	(0.54- 2.24)

Table 1G1 - 2

IESLC - Meta-analysis of Ever Smoking by Amount, Overview, Any product (or Cigarettes if Any not available)
All LC types
Most adjusted

REF	NRR	SEX	AD	Number Exposed Case	Number Exposed Cont	Non-exposed Case	Non-exposed Cont	RR	95.00%CI
ESAKI	2	m	0	74	58	16	28	2.23	(1.10- 4.51)
ESAKI	3	m	0	34	10	16	28	5.95	(2.34- 15.16)
Subtotal ESAKI								2.12	(1.36- 3.29)
FAN	6	m	0	13	121	36	236	0.70	(0.36- 1.38)
FAN	7	m	0	53	171	36	236	2.03	(1.27- 3.24)
FAN	8	m	0	111	183	36	236	3.98	(2.61- 6.07)
FAN	9	m	0	39	23	36	236	11.12	(5.96- 20.73)
FAN	10	f	0	17	48	69	320	1.64	(0.89- 3.03)
FAN	11	f	0	30	37	69	320	3.76	(2.18- 6.50)
FAN	12	f	0	31	12	69	320	11.98	(5.86- 24.50)
FAN	13	f	0	4	1	69	320	18.55	(2.04- 168.54)
Subtotal FAN								3.29	(2.68- 4.05)
GAO	21	f	2	-	-	-	-	1.74	(1.22- 2.48)
GAO	22	f	2	-	-	-	-	3.04	(2.02- 4.58)
GAO	23	f	2	-	-	-	-	13.18	(7.04- 24.68)
Subtotal GAO								2.91	(2.28- 3.73)
GARSHI	26	m	1	-	-	-	-	3.29	(2.22- 4.87)
GARSHI	27	m	1	-	-	-	-	5.72	(4.04- 8.10)
GARSHI	28	m	1	-	-	-	-	7.69	(5.20- 11.37)
GARSHI	29	m	1	-	-	-	-	5.24	(3.61- 7.60)
Subtotal GARSHI								5.28	(4.38- 6.37)
GER	22	c	14	-	-	-	-	1.26	(0.61- 2.61)
GER	23	c	14	-	-	-	-	1.90	(0.98- 3.70)
GER	24	c	14	-	-	-	-	3.00	(1.40- 6.40)
Subtotal GER								1.90	(1.26- 2.88)
GOLLED	1	m	1	-	-	-	-	4.45	(2.54- 7.81)
GOLLED	2	m	1	-	-	-	-	6.37	(3.67- 11.03)
GOLLED	3	m	1	-	-	-	-	18.28	(10.52- 31.74)
Subtotal GOLLED								8.08	(5.87- 11.13)
GSELL	1	m	0	11	36	2	29	4.43	(0.91- 21.60)
GSELL	2	m	0	10	37	2	29	3.92	(0.80- 19.30)
GSELL	3	m	0	27	26	2	29	15.06	(3.26- 69.59)
GSELL	4	m	0	49	9	2	29	78.94	(15.95- 390.82)
GSELL	5	m	0	51	13	2	29	56.88	(11.99- 269.87)
Subtotal GSELL								16.45	(8.14- 33.23)
*HAMMON	153	m	1	-	-	-	-	7.38	(3.72- 14.63)
*HAMMON	154	m	1	-	-	-	-	8.32	(4.66- 14.84)
*HAMMON	155	m	1	-	-	-	-	17.06	(9.44- 30.82)
Subtotal HAMMON								10.42	(7.31- 14.85)
*HANSEN	1	m	2	-	-	-	-	1.37	(0.63- 3.54)
*HANSEN	2	m	2	-	-	-	-	2.90	(1.14- 8.27)
Subtotal HANSEN								1.89	(0.99- 3.63)
HU	1	m	0	36	38	41	67	1.55	(0.85- 2.82)
HU	2	m	0	55	43	41	67	2.09	(1.20- 3.65)
HU	3	m	0	29	13	41	67	3.65	(1.70- 7.80)
HU	4	f	0	19	10	40	48	2.28	(0.95- 5.46)
HU	5	f	0	6	6	40	48	1.20	(0.36- 4.01)
HU	6	f	0	1	2	40	48	0.60	(0.05- 6.86)
Subtotal HU								2.02	(1.47- 2.77)
HU2	2	c	0	16	33	121	213	0.85	(0.45- 1.61)
HU2	3	c	0	44	58	121	213	1.34	(0.85- 2.10)
HU2	4	c	0	65	59	121	213	1.94	(1.28- 2.94)
HU2	5	c	0	64	54	121	213	2.09	(1.36- 3.19)
HU2	6	c	0	149	87	121	213	3.01	(2.13- 4.26)
HU2	7	c	0	64	19	121	213	5.93	(3.39- 10.37)
Subtotal HU2								2.20	(1.83- 2.64)
JARUP	4	m	2	-	-	-	-	6.90	(2.40- 22.70)
JARUP	5	m	2	-	-	-	-	8.00	(3.00- 24.80)
Subtotal JARUP								7.46	(3.46- 16.11)
JEDRYC	45	m	4	-	-	-	-	3.48	(2.33- 5.19)
JEDRYC	46	m	4	-	-	-	-	6.16	(4.25- 8.90)
JEDRYC	47	m	4	-	-	-	-	7.69	(5.15- 11.47)
JEDRYC	48	f	4	-	-	-	-	6.37	(2.66- 15.24)
JEDRYC	49	f	4	-	-	-	-	2.38	(1.17- 6.86)
JEDRYC	50	f	4	-	-	-	-	7.37	(2.20- 24.69)
Subtotal JEDRYC								5.36	(4.35- 6.60)
JOLY	7	m	0	16	54	12	218	5.38	(2.41- 12.05)
JOLY	8	m	0	217	318	12	218	12.40	(6.76- 22.73)
JOLY	9	m	0	126	175	12	218	13.08	(7.00- 24.43)
JOLY	10	m	0	193	161	12	218	21.78	(11.74- 40.39)
JOLY	3	f	0	33	38	52	283	4.73	(2.72- 8.21)

Table 1G1 - 2

IESLC - Meta-analysis of Ever Smoking by Amount, Overview, Any product (or Cigarettes if Any not available)
All LC types
Most adjusted

REF	NRR	SEX	AD	Number Exposed Case	Cont	Non-exposed Case	Cont	RR	95.00%CI
JOLY	4	f	0	72	49	52	283	8.00	(5.01- 12.77)
JOLY	5	f	0	28	22	52	283	6.93	(3.68- 13.03)
JOLY	6	f	0	32	13	52	283	13.40	(6.59- 27.23)
Subtotal JOLY								9.35	(7.55- 11.59)
JUSSAW	34	m	2	-	-	-	-	5.57	(2.46- 14.76)
JUSSAW	35	m	2	-	-	-	-	14.00	(5.17- 53.16)
Subtotal JUSSAW								7.84	(3.86- 15.96)
KHUDER	1	m	0	81	434	23	309	2.51	(1.54- 4.07)
KHUDER	2	m	0	224	288	23	309	10.45	(6.61- 16.52)
KHUDER	3	m	0	154	63	23	309	32.84	(19.62- 54.97)
Subtotal KHUDER								9.12	(6.89- 12.06)
KOULUM	6	m	0	37	77	5	54	5.19	(1.92- 14.06)
KOULUM	5	m	0	208	94	5	54	23.90	(9.26- 61.67)
KOULUM	4	m	0	478	75	5	54	68.83	(26.67- 177.62)
Subtotal KOULUM								21.38	(12.26- 37.28)
KREUZE	19	m	3	-	-	-	-	2.50	(0.70- 8.20)
KREUZE	20	m	3	-	-	-	-	8.70	(3.50- 21.90)
KREUZE	21	m	3	-	-	-	-	19.50	(7.50- 50.30)
KREUZE	22	m	3	-	-	-	-	20.80	(7.20- 60.50)
KREUZE	30	m	3	-	-	-	-	8.20	(5.20- 13.00)
KREUZE	31	m	3	-	-	-	-	25.10	(16.20- 38.70)
KREUZE	32	m	3	-	-	-	-	32.80	(20.90- 51.40)
KREUZE	33	m	3	-	-	-	-	33.30	(20.50- 54.00)
KREUZE	25	f	3	-	-	-	-	5.70	(1.60- 16.60)
KREUZE	26	f	3	-	-	-	-	11.80	(3.50- 29.00)
KREUZE	27	f	3	-	-	-	-	12.10	(3.00- 48.00)
KREUZE	36	f	3	-	-	-	-	2.00	(1.20- 3.30)
KREUZE	37	f	3	-	-	-	-	5.40	(3.50- 8.60)
KREUZE	38	f	3	-	-	-	-	7.70	(3.50- 17.30)
Subtotal KREUZE								11.60	(9.81- 13.72)
KREYBE	9	m	1	-	-	-	-	5.82	(2.56- 13.23)
KREYBE	10	m	1	-	-	-	-	6.23	(2.66- 14.56)
KREYBE	11	m	1	-	-	-	-	14.31	(5.99- 34.17)
KREYBE	28	f	1	-	-	-	-	1.36	(0.65- 2.82)
KREYBE	29	f	1	-	-	-	-	1.91	(0.48- 7.55)
Subtotal KREYBE								4.29	(2.90- 6.34)
LAMTH	7	f	0	101	63	202	337	2.67	(1.87- 3.83)
LAMTH	2	f	0	90	28	202	337	5.36	(3.39- 8.48)
LAMTH	9	f	0	39	9	202	337	7.23	(3.43- 15.24)
Subtotal LAMTH								3.82	(2.93- 4.98)
LAUSSM	18	m	3	-	-	-	-	3.29	(2.31- 4.66)
LAUSSM	19	m	3	-	-	-	-	5.81	(4.16- 8.11)
LAUSSM	20	m	3	-	-	-	-	9.62	(6.05- 15.23)
Subtotal LAUSSM								5.24	(4.23- 6.49)
LETOUR	2	c	0	271	266	24	224	9.51	(6.04- 14.97)
LETOUR	3	c	0	367	198	24	224	17.30	(10.98- 27.27)
LETOUR	4	c	0	65	23	24	224	26.38	(13.98- 49.78)
Subtotal LETOUR								14.84	(11.14- 19.77)
LIU2	8	m	3	-	-	-	-	1.20	(0.43- 3.50)
LIU2	9	m	3	-	-	-	-	7.10	(2.60- 19.50)
LIU2	10	m	3	-	-	-	-	21.40	(7.10- 64.00)
LIU2	14	f	3	-	-	-	-	1.80	(0.57- 5.90)
LIU2	15	f	3	-	-	-	-	3.50	(1.20- 9.80)
LIU2	16	f	3	-	-	-	-	17.90	(4.00- 80.60)
Subtotal LIU2								4.74	(3.00- 7.48)
LIU3	6	m	2	-	-	-	-	1.41	(0.33- 6.09)
LIU3	7	m	2	-	-	-	-	1.09	(0.24- 4.82)
LIU3	8	m	2	-	-	-	-	1.91	(0.32- 11.40)
Subtotal LIU3								1.39	(0.56- 3.42)
LIU4	7	m	2	-	-	-	-	2.11	(2.02- 2.20)
LIU4	8	m	2	-	-	-	-	3.60	(3.49- 3.71)
LIU4	9	m	2	-	-	-	-	6.98	(6.73- 7.23)
Subtotal LIU4								3.95	(3.87- 4.03)
LIU5	2	c	0	14	27	26	41	0.82	(0.36- 1.84)
LIU5	3	c	0	21	21	26	41	1.58	(0.72- 3.44)
LIU5	4	c	0	50	22	26	41	3.58	(1.78- 7.23)
Subtotal LIU5								1.79	(1.16- 2.78)
LUBIN	11	m	4	-	-	-	-	0.72	(0.20- 2.54)
LUBIN	12	m	4	-	-	-	-	1.25	(0.43- 3.65)
LUBIN	13	m	4	-	-	-	-	6.50	(2.38- 17.78)
LUBIN	14	m	4	-	-	-	-	8.00	(3.42- 18.72)

Table 1G1 - 2

IESLC - Meta-analysis of Ever Smoking by Amount, Overview, Any product (or Cigarettes if Any not available)
All LC types
Most adjusted

REF	NRR	SEX	AD	Number Exposed Case	Number Exposed Cont	Non-exposed Case	Non-exposed Cont	RR	95.00%CI
Subtotal LUBIN								3.39	(2.04- 5.64)
LUBIN2	273	m	0	1887	3759	190	2616	6.91	(5.91- 8.09)
LUBIN2	274	m	0	1529	2771	190	2616	7.60	(6.47- 8.92)
LUBIN2	275	m	0	1963	2547	190	2616	10.61	(9.06- 12.44)
LUBIN2	276	m	0	1261	1394	190	2616	12.45	(10.55- 14.70)
LUBIN2	281	f	0	151	218	336	1188	2.45	(1.93- 3.11)
LUBIN2	282	f	0	221	213	336	1188	3.67	(2.93- 4.59)
LUBIN2	283	f	0	134	103	336	1188	4.60	(3.46- 6.11)
LUBIN2	284	f	0	45	33	336	1188	4.82	(3.03- 7.68)
Subtotal LUBIN2								7.07	(6.60- 7.58)
MACLEN	36	c	2	-	-	-	-	1.35	(0.64- 2.84)
MACLEN	37	c	2	-	-	-	-	2.66	(1.46- 4.81)
MACLEN	38	c	2	-	-	-	-	2.93	(1.57- 5.45)
MACLEN	39	c	2	-	-	-	-	4.10	(2.07- 8.15)
Subtotal MACLEN								2.65	(1.91- 3.67)
MARTIS	1	m	0	31	39	4	25	4.97	(1.56- 15.78)
MARTIS	2	m	0	91	87	4	25	6.54	(2.19- 19.55)
MARTIS	3	m	0	75	50	4	25	9.38	(3.08- 28.57)
Subtotal MARTIS								6.77	(3.55- 12.94)
MATOS	29	m	2	-	-	-	-	2.00	(0.90- 4.50)
MATOS	31	m	2	-	-	-	-	7.50	(3.70- 15.00)
MATOS	33	m	2	-	-	-	-	10.40	(5.30- 20.70)
Subtotal MATOS								5.94	(3.91- 9.02)
MATSUD	1	m	0	37	1237	3	1255	12.51	(3.85- 40.69)
MATSUD	2	m	0	75	1607	3	1255	19.52	(6.14- 62.05)
MATSUD	3	m	0	58	470	3	1255	51.62	(16.10- 165.55)
Subtotal MATSUD								23.37	(11.91- 45.84)
MCCONN	26	c	0	7	43	9	23	0.42	(0.14- 1.26)
MCCONN	25	c	0	49	92	9	23	1.36	(0.58- 3.17)
MCCONN	24	c	0	35	42	9	23	2.13	(0.87- 5.19)
Subtotal MCCONN								1.21	(0.71- 2.08)
NOTAN2	8	m	0	6	42	134	544	0.58	(0.24- 1.39)
NOTAN2	9	m	0	28	47	134	544	2.42	(1.46- 4.01)
NOTAN2	10	m	0	44	40	134	544	4.47	(2.80- 7.13)
Subtotal NOTAN2								2.66	(1.93- 3.66)
ORMOS	1	m	0	32	329	7	777	10.80	(4.72- 24.71)
ORMOS	2	m	0	40	577	7	777	7.69	(3.42- 17.30)
ORMOS	3	m	0	15	128	7	777	13.01	(5.20- 32.52)
Subtotal ORMOS								10.06	(6.17- 16.42)
OSANN	49	m	2	-	-	-	-	17.70	(12.60- 24.80)
OSANN	57	m	2	-	-	-	-	42.80	(30.50- 60.10)
OSANN	50	f	2	-	-	-	-	14.40	(11.00- 18.90)
OSANN	58	f	2	-	-	-	-	40.90	(29.30- 57.10)
Subtotal OSANN								24.11	(20.59- 28.24)
OSANN2	22	f	1	-	-	-	-	2.50	(1.20- 5.20)
OSANN2	23	f	1	-	-	-	-	12.60	(6.20- 25.60)
Subtotal OSANN2								5.77	(3.46- 9.60)
PASTOR	6	m	1	-	-	-	-	2.33	(0.78- 6.95)
PASTOR	7	m	1	-	-	-	-	6.42	(2.96- 13.92)
PASTOR	8	m	1	-	-	-	-	8.02	(3.84- 16.73)
PASTOR	9	m	1	-	-	-	-	8.61	(3.86- 19.19)
Subtotal PASTOR								6.44	(4.27- 9.72)
PERNU	17	m	0	15	15	97	275	2.84	(1.34- 6.01)
PERNU	18	m	0	61	31	97	275	5.58	(3.42- 9.11)
PERNU	19	m	0	224	96	97	275	6.62	(4.74- 9.23)
PERNU	20	m	0	127	67	97	275	5.37	(3.69- 7.82)
PERNU	21	m	0	478	138	97	275	9.82	(7.28- 13.24)
PERNU	22	m	0	361	54	97	275	18.95	(13.12- 27.38)
PERNU	23	m	0	40	23	97	275	4.93	(2.81- 8.66)
PERNU	24	m	0	74	14	97	275	14.99	(8.09- 27.75)
PERNU	11	f	0	3	14	110	971	1.89	(0.54- 6.68)
PERNU	12	f	0	5	13	110	971	3.40	(1.19- 9.70)
PERNU	13	f	0	4	30	110	971	1.18	(0.41- 3.40)
PERNU	14	f	0	1	14	110	971	0.63	(0.08- 4.84)
PERNU	15	f	0	1	11	110	971	0.80	(0.10- 6.27)
PERNU	16	f	0	5	7	110	971	6.31	(1.97- 20.20)
Subtotal PERNU								7.42	(6.44- 8.54)
PIKE	1	m	0	181	168	18	69	4.13	(2.36- 7.23)
PIKE	2	m	0	228	109	18	69	8.02	(4.55- 14.13)
PIKE	3	m	0	66	37	18	69	6.84	(3.55- 13.18)
PIKE	5	f	0	73	60	36	96	3.24	(1.94- 5.42)

Table 1G1 - 2

IESLC - Meta-analysis of Ever Smoking by Amount, Overview, Any product (or Cigarettes if Any not available)
All LC types
Most adjusted

REF	NRR	SEX	AD	Number Exposed Case	Number Exposed Cont	Non-exposed Case	Non-exposed Cont	RR	95.00%CI
PIKE	6	f	0	67	26	36	96	6.87	(3.80- 12.44)
PIKE	7	f	0	16	3	36	96	14.22	(3.91- 51.73)
Subtotal PIKE								5.49	(4.27- 7.05)
POLEDN	2	c	0	53	103	12	139	5.96	(3.03- 11.72)
POLEDN	4	c	0	143	168	12	139	9.86	(5.25- 18.52)
Subtotal POLEDN								7.80	(4.92- 12.37)
RACHTA	10	f	1	-	-	-	-	3.64	(1.08- 12.32)
RACHTA	11	f	1	-	-	-	-	3.55	(1.75- 7.23)
RACHTA	12	f	1	-	-	-	-	13.77	(6.50- 29.16)
Subtotal RACHTA								6.13	(3.81- 9.85)
RANDIG	1	m	0	13	28	5	22	2.04	(0.63- 6.60)
RANDIG	2	m	0	65	99	5	22	2.89	(1.04- 8.01)
RANDIG	3	m	0	190	164	5	22	5.10	(1.89- 13.76)
RANDIG	4	m	0	142	68	5	22	9.19	(3.34- 25.31)
RANDIG	5	f	0	1	21	17	92	0.26	(0.03- 2.05)
RANDIG	6	f	0	12	13	17	92	5.00	(1.95- 12.79)
RANDIG	7	f	0	3	5	17	92	3.25	(0.71- 14.88)
Subtotal RANDIG								3.84	(2.50- 5.89)
SHAW	10	c	0	46	90	11	107	4.97	(2.43- 10.16)
SHAW	11	c	0	278	176	11	107	15.36	(8.03- 29.39)
Subtotal SHAW								9.23	(5.71- 14.93)
SIEMIA	13	m	0	-	-	-	-	3.00	(1.00- 9.90)
SIEMIA	14	m	0	-	-	-	-	4.50	(1.80- 13.20)
SIEMIA	15	m	0	-	-	-	-	7.90	(3.00- 24.10)
Subtotal SIEMIA								4.87	(2.64- 8.95)
SPITZ	5	c	0	27	88	10	96	2.95	(1.35- 6.43)
SPITZ	6	c	0	95	48	10	96	19.00	(9.08- 39.74)
Subtotal SPITZ								7.89	(4.61- 13.49)
STOCKS	41	m	2	-	-	-	-	4.64	(3.25- 6.61)
STOCKS	42	m	2	-	-	-	-	7.89	(5.51- 11.29)
STOCKS	43	m	2	-	-	-	-	10.95	(7.09- 16.91)
STOCKS	44	m	2	-	-	-	-	10.03	(6.51- 15.46)
STOCKS	45	m	2	-	-	-	-	13.91	(8.96- 21.60)
STOCKS	48	f	1	-	-	-	-	2.24	(1.64- 3.03)
STOCKS	49	f	1	-	-	-	-	6.34	(4.42- 8.93)
Subtotal STOCKS								6.04	(5.25- 6.96)
TIZZAN	7	m	0	130	238	180	305	0.93	(0.70- 1.23)
TIZZAN	8	m	0	468	470	180	305	1.69	(1.35- 2.11)
TIZZAN	9	m	0	301	108	180	305	4.72	(3.54- 6.29)
TIZZAN	10	m	0	83	20	180	305	7.03	(4.17- 11.85)
TIZZAN	15	f	0	11	14	25	114	3.58	(1.46- 8.82)
TIZZAN	16	f	0	14	14	25	114	4.56	(1.93- 10.75)
Subtotal TIZZAN								2.16	(1.87- 2.48)
WANG2	9	c	4	-	-	-	-	1.40	(0.43- 4.54)
WANG2	10	c	4	-	-	-	-	1.22	(0.31- 4.83)
WANG2	11	c	4	-	-	-	-	1.41	(0.43- 4.61)
WANG2	12	c	4	-	-	-	-	1.16	(0.33- 4.03)
WANG2	13	c	4	-	-	-	-	3.19	(1.39- 7.29)
WANG2	14	c	4	-	-	-	-	2.39	(0.78- 7.34)
WANG2	15	c	4	-	-	-	-	7.25	(2.05- 25.65)
Subtotal WANG2								2.20	(1.43- 3.37)
WUWILL	12	f	3	-	-	-	-	2.13	(1.75- 2.58)
WUWILL	13	f	3	-	-	-	-	3.37	(2.28- 4.97)
Subtotal WUWILL								2.33	(1.96- 2.78)
WYNDE2	17	m	0	17	114	8	105	1.96	(0.81- 4.72)
WYNDE2	18	m	0	122	203	8	105	7.89	(3.71- 16.75)
WYNDE2	19	m	0	88	83	8	105	13.92	(6.39- 30.32)
WYNDE2	20	m	0	155	112	8	105	18.16	(8.50- 38.80)
Subtotal WYNDE2								8.64	(5.83- 12.82)
WYNDE3	44	m	0	8	42	9	88	1.86	(0.67- 5.17)
WYNDE3	45	m	0	77	114	9	88	6.60	(3.14- 13.90)
WYNDE3	46	m	0	108	82	9	88	12.88	(6.12- 27.09)
WYNDE3	47	m	0	68	26	9	88	25.57	(11.25- 58.14)
WYNDE3	79	f	0	3	19	20	76	0.60	(0.16- 2.23)
WYNDE3	80	f	0	24	24	20	76	3.80	(1.79- 8.05)
WYNDE3	81	f	0	15	10	20	76	5.70	(2.23- 14.59)
WYNDE3	82	f	0	4	3	20	76	5.07	(1.05- 24.50)
Subtotal WYNDE3								6.19	(4.51- 8.50)
WYNDE4	43	m	0	17	82	12	115	1.99	(0.90- 4.38)
WYNDE4	44	m	0	67	147	12	115	4.37	(2.26- 8.46)
WYNDE4	45	m	0	228	274	12	115	7.97	(4.29- 14.82)

Table 1G1 - 2

IESLC - Meta-analysis of Ever Smoking by Amount, Overview, Any product (or Cigarettes if Any not available)
All LC types
Most adjusted

REF	NRR	SEX	AD	Number Exposed Case	Number Exposed Cont	Non-exposed Case	Non-exposed Cont	RR	95.00%CI
WYNDE4	46	m	0	190	98	12	115	18.58	(9.77- 35.33)
WYNDE4	47	m	0	130	64	12	115	19.47	(10.00- 37.88)
WYNDE4	57	f	2	-	-	-	-	1.13	(0.33- 3.89)
WYNDE4	58	f	2	-	-	-	-	2.01	(0.66- 6.10)
WYNDE4	59	f	2	-	-	-	-	6.49	(2.35- 17.93)
WYNDE4	60	f	2	-	-	-	-	11.54	(1.90- 70.11)
WYNDE4	61	f	2	-	-	-	-	11.54	(1.90- 70.11)
Subtotal WYNDE4								6.97	(5.35- 9.09)
XU3	9	m	1	-	-	-	-	1.66	(0.55- 4.97)
XU3	10	m	1	-	-	-	-	2.98	(1.09- 8.17)
XU3	11	m	1	-	-	-	-	14.78	(5.30- 41.18)
XU3	12	m	1	-	-	-	-	27.72	(5.15- 149.23)
XU3	16	f	1	-	-	-	-	2.18	(0.61- 7.84)
XU3	17	f	1	-	-	-	-	4.41	(1.16- 16.83)
XU3	18	f	1	-	-	-	-	8.19	(0.89- 75.45)
Subtotal XU3								4.71	(2.94- 7.56)
*YUAN	2	m	2	-	-	-	-	3.60	(1.88- 6.91)
*YUAN	3	m	2	-	-	-	-	9.40	(5.21- 16.97)
Subtotal YUAN								6.09	(3.94- 9.44)
ZHENG	11	m	0	25	40	33	94	1.78	(0.94- 3.37)
ZHENG	12	m	0	60	66	33	94	2.59	(1.53- 4.39)
ZHENG	13	m	0	128	89	33	94	4.10	(2.53- 6.62)
ZHENG	14	m	0	66	23	33	94	8.17	(4.40- 15.17)
ZHENG	22	f	0	24	29	152	184	1.00	(0.56- 1.79)
ZHENG	23	f	0	52	15	152	184	4.20	(2.27- 7.75)
Subtotal ZHENG								2.97	(2.36- 3.75)
ZHOU	4	c	0	61	5	507	68	1.64	(0.64- 4.22)
ZHOU	5	c	0	211	14	507	68	2.02	(1.11- 3.67)
ZHOU	6	c	0	581	29	507	68	2.69	(1.71- 4.22)
Subtotal ZHOU								2.31	(1.65- 3.23)
Partial Totals				23450	84905	11175	123308		

*prospective study ~ With 0.5 adjustment for zero

REF	NRR	SEX	AD	Ys	Ws	Qs	Ps
AGUDO	4	f	3	0.45	3.17	4.08	0.4219
AGUDO	5	f	3	1.60	4.04	0.00	0.0013
Subtotal AGUDO				1.09	7.21	4.08	
ALDERS	18	m	1	1.27	10.54	1.07	0.0000
ALDERS	19	m	1	2.07	13.07	3.12	0.0000
ALDERS	20	m	1	2.14	14.23	4.41	0.0000
ALDERS	21	f	1	0.96	34.91	13.53	0.0000
ALDERS	22	f	1	1.66	34.88	0.21	0.0000
ALDERS	23	f	1	1.93	25.78	3.08	0.0000
Subtotal ALDERS				1.59	133.42	25.43	
ARMADA	46	m	0	1.79	3.37	0.15	0.0010
ARMADA	47	m	0	3.02	3.54	7.24	0.0000
ARMADA	48	m	0	4.24	3.29	23.19	0.0000
Subtotal ARMADA				3.01	10.20	30.58	
AUVINE	13	c	2	3.00	7.37	14.65	0.0000
AUVINE	14	c	2	3.52	8.24	30.94	0.0000
AUVINE	15	c	2	4.20	4.27	29.12	0.0000
Subtotal AUVINE				3.47	19.88	74.70	
AXELSS	5	m	6	1.34	8.91	0.54	0.0001
AXELSS	6	m	6	2.19	10.87	3.92	0.0000
AXELSS	7	m	6	2.34	11.25	6.43	0.0000
AXELSS	13	f	0	1.10	6.02	1.42	0.0069
AXELSS	14	f	0	2.38	10.21	6.40	0.0000
AXELSS	15	f	0	2.77	6.08	8.54	0.0000
AXELSS	16	f	0	1.99	2.69	0.45	0.0011
Subtotal AXELSS				2.06	56.05	27.70	
BARBON	82	m	3	1.90	16.58	1.66	0.0000
BARBON	83	m	3	2.55	16.08	14.93	0.0000
BARBON	84	m	3	3.06	14.81	32.13	0.0000
Subtotal BARBON				2.48	47.46	48.72	
*BOUCOT	99	m	0	3.15	0.49	1.20	0.0271
*BOUCOT	100	m	0	3.83	0.49	2.49	0.0070
Subtotal BOUCOT				3.49	0.99	3.70	
BRESLO	13	m	0	0.58	7.18	7.33	0.1231
BRESLO	14	m	0	1.19	12.73	2.00	0.0000
BRESLO	15	m	0	2.04	15.85	3.23	0.0000

Table 1G1 - 2

IESLC - Meta-analysis of Ever Smoking by Amount, Overview, Any product (or Cigarettes if Any not available)
All LC types
Most adjusted

REF	NRR	SEX	AD	Ys	Ws	Qs	Ps
BRESLO	16	m	0	2.90	8.89	15.36	0.0000
BRESLO	29	f	0	0.15	1.80	3.69	0.8360
BRESLO	30	f	0	0.44	2.24	2.93	0.5084
Subtotal BRESLO				1.61	48.69	34.55	
BROWN2	32	m	2	1.81	220.78	10.93	0.0000
BROWN2	42	m	2	2.65	387.09	435.28	0.0000
BROWN2	31	f	2	2.13	172.98	50.90	0.0000
BROWN2	41	f	2	2.84	312.24	490.48	0.0000
Subtotal BROWN2				2.45	1093.09	987.59	
BUFFLE	28	f	0	1.54	6.11	0.01	0.0001
BUFFLE	29	f	0	2.47	8.19	6.40	0.0000
BUFFLE	35	f	0	3.06	8.66	18.70	0.0000
Subtotal BUFFLE				2.44	22.96	25.11	
CHATZI	1	c	0	0.94	14.84	6.20	0.0003
CHATZI	2	c	0	1.04	15.01	4.43	0.0001
CHATZI	3	c	0	1.56	16.06	0.01	0.0000
Subtotal CHATZI				1.19	45.92	10.65	
CHEN2	3	m	0	0.87	4.19	2.12	0.0735
CHEN2	4	m	0	1.17	5.43	0.93	0.0063
CHEN2	5	m	0	2.63	3.55	3.86	0.0000
CHEN2	6	m	0	2.07	3.80	0.90	0.0001
CHEN2	7	f	0	-0.95	3.04	19.48	0.0991
CHEN2	8	f	0	1.07	4.63	1.25	0.0217
CHEN2	9	f	0	2.22	0.82	0.34	0.0435
CHEN2	10	f	0	0.97	1.22	0.46	0.2838
Subtotal CHEN2				1.21	26.68	29.33	
CHOI	12	m	0	0.48	6.73	8.16	0.2084
CHOI	13	m	0	1.32	10.21	0.72	0.0000
CHOI	14	m	0	2.01	7.82	1.40	0.0000
CHOI	15	m	0	1.94	7.14	0.88	0.0000
CHOI	16	m	0	2.97	3.16	6.05	0.0000
CHOI	17	f	0	0.19	5.18	10.05	0.6591
CHOI	18	f	0	0.52	3.66	4.17	0.3219
CHOI	20	f	0	1.87	0.74	0.06	0.1083
Subtotal CHOI				1.34	44.64	31.49	
COOKSO	1	c	0	1.63	11.70	0.03	0.0000
COOKSO	2	c	0	2.31	7.06	3.73	0.0000
Subtotal COOKSO				1.89	18.76	3.76	
*CPSI	243	m	1	1.76	44.36	1.34	0.0000
*CPSI	246	m	1	2.61	55.90	58.65	0.0000
Subtotal CPSI				2.23	100.25	59.99	
*CPSII	102	m	1	2.30	75.60	38.74	0.0000
*CPSII	103	m	1	2.87	75.65	124.36	0.0000
*CPSII	105	f	1	1.43	95.77	2.46	0.0000
*CPSII	106	f	1	2.59	140.12	141.53	0.0000
Subtotal CPSII				2.30	387.13	307.08	
DAMBER	6	m	1	0.83	10.34	5.86	0.0074
DAMBER	7	m	1	1.99	13.68	2.21	0.0000
DAMBER	8	m	1	2.21	14.68	5.69	0.0000
DAMBER	9	m	1	2.70	5.93	7.38	0.0000
Subtotal DAMBER				1.89	44.62	21.14	
DAVEYS	1	m	0	0.20	2.07	3.98	0.7726
DAVEYS	2	m	0	2.01	2.27	0.40	0.0025
DAVEYS	3	m	0	1.89	2.11	0.20	0.0061
DAVEYS	4	m	0	2.36	2.18	1.31	0.0005
Subtotal DAVEYS				1.63	8.63	5.88	
DEAN	1	m	0	0.79	8.38	5.27	0.0218
DEAN	2	m	0	1.91	9.10	0.94	0.0000
DEAN	3	m	0	2.44	7.59	5.59	0.0000
Subtotal DEAN				1.70	25.06	11.81	
DEAN2	25	m	0	1.17	22.52	3.84	0.0000
DEAN2	26	m	0	2.03	19.18	3.84	0.0000
DEAN2	29	f	0	0.92	11.90	5.20	0.0014
DEAN2	30	f	0	1.60	3.63	0.00	0.0023
Subtotal DEAN2				1.44	57.24	12.88	
DESTEF	6	m	4	1.06	11.84	3.21	0.0002
DESTEF	7	m	4	2.13	16.62	4.89	0.0000
DESTEF	8	m	4	2.34	16.30	9.32	0.0000
DESTEF	9	m	4	3.17	11.72	29.26	0.0000
Subtotal DESTEF				2.18	56.48	46.68	
DOLL	1	m	0	1.31	5.40	0.40	0.0023

Table 1G1 - 2

IESLC - Meta-analysis of Ever Smoking by Amount, Overview, Any product (or Cigarettes if Any not available)
All LC types
Most adjusted

REF	NRR	SEX	AD	Ys	Ws	Qs	Ps
DOLL	2	m	0	2.01	6.13	1.11	0.0000
DOLL	3	m	0	2.26	6.11	2.80	0.0000
DOLL	4	m	0	2.81	5.91	8.83	0.0000
DOLL	5	m	0	3.32	3.72	11.15	0.0000
DOLL	7	f	0	-0.06	6.92	18.70	0.8795
DOLL	8	f	0	0.68	7.19	5.94	0.0698
DOLL	9	f	0	1.24	3.57	0.44	0.0195
DOLL	10	f	0	3.75	0.47	2.22	0.0098
Subtotal DOLL				1.60	45.43	51.60	
*DOLL2	46	m	1	1.90	0.96	0.10	0.0623
*DOLL2	47	m	1	2.51	0.96	0.82	0.0137
*DOLL2	48	m	1	3.17	0.97	2.43	0.0018
Subtotal DOLL2				2.53	2.90	3.35	
DORGAN	108	m	2	1.93	10.33	1.21	0.0000
DORGAN	109	m	2	2.64	11.27	12.50	0.0000
DORGAN	96	f	3	1.74	56.05	1.25	0.0000
DORGAN	97	f	3	2.50	51.92	43.69	0.0000
Subtotal DORGAN				2.14	129.57	58.66	
DOSEME	5	m	2	0.79	20.90	13.29	0.0003
DOSEME	9	m	2	1.13	45.98	9.49	0.0000
DOSEME	13	m	2	1.89	21.74	1.97	0.0000
Subtotal DOSEME				1.24	88.62	24.75	
*DUNN	1	m	0	2.12	1.20	0.35	0.0199
*DUNN	2	m	0	2.20	1.71	0.65	0.0040
*DUNN	3	m	0	2.95	1.95	3.64	0.0000
*DUNN	4	m	0	3.22	1.88	5.05	0.0000
*DUNN	5	m	0	3.36	1.73	5.44	0.0000
Subtotal DUNN				2.83	8.48	15.13	
EBELIN	2	m	0	1.00	6.42	2.23	0.0116
EBELIN	3	m	0	1.96	5.47	0.78	0.0000
EBELIN	4	m	0	2.52	7.13	6.18	0.0000
EBELIN	5	m	0	2.79	1.60	2.31	0.0004
EBELIN	6	m	0	2.28	1.69	0.81	0.0031
Subtotal EBELIN				1.94	22.31	12.31	
ESAKI	1	m	0	0.09	7.53	16.79	0.8001
ESAKI	2	m	0	0.80	7.75	4.75	0.0253
ESAKI	3	m	0	1.78	4.39	0.17	0.0002
Subtotal ESAKI				0.75	19.68	21.71	
FAN	6	m	0	-0.35	8.53	31.99	0.3059
FAN	7	m	0	0.71	17.63	13.55	0.0029
FAN	8	m	0	1.38	21.51	0.91	0.0000
FAN	9	m	0	2.41	9.89	6.69	0.0000
FAN	10	f	0	0.50	10.28	12.20	0.1116
FAN	11	f	0	1.32	12.82	0.88	0.0000
FAN	12	f	0	2.48	7.51	6.05	0.0000
FAN	13	f	0	2.92	0.79	1.41	0.0095
Subtotal FAN				1.19	88.96	73.67	
GAO	21	f	2	0.55	30.53	32.51	0.0022
GAO	22	f	2	1.11	22.93	5.15	0.0000
GAO	23	f	2	2.58	9.77	9.63	0.0000
Subtotal GAO				1.07	63.23	47.29	
GARSHI	26	m	1	1.19	24.90	3.88	0.0000
GARSHI	27	m	1	1.74	31.76	0.79	0.0000
GARSHI	28	m	1	2.04	25.11	5.18	0.0000
GARSHI	29	m	1	1.66	27.73	0.14	0.0000
Subtotal GARSHI				1.66	109.49	9.99	
GER	22	c	14	0.23	7.27	13.34	0.5331
GER	23	c	14	0.64	8.71	7.76	0.0582
GER	24	c	14	1.10	6.65	1.58	0.0046
Subtotal GER				0.64	22.63	22.68	
GOLLED	1	m	1	1.49	12.18	0.11	0.0000
GOLLED	2	m	1	1.85	12.69	0.90	0.0000
GOLLED	3	m	1	2.91	12.60	21.96	0.0000
Subtotal GOLLED				2.09	37.47	22.96	
GSELL	1	m	0	1.49	1.53	0.01	0.0655
GSELL	2	m	0	1.37	1.51	0.07	0.0931
GSELL	3	m	0	2.71	1.64	2.08	0.0005
GSELL	4	m	0	4.37	1.50	11.63	0.0000
GSELL	5	m	0	4.04	1.58	9.55	0.0000
Subtotal GSELL				2.80	7.77	23.35	
*HAMMON	153	m	1	2.00	8.19	1.40	0.0000

Table 1G1 - 2

IESLC - Meta-analysis of Ever Smoking by Amount, Overview, Any product (or Cigarettes if Any not available)
All LC types
Most adjusted

REF	NRR	SEX	AD	Ys	Ws	Qs	Ps
*HAMMON	154	m	1	2.12	11.45	3.25	0.0000
*HAMMON	155	m	1	2.84	10.98	17.18	0.0000
Subtotal HAMMON				2.34	30.62	21.83	
*HANSEN	1	m	2	0.31	5.16	8.33	0.4747
*HANSEN	2	m	2	1.06	3.91	1.06	0.0352
Subtotal HANSEN				0.64	9.07	9.39	
HU	1	m	0	0.44	10.71	14.13	0.1527
HU	2	m	0	0.74	12.38	8.92	0.0095
HU	3	m	0	1.29	6.63	0.57	0.0009
HU	4	f	0	0.82	5.04	2.92	0.0643
HU	5	f	0	0.18	2.64	5.19	0.7672
HU	6	f	0	-0.51	0.65	2.84	0.6812
Subtotal HU				0.70	38.05	34.57	
HU2	2	c	0	-0.16	9.46	28.76	0.6262
HU2	3	c	0	0.29	18.89	31.76	0.2087
HU2	4	c	0	0.66	22.08	18.83	0.0019
HU2	5	c	0	0.74	21.23	15.35	0.0007
HU2	6	c	0	1.10	32.09	7.46	0.0000
HU2	7	c	0	1.78	12.31	0.46	0.0000
Subtotal HU2				0.79	116.06	102.63	
JARUP	4	m	2	1.93	3.04	0.36	0.0008
JARUP	5	m	2	2.08	3.44	0.84	0.0001
Subtotal JARUP				2.01	6.49	1.20	
JEDRYC	45	m	4	1.25	23.96	2.75	0.0000
JEDRYC	46	m	4	1.82	28.13	1.52	0.0000
JEDRYC	47	m	4	2.04	23.96	4.94	0.0000
JEDRYC	48	f	4	1.85	5.04	0.36	0.0000
JEDRYC	49	f	4	0.87	4.91	2.54	0.0546
JEDRYC	50	f	4	2.00	2.63	0.45	0.0012
Subtotal JEDRYC				1.68	88.63	12.55	
JOLY	7	m	0	1.68	5.92	0.06	0.0000
JOLY	8	m	0	2.52	10.45	9.07	0.0000
JOLY	9	m	0	2.57	9.85	9.56	0.0000
JOLY	10	m	0	3.08	10.07	22.51	0.0000
JOLY	3	f	0	1.55	12.60	0.01	0.0000
JOLY	4	f	0	2.08	17.52	4.26	0.0000
JOLY	5	f	0	1.94	9.62	1.18	0.0000
JOLY	6	f	0	2.59	7.64	7.78	0.0000
Subtotal JOLY				2.24	83.67	54.43	
JUSSAW	34	m	2	1.72	4.79	0.08	0.0002
JUSSAW	35	m	2	2.64	2.83	3.14	0.0000
Subtotal JUSSAW				2.06	7.62	3.22	
KHUDER	1	m	0	0.92	16.30	7.24	0.0002
KHUDER	2	m	0	2.35	18.30	10.59	0.0000
KHUDER	3	m	0	3.49	14.48	52.58	0.0000
Subtotal KHUDER				2.21	49.07	70.41	
KOULUM	6	m	0	1.65	3.87	0.01	0.0012
KOULUM	5	m	0	3.17	4.27	10.78	0.0000
KOULUM	4	m	0	4.23	4.27	29.93	0.0000
Subtotal KOULUM				3.06	12.42	40.72	
KREUZE	19	m	3	0.92	2.54	1.14	0.1444
KREUZE	20	m	3	2.16	4.57	1.52	0.0000
KREUZE	21	m	3	2.97	4.24	8.13	0.0000
KREUZE	22	m	3	3.03	3.39	7.12	0.0000
KREUZE	30	m	3	2.10	18.30	4.92	0.0000
KREUZE	31	m	3	3.22	20.26	54.31	0.0000
KREUZE	32	m	3	3.49	18.97	68.84	0.0000
KREUZE	33	m	3	3.51	16.38	60.37	0.0000
KREUZE	25	f	3	1.74	2.81	0.07	0.0035
KREUZE	26	f	3	2.47	3.44	2.68	0.0000
KREUZE	27	f	3	2.49	2.00	1.65	0.0004
KREUZE	36	f	3	0.69	15.02	11.96	0.0072
KREUZE	37	f	3	1.69	19.01	0.19	0.0000
KREUZE	38	f	3	2.04	6.02	1.25	0.0000
Subtotal KREUZE				2.45	136.95	224.14	
KREYBE	9	m	1	1.76	5.70	0.18	0.0000
KREYBE	10	m	1	1.83	5.32	0.32	0.0000
KREYBE	11	m	1	2.66	5.07	5.86	0.0000
KREYBE	28	f	1	0.31	7.13	11.66	0.4115
KREYBE	29	f	1	0.65	2.02	1.78	0.3573
Subtotal KREYBE				1.46	25.24	19.79	

Table 1G1 - 2

IESLC - Meta-analysis of Ever Smoking by Amount, Overview, Any product (or Cigarettes if Any not available)
All LC types
Most adjusted

REF	NRR	SEX	AD	Ys	Ws	Qs	Ps
LAMTH	7	f	0	0.98	29.68	10.75	0.0000
LAMTH	2	f	0	1.68	18.27	0.16	0.0000
LAMTH	9	f	0	1.98	6.91	1.06	0.0000
Subtotal LAMTH				1.34	54.86	11.98	
LAUSSM	18	m	3	1.19	31.20	4.86	0.0000
LAUSSM	19	m	3	1.76	34.48	1.04	0.0000
LAUSSM	20	m	3	2.26	18.03	8.29	0.0000
Subtotal LAUSSM				1.66	83.71	14.20	
LETOUR	2	c	0	2.25	18.66	8.29	0.0000
LETOUR	3	c	0	2.85	18.55	29.68	0.0000
LETOUR	4	c	0	3.27	9.52	27.10	0.0000
Subtotal LETOUR				2.70	46.74	65.07	
LIU2	8	m	3	0.18	3.50	6.88	0.7332
LIU2	9	m	3	1.96	3.78	0.53	0.0001
LIU2	10	m	3	3.06	3.18	6.94	0.0000
LIU2	14	f	3	0.59	2.81	2.80	0.3242
LIU2	15	f	3	1.25	3.48	0.39	0.0194
LIU2	16	f	3	2.88	1.70	2.87	0.0002
Subtotal LIU2				1.56	18.46	20.42	
LIU3	6	m	2	0.34	1.81	2.79	0.6441
LIU3	7	m	2	0.09	1.71	3.84	0.9103
LIU3	8	m	2	0.65	1.20	1.06	0.4778
Subtotal LIU3				0.33	4.72	7.69	
LIU4	7	m	2	0.75	2108.86	1484.72	0.0000
LIU4	8	m	2	1.28	4111.92	382.07	0.0000
LIU4	9	m	2	1.94	2991.95	381.95	0.0000
Subtotal LIU4				1.37	9212.74	2248.73	
LIU5	2	c	0	-0.20	5.84	18.64	0.6267
LIU5	3	c	0	0.46	6.33	8.08	0.2520
LIU5	4	c	0	1.28	7.79	0.75	0.0004
Subtotal LIU5				0.58	19.96	27.47	
LUBIN	11	m	4	-0.33	2.38	8.72	0.6124
LUBIN	12	m	4	0.22	3.36	6.24	0.6825
LUBIN	13	m	4	1.87	3.80	0.31	0.0003
LUBIN	14	m	4	2.08	5.32	1.30	0.0000
Subtotal LUBIN				1.22	14.85	16.56	
LUBIN2	273	m	0	1.93	155.25	18.74	0.0000
LUBIN2	274	m	0	2.03	150.14	29.34	0.0000
LUBIN2	275	m	0	2.36	152.73	92.01	0.0000
LUBIN2	276	m	0	2.52	139.75	122.52	0.0000
LUBIN2	281	f	0	0.90	66.54	31.69	0.0000
LUBIN2	282	f	0	1.30	76.70	6.27	0.0000
LUBIN2	283	f	0	1.53	47.64	0.17	0.0000
LUBIN2	284	f	0	1.57	17.75	0.00	0.0000
Subtotal LUBIN2				1.96	806.50	300.75	
MACLEN	36	c	2	0.30	6.92	11.44	0.4298
MACLEN	37	c	2	0.98	10.81	3.99	0.0013
MACLEN	38	c	2	1.08	9.92	2.59	0.0007
MACLEN	39	c	2	1.41	8.18	0.25	0.0001
Subtotal MACLEN				0.97	35.83	18.27	
MARTIS	1	m	0	1.60	2.87	0.00	0.0066
MARTIS	2	m	0	1.88	3.20	0.27	0.0008
MARTIS	3	m	0	2.24	3.09	1.32	0.0001
Subtotal MARTIS				1.91	9.17	1.59	
MATOS	29	m	2	0.69	5.93	4.73	0.0914
MATOS	31	m	2	2.01	7.84	1.44	0.0000
MATOS	33	m	2	2.34	8.28	4.73	0.0000
Subtotal MATOS				1.78	22.05	10.90	
MATSUD	1	m	0	2.53	2.76	2.45	0.0000
MATSUD	2	m	0	2.97	2.87	5.52	0.0000
MATSUD	3	m	0	3.94	2.83	15.73	0.0000
Subtotal MATSUD				3.15	8.46	23.70	
MCCONN	26	c	0	-0.88	3.12	18.91	0.1215
MCCONN	25	c	0	0.31	5.38	8.78	0.4745
MCCONN	24	c	0	0.76	4.83	3.33	0.0966
Subtotal MCCONN				0.19	13.33	31.02	
NOTAN2	8	m	0	-0.54	5.01	22.72	0.2229
NOTAN2	9	m	0	0.88	15.08	7.45	0.0006
NOTAN2	10	m	0	1.50	17.54	0.14	0.0000
Subtotal NOTAN2				0.98	37.63	30.31	
ORMOS	1	m	0	2.38	5.60	3.53	0.0000

Table 1G1 - 2

IESLC - Meta-analysis of Ever Smoking by Amount, Overview, Any product (or Cigarettes if Any not available)
All LC types
Most adjusted

REF	NRR	SEX	AD	Ys	Ws	Qs	Ps
ORMOS	2	m	0	2.04	5.85	1.21	0.0000
ORMOS	3	m	0	2.57	4.57	4.39	0.0000
Subtotal ORMOS				2.31	16.03	9.13	
OSANN	49	m	2	2.87	33.51	55.58	0.0000
OSANN	57	m	2	3.76	33.40	157.39	0.0000
OSANN	50	f	2	2.67	52.45	61.34	0.0000
OSANN	58	f	2	3.71	34.52	155.92	0.0000
Subtotal OSANN				3.18	153.87	430.22	
OSANN2	22	f	1	0.92	7.15	3.20	0.0143
OSANN2	23	f	1	2.53	7.64	6.87	0.0000
Subtotal OSANN2				1.75	14.79	10.07	
PASTOR	6	m	1	0.85	3.21	1.76	0.1295
PASTOR	7	m	1	1.86	6.41	0.48	0.0000
PASTOR	8	m	1	2.08	7.09	1.75	0.0000
PASTOR	9	m	1	2.15	5.97	1.92	0.0000
Subtotal PASTOR				1.86	22.69	5.91	
PERNU	17	m	0	1.04	6.79	2.01	0.0066
PERNU	18	m	0	1.72	15.98	0.28	0.0000
PERNU	19	m	0	1.89	34.69	3.20	0.0000
PERNU	20	m	0	1.68	27.21	0.25	0.0000
PERNU	21	m	0	2.28	42.95	20.96	0.0000
PERNU	22	m	0	2.94	28.38	52.20	0.0000
PERNU	23	m	0	1.60	12.13	0.00	0.0000
PERNU	24	m	0	2.71	10.11	12.71	0.0000
PERNU	11	f	0	0.64	2.41	2.17	0.3224
PERNU	12	f	0	1.22	3.48	0.46	0.0225
PERNU	13	f	0	0.16	3.41	6.90	0.7636
PERNU	14	f	0	-0.46	0.92	3.87	0.6574
PERNU	15	f	0	-0.22	0.91	2.96	0.8339
PERNU	16	f	0	1.84	2.83	0.19	0.0019
Subtotal PERNU				2.00	192.21	108.17	
PIKE	1	m	0	1.42	12.27	0.34	0.0000
PIKE	2	m	0	2.08	11.96	2.94	0.0000
PIKE	3	m	0	1.92	8.91	1.01	0.0000
PIKE	5	f	0	1.18	14.59	2.44	0.0000
PIKE	6	f	0	1.93	10.92	1.27	0.0000
PIKE	7	f	0	2.65	2.30	2.63	0.0001
Subtotal PIKE				1.70	60.95	10.64	
POLEDN	2	c	0	1.79	8.40	0.33	0.0000
POLEDN	4	c	0	2.29	9.66	4.77	0.0000
Subtotal POLEDN				2.05	18.06	5.11	
RACHTA	10	f	1	1.29	2.59	0.22	0.0375
RACHTA	11	f	1	1.27	7.64	0.78	0.0005
RACHTA	12	f	1	2.62	6.82	7.33	0.0000
Subtotal RACHTA				1.81	17.05	8.33	
RANDIG	1	m	0	0.71	2.79	2.12	0.2326
RANDIG	2	m	0	1.06	3.69	1.02	0.0415
RANDIG	3	m	0	1.63	3.89	0.01	0.0013
RANDIG	4	m	0	2.22	3.74	1.50	0.0000
RANDIG	5	f	0	-1.36	0.90	7.75	0.1996
RANDIG	6	f	0	1.61	4.35	0.00	0.0008
RANDIG	7	f	0	1.18	1.66	0.28	0.1294
Subtotal RANDIG				1.35	21.02	12.66	
SHAW	10	c	0	1.60	7.51	0.00	0.0000
SHAW	11	c	0	2.73	9.13	12.00	0.0000
Subtotal SHAW				2.22	16.64	12.00	
SIEMIA	13	m	0	1.10	2.92	0.69	0.0603
SIEMIA	14	m	0	1.50	3.87	0.03	0.0031
SIEMIA	15	m	0	2.07	3.54	0.82	0.0001
Subtotal SIEMIA				1.58	10.33	1.54	
SPITZ	5	c	0	1.08	6.30	1.61	0.0067
SPITZ	6	c	0	2.94	7.05	13.02	0.0000
Subtotal SPITZ				2.07	13.35	14.63	
STOCKS	41	m	2	1.53	30.49	0.08	0.0000
STOCKS	42	m	2	2.07	29.86	6.88	0.0000
STOCKS	43	m	2	2.39	20.34	13.26	0.0000
STOCKS	44	m	2	2.31	20.54	10.64	0.0000
STOCKS	45	m	2	2.63	19.85	21.75	0.0000
STOCKS	48	f	1	0.81	40.78	24.76	0.0000
STOCKS	49	f	1	1.85	31.07	2.12	0.0000
Subtotal STOCKS				1.80	192.91	79.49	

Table 1G1 - 2

IESLC - Meta-analysis of Ever Smoking by Amount, Overview, Any product (or Cigarettes if Any not available)
All LC types
Most adjusted

REF	NRR	SEX	AD	Ys	Ws	Qs	Ps
TIZZAN	7	m	0	-0.08	48.24	133.44	0.5909
TIZZAN	8	m	0	0.52	76.34	86.21	0.0000
TIZZAN	9	m	0	1.55	46.69	0.05	0.0000
TIZZAN	10	m	0	1.95	14.11	1.88	0.0000
TIZZAN	15	f	0	1.28	4.74	0.45	0.0055
TIZZAN	16	f	0	1.52	5.22	0.02	0.0005
Subtotal TIZZAN				0.77	195.34	222.06	
WANG2	9	c	4	0.34	2.77	4.32	0.5757
WANG2	10	c	4	0.20	2.04	3.92	0.7765
WANG2	11	c	4	0.34	2.73	4.21	0.5702
WANG2	12	c	4	0.15	2.45	5.07	0.8162
WANG2	13	c	4	1.16	5.60	1.01	0.0061
WANG2	14	c	4	0.87	3.06	1.56	0.1276
WANG2	15	c	4	1.98	2.41	0.38	0.0021
Subtotal WANG2				0.79	21.05	20.47	
WUWILL	12	f	3	0.76	101.98	70.19	0.0000
WUWILL	13	f	3	1.21	25.31	3.48	0.0000
Subtotal WUWILL				0.85	127.28	73.67	
WYNDE2	17	m	0	0.67	4.95	4.14	0.1353
WYNDE2	18	m	0	2.07	6.77	1.56	0.0000
WYNDE2	19	m	0	2.63	6.33	6.94	0.0000
WYNDE2	20	m	0	2.90	6.67	11.51	0.0000
Subtotal WYNDE2				2.16	24.72	24.15	
WYNDE3	44	m	0	0.62	3.69	3.42	0.2325
WYNDE3	45	m	0	1.89	6.93	0.63	0.0000
WYNDE3	46	m	0	2.56	6.95	6.53	0.0000
WYNDE3	47	m	0	3.24	5.69	15.61	0.0000
WYNDE3	79	f	0	-0.51	2.23	9.79	0.4459
WYNDE3	80	f	0	1.34	6.83	0.43	0.0005
WYNDE3	81	f	0	1.74	4.35	0.10	0.0003
WYNDE3	82	f	0	1.62	1.55	0.00	0.0436
Subtotal WYNDE3				1.82	38.21	36.52	
WYNDE4	43	m	0	0.69	6.13	4.96	0.0891
WYNDE4	44	m	0	1.47	8.79	0.11	0.0000
WYNDE4	45	m	0	2.08	9.99	2.40	0.0000
WYNDE4	46	m	0	2.92	9.30	16.61	0.0000
WYNDE4	47	m	0	2.97	8.67	16.58	0.0000
WYNDE4	57	f	2	0.12	2.52	5.41	0.8460
WYNDE4	58	f	2	0.70	3.11	2.45	0.2185
WYNDE4	59	f	2	1.87	3.72	0.30	0.0003
WYNDE4	60	f	2	2.45	1.18	0.87	0.0079
WYNDE4	61	f	2	2.45	1.18	0.87	0.0079
Subtotal WYNDE4				1.94	54.60	50.57	
XU3	9	m	1	0.51	3.17	3.69	0.3668
XU3	10	m	1	1.09	3.79	0.92	0.0336
XU3	11	m	1	2.69	3.66	4.48	0.0000
XU3	12	m	1	3.32	1.36	4.09	0.0001
XU3	16	f	1	0.78	2.36	1.53	0.2316
XU3	17	f	1	1.48	2.15	0.02	0.0297
XU3	18	f	1	2.10	0.78	0.21	0.0634
Subtotal XU3				1.55	17.25	14.95	
*YUAN	2	m	2	1.28	9.07	0.84	0.0001
*YUAN	3	m	2	2.24	11.02	4.73	0.0000
Subtotal YUAN				1.81	20.09	5.57	
ZHENG	11	m	0	0.58	9.44	9.61	0.0764
ZHENG	12	m	0	0.95	13.74	5.53	0.0004
ZHENG	13	m	0	1.41	16.67	0.51	0.0000
ZHENG	14	m	0	2.10	10.04	2.67	0.0000
ZHENG	22	f	0	0.00	11.34	28.46	0.9951
ZHENG	23	f	0	1.43	10.21	0.23	0.0000
Subtotal ZHENG				1.09	71.45	47.01	
ZHOU	4	c	0	0.49	4.29	5.13	0.3077
ZHOU	5	c	0	0.70	10.77	8.38	0.0209
ZHOU	6	c	0	0.99	18.91	6.75	0.0000
Subtotal ZHOU				0.84	33.97	20.25	

Table 1G1 - 2

IESLC - Meta-analysis of Ever Smoking by Amount, Overview, Any product (or Cigarettes if Any not available)
All LC types
Most adjusted

N	372
NS	84

Table 1G1 - 3

IESLC - Meta-analysis of Ever Smoking by Amount, Overview, Any product (or Cigarettes if Any not available)
All LC types
Most adjusted

	Sex			
	combined	male	female	Total
N	46	219	107	372
NS	14	63	35	112

In this overview table, other than the "N" rows, entries in the "absent" and "Total" columns may be invalid and should be ignored

	Amount smoked (broad categories)				
	absent	<20k5	6-44k20	>20k45	Total
N	123	104	69	76	372
NS	60	78	54	60	252
Wt	2450.22	3894.15	5097.33	3806.95	15248.64
Het Chi	1226.44	1124.81	722.91	614.07	6836.92
Het df	122	103	68	75	371
Het P	***	***	***	***	***
Fixed RR	8.59	2.76	4.00	7.94	4.88
RRl	8.26	2.68	3.89	7.69	4.81
RRu	8.94	2.85	4.11	8.20	4.96
P	+++	+++	+++	+++	+++
Random RR	5.99	2.85	6.26	11.76	5.56
RRl	5.20	2.48	5.29	9.91	5.12
RRu	6.89	3.28	7.42	13.94	6.04
P	+++	+++	+++	+++	+++

	Amount smoked (narrow categories)							
	absent	<10k1	2-19k10	11-29k20	21-39k30	31-98k40	>40k99	Total
N	201	51	41	54	12	1	12	372
NS	84	35	30	42	10	1	10	212
Wt	8941.10	598.53	628.26	4896.46	82.70	7.14	94.45	15248.64
Het Chi	4979.03	359.75	239.15	557.78	21.69	0.00	40.33	6836.92
Het df	200	50	40	53	11	0	11	371
Het P	***	***	***	***	*	N.S.	***	***
Fixed RR	5.55	2.85	5.11	3.99	10.19	6.93	11.74	4.88
RRl	5.43	2.63	4.73	3.88	8.21	3.33	9.60	4.81
RRu	5.66	3.09	5.53	4.10	12.64	14.44	14.36	4.96
P	+++	+++	+++	+++	+++	+++	+++	+++
Random RR	6.34	2.10	4.55	6.70	11.18	6.93	12.99	5.56
RRl	5.59	1.65	3.67	5.56	7.83	3.33	8.64	5.12
RRu	7.18	2.68	5.64	8.08	15.97	14.44	19.53	6.04
P	+++	+++	+++	+++	+++	+++	+++	+++

MALES

	Amount smoked (broad categories)				
	absent	<20k5	6-44k20	>20k45	Total
N	65	58	45	51	219
NS	43	57	44	50	194
Wt	1350.47	3022.62	4837.76	3636.83	12847.69
Het Chi	441.15	668.70	574.41	442.89	4928.95
Het df	64	57	44	50	218
Het P	***	***	***	***	***
Fixed RR	9.32	2.59	3.94	7.83	4.75
RRl	8.84	2.50	3.83	7.58	4.67
RRu	9.83	2.69	4.05	8.09	4.83
P	+++	+++	+++	+++	+++
Random RR	7.88	3.12	7.05	13.24	6.81
RRl	6.69	2.57	5.70	10.90	6.15
RRu	9.27	3.79	8.72	16.08	7.55
P	+++	+++	+++	+++	+++

Table 1G1 - 3

IESLC - Meta-analysis of Ever Smoking by Amount, Overview, Any product (or Cigarettes if Any not available)
All LC types
Most adjusted

MALES

		Amount smoked (narrow categories)						
	absent	<10k1	2-19k10	11-29k20	21-39k30	31-98k40	>40k99	Total
N	116	27	23	35	9	1	8	219
NS	63	26	22	34	9	1	8	162
Wt	7264.11	371.90	385.94	4675.95	77.63	7.14	65.02	12847.69
Het Chi	3649.63	241.76	124.30	458.76	15.00	0.00	18.76	4928.95
Het df	115	26	22	34	8	0	7	218
Het P	***	***	***	***	(*)	N.S.	**	***
Fixed RR	5.24	3.57	6.46	3.94	10.78	6.93	13.22	4.75
RRl	5.12	3.22	5.85	3.83	8.63	3.33	10.37	4.67
RRu	5.36	3.95	7.14	4.06	13.46	14.44	16.86	4.83
P	+++	+++	+++	+++	+++	+++	+++	+++
Random RR	7.62	2.52	5.85	7.57	12.34	6.93	14.23	6.81
RRl	6.47	1.75	4.43	5.94	8.71	3.33	9.42	6.15
RRu	8.98	3.64	7.74	9.66	17.48	14.44	21.49	7.55
P	+++	+++	+++	+++	+++	+++	+++	+++

FEMALES

	Amount smoked (broad categories)				
	absent	<20k5	6-44k20	>20k45	Total
N	39	33	17	18	107
NS	29	32	16	18	95
Wt	902.55	763.22	171.08	121.94	1958.78
Het Chi	451.13	290.52	41.81	95.51	1353.64
Het df	38	32	16	17	106
Het P	***	***	***	***	***
Fixed RR	9.52	3.51	5.29	12.22	6.22
RRl	8.92	3.27	4.55	10.23	5.96
RRu	10.17	3.76	6.14	14.59	6.51
P	+++	+++	+++	+++	+++
Random RR	5.08	2.48	5.22	8.81	4.27
RRl	3.87	1.94	3.95	5.32	3.57
RRu	6.67	3.18	6.90	14.56	5.11
P	+++	+++	+++	+++	+++

		Amount smoked (narrow categories)						
	absent	<10k1	2-19k10	11-29k20	21-39k30	31-98k40	>40k99	Total
N	58	18	13	14	2		2	107
NS	34	17	12	13	2		2	80
Wt	1421.70	185.66	189.61	155.96	2.00		3.85	1958.78
Het Chi	907.14	44.94	36.76	34.03	0.02		0.99	1353.64
Het df	57	17	12	13	1		1	106
Het P	***	***	***	**	N.S.		N.S.	***
Fixed RR	7.68	2.04	4.12	5.61	10.53		9.40	6.22
RRl	7.29	1.77	3.57	4.79	2.64		3.46	5.96
RRu	8.09	2.35	4.74	6.56	42.05		25.51	6.51
P	+++	+++	+++	+++	+++		+++	+++
Random RR	5.12	1.84	4.15	5.90	10.53		9.40	4.27
RRl	4.03	1.39	3.09	4.38	2.64		3.46	3.57
RRu	6.52	2.44	5.57	7.94	42.05		25.51	5.11
P	+++	+++	+++	+++	+++		+++	+++

Table 1G1 - 4

IESLC - Meta-analysis of Ever Smoking by Amount, Overview, Any product (or Cigarettes if Any not available)
All LC types
Least adjusted

REF	NRR	X	SEX	AGEL	AGEH	RACE	YF	LC TYPE	LOC	START	ST	NLC	R	VB	P	H	AD	PRODUCT	exL	exH	S1	S2	DENOM	De
AGUDO	11	x	f	0	0	all	-	all	Eu:wst	1989	CC	103	n	bl	n	n	0	cig only	1	10	1	0	nev any	st
AGUDO	12	x	f	0	0	all	-	all	Eu:wst	1989	CC	103	n	bl	n	n	0	cig only	11	99	0	0	nev any	st
ALDERS	18		m	0	0	all	-	all	Eu:UK	1977	CC	1448	n	V	n	n	1	cig only	1	17	1	0	nev+2	ot
ALDERS	19		m	0	0	all	-	all	Eu:UK	1977	CC	1448	n	V	n	n	1	cig only	18	27	2	3	nev+2	ot
ALDERS	20		m	0	0	all	-	all	Eu:UK	1977	CC	1448	n	V	n	n	1	cig only	28	99	3	0	nev+2	ot
ALDERS	21		f	0	0	all	-	all	Eu:UK	1977	CC	1448	n	V	n	n	1	cig only	1	17	1	0	nev+2	ot
ALDERS	22		f	0	0	all	-	all	Eu:UK	1977	CC	1448	n	V	n	n	1	cig only	18	27	2	3	nev+2	ot
ALDERS	23		f	0	0	all	-	all	Eu:UK	1977	CC	1448	n	V	n	n	1	cig only	28	99	3	0	nev+2	ot
ARMADA	46		m	0	0	all	-	all	Eu:wst	1986	CC	325	n	bl	n	y	0	cig+/-ot	1	14	1	0	nev any	st
ARMADA	47		m	0	0	all	-	all	Eu:wst	1986	CC	325	n	bl	n	y	0	cig+/-ot	15	24	2	3	nev any	st
ARMADA	48		m	0	0	all	-	all	Eu:wst	1986	CC	325	n	bl	n	y	0	cig+/-ot	25	99	3	0	nev any	st
AUVINE	5	x	c	0	0	all	-	all	Eu:Sca	1986	CC	517	n	bl	y	n	0	cig+/-ot	1	10	1	0	nev cigs	st
AUVINE	6	x	c	0	0	all	-	all	Eu:Sca	1986	CC	517	n	bl	y	n	0	cig+/-ot	11	20	2	3	nev cigs	st
AUVINE	7	x	c	0	0	all	-	all	Eu:Sca	1986	CC	517	n	bl	y	n	0	cig+/-ot	21	99	3	0	nev cigs	st
AXELSS	19	x	m	0	0	sca	-	all	Eu:Sca	1989	CC	436	n	bl	n	n	0	all/unsp	1	9	1	1	nev any	st
AXELSS	20	x	m	0	0	sca	-	all	Eu:Sca	1989	CC	436	n	bl	n	n	0	all/unsp	10	19	0	2	nev any	st
AXELSS	21	x	m	0	0	sca	-	all	Eu:Sca	1989	CC	436	n	bl	n	n	0	all/unsp	20	99	0	0	nev any	st
AXELSS	13		f	0	0	sca	-	all	Eu:Sca	1989	CC	436	n	bl	n	n	0	all/unsp	1	9	1	1	nev any	st
AXELSS	14		f	0	0	sca	-	all	Eu:Sca	1989	CC	436	n	bl	n	n	0	all/unsp	10	19	0	2	nev any	st
AXELSS	15		f	0	0	sca	-	all	Eu:Sca	1989	CC	436	n	bl	n	n	0	all/unsp	20	29	2	3	nev any	st
AXELSS	16		f	0	0	sca	-	all	Eu:Sca	1989	CC	436	n	bl	n	n	0	all/unsp	30	99	3	0	nev any	st
BARBON	5	x	m	0	0	all	-	all	Eu:wst	1979	CC	755	n	bl	y	y	0	all/unsp	1	9	1	1	nev any	st
BARBON	7	x	m	0	0	all	-	all	Eu:wst	1979	CC	755	n	bl	y	y	0	all/unsp	10	19	0	2	nev any	st
BARBON	9	x	m	0	0	all	-	all	Eu:wst	1979	CC	755	n	bl	y	y	0	all/unsp	20	29	2	3	nev any	st
BARBON	11	x	m	0	0	all	-	all	Eu:wst	1979	CC	755	n	bl	y	y	0	all/unsp	30	39	0	4	nev any	st
BARBON	13	x	m	0	0	all	-	all	Eu:wst	1979	CC	755	n	bl	y	y	0	all/unsp	40	99	3	0	nev any	st
BOUCOT	99		m	0	0	all	9	all	NAmer	1951	pr	121	n	bl	n	n	0	cig+/-ot	1	20	0	0	nev any	ot
BOUCOT	100		m	0	0	all	9	all	NAmer	1951	pr	121	n	bl	n	n	0	cig+/-ot	21	99	3	0	nev any	ot
BRESLO	13		m	0	0	all	-	all	NAmer	1949	CC	518	n	bl	n	y	0	cig+/-ot	1	9	1	1	nev+3	st
BRESLO	14		m	0	0	all	-	all	NAmer	1949	CC	518	n	bl	n	y	0	cig+/-ot	10	19	0	2	nev+3	st
BRESLO	15		m	0	0	all	-	all	NAmer	1949	CC	518	n	bl	n	y	0	cig+/-ot	20	39	2	0	nev+3	st
BRESLO	16		m	0	0	all	-	all	NAmer	1949	CC	518	n	bl	n	y	0	cig+/-ot	40	99	3	0	nev+3	st
BRESLO	29		f	0	0	all	-	all	NAmer	1949	CC	518	n	bl	n	y	0	cig+/-ot	1	19	1	0	nev+3	st
BRESLO	30		f	0	0	all	-	all	NAmer	1949	CC	518	n	bl	n	y	0	cig+/-ot	20	99	0	0	nev+3	st
BROWN2	32		m	0	0	wh	-	all	NAmer	1984	CC	14596	n	bl	n	y	2	cig+/-ot	1	19	1	0	nev cigs	or
BROWN2	42		m	0	0	wh	-	all	NAmer	1984	CC	14596	n	bl	n	y	2	cig+/-ot	20	99	0	0	nev cigs	or
BROWN2	31		f	0	0	wh	-	all	NAmer	1984	CC	14596	n	bl	n	y	2	cig+/-ot	1	19	1	0	nev cigs	or
BROWN2	41		f	0	0	wh	-	all	NAmer	1984	CC	14596	n	bl	n	y	2	cig+/-ot	20	99	0	0	nev cigs	or
BUFFLE	28		f	0	0	w-hi	-	all	NAmer	1976	CC	943	n	bl	y	n	0	cig+/-ot	1	19	1	0	nev cigs	or
BUFFLE	29		f	0	0	w-hi	-	all	NAmer	1976	CC	943	n	bl	y	n	0	cig+/-ot	20	20	2	3	nev cigs	or
BUFFLE	35		f	0	0	w-hi	-	all	NAmer	1976	CC	943	n	bl	y	n	0	cig+/-ot	21	99	3	0	nev cigs	or
CHATZI	1		c	0	0	all	-	all	Eu:bal	1987	CC	282	n	bl	n	y	0	all/unsp	1	45	0	0	nev any	st
CHATZI	2		c	0	0	all	-	all	Eu:bal	1987	CC	282	n	bl	n	y	0	all/unsp	46	74	0	0	nev any	st
CHATZI	3		c	0	0	all	-	all	Eu:bal	1987	CC	282	n	bl	n	y	0	all/unsp	75	99	0	6	nev any	st
CHEN2	3		m	0	0	all	-	all	As:Chi	1983	CC	193	n	ot	y	n	0	all/unsp	1	9	1	1	nev any	st
CHEN2	4		m	0	0	all	-	all	As:Chi	1983	CC	193	n	ot	y	n	0	all/unsp	10	20	2	0	nev any	st
CHEN2	5		m	0	0	all	-	all	As:Chi	1983	CC	193	n	ot	y	n	0	all/unsp	21	30	0	4	nev any	st
CHEN2	6		m	0	0	all	-	all	As:Chi	1983	CC	193	n	ot	y	n	0	all/unsp	31	99	3	0	nev any	st
CHEN2	7		f	0	0	all	-	all	As:Chi	1983	CC	193	n	ot	y	n	0	all/unsp	1	9	1	1	nev any	st
CHEN2	8		f	0	0	all	-	all	As:Chi	1983	CC	193	n	ot	y	n	0	all/unsp	10	20	2	0	nev any	st
CHEN2	9		f	0	0	all	-	all	As:Chi	1983	CC	193	n	ot	y	n	0	all/unsp	21	30	0	4	nev any	st
CHEN2	10		f	0	0	all	-	all	As:Chi	1983	CC	193	n	ot	y	n	0	all/unsp	31	99	3	0	nev any	st
CHOI	12		m	0	0	all	-	all	As:oth	1985	CC	375	n	bl	n	n	0	cig+/-ot	1	10	1	0	nev cigs	st
CHOI	13		m	0	0	all	-	all	As:oth	1985	CC	375	n	bl	n	n	0	cig+/-ot	11	20	2	3	nev cigs	st
CHOI	14		m	0	0	all	-	all	As:oth	1985	CC	375	n	bl	n	n	0	cig+/-ot	21	30	0	4	nev cigs	st
CHOI	15		m	0	0	all	-	all	As:oth	1985	CC	375	n	bl	n	n	0	cig+/-ot	31	40	0	5	nev cigs	st
CHOI	16		m	0	0	all	-	all	As:oth	1985	CC	375	n	bl	n	n	0	cig+/-ot	41	99	3	6	nev cigs	st
CHOI	17		f	0	0	all	-	all	As:oth	1985	CC	375	n	bl	n	n	0	cig+/-ot	1	10	1	0	nev cigs	st
CHOI	18		f	0	0	all	-	all	As:oth	1985	CC	375	n	bl	n	n	0	cig+/-ot	11	30	2	0	nev cigs	st
CHOI	20		f	0	0	all	-	all	As:oth	1985	CC	375	n	bl	n	n	0	cig+/-ot	31	99	3	0	nev cigs	st
COOKSO	1		c	0	0	bl	-	all	Africa	1961	CC	234	n	V	n	y	0	cig+/-ot	1	9	1	1	nev any	st
COOKSO	2		c	0	0	bl	-	all	Africa	1961	CC	234	n	V	n	y	0	cig+/-ot	10	99	0	0	nev any	st
CPSI	243		m	50	74	all	6	all	NAmer	1959	pr	5138	n	bl	n	n	1	cig only	1	19	1	0	nev any	ot
CPSI	246		m	50	74	all	6	all	NAmer	1959	pr	5138	n	bl	n	n	1	cig only	20	99	0	0	nev any	ot
CPSII	102		m	35	99	all	4	all	NAmer	1982	pr	3229	n	bl	n	n	1	cig only	1	20	0	0	nev any	ot
CPSII	103		m	35	99	all	4	all	NAmer	1982	pr	3229	n	bl	n	n	1	cig only	21	99	3	0	nev any	ot
CPSII	105		f	35	99	all	4	all	NAmer	1982	pr	3229	n	bl	n	n	1	cig+/-ot	1	19	1	0	nev cigs	ot
CPSII	106		f	35	99	all	4	all	NAmer	1982	pr	3229	n	bl	n	n	1	cig+/-ot	20	99	0	0	nev cigs	ot
DAMBER	6		m	0	0	all	-	all	Eu:Sca	1972	CC	579	n	bl	y	n	1	cig only	1	7	1	1	nev any	ot
DAMBER	7		m	0	0	all	-	all	Eu:Sca	1972	CC	579	n	bl	y	n	1	cig only	8	15	0	2	nev any	ot
DAMBER	8		m	0	0	all	-	all	Eu:Sca	1972	CC	579	n	bl	y	n	1	cig only	16	25	2	3	nev any	ot
DAMBER	9		m	0	0	all	-	all	Eu:Sca	1972	CC	579	n	bl	y	n	1	cig only	26	99	3	0	nev any	ot

Table 1G1 - 4

IESLC - Meta-analysis of Ever Smoking by Amount, Overview, Any product (or Cigarettes if Any not available)
All LC types
Least adjusted

REF	NRR	X	SEX	AGEL	AGEH	RACE	YF	LC TYPE	LOC	START	ST	NLC	R	VB	P	H	AD	PRODUCT	exL	exH	S1	S2	DENOM	De
DAVEYS	1		m	0	0	all	-	all	Eu:Ger	1930	CC	109	n	bl	y	n	0	all/unsp	1	5	1	1	nev any	st
DAVEYS	2		m	0	0	all	-	all	Eu:Ger	1930	CC	109	n	bl	y	n	0	all/unsp	6	10	0	2	nev any	st
DAVEYS	3		m	0	0	all	-	all	Eu:Ger	1930	CC	109	n	bl	y	n	0	all/unsp	11	20	2	3	nev any	st
DAVEYS	4		m	0	0	all	-	all	Eu:Ger	1930	CC	109	n	bl	y	n	0	all/unsp	21	99	3	0	nev any	st
DEAN	1		m	0	0	wh	-	all	Africa	1947	CC	603	n	V	y	n	0	cig only	1	20	0	0	nev any	st
DEAN	2		m	0	0	wh	-	all	Africa	1947	CC	603	n	V	y	n	0	cig only	25	45	3	0	nev any	st
DEAN	3		m	0	0	wh	-	all	Africa	1947	CC	603	n	V	y	n	0	cig only	50	99	0	6	nev any	st
DEAN2	25		m	0	0	all	-	all	Eu:UK	1960	CC	954	n	V	y	n	0	cig only	1	22	0	0	nev any	st
DEAN2	26		m	0	0	all	-	all	Eu:UK	1960	CC	954	n	V	y	n	0	cig only	23	99	3	0	nev any	st
DEAN2	29		f	0	0	all	-	all	Eu:UK	1960	CC	954	n	V	y	n	0	cig only	1	22	0	0	nev any	st
DEAN2	30		f	0	0	all	-	all	Eu:UK	1960	CC	954	n	V	y	n	0	cig only	23	99	3	0	nev any	st
DESTEF	1	x	m	0	0	all	-	all	SCAmer	1988	CC	497	n	bl	n	y	0	all/unsp	1	10	1	0	nev any	st
DESTEF	2	x	m	0	0	all	-	all	SCAmer	1988	CC	497	n	bl	n	y	0	all/unsp	11	20	2	3	nev any	st
DESTEF	3	x	m	0	0	all	-	all	SCAmer	1988	CC	497	n	bl	n	y	0	all/unsp	21	40	0	0	nev any	st
DESTEF	4	x	m	0	0	all	-	all	SCAmer	1988	CC	497	n	bl	n	y	0	all/unsp	41	99	3	6	nev any	st
DOLL	1		m	0	0	all	-	all	Eu:UK	1948	CC	1465	n	V	n	n	0	all/unsp	1	4	0	1	nev any	st
DOLL	2		m	0	0	all	-	all	Eu:UK	1948	CC	1465	n	V	n	n	0	all/unsp	5	14	1	2	nev any	st
DOLL	3		m	0	0	all	-	all	Eu:UK	1948	CC	1465	n	V	n	n	0	all/unsp	15	24	2	3	nev any	st
DOLL	4		m	0	0	all	-	all	Eu:UK	1948	CC	1465	n	V	n	n	0	all/unsp	25	49	3	0	nev any	st
DOLL	5		m	0	0	all	-	all	Eu:UK	1948	CC	1465	n	V	n	n	0	all/unsp	50	99	0	6	nev any	st
DOLL	7		f	0	0	all	-	all	Eu:UK	1948	CC	1465	n	V	n	n	0	all/unsp	1	4	0	1	nev any	st
DOLL	8		f	0	0	all	-	all	Eu:UK	1948	CC	1465	n	V	n	n	0	all/unsp	5	14	1	2	nev any	st
DOLL	9		f	0	0	all	-	all	Eu:UK	1948	CC	1465	n	V	n	n	0	all/unsp	15	24	2	3	nev any	st
DOLL	10		f	0	0	all	-	all	Eu:UK	1948	CC	1465	n	V	n	n	0	all/unsp	25	49	3	0	nev any	ot
DOLL2	46		m	35	99	all	5	all	Eu:UK	1951	pr	920	n	V	n	n	1	all/unsp	1	14	1	0	nev any	ot
DOLL2	47		m	35	99	all	5	all	Eu:UK	1951	pr	920	n	V	n	n	1	all/unsp	15	24	2	3	nev any	ot
DOLL2	48		m	35	99	all	5	all	Eu:UK	1951	pr	920	n	V	n	n	1	all/unsp	25	99	3	0	nev any	ot
DORGAN	10	x	m	0	0	wh	-	all	NAmer	1980	CC	2026	n	bl	y	y	0	cig+/-ot	1	19	1	0	nev any	st
DORGAN	11	x	m	0	0	wh	-	all	NAmer	1980	CC	2026	n	bl	y	y	0	cig+/-ot	20	99	0	0	nev any	st
DORGAN	34	x	m	0	0	bl	-	all	NAmer	1980	CC	2026	n	bl	y	y	0	cig+/-ot	1	19	1	0	nev any	st
DORGAN	35	x	m	0	0	bl	-	all	NAmer	1980	CC	2026	n	bl	y	y	0	cig+/-ot	20	99	0	0	nev any	st
DORGAN	96		f	0	0	all	-	all	NAmer	1980	CC	2026	n	bl	y	y	3	cig+/-ot	1	19	1	0	nev any	ot
DORGAN	97		f	0	0	all	-	all	NAmer	1980	CC	2026	n	bl	y	y	3	cig+/-ot	20	99	0	0	nev any	ot
DOSEME	5		m	0	0	all	-	all	Eu:bal	1979	CC	1210	n	bl	n	n	2	cig+/-ot	1	10	1	0	nev cigs	or
DOSEME	9		m	0	0	all	-	all	Eu:bal	1979	CC	1210	n	bl	n	n	2	cig+/-ot	11	20	2	3	nev cigs	or
DOSEME	13		m	0	0	all	-	all	Eu:bal	1979	CC	1210	n	bl	n	n	2	cig+/-ot	21	99	3	0	nev cigs	or
DUNN	1		m	0	0	all	0	all	NAmer	1954	pr	139	o	bl	n	n	0	cig+/-ot	1	4	0	1	nev cigs	st
DUNN	2		m	0	0	all	0	all	NAmer	1954	pr	139	o	bl	n	n	0	cig+/-ot	5	14	1	2	nev cigs	st
DUNN	3		m	0	0	all	0	all	NAmer	1954	pr	139	o	bl	n	n	0	cig+/-ot	15	24	2	3	nev cigs	st
DUNN	4		m	0	0	all	0	all	NAmer	1954	pr	139	o	bl	n	n	0	cig+/-ot	25	34	0	4	nev cigs	st
DUNN	5		m	0	0	all	0	all	NAmer	1954	pr	139	o	bl	n	n	0	cig+/-ot	35	99	3	0	nev cigs	st
EBELIN	2		m	0	0	all	-	all	Eu:Ger	1980	CC	130	n	bl	n	n	0	all/unsp	1	9	1	1	nev any	st
EBELIN	3		m	0	0	all	-	all	Eu:Ger	1980	CC	130	n	bl	n	n	0	all/unsp	10	19	0	2	nev any	st
EBELIN	4		m	0	0	all	-	all	Eu:Ger	1980	CC	130	n	bl	n	n	0	all/unsp	20	29	2	3	nev any	st
EBELIN	5		m	0	0	all	-	all	Eu:Ger	1980	CC	130	n	bl	n	n	0	all/unsp	30	39	0	4	nev any	st
EBELIN	6		m	0	0	all	-	all	Eu:Ger	1980	CC	130	n	bl	n	n	0	all/unsp	40	99	3	0	nev any	st
ESAKI	1		m	0	0	all	-	all	As:Jap	1961	CC	245	n	bl	y	n	0	cig+/-ot	1	14	1	0	nev cigs	st
ESAKI	2		m	0	0	all	-	all	As:Jap	1961	CC	245	n	bl	y	n	0	cig+/-ot	15	29	2	3	nev cigs	st
ESAKI	3		m	0	0	all	-	all	As:Jap	1961	CC	245	n	bl	y	n	0	cig+/-ot	30	99	3	0	nev cigs	st
FAN	6		m	0	0	all	-	all	As:Chi	1990	CC	403	n	ot	y	n	0	cig+/-ot	1	9	1	1	nev cigs	st
FAN	7		m	0	0	all	-	all	As:Chi	1990	CC	403	n	ot	y	n	0	cig+/-ot	10	19	0	2	nev cigs	st
FAN	8		m	0	0	all	-	all	As:Chi	1990	CC	403	n	ot	y	n	0	cig+/-ot	20	29	2	3	nev cigs	st
FAN	9		m	0	0	all	-	all	As:Chi	1990	CC	403	n	ot	y	n	0	cig+/-ot	30	99	3	0	nev cigs	st
FAN	10		f	0	0	all	-	all	As:Chi	1990	CC	403	n	ot	y	n	0	cig+/-ot	1	9	1	1	nev cigs	st
FAN	11		f	0	0	all	-	all	As:Chi	1990	CC	403	n	ot	y	n	0	cig+/-ot	10	19	0	2	nev cigs	st
FAN	12		f	0	0	all	-	all	As:Chi	1990	CC	403	n	ot	y	n	0	cig+/-ot	20	29	2	3	nev cigs	st
FAN	13		f	0	0	all	-	all	As:Chi	1990	CC	403	n	ot	y	n	0	cig+/-ot	30	99	3	0	nev cigs	st
GAO	24	x	f	0	0	all	-	all	As:Chi	1984	CC	1405	n	ot	n	n	0	cig+/-ot	1	9	1	1	nev cigs	st
GAO	25	x	f	0	0	all	-	all	As:Chi	1984	CC	1405	n	ot	n	n	0	cig+/-ot	10	19	0	2	nev cigs	st
GAO	26	x	f	0	0	all	-	all	As:Chi	1984	CC	1405	n	ot	n	n	0	cig+/-ot	20	99	0	0	nev cigs	st
GARSHI	18	x	m	0	0	all	-	all	NAmer	1981	CC	1081	o	bl	y	n	0	all/unsp	1	15	1	0	nev any	st
GARSHI	19	x	m	0	0	all	-	all	NAmer	1981	CC	1081	o	bl	y	n	0	all/unsp	16	25	2	3	nev any	st
GARSHI	20	x	m	0	0	all	-	all	NAmer	1981	CC	1081	o	bl	y	n	0	all/unsp	26	35	0	4	nev any	st
GARSHI	21	x	m	0	0	all	-	all	NAmer	1981	CC	1081	o	bl	y	n	0	all/unsp	36	99	3	0	nev any	st
GER	18	x	c	0	0	all	-	all	As:oth	1990	CC	141	n	ot	y	n	0	all/unsp	1	10	1	0	nev any	st
GER	19	x	c	0	0	all	-	all	As:oth	1990	CC	141	n	ot	y	n	0	all/unsp	11	20	2	3	nev any	st
GER	20	x	c	0	0	all	-	all	As:oth	1990	CC	141	n	ot	y	n	0	all/unsp	21	99	3	0	nev any	st
GOLLED	15	x	m	35	99	all	-	all	Eu:UK	1952	CC	443	n	V	y	n	0	cig only	1	10	1	0	nev any	st
GOLLED	16	x	m	35	99	all	-	all	Eu:UK	1952	CC	443	n	V	y	n	0	cig only	11	22	2	3	nev any	st
GOLLED	17	x	m	35	99	all	-	all	Eu:UK	1952	CC	443	n	V	y	n	0	cig only	23	99	3	0	nev any	st
GSELL	1		m	0	0	all	-	all	Eu:wst	1937	CC	150	n	bl	n	y	0	all/unsp	1	9	1	1	nev any	st
GSELL	2		m	0	0	all	-	all	Eu:wst	1937	CC	150	n	bl	n	y	0	all/unsp	10	14	0	2	nev any	st

Table 1G1 - 4

IESLC - Meta-analysis of Ever Smoking by Amount, Overview, Any product (or Cigarettes if Any not available)
All LC types
Least adjusted

REF	NRR	X	SEX	AGEL	AGEH	RACE	YF	LC TYPE	LOC	START	ST	NLC	R	VB	P	H	AD	PRODUCT	exL	exH	S1	S2	DENOM	De
GSELL	3		m	0	0	all	-	all	Eu:wst	1937	CC	150	n	bl	n	y	0	all/unsp	15	20	2	3	nev any	st
GSELL	4		m	0	0	all	-	all	Eu:wst	1937	CC	150	n	bl	n	y	0	all/unsp	21	35	0	4	nev any	st
GSELL	5		m	0	0	all	-	all	Eu:wst	1937	CC	150	n	bl	n	y	0	all/unsp	36	99	3	0	nev any	st
HAMMON	153		m	0	0	wh	0	all	NAmer	1952	pr	448	n	bl	n	n	1	cig only	1	9	1	1	nev any	ot
HAMMON	154		m	0	0	wh	0	all	NAmer	1952	pr	448	n	bl	n	n	1	cig only	10	20	2	0	nev any	ot
HAMMON	155		m	0	0	wh	0	all	NAmer	1952	pr	448	n	bl	n	n	1	cig only	21	99	3	0	nev any	ot
HANSEN	1		m	0	0	all	0	all	Eu:Sca	1968	pr	105	o	bl	y	n	2	all/unsp	1	19	1	0	nev any	ot
HANSEN	2		m	0	0	all	0	all	Eu:Sca	1968	pr	105	o	bl	y	n	2	all/unsp	20	99	0	0	nev any	ot
HU	1		m	0	0	all	-	all	As:Chi	1985	CC	227	n	ot	n	y	0	cig+/-ot	1	14	1	0	nev any	st
HU	2		m	0	0	all	-	all	As:Chi	1985	CC	227	n	ot	n	y	0	cig+/-ot	14	24	2	3	nev any	st
HU	3		m	0	0	all	-	all	As:Chi	1985	CC	227	n	ot	n	y	0	cig+/-ot	25	99	3	0	nev any	st
HU	4		f	0	0	all	-	all	As:Chi	1985	CC	227	n	ot	n	y	0	cig+/-ot	1	14	1	0	nev any	st
HU	5		f	0	0	all	-	all	As:Chi	1985	CC	227	n	ot	n	y	0	cig+/-ot	14	24	2	3	nev any	st
HU	6		f	0	0	all	-	all	As:Chi	1985	CC	227	n	ot	n	y	0	cig+/-ot	25	99	3	0	nev any	st
HU2	2		c	0	0	all	-	all	As:Chi	1977	CC	523	n	ot	y	n	0	cig+/-ot	1	4	0	1	nev cigs	st
HU2	3		c	0	0	all	-	all	As:Chi	1977	CC	523	n	ot	y	n	0	cig+/-ot	5	9	1	0	nev cigs	st
HU2	4		c	0	0	all	-	all	As:Chi	1977	CC	523	n	ot	y	n	0	cig+/-ot	10	14	0	2	nev cigs	st
HU2	5		c	0	0	all	-	all	As:Chi	1977	CC	523	n	ot	y	n	0	cig+/-ot	15	19	0	0	nev cigs	or
HU2	6		c	0	0	all	-	all	As:Chi	1977	CC	523	n	ot	y	n	0	cig+/-ot	20	29	2	3	nev cigs	st
HU2	7		c	0	0	all	-	all	As:Chi	1977	CC	523	n	ot	y	n	0	cig+/-ot	30	99	3	0	nev cigs	st
JARUP	1	x	m	0	0	all	-	all	Eu:Sca	1928	CC	102	o	bl	y	n	0	all/unsp	1	10	1	0	nev any	st
JARUP	2	x	m	0	0	all	-	all	Eu:Sca	1928	CC	102	o	bl	y	n	0	all/unsp	11	99	0	0	nev any	st
JEDRYC	60	x	m	0	0	all	-	all	Eu:est	1980	CC	1630	n	bl	y	n	0	cig+/-ot	1	19	1	0	nev any	st
JEDRYC	61	x	m	0	0	all	-	all	Eu:est	1980	CC	1630	n	bl	y	n	0	cig+/-ot	20	29	2	3	nev any	st
JEDRYC	62	x	m	0	0	all	-	all	Eu:est	1980	CC	1630	n	bl	y	n	0	cig+/-ot	30	99	3	0	nev any	st
JEDRYC	65	x	f	0	0	all	-	all	Eu:est	1980	CC	1630	n	bl	y	n	0	cig+/-ot	1	19	1	0	nev any	st
JEDRYC	66	x	f	0	0	all	-	all	Eu:est	1980	CC	1630	n	bl	y	n	0	cig+/-ot	20	29	2	3	nev any	st
JEDRYC	67	x	f	0	0	all	-	all	Eu:est	1980	CC	1630	n	bl	y	n	0	cig+/-ot	30	99	3	0	nev any	st
JOLY	7		m	0	0	all	-	all	SCAmer	1978	CC	826	n	bl	n	n	0	cig+/-ot	1	9	1	1	nev any	st
JOLY	8		m	0	0	all	-	all	SCAmer	1978	CC	826	n	bl	n	n	0	cig+/-ot	10	19	0	2	nev any	st
JOLY	9		m	0	0	all	-	all	SCAmer	1978	CC	826	n	bl	n	n	0	cig+/-ot	20	29	2	3	nev any	st
JOLY	10		m	0	0	all	-	all	SCAmer	1978	CC	826	n	bl	n	n	0	cig+/-ot	30	99	3	0	nev any	st
JOLY	3		f	0	0	all	-	all	SCAmer	1978	CC	826	n	bl	n	n	0	cig+/-ot	1	9	1	1	nev any	st
JOLY	4		f	0	0	all	-	all	SCAmer	1978	CC	826	n	bl	n	n	0	cig+/-ot	10	19	0	2	nev any	st
JOLY	5		f	0	0	all	-	all	SCAmer	1978	CC	826	n	bl	n	n	0	cig+/-ot	20	29	2	3	nev any	st
JOLY	6		f	0	0	all	-	all	SCAmer	1978	CC	826	n	bl	n	n	0	cig+/-ot	30	99	3	0	nev any	st
JUSSAW	10	x	m	0	0	all	-	all	As:Ind	1964	CC	792	n	V	n	n	0	cig only	1	4	0	1	nev any	st
JUSSAW	11	x	m	0	0	all	-	all	As:Ind	1964	CC	792	n	V	n	n	0	cig only	5	9	1	0	nev any	st
JUSSAW	12	x	m	0	0	all	-	all	As:Ind	1964	CC	792	n	V	n	n	0	cig only	10	14	0	2	nev any	st
JUSSAW	13	x	m	0	0	all	-	all	As:Ind	1964	CC	792	n	V	n	n	0	cig only	15	19	0	0	nev any	st
JUSSAW	14	x	m	0	0	all	-	all	As:Ind	1964	CC	792	n	V	n	n	0	cig only	20	24	2	3	nev any	st
JUSSAW	15	x	m	0	0	all	-	all	As:Ind	1964	CC	792	n	V	n	n	0	cig only	25	99	3	0	nev any	st
KHUDER	1		m	0	0	all	-	all	NAmer	1985	CC	482	n	bl	n	y	0	cig+/-ot	1	19	1	0	nev cigs	st
KHUDER	2		m	0	0	all	-	all	NAmer	1985	CC	482	n	bl	n	y	0	cig+/-ot	20	39	2	0	nev cigs	st
KHUDER	3		m	0	0	all	-	all	NAmer	1985	CC	482	n	bl	n	y	0	cig+/-ot	40	99	3	0	nev cigs	st
KOULUM	6		m	0	0	all	-	all	Eu:Sca	1936	CC	812	n	bl	n	n	0	all/unsp	1	9	1	1	nev any	st
KOULUM	5		m	0	0	all	-	all	Eu:Sca	1936	CC	812	n	bl	n	n	0	all/unsp	10	19	0	2	nev any	st
KOULUM	4		m	0	0	all	-	all	Eu:Sca	1936	CC	812	n	bl	n	n	0	all/unsp	20	99	0	0	nev any	st
KREUZE	19		m	1	45	all	-	all	Eu:Ger	1990	CC	2260	n	bl	n	n	3	cig+/-ot	1	9	1	1	nev any	or
KREUZE	20		m	1	45	all	-	all	Eu:Ger	1990	CC	2260	n	bl	n	n	3	cig+/-ot	10	19	0	2	nev any	or
KREUZE	21		m	1	45	all	-	all	Eu:Ger	1990	CC	2260	n	bl	n	n	3	cig+/-ot	20	29	2	3	nev any	or
KREUZE	22		m	1	45	all	-	all	Eu:Ger	1990	CC	2260	n	bl	n	n	3	cig+/-ot	30	99	3	0	nev any	or
KREUZE	30		m	55	69	all	-	all	Eu:Ger	1990	CC	2260	n	bl	n	n	3	cig+/-ot	1	9	1	1	nev any	or
KREUZE	31		m	55	69	all	-	all	Eu:Ger	1990	CC	2260	n	bl	n	n	3	cig+/-ot	10	19	0	2	nev any	or
KREUZE	32		m	55	69	all	-	all	Eu:Ger	1990	CC	2260	n	bl	n	n	3	cig+/-ot	20	29	2	3	nev any	or
KREUZE	33		m	55	69	all	-	all	Eu:Ger	1990	CC	2260	n	bl	n	n	3	cig+/-ot	30	99	3	0	nev any	or
KREUZE	25		f	1	45	all	-	all	Eu:Ger	1990	CC	2260	n	bl	n	n	3	cig+/-ot	1	9	1	1	nev any	or
KREUZE	26		f	1	45	all	-	all	Eu:Ger	1990	CC	2260	n	bl	n	n	3	cig+/-ot	10	19	0	2	nev any	or
KREUZE	27		f	1	45	all	-	all	Eu:Ger	1990	CC	2260	n	bl	n	n	3	cig+/-ot	20	29	2	3	nev any	or
KREUZE	36		f	55	69	all	-	all	Eu:Ger	1990	CC	2260	n	bl	n	n	3	cig+/-ot	1	9	1	1	nev any	or
KREUZE	37		f	55	69	all	-	all	Eu:Ger	1990	CC	2260	n	bl	n	n	3	cig+/-ot	10	19	0	2	nev any	or
KREUZE	38		f	55	69	all	-	all	Eu:Ger	1990	CC	2260	n	bl	n	n	3	cig+/-ot	20	29	2	3	nev any	or
KREYBE	21	x	m	0	0	all	-	all	Eu:Sca	1948	CC	300	n	bl	n	y	0	all/unsp	1	14	1	0	nev any	st
KREYBE	22	x	m	0	0	all	-	all	Eu:Sca	1948	CC	300	n	bl	n	y	0	all/unsp	15	24	2	3	nev any	st
KREYBE	23	x	m	0	0	all	-	all	Eu:Sca	1948	CC	300	n	bl	n	y	0	all/unsp	25	99	3	0	nev any	st
KREYBE	37	x	f	0	0	all	-	all	Eu:Sca	1948	CC	300	n	bl	n	y	0	all/unsp	1	14	1	0	nev any	st
KREYBE	38	x	f	0	0	all	-	all	Eu:Sca	1948	CC	300	n	bl	n	y	0	all/unsp	15	99	0	0	nev any	st
LAMTH	7		f	0	0	ch	-	all	As:HK	1983	CC	445	n	bl	n	n	0	all/unsp	1	10	1	0	nev any	or
LAMTH	2		f	0	0	ch	-	all	As:HK	1983	CC	445	n	bl	n	n	0	all/unsp	11	20	2	3	nev any	or
LAMTH	9		f	0	0	ch	-	all	As:HK	1983	CC	445	n	bl	n	n	0	all/unsp	21	99	3	0	nev any	or
LAUSSM	3	x	m	0	0	all	-	all	Eu:Ger	1982	CC	432	n	bl	n	n	0	all/unsp	1	9	1	1	nev any	st
LAUSSM	2	x	m	0	0	all	-	all	Eu:Ger	1982	CC	432	n	bl	n	n	0	all/unsp	10	19	0	2	nev any	st

Table 1G1 - 4

IESLC - Meta-analysis of Ever Smoking by Amount, Overview, Any product (or Cigarettes if Any not available)
All LC types
Least adjusted

REF	NRR	X	SEX	AGEL	AGEH	RACE	YF	LC TYPE	LOC	START	ST	NLC	R	VB	P	H	AD	PRODUCT	exL	exH	S1	S2	DENOM	De
LAUSSM	1	x	m	0	0	all	-	all	Eu:Ger	1982	CC	432	n	bl	n	n	0	all/unsp	20	99	0	0	nev any	st
LETOUR	2		c	0	0	all	-	all	NAmer	1983	CC	738	n	V	y	y	0	cig+/-ot	1	19	1	0	nev cigs	st
LETOUR	3		c	0	0	all	-	all	NAmer	1983	CC	738	n	V	y	y	0	cig+/-ot	20	40	2	0	nev cigs	st
LETOUR	4		c	0	0	all	-	all	NAmer	1983	CC	738	n	V	y	y	0	cig+/-ot	41	99	3	6	nev cigs	st
LIU2	5	x	m	0	0	all	-	all	As:Chi	1983	CC	316	n	ot	n	n	0	all/unsp	1	19	1	0	nev any	st
LIU2	6	x	m	0	0	all	-	all	As:Chi	1983	CC	316	n	ot	n	n	0	all/unsp	20	29	2	3	nev any	st
LIU2	7	x	m	0	0	all	-	all	As:Chi	1983	CC	316	n	ot	n	n	0	all/unsp	30	99	3	0	nev any	st
LIU2	11	x	f	0	0	all	-	all	As:Chi	1983	CC	316	n	ot	n	n	0	all/unsp	1	9	1	1	nev any	st
LIU2	12	x	f	0	0	all	-	all	As:Chi	1983	CC	316	n	ot	n	n	0	all/unsp	10	19	0	2	nev any	st
LIU2	13	x	f	0	0	all	-	all	As:Chi	1983	CC	316	n	ot	n	n	0	all/unsp	20	99	0	0	nev any	st
LIU3	3	x	m	0	0	all	-	all	As:Chi	1985	CC	110	n	ot	n	n	0	all/unsp	1	15	1	0	nev any	st
LIU3	4	x	m	0	0	all	-	all	As:Chi	1985	CC	110	n	ot	n	n	0	all/unsp	16	30	2	0	nev any	st
LIU3	5	x	m	0	0	all	-	all	As:Chi	1985	CC	110	n	ot	n	n	0	all/unsp	31	99	3	0	nev any	st
LIU4	7		m	35	69	all	-	all	As:Chi	1986	CC	1000-00	n	ot	y	n	2	cig only	1	19	1	0	nev any	ot
LIU4	8		m	35	69	all	-	all	As:Chi	1986	CC	1000-00	n	ot	y	n	2	cig only	20	20	2	3	nev any	ot
LIU4	9		m	35	69	all	-	all	As:Chi	1986	CC	1000-00	n	ot	y	n	2	cig only	21	99	3	0	nev any	ot
LIU5	2		c	0	0	all	-	all	As:Chi	1978	CC	111	n	ot	y	n	0	all/unsp	1	9	1	1	nev any	st
LIU5	3		c	0	0	all	-	all	As:Chi	1978	CC	111	n	ot	y	n	0	all/unsp	10	19	0	2	nev any	st
LIU5	4		c	0	0	all	-	all	As:Chi	1978	CC	111	n	ot	y	n	0	all/unsp	20	99	0	0	nev any	st
LUBIN	7	x	m	0	0	all	-	all	As:Chi	1984	CC	427	m	ot	y	n	0	cig only	1	6	1	1	nev any	st
LUBIN	8	x	m	0	0	all	-	all	As:Chi	1984	CC	427	m	ot	y	n	0	cig only	7	14	0	2	nev any	st
LUBIN	9	x	m	0	0	all	-	all	As:Chi	1984	CC	427	m	ot	y	n	0	cig only	15	19	0	0	nev any	st
LUBIN	10	x	m	0	0	all	-	all	As:Chi	1984	CC	427	m	ot	y	n	0	cig only	20	99	0	0	nev any	st
LUBIN2	273		m	0	0	all	-	all	Eu:mul	1976	CC	7804	n	bl	n	y	0	cig+/-ot	1	9	1	1	nev any	st
LUBIN2	274		m	0	0	all	-	all	Eu:mul	1976	CC	7804	n	bl	n	y	0	cig+/-ot	10	19	0	2	nev any	st
LUBIN2	275		m	0	0	all	-	all	Eu:mul	1976	CC	7804	n	bl	n	y	0	cig+/-ot	20	29	2	3	nev any	st
LUBIN2	276		m	0	0	all	-	all	Eu:mul	1976	CC	7804	n	bl	n	y	0	cig+/-ot	30	99	3	0	nev any	st
LUBIN2	281		f	0	0	all	-	all	Eu:mul	1976	CC	7804	n	bl	n	y	0	cig+/-ot	1	9	1	1	nev any	st
LUBIN2	282		f	0	0	all	-	all	Eu:mul	1976	CC	7804	n	bl	n	y	0	cig+/-ot	10	19	0	2	nev any	st
LUBIN2	283		f	0	0	all	-	all	Eu:mul	1976	CC	7804	n	bl	n	y	0	cig+/-ot	20	29	2	3	nev any	st
LUBIN2	284		f	0	0	all	-	all	Eu:mul	1976	CC	7804	n	bl	n	y	0	cig+/-ot	30	99	3	0	nev any	st
MACLEN	36		c	0	0	ch	-	all	As:oth	1972	CC	233	n	bl	n	n	2	cig+/-ot	1	9	1	1	nev cigs	or
MACLEN	37		c	0	0	ch	-	all	As:oth	1972	CC	233	n	bl	n	n	2	cig+/-ot	10	19	0	2	nev cigs	or
MACLEN	38		c	0	0	ch	-	all	As:oth	1972	CC	233	n	bl	n	n	2	cig+/-ot	20	29	2	3	nev cigs	or
MACLEN	39		c	0	0	ch	-	all	As:oth	1972	CC	233	n	bl	n	n	2	cig+/-ot	30	99	3	0	nev cigs	or
MARTIS	1		m	0	0	all	-	all	Eu:UK	1972	CC	201	n	V	n	n	0	cig+/-ot	1	14	1	0	nev cigs	st
MARTIS	2		m	0	0	all	-	all	Eu:UK	1972	CC	201	n	V	n	n	0	cig+/-ot	15	24	2	3	nev cigs	st
MARTIS	3		m	0	0	all	-	all	Eu:UK	1972	CC	201	n	V	n	n	0	cig+/-ot	25	99	3	0	nev cigs	st
MATOS	28	x	m	0	0	all	-	all	SCAmer	1994	CC	200	n	bl	n	n	0	cig+/-ot	1	14	1	0	nev any	st
MATOS	30	x	m	0	0	all	-	all	SCAmer	1994	CC	200	n	bl	n	n	0	cig+/-ot	15	24	2	3	nev any	st
MATOS	32	x	m	0	0	all	-	all	SCAmer	1994	CC	200	n	bl	n	n	0	cig+/-ot	25	99	3	0	nev any	st
MATSUD	1		m	0	0	all	-	all	As:Jap	1965	CC	179	n	bl	n	n	0	cig+/-ot	1	10	1	0	nev cigs	st
MATSUD	2		m	0	0	all	-	all	As:Jap	1965	CC	179	n	bl	n	n	0	cig+/-ot	11	20	2	3	nev cigs	st
MATSUD	3		m	0	0	all	-	all	As:Jap	1965	CC	179	n	bl	n	n	0	cig+/-ot	21	99	3	0	nev cigs	st
MCCONN	26		c	0	0	all	-	all	Eu:UK	1946	CC	100	n	V	n	y	0	all/unsp	1	10	1	0	nev any	st
MCCONN	25		c	0	0	all	-	all	Eu:UK	1946	CC	100	n	V	n	y	0	all/unsp	10	20	2	0	nev any	st
MCCONN	24		c	0	0	all	-	all	Eu:UK	1946	CC	100	n	V	n	y	0	all/unsp	21	99	3	0	nev any	st
NOTAN2	8		m	0	0	all	-	all	As:Ind	1963	CC	683	n	V	n	n	0	cig only	1	9	1	1	nev any	st
NOTAN2	9		m	0	0	all	-	all	As:Ind	1963	CC	683	n	V	n	n	0	cig only	10	19	0	2	nev any	st
NOTAN2	10		m	0	0	all	-	all	As:Ind	1963	CC	683	n	V	n	n	0	cig only	20	99	0	0	nev any	st
ORMOS	1		m	0	0	all	-	all	Eu:est	1947	CC	119	n	bl	y	y	0	cig+/-ot	1	15	1	0	nev any	st
ORMOS	2		m	0	0	all	-	all	Eu:est	1947	CC	119	n	bl	y	y	0	cig+/-ot	16	30	2	0	nev any	st
ORMOS	3		m	0	0	all	-	all	Eu:est	1947	CC	119	n	bl	y	y	0	cig+/-ot	31	99	3	0	nev any	st
OSANN	49		m	0	0	all	-	all	NAmer	1984	CC	1986	n	bl	n	n	2	cig+/-ot	1	39	0	0	nev cigs	or
OSANN	57		m	0	0	all	-	all	NAmer	1984	CC	1986	n	bl	n	n	2	cig+/-ot	40	99	3	0	nev cigs	or
OSANN	50		f	0	0	all	-	all	NAmer	1984	CC	1986	n	bl	n	n	2	cig+/-ot	1	39	0	0	nev cigs	or
OSANN	58		f	0	0	all	-	all	NAmer	1984	CC	1986	n	bl	n	n	2	cig+/-ot	40	99	3	0	nev cigs	or
OSANN2	4	x	f	0	0	all	-	all	NAmer	1964	ot	217	n	bl	n	y	0	cig+/-ot	1	19	1	0	nev cigs	st
OSANN2	5	x	f	0	0	all	-	all	NAmer	1964	ot	217	n	bl	n	y	0	cig+/-ot	20	99	0	0	nev cigs	st
PASTOR	1	x	m	0	0	all	-	all	Eu:wst	1976	CC	204	n	bl	y	n	0	all/unsp	1	9	1	1	nev any	st
PASTOR	2	x	m	0	0	all	-	all	Eu:wst	1976	CC	204	n	bl	y	n	0	all/unsp	10	19	0	2	nev any	st
PASTOR	3	x	m	0	0	all	-	all	Eu:wst	1976	CC	204	n	bl	y	n	0	all/unsp	20	29	2	3	nev any	st
PASTOR	4	x	m	0	0	all	-	all	Eu:wst	1976	CC	204	n	bl	y	n	0	all/unsp	30	99	3	0	nev any	st
PERNU	17		m	0	0	all	-	all	Eu:Sca	1944	CC	1606	n	bl	n	n	0	all/unsp	1	4	0	1	nev any	st
PERNU	18		m	0	0	all	-	all	Eu:Sca	1944	CC	1606	n	bl	n	n	0	all/unsp	5	9	1	0	nev any	st
PERNU	19		m	0	0	all	-	all	Eu:Sca	1944	CC	1606	n	bl	n	n	0	all/unsp	10	14	0	2	nev any	st
PERNU	20		m	0	0	all	-	all	Eu:Sca	1944	CC	1606	n	bl	n	n	0	all/unsp	15	19	0	0	nev any	st
PERNU	21		m	0	0	all	-	all	Eu:Sca	1944	CC	1606	n	bl	n	n	0	all/unsp	20	24	2	3	nev any	st
PERNU	22		m	0	0	all	-	all	Eu:Sca	1944	CC	1606	n	bl	n	n	0	all/unsp	25	29	0	0	nev any	st

Table 1G1 - 4

IESLC - Meta-analysis of Ever Smoking by Amount, Overview, Any product (or Cigarettes if Any not available)
All LC types
Least adjusted

REF	NRR	X	SEX	AGEL	AGEH	RACE	YF	LC TYPE	LOC	START	ST	NLC	R	VB	P	H	AD	PRODUCT	exL	exH	S1	S2	DENOM	De
PERNU	23		m	0	0	all	-	all	Eu:Sca	1944	CC	1606	n	bl	n	n	0	all/unsp	30	49	3	0	nev any	st
PERNU	24		m	0	0	all	-	all	Eu:Sca	1944	CC	1606	n	bl	n	n	0	all/unsp	50	99	0	6	nev any	st
PERNU	11		f	0	0	all	-	all	Eu:Sca	1944	CC	1606	n	bl	n	n	0	all/unsp	1	4	0	1	nev any	st
PERNU	12		f	0	0	all	-	all	Eu:Sca	1944	CC	1606	n	bl	n	n	0	all/unsp	5	9	1	0	nev any	st
PERNU	13		f	0	0	all	-	all	Eu:Sca	1944	CC	1606	n	bl	n	n	0	all/unsp	10	14	0	2	nev any	st
PERNU	14		f	0	0	all	-	all	Eu:Sca	1944	CC	1606	n	bl	n	n	0	all/unsp	15	19	0	0	nev any	st
PERNU	15		f	0	0	all	-	all	Eu:Sca	1944	CC	1606	n	bl	n	n	0	all/unsp	20	24	2	3	nev any	st
PERNU	16		f	0	0	all	-	all	Eu:Sca	1944	CC	1606	n	bl	n	n	0	all/unsp	25	99	3	0	nev any	st
PIKE	1		m	0	0	w-hi	-	all	NAmer	1972	CC	731	n	bl	y	n	0	all/unsp	1	20	0	0	nev any	st
PIKE	2		m	0	0	w-hi	-	all	NAmer	1972	CC	731	n	bl	y	n	0	all/unsp	21	40	0	0	nev any	st
PIKE	3		m	0	0	w-hi	-	all	NAmer	1972	CC	731	n	bl	y	n	0	all/unsp	41	99	3	6	nev any	st
PIKE	5		f	0	0	w-hi	-	all	NAmer	1972	CC	731	n	bl	y	n	0	all/unsp	1	20	0	0	nev any	st
PIKE	6		f	0	0	w-hi	-	all	NAmer	1972	CC	731	n	bl	y	n	0	all/unsp	21	40	0	0	nev any	st
PIKE	7		f	0	0	w-hi	-	all	NAmer	1972	CC	731	n	bl	y	n	0	all/unsp	41	99	3	6	nev any	st
POLEDN	2		c	0	0	all	-	all	NAmer	1978	CC	209	n	bl	y	n	0	cig+/-ot	1	19	1	0	nev cigs	st
POLEDN	4		c	0	0	all	-	all	NAmer	1978	CC	209	n	bl	y	n	0	cig+/-ot	20	99	0	0	nev cigs	st
RACHTA	5	x	f	0	0	all	-	all	Eu:est	1991	CC	118	n	bl	n	y	0	cig+/-ot	1	9	1	1	nev cigs	st
RACHTA	6	x	f	0	0	all	-	all	Eu:est	1991	CC	118	n	bl	n	y	0	cig+/-ot	10	19	0	2	nev cigs	st
RACHTA	7	x	f	0	0	all	-	all	Eu:est	1991	CC	118	n	bl	n	y	0	cig+/-ot	20	99	0	0	nev cigs	st
RANDIG	1		m	0	0	all	-	all	Eu:Ger	1951	CC	448	n	bl	n	n	0	all/unsp	1	4	0	1	nev any	st
RANDIG	2		m	0	0	all	-	all	Eu:Ger	1951	CC	448	n	bl	n	n	0	all/unsp	5	9	1	0	nev any	st
RANDIG	3		m	0	0	all	-	all	Eu:Ger	1951	CC	448	n	bl	n	n	0	all/unsp	10	19	0	2	nev any	st
RANDIG	4		m	0	0	all	-	all	Eu:Ger	1951	CC	448	n	bl	n	n	0	all/unsp	20	99	0	0	nev any	st
RANDIG	5		f	0	0	all	-	all	Eu:Ger	1951	CC	448	n	bl	n	n	0	all/unsp	1	4	0	1	nev any	st
RANDIG	6		f	0	0	all	-	all	Eu:Ger	1951	CC	448	n	bl	n	n	0	all/unsp	5	9	1	0	nev any	st
RANDIG	7		f	0	0	all	-	all	Eu:Ger	1951	CC	448	n	bl	n	n	0	all/unsp	10	99	0	0	nev any	st
SHAW	10		c	0	0	wh	-	all	NAmer	1988	CC	335	n	V	n	y	0	all/unsp	1	19	1	0	nev any	st
SHAW	11		c	0	0	wh	-	all	NAmer	1988	CC	335	n	V	n	y	0	all/unsp	20	99	0	0	nev any	st
SIEMIA	13		m	0	0	all	-	all	NAmer	1979	CC	857	n	V	y	y	0	cig+/-ot	1	19	1	0	nev cigs	or
SIEMIA	14		m	0	0	all	-	all	NAmer	1979	CC	857	n	V	y	y	0	cig+/-ot	20	39	2	0	nev cigs	or
SIEMIA	15		m	0	0	all	-	all	NAmer	1979	CC	857	n	V	y	y	0	cig+/-ot	40	99	3	0	nev cigs	or
SPITZ	5		c	0	0	b+hi	-	all	NAmer	1992	CC	177	n	bl	n	y	0	cig+/-ot	1	19	1	0	nev cigs	st
SPITZ	6		c	0	0	b+hi	-	all	NAmer	1992	CC	177	n	bl	n	y	0	cig+/-ot	20	99	0	0	nev cigs	st
STOCKS	25	x	m	0	0	all	-	all	Eu:UK	1952	CC	2932	n	V	y	n	0	cig+/-ot	1	14	1	0	nev any	st
STOCKS	26	x	m	0	0	all	-	all	Eu:UK	1952	CC	2932	n	V	y	n	0	cig+/-ot	15	21	2	3	nev any	st
STOCKS	27	x	m	0	0	all	-	all	Eu:UK	1952	CC	2932	n	V	y	n	0	cig+/-ot	22	28	0	0	nev any	st
STOCKS	28	x	m	0	0	all	-	all	Eu:UK	1952	CC	2932	n	V	y	n	0	cig+/-ot	29	36	0	4	nev any	st
STOCKS	29	x	m	0	0	all	-	all	Eu:UK	1952	CC	2932	n	V	y	n	0	cig+/-ot	37	99	3	0	nev any	st
STOCKS	48		f	0	0	all	-	all	Eu:UK	1952	CC	2932	n	V	y	n	1	cig+/-ot	1	14	1	0	nev any	ot
STOCKS	49		f	0	0	all	-	all	Eu:UK	1952	CC	2932	n	V	y	n	1	cig+/-ot	15	99	0	0	nev any	ot
TIZZAN	7		m	0	0	all	-	all	Eu:wst	1959	CC	1358	n	bl	n	n	0	cig only	1	9	1	1	nev any	st
TIZZAN	8		m	0	0	all	-	all	Eu:wst	1959	CC	1358	n	bl	n	n	0	cig only	10	20	2	0	nev any	st
TIZZAN	9		m	0	0	all	-	all	Eu:wst	1959	CC	1358	n	bl	n	n	0	cig only	21	40	0	0	nev any	st
TIZZAN	10		m	0	0	all	-	all	Eu:wst	1959	CC	1358	n	bl	n	n	0	cig only	41	99	3	6	nev any	st
TIZZAN	15		f	0	0	all	-	all	Eu:wst	1959	CC	1358	n	bl	n	n	0	cig only	1	9	1	1	nev any	st
TIZZAN	16		f	0	0	all	-	all	Eu:wst	1959	CC	1358	n	bl	n	n	0	cig only	10	99	0	0	nev any	st
WANG2	1	x	c	0	0	all	-	all	As:Chi	1980	CC	103	n	ot	n	n	0	cig+/-ot	1	4	0	1	nev cigs	st
WANG2	2	x	c	0	0	all	-	all	As:Chi	1980	CC	103	n	ot	n	n	0	cig+/-ot	5	9	1	0	nev cigs	st
WANG2	3	x	c	0	0	all	-	all	As:Chi	1980	CC	103	n	ot	n	n	0	cig+/-ot	10	14	0	2	nev cigs	st
WANG2	4	x	c	0	0	all	-	all	As:Chi	1980	CC	103	n	ot	n	n	0	cig+/-ot	15	19	0	0	nev cigs	st
WANG2	5	x	c	0	0	all	-	all	As:Chi	1980	CC	103	n	ot	n	n	0	cig+/-ot	20	29	2	3	nev cigs	st
WANG2	6	x	c	0	0	all	-	all	As:Chi	1980	CC	103	n	ot	n	n	0	cig+/-ot	30	39	0	4	nev cigs	st
WANG2	7	x	c	0	0	all	-	all	As:Chi	1980	CC	103	n	ot	n	n	0	cig+/-ot	40	99	3	0	nev cigs	st
WUWILL	18	x	f	0	0	all	-	all	As:Chi	1985	CC	965	n	ot	n	n	0	cig+/-ot	1	19	1	0	nev cigs	st
WUWILL	19	x	f	0	0	all	-	all	As:Chi	1985	CC	965	n	ot	n	n	0	cig+/-ot	20	99	0	0	nev cigs	st
WYNDE2	17		m	0	0	all	-	all	NAmer	1962	CC	404	n	bl	n	y	0	cig+/-ot	1	10	1	0	nev any	st
WYNDE2	18		m	0	0	all	-	all	NAmer	1962	CC	404	n	bl	n	y	0	cig+/-ot	11	20	2	3	nev any	st
WYNDE2	19		m	0	0	all	-	all	NAmer	1962	CC	404	n	bl	n	y	0	cig+/-ot	21	34	0	4	nev any	st
WYNDE2	20		m	0	0	all	-	all	NAmer	1962	CC	404	n	bl	n	y	0	cig+/-ot	35	99	3	0	nev any	st
WYNDE3	44		m	0	0	all	-	all	NAmer	1966	CC	350	n	bl	n	y	0	cig+/-ot	1	9	1	1	nev any	st
WYNDE3	45		m	0	0	all	-	all	NAmer	1966	CC	350	n	bl	n	y	0	cig+/-ot	10	20	2	0	nev any	st
WYNDE3	46		m	0	0	all	-	all	NAmer	1966	CC	350	n	bl	n	y	0	cig+/-ot	21	40	0	0	nev any	st
WYNDE3	47		m	0	0	all	-	all	NAmer	1966	CC	350	n	bl	n	y	0	cig+/-ot	41	99	3	6	nev any	st
WYNDE3	79		f	0	0	all	-	all	NAmer	1966	CC	350	n	bl	n	y	0	cig+/-ot	1	9	1	1	nev any	st
WYNDE3	80		f	0	0	all	-	all	NAmer	1966	CC	350	n	bl	n	y	0	cig+/-ot	10	20	2	0	nev any	st
WYNDE3	81		f	0	0	all	-	all	NAmer	1966	CC	350	n	bl	n	y	0	cig+/-ot	21	40	0	0	nev any	st
WYNDE3	82		f	0	0	all	-	all	NAmer	1966	CC	350	n	bl	n	y	0	cig+/-ot	41	99	3	6	nev any	st
WYNDE4	43		m	0	0	all	-	all	NAmer	1948	CC	684	n	bl	y	n	0	all/unsp	1	9	1	1	nev any	st
WYNDE4	44		m	0	0	all	-	all	NAmer	1948	CC	684	n	bl	y	n	0	all/unsp	10	15	0	2	nev any	st
WYNDE4	45		m	0	0	all	-	all	NAmer	1948	CC	684	n	bl	y	n	0	all/unsp	16	20	2	3	nev any	st
WYNDE4	46		m	0	0	all	-	all	NAmer	1948	CC	684	n	bl	y	n	0	all/unsp	21	34	0	4	nev any	st
WYNDE4	47		m	0	0	all	-	all	NAmer	1948	CC	684	n	bl	y	n	0	all/unsp	35	99	3	0	nev any	st

Table 1G1 - 4

IESLC - Meta-analysis of Ever Smoking by Amount, Overview, Any product (or Cigarettes if Any not available)
All LC types
Least adjusted

REF	NRR	X	SEX	AGEL	AGEH	RACE	YF	LC TYPE	LOC	START	ST	NLC	R	VB	P	H	AD	PRODUCT	exL	exH	S1	S2	DENOM	De
WYNDE4	57		f	0	0	all	-	all	NAmer	1948	CC	684	n	bl	y	n	2	all/unsp	1	9	1	1	nev any	ot
WYNDE4	58		f	0	0	all	-	all	NAmer	1948	CC	684	n	bl	y	n	2	all/unsp	10	15	0	2	nev any	ot
WYNDE4	59		f	0	0	all	-	all	NAmer	1948	CC	684	n	bl	y	n	2	all/unsp	16	20	2	3	nev any	ot
WYNDE4	60		f	0	0	all	-	all	NAmer	1948	CC	684	n	bl	y	n	2	all/unsp	21	34	0	4	nev any	ot
WYNDE4	61		f	0	0	all	-	all	NAmer	1948	CC	684	n	bl	y	n	2	all/unsp	35	99	3	0	nev any	ot
XU3	5	x	m	0	0	all	-	all	As:Chi	1981	CC	135	n	ot	n	n	0	all/unsp	1	9	1	1	nev any	st
XU3	6	x	m	0	0	all	-	all	As:Chi	1981	CC	135	n	ot	n	n	0	all/unsp	10	19	0	2	nev any	st
XU3	7	x	m	0	0	all	-	all	As:Chi	1981	CC	135	n	ot	n	n	0	all/unsp	20	29	2	3	nev any	st
XU3	8	x	m	0	0	all	-	all	As:Chi	1981	CC	135	n	ot	n	n	0	all/unsp	30	99	3	0	nev any	st
XU3	13	x	f	0	0	all	-	all	As:Chi	1981	CC	135	n	ot	n	n	0	all/unsp	1	9	1	1	nev any	st
XU3	14	x	f	0	0	all	-	all	As:Chi	1981	CC	135	n	ot	n	n	0	all/unsp	10	19	0	2	nev any	st
XU3	15	x	f	0	0	all	-	all	As:Chi	1981	CC	135	n	ot	n	n	0	all/unsp	20	99	0	0	nev any	st
YUAN	2		m	0	0	all	0	all	As:Chi	1986	pr	142	n	ot	n	n	2	cig+/-ot	1	19	1	0	nev cigs	ot
YUAN	3		m	0	0	all	0	all	As:Chi	1986	pr	142	n	ot	n	n	2	cig+/-ot	20	99	0	0	nev cigs	ot
ZHENG	11		m	0	0	all	-	all	As:Chi	1982	CC	540	n	ot	*	y	0	cig+/-ot	1	9	1	1	nev cigs	st
ZHENG	12		m	0	0	all	-	all	As:Chi	1982	CC	540	n	ot	*	y	0	cig+/-ot	10	19	0	2	nev cigs	st
ZHENG	13		m	0	0	all	-	all	As:Chi	1982	CC	540	n	ot	*	y	0	cig+/-ot	20	29	2	3	nev cigs	st
ZHENG	14		m	0	0	all	-	all	As:Chi	1982	CC	540	n	ot	*	y	0	cig+/-ot	30	99	3	0	nev cigs	st
ZHENG	22		f	0	0	all	-	all	As:Chi	1982	CC	540	n	ot	*	y	0	cig+/-ot	1	9	1	1	nev cigs	st
ZHENG	23		f	0	0	all	-	all	As:Chi	1982	CC	540	n	ot	*	y	0	cig+/-ot	10	99	0	0	nev cigs	st
ZHOU	4		c	0	0	all	-	all	As:Chi	1978	CC	1360	n	ot	n	n	0	all/unsp	1	9	1	1	nev any	st
ZHOU	5		c	0	0	all	-	all	As:Chi	1978	CC	1360	n	ot	n	n	0	all/unsp	10	19	0	2	nev any	st
ZHOU	6		c	0	0	all	-	all	As:Chi	1978	CC	1360	n	ot	n	n	0	all/unsp	20	99	0	0	nev any	st

Cigarette type is all/unspec for all RRs
except for the following:

REF	NRR	CIGTYPE
ALDERS	18	MC only
ALDERS	19	MC only
ALDERS	20	MC only
ALDERS	21	MC only
ALDERS	22	MC only
ALDERS	23	MC only
JUSSAW	10	MC only
JUSSAW	11	MC only
JUSSAW	12	MC only
JUSSAW	13	MC only
JUSSAW	14	MC only
JUSSAW	15	MC only
NOTAN2	8	MC only
NOTAN2	9	MC only
NOTAN2	10	MC only

REF	NRR	Cigarette equivalent
AGUDO	11	-
AGUDO	12	-
ALDERS	18	-
ALDERS	19	-
ALDERS	20	-
ALDERS	21	-
ALDERS	22	-
ALDERS	23	-
ARMADA	46	*
ARMADA	47	*
ARMADA	48	*
AUVINE	5	*
AUVINE	6	*
AUVINE	7	*
AXELSS	19	inc 1 g pipe = 1 cig
AXELSS	20	inc 1 g pipe = 1 cig
AXELSS	21	inc 1 g pipe = 1 cig
AXELSS	13	includes 1 g pipe tob = 1 cig
AXELSS	14	includes 1 g pipe tob = 1 cig
AXELSS	15	includes 1 g pipe tob = 1 cig
AXELSS	16	includes 1 g pipe tob = 1 cig
BARBON	5	*
BARBON	7	*
BARBON	9	*

Table 1G1 - 4

IESLC - Meta-analysis of Ever Smoking by Amount, Overview, Any product (or Cigarettes if Any not available)
All LC types
Least adjusted

REF	NRR	Cigarette equivalent
BARBON	11	*
BARBON	13	*
BOUCOT	99	up to 1 pk cigs, 4 cigars or 10 pipes
BOUCOT	100	> 1 pk cigs, 4 cigars or 10 pipes
BRESLO	13	*
BRESLO	14	*
BRESLO	15	*
BRESLO	16	*
BRESLO	29	*
BRESLO	30	*
BROWN2	32	*
BROWN2	42	*
BROWN2	31	*
BROWN2	41	*
BUFFLE	28	*
BUFFLE	29	*
BUFFLE	35	*
CHATZI	1	*
CHATZI	2	*
CHATZI	3	*
CHEN2	3	*
CHEN2	4	*
CHEN2	5	*
CHEN2	6	*
CHEN2	7	*
CHEN2	8	*
CHEN2	9	*
CHEN2	10	*
CHOI	12	*
CHOI	13	*
CHOI	14	*
CHOI	15	*
CHOI	16	*
CHOI	17	*
CHOI	18	*
CHOI	20	*
COOKSO	1	*
COOKSO	2	*
CPSI	243	-
CPSI	246	-
CPSII	102	-
CPSII	103	-
CPSII	105	*
CPSII	106	*
DAMBER	6	-
DAMBER	7	-
DAMBER	8	-
DAMBER	9	-
DAVEYS	1	1 cigar or up to 5 cigs
DAVEYS	2	2 cigars or 6-10 cigs
DAVEYS	3	3-4 cigars or 11-20 cigs
DAVEYS	4	>4 cigars or >20 cigarettes
DEAN	1	-
DEAN	2	-
DEAN	3	-
DEAN2	25	-
DEAN2	26	-
DEAN2	29	-
DEAN2	30	-
DESTEF	1	*
DESTEF	2	*
DESTEF	3	*
DESTEF	4	*
DOLL	1	*
DOLL	2	*
DOLL	3	*
DOLL	4	*
DOLL	5	*
DOLL	7	*
DOLL	8	*
DOLL	9	*
DOLL	10	*

Table 1G1 - 4

IESLC - Meta-analysis of Ever Smoking by Amount, Overview, Any product (or Cigarettes if Any not available)
All LC types
Least adjusted

REF	NRR	Cigarette equivalent
DOLL2	46	grams
DOLL2	47	grams
DOLL2	48	grams
DORGAN	10	*
DORGAN	11	*
DORGAN	34	*
DORGAN	35	*
DORGAN	96	*
DORGAN	97	*
DOSEME	5	*
DOSEME	9	*
DOSEME	13	*
DUNN	1	*
DUNN	2	*
DUNN	3	*
DUNN	4	*
DUNN	5	*
EBELIN	2	*
EBELIN	3	*
EBELIN	4	*
EBELIN	5	*
EBELIN	6	*
ESAKI	1	*
ESAKI	2	*
ESAKI	3	*
FAN	6	*
FAN	7	*
FAN	8	*
FAN	9	*
FAN	10	*
FAN	11	*
FAN	12	*
FAN	13	*
GAO	24	*
GAO	25	*
GAO	26	*
GARSHI	18	*
GARSHI	19	*
GARSHI	20	*
GARSHI	21	*
GER	18	*
GER	19	*
GER	20	*
GOLLED	15	-
GOLLED	16	-
GOLLED	17	-
GSELL	1	inc cigar = 5, cheroot = 4, pipe = 2.5
GSELL	2	inc cigar = 5, cheroot = 4, pipe = 2.5
GSELL	3	inc cigar = 5, cheroot = 4, pipe = 2.5
GSELL	4	inc cigar = 5, cheroot = 4, pipe = 2.5
GSELL	5	inc cigar = 5, cheroot = 4, pipe = 2.5
HAMMON	153	-
HAMMON	154	-
HAMMON	155	-
HANSEN	1	cig equivalents (not defined)
HANSEN	2	cig equivalents (not defined)
HU	1	*
HU	2	*
HU	3	*
HU	4	*
HU	5	*
HU	6	*
HU2	2	*
HU2	3	*
HU2	4	*
HU2	5	*
HU2	6	*
HU2	7	*
JARUP	1	gms, inc 1 pk pipe/wk = 7/d, 1 cigar = 4
JARUP	2	gms, inc 1 pk pipe/wk = 7/d, 1 cigar = 4
JEDRYC	60	*
JEDRYC	61	*

Table 1G1 - 4

IESLC - Meta-analysis of Ever Smoking by Amount, Overview, Any product (or Cigarettes if Any not available)
All LC types
Least adjusted

REF	NRR	Cigarette equivalent
JEDRYC	62	*
JEDRYC	65	*
JEDRYC	66	*
JEDRYC	67	*
JOLY	7	*
JOLY	8	*
JOLY	9	*
JOLY	10	*
JOLY	3	*
JOLY	4	*
JOLY	5	*
JOLY	6	*
JUSSAW	10	-
JUSSAW	11	-
JUSSAW	12	-
JUSSAW	13	-
JUSSAW	14	-
JUSSAW	15	-
KHUDER	1	*
KHUDER	2	*
KHUDER	3	*
KOULUM	6	*
KOULUM	5	*
KOULUM	4	*
KREUZE	19	*
KREUZE	20	*
KREUZE	21	*
KREUZE	22	*
KREUZE	30	*
KREUZE	31	*
KREUZE	32	*
KREUZE	33	*
KREUZE	25	*
KREUZE	26	*
KREUZE	27	*
KREUZE	36	*
KREUZE	37	*
KREUZE	38	*
KREYBE	21	grams inc 1 cig=1
KREYBE	22	grams inc 1 cig=1
KREYBE	23	grams inc 1 cig=1
KREYBE	37	grams inc 1 cig=1
KREYBE	38	grams inc 1 cig=1
LAMTH	7	*
LAMTH	2	*
LAMTH	9	*
LAUSSM	3	inc 1 cigar = 1 cig, 1 pipe = 1 cig
LAUSSM	2	inc 1 cigar = 1 cig, 1 pipe = 1 cig
LAUSSM	1	inc 1 cigar = 1 cig, 1 pipe = 1 cig
LETOUR	2	*
LETOUR	3	*
LETOUR	4	*
LIU2	5	*
LIU2	6	*
LIU2	7	*
LIU2	11	*
LIU2	12	*
LIU2	13	*
LIU3	3	Converted from kg/month
LIU3	4	Converted from kg/month
LIU3	5	Converted from kg/month
LIU4	7	-
LIU4	8	-
LIU4	9	-
LIU5	2	*
LIU5	3	*
LIU5	4	*
LUBIN	7	-
LUBIN	8	-
LUBIN	9	-
LUBIN	10	-
LUBIN2	273	*

Table 1G1 - 4

IESLC - Meta-analysis of Ever Smoking by Amount, Overview, Any product (or Cigarettes if Any not available)
All LC types
Least adjusted

REF	NRR	Cigarette equivalent
LUBIN2	274	*
LUBIN2	275	*
LUBIN2	276	*
LUBIN2	281	*
LUBIN2	282	*
LUBIN2	283	*
LUBIN2	284	*
MACLEN	36	*
MACLEN	37	*
MACLEN	38	*
MACLEN	39	*
MARTIS	1	*
MARTIS	2	*
MARTIS	3	*
MATOS	28	*
MATOS	30	*
MATOS	32	*
MATSUD	1	*
MATSUD	2	*
MATSUD	3	*
MCCONN	26	N cigs exc mixed pipe, or <2oz pure pipe
MCCONN	25	N cigs exc mixed pipe, or 2-4oz pure pip
MCCONN	24	N cigs exc mixed pipe, or >4oz pure pipe
NOTAN2	8	-
NOTAN2	9	-
NOTAN2	10	-
ORMOS	1	*
ORMOS	2	*
ORMOS	3	*
OSANN	49	*
OSANN	57	*
OSANN	50	*
OSANN	58	*
OSANN2	4	*
OSANN2	5	*
PASTOR	1	*
PASTOR	2	*
PASTOR	3	*
PASTOR	4	*
PERNU	17	grams
PERNU	18	grams
PERNU	19	grams
PERNU	20	grams
PERNU	21	grams
PERNU	22	grams
PERNU	23	grams
PERNU	24	grams
PERNU	11	grams
PERNU	12	grams
PERNU	13	grams
PERNU	14	grams
PERNU	15	grams
PERNU	16	grams
PIKE	1	*
PIKE	2	*
PIKE	3	*
PIKE	5	*
PIKE	6	*
PIKE	7	*
POLEDN	2	*
POLEDN	4	*
RACHTA	5	*
RACHTA	6	*
RACHTA	7	*
RANDIG	1	inc 1g pip=1, cgr=5, chrt=4, cigarillo=3
RANDIG	2	inc 1g pip=1, cgr=5, chrt=4, cigarillo=3
RANDIG	3	inc 1g pip=1, cgr=5, chrt=4, cigarillo=3
RANDIG	4	inc 1g pip=1, cgr=5, chrt=4, cigarillo=3
RANDIG	5	inc 1g pip=1, cgr=5, chrt=4, cigarillo=3
RANDIG	6	inc 1g pip=1, cgr=5, chrt=4, cigarillo=3
RANDIG	7	inc 1g pip=1, cgr=5, chrt=4, cigarillo=3
SHAW	10	*

Table 1G1 - 4

IESLC - Meta-analysis of Ever Smoking by Amount, Overview, Any product (or Cigarettes if Any not available)
All LC types
Least adjusted

REF	NRR	Cigarette equivalent
SHAW	11	*
SIEMIA	13	*
SIEMIA	14	*
SIEMIA	15	*
SPITZ	5	*
SPITZ	6	*
STOCKS	25	*
STOCKS	26	*
STOCKS	27	*
STOCKS	28	*
STOCKS	29	*
STOCKS	48	*
STOCKS	49	*
TIZZAN	7	-
TIZZAN	8	-
TIZZAN	9	-
TIZZAN	10	-
TIZZAN	15	-
TIZZAN	16	-
WANG2	1	*
WANG2	2	*
WANG2	3	*
WANG2	4	*
WANG2	5	*
WANG2	6	*
WANG2	7	*
WUWILL	18	*
WUWILL	19	*
WYNDE2	17	*
WYNDE2	18	*
WYNDE2	19	*
WYNDE2	20	*
WYNDE3	44	*
WYNDE3	45	*
WYNDE3	46	*
WYNDE3	47	*
WYNDE3	79	*
WYNDE3	80	*
WYNDE3	81	*
WYNDE3	82	*
WYNDE4	43	inc 1 cigar = 5 cigs, 1 pipe = 2.5 cigs
WYNDE4	44	inc 1 cigar = 5 cigs, 1 pipe = 2.5 cigs
WYNDE4	45	inc 1 cigar = 5 cigs, 1 pipe = 2.5 cigs
WYNDE4	46	inc 1 cigar = 5 cigs, 1 pipe = 2.5 cigs
WYNDE4	47	inc 1 cigar = 5 cigs, 1 pipe = 2.5 cigs
WYNDE4	57	inc 1 cigar = 5 cigs, 1 pipe = 2.5 cigs
WYNDE4	58	inc 1 cigar = 5 cigs, 1 pipe = 2.5 cigs
WYNDE4	59	inc 1 cigar = 5 cigs, 1 pipe = 2.5 cigs
WYNDE4	60	inc 1 cigar = 5 cigs, 1 pipe = 2.5 cigs
WYNDE4	61	inc 1 cigar = 5 cigs, 1 pipe = 2.5 cigs
XU3	5	*
XU3	6	*
XU3	7	*
XU3	8	*
XU3	13	*
XU3	14	*
XU3	15	*
YUAN	2	*
YUAN	3	*
ZHENG	11	*
ZHENG	12	*
ZHENG	13	*
ZHENG	14	*
ZHENG	22	*
ZHENG	23	*
ZHOU	4	*
ZHOU	5	*
ZHOU	6	*

In this overview table, subtotals and Qs values may be invalid and should be ignored

Table 1G1 - 5

IESLC - Meta-analysis of Ever Smoking by Amount, Overview, Any product (or Cigarettes if Any not available)
All LC types
Least adjusted

REF	NRR	SEX	AD	Number Exposed Case	Number Exposed Cont	Non-exposed Case	Non-exposed Cont	RR	95.00%CI
AGUDO	11	f	0	6	12	80	183	1.14	(0.41- 3.15)
AGUDO	12	f	0	17	11	80	183	3.54	(1.58- 7.89)
Subtotal AGUDO								2.29	(1.22- 4.30)
ALDERS	18	m	1	-	-	-	-	3.55	(1.94- 6.49)
ALDERS	19	m	1	-	-	-	-	7.96	(4.63- 13.69)
ALDERS	20	m	1	-	-	-	-	8.52	(5.07- 14.33)
ALDERS	21	f	1	-	-	-	-	2.62	(1.88- 3.65)
ALDERS	22	f	1	-	-	-	-	5.28	(3.79- 7.36)
ALDERS	23	f	1	-	-	-	-	6.90	(4.69- 10.15)
Subtotal ALDERS								4.91	(4.15- 5.82)
ARMADA	46	m	0	44	117	4	64	6.02	(2.07- 17.51)
ARMADA	47	m	0	134	105	4	64	20.42	(7.20- 57.88)
ARMADA	48	m	0	139	32	4	64	69.50	(23.58- 204.81)
Subtotal ARMADA								20.25	(10.96- 37.40)
AUVINE	5	c	0	57	33	44	229	8.99	(5.26- 15.37)
AUVINE	6	c	0	148	39	44	229	19.75	(12.25- 31.86)
AUVINE	7	c	0	61	8	44	229	39.68	(17.75- 88.72)
Subtotal AUVINE								16.56	(11.95- 22.95)
AXELSS	19	m	0	39	96	16	160	4.06	(2.15- 7.66)
AXELSS	20	m	0	111	122	16	160	9.10	(5.12- 16.16)
AXELSS	21	m	0	142	126	16	160	11.27	(6.39- 19.87)
AXELSS	13	f	0	13	37	18	154	3.01	(1.35- 6.68)
AXELSS	14	f	0	63	50	18	154	10.78	(5.84- 19.91)
AXELSS	15	f	0	28	15	18	154	15.97	(7.21- 35.36)
AXELSS	16	f	0	6	7	18	154	7.33	(2.22- 24.22)
Subtotal AXELSS								8.03	(6.21- 10.39)
BARBON	5	m	0	28	87	22	188	2.75	(1.49- 5.08)
BARBON	7	m	0	126	111	22	188	9.70	(5.82- 16.15)
BARBON	9	m	0	223	176	22	188	10.83	(6.67- 17.57)
BARBON	11	m	0	129	82	22	188	13.44	(7.98- 22.64)
BARBON	13	m	0	227	111	22	188	17.48	(10.64- 28.71)
Subtotal BARBON								10.08	(7.99- 12.72)
*BOUCOT	99	m	0	38	2670	0	805	23.23~	(1.43- 377.62)
*BOUCOT	100	m	0	43	1519	0	805	46.12~	(2.84- 748.16)
Subtotal BOUCOT								32.74	(4.56- 235.00)
BRESLO	13	m	0	16	45	22	110	1.78	(0.86- 3.69)
BRESLO	14	m	0	69	105	22	110	3.29	(1.90- 5.69)
BRESLO	15	m	0	296	193	22	110	7.67	(4.69- 12.55)
BRESLO	16	m	0	80	22	22	110	18.18	(9.42- 35.09)
BRESLO	29	f	0	5	5	12	14	1.17	(0.27- 5.02)
BRESLO	30	f	0	8	6	12	14	1.56	(0.42- 5.76)
Subtotal BRESLO								5.03	(3.79- 6.65)
BROWN2	32	m	2	-	-	-	-	6.10	(5.30- 6.90)
BROWN2	42	m	2	-	-	-	-	14.10	(12.70- 15.50)
BROWN2	31	f	2	-	-	-	-	8.40	(7.20- 9.70)
BROWN2	41	f	2	-	-	-	-	17.10	(15.30- 19.10)
Subtotal BROWN2								11.59	(10.92- 12.30)
BUFFLE	28	f	0	21	42	12	112	4.67	(2.11- 10.31)
BUFFLE	29	f	0	76	60	12	112	11.82	(5.96- 23.45)
BUFFLE	35	f	0	141	62	12	112	21.23	(10.90- 41.32)
Subtotal BUFFLE								11.51	(7.65- 17.33)
CHATZI	1	c	0	68	127	27	129	2.56	(1.54- 4.25)
CHATZI	2	c	0	73	123	27	129	2.84	(1.71- 4.70)
CHATZI	3	c	0	114	115	27	129	4.74	(2.90- 7.72)
Subtotal CHATZI								3.28	(2.46- 4.38)
CHEN2	3	m	0	17	26	9	33	2.40	(0.92- 6.25)
CHEN2	4	m	0	44	50	9	33	3.23	(1.39- 7.48)
CHEN2	5	m	0	34	9	9	33	13.85	(4.89- 39.22)
CHEN2	6	m	0	26	12	9	33	7.94	(2.91- 21.72)
CHEN2	7	f	0	5	17	25	33	0.39	(0.13- 1.20)
CHEN2	8	f	0	22	10	25	33	2.90	(1.17- 7.22)
CHEN2	9	f	0	7	1	25	33	9.24	(1.07- 80.02)
CHEN2	10	f	0	4	2	25	33	2.64	(0.45- 15.58)
Subtotal CHEN2								3.36	(2.30- 4.90)
CHOI	12	m	0	20	90	13	95	1.62	(0.76- 3.46)
CHOI	13	m	0	144	281	13	95	3.74	(2.03- 6.92)
CHOI	14	m	0	50	49	13	95	7.46	(3.70- 15.03)
CHOI	15	m	0	37	39	13	95	6.93	(3.33- 14.44)
CHOI	16	m	0	16	6	13	95	19.49	(6.47- 58.71)
CHOI	17	f	0	9	16	76	164	1.21	(0.51- 2.87)
CHOI	18	f	0	7	9	76	164	1.68	(0.60- 4.68)

Table 1G1 - 5

IESLC - Meta-analysis of Ever Smoking by Amount, Overview, Any product (or Cigarettes if Any not available)
All LC types
Least adjusted

REF	NRR	SEX	AD	Number Exposed Case	Number Exposed Cont	Non-exposed Case	Non-exposed Cont	RR	95.00%CI
CHOI	20	f	0	3	1	76	164	6.47	(0.66- 63.26)
Subtotal CHOI								3.83	(2.86- 5.13)
COOKSO	1	c	0	102	27	45	61	5.12	(2.89- 9.08)
COOKSO	2	c	0	82	11	45	61	10.11	(4.83- 21.13)
Subtotal COOKSO								6.61	(4.21- 10.40)
*CPSI	243	m	1	-	-	-	-	5.81	(4.33- 7.80)
*CPSI	246	m	1	-	-	-	-	13.60	(10.46- 17.67)
Subtotal CPSI								9.33	(7.68- 11.35)
*CPSII	102	m	1	-	-	-	-	9.99	(7.97- 12.51)
*CPSII	103	m	1	-	-	-	-	17.60	(14.05- 22.05)
*CPSII	105	f	1	-	-	-	-	4.16	(3.41- 5.09)
*CPSII	106	f	1	-	-	-	-	13.34	(11.31- 15.75)
Subtotal CPSII								9.98	(9.03- 11.02)
DAMBER	6	m	1	-	-	-	-	2.30	(1.30- 4.40)
DAMBER	7	m	1	-	-	-	-	7.30	(4.40- 12.70)
DAMBER	8	m	1	-	-	-	-	9.10	(5.50- 15.30)
DAMBER	9	m	1	-	-	-	-	14.90	(6.70- 33.50)
Subtotal DAMBER								6.60	(4.92- 8.86)
DAVEYS	1	m	0	11	69	3	23	1.22	(0.31- 4.77)
DAVEYS	2	m	0	31	32	3	23	7.43	(2.02- 27.27)
DAVEYS	3	m	0	19	22	3	23	6.62	(1.72- 25.56)
DAVEYS	4	m	0	29	21	3	23	10.59	(2.81- 39.94)
Subtotal DAVEYS								5.12	(2.63- 9.98)
DEAN	1	m	0	73	168	12	61	2.21	(1.12- 4.35)
DEAN	2	m	0	228	172	12	61	6.74	(3.52- 12.91)
DEAN	3	m	0	102	45	12	61	11.52	(5.66- 23.47)
Subtotal DEAN								5.46	(3.69- 8.08)
DEAN2	25	m	0	377	396	33	112	3.23	(2.14- 4.88)
DEAN2	26	m	0	252	112	33	112	7.64	(4.88- 11.95)
DEAN2	29	f	0	44	24	88	121	2.52	(1.43- 4.45)
DEAN2	30	f	0	18	5	88	121	4.95	(1.77- 13.84)
Subtotal DEAN2								4.21	(3.25- 5.45)
DESTEF	1	m	0	38	84	27	163	2.73	(1.56- 4.78)
DESTEF	2	m	0	155	119	27	163	7.86	(4.90- 12.61)
DESTEF	3	m	0	161	100	27	163	9.72	(6.03- 15.67)
DESTEF	4	m	0	116	31	27	163	22.59	(12.80- 39.87)
Subtotal DESTEF								8.30	(6.42- 10.73)
DOLL	1	m	0	55	129	7	61	3.72	(1.60- 8.64)
DOLL	2	m	0	489	570	7	61	7.48	(3.39- 16.50)
DOLL	3	m	0	475	431	7	61	9.60	(4.35- 21.22)
DOLL	4	m	0	293	154	7	61	16.58	(7.40- 37.13)
DOLL	5	m	0	38	12	7	61	27.60	(9.99- 76.25)
DOLL	7	f	0	16	25	40	59	0.94	(0.45- 1.99)
DOLL	8	f	0	24	18	40	59	1.97	(0.95- 4.09)
DOLL	9	f	0	14	6	40	59	3.44	(1.22- 9.71)
DOLL	10	f	0	14	0	40	59	42.60~	(2.47- 734.58)
Subtotal DOLL								4.97	(3.72- 6.65)
*DOLL2	46	m	1	-	-	-	-	6.71	(0.91- 49.81)
*DOLL2	47	m	1	-	-	-	-	12.29	(1.67- 90.41)
*DOLL2	48	m	1	-	-	-	-	23.71	(3.25- 173.24)
Subtotal DOLL2								12.54	(3.96- 39.67)
DORGAN	10	m	0	125	148	15	93	5.24	(2.89- 9.49)
DORGAN	11	m	0	574	252	15	93	14.12	(8.03- 24.85)
DORGAN	34	m	0	118	84	3	35	16.39	(4.88- 55.06)
DORGAN	35	m	0	145	32	3	35	52.86	(15.30- 182.62)
DORGAN	96	f	3	-	-	-	-	5.67	(4.36- 7.36)
DORGAN	97	f	3	-	-	-	-	12.22	(9.31- 16.04)
Subtotal DORGAN								8.71	(7.36- 10.30)
DOSEME	5	m	2	-	-	-	-	2.20	(1.40- 3.30)
DOSEME	9	m	2	-	-	-	-	3.10	(2.30- 4.10)
DOSEME	13	m	2	-	-	-	-	6.60	(4.40- 10.20)
Subtotal DOSEME								3.44	(2.79- 4.24)
*DUNN	1	m	0	3	2538	2	14160	8.37	(1.40- 50.06)
*DUNN	2	m	0	12	9418	2	14160	9.02	(2.02- 40.30)
*DUNN	3	m	0	75	27720	2	14160	19.16	(4.70- 78.00)
*DUNN	4	m	0	32	9017	2	14160	25.13	(6.02- 104.82)
*DUNN	5	m	0	13	3206	2	14160	28.71	(6.48- 127.15)
Subtotal DUNN								16.88	(8.61- 33.09)
EBELIN	2	m	0	20	72	12	117	2.71	(1.25- 5.87)
EBELIN	3	m	0	19	26	12	117	7.13	(3.08- 16.48)
EBELIN	4	m	0	47	37	12	117	12.39	(5.95- 25.80)

Table 1G1 - 5

IESLC - Meta-analysis of Ever Smoking by Amount, Overview, Any product (or Cigarettes if Any not available)
All LC types
Least adjusted

REF	NRR	SEX	AD	Number Exposed Case	Number Exposed Cont	Non-exposed Case	Non-exposed Cont	RR	95.00%CI
EBELIN	5	m	0	5	3	12	117	16.25	(3.45- 76.54)
EBELIN	6	m	0	4	4	12	117	9.75	(2.16- 44.04)
Subtotal EBELIN								6.99	(4.62- 10.59)
ESAKI	1	m	0	47	75	16	28	1.10	(0.54- 2.24)
ESAKI	2	m	0	74	58	16	28	2.23	(1.10- 4.51)
ESAKI	3	m	0	34	10	16	28	5.95	(2.34- 15.16)
Subtotal ESAKI								2.12	(1.36- 3.29)
FAN	6	m	0	13	121	36	236	0.70	(0.36- 1.38)
FAN	7	m	0	53	171	36	236	2.03	(1.27- 3.24)
FAN	8	m	0	111	183	36	236	3.98	(2.61- 6.07)
FAN	9	m	0	39	23	36	236	11.12	(5.96- 20.73)
FAN	10	f	0	17	48	69	320	1.64	(0.89- 3.03)
FAN	11	f	0	30	37	69	320	3.76	(2.18- 6.50)
FAN	12	f	0	31	12	69	320	11.98	(5.86- 24.50)
FAN	13	f	0	4	1	69	320	18.55	(2.04- 168.54)
Subtotal FAN								3.29	(2.68- 4.05)
GAO	24	f	0	70	74	435	605	1.32	(0.93- 1.87)
GAO	25	f	0	75	44	435	605	2.37	(1.60- 3.51)
GAO	26	f	0	91	12	435	605	10.55	(5.71- 19.50)
Subtotal GAO								2.25	(1.77- 2.87)
GARSHI	18	m	0	105	282	41	363	3.30	(2.23- 4.88)
GARSHI	19	m	0	387	601	41	363	5.70	(4.03- 8.07)
GARSHI	20	m	0	153	182	41	363	7.44	(5.05- 10.97)
GARSHI	21	m	0	223	350	41	363	5.64	(3.92- 8.12)
Subtotal GARSHI								5.35	(4.44- 6.44)
GER	18	c	0	21	108	51	246	0.94	(0.54- 1.64)
GER	19	c	0	45	168	51	246	1.29	(0.83- 2.02)
GER	20	c	0	24	42	51	246	2.76	(1.54- 4.95)
Subtotal GER								1.44	(1.06- 1.94)
GOLLED	15	m	0	83	679	15	490	3.99	(2.28- 7.00)
GOLLED	16	m	0	118	779	15	490	4.95	(2.86- 8.57)
GOLLED	17	m	0	143	333	15	490	14.03	(8.09- 24.31)
Subtotal GOLLED								6.56	(4.77- 9.04)
GSELL	1	m	0	11	36	2	29	4.43	(0.91- 21.60)
GSELL	2	m	0	10	37	2	29	3.92	(0.80- 19.30)
GSELL	3	m	0	27	26	2	29	15.06	(3.26- 69.59)
GSELL	4	m	0	49	9	2	29	78.94	(15.95- 390.82)
GSELL	5	m	0	51	13	2	29	56.88	(11.99- 269.87)
Subtotal GSELL								16.45	(8.14- 33.23)
*HAMMON	153	m	1	-	-	-	-	7.38	(3.72- 14.63)
*HAMMON	154	m	1	-	-	-	-	8.32	(4.66- 14.84)
*HAMMON	155	m	1	-	-	-	-	17.06	(9.44- 30.82)
Subtotal HAMMON								10.42	(7.31- 14.85)
*HANSEN	1	m	2	-	-	-	-	1.37	(0.63- 3.54)
*HANSEN	2	m	2	-	-	-	-	2.90	(1.14- 8.27)
Subtotal HANSEN								1.89	(0.99- 3.63)
HU	1	m	0	36	38	41	67	1.55	(0.85- 2.82)
HU	2	m	0	55	43	41	67	2.09	(1.20- 3.65)
HU	3	m	0	29	13	41	67	3.65	(1.70- 7.80)
HU	4	f	0	19	10	40	48	2.28	(0.95- 5.46)
HU	5	f	0	6	6	40	48	1.20	(0.36- 4.01)
HU	6	f	0	1	2	40	48	0.60	(0.05- 6.86)
Subtotal HU								2.02	(1.47- 2.77)
HU2	2	c	0	16	33	121	213	0.85	(0.45- 1.61)
HU2	3	c	0	44	58	121	213	1.34	(0.85- 2.10)
HU2	4	c	0	65	59	121	213	1.94	(1.28- 2.94)
HU2	5	c	0	64	54	121	213	2.09	(1.36- 3.19)
HU2	6	c	0	149	87	121	213	3.01	(2.13- 4.26)
HU2	7	c	0	64	19	121	213	5.93	(3.39- 10.37)
Subtotal HU2								2.20	(1.83- 2.64)
JARUP	1	m	0	42	25	11	42	6.41	(2.80- 14.68)
JARUP	2	m	0	49	27	11	42	6.93	(3.07- 15.63)
Subtotal JARUP								6.67	(3.73- 11.92)
JEDRYC	60	m	0	131	180	49	219	3.25	(2.22- 4.77)
JEDRYC	61	m	0	426	295	49	219	6.45	(4.58- 9.10)
JEDRYC	62	m	0	278	143	49	219	8.69	(6.00- 12.57)
JEDRYC	65	f	0	37	10	78	166	7.87	(3.72- 16.65)
JEDRYC	66	f	0	52	16	78	166	6.92	(3.71- 12.88)
JEDRYC	67	f	0	28	4	78	166	14.90	(5.05- 43.94)
Subtotal JEDRYC								6.17	(5.11- 7.46)
JOLY	7	m	0	16	54	12	218	5.38	(2.41- 12.05)

Table 1G1 - 5

IESLC - Meta-analysis of Ever Smoking by Amount, Overview, Any product (or Cigarettes if Any not available)
All LC types
Least adjusted

REF	NRR	SEX	AD	Number Exposed Case	Number Exposed Cont	Non-exposed Case	Non-exposed Cont	RR	95.00%CI
JOLY	8	m	0	217	318	12	218	12.40	(6.76- 22.73)
JOLY	9	m	0	126	175	12	218	13.08	(7.00- 24.43)
JOLY	10	m	0	193	161	12	218	21.78	(11.74- 40.39)
JOLY	3	f	0	33	38	52	283	4.73	(2.72- 8.21)
JOLY	4	f	0	72	49	52	283	8.00	(5.01- 12.77)
JOLY	5	f	0	28	22	52	283	6.93	(3.68- 13.03)
JOLY	6	f	0	32	13	52	283	13.40	(6.59- 27.23)
Subtotal JOLY								9.35	(7.55- 11.59)
JUSSAW	10	m	0	11	8	149	624	5.76	(2.28- 14.57)
JUSSAW	11	m	0	6	9	149	624	2.79	(0.98- 7.97)
JUSSAW	12	m	0	34	37	149	624	3.85	(2.34- 6.34)
JUSSAW	13	m	0	3	11	149	624	1.14	(0.31- 4.15)
JUSSAW	14	m	0	24	8	149	624	12.56	(5.53- 28.52)
JUSSAW	15	m	0	48	4	149	624	50.26	(17.84- 141.55)
Subtotal JUSSAW								5.71	(4.10- 7.96)
KHUDER	1	m	0	81	434	23	309	2.51	(1.54- 4.07)
KHUDER	2	m	0	224	288	23	309	10.45	(6.61- 16.52)
KHUDER	3	m	0	154	63	23	309	32.84	(19.62- 54.97)
Subtotal KHUDER								9.12	(6.89- 12.06)
KOULUM	6	m	0	37	77	5	54	5.19	(1.92- 14.06)
KOULUM	5	m	0	208	94	5	54	23.90	(9.26- 61.67)
KOULUM	4	m	0	478	75	5	54	68.83	(26.67- 177.62)
Subtotal KOULUM								21.38	(12.26- 37.28)
KREUZE	19	m	3	-	-	-	-	2.50	(0.70- 8.20)
KREUZE	20	m	3	-	-	-	-	8.70	(3.50- 21.90)
KREUZE	21	m	3	-	-	-	-	19.50	(7.50- 50.30)
KREUZE	22	m	3	-	-	-	-	20.80	(7.20- 60.50)
KREUZE	30	m	3	-	-	-	-	8.20	(5.20- 13.00)
KREUZE	31	m	3	-	-	-	-	25.10	(16.20- 38.70)
KREUZE	32	m	3	-	-	-	-	32.80	(20.90- 51.40)
KREUZE	33	m	3	-	-	-	-	33.30	(20.50- 54.00)
KREUZE	25	f	3	-	-	-	-	5.70	(1.60- 16.60)
KREUZE	26	f	3	-	-	-	-	11.80	(3.50- 29.00)
KREUZE	27	f	3	-	-	-	-	12.10	(3.00- 48.00)
KREUZE	36	f	3	-	-	-	-	2.00	(1.20- 3.30)
KREUZE	37	f	3	-	-	-	-	5.40	(3.50- 8.60)
KREUZE	38	f	3	-	-	-	-	7.70	(3.50- 17.30)
Subtotal KREUZE								11.60	(9.81- 13.72)
KREYBE	21	m	0	154	2341	6	644	7.06	(3.11- 16.04)
KREYBE	22	m	0	55	925	6	644	6.38	(2.73- 14.91)
KREYBE	23	m	0	43	248	6	644	18.61	(7.82- 44.27)
KREYBE	37	f	0	10	286	30	657	0.77	(0.37- 1.59)
KREYBE	38	f	0	2	42	30	657	1.04	(0.24- 4.51)
Subtotal KREYBE								3.88	(2.63- 5.74)
LAMTH	7	f	0	101	63	202	337	2.67	(1.87- 3.83)
LAMTH	2	f	0	90	28	202	337	5.36	(3.39- 8.48)
LAMTH	9	f	0	39	9	202	337	7.23	(3.43- 15.24)
Subtotal LAMTH								3.82	(2.93- 4.98)
LAUSSM	3	m	0	75	63	85	226	3.17	(2.08- 4.81)
LAUSSM	2	m	0	138	73	85	226	5.03	(3.45- 7.33)
LAUSSM	1	m	0	105	37	85	226	7.55	(4.81- 11.83)
Subtotal LAUSSM								4.85	(3.82- 6.15)
LETOUR	2	c	0	271	266	24	224	9.51	(6.04- 14.97)
LETOUR	3	c	0	367	198	24	224	17.30	(10.98- 27.27)
LETOUR	4	c	0	65	23	24	224	26.38	(13.98- 49.78)
Subtotal LETOUR								14.84	(11.14- 19.77)
LIU2	5	m	0	21	93	12	44	0.83	(0.37- 1.83)
LIU2	6	m	0	97	66	12	44	5.39	(2.65- 10.97)
LIU2	7	m	0	94	21	12	44	16.41	(7.42- 36.33)
LIU2	11	f	0	8	10	38	69	1.45	(0.53- 3.99)
LIU2	12	f	0	16	9	38	69	3.23	(1.30- 8.00)
LIU2	13	f	0	30	4	38	69	13.62	(4.46- 41.57)
Subtotal LIU2								4.03	(2.84- 5.72)
LIU3	3	m	0	25	93	4	19	1.28	(0.40- 4.09)
LIU3	4	m	0	20	93	4	19	1.02	(0.31- 3.33)
LIU3	5	m	0	7	19	4	19	1.75	(0.44- 6.98)
Subtotal LIU3								1.28	(0.63- 2.61)
LIU4	7	m	2	-	-	-	-	2.11	(2.02- 2.20)
LIU4	8	m	2	-	-	-	-	3.60	(3.49- 3.71)
LIU4	9	m	2	-	-	-	-	6.98	(6.73- 7.23)
Subtotal LIU4								3.95	(3.87- 4.03)

Table 1G1 - 5

IESLC - Meta-analysis of Ever Smoking by Amount, Overview, Any product (or Cigarettes if Any not available)
All LC types
Least adjusted

REF	NRR	SEX	AD	Number Exposed Case	Number Exposed Cont	Non-exposed Case	Non-exposed Cont	RR	95.00%CI
LIU5	2	c	0	14	27	26	41	0.82	(0.36- 1.84)
LIU5	3	c	0	21	21	26	41	1.58	(0.72- 3.44)
LIU5	4	c	0	50	22	26	41	3.58	(1.78- 7.23)
Subtotal LIU5								1.79	(1.16- 2.78)
LUBIN	7	m	0	4	41	8	73	0.89	(0.25- 3.14)
LUBIN	8	m	0	7	59	8	73	1.08	(0.37- 3.16)
LUBIN	9	m	0	12	24	8	73	4.56	(1.67- 12.48)
LUBIN	10	m	0	29	67	8	73	3.95	(1.69- 9.24)
Subtotal LUBIN								2.40	(1.45- 3.99)
LUBIN2	273	m	0	1887	3759	190	2616	6.91	(5.91- 8.09)
LUBIN2	274	m	0	1529	2771	190	2616	7.60	(6.47- 8.92)
LUBIN2	275	m	0	1963	2547	190	2616	10.61	(9.06- 12.44)
LUBIN2	276	m	0	1261	1394	190	2616	12.45	(10.55- 14.70)
LUBIN2	281	f	0	151	218	336	1188	2.45	(1.93- 3.11)
LUBIN2	282	f	0	221	213	336	1188	3.67	(2.93- 4.59)
LUBIN2	283	f	0	134	103	336	1188	4.60	(3.46- 6.11)
LUBIN2	284	f	0	45	33	336	1188	4.82	(3.03- 7.68)
Subtotal LUBIN2								7.07	(6.60- 7.58)
MACLEN	36	c	2	-	-	-	-	1.35	(0.64- 2.84)
MACLEN	37	c	2	-	-	-	-	2.66	(1.46- 4.81)
MACLEN	38	c	2	-	-	-	-	2.93	(1.57- 5.45)
MACLEN	39	c	2	-	-	-	-	4.10	(2.07- 8.15)
Subtotal MACLEN								2.65	(1.91- 3.67)
MARTIS	1	m	0	31	39	4	25	4.97	(1.56- 15.78)
MARTIS	2	m	0	91	87	4	25	6.54	(2.19- 19.55)
MARTIS	3	m	0	75	50	4	25	9.38	(3.08- 28.57)
Subtotal MARTIS								6.77	(3.55- 12.94)
MATOS	28	m	0	17	88	11	110	1.93	(0.86- 4.34)
MATOS	30	m	0	65	90	11	110	7.22	(3.60- 14.50)
MATOS	32	m	0	106	105	11	110	10.10	(5.13- 19.85)
Subtotal MATOS								5.78	(3.81- 8.77)
MATSUD	1	m	0	37	1237	3	1255	12.51	(3.85- 40.69)
MATSUD	2	m	0	75	1607	3	1255	19.52	(6.14- 62.05)
MATSUD	3	m	0	58	470	3	1255	51.62	(16.10- 165.55)
Subtotal MATSUD								23.37	(11.91- 45.84)
MCCONN	26	c	0	7	43	9	23	0.42	(0.14- 1.26)
MCCONN	25	c	0	49	92	9	23	1.36	(0.58- 3.17)
MCCONN	24	c	0	35	42	9	23	2.13	(0.87- 5.19)
Subtotal MCCONN								1.21	(0.71- 2.08)
NOTAN2	8	m	0	6	42	134	544	0.58	(0.24- 1.39)
NOTAN2	9	m	0	28	47	134	544	2.42	(1.46- 4.01)
NOTAN2	10	m	0	44	40	134	544	4.47	(2.80- 7.13)
Subtotal NOTAN2								2.66	(1.93- 3.66)
ORMOS	1	m	0	32	329	7	777	10.80	(4.72- 24.71)
ORMOS	2	m	0	40	577	7	777	7.69	(3.42- 17.30)
ORMOS	3	m	0	15	128	7	777	13.01	(5.20- 32.52)
Subtotal ORMOS								10.06	(6.17- 16.42)
OSANN	49	m	2	-	-	-	-	17.70	(12.60- 24.80)
OSANN	57	m	2	-	-	-	-	42.80	(30.50- 60.10)
OSANN	50	f	2	-	-	-	-	14.40	(11.00- 18.90)
OSANN	58	f	2	-	-	-	-	40.90	(29.30- 57.10)
Subtotal OSANN								24.11	(20.59- 28.24)
OSANN2	4	f	0	35	55	33	109	2.10	(1.18- 3.74)
OSANN2	5	f	0	149	47	33	109	10.47	(6.29- 17.42)
Subtotal OSANN2								5.17	(3.53- 7.58)
PASTOR	1	m	0	8	39	10	89	1.83	(0.67- 4.98)
PASTOR	2	m	0	51	78	10	89	5.82	(2.77- 12.23)
PASTOR	3	m	0	89	100	10	89	7.92	(3.88- 16.17)
PASTOR	4	m	0	46	45	10	89	9.10	(4.20- 19.69)
Subtotal PASTOR								6.01	(4.05- 8.90)
PERNU	17	m	0	15	15	97	275	2.84	(1.34- 6.01)
PERNU	18	m	0	61	31	97	275	5.58	(3.42- 9.11)
PERNU	19	m	0	224	96	97	275	6.62	(4.74- 9.23)
PERNU	20	m	0	127	67	97	275	5.37	(3.69- 7.82)
PERNU	21	m	0	478	138	97	275	9.82	(7.28- 13.24)
PERNU	22	m	0	361	54	97	275	18.95	(13.12- 27.38)
PERNU	23	m	0	40	23	97	275	4.93	(2.81- 8.66)
PERNU	24	m	0	74	14	97	275	14.99	(8.09- 27.75)
PERNU	11	f	0	3	14	110	971	1.89	(0.54- 6.68)
PERNU	12	f	0	5	13	110	971	3.40	(1.19- 9.70)
PERNU	13	f	0	4	30	110	971	1.18	(0.41- 3.40)

Table 1G1 - 5

IESLC - Meta-analysis of Ever Smoking by Amount, Overview, Any product (or Cigarettes if Any not available)
All LC types
Least adjusted

REF	NRR	SEX	AD	Number Exposed Case	Number Exposed Cont	Non-exposed Case	Non-exposed Cont	RR	95.00%CI
PERNU	14	f	0	1	14	110	971	0.63	(0.08- 4.84)
PERNU	15	f	0	1	11	110	971	0.80	(0.10- 6.27)
PERNU	16	f	0	5	7	110	971	6.31	(1.97- 20.20)
Subtotal PERNU								7.42	(6.44- 8.54)
PIKE	1	m	0	181	168	18	69	4.13	(2.36- 7.23)
PIKE	2	m	0	228	109	18	69	8.02	(4.55- 14.13)
PIKE	3	m	0	66	37	18	69	6.84	(3.55- 13.18)
PIKE	5	f	0	73	60	36	96	3.24	(1.94- 5.42)
PIKE	6	f	0	67	26	36	96	6.87	(3.80- 12.44)
PIKE	7	f	0	16	3	36	96	14.22	(3.91- 51.73)
Subtotal PIKE								5.49	(4.27- 7.05)
POLEDN	2	c	0	53	103	12	139	5.96	(3.03- 11.72)
POLEDN	4	c	0	143	168	12	139	9.86	(5.25- 18.52)
Subtotal POLEDN								7.80	(4.92- 12.37)
RACHTA	5	f	0	6	7	33	98	2.55	(0.80- 8.12)
RACHTA	6	f	0	25	24	33	98	3.09	(1.56- 6.14)
RACHTA	7	f	0	54	12	33	98	13.36	(6.38- 28.00)
Subtotal RACHTA								5.30	(3.34- 8.40)
RANDIG	1	m	0	13	28	5	22	2.04	(0.63- 6.60)
RANDIG	2	m	0	65	99	5	22	2.89	(1.04- 8.01)
RANDIG	3	m	0	190	164	5	22	5.10	(1.89- 13.76)
RANDIG	4	m	0	142	68	5	22	9.19	(3.34- 25.31)
RANDIG	5	f	0	1	21	17	92	0.26	(0.03- 2.05)
RANDIG	6	f	0	12	13	17	92	5.00	(1.95- 12.79)
RANDIG	7	f	0	3	5	17	92	3.25	(0.71- 14.88)
Subtotal RANDIG								3.84	(2.50- 5.89)
SHAW	10	c	0	46	90	11	107	4.97	(2.43- 10.16)
SHAW	11	c	0	278	176	11	107	15.36	(8.03- 29.39)
Subtotal SHAW								9.23	(5.71- 14.93)
SIEMIA	13	m	0	-	-	-	-	3.00	(1.00- 9.90)
SIEMIA	14	m	0	-	-	-	-	4.50	(1.80- 13.20)
SIEMIA	15	m	0	-	-	-	-	7.90	(3.00- 24.10)
Subtotal SIEMIA								4.87	(2.64- 8.95)
SPITZ	5	c	0	27	88	10	96	2.95	(1.35- 6.43)
SPITZ	6	c	0	95	48	10	96	19.00	(9.08- 39.74)
Subtotal SPITZ								7.89	(4.61- 13.49)
STOCKS	25	m	0	691	1743	45	638	5.62	(4.10- 7.70)
STOCKS	26	m	0	919	2257	45	638	5.77	(4.23- 7.89)
STOCKS	27	m	0	232	380	45	638	8.66	(6.14- 12.20)
STOCKS	28	m	0	277	635	45	638	6.18	(4.43- 8.63)
STOCKS	29	m	0	302	468	45	638	9.15	(6.54- 12.79)
STOCKS	48	f	1	-	-	-	-	2.24	(1.64- 3.03)
STOCKS	49	f	1	-	-	-	-	6.34	(4.42- 8.93)
Subtotal STOCKS								5.65	(4.99- 6.39)
TIZZAN	7	m	0	130	238	180	305	0.93	(0.70- 1.23)
TIZZAN	8	m	0	468	470	180	305	1.69	(1.35- 2.11)
TIZZAN	9	m	0	301	108	180	305	4.72	(3.54- 6.29)
TIZZAN	10	m	0	83	20	180	305	7.03	(4.17- 11.85)
TIZZAN	15	f	0	11	14	25	114	3.58	(1.46- 8.82)
TIZZAN	16	f	0	14	14	25	114	4.56	(1.93- 10.75)
Subtotal TIZZAN								2.16	(1.87- 2.48)
WANG2	1	c	0	5	14	11	43	1.40	(0.41- 4.71)
WANG2	2	c	0	3	10	11	43	1.17	(0.28- 5.00)
WANG2	3	c	0	5	14	11	43	1.40	(0.41- 4.71)
WANG2	4	c	0	4	14	11	43	1.12	(0.31- 4.07)
WANG2	5	c	0	27	32	11	43	3.30	(1.43- 7.62)
WANG2	6	c	0	7	11	11	43	2.49	(0.78- 7.90)
WANG2	7	c	0	9	4	11	43	8.80	(2.28- 33.97)
Subtotal WANG2								2.25	(1.45- 3.49)
WUWILL	18	f	0	451	311	417	601	2.09	(1.73- 2.53)
WUWILL	19	f	0	88	40	417	601	3.17	(2.14- 4.70)
Subtotal WUWILL								2.26	(1.91- 2.69)
WYNDE2	17	m	0	17	114	8	105	1.96	(0.81- 4.72)
WYNDE2	18	m	0	122	203	8	105	7.89	(3.71- 16.75)
WYNDE2	19	m	0	88	83	8	105	13.92	(6.39- 30.32)
WYNDE2	20	m	0	155	112	8	105	18.16	(8.50- 38.80)
Subtotal WYNDE2								8.64	(5.83- 12.82)
WYNDE3	44	m	0	8	42	9	88	1.86	(0.67- 5.17)
WYNDE3	45	m	0	77	114	9	88	6.60	(3.14- 13.90)
WYNDE3	46	m	0	108	82	9	88	12.88	(6.12- 27.09)
WYNDE3	47	m	0	68	26	9	88	25.57	(11.25- 58.14)

Table 1G1 - 5

IESLC - Meta-analysis of Ever Smoking by Amount, Overview, Any product (or Cigarettes if Any not available)
All LC types
Least adjusted

REF	NRR	SEX	AD	Number Exposed Case	Number Exposed Cont	Non-exposed Case	Non-exposed Cont	RR	95.00%CI
WYNDE3	79	f	0	3	19	20	76	0.60	(0.16- 2.23)
WYNDE3	80	f	0	24	24	20	76	3.80	(1.79- 8.05)
WYNDE3	81	f	0	15	10	20	76	5.70	(2.23- 14.59)
WYNDE3	82	f	0	4	3	20	76	5.07	(1.05- 24.50)
Subtotal WYNDE3								6.19	(4.51- 8.50)
WYNDE4	43	m	0	17	82	12	115	1.99	(0.90- 4.38)
WYNDE4	44	m	0	67	147	12	115	4.37	(2.26- 8.46)
WYNDE4	45	m	0	228	274	12	115	7.97	(4.29- 14.82)
WYNDE4	46	m	0	190	98	12	115	18.58	(9.77- 35.33)
WYNDE4	47	m	0	130	64	12	115	19.47	(10.00- 37.88)
WYNDE4	57	f	2	-	-	-	-	1.13	(0.33- 3.89)
WYNDE4	58	f	2	-	-	-	-	2.01	(0.66- 6.10)
WYNDE4	59	f	2	-	-	-	-	6.49	(2.35- 17.93)
WYNDE4	60	f	2	-	-	-	-	11.54	(1.90- 70.11)
WYNDE4	61	f	2	-	-	-	-	11.54	(1.90- 70.11)
Subtotal WYNDE4								6.97	(5.35- 9.09)
XU3	5	m	0	10	26	7	31	1.70	(0.57- 5.10)
XU3	6	m	0	19	27	7	31	3.12	(1.14- 8.54)
XU3	7	m	0	47	13	7	31	16.01	(5.75- 44.61)
XU3	8	m	0	16	2	7	31	35.43	(6.58- 190.72)
XU3	13	f	0	7	6	13	25	2.24	(0.62- 8.07)
XU3	14	f	0	10	4	13	25	4.81	(1.26- 18.35)
XU3	15	f	0	6	1	13	25	11.54	(1.25- 106.30)
Subtotal XU3								5.11	(3.19- 8.19)
*YUAN	2	m	2	-	-	-	-	3.60	(1.88- 6.91)
*YUAN	3	m	2	-	-	-	-	9.40	(5.21- 16.97)
Subtotal YUAN								6.09	(3.94- 9.44)
ZHENG	11	m	0	25	40	33	94	1.78	(0.94- 3.37)
ZHENG	12	m	0	60	66	33	94	2.59	(1.53- 4.39)
ZHENG	13	m	0	128	89	33	94	4.10	(2.53- 6.62)
ZHENG	14	m	0	66	23	33	94	8.17	(4.40- 15.17)
ZHENG	22	f	0	24	29	152	184	1.00	(0.56- 1.79)
ZHENG	23	f	0	52	15	152	184	4.20	(2.27- 7.75)
Subtotal ZHENG								2.97	(2.36- 3.75)
ZHOU	4	c	0	61	5	507	68	1.64	(0.64- 4.22)
ZHOU	5	c	0	211	14	507	68	2.02	(1.11- 3.67)
ZHOU	6	c	0	581	29	507	68	2.69	(1.71- 4.22)
Subtotal ZHOU								2.31	(1.65- 3.23)
Partial Totals				33641	102516	16701	147849		

*prospective study ~ With 0.5 adjustment for zero

REF	NRR	SEX	AD	Ys	Ws	Qs	Ps
AGUDO	11	f	0	0.13	3.73	7.82	0.7953
AGUDO	12	f	0	1.26	5.96	0.61	0.0020
Subtotal AGUDO				0.83	9.69	8.43	
ALDERS	18	m	1	1.27	10.54	1.05	0.0000
ALDERS	19	m	1	2.07	13.07	3.17	0.0000
ALDERS	20	m	1	2.14	14.23	4.47	0.0000
ALDERS	21	f	1	0.96	34.91	13.38	0.0000
ALDERS	22	f	1	1.66	34.88	0.23	0.0000
ALDERS	23	f	1	1.93	25.78	3.15	0.0000
Subtotal ALDERS				1.59	133.42	25.44	
ARMADA	46	m	0	1.79	3.37	0.15	0.0010
ARMADA	47	m	0	3.02	3.54	7.28	0.0000
ARMADA	48	m	0	4.24	3.29	23.25	0.0000
Subtotal ARMADA				3.01	10.20	30.68	
AUVINE	5	c	0	2.20	13.34	5.03	0.0000
AUVINE	6	c	0	2.98	16.81	32.99	0.0000
AUVINE	7	c	0	3.68	5.94	26.14	0.0000
Subtotal AUVINE				2.81	36.09	64.16	
AXELSS	19	m	0	1.40	9.54	0.31	0.0000
AXELSS	20	m	0	2.21	11.63	4.56	0.0000
AXELSS	21	m	0	2.42	11.94	8.42	0.0000
AXELSS	13	f	0	1.10	6.02	1.40	0.0069
AXELSS	14	f	0	2.38	10.21	6.46	0.0000
AXELSS	15	f	0	2.77	6.08	8.59	0.0000
AXELSS	16	f	0	1.99	2.69	0.45	0.0011
Subtotal AXELSS				2.08	58.13	30.19	
BARBON	5	m	0	1.01	10.21	3.32	0.0012
BARBON	7	m	0	2.27	14.77	7.03	0.0000

Table 1G1 - 5

IESLC - Meta-analysis of Ever Smoking by Amount, Overview, Any product (or Cigarettes if Any not available)
All LC types
Least adjusted

REF	NRR	SEX	AD	Ys	Ws	Qs	Ps
BARBON	9	m	0	2.38	16.41	10.50	0.0000
BARBON	11	m	0	2.60	14.14	14.60	0.0000
BARBON	13	m	0	2.86	15.58	25.47	0.0000
Subtotal BARBON				2.31	71.10	60.92	
*BOUCOT	99	m	0	3.15	0.49	1.21	0.0271
*BOUCOT	100	m	0	3.83	0.49	2.50	0.0070
Subtotal BOUCOT				3.49	0.99	3.71	
BRESLO	13	m	0	0.58	7.18	7.28	0.1231
BRESLO	14	m	0	1.19	12.73	1.96	0.0000
BRESLO	15	m	0	2.04	15.85	3.28	0.0000
BRESLO	16	m	0	2.90	8.89	15.45	0.0000
BRESLO	29	f	0	0.15	1.80	3.68	0.8360
BRESLO	30	f	0	0.44	2.24	2.91	0.5084
Subtotal BRESLO				1.61	48.69	34.56	
BROWN2	32	m	2	1.81	220.78	11.28	0.0000
BROWN2	42	m	2	2.65	387.09	438.17	0.0000
BROWN2	31	f	2	2.13	172.98	51.57	0.0000
BROWN2	41	f	2	2.84	312.24	493.23	0.0000
Subtotal BROWN2				2.45	1093.09	994.25	
BUFFLE	28	f	0	1.54	6.11	0.01	0.0001
BUFFLE	29	f	0	2.47	8.19	6.46	0.0000
BUFFLE	35	f	0	3.06	8.66	18.79	0.0000
Subtotal BUFFLE				2.44	22.96	25.25	
CHATZI	1	c	0	0.94	14.84	6.14	0.0003
CHATZI	2	c	0	1.04	15.01	4.38	0.0001
CHATZI	3	c	0	1.56	16.06	0.01	0.0000
Subtotal CHATZI				1.19	45.92	10.52	
CHEN2	3	m	0	0.87	4.19	2.10	0.0735
CHEN2	4	m	0	1.17	5.43	0.92	0.0063
CHEN2	5	m	0	2.63	3.55	3.88	0.0000
CHEN2	6	m	0	2.07	3.80	0.91	0.0001
CHEN2	7	f	0	-0.95	3.04	19.42	0.0991
CHEN2	8	f	0	1.07	4.63	1.23	0.0217
CHEN2	9	f	0	2.22	0.82	0.34	0.0435
CHEN2	10	f	0	0.97	1.22	0.46	0.2838
Subtotal CHEN2				1.21	26.68	29.26	
CHOI	12	m	0	0.48	6.73	8.11	0.2084
CHOI	13	m	0	1.32	10.21	0.70	0.0000
CHOI	14	m	0	2.01	7.82	1.43	0.0000
CHOI	15	m	0	1.94	7.14	0.89	0.0000
CHOI	16	m	0	2.97	3.16	6.08	0.0000
CHOI	17	f	0	0.19	5.18	10.00	0.6591
CHOI	18	f	0	0.52	3.66	4.15	0.3219
CHOI	20	f	0	1.87	0.74	0.06	0.1083
Subtotal CHOI				1.34	44.64	31.41	
COOKSO	1	c	0	1.63	11.70	0.03	0.0000
COOKSO	2	c	0	2.31	7.06	3.77	0.0000
Subtotal COOKSO				1.89	18.76	3.80	
*CPSI	243	m	1	1.76	44.36	1.40	0.0000
*CPSI	246	m	1	2.61	55.90	59.05	0.0000
Subtotal CPSI				2.23	100.25	60.45	
*CPSII	102	m	1	2.30	75.60	39.12	0.0000
*CPSII	103	m	1	2.87	75.65	125.04	0.0000
*CPSII	105	f	1	1.43	95.77	2.35	0.0000
*CPSII	106	f	1	2.59	140.12	142.52	0.0000
Subtotal CPSII				2.30	387.13	309.03	
DAMBER	6	m	1	0.83	10.34	5.80	0.0074
DAMBER	7	m	1	1.99	13.68	2.25	0.0000
DAMBER	8	m	1	2.21	14.68	5.75	0.0000
DAMBER	9	m	1	2.70	5.93	7.43	0.0000
Subtotal DAMBER				1.89	44.62	21.24	
DAVEYS	1	m	0	0.20	2.07	3.96	0.7726
DAVEYS	2	m	0	2.01	2.27	0.41	0.0025
DAVEYS	3	m	0	1.89	2.11	0.20	0.0061
DAVEYS	4	m	0	2.36	2.18	1.32	0.0005
Subtotal DAVEYS				1.63	8.63	5.88	
DEAN	1	m	0	0.79	8.38	5.23	0.0218
DEAN	2	m	0	1.91	9.10	0.96	0.0000
DEAN	3	m	0	2.44	7.59	5.64	0.0000
Subtotal DEAN				1.70	25.06	11.83	
DEAN2	25	m	0	1.17	22.52	3.77	0.0000

Table 1G1 - 5

IESLC - Meta-analysis of Ever Smoking by Amount, Overview, Any product (or Cigarettes if Any not available)
All LC types
Least adjusted

REF	NRR	SEX	AD	Ys	Ws	Qs	Ps
DEAN2	26	m	0	2.03	19.18	3.90	0.0000
DEAN2	29	f	0	0.92	11.90	5.15	0.0014
DEAN2	30	f	0	1.60	3.63	0.00	0.0023
Subtotal DEAN2				1.44	57.24	12.82	
DESTEF	1	m	0	1.00	12.29	4.10	0.0004
DESTEF	2	m	0	2.06	17.23	3.97	0.0000
DESTEF	3	m	0	2.27	16.84	8.06	0.0000
DESTEF	4	m	0	3.12	11.90	28.04	0.0000
Subtotal DESTEF				2.12	58.26	44.17	
DOLL	1	m	0	1.31	5.40	0.39	0.0023
DOLL	2	m	0	2.01	6.13	1.13	0.0000
DOLL	3	m	0	2.26	6.11	2.82	0.0000
DOLL	4	m	0	2.81	5.91	8.88	0.0000
DOLL	5	m	0	3.32	3.72	11.20	0.0000
DOLL	7	f	0	-0.06	6.92	18.62	0.8795
DOLL	8	f	0	0.68	7.19	5.90	0.0698
DOLL	9	f	0	1.24	3.57	0.43	0.0195
DOLL	10	f	0	3.75	0.47	2.23	0.0098
Subtotal DOLL				1.60	45.43	51.61	
*DOLL2	46	m	1	1.90	0.96	0.10	0.0623
*DOLL2	47	m	1	2.51	0.96	0.83	0.0137
*DOLL2	48	m	1	3.17	0.97	2.44	0.0018
Subtotal DOLL2				2.53	2.90	3.36	
DORGAN	10	m	0	1.66	10.85	0.06	0.0000
DORGAN	11	m	0	2.65	12.03	13.66	0.0000
DORGAN	34	m	0	2.80	2.62	3.86	0.0000
DORGAN	35	m	0	3.97	2.50	14.22	0.0000
DORGAN	96	f	3	1.74	56.05	1.31	0.0000
DORGAN	97	f	3	2.50	51.92	44.03	0.0000
Subtotal DORGAN				2.16	135.97	77.14	
DOSEME	5	m	2	0.79	20.90	13.17	0.0003
DOSEME	9	m	2	1.13	45.98	9.35	0.0000
DOSEME	13	m	2	1.89	21.74	2.02	0.0000
Subtotal DOSEME				1.24	88.62	24.53	
*DUNN	1	m	0	2.12	1.20	0.35	0.0199
*DUNN	2	m	0	2.20	1.71	0.65	0.0040
*DUNN	3	m	0	2.95	1.95	3.66	0.0000
*DUNN	4	m	0	3.22	1.88	5.07	0.0000
*DUNN	5	m	0	3.36	1.73	5.46	0.0000
Subtotal DUNN				2.83	8.48	15.20	
EBELIN	2	m	0	1.00	6.42	2.20	0.0116
EBELIN	3	m	0	1.96	5.47	0.79	0.0000
EBELIN	4	m	0	2.52	7.13	6.23	0.0000
EBELIN	5	m	0	2.79	1.60	2.33	0.0004
EBELIN	6	m	0	2.28	1.69	0.82	0.0031
Subtotal EBELIN				1.94	22.31	12.37	
ESAKI	1	m	0	0.09	7.53	16.71	0.8001
ESAKI	2	m	0	0.80	7.75	4.71	0.0253
ESAKI	3	m	0	1.78	4.39	0.18	0.0002
Subtotal ESAKI				0.75	19.68	21.60	
FAN	6	m	0	-0.35	8.53	31.87	0.3059
FAN	7	m	0	0.71	17.63	13.44	0.0029
FAN	8	m	0	1.38	21.51	0.88	0.0000
FAN	9	m	0	2.41	9.89	6.75	0.0000
FAN	10	f	0	0.50	10.28	12.12	0.1116
FAN	11	f	0	1.32	12.82	0.85	0.0000
FAN	12	f	0	2.48	7.51	6.09	0.0000
FAN	13	f	0	2.92	0.79	1.41	0.0095
Subtotal FAN				1.19	88.96	73.43	
GAO	24	f	0	0.27	31.50	53.88	0.1237
GAO	25	f	0	0.86	24.99	12.92	0.0000
GAO	26	f	0	2.36	10.18	6.09	0.0000
Subtotal GAO				0.81	66.66	72.89	
GARSHI	18	m	0	1.19	24.87	3.77	0.0000
GARSHI	19	m	0	1.74	31.85	0.80	0.0000
GARSHI	20	m	0	2.01	25.53	4.61	0.0000
GARSHI	21	m	0	1.73	29.00	0.63	0.0000
Subtotal GARSHI				1.68	111.24	9.81	
GER	18	c	0	-0.06	12.41	33.65	0.8213
GER	19	c	0	0.26	19.29	33.91	0.2605
GER	20	c	0	1.01	11.22	3.62	0.0007

Table 1G1 - 5

IESLC - Meta-analysis of Ever Smoking by Amount, Overview, Any product (or Cigarettes if Any not available)
All LC types
Least adjusted

REF	NRR	SEX	AD	Ys	Ws	Qs	Ps
Subtotal GER				0.36	42.92	71.19	
GOLLED	15	m	0	1.38	12.16	0.48	0.0000
GOLLED	16	m	0	1.60	12.74	0.00	0.0000
GOLLED	17	m	0	2.64	12.71	14.24	0.0000
Subtotal GOLLED				1.88	37.61	14.72	
GSELL	1	m	0	1.49	1.53	0.01	0.0655
GSELL	2	m	0	1.37	1.51	0.07	0.0931
GSELL	3	m	0	2.71	1.64	2.09	0.0005
GSELL	4	m	0	4.37	1.50	11.66	0.0000
GSELL	5	m	0	4.04	1.58	9.58	0.0000
Subtotal GSELL				2.80	7.77	23.42	
*HAMMON	153	m	1	2.00	8.19	1.42	0.0000
*HAMMON	154	m	1	2.12	11.45	3.30	0.0000
*HAMMON	155	m	1	2.84	10.98	17.27	0.0000
Subtotal HAMMON				2.34	30.62	21.99	
*HANSEN	1	m	2	0.31	5.16	8.28	0.4747
*HANSEN	2	m	2	1.06	3.91	1.05	0.0352
Subtotal HANSEN				0.64	9.07	9.33	
HU	1	m	0	0.44	10.71	14.04	0.1527
HU	2	m	0	0.74	12.38	8.84	0.0095
HU	3	m	0	1.29	6.63	0.55	0.0009
HU	4	f	0	0.82	5.04	2.90	0.0643
HU	5	f	0	0.18	2.64	5.17	0.7672
HU	6	f	0	-0.51	0.65	2.83	0.6812
Subtotal HU				0.70	38.05	34.33	
HU2	2	c	0	-0.16	9.46	28.65	0.6262
HU2	3	c	0	0.29	18.89	31.59	0.2087
HU2	4	c	0	0.66	22.08	18.68	0.0019
HU2	5	c	0	0.74	21.23	15.22	0.0007
HU2	6	c	0	1.10	32.09	7.35	0.0000
HU2	7	c	0	1.78	12.31	0.48	0.0000
Subtotal HU2				0.79	116.06	101.98	
JARUP	1	m	0	1.86	5.60	0.43	0.0000
JARUP	2	m	0	1.94	5.81	0.73	0.0000
Subtotal JARUP				1.90	11.41	1.15	
JEDRYC	60	m	0	1.18	26.20	4.25	0.0000
JEDRYC	61	m	0	1.86	32.56	2.60	0.0000
JEDRYC	62	m	0	2.16	28.12	9.45	0.0000
JEDRYC	65	f	0	2.06	6.86	1.59	0.0000
JEDRYC	66	f	0	1.93	9.94	1.23	0.0000
JEDRYC	67	f	0	2.70	3.28	4.11	0.0000
Subtotal JEDRYC				1.82	106.96	23.23	
JOLY	7	m	0	1.68	5.92	0.06	0.0000
JOLY	8	m	0	2.52	10.45	9.14	0.0000
JOLY	9	m	0	2.57	9.85	9.63	0.0000
JOLY	10	m	0	3.08	10.07	22.61	0.0000
JOLY	3	f	0	1.55	12.60	0.01	0.0000
JOLY	4	f	0	2.08	17.52	4.33	0.0000
JOLY	5	f	0	1.94	9.62	1.20	0.0000
JOLY	6	f	0	2.59	7.64	7.83	0.0000
Subtotal JOLY				2.24	83.67	54.81	
JUSSAW	10	m	0	1.75	4.46	0.13	0.0002
JUSSAW	11	m	0	1.03	3.50	1.08	0.0549
JUSSAW	12	m	0	1.35	15.44	0.85	0.0000
JUSSAW	13	m	0	0.13	2.31	4.86	0.8398
JUSSAW	14	m	0	2.53	5.71	5.14	0.0000
JUSSAW	15	m	0	3.92	3.58	19.53	0.0000
Subtotal JUSSAW				1.74	35.01	31.58	
KHUDER	1	m	0	0.92	16.30	7.16	0.0002
KHUDER	2	m	0	2.35	18.30	10.69	0.0000
KHUDER	3	m	0	3.49	14.48	52.78	0.0000
Subtotal KHUDER				2.21	49.07	70.63	
KOULUM	6	m	0	1.65	3.87	0.02	0.0012
KOULUM	5	m	0	3.17	4.27	10.83	0.0000
KOULUM	4	m	0	4.23	4.27	30.00	0.0000
Subtotal KOULUM				3.06	12.42	40.85	
KREUZE	19	m	3	0.92	2.54	1.13	0.1444
KREUZE	20	m	3	2.16	4.57	1.54	0.0000
KREUZE	21	m	3	2.97	4.24	8.18	0.0000
KREUZE	22	m	3	3.03	3.39	7.16	0.0000
KREUZE	30	m	3	2.10	18.30	4.98	0.0000

Table 1G1 - 5

IESLC - Meta-analysis of Ever Smoking by Amount, Overview, Any product (or Cigarettes if Any not available)
All LC types
Least adjusted

REF	NRR	SEX	AD	Ys	Ws	Qs	Ps
KREUZE	31	m	3	3.22	20.26	54.54	0.0000
KREUZE	32	m	3	3.49	18.97	69.09	0.0000
KREUZE	33	m	3	3.51	16.38	60.59	0.0000
KREUZE	25	f	3	1.74	2.81	0.07	0.0035
KREUZE	26	f	3	2.47	3.44	2.70	0.0000
KREUZE	27	f	3	2.49	2.00	1.66	0.0004
KREUZE	36	f	3	0.69	15.02	11.87	0.0072
KREUZE	37	f	3	1.69	19.01	0.21	0.0000
KREUZE	38	f	3	2.04	6.02	1.27	0.0000
Subtotal KREUZE				2.45	136.95	224.98	
KREYBE	21	m	0	1.95	5.71	0.79	0.0000
KREYBE	22	m	0	1.85	5.33	0.39	0.0000
KREYBE	23	m	0	2.92	5.11	9.20	0.0000
KREYBE	37	f	0	-0.27	7.23	24.72	0.4730
KREYBE	38	f	0	0.04	1.79	4.25	0.9552
Subtotal KREYBE				1.36	25.18	39.35	
LAMTH	7	f	0	0.98	29.68	10.63	0.0000
LAMTH	2	f	0	1.68	18.27	0.17	0.0000
LAMTH	9	f	0	1.98	6.91	1.08	0.0000
Subtotal LAMTH				1.34	54.86	11.89	
LAUSSM	3	m	0	1.15	22.03	4.07	0.0000
LAUSSM	2	m	0	1.61	26.93	0.03	0.0000
LAUSSM	1	m	0	2.02	18.96	3.65	0.0000
Subtotal LAUSSM				1.58	67.92	7.75	
LETOUR	2	c	0	2.25	18.66	8.38	0.0000
LETOUR	3	c	0	2.85	18.55	29.85	0.0000
LETOUR	4	c	0	3.27	9.52	27.21	0.0000
Subtotal LETOUR				2.70	46.74	65.44	
LIU2	5	m	0	-0.19	6.08	19.08	0.6415
LIU2	6	m	0	1.68	7.60	0.08	0.0000
LIU2	7	m	0	2.80	6.09	9.00	0.0000
LIU2	11	f	0	0.37	3.76	5.50	0.4689
LIU2	12	f	0	1.17	4.66	0.79	0.0114
LIU2	13	f	0	2.61	3.09	3.27	0.0000
Subtotal LIU2				1.39	31.28	37.70	
LIU3	3	m	0	0.24	2.83	5.06	0.6810
LIU3	4	m	0	0.02	2.75	6.71	0.9718
LIU3	5	m	0	0.56	2.01	2.10	0.4278
Subtotal LIU3				0.25	7.59	13.87	
LIU4	7	m	2	0.75	2108.86	1472.30	0.0000
LIU4	8	m	2	1.28	4111.92	373.31	0.0000
LIU4	9	m	2	1.94	2991.95	389.50	0.0000
Subtotal LIU4				1.37	9212.74	2235.11	
LIU5	2	c	0	-0.20	5.84	18.57	0.6267
LIU5	3	c	0	0.46	6.33	8.03	0.2520
LIU5	4	c	0	1.28	7.79	0.73	0.0004
Subtotal LIU5				0.58	19.96	27.33	
LUBIN	7	m	0	-0.12	2.42	6.98	0.8565
LUBIN	8	m	0	0.08	3.35	7.57	0.8845
LUBIN	9	m	0	1.52	3.79	0.02	0.0031
LUBIN	10	m	0	1.37	5.32	0.23	0.0015
Subtotal LUBIN				0.88	14.88	14.80	
LUBIN2	273	m	0	1.93	155.25	19.12	0.0000
LUBIN2	274	m	0	2.03	150.14	29.80	0.0000
LUBIN2	275	m	0	2.36	152.73	92.85	0.0000
LUBIN2	276	m	0	2.52	139.75	123.44	0.0000
LUBIN2	281	f	0	0.90	66.54	31.36	0.0000
LUBIN2	282	f	0	1.30	76.70	6.12	0.0000
LUBIN2	283	f	0	1.53	47.64	0.15	0.0000
LUBIN2	284	f	0	1.57	17.75	0.00	0.0000
Subtotal LUBIN2				1.96	806.50	302.86	
MACLEN	36	c	2	0.30	6.92	11.38	0.4298
MACLEN	37	c	2	0.98	10.81	3.94	0.0013
MACLEN	38	c	2	1.08	9.92	2.55	0.0007
MACLEN	39	c	2	1.41	8.18	0.24	0.0001
Subtotal MACLEN				0.97	35.83	18.11	
MARTIS	1	m	0	1.60	2.87	0.00	0.0066
MARTIS	2	m	0	1.88	3.20	0.28	0.0008
MARTIS	3	m	0	2.24	3.09	1.33	0.0001
Subtotal MARTIS				1.91	9.17	1.61	
MATOS	28	m	0	0.66	5.88	5.01	0.1105

Table 1G1 - 5

IESLC - Meta-analysis of Ever Smoking by Amount, Overview, Any product (or Cigarettes if Any not available)
All LC types
Least adjusted

REF	NRR	SEX	AD	Ys	Ws	Qs	Ps
MATOS	30	m	0	1.98	7.91	1.23	0.0000
MATOS	32	m	0	2.31	8.41	4.48	0.0000
Subtotal MATOS				1.75	22.19	10.72	
MATSUD	1	m	0	2.53	2.76	2.46	0.0000
MATSUD	2	m	0	2.97	2.87	5.55	0.0000
MATSUD	3	m	0	3.94	2.83	15.78	0.0000
Subtotal MATSUD				3.15	8.46	23.79	
MCCONN	26	c	0	-0.88	3.12	18.86	0.1215
MCCONN	25	c	0	0.31	5.38	8.73	0.4745
MCCONN	24	c	0	0.76	4.83	3.30	0.0966
Subtotal MCCONN				0.19	13.33	30.89	
NOTAN2	8	m	0	-0.54	5.01	22.65	0.2229
NOTAN2	9	m	0	0.88	15.08	7.37	0.0006
NOTAN2	10	m	0	1.50	17.54	0.13	0.0000
Subtotal NOTAN2				0.98	37.63	30.15	
ORMOS	1	m	0	2.38	5.60	3.56	0.0000
ORMOS	2	m	0	2.04	5.85	1.23	0.0000
ORMOS	3	m	0	2.57	4.57	4.42	0.0000
Subtotal ORMOS				2.31	16.03	9.21	
OSANN	49	m	2	2.87	33.51	55.88	0.0000
OSANN	57	m	2	3.76	33.40	157.90	0.0000
OSANN	50	f	2	2.67	52.45	61.74	0.0000
OSANN	58	f	2	3.71	34.52	156.43	0.0000
Subtotal OSANN				3.18	153.87	431.95	
OSANN2	4	f	0	0.74	11.60	8.17	0.0114
OSANN2	5	f	0	2.35	14.82	8.71	0.0000
Subtotal OSANN2				1.64	26.42	16.88	
PASTOR	1	m	0	0.60	3.82	3.67	0.2395
PASTOR	2	m	0	1.76	6.96	0.22	0.0000
PASTOR	3	m	0	2.07	7.55	1.79	0.0000
PASTOR	4	m	0	2.21	6.44	2.52	0.0000
Subtotal PASTOR				1.79	24.77	8.21	
PERNU	17	m	0	1.04	6.79	1.98	0.0066
PERNU	18	m	0	1.72	15.98	0.30	0.0000
PERNU	19	m	0	1.89	34.69	3.27	0.0000
PERNU	20	m	0	1.68	27.21	0.27	0.0000
PERNU	21	m	0	2.28	42.95	21.18	0.0000
PERNU	22	m	0	2.94	28.38	52.47	0.0000
PERNU	23	m	0	1.60	12.13	0.00	0.0000
PERNU	24	m	0	2.71	10.11	12.79	0.0000
PERNU	11	f	0	0.64	2.41	2.15	0.3224
PERNU	12	f	0	1.22	3.48	0.45	0.0225
PERNU	13	f	0	0.16	3.41	6.86	0.7636
PERNU	14	f	0	-0.46	0.92	3.86	0.6574
PERNU	15	f	0	-0.22	0.91	2.95	0.8339
PERNU	16	f	0	1.84	2.83	0.19	0.0019
Subtotal PERNU				2.00	192.21	108.73	
PIKE	1	m	0	1.42	12.27	0.33	0.0000
PIKE	2	m	0	2.08	11.96	2.98	0.0000
PIKE	3	m	0	1.92	8.91	1.03	0.0000
PIKE	5	f	0	1.18	14.59	2.40	0.0000
PIKE	6	f	0	1.93	10.92	1.30	0.0000
PIKE	7	f	0	2.65	2.30	2.65	0.0001
Subtotal PIKE				1.70	60.95	10.69	
POLEDN	2	c	0	1.79	8.40	0.35	0.0000
POLEDN	4	c	0	2.29	9.66	4.82	0.0000
Subtotal POLEDN				2.05	18.06	5.17	
RACHTA	5	f	0	0.93	2.86	1.20	0.1143
RACHTA	6	f	0	1.13	8.19	1.68	0.0012
RACHTA	7	f	0	2.59	7.02	7.17	0.0000
Subtotal RACHTA				1.67	18.07	10.05	
RANDIG	1	m	0	0.71	2.79	2.10	0.2326
RANDIG	2	m	0	1.06	3.69	1.00	0.0415
RANDIG	3	m	0	1.63	3.89	0.01	0.0013
RANDIG	4	m	0	2.22	3.74	1.51	0.0000
RANDIG	5	f	0	-1.36	0.90	7.73	0.1996
RANDIG	6	f	0	1.61	4.35	0.00	0.0008
RANDIG	7	f	0	1.18	1.66	0.27	0.1294
Subtotal RANDIG				1.35	21.02	12.63	
SHAW	10	c	0	1.60	7.51	0.00	0.0000
SHAW	11	c	0	2.73	9.13	12.07	0.0000

Table 1G1 - 5

IESLC - Meta-analysis of Ever Smoking by Amount, Overview, Any product (or Cigarettes if Any not available)
All LC types
Least adjusted

REF	NRR	SEX	AD	Ys	Ws	Qs	Ps
Subtotal SHAW				2.22	16.64	12.07	
SIEMIA	13	m	0	1.10	2.92	0.68	0.0603
SIEMIA	14	m	0	1.50	3.87	0.02	0.0031
SIEMIA	15	m	0	2.07	3.54	0.83	0.0001
Subtotal SIEMIA				1.58	10.33	1.54	
SPITZ	5	c	0	1.08	6.30	1.59	0.0067
SPITZ	6	c	0	2.94	7.05	13.09	0.0000
Subtotal SPITZ				2.07	13.35	14.67	
STOCKS	25	m	0	1.73	38.74	0.81	0.0000
STOCKS	26	m	0	1.75	39.49	1.15	0.0000
STOCKS	27	m	0	2.16	32.54	10.80	0.0000
STOCKS	28	m	0	1.82	34.51	1.99	0.0000
STOCKS	29	m	0	2.21	34.20	13.64	0.0000
STOCKS	48	f	1	0.81	40.78	24.54	0.0000
STOCKS	49	f	1	1.85	31.07	2.18	0.0000
Subtotal STOCKS				1.73	251.34	55.09	
TIZZAN	7	m	0	-0.08	48.24	132.88	0.5909
TIZZAN	8	m	0	0.52	76.34	85.64	0.0000
TIZZAN	9	m	0	1.55	46.69	0.04	0.0000
TIZZAN	10	m	0	1.95	14.11	1.91	0.0000
TIZZAN	15	f	0	1.28	4.74	0.44	0.0055
TIZZAN	16	f	0	1.52	5.22	0.02	0.0005
Subtotal TIZZAN				0.77	195.34	220.94	
WANG2	1	c	0	0.33	2.59	4.04	0.5910
WANG2	2	c	0	0.16	1.83	3.70	0.8295
WANG2	3	c	0	0.33	2.59	4.04	0.5910
WANG2	4	c	0	0.11	2.30	4.97	0.8670
WANG2	5	c	0	1.19	5.48	0.83	0.0052
WANG2	6	c	0	0.91	2.87	1.29	0.1224
WANG2	7	c	0	2.17	2.10	0.74	0.0016
Subtotal WANG2				0.81	19.77	19.62	
WUWILL	18	f	0	0.74	105.32	75.21	0.0000
WUWILL	19	f	0	1.15	24.74	4.54	0.0000
Subtotal WUWILL				0.82	130.06	79.75	
WYNDE2	17	m	0	0.67	4.95	4.10	0.1353
WYNDE2	18	m	0	2.07	6.77	1.58	0.0000
WYNDE2	19	m	0	2.63	6.33	6.99	0.0000
WYNDE2	20	m	0	2.90	6.67	11.57	0.0000
Subtotal WYNDE2				2.16	24.72	24.25	
WYNDE3	44	m	0	0.62	3.69	3.40	0.2325
WYNDE3	45	m	0	1.89	6.93	0.65	0.0000
WYNDE3	46	m	0	2.56	6.95	6.58	0.0000
WYNDE3	47	m	0	3.24	5.69	15.68	0.0000
WYNDE3	79	f	0	-0.51	2.23	9.75	0.4459
WYNDE3	80	f	0	1.34	6.83	0.42	0.0005
WYNDE3	81	f	0	1.74	4.35	0.11	0.0003
WYNDE3	82	f	0	1.62	1.55	0.00	0.0436
Subtotal WYNDE3				1.82	38.21	36.59	
WYNDE4	43	m	0	0.69	6.13	4.92	0.0891
WYNDE4	44	m	0	1.47	8.79	0.10	0.0000
WYNDE4	45	m	0	2.08	9.99	2.44	0.0000
WYNDE4	46	m	0	2.92	9.30	16.70	0.0000
WYNDE4	47	m	0	2.97	8.67	16.66	0.0000
WYNDE4	57	f	2	0.12	2.52	5.38	0.8460
WYNDE4	58	f	2	0.70	3.11	2.43	0.2185
WYNDE4	59	f	2	1.87	3.72	0.31	0.0003
WYNDE4	60	f	2	2.45	1.18	0.88	0.0079
WYNDE4	61	f	2	2.45	1.18	0.88	0.0079
Subtotal WYNDE4				1.94	54.60	50.71	
XU3	5	m	0	0.53	3.19	3.51	0.3416
XU3	6	m	0	1.14	3.78	0.75	0.0272
XU3	7	m	0	2.77	3.66	5.19	0.0000
XU3	8	m	0	3.57	1.36	5.34	0.0000
XU3	13	f	0	0.81	2.34	1.41	0.2159
XU3	14	f	0	1.57	2.14	0.00	0.0216
XU3	15	f	0	2.45	0.78	0.58	0.0309
Subtotal XU3				1.63	17.25	16.78	
*YUAN	2	m	2	1.28	9.07	0.82	0.0001
*YUAN	3	m	2	2.24	11.02	4.78	0.0000
Subtotal YUAN				1.81	20.09	5.60	
ZHENG	11	m	0	0.58	9.44	9.54	0.0764

Table 1G1 - 5

IESLC - Meta-analysis of Ever Smoking by Amount, Overview, Any product (or Cigarettes if Any not available)
All LC types
Least adjusted

REF	NRR	SEX	AD	Ys	Ws	Qs	Ps
ZHENG	12	m	0	0.95	13.74	5.47	0.0004
ZHENG	13	m	0	1.41	16.67	0.49	0.0000
ZHENG	14	m	0	2.10	10.04	2.70	0.0000
ZHENG	22	f	0	0.00	11.34	28.33	0.9951
ZHENG	23	f	0	1.43	10.21	0.22	0.0000
Subtotal ZHENG				1.09	71.45	46.76	
ZHOU	4	c	0	0.49	4.29	5.10	0.3077
ZHOU	5	c	0	0.70	10.77	8.31	0.0209
ZHOU	6	c	0	0.99	18.91	6.67	0.0000
Subtotal ZHOU				0.84	33.97	20.07	

N	380
NS	84

Table 1G1 - 6

IESLC - Meta-analysis of Ever Smoking by Amount, Overview, Any product (or Cigarettes if Any not available)
All LC types
Least adjusted

	Sex			
	combined	male	female	Total
N	46	227	107	380
NS	14	63	35	112

In this overview table, other than the "N" rows, entries in the "absent" and "Total" columns may be invalid and should be ignored

	Amount smoked (broad categories)				
	absent	<20k5	6-44k20	>20k45	Total
N	128	105	70	77	380
NS	61	78	55	61	255
Wt	2540.07	3922.10	5147.67	3842.30	15452.14
Het Chi	1293.83	1160.46	744.10	617.82	6993.45
Het df	127	104	69	76	379
Het P	***	***	***	***	***
Fixed RR	8.41	2.74	4.00	7.94	4.87
RRl	8.09	2.65	3.89	7.69	4.79
RRu	8.74	2.83	4.11	8.20	4.94
P	+++	+++	+++	+++	+++
Random RR	5.83	2.74	6.19	11.79	5.46
RRl	5.08	2.38	5.24	9.98	5.03
RRu	6.70	3.16	7.30	13.93	5.92
P	+++	+++	+++	+++	+++

	Amount smoked (narrow categories)							
	absent	<10k1	2-19k10	11-29k20	21-39k30	31-98k40	>40k99	Total
N	202	53	43	56	13	1	12	380
NS	84	37	32	44	11	1	10	219
Wt	9014.32	607.31	655.86	4961.84	111.04	7.14	94.63	15452.14
Het Chi	5063.72	373.41	257.21	598.53	29.53	0.00	39.64	6993.45
Het df	201	52	42	55	12	0	11	379
Het P	***	***	***	***	**	N.S.	***	***
Fixed RR	5.51	2.80	5.04	4.00	9.02	6.93	11.68	4.87
RRl	5.40	2.59	4.67	3.89	7.49	3.33	9.55	4.79
RRu	5.63	3.03	5.44	4.12	10.86	14.44	14.29	4.94
P	+++	+++	+++	+++	+++	+++	+++	+++
Random RR	6.15	2.12	4.53	6.72	10.74	6.93	12.92	5.46
RRl	5.43	1.67	3.68	5.60	7.57	3.33	8.63	5.03
RRu	6.97	2.69	5.60	8.06	15.25	14.44	19.36	5.92
P	+++	+++	+++	+++	+++	+++	+++	+++

MALES

	Amount smoked (broad categories)				
	absent	<20k5	6-44k20	>20k45	Total
N	70	59	46	52	227
NS	44	57	45	51	197
Wt	1426.88	3027.24	4864.04	3665.60	12983.77
Het Chi	489.29	675.45	568.90	443.04	4979.70
Het df	69	58	45	51	226
Het P	***	***	***	***	***
Fixed RR	9.03	2.58	3.94	7.83	4.75
RRl	8.57	2.49	3.83	7.59	4.67
RRu	9.51	2.67	4.05	8.09	4.83
P	+++	+++	+++	+++	+++
Random RR	7.53	3.05	6.90	13.22	6.67
RRl	6.42	2.52	5.61	10.95	6.04
RRu	8.83	3.69	8.48	15.97	7.38
P	+++	+++	+++	+++	+++

Table 1G1 - 6

IESLC - Meta-analysis of Ever Smoking by Amount, Overview, Any product (or Cigarettes if Any not available)
All LC types
Least adjusted

MALES

	Amount smoked (narrow categories)							
	absent	<10k1	2-19k10	11-29k20	21-39k30	31-98k40	>40k99	Total
N	117	29	25	37	10	1	8	227
NS	63	28	24	36	10	1	8	169
Wt	7299.43	378.68	409.89	4717.27	106.17	7.14	65.19	12983.77
Het Chi	3679.56	243.29	133.81	473.27	24.58	0.00	18.18	4979.70
Het df	116	28	24	36	9	0	7	226
Het P	***	***	***	***	**	N.S.	*	***
Fixed RR	5.23	3.56	6.38	3.96	9.31	6.93	13.13	4.75
RRl	5.11	3.22	5.79	3.85	7.70	3.33	10.30	4.67
RRu	5.35	3.94	7.03	4.07	11.26	14.44	16.73	4.83
P	+++	+++	+++	+++	+++	+++	+++	+++
Random RR	7.42	2.60	5.80	7.55	11.80	6.93	14.11	6.67
RRl	6.30	1.84	4.46	5.99	8.19	3.33	9.41	6.04
RRu	8.74	3.68	7.56	9.52	17.02	14.44	21.16	7.38
P	+++	+++	+++	+++	+++	+++	+++	+++

FEMALES

	Amount smoked (broad categories)				
	absent	<20k5	6-44k20	>20k45	Total
N	39	33	17	18	107
NS	29	32	16	18	95
Wt	916.63	775.64	176.11	122.59	1990.98
Het Chi	482.24	327.78	39.15	94.93	1436.46
Het df	38	32	16	17	106
Het P	***	***	**	***	***
Fixed RR	9.33	3.41	5.49	12.42	6.12
RRl	8.74	3.18	4.74	10.40	5.85
RRu	9.95	3.65	6.36	14.82	6.39
P	+++	+++	+++	+++	+++
Random RR	4.86	2.36	5.57	9.19	4.20
RRl	3.69	1.82	4.27	5.58	3.50
RRu	6.41	3.05	7.26	15.15	5.04
P	+++	+++	+++	+++	+++

	Amount smoked (narrow categories)							
	absent	<10k1	2-19k10	11-29k20	21-39k30	31-98k40	>40k99	Total
N	58	18	13	14	2		2	107
NS	34	17	12	13	2		2	80
Wt	1442.92	187.82	193.39	160.99	2.00		3.85	1990.98
Het Chi	957.49	49.28	42.62	30.61	0.02		0.99	1436.46
Het df	57	17	12	13	1		1	106
Het P	***	***	***	**	N.S.		N.S.	***
Fixed RR	7.57	1.93	3.94	5.83	10.53		9.40	6.12
RRl	7.19	1.67	3.42	5.00	2.64		3.46	5.85
RRu	7.97	2.22	4.54	6.81	42.05		25.51	6.39
P	+++	+++	+++	+++	+++		+++	+++
Random RR	4.99	1.74	3.97	6.33	10.53		9.40	4.20
RRl	3.91	1.30	2.91	4.80	2.64		3.46	3.50
RRu	6.36	2.34	5.41	8.33	42.05		25.51	5.04
P	+++	+++	+++	+++	+++		+++	+++

Table 1G1 - 7

IESLC - Meta-analysis of Ever Smoking by Amount, Overview, Any product (or Cigarettes if Any not available)
All LC types
Excluded studies (and stage at which they were excluded)

1	ABELIN ABRAHA AMANDU AMES ANDERS AUSTIN AXELSO BAND BECHER BERRIN BLOHMK BLOT4 BROCKM BROWN1 BYERS1 BYERS2 CARPEN CASCO2 CASCOR CHAN CHEN3 CHIAZZ CHYOU DESTE2 DOCKER DROSTE DU GARCIA GARDIN GENG GODLEY GOODMA GRAHAM GREGOR HEGMAN HEIN HENNEK HINDS HIRAOK HOROWI HORWIT HUANG ISHIMA JAHN JAIN JARVHO JIANG KELLER KIHARA KJUUS KO KOHLME KUBIK LAMWK LAMWK2 LANGE LEI LEMARC LEVIN LIU LOMBA2 LOMBAR MAGNUS MARSH MARSH2 MCDUFF MCLAUG MILLER MILLS NOTANI NOU ODRISC PAWLEG PERSHA POFFIJ QIAO QIAO2 RADZIK REN RONCO ROOTS ROTHSC SAARIK SANKAR SCHWAR SEGI SEOW SHIMIZ SIMARA SIMONA SITAS SOBUE2 STASZE STAYNE STUCKE SUN SUZUK2 SUZUKI TANG TAO TOKARS TOUSEY ULMER VEIERO VUTUC WALD WANG WANG3 WANG4 WICKLU WIGLE WILKIN WU2 WUNSCH WYNDE8 XIANGZ XU XU2 XU4 YONG ZHANG
2	AKIBA ARCHER BENSHL BRETT BROSS CEDERL CHANG CHOW COMSTO DARBY DEAN3 DEKLER DORANT DORN ENGELA ENSTRO GAO2 GILLIS HAENSZ HAMMO2 HIRAY2 HIRAYA HITOSU HOLE HUMBLE KAISE2 KAISER KANELL KATSOU KAUFMA KINLEN KNEKT KOO LAURIL LIAW LICKIN LIDDEL MIGRAN MRFIT MRFITR MURATA NAM PARKIN PERSH2 PETO PEZZO2 PEZZOT PISANI PRESCO RIMING SEGI2 SOBUE SPEIZE STOCKW SVENSS TANG2 TENKAN TSUGAN TULINI TVERDA WAKAI WARSIN WATSON WU WYNDE5 WYNDER YAMAGU
3	BUELL CHEN MASTRA MZILEN RESTRE SADOWS
4	BEST BOFFET WYNDE7
6	BLOT1 BLOT2 BLOT3 BOUCHA JONES MOLLO SCHWA2 VANDER
8	CORREA LUO WYNDE6
10	BENHAM

Table 1G1 - 8

Potentially overlapping studies

REF	REFGP	PRINC	.	OVERLAP/LINK
LUBIN2	LUBIN2	1		Lubin-combined
LAMTH	LAMTH	1		KOO/LAMTH/LAMWK
OSANN2	KAISER	2		KAISER/OSANN2
CPSI	CPSI	1		CPSI overall
MATSUD	MATSUD	1		SOBUE2/MATSUD
LUBIN	XIANGZ	2		LUBIN/XIANGZ/QIAO

Table 1G1 - 9

Most adjusted - insufficient data for metaanalysis

REF	NRR	SEX	AGEL	AGEH	RACE	YF	LC TYPE	LOC	START	ST	NLC	R	VB	P	H	AD	PRODUCT	exL	exH	S1	S2	DENOM	De
BLOT1	5	m	0	0	all	-	all	NAmer	1970	CC	458	n	bl	y	n	0	cig+/-ot	1	9	1	1	nev cigs	or
BLOT1	6	m	0	0	all	-	all	NAmer	1970	CC	458	n	bl	y	n	0	cig+/-ot	10	39	2	0	nev cigs	or
BLOT1	7	m	0	0	all	-	all	NAmer	1970	CC	458	n	bl	y	n	0	cig+/-ot	40	99	3	0	nev cigs	or
BUELL	1	m	0	0	all	0	all	NAmer	1957	pr	304	n	bl	n	n	0	cig+/-ot	1	18	1	0	nev cigs	st
BUELL	2	m	0	0	all	0	all	NAmer	1957	pr	304	n	bl	n	n	0	cig+/-ot	19	21	2	3	nev cigs	st
BUELL	3	m	0	0	all	0	all	NAmer	1957	pr	304	n	bl	n	n	0	cig+/-ot	22	99	3	0	nev cigs	st
DOLL	11	f	0	0	all	-	all	Eu:UK	1948	CC	1465	n	V	n	n	0	all/unsp	50	99	0	6	nev any	or
MASTRA	3	m	0	0	all	-	all	Eu:wst	1973	CC	309	n	bl	n	n	1	all/unsp	1	10	1	0	nev any	or
MASTRA	4	m	0	0	all	-	all	Eu:wst	1973	CC	309	n	bl	n	n	1	all/unsp	20	99	0	0	nev any	or
MZILEN	1	m	0	0	bl	-	all	Africa	1994	CC	374	n	V	*	n	3	all/unsp	1	14	1	0	nev any	or
MZILEN	2	m	0	0	bl	-	all	Africa	1994	CC	374	n	V	*	n	3	all/unsp	15	99	0	0	nev any	or
MZILEN	3	f	0	0	bl	-	all	Africa	1994	CC	374	n	V	*	n	3	all/unsp	1	14	1	0	nev any	or
MZILEN	4	f	0	0	bl	-	all	Africa	1994	CC	374	n	V	*	n	3	all/unsp	15	99	0	0	nev any	or
RESTRE	6	c	0	0	all	-	all	SCAmer	1978	CC	102	n	bl	n	n	5	cig+/-ot	1	10	1	0	nev cigs	or
RESTRE	7	c	0	0	all	-	all	SCAmer	1978	CC	102	n	bl	n	n	5	cig+/-ot	11	20	2	3	nev cigs	or
RESTRE	8	c	0	0	all	-	all	SCAmer	1978	CC	102	n	bl	n	n	5	cig+/-ot	21	40	0	0	nev cigs	or
RESTRE	9	c	0	0	all	-	all	SCAmer	1978	CC	102	n	bl	n	n	5	cig+/-ot	41	99	3	6	nev cigs	or
SADOWS	85	m	0	0	wh	-	all	NAmer	1938	CC	477	n	bl	n	n	1	all/unsp	1	10	1	0	nev any	st
SADOWS	86	m	0	0	wh	-	all	NAmer	1938	CC	477	n	bl	n	n	1	all/unsp	11	20	2	3	nev any	st
SADOWS	87	m	0	0	wh	-	all	NAmer	1938	CC	477	n	bl	n	n	1	all/unsp	21	30	0	4	nev any	st
SADOWS	88	m	0	0	wh	-	all	NAmer	1938	CC	477	n	bl	n	n	1	all/unsp	31	40	0	5	nev any	st
SADOWS	89	m	0	0	wh	-	all	NAmer	1938	CC	477	n	bl	n	n	1	all/unsp	41	99	3	6	nev any	st

REF	NRR	RR	SIG	Cigarette equivalent	RRDATA comment
BLOT1	5	0.40		*	0
BLOT1	6	4.50		*	0
BLOT1	7	8.60		*	0
BUELL	1	2.32		*	0
BUELL	2	3.52		*	0
BUELL	3	4.93		*	0

Table 1G1 - 9

IESLC - Meta-analysis of Ever Smoking by Amount, Overview, Any product (or Cigarettes if Any not available)
All LC types
Most adjusted - insufficient data for metaanalysis

REF	NRR	RR	SIG	Cigarette equivalent	RRDATA comment
DOLL	11	*			* RR/CI not estimable because no cases or controls in exposed group
MASTRA	3	6.80		*	0
MASTRA	4	13.90		*	0
MZILEN	1	9.60		*	0
MZILEN	2	11.60		*	0
MZILEN	3	13.60		*	0
MZILEN	4	14.90		*	0
RESTRE	6	1.29		*	0
RESTRE	7	12.40		*	0
RESTRE	8	42.49		*	0
RESTRE	9	169.90		*	0
SADOWS	85	2.32		inc 1 cigar = 10, qtr can pipe = 10 cigs	0
SADOWS	86	4.70		inc 1 cigar = 10, qtr can pipe = 10 cigs	0
SADOWS	87	8.00		inc 1 cigar = 10, qtr can pipe = 10 cigs	0
SADOWS	88	5.33		inc 1 cigar = 10, qtr can pipe = 10 cigs	0
SADOWS	89	6.28		inc 1 cigar = 10, qtr can pipe = 10 cigs	0

Least adjusted - insufficient data for meta-analysis: as for adjusted plus the following

REF	NRR	SEX	AGEL	AGEH	RACE	YF	LC TYPE	LOC	START	ST	NLC	R	VB	P	H	AD	PRODUCT	exL	exH	S1	S2	DENOM	De
SADOWS	80	m	0	0	wh	-	all	NAmer	1938	CC	477	n	bl	n	n	0	all/unsp	1	10	1	0	nev any	st
SADOWS	81	m	0	0	wh	-	all	NAmer	1938	CC	477	n	bl	n	n	0	all/unsp	11	20	2	3	nev any	st
SADOWS	82	m	0	0	wh	-	all	NAmer	1938	CC	477	n	bl	n	n	0	all/unsp	21	30	0	4	nev any	st
SADOWS	83	m	0	0	wh	-	all	NAmer	1938	CC	477	n	bl	n	n	0	all/unsp	31	40	0	5	nev any	st
SADOWS	84	m	0	0	wh	-	all	NAmer	1938	CC	477	n	bl	n	n	0	all/unsp	41	99	3	6	nev any	st

REF	NRR	RR	SIG	Cigarette equivalent	RRDATA comment
SADOWS	80	1.83		inc 1 cigar = 10, qtr can pipe = 10 cigs	0
SADOWS	81	3.18		inc 1 cigar = 10, qtr can pipe = 10 cigs	0
SADOWS	82	4.28		inc 1 cigar = 10, qtr can pipe = 10 cigs	0
SADOWS	83	5.23		inc 1 cigar = 10, qtr can pipe = 10 cigs	0
SADOWS	84	5.28		inc 1 cigar = 10, qtr can pipe = 10 cigs	0

Table 1G2 -

IESLC - Meta-analysis of Ever Smoking, Amount smoked, "Low", Any product (or Cigarettes if Any not available)
All LC types

This analysis is restricted to results for:
1) Results by Amount smoked
2) Ever smokers
3) Results complete enough for use in metaanalysis

Within each study, results are then selected (in the following order of preference, within each sex) for:
4) PRODUCT: all/unspec, cigarettes regardless of other products, cigarettes only
5) CIGTYPE: all/unspecified, MC regardless of HR, MC only
6) DENOM: never smoked anything, never smoked cigarettes, (never +1 = +long term ex, +2 = +amount unknown,
+3 = never cigs+long term ex)
7) Followup period (YF, prospective studies): whole study (coded as 0) or longest available
8) LCtype: all or nearest available, at least Squamous and Adeno. (q = squamous, s = small,
l = large, a = adeno, mix = mixed, alv = alveolar)
9) Race: all or nearest available, otherwise by race (wh or w = white, bl or b = black, hi = hispanic
ch = chinese, jap = japanese, haw = hawaiian, w+o = white + oriental, sca = scandinavian, as = asian)
10) Amount smoked "low" in key scheme 1 (key value 5, maximum range <20, in numbers of
cigarettes or cigarette equivalents)
11) For overlapping studies: principal rather than subsidiary studies
Finally by Age: whole study (coded as 0) if available, otherwise by widest available age group
and then for single sex results (m, f) in preference to combined sex results (c).

Results adjusted (AD) for the most potential confounders are then chosen in Sections -1 to -3
and results adjusted for the least confounders in Sections -4 to -6. (Those least adjusted results which
actually differ from the most adjusted as marked 'x' in column X in Section -4)
(Results adjusted for an unknown number of confounder(s) are coded as 20.)

Section -7 shows excluded studies, together with the stage (as above) at which no qualifying
results were found.

Section -8 lists the potentially overlapping studies which have been included (1=principal, 2=subsidiary).

Section -9 lists any results which would have been included in preference except that they had data not complete
enough for use in meta-analysis, with their significance (yes/no), if known, and any further comment as entered
on the database.

In addition to those mentioned above, the following fields, levels and abbreviations are used:
* or nk = not known, n = no, y = yes, ot = other
nev = never
all/unspec = all or unspecified, cig+/-ot = cigarettes irrespective of other products (cigar, pipe etc)
MC = manufactured cigarettes, HR = hand-rolled cigarettes
exL, exH = range of exposure (low and high) in the smoking group, in terms of Amount smoked,
cigarettes or cigarette equivalents
REF: 6-character study reference
NRR: number of the RR on the database within the study
ST : study type (CC = case control, pr or prosp = prospective)
NLC: number of lung cancer cases in whole study
R : risky occupational population (n = no, m = mining, o = other risky)
VB : national cigarette type (V = at least 75% Virginia, bl = at least 75% blended, ot = other)
P : any proxy use
H : full histological confirmation
De : derivation of RR/CI (or = original, st = standard method, ot = other method of estimation)

Table 1G2 - 1

IESLC - Meta-analysis of Ever Smoking, Amount smoked, "Low", Any product (or Cigarettes if Any not available)
All LC types
Most adjusted

REF	NRR	SEX	AGEL	AGEH	RACE	YF	LC TYPE	LOC	START	ST	NLC	R	VB	P	H	AD	PRODUCT	exL	exH	DENOM	De
AGUDO	4	f	0	0	all	-	all	Eu:wst	1989	CC	103	n	bl	n	n	3	cig only	1	10	nev any	or
ALDERS	18	m	0	0	all	-	all	Eu:UK	1977	CC	1448	n	V	n	n	1	cig only	1	17	nev+2	ot
ALDERS	21	f	0	0	all	-	all	Eu:UK	1977	CC	1448	n	V	n	n	1	cig only	1	17	nev+2	ot
ARMADA	46	m	0	0	all	-	all	Eu:wst	1986	CC	325	n	bl	n	y	0	cig+/-ot	1	14	nev any	st
AUVINE	13	c	0	0	all	-	all	Eu:Sca	1986	CC	517	n	bl	y	n	2	cig+/-ot	1	10	nev cigs	or
AXELSS	5	m	0	0	sca	-	all	Eu:Sca	1989	CC	436	n	bl	n	n	6	all/unsp	1	9	nev any	ot
AXELSS	13	f	0	0	sca	-	all	Eu:Sca	1989	CC	436	n	bl	n	n	0	all/unsp	1	9	nev any	st
BARBON	82	m	0	0	all	-	all	Eu:wst	1979	CC	755	n	bl	y	y	3	all/unsp	1	19	nev any	or
BRESLO	13	m	0	0	all	-	all	NAmer	1949	CC	518	n	bl	n	y	0	cig+/-ot	1	9	nev+3	st
BRESLO	29	f	0	0	all	-	all	NAmer	1949	CC	518	n	bl	n	y	0	cig+/-ot	1	19	nev+3	st
BROWN2	32	m	0	0	wh	-	all	NAmer	1984	CC	14596	n	bl	n	y	2	cig+/-ot	1	19	nev cigs	or
BROWN2	31	f	0	0	wh	-	all	NAmer	1984	CC	14596	n	bl	n	y	2	cig+/-ot	1	19	nev cigs	or
BUFFLE	28	f	0	0	w-hi	-	all	NAmer	1976	CC	943	n	bl	y	n	0	cig+/-ot	1	19	nev cigs	or
CHEN2	3	m	0	0	all	-	all	As:Chi	1983	CC	193	n	ot	y	n	0	all/unsp	1	9	nev any	st
CHEN2	7	f	0	0	all	-	all	As:Chi	1983	CC	193	n	ot	y	n	0	all/unsp	1	9	nev any	st
CHOI	12	m	0	0	all	-	all	As:oth	1985	CC	375	n	bl	n	n	0	cig+/-ot	1	10	nev cigs	st
CHOI	17	f	0	0	all	-	all	As:oth	1985	CC	375	n	bl	n	n	0	cig+/-ot	1	10	nev cigs	st
COOKSO	1	c	0	0	bl	-	all	Africa	1961	CC	234	n	V	n	y	0	cig+/-ot	1	9	nev any	st
CPSI	243	m	50	74	all	6	all	NAmer	1959	pr	5138	n	bl	n	n	1	cig only	1	19	nev any	ot
CPSII	105	f	35	99	all	4	all	NAmer	1982	pr	3229	n	bl	n	n	1	cig+/-ot	1	19	nev cigs	ot
DAMBER	6	m	0	0	all	-	all	Eu:Sca	1972	CC	579	n	bl	y	n	1	cig only	1	7	nev any	ot
DAVEYS	1	m	0	0	all	-	all	Eu:Ger	1930	CC	109	n	bl	y	n	0	all/unsp	1	5	nev any	st
DESTEF	6	m	0	0	all	-	all	SCAmer	1988	CC	497	n	bl	n	y	4	all/unsp	1	10	nev any	or
DOLL	2	m	0	0	all	-	all	Eu:UK	1948	CC	1465	n	V	n	n	0	all/unsp	5	14	nev any	st
DOLL	8	f	0	0	all	-	all	Eu:UK	1948	CC	1465	n	V	n	n	0	all/unsp	5	14	nev any	st
DOLL2	46	m	35	99	all	5	all	Eu:UK	1951	pr	920	n	V	n	n	1	all/unsp	1	14	nev any	ot
DORGAN	108	m	0	0	wh	-	all	NAmer	1980	CC	2026	n	bl	y	y	2	cig+/-ot	1	19	nev any	ot
DORGAN	96	f	0	0	all	-	all	NAmer	1980	CC	2026	n	bl	y	y	3	cig+/-ot	1	19	nev any	ot
DOSEME	5	m	0	0	all	-	all	Eu:bal	1979	CC	1210	n	bl	n	n	2	cig+/-ot	1	10	nev cigs	or
DUNN	2	m	0	0	all	0	all	NAmer	1954	pr	139	o	bl	n	n	0	cig+/-ot	5	14	nev cigs	st
EBELIN	2	m	0	0	all	-	all	Eu:Ger	1980	CC	130	n	bl	n	n	0	all/unsp	1	9	nev any	st
ESAKI	1	m	0	0	all	-	all	As:Jap	1961	CC	245	n	bl	y	n	0	cig+/-ot	1	14	nev cigs	st
FAN	6	m	0	0	all	-	all	As:Chi	1990	CC	403	n	ot	y	n	0	cig+/-ot	1	9	nev cigs	st
FAN	10	f	0	0	all	-	all	As:Chi	1990	CC	403	n	ot	y	n	0	cig+/-ot	1	9	nev cigs	st
GAO	21	f	0	0	all	-	all	As:Chi	1984	CC	1405	n	ot	n	n	2	cig+/-ot	1	9	nev cigs	st
GARSHI	26	m	0	0	all	-	all	NAmer	1981	CC	1081	o	bl	y	n	1	all/unsp	1	15	nev any	st
GER	22	c	0	0	all	-	all	As:oth	1990	CC	141	n	ot	y	n	14	all/unsp	1	10	nev any	ot
GOLLED	1	m	35	99	all	-	all	Eu:UK	1952	CC	443	n	V	y	n	1	cig only	1	10	nev any	ot
GSELL	1	m	0	0	all	-	all	Eu:wst	1937	CC	150	n	bl	n	y	0	all/unsp	1	9	nev any	st
HAMMON	153	m	0	0	wh	0	all	NAmer	1952	pr	448	n	bl	n	n	1	cig only	1	9	nev any	ot
HANSEN	1	m	0	0	all	0	all	Eu:Sca	1968	pr	105	o	bl	y	n	2	all/unsp	1	19	nev any	ot
HU	1	m	0	0	all	-	all	As:Chi	1985	CC	227	n	ot	n	y	0	cig+/-ot	1	14	nev any	st
HU	4	f	0	0	all	-	all	As:Chi	1985	CC	227	n	ot	n	y	0	cig+/-ot	1	14	nev any	st
HU2	3	c	0	0	all	-	all	As:Chi	1977	CC	523	n	ot	y	n	0	cig+/-ot	5	9	nev cigs	st
JARUP	4	m	0	0	all	-	all	Eu:Sca	1928	CC	102	o	bl	y	n	2	all/unsp	1	10	nev any	or
JEDRYC	45	m	0	0	all	-	all	Eu:est	1980	CC	1630	n	bl	y	n	4	cig+/-ot	1	19	nev any	or
JEDRYC	48	f	0	0	all	-	all	Eu:est	1980	CC	1630	n	bl	y	n	4	cig+/-ot	1	19	nev any	or
JOLY	7	m	0	0	all	-	all	SCAmer	1978	CC	826	n	bl	n	n	0	cig+/-ot	1	9	nev any	st
JOLY	3	f	0	0	all	-	all	SCAmer	1978	CC	826	n	bl	n	n	0	cig+/-ot	1	9	nev any	st
JUSSAW	34	m	0	0	all	-	all	As:Ind	1964	CC	792	n	V	n	n	2	cig only	1	19	nev any	st
KHUDER	1	m	0	0	all	-	all	NAmer	1985	CC	482	n	bl	n	y	0	cig+/-ot	1	19	nev cigs	st
KOULUM	6	m	0	0	all	-	all	Eu:Sca	1936	CC	812	n	bl	n	n	0	all/unsp	1	9	nev any	st
KREUZE	19	m	1	45	all	-	all	Eu:Ger	1990	CC	2260	n	bl	n	n	3	cig+/-ot	1	9	nev any	or
KREUZE	30	m	55	69	all	-	all	Eu:Ger	1990	CC	2260	n	bl	n	n	3	cig+/-ot	1	9	nev any	or
KREUZE	25	f	1	45	all	-	all	Eu:Ger	1990	CC	2260	n	bl	n	n	3	cig+/-ot	1	9	nev any	or
KREUZE	36	f	55	69	all	-	all	Eu:Ger	1990	CC	2260	n	bl	n	n	3	cig+/-ot	1	9	nev any	or
KREYBE	9	m	0	0	all	-	all	Eu:Sca	1948	CC	300	n	bl	n	y	1	all/unsp	1	14	nev any	ot
KREYBE	28	f	0	0	all	-	all	Eu:Sca	1948	CC	300	n	bl	n	y	1	all/unsp	1	14	nev any	ot
LAMTH	7	f	0	0	ch	-	all	As:HK	1983	CC	445	n	bl	n	n	0	all/unsp	1	10	nev any	or
LAUSSM	18	m	0	0	all	-	all	Eu:Ger	1982	CC	432	n	bl	n	n	3	all/unsp	1	9	nev any	ot
LETOUR	2	c	0	0	all	-	all	NAmer	1983	CC	738	n	V	y	y	0	cig+/-ot	1	19	nev cigs	st
LIU2	8	m	0	0	all	-	all	As:Chi	1983	CC	316	n	ot	n	n	3	all/unsp	1	19	nev any	or
LIU2	14	f	0	0	all	-	all	As:Chi	1983	CC	316	n	ot	n	n	3	all/unsp	1	9	nev any	or
LIU3	6	m	0	0	all	-	all	As:Chi	1985	CC	110	n	ot	n	n	2	all/unsp	1	15	nev any	or
LIU4	7	m	35	69	all	-	all	As:Chi	1986	CC	1000-00	n	ot	y	n	2	cig only	1	19	nev any	ot
LIU5	2	c	0	0	all	-	all	As:Chi	1978	CC	111	n	ot	y	n	0	all/unsp	1	9	nev any	st
LUBIN	11	m	0	0	all	-	all	As:Chi	1984	CC	427	m	ot	y	n	4	cig only	1	6	nev any	ot
LUBIN2	273	m	0	0	all	-	all	Eu:mul	1976	CC	7804	n	bl	n	y	0	cig+/-ot	1	9	nev any	st
LUBIN2	281	f	0	0	all	-	all	Eu:mul	1976	CC	7804	n	bl	n	y	0	cig+/-ot	1	9	nev any	st
MACLEN	36	c	0	0	ch	-	all	As:oth	1972	CC	233	n	bl	n	n	2	cig+/-ot	1	9	nev cigs	or
MARTIS	1	m	0	0	all	-	all	Eu:UK	1972	CC	201	n	V	n	n	0	cig+/-ot	1	14	nev cigs	st

Table 1G2 - 1

IESLC - Meta-analysis of Ever Smoking, Amount smoked, "Low", Any product (or Cigarettes if Any not available)
All LC types
Most adjusted

REF	NRR	SEX	AGEL	AGEH	RACE	YF	LC TYPE	LOC	START	ST	NLC	R	VB	P	H	AD	PRODUCT	exL	exH	DENOM	De
MATOS	29	m	0	0	all	-	all	SCAmer	1994	CC	200	n	bl	n	n	2	cig+/-ot	1	14	nev any	or
MATSUD	1	m	0	0	all	-	all	As:Jap	1965	CC	179	n	bl	n	n	0	cig+/-ot	1	10	nev cigs	st
MCCONN	26	c	0	0	all	-	all	Eu:UK	1946	CC	100	n	V	n	y	0	all/unsp	1	10	nev any	st
NOTAN2	8	m	0	0	all	-	all	As:Ind	1963	CC	683	n	V	n	n	0	cig only	1	9	nev any	st
ORMOS	1	m	0	0	all	-	all	Eu:est	1947	CC	119	n	bl	y	y	0	cig+/-ot	1	15	nev any	st
OSANN2	22	f	0	0	all	-	all	NAmer	1964	ot	217	n	bl	n	y	1	cig+/-ot	1	19	nev cigs	or
PASTOR	6	m	0	0	all	-	all	Eu:wst	1976	CC	204	n	bl	y	n	1	all/unsp	1	9	nev any	st
PERNU	18	m	0	0	all	-	all	Eu:Sca	1944	CC	1606	n	bl	n	n	0	all/unsp	5	9	nev any	st
PERNU	12	f	0	0	all	-	all	Eu:Sca	1944	CC	1606	n	bl	n	n	0	all/unsp	5	9	nev any	st
POLEDN	2	c	0	0	all	-	all	NAmer	1978	CC	209	n	bl	y	n	0	cig+/-ot	1	19	nev cigs	st
RACHTA	10	f	0	0	all	-	all	Eu:est	1991	CC	118	n	bl	n	y	1	cig+/-ot	1	9	nev cigs	or
RANDIG	2	m	0	0	all	-	all	Eu:Ger	1951	CC	448	n	bl	n	n	0	all/unsp	5	9	nev any	st
RANDIG	6	f	0	0	all	-	all	Eu:Ger	1951	CC	448	n	bl	n	n	0	all/unsp	5	9	nev any	st
SHAW	10	c	0	0	wh	-	all	NAmer	1988	CC	335	n	V	n	y	0	all/unsp	1	19	nev any	st
SIEMIA	13	m	0	0	all	-	all	NAmer	1979	CC	857	n	V	y	y	0	cig+/-ot	1	19	nev cigs	or
SPITZ	5	c	0	0	b+hi	-	all	NAmer	1992	CC	177	n	bl	n	y	0	cig+/-ot	1	19	nev cigs	st
STOCKS	41	m	0	0	all	-	all	Eu:UK	1952	CC	2932	n	V	y	n	2	cig+/-ot	1	14	nev any	st
STOCKS	48	f	0	0	all	-	all	Eu:UK	1952	CC	2932	n	V	y	n	1	cig+/-ot	1	14	nev any	ot
TIZZAN	7	m	0	0	all	-	all	Eu:wst	1959	CC	1358	n	bl	n	n	0	cig only	1	9	nev any	st
TIZZAN	15	f	0	0	all	-	all	Eu:wst	1959	CC	1358	n	bl	n	n	0	cig only	1	9	nev any	st
WANG2	10	c	0	0	all	-	all	As:Chi	1980	CC	103	n	ot	n	n	4	cig+/-ot	5	9	nev cigs	ot
WUWILL	12	f	0	0	all	-	all	As:Chi	1985	CC	965	n	ot	n	n	3	cig+/-ot	1	19	nev cigs	ot
WYNDE2	17	m	0	0	all	-	all	NAmer	1962	CC	404	n	bl	n	y	0	cig+/-ot	1	10	nev any	st
WYNDE3	44	m	0	0	all	-	all	NAmer	1966	CC	350	n	bl	n	y	0	cig+/-ot	1	9	nev any	st
WYNDE3	79	f	0	0	all	-	all	NAmer	1966	CC	350	n	bl	n	y	0	cig+/-ot	1	9	nev any	st
WYNDE4	43	m	0	0	all	-	all	NAmer	1948	CC	684	n	bl	y	n	0	all/unsp	1	9	nev any	st
WYNDE4	57	f	0	0	all	-	all	NAmer	1948	CC	684	n	bl	y	n	2	all/unsp	1	9	nev any	ot
XU3	9	m	0	0	all	-	all	As:Chi	1981	CC	135	n	ot	n	n	1	all/unsp	1	9	nev any	ot
XU3	16	f	0	0	all	-	all	As:Chi	1981	CC	135	n	ot	n	n	1	all/unsp	1	9	nev any	ot
YUAN	2	m	0	0	all	0	all	As:Chi	1986	pr	142	n	ot	n	n	2	cig+/-ot	1	19	nev cigs	ot
ZHENG	11	m	0	0	all	-	all	As:Chi	1982	CC	540	n	ot	*	y	0	cig+/-ot	1	9	nev cigs	st
ZHENG	22	f	0	0	all	-	all	As:Chi	1982	CC	540	n	ot	*	y	0	cig+/-ot	1	9	nev cigs	st
ZHOU	4	c	0	0	all	-	all	As:Chi	1978	CC	1360	n	ot	n	n	0	all/unsp	1	9	nev any	st

Cigarette type is all/unspec for all RRs
except for the following:

REF	NRR	CIGTYPE
ALDERS	18	MC only
ALDERS	21	MC only
JUSSAW	34	MC only
NOTAN2	8	MC only

Table 1G2 - 2

IESLC - Meta-analysis of Ever Smoking, Amount smoked, "Low", Any product (or Cigarettes if Any not available)
All LC types
Most adjusted

REF	NRR	SEX	AD	Number Exposed Case	Number Exposed Cont	Non-exposed Case	Non-exposed Cont	RR	95.00%CI
AGUDO	4	f	3	-	-	-	-	1.57	(0.52- 4.70)
ALDERS	18	m	1	-	-	-	-	3.55	(1.94- 6.49)
ALDERS	21	f	1	-	-	-	-	2.62	(1.88- 3.65)
Subtotal ALDERS								2.81	(2.10- 3.76)
ARMADA	46	m	0	44	117	4	64	6.02	(2.07- 17.51)
AUVINE	13	c	2	-	-	-	-	20.00	(9.72- 41.20)
AXELSS	5	m	6	-	-	-	-	3.82	(1.98- 7.36)
AXELSS	13	f	0	13	37	18	154	3.01	(1.35- 6.68)
Subtotal AXELSS								3.47	(2.09- 5.76)
BARBON	82	m	3	-	-	-	-	6.70	(4.20- 11.00)
BRESLO	13	m	0	16	45	22	110	1.78	(0.86- 3.69)
BRESLO	29	f	0	5	5	12	14	1.17	(0.27- 5.02)
Subtotal BRESLO								1.63	(0.85- 3.14)
BROWN2	32	m	2	-	-	-	-	6.10	(5.30- 6.90)
BROWN2	31	f	2	-	-	-	-	8.40	(7.20- 9.70)
Subtotal BROWN2								7.02	(6.36- 7.75)
BUFFLE	28	f	0	21	42	12	112	4.67	(2.11- 10.31)
CHEN2	3	m	0	17	26	9	33	2.40	(0.92- 6.25)
CHEN2	7	f	0	5	17	25	33	0.39	(0.13- 1.20)
Subtotal CHEN2								1.12	(0.54- 2.31)
CHOI	12	m	0	20	90	13	95	1.62	(0.76- 3.46)
CHOI	17	f	0	9	16	76	164	1.21	(0.51- 2.87)
Subtotal CHOI								1.43	(0.81- 2.52)
COOKSO	1	c	0	102	27	45	61	5.12	(2.89- 9.08)
*CPSI	243	m	1	-	-	-	-	5.81	(4.33- 7.80)
*CPSII	105	f	1	-	-	-	-	4.16	(3.41- 5.09)
DAMBER	6	m	1	-	-	-	-	2.30	(1.30- 4.40)
DAVEYS	1	m	0	11	69	3	23	1.22	(0.31- 4.77)
DESTEF	6	m	4	-	-	-	-	2.90	(1.60- 5.00)
DOLL	2	m	0	489	570	7	61	7.48	(3.39- 16.50)
DOLL	8	f	0	24	18	40	59	1.97	(0.95- 4.09)
Subtotal DOLL								3.64	(2.13- 6.22)
*DOLL2	46	m	1	-	-	-	-	6.71	(0.91- 49.81)
DORGAN	108	m	2	-	-	-	-	6.88	(3.74- 12.66)
DORGAN	96	f	3	-	-	-	-	5.67	(4.36- 7.36)
Subtotal DORGAN								5.84	(4.59- 7.43)
DOSEME	5	m	2	-	-	-	-	2.20	(1.40- 3.30)
*DUNN	2	m	0	12	9418	2	14160	9.02	(2.02- 40.30)
EBELIN	2	m	0	20	72	12	117	2.71	(1.25- 5.87)
ESAKI	1	m	0	47	75	16	28	1.10	(0.54- 2.24)
FAN	6	m	0	13	121	36	236	0.70	(0.36- 1.38)
FAN	10	f	0	17	48	69	320	1.64	(0.89- 3.03)
Subtotal FAN								1.12	(0.71- 1.76)
GAO	21	f	2	-	-	-	-	1.74	(1.22- 2.48)
GARSHI	26	m	1	-	-	-	-	3.29	(2.22- 4.87)
GER	22	c	14	-	-	-	-	1.26	(0.61- 2.61)
GOLLED	1	m	1	-	-	-	-	4.45	(2.54- 7.81)
GSELL	1	m	0	11	36	2	29	4.43	(0.91- 21.60)
*HAMMON	153	m	1	-	-	-	-	7.38	(3.72- 14.63)
*HANSEN	1	m	2	-	-	-	-	1.37	(0.63- 3.54)
HU	1	m	0	36	38	41	67	1.55	(0.85- 2.82)
HU	4	f	0	19	10	40	48	2.28	(0.95- 5.46)
Subtotal HU								1.75	(1.07- 2.87)
HU2	3	c	0	44	58	121	213	1.34	(0.85- 2.10)
JARUP	4	m	2	-	-	-	-	6.90	(2.40- 22.70)
JEDRYC	45	m	4	-	-	-	-	3.48	(2.33- 5.19)
JEDRYC	48	f	4	-	-	-	-	6.37	(2.66- 15.24)
Subtotal JEDRYC								3.87	(2.69- 5.56)
JOLY	7	m	0	16	54	12	218	5.38	(2.41- 12.05)
JOLY	3	f	0	33	38	52	283	4.73	(2.72- 8.21)
Subtotal JOLY								4.93	(3.12- 7.77)
JUSSAW	34	m	2	-	-	-	-	5.57	(2.46- 14.76)
KHUDER	1	m	0	81	434	23	309	2.51	(1.54- 4.07)
KOULUM	6	m	0	37	77	5	54	5.19	(1.92- 14.06)
KREUZE	19	m	3	-	-	-	-	2.50	(0.70- 8.20)
KREUZE	30	m	3	-	-	-	-	8.20	(5.20- 13.00)
KREUZE	25	f	3	-	-	-	-	5.70	(1.60- 16.60)
KREUZE	36	f	3	-	-	-	-	2.00	(1.20- 3.30)
Subtotal KREUZE								4.27	(3.12- 5.85)
KREYBE	9	m	1	-	-	-	-	5.82	(2.56- 13.23)
KREYBE	28	f	1	-	-	-	-	1.36	(0.65- 2.82)

Table 1G2 - 2

IESLC - Meta-analysis of Ever Smoking, Amount smoked, "Low", Any product (or Cigarettes if Any not available)
All LC types
Most adjusted

REF	NRR	SEX	AD	Number Exposed Case	Number Exposed Cont	Non-exposed Case	Non-exposed Cont	RR	95.00%CI
Subtotal KREYBE								2.59	(1.50- 4.48)
LAMTH	7	f	0	101	63	202	337	2.67	(1.87- 3.83)
LAUSSM	18	m	3	-	-	-	-	3.29	(2.31- 4.66)
LETOUR	2	c	0	271	266	24	224	9.51	(6.04- 14.97)
LIU2	8	m	3	-	-	-	-	1.20	(0.43- 3.50)
LIU2	14	f	3	-	-	-	-	1.80	(0.57- 5.90)
Subtotal LIU2								1.44	(0.66- 3.14)
LIU3	6	m	2	-	-	-	-	1.41	(0.33- 6.09)
LIU4	7	m	2	-	-	-	-	2.11	(2.02- 2.20)
LIU5	2	c	0	14	27	26	41	0.82	(0.36- 1.84)
LUBIN	11	m	4	-	-	-	-	0.72	(0.20- 2.54)
LUBIN2	273	m	0	1887	3759	190	2616	6.91	(5.91- 8.09)
LUBIN2	281	f	0	151	218	336	1188	2.45	(1.93- 3.11)
Subtotal LUBIN2								5.06	(4.44- 5.77)
MACLEN	36	c	2	-	-	-	-	1.35	(0.64- 2.84)
MARTIS	1	m	0	31	39	4	25	4.97	(1.56- 15.78)
MATOS	29	m	2	-	-	-	-	2.00	(0.90- 4.50)
MATSUD	1	m	0	37	1237	3	1255	12.51	(3.85- 40.69)
MCCONN	26	c	0	7	43	9	23	0.42	(0.14- 1.26)
NOTAN2	8	m	0	6	42	134	544	0.58	(0.24- 1.39)
ORMOS	1	m	0	32	329	7	777	10.80	(4.72- 24.71)
OSANN2	22	f	1	-	-	-	-	2.50	(1.20- 5.20)
PASTOR	6	m	1	-	-	-	-	2.33	(0.78- 6.95)
PERNU	18	m	0	61	31	97	275	5.58	(3.42- 9.11)
PERNU	12	f	0	5	13	110	971	3.40	(1.19- 9.70)
Subtotal PERNU								5.10	(3.27- 7.96)
POLEDN	2	c	0	53	103	12	139	5.96	(3.03- 11.72)
RACHTA	10	f	1	-	-	-	-	3.64	(1.08- 12.32)
RANDIG	2	m	0	65	99	5	22	2.89	(1.04- 8.01)
RANDIG	6	f	0	12	13	17	92	5.00	(1.95- 12.79)
Subtotal RANDIG								3.88	(1.95- 7.76)
SHAW	10	c	0	46	90	11	107	4.97	(2.43- 10.16)
SIEMIA	13	m	0	-	-	-	-	3.00	(1.00- 9.90)
SPITZ	5	c	0	27	88	10	96	2.95	(1.35- 6.43)
STOCKS	41	m	2	-	-	-	-	4.64	(3.25- 6.61)
STOCKS	48	f	1	-	-	-	-	2.24	(1.64- 3.03)
Subtotal STOCKS								3.06	(2.43- 3.86)
TIZZAN	7	m	0	130	238	180	305	0.93	(0.70- 1.23)
TIZZAN	15	f	0	11	14	25	114	3.58	(1.46- 8.82)
Subtotal TIZZAN								1.04	(0.80- 1.37)
WANG2	10	c	4	-	-	-	-	1.22	(0.31- 4.83)
WUWILL	12	f	3	-	-	-	-	2.13	(1.75- 2.58)
WYNDE2	17	m	0	17	114	8	105	1.96	(0.81- 4.72)
WYNDE3	44	m	0	8	42	9	88	1.86	(0.67- 5.17)
WYNDE3	79	f	0	3	19	20	76	0.60	(0.16- 2.23)
Subtotal WYNDE3								1.22	(0.54- 2.72)
WYNDE4	43	m	0	17	82	12	115	1.99	(0.90- 4.38)
WYNDE4	57	f	2	-	-	-	-	1.13	(0.33- 3.89)
Subtotal WYNDE4								1.69	(0.87- 3.28)
XU3	9	m	1	-	-	-	-	1.66	(0.55- 4.97)
XU3	16	f	1	-	-	-	-	2.18	(0.61- 7.84)
Subtotal XU3								1.86	(0.81- 4.29)
*YUAN	2	m	2	-	-	-	-	3.60	(1.88- 6.91)
ZHENG	11	m	0	25	40	33	94	1.78	(0.94- 3.37)
ZHENG	22	f	0	24	29	152	184	1.00	(0.56- 1.79)
Subtotal ZHENG								1.30	(0.85- 2.00)
ZHOU	4	c	0	61	5	507	68	1.64	(0.64- 4.22)
Partial Totals				4264	18601	2860	26946		

*prospective study

Table 1G2 - 2

IESLC - Meta-analysis of Ever Smoking, Amount smoked, "Low", Any product (or Cigarettes if Any not available)
All LC types
Most adjusted

REF	NRR	SEX	AD	Ys	Ws	Qs	Ps
AGUDO	4	f	3	0.45	3.17	1.02	0.4219
ALDERS	18	m	1	1.27	10.54	0.66	0.0000
ALDERS	21	f	1	0.96	34.91	0.10	0.0000
Subtotal ALDERS				1.03	45.45	0.76	
ARMADA	46	m	0	1.79	3.37	2.04	0.0010
AUVINE	13	c	2	3.00	7.37	28.84	0.0000
AXELSS	5	m	6	1.34	8.91	0.93	0.0001
AXELSS	13	f	0	1.10	6.02	0.04	0.0069
Subtotal AXELSS				1.24	14.94	0.97	
BARBON	82	m	3	1.90	16.58	12.99	0.0000
BRESLO	13	m	0	0.58	7.18	1.40	0.1231
BRESLO	29	f	0	0.15	1.80	1.34	0.8360
Subtotal BRESLO				0.49	8.98	2.74	
BROWN2	32	m	2	1.81	220.78	138.25	0.0000
BROWN2	31	f	2	2.13	172.98	213.61	0.0000
Subtotal BROWN2				1.95	393.76	351.87	
BUFFLE	28	f	0	1.54	6.11	1.67	0.0001
CHEN2	3	m	0	0.87	4.19	0.09	0.0735
CHEN2	7	f	0	-0.95	3.04	11.71	0.0991
Subtotal CHEN2				0.11	7.23	11.79	
CHOI	12	m	0	0.48	6.73	1.91	0.2084
CHOI	17	f	0	0.19	5.18	3.51	0.6591
Subtotal CHOI				0.36	11.92	5.42	
COOKSO	1	c	0	1.63	11.70	4.45	0.0000
*CPSI	243	m	1	1.76	44.36	24.46	0.0000
*CPSII	105	f	1	1.43	95.77	15.98	0.0000
DAMBER	6	m	1	0.83	10.34	0.35	0.0074
DAVEYS	1	m	0	0.20	2.07	1.38	0.7726
DESTEF	6	m	4	1.06	11.84	0.03	0.0002
DOLL	2	m	0	2.01	6.13	6.07	0.0000
DOLL	8	f	0	0.68	7.19	0.83	0.0698
Subtotal DOLL				1.29	13.32	6.90	
*DOLL2	46	m	1	1.90	0.96	0.75	0.0623
DORGAN	108	m	2	1.93	10.33	8.59	0.0000
DORGAN	96	f	3	1.74	56.05	28.91	0.0000
Subtotal DORGAN				1.77	66.38	37.50	
DOSEME	5	m	2	0.79	20.90	1.09	0.0003
*DUNN	2	m	0	2.20	1.71	2.40	0.0040
EBELIN	2	m	0	1.00	6.42	0.00	0.0116
ESAKI	1	m	0	0.09	7.53	6.44	0.8001
FAN	6	m	0	-0.35	8.53	15.96	0.3059
FAN	10	f	0	0.50	10.28	2.79	0.1116
Subtotal FAN				0.11	18.81	18.74	
GAO	21	f	2	0.55	30.53	6.55	0.0022
GARSHI	26	m	1	1.19	24.90	0.75	0.0000
GER	22	c	14	0.23	7.27	4.49	0.5331
GOLLED	1	m	1	1.49	12.18	2.76	0.0000
GSELL	1	m	0	1.49	1.53	0.34	0.0655
*HAMMON	153	m	1	2.00	8.19	7.90	0.0000
*HANSEN	1	m	2	0.31	5.16	2.54	0.4747
HU	1	m	0	0.44	10.71	3.60	0.1527
HU	4	f	0	0.82	5.04	0.19	0.0643
Subtotal HU				0.56	15.74	3.79	
HU2	3	c	0	0.29	18.89	10.01	0.2087
JARUP	4	m	2	1.93	3.04	2.55	0.0008
JEDRYC	45	m	4	1.25	23.96	1.27	0.0000
JEDRYC	48	f	4	1.85	5.04	3.51	0.0000
Subtotal JEDRYC				1.35	29.00	4.78	
JOLY	7	m	0	1.68	5.92	2.63	0.0000
JOLY	3	f	0	1.55	12.60	3.62	0.0000
Subtotal JOLY				1.59	18.52	6.25	
JUSSAW	34	m	2	1.72	4.79	2.35	0.0002
KHUDER	1	m	0	0.92	16.30	0.16	0.0002
KOULUM	6	m	0	1.65	3.87	1.53	0.0012
KREUZE	19	m	3	0.92	2.54	0.03	0.1444
KREUZE	30	m	3	2.10	18.30	21.63	0.0000
KREUZE	25	f	3	1.74	2.81	1.47	0.0035
KREUZE	36	f	3	0.69	15.02	1.57	0.0072
Subtotal KREUZE				1.45	38.66	24.70	
KREYBE	9	m	1	1.76	5.70	3.16	0.0000
KREYBE	28	f	1	0.31	7.13	3.59	0.4115

Table 1G2 - 2

IESLC - Meta-analysis of Ever Smoking, Amount smoked, "Low", Any product (or Cigarettes if Any not available)
All LC types
Most adjusted

REF	NRR	SEX	AD	Ys	Ws	Qs	Ps
Subtotal KREYBE				0.95	12.83	6.75	
LAMTH	7	f	0	0.98	29.68	0.03	0.0000
LAUSSM	18	m	3	1.19	31.20	0.94	0.0000
LETOUR	2	c	0	2.25	18.66	28.48	0.0000
LIU2	8	m	3	0.18	3.50	2.43	0.7332
LIU2	14	f	3	0.59	2.81	0.52	0.3242
Subtotal LIU2				0.36	6.31	2.95	
LIU3	6	m	2	0.34	1.81	0.82	0.6441
LIU4	7	m	2	0.75	2108.86	154.05	0.0000
LIU5	2	c	0	-0.20	5.84	8.66	0.6267
LUBIN	11	m	4	-0.33	2.38	4.31	0.6124
LUBIN2	273	m	0	1.93	155.25	130.33	0.0000
LUBIN2	281	f	0	0.90	66.54	0.98	0.0000
Subtotal LUBIN2				1.62	221.79	131.31	
MACLEN	36	c	2	0.30	6.92	3.56	0.4298
MARTIS	1	m	0	1.60	2.87	0.99	0.0066
MATOS	29	m	2	0.69	5.93	0.62	0.0914
MATSUD	1	m	0	2.53	2.76	6.30	0.0000
MCCONN	26	c	0	-0.88	3.12	11.19	0.1215
NOTAN2	8	m	0	-0.54	5.01	12.21	0.2229
ORMOS	1	m	0	2.38	5.60	10.40	0.0000
OSANN2	22	f	1	0.92	7.15	0.07	0.0143
PASTOR	6	m	1	0.85	3.21	0.09	0.1295
PERNU	18	m	0	1.72	15.98	7.87	0.0000
PERNU	12	f	0	1.22	3.48	0.15	0.0225
Subtotal PERNU				1.63	19.46	8.02	
POLEDN	2	c	0	1.79	8.40	4.95	0.0000
RACHTA	10	f	1	1.29	2.59	0.20	0.0375
RANDIG	2	m	0	1.06	3.69	0.01	0.0415
RANDIG	6	f	0	1.61	4.35	1.52	0.0008
Subtotal RANDIG				1.36	8.04	1.53	
SHAW	10	c	0	1.60	7.51	2.59	0.0000
SIEMIA	13	m	0	1.10	2.92	0.02	0.0603
SPITZ	5	c	0	1.08	6.30	0.03	0.0067
STOCKS	41	m	2	1.53	30.49	8.17	0.0000
STOCKS	48	f	1	0.81	40.78	1.81	0.0000
Subtotal STOCKS				1.12	71.26	9.98	
TIZZAN	7	m	0	-0.08	48.24	57.78	0.5909
TIZZAN	15	f	0	1.28	4.74	0.32	0.0055
Subtotal TIZZAN				0.04	52.98	58.09	
WANG2	10	c	4	0.20	2.04	1.36	0.7765
WUWILL	12	f	3	0.76	101.98	6.94	0.0000
WYNDE2	17	m	0	0.67	4.95	0.59	0.1353
WYNDE3	44	m	0	0.62	3.69	0.58	0.2325
WYNDE3	79	f	0	-0.51	2.23	5.20	0.4459
Subtotal WYNDE3				0.20	5.91	5.77	
WYNDE4	43	m	0	0.69	6.13	0.67	0.0891
WYNDE4	57	f	2	0.12	2.52	2.02	0.8460
Subtotal WYNDE4				0.52	8.66	2.69	
XU3	9	m	1	0.51	3.17	0.83	0.3668
XU3	16	f	1	0.78	2.36	0.13	0.2316
Subtotal XU3				0.62	5.53	0.96	
*YUAN	2	m	2	1.28	9.07	0.63	0.0001
ZHENG	11	m	0	0.58	9.44	1.83	0.0764
ZHENG	22	f	0	0.00	11.34	11.69	0.9951
Subtotal ZHENG				0.26	20.78	13.52	
ZHOU	4	c	0	0.49	4.29	1.18	0.3077

Table 1G2 - 2

IESLC - Meta-analysis of Ever Smoking, Amount smoked, "Low", Any product (or Cigarettes if Any not available)
All LC types
Most adjusted

	N	104
	NS	78
	Wt	3894.15
Het	Chi	1124.81
Het	df	103
Het	P	***
Fixed	RR	2.76
	RRl	2.68
	RRu	2.85
	P	+++
Random	RR	2.85
	RRl	2.48
	RRu	3.28
	P	+++
Asymm	P	N.S.

Table 1G2 - 3

IESLC - Meta-analysis of Ever Smoking, Amount smoked, "Low", Any product (or Cigarettes if Any not available)
All LC types
Most adjusted

	Sex: combined	male	female	Total
N	13	58	33	104
NS	13	57	32	102
Wt	108.31	3022.62	763.22	3894.15
Het Chi	107.77	668.70	290.52	1124.81
Het df	12	57	32	103
Het P	***	***	***	***
Fixed RR	3.17	2.59	3.51	2.76
RRl	2.62	2.50	3.27	2.68
RRu	3.82	2.69	3.76	2.85
P	+++	+++	+++	+++
Random RR	2.59	3.12	2.48	2.85
RRl	1.45	2.57	1.94	2.48
RRu	4.65	3.79	3.18	3.28
P	++	+++	+++	+++
Between Chi				57.81
Between df				2
Between P				***
Btwn(F) P				(*)
Btwn(R) P				N.S.

	Lung cancer type: all	other	Total
N	104		104
NS	78		78
Wt	3894.15		3894.15
Het Chi	1124.81		1124.81
Het df	103		103
Het P	***		***
Fixed RR	2.76		2.76
RRl	2.68		2.68
RRu	2.85		2.85
P	+++		+++
Random RR	2.85		2.85
RRl	2.48		2.48
RRu	3.28		3.28
P	+++		+++
Between Chi			
Between df			
Between P			N.S.
Btwn(F) P			N.S.
Btwn(R) P			N.S.

	Location: NAmer	UK	Scand	othEur	China	Japan	othAs	other	Total
N	24	10	11	23	22	2	7	5	104
NS	19	7	8	16	16	2	6	4	78
Wt	736.92	149.16	77.00	447.12	2360.09	10.29	65.58	47.99	3894.15
Het Chi	119.53	32.01	40.81	210.31	44.39	11.98	19.29	5.63	1124.81
Het df	23	9	10	22	21	1	6	4	103
Het P	***	***	***	***	**	***	**	N.S.	***
Fixed RR	5.62	3.04	4.02	3.74	2.06	2.11	1.92	3.90	2.76
RRl	5.23	2.59	3.21	3.41	1.98	1.14	1.51	2.94	2.68
RRu	6.04	3.57	5.02	4.10	2.15	3.88	2.44	5.18	2.85
P	+++	+++	+++	+++	+++	+	+++	+++	+++
Random RR	4.07	3.10	3.95	3.48	1.61	3.53	1.66	3.87	2.85
RRl	3.30	2.19	2.48	2.48	1.36	0.33	1.03	2.75	2.48
RRu	5.02	4.40	6.30	4.87	1.91	38.34	2.67	5.44	3.28
P	+++	+++	+++	+++	+++	N.S.	+	+++	+++
Between Chi									640.86
Between df									7
Between P									***
Btwn(F) P									***
Btwn(R) P									***

Table 1G2 - 3

IESLC - Meta-analysis of Ever Smoking, Amount smoked, "Low", Any product (or Cigarettes if Any not available)
All LC types
Most adjusted

	Detailed Country in "other Europe"					
	multi	Germany	othWest	East	Balkans	Total
N	2	9	7	4	1	23
NS	1	5	6	3	1	16
Wt	221.79	86.40	80.84	37.20	20.90	447.12
Het Chi	50.14	22.36	58.63	6.60	0.00	210.31
Het df	1	8	6	3	0	22
Het P	***	**	***	(*)	N.S.	***
Fixed RR	5.06	3.61	1.77	4.49	2.20	3.74
RRl	4.44	2.93	1.43	3.26	1.43	3.41
RRu	5.77	4.46	2.21	6.20	3.38	4.10
P	+++	+++	+++	+++	+++	+++
Random RR	4.13	3.42	2.92	5.26	2.20	3.48
RRl	1.49	2.28	1.24	2.97	1.43	2.48
RRu	11.42	5.13	6.89	9.32	3.38	4.87
P	++	+++	+	+++	+++	+++
Between Chi						72.59
Between df						4
Between P						***
Btwn(F) P						(*)
Btwn(R) P						N.S.

	Detailed Country in "other Asia"			
	India	HongKong	other	Total
N	2	1	4	7
NS	2	1	3	6
Wt	9.79	29.68	26.11	65.58
Het Chi	12.52	0.00	0.32	19.29
Het df	1	0	3	6
Het P	***	N.S.	N.S.	**
Fixed RR	1.75	2.67	1.36	1.92
RRl	0.94	1.87	0.93	1.51
RRu	3.28	3.83	2.00	2.44
P	(+)	+++	N.S.	+++
Random RR	1.79	2.67	1.36	1.66
RRl	0.20	1.87	0.93	1.03
RRu	16.46	3.83	2.00	2.67
P	N.S.	+++	N.S.	+
Between Chi				6.45
Between df				2
Between P				*
Btwn(F) P				N.S.
Btwn(R) P				*

	Detailed other continent			
	SCAmer	Auslia	Africa	Total
N	4		1	5
NS	3		1	4
Wt	36.28		11.70	47.99
Het Chi	4.49		0.00	5.63
Het df	3		0	4
Het P	N.S.		N.S.	N.S.
Fixed RR	3.58		5.12	3.90
RRl	2.58		2.89	2.94
RRu	4.95		9.08	5.18
P	+++		+++	+++
Random RR	3.55		5.12	3.87
RRl	2.36		2.89	2.75
RRu	5.32		9.08	5.44
P	+++		+++	+++
Between Chi				1.14
Between df				1
Between P				N.S.
Btwn(F) P				N.S.
Btwn(R) P				N.S.

Table 1G2 - 3

IESLC - Meta-analysis of Ever Smoking, Amount smoked, "Low", Any product (or Cigarettes if Any not available)
All LC types
Most adjusted

	Start year of study<br><1960	1960-69	1970-79	1980-89	1990+	Total
N	27	10	18	39	10	104
NS	19	9	15	29	6	78
Wt	282.18	54.95	393.02	3084.43	79.57	3894.15
Het Chi	160.98	38.53	136.42	682.84	48.21	1124.81
Het df	26	9	17	38	9	103
Het P	***	***	***	***	***	***
Fixed RR	2.97	2.27	3.91	2.64	2.54	2.76
RRl	2.64	1.74	3.55	2.55	2.04	2.68
RRu	3.33	2.95	4.32	2.74	3.16	2.85
P	+++	+++	+++	+++	+++	+++
Random RR	3.23	2.12	3.00	2.87	2.38	2.85
RRl	2.33	1.21	2.17	2.28	1.39	2.48
RRu	4.46	3.73	4.16	3.61	4.06	3.28
P	+++	++	+++	+++	++	+++
Between Chi						57.83
Between df						4
Between P						***
Btwn(F) P						N.S.
Btwn(R) P						N.S.

	Study type (1)<br>CC	other	Total
N	96	8	104
NS	70	8	78
Wt	3721.78	172.36	3894.15
Het Chi	1068.30	16.64	1124.81
Het df	95	7	103
Het P	***	*	***
Fixed RR	2.71	4.42	2.76
RRl	2.62	3.81	2.68
RRu	2.79	5.14	2.85
P	+++	+++	+++
Random RR	2.76	4.20	2.85
RRl	2.38	3.07	2.48
RRu	3.21	5.76	3.28
P	+++	+++	+++
Between Chi			39.87
Between df			1
Between P			***
Btwn(F) P			(*)
Btwn(R) P			*

	Study type (2)<br>CC	prosp	other	Total
N	96	7	1	104
NS	70	7	1	78
Wt	3721.78	165.22	7.15	3894.15
Het Chi	1068.30	14.21	0.00	1124.81
Het df	95	6	0	103
Het P	***	*	N.S.	***
Fixed RR	2.71	4.54	2.50	2.76
RRl	2.62	3.89	1.20	2.68
RRu	2.79	5.28	5.20	2.85
P	+++	+++	+	+++
Random RR	2.76	4.49	2.50	2.85
RRl	2.38	3.24	1.20	2.48
RRu	3.21	6.23	5.20	3.28
P	+++	+++	+	+++
Between Chi				42.30
Between df				2
Between P				***
Btwn(F) P				N.S.
Btwn(R) P				*

Table 1G2 - 3

IESLC - Meta-analysis of Ever Smoking, Amount smoked, "Low", Any product (or Cigarettes if Any not available)
All LC types
Most adjusted

	Study size (number of LC cases)				
	100-249	250-499	500-999	1000+	Total
N	31	24	20	29	104
NS	28	17	16	17	78
Wt	151.72	206.35	254.40	3281.67	3894.15
Het Chi	88.31	63.07	131.33	837.62	1124.81
Het df	30	23	19	28	103
Het P	***	***	***	***	***
Fixed RR	2.36	2.64	2.75	2.79	2.76
RRl	2.02	2.31	2.43	2.70	2.68
RRu	2.77	3.03	3.11	2.89	2.85
P	+++	+++	+++	+++	+++
Random RR	2.32	2.48	2.98	3.63	2.85
RRl	1.74	1.94	2.06	2.80	2.48
RRu	3.07	3.16	4.32	4.70	3.28
P	+++	+++	+++	+++	+++
Between Chi					4.48
Between df					3
Between P					N.S.
Btwn(F) P					N.S.
Btwn(R) P					(*)

	Risky occupational population			
	no	mining	othRisky	Total
N	99	1	4	104
NS	73	1	4	78
Wt	3856.95	2.38	34.81	3894.15
Het Chi	1112.26	0.00	7.36	1124.81
Het df	98	0	3	103
Het P	***	N.S.	(*)	***
Fixed RR	2.76	0.72	3.24	2.76
RRl	2.68	0.20	2.32	2.68
RRu	2.85	2.57	4.52	2.85
P	+++	N.S.	+++	+++
Random RR	2.85	0.72	3.51	2.85
RRl	2.47	0.20	1.77	2.48
RRu	3.30	2.57	6.98	3.28
P	+++	N.S.	+++	+++
Between Chi				5.18
Between df				2
Between P				(*)
Btwn(F) P				N.S.
Btwn(R) P				(*)

	National cigarette tobacco type			
	Virginia	blended	other	Total
N	16	65	23	104
NS	13	48	17	78
Wt	199.75	1327.03	2367.36	3894.15
Het Chi	73.51	470.82	46.15	1124.81
Het df	15	64	22	103
Het P	***	***	**	***
Fixed RR	3.45	4.52	2.06	2.76
RRl	3.01	4.28	1.98	2.68
RRu	3.97	4.77	2.15	2.85
P	+++	+++	+++	+++
Random RR	3.38	3.43	1.59	2.85
RRl	2.41	2.89	1.35	2.48
RRu	4.75	4.05	1.89	3.28
P	+++	+++	+++	+++
Between Chi				534.34
Between df				2
Between P				***
Btwn(F) P				***
Btwn(R) P				***

Table 1G2 - 3

IESLC - Meta-analysis of Ever Smoking, Amount smoked, "Low", Any product (or Cigarettes if Any not available)
All LC types
Most adjusted

	Any proxy use No/nk	Yes	Total
N	72	32	104
NS	52	26	78
Wt	1415.49	2478.66	3894.15
Het Chi	581.87	273.17	1124.81
Het df	71	31	103
Het P	***	***	***
Fixed RR	3.92	2.27	2.76
RRl	3.72	2.18	2.68
RRu	4.13	2.36	2.85
P	+++	+++	+++
Random RR	2.85	2.86	2.85
RRl	2.40	2.24	2.48
RRu	3.38	3.65	3.28
P	+++	+++	+++
Between Chi			269.77
Between df			1
Between P			***
Btwn(F) P			***
Btwn(R) P			N.S.

	Full histological confirmation No	Yes	Total
N	73	31	104
NS	55	23	78
Wt	3027.85	866.30	3894.15
Het Chi	390.77	251.95	1124.81
Het df	72	30	103
Het P	***	***	***
Fixed RR	2.29	5.34	2.76
RRl	2.21	4.99	2.68
RRu	2.37	5.71	2.85
P	+++	+++	+++
Random RR	2.69	3.33	2.85
RRl	2.35	2.64	2.48
RRu	3.09	4.20	3.28
P	+++	+++	+++
Between Chi			482.08
Between df			1
Between P			***
Btwn(F) P			***
Btwn(R) P			N.S.

	Number of adjustment variables (1) 0	1	2+/+nk	Total
N	53	17	34	104
NS	40	14	27	81
Wt	623.06	314.22	2956.86	3894.15
Het Chi	386.33	44.33	661.93	1124.81
Het df	52	16	33	103
Het P	***	***	***	***
Fixed RR	3.06	3.54	2.64	2.76
RRl	2.83	3.17	2.54	2.68
RRu	3.31	3.96	2.73	2.85
P	+++	+++	+++	+++
Random RR	2.51	3.31	3.18	2.85
RRl	1.98	2.65	2.46	2.48
RRu	3.18	4.13	4.11	3.28
P	+++	+++	+++	+++
Between Chi				32.22
Between df				2
Between P				***
Btwn(F) P				N.S.
Btwn(R) P				N.S.

Table 1G2 - 3

IESLC - Meta-analysis of Ever Smoking, Amount smoked, "Low", Any product (or Cigarettes if Any not available)
All LC types
Most adjusted

	Number of adjustment variables (2) 0	1	2	3-5	6+/+nk	Total
N	53	17	16	16	2	104
NS	40	14	15	11	2	82
Wt	623.06	314.22	2641.48	299.20	16.19	3894.15
Het Chi	386.33	44.33	560.92	80.55	4.93	1124.81
Het df	52	16	15	15	1	103
Het P	***	***	***	***	*	***
Fixed RR	3.06	3.54	2.58	3.27	2.32	2.76
RRl	2.83	3.17	2.48	2.92	1.43	2.68
RRu	3.31	3.96	2.68	3.66	3.78	2.85
P	+++	+++	+++	+++	+++	+++
Random RR	2.51	3.31	3.46	3.12	2.22	2.85
RRl	1.98	2.65	2.28	2.28	0.75	2.48
RRu	3.18	4.13	5.23	4.28	6.58	3.28
P	+++	+++	+++	+++	N.S.	+++
Between Chi						47.75
Between df						4
Between P						***
Btwn(F) P						N.S.
Btwn(R) P						N.S.

	Product all/unsp	cig+/-ot	cig only	Total
N	37	54	13	104
NS	28	39	11	78
Wt	266.60	1329.85	2297.70	3894.15
Het Chi	95.00	512.84	119.13	1124.81
Het df	36	53	12	103
Het P	***	***	***	***
Fixed RR	2.88	4.26	2.14	2.76
RRl	2.55	4.04	2.06	2.68
RRu	3.25	4.49	2.23	2.85
P	+++	+++	+++	+++
Random RR	2.56	3.16	2.56	2.85
RRl	2.07	2.61	1.81	2.48
RRu	3.17	3.82	3.62	3.28
P	+++	+++	+++	+++
Between Chi				397.85
Between df				2
Between P				***
Btwn(F) P				***
Btwn(R) P				N.S.

	Denominator nev any	nev cigs	Total
N	73	31	104
NS	52	26	78
Wt	3063.13	831.02	3894.15
Het Chi	567.11	376.11	1124.81
Het df	72	30	103
Het P	***	***	***
Fixed RR	2.47	4.19	2.76
RRl	2.38	3.91	2.68
RRu	2.56	4.48	2.85
P	+++	+++	+++
Random RR	2.89	2.79	2.85
RRl	2.47	2.11	2.48
RRu	3.39	3.70	3.28
P	+++	+++	+++
Between Chi			181.59
Between df			1
Between P			***
Btwn(F) P			***
Btwn(R) P			N.S.

Table 1G2 - 3

IESLC - Meta-analysis of Ever Smoking, Amount smoked, "Low", Any product (or Cigarettes if Any not available)
All LC types
Most adjusted

	Derivation of RR/CI Orig	StdCalc	Other	Total
N	24	54	26	104
NS	18	41	22	81
Wt	593.73	647.73	2652.68	3894.15
Het Chi	155.07	391.61	193.21	1124.81
Het df	23	53	25	103
Het P	***	***	***	***
Fixed RR	5.51	3.14	2.30	2.76
RRl	5.09	2.91	2.21	2.68
RRu	5.97	3.39	2.39	2.85
P	+++	+++	+++	+++
Random RR	3.67	2.55	2.90	2.85
RRl	2.81	2.03	2.37	2.48
RRu	4.78	3.22	3.56	3.28
P	+++	+++	+++	+++
Between Chi				384.91
Between df				2
Between P				***
Btwn(F) P				***
Btwn(R) P				N.S.

	Study LIU4 LIU4	others	Total
N	1	103	104
NS	1	77	78
Wt	2108.86	1785.28	3894.15
Het Chi	0.00	788.79	1124.81
Het df	0	102	103
Het P	N.S.	***	***
Fixed RR	2.11	3.80	2.76
RRl	2.02	3.63	2.68
RRu	2.20	3.99	2.85
P	+++	+++	+++
Random RR	2.11	2.86	2.85
RRl	2.02	2.47	2.48
RRu	2.20	3.30	3.28
P	+++	+++	+++
Between Chi			336.02
Between df			1
Between P			***
Btwn(F) P			***
Btwn(R) P			***

Table 1G2 - 4

IESLC - Meta-analysis of Ever Smoking, Amount smoked, "Low", Any product (or Cigarettes if Any not available)
All LC types
Least adjusted

REF	NRR	X	SEX	AGEL	AGEH	RACE	YF	LC TYPE	LOC	START	ST	NLC	R	VB	P	H	AD	PRODUCT	exL	exH	DENOM	De
AGUDO	11	x	f	0	0	all	-	all	Eu:wst	1989	CC	103	n	bl	n	n	0	cig only	1	10	nev any	st
ALDERS	18		m	0	0	all	-	all	Eu:UK	1977	CC	1448	n	V	n	n	1	cig only	1	17	nev+2	ot
ALDERS	21		f	0	0	all	-	all	Eu:UK	1977	CC	1448	n	V	n	n	1	cig only	1	17	nev+2	ot
ARMADA	46		m	0	0	all	-	all	Eu:wst	1986	CC	325	n	bl	n	y	0	cig+/-ot	1	14	nev any	st
AUVINE	5	x	c	0	0	all	-	all	Eu:Sca	1986	CC	517	n	bl	y	n	0	cig+/-ot	1	10	nev cigs	st
AXELSS	19	x	m	0	0	sca	-	all	Eu:Sca	1989	CC	436	n	bl	n	n	0	all/unsp	1	9	nev any	st
AXELSS	13		f	0	0	sca	-	all	Eu:Sca	1989	CC	436	n	bl	n	n	0	all/unsp	1	9	nev any	st
BARBON	5	x	m	0	0	all	-	all	Eu:wst	1979	CC	755	n	bl	y	y	0	all/unsp	1	9	nev any	st
BRESLO	13		m	0	0	all	-	all	NAmer	1949	CC	518	n	bl	n	y	0	cig+/-ot	1	9	nev+3	st
BRESLO	29		f	0	0	all	-	all	NAmer	1949	CC	518	n	bl	n	y	0	cig+/-ot	1	19	nev+3	st
BROWN2	32		m	0	0	wh	-	all	NAmer	1984	CC	14596	n	bl	n	y	2	cig+/-ot	1	19	nev cigs	or
BROWN2	31		f	0	0	wh	-	all	NAmer	1984	CC	14596	n	bl	n	y	2	cig+/-ot	1	19	nev cigs	or
BUFFLE	28		f	0	0	w-hi	-	all	NAmer	1976	CC	943	n	bl	y	n	0	cig+/-ot	1	19	nev cigs	or
CHEN2	3		m	0	0	all	-	all	As:Chi	1983	CC	193	n	ot	y	n	0	all/unsp	1	9	nev any	st
CHEN2	7		f	0	0	all	-	all	As:Chi	1983	CC	193	n	ot	y	n	0	all/unsp	1	9	nev any	st
CHOI	12		m	0	0	all	-	all	As:oth	1985	CC	375	n	bl	n	n	0	cig+/-ot	1	10	nev cigs	st
CHOI	17		f	0	0	all	-	all	As:oth	1985	CC	375	n	bl	n	n	0	cig+/-ot	1	10	nev cigs	st
COOKSO	1		c	0	0	bl	-	all	Africa	1961	CC	234	n	V	n	y	0	cig+/-ot	1	9	nev any	st
CPSI	243		m	50	74	all	6	all	NAmer	1959	pr	5138	n	bl	n	n	1	cig only	1	19	nev any	ot
CPSII	105		f	35	99	all	4	all	NAmer	1982	pr	3229	n	bl	n	n	1	cig+/-ot	1	19	nev cigs	ot
DAMBER	6		m	0	0	all	-	all	Eu:Sca	1972	CC	579	n	bl	y	n	1	cig only	1	7	nev any	ot
DAVEYS	1		m	0	0	all	-	all	Eu:Ger	1930	CC	109	n	bl	y	n	0	all/unsp	1	5	nev any	st
DESTEF	1	x	m	0	0	all	-	all	SCAmer	1988	CC	497	n	bl	n	y	0	all/unsp	1	10	nev any	st
DOLL	2		m	0	0	all	-	all	Eu:UK	1948	CC	1465	n	V	n	n	0	all/unsp	5	14	nev any	st
DOLL	8		f	0	0	all	-	all	Eu:UK	1948	CC	1465	n	V	n	n	0	all/unsp	5	14	nev any	st
DOLL2	46		m	35	99	all	5	all	Eu:UK	1951	pr	920	n	V	n	n	1	all/unsp	1	14	nev any	ot
DORGAN	10	x	m	0	0	wh	-	all	NAmer	1980	CC	2026	n	bl	y	y	0	cig+/-ot	1	19	nev any	st
DORGAN	34	x	m	0	0	bl	-	all	NAmer	1980	CC	2026	n	bl	y	y	0	cig+/-ot	1	19	nev any	st
DORGAN	96		f	0	0	all	-	all	NAmer	1980	CC	2026	n	bl	y	y	3	cig+/-ot	1	19	nev any	ot
DOSEME	5		m	0	0	all	-	all	Eu:bal	1979	CC	1210	n	bl	n	n	2	cig+/-ot	1	10	nev cigs	or
DUNN	2		m	0	0	all	0	all	NAmer	1954	pr	139	o	bl	n	n	0	cig+/-ot	5	14	nev cigs	st
EBELIN	2		m	0	0	all	-	all	Eu:Ger	1980	CC	130	n	bl	n	n	0	all/unsp	1	9	nev any	st
ESAKI	1		m	0	0	all	-	all	As:Jap	1961	CC	245	n	bl	y	n	0	cig+/-ot	1	14	nev cigs	st
FAN	6		m	0	0	all	-	all	As:Chi	1990	CC	403	n	ot	y	n	0	cig+/-ot	1	9	nev cigs	st
FAN	10		f	0	0	all	-	all	As:Chi	1990	CC	403	n	ot	y	n	0	cig+/-ot	1	9	nev cigs	st
GAO	24	x	f	0	0	all	-	all	As:Chi	1984	CC	1405	n	ot	n	n	0	cig+/-ot	1	9	nev cigs	st
GARSHI	18	x	m	0	0	all	-	all	NAmer	1981	CC	1081	o	bl	y	n	0	all/unsp	1	15	nev any	st
GER	18	x	c	0	0	all	-	all	As:oth	1990	CC	141	n	ot	y	n	0	all/unsp	1	10	nev any	st
GOLLED	15	x	m	35	99	all	-	all	Eu:UK	1952	CC	443	n	V	y	n	0	cig only	1	10	nev any	st
GSELL	1		m	0	0	all	-	all	Eu:wst	1937	CC	150	n	bl	n	y	0	all/unsp	1	9	nev any	st
HAMMON	153		m	0	0	wh	0	all	NAmer	1952	pr	448	n	bl	n	n	1	cig only	1	9	nev any	ot
HANSEN	1		m	0	0	all	0	all	Eu:Sca	1968	pr	105	o	bl	y	n	2	all/unsp	1	19	nev any	ot
HU	1		m	0	0	all	-	all	As:Chi	1985	CC	227	n	ot	n	y	0	cig+/-ot	1	14	nev any	st
HU	4		f	0	0	all	-	all	As:Chi	1985	CC	227	n	ot	n	y	0	cig+/-ot	1	14	nev any	st
HU2	3		c	0	0	all	-	all	As:Chi	1977	CC	523	n	ot	y	n	0	cig+/-ot	5	9	nev cigs	st
JARUP	1	x	m	0	0	all	-	all	Eu:Sca	1928	CC	102	o	bl	y	n	0	all/unsp	1	10	nev any	st
JEDRYC	60	x	m	0	0	all	-	all	Eu:est	1980	CC	1630	n	bl	y	n	0	cig+/-ot	1	19	nev any	st
JEDRYC	65	x	f	0	0	all	-	all	Eu:est	1980	CC	1630	n	bl	y	n	0	cig+/-ot	1	19	nev any	st
JOLY	7		m	0	0	all	-	all	SCAmer	1978	CC	826	n	bl	n	n	0	cig+/-ot	1	9	nev any	st
JOLY	3		f	0	0	all	-	all	SCAmer	1978	CC	826	n	bl	n	n	0	cig+/-ot	1	9	nev any	st
JUSSAW	11	x	m	0	0	all	-	all	As:Ind	1964	CC	792	n	V	n	n	0	cig only	5	9	nev any	st
KHUDER	1		m	0	0	all	-	all	NAmer	1985	CC	482	n	bl	n	y	0	cig+/-ot	1	19	nev cigs	st
KOULUM	6		m	0	0	all	-	all	Eu:Sca	1936	CC	812	n	bl	n	n	0	all/unsp	1	9	nev any	st
KREUZE	19		m	1	45	all	-	all	Eu:Ger	1990	CC	2260	n	bl	n	n	3	cig+/-ot	1	9	nev any	or
KREUZE	30		m	55	69	all	-	all	Eu:Ger	1990	CC	2260	n	bl	n	n	3	cig+/-ot	1	9	nev any	or
KREUZE	25		f	1	45	all	-	all	Eu:Ger	1990	CC	2260	n	bl	n	n	3	cig+/-ot	1	9	nev any	or
KREUZE	36		f	55	69	all	-	all	Eu:Ger	1990	CC	2260	n	bl	n	n	3	cig+/-ot	1	9	nev any	or
KREYBE	21	x	m	0	0	all	-	all	Eu:Sca	1948	CC	300	n	bl	n	y	0	all/unsp	1	14	nev any	st
KREYBE	37	x	f	0	0	all	-	all	Eu:Sca	1948	CC	300	n	bl	n	y	0	all/unsp	1	14	nev any	st
LAMTH	7		f	0	0	ch	-	all	As:HK	1983	CC	445	n	bl	n	n	0	all/unsp	1	10	nev any	or
LAUSSM	3	x	m	0	0	all	-	all	Eu:Ger	1982	CC	432	n	bl	n	n	0	all/unsp	1	9	nev any	st
LETOUR	2		c	0	0	all	-	all	NAmer	1983	CC	738	n	V	y	y	0	cig+/-ot	1	19	nev cigs	st
LIU2	5	x	m	0	0	all	-	all	As:Chi	1983	CC	316	n	ot	n	n	0	all/unsp	1	19	nev any	st
LIU2	11	x	f	0	0	all	-	all	As:Chi	1983	CC	316	n	ot	n	n	0	all/unsp	1	9	nev any	st
LIU3	3	x	m	0	0	all	-	all	As:Chi	1985	CC	110	n	ot	n	n	0	all/unsp	1	15	nev any	st
LIU4	7		m	35	69	all	-	all	As:Chi	1986	CC	1000-00	n	ot	y	n	2	cig only	1	19	nev any	ot
LIU5	2		c	0	0	all	-	all	As:Chi	1978	CC	111	n	ot	y	n	0	all/unsp	1	9	nev any	st
LUBIN	7	x	m	0	0	all	-	all	As:Chi	1984	CC	427	m	ot	y	n	0	cig only	1	6	nev any	st
LUBIN2	273		m	0	0	all	-	all	Eu:mul	1976	CC	7804	n	bl	n	y	0	cig+/-ot	1	9	nev any	st
LUBIN2	281		f	0	0	all	-	all	Eu:mul	1976	CC	7804	n	bl	n	y	0	cig+/-ot	1	9	nev any	st
MACLEN	36		c	0	0	ch	-	all	As:oth	1972	CC	233	n	bl	n	n	2	cig+/-ot	1	9	nev cigs	or

Table 1G2 - 4

IESLC - Meta-analysis of Ever Smoking, Amount smoked, "Low", Any product (or Cigarettes if Any not available)
All LC types
Least adjusted

REF	NRR	X	SEX	AGEL	AGEH	RACE	YF	LC TYPE	LOC	START	ST	NLC	R	VB	P	H	AD	PRODUCT	exL	exH	DENOM	De
MARTIS	1		m	0	0	all	-	all	Eu:UK	1972	CC	201	n	V	n	n	0	cig+/-ot	1	14	nev cigs	st
MATOS	28	x	m	0	0	all	-	all	SCAmer	1994	CC	200	n	bl	n	n	0	cig+/-ot	1	14	nev any	st
MATSUD	1		m	0	0	all	-	all	As:Jap	1965	CC	179	n	bl	n	n	0	cig+/-ot	1	10	nev cigs	st
MCCONN	26		c	0	0	all	-	all	Eu:UK	1946	CC	100	n	V	n	y	0	all/unsp	1	10	nev any	st
NOTAN2	8		m	0	0	all	-	all	As:Ind	1963	CC	683	n	V	n	n	0	cig only	1	9	nev any	st
ORMOS	1		m	0	0	all	-	all	Eu:est	1947	CC	119	n	bl	y	y	0	cig+/-ot	1	15	nev any	st
OSANN2	4	x	f	0	0	all	-	all	NAmer	1964	ot	217	n	bl	n	y	0	cig+/-ot	1	19	nev cigs	st
PASTOR	1	x	m	0	0	all	-	all	Eu:wst	1976	CC	204	n	bl	y	n	0	all/unsp	1	9	nev any	st
PERNU	18		m	0	0	all	-	all	Eu:Sca	1944	CC	1606	n	bl	n	n	0	all/unsp	5	9	nev any	st
PERNU	12		f	0	0	all	-	all	Eu:Sca	1944	CC	1606	n	bl	n	n	0	all/unsp	5	9	nev any	st
POLEDN	2		c	0	0	all	-	all	NAmer	1978	CC	209	n	bl	y	n	0	cig+/-ot	1	19	nev cigs	st
RACHTA	5	x	f	0	0	all	-	all	Eu:est	1991	CC	118	n	bl	n	y	0	cig+/-ot	1	9	nev cigs	st
RANDIG	2		m	0	0	all	-	all	Eu:Ger	1951	CC	448	n	bl	n	n	0	all/unsp	5	9	nev any	st
RANDIG	6		f	0	0	all	-	all	Eu:Ger	1951	CC	448	n	bl	n	n	0	all/unsp	5	9	nev any	st
SHAW	10		c	0	0	wh	-	all	NAmer	1988	CC	335	n	V	n	y	0	all/unsp	1	19	nev any	st
SIEMIA	13		m	0	0	all	-	all	NAmer	1979	CC	857	n	V	y	y	0	cig+/-ot	1	19	nev cigs	or
SPITZ	5		c	0	0	b+hi	-	all	NAmer	1992	CC	177	n	bl	n	y	0	cig+/-ot	1	19	nev cigs	st
STOCKS	25	x	m	0	0	all	-	all	Eu:UK	1952	CC	2932	n	V	y	n	0	cig+/-ot	1	14	nev any	st
STOCKS	48		f	0	0	all	-	all	Eu:UK	1952	CC	2932	n	V	y	n	1	cig+/-ot	1	14	nev any	ot
TIZZAN	7		m	0	0	all	-	all	Eu:wst	1959	CC	1358	n	bl	n	n	0	cig only	1	9	nev any	st
TIZZAN	15		f	0	0	all	-	all	Eu:wst	1959	CC	1358	n	bl	n	n	0	cig only	1	9	nev any	st
WANG2	2	x	c	0	0	all	-	all	As:Chi	1980	CC	103	n	ot	n	n	0	cig+/-ot	5	9	nev cigs	st
WUWILL	18	x	f	0	0	all	-	all	As:Chi	1985	CC	965	n	ot	n	n	0	cig+/-ot	1	19	nev cigs	st
WYNDE2	17		m	0	0	all	-	all	NAmer	1962	CC	404	n	bl	n	y	0	cig+/-ot	1	10	nev any	st
WYNDE3	44		m	0	0	all	-	all	NAmer	1966	CC	350	n	bl	n	y	0	cig+/-ot	1	9	nev any	st
WYNDE3	79		f	0	0	all	-	all	NAmer	1966	CC	350	n	bl	n	y	0	cig+/-ot	1	9	nev any	st
WYNDE4	43		m	0	0	all	-	all	NAmer	1948	CC	684	n	bl	y	n	0	all/unsp	1	9	nev any	st
WYNDE4	57		f	0	0	all	-	all	NAmer	1948	CC	684	n	bl	y	n	2	all/unsp	1	9	nev any	ot
XU3	5	x	m	0	0	all	-	all	As:Chi	1981	CC	135	n	ot	n	n	0	all/unsp	1	9	nev any	st
XU3	13	x	f	0	0	all	-	all	As:Chi	1981	CC	135	n	ot	n	n	0	all/unsp	1	9	nev any	st
YUAN	2		m	0	0	all	0	all	As:Chi	1986	pr	142	n	ot	n	n	2	cig+/-ot	1	19	nev cigs	ot
ZHENG	11		m	0	0	all	-	all	As:Chi	1982	CC	540	n	ot	*	y	0	cig+/-ot	1	9	nev cigs	st
ZHENG	22		f	0	0	all	-	all	As:Chi	1982	CC	540	n	ot	*	y	0	cig+/-ot	1	9	nev cigs	st
ZHOU	4		c	0	0	all	-	all	As:Chi	1978	CC	1360	n	ot	n	n	0	all/unsp	1	9	nev any	st

Cigarette type is all/unspec for all RRs
except for the following:

REF	NRR	CIGTYPE
ALDERS	18	MC only
ALDERS	21	MC only
JUSSAW	11	MC only
NOTAN2	8	MC only

Table 1G2 - 5

IESLC - Meta-analysis of Ever Smoking, Amount smoked, "Low", Any product (or Cigarettes if Any not available)
All LC types
Least adjusted

REF	NRR	SEX	AD	Number Exposed Case	Number Exposed Cont	Non-exposed Case	Non-exposed Cont	RR	95.00%CI
AGUDO	11	f	0	6	12	80	183	1.14	(0.41- 3.15)
ALDERS	18	m	1	-	-	-	-	3.55	(1.94- 6.49)
ALDERS	21	f	1	-	-	-	-	2.62	(1.88- 3.65)
Subtotal ALDERS								2.81	(2.10- 3.76)
ARMADA	46	m	0	44	117	4	64	6.02	(2.07- 17.51)
AUVINE	5	c	0	57	33	44	229	8.99	(5.26- 15.37)
AXELSS	19	m	0	39	96	16	160	4.06	(2.15- 7.66)
AXELSS	13	f	0	13	37	18	154	3.01	(1.35- 6.68)
Subtotal AXELSS								3.62	(2.20- 5.94)
BARBON	5	m	0	28	87	22	188	2.75	(1.49- 5.08)
BRESLO	13	m	0	16	45	22	110	1.78	(0.86- 3.69)
BRESLO	29	f	0	5	5	12	14	1.17	(0.27- 5.02)
Subtotal BRESLO								1.63	(0.85- 3.14)
BROWN2	32	m	2	-	-	-	-	6.10	(5.30- 6.90)
BROWN2	31	f	2	-	-	-	-	8.40	(7.20- 9.70)
Subtotal BROWN2								7.02	(6.36- 7.75)
BUFFLE	28	f	0	21	42	12	112	4.67	(2.11- 10.31)
CHEN2	3	m	0	17	26	9	33	2.40	(0.92- 6.25)
CHEN2	7	f	0	5	17	25	33	0.39	(0.13- 1.20)
Subtotal CHEN2								1.12	(0.54- 2.31)
CHOI	12	m	0	20	90	13	95	1.62	(0.76- 3.46)
CHOI	17	f	0	9	16	76	164	1.21	(0.51- 2.87)
Subtotal CHOI								1.43	(0.81- 2.52)
COOKSO	1	c	0	102	27	45	61	5.12	(2.89- 9.08)
*CPSI	243	m	1	-	-	-	-	5.81	(4.33- 7.80)
*CPSII	105	f	1	-	-	-	-	4.16	(3.41- 5.09)
DAMBER	6	m	1	-	-	-	-	2.30	(1.30- 4.40)
DAVEYS	1	m	0	11	69	3	23	1.22	(0.31- 4.77)
DESTEF	1	m	0	38	84	27	163	2.73	(1.56- 4.78)
DOLL	2	m	0	489	570	7	61	7.48	(3.39- 16.50)
DOLL	8	f	0	24	18	40	59	1.97	(0.95- 4.09)
Subtotal DOLL								3.64	(2.13- 6.22)
*DOLL2	46	m	1	-	-	-	-	6.71	(0.91- 49.81)
DORGAN	10	m	0	125	148	15	93	5.24	(2.89- 9.49)
DORGAN	34	m	0	118	84	3	35	16.39	(4.88- 55.06)
DORGAN	96	f	3	-	-	-	-	5.67	(4.36- 7.36)
Subtotal DORGAN								5.83	(4.61- 7.37)
DOSEME	5	m	2	-	-	-	-	2.20	(1.40- 3.30)
*DUNN	2	m	0	12	9418	2	14160	9.02	(2.02- 40.30)
EBELIN	2	m	0	20	72	12	117	2.71	(1.25- 5.87)
ESAKI	1	m	0	47	75	16	28	1.10	(0.54- 2.24)
FAN	6	m	0	13	121	36	236	0.70	(0.36- 1.38)
FAN	10	f	0	17	48	69	320	1.64	(0.89- 3.03)
Subtotal FAN								1.12	(0.71- 1.76)
GAO	24	f	0	70	74	435	605	1.32	(0.93- 1.87)
GARSHI	18	m	0	105	282	41	363	3.30	(2.23- 4.88)
GER	18	c	0	21	108	51	246	0.94	(0.54- 1.64)
GOLLED	15	m	0	83	679	15	490	3.99	(2.28- 7.00)
GSELL	1	m	0	11	36	2	29	4.43	(0.91- 21.60)
*HAMMON	153	m	1	-	-	-	-	7.38	(3.72- 14.63)
*HANSEN	1	m	2	-	-	-	-	1.37	(0.63- 3.54)
HU	1	m	0	36	38	41	67	1.55	(0.85- 2.82)
HU	4	f	0	19	10	40	48	2.28	(0.95- 5.46)
Subtotal HU								1.75	(1.07- 2.87)
HU2	3	c	0	44	58	121	213	1.34	(0.85- 2.10)
JARUP	1	m	0	42	25	11	42	6.41	(2.80- 14.68)
JEDRYC	60	m	0	131	180	49	219	3.25	(2.22- 4.77)
JEDRYC	65	f	0	37	10	78	166	7.87	(3.72- 16.65)
Subtotal JEDRYC								3.91	(2.78- 5.49)
JOLY	7	m	0	16	54	12	218	5.38	(2.41- 12.05)
JOLY	3	f	0	33	38	52	283	4.73	(2.72- 8.21)
Subtotal JOLY								4.93	(3.12- 7.77)
JUSSAW	11	m	0	6	9	149	624	2.79	(0.98- 7.97)
KHUDER	1	m	0	81	434	23	309	2.51	(1.54- 4.07)
KOULUM	6	m	0	37	77	5	54	5.19	(1.92- 14.06)
KREUZE	19	m	3	-	-	-	-	2.50	(0.70- 8.20)
KREUZE	30	m	3	-	-	-	-	8.20	(5.20- 13.00)
KREUZE	25	f	3	-	-	-	-	5.70	(1.60- 16.60)
KREUZE	36	f	3	-	-	-	-	2.00	(1.20- 3.30)
Subtotal KREUZE								4.27	(3.12- 5.85)
KREYBE	21	m	0	154	2341	6	644	7.06	(3.11- 16.04)

Table 1G2 - 5

IESLC - Meta-analysis of Ever Smoking, Amount smoked, "Low", Any product (or Cigarettes if Any not available)
All LC types
Least adjusted

REF	NRR	SEX	AD	Number Exposed Case	Number Exposed Cont	Non-exposed Case	Non-exposed Cont	RR	95.00%CI
KREYBE	37	f	0	10	286	30	657	0.77	(0.37- 1.59)
Subtotal KREYBE								2.04	(1.18- 3.52)
LAMTH	7	f	0	101	63	202	337	2.67	(1.87- 3.83)
LAUSSM	3	m	0	75	63	85	226	3.17	(2.08- 4.81)
LETOUR	2	c	0	271	266	24	224	9.51	(6.04- 14.97)
LIU2	5	m	0	21	93	12	44	0.83	(0.37- 1.83)
LIU2	11	f	0	8	10	38	69	1.45	(0.53- 3.99)
Subtotal LIU2								1.03	(0.55- 1.92)
LIU3	3	m	0	25	93	4	19	1.28	(0.40- 4.09)
LIU4	7	m	2	-	-	-	-	2.11	(2.02- 2.20)
LIU5	2	c	0	14	27	26	41	0.82	(0.36- 1.84)
LUBIN	7	m	0	4	41	8	73	0.89	(0.25- 3.14)
LUBIN2	273	m	0	1887	3759	190	2616	6.91	(5.91- 8.09)
LUBIN2	281	f	0	151	218	336	1188	2.45	(1.93- 3.11)
Subtotal LUBIN2								5.06	(4.44- 5.77)
MACLEN	36	c	2	-	-	-	-	1.35	(0.64- 2.84)
MARTIS	1	m	0	31	39	4	25	4.97	(1.56- 15.78)
MATOS	28	m	0	17	88	11	110	1.93	(0.86- 4.34)
MATSUD	1	m	0	37	1237	3	1255	12.51	(3.85- 40.69)
MCCONN	26	c	0	7	43	9	23	0.42	(0.14- 1.26)
NOTAN2	8	m	0	6	42	134	544	0.58	(0.24- 1.39)
ORMOS	1	m	0	32	329	7	777	10.80	(4.72- 24.71)
OSANN2	4	f	0	35	55	33	109	2.10	(1.18- 3.74)
PASTOR	1	m	0	8	39	10	89	1.83	(0.67- 4.98)
PERNU	18	m	0	61	31	97	275	5.58	(3.42- 9.11)
PERNU	12	f	0	5	13	110	971	3.40	(1.19- 9.70)
Subtotal PERNU								5.10	(3.27- 7.96)
POLEDN	2	c	0	53	103	12	139	5.96	(3.03- 11.72)
RACHTA	5	f	0	6	7	33	98	2.55	(0.80- 8.12)
RANDIG	2	m	0	65	99	5	22	2.89	(1.04- 8.01)
RANDIG	6	f	0	12	13	17	92	5.00	(1.95- 12.79)
Subtotal RANDIG								3.88	(1.95- 7.76)
SHAW	10	c	0	46	90	11	107	4.97	(2.43- 10.16)
SIEMIA	13	m	0	-	-	-	-	3.00	(1.00- 9.90)
SPITZ	5	c	0	27	88	10	96	2.95	(1.35- 6.43)
STOCKS	25	m	0	691	1743	45	638	5.62	(4.10- 7.70)
STOCKS	48	f	1	-	-	-	-	2.24	(1.64- 3.03)
Subtotal STOCKS								3.51	(2.81- 4.37)
TIZZAN	7	m	0	130	238	180	305	0.93	(0.70- 1.23)
TIZZAN	15	f	0	11	14	25	114	3.58	(1.46- 8.82)
Subtotal TIZZAN								1.04	(0.80- 1.37)
WANG2	2	c	0	3	10	11	43	1.17	(0.28- 5.00)
WUWILL	18	f	0	451	311	417	601	2.09	(1.73- 2.53)
WYNDE2	17	m	0	17	114	8	105	1.96	(0.81- 4.72)
WYNDE3	44	m	0	8	42	9	88	1.86	(0.67- 5.17)
WYNDE3	79	f	0	3	19	20	76	0.60	(0.16- 2.23)
Subtotal WYNDE3								1.22	(0.54- 2.72)
WYNDE4	43	m	0	17	82	12	115	1.99	(0.90- 4.38)
WYNDE4	57	f	2	-	-	-	-	1.13	(0.33- 3.89)
Subtotal WYNDE4								1.69	(0.87- 3.28)
XU3	5	m	0	10	26	7	31	1.70	(0.57- 5.10)
XU3	13	f	0	7	6	13	25	2.24	(0.62- 8.07)
Subtotal XU3								1.91	(0.83- 4.40)
*YUAN	2	m	2	-	-	-	-	3.60	(1.88- 6.91)
ZHENG	11	m	0	25	40	33	94	1.78	(0.94- 3.37)
ZHENG	22	f	0	24	29	152	184	1.00	(0.56- 1.79)
Subtotal ZHENG								1.30	(0.85- 2.00)
ZHOU	4	c	0	61	5	507	68	1.64	(0.64- 4.22)
Partial Totals				6695	25724	4659	34228		

*prospective study

Table 1G2 - 5

IESLC - Meta-analysis of Ever Smoking, Amount smoked, "Low", Any product (or Cigarettes if Any not available)
All LC types
Least adjusted

REF	NRR	SEX	AD	Ys	Ws	Qs	Ps
AGUDO	11	f	0	0.13	3.73	2.84	0.7953
ALDERS	18	m	1	1.27	10.54	0.71	0.0000
ALDERS	21	f	1	0.96	34.91	0.07	0.0000
Subtotal ALDERS				1.03	45.45	0.78	
ARMADA	46	m	0	1.79	3.37	2.09	0.0010
AUVINE	5	c	0	2.20	13.34	18.86	0.0000
AXELSS	19	m	0	1.40	9.54	1.49	0.0000
AXELSS	13	f	0	1.10	6.02	0.05	0.0069
Subtotal AXELSS				1.29	15.57	1.54	
BARBON	5	m	0	1.01	10.21	0.00	0.0012
BRESLO	13	m	0	0.58	7.18	1.34	0.1231
BRESLO	29	f	0	0.15	1.80	1.31	0.8360
Subtotal BRESLO				0.49	8.98	2.65	
BROWN2	32	m	2	1.81	220.78	141.66	0.0000
BROWN2	31	f	2	2.13	172.98	217.35	0.0000
Subtotal BROWN2				1.95	393.76	359.01	
BUFFLE	28	f	0	1.54	6.11	1.74	0.0001
CHEN2	3	m	0	0.87	4.19	0.07	0.0735
CHEN2	7	f	0	-0.95	3.04	11.59	0.0991
Subtotal CHEN2				0.11	7.23	11.67	
CHOI	12	m	0	0.48	6.73	1.84	0.2084
CHOI	17	f	0	0.19	5.18	3.43	0.6591
Subtotal CHOI				0.36	11.92	5.27	
COOKSO	1	c	0	1.63	11.70	4.59	0.0000
*CPSI	243	m	1	1.76	44.36	25.11	0.0000
*CPSII	105	f	1	1.43	95.77	16.75	0.0000
DAMBER	6	m	1	0.83	10.34	0.31	0.0074
DAVEYS	1	m	0	0.20	2.07	1.35	0.7726
DESTEF	1	m	0	1.00	12.29	0.00	0.0004
DOLL	2	m	0	2.01	6.13	6.19	0.0000
DOLL	8	f	0	0.68	7.19	0.79	0.0698
Subtotal DOLL				1.29	13.32	6.97	
*DOLL2	46	m	1	1.90	0.96	0.77	0.0623
DORGAN	10	m	0	1.66	10.85	4.56	0.0000
DORGAN	34	m	0	2.80	2.62	8.38	0.0000
DORGAN	96	f	3	1.74	56.05	29.70	0.0000
Subtotal DORGAN				1.76	69.51	42.63	
DOSEME	5	m	2	0.79	20.90	1.00	0.0003
*DUNN	2	m	0	2.20	1.71	2.44	0.0040
EBELIN	2	m	0	1.00	6.42	0.00	0.0116
ESAKI	1	m	0	0.09	7.53	6.30	0.8001
FAN	6	m	0	-0.35	8.53	15.73	0.3059
FAN	10	f	0	0.50	10.28	2.68	0.1116
Subtotal FAN				0.11	18.81	18.42	
GAO	24	f	0	0.27	31.50	16.92	0.1237
GARSHI	18	m	0	1.19	24.87	0.86	0.0000
GER	18	c	0	-0.06	12.41	14.25	0.8213
GOLLED	15	m	0	1.38	12.16	1.73	0.0000
GSELL	1	m	0	1.49	1.53	0.35	0.0655
*HAMMON	153	m	1	2.00	8.19	8.06	0.0000
*HANSEN	1	m	2	0.31	5.16	2.47	0.4747
HU	1	m	0	0.44	10.71	3.48	0.1527
HU	4	f	0	0.82	5.04	0.17	0.0643
Subtotal HU				0.56	15.74	3.65	
HU2	3	c	0	0.29	18.89	9.74	0.2087
JARUP	1	m	0	1.86	5.60	4.06	0.0000
JEDRYC	60	m	0	1.18	26.20	0.78	0.0000
JEDRYC	65	f	0	2.06	6.86	7.65	0.0000
Subtotal JEDRYC				1.36	33.06	8.43	
JOLY	7	m	0	1.68	5.92	2.70	0.0000
JOLY	3	f	0	1.55	12.60	3.75	0.0000
Subtotal JOLY				1.59	18.52	6.46	
JUSSAW	11	m	0	1.03	3.50	0.00	0.0549
KHUDER	1	m	0	0.92	16.30	0.13	0.0002
KOULUM	6	m	0	1.65	3.87	1.58	0.0012
KREUZE	19	m	3	0.92	2.54	0.02	0.1444
KREUZE	30	m	3	2.10	18.30	22.02	0.0000
KREUZE	25	f	3	1.74	2.81	1.51	0.0035
KREUZE	36	f	3	0.69	15.02	1.48	0.0072
Subtotal KREUZE				1.45	38.66	25.03	
KREYBE	21	m	0	1.95	5.71	5.12	0.0000

Table 1G2 - 5

IESLC - Meta-analysis of Ever Smoking, Amount smoked, "Low", Any product (or Cigarettes if Any not available)
All LC types
Least adjusted

REF	NRR	SEX	AD	Ys	Ws	Qs	Ps
KREYBE	37	f	0	-0.27	7.23	11.74	0.4730
Subtotal KREYBE				0.71	12.94	16.86	
LAMTH	7	f	0	0.98	29.68	0.02	0.0000
LAUSSM	3	m	0	1.15	22.03	0.46	0.0000
LETOUR	2	c	0	2.25	18.66	28.93	0.0000
LIU2	5	m	0	-0.19	6.08	8.70	0.6415
LIU2	11	f	0	0.37	3.76	1.51	0.4689
Subtotal LIU2				0.03	9.84	10.21	
LIU3	3	m	0	0.24	2.83	1.65	0.6810
LIU4	7	m	2	0.75	2108.86	143.21	0.0000
LIU5	2	c	0	-0.20	5.84	8.53	0.6267
LUBIN	7	m	0	-0.12	2.42	3.06	0.8565
LUBIN2	273	m	0	1.93	155.25	133.10	0.0000
LUBIN2	281	f	0	0.90	66.54	0.83	0.0000
Subtotal LUBIN2				1.62	221.79	133.93	
MACLEN	36	c	2	0.30	6.92	3.46	0.4298
MARTIS	1	m	0	1.60	2.87	1.02	0.0066
MATOS	28	m	0	0.66	5.88	0.71	0.1105
MATSUD	1	m	0	2.53	2.76	6.38	0.0000
MCCONN	26	c	0	-0.88	3.12	11.07	0.1215
NOTAN2	8	m	0	-0.54	5.01	12.06	0.2229
ORMOS	1	m	0	2.38	5.60	10.55	0.0000
OSANN2	4	f	0	0.74	11.60	0.81	0.0114
PASTOR	1	m	0	0.60	3.82	0.63	0.2395
PERNU	18	m	0	1.72	15.98	8.09	0.0000
PERNU	12	f	0	1.22	3.48	0.16	0.0225
Subtotal PERNU				1.63	19.46	8.25	
POLEDN	2	c	0	1.79	8.40	5.08	0.0000
RACHTA	5	f	0	0.93	2.86	0.02	0.1143
RANDIG	2	m	0	1.06	3.69	0.01	0.0415
RANDIG	6	f	0	1.61	4.35	1.57	0.0008
Subtotal RANDIG				1.36	8.04	1.58	
SHAW	10	c	0	1.60	7.51	2.67	0.0000
SIEMIA	13	m	0	1.10	2.92	0.02	0.0603
SPITZ	5	c	0	1.08	6.30	0.03	0.0067
STOCKS	25	m	0	1.73	38.74	20.04	0.0000
STOCKS	48	f	1	0.81	40.78	1.64	0.0000
Subtotal STOCKS				1.25	79.52	21.68	
TIZZAN	7	m	0	-0.08	48.24	56.76	0.5909
TIZZAN	15	f	0	1.28	4.74	0.34	0.0055
Subtotal TIZZAN				0.04	52.98	57.10	
WANG2	2	c	0	0.16	1.83	1.31	0.8295
WUWILL	18	f	0	0.74	105.32	7.68	0.0000
WYNDE2	17	m	0	0.67	4.95	0.56	0.1353
WYNDE3	44	m	0	0.62	3.69	0.55	0.2325
WYNDE3	79	f	0	-0.51	2.23	5.13	0.4459
Subtotal WYNDE3				0.20	5.91	5.68	
WYNDE4	43	m	0	0.69	6.13	0.63	0.0891
WYNDE4	57	f	2	0.12	2.52	1.98	0.8460
Subtotal WYNDE4				0.52	8.66	2.61	
XU3	5	m	0	0.53	3.19	0.72	0.3416
XU3	13	f	0	0.81	2.34	0.09	0.2159
Subtotal XU3				0.65	5.53	0.81	
*YUAN	2	m	2	1.28	9.07	0.68	0.0001
ZHENG	11	m	0	0.58	9.44	1.75	0.0764
ZHENG	22	f	0	0.00	11.34	11.47	0.9951
Subtotal ZHENG				0.26	20.78	13.22	
ZHOU	4	c	0	0.49	4.29	1.14	0.3077

Table 1G2 - 5

IESLC - Meta-analysis of Ever Smoking, Amount smoked, "Low", Any product (or Cigarettes if Any not available)
All LC types
Least adjusted

	N	105
	NS	78
	Wt	3922.10
Het	Chi	1160.46
Het	df	104
Het	P	***
Fixed	RR	2.74
	RRl	2.65
	RRu	2.83
	P	+++
Random	RR	2.74
	RRl	2.38
	RRu	3.16
	P	+++
Asymm	P	N.S.

Table 1G2 - 6

IESLC - Meta-analysis of Ever Smoking, Amount smoked, "Low", Any product (or Cigarettes if Any not available)
All LC types
Least adjusted

	Sex: combined	male	female	Total
N	13	59	33	105
NS	13	57	32	102
Wt	119.22	3027.24	775.64	3922.10
Het Chi	108.91	675.45	327.78	1160.46
Het df	12	58	32	104
Het P	***	***	***	***
Fixed RR	2.96	2.58	3.41	2.74
RRl	2.48	2.49	3.18	2.65
RRu	3.55	2.67	3.65	2.83
P	+++	+++	+++	+++
Random RR	2.39	3.05	2.36	2.74
RRl	1.37	2.52	1.82	2.38
RRu	4.19	3.69	3.05	3.16
P	++	+++	+++	+++
Between Chi				48.33
Between df				2
Between P				***
Btwn(F) P				N.S.
Btwn(R) P				N.S.

Table 1G2 - 7

IESLC - Meta-analysis of Ever Smoking, Amount smoked, "Low", Any product (or Cigarettes if Any not available)
All LC types
Excluded studies (and stage at which they were excluded)

1	ABELIN ABRAHA AMANDU AMES ANDERS AUSTIN AXELSO BAND BECHER BERRIN BLOHMK BLOT4 BROCKM BROWN1 BYERS1 BYERS2 CARPEN CASCO2 CASCOR CHAN CHEN3 CHIAZZ CHYOU DESTE2 DOCKER DROSTE DU GARCIA GARDIN GENG GODLEY GOODMA GRAHAM GREGOR HEGMAN HEIN HENNEK HINDS HIRAOK HOROWI HORWIT HUANG ISHIMA JAHN JAIN JARVHO JIANG KELLER KIHARA KJUUS KO KOHLME KUBIK LAMWK LAMWK2 LANGE LEI LEMARC LEVIN LIU LOMBA2 LOMBAR MAGNUS MARSH MARSH2 MCDUFF MCLAUG MILLER MILLS NOTANI NOU ODRISC PAWLEG PERSHA POFFIJ QIAO QIAO2 RADZIK REN RONCO ROOTS ROTHSC SAARIK SANKAR SCHWAR SEGI SEOW SHIMIZ SIMARA SIMONA SITAS SOBUE2 STASZE STAYNE STUCKE SUN SUZUK2 SUZUKI TANG TAO TOKARS TOUSEY ULMER VEIERO VUTUC WALD WANG WANG3 WANG4 WICKLU WIGLE WILKIN WU2 WUNSCH WYNDE8 XIANGZ XU XU2 XU4 YONG ZHANG
2	AKIBA ARCHER BENSHL BRETT BROSS CEDERL CHANG CHOW COMSTO DARBY DEAN3 DEKLER DORANT DORN ENGELA ENSTRO GAO2 GILLIS HAENSZ HAMMO2 HIRAY2 HIRAYA HITOSU HOLE HUMBLE KAISE2 KAISER KANELL KATSOU KAUFMA KINLEN KNEKT KOO LAURIL LIAW LICKIN LIDDEL MIGRAN MRFIT MRFITR MURATA NAM PARKIN PERSH2 PETO PEZZO2 PEZZOT PISANI PRESCO RIMING SEGI2 SOBUE SPEIZE STOCKW SVENSS TANG2 TENKAN TSUGAN TULINI TVERDA WAKAI WARSIN WATSON WU WYNDE5 WYNDER YAMAGU
3	BUELL CHEN MASTRA MZILEN RESTRE SADOWS
4	BEST BOFFET WYNDE7
6	BLOT1 BLOT2 BLOT3 BOUCHA JONES MOLLO SCHWA2 VANDER
8	CORREA LUO WYNDE6
10	BOUCOT CHATZI DEAN DEAN2 OSANN PIKE
11	BENHAM

Table 1G2 - 8
Potentially overlapping studies

REF	REFGP	PRINC	.	OVERLAP/LINK
LUBIN2	LUBIN2	1		Lubin-combined
LAMTH	LAMTH	1		KOO/LAMTH/LAMWK
OSANN2	KAISER	2		KAISER/OSANN2
CPSI	CPSI	1		CPSI overall
MATSUD	MATSUD	1		SOBUE2/MATSUD
LUBIN	XIANGZ	2		LUBIN/XIANGZ/QIAO

Table 1G2 - 9
Most adjusted - insufficient data for metaanalysis

REF	NRR	SEX	AGEL	AGEH	RACE	YF	LC TYPE	LOC	START	ST	NLC	R	VB	P	H	AD	PRODUCT	exL	exH	DENOM	De
BLOT1	5	m	0	0	all	-	all	NAmer	1970	CC	458	n	bl	y	n	0	cig+/-ot	1	9	nev cigs	or
BUELL	1	m	0	0	all	0	all	NAmer	1957	pr	304	n	bl	n	n	0	cig+/-ot	1	18	nev cigs	st
MASTRA	3	m	0	0	all	-	all	Eu:wst	1973	CC	309	n	bl	n	n	1	all/unsp	1	10	nev any	or
MZILEN	1	m	0	0	bl	-	all	Africa	1994	CC	374	n	V	*	n	3	all/unsp	1	14	nev any	or
MZILEN	3	f	0	0	bl	-	all	Africa	1994	CC	374	n	V	*	n	3	all/unsp	1	14	nev any	or
RESTRE	6	c	0	0	all	-	all	SCAmer	1978	CC	102	n	bl	n	n	5	cig+/-ot	1	10	nev cigs	or
SADOWS	85	m	0	0	wh	-	all	NAmer	1938	CC	477	n	bl	n	n	1	all/unsp	1	10	nev any	st

REF	NRR	RR	SIG	RRDATA comment
BLOT1	5	0.40		0
BUELL	1	2.32		0
MASTRA	3	6.80		0
MZILEN	1	9.60		0
MZILEN	3	13.60		0
RESTRE	6	1.29		0
SADOWS	85	2.32		0

Least adjusted - insufficient data for meta-analysis: as for adjusted plus the following

REF	NRR	SEX	AGEL	AGEH	RACE	YF	LC TYPE	LOC	START	ST	NLC	R	VB	P	H	AD	PRODUCT	exL	exH	DENOM	De
SADOWS	80	m	0	0	wh	-	all	NAmer	1938	CC	477	n	bl	n	n	0	all/unsp	1	10	nev any	st

REF	NRR	RR	SIG	RRDATA comment
SADOWS	80	1.83		0

Table 1G3 -

IESLC - Meta-analysis of Ever Smoking, Amount smoked, "Mid", Any product (or Cigarettes if Any not available)
All LC types

This analysis is restricted to results for:
1) Results by Amount smoked
2) Ever smokers
3) Results complete enough for use in metaanalysis

Within each study, results are then selected (in the following order of preference, within each sex) for:
4) PRODUCT: all/unspec, cigarettes regardless of other products, cigarettes only
5) CIGTYPE: all/unspecified, MC regardless of HR, MC only
6) DENOM: never smoked anything, never smoked cigarettes, (never +1 = +long term ex, +2 = +amount unknown, +3 = never cigs+long term ex)
7) Followup period (YF, prospective studies): whole study (coded as 0) or longest available
8) LCtype: all or nearest available, at least Squamous and Adeno. (q = squamous, s = small, l = large, a = adeno, mix = mixed, alv = alveolar)
9) Race: all or nearest available, otherwise by race (wh or w = white, bl or b = black, hi = hispanic ch = chinese, jap = japanese, haw = hawaiian, w+o = white + oriental, sca = scandinavian, as = asian)
10) Amount smoked "mid" in key scheme 1 (key value 20, maximum range 6-44, in numbers of cigarettes or cigarette equivalents)
11) For overlapping studies: principal rather than subsidiary studies
Finally by Age: whole study (coded as 0) if available, otherwise by widest available age group
and then for single sex results (m, f) in preference to combined sex results (c).

Results adjusted (AD) for the most potential confounders are then chosen in Sections -1 to -3
and results adjusted for the least confounders in Sections -4 to -6. (Those least adjusted results which actually differ from the most adjusted as marked 'x' in column X in Section -4)
(Results adjusted for an unknown number of confounder(s) are coded as 20.)

Section -7 shows excluded studies, together with the stage (as above) at which no qualifying results were found.

Section -8 lists the potentially overlapping studies which have been included (1=principal, 2=subsidiary).

Section -9 lists any results which would have been included in preference except that they had data not complete enough for use in meta-analysis, with their significance (yes/no), if known, and any further comment as entered on the database.

In addition to those mentioned above, the following fields, levels and abbreviations are used:
* or nk = not known, n = no, y = yes, ot = other
nev = never
all/unspec = all or unspecified, cig+/-ot = cigarettes irrespective of other products (cigar, pipe etc)
MC = manufactured cigarettes, HR = hand-rolled cigarettes
exL, exH = range of exposure (low and high) in the smoking group, in terms of Amount smoked, cigarettes or cigarette equivalents
REF: 6-character study reference
NRR: number of the RR on the database within the study
ST : study type (CC = case control, pr or prosp = prospective)
NLC: number of lung cancer cases in whole study
R : risky occupational population (n = no, m = mining, o = other risky)
VB : national cigarette type (V = at least 75% Virginia, bl = at least 75% blended, ot = other)
P : any proxy use
H : full histological confirmation
De : derivation of RR/CI (or = original, st = standard method, ot = other method of estimation)

Table 1G3 - 1

IESLC - Meta-analysis of Ever Smoking, Amount smoked, "Mid", Any product (or Cigarettes if Any not available)
All LC types
Most adjusted

REF	NRR	SEX	AGEL	AGEH	RACE	YF	LC TYPE	LOC	START	ST	NLC	R	VB	P	H	AD	PRODUCT	exL	exH	DENOM	De
ALDERS	19	m	0	0	all	-	all	Eu:UK	1977	CC	1448	n	V	n	n	1	cig only	18	27	nev+2	ot
ALDERS	22	f	0	0	all	-	all	Eu:UK	1977	CC	1448	n	V	n	n	1	cig only	18	27	nev+2	ot
ARMADA	47	m	0	0	all	-	all	Eu:wst	1986	CC	325	n	bl	n	y	0	cig+/-ot	15	24	nev any	st
AUVINE	14	c	0	0	all	-	all	Eu:Sca	1986	CC	517	n	bl	y	n	2	cig+/-ot	11	20	nev cigs	or
AXELSS	15	f	0	0	sca	-	all	Eu:Sca	1989	CC	436	n	bl	n	n	0	all/unsp	20	29	nev any	st
BARBON	83	m	0	0	all	-	all	Eu:wst	1979	CC	755	n	bl	y	y	3	all/unsp	20	39	nev any	or
BRESLO	15	m	0	0	all	-	all	NAmer	1949	CC	518	n	bl	n	y	0	cig+/-ot	20	39	nev+3	st
BUFFLE	29	f	0	0	w-hi	-	all	NAmer	1976	CC	943	n	bl	y	n	0	cig+/-ot	20	20	nev cigs	or
CHEN2	4	m	0	0	all	-	all	As:Chi	1983	CC	193	n	ot	y	n	0	all/unsp	10	20	nev any	st
CHEN2	8	f	0	0	all	-	all	As:Chi	1983	CC	193	n	ot	y	n	0	all/unsp	10	20	nev any	st
CHOI	13	m	0	0	all	-	all	As:oth	1985	CC	375	n	bl	n	n	0	cig+/-ot	11	20	nev cigs	st
CHOI	18	f	0	0	all	-	all	As:oth	1985	CC	375	n	bl	n	n	0	cig+/-ot	11	30	nev cigs	st
DAMBER	8	m	0	0	all	-	all	Eu:Sca	1972	CC	579	n	bl	y	n	1	cig only	16	25	nev any	ot
DAVEYS	3	m	0	0	all	-	all	Eu:Ger	1930	CC	109	n	bl	y	n	0	all/unsp	11	20	nev any	st
DESTEF	7	m	0	0	all	-	all	SCAmer	1988	CC	497	n	bl	n	y	4	all/unsp	11	20	nev any	or
DOLL	3	m	0	0	all	-	all	Eu:UK	1948	CC	1465	n	V	n	n	0	all/unsp	15	24	nev any	st
DOLL	9	f	0	0	all	-	all	Eu:UK	1948	CC	1465	n	V	n	n	0	all/unsp	15	24	nev any	st
DOLL2	47	m	35	99	all	5	all	Eu:UK	1951	pr	920	n	V	n	n	1	all/unsp	15	24	nev any	ot
DOSEME	9	m	0	0	all	-	all	Eu:bal	1979	CC	1210	n	bl	n	n	2	cig+/-ot	11	20	nev cigs	or
DUNN	3	m	0	0	all	0	all	NAmer	1954	pr	139	o	bl	n	n	0	cig+/-ot	15	24	nev cigs	st
EBELIN	4	m	0	0	all	-	all	Eu:Ger	1980	CC	130	n	bl	n	n	0	all/unsp	20	29	nev any	st
ESAKI	2	m	0	0	all	-	all	As:Jap	1961	CC	245	n	bl	y	n	0	cig+/-ot	15	29	nev cigs	st
FAN	8	m	0	0	all	-	all	As:Chi	1990	CC	403	n	ot	y	n	0	cig+/-ot	20	29	nev cigs	st
FAN	12	f	0	0	all	-	all	As:Chi	1990	CC	403	n	ot	y	n	0	cig+/-ot	20	29	nev cigs	st
GARSHI	27	m	0	0	all	-	all	NAmer	1981	CC	1081	o	bl	y	n	1	all/unsp	16	25	nev any	st
GER	23	c	0	0	all	-	all	As:oth	1990	CC	141	n	ot	y	n	14	all/unsp	11	20	nev any	ot
GOLLED	2	m	35	99	all	-	all	Eu:UK	1952	CC	443	n	V	y	n	1	cig only	11	22	nev any	ot
GSELL	3	m	0	0	all	-	all	Eu:wst	1937	CC	150	n	bl	n	y	0	all/unsp	15	20	nev any	st
HAMMON	154	m	0	0	wh	0	all	NAmer	1952	pr	448	n	bl	n	n	1	cig only	10	20	nev any	ot
HU	2	m	0	0	all	-	all	As:Chi	1985	CC	227	n	ot	n	y	0	cig+/-ot	14	24	nev any	st
HU	5	f	0	0	all	-	all	As:Chi	1985	CC	227	n	ot	n	y	0	cig+/-ot	14	24	nev any	st
HU2	6	c	0	0	all	-	all	As:Chi	1977	CC	523	n	ot	y	n	0	cig+/-ot	20	29	nev cigs	st
JEDRYC	46	m	0	0	all	-	all	Eu:est	1980	CC	1630	n	bl	y	n	4	cig+/-ot	20	29	nev any	or
JEDRYC	49	f	0	0	all	-	all	Eu:est	1980	CC	1630	n	bl	y	n	4	cig+/-ot	20	29	nev any	or
JOLY	9	m	0	0	all	-	all	SCAmer	1978	CC	826	n	bl	n	n	0	cig+/-ot	20	29	nev any	st
JOLY	5	f	0	0	all	-	all	SCAmer	1978	CC	826	n	bl	n	n	0	cig+/-ot	20	29	nev any	st
JUSSAW	14	m	0	0	all	-	all	As:Ind	1964	CC	792	n	V	n	n	0	cig only	20	24	nev any	st
KHUDER	2	m	0	0	all	-	all	NAmer	1985	CC	482	n	bl	n	y	0	cig+/-ot	20	39	nev cigs	st
KREUZE	21	m	1	45	all	-	all	Eu:Ger	1990	CC	2260	n	bl	n	n	3	cig+/-ot	20	29	nev any	or
KREUZE	32	m	55	69	all	-	all	Eu:Ger	1990	CC	2260	n	bl	n	n	3	cig+/-ot	20	29	nev any	or
KREUZE	27	f	1	45	all	-	all	Eu:Ger	1990	CC	2260	n	bl	n	n	3	cig+/-ot	20	29	nev any	or
KREUZE	38	f	55	69	all	-	all	Eu:Ger	1990	CC	2260	n	bl	n	n	3	cig+/-ot	20	29	nev any	or
KREYBE	10	m	0	0	all	-	all	Eu:Sca	1948	CC	300	n	bl	n	y	1	all/unsp	15	24	nev any	ot
LAMTH	2	f	0	0	ch	-	all	As:HK	1983	CC	445	n	bl	n	n	0	all/unsp	11	20	nev any	or
LETOUR	3	c	0	0	all	-	all	NAmer	1983	CC	738	n	V	y	y	0	cig+/-ot	20	40	nev cigs	st
LIU2	9	m	0	0	all	-	all	As:Chi	1983	CC	316	n	ot	n	n	3	all/unsp	20	29	nev any	or
LIU3	7	m	0	0	all	-	all	As:Chi	1985	CC	110	n	ot	n	n	2	all/unsp	16	30	nev any	or
LIU4	8	m	35	69	all	-	all	As:Chi	1986	CC	1000-00	n	ot	y	n	2	cig only	20	20	nev any	ot
LUBIN2	275	m	0	0	all	-	all	Eu:mul	1976	CC	7804	n	bl	n	y	0	cig+/-ot	20	29	nev any	st
LUBIN2	283	f	0	0	all	-	all	Eu:mul	1976	CC	7804	n	bl	n	y	0	cig+/-ot	20	29	nev any	st
MACLEN	38	c	0	0	ch	-	all	As:oth	1972	CC	233	n	bl	n	n	2	cig+/-ot	20	29	nev cigs	or
MARTIS	2	m	0	0	all	-	all	Eu:UK	1972	CC	201	n	V	n	n	0	cig+/-ot	15	24	nev cigs	st
MATOS	31	m	0	0	all	-	all	SCAmer	1994	CC	200	n	bl	n	n	2	cig+/-ot	15	24	nev any	or
MATSUD	2	m	0	0	all	-	all	As:Jap	1965	CC	179	n	bl	n	n	0	cig+/-ot	11	20	nev cigs	st
MCCONN	25	c	0	0	all	-	all	Eu:UK	1946	CC	100	n	V	n	y	0	all/unsp	10	20	nev any	st
ORMOS	2	m	0	0	all	-	all	Eu:est	1947	CC	119	n	bl	y	y	0	cig+/-ot	16	30	nev any	st
PASTOR	8	m	0	0	all	-	all	Eu:wst	1976	CC	204	n	bl	y	n	1	all/unsp	20	29	nev any	st
PERNU	21	m	0	0	all	-	all	Eu:Sca	1944	CC	1606	n	bl	n	n	0	all/unsp	20	24	nev any	st
PERNU	15	f	0	0	all	-	all	Eu:Sca	1944	CC	1606	n	bl	n	n	0	all/unsp	20	24	nev any	st
SIEMIA	14	m	0	0	all	-	all	NAmer	1979	CC	857	n	V	y	y	0	cig+/-ot	20	39	nev cigs	or
STOCKS	42	m	0	0	all	-	all	Eu:UK	1952	CC	2932	n	V	y	n	2	cig+/-ot	15	21	nev any	st
TIZZAN	8	m	0	0	all	-	all	Eu:wst	1959	CC	1358	n	bl	n	n	0	cig only	10	20	nev any	st
WANG2	13	c	0	0	all	-	all	As:Chi	1980	CC	103	n	ot	n	n	4	cig+/-ot	20	29	nev cigs	ot
WYNDE2	18	m	0	0	all	-	all	NAmer	1962	CC	404	n	bl	n	y	0	cig+/-ot	11	20	nev any	st
WYNDE3	45	m	0	0	all	-	all	NAmer	1966	CC	350	n	bl	n	y	0	cig+/-ot	10	20	nev any	st
WYNDE3	80	f	0	0	all	-	all	NAmer	1966	CC	350	n	bl	n	y	0	cig+/-ot	10	20	nev any	st
WYNDE4	45	m	0	0	all	-	all	NAmer	1948	CC	684	n	bl	y	n	0	all/unsp	16	20	nev any	st
WYNDE4	59	f	0	0	all	-	all	NAmer	1948	CC	684	n	bl	y	n	2	all/unsp	16	20	nev any	ot
XU3	11	m	0	0	all	-	all	As:Chi	1981	CC	135	n	ot	n	n	1	all/unsp	20	29	nev any	ot
ZHENG	13	m	0	0	all	-	all	As:Chi	1982	CC	540	n	ot	*	y	0	cig+/-ot	20	29	nev cigs	st

Table 1G3 - 1

IESLC - Meta-analysis of Ever Smoking, Amount smoked, "Mid", Any product (or Cigarettes if Any not available)
All LC types
Most adjusted

Cigarette type is all/unspec for all RRs
except for the following:

REF	NRR	CIGTYPE
ALDERS	19	MC only
ALDERS	22	MC only
JUSSAW	14	MC only

Table 1G3 - 2

IESLC - Meta-analysis of Ever Smoking, Amount smoked, "Mid", Any product (or Cigarettes if Any not available)
All LC types
Most adjusted

REF	NRR	SEX	AD	Number Exposed Case	Number Exposed Cont	Non-exposed Case	Non-exposed Cont	RR	95.00%CI
ALDERS	19	m	1	-	-	-	-	7.96	(4.63- 13.69)
ALDERS	22	f	1	-	-	-	-	5.28	(3.79- 7.36)
Subtotal ALDERS								5.91	(4.45- 7.84)
ARMADA	47	m	0	134	105	4	64	20.42	(7.20- 57.88)
AUVINE	14	c	2	-	-	-	-	33.90	(17.10- 67.00)
AXELSS	15	f	0	28	15	18	154	15.97	(7.21- 35.36)
BARBON	83	m	3	-	-	-	-	12.80	(7.90- 21.00)
BRESLO	15	m	0	296	193	22	110	7.67	(4.69- 12.55)
BUFFLE	29	f	0	76	60	12	112	11.82	(5.96- 23.45)
CHEN2	4	m	0	44	50	9	33	3.23	(1.39- 7.48)
CHEN2	8	f	0	22	10	25	33	2.90	(1.17- 7.22)
Subtotal CHEN2								3.07	(1.66- 5.70)
CHOI	13	m	0	144	281	13	95	3.74	(2.03- 6.92)
CHOI	18	f	0	7	9	76	164	1.68	(0.60- 4.68)
Subtotal CHOI								3.03	(1.79- 5.13)
DAMBER	8	m	1	-	-	-	-	9.10	(5.50- 15.30)
DAVEYS	3	m	0	19	22	3	23	6.62	(1.72- 25.56)
DESTEF	7	m	4	-	-	-	-	8.40	(5.20- 13.60)
DOLL	3	m	0	475	431	7	61	9.60	(4.35- 21.22)
DOLL	9	f	0	14	6	40	59	3.44	(1.22- 9.71)
Subtotal DOLL								6.58	(3.50- 12.35)
*DOLL2	47	m	1	-	-	-	-	12.29	(1.67- 90.41)
DOSEME	9	m	2	-	-	-	-	3.10	(2.30- 4.10)
*DUNN	3	m	0	75	27720	2	14160	19.16	(4.70- 78.00)
EBELIN	4	m	0	47	37	12	117	12.39	(5.95- 25.80)
ESAKI	2	m	0	74	58	16	28	2.23	(1.10- 4.51)
FAN	8	m	0	111	183	36	236	3.98	(2.61- 6.07)
FAN	12	f	0	31	12	69	320	11.98	(5.86- 24.50)
Subtotal FAN								5.29	(3.68- 7.61)
GARSHI	27	m	1	-	-	-	-	5.72	(4.04- 8.10)
GER	23	c	14	-	-	-	-	1.90	(0.98- 3.70)
GOLLED	2	m	1	-	-	-	-	6.37	(3.67- 11.03)
GSELL	3	m	0	27	26	2	29	15.06	(3.26- 69.59)
*HAMMON	154	m	1	-	-	-	-	8.32	(4.66- 14.84)
HU	2	m	0	55	43	41	67	2.09	(1.20- 3.65)
HU	5	f	0	6	6	40	48	1.20	(0.36- 4.01)
Subtotal HU								1.90	(1.14- 3.14)
HU2	6	c	0	149	87	121	213	3.01	(2.13- 4.26)
JEDRYC	46	m	4	-	-	-	-	6.16	(4.25- 8.90)
JEDRYC	49	f	4	-	-	-	-	2.38	(1.17- 6.86)
Subtotal JEDRYC								5.35	(3.80- 7.52)
JOLY	9	m	0	126	175	12	218	13.08	(7.00- 24.43)
JOLY	5	f	0	28	22	52	283	6.93	(3.68- 13.03)
Subtotal JOLY								9.55	(6.13- 14.90)
JUSSAW	14	m	0	24	8	149	624	12.56	(5.53- 28.52)
KHUDER	2	m	0	224	288	23	309	10.45	(6.61- 16.52)
KREUZE	21	m	3	-	-	-	-	19.50	(7.50- 50.30)
KREUZE	32	m	3	-	-	-	-	32.80	(20.90- 51.40)
KREUZE	27	f	3	-	-	-	-	12.10	(3.00- 48.00)
KREUZE	38	f	3	-	-	-	-	7.70	(3.50- 17.30)
Subtotal KREUZE								21.69	(15.27- 30.80)
KREYBE	10	m	1	-	-	-	-	6.23	(2.66- 14.56)
LAMTH	2	f	0	90	28	202	337	5.36	(3.39- 8.48)
LETOUR	3	c	0	367	198	24	224	17.30	(10.98- 27.27)
LIU2	9	m	3	-	-	-	-	7.10	(2.60- 19.50)
LIU3	7	m	2	-	-	-	-	1.09	(0.24- 4.82)
LIU4	8	m	2	-	-	-	-	3.60	(3.49- 3.71)
LUBIN2	275	m	0	1963	2547	190	2616	10.61	(9.06- 12.44)
LUBIN2	283	f	0	134	103	336	1188	4.60	(3.46- 6.11)
Subtotal LUBIN2								8.70	(7.57- 9.99)
MACLEN	38	c	2	-	-	-	-	2.93	(1.57- 5.45)
MARTIS	2	m	0	91	87	4	25	6.54	(2.19- 19.55)
MATOS	31	m	2	-	-	-	-	7.50	(3.70- 15.00)
MATSUD	2	m	0	75	1607	3	1255	19.52	(6.14- 62.05)
MCCONN	25	c	0	49	92	9	23	1.36	(0.58- 3.17)
ORMOS	2	m	0	40	577	7	777	7.69	(3.42- 17.30)
PASTOR	8	m	1	-	-	-	-	8.02	(3.84- 16.73)
PERNU	21	m	0	478	138	97	275	9.82	(7.28- 13.24)
PERNU	15	f	0	1	11	110	971	0.80	(0.10- 6.27)
Subtotal PERNU								9.32	(6.94- 12.53)
SIEMIA	14	m	0	-	-	-	-	4.50	(1.80- 13.20)

Table 1G3 - 2

IESLC - Meta-analysis of Ever Smoking, Amount smoked, "Mid", Any product (or Cigarettes if Any not available)
All LC types
Most adjusted

REF	NRR	SEX	AD	Number Exposed Case	Number Exposed Cont	Non-exposed Case	Non-exposed Cont	RR	95.00%CI
STOCKS	42	m	2	-	-	-	-	7.89	(5.51- 11.29)
TIZZAN	8	m	0	468	470	180	305	1.69	(1.35- 2.11)
WANG2	13	c	4	-	-	-	-	3.19	(1.39- 7.29)
WYNDE2	18	m	0	122	203	8	105	7.89	(3.71- 16.75)
WYNDE3	45	m	0	77	114	9	88	6.60	(3.14- 13.90)
WYNDE3	80	f	0	24	24	20	76	3.80	(1.79- 8.05)
Subtotal WYNDE3								5.02	(2.96- 8.52)
WYNDE4	45	m	0	228	274	12	115	7.97	(4.29- 14.82)
WYNDE4	59	f	2	-	-	-	-	6.49	(2.35- 17.93)
Subtotal WYNDE4								7.54	(4.44- 12.80)
XU3	11	m	1	-	-	-	-	14.78	(5.30- 41.18)
ZHENG	13	m	0	128	89	33	94	4.10	(2.53- 6.62)
Partial Totals				6571	36409	2048	26034		

*prospective study

REF	NRR	SEX	AD	Ys	Ws	Qs	Ps
ALDERS	19	m	1	2.07	13.07	6.19	0.0000
ALDERS	22	f	1	1.66	34.88	2.68	0.0000
Subtotal ALDERS				1.78	47.96	8.87	
ARMADA	47	m	0	3.02	3.54	9.40	0.0000
AUVINE	14	c	2	3.52	8.24	37.62	0.0000
AXELSS	15	f	0	2.77	6.08	11.65	0.0000
BARBON	83	m	3	2.55	16.08	21.74	0.0000
BRESLO	15	m	0	2.04	15.85	6.71	0.0000
BUFFLE	29	f	0	2.47	8.19	9.62	0.0000
CHEN2	4	m	0	1.17	5.43	0.25	0.0063
CHEN2	8	f	0	1.07	4.63	0.48	0.0217
Subtotal CHEN2				1.12	10.07	0.73	
CHOI	13	m	0	1.32	10.21	0.04	0.0000
CHOI	18	f	0	0.52	3.66	2.76	0.3219
Subtotal CHOI				1.11	13.87	2.81	
DAMBER	8	m	1	2.21	14.68	9.91	0.0000
DAVEYS	3	m	0	1.89	2.11	0.53	0.0061
DESTEF	7	m	4	2.13	16.62	9.15	0.0000
DOLL	3	m	0	2.26	6.11	4.68	0.0000
DOLL	9	f	0	1.24	3.57	0.08	0.0195
Subtotal DOLL				1.88	9.68	4.77	
*DOLL2	47	m	1	2.51	0.96	1.21	0.0137
DOSEME	9	m	2	1.13	45.98	2.99	0.0000
*DUNN	3	m	0	2.95	1.95	4.78	0.0000
EBELIN	4	m	0	2.52	7.13	9.11	0.0000
ESAKI	2	m	0	0.80	7.75	2.64	0.0253
FAN	8	m	0	1.38	21.51	0.00	0.0000
FAN	12	f	0	2.48	7.51	9.03	0.0000
Subtotal FAN				1.67	29.02	9.03	
GARSHI	27	m	1	1.74	31.76	4.06	0.0000
GER	23	c	14	0.64	8.71	4.83	0.0582
GOLLED	2	m	1	1.85	12.69	2.74	0.0000
GSELL	3	m	0	2.71	1.64	2.88	0.0005
*HAMMON	154	m	1	2.12	11.45	6.14	0.0000
HU	2	m	0	0.74	12.38	5.22	0.0095
HU	5	f	0	0.18	2.64	3.82	0.7672
Subtotal HU				0.64	15.02	9.04	
HU2	6	c	0	1.10	32.09	2.57	0.0000
JEDRYC	46	m	4	1.82	28.13	5.24	0.0000
JEDRYC	49	f	4	0.87	4.91	1.33	0.0546
Subtotal JEDRYC				1.68	33.04	6.56	
JOLY	9	m	0	2.57	9.85	13.81	0.0000
JOLY	5	f	0	1.94	9.62	2.90	0.0000
Subtotal JOLY				2.26	19.47	16.71	
JUSSAW	14	m	0	2.53	5.71	7.48	0.0000
KHUDER	2	m	0	2.35	18.30	16.86	0.0000
KREUZE	21	m	3	2.97	4.24	10.64	0.0000
KREUZE	32	m	3	3.49	18.97	83.99	0.0000
KREUZE	27	f	3	2.49	2.00	2.45	0.0004
KREUZE	38	f	3	2.04	6.02	2.58	0.0000
Subtotal KREUZE				3.08	31.23	99.66	
KREYBE	10	m	1	1.83	5.32	1.04	0.0000
LAMTH	2	f	0	1.68	18.27	1.57	0.0000
LETOUR	3	c	0	2.85	18.55	39.77	0.0000

Table 1G3 - 2

IESLC - Meta-analysis of Ever Smoking, Amount smoked, "Mid", Any product (or Cigarettes if Any not available)
All LC types
Most adjusted

REF	NRR	SEX	AD	Ys	Ws	Qs	Ps
LIU2	9	m	3	1.96	3.78	1.25	0.0001
LIU3	7	m	2	0.09	1.71	2.89	0.9103
LIU4	8	m	2	1.28	4111.92	45.85	0.0000
LUBIN2	275	m	0	2.36	152.73	145.31	0.0000
LUBIN2	283	f	0	1.53	47.64	0.93	0.0000
Subtotal LUBIN2				2.16	200.37	146.24	
MACLEN	38	c	2	1.08	9.92	0.96	0.0007
MARTIS	2	m	0	1.88	3.20	0.77	0.0008
MATOS	31	m	2	2.01	7.84	3.10	0.0000
MATSUD	2	m	0	2.97	2.87	7.22	0.0000
MCCONN	25	c	0	0.31	5.38	6.25	0.4745
ORMOS	2	m	0	2.04	5.85	2.50	0.0000
PASTOR	8	m	1	2.08	7.09	3.43	0.0000
PERNU	21	m	0	2.28	42.95	34.62	0.0000
PERNU	15	f	0	-0.22	0.91	2.34	0.8339
Subtotal PERNU				2.23	43.86	36.97	
SIEMIA	14	m	0	1.50	3.87	0.05	0.0031
STOCKS	42	m	2	2.07	29.86	13.77	0.0000
TIZZAN	8	m	0	0.52	76.34	56.92	0.0000
WANG2	13	c	4	1.16	5.60	0.29	0.0061
WYNDE2	18	m	0	2.07	6.77	3.12	0.0000
WYNDE3	45	m	0	1.89	6.93	1.74	0.0000
WYNDE3	80	f	0	1.34	6.83	0.02	0.0005
Subtotal WYNDE3				1.61	13.76	1.76	
WYNDE4	45	m	0	2.08	9.99	4.75	0.0000
WYNDE4	59	f	2	1.87	3.72	0.87	0.0003
Subtotal WYNDE4				2.02	13.71	5.62	
XU3	11	m	1	2.69	3.66	6.24	0.0000
ZHENG	13	m	0	1.41	16.67	0.01	0.0000

	N	70
	NS	55
	Wt	5103.04
Het	Chi	730.40
Het	df	69
Het	P	***
Fixed	RR	4.00
	RRl	3.89
	RRu	4.11
	P	+++
Random	RR	6.32
	RRl	5.34
	RRu	7.48
	P	+++
Asymm	P	***

Table 1G3 - 3

IESLC - Meta-analysis of Ever Smoking, Amount smoked, "Mid", Any product (or Cigarettes if Any not available)
All LC types
Most adjusted

	Sex			
	combined	male	female	Total
N	7	46	17	70
NS	7	45	16	68
Wt	88.48	4843.48	171.08	5103.04
Het Chi	88.19	582.08	41.81	730.40
Het df	6	45	16	69
Het P	***	***	***	***
Fixed RR	4.96	3.95	5.29	4.00
RRl	4.03	3.84	4.55	3.89
RRu	6.11	4.06	6.14	4.11
P	+++	+++	+++	+++
Random RR	4.65	7.13	5.22	6.32
RRl	2.00	5.77	3.95	5.34
RRu	10.80	8.81	6.90	7.48
P	+++	+++	+++	+++
Between Chi				18.31
Between df				2
Between P				***
Btwn(F) P				N.S.
Btwn(R) P				N.S.

	Lung cancer type		
	all	other	Total
N	70		70
NS	55		55
Wt	5103.04		5103.04
Het Chi	730.40		730.40
Het df	69		69
Het P	***		***
Fixed RR	4.00		4.00
RRl	3.89		3.89
RRu	4.11		4.11
P	+++		+++
Random RR	6.32		6.32
RRl	5.34		5.34
RRu	7.48		7.48
P	+++		+++
Between Chi			
Between df			
Between P			N.S.
Btwn(F) P			N.S.
Btwn(R) P			N.S.

	Location								
	NAmer	UK	Scand	othEur	China	Japan	othAs	other	Total
N	13	9	6	17	13	2	6	4	70
NS	11	7	5	12	10	2	5	3	55
Wt	144.16	109.73	78.17	430.41	4229.53	10.63	56.48	43.93	5103.04
Het Chi	24.08	18.68	20.28	282.57	31.03	9.86	17.61	2.33	730.40
Het df	12	8	5	16	12	1	5	3	69
Het P	*	*	**	***	**	**	**	N.S.	***
Fixed RR	8.21	6.11	10.79	6.15	3.60	4.01	3.89	8.71	4.00
RRl	6.97	5.07	8.64	5.59	3.49	2.20	3.00	6.48	3.89
RRu	9.66	7.37	13.47	6.76	3.71	7.32	5.05	11.71	4.11
P	+++	+++	+++	+++	+++	+++	+++	+++	+++
Random RR	8.17	5.81	10.64	8.00	3.74	6.28	3.72	8.71	6.32
RRl	6.38	4.18	6.17	5.07	2.94	0.75	2.24	6.48	5.34
RRu	10.47	8.09	18.34	12.62	4.75	52.44	6.20	11.71	7.48
P	+++	+++	+++	+++	+++	(+)	+++	+++	+++
Between Chi									323.97
Between df									7
Between P									***
Btwn(F) P									***
Btwn(R) P									***

Table 1G3 - 3

IESLC - Meta-analysis of Ever Smoking, Amount smoked, "Mid", Any product (or Cigarettes if Any not available)
All LC types
Most adjusted

	Detailed Country in "other Europe"					
	multi	Germany	othWest	East	Balkans	Total
N	2	6	5	3	1	17
NS	1	3	5	2	1	12
Wt	200.37	40.47	104.69	38.89	45.98	430.41
Het Chi	25.37	14.59	83.10	4.44	0.00	282.57
Het df	1	5	4	2	0	16
Het P	***	*	***	N.S.	N.S.	***
Fixed RR	8.70	18.47	2.88	5.65	3.10	6.15
RRl	7.57	13.57	2.38	4.13	2.32	5.59
RRu	9.99	25.14	3.49	7.73	4.14	6.76
P	+++	+++	+++	+++	+++	+++
Random RR	7.05	14.30	8.33	5.17	3.10	8.00
RRl	3.11	7.90	2.47	2.91	2.32	5.07
RRu	15.98	25.89	28.10	9.18	4.14	12.62
P	+++	+++	+++	+++	+++	+++
Between Chi						155.06
Between df						4
Between P						***
Btwn(F) P						*
Btwn(R) P						***

	Detailed Country in "other Asia"			
	India	HongKong	other	Total
N	1	1	4	6
NS	1	1	3	5
Wt	5.71	18.27	32.50	56.48
Het Chi	0.00	0.00	3.05	17.61
Het df	0	0	3	5
Het P	N.S.	N.S.	N.S.	**
Fixed RR	12.56	5.36	2.65	3.89
RRl	5.53	3.39	1.88	3.00
RRu	28.52	8.48	3.73	5.05
P	+++	+++	+++	+++
Random RR	12.56	5.36	2.64	3.72
RRl	5.53	3.39	1.87	2.24
RRu	28.52	8.48	3.74	6.20
P	+++	+++	+++	+++
Between Chi				14.56
Between df				2
Between P				***
Btwn(F) P				(*)
Btwn(R) P				***

	Detailed other continent			
	SCAmer	Auslia	Africa	Total
N	4			4
NS	3			3
Wt	43.93			43.93
Het Chi	2.33			2.33
Het df	3			3
Het P	N.S.			N.S.
Fixed RR	8.71			8.71
RRl	6.48			6.48
RRu	11.71			11.71
P	+++			+++
Random RR	8.71			8.71
RRl	6.48			6.48
RRu	11.71			11.71
P	+++			+++
Between Chi				
Between df				
Between P				N.S.
Btwn(F) P				N.S.
Btwn(R) P				N.S.

Table 1G3 - 3

IESLC - Meta-analysis of Ever Smoking, Amount smoked, "Mid", Any product (or Cigarettes if Any not available)
All LC types
Most adjusted

	<1960	1960-69	1970-79	1980-89	1990+	Total
	Start year of study					
N	18	6	15	23	8	70
NS	15	5	12	19	4	55
Wt	236.65	36.87	408.90	4343.82	76.80	5103.04
Het Chi	146.96	16.68	111.95	190.55	70.77	730.40
Het df	17	5	14	22	7	69
Het P	***	**	***	***	***	***
Fixed RR	4.68	5.89	6.75	3.71	8.66	4.00
RRl	4.12	4.27	6.13	3.60	6.93	3.89
RRu	5.32	8.14	7.44	3.82	10.84	4.11
P	+++	+++	+++	+++	+++	+++
Random RR	6.06	6.49	6.46	5.73	8.79	6.32
RRl	3.93	3.57	4.73	4.28	4.15	5.34
RRu	9.35	11.80	8.81	7.68	18.62	7.48
P	+++	+++	+++	+++	+++	+++
Between Chi						193.48
Between df						4
Between P						***
Btwn(F) P						***
Btwn(R) P						N.S.

	CC	other	Total
	Study type (1)		
N	67	3	70
NS	52	3	55
Wt	5088.67	14.37	5103.04
Het Chi	718.24	1.22	730.40
Het df	66	2	69
Het P	***	N.S.	***
Fixed RR	3.99	9.56	4.00
RRl	3.88	5.70	3.89
RRu	4.10	16.04	4.11
P	+++	+++	+++
Random RR	6.21	9.56	6.32
RRl	5.24	5.70	5.34
RRu	7.37	16.04	7.48
P	+++	+++	+++
Between Chi			10.94
Between df			1
Between P			***
Btwn(F) P			N.S.
Btwn(R) P			N.S.

	CC	prosp	other	Total
	Study type (2)			
N	67	3		70
NS	52	3		55
Wt	5088.67	14.37		5103.04
Het Chi	718.24	1.22		730.40
Het df	66	2		69
Het P	***	N.S.		***
Fixed RR	3.99	9.56		4.00
RRl	3.88	5.70		3.89
RRu	4.10	16.04		4.11
P	+++	+++		+++
Random RR	6.21	9.56		6.32
RRl	5.24	5.70		5.34
RRu	7.37	16.04		7.48
P	+++	+++		+++
Between Chi				10.94
Between df				1
Between P				***
Btwn(F) P				N.S.
Btwn(R) P				N.S.

Table 1G3 - 3

IESLC - Meta-analysis of Ever Smoking, Amount smoked, "Mid", Any product (or Cigarettes if Any not available)
All LC types
Most adjusted

	Study size (number of LC cases)				
	100-249	250-499	500-999	1000+	Total
N	20	16	15	19	70
NS	18	13	13	11	55
Wt	107.49	159.47	174.07	4662.00	5103.04
Het Chi	68.17	36.17	76.67	417.93	730.40
Het df	19	15	14	18	69
Het P	***	**	***	***	***
Fixed RR	4.06	6.62	7.96	3.83	4.00
RRl	3.36	5.66	6.86	3.72	3.89
RRu	4.91	7.73	9.23	3.94	4.11
P	+++	+++	+++	+++	+++
Random RR	4.50	6.75	8.98	6.14	6.32
RRl	3.10	5.25	6.22	4.48	5.34
RRu	6.53	8.69	12.96	8.40	7.48
P	+++	+++	+++	+++	+++
Between Chi					131.45
Between df					3
Between P					***
Btwn(F) P					**
Btwn(R) P					(*)

	Risky occupational population			
	no	mining	othRisky	Total
N	68		2	70
NS	53		2	55
Wt	5069.34		33.70	5103.04
Het Chi	721.52		2.68	730.40
Het df	67		1	69
Het P	***		N.S.	***
Fixed RR	3.99		6.13	4.00
RRl	3.88		4.38	3.89
RRu	4.10		8.60	4.11
P	+++		+++	+++
Random RR	6.27		8.58	6.32
RRl	5.28		2.80	5.34
RRu	7.45		26.24	7.48
P	+++		+++	+++
Between Chi				6.20
Between df				1
Between P				*
Btwn(F) P				N.S.
Btwn(R) P				N.S.

	National cigarette tobacco type			
	Virginia	blended	other	Total
N	12	44	14	70
NS	10	34	11	55
Wt	137.87	726.94	4238.23	5103.04
Het Chi	38.50	372.64	34.58	730.40
Het df	11	43	13	69
Het P	***	***	***	***
Fixed RR	7.18	6.67	3.60	4.00
RRl	6.08	6.20	3.49	3.89
RRu	8.49	7.17	3.71	4.11
P	+++	+++	+++	+++
Random RR	6.78	7.46	3.57	6.32
RRl	4.77	5.90	2.82	5.34
RRu	9.63	9.44	4.51	7.48
P	+++	+++	+++	+++
Between Chi				284.68
Between df				2
Between P				***
Btwn(F) P				***
Btwn(R) P				***

Table 1G3 - 3

IESLC - Meta-analysis of Ever Smoking, Amount smoked, "Mid", Any product (or Cigarettes if Any not available)
All LC types
Most adjusted

	Any proxy use No/nk	Yes	Total
N	46	24	70
NS	35	20	55
Wt	697.77	4405.27	5103.04
Het Chi	379.25	203.38	730.40
Het df	45	23	69
Het P	***	***	***
Fixed RR	6.14	3.74	4.00
RRl	5.70	3.63	3.89
RRu	6.61	3.85	4.11
P	+++	+++	+++
Random RR	6.43	6.16	6.32
RRl	5.05	4.74	5.34
RRu	8.17	8.00	7.48
P	+++	+++	+++
Between Chi			147.77
Between df			1
Between P			***
Btwn(F) P			***
Btwn(R) P			N.S.

	Full histological confirmation No	Yes	Total
N	51	19	70
NS	39	16	55
Wt	4739.45	363.59	5103.04
Het Chi	440.96	108.25	730.40
Het df	50	18	69
Het P	***	***	***
Fixed RR	3.80	7.90	4.00
RRl	3.69	7.13	3.89
RRu	3.91	8.76	4.11
P	+++	+++	+++
Random RR	6.29	6.42	6.32
RRl	5.20	4.76	5.34
RRu	7.61	8.64	7.48
P	+++	+++	+++
Between Chi			181.19
Between df			1
Between P			***
Btwn(F) P			***
Btwn(R) P			N.S.

	Number of adjustment variables (1) 0	1	2+/+nk	Total
N	41	10	19	70
NS	32	9	15	56
Wt	633.22	135.57	4334.25	5103.04
Het Chi	337.09	8.01	228.07	730.40
Het df	40	9	18	69
Het P	***	N.S.	***	***
Fixed RR	5.93	6.68	3.72	4.00
RRl	5.49	5.65	3.61	3.89
RRu	6.41	7.91	3.83	4.11
P	+++	+++	+++	+++
Random RR	5.94	6.68	6.54	6.32
RRl	4.62	5.65	4.59	5.34
RRu	7.64	7.91	9.33	7.48
P	+++	+++	+++	+++
Between Chi				157.23
Between df				2
Between P				***
Btwn(F) P				***
Btwn(R) P				N.S.

Table 1G3 - 3

IESLC - Meta-analysis of Ever Smoking, Amount smoked, "Mid", Any product (or Cigarettes if Any not available)
All LC types
Most adjusted

	Number of adjustment variables (2) 0	1	2	3-5	6+/+nk	Total
N	41	10	8	10	1	70
NS	32	9	8	6	1	56
Wt	633.22	135.57	4219.20	106.35	8.71	5103.04
Het Chi	337.09	8.01	68.86	55.01	0.00	730.40
Het df	40	9	7	9	0	69
Het P	***	N.S.	***	***	N.S.	***
Fixed RR	5.93	6.68	3.63	9.71	1.90	4.00
RRl	5.49	5.65	3.53	8.03	0.98	3.89
RRu	6.41	7.91	3.74	11.74	3.69	4.11
P	+++	+++	+++	+++	(+)	+++
Random RR	5.94	6.68	5.45	8.72	1.90	6.32
RRl	4.62	5.65	3.51	5.25	0.98	5.34
RRu	7.64	7.91	8.46	14.47	3.69	7.48
P	+++	+++	+++	+++	(+)	+++
Between Chi						261.43
Between df						4
Between P						***
Btwn(F) P						***
Btwn(R) P						**

	Product all/unsp	cig+/-ot	cig only	Total
N	24	38	8	70
NS	20	28	7	55
Wt	213.61	608.67	4280.76	5103.04
Het Chi	72.70	275.10	90.91	730.40
Het df	23	37	7	69
Het P	***	***	***	***
Fixed RR	6.89	6.91	3.60	4.00
RRl	6.03	6.38	3.50	3.89
RRu	7.88	7.48	3.71	4.11
P	+++	+++	+++	+++
Random RR	6.18	6.64	5.51	6.32
RRl	4.72	5.23	3.74	5.34
RRu	8.07	8.43	8.12	7.48
P	+++	+++	+++	+++
Between Chi				291.69
Between df				2
Between P				***
Btwn(F) P				***
Btwn(R) P				N.S.

	Denominator nev any	nev cigs	Total
N	51	19	70
NS	38	17	55
Wt	4861.13	241.91	5103.04
Het Chi	584.80	126.64	730.40
Het df	50	18	69
Het P	***	***	***
Fixed RR	3.95	5.26	4.00
RRl	3.84	4.64	3.89
RRu	4.06	5.96	4.11
P	+++	+++	+++
Random RR	6.43	6.09	6.32
RRl	5.25	4.27	5.34
RRu	7.86	8.70	7.48
P	+++	+++	+++
Between Chi			18.96
Between df			1
Between P			***
Btwn(F) P			N.S.
Btwn(R) P			N.S.

Table 1G3 - 3

IESLC - Meta-analysis of Ever Smoking, Amount smoked, "Mid", Any product (or Cigarettes if Any not available)
All LC types
Most adjusted

	Derivation of RR/CI			
	Orig	StdCalc	Other	Total
N	17	41	12	70
NS	13	32	11	56
Wt	204.78	671.60	4226.66	5103.04
Het Chi	131.73	335.76	52.58	730.40
Het df	16	40	11	69
Het P	***	***	***	***
Fixed RR	7.04	5.99	3.65	4.00
RRl	6.14	5.55	3.54	3.89
RRu	8.07	6.46	3.76	4.11
P	+++	+++	+++	+++
Random RR	7.58	5.98	5.71	6.32
RRl	4.98	4.69	4.18	5.34
RRu	11.55	7.62	7.80	7.48
P	+++	+++	+++	+++
Between Chi				210.33
Between df				2
Between P				***
Btwn(F) P				***
Btwn(R) P				N.S.

	Study LIU4		
	LIU4	others	Total
N	1	69	70
NS	1	54	55
Wt	4111.92	991.12	5103.04
Het Chi	0.00	494.34	730.40
Het df	0	68	69
Het P	N.S.	***	***
Fixed RR	3.60	6.20	4.00
RRl	3.49	5.83	3.89
RRu	3.71	6.60	4.11
P	+++	+++	+++
Random RR	3.60	6.39	6.32
RRl	3.49	5.32	5.34
RRu	3.71	7.67	7.48
P	+++	+++	+++
Between Chi			236.06
Between df			1
Between P			***
Btwn(F) P			***
Btwn(R) P			***

Table 1G3 - 4

IESLC - Meta-analysis of Ever Smoking, Amount smoked, "Mid", Any product (or Cigarettes if Any not available)
All LC types
Least adjusted

REF	NRR	X	SEX	AGEL	AGEH	RACE	YF	LC TYPE	LOC	START	ST	NLC	R	VB	P	H	AD	PRODUCT	exL	exH	DENOM	De
ALDERS	19		m	0	0	all	-	all	Eu:UK	1977	CC	1448	n	V	n	n	1	cig only	18	27	nev+2	ot
ALDERS	22		f	0	0	all	-	all	Eu:UK	1977	CC	1448	n	V	n	n	1	cig only	18	27	nev+2	ot
ARMADA	47		m	0	0	all	-	all	Eu:wst	1986	CC	325	n	bl	n	y	0	cig+/-ot	15	24	nev any	st
AUVINE	6	x	c	0	0	all	-	all	Eu:Sca	1986	CC	517	n	bl	y	n	0	cig+/-ot	11	20	nev cigs	st
AXELSS	15		f	0	0	sca	-	all	Eu:Sca	1989	CC	436	n	bl	n	n	0	all/unsp	20	29	nev any	st
BARBON	9	x	m	0	0	all	-	all	Eu:wst	1979	CC	755	n	bl	y	y	0	all/unsp	20	29	nev any	st
BRESLO	15		m	0	0	all	-	all	NAmer	1949	CC	518	n	bl	n	y	0	cig+/-ot	20	39	nev+3	st
BUFFLE	29		f	0	0	w-hi	-	all	NAmer	1976	CC	943	n	bl	y	n	0	cig+/-ot	20	20	nev cigs	or
CHEN2	4		m	0	0	all	-	all	As:Chi	1983	CC	193	n	ot	y	n	0	all/unsp	10	20	nev any	st
CHEN2	8		f	0	0	all	-	all	As:Chi	1983	CC	193	n	ot	y	n	0	all/unsp	10	20	nev any	st
CHOI	13		m	0	0	all	-	all	As:oth	1985	CC	375	n	bl	n	n	0	cig+/-ot	11	20	nev cigs	st
CHOI	18		f	0	0	all	-	all	As:oth	1985	CC	375	n	bl	n	n	0	cig+/-ot	11	30	nev cigs	st
DAMBER	8		m	0	0	all	-	all	Eu:Sca	1972	CC	579	n	bl	y	n	1	cig only	16	25	nev any	ot
DAVEYS	3		m	0	0	all	-	all	Eu:Ger	1930	CC	109	n	bl	y	n	0	all/unsp	11	20	nev any	st
DESTEF	2	x	m	0	0	all	-	all	SCAmer	1988	CC	497	n	bl	n	y	0	all/unsp	11	20	nev any	st
DOLL	3		m	0	0	all	-	all	Eu:UK	1948	CC	1465	n	V	n	n	0	all/unsp	15	24	nev any	st
DOLL	9		f	0	0	all	-	all	Eu:UK	1948	CC	1465	n	V	n	n	0	all/unsp	15	24	nev any	st
DOLL2	47		m	35	99	all	5	all	Eu:UK	1951	pr	920	n	V	n	n	1	all/unsp	15	24	nev any	ot
DOSEME	9		m	0	0	all	-	all	Eu:bal	1979	CC	1210	n	bl	n	n	2	cig+/-ot	11	20	nev cigs	or
DUNN	3		m	0	0	all	0	all	NAmer	1954	pr	139	o	bl	n	n	0	cig+/-ot	15	24	nev cigs	st
EBELIN	4		m	0	0	all	-	all	Eu:Ger	1980	CC	130	n	bl	n	n	0	all/unsp	20	29	nev any	st
ESAKI	2		m	0	0	all	-	all	As:Jap	1961	CC	245	n	bl	y	n	0	cig+/-ot	15	29	nev cigs	st
FAN	8		m	0	0	all	-	all	As:Chi	1990	CC	403	n	ot	y	n	0	cig+/-ot	20	29	nev cigs	st
FAN	12		f	0	0	all	-	all	As:Chi	1990	CC	403	n	ot	y	n	0	cig+/-ot	20	29	nev cigs	st
GARSHI	19	x	m	0	0	all	-	all	NAmer	1981	CC	1081	o	bl	y	n	0	all/unsp	16	25	nev any	st
GER	19	x	c	0	0	all	-	all	As:oth	1990	CC	141	n	ot	y	n	0	all/unsp	11	20	nev any	st
GOLLED	16	x	m	35	99	all	-	all	Eu:UK	1952	CC	443	n	V	y	n	0	cig only	11	22	nev any	st
GSELL	3		m	0	0	all	-	all	Eu:wst	1937	CC	150	n	bl	n	y	0	all/unsp	15	20	nev any	st
HAMMON	154		m	0	0	wh	0	all	NAmer	1952	pr	448	n	bl	n	n	1	cig only	10	20	nev any	ot
HU	2		m	0	0	all	-	all	As:Chi	1985	CC	227	n	ot	n	y	0	cig+/-ot	14	24	nev any	st
HU	5		f	0	0	all	-	all	As:Chi	1985	CC	227	n	ot	n	y	0	cig+/-ot	14	24	nev any	st
HU2	6		c	0	0	all	-	all	As:Chi	1977	CC	523	n	ot	y	n	0	cig+/-ot	20	29	nev cigs	st
JEDRYC	61	x	m	0	0	all	-	all	Eu:est	1980	CC	1630	n	bl	y	n	0	cig+/-ot	20	29	nev any	st
JEDRYC	66	x	f	0	0	all	-	all	Eu:est	1980	CC	1630	n	bl	y	n	0	cig+/-ot	20	29	nev any	st
JOLY	9		m	0	0	all	-	all	SCAmer	1978	CC	826	n	bl	n	n	0	cig+/-ot	20	29	nev any	st
JOLY	5		f	0	0	all	-	all	SCAmer	1978	CC	826	n	bl	n	n	0	cig+/-ot	20	29	nev any	st
JUSSAW	14		m	0	0	all	-	all	As:Ind	1964	CC	792	n	V	n	n	0	cig only	20	24	nev any	st
KHUDER	2		m	0	0	all	-	all	NAmer	1985	CC	482	n	bl	n	y	0	cig+/-ot	20	39	nev cigs	st
KREUZE	21		m	1	45	all	-	all	Eu:Ger	1990	CC	2260	n	bl	n	n	3	cig+/-ot	20	29	nev any	or
KREUZE	32		m	55	69	all	-	all	Eu:Ger	1990	CC	2260	n	bl	n	n	3	cig+/-ot	20	29	nev any	or
KREUZE	27		f	1	45	all	-	all	Eu:Ger	1990	CC	2260	n	bl	n	n	3	cig+/-ot	20	29	nev any	or
KREUZE	38		f	55	69	all	-	all	Eu:Ger	1990	CC	2260	n	bl	n	n	3	cig+/-ot	20	29	nev any	or
KREYBE	22	x	m	0	0	all	-	all	Eu:Sca	1948	CC	300	n	bl	n	y	0	all/unsp	15	24	nev any	st
LAMTH	2		f	0	0	ch	-	all	As:HK	1983	CC	445	n	bl	n	n	0	all/unsp	11	20	nev any	or
LETOUR	3		c	0	0	all	-	all	NAmer	1983	CC	738	n	V	y	y	0	cig+/-ot	20	40	nev cigs	st
LIU2	6	x	m	0	0	all	-	all	As:Chi	1983	CC	316	n	ot	n	n	0	all/unsp	20	29	nev any	st
LIU3	4	x	m	0	0	all	-	all	As:Chi	1985	CC	110	n	ot	n	n	0	all/unsp	16	30	nev any	st
LIU4	8		m	35	69	all	-	all	As:Chi	1986	CC	1000-00	n	ot	y	n	2	cig only	20	20	nev any	ot
LUBIN2	275		m	0	0	all	-	all	Eu:mul	1976	CC	7804	n	bl	n	y	0	cig+/-ot	20	29	nev any	st
LUBIN2	283		f	0	0	all	-	all	Eu:mul	1976	CC	7804	n	bl	n	y	0	cig+/-ot	20	29	nev any	st
MACLEN	38		c	0	0	ch	-	all	As:oth	1972	CC	233	n	bl	n	n	2	cig+/-ot	20	29	nev cigs	or
MARTIS	2		m	0	0	all	-	all	Eu:UK	1972	CC	201	n	V	n	n	0	cig+/-ot	15	24	nev cigs	st
MATOS	30	x	m	0	0	all	-	all	SCAmer	1994	CC	200	n	bl	n	n	0	cig+/-ot	15	24	nev any	st
MATSUD	2		m	0	0	all	-	all	As:Jap	1965	CC	179	n	bl	n	n	0	cig+/-ot	11	20	nev cigs	st
MCCONN	25		c	0	0	all	-	all	Eu:UK	1946	CC	100	n	V	n	y	0	all/unsp	10	20	nev any	st
ORMOS	2		m	0	0	all	-	all	Eu:est	1947	CC	119	n	bl	y	y	0	cig+/-ot	16	30	nev any	st
PASTOR	3	x	m	0	0	all	-	all	Eu:wst	1976	CC	204	n	bl	y	n	0	all/unsp	20	29	nev any	st
PERNU	21		m	0	0	all	-	all	Eu:Sca	1944	CC	1606	n	bl	n	n	0	all/unsp	20	24	nev any	st
PERNU	15		f	0	0	all	-	all	Eu:Sca	1944	CC	1606	n	bl	n	n	0	all/unsp	20	24	nev any	st
SIEMIA	14		m	0	0	all	-	all	NAmer	1979	CC	857	n	V	y	y	0	cig+/-ot	20	39	nev cigs	or
STOCKS	26	x	m	0	0	all	-	all	Eu:UK	1952	CC	2932	n	V	y	n	0	cig+/-ot	15	21	nev any	st
TIZZAN	8		m	0	0	all	-	all	Eu:wst	1959	CC	1358	n	bl	n	n	0	cig only	10	20	nev any	st
WANG2	5	x	c	0	0	all	-	all	As:Chi	1980	CC	103	n	ot	n	n	0	cig+/-ot	20	29	nev cigs	st
WYNDE2	18		m	0	0	all	-	all	NAmer	1962	CC	404	n	bl	n	y	0	cig+/-ot	11	20	nev any	st
WYNDE3	45		m	0	0	all	-	all	NAmer	1966	CC	350	n	bl	n	y	0	cig+/-ot	10	20	nev any	st
WYNDE3	80		f	0	0	all	-	all	NAmer	1966	CC	350	n	bl	n	y	0	cig+/-ot	10	20	nev any	st
WYNDE4	45		m	0	0	all	-	all	NAmer	1948	CC	684	n	bl	y	n	0	all/unsp	16	20	nev any	st
WYNDE4	59		f	0	0	all	-	all	NAmer	1948	CC	684	n	bl	y	n	2	all/unsp	16	20	nev any	ot
XU3	7	x	m	0	0	all	-	all	As:Chi	1981	CC	135	n	ot	n	n	0	all/unsp	20	29	nev any	st
ZHENG	13		m	0	0	all	-	all	As:Chi	1982	CC	540	n	ot	*	y	0	cig+/-ot	20	29	nev cigs	st

Table 1G3 - 4

IESLC - Meta-analysis of Ever Smoking, Amount smoked, "Mid", Any product (or Cigarettes if Any not available)
All LC types
Least adjusted

Cigarette type is all/unspec for all RRs
except for the following:

REF	NRR	CIGTYPE
ALDERS	19	MC only
ALDERS	22	MC only
JUSSAW	14	MC only

Table 1G3 - 5

IESLC - Meta-analysis of Ever Smoking, Amount smoked, "Mid", Any product (or Cigarettes if Any not available)
All LC types
Least adjusted

REF	NRR	SEX	AD	Number Exposed Case	Number Exposed Cont	Non-exposed Case	Non-exposed Cont	RR	95.00%CI
ALDERS	19	m	1	-	-	-	-	7.96	(4.63- 13.69)
ALDERS	22	f	1	-	-	-	-	5.28	(3.79- 7.36)
Subtotal ALDERS								5.91	(4.45- 7.84)
ARMADA	47	m	0	134	105	4	64	20.42	(7.20- 57.88)
AUVINE	6	c	0	148	39	44	229	19.75	(12.25- 31.86)
AXELSS	15	f	0	28	15	18	154	15.97	(7.21- 35.36)
BARBON	9	m	0	223	176	22	188	10.83	(6.67- 17.57)
BRESLO	15	m	0	296	193	22	110	7.67	(4.69- 12.55)
BUFFLE	29	f	0	76	60	12	112	11.82	(5.96- 23.45)
CHEN2	4	m	0	44	50	9	33	3.23	(1.39- 7.48)
CHEN2	8	f	0	22	10	25	33	2.90	(1.17- 7.22)
Subtotal CHEN2								3.07	(1.66- 5.70)
CHOI	13	m	0	144	281	13	95	3.74	(2.03- 6.92)
CHOI	18	f	0	7	9	76	164	1.68	(0.60- 4.68)
Subtotal CHOI								3.03	(1.79- 5.13)
DAMBER	8	m	1	-	-	-	-	9.10	(5.50- 15.30)
DAVEYS	3	m	0	19	22	3	23	6.62	(1.72- 25.56)
DESTEF	2	m	0	155	119	27	163	7.86	(4.90- 12.61)
DOLL	3	m	0	475	431	7	61	9.60	(4.35- 21.22)
DOLL	9	f	0	14	6	40	59	3.44	(1.22- 9.71)
Subtotal DOLL								6.58	(3.50- 12.35)
*DOLL2	47	m	1	-	-	-	-	12.29	(1.67- 90.41)
DOSEME	9	m	2	-	-	-	-	3.10	(2.30- 4.10)
*DUNN	3	m	0	75	27720	2	14160	19.16	(4.70- 78.00)
EBELIN	4	m	0	47	37	12	117	12.39	(5.95- 25.80)
ESAKI	2	m	0	74	58	16	28	2.23	(1.10- 4.51)
FAN	8	m	0	111	183	36	236	3.98	(2.61- 6.07)
FAN	12	f	0	31	12	69	320	11.98	(5.86- 24.50)
Subtotal FAN								5.29	(3.68- 7.61)
GARSHI	19	m	0	387	601	41	363	5.70	(4.03- 8.07)
GER	19	c	0	45	168	51	246	1.29	(0.83- 2.02)
GOLLED	16	m	0	118	779	15	490	4.95	(2.86- 8.57)
GSELL	3	m	0	27	26	2	29	15.06	(3.26- 69.59)
*HAMMON	154	m	1	-	-	-	-	8.32	(4.66- 14.84)
HU	2	m	0	55	43	41	67	2.09	(1.20- 3.65)
HU	5	f	0	6	6	40	48	1.20	(0.36- 4.01)
Subtotal HU								1.90	(1.14- 3.14)
HU2	6	c	0	149	87	121	213	3.01	(2.13- 4.26)
JEDRYC	61	m	0	426	295	49	219	6.45	(4.58- 9.10)
JEDRYC	66	f	0	52	16	78	166	6.92	(3.71- 12.88)
Subtotal JEDRYC								6.56	(4.86- 8.86)
JOLY	9	m	0	126	175	12	218	13.08	(7.00- 24.43)
JOLY	5	f	0	28	22	52	283	6.93	(3.68- 13.03)
Subtotal JOLY								9.55	(6.13- 14.90)
JUSSAW	14	m	0	24	8	149	624	12.56	(5.53- 28.52)
KHUDER	2	m	0	224	288	23	309	10.45	(6.61- 16.52)
KREUZE	21	m	3	-	-	-	-	19.50	(7.50- 50.30)
KREUZE	32	m	3	-	-	-	-	32.80	(20.90- 51.40)
KREUZE	27	f	3	-	-	-	-	12.10	(3.00- 48.00)
KREUZE	38	f	3	-	-	-	-	7.70	(3.50- 17.30)
Subtotal KREUZE								21.69	(15.27- 30.80)
KREYBE	22	m	0	55	925	6	644	6.38	(2.73- 14.91)
LAMTH	2	f	0	90	28	202	337	5.36	(3.39- 8.48)
LETOUR	3	c	0	367	198	24	224	17.30	(10.98- 27.27)
LIU2	6	m	0	97	66	12	44	5.39	(2.65- 10.97)
LIU3	4	m	0	20	93	4	19	1.02	(0.31- 3.33)
LIU4	8	m	2	-	-	-	-	3.60	(3.49- 3.71)
LUBIN2	275	m	0	1963	2547	190	2616	10.61	(9.06- 12.44)
LUBIN2	283	f	0	134	103	336	1188	4.60	(3.46- 6.11)
Subtotal LUBIN2								8.70	(7.57- 9.99)
MACLEN	38	c	2	-	-	-	-	2.93	(1.57- 5.45)
MARTIS	2	m	0	91	87	4	25	6.54	(2.19- 19.55)
MATOS	30	m	0	65	90	11	110	7.22	(3.60- 14.50)
MATSUD	2	m	0	75	1607	3	1255	19.52	(6.14- 62.05)
MCCONN	25	c	0	49	92	9	23	1.36	(0.58- 3.17)
ORMOS	2	m	0	40	577	7	777	7.69	(3.42- 17.30)
PASTOR	3	m	0	89	100	10	89	7.92	(3.88- 16.17)
PERNU	21	m	0	478	138	97	275	9.82	(7.28- 13.24)
PERNU	15	f	0	1	11	110	971	0.80	(0.10- 6.27)
Subtotal PERNU								9.32	(6.94- 12.53)
SIEMIA	14	m	0	-	-	-	-	4.50	(1.80- 13.20)

Table 1G3 - 5

IESLC - Meta-analysis of Ever Smoking, Amount smoked, "Mid", Any product (or Cigarettes if Any not available)
All LC types
Least adjusted

REF	NRR	SEX	AD	Number Exposed Case	Cont	Non-exposed Case	Cont	RR	95.00%CI
STOCKS	26	m	0	919	2257	45	638	5.77	(4.23- 7.89)
TIZZAN	8	m	0	468	470	180	305	1.69	(1.35- 2.11)
WANG2	5	c	0	27	32	11	43	3.30	(1.43- 7.62)
WYNDE2	18	m	0	122	203	8	105	7.89	(3.71- 16.75)
WYNDE3	45	m	0	77	114	9	88	6.60	(3.14- 13.90)
WYNDE3	80	f	0	24	24	20	76	3.80	(1.79- 8.05)
Subtotal WYNDE3								5.02	(2.96- 8.52)
WYNDE4	45	m	0	228	274	12	115	7.97	(4.29- 14.82)
WYNDE4	59	f	2	-	-	-	-	6.49	(2.35- 17.93)
Subtotal WYNDE4								7.54	(4.44- 12.80)
XU3	7	m	0	47	13	7	31	16.01	(5.75- 44.61)
ZHENG	13	m	0	128	89	33	94	4.10	(2.53- 6.62)
Partial Totals				9444	42178	2481	29716		

*prospective study

REF	NRR	SEX	AD	Ys	Ws	Qs	Ps
ALDERS	19	m	1	2.07	13.07	6.21	0.0000
ALDERS	22	f	1	1.66	34.88	2.71	0.0000
Subtotal ALDERS				1.78	47.96	8.93	
ARMADA	47	m	0	3.02	3.54	9.42	0.0000
AUVINE	6	c	0	2.98	16.81	42.93	0.0000
AXELSS	15	f	0	2.77	6.08	11.68	0.0000
BARBON	9	m	0	2.38	16.41	16.31	0.0000
BRESLO	15	m	0	2.04	15.85	6.74	0.0000
BUFFLE	29	f	0	2.47	8.19	9.64	0.0000
CHEN2	4	m	0	1.17	5.43	0.25	0.0063
CHEN2	8	f	0	1.07	4.63	0.47	0.0217
Subtotal CHEN2				1.12	10.07	0.72	
CHOI	13	m	0	1.32	10.21	0.04	0.0000
CHOI	18	f	0	0.52	3.66	2.75	0.3219
Subtotal CHOI				1.11	13.87	2.80	
DAMBER	8	m	1	2.21	14.68	9.95	0.0000
DAVEYS	3	m	0	1.89	2.11	0.54	0.0061
DESTEF	2	m	0	2.06	17.23	7.90	0.0000
DOLL	3	m	0	2.26	6.11	4.70	0.0000
DOLL	9	f	0	1.24	3.57	0.08	0.0195
Subtotal DOLL				1.88	9.68	4.78	
*DOLL2	47	m	1	2.51	0.96	1.22	0.0137
DOSEME	9	m	2	1.13	45.98	2.96	0.0000
*DUNN	3	m	0	2.95	1.95	4.79	0.0000
EBELIN	4	m	0	2.52	7.13	9.13	0.0000
ESAKI	2	m	0	0.80	7.75	2.62	0.0253
FAN	8	m	0	1.38	21.51	0.00	0.0000
FAN	12	f	0	2.48	7.51	9.05	0.0000
Subtotal FAN				1.67	29.02	9.05	
GARSHI	19	m	0	1.74	31.85	4.03	0.0000
GER	19	c	0	0.26	19.29	24.58	0.2605
GOLLED	16	m	0	1.60	12.74	0.58	0.0000
GSELL	3	m	0	2.71	1.64	2.89	0.0005
*HAMMON	154	m	1	2.12	11.45	6.16	0.0000
HU	2	m	0	0.74	12.38	5.20	0.0095
HU	5	f	0	0.18	2.64	3.82	0.7672
Subtotal HU				0.64	15.02	9.01	
HU2	6	c	0	1.10	32.09	2.54	0.0000
JEDRYC	61	m	0	1.86	32.56	7.49	0.0000
JEDRYC	66	f	0	1.93	9.94	3.00	0.0000
Subtotal JEDRYC				1.88	42.50	10.49	
JOLY	9	m	0	2.57	9.85	13.85	0.0000
JOLY	5	f	0	1.94	9.62	2.91	0.0000
Subtotal JOLY				2.26	19.47	16.76	
JUSSAW	14	m	0	2.53	5.71	7.50	0.0000
KHUDER	2	m	0	2.35	18.30	16.92	0.0000
KREUZE	21	m	3	2.97	4.24	10.66	0.0000
KREUZE	32	m	3	3.49	18.97	84.11	0.0000
KREUZE	27	f	3	2.49	2.00	2.45	0.0004
KREUZE	38	f	3	2.04	6.02	2.59	0.0000
Subtotal KREUZE				3.08	31.23	99.82	
KREYBE	22	m	0	1.85	5.33	1.17	0.0000
LAMTH	2	f	0	1.68	18.27	1.58	0.0000
LETOUR	3	c	0	2.85	18.55	39.85	0.0000

Table 1G3 - 5

IESLC - Meta-analysis of Ever Smoking, Amount smoked, "Mid", Any product (or Cigarettes if Any not available)
All LC types
Least adjusted

REF	NRR	SEX	AD	Ys	Ws	Qs	Ps
LIU2	6	m	0	1.68	7.60	0.68	0.0000
LIU3	4	m	0	0.02	2.75	5.12	0.9718
LIU4	8	m	2	1.28	4111.92	44.57	0.0000
LUBIN2	275	m	0	2.36	152.73	145.75	0.0000
LUBIN2	283	f	0	1.53	47.64	0.95	0.0000
Subtotal LUBIN2				2.16	200.37	146.70	
MACLEN	38	c	2	1.08	9.92	0.95	0.0007
MARTIS	2	m	0	1.88	3.20	0.78	0.0008
MATOS	30	m	0	1.98	7.91	2.77	0.0000
MATSUD	2	m	0	2.97	2.87	7.23	0.0000
MCCONN	25	c	0	0.31	5.38	6.24	0.4745
ORMOS	2	m	0	2.04	5.85	2.51	0.0000
PASTOR	3	m	0	2.07	7.55	3.54	0.0000
PERNU	21	m	0	2.28	42.95	34.74	0.0000
PERNU	15	f	0	-0.22	0.91	2.34	0.8339
Subtotal PERNU				2.23	43.86	37.08	
SIEMIA	14	m	0	1.50	3.87	0.05	0.0031
STOCKS	26	m	0	1.75	39.49	5.35	0.0000
TIZZAN	8	m	0	0.52	76.34	56.72	0.0000
WANG2	5	c	0	1.19	5.48	0.20	0.0052
WYNDE2	18	m	0	2.07	6.77	3.13	0.0000
WYNDE3	45	m	0	1.89	6.93	1.75	0.0000
WYNDE3	80	f	0	1.34	6.83	0.02	0.0005
Subtotal WYNDE3				1.61	13.76	1.77	
WYNDE4	45	m	0	2.08	9.99	4.77	0.0000
WYNDE4	59	f	2	1.87	3.72	0.88	0.0003
Subtotal WYNDE4				2.02	13.71	5.65	
XU3	7	m	0	2.77	3.66	7.05	0.0000
ZHENG	13	m	0	1.41	16.67	0.01	0.0000

	N	70
	NS	55
	Wt	5147.67
Het	Chi	744.10
Het	df	69
Het	P	***
Fixed	RR	4.00
	RRl	3.89
	RRu	4.11
	P	+++
Random	RR	6.19
	RRl	5.24
	RRu	7.30
	P	+++
Asymm	P	***

Table 1G3 - 6

IESLC - Meta-analysis of Ever Smoking, Amount smoked, "Mid", Any product (or Cigarettes if Any not available)
All LC types
Least adjusted

		Sex		
	combined	male	female	Total
N	7	46	17	70
NS	7	45	16	68
Wt	107.52	4864.04	176.11	5147.67
Het Chi	115.64	568.90	39.15	744.10
Het df	6	45	16	69
Het P	***	***	**	***
Fixed RR	4.52	3.94	5.49	4.00
RRl	3.74	3.83	4.74	3.89
RRu	5.46	4.05	6.36	4.11
P	+++	+++	+++	+++
Random RR	4.11	6.90	5.57	6.19
RRl	1.74	5.61	4.27	5.24
RRu	9.71	8.48	7.26	7.30
P	++	+++	+++	+++
Between Chi				20.41
Between df				2
Between P				***
Btwn(F) P				N.S.
Btwn(R) P				N.S.

Table 1G3 - 7

IESLC - Meta-analysis of Ever Smoking, Amount smoked, "Mid", Any product (or Cigarettes if Any not available)
All LC types
Excluded studies (and stage at which they were excluded)

1 ABELIN ABRAHA AMANDU AMES ANDERS AUSTIN AXELSO BAND BECHER BERRIN BLOHMK BLOT4 BROCKM BROWN1 BYERS1 BYERS2
CARPEN CASCO2 CASCOR CHAN CHEN3 CHIAZZ CHYOU DESTE2 DOCKER DROSTE DU GARCIA GARDIN GENG GODLEY GOODMA
GRAHAM GREGOR HEGMAN HEIN HENNEK HINDS HIRAOK HOROWI HORWIT HUANG ISHIMA JAHN JAIN JARVHO JIANG KELLER
KIHARA KJUUS KO KOHLME KUBIK LAMWK LAMWK2 LANGE LEI LEMARC LEVIN LIU LOMBA2 LOMBAR MAGNUS MARSH
MARSH2 MCDUFF MCLAUG MILLER MILLS NOTANI NOU ODRISC PAWLEG PERSHA POFFIJ QIAO QIAO2 RADZIK REN RONCO
ROOTS ROTHSC SAARIK SANKAR SCHWAR SEGI SEOW SHIMIZ SIMARA SIMONA SITAS SOBUE2 STASZE STAYNE STUCKE SUN
SUZUK2 SUZUKI TANG TAO TOKARS TOUSEY ULMER VEIERO VUTUC WALD WANG WANG3 WANG4 WICKLU WIGLE WILKIN
WU2 WUNSCH WYNDE8 XIANGZ XU XU2 XU4 YONG ZHANG

2 AKIBA ARCHER BENSHL BRETT BROSS CEDERL CHANG CHOW COMSTO DARBY DEAN3 DEKLER DORANT DORN ENGELA ENSTRO
GAO2 GILLIS HAENSZ HAMMO2 HIRAY2 HIRAYA HITOSU HOLE HUMBLE KAISE2 KAISER KANELL KATSOU KAUFMA KINLEN KNEKT
KOO LAURIL LIAW LICKIN LIDDEL MIGRAN MRFIT MRFITR MURATA NAM PARKIN PERSH2 PETO PEZZO2 PEZZOT PISANI
PRESCO RIMING SEGI2 SOBUE SPEIZE STOCKW SVENSS TANG2 TENKAN TSUGAN TULINI TVERDA WAKAI WARSIN WATSON WU
WYNDE5 WYNDER YAMAGU

3 BUELL CHEN MASTRA MZILEN RESTRE SADOWS

4 BEST BOFFET WYNDE7

6 BLOT1 BLOT2 BLOT3 BOUCHA JONES MOLLO SCHWA2 VANDER

8 CORREA LUO WYNDE6

10 AGUDO BOUCOT BROWN2 CHATZI COOKSO CPSI CPSII DEAN DEAN2 DORGAN GAO HANSEN JARUP KOULUM LAUSSM LIU5
LUBIN NOTAN2 OSANN OSANN2 PIKE POLEDN RACHTA RANDIG SHAW SPITZ WUWILL YUAN ZHOU

11 BENHAM

Table 1G3 - 8

Potentially overlapping studies

REF	REFGP	PRINC	.	OVERLAP/LINK
LUBIN2	LUBIN2	1		Lubin-combined
LAMTH	LAMTH	1		KOO/LAMTH/LAMWK
MATSUD	MATSUD	1		SOBUE2/MATSUD

Table 1G3 - 9

Most adjusted - insufficient data for metaanalysis

REF	NRR	SEX	AGEL	AGEH	RACE	YF	LC TYPE	LOC	START	ST	NLC	R	VB	P	H	AD	PRODUCT	exL	exH	DENOM	De
BLOT1	6	m	0	0	all	-	all	NAmer	1970	CC	458	n	bl	y	n	0	cig+/-ot	10	39	nev cigs	or
BUELL	2	m	0	0	all	0	all	NAmer	1957	pr	304	n	bl	n	n	0	cig+/-ot	19	21	nev cigs	st
RESTRE	7	c	0	0	all	-	all	SCAmer	1978	CC	102	n	bl	n	n	5	cig+/-ot	11	20	nev cigs	or
SADOWS	86	m	0	0	wh	-	all	NAmer	1938	CC	477	n	bl	n	n	1	all/unsp	11	20	nev any	st

REF	NRR	RR	SIG	RRDATA comment
BLOT1	6	4.50		0
BUELL	2	3.52		0
RESTRE	7	12.40		0
SADOWS	86	4.70		0

Least adjusted - insufficient data for meta-analysis: as for adjusted plus the following

REF	NRR	SEX	AGEL	AGEH	RACE	YF	LC TYPE	LOC	START	ST	NLC	R	VB	P	H	AD	PRODUCT	exL	exH	DENOM	De
SADOWS	81	m	0	0	wh	-	all	NAmer	1938	CC	477	n	bl	n	n	0	all/unsp	11	20	nev any	st

REF	NRR	RR	SIG	RRDATA comment
SADOWS	81	3.18		0

Table 1G4 -

IESLC - Meta-analysis of Ever Smoking, Amount smoked, "High", Any product (or Cigarettes if Any not available)
All LC types

This analysis is restricted to results for:
1) Results by Amount smoked
2) Ever smokers
3) Results complete enough for use in metaanalysis

Within each study, results are then selected (in the following order of preference, within each sex) for:
4) PRODUCT: all/unspec, cigarettes regardless of other products, cigarettes only
5) CIGTYPE: all/unspecified, MC regardless of HR, MC only
6) DENOM: never smoked anything, never smoked cigarettes, (never +1 = +long term ex, +2 = +amount unknown,
+3 = never cigs+long term ex)
7) Followup period (YF, prospective studies): whole study (coded as 0) or longest available
8) LCtype: all or nearest available, at least Squamous and Adeno. (q = squamous, s = small,
l = large, a = adeno, mix = mixed, alv = alveolar)
9) Race: all or nearest available, otherwise by race (wh or w = white, bl or b = black, hi = hispanic
ch = chinese, jap = japanese, haw = hawaiian, w+o = white + oriental, sca = scandinavian, as = asian)
10) Amount smoked "high" in key scheme 1 (key value 45, maximum range >20, in numbers of
cigarettes or cigarette equivalents)
11) For overlapping studies: principal rather than subsidiary studies
Finally by Age: whole study (coded as 0) if available, otherwise by widest available age group
and then for single sex results (m, f) in preference to combined sex results (c).

Results adjusted (AD) for the most potential confounders are then chosen in Sections -1 to -3
and results adjusted for the least confounders in Sections -4 to -6. (Those least adjusted results which
actually differ from the most adjusted as marked 'x' in column X in Section -4)
(Results adjusted for an unknown number of confounder(s) are coded as 20.)

Section -7 shows excluded studies, together with the stage (as above) at which no qualifying
results were found.

Section -8 lists the potentially overlapping studies which have been included (1=principal, 2=subsidiary).

Section -9 lists any results which would have been included in preference except that they had data not complete
enough for use in meta-analysis, with their significance (yes/no), if known, and any further comment as entered
on the database.

In addition to those mentioned above, the following fields, levels and abbreviations are used:
* or nk = not known, n = no, y = yes, ot = other
nev = never
all/unspec = all or unspecified, cig+/-ot = cigarettes irrespective of other products (cigar, pipe etc)
MC = manufactured cigarettes, HR = hand-rolled cigarettes
exL, exH = range of exposure (low and high) in the smoking group, in terms of Amount smoked,
cigarettes or cigarette equivalents
REF: 6-character study reference
NRR: number of the RR on the database within the study
ST : study type (CC = case control, pr or prosp = prospective)
NLC: number of lung cancer cases in whole study
R : risky occupational population (n = no, m = mining, o = other risky)
VB : national cigarette type (V = at least 75% Virginia, bl = at least 75% blended, ot = other)
P : any proxy use
H : full histological confirmation
De : derivation of RR/CI (or = original, st = standard method, ot = other method of estimation)

Table 1G4 - 1

IESLC - Meta-analysis of Ever Smoking, Amount smoked, "High", Any product (or Cigarettes if Any not available)
All LC types
Most adjusted

REF	NRR	SEX	AGEL	AGEH	RACE	YF	LC TYPE	LOC	START	ST	NLC	R	VB	P	H	AD	PRODUCT	exL	exH	DENOM	De
ALDERS	20	m	0	0	all	-	all	Eu:UK	1977	CC	1448	n	V	n	n	1	cig only	28	99	nev+2	ot
ALDERS	23	f	0	0	all	-	all	Eu:UK	1977	CC	1448	n	V	n	n	1	cig only	28	99	nev+2	ot
ARMADA	48	m	0	0	all	-	all	Eu:wst	1986	CC	325	n	bl	n	y	0	cig+/-ot	25	99	nev any	st
AUVINE	15	c	0	0	all	-	all	Eu:Sca	1986	CC	517	n	bl	y	n	2	cig+/-ot	21	99	nev cigs	or
AXELSS	16	f	0	0	sca	-	all	Eu:Sca	1989	CC	436	n	bl	n	n	0	all/unsp	30	99	nev any	st
BARBON	84	m	0	0	all	-	all	Eu:wst	1979	CC	755	n	bl	y	y	3	all/unsp	40	99	nev any	or
BOUCOT	100	m	0	0	all	9	all	NAmer	1951	pr	121	n	bl	n	n	0	cig+/-ot	21	99	nev any	ot
BRESLO	16	m	0	0	all	-	all	NAmer	1949	CC	518	n	bl	n	y	0	cig+/-ot	40	99	nev+3	st
BUFFLE	35	f	0	0	w-hi	-	all	NAmer	1976	CC	943	n	bl	y	n	0	cig+/-ot	21	99	nev cigs	or
CHEN2	6	m	0	0	all	-	all	As:Chi	1983	CC	193	n	ot	y	n	0	all/unsp	31	99	nev any	st
CHEN2	10	f	0	0	all	-	all	As:Chi	1983	CC	193	n	ot	y	n	0	all/unsp	31	99	nev any	st
CHOI	16	m	0	0	all	-	all	As:oth	1985	CC	375	n	bl	n	n	0	cig+/-ot	41	99	nev cigs	st
CHOI	20	f	0	0	all	-	all	As:oth	1985	CC	375	n	bl	n	n	0	cig+/-ot	31	99	nev cigs	st
CPSII	103	m	35	99	all	4	all	NAmer	1982	pr	3229	n	bl	n	n	1	cig only	21	99	nev any	ot
DAMBER	9	m	0	0	all	-	all	Eu:Sca	1972	CC	579	n	bl	y	n	1	cig only	26	99	nev any	ot
DAVEYS	4	m	0	0	all	-	all	Eu:Ger	1930	CC	109	n	bl	y	n	0	all/unsp	21	99	nev any	st
DEAN	2	m	0	0	wh	-	all	Africa	1947	CC	603	n	V	y	n	0	cig only	25	45	nev any	st
DEAN2	26	m	0	0	all	-	all	Eu:UK	1960	CC	954	n	V	y	n	0	cig only	23	99	nev any	st
DEAN2	30	f	0	0	all	-	all	Eu:UK	1960	CC	954	n	V	y	n	0	cig only	23	99	nev any	st
DESTEF	9	m	0	0	all	-	all	SCAmer	1988	CC	497	n	bl	n	y	4	all/unsp	41	99	nev any	or
DOLL	4	m	0	0	all	-	all	Eu:UK	1948	CC	1465	n	V	n	n	0	all/unsp	25	49	nev any	st
DOLL	10	f	0	0	all	-	all	Eu:UK	1948	CC	1465	n	V	n	n	0	all/unsp	25	49	nev any	ot
DOLL2	48	m	35	99	all	5	all	Eu:UK	1951	pr	920	n	V	n	n	1	all/unsp	25	99	nev any	ot
DOSEME	13	m	0	0	all	-	all	Eu:bal	1979	CC	1210	n	bl	n	n	2	cig+/-ot	21	99	nev cigs	or
DUNN	5	m	0	0	all	0	all	NAmer	1954	pr	139	o	bl	n	n	0	cig+/-ot	35	99	nev cigs	st
EBELIN	6	m	0	0	all	-	all	Eu:Ger	1980	CC	130	n	bl	n	n	0	all/unsp	40	99	nev any	st
ESAKI	3	m	0	0	all	-	all	As:Jap	1961	CC	245	n	bl	y	n	0	cig+/-ot	30	99	nev cigs	st
FAN	9	m	0	0	all	-	all	As:Chi	1990	CC	403	n	ot	y	n	0	cig+/-ot	30	99	nev cigs	st
FAN	13	f	0	0	all	-	all	As:Chi	1990	CC	403	n	ot	y	n	0	cig+/-ot	30	99	nev cigs	st
GARSHI	29	m	0	0	all	-	all	NAmer	1981	CC	1081	o	bl	y	n	1	all/unsp	36	99	nev any	st
GER	24	c	0	0	all	-	all	As:oth	1990	CC	141	n	ot	y	n	14	all/unsp	21	99	nev any	ot
GOLLED	3	m	35	99	all	-	all	Eu:UK	1952	CC	443	n	V	y	n	1	cig only	23	99	nev any	ot
GSELL	5	m	0	0	all	-	all	Eu:wst	1937	CC	150	n	bl	n	y	0	all/unsp	36	99	nev any	st
HAMMON	155	m	0	0	wh	0	all	NAmer	1952	pr	448	n	bl	n	n	1	cig only	21	99	nev any	ot
HU	3	m	0	0	all	-	all	As:Chi	1985	CC	227	n	ot	n	y	0	cig+/-ot	25	99	nev any	st
HU	6	f	0	0	all	-	all	As:Chi	1985	CC	227	n	ot	n	y	0	cig+/-ot	25	99	nev any	st
HU2	7	c	0	0	all	-	all	As:Chi	1977	CC	523	n	ot	y	n	0	cig+/-ot	30	99	nev cigs	st
JEDRYC	47	m	0	0	all	-	all	Eu:est	1980	CC	1630	n	bl	y	n	4	cig+/-ot	30	99	nev any	or
JEDRYC	50	f	0	0	all	-	all	Eu:est	1980	CC	1630	n	bl	y	n	4	cig+/-ot	30	99	nev any	or
JOLY	10	m	0	0	all	-	all	SCAmer	1978	CC	826	n	bl	n	n	0	cig+/-ot	30	99	nev any	st
JOLY	6	f	0	0	all	-	all	SCAmer	1978	CC	826	n	bl	n	n	0	cig+/-ot	30	99	nev any	st
JUSSAW	15	m	0	0	all	-	all	As:Ind	1964	CC	792	n	V	n	n	0	cig only	25	99	nev any	st
KHUDER	3	m	0	0	all	-	all	NAmer	1985	CC	482	n	bl	n	y	0	cig+/-ot	40	99	nev cigs	st
KREUZE	22	m	1	45	all	-	all	Eu:Ger	1990	CC	2260	n	bl	n	n	3	cig+/-ot	30	99	nev any	or
KREUZE	33	m	55	69	all	-	all	Eu:Ger	1990	CC	2260	n	bl	n	n	3	cig+/-ot	30	99	nev any	or
KREYBE	11	m	0	0	all	-	all	Eu:Sca	1948	CC	300	n	bl	n	y	1	all/unsp	25	99	nev any	ot
LAMTH	9	f	0	0	ch	-	all	As:HK	1983	CC	445	n	bl	n	n	0	all/unsp	21	99	nev any	or
LETOUR	4	c	0	0	all	-	all	NAmer	1983	CC	738	n	V	y	y	0	cig+/-ot	41	99	nev cigs	st
LIU2	10	m	0	0	all	-	all	As:Chi	1983	CC	316	n	ot	n	n	3	all/unsp	30	99	nev any	or
LIU3	8	m	0	0	all	-	all	As:Chi	1985	CC	110	n	ot	n	n	2	all/unsp	31	99	nev any	or
LIU4	9	m	35	69	all	-	all	As:Chi	1986	CC	1000-00	n	ot	y	n	2	cig only	21	99	nev any	ot
LUBIN2	276	m	0	0	all	-	all	Eu:mul	1976	CC	7804	n	bl	n	y	0	cig+/-ot	30	99	nev any	st
LUBIN2	284	f	0	0	all	-	all	Eu:mul	1976	CC	7804	n	bl	n	y	0	cig+/-ot	30	99	nev any	st
MACLEN	39	c	0	0	ch	-	all	As:oth	1972	CC	233	n	bl	n	n	2	cig+/-ot	30	99	nev cigs	or
MARTIS	3	m	0	0	all	-	all	Eu:UK	1972	CC	201	n	V	n	n	0	cig+/-ot	25	99	nev cigs	st
MATOS	33	m	0	0	all	-	all	SCAmer	1994	CC	200	n	bl	n	n	2	cig+/-ot	25	99	nev any	or
MATSUD	3	m	0	0	all	-	all	As:Jap	1965	CC	179	n	bl	n	n	0	cig+/-ot	21	99	nev cigs	st
MCCONN	24	c	0	0	all	-	all	Eu:UK	1946	CC	100	n	V	n	y	0	all/unsp	21	99	nev any	st
ORMOS	3	m	0	0	all	-	all	Eu:est	1947	CC	119	n	bl	y	y	0	cig+/-ot	31	99	nev any	st
OSANN	57	m	0	0	all	-	all	NAmer	1984	CC	1986	n	bl	n	n	2	cig+/-ot	40	99	nev cigs	or
OSANN	58	f	0	0	all	-	all	NAmer	1984	CC	1986	n	bl	n	n	2	cig+/-ot	40	99	nev cigs	or
PASTOR	9	m	0	0	all	-	all	Eu:wst	1976	CC	204	n	bl	y	n	1	all/unsp	30	99	nev any	st
PERNU	23	m	0	0	all	-	all	Eu:Sca	1944	CC	1606	n	bl	n	n	0	all/unsp	30	49	nev any	st
PERNU	16	f	0	0	all	-	all	Eu:Sca	1944	CC	1606	n	bl	n	n	0	all/unsp	25	99	nev any	st
PIKE	3	m	0	0	w-hi	-	all	NAmer	1972	CC	731	n	bl	y	n	0	all/unsp	41	99	nev any	st
PIKE	7	f	0	0	w-hi	-	all	NAmer	1972	CC	731	n	bl	y	n	0	all/unsp	41	99	nev any	st
SIEMIA	15	m	0	0	all	-	all	NAmer	1979	CC	857	n	V	y	y	0	cig+/-ot	40	99	nev cigs	or
STOCKS	45	m	0	0	all	-	all	Eu:UK	1952	CC	2932	n	V	y	n	2	cig+/-ot	37	99	nev any	st
TIZZAN	10	m	0	0	all	-	all	Eu:wst	1959	CC	1358	n	bl	n	n	0	cig only	41	99	nev any	st
WANG2	15	c	0	0	all	-	all	As:Chi	1980	CC	103	n	ot	n	n	4	cig+/-ot	40	99	nev cigs	ot
WYNDE2	20	m	0	0	all	-	all	NAmer	1962	CC	404	n	bl	n	y	0	cig+/-ot	35	99	nev any	st

Table 1G4 - 1

IESLC - Meta-analysis of Ever Smoking, Amount smoked, "High", Any product (or Cigarettes if Any not available)
All LC types
Most adjusted

REF	NRR	SEX	AGEL	AGEH	RACE	YF	LC TYPE	LOC	START	ST	NLC	R	VB	P	H	AD	PRODUCT	exL	exH	DENOM	De
WYNDE3	47	m	0	0	all	-	all	NAmer	1966	CC	350	n	bl	n	y	0	cig+/-ot	41	99	nev any	st
WYNDE3	82	f	0	0	all	-	all	NAmer	1966	CC	350	n	bl	n	y	0	cig+/-ot	41	99	nev any	st
WYNDE4	47	m	0	0	all	-	all	NAmer	1948	CC	684	n	bl	y	n	0	all/unsp	35	99	nev any	st
WYNDE4	61	f	0	0	all	-	all	NAmer	1948	CC	684	n	bl	y	n	2	all/unsp	35	99	nev any	ot
XU3	12	m	0	0	all	-	all	As:Chi	1981	CC	135	n	ot	n	n	1	all/unsp	30	99	nev any	ot
ZHENG	14	m	0	0	all	-	all	As:Chi	1982	CC	540	n	ot	*	y	0	cig+/-ot	30	99	nev cigs	st

Cigarette type is all/unspec for all RRs
except for the following:

REF	NRR	CIGTYPE
ALDERS	20	MC only
ALDERS	23	MC only
JUSSAW	15	MC only

Table 1G4 - 2

IESLC - Meta-analysis of Ever Smoking, Amount smoked, "High", Any product (or Cigarettes if Any not available)
All LC types
Most adjusted

REF	NRR	SEX	AD	Number Exposed Case	Number Exposed Cont	Non-exposed Case	Non-exposed Cont	RR	95.00%CI
ALDERS	20	m	1	-	-	-	-	8.52	(5.07- 14.33)
ALDERS	23	f	1	-	-	-	-	6.90	(4.69- 10.15)
Subtotal ALDERS								7.44	(5.46- 10.14)
ARMADA	48	m	0	139	32	4	64	69.50	(23.58- 204.81)
AUVINE	15	c	2	-	-	-	-	66.50	(25.80- 172.00)
AXELSS	16	f	0	6	7	18	154	7.33	(2.22- 24.22)
BARBON	84	m	3	-	-	-	-	21.30	(13.00- 36.00)
*BOUCOT	100	m	0	43	1519	0	805	46.12~	(2.84- 748.16)
BRESLO	16	m	0	80	22	22	110	18.18	(9.42- 35.09)
BUFFLE	35	f	0	141	62	12	112	21.23	(10.90- 41.32)
CHEN2	6	m	0	26	12	9	33	7.94	(2.91- 21.72)
CHEN2	10	f	0	4	2	25	33	2.64	(0.45- 15.58)
Subtotal CHEN2								6.08	(2.53- 14.58)
CHOI	16	m	0	16	6	13	95	19.49	(6.47- 58.71)
CHOI	20	f	0	3	1	76	164	6.47	(0.66- 63.26)
Subtotal CHOI								15.81	(5.86- 42.67)
*CPSII	103	m	1	-	-	-	-	17.60	(14.05- 22.05)
DAMBER	9	m	1	-	-	-	-	14.90	(6.70- 33.50)
DAVEYS	4	m	0	29	21	3	23	10.59	(2.81- 39.94)
DEAN	2	m	0	228	172	12	61	6.74	(3.52- 12.91)
DEAN2	26	m	0	252	112	33	112	7.64	(4.88- 11.95)
DEAN2	30	f	0	18	5	88	121	4.95	(1.77- 13.84)
Subtotal DEAN2								7.13	(4.73- 10.74)
DESTEF	9	m	4	-	-	-	-	23.70	(13.40- 42.10)
DOLL	4	m	0	293	154	7	61	16.58	(7.40- 37.13)
DOLL	10	f	0	14	0	40	59	42.60~	(2.47- 734.58)
Subtotal DOLL								17.78	(8.19- 38.62)
*DOLL2	48	m	1	-	-	-	-	23.71	(3.25- 173.24)
DOSEME	13	m	2	-	-	-	-	6.60	(4.40- 10.20)
*DUNN	5	m	0	13	3206	2	14160	28.71	(6.48- 127.15)
EBELIN	6	m	0	4	4	12	117	9.75	(2.16- 44.04)
ESAKI	3	m	0	34	10	16	28	5.95	(2.34- 15.16)
FAN	9	m	0	39	23	36	236	11.12	(5.96- 20.73)
FAN	13	f	0	4	1	69	320	18.55	(2.04- 168.54)
Subtotal FAN								11.54	(6.34- 21.03)
GARSHI	29	m	1	-	-	-	-	5.24	(3.61- 7.60)
GER	24	c	14	-	-	-	-	3.00	(1.40- 6.40)
GOLLED	3	m	1	-	-	-	-	18.28	(10.52- 31.74)
GSELL	5	m	0	51	13	2	29	56.88	(11.99- 269.87)
*HAMMON	155	m	1	-	-	-	-	17.06	(9.44- 30.82)
HU	3	m	0	29	13	41	67	3.65	(1.70- 7.80)
HU	6	f	0	1	2	40	48	0.60	(0.05- 6.86)
Subtotal HU								3.11	(1.50- 6.42)
HU2	7	c	0	64	19	121	213	5.93	(3.39- 10.37)
JEDRYC	47	m	4	-	-	-	-	7.69	(5.15- 11.47)
JEDRYC	50	f	4	-	-	-	-	7.37	(2.20- 24.69)
Subtotal JEDRYC								7.66	(5.24- 11.20)
JOLY	10	m	0	193	161	12	218	21.78	(11.74- 40.39)
JOLY	6	f	0	32	13	52	283	13.40	(6.59- 27.23)
Subtotal JOLY								17.66	(11.08- 28.14)
JUSSAW	15	m	0	48	4	149	624	50.26	(17.84- 141.55)
KHUDER	3	m	0	154	63	23	309	32.84	(19.62- 54.97)
KREUZE	22	m	3	-	-	-	-	20.80	(7.20- 60.50)
KREUZE	33	m	3	-	-	-	-	33.30	(20.50- 54.00)
Subtotal KREUZE								30.72	(19.77- 47.73)
KREYBE	11	m	1	-	-	-	-	14.31	(5.99- 34.17)
LAMTH	9	f	0	39	9	202	337	7.23	(3.43- 15.24)
LETOUR	4	c	0	65	23	24	224	26.38	(13.98- 49.78)
LIU2	10	m	3	-	-	-	-	21.40	(7.10- 64.00)
LIU3	8	m	2	-	-	-	-	1.91	(0.32- 11.40)
LIU4	9	m	2	-	-	-	-	6.98	(6.73- 7.23)
LUBIN2	276	m	0	1261	1394	190	2616	12.45	(10.55- 14.70)
LUBIN2	284	f	0	45	33	336	1188	4.82	(3.03- 7.68)
Subtotal LUBIN2								11.19	(9.57- 13.08)
MACLEN	39	c	2	-	-	-	-	4.10	(2.07- 8.15)
MARTIS	3	m	0	75	50	4	25	9.38	(3.08- 28.57)
MATOS	33	m	2	-	-	-	-	10.40	(5.30- 20.70)
MATSUD	3	m	0	58	470	3	1255	51.62	(16.10- 165.55)
MCCONN	24	c	0	35	42	9	23	2.13	(0.87- 5.19)
ORMOS	3	m	0	15	128	7	777	13.01	(5.20- 32.52)
OSANN	57	m	2	-	-	-	-	42.80	(30.50- 60.10)

Table 1G4 - 2

IESLC - Meta-analysis of Ever Smoking, Amount smoked, "High", Any product (or Cigarettes if Any not available)
All LC types
Most adjusted

REF	NRR	SEX	AD	Number Exposed Case	Number Exposed Cont	Non-exposed Case	Non-exposed Cont	RR	95.00%CI
OSANN	58	f	2	-	-	-	-	40.90	(29.30- 57.10)
Subtotal OSANN								41.82	(32.97- 53.05)
PASTOR	9	m	1	-	-	-	-	8.61	(3.86- 19.19)
PERNU	23	m	0	40	23	97	275	4.93	(2.81- 8.66)
PERNU	16	f	0	5	7	110	971	6.31	(1.97- 20.20)
Subtotal PERNU								5.17	(3.11- 8.57)
PIKE	3	m	0	66	37	18	69	6.84	(3.55- 13.18)
PIKE	7	f	0	16	3	36	96	14.22	(3.91- 51.73)
Subtotal PIKE								7.95	(4.43- 14.27)
SIEMIA	15	m	0	-	-	-	-	7.90	(3.00- 24.10)
STOCKS	45	m	2	-	-	-	-	13.91	(8.96- 21.60)
TIZZAN	10	m	0	83	20	180	305	7.03	(4.17- 11.85)
WANG2	15	c	4	-	-	-	-	7.25	(2.05- 25.65)
WYNDE2	20	m	0	155	112	8	105	18.16	(8.50- 38.80)
WYNDE3	47	m	0	68	26	9	88	25.57	(11.25- 58.14)
WYNDE3	82	f	0	4	3	20	76	5.07	(1.05- 24.50)
Subtotal WYNDE3								18.10	(8.73- 37.49)
WYNDE4	47	m	0	130	64	12	115	19.47	(10.00- 37.88)
WYNDE4	61	f	2	-	-	-	-	11.54	(1.90- 70.11)
Subtotal WYNDE4								18.28	(9.79- 34.14)
XU3	12	m	1	-	-	-	-	27.72	(5.15- 149.23)
ZHENG	14	m	0	66	23	33	94	8.17	(4.40- 15.17)
Partial Totals				4184	8126	2235	27298		

*prospective study ~ With 0.5 adjustment for zero

REF	NRR	SEX	AD	Ys	Ws	Qs	Ps
ALDERS	20	m	1	2.14	14.23	0.07	0.0000
ALDERS	23	f	1	1.93	25.78	0.52	0.0000
Subtotal ALDERS				2.01	40.01	0.59	
ARMADA	48	m	0	4.24	3.29	15.45	0.0000
AUVINE	15	c	2	4.20	4.27	19.25	0.0000
AXELSS	16	f	0	1.99	2.69	0.02	0.0011
BARBON	84	m	3	3.06	14.81	14.36	0.0000
*BOUCOT	100	m	0	3.83	0.49	1.53	0.0070
BRESLO	16	m	0	2.90	8.89	6.07	0.0000
BUFFLE	35	f	0	3.06	8.66	8.34	0.0000
CHEN2	6	m	0	2.07	3.80	0.00	0.0001
CHEN2	10	f	0	0.97	1.22	1.48	0.2838
Subtotal CHEN2				1.80	5.02	1.48	
CHOI	16	m	0	2.97	3.16	2.53	0.0000
CHOI	20	f	0	1.87	0.74	0.03	0.1083
Subtotal CHOI				2.76	3.90	2.57	
*CPSII	103	m	1	2.87	75.65	47.69	0.0000
DAMBER	9	m	1	2.70	5.93	2.34	0.0000
DAVEYS	4	m	0	2.36	2.18	0.18	0.0005
DEAN	2	m	0	1.91	9.10	0.25	0.0000
DEAN2	26	m	0	2.03	19.18	0.03	0.0000
DEAN2	30	f	0	1.60	3.63	0.82	0.0023
Subtotal DEAN2				1.96	22.82	0.85	
DESTEF	9	m	4	3.17	11.72	13.97	0.0000
DOLL	4	m	0	2.81	5.91	3.19	0.0000
DOLL	10	f	0	3.75	0.47	1.33	0.0098
Subtotal DOLL				2.88	6.39	4.52	
*DOLL2	48	m	1	3.17	0.97	1.16	0.0018
DOSEME	13	m	2	1.89	21.74	0.76	0.0000
*DUNN	5	m	0	3.36	1.73	2.86	0.0000
EBELIN	6	m	0	2.28	1.69	0.07	0.0031
ESAKI	3	m	0	1.78	4.39	0.37	0.0002
FAN	9	m	0	2.41	9.89	1.11	0.0000
FAN	13	f	0	2.92	0.79	0.57	0.0095
Subtotal FAN				2.45	10.68	1.67	
GARSHI	29	m	1	1.66	27.73	4.84	0.0000
GER	24	c	14	1.10	6.65	6.33	0.0046
GOLLED	3	m	1	2.91	12.60	8.72	0.0000
GSELL	5	m	0	4.04	1.58	6.13	0.0000
*HAMMON	155	m	1	2.84	10.98	6.39	0.0000
HU	3	m	0	1.29	6.63	4.04	0.0009
HU	6	f	0	-0.51	0.65	4.32	0.6812
Subtotal HU				1.13	7.28	8.36	
HU2	7	c	0	1.78	12.31	1.06	0.0000

Table 1G4 - 2

IESLC - Meta-analysis of Ever Smoking, Amount smoked, "High", Any product (or Cigarettes if Any not available)
All LC types
Most adjusted

REF	NRR	SEX	AD	Ys	Ws	Qs	Ps
JEDRYC	47	m	4	2.04	23.96	0.03	0.0000
JEDRYC	50	f	4	2.00	2.63	0.02	0.0012
Subtotal JEDRYC				2.04	26.59	0.04	
JOLY	10	m	0	3.08	10.07	10.21	0.0000
JOLY	6	f	0	2.59	7.64	2.07	0.0000
Subtotal JOLY				2.87	17.71	12.28	
JUSSAW	15	m	0	3.92	3.58	12.17	0.0000
KHUDER	3	m	0	3.49	14.48	29.10	0.0000
KREUZE	22	m	3	3.03	3.39	3.13	0.0000
KREUZE	33	m	3	3.51	16.38	33.57	0.0000
Subtotal KREUZE				3.42	19.77	36.70	
KREYBE	11	m	1	2.66	5.07	1.75	0.0000
LAMTH	9	f	0	1.98	6.91	0.06	0.0000
LETOUR	4	c	0	3.27	9.52	13.68	0.0000
LIU2	10	m	3	3.06	3.18	3.11	0.0000
LIU3	8	m	2	0.65	1.20	2.45	0.4778
LIU4	9	m	2	1.94	2991.95	51.26	0.0000
LUBIN2	276	m	0	2.52	139.75	28.07	0.0000
LUBIN2	284	f	0	1.57	17.75	4.45	0.0000
Subtotal LUBIN2				2.42	157.50	32.52	
MACLEN	39	c	2	1.41	8.18	3.60	0.0001
MARTIS	3	m	0	2.24	3.09	0.08	0.0001
MATOS	33	m	2	2.34	8.28	0.59	0.0000
MATSUD	3	m	0	3.94	2.83	9.89	0.0000
MCCONN	24	c	0	0.76	4.83	8.39	0.0966
ORMOS	3	m	0	2.57	4.57	1.11	0.0000
OSANN	57	m	2	3.76	33.40	94.56	0.0000
OSANN	58	f	2	3.71	34.52	92.52	0.0000
Subtotal OSANN				3.73	67.92	187.07	
PASTOR	9	m	1	2.15	5.97	0.04	0.0000
PERNU	23	m	0	1.60	12.13	2.78	0.0000
PERNU	16	f	0	1.84	2.83	0.15	0.0019
Subtotal PERNU				1.64	14.97	2.93	
PIKE	3	m	0	1.92	8.91	0.20	0.0000
PIKE	7	f	0	2.65	2.30	0.78	0.0001
Subtotal PIKE				2.07	11.21	0.98	
SIEMIA	15	m	0	2.07	3.54	0.00	0.0001
STOCKS	45	m	2	2.63	19.85	6.19	0.0000
TIZZAN	10	m	0	1.95	14.11	0.22	0.0000
WANG2	15	c	4	1.98	2.41	0.02	0.0021
WYNDE2	20	m	0	2.90	6.67	4.55	0.0000
WYNDE3	47	m	0	3.24	5.69	7.76	0.0000
WYNDE3	82	f	0	1.62	1.55	0.31	0.0436
Subtotal WYNDE3				2.90	7.24	8.08	
WYNDE4	47	m	0	2.97	8.67	6.94	0.0000
WYNDE4	61	f	2	2.45	1.18	0.16	0.0079
Subtotal WYNDE4				2.91	9.85	7.10	
XU3	12	m	1	3.32	1.36	2.11	0.0001
ZHENG	14	m	0	2.10	10.04	0.01	0.0000

	N	77
	NS	61
	Wt	3810.53
Het	Chi	626.25
Het	df	76
Het	P	***
Fixed	RR	7.96
	RRl	7.71
	RRu	8.21
	P	+++
Random	RR	11.96
	RRl	10.08
	RRu	14.18
	P	+++
Asymm	P	***

Table 1G4 - 3

IESLC - Meta-analysis of Ever Smoking, Amount smoked, "High", Any product (or Cigarettes if Any not available)
All LC types
Most adjusted

	Sex			
	combined	male	female	Total
N	7	52	18	77
NS	7	51	18	76
Wt	48.18	3640.42	121.94	3810.53
Het Chi	52.28	455.25	95.51	626.25
Het df	6	51	17	76
Het P	***	***	***	***
Fixed RR	7.69	7.85	12.22	7.96
RRl	5.80	7.60	10.23	7.71
RRu	10.20	8.11	14.59	8.21
P	+++	+++	+++	+++
Random RR	7.88	13.53	8.81	11.96
RRl	3.34	11.14	5.32	10.08
RRu	18.58	16.43	14.56	14.18
P	+++	+++	+++	+++
Between Chi				23.22
Between df				2
Between P				***
Btwn(F) P				N.S.
Btwn(R) P				N.S.

	Lung cancer type		
	all	other	Total
N	77		77
NS	61		61
Wt	3810.53		3810.53
Het Chi	626.25		626.25
Het df	76		76
Het P	***		***
Fixed RR	7.96		7.96
RRl	7.71		7.71
RRu	8.21		8.21
P	+++		+++
Random RR	11.96		11.96
RRl	10.08		10.08
RRu	14.18		14.18
P	+++		+++
Between Chi			
Between df			
Between P			N.S.
Btwn(F) P			N.S.
Btwn(R) P			N.S.

	Location								
	NAmer	UK	Scand	othEur	China	Japan	othAs	other	Total
N	19	11	6	15	13	2	6	5	77
NS	15	8	5	12	10	2	5	4	61
Wt	264.56	110.56	32.93	273.80	3045.43	7.22	29.23	46.81	3810.53
Het Chi	110.44	27.99	23.77	69.47	19.97	8.03	24.35	10.80	626.25
Het df	18	10	5	14	12	1	5	4	76
Het P	***	**	***	***	(*)	**	***	*	***
Fixed RR	19.71	9.25	10.48	11.55	6.99	13.87	7.11	14.35	7.96
RRl	17.48	7.68	7.45	10.26	6.74	6.69	4.95	10.78	7.71
RRu	22.24	11.15	14.75	13.01	7.24	28.76	10.21	19.12	8.21
P	+++	+++	+++	+++	+++	+++	+++	+++	+++
Random RR	18.00	9.21	12.14	12.47	7.18	17.02	8.84	13.93	11.96
RRl	12.76	6.47	5.55	8.89	5.41	2.05	3.76	8.68	10.08
RRu	25.40	13.13	26.55	17.47	9.53	141.34	20.76	22.35	14.18
P	+++	+++	+++	+++	+++	++	+++	+++	+++
Between Chi									331.42
Between df									7
Between P									***
Btwn(F) P									***
Btwn(R) P									**

Table 1G4 - 3

IESLC - Meta-analysis of Ever Smoking, Amount smoked, "High", Any product (or Cigarettes if Any not available)
All LC types
Most adjusted

	Detailed Country in "other Europe"					
	multi	Germany	othWest	East	Balkans	Total
N	2	4	5	3	1	15
NS	1	3	5	2	1	12
Wt	157.50	23.64	39.77	31.17	21.74	273.80
Het Chi	14.18	4.55	22.24	1.10	0.00	69.47
Het df	1	3	4	2	0	14
Het P	***	N.S.	***	N.S.	N.S.	***
Fixed RR	11.19	25.65	14.39	8.28	6.60	11.55
RRl	9.57	17.14	10.54	5.83	4.33	10.26
RRu	13.08	38.39	19.63	11.76	10.05	13.01
P	+++	+++	+++	+++	+++	+++
Random RR	7.95	21.24	19.09	8.28	6.60	12.47
RRl	3.14	11.59	8.48	5.83	4.33	8.89
RRu	20.13	38.91	43.01	11.76	10.05	17.47
P	+++	+++	+++	+++	+++	+++
Between Chi						27.40
Between df						4
Between P						***
Btwn(F) P						N.S.
Btwn(R) P						*

	Detailed Country in "other Asia"			
	India	HongKong	other	Total
N	1	1	4	6
NS	1	1	3	5
Wt	3.58	6.91	18.73	29.23
Het Chi	0.00	0.00	7.94	24.35
Het df	0	0	3	5
Het P	N.S.	N.S.	*	***
Fixed RR	50.26	7.23	4.86	7.11
RRl	17.84	3.43	3.09	4.95
RRu	141.55	15.24	7.64	10.21
P	+++	+++	+++	+++
Random RR	50.26	7.23	5.69	8.84
RRl	17.84	3.43	2.50	3.76
RRu	141.55	15.24	12.97	20.76
P	+++	+++	+++	+++
Between Chi				16.42
Between df				2
Between P				***
Btwn(F) P				N.S.
Btwn(R) P				**

	Detailed other continent			
	SCAmer	Auslia	Africa	Total
N	4		1	5
NS	3		1	4
Wt	37.71		9.10	46.81
Het Chi	4.34		0.00	10.80
Het df	3		0	4
Het P	N.S.		N.S.	*
Fixed RR	17.23		6.74	14.35
RRl	12.52		3.52	10.78
RRu	23.71		12.91	19.12
P	+++		+++	+++
Random RR	16.96		6.74	13.93
RRl	11.53		3.52	8.68
RRu	24.93		12.91	22.35
P	+++		+++	+++
Between Chi				6.46
Between df				1
Between P				*
Btwn(F) P				N.S.
Btwn(R) P				*

Table 1G4 - 3

IESLC - Meta-analysis of Ever Smoking, Amount smoked, "High", Any product (or Cigarettes if Any not available)
All LC types
Most adjusted

	Start year of study <1960	1960-69	1970-79	1980-89	1990+	Total
N	20	8	17	26	6	77
NS	17	6	13	21	4	61
Wt	128.15	47.53	310.67	3278.80	45.38	3810.53
Het Chi	47.77	27.86	56.45	393.44	29.92	626.25
Het df	19	7	16	25	5	76
Het P	***	***	***	***	***	***
Fixed RR	11.54	12.01	10.29	7.55	14.24	7.96
RRl	9.71	9.04	9.20	7.29	10.64	7.71
RRu	13.72	15.95	11.50	7.81	19.05	8.21
P	+++	+++	+++	+++	+++	+++
Random RR	12.02	13.96	9.60	12.74	12.41	11.96
RRl	8.85	7.50	7.48	8.80	5.69	10.08
RRu	16.31	26.00	12.33	18.45	27.06	14.18
P	+++	+++	+++	+++	+++	+++
Between Chi						70.82
Between df						4
Between P						***
Btwn(F) P						(*)
Btwn(R) P						N.S.

	Study type (1) CC	other	Total
N	72	5	77
NS	56	5	61
Wt	3720.71	89.83	3810.53
Het Chi	565.22	0.95	626.25
Het df	71	4	76
Het P	***	N.S.	***
Fixed RR	7.80	17.85	7.96
RRl	7.56	14.52	7.71
RRu	8.06	21.95	8.21
P	+++	+++	+++
Random RR	11.61	17.85	11.96
RRl	9.74	14.52	10.08
RRu	13.83	21.95	14.18
P	+++	+++	+++
Between Chi			60.08
Between df			1
Between P			***
Btwn(F) P			**
Btwn(R) P			**

	Study type (2) CC	prosp	other	Total
N	72	5		77
NS	56	5		61
Wt	3720.71	89.83		3810.53
Het Chi	565.22	0.95		626.25
Het df	71	4		76
Het P	***	N.S.		***
Fixed RR	7.80	17.85		7.96
RRl	7.56	14.52		7.71
RRu	8.06	21.95		8.21
P	+++	+++		+++
Random RR	11.61	17.85		11.96
RRl	9.74	14.52		10.08
RRu	13.83	21.95		14.18
P	+++	+++		+++
Between Chi				60.08
Between df				1
Between P				***
Btwn(F) P				**
Btwn(R) P				**

Table 1G4 - 3

IESLC - Meta-analysis of Ever Smoking, Amount smoked, "High", Any product (or Cigarettes if Any not available)
All LC types
Most adjusted

	Study size (number of LC cases) 100-249	250-499	500-999	1000+	Total
N	21	16	20	20	77
NS	19	13	16	13	61
Wt	73.76	99.40	153.22	3484.16	3810.53
Het Chi	54.21	26.70	61.01	368.90	626.25
Het df	20	15	19	19	76
Het P	***	*	***	***	***
Fixed RR	6.94	18.27	13.17	7.62	7.96
RRl	5.52	15.01	11.24	7.37	7.71
RRu	8.72	22.24	15.43	7.88	8.21
P	+++	+++	+++	+++	+++
Random RR	7.77	17.56	14.00	11.19	11.96
RRl	5.20	13.24	10.38	8.25	10.08
RRu	11.62	23.27	18.89	15.17	14.18
P	+++	+++	+++	+++	+++
Between Chi					115.44
Between df					3
Between P					***
Btwn(F) P					**
Btwn(R) P					**

	Risky occupational population no	mining	othRisky	Total
N	75		2	77
NS	59		2	61
Wt	3781.07		29.46	3810.53
Het Chi	618.54		4.72	626.25
Het df	74		1	76
Het P	***		*	***
Fixed RR	7.98		5.79	7.96
RRl	7.73		4.04	7.71
RRu	8.23		8.31	8.21
P	+++		+++	+++
Random RR	12.05		10.46	11.96
RRl	10.13		2.03	10.08
RRu	14.35		53.80	14.18
P	+++		++	+++
Between Chi				2.99
Between df				1
Between P				(*)
Btwn(F) P				N.S.
Btwn(R) P				N.S.

	National cigarette tobacco type Virginia	blended	other	Total
N	15	48	14	77
NS	12	38	11	61
Wt	136.30	622.15	3052.08	3810.53
Het Chi	48.54	272.17	24.72	626.25
Het df	14	47	13	76
Het P	***	***	*	***
Fixed RR	10.15	14.41	6.97	7.96
RRl	8.58	13.32	6.73	7.71
RRu	12.00	15.59	7.22	8.21
P	+++	+++	+++	+++
Random RR	10.66	14.32	6.65	11.96
RRl	7.54	11.54	4.97	10.08
RRu	15.07	17.77	8.89	14.18
P	+++	+++	+++	+++
Between Chi				280.83
Between df				2
Between P				***
Btwn(F) P				***
Btwn(R) P				***

Table 1G4 - 3

IESLC - Meta-analysis of Ever Smoking, Amount smoked, "High", Any product (or Cigarettes if Any not available)
All LC types
Most adjusted

	Any proxy use No/nk	Yes	Total
N	48	29	77
NS	38	23	61
Wt	580.32	3230.21	3810.53
Het Chi	280.50	115.36	626.25
Het df	47	28	76
Het P	***	***	***
Fixed RR	14.21	7.17	7.96
RRl	13.10	6.93	7.71
RRu	15.42	7.42	8.21
P	+++	+++	+++
Random RR	13.26	10.15	11.96
RRl	10.52	8.18	10.08
RRu	16.73	12.58	14.18
P	+++	+++	+++
Between Chi			230.39
Between df			1
Between P			***
Btwn(F) P			***
Btwn(R) P			(*)

	Full histological confirmation No	Yes	Total
N	58	19	77
NS	45	16	61
Wt	3539.49	271.04	3810.53
Het Chi	457.63	96.92	626.25
Het df	57	18	76
Het P	***	***	***
Fixed RR	7.66	13.06	7.96
RRl	7.41	11.60	7.71
RRu	7.92	14.71	8.21
P	+++	+++	+++
Random RR	11.57	13.15	11.96
RRl	9.46	9.30	10.08
RRu	14.15	18.58	14.18
P	+++	+++	+++
Between Chi			71.70
Between df			1
Between P			***
Btwn(F) P			**
Btwn(R) P			N.S.

	Number of adjustment variables (1) 0	1	2+/+nk	Total
N	47	11	19	77
NS	36	10	16	62
Wt	414.57	186.27	3209.70	3810.53
Het Chi	155.27	45.76	331.10	626.25
Het df	46	10	18	76
Het P	***	***	***	***
Fixed RR	11.24	11.87	7.43	7.96
RRl	10.21	10.28	7.18	7.71
RRu	12.38	13.71	7.70	8.21
P	+++	+++	+++	+++
Random RR	11.27	11.74	13.19	11.96
RRl	9.13	8.15	8.58	10.08
RRu	13.90	16.90	20.29	14.18
P	+++	+++	+++	+++
Between Chi				94.13
Between df				2
Between P				***
Btwn(F) P				**
Btwn(R) P				N.S.

Table 1G4 - 3

IESLC - Meta-analysis of Ever Smoking, Amount smoked, "High", Any product (or Cigarettes if Any not available)
All LC types
Most adjusted

	Number of adjustment variables (2) 0	1	2	3-5	6+/+nk	Total
N	47	11	10	8	1	77
NS	36	10	9	6	1	62
Wt	414.57	186.27	3124.56	78.48	6.65	3810.53
Het Chi	155.27	45.76	248.47	28.26	0.00	626.25
Het df	46	10	9	7	0	76
Het P	***	***	***	***	N.S.	***
Fixed RR	11.24	11.87	7.30	16.24	3.00	7.96
RRl	10.21	10.28	7.05	13.02	1.40	7.71
RRu	12.38	13.71	7.56	20.26	6.41	8.21
P	+++	+++	+++	+++	++	+++
Random RR	11.27	11.74	13.12	16.28	3.00	11.96
RRl	9.13	8.15	6.86	9.94	1.40	10.08
RRu	13.90	16.90	25.09	26.66	6.41	14.18
P	+++	+++	+++	+++	++	+++
Between Chi						148.50
Between df						4
Between P						***
Btwn(F) P						***
Btwn(R) P						**

	Product all/unsp	cig+/-ot	cig only	Total
N	26	39	12	77
NS	21	30	10	61
Wt	145.99	477.82	3186.73	3810.53
Het Chi	79.44	235.87	100.14	626.25
Het df	25	38	11	76
Het P	***	***	***	***
Fixed RR	9.08	14.65	7.22	7.96
RRl	7.72	13.39	6.97	7.71
RRu	10.68	16.02	7.47	8.21
P	+++	+++	+++	+++
Random RR	9.61	14.12	10.53	11.96
RRl	6.96	10.92	7.64	10.08
RRu	13.26	18.26	14.53	14.18
P	+++	+++	+++	+++
Between Chi				210.79
Between df				2
Between P				***
Btwn(F) P				***
Btwn(R) P				N.S.

	Denominator nev any	nev cigs	Total
N	56	21	77
NS	43	18	61
Wt	3611.96	198.58	3810.53
Het Chi	331.87	141.08	626.25
Het df	55	20	76
Het P	***	***	***
Fixed RR	7.59	18.72	7.96
RRl	7.35	16.29	7.71
RRu	7.84	21.51	8.21
P	+++	+++	+++
Random RR	10.78	15.45	11.96
RRl	9.07	10.32	10.08
RRu	12.82	23.14	14.18
P	+++	+++	+++
Between Chi			153.30
Between df			1
Between P			***
Btwn(F) P			***
Btwn(R) P			N.S.

Table 1G4 - 3

IESLC - Meta-analysis of Ever Smoking, Amount smoked, "High", Any product (or Cigarettes if Any not available)
All LC types
Most adjusted

	Derivation of RR/CI Orig	StdCalc	Other	Total
N	17	45	15	77
NS	14	35	14	63
Wt	206.77	448.03	3155.73	3810.53
Het Chi	139.11	164.64	100.81	626.25
Het df	16	44	14	76
Het P	***	***	***	***
Fixed RR	18.71	10.72	7.21	7.96
RRl	16.33	9.78	6.96	7.71
RRu	21.44	11.76	7.47	8.21
P	+++	+++	+++	+++
Random RR	15.07	10.85	11.26	11.96
RRl	9.80	8.80	7.89	10.08
RRu	23.17	13.38	16.05	14.18
P	+++	+++	+++	+++
Between Chi				221.70
Between df				2
Between P				***
Btwn(F) P				***
Btwn(R) P				N.S.

	Study LIU4 LIU4	others	Total
N	1	76	77
NS	1	60	61
Wt	2991.95	818.58	3810.53
Het Chi	0.00	387.62	626.25
Het df	0	75	76
Het P	N.S.	***	***
Fixed RR	6.98	12.84	7.96
RRl	6.73	11.99	7.71
RRu	7.23	13.75	8.21
P	+++	+++	+++
Random RR	6.98	12.08	11.96
RRl	6.73	10.13	10.08
RRu	7.23	14.41	14.18
P	+++	+++	+++
Between Chi			238.63
Between df			1
Between P			***
Btwn(F) P			***
Btwn(R) P			***

Table 1G4 - 4

IESLC - Meta-analysis of Ever Smoking, Amount smoked, "High", Any product (or Cigarettes if Any not available)
All LC types
Least adjusted

REF	NRR	X	SEX	AGEL	AGEH	RACE	YF	LC TYPE	LOC	START	ST	NLC	R	VB	P	H	AD	PRODUCT	exL	exH	DENOM	De
ALDERS	20		m	0	0	all	-	all	Eu:UK	1977	CC	1448	n	V	n	n	1	cig only	28	99	nev+2	ot
ALDERS	23		f	0	0	all	-	all	Eu:UK	1977	CC	1448	n	V	n	n	1	cig only	28	99	nev+2	ot
ARMADA	48		m	0	0	all	-	all	Eu:wst	1986	CC	325	n	bl	n	y	0	cig+/-ot	25	99	nev any	st
AUVINE	7	x	c	0	0	all	-	all	Eu:Sca	1986	CC	517	n	bl	y	n	0	cig+/-ot	21	99	nev cigs	st
AXELSS	16		f	0	0	sca	-	all	Eu:Sca	1989	CC	436	n	bl	n	n	0	all/unsp	30	99	nev any	st
BARBON	13	x	m	0	0	all	-	all	Eu:wst	1979	CC	755	n	bl	y	y	0	all/unsp	40	99	nev any	st
BOUCOT	100		m	0	0	all	9	all	NAmer	1951	pr	121	n	bl	n	n	0	cig+/-ot	21	99	nev any	ot
BRESLO	16		m	0	0	all	-	all	NAmer	1949	CC	518	n	bl	n	y	0	cig+/-ot	40	99	nev+3	st
BUFFLE	35		f	0	0	w-hi	-	all	NAmer	1976	CC	943	n	bl	y	n	0	cig+/-ot	21	99	nev cigs	or
CHEN2	6		m	0	0	all	-	all	As:Chi	1983	CC	193	n	ot	y	n	0	all/unsp	31	99	nev any	st
CHEN2	10		f	0	0	all	-	all	As:Chi	1983	CC	193	n	ot	y	n	0	all/unsp	31	99	nev any	st
CHOI	16		m	0	0	all	-	all	As:oth	1985	CC	375	n	bl	n	n	0	cig+/-ot	41	99	nev cigs	st
CHOI	20		f	0	0	all	-	all	As:oth	1985	CC	375	n	bl	n	n	0	cig+/-ot	31	99	nev cigs	st
CPSII	103		m	35	99	all	4	all	NAmer	1982	pr	3229	n	bl	n	n	1	cig only	21	99	nev any	ot
DAMBER	9		m	0	0	all	-	all	Eu:Sca	1972	CC	579	n	bl	y	n	1	cig only	26	99	nev any	ot
DAVEYS	4		m	0	0	all	-	all	Eu:Ger	1930	CC	109	n	bl	y	n	0	all/unsp	21	99	nev any	st
DEAN	2		m	0	0	wh	-	all	Africa	1947	CC	603	n	V	y	n	0	cig only	25	45	nev any	st
DEAN2	26		m	0	0	all	-	all	Eu:UK	1960	CC	954	n	V	y	n	0	cig only	23	99	nev any	st
DEAN2	30		f	0	0	all	-	all	Eu:UK	1960	CC	954	n	V	y	n	0	cig only	23	99	nev any	st
DESTEF	4	x	m	0	0	all	-	all	SCAmer	1988	CC	497	n	bl	n	y	0	all/unsp	41	99	nev any	st
DOLL	4		m	0	0	all	-	all	Eu:UK	1948	CC	1465	n	V	n	n	0	all/unsp	25	49	nev any	st
DOLL	10		f	0	0	all	-	all	Eu:UK	1948	CC	1465	n	V	n	n	0	all/unsp	25	49	nev any	st
DOLL2	48		m	35	99	all	5	all	Eu:UK	1951	pr	920	n	V	n	n	1	all/unsp	25	99	nev any	ot
DOSEME	13		m	0	0	all	-	all	Eu:bal	1979	CC	1210	n	bl	n	n	2	cig+/-ot	21	99	nev cigs	or
DUNN	5		m	0	0	all	0	all	NAmer	1954	pr	139	o	bl	n	n	0	cig+/-ot	35	99	nev cigs	st
EBELIN	6		m	0	0	all	-	all	Eu:Ger	1980	CC	130	n	bl	n	n	0	all/unsp	40	99	nev any	st
ESAKI	3		m	0	0	all	-	all	As:Jap	1961	CC	245	n	bl	y	n	0	cig+/-ot	30	99	nev cigs	st
FAN	9		m	0	0	all	-	all	As:Chi	1990	CC	403	n	ot	y	n	0	cig+/-ot	30	99	nev cigs	st
FAN	13		f	0	0	all	-	all	As:Chi	1990	CC	403	n	ot	y	n	0	cig+/-ot	30	99	nev cigs	st
GARSHI	21	x	m	0	0	all	-	all	NAmer	1981	CC	1081	o	bl	y	n	0	all/unsp	36	99	nev any	st
GER	20	x	c	0	0	all	-	all	As:oth	1990	CC	141	n	ot	y	n	0	all/unsp	21	99	nev any	st
GOLLED	17	x	m	35	99	all	-	all	Eu:UK	1952	CC	443	n	V	y	n	0	cig only	23	99	nev any	st
GSELL	5		m	0	0	all	-	all	Eu:wst	1937	CC	150	n	bl	n	y	0	all/unsp	36	99	nev any	st
HAMMON	155		m	0	0	wh	0	all	NAmer	1952	pr	448	n	bl	n	n	1	cig only	21	99	nev any	ot
HU	3		m	0	0	all	-	all	As:Chi	1985	CC	227	n	ot	n	y	0	cig+/-ot	25	99	nev any	st
HU	6		f	0	0	all	-	all	As:Chi	1985	CC	227	n	ot	n	y	0	cig+/-ot	25	99	nev any	st
HU2	7		c	0	0	all	-	all	As:Chi	1977	CC	523	n	ot	y	n	0	cig+/-ot	30	99	nev cigs	st
JEDRYC	62	x	m	0	0	all	-	all	Eu:est	1980	CC	1630	n	bl	y	n	0	cig+/-ot	30	99	nev any	st
JEDRYC	67	x	f	0	0	all	-	all	Eu:est	1980	CC	1630	n	bl	y	n	0	cig+/-ot	30	99	nev any	st
JOLY	10		m	0	0	all	-	all	SCAmer	1978	CC	826	n	bl	n	n	0	cig+/-ot	30	99	nev any	st
JOLY	6		f	0	0	all	-	all	SCAmer	1978	CC	826	n	bl	n	n	0	cig+/-ot	30	99	nev any	st
JUSSAW	15		m	0	0	all	-	all	As:Ind	1964	CC	792	n	V	n	n	0	cig only	25	99	nev any	st
KHUDER	3		m	0	0	all	-	all	NAmer	1985	CC	482	n	bl	n	y	0	cig+/-ot	40	99	nev cigs	st
KREUZE	22		m	1	45	all	-	all	Eu:Ger	1990	CC	2260	n	bl	n	n	3	cig+/-ot	30	99	nev any	or
KREUZE	33		m	55	69	all	-	all	Eu:Ger	1990	CC	2260	n	bl	n	n	3	cig+/-ot	30	99	nev any	or
KREYBE	23	x	m	0	0	all	-	all	Eu:Sca	1948	CC	300	n	bl	n	y	0	all/unsp	25	99	nev any	st
LAMTH	9		f	0	0	ch	-	all	As:HK	1983	CC	445	n	bl	n	n	0	all/unsp	21	99	nev any	or
LETOUR	4		c	0	0	all	-	all	NAmer	1983	CC	738	n	V	y	y	0	cig+/-ot	41	99	nev cigs	st
LIU2	7	x	m	0	0	all	-	all	As:Chi	1983	CC	316	n	ot	n	n	0	all/unsp	30	99	nev any	st
LIU3	5	x	m	0	0	all	-	all	As:Chi	1985	CC	110	n	ot	n	n	0	all/unsp	31	99	nev any	st
LIU4	9		m	35	69	all	-	all	As:Chi	1986	CC	1000-00	n	ot	y	n	2	cig only	21	99	nev any	ot
LUBIN2	276		m	0	0	all	-	all	Eu:mul	1976	CC	7804	n	bl	n	y	0	cig+/-ot	30	99	nev any	st
LUBIN2	284		f	0	0	all	-	all	Eu:mul	1976	CC	7804	n	bl	n	y	0	cig+/-ot	30	99	nev any	st
MACLEN	39		c	0	0	ch	-	all	As:oth	1972	CC	233	n	bl	n	n	2	cig+/-ot	30	99	nev cigs	or
MARTIS	3		m	0	0	all	-	all	Eu:UK	1972	CC	201	n	V	n	n	0	cig+/-ot	25	99	nev cigs	st
MATOS	32	x	m	0	0	all	-	all	SCAmer	1994	CC	200	n	bl	n	n	0	cig+/-ot	25	99	nev any	st
MATSUD	3		m	0	0	all	-	all	As:Jap	1965	CC	179	n	bl	n	n	0	cig+/-ot	21	99	nev cigs	st
MCCONN	24		c	0	0	all	-	all	Eu:UK	1946	CC	100	n	V	n	y	0	all/unsp	21	99	nev any	st
ORMOS	3		m	0	0	all	-	all	Eu:est	1947	CC	119	n	bl	y	y	0	cig+/-ot	31	99	nev any	st
OSANN	57		m	0	0	all	-	all	NAmer	1984	CC	1986	n	bl	n	n	2	cig+/-ot	40	99	nev cigs	or
OSANN	58		f	0	0	all	-	all	NAmer	1984	CC	1986	n	bl	n	n	2	cig+/-ot	40	99	nev cigs	or
PASTOR	4	x	m	0	0	all	-	all	Eu:wst	1976	CC	204	n	bl	y	n	0	all/unsp	30	99	nev any	st
PERNU	23		m	0	0	all	-	all	Eu:Sca	1944	CC	1606	n	bl	n	n	0	all/unsp	30	49	nev any	st
PERNU	16		f	0	0	all	-	all	Eu:Sca	1944	CC	1606	n	bl	n	n	0	all/unsp	25	99	nev any	st
PIKE	3		m	0	0	w-hi	-	all	NAmer	1972	CC	731	n	bl	y	n	0	all/unsp	41	99	nev any	st
PIKE	7		f	0	0	w-hi	-	all	NAmer	1972	CC	731	n	bl	y	n	0	all/unsp	41	99	nev any	st
SIEMIA	15		m	0	0	all	-	all	NAmer	1979	CC	857	n	V	y	y	0	cig+/-ot	40	99	nev cigs	or
STOCKS	29	x	m	0	0	all	-	all	Eu:UK	1952	CC	2932	n	V	y	n	0	cig+/-ot	37	99	nev any	st
TIZZAN	10		m	0	0	all	-	all	Eu:wst	1959	CC	1358	n	bl	n	n	0	cig only	41	99	nev any	st
WANG2	7	x	c	0	0	all	-	all	As:Chi	1980	CC	103	n	ot	n	n	0	cig+/-ot	40	99	nev cigs	st
WYNDE2	20		m	0	0	all	-	all	NAmer	1962	CC	404	n	bl	n	y	0	cig+/-ot	35	99	nev any	st

Table 1G4 - 4

IESLC - Meta-analysis of Ever Smoking, Amount smoked, "High", Any product (or Cigarettes if Any not available)
All LC types
Least adjusted

REF	NRR	X	SEX	AGEL	AGEH	RACE	YF	LC TYPE	LOC	START	ST	NLC	R	VB	P	H	AD	PRODUCT	exL	exH	DENOM	De
WYNDE3	47		m	0	0	all	-	all	NAmer	1966	CC	350	n	bl	n	y	0	cig+/-ot	41	99	nev any	st
WYNDE3	82		f	0	0	all	-	all	NAmer	1966	CC	350	n	bl	n	y	0	cig+/-ot	41	99	nev any	st
WYNDE4	47		m	0	0	all	-	all	NAmer	1948	CC	684	n	bl	y	n	0	all/unsp	35	99	nev any	st
WYNDE4	61		f	0	0	all	-	all	NAmer	1948	CC	684	n	bl	y	n	2	all/unsp	35	99	nev any	ot
XU3	8	x	m	0	0	all	-	all	As:Chi	1981	CC	135	n	ot	n	n	0	all/unsp	30	99	nev any	st
ZHENG	14		m	0	0	all	-	all	As:Chi	1982	CC	540	n	ot	*	y	0	cig+/-ot	30	99	nev cigs	st

Cigarette type is all/unspec for all RRs
except for the following:

REF	NRR	CIGTYPE
ALDERS	20	MC only
ALDERS	23	MC only
JUSSAW	15	MC only

Table 1G4 - 5

IESLC - Meta-analysis of Ever Smoking, Amount smoked, "High", Any product (or Cigarettes if Any not available)
All LC types
Least adjusted

REF	NRR	SEX	AD	Number Exposed Case	Number Exposed Cont	Non-exposed Case	Non-exposed Cont	RR	95.00%CI
ALDERS	20	m	1	-	-	-	-	8.52	(5.07- 14.33)
ALDERS	23	f	1	-	-	-	-	6.90	(4.69- 10.15)
Subtotal ALDERS								7.44	(5.46- 10.14)
ARMADA	48	m	0	139	32	4	64	69.50	(23.58- 204.81)
AUVINE	7	c	0	61	8	44	229	39.68	(17.75- 88.72)
AXELSS	16	f	0	6	7	18	154	7.33	(2.22- 24.22)
BARBON	13	m	0	227	111	22	188	17.48	(10.64- 28.71)
*BOUCOT	100	m	0	43	1519	0	805	46.12~	(2.84- 748.16)
BRESLO	16	m	0	80	22	22	110	18.18	(9.42- 35.09)
BUFFLE	35	f	0	141	62	12	112	21.23	(10.90- 41.32)
CHEN2	6	m	0	26	12	9	33	7.94	(2.91- 21.72)
CHEN2	10	f	0	4	2	25	33	2.64	(0.45- 15.58)
Subtotal CHEN2								6.08	(2.53- 14.58)
CHOI	16	m	0	16	6	13	95	19.49	(6.47- 58.71)
CHOI	20	f	0	3	1	76	164	6.47	(0.66- 63.26)
Subtotal CHOI								15.81	(5.86- 42.67)
*CPSII	103	m	1	-	-	-	-	17.60	(14.05- 22.05)
DAMBER	9	m	1	-	-	-	-	14.90	(6.70- 33.50)
DAVEYS	4	m	0	29	21	3	23	10.59	(2.81- 39.94)
DEAN	2	m	0	228	172	12	61	6.74	(3.52- 12.91)
DEAN2	26	m	0	252	112	33	112	7.64	(4.88- 11.95)
DEAN2	30	f	0	18	5	88	121	4.95	(1.77- 13.84)
Subtotal DEAN2								7.13	(4.73- 10.74)
DESTEF	4	m	0	116	31	27	163	22.59	(12.80- 39.87)
DOLL	4	m	0	293	154	7	61	16.58	(7.40- 37.13)
DOLL	10	f	0	14	0	40	59	42.60~	(2.47- 734.58)
Subtotal DOLL								17.78	(8.19- 38.62)
*DOLL2	48	m	1	-	-	-	-	23.71	(3.25- 173.24)
DOSEME	13	m	2	-	-	-	-	6.60	(4.40- 10.20)
*DUNN	5	m	0	13	3206	2	14160	28.71	(6.48- 127.15)
EBELIN	6	m	0	4	4	12	117	9.75	(2.16- 44.04)
ESAKI	3	m	0	34	10	16	28	5.95	(2.34- 15.16)
FAN	9	m	0	39	23	36	236	11.12	(5.96- 20.73)
FAN	13	f	0	4	1	69	320	18.55	(2.04- 168.54)
Subtotal FAN								11.54	(6.34- 21.03)
GARSHI	21	m	0	223	350	41	363	5.64	(3.92- 8.12)
GER	20	c	0	24	42	51	246	2.76	(1.54- 4.95)
GOLLED	17	m	0	143	333	15	490	14.03	(8.09- 24.31)
GSELL	5	m	0	51	13	2	29	56.88	(11.99- 269.87)
*HAMMON	155	m	1	-	-	-	-	17.06	(9.44- 30.82)
HU	3	m	0	29	13	41	67	3.65	(1.70- 7.80)
HU	6	f	0	1	2	40	48	0.60	(0.05- 6.86)
Subtotal HU								3.11	(1.50- 6.42)
HU2	7	c	0	64	19	121	213	5.93	(3.39- 10.37)
JEDRYC	62	m	0	278	143	49	219	8.69	(6.00- 12.57)
JEDRYC	67	f	0	28	4	78	166	14.90	(5.05- 43.94)
Subtotal JEDRYC								9.19	(6.48- 13.04)
JOLY	10	m	0	193	161	12	218	21.78	(11.74- 40.39)
JOLY	6	f	0	32	13	52	283	13.40	(6.59- 27.23)
Subtotal JOLY								17.66	(11.08- 28.14)
JUSSAW	15	m	0	48	4	149	624	50.26	(17.84- 141.55)
KHUDER	3	m	0	154	63	23	309	32.84	(19.62- 54.97)
KREUZE	22	m	3	-	-	-	-	20.80	(7.20- 60.50)
KREUZE	33	m	3	-	-	-	-	33.30	(20.50- 54.00)
Subtotal KREUZE								30.72	(19.77- 47.73)
KREYBE	23	m	0	43	248	6	644	18.61	(7.82- 44.27)
LAMTH	9	f	0	39	9	202	337	7.23	(3.43- 15.24)
LETOUR	4	c	0	65	23	24	224	26.38	(13.98- 49.78)
LIU2	7	m	0	94	21	12	44	16.41	(7.42- 36.33)
LIU3	5	m	0	7	19	4	19	1.75	(0.44- 6.98)
LIU4	9	m	2	-	-	-	-	6.98	(6.73- 7.23)
LUBIN2	276	m	0	1261	1394	190	2616	12.45	(10.55- 14.70)
LUBIN2	284	f	0	45	33	336	1188	4.82	(3.03- 7.68)
Subtotal LUBIN2								11.19	(9.57- 13.08)
MACLEN	39	c	2	-	-	-	-	4.10	(2.07- 8.15)
MARTIS	3	m	0	75	50	4	25	9.38	(3.08- 28.57)
MATOS	32	m	0	106	105	11	110	10.10	(5.13- 19.85)
MATSUD	3	m	0	58	470	3	1255	51.62	(16.10- 165.55)
MCCONN	24	c	0	35	42	9	23	2.13	(0.87- 5.19)
ORMOS	3	m	0	15	128	7	777	13.01	(5.20- 32.52)
OSANN	57	m	2	-	-	-	-	42.80	(30.50- 60.10)

Table 1G4 - 5

IESLC - Meta-analysis of Ever Smoking, Amount smoked, "High", Any product (or Cigarettes if Any not available)
All LC types
Least adjusted

REF	NRR	SEX	AD	Number Exposed Case	Number Exposed Cont	Non-exposed Case	Non-exposed Cont	RR	95.00%CI
OSANN	58	f	2	-	-	-	-	40.90	(29.30- 57.10)
Subtotal OSANN								41.82	(32.97- 53.05)
PASTOR	4	m	0	46	45	10	89	9.10	(4.20- 19.69)
PERNU	23	m	0	40	23	97	275	4.93	(2.81- 8.66)
PERNU	16	f	0	5	7	110	971	6.31	(1.97- 20.20)
Subtotal PERNU								5.17	(3.11- 8.57)
PIKE	3	m	0	66	37	18	69	6.84	(3.55- 13.18)
PIKE	7	f	0	16	3	36	96	14.22	(3.91- 51.73)
Subtotal PIKE								7.95	(4.43- 14.27)
SIEMIA	15	m	0	-	-	-	-	7.90	(3.00- 24.10)
STOCKS	29	m	0	302	468	45	638	9.15	(6.54- 12.79)
TIZZAN	10	m	0	83	20	180	305	7.03	(4.17- 11.85)
WANG2	7	c	0	9	4	11	43	8.80	(2.28- 33.97)
WYNDE2	20	m	0	155	112	8	105	18.16	(8.50- 38.80)
WYNDE3	47	m	0	68	26	9	88	25.57	(11.25- 58.14)
WYNDE3	82	f	0	4	3	20	76	5.07	(1.05- 24.50)
Subtotal WYNDE3								18.10	(8.73- 37.49)
WYNDE4	47	m	0	130	64	12	115	19.47	(10.00- 37.88)
WYNDE4	61	f	2	-	-	-	-	11.54	(1.90- 70.11)
Subtotal WYNDE4								18.28	(9.79- 34.14)
XU3	8	m	0	16	2	7	31	35.43	(6.58- 190.72)
ZHENG	14	m	0	66	23	33	94	8.17	(4.40- 15.17)
Partial Totals				5907	10060	2668	30980		

*prospective study ~ With 0.5 adjustment for zero

REF	NRR	SEX	AD	Ys	Ws	Qs	Ps
ALDERS	20	m	1	2.14	14.23	0.07	0.0000
ALDERS	23	f	1	1.93	25.78	0.51	0.0000
Subtotal ALDERS				2.01	40.01	0.58	
ARMADA	48	m	0	4.24	3.29	15.48	0.0000
AUVINE	7	c	0	3.68	5.94	15.37	0.0000
AXELSS	16	f	0	1.99	2.69	0.02	0.0011
BARBON	13	m	0	2.86	15.58	9.70	0.0000
*BOUCOT	100	m	0	3.83	0.49	1.53	0.0070
BRESLO	16	m	0	2.90	8.89	6.10	0.0000
BUFFLE	35	f	0	3.06	8.66	8.37	0.0000
CHEN2	6	m	0	2.07	3.80	0.00	0.0001
CHEN2	10	f	0	0.97	1.22	1.48	0.2838
Subtotal CHEN2				1.80	5.02	1.48	
CHOI	16	m	0	2.97	3.16	2.55	0.0000
CHOI	20	f	0	1.87	0.74	0.03	0.1083
Subtotal CHOI				2.76	3.90	2.58	
*CPSII	103	m	1	2.87	75.65	47.93	0.0000
DAMBER	9	m	1	2.70	5.93	2.35	0.0000
DAVEYS	4	m	0	2.36	2.18	0.18	0.0005
DEAN	2	m	0	1.91	9.10	0.24	0.0000
DEAN2	26	m	0	2.03	19.18	0.03	0.0000
DEAN2	30	f	0	1.60	3.63	0.81	0.0023
Subtotal DEAN2				1.96	22.82	0.84	
DESTEF	4	m	0	3.12	11.90	13.01	0.0000
DOLL	4	m	0	2.81	5.91	3.20	0.0000
DOLL	10	f	0	3.75	0.47	1.34	0.0098
Subtotal DOLL				2.88	6.39	4.54	
*DOLL2	48	m	1	3.17	0.97	1.16	0.0018
DOSEME	13	m	2	1.89	21.74	0.74	0.0000
*DUNN	5	m	0	3.36	1.73	2.87	0.0000
EBELIN	6	m	0	2.28	1.69	0.07	0.0031
ESAKI	3	m	0	1.78	4.39	0.37	0.0002
FAN	9	m	0	2.41	9.89	1.12	0.0000
FAN	13	f	0	2.92	0.79	0.57	0.0095
Subtotal FAN				2.45	10.68	1.69	
GARSHI	21	m	0	1.73	29.00	3.39	0.0000
GER	20	c	0	1.01	11.22	12.56	0.0007
GOLLED	17	m	0	2.64	12.71	4.12	0.0000
GSELL	5	m	0	4.04	1.58	6.14	0.0000
*HAMMON	155	m	1	2.84	10.98	6.42	0.0000
HU	3	m	0	1.29	6.63	4.02	0.0009
HU	6	f	0	-0.51	0.65	4.32	0.6812
Subtotal HU				1.13	7.28	8.34	
HU2	7	c	0	1.78	12.31	1.05	0.0000

Table 1G4 - 5

IESLC - Meta-analysis of Ever Smoking, Amount smoked, "High", Any product (or Cigarettes if Any not available)
All LC types
Least adjusted

REF	NRR	SEX	AD	Ys	Ws	Qs	Ps
JEDRYC	62	m	0	2.16	28.12	0.23	0.0000
JEDRYC	67	f	0	2.70	3.28	1.30	0.0000
Subtotal JEDRYC				2.22	31.40	1.53	
JOLY	10	m	0	3.08	10.07	10.25	0.0000
JOLY	6	f	0	2.59	7.64	2.09	0.0000
Subtotal JOLY				2.87	17.71	12.34	
JUSSAW	15	m	0	3.92	3.58	12.20	0.0000
KHUDER	3	m	0	3.49	14.48	29.18	0.0000
KREUZE	22	m	3	3.03	3.39	3.15	0.0000
KREUZE	33	m	3	3.51	16.38	33.67	0.0000
Subtotal KREUZE				3.42	19.77	36.81	
KREYBE	23	m	0	2.92	5.11	3.71	0.0000
LAMTH	9	f	0	1.98	6.91	0.06	0.0000
LETOUR	4	c	0	3.27	9.52	13.73	0.0000
LIU2	7	m	0	2.80	6.09	3.21	0.0000
LIU3	5	m	0	0.56	2.01	4.59	0.4278
LIU4	9	m	2	1.94	2991.95	49.68	0.0000
LUBIN2	276	m	0	2.52	139.75	28.32	0.0000
LUBIN2	284	f	0	1.57	17.75	4.42	0.0000
Subtotal LUBIN2				2.42	157.50	32.74	
MACLEN	39	c	2	1.41	8.18	3.57	0.0001
MARTIS	3	m	0	2.24	3.09	0.09	0.0001
MATOS	32	m	0	2.31	8.41	0.48	0.0000
MATSUD	3	m	0	3.94	2.83	9.91	0.0000
MCCONN	24	c	0	0.76	4.83	8.37	0.0966
ORMOS	3	m	0	2.57	4.57	1.11	0.0000
OSANN	57	m	2	3.76	33.40	94.79	0.0000
OSANN	58	f	2	3.71	34.52	92.75	0.0000
Subtotal OSANN				3.73	67.92	187.53	
PASTOR	4	m	0	2.21	6.44	0.12	0.0000
PERNU	23	m	0	1.60	12.13	2.75	0.0000
PERNU	16	f	0	1.84	2.83	0.15	0.0019
Subtotal PERNU				1.64	14.97	2.90	
PIKE	3	m	0	1.92	8.91	0.20	0.0000
PIKE	7	f	0	2.65	2.30	0.78	0.0001
Subtotal PIKE				2.07	11.21	0.98	
SIEMIA	15	m	0	2.07	3.54	0.00	0.0001
STOCKS	29	m	0	2.21	34.20	0.69	0.0000
TIZZAN	10	m	0	1.95	14.11	0.21	0.0000
WANG2	7	c	0	2.17	2.10	0.02	0.0016
WYNDE2	20	m	0	2.90	6.67	4.57	0.0000
WYNDE3	47	m	0	3.24	5.69	7.79	0.0000
WYNDE3	82	f	0	1.62	1.55	0.31	0.0436
Subtotal WYNDE3				2.90	7.24	8.10	
WYNDE4	47	m	0	2.97	8.67	6.97	0.0000
WYNDE4	61	f	2	2.45	1.18	0.17	0.0079
Subtotal WYNDE4				2.91	9.85	7.14	
XU3	8	m	0	3.57	1.36	3.03	0.0000
ZHENG	14	m	0	2.10	10.04	0.01	0.0000

	N	77
	NS	61
	Wt	3842.30
Het	Chi	617.82
Het	df	76
Het	P	***
Fixed	RR	7.94
	RRl	7.69
	RRu	8.20
	P	+++
Random	RR	11.79
	RRl	9.98
	RRu	13.93
	P	+++
Asymm	P	***

Table 1G4 - 6

IESLC - Meta-analysis of Ever Smoking, Amount smoked, "High", Any product (or Cigarettes if Any not available)
All LC types
Least adjusted

		Sex			
		combined	male	female	Total
	N	7	52	18	77
	NS	7	51	18	76
	Wt	54.11	3665.60	122.59	3842.30
Het	Chi	53.99	443.04	94.93	617.82
Het	df	6	51	17	76
Het	P	***	***	***	***
Fixed	RR	7.10	7.83	12.42	7.94
	RRl	5.44	7.59	10.40	7.69
	RRu	9.27	8.09	14.82	8.20
	P	+++	+++	+++	+++
Random	RR	7.45	13.22	9.19	11.79
	RRl	3.27	10.95	5.58	9.98
	RRu	16.96	15.97	15.15	13.93
	P	+++	+++	+++	+++
Between	Chi				25.85
Between	df				2
Between	P				***
Btwn(F)	P				N.S.
Btwn(R)	P				N.S.

Table 1G4 - 7

IESLC - Meta-analysis of Ever Smoking, Amount smoked, "High", Any product (or Cigarettes if Any not available)
All LC types
Excluded studies (and stage at which they were excluded)

1 ABELIN ABRAHA AMANDU AMES ANDERS AUSTIN AXELSO BAND BECHER BERRIN BLOHMK BLOT4 BROCKM BROWN1 BYERS1 BYERS2
CARPEN CASCO2 CASCOR CHAN CHEN3 CHIAZZ CHYOU DESTE2 DOCKER DROSTE DU GARCIA GARDIN GENG GODLEY GOODMA
GRAHAM GREGOR HEGMAN HEIN HENNEK HINDS HIRAOK HOROWI HORWIT HUANG ISHIMA JAHN JAIN JARVHO JIANG KELLER
KIHARA KJUUS KO KOHLME KUBIK LAMWK LAMWK2 LANGE LEI LEMARC LEVIN LIU LOMBA2 LOMBAR MAGNUS MARSH
MARSH2 MCDUFF MCLAUG MILLER MILLS NOTANI NOU ODRISC PAWLEG PERSHA POFFIJ QIAO QIAO2 RADZIK REN RONCO
ROOTS ROTHSC SAARIK SANKAR SCHWAR SEGI SEOW SHIMIZ SIMARA SIMONA SITAS SOBUE2 STASZE STAYNE STUCKE SUN
SUZUK2 SUZUKI TANG TAO TOKARS TOUSEY ULMER VEIERO VUTUC WALD WANG WANG3 WANG4 WICKLU WIGLE WILKIN
WU2 WUNSCH WYNDE8 XIANGZ XU XU2 XU4 YONG ZHANG

2 AKIBA ARCHER BENSHL BRETT BROSS CEDERL CHANG CHOW COMSTO DARBY DEAN3 DEKLER DORANT DORN ENGELA ENSTRO
GAO2 GILLIS HAENSZ HAMMO2 HIRAY2 HIRAYA HITOSU HOLE HUMBLE KAISE2 KAISER KANELL KATSOU KAUFMA KINLEN KNEKT
KOO LAURIL LIAW LICKIN LIDDEL MIGRAN MRFIT MRFITR MURATA NAM PARKIN PERSH2 PETO PEZZO2 PEZZOT PISANI
PRESCO RIMING SEGI2 SOBUE SPEIZE STOCKW SVENSS TANG2 TENKAN TSUGAN TULINI TVERDA WAKAI WARSIN WATSON WU
WYNDE5 WYNDER YAMAGU

3 BUELL CHEN MASTRA MZILEN RESTRE SADOWS

4 BEST BOFFET WYNDE7

6 BLOT1 BLOT2 BLOT3 BOUCHA JONES MOLLO SCHWA2 VANDER

8 CORREA LUO WYNDE6

10 AGUDO BROWN2 CHATZI COOKSO CPSI DORGAN GAO HANSEN JARUP KOULUM LAUSSM LIU5 LUBIN NOTAN2 OSANN2 POLEDN
RACHTA RANDIG SHAW SPITZ WUWILL YUAN ZHOU

11 BENHAM

Table 1G4 - 8

Potentially overlapping studies

REF	REFGP	PRINC	.	OVERLAP/LINK
LUBIN2	LUBIN2	1		Lubin-combined
LAMTH	LAMTH	1		KOO/LAMTH/LAMWK
MATSUD	MATSUD	1		SOBUE2/MATSUD

Table 1G4 - 9

Most adjusted - insufficient data for metaanalysis

REF	NRR	SEX	AGEL	AGEH	RACE	YF	LC TYPE	LOC	START	ST	NLC	R	VB	P	H	AD	PRODUCT	exL	exH	DENOM	De
BLOT1	7	m	0	0	all	-	all	NAmer	1970	CC	458	n	bl	y	n	0	cig+/-ot	40	99	nev cigs	or
BUELL	3	m	0	0	all	0	all	NAmer	1957	pr	304	n	bl	n	n	0	cig+/-ot	22	99	nev cigs	st
RESTRE	9	c	0	0	all	-	all	SCAmer	1978	CC	102	n	bl	n	n	5	cig+/-ot	41	99	nev cigs	or
SADOWS	89	m	0	0	wh	-	all	NAmer	1938	CC	477	n	bl	n	n	1	all/unsp	41	99	nev any	st

REF	NRR	RR	SIG	RRDATA comment
BLOT1	7	8.60		0
BUELL	3	4.93		0
RESTRE	9	169.90		0
SADOWS	89	6.28		0

Least adjusted - insufficient data for meta-analysis: as for adjusted plus the following

REF	NRR	SEX	AGEL	AGEH	RACE	YF	LC TYPE	LOC	START	ST	NLC	R	VB	P	H	AD	PRODUCT	exL	exH	DENOM	De
SADOWS	84	m	0	0	wh	-	all	NAmer	1938	CC	477	n	bl	n	n	0	all/unsp	41	99	nev any	st

REF	NRR	RR	SIG	RRDATA comment
SADOWS	84	5.28		0

Table 1G6 -

IESLC - Meta-analysis of Current Smoking by Amount, Overview, Any product (or Cigarettes if Any not available)
All LC types

This analysis is restricted to results for:
1) Results by Amount smoked
Results by Amount smoked (in numbers of cigarettes or cigarette equivalents) are grouped under 2 schemes (S1, S2).
Each scheme has a set of "key values". An interval is allocated to the category whose key value
it includes and intervals which include none or more than one of the key values are excluded.
(Open-ended intervals are coded as 99.)

S1	key value	maximum range	S2	key value	maximum range
1	5	1-19	1	1	1-9
2	20	6-44	2	10	2-19
3	45	21+	3	20	11-29
			4	30	21-39
			5	40	31-98
			6	99	41+

For all/unspec product, the definition of cigarette equivalents is shown at the end of Sections -1 and -4.
2) Current smokers
3) Results complete enough for use in metaanalysis

Within each study, results are then selected (in the following order of preference, within each sex) for:
4) PRODUCT: all/unspec, cigarettes regardless of other products, cigarettes only
5) CIGTYPE: all/unspecified, MC regardless of HR, MC only
6) DENOM: never smoked anything, never smoked cigarettes, (never +1 = +long term ex, +2 = +amount unknown,
+3 = never cigs+long term ex)
7) Followup period (YF, prospective studies): whole study (coded as 0) or longest available
8) LCtype: all or nearest available, at least Squamous and Adeno. (q = squamous, s = small,
l = large, a = adeno, mix = mixed, alv = alveolar)
9) Race: all or nearest available, otherwise by race (wh or w = white, bl or b = black, hi = hispanic
ch = chinese, jap = japanese, haw = hawaiian, w+o = white + oriental, sca = scandinavian, as = asian)
10) For overlapping studies: principal rather than subsidiary studies
Finally by Age: whole study (coded as 0) if available, otherwise by widest available age group
and then for single sex results (m, f) in preference to combined sex results (c).

Results adjusted (AD) for the most potential confounders are then chosen in Sections -1 to -3
and results adjusted for the least confounders in Sections -4 to -6. (Those least adjusted results which
actually differ from the most adjusted as marked 'x' in column X in Section -4)
(Results adjusted for an unknown number of confounder(s) are coded as 20.)

Section -7 shows excluded studies, together with the stage (as above) at which no qualifying
results were found.

Section -8 lists the potentially overlapping studies which have been included (1=principal, 2=subsidiary).

Section -9 lists any results which would have been included in preference except that they had data not complete
enough for use in meta-analysis, with their significance (yes/no), if known, and any further comment as entered
on the database.

In addition to those mentioned above, the following fields, levels and abbreviations are used:
* or nk = not known, n = no, y = yes, ot = other
nev = never
all/unspec = all or unspecified, cig+/-ot = cigarettes irrespective of other products (cigar, pipe etc)
MC = manufactured cigarettes, HR = hand-rolled cigarettes
exL, exH = range of exposure (low and high) in the smoking group, in terms of Amount smoked,
cigarettes or cigarette equivalents
REF: 6-character study reference
NRR: number of the RR on the database within the study
ST : study type (CC = case control, pr or prosp = prospective)
NLC: number of lung cancer cases in whole study
R : risky occupational population (n = no, m = mining, o = other risky)
VB : national cigarette type (V = at least 75% Virginia, bl = at least 75% blended, ot = other)
P : any proxy use
H : full histological confirmation
De : derivation of RR/CI (or = original, st = standard method, ot = other method of estimation)

Table 1G6 - 1

IESLC - Meta-analysis of Current Smoking by Amount, Overview, Any product (or Cigarettes if Any not available)
All LC types
Most adjusted

REF	NRR	SEX	AGEL	AGEH	RACE	YF	LC TYPE	LOC	START	ST	NLC	R	VB	P	H	AD	PRODUCT	exL	exH	S1	S2	DENOM	De
AKIBA	27	m	0	0	all	0	all	As:Jap	1963	pr	610	n	bl	n	n	5	cig+/-ot	1	14	1	0	nev cigs	or
AKIBA	28	m	0	0	all	0	all	As:Jap	1963	pr	610	n	bl	n	n	5	cig+/-ot	15	24	2	3	nev cigs	ot
AKIBA	29	m	0	0	all	0	all	As:Jap	1963	pr	610	n	bl	n	n	5	cig+/-ot	25	99	3	0	nev cigs	or
AKIBA	33	f	0	0	all	0	all	As:Jap	1963	pr	610	n	bl	n	n	5	cig+/-ot	1	14	1	0	nev cigs	or
AKIBA	34	f	0	0	all	0	all	As:Jap	1963	pr	610	n	bl	n	n	5	cig+/-ot	15	99	0	0	nev cigs	or
ARCHER	1	m	0	0	wh	0	all	NAmer	1950	pr	146	m	bl	n	n	0	cig+/-ot	1	19	1	0	nev cigs	st
ARCHER	2	m	0	0	wh	0	all	NAmer	1950	pr	146	m	bl	n	n	0	cig+/-ot	20	20	2	3	nev cigs	st
ARCHER	3	m	0	0	wh	0	all	NAmer	1950	pr	146	m	bl	n	n	0	cig+/-ot	21	99	3	0	nev cigs	st
AXELSS	18	f	0	0	sca	-	all	Eu:Sca	1989	CC	436	n	bl	n	n	1	all/unsp	20	20	2	3	nev any	or
BENSHL	11	m	40	64	all	10	all	Eu:UK	1967	pr	486	n	V	n	n	1	cig+/-ot	1	9	1	1	nev any	ot
BENSHL	12	m	40	64	all	10	all	Eu:UK	1967	pr	486	n	V	n	n	1	cig+/-ot	10	19	0	2	nev any	ot
BENSHL	13	m	40	64	all	10	all	Eu:UK	1967	pr	486	n	V	n	n	1	cig+/-ot	20	99	0	0	nev any	ot
BEST	13	m	0	0	all	0	all	NAmer	1955	pr	381	n	V	n	n	1	cig only	1	9	1	1	nev any	ot
BEST	14	m	0	0	all	0	all	NAmer	1955	pr	381	n	V	n	n	1	cig only	10	20	2	0	nev any	ot
BEST	15	m	0	0	all	0	all	NAmer	1955	pr	381	n	V	n	n	1	cig only	21	99	3	0	nev any	ot
BOUCOT	124	m	0	0	all	0	all	NAmer	1951	pr	121	n	bl	n	n	2	cig only	1	20	0	0	nev any	ot
BOUCOT	125	m	0	0	all	0	all	NAmer	1951	pr	121	n	bl	n	n	2	cig only	21	40	0	0	nev any	ot
BOUCOT	126	m	0	0	all	0	all	NAmer	1951	pr	121	n	bl	n	n	2	cig only	41	99	3	6	nev any	ot
BRETT	1	m	0	0	all	0	all	Eu:UK	1960	pr	150	n	V	n	n	0	cig+/-ot	1	14	1	0	nev cigs	st
BRETT	2	m	0	0	all	0	all	Eu:UK	1960	pr	150	n	V	n	n	0	cig+/-ot	15	24	2	3	nev cigs	st
BRETT	3	m	0	0	all	0	all	Eu:UK	1960	pr	150	n	V	n	n	0	cig+/-ot	25	99	3	0	nev cigs	st
BROSS	18	m	0	0	wh	-	all	NAmer	1960	CC	974	n	bl	n	n	0	cig+/-ot	1	20	0	0	nev any	st
BROSS	19	m	0	0	wh	-	all	NAmer	1960	CC	974	n	bl	n	n	0	cig+/-ot	21	99	3	0	nev any	st
BUFFLE	30	f	0	0	w-hi	-	all	NAmer	1976	CC	943	n	bl	y	n	0	cig+/-ot	1	19	1	0	nev cigs	or
BUFFLE	31	f	0	0	w-hi	-	all	NAmer	1976	CC	943	n	bl	y	n	0	cig+/-ot	20	20	2	3	nev cigs	or
BUFFLE	36	f	0	0	w-hi	-	all	NAmer	1976	CC	943	n	bl	y	n	0	cig+/-ot	21	99	3	0	nev cigs	or
CEDERL	80	m	0	0	all	16	all	Eu:Sca	1963	pr	491	n	bl	n	n	2	all/unsp	1	7	1	1	nev any	ot
CEDERL	81	m	0	0	all	16	all	Eu:Sca	1963	pr	491	n	bl	n	n	2	all/unsp	8	15	0	2	nev any	ot
CEDERL	82	m	0	0	all	16	all	Eu:Sca	1963	pr	491	n	bl	n	n	2	all/unsp	16	99	0	0	nev any	ot
CEDERL	76	f	0	0	all	0	all	Eu:Sca	1963	pr	491	n	bl	n	n	2	all/unsp	1	7	1	1	nev any	or
CEDERL	77	f	0	0	all	0	all	Eu:Sca	1963	pr	491	n	bl	n	n	2	all/unsp	8	15	0	2	nev any	or
CEDERL	78	f	0	0	all	0	all	Eu:Sca	1963	pr	491	n	bl	n	n	2	all/unsp	16	99	0	0	nev any	or
CHANG	2	m	0	0	all	0	all	NAmer	1972	pr	136	n	bl	n	n	0	cig+/-ot	1	10	1	0	nev cigs	st
CHANG	3	m	0	0	all	0	all	NAmer	1972	pr	136	n	bl	n	n	0	cig+/-ot	11	20	2	3	nev cigs	st
CHANG	4	m	0	0	all	0	all	NAmer	1972	pr	136	n	bl	n	n	0	cig+/-ot	21	99	3	0	nev cigs	st
CHANG	8	f	0	0	all	0	all	NAmer	1972	pr	136	n	bl	n	n	0	cig+/-ot	1	10	1	0	nev cigs	st
CHANG	9	f	0	0	all	0	all	NAmer	1972	pr	136	n	bl	n	n	0	cig+/-ot	11	20	2	3	nev cigs	st
CHANG	10	f	0	0	all	0	all	NAmer	1972	pr	136	n	bl	n	n	0	cig+/-ot	21	99	3	0	nev cigs	st
CHOW	10	m	0	0	wh	0	all	NAmer	1966	pr	219	n	bl	n	n	2	cig+/-ot	1	19	1	0	nev any	ot
CHOW	11	m	0	0	wh	0	all	NAmer	1966	pr	219	n	bl	n	n	2	cig+/-ot	20	29	2	3	nev any	ot
CHOW	12	m	0	0	wh	0	all	NAmer	1966	pr	219	n	bl	n	n	2	cig+/-ot	30	99	3	0	nev any	ot
COMSTO	4	m	0	0	all	-	all	NAmer	1975	ot	258	n	bl	n	n	0	cig+/-ot	1	19	1	0	nev any	st
COMSTO	5	m	0	0	all	-	all	NAmer	1975	ot	258	n	bl	n	n	0	cig+/-ot	20	39	2	0	nev any	st
COMSTO	6	m	0	0	all	-	all	NAmer	1975	ot	258	n	bl	n	n	0	cig+/-ot	40	99	3	0	nev any	st
COMSTO	9	f	0	0	all	-	all	NAmer	1975	ot	258	n	bl	n	n	0	cig+/-ot	1	19	1	0	nev any	st
COMSTO	10	f	0	0	all	-	all	NAmer	1975	ot	258	n	bl	n	n	0	cig+/-ot	20	39	2	0	nev any	st
COMSTO	11	f	0	0	all	-	all	NAmer	1975	ot	258	n	bl	n	n	0	cig+/-ot	40	99	3	0	nev any	st
CORREA	46	c	0	0	all	-	all	NAmer	1979	CC	1359	n	bl	y	n	1	cig+/-ot	1	20	0	0	nev cigs	or
CORREA	50	c	0	0	all	-	all	NAmer	1979	CC	1359	n	bl	y	n	1	cig+/-ot	21	99	3	0	nev cigs	or
CPSI	216	m	35	84	all	6	all	NAmer	1959	pr	5138	n	bl	n	n	1	cig+/-ot	1	9	1	1	nev any	ot
CPSI	217	m	35	84	all	6	all	NAmer	1959	pr	5138	n	bl	n	n	1	cig+/-ot	10	19	0	2	nev any	ot
CPSI	218	m	35	84	all	6	all	NAmer	1959	pr	5138	n	bl	n	n	1	cig+/-ot	20	39	2	0	nev any	ot
CPSI	219	m	35	84	all	6	all	NAmer	1959	pr	5138	n	bl	n	n	1	cig+/-ot	40	99	3	0	nev any	ot
CPSI	275	f	40	74	all	6	all	NAmer	1959	pr	5138	n	bl	n	n	1	cig+/-ot	1	9	1	1	nev cigs	ot
CPSI	276	f	40	74	all	6	all	NAmer	1959	pr	5138	n	bl	n	n	1	cig+/-ot	10	19	0	2	nev cigs	ot
CPSI	277	f	40	74	all	6	all	NAmer	1959	pr	5138	n	bl	n	n	1	cig+/-ot	20	39	2	0	nev cigs	ot
CPSI	278	f	40	74	all	6	all	NAmer	1959	pr	5138	n	bl	n	n	1	cig+/-ot	40	99	3	0	nev cigs	ot
CPSII	120	m	0	0	all	6	all	NAmer	1982	pr	3229	n	bl	n	n	1	cig only	1	9	1	1	nev any	ot
CPSII	121	m	0	0	all	6	all	NAmer	1982	pr	3229	n	bl	n	n	1	cig only	10	19	0	2	nev any	ot
CPSII	122	m	0	0	all	6	all	NAmer	1982	pr	3229	n	bl	n	n	1	cig only	20	20	2	3	nev any	ot
CPSII	123	m	0	0	all	6	all	NAmer	1982	pr	3229	n	bl	n	n	1	cig only	21	39	0	4	nev any	ot
CPSII	124	m	0	0	all	6	all	NAmer	1982	pr	3229	n	bl	n	n	1	cig only	40	40	0	5	nev any	ot
CPSII	125	m	0	0	all	6	all	NAmer	1982	pr	3229	n	bl	n	n	1	cig only	41	99	3	6	nev any	ot
CPSII	127	f	0	0	all	6	all	NAmer	1982	pr	3229	n	bl	n	n	1	cig+/-ot	1	9	1	1	nev cigs	ot
CPSII	128	f	0	0	all	6	all	NAmer	1982	pr	3229	n	bl	n	n	1	cig+/-ot	10	19	0	2	nev cigs	ot
CPSII	129	f	0	0	all	6	all	NAmer	1982	pr	3229	n	bl	n	n	1	cig+/-ot	20	20	2	3	nev cigs	ot
CPSII	130	f	0	0	all	6	all	NAmer	1982	pr	3229	n	bl	n	n	1	cig+/-ot	21	39	0	4	nev cigs	ot
CPSII	131	f	0	0	all	6	all	NAmer	1982	pr	3229	n	bl	n	n	1	cig+/-ot	40	40	0	5	nev cigs	ot
CPSII	132	f	0	0	all	6	all	NAmer	1982	pr	3229	n	bl	n	n	1	cig+/-ot	41	99	3	6	nev cigs	ot
DARBY	1	m	0	0	wh	-	all	Eu:UK	1988	CC	982	n	V	n	n	0	cig+/-ot	1	14	1	0	nev any	st
DARBY	2	m	0	0	wh	-	all	Eu:UK	1988	CC	982	n	V	n	n	0	cig+/-ot	15	24	2	3	nev any	st
DARBY	3	m	0	0	wh	-	all	Eu:UK	1988	CC	982	n	V	n	n	0	cig+/-ot	25	99	3	0	nev any	st

Table 1G6 - 1

IESLC - Meta-analysis of Current Smoking by Amount, Overview, Any product (or Cigarettes if Any not available)
All LC types
Most adjusted

REF	NRR	SEX	AGEL	AGEH	RACE	YF	LC TYPE	LOC	START	ST	NLC	R	VB	P	H	AD	PRODUCT	exL	exH	S1	S2	DENOM	De
DARBY	8	f	0	0	wh	-	all	Eu:UK	1988	CC	982	n	V	n	n	0	cig+/-ot	1	14	1	0	nev any	st
DARBY	9	f	0	0	wh	-	all	Eu:UK	1988	CC	982	n	V	n	n	0	cig+/-ot	15	24	2	3	nev any	st
DARBY	10	f	0	0	wh	-	all	Eu:UK	1988	CC	982	n	V	n	n	0	cig+/-ot	25	99	3	0	nev any	st
DEAN3	7	m	0	0	all	-	all	Eu:UK	1969	CC	766	n	V	y	n	3	cig only	1	12	1	0	nev any	ot
DEAN3	14	m	0	0	all	-	all	Eu:UK	1969	CC	766	n	V	y	n	3	cig only	13	22	2	3	nev any	ot
DEAN3	21	m	0	0	all	-	all	Eu:UK	1969	CC	766	n	V	y	n	3	cig only	23	99	3	0	nev any	ot
DEAN3	91	f	0	0	all	-	all	Eu:UK	1969	CC	766	n	V	y	n	3	cig only	1	12	1	0	nev any	ot
DEAN3	98	f	0	0	all	-	all	Eu:UK	1969	CC	766	n	V	y	n	3	cig only	13	22	2	3	nev any	ot
DEAN3	105	f	0	0	all	-	all	Eu:UK	1969	CC	766	n	V	y	n	3	cig only	23	99	3	0	nev any	ot
DEKLER	2	m	0	0	all	0	all	Auslia	1961	pr	138	m	V	n	n	2	cig+/-ot	1	14	1	0	nev any	or
DEKLER	3	m	0	0	all	0	all	Auslia	1961	pr	138	m	V	n	n	2	cig+/-ot	15	24	2	3	nev any	or
DEKLER	4	m	0	0	all	0	all	Auslia	1961	pr	138	m	V	n	n	2	cig+/-ot	25	99	3	0	nev any	or
DOLL2	16	m	0	0	all	20	all	Eu:UK	1951	pr	920	n	V	n	n	1	all/unsp	1	14	1	0	nev any	ot
DOLL2	17	m	0	0	all	20	all	Eu:UK	1951	pr	920	n	V	n	n	1	all/unsp	15	24	2	3	nev any	ot
DOLL2	18	m	0	0	all	20	all	Eu:UK	1951	pr	920	n	V	n	n	1	all/unsp	25	99	3	0	nev any	ot
DOLL2	10	f	0	0	all	22	all	Eu:UK	1951	pr	920	n	V	n	n	1	cig only	1	14	1	0	nev any	ot
DOLL2	11	f	0	0	all	22	all	Eu:UK	1951	pr	920	n	V	n	n	1	cig only	15	24	2	3	nev any	ot
DOLL2	12	f	0	0	all	22	all	Eu:UK	1951	pr	920	n	V	n	n	1	cig only	25	99	3	0	nev any	ot
DORANT	6	c	0	0	all	0	all	Eu:wst	1986	ot	550	n	bl	n	y	0	cig+/-ot	1	9	1	1	nev any	st
DORANT	7	c	0	0	all	0	all	Eu:wst	1986	ot	550	n	bl	n	y	0	cig+/-ot	10	19	0	2	nev any	st
DORANT	8	c	0	0	all	0	all	Eu:wst	1986	ot	550	n	bl	n	y	0	cig+/-ot	20	99	0	0	nev any	st
DORGAN	4	m	0	0	wh	-	all	NAmer	1980	CC	2026	n	bl	y	y	0	cig+/-ot	1	19	1	0	nev any	st
DORGAN	5	m	0	0	wh	-	all	NAmer	1980	CC	2026	n	bl	y	y	0	cig+/-ot	20	99	0	0	nev any	st
DORGAN	28	m	0	0	bl	-	all	NAmer	1980	CC	2026	n	bl	y	y	0	cig+/-ot	1	19	1	0	nev any	st
DORGAN	29	m	0	0	bl	-	all	NAmer	1980	CC	2026	n	bl	y	y	0	cig+/-ot	20	99	0	0	nev any	st
DORGAN	51	f	0	0	wh	-	all	NAmer	1980	CC	2026	n	bl	y	y	0	cig+/-ot	1	19	1	0	nev any	st
DORGAN	52	f	0	0	wh	-	all	NAmer	1980	CC	2026	n	bl	y	y	0	cig+/-ot	20	99	0	0	nev any	st
DORGAN	74	f	0	0	bl	-	all	NAmer	1980	CC	2026	n	bl	y	y	0	cig+/-ot	1	19	1	0	nev any	st
DORGAN	75	f	0	0	bl	-	all	NAmer	1980	CC	2026	n	bl	y	y	0	cig+/-ot	20	99	0	0	nev any	st
DORN	408	m	0	0	wh	25	all	NAmer	1954	pr	5097	n	bl	n	n	1	cig+/-ot	1	9	1	1	nev any	or
DORN	409	m	0	0	wh	25	all	NAmer	1954	pr	5097	n	bl	n	n	1	cig+/-ot	10	20	2	0	nev any	or
DORN	410	m	0	0	wh	25	all	NAmer	1954	pr	5097	n	bl	n	n	1	cig+/-ot	21	39	0	4	nev any	or
DORN	411	m	0	0	wh	25	all	NAmer	1954	pr	5097	n	bl	n	n	1	cig+/-ot	40	99	3	0	nev any	or
ENGELA	31	m	0	0	all	0	all	Eu:Sca	1964	pr	435	n	bl	n	n	7	cig+/-ot	1	4	0	1	nev cigs	or
ENGELA	32	m	0	0	all	0	all	Eu:Sca	1964	pr	435	n	bl	n	n	7	cig+/-ot	5	9	1	0	nev cigs	or
ENGELA	33	m	0	0	all	0	all	Eu:Sca	1964	pr	435	n	bl	n	n	7	cig+/-ot	10	14	0	2	nev cigs	or
ENGELA	34	m	0	0	all	0	all	Eu:Sca	1964	pr	435	n	bl	n	n	7	cig+/-ot	15	19	0	0	nev cigs	or
ENGELA	35	m	0	0	all	0	all	Eu:Sca	1964	pr	435	n	bl	n	n	7	cig+/-ot	20	99	0	0	nev cigs	or
ENGELA	45	f	0	0	all	0	all	Eu:Sca	1964	pr	435	n	bl	n	n	5	cig+/-ot	1	4	0	1	nev cigs	or
ENGELA	46	f	0	0	all	0	all	Eu:Sca	1964	pr	435	n	bl	n	n	5	cig+/-ot	5	9	1	0	nev cigs	or
ENGELA	47	f	0	0	all	0	all	Eu:Sca	1964	pr	435	n	bl	n	n	5	cig+/-ot	10	14	0	2	nev cigs	or
ENGELA	48	f	0	0	all	0	all	Eu:Sca	1964	pr	435	n	bl	n	n	5	cig+/-ot	15	99	0	0	nev cigs	or
ENSTRO	7	m	0	0	all	0	all	NAmer	1959	pr	2879	n	bl	n	n	1	cig only	1	9	1	1	nev any	ot
ENSTRO	6	m	0	0	all	0	all	NAmer	1959	pr	2879	n	bl	n	n	1	cig only	10	19	0	2	nev any	ot
ENSTRO	5	m	0	0	all	0	all	NAmer	1959	pr	2879	n	bl	n	n	1	cig only	20	20	2	3	nev any	ot
ENSTRO	4	m	0	0	all	0	all	NAmer	1959	pr	2879	n	bl	n	n	1	cig only	21	39	0	4	nev any	ot
ENSTRO	3	m	0	0	all	0	all	NAmer	1959	pr	2879	n	bl	n	n	1	cig only	40	99	3	0	nev any	ot
ENSTRO	11	f	0	0	all	0	all	NAmer	1959	pr	2879	n	bl	n	n	1	cig only	1	9	1	1	nev any	ot
ENSTRO	10	f	0	0	all	0	all	NAmer	1959	pr	2879	n	bl	n	n	1	cig only	10	19	0	2	nev any	ot
ENSTRO	9	f	0	0	all	0	all	NAmer	1959	pr	2879	n	bl	n	n	1	cig only	20	20	2	3	nev any	ot
ENSTRO	8	f	0	0	all	0	all	NAmer	1959	pr	2879	n	bl	n	n	1	cig only	21	99	3	0	nev any	ot
GAO2	2	m	0	0	all	-	all	As:Jap	1988	CC	282	n	bl	n	n	0	cig+/-ot	1	19	1	0	nev cigs	st
GAO2	3	m	0	0	all	-	all	As:Jap	1988	CC	282	n	bl	n	n	0	cig+/-ot	20	29	2	3	nev cigs	st
GAO2	4	m	0	0	all	-	all	As:Jap	1988	CC	282	n	bl	n	n	0	cig+/-ot	30	99	3	0	nev cigs	or
GILLIS	21	m	0	0	all	-	all	Eu:UK	1977	CC	656	n	V	n	n	3	cig+/-ot	1	14	1	0	nev any	or
GILLIS	22	m	0	0	all	-	all	Eu:UK	1977	CC	656	n	V	n	n	3	cig+/-ot	15	24	2	3	nev any	or
GILLIS	23	m	0	0	all	-	all	Eu:UK	1977	CC	656	n	V	n	n	3	cig+/-ot	25	34	0	4	nev any	or
GILLIS	24	m	0	0	all	-	all	Eu:UK	1977	CC	656	n	V	n	n	3	cig+/-ot	35	49	3	5	nev any	or
GILLIS	25	m	0	0	all	-	all	Eu:UK	1977	CC	656	n	V	n	n	3	cig+/-ot	50	99	0	6	nev any	or
HAENSZ	52	f	0	0	all	-	not alv	NAmer	1955	CC	158	n	bl	n	y	0	cig+/-ot	1	20	0	0	nev any	st
HAENSZ	51	f	0	0	all	-	not alv	NAmer	1955	CC	158	n	bl	n	y	0	cig+/-ot	21	99	3	0	nev any	st
HAMMO2	7	m	0	0	all	0	all	NAmer	1967	pr	450	o	bl	n	n	1	cig+/-ot	1	19	1	0	nev any	ot
HAMMO2	6	m	0	0	all	0	all	NAmer	1967	pr	450	o	bl	n	n	1	cig+/-ot	20	99	0	0	nev any	ot
HAMMON	135	m	0	0	wh	0	all	NAmer	1952	pr	448	n	bl	n	n	1	cig only	1	9	1	1	nev any	ot
HAMMON	136	m	0	0	wh	0	all	NAmer	1952	pr	448	n	bl	n	n	1	cig only	10	20	2	0	nev any	ot
HAMMON	137	m	0	0	wh	0	all	NAmer	1952	pr	448	n	bl	n	n	1	cig only	21	39	0	4	nev any	ot
HAMMON	138	m	0	0	wh	0	all	NAmer	1952	pr	448	n	bl	n	n	1	cig only	40	99	3	0	nev any	ot
HIRAYA	23	m	0	0	all	0	all	As:Jap	1965	pr	1917	n	bl	n	n	1	cig+/-ot	1	9	1	1	nev any	st
HIRAYA	24	m	0	0	all	0	all	As:Jap	1965	pr	1917	n	bl	n	n	1	cig+/-ot	10	19	0	2	nev any	st
HIRAYA	25	m	0	0	all	0	all	As:Jap	1965	pr	1917	n	bl	n	n	1	cig+/-ot	20	99	0	0	nev any	st
HIRAYA	26	f	0	0	all	0	all	As:Jap	1965	pr	1917	n	bl	n	n	1	cig+/-ot	1	9	1	1	nev any	st
HIRAYA	27	f	0	0	all	0	all	As:Jap	1965	pr	1917	n	bl	n	n	1	cig+/-ot	10	19	0	2	nev any	st

Table 1G6 - 1

IESLC - Meta-analysis of Current Smoking by Amount, Overview, Any product (or Cigarettes if Any not available)
All LC types
Most adjusted

REF	NRR	SEX	AGEL	AGEH	RACE	YF	LC TYPE	LOC	START	ST	NLC	R	VB	P	H	AD	PRODUCT	exL	exH	S1	S2	DENOM	De
HIRAYA	28	f	0	0	all	0	all	As:Jap	1965	pr	1917	n	bl	n	n	1	cig+/-ot	20	99	0	0	nev any	st
HITOSU	35	m	0	0	all	-	all	As:Jap	1960	CC	216	n	bl	y	n	1	all/unsp	1	14	1	0	nev any	st
HITOSU	36	m	0	0	all	-	all	As:Jap	1960	CC	216	n	bl	y	n	1	all/unsp	15	24	2	3	nev any	st
HITOSU	37	m	0	0	all	-	all	As:Jap	1960	CC	216	n	bl	y	n	1	all/unsp	25	99	3	0	nev any	st
HITOSU	60	f	0	0	all	-	all	As:Jap	1960	CC	216	n	bl	y	n	1	all/unsp	1	14	1	0	nev any	st
HITOSU	61	f	0	0	all	-	all	As:Jap	1960	CC	216	n	bl	y	n	1	all/unsp	15	99	0	0	nev any	st
HOLE	1	m	0	0	all	0	all	Eu:UK	1972	pr	225	n	V	n	n	1	cig+/-ot	1	14	1	0	nev any	ot
HOLE	3	m	0	0	all	0	all	Eu:UK	1972	pr	225	n	V	n	n	1	cig+/-ot	15	24	2	3	nev any	ot
HOLE	4	m	0	0	all	0	all	Eu:UK	1972	pr	225	n	V	n	n	1	cig+/-ot	25	34	0	4	nev any	ot
HOLE	5	m	0	0	all	0	all	Eu:UK	1972	pr	225	n	V	n	n	1	cig+/-ot	35	99	3	0	nev any	ot
HUMBLE	2	m	0	0	w-hi	-	all	NAmer	1980	CC	521	n	bl	y	n	1	cig+/-ot	1	19	1	0	nev cigs	or
HUMBLE	3	m	0	0	w-hi	-	all	NAmer	1980	CC	521	n	bl	y	n	1	cig+/-ot	20	99	0	0	nev cigs	or
HUMBLE	5	m	0	0	hi	-	all	NAmer	1980	CC	521	n	bl	y	n	1	cig+/-ot	1	19	1	0	nev cigs	or
HUMBLE	6	m	0	0	hi	-	all	NAmer	1980	CC	521	n	bl	y	n	1	cig+/-ot	20	99	0	0	nev cigs	or
HUMBLE	8	f	0	0	w-hi	-	all	NAmer	1980	CC	521	n	bl	y	n	1	cig+/-ot	1	19	1	0	nev cigs	or
HUMBLE	9	f	0	0	w-hi	-	all	NAmer	1980	CC	521	n	bl	y	n	1	cig+/-ot	20	99	0	0	nev cigs	or
HUMBLE	11	f	0	0	hi	-	all	NAmer	1980	CC	521	n	bl	y	n	1	cig+/-ot	1	19	1	0	nev cigs	or
HUMBLE	12	f	0	0	hi	-	all	NAmer	1980	CC	521	n	bl	y	n	1	cig+/-ot	20	99	0	0	nev cigs	or
KAISE2	66	m	35	99	all	9	all	NAmer	1979	pr	318	n	bl	n	n	1	cig only	1	19	1	0	nev any	st
KAISE2	67	m	35	99	all	9	all	NAmer	1979	pr	318	n	bl	n	n	1	cig only	20	99	0	0	nev any	st
KAISE2	58	f	35	99	all	9	all	NAmer	1979	pr	318	n	bl	n	n	1	cig only	1	19	1	0	nev any	st
KAISE2	59	f	35	99	all	9	all	NAmer	1979	pr	318	n	bl	n	n	1	cig only	20	99	0	0	nev any	st
KAISER	6	m	0	0	all	0	all	NAmer	1964	pr	714	n	bl	n	n	2	cig+/-ot	1	19	1	0	nev cigs	or
KAISER	7	m	0	0	all	0	all	NAmer	1964	pr	714	n	bl	n	n	2	cig+/-ot	20	40	2	0	nev cigs	or
KAISER	8	m	0	0	all	0	all	NAmer	1964	pr	714	n	bl	n	n	2	cig+/-ot	41	99	3	6	nev cigs	or
KAISER	2	f	0	0	all	0	all	NAmer	1964	pr	714	n	bl	n	n	2	cig+/-ot	1	19	1	0	nev cigs	or
KAISER	3	f	0	0	all	0	all	NAmer	1964	pr	714	n	bl	n	n	2	cig+/-ot	20	40	2	0	nev cigs	or
KAISER	4	f	0	0	all	0	all	NAmer	1964	pr	714	n	bl	n	n	2	cig+/-ot	41	99	3	6	nev cigs	or
KANELL	26	m	0	0	all	-	all	Eu:bal	1950	CC	862	n	bl	n	n	1	cig+/-ot	1	10	1	0	nev any	st
KANELL	27	m	0	0	all	-	all	Eu:bal	1950	CC	862	n	bl	n	n	1	cig+/-ot	11	20	2	3	nev any	st
KANELL	28	m	0	0	all	-	all	Eu:bal	1950	CC	862	n	bl	n	n	1	cig+/-ot	21	35	0	4	nev any	st
KANELL	29	m	0	0	all	-	all	Eu:bal	1950	CC	862	n	bl	n	n	1	cig+/-ot	36	99	3	0	nev any	st
KATSOU	3	f	0	0	all	-	all	Eu:bal	1987	CC	101	n	bl	n	n	1	all/unsp	1	20	0	0	nev any	or
KATSOU	4	f	0	0	all	-	all	Eu:bal	1987	CC	101	n	bl	n	n	1	all/unsp	21	99	3	0	nev any	or
KAUFMA	11	c	0	0	all	-	all	NAmer	1981	CC	881	n	bl	n	n	6	cig+/-ot	1	14	1	0	nev cigs	or
KAUFMA	12	c	0	0	all	-	all	NAmer	1981	CC	881	n	bl	n	n	6	cig+/-ot	15	24	2	3	nev cigs	or
KAUFMA	13	c	0	0	all	-	all	NAmer	1981	CC	881	n	bl	n	n	6	cig+/-ot	25	34	0	4	nev cigs	or
KAUFMA	14	c	0	0	all	-	all	NAmer	1981	CC	881	n	bl	n	n	6	cig+/-ot	35	44	0	5	nev cigs	or
KAUFMA	15	c	0	0	all	-	all	NAmer	1981	CC	881	n	bl	n	n	6	cig+/-ot	45	99	3	6	nev cigs	or
KINLEN	14	m	0	0	all	0	all	Eu:UK	1967	pr	718	n	V	n	n	2	cig+/-ot	1	14	1	0	nev any	ot
KINLEN	15	m	0	0	all	0	all	Eu:UK	1967	pr	718	n	V	n	n	2	cig+/-ot	15	24	2	3	nev any	ot
KINLEN	16	m	0	0	all	0	all	Eu:UK	1967	pr	718	n	V	n	n	2	cig+/-ot	25	99	3	0	nev any	ot
KNEKT	29	m	20	69	all	21	all	Eu:Sca	1966	pr	515	n	bl	n	n	1	cig+/-ot	1	14	1	0	nev any	or
KNEKT	30	m	20	69	all	21	all	Eu:Sca	1966	pr	515	n	bl	n	n	1	cig+/-ot	15	99	0	0	nev any	or
KOO	11	f	0	0	all	-	all	As:HK	1981	CC	200	n	bl	n	n	0	all/unsp	1	10	1	0	nev any	st
KOO	12	f	0	0	all	-	all	As:HK	1981	CC	200	n	bl	n	n	0	all/unsp	11	20	2	3	nev any	st
KOO	13	f	0	0	all	-	all	As:HK	1981	CC	200	n	bl	n	n	0	all/unsp	21	30	0	4	nev any	st
LIAW	3	c	0	0	all	0	all	As:oth	1982	pr	127	n	ot	n	n	2	all/unsp	1	10	1	0	nev any	or
LIAW	4	c	0	0	all	0	all	As:oth	1982	pr	127	n	ot	n	n	2	all/unsp	11	20	2	3	nev any	or
LIAW	5	c	0	0	all	0	all	As:oth	1982	pr	127	n	ot	n	n	2	all/unsp	21	99	3	0	nev any	or
LIDDEL	2	m	0	0	all	18	all	NAmer	1970	pr	304	m	V	n	n	1	cig+/-ot	1	19	1	0	nev cigs	ot
LIDDEL	3	m	0	0	all	18	all	NAmer	1970	pr	304	m	V	n	n	1	cig+/-ot	20	99	0	0	nev cigs	ot
MACLEN	20	m	0	0	ch	-	all	As:oth	1972	CC	233	n	bl	n	n	0	cig+/-ot	1	9	1	1	nev cigs	st
MACLEN	21	m	0	0	ch	-	all	As:oth	1972	CC	233	n	bl	n	n	0	cig+/-ot	10	19	0	2	nev cigs	st
MACLEN	22	m	0	0	ch	-	all	As:oth	1972	CC	233	n	bl	n	n	0	cig+/-ot	20	29	2	3	nev cigs	st
MACLEN	23	m	0	0	ch	-	all	As:oth	1972	CC	233	n	bl	n	n	0	cig+/-ot	30	99	3	0	nev cigs	st
MACLEN	33	f	0	0	ch	-	all	As:oth	1972	CC	233	n	bl	n	n	0	cig+/-ot	1	9	1	1	nev cigs	st
MACLEN	34	f	0	0	ch	-	all	As:oth	1972	CC	233	n	bl	n	n	0	cig+/-ot	10	19	0	2	nev cigs	st
MACLEN	35	f	0	0	ch	-	all	As:oth	1972	CC	233	n	bl	n	n	0	cig+/-ot	20	99	0	0	nev cigs	st
MATOS	5	m	0	0	all	-	all	SCAmer	1994	CC	200	n	bl	n	n	2	cig+/-ot	1	14	1	0	nev any	or
MATOS	7	m	0	0	all	-	all	SCAmer	1994	CC	200	n	bl	n	n	2	cig+/-ot	15	24	2	3	nev any	or
MATOS	9	m	0	0	all	-	all	SCAmer	1994	CC	200	n	bl	n	n	2	cig+/-ot	25	99	3	0	nev any	or
MIGRAN	2	m	0	0	all	0	all	Eu:UK	1964	pr	259	n	V	n	n	2	cig only	1	9	1	1	nev any	ot
MIGRAN	4	m	0	0	all	0	all	Eu:UK	1964	pr	259	n	V	n	n	2	cig only	10	19	0	2	nev any	ot
MIGRAN	6	m	0	0	all	0	all	Eu:UK	1964	pr	259	n	V	n	n	2	cig only	20	20	2	3	nev any	ot
MIGRAN	8	m	0	0	all	0	all	Eu:UK	1964	pr	259	n	V	n	n	2	cig only	21	99	3	0	nev any	ot
MIGRAN	29	f	0	0	all	0	all	Eu:UK	1964	pr	259	n	V	n	n	2	cig only	1	9	1	1	nev any	ot
MIGRAN	31	f	0	0	all	0	all	Eu:UK	1964	pr	259	n	V	n	n	2	cig only	10	19	0	2	nev any	ot
MIGRAN	33	f	0	0	all	0	all	Eu:UK	1964	pr	259	n	V	n	n	2	cig only	20	20	2	3	nev any	ot
MRFITR	3	m	0	0	all	0	all	NAmer	1973	pr	119	n	bl	n	n	0	cig+/-ot	1	19	1	0	nev cigs	ot
MRFITR	4	m	0	0	all	0	all	NAmer	1973	pr	119	n	bl	n	n	0	cig+/-ot	20	39	2	0	nev cigs	ot
MRFITR	5	m	0	0	all	0	all	NAmer	1973	pr	119	n	bl	n	n	0	cig+/-ot	40	99	3	0	nev cigs	ot

Table 1G6 - 1

IESLC - Meta-analysis of Current Smoking by Amount, Overview, Any product (or Cigarettes if Any not available)
All LC types
Most adjusted

REF	NRR	SEX	AGEL	AGEH	RACE	YF	LC TYPE	LOC	START	ST	NLC	R	VB	P	H	AD	PRODUCT	exL	exH	S1	S2	DENOM	De
NAM	74	m	0	0	all	-	all	NAmer	1986	CC	1199	n	bl	y	n	1	cig+/-ot	1	24	0	0	nev cigs	ot
NAM	75	m	0	0	all	-	all	NAmer	1986	CC	1199	n	bl	y	n	1	cig+/-ot	25	99	3	0	nev cigs	ot
NAM	90	f	0	0	all	-	all	NAmer	1986	CC	1199	n	bl	y	n	1	cig+/-ot	1	24	0	0	nev cigs	ot
NAM	91	f	0	0	all	-	all	NAmer	1986	CC	1199	n	bl	y	n	1	cig+/-ot	25	99	3	0	nev cigs	ot
PARKIN	14	m	0	0	bl	-	all	Africa	1963	CC	877	n	V	y	n	6	all/unsp	1	14	1	0	nev any	or
PARKIN	15	m	0	0	bl	-	all	Africa	1963	CC	877	n	V	y	n	6	all/unsp	15	99	0	0	nev any	or
PERSH2	8	c	0	0	all	-	all	Eu:Sca	1980	CC	1022	n	bl	y	n	4	all/unsp	1	9	1	1	nev any	ot
PERSH2	9	c	0	0	all	-	all	Eu:Sca	1980	CC	1022	n	bl	y	n	4	all/unsp	10	99	0	0	nev any	ot
PETO	2	m	0	0	all	0	all	Eu:UK	1954	pr	103	n	V	n	n	0	all/unsp	1	14	1	0	nev any	st
PETO	3	m	0	0	all	0	all	Eu:UK	1954	pr	103	n	V	n	n	0	all/unsp	15	99	0	0	nev any	st
PEZZO2	3	m	0	0	all	-	all	SCAmer	1992	CC	367	n	bl	n	y	0	cig+/-ot	1	20	0	0	nev cigs	st
PEZZO2	4	m	0	0	all	-	all	SCAmer	1992	CC	367	n	bl	n	y	0	cig+/-ot	21	40	0	0	nev cigs	st
PEZZO2	5	m	0	0	all	-	all	SCAmer	1992	CC	367	n	bl	n	y	0	cig+/-ot	41	99	3	6	nev cigs	st
PEZZOT	2	m	0	0	all	-	all	SCAmer	1987	CC	215	n	bl	n	y	0	cig only	1	20	0	0	nev cigs	st
PEZZOT	3	m	0	0	all	-	all	SCAmer	1987	CC	215	n	bl	n	y	0	cig only	21	40	0	0	nev cigs	st
PEZZOT	4	m	0	0	all	-	all	SCAmer	1987	CC	215	n	bl	n	y	0	cig only	41	99	3	6	nev cigs	st
PRESCO	2	m	0	0	all	0	all	Eu:Sca	1964	pr	867	n	bl	n	n	1	all/unsp	1	14	1	0	nev any	st
PRESCO	4	m	0	0	all	0	all	Eu:Sca	1964	pr	867	n	bl	n	n	1	all/unsp	15	99	0	0	nev any	st
PRESCO	1	f	0	0	all	0	all	Eu:Sca	1964	pr	867	n	bl	n	n	1	all/unsp	1	14	1	0	nev any	st
PRESCO	3	f	0	0	all	0	all	Eu:Sca	1964	pr	867	n	bl	n	n	1	all/unsp	15	99	0	0	nev any	st
SEGI2	10	m	0	0	all	-	all	As:Jap	1962	CC	378	n	bl	n	n	1	cig+/-ot	1	9	1	1	nev any	ot
SEGI2	12	m	0	0	all	-	all	As:Jap	1962	CC	378	n	bl	n	n	1	cig+/-ot	10	19	0	2	nev any	ot
SEGI2	14	m	0	0	all	-	all	As:Jap	1962	CC	378	n	bl	n	n	1	cig+/-ot	20	29	2	3	nev any	ot
SEGI2	16	m	0	0	all	-	all	As:Jap	1962	CC	378	n	bl	n	n	1	cig+/-ot	30	39	0	4	nev any	ot
SEGI2	18	m	0	0	all	-	all	As:Jap	1962	CC	378	n	bl	n	n	1	cig+/-ot	40	99	3	0	nev any	ot
SEGI2	22	f	0	0	all	-	all	As:Jap	1962	CC	378	n	bl	n	n	1	cig+/-ot	1	9	1	1	nev any	ot
SEGI2	24	f	0	0	all	-	all	As:Jap	1962	CC	378	n	bl	n	n	1	cig+/-ot	10	19	0	2	nev any	ot
SEGI2	26	f	0	0	all	-	all	As:Jap	1962	CC	378	n	bl	n	n	1	cig+/-ot	20	99	0	0	nev any	ot
SHAW	4	c	0	0	wh	-	all	NAmer	1988	CC	335	n	V	n	y	0	all/unsp	1	19	1	0	nev any	st
SHAW	5	c	0	0	wh	-	all	NAmer	1988	CC	335	n	V	n	y	0	all/unsp	20	99	0	0	nev any	st
SOBUE	117	m	0	0	all	-	q+s+l+a	As:Jap	1986	CC	1376	n	bl	n	y	0	cig+/-ot	1	19	1	0	nev cigs	st
SOBUE	118	m	0	0	all	-	q+s+l+a	As:Jap	1986	CC	1376	n	bl	n	y	0	cig+/-ot	20	29	2	3	nev cigs	st
SOBUE	119	m	0	0	all	-	q+s+l+a	As:Jap	1986	CC	1376	n	bl	n	y	0	cig+/-ot	30	99	3	0	nev cigs	st
SPEIZE	1	f	0	0	all	0	all	NAmer	1976	pr	593	n	bl	n	y	1	cig+/-ot	1	4	0	1	nev cigs	ot
SPEIZE	2	f	0	0	all	0	all	NAmer	1976	pr	593	n	bl	n	y	1	cig+/-ot	5	14	1	2	nev cigs	ot
SPEIZE	3	f	0	0	all	0	all	NAmer	1976	pr	593	n	bl	n	y	1	cig+/-ot	15	24	2	3	nev cigs	ot
SPEIZE	4	f	0	0	all	0	all	NAmer	1976	pr	593	n	bl	n	y	1	cig+/-ot	25	34	0	4	nev cigs	ot
SPEIZE	5	f	0	0	all	0	all	NAmer	1976	pr	593	n	bl	n	y	1	cig+/-ot	35	99	3	0	nev cigs	ot
STOCKW	1	c	0	0	all	-	all	NAmer	1981	CC	22161	n	bl	n	n	0	cig+/-ot	1	19	1	0	nev any	st
STOCKW	2	c	0	0	all	-	all	NAmer	1981	CC	22161	n	bl	n	n	0	cig+/-ot	20	40	2	0	nev any	st
STOCKW	3	c	0	0	all	-	all	NAmer	1981	CC	22161	n	bl	n	n	0	cig+/-ot	41	99	3	6	nev any	st
SVENSS	6	f	0	0	all	-	all	Eu:Sca	1983	CC	210	n	bl	n	n	1	all/unsp	1	10	1	0	nev any	or
SVENSS	11	f	0	0	all	-	all	Eu:Sca	1983	CC	210	n	bl	n	n	1	all/unsp	11	20	2	3	nev any	or
SVENSS	16	f	0	0	all	-	all	Eu:Sca	1983	CC	210	n	bl	n	n	1	all/unsp	21	99	3	0	nev any	ot
TENKAN	10	m	0	0	all	17	all	Eu:Sca	1962	pr	242	n	bl	n	n	1	all/unsp	1	14	1	0	nev any	ot
TENKAN	11	m	0	0	all	17	all	Eu:Sca	1962	pr	242	n	bl	n	n	1	all/unsp	15	24	2	3	nev any	ot
TENKAN	12	m	0	0	all	17	all	Eu:Sca	1962	pr	242	n	bl	n	n	1	all/unsp	25	99	3	0	nev any	ot
TSUGAN	29	m	0	0	all	-	q+a	As:Jap	1976	CC	134	n	bl	n	y	0	all/unsp	1	15	1	0	nev any	st
TSUGAN	30	m	0	0	all	-	q+a	As:Jap	1976	CC	134	n	bl	n	y	0	all/unsp	16	35	2	0	nev any	st
TSUGAN	31	m	0	0	all	-	q+a	As:Jap	1976	CC	134	n	bl	n	y	0	all/unsp	36	99	3	0	nev any	st
TULINI	27	m	0	0	all	0	all	Eu:Sca	1967	pr	472	n	bl	n	n	3	cig+/-ot	1	14	1	0	nev any	or
TULINI	28	m	0	0	all	0	all	Eu:Sca	1967	pr	472	n	bl	n	n	3	cig+/-ot	15	24	2	3	nev any	or
TULINI	29	m	0	0	all	0	all	Eu:Sca	1967	pr	472	n	bl	n	n	3	cig+/-ot	25	99	3	0	nev any	or
TULINI	32	f	0	0	all	0	all	Eu:Sca	1967	pr	472	n	bl	n	n	3	cig+/-ot	1	14	1	0	nev any	or
TULINI	33	f	0	0	all	0	all	Eu:Sca	1967	pr	472	n	bl	n	n	3	cig+/-ot	15	24	2	3	nev any	or
TULINI	34	f	0	0	all	0	all	Eu:Sca	1967	pr	472	n	bl	n	n	3	cig+/-ot	25	99	3	0	nev any	or
TVERDA	9	m	0	0	all	0	all	Eu:Sca	1972	pr	238	n	bl	n	n	2	cig only	1	9	1	1	nev cigs	ot
TVERDA	10	m	0	0	all	0	all	Eu:Sca	1972	pr	238	n	bl	n	n	2	cig only	10	19	0	2	nev cigs	ot
TVERDA	11	m	0	0	all	0	all	Eu:Sca	1972	pr	238	n	bl	n	n	2	cig only	20	99	0	0	nev cigs	ot
TVERDA	16	f	0	0	all	0	all	Eu:Sca	1972	pr	238	n	bl	n	n	2	cig only	1	9	1	1	nev cigs	ot
TVERDA	17	f	0	0	all	0	all	Eu:Sca	1972	pr	238	n	bl	n	n	2	cig only	20	99	0	0	nev cigs	ot
WAKAI	40	m	0	0	all	-	all	As:Jap	1988	CC	333	n	bl	n	y	2	cig+/-ot	1	19	1	0	nev any	or
WAKAI	41	m	0	0	all	-	all	As:Jap	1988	CC	333	n	bl	n	y	2	cig+/-ot	20	29	2	3	nev any	or
WAKAI	42	m	0	0	all	-	all	As:Jap	1988	CC	333	n	bl	n	y	2	cig+/-ot	30	99	3	0	nev any	or
WU	43	f	0	0	wh	-	q+a	NAmer	1981	CC	220	n	bl	n	y	2	all/unsp	1	20	0	0	nev any	st
WU	44	f	0	0	wh	-	q+a	NAmer	1981	CC	220	n	bl	n	y	2	all/unsp	21	99	3	0	nev any	st
WYNDE6	27	m	0	0	all	-	all	NAmer	1969	CC	4423	n	bl	n	y	0	cig+/-ot	1	10	1	0	nev any	st
WYNDE6	36	m	0	0	all	-	all	NAmer	1969	CC	4423	n	bl	n	y	0	cig+/-ot	11	20	2	3	nev any	st
WYNDE6	45	m	0	0	all	-	all	NAmer	1969	CC	4423	n	bl	n	y	0	cig+/-ot	21	30	0	4	nev any	st
WYNDE6	54	m	0	0	all	-	all	NAmer	1969	CC	4423	n	bl	n	y	0	cig+/-ot	31	99	3	0	nev any	st
WYNDE6	216	f	0	0	all	-	all	NAmer	1969	CC	4423	n	bl	n	y	0	cig+/-ot	1	10	1	0	nev cigs	st
WYNDE6	225	f	0	0	all	-	all	NAmer	1969	CC	4423	n	bl	n	y	0	cig+/-ot	11	20	2	3	nev cigs	st

Table 1G6 - 1

IESLC - Meta-analysis of Current Smoking by Amount, Overview, Any product (or Cigarettes if Any not available)
All LC types
Most adjusted

REF	NRR	SEX	AGEL	AGEH	RACE	YF	LC TYPE	LOC	START	ST	NLC	R	VB	P	H	AD	PRODUCT	exL	exH	S1	S2	DENOM	De
WYNDE6	234	f	0	0	all	-	all	NAmer	1969	CC	4423	n	bl	n	y	0	cig+/-ot	21	30	0	4	nev cigs	st
WYNDE6	243	f	0	0	all	-	all	NAmer	1969	CC	4423	n	bl	n	y	0	cig+/-ot	30	99	3	0	nev cigs	st
YAMAGU	8	c	0	0	all	-	all	As:Jap	1989	CC	144	n	bl	n	y	1	all/unsp	1	20	0	0	nev any	or
YAMAGU	7	c	0	0	all	-	all	As:Jap	1989	CC	144	n	bl	n	y	1	all/unsp	21	99	3	0	nev any	or

Cigarette type is all/unspec for all RRs
except for the following:

REF	NRR	CIGTYPE
DEAN3	7	MC only
DEAN3	14	MC only
DEAN3	21	MC only
DEAN3	91	MC only
DEAN3	98	MC only
DEAN3	105	MC only

REF	NRR	Cigarette equivalent
AKIBA	27	*
AKIBA	28	*
AKIBA	29	*
AKIBA	33	*
AKIBA	34	*
ARCHER	1	*
ARCHER	2	*
ARCHER	3	*
AXELSS	18	includes 1 g pipe tob = 1 cig
BENSHL	11	*
BENSHL	12	*
BENSHL	13	*
BEST	13	-
BEST	14	-
BEST	15	-
BOUCOT	124	-
BOUCOT	125	-
BOUCOT	126	-
BRETT	1	*
BRETT	2	*
BRETT	3	*
BROSS	18	*
BROSS	19	*
BUFFLE	30	*
BUFFLE	31	*
BUFFLE	36	*
CEDERL	80	gms inc cig = 1, sm cgr = 3, lge cgr = 5
CEDERL	81	gms inc cig = 1, sm cgr = 3, lge cgr = 5
CEDERL	82	gms inc cig = 1, sm cgr = 3, lge cgr = 5
CEDERL	76	inc 1 g other tob = 1 cig
CEDERL	77	inc 1 g other tob = 1 cig
CEDERL	78	inc 1 g other tob = 1 cig
CHANG	2	*
CHANG	3	*
CHANG	4	*
CHANG	8	*
CHANG	9	*
CHANG	10	*
CHOW	10	*
CHOW	11	*
CHOW	12	*
COMSTO	4	*
COMSTO	5	*
COMSTO	6	*
COMSTO	9	*
COMSTO	10	*
COMSTO	11	*
CORREA	46	*
CORREA	50	*
CPSI	216	*
CPSI	217	*
CPSI	218	*

Table 1G6 - 1

IESLC - Meta-analysis of Current Smoking by Amount, Overview, Any product (or Cigarettes if Any not available)
All LC types
Most adjusted

REF	NRR	Cigarette equivalent
CPSI	219	*
CPSI	275	*
CPSI	276	*
CPSI	277	*
CPSI	278	*
CPSII	120	-
CPSII	121	-
CPSII	122	-
CPSII	123	-
CPSII	124	-
CPSII	125	-
CPSII	127	*
CPSII	128	*
CPSII	129	*
CPSII	130	*
CPSII	131	*
CPSII	132	*
DARBY	1	inc 1oz pipe/wk=2 cigs/d, excl cigar/llo
DARBY	2	inc 1oz pipe/wk=2 cigs/d, excl cigar/llo
DARBY	3	inc 1oz pipe/wk=2 cigs/d, excl cigar/llo
DARBY	8	inc 1oz pipe/wk=2 cigs/d, excl cigar/llo
DARBY	9	inc 1oz pipe/wk=2 cigs/d, excl cigar/llo
DARBY	10	inc 1oz pipe/wk=2 cigs/d, excl cigar/llo
DEAN3	7	-
DEAN3	14	-
DEAN3	21	-
DEAN3	91	-
DEAN3	98	-
DEAN3	105	-
DEKLER	2	*
DEKLER	3	*
DEKLER	4	*
DOLL2	16	grams
DOLL2	17	grams
DOLL2	18	grams
DOLL2	10	-
DOLL2	11	-
DOLL2	12	-
DORANT	6	*
DORANT	7	*
DORANT	8	*
DORGAN	4	*
DORGAN	5	*
DORGAN	28	*
DORGAN	29	*
DORGAN	51	*
DORGAN	52	*
DORGAN	74	*
DORGAN	75	*
DORN	408	*
DORN	409	*
DORN	410	*
DORN	411	*
ENGELA	31	*
ENGELA	32	*
ENGELA	33	*
ENGELA	34	*
ENGELA	35	*
ENGELA	45	*
ENGELA	46	*
ENGELA	47	*
ENGELA	48	*
ENSTRO	7	-
ENSTRO	6	-
ENSTRO	5	-
ENSTRO	4	-
ENSTRO	3	-
ENSTRO	11	-
ENSTRO	10	-
ENSTRO	9	-
ENSTRO	8	-
GAO2	2	*

Table 1G6 - 1

IESLC - Meta-analysis of Current Smoking by Amount, Overview, Any product (or Cigarettes if Any not available)
All LC types
Most adjusted

REF	NRR	Cigarette equivalent
GAO2	3	*
GAO2	4	*
GILLIS	21	*
GILLIS	22	*
GILLIS	23	*
GILLIS	24	*
GILLIS	25	*
HAENSZ	52	*
HAENSZ	51	*
HAMMO2	7	*
HAMMO2	6	*
HAMMON	135	-
HAMMON	136	-
HAMMON	137	-
HAMMON	138	-
HIRAYA	23	*
HIRAYA	24	*
HIRAYA	25	*
HIRAYA	26	*
HIRAYA	27	*
HIRAYA	28	*
HITOSU	35	*
HITOSU	36	*
HITOSU	37	*
HITOSU	60	*
HITOSU	61	*
HOLE	1	*
HOLE	3	*
HOLE	4	*
HOLE	5	*
HUMBLE	2	*
HUMBLE	3	*
HUMBLE	5	*
HUMBLE	6	*
HUMBLE	8	*
HUMBLE	9	*
HUMBLE	11	*
HUMBLE	12	*
KAISE2	66	-
KAISE2	67	-
KAISE2	58	-
KAISE2	59	-
KAISER	6	*
KAISER	7	*
KAISER	8	*
KAISER	2	*
KAISER	3	*
KAISER	4	*
KANELL	26	*
KANELL	27	*
KANELL	28	*
KANELL	29	*
KATSOU	3	*
KATSOU	4	*
KAUFMA	11	*
KAUFMA	12	*
KAUFMA	13	*
KAUFMA	14	*
KAUFMA	15	*
KINLEN	14	*
KINLEN	15	*
KINLEN	16	*
KNEKT	29	*
KNEKT	30	*
KOO	11	*
KOO	12	*
KOO	13	*
LIAW	3	*
LIAW	4	*
LIAW	5	*
LIDDEL	2	*
LIDDEL	3	*

Table 1G6 - 1

IESLC - Meta-analysis of Current Smoking by Amount, Overview, Any product (or Cigarettes if Any not available)
All LC types
Most adjusted

REF	NRR	Cigarette equivalent
MACLEN	20	*
MACLEN	21	*
MACLEN	22	*
MACLEN	23	*
MACLEN	33	*
MACLEN	34	*
MACLEN	35	*
MATOS	5	*
MATOS	7	*
MATOS	9	*
MIGRAN	2	-
MIGRAN	4	-
MIGRAN	6	-
MIGRAN	8	-
MIGRAN	29	-
MIGRAN	31	-
MIGRAN	33	-
MRFITR	3	*
MRFITR	4	*
MRFITR	5	*
NAM	74	*
NAM	75	*
NAM	90	*
NAM	91	*
PARKIN	14	grams inc 1 cig=1g, 1 pipe=0.65g
PARKIN	15	grams inc 1 cig=1g, 1 pipe=0.65g
PERSH2	8	inc 50g pipe/wk= 7 cigs/day
PERSH2	9	inc 50g pipe/wk= 7 cigs/day
PETO	2	*
PETO	3	*
PEZZO2	3	*
PEZZO2	4	*
PEZZO2	5	*
PEZZOT	2	-
PEZZOT	3	-
PEZZOT	4	-
PRESCO	2	gms, inc cig = 1, cheroot = 3, cigar = 5
PRESCO	4	gms, inc cig = 1, cheroot = 3, cigar = 5
PRESCO	1	gms, inc cig = 1, cheroot = 3, cigar = 5
PRESCO	3	gms, inc cig = 1, cheroot = 3, cigar = 5
SEGI2	10	*
SEGI2	12	*
SEGI2	14	*
SEGI2	16	*
SEGI2	18	*
SEGI2	22	*
SEGI2	24	*
SEGI2	26	*
SHAW	4	*
SHAW	5	*
SOBUE	117	*
SOBUE	118	*
SOBUE	119	*
SPEIZE	1	*
SPEIZE	2	*
SPEIZE	3	*
SPEIZE	4	*
SPEIZE	5	*
STOCKW	1	*
STOCKW	2	*
STOCKW	3	*
SVENSS	6	*
SVENSS	11	*
SVENSS	16	*
TENKAN	10	grams
TENKAN	11	grams
TENKAN	12	grams
TSUGAN	29	*
TSUGAN	30	*
TSUGAN	31	*
TULINI	27	*
TULINI	28	*

Table 1G6 - 1

IESLC - Meta-analysis of Current Smoking by Amount, Overview, Any product (or Cigarettes if Any not available)
All LC types
Most adjusted

REF	NRR	Cigarette equivalent
TULINI	29	*
TULINI	32	*
TULINI	33	*
TULINI	34	*
TVERDA	9	-
TVERDA	10	-
TVERDA	11	-
TVERDA	16	-
TVERDA	17	-
WAKAI	40	*
WAKAI	41	*
WAKAI	42	*
WU	43	*
WU	44	*
WYNDE6	27	*
WYNDE6	36	*
WYNDE6	45	*
WYNDE6	54	*
WYNDE6	216	*
WYNDE6	225	*
WYNDE6	234	*
WYNDE6	243	*
YAMAGU	8	*
YAMAGU	7	*

In this overview table, subtotals and Qs values may be invalid and should be ignored

Table 1G6 - 2

IESLC - Meta-analysis of Current Smoking by Amount, Overview, Any product (or Cigarettes if Any not available)
All LC types
Most adjusted

REF	NRR	SEX	AD	Number Exposed Case	Cont	Non-exposed Case	Cont	RR	95.00%CI
*AKIBA	27	m	5	-	-	-	-	3.50	(2.20- 6.00)
*AKIBA	28	m	5	-	-	-	-	6.10	(3.90- 9.50)
*AKIBA	29	m	5	-	-	-	-	9.10	(5.40- 15.90)
*AKIBA	33	f	5	-	-	-	-	3.60	(2.60- 5.00)
*AKIBA	34	f	5	-	-	-	-	5.80	(3.30- 9.50)
Subtotal AKIBA								4.83	(3.96- 5.88)
*ARCHER	1	m	0	14	6504	6	9842	3.53	(1.36- 9.18)
*ARCHER	2	m	0	68	18320	6	9842	6.09	(2.64- 14.02)
*ARCHER	3	m	0	40	7705	6	9842	8.52	(3.61- 20.07)
Subtotal ARCHER								5.87	(3.54- 9.75)
AXELSS	18	f	1	-	-	-	-	43.30	(12.60- 148.80)
*BENSHL	11	m	1	-	-	-	-	4.00	(1.55- 10.31)
*BENSHL	12	m	1	-	-	-	-	9.05	(3.91- 20.94)
*BENSHL	13	m	1	-	-	-	-	10.95	(4.76- 25.22)
Subtotal BENSHL								7.71	(4.67- 12.74)
*BEST	13	m	1	-	-	-	-	10.00	(4.56- 21.92)
*BEST	14	m	1	-	-	-	-	16.41	(7.73- 34.86)
*BEST	15	m	1	-	-	-	-	17.31	(7.93- 37.79)
Subtotal BEST								14.23	(9.11- 22.23)
*BOUCOT	124	m	2	-	-	-	-	54.09	(3.86-1013.32)
*BOUCOT	125	m	2	-	-	-	-	78.56	(4.78-1290.69)
*BOUCOT	126	m	2	-	-	-	-	161.70	(9.23-2834.24)
Subtotal BOUCOT								87.30	(17.18- 443.54)
*BRETT	1	m	0	40	17090	6	6530	2.55	(1.08- 6.01)
*BRETT	2	m	0	62	15868	6	6530	4.25	(1.84- 9.83)
*BRETT	3	m	0	33	4490	6	6530	8.00	(3.35- 19.07)
Subtotal BRETT								4.40	(2.69- 7.21)
BROSS	18	m	0	170	155	38	170	4.91	(3.24- 7.42)
BROSS	19	m	0	95	59	38	170	7.20	(4.46- 11.63)
Subtotal BROSS								5.78	(4.23- 7.91)
BUFFLE	30	f	0	12	20	12	112	5.60	(2.21- 14.21)
BUFFLE	31	f	0	52	41	12	112	11.84	(5.75- 24.38)
BUFFLE	36	f	0	116	49	12	112	22.10	(11.16- 43.73)
Subtotal BUFFLE								12.97	(8.37- 20.09)
*CEDERL	80	m	2	-	-	-	-	3.40	(1.96- 5.90)
*CEDERL	81	m	2	-	-	-	-	7.50	(4.79- 11.74)
*CEDERL	82	m	2	-	-	-	-	11.90	(7.55- 18.75)
*CEDERL	76	f	2	-	-	-	-	2.83	(1.72- 4.67)
*CEDERL	77	f	2	-	-	-	-	7.74	(4.96- 12.08)
*CEDERL	78	f	2	-	-	-	-	7.75	(4.03- 14.91)
Subtotal CEDERL								6.36	(5.20- 7.78)
*CHANG	2	m	0	5	100	5	502	5.02	(1.48- 17.02)
*CHANG	3	m	0	17	161	5	502	10.60	(3.97- 28.28)
*CHANG	4	m	0	13	158	5	502	8.26	(2.99- 22.81)
*CHANG	8	f	0	6	205	11	1139	3.03	(1.13- 8.10)
*CHANG	9	f	0	11	234	11	1139	4.87	(2.14- 11.09)
*CHANG	10	f	0	13	164	11	1139	8.21	(3.74- 18.01)
Subtotal CHANG								6.25	(4.26- 9.17)
*CHOW	10	m	2	-	-	-	-	13.88	(5.81- 33.12)
*CHOW	11	m	2	-	-	-	-	21.87	(9.35- 51.14)
*CHOW	12	m	2	-	-	-	-	44.48	(18.63- 106.19)
Subtotal CHOW								23.78	(14.45- 39.14)
COMSTO	4	m	0	18	25	4	69	12.42	(3.83- 40.26)
COMSTO	5	m	0	60	57	4	69	18.16	(6.22- 53.00)
COMSTO	6	m	0	26	18	4	69	24.92	(7.71- 80.57)
COMSTO	9	f	0	16	19	13	115	7.45	(3.10- 17.93)
COMSTO	10	f	0	51	26	13	115	17.35	(8.25- 36.48)
COMSTO	11	f	0	9	6	13	115	13.27	(4.07- 43.25)
Subtotal COMSTO								14.21	(9.49- 21.27)
CORREA	46	c	1	-	-	-	-	9.30	(6.80- 12.70)
CORREA	50	c	1	-	-	-	-	25.30	(18.50- 34.60)
Subtotal CORREA								15.32	(12.28- 19.11)
*CPSI	216	m	1	-	-	-	-	4.51	(3.10- 6.55)
*CPSI	217	m	1	-	-	-	-	8.41	(6.41- 11.03)
*CPSI	218	m	1	-	-	-	-	14.30	(11.33- 18.05)
*CPSI	219	m	1	-	-	-	-	17.49	(13.29- 23.03)
*CPSI	275	f	1	-	-	-	-	1.25	(0.73- 2.13)
*CPSI	276	f	1	-	-	-	-	2.44	(1.67- 3.56)
*CPSI	277	f	1	-	-	-	-	5.03	(3.82- 6.63)
*CPSI	278	f	1	-	-	-	-	11.10	(6.00- 20.53)
Subtotal CPSI								7.76	(6.94- 8.69)

Table 1G6 - 2

IESLC - Meta-analysis of Current Smoking by Amount, Overview, Any product (or Cigarettes if Any not available)
All LC types
Most adjusted

REF	NRR	SEX	AD	Number Exposed Case	Number Exposed Cont	Non-exposed Case	Non-exposed Cont	RR	95.00%CI
*CPSII	120	m	1	-	-	-	-	12.22	(9.12- 16.36)
*CPSII	121	m	1	-	-	-	-	14.52	(11.19- 18.85)
*CPSII	122	m	1	-	-	-	-	21.59	(16.98- 27.46)
*CPSII	123	m	1	-	-	-	-	22.72	(16.44- 31.40)
*CPSII	124	m	1	-	-	-	-	24.14	(18.64- 31.28)
*CPSII	125	m	1	-	-	-	-	45.52	(29.35- 70.60)
*CPSII	127	f	1	-	-	-	-	3.89	(2.77- 5.47)
*CPSII	128	f	1	-	-	-	-	8.33	(6.47- 10.72)
*CPSII	129	f	1	-	-	-	-	14.21	(11.67- 17.31)
*CPSII	130	f	1	-	-	-	-	21.40	(15.68- 29.22)
*CPSII	131	f	1	-	-	-	-	19.31	(14.77- 25.25)
*CPSII	132	f	1	-	-	-	-	18.22	(10.78- 30.82)
Subtotal CPSII								15.59	(14.38- 16.91)
DARBY	1	m	0	128	223	3	384	73.47	(23.11- 233.57)
DARBY	2	m	0	126	169	3	384	95.43	(29.94- 304.17)
DARBY	3	m	0	68	61	3	384	142.69	(43.52- 467.82)
DARBY	8	f	0	71	104	23	529	15.70	(9.38- 26.28)
DARBY	9	f	0	86	92	23	529	21.50	(12.90- 35.82)
DARBY	10	f	0	38	21	23	529	41.62	(21.15- 81.90)
Subtotal DARBY								29.10	(21.80- 38.84)
DEAN3	7	m	3	-	-	-	-	5.46	(3.27- 9.10)
DEAN3	14	m	3	-	-	-	-	7.42	(4.56- 12.06)
DEAN3	21	m	3	-	-	-	-	21.66	(12.78- 36.72)
DEAN3	91	f	3	-	-	-	-	3.16	(1.92- 5.21)
DEAN3	98	f	3	-	-	-	-	8.42	(5.14- 13.78)
DEAN3	105	f	3	-	-	-	-	24.24	(13.08- 44.93)
Subtotal DEAN3								8.44	(6.83- 10.42)
*DEKLER	2	m	2	-	-	-	-	19.40	(2.60- 143.70)
*DEKLER	3	m	2	-	-	-	-	23.00	(3.20- 167.60)
*DEKLER	4	m	2	-	-	-	-	32.50	(4.40- 241.20)
Subtotal DEKLER								24.38	(7.70- 77.17)
*DOLL2	16	m	1	-	-	-	-	5.20	(2.41- 11.22)
*DOLL2	17	m	1	-	-	-	-	10.60	(4.94- 22.73)
*DOLL2	18	m	1	-	-	-	-	22.40	(10.46- 47.99)
*DOLL2	10	f	1	-	-	-	-	1.29	(0.14- 11.50)
*DOLL2	11	f	1	-	-	-	-	6.43	(1.81- 22.78)
*DOLL2	12	f	1	-	-	-	-	29.71	(9.46- 93.32)
Subtotal DOLL2								10.80	(7.35- 15.88)
DORANT	6	c	0	21	192	14	1090	8.52	(4.26- 17.04)
DORANT	7	c	0	143	409	14	1090	27.22	(15.54- 47.68)
DORANT	8	c	0	128	275	14	1090	36.24	(20.55- 63.91)
Subtotal DORANT								22.68	(16.05- 32.04)
DORGAN	4	m	0	81	55	15	93	9.13	(4.80- 17.39)
DORGAN	5	m	0	383	115	15	93	20.65	(11.52- 37.02)
DORGAN	28	m	0	96	42	3	35	26.67	(7.77- 91.56)
DORGAN	29	m	0	118	19	3	35	72.46	(20.25- 259.23)
DORGAN	51	f	0	224	81	103	244	6.55	(4.65- 9.23)
DORGAN	52	f	0	387	38	103	244	24.13	(16.09- 36.17)
DORGAN	74	f	0	39	15	7	20	7.43	(2.61- 21.16)
DORGAN	75	f	0	29	2	7	20	41.43	(7.79- 220.41)
Subtotal DORGAN								12.93	(10.47- 15.97)
*DORN	408	m	1	-	-	-	-	4.02	(3.43- 4.71)
*DORN	409	m	1	-	-	-	-	9.92	(8.84- 11.14)
*DORN	410	m	1	-	-	-	-	17.19	(15.28- 19.33)
*DORN	411	m	1	-	-	-	-	22.75	(19.63- 26.37)
Subtotal DORN								11.88	(11.13- 12.68)
*ENGELA	31	m	7	-	-	-	-	1.40	(0.60- 3.70)
*ENGELA	32	m	7	-	-	-	-	4.10	(1.70- 10.00)
*ENGELA	33	m	7	-	-	-	-	7.00	(2.90- 17.00)
*ENGELA	34	m	7	-	-	-	-	11.00	(4.20- 28.00)
*ENGELA	35	m	7	-	-	-	-	15.00	(6.10- 37.00)
*ENGELA	45	f	5	-	-	-	-	12.00	(4.50- 32.00)
*ENGELA	46	f	5	-	-	-	-	12.00	(4.40- 30.00)
*ENGELA	47	f	5	-	-	-	-	24.00	(9.50- 59.00)
*ENGELA	48	f	5	-	-	-	-	26.00	(9.20- 73.00)
Subtotal ENGELA								9.05	(6.63- 12.35)
*ENSTRO	7	m	1	-	-	-	-	4.74	(3.34- 6.73)
*ENSTRO	6	m	1	-	-	-	-	7.68	(5.95- 9.90)
*ENSTRO	5	m	1	-	-	-	-	13.65	(10.88- 17.13)
*ENSTRO	4	m	1	-	-	-	-	16.08	(12.77- 20.23)
*ENSTRO	3	m	1	-	-	-	-	19.41	(15.22- 24.75)

Table 1G6 - 2

IESLC - Meta-analysis of Current Smoking by Amount, Overview, Any product (or Cigarettes if Any not available)
All LC types
Most adjusted

REF	NRR	SEX	AD	Number Exposed Case	Number Exposed Cont	Non-exposed Case	Non-exposed Cont	RR	95.00%CI
*ENSTRO	11	f	1	-	-	-	-	2.15	(1.62- 2.84)
*ENSTRO	10	f	1	-	-	-	-	4.31	(3.56- 5.22)
*ENSTRO	9	f	1	-	-	-	-	9.48	(8.04- 11.18)
*ENSTRO	8	f	1	-	-	-	-	16.47	(13.74- 19.75)
Subtotal ENSTRO								9.31	(8.66- 10.02)
GAO2	2	m	0	32	41	13	56	3.36	(1.57- 7.19)
GAO2	3	m	0	77	44	13	56	7.54	(3.71- 15.30)
GAO2	4	m	0	74	30	13	56	10.63	(5.08- 22.22)
Subtotal GAO2								6.57	(4.30- 10.04)
GILLIS	21	m	3	-	-	-	-	4.50	(2.50- 8.10)
GILLIS	22	m	3	-	-	-	-	7.60	(4.20- 13.80)
GILLIS	23	m	3	-	-	-	-	8.60	(4.60- 16.10)
GILLIS	24	m	3	-	-	-	-	9.70	(5.10- 18.40)
GILLIS	25	m	3	-	-	-	-	7.80	(3.70- 16.40)
Subtotal GILLIS								7.27	(5.48- 9.64)
HAENSZ	52	f	0	40	66	81	236	1.77	(1.11- 2.82)
HAENSZ	51	f	0	23	13	81	236	5.15	(2.50- 10.65)
Subtotal HAENSZ								2.42	(1.63- 3.58)
*HAMMO2	7	m	1	-	-	-	-	9.15	(3.62- 23.12)
*HAMMO2	6	m	1	-	-	-	-	10.39	(4.28- 25.22)
Subtotal HAMMO2								9.78	(5.15- 18.56)
*HAMMON	135	m	1	-	-	-	-	7.44	(3.90- 14.18)
*HAMMON	136	m	1	-	-	-	-	8.42	(4.86- 14.59)
*HAMMON	137	m	1	-	-	-	-	17.91	(10.37- 30.93)
*HAMMON	138	m	1	-	-	-	-	20.64	(10.98- 38.80)
Subtotal HAMMON								12.40	(9.24- 16.64)
*HIRAYA	23	m	1	-	-	-	-	2.06	(1.49- 2.85)
*HIRAYA	24	m	1	-	-	-	-	4.00	(3.20- 4.99)
*HIRAYA	25	m	1	-	-	-	-	6.24	(5.07- 7.68)
*HIRAYA	26	f	1	-	-	-	-	2.25	(1.64- 3.08)
*HIRAYA	27	f	1	-	-	-	-	2.56	(1.85- 3.54)
*HIRAYA	28	f	1	-	-	-	-	4.47	(2.73- 7.33)
Subtotal HIRAYA								3.72	(3.32- 4.17)
HITOSU	35	m	1	-	-	-	-	2.08	(0.90- 4.83)
HITOSU	36	m	1	-	-	-	-	2.82	(1.25- 6.36)
HITOSU	37	m	1	-	-	-	-	4.68	(1.97- 11.11)
HITOSU	60	f	1	-	-	-	-	3.11	(1.77- 5.46)
HITOSU	61	f	1	-	-	-	-	3.17	(1.07- 9.36)
Subtotal HITOSU								3.05	(2.16- 4.32)
*HOLE	1	m	1	-	-	-	-	5.47	(2.35- 12.75)
*HOLE	3	m	1	-	-	-	-	8.90	(4.12- 19.23)
*HOLE	4	m	1	-	-	-	-	10.75	(4.80- 24.06)
*HOLE	5	m	1	-	-	-	-	7.49	(2.99- 18.77)
Subtotal HOLE								8.03	(5.31- 12.17)
HUMBLE	2	m	1	-	-	-	-	9.20	(3.30- 25.80)
HUMBLE	3	m	1	-	-	-	-	24.70	(10.00- 59.90)
HUMBLE	5	m	1	-	-	-	-	11.60	(2.70- 61.50)
HUMBLE	6	m	1	-	-	-	-	26.10	(5.60- 146.60)
HUMBLE	8	f	1	-	-	-	-	19.20	(6.50- 60.80)
HUMBLE	9	f	1	-	-	-	-	16.00	(6.70- 36.30)
HUMBLE	11	f	1	-	-	-	-	18.50	(4.90- 72.40)
HUMBLE	12	f	1	-	-	-	-	36.90	(7.60- 217.10)
Subtotal HUMBLE								17.64	(11.76- 26.46)
*KAISE2	66	m	1	-	-	-	-	4.47	(2.00- 9.99)
*KAISE2	67	m	1	-	-	-	-	10.34	(5.56- 19.23)
*KAISE2	58	f	1	-	-	-	-	7.61	(3.26- 17.75)
*KAISE2	59	f	1	-	-	-	-	22.12	(11.22- 43.61)
Subtotal KAISE2								10.24	(7.14- 14.68)
*KAISER	6	m	2	-	-	-	-	6.58	(3.87- 11.20)
*KAISER	7	m	2	-	-	-	-	17.24	(10.71- 27.73)
*KAISER	8	m	2	-	-	-	-	20.91	(12.78- 27.73)
*KAISER	2	f	2	-	-	-	-	3.42	(2.17- 5.40)
*KAISER	3	f	2	-	-	-	-	7.98	(5.35- 11.90)
*KAISER	4	f	2	-	-	-	-	12.63	(8.06- 19.80)
Subtotal KAISER								10.14	(8.46- 12.15)
KANELL	26	m	1	-	-	-	-	1.71	(1.15- 2.56)
KANELL	27	m	1	-	-	-	-	7.06	(4.76- 10.48)
KANELL	28	m	1	-	-	-	-	20.39	(10.37- 40.09)
KANELL	29	m	1	-	-	-	-	34.22	(18.86- 62.11)
Subtotal KANELL								6.27	(4.94- 7.96)
KATSOU	3	f	1	-	-	-	-	2.26	(1.06- 4.85)

Table 1G6 - 2

IESLC - Meta-analysis of Current Smoking by Amount, Overview, Any product (or Cigarettes if Any not available)
All LC types
Most adjusted

REF	NRR	SEX	AD	Number Exposed Case	Number Exposed Cont	Non-exposed Case	Non-exposed Cont	RR	95.00%CI
KATSOU	4	f	1	-	-	-	-	7.46	(2.40- 23.17)
Subtotal KATSOU								3.27	(1.74- 6.16)
KAUFMA	11	c	6	-	-	-	-	8.00	(5.00- 13.00)
KAUFMA	12	c	6	-	-	-	-	15.00	(10.00- 23.00)
KAUFMA	13	c	6	-	-	-	-	28.00	(17.00- 44.00)
KAUFMA	14	c	6	-	-	-	-	43.00	(27.00- 68.00)
KAUFMA	15	c	6	-	-	-	-	60.00	(35.00- 102.00)
Subtotal KAUFMA								23.07	(18.70- 28.44)
*KINLEN	14	m	2	-	-	-	-	10.61	(5.01- 22.48)
*KINLEN	15	m	2	-	-	-	-	14.14	(6.68- 29.91)
*KINLEN	16	m	2	-	-	-	-	21.74	(10.23- 46.19)
Subtotal KINLEN								14.81	(9.60- 22.86)
*KNEKT	29	m	1	-	-	-	-	5.00	(2.00- 12.30)
*KNEKT	30	m	1	-	-	-	-	12.70	(5.50- 29.40)
Subtotal KNEKT								8.27	(4.47- 15.31)
KOO	11	f	0	17	19	56	85	1.36	(0.65- 2.84)
KOO	12	f	0	24	5	56	85	7.29	(2.62- 20.22)
KOO	13	f	0	1	1	56	85	1.52	(0.09- 24.77)
Subtotal KOO								2.36	(1.32- 4.24)
*LIAW	3	c	2	-	-	-	-	3.10	(1.70- 5.60)
*LIAW	4	c	2	-	-	-	-	3.60	(2.00- 6.40)
*LIAW	5	c	2	-	-	-	-	8.30	(4.00- 17.30)
Subtotal LIAW								4.18	(2.91- 6.00)
*LIDDEL	2	m	1	-	-	-	-	3.33	(2.05- 5.64)
*LIDDEL	3	m	1	-	-	-	-	5.02	(3.21- 8.22)
Subtotal LIDDEL								4.15	(2.94- 5.86)
MACLEN	20	m	0	5	11	5	15	1.36	(0.32- 5.89)
MACLEN	21	m	0	42	37	5	15	3.41	(1.13- 10.28)
MACLEN	22	m	0	43	31	5	15	4.16	(1.37- 12.66)
MACLEN	23	m	0	45	27	5	15	5.00	(1.63- 15.31)
MACLEN	33	f	0	6	21	41	109	0.76	(0.29- 2.02)
MACLEN	34	f	0	22	17	41	109	3.44	(1.66- 7.12)
MACLEN	35	f	0	13	9	41	109	3.84	(1.53- 9.66)
Subtotal MACLEN								2.80	(1.92- 4.08)
MATOS	5	m	2	-	-	-	-	1.60	(0.50- 5.00)
MATOS	7	m	2	-	-	-	-	8.00	(3.40- 16.80)
MATOS	9	m	2	-	-	-	-	15.00	(7.10- 31.90)
Subtotal MATOS								7.81	(4.76- 12.80)
*MIGRAN	2	m	2	-	-	-	-	4.01	(1.19- 13.47)
*MIGRAN	4	m	2	-	-	-	-	4.24	(1.53- 11.74)
*MIGRAN	6	m	2	-	-	-	-	5.14	(1.79- 14.81)
*MIGRAN	8	m	2	-	-	-	-	5.93	(2.03- 17.29)
*MIGRAN	29	f	2	-	-	-	-	4.88	(1.17- 20.43)
*MIGRAN	31	f	2	-	-	-	-	6.53	(2.11- 20.22)
*MIGRAN	33	f	2	-	-	-	-	7.48	(1.64- 34.03)
Subtotal MIGRAN								5.24	(3.37- 8.15)
*MRFITR	3	m	0	2	856	0	1859	10.86~	(0.52- 225.86)
*MRFITR	4	m	0	50	3747	0	1859	50.12~	(3.09- 811.82)
*MRFITR	5	m	0	54	3591	0	1859	56.43~	(3.49- 913.25)
Subtotal MRFITR								33.22	(6.37- 173.28)
NAM	74	m	1	-	-	-	-	6.70	(4.19- 10.71)
NAM	75	m	1	-	-	-	-	10.27	(6.42- 16.43)
NAM	90	f	1	-	-	-	-	9.06	(5.83- 14.06)
NAM	91	f	1	-	-	-	-	16.65	(10.20- 27.19)
Subtotal NAM								9.95	(7.88- 12.57)
PARKIN	14	m	6	-	-	-	-	3.90	(3.00- 5.00)
PARKIN	15	m	6	-	-	-	-	5.20	(3.50- 7.70)
Subtotal PARKIN								4.25	(3.43- 5.26)
PERSH2	8	c	4	-	-	-	-	5.76	(4.61- 7.19)
PERSH2	9	c	4	-	-	-	-	11.34	(9.14- 14.07)
Subtotal PERSH2								8.16	(6.99- 9.53)
*PETO	2	m	0	44	1181	2	295	5.50	(1.34- 22.54)
*PETO	3	m	0	55	855	2	295	9.49	(2.33- 38.66)
Subtotal PETO								7.23	(2.67- 19.57)
PEZZO2	3	m	0	57	139	6	117	8.00	(3.33- 19.21)
PEZZO2	4	m	0	107	47	6	117	44.39	(18.24- 108.02)
PEZZO2	5	m	0	69	12	6	117	112.13	(40.26- 312.24)
Subtotal PEZZO2								30.27	(17.76- 51.58)
PEZZOT	2	m	0	24	94	4	116	7.40	(2.48- 22.09)
PEZZOT	3	m	0	70	29	4	116	70.00	(23.61- 207.50)
PEZZOT	4	m	0	51	6	4	116	246.50	(66.69- 911.11)

Table 1G6 - 2

IESLC - Meta-analysis of Current Smoking by Amount, Overview, Any product (or Cigarettes if Any not available)
All LC types
Most adjusted

REF	NRR	SEX	AD	Number Exposed Case	Cont	Non-exposed Case	Cont	RR	95.00%CI
Subtotal PEZZOT								42.28	(21.77- 82.11)
*PRESCO	2	m	1	-	-	-	-	10.20	(4.49- 23.15)
*PRESCO	4	m	1	-	-	-	-	19.96	(8.92- 44.67)
*PRESCO	1	f	1	-	-	-	-	6.36	(3.60- 11.24)
*PRESCO	3	f	1	-	-	-	-	10.08	(5.72- 17.75)
Subtotal PRESCO								9.70	(6.98- 13.49)
SEGI2	10	m	1	-	-	-	-	2.10	(0.86- 5.16)
SEGI2	12	m	1	-	-	-	-	3.10	(1.40- 6.84)
SEGI2	14	m	1	-	-	-	-	3.40	(1.55- 7.45)
SEGI2	16	m	1	-	-	-	-	6.90	(2.78- 17.14)
SEGI2	18	m	1	-	-	-	-	7.90	(3.40- 18.37)
SEGI2	22	f	1	-	-	-	-	2.90	(1.09- 7.70)
SEGI2	24	f	1	-	-	-	-	1.44	(0.61- 3.40)
SEGI2	26	f	1	-	-	-	-	1.03	(0.34- 3.15)
Subtotal SEGI2								3.08	(2.26- 4.21)
SHAW	4	c	0	24	37	11	107	6.31	(2.82- 14.12)
SHAW	5	c	0	188	60	11	107	30.48	(15.36- 60.48)
Subtotal SHAW								15.73	(9.34- 26.52)
SOBUE	117	m	0	147	157	34	128	3.52	(2.27- 5.47)
SOBUE	118	m	0	236	222	34	128	4.00	(2.63- 6.09)
SOBUE	119	m	0	226	187	34	128	4.55	(2.97- 6.96)
Subtotal SOBUE								4.02	(3.14- 5.14)
*SPEIZE	1	f	1	-	-	-	-	2.70	(0.90- 5.40)
*SPEIZE	2	f	1	-	-	-	-	5.20	(3.60- 8.10)
*SPEIZE	3	f	1	-	-	-	-	12.60	(9.90- 16.70)
*SPEIZE	4	f	1	-	-	-	-	15.70	(12.10- 20.20)
*SPEIZE	5	f	1	-	-	-	-	22.00	(14.80- 32.30)
Subtotal SPEIZE								12.52	(10.76- 14.56)
STOCKW	1	c	0	2090	1194	2791	10641	6.67	(6.15- 7.25)
STOCKW	2	c	0	6053	1591	2791	10641	14.51	(13.54- 15.54)
STOCKW	3	c	0	4327	572	2791	10641	28.84	(26.18- 31.77)
Subtotal STOCKW								13.28	(12.68- 13.92)
SVENSS	6	f	1	-	-	-	-	4.60	(2.50- 9.30)
SVENSS	11	f	1	-	-	-	-	12.60	(6.50- 25.20)
SVENSS	16	f	1	-	-	-	-	59.00	(7.60- 458.03)
Subtotal SVENSS								8.31	(5.25- 13.17)
*TENKAN	10	m	1	-	-	-	-	15.86	(6.80- 37.00)
*TENKAN	11	m	1	-	-	-	-	20.25	(8.20- 50.00)
*TENKAN	12	m	1	-	-	-	-	24.97	(9.90- 63.00)
Subtotal TENKAN								19.74	(11.81- 33.01)
TSUGAN	29	m	0	14	19	18	22	0.90	(0.36- 2.28)
TSUGAN	30	m	0	30	30	18	22	1.22	(0.55- 2.73)
TSUGAN	31	m	0	19	14	18	22	1.66	(0.65- 4.20)
Subtotal TSUGAN								1.22	(0.73- 2.03)
*TULINI	27	m	3	-	-	-	-	6.02	(3.01- 12.00)
*TULINI	28	m	3	-	-	-	-	12.00	(6.31- 22.90)
*TULINI	29	m	3	-	-	-	-	27.30	(14.20- 52.40)
*TULINI	32	f	3	-	-	-	-	8.17	(4.33- 15.40)
*TULINI	33	f	3	-	-	-	-	26.30	(14.40- 48.10)
*TULINI	34	f	3	-	-	-	-	38.70	(18.50- 80.80)
Subtotal TULINI								15.82	(12.10- 20.68)
*TVERDA	9	m	2	-	-	-	-	2.14	(1.15- 3.96)
*TVERDA	10	m	2	-	-	-	-	3.32	(2.07- 5.32)
*TVERDA	11	m	2	-	-	-	-	6.56	(4.04- 10.64)
*TVERDA	16	f	2	-	-	-	-	4.53	(1.08- 18.94)
*TVERDA	17	f	2	-	-	-	-	18.00	(5.33- 60.83)
Subtotal TVERDA								4.23	(3.19- 5.62)
WAKAI	40	m	2	-	-	-	-	1.80	(0.81- 4.02)
WAKAI	41	m	2	-	-	-	-	4.01	(1.91- 8.41)
WAKAI	42	m	2	-	-	-	-	9.19	(4.20- 20.10)
Subtotal WAKAI								4.09	(2.62- 6.40)
WU	43	f	2	-	-	-	-	3.25	(1.71- 6.16)
WU	44	f	2	-	-	-	-	8.48	(4.16- 17.29)
Subtotal WU								4.99	(3.10- 8.04)
WYNDE6	27	m	0	117	122	87	617	6.80	(4.85- 9.54)
WYNDE6	36	m	0	461	293	87	617	11.16	(8.54- 14.59)
WYNDE6	45	m	0	315	129	87	617	17.32	(12.78- 23.47)
WYNDE6	54	m	0	784	197	87	617	28.22	(21.47- 37.10)
WYNDE6	216	f	0	76	109	159	856	3.75	(2.68- 5.26)
WYNDE6	225	f	0	367	165	159	856	11.97	(9.33- 15.37)
WYNDE6	234	f	0	201	50	159	856	21.64	(15.21- 30.80)

Table 1G6 - 2

IESLC - Meta-analysis of Current Smoking by Amount, Overview, Any product (or Cigarettes if Any not available)
All LC types
Most adjusted

REF	NRR	SEX	AD	Number Exposed Case	Number Exposed Cont	Non-exposed Case	Non-exposed Cont	RR	95.00%CI
WYNDE6	243	f	0	378	52	159	856	39.13	(27.98- 54.75)
Subtotal WYNDE6								14.13	(12.71- 15.72)
YAMAGU	8	c	1	-	-	-	-	3.75	(1.89- 7.47)
YAMAGU	7	c	1	-	-	-	-	12.14	(5.10- 28.90)
Subtotal YAMAGU								5.90	(3.44- 10.11)
Partial Totals				20213	89516	10704	108687		

*prospective study ~ With 0.5 adjustment for zero

REF	NRR	SEX	AD	Ys	Ws	Qs	Ps
*AKIBA	27	m	5	1.25	15.26	17.84	0.0000
*AKIBA	28	m	5	1.81	19.39	5.35	0.0000
*AKIBA	29	m	5	2.21	13.18	0.21	0.0000
*AKIBA	33	f	5	1.28	35.93	39.83	0.0000
*AKIBA	34	f	5	1.76	13.74	4.56	0.0000
Subtotal AKIBA				1.57	97.50	67.78	
*ARCHER	1	m	0	1.26	4.20	4.83	0.0097
*ARCHER	2	m	0	1.81	5.52	1.53	0.0000
*ARCHER	3	m	0	2.14	5.22	0.19	0.0000
Subtotal ARCHER				1.77	14.95	6.56	
AXELSS	18	f	1	3.77	2.52	5.19	0.0000
*BENSHL	11	m	1	1.39	4.28	3.84	0.0041
*BENSHL	12	m	1	2.20	5.46	0.09	0.0000
*BENSHL	13	m	1	2.39	5.53	0.02	0.0000
Subtotal BENSHL				2.04	15.26	3.95	
*BEST	13	m	1	2.30	6.23	0.01	0.0000
*BEST	14	m	1	2.80	6.77	1.46	0.0000
*BEST	15	m	1	2.85	6.30	1.69	0.0000
Subtotal BEST				2.66	19.31	3.15	
*BOUCOT	124	m	2	3.99	0.50	1.36	0.0050
*BOUCOT	125	m	2	4.36	0.49	2.02	0.0022
*BOUCOT	126	m	2	5.09	0.47	3.55	0.0005
Subtotal BOUCOT				4.47	1.45	6.93	
*BRETT	1	m	0	0.94	5.22	10.22	0.0326
*BRETT	2	m	0	1.45	5.48	4.30	0.0007
*BRETT	3	m	0	2.08	5.09	0.33	0.0000
Subtotal BRETT				1.48	15.79	14.85	
BROSS	18	m	0	1.59	22.46	12.40	0.0000
BROSS	19	m	0	1.97	16.76	2.16	0.0000
Subtotal BROSS				1.75	39.21	14.56	
BUFFLE	30	f	0	1.72	4.43	1.65	0.0003
BUFFLE	31	f	0	2.47	7.36	0.14	0.0000
BUFFLE	36	f	0	3.10	8.24	4.78	0.0000
Subtotal BUFFLE				2.56	20.04	6.58	
*CEDERL	80	m	2	1.22	12.65	15.59	0.0000
*CEDERL	81	m	2	2.01	19.12	1.94	0.0000
*CEDERL	82	m	2	2.48	18.57	0.38	0.0000
*CEDERL	76	f	2	1.04	15.40	25.77	0.0000
*CEDERL	77	f	2	2.05	19.39	1.60	0.0000
*CEDERL	78	f	2	2.05	8.98	0.73	0.0000
Subtotal CEDERL				1.85	94.11	46.01	
*CHANG	2	m	0	1.61	2.58	1.34	0.0096
*CHANG	3	m	0	2.36	3.99	0.00	0.0000
*CHANG	4	m	0	2.11	3.72	0.18	0.0000
*CHANG	8	f	0	1.11	3.97	5.96	0.0271
*CHANG	9	f	0	1.58	5.66	3.19	0.0002
*CHANG	10	f	0	2.11	6.22	0.32	0.0000
Subtotal CHANG				1.83	26.14	11.00	
*CHOW	10	m	2	2.63	5.07	0.45	0.0000
*CHOW	11	m	2	3.09	5.32	3.00	0.0000
*CHOW	12	m	2	3.80	5.07	10.83	0.0000
Subtotal CHOW				3.17	15.47	14.28	
COMSTO	4	m	0	2.52	2.78	0.10	0.0000
COMSTO	5	m	0	2.90	3.35	1.07	0.0000
COMSTO	6	m	0	3.22	2.79	2.17	0.0000
COMSTO	9	f	0	2.01	4.98	0.53	0.0000
COMSTO	10	f	0	2.85	6.96	1.88	0.0000
COMSTO	11	f	0	2.59	2.75	0.17	0.0000
Subtotal COMSTO				2.65	23.61	5.92	
CORREA	46	c	1	2.23	39.38	0.42	0.0000
CORREA	50	c	1	3.23	39.20	31.55	0.0000

Table 1G6 - 2

IESLC - Meta-analysis of Current Smoking by Amount, Overview, Any product (or Cigarettes if Any not available)
All LC types
Most adjusted

REF	NRR	SEX	AD	Ys	Ws	Qs	Ps
Subtotal CORREA				2.73	78.58	31.97	
*CPSI	216	m	1	1.51	27.46	18.80	0.0000
*CPSI	217	m	1	2.13	52.16	2.18	0.0000
*CPSI	218	m	1	2.66	70.85	7.56	0.0000
*CPSI	219	m	1	2.86	50.84	14.17	0.0000
*CPSI	275	f	1	0.22	13.40	59.69	0.4140
*CPSI	276	f	1	0.89	26.82	55.74	0.0000
*CPSI	277	f	1	1.62	50.55	26.08	0.0000
*CPSI	278	f	1	2.41	10.15	0.05	0.0000
Subtotal CPSI				2.05	302.23	184.26	
*CPSII	120	m	1	2.50	45.00	1.29	0.0000
*CPSII	121	m	1	2.68	56.50	6.60	0.0000
*CPSII	122	m	1	3.07	66.50	36.27	0.0000
*CPSII	123	m	1	3.12	36.70	22.88	0.0000
*CPSII	124	m	1	3.18	57.34	41.44	0.0000
*CPSII	125	m	1	3.82	19.94	43.95	0.0000
*CPSII	127	f	1	1.36	33.19	31.57	0.0000
*CPSII	128	f	1	2.12	60.27	2.76	0.0000
*CPSII	129	f	1	2.65	98.85	10.14	0.0000
*CPSII	130	f	1	3.06	39.66	21.12	0.0000
*CPSII	131	f	1	2.96	53.44	21.00	0.0000
*CPSII	132	f	1	2.90	13.92	4.51	0.0000
Subtotal CPSII				2.75	581.31	243.52	
DARBY	1	m	0	4.30	2.87	11.07	0.0000
DARBY	2	m	0	4.56	2.86	14.15	0.0000
DARBY	3	m	0	4.96	2.72	18.80	0.0000
DARBY	8	f	0	2.75	14.48	2.56	0.0000
DARBY	9	f	0	3.07	14.73	7.95	0.0000
DARBY	10	f	0	3.73	8.38	16.31	0.0000
Subtotal DARBY				3.37	46.05	70.83	
DEAN3	7	m	3	1.70	14.67	5.94	0.0000
DEAN3	14	m	3	2.00	16.24	1.76	0.0000
DEAN3	21	m	3	3.08	13.79	7.59	0.0000
DEAN3	91	f	3	1.15	15.42	21.58	0.0000
DEAN3	98	f	3	2.13	15.80	0.65	0.0000
DEAN3	105	f	3	3.19	10.09	7.36	0.0000
Subtotal DEAN3				2.13	86.02	44.89	
*DEKLER	2	m	2	2.97	0.95	0.38	0.0038
*DEKLER	3	m	2	3.14	0.98	0.63	0.0019
*DEKLER	4	m	2	3.48	0.96	1.26	0.0007
Subtotal DEKLER				3.19	2.89	2.27	
*DOLL2	16	m	1	1.65	6.50	3.05	0.0000
*DOLL2	17	m	1	2.36	6.60	0.00	0.0000
*DOLL2	18	m	1	3.11	6.62	3.98	0.0000
*DOLL2	10	f	1	0.25	0.79	3.42	0.8209
*DOLL2	11	f	1	1.86	2.40	0.54	0.0040
*DOLL2	12	f	1	3.39	2.93	3.28	0.0000
Subtotal DOLL2				2.38	25.83	14.27	
DORANT	6	c	0	2.14	7.99	0.29	0.0000
DORANT	7	c	0	3.30	12.23	11.51	0.0000
DORANT	8	c	0	3.59	11.93	18.84	0.0000
Subtotal DORANT				3.12	32.15	30.65	
DORGAN	4	m	0	2.21	9.26	0.14	0.0000
DORGAN	5	m	0	3.03	11.27	5.43	0.0000
DORGAN	28	m	0	3.28	2.52	2.28	0.0000
DORGAN	29	m	0	4.28	2.36	8.98	0.0000
DORGAN	51	f	0	1.88	32.66	6.73	0.0000
DORGAN	52	f	0	3.18	23.42	16.90	0.0000
DORGAN	74	f	0	2.01	3.51	0.38	0.0002
DORGAN	75	f	0	3.72	1.37	2.66	0.0000
Subtotal DORGAN				2.56	86.38	43.50	
*DORN	408	m	1	1.39	152.79	135.70	0.0000
*DORN	409	m	1	2.29	287.32	0.44	0.0000
*DORN	410	m	1	2.84	277.97	72.48	0.0000
*DORN	411	m	1	3.12	176.37	110.31	0.0000
Subtotal DORN				2.47	894.45	318.93	
*ENGELA	31	m	7	0.34	4.64	18.52	0.4684
*ENGELA	32	m	7	1.41	4.89	4.17	0.0018
*ENGELA	33	m	7	1.95	4.91	0.74	0.0000
*ENGELA	34	m	7	2.40	4.27	0.02	0.0000
*ENGELA	35	m	7	2.71	4.73	0.66	0.0000

Table 1G6 - 2

IESLC - Meta-analysis of Current Smoking by Amount, Overview, Any product (or Cigarettes if Any not available)
All LC types
Most adjusted

REF	NRR	SEX	AD	Ys	Ws	Qs	Ps
*ENGELA	45	f	5	2.48	3.99	0.09	0.0000
*ENGELA	46	f	5	2.48	4.17	0.10	0.0000
*ENGELA	47	f	5	3.18	4.61	3.28	0.0000
*ENGELA	48	f	5	3.26	3.58	3.06	0.0000
Subtotal ENGELA				2.20	39.80	30.64	
*ENSTRO	7	m	1	1.56	31.30	18.93	0.0000
*ENSTRO	6	m	1	2.04	59.28	5.16	0.0000
*ENSTRO	5	m	1	2.61	74.58	5.85	0.0000
*ENSTRO	4	m	1	2.78	72.60	14.30	0.0000
*ENSTRO	3	m	1	2.97	65.00	25.97	0.0000
*ENSTRO	11	f	1	0.77	48.76	119.91	0.0000
*ENSTRO	10	f	1	1.46	104.90	79.90	0.0000
*ENSTRO	9	f	1	2.25	141.36	1.01	0.0000
*ENSTRO	8	f	1	2.80	116.71	25.55	0.0000
Subtotal ENSTRO				2.23	714.48	296.58	
GAO2	2	m	0	1.21	6.65	8.36	0.0018
GAO2	3	m	0	2.02	7.66	0.75	0.0000
GAO2	4	m	0	2.36	7.06	0.01	0.0000
Subtotal GAO2				1.88	21.37	9.12	
GILLIS	21	m	3	1.50	11.12	7.65	0.0000
GILLIS	22	m	3	2.03	10.86	1.01	0.0000
GILLIS	23	m	3	2.15	9.79	0.32	0.0000
GILLIS	24	m	3	2.27	9.33	0.04	0.0000
GILLIS	25	m	3	2.05	6.93	0.54	0.0000
Subtotal GILLIS				1.98	48.03	9.57	
HAENSZ	52	f	0	0.57	17.63	54.91	0.0170
HAENSZ	51	f	0	1.64	7.30	3.51	0.0000
Subtotal HAENSZ				0.88	24.93	58.43	
*HAMMO2	7	m	1	2.21	4.47	0.06	0.0000
*HAMMO2	6	m	1	2.34	4.88	0.00	0.0000
Subtotal HAMMO2				2.28	9.35	0.06	
*HAMMON	135	m	1	2.01	9.22	0.99	0.0000
*HAMMON	136	m	1	2.13	12.72	0.52	0.0000
*HAMMON	137	m	1	2.89	12.87	3.92	0.0000
*HAMMON	138	m	1	3.03	9.64	4.64	0.0000
Subtotal HAMMON				2.52	44.45	10.06	
*HIRAYA	23	m	1	0.72	36.53	94.81	0.0000
*HIRAYA	24	m	1	1.39	77.85	69.87	0.0000
*HIRAYA	25	m	1	1.83	89.10	22.52	0.0000
*HIRAYA	26	f	1	0.81	38.69	89.71	0.0000
*HIRAYA	27	f	1	0.94	36.49	70.87	0.0000
*HIRAYA	28	f	1	1.50	15.75	11.02	0.0000
Subtotal HIRAYA				1.31	294.40	358.80	
HITOSU	35	m	1	0.73	5.44	13.96	0.0875
HITOSU	36	m	1	1.04	5.81	9.77	0.0125
HITOSU	37	m	1	1.54	5.14	3.21	0.0005
HITOSU	60	f	1	1.13	12.11	17.41	0.0001
HITOSU	61	f	1	1.15	3.27	4.55	0.0370
Subtotal HITOSU				1.12	31.76	48.89	
*HOLE	1	m	1	1.70	5.37	2.16	0.0001
*HOLE	3	m	1	2.19	6.47	0.14	0.0000
*HOLE	4	m	1	2.37	5.91	0.01	0.0000
*HOLE	5	m	1	2.01	4.55	0.47	0.0000
Subtotal HOLE				2.08	22.31	2.78	
HUMBLE	2	m	1	2.22	3.63	0.05	0.0000
HUMBLE	3	m	1	3.21	4.80	3.66	0.0000
HUMBLE	5	m	1	2.45	1.57	0.02	0.0021
HUMBLE	6	m	1	3.26	1.44	1.24	0.0001
HUMBLE	8	f	1	2.95	3.07	1.19	0.0000
HUMBLE	9	f	1	2.77	5.38	1.04	0.0000
HUMBLE	11	f	1	2.92	2.12	0.72	0.0000
HUMBLE	12	f	1	3.61	1.37	2.22	0.0000
Subtotal HUMBLE				2.87	23.38	10.13	
*KAISE2	66	m	1	1.50	5.94	4.15	0.0003
*KAISE2	67	m	1	2.34	9.98	0.00	0.0000
*KAISE2	58	f	1	2.03	5.35	0.50	0.0000
*KAISE2	59	f	1	3.10	8.34	4.85	0.0000
Subtotal KAISE2				2.33	29.61	9.50	
*KAISER	6	m	2	1.88	13.61	2.75	0.0000
*KAISER	7	m	2	2.85	16.98	4.48	0.0000
*KAISER	8	m	2	3.04	25.61	12.78	0.0000

Table 1G6 - 2

IESLC - Meta-analysis of Current Smoking by Amount, Overview, Any product (or Cigarettes if Any not available)
All LC types
Most adjusted

REF	NRR	SEX	AD	Ys	Ws	Qs	Ps
*KAISER	2	f	2	1.23	18.49	22.54	0.0000
*KAISER	3	f	2	2.08	24.04	1.59	0.0000
*KAISER	4	f	2	2.54	19.02	0.78	0.0000
Subtotal KAISER				2.32	117.74	44.91	
KANELL	26	m	1	0.54	23.99	77.50	0.0086
KANELL	27	m	1	1.95	24.67	3.55	0.0000
KANELL	28	m	1	3.02	8.40	3.90	0.0000
KANELL	29	m	1	3.53	10.82	15.55	0.0000
Subtotal KANELL				1.84	67.88	100.50	
KATSOU	3	f	1	0.82	6.64	15.32	0.0356
KATSOU	4	f	1	2.01	2.99	0.31	0.0005
Subtotal KATSOU				1.19	9.63	15.63	
KAUFMA	11	c	6	2.08	16.83	1.09	0.0000
KAUFMA	12	c	6	2.71	22.15	3.10	0.0000
KAUFMA	13	c	6	3.33	16.99	16.94	0.0000
KAUFMA	14	c	6	3.76	18.01	36.70	0.0000
KAUFMA	15	c	6	4.09	13.43	41.63	0.0000
Subtotal KAUFMA				3.14	87.41	99.47	
*KINLEN	14	m	2	2.36	6.82	0.01	0.0000
*KINLEN	15	m	2	2.65	6.84	0.68	0.0000
*KINLEN	16	m	2	3.08	6.76	3.76	0.0000
Subtotal KINLEN				2.70	20.42	4.44	
*KNEKT	29	m	1	1.61	4.66	2.44	0.0005
*KNEKT	30	m	1	2.54	5.47	0.24	0.0000
Subtotal KNEKT				2.11	10.13	2.68	
KOO	11	f	0	0.31	7.09	29.14	0.4151
KOO	12	f	0	1.99	3.69	0.45	0.0001
KOO	13	f	0	0.42	0.49	1.81	0.7696
Subtotal KOO				0.86	11.27	31.40	
*LIAW	3	c	2	1.13	10.81	15.63	0.0002
*LIAW	4	c	2	1.28	11.36	12.59	0.0000
*LIAW	5	c	2	2.12	7.17	0.34	0.0000
Subtotal LIAW				1.43	29.33	28.56	
*LIDDEL	2	m	1	1.20	15.00	19.18	0.0000
*LIDDEL	3	m	1	1.61	17.38	9.02	0.0000
Subtotal LIDDEL				1.42	32.38	28.20	
MACLEN	20	m	0	0.31	1.79	7.34	0.6779
MACLEN	21	m	0	1.23	3.15	3.87	0.0297
MACLEN	22	m	0	1.43	3.10	2.56	0.0120
MACLEN	23	m	0	1.61	3.07	1.61	0.0048
MACLEN	33	f	0	-0.27	4.03	27.46	0.5807
MACLEN	34	f	0	1.24	7.25	8.75	0.0009
MACLEN	35	f	0	1.35	4.51	4.41	0.0043
Subtotal MACLEN				1.03	26.92	55.99	
MATOS	5	m	2	0.47	2.90	10.07	0.4236
MATOS	7	m	2	2.08	6.02	0.39	0.0000
MATOS	9	m	2	2.71	6.81	0.95	0.0000
Subtotal MATOS				2.05	15.72	11.41	
*MIGRAN	2	m	2	1.39	2.61	2.33	0.0249
*MIGRAN	4	m	2	1.44	3.70	2.93	0.0055
*MIGRAN	6	m	2	1.64	3.44	1.67	0.0024
*MIGRAN	8	m	2	1.78	3.35	1.03	0.0011
*MIGRAN	29	f	2	1.59	1.88	1.05	0.0298
*MIGRAN	31	f	2	1.88	3.01	0.63	0.0011
*MIGRAN	33	f	2	2.01	1.67	0.17	0.0093
Subtotal MIGRAN				1.66	19.66	9.81	
*MRFITR	3	m	0	2.38	0.42	0.00	0.1236
*MRFITR	4	m	0	3.91	0.50	1.24	0.0059
*MRFITR	5	m	0	4.03	0.50	1.43	0.0045
Subtotal MRFITR				3.50	1.41	2.67	
NAM	74	m	1	1.90	17.45	3.25	0.0000
NAM	75	m	1	2.33	17.40	0.00	0.0000
NAM	90	f	1	2.20	19.83	0.33	0.0000
NAM	91	f	1	2.81	15.98	3.66	0.0000
Subtotal NAM				2.30	70.66	7.25	
PARKIN	14	m	6	1.36	58.89	55.72	0.0000
PARKIN	15	m	6	1.65	24.72	11.60	0.0000
Subtotal PARKIN				1.45	83.60	67.32	
PERSH2	8	c	4	1.75	77.78	26.42	0.0000
PERSH2	9	c	4	2.43	82.57	0.74	0.0000
Subtotal PERSH2				2.10	160.35	27.16	

Table 1G6 - 2

IESLC - Meta-analysis of Current Smoking by Amount, Overview, Any product (or Cigarettes if Any not available)
All LC types
Most adjusted

REF	NRR	SEX	AD	Ys	Ws	Qs	Ps
*PETO	2	m	0	1.70	1.93	0.76	0.0180
*PETO	3	m	0	2.25	1.95	0.01	0.0017
Subtotal PETO				1.98	3.88	0.78	
PEZZO2	3	m	0	2.08	5.00	0.32	0.0000
PEZZO2	4	m	0	3.79	4.86	10.35	0.0000
PEZZO2	5	m	0	4.72	3.66	20.85	0.0000
Subtotal PEZZO2				3.41	13.52	31.52	
PEZZOT	2	m	0	2.00	3.22	0.35	0.0003
PEZZOT	3	m	0	4.25	3.25	11.93	0.0000
PEZZOT	4	m	0	5.51	2.25	22.64	0.0000
Subtotal PEZZOT				3.74	8.72	34.92	
*PRESCO	2	m	1	2.32	5.71	0.00	0.0000
*PRESCO	4	m	1	2.99	5.92	2.58	0.0000
*PRESCO	1	f	1	1.85	11.85	2.77	0.0000
*PRESCO	3	f	1	2.31	11.98	0.01	0.0000
Subtotal PRESCO				2.27	35.47	5.36	
SEGI2	10	m	1	0.74	4.79	12.13	0.1046
SEGI2	12	m	1	1.13	6.11	8.83	0.0052
SEGI2	14	m	1	1.22	6.23	7.68	0.0022
SEGI2	16	m	1	1.93	4.64	0.75	0.0000
SEGI2	18	m	1	2.07	5.40	0.38	0.0000
SEGI2	22	f	1	1.06	4.02	6.47	0.0328
SEGI2	24	f	1	0.36	5.21	20.18	0.4054
SEGI2	26	f	1	0.03	3.10	16.46	0.9585
Subtotal SEGI2				1.13	39.50	72.89	
SHAW	4	c	0	1.84	5.92	1.43	0.0000
SHAW	5	c	0	3.42	8.18	9.60	0.0000
Subtotal SHAW				2.76	14.10	11.03	
SOBUE	117	m	0	1.26	19.84	22.88	0.0000
SOBUE	118	m	0	1.39	21.76	19.51	0.0000
SOBUE	119	m	0	1.52	21.28	14.26	0.0000
Subtotal SOBUE				1.39	62.88	56.65	
*SPEIZE	1	f	1	0.99	4.79	8.60	0.0298
*SPEIZE	2	f	1	1.65	23.37	10.97	0.0000
*SPEIZE	3	f	1	2.53	56.20	2.25	0.0000
*SPEIZE	4	f	1	2.75	58.51	10.32	0.0000
*SPEIZE	5	f	1	3.09	25.23	14.47	0.0000
Subtotal SPEIZE				2.53	168.09	46.60	
STOCKW	1	c	0	1.90	565.53	107.27	0.0000
STOCKW	2	c	0	2.67	802.56	93.22	0.0000
STOCKW	3	c	0	3.36	411.25	434.69	0.0000
Subtotal STOCKW				2.59	1779.33	635.18	
SVENSS	6	f	1	1.53	8.90	5.81	0.0000
SVENSS	11	f	1	2.53	8.37	0.33	0.0000
SVENSS	16	f	1	4.08	0.91	2.78	0.0001
Subtotal SVENSS				2.12	18.19	8.92	
*TENKAN	10	m	1	2.76	5.35	0.99	0.0000
*TENKAN	11	m	1	3.01	4.70	2.14	0.0000
*TENKAN	12	m	1	3.22	4.49	3.51	0.0000
Subtotal TENKAN				2.98	14.54	6.64	
TSUGAN	29	m	0	-0.10	4.44	26.42	0.8253
TSUGAN	30	m	0	0.20	5.96	27.13	0.6241
TSUGAN	31	m	0	0.51	4.44	14.84	0.2861
Subtotal TSUGAN				0.20	14.85	68.39	
*TULINI	27	m	3	1.80	8.03	2.33	0.0000
*TULINI	28	m	3	2.48	9.25	0.21	0.0000
*TULINI	29	m	3	3.31	9.01	8.54	0.0000
*TULINI	32	f	3	2.10	9.54	0.52	0.0000
*TULINI	33	f	3	3.27	10.56	9.25	0.0000
*TULINI	34	f	3	3.66	7.07	12.36	0.0000
Subtotal TULINI				2.76	53.47	33.21	
*TVERDA	9	m	2	0.76	10.05	24.86	0.0159
*TVERDA	10	m	2	1.20	17.25	22.17	0.0000
*TVERDA	11	m	2	1.88	16.39	3.36	0.0000
*TVERDA	16	f	2	1.51	1.87	1.27	0.0387
*TVERDA	17	f	2	2.89	2.59	0.80	0.0000
Subtotal TVERDA				1.44	48.15	52.46	
WAKAI	40	m	2	0.59	5.99	18.25	0.1504
WAKAI	41	m	2	1.39	6.99	6.24	0.0002
WAKAI	42	m	2	2.22	6.27	0.08	0.0000
Subtotal WAKAI				1.41	19.25	24.58	

Table 1G6 - 2

IESLC - Meta-analysis of Current Smoking by Amount, Overview, Any product (or Cigarettes if Any not available)
All LC types
Most adjusted

REF	NRR	SEX	AD	Ys	Ws	Qs	Ps
WU	43	f	2	1.18	9.36	12.48	0.0003
WU	44	f	2	2.14	7.57	0.29	0.0000
Subtotal WU				1.61	16.93	12.77	
WYNDE6	27	m	0	1.92	33.49	5.81	0.0000
WYNDE6	36	m	0	2.41	53.48	0.33	0.0000
WYNDE6	45	m	0	2.85	41.59	11.16	0.0000
WYNDE6	54	m	0	3.34	51.37	52.04	0.0000
WYNDE6	216	f	0	1.32	33.57	34.31	0.0000
WYNDE6	225	f	0	2.48	61.57	1.37	0.0000
WYNDE6	234	f	0	3.07	30.83	16.93	0.0000
WYNDE6	243	f	0	3.67	34.09	60.60	0.0000
Subtotal WYNDE6				2.65	340.00	182.55	
YAMAGU	8	c	1	1.32	8.14	8.33	0.0002
YAMAGU	7	c	1	2.50	5.11	0.14	0.0000
Subtotal YAMAGU				1.77	13.24	8.47	

N	292
NS	69

Table 1G6 - 3

IESLC - Meta-analysis of Current Smoking by Amount, Overview, Any product (or Cigarettes if Any not available)
All LC types
Most adjusted

	Sex combined	male	female	Total
N	22	171	99	292
NS	8	53	33	94

In this overview table, other than the "N" rows, entries in the "absent" and "Total" columns may be invalid and should be ignored

	Amount smoked (broad categories) absent	<20k5	6-44k20	>20k45	Total
N	90	86	54	62	292
NS	44	58	42	50	194
Wt	1948.74	1778.68	2137.55	1388.77	7253.74
Het Chi	1009.18	460.10	311.39	371.22	3866.70
Het df	89	85	53	61	291
Het P	***	***	***	***	***
Fixed RR	10.24	4.98	11.89	21.28	10.32
RRl	9.80	4.75	11.40	20.19	10.08
RRu	10.71	5.22	12.40	22.43	10.56
P	+++	+++	+++	+++	+++
Random RR	9.62	4.71	9.83	17.10	8.90
RRl	8.16	4.14	8.60	14.62	8.11
RRu	11.34	5.37	11.24	19.99	9.76
P	+++	+++	+++	+++	+++

	absent	<10k1	2-19k10	Amount smoked (narrow categories) 11-29k20	21-39k30	31-98k40	>40k99	Total
N	173	27	22	42	14	4	10	292
NS	67	17	13	33	12	3	8	153
Wt	3919.04	605.14	609.00	848.99	616.96	138.12	516.49	7253.74
Het Chi	1651.38	179.02	195.87	183.89	24.07	15.69	60.07	3866.70
Het df	172	26	21	41	13	3	9	291
Het P	***	***	***	***	*	**	***	***
Fixed RR	10.11	3.89	5.86	11.04	17.51	22.45	28.30	10.32
RRl	9.80	3.60	5.41	10.32	16.18	19.00	25.96	10.08
RRu	10.43	4.22	6.35	11.81	18.95	26.52	30.84	10.56
P	+++	+++	+++	+++	+++	+++	+++	+++
Random RR	9.20	3.55	5.80	9.73	17.58	21.88	30.88	8.90
RRl	8.22	2.79	4.44	8.24	15.37	14.38	20.81	8.11
RRu	10.30	4.52	7.57	11.48	20.10	33.29	45.83	9.76
P	+++	+++	+++	+++	+++	+++	+++	+++

MALES

	Amount smoked (broad categories) absent	<20k5	6-44k20	>20k45	Total
N	48	50	34	39	171
NS	31	48	34	39	152
Wt	1130.83	676.77	789.19	613.04	3209.83
Het Chi	420.42	219.05	173.08	226.67	1705.92
Het df	47	49	33	38	170
Het P	***	***	***	***	***
Fixed RR	11.09	4.52	10.52	18.35	9.97
RRl	10.46	4.19	9.81	16.95	9.63
RRu	11.75	4.87	11.28	19.86	10.33
P	+++	+++	+++	+++	+++
Random RR	10.28	4.92	9.08	16.16	9.06
RRl	8.42	4.10	7.46	12.90	8.02
RRu	12.57	5.91	11.05	20.26	10.23
P	+++	+++	+++	+++	+++

Table 1G6 - 3

IESLC - Meta-analysis of Current Smoking by Amount, Overview, Any product (or Cigarettes if Any not available)
All LC types
Most adjusted

MALES

	absent	<10k1	2-19k10	11-29k20	21-39k30	31-98k40	>40k99	Total
		Amount smoked (narrow categories)						
N	103	14	11	26	9	2	6	171
NS	52	14	11	26	9	2	6	120
Wt	1574.26	349.35	305.47	384.74	470.48	66.67	58.86	3209.83
Het Chi	824.45	93.40	73.25	129.44	13.24	6.67	37.05	1705.92
Het df	102	13	10	25	8	1	5	170
Het P	***	***	***	***	N.S.	**	***	***
Fixed RR	10.12	4.34	6.85	10.44	16.97	21.25	30.04	9.97
RRl	9.63	3.91	6.13	9.45	15.50	16.71	23.27	9.63
RRu	10.63	4.82	7.67	11.54	18.57	27.01	38.79	10.33
P	+++	+++	+++	+++	+++	+++	+++	+++
Random RR	9.44	3.91	6.15	8.72	16.52	16.07	44.20	9.06
RRl	8.05	2.79	4.35	6.75	14.14	6.61	19.13	8.02
RRu	11.07	5.49	8.69	11.26	19.31	39.07	102.12	10.23
P	+++	+++	+++	+++	+++	+++	+++	+++

FEMALES

	absent	<20k5	6-44k20	>20k45	Total
	Amount smoked (broad categories)				
N	34	30	17	18	99
NS	23	28	17	18	86
Wt	620.49	417.05	512.29	299.58	1849.41
Het Chi	433.82	141.29	71.55	62.69	1196.82
Het df	33	29	16	17	98
Het P	***	***	***	***	***
Fixed RR	7.86	3.74	10.68	18.30	8.30
RRl	7.26	3.39	9.80	16.34	7.93
RRu	8.50	4.11	11.65	20.49	8.68
P	+++	+++	+++	+++	+++
Random RR	7.26	4.18	11.13	17.55	7.84
RRl	5.32	3.31	8.92	13.57	6.60
RRu	9.90	5.28	13.89	22.69	9.30
P	+++	+++	+++	+++	+++

	absent	<10k1	2-19k10	11-29k20	21-39k30	31-98k40	>40k99	Total
		Amount smoked (narrow categories)						
N	57	11	10	14	4	1	2	99
NS	30	11	10	14	4	1	2	71
Wt	741.47	170.02	291.30	430.74	129.49	53.44	32.95	1849.41
Het Chi	523.32	31.87	71.13	35.15	6.26	0.00	1.08	1196.82
Het df	56	10	9	13	3	0	1	98
Het P	***	***	***	***	(*)	N.S.	N.S.	***
Fixed RR	8.91	2.51	4.66	11.77	18.47	19.31	14.75	8.30
RRl	8.29	2.16	4.16	10.71	15.55	14.77	10.48	7.93
RRu	9.57	2.92	5.23	12.93	21.94	25.25	20.75	8.68
P	+++	+++	+++	+++	+++	+++	+++	+++
Random RR	8.62	2.62	4.69	12.23	18.63	19.31	14.78	7.84
RRl	6.81	1.91	3.25	10.02	14.03	14.77	10.36	6.60
RRu	10.92	3.60	6.78	14.93	24.72	25.25	21.08	9.30
P	+++	+++	+++	+++	+++	+++	+++	+++

Table 1G6 - 4

IESLC - Meta-analysis of Current Smoking by Amount, Overview, Any product (or Cigarettes if Any not available)
All LC types
Least adjusted

REF	NRR	X	SEX	AGEL	AGEH	RACE	YF	LC TYPE	LOC	START	ST	NLC	R	VB	P	H	AD	PRODUCT	exL	exH	S1	S2	DENOM	De
AKIBA	17	x	m	0	0	all	0	all	As:Jap	1963	pr	610	n	bl	n	n	0	cig+/-ot	1	14	1	0	nev cigs	or
AKIBA	18	x	m	0	0	all	0	all	As:Jap	1963	pr	610	n	bl	n	n	0	cig+/-ot	15	24	2	3	nev cigs	or
AKIBA	19	x	m	0	0	all	0	all	As:Jap	1963	pr	610	n	bl	n	n	0	cig+/-ot	25	99	3	0	nev cigs	or
AKIBA	23	x	f	0	0	all	0	all	As:Jap	1963	pr	610	n	bl	n	n	0	cig+/-ot	1	14	1	0	nev cigs	or
AKIBA	24	x	f	0	0	all	0	all	As:Jap	1963	pr	610	n	bl	n	n	0	cig+/-ot	15	99	0	0	nev cigs	or
ARCHER	1		m	0	0	wh	0	all	NAmer	1950	pr	146	m	bl	n	n	0	cig+/-ot	1	19	1	0	nev cigs	st
ARCHER	2		m	0	0	wh	0	all	NAmer	1950	pr	146	m	bl	n	n	0	cig+/-ot	20	20	2	3	nev cigs	st
ARCHER	3		m	0	0	wh	0	all	NAmer	1950	pr	146	m	bl	n	n	0	cig+/-ot	21	99	3	0	nev cigs	st
AXELSS	18		f	0	0	sca	-	all	Eu:Sca	1989	CC	436	n	bl	n	n	1	all/unsp	20	20	2	3	nev any	or
BENSHL	11		m	40	64	all	10	all	Eu:UK	1967	pr	486	n	V	n	n	1	cig+/-ot	1	9	1	1	nev any	ot
BENSHL	12		m	40	64	all	10	all	Eu:UK	1967	pr	486	n	V	n	n	1	cig+/-ot	10	19	0	2	nev any	ot
BENSHL	13		m	40	64	all	10	all	Eu:UK	1967	pr	486	n	V	n	n	1	cig+/-ot	20	99	0	0	nev any	ot
BEST	13		m	0	0	all	0	all	NAmer	1955	pr	381	n	V	n	n	1	cig only	1	9	1	1	nev any	ot
BEST	14		m	0	0	all	0	all	NAmer	1955	pr	381	n	V	n	n	1	cig only	10	20	2	0	nev any	ot
BEST	15		m	0	0	all	0	all	NAmer	1955	pr	381	n	V	n	n	1	cig only	21	99	3	0	nev any	ot
BOUCOT	11	x	m	0	0	all	0	all	NAmer	1951	pr	121	n	bl	n	n	0	cig only	1	20	0	0	nev any	ot
BOUCOT	12	x	m	0	0	all	0	all	NAmer	1951	pr	121	n	bl	n	n	0	cig only	21	40	0	0	nev any	ot
BOUCOT	13	x	m	0	0	all	0	all	NAmer	1951	pr	121	n	bl	n	n	0	cig only	41	99	3	6	nev any	ot
BRETT	1		m	0	0	all	0	all	Eu:UK	1960	pr	150	n	V	n	n	0	cig+/-ot	1	14	1	0	nev cigs	st
BRETT	2		m	0	0	all	0	all	Eu:UK	1960	pr	150	n	V	n	n	0	cig+/-ot	15	24	2	3	nev cigs	st
BRETT	3		m	0	0	all	0	all	Eu:UK	1960	pr	150	n	V	n	n	0	cig+/-ot	25	99	3	0	nev cigs	st
BROSS	18		m	0	0	wh	-	all	NAmer	1960	CC	974	n	bl	n	n	0	cig+/-ot	1	20	0	0	nev any	st
BROSS	19		m	0	0	wh	-	all	NAmer	1960	CC	974	n	bl	n	n	0	cig+/-ot	21	99	3	0	nev any	st
BUFFLE	30		f	0	0	w-hi	-	all	NAmer	1976	CC	943	n	bl	y	n	0	cig+/-ot	1	19	1	0	nev cigs	or
BUFFLE	31		f	0	0	w-hi	-	all	NAmer	1976	CC	943	n	bl	y	n	0	cig+/-ot	20	20	2	3	nev cigs	or
BUFFLE	36		f	0	0	w-hi	-	all	NAmer	1976	CC	943	n	bl	y	n	0	cig+/-ot	21	99	3	0	nev cigs	or
CEDERL	80		m	0	0	all	16	all	Eu:Sca	1963	pr	491	n	bl	n	n	2	all/unsp	1	7	1	1	nev any	ot
CEDERL	81		m	0	0	all	16	all	Eu:Sca	1963	pr	491	n	bl	n	n	2	all/unsp	8	15	0	2	nev any	ot
CEDERL	82		m	0	0	all	16	all	Eu:Sca	1963	pr	491	n	bl	n	n	2	all/unsp	16	99	0	0	nev any	ot
CEDERL	76		f	0	0	all	0	all	Eu:Sca	1963	pr	491	n	bl	n	n	2	all/unsp	1	7	1	1	nev any	or
CEDERL	77		f	0	0	all	0	all	Eu:Sca	1963	pr	491	n	bl	n	n	2	all/unsp	8	15	0	2	nev any	or
CEDERL	78		f	0	0	all	0	all	Eu:Sca	1963	pr	491	n	bl	n	n	2	all/unsp	16	99	0	0	nev any	or
CHANG	2		m	0	0	all	0	all	NAmer	1972	pr	136	n	bl	n	n	0	cig+/-ot	1	10	1	0	nev cigs	st
CHANG	3		m	0	0	all	0	all	NAmer	1972	pr	136	n	bl	n	n	0	cig+/-ot	11	20	2	3	nev cigs	st
CHANG	4		m	0	0	all	0	all	NAmer	1972	pr	136	n	bl	n	n	0	cig+/-ot	21	99	3	0	nev cigs	st
CHANG	8		f	0	0	all	0	all	NAmer	1972	pr	136	n	bl	n	n	0	cig+/-ot	1	10	1	0	nev cigs	st
CHANG	9		f	0	0	all	0	all	NAmer	1972	pr	136	n	bl	n	n	0	cig+/-ot	11	20	2	3	nev cigs	st
CHANG	10		f	0	0	all	0	all	NAmer	1972	pr	136	n	bl	n	n	0	cig+/-ot	21	99	3	0	nev cigs	st
CHOW	3	x	m	0	0	wh	0	all	NAmer	1966	pr	219	n	bl	n	n	0	cig+/-ot	1	19	1	0	nev any	st
CHOW	4	x	m	0	0	wh	0	all	NAmer	1966	pr	219	n	bl	n	n	0	cig+/-ot	20	29	2	3	nev any	st
CHOW	5	x	m	0	0	wh	0	all	NAmer	1966	pr	219	n	bl	n	n	0	cig+/-ot	30	99	3	0	nev any	st
COMSTO	4		m	0	0	all	-	all	NAmer	1975	ot	258	n	bl	n	n	0	cig+/-ot	1	19	1	0	nev any	st
COMSTO	5		m	0	0	all	-	all	NAmer	1975	ot	258	n	bl	n	n	0	cig+/-ot	20	39	2	0	nev any	st
COMSTO	6		m	0	0	all	-	all	NAmer	1975	ot	258	n	bl	n	n	0	cig+/-ot	40	99	3	0	nev any	st
COMSTO	9		f	0	0	all	-	all	NAmer	1975	ot	258	n	bl	n	n	0	cig+/-ot	1	19	1	0	nev any	st
COMSTO	10		f	0	0	all	-	all	NAmer	1975	ot	258	n	bl	n	n	0	cig+/-ot	20	39	2	0	nev any	st
COMSTO	11		f	0	0	all	-	all	NAmer	1975	ot	258	n	bl	n	n	0	cig+/-ot	40	99	3	0	nev any	st
CORREA	45	x	c	0	0	all	-	all	NAmer	1979	CC	1359	n	bl	y	n	0	cig+/-ot	1	20	0	0	nev cigs	st
CORREA	49	x	c	0	0	all	-	all	NAmer	1979	CC	1359	n	bl	y	n	0	cig+/-ot	21	99	3	0	nev cigs	st
CPSI	216		m	35	84	all	6	all	NAmer	1959	pr	5138	n	bl	n	n	1	cig+/-ot	1	9	1	1	nev any	ot
CPSI	217		m	35	84	all	6	all	NAmer	1959	pr	5138	n	bl	n	n	1	cig+/-ot	10	19	0	2	nev any	ot
CPSI	218		m	35	84	all	6	all	NAmer	1959	pr	5138	n	bl	n	n	1	cig+/-ot	20	39	2	0	nev any	ot
CPSI	219		m	35	84	all	6	all	NAmer	1959	pr	5138	n	bl	n	n	1	cig+/-ot	40	99	3	0	nev any	ot
CPSI	275		f	40	74	all	6	all	NAmer	1959	pr	5138	n	bl	n	n	1	cig+/-ot	1	9	1	1	nev cigs	ot
CPSI	276		f	40	74	all	6	all	NAmer	1959	pr	5138	n	bl	n	n	1	cig+/-ot	10	19	0	2	nev cigs	ot
CPSI	277		f	40	74	all	6	all	NAmer	1959	pr	5138	n	bl	n	n	1	cig+/-ot	20	39	2	0	nev cigs	ot
CPSI	278		f	40	74	all	6	all	NAmer	1959	pr	5138	n	bl	n	n	1	cig+/-ot	40	99	3	0	nev cigs	ot
CPSII	30	x	m	0	0	all	6	all	NAmer	1982	pr	3229	n	bl	n	n	0	cig only	1	9	1	1	nev any	st
CPSII	31	x	m	0	0	all	6	all	NAmer	1982	pr	3229	n	bl	n	n	0	cig only	10	19	0	2	nev any	st
CPSII	32	x	m	0	0	all	6	all	NAmer	1982	pr	3229	n	bl	n	n	0	cig only	20	20	2	3	nev any	st
CPSII	33	x	m	0	0	all	6	all	NAmer	1982	pr	3229	n	bl	n	n	0	cig only	21	39	0	4	nev any	st
CPSII	34	x	m	0	0	all	6	all	NAmer	1982	pr	3229	n	bl	n	n	0	cig only	40	40	0	5	nev any	st
CPSII	35	x	m	0	0	all	6	all	NAmer	1982	pr	3229	n	bl	n	n	0	cig only	41	99	3	6	nev any	st
CPSII	65	x	f	0	0	all	6	all	NAmer	1982	pr	3229	n	bl	n	n	0	cig+/-ot	1	9	1	1	nev cigs	st
CPSII	66	x	f	0	0	all	6	all	NAmer	1982	pr	3229	n	bl	n	n	0	cig+/-ot	10	19	0	2	nev cigs	st
CPSII	67	x	f	0	0	all	6	all	NAmer	1982	pr	3229	n	bl	n	n	0	cig+/-ot	20	20	2	3	nev cigs	st
CPSII	68	x	f	0	0	all	6	all	NAmer	1982	pr	3229	n	bl	n	n	0	cig+/-ot	21	39	0	4	nev cigs	st
CPSII	69	x	f	0	0	all	6	all	NAmer	1982	pr	3229	n	bl	n	n	0	cig+/-ot	40	40	0	5	nev cigs	st
CPSII	70	x	f	0	0	all	6	all	NAmer	1982	pr	3229	n	bl	n	n	0	cig+/-ot	41	99	3	6	nev cigs	st
DARBY	1		m	0	0	wh	-	all	Eu:UK	1988	CC	982	n	V	n	n	0	cig+/-ot	1	14	1	0	nev any	st
DARBY	2		m	0	0	wh	-	all	Eu:UK	1988	CC	982	n	V	n	n	0	cig+/-ot	15	24	2	3	nev any	st
DARBY	3		m	0	0	wh	-	all	Eu:UK	1988	CC	982	n	V	n	n	0	cig+/-ot	25	99	3	0	nev any	st

Table 1G6 - 4

IESLC - Meta-analysis of Current Smoking by Amount, Overview, Any product (or Cigarettes if Any not available)
All LC types
Least adjusted

REF	NRR	X	SEX	AGEL	AGEH	RACE	YF	LC TYPE	LOC	START	ST	NLC	R	VB	P	H	AD	PRODUCT	exL	exH	S1	S2	DENOM	De
DARBY	8		f	0	0	wh	-	all	Eu:UK	1988	CC	982	n	V	n	n	0	cig+/-ot	1	14	1	0	nev any	st
DARBY	9		f	0	0	wh	-	all	Eu:UK	1988	CC	982	n	V	n	n	0	cig+/-ot	15	24	2	3	nev any	st
DARBY	10		f	0	0	wh	-	all	Eu:UK	1988	CC	982	n	V	n	n	0	cig+/-ot	25	99	3	0	nev any	st
DEAN3	5	x	m	0	0	all	-	all	Eu:UK	1969	CC	766	n	V	y	n	0	cig only	1	12	1	0	nev any	st
DEAN3	12	x	m	0	0	all	-	all	Eu:UK	1969	CC	766	n	V	y	n	0	cig only	13	22	2	3	nev any	st
DEAN3	19	x	m	0	0	all	-	all	Eu:UK	1969	CC	766	n	V	y	n	0	cig only	23	99	3	0	nev any	st
DEAN3	89	x	f	0	0	all	-	all	Eu:UK	1969	CC	766	n	V	y	n	0	cig only	1	12	1	0	nev any	st
DEAN3	96	x	f	0	0	all	-	all	Eu:UK	1969	CC	766	n	V	y	n	0	cig only	13	22	2	3	nev any	st
DEAN3	103	x	f	0	0	all	-	all	Eu:UK	1969	CC	766	n	V	y	n	0	cig only	23	99	3	0	nev any	st
DEKLER	2		m	0	0	all	0	all	Auslia	1961	pr	138	m	V	n	n	2	cig+/-ot	1	14	1	0	nev any	or
DEKLER	3		m	0	0	all	0	all	Auslia	1961	pr	138	m	V	n	n	2	cig+/-ot	15	24	2	3	nev any	or
DEKLER	4		m	0	0	all	0	all	Auslia	1961	pr	138	m	V	n	n	2	cig+/-ot	25	99	3	0	nev any	or
DOLL2	16		m	0	0	all	20	all	Eu:UK	1951	pr	920	n	V	n	n	1	all/unsp	1	14	1	0	nev any	ot
DOLL2	17		m	0	0	all	20	all	Eu:UK	1951	pr	920	n	V	n	n	1	all/unsp	15	24	2	3	nev any	ot
DOLL2	18		m	0	0	all	20	all	Eu:UK	1951	pr	920	n	V	n	n	1	all/unsp	25	99	3	0	nev any	ot
DOLL2	10		f	0	0	all	22	all	Eu:UK	1951	pr	920	n	V	n	n	1	cig only	1	14	1	0	nev any	ot
DOLL2	11		f	0	0	all	22	all	Eu:UK	1951	pr	920	n	V	n	n	1	cig only	15	24	2	3	nev any	ot
DOLL2	12		f	0	0	all	22	all	Eu:UK	1951	pr	920	n	V	n	n	1	cig only	25	99	3	0	nev any	ot
DORANT	6		c	0	0	all	0	all	Eu:wst	1986	ot	550	n	bl	n	y	0	cig+/-ot	1	9	1	1	nev any	st
DORANT	7		c	0	0	all	0	all	Eu:wst	1986	ot	550	n	bl	n	y	0	cig+/-ot	10	19	0	2	nev any	st
DORANT	8		c	0	0	all	0	all	Eu:wst	1986	ot	550	n	bl	n	y	0	cig+/-ot	20	99	0	0	nev any	st
DORGAN	4		m	0	0	wh	-	all	NAmer	1980	CC	2026	n	bl	y	y	0	cig+/-ot	1	19	1	0	nev any	st
DORGAN	5		m	0	0	wh	-	all	NAmer	1980	CC	2026	n	bl	y	y	0	cig+/-ot	20	99	0	0	nev any	st
DORGAN	28		m	0	0	bl	-	all	NAmer	1980	CC	2026	n	bl	y	y	0	cig+/-ot	1	19	1	0	nev any	st
DORGAN	29		m	0	0	bl	-	all	NAmer	1980	CC	2026	n	bl	y	y	0	cig+/-ot	20	99	0	0	nev any	st
DORGAN	51		f	0	0	wh	-	all	NAmer	1980	CC	2026	n	bl	y	y	0	cig+/-ot	1	19	1	0	nev any	st
DORGAN	52		f	0	0	wh	-	all	NAmer	1980	CC	2026	n	bl	y	y	0	cig+/-ot	20	99	0	0	nev any	st
DORGAN	74		f	0	0	bl	-	all	NAmer	1980	CC	2026	n	bl	y	y	0	cig+/-ot	1	19	1	0	nev any	st
DORGAN	75		f	0	0	bl	-	all	NAmer	1980	CC	2026	n	bl	y	y	0	cig+/-ot	20	99	0	0	nev any	st
DORN	408		m	0	0	wh	25	all	NAmer	1954	pr	5097	n	bl	n	n	1	cig+/-ot	1	9	1	1	nev any	or
DORN	409		m	0	0	wh	25	all	NAmer	1954	pr	5097	n	bl	n	n	1	cig+/-ot	10	20	2	0	nev any	or
DORN	410		m	0	0	wh	25	all	NAmer	1954	pr	5097	n	bl	n	n	1	cig+/-ot	21	39	0	4	nev any	or
DORN	411		m	0	0	wh	25	all	NAmer	1954	pr	5097	n	bl	n	n	1	cig+/-ot	40	99	3	0	nev any	or
ENGELA	3	x	m	0	0	all	0	all	Eu:Sca	1964	pr	435	n	bl	n	n	0	cig+/-ot	1	4	0	1	nev cigs	st
ENGELA	4	x	m	0	0	all	0	all	Eu:Sca	1964	pr	435	n	bl	n	n	0	cig+/-ot	5	9	1	0	nev cigs	st
ENGELA	5	x	m	0	0	all	0	all	Eu:Sca	1964	pr	435	n	bl	n	n	0	cig+/-ot	10	14	0	2	nev cigs	st
ENGELA	6	x	m	0	0	all	0	all	Eu:Sca	1964	pr	435	n	bl	n	n	0	cig+/-ot	15	19	0	0	nev cigs	st
ENGELA	7	x	m	0	0	all	0	all	Eu:Sca	1964	pr	435	n	bl	n	n	0	cig+/-ot	20	99	0	0	nev cigs	st
ENGELA	17	x	f	0	0	all	0	all	Eu:Sca	1964	pr	435	n	bl	n	n	0	cig+/-ot	1	4	0	1	nev cigs	st
ENGELA	18	x	f	0	0	all	0	all	Eu:Sca	1964	pr	435	n	bl	n	n	0	cig+/-ot	5	9	1	0	nev cigs	st
ENGELA	19	x	f	0	0	all	0	all	Eu:Sca	1964	pr	435	n	bl	n	n	0	cig+/-ot	10	14	0	2	nev cigs	st
ENGELA	20	x	f	0	0	all	0	all	Eu:Sca	1964	pr	435	n	bl	n	n	0	cig+/-ot	15	19	0	0	nev cigs	st
ENGELA	21	x	f	0	0	all	0	all	Eu:Sca	1964	pr	435	n	bl	n	n	0	cig+/-ot	20	99	0	0	nev cigs	st
ENSTRO	7		m	0	0	all	0	all	NAmer	1959	pr	2879	n	bl	n	n	1	cig only	1	9	1	1	nev any	ot
ENSTRO	6		m	0	0	all	0	all	NAmer	1959	pr	2879	n	bl	n	n	1	cig only	10	19	0	2	nev any	ot
ENSTRO	5		m	0	0	all	0	all	NAmer	1959	pr	2879	n	bl	n	n	1	cig only	20	20	2	3	nev any	ot
ENSTRO	4		m	0	0	all	0	all	NAmer	1959	pr	2879	n	bl	n	n	1	cig only	21	39	0	4	nev any	ot
ENSTRO	3		m	0	0	all	0	all	NAmer	1959	pr	2879	n	bl	n	n	1	cig only	40	99	3	0	nev any	ot
ENSTRO	11		f	0	0	all	0	all	NAmer	1959	pr	2879	n	bl	n	n	1	cig only	1	9	1	1	nev any	ot
ENSTRO	10		f	0	0	all	0	all	NAmer	1959	pr	2879	n	bl	n	n	1	cig only	10	19	0	2	nev any	ot
ENSTRO	9		f	0	0	all	0	all	NAmer	1959	pr	2879	n	bl	n	n	1	cig only	20	20	2	3	nev any	ot
ENSTRO	8		f	0	0	all	0	all	NAmer	1959	pr	2879	n	bl	n	n	1	cig only	21	99	3	0	nev any	ot
GAO2	2		m	0	0	all	-	all	As:Jap	1988	CC	282	n	bl	n	n	0	cig+/-ot	1	19	1	0	nev cigs	st
GAO2	3		m	0	0	all	-	all	As:Jap	1988	CC	282	n	bl	n	n	0	cig+/-ot	20	29	2	3	nev cigs	st
GAO2	4		m	0	0	all	-	all	As:Jap	1988	CC	282	n	bl	n	n	0	cig+/-ot	30	99	3	0	nev cigs	or
GILLIS	1	x	m	0	0	all	-	all	Eu:UK	1977	CC	656	n	V	n	n	0	cig+/-ot	1	14	1	0	nev any	st
GILLIS	2	x	m	0	0	all	-	all	Eu:UK	1977	CC	656	n	V	n	n	0	cig+/-ot	15	24	2	3	nev any	st
GILLIS	3	x	m	0	0	all	-	all	Eu:UK	1977	CC	656	n	V	n	n	0	cig+/-ot	25	34	0	4	nev any	st
GILLIS	4	x	m	0	0	all	-	all	Eu:UK	1977	CC	656	n	V	n	n	0	cig+/-ot	35	49	3	5	nev any	st
GILLIS	5	x	m	0	0	all	-	all	Eu:UK	1977	CC	656	n	V	n	n	0	cig+/-ot	50	99	0	6	nev any	st
HAENSZ	52		f	0	0	all	-	not alv	NAmer	1955	CC	158	n	bl	n	y	0	cig+/-ot	1	20	0	0	nev any	st
HAENSZ	51		f	0	0	all	-	not alv	NAmer	1955	CC	158	n	bl	n	y	0	cig+/-ot	21	99	3	0	nev any	st
HAMMO2	21	x	m	0	0	all	0	all	NAmer	1967	pr	450	o	bl	n	n	0	cig+/-ot	1	19	1	0	nev any	st
HAMMO2	20	x	m	0	0	all	0	all	NAmer	1967	pr	450	o	bl	n	n	0	cig+/-ot	20	99	0	0	nev any	st
HAMMON	135		m	0	0	wh	0	all	NAmer	1952	pr	448	n	bl	n	n	1	cig only	1	9	1	1	nev any	ot
HAMMON	136		m	0	0	wh	0	all	NAmer	1952	pr	448	n	bl	n	n	1	cig only	10	20	2	0	nev any	ot
HAMMON	137		m	0	0	wh	0	all	NAmer	1952	pr	448	n	bl	n	n	1	cig only	21	39	0	4	nev any	ot
HAMMON	138		m	0	0	wh	0	all	NAmer	1952	pr	448	n	bl	n	n	1	cig only	40	99	3	0	nev any	ot
HIRAYA	23		m	0	0	all	0	all	As:Jap	1965	pr	1917	n	bl	n	n	1	cig+/-ot	1	9	1	1	nev any	st
HIRAYA	24		m	0	0	all	0	all	As:Jap	1965	pr	1917	n	bl	n	n	1	cig+/-ot	10	19	0	2	nev any	st
HIRAYA	25		m	0	0	all	0	all	As:Jap	1965	pr	1917	n	bl	n	n	1	cig+/-ot	20	99	0	0	nev any	st
HIRAYA	26		f	0	0	all	0	all	As:Jap	1965	pr	1917	n	bl	n	n	1	cig+/-ot	1	9	1	1	nev any	st

Table 1G6 - 4

IESLC - Meta-analysis of Current Smoking by Amount, Overview, Any product (or Cigarettes if Any not available)
All LC types
Least adjusted

REF	NRR	X	SEX	AGEL	AGEH	RACE	YF	LC TYPE	LOC	START	ST	NLC	R	VB	P	H	AD	PRODUCT	exL	exH	S1	S2	DENOM	De
HIRAYA	27		f	0	0	all	0	all	As:Jap	1965	pr	1917	n	bl	n	n	1	cig+/-ot	10	19	0	2	nev any	st
HIRAYA	28		f	0	0	all	0	all	As:Jap	1965	pr	1917	n	bl	n	n	1	cig+/-ot	20	99	0	0	nev any	st
HITOSU	3	x	m	0	0	all	-	all	As:Jap	1960	CC	216	n	bl	y	n	0	all/unsp	1	14	1	0	nev any	st
HITOSU	4	x	m	0	0	all	-	all	As:Jap	1960	CC	216	n	bl	y	n	0	all/unsp	15	24	2	3	nev any	st
HITOSU	5	x	m	0	0	all	-	all	As:Jap	1960	CC	216	n	bl	y	n	0	all/unsp	25	99	3	0	nev any	st
HITOSU	10	x	f	0	0	all	-	all	As:Jap	1960	CC	216	n	bl	y	n	0	all/unsp	1	14	1	0	nev any	st
HITOSU	11	x	f	0	0	all	-	all	As:Jap	1960	CC	216	n	bl	y	n	0	all/unsp	15	99	0	0	nev any	st
HOLE	9	x	m	0	0	all	0	all	Eu:UK	1972	pr	225	n	V	n	n	0	cig+/-ot	1	14	1	0	nev any	st
HOLE	10	x	m	0	0	all	0	all	Eu:UK	1972	pr	225	n	V	n	n	0	cig+/-ot	15	24	2	3	nev any	st
HOLE	11	x	m	0	0	all	0	all	Eu:UK	1972	pr	225	n	V	n	n	0	cig+/-ot	25	34	0	4	nev any	st
HOLE	12	x	m	0	0	all	0	all	Eu:UK	1972	pr	225	n	V	n	n	0	cig+/-ot	35	99	3	0	nev any	st
HUMBLE	2		m	0	0	w-hi	-	all	NAmer	1980	CC	521	n	bl	y	n	1	cig+/-ot	1	19	1	0	nev cigs	or
HUMBLE	3		m	0	0	w-hi	-	all	NAmer	1980	CC	521	n	bl	y	n	1	cig+/-ot	20	99	0	0	nev cigs	or
HUMBLE	5		m	0	0	hi	-	all	NAmer	1980	CC	521	n	bl	y	n	1	cig+/-ot	1	19	1	0	nev cigs	or
HUMBLE	6		m	0	0	hi	-	all	NAmer	1980	CC	521	n	bl	y	n	1	cig+/-ot	20	99	0	0	nev cigs	or
HUMBLE	8		f	0	0	w-hi	-	all	NAmer	1980	CC	521	n	bl	y	n	1	cig+/-ot	1	19	1	0	nev cigs	or
HUMBLE	9		f	0	0	w-hi	-	all	NAmer	1980	CC	521	n	bl	y	n	1	cig+/-ot	20	99	0	0	nev cigs	or
HUMBLE	11		f	0	0	hi	-	all	NAmer	1980	CC	521	n	bl	y	n	1	cig+/-ot	1	19	1	0	nev cigs	or
HUMBLE	12		f	0	0	hi	-	all	NAmer	1980	CC	521	n	bl	y	n	1	cig+/-ot	20	99	0	0	nev cigs	or
KAISE2	66		m	35	99	all	9	all	NAmer	1979	pr	318	n	bl	n	n	1	cig only	1	19	1	0	nev any	st
KAISE2	67		m	35	99	all	9	all	NAmer	1979	pr	318	n	bl	n	n	1	cig only	20	99	0	0	nev any	st
KAISE2	58		f	35	99	all	9	all	NAmer	1979	pr	318	n	bl	n	n	1	cig only	1	19	1	0	nev any	st
KAISE2	59		f	35	99	all	9	all	NAmer	1979	pr	318	n	bl	n	n	1	cig only	20	99	0	0	nev any	st
KAISER	6		m	0	0	all	0	all	NAmer	1964	pr	714	n	bl	n	n	2	cig+/-ot	1	19	1	0	nev cigs	or
KAISER	7		m	0	0	all	0	all	NAmer	1964	pr	714	n	bl	n	n	2	cig+/-ot	20	40	2	0	nev cigs	or
KAISER	8		m	0	0	all	0	all	NAmer	1964	pr	714	n	bl	n	n	2	cig+/-ot	41	99	3	6	nev cigs	or
KAISER	2		f	0	0	all	0	all	NAmer	1964	pr	714	n	bl	n	n	2	cig+/-ot	1	19	1	0	nev cigs	or
KAISER	3		f	0	0	all	0	all	NAmer	1964	pr	714	n	bl	n	n	2	cig+/-ot	20	40	2	0	nev cigs	or
KAISER	4		f	0	0	all	0	all	NAmer	1964	pr	714	n	bl	n	n	2	cig+/-ot	41	99	3	6	nev cigs	or
KANELL	1	x	m	0	0	all	-	all	Eu:bal	1950	CC	862	n	bl	n	n	0	cig+/-ot	1	10	1	0	nev any	st
KANELL	2	x	m	0	0	all	-	all	Eu:bal	1950	CC	862	n	bl	n	n	0	cig+/-ot	11	20	2	3	nev any	st
KANELL	3	x	m	0	0	all	-	all	Eu:bal	1950	CC	862	n	bl	n	n	0	cig+/-ot	21	35	0	4	nev any	st
KANELL	4	x	m	0	0	all	-	all	Eu:bal	1950	CC	862	n	bl	n	n	0	cig+/-ot	36	99	3	0	nev any	st
KATSOU	7	x	f	0	0	all	-	all	Eu:bal	1987	CC	101	n	bl	n	n	0	all/unsp	1	10	1	0	nev any	st
KATSOU	8	x	f	0	0	all	-	all	Eu:bal	1987	CC	101	n	bl	n	n	0	all/unsp	11	20	2	3	nev any	st
KATSOU	9	x	f	0	0	all	-	all	Eu:bal	1987	CC	101	n	bl	n	n	0	all/unsp	21	30	0	4	nev any	st
KATSOU	10	x	f	0	0	all	-	all	Eu:bal	1987	CC	101	n	bl	n	n	0	all/unsp	31	99	3	0	nev any	st
KAUFMA	2	x	c	0	0	all	-	all	NAmer	1981	CC	881	n	bl	n	n	0	cig+/-ot	1	14	1	0	nev cigs	st
KAUFMA	3	x	c	0	0	all	-	all	NAmer	1981	CC	881	n	bl	n	n	0	cig+/-ot	15	24	2	3	nev cigs	st
KAUFMA	4	x	c	0	0	all	-	all	NAmer	1981	CC	881	n	bl	n	n	0	cig+/-ot	25	34	0	4	nev cigs	st
KAUFMA	5	x	c	0	0	all	-	all	NAmer	1981	CC	881	n	bl	n	n	0	cig+/-ot	35	44	0	5	nev cigs	st
KAUFMA	6	x	c	0	0	all	-	all	NAmer	1981	CC	881	n	bl	n	n	0	cig+/-ot	45	99	3	6	nev cigs	st
KINLEN	3	x	m	0	0	all	0	all	Eu:UK	1967	pr	718	n	V	n	n	0	cig+/-ot	1	14	1	0	nev any	st
KINLEN	4	x	m	0	0	all	0	all	Eu:UK	1967	pr	718	n	V	n	n	0	cig+/-ot	15	24	2	3	nev any	st
KINLEN	5	x	m	0	0	all	0	all	Eu:UK	1967	pr	718	n	V	n	n	0	cig+/-ot	25	99	3	0	nev any	st
KNEKT	25	x	m	20	69	all	21	all	Eu:Sca	1966	pr	515	n	bl	n	n	0	cig+/-ot	1	14	1	0	nev any	st
KNEKT	26	x	m	20	69	all	21	all	Eu:Sca	1966	pr	515	n	bl	n	n	0	cig+/-ot	15	99	0	0	nev any	st
KOO	11		f	0	0	all	-	all	As:HK	1981	CC	200	n	bl	n	n	0	all/unsp	1	10	1	0	nev any	st
KOO	12		f	0	0	all	-	all	As:HK	1981	CC	200	n	bl	n	n	0	all/unsp	11	20	2	3	nev any	st
KOO	13		f	0	0	all	-	all	As:HK	1981	CC	200	n	bl	n	n	0	all/unsp	21	30	0	4	nev any	st
LIAW	3		c	0	0	all	0	all	As:oth	1982	pr	127	n	ot	n	n	2	all/unsp	1	10	1	0	nev any	or
LIAW	4		c	0	0	all	0	all	As:oth	1982	pr	127	n	ot	n	n	2	all/unsp	11	20	2	3	nev any	or
LIAW	5		c	0	0	all	0	all	As:oth	1982	pr	127	n	ot	n	n	2	all/unsp	21	99	3	0	nev any	or
LIDDEL	2		m	0	0	all	18	all	NAmer	1970	pr	304	m	V	n	n	1	cig+/-ot	1	19	1	0	nev cigs	ot
LIDDEL	3		m	0	0	all	18	all	NAmer	1970	pr	304	m	V	n	n	1	cig+/-ot	20	99	0	0	nev cigs	ot
MACLEN	20		m	0	0	ch	-	all	As:oth	1972	CC	233	n	bl	n	n	0	cig+/-ot	1	9	1	1	nev cigs	st
MACLEN	21		m	0	0	ch	-	all	As:oth	1972	CC	233	n	bl	n	n	0	cig+/-ot	10	19	0	2	nev cigs	st
MACLEN	22		m	0	0	ch	-	all	As:oth	1972	CC	233	n	bl	n	n	0	cig+/-ot	20	29	2	3	nev cigs	st
MACLEN	23		m	0	0	ch	-	all	As:oth	1972	CC	233	n	bl	n	n	0	cig+/-ot	30	99	3	0	nev cigs	st
MACLEN	33		f	0	0	ch	-	all	As:oth	1972	CC	233	n	bl	n	n	0	cig+/-ot	1	9	1	1	nev cigs	st
MACLEN	34		f	0	0	ch	-	all	As:oth	1972	CC	233	n	bl	n	n	0	cig+/-ot	10	19	0	2	nev cigs	st
MACLEN	35		f	0	0	ch	-	all	As:oth	1972	CC	233	n	bl	n	n	0	cig+/-ot	20	99	0	0	nev cigs	st
MATOS	4	x	m	0	0	all	-	all	SCAmer	1994	CC	200	n	bl	n	n	0	cig+/-ot	1	14	1	0	nev any	st
MATOS	6	x	m	0	0	all	-	all	SCAmer	1994	CC	200	n	bl	n	n	0	cig+/-ot	15	24	2	3	nev any	st
MATOS	8	x	m	0	0	all	-	all	SCAmer	1994	CC	200	n	bl	n	n	0	cig+/-ot	25	99	3	0	nev any	st
MIGRAN	1	x	m	0	0	all	0	all	Eu:UK	1964	pr	259	n	V	n	n	0	cig only	1	9	1	1	nev any	st
MIGRAN	3	x	m	0	0	all	0	all	Eu:UK	1964	pr	259	n	V	n	n	0	cig only	10	19	0	2	nev any	st
MIGRAN	5	x	m	0	0	all	0	all	Eu:UK	1964	pr	259	n	V	n	n	0	cig only	20	20	2	3	nev any	st
MIGRAN	7	x	m	0	0	all	0	all	Eu:UK	1964	pr	259	n	V	n	n	0	cig only	21	99	3	0	nev any	st
MIGRAN	28	x	f	0	0	all	0	all	Eu:UK	1964	pr	259	n	V	n	n	0	cig only	1	9	1	1	nev any	st
MIGRAN	30	x	f	0	0	all	0	all	Eu:UK	1964	pr	259	n	V	n	n	0	cig only	10	19	0	2	nev any	st
MIGRAN	32	x	f	0	0	all	0	all	Eu:UK	1964	pr	259	n	V	n	n	0	cig only	20	20	2	3	nev any	st

Table 1G6 - 4

IESLC - Meta-analysis of Current Smoking by Amount, Overview, Any product (or Cigarettes if Any not available)
All LC types
Least adjusted

REF	NRR	X	SEX	AGEL	AGEH	RACE	YF	LC TYPE	LOC	START	ST	NLC	R	VB	P	H	AD	PRODUCT	exL	exH	S1	S2	DENOM	De
MIGRAN	34	x	f	0	0	all	0	all	Eu:UK	1964	pr	259	n	V	n	n	0	cig only	21	99	3	0	nev any	st
MRFITR	3		m	0	0	all	0	all	NAmer	1973	pr	119	n	bl	n	n	0	cig+/-ot	1	19	1	0	nev cigs	ot
MRFITR	4		m	0	0	all	0	all	NAmer	1973	pr	119	n	bl	n	n	0	cig+/-ot	20	39	2	0	nev cigs	ot
MRFITR	5		m	0	0	all	0	all	NAmer	1973	pr	119	n	bl	n	n	0	cig+/-ot	40	99	3	0	nev cigs	ot
NAM	66	x	m	0	0	all	-	all	NAmer	1986	CC	1199	n	bl	y	n	0	cig+/-ot	1	24	0	0	nev cigs	ot
NAM	67	x	m	0	0	all	-	all	NAmer	1986	CC	1199	n	bl	y	n	0	cig+/-ot	25	99	3	0	nev cigs	ot
NAM	82	x	f	0	0	all	-	all	NAmer	1986	CC	1199	n	bl	y	n	0	cig+/-ot	1	24	0	0	nev cigs	ot
NAM	83	x	f	0	0	all	-	all	NAmer	1986	CC	1199	n	bl	y	n	0	cig+/-ot	25	99	3	0	nev cigs	ot
PARKIN	17	x	m	0	0	bl	-	all	Africa	1963	CC	877	n	V	y	n	0	all/unsp	1	14	1	0	nev any	st
PARKIN	18	x	m	0	0	bl	-	all	Africa	1963	CC	877	n	V	y	n	0	all/unsp	15	99	0	0	nev any	st
PERSH2	2	x	c	0	0	all	-	all	Eu:Sca	1980	CC	1022	n	bl	y	n	0	all/unsp	1	9	1	1	nev any	st
PERSH2	3	x	c	0	0	all	-	all	Eu:Sca	1980	CC	1022	n	bl	y	n	0	all/unsp	10	99	0	0	nev any	st
PETO	2		m	0	0	all	0	all	Eu:UK	1954	pr	103	n	V	n	n	0	all/unsp	1	14	1	0	nev any	st
PETO	3		m	0	0	all	0	all	Eu:UK	1954	pr	103	n	V	n	n	0	all/unsp	15	99	0	0	nev any	st
PEZZO2	3		m	0	0	all	-	all	SCAmer	1992	CC	367	n	bl	n	y	0	cig+/-ot	1	20	0	0	nev cigs	st
PEZZO2	4		m	0	0	all	-	all	SCAmer	1992	CC	367	n	bl	n	y	0	cig+/-ot	21	40	0	0	nev cigs	st
PEZZO2	5		m	0	0	all	-	all	SCAmer	1992	CC	367	n	bl	n	y	0	cig+/-ot	41	99	3	6	nev cigs	st
PEZZOT	2		m	0	0	all	-	all	SCAmer	1987	CC	215	n	bl	n	y	0	cig only	1	20	0	0	nev cigs	st
PEZZOT	3		m	0	0	all	-	all	SCAmer	1987	CC	215	n	bl	n	y	0	cig only	21	40	0	0	nev cigs	st
PEZZOT	4		m	0	0	all	-	all	SCAmer	1987	CC	215	n	bl	n	y	0	cig only	41	99	3	6	nev cigs	st
PRESCO	2		m	0	0	all	0	all	Eu:Sca	1964	pr	867	n	bl	n	n	1	all/unsp	1	14	1	0	nev any	st
PRESCO	4		m	0	0	all	0	all	Eu:Sca	1964	pr	867	n	bl	n	n	1	all/unsp	15	99	0	0	nev any	st
PRESCO	1		f	0	0	all	0	all	Eu:Sca	1964	pr	867	n	bl	n	n	1	all/unsp	1	14	1	0	nev any	st
PRESCO	3		f	0	0	all	0	all	Eu:Sca	1964	pr	867	n	bl	n	n	1	all/unsp	15	99	0	0	nev any	st
SEGI2	9	x	m	0	0	all	-	all	As:Jap	1962	CC	378	n	bl	n	n	0	cig+/-ot	1	9	1	1	nev any	st
SEGI2	11	x	m	0	0	all	-	all	As:Jap	1962	CC	378	n	bl	n	n	0	cig+/-ot	10	19	0	2	nev any	st
SEGI2	13	x	m	0	0	all	-	all	As:Jap	1962	CC	378	n	bl	n	n	0	cig+/-ot	20	29	2	3	nev any	st
SEGI2	15	x	m	0	0	all	-	all	As:Jap	1962	CC	378	n	bl	n	n	0	cig+/-ot	30	39	0	4	nev any	st
SEGI2	17	x	m	0	0	all	-	all	As:Jap	1962	CC	378	n	bl	n	n	0	cig+/-ot	40	99	3	0	nev any	st
SEGI2	21	x	f	0	0	all	-	all	As:Jap	1962	CC	378	n	bl	n	n	0	cig+/-ot	1	9	1	1	nev any	st
SEGI2	23	x	f	0	0	all	-	all	As:Jap	1962	CC	378	n	bl	n	n	0	cig+/-ot	10	19	0	2	nev any	st
SEGI2	25	x	f	0	0	all	-	all	As:Jap	1962	CC	378	n	bl	n	n	0	cig+/-ot	20	99	0	0	nev any	st
SHAW	4		c	0	0	wh	-	all	NAmer	1988	CC	335	n	V	n	y	0	all/unsp	1	19	1	0	nev any	st
SHAW	5		c	0	0	wh	-	all	NAmer	1988	CC	335	n	V	n	y	0	all/unsp	20	99	0	0	nev any	st
SOBUE	117		m	0	0	all	-	q+s+l+a	As:Jap	1986	CC	1376	n	bl	n	y	0	cig+/-ot	1	19	1	0	nev cigs	st
SOBUE	118		m	0	0	all	-	q+s+l+a	As:Jap	1986	CC	1376	n	bl	n	y	0	cig+/-ot	20	29	2	3	nev cigs	st
SOBUE	119		m	0	0	all	-	q+s+l+a	As:Jap	1986	CC	1376	n	bl	n	y	0	cig+/-ot	30	99	3	0	nev cigs	st
SPEIZE	1		f	0	0	all	0	all	NAmer	1976	pr	593	n	bl	n	y	1	cig+/-ot	1	4	0	1	nev cigs	ot
SPEIZE	2		f	0	0	all	0	all	NAmer	1976	pr	593	n	bl	n	y	1	cig+/-ot	5	14	1	2	nev cigs	ot
SPEIZE	3		f	0	0	all	0	all	NAmer	1976	pr	593	n	bl	n	y	1	cig+/-ot	15	24	2	3	nev cigs	ot
SPEIZE	4		f	0	0	all	0	all	NAmer	1976	pr	593	n	bl	n	y	1	cig+/-ot	25	34	0	4	nev cigs	ot
SPEIZE	5		f	0	0	all	0	all	NAmer	1976	pr	593	n	bl	n	y	1	cig+/-ot	35	99	3	0	nev cigs	ot
STOCKW	1		c	0	0	all	-	all	NAmer	1981	CC	22161	n	bl	n	n	0	cig+/-ot	1	19	1	0	nev any	st
STOCKW	2		c	0	0	all	-	all	NAmer	1981	CC	22161	n	bl	n	n	0	cig+/-ot	20	40	2	0	nev any	st
STOCKW	3		c	0	0	all	-	all	NAmer	1981	CC	22161	n	bl	n	n	0	cig+/-ot	41	99	3	6	nev any	st
SVENSS	26	x	f	0	0	all	-	all	Eu:Sca	1983	CC	210	n	bl	n	n	0	all/unsp	1	10	1	0	nev any	st
SVENSS	31	x	f	0	0	all	-	all	Eu:Sca	1983	CC	210	n	bl	n	n	0	all/unsp	11	20	2	3	nev any	st
SVENSS	36	x	f	0	0	all	-	all	Eu:Sca	1983	CC	210	n	bl	n	n	0	all/unsp	21	99	3	0	nev any	st
TENKAN	10		m	0	0	all	17	all	Eu:Sca	1962	pr	242	n	bl	n	n	1	all/unsp	1	14	1	0	nev any	ot
TENKAN	11		m	0	0	all	17	all	Eu:Sca	1962	pr	242	n	bl	n	n	1	all/unsp	15	24	2	3	nev any	ot
TENKAN	12		m	0	0	all	17	all	Eu:Sca	1962	pr	242	n	bl	n	n	1	all/unsp	25	99	3	0	nev any	ot
TSUGAN	29		m	0	0	all	-	q+a	As:Jap	1976	CC	134	n	bl	n	y	0	all/unsp	1	15	1	0	nev any	st
TSUGAN	30		m	0	0	all	-	q+a	As:Jap	1976	CC	134	n	bl	n	y	0	all/unsp	16	35	2	0	nev any	st
TSUGAN	31		m	0	0	all	-	q+a	As:Jap	1976	CC	134	n	bl	n	y	0	all/unsp	36	99	3	0	nev any	st
TULINI	4	x	m	0	0	all	0	all	Eu:Sca	1967	pr	472	n	bl	n	n	1	cig+/-ot	1	14	1	0	nev any	or
TULINI	5	x	m	0	0	all	0	all	Eu:Sca	1967	pr	472	n	bl	n	n	1	cig+/-ot	15	24	2	3	nev any	or
TULINI	6	x	m	0	0	all	0	all	Eu:Sca	1967	pr	472	n	bl	n	n	1	cig+/-ot	25	99	3	0	nev any	or
TULINI	9	x	f	0	0	all	0	all	Eu:Sca	1967	pr	472	n	bl	n	n	1	cig+/-ot	1	14	1	0	nev any	or
TULINI	10	x	f	0	0	all	0	all	Eu:Sca	1967	pr	472	n	bl	n	n	1	cig+/-ot	15	24	2	3	nev any	or
TULINI	11	x	f	0	0	all	0	all	Eu:Sca	1967	pr	472	n	bl	n	n	1	cig+/-ot	25	99	3	0	nev any	or
TVERDA	9		m	0	0	all	0	all	Eu:Sca	1972	pr	238	n	bl	n	n	2	cig only	1	9	1	1	nev cigs	ot
TVERDA	10		m	0	0	all	0	all	Eu:Sca	1972	pr	238	n	bl	n	n	2	cig only	10	19	0	2	nev cigs	ot
TVERDA	11		m	0	0	all	0	all	Eu:Sca	1972	pr	238	n	bl	n	n	2	cig only	20	99	0	0	nev cigs	ot
TVERDA	16		f	0	0	all	0	all	Eu:Sca	1972	pr	238	n	bl	n	n	2	cig only	1	9	1	1	nev cigs	ot
TVERDA	17		f	0	0	all	0	all	Eu:Sca	1972	pr	238	n	bl	n	n	2	cig only	20	99	0	0	nev cigs	ot
WAKAI	37	x	m	0	0	all	-	all	As:Jap	1988	CC	333	n	bl	n	y	0	cig+/-ot	1	19	1	0	nev any	st
WAKAI	38	x	m	0	0	all	-	all	As:Jap	1988	CC	333	n	bl	n	y	0	cig+/-ot	20	29	2	3	nev any	st
WAKAI	39	x	m	0	0	all	-	all	As:Jap	1988	CC	333	n	bl	n	y	0	cig+/-ot	30	99	3	0	nev any	st
WU	35	x	f	0	0	wh	-	q+a	NAmer	1981	CC	220	n	bl	n	y	0	all/unsp	1	20	0	0	nev any	st
WU	36	x	f	0	0	wh	-	q+a	NAmer	1981	CC	220	n	bl	n	y	0	all/unsp	21	99	3	0	nev any	st
WYNDE6	27		m	0	0	all	-	all	NAmer	1969	CC	4423	n	bl	n	y	0	cig+/-ot	1	10	1	0	nev any	st
WYNDE6	36		m	0	0	all	-	all	NAmer	1969	CC	4423	n	bl	n	y	0	cig+/-ot	11	20	2	3	nev any	st

Table 1G6 - 4

IESLC - Meta-analysis of Current Smoking by Amount, Overview, Any product (or Cigarettes if Any not available)
All LC types
Least adjusted

REF	NRR	X	SEX	AGEL	AGEH	RACE	YF	LC TYPE	LOC	START	ST	NLC	R	VB	P	H	AD	PRODUCT	exL	exH	S1	S2	DENOM	De
WYNDE6	45		m	0	0	all	-	all	NAmer	1969	CC	4423	n	bl	n	y	0	cig+/-ot	21	30	0	4	nev any	st
WYNDE6	54		m	0	0	all	-	all	NAmer	1969	CC	4423	n	bl	n	y	0	cig+/-ot	31	99	3	0	nev any	st
WYNDE6	216		f	0	0	all	-	all	NAmer	1969	CC	4423	n	bl	n	y	0	cig+/-ot	1	10	1	0	nev cigs	st
WYNDE6	225		f	0	0	all	-	all	NAmer	1969	CC	4423	n	bl	n	y	0	cig+/-ot	11	20	2	3	nev cigs	st
WYNDE6	234		f	0	0	all	-	all	NAmer	1969	CC	4423	n	bl	n	y	0	cig+/-ot	21	30	0	4	nev cigs	st
WYNDE6	243		f	0	0	all	-	all	NAmer	1969	CC	4423	n	bl	n	y	0	cig+/-ot	30	99	3	0	nev cigs	st
YAMAGU	3	x	c	0	0	all	-	all	As:Jap	1989	CC	144	n	bl	n	y	0	all/unsp	1	20	0	0	nev any	st
YAMAGU	2	x	c	0	0	all	-	all	As:Jap	1989	CC	144	n	bl	n	y	0	all/unsp	21	99	3	0	nev any	st

Cigarette type is all/unspec for all RRs
except for the following:

REF	NRR	CIGTYPE
DEAN3	5	MC only
DEAN3	12	MC only
DEAN3	19	MC only
DEAN3	89	MC only
DEAN3	96	MC only
DEAN3	103	MC only

REF	NRR	Cigarette equivalent
AKIBA	17	*
AKIBA	18	*
AKIBA	19	*
AKIBA	23	*
AKIBA	24	*
ARCHER	1	*
ARCHER	2	*
ARCHER	3	*
AXELSS	18	includes 1 g pipe tob = 1 cig
BENSHL	11	*
BENSHL	12	*
BENSHL	13	*
BEST	13	-
BEST	14	-
BEST	15	-
BOUCOT	11	-
BOUCOT	12	-
BOUCOT	13	-
BRETT	1	*
BRETT	2	*
BRETT	3	*
BROSS	18	*
BROSS	19	*
BUFFLE	30	*
BUFFLE	31	*
BUFFLE	36	*
CEDERL	80	gms inc cig = 1, sm cgr = 3, lge cgr = 5
CEDERL	81	gms inc cig = 1, sm cgr = 3, lge cgr = 5
CEDERL	82	gms inc cig = 1, sm cgr = 3, lge cgr = 5
CEDERL	76	inc 1 g other tob = 1 cig
CEDERL	77	inc 1 g other tob = 1 cig
CEDERL	78	inc 1 g other tob = 1 cig
CHANG	2	*
CHANG	3	*
CHANG	4	*
CHANG	8	*
CHANG	9	*
CHANG	10	*
CHOW	3	*
CHOW	4	*
CHOW	5	*
COMSTO	4	*
COMSTO	5	*
COMSTO	6	*
COMSTO	9	*
COMSTO	10	*
COMSTO	11	*
CORREA	45	*

Table 1G6 - 4

IESLC - Meta-analysis of Current Smoking by Amount, Overview, Any product (or Cigarettes if Any not available)
All LC types
Least adjusted

REF	NRR	Cigarette equivalent
CORREA	49	*
CPSI	216	*
CPSI	217	*
CPSI	218	*
CPSI	219	*
CPSI	275	*
CPSI	276	*
CPSI	277	*
CPSI	278	*
CPSII	30	-
CPSII	31	-
CPSII	32	-
CPSII	33	-
CPSII	34	-
CPSII	35	-
CPSII	65	*
CPSII	66	*
CPSII	67	*
CPSII	68	*
CPSII	69	*
CPSII	70	*
DARBY	1	inc 1oz pipe/wk=2 cigs/d, excl cigar/llo
DARBY	2	inc 1oz pipe/wk=2 cigs/d, excl cigar/llo
DARBY	3	inc 1oz pipe/wk=2 cigs/d, excl cigar/llo
DARBY	8	inc 1oz pipe/wk=2 cigs/d, excl cigar/llo
DARBY	9	inc 1oz pipe/wk=2 cigs/d, excl cigar/llo
DARBY	10	inc 1oz pipe/wk=2 cigs/d, excl cigar/llo
DEAN3	5	-
DEAN3	12	-
DEAN3	19	-
DEAN3	89	-
DEAN3	96	-
DEAN3	103	-
DEKLER	2	*
DEKLER	3	*
DEKLER	4	*
DOLL2	16	grams
DOLL2	17	grams
DOLL2	18	grams
DOLL2	10	-
DOLL2	11	-
DOLL2	12	-
DORANT	6	*
DORANT	7	*
DORANT	8	*
DORGAN	4	*
DORGAN	5	*
DORGAN	28	*
DORGAN	29	*
DORGAN	51	*
DORGAN	52	*
DORGAN	74	*
DORGAN	75	*
DORN	408	*
DORN	409	*
DORN	410	*
DORN	411	*
ENGELA	3	*
ENGELA	4	*
ENGELA	5	*
ENGELA	6	*
ENGELA	7	*
ENGELA	17	*
ENGELA	18	*
ENGELA	19	*
ENGELA	20	*
ENGELA	21	*
ENSTRO	7	-
ENSTRO	6	-
ENSTRO	5	-
ENSTRO	4	-
ENSTRO	3	-

Table 1G6 - 4

IESLC - Meta-analysis of Current Smoking by Amount, Overview, Any product (or Cigarettes if Any not available)
All LC types
Least adjusted

REF	NRR	Cigarette equivalent
ENSTRO	11	-
ENSTRO	10	-
ENSTRO	9	-
ENSTRO	8	-
GAO2	2	*
GAO2	3	*
GAO2	4	*
GILLIS	1	*
GILLIS	2	*
GILLIS	3	*
GILLIS	4	*
GILLIS	5	*
HAENSZ	52	*
HAENSZ	51	*
HAMMO2	21	*
HAMMO2	20	*
HAMMON	135	-
HAMMON	136	-
HAMMON	137	-
HAMMON	138	-
HIRAYA	23	*
HIRAYA	24	*
HIRAYA	25	*
HIRAYA	26	*
HIRAYA	27	*
HIRAYA	28	*
HITOSU	3	*
HITOSU	4	*
HITOSU	5	*
HITOSU	10	*
HITOSU	11	*
HOLE	9	*
HOLE	10	*
HOLE	11	*
HOLE	12	*
HUMBLE	2	*
HUMBLE	3	*
HUMBLE	5	*
HUMBLE	6	*
HUMBLE	8	*
HUMBLE	9	*
HUMBLE	11	*
HUMBLE	12	*
KAISE2	66	-
KAISE2	67	-
KAISE2	58	-
KAISE2	59	-
KAISER	6	*
KAISER	7	*
KAISER	8	*
KAISER	2	*
KAISER	3	*
KAISER	4	*
KANELL	1	*
KANELL	2	*
KANELL	3	*
KANELL	4	*
KATSOU	7	*
KATSOU	8	*
KATSOU	9	*
KATSOU	10	*
KAUFMA	2	*
KAUFMA	3	*
KAUFMA	4	*
KAUFMA	5	*
KAUFMA	6	*
KINLEN	3	*
KINLEN	4	*
KINLEN	5	*
KNEKT	25	*
KNEKT	26	*
KOO	11	*

Table 1G6 - 4

IESLC - Meta-analysis of Current Smoking by Amount, Overview, Any product (or Cigarettes if Any not available)
All LC types
Least adjusted

REF	NRR	Cigarette equivalent
KOO	12	*
KOO	13	*
LIAW	3	*
LIAW	4	*
LIAW	5	*
LIDDEL	2	*
LIDDEL	3	*
MACLEN	20	*
MACLEN	21	*
MACLEN	22	*
MACLEN	23	*
MACLEN	33	*
MACLEN	34	*
MACLEN	35	*
MATOS	4	*
MATOS	6	*
MATOS	8	*
MIGRAN	1	-
MIGRAN	3	-
MIGRAN	5	-
MIGRAN	7	-
MIGRAN	28	-
MIGRAN	30	-
MIGRAN	32	-
MIGRAN	34	-
MRFITR	3	*
MRFITR	4	*
MRFITR	5	*
NAM	66	*
NAM	67	*
NAM	82	*
NAM	83	*
PARKIN	17	grams inc 1 cig=1g, 1 pipe=0.65g
PARKIN	18	grams inc 1 cig=1g, 1 pipe=0.65g
PERSH2	2	includes 50g pipe/wk = 7 cigs/day
PERSH2	3	includes 50g pipe/wk = 7 cigs/day
PETO	2	*
PETO	3	*
PEZZO2	3	*
PEZZO2	4	*
PEZZO2	5	*
PEZZOT	2	-
PEZZOT	3	-
PEZZOT	4	-
PRESCO	2	gms, inc cig = 1, cheroot = 3, cigar = 5
PRESCO	4	gms, inc cig = 1, cheroot = 3, cigar = 5
PRESCO	1	gms, inc cig = 1, cheroot = 3, cigar = 5
PRESCO	3	gms, inc cig = 1, cheroot = 3, cigar = 5
SEGI2	9	*
SEGI2	11	*
SEGI2	13	*
SEGI2	15	*
SEGI2	17	*
SEGI2	21	*
SEGI2	23	*
SEGI2	25	*
SHAW	4	*
SHAW	5	*
SOBUE	117	*
SOBUE	118	*
SOBUE	119	*
SPEIZE	1	*
SPEIZE	2	*
SPEIZE	3	*
SPEIZE	4	*
SPEIZE	5	*
STOCKW	1	*
STOCKW	2	*
STOCKW	3	*
SVENSS	26	*
SVENSS	31	*
SVENSS	36	*

Table 1G6 - 4

IESLC - Meta-analysis of Current Smoking by Amount, Overview, Any product (or Cigarettes if Any not available)
All LC types
Least adjusted

REF	NRR	Cigarette equivalent
TENKAN	10	grams
TENKAN	11	grams
TENKAN	12	grams
TSUGAN	29	*
TSUGAN	30	*
TSUGAN	31	*
TULINI	4	*
TULINI	5	*
TULINI	6	*
TULINI	9	*
TULINI	10	*
TULINI	11	*
TVERDA	9	-
TVERDA	10	-
TVERDA	11	-
TVERDA	16	-
TVERDA	17	-
WAKAI	37	*
WAKAI	38	*
WAKAI	39	*
WU	35	*
WU	36	*
WYNDE6	27	*
WYNDE6	36	*
WYNDE6	45	*
WYNDE6	54	*
WYNDE6	216	*
WYNDE6	225	*
WYNDE6	234	*
WYNDE6	243	*
YAMAGU	3	*
YAMAGU	2	*

In this overview table, subtotals and Qs values may be invalid and should be ignored

Table 1G6 - 5

IESLC - Meta-analysis of Current Smoking by Amount, Overview, Any product (or Cigarettes if Any not available)
All LC types
Least adjusted

REF	NRR	SEX	AD	Number Exposed Case	Number Exposed Cont	Non-exposed Case	Non-exposed Cont	RR	95.00%CI
*AKIBA	17	m	0	104	59893	18	35833	3.46	(2.10- 5.70)
*AKIBA	18	m	0	178	82289	18	35833	4.31	(2.65- 6.99)
*AKIBA	19	m	0	63	28351	18	35833	4.42	(2.62- 7.47)
*AKIBA	23	f	0	54	38968	116	359850	4.30	(3.11- 5.94)
*AKIBA	24	f	0	17	10284	116	359850	5.13	(3.08- 8.53)
Subtotal AKIBA								4.29	(3.51- 5.23)
*ARCHER	1	m	0	14	6504	6	9842	3.53	(1.36- 9.18)
*ARCHER	2	m	0	68	18320	6	9842	6.09	(2.64- 14.02)
*ARCHER	3	m	0	40	7705	6	9842	8.52	(3.61- 20.07)
Subtotal ARCHER								5.87	(3.54- 9.75)
AXELSS	18	f	1	-	-	-	-	43.30	(12.60- 148.80)
*BENSHL	11	m	1	-	-	-	-	4.00	(1.55- 10.31)
*BENSHL	12	m	1	-	-	-	-	9.05	(3.91- 20.94)
*BENSHL	13	m	1	-	-	-	-	10.95	(4.76- 25.22)
Subtotal BENSHL								7.71	(4.67- 12.74)
*BEST	13	m	1	-	-	-	-	10.00	(4.56- 21.92)
*BEST	14	m	1	-	-	-	-	16.41	(7.73- 34.86)
*BEST	15	m	1	-	-	-	-	17.31	(7.93- 37.79)
Subtotal BEST								14.23	(9.11- 22.23)
*BOUCOT	11	m	0	53	15208	0	7551	53.13~	(3.28- 860.33)
*BOUCOT	12	m	0	25	6054	0	7551	63.61~	(3.87-1044.63)
*BOUCOT	13	m	0	7	886	0	7551	127.77~	(7.30-2235.31)
Subtotal BOUCOT								74.91	(14.75- 380.40)
*BRETT	1	m	0	40	17090	6	6530	2.55	(1.08- 6.01)
*BRETT	2	m	0	62	15868	6	6530	4.25	(1.84- 9.83)
*BRETT	3	m	0	33	4490	6	6530	8.00	(3.35- 19.07)
Subtotal BRETT								4.40	(2.69- 7.21)
BROSS	18	m	0	170	155	38	170	4.91	(3.24- 7.42)
BROSS	19	m	0	95	59	38	170	7.20	(4.46- 11.63)
Subtotal BROSS								5.78	(4.23- 7.91)
BUFFLE	30	f	0	12	20	12	112	5.60	(2.21- 14.21)
BUFFLE	31	f	0	52	41	12	112	11.84	(5.75- 24.38)
BUFFLE	36	f	0	116	49	12	112	22.10	(11.16- 43.73)
Subtotal BUFFLE								12.97	(8.37- 20.09)
*CEDERL	80	m	2	-	-	-	-	3.40	(1.96- 5.90)
*CEDERL	81	m	2	-	-	-	-	7.50	(4.79- 11.74)
*CEDERL	82	m	2	-	-	-	-	11.90	(7.55- 18.75)
*CEDERL	76	f	2	-	-	-	-	2.83	(1.72- 4.67)
*CEDERL	77	f	2	-	-	-	-	7.74	(4.96- 12.08)
*CEDERL	78	f	2	-	-	-	-	7.75	(4.03- 14.91)
Subtotal CEDERL								6.36	(5.20- 7.78)
*CHANG	2	m	0	5	100	5	502	5.02	(1.48- 17.02)
*CHANG	3	m	0	17	161	5	502	10.60	(3.97- 28.28)
*CHANG	4	m	0	13	158	5	502	8.26	(2.99- 22.81)
*CHANG	8	f	0	6	205	11	1139	3.03	(1.13- 8.10)
*CHANG	9	f	0	11	234	11	1139	4.87	(2.14- 11.09)
*CHANG	10	f	0	13	164	11	1139	8.21	(3.74- 18.01)
Subtotal CHANG								6.25	(4.26- 9.17)
*CHOW	3	m	0	38	29404	6	62913	13.55	(5.73- 32.05)
*CHOW	4	m	0	60	36589	6	62913	17.19	(7.43- 39.79)
*CHOW	5	m	0	40	15732	6	62913	26.66	(11.31- 62.87)
Subtotal CHOW								18.38	(11.23- 30.06)
COMSTO	4	m	0	18	25	4	69	12.42	(3.83- 40.26)
COMSTO	5	m	0	60	57	4	69	18.16	(6.22- 53.00)
COMSTO	6	m	0	26	18	4	69	24.92	(7.71- 80.57)
COMSTO	9	f	0	16	19	13	115	7.45	(3.10- 17.93)
COMSTO	10	f	0	51	26	13	115	17.35	(8.25- 36.48)
COMSTO	11	f	0	9	6	13	115	13.27	(4.07- 43.25)
Subtotal COMSTO								14.21	(9.49- 21.27)
CORREA	45	c	0	371	329	51	388	8.58	(6.18- 11.90)
CORREA	49	c	0	514	195	51	388	20.05	(14.34- 28.04)
Subtotal CORREA								12.99	(10.27- 16.41)
*CPSI	216	m	1	-	-	-	-	4.51	(3.10- 6.55)
*CPSI	217	m	1	-	-	-	-	8.41	(6.41- 11.03)
*CPSI	218	m	1	-	-	-	-	14.30	(11.33- 18.05)
*CPSI	219	m	1	-	-	-	-	17.49	(13.29- 23.03)
*CPSI	275	f	1	-	-	-	-	1.25	(0.73- 2.13)
*CPSI	276	f	1	-	-	-	-	2.44	(1.67- 3.56)
*CPSI	277	f	1	-	-	-	-	5.03	(3.82- 6.63)
*CPSI	278	f	1	-	-	-	-	11.10	(6.00- 20.53)
Subtotal CPSI								7.76	(6.94- 8.69)

Table 1G6 - 5

IESLC - Meta-analysis of Current Smoking by Amount, Overview, Any product (or Cigarettes if Any not available)
All LC types
Least adjusted

REF	NRR	SEX	AD	Number Exposed Case	Number Exposed Cont	Non-exposed Case	Non-exposed Cont	RR	95.00%CI
*CPSII	30	m	0	115	53096	124	742207	12.96	(10.06- 16.71)
*CPSII	31	m	0	214	84312	124	742207	15.19	(12.18- 18.95)
*CPSII	32	m	0	564	171438	124	742207	19.69	(16.21- 23.92)
*CPSII	33	m	0	397	138601	124	742207	17.14	(14.02- 20.97)
*CPSII	34	m	0	346	100084	124	742207	20.69	(16.86- 25.40)
*CPSII	35	m	0	145	36115	124	742207	24.03	(18.91- 30.53)
*CPSII	65	f	0	55	113074	310	2091302	3.28	(2.46- 4.37)
*CPSII	66	f	0	173	176246	310	2091302	6.62	(5.50- 7.98)
*CPSII	67	f	0	395	251336	310	2091302	10.60	(9.14- 12.30)
*CPSII	68	f	0	222	122002	310	2091302	12.28	(10.33- 14.58)
*CPSII	69	f	0	144	68066	310	2091302	14.27	(11.71- 17.39)
*CPSII	70	f	0	25	13493	310	2091302	12.50	(8.32- 18.78)
Subtotal CPSII								12.88	(12.14- 13.68)
DARBY	1	m	0	128	223	3	384	73.47	(23.11- 233.57)
DARBY	2	m	0	126	169	3	384	95.43	(29.94- 304.17)
DARBY	3	m	0	68	61	3	384	142.69	(43.52- 467.82)
DARBY	8	f	0	71	104	23	529	15.70	(9.38- 26.28)
DARBY	9	f	0	86	92	23	529	21.50	(12.90- 35.82)
DARBY	10	f	0	38	21	23	529	41.62	(21.15- 81.90)
Subtotal DARBY								29.10	(21.80- 38.84)
DEAN3	5	m	0	81	264	25	510	6.26	(3.90- 10.04)
DEAN3	12	m	0	125	429	25	510	5.94	(3.80- 9.31)
DEAN3	19	m	0	131	237	25	510	11.28	(7.16- 17.77)
DEAN3	89	f	0	31	486	41	1538	2.39	(1.48- 3.86)
DEAN3	96	f	0	44	521	41	1538	3.17	(2.05- 4.90)
DEAN3	103	f	0	27	151	41	1538	6.71	(4.01- 11.21)
Subtotal DEAN3								5.24	(4.33- 6.33)
*DEKLER	2	m	2	-	-	-	-	19.40	(2.60- 143.70)
*DEKLER	3	m	2	-	-	-	-	23.00	(3.20- 167.60)
*DEKLER	4	m	2	-	-	-	-	32.50	(4.40- 241.20)
Subtotal DEKLER								24.38	(7.70- 77.17)
*DOLL2	16	m	1	-	-	-	-	5.20	(2.41- 11.22)
*DOLL2	17	m	1	-	-	-	-	10.60	(4.94- 22.73)
*DOLL2	18	m	1	-	-	-	-	22.40	(10.46- 47.99)
*DOLL2	10	f	1	-	-	-	-	1.29	(0.14- 11.50)
*DOLL2	11	f	1	-	-	-	-	6.43	(1.81- 22.78)
*DOLL2	12	f	1	-	-	-	-	29.71	(9.46- 93.32)
Subtotal DOLL2								10.80	(7.35- 15.88)
DORANT	6	c	0	21	192	14	1090	8.52	(4.26- 17.04)
DORANT	7	c	0	143	409	14	1090	27.22	(15.54- 47.68)
DORANT	8	c	0	128	275	14	1090	36.24	(20.55- 63.91)
Subtotal DORANT								22.68	(16.05- 32.04)
DORGAN	4	m	0	81	55	15	93	9.13	(4.80- 17.39)
DORGAN	5	m	0	383	115	15	93	20.65	(11.52- 37.02)
DORGAN	28	m	0	96	42	3	35	26.67	(7.77- 91.56)
DORGAN	29	m	0	118	19	3	35	72.46	(20.25- 259.23)
DORGAN	51	f	0	224	81	103	244	6.55	(4.65- 9.23)
DORGAN	52	f	0	387	38	103	244	24.13	(16.09- 36.17)
DORGAN	74	f	0	39	15	7	20	7.43	(2.61- 21.16)
DORGAN	75	f	0	29	2	7	20	41.43	(7.79- 220.41)
Subtotal DORGAN								12.93	(10.47- 15.97)
*DORN	408	m	1	-	-	-	-	4.02	(3.43- 4.71)
*DORN	409	m	1	-	-	-	-	9.92	(8.84- 11.14)
*DORN	410	m	1	-	-	-	-	17.19	(15.28- 19.33)
*DORN	411	m	1	-	-	-	-	22.75	(19.63- 26.37)
Subtotal DORN								11.88	(11.13- 12.68)
*ENGELA	3	m	0	28	28761	27	58716	2.12	(1.25- 3.59)
*ENGELA	4	m	0	49	25801	27	58716	4.13	(2.58- 6.60)
*ENGELA	5	m	0	97	32257	27	58716	6.54	(4.27- 10.02)
*ENGELA	6	m	0	27	6424	27	58716	9.14	(5.36- 15.57)
*ENGELA	7	m	0	57	11329	27	58716	10.94	(6.92- 17.29)
*ENGELA	17	f	0	12	31450	31	207789	2.56	(1.31- 4.98)
*ENGELA	18	f	0	20	28450	31	207789	4.71	(2.69- 8.27)
*ENGELA	19	f	0	24	14820	31	207789	10.85	(6.37- 18.49)
*ENGELA	20	f	0	6	3030	31	207789	13.27	(5.54- 31.79)
*ENGELA	21	f	0	3	2445	31	207789	8.22	(2.52- 26.88)
Subtotal ENGELA								6.01	(5.05- 7.16)
*ENSTRO	7	m	1	-	-	-	-	4.74	(3.34- 6.73)
*ENSTRO	6	m	1	-	-	-	-	7.68	(5.95- 9.90)
*ENSTRO	5	m	1	-	-	-	-	13.65	(10.88- 17.13)
*ENSTRO	4	m	1	-	-	-	-	16.08	(12.77- 20.23)

Table 1G6 - 5

IESLC - Meta-analysis of Current Smoking by Amount, Overview, Any product (or Cigarettes if Any not available)
All LC types
Least adjusted

REF	NRR	SEX	AD	Number Exposed Case	Number Exposed Cont	Non-exposed Case	Non-exposed Cont	RR	95.00%CI
*ENSTRO	3	m	1	-	-	-	-	19.41	(15.22- 24.75)
*ENSTRO	11	f	1	-	-	-	-	2.15	(1.62- 2.84)
*ENSTRO	10	f	1	-	-	-	-	4.31	(3.56- 5.22)
*ENSTRO	9	f	1	-	-	-	-	9.48	(8.04- 11.18)
*ENSTRO	8	f	1	-	-	-	-	16.47	(13.74- 19.75)
Subtotal ENSTRO								9.31	(8.66- 10.02)
GAO2	2	m	0	32	41	13	56	3.36	(1.57- 7.19)
GAO2	3	m	0	77	44	13	56	7.54	(3.71- 15.30)
GAO2	4	m	0	74	30	13	56	10.63	(5.08- 22.22)
Subtotal GAO2								6.57	(4.30- 10.04)
GILLIS	1	m	0	82	205	13	145	4.46	(2.39- 8.31)
GILLIS	2	m	0	248	361	13	145	7.66	(4.25- 13.82)
GILLIS	3	m	0	76	113	13	145	7.50	(3.97- 14.19)
GILLIS	4	m	0	59	66	13	145	9.97	(5.12- 19.43)
GILLIS	5	m	0	25	26	13	145	10.72	(4.87- 23.62)
Subtotal GILLIS								7.46	(5.57- 9.98)
HAENSZ	52	f	0	40	66	81	236	1.77	(1.11- 2.82)
HAENSZ	51	f	0	23	13	81	236	5.15	(2.50- 10.65)
Subtotal HAENSZ								2.42	(1.63- 3.58)
*HAMMO2	21	m	0	38	764	5	891	8.86	(3.51- 22.41)
*HAMMO2	20	m	0	171	3708	5	891	8.22	(3.39- 19.94)
Subtotal HAMMO2								8.52	(4.49- 16.17)
*HAMMON	135	m	1	-	-	-	-	7.44	(3.90- 14.18)
*HAMMON	136	m	1	-	-	-	-	8.42	(4.86- 14.59)
*HAMMON	137	m	1	-	-	-	-	17.91	(10.37- 30.93)
*HAMMON	138	m	1	-	-	-	-	20.64	(10.98- 38.80)
Subtotal HAMMON								12.40	(9.24- 16.64)
*HIRAYA	23	m	1	-	-	-	-	2.06	(1.49- 2.85)
*HIRAYA	24	m	1	-	-	-	-	4.00	(3.20- 4.99)
*HIRAYA	25	m	1	-	-	-	-	6.24	(5.07- 7.68)
*HIRAYA	26	f	1	-	-	-	-	2.25	(1.64- 3.08)
*HIRAYA	27	f	1	-	-	-	-	2.56	(1.85- 3.54)
*HIRAYA	28	f	1	-	-	-	-	4.47	(2.73- 7.33)
Subtotal HIRAYA								3.72	(3.32- 4.17)
HITOSU	3	m	0	33	585	7	242	1.95	(0.85- 4.47)
HITOSU	4	m	0	52	747	7	242	2.41	(1.08- 5.37)
HITOSU	5	m	0	32	265	7	242	4.17	(1.81- 9.63)
HITOSU	10	f	0	24	391	33	1893	3.52	(2.06- 6.02)
HITOSU	11	f	0	4	68	33	1893	3.37	(1.16- 9.79)
Subtotal HITOSU								3.06	(2.18- 4.28)
*HOLE	9	m	0	23	840	7	1189	4.65	(2.00- 10.79)
*HOLE	10	m	0	87	2056	7	1189	7.19	(3.34- 15.47)
*HOLE	11	m	0	38	782	7	1189	8.25	(3.70- 18.39)
*HOLE	12	m	0	13	311	7	1189	7.10	(2.86- 17.64)
Subtotal HOLE								6.70	(4.43- 10.12)
HUMBLE	2	m	1	-	-	-	-	9.20	(3.30- 25.80)
HUMBLE	3	m	1	-	-	-	-	24.70	(10.00- 59.90)
HUMBLE	5	m	1	-	-	-	-	11.60	(2.70- 61.50)
HUMBLE	6	m	1	-	-	-	-	26.10	(5.60- 146.60)
HUMBLE	8	f	1	-	-	-	-	19.20	(6.50- 60.80)
HUMBLE	9	f	1	-	-	-	-	16.00	(6.70- 36.30)
HUMBLE	11	f	1	-	-	-	-	18.50	(4.90- 72.40)
HUMBLE	12	f	1	-	-	-	-	36.90	(7.60- 217.10)
Subtotal HUMBLE								17.64	(11.76- 26.46)
*KAISE2	66	m	1	-	-	-	-	4.47	(2.00- 9.99)
*KAISE2	67	m	1	-	-	-	-	10.34	(5.56- 19.23)
*KAISE2	58	f	1	-	-	-	-	7.61	(3.26- 17.75)
*KAISE2	59	f	1	-	-	-	-	22.12	(11.22- 43.61)
Subtotal KAISE2								10.24	(7.14- 14.68)
*KAISER	6	m	2	-	-	-	-	6.58	(3.87- 11.20)
*KAISER	7	m	2	-	-	-	-	17.24	(10.71- 27.73)
*KAISER	8	m	2	-	-	-	-	20.91	(12.78- 27.73)
*KAISER	2	f	2	-	-	-	-	3.42	(2.17- 5.40)
*KAISER	3	f	2	-	-	-	-	7.98	(5.35- 11.90)
*KAISER	4	f	2	-	-	-	-	12.63	(8.06- 19.80)
Subtotal KAISER								10.14	(8.46- 12.15)
KANELL	1	m	0	162	272	48	172	2.13	(1.47- 3.10)
KANELL	2	m	0	321	134	48	172	8.58	(5.88- 12.53)
KANELL	3	m	0	111	15	48	172	26.52	(14.17- 49.64)
KANELL	4	m	0	220	20	48	172	39.42	(22.55- 68.90)
Subtotal KANELL								7.70	(6.15- 9.63)

Table 1G6 - 5

IESLC - Meta-analysis of Current Smoking by Amount, Overview, Any product (or Cigarettes if Any not available)
All LC types
Least adjusted

REF	NRR	SEX	AD	Number Exposed Case	Number Exposed Cont	Non-exposed Case	Non-exposed Cont	RR	95.00%CI
KATSOU	7	f	0	8	9	48	67	1.24	(0.45- 3.45)
KATSOU	8	f	0	15	5	48	67	4.19	(1.43- 12.30)
KATSOU	9	f	0	12	3	48	67	5.58	(1.49- 20.87)
KATSOU	10	f	0	10	1	48	67	13.96	(1.73- 112.71)
Subtotal KATSOU								3.18	(1.71- 5.89)
KAUFMA	2	c	0	66	214	35	925	8.15	(5.27- 12.60)
KAUFMA	3	c	0	207	363	35	925	15.07	(10.32- 22.00)
KAUFMA	4	c	0	96	118	35	925	21.50	(13.96- 33.11)
KAUFMA	5	c	0	149	110	35	925	35.80	(23.57- 54.37)
KAUFMA	6	c	0	96	47	35	925	53.98	(33.22- 87.71)
Subtotal KAUFMA								20.92	(17.29- 25.30)
*KINLEN	3	m	0	200	3659	7	1333	10.41	(4.91- 22.06)
*KINLEN	4	m	0	232	3369	7	1333	13.11	(6.20- 27.74)
*KINLEN	5	m	0	157	1484	7	1333	20.15	(9.48- 42.80)
Subtotal KINLEN								14.00	(9.07- 21.60)
*KNEKT	25	m	0	23	14798	6	17814	4.61	(1.88- 11.33)
*KNEKT	26	m	0	63	18869	6	17814	9.91	(4.29- 22.90)
Subtotal KNEKT								6.95	(3.77- 12.82)
KOO	11	f	0	17	19	56	85	1.36	(0.65- 2.84)
KOO	12	f	0	24	5	56	85	7.29	(2.62- 20.22)
KOO	13	f	0	1	1	56	85	1.52	(0.09- 24.77)
Subtotal KOO								2.36	(1.32- 4.24)
*LIAW	3	c	2	-	-	-	-	3.10	(1.70- 5.60)
*LIAW	4	c	2	-	-	-	-	3.60	(2.00- 6.40)
*LIAW	5	c	2	-	-	-	-	8.30	(4.00- 17.30)
Subtotal LIAW								4.18	(2.91- 6.00)
*LIDDEL	2	m	1	-	-	-	-	3.33	(2.05- 5.64)
*LIDDEL	3	m	1	-	-	-	-	5.02	(3.21- 8.22)
Subtotal LIDDEL								4.15	(2.94- 5.86)
MACLEN	20	m	0	5	11	5	15	1.36	(0.32- 5.89)
MACLEN	21	m	0	42	37	5	15	3.41	(1.13- 10.28)
MACLEN	22	m	0	43	31	5	15	4.16	(1.37- 12.66)
MACLEN	23	m	0	45	27	5	15	5.00	(1.63- 15.31)
MACLEN	33	f	0	6	21	41	109	0.76	(0.29- 2.02)
MACLEN	34	f	0	22	17	41	109	3.44	(1.66- 7.12)
MACLEN	35	f	0	13	9	41	109	3.84	(1.53- 9.66)
Subtotal MACLEN								2.80	(1.92- 4.08)
MATOS	4	m	0	5	32	11	110	1.56	(0.51- 4.83)
MATOS	6	m	0	42	54	11	110	7.78	(3.71- 16.29)
MATOS	8	m	0	65	46	11	110	14.13	(6.84- 29.20)
Subtotal MATOS								7.56	(4.72- 12.11)
*MIGRAN	1	m	0	11	508	4	867	4.69	(1.50- 14.66)
*MIGRAN	3	m	0	59	1696	4	867	7.54	(2.75- 20.69)
*MIGRAN	5	m	0	36	851	4	867	9.17	(3.28- 25.65)
*MIGRAN	7	m	0	30	604	4	867	10.77	(3.81- 30.40)
*MIGRAN	28	f	0	4	772	4	3814	4.94	(1.24- 19.71)
*MIGRAN	30	f	0	14	1324	4	3814	10.08	(3.32- 30.58)
*MIGRAN	32	f	0	3	471	4	3814	6.07	(1.36- 27.05)
*MIGRAN	34	f	0	2	149	4	3814	12.80	(2.36- 69.33)
Subtotal MIGRAN								7.88	(5.19- 11.95)
*MRFITR	3	m	0	2	856	0	1859	10.86~	(0.52- 225.86)
*MRFITR	4	m	0	50	3747	0	1859	50.12~	(3.09- 811.82)
*MRFITR	5	m	0	54	3591	0	1859	56.43~	(3.49- 913.25)
Subtotal MRFITR								33.22	(6.37- 173.28)
NAM	66	m	0	92	346	30	520	4.61	(2.99- 7.11)
NAM	67	m	0	149	243	30	520	10.63	(6.98- 16.19)
NAM	82	f	0	68	150	52	885	7.72	(5.17- 11.51)
NAM	83	f	0	65	84	52	885	13.17	(8.59- 20.20)
Subtotal NAM								8.43	(6.83- 10.39)
PARKIN	17	m	0	314	818	107	1248	4.48	(3.53- 5.67)
PARKIN	18	m	0	61	128	107	1248	5.56	(3.87- 7.99)
Subtotal PARKIN								4.77	(3.92- 5.82)
PERSH2	2	c	0	290	349	178	1164	5.43	(4.35- 6.78)
PERSH2	3	c	0	446	282	178	1164	10.34	(8.32- 12.85)
Subtotal PERSH2								7.55	(6.46- 8.81)
*PETO	2	m	0	44	1181	2	295	5.50	(1.34- 22.54)
*PETO	3	m	0	55	855	2	295	9.49	(2.33- 38.66)
Subtotal PETO								7.23	(2.67- 19.57)
PEZZO2	3	m	0	57	139	6	117	8.00	(3.33- 19.21)
PEZZO2	4	m	0	107	47	6	117	44.39	(18.24- 108.02)
PEZZO2	5	m	0	69	12	6	117	112.13	(40.26- 312.24)

Table 1G6 - 5

IESLC - Meta-analysis of Current Smoking by Amount, Overview, Any product (or Cigarettes if Any not available)
All LC types
Least adjusted

REF	NRR	SEX	AD	Number Exposed Case	Number Exposed Cont	Non-exposed Case	Non-exposed Cont	RR	95.00%CI
Subtotal PEZZO2								30.27	(17.76- 51.58)
PEZZOT	2	m	0	24	94	4	116	7.40	(2.48- 22.09)
PEZZOT	3	m	0	70	29	4	116	70.00	(23.61- 207.50)
PEZZOT	4	m	0	51	6	4	116	246.50	(66.69- 911.11)
Subtotal PEZZOT								42.28	(21.77- 82.11)
*PRESCO	2	m	1	-	-	-	-	10.20	(4.49- 23.15)
*PRESCO	4	m	1	-	-	-	-	19.96	(8.92- 44.67)
*PRESCO	1	f	1	-	-	-	-	6.36	(3.60- 11.24)
*PRESCO	3	f	1	-	-	-	-	10.08	(5.72- 17.75)
Subtotal PRESCO								9.70	(6.98- 13.49)
SEGI2	9	m	0	20	62	8	53	2.14	(0.87- 5.25)
SEGI2	11	m	0	76	166	8	53	3.03	(1.37- 6.69)
SEGI2	13	m	0	92	183	8	53	3.33	(1.52- 7.30)
SEGI2	15	m	0	27	29	8	53	6.17	(2.48- 15.32)
SEGI2	17	m	0	52	45	8	53	7.66	(3.29- 17.80)
SEGI2	21	f	0	9	9	56	126	2.25	(0.85- 5.97)
SEGI2	23	f	0	10	15	56	126	1.50	(0.63- 3.54)
SEGI2	25	f	0	5	10	56	126	1.13	(0.37- 3.44)
Subtotal SEGI2								2.98	(2.18- 4.07)
SHAW	4	c	0	24	37	11	107	6.31	(2.82- 14.12)
SHAW	5	c	0	188	60	11	107	30.48	(15.36- 60.48)
Subtotal SHAW								15.73	(9.34- 26.52)
SOBUE	117	m	0	147	157	34	128	3.52	(2.27- 5.47)
SOBUE	118	m	0	236	222	34	128	4.00	(2.63- 6.09)
SOBUE	119	m	0	226	187	34	128	4.55	(2.97- 6.96)
Subtotal SOBUE								4.02	(3.14- 5.14)
*SPEIZE	1	f	1	-	-	-	-	2.70	(0.90- 5.40)
*SPEIZE	2	f	1	-	-	-	-	5.20	(3.60- 8.10)
*SPEIZE	3	f	1	-	-	-	-	12.60	(9.90- 16.70)
*SPEIZE	4	f	1	-	-	-	-	15.70	(12.10- 20.20)
*SPEIZE	5	f	1	-	-	-	-	22.00	(14.80- 32.30)
Subtotal SPEIZE								12.52	(10.76- 14.56)
STOCKW	1	c	0	2090	1194	2791	10641	6.67	(6.15- 7.25)
STOCKW	2	c	0	6053	1591	2791	10641	14.51	(13.54- 15.54)
STOCKW	3	c	0	4327	572	2791	10641	28.84	(26.18- 31.77)
Subtotal STOCKW								13.28	(12.68- 13.92)
SVENSS	26	f	0	42	30	38	120	4.42	(2.44- 8.01)
SVENSS	31	f	0	81	22	38	120	11.63	(6.41- 21.10)
SVENSS	36	f	0	19	1	38	120	60.00	(7.77- 463.15)
Subtotal SVENSS								7.80	(5.17- 11.78)
*TENKAN	10	m	1	-	-	-	-	15.86	(6.80- 37.00)
*TENKAN	11	m	1	-	-	-	-	20.25	(8.20- 50.00)
*TENKAN	12	m	1	-	-	-	-	24.97	(9.90- 63.00)
Subtotal TENKAN								19.74	(11.81- 33.01)
TSUGAN	29	m	0	14	19	18	22	0.90	(0.36- 2.28)
TSUGAN	30	m	0	30	30	18	22	1.22	(0.55- 2.73)
TSUGAN	31	m	0	19	14	18	22	1.66	(0.65- 4.20)
Subtotal TSUGAN								1.22	(0.73- 2.03)
*TULINI	4	m	1	-	-	-	-	6.49	(3.25- 13.00)
*TULINI	5	m	1	-	-	-	-	13.50	(7.08- 25.60)
*TULINI	6	m	1	-	-	-	-	28.70	(14.90- 55.10)
*TULINI	9	f	1	-	-	-	-	9.39	(4.99- 17.70)
*TULINI	10	f	1	-	-	-	-	30.70	(16.80- 56.00)
*TULINI	11	f	1	-	-	-	-	44.10	(21.10- 91.80)
Subtotal TULINI								17.72	(13.55- 23.16)
*TVERDA	9	m	2	-	-	-	-	2.14	(1.15- 3.96)
*TVERDA	10	m	2	-	-	-	-	3.32	(2.07- 5.32)
*TVERDA	11	m	2	-	-	-	-	6.56	(4.04- 10.64)
*TVERDA	16	f	2	-	-	-	-	4.53	(1.08- 18.94)
*TVERDA	17	f	2	-	-	-	-	18.00	(5.33- 60.83)
Subtotal TVERDA								4.23	(3.19- 5.62)
WAKAI	37	m	0	30	105	10	65	1.86	(0.85- 4.05)
WAKAI	38	m	0	79	129	10	65	3.98	(1.93- 8.20)
WAKAI	39	m	0	69	48	10	65	9.34	(4.37- 20.00)
Subtotal WAKAI								4.15	(2.69- 6.41)
WU	35	f	0	57	42	31	92	4.03	(2.28- 7.12)
WU	36	f	0	103	31	31	92	9.86	(5.57- 17.47)
Subtotal WU								6.29	(4.20- 9.42)
WYNDE6	27	m	0	117	122	87	617	6.80	(4.85- 9.54)
WYNDE6	36	m	0	461	293	87	617	11.16	(8.54- 14.59)
WYNDE6	45	m	0	315	129	87	617	17.32	(12.78- 23.47)

Table 1G6 - 5

IESLC - Meta-analysis of Current Smoking by Amount, Overview, Any product (or Cigarettes if Any not available)
All LC types
Least adjusted

REF	NRR	SEX	AD	Number Exposed Case	Number Exposed Cont	Non-exposed Case	Non-exposed Cont	RR	95.00%CI
WYNDE6	54	m	0	784	197	87	617	28.22	(21.47- 37.10)
WYNDE6	216	f	0	76	109	159	856	3.75	(2.68- 5.26)
WYNDE6	225	f	0	367	165	159	856	11.97	(9.33- 15.37)
WYNDE6	234	f	0	201	50	159	856	21.64	(15.21- 30.80)
WYNDE6	243	f	0	378	52	159	856	39.13	(27.98- 54.75)
Subtotal WYNDE6								14.13	(12.71- 15.72)
YAMAGU	3	c	0	48	191	24	267	2.80	(1.66- 4.72)
YAMAGU	2	c	0	28	56	24	267	5.56	(3.00- 10.31)
Subtotal YAMAGU								3.73	(2.50- 5.56)
Partial Totals				31050	1993275	16245	19573364		

*prospective study ~ With 0.5 adjustment for zero

REF	NRR	SEX	AD	Ys	Ws	Qs	Ps
*AKIBA	17	m	0	1.24	15.35	17.60	0.0000
*AKIBA	18	m	0	1.46	16.36	11.85	0.0000
*AKIBA	19	m	0	1.49	14.01	9.51	0.0000
*AKIBA	23	f	0	1.46	36.89	26.82	0.0000
*AKIBA	24	f	0	1.63	14.85	6.79	0.0000
Subtotal AKIBA				1.46	97.46	72.57	
*ARCHER	1	m	0	1.26	4.20	4.63	0.0097
*ARCHER	2	m	0	1.81	5.52	1.41	0.0000
*ARCHER	3	m	0	2.14	5.22	0.15	0.0000
Subtotal ARCHER				1.77	14.95	6.19	
AXELSS	18	f	1	3.77	2.52	5.35	0.0000
*BENSHL	11	m	1	1.39	4.28	3.66	0.0041
*BENSHL	12	m	1	2.20	5.46	0.06	0.0000
*BENSHL	13	m	1	2.39	5.53	0.04	0.0000
Subtotal BENSHL				2.04	15.26	3.76	
*BEST	13	m	1	2.30	6.23	0.00	0.0000
*BEST	14	m	1	2.80	6.77	1.61	0.0000
*BEST	15	m	1	2.85	6.30	1.84	0.0000
Subtotal BEST				2.66	19.31	3.45	
*BOUCOT	11	m	0	3.97	0.50	1.37	0.0052
*BOUCOT	12	m	0	4.15	0.49	1.66	0.0036
*BOUCOT	13	m	0	4.85	0.47	3.02	0.0009
Subtotal BOUCOT				4.32	1.45	6.06	
*BRETT	1	m	0	0.94	5.22	9.89	0.0326
*BRETT	2	m	0	1.45	5.48	4.08	0.0007
*BRETT	3	m	0	2.08	5.09	0.27	0.0000
Subtotal BRETT				1.48	15.79	14.25	
BROSS	18	m	0	1.59	22.46	11.65	0.0000
BROSS	19	m	0	1.97	16.76	1.90	0.0000
Subtotal BROSS				1.75	39.21	13.55	
BUFFLE	30	f	0	1.72	4.43	1.53	0.0003
BUFFLE	31	f	0	2.47	7.36	0.19	0.0000
BUFFLE	36	f	0	3.10	8.24	5.07	0.0000
Subtotal BUFFLE				2.56	20.04	6.80	
*CEDERL	80	m	2	1.22	12.65	14.96	0.0000
*CEDERL	81	m	2	2.01	19.12	1.68	0.0000
*CEDERL	82	m	2	2.48	18.57	0.51	0.0000
*CEDERL	76	f	2	1.04	15.40	24.87	0.0000
*CEDERL	77	f	2	2.05	19.39	1.36	0.0000
*CEDERL	78	f	2	2.05	8.98	0.62	0.0000
Subtotal CEDERL				1.85	94.11	43.99	
*CHANG	2	m	0	1.61	2.58	1.25	0.0096
*CHANG	3	m	0	2.36	3.99	0.01	0.0000
*CHANG	4	m	0	2.11	3.72	0.15	0.0000
*CHANG	8	f	0	1.11	3.97	5.74	0.0271
*CHANG	9	f	0	1.58	5.66	3.00	0.0002
*CHANG	10	f	0	2.11	6.22	0.26	0.0000
Subtotal CHANG				1.83	26.14	10.42	
*CHOW	3	m	0	2.61	5.18	0.45	0.0000
*CHOW	4	m	0	2.84	5.46	1.55	0.0000
*CHOW	5	m	0	3.28	5.22	4.93	0.0000
Subtotal CHOW				2.91	15.86	6.94	
COMSTO	4	m	0	2.52	2.78	0.12	0.0000
COMSTO	5	m	0	2.90	3.35	1.16	0.0000
COMSTO	6	m	0	3.22	2.79	2.28	0.0000
COMSTO	9	f	0	2.01	4.98	0.46	0.0000
COMSTO	10	f	0	2.85	6.96	2.05	0.0000

Table 1G6 - 5

IESLC - Meta-analysis of Current Smoking by Amount, Overview, Any product (or Cigarettes if Any not available)
All LC types
Least adjusted

REF	NRR	SEX	AD	Ys	Ws	Qs	Ps
COMSTO	11	f	0	2.59	2.75	0.21	0.0000
Subtotal COMSTO				2.65	23.61	6.27	
CORREA	45	c	0	2.15	35.82	0.94	0.0000
CORREA	49	c	0	3.00	34.18	16.15	0.0000
Subtotal CORREA				2.56	69.99	17.09	
*CPSI	216	m	1	1.51	27.46	17.78	0.0000
*CPSI	217	m	1	2.13	52.16	1.72	0.0000
*CPSI	218	m	1	2.66	70.85	8.64	0.0000
*CPSI	219	m	1	2.86	50.84	15.41	0.0000
*CPSI	275	f	1	0.22	13.40	58.41	0.4140
*CPSI	276	f	1	0.89	26.82	54.00	0.0000
*CPSI	277	f	1	1.62	50.55	24.46	0.0000
*CPSI	278	f	1	2.41	10.15	0.09	0.0000
Subtotal CPSI				2.05	302.23	180.52	
*CPSII	30	m	0	2.56	59.74	3.77	0.0000
*CPSII	31	m	0	2.72	78.59	13.20	0.0000
*CPSII	32	m	0	2.98	101.73	45.55	0.0000
*CPSII	33	m	0	2.84	94.56	26.63	0.0000
*CPSII	34	m	0	3.03	91.38	47.21	0.0000
*CPSII	35	m	0	3.18	66.97	50.50	0.0000
*CPSII	65	f	0	1.19	46.73	58.91	0.0000
*CPSII	66	f	0	1.89	111.11	19.66	0.0000
*CPSII	67	f	0	2.36	173.82	0.44	0.0000
*CPSII	68	f	0	2.51	129.51	5.01	0.0000
*CPSII	69	f	0	2.66	98.47	11.88	0.0000
*CPSII	70	f	0	2.53	23.17	1.07	0.0000
Subtotal CPSII				2.56	1075.79	283.81	
DARBY	1	m	0	4.30	2.87	11.32	0.0000
DARBY	2	m	0	4.56	2.86	14.44	0.0000
DARBY	3	m	0	4.96	2.72	19.13	0.0000
DARBY	8	f	0	2.75	14.48	2.84	0.0000
DARBY	9	f	0	3.07	14.73	8.44	0.0000
DARBY	10	f	0	3.73	8.38	16.84	0.0000
Subtotal DARBY				3.37	46.05	73.02	
DEAN3	5	m	0	1.83	17.21	3.92	0.0000
DEAN3	12	m	0	1.78	19.12	5.34	0.0000
DEAN3	19	m	0	2.42	18.58	0.23	0.0000
DEAN3	89	f	0	0.87	16.85	34.87	0.0003
DEAN3	96	f	0	1.15	20.13	26.98	0.0000
DEAN3	103	f	0	1.90	14.56	2.42	0.0000
Subtotal DEAN3				1.66	106.45	73.76	
*DEKLER	2	m	2	2.97	0.95	0.41	0.0038
*DEKLER	3	m	2	3.14	0.98	0.67	0.0019
*DEKLER	4	m	2	3.48	0.96	1.31	0.0007
Subtotal DEKLER				3.19	2.89	2.39	
*DOLL2	16	m	1	1.65	6.50	2.85	0.0000
*DOLL2	17	m	1	2.36	6.60	0.02	0.0000
*DOLL2	18	m	1	3.11	6.62	4.22	0.0000
*DOLL2	10	f	1	0.25	0.79	3.34	0.8209
*DOLL2	11	f	1	1.86	2.40	0.49	0.0040
*DOLL2	12	f	1	3.39	2.93	3.42	0.0000
Subtotal DOLL2				2.38	25.83	14.33	
DORANT	6	c	0	2.14	7.99	0.23	0.0000
DORANT	7	c	0	3.30	12.23	12.06	0.0000
DORANT	8	c	0	3.59	11.93	19.53	0.0000
Subtotal DORANT				3.12	32.15	31.81	
DORGAN	4	m	0	2.21	9.26	0.09	0.0000
DORGAN	5	m	0	3.03	11.27	5.79	0.0000
DORGAN	28	m	0	3.28	2.52	2.39	0.0000
DORGAN	29	m	0	4.28	2.36	9.19	0.0000
DORGAN	51	f	0	1.88	32.66	6.08	0.0000
DORGAN	52	f	0	3.18	23.42	17.82	0.0000
DORGAN	74	f	0	2.01	3.51	0.33	0.0002
DORGAN	75	f	0	3.72	1.37	2.74	0.0000
Subtotal DORGAN				2.56	86.38	44.43	
*DORN	408	m	1	1.39	152.79	129.24	0.0000
*DORN	409	m	1	2.29	287.32	0.08	0.0000
*DORN	410	m	1	2.84	277.97	79.06	0.0000
*DORN	411	m	1	3.12	176.37	116.73	0.0000
Subtotal DORN				2.47	894.45	325.12	
*ENGELA	3	m	0	0.75	13.76	33.52	0.0054

Table 1G6 - 5

IESLC - Meta-analysis of Current Smoking by Amount, Overview, Any product (or Cigarettes if Any not available)
All LC types
Least adjusted

REF	NRR	SEX	AD	Ys	Ws	Qs	Ps
*ENGELA	4	m	0	1.42	17.42	13.89	0.0000
*ENGELA	5	m	0	1.88	21.14	3.97	0.0000
*ENGELA	6	m	0	2.21	13.53	0.13	0.0000
*ENGELA	7	m	0	2.39	18.36	0.12	0.0000
*ENGELA	17	f	0	0.94	8.65	16.29	0.0057
*ENGELA	18	f	0	1.55	12.16	7.04	0.0000
*ENGELA	19	f	0	2.38	13.54	0.07	0.0000
*ENGELA	20	f	0	2.59	5.04	0.38	0.0000
*ENGELA	21	f	0	2.11	2.74	0.11	0.0005
Subtotal ENGELA				1.79	126.34	75.52	
*ENSTRO	7	m	1	1.56	31.30	17.84	0.0000
*ENSTRO	6	m	1	2.04	59.28	4.40	0.0000
*ENSTRO	5	m	1	2.61	74.58	6.84	0.0000
*ENSTRO	4	m	1	2.78	72.60	15.80	0.0000
*ENSTRO	3	m	1	2.97	65.00	27.87	0.0000
*ENSTRO	11	f	1	0.77	48.76	116.47	0.0000
*ENSTRO	10	f	1	1.46	104.90	75.80	0.0000
*ENSTRO	9	f	1	2.25	141.36	0.54	0.0000
*ENSTRO	8	f	1	2.80	116.71	28.08	0.0000
Subtotal ENSTRO				2.23	714.48	293.64	
GAO2	2	m	0	1.21	6.65	8.02	0.0018
GAO2	3	m	0	2.02	7.66	0.65	0.0000
GAO2	4	m	0	2.36	7.06	0.02	0.0000
Subtotal GAO2				1.88	21.37	8.69	
GILLIS	1	m	0	1.50	9.91	6.59	0.0000
GILLIS	2	m	0	2.04	11.03	0.83	0.0000
GILLIS	3	m	0	2.02	9.45	0.83	0.0000
GILLIS	4	m	0	2.30	8.63	0.00	0.0000
GILLIS	5	m	0	2.37	6.16	0.02	0.0000
Subtotal GILLIS				2.01	45.18	8.28	
HAENSZ	52	f	0	0.57	17.63	53.51	0.0170
HAENSZ	51	f	0	1.64	7.30	3.29	0.0000
Subtotal HAENSZ				0.88	24.93	56.80	
*HAMMO2	21	m	0	2.18	4.47	0.07	0.0000
*HAMMO2	20	m	0	2.11	4.89	0.20	0.0000
Subtotal HAMMO2				2.14	9.36	0.28	
*HAMMON	135	m	1	2.01	9.22	0.85	0.0000
*HAMMON	136	m	1	2.13	12.72	0.41	0.0000
*HAMMON	137	m	1	2.89	12.87	4.24	0.0000
*HAMMON	138	m	1	3.03	9.64	4.95	0.0000
Subtotal HAMMON				2.52	44.45	10.46	
*HIRAYA	23	m	1	0.72	36.53	92.16	0.0000
*HIRAYA	24	m	1	1.39	77.85	66.57	0.0000
*HIRAYA	25	m	1	1.83	89.10	20.53	0.0000
*HIRAYA	26	f	1	0.81	38.69	87.05	0.0000
*HIRAYA	27	f	1	0.94	36.49	68.58	0.0000
*HIRAYA	28	f	1	1.50	15.75	10.43	0.0000
Subtotal HIRAYA				1.31	294.40	345.32	
HITOSU	3	m	0	0.67	5.59	15.08	0.1144
HITOSU	4	m	0	0.88	5.97	12.25	0.0319
HITOSU	5	m	0	1.43	5.49	4.27	0.0008
HITOSU	10	f	0	1.26	13.32	14.75	0.0000
HITOSU	11	f	0	1.22	3.38	4.06	0.0253
Subtotal HITOSU				1.12	33.76	50.42	
*HOLE	9	m	0	1.54	5.43	3.25	0.0003
*HOLE	10	m	0	1.97	6.53	0.75	0.0000
*HOLE	11	m	0	2.11	5.99	0.24	0.0000
*HOLE	12	m	0	1.96	4.64	0.57	0.0000
Subtotal HOLE				1.90	22.58	4.81	
HUMBLE	2	m	1	2.22	3.63	0.03	0.0000
HUMBLE	3	m	1	3.21	4.80	3.85	0.0000
HUMBLE	5	m	1	2.45	1.57	0.03	0.0021
HUMBLE	6	m	1	3.26	1.44	1.30	0.0001
HUMBLE	8	f	1	2.95	3.07	1.27	0.0000
HUMBLE	9	f	1	2.77	5.38	1.15	0.0000
HUMBLE	11	f	1	2.92	2.12	0.78	0.0000
HUMBLE	12	f	1	3.61	1.37	2.30	0.0000
Subtotal HUMBLE				2.87	23.38	10.71	
*KAISE2	66	m	1	1.50	5.94	3.93	0.0003
*KAISE2	67	m	1	2.34	9.98	0.01	0.0000
*KAISE2	58	f	1	2.03	5.35	0.42	0.0000

Table 1G6 - 5

IESLC - Meta-analysis of Current Smoking by Amount, Overview, Any product (or Cigarettes if Any not available)
All LC types
Least adjusted

REF	NRR	SEX	AD	Ys	Ws	Qs	Ps
*KAISE2	59	f	1	3.10	8.34	5.14	0.0000
Subtotal KAISE2				2.33	29.61	9.51	
*KAISER	6	m	2	1.88	13.61	2.48	0.0000
*KAISER	7	m	2	2.85	16.98	4.88	0.0000
*KAISER	8	m	2	3.04	25.61	13.62	0.0000
*KAISER	2	f	2	1.23	18.49	21.62	0.0000
*KAISER	3	f	2	2.08	24.04	1.32	0.0000
*KAISER	4	f	2	2.54	19.02	0.96	0.0000
Subtotal KAISER				2.32	117.74	44.88	
KANELL	1	m	0	0.76	27.40	66.08	0.0001
KANELL	2	m	0	2.15	26.86	0.70	0.0000
KANELL	3	m	0	3.28	9.77	9.13	0.0000
KANELL	4	m	0	3.67	12.32	22.89	0.0000
Subtotal KANELL				2.04	76.35	98.79	
KATSOU	7	f	0	0.22	3.68	16.15	0.6791
KATSOU	8	f	0	1.43	3.31	2.55	0.0092
KATSOU	9	f	0	1.72	2.21	0.77	0.0106
KATSOU	10	f	0	2.64	0.88	0.09	0.0134
Subtotal KATSOU				1.16	10.08	19.57	
KAUFMA	2	c	0	2.10	20.21	0.92	0.0000
KAUFMA	3	c	0	2.71	26.85	4.33	0.0000
KAUFMA	4	c	0	3.07	20.60	11.81	0.0000
KAUFMA	5	c	0	3.58	22.00	35.31	0.0000
KAUFMA	6	c	0	3.99	16.30	45.88	0.0000
Subtotal KAUFMA				3.04	105.97	98.25	
*KINLEN	3	m	0	2.34	6.81	0.01	0.0000
*KINLEN	4	m	0	2.57	6.84	0.47	0.0000
*KINLEN	5	m	0	3.00	6.77	3.24	0.0000
Subtotal KINLEN				2.64	20.42	3.72	
*KNEKT	25	m	0	1.53	4.76	2.91	0.0008
*KNEKT	26	m	0	2.29	5.48	0.00	0.0000
Subtotal KNEKT				1.94	10.24	2.91	
KOO	11	f	0	0.31	7.09	28.49	0.4151
KOO	12	f	0	1.99	3.69	0.39	0.0001
KOO	13	f	0	0.42	0.49	1.77	0.7696
Subtotal KOO				0.86	11.27	30.65	
*LIAW	3	c	2	1.13	10.81	15.04	0.0002
*LIAW	4	c	2	1.28	11.36	12.05	0.0000
*LIAW	5	c	2	2.12	7.17	0.27	0.0000
Subtotal LIAW				1.43	29.33	27.37	
*LIDDEL	2	m	1	1.20	15.00	18.42	0.0000
*LIDDEL	3	m	1	1.61	17.38	8.46	0.0000
Subtotal LIDDEL				1.42	32.38	26.88	
MACLEN	20	m	0	0.31	1.79	7.18	0.6779
MACLEN	21	m	0	1.23	3.15	3.71	0.0297
MACLEN	22	m	0	1.43	3.10	2.43	0.0120
MACLEN	23	m	0	1.61	3.07	1.51	0.0048
MACLEN	33	f	0	-0.27	4.03	26.98	0.5807
MACLEN	34	f	0	1.24	7.25	8.39	0.0009
MACLEN	35	f	0	1.35	4.51	4.21	0.0043
Subtotal MACLEN				1.03	26.92	54.41	
MATOS	4	m	0	0.45	3.02	10.50	0.4381
MATOS	6	m	0	2.05	7.03	0.47	0.0000
MATOS	8	m	0	2.65	7.29	0.83	0.0000
Subtotal MATOS				2.02	17.34	11.80	
*MIGRAN	1	m	0	1.55	2.96	1.73	0.0078
*MIGRAN	3	m	0	2.02	3.77	0.32	0.0001
*MIGRAN	5	m	0	2.22	3.63	0.03	0.0000
*MIGRAN	7	m	0	2.38	3.56	0.02	0.0000
*MIGRAN	28	f	0	1.60	2.01	1.02	0.0237
*MIGRAN	30	f	0	2.31	3.12	0.00	0.0000
*MIGRAN	32	f	0	1.80	1.72	0.44	0.0179
*MIGRAN	34	f	0	2.55	1.35	0.08	0.0031
Subtotal MIGRAN				2.06	22.12	3.64	
*MRFITR	3	m	0	2.38	0.42	0.00	0.1236
*MRFITR	4	m	0	3.91	0.50	1.27	0.0059
*MRFITR	5	m	0	4.03	0.50	1.47	0.0045
Subtotal MRFITR				3.50	1.41	2.75	
NAM	66	m	0	1.53	20.40	12.51	0.0000
NAM	67	m	0	2.36	21.70	0.06	0.0000
NAM	82	f	0	2.04	23.96	1.72	0.0000

Table 1G6 - 5

IESLC - Meta-analysis of Current Smoking by Amount, Overview, Any product (or Cigarettes if Any not available)
All LC types
Least adjusted

REF	NRR	SEX	AD	Ys	Ws	Qs	Ps
NAM	83	f	0	2.58	20.99	1.50	0.0000
Subtotal NAM				2.13	87.05	15.78	
PARKIN	17	m	0	1.50	68.71	45.30	0.0000
PARKIN	18	m	0	1.72	29.11	10.33	0.0000
Subtotal PARKIN				1.56	97.82	55.63	
PERSH2	2	c	0	1.69	78.18	29.89	0.0000
PERSH2	3	c	0	2.34	81.53	0.05	0.0000
Subtotal PERSH2				2.02	159.71	29.95	
*PETO	2	m	0	1.70	1.93	0.71	0.0180
*PETO	3	m	0	2.25	1.95	0.01	0.0017
Subtotal PETO				1.98	3.88	0.72	
PEZZO2	3	m	0	2.08	5.00	0.27	0.0000
PEZZO2	4	m	0	3.79	4.86	10.67	0.0000
PEZZO2	5	m	0	4.72	3.66	21.25	0.0000
Subtotal PEZZO2				3.41	13.52	32.19	
PEZZOT	2	m	0	2.00	3.22	0.31	0.0003
PEZZOT	3	m	0	4.25	3.25	12.21	0.0000
PEZZOT	4	m	0	5.51	2.25	22.96	0.0000
Subtotal PEZZOT				3.74	8.72	35.48	
*PRESCO	2	m	1	2.32	5.71	0.00	0.0000
*PRESCO	4	m	1	2.99	5.92	2.76	0.0000
*PRESCO	1	f	1	1.85	11.85	2.52	0.0000
*PRESCO	3	f	1	2.31	11.98	0.00	0.0000
Subtotal PRESCO				2.27	35.47	5.28	
SEGI2	9	m	0	0.76	4.76	11.46	0.0975
SEGI2	11	m	0	1.11	6.13	8.85	0.0060
SEGI2	13	m	0	1.20	6.24	7.66	0.0026
SEGI2	15	m	0	1.82	4.64	1.12	0.0001
SEGI2	17	m	0	2.04	5.40	0.41	0.0000
SEGI2	21	f	0	0.81	4.03	9.07	0.1035
SEGI2	23	f	0	0.41	5.20	18.87	0.3554
SEGI2	25	f	0	0.12	3.07	14.76	0.8365
Subtotal SEGI2				1.09	39.47	72.21	
SHAW	4	c	0	1.84	5.92	1.30	0.0000
SHAW	5	c	0	3.42	8.18	10.01	0.0000
Subtotal SHAW				2.76	14.10	11.31	
SOBUE	117	m	0	1.26	19.84	21.92	0.0000
SOBUE	118	m	0	1.39	21.76	18.58	0.0000
SOBUE	119	m	0	1.52	21.28	13.48	0.0000
Subtotal SOBUE				1.39	62.88	53.99	
*SPEIZE	1	f	1	0.99	4.79	8.31	0.0298
*SPEIZE	2	f	1	1.65	23.37	10.25	0.0000
*SPEIZE	3	f	1	2.53	56.20	2.79	0.0000
*SPEIZE	4	f	1	2.75	58.51	11.46	0.0000
*SPEIZE	5	f	1	3.09	25.23	15.35	0.0000
Subtotal SPEIZE				2.53	168.09	48.16	
STOCKW	1	c	0	1.90	565.53	96.39	0.0000
STOCKW	2	c	0	2.67	802.56	106.04	0.0000
STOCKW	3	c	0	3.36	411.25	454.09	0.0000
Subtotal STOCKW				2.59	1779.33	656.52	
SVENSS	26	f	0	1.49	10.89	7.41	0.0000
SVENSS	31	f	0	2.45	10.82	0.22	0.0000
SVENSS	36	f	0	4.09	0.92	2.92	0.0001
Subtotal SVENSS				2.05	22.63	10.55	
*TENKAN	10	m	1	2.76	5.35	1.10	0.0000
*TENKAN	11	m	1	3.01	4.70	2.28	0.0000
*TENKAN	12	m	1	3.22	4.49	3.69	0.0000
Subtotal TENKAN				2.98	14.54	7.07	
TSUGAN	29	m	0	-0.10	4.44	25.93	0.8253
TSUGAN	30	m	0	0.20	5.96	26.56	0.6241
TSUGAN	31	m	0	0.51	4.44	14.47	0.2861
Subtotal TSUGAN				0.20	14.85	66.96	
*TULINI	4	m	1	1.87	8.00	1.55	0.0000
*TULINI	5	m	1	2.60	9.30	0.79	0.0000
*TULINI	6	m	1	3.36	8.98	9.83	0.0000
*TULINI	9	f	1	2.24	9.59	0.05	0.0000
*TULINI	10	f	1	3.42	10.60	13.14	0.0000
*TULINI	11	f	1	3.79	7.11	15.47	0.0000
Subtotal TULINI				2.87	53.57	40.83	
*TVERDA	9	m	2	0.76	10.05	24.15	0.0159
*TVERDA	10	m	2	1.20	17.25	21.29	0.0000

Table 1G6 - 5

IESLC - Meta-analysis of Current Smoking by Amount, Overview, Any product (or Cigarettes if Any not available)
All LC types
Least adjusted

REF	NRR	SEX	AD	Ys	Ws	Qs	Ps
*TVERDA	11	m	2	1.88	16.39	3.03	0.0000
*TVERDA	16	f	2	1.51	1.87	1.20	0.0387
*TVERDA	17	f	2	2.89	2.59	0.87	0.0000
Subtotal TVERDA				1.44	48.15	50.54	
WAKAI	37	m	0	0.62	6.32	18.09	0.1197
WAKAI	38	m	0	1.38	7.36	6.36	0.0002
WAKAI	39	m	0	2.23	6.64	0.04	0.0000
Subtotal WAKAI				1.42	20.32	24.49	
WU	35	f	0	1.39	11.84	9.97	0.0000
WU	36	f	0	2.29	11.75	0.01	0.0000
Subtotal WU				1.84	23.59	9.98	
WYNDE6	27	m	0	1.92	33.49	5.20	0.0000
WYNDE6	36	m	0	2.41	53.48	0.55	0.0000
WYNDE6	45	m	0	2.85	41.59	12.16	0.0000
WYNDE6	54	m	0	3.34	51.37	54.41	0.0000
WYNDE6	216	f	0	1.32	33.57	32.78	0.0000
WYNDE6	225	f	0	2.48	61.57	1.82	0.0000
WYNDE6	234	f	0	3.07	30.83	17.98	0.0000
WYNDE6	243	f	0	3.67	34.09	62.68	0.0000
Subtotal WYNDE6				2.65	340.00	187.58	
YAMAGU	3	c	0	1.03	13.99	23.02	0.0001
YAMAGU	2	c	0	1.72	10.10	3.58	0.0000
Subtotal YAMAGU				1.32	24.09	26.60	

N	296
NS	69

Table 1G6 - 6

IESLC - Meta-analysis of Current Smoking by Amount, Overview, Any product (or Cigarettes if Any not available)
All LC types
Least adjusted

	Sex			
	combined	male	female	Total
N	22	171	103	296
NS	8	53	33	94

In this overview table, other than the "N" rows, entries in the "absent" and "Total" columns may be invalid and should be ignored

	Amount smoked (broad categories)				
	absent	<20k5	6-44k20	>20k45	Total
N	91	87	55	63	296
NS	44	59	43	50	196
Wt	2334.60	1856.51	2266.83	1473.17	7931.11
Het Chi	1041.85	484.79	363.92	432.46	3987.73
Het df	90	86	54	62	295
Het P	***	***	***	***	***
Fixed RR	9.97	4.99	11.60	20.10	10.08
RRl	9.57	4.77	11.13	19.10	9.87
RRu	10.38	5.22	12.08	21.15	10.31
P	+++	+++	+++	+++	+++
Random RR	9.08	4.63	9.32	15.63	8.44
RRl	7.79	4.07	8.12	13.30	7.72
RRu	10.57	5.27	10.69	18.36	9.24
P	+++	+++	+++	+++	+++

	Amount smoked (narrow categories)							
	absent	<10k1	2-19k10	11-29k20	21-39k30	31-98k40	>40k99	Total
N	175	27	22	43	15	4	10	296
NS	67	17	13	34	13	3	8	155
Wt	4030.54	648.06	707.30	978.27	771.59	220.48	574.86	7931.11
Het Chi	1657.67	202.28	210.54	227.95	35.97	20.49	61.71	3987.73
Het df	174	26	21	42	14	3	9	295
Het P	***	***	***	***	**	***	***	***
Fixed RR	9.83	3.87	6.05	10.53	15.95	17.99	26.86	10.08
RRl	9.53	3.58	5.62	9.89	14.86	15.77	24.75	9.87
RRu	10.13	4.18	6.52	11.21	17.11	20.53	29.15	10.31
P	+++	+++	+++	+++	+++	+++	+++	+++
Random RR	8.81	3.38	5.82	9.07	15.60	18.65	26.74	8.44
RRl	7.89	2.65	4.51	7.64	13.52	12.52	19.02	7.72
RRu	9.84	4.31	7.51	10.77	17.99	27.78	37.59	9.24
P	+++	+++	+++	+++	+++	+++	+++	+++

MALES

	Amount smoked (broad categories)				
	absent	<20k5	6-44k20	>20k45	Total
N	48	50	34	39	171
NS	31	48	34	39	152
Wt	1300.85	719.84	828.63	672.42	3521.74
Het Chi	460.65	232.96	188.83	239.97	1768.09
Het df	47	49	33	38	170
Het P	***	***	***	***	***
Fixed RR	11.10	4.71	10.64	17.72	10.08
RRl	10.51	4.38	9.94	16.43	9.76
RRu	11.72	5.06	11.39	19.11	10.42
P	+++	+++	+++	+++	+++
Random RR	9.96	4.97	8.88	15.12	8.84
RRl	8.20	4.14	7.27	12.11	7.85
RRu	12.09	5.97	10.84	18.87	9.95
P	+++	+++	+++	+++	+++

Table 1G6 - 6

IESLC - Meta-analysis of Current Smoking by Amount, Overview, Any product (or Cigarettes if Any not available)
All LC types
Least adjusted

MALES

		Amount smoked (narrow categories)						
	absent	<10k1	2-19k10	11-29k20	21-39k30	31-98k40	>40k99	Total
N	103	14	11	26	9	2	6	171
NS	52	14	11	26	9	2	6	120
Wt	1645.57	373.53	343.89	424.18	529.44	100.01	105.12	3521.74
Het Chi	828.78	117.14	89.38	145.23	16.23	4.20	26.59	1768.09
Het df	102	13	10	25	8	1	5	170
Het P	***	***	***	***	*	*	***	***
Fixed RR	9.86	4.52	7.28	10.67	16.65	19.43	24.76	10.08
RRl	9.39	4.08	6.55	9.70	15.29	15.97	20.45	9.76
RRu	10.34	5.00	8.10	11.73	18.13	23.64	29.97	10.42
P	+++	+++	+++	+++	+++	+++	+++	+++
Random RR	9.16	4.00	6.36	8.49	15.82	15.43	36.20	8.84
RRl	7.84	2.80	4.46	6.54	13.48	7.65	19.33	7.85
RRu	10.70	5.72	9.05	11.02	18.57	31.13	67.81	9.95
P	+++	+++	+++	+++	+++	+++	+++	+++

FEMALES

	Amount smoked (broad categories)				
	absent	<20k5	6-44k20	>20k45	Total
N	35	31	18	19	103
NS	23	29	18	19	89
Wt	827.47	448.03	597.43	321.76	2194.69
Het Chi	403.67	147.49	94.06	78.65	1197.02
Het df	34	30	17	18	102
Het P	***	***	***	***	***
Fixed RR	7.83	3.65	9.78	16.66	7.95
RRl	7.31	3.33	9.03	14.94	7.63
RRu	8.38	4.01	10.60	18.59	8.29
P	+++	+++	+++	+++	+++
Random RR	6.75	3.93	9.87	15.93	7.20
RRl	5.18	3.13	7.83	12.15	6.15
RRu	8.79	4.93	12.45	20.89	8.42
P	+++	+++	+++	+++	+++

		Amount smoked (narrow categories)						
	absent	<10k1	2-19k10	11-29k20	21-39k30	31-98k40	>40k99	Total
N	59	11	10	15	5	1	2	103
NS	31	11	10	15	5	1	2	74
Wt	777.05	188.36	351.18	515.88	221.55	98.47	42.20	2194.69
Het Chi	517.76	18.70	62.56	66.09	13.16	0.00	0.00	1197.02
Het df	58	10	9	14	4	0	1	102
Het P	***	*	***	***	*	N.S.	N.S.	***
Fixed RR	8.60	2.39	4.79	10.46	14.00	14.27	12.56	7.95
RRl	8.01	2.07	4.32	9.60	12.27	11.71	9.29	7.63
RRu	9.23	2.76	5.32	11.40	15.97	17.39	16.98	8.29
P	+++	+++	+++	+++	+++	+++	+++	+++
Random RR	8.24	2.32	4.56	10.43	14.39	14.27	12.56	7.20
RRl	6.57	1.84	3.32	8.18	10.36	11.71	9.29	6.15
RRu	10.34	2.91	6.26	13.30	20.00	17.39	16.98	8.42
P	+++	+++	+++	+++	+++	+++	+++	+++

Table 1G6 - 7

IESLC - Meta-analysis of Current Smoking by Amount, Overview, Any product (or Cigarettes if Any not available)
All LC types
Excluded studies (and stage at which they were excluded)

1	ABELIN ABRAHA AMANDU AMES ANDERS AUSTIN AXELSO BAND BECHER BERRIN BLOHMK BLOT4 BROCKM BROWN1 BYERS1 BYERS2 CARPEN CASCO2 CASCOR CHAN CHEN3 CHIAZZ CHYOU DESTE2 DOCKER DROSTE DU GARCIA GARDIN GENG GODLEY GOODMA GRAHAM GREGOR HEGMAN HEIN HENNEK HINDS HIRAOK HOROWI HORWIT HUANG ISHIMA JAHN JAIN JARVHO JIANG KELLER KIHARA KJUUS KO KOHLME KUBIK LAMWK LAMWK2 LANGE LEI LEMARC LEVIN LIU LOMBA2 LOMBAR MAGNUS MARSH MARSH2 MCDUFF MCLAUG MILLER MILLS NOTANI NOU ODRISC PAWLEG PERSHA POFFIJ QIAO QIAO2 RADZIK REN RONCO ROOTS ROTHSC SAARIK SANKAR SCHWAR SEGI SEOW SHIMIZ SIMARA SIMONA SITAS SOBUE2 STASZE STAYNE STUCKE SUN SUZUK2 SUZUKI TANG TAO TOKARS TOUSEY ULMER VEIERO VUTUC WALD WANG WANG3 WANG4 WICKLU WIGLE WILKIN WU2 WUNSCH WYNDE8 XIANGZ XU XU2 XU4 YONG ZHANG
2	AGUDO ALDERS ARMADA AUVINE BARBON BENHAM BLOT1 BLOT2 BLOT3 BOFFET BOUCHA BRESLO BROWN2 BUELL CHATZI CHEN CHEN2 CHOI COOKSO DAMBER DAVEYS DEAN DEAN2 DOLL DOSEME DUNN EBELIN ESAKI FAN GAO GARSHI GER GOLLED GSELL HANSEN HU HU2 JARUP JEDRYC JOLY JONES JUSSAW KHUDER KOULUM KREUZE KREYBE LAMTH LAUSSM LETOUR LIU2 LIU3 LIU4 LIU5 LUBIN LUBIN2 LUO MARTIS MASTRA MATSUD MCCONN MOLLO MZILEN NOTAN2 ORMOS OSANN OSANN2 PASTOR PERNU PIKE POLEDN RACHTA RANDIG RESTRE SADOWS SCHWA2 SIEMIA SPITZ STOCKS TIZZAN VANDER WANG2 WUWILL WYNDE2 WYNDE3 WYNDE4 XU3 YUAN ZHENG ZHOU
3	PISANI
4	WYNDE7
5	RIMING TANG2 WYNDE5
6	DESTEF HIRAY2 LAURIL LICKIN MRFIT MURATA WARSIN WATSON WYNDER

Table 1G6 - 8
Potentially overlapping studies

REF	REFGP	PRINC	.	OVERLAP/LINK
GILLIS	LUBIN2	2		Subset of Lubin2
KOO	KOO	1		KOO/LAMTH/LAMWK
TVERDA	TVERDA	1		VEIERO/TVERDAL
AKIBA	AKIBA	1		AKIBA/ISHIMA
PRESCO	PRESCO	2		PRESCO-combined
BROSS	BYERS1	1		GRAHAM/BROSS/BYERS1
HOLE	TANG2	1		Subset of TANG2
BENSHL	TANG2	1		Subset of TANG2
KAISER	KAISER	1		KAISER/OSANN2
MRFITR	MRFIT	2		Subset of MRFIT
WYNDE6	WYNDE6	1		WYNDE5/6/7/8
CPSI	CPSI	1		CPSI overall
ENSTRO	ENSTRO	1		Subset of CPSI

Table 1G6 - 9
Most adjusted - insufficient data for metaanalysis

REF	NRR	SEX	AGEL	AGEH	RACE	YF	LC TYPE	LOC	START	ST	NLC	R	VB	P	H	AD	PRODUCT	exL	exH	S1	S2	DENOM	De
HITOSU	14	m	0	0	all	-	all	As:Jap	1960	CC	216	n	bl	y	n	2	all/unsp	1	14	1	0	nev any	or
HITOSU	15	m	0	0	all	-	all	As:Jap	1960	CC	216	n	bl	y	n	2	all/unsp	15	24	2	3	nev any	or
HITOSU	16	m	0	0	all	-	all	As:Jap	1960	CC	216	n	bl	y	n	2	all/unsp	25	99	3	0	nev any	or
HITOSU	17	f	0	0	all	-	all	As:Jap	1960	CC	216	n	bl	y	n	2	all/unsp	1	14	1	0	nev any	or
HITOSU	18	f	0	0	all	-	all	As:Jap	1960	CC	216	n	bl	y	n	2	all/unsp	15	99	0	0	nev any	or

REF	NRR	RR	SIG	Cigarette equivalent	RRDATA comment
HITOSU	14	1.80		*	0
HITOSU	15	2.40		*	0
HITOSU	16	6.40		*	0
HITOSU	17	3.10		*	0
HITOSU	18	3.70		*	0

Table 1G7 -

IESLC - Meta-analysis of Current Smoking, Amount smoked, "Low", Any product (or Cigarettes if Any not available)
All LC types

This analysis is restricted to results for:
1) Results by Amount smoked
2) Current smokers
3) Results complete enough for use in metaanalysis

Within each study, results are then selected (in the following order of preference, within each sex) for:
4) PRODUCT: all/unspec, cigarettes regardless of other products, cigarettes only
5) CIGTYPE: all/unspecified, MC regardless of HR, MC only
6) DENOM: never smoked anything, never smoked cigarettes, (never +1 = +long term ex, +2 = +amount unknown,
+3 = never cigs+long term ex)
7) Followup period (YF, prospective studies): whole study (coded as 0) or longest available
8) LCtype: all or nearest available, at least Squamous and Adeno. (q = squamous, s = small,
l = large, a = adeno, mix = mixed, alv = alveolar)
9) Race: all or nearest available, otherwise by race (wh or w = white, bl or b = black, hi = hispanic
ch = chinese, jap = japanese, haw = hawaiian, w+o = white + oriental, sca = scandinavian, as = asian)
10) Amount smoked "low" in key scheme 1 (key value 5, maximum range <20, in numbers of
cigarettes or cigarette equivalents)
11) For overlapping studies: principal rather than subsidiary studies
Finally by Age: whole study (coded as 0) if available, otherwise by widest available age group
and then for single sex results (m, f) in preference to combined sex results (c).

Results adjusted (AD) for the most potential confounders are then chosen in Sections -1 to -3
and results adjusted for the least confounders in Sections -4 to -6. (Those least adjusted results which
actually differ from the most adjusted as marked 'x' in column X in Section -4)
(Results adjusted for an unknown number of confounder(s) are coded as 20.)

Section -7 shows excluded studies, together with the stage (as above) at which no qualifying
results were found.

Section -8 lists the potentially overlapping studies which have been included (1=principal, 2=subsidiary).

Section -9 lists any results which would have been included in preference except that they had data not complete
enough for use in meta-analysis, with their significance (yes/no), if known, and any further comment as entered
on the database.

In addition to those mentioned above, the following fields, levels and abbreviations are used:
* or nk = not known, n = no, y = yes, ot = other
nev = never
all/unspec = all or unspecified, cig+/-ot = cigarettes irrespective of other products (cigar, pipe etc)
MC = manufactured cigarettes, HR = hand-rolled cigarettes
exL, exH = range of exposure (low and high) in the smoking group, in terms of Amount smoked,
cigarettes or cigarette equivalents
REF: 6-character study reference
NRR: number of the RR on the database within the study
ST : study type (CC = case control, pr or prosp = prospective)
NLC: number of lung cancer cases in whole study
R : risky occupational population (n = no, m = mining, o = other risky)
VB : national cigarette type (V = at least 75% Virginia, bl = at least 75% blended, ot = other)
P : any proxy use
H : full histological confirmation
De : derivation of RR/CI (or = original, st = standard method, ot = other method of estimation)

Table 1G7 - 1

IESLC - Meta-analysis of Current Smoking, Amount smoked, "Low", Any product (or Cigarettes if Any not available)
All LC types
Most adjusted

REF	NRR	SEX	AGEL	AGEH	RACE	YF	LC TYPE	LOC	START	ST	NLC	R	VB	P	H	AD	PRODUCT	exL	exH	DENOM	De
AKIBA	27	m	0	0	all	0	all	As:Jap	1963	pr	610	n	bl	n	n	5	cig+/-ot	1	14	nev cigs	or
AKIBA	33	f	0	0	all	0	all	As:Jap	1963	pr	610	n	bl	n	n	5	cig+/-ot	1	14	nev cigs	or
ARCHER	1	m	0	0	wh	0	all	NAmer	1950	pr	146	m	bl	n	n	0	cig+/-ot	1	19	nev cigs	st
BENSHL	11	m	40	64	all	10	all	Eu:UK	1967	pr	486	n	V	n	n	1	cig+/-ot	1	9	nev any	ot
BEST	13	m	0	0	all	0	all	NAmer	1955	pr	381	n	V	n	n	1	cig only	1	9	nev any	ot
BRETT	1	m	0	0	all	0	all	Eu:UK	1960	pr	150	n	V	n	n	0	cig+/-ot	1	14	nev cigs	st
BUFFLE	30	f	0	0	w-hi	-	all	NAmer	1976	CC	943	n	bl	y	n	0	cig+/-ot	1	19	nev cigs	or
CEDERL	80	m	0	0	all	16	all	Eu:Sca	1963	pr	491	n	bl	n	n	2	all/unsp	1	7	nev any	ot
CEDERL	76	f	0	0	all	0	all	Eu:Sca	1963	pr	491	n	bl	n	n	2	all/unsp	1	7	nev any	or
CHANG	2	m	0	0	all	0	all	NAmer	1972	pr	136	n	bl	n	n	0	cig+/-ot	1	10	nev cigs	st
CHANG	8	f	0	0	all	0	all	NAmer	1972	pr	136	n	bl	n	n	0	cig+/-ot	1	10	nev cigs	st
CHOW	10	m	0	0	wh	0	all	NAmer	1966	pr	219	n	bl	n	n	2	cig+/-ot	1	19	nev any	ot
COMSTO	4	m	0	0	all	-	all	NAmer	1975	ot	258	n	bl	n	n	0	cig+/-ot	1	19	nev any	st
COMSTO	9	f	0	0	all	-	all	NAmer	1975	ot	258	n	bl	n	n	0	cig+/-ot	1	19	nev any	st
CPSI	216	m	35	84	all	6	all	NAmer	1959	pr	5138	n	bl	n	n	1	cig+/-ot	1	9	nev any	ot
CPSI	275	f	40	74	all	6	all	NAmer	1959	pr	5138	n	bl	n	n	1	cig+/-ot	1	9	nev cigs	ot
CPSII	120	m	0	0	all	6	all	NAmer	1982	pr	3229	n	bl	n	n	1	cig only	1	9	nev any	ot
CPSII	127	f	0	0	all	6	all	NAmer	1982	pr	3229	n	bl	n	n	1	cig+/-ot	1	9	nev cigs	ot
DARBY	1	m	0	0	wh	-	all	Eu:UK	1988	CC	982	n	V	n	n	0	cig+/-ot	1	14	nev any	st
DARBY	8	f	0	0	wh	-	all	Eu:UK	1988	CC	982	n	V	n	n	0	cig+/-ot	1	14	nev any	st
DEAN3	7	m	0	0	all	-	all	Eu:UK	1969	CC	766	n	V	y	n	3	cig only	1	12	nev any	ot
DEAN3	91	f	0	0	all	-	all	Eu:UK	1969	CC	766	n	V	y	n	3	cig only	1	12	nev any	ot
DEKLER	2	m	0	0	all	0	all	Auslia	1961	pr	138	m	V	n	n	2	cig+/-ot	1	14	nev any	or
DOLL2	16	m	0	0	all	20	all	Eu:UK	1951	pr	920	n	V	n	n	1	all/unsp	1	14	nev any	ot
DOLL2	10	f	0	0	all	22	all	Eu:UK	1951	pr	920	n	V	n	n	1	cig only	1	14	nev any	ot
DORANT	6	c	0	0	all	0	all	Eu:wst	1986	ot	550	n	bl	n	y	0	cig+/-ot	1	9	nev any	st
DORGAN	4	m	0	0	wh	-	all	NAmer	1980	CC	2026	n	bl	y	y	0	cig+/-ot	1	19	nev any	st
DORGAN	28	m	0	0	bl	-	all	NAmer	1980	CC	2026	n	bl	y	y	0	cig+/-ot	1	19	nev any	st
DORGAN	51	f	0	0	wh	-	all	NAmer	1980	CC	2026	n	bl	y	y	0	cig+/-ot	1	19	nev any	st
DORGAN	74	f	0	0	bl	-	all	NAmer	1980	CC	2026	n	bl	y	y	0	cig+/-ot	1	19	nev any	st
DORN	408	m	0	0	wh	25	all	NAmer	1954	pr	5097	n	bl	n	n	1	cig+/-ot	1	9	nev any	or
ENGELA	32	m	0	0	all	0	all	Eu:Sca	1964	pr	435	n	bl	n	n	7	cig+/-ot	5	9	nev cigs	or
ENGELA	46	f	0	0	all	0	all	Eu:Sca	1964	pr	435	n	bl	n	n	5	cig+/-ot	5	9	nev cigs	or
ENSTRO	7	m	0	0	all	0	all	NAmer	1959	pr	2879	n	bl	n	n	1	cig only	1	9	nev any	ot
ENSTRO	11	f	0	0	all	0	all	NAmer	1959	pr	2879	n	bl	n	n	1	cig only	1	9	nev any	ot
GAO2	2	m	0	0	all	-	all	As:Jap	1988	CC	282	n	bl	n	n	0	cig+/-ot	1	19	nev cigs	st
GILLIS	21	m	0	0	all	-	all	Eu:UK	1977	CC	656	n	V	n	n	3	cig+/-ot	1	14	nev any	or
HAMMO2	7	m	0	0	all	0	all	NAmer	1967	pr	450	o	bl	n	n	1	cig+/-ot	1	19	nev any	ot
HAMMON	135	m	0	0	wh	0	all	NAmer	1952	pr	448	n	bl	n	n	1	cig only	1	9	nev any	ot
HIRAYA	23	m	0	0	all	0	all	As:Jap	1965	pr	1917	n	bl	n	n	1	cig+/-ot	1	9	nev any	st
HIRAYA	26	f	0	0	all	0	all	As:Jap	1965	pr	1917	n	bl	n	n	1	cig+/-ot	1	9	nev any	st
HITOSU	35	m	0	0	all	-	all	As:Jap	1960	CC	216	n	bl	y	n	1	all/unsp	1	14	nev any	st
HITOSU	60	f	0	0	all	-	all	As:Jap	1960	CC	216	n	bl	y	n	1	all/unsp	1	14	nev any	st
HOLE	1	m	0	0	all	0	all	Eu:UK	1972	pr	225	n	V	n	n	1	cig+/-ot	1	14	nev any	ot
HUMBLE	2	m	0	0	w-hi	-	all	NAmer	1980	CC	521	n	bl	y	n	1	cig+/-ot	1	19	nev cigs	or
HUMBLE	5	m	0	0	hi	-	all	NAmer	1980	CC	521	n	bl	y	n	1	cig+/-ot	1	19	nev cigs	or
HUMBLE	8	f	0	0	w-hi	-	all	NAmer	1980	CC	521	n	bl	y	n	1	cig+/-ot	1	19	nev cigs	or
HUMBLE	11	f	0	0	hi	-	all	NAmer	1980	CC	521	n	bl	y	n	1	cig+/-ot	1	19	nev cigs	or
KAISE2	66	m	35	99	all	9	all	NAmer	1979	pr	318	n	bl	n	n	1	cig only	1	19	nev any	st
KAISE2	58	f	35	99	all	9	all	NAmer	1979	pr	318	n	bl	n	n	1	cig only	1	19	nev any	st
KAISER	6	m	0	0	all	0	all	NAmer	1964	pr	714	n	bl	n	n	2	cig+/-ot	1	19	nev cigs	or
KAISER	2	f	0	0	all	0	all	NAmer	1964	pr	714	n	bl	n	n	2	cig+/-ot	1	19	nev cigs	or
KANELL	26	m	0	0	all	-	all	Eu:bal	1950	CC	862	n	bl	n	n	1	cig+/-ot	1	10	nev any	st
KATSOU	7	f	0	0	all	-	all	Eu:bal	1987	CC	101	n	bl	n	n	0	all/unsp	1	10	nev any	st
KAUFMA	11	c	0	0	all	-	all	NAmer	1981	CC	881	n	bl	n	n	6	cig+/-ot	1	14	nev cigs	or
KINLEN	14	m	0	0	all	0	all	Eu:UK	1967	pr	718	n	V	n	n	2	cig+/-ot	1	14	nev any	ot
KNEKT	29	m	20	69	all	21	all	Eu:Sca	1966	pr	515	n	bl	n	n	1	cig+/-ot	1	14	nev any	or
KOO	11	f	0	0	all	-	all	As:HK	1981	CC	200	n	bl	n	n	0	all/unsp	1	10	nev any	st
LIAW	3	c	0	0	all	0	all	As:oth	1982	pr	127	n	ot	n	n	2	all/unsp	1	10	nev any	or
LIDDEL	2	m	0	0	all	18	all	NAmer	1970	pr	304	m	V	n	n	1	cig+/-ot	1	19	nev cigs	ot
MACLEN	20	m	0	0	ch	-	all	As:oth	1972	CC	233	n	bl	n	n	0	cig+/-ot	1	9	nev cigs	st
MACLEN	33	f	0	0	ch	-	all	As:oth	1972	CC	233	n	bl	n	n	0	cig+/-ot	1	9	nev cigs	st
MATOS	5	m	0	0	all	-	all	SCAmer	1994	CC	200	n	bl	n	n	2	cig+/-ot	1	14	nev any	or
MIGRAN	2	m	0	0	all	0	all	Eu:UK	1964	pr	259	n	V	n	n	2	cig only	1	9	nev any	ot
MIGRAN	29	f	0	0	all	0	all	Eu:UK	1964	pr	259	n	V	n	n	2	cig only	1	9	nev any	ot
MRFITR	3	m	0	0	all	0	all	NAmer	1973	pr	119	n	bl	n	n	0	cig+/-ot	1	19	nev cigs	ot
PARKIN	14	m	0	0	bl	-	all	Africa	1963	CC	877	n	V	y	n	6	all/unsp	1	14	nev any	or
PERSH2	8	c	0	0	all	-	all	Eu:Sca	1980	CC	1022	n	bl	y	n	4	all/unsp	1	9	nev any	ot
PETO	2	m	0	0	all	0	all	Eu:UK	1954	pr	103	n	V	n	n	0	all/unsp	1	14	nev any	st
PRESCO	2	m	0	0	all	0	all	Eu:Sca	1964	pr	867	n	bl	n	n	1	all/unsp	1	14	nev any	st
PRESCO	1	f	0	0	all	0	all	Eu:Sca	1964	pr	867	n	bl	n	n	1	all/unsp	1	14	nev any	st
SEGI2	10	m	0	0	all	-	all	As:Jap	1962	CC	378	n	bl	n	n	1	cig+/-ot	1	9	nev any	ot

Table 1G7 - 1

IESLC - Meta-analysis of Current Smoking, Amount smoked, "Low", Any product (or Cigarettes if Any not available)
All LC types
Most adjusted

REF	NRR	SEX	AGEL	AGEH	RACE	YF	LC TYPE	LOC	START	ST	NLC	R	VB	P	H	AD	PRODUCT	exL	exH	DENOM	De
SEGI2	22	f	0	0	all	-	all	As:Jap	1962	CC	378	n	bl	n	n	1	cig+/-ot	1	9	nev any	ot
SHAW	4	c	0	0	wh	-	all	NAmer	1988	CC	335	n	V	n	y	0	all/unsp	1	19	nev any	st
SOBUE	117	m	0	0	all	-	q+s+l+a	As:Jap	1986	CC	1376	n	bl	n	y	0	cig+/-ot	1	19	nev cigs	st
SPEIZE	2	f	0	0	all	0	all	NAmer	1976	pr	593	n	bl	n	y	1	cig+/-ot	5	14	nev cigs	ot
STOCKW	1	c	0	0	all	-	all	NAmer	1981	CC	22161	n	bl	n	n	0	cig+/-ot	1	19	nev any	st
SVENSS	6	f	0	0	all	-	all	Eu:Sca	1983	CC	210	n	bl	n	n	1	all/unsp	1	10	nev any	or
TENKAN	10	m	0	0	all	17	all	Eu:Sca	1962	pr	242	n	bl	n	n	1	all/unsp	1	14	nev any	ot
TSUGAN	29	m	0	0	all	-	q+a	As:Jap	1976	CC	134	n	bl	n	y	0	all/unsp	1	15	nev any	st
TULINI	27	m	0	0	all	0	all	Eu:Sca	1967	pr	472	n	bl	n	n	3	cig+/-ot	1	14	nev any	or
TULINI	32	f	0	0	all	0	all	Eu:Sca	1967	pr	472	n	bl	n	n	3	cig+/-ot	1	14	nev any	or
TVERDA	9	m	0	0	all	0	all	Eu:Sca	1972	pr	238	n	bl	n	n	2	cig only	1	9	nev cigs	ot
TVERDA	16	f	0	0	all	0	all	Eu:Sca	1972	pr	238	n	bl	n	n	2	cig only	1	9	nev cigs	ot
WAKAI	40	m	0	0	all	-	all	As:Jap	1988	CC	333	n	bl	n	y	2	cig+/-ot	1	19	nev any	or
WYNDE6	27	m	0	0	all	-	all	NAmer	1969	CC	4423	n	bl	n	y	0	cig+/-ot	1	10	nev any	st
WYNDE6	216	f	0	0	all	-	all	NAmer	1969	CC	4423	n	bl	n	y	0	cig+/-ot	1	10	nev cigs	st

Cigarette type is all/unspec for all RRs
except for the following:

REF	NRR	CIGTYPE
DEAN3	7	MC only
DEAN3	91	MC only

Table 1G7 - 2

IESLC - Meta-analysis of Current Smoking, Amount smoked, "Low", Any product (or Cigarettes if Any not available)
All LC types
Most adjusted

REF	NRR	SEX	AD	Number Exposed Case	Number Exposed Cont	Non-exposed Case	Non-exposed Cont	RR	95.00%CI
*AKIBA	27	m	5	-	-	-	-	3.50	(2.20- 6.00)
*AKIBA	33	f	5	-	-	-	-	3.60	(2.60- 5.00)
Subtotal AKIBA								3.57	(2.71- 4.69)
*ARCHER	1	m	0	14	6504	6	9842	3.53	(1.36- 9.18)
*BENSHL	11	m	1	-	-	-	-	4.00	(1.55- 10.31)
*BEST	13	m	1	-	-	-	-	10.00	(4.56- 21.92)
*BRETT	1	m	0	40	17090	6	6530	2.55	(1.08- 6.01)
BUFFLE	30	f	0	12	20	12	112	5.60	(2.21- 14.21)
*CEDERL	80	m	2	-	-	-	-	3.40	(1.96- 5.90)
*CEDERL	76	f	2	-	-	-	-	2.83	(1.72- 4.67)
Subtotal CEDERL								3.07	(2.12- 4.45)
*CHANG	2	m	0	5	100	5	502	5.02	(1.48- 17.02)
*CHANG	8	f	0	6	205	11	1139	3.03	(1.13- 8.10)
Subtotal CHANG								3.70	(1.72- 7.95)
*CHOW	10	m	2	-	-	-	-	13.88	(5.81- 33.12)
COMSTO	4	m	0	18	25	4	69	12.42	(3.83- 40.26)
COMSTO	9	f	0	16	19	13	115	7.45	(3.10- 17.93)
Subtotal COMSTO								8.95	(4.43- 18.08)
*CPSI	216	m	1	-	-	-	-	4.51	(3.10- 6.55)
*CPSI	275	f	1	-	-	-	-	1.25	(0.73- 2.13)
Subtotal CPSI								2.96	(2.18- 4.02)
*CPSII	120	m	1	-	-	-	-	12.22	(9.12- 16.36)
*CPSII	127	f	1	-	-	-	-	3.89	(2.77- 5.47)
Subtotal CPSII								7.52	(6.02- 9.38)
DARBY	1	m	0	128	223	3	384	73.47	(23.11- 233.57)
DARBY	8	f	0	71	104	23	529	15.70	(9.38- 26.28)
Subtotal DARBY								20.27	(12.66- 32.45)
DEAN3	7	m	3	-	-	-	-	5.46	(3.27- 9.10)
DEAN3	91	f	3	-	-	-	-	3.16	(1.92- 5.21)
Subtotal DEAN3								4.13	(2.89- 5.90)
*DEKLER	2	m	2	-	-	-	-	19.40	(2.60- 143.70)
*DOLL2	16	m	1	-	-	-	-	5.20	(2.41- 11.22)
*DOLL2	10	f	1	-	-	-	-	1.29	(0.14- 11.50)
Subtotal DOLL2								4.47	(2.16- 9.24)
DORANT	6	c	0	21	192	14	1090	8.52	(4.26- 17.04)
DORGAN	4	m	0	81	55	15	93	9.13	(4.80- 17.39)
DORGAN	28	m	0	96	42	3	35	26.67	(7.77- 91.56)
DORGAN	51	f	0	224	81	103	244	6.55	(4.65- 9.23)
DORGAN	74	f	0	39	15	7	20	7.43	(2.61- 21.16)
Subtotal DORGAN								7.59	(5.72- 10.07)
*DORN	408	m	1	-	-	-	-	4.02	(3.43- 4.71)
*ENGELA	32	m	7	-	-	-	-	4.10	(1.70- 10.00)
*ENGELA	46	f	5	-	-	-	-	12.00	(4.40- 30.00)
Subtotal ENGELA								6.72	(3.50- 12.89)
*ENSTRO	7	m	1	-	-	-	-	4.74	(3.34- 6.73)
*ENSTRO	11	f	1	-	-	-	-	2.15	(1.62- 2.84)
Subtotal ENSTRO								2.93	(2.35- 3.65)
GAO2	2	m	0	32	41	13	56	3.36	(1.57- 7.19)
GILLIS	21	m	3	-	-	-	-	4.50	(2.50- 8.10)
*HAMMO2	7	m	1	-	-	-	-	9.15	(3.62- 23.12)
*HAMMON	135	m	1	-	-	-	-	7.44	(3.90- 14.18)
*HIRAYA	23	m	1	-	-	-	-	2.06	(1.49- 2.85)
*HIRAYA	26	f	1	-	-	-	-	2.25	(1.64- 3.08)
Subtotal HIRAYA								2.16	(1.72- 2.70)
HITOSU	35	m	1	-	-	-	-	2.08	(0.90- 4.83)
HITOSU	60	f	1	-	-	-	-	3.11	(1.77- 5.46)
Subtotal HITOSU								2.75	(1.72- 4.38)
*HOLE	1	m	1	-	-	-	-	5.47	(2.35- 12.75)
HUMBLE	2	m	1	-	-	-	-	9.20	(3.30- 25.80)
HUMBLE	5	m	1	-	-	-	-	11.60	(2.70- 61.50)
HUMBLE	8	f	1	-	-	-	-	19.20	(6.50- 60.80)
HUMBLE	11	f	1	-	-	-	-	18.50	(4.90- 72.40)
Subtotal HUMBLE								13.65	(7.44- 25.07)
*KAISE2	66	m	1	-	-	-	-	4.47	(2.00- 9.99)
*KAISE2	58	f	1	-	-	-	-	7.61	(3.26- 17.75)
Subtotal KAISE2								5.75	(3.21- 10.31)
*KAISER	6	m	2	-	-	-	-	6.58	(3.87- 11.20)
*KAISER	2	f	2	-	-	-	-	3.42	(2.17- 5.40)
Subtotal KAISER								4.51	(3.19- 6.38)
KANELL	26	m	1	-	-	-	-	1.71	(1.15- 2.56)
KATSOU	7	f	0	8	9	48	67	1.24	(0.45- 3.45)

Table 1G7 - 2

IESLC - Meta-analysis of Current Smoking, Amount smoked, "Low", Any product (or Cigarettes if Any not available)
All LC types
Most adjusted

REF	NRR	SEX	AD	Number Exposed Case	Number Exposed Cont	Non-exposed Case	Non-exposed Cont	RR	95.00%CI
KAUFMA	11	c	6	-	-	-	-	8.00	(5.00- 13.00)
*KINLEN	14	m	2	-	-	-	-	10.61	(5.01- 22.48)
*KNEKT	29	m	1	-	-	-	-	5.00	(2.00- 12.30)
KOO	11	f	0	17	19	56	85	1.36	(0.65- 2.84)
*LIAW	3	c	2	-	-	-	-	3.10	(1.70- 5.60)
*LIDDEL	2	m	1	-	-	-	-	3.33	(2.05- 5.64)
MACLEN	20	m	0	5	11	5	15	1.36	(0.32- 5.89)
MACLEN	33	f	0	6	21	41	109	0.76	(0.29- 2.02)
Subtotal MACLEN								0.91	(0.40- 2.05)
MATOS	5	m	2	-	-	-	-	1.60	(0.50- 5.00)
*MIGRAN	2	m	2	-	-	-	-	4.01	(1.19- 13.47)
*MIGRAN	29	f	2	-	-	-	-	4.88	(1.17- 20.43)
Subtotal MIGRAN								4.35	(1.73- 10.98)
*MRFITR	3	m	0	2	856	0	1859	10.86~	(0.52- 225.86)
PARKIN	14	m	6	-	-	-	-	3.90	(3.00- 5.00)
PERSH2	8	c	4	-	-	-	-	5.76	(4.61- 7.19)
*PETO	2	m	0	44	1181	2	295	5.50	(1.34- 22.54)
*PRESCO	2	m	1	-	-	-	-	10.20	(4.49- 23.15)
*PRESCO	1	f	1	-	-	-	-	6.36	(3.60- 11.24)
Subtotal PRESCO								7.42	(4.65- 11.84)
SEGI2	10	m	1	-	-	-	-	2.10	(0.86- 5.16)
SEGI2	22	f	1	-	-	-	-	2.90	(1.09- 7.70)
Subtotal SEGI2								2.43	(1.26- 4.71)
SHAW	4	c	0	24	37	11	107	6.31	(2.82- 14.12)
SOBUE	117	m	0	147	157	34	128	3.52	(2.27- 5.47)
*SPEIZE	2	f	1	-	-	-	-	5.20	(3.60- 8.10)
STOCKW	1	c	0	2090	1194	2791	10641	6.67	(6.15- 7.25)
SVENSS	6	f	1	-	-	-	-	4.60	(2.50- 9.30)
*TENKAN	10	m	1	-	-	-	-	15.86	(6.80- 37.00)
TSUGAN	29	m	0	14	19	18	22	0.90	(0.36- 2.28)
*TULINI	27	m	3	-	-	-	-	6.02	(3.01- 12.00)
*TULINI	32	f	3	-	-	-	-	8.17	(4.33- 15.40)
Subtotal TULINI								7.11	(4.45- 11.34)
*TVERDA	9	m	2	-	-	-	-	2.14	(1.15- 3.96)
*TVERDA	16	f	2	-	-	-	-	4.53	(1.08- 18.94)
Subtotal TVERDA								2.41	(1.36- 4.25)
WAKAI	40	m	2	-	-	-	-	1.80	(0.81- 4.02)
WYNDE6	27	m	0	117	122	87	617	6.80	(4.85- 9.54)
WYNDE6	216	f	0	76	109	159	856	3.75	(2.68- 5.26)
Subtotal WYNDE6								5.05	(3.98- 6.42)
Partial Totals				3353	28451	3490	35561		

*prospective study ~ With 0.5 adjustment for zero

REF	NRR	SEX	AD	Ys	Ws	Qs	Ps
*AKIBA	27	m	5	1.25	15.26	1.87	0.0000
*AKIBA	33	f	5	1.28	35.93	3.72	0.0000
Subtotal AKIBA				1.27	51.20	5.58	
*ARCHER	1	m	0	1.26	4.20	0.49	0.0097
*BENSHL	11	m	1	1.39	4.28	0.20	0.0041
*BEST	13	m	1	2.30	6.23	3.05	0.0000
*BRETT	1	m	0	0.94	5.22	2.33	0.0326
BUFFLE	30	f	0	1.72	4.43	0.06	0.0003
*CEDERL	80	m	2	1.22	12.65	1.82	0.0000
*CEDERL	76	f	2	1.04	15.40	4.87	0.0000
Subtotal CEDERL				1.12	28.05	6.68	
*CHANG	2	m	0	1.61	2.58	0.00	0.0096
*CHANG	8	f	0	1.11	3.97	0.97	0.0271
Subtotal CHANG				1.31	6.55	0.97	
*CHOW	10	m	2	2.63	5.07	5.36	0.0000
COMSTO	4	m	0	2.52	2.78	2.33	0.0000
COMSTO	9	f	0	2.01	4.98	0.82	0.0000
Subtotal COMSTO				2.19	7.76	3.15	
*CPSI	216	m	1	1.51	27.46	0.25	0.0000
*CPSI	275	f	1	0.22	13.40	25.50	0.4140
Subtotal CPSI				1.09	40.86	25.75	
*CPSII	120	m	1	2.50	45.00	36.49	0.0000
*CPSII	127	f	1	1.36	33.19	1.98	0.0000
Subtotal CPSII				2.02	78.19	38.47	
DARBY	1	m	0	4.30	2.87	20.85	0.0000
DARBY	8	f	0	2.75	14.48	19.19	0.0000

Table 1G7 - 2

IESLC - Meta-analysis of Current Smoking, Amount smoked, "Low", Any product (or Cigarettes if Any not available)
All LC types
Most adjusted

REF	NRR	SEX	AD	Ys	Ws	Qs	Ps
Subtotal DARBY				3.01	17.35	40.04	
DEAN3	7	m	3	1.70	14.67	0.13	0.0000
DEAN3	91	f	3	1.15	15.42	3.15	0.0000
Subtotal DEAN3				1.42	30.09	3.28	
*DEKLER	2	m	2	2.97	0.95	1.77	0.0038
*DOLL2	16	m	1	1.65	6.50	0.01	0.0000
*DOLL2	10	f	1	0.25	0.79	1.44	0.8209
Subtotal DOLL2				1.50	7.29	1.45	
DORANT	6	c	0	2.14	7.99	2.32	0.0000
DORGAN	4	m	0	2.21	9.26	3.44	0.0000
DORGAN	28	m	0	3.28	2.52	7.13	0.0000
DORGAN	51	f	0	1.88	32.66	2.51	0.0000
DORGAN	74	f	0	2.01	3.51	0.57	0.0002
Subtotal DORGAN				2.03	47.96	13.65	
*DORN	408	m	1	1.39	152.79	6.82	0.0000
*ENGELA	32	m	7	1.41	4.89	0.18	0.0018
*ENGELA	46	f	5	2.48	4.17	3.25	0.0000
Subtotal ENGELA				1.91	9.06	3.43	
*ENSTRO	7	m	1	1.56	31.30	0.07	0.0000
*ENSTRO	11	f	1	0.77	48.76	34.16	0.0000
Subtotal ENSTRO				1.07	80.06	34.23	
GAO2	2	m	0	1.21	6.65	1.01	0.0018
GILLIS	21	m	3	1.50	11.12	0.11	0.0000
*HAMMO2	7	m	1	2.21	4.47	1.67	0.0000
*HAMMON	135	m	1	2.01	9.22	1.51	0.0000
*HIRAYA	23	m	1	0.72	36.53	28.28	0.0000
*HIRAYA	26	f	1	0.81	38.69	24.24	0.0000
Subtotal HIRAYA				0.77	75.22	52.52	
HITOSU	35	m	1	0.73	5.44	4.12	0.0875
HITOSU	60	f	1	1.13	12.11	2.65	0.0001
Subtotal HITOSU				1.01	17.55	6.77	
*HOLE	1	m	1	1.70	5.37	0.05	0.0001
HUMBLE	2	m	1	2.22	3.63	1.38	0.0000
HUMBLE	5	m	1	2.45	1.57	1.13	0.0021
HUMBLE	8	f	1	2.95	3.07	5.62	0.0000
HUMBLE	11	f	1	2.92	2.12	3.67	0.0000
Subtotal HUMBLE				2.61	10.40	11.80	
*KAISE2	66	m	1	1.50	5.94	0.07	0.0003
*KAISE2	58	f	1	2.03	5.35	0.98	0.0000
Subtotal KAISE2				1.75	11.29	1.04	
*KAISER	6	m	2	1.88	13.61	1.08	0.0000
*KAISER	2	f	2	1.23	18.49	2.57	0.0000
Subtotal KAISER				1.51	32.09	3.65	
KANELL	26	m	1	0.54	23.99	27.27	0.0086
KATSOU	7	f	0	0.22	3.68	7.07	0.6791
KAUFMA	11	c	6	2.08	16.83	3.83	0.0000
*KINLEN	14	m	2	2.36	6.82	3.93	0.0000
*KNEKT	29	m	1	1.61	4.66	0.00	0.0005
KOO	11	f	0	0.31	7.09	11.91	0.4151
*LIAW	3	c	2	1.13	10.81	2.40	0.0002
*LIDDEL	2	m	1	1.20	15.00	2.40	0.0000
MACLEN	20	m	0	0.31	1.79	3.00	0.6779
MACLEN	33	f	0	-0.27	4.03	14.22	0.5807
Subtotal MACLEN				-0.09	5.83	17.22	
MATOS	5	m	2	0.47	2.90	3.72	0.4236
*MIGRAN	2	m	2	1.39	2.61	0.12	0.0249
*MIGRAN	29	f	2	1.59	1.88	0.00	0.0298
Subtotal MIGRAN				1.47	4.49	0.12	
*MRFITR	3	m	0	2.38	0.42	0.26	0.1236
PARKIN	14	m	6	1.36	58.89	3.44	0.0000
PERSH2	8	c	4	1.75	77.78	1.71	0.0000
*PETO	2	m	0	1.70	1.93	0.02	0.0180
*PRESCO	2	m	1	2.32	5.71	2.96	0.0000
*PRESCO	1	f	1	1.85	11.85	0.73	0.0000
Subtotal PRESCO				2.00	17.57	3.69	
SEGI2	10	m	1	0.74	4.79	3.54	0.1046
SEGI2	22	f	1	1.06	4.02	1.16	0.0328
Subtotal SEGI2				0.89	8.81	4.71	
SHAW	4	c	0	1.84	5.92	0.34	0.0000
SOBUE	117	m	0	1.26	19.84	2.33	0.0000
*SPEIZE	2	f	1	1.65	23.37	0.05	0.0000

Table 1G7 - 2

IESLC - Meta-analysis of Current Smoking, Amount smoked, "Low", Any product (or Cigarettes if Any not available)
All LC types
Most adjusted

REF	NRR	SEX	AD	Ys	Ws	Qs	Ps
STOCKW	1	c	0	1.90	565.53	49.43	0.0000
SVENSS	6	f	1	1.53	8.90	0.05	0.0000
*TENKAN	10	m	1	2.76	5.35	7.22	0.0000
TSUGAN	29	m	0	-0.10	4.44	12.95	0.8253
*TULINI	27	m	3	1.80	8.03	0.30	0.0000
*TULINI	32	f	3	2.10	9.54	2.37	0.0000
Subtotal TULINI				1.96	17.58	2.66	
*TVERDA	9	m	2	0.76	10.05	7.12	0.0159
*TVERDA	16	f	2	1.51	1.87	0.02	0.0387
Subtotal TVERDA				0.88	11.92	7.14	
WAKAI	40	m	2	0.59	5.99	6.17	0.1504
WYNDE6	27	m	0	1.92	33.49	3.31	0.0000
WYNDE6	216	f	0	1.32	33.57	2.63	0.0000
Subtotal WYNDE6				1.62	67.06	5.94	

	N	87
	NS	59
	Wt	1782.36
Het	Chi	467.18
Het	df	86
Het	P	***
Fixed	RR	4.97
	RRl	4.74
	RRu	5.20
	P	+++
Random	RR	4.66
	RRl	4.09
	RRu	5.31
	P	+++
Asymm	P	N.S.

Table 1G7 - 3

IESLC - Meta-analysis of Current Smoking, Amount smoked, "Low", Any product (or Cigarettes if Any not available)
All LC types
Most adjusted

		Sex		
	combined	male	female	Total
N	6	50	31	87
NS	6	48	29	83
Wt	684.86	676.77	420.73	1782.36
Het Chi	8.76	219.05	145.72	467.18
Het df	5	49	30	86
Het P	N.S.	***	***	***
Fixed RR	6.53	4.52	3.70	4.97
RRl	6.06	4.19	3.36	4.74
RRu	7.04	4.87	4.07	5.20
P	+++	+++	+++	+++
Random RR	6.24	4.92	4.06	4.66
RRl	5.20	4.10	3.22	4.09
RRu	7.47	5.91	5.12	5.31
P	+++	+++	+++	+++
Between Chi				93.66
Between df				2
Between P				***
Btwn(F) P				***
Btwn(R) P				*

	Lung cancer type		
	all	other	Total
N	85	2	87
NS	57	2	59
Wt	1758.07	24.29	1782.36
Het Chi	451.79	6.76	467.18
Het df	84	1	86
Het P	***	**	***
Fixed RR	5.01	2.75	4.97
RRl	4.78	1.85	4.74
RRu	5.25	4.09	5.20
P	+++	+++	+++
Random RR	4.75	1.90	4.66
RRl	4.17	0.50	4.09
RRu	5.42	7.19	5.31
P	+++	N.S.	+++
Between Chi			8.64
Between df			1
Between P			**
Btwn(F) P			N.S.
Btwn(R) P			N.S.

				Location					
	NAmer	UK	Scand	othEur	China	Japan	othAs	other	Total
N	37	14	14	3		12	4	3	87
NS	23	10	9	3		8	3	3	59
Wt	1195.70	93.95	180.88	35.66		189.70	23.73	62.74	1782.36
Het Chi	193.25	48.13	31.90	17.17		16.89	6.90	4.74	467.18
Het df	36	13	13	2		11	3	2	86
Het P	***	***	**	***		N.S.	(*)	(*)	***
Fixed RR	5.66	6.01	5.28	2.37		2.65	1.79	3.84	4.97
RRl	5.35	4.91	4.56	1.71		2.30	1.20	2.99	4.74
RRu	5.99	7.35	6.11	3.29		3.05	2.68	4.91	5.20
P	+++	+++	+++	+++		+++	++	+++	+++
Random RR	5.78	6.00	5.36	2.67		2.63	1.57	3.81	4.66
RRl	4.85	3.94	4.10	0.86		2.17	0.81	1.53	4.09
RRu	6.89	9.14	6.99	8.35		3.18	3.04	9.45	5.31
P	+++	+++	+++	(+)		+++	N.S.	++	+++
Between Chi									148.21
Between df									6
Between P									***
Btwn(F) P									***
Btwn(R) P									***

Table 1G7 - 3

IESLC - Meta-analysis of Current Smoking, Amount smoked, "Low", Any product (or Cigarettes if Any not available)
All LC types
Most adjusted

	Detailed Country in "other Europe"					
	multi	Germany	othWest	East	Balkans	Total
N			1		2	3
NS			1		2	3
Wt			7.99		27.67	35.66
Het Chi			0.00		0.33	17.17
Het df			0		1	2
Het P			N.S.		N.S.	***
Fixed RR			8.52		1.64	2.37
RRl			4.26		1.13	1.71
RRu			17.04		2.38	3.29
P			+++		++	+++
Random RR			8.52		1.64	2.67
RRl			4.26		1.13	0.86
RRu			17.04		2.38	8.35
P			+++		++	(+)
Between Chi						16.84
Between df						1
Between P						***
Btwn(F) P						(*)
Btwn(R) P						***

	Detailed Country in "other Asia"			
	India	HongKong	other	Total
N		1	3	4
NS		1	2	3
Wt		7.09	16.64	23.73
Het Chi		0.00	6.12	6.90
Het df		0	2	3
Het P		N.S.	*	(*)
Fixed RR		1.36	2.02	1.79
RRl		0.65	1.25	1.20
RRu		2.84	3.26	2.68
P		N.S.	++	++
Random RR		1.36	1.59	1.57
RRl		0.65	0.60	0.81
RRu		2.84	4.22	3.04
P		N.S.	N.S.	N.S.
Between Chi				0.78
Between df				1
Between P				N.S.
Btwn(F) P				N.S.
Btwn(R) P				N.S.

	Detailed other continent			
	SCAmer	Auslia	Africa	Total
N	1	1	1	3
NS	1	1	1	3
Wt	2.90	0.95	58.89	62.74
Het Chi	0.00	0.00	0.00	4.74
Het df	0	0	0	2
Het P	N.S.	N.S.	N.S.	(*)
Fixed RR	1.60	19.40	3.90	3.84
RRl	0.51	2.61	3.02	2.99
RRu	5.06	144.23	5.03	4.91
P	N.S.	++	+++	+++
Random RR	1.60	19.40	3.90	3.81
RRl	0.51	2.61	3.02	1.53
RRu	5.06	144.23	5.03	9.45
P	N.S.	++	+++	++
Between Chi				4.74
Between df				2
Between P				(*)
Btwn(F) P				N.S.
Btwn(R) P				(*)

Table 1G7 - 3

IESLC - Meta-analysis of Current Smoking, Amount smoked, "Low", Any product (or Cigarettes if Any not available)
All LC types
Most adjusted

	Start year of study <1960	1960-69	1970-79	1980-89	1990+	Total
N	12	33	17	24	1	87
NS	9	21	12	16	1	59
Wt	326.58	454.48	107.50	890.90	2.90	1782.36
Het Chi	59.98	103.06	38.88	125.35	0.00	467.18
Het df	11	32	16	23	0	86
Het P	***	***	**	***	N.S.	***
Fixed RR	3.49	3.99	3.88	6.53	1.60	4.97
RRl	3.13	3.64	3.21	6.12	0.51	4.74
RRu	3.89	4.37	4.69	6.98	5.06	5.20
P	+++	+++	+++	+++	N.S.	+++
Random RR	3.55	4.50	3.76	6.38	1.60	4.66
RRl	2.59	3.75	2.73	5.08	0.51	4.09
RRu	4.88	5.40	5.19	8.02	5.06	5.31
P	+++	+++	+++	+++	N.S.	+++
Between Chi						139.91
Between df						4
Between P						***
Btwn(F) P						***
Btwn(R) P						**

	Study type (1) CC	other	Total
N	36	51	87
NS	24	35	59
Wt	1029.02	753.34	1782.36
Het Chi	218.19	209.68	467.18
Het df	35	50	86
Het P	***	***	***
Fixed RR	5.64	4.17	4.97
RRl	5.30	3.89	4.74
RRu	5.99	4.48	5.20
P	+++	+++	+++
Random RR	4.49	4.77	4.66
RRl	3.65	4.04	4.09
RRu	5.52	5.64	5.31
P	+++	+++	+++
Between Chi			39.32
Between df			1
Between P			***
Btwn(F) P			**
Btwn(R) P			N.S.

	Study type (2) CC	prosp	other	Total
N	36	48	3	87
NS	24	33	2	59
Wt	1029.02	737.59	15.75	1782.36
Het Chi	218.19	200.46	0.48	467.18
Het df	35	47	2	86
Het P	***	***	N.S.	***
Fixed RR	5.64	4.11	8.72	4.97
RRl	5.30	3.82	5.32	4.74
RRu	5.99	4.42	14.30	5.20
P	+++	+++	+++	+++
Random RR	4.49	4.62	8.72	4.66
RRl	3.65	3.89	5.32	4.09
RRu	5.52	5.48	14.30	5.31
P	+++	+++	+++	+++
Between Chi				48.06
Between df				2
Between P				***
Btwn(F) P				*
Btwn(R) P				*

Table 1G7 - 3

IESLC - Meta-analysis of Current Smoking, Amount smoked, "Low", Any product (or Cigarettes if Any not available)
All LC types
Most adjusted

	Study size (number of LC cases)				
	100-249	250-499	500-999	1000+	Total
N	22	22	25	18	87
NS	18	15	16	10	59
Wt	108.20	144.80	324.07	1205.28	1782.36
Het Chi	61.80	37.65	110.38	227.08	467.18
Het df	21	21	24	17	86
Het P	***	*	***	***	***
Fixed RR	3.04	4.69	4.87	5.25	4.97
RRl	2.52	3.98	4.37	4.96	4.74
RRu	3.68	5.51	5.43	5.56	5.20
P	+++	+++	+++	+++	+++
Random RR	3.10	4.89	6.10	4.52	4.66
RRl	2.20	3.90	4.72	3.54	4.09
RRu	4.37	6.13	7.88	5.78	5.31
P	+++	+++	+++	+++	+++
Between Chi					30.27
Between df					3
Between P					***
Btwn(F) P					N.S.
Btwn(R) P					*

	Risky occupational population			
	no	mining	othRisky	Total
N	83	3	1	87
NS	55	3	1	59
Wt	1757.73	20.16	4.47	1782.36
Het Chi	460.85	2.79	0.00	467.18
Het df	82	2	0	86
Het P	***	N.S.	N.S.	***
Fixed RR	4.98	3.66	9.15	4.97
RRl	4.75	2.37	3.62	4.74
RRu	5.21	5.67	23.12	5.20
P	+++	+++	+++	+++
Random RR	4.64	3.96	9.15	4.66
RRl	4.06	2.12	3.62	4.09
RRu	5.31	7.38	23.12	5.31
P	+++	+++	+++	+++
Between Chi				3.54
Between df				2
Between P				N.S.
Btwn(F) P				N.S.
Btwn(R) P				N.S.

	National cigarette tobacco type			
	Virginia	blended	other	Total
N	19	67	1	87
NS	15	43	1	59
Wt	180.95	1590.60	10.81	1782.36
Het Chi	62.40	402.26	0.00	467.18
Het df	18	66	0	86
Het P	***	***	N.S.	***
Fixed RR	5.10	4.97	3.10	4.97
RRl	4.41	4.73	1.71	4.74
RRu	5.90	5.22	5.63	5.20
P	+++	+++	+++	+++
Random RR	5.87	4.44	3.10	4.66
RRl	4.29	3.83	1.71	4.09
RRu	8.02	5.15	5.63	5.31
P	+++	+++	+++	+++
Between Chi				2.52
Between df				2
Between P				N.S.
Btwn(F) P				N.S.
Btwn(R) P				N.S.

Table 1G7 - 3

IESLC - Meta-analysis of Current Smoking, Amount smoked, "Low", Any product (or Cigarettes if Any not available)
All LC types
Most adjusted

	Any proxy use No/nk	Yes	Total
N	72	15	87
NS	52	7	59
Wt	1535.26	247.10	1782.36
Het Chi	426.36	40.06	467.18
Het df	71	14	86
Het P	***	***	***
Fixed RR	4.92	5.23	4.97
RRl	4.68	4.62	4.74
RRu	5.18	5.92	5.20
P	+++	+++	+++
Random RR	4.42	5.83	4.66
RRl	3.80	4.50	4.09
RRu	5.13	7.55	5.31
P	+++	+++	+++
Between Chi			0.76
Between df			1
Between P			N.S.
Btwn(F) P			N.S.
Btwn(R) P			(*)

	Full histological confirmation No	Yes	Total
N	75	12	87
NS	51	8	59
Wt	1599.80	182.56	1782.36
Het Chi	423.40	43.41	467.18
Het df	74	11	86
Het P	***	***	***
Fixed RR	4.94	5.18	4.97
RRl	4.71	4.48	4.74
RRu	5.19	5.99	5.20
P	+++	+++	+++
Random RR	4.59	5.14	4.66
RRl	3.97	3.74	4.09
RRu	5.30	7.05	5.31
P	+++	+++	+++
Between Chi			0.37
Between df			1
Between P			N.S.
Btwn(F) P			N.S.
Btwn(R) P			N.S.

	Number of adjustment variables (1) 0	1	2+/+nk	Total
N	27	34	26	87
NS	19	22	18	59
Wt	789.84	610.87	381.65	1782.36
Het Chi	132.08	199.20	61.26	467.18
Het df	26	33	25	86
Het P	***	***	***	***
Fixed RR	6.21	3.96	4.50	4.97
RRl	5.79	3.65	4.07	4.74
RRu	6.66	4.28	4.97	5.20
P	+++	+++	+++	+++
Random RR	4.98	4.56	4.51	4.66
RRl	3.85	3.66	3.77	4.09
RRu	6.45	5.69	5.39	5.31
P	+++	+++	+++	+++
Between Chi				74.64
Between df				2
Between P				***
Btwn(F) P				***
Btwn(R) P				N.S.

Table 1G7 - 3

IESLC - Meta-analysis of Current Smoking, Amount smoked, "Low", Any product (or Cigarettes if Any not available)
All LC types
Most adjusted

	Number of adjustment variables (2) 0	1	2	3-5	6+/+nk	Total
N	27	34	14	9	3	87
NS	19	22	10	6	3	60
Wt	789.84	610.87	109.10	191.94	80.61	1782.36
Het Chi	132.08	199.20	33.49	16.58	6.81	467.18
Het df	26	33	13	8	2	86
Het P	***	***	**	*	*	***
Fixed RR	6.21	3.96	3.82	4.91	4.54	4.97
RRl	5.79	3.65	3.17	4.26	3.65	4.74
RRu	6.66	4.28	4.61	5.66	5.65	5.20
P	+++	+++	+++	+++	+++	+++
Random RR	4.98	4.56	4.02	4.90	5.07	4.66
RRl	3.85	3.66	2.90	3.89	3.01	4.09
RRu	6.45	5.69	5.58	6.17	8.54	5.31
P	+++	+++	+++	+++	+++	+++
Between Chi						79.02
Between df						4
Between P						***
Btwn(F) P						**
Btwn(R) P						N.S.

	Product all/unsp	cig+/-ot	cig only	Total
N	17	56	14	87
NS	14	38	9	61
Wt	254.46	1328.80	199.09	1782.36
Het Chi	58.30	312.96	87.99	467.18
Het df	16	55	13	86
Het P	***	***	***	***
Fixed RR	4.26	5.14	4.77	4.97
RRl	3.77	4.87	4.15	4.74
RRu	4.82	5.43	5.48	5.20
P	+++	+++	+++	+++
Random RR	3.82	4.94	4.75	4.66
RRl	2.92	4.19	3.15	4.09
RRu	5.01	5.82	7.18	5.31
P	+++	+++	+++	+++
Between Chi				7.94
Between df				2
Between P				*
Btwn(F) P				N.S.
Btwn(R) P				N.S.

	Denominator nev any	nev cigs	Total
N	59	28	87
NS	43	19	62
Wt	1479.18	303.18	1782.36
Het Chi	370.90	74.83	467.18
Het df	58	27	86
Het P	***	***	***
Fixed RR	5.22	3.90	4.97
RRl	4.96	3.48	4.74
RRu	5.49	4.36	5.20
P	+++	+++	+++
Random RR	4.95	4.03	4.66
RRl	4.22	3.27	4.09
RRu	5.80	4.98	5.31
P	+++	+++	+++
Between Chi			21.46
Between df			1
Between P			***
Btwn(F) P			*
Btwn(R) P			N.S.

Table 1G7 - 3

IESLC - Meta-analysis of Current Smoking, Amount smoked, "Low", Any product (or Cigarettes if Any not available)
All LC types
Most adjusted

	Derivation of RR/CI			
	Orig	StdCalc	Other	Total
N	24	34	29	87
NS	17	22	21	60
Wt	414.00	930.61	437.74	1782.36
Het Chi	51.10	254.60	143.09	467.18
Het df	23	33	28	86
Het P	***	***	***	***
Fixed RR	4.28	5.44	4.70	4.97
RRl	3.88	5.10	4.28	4.74
RRu	4.71	5.80	5.17	5.20
P	+++	+++	+++	+++
Random RR	4.73	4.43	4.73	4.66
RRl	3.95	3.46	3.72	4.09
RRu	5.65	5.67	6.00	5.31
P	+++	+++	+++	+++
Between Chi				18.39
Between df				2
Between P				***
Btwn(F) P				N.S.
Btwn(R) P				N.S.

Table 1G7 - 4

IESLC - Meta-analysis of Current Smoking, Amount smoked, "Low", Any product (or Cigarettes if Any not available)
All LC types
Least adjusted

REF	NRR	X	SEX	AGEL	AGEH	RACE	YF	LC TYPE	LOC	START	ST	NLC	R	VB	P	H	AD	PRODUCT	exL	exH	DENOM	De
AKIBA	17	x	m	0	0	all	0	all	As:Jap	1963	pr	610	n	bl	n	n	0	cig+/-ot	1	14	nev cigs	or
AKIBA	23	x	f	0	0	all	0	all	As:Jap	1963	pr	610	n	bl	n	n	0	cig+/-ot	1	14	nev cigs	or
ARCHER	1		m	0	0	wh	0	all	NAmer	1950	pr	146	m	bl	n	n	0	cig+/-ot	1	19	nev cigs	st
BENSHL	11		m	40	64	all	10	all	Eu:UK	1967	pr	486	n	V	n	n	1	cig+/-ot	1	9	nev any	ot
BEST	13		m	0	0	all	0	all	NAmer	1955	pr	381	n	V	n	n	1	cig only	1	9	nev any	ot
BRETT	1		m	0	0	all	0	all	Eu:UK	1960	pr	150	n	V	n	n	0	cig+/-ot	1	14	nev cigs	st
BUFFLE	30		f	0	0	w-hi	-	all	NAmer	1976	CC	943	n	bl	y	n	0	cig+/-ot	1	19	nev cigs	or
CEDERL	80		m	0	0	all	16	all	Eu:Sca	1963	pr	491	n	bl	n	n	2	all/unsp	1	7	nev any	ot
CEDERL	76		f	0	0	all	0	all	Eu:Sca	1963	pr	491	n	bl	n	n	2	all/unsp	1	7	nev any	or
CHANG	2		m	0	0	all	0	all	NAmer	1972	pr	136	n	bl	n	n	0	cig+/-ot	1	10	nev cigs	st
CHANG	8		f	0	0	all	0	all	NAmer	1972	pr	136	n	bl	n	n	0	cig+/-ot	1	10	nev cigs	st
CHOW	3	x	m	0	0	wh	0	all	NAmer	1966	pr	219	n	bl	n	n	0	cig+/-ot	1	19	nev any	st
COMSTO	4		m	0	0	all	-	all	NAmer	1975	ot	258	n	bl	n	n	0	cig+/-ot	1	19	nev any	st
COMSTO	9		f	0	0	all	-	all	NAmer	1975	ot	258	n	bl	n	n	0	cig+/-ot	1	19	nev any	st
CPSI	216		m	35	84	all	6	all	NAmer	1959	pr	5138	n	bl	n	n	1	cig+/-ot	1	9	nev any	ot
CPSI	275		f	40	74	all	6	all	NAmer	1959	pr	5138	n	bl	n	n	1	cig+/-ot	1	9	nev cigs	ot
CPSII	30	x	m	0	0	all	6	all	NAmer	1982	pr	3229	n	bl	n	n	0	cig only	1	9	nev any	st
CPSII	65	x	f	0	0	all	6	all	NAmer	1982	pr	3229	n	bl	n	n	0	cig+/-ot	1	9	nev cigs	st
DARBY	1		m	0	0	wh	-	all	Eu:UK	1988	CC	982	n	V	n	n	0	cig+/-ot	1	14	nev any	st
DARBY	8		f	0	0	wh	-	all	Eu:UK	1988	CC	982	n	V	n	n	0	cig+/-ot	1	14	nev any	st
DEAN3	5	x	m	0	0	all	-	all	Eu:UK	1969	CC	766	n	V	y	n	0	cig only	1	12	nev any	st
DEAN3	89	x	f	0	0	all	-	all	Eu:UK	1969	CC	766	n	V	y	n	0	cig only	1	12	nev any	st
DEKLER	2		m	0	0	all	0	all	Auslia	1961	pr	138	m	V	n	n	2	cig+/-ot	1	14	nev any	or
DOLL2	16		m	0	0	all	20	all	Eu:UK	1951	pr	920	n	V	n	n	1	all/unsp	1	14	nev any	ot
DOLL2	10		f	0	0	all	22	all	Eu:UK	1951	pr	920	n	V	n	n	1	cig only	1	14	nev any	ot
DORANT	6		c	0	0	all	0	all	Eu:wst	1986	ot	550	n	bl	n	y	0	cig+/-ot	1	9	nev any	st
DORGAN	4		m	0	0	wh	-	all	NAmer	1980	CC	2026	n	bl	y	y	0	cig+/-ot	1	19	nev any	st
DORGAN	28		m	0	0	bl	-	all	NAmer	1980	CC	2026	n	bl	y	y	0	cig+/-ot	1	19	nev any	st
DORGAN	51		f	0	0	wh	-	all	NAmer	1980	CC	2026	n	bl	y	y	0	cig+/-ot	1	19	nev any	st
DORGAN	74		f	0	0	bl	-	all	NAmer	1980	CC	2026	n	bl	y	y	0	cig+/-ot	1	19	nev any	st
DORN	408		m	0	0	wh	25	all	NAmer	1954	pr	5097	n	bl	n	n	1	cig+/-ot	1	9	nev any	or
ENGELA	4	x	m	0	0	all	0	all	Eu:Sca	1964	pr	435	n	bl	n	n	0	cig+/-ot	5	9	nev cigs	st
ENGELA	18	x	f	0	0	all	0	all	Eu:Sca	1964	pr	435	n	bl	n	n	0	cig+/-ot	5	9	nev cigs	st
ENSTRO	7		m	0	0	all	0	all	NAmer	1959	pr	2879	n	bl	n	n	1	cig only	1	9	nev any	ot
ENSTRO	11		f	0	0	all	0	all	NAmer	1959	pr	2879	n	bl	n	n	1	cig only	1	9	nev any	ot
GAO2	2		m	0	0	all	-	all	As:Jap	1988	CC	282	n	bl	n	n	0	cig+/-ot	1	19	nev cigs	st
GILLIS	1	x	m	0	0	all	-	all	Eu:UK	1977	CC	656	n	V	n	n	0	cig+/-ot	1	14	nev any	st
HAMMO2	21	x	m	0	0	all	0	all	NAmer	1967	pr	450	o	bl	n	n	0	cig+/-ot	1	19	nev any	st
HAMMON	135		m	0	0	wh	0	all	NAmer	1952	pr	448	n	bl	n	n	1	cig only	1	9	nev any	ot
HIRAYA	23		m	0	0	all	0	all	As:Jap	1965	pr	1917	n	bl	n	n	1	cig+/-ot	1	9	nev any	st
HIRAYA	26		f	0	0	all	0	all	As:Jap	1965	pr	1917	n	bl	n	n	1	cig+/-ot	1	9	nev any	st
HITOSU	3	x	m	0	0	all	-	all	As:Jap	1960	CC	216	n	bl	y	n	0	all/unsp	1	14	nev any	st
HITOSU	10	x	f	0	0	all	-	all	As:Jap	1960	CC	216	n	bl	y	n	0	all/unsp	1	14	nev any	st
HOLE	9	x	m	0	0	all	0	all	Eu:UK	1972	pr	225	n	V	n	n	0	cig+/-ot	1	14	nev any	st
HUMBLE	2		m	0	0	w-hi	-	all	NAmer	1980	CC	521	n	bl	y	n	1	cig+/-ot	1	19	nev cigs	or
HUMBLE	5		m	0	0	hi	-	all	NAmer	1980	CC	521	n	bl	y	n	1	cig+/-ot	1	19	nev cigs	or
HUMBLE	8		f	0	0	w-hi	-	all	NAmer	1980	CC	521	n	bl	y	n	1	cig+/-ot	1	19	nev cigs	or
HUMBLE	11		f	0	0	hi	-	all	NAmer	1980	CC	521	n	bl	y	n	1	cig+/-ot	1	19	nev cigs	or
KAISE2	66		m	35	99	all	9	all	NAmer	1979	pr	318	n	bl	n	n	1	cig only	1	19	nev any	st
KAISE2	58		f	35	99	all	9	all	NAmer	1979	pr	318	n	bl	n	n	1	cig only	1	19	nev any	st
KAISER	6		m	0	0	all	0	all	NAmer	1964	pr	714	n	bl	n	n	2	cig+/-ot	1	19	nev cigs	or
KAISER	2		f	0	0	all	0	all	NAmer	1964	pr	714	n	bl	n	n	2	cig+/-ot	1	19	nev cigs	or
KANELL	1	x	m	0	0	all	-	all	Eu:bal	1950	CC	862	n	bl	n	n	0	cig+/-ot	1	10	nev any	st
KATSOU	7		f	0	0	all	-	all	Eu:bal	1987	CC	101	n	bl	n	n	0	all/unsp	1	10	nev any	st
KAUFMA	2	x	c	0	0	all	-	all	NAmer	1981	CC	881	n	bl	n	n	0	cig+/-ot	1	14	nev cigs	st
KINLEN	3	x	m	0	0	all	0	all	Eu:UK	1967	pr	718	n	V	n	n	0	cig+/-ot	1	14	nev any	st
KNEKT	25	x	m	20	69	all	21	all	Eu:Sca	1966	pr	515	n	bl	n	n	0	cig+/-ot	1	14	nev any	st
KOO	11		f	0	0	all	-	all	As:HK	1981	CC	200	n	bl	n	n	0	all/unsp	1	10	nev any	st
LIAW	3		c	0	0	all	0	all	As:oth	1982	pr	127	n	ot	n	n	2	all/unsp	1	10	nev any	or
LIDDEL	2		m	0	0	all	18	all	NAmer	1970	pr	304	m	V	n	n	1	cig+/-ot	1	19	nev cigs	ot
MACLEN	20		m	0	0	ch	-	all	As:oth	1972	CC	233	n	bl	n	n	0	cig+/-ot	1	9	nev cigs	st
MACLEN	33		f	0	0	ch	-	all	As:oth	1972	CC	233	n	bl	n	n	0	cig+/-ot	1	9	nev cigs	st
MATOS	4	x	m	0	0	all	-	all	SCAmer	1994	CC	200	n	bl	n	n	0	cig+/-ot	1	14	nev any	st
MIGRAN	1	x	m	0	0	all	0	all	Eu:UK	1964	pr	259	n	V	n	n	0	cig only	1	9	nev any	st
MIGRAN	28	x	f	0	0	all	0	all	Eu:UK	1964	pr	259	n	V	n	n	0	cig only	1	9	nev any	st
MRFITR	3		m	0	0	all	0	all	NAmer	1973	pr	119	n	bl	n	n	0	cig+/-ot	1	19	nev cigs	ot
PARKIN	17	x	m	0	0	bl	-	all	Africa	1963	CC	877	n	V	y	n	0	all/unsp	1	14	nev any	st
PERSH2	2	x	c	0	0	all	-	all	Eu:Sca	1980	CC	1022	n	bl	y	n	0	all/unsp	1	9	nev any	st
PETO	2		m	0	0	all	0	all	Eu:UK	1954	pr	103	n	V	n	n	0	all/unsp	1	14	nev any	st
PRESCO	2		m	0	0	all	0	all	Eu:Sca	1964	pr	867	n	bl	n	n	1	all/unsp	1	14	nev any	st
PRESCO	1		f	0	0	all	0	all	Eu:Sca	1964	pr	867	n	bl	n	n	1	all/unsp	1	14	nev any	st
SEGI2	9	x	m	0	0	all	-	all	As:Jap	1962	CC	378	n	bl	n	n	0	cig+/-ot	1	9	nev any	st

Table 1G7 - 4

IESLC - Meta-analysis of Current Smoking, Amount smoked, "Low", Any product (or Cigarettes if Any not available)
All LC types
Least adjusted

REF	NRR	X	SEX	AGEL	AGEH	RACE	YF	LC TYPE	LOC	START	ST	NLC	R	VB	P	H	AD	PRODUCT	exL	exH	DENOM	De
SEGI2	21	x	f	0	0	all	-	all	As:Jap	1962	CC	378	n	bl	n	n	0	cig+/-ot	1	9	nev any	st
SHAW	4		c	0	0	wh	-	all	NAmer	1988	CC	335	n	V	n	y	0	all/unsp	1	19	nev any	st
SOBUE	117		m	0	0	all	-	q+s+l+a	As:Jap	1986	CC	1376	n	bl	n	y	0	cig+/-ot	1	19	nev cigs	st
SPEIZE	2		f	0	0	all	0	all	NAmer	1976	pr	593	n	bl	n	y	1	cig+/-ot	5	14	nev cigs	ot
STOCKW	1		c	0	0	all	-	all	NAmer	1981	CC	22161	n	bl	n	n	0	cig+/-ot	1	19	nev any	st
SVENSS	26	x	f	0	0	all	-	all	Eu:Sca	1983	CC	210	n	bl	n	n	0	all/unsp	1	10	nev any	st
TENKAN	10		m	0	0	all	17	all	Eu:Sca	1962	pr	242	n	bl	n	n	1	all/unsp	1	14	nev any	ot
TSUGAN	29		m	0	0	all	-	q+a	As:Jap	1976	CC	134	n	bl	n	y	0	all/unsp	1	15	nev any	st
TULINI	4	x	m	0	0	all	0	all	Eu:Sca	1967	pr	472	n	bl	n	n	1	cig+/-ot	1	14	nev any	or
TULINI	9	x	f	0	0	all	0	all	Eu:Sca	1967	pr	472	n	bl	n	n	1	cig+/-ot	1	14	nev any	or
TVERDA	9		m	0	0	all	0	all	Eu:Sca	1972	pr	238	n	bl	n	n	2	cig only	1	9	nev cigs	ot
TVERDA	16		f	0	0	all	0	all	Eu:Sca	1972	pr	238	n	bl	n	n	2	cig only	1	9	nev cigs	ot
WAKAI	37	x	m	0	0	all	-	all	As:Jap	1988	CC	333	n	bl	n	y	0	cig+/-ot	1	19	nev any	st
WYNDE6	27		m	0	0	all	-	all	NAmer	1969	CC	4423	n	bl	n	y	0	cig+/-ot	1	10	nev any	st
WYNDE6	216		f	0	0	all	-	all	NAmer	1969	CC	4423	n	bl	n	y	0	cig+/-ot	1	10	nev cigs	st

Cigarette type is all/unspec for all RRs
except for the following:

REF	NRR	CIGTYPE
DEAN3	5	MC only
DEAN3	89	MC only

Table 1G7 - 5

IESLC - Meta-analysis of Current Smoking, Amount smoked, "Low", Any product (or Cigarettes if Any not available)
All LC types
Least adjusted

REF	NRR	SEX	AD	Number Exposed Case	Cont	Non-exposed Case	Cont	RR	95.00%CI
*AKIBA	17	m	0	104	59893	18	35833	3.46	(2.10- 5.70)
*AKIBA	23	f	0	54	38968	116	359850	4.30	(3.11- 5.94)
Subtotal AKIBA								4.03	(3.07- 5.29)
*ARCHER	1	m	0	14	6504	6	9842	3.53	(1.36- 9.18)
*BENSHL	11	m	1	-	-	-	-	4.00	(1.55- 10.31)
*BEST	13	m	1	-	-	-	-	10.00	(4.56- 21.92)
*BRETT	1	m	0	40	17090	6	6530	2.55	(1.08- 6.01)
BUFFLE	30	f	0	12	20	12	112	5.60	(2.21- 14.21)
*CEDERL	80	m	2	-	-	-	-	3.40	(1.96- 5.90)
*CEDERL	76	f	2	-	-	-	-	2.83	(1.72- 4.67)
Subtotal CEDERL								3.07	(2.12- 4.45)
*CHANG	2	m	0	5	100	5	502	5.02	(1.48- 17.02)
*CHANG	8	f	0	6	205	11	1139	3.03	(1.13- 8.10)
Subtotal CHANG								3.70	(1.72- 7.95)
*CHOW	3	m	0	38	29404	6	62913	13.55	(5.73- 32.05)
COMSTO	4	m	0	18	25	4	69	12.42	(3.83- 40.26)
COMSTO	9	f	0	16	19	13	115	7.45	(3.10- 17.93)
Subtotal COMSTO								8.95	(4.43- 18.08)
*CPSI	216	m	1	-	-	-	-	4.51	(3.10- 6.55)
*CPSI	275	f	1	-	-	-	-	1.25	(0.73- 2.13)
Subtotal CPSI								2.96	(2.18- 4.02)
*CPSII	30	m	0	115	53096	124	742207	12.96	(10.06- 16.71)
*CPSII	65	f	0	55	113074	310	2091302	3.28	(2.46- 4.37)
Subtotal CPSII								7.09	(5.87- 8.58)
DARBY	1	m	0	128	223	3	384	73.47	(23.11- 233.57)
DARBY	8	f	0	71	104	23	529	15.70	(9.38- 26.28)
Subtotal DARBY								20.27	(12.66- 32.45)
DEAN3	5	m	0	81	264	25	510	6.26	(3.90- 10.04)
DEAN3	89	f	0	31	486	41	1538	2.39	(1.48- 3.86)
Subtotal DEAN3								3.89	(2.78- 5.44)
*DEKLER	2	m	2	-	-	-	-	19.40	(2.60- 143.70)
*DOLL2	16	m	1	-	-	-	-	5.20	(2.41- 11.22)
*DOLL2	10	f	1	-	-	-	-	1.29	(0.14- 11.50)
Subtotal DOLL2								4.47	(2.16- 9.24)
DORANT	6	c	0	21	192	14	1090	8.52	(4.26- 17.04)
DORGAN	4	m	0	81	55	15	93	9.13	(4.80- 17.39)
DORGAN	28	m	0	96	42	3	35	26.67	(7.77- 91.56)
DORGAN	51	f	0	224	81	103	244	6.55	(4.65- 9.23)
DORGAN	74	f	0	39	15	7	20	7.43	(2.61- 21.16)
Subtotal DORGAN								7.59	(5.72- 10.07)
*DORN	408	m	1	-	-	-	-	4.02	(3.43- 4.71)
*ENGELA	4	m	0	49	25801	27	58716	4.13	(2.58- 6.60)
*ENGELA	18	f	0	20	28450	31	207789	4.71	(2.69- 8.27)
Subtotal ENGELA								4.36	(3.04- 6.25)
*ENSTRO	7	m	1	-	-	-	-	4.74	(3.34- 6.73)
*ENSTRO	11	f	1	-	-	-	-	2.15	(1.62- 2.84)
Subtotal ENSTRO								2.93	(2.35- 3.65)
GAO2	2	m	0	32	41	13	56	3.36	(1.57- 7.19)
GILLIS	1	m	0	82	205	13	145	4.46	(2.39- 8.31)
*HAMMO2	21	m	0	38	764	5	891	8.86	(3.51- 22.41)
*HAMMON	135	m	1	-	-	-	-	7.44	(3.90- 14.18)
*HIRAYA	23	m	1	-	-	-	-	2.06	(1.49- 2.85)
*HIRAYA	26	f	1	-	-	-	-	2.25	(1.64- 3.08)
Subtotal HIRAYA								2.16	(1.72- 2.70)
HITOSU	3	m	0	33	585	7	242	1.95	(0.85- 4.47)
HITOSU	10	f	0	24	391	33	1893	3.52	(2.06- 6.02)
Subtotal HITOSU								2.96	(1.88- 4.64)
*HOLE	9	m	0	23	840	7	1189	4.65	(2.00- 10.79)
HUMBLE	2	m	1	-	-	-	-	9.20	(3.30- 25.80)
HUMBLE	5	m	1	-	-	-	-	11.60	(2.70- 61.50)
HUMBLE	8	f	1	-	-	-	-	19.20	(6.50- 60.80)
HUMBLE	11	f	1	-	-	-	-	18.50	(4.90- 72.40)
Subtotal HUMBLE								13.65	(7.44- 25.07)
*KAISE2	66	m	1	-	-	-	-	4.47	(2.00- 9.99)
*KAISE2	58	f	1	-	-	-	-	7.61	(3.26- 17.75)
Subtotal KAISE2								5.75	(3.21- 10.31)
*KAISER	6	m	2	-	-	-	-	6.58	(3.87- 11.20)
*KAISER	2	f	2	-	-	-	-	3.42	(2.17- 5.40)
Subtotal KAISER								4.51	(3.19- 6.38)
KANELL	1	m	0	162	272	48	172	2.13	(1.47- 3.10)
KATSOU	7	f	0	8	9	48	67	1.24	(0.45- 3.45)

Table 1G7 - 5

IESLC - Meta-analysis of Current Smoking, Amount smoked, "Low", Any product (or Cigarettes if Any not available)
All LC types
Least adjusted

REF	NRR	SEX	AD	Number Exposed Case	Number Exposed Cont	Non-exposed Case	Non-exposed Cont	RR	95.00%CI
KAUFMA	2	c	0	66	214	35	925	8.15	(5.27- 12.60)
*KINLEN	3	m	0	200	3659	7	1333	10.41	(4.91- 22.06)
*KNEKT	25	m	0	23	14798	6	17814	4.61	(1.88- 11.33)
KOO	11	f	0	17	19	56	85	1.36	(0.65- 2.84)
*LIAW	3	c	2	-	-	-	-	3.10	(1.70- 5.60)
*LIDDEL	2	m	1	-	-	-	-	3.33	(2.05- 5.64)
MACLEN	20	m	0	5	11	5	15	1.36	(0.32- 5.89)
MACLEN	33	f	0	6	21	41	109	0.76	(0.29- 2.02)
Subtotal MACLEN								0.91	(0.40- 2.05)
MATOS	4	m	0	5	32	11	110	1.56	(0.51- 4.83)
*MIGRAN	1	m	0	11	508	4	867	4.69	(1.50- 14.66)
*MIGRAN	28	f	0	4	772	4	3814	4.94	(1.24- 19.71)
Subtotal MIGRAN								4.79	(1.99- 11.55)
*MRFITR	3	m	0	2	856	0	1859	10.86~	(0.52- 225.86)
PARKIN	17	m	0	314	818	107	1248	4.48	(3.53- 5.67)
PERSH2	2	c	0	290	349	178	1164	5.43	(4.35- 6.78)
*PETO	2	m	0	44	1181	2	295	5.50	(1.34- 22.54)
*PRESCO	2	m	1	-	-	-	-	10.20	(4.49- 23.15)
*PRESCO	1	f	1	-	-	-	-	6.36	(3.60- 11.24)
Subtotal PRESCO								7.42	(4.65- 11.84)
SEGI2	9	m	0	20	62	8	53	2.14	(0.87- 5.25)
SEGI2	21	f	0	9	9	56	126	2.25	(0.85- 5.97)
Subtotal SEGI2								2.19	(1.13- 4.24)
SHAW	4	c	0	24	37	11	107	6.31	(2.82- 14.12)
SOBUE	117	m	0	147	157	34	128	3.52	(2.27- 5.47)
*SPEIZE	2	f	1	-	-	-	-	5.20	(3.60- 8.10)
STOCKW	1	c	0	2090	1194	2791	10641	6.67	(6.15- 7.25)
SVENSS	26	f	0	42	30	38	120	4.42	(2.44- 8.01)
*TENKAN	10	m	1	-	-	-	-	15.86	(6.80- 37.00)
TSUGAN	29	m	0	14	19	18	22	0.90	(0.36- 2.28)
*TULINI	4	m	1	-	-	-	-	6.49	(3.25- 13.00)
*TULINI	9	f	1	-	-	-	-	9.39	(4.99- 17.70)
Subtotal TULINI								7.94	(4.97- 12.67)
*TVERDA	9	m	2	-	-	-	-	2.14	(1.15- 3.96)
*TVERDA	16	f	2	-	-	-	-	4.53	(1.08- 18.94)
Subtotal TVERDA								2.41	(1.36- 4.25)
WAKAI	37	m	0	30	105	10	65	1.86	(0.85- 4.05)
WYNDE6	27	m	0	117	122	87	617	6.80	(4.85- 9.54)
WYNDE6	216	f	0	76	109	159	856	3.75	(2.68- 5.26)
Subtotal WYNDE6								5.05	(3.98- 6.42)
Partial Totals				5276	402300	4765	3628390		

*prospective study ~ With 0.5 adjustment for zero

REF	NRR	SEX	AD	Ys	Ws	Qs	Ps
*AKIBA	17	m	0	1.24	15.35	2.07	0.0000
*AKIBA	23	f	0	1.46	36.89	0.82	0.0000
Subtotal AKIBA				1.39	52.24	2.89	
*ARCHER	1	m	0	1.26	4.20	0.50	0.0097
*BENSHL	11	m	1	1.39	4.28	0.21	0.0041
*BEST	13	m	1	2.30	6.23	3.01	0.0000
*BRETT	1	m	0	0.94	5.22	2.36	0.0326
BUFFLE	30	f	0	1.72	4.43	0.06	0.0003
*CEDERL	80	m	2	1.22	12.65	1.86	0.0000
*CEDERL	76	f	2	1.04	15.40	4.95	0.0000
Subtotal CEDERL				1.12	28.05	6.81	
*CHANG	2	m	0	1.61	2.58	0.00	0.0096
*CHANG	8	f	0	1.11	3.97	0.99	0.0271
Subtotal CHANG				1.31	6.55	0.99	
*CHOW	3	m	0	2.61	5.18	5.17	0.0000
COMSTO	4	m	0	2.52	2.78	2.31	0.0000
COMSTO	9	f	0	2.01	4.98	0.80	0.0000
Subtotal COMSTO				2.19	7.76	3.11	
*CPSI	216	m	1	1.51	27.46	0.28	0.0000
*CPSI	275	f	1	0.22	13.40	25.67	0.4140
Subtotal CPSI				1.09	40.86	25.95	
*CPSII	30	m	0	2.56	59.74	54.47	0.0000
*CPSII	65	f	0	1.19	46.73	8.21	0.0000
Subtotal CPSII				1.96	106.47	62.67	
DARBY	1	m	0	4.30	2.87	20.77	0.0000
DARBY	8	f	0	2.75	14.48	19.03	0.0000

Table 1G7 - 5

IESLC - Meta-analysis of Current Smoking, Amount smoked, "Low", Any product (or Cigarettes if Any not available)
All LC types
Least adjusted

REF	NRR	SEX	AD	Ys	Ws	Qs	Ps
Subtotal DARBY				3.01	17.35	39.80	
DEAN3	5	m	0	1.83	17.21	0.88	0.0000
DEAN3	89	f	0	0.87	16.85	9.10	0.0003
Subtotal DEAN3				1.36	34.06	9.98	
*DEKLER	2	m	2	2.97	0.95	1.76	0.0038
*DOLL2	16	m	1	1.65	6.50	0.01	0.0000
*DOLL2	10	f	1	0.25	0.79	1.45	0.8209
Subtotal DOLL2				1.50	7.29	1.46	
DORANT	6	c	0	2.14	7.99	2.28	0.0000
DORGAN	4	m	0	2.21	9.26	3.38	0.0000
DORGAN	28	m	0	3.28	2.52	7.09	0.0000
DORGAN	51	f	0	1.88	32.66	2.42	0.0000
DORGAN	74	f	0	2.01	3.51	0.56	0.0002
Subtotal DORGAN				2.03	47.96	13.45	
*DORN	408	m	1	1.39	152.79	7.13	0.0000
*ENGELA	4	m	0	1.42	17.42	0.62	0.0000
*ENGELA	18	f	0	1.55	12.16	0.04	0.0000
Subtotal ENGELA				1.47	29.59	0.66	
*ENSTRO	7	m	1	1.56	31.30	0.08	0.0000
*ENSTRO	11	f	1	0.77	48.76	34.55	0.0000
Subtotal ENSTRO				1.07	80.06	34.64	
GAO2	2	m	0	1.21	6.65	1.04	0.0018
GILLIS	1	m	0	1.50	9.91	0.12	0.0000
*HAMMO2	21	m	0	2.18	4.47	1.47	0.0000
*HAMMON	135	m	1	2.01	9.22	1.47	0.0000
*HIRAYA	23	m	1	0.72	36.53	28.59	0.0000
*HIRAYA	26	f	1	0.81	38.69	24.54	0.0000
Subtotal HIRAYA				0.77	75.22	53.12	
HITOSU	3	m	0	0.67	5.59	4.93	0.1144
HITOSU	10	f	0	1.26	13.32	1.62	0.0000
Subtotal HITOSU				1.08	18.91	6.55	
*HOLE	9	m	0	1.54	5.43	0.03	0.0003
HUMBLE	2	m	1	2.22	3.63	1.36	0.0000
HUMBLE	5	m	1	2.45	1.57	1.12	0.0021
HUMBLE	8	f	1	2.95	3.07	5.58	0.0000
HUMBLE	11	f	1	2.92	2.12	3.64	0.0000
Subtotal HUMBLE				2.61	10.40	11.70	
*KAISE2	66	m	1	1.50	5.94	0.07	0.0003
*KAISE2	58	f	1	2.03	5.35	0.95	0.0000
Subtotal KAISE2				1.75	11.29	1.03	
*KAISER	6	m	2	1.88	13.61	1.04	0.0000
*KAISER	2	f	2	1.23	18.49	2.64	0.0000
Subtotal KAISER				1.51	32.09	3.68	
KANELL	1	m	0	0.76	27.40	19.76	0.0001
KATSOU	7	f	0	0.22	3.68	7.12	0.6791
KAUFMA	2	c	0	2.10	20.21	4.87	0.0000
*KINLEN	3	m	0	2.34	6.81	3.68	0.0000
*KNEKT	25	m	0	1.53	4.76	0.03	0.0008
KOO	11	f	0	0.31	7.09	12.00	0.4151
*LIAW	3	c	2	1.13	10.81	2.45	0.0002
*LIDDEL	2	m	1	1.20	15.00	2.45	0.0000
MACLEN	20	m	0	0.31	1.79	3.02	0.6779
MACLEN	33	f	0	-0.27	4.03	14.30	0.5807
Subtotal MACLEN				-0.09	5.83	17.31	
MATOS	4	m	0	0.45	3.02	4.07	0.4381
*MIGRAN	1	m	0	1.55	2.96	0.01	0.0078
*MIGRAN	28	f	0	1.60	2.01	0.00	0.0237
Subtotal MIGRAN				1.57	4.97	0.01	
*MRFITR	3	m	0	2.38	0.42	0.25	0.1236
PARKIN	17	m	0	1.50	68.71	0.81	0.0000
PERSH2	2	c	0	1.69	78.18	0.57	0.0000
*PETO	2	m	0	1.70	1.93	0.02	0.0180
*PRESCO	2	m	1	2.32	5.71	2.92	0.0000
*PRESCO	1	f	1	1.85	11.85	0.70	0.0000
Subtotal PRESCO				2.00	17.57	3.62	
SEGI2	9	m	0	0.76	4.76	3.42	0.0975
SEGI2	21	f	0	0.81	4.03	2.56	0.1035
Subtotal SEGI2				0.78	8.79	5.98	
SHAW	4	c	0	1.84	5.92	0.33	0.0000
SOBUE	117	m	0	1.26	19.84	2.40	0.0000
*SPEIZE	2	f	1	1.65	23.37	0.04	0.0000

Table 1G7 - 5

IESLC - Meta-analysis of Current Smoking, Amount smoked, "Low", Any product (or Cigarettes if Any not available)
All LC types
Least adjusted

REF	NRR	SEX	AD	Ys	Ws	Qs	Ps
STOCKW	1	c	0	1.90	565.53	47.84	0.0000
SVENSS	26	f	0	1.49	10.89	0.16	0.0000
*TENKAN	10	m	1	2.76	5.35	7.16	0.0000
TSUGAN	29	m	0	-0.10	4.44	13.02	0.8253
*TULINI	4	m	1	1.87	8.00	0.55	0.0000
*TULINI	9	f	1	2.24	9.59	3.83	0.0000
Subtotal TULINI				2.07	17.58	4.39	
*TVERDA	9	m	2	0.76	10.05	7.20	0.0159
*TVERDA	16	f	2	1.51	1.87	0.02	0.0387
Subtotal TVERDA				0.88	11.92	7.22	
WAKAI	37	m	0	0.62	6.32	6.17	0.1197
WYNDE6	27	m	0	1.92	33.49	3.21	0.0000
WYNDE6	216	f	0	1.32	33.57	2.72	0.0000
Subtotal WYNDE6				1.62	67.06	5.93	

	N	87
	NS	59
	Wt	1856.51
Het	Chi	484.79
Het	df	86
Het	P	***
Fixed	RR	4.99
	RRl	4.77
	RRu	5.22
	P	+++
Random	RR	4.63
	RRl	4.07
	RRu	5.27
	P	+++
Asymm	P	N.S.

Table 1G7 - 6

IESLC - Meta-analysis of Current Smoking, Amount smoked, "Low", Any product (or Cigarettes if Any not available)
All LC types
Least adjusted

	combined	Sex male	female	Total
N	6	50	31	87
NS	6	48	29	83
Wt	688.64	719.84	448.03	1856.51
Het Chi	10.45	232.96	147.49	484.79
Het df	5	49	30	86
Het P	(*)	***	***	***
Fixed RR	6.49	4.71	3.65	4.99
RRl	6.03	4.38	3.33	4.77
RRu	7.00	5.06	4.01	5.22
P	+++	+++	+++	+++
Random RR	6.15	4.97	3.93	4.63
RRl	5.04	4.14	3.13	4.07
RRu	7.51	5.97	4.93	5.27
P	+++	+++	+++	+++
Between Chi				93.89
Between df				2
Between P				***
Btwn(F) P				***
Btwn(R) P				*

Table 1G7 - 7

IESLC - Meta-analysis of Current Smoking, Amount smoked, "Low", Any product (or Cigarettes if Any not available)
All LC types
Excluded studies (and stage at which they were excluded)

1	ABELIN ABRAHA AMANDU AMES ANDERS AUSTIN AXELSO BAND BECHER BERRIN BLOHMK BLOT4 BROCKM BROWN1 BYERS1 BYERS2 CARPEN CASCO2 CASCOR CHAN CHEN3 CHIAZZ CHYOU DESTE2 DOCKER DROSTE DU GARCIA GARDIN GENG GODLEY GOODMA GRAHAM GREGOR HEGMAN HEIN HENNEK HINDS HIRAOK HOROWI HORWIT HUANG ISHIMA JAHN JAIN JARVHO JIANG KELLER KIHARA KJUUS KO KOHLME KUBIK LAMWK LAMWK2 LANGE LEI LEMARC LEVIN LIU LOMBA2 LOMBAR MAGNUS MARSH MARSH2 MCDUFF MCLAUG MILLER MILLS NOTANI NOU ODRISC PAWLEG PERSHA POFFIJ QIAO QIAO2 RADZIK REN RONCO ROOTS ROTHSC SAARIK SANKAR SCHWAR SEGI SEOW SHIMIZ SIMARA SIMONA SITAS SOBUE2 STASZE STAYNE STUCKE SUN SUZUK2 SUZUKI TANG TAO TOKARS TOUSEY ULMER VEIERO VUTUC WALD WANG WANG3 WANG4 WICKLU WIGLE WILKIN WU2 WUNSCH WYNDE8 XIANGZ XU XU2 XU4 YONG ZHANG
2	AGUDO ALDERS ARMADA AUVINE BARBON BENHAM BLOT1 BLOT2 BLOT3 BOFFET BOUCHA BRESLO BROWN2 BUELL CHATZI CHEN CHEN2 CHOI COOKSO DAMBER DAVEYS DEAN DEAN2 DOLL DOSEME DUNN EBELIN ESAKI FAN GAO GARSHI GER GOLLED GSELL HANSEN HU HU2 JARUP JEDRYC JOLY JONES JUSSAW KHUDER KOULUM KREUZE KREYBE LAMTH LAUSSM LETOUR LIU2 LIU3 LIU4 LIU5 LUBIN LUBIN2 LUO MARTIS MASTRA MATSUD MCCONN MOLLO MZILEN NOTAN2 ORMOS OSANN OSANN2 PASTOR PERNU PIKE POLEDN RACHTA RANDIG RESTRE SADOWS SCHWA2 SIEMIA SPITZ STOCKS TIZZAN VANDER WANG2 WUWILL WYNDE2 WYNDE3 WYNDE4 XU3 YUAN ZHENG ZHOU
3	PISANI
4	WYNDE7
5	RIMING TANG2 WYNDE5
6	DESTEF HIRAY2 LAURIL LICKIN MRFIT MURATA WARSIN WATSON WYNDER
10	AXELSS BOUCOT BROSS CORREA HAENSZ NAM PEZZO2 PEZZOT WU YAMAGU

Table 1G7 - 8

Potentially overlapping studies

REF	REFGP	PRINC	. OVERLAP/LINK
GILLIS	LUBIN2	2	Subset of Lubin2
KOO	KOO	1	KOO/LAMTH/LAMWK
TVERDA	TVERDA	1	VEIERO/TVERDAL
AKIBA	AKIBA	1	AKIBA/ISHIMA
PRESCO	PRESCO	2	PRESCO-combined
HOLE	TANG2	1	Subset of TANG2
BENSHL	TANG2	1	Subset of TANG2
KAISER	KAISER	1	KAISER/OSANN2
MRFITR	MRFIT	2	Subset of MRFIT
WYNDE6	WYNDE6	1	WYNDE5/6/7/8
CPSI	CPSI	1	CPSI overall
ENSTRO	ENSTRO	1	Subset of CPSI

Table 1G7 - 9

Most adjusted - insufficient data for metaanalysis

REF	NRR	SEX	AGEL	AGEH	RACE	YF	LC TYPE	LOC	START	ST	NLC	R	VB	P	H	AD	PRODUCT	exL	exH	DENOM	De
HITOSU	14	m	0	0	all	-	all	As:Jap	1960	CC	216	n	bl	y	n	2	all/unsp	1	14	nev any	or
HITOSU	17	f	0	0	all	-	all	As:Jap	1960	CC	216	n	bl	y	n	2	all/unsp	1	14	nev any	or

REF	NRR	RR	SIG	RRDATA comment
HITOSU	14	1.80		0
HITOSU	17	3.10		0

Table 1G8 -

IESLC - Meta-analysis of Current Smoking, Amount smoked, "Mid", Any product (or Cigarettes if Any not available)
All LC types

This analysis is restricted to results for:
1) Results by Amount smoked
2) Current smokers
3) Results complete enough for use in metaanalysis

Within each study, results are then selected (in the following order of preference, within each sex) for:
4) PRODUCT: all/unspec, cigarettes regardless of other products, cigarettes only
5) CIGTYPE: all/unspecified, MC regardless of HR, MC only
6) DENOM: never smoked anything, never smoked cigarettes, (never +1 = +long term ex, +2 = +amount unknown,
+3 = never cigs+long term ex)
7) Followup period (YF, prospective studies): whole study (coded as 0) or longest available
8) LCtype: all or nearest available, at least Squamous and Adeno. (q = squamous, s = small,
l = large, a = adeno, mix = mixed, alv = alveolar)
9) Race: all or nearest available, otherwise by race (wh or w = white, bl or b = black, hi = hispanic
ch = chinese, jap = japanese, haw = hawaiian, w+o = white + oriental, sca = scandinavian, as = asian)
10) Amount smoked "mid" in key scheme 1 (key value 20, maximum range 6-44, in numbers of
cigarettes or cigarette equivalents)
11) For overlapping studies: principal rather than subsidiary studies
Finally by Age: whole study (coded as 0) if available, otherwise by widest available age group
and then for single sex results (m, f) in preference to combined sex results (c).

Results adjusted (AD) for the most potential confounders are then chosen in Sections -1 to -3
and results adjusted for the least confounders in Sections -4 to -6. (Those least adjusted results which
actually differ from the most adjusted as marked 'x' in column X in Section -4)
(Results adjusted for an unknown number of confounder(s) are coded as 20.)

Section -7 shows excluded studies, together with the stage (as above) at which no qualifying
results were found.

Section -8 lists the potentially overlapping studies which have been included (1=principal, 2=subsidiary).

Section -9 lists any results which would have been included in preference except that they had data not complete
enough for use in meta-analysis, with their significance (yes/no), if known, and any further comment as entered
on the database.

In addition to those mentioned above, the following fields, levels and abbreviations are used:
* or nk = not known, n = no, y = yes, ot = other
nev = never
all/unspec = all or unspecified, cig+/-ot = cigarettes irrespective of other products (cigar, pipe etc)
MC = manufactured cigarettes, HR = hand-rolled cigarettes
exL, exH = range of exposure (low and high) in the smoking group, in terms of Amount smoked,
cigarettes or cigarette equivalents
REF: 6-character study reference
NRR: number of the RR on the database within the study
ST : study type (CC = case control, pr or prosp = prospective)
NLC: number of lung cancer cases in whole study
R : risky occupational population (n = no, m = mining, o = other risky)
VB : national cigarette type (V = at least 75% Virginia, bl = at least 75% blended, ot = other)
P : any proxy use
H : full histological confirmation
De : derivation of RR/CI (or = original, st = standard method, ot = other method of estimation)

Table 1G8 - 1

IESLC - Meta-analysis of Current Smoking, Amount smoked, "Mid", Any product (or Cigarettes if Any not available)
All LC types
Most adjusted

REF	NRR	SEX	AGEL	AGEH	RACE	YF	LC TYPE	LOC	START	ST	NLC	R	VB	P	H	AD	PRODUCT	exL	exH	DENOM	De
AKIBA	28	m	0	0	all	0	all	As:Jap	1963	pr	610	n	bl	n	n	5	cig+/-ot	15	24	nev cigs	ot
ARCHER	2	m	0	0	wh	0	all	NAmer	1950	pr	146	m	bl	n	n	0	cig+/-ot	20	20	nev cigs	st
AXELSS	18	f	0	0	sca	-	all	Eu:Sca	1989	CC	436	n	bl	n	n	1	all/unsp	20	20	nev any	or
BEST	14	m	0	0	all	0	all	NAmer	1955	pr	381	n	V	n	n	1	cig only	10	20	nev any	ot
BRETT	2	m	0	0	all	0	all	Eu:UK	1960	pr	150	n	V	n	n	0	cig+/-ot	15	24	nev cigs	st
BUFFLE	31	f	0	0	w-hi	-	all	NAmer	1976	CC	943	n	bl	y	n	0	cig+/-ot	20	20	nev cigs	or
CHANG	3	m	0	0	all	0	all	NAmer	1972	pr	136	n	bl	n	n	0	cig+/-ot	11	20	nev cigs	st
CHANG	9	f	0	0	all	0	all	NAmer	1972	pr	136	n	bl	n	n	0	cig+/-ot	11	20	nev cigs	st
CHOW	11	m	0	0	wh	0	all	NAmer	1966	pr	219	n	bl	n	n	2	cig+/-ot	20	29	nev any	ot
COMSTO	5	m	0	0	all	-	all	NAmer	1975	ot	258	n	bl	n	n	0	cig+/-ot	20	39	nev any	st
COMSTO	10	f	0	0	all	-	all	NAmer	1975	ot	258	n	bl	n	n	0	cig+/-ot	20	39	nev any	st
CPSI	218	m	35	84	all	6	all	NAmer	1959	pr	5138	n	bl	n	n	1	cig+/-ot	20	39	nev any	ot
CPSI	277	f	40	74	all	6	all	NAmer	1959	pr	5138	n	bl	n	n	1	cig+/-ot	20	39	nev cigs	ot
CPSII	122	m	0	0	all	6	all	NAmer	1982	pr	3229	n	bl	n	n	1	cig only	20	20	nev any	ot
CPSII	129	f	0	0	all	6	all	NAmer	1982	pr	3229	n	bl	n	n	1	cig+/-ot	20	20	nev cigs	ot
DARBY	2	m	0	0	wh	-	all	Eu:UK	1988	CC	982	n	V	n	n	0	cig+/-ot	15	24	nev any	st
DARBY	9	f	0	0	wh	-	all	Eu:UK	1988	CC	982	n	V	n	n	0	cig+/-ot	15	24	nev any	st
DEAN3	14	m	0	0	all	-	all	Eu:UK	1969	CC	766	n	V	y	n	3	cig only	13	22	nev any	ot
DEAN3	98	f	0	0	all	-	all	Eu:UK	1969	CC	766	n	V	y	n	3	cig only	13	22	nev any	ot
DEKLER	3	m	0	0	all	0	all	Auslia	1961	pr	138	m	V	n	n	2	cig+/-ot	15	24	nev any	or
DOLL2	17	m	0	0	all	20	all	Eu:UK	1951	pr	920	n	V	n	n	1	all/unsp	15	24	nev any	ot
DOLL2	11	f	0	0	all	22	all	Eu:UK	1951	pr	920	n	V	n	n	1	cig only	15	24	nev any	ot
DORN	409	m	0	0	wh	25	all	NAmer	1954	pr	5097	n	bl	n	n	1	cig+/-ot	10	20	nev any	or
ENSTRO	5	m	0	0	all	0	all	NAmer	1959	pr	2879	n	bl	n	n	1	cig only	20	20	nev any	ot
ENSTRO	9	f	0	0	all	0	all	NAmer	1959	pr	2879	n	bl	n	n	1	cig only	20	20	nev any	ot
GAO2	3	m	0	0	all	-	all	As:Jap	1988	CC	282	n	bl	n	n	0	cig+/-ot	20	29	nev cigs	st
GILLIS	22	m	0	0	all	-	all	Eu:UK	1977	CC	656	n	V	n	n	3	cig+/-ot	15	24	nev any	or
HAMMON	136	m	0	0	wh	0	all	NAmer	1952	pr	448	n	bl	n	n	1	cig only	10	20	nev any	ot
HIRAYA	74	m	0	0	all	16	all	As:Jap	1965	pr	1917	n	bl	n	n	4	cig+/-ot	15	24	nev any	or
HITOSU	36	m	0	0	all	-	all	As:Jap	1960	CC	216	n	bl	y	n	1	all/unsp	15	24	nev any	st
HOLE	3	m	0	0	all	0	all	Eu:UK	1972	pr	225	n	V	n	n	1	cig+/-ot	15	24	nev any	ot
HUMBLE	76	c	0	0	wh	-	not alv	NAmer	1980	CC	521	n	bl	y	n	3	cig+/-ot	16	20	nev cigs	ot
KAISER	7	m	0	0	all	0	all	NAmer	1964	pr	714	n	bl	n	n	2	cig+/-ot	20	40	nev cigs	or
KAISER	3	f	0	0	all	0	all	NAmer	1964	pr	714	n	bl	n	n	2	cig+/-ot	20	40	nev cigs	or
KANELL	27	m	0	0	all	-	all	Eu:bal	1950	CC	862	n	bl	n	n	1	cig+/-ot	11	20	nev any	st
KATSOU	8	f	0	0	all	-	all	Eu:bal	1987	CC	101	n	bl	n	n	0	all/unsp	11	20	nev any	st
KAUFMA	12	c	0	0	all	-	all	NAmer	1981	CC	881	n	bl	n	n	6	cig+/-ot	15	24	nev cigs	or
KINLEN	15	m	0	0	all	0	all	Eu:UK	1967	pr	718	n	V	n	n	2	cig+/-ot	15	24	nev any	ot
KOO	12	f	0	0	all	-	all	As:HK	1981	CC	200	n	bl	n	n	0	all/unsp	11	20	nev any	st
LIAW	4	c	0	0	all	0	all	As:oth	1982	pr	127	n	ot	n	n	2	all/unsp	11	20	nev any	or
MACLEN	22	m	0	0	ch	-	all	As:oth	1972	CC	233	n	bl	n	n	0	cig+/-ot	20	29	nev cigs	st
MATOS	7	m	0	0	all	-	all	SCAmer	1994	CC	200	n	bl	n	n	2	cig+/-ot	15	24	nev any	or
MIGRAN	6	m	0	0	all	0	all	Eu:UK	1964	pr	259	n	V	n	n	2	cig only	20	20	nev any	ot
MIGRAN	33	f	0	0	all	0	all	Eu:UK	1964	pr	259	n	V	n	n	2	cig only	20	20	nev any	ot
MRFITR	4	m	0	0	all	0	all	NAmer	1973	pr	119	n	bl	n	n	0	cig+/-ot	20	39	nev cigs	ot
SEGI2	14	m	0	0	all	-	all	As:Jap	1962	CC	378	n	bl	n	n	1	cig+/-ot	20	29	nev any	ot
SOBUE	118	m	0	0	all	-	q+s+l+a	As:Jap	1986	CC	1376	n	bl	n	y	0	cig+/-ot	20	29	nev cigs	st
SPEIZE	3	f	0	0	all	0	all	NAmer	1976	pr	593	n	bl	n	y	1	cig+/-ot	15	24	nev cigs	ot
STOCKW	2	c	0	0	all	-	all	NAmer	1981	CC	22161	n	bl	n	n	0	cig+/-ot	20	40	nev any	st
SVENSS	11	f	0	0	all	-	all	Eu:Sca	1983	CC	210	n	bl	n	n	1	all/unsp	11	20	nev any	or
TENKAN	11	m	0	0	all	17	all	Eu:Sca	1962	pr	242	n	bl	n	n	1	all/unsp	15	24	nev any	ot
TSUGAN	30	m	0	0	all	-	q+a	As:Jap	1976	CC	134	n	bl	n	y	0	all/unsp	16	35	nev any	st
TULINI	28	m	0	0	all	0	all	Eu:Sca	1967	pr	472	n	bl	n	n	3	cig+/-ot	15	24	nev any	or
TULINI	33	f	0	0	all	0	all	Eu:Sca	1967	pr	472	n	bl	n	n	3	cig+/-ot	15	24	nev any	or
WAKAI	41	m	0	0	all	-	all	As:Jap	1988	CC	333	n	bl	n	y	2	cig+/-ot	20	29	nev any	or
WYNDE6	36	m	0	0	all	-	all	NAmer	1969	CC	4423	n	bl	n	y	0	cig+/-ot	11	20	nev any	st
WYNDE6	225	f	0	0	all	-	all	NAmer	1969	CC	4423	n	bl	n	y	0	cig+/-ot	11	20	nev cigs	st

Cigarette type is all/unspec for all RRs
except for the following:

REF	NRR	CIGTYPE
DEAN3	14	MC only
DEAN3	98	MC only

Table 1G8 - 2

IESLC - Meta-analysis of Current Smoking, Amount smoked, "Mid", Any product (or Cigarettes if Any not available)
All LC types
Most adjusted

REF	NRR	SEX	AD	Number Exposed Case	Cont	Non-exposed Case	Cont	RR	95.00%CI
*AKIBA	28	m	5	-	-	-	-	6.10	(3.90- 9.50)
*ARCHER	2	m	0	68	18320	6	9842	6.09	(2.64- 14.02)
AXELSS	18	f	1	-	-	-	-	43.30	(12.60- 148.80)
*BEST	14	m	1	-	-	-	-	16.41	(7.73- 34.86)
*BRETT	2	m	0	62	15868	6	6530	4.25	(1.84- 9.83)
BUFFLE	31	f	0	52	41	12	112	11.84	(5.75- 24.38)
*CHANG	3	m	0	17	161	5	502	10.60	(3.97- 28.28)
*CHANG	9	f	0	11	234	11	1139	4.87	(2.14- 11.09)
Subtotal CHANG								6.72	(3.57- 12.62)
*CHOW	11	m	2	-	-	-	-	21.87	(9.35- 51.14)
COMSTO	5	m	0	60	57	4	69	18.16	(6.22- 53.00)
COMSTO	10	f	0	51	26	13	115	17.35	(8.25- 36.48)
Subtotal COMSTO								17.61	(9.56- 32.43)
*CPSI	218	m	1	-	-	-	-	14.30	(11.33- 18.05)
*CPSI	277	f	1	-	-	-	-	5.03	(3.82- 6.63)
Subtotal CPSI								9.26	(7.75- 11.06)
*CPSII	122	m	1	-	-	-	-	21.59	(16.98- 27.46)
*CPSII	129	f	1	-	-	-	-	14.21	(11.67- 17.31)
Subtotal CPSII								16.81	(14.44- 19.58)
DARBY	2	m	0	126	169	3	384	95.43	(29.94- 304.17)
DARBY	9	f	0	86	92	23	529	21.50	(12.90- 35.82)
Subtotal DARBY								27.39	(17.17- 43.71)
DEAN3	14	m	3	-	-	-	-	7.42	(4.56- 12.06)
DEAN3	98	f	3	-	-	-	-	8.42	(5.14- 13.78)
Subtotal DEAN3								7.90	(5.59- 11.16)
*DEKLER	3	m	2	-	-	-	-	23.00	(3.20- 167.60)
*DOLL2	17	m	1	-	-	-	-	10.60	(4.94- 22.73)
*DOLL2	11	f	1	-	-	-	-	6.43	(1.81- 22.78)
Subtotal DOLL2								9.28	(4.83- 17.84)
*DORN	409	m	1	-	-	-	-	9.92	(8.84- 11.14)
*ENSTRO	5	m	1	-	-	-	-	13.65	(10.88- 17.13)
*ENSTRO	9	f	1	-	-	-	-	9.48	(8.04- 11.18)
Subtotal ENSTRO								10.75	(9.41- 12.29)
GAO2	3	m	0	77	44	13	56	7.54	(3.71- 15.30)
GILLIS	22	m	3	-	-	-	-	7.60	(4.20- 13.80)
*HAMMON	136	m	1	-	-	-	-	8.42	(4.86- 14.59)
*HIRAYA	74	m	4	-	-	-	-	5.40	(4.30- 6.90)
HITOSU	36	m	1	-	-	-	-	2.82	(1.25- 6.36)
*HOLE	3	m	1	-	-	-	-	8.90	(4.12- 19.23)
HUMBLE	76	c	3	-	-	-	-	15.16	(9.18- 25.03)
*KAISER	7	m	2	-	-	-	-	17.24	(10.71- 27.73)
*KAISER	3	f	2	-	-	-	-	7.98	(5.35- 11.90)
Subtotal KAISER								10.98	(8.08- 14.91)
KANELL	27	m	1	-	-	-	-	7.06	(4.76- 10.48)
KATSOU	8	f	0	15	5	48	67	4.19	(1.43- 12.30)
KAUFMA	12	c	6	-	-	-	-	15.00	(10.00- 23.00)
*KINLEN	15	m	2	-	-	-	-	14.14	(6.68- 29.91)
KOO	12	f	0	24	5	56	85	7.29	(2.62- 20.22)
*LIAW	4	c	2	-	-	-	-	3.60	(2.00- 6.40)
MACLEN	22	m	0	43	31	5	15	4.16	(1.37- 12.66)
MATOS	7	m	2	-	-	-	-	8.00	(3.40- 16.80)
*MIGRAN	6	m	2	-	-	-	-	5.14	(1.79- 14.81)
*MIGRAN	33	f	2	-	-	-	-	7.48	(1.64- 34.03)
Subtotal MIGRAN								5.81	(2.44- 13.83)
*MRFITR	4	m	0	50	3747	0	1859	50.12~	(3.09- 811.82)
SEGI2	14	m	1	-	-	-	-	3.40	(1.55- 7.45)
SOBUE	118	m	0	236	222	34	128	4.00	(2.63- 6.09)
*SPEIZE	3	f	1	-	-	-	-	12.60	(9.90- 16.70)
STOCKW	2	c	0	6053	1591	2791	10641	14.51	(13.54- 15.54)
SVENSS	11	f	1	-	-	-	-	12.60	(6.50- 25.20)
*TENKAN	11	m	1	-	-	-	-	20.25	(8.20- 50.00)
TSUGAN	30	m	0	30	30	18	22	1.22	(0.55- 2.73)
*TULINI	28	m	3	-	-	-	-	12.00	(6.31- 22.90)
*TULINI	33	f	3	-	-	-	-	26.30	(14.40- 48.10)
Subtotal TULINI								18.23	(11.74- 28.32)
WAKAI	41	m	2	-	-	-	-	4.01	(1.91- 8.41)
WYNDE6	36	m	0	461	293	87	617	11.16	(8.54- 14.59)
WYNDE6	225	f	0	367	165	159	856	11.97	(9.33- 15.37)
Subtotal WYNDE6								11.59	(9.65- 13.91)
Partial Totals				7889	41101	3294	33568		

*prospective study ~ With 0.5 adjustment for zero

Table 1G8 - 2

IESLC - Meta-analysis of Current Smoking, Amount smoked, "Mid", Any product (or Cigarettes if Any not available)
All LC types
Most adjusted

REF	NRR	SEX	AD	Ys	Ws	Qs	Ps
*AKIBA	28	m	5	1.81	19.39	8.02	0.0000
*ARCHER	2	m	0	1.81	5.52	2.30	0.0000
AXELSS	18	f	1	3.77	2.52	4.37	0.0000
*BEST	14	m	1	2.80	6.77	0.81	0.0000
*BRETT	2	m	0	1.45	5.48	5.52	0.0007
BUFFLE	31	f	0	2.47	7.36	0.00	0.0000
*CHANG	3	m	0	2.36	3.99	0.03	0.0000
*CHANG	9	f	0	1.58	5.66	4.27	0.0002
Subtotal CHANG				1.90	9.65	4.31	
*CHOW	11	m	2	3.09	5.32	2.14	0.0000
COMSTO	5	m	0	2.90	3.35	0.67	0.0000
COMSTO	10	f	0	2.85	6.96	1.13	0.0000
Subtotal COMSTO				2.87	10.31	1.80	
*CPSI	218	m	1	2.66	70.85	3.09	0.0000
*CPSI	277	f	1	1.62	50.55	35.33	0.0000
Subtotal CPSI				2.23	121.40	38.42	
*CPSII	122	m	1	3.07	66.50	25.63	0.0000
*CPSII	129	f	1	2.65	98.85	4.06	0.0000
Subtotal CPSII				2.82	165.35	29.69	
DARBY	2	m	0	4.56	2.86	12.69	0.0000
DARBY	9	f	0	3.07	14.73	5.60	0.0000
Subtotal DARBY				3.31	17.59	18.29	
DEAN3	14	m	3	2.00	16.24	3.25	0.0000
DEAN3	98	f	3	2.13	15.80	1.63	0.0000
Subtotal DEAN3				2.07	32.04	4.88	
*DEKLER	3	m	2	3.14	0.98	0.46	0.0019
*DOLL2	17	m	1	2.36	6.60	0.05	0.0000
*DOLL2	11	f	1	1.86	2.40	0.84	0.0040
Subtotal DOLL2				2.23	8.99	0.89	
*DORN	409	m	1	2.29	287.32	7.07	0.0000
*ENSTRO	5	m	1	2.61	74.58	1.97	0.0000
*ENSTRO	9	f	1	2.25	141.36	5.78	0.0000
Subtotal ENSTRO				2.38	215.94	7.75	
GAO2	3	m	0	2.02	7.66	1.43	0.0000
GILLIS	22	m	3	2.03	10.86	1.95	0.0000
*HAMMON	136	m	1	2.13	12.72	1.31	0.0000
*HIRAYA	74	m	4	1.69	68.71	40.21	0.0000
HITOSU	36	m	1	1.04	5.81	11.62	0.0125
*HOLE	3	m	1	2.19	6.47	0.46	0.0000
HUMBLE	76	c	3	2.72	15.27	1.09	0.0000
*KAISER	7	m	2	2.85	16.98	2.66	0.0000
*KAISER	3	f	2	2.08	24.04	3.37	0.0000
Subtotal KAISER				2.40	41.02	6.03	
KANELL	27	m	1	1.95	24.67	6.09	0.0000
KATSOU	8	f	0	1.43	3.31	3.44	0.0092
KAUFMA	12	c	6	2.71	22.15	1.46	0.0000
*KINLEN	15	m	2	2.65	6.84	0.27	0.0000
KOO	12	f	0	1.99	3.69	0.80	0.0001
*LIAW	4	c	2	1.28	11.36	15.56	0.0000
MACLEN	22	m	0	1.43	3.10	3.26	0.0120
MATOS	7	m	2	2.08	6.02	0.83	0.0000
*MIGRAN	6	m	2	1.64	3.44	2.28	0.0024
*MIGRAN	33	f	2	2.01	1.67	0.32	0.0093
Subtotal MIGRAN				1.76	5.11	2.60	
*MRFITR	4	m	0	3.91	0.50	1.06	0.0059
SEGI2	14	m	1	1.22	6.23	9.40	0.0022
SOBUE	118	m	0	1.39	21.76	24.66	0.0000
*SPEIZE	3	f	1	2.53	56.20	0.38	0.0000
STOCKW	2	c	0	2.67	802.56	39.95	0.0000
SVENSS	11	f	1	2.53	8.37	0.06	0.0000
*TENKAN	11	m	1	3.01	4.70	1.46	0.0000
TSUGAN	30	m	0	0.20	5.96	30.21	0.6241
*TULINI	28	m	3	2.48	9.25	0.01	0.0000
*TULINI	33	f	3	3.27	10.56	7.07	0.0000
Subtotal TULINI				2.90	19.81	7.08	
WAKAI	41	m	2	1.39	6.99	7.90	0.0002
WYNDE6	36	m	0	2.41	53.48	0.08	0.0000
WYNDE6	225	f	0	2.48	61.57	0.06	0.0000
Subtotal WYNDE6				2.45	115.05	0.14	

Table 1G8 - 2

IESLC - Meta-analysis of Current Smoking, Amount smoked, "Mid", Any product (or Cigarettes if Any not available)
All LC types
Most adjusted

	N	57
	NS	45
	Wt	2224.83
Het	Chi	357.38
Het	df	56
Het	P	***
Fixed	RR	11.60
	RRl	11.13
	RRu	12.10
	P	+++
Random	RR	9.67
	RRl	8.45
	RRu	11.05
	P	+++
Asymm	P	*

Table 1G8 - 3

IESLC - Meta-analysis of Current Smoking, Amount smoked, "Mid", Any product (or Cigarettes if Any not available)
All LC types
Most adjusted

	Sex			
	combined	male	female	Total
N	4	35	18	57
NS	4	35	18	57
Wt	851.34	857.90	515.60	2224.83
Het Chi	21.87	201.22	74.43	357.38
Het df	3	34	17	56
Het P	***	***	***	***
Fixed RR	14.26	9.98	10.62	11.60
RRl	13.34	9.33	9.74	11.13
RRu	15.25	10.67	11.58	12.10
P	+++	+++	+++	+++
Random RR	10.98	8.88	10.82	9.67
RRl	6.75	7.29	8.68	8.45
RRu	17.86	10.82	13.49	11.05
P	+++	+++	+++	+++
Between Chi				59.87
Between df				2
Between P				***
Btwn(F) P				**
Btwn(R) P				N.S.

	Lung cancer type		
	all	other	Total
N	54	3	57
NS	42	3	45
Wt	2181.84	42.99	2224.83
Het Chi	300.94	31.39	357.38
Het df	53	2	56
Het P	***	***	***
Fixed RR	11.78	5.45	11.60
RRl	11.29	4.04	11.13
RRu	12.28	7.35	12.10
P	+++	+++	+++
Random RR	10.10	4.34	9.67
RRl	8.87	1.24	8.45
RRu	11.51	15.25	11.05
P	+++	+	+++
Between Chi			25.06
Between df			1
Between P			***
Btwn(F) P			*
Btwn(R) P			N.S.

	Location								
	NAmer	UK	Scand	othEur	China	Japan	othAs	other	Total
N	25	12	5	2		8	3	2	57
NS	18	8	4	2		8	3	2	45
Wt	1900.41	93.39	35.40	27.98		142.51	18.15	7.00	2224.83
Het Chi	132.00	32.98	6.11	0.80		18.08	1.39	0.94	357.38
Het df	24	11	4	1		7	2	1	56
Het P	***	***	N.S.	N.S.		*	N.S.	N.S.	***
Fixed RR	12.63	10.07	18.02	6.64		4.72	4.26	9.28	11.60
RRl	12.08	8.22	12.96	4.58		4.00	2.69	4.42	11.13
RRu	13.21	12.34	25.05	9.61		5.56	6.75	19.46	12.10
P	+++	+++	+++	+++		+++	+++	+++	+++
Random RR	12.04	10.14	18.39	6.64		4.16	4.26	9.28	9.67
RRl	10.48	6.97	12.08	4.58		3.05	2.69	4.42	8.45
RRu	13.82	14.76	27.99	9.61		5.68	6.75	19.46	11.05
P	+++	+++	+++	+++		+++	+++	+++	+++
Between Chi									165.09
Between df									6
Between P									***
Btwn(F) P									***
Btwn(R) P									***

Table 1G8 - 3

IESLC - Meta-analysis of Current Smoking, Amount smoked, "Mid", Any product (or Cigarettes if Any not available)
All LC types
Most adjusted

	Detailed Country in "other Europe"					
	multi	Germany	othWest	East	Balkans	Total
N					2	2
NS					2	2
Wt					27.98	27.98
Het Chi					0.80	0.80
Het df					1	1
Het P					N.S.	N.S.
Fixed RR					6.64	6.64
RRl					4.58	4.58
RRu					9.61	9.61
P					+++	+++
Random RR					6.64	6.64
RRl					4.58	4.58
RRu					9.61	9.61
P					+++	+++
Between Chi						
Between df						
Between P						N.S.
Btwn(F) P						N.S.
Btwn(R) P						N.S.

	Detailed Country in "other Asia"			
	India	HongKong	other	Total
N		1	2	3
NS		1	2	3
Wt		3.69	14.46	18.15
Het Chi		0.00	0.05	1.39
Het df		0	1	2
Het P		N.S.	N.S.	N.S.
Fixed RR		7.29	3.71	4.26
RRl		2.62	2.22	2.69
RRu		20.22	6.22	6.75
P		+++	+++	+++
Random RR		7.29	3.71	4.26
RRl		2.62	2.22	2.69
RRu		20.22	6.22	6.75
P		+++	+++	+++
Between Chi				1.33
Between df				1
Between P				N.S.
Btwn(F) P				N.S.
Btwn(R) P				N.S.

	Detailed other continent			
	SCAmer	Auslia	Africa	Total
N	1	1		2
NS	1	1		2
Wt	6.02	0.98		7.00
Het Chi	0.00	0.00		0.94
Het df	0	0		1
Het P	N.S.	N.S.		N.S.
Fixed RR	8.00	23.00		9.28
RRl	3.60	3.18		4.42
RRu	17.78	166.45		19.46
P	+++	++		+++
Random RR	8.00	23.00		9.28
RRl	3.60	3.18		4.42
RRu	17.78	166.45		19.46
P	+++	++		+++
Between Chi				0.94
Between df				1
Between P				N.S.
Btwn(F) P				N.S.
Btwn(R) P				N.S.

Table 1G8 - 3

IESLC - Meta-analysis of Current Smoking, Amount smoked, "Mid", Any product (or Cigarettes if Any not available)
All LC types
Most adjusted

	Start year of study					
	<1960	1960-69	1970-79	1980-89	1990+	Total
N	11	19	11	15	1	57
NS	8	14	9	13	1	45
Wt	683.33	336.49	110.42	1088.58	6.02	2224.83
Het Chi	47.38	76.94	40.36	105.43	0.00	357.38
Het df	10	18	10	14	0	56
Het P	***	***	***	***	N.S.	***
Fixed RR	9.90	8.94	9.82	14.16	8.00	11.60
RRl	9.18	8.03	8.15	13.35	3.60	11.13
RRu	10.67	9.95	11.84	15.03	17.78	12.10
P	+++	+++	+++	+++	+++	+++
Random RR	9.46	9.17	8.27	11.56	8.00	9.67
RRl	7.69	7.13	5.26	8.82	3.60	8.45
RRu	11.63	11.80	13.03	15.17	17.78	11.05
P	+++	+++	+++	+++	+++	+++
Between Chi						87.28
Between df						4
Between P						***
Btwn(F) P						**
Btwn(R) P						N.S.

	Study type (1)		
	CC	other	Total
N	24	33	57
NS	21	24	45
Wt	1128.98	1095.86	2224.83
Het Chi	162.04	175.50	357.38
Het df	23	32	56
Het P	***	***	***
Fixed RR	12.74	10.54	11.60
RRl	12.01	9.94	11.13
RRu	13.50	11.19	12.10
P	+++	+++	+++
Random RR	8.62	10.43	9.67
RRl	6.76	8.79	8.45
RRu	10.98	12.38	11.05
P	+++	+++	+++
Between Chi			19.84
Between df			1
Between P			***
Btwn(F) P			(*)
Btwn(R) P			N.S.

	Study type (2)			
	CC	prosp	other	Total
N	24	31	2	57
NS	21	23	1	45
Wt	1128.98	1085.55	10.31	2224.83
Het Chi	162.04	172.76	0.00	357.38
Het df	23	30	1	56
Het P	***	***	N.S.	***
Fixed RR	12.74	10.49	17.61	11.60
RRl	12.01	9.89	9.56	11.13
RRu	13.50	11.14	32.43	12.10
P	+++	+++	+++	+++
Random RR	8.62	10.19	17.61	9.67
RRl	6.76	8.55	9.56	8.45
RRu	10.98	12.14	32.43	11.05
P	+++	+++	+++	+++
Between Chi				22.57
Between df				2
Between P				***
Btwn(F) P				N.S.
Btwn(R) P				(*)

Table 1G8 - 3

IESLC - Meta-analysis of Current Smoking, Amount smoked, "Mid", Any product (or Cigarettes if Any not available)
All LC types
Most adjusted

	Study size (number of LC cases)				
	100-249	250-499	500-999	1000+	Total
N	17	12	16	12	57
NS	16	9	12	8	45
Wt	86.23	78.13	262.39	1798.09	2224.83
Het Chi	50.38	36.05	48.49	184.71	357.38
Het df	16	11	15	11	56
Het P	***	***	***	***	***
Fixed RR	6.25	10.60	10.96	12.10	11.60
RRl	5.06	8.49	9.71	11.56	11.13
RRu	7.71	13.23	12.37	12.67	12.10
P	+++	+++	+++	+++	+++
Random RR	6.66	10.58	11.30	10.34	9.67
RRl	4.51	6.97	8.95	8.36	8.45
RRu	9.84	16.07	14.26	12.80	11.05
P	+++	+++	+++	+++	+++
Between Chi					37.75
Between df					3
Between P					***
Btwn(F) P					N.S.
Btwn(R) P					N.S.

	Risky occupational population			
	no	mining	othRisky	Total
N	55	2		57
NS	43	2		45
Wt	2218.34	6.50		2224.83
Het Chi	354.62	1.47		357.38
Het df	54	1		56
Het P	***	N.S.		***
Fixed RR	11.62	7.44		11.60
RRl	11.15	3.45		11.13
RRu	12.11	16.05		12.10
P	+++	+++		+++
Random RR	9.69	8.63		9.67
RRl	8.47	2.74		8.45
RRu	11.10	27.16		11.05
P	+++	+++		+++
Between Chi				1.29
Between df				1
Between P				N.S.
Btwn(F) P				N.S.
Btwn(R) P				N.S.

	National cigarette tobacco type			
	Virginia	blended	other	Total
N	14	42	1	57
NS	10	34	1	45
Wt	101.14	2112.33	11.36	2224.83
Het Chi	35.09	305.44	0.00	357.38
Het df	13	41	0	56
Het P	***	***	N.S.	***
Fixed RR	10.49	11.73	3.60	11.60
RRl	8.63	11.24	2.01	11.13
RRu	12.75	12.25	6.44	12.10
P	+++	+++	+++	+++
Random RR	10.74	9.67	3.60	9.67
RRl	7.60	8.34	2.01	8.45
RRu	15.17	11.21	6.44	11.05
P	+++	+++	+++	+++
Between Chi				16.85
Between df				2
Between P				***
Btwn(F) P				N.S.
Btwn(R) P				**

Table 1G8 - 3

IESLC - Meta-analysis of Current Smoking, Amount smoked, "Mid", Any product (or Cigarettes if Any not available)
All LC types
Most adjusted

	Any proxy use No/nk	Yes	Total
N	52	5	57
NS	41	4	45
Wt	2164.35	60.48	2224.83
Het Chi	339.67	13.18	357.38
Het df	51	4	56
Het P	***	*	***
Fixed RR	11.69	8.86	11.60
RRl	11.21	6.89	11.13
RRu	12.20	11.40	12.10
P	+++	+++	+++
Random RR	9.81	8.33	9.67
RRl	8.52	5.20	8.45
RRu	11.29	13.35	11.05
P	+++	+++	+++
Between Chi			4.53
Between df			1
Between P			*
Btwn(F) P			N.S.
Btwn(R) P			N.S.

	Full histological confirmation No	Yes	Total
N	51	6	57
NS	40	5	45
Wt	2018.87	205.96	2224.83
Het Chi	293.32	55.69	357.38
Het df	50	5	56
Het P	***	***	***
Fixed RR	11.83	9.58	11.60
RRl	11.33	8.35	11.13
RRu	12.36	10.98	12.10
P	+++	+++	+++
Random RR	10.20	6.32	9.67
RRl	8.87	3.85	8.45
RRu	11.74	10.38	11.05
P	+++	+++	+++
Between Chi			8.37
Between df			1
Between P			**
Btwn(F) P			N.S.
Btwn(R) P			(*)

	Number of adjustment variables (1) 0	1	2+/+nk	Total
N	19	19	19	57
NS	15	15	15	45
Wt	1019.49	933.47	271.87	2224.83
Het Chi	118.84	117.07	72.87	357.38
Het df	18	18	18	56
Het P	***	***	***	***
Fixed RR	13.27	11.00	8.44	11.60
RRl	12.48	10.32	7.49	11.13
RRu	14.11	11.73	9.50	12.10
P	+++	+++	+++	+++
Random RR	8.99	10.52	9.35	9.67
RRl	6.71	8.63	7.20	8.45
RRu	12.06	12.83	12.16	11.05
P	+++	+++	+++	+++
Between Chi				48.61
Between df				2
Between P				***
Btwn(F) P				*
Btwn(R) P				N.S.

Table 1G8 - 3

IESLC - Meta-analysis of Current Smoking, Amount smoked, "Mid", Any product (or Cigarettes if Any not available)
All LC types
Most adjusted

	Number of adjustment variables (2) 0	1	2	3-5	6+/+nk	Total
N	19	19	10	8	1	57
NS	15	15	8	6	1	45
Wt	1019.49	933.47	83.64	166.08	22.15	2224.83
Het Chi	118.84	117.07	29.25	34.62	0.00	357.38
Het df	18	18	9	7	0	56
Het P	***	***	***	***	N.S.	***
Fixed RR	13.27	11.00	8.77	7.66	15.00	11.60
RRl	12.48	10.32	7.08	6.58	9.89	11.13
RRu	14.11	11.73	10.87	8.92	22.75	12.10
P	+++	+++	+++	+++	+++	+++
Random RR	8.99	10.52	8.74	9.33	15.00	9.67
RRl	6.71	8.63	5.70	6.47	9.89	8.45
RRu	12.06	12.83	13.41	13.47	22.75	11.05
P	+++	+++	+++	+++	+++	+++
Between Chi						57.61
Between df						4
Between P						***
Btwn(F) P						(*)
Btwn(R) P						N.S.

	Product all/unsp	cig+/-ot	cig only	Total
N	9	38	10	57
NS	9	31	7	47
Wt	52.31	1831.05	341.48	2224.83
Het Chi	45.48	245.57	43.71	357.38
Het df	8	37	9	56
Het P	***	***	***	***
Fixed RR	6.06	11.78	11.80	11.60
RRl	4.62	11.26	10.62	11.13
RRu	7.95	12.34	13.12	12.10
P	+++	+++	+++	+++
Random RR	6.80	10.14	10.61	9.67
RRl	3.51	8.68	7.88	8.45
RRu	13.19	11.85	14.27	11.05
P	+++	+++	+++	+++
Between Chi				22.62
Between df				2
Between P				***
Btwn(F) P				N.S.
Btwn(R) P				N.S.

	Denominator nev any	nev cigs	Total
N	39	18	57
NS	32	16	48
Wt	1798.82	426.02	2224.83
Het Chi	256.17	89.40	357.38
Het df	38	17	56
Het P	***	***	***
Fixed RR	12.02	9.99	11.60
RRl	11.48	9.09	11.13
RRu	12.59	10.99	12.10
P	+++	+++	+++
Random RR	10.08	8.85	9.67
RRl	8.56	6.91	8.45
RRu	11.88	11.34	11.05
P	+++	+++	+++
Between Chi			11.81
Between df			1
Between P			***
Btwn(F) P			N.S.
Btwn(R) P			N.S.

Table 1G8 - 3

IESLC - Meta-analysis of Current Smoking, Amount smoked, "Mid", Any product (or Cigarettes if Any not available)
All LC types
Most adjusted

	Derivation of RR/CI			
	Orig	StdCalc	Other	Total
N	15	19	23	57
NS	13	15	17	45
Wt	493.47	1042.11	689.25	2224.83
Het Chi	69.33	141.04	110.52	357.38
Het df	14	18	22	56
Het P	***	***	***	***
Fixed RR	9.32	12.96	11.48	11.60
RRl	8.54	12.20	10.66	11.13
RRu	10.18	13.77	12.37	12.10
P	+++	+++	+++	+++
Random RR	10.04	8.09	10.66	9.67
RRl	7.69	6.02	8.73	8.45
RRu	13.11	10.88	13.00	11.05
P	+++	+++	+++	+++
Between Chi				36.48
Between df				2
Between P				***
Btwn(F) P				(*)
Btwn(R) P				N.S.

Table 1G8 - 4

IESLC - Meta-analysis of Current Smoking, Amount smoked, "Mid", Any product (or Cigarettes if Any not available)
All LC types
Least adjusted

REF	NRR	X	SEX	AGEL	AGEH	RACE	YF	LC TYPE	LOC	START	ST	NLC	R	VB	P	H	AD	PRODUCT	exL	exH	DENOM	De
AKIBA	18	x	m	0	0	all	0	all	As:Jap	1963	pr	610	n	bl	n	n	0	cig+/-ot	15	24	nev cigs	or
ARCHER	2		m	0	0	wh	0	all	NAmer	1950	pr	146	m	bl	n	n	0	cig+/-ot	20	20	nev cigs	st
AXELSS	18		f	0	0	sca	-	all	Eu:Sca	1989	CC	436	n	bl	n	n	1	all/unsp	20	20	nev any	or
BEST	14		m	0	0	all	0	all	NAmer	1955	pr	381	n	V	n	n	1	cig only	10	20	nev any	ot
BRETT	2		m	0	0	all	0	all	Eu:UK	1960	pr	150	n	V	n	n	0	cig+/-ot	15	24	nev cigs	st
BUFFLE	31		f	0	0	w-hi	-	all	NAmer	1976	CC	943	n	bl	y	n	0	cig+/-ot	20	20	nev cigs	or
CHANG	3		m	0	0	all	0	all	NAmer	1972	pr	136	n	bl	n	n	0	cig+/-ot	11	20	nev cigs	st
CHANG	9		f	0	0	all	0	all	NAmer	1972	pr	136	n	bl	n	n	0	cig+/-ot	11	20	nev cigs	st
CHOW	4	x	m	0	0	wh	0	all	NAmer	1966	pr	219	n	bl	n	n	0	cig+/-ot	20	29	nev any	st
COMSTO	5		m	0	0	all	-	all	NAmer	1975	ot	258	n	bl	n	n	0	cig+/-ot	20	39	nev any	st
COMSTO	10		f	0	0	all	-	all	NAmer	1975	ot	258	n	bl	n	n	0	cig+/-ot	20	39	nev any	st
CPSI	218		m	35	84	all	6	all	NAmer	1959	pr	5138	n	bl	n	n	1	cig+/-ot	20	39	nev any	ot
CPSI	277		f	40	74	all	6	all	NAmer	1959	pr	5138	n	bl	n	n	1	cig+/-ot	20	39	nev cigs	ot
CPSII	32	x	m	0	0	all	6	all	NAmer	1982	pr	3229	n	bl	n	n	0	cig only	20	20	nev any	st
CPSII	67	x	f	0	0	all	6	all	NAmer	1982	pr	3229	n	bl	n	n	0	cig+/-ot	20	20	nev cigs	st
DARBY	2		m	0	0	wh	-	all	Eu:UK	1988	CC	982	n	V	n	n	0	cig+/-ot	15	24	nev any	st
DARBY	9		f	0	0	wh	-	all	Eu:UK	1988	CC	982	n	V	n	n	0	cig+/-ot	15	24	nev any	st
DEAN3	12	x	m	0	0	all	-	all	Eu:UK	1969	CC	766	n	V	y	n	0	cig only	13	22	nev any	st
DEAN3	96	x	f	0	0	all	-	all	Eu:UK	1969	CC	766	n	V	y	n	0	cig only	13	22	nev any	st
DEKLER	3		m	0	0	all	0	all	Auslia	1961	pr	138	m	V	n	n	2	cig+/-ot	15	24	nev any	or
DOLL2	17		m	0	0	all	20	all	Eu:UK	1951	pr	920	n	V	n	n	1	all/unsp	15	24	nev any	ot
DOLL2	11		f	0	0	all	22	all	Eu:UK	1951	pr	920	n	V	n	n	1	cig only	15	24	nev any	ot
DORN	409		m	0	0	wh	25	all	NAmer	1954	pr	5097	n	bl	n	n	1	cig+/-ot	10	20	nev any	or
ENSTRO	5		m	0	0	all	0	all	NAmer	1959	pr	2879	n	bl	n	n	1	cig only	20	20	nev any	ot
ENSTRO	9		f	0	0	all	0	all	NAmer	1959	pr	2879	n	bl	n	n	1	cig only	20	20	nev any	ot
GAO2	3		m	0	0	all	-	all	As:Jap	1988	CC	282	n	bl	n	n	0	cig+/-ot	20	29	nev cigs	st
GILLIS	2	x	m	0	0	all	-	all	Eu:UK	1977	CC	656	n	V	n	n	0	cig+/-ot	15	24	nev any	st
HAMMON	136		m	0	0	wh	0	all	NAmer	1952	pr	448	n	bl	n	n	1	cig only	10	20	nev any	ot
HIRAYA	74		m	0	0	all	16	all	As:Jap	1965	pr	1917	n	bl	n	n	4	cig+/-ot	15	24	nev any	or
HITOSU	4	x	m	0	0	all	-	all	As:Jap	1960	CC	216	n	bl	y	n	0	all/unsp	15	24	nev any	st
HOLE	10	x	m	0	0	all	0	all	Eu:UK	1972	pr	225	n	V	n	n	0	cig+/-ot	15	24	nev any	st
HUMBLE	76		c	0	0	wh	-	not alv	NAmer	1980	CC	521	n	bl	y	n	3	cig+/-ot	16	20	nev cigs	ot
KAISER	7		m	0	0	all	0	all	NAmer	1964	pr	714	n	bl	n	n	2	cig+/-ot	20	40	nev cigs	or
KAISER	3		f	0	0	all	0	all	NAmer	1964	pr	714	n	bl	n	n	2	cig+/-ot	20	40	nev cigs	or
KANELL	2	x	m	0	0	all	-	all	Eu:bal	1950	CC	862	n	bl	n	n	0	cig+/-ot	11	20	nev any	st
KATSOU	8		f	0	0	all	-	all	Eu:bal	1987	CC	101	n	bl	n	n	0	all/unsp	11	20	nev any	st
KAUFMA	3	x	c	0	0	all	-	all	NAmer	1981	CC	881	n	bl	n	n	0	cig+/-ot	15	24	nev cigs	st
KINLEN	4	x	m	0	0	all	0	all	Eu:UK	1967	pr	718	n	V	n	n	0	cig+/-ot	15	24	nev any	st
KOO	12		f	0	0	all	-	all	As:HK	1981	CC	200	n	bl	n	n	0	all/unsp	11	20	nev any	st
LIAW	4		c	0	0	all	0	all	As:oth	1982	pr	127	n	ot	n	n	2	all/unsp	11	20	nev any	or
MACLEN	22		m	0	0	ch	-	all	As:oth	1972	CC	233	n	bl	n	n	0	cig+/-ot	20	29	nev cigs	st
MATOS	6	x	m	0	0	all	-	all	SCAmer	1994	CC	200	n	bl	n	n	0	cig+/-ot	15	24	nev any	st
MIGRAN	5	x	m	0	0	all	0	all	Eu:UK	1964	pr	259	n	V	n	n	0	cig only	20	20	nev any	st
MIGRAN	32	x	f	0	0	all	0	all	Eu:UK	1964	pr	259	n	V	n	n	0	cig only	20	20	nev any	st
MRFITR	4		m	0	0	all	0	all	NAmer	1973	pr	119	n	bl	n	n	0	cig+/-ot	20	39	nev cigs	ot
SEGI2	13	x	m	0	0	all	-	all	As:Jap	1962	CC	378	n	bl	n	n	0	cig+/-ot	20	29	nev any	st
SOBUE	118		m	0	0	all	-	q+s+l+a	As:Jap	1986	CC	1376	n	bl	n	y	0	cig+/-ot	20	29	nev cigs	st
SPEIZE	3		f	0	0	all	0	all	NAmer	1976	pr	593	n	bl	n	y	1	cig+/-ot	15	24	nev cigs	ot
STOCKW	2		c	0	0	all	-	all	NAmer	1981	CC	22161	n	bl	n	n	0	cig+/-ot	20	40	nev any	st
SVENSS	31	x	f	0	0	all	-	all	Eu:Sca	1983	CC	210	n	bl	n	n	0	all/unsp	11	20	nev any	st
TENKAN	11		m	0	0	all	17	all	Eu:Sca	1962	pr	242	n	bl	n	n	1	all/unsp	15	24	nev any	ot
TSUGAN	30		m	0	0	all	-	q+a	As:Jap	1976	CC	134	n	bl	n	y	0	all/unsp	16	35	nev any	st
TULINI	5	x	m	0	0	all	0	all	Eu:Sca	1967	pr	472	n	bl	n	n	1	cig+/-ot	15	24	nev any	or
TULINI	10	x	f	0	0	all	0	all	Eu:Sca	1967	pr	472	n	bl	n	n	1	cig+/-ot	15	24	nev any	or
WAKAI	38	x	m	0	0	all	-	all	As:Jap	1988	CC	333	n	bl	n	y	0	cig+/-ot	20	29	nev any	st
WYNDE6	36		m	0	0	all	-	all	NAmer	1969	CC	4423	n	bl	n	y	0	cig+/-ot	11	20	nev any	st
WYNDE6	225		f	0	0	all	-	all	NAmer	1969	CC	4423	n	bl	n	y	0	cig+/-ot	11	20	nev cigs	st

Cigarette type is all/unspec for all RRs
except for the following:

REF	NRR	CIGTYPE
DEAN3	12	MC only
DEAN3	96	MC only

Table 1G8 - 5

IESLC - Meta-analysis of Current Smoking, Amount smoked, "Mid", Any product (or Cigarettes if Any not available)
All LC types
Least adjusted

REF	NRR	SEX	AD	Number Exposed Case	Number Exposed Cont	Non-exposed Case	Non-exposed Cont	RR	95.00%CI
*AKIBA	18	m	0	178	82289	18	35833	4.31	(2.65- 6.99)
*ARCHER	2	m	0	68	18320	6	9842	6.09	(2.64- 14.02)
AXELSS	18	f	1	-	-	-	-	43.30	(12.60- 148.80)
*BEST	14	m	1	-	-	-	-	16.41	(7.73- 34.86)
*BRETT	2	m	0	62	15868	6	6530	4.25	(1.84- 9.83)
BUFFLE	31	f	0	52	41	12	112	11.84	(5.75- 24.38)
*CHANG	3	m	0	17	161	5	502	10.60	(3.97- 28.28)
*CHANG	9	f	0	11	234	11	1139	4.87	(2.14- 11.09)
Subtotal CHANG								6.72	(3.57- 12.62)
*CHOW	4	m	0	60	36589	6	62913	17.19	(7.43- 39.79)
COMSTO	5	m	0	60	57	4	69	18.16	(6.22- 53.00)
COMSTO	10	f	0	51	26	13	115	17.35	(8.25- 36.48)
Subtotal COMSTO								17.61	(9.56- 32.43)
*CPSI	218	m	1	-	-	-	-	14.30	(11.33- 18.05)
*CPSI	277	f	1	-	-	-	-	5.03	(3.82- 6.63)
Subtotal CPSI								9.26	(7.75- 11.06)
*CPSII	32	m	0	564	171438	124	742207	19.69	(16.21- 23.92)
*CPSII	67	f	0	395	251336	310	2091302	10.60	(9.14- 12.30)
Subtotal CPSII								13.32	(11.84- 14.99)
DARBY	2	m	0	126	169	3	384	95.43	(29.94- 304.17)
DARBY	9	f	0	86	92	23	529	21.50	(12.90- 35.82)
Subtotal DARBY								27.39	(17.17- 43.71)
DEAN3	12	m	0	125	429	25	510	5.94	(3.80- 9.31)
DEAN3	96	f	0	44	521	41	1538	3.17	(2.05- 4.90)
Subtotal DEAN3								4.30	(3.15- 5.89)
*DEKLER	3	m	2	-	-	-	-	23.00	(3.20- 167.60)
*DOLL2	17	m	1	-	-	-	-	10.60	(4.94- 22.73)
*DOLL2	11	f	1	-	-	-	-	6.43	(1.81- 22.78)
Subtotal DOLL2								9.28	(4.83- 17.84)
*DORN	409	m	1	-	-	-	-	9.92	(8.84- 11.14)
*ENSTRO	5	m	1	-	-	-	-	13.65	(10.88- 17.13)
*ENSTRO	9	f	1	-	-	-	-	9.48	(8.04- 11.18)
Subtotal ENSTRO								10.75	(9.41- 12.29)
GAO2	3	m	0	77	44	13	56	7.54	(3.71- 15.30)
GILLIS	2	m	0	248	361	13	145	7.66	(4.25- 13.82)
*HAMMON	136	m	1	-	-	-	-	8.42	(4.86- 14.59)
*HIRAYA	74	m	4	-	-	-	-	5.40	(4.30- 6.90)
HITOSU	4	m	0	52	747	7	242	2.41	(1.08- 5.37)
*HOLE	10	m	0	87	2056	7	1189	7.19	(3.34- 15.47)
HUMBLE	76	c	3	-	-	-	-	15.16	(9.18- 25.03)
*KAISER	7	m	2	-	-	-	-	17.24	(10.71- 27.73)
*KAISER	3	f	2	-	-	-	-	7.98	(5.35- 11.90)
Subtotal KAISER								10.98	(8.08- 14.91)
KANELL	2	m	0	321	134	48	172	8.58	(5.88- 12.53)
KATSOU	8	f	0	15	5	48	67	4.19	(1.43- 12.30)
KAUFMA	3	c	0	207	363	35	925	15.07	(10.32- 22.00)
*KINLEN	4	m	0	232	3369	7	1333	13.11	(6.20- 27.74)
KOO	12	f	0	24	5	56	85	7.29	(2.62- 20.22)
*LIAW	4	c	2	-	-	-	-	3.60	(2.00- 6.40)
MACLEN	22	m	0	43	31	5	15	4.16	(1.37- 12.66)
MATOS	6	m	0	42	54	11	110	7.78	(3.71- 16.29)
*MIGRAN	5	m	0	36	851	4	867	9.17	(3.28- 25.65)
*MIGRAN	32	f	0	3	471	4	3814	6.07	(1.36- 27.05)
Subtotal MIGRAN								8.03	(3.44- 18.74)
*MRFITR	4	m	0	50	3747	0	1859	50.12~	(3.09- 811.82)
SEGI2	13	m	0	92	183	8	53	3.33	(1.52- 7.30)
SOBUE	118	m	0	236	222	34	128	4.00	(2.63- 6.09)
*SPEIZE	3	f	1	-	-	-	-	12.60	(9.90- 16.70)
STOCKW	2	c	0	6053	1591	2791	10641	14.51	(13.54- 15.54)
SVENSS	31	f	0	81	22	38	120	11.63	(6.41- 21.10)
*TENKAN	11	m	1	-	-	-	-	20.25	(8.20- 50.00)
TSUGAN	30	m	0	30	30	18	22	1.22	(0.55- 2.73)
*TULINI	5	m	1	-	-	-	-	13.50	(7.08- 25.60)
*TULINI	10	f	1	-	-	-	-	30.70	(16.80- 56.00)
Subtotal TULINI								20.91	(13.48- 32.45)
WAKAI	38	m	0	79	129	10	65	3.98	(1.93- 8.20)
WYNDE6	36	m	0	461	293	87	617	11.16	(8.54- 14.59)
WYNDE6	225	f	0	367	165	159	856	11.97	(9.33- 15.37)
Subtotal WYNDE6								11.59	(9.65- 13.91)
Partial Totals				10735	592443	4010	2976906		

*prospective study ~ With 0.5 adjustment for zero

Table 1G8 - 5

IESLC - Meta-analysis of Current Smoking, Amount smoked, "Mid", Any product (or Cigarettes if Any not available)
All LC types
Least adjusted

REF	NRR	SEX	AD	Ys	Ws	Qs	Ps
*AKIBA	18	m	0	1.46	16.36	15.40	0.0000
*ARCHER	2	m	0	1.81	5.52	2.15	0.0000
AXELSS	18	f	1	3.77	2.52	4.51	0.0000
*BEST	14	m	1	2.80	6.77	0.92	0.0000
*BRETT	2	m	0	1.45	5.48	5.29	0.0007
BUFFLE	31	f	0	2.47	7.36	0.01	0.0000
*CHANG	3	m	0	2.36	3.99	0.02	0.0000
*CHANG	9	f	0	1.58	5.66	4.07	0.0002
Subtotal CHANG				1.90	9.65	4.09	
*CHOW	4	m	0	2.84	5.46	0.94	0.0000
COMSTO	5	m	0	2.90	3.35	0.74	0.0000
COMSTO	10	f	0	2.85	6.96	1.25	0.0000
Subtotal COMSTO				2.87	10.31	1.98	
*CPSI	218	m	1	2.66	70.85	3.75	0.0000
*CPSI	277	f	1	1.62	50.55	33.56	0.0000
Subtotal CPSI				2.23	121.40	37.31	
*CPSII	32	m	0	2.98	101.73	30.77	0.0000
*CPSII	67	f	0	2.36	173.82	0.83	0.0000
Subtotal CPSII				2.59	275.55	31.60	
DARBY	2	m	0	4.56	2.86	12.95	0.0000
DARBY	9	f	0	3.07	14.73	6.00	0.0000
Subtotal DARBY				3.31	17.59	18.94	
DEAN3	12	m	0	1.78	19.12	8.03	0.0000
DEAN3	96	f	0	1.15	20.13	32.82	0.0000
Subtotal DEAN3				1.46	39.25	40.85	
*DEKLER	3	m	2	3.14	0.98	0.49	0.0019
*DOLL2	17	m	1	2.36	6.60	0.03	0.0000
*DOLL2	11	f	1	1.86	2.40	0.78	0.0040
Subtotal DOLL2				2.23	8.99	0.81	
*DORN	409	m	1	2.29	287.32	5.29	0.0000
*ENSTRO	5	m	1	2.61	74.58	2.51	0.0000
*ENSTRO	9	f	1	2.25	141.36	4.63	0.0000
Subtotal ENSTRO				2.38	215.94	7.14	
GAO2	3	m	0	2.02	7.66	1.29	0.0000
GILLIS	2	m	0	2.04	11.03	1.71	0.0000
*HAMMON	136	m	1	2.13	12.72	1.14	0.0000
*HIRAYA	74	m	4	1.69	68.71	38.01	0.0000
HITOSU	4	m	0	0.88	5.97	14.38	0.0319
*HOLE	10	m	0	1.97	6.53	1.37	0.0000
HUMBLE	76	c	3	2.72	15.27	1.27	0.0000
*KAISER	7	m	2	2.85	16.98	2.95	0.0000
*KAISER	3	f	2	2.08	24.04	3.00	0.0000
Subtotal KAISER				2.40	41.02	5.95	
KANELL	2	m	0	2.15	26.86	2.11	0.0000
KATSOU	8	f	0	1.43	3.31	3.29	0.0092
KAUFMA	3	c	0	2.71	26.85	2.14	0.0000
*KINLEN	4	m	0	2.57	6.84	0.14	0.0000
KOO	12	f	0	1.99	3.69	0.73	0.0001
*LIAW	4	c	2	1.28	11.36	15.00	0.0000
MACLEN	22	m	0	1.43	3.10	3.13	0.0120
MATOS	6	m	0	2.05	7.03	1.01	0.0000
*MIGRAN	5	m	0	2.22	3.63	0.17	0.0000
*MIGRAN	32	f	0	1.80	1.72	0.68	0.0179
Subtotal MIGRAN				2.08	5.35	0.84	
*MRFITR	4	m	0	3.91	0.50	1.09	0.0059
SEGI2	13	m	0	1.20	6.24	9.40	0.0026
SOBUE	118	m	0	1.39	21.76	23.68	0.0000
*SPEIZE	3	f	1	2.53	56.20	0.60	0.0000
STOCKW	2	c	0	2.67	802.56	47.90	0.0000
SVENSS	31	f	0	2.45	10.82	0.01	0.0000
*TENKAN	11	m	1	3.01	4.70	1.57	0.0000
TSUGAN	30	m	0	0.20	5.96	29.64	0.6241
*TULINI	5	m	1	2.60	9.30	0.28	0.0000
*TULINI	10	f	1	3.42	10.60	10.48	0.0000
Subtotal TULINI				3.04	19.90	10.75	
WAKAI	38	m	0	1.38	7.36	8.10	0.0002
WYNDE6	36	m	0	2.41	53.48	0.02	0.0000
WYNDE6	225	f	0	2.48	61.57	0.17	0.0000
Subtotal WYNDE6				2.45	115.05	0.19	

Table 1G8 - 5

IESLC - Meta-analysis of Current Smoking, Amount smoked, "Mid", Any product (or Cigarettes if Any not available)
All LC types
Least adjusted

	N	57
	NS	45
	Wt	2350.81
Het	Chi	404.17
Het	df	56
Het	P	***
Fixed	RR	11.36
	RRl	10.91
	RRu	11.83
	P	+++
Random	RR	9.27
	RRl	8.08
	RRu	10.63
	P	+++
Asymm	P	*

Table 1G8 - 6

IESLC - Meta-analysis of Current Smoking, Amount smoked, "Mid", Any product (or Cigarettes if Any not available)
All LC types
Least adjusted

	Sex: combined	male	female	Total
N	4	35	18	57
NS	4	35	18	57
Wt	856.04	897.34	597.43	2350.81
Het Chi	21.89	217.99	94.06	404.17
Het df	3	34	17	56
Het P	***	***	***	***
Fixed RR	14.27	10.10	9.78	11.36
RRl	13.34	9.46	9.03	10.91
RRu	15.26	10.78	10.60	11.83
P	+++	+++	+++	+++
Random RR	11.06	8.70	9.87	9.27
RRl	6.93	7.12	7.83	8.08
RRu	17.65	10.63	12.45	10.63
P	+++	+++	+++	+++
Between Chi				70.23
Between df				2
Between P				***
Btwn(F) P				**
Btwn(R) P				N.S.

Table 1G8 - 7

IESLC - Meta-analysis of Current Smoking, Amount smoked, "Mid", Any product (or Cigarettes if Any not available)
All LC types
Excluded studies (and stage at which they were excluded)

1	ABELIN ABRAHA AMANDU AMES ANDERS AUSTIN AXELSO BAND BECHER BERRIN BLOHMK BLOT4 BROCKM BROWN1 BYERS1 BYERS2 CARPEN CASCO2 CASCOR CHAN CHEN3 CHIAZZ CHYOU DESTE2 DOCKER DROSTE DU GARCIA GARDIN GENG GODLEY GOODMA GRAHAM GREGOR HEGMAN HEIN HENNEK HINDS HIRAOK HOROWI HORWIT HUANG ISHIMA JAHN JAIN JARVHO JIANG KELLER KIHARA KJUUS KO KOHLME KUBIK LAMWK LAMWK2 LANGE LEI LEMARC LEVIN LIU LOMBA2 LOMBAR MAGNUS MARSH MARSH2 MCDUFF MCLAUG MILLER MILLS NOTANI NOU ODRISC PAWLEG PERSHA POFFIJ QIAO QIAO2 RADZIK REN RONCO ROOTS ROTHSC SAARIK SANKAR SCHWAR SEGI SEOW SHIMIZ SIMARA SIMONA SITAS SOBUE2 STASZE STAYNE STUCKE SUN SUZUK2 SUZUKI TANG TAO TOKARS TOUSEY ULMER VEIERO VUTUC WALD WANG WANG3 WANG4 WICKLU WIGLE WILKIN WU2 WUNSCH WYNDE8 XIANGZ XU XU2 XU4 YONG ZHANG
2	AGUDO ALDERS ARMADA AUVINE BARBON BENHAM BLOT1 BLOT2 BLOT3 BOFFET BOUCHA BRESLO BROWN2 BUELL CHATZI CHEN CHEN2 CHOI COOKSO DAMBER DAVEYS DEAN DEAN2 DOLL DOSEME DUNN EBELIN ESAKI FAN GAO GARSHI GER GOLLED GSELL HANSEN HU HU2 JARUP JEDRYC JOLY JONES JUSSAW KHUDER KOULUM KREUZE KREYBE LAMTH LAUSSM LETOUR LIU2 LIU3 LIU4 LIU5 LUBIN LUBIN2 LUO MARTIS MASTRA MATSUD MCCONN MOLLO MZILEN NOTAN2 ORMOS OSANN OSANN2 PASTOR PERNU PIKE POLEDN RACHTA RANDIG RESTRE SADOWS SCHWA2 SIEMIA SPITZ STOCKS TIZZAN VANDER WANG2 WUWILL WYNDE2 WYNDE3 WYNDE4 XU3 YUAN ZHENG ZHOU
3	PISANI
4	WYNDE7
5	RIMING TANG2 WYNDE5
6	DESTEF HIRAY2 LAURIL LICKIN MRFIT MURATA WARSIN WATSON WYNDER
10	BENSHL BOUCOT BROSS CEDERL CORREA DORANT DORGAN ENGELA HAENSZ HAMMO2 KAISE2 KNEKT LIDDEL NAM PARKIN PERSH2 PETO PEZZO2 PEZZOT PRESCO SHAW TVERDA WU YAMAGU

Table 1G8 - 8

Potentially overlapping studies

REF	REFGP	PRINC	.	OVERLAP/LINK
GILLIS	LUBIN2	2		Subset of Lubin2
KOO	KOO	1		KOO/LAMTH/LAMWK
AKIBA	AKIBA	1		AKIBA/ISHIMA
HOLE	TANG2	1		Subset of TANG2
KAISER	KAISER	1		KAISER/OSANN2
MRFITR	MRFIT	2		Subset of MRFIT
WYNDE6	WYNDE6	1		WYNDE5/6/7/8
CPSI	CPSI	1		CPSI overall
ENSTRO	ENSTRO	1		Subset of CPSI

Table 1G8 - 9

Most adjusted - insufficient data for metaanalysis

REF	NRR	SEX	AGEL	AGEH	RACE	YF	LC TYPE	LOC	START	ST	NLC	R	VB	P	H	AD	PRODUCT	exL	exH	DENOM	De
HITOSU	15	m	0	0	all	-	all	As:Jap	1960	CC	216	n	bl	y	n	2	all/unsp	15	24	nev any	or

REF	NRR	RR	SIG	RRDATA comment
HITOSU	15	2.40		0

Table 1G9 -

IESLC - Meta-analysis of Current Smoking, Amount smoked, "High", Any product (or Cigarettes if Any not available)
All LC types

This analysis is restricted to results for:
1) Results by Amount smoked
2) Current smokers
3) Results complete enough for use in metaanalysis

Within each study, results are then selected (in the following order of preference, within each sex) for:
4) PRODUCT: all/unspec, cigarettes regardless of other products, cigarettes only
5) CIGTYPE: all/unspecified, MC regardless of HR, MC only
6) DENOM: never smoked anything, never smoked cigarettes, (never +1 = +long term ex, +2 = +amount unknown,
+3 = never cigs+long term ex)
7) Followup period (YF, prospective studies): whole study (coded as 0) or longest available
8) LCtype: all or nearest available, at least Squamous and Adeno. (q = squamous, s = small,
l = large, a = adeno, mix = mixed, alv = alveolar)
9) Race: all or nearest available, otherwise by race (wh or w = white, bl or b = black, hi = hispanic
ch = chinese, jap = japanese, haw = hawaiian, w+o = white + oriental, sca = scandinavian, as = asian)
10) Amount smoked "high" in key scheme 1 (key value 45, maximum range >20, in numbers of
cigarettes or cigarette equivalents)
11) For overlapping studies: principal rather than subsidiary studies
Finally by Age: whole study (coded as 0) if available, otherwise by widest available age group
and then for single sex results (m, f) in preference to combined sex results (c).

Results adjusted (AD) for the most potential confounders are then chosen in Sections -1 to -3
and results adjusted for the least confounders in Sections -4 to -6. (Those least adjusted results which
actually differ from the most adjusted as marked 'x' in column X in Section -4)
(Results adjusted for an unknown number of confounder(s) are coded as 20.)

Section -7 shows excluded studies, together with the stage (as above) at which no qualifying
results were found.

Section -8 lists the potentially overlapping studies which have been included (1=principal, 2=subsidiary).

Section -9 lists any results which would have been included in preference except that they had data not complete
enough for use in meta-analysis, with their significance (yes/no), if known, and any further comment as entered
on the database.

In addition to those mentioned above, the following fields, levels and abbreviations are used:
* or nk = not known, n = no, y = yes, ot = other
nev = never
all/unspec = all or unspecified, cig+/-ot = cigarettes irrespective of other products (cigar, pipe etc)
MC = manufactured cigarettes, HR = hand-rolled cigarettes
exL, exH = range of exposure (low and high) in the smoking group, in terms of Amount smoked,
cigarettes or cigarette equivalents
REF: 6-character study reference
NRR: number of the RR on the database within the study
ST : study type (CC = case control, pr or prosp = prospective)
NLC: number of lung cancer cases in whole study
R : risky occupational population (n = no, m = mining, o = other risky)
VB : national cigarette type (V = at least 75% Virginia, bl = at least 75% blended, ot = other)
P : any proxy use
H : full histological confirmation
De : derivation of RR/CI (or = original, st = standard method, ot = other method of estimation)

Table 1G9 - 1

IESLC - Meta-analysis of Current Smoking, Amount smoked, "High", Any product (or Cigarettes if Any not available)
All LC types
Most adjusted

REF	NRR	SEX	AGEL	AGEH	RACE	YF	LC TYPE	LOC	START	ST	NLC	R	VB	P	H	AD	PRODUCT	exL	exH	DENOM	De
AKIBA	29	m	0	0	all	0	all	As:Jap	1963	pr	610	n	bl	n	n	5	cig+/-ot	25	99	nev cigs	or
ARCHER	3	m	0	0	wh	0	all	NAmer	1950	pr	146	m	bl	n	n	0	cig+/-ot	21	99	nev cigs	st
BEST	15	m	0	0	all	0	all	NAmer	1955	pr	381	n	V	n	n	1	cig only	21	99	nev any	ot
BOUCOT	126	m	0	0	all	0	all	NAmer	1951	pr	121	n	bl	n	n	2	cig only	41	99	nev any	ot
BRETT	3	m	0	0	all	0	all	Eu:UK	1960	pr	150	n	V	n	n	0	cig+/-ot	25	99	nev cigs	st
BROSS	19	m	0	0	wh	-	all	NAmer	1960	CC	974	n	bl	n	n	0	cig+/-ot	21	99	nev any	st
BUFFLE	36	f	0	0	w-hi	-	all	NAmer	1976	CC	943	n	bl	y	n	0	cig+/-ot	21	99	nev cigs	or
CHANG	4	m	0	0	all	0	all	NAmer	1972	pr	136	n	bl	n	n	0	cig+/-ot	21	99	nev cigs	st
CHANG	10	f	0	0	all	0	all	NAmer	1972	pr	136	n	bl	n	n	0	cig+/-ot	21	99	nev cigs	st
CHOW	12	m	0	0	wh	0	all	NAmer	1966	pr	219	n	bl	n	n	2	cig+/-ot	30	99	nev any	ot
COMSTO	6	m	0	0	all	-	all	NAmer	1975	ot	258	n	bl	n	n	0	cig+/-ot	40	99	nev any	st
COMSTO	11	f	0	0	all	-	all	NAmer	1975	ot	258	n	bl	n	n	0	cig+/-ot	40	99	nev any	st
CORREA	50	c	0	0	all	-	all	NAmer	1979	CC	1359	n	bl	y	n	1	cig+/-ot	21	99	nev cigs	or
CPSI	219	m	35	84	all	6	all	NAmer	1959	pr	5138	n	bl	n	n	1	cig+/-ot	40	99	nev any	ot
CPSI	278	f	40	74	all	6	all	NAmer	1959	pr	5138	n	bl	n	n	1	cig+/-ot	40	99	nev cigs	ot
CPSII	125	m	0	0	all	6	all	NAmer	1982	pr	3229	n	bl	n	n	1	cig only	41	99	nev any	ot
CPSII	132	f	0	0	all	6	all	NAmer	1982	pr	3229	n	bl	n	n	1	cig+/-ot	41	99	nev cigs	ot
DARBY	3	m	0	0	wh	-	all	Eu:UK	1988	CC	982	n	V	n	n	0	cig+/-ot	25	99	nev any	st
DARBY	10	f	0	0	wh	-	all	Eu:UK	1988	CC	982	n	V	n	n	0	cig+/-ot	25	99	nev any	st
DEAN3	21	m	0	0	all	-	all	Eu:UK	1969	CC	766	n	V	y	n	3	cig only	23	99	nev any	ot
DEAN3	105	f	0	0	all	-	all	Eu:UK	1969	CC	766	n	V	y	n	3	cig only	23	99	nev any	ot
DEKLER	4	m	0	0	all	0	all	Auslia	1961	pr	138	m	V	n	n	2	cig+/-ot	25	99	nev any	or
DOLL2	18	m	0	0	all	20	all	Eu:UK	1951	pr	920	n	V	n	n	1	all/unsp	25	99	nev any	ot
DOLL2	12	f	0	0	all	22	all	Eu:UK	1951	pr	920	n	V	n	n	1	cig only	25	99	nev any	ot
DORN	411	m	0	0	wh	25	all	NAmer	1954	pr	5097	n	bl	n	n	1	cig+/-ot	40	99	nev any	or
ENSTRO	3	m	0	0	all	0	all	NAmer	1959	pr	2879	n	bl	n	n	1	cig only	40	99	nev any	ot
ENSTRO	8	f	0	0	all	0	all	NAmer	1959	pr	2879	n	bl	n	n	1	cig only	21	99	nev any	ot
GAO2	4	m	0	0	all	-	all	As:Jap	1988	CC	282	n	bl	n	n	0	cig+/-ot	30	99	nev cigs	or
GILLIS	24	m	0	0	all	-	all	Eu:UK	1977	CC	656	n	V	n	n	3	cig+/-ot	35	49	nev any	or
HAENSZ	51	f	0	0	all	-	not alv	NAmer	1955	CC	158	n	bl	n	y	0	cig+/-ot	21	99	nev any	st
HAMMON	138	m	0	0	wh	0	all	NAmer	1952	pr	448	n	bl	n	n	1	cig only	40	99	nev any	ot
HIRAYA	76	m	0	0	all	16	all	As:Jap	1965	pr	1917	n	bl	n	n	4	cig+/-ot	35	99	nev any	or
HITOSU	37	m	0	0	all	-	all	As:Jap	1960	CC	216	n	bl	y	n	1	all/unsp	25	99	nev any	st
HOLE	5	m	0	0	all	0	all	Eu:UK	1972	pr	225	n	V	n	n	1	cig+/-ot	35	99	nev any	ot
HUMBLE	78	c	0	0	wh	-	not alv	NAmer	1980	CC	521	n	bl	y	n	3	cig+/-ot	31	99	nev cigs	or
KAISER	8	m	0	0	all	0	all	NAmer	1964	pr	714	n	bl	n	n	2	cig+/-ot	41	99	nev cigs	or
KAISER	4	f	0	0	all	0	all	NAmer	1964	pr	714	n	bl	n	n	2	cig+/-ot	41	99	nev cigs	or
KANELL	29	m	0	0	all	-	all	Eu:bal	1950	CC	862	n	bl	n	n	1	cig+/-ot	36	99	nev any	st
KATSOU	4	f	0	0	all	-	all	Eu:bal	1987	CC	101	n	bl	n	n	1	all/unsp	21	99	nev any	or
KAUFMA	15	c	0	0	all	-	all	NAmer	1981	CC	881	n	bl	n	n	6	cig+/-ot	45	99	nev cigs	or
KINLEN	16	m	0	0	all	0	all	Eu:UK	1967	pr	718	n	V	n	n	2	cig+/-ot	25	99	nev any	ot
LIAW	5	c	0	0	all	0	all	As:oth	1982	pr	127	n	ot	n	n	2	all/unsp	21	99	nev any	or
MACLEN	23	m	0	0	ch	-	all	As:oth	1972	CC	233	n	bl	n	n	0	cig+/-ot	30	99	nev cigs	st
MATOS	9	m	0	0	all	-	all	SCAmer	1994	CC	200	n	bl	n	n	2	cig+/-ot	25	99	nev any	or
MIGRAN	8	m	0	0	all	0	all	Eu:UK	1964	pr	259	n	V	n	n	2	cig only	21	99	nev any	ot
MIGRAN	34	f	0	0	all	0	all	Eu:UK	1964	pr	259	n	V	n	n	0	cig only	21	99	nev any	st
MRFITR	5	m	0	0	all	0	all	NAmer	1973	pr	119	n	bl	n	n	0	cig+/-ot	40	99	nev cigs	ot
NAM	75	m	0	0	all	-	all	NAmer	1986	CC	1199	n	bl	y	n	1	cig+/-ot	25	99	nev cigs	ot
NAM	91	f	0	0	all	-	all	NAmer	1986	CC	1199	n	bl	y	n	1	cig+/-ot	25	99	nev cigs	ot
PEZZO2	5	m	0	0	all	-	all	SCAmer	1992	CC	367	n	bl	n	y	0	cig+/-ot	41	99	nev cigs	st
PEZZOT	4	m	0	0	all	-	all	SCAmer	1987	CC	215	n	bl	n	y	0	cig only	41	99	nev cigs	st
SEGI2	18	m	0	0	all	-	all	As:Jap	1962	CC	378	n	bl	n	n	1	cig+/-ot	40	99	nev any	ot
SOBUE	119	m	0	0	all	-	q+s+l+a	As:Jap	1986	CC	1376	n	bl	n	y	0	cig+/-ot	30	99	nev cigs	st
SPEIZE	5	f	0	0	all	0	all	NAmer	1976	pr	593	n	bl	n	y	1	cig+/-ot	35	99	nev cigs	ot
STOCKW	3	c	0	0	all	-	all	NAmer	1981	CC	22161	n	bl	n	n	0	cig+/-ot	41	99	nev any	st
SVENSS	16	f	0	0	all	-	all	Eu:Sca	1983	CC	210	n	bl	n	n	1	all/unsp	21	99	nev any	ot
TENKAN	12	m	0	0	all	17	all	Eu:Sca	1962	pr	242	n	bl	n	n	1	all/unsp	25	99	nev any	ot
TSUGAN	31	m	0	0	all	-	q+a	As:Jap	1976	CC	134	n	bl	n	y	0	all/unsp	36	99	nev any	st
TULINI	29	m	0	0	all	0	all	Eu:Sca	1967	pr	472	n	bl	n	n	3	cig+/-ot	25	99	nev any	or
TULINI	34	f	0	0	all	0	all	Eu:Sca	1967	pr	472	n	bl	n	n	3	cig+/-ot	25	99	nev any	or
WAKAI	42	m	0	0	all	-	all	As:Jap	1988	CC	333	n	bl	n	y	2	cig+/-ot	30	99	nev any	or
WU	44	f	0	0	wh	-	q+a	NAmer	1981	CC	220	n	bl	n	y	2	all/unsp	21	99	nev any	st
WYNDE6	54	m	0	0	all	-	all	NAmer	1969	CC	4423	n	bl	n	y	0	cig+/-ot	31	99	nev any	st
WYNDE6	243	f	0	0	all	-	all	NAmer	1969	CC	4423	n	bl	n	y	0	cig+/-ot	30	99	nev cigs	st
YAMAGU	7	c	0	0	all	-	all	As:Jap	1989	CC	144	n	bl	n	y	1	all/unsp	21	99	nev any	or

Cigarette type is all/unspec for all RRs
except for the following:

Table 1G9 - 1

IESLC - Meta-analysis of Current Smoking, Amount smoked, "High", Any product (or Cigarettes if Any not available)
All LC types
Most adjusted

REF	NRR	CIGTYPE
DEAN3	21	MC only
DEAN3	105	MC only

Table 1G9 - 2

IESLC - Meta-analysis of Current Smoking, Amount smoked, "High", Any product (or Cigarettes if Any not available)
All LC types
Most adjusted

REF	NRR	SEX	AD	Number Exposed Case	Number Exposed Cont	Non-exposed Case	Non-exposed Cont	RR	95.00%CI
*AKIBA	29	m	5	-	-	-	-	9.10	(5.40- 15.90)
*ARCHER	3	m	0	40	7705	6	9842	8.52	(3.61- 20.07)
*BEST	15	m	1	-	-	-	-	17.31	(7.93- 37.79)
*BOUCOT	126	m	2	-	-	-	-	161.70	(9.23-2834.24)
*BRETT	3	m	0	33	4490	6	6530	8.00	(3.35- 19.07)
BROSS	19	m	0	95	59	38	170	7.20	(4.46- 11.63)
BUFFLE	36	f	0	116	49	12	112	22.10	(11.16- 43.73)
*CHANG	4	m	0	13	158	5	502	8.26	(2.99- 22.81)
*CHANG	10	f	0	13	164	11	1139	8.21	(3.74- 18.01)
Subtotal CHANG								8.23	(4.42- 15.32)
*CHOW	12	m	2	-	-	-	-	44.48	(18.63- 106.19)
COMSTO	6	m	0	26	18	4	69	24.92	(7.71- 80.57)
COMSTO	11	f	0	9	6	13	115	13.27	(4.07- 43.25)
Subtotal COMSTO								18.22	(7.92- 41.90)
CORREA	50	c	1	-	-	-	-	25.30	(18.50- 34.60)
*CPSI	219	m	1	-	-	-	-	17.49	(13.29- 23.03)
*CPSI	278	f	1	-	-	-	-	11.10	(6.00- 20.53)
Subtotal CPSI								16.21	(12.62- 20.84)
*CPSII	125	m	1	-	-	-	-	45.52	(29.35- 70.60)
*CPSII	132	f	1	-	-	-	-	18.22	(10.78- 30.82)
Subtotal CPSII								31.24	(22.31- 43.75)
DARBY	3	m	0	68	61	3	384	142.69	(43.52- 467.82)
DARBY	10	f	0	38	21	23	529	41.62	(21.15- 81.90)
Subtotal DARBY								56.31	(31.27- 101.38)
DEAN3	21	m	3	-	-	-	-	21.66	(12.78- 36.72)
DEAN3	105	f	3	-	-	-	-	24.24	(13.08- 44.93)
Subtotal DEAN3								22.71	(15.21- 33.92)
*DEKLER	4	m	2	-	-	-	-	32.50	(4.40- 241.20)
*DOLL2	18	m	1	-	-	-	-	22.40	(10.46- 47.99)
*DOLL2	12	f	1	-	-	-	-	29.71	(9.46- 93.32)
Subtotal DOLL2								24.43	(12.96- 46.06)
*DORN	411	m	1	-	-	-	-	22.75	(19.63- 26.37)
*ENSTRO	3	m	1	-	-	-	-	19.41	(15.22- 24.75)
*ENSTRO	8	f	1	-	-	-	-	16.47	(13.74- 19.75)
Subtotal ENSTRO								17.47	(15.10- 20.20)
GAO2	4	m	0	74	30	13	56	10.63	(5.08- 22.22)
GILLIS	24	m	3	-	-	-	-	9.70	(5.10- 18.40)
HAENSZ	51	f	0	23	13	81	236	5.15	(2.50- 10.65)
*HAMMON	138	m	1	-	-	-	-	20.64	(10.98- 38.80)
*HIRAYA	76	m	4	-	-	-	-	8.40	(5.70- 12.30)
HITOSU	37	m	1	-	-	-	-	4.68	(1.97- 11.11)
*HOLE	5	m	1	-	-	-	-	7.49	(2.99- 18.77)
HUMBLE	78	c	3	-	-	-	-	39.70	(21.02- 74.98)
*KAISER	8	m	2	-	-	-	-	20.91	(12.78- 27.73)
*KAISER	4	f	2	-	-	-	-	12.63	(8.06- 19.80)
Subtotal KAISER								16.87	(12.58- 22.62)
KANELL	29	m	1	-	-	-	-	34.22	(18.86- 62.11)
KATSOU	4	f	1	-	-	-	-	7.46	(2.40- 23.17)
KAUFMA	15	c	6	-	-	-	-	60.00	(35.00- 102.00)
*KINLEN	16	m	2	-	-	-	-	21.74	(10.23- 46.19)
*LIAW	5	c	2	-	-	-	-	8.30	(4.00- 17.30)
MACLEN	23	m	0	45	27	5	15	5.00	(1.63- 15.31)
MATOS	9	m	2	-	-	-	-	15.00	(7.10- 31.90)
*MIGRAN	8	m	2	-	-	-	-	5.93	(2.03- 17.29)
*MIGRAN	34	f	0	2	149	4	3814	12.80	(2.36- 69.33)
Subtotal MIGRAN								7.39	(2.99- 18.27)
*MRFITR	5	m	0	54	3591	0	1859	56.43~	(3.49- 913.25)
NAM	75	m	1	-	-	-	-	10.27	(6.42- 16.43)
NAM	91	f	1	-	-	-	-	16.65	(10.20- 27.19)
Subtotal NAM								12.94	(9.22- 18.17)
PEZZO2	5	m	0	69	12	6	117	112.13	(40.26- 312.24)
PEZZOT	4	m	0	51	6	4	116	246.50	(66.69- 911.11)
SEGI2	18	m	1	-	-	-	-	7.90	(3.40- 18.37)
SOBUE	119	m	0	226	187	34	128	4.55	(2.97- 6.96)
*SPEIZE	5	f	1	-	-	-	-	22.00	(14.80- 32.30)
STOCKW	3	c	0	4327	572	2791	10641	28.84	(26.18- 31.77)
SVENSS	16	f	1	-	-	-	-	59.00	(7.60- 458.03)
*TENKAN	12	m	1	-	-	-	-	24.97	(9.90- 63.00)
TSUGAN	31	m	0	19	14	18	22	1.66	(0.65- 4.20)
*TULINI	29	m	3	-	-	-	-	27.30	(14.20- 52.40)
*TULINI	34	f	3	-	-	-	-	38.70	(18.50- 80.80)

Table 1G9 - 2

IESLC - Meta-analysis of Current Smoking, Amount smoked, "High", Any product (or Cigarettes if Any not available)
All LC types
Most adjusted

REF	NRR	SEX	AD	Number Exposed Case	Cont	Non-exposed Case	Cont	RR	95.00%CI
Subtotal TULINI								31.83	(19.52- 51.88)
WAKAI	42	m	2	-	-	-	-	9.19	(4.20- 20.10)
WU	44	f	2	-	-	-	-	8.48	(4.16- 17.29)
WYNDE6	54	m	0	784	197	87	617	28.22	(21.47- 37.10)
WYNDE6	243	f	0	378	52	159	856	39.13	(27.98- 54.75)
Subtotal WYNDE6								32.15	(26.01- 39.75)
YAMAGU	7	c	1	-	-	-	-	12.14	(5.10- 28.90)
Partial Totals				6503	17581	3323	37869		

*prospective study ~ With 0.5 adjustment for zero

REF	NRR	SEX	AD	Ys	Ws	Qs	Ps
*AKIBA	29	m	5	2.21	13.18	9.21	0.0000
*ARCHER	3	m	0	2.14	5.22	4.26	0.0000
*BEST	15	m	1	2.85	6.30	0.24	0.0000
*BOUCOT	126	m	2	5.09	0.47	1.95	0.0005
*BRETT	3	m	0	2.08	5.09	4.74	0.0000
BROSS	19	m	0	1.97	16.76	19.19	0.0000
BUFFLE	36	f	0	3.10	8.24	0.02	0.0000
*CHANG	4	m	0	2.11	3.72	3.24	0.0000
*CHANG	10	f	0	2.11	6.22	5.49	0.0000
Subtotal CHANG				2.11	9.94	8.73	
*CHOW	12	m	2	3.80	5.07	2.86	0.0000
COMSTO	6	m	0	3.22	2.79	0.08	0.0000
COMSTO	11	f	0	2.59	2.75	0.58	0.0000
Subtotal COMSTO				2.90	5.54	0.66	
CORREA	50	c	1	3.23	39.20	1.36	0.0000
*CPSI	219	m	1	2.86	50.84	1.70	0.0000
*CPSI	278	f	1	2.41	10.15	4.13	0.0000
Subtotal CPSI				2.79	60.99	5.83	
*CPSII	125	m	1	3.82	19.94	11.94	0.0000
*CPSII	132	f	1	2.90	13.92	0.28	0.0000
Subtotal CPSII				3.44	33.87	12.22	
DARBY	3	m	0	4.96	2.72	10.00	0.0000
DARBY	10	f	0	3.73	8.38	3.92	0.0000
Subtotal DARBY				4.03	11.11	13.92	
DEAN3	21	m	3	3.08	13.79	0.01	0.0000
DEAN3	105	f	3	3.19	10.09	0.21	0.0000
Subtotal DEAN3				3.12	23.88	0.22	
*DEKLER	4	m	2	3.48	0.96	0.18	0.0007
*DOLL2	18	m	1	3.11	6.62	0.03	0.0000
*DOLL2	12	f	1	3.39	2.93	0.35	0.0000
Subtotal DOLL2				3.20	9.55	0.38	
*DORN	411	m	1	3.12	176.37	1.13	0.0000
*ENSTRO	3	m	1	2.97	65.00	0.40	0.0000
*ENSTRO	8	f	1	2.80	116.71	6.89	0.0000
Subtotal ENSTRO				2.86	181.71	7.30	
GAO2	4	m	0	2.36	7.06	3.28	0.0000
GILLIS	24	m	3	2.27	9.33	5.57	0.0000
HAENSZ	51	f	0	1.64	7.30	14.40	0.0000
*HAMMON	138	m	1	3.03	9.64	0.00	0.0000
*HIRAYA	76	m	4	2.13	25.97	21.81	0.0000
HITOSU	37	m	1	1.54	5.14	11.57	0.0005
*HOLE	5	m	1	2.01	4.55	4.84	0.0000
HUMBLE	78	c	3	3.68	9.50	3.85	0.0000
*KAISER	8	m	2	3.04	25.61	0.00	0.0000
*KAISER	4	f	2	2.54	19.02	4.92	0.0000
Subtotal KAISER				2.83	44.63	4.92	
KANELL	29	m	1	3.53	10.82	2.58	0.0000
KATSOU	4	f	1	2.01	2.99	3.20	0.0005
KAUFMA	15	c	6	4.09	13.43	14.80	0.0000
*KINLEN	16	m	2	3.08	6.76	0.01	0.0000
*LIAW	5	c	2	2.12	7.17	6.17	0.0000
MACLEN	23	m	0	1.61	3.07	6.32	0.0048
MATOS	9	m	2	2.71	6.81	0.77	0.0000
*MIGRAN	8	m	2	1.78	3.35	5.35	0.0011
*MIGRAN	34	f	0	2.55	1.35	0.33	0.0031
Subtotal MIGRAN				2.00	4.69	5.68	
*MRFITR	5	m	0	4.03	0.50	0.48	0.0045
NAM	75	m	1	2.33	17.40	8.90	0.0000
NAM	91	f	1	2.81	15.98	0.86	0.0000

Table 1G9 - 2

IESLC - Meta-analysis of Current Smoking, Amount smoked, "High", Any product (or Cigarettes if Any not available)
All LC types
Most adjusted

REF	NRR	SEX	AD	Ys	Ws	Qs	Ps
Subtotal NAM				2.56	33.39	9.77	
PEZZO2	5	m	0	4.72	3.66	10.28	0.0000
PEZZOT	4	m	0	5.51	2.25	13.63	0.0000
SEGI2	18	m	1	2.07	5.40	5.16	0.0000
SOBUE	119	m	0	1.52	21.28	49.77	0.0000
*SPEIZE	5	f	1	3.09	25.23	0.05	0.0000
STOCKW	3	c	0	3.36	411.25	41.39	0.0000
SVENSS	16	f	1	4.08	0.91	0.98	0.0001
*TENKAN	12	m	1	3.22	4.49	0.13	0.0000
TSUGAN	31	m	0	0.51	4.44	28.63	0.2861
*TULINI	29	m	3	3.31	9.01	0.62	0.0000
*TULINI	34	f	3	3.66	7.07	2.64	0.0000
Subtotal TULINI				3.46	16.08	3.26	
WAKAI	42	m	2	2.22	6.27	4.28	0.0000
WU	44	f	2	2.14	7.57	6.23	0.0000
WYNDE6	54	m	0	3.34	51.37	4.49	0.0000
WYNDE6	243	f	0	3.67	34.09	13.21	0.0000
Subtotal WYNDE6				3.47	85.46	17.70	
YAMAGU	7	c	1	2.50	5.11	1.53	0.0000

	N	65
	NS	52
	Wt	1425.60
Het	Chi	397.46
Het	df	64
Het	P	***
Fixed	RR	21.00
	RRl	19.94
	RRu	22.12
	P	+++
Random	RR	17.05
	RRl	14.60
	RRu	19.92
	P	+++
Asymm	P	*

Table 1G9 - 3

IESLC - Meta-analysis of Current Smoking, Amount smoked, "High", Any product (or Cigarettes if Any not available)
All LC types
Most adjusted

	Sex: combined	male	female	Total
N	6	40	19	65
NS	6	40	19	65
Wt	485.65	639.02	300.93	1425.60
Het Chi	23.72	241.88	62.86	397.46
Het df	5	39	18	64
Het P	***	***	***	***
Fixed RR	28.51	17.77	18.27	21.00
RRl	26.08	16.45	16.32	19.94
RRu	31.16	19.21	20.46	22.12
P	+++	+++	+++	+++
Random RR	25.85	15.83	17.45	17.05
RRl	17.89	12.65	13.56	14.60
RRu	37.36	19.79	22.47	19.92
P	+++	+++	+++	+++
Between Chi				69.00
Between df				2
Between P				***
Btwn(F) P				**
Btwn(R) P				(*)

	Lung cancer type: all	other	Total
N	60	5	65
NS	47	5	52
Wt	1375.50	50.09	1425.60
Het Chi	292.38	42.74	397.46
Het df	59	4	64
Het P	***	***	***
Fixed RR	21.86	7.02	21.00
RRl	20.73	5.32	19.94
RRu	23.04	9.26	22.12
P	+++	+++	+++
Random RR	18.46	6.81	17.05
RRl	15.96	2.63	14.60
RRu	21.35	17.68	19.92
P	+++	+++	+++
Between Chi			62.34
Between df			1
Between P			***
Btwn(F) P			**
Btwn(R) P			*

	Location: NAmer	UK	Scand	othEur	China	Japan	othAs	other	Total
N	32	12	4	2		9	2	4	65
NS	24	8	3	2		9	2	4	52
Wt	1197.58	74.97	21.49	13.81		93.84	10.23	13.68	1425.60
Het Chi	170.59	34.67	1.08	5.43		19.07	0.55	17.84	397.46
Het df	31	11	3	1		8	1	3	64
Het P	***	***	N.S.	*		*	N.S.	***	***
Fixed RR	22.87	19.08	31.06	24.61		6.90	7.13	43.00	21.00
RRl	21.61	15.21	20.35	14.52		5.64	3.86	25.31	19.94
RRu	24.21	23.92	47.41	41.70		8.45	13.16	73.05	22.12
P	+++	+++	+++	+++		+++	+++	+++	+++
Random RR	19.26	18.77	31.06	17.30		6.84	7.13	60.03	17.05
RRl	16.34	12.31	20.35	3.92		4.88	3.86	14.50	14.60
RRu	22.71	28.62	47.41	76.35		9.57	13.16	248.53	19.92
P	+++	+++	+++	+++		+++	+++	+++	+++
Between Chi									148.22
Between df									6
Between P									***
Btwn(F) P									***
Btwn(R) P									***

Table 1G9 - 3

IESLC - Meta-analysis of Current Smoking, Amount smoked, "High", Any product (or Cigarettes if Any not available)
All LC types
Most adjusted

Detailed Country in "other Europe"

	multi	Germany	othWest	East	Balkans	Total
N					2	2
NS					2	2
Wt					13.81	13.81
Het Chi					5.43	5.43
Het df					1	1
Het P					*	*
Fixed RR					24.61	24.61
RRl					14.52	14.52
RRu					41.70	41.70
P					+++	+++
Random RR					17.30	17.30
RRl					3.92	3.92
RRu					76.35	76.35
P					+++	+++
Between Chi						
Between df						
Between P						N.S.
Btwn(F) P						N.S.
Btwn(R) P						N.S.

Detailed Country in "other Asia"

	India	HongKong	other	Total
N			2	2
NS			2	2
Wt			10.23	10.23
Het Chi			0.55	0.55
Het df			1	1
Het P			N.S.	N.S.
Fixed RR			7.13	7.13
RRl			3.86	3.86
RRu			13.16	13.16
P			+++	+++
Random RR			7.13	7.13
RRl			3.86	3.86
RRu			13.16	13.16
P			+++	+++
Between Chi				
Between df				
Between P				N.S.
Btwn(F) P				N.S.
Btwn(R) P				N.S.

Detailed other continent

	SCAmer	Auslia	Africa	Total
N	3	1		4
NS	3	1		4
Wt	12.72	0.96		13.68
Het Chi	17.76	0.00		17.84
Het df	2	0		3
Het P	***	N.S.		***
Fixed RR	43.91	32.50		43.00
RRl	25.34	4.39		25.31
RRu	76.08	240.63		73.05
P	+++	+++		+++
Random RR	70.37	32.50		60.03
RRl	12.42	4.39		14.50
RRu	398.80	240.63		248.53
P	+++	+++		+++
Between Chi				0.08
Between df				1
Between P				N.S.
Btwn(F) P				N.S.
Btwn(R) P				N.S.

Table 1G9 - 3

IESLC - Meta-analysis of Current Smoking, Amount smoked, "High", Any product (or Cigarettes if Any not available)
All LC types
Most adjusted

	Start year of study <1960	1960-69	1970-79	1980-89	1990+	Total
N	13	20	12	18	2	65
NS	10	15	10	15	2	52
Wt	468.38	263.56	110.05	573.14	10.47	1425.60
Het Chi	33.96	101.97	49.39	166.38	9.64	397.46
Het df	12	19	11	17	1	64
Het P	***	***	***	***	**	***
Fixed RR	19.13	18.38	16.24	25.15	30.32	21.00
RRl	17.47	16.29	13.47	23.18	16.54	19.94
RRu	20.94	20.74	19.57	27.30	55.56	22.12
P	+++	+++	+++	+++	+++	+++
Random RR	17.75	16.34	11.51	20.71	39.74	17.05
RRl	14.51	12.03	7.27	14.08	5.54	14.60
RRu	21.71	22.19	18.22	30.46	285.09	19.92
P	+++	+++	+++	+++	+++	+++
Between Chi						36.13
Between df						4
Between P						***
Btwn(F) P						N.S.
Btwn(R) P						N.S.

	Study type (1) CC	other	Total
N	31	34	65
NS	27	25	52
Wt	761.62	663.98	1425.60
Het Chi	280.32	95.48	397.46
Het df	30	33	64
Het P	***	***	***
Fixed RR	23.56	18.40	21.00
RRl	21.95	17.06	19.94
RRu	25.30	19.86	22.12
P	+++	+++	+++
Random RR	17.26	17.00	17.05
RRl	13.10	14.45	14.60
RRu	22.74	19.99	19.92
P	+++	+++	+++
Between Chi			21.66
Between df			1
Between P			***
Btwn(F) P			(*)
Btwn(R) P			N.S.

	Study type (2) CC	prosp	other	Total
N	31	32	2	65
NS	27	24	1	52
Wt	761.62	658.44	5.54	1425.60
Het Chi	280.32	94.93	0.55	397.46
Het df	30	31	1	64
Het P	***	***	N.S.	***
Fixed RR	23.56	18.40	18.22	21.00
RRl	21.95	17.05	7.92	19.94
RRu	25.30	19.87	41.90	22.12
P	+++	+++	+++	+++
Random RR	17.26	16.95	18.22	17.05
RRl	13.10	14.35	7.92	14.60
RRu	22.74	20.02	41.90	19.92
P	+++	+++	+++	+++
Between Chi				21.67
Between df				2
Between P				***
Btwn(F) P				N.S.
Btwn(R) P				N.S.

Table 1G9 - 3

IESLC - Meta-analysis of Current Smoking, Amount smoked, "High", Any product (or Cigarettes if Any not available)
All LC types
Most adjusted

	Study size (number of LC cases) 100-249	250-499	500-999	1000+	Total
N	21	12	17	15	65
NS	20	9	13	10	52
Wt	89.03	64.66	202.42	1069.48	1425.60
Het Chi	72.62	31.44	74.63	162.29	397.46
Het df	20	11	16	14	64
Het P	***	***	***	***	***
Fixed RR	10.00	18.13	20.55	22.63	21.00
RRl	8.13	14.20	17.90	21.31	19.94
RRu	12.31	23.13	23.58	24.03	22.12
P	+++	+++	+++	+++	+++
Random RR	11.65	17.74	22.62	18.36	17.05
RRl	7.68	11.57	16.61	14.59	14.60
RRu	17.67	27.20	30.79	23.12	19.92
P	+++	+++	+++	+++	+++
Between Chi					56.48
Between df					3
Between P					***
Btwn(F) P					*
Btwn(R) P					(*)

	Risky occupational population no	mining	othRisky	Total
N	63	2		65
NS	50	2		52
Wt	1419.41	6.18		1425.60
Het Chi	393.01	1.45		397.46
Het df	62	1		64
Het P	***	N.S.		***
Fixed RR	21.06	10.48		21.00
RRl	20.00	4.76		19.94
RRu	22.19	23.05		22.12
P	+++	+++		+++
Random RR	17.17	12.10		17.05
RRl	14.68	3.81		14.60
RRu	20.07	38.42		19.92
P	+++	+++		+++
Between Chi				3.00
Between df				1
Between P				(*)
Btwn(F) P				N.S.
Btwn(R) P				N.S.

	National cigarette tobacco type Virginia	blended	other	Total
N	14	50	1	65
NS	10	41	1	52
Wt	82.24	1336.20	7.17	1425.60
Het Chi	35.01	355.34	0.00	397.46
Het df	13	49	0	64
Het P	***	***	N.S.	***
Fixed RR	19.05	21.23	8.30	21.00
RRl	15.35	20.12	3.99	19.94
RRu	23.65	22.40	17.26	22.12
P	+++	+++	+++	+++
Random RR	18.93	16.89	8.30	17.05
RRl	13.01	14.20	3.99	14.60
RRu	27.56	20.08	17.26	19.92
P	+++	+++	+++	+++
Between Chi				7.12
Between df				2
Between P				*
Btwn(F) P				N.S.
Btwn(R) P				N.S.

Table 1G9 - 3

IESLC - Meta-analysis of Current Smoking, Amount smoked, "High", Any product (or Cigarettes if Any not available)
All LC types
Most adjusted

	Any proxy use No/nk	Yes	Total
N	57	8	65
NS	46	6	52
Wt	1306.24	119.35	1425.60
Het Chi	370.61	26.23	397.46
Het df	56	7	64
Het P	***	***	***
Fixed RR	21.13	19.60	21.00
RRl	20.02	16.38	19.94
RRu	22.31	23.45	22.12
P	+++	+++	+++
Random RR	16.86	18.35	17.05
RRl	14.22	12.70	14.60
RRu	20.00	26.50	19.92
P	+++	+++	+++
Between Chi			0.62
Between df			1
Between P			N.S.
Btwn(F) P			N.S.
Btwn(R) P			N.S.

	Full histological confirmation No	Yes	Total
N	54	11	65
NS	42	10	52
Wt	1257.03	168.57	1425.60
Het Chi	250.64	144.22	397.46
Het df	53	10	64
Het P	***	***	***
Fixed RR	21.33	18.69	21.00
RRl	20.18	16.07	19.94
RRu	22.54	21.73	22.12
P	+++	+++	+++
Random RR	17.54	15.72	17.05
RRl	15.08	8.43	14.60
RRu	20.40	29.32	19.92
P	+++	+++	+++
Between Chi			2.60
Between df			1
Between P			N.S.
Btwn(F) P			N.S.
Btwn(R) P			N.S.

	Number of adjustment variables (1) 0	1	2+/+nk	Total
N	22	23	20	65
NS	18	18	17	53
Wt	609.51	615.65	200.43	1425.60
Het Chi	224.80	64.85	82.27	397.46
Het df	21	22	19	64
Het P	***	***	***	***
Fixed RR	24.29	19.49	16.95	21.00
RRl	22.44	18.01	14.76	19.94
RRu	26.30	21.09	19.47	22.12
P	+++	+++	+++	+++
Random RR	16.16	18.13	17.78	17.05
RRl	11.01	15.33	13.09	14.60
RRu	23.73	21.43	24.15	19.92
P	+++	+++	+++	+++
Between Chi				25.54
Between df				2
Between P				***
Btwn(F) P				N.S.
Btwn(R) P				N.S.

Table 1G9 - 3

IESLC - Meta-analysis of Current Smoking, Amount smoked, "High", Any product (or Cigarettes if Any not available)
All LC types
Most adjusted

	Number of adjustment variables (2) 0	1	2	3-5	6+/+nk	Total
N	22	23	11	8	1	65
NS	18	18	10	6	1	53
Wt	609.51	615.65	89.05	97.95	13.43	1425.60
Het Chi	224.80	64.85	22.89	36.25	0.00	397.46
Het df	21	22	10	7	0	64
Het P	***	***	*	***	N.S.	***
Fixed RR	24.29	19.49	15.06	15.87	60.00	21.00
RRl	22.44	18.01	12.24	13.02	35.15	19.94
RRu	26.30	21.09	18.54	19.35	102.43	22.12
P	+++	+++	+++	+++	+++	+++
Random RR	16.16	18.13	14.69	18.39	60.00	17.05
RRl	11.01	15.33	10.33	11.59	35.15	14.60
RRu	23.73	21.43	20.90	29.17	102.43	19.92
P	+++	+++	+++	+++	+++	+++
Between Chi						48.67
Between df						4
Between P						***
Btwn(F) P						(*)
Btwn(R) P						***

	Product all/unsp	cig+/-ot	cig only	Total
N	9	44	12	65
NS	9	36	9	54
Wt	44.43	1129.33	251.83	1425.60
Het Chi	28.91	295.35	40.43	397.46
Het df	8	43	11	64
Het P	***	***	***	***
Fixed RR	9.29	21.97	19.79	21.00
RRl	6.92	20.73	17.49	19.94
RRu	12.46	23.29	22.39	22.12
P	+++	+++	+++	+++
Random RR	9.56	17.32	23.40	17.05
RRl	5.36	14.39	17.02	14.60
RRu	17.04	20.83	32.16	19.92
P	+++	+++	+++	+++
Between Chi				32.78
Between df				2
Between P				***
Btwn(F) P				(*)
Btwn(R) P				*

	Denominator nev any	nev cigs	Total
N	42	23	65
NS	35	20	55
Wt	1122.57	303.03	1425.60
Het Chi	232.67	156.80	397.46
Het df	41	22	64
Het P	***	***	***
Fixed RR	21.83	18.18	21.00
RRl	20.59	16.25	19.94
RRu	23.15	20.35	22.12
P	+++	+++	+++
Random RR	16.94	17.49	17.05
RRl	14.14	12.70	14.60
RRu	20.29	24.10	19.92
P	+++	+++	+++
Between Chi			8.00
Between df			1
Between P			**
Btwn(F) P			N.S.
Btwn(R) P			N.S.

Table 1G9 - 3

IESLC - Meta-analysis of Current Smoking, Amount smoked, "High", Any product (or Cigarettes if Any not available)
All LC types
Most adjusted

	Derivation of RR/CI Orig	StdCalc	Other	Total
N	18	22	25	65
NS	16	18	19	53
Wt	382.79	617.23	425.57	1425.60
Het Chi	78.56	243.06	55.79	397.46
Het df	17	21	24	64
Het P	***	***	***	***
Fixed RR	19.24	24.04	18.68	21.00
RRl	17.40	22.22	16.99	19.94
RRu	21.26	26.01	20.54	22.12
P	+++	+++	+++	+++
Random RR	16.33	15.21	19.39	17.05
RRl	12.57	10.34	16.28	14.60
RRu	21.23	22.36	23.08	19.92
P	+++	+++	+++	+++
Between Chi				20.05
Between df				2
Between P				***
Btwn(F) P				N.S.
Btwn(R) P				N.S.

Table 1G9 - 4

IESLC - Meta-analysis of Current Smoking, Amount smoked, "High", Any product (or Cigarettes if Any not available)
All LC types
Least adjusted

REF	NRR	X	SEX	AGEL	AGEH	RACE	YF	LC TYPE	LOC	START	ST	NLC	R	VB	P	H	AD	PRODUCT	exL	exH	DENOM	De
AKIBA	19	x	m	0	0	all	0	all	As:Jap	1963	pr	610	n	bl	n	n	0	cig+/-ot	25	99	nev cigs	or
ARCHER	3		m	0	0	wh	0	all	NAmer	1950	pr	146	m	bl	n	n	0	cig+/-ot	21	99	nev cigs	st
BEST	15		m	0	0	all	0	all	NAmer	1955	pr	381	n	V	n	n	1	cig only	21	99	nev any	ot
BOUCOT	13	x	m	0	0	all	0	all	NAmer	1951	pr	121	n	bl	n	n	0	cig only	41	99	nev any	ot
BRETT	3		m	0	0	all	0	all	Eu:UK	1960	pr	150	n	V	n	n	0	cig+/-ot	25	99	nev cigs	st
BROSS	19		m	0	0	wh	-	all	NAmer	1960	CC	974	n	bl	n	n	0	cig+/-ot	21	99	nev any	st
BUFFLE	36		f	0	0	w-hi	-	all	NAmer	1976	CC	943	n	bl	y	n	0	cig+/-ot	21	99	nev cigs	or
CHANG	4		m	0	0	all	0	all	NAmer	1972	pr	136	n	bl	n	n	0	cig+/-ot	21	99	nev cigs	st
CHANG	10		f	0	0	all	0	all	NAmer	1972	pr	136	n	bl	n	n	0	cig+/-ot	21	99	nev cigs	st
CHOW	5	x	m	0	0	wh	0	all	NAmer	1966	pr	219	n	bl	n	n	0	cig+/-ot	30	99	nev any	st
COMSTO	6		m	0	0	all	-	all	NAmer	1975	ot	258	n	bl	n	n	0	cig+/-ot	40	99	nev any	st
COMSTO	11		f	0	0	all	-	all	NAmer	1975	ot	258	n	bl	n	n	0	cig+/-ot	40	99	nev any	st
CORREA	49	x	c	0	0	all	-	all	NAmer	1979	CC	1359	n	bl	y	n	0	cig+/-ot	21	99	nev cigs	st
CPSI	219		m	35	84	all	6	all	NAmer	1959	pr	5138	n	bl	n	n	1	cig+/-ot	40	99	nev any	ot
CPSI	278		f	40	74	all	6	all	NAmer	1959	pr	5138	n	bl	n	n	1	cig+/-ot	40	99	nev cigs	ot
CPSII	35	x	m	0	0	all	6	all	NAmer	1982	pr	3229	n	bl	n	n	0	cig only	41	99	nev any	st
CPSII	70	x	f	0	0	all	6	all	NAmer	1982	pr	3229	n	bl	n	n	0	cig+/-ot	41	99	nev cigs	st
DARBY	3		m	0	0	wh	-	all	Eu:UK	1988	CC	982	n	V	n	n	0	cig+/-ot	25	99	nev any	st
DARBY	10		f	0	0	wh	-	all	Eu:UK	1988	CC	982	n	V	n	n	0	cig+/-ot	25	99	nev any	st
DEAN3	19	x	m	0	0	all	-	all	Eu:UK	1969	CC	766	n	V	y	n	0	cig only	23	99	nev any	st
DEAN3	103	x	f	0	0	all	-	all	Eu:UK	1969	CC	766	n	V	y	n	0	cig only	23	99	nev any	st
DEKLER	4		m	0	0	all	0	all	Auslia	1961	pr	138	m	V	n	n	2	cig+/-ot	25	99	nev any	or
DOLL2	18		m	0	0	all	20	all	Eu:UK	1951	pr	920	n	V	n	n	1	all/unsp	25	99	nev any	ot
DOLL2	12		f	0	0	all	22	all	Eu:UK	1951	pr	920	n	V	n	n	1	cig only	25	99	nev any	ot
DORN	411		m	0	0	wh	25	all	NAmer	1954	pr	5097	n	bl	n	n	1	cig+/-ot	40	99	nev any	or
ENSTRO	3		m	0	0	all	0	all	NAmer	1959	pr	2879	n	bl	n	n	1	cig only	40	99	nev any	ot
ENSTRO	8		f	0	0	all	0	all	NAmer	1959	pr	2879	n	bl	n	n	1	cig only	21	99	nev any	ot
GAO2	4		m	0	0	all	-	all	As:Jap	1988	CC	282	n	bl	n	n	0	cig+/-ot	30	99	nev cigs	or
GILLIS	4	x	m	0	0	all	-	all	Eu:UK	1977	CC	656	n	V	n	n	0	cig+/-ot	35	49	nev any	st
HAENSZ	51		f	0	0	all	-	not alv	NAmer	1955	CC	158	n	bl	n	y	0	cig+/-ot	21	99	nev any	st
HAMMON	138		m	0	0	wh	0	all	NAmer	1952	pr	448	n	bl	n	n	1	cig only	40	99	nev any	ot
HIRAYA	76		m	0	0	all	16	all	As:Jap	1965	pr	1917	n	bl	n	n	4	cig+/-ot	35	99	nev any	or
HITOSU	5	x	m	0	0	all	-	all	As:Jap	1960	CC	216	n	bl	y	n	0	all/unsp	25	99	nev any	st
HOLE	12	x	m	0	0	all	0	all	Eu:UK	1972	pr	225	n	V	n	n	0	cig+/-ot	35	99	nev any	st
HUMBLE	78		c	0	0	wh	-	not alv	NAmer	1980	CC	521	n	bl	y	n	3	cig+/-ot	31	99	nev cigs	ot
KAISER	8		m	0	0	all	0	all	NAmer	1964	pr	714	n	bl	n	n	2	cig+/-ot	41	99	nev cigs	or
KAISER	4		f	0	0	all	0	all	NAmer	1964	pr	714	n	bl	n	n	2	cig+/-ot	41	99	nev cigs	or
KANELL	4	x	m	0	0	all	-	all	Eu:bal	1950	CC	862	n	bl	n	n	0	cig+/-ot	36	99	nev any	st
KATSOU	10	x	f	0	0	all	-	all	Eu:bal	1987	CC	101	n	bl	n	n	0	all/unsp	31	99	nev any	st
KAUFMA	6	x	c	0	0	all	-	all	NAmer	1981	CC	881	n	bl	n	n	0	cig+/-ot	45	99	nev cigs	st
KINLEN	5	x	m	0	0	all	0	all	Eu:UK	1967	pr	718	n	V	n	n	0	cig+/-ot	25	99	nev any	st
LIAW	5		c	0	0	all	0	all	As:oth	1982	pr	127	n	ot	n	n	2	all/unsp	21	99	nev any	or
MACLEN	23		m	0	0	ch	-	all	As:oth	1972	CC	233	n	bl	n	n	0	cig+/-ot	30	99	nev cigs	st
MATOS	8	x	m	0	0	all	-	all	SCAmer	1994	CC	200	n	bl	n	n	0	cig+/-ot	25	99	nev any	st
MIGRAN	7	x	m	0	0	all	0	all	Eu:UK	1964	pr	259	n	V	n	n	0	cig only	21	99	nev any	st
MIGRAN	34		f	0	0	all	0	all	Eu:UK	1964	pr	259	n	V	n	n	0	cig only	21	99	nev any	st
MRFITR	5		m	0	0	all	0	all	NAmer	1973	pr	119	n	bl	n	n	0	cig+/-ot	40	99	nev cigs	ot
NAM	67	x	m	0	0	all	-	all	NAmer	1986	CC	1199	n	bl	y	n	0	cig+/-ot	25	99	nev cigs	ot
NAM	83	x	f	0	0	all	-	all	NAmer	1986	CC	1199	n	bl	y	n	0	cig+/-ot	25	99	nev cigs	ot
PEZZO2	5		m	0	0	all	-	all	SCAmer	1992	CC	367	n	bl	n	y	0	cig+/-ot	41	99	nev cigs	st
PEZZOT	4		m	0	0	all	-	all	SCAmer	1987	CC	215	n	bl	n	y	0	cig only	41	99	nev cigs	st
SEGI2	17	x	m	0	0	all	-	all	As:Jap	1962	CC	378	n	bl	n	n	0	cig+/-ot	40	99	nev any	st
SOBUE	119		m	0	0	all	-	q+s+l+a	As:Jap	1986	CC	1376	n	bl	n	y	0	cig+/-ot	30	99	nev cigs	st
SPEIZE	5		f	0	0	all	0	all	NAmer	1976	pr	593	n	bl	n	y	1	cig+/-ot	35	99	nev cigs	ot
STOCKW	3		c	0	0	all	-	all	NAmer	1981	CC	22161	n	bl	n	n	0	cig+/-ot	41	99	nev any	st
SVENSS	36	x	f	0	0	all	-	all	Eu:Sca	1983	CC	210	n	bl	n	n	0	all/unsp	21	99	nev any	st
TENKAN	12		m	0	0	all	17	all	Eu:Sca	1962	pr	242	n	bl	n	n	1	all/unsp	25	99	nev any	ot
TSUGAN	31		m	0	0	all	-	q+a	As:Jap	1976	CC	134	n	bl	n	y	0	all/unsp	36	99	nev any	st
TULINI	6	x	m	0	0	all	0	all	Eu:Sca	1967	pr	472	n	bl	n	n	1	cig+/-ot	25	99	nev any	or
TULINI	11	x	f	0	0	all	0	all	Eu:Sca	1967	pr	472	n	bl	n	n	1	cig+/-ot	25	99	nev any	or
WAKAI	39	x	m	0	0	all	-	all	As:Jap	1988	CC	333	n	bl	n	y	0	cig+/-ot	30	99	nev any	st
WU	36	x	f	0	0	wh	-	q+a	NAmer	1981	CC	220	n	bl	n	y	0	all/unsp	21	99	nev any	st
WYNDE6	54		m	0	0	all	-	all	NAmer	1969	CC	4423	n	bl	n	y	0	cig+/-ot	31	99	nev any	st
WYNDE6	243		f	0	0	all	-	all	NAmer	1969	CC	4423	n	bl	n	y	0	cig+/-ot	30	99	nev cigs	st
YAMAGU	2	x	c	0	0	all	-	all	As:Jap	1989	CC	144	n	bl	n	y	0	all/unsp	21	99	nev any	st

Cigarette type is all/unspec for all RRs
except for the following:

Table 1G9 - 4

IESLC - Meta-analysis of Current Smoking, Amount smoked, "High", Any product (or Cigarettes if Any not available)
All LC types
Least adjusted

REF	NRR	CIGTYPE
DEAN3	19	MC only
DEAN3	103	MC only

Table 1G9 - 5

IESLC - Meta-analysis of Current Smoking, Amount smoked, "High", Any product (or Cigarettes if Any not available)
All LC types
Least adjusted

				Number Exposed		Non-exposed			
REF	NRR	SEX	AD	Case	Cont	Case	Cont	RR	95.00%CI
*AKIBA	19	m	0	63	28351	18	35833	4.42	(2.62- 7.47)
*ARCHER	3	m	0	40	7705	6	9842	8.52	(3.61- 20.07)
*BEST	15	m	1	-	-	-	-	17.31	(7.93- 37.79)
*BOUCOT	13	m	0	7	886	0	7551	127.77~	(7.30-2235.31)
*BRETT	3	m	0	33	4490	6	6530	8.00	(3.35- 19.07)
BROSS	19	m	0	95	59	38	170	7.20	(4.46- 11.63)
BUFFLE	36	f	0	116	49	12	112	22.10	(11.16- 43.73)
*CHANG	4	m	0	13	158	5	502	8.26	(2.99- 22.81)
*CHANG	10	f	0	13	164	11	1139	8.21	(3.74- 18.01)
Subtotal CHANG								8.23	(4.42- 15.32)
*CHOW	5	m	0	40	15732	6	62913	26.66	(11.31- 62.87)
COMSTO	6	m	0	26	18	4	69	24.92	(7.71- 80.57)
COMSTO	11	f	0	9	6	13	115	13.27	(4.07- 43.25)
Subtotal COMSTO								18.22	(7.92- 41.90)
CORREA	49	c	0	514	195	51	388	20.05	(14.34- 28.04)
*CPSI	219	m	1	-	-	-	-	17.49	(13.29- 23.03)
*CPSI	278	f	1	-	-	-	-	11.10	(6.00- 20.53)
Subtotal CPSI								16.21	(12.62- 20.84)
*CPSII	35	m	0	145	36115	124	742207	24.03	(18.91- 30.53)
*CPSII	70	f	0	25	13493	310	2091302	12.50	(8.32- 18.78)
Subtotal CPSII								20.31	(16.53- 24.97)
DARBY	3	m	0	68	61	3	384	142.69	(43.52- 467.82)
DARBY	10	f	0	38	21	23	529	41.62	(21.15- 81.90)
Subtotal DARBY								56.31	(31.27- 101.38)
DEAN3	19	m	0	131	237	25	510	11.28	(7.16- 17.77)
DEAN3	103	f	0	27	151	41	1538	6.71	(4.01- 11.21)
Subtotal DEAN3								8.98	(6.39- 12.62)
*DEKLER	4	m	2	-	-	-	-	32.50	(4.40- 241.20)
*DOLL2	18	m	1	-	-	-	-	22.40	(10.46- 47.99)
*DOLL2	12	f	1	-	-	-	-	29.71	(9.46- 93.32)
Subtotal DOLL2								24.43	(12.96- 46.06)
*DORN	411	m	1	-	-	-	-	22.75	(19.63- 26.37)
*ENSTRO	3	m	1	-	-	-	-	19.41	(15.22- 24.75)
*ENSTRO	8	f	1	-	-	-	-	16.47	(13.74- 19.75)
Subtotal ENSTRO								17.47	(15.10- 20.20)
GAO2	4	m	0	74	30	13	56	10.63	(5.08- 22.22)
GILLIS	4	m	0	59	66	13	145	9.97	(5.12- 19.43)
HAENSZ	51	f	0	23	13	81	236	5.15	(2.50- 10.65)
*HAMMON	138	m	1	-	-	-	-	20.64	(10.98- 38.80)
*HIRAYA	76	m	4	-	-	-	-	8.40	(5.70- 12.30)
HITOSU	5	m	0	32	265	7	242	4.17	(1.81- 9.63)
*HOLE	12	m	0	13	311	7	1189	7.10	(2.86- 17.64)
HUMBLE	78	c	3	-	-	-	-	39.70	(21.02- 74.98)
*KAISER	8	m	2	-	-	-	-	20.91	(12.78- 27.73)
*KAISER	4	f	2	-	-	-	-	12.63	(8.06- 19.80)
Subtotal KAISER								16.87	(12.58- 22.62)
KANELL	4	m	0	220	20	48	172	39.42	(22.55- 68.90)
KATSOU	10	f	0	10	1	48	67	13.96	(1.73- 112.71)
KAUFMA	6	c	0	96	47	35	925	53.98	(33.22- 87.71)
*KINLEN	5	m	0	157	1484	7	1333	20.15	(9.48- 42.80)
*LIAW	5	c	2	-	-	-	-	8.30	(4.00- 17.30)
MACLEN	23	m	0	45	27	5	15	5.00	(1.63- 15.31)
MATOS	8	m	0	65	46	11	110	14.13	(6.84- 29.20)
*MIGRAN	7	m	0	30	604	4	867	10.77	(3.81- 30.40)
*MIGRAN	34	f	0	2	149	4	3814	12.80	(2.36- 69.33)
Subtotal MIGRAN								11.29	(4.66- 27.34)
*MRFITR	5	m	0	54	3591	0	1859	56.43~	(3.49- 913.25)
NAM	67	m	0	149	243	30	520	10.63	(6.98- 16.19)
NAM	83	f	0	65	84	52	885	13.17	(8.59- 20.20)
Subtotal NAM								11.81	(8.75- 15.94)
PEZZO2	5	m	0	69	12	6	117	112.13	(40.26- 312.24)
PEZZOT	4	m	0	51	6	4	116	246.50	(66.69- 911.11)
SEGI2	17	m	0	52	45	8	53	7.66	(3.29- 17.80)
SOBUE	119	m	0	226	187	34	128	4.55	(2.97- 6.96)
*SPEIZE	5	f	1	-	-	-	-	22.00	(14.80- 32.30)
STOCKW	3	c	0	4327	572	2791	10641	28.84	(26.18- 31.77)
SVENSS	36	f	0	19	1	38	120	60.00	(7.77- 463.15)
*TENKAN	12	m	1	-	-	-	-	24.97	(9.90- 63.00)
TSUGAN	31	m	0	19	14	18	22	1.66	(0.65- 4.20)
*TULINI	6	m	1	-	-	-	-	28.70	(14.90- 55.10)
*TULINI	11	f	1	-	-	-	-	44.10	(21.10- 91.80)

Table 1G9 - 5

IESLC - Meta-analysis of Current Smoking, Amount smoked, "High", Any product (or Cigarettes if Any not available)
All LC types
Least adjusted

REF	NRR	SEX	AD	Number Exposed Case	Cont	Non-exposed Case	Cont	RR	95.00%CI
Subtotal TULINI								34.70	(21.29- 56.56)
WAKAI	39	m	0	69	48	10	65	9.34	(4.37- 20.00)
WU	36	f	0	103	31	31	92	9.86	(5.57- 17.47)
WYNDE6	54	m	0	784	197	87	617	28.22	(21.47- 37.10)
WYNDE6	243	f	0	378	52	159	856	39.13	(27.98- 54.75)
Subtotal WYNDE6								32.15	(26.01- 39.75)
YAMAGU	2	c	0	28	56	24	267	5.56	(3.00- 10.31)
Partial Totals				8622	116093	4271	2987163		

*prospective study ~ With 0.5 adjustment for zero

REF	NRR	SEX	AD	Ys	Ws	Qs	Ps
*AKIBA	19	m	0	1.49	14.01	31.65	0.0000
*ARCHER	3	m	0	2.14	5.22	3.76	0.0000
*BEST	15	m	1	2.85	6.30	0.12	0.0000
*BOUCOT	13	m	0	4.85	0.47	1.62	0.0009
*BRETT	3	m	0	2.08	5.09	4.22	0.0000
BROSS	19	m	0	1.97	16.76	17.28	0.0000
BUFFLE	36	f	0	3.10	8.24	0.09	0.0000
*CHANG	4	m	0	2.11	3.72	2.87	0.0000
*CHANG	10	f	0	2.11	6.22	4.87	0.0000
Subtotal CHANG				2.11	9.94	7.74	
*CHOW	5	m	0	3.28	5.22	0.45	0.0000
COMSTO	6	m	0	3.22	2.79	0.14	0.0000
COMSTO	11	f	0	2.59	2.75	0.45	0.0000
Subtotal COMSTO				2.90	5.54	0.59	
CORREA	49	c	0	3.00	34.18	0.00	0.0000
*CPSI	219	m	1	2.86	50.84	0.84	0.0000
*CPSI	278	f	1	2.41	10.15	3.45	0.0000
Subtotal CPSI				2.79	60.99	4.29	
*CPSII	35	m	0	3.18	66.97	2.40	0.0000
*CPSII	70	f	0	2.53	23.17	5.00	0.0000
Subtotal CPSII				3.01	90.14	7.40	
DARBY	3	m	0	4.96	2.72	10.58	0.0000
DARBY	10	f	0	3.73	8.38	4.57	0.0000
Subtotal DARBY				4.03	11.11	15.15	
DEAN3	19	m	0	2.42	18.58	5.98	0.0000
DEAN3	103	f	0	1.90	14.56	17.19	0.0000
Subtotal DEAN3				2.19	33.14	23.17	
*DEKLER	4	m	2	3.48	0.96	0.23	0.0007
*DOLL2	18	m	1	3.11	6.62	0.09	0.0000
*DOLL2	12	f	1	3.39	2.93	0.47	0.0000
Subtotal DOLL2				3.20	9.55	0.57	
*DORN	411	m	1	3.12	176.37	3.19	0.0000
*ENSTRO	3	m	1	2.97	65.00	0.04	0.0000
*ENSTRO	8	f	1	2.80	116.71	4.14	0.0000
Subtotal ENSTRO				2.86	181.71	4.18	
GAO2	4	m	0	2.36	7.06	2.77	0.0000
GILLIS	4	m	0	2.30	8.63	4.11	0.0000
HAENSZ	51	f	0	1.64	7.30	13.31	0.0000
*HAMMON	138	m	1	3.03	9.64	0.01	0.0000
*HIRAYA	76	m	4	2.13	25.97	19.29	0.0000
HITOSU	5	m	0	1.43	5.49	13.39	0.0008
*HOLE	12	m	0	1.96	4.64	4.92	0.0000
HUMBLE	78	c	3	3.68	9.50	4.54	0.0000
*KAISER	8	m	2	3.04	25.61	0.06	0.0000
*KAISER	4	f	2	2.54	19.02	3.92	0.0000
Subtotal KAISER				2.83	44.63	3.98	
KANELL	4	m	0	3.67	12.32	5.77	0.0000
KATSOU	10	f	0	2.64	0.88	0.11	0.0134
KAUFMA	6	c	0	3.99	16.30	16.26	0.0000
*KINLEN	5	m	0	3.00	6.77	0.00	0.0000
*LIAW	5	c	2	2.12	7.17	5.47	0.0000
MACLEN	23	m	0	1.61	3.07	5.85	0.0048
MATOS	8	m	0	2.65	7.29	0.85	0.0000
*MIGRAN	7	m	0	2.38	3.56	1.34	0.0000
*MIGRAN	34	f	0	2.55	1.35	0.26	0.0031
Subtotal MIGRAN				2.42	4.91	1.60	
*MRFITR	5	m	0	4.03	0.50	0.54	0.0045
NAM	67	m	0	2.36	21.70	8.52	0.0000
NAM	83	f	0	2.58	20.99	3.56	0.0000

Table 1G9 - 5

IESLC - Meta-analysis of Current Smoking, Amount smoked, "High", Any product (or Cigarettes if Any not available)
All LC types
Least adjusted

REF	NRR	SEX	AD	Ys	Ws	Qs	Ps
Subtotal NAM				2.47	42.69	12.08	
PEZZO2	5	m	0	4.72	3.66	10.96	0.0000
PEZZOT	4	m	0	5.51	2.25	14.24	0.0000
SEGI2	17	m	0	2.04	5.40	4.92	0.0000
SOBUE	119	m	0	1.52	21.28	46.29	0.0000
*SPEIZE	5	f	1	3.09	25.23	0.26	0.0000
STOCKW	3	c	0	3.36	411.25	56.86	0.0000
SVENSS	36	f	0	4.09	0.92	1.12	0.0001
*TENKAN	12	m	1	3.22	4.49	0.23	0.0000
TSUGAN	31	m	0	0.51	4.44	27.41	0.2861
*TULINI	6	m	1	3.36	8.98	1.21	0.0000
*TULINI	11	f	1	3.79	7.11	4.51	0.0000
Subtotal TULINI				3.55	16.09	5.72	
WAKAI	39	m	0	2.23	6.64	3.78	0.0000
WU	36	f	0	2.29	11.75	5.78	0.0000
WYNDE6	54	m	0	3.34	51.37	6.30	0.0000
WYNDE6	243	f	0	3.67	34.09	15.63	0.0000
Subtotal WYNDE6				3.47	85.46	21.93	
YAMAGU	2	c	0	1.72	10.10	16.40	0.0000

	N	65
	NS	52
	Wt	1508.64
Het	Chi	456.46
Het	df	64
Het	P	***
Fixed	RR	19.89
	RRl	18.91
	RRu	20.91
	P	+++
Random	RR	15.68
	RRl	13.37
	RRu	18.40
	P	+++
Asymm	P	**

Table 1G9 - 6

IESLC - Meta-analysis of Current Smoking, Amount smoked, "High", Any product (or Cigarettes if Any not available)
All LC types
Least adjusted

	Sex combined	male	female	Total
N	6	40	19	65
NS	6	40	19	65
Wt	488.49	698.39	321.76	1508.64
Het Chi	49.12	253.90	78.65	456.46
Het df	5	39	18	64
Het P	***	***	***	***
Fixed RR	27.42	17.23	16.66	19.89
RRl	25.09	16.00	14.94	18.91
RRu	29.96	18.56	18.59	20.91
P	+++	+++	+++	+++
Random RR	20.52	14.84	15.93	15.68
RRl	12.49	11.91	12.15	13.37
RRu	33.70	18.47	20.89	18.40
P	+++	+++	+++	+++
Between Chi				74.79
Between df				2
Between P				***
Btwn(F) P				**
Btwn(R) P				N.S.

Table 1G9 - 7

IESLC - Meta-analysis of Current Smoking, Amount smoked, "High", Any product (or Cigarettes if Any not available)
All LC types
Excluded studies (and stage at which they were excluded)

1 ABELIN ABRAHA AMANDU AMES ANDERS AUSTIN AXELSO BAND BECHER BERRIN BLOHMK BLOT4 BROCKM BROWN1 BYERS1 BYERS2
CARPEN CASCO2 CASCOR CHAN CHEN3 CHIAZZ CHYOU DESTE2 DOCKER DROSTE DU GARCIA GARDIN GENG GODLEY GOODMA
GRAHAM GREGOR HEGMAN HEIN HENNEK HINDS HIRAOK HOROWI HORWIT HUANG ISHIMA JAHN JAIN JARVHO JIANG KELLER
KIHARA KJUUS KO KOHLME KUBIK LAMWK LAMWK2 LANGE LEI LEMARC LEVIN LIU LOMBA2 LOMBAR MAGNUS MARSH
MARSH2 MCDUFF MCLAUG MILLER MILLS NOTANI NOU ODRISC PAWLEG PERSHA POFFIJ QIAO QIAO2 RADZIK REN RONCO
ROOTS ROTHSC SAARIK SANKAR SCHWAR SEGI SEOW SHIMIZ SIMARA SIMONA SITAS SOBUE2 STASZE STAYNE STUCKE SUN
SUZUK2 SUZUKI TANG TAO TOKARS TOUSEY ULMER VEIERO VUTUC WALD WANG WANG3 WANG4 WICKLU WIGLE WILKIN
WU2 WUNSCH WYNDE8 XIANGZ XU XU2 XU4 YONG ZHANG

2 AGUDO ALDERS ARMADA AUVINE BARBON BENHAM BLOT1 BLOT2 BLOT3 BOFFET BOUCHA BRESLO BROWN2 BUELL CHATZI CHEN
CHEN2 CHOI COOKSO DAMBER DAVEYS DEAN DEAN2 DOLL DOSEME DUNN EBELIN ESAKI FAN GAO GARSHI GER
GOLLED GSELL HANSEN HU HU2 JARUP JEDRYC JOLY JONES JUSSAW KHUDER KOULUM KREUZE KREYBE LAMTH LAUSSM
LETOUR LIU2 LIU3 LIU4 LIU5 LUBIN LUBIN2 LUO MARTIS MASTRA MATSUD MCCONN MOLLO MZILEN NOTAN2 ORMOS
OSANN OSANN2 PASTOR PERNU PIKE POLEDN RACHTA RANDIG RESTRE SADOWS SCHWA2 SIEMIA SPITZ STOCKS TIZZAN VANDER
WANG2 WUWILL WYNDE2 WYNDE3 WYNDE4 XU3 YUAN ZHENG ZHOU

3 PISANI

4 WYNDE7

5 RIMING TANG2 WYNDE5

6 DESTEF HIRAY2 LAURIL LICKIN MRFIT MURATA WARSIN WATSON WYNDER

10 AXELSS BENSHL CEDERL DORANT DORGAN ENGELA HAMMO2 KAISE2 KNEKT KOO LIDDEL PARKIN PERSH2 PETO PRESCO SHAW
TVERDA

Table 1G9 - 8

Potentially overlapping studies

REF	REFGP	PRINC	.	OVERLAP/LINK
GILLIS	LUBIN2	2		Subset of Lubin2
AKIBA	AKIBA	1		AKIBA/ISHIMA
BROSS	BYERS1	1		GRAHAM/BROSS/BYERS1
HOLE	TANG2	1		Subset of TANG2
KAISER	KAISER	1		KAISER/OSANN2
MRFITR	MRFIT	2		Subset of MRFIT
WYNDE6	WYNDE6	1		WYNDE5/6/7/8
CPSI	CPSI	1		CPSI overall
ENSTRO	ENSTRO	1		Subset of CPSI

Table 1G9 - 9

Most adjusted - insufficient data for metaanalysis

REF	NRR	SEX	AGEL	AGEH	RACE	YF	LC TYPE	LOC	START	ST	NLC	R	VB	P	H	AD	PRODUCT	exL	exH	DENOM	De
HITOSU	16	m	0	0	all	-	all	As:Jap	1960	CC	216	n	bl	y	n	2	all/unsp	25	99	nev any	or

REF	NRR	RR	SIG	RRDATA comment
HITOSU	16	6.40		0

Table 1G11 -

IESLC - Meta-anal of Ever Smoking (or Curr if Ever not avail) by Amount, Overview, Any prod (or Cigs if Any not avail)
All LC types

This analysis is restricted to results for:
1) Results by Amount smoked
Results by Amount smoked (in numbers of cigarettes or cigarette equivalents) are grouped under 2 schemes (S1, S2).
Each scheme has a set of "key values". An interval is allocated to the category whose key value
it includes and intervals which include none or more than one of the key values are excluded.
(Open-ended intervals are coded as 99.)

S1	key value	maximum range	S2	key value	maximum range
1	5	1-19	1	1	1-9
2	20	6-44	2	10	2-19
3	45	21+	3	20	11-29
			4	30	21-39
			5	40	31-98
			6	99	41+

For all/unspec product, the definition of cigarette equivalents is shown at the end of Sections -1 and -4.
2) Results complete enough for use in metaanalysis

Within each study, results are then selected (in the following order of preference, within each sex) for:
3) SMKSTA: ever smokers, current smokers
4) PRODUCT: all/unspec, cigarettes regardless of other products, cigarettes only
5) CIGTYPE: all/unspecified, MC regardless of HR, MC only
6) DENOM: never smoked anything, never smoked cigarettes, (never +1 = +long term ex, +2 = +amount unknown,
+3 = never cigs+long term ex)
7) Followup period (YF, prospective studies): whole study (coded as 0) or longest available
8) LCtype: all or nearest available, at least Squamous and Adeno. (q = squamous, s = small,
l = large, a = adeno, mix = mixed, alv = alveolar)
9) Race: all or nearest available, otherwise by race (wh or w = white, bl or b = black, hi = hispanic
ch = chinese, jap = japanese, haw = hawaiian, w+o = white + oriental, sca = scandinavian, as = asian)
10) For overlapping studies: principal rather than subsidiary studies
Finally by Age: whole study (coded as 0) if available, otherwise by widest available age group
and then for single sex results (m, f) in preference to combined sex results (c).

Results adjusted (AD) for the most potential confounders are then chosen in Sections -1 to -3
and results adjusted for the least confounders in Sections -4 to -6. (Those least adjusted results which
actually differ from the most adjusted as marked 'x' in column X in Section -4)
(Results adjusted for an unknown number of confounder(s) are coded as 20.)

Section -7 shows excluded studies, together with the stage (as above) at which no qualifying
results were found.

Section -8 lists the potentially overlapping studies which have been included (1=principal, 2=subsidiary).

Section -9 lists any results which would have been included in preference except that they had data not complete
enough for use in meta-analysis, with their significance (yes/no), if known, and any further comment as entered
on the database.

In addition to those mentioned above, the following fields, levels and abbreviations are used:
* or nk = not known, n = no, y = yes, ot = other
ev = ever, cu = current, nev = never
all/unspec = all or unspecified, cig+/-ot = cigarettes irrespective of other products (cigar, pipe etc)
MC = manufactured cigarettes, HR = hand-rolled cigarettes
exL, exH = range of exposure (low and high) in the smoking group, in terms of Amount smoked,
cigarettes or cigarette equivalents
REF: 6-character study reference
NRR: number of the RR on the database within the study
ST : study type (CC = case control, pr or prosp = prospective)
NLC: number of lung cancer cases in whole study
R : risky occupational population (n = no, m = mining, o = other risky)
VB : national cigarette type (V = at least 75% Virginia, bl = at least 75% blended, ot = other)
P : any proxy use
H : full histological confirmation
De : derivation of RR/CI (or = original, st = standard method, ot = other method of estimation)

Table 1G11 - 1

IESLC - Meta-anal of Ever Smoking (or Curr if Ever not avail) by Amount, Overview, Any prod (or Cigs if Any not avail)
All LC types
Most adjusted

REF	NRR	SEX	AGEL	AGEH	RACE	YF	LC TYPE	LOC	START	ST	NLC	R	VB	P	H	AD	SM	PRODUCT	exL	exH	S1	S2	DENOM	De
AGUDO	4	f	0	0	all	-	all	Eu:wst	1989	CC	103	n	bl	n	n	3	ev	cig only	1	10	1	0	nev any	or
AGUDO	5	f	0	0	all	-	all	Eu:wst	1989	CC	103	n	bl	n	n	3	ev	cig only	11	99	0	0	nev any	or
AKIBA	27	m	0	0	all	0	all	As:Jap	1963	pr	610	n	bl	n	n	5	cu	cig+/-ot	1	14	1	0	nev cigs	or
AKIBA	28	m	0	0	all	0	all	As:Jap	1963	pr	610	n	bl	n	n	5	cu	cig+/-ot	15	24	2	3	nev cigs	ot
AKIBA	29	m	0	0	all	0	all	As:Jap	1963	pr	610	n	bl	n	n	5	cu	cig+/-ot	25	99	3	0	nev cigs	or
AKIBA	33	f	0	0	all	0	all	As:Jap	1963	pr	610	n	bl	n	n	5	cu	cig+/-ot	1	14	1	0	nev cigs	or
AKIBA	34	f	0	0	all	0	all	As:Jap	1963	pr	610	n	bl	n	n	5	cu	cig+/-ot	15	99	0	0	nev cigs	or
ALDERS	18	m	0	0	all	-	all	Eu:UK	1977	CC	1448	n	V	n	n	1	ev	cig only	1	17	1	0	nev+2	ot
ALDERS	19	m	0	0	all	-	all	Eu:UK	1977	CC	1448	n	V	n	n	1	ev	cig only	18	27	2	3	nev+2	ot
ALDERS	20	m	0	0	all	-	all	Eu:UK	1977	CC	1448	n	V	n	n	1	ev	cig only	28	99	3	0	nev+2	ot
ALDERS	21	f	0	0	all	-	all	Eu:UK	1977	CC	1448	n	V	n	n	1	ev	cig only	1	17	1	0	nev+2	ot
ALDERS	22	f	0	0	all	-	all	Eu:UK	1977	CC	1448	n	V	n	n	1	ev	cig only	18	27	2	3	nev+2	ot
ALDERS	23	f	0	0	all	-	all	Eu:UK	1977	CC	1448	n	V	n	n	1	ev	cig only	28	99	3	0	nev+2	ot
ARCHER	1	m	0	0	wh	0	all	NAmer	1950	pr	146	m	bl	n	n	0	cu	cig+/-ot	1	19	1	0	nev cigs	st
ARCHER	2	m	0	0	wh	0	all	NAmer	1950	pr	146	m	bl	n	n	0	cu	cig+/-ot	20	20	2	3	nev cigs	st
ARCHER	3	m	0	0	wh	0	all	NAmer	1950	pr	146	m	bl	n	n	0	cu	cig+/-ot	21	99	3	0	nev cigs	st
ARMADA	46	m	0	0	all	-	all	Eu:wst	1986	CC	325	n	bl	n	y	0	ev	cig+/-ot	1	14	1	0	nev any	st
ARMADA	47	m	0	0	all	-	all	Eu:wst	1986	CC	325	n	bl	n	y	0	ev	cig+/-ot	15	24	2	3	nev any	st
ARMADA	48	m	0	0	all	-	all	Eu:wst	1986	CC	325	n	bl	n	y	0	ev	cig+/-ot	25	99	3	0	nev any	st
AUVINE	13	c	0	0	all	-	all	Eu:Sca	1986	CC	517	n	bl	y	n	2	ev	cig+/-ot	1	10	1	0	nev cigs	or
AUVINE	14	c	0	0	all	-	all	Eu:Sca	1986	CC	517	n	bl	y	n	2	ev	cig+/-ot	11	20	2	3	nev cigs	or
AUVINE	15	c	0	0	all	-	all	Eu:Sca	1986	CC	517	n	bl	y	n	2	ev	cig+/-ot	21	99	3	0	nev cigs	or
AXELSS	5	m	0	0	sca	-	all	Eu:Sca	1989	CC	436	n	bl	n	n	6	ev	all/unsp	1	9	1	1	nev any	ot
AXELSS	6	m	0	0	sca	-	all	Eu:Sca	1989	CC	436	n	bl	n	n	6	ev	all/unsp	10	19	0	2	nev any	ot
AXELSS	7	m	0	0	sca	-	all	Eu:Sca	1989	CC	436	n	bl	n	n	6	ev	all/unsp	20	99	0	0	nev any	ot
AXELSS	13	f	0	0	sca	-	all	Eu:Sca	1989	CC	436	n	bl	n	n	0	ev	all/unsp	1	9	1	1	nev any	st
AXELSS	14	f	0	0	sca	-	all	Eu:Sca	1989	CC	436	n	bl	n	n	0	ev	all/unsp	10	19	0	2	nev any	st
AXELSS	15	f	0	0	sca	-	all	Eu:Sca	1989	CC	436	n	bl	n	n	0	ev	all/unsp	20	29	2	3	nev any	st
AXELSS	16	f	0	0	sca	-	all	Eu:Sca	1989	CC	436	n	bl	n	n	0	ev	all/unsp	30	99	3	0	nev any	st
BARBON	82	m	0	0	all	-	all	Eu:wst	1979	CC	755	n	bl	y	y	3	ev	all/unsp	1	19	1	0	nev any	or
BARBON	83	m	0	0	all	-	all	Eu:wst	1979	CC	755	n	bl	y	y	3	ev	all/unsp	20	39	2	0	nev any	or
BARBON	84	m	0	0	all	-	all	Eu:wst	1979	CC	755	n	bl	y	y	3	ev	all/unsp	40	99	3	0	nev any	or
BENSHL	11	m	40	64	all	10	all	Eu:UK	1967	pr	486	n	V	n	n	1	cu	cig+/-ot	1	9	1	1	nev any	ot
BENSHL	12	m	40	64	all	10	all	Eu:UK	1967	pr	486	n	V	n	n	1	cu	cig+/-ot	10	19	0	2	nev any	ot
BENSHL	13	m	40	64	all	10	all	Eu:UK	1967	pr	486	n	V	n	n	1	cu	cig+/-ot	20	99	0	0	nev any	ot
BEST	13	m	0	0	all	0	all	NAmer	1955	pr	381	n	V	n	n	1	cu	cig only	1	9	1	1	nev any	ot
BEST	14	m	0	0	all	0	all	NAmer	1955	pr	381	n	V	n	n	1	cu	cig only	10	20	2	0	nev any	ot
BEST	15	m	0	0	all	0	all	NAmer	1955	pr	381	n	V	n	n	1	cu	cig only	21	99	3	0	nev any	ot
BOUCOT	99	m	0	0	all	9	all	NAmer	1951	pr	121	n	bl	n	n	0	ev	cig+/-ot	1	20	0	0	nev any	ot
BOUCOT	100	m	0	0	all	9	all	NAmer	1951	pr	121	n	bl	n	n	0	ev	cig+/-ot	21	99	3	0	nev any	ot
BRESLO	13	m	0	0	all	-	all	NAmer	1949	CC	518	n	bl	n	y	0	ev	cig+/-ot	1	9	1	1	nev+3	st
BRESLO	14	m	0	0	all	-	all	NAmer	1949	CC	518	n	bl	n	y	0	ev	cig+/-ot	10	19	0	2	nev+3	st
BRESLO	15	m	0	0	all	-	all	NAmer	1949	CC	518	n	bl	n	y	0	ev	cig+/-ot	20	39	2	0	nev+3	st
BRESLO	16	m	0	0	all	-	all	NAmer	1949	CC	518	n	bl	n	y	0	ev	cig+/-ot	40	99	3	0	nev+3	st
BRESLO	29	f	0	0	all	-	all	NAmer	1949	CC	518	n	bl	n	y	0	ev	cig+/-ot	1	19	1	0	nev+3	st
BRESLO	30	f	0	0	all	-	all	NAmer	1949	CC	518	n	bl	n	y	0	ev	cig+/-ot	20	99	0	0	nev+3	st
BRETT	1	m	0	0	all	0	all	Eu:UK	1960	pr	150	n	V	n	n	0	cu	cig+/-ot	1	14	1	0	nev cigs	st
BRETT	2	m	0	0	all	0	all	Eu:UK	1960	pr	150	n	V	n	n	0	cu	cig+/-ot	15	24	2	3	nev cigs	st
BRETT	3	m	0	0	all	0	all	Eu:UK	1960	pr	150	n	V	n	n	0	cu	cig+/-ot	25	99	3	0	nev cigs	st
BROSS	18	m	0	0	wh	-	all	NAmer	1960	CC	974	n	bl	n	n	0	cu	cig+/-ot	1	20	0	0	nev any	st
BROSS	19	m	0	0	wh	-	all	NAmer	1960	CC	974	n	bl	n	n	0	cu	cig+/-ot	21	99	3	0	nev any	st
BROWN2	32	m	0	0	wh	-	all	NAmer	1984	CC	14596	n	bl	n	y	2	ev	cig+/-ot	1	19	1	0	nev cigs	or
BROWN2	42	m	0	0	wh	-	all	NAmer	1984	CC	14596	n	bl	n	y	2	ev	cig+/-ot	20	99	0	0	nev cigs	or
BROWN2	31	f	0	0	wh	-	all	NAmer	1984	CC	14596	n	bl	n	y	2	ev	cig+/-ot	1	19	1	0	nev cigs	or
BROWN2	41	f	0	0	wh	-	all	NAmer	1984	CC	14596	n	bl	n	y	2	ev	cig+/-ot	20	99	0	0	nev cigs	or
BUFFLE	28	f	0	0	w-hi	-	all	NAmer	1976	CC	943	n	bl	y	n	0	ev	cig+/-ot	1	19	1	0	nev cigs	or
BUFFLE	29	f	0	0	w-hi	-	all	NAmer	1976	CC	943	n	bl	y	n	0	ev	cig+/-ot	20	20	2	3	nev cigs	or
BUFFLE	35	f	0	0	w-hi	-	all	NAmer	1976	CC	943	n	bl	y	n	0	ev	cig+/-ot	21	99	3	0	nev cigs	or
CEDERL	80	m	0	0	all	16	all	Eu:Sca	1963	pr	491	n	bl	n	n	2	cu	all/unsp	1	7	1	1	nev any	ot
CEDERL	81	m	0	0	all	16	all	Eu:Sca	1963	pr	491	n	bl	n	n	2	cu	all/unsp	8	15	0	2	nev any	ot
CEDERL	82	m	0	0	all	16	all	Eu:Sca	1963	pr	491	n	bl	n	n	2	cu	all/unsp	16	99	0	0	nev any	ot
CEDERL	76	f	0	0	all	0	all	Eu:Sca	1963	pr	491	n	bl	n	n	2	cu	all/unsp	1	7	1	1	nev any	or
CEDERL	77	f	0	0	all	0	all	Eu:Sca	1963	pr	491	n	bl	n	n	2	cu	all/unsp	8	15	0	2	nev any	or
CEDERL	78	f	0	0	all	0	all	Eu:Sca	1963	pr	491	n	bl	n	n	2	cu	all/unsp	16	99	0	0	nev any	or
CHANG	2	m	0	0	all	0	all	NAmer	1972	pr	136	n	bl	n	n	0	cu	cig+/-ot	1	10	1	0	nev cigs	st
CHANG	3	m	0	0	all	0	all	NAmer	1972	pr	136	n	bl	n	n	0	cu	cig+/-ot	11	20	2	3	nev cigs	st
CHANG	4	m	0	0	all	0	all	NAmer	1972	pr	136	n	bl	n	n	0	cu	cig+/-ot	21	99	3	0	nev cigs	st
CHANG	8	f	0	0	all	0	all	NAmer	1972	pr	136	n	bl	n	n	0	cu	cig+/-ot	1	10	1	0	nev cigs	st
CHANG	9	f	0	0	all	0	all	NAmer	1972	pr	136	n	bl	n	n	0	cu	cig+/-ot	11	20	2	3	nev cigs	st
CHANG	10	f	0	0	all	0	all	NAmer	1972	pr	136	n	bl	n	n	0	cu	cig+/-ot	21	99	3	0	nev cigs	st
CHATZI	1	c	0	0	all	-	all	Eu:bal	1987	CC	282	n	bl	n	y	0	ev	all/unsp	1	45	0	0	nev any	st
CHATZI	2	c	0	0	all	-	all	Eu:bal	1987	CC	282	n	bl	n	y	0	ev	all/unsp	46	74	0	0	nev any	st

Table 1G11 - 1

IESLC - Meta-anal of Ever Smoking (or Curr if Ever not avail) by Amount, Overview, Any prod (or Cigs if Any not avail)
All LC types
Most adjusted

REF	NRR	SEX	AGEL	AGEH	RACE	YF	LC TYPE	LOC	START	ST	NLC	R	VB	P	H	AD	SM	PRODUCT	exL	exH	S1	S2	DENOM	De
CHATZI	3	c	0	0	all	-	all	Eu:bal	1987	CC	282	n	bl	n	y	0	ev	all/unsp	75	99	0	6	nev any	st
CHEN2	3	m	0	0	all	-	all	As:Chi	1983	CC	193	n	ot	y	n	0	ev	all/unsp	1	9	1	1	nev any	st
CHEN2	4	m	0	0	all	-	all	As:Chi	1983	CC	193	n	ot	y	n	0	ev	all/unsp	10	20	2	0	nev any	st
CHEN2	5	m	0	0	all	-	all	As:Chi	1983	CC	193	n	ot	y	n	0	ev	all/unsp	21	30	0	4	nev any	st
CHEN2	6	m	0	0	all	-	all	As:Chi	1983	CC	193	n	ot	y	n	0	ev	all/unsp	31	99	3	0	nev any	st
CHEN2	7	f	0	0	all	-	all	As:Chi	1983	CC	193	n	ot	y	n	0	ev	all/unsp	1	9	1	1	nev any	st
CHEN2	8	f	0	0	all	-	all	As:Chi	1983	CC	193	n	ot	y	n	0	ev	all/unsp	10	20	2	0	nev any	st
CHEN2	9	f	0	0	all	-	all	As:Chi	1983	CC	193	n	ot	y	n	0	ev	all/unsp	21	30	0	4	nev any	st
CHEN2	10	f	0	0	all	-	all	As:Chi	1983	CC	193	n	ot	y	n	0	ev	all/unsp	31	99	3	0	nev any	st
CHOI	12	m	0	0	all	-	all	As:oth	1985	CC	375	n	bl	n	n	0	ev	cig+/-ot	1	10	1	0	nev cigs	st
CHOI	13	m	0	0	all	-	all	As:oth	1985	CC	375	n	bl	n	n	0	ev	cig+/-ot	11	20	2	3	nev cigs	st
CHOI	14	m	0	0	all	-	all	As:oth	1985	CC	375	n	bl	n	n	0	ev	cig+/-ot	21	30	0	4	nev cigs	st
CHOI	15	m	0	0	all	-	all	As:oth	1985	CC	375	n	bl	n	n	0	ev	cig+/-ot	31	40	0	5	nev cigs	st
CHOI	16	m	0	0	all	-	all	As:oth	1985	CC	375	n	bl	n	n	0	ev	cig+/-ot	41	99	3	6	nev cigs	st
CHOI	17	f	0	0	all	-	all	As:oth	1985	CC	375	n	bl	n	n	0	ev	cig+/-ot	1	10	1	0	nev cigs	st
CHOI	18	f	0	0	all	-	all	As:oth	1985	CC	375	n	bl	n	n	0	ev	cig+/-ot	11	30	2	0	nev cigs	st
CHOI	20	f	0	0	all	-	all	As:oth	1985	CC	375	n	bl	n	n	0	ev	cig+/-ot	31	99	3	0	nev cigs	st
CHOW	10	m	0	0	wh	0	all	NAmer	1966	pr	219	n	bl	n	n	2	cu	cig+/-ot	1	19	1	0	nev any	ot
CHOW	11	m	0	0	wh	0	all	NAmer	1966	pr	219	n	bl	n	n	2	cu	cig+/-ot	20	29	2	3	nev any	ot
CHOW	12	m	0	0	wh	0	all	NAmer	1966	pr	219	n	bl	n	n	2	cu	cig+/-ot	30	99	3	0	nev any	ot
COMSTO	4	m	0	0	all	-	all	NAmer	1975	ot	258	n	bl	n	n	0	cu	cig+/-ot	1	19	1	0	nev any	st
COMSTO	5	m	0	0	all	-	all	NAmer	1975	ot	258	n	bl	n	n	0	cu	cig+/-ot	20	39	2	0	nev any	st
COMSTO	6	m	0	0	all	-	all	NAmer	1975	ot	258	n	bl	n	n	0	cu	cig+/-ot	40	99	3	0	nev any	st
COMSTO	9	f	0	0	all	-	all	NAmer	1975	ot	258	n	bl	n	n	0	cu	cig+/-ot	1	19	1	0	nev any	st
COMSTO	10	f	0	0	all	-	all	NAmer	1975	ot	258	n	bl	n	n	0	cu	cig+/-ot	20	39	2	0	nev any	st
COMSTO	11	f	0	0	all	-	all	NAmer	1975	ot	258	n	bl	n	n	0	cu	cig+/-ot	40	99	3	0	nev any	st
COOKSO	1	c	0	0	bl	-	all	Africa	1961	CC	234	n	V	n	y	0	ev	cig+/-ot	1	9	1	1	nev any	st
COOKSO	2	c	0	0	bl	-	all	Africa	1961	CC	234	n	V	n	y	0	ev	cig+/-ot	10	99	0	0	nev any	st
CORREA	46	c	0	0	all	-	all	NAmer	1979	CC	1359	n	bl	y	n	1	cu	cig+/-ot	1	20	0	0	nev cigs	or
CORREA	50	c	0	0	all	-	all	NAmer	1979	CC	1359	n	bl	y	n	1	cu	cig+/-ot	21	99	3	0	nev cigs	or
CPSI	243	m	50	74	all	6	all	NAmer	1959	pr	5138	n	bl	n	n	1	ev	cig only	1	19	1	0	nev any	ot
CPSI	246	m	50	74	all	6	all	NAmer	1959	pr	5138	n	bl	n	n	1	ev	cig only	20	99	0	0	nev any	ot
CPSI	275	f	40	74	all	6	all	NAmer	1959	pr	5138	n	bl	n	n	1	cu	cig+/-ot	1	9	1	1	nev cigs	ot
CPSI	276	f	40	74	all	6	all	NAmer	1959	pr	5138	n	bl	n	n	1	cu	cig+/-ot	10	19	0	2	nev cigs	ot
CPSI	277	f	40	74	all	6	all	NAmer	1959	pr	5138	n	bl	n	n	1	cu	cig+/-ot	20	39	2	0	nev cigs	ot
CPSI	278	f	40	74	all	6	all	NAmer	1959	pr	5138	n	bl	n	n	1	cu	cig+/-ot	40	99	3	0	nev cigs	ot
CPSII	102	m	35	99	all	4	all	NAmer	1982	pr	3229	n	bl	n	n	1	ev	cig only	1	20	0	0	nev any	ot
CPSII	103	m	35	99	all	4	all	NAmer	1982	pr	3229	n	bl	n	n	1	ev	cig only	21	99	3	0	nev any	ot
CPSII	105	f	35	99	all	4	all	NAmer	1982	pr	3229	n	bl	n	n	1	ev	cig+/-ot	1	19	1	0	nev cigs	ot
CPSII	106	f	35	99	all	4	all	NAmer	1982	pr	3229	n	bl	n	n	1	ev	cig+/-ot	20	99	0	0	nev cigs	ot
DAMBER	6	m	0	0	all	-	all	Eu:Sca	1972	CC	579	n	bl	y	n	1	ev	cig only	1	7	1	1	nev any	ot
DAMBER	7	m	0	0	all	-	all	Eu:Sca	1972	CC	579	n	bl	y	n	1	ev	cig only	8	15	0	2	nev any	ot
DAMBER	8	m	0	0	all	-	all	Eu:Sca	1972	CC	579	n	bl	y	n	1	ev	cig only	16	25	2	3	nev any	ot
DAMBER	9	m	0	0	all	-	all	Eu:Sca	1972	CC	579	n	bl	y	n	1	ev	cig only	26	99	3	0	nev any	ot
DARBY	1	m	0	0	wh	-	all	Eu:UK	1988	CC	982	n	V	n	n	0	cu	cig+/-ot	1	14	1	0	nev any	st
DARBY	2	m	0	0	wh	-	all	Eu:UK	1988	CC	982	n	V	n	n	0	cu	cig+/-ot	15	24	2	3	nev any	st
DARBY	3	m	0	0	wh	-	all	Eu:UK	1988	CC	982	n	V	n	n	0	cu	cig+/-ot	25	99	3	0	nev any	st
DARBY	8	f	0	0	wh	-	all	Eu:UK	1988	CC	982	n	V	n	n	0	cu	cig+/-ot	1	14	1	0	nev any	st
DARBY	9	f	0	0	wh	-	all	Eu:UK	1988	CC	982	n	V	n	n	0	cu	cig+/-ot	15	24	2	3	nev any	st
DARBY	10	f	0	0	wh	-	all	Eu:UK	1988	CC	982	n	V	n	n	0	cu	cig+/-ot	25	99	3	0	nev any	st
DAVEYS	1	m	0	0	all	-	all	Eu:Ger	1930	CC	109	n	bl	y	n	0	ev	all/unsp	1	5	1	1	nev any	st
DAVEYS	2	m	0	0	all	-	all	Eu:Ger	1930	CC	109	n	bl	y	n	0	ev	all/unsp	6	10	0	2	nev any	st
DAVEYS	3	m	0	0	all	-	all	Eu:Ger	1930	CC	109	n	bl	y	n	0	ev	all/unsp	11	20	2	3	nev any	st
DAVEYS	4	m	0	0	all	-	all	Eu:Ger	1930	CC	109	n	bl	y	n	0	ev	all/unsp	21	99	3	0	nev any	st
DEAN	1	m	0	0	wh	-	all	Africa	1947	CC	603	n	V	y	n	0	ev	cig only	1	20	0	0	nev any	st
DEAN	2	m	0	0	wh	-	all	Africa	1947	CC	603	n	V	y	n	0	ev	cig only	25	45	3	0	nev any	st
DEAN	3	m	0	0	wh	-	all	Africa	1947	CC	603	n	V	y	n	0	ev	cig only	50	99	0	6	nev any	st
DEAN2	25	m	0	0	all	-	all	Eu:UK	1960	CC	954	n	V	y	n	0	ev	cig only	1	22	0	0	nev any	st
DEAN2	26	m	0	0	all	-	all	Eu:UK	1960	CC	954	n	V	y	n	0	ev	cig only	23	99	3	0	nev any	st
DEAN2	29	f	0	0	all	-	all	Eu:UK	1960	CC	954	n	V	y	n	0	ev	cig only	1	22	0	0	nev any	st
DEAN2	30	f	0	0	all	-	all	Eu:UK	1960	CC	954	n	V	y	n	0	ev	cig only	23	99	3	0	nev any	st
DEAN3	7	m	0	0	all	-	all	Eu:UK	1969	CC	766	n	V	y	n	3	cu	cig only	1	12	1	0	nev any	ot
DEAN3	14	m	0	0	all	-	all	Eu:UK	1969	CC	766	n	V	y	n	3	cu	cig only	13	22	2	3	nev any	ot
DEAN3	21	m	0	0	all	-	all	Eu:UK	1969	CC	766	n	V	y	n	3	cu	cig only	23	99	3	0	nev any	ot
DEAN3	91	f	0	0	all	-	all	Eu:UK	1969	CC	766	n	V	y	n	3	cu	cig only	1	12	1	0	nev any	ot
DEAN3	98	f	0	0	all	-	all	Eu:UK	1969	CC	766	n	V	y	n	3	cu	cig only	13	22	2	3	nev any	ot
DEAN3	105	f	0	0	all	-	all	Eu:UK	1969	CC	766	n	V	y	n	3	cu	cig only	23	99	3	0	nev any	ot
DEKLER	2	m	0	0	all	0	all	Auslia	1961	pr	138	m	V	n	n	2	cu	cig+/-ot	1	14	1	0	nev any	or
DEKLER	3	m	0	0	all	0	all	Auslia	1961	pr	138	m	V	n	n	2	cu	cig+/-ot	15	24	2	3	nev any	or
DEKLER	4	m	0	0	all	0	all	Auslia	1961	pr	138	m	V	n	n	2	cu	cig+/-ot	25	99	3	0	nev any	or
DESTEF	6	m	0	0	all	-	all	SCAmer	1988	CC	497	n	bl	n	y	4	ev	all/unsp	1	10	1	0	nev any	or
DESTEF	7	m	0	0	all	-	all	SCAmer	1988	CC	497	n	bl	n	y	4	ev	all/unsp	11	20	2	3	nev any	or

Table 1G11 - 1

IESLC - Meta-anal of Ever Smoking (or Curr if Ever not avail) by Amount, Overview, Any prod (or Cigs if Any not avail)
All LC types
Most adjusted

REF	NRR	SEX	AGEL	AGEH	RACE	YF	LC TYPE	LOC	START	ST	NLC	R	VB	P	H	AD	SM	PRODUCT	exL	exH	S1	S2	DENOM	De
DESTEF	8	m	0	0	all	-	all	SCAmer	1988	CC	497	n	bl	n	y	4	ev	all/unsp	21	40	0	0	nev any	or
DESTEF	9	m	0	0	all	-	all	SCAmer	1988	CC	497	n	bl	n	y	4	ev	all/unsp	41	99	3	6	nev any	or
DOLL	1	m	0	0	all	-	all	Eu:UK	1948	CC	1465	n	V	n	n	0	ev	all/unsp	1	4	0	1	nev any	st
DOLL	2	m	0	0	all	-	all	Eu:UK	1948	CC	1465	n	V	n	n	0	ev	all/unsp	5	14	1	2	nev any	st
DOLL	3	m	0	0	all	-	all	Eu:UK	1948	CC	1465	n	V	n	n	0	ev	all/unsp	15	24	2	3	nev any	st
DOLL	4	m	0	0	all	-	all	Eu:UK	1948	CC	1465	n	V	n	n	0	ev	all/unsp	25	49	3	0	nev any	st
DOLL	5	m	0	0	all	-	all	Eu:UK	1948	CC	1465	n	V	n	n	0	ev	all/unsp	50	99	0	6	nev any	st
DOLL	7	f	0	0	all	-	all	Eu:UK	1948	CC	1465	n	V	n	n	0	ev	all/unsp	1	4	0	1	nev any	st
DOLL	8	f	0	0	all	-	all	Eu:UK	1948	CC	1465	n	V	n	n	0	ev	all/unsp	5	14	1	2	nev any	st
DOLL	9	f	0	0	all	-	all	Eu:UK	1948	CC	1465	n	V	n	n	0	ev	all/unsp	15	24	2	3	nev any	st
DOLL	10	f	0	0	all	-	all	Eu:UK	1948	CC	1465	n	V	n	n	0	ev	all/unsp	25	49	3	0	nev any	ot
DOLL2	46	m	35	99	all	5	all	Eu:UK	1951	pr	920	n	V	n	n	1	ev	all/unsp	1	14	1	0	nev any	ot
DOLL2	47	m	35	99	all	5	all	Eu:UK	1951	pr	920	n	V	n	n	1	ev	all/unsp	15	24	2	3	nev any	ot
DOLL2	48	m	35	99	all	5	all	Eu:UK	1951	pr	920	n	V	n	n	1	ev	all/unsp	25	99	3	0	nev any	ot
DOLL2	10	f	0	0	all	22	all	Eu:UK	1951	pr	920	n	V	n	n	1	cu	cig only	1	14	1	0	nev any	ot
DOLL2	11	f	0	0	all	22	all	Eu:UK	1951	pr	920	n	V	n	n	1	cu	cig only	15	24	2	3	nev any	ot
DOLL2	12	f	0	0	all	22	all	Eu:UK	1951	pr	920	n	V	n	n	1	cu	cig only	25	99	3	0	nev any	ot
DORANT	6	c	0	0	all	0	all	Eu:wst	1986	ot	550	n	bl	n	y	0	cu	cig+/-ot	1	9	1	1	nev any	st
DORANT	7	c	0	0	all	0	all	Eu:wst	1986	ot	550	n	bl	n	y	0	cu	cig+/-ot	10	19	0	2	nev any	st
DORANT	8	c	0	0	all	0	all	Eu:wst	1986	ot	550	n	bl	n	y	0	cu	cig+/-ot	20	99	0	0	nev any	st
DORGAN	108	m	0	0	wh	-	all	NAmer	1980	CC	2026	n	bl	y	y	2	ev	cig+/-ot	1	19	1	0	nev any	ot
DORGAN	109	m	0	0	wh	-	all	NAmer	1980	CC	2026	n	bl	y	y	2	ev	cig+/-ot	20	99	0	0	nev any	ot
DORGAN	96	f	0	0	all	-	all	NAmer	1980	CC	2026	n	bl	y	y	3	ev	cig+/-ot	1	19	1	0	nev any	ot
DORGAN	97	f	0	0	all	-	all	NAmer	1980	CC	2026	n	bl	y	y	3	ev	cig+/-ot	20	99	0	0	nev any	ot
DORN	408	m	0	0	wh	25	all	NAmer	1954	pr	5097	n	bl	n	n	1	cu	cig+/-ot	1	9	1	1	nev any	or
DORN	409	m	0	0	wh	25	all	NAmer	1954	pr	5097	n	bl	n	n	1	cu	cig+/-ot	10	20	2	0	nev any	or
DORN	410	m	0	0	wh	25	all	NAmer	1954	pr	5097	n	bl	n	n	1	cu	cig+/-ot	21	39	0	4	nev any	or
DORN	411	m	0	0	wh	25	all	NAmer	1954	pr	5097	n	bl	n	n	1	cu	cig+/-ot	40	99	3	0	nev any	or
DOSEME	5	m	0	0	all	-	all	Eu:bal	1979	CC	1210	n	bl	n	n	2	ev	cig+/-ot	1	10	1	0	nev cigs	or
DOSEME	9	m	0	0	all	-	all	Eu:bal	1979	CC	1210	n	bl	n	n	2	ev	cig+/-ot	11	20	2	3	nev cigs	or
DOSEME	13	m	0	0	all	-	all	Eu:bal	1979	CC	1210	n	bl	n	n	2	ev	cig+/-ot	21	99	3	0	nev cigs	or
DUNN	1	m	0	0	all	0	all	NAmer	1954	pr	139	o	bl	n	n	0	ev	cig+/-ot	1	4	0	1	nev cigs	st
DUNN	2	m	0	0	all	0	all	NAmer	1954	pr	139	o	bl	n	n	0	ev	cig+/-ot	5	14	1	2	nev cigs	st
DUNN	3	m	0	0	all	0	all	NAmer	1954	pr	139	o	bl	n	n	0	ev	cig+/-ot	15	24	2	3	nev cigs	st
DUNN	4	m	0	0	all	0	all	NAmer	1954	pr	139	o	bl	n	n	0	ev	cig+/-ot	25	34	0	4	nev cigs	st
DUNN	5	m	0	0	all	0	all	NAmer	1954	pr	139	o	bl	n	n	0	ev	cig+/-ot	35	99	3	0	nev cigs	st
EBELIN	2	m	0	0	all	-	all	Eu:Ger	1980	CC	130	n	bl	n	n	0	ev	all/unsp	1	9	1	1	nev any	st
EBELIN	3	m	0	0	all	-	all	Eu:Ger	1980	CC	130	n	bl	n	n	0	ev	all/unsp	10	19	0	2	nev any	st
EBELIN	4	m	0	0	all	-	all	Eu:Ger	1980	CC	130	n	bl	n	n	0	ev	all/unsp	20	29	2	3	nev any	st
EBELIN	5	m	0	0	all	-	all	Eu:Ger	1980	CC	130	n	bl	n	n	0	ev	all/unsp	30	39	0	4	nev any	st
EBELIN	6	m	0	0	all	-	all	Eu:Ger	1980	CC	130	n	bl	n	n	0	ev	all/unsp	40	99	3	0	nev any	st
ENGELA	31	m	0	0	all	0	all	Eu:Sca	1964	pr	435	n	bl	n	n	7	cu	cig+/-ot	1	4	0	1	nev cigs	or
ENGELA	32	m	0	0	all	0	all	Eu:Sca	1964	pr	435	n	bl	n	n	7	cu	cig+/-ot	5	9	1	0	nev cigs	or
ENGELA	33	m	0	0	all	0	all	Eu:Sca	1964	pr	435	n	bl	n	n	7	cu	cig+/-ot	10	14	0	2	nev cigs	or
ENGELA	34	m	0	0	all	0	all	Eu:Sca	1964	pr	435	n	bl	n	n	7	cu	cig+/-ot	15	19	0	0	nev cigs	or
ENGELA	35	m	0	0	all	0	all	Eu:Sca	1964	pr	435	n	bl	n	n	7	cu	cig+/-ot	20	99	0	0	nev cigs	or
ENGELA	45	f	0	0	all	0	all	Eu:Sca	1964	pr	435	n	bl	n	n	5	cu	cig+/-ot	1	4	0	1	nev cigs	or
ENGELA	46	f	0	0	all	0	all	Eu:Sca	1964	pr	435	n	bl	n	n	5	cu	cig+/-ot	5	9	1	0	nev cigs	or
ENGELA	47	f	0	0	all	0	all	Eu:Sca	1964	pr	435	n	bl	n	n	5	cu	cig+/-ot	10	14	0	2	nev cigs	or
ENGELA	48	f	0	0	all	0	all	Eu:Sca	1964	pr	435	n	bl	n	n	5	cu	cig+/-ot	15	99	0	0	nev cigs	or
ENSTRO	7	m	0	0	all	0	all	NAmer	1959	pr	2879	n	bl	n	n	1	cu	cig only	1	9	1	1	nev any	ot
ENSTRO	6	m	0	0	all	0	all	NAmer	1959	pr	2879	n	bl	n	n	1	cu	cig only	10	19	0	2	nev any	ot
ENSTRO	5	m	0	0	all	0	all	NAmer	1959	pr	2879	n	bl	n	n	1	cu	cig only	20	20	2	3	nev any	ot
ENSTRO	4	m	0	0	all	0	all	NAmer	1959	pr	2879	n	bl	n	n	1	cu	cig only	21	39	0	4	nev any	ot
ENSTRO	3	m	0	0	all	0	all	NAmer	1959	pr	2879	n	bl	n	n	1	cu	cig only	40	99	3	0	nev any	ot
ENSTRO	11	f	0	0	all	0	all	NAmer	1959	pr	2879	n	bl	n	n	1	cu	cig only	1	9	1	1	nev any	ot
ENSTRO	10	f	0	0	all	0	all	NAmer	1959	pr	2879	n	bl	n	n	1	cu	cig only	10	19	0	2	nev any	ot
ENSTRO	9	f	0	0	all	0	all	NAmer	1959	pr	2879	n	bl	n	n	1	cu	cig only	20	20	2	3	nev any	ot
ENSTRO	8	f	0	0	all	0	all	NAmer	1959	pr	2879	n	bl	n	n	1	cu	cig only	21	99	3	0	nev any	ot
ESAKI	1	m	0	0	all	-	all	As:Jap	1961	CC	245	n	bl	y	n	0	ev	cig+/-ot	1	14	1	0	nev cigs	st
ESAKI	2	m	0	0	all	-	all	As:Jap	1961	CC	245	n	bl	y	n	0	ev	cig+/-ot	15	29	2	3	nev cigs	st
ESAKI	3	m	0	0	all	-	all	As:Jap	1961	CC	245	n	bl	y	n	0	ev	cig+/-ot	30	99	3	0	nev cigs	st
FAN	6	m	0	0	all	-	all	As:Chi	1990	CC	403	n	ot	y	n	0	ev	cig+/-ot	1	9	1	1	nev cigs	st
FAN	7	m	0	0	all	-	all	As:Chi	1990	CC	403	n	ot	y	n	0	ev	cig+/-ot	10	19	0	2	nev cigs	st
FAN	8	m	0	0	all	-	all	As:Chi	1990	CC	403	n	ot	y	n	0	ev	cig+/-ot	20	29	2	3	nev cigs	st
FAN	9	m	0	0	all	-	all	As:Chi	1990	CC	403	n	ot	y	n	0	ev	cig+/-ot	30	99	3	0	nev cigs	st
FAN	10	f	0	0	all	-	all	As:Chi	1990	CC	403	n	ot	y	n	0	ev	cig+/-ot	1	9	1	1	nev cigs	st
FAN	11	f	0	0	all	-	all	As:Chi	1990	CC	403	n	ot	y	n	0	ev	cig+/-ot	10	19	0	2	nev cigs	st
FAN	12	f	0	0	all	-	all	As:Chi	1990	CC	403	n	ot	y	n	0	ev	cig+/-ot	20	29	2	3	nev cigs	st
FAN	13	f	0	0	all	-	all	As:Chi	1990	CC	403	n	ot	y	n	0	ev	cig+/-ot	30	99	3	0	nev cigs	st
GAO	21	f	0	0	all	-	all	As:Chi	1984	CC	1405	n	ot	n	n	2	ev	cig+/-ot	1	9	1	1	nev cigs	ot
GAO	22	f	0	0	all	-	all	As:Chi	1984	CC	1405	n	ot	n	n	2	ev	cig+/-ot	10	19	0	2	nev cigs	ot

Table 1G11 - 1

IESLC - Meta-anal of Ever Smoking (or Curr if Ever not avail) by Amount, Overview, Any prod (or Cigs if Any not avail)
All LC types
Most adjusted

REF	NRR	SEX	AGEL	AGEH	RACE	YF	LC TYPE	LOC	START	ST	NLC	R	VB	P	H	AD	SM	PRODUCT	exL	exH	S1	S2	DENOM	De
GAO	23	f	0	0	all	-	all	As:Chi	1984	CC	1405	n	ot	n	n	2	ev	cig+/-ot	20	99	0	0	nev cigs	ot
GAO2	2	m	0	0	all	-	all	As:Jap	1988	CC	282	n	bl	n	n	0	cu	cig+/-ot	1	19	1	0	nev cigs	st
GAO2	3	m	0	0	all	-	all	As:Jap	1988	CC	282	n	bl	n	n	0	cu	cig+/-ot	20	29	2	3	nev cigs	st
GAO2	4	m	0	0	all	-	all	As:Jap	1988	CC	282	n	bl	n	n	0	cu	cig+/-ot	30	99	3	0	nev cigs	or
GARSHI	26	m	0	0	all	-	all	NAmer	1981	CC	1081	o	bl	y	n	1	ev	all/unsp	1	15	1	0	nev any	st
GARSHI	27	m	0	0	all	-	all	NAmer	1981	CC	1081	o	bl	y	n	1	ev	all/unsp	16	25	2	3	nev any	st
GARSHI	28	m	0	0	all	-	all	NAmer	1981	CC	1081	o	bl	y	n	1	ev	all/unsp	26	35	0	4	nev any	st
GARSHI	29	m	0	0	all	-	all	NAmer	1981	CC	1081	o	bl	y	n	1	ev	all/unsp	36	99	3	0	nev any	st
GER	22	c	0	0	all	-	all	As:oth	1990	CC	141	n	ot	y	n	14	ev	all/unsp	1	10	1	0	nev any	ot
GER	23	c	0	0	all	-	all	As:oth	1990	CC	141	n	ot	y	n	14	ev	all/unsp	11	20	2	3	nev any	ot
GER	24	c	0	0	all	-	all	As:oth	1990	CC	141	n	ot	y	n	14	ev	all/unsp	21	99	3	0	nev any	ot
GOLLED	1	m	35	99	all	-	all	Eu:UK	1952	CC	443	n	V	y	n	1	ev	cig only	1	10	1	0	nev any	ot
GOLLED	2	m	35	99	all	-	all	Eu:UK	1952	CC	443	n	V	y	n	1	ev	cig only	11	22	2	3	nev any	ot
GOLLED	3	m	35	99	all	-	all	Eu:UK	1952	CC	443	n	V	y	n	1	ev	cig only	23	99	3	0	nev any	ot
GSELL	1	m	0	0	all	-	all	Eu:wst	1937	CC	150	n	bl	n	y	0	ev	all/unsp	1	9	1	1	nev any	st
GSELL	2	m	0	0	all	-	all	Eu:wst	1937	CC	150	n	bl	n	y	0	ev	all/unsp	10	14	0	2	nev any	st
GSELL	3	m	0	0	all	-	all	Eu:wst	1937	CC	150	n	bl	n	y	0	ev	all/unsp	15	20	2	3	nev any	st
GSELL	4	m	0	0	all	-	all	Eu:wst	1937	CC	150	n	bl	n	y	0	ev	all/unsp	21	35	0	4	nev any	st
GSELL	5	m	0	0	all	-	all	Eu:wst	1937	CC	150	n	bl	n	y	0	ev	all/unsp	36	99	3	0	nev any	st
HAENSZ	52	f	0	0	all	-	not alv	NAmer	1955	CC	158	n	bl	n	y	0	cu	cig+/-ot	1	20	0	0	nev any	st
HAENSZ	51	f	0	0	all	-	not alv	NAmer	1955	CC	158	n	bl	n	y	0	cu	cig+/-ot	21	99	3	0	nev any	st
HAMMO2	7	m	0	0	all	0	all	NAmer	1967	pr	450	o	bl	n	n	1	cu	cig+/-ot	1	19	1	0	nev any	ot
HAMMO2	6	m	0	0	all	0	all	NAmer	1967	pr	450	o	bl	n	n	1	cu	cig+/-ot	20	99	0	0	nev any	ot
HAMMON	153	m	0	0	wh	0	all	NAmer	1952	pr	448	n	bl	n	n	1	ev	cig only	1	9	1	1	nev any	ot
HAMMON	154	m	0	0	wh	0	all	NAmer	1952	pr	448	n	bl	n	n	1	ev	cig only	10	20	2	0	nev any	ot
HAMMON	155	m	0	0	wh	0	all	NAmer	1952	pr	448	n	bl	n	n	1	ev	cig only	21	99	3	0	nev any	ot
HANSEN	1	m	0	0	all	0	all	Eu:Sca	1968	pr	105	o	bl	y	n	2	ev	all/unsp	1	19	1	0	nev any	ot
HANSEN	2	m	0	0	all	0	all	Eu:Sca	1968	pr	105	o	bl	y	n	2	ev	all/unsp	20	99	0	0	nev any	ot
HIRAYA	23	m	0	0	all	0	all	As:Jap	1965	pr	1917	n	bl	n	n	1	cu	cig+/-ot	1	9	1	1	nev any	st
HIRAYA	24	m	0	0	all	0	all	As:Jap	1965	pr	1917	n	bl	n	n	1	cu	cig+/-ot	10	19	0	2	nev any	st
HIRAYA	25	m	0	0	all	0	all	As:Jap	1965	pr	1917	n	bl	n	n	1	cu	cig+/-ot	20	99	0	0	nev any	st
HIRAYA	26	f	0	0	all	0	all	As:Jap	1965	pr	1917	n	bl	n	n	1	cu	cig+/-ot	1	9	1	1	nev any	st
HIRAYA	27	f	0	0	all	0	all	As:Jap	1965	pr	1917	n	bl	n	n	1	cu	cig+/-ot	10	19	0	2	nev any	st
HIRAYA	28	f	0	0	all	0	all	As:Jap	1965	pr	1917	n	bl	n	n	1	cu	cig+/-ot	20	99	0	0	nev any	st
HITOSU	35	m	0	0	all	-	all	As:Jap	1960	CC	216	n	bl	y	n	1	cu	all/unsp	1	14	1	0	nev any	st
HITOSU	36	m	0	0	all	-	all	As:Jap	1960	CC	216	n	bl	y	n	1	cu	all/unsp	15	24	2	3	nev any	st
HITOSU	37	m	0	0	all	-	all	As:Jap	1960	CC	216	n	bl	y	n	1	cu	all/unsp	25	99	3	0	nev any	st
HITOSU	60	f	0	0	all	-	all	As:Jap	1960	CC	216	n	bl	y	n	1	cu	all/unsp	1	14	1	0	nev any	st
HITOSU	61	f	0	0	all	-	all	As:Jap	1960	CC	216	n	bl	y	n	1	cu	all/unsp	15	99	0	0	nev any	st
HOLE	1	m	0	0	all	0	all	Eu:UK	1972	pr	225	n	V	n	n	1	cu	cig+/-ot	1	14	1	0	nev any	ot
HOLE	3	m	0	0	all	0	all	Eu:UK	1972	pr	225	n	V	n	n	1	cu	cig+/-ot	15	24	2	3	nev any	ot
HOLE	4	m	0	0	all	0	all	Eu:UK	1972	pr	225	n	V	n	n	1	cu	cig+/-ot	25	34	0	4	nev any	ot
HOLE	5	m	0	0	all	0	all	Eu:UK	1972	pr	225	n	V	n	n	1	cu	cig+/-ot	35	99	3	0	nev any	ot
HU	1	m	0	0	all	-	all	As:Chi	1985	CC	227	n	ot	n	y	0	ev	cig+/-ot	1	14	1	0	nev any	st
HU	2	m	0	0	all	-	all	As:Chi	1985	CC	227	n	ot	n	y	0	ev	cig+/-ot	14	24	2	3	nev any	st
HU	3	m	0	0	all	-	all	As:Chi	1985	CC	227	n	ot	n	y	0	ev	cig+/-ot	25	99	3	0	nev any	st
HU	4	f	0	0	all	-	all	As:Chi	1985	CC	227	n	ot	n	y	0	ev	cig+/-ot	1	14	1	0	nev any	st
HU	5	f	0	0	all	-	all	As:Chi	1985	CC	227	n	ot	n	y	0	ev	cig+/-ot	14	24	2	3	nev any	st
HU	6	f	0	0	all	-	all	As:Chi	1985	CC	227	n	ot	n	y	0	ev	cig+/-ot	25	99	3	0	nev any	st
HU2	2	c	0	0	all	-	all	As:Chi	1977	CC	523	n	ot	y	n	0	ev	cig+/-ot	1	4	0	1	nev cigs	st
HU2	3	c	0	0	all	-	all	As:Chi	1977	CC	523	n	ot	y	n	0	ev	cig+/-ot	5	9	1	0	nev cigs	st
HU2	4	c	0	0	all	-	all	As:Chi	1977	CC	523	n	ot	y	n	0	ev	cig+/-ot	10	14	0	2	nev cigs	st
HU2	5	c	0	0	all	-	all	As:Chi	1977	CC	523	n	ot	y	n	0	ev	cig+/-ot	15	19	0	0	nev cigs	or
HU2	6	c	0	0	all	-	all	As:Chi	1977	CC	523	n	ot	y	n	0	ev	cig+/-ot	20	29	2	3	nev cigs	st
HU2	7	c	0	0	all	-	all	As:Chi	1977	CC	523	n	ot	y	n	0	ev	cig+/-ot	30	99	3	0	nev cigs	st
HUMBLE	2	m	0	0	w-hi	-	all	NAmer	1980	CC	521	n	bl	y	n	1	cu	cig+/-ot	1	19	1	0	nev cigs	or
HUMBLE	3	m	0	0	w-hi	-	all	NAmer	1980	CC	521	n	bl	y	n	1	cu	cig+/-ot	20	99	0	0	nev cigs	or
HUMBLE	5	m	0	0	hi	-	all	NAmer	1980	CC	521	n	bl	y	n	1	cu	cig+/-ot	1	19	1	0	nev cigs	or
HUMBLE	6	m	0	0	hi	-	all	NAmer	1980	CC	521	n	bl	y	n	1	cu	cig+/-ot	20	99	0	0	nev cigs	or
HUMBLE	8	f	0	0	w-hi	-	all	NAmer	1980	CC	521	n	bl	y	n	1	cu	cig+/-ot	1	19	1	0	nev cigs	or
HUMBLE	9	f	0	0	w-hi	-	all	NAmer	1980	CC	521	n	bl	y	n	1	cu	cig+/-ot	20	99	0	0	nev cigs	or
HUMBLE	11	f	0	0	hi	-	all	NAmer	1980	CC	521	n	bl	y	n	1	cu	cig+/-ot	1	19	1	0	nev cigs	or
HUMBLE	12	f	0	0	hi	-	all	NAmer	1980	CC	521	n	bl	y	n	1	cu	cig+/-ot	20	99	0	0	nev cigs	or
JARUP	4	m	0	0	all	-	all	Eu:Sca	1928	CC	102	o	bl	y	n	2	ev	all/unsp	1	10	1	0	nev any	or
JARUP	5	m	0	0	all	-	all	Eu:Sca	1928	CC	102	o	bl	y	n	2	ev	all/unsp	11	99	0	0	nev any	or
JEDRYC	45	m	0	0	all	-	all	Eu:est	1980	CC	1630	n	bl	y	n	4	ev	cig+/-ot	1	19	1	0	nev any	or
JEDRYC	46	m	0	0	all	-	all	Eu:est	1980	CC	1630	n	bl	y	n	4	ev	cig+/-ot	20	29	2	3	nev any	or
JEDRYC	47	m	0	0	all	-	all	Eu:est	1980	CC	1630	n	bl	y	n	4	ev	cig+/-ot	30	99	3	0	nev any	or
JEDRYC	48	f	0	0	all	-	all	Eu:est	1980	CC	1630	n	bl	y	n	4	ev	cig+/-ot	1	19	1	0	nev any	or
JEDRYC	49	f	0	0	all	-	all	Eu:est	1980	CC	1630	n	bl	y	n	4	ev	cig+/-ot	20	29	2	3	nev any	or
JEDRYC	50	f	0	0	all	-	all	Eu:est	1980	CC	1630	n	bl	y	n	4	ev	cig+/-ot	30	99	3	0	nev any	or
JOLY	7	m	0	0	all	-	all	SCAmer	1978	CC	826	n	bl	n	n	0	ev	cig+/-ot	1	9	1	1	nev any	st

Table 1G11 - 1

IESLC - Meta-anal of Ever Smoking (or Curr if Ever not avail) by Amount, Overview, Any prod (or Cigs if Any not avail)
All LC types
Most adjusted

REF	NRR	SEX	AGEL	AGEH	RACE	YF	LC TYPE	LOC	START	ST	NLC	R	VB	P	H	AD	SM	PRODUCT	exL	exH	S1	S2	DENOM	De
JOLY	8	m	0	0	all	-	all	SCAmer	1978	CC	826	n	bl	n	n	0	ev	cig+/-ot	10	19	0	2	nev any	st
JOLY	9	m	0	0	all	-	all	SCAmer	1978	CC	826	n	bl	n	n	0	ev	cig+/-ot	20	29	2	3	nev any	st
JOLY	10	m	0	0	all	-	all	SCAmer	1978	CC	826	n	bl	n	n	0	ev	cig+/-ot	30	99	3	0	nev any	st
JOLY	3	f	0	0	all	-	all	SCAmer	1978	CC	826	n	bl	n	n	0	ev	cig+/-ot	1	9	1	1	nev any	st
JOLY	4	f	0	0	all	-	all	SCAmer	1978	CC	826	n	bl	n	n	0	ev	cig+/-ot	10	19	0	2	nev any	st
JOLY	5	f	0	0	all	-	all	SCAmer	1978	CC	826	n	bl	n	n	0	ev	cig+/-ot	20	29	2	3	nev any	st
JOLY	6	f	0	0	all	-	all	SCAmer	1978	CC	826	n	bl	n	n	0	ev	cig+/-ot	30	99	3	0	nev any	st
JUSSAW	34	m	0	0	all	-	all	As:Ind	1964	CC	792	n	V	n	n	2	ev	cig only	1	19	1	0	nev any	st
JUSSAW	35	m	0	0	all	-	all	As:Ind	1964	CC	792	n	V	n	n	2	ev	cig only	20	99	0	0	nev any	st
KAISE2	66	m	35	99	all	9	all	NAmer	1979	pr	318	n	bl	n	n	1	cu	cig only	1	19	1	0	nev any	st
KAISE2	67	m	35	99	all	9	all	NAmer	1979	pr	318	n	bl	n	n	1	cu	cig only	20	99	0	0	nev any	st
KAISE2	58	f	35	99	all	9	all	NAmer	1979	pr	318	n	bl	n	n	1	cu	cig only	1	19	1	0	nev any	st
KAISE2	59	f	35	99	all	9	all	NAmer	1979	pr	318	n	bl	n	n	1	cu	cig only	20	99	0	0	nev any	st
KAISER	6	m	0	0	all	0	all	NAmer	1964	pr	714	n	bl	n	n	2	cu	cig+/-ot	1	19	1	0	nev cigs	or
KAISER	7	m	0	0	all	0	all	NAmer	1964	pr	714	n	bl	n	n	2	cu	cig+/-ot	20	40	2	0	nev cigs	or
KAISER	8	m	0	0	all	0	all	NAmer	1964	pr	714	n	bl	n	n	2	cu	cig+/-ot	41	99	3	6	nev cigs	or
KAISER	2	f	0	0	all	0	all	NAmer	1964	pr	714	n	bl	n	n	2	cu	cig+/-ot	1	19	1	0	nev cigs	or
KAISER	3	f	0	0	all	0	all	NAmer	1964	pr	714	n	bl	n	n	2	cu	cig+/-ot	20	40	2	0	nev cigs	or
KAISER	4	f	0	0	all	0	all	NAmer	1964	pr	714	n	bl	n	n	2	cu	cig+/-ot	41	99	3	6	nev cigs	or
KANELL	26	m	0	0	all	-	all	Eu:bal	1950	CC	862	n	bl	n	n	1	cu	cig+/-ot	1	10	1	0	nev any	st
KANELL	27	m	0	0	all	-	all	Eu:bal	1950	CC	862	n	bl	n	n	1	cu	cig+/-ot	11	20	2	3	nev any	st
KANELL	28	m	0	0	all	-	all	Eu:bal	1950	CC	862	n	bl	n	n	1	cu	cig+/-ot	21	35	0	4	nev any	st
KANELL	29	m	0	0	all	-	all	Eu:bal	1950	CC	862	n	bl	n	n	1	cu	cig+/-ot	36	99	3	0	nev any	st
KATSOU	3	f	0	0	all	-	all	Eu:bal	1987	CC	101	n	bl	n	n	1	cu	all/unsp	1	20	0	0	nev any	or
KATSOU	4	f	0	0	all	-	all	Eu:bal	1987	CC	101	n	bl	n	n	1	cu	all/unsp	21	99	3	0	nev any	or
KAUFMA	11	c	0	0	all	-	all	NAmer	1981	CC	881	n	bl	n	n	6	cu	cig+/-ot	1	14	1	0	nev cigs	or
KAUFMA	12	c	0	0	all	-	all	NAmer	1981	CC	881	n	bl	n	n	6	cu	cig+/-ot	15	24	2	3	nev cigs	or
KAUFMA	13	c	0	0	all	-	all	NAmer	1981	CC	881	n	bl	n	n	6	cu	cig+/-ot	25	34	0	4	nev cigs	or
KAUFMA	14	c	0	0	all	-	all	NAmer	1981	CC	881	n	bl	n	n	6	cu	cig+/-ot	35	44	0	5	nev cigs	or
KAUFMA	15	c	0	0	all	-	all	NAmer	1981	CC	881	n	bl	n	n	6	cu	cig+/-ot	45	99	3	6	nev cigs	or
KHUDER	1	m	0	0	all	-	all	NAmer	1985	CC	482	n	bl	n	y	0	ev	cig+/-ot	1	19	1	0	nev cigs	st
KHUDER	2	m	0	0	all	-	all	NAmer	1985	CC	482	n	bl	n	y	0	ev	cig+/-ot	20	39	2	0	nev cigs	st
KHUDER	3	m	0	0	all	-	all	NAmer	1985	CC	482	n	bl	n	y	0	ev	cig+/-ot	40	99	3	0	nev cigs	st
KINLEN	14	m	0	0	all	0	all	Eu:UK	1967	pr	718	n	V	n	n	2	cu	cig+/-ot	1	14	1	0	nev any	ot
KINLEN	15	m	0	0	all	0	all	Eu:UK	1967	pr	718	n	V	n	n	2	cu	cig+/-ot	15	24	2	3	nev any	ot
KINLEN	16	m	0	0	all	0	all	Eu:UK	1967	pr	718	n	V	n	n	2	cu	cig+/-ot	25	99	3	0	nev any	ot
KNEKT	29	m	20	69	all	21	all	Eu:Sca	1966	pr	515	n	bl	n	n	1	cu	cig+/-ot	1	14	1	0	nev any	or
KNEKT	30	m	20	69	all	21	all	Eu:Sca	1966	pr	515	n	bl	n	n	1	cu	cig+/-ot	15	99	0	0	nev any	or
KOO	11	f	0	0	all	-	all	As:HK	1981	CC	200	n	bl	n	n	0	cu	all/unsp	1	10	1	0	nev any	st
KOO	12	f	0	0	all	-	all	As:HK	1981	CC	200	n	bl	n	n	0	cu	all/unsp	11	20	2	3	nev any	st
KOO	13	f	0	0	all	-	all	As:HK	1981	CC	200	n	bl	n	n	0	cu	all/unsp	21	30	0	4	nev any	st
KOULUM	6	m	0	0	all	-	all	Eu:Sca	1936	CC	812	n	bl	n	n	0	ev	all/unsp	1	9	1	1	nev any	st
KOULUM	5	m	0	0	all	-	all	Eu:Sca	1936	CC	812	n	bl	n	n	0	ev	all/unsp	10	19	0	2	nev any	st
KOULUM	4	m	0	0	all	-	all	Eu:Sca	1936	CC	812	n	bl	n	n	0	ev	all/unsp	20	99	0	0	nev any	st
KREUZE	19	m	1	45	all	-	all	Eu:Ger	1990	CC	2260	n	bl	n	n	3	ev	cig+/-ot	1	9	1	1	nev any	or
KREUZE	20	m	1	45	all	-	all	Eu:Ger	1990	CC	2260	n	bl	n	n	3	ev	cig+/-ot	10	19	0	2	nev any	or
KREUZE	21	m	1	45	all	-	all	Eu:Ger	1990	CC	2260	n	bl	n	n	3	ev	cig+/-ot	20	29	2	3	nev any	or
KREUZE	22	m	1	45	all	-	all	Eu:Ger	1990	CC	2260	n	bl	n	n	3	ev	cig+/-ot	30	99	3	0	nev any	or
KREUZE	30	m	55	69	all	-	all	Eu:Ger	1990	CC	2260	n	bl	n	n	3	ev	cig+/-ot	1	9	1	1	nev any	or
KREUZE	31	m	55	69	all	-	all	Eu:Ger	1990	CC	2260	n	bl	n	n	3	ev	cig+/-ot	10	19	0	2	nev any	or
KREUZE	32	m	55	69	all	-	all	Eu:Ger	1990	CC	2260	n	bl	n	n	3	ev	cig+/-ot	20	29	2	3	nev any	or
KREUZE	33	m	55	69	all	-	all	Eu:Ger	1990	CC	2260	n	bl	n	n	3	ev	cig+/-ot	30	99	3	0	nev any	or
KREUZE	25	f	1	45	all	-	all	Eu:Ger	1990	CC	2260	n	bl	n	n	3	ev	cig+/-ot	1	9	1	1	nev any	or
KREUZE	26	f	1	45	all	-	all	Eu:Ger	1990	CC	2260	n	bl	n	n	3	ev	cig+/-ot	10	19	0	2	nev any	or
KREUZE	27	f	1	45	all	-	all	Eu:Ger	1990	CC	2260	n	bl	n	n	3	ev	cig+/-ot	20	29	2	3	nev any	or
KREUZE	36	f	55	69	all	-	all	Eu:Ger	1990	CC	2260	n	bl	n	n	3	ev	cig+/-ot	1	9	1	1	nev any	or
KREUZE	37	f	55	69	all	-	all	Eu:Ger	1990	CC	2260	n	bl	n	n	3	ev	cig+/-ot	10	19	0	2	nev any	or
KREUZE	38	f	55	69	all	-	all	Eu:Ger	1990	CC	2260	n	bl	n	n	3	ev	cig+/-ot	20	29	2	3	nev any	or
KREYBE	9	m	0	0	all	-	all	Eu:Sca	1948	CC	300	n	bl	n	y	1	ev	all/unsp	1	14	1	0	nev any	ot
KREYBE	10	m	0	0	all	-	all	Eu:Sca	1948	CC	300	n	bl	n	y	1	ev	all/unsp	15	24	2	3	nev any	ot
KREYBE	11	m	0	0	all	-	all	Eu:Sca	1948	CC	300	n	bl	n	y	1	ev	all/unsp	25	99	3	0	nev any	ot
KREYBE	28	f	0	0	all	-	all	Eu:Sca	1948	CC	300	n	bl	n	y	1	ev	all/unsp	1	14	1	0	nev any	ot
KREYBE	29	f	0	0	all	-	all	Eu:Sca	1948	CC	300	n	bl	n	y	1	ev	all/unsp	15	99	0	0	nev any	ot
LAMTH	7	f	0	0	ch	-	all	As:HK	1983	CC	445	n	bl	n	n	0	ev	all/unsp	1	10	1	0	nev any	or
LAMTH	2	f	0	0	ch	-	all	As:HK	1983	CC	445	n	bl	n	n	0	ev	all/unsp	11	20	2	3	nev any	or
LAMTH	9	f	0	0	ch	-	all	As:HK	1983	CC	445	n	bl	n	n	0	ev	all/unsp	21	99	3	0	nev any	or
LAUSSM	18	m	0	0	all	-	all	Eu:Ger	1982	CC	432	n	bl	n	n	3	ev	all/unsp	1	9	1	1	nev any	ot
LAUSSM	19	m	0	0	all	-	all	Eu:Ger	1982	CC	432	n	bl	n	n	3	ev	all/unsp	10	19	0	2	nev any	ot
LAUSSM	20	m	0	0	all	-	all	Eu:Ger	1982	CC	432	n	bl	n	n	3	ev	all/unsp	20	99	0	0	nev any	ot
LETOUR	2	c	0	0	all	-	all	NAmer	1983	CC	738	n	V	y	y	0	ev	cig+/-ot	1	19	1	0	nev cigs	st
LETOUR	3	c	0	0	all	-	all	NAmer	1983	CC	738	n	V	y	y	0	ev	cig+/-ot	20	40	2	0	nev cigs	st
LETOUR	4	c	0	0	all	-	all	NAmer	1983	CC	738	n	V	y	y	0	ev	cig+/-ot	41	99	3	6	nev cigs	st

Table 1G11 - 1

IESLC - Meta-anal of Ever Smoking (or Curr if Ever not avail) by Amount, Overview, Any prod (or Cigs if Any not avail)
All LC types
Most adjusted

REF	NRR	SEX	AGEL	AGEH	RACE	YF	LC TYPE	LOC	START	ST	NLC	R	VB	P	H	AD	SM	PRODUCT	exL	exH	S1	S2	DENOM	De
LIAW	3	c	0	0	all	0	all	As:oth	1982	pr	127	n	ot	n	n	2	cu	all/unsp	1	10	1	0	nev any	or
LIAW	4	c	0	0	all	0	all	As:oth	1982	pr	127	n	ot	n	n	2	cu	all/unsp	11	20	2	3	nev any	or
LIAW	5	c	0	0	all	0	all	As:oth	1982	pr	127	n	ot	n	n	2	cu	all/unsp	21	99	3	0	nev any	or
LIDDEL	2	m	0	0	all	18	all	NAmer	1970	pr	304	m	V	n	n	1	cu	cig+/-ot	1	19	1	0	nev cigs	ot
LIDDEL	3	m	0	0	all	18	all	NAmer	1970	pr	304	m	V	n	n	1	cu	cig+/-ot	20	99	0	0	nev cigs	ot
LIU2	8	m	0	0	all	-	all	As:Chi	1983	CC	316	n	ot	n	n	3	ev	all/unsp	1	19	1	0	nev any	or
LIU2	9	m	0	0	all	-	all	As:Chi	1983	CC	316	n	ot	n	n	3	ev	all/unsp	20	29	2	3	nev any	or
LIU2	10	m	0	0	all	-	all	As:Chi	1983	CC	316	n	ot	n	n	3	ev	all/unsp	30	99	3	0	nev any	or
LIU2	14	f	0	0	all	-	all	As:Chi	1983	CC	316	n	ot	n	n	3	ev	all/unsp	1	9	1	1	nev any	or
LIU2	15	f	0	0	all	-	all	As:Chi	1983	CC	316	n	ot	n	n	3	ev	all/unsp	10	19	0	2	nev any	or
LIU2	16	f	0	0	all	-	all	As:Chi	1983	CC	316	n	ot	n	n	3	ev	all/unsp	20	99	0	0	nev any	or
LIU3	6	m	0	0	all	-	all	As:Chi	1985	CC	110	n	ot	n	n	2	ev	all/unsp	1	15	1	0	nev any	or
LIU3	7	m	0	0	all	-	all	As:Chi	1985	CC	110	n	ot	n	n	2	ev	all/unsp	16	30	2	0	nev any	or
LIU3	8	m	0	0	all	-	all	As:Chi	1985	CC	110	n	ot	n	n	2	ev	all/unsp	31	99	3	0	nev any	or
LIU4	7	m	35	69	all	-	all	As:Chi	1986	CC	1000-00	n	ot	y	n	2	ev	cig only	1	19	1	0	nev any	ot
LIU4	8	m	35	69	all	-	all	As:Chi	1986	CC	1000-00	n	ot	y	n	2	ev	cig only	20	20	2	3	nev any	ot
LIU4	9	m	35	69	all	-	all	As:Chi	1986	CC	1000-00	n	ot	y	n	2	ev	cig only	21	99	3	0	nev any	ot
LIU5	2	c	0	0	all	-	all	As:Chi	1978	CC	111	n	ot	y	n	0	ev	all/unsp	1	9	1	1	nev any	st
LIU5	3	c	0	0	all	-	all	As:Chi	1978	CC	111	n	ot	y	n	0	ev	all/unsp	10	19	0	2	nev any	st
LIU5	4	c	0	0	all	-	all	As:Chi	1978	CC	111	n	ot	y	n	0	ev	all/unsp	20	99	0	0	nev any	st
LUBIN	11	m	0	0	all	-	all	As:Chi	1984	CC	427	m	ot	y	n	4	ev	cig only	1	6	1	1	nev any	ot
LUBIN	12	m	0	0	all	-	all	As:Chi	1984	CC	427	m	ot	y	n	4	ev	cig only	7	14	0	2	nev any	ot
LUBIN	13	m	0	0	all	-	all	As:Chi	1984	CC	427	m	ot	y	n	4	ev	cig only	15	19	0	0	nev any	ot
LUBIN	14	m	0	0	all	-	all	As:Chi	1984	CC	427	m	ot	y	n	4	ev	cig only	20	99	0	0	nev any	ot
LUBIN2	273	m	0	0	all	-	all	Eu:mul	1976	CC	7804	n	bl	n	y	0	ev	cig+/-ot	1	9	1	1	nev any	st
LUBIN2	274	m	0	0	all	-	all	Eu:mul	1976	CC	7804	n	bl	n	y	0	ev	cig+/-ot	10	19	0	2	nev any	st
LUBIN2	275	m	0	0	all	-	all	Eu:mul	1976	CC	7804	n	bl	n	y	0	ev	cig+/-ot	20	29	2	3	nev any	st
LUBIN2	276	m	0	0	all	-	all	Eu:mul	1976	CC	7804	n	bl	n	y	0	ev	cig+/-ot	30	99	3	0	nev any	st
LUBIN2	281	f	0	0	all	-	all	Eu:mul	1976	CC	7804	n	bl	n	y	0	ev	cig+/-ot	1	9	1	1	nev any	st
LUBIN2	282	f	0	0	all	-	all	Eu:mul	1976	CC	7804	n	bl	n	y	0	ev	cig+/-ot	10	19	0	2	nev any	st
LUBIN2	283	f	0	0	all	-	all	Eu:mul	1976	CC	7804	n	bl	n	y	0	ev	cig+/-ot	20	29	2	3	nev any	st
LUBIN2	284	f	0	0	all	-	all	Eu:mul	1976	CC	7804	n	bl	n	y	0	ev	cig+/-ot	30	99	3	0	nev any	st
MACLEN	36	c	0	0	ch	-	all	As:oth	1972	CC	233	n	bl	n	n	2	ev	cig+/-ot	1	9	1	1	nev cigs	or
MACLEN	37	c	0	0	ch	-	all	As:oth	1972	CC	233	n	bl	n	n	2	ev	cig+/-ot	10	19	0	2	nev cigs	or
MACLEN	38	c	0	0	ch	-	all	As:oth	1972	CC	233	n	bl	n	n	2	ev	cig+/-ot	20	29	2	3	nev cigs	or
MACLEN	39	c	0	0	ch	-	all	As:oth	1972	CC	233	n	bl	n	n	2	ev	cig+/-ot	30	99	3	0	nev cigs	or
MARTIS	1	m	0	0	all	-	all	Eu:UK	1972	CC	201	n	V	n	n	0	ev	cig+/-ot	1	14	1	0	nev cigs	st
MARTIS	2	m	0	0	all	-	all	Eu:UK	1972	CC	201	n	V	n	n	0	ev	cig+/-ot	15	24	2	3	nev cigs	st
MARTIS	3	m	0	0	all	-	all	Eu:UK	1972	CC	201	n	V	n	n	0	ev	cig+/-ot	25	99	3	0	nev cigs	st
MATOS	29	m	0	0	all	-	all	SCAmer	1994	CC	200	n	bl	n	n	2	ev	cig+/-ot	1	14	1	0	nev any	or
MATOS	31	m	0	0	all	-	all	SCAmer	1994	CC	200	n	bl	n	n	2	ev	cig+/-ot	15	24	2	3	nev any	or
MATOS	33	m	0	0	all	-	all	SCAmer	1994	CC	200	n	bl	n	n	2	ev	cig+/-ot	25	99	3	0	nev any	or
MATSUD	1	m	0	0	all	-	all	As:Jap	1965	CC	179	n	bl	n	n	0	ev	cig+/-ot	1	10	1	0	nev cigs	st
MATSUD	2	m	0	0	all	-	all	As:Jap	1965	CC	179	n	bl	n	n	0	ev	cig+/-ot	11	20	2	3	nev cigs	st
MATSUD	3	m	0	0	all	-	all	As:Jap	1965	CC	179	n	bl	n	n	0	ev	cig+/-ot	21	99	3	0	nev cigs	st
MCCONN	26	c	0	0	all	-	all	Eu:UK	1946	CC	100	n	V	n	y	0	ev	all/unsp	1	10	1	0	nev any	st
MCCONN	25	c	0	0	all	-	all	Eu:UK	1946	CC	100	n	V	n	y	0	ev	all/unsp	10	20	2	0	nev any	st
MCCONN	24	c	0	0	all	-	all	Eu:UK	1946	CC	100	n	V	n	y	0	ev	all/unsp	21	99	3	0	nev any	st
MIGRAN	2	m	0	0	all	0	all	Eu:UK	1964	pr	259	n	V	n	n	2	cu	cig only	1	9	1	1	nev any	ot
MIGRAN	4	m	0	0	all	0	all	Eu:UK	1964	pr	259	n	V	n	n	2	cu	cig only	10	19	0	2	nev any	ot
MIGRAN	6	m	0	0	all	0	all	Eu:UK	1964	pr	259	n	V	n	n	2	cu	cig only	20	20	2	3	nev any	ot
MIGRAN	8	m	0	0	all	0	all	Eu:UK	1964	pr	259	n	V	n	n	2	cu	cig only	21	99	3	0	nev any	ot
MIGRAN	29	f	0	0	all	0	all	Eu:UK	1964	pr	259	n	V	n	n	2	cu	cig only	1	9	1	1	nev any	ot
MIGRAN	31	f	0	0	all	0	all	Eu:UK	1964	pr	259	n	V	n	n	2	cu	cig only	10	19	0	2	nev any	ot
MIGRAN	33	f	0	0	all	0	all	Eu:UK	1964	pr	259	n	V	n	n	2	cu	cig only	20	20	2	3	nev any	ot
MRFITR	3	m	0	0	all	0	all	NAmer	1973	pr	119	n	bl	n	n	0	cu	cig+/-ot	1	19	1	0	nev cigs	ot
MRFITR	4	m	0	0	all	0	all	NAmer	1973	pr	119	n	bl	n	n	0	cu	cig+/-ot	20	39	2	0	nev cigs	ot
MRFITR	5	m	0	0	all	0	all	NAmer	1973	pr	119	n	bl	n	n	0	cu	cig+/-ot	40	99	3	0	nev cigs	ot
NAM	74	m	0	0	all	-	all	NAmer	1986	CC	1199	n	bl	y	n	1	cu	cig+/-ot	1	24	0	0	nev cigs	ot
NAM	75	m	0	0	all	-	all	NAmer	1986	CC	1199	n	bl	y	n	1	cu	cig+/-ot	25	99	3	0	nev cigs	ot
NAM	90	f	0	0	all	-	all	NAmer	1986	CC	1199	n	bl	y	n	1	cu	cig+/-ot	1	24	0	0	nev cigs	ot
NAM	91	f	0	0	all	-	all	NAmer	1986	CC	1199	n	bl	y	n	1	cu	cig+/-ot	25	99	3	0	nev cigs	ot
NOTAN2	8	m	0	0	all	-	all	As:Ind	1963	CC	683	n	V	n	n	0	ev	cig only	1	9	1	1	nev any	st
NOTAN2	9	m	0	0	all	-	all	As:Ind	1963	CC	683	n	V	n	n	0	ev	cig only	10	19	0	2	nev any	st
NOTAN2	10	m	0	0	all	-	all	As:Ind	1963	CC	683	n	V	n	n	0	ev	cig only	20	99	0	0	nev any	st
ORMOS	1	m	0	0	all	-	all	Eu:est	1947	CC	119	n	bl	y	y	0	ev	cig+/-ot	1	15	1	0	nev any	st
ORMOS	2	m	0	0	all	-	all	Eu:est	1947	CC	119	n	bl	y	y	0	ev	cig+/-ot	16	30	2	0	nev any	st
ORMOS	3	m	0	0	all	-	all	Eu:est	1947	CC	119	n	bl	y	y	0	ev	cig+/-ot	31	99	3	0	nev any	st
OSANN	49	m	0	0	all	-	all	NAmer	1984	CC	1986	n	bl	n	n	2	ev	cig+/-ot	1	39	0	0	nev cigs	or

Table 1G11 - 1

IESLC - Meta-anal of Ever Smoking (or Curr if Ever not avail) by Amount, Overview, Any prod (or Cigs if Any not avail)
All LC types
Most adjusted

REF	NRR	SEX	AGEL	AGEH	RACE	YF	LC TYPE	LOC	START	ST	NLC	R	VB	P	H	AD	SM	PRODUCT	exL	exH	S1	S2	DENOM	De
OSANN	57	m	0	0	all	-	all	NAmer	1984	CC	1986	n	bl	n	n	2	ev	cig+/-ot	40	99	3	0	nev cigs	or
OSANN	50	f	0	0	all	-	all	NAmer	1984	CC	1986	n	bl	n	n	2	ev	cig+/-ot	1	39	0	0	nev cigs	or
OSANN	58	f	0	0	all	-	all	NAmer	1984	CC	1986	n	bl	n	n	2	ev	cig+/-ot	40	99	3	0	nev cigs	or
PARKIN	14	m	0	0	bl	-	all	Africa	1963	CC	877	n	V	y	n	6	cu	all/unsp	1	14	1	0	nev any	or
PARKIN	15	m	0	0	bl	-	all	Africa	1963	CC	877	n	V	y	n	6	cu	all/unsp	15	99	0	0	nev any	or
PASTOR	6	m	0	0	all	-	all	Eu:wst	1976	CC	204	n	bl	y	n	1	ev	all/unsp	1	9	1	1	nev any	st
PASTOR	7	m	0	0	all	-	all	Eu:wst	1976	CC	204	n	bl	y	n	1	ev	all/unsp	10	19	0	2	nev any	st
PASTOR	8	m	0	0	all	-	all	Eu:wst	1976	CC	204	n	bl	y	n	1	ev	all/unsp	20	29	2	3	nev any	st
PASTOR	9	m	0	0	all	-	all	Eu:wst	1976	CC	204	n	bl	y	n	1	ev	all/unsp	30	99	3	0	nev any	st
PERNU	17	m	0	0	all	-	all	Eu:Sca	1944	CC	1606	n	bl	n	n	0	ev	all/unsp	1	4	0	1	nev any	st
PERNU	18	m	0	0	all	-	all	Eu:Sca	1944	CC	1606	n	bl	n	n	0	ev	all/unsp	5	9	1	0	nev any	st
PERNU	19	m	0	0	all	-	all	Eu:Sca	1944	CC	1606	n	bl	n	n	0	ev	all/unsp	10	14	0	2	nev any	st
PERNU	20	m	0	0	all	-	all	Eu:Sca	1944	CC	1606	n	bl	n	n	0	ev	all/unsp	15	19	0	0	nev any	st
PERNU	21	m	0	0	all	-	all	Eu:Sca	1944	CC	1606	n	bl	n	n	0	ev	all/unsp	20	24	2	3	nev any	st
PERNU	22	m	0	0	all	-	all	Eu:Sca	1944	CC	1606	n	bl	n	n	0	ev	all/unsp	25	29	0	0	nev any	st
PERNU	23	m	0	0	all	-	all	Eu:Sca	1944	CC	1606	n	bl	n	n	0	ev	all/unsp	30	49	3	0	nev any	st
PERNU	24	m	0	0	all	-	all	Eu:Sca	1944	CC	1606	n	bl	n	n	0	ev	all/unsp	50	99	0	6	nev any	st
PERNU	11	f	0	0	all	-	all	Eu:Sca	1944	CC	1606	n	bl	n	n	0	ev	all/unsp	1	4	0	1	nev any	st
PERNU	12	f	0	0	all	-	all	Eu:Sca	1944	CC	1606	n	bl	n	n	0	ev	all/unsp	5	9	1	0	nev any	st
PERNU	13	f	0	0	all	-	all	Eu:Sca	1944	CC	1606	n	bl	n	n	0	ev	all/unsp	10	14	0	2	nev any	st
PERNU	14	f	0	0	all	-	all	Eu:Sca	1944	CC	1606	n	bl	n	n	0	ev	all/unsp	15	19	0	0	nev any	st
PERNU	15	f	0	0	all	-	all	Eu:Sca	1944	CC	1606	n	bl	n	n	0	ev	all/unsp	20	24	2	3	nev any	st
PERNU	16	f	0	0	all	-	all	Eu:Sca	1944	CC	1606	n	bl	n	n	0	ev	all/unsp	25	99	3	0	nev any	st
PERSH2	8	c	0	0	all	-	all	Eu:Sca	1980	CC	1022	n	bl	y	n	4	cu	all/unsp	1	9	1	1	nev any	ot
PERSH2	9	c	0	0	all	-	all	Eu:Sca	1980	CC	1022	n	bl	y	n	4	cu	all/unsp	10	99	0	0	nev any	ot
PETO	2	m	0	0	all	0	all	Eu:UK	1954	pr	103	n	V	n	n	0	cu	all/unsp	1	14	1	0	nev any	st
PETO	3	m	0	0	all	0	all	Eu:UK	1954	pr	103	n	V	n	n	0	cu	all/unsp	15	99	0	0	nev any	st
PEZZO2	3	m	0	0	all	-	all	SCAmer	1992	CC	367	n	bl	n	y	0	cu	cig+/-ot	1	20	0	0	nev cigs	st
PEZZO2	4	m	0	0	all	-	all	SCAmer	1992	CC	367	n	bl	n	y	0	cu	cig+/-ot	21	40	0	0	nev cigs	st
PEZZO2	5	m	0	0	all	-	all	SCAmer	1992	CC	367	n	bl	n	y	0	cu	cig+/-ot	41	99	3	6	nev cigs	st
PEZZOT	2	m	0	0	all	-	all	SCAmer	1987	CC	215	n	bl	n	y	0	cu	cig only	1	20	0	0	nev cigs	st
PEZZOT	3	m	0	0	all	-	all	SCAmer	1987	CC	215	n	bl	n	y	0	cu	cig only	21	40	0	0	nev cigs	st
PEZZOT	4	m	0	0	all	-	all	SCAmer	1987	CC	215	n	bl	n	y	0	cu	cig only	41	99	3	6	nev cigs	st
PIKE	1	m	0	0	w-hi	-	all	NAmer	1972	CC	731	n	bl	y	n	0	ev	all/unsp	1	20	0	0	nev any	st
PIKE	2	m	0	0	w-hi	-	all	NAmer	1972	CC	731	n	bl	y	n	0	ev	all/unsp	21	40	0	0	nev any	st
PIKE	3	m	0	0	w-hi	-	all	NAmer	1972	CC	731	n	bl	y	n	0	ev	all/unsp	41	99	3	6	nev any	st
PIKE	5	f	0	0	w-hi	-	all	NAmer	1972	CC	731	n	bl	y	n	0	ev	all/unsp	1	20	0	0	nev any	st
PIKE	6	f	0	0	w-hi	-	all	NAmer	1972	CC	731	n	bl	y	n	0	ev	all/unsp	21	40	0	0	nev any	st
PIKE	7	f	0	0	w-hi	-	all	NAmer	1972	CC	731	n	bl	y	n	0	ev	all/unsp	41	99	3	6	nev any	st
POLEDN	2	c	0	0	all	-	all	NAmer	1978	CC	209	n	bl	y	n	0	ev	cig+/-ot	1	19	1	0	nev cigs	st
POLEDN	4	c	0	0	all	-	all	NAmer	1978	CC	209	n	bl	y	n	0	ev	cig+/-ot	20	99	0	0	nev cigs	st
PRESCO	2	m	0	0	all	0	all	Eu:Sca	1964	pr	867	n	bl	n	n	1	cu	all/unsp	1	14	1	0	nev any	st
PRESCO	4	m	0	0	all	0	all	Eu:Sca	1964	pr	867	n	bl	n	n	1	cu	all/unsp	15	99	0	0	nev any	st
PRESCO	1	f	0	0	all	0	all	Eu:Sca	1964	pr	867	n	bl	n	n	1	cu	all/unsp	1	14	1	0	nev any	st
PRESCO	3	f	0	0	all	0	all	Eu:Sca	1964	pr	867	n	bl	n	n	1	cu	all/unsp	15	99	0	0	nev any	st
RACHTA	10	f	0	0	all	-	all	Eu:est	1991	CC	118	n	bl	n	y	1	ev	cig+/-ot	1	9	1	1	nev cigs	or
RACHTA	11	f	0	0	all	-	all	Eu:est	1991	CC	118	n	bl	n	y	1	ev	cig+/-ot	10	19	0	2	nev cigs	or
RACHTA	12	f	0	0	all	-	all	Eu:est	1991	CC	118	n	bl	n	y	1	ev	cig+/-ot	20	99	0	0	nev cigs	or
RANDIG	1	m	0	0	all	-	all	Eu:Ger	1951	CC	448	n	bl	n	n	0	ev	all/unsp	1	4	0	1	nev any	st
RANDIG	2	m	0	0	all	-	all	Eu:Ger	1951	CC	448	n	bl	n	n	0	ev	all/unsp	5	9	1	0	nev any	st
RANDIG	3	m	0	0	all	-	all	Eu:Ger	1951	CC	448	n	bl	n	n	0	ev	all/unsp	10	19	0	2	nev any	st
RANDIG	4	m	0	0	all	-	all	Eu:Ger	1951	CC	448	n	bl	n	n	0	ev	all/unsp	20	99	0	0	nev any	st
RANDIG	5	f	0	0	all	-	all	Eu:Ger	1951	CC	448	n	bl	n	n	0	ev	all/unsp	1	4	0	1	nev any	st
RANDIG	6	f	0	0	all	-	all	Eu:Ger	1951	CC	448	n	bl	n	n	0	ev	all/unsp	5	9	1	0	nev any	st
RANDIG	7	f	0	0	all	-	all	Eu:Ger	1951	CC	448	n	bl	n	n	0	ev	all/unsp	10	99	0	0	nev any	st
SEGI2	10	m	0	0	all	-	all	As:Jap	1962	CC	378	n	bl	n	n	1	cu	cig+/-ot	1	9	1	1	nev any	ot
SEGI2	12	m	0	0	all	-	all	As:Jap	1962	CC	378	n	bl	n	n	1	cu	cig+/-ot	10	19	0	2	nev any	ot
SEGI2	14	m	0	0	all	-	all	As:Jap	1962	CC	378	n	bl	n	n	1	cu	cig+/-ot	20	29	2	3	nev any	ot
SEGI2	16	m	0	0	all	-	all	As:Jap	1962	CC	378	n	bl	n	n	1	cu	cig+/-ot	30	39	0	4	nev any	ot
SEGI2	18	m	0	0	all	-	all	As:Jap	1962	CC	378	n	bl	n	n	1	cu	cig+/-ot	40	99	3	0	nev any	ot
SEGI2	22	f	0	0	all	-	all	As:Jap	1962	CC	378	n	bl	n	n	1	cu	cig+/-ot	1	9	1	1	nev any	ot
SEGI2	24	f	0	0	all	-	all	As:Jap	1962	CC	378	n	bl	n	n	1	cu	cig+/-ot	10	19	0	2	nev any	ot
SEGI2	26	f	0	0	all	-	all	As:Jap	1962	CC	378	n	bl	n	n	1	cu	cig+/-ot	20	99	0	0	nev any	ot
SHAW	10	c	0	0	wh	-	all	NAmer	1988	CC	335	n	V	n	y	0	ev	all/unsp	1	19	1	0	nev any	st
SHAW	11	c	0	0	wh	-	all	NAmer	1988	CC	335	n	V	n	y	0	ev	all/unsp	20	99	0	0	nev any	st
SIEMIA	13	m	0	0	all	-	all	NAmer	1979	CC	857	n	V	y	y	0	ev	cig+/-ot	1	19	1	0	nev cigs	or
SIEMIA	14	m	0	0	all	-	all	NAmer	1979	CC	857	n	V	y	y	0	ev	cig+/-ot	20	39	2	0	nev cigs	or
SIEMIA	15	m	0	0	all	-	all	NAmer	1979	CC	857	n	V	y	y	0	ev	cig+/-ot	40	99	3	0	nev cigs	or
SOBUE	117	m	0	0	all	-	q+s+l+a	As:Jap	1986	CC	1376	n	bl	n	y	0	cu	cig+/-ot	1	19	1	0	nev cigs	st
SOBUE	118	m	0	0	all	-	q+s+l+a	As:Jap	1986	CC	1376	n	bl	n	y	0	cu	cig+/-ot	20	29	2	3	nev cigs	st
SOBUE	119	m	0	0	all	-	q+s+l+a	As:Jap	1986	CC	1376	n	bl	n	y	0	cu	cig+/-ot	30	99	3	0	nev cigs	st
SPEIZE	1	f	0	0	all	0	all	NAmer	1976	pr	593	n	bl	n	y	1	cu	cig+/-ot	1	4	0	1	nev cigs	ot

Table 1G11 - 1

IESLC - Meta-anal of Ever Smoking (or Curr if Ever not avail) by Amount, Overview, Any prod (or Cigs if Any not avail)
All LC types
Most adjusted

REF	NRR	SEX	AGEL	AGEH	RACE	YF	LC TYPE	LOC	START	ST	NLC	R	VB	P	H	AD	SM	PRODUCT	exL	exH	S1	S2	DENOM	De
SPEIZE	2	f	0	0	all	0	all	NAmer	1976	pr	593	n	bl	n	y	1	cu	cig+/-ot	5	14	1	2	nev cigs	ot
SPEIZE	3	f	0	0	all	0	all	NAmer	1976	pr	593	n	bl	n	y	1	cu	cig+/-ot	15	24	2	3	nev cigs	ot
SPEIZE	4	f	0	0	all	0	all	NAmer	1976	pr	593	n	bl	n	y	1	cu	cig+/-ot	25	34	0	4	nev cigs	ot
SPEIZE	5	f	0	0	all	0	all	NAmer	1976	pr	593	n	bl	n	y	1	cu	cig+/-ot	35	99	3	0	nev cigs	ot
SPITZ	5	c	0	0	b+hi	-	all	NAmer	1992	CC	177	n	bl	n	y	0	ev	cig+/-ot	1	19	1	0	nev cigs	st
SPITZ	6	c	0	0	b+hi	-	all	NAmer	1992	CC	177	n	bl	n	y	0	ev	cig+/-ot	20	99	0	0	nev cigs	st
STOCKS	41	m	0	0	all	-	all	Eu:UK	1952	CC	2932	n	V	y	n	2	ev	cig+/-ot	1	14	1	0	nev any	st
STOCKS	42	m	0	0	all	-	all	Eu:UK	1952	CC	2932	n	V	y	n	2	ev	cig+/-ot	15	21	2	3	nev any	st
STOCKS	43	m	0	0	all	-	all	Eu:UK	1952	CC	2932	n	V	y	n	2	ev	cig+/-ot	22	28	0	0	nev any	st
STOCKS	44	m	0	0	all	-	all	Eu:UK	1952	CC	2932	n	V	y	n	2	ev	cig+/-ot	29	36	0	4	nev any	st
STOCKS	45	m	0	0	all	-	all	Eu:UK	1952	CC	2932	n	V	y	n	2	ev	cig+/-ot	37	99	3	0	nev any	st
STOCKS	48	f	0	0	all	-	all	Eu:UK	1952	CC	2932	n	V	y	n	1	ev	cig+/-ot	1	14	1	0	nev any	ot
STOCKS	49	f	0	0	all	-	all	Eu:UK	1952	CC	2932	n	V	y	n	1	ev	cig+/-ot	15	99	0	0	nev any	ot
STOCKW	1	c	0	0	all	-	all	NAmer	1981	CC	22161	n	bl	n	n	0	cu	cig+/-ot	1	19	1	0	nev any	st
STOCKW	2	c	0	0	all	-	all	NAmer	1981	CC	22161	n	bl	n	n	0	cu	cig+/-ot	20	40	2	0	nev any	st
STOCKW	3	c	0	0	all	-	all	NAmer	1981	CC	22161	n	bl	n	n	0	cu	cig+/-ot	41	99	3	6	nev any	st
SVENSS	6	f	0	0	all	-	all	Eu:Sca	1983	CC	210	n	bl	n	n	1	cu	all/unsp	1	10	1	0	nev any	or
SVENSS	11	f	0	0	all	-	all	Eu:Sca	1983	CC	210	n	bl	n	n	1	cu	all/unsp	11	20	2	3	nev any	or
SVENSS	16	f	0	0	all	-	all	Eu:Sca	1983	CC	210	n	bl	n	n	1	cu	all/unsp	21	99	3	0	nev any	ot
TENKAN	10	m	0	0	all	17	all	Eu:Sca	1962	pr	242	n	bl	n	n	1	cu	all/unsp	1	14	1	0	nev any	ot
TENKAN	11	m	0	0	all	17	all	Eu:Sca	1962	pr	242	n	bl	n	n	1	cu	all/unsp	15	24	2	3	nev any	ot
TENKAN	12	m	0	0	all	17	all	Eu:Sca	1962	pr	242	n	bl	n	n	1	cu	all/unsp	25	99	3	0	nev any	ot
TIZZAN	7	m	0	0	all	-	all	Eu:wst	1959	CC	1358	n	bl	n	n	0	ev	cig only	1	9	1	1	nev any	st
TIZZAN	8	m	0	0	all	-	all	Eu:wst	1959	CC	1358	n	bl	n	n	0	ev	cig only	10	20	2	0	nev any	st
TIZZAN	9	m	0	0	all	-	all	Eu:wst	1959	CC	1358	n	bl	n	n	0	ev	cig only	21	40	0	0	nev any	st
TIZZAN	10	m	0	0	all	-	all	Eu:wst	1959	CC	1358	n	bl	n	n	0	ev	cig only	41	99	3	6	nev any	st
TIZZAN	15	f	0	0	all	-	all	Eu:wst	1959	CC	1358	n	bl	n	n	0	ev	cig only	1	9	1	1	nev any	st
TIZZAN	16	f	0	0	all	-	all	Eu:wst	1959	CC	1358	n	bl	n	n	0	ev	cig only	10	99	0	0	nev any	st
TSUGAN	29	m	0	0	all	-	q+a	As:Jap	1976	CC	134	n	bl	n	y	0	cu	all/unsp	1	15	1	0	nev any	st
TSUGAN	30	m	0	0	all	-	q+a	As:Jap	1976	CC	134	n	bl	n	y	0	cu	all/unsp	16	35	2	0	nev any	st
TSUGAN	31	m	0	0	all	-	q+a	As:Jap	1976	CC	134	n	bl	n	y	0	cu	all/unsp	36	99	3	0	nev any	st
TULINI	27	m	0	0	all	0	all	Eu:Sca	1967	pr	472	n	bl	n	n	3	cu	cig+/-ot	1	14	1	0	nev any	or
TULINI	28	m	0	0	all	0	all	Eu:Sca	1967	pr	472	n	bl	n	n	3	cu	cig+/-ot	15	24	2	3	nev any	or
TULINI	29	m	0	0	all	0	all	Eu:Sca	1967	pr	472	n	bl	n	n	3	cu	cig+/-ot	25	99	3	0	nev any	or
TULINI	32	f	0	0	all	0	all	Eu:Sca	1967	pr	472	n	bl	n	n	3	cu	cig+/-ot	1	14	1	0	nev any	or
TULINI	33	f	0	0	all	0	all	Eu:Sca	1967	pr	472	n	bl	n	n	3	cu	cig+/-ot	15	24	2	3	nev any	or
TULINI	34	f	0	0	all	0	all	Eu:Sca	1967	pr	472	n	bl	n	n	3	cu	cig+/-ot	25	99	3	0	nev any	or
TVERDA	9	m	0	0	all	0	all	Eu:Sca	1972	pr	238	n	bl	n	n	2	cu	cig only	1	9	1	1	nev cigs	ot
TVERDA	10	m	0	0	all	0	all	Eu:Sca	1972	pr	238	n	bl	n	n	2	cu	cig only	10	19	0	2	nev cigs	ot
TVERDA	11	m	0	0	all	0	all	Eu:Sca	1972	pr	238	n	bl	n	n	2	cu	cig only	20	99	0	0	nev cigs	ot
TVERDA	16	f	0	0	all	0	all	Eu:Sca	1972	pr	238	n	bl	n	n	2	cu	cig only	1	9	1	1	nev cigs	ot
TVERDA	17	f	0	0	all	0	all	Eu:Sca	1972	pr	238	n	bl	n	n	2	cu	cig only	20	99	0	0	nev cigs	ot
WAKAI	40	m	0	0	all	-	all	As:Jap	1988	CC	333	n	bl	n	y	2	cu	cig+/-ot	1	19	1	0	nev any	or
WAKAI	41	m	0	0	all	-	all	As:Jap	1988	CC	333	n	bl	n	y	2	cu	cig+/-ot	20	29	2	3	nev any	or
WAKAI	42	m	0	0	all	-	all	As:Jap	1988	CC	333	n	bl	n	y	2	cu	cig+/-ot	30	99	3	0	nev any	or
WANG2	9	c	0	0	all	-	all	As:Chi	1980	CC	103	n	ot	n	n	4	ev	cig+/-ot	1	4	0	1	nev cigs	ot
WANG2	10	c	0	0	all	-	all	As:Chi	1980	CC	103	n	ot	n	n	4	ev	cig+/-ot	5	9	1	0	nev cigs	ot
WANG2	11	c	0	0	all	-	all	As:Chi	1980	CC	103	n	ot	n	n	4	ev	cig+/-ot	10	14	0	2	nev cigs	ot
WANG2	12	c	0	0	all	-	all	As:Chi	1980	CC	103	n	ot	n	n	4	ev	cig+/-ot	15	19	0	0	nev cigs	ot
WANG2	13	c	0	0	all	-	all	As:Chi	1980	CC	103	n	ot	n	n	4	ev	cig+/-ot	20	29	2	3	nev cigs	ot
WANG2	14	c	0	0	all	-	all	As:Chi	1980	CC	103	n	ot	n	n	4	ev	cig+/-ot	30	39	0	4	nev cigs	ot
WANG2	15	c	0	0	all	-	all	As:Chi	1980	CC	103	n	ot	n	n	4	ev	cig+/-ot	40	99	3	0	nev cigs	ot
WU	43	f	0	0	wh	-	q+a	NAmer	1981	CC	220	n	bl	n	y	2	cu	all/unsp	1	20	0	0	nev any	st
WU	44	f	0	0	wh	-	q+a	NAmer	1981	CC	220	n	bl	n	y	2	cu	all/unsp	21	99	3	0	nev any	st
WUWILL	12	f	0	0	all	-	all	As:Chi	1985	CC	965	n	ot	n	n	3	ev	cig+/-ot	1	19	1	0	nev cigs	ot
WUWILL	13	f	0	0	all	-	all	As:Chi	1985	CC	965	n	ot	n	n	3	ev	cig+/-ot	20	99	0	0	nev cigs	ot
WYNDE2	17	m	0	0	all	-	all	NAmer	1962	CC	404	n	bl	n	y	0	ev	cig+/-ot	1	10	1	0	nev any	st
WYNDE2	18	m	0	0	all	-	all	NAmer	1962	CC	404	n	bl	n	y	0	ev	cig+/-ot	11	20	2	3	nev any	st
WYNDE2	19	m	0	0	all	-	all	NAmer	1962	CC	404	n	bl	n	y	0	ev	cig+/-ot	21	34	0	4	nev any	st
WYNDE2	20	m	0	0	all	-	all	NAmer	1962	CC	404	n	bl	n	y	0	ev	cig+/-ot	35	99	3	0	nev any	st
WYNDE3	44	m	0	0	all	-	all	NAmer	1966	CC	350	n	bl	n	y	0	ev	cig+/-ot	1	9	1	1	nev any	st
WYNDE3	45	m	0	0	all	-	all	NAmer	1966	CC	350	n	bl	n	y	0	ev	cig+/-ot	10	20	2	0	nev any	st
WYNDE3	46	m	0	0	all	-	all	NAmer	1966	CC	350	n	bl	n	y	0	ev	cig+/-ot	21	40	0	0	nev any	st
WYNDE3	47	m	0	0	all	-	all	NAmer	1966	CC	350	n	bl	n	y	0	ev	cig+/-ot	41	99	3	6	nev any	st
WYNDE3	79	f	0	0	all	-	all	NAmer	1966	CC	350	n	bl	n	y	0	ev	cig+/-ot	1	9	1	1	nev any	st
WYNDE3	80	f	0	0	all	-	all	NAmer	1966	CC	350	n	bl	n	y	0	ev	cig+/-ot	10	20	2	0	nev any	st
WYNDE3	81	f	0	0	all	-	all	NAmer	1966	CC	350	n	bl	n	y	0	ev	cig+/-ot	21	40	0	0	nev any	st
WYNDE3	82	f	0	0	all	-	all	NAmer	1966	CC	350	n	bl	n	y	0	ev	cig+/-ot	41	99	3	6	nev any	st
WYNDE4	43	m	0	0	all	-	all	NAmer	1948	CC	684	n	bl	y	n	0	ev	all/unsp	1	9	1	1	nev any	st
WYNDE4	44	m	0	0	all	-	all	NAmer	1948	CC	684	n	bl	y	n	0	ev	all/unsp	10	15	0	2	nev any	st
WYNDE4	45	m	0	0	all	-	all	NAmer	1948	CC	684	n	bl	y	n	0	ev	all/unsp	16	20	2	3	nev any	st
WYNDE4	46	m	0	0	all	-	all	NAmer	1948	CC	684	n	bl	y	n	0	ev	all/unsp	21	34	0	4	nev any	st

Table 1G11 - 1

IESLC - Meta-anal of Ever Smoking (or Curr if Ever not avail) by Amount, Overview, Any prod (or Cigs if Any not avail)
All LC types
Most adjusted

REF	NRR	SEX	AGEL	AGEH	RACE	YF	LC TYPE	LOC	START	ST	NLC	R	VB	P	H	AD	SM	PRODUCT	exL	exH	S1	S2	DENOM	De
WYNDE4	47	m	0	0	all	-	all	NAmer	1948	CC	684	n	bl	y	n	0	ev	all/unsp	35	99	3	0	nev any	st
WYNDE4	57	f	0	0	all	-	all	NAmer	1948	CC	684	n	bl	y	n	2	ev	all/unsp	1	9	1	1	nev any	ot
WYNDE4	58	f	0	0	all	-	all	NAmer	1948	CC	684	n	bl	y	n	2	ev	all/unsp	10	15	0	2	nev any	ot
WYNDE4	59	f	0	0	all	-	all	NAmer	1948	CC	684	n	bl	y	n	2	ev	all/unsp	16	20	2	3	nev any	ot
WYNDE4	60	f	0	0	all	-	all	NAmer	1948	CC	684	n	bl	y	n	2	ev	all/unsp	21	34	0	4	nev any	ot
WYNDE4	61	f	0	0	all	-	all	NAmer	1948	CC	684	n	bl	y	n	2	ev	all/unsp	35	99	3	0	nev any	ot
WYNDE6	27	m	0	0	all	-	all	NAmer	1969	CC	4423	n	bl	n	y	0	cu	cig+/-ot	1	10	1	0	nev any	st
WYNDE6	36	m	0	0	all	-	all	NAmer	1969	CC	4423	n	bl	n	y	0	cu	cig+/-ot	11	20	2	3	nev any	st
WYNDE6	45	m	0	0	all	-	all	NAmer	1969	CC	4423	n	bl	n	y	0	cu	cig+/-ot	21	30	0	4	nev any	st
WYNDE6	54	m	0	0	all	-	all	NAmer	1969	CC	4423	n	bl	n	y	0	cu	cig+/-ot	31	99	3	0	nev any	st
WYNDE6	216	f	0	0	all	-	all	NAmer	1969	CC	4423	n	bl	n	y	0	cu	cig+/-ot	1	10	1	0	nev cigs	st
WYNDE6	225	f	0	0	all	-	all	NAmer	1969	CC	4423	n	bl	n	y	0	cu	cig+/-ot	11	20	2	3	nev cigs	st
WYNDE6	234	f	0	0	all	-	all	NAmer	1969	CC	4423	n	bl	n	y	0	cu	cig+/-ot	21	30	0	4	nev cigs	st
WYNDE6	243	f	0	0	all	-	all	NAmer	1969	CC	4423	n	bl	n	y	0	cu	cig+/-ot	30	99	3	0	nev cigs	st
XU3	9	m	0	0	all	-	all	As:Chi	1981	CC	135	n	ot	n	n	1	ev	all/unsp	1	9	1	1	nev any	ot
XU3	10	m	0	0	all	-	all	As:Chi	1981	CC	135	n	ot	n	n	1	ev	all/unsp	10	19	0	2	nev any	ot
XU3	11	m	0	0	all	-	all	As:Chi	1981	CC	135	n	ot	n	n	1	ev	all/unsp	20	29	2	3	nev any	ot
XU3	12	m	0	0	all	-	all	As:Chi	1981	CC	135	n	ot	n	n	1	ev	all/unsp	30	99	3	0	nev any	ot
XU3	16	f	0	0	all	-	all	As:Chi	1981	CC	135	n	ot	n	n	1	ev	all/unsp	1	9	1	1	nev any	ot
XU3	17	f	0	0	all	-	all	As:Chi	1981	CC	135	n	ot	n	n	1	ev	all/unsp	10	19	0	2	nev any	ot
XU3	18	f	0	0	all	-	all	As:Chi	1981	CC	135	n	ot	n	n	1	ev	all/unsp	20	99	0	0	nev any	ot
YAMAGU	8	c	0	0	all	-	all	As:Jap	1989	CC	144	n	bl	n	y	1	cu	all/unsp	1	20	0	0	nev any	or
YAMAGU	7	c	0	0	all	-	all	As:Jap	1989	CC	144	n	bl	n	y	1	cu	all/unsp	21	99	3	0	nev any	or
YUAN	2	m	0	0	all	0	all	As:Chi	1986	pr	142	n	ot	n	n	2	ev	cig+/-ot	1	19	1	0	nev cigs	ot
YUAN	3	m	0	0	all	0	all	As:Chi	1986	pr	142	n	ot	n	n	2	ev	cig+/-ot	20	99	0	0	nev cigs	ot
ZHENG	11	m	0	0	all	-	all	As:Chi	1982	CC	540	n	ot	*	y	0	ev	cig+/-ot	1	9	1	1	nev cigs	st
ZHENG	12	m	0	0	all	-	all	As:Chi	1982	CC	540	n	ot	*	y	0	ev	cig+/-ot	10	19	0	2	nev cigs	st
ZHENG	13	m	0	0	all	-	all	As:Chi	1982	CC	540	n	ot	*	y	0	ev	cig+/-ot	20	29	2	3	nev cigs	st
ZHENG	14	m	0	0	all	-	all	As:Chi	1982	CC	540	n	ot	*	y	0	ev	cig+/-ot	30	99	3	0	nev cigs	st
ZHENG	22	f	0	0	all	-	all	As:Chi	1982	CC	540	n	ot	*	y	0	ev	cig+/-ot	1	9	1	1	nev cigs	st
ZHENG	23	f	0	0	all	-	all	As:Chi	1982	CC	540	n	ot	*	y	0	ev	cig+/-ot	10	99	0	0	nev cigs	st
ZHOU	4	c	0	0	all	-	all	As:Chi	1978	CC	1360	n	ot	n	n	0	ev	all/unsp	1	9	1	1	nev any	st
ZHOU	5	c	0	0	all	-	all	As:Chi	1978	CC	1360	n	ot	n	n	0	ev	all/unsp	10	19	0	2	nev any	st
ZHOU	6	c	0	0	all	-	all	As:Chi	1978	CC	1360	n	ot	n	n	0	ev	all/unsp	20	99	0	0	nev any	st

Cigarette type is all/unspec for all RRs
except for the following:

REF	NRR	CIGTYPE
ALDERS	18	MC only
ALDERS	19	MC only
ALDERS	20	MC only
ALDERS	21	MC only
ALDERS	22	MC only
ALDERS	23	MC only
DEAN3	7	MC only
DEAN3	14	MC only
DEAN3	21	MC only
DEAN3	91	MC only
DEAN3	98	MC only
DEAN3	105	MC only
JUSSAW	34	MC only
JUSSAW	35	MC only
NOTAN2	8	MC only
NOTAN2	9	MC only
NOTAN2	10	MC only

REF	NRR	Cigarette equivalent
AGUDO	4	-
AGUDO	5	-
AKIBA	27	*
AKIBA	28	*
AKIBA	29	*
AKIBA	33	*
AKIBA	34	*
ALDERS	18	-
ALDERS	19	-
ALDERS	20	-
ALDERS	21	-

Table 1G11 - 1

IESLC - Meta-anal of Ever Smoking (or Curr if Ever not avail) by Amount, Overview, Any prod (or Cigs if Any not avail)
All LC types
Most adjusted

REF	NRR	Cigarette equivalent
ALDERS	22	-
ALDERS	23	-
ARCHER	1	*
ARCHER	2	*
ARCHER	3	*
ARMADA	46	*
ARMADA	47	*
ARMADA	48	*
AUVINE	13	*
AUVINE	14	*
AUVINE	15	*
AXELSS	5	includes 1 g pipe tob = 1 cig
AXELSS	6	includes 1 g pipe tob = 1 cig
AXELSS	7	includes 1 g pipe tob = 1 cig
AXELSS	13	includes 1 g pipe tob = 1 cig
AXELSS	14	includes 1 g pipe tob = 1 cig
AXELSS	15	includes 1 g pipe tob = 1 cig
AXELSS	16	includes 1 g pipe tob = 1 cig
BARBON	82	*
BARBON	83	*
BARBON	84	*
BENSHL	11	*
BENSHL	12	*
BENSHL	13	*
BEST	13	-
BEST	14	-
BEST	15	-
BOUCOT	99	up to 1 pk cigs, 4 cigars or 10 pipes
BOUCOT	100	> 1 pk cigs, 4 cigars or 10 pipes
BRESLO	13	*
BRESLO	14	*
BRESLO	15	*
BRESLO	16	*
BRESLO	29	*
BRESLO	30	*
BRETT	1	*
BRETT	2	*
BRETT	3	*
BROSS	18	*
BROSS	19	*
BROWN2	32	*
BROWN2	42	*
BROWN2	31	*
BROWN2	41	*
BUFFLE	28	*
BUFFLE	29	*
BUFFLE	35	*
CEDERL	80	gms inc cig = 1, sm cgr = 3, lge cgr = 5
CEDERL	81	gms inc cig = 1, sm cgr = 3, lge cgr = 5
CEDERL	82	gms inc cig = 1, sm cgr = 3, lge cgr = 5
CEDERL	76	inc 1 g other tob = 1 cig
CEDERL	77	inc 1 g other tob = 1 cig
CEDERL	78	inc 1 g other tob = 1 cig
CHANG	2	*
CHANG	3	*
CHANG	4	*
CHANG	8	*
CHANG	9	*
CHANG	10	*
CHATZI	1	*
CHATZI	2	*
CHATZI	3	*
CHEN2	3	*
CHEN2	4	*
CHEN2	5	*
CHEN2	6	*
CHEN2	7	*
CHEN2	8	*
CHEN2	9	*
CHEN2	10	*
CHOI	12	*
CHOI	13	*

Table 1G11 - 1

IESLC - Meta-anal of Ever Smoking (or Curr if Ever not avail) by Amount, Overview, Any prod (or Cigs if Any not avail)
All LC types
Most adjusted

REF	NRR	Cigarette equivalent
CHOI	14	*
CHOI	15	*
CHOI	16	*
CHOI	17	*
CHOI	18	*
CHOI	20	*
CHOW	10	*
CHOW	11	*
CHOW	12	*
COMSTO	4	*
COMSTO	5	*
COMSTO	6	*
COMSTO	9	*
COMSTO	10	*
COMSTO	11	*
COOKSO	1	*
COOKSO	2	*
CORREA	46	*
CORREA	50	*
CPSI	243	-
CPSI	246	-
CPSI	275	*
CPSI	276	*
CPSI	277	*
CPSI	278	*
CPSII	102	-
CPSII	103	-
CPSII	105	*
CPSII	106	*
DAMBER	6	-
DAMBER	7	-
DAMBER	8	-
DAMBER	9	-
DARBY	1	inc 1oz pipe/wk=2 cigs/d, excl cigar/llo
DARBY	2	inc 1oz pipe/wk=2 cigs/d, excl cigar/llo
DARBY	3	inc 1oz pipe/wk=2 cigs/d, excl cigar/llo
DARBY	8	inc 1oz pipe/wk=2 cigs/d, excl cigar/llo
DARBY	9	inc 1oz pipe/wk=2 cigs/d, excl cigar/llo
DARBY	10	inc 1oz pipe/wk=2 cigs/d, excl cigar/llo
DAVEYS	1	1 cigar or up to 5 cigs
DAVEYS	2	2 cigars or 6-10 cigs
DAVEYS	3	3-4 cigars or 11-20 cigs
DAVEYS	4	>4 cigars or >20 cigarettes
DEAN	1	-
DEAN	2	-
DEAN	3	-
DEAN2	25	-
DEAN2	26	-
DEAN2	29	-
DEAN2	30	-
DEAN3	7	-
DEAN3	14	-
DEAN3	21	-
DEAN3	91	-
DEAN3	98	-
DEAN3	105	-
DEKLER	2	*
DEKLER	3	*
DEKLER	4	*
DESTEF	6	*
DESTEF	7	*
DESTEF	8	*
DESTEF	9	*
DOLL	1	*
DOLL	2	*
DOLL	3	*
DOLL	4	*
DOLL	5	*
DOLL	7	*
DOLL	8	*
DOLL	9	*
DOLL	10	*

Table 1G11 - 1

IESLC - Meta-anal of Ever Smoking (or Curr if Ever not avail) by Amount, Overview, Any prod (or Cigs if Any not avail)
All LC types
Most adjusted

REF	NRR	Cigarette equivalent
DOLL2	46	grams
DOLL2	47	grams
DOLL2	48	grams
DOLL2	10	-
DOLL2	11	-
DOLL2	12	-
DORANT	6	*
DORANT	7	*
DORANT	8	*
DORGAN	108	*
DORGAN	109	*
DORGAN	96	*
DORGAN	97	*
DORN	408	*
DORN	409	*
DORN	410	*
DORN	411	*
DOSEME	5	*
DOSEME	9	*
DOSEME	13	*
DUNN	1	*
DUNN	2	*
DUNN	3	*
DUNN	4	*
DUNN	5	*
EBELIN	2	*
EBELIN	3	*
EBELIN	4	*
EBELIN	5	*
EBELIN	6	*
ENGELA	31	*
ENGELA	32	*
ENGELA	33	*
ENGELA	34	*
ENGELA	35	*
ENGELA	45	*
ENGELA	46	*
ENGELA	47	*
ENGELA	48	*
ENSTRO	7	-
ENSTRO	6	-
ENSTRO	5	-
ENSTRO	4	-
ENSTRO	3	-
ENSTRO	11	-
ENSTRO	10	-
ENSTRO	9	-
ENSTRO	8	-
ESAKI	1	*
ESAKI	2	*
ESAKI	3	*
FAN	6	*
FAN	7	*
FAN	8	*
FAN	9	*
FAN	10	*
FAN	11	*
FAN	12	*
FAN	13	*
GAO	21	*
GAO	22	*
GAO	23	*
GAO2	2	*
GAO2	3	*
GAO2	4	*
GARSHI	26	*
GARSHI	27	*
GARSHI	28	*
GARSHI	29	*
GER	22	*
GER	23	*
GER	24	*

Table 1G11 - 1

IESLC - Meta-anal of Ever Smoking (or Curr if Ever not avail) by Amount, Overview, Any prod (or Cigs if Any not avail)
All LC types
Most adjusted

REF	NRR	Cigarette equivalent
GOLLED	1	-
GOLLED	2	-
GOLLED	3	-
GSELL	1	inc cigar = 5, cheroot = 4, pipe = 2.5
GSELL	2	inc cigar = 5, cheroot = 4, pipe = 2.5
GSELL	3	inc cigar = 5, cheroot = 4, pipe = 2.5
GSELL	4	inc cigar = 5, cheroot = 4, pipe = 2.5
GSELL	5	inc cigar = 5, cheroot = 4, pipe = 2.5
HAENSZ	52	*
HAENSZ	51	*
HAMMO2	7	*
HAMMO2	6	*
HAMMON	153	-
HAMMON	154	-
HAMMON	155	-
HANSEN	1	cig equivalents (not defined)
HANSEN	2	cig equivalents (not defined)
HIRAYA	23	*
HIRAYA	24	*
HIRAYA	25	*
HIRAYA	26	*
HIRAYA	27	*
HIRAYA	28	*
HITOSU	35	*
HITOSU	36	*
HITOSU	37	*
HITOSU	60	*
HITOSU	61	*
HOLE	1	*
HOLE	3	*
HOLE	4	*
HOLE	5	*
HU	1	*
HU	2	*
HU	3	*
HU	4	*
HU	5	*
HU	6	*
HU2	2	*
HU2	3	*
HU2	4	*
HU2	5	*
HU2	6	*
HU2	7	*
HUMBLE	2	*
HUMBLE	3	*
HUMBLE	5	*
HUMBLE	6	*
HUMBLE	8	*
HUMBLE	9	*
HUMBLE	11	*
HUMBLE	12	*
JARUP	4	gms, inc 1 pk pipe/wk = 7/d, 1 cigar = 4
JARUP	5	gms, inc 1 pk pipe/wk = 7/d, 1 cigar = 4
JEDRYC	45	*
JEDRYC	46	*
JEDRYC	47	*
JEDRYC	48	*
JEDRYC	49	*
JEDRYC	50	*
JOLY	7	*
JOLY	8	*
JOLY	9	*
JOLY	10	*
JOLY	3	*
JOLY	4	*
JOLY	5	*
JOLY	6	*
JUSSAW	34	-
JUSSAW	35	-
KAISE2	66	-
KAISE2	67	-

Table 1G11 - 1

IESLC - Meta-anal of Ever Smoking (or Curr if Ever not avail) by Amount, Overview, Any prod (or Cigs if Any not avail)
All LC types
Most adjusted

REF	NRR	Cigarette equivalent
KAISE2	58	-
KAISE2	59	-
KAISER	6	*
KAISER	7	*
KAISER	8	*
KAISER	2	*
KAISER	3	*
KAISER	4	*
KANELL	26	*
KANELL	27	*
KANELL	28	*
KANELL	29	*
KATSOU	3	*
KATSOU	4	*
KAUFMA	11	*
KAUFMA	12	*
KAUFMA	13	*
KAUFMA	14	*
KAUFMA	15	*
KHUDER	1	*
KHUDER	2	*
KHUDER	3	*
KINLEN	14	*
KINLEN	15	*
KINLEN	16	*
KNEKT	29	*
KNEKT	30	*
KOO	11	*
KOO	12	*
KOO	13	*
KOULUM	6	*
KOULUM	5	*
KOULUM	4	*
KREUZE	19	*
KREUZE	20	*
KREUZE	21	*
KREUZE	22	*
KREUZE	30	*
KREUZE	31	*
KREUZE	32	*
KREUZE	33	*
KREUZE	25	*
KREUZE	26	*
KREUZE	27	*
KREUZE	36	*
KREUZE	37	*
KREUZE	38	*
KREYBE	9	grams inc 1 cig=1
KREYBE	10	grams inc 1 cig=1
KREYBE	11	grams inc 1 cig=1
KREYBE	28	grams inc 1 cig=1
KREYBE	29	grams inc 1 cig=1
LAMTH	7	*
LAMTH	2	*
LAMTH	9	*
LAUSSM	18	inc cigars and pipes in grams
LAUSSM	19	inc cigars and pipes in grams
LAUSSM	20	inc cigars and pipes in grams
LETOUR	2	*
LETOUR	3	*
LETOUR	4	*
LIAW	3	*
LIAW	4	*
LIAW	5	*
LIDDEL	2	*
LIDDEL	3	*
LIU2	8	*
LIU2	9	*
LIU2	10	*
LIU2	14	*
LIU2	15	*
LIU2	16	*

Table 1G11 - 1

IESLC - Meta-anal of Ever Smoking (or Curr if Ever not avail) by Amount, Overview, Any prod (or Cigs if Any not avail)
All LC types
Most adjusted

REF	NRR	Cigarette equivalent
LIU3	6	Converted from kg/month
LIU3	7	Converted from kg/month
LIU3	8	Converted from kg/month
LIU4	7	-
LIU4	8	-
LIU4	9	-
LIU5	2	*
LIU5	3	*
LIU5	4	*
LUBIN	11	-
LUBIN	12	-
LUBIN	13	-
LUBIN	14	-
LUBIN2	273	*
LUBIN2	274	*
LUBIN2	275	*
LUBIN2	276	*
LUBIN2	281	*
LUBIN2	282	*
LUBIN2	283	*
LUBIN2	284	*
MACLEN	36	*
MACLEN	37	*
MACLEN	38	*
MACLEN	39	*
MARTIS	1	*
MARTIS	2	*
MARTIS	3	*
MATOS	29	*
MATOS	31	*
MATOS	33	*
MATSUD	1	*
MATSUD	2	*
MATSUD	3	*
MCCONN	26	N cigs exc mixed pipe, or <2oz pure pipe
MCCONN	25	N cigs exc mixed pipe, or 2-4oz pure pip
MCCONN	24	N cigs exc mixed pipe, or >4oz pure pipe
MIGRAN	2	-
MIGRAN	4	-
MIGRAN	6	-
MIGRAN	8	-
MIGRAN	29	-
MIGRAN	31	-
MIGRAN	33	-
MRFITR	3	*
MRFITR	4	*
MRFITR	5	*
NAM	74	*
NAM	75	*
NAM	90	*
NAM	91	*
NOTAN2	8	-
NOTAN2	9	-
NOTAN2	10	-
ORMOS	1	*
ORMOS	2	*
ORMOS	3	*
OSANN	49	*
OSANN	57	*
OSANN	50	*
OSANN	58	*
PARKIN	14	grams inc 1 cig=1g, 1 pipe=0.65g
PARKIN	15	grams inc 1 cig=1g, 1 pipe=0.65g
PASTOR	6	*
PASTOR	7	*
PASTOR	8	*
PASTOR	9	*
PERNU	17	grams
PERNU	18	grams
PERNU	19	grams
PERNU	20	grams
PERNU	21	grams

Table 1G11 - 1

IESLC - Meta-anal of Ever Smoking (or Curr if Ever not avail) by Amount, Overview, Any prod (or Cigs if Any not avail)
All LC types
Most adjusted

REF	NRR	Cigarette equivalent
PERNU	22	grams
PERNU	23	grams
PERNU	24	grams
PERNU	11	grams
PERNU	12	grams
PERNU	13	grams
PERNU	14	grams
PERNU	15	grams
PERNU	16	grams
PERSH2	8	inc 50g pipe/wk= 7 cigs/day
PERSH2	9	inc 50g pipe/wk= 7 cigs/day
PETO	2	*
PETO	3	*
PEZZO2	3	*
PEZZO2	4	*
PEZZO2	5	*
PEZZOT	2	-
PEZZOT	3	-
PEZZOT	4	-
PIKE	1	*
PIKE	2	*
PIKE	3	*
PIKE	5	*
PIKE	6	*
PIKE	7	*
POLEDN	2	*
POLEDN	4	*
PRESCO	2	gms, inc cig = 1, cheroot = 3, cigar = 5
PRESCO	4	gms, inc cig = 1, cheroot = 3, cigar = 5
PRESCO	1	gms, inc cig = 1, cheroot = 3, cigar = 5
PRESCO	3	gms, inc cig = 1, cheroot = 3, cigar = 5
RACHTA	10	*
RACHTA	11	*
RACHTA	12	*
RANDIG	1	inc 1g pip=1, cgr=5, chrt=4, cigarillo=3
RANDIG	2	inc 1g pip=1, cgr=5, chrt=4, cigarillo=3
RANDIG	3	inc 1g pip=1, cgr=5, chrt=4, cigarillo=3
RANDIG	4	inc 1g pip=1, cgr=5, chrt=4, cigarillo=3
RANDIG	5	inc 1g pip=1, cgr=5, chrt=4, cigarillo=3
RANDIG	6	inc 1g pip=1, cgr=5, chrt=4, cigarillo=3
RANDIG	7	inc 1g pip=1, cgr=5, chrt=4, cigarillo=3
SEGI2	10	*
SEGI2	12	*
SEGI2	14	*
SEGI2	16	*
SEGI2	18	*
SEGI2	22	*
SEGI2	24	*
SEGI2	26	*
SHAW	10	*
SHAW	11	*
SIEMIA	13	*
SIEMIA	14	*
SIEMIA	15	*
SOBUE	117	*
SOBUE	118	*
SOBUE	119	*
SPEIZE	1	*
SPEIZE	2	*
SPEIZE	3	*
SPEIZE	4	*
SPEIZE	5	*
SPITZ	5	*
SPITZ	6	*
STOCKS	41	*
STOCKS	42	*
STOCKS	43	*
STOCKS	44	*
STOCKS	45	*
STOCKS	48	*
STOCKS	49	*
STOCKW	1	*

Table 1G11 - 1

IESLC - Meta-anal of Ever Smoking (or Curr if Ever not avail) by Amount, Overview, Any prod (or Cigs if Any not avail)
All LC types
Most adjusted

REF	NRR	Cigarette equivalent
STOCKW	2	*
STOCKW	3	*
SVENSS	6	*
SVENSS	11	*
SVENSS	16	*
TENKAN	10	grams
TENKAN	11	grams
TENKAN	12	grams
TIZZAN	7	-
TIZZAN	8	-
TIZZAN	9	-
TIZZAN	10	-
TIZZAN	15	-
TIZZAN	16	-
TSUGAN	29	*
TSUGAN	30	*
TSUGAN	31	*
TULINI	27	*
TULINI	28	*
TULINI	29	*
TULINI	32	*
TULINI	33	*
TULINI	34	*
TVERDA	9	-
TVERDA	10	-
TVERDA	11	-
TVERDA	16	-
TVERDA	17	-
WAKAI	40	*
WAKAI	41	*
WAKAI	42	*
WANG2	9	*
WANG2	10	*
WANG2	11	*
WANG2	12	*
WANG2	13	*
WANG2	14	*
WANG2	15	*
WU	43	*
WU	44	*
WUWILL	12	*
WUWILL	13	*
WYNDE2	17	*
WYNDE2	18	*
WYNDE2	19	*
WYNDE2	20	*
WYNDE3	44	*
WYNDE3	45	*
WYNDE3	46	*
WYNDE3	47	*
WYNDE3	79	*
WYNDE3	80	*
WYNDE3	81	*
WYNDE3	82	*
WYNDE4	43	inc 1 cigar = 5 cigs, 1 pipe = 2.5 cigs
WYNDE4	44	inc 1 cigar = 5 cigs, 1 pipe = 2.5 cigs
WYNDE4	45	inc 1 cigar = 5 cigs, 1 pipe = 2.5 cigs
WYNDE4	46	inc 1 cigar = 5 cigs, 1 pipe = 2.5 cigs
WYNDE4	47	inc 1 cigar = 5 cigs, 1 pipe = 2.5 cigs
WYNDE4	57	inc 1 cigar = 5 cigs, 1 pipe = 2.5 cigs
WYNDE4	58	inc 1 cigar = 5 cigs, 1 pipe = 2.5 cigs
WYNDE4	59	inc 1 cigar = 5 cigs, 1 pipe = 2.5 cigs
WYNDE4	60	inc 1 cigar = 5 cigs, 1 pipe = 2.5 cigs
WYNDE4	61	inc 1 cigar = 5 cigs, 1 pipe = 2.5 cigs
WYNDE6	27	*
WYNDE6	36	*
WYNDE6	45	*
WYNDE6	54	*
WYNDE6	216	*
WYNDE6	225	*
WYNDE6	234	*
WYNDE6	243	*

Table 1G11 - 1

IESLC - Meta-anal of Ever Smoking (or Curr if Ever not avail) by Amount, Overview, Any prod (or Cigs if Any not avail)
All LC types
Most adjusted

REF	NRR	Cigarette equivalent
XU3	9	*
XU3	10	*
XU3	11	*
XU3	12	*
XU3	16	*
XU3	17	*
XU3	18	*
YAMAGU	8	*
YAMAGU	7	*
YUAN	2	*
YUAN	3	*
ZHENG	11	*
ZHENG	12	*
ZHENG	13	*
ZHENG	14	*
ZHENG	22	*
ZHENG	23	*
ZHOU	4	*
ZHOU	5	*
ZHOU	6	*

In this overview table, subtotals and Qs values may be invalid and should be ignored

Table 1G11 - 2

IESLC - Meta-anal of Ever Smoking (or Curr if Ever not avail) by Amount, Overview, Any prod (or Cigs if Any not avail)
All LC types
Most adjusted

REF	NRR	SEX	AD	Number Exposed Case	Number Exposed Cont	Non-exposed Case	Non-exposed Cont	RR	95.00%CI
AGUDO	4	f	3	-	-	-	-	1.57	(0.52- 4.70)
AGUDO	5	f	3	-	-	-	-	4.94	(1.86- 13.09)
Subtotal AGUDO								2.98	(1.44- 6.19)
*AKIBA	27	m	5	-	-	-	-	3.50	(2.20- 6.00)
*AKIBA	28	m	5	-	-	-	-	6.10	(3.90- 9.50)
*AKIBA	29	m	5	-	-	-	-	9.10	(5.40- 15.90)
*AKIBA	33	f	5	-	-	-	-	3.60	(2.60- 5.00)
*AKIBA	34	f	5	-	-	-	-	5.80	(3.30- 9.50)
Subtotal AKIBA								4.83	(3.96- 5.88)
ALDERS	18	m	1	-	-	-	-	3.55	(1.94- 6.49)
ALDERS	19	m	1	-	-	-	-	7.96	(4.63- 13.69)
ALDERS	20	m	1	-	-	-	-	8.52	(5.07- 14.33)
ALDERS	21	f	1	-	-	-	-	2.62	(1.88- 3.65)
ALDERS	22	f	1	-	-	-	-	5.28	(3.79- 7.36)
ALDERS	23	f	1	-	-	-	-	6.90	(4.69- 10.15)
Subtotal ALDERS								4.91	(4.15- 5.82)
*ARCHER	1	m	0	14	6504	6	9842	3.53	(1.36- 9.18)
*ARCHER	2	m	0	68	18320	6	9842	6.09	(2.64- 14.02)
*ARCHER	3	m	0	40	7705	6	9842	8.52	(3.61- 20.07)
Subtotal ARCHER								5.87	(3.54- 9.75)
ARMADA	46	m	0	44	117	4	64	6.02	(2.07- 17.51)
ARMADA	47	m	0	134	105	4	64	20.42	(7.20- 57.88)
ARMADA	48	m	0	139	32	4	64	69.50	(23.58- 204.81)
Subtotal ARMADA								20.25	(10.96- 37.40)
AUVINE	13	c	2	-	-	-	-	20.00	(9.72- 41.20)
AUVINE	14	c	2	-	-	-	-	33.90	(17.10- 67.00)
AUVINE	15	c	2	-	-	-	-	66.50	(25.80- 172.00)
Subtotal AUVINE								32.22	(20.76- 50.01)
AXELSS	5	m	6	-	-	-	-	3.82	(1.98- 7.36)
AXELSS	6	m	6	-	-	-	-	8.90	(4.91- 16.12)
AXELSS	7	m	6	-	-	-	-	10.40	(5.80- 18.66)
AXELSS	13	f	0	13	37	18	154	3.01	(1.35- 6.68)
AXELSS	14	f	0	63	50	18	154	10.78	(5.84- 19.91)
AXELSS	15	f	0	28	15	18	154	15.97	(7.21- 35.36)
AXELSS	16	f	0	6	7	18	154	7.33	(2.22- 24.22)
Subtotal AXELSS								7.81	(6.01- 10.15)
BARBON	82	m	3	-	-	-	-	6.70	(4.20- 11.00)
BARBON	83	m	3	-	-	-	-	12.80	(7.90- 21.00)
BARBON	84	m	3	-	-	-	-	21.30	(13.00- 36.00)
Subtotal BARBON								11.97	(9.01- 15.91)
*BENSHL	11	m	1	-	-	-	-	4.00	(1.55- 10.31)
*BENSHL	12	m	1	-	-	-	-	9.05	(3.91- 20.94)
*BENSHL	13	m	1	-	-	-	-	10.95	(4.76- 25.22)
Subtotal BENSHL								7.71	(4.67- 12.74)
*BEST	13	m	1	-	-	-	-	10.00	(4.56- 21.92)
*BEST	14	m	1	-	-	-	-	16.41	(7.73- 34.86)
*BEST	15	m	1	-	-	-	-	17.31	(7.93- 37.79)
Subtotal BEST								14.23	(9.11- 22.23)
*BOUCOT	99	m	0	38	2670	0	805	23.23~	(1.43- 377.62)
*BOUCOT	100	m	0	43	1519	0	805	46.12~	(2.84- 748.16)
Subtotal BOUCOT								32.74	(4.56- 235.00)
BRESLO	13	m	0	16	45	22	110	1.78	(0.86- 3.69)
BRESLO	14	m	0	69	105	22	110	3.29	(1.90- 5.69)
BRESLO	15	m	0	296	193	22	110	7.67	(4.69- 12.55)
BRESLO	16	m	0	80	22	22	110	18.18	(9.42- 35.09)
BRESLO	29	f	0	5	5	12	14	1.17	(0.27- 5.02)
BRESLO	30	f	0	8	6	12	14	1.56	(0.42- 5.76)
Subtotal BRESLO								5.03	(3.79- 6.65)
*BRETT	1	m	0	40	17090	6	6530	2.55	(1.08- 6.01)
*BRETT	2	m	0	62	15868	6	6530	4.25	(1.84- 9.83)
*BRETT	3	m	0	33	4490	6	6530	8.00	(3.35- 19.07)
Subtotal BRETT								4.40	(2.69- 7.21)
BROSS	18	m	0	170	155	38	170	4.91	(3.24- 7.42)
BROSS	19	m	0	95	59	38	170	7.20	(4.46- 11.63)
Subtotal BROSS								5.78	(4.23- 7.91)
BROWN2	32	m	2	-	-	-	-	6.10	(5.30- 6.90)
BROWN2	42	m	2	-	-	-	-	14.10	(12.70- 15.50)
BROWN2	31	f	2	-	-	-	-	8.40	(7.20- 9.70)
BROWN2	41	f	2	-	-	-	-	17.10	(15.30- 19.10)
Subtotal BROWN2								11.59	(10.92- 12.30)
BUFFLE	28	f	0	21	42	12	112	4.67	(2.11- 10.31)

Table 1G11 - 2

IESLC - Meta-anal of Ever Smoking (or Curr if Ever not avail) by Amount, Overview, Any prod (or Cigs if Any not avail)
All LC types
Most adjusted

				Number Exposed		Non-exposed			
REF	NRR	SEX	AD	Case	Cont	Case	Cont	RR	95.00%CI
BUFFLE	29	f	0	76	60	12	112	11.82	(5.96- 23.45)
BUFFLE	35	f	0	141	62	12	112	21.23	(10.90- 41.32)
Subtotal BUFFLE								11.51	(7.65- 17.33)
*CEDERL	80	m	2	-	-	-	-	3.40	(1.96- 5.90)
*CEDERL	81	m	2	-	-	-	-	7.50	(4.79- 11.74)
*CEDERL	82	m	2	-	-	-	-	11.90	(7.55- 18.75)
*CEDERL	76	f	2	-	-	-	-	2.83	(1.72- 4.67)
*CEDERL	77	f	2	-	-	-	-	7.74	(4.96- 12.08)
*CEDERL	78	f	2	-	-	-	-	7.75	(4.03- 14.91)
Subtotal CEDERL								6.36	(5.20- 7.78)
*CHANG	2	m	0	5	100	5	502	5.02	(1.48- 17.02)
*CHANG	3	m	0	17	161	5	502	10.60	(3.97- 28.28)
*CHANG	4	m	0	13	158	5	502	8.26	(2.99- 22.81)
*CHANG	8	f	0	6	205	11	1139	3.03	(1.13- 8.10)
*CHANG	9	f	0	11	234	11	1139	4.87	(2.14- 11.09)
*CHANG	10	f	0	13	164	11	1139	8.21	(3.74- 18.01)
Subtotal CHANG								6.25	(4.26- 9.17)
CHATZI	1	c	0	68	127	27	129	2.56	(1.54- 4.25)
CHATZI	2	c	0	73	123	27	129	2.84	(1.71- 4.70)
CHATZI	3	c	0	114	115	27	129	4.74	(2.90- 7.72)
Subtotal CHATZI								3.28	(2.46- 4.38)
CHEN2	3	m	0	17	26	9	33	2.40	(0.92- 6.25)
CHEN2	4	m	0	44	50	9	33	3.23	(1.39- 7.48)
CHEN2	5	m	0	34	9	9	33	13.85	(4.89- 39.22)
CHEN2	6	m	0	26	12	9	33	7.94	(2.91- 21.72)
CHEN2	7	f	0	5	17	25	33	0.39	(0.13- 1.20)
CHEN2	8	f	0	22	10	25	33	2.90	(1.17- 7.22)
CHEN2	9	f	0	7	1	25	33	9.24	(1.07- 80.02)
CHEN2	10	f	0	4	2	25	33	2.64	(0.45- 15.58)
Subtotal CHEN2								3.36	(2.30- 4.90)
CHOI	12	m	0	20	90	13	95	1.62	(0.76- 3.46)
CHOI	13	m	0	144	281	13	95	3.74	(2.03- 6.92)
CHOI	14	m	0	50	49	13	95	7.46	(3.70- 15.03)
CHOI	15	m	0	37	39	13	95	6.93	(3.33- 14.44)
CHOI	16	m	0	16	6	13	95	19.49	(6.47- 58.71)
CHOI	17	f	0	9	16	76	164	1.21	(0.51- 2.87)
CHOI	18	f	0	7	9	76	164	1.68	(0.60- 4.68)
CHOI	20	f	0	3	1	76	164	6.47	(0.66- 63.26)
Subtotal CHOI								3.83	(2.86- 5.13)
*CHOW	10	m	2	-	-	-	-	13.88	(5.81- 33.12)
*CHOW	11	m	2	-	-	-	-	21.87	(9.35- 51.14)
*CHOW	12	m	2	-	-	-	-	44.48	(18.63- 106.19)
Subtotal CHOW								23.78	(14.45- 39.14)
COMSTO	4	m	0	18	25	4	69	12.42	(3.83- 40.26)
COMSTO	5	m	0	60	57	4	69	18.16	(6.22- 53.00)
COMSTO	6	m	0	26	18	4	69	24.92	(7.71- 80.57)
COMSTO	9	f	0	16	19	13	115	7.45	(3.10- 17.93)
COMSTO	10	f	0	51	26	13	115	17.35	(8.25- 36.48)
COMSTO	11	f	0	9	6	13	115	13.27	(4.07- 43.25)
Subtotal COMSTO								14.21	(9.49- 21.27)
COOKSO	1	c	0	102	27	45	61	5.12	(2.89- 9.08)
COOKSO	2	c	0	82	11	45	61	10.11	(4.83- 21.13)
Subtotal COOKSO								6.61	(4.21- 10.40)
CORREA	46	c	1	-	-	-	-	9.30	(6.80- 12.70)
CORREA	50	c	1	-	-	-	-	25.30	(18.50- 34.60)
Subtotal CORREA								15.32	(12.28- 19.11)
*CPSI	243	m	1	-	-	-	-	5.81	(4.33- 7.80)
*CPSI	246	m	1	-	-	-	-	13.60	(10.46- 17.67)
*CPSI	275	f	1	-	-	-	-	1.25	(0.73- 2.13)
*CPSI	276	f	1	-	-	-	-	2.44	(1.67- 3.56)
*CPSI	277	f	1	-	-	-	-	5.03	(3.82- 6.63)
*CPSI	278	f	1	-	-	-	-	11.10	(6.00- 20.53)
Subtotal CPSI								5.90	(5.14- 6.77)
*CPSII	102	m	1	-	-	-	-	9.99	(7.97- 12.51)
*CPSII	103	m	1	-	-	-	-	17.60	(14.05- 22.05)
*CPSII	105	f	1	-	-	-	-	4.16	(3.41- 5.09)
*CPSII	106	f	1	-	-	-	-	13.34	(11.31- 15.75)
Subtotal CPSII								9.98	(9.03- 11.02)
DAMBER	6	m	1	-	-	-	-	2.30	(1.30- 4.40)
DAMBER	7	m	1	-	-	-	-	7.30	(4.40- 12.70)
DAMBER	8	m	1	-	-	-	-	9.10	(5.50- 15.30)

Table 1G11 - 2

IESLC - Meta-anal of Ever Smoking (or Curr if Ever not avail) by Amount, Overview, Any prod (or Cigs if Any not avail)
All LC types
Most adjusted

REF	NRR	SEX	AD	Number Exposed Case	Cont	Non-exposed Case	Cont	RR	95.00%CI
DAMBER	9	m	1	-	-	-	-	14.90	(6.70- 33.50)
Subtotal DAMBER								6.60	(4.92- 8.86)
DARBY	1	m	0	128	223	3	384	73.47	(23.11- 233.57)
DARBY	2	m	0	126	169	3	384	95.43	(29.94- 304.17)
DARBY	3	m	0	68	61	3	384	142.69	(43.52- 467.82)
DARBY	8	f	0	71	104	23	529	15.70	(9.38- 26.28)
DARBY	9	f	0	86	92	23	529	21.50	(12.90- 35.82)
DARBY	10	f	0	38	21	23	529	41.62	(21.15- 81.90)
Subtotal DARBY								29.10	(21.80- 38.84)
DAVEYS	1	m	0	11	69	3	23	1.22	(0.31- 4.77)
DAVEYS	2	m	0	31	32	3	23	7.43	(2.02- 27.27)
DAVEYS	3	m	0	19	22	3	23	6.62	(1.72- 25.56)
DAVEYS	4	m	0	29	21	3	23	10.59	(2.81- 39.94)
Subtotal DAVEYS								5.12	(2.63- 9.98)
DEAN	1	m	0	73	168	12	61	2.21	(1.12- 4.35)
DEAN	2	m	0	228	172	12	61	6.74	(3.52- 12.91)
DEAN	3	m	0	102	45	12	61	11.52	(5.66- 23.47)
Subtotal DEAN								5.46	(3.69- 8.08)
DEAN2	25	m	0	377	396	33	112	3.23	(2.14- 4.88)
DEAN2	26	m	0	252	112	33	112	7.64	(4.88- 11.95)
DEAN2	29	f	0	44	24	88	121	2.52	(1.43- 4.45)
DEAN2	30	f	0	18	5	88	121	4.95	(1.77- 13.84)
Subtotal DEAN2								4.21	(3.25- 5.45)
DEAN3	7	m	3	-	-	-	-	5.46	(3.27- 9.10)
DEAN3	14	m	3	-	-	-	-	7.42	(4.56- 12.06)
DEAN3	21	m	3	-	-	-	-	21.66	(12.78- 36.72)
DEAN3	91	f	3	-	-	-	-	3.16	(1.92- 5.21)
DEAN3	98	f	3	-	-	-	-	8.42	(5.14- 13.78)
DEAN3	105	f	3	-	-	-	-	24.24	(13.08- 44.93)
Subtotal DEAN3								8.44	(6.83- 10.42)
*DEKLER	2	m	2	-	-	-	-	19.40	(2.60- 143.70)
*DEKLER	3	m	2	-	-	-	-	23.00	(3.20- 167.60)
*DEKLER	4	m	2	-	-	-	-	32.50	(4.40- 241.20)
Subtotal DEKLER								24.38	(7.70- 77.17)
DESTEF	6	m	4	-	-	-	-	2.90	(1.60- 5.00)
DESTEF	7	m	4	-	-	-	-	8.40	(5.20- 13.60)
DESTEF	8	m	4	-	-	-	-	10.40	(6.40- 16.90)
DESTEF	9	m	4	-	-	-	-	23.70	(13.40- 42.10)
Subtotal DESTEF								8.87	(6.83- 11.51)
DOLL	1	m	0	55	129	7	61	3.72	(1.60- 8.64)
DOLL	2	m	0	489	570	7	61	7.48	(3.39- 16.50)
DOLL	3	m	0	475	431	7	61	9.60	(4.35- 21.22)
DOLL	4	m	0	293	154	7	61	16.58	(7.40- 37.13)
DOLL	5	m	0	38	12	7	61	27.60	(9.99- 76.25)
DOLL	7	f	0	16	25	40	59	0.94	(0.45- 1.99)
DOLL	8	f	0	24	18	40	59	1.97	(0.95- 4.09)
DOLL	9	f	0	14	6	40	59	3.44	(1.22- 9.71)
DOLL	10	f	0	14	0	40	59	42.60~	(2.47- 734.58)
Subtotal DOLL								4.97	(3.72- 6.65)
*DOLL2	46	m	1	-	-	-	-	6.71	(0.91- 49.81)
*DOLL2	47	m	1	-	-	-	-	12.29	(1.67- 90.41)
*DOLL2	48	m	1	-	-	-	-	23.71	(3.25- 173.24)
*DOLL2	10	f	1	-	-	-	-	1.29	(0.14- 11.50)
*DOLL2	11	f	1	-	-	-	-	6.43	(1.81- 22.78)
*DOLL2	12	f	1	-	-	-	-	29.71	(9.46- 93.32)
Subtotal DOLL2								11.39	(5.93- 21.87)
DORANT	6	c	0	21	192	14	1090	8.52	(4.26- 17.04)
DORANT	7	c	0	143	409	14	1090	27.22	(15.54- 47.68)
DORANT	8	c	0	128	275	14	1090	36.24	(20.55- 63.91)
Subtotal DORANT								22.68	(16.05- 32.04)
DORGAN	108	m	2	-	-	-	-	6.88	(3.74- 12.66)
DORGAN	109	m	2	-	-	-	-	14.00	(7.81- 25.11)
DORGAN	96	f	3	-	-	-	-	5.67	(4.36- 7.36)
DORGAN	97	f	3	-	-	-	-	12.22	(9.31- 16.04)
Subtotal DORGAN								8.47	(7.13- 10.07)
*DORN	408	m	1	-	-	-	-	4.02	(3.43- 4.71)
*DORN	409	m	1	-	-	-	-	9.92	(8.84- 11.14)
*DORN	410	m	1	-	-	-	-	17.19	(15.28- 19.33)
*DORN	411	m	1	-	-	-	-	22.75	(19.63- 26.37)
Subtotal DORN								11.88	(11.13- 12.68)
DOSEME	5	m	2	-	-	-	-	2.20	(1.40- 3.30)

Table 1G11 - 2

IESLC - Meta-anal of Ever Smoking (or Curr if Ever not avail) by Amount, Overview, Any prod (or Cigs if Any not avail)
All LC types
Most adjusted

REF	NRR	SEX	AD	Number Exposed Case	Number Exposed Cont	Non-exposed Case	Non-exposed Cont	RR	95.00%CI
DOSEME	9	m	2	-	-	-	-	3.10	(2.30- 4.10)
DOSEME	13	m	2	-	-	-	-	6.60	(4.40- 10.20)
Subtotal DOSEME								3.44	(2.79- 4.24)
*DUNN	1	m	0	3	2538	2	14160	8.37	(1.40- 50.06)
*DUNN	2	m	0	12	9418	2	14160	9.02	(2.02- 40.30)
*DUNN	3	m	0	75	27720	2	14160	19.16	(4.70- 78.00)
*DUNN	4	m	0	32	9017	2	14160	25.13	(6.02- 104.82)
*DUNN	5	m	0	13	3206	2	14160	28.71	(6.48- 127.15)
Subtotal DUNN								16.88	(8.61- 33.09)
EBELIN	2	m	0	20	72	12	117	2.71	(1.25- 5.87)
EBELIN	3	m	0	19	26	12	117	7.13	(3.08- 16.48)
EBELIN	4	m	0	47	37	12	117	12.39	(5.95- 25.80)
EBELIN	5	m	0	5	3	12	117	16.25	(3.45- 76.54)
EBELIN	6	m	0	4	4	12	117	9.75	(2.16- 44.04)
Subtotal EBELIN								6.99	(4.62- 10.59)
*ENGELA	31	m	7	-	-	-	-	1.40	(0.60- 3.70)
*ENGELA	32	m	7	-	-	-	-	4.10	(1.70- 10.00)
*ENGELA	33	m	7	-	-	-	-	7.00	(2.90- 17.00)
*ENGELA	34	m	7	-	-	-	-	11.00	(4.20- 28.00)
*ENGELA	35	m	7	-	-	-	-	15.00	(6.10- 37.00)
*ENGELA	45	f	5	-	-	-	-	12.00	(4.50- 32.00)
*ENGELA	46	f	5	-	-	-	-	12.00	(4.40- 30.00)
*ENGELA	47	f	5	-	-	-	-	24.00	(9.50- 59.00)
*ENGELA	48	f	5	-	-	-	-	26.00	(9.20- 73.00)
Subtotal ENGELA								9.05	(6.63- 12.35)
*ENSTRO	7	m	1	-	-	-	-	4.74	(3.34- 6.73)
*ENSTRO	6	m	1	-	-	-	-	7.68	(5.95- 9.90)
*ENSTRO	5	m	1	-	-	-	-	13.65	(10.88- 17.13)
*ENSTRO	4	m	1	-	-	-	-	16.08	(12.77- 20.23)
*ENSTRO	3	m	1	-	-	-	-	19.41	(15.22- 24.75)
*ENSTRO	11	f	1	-	-	-	-	2.15	(1.62- 2.84)
*ENSTRO	10	f	1	-	-	-	-	4.31	(3.56- 5.22)
*ENSTRO	9	f	1	-	-	-	-	9.48	(8.04- 11.18)
*ENSTRO	8	f	1	-	-	-	-	16.47	(13.74- 19.75)
Subtotal ENSTRO								9.31	(8.66- 10.02)
ESAKI	1	m	0	47	75	16	28	1.10	(0.54- 2.24)
ESAKI	2	m	0	74	58	16	28	2.23	(1.10- 4.51)
ESAKI	3	m	0	34	10	16	28	5.95	(2.34- 15.16)
Subtotal ESAKI								2.12	(1.36- 3.29)
FAN	6	m	0	13	121	36	236	0.70	(0.36- 1.38)
FAN	7	m	0	53	171	36	236	2.03	(1.27- 3.24)
FAN	8	m	0	111	183	36	236	3.98	(2.61- 6.07)
FAN	9	m	0	39	23	36	236	11.12	(5.96- 20.73)
FAN	10	f	0	17	48	69	320	1.64	(0.89- 3.03)
FAN	11	f	0	30	37	69	320	3.76	(2.18- 6.50)
FAN	12	f	0	31	12	69	320	11.98	(5.86- 24.50)
FAN	13	f	0	4	1	69	320	18.55	(2.04- 168.54)
Subtotal FAN								3.29	(2.68- 4.05)
GAO	21	f	2	-	-	-	-	1.74	(1.22- 2.48)
GAO	22	f	2	-	-	-	-	3.04	(2.02- 4.58)
GAO	23	f	2	-	-	-	-	13.18	(7.04- 24.68)
Subtotal GAO								2.91	(2.28- 3.73)
GAO2	2	m	0	32	41	13	56	3.36	(1.57- 7.19)
GAO2	3	m	0	77	44	13	56	7.54	(3.71- 15.30)
GAO2	4	m	0	74	30	13	56	10.63	(5.08- 22.22)
Subtotal GAO2								6.57	(4.30- 10.04)
GARSHI	26	m	1	-	-	-	-	3.29	(2.22- 4.87)
GARSHI	27	m	1	-	-	-	-	5.72	(4.04- 8.10)
GARSHI	28	m	1	-	-	-	-	7.69	(5.20- 11.37)
GARSHI	29	m	1	-	-	-	-	5.24	(3.61- 7.60)
Subtotal GARSHI								5.28	(4.38- 6.37)
GER	22	c	14	-	-	-	-	1.26	(0.61- 2.61)
GER	23	c	14	-	-	-	-	1.90	(0.98- 3.70)
GER	24	c	14	-	-	-	-	3.00	(1.40- 6.40)
Subtotal GER								1.90	(1.26- 2.88)
GOLLED	1	m	1	-	-	-	-	4.45	(2.54- 7.81)
GOLLED	2	m	1	-	-	-	-	6.37	(3.67- 11.03)
GOLLED	3	m	1	-	-	-	-	18.28	(10.52- 31.74)
Subtotal GOLLED								8.08	(5.87- 11.13)
GSELL	1	m	0	11	36	2	29	4.43	(0.91- 21.60)
GSELL	2	m	0	10	37	2	29	3.92	(0.80- 19.30)

Table 1G11 - 2

IESLC - Meta-anal of Ever Smoking (or Curr if Ever not avail) by Amount, Overview, Any prod (or Cigs if Any not avail)
All LC types
Most adjusted

REF	NRR	SEX	AD	Number Exposed Case	Number Exposed Cont	Non-exposed Case	Non-exposed Cont	RR	95.00%CI
GSELL	3	m	0	27	26	2	29	15.06	(3.26- 69.59)
GSELL	4	m	0	49	9	2	29	78.94	(15.95- 390.82)
GSELL	5	m	0	51	13	2	29	56.88	(11.99- 269.87)
Subtotal GSELL								16.45	(8.14- 33.23)
HAENSZ	52	f	0	40	66	81	236	1.77	(1.11- 2.82)
HAENSZ	51	f	0	23	13	81	236	5.15	(2.50- 10.65)
Subtotal HAENSZ								2.42	(1.63- 3.58)
*HAMMO2	7	m	1	-	-	-	-	9.15	(3.62- 23.12)
*HAMMO2	6	m	1	-	-	-	-	10.39	(4.28- 25.22)
Subtotal HAMMO2								9.78	(5.15- 18.56)
*HAMMON	153	m	1	-	-	-	-	7.38	(3.72- 14.63)
*HAMMON	154	m	1	-	-	-	-	8.32	(4.66- 14.84)
*HAMMON	155	m	1	-	-	-	-	17.06	(9.44- 30.82)
Subtotal HAMMON								10.42	(7.31- 14.85)
*HANSEN	1	m	2	-	-	-	-	1.37	(0.63- 3.54)
*HANSEN	2	m	2	-	-	-	-	2.90	(1.14- 8.27)
Subtotal HANSEN								1.89	(0.99- 3.63)
*HIRAYA	23	m	1	-	-	-	-	2.06	(1.49- 2.85)
*HIRAYA	24	m	1	-	-	-	-	4.00	(3.20- 4.99)
*HIRAYA	25	m	1	-	-	-	-	6.24	(5.07- 7.68)
*HIRAYA	26	f	1	-	-	-	-	2.25	(1.64- 3.08)
*HIRAYA	27	f	1	-	-	-	-	2.56	(1.85- 3.54)
*HIRAYA	28	f	1	-	-	-	-	4.47	(2.73- 7.33)
Subtotal HIRAYA								3.72	(3.32- 4.17)
HITOSU	35	m	1	-	-	-	-	2.08	(0.90- 4.83)
HITOSU	36	m	1	-	-	-	-	2.82	(1.25- 6.36)
HITOSU	37	m	1	-	-	-	-	4.68	(1.97- 11.11)
HITOSU	60	f	1	-	-	-	-	3.11	(1.77- 5.46)
HITOSU	61	f	1	-	-	-	-	3.17	(1.07- 9.36)
Subtotal HITOSU								3.05	(2.16- 4.32)
*HOLE	1	m	1	-	-	-	-	5.47	(2.35- 12.75)
*HOLE	3	m	1	-	-	-	-	8.90	(4.12- 19.23)
*HOLE	4	m	1	-	-	-	-	10.75	(4.80- 24.06)
*HOLE	5	m	1	-	-	-	-	7.49	(2.99- 18.77)
Subtotal HOLE								8.03	(5.31- 12.17)
HU	1	m	0	36	38	41	67	1.55	(0.85- 2.82)
HU	2	m	0	55	43	41	67	2.09	(1.20- 3.65)
HU	3	m	0	29	13	41	67	3.65	(1.70- 7.80)
HU	4	f	0	19	10	40	48	2.28	(0.95- 5.46)
HU	5	f	0	6	6	40	48	1.20	(0.36- 4.01)
HU	6	f	0	1	2	40	48	0.60	(0.05- 6.86)
Subtotal HU								2.02	(1.47- 2.77)
HU2	2	c	0	16	33	121	213	0.85	(0.45- 1.61)
HU2	3	c	0	44	58	121	213	1.34	(0.85- 2.10)
HU2	4	c	0	65	59	121	213	1.94	(1.28- 2.94)
HU2	5	c	0	64	54	121	213	2.09	(1.36- 3.19)
HU2	6	c	0	149	87	121	213	3.01	(2.13- 4.26)
HU2	7	c	0	64	19	121	213	5.93	(3.39- 10.37)
Subtotal HU2								2.20	(1.83- 2.64)
HUMBLE	2	m	1	-	-	-	-	9.20	(3.30- 25.80)
HUMBLE	3	m	1	-	-	-	-	24.70	(10.00- 59.90)
HUMBLE	5	m	1	-	-	-	-	11.60	(2.70- 61.50)
HUMBLE	6	m	1	-	-	-	-	26.10	(5.60- 146.60)
HUMBLE	8	f	1	-	-	-	-	19.20	(6.50- 60.80)
HUMBLE	9	f	1	-	-	-	-	16.00	(6.70- 36.30)
HUMBLE	11	f	1	-	-	-	-	18.50	(4.90- 72.40)
HUMBLE	12	f	1	-	-	-	-	36.90	(7.60- 217.10)
Subtotal HUMBLE								17.64	(11.76- 26.46)
JARUP	4	m	2	-	-	-	-	6.90	(2.40- 22.70)
JARUP	5	m	2	-	-	-	-	8.00	(3.00- 24.80)
Subtotal JARUP								7.46	(3.46- 16.11)
JEDRYC	45	m	4	-	-	-	-	3.48	(2.33- 5.19)
JEDRYC	46	m	4	-	-	-	-	6.16	(4.25- 8.90)
JEDRYC	47	m	4	-	-	-	-	7.69	(5.15- 11.47)
JEDRYC	48	f	4	-	-	-	-	6.37	(2.66- 15.24)
JEDRYC	49	f	4	-	-	-	-	2.38	(1.17- 6.86)
JEDRYC	50	f	4	-	-	-	-	7.37	(2.20- 24.69)
Subtotal JEDRYC								5.36	(4.35- 6.60)
JOLY	7	m	0	16	54	12	218	5.38	(2.41- 12.05)
JOLY	8	m	0	217	318	12	218	12.40	(6.76- 22.73)
JOLY	9	m	0	126	175	12	218	13.08	(7.00- 24.43)

Table 1G11 - 2

IESLC - Meta-anal of Ever Smoking (or Curr if Ever not avail) by Amount, Overview, Any prod (or Cigs if Any not avail)
All LC types
Most adjusted

REF	NRR	SEX	AD	Number Exposed Case	Cont	Non-exposed Case	Cont	RR	95.00%CI
JOLY	10	m	0	193	161	12	218	21.78	(11.74- 40.39)
JOLY	3	f	0	33	38	52	283	4.73	(2.72- 8.21)
JOLY	4	f	0	72	49	52	283	8.00	(5.01- 12.77)
JOLY	5	f	0	28	22	52	283	6.93	(3.68- 13.03)
JOLY	6	f	0	32	13	52	283	13.40	(6.59- 27.23)
Subtotal JOLY								9.35	(7.55- 11.59)
JUSSAW	34	m	2	-	-	-	-	5.57	(2.46- 14.76)
JUSSAW	35	m	2	-	-	-	-	14.00	(5.17- 53.16)
Subtotal JUSSAW								7.84	(3.86- 15.96)
*KAISE2	66	m	1	-	-	-	-	4.47	(2.00- 9.99)
*KAISE2	67	m	1	-	-	-	-	10.34	(5.56- 19.23)
*KAISE2	58	f	1	-	-	-	-	7.61	(3.26- 17.75)
*KAISE2	59	f	1	-	-	-	-	22.12	(11.22- 43.61)
Subtotal KAISE2								10.24	(7.14- 14.68)
*KAISER	6	m	2	-	-	-	-	6.58	(3.87- 11.20)
*KAISER	7	m	2	-	-	-	-	17.24	(10.71- 27.73)
*KAISER	8	m	2	-	-	-	-	20.91	(12.78- 27.73)
*KAISER	2	f	2	-	-	-	-	3.42	(2.17- 5.40)
*KAISER	3	f	2	-	-	-	-	7.98	(5.35- 11.90)
*KAISER	4	f	2	-	-	-	-	12.63	(8.06- 19.80)
Subtotal KAISER								10.14	(8.46- 12.15)
KANELL	26	m	1	-	-	-	-	1.71	(1.15- 2.56)
KANELL	27	m	1	-	-	-	-	7.06	(4.76- 10.48)
KANELL	28	m	1	-	-	-	-	20.39	(10.37- 40.09)
KANELL	29	m	1	-	-	-	-	34.22	(18.86- 62.11)
Subtotal KANELL								6.27	(4.94- 7.96)
KATSOU	3	f	1	-	-	-	-	2.26	(1.06- 4.85)
KATSOU	4	f	1	-	-	-	-	7.46	(2.40- 23.17)
Subtotal KATSOU								3.27	(1.74- 6.16)
KAUFMA	11	c	6	-	-	-	-	8.00	(5.00- 13.00)
KAUFMA	12	c	6	-	-	-	-	15.00	(10.00- 23.00)
KAUFMA	13	c	6	-	-	-	-	28.00	(17.00- 44.00)
KAUFMA	14	c	6	-	-	-	-	43.00	(27.00- 68.00)
KAUFMA	15	c	6	-	-	-	-	60.00	(35.00- 102.00)
Subtotal KAUFMA								23.07	(18.70- 28.44)
KHUDER	1	m	0	81	434	23	309	2.51	(1.54- 4.07)
KHUDER	2	m	0	224	288	23	309	10.45	(6.61- 16.52)
KHUDER	3	m	0	154	63	23	309	32.84	(19.62- 54.97)
Subtotal KHUDER								9.12	(6.89- 12.06)
*KINLEN	14	m	2	-	-	-	-	10.61	(5.01- 22.48)
*KINLEN	15	m	2	-	-	-	-	14.14	(6.68- 29.91)
*KINLEN	16	m	2	-	-	-	-	21.74	(10.23- 46.19)
Subtotal KINLEN								14.81	(9.60- 22.86)
*KNEKT	29	m	1	-	-	-	-	5.00	(2.00- 12.30)
*KNEKT	30	m	1	-	-	-	-	12.70	(5.50- 29.40)
Subtotal KNEKT								8.27	(4.47- 15.31)
KOO	11	f	0	17	19	56	85	1.36	(0.65- 2.84)
KOO	12	f	0	24	5	56	85	7.29	(2.62- 20.22)
KOO	13	f	0	1	1	56	85	1.52	(0.09- 24.77)
Subtotal KOO								2.36	(1.32- 4.24)
KOULUM	6	m	0	37	77	5	54	5.19	(1.92- 14.06)
KOULUM	5	m	0	208	94	5	54	23.90	(9.26- 61.67)
KOULUM	4	m	0	478	75	5	54	68.83	(26.67- 177.62)
Subtotal KOULUM								21.38	(12.26- 37.28)
KREUZE	19	m	3	-	-	-	-	2.50	(0.70- 8.20)
KREUZE	20	m	3	-	-	-	-	8.70	(3.50- 21.90)
KREUZE	21	m	3	-	-	-	-	19.50	(7.50- 50.30)
KREUZE	22	m	3	-	-	-	-	20.80	(7.20- 60.50)
KREUZE	30	m	3	-	-	-	-	8.20	(5.20- 13.00)
KREUZE	31	m	3	-	-	-	-	25.10	(16.20- 38.70)
KREUZE	32	m	3	-	-	-	-	32.80	(20.90- 51.40)
KREUZE	33	m	3	-	-	-	-	33.30	(20.50- 54.00)
KREUZE	25	f	3	-	-	-	-	5.70	(1.60- 16.60)
KREUZE	26	f	3	-	-	-	-	11.80	(3.50- 29.00)
KREUZE	27	f	3	-	-	-	-	12.10	(3.00- 48.00)
KREUZE	36	f	3	-	-	-	-	2.00	(1.20- 3.30)
KREUZE	37	f	3	-	-	-	-	5.40	(3.50- 8.60)
KREUZE	38	f	3	-	-	-	-	7.70	(3.50- 17.30)
Subtotal KREUZE								11.60	(9.81- 13.72)
KREYBE	9	m	1	-	-	-	-	5.82	(2.56- 13.23)
KREYBE	10	m	1	-	-	-	-	6.23	(2.66- 14.56)

Table 1G11 - 2

IESLC - Meta-anal of Ever Smoking (or Curr if Ever not avail) by Amount, Overview, Any prod (or Cigs if Any not avail)
All LC types
Most adjusted

REF	NRR	SEX	AD	Number Exposed Case	Number Exposed Cont	Non-exposed Case	Non-exposed Cont	RR	95.00%CI
KREYBE	11	m	1	-	-	-	-	14.31	(5.99- 34.17)
KREYBE	28	f	1	-	-	-	-	1.36	(0.65- 2.82)
KREYBE	29	f	1	-	-	-	-	1.91	(0.48- 7.55)
Subtotal KREYBE								4.29	(2.90- 6.34)
LAMTH	7	f	0	101	63	202	337	2.67	(1.87- 3.83)
LAMTH	2	f	0	90	28	202	337	5.36	(3.39- 8.48)
LAMTH	9	f	0	39	9	202	337	7.23	(3.43- 15.24)
Subtotal LAMTH								3.82	(2.93- 4.98)
LAUSSM	18	m	3	-	-	-	-	3.29	(2.31- 4.66)
LAUSSM	19	m	3	-	-	-	-	5.81	(4.16- 8.11)
LAUSSM	20	m	3	-	-	-	-	9.62	(6.05- 15.23)
Subtotal LAUSSM								5.24	(4.23- 6.49)
LETOUR	2	c	0	271	266	24	224	9.51	(6.04- 14.97)
LETOUR	3	c	0	367	198	24	224	17.30	(10.98- 27.27)
LETOUR	4	c	0	65	23	24	224	26.38	(13.98- 49.78)
Subtotal LETOUR								14.84	(11.14- 19.77)
*LIAW	3	c	2	-	-	-	-	3.10	(1.70- 5.60)
*LIAW	4	c	2	-	-	-	-	3.60	(2.00- 6.40)
*LIAW	5	c	2	-	-	-	-	8.30	(4.00- 17.30)
Subtotal LIAW								4.18	(2.91- 6.00)
*LIDDEL	2	m	1	-	-	-	-	3.33	(2.05- 5.64)
*LIDDEL	3	m	1	-	-	-	-	5.02	(3.21- 8.22)
Subtotal LIDDEL								4.15	(2.94- 5.86)
LIU2	8	m	3	-	-	-	-	1.20	(0.43- 3.50)
LIU2	9	m	3	-	-	-	-	7.10	(2.60- 19.50)
LIU2	10	m	3	-	-	-	-	21.40	(7.10- 64.00)
LIU2	14	f	3	-	-	-	-	1.80	(0.57- 5.90)
LIU2	15	f	3	-	-	-	-	3.50	(1.20- 9.80)
LIU2	16	f	3	-	-	-	-	17.90	(4.00- 80.60)
Subtotal LIU2								4.74	(3.00- 7.48)
LIU3	6	m	2	-	-	-	-	1.41	(0.33- 6.09)
LIU3	7	m	2	-	-	-	-	1.09	(0.24- 4.82)
LIU3	8	m	2	-	-	-	-	1.91	(0.32- 11.40)
Subtotal LIU3								1.39	(0.56- 3.42)
LIU4	7	m	2	-	-	-	-	2.11	(2.02- 2.20)
LIU4	8	m	2	-	-	-	-	3.60	(3.49- 3.71)
LIU4	9	m	2	-	-	-	-	6.98	(6.73- 7.23)
Subtotal LIU4								3.95	(3.87- 4.03)
LIU5	2	c	0	14	27	26	41	0.82	(0.36- 1.84)
LIU5	3	c	0	21	21	26	41	1.58	(0.72- 3.44)
LIU5	4	c	0	50	22	26	41	3.58	(1.78- 7.23)
Subtotal LIU5								1.79	(1.16- 2.78)
LUBIN	11	m	4	-	-	-	-	0.72	(0.20- 2.54)
LUBIN	12	m	4	-	-	-	-	1.25	(0.43- 3.65)
LUBIN	13	m	4	-	-	-	-	6.50	(2.38- 17.78)
LUBIN	14	m	4	-	-	-	-	8.00	(3.42- 18.72)
Subtotal LUBIN								3.39	(2.04- 5.64)
LUBIN2	273	m	0	1887	3759	190	2616	6.91	(5.91- 8.09)
LUBIN2	274	m	0	1529	2771	190	2616	7.60	(6.47- 8.92)
LUBIN2	275	m	0	1963	2547	190	2616	10.61	(9.06- 12.44)
LUBIN2	276	m	0	1261	1394	190	2616	12.45	(10.55- 14.70)
LUBIN2	281	f	0	151	218	336	1188	2.45	(1.93- 3.11)
LUBIN2	282	f	0	221	213	336	1188	3.67	(2.93- 4.59)
LUBIN2	283	f	0	134	103	336	1188	4.60	(3.46- 6.11)
LUBIN2	284	f	0	45	33	336	1188	4.82	(3.03- 7.68)
Subtotal LUBIN2								7.07	(6.60- 7.58)
MACLEN	36	c	2	-	-	-	-	1.35	(0.64- 2.84)
MACLEN	37	c	2	-	-	-	-	2.66	(1.46- 4.81)
MACLEN	38	c	2	-	-	-	-	2.93	(1.57- 5.45)
MACLEN	39	c	2	-	-	-	-	4.10	(2.07- 8.15)
Subtotal MACLEN								2.65	(1.91- 3.67)
MARTIS	1	m	0	31	39	4	25	4.97	(1.56- 15.78)
MARTIS	2	m	0	91	87	4	25	6.54	(2.19- 19.55)
MARTIS	3	m	0	75	50	4	25	9.38	(3.08- 28.57)
Subtotal MARTIS								6.77	(3.55- 12.94)
MATOS	29	m	2	-	-	-	-	2.00	(0.90- 4.50)
MATOS	31	m	2	-	-	-	-	7.50	(3.70- 15.00)
MATOS	33	m	2	-	-	-	-	10.40	(5.30- 20.70)
Subtotal MATOS								5.94	(3.91- 9.02)
MATSUD	1	m	0	37	1237	3	1255	12.51	(3.85- 40.69)
MATSUD	2	m	0	75	1607	3	1255	19.52	(6.14- 62.05)

Table 1G11 - 2

IESLC - Meta-anal of Ever Smoking (or Curr if Ever not avail) by Amount, Overview, Any prod (or Cigs if Any not avail)
All LC types
Most adjusted

REF	NRR	SEX	AD	Number Exposed Case	Cont	Non-exposed Case	Cont	RR	95.00%CI
MATSUD	3	m	0	58	470	3	1255	51.62	(16.10- 165.55)
Subtotal MATSUD								23.37	(11.91- 45.84)
MCCONN	26	c	0	7	43	9	23	0.42	(0.14- 1.26)
MCCONN	25	c	0	49	92	9	23	1.36	(0.58- 3.17)
MCCONN	24	c	0	35	42	9	23	2.13	(0.87- 5.19)
Subtotal MCCONN								1.21	(0.71- 2.08)
*MIGRAN	2	m	2	-	-	-	-	4.01	(1.19- 13.47)
*MIGRAN	4	m	2	-	-	-	-	4.24	(1.53- 11.74)
*MIGRAN	6	m	2	-	-	-	-	5.14	(1.79- 14.81)
*MIGRAN	8	m	2	-	-	-	-	5.93	(2.03- 17.29)
*MIGRAN	29	f	2	-	-	-	-	4.88	(1.17- 20.43)
*MIGRAN	31	f	2	-	-	-	-	6.53	(2.11- 20.22)
*MIGRAN	33	f	2	-	-	-	-	7.48	(1.64- 34.03)
Subtotal MIGRAN								5.24	(3.37- 8.15)
*MRFITR	3	m	0	2	856	0	1859	10.86~	(0.52- 225.86)
*MRFITR	4	m	0	50	3747	0	1859	50.12~	(3.09- 811.82)
*MRFITR	5	m	0	54	3591	0	1859	56.43~	(3.49- 913.25)
Subtotal MRFITR								33.22	(6.37- 173.28)
NAM	74	m	1	-	-	-	-	6.70	(4.19- 10.71)
NAM	75	m	1	-	-	-	-	10.27	(6.42- 16.43)
NAM	90	f	1	-	-	-	-	9.06	(5.83- 14.06)
NAM	91	f	1	-	-	-	-	16.65	(10.20- 27.19)
Subtotal NAM								9.95	(7.88- 12.57)
NOTAN2	8	m	0	6	42	134	544	0.58	(0.24- 1.39)
NOTAN2	9	m	0	28	47	134	544	2.42	(1.46- 4.01)
NOTAN2	10	m	0	44	40	134	544	4.47	(2.80- 7.13)
Subtotal NOTAN2								2.66	(1.93- 3.66)
ORMOS	1	m	0	32	329	7	777	10.80	(4.72- 24.71)
ORMOS	2	m	0	40	577	7	777	7.69	(3.42- 17.30)
ORMOS	3	m	0	15	128	7	777	13.01	(5.20- 32.52)
Subtotal ORMOS								10.06	(6.17- 16.42)
OSANN	49	m	2	-	-	-	-	17.70	(12.60- 24.80)
OSANN	57	m	2	-	-	-	-	42.80	(30.50- 60.10)
OSANN	50	f	2	-	-	-	-	14.40	(11.00- 18.90)
OSANN	58	f	2	-	-	-	-	40.90	(29.30- 57.10)
Subtotal OSANN								24.11	(20.59- 28.24)
PARKIN	14	m	6	-	-	-	-	3.90	(3.00- 5.00)
PARKIN	15	m	6	-	-	-	-	5.20	(3.50- 7.70)
Subtotal PARKIN								4.25	(3.43- 5.26)
PASTOR	6	m	1	-	-	-	-	2.33	(0.78- 6.95)
PASTOR	7	m	1	-	-	-	-	6.42	(2.96- 13.92)
PASTOR	8	m	1	-	-	-	-	8.02	(3.84- 16.73)
PASTOR	9	m	1	-	-	-	-	8.61	(3.86- 19.19)
Subtotal PASTOR								6.44	(4.27- 9.72)
PERNU	17	m	0	15	15	97	275	2.84	(1.34- 6.01)
PERNU	18	m	0	61	31	97	275	5.58	(3.42- 9.11)
PERNU	19	m	0	224	96	97	275	6.62	(4.74- 9.23)
PERNU	20	m	0	127	67	97	275	5.37	(3.69- 7.82)
PERNU	21	m	0	478	138	97	275	9.82	(7.28- 13.24)
PERNU	22	m	0	361	54	97	275	18.95	(13.12- 27.38)
PERNU	23	m	0	40	23	97	275	4.93	(2.81- 8.66)
PERNU	24	m	0	74	14	97	275	14.99	(8.09- 27.75)
PERNU	11	f	0	3	14	110	971	1.89	(0.54- 6.68)
PERNU	12	f	0	5	13	110	971	3.40	(1.19- 9.70)
PERNU	13	f	0	4	30	110	971	1.18	(0.41- 3.40)
PERNU	14	f	0	1	14	110	971	0.63	(0.08- 4.84)
PERNU	15	f	0	1	11	110	971	0.80	(0.10- 6.27)
PERNU	16	f	0	5	7	110	971	6.31	(1.97- 20.20)
Subtotal PERNU								7.42	(6.44- 8.54)
PERSH2	8	c	4	-	-	-	-	5.76	(4.61- 7.19)
PERSH2	9	c	4	-	-	-	-	11.34	(9.14- 14.07)
Subtotal PERSH2								8.16	(6.99- 9.53)
*PETO	2	m	0	44	1181	2	295	5.50	(1.34- 22.54)
*PETO	3	m	0	55	855	2	295	9.49	(2.33- 38.66)
Subtotal PETO								7.23	(2.67- 19.57)
PEZZO2	3	m	0	57	139	6	117	8.00	(3.33- 19.21)
PEZZO2	4	m	0	107	47	6	117	44.39	(18.24- 108.02)
PEZZO2	5	m	0	69	12	6	117	112.13	(40.26- 312.24)
Subtotal PEZZO2								30.27	(17.76- 51.58)
PEZZOT	2	m	0	24	94	4	116	7.40	(2.48- 22.09)
PEZZOT	3	m	0	70	29	4	116	70.00	(23.61- 207.50)

Table 1G11 - 2

IESLC - Meta-anal of Ever Smoking (or Curr if Ever not avail) by Amount, Overview, Any prod (or Cigs if Any not avail)
All LC types
Most adjusted

REF	NRR	SEX	AD	Number Exposed Case	Number Exposed Cont	Non-exposed Case	Non-exposed Cont	RR	95.00%CI
PEZZOT	4	m	0	51	6	4	116	246.50	(66.69- 911.11)
Subtotal PEZZOT								42.28	(21.77- 82.11)
PIKE	1	m	0	181	168	18	69	4.13	(2.36- 7.23)
PIKE	2	m	0	228	109	18	69	8.02	(4.55- 14.13)
PIKE	3	m	0	66	37	18	69	6.84	(3.55- 13.18)
PIKE	5	f	0	73	60	36	96	3.24	(1.94- 5.42)
PIKE	6	f	0	67	26	36	96	6.87	(3.80- 12.44)
PIKE	7	f	0	16	3	36	96	14.22	(3.91- 51.73)
Subtotal PIKE								5.49	(4.27- 7.05)
POLEDN	2	c	0	53	103	12	139	5.96	(3.03- 11.72)
POLEDN	4	c	0	143	168	12	139	9.86	(5.25- 18.52)
Subtotal POLEDN								7.80	(4.92- 12.37)
*PRESCO	2	m	1	-	-	-	-	10.20	(4.49- 23.15)
*PRESCO	4	m	1	-	-	-	-	19.96	(8.92- 44.67)
*PRESCO	1	f	1	-	-	-	-	6.36	(3.60- 11.24)
*PRESCO	3	f	1	-	-	-	-	10.08	(5.72- 17.75)
Subtotal PRESCO								9.70	(6.98- 13.49)
RACHTA	10	f	1	-	-	-	-	3.64	(1.08- 12.32)
RACHTA	11	f	1	-	-	-	-	3.55	(1.75- 7.23)
RACHTA	12	f	1	-	-	-	-	13.77	(6.50- 29.16)
Subtotal RACHTA								6.13	(3.81- 9.85)
RANDIG	1	m	0	13	28	5	22	2.04	(0.63- 6.60)
RANDIG	2	m	0	65	99	5	22	2.89	(1.04- 8.01)
RANDIG	3	m	0	190	164	5	22	5.10	(1.89- 13.76)
RANDIG	4	m	0	142	68	5	22	9.19	(3.34- 25.31)
RANDIG	5	f	0	1	21	17	92	0.26	(0.03- 2.05)
RANDIG	6	f	0	12	13	17	92	5.00	(1.95- 12.79)
RANDIG	7	f	0	3	5	17	92	3.25	(0.71- 14.88)
Subtotal RANDIG								3.84	(2.50- 5.89)
SEGI2	10	m	1	-	-	-	-	2.10	(0.86- 5.16)
SEGI2	12	m	1	-	-	-	-	3.10	(1.40- 6.84)
SEGI2	14	m	1	-	-	-	-	3.40	(1.55- 7.45)
SEGI2	16	m	1	-	-	-	-	6.90	(2.78- 17.14)
SEGI2	18	m	1	-	-	-	-	7.90	(3.40- 18.37)
SEGI2	22	f	1	-	-	-	-	2.90	(1.09- 7.70)
SEGI2	24	f	1	-	-	-	-	1.44	(0.61- 3.40)
SEGI2	26	f	1	-	-	-	-	1.03	(0.34- 3.15)
Subtotal SEGI2								3.08	(2.26- 4.21)
SHAW	10	c	0	46	90	11	107	4.97	(2.43- 10.16)
SHAW	11	c	0	278	176	11	107	15.36	(8.03- 29.39)
Subtotal SHAW								9.23	(5.71- 14.93)
SIEMIA	13	m	0	-	-	-	-	3.00	(1.00- 9.90)
SIEMIA	14	m	0	-	-	-	-	4.50	(1.80- 13.20)
SIEMIA	15	m	0	-	-	-	-	7.90	(3.00- 24.10)
Subtotal SIEMIA								4.87	(2.64- 8.95)
SOBUE	117	m	0	147	157	34	128	3.52	(2.27- 5.47)
SOBUE	118	m	0	236	222	34	128	4.00	(2.63- 6.09)
SOBUE	119	m	0	226	187	34	128	4.55	(2.97- 6.96)
Subtotal SOBUE								4.02	(3.14- 5.14)
*SPEIZE	1	f	1	-	-	-	-	2.70	(0.90- 5.40)
*SPEIZE	2	f	1	-	-	-	-	5.20	(3.60- 8.10)
*SPEIZE	3	f	1	-	-	-	-	12.60	(9.90- 16.70)
*SPEIZE	4	f	1	-	-	-	-	15.70	(12.10- 20.20)
*SPEIZE	5	f	1	-	-	-	-	22.00	(14.80- 32.30)
Subtotal SPEIZE								12.52	(10.76- 14.56)
SPITZ	5	c	0	27	88	10	96	2.95	(1.35- 6.43)
SPITZ	6	c	0	95	48	10	96	19.00	(9.08- 39.74)
Subtotal SPITZ								7.89	(4.61- 13.49)
STOCKS	41	m	2	-	-	-	-	4.64	(3.25- 6.61)
STOCKS	42	m	2	-	-	-	-	7.89	(5.51- 11.29)
STOCKS	43	m	2	-	-	-	-	10.95	(7.09- 16.91)
STOCKS	44	m	2	-	-	-	-	10.03	(6.51- 15.46)
STOCKS	45	m	2	-	-	-	-	13.91	(8.96- 21.60)
STOCKS	48	f	1	-	-	-	-	2.24	(1.64- 3.03)
STOCKS	49	f	1	-	-	-	-	6.34	(4.42- 8.93)
Subtotal STOCKS								6.04	(5.25- 6.96)
STOCKW	1	c	0	2090	1194	2791	10641	6.67	(6.15- 7.25)
STOCKW	2	c	0	6053	1591	2791	10641	14.51	(13.54- 15.54)
STOCKW	3	c	0	4327	572	2791	10641	28.84	(26.18- 31.77)
Subtotal STOCKW								13.28	(12.68- 13.92)

Table 1G11 - 2

IESLC - Meta-anal of Ever Smoking (or Curr if Ever not avail) by Amount, Overview, Any prod (or Cigs if Any not avail)
All LC types
Most adjusted

REF	NRR	SEX	AD	Number Exposed Case	Number Exposed Cont	Non-exposed Case	Non-exposed Cont	RR	95.00%CI
SVENSS	6	f	1	-	-	-	-	4.60	(2.50- 9.30)
SVENSS	11	f	1	-	-	-	-	12.60	(6.50- 25.20)
SVENSS	16	f	1	-	-	-	-	59.00	(7.60- 458.03)
Subtotal SVENSS								8.31	(5.25- 13.17)
*TENKAN	10	m	1	-	-	-	-	15.86	(6.80- 37.00)
*TENKAN	11	m	1	-	-	-	-	20.25	(8.20- 50.00)
*TENKAN	12	m	1	-	-	-	-	24.97	(9.90- 63.00)
Subtotal TENKAN								19.74	(11.81- 33.01)
TIZZAN	7	m	0	130	238	180	305	0.93	(0.70- 1.23)
TIZZAN	8	m	0	468	470	180	305	1.69	(1.35- 2.11)
TIZZAN	9	m	0	301	108	180	305	4.72	(3.54- 6.29)
TIZZAN	10	m	0	83	20	180	305	7.03	(4.17- 11.85)
TIZZAN	15	f	0	11	14	25	114	3.58	(1.46- 8.82)
TIZZAN	16	f	0	14	14	25	114	4.56	(1.93- 10.75)
Subtotal TIZZAN								2.16	(1.87- 2.48)
TSUGAN	29	m	0	14	19	18	22	0.90	(0.36- 2.28)
TSUGAN	30	m	0	30	30	18	22	1.22	(0.55- 2.73)
TSUGAN	31	m	0	19	14	18	22	1.66	(0.65- 4.20)
Subtotal TSUGAN								1.22	(0.73- 2.03)
*TULINI	27	m	3	-	-	-	-	6.02	(3.01- 12.00)
*TULINI	28	m	3	-	-	-	-	12.00	(6.31- 22.90)
*TULINI	29	m	3	-	-	-	-	27.30	(14.20- 52.40)
*TULINI	32	f	3	-	-	-	-	8.17	(4.33- 15.40)
*TULINI	33	f	3	-	-	-	-	26.30	(14.40- 48.10)
*TULINI	34	f	3	-	-	-	-	38.70	(18.50- 80.80)
Subtotal TULINI								15.82	(12.10- 20.68)
*TVERDA	9	m	2	-	-	-	-	2.14	(1.15- 3.96)
*TVERDA	10	m	2	-	-	-	-	3.32	(2.07- 5.32)
*TVERDA	11	m	2	-	-	-	-	6.56	(4.04- 10.64)
*TVERDA	16	f	2	-	-	-	-	4.53	(1.08- 18.94)
*TVERDA	17	f	2	-	-	-	-	18.00	(5.33- 60.83)
Subtotal TVERDA								4.23	(3.19- 5.62)
WAKAI	40	m	2	-	-	-	-	1.80	(0.81- 4.02)
WAKAI	41	m	2	-	-	-	-	4.01	(1.91- 8.41)
WAKAI	42	m	2	-	-	-	-	9.19	(4.20- 20.10)
Subtotal WAKAI								4.09	(2.62- 6.40)
WANG2	9	c	4	-	-	-	-	1.40	(0.43- 4.54)
WANG2	10	c	4	-	-	-	-	1.22	(0.31- 4.83)
WANG2	11	c	4	-	-	-	-	1.41	(0.43- 4.61)
WANG2	12	c	4	-	-	-	-	1.16	(0.33- 4.03)
WANG2	13	c	4	-	-	-	-	3.19	(1.39- 7.29)
WANG2	14	c	4	-	-	-	-	2.39	(0.78- 7.34)
WANG2	15	c	4	-	-	-	-	7.25	(2.05- 25.65)
Subtotal WANG2								2.20	(1.43- 3.37)
WU	43	f	2	-	-	-	-	3.25	(1.71- 6.16)
WU	44	f	2	-	-	-	-	8.48	(4.16- 17.29)
Subtotal WU								4.99	(3.10- 8.04)
WUWILL	12	f	3	-	-	-	-	2.13	(1.75- 2.58)
WUWILL	13	f	3	-	-	-	-	3.37	(2.28- 4.97)
Subtotal WUWILL								2.33	(1.96- 2.78)
WYNDE2	17	m	0	17	114	8	105	1.96	(0.81- 4.72)
WYNDE2	18	m	0	122	203	8	105	7.89	(3.71- 16.75)
WYNDE2	19	m	0	88	83	8	105	13.92	(6.39- 30.32)
WYNDE2	20	m	0	155	112	8	105	18.16	(8.50- 38.80)
Subtotal WYNDE2								8.64	(5.83- 12.82)
WYNDE3	44	m	0	8	42	9	88	1.86	(0.67- 5.17)
WYNDE3	45	m	0	77	114	9	88	6.60	(3.14- 13.90)
WYNDE3	46	m	0	108	82	9	88	12.88	(6.12- 27.09)
WYNDE3	47	m	0	68	26	9	88	25.57	(11.25- 58.14)
WYNDE3	79	f	0	3	19	20	76	0.60	(0.16- 2.23)
WYNDE3	80	f	0	24	24	20	76	3.80	(1.79- 8.05)
WYNDE3	81	f	0	15	10	20	76	5.70	(2.23- 14.59)
WYNDE3	82	f	0	4	3	20	76	5.07	(1.05- 24.50)
Subtotal WYNDE3								6.19	(4.51- 8.50)
WYNDE4	43	m	0	17	82	12	115	1.99	(0.90- 4.38)
WYNDE4	44	m	0	67	147	12	115	4.37	(2.26- 8.46)
WYNDE4	45	m	0	228	274	12	115	7.97	(4.29- 14.82)
WYNDE4	46	m	0	190	98	12	115	18.58	(9.77- 35.33)
WYNDE4	47	m	0	130	64	12	115	19.47	(10.00- 37.88)
WYNDE4	57	f	2	-	-	-	-	1.13	(0.33- 3.89)
WYNDE4	58	f	2	-	-	-	-	2.01	(0.66- 6.10)

Table 1G11 - 2

IESLC - Meta-anal of Ever Smoking (or Curr if Ever not avail) by Amount, Overview, Any prod (or Cigs if Any not avail)
All LC types
Most adjusted

REF	NRR	SEX	AD	Number Exposed Case	Number Exposed Cont	Non-exposed Case	Non-exposed Cont	RR	95.00%CI
WYNDE4	59	f	2	-	-	-	-	6.49	(2.35- 17.93)
WYNDE4	60	f	2	-	-	-	-	11.54	(1.90- 70.11)
WYNDE4	61	f	2	-	-	-	-	11.54	(1.90- 70.11)
Subtotal WYNDE4								6.97	(5.35- 9.09)
WYNDE6	27	m	0	117	122	87	617	6.80	(4.85- 9.54)
WYNDE6	36	m	0	461	293	87	617	11.16	(8.54- 14.59)
WYNDE6	45	m	0	315	129	87	617	17.32	(12.78- 23.47)
WYNDE6	54	m	0	784	197	87	617	28.22	(21.47- 37.10)
WYNDE6	216	f	0	76	109	159	856	3.75	(2.68- 5.26)
WYNDE6	225	f	0	367	165	159	856	11.97	(9.33- 15.37)
WYNDE6	234	f	0	201	50	159	856	21.64	(15.21- 30.80)
WYNDE6	243	f	0	378	52	159	856	39.13	(27.98- 54.75)
Subtotal WYNDE6								14.13	(12.71- 15.72)
XU3	9	m	1	-	-	-	-	1.66	(0.55- 4.97)
XU3	10	m	1	-	-	-	-	2.98	(1.09- 8.17)
XU3	11	m	1	-	-	-	-	14.78	(5.30- 41.18)
XU3	12	m	1	-	-	-	-	27.72	(5.15- 149.23)
XU3	16	f	1	-	-	-	-	2.18	(0.61- 7.84)
XU3	17	f	1	-	-	-	-	4.41	(1.16- 16.83)
XU3	18	f	1	-	-	-	-	8.19	(0.89- 75.45)
Subtotal XU3								4.71	(2.94- 7.56)
YAMAGU	8	c	1	-	-	-	-	3.75	(1.89- 7.47)
YAMAGU	7	c	1	-	-	-	-	12.14	(5.10- 28.90)
Subtotal YAMAGU								5.90	(3.44- 10.11)
*YUAN	2	m	2	-	-	-	-	3.60	(1.88- 6.91)
*YUAN	3	m	2	-	-	-	-	9.40	(5.21- 16.97)
Subtotal YUAN								6.09	(3.94- 9.44)
ZHENG	11	m	0	25	40	33	94	1.78	(0.94- 3.37)
ZHENG	12	m	0	60	66	33	94	2.59	(1.53- 4.39)
ZHENG	13	m	0	128	89	33	94	4.10	(2.53- 6.62)
ZHENG	14	m	0	66	23	33	94	8.17	(4.40- 15.17)
ZHENG	22	f	0	24	29	152	184	1.00	(0.56- 1.79)
ZHENG	23	f	0	52	15	152	184	4.20	(2.27- 7.75)
Subtotal ZHENG								2.97	(2.36- 3.75)
ZHOU	4	c	0	61	5	507	68	1.64	(0.64- 4.22)
ZHOU	5	c	0	211	14	507	68	2.02	(1.11- 3.67)
ZHOU	6	c	0	581	29	507	68	2.69	(1.71- 4.22)
Subtotal ZHOU								2.31	(1.65- 3.23)
Partial Totals				41738	173694	21422	230274		

*prospective study ~ With 0.5 adjustment for zero

REF	NRR	SEX	AD	Ys	Ws	Qs	Ps
AGUDO	4	f	3	0.45	3.17	5.69	0.4219
AGUDO	5	f	3	1.60	4.04	0.15	0.0013
Subtotal AGUDO				1.09	7.21	5.84	
*AKIBA	27	m	5	1.25	15.26	4.41	0.0000
*AKIBA	28	m	5	1.81	19.39	0.01	0.0000
*AKIBA	29	m	5	2.21	13.18	2.30	0.0000
*AKIBA	33	f	5	1.28	35.93	9.32	0.0000
*AKIBA	34	f	5	1.76	13.74	0.01	0.0000
Subtotal AKIBA				1.57	97.50	16.06	
ALDERS	18	m	1	1.27	10.54	2.89	0.0000
ALDERS	19	m	1	2.07	13.07	1.06	0.0000
ALDERS	20	m	1	2.14	14.23	1.76	0.0000
ALDERS	21	f	1	0.96	34.91	23.88	0.0000
ALDERS	22	f	1	1.66	34.88	0.56	0.0000
ALDERS	23	f	1	1.93	25.78	0.51	0.0000
Subtotal ALDERS				1.59	133.42	30.66	
*ARCHER	1	m	0	1.26	4.20	1.18	0.0097
*ARCHER	2	m	0	1.81	5.52	0.00	0.0000
*ARCHER	3	m	0	2.14	5.22	0.65	0.0000
Subtotal ARCHER				1.77	14.95	1.82	
ARMADA	46	m	0	1.79	3.37	0.00	0.0010
ARMADA	47	m	0	3.02	3.54	5.32	0.0000
ARMADA	48	m	0	4.24	3.29	19.76	0.0000
Subtotal ARMADA				3.01	10.20	25.08	
AUVINE	13	c	2	3.00	7.37	10.70	0.0000
AUVINE	14	c	2	3.52	8.24	24.75	0.0000
AUVINE	15	c	2	4.20	4.27	24.73	0.0000
Subtotal AUVINE				3.47	19.88	60.19	

Table 1G11 - 2

IESLC - Meta-anal of Ever Smoking (or Curr if Ever not avail) by Amount, Overview, Any prod (or Cigs if Any not avail)
All LC types
Most adjusted

REF	NRR	SEX	AD	Ys	Ws	Qs	Ps
AXELSS	5	m	6	1.34	8.91	1.81	0.0001
AXELSS	6	m	6	2.19	10.87	1.70	0.0000
AXELSS	7	m	6	2.34	11.25	3.42	0.0000
AXELSS	13	f	0	1.10	6.02	2.87	0.0069
AXELSS	14	f	0	2.38	10.21	3.52	0.0000
AXELSS	15	f	0	2.77	6.08	5.85	0.0000
AXELSS	16	f	0	1.99	2.69	0.11	0.0011
Subtotal AXELSS				2.06	56.05	19.28	
BARBON	82	m	3	1.90	16.58	0.21	0.0000
BARBON	83	m	3	2.55	16.08	9.26	0.0000
BARBON	84	m	3	3.06	14.81	23.83	0.0000
Subtotal BARBON				2.48	47.46	33.30	
*BENSHL	11	m	1	1.39	4.28	0.70	0.0041
*BENSHL	12	m	1	2.20	5.46	0.93	0.0000
*BENSHL	13	m	1	2.39	5.53	2.01	0.0000
Subtotal BENSHL				2.04	15.26	3.64	
*BEST	13	m	1	2.30	6.23	1.64	0.0000
*BEST	14	m	1	2.80	6.77	6.88	0.0000
*BEST	15	m	1	2.85	6.30	7.09	0.0000
Subtotal BEST				2.66	19.31	15.61	
*BOUCOT	99	m	0	3.15	0.49	0.91	0.0271
*BOUCOT	100	m	0	3.83	0.49	2.06	0.0070
Subtotal BOUCOT				3.49	0.99	2.97	
BRESLO	13	m	0	0.58	7.18	10.60	0.1231
BRESLO	14	m	0	1.19	12.73	4.59	0.0000
BRESLO	15	m	0	2.04	15.85	0.97	0.0000
BRESLO	16	m	0	2.90	8.89	10.95	0.0000
BRESLO	29	f	0	0.15	1.80	4.83	0.8360
BRESLO	30	f	0	0.44	2.24	4.07	0.5084
Subtotal BRESLO				1.61	48.69	36.01	
*BRETT	1	m	0	0.94	5.22	3.82	0.0326
*BRETT	2	m	0	1.45	5.48	0.64	0.0007
*BRETT	3	m	0	2.08	5.09	0.42	0.0000
Subtotal BRETT				1.48	15.79	4.89	
BROSS	18	m	0	1.59	22.46	0.90	0.0000
BROSS	19	m	0	1.97	16.76	0.57	0.0000
Subtotal BROSS				1.75	39.21	1.46	
BROWN2	32	m	2	1.81	220.78	0.07	0.0000
BROWN2	42	m	2	2.65	387.09	283.55	0.0000
BROWN2	31	f	2	2.13	172.98	19.75	0.0000
BROWN2	41	f	2	2.84	312.24	343.45	0.0000
Subtotal BROWN2				2.45	1093.09	646.83	
BUFFLE	28	f	0	1.54	6.11	0.38	0.0001
BUFFLE	29	f	0	2.47	8.19	3.78	0.0000
BUFFLE	35	f	0	3.06	8.66	13.86	0.0000
Subtotal BUFFLE				2.44	22.96	18.02	
*CEDERL	80	m	2	1.22	12.65	4.06	0.0000
*CEDERL	81	m	2	2.01	19.12	0.96	0.0000
*CEDERL	82	m	2	2.48	18.57	8.75	0.0000
*CEDERL	76	f	2	1.04	15.40	8.66	0.0000
*CEDERL	77	f	2	2.05	19.39	1.27	0.0000
*CEDERL	78	f	2	2.05	8.98	0.59	0.0000
Subtotal CEDERL				1.85	94.11	24.30	
*CHANG	2	m	0	1.61	2.58	0.08	0.0096
*CHANG	3	m	0	2.36	3.99	1.30	0.0000
*CHANG	4	m	0	2.11	3.72	0.38	0.0000
*CHANG	8	f	0	1.11	3.97	1.84	0.0271
*CHANG	9	f	0	1.58	5.66	0.24	0.0002
*CHANG	10	f	0	2.11	6.22	0.62	0.0000
Subtotal CHANG				1.83	26.14	4.47	
CHATZI	1	c	0	0.94	14.84	10.75	0.0003
CHATZI	2	c	0	1.04	15.01	8.40	0.0001
CHATZI	3	c	0	1.56	16.06	0.89	0.0000
Subtotal CHATZI				1.19	45.92	20.04	
CHEN2	3	m	0	0.87	4.19	3.51	0.0735
CHEN2	4	m	0	1.17	5.43	2.08	0.0063
CHEN2	5	m	0	2.63	3.55	2.49	0.0000
CHEN2	6	m	0	2.07	3.80	0.30	0.0001
CHEN2	7	f	0	-0.95	3.04	22.75	0.0991
CHEN2	8	f	0	1.07	4.63	2.43	0.0217
CHEN2	9	f	0	2.22	0.82	0.15	0.0435

Table 1G11 - 2

IESLC - Meta-anal of Ever Smoking (or Curr if Ever not avail) by Amount, Overview, Any prod (or Cigs if Any not avail)
All LC types
Most adjusted

REF	NRR	SEX	AD	Ys	Ws	Qs	Ps
CHEN2	10	f	0	0.97	1.22	0.82	0.2838
Subtotal CHEN2				1.21	26.68	34.54	
CHOI	12	m	0	0.48	6.73	11.47	0.2084
CHOI	13	m	0	1.32	10.21	2.25	0.0000
CHOI	14	m	0	2.01	7.82	0.37	0.0000
CHOI	15	m	0	1.94	7.14	0.15	0.0000
CHOI	16	m	0	2.97	3.16	4.39	0.0000
CHOI	17	f	0	0.19	5.18	13.22	0.6591
CHOI	18	f	0	0.52	3.66	5.93	0.3219
CHOI	20	f	0	1.87	0.74	0.00	0.1083
Subtotal CHOI				1.34	44.64	37.79	
*CHOW	10	m	2	2.63	5.07	3.58	0.0000
*CHOW	11	m	2	3.09	5.32	8.92	0.0000
*CHOW	12	m	2	3.80	5.07	20.39	0.0000
Subtotal CHOW				3.17	15.47	32.89	
COMSTO	4	m	0	2.52	2.78	1.48	0.0000
COMSTO	5	m	0	2.90	3.35	4.12	0.0000
COMSTO	6	m	0	3.22	2.79	5.67	0.0000
COMSTO	9	f	0	2.01	4.98	0.24	0.0000
COMSTO	10	f	0	2.85	6.96	7.87	0.0000
COMSTO	11	f	0	2.59	2.75	1.74	0.0000
Subtotal COMSTO				2.65	23.61	21.10	
COOKSO	1	c	0	1.63	11.70	0.29	0.0000
COOKSO	2	c	0	2.31	7.06	1.93	0.0000
Subtotal COOKSO				1.89	18.76	2.22	
CORREA	46	c	1	2.23	39.38	7.61	0.0000
CORREA	50	c	1	3.23	39.20	81.34	0.0000
Subtotal CORREA				2.73	78.58	88.96	
*CPSI	243	m	1	1.76	44.36	0.04	0.0000
*CPSI	246	m	1	2.61	55.90	37.56	0.0000
*CPSI	275	f	1	0.22	13.40	32.91	0.4140
*CPSI	276	f	1	0.89	26.82	21.64	0.0000
*CPSI	277	f	1	1.62	50.55	1.55	0.0000
*CPSI	278	f	1	2.41	10.15	3.86	0.0000
Subtotal CPSI				1.77	201.17	97.56	
*CPSII	102	m	1	2.30	75.60	19.76	0.0000
*CPSII	103	m	1	2.87	75.65	87.84	0.0000
*CPSII	105	f	1	1.43	95.77	12.74	0.0000
*CPSII	106	f	1	2.59	140.12	89.78	0.0000
Subtotal CPSII				2.30	387.13	210.13	
DAMBER	6	m	1	0.83	10.34	9.47	0.0074
DAMBER	7	m	1	1.99	13.68	0.53	0.0000
DAMBER	8	m	1	2.21	14.68	2.56	0.0000
DAMBER	9	m	1	2.70	5.93	4.92	0.0000
Subtotal DAMBER				1.89	44.62	17.50	
DARBY	1	m	0	4.30	2.87	18.04	0.0000
DARBY	2	m	0	4.56	2.86	21.91	0.0000
DARBY	3	m	0	4.96	2.72	27.38	0.0000
DARBY	8	f	0	2.75	14.48	13.44	0.0000
DARBY	9	f	0	3.07	14.73	24.06	0.0000
DARBY	10	f	0	3.73	8.38	31.49	0.0000
Subtotal DARBY				3.37	46.05	136.32	
DAVEYS	1	m	0	0.20	2.07	5.24	0.7726
DAVEYS	2	m	0	2.01	2.27	0.10	0.0025
DAVEYS	3	m	0	1.89	2.11	0.02	0.0061
DAVEYS	4	m	0	2.36	2.18	0.71	0.0005
Subtotal DAVEYS				1.63	8.63	6.07	
DEAN	1	m	0	0.79	8.38	8.34	0.0218
DEAN	2	m	0	1.91	9.10	0.13	0.0000
DEAN	3	m	0	2.44	7.59	3.25	0.0000
Subtotal DEAN				1.70	25.06	11.71	
DEAN2	25	m	0	1.17	22.52	8.59	0.0000
DEAN2	26	m	0	2.03	19.18	1.13	0.0000
DEAN2	29	f	0	0.92	11.90	8.92	0.0014
DEAN2	30	f	0	1.60	3.63	0.13	0.0023
Subtotal DEAN2				1.44	57.24	18.77	
DEAN3	7	m	3	1.70	14.67	0.13	0.0000
DEAN3	14	m	3	2.00	16.24	0.74	0.0000
DEAN3	21	m	3	3.08	13.79	22.78	0.0000
DEAN3	91	f	3	1.15	15.42	6.31	0.0000
DEAN3	98	f	3	2.13	15.80	1.83	0.0000

Table 1G11 - 2

IESLC - Meta-anal of Ever Smoking (or Curr if Ever not avail) by Amount, Overview, Any prod (or Cigs if Any not avail)
All LC types
Most adjusted

REF	NRR	SEX	AD	Ys	Ws	Qs	Ps
DEAN3	105	f	3	3.19	10.09	19.71	0.0000
Subtotal DEAN3				2.13	86.02	51.51	
*DEKLER	2	m	2	2.97	0.95	1.32	0.0038
*DEKLER	3	m	2	3.14	0.98	1.77	0.0019
*DEKLER	4	m	2	3.48	0.96	2.74	0.0007
Subtotal DEKLER				3.19	2.89	5.83	
DESTEF	6	m	4	1.06	11.84	6.23	0.0002
DESTEF	7	m	4	2.13	16.62	1.90	0.0000
DESTEF	8	m	4	2.34	16.30	4.96	0.0000
DESTEF	9	m	4	3.17	11.72	22.17	0.0000
Subtotal DESTEF				2.18	56.48	35.26	
DOLL	1	m	0	1.31	5.40	1.23	0.0023
DOLL	2	m	0	2.01	6.13	0.30	0.0000
DOLL	3	m	0	2.26	6.11	1.36	0.0000
DOLL	4	m	0	2.81	5.91	6.13	0.0000
DOLL	5	m	0	3.32	3.72	8.68	0.0000
DOLL	7	f	0	-0.06	6.92	23.64	0.8795
DOLL	8	f	0	0.68	7.19	8.92	0.0698
DOLL	9	f	0	1.24	3.57	1.10	0.0195
DOLL	10	f	0	3.75	0.47	1.82	0.0098
Subtotal DOLL				1.60	45.43	53.17	
*DOLL2	46	m	1	1.90	0.96	0.01	0.0623
*DOLL2	47	m	1	2.51	0.96	0.50	0.0137
*DOLL2	48	m	1	3.17	0.97	1.84	0.0018
*DOLL2	10	f	1	0.25	0.79	1.86	0.8209
*DOLL2	11	f	1	1.86	2.40	0.01	0.0040
*DOLL2	12	f	1	3.39	2.93	7.52	0.0000
Subtotal DOLL2				2.43	9.01	11.74	
DORANT	6	c	0	2.14	7.99	0.99	0.0000
DORANT	7	c	0	3.30	12.23	28.02	0.0000
DORANT	8	c	0	3.59	11.93	38.66	0.0000
Subtotal DORANT				3.12	32.15	67.66	
DORGAN	108	m	2	1.93	10.33	0.20	0.0000
DORGAN	109	m	2	2.64	11.27	8.12	0.0000
DORGAN	96	f	3	1.74	56.05	0.17	0.0000
DORGAN	97	f	3	2.50	51.92	26.38	0.0000
Subtotal DORGAN				2.14	129.57	34.86	
*DORN	408	m	1	1.39	152.79	24.33	0.0000
*DORN	409	m	1	2.29	287.32	73.06	0.0000
*DORN	410	m	1	2.84	277.97	308.82	0.0000
*DORN	411	m	1	3.12	176.37	313.98	0.0000
Subtotal DORN				2.47	894.45	720.19	
DOSEME	5	m	2	0.79	20.90	20.98	0.0003
DOSEME	9	m	2	1.13	45.98	19.96	0.0000
DOSEME	13	m	2	1.89	21.74	0.20	0.0000
Subtotal DOSEME				1.24	88.62	41.14	
*DUNN	1	m	0	2.12	1.20	0.13	0.0199
*DUNN	2	m	0	2.20	1.71	0.29	0.0040
*DUNN	3	m	0	2.95	1.95	2.63	0.0000
*DUNN	4	m	0	3.22	1.88	3.87	0.0000
*DUNN	5	m	0	3.36	1.73	4.26	0.0000
Subtotal DUNN				2.83	8.48	11.18	
EBELIN	2	m	0	1.00	6.42	4.05	0.0116
EBELIN	3	m	0	1.96	5.47	0.16	0.0000
EBELIN	4	m	0	2.52	7.13	3.76	0.0000
EBELIN	5	m	0	2.79	1.60	1.59	0.0004
EBELIN	6	m	0	2.28	1.69	0.40	0.0031
Subtotal EBELIN				1.94	22.31	9.97	
*ENGELA	31	m	7	0.34	4.64	9.81	0.4684
*ENGELA	32	m	7	1.41	4.89	0.70	0.0018
*ENGELA	33	m	7	1.95	4.91	0.12	0.0000
*ENGELA	34	m	7	2.40	4.27	1.58	0.0000
*ENGELA	35	m	7	2.71	4.73	3.98	0.0000
*ENGELA	45	f	5	2.48	3.99	1.93	0.0000
*ENGELA	46	f	5	2.48	4.17	2.01	0.0000
*ENGELA	47	f	5	3.18	4.61	8.87	0.0000
*ENGELA	48	f	5	3.26	3.58	7.72	0.0000
Subtotal ENGELA				2.20	39.80	36.72	
*ENSTRO	7	m	1	1.56	31.30	1.72	0.0000
*ENSTRO	6	m	1	2.04	59.28	3.66	0.0000
*ENSTRO	5	m	1	2.61	74.58	50.57	0.0000

Table 1G11 - 2

IESLC - Meta-anal of Ever Smoking (or Curr if Ever not avail) by Amount, Overview, Any prod (or Cigs if Any not avail)
All LC types
Most adjusted

REF	NRR	SEX	AD	Ys	Ws	Qs	Ps
*ENSTRO	4	m	1	2.78	72.60	70.76	0.0000
*ENSTRO	3	m	1	2.97	65.00	89.81	0.0000
*ENSTRO	11	f	1	0.77	48.76	51.21	0.0000
*ENSTRO	10	f	1	1.46	104.90	11.38	0.0000
*ENSTRO	9	f	1	2.25	141.36	29.77	0.0000
*ENSTRO	8	f	1	2.80	116.71	119.35	0.0000
Subtotal ENSTRO				2.23	714.48	428.22	
ESAKI	1	m	0	0.09	7.53	21.71	0.8001
ESAKI	2	m	0	0.80	7.75	7.55	0.0253
ESAKI	3	m	0	1.78	4.39	0.00	0.0002
Subtotal ESAKI				0.75	19.68	29.26	
FAN	6	m	0	-0.35	8.53	39.10	0.3059
FAN	7	m	0	0.71	17.63	20.61	0.0029
FAN	8	m	0	1.38	21.51	3.61	0.0000
FAN	9	m	0	2.41	9.89	3.78	0.0000
FAN	10	f	0	0.50	10.28	17.22	0.1116
FAN	11	f	0	1.32	12.82	2.78	0.0000
FAN	12	f	0	2.48	7.51	3.61	0.0000
FAN	13	f	0	2.92	0.79	1.01	0.0095
Subtotal FAN				1.19	88.96	91.72	
GAO	21	f	2	0.55	30.53	46.68	0.0022
GAO	22	f	2	1.11	22.93	10.55	0.0000
GAO	23	f	2	2.58	9.77	6.07	0.0000
Subtotal GAO				1.07	63.23	63.30	
GAO2	2	m	0	1.21	6.65	2.22	0.0018
GAO2	3	m	0	2.02	7.66	0.40	0.0000
GAO2	4	m	0	2.36	7.06	2.32	0.0000
Subtotal GAO2				1.88	21.37	4.94	
GARSHI	26	m	1	1.19	24.90	8.95	0.0000
GARSHI	27	m	1	1.74	31.76	0.07	0.0000
GARSHI	28	m	1	2.04	25.11	1.56	0.0000
GARSHI	29	m	1	1.66	27.73	0.50	0.0000
Subtotal GARSHI				1.66	109.49	11.08	
GER	22	c	14	0.23	7.27	17.68	0.5331
GER	23	c	14	0.64	8.71	11.48	0.0582
GER	24	c	14	1.10	6.65	3.18	0.0046
Subtotal GER				0.64	22.63	32.34	
GOLLED	1	m	1	1.49	12.18	1.08	0.0000
GOLLED	2	m	1	1.85	12.69	0.05	0.0000
GOLLED	3	m	1	2.91	12.60	15.68	0.0000
Subtotal GOLLED				2.09	37.47	16.80	
GSELL	1	m	0	1.49	1.53	0.14	0.0655
GSELL	2	m	0	1.37	1.51	0.27	0.0931
GSELL	3	m	0	2.71	1.64	1.39	0.0005
GSELL	4	m	0	4.37	1.50	9.98	0.0000
GSELL	5	m	0	4.04	1.58	8.03	0.0000
Subtotal GSELL				2.80	7.77	19.81	
HAENSZ	52	f	0	0.57	17.63	26.31	0.0170
HAENSZ	51	f	0	1.64	7.30	0.17	0.0000
Subtotal HAENSZ				0.88	24.93	26.47	
*HAMMO2	7	m	1	2.21	4.47	0.80	0.0000
*HAMMO2	6	m	1	2.34	4.88	1.48	0.0000
Subtotal HAMMO2				2.28	9.35	2.28	
*HAMMON	153	m	1	2.00	8.19	0.36	0.0000
*HAMMON	154	m	1	2.12	11.45	1.23	0.0000
*HAMMON	155	m	1	2.84	10.98	12.02	0.0000
Subtotal HAMMON				2.34	30.62	13.61	
*HANSEN	1	m	2	0.31	5.16	11.23	0.4747
*HANSEN	2	m	2	1.06	3.91	2.06	0.0352
Subtotal HANSEN				0.64	9.07	13.29	
*HIRAYA	23	m	1	0.72	36.53	41.64	0.0000
*HIRAYA	24	m	1	1.39	77.85	12.71	0.0000
*HIRAYA	25	m	1	1.83	89.10	0.15	0.0000
*HIRAYA	26	f	1	0.81	38.69	37.11	0.0000
*HIRAYA	27	f	1	0.94	36.49	26.38	0.0000
*HIRAYA	28	f	1	1.50	15.75	1.35	0.0000
Subtotal HIRAYA				1.31	294.40	119.33	
HITOSU	35	m	1	0.73	5.44	6.09	0.0875
HITOSU	36	m	1	1.04	5.81	3.30	0.0125
HITOSU	37	m	1	1.54	5.14	0.31	0.0005
HITOSU	60	f	1	1.13	12.11	5.21	0.0001

Table 1G11 - 2

IESLC - Meta-anal of Ever Smoking (or Curr if Ever not avail) by Amount, Overview, Any prod (or Cigs if Any not avail)
All LC types
Most adjusted

REF	NRR	SEX	AD	Ys	Ws	Qs	Ps
HITOSU	61	f	1	1.15	3.27	1.32	0.0370
Subtotal HITOSU				1.12	31.76	16.23	
*HOLE	1	m	1	1.70	5.37	0.04	0.0001
*HOLE	3	m	1	2.19	6.47	1.01	0.0000
*HOLE	4	m	1	2.37	5.91	2.02	0.0000
*HOLE	5	m	1	2.01	4.55	0.23	0.0000
Subtotal HOLE				2.08	22.31	3.31	
HU	1	m	0	0.44	10.71	19.60	0.1527
HU	2	m	0	0.74	12.38	13.73	0.0095
HU	3	m	0	1.29	6.63	1.64	0.0009
HU	4	f	0	0.82	5.04	4.70	0.0643
HU	5	f	0	0.18	2.64	6.82	0.7672
HU	6	f	0	-0.51	0.65	3.43	0.6812
Subtotal HU				0.70	38.05	49.92	
HU2	2	c	0	-0.16	9.46	35.91	0.6262
HU2	3	c	0	0.29	18.89	42.57	0.2087
HU2	4	c	0	0.66	22.08	28.09	0.0019
HU2	5	c	0	0.74	21.23	23.63	0.0007
HU2	6	c	0	1.10	32.09	15.13	0.0000
HU2	7	c	0	1.78	12.31	0.00	0.0000
Subtotal HU2				0.79	116.06	145.33	
HUMBLE	2	m	1	2.22	3.63	0.67	0.0000
HUMBLE	3	m	1	3.21	4.80	9.62	0.0000
HUMBLE	5	m	1	2.45	1.57	0.69	0.0021
HUMBLE	6	m	1	3.26	1.44	3.12	0.0001
HUMBLE	8	f	1	2.95	3.07	4.17	0.0000
HUMBLE	9	f	1	2.77	5.38	5.19	0.0000
HUMBLE	11	f	1	2.92	2.12	2.69	0.0000
HUMBLE	12	f	1	3.61	1.37	4.52	0.0000
Subtotal HUMBLE				2.87	23.38	30.67	
JARUP	4	m	2	1.93	3.04	0.06	0.0008
JARUP	5	m	2	2.08	3.44	0.29	0.0001
Subtotal JARUP				2.01	6.49	0.35	
JEDRYC	45	m	4	1.25	23.96	7.07	0.0000
JEDRYC	46	m	4	1.82	28.13	0.02	0.0000
JEDRYC	47	m	4	2.04	23.96	1.49	0.0000
JEDRYC	48	f	4	1.85	5.04	0.02	0.0000
JEDRYC	49	f	4	0.87	4.91	4.19	0.0546
JEDRYC	50	f	4	2.00	2.63	0.11	0.0012
Subtotal JEDRYC				1.68	88.63	12.90	
JOLY	7	m	0	1.68	5.92	0.07	0.0000
JOLY	8	m	0	2.52	10.45	5.53	0.0000
JOLY	9	m	0	2.57	9.85	6.00	0.0000
JOLY	10	m	0	3.08	10.07	16.77	0.0000
JOLY	3	f	0	1.55	12.60	0.71	0.0000
JOLY	4	f	0	2.08	17.52	1.46	0.0000
JOLY	5	f	0	1.94	9.62	0.20	0.0000
JOLY	6	f	0	2.59	7.64	4.95	0.0000
Subtotal JOLY				2.24	83.67	35.68	
JUSSAW	34	m	2	1.72	4.79	0.03	0.0002
JUSSAW	35	m	2	2.64	2.83	2.04	0.0000
Subtotal JUSSAW				2.06	7.62	2.06	
*KAISE2	66	m	1	1.50	5.94	0.51	0.0003
*KAISE2	67	m	1	2.34	9.98	2.97	0.0000
*KAISE2	58	f	1	2.03	5.35	0.31	0.0000
*KAISE2	59	f	1	3.10	8.34	14.22	0.0000
Subtotal KAISE2				2.33	29.61	18.01	
*KAISER	6	m	2	1.88	13.61	0.12	0.0000
*KAISER	7	m	2	2.85	16.98	18.97	0.0000
*KAISER	8	m	2	3.04	25.61	40.01	0.0000
*KAISER	2	f	2	1.23	18.49	5.81	0.0000
*KAISER	3	f	2	2.08	24.04	1.98	0.0000
*KAISER	4	f	2	2.54	19.02	10.58	0.0000
Subtotal KAISER				2.32	117.74	77.46	
KANELL	26	m	1	0.54	23.99	37.72	0.0086
KANELL	27	m	1	1.95	24.67	0.66	0.0000
KANELL	28	m	1	3.02	8.40	12.61	0.0000
KANELL	29	m	1	3.53	10.82	32.84	0.0000
Subtotal KANELL				1.84	67.88	83.83	
KATSOU	3	f	1	0.82	6.64	6.32	0.0356
KATSOU	4	f	1	2.01	2.99	0.14	0.0005

Table 1G11 - 2

IESLC - Meta-anal of Ever Smoking (or Curr if Ever not avail) by Amount, Overview, Any prod (or Cigs if Any not avail)
All LC types
Most adjusted

REF	NRR	SEX	AD	Ys	Ws	Qs	Ps
Subtotal KATSOU				1.19	9.63	6.46	
KAUFMA	11	c	6	2.08	16.83	1.41	0.0000
KAUFMA	12	c	6	2.71	22.15	18.66	0.0000
KAUFMA	13	c	6	3.33	16.99	40.40	0.0000
KAUFMA	14	c	6	3.76	18.01	69.96	0.0000
KAUFMA	15	c	6	4.09	13.43	71.30	0.0000
Subtotal KAUFMA				3.14	87.41	201.72	
KHUDER	1	m	0	0.92	16.30	12.36	0.0002
KHUDER	2	m	0	2.35	18.30	5.66	0.0000
KHUDER	3	m	0	3.49	14.48	41.90	0.0000
Subtotal KHUDER				2.21	49.07	59.93	
*KINLEN	14	m	2	2.36	6.82	2.23	0.0000
*KINLEN	15	m	2	2.65	6.84	5.04	0.0000
*KINLEN	16	m	2	3.08	6.76	11.23	0.0000
Subtotal KINLEN				2.70	20.42	18.50	
*KNEKT	29	m	1	1.61	4.66	0.15	0.0005
*KNEKT	30	m	1	2.54	5.47	3.09	0.0000
Subtotal KNEKT				2.11	10.13	3.24	
KOO	11	f	0	0.31	7.09	15.62	0.4151
KOO	12	f	0	1.99	3.69	0.14	0.0001
KOO	13	f	0	0.42	0.49	0.93	0.7696
Subtotal KOO				0.86	11.27	16.68	
KOULUM	6	m	0	1.65	3.87	0.08	0.0012
KOULUM	5	m	0	3.17	4.27	8.18	0.0000
KOULUM	4	m	0	4.23	4.27	25.48	0.0000
Subtotal KOULUM				3.06	12.42	33.74	
KREUZE	19	m	3	0.92	2.54	1.94	0.1444
KREUZE	20	m	3	2.16	4.57	0.64	0.0000
KREUZE	21	m	3	2.97	4.24	5.91	0.0000
KREUZE	22	m	3	3.03	3.39	5.25	0.0000
KREUZE	30	m	3	2.10	18.30	1.80	0.0000
KREUZE	31	m	3	3.22	20.26	41.58	0.0000
KREUZE	32	m	3	3.49	18.97	54.85	0.0000
KREUZE	33	m	3	3.51	16.38	48.19	0.0000
KREUZE	25	f	3	1.74	2.81	0.01	0.0035
KREUZE	26	f	3	2.47	3.44	1.58	0.0000
KREUZE	27	f	3	2.49	2.00	0.99	0.0004
KREUZE	36	f	3	0.69	15.02	18.07	0.0072
KREUZE	37	f	3	1.69	19.01	0.21	0.0000
KREUZE	38	f	3	2.04	6.02	0.38	0.0000
Subtotal KREUZE				2.45	136.95	181.39	
KREYBE	9	m	1	1.76	5.70	0.00	0.0000
KREYBE	10	m	1	1.83	5.32	0.01	0.0000
KREYBE	11	m	1	2.66	5.07	3.84	0.0000
KREYBE	28	f	1	0.31	7.13	15.69	0.4115
KREYBE	29	f	1	0.65	2.02	2.64	0.3573
Subtotal KREYBE				1.46	25.24	22.19	
LAMTH	7	f	0	0.98	29.68	19.31	0.0000
LAMTH	2	f	0	1.68	18.27	0.22	0.0000
LAMTH	9	f	0	1.98	6.91	0.24	0.0000
Subtotal LAMTH				1.34	54.86	19.77	
LAUSSM	18	m	3	1.19	31.20	11.21	0.0000
LAUSSM	19	m	3	1.76	34.48	0.03	0.0000
LAUSSM	20	m	3	2.26	18.03	4.04	0.0000
Subtotal LAUSSM				1.66	83.71	15.29	
LETOUR	2	c	0	2.25	18.66	3.98	0.0000
LETOUR	3	c	0	2.85	18.55	20.86	0.0000
LETOUR	4	c	0	3.27	9.52	20.92	0.0000
Subtotal LETOUR				2.70	46.74	45.77	
*LIAW	3	c	2	1.13	10.81	4.69	0.0002
*LIAW	4	c	2	1.28	11.36	2.95	0.0000
*LIAW	5	c	2	2.12	7.17	0.76	0.0000
Subtotal LIAW				1.43	29.33	8.40	
*LIDDEL	2	m	1	1.20	15.00	5.18	0.0000
*LIDDEL	3	m	1	1.61	17.38	0.54	0.0000
Subtotal LIDDEL				1.42	32.38	5.72	
LIU2	8	m	3	0.18	3.50	9.04	0.7332
LIU2	9	m	3	1.96	3.78	0.11	0.0001
LIU2	10	m	3	3.06	3.18	5.15	0.0000
LIU2	14	f	3	0.59	2.81	4.07	0.3242
LIU2	15	f	3	1.25	3.48	1.01	0.0194

Table 1G11 - 2

IESLC - Meta-anal of Ever Smoking (or Curr if Ever not avail) by Amount, Overview, Any prod (or Cigs if Any not avail)
All LC types
Most adjusted

REF	NRR	SEX	AD	Ys	Ws	Qs	Ps
LIU2	16	f	3	2.88	1.70	2.04	0.0002
Subtotal LIU2				1.56	18.46	21.41	
LIU3	6	m	2	0.34	1.81	3.78	0.6441
LIU3	7	m	2	0.09	1.71	4.96	0.9103
LIU3	8	m	2	0.65	1.20	1.57	0.4778
Subtotal LIU3				0.33	4.72	10.32	
LIU4	7	m	2	0.75	2108.86	2296.81	0.0000
LIU4	8	m	2	1.28	4111.92	1066.84	0.0000
LIU4	9	m	2	1.94	2991.95	69.81	0.0000
Subtotal LIU4				1.37	9212.74	3433.46	
LIU5	2	c	0	-0.20	5.84	23.15	0.6267
LIU5	3	c	0	0.46	6.33	11.27	0.2520
LIU5	4	c	0	1.28	7.79	2.06	0.0004
Subtotal LIU5				0.58	19.96	36.48	
LUBIN	11	m	4	-0.33	2.38	10.68	0.6124
LUBIN	12	m	4	0.22	3.36	8.25	0.6825
LUBIN	13	m	4	1.87	3.80	0.03	0.0003
LUBIN	14	m	4	2.08	5.32	0.44	0.0000
Subtotal LUBIN				1.22	14.85	19.40	
LUBIN2	273	m	0	1.93	155.25	3.17	0.0000
LUBIN2	274	m	0	2.03	150.14	8.47	0.0000
LUBIN2	275	m	0	2.36	152.73	49.91	0.0000
LUBIN2	276	m	0	2.52	139.75	74.84	0.0000
LUBIN2	281	f	0	0.90	66.54	53.26	0.0000
LUBIN2	282	f	0	1.30	76.70	18.45	0.0000
LUBIN2	283	f	0	1.53	47.64	3.33	0.0000
LUBIN2	284	f	0	1.57	17.75	0.84	0.0000
Subtotal LUBIN2				1.96	806.50	212.26	
MACLEN	36	c	2	0.30	6.92	15.37	0.4298
MACLEN	37	c	2	0.98	10.81	7.13	0.0013
MACLEN	38	c	2	1.08	9.92	5.08	0.0007
MACLEN	39	c	2	1.41	8.18	1.18	0.0001
Subtotal MACLEN				0.97	35.83	28.75	
MARTIS	1	m	0	1.60	2.87	0.10	0.0066
MARTIS	2	m	0	1.88	3.20	0.02	0.0008
MARTIS	3	m	0	2.24	3.09	0.62	0.0001
Subtotal MARTIS				1.91	9.17	0.75	
MATOS	29	m	2	0.69	5.93	7.14	0.0914
MATOS	31	m	2	2.01	7.84	0.40	0.0000
MATOS	33	m	2	2.34	8.28	2.52	0.0000
Subtotal MATOS				1.78	22.05	10.05	
MATSUD	1	m	0	2.53	2.76	1.50	0.0000
MATSUD	2	m	0	2.97	2.87	4.01	0.0000
MATSUD	3	m	0	3.94	2.83	13.12	0.0000
Subtotal MATSUD				3.15	8.46	18.63	
MCCONN	26	c	0	-0.88	3.12	22.18	0.1215
MCCONN	25	c	0	0.31	5.38	11.82	0.4745
MCCONN	24	c	0	0.76	4.83	5.17	0.0966
Subtotal MCCONN				0.19	13.33	39.17	
*MIGRAN	2	m	2	1.39	2.61	0.42	0.0249
*MIGRAN	4	m	2	1.44	3.70	0.44	0.0055
*MIGRAN	6	m	2	1.64	3.44	0.08	0.0024
*MIGRAN	8	m	2	1.78	3.35	0.00	0.0011
*MIGRAN	29	f	2	1.59	1.88	0.08	0.0298
*MIGRAN	31	f	2	1.88	3.01	0.02	0.0011
*MIGRAN	33	f	2	2.01	1.67	0.08	0.0093
Subtotal MIGRAN				1.66	19.66	1.13	
*MRFITR	3	m	0	2.38	0.42	0.15	0.1236
*MRFITR	4	m	0	3.91	0.50	2.23	0.0059
*MRFITR	5	m	0	4.03	0.50	2.49	0.0045
Subtotal MRFITR				3.50	1.41	4.88	
NAM	74	m	1	1.90	17.45	0.22	0.0000
NAM	75	m	1	2.33	17.40	5.05	0.0000
NAM	90	f	1	2.20	19.83	3.39	0.0000
NAM	91	f	1	2.81	15.98	16.70	0.0000
Subtotal NAM				2.30	70.66	25.36	
NOTAN2	8	m	0	-0.54	5.01	27.29	0.2229
NOTAN2	9	m	0	0.88	15.08	12.41	0.0006
NOTAN2	10	m	0	1.50	17.54	1.51	0.0000
Subtotal NOTAN2				0.98	37.63	41.22	
ORMOS	1	m	0	2.38	5.60	1.94	0.0000

Table 1G11 - 2

IESLC - Meta-anal of Ever Smoking (or Curr if Ever not avail) by Amount, Overview, Any prod (or Cigs if Any not avail)
All LC types
Most adjusted

REF	NRR	SEX	AD	Ys	Ws	Qs	Ps
ORMOS	2	m	0	2.04	5.85	0.37	0.0000
ORMOS	3	m	0	2.57	4.57	2.75	0.0000
Subtotal ORMOS				2.31	16.03	5.06	
OSANN	49	m	2	2.87	33.51	39.32	0.0000
OSANN	57	m	2	3.76	33.40	129.12	0.0000
OSANN	50	f	2	2.67	52.45	40.33	0.0000
OSANN	58	f	2	3.71	34.52	127.35	0.0000
Subtotal OSANN				3.18	153.87	336.13	
PARKIN	14	m	6	1.36	58.89	10.85	0.0000
PARKIN	15	m	6	1.65	24.72	0.50	0.0000
Subtotal PARKIN				1.45	83.60	11.35	
PASTOR	6	m	1	0.85	3.21	2.86	0.1295
PASTOR	7	m	1	1.86	6.41	0.03	0.0000
PASTOR	8	m	1	2.08	7.09	0.60	0.0000
PASTOR	9	m	1	2.15	5.97	0.79	0.0000
Subtotal PASTOR				1.86	22.69	4.28	
PERNU	17	m	0	1.04	6.79	3.80	0.0066
PERNU	18	m	0	1.72	15.98	0.08	0.0000
PERNU	19	m	0	1.89	34.69	0.34	0.0000
PERNU	20	m	0	1.68	27.21	0.32	0.0000
PERNU	21	m	0	2.28	42.95	10.49	0.0000
PERNU	22	m	0	2.94	28.38	37.64	0.0000
PERNU	23	m	0	1.60	12.13	0.46	0.0000
PERNU	24	m	0	2.71	10.11	8.50	0.0000
PERNU	11	f	0	0.64	2.41	3.20	0.3224
PERNU	12	f	0	1.22	3.48	1.12	0.0225
PERNU	13	f	0	0.16	3.41	9.02	0.7636
PERNU	14	f	0	-0.46	0.92	4.69	0.6574
PERNU	15	f	0	-0.22	0.91	3.67	0.8339
PERNU	16	f	0	1.84	2.83	0.01	0.0019
Subtotal PERNU				2.00	192.21	83.35	
PERSH2	8	c	4	1.75	77.78	0.12	0.0000
PERSH2	9	c	4	2.43	82.57	33.61	0.0000
Subtotal PERSH2				2.10	160.35	33.73	
*PETO	2	m	0	1.70	1.93	0.01	0.0180
*PETO	3	m	0	2.25	1.95	0.41	0.0017
Subtotal PETO				1.98	3.88	0.43	
PEZZO2	3	m	0	2.08	5.00	0.42	0.0000
PEZZO2	4	m	0	3.79	4.86	19.49	0.0000
PEZZO2	5	m	0	4.72	3.66	31.43	0.0000
Subtotal PEZZO2				3.41	13.52	51.33	
PEZZOT	2	m	0	2.00	3.22	0.14	0.0003
PEZZOT	3	m	0	4.25	3.25	19.66	0.0000
PEZZOT	4	m	0	5.51	2.25	31.06	0.0000
Subtotal PEZZOT				3.74	8.72	50.86	
PIKE	1	m	0	1.42	12.27	1.70	0.0000
PIKE	2	m	0	2.08	11.96	1.02	0.0000
PIKE	3	m	0	1.92	8.91	0.16	0.0000
PIKE	5	f	0	1.18	14.59	5.49	0.0000
PIKE	6	f	0	1.93	10.92	0.21	0.0000
PIKE	7	f	0	2.65	2.30	1.72	0.0001
Subtotal PIKE				1.70	60.95	10.28	
POLEDN	2	c	0	1.79	8.40	0.00	0.0000
POLEDN	4	c	0	2.29	9.66	2.40	0.0000
Subtotal POLEDN				2.05	18.06	2.40	
*PRESCO	2	m	1	2.32	5.71	1.62	0.0000
*PRESCO	4	m	1	2.99	5.92	8.57	0.0000
*PRESCO	1	f	1	1.85	11.85	0.04	0.0000
*PRESCO	3	f	1	2.31	11.98	3.24	0.0000
Subtotal PRESCO				2.27	35.47	13.48	
RACHTA	10	f	1	1.29	2.59	0.64	0.0375

Table 1G11 - 2

IESLC - Meta-anal of Ever Smoking (or Curr if Ever not avail) by Amount, Overview, Any prod (or Cigs if Any not avail)
All LC types
Most adjusted

REF	NRR	SEX	AD	Ys	Ws	Qs	Ps
RACHTA	11	f	1	1.27	7.64	2.09	0.0005
RACHTA	12	f	1	2.62	6.82	4.72	0.0000
Subtotal RACHTA				1.81	17.05	7.46	
RANDIG	1	m	0	0.71	2.79	3.23	0.2326
RANDIG	2	m	0	1.06	3.69	1.96	0.0415
RANDIG	3	m	0	1.63	3.89	0.10	0.0013
RANDIG	4	m	0	2.22	3.74	0.68	0.0000
RANDIG	5	f	0	-1.36	0.90	8.86	0.1996
RANDIG	6	f	0	1.61	4.35	0.14	0.0008
RANDIG	7	f	0	1.18	1.66	0.62	0.1294
Subtotal RANDIG				1.35	21.02	15.61	
SEGI2	10	m	1	0.74	4.79	5.26	0.1046
SEGI2	12	m	1	1.13	6.11	2.65	0.0052
SEGI2	14	m	1	1.22	6.23	2.00	0.0022
SEGI2	16	m	1	1.93	4.64	0.09	0.0000
SEGI2	18	m	1	2.07	5.40	0.41	0.0000
SEGI2	22	f	1	1.06	4.02	2.12	0.0328
SEGI2	24	f	1	0.36	5.21	10.58	0.4054
SEGI2	26	f	1	0.03	3.10	9.61	0.9585
Subtotal SEGI2				1.13	39.50	32.73	
SHAW	10	c	0	1.60	7.51	0.26	0.0000
SHAW	11	c	0	2.73	9.13	8.10	0.0000
Subtotal SHAW				2.22	16.64	8.36	
SIEMIA	13	m	0	1.10	2.92	1.40	0.0603
SIEMIA	14	m	0	1.50	3.87	0.32	0.0031
SIEMIA	15	m	0	2.07	3.54	0.27	0.0001
Subtotal SIEMIA				1.58	10.33	1.99	
SOBUE	117	m	0	1.26	19.84	5.58	0.0000
SOBUE	118	m	0	1.39	21.76	3.54	0.0000
SOBUE	119	m	0	1.52	21.28	1.61	0.0000
Subtotal SOBUE				1.39	62.88	10.74	
*SPEIZE	1	f	1	0.99	4.79	3.04	0.0298
*SPEIZE	2	f	1	1.65	23.37	0.47	0.0000
*SPEIZE	3	f	1	2.53	56.20	31.06	0.0000
*SPEIZE	4	f	1	2.75	58.51	54.30	0.0000
*SPEIZE	5	f	1	3.09	25.23	42.68	0.0000
Subtotal SPEIZE				2.53	168.09	131.55	
SPITZ	5	c	0	1.08	6.30	3.17	0.0067
SPITZ	6	c	0	2.94	7.05	9.40	0.0000
Subtotal SPITZ				2.07	13.35	12.57	
STOCKS	41	m	2	1.53	30.49	1.99	0.0000
STOCKS	42	m	2	2.07	29.86	2.26	0.0000
STOCKS	43	m	2	2.39	20.34	7.40	0.0000
STOCKS	44	m	2	2.31	20.54	5.45	0.0000
STOCKS	45	m	2	2.63	19.85	14.08	0.0000
STOCKS	48	f	1	0.81	40.78	39.47	0.0000
STOCKS	49	f	1	1.85	31.07	0.10	0.0000
Subtotal STOCKS				1.80	192.91	70.75	
STOCKW	1	c	0	1.90	565.53	6.58	0.0000
STOCKW	2	c	0	2.67	802.56	627.46	0.0000
STOCKW	3	c	0	3.36	411.25	1015.63	0.0000
Subtotal STOCKW				2.59	1779.33	1649.67	
SVENSS	6	f	1	1.53	8.90	0.62	0.0000
SVENSS	11	f	1	2.53	8.37	4.62	0.0000
SVENSS	16	f	1	4.08	0.91	4.78	0.0001
Subtotal SVENSS				2.12	18.19	10.03	
*TENKAN	10	m	1	2.76	5.35	5.07	0.0000
*TENKAN	11	m	1	3.01	4.70	6.97	0.0000
*TENKAN	12	m	1	3.22	4.49	9.14	0.0000
Subtotal TENKAN				2.98	14.54	21.19	
TIZZAN	7	m	0	-0.08	48.24	168.28	0.5909
TIZZAN	8	m	0	0.52	76.34	122.59	0.0000
TIZZAN	9	m	0	1.55	46.69	2.64	0.0000
TIZZAN	10	m	0	1.95	14.11	0.36	0.0000
TIZZAN	15	f	0	1.28	4.74	1.25	0.0055
TIZZAN	16	f	0	1.52	5.22	0.39	0.0005
Subtotal TIZZAN				0.77	195.34	295.52	
TSUGAN	29	m	0	-0.10	4.44	15.96	0.8253
TSUGAN	30	m	0	0.20	5.96	15.07	0.6241
TSUGAN	31	m	0	0.51	4.44	7.33	0.2861
Subtotal TSUGAN				0.20	14.85	38.35	

Table 1G11 - 2

IESLC - Meta-anal of Ever Smoking (or Curr if Ever not avail) by Amount, Overview, Any prod (or Cigs if Any not avail)
All LC types
Most adjusted

REF	NRR	SEX	AD	Ys	Ws	Qs	Ps
*TULINI	27	m	3	1.80	8.03	0.00	0.0000
*TULINI	28	m	3	2.48	9.25	4.46	0.0000
*TULINI	29	m	3	3.31	9.01	20.73	0.0000
*TULINI	32	f	3	2.10	9.54	0.92	0.0000
*TULINI	33	f	3	3.27	10.56	23.12	0.0000
*TULINI	34	f	3	3.66	7.07	24.61	0.0000
Subtotal TULINI				2.76	53.47	73.83	
*TVERDA	9	m	2	0.76	10.05	10.65	0.0159
*TVERDA	10	m	2	1.20	17.25	6.01	0.0000
*TVERDA	11	m	2	1.88	16.39	0.13	0.0000
*TVERDA	16	f	2	1.51	1.87	0.15	0.0387
*TVERDA	17	f	2	2.89	2.59	3.14	0.0000
Subtotal TVERDA				1.44	48.15	20.08	
WAKAI	40	m	2	0.59	5.99	8.66	0.1504
WAKAI	41	m	2	1.39	6.99	1.13	0.0002
WAKAI	42	m	2	2.22	6.27	1.15	0.0000
Subtotal WAKAI				1.41	19.25	10.93	
WANG2	9	c	4	0.34	2.77	5.85	0.5757
WANG2	10	c	4	0.20	2.04	5.16	0.7765
WANG2	11	c	4	0.34	2.73	5.71	0.5702
WANG2	12	c	4	0.15	2.45	6.61	0.8162
WANG2	13	c	4	1.16	5.60	2.22	0.0061
WANG2	14	c	4	0.87	3.06	2.58	0.1276
WANG2	15	c	4	1.98	2.41	0.09	0.0021
Subtotal WANG2				0.79	21.05	28.23	
WU	43	f	2	1.18	9.36	3.50	0.0003
WU	44	f	2	2.14	7.57	0.91	0.0000
Subtotal WU				1.61	16.93	4.41	
WUWILL	12	f	3	0.76	101.98	109.07	0.0000
WUWILL	13	f	3	1.21	25.31	8.38	0.0000
Subtotal WUWILL				0.85	127.28	117.44	
WYNDE2	17	m	0	0.67	4.95	6.19	0.1353
WYNDE2	18	m	0	2.07	6.77	0.51	0.0000
WYNDE2	19	m	0	2.63	6.33	4.50	0.0000
WYNDE2	20	m	0	2.90	6.67	8.21	0.0000
Subtotal WYNDE2				2.16	24.72	19.41	
WYNDE3	44	m	0	0.62	3.69	5.03	0.2325
WYNDE3	45	m	0	1.89	6.93	0.07	0.0000
WYNDE3	46	m	0	2.56	6.95	4.07	0.0000
WYNDE3	47	m	0	3.24	5.69	11.99	0.0000
WYNDE3	79	f	0	-0.51	2.23	11.79	0.4459
WYNDE3	80	f	0	1.34	6.83	1.42	0.0005
WYNDE3	81	f	0	1.74	4.35	0.01	0.0003
WYNDE3	82	f	0	1.62	1.55	0.04	0.0436
Subtotal WYNDE3				1.82	38.21	34.42	
WYNDE4	43	m	0	0.69	6.13	7.47	0.0891
WYNDE4	44	m	0	1.47	8.79	0.88	0.0000
WYNDE4	45	m	0	2.08	9.99	0.82	0.0000
WYNDE4	46	m	0	2.92	9.30	11.92	0.0000
WYNDE4	47	m	0	2.97	8.67	12.04	0.0000
WYNDE4	57	f	2	0.12	2.52	7.02	0.8460
WYNDE4	58	f	2	0.70	3.11	3.71	0.2185
WYNDE4	59	f	2	1.87	3.72	0.02	0.0003
WYNDE4	60	f	2	2.45	1.18	0.51	0.0079
WYNDE4	61	f	2	2.45	1.18	0.51	0.0079
Subtotal WYNDE4				1.94	54.60	44.89	
WYNDE6	27	m	0	1.92	33.49	0.54	0.0000
WYNDE6	36	m	0	2.41	53.48	20.68	0.0000
WYNDE6	45	m	0	2.85	41.59	46.86	0.0000
WYNDE6	54	m	0	3.34	51.37	123.40	0.0000
WYNDE6	216	f	0	1.32	33.57	7.34	0.0000
WYNDE6	225	f	0	2.48	61.57	29.52	0.0000
WYNDE6	234	f	0	3.07	30.83	50.86	0.0000
WYNDE6	243	f	0	3.67	34.09	120.07	0.0000
Subtotal WYNDE6				2.65	340.00	399.27	
XU3	9	m	1	0.51	3.17	5.22	0.3668
XU3	10	m	1	1.09	3.79	1.85	0.0336
XU3	11	m	1	2.69	3.66	2.98	0.0000
XU3	12	m	1	3.32	1.36	3.18	0.0001
XU3	16	f	1	0.78	2.36	2.41	0.2316
XU3	17	f	1	1.48	2.15	0.20	0.0297

Table 1G11 - 2

IESLC - Meta-anal of Ever Smoking (or Curr if Ever not avail) by Amount, Overview, Any prod (or Cigs if Any not avail)
All LC types
Most adjusted

REF	NRR	SEX	AD	Ys	Ws	Qs	Ps
XU3	18	f	1	2.10	0.78	0.08	0.0634
Subtotal XU3				1.55	17.25	15.92	
YAMAGU	8	c	1	1.32	8.14	1.79	0.0002
YAMAGU	7	c	1	2.50	5.11	2.55	0.0000
Subtotal YAMAGU				1.77	13.24	4.33	
*YUAN	2	m	2	1.28	9.07	2.35	0.0001
*YUAN	3	m	2	2.24	11.02	2.24	0.0000
Subtotal YUAN				1.81	20.09	4.59	
ZHENG	11	m	0	0.58	9.44	13.90	0.0764
ZHENG	12	m	0	0.95	13.74	9.67	0.0004
ZHENG	13	m	0	1.41	16.67	2.41	0.0000
ZHENG	14	m	0	2.10	10.04	0.97	0.0000
ZHENG	22	f	0	0.00	11.34	36.28	0.9951
ZHENG	23	f	0	1.43	10.21	1.29	0.0000
Subtotal ZHENG				1.09	71.45	64.52	
ZHOU	4	c	0	0.49	4.29	7.23	0.3077
ZHOU	5	c	0	0.70	10.77	12.71	0.0209
ZHOU	6	c	0	0.99	18.91	12.16	0.0000
Subtotal ZHOU				0.84	33.97	32.10	

N	607
NS	140

Table 1G11 - 3

IESLC - Meta-anal of Ever Smoking (or Curr if Ever not avail) by Amount, Overview, Any prod (or Cigs if Any not avail)
All LC types
Most adjusted

		Sex		
	combined	male	female	Total
N	66	354	187	607
NS	21	107	62	190

In this overview table, other than the "N" rows, entries in the "absent" and "Total" columns may be invalid and should be ignored

	Amount smoked (broad categories)				
	absent	<20k5	6-44k20	>20k45	Total
N	192	174	113	128	607
NS	95	125	88	102	410
Wt	3943.16	5466.17	6949.50	5066.84	21425.66
Het Chi	2029.43	1863.48	2514.85	1890.56	12439.13
Het df	191	173	112	127	606
Het P	***	***	***	***	***
Fixed RR	8.79	3.25	5.30	10.17	5.99
RRl	8.52	3.17	5.18	9.89	5.91
RRu	9.07	3.34	5.43	10.45	6.07
P	+++	+++	+++	+++	+++
Random RR	6.82	3.49	7.33	13.69	6.57
RRl	6.09	3.13	6.29	11.80	6.13
RRu	7.63	3.89	8.54	15.89	7.04
P	+++	+++	+++	+++	+++

			Amount smoked (narrow categories)					
	absent	<10k1	2-19k10	11-29k20	21-39k30	31-98k40	>40k99	Total
N	347	72	58	88	22	2	18	607
NS	140	49	41	68	19	2	15	334
Wt	12545.64	1082.98	1057.94	5543.64	600.64	25.15	569.67	21425.66
Het Chi	7360.90	467.94	355.19	1172.27	55.85	17.02	142.60	12439.13
Het df	346	71	57	87	21	1	17	606
Het P	***	***	***	***	***	***	***	***
Fixed RR	6.56	3.12	4.98	4.44	15.96	25.62	24.66	5.99
RRl	6.44	2.94	4.69	4.32	14.74	17.33	22.72	5.91
RRu	6.67	3.31	5.29	4.56	17.29	37.87	26.77	6.07
P	+++	+++	+++	+++	+++	+++	+++	+++
Random RR	7.33	2.45	4.81	7.59	14.37	17.67	19.40	6.57
RRl	6.68	2.06	4.07	6.48	11.98	2.96	13.48	6.13
RRu	8.05	2.92	5.68	8.90	17.24	105.61	27.91	7.04
P	+++	+++	+++	+++	+++	++	+++	+++

MALES

	Amount smoked (broad categories)				
	absent	<20k5	6-44k20	>20k45	Total
N	101	99	72	82	354
NS	67	97	71	81	316
Wt	2231.24	3583.62	5450.31	4143.16	15408.33
Het Chi	770.90	915.65	1123.25	951.49	7257.11
Het df	100	98	71	81	353
Het P	***	***	***	***	***
Fixed RR	9.62	2.78	4.35	8.67	5.30
RRl	9.23	2.69	4.24	8.41	5.21
RRu	10.03	2.88	4.47	8.94	5.38
P	+++	+++	+++	+++	+++
Random RR	8.29	3.70	7.66	14.19	7.41
RRl	7.27	3.21	6.40	11.97	6.81
RRu	9.46	4.27	9.16	16.82	8.08
P	+++	+++	+++	+++	+++

Table 1G11 - 3

IESLC - Meta-anal of Ever Smoking (or Curr if Ever not avail) by Amount, Overview, Any prod (or Cigs if Any not avail)
All LC types
Most adjusted

MALES

Amount smoked (narrow categories)

	absent	<10k1	2-19k10	11-29k20	21-39k30	31-98k40	>40k99	Total
N	203	37	31	56	15	1	11	354
NS	107	36	30	55	15	1	11	254
Wt	8630.91	637.78	579.60	4967.62	488.76	7.14	96.53	15408.33
Het Chi	4827.64	272.25	153.97	734.53	33.28	0.00	53.04	7257.11
Het df	202	36	30	55	14	0	10	353
Het P	***	***	***	***	**	N.S.	***	***
Fixed RR	5.78	3.58	6.04	4.14	15.63	6.93	17.33	5.30
RRl	5.66	3.31	5.57	4.03	14.31	3.33	14.20	5.21
RRu	5.90	3.87	6.55	4.26	17.08	14.44	21.16	5.38
P	+++	+++	+++	+++	+++	+++	+++	+++
Random RR	8.20	2.74	5.71	7.92	13.83	6.93	21.40	7.41
RRl	7.23	2.13	4.61	6.49	11.32	3.33	13.13	6.81
RRu	9.29	3.51	7.06	9.67	16.89	14.44	34.87	8.08
P	+++	+++	+++	+++	+++	+++	+++	+++

FEMALES

Amount smoked (broad categories)

	absent	<20k5	6-44k20	>20k45	Total
N	65	57	31	34	187
NS	48	55	30	34	167
Wt	1325.47	1095.30	574.64	399.35	3394.75
Het Chi	790.23	407.12	144.27	171.34	2349.48
Het df	64	56	30	33	186
Het P	***	***	***	***	***
Fixed RR	8.22	3.53	8.19	16.12	6.77
RRl	7.79	3.32	7.54	14.61	6.54
RRu	8.67	3.74	8.89	17.78	7.00
P	+++	+++	+++	+++	+++
Random RR	5.48	3.15	7.35	12.94	5.50
RRl	4.41	2.63	5.96	9.90	4.82
RRu	6.80	3.77	9.08	16.90	6.27
P	+++	+++	+++	+++	+++

Amount smoked (narrow categories)

	absent	<10k1	2-19k10	11-29k20	21-39k30	31-98k40	>40k99	Total
N	106	27	21	25	5		3	187
NS	60	26	20	24	5		3	138
Wt	2070.23	318.46	413.39	477.97	91.84		22.87	3394.75
Het Chi	1385.00	65.37	82.21	107.44	5.52		1.27	2349.48
Het df	105	26	20	24	4		2	186
Het P	***	***	***	***	N.S.		N.S.	***
Fixed RR	8.01	2.16	4.07	8.83	17.12		12.02	6.77
RRl	7.67	1.93	3.69	8.07	13.95		7.98	6.54
RRu	8.36	2.41	4.48	9.65	21.00		18.10	7.00
P	+++	+++	+++	+++	+++		+++	+++
Random RR	6.50	2.13	4.24	8.03	16.99		12.02	5.50
RRl	5.46	1.73	3.38	6.39	12.29		7.98	4.82
RRu	7.74	2.61	5.31	10.10	23.47		18.10	6.27
P	+++	+++	+++	+++	+++		+++	+++

Table 1G11 - 4

IESLC - Meta-anal of Ever Smoking (or Curr if Ever not avail) by Amount, Overview, Any prod (or Cigs if Any not avail)
All LC types
Least adjusted

REF	NRR	X	SEX	AGEL	AGEH	RACE	YF	LC TYPE	LOC	START	ST	NLC	R	VB	P	H	AD	SM	PRODUCT	exL	exH	S1	S2	DENOM	De
AGUDO	11	x	f	0	0	all	-	all	Eu:wst	1989	CC	103	n	bl	n	n	0	ev	cig only	1	10	1	0	nev any	st
AGUDO	12	x	f	0	0	all	-	all	Eu:wst	1989	CC	103	n	bl	n	n	0	ev	cig only	11	99	0	0	nev any	st
AKIBA	17	x	m	0	0	all	0	all	As:Jap	1963	pr	610	n	bl	n	n	0	cu	cig+/-ot	1	14	1	0	nev cigs	or
AKIBA	18	x	m	0	0	all	0	all	As:Jap	1963	pr	610	n	bl	n	n	0	cu	cig+/-ot	15	24	2	3	nev cigs	or
AKIBA	19	x	m	0	0	all	0	all	As:Jap	1963	pr	610	n	bl	n	n	0	cu	cig+/-ot	25	99	3	0	nev cigs	or
AKIBA	23	x	f	0	0	all	0	all	As:Jap	1963	pr	610	n	bl	n	n	0	cu	cig+/-ot	1	14	1	0	nev cigs	or
AKIBA	24	x	f	0	0	all	0	all	As:Jap	1963	pr	610	n	bl	n	n	0	cu	cig+/-ot	15	99	0	0	nev cigs	or
ALDERS	18		m	0	0	all	-	all	Eu:UK	1977	CC	1448	n	V	n	n	1	ev	cig only	1	17	1	0	nev+2	ot
ALDERS	19		m	0	0	all	-	all	Eu:UK	1977	CC	1448	n	V	n	n	1	ev	cig only	18	27	2	3	nev+2	ot
ALDERS	20		m	0	0	all	-	all	Eu:UK	1977	CC	1448	n	V	n	n	1	ev	cig only	28	99	3	0	nev+2	ot
ALDERS	21		f	0	0	all	-	all	Eu:UK	1977	CC	1448	n	V	n	n	1	ev	cig only	1	17	1	0	nev+2	ot
ALDERS	22		f	0	0	all	-	all	Eu:UK	1977	CC	1448	n	V	n	n	1	ev	cig only	18	27	2	3	nev+2	ot
ALDERS	23		f	0	0	all	-	all	Eu:UK	1977	CC	1448	n	V	n	n	1	ev	cig only	28	99	3	0	nev+2	ot
ARCHER	1		m	0	0	wh	0	all	NAmer	1950	pr	146	m	bl	n	n	0	cu	cig+/-ot	1	19	1	0	nev cigs	st
ARCHER	2		m	0	0	wh	0	all	NAmer	1950	pr	146	m	bl	n	n	0	cu	cig+/-ot	20	20	2	3	nev cigs	st
ARCHER	3		m	0	0	wh	0	all	NAmer	1950	pr	146	m	bl	n	n	0	cu	cig+/-ot	21	99	3	0	nev cigs	st
ARMADA	46		m	0	0	all	-	all	Eu:wst	1986	CC	325	n	bl	n	y	0	ev	cig+/-ot	1	14	1	0	nev any	st
ARMADA	47		m	0	0	all	-	all	Eu:wst	1986	CC	325	n	bl	n	y	0	ev	cig+/-ot	15	24	2	3	nev any	st
ARMADA	48		m	0	0	all	-	all	Eu:wst	1986	CC	325	n	bl	n	y	0	ev	cig+/-ot	25	99	3	0	nev any	st
AUVINE	5	x	c	0	0	all	-	all	Eu:Sca	1986	CC	517	n	bl	y	n	0	ev	cig+/-ot	1	10	1	0	nev cigs	st
AUVINE	6	x	c	0	0	all	-	all	Eu:Sca	1986	CC	517	n	bl	y	n	0	ev	cig+/-ot	11	20	2	3	nev cigs	st
AUVINE	7	x	c	0	0	all	-	all	Eu:Sca	1986	CC	517	n	bl	y	n	0	ev	cig+/-ot	21	99	3	0	nev cigs	st
AXELSS	19	x	m	0	0	sca	-	all	Eu:Sca	1989	CC	436	n	bl	n	n	0	ev	all/unsp	1	9	1	1	nev any	st
AXELSS	20	x	m	0	0	sca	-	all	Eu:Sca	1989	CC	436	n	bl	n	n	0	ev	all/unsp	10	19	0	2	nev any	st
AXELSS	21	x	m	0	0	sca	-	all	Eu:Sca	1989	CC	436	n	bl	n	n	0	ev	all/unsp	20	99	0	0	nev any	st
AXELSS	13		f	0	0	sca	-	all	Eu:Sca	1989	CC	436	n	bl	n	n	0	ev	all/unsp	1	9	1	1	nev any	st
AXELSS	14		f	0	0	sca	-	all	Eu:Sca	1989	CC	436	n	bl	n	n	0	ev	all/unsp	10	19	0	2	nev any	st
AXELSS	15		f	0	0	sca	-	all	Eu:Sca	1989	CC	436	n	bl	n	n	0	ev	all/unsp	20	29	2	3	nev any	st
AXELSS	16		f	0	0	sca	-	all	Eu:Sca	1989	CC	436	n	bl	n	n	0	ev	all/unsp	30	99	3	0	nev any	st
BARBON	5	x	m	0	0	all	-	all	Eu:wst	1979	CC	755	n	bl	y	y	0	ev	all/unsp	1	9	1	1	nev any	st
BARBON	7	x	m	0	0	all	-	all	Eu:wst	1979	CC	755	n	bl	y	y	0	ev	all/unsp	10	19	0	2	nev any	st
BARBON	9	x	m	0	0	all	-	all	Eu:wst	1979	CC	755	n	bl	y	y	0	ev	all/unsp	20	29	2	3	nev any	st
BARBON	11	x	m	0	0	all	-	all	Eu:wst	1979	CC	755	n	bl	y	y	0	ev	all/unsp	30	39	0	4	nev any	st
BARBON	13	x	m	0	0	all	-	all	Eu:wst	1979	CC	755	n	bl	y	y	0	ev	all/unsp	40	99	3	0	nev any	st
BENSHL	11		m	40	64	all	10	all	Eu:UK	1967	pr	486	n	V	n	n	1	cu	cig+/-ot	1	9	1	1	nev any	ot
BENSHL	12		m	40	64	all	10	all	Eu:UK	1967	pr	486	n	V	n	n	1	cu	cig+/-ot	10	19	0	2	nev any	ot
BENSHL	13		m	40	64	all	10	all	Eu:UK	1967	pr	486	n	V	n	n	1	cu	cig+/-ot	20	99	0	0	nev any	ot
BEST	13		m	0	0	all	0	all	NAmer	1955	pr	381	n	V	n	n	1	cu	cig only	1	9	1	1	nev any	ot
BEST	14		m	0	0	all	0	all	NAmer	1955	pr	381	n	V	n	n	1	cu	cig only	10	20	2	0	nev any	ot
BEST	15		m	0	0	all	0	all	NAmer	1955	pr	381	n	V	n	n	1	cu	cig only	21	99	3	0	nev any	ot
BOUCOT	99		m	0	0	all	9	all	NAmer	1951	pr	121	n	bl	n	n	0	ev	cig+/-ot	1	20	0	0	nev any	ot
BOUCOT	100		m	0	0	all	9	all	NAmer	1951	pr	121	n	bl	n	n	0	ev	cig+/-ot	21	99	3	0	nev any	ot
BRESLO	13		m	0	0	all	-	all	NAmer	1949	CC	518	n	bl	n	y	0	ev	cig+/-ot	1	9	1	1	nev+3	st
BRESLO	14		m	0	0	all	-	all	NAmer	1949	CC	518	n	bl	n	y	0	ev	cig+/-ot	10	19	0	2	nev+3	st
BRESLO	15		m	0	0	all	-	all	NAmer	1949	CC	518	n	bl	n	y	0	ev	cig+/-ot	20	39	2	0	nev+3	st
BRESLO	16		m	0	0	all	-	all	NAmer	1949	CC	518	n	bl	n	y	0	ev	cig+/-ot	40	99	3	0	nev+3	st
BRESLO	29		f	0	0	all	-	all	NAmer	1949	CC	518	n	bl	n	y	0	ev	cig+/-ot	1	19	1	0	nev+3	st
BRESLO	30		f	0	0	all	-	all	NAmer	1949	CC	518	n	bl	n	y	0	ev	cig+/-ot	20	99	0	0	nev+3	st
BRETT	1		m	0	0	all	0	all	Eu:UK	1960	pr	150	n	V	n	n	0	cu	cig+/-ot	1	14	1	0	nev cigs	st
BRETT	2		m	0	0	all	0	all	Eu:UK	1960	pr	150	n	V	n	n	0	cu	cig+/-ot	15	24	2	3	nev cigs	st
BRETT	3		m	0	0	all	0	all	Eu:UK	1960	pr	150	n	V	n	n	0	cu	cig+/-ot	25	99	3	0	nev cigs	st
BROSS	18		m	0	0	wh	-	all	NAmer	1960	CC	974	n	bl	n	n	0	cu	cig+/-ot	1	20	0	0	nev any	st
BROSS	19		m	0	0	wh	-	all	NAmer	1960	CC	974	n	bl	n	n	0	cu	cig+/-ot	21	99	3	0	nev any	st
BROWN2	32		m	0	0	wh	-	all	NAmer	1984	CC	14596	n	bl	n	y	2	ev	cig+/-ot	1	19	1	0	nev cigs	or
BROWN2	42		m	0	0	wh	-	all	NAmer	1984	CC	14596	n	bl	n	y	2	ev	cig+/-ot	20	99	0	0	nev cigs	or
BROWN2	31		f	0	0	wh	-	all	NAmer	1984	CC	14596	n	bl	n	y	2	ev	cig+/-ot	1	19	1	0	nev cigs	or
BROWN2	41		f	0	0	wh	-	all	NAmer	1984	CC	14596	n	bl	n	y	2	ev	cig+/-ot	20	99	0	0	nev cigs	or
BUFFLE	28		f	0	0	w-hi	-	all	NAmer	1976	CC	943	n	bl	y	n	0	ev	cig+/-ot	1	19	1	0	nev cigs	or
BUFFLE	29		f	0	0	w-hi	-	all	NAmer	1976	CC	943	n	bl	y	n	0	ev	cig+/-ot	20	20	2	3	nev cigs	or
BUFFLE	35		f	0	0	w-hi	-	all	NAmer	1976	CC	943	n	bl	y	n	0	ev	cig+/-ot	21	99	3	0	nev cigs	or
CEDERL	80		m	0	0	all	16	all	Eu:Sca	1963	pr	491	n	bl	n	n	2	cu	all/unsp	1	7	1	1	nev any	ot
CEDERL	81		m	0	0	all	16	all	Eu:Sca	1963	pr	491	n	bl	n	n	2	cu	all/unsp	8	15	0	2	nev any	ot
CEDERL	82		m	0	0	all	16	all	Eu:Sca	1963	pr	491	n	bl	n	n	2	cu	all/unsp	16	99	0	0	nev any	ot
CEDERL	76		f	0	0	all	0	all	Eu:Sca	1963	pr	491	n	bl	n	n	2	cu	all/unsp	1	7	1	1	nev any	or
CEDERL	77		f	0	0	all	0	all	Eu:Sca	1963	pr	491	n	bl	n	n	2	cu	all/unsp	8	15	0	2	nev any	or
CEDERL	78		f	0	0	all	0	all	Eu:Sca	1963	pr	491	n	bl	n	n	2	cu	all/unsp	16	99	0	0	nev any	or
CHANG	2		m	0	0	all	0	all	NAmer	1972	pr	136	n	bl	n	n	0	cu	cig+/-ot	1	10	1	0	nev cigs	st
CHANG	3		m	0	0	all	0	all	NAmer	1972	pr	136	n	bl	n	n	0	cu	cig+/-ot	11	20	2	3	nev cigs	st
CHANG	4		m	0	0	all	0	all	NAmer	1972	pr	136	n	bl	n	n	0	cu	cig+/-ot	21	99	3	0	nev cigs	st
CHANG	8		f	0	0	all	0	all	NAmer	1972	pr	136	n	bl	n	n	0	cu	cig+/-ot	1	10	1	0	nev cigs	st
CHANG	9		f	0	0	all	0	all	NAmer	1972	pr	136	n	bl	n	n	0	cu	cig+/-ot	11	20	2	3	nev cigs	st
CHANG	10		f	0	0	all	0	all	NAmer	1972	pr	136	n	bl	n	n	0	cu	cig+/-ot	21	99	3	0	nev cigs	st

Table 1G11 - 4

IESLC - Meta-anal of Ever Smoking (or Curr if Ever not avail) by Amount, Overview, Any prod (or Cigs if Any not avail)
All LC types
Least adjusted

REF	NRR	X	SEX	AGEL	AGEH	RACE	YF	LC TYPE	LOC	START	ST	NLC	R	VB	P	H	AD	SM	PRODUCT	exL	exH	S1	S2	DENOM	De
CHATZI	1		c	0	0	all	-	all	Eu:bal	1987	CC	282	n	bl	n	y	0	ev	all/unsp	1	45	0	0	nev any	st
CHATZI	2		c	0	0	all	-	all	Eu:bal	1987	CC	282	n	bl	n	y	0	ev	all/unsp	46	74	0	0	nev any	st
CHATZI	3		c	0	0	all	-	all	Eu:bal	1987	CC	282	n	bl	n	y	0	ev	all/unsp	75	99	0	6	nev any	st
CHEN2	3		m	0	0	all	-	all	As:Chi	1983	CC	193	n	ot	y	n	0	ev	all/unsp	1	9	1	1	nev any	st
CHEN2	4		m	0	0	all	-	all	As:Chi	1983	CC	193	n	ot	y	n	0	ev	all/unsp	10	20	2	0	nev any	st
CHEN2	5		m	0	0	all	-	all	As:Chi	1983	CC	193	n	ot	y	n	0	ev	all/unsp	21	30	0	4	nev any	st
CHEN2	6		m	0	0	all	-	all	As:Chi	1983	CC	193	n	ot	y	n	0	ev	all/unsp	31	99	3	0	nev any	st
CHEN2	7		f	0	0	all	-	all	As:Chi	1983	CC	193	n	ot	y	n	0	ev	all/unsp	1	9	1	1	nev any	st
CHEN2	8		f	0	0	all	-	all	As:Chi	1983	CC	193	n	ot	y	n	0	ev	all/unsp	10	20	2	0	nev any	st
CHEN2	9		f	0	0	all	-	all	As:Chi	1983	CC	193	n	ot	y	n	0	ev	all/unsp	21	30	0	4	nev any	st
CHEN2	10		f	0	0	all	-	all	As:Chi	1983	CC	193	n	ot	y	n	0	ev	all/unsp	31	99	3	0	nev any	st
CHOI	12		m	0	0	all	-	all	As:oth	1985	CC	375	n	bl	n	n	0	ev	cig+/-ot	1	10	1	0	nev cigs	st
CHOI	13		m	0	0	all	-	all	As:oth	1985	CC	375	n	bl	n	n	0	ev	cig+/-ot	11	20	2	3	nev cigs	st
CHOI	14		m	0	0	all	-	all	As:oth	1985	CC	375	n	bl	n	n	0	ev	cig+/-ot	21	30	0	4	nev cigs	st
CHOI	15		m	0	0	all	-	all	As:oth	1985	CC	375	n	bl	n	n	0	ev	cig+/-ot	31	40	0	5	nev cigs	st
CHOI	16		m	0	0	all	-	all	As:oth	1985	CC	375	n	bl	n	n	0	ev	cig+/-ot	41	99	3	6	nev cigs	st
CHOI	17		f	0	0	all	-	all	As:oth	1985	CC	375	n	bl	n	n	0	ev	cig+/-ot	1	10	1	0	nev cigs	st
CHOI	18		f	0	0	all	-	all	As:oth	1985	CC	375	n	bl	n	n	0	ev	cig+/-ot	11	30	2	0	nev cigs	st
CHOI	20		f	0	0	all	-	all	As:oth	1985	CC	375	n	bl	n	n	0	ev	cig+/-ot	31	99	3	0	nev cigs	st
CHOW	3	x	m	0	0	wh	0	all	NAmer	1966	pr	219	n	bl	n	n	0	cu	cig+/-ot	1	19	1	0	nev any	st
CHOW	4	x	m	0	0	wh	0	all	NAmer	1966	pr	219	n	bl	n	n	0	cu	cig+/-ot	20	29	2	3	nev any	st
CHOW	5	x	m	0	0	wh	0	all	NAmer	1966	pr	219	n	bl	n	n	0	cu	cig+/-ot	30	99	3	0	nev any	st
COMSTO	4		m	0	0	all	-	all	NAmer	1975	ot	258	n	bl	n	n	0	cu	cig+/-ot	1	19	1	0	nev any	st
COMSTO	5		m	0	0	all	-	all	NAmer	1975	ot	258	n	bl	n	n	0	cu	cig+/-ot	20	39	2	0	nev any	st
COMSTO	6		m	0	0	all	-	all	NAmer	1975	ot	258	n	bl	n	n	0	cu	cig+/-ot	40	99	3	0	nev any	st
COMSTO	9		f	0	0	all	-	all	NAmer	1975	ot	258	n	bl	n	n	0	cu	cig+/-ot	1	19	1	0	nev any	st
COMSTO	10		f	0	0	all	-	all	NAmer	1975	ot	258	n	bl	n	n	0	cu	cig+/-ot	20	39	2	0	nev any	st
COMSTO	11		f	0	0	all	-	all	NAmer	1975	ot	258	n	bl	n	n	0	cu	cig+/-ot	40	99	3	0	nev any	st
COOKSO	1		c	0	0	bl	-	all	Africa	1961	CC	234	n	V	n	y	0	ev	cig+/-ot	1	9	1	1	nev any	st
COOKSO	2		c	0	0	bl	-	all	Africa	1961	CC	234	n	V	n	y	0	ev	cig+/-ot	10	99	0	0	nev any	st
CORREA	45	x	c	0	0	all	-	all	NAmer	1979	CC	1359	n	bl	y	n	0	cu	cig+/-ot	1	20	0	0	nev cigs	st
CORREA	49	x	c	0	0	all	-	all	NAmer	1979	CC	1359	n	bl	y	n	0	cu	cig+/-ot	21	99	3	0	nev cigs	st
CPSI	243		m	50	74	all	6	all	NAmer	1959	pr	5138	n	bl	n	n	1	ev	cig only	1	19	1	0	nev any	ot
CPSI	246		m	50	74	all	6	all	NAmer	1959	pr	5138	n	bl	n	n	1	ev	cig only	20	99	0	0	nev any	ot
CPSI	275		f	40	74	all	6	all	NAmer	1959	pr	5138	n	bl	n	n	1	cu	cig+/-ot	1	9	1	1	nev cigs	ot
CPSI	276		f	40	74	all	6	all	NAmer	1959	pr	5138	n	bl	n	n	1	cu	cig+/-ot	10	19	0	2	nev cigs	ot
CPSI	277		f	40	74	all	6	all	NAmer	1959	pr	5138	n	bl	n	n	1	cu	cig+/-ot	20	39	2	0	nev cigs	ot
CPSI	278		f	40	74	all	6	all	NAmer	1959	pr	5138	n	bl	n	n	1	cu	cig+/-ot	40	99	3	0	nev cigs	ot
CPSII	102		m	35	99	all	4	all	NAmer	1982	pr	3229	n	bl	n	n	1	ev	cig only	1	20	0	0	nev any	ot
CPSII	103		m	35	99	all	4	all	NAmer	1982	pr	3229	n	bl	n	n	1	ev	cig only	21	99	3	0	nev any	ot
CPSII	105		f	35	99	all	4	all	NAmer	1982	pr	3229	n	bl	n	n	1	ev	cig+/-ot	1	19	1	0	nev cigs	ot
CPSII	106		f	35	99	all	4	all	NAmer	1982	pr	3229	n	bl	n	n	1	ev	cig+/-ot	20	99	0	0	nev cigs	ot
DAMBER	6		m	0	0	all	-	all	Eu:Sca	1972	CC	579	n	bl	y	n	1	ev	cig only	1	7	1	1	nev any	ot
DAMBER	7		m	0	0	all	-	all	Eu:Sca	1972	CC	579	n	bl	y	n	1	ev	cig only	8	15	0	2	nev any	ot
DAMBER	8		m	0	0	all	-	all	Eu:Sca	1972	CC	579	n	bl	y	n	1	ev	cig only	16	25	2	3	nev any	ot
DAMBER	9		m	0	0	all	-	all	Eu:Sca	1972	CC	579	n	bl	y	n	1	ev	cig only	26	99	3	0	nev any	ot
DARBY	1		m	0	0	wh	-	all	Eu:UK	1988	CC	982	n	V	n	n	0	cu	cig+/-ot	1	14	1	0	nev any	st
DARBY	2		m	0	0	wh	-	all	Eu:UK	1988	CC	982	n	V	n	n	0	cu	cig+/-ot	15	24	2	3	nev any	st
DARBY	3		m	0	0	wh	-	all	Eu:UK	1988	CC	982	n	V	n	n	0	cu	cig+/-ot	25	99	3	0	nev any	st
DARBY	8		f	0	0	wh	-	all	Eu:UK	1988	CC	982	n	V	n	n	0	cu	cig+/-ot	1	14	1	0	nev any	st
DARBY	9		f	0	0	wh	-	all	Eu:UK	1988	CC	982	n	V	n	n	0	cu	cig+/-ot	15	24	2	3	nev any	st
DARBY	10		f	0	0	wh	-	all	Eu:UK	1988	CC	982	n	V	n	n	0	cu	cig+/-ot	25	99	3	0	nev any	st
DAVEYS	1		m	0	0	all	-	all	Eu:Ger	1930	CC	109	n	bl	y	n	0	ev	all/unsp	1	5	1	1	nev any	st
DAVEYS	2		m	0	0	all	-	all	Eu:Ger	1930	CC	109	n	bl	y	n	0	ev	all/unsp	6	10	0	2	nev any	st
DAVEYS	3		m	0	0	all	-	all	Eu:Ger	1930	CC	109	n	bl	y	n	0	ev	all/unsp	11	20	2	3	nev any	st
DAVEYS	4		m	0	0	all	-	all	Eu:Ger	1930	CC	109	n	bl	y	n	0	ev	all/unsp	21	99	3	0	nev any	st
DEAN	1		m	0	0	wh	-	all	Africa	1947	CC	603	n	V	y	n	0	ev	cig only	1	20	0	0	nev any	st
DEAN	2		m	0	0	wh	-	all	Africa	1947	CC	603	n	V	y	n	0	ev	cig only	25	45	3	0	nev any	st
DEAN	3		m	0	0	wh	-	all	Africa	1947	CC	603	n	V	y	n	0	ev	cig only	50	99	0	6	nev any	st
DEAN2	25		m	0	0	all	-	all	Eu:UK	1960	CC	954	n	V	y	n	0	ev	cig only	1	22	0	0	nev any	st
DEAN2	26		m	0	0	all	-	all	Eu:UK	1960	CC	954	n	V	y	n	0	ev	cig only	23	99	3	0	nev any	st
DEAN2	29		f	0	0	all	-	all	Eu:UK	1960	CC	954	n	V	y	n	0	ev	cig only	1	22	0	0	nev any	st
DEAN2	30		f	0	0	all	-	all	Eu:UK	1960	CC	954	n	V	y	n	0	ev	cig only	23	99	3	0	nev any	st
DEAN3	5	x	m	0	0	all	-	all	Eu:UK	1969	CC	766	n	V	y	n	0	cu	cig only	1	12	1	0	nev any	st
DEAN3	12	x	m	0	0	all	-	all	Eu:UK	1969	CC	766	n	V	y	n	0	cu	cig only	13	22	2	3	nev any	st
DEAN3	19	x	m	0	0	all	-	all	Eu:UK	1969	CC	766	n	V	y	n	0	cu	cig only	23	99	3	0	nev any	st
DEAN3	89	x	f	0	0	all	-	all	Eu:UK	1969	CC	766	n	V	y	n	0	cu	cig only	1	12	1	0	nev any	st
DEAN3	96	x	f	0	0	all	-	all	Eu:UK	1969	CC	766	n	V	y	n	0	cu	cig only	13	22	2	3	nev any	st
DEAN3	103	x	f	0	0	all	-	all	Eu:UK	1969	CC	766	n	V	y	n	0	cu	cig only	23	99	3	0	nev any	st
DEKLER	2		m	0	0	all	0	all	Auslia	1961	pr	138	m	V	n	n	2	cu	cig+/-ot	1	14	1	0	nev any	or
DEKLER	3		m	0	0	all	0	all	Auslia	1961	pr	138	m	V	n	n	2	cu	cig+/-ot	15	24	2	3	nev any	or
DEKLER	4		m	0	0	all	0	all	Auslia	1961	pr	138	m	V	n	n	2	cu	cig+/-ot	25	99	3	0	nev any	or

Table 1G11 - 4

IESLC - Meta-anal of Ever Smoking (or Curr if Ever not avail) by Amount, Overview, Any prod (or Cigs if Any not avail)
All LC types
Least adjusted

REF	NRR	X	SEX	AGEL	AGEH	RACE	YF	LC TYPE	LOC	START	ST	NLC	R	VB	P	H	AD	SM	PRODUCT	exL	exH	S1	S2	DENOM	De
DESTEF	1	x	m	0	0	all	-	all	SCAmer	1988	CC	497	n	bl	n	y	0	ev	all/unsp	1	10	1	0	nev any	st
DESTEF	2	x	m	0	0	all	-	all	SCAmer	1988	CC	497	n	bl	n	y	0	ev	all/unsp	11	20	2	3	nev any	st
DESTEF	3	x	m	0	0	all	-	all	SCAmer	1988	CC	497	n	bl	n	y	0	ev	all/unsp	21	40	0	0	nev any	st
DESTEF	4	x	m	0	0	all	-	all	SCAmer	1988	CC	497	n	bl	n	y	0	ev	all/unsp	41	99	3	6	nev any	st
DOLL	1		m	0	0	all	-	all	Eu:UK	1948	CC	1465	n	V	n	n	0	ev	all/unsp	1	4	0	1	nev any	st
DOLL	2		m	0	0	all	-	all	Eu:UK	1948	CC	1465	n	V	n	n	0	ev	all/unsp	5	14	1	2	nev any	st
DOLL	3		m	0	0	all	-	all	Eu:UK	1948	CC	1465	n	V	n	n	0	ev	all/unsp	15	24	2	3	nev any	st
DOLL	4		m	0	0	all	-	all	Eu:UK	1948	CC	1465	n	V	n	n	0	ev	all/unsp	25	49	3	0	nev any	st
DOLL	5		m	0	0	all	-	all	Eu:UK	1948	CC	1465	n	V	n	n	0	ev	all/unsp	50	99	0	6	nev any	st
DOLL	7		f	0	0	all	-	all	Eu:UK	1948	CC	1465	n	V	n	n	0	ev	all/unsp	1	4	0	1	nev any	st
DOLL	8		f	0	0	all	-	all	Eu:UK	1948	CC	1465	n	V	n	n	0	ev	all/unsp	5	14	1	2	nev any	st
DOLL	9		f	0	0	all	-	all	Eu:UK	1948	CC	1465	n	V	n	n	0	ev	all/unsp	15	24	2	3	nev any	st
DOLL	10		f	0	0	all	-	all	Eu:UK	1948	CC	1465	n	V	n	n	0	ev	all/unsp	25	49	3	0	nev any	ot
DOLL2	46		m	35	99	all	5	all	Eu:UK	1951	pr	920	n	V	n	n	1	ev	all/unsp	1	14	1	0	nev any	ot
DOLL2	47		m	35	99	all	5	all	Eu:UK	1951	pr	920	n	V	n	n	1	ev	all/unsp	15	24	2	3	nev any	ot
DOLL2	48		m	35	99	all	5	all	Eu:UK	1951	pr	920	n	V	n	n	1	ev	all/unsp	25	99	3	0	nev any	ot
DOLL2	10		f	0	0	all	22	all	Eu:UK	1951	pr	920	n	V	n	n	1	cu	cig only	1	14	1	0	nev any	ot
DOLL2	11		f	0	0	all	22	all	Eu:UK	1951	pr	920	n	V	n	n	1	cu	cig only	15	24	2	3	nev any	ot
DOLL2	12		f	0	0	all	22	all	Eu:UK	1951	pr	920	n	V	n	n	1	cu	cig only	25	99	3	0	nev any	ot
DORANT	6		c	0	0	all	0	all	Eu:wst	1986	ot	550	n	bl	n	y	0	cu	cig+/-ot	1	9	1	1	nev any	st
DORANT	7		c	0	0	all	0	all	Eu:wst	1986	ot	550	n	bl	n	y	0	cu	cig+/-ot	10	19	0	2	nev any	st
DORANT	8		c	0	0	all	0	all	Eu:wst	1986	ot	550	n	bl	n	y	0	cu	cig+/-ot	20	99	0	0	nev any	st
DORGAN	10	x	m	0	0	wh	-	all	NAmer	1980	CC	2026	n	bl	y	y	0	ev	cig+/-ot	1	19	1	0	nev any	st
DORGAN	11	x	m	0	0	wh	-	all	NAmer	1980	CC	2026	n	bl	y	y	0	ev	cig+/-ot	20	99	0	0	nev any	st
DORGAN	34	x	m	0	0	bl	-	all	NAmer	1980	CC	2026	n	bl	y	y	0	ev	cig+/-ot	1	19	1	0	nev any	st
DORGAN	35	x	m	0	0	bl	-	all	NAmer	1980	CC	2026	n	bl	y	y	0	ev	cig+/-ot	20	99	0	0	nev any	st
DORGAN	96		f	0	0	all	-	all	NAmer	1980	CC	2026	n	bl	y	y	3	ev	cig+/-ot	1	19	1	0	nev any	ot
DORGAN	97		f	0	0	all	-	all	NAmer	1980	CC	2026	n	bl	y	y	3	ev	cig+/-ot	20	99	0	0	nev any	ot
DORN	408		m	0	0	wh	25	all	NAmer	1954	pr	5097	n	bl	n	n	1	cu	cig+/-ot	1	9	1	1	nev any	or
DORN	409		m	0	0	wh	25	all	NAmer	1954	pr	5097	n	bl	n	n	1	cu	cig+/-ot	10	20	2	0	nev any	or
DORN	410		m	0	0	wh	25	all	NAmer	1954	pr	5097	n	bl	n	n	1	cu	cig+/-ot	21	39	0	4	nev any	or
DORN	411		m	0	0	wh	25	all	NAmer	1954	pr	5097	n	bl	n	n	1	cu	cig+/-ot	40	99	3	0	nev any	or
DOSEME	5		m	0	0	all	-	all	Eu:bal	1979	CC	1210	n	bl	n	n	2	ev	cig+/-ot	1	10	1	0	nev cigs	or
DOSEME	9		m	0	0	all	-	all	Eu:bal	1979	CC	1210	n	bl	n	n	2	ev	cig+/-ot	11	20	2	3	nev cigs	or
DOSEME	13		m	0	0	all	-	all	Eu:bal	1979	CC	1210	n	bl	n	n	2	ev	cig+/-ot	21	99	3	0	nev cigs	or
DUNN	1		m	0	0	all	0	all	NAmer	1954	pr	139	o	bl	n	n	0	ev	cig+/-ot	1	4	0	1	nev cigs	st
DUNN	2		m	0	0	all	0	all	NAmer	1954	pr	139	o	bl	n	n	0	ev	cig+/-ot	5	14	1	2	nev cigs	st
DUNN	3		m	0	0	all	0	all	NAmer	1954	pr	139	o	bl	n	n	0	ev	cig+/-ot	15	24	2	3	nev cigs	st
DUNN	4		m	0	0	all	0	all	NAmer	1954	pr	139	o	bl	n	n	0	ev	cig+/-ot	25	34	0	4	nev cigs	st
DUNN	5		m	0	0	all	0	all	NAmer	1954	pr	139	o	bl	n	n	0	ev	cig+/-ot	35	99	3	0	nev cigs	st
EBELIN	2		m	0	0	all	-	all	Eu:Ger	1980	CC	130	n	bl	n	n	0	ev	all/unsp	1	9	1	1	nev any	st
EBELIN	3		m	0	0	all	-	all	Eu:Ger	1980	CC	130	n	bl	n	n	0	ev	all/unsp	10	19	0	2	nev any	st
EBELIN	4		m	0	0	all	-	all	Eu:Ger	1980	CC	130	n	bl	n	n	0	ev	all/unsp	20	29	2	3	nev any	st
EBELIN	5		m	0	0	all	-	all	Eu:Ger	1980	CC	130	n	bl	n	n	0	ev	all/unsp	30	39	0	4	nev any	st
EBELIN	6		m	0	0	all	-	all	Eu:Ger	1980	CC	130	n	bl	n	n	0	ev	all/unsp	40	99	3	0	nev any	st
ENGELA	3	x	m	0	0	all	0	all	Eu:Sca	1964	pr	435	n	bl	n	n	0	cu	cig+/-ot	1	4	0	1	nev cigs	st
ENGELA	4	x	m	0	0	all	0	all	Eu:Sca	1964	pr	435	n	bl	n	n	0	cu	cig+/-ot	5	9	1	0	nev cigs	st
ENGELA	5	x	m	0	0	all	0	all	Eu:Sca	1964	pr	435	n	bl	n	n	0	cu	cig+/-ot	10	14	0	2	nev cigs	st
ENGELA	6	x	m	0	0	all	0	all	Eu:Sca	1964	pr	435	n	bl	n	n	0	cu	cig+/-ot	15	19	0	0	nev cigs	st
ENGELA	7	x	m	0	0	all	0	all	Eu:Sca	1964	pr	435	n	bl	n	n	0	cu	cig+/-ot	20	99	0	0	nev cigs	st
ENGELA	17	x	f	0	0	all	0	all	Eu:Sca	1964	pr	435	n	bl	n	n	0	cu	cig+/-ot	1	4	0	1	nev cigs	st
ENGELA	18	x	f	0	0	all	0	all	Eu:Sca	1964	pr	435	n	bl	n	n	0	cu	cig+/-ot	5	9	1	0	nev cigs	st
ENGELA	19	x	f	0	0	all	0	all	Eu:Sca	1964	pr	435	n	bl	n	n	0	cu	cig+/-ot	10	14	0	2	nev cigs	st
ENGELA	20	x	f	0	0	all	0	all	Eu:Sca	1964	pr	435	n	bl	n	n	0	cu	cig+/-ot	15	19	0	0	nev cigs	st
ENGELA	21	x	f	0	0	all	0	all	Eu:Sca	1964	pr	435	n	bl	n	n	0	cu	cig+/-ot	20	99	0	0	nev cigs	st
ENSTRO	7		m	0	0	all	0	all	NAmer	1959	pr	2879	n	bl	n	n	1	cu	cig only	1	9	1	1	nev any	ot
ENSTRO	6		m	0	0	all	0	all	NAmer	1959	pr	2879	n	bl	n	n	1	cu	cig only	10	19	0	2	nev any	ot
ENSTRO	5		m	0	0	all	0	all	NAmer	1959	pr	2879	n	bl	n	n	1	cu	cig only	20	20	2	3	nev any	ot
ENSTRO	4		m	0	0	all	0	all	NAmer	1959	pr	2879	n	bl	n	n	1	cu	cig only	21	39	0	4	nev any	ot
ENSTRO	3		m	0	0	all	0	all	NAmer	1959	pr	2879	n	bl	n	n	1	cu	cig only	40	99	3	0	nev any	ot
ENSTRO	11		f	0	0	all	0	all	NAmer	1959	pr	2879	n	bl	n	n	1	cu	cig only	1	9	1	1	nev any	ot
ENSTRO	10		f	0	0	all	0	all	NAmer	1959	pr	2879	n	bl	n	n	1	cu	cig only	10	19	0	2	nev any	ot
ENSTRO	9		f	0	0	all	0	all	NAmer	1959	pr	2879	n	bl	n	n	1	cu	cig only	20	20	2	3	nev any	ot
ENSTRO	8		f	0	0	all	0	all	NAmer	1959	pr	2879	n	bl	n	n	1	cu	cig only	21	99	3	0	nev any	ot
ESAKI	1		m	0	0	all	-	all	As:Jap	1961	CC	245	n	bl	y	n	0	ev	cig+/-ot	1	14	1	0	nev cigs	st
ESAKI	2		m	0	0	all	-	all	As:Jap	1961	CC	245	n	bl	y	n	0	ev	cig+/-ot	15	29	2	3	nev cigs	st
ESAKI	3		m	0	0	all	-	all	As:Jap	1961	CC	245	n	bl	y	n	0	ev	cig+/-ot	30	99	3	0	nev cigs	st
FAN	6		m	0	0	all	-	all	As:Chi	1990	CC	403	n	ot	y	n	0	ev	cig+/-ot	1	9	1	1	nev cigs	st
FAN	7		m	0	0	all	-	all	As:Chi	1990	CC	403	n	ot	y	n	0	ev	cig+/-ot	10	19	0	2	nev cigs	st
FAN	8		m	0	0	all	-	all	As:Chi	1990	CC	403	n	ot	y	n	0	ev	cig+/-ot	20	29	2	3	nev cigs	st
FAN	9		m	0	0	all	-	all	As:Chi	1990	CC	403	n	ot	y	n	0	ev	cig+/-ot	30	99	3	0	nev cigs	st
FAN	10		f	0	0	all	-	all	As:Chi	1990	CC	403	n	ot	y	n	0	ev	cig+/-ot	1	9	1	1	nev cigs	st

Table 1G11 - 4

IESLC - Meta-anal of Ever Smoking (or Curr if Ever not avail) by Amount, Overview, Any prod (or Cigs if Any not avail)
All LC types
Least adjusted

REF	NRR	X	SEX	AGEL	AGEH	RACE	YF	LC TYPE	LOC	START	ST	NLC	R	VB	P	H	AD	SM	PRODUCT	exL	exH	S1	S2	DENOM	De
FAN	11		f	0	0	all	-	all	As:Chi	1990	CC	403	n	ot	y	n	0	ev	cig+/-ot	10	19	0	2	nev cigs	st
FAN	12		f	0	0	all	-	all	As:Chi	1990	CC	403	n	ot	y	n	0	ev	cig+/-ot	20	29	2	3	nev cigs	st
FAN	13		f	0	0	all	-	all	As:Chi	1990	CC	403	n	ot	y	n	0	ev	cig+/-ot	30	99	3	0	nev cigs	st
GAO	24	x	f	0	0	all	-	all	As:Chi	1984	CC	1405	n	ot	n	n	0	ev	cig+/-ot	1	9	1	1	nev cigs	st
GAO	25	x	f	0	0	all	-	all	As:Chi	1984	CC	1405	n	ot	n	n	0	ev	cig+/-ot	10	19	0	2	nev cigs	st
GAO	26	x	f	0	0	all	-	all	As:Chi	1984	CC	1405	n	ot	n	n	0	ev	cig+/-ot	20	99	0	0	nev cigs	st
GAO2	2		m	0	0	all	-	all	As:Jap	1988	CC	282	n	bl	n	n	0	cu	cig+/-ot	1	19	1	0	nev cigs	st
GAO2	3		m	0	0	all	-	all	As:Jap	1988	CC	282	n	bl	n	n	0	cu	cig+/-ot	20	29	2	3	nev cigs	st
GAO2	4		m	0	0	all	-	all	As:Jap	1988	CC	282	n	bl	n	n	0	cu	cig+/-ot	30	99	3	0	nev cigs	or
GARSHI	18	x	m	0	0	all	-	all	NAmer	1981	CC	1081	o	bl	y	n	0	ev	all/unsp	1	15	1	0	nev any	st
GARSHI	19	x	m	0	0	all	-	all	NAmer	1981	CC	1081	o	bl	y	n	0	ev	all/unsp	16	25	2	3	nev any	st
GARSHI	20	x	m	0	0	all	-	all	NAmer	1981	CC	1081	o	bl	y	n	0	ev	all/unsp	26	35	0	4	nev any	st
GARSHI	21	x	m	0	0	all	-	all	NAmer	1981	CC	1081	o	bl	y	n	0	ev	all/unsp	36	99	3	0	nev any	st
GER	18	x	c	0	0	all	-	all	As:oth	1990	CC	141	n	ot	y	n	0	ev	all/unsp	1	10	1	0	nev any	st
GER	19	x	c	0	0	all	-	all	As:oth	1990	CC	141	n	ot	y	n	0	ev	all/unsp	11	20	2	3	nev any	st
GER	20	x	c	0	0	all	-	all	As:oth	1990	CC	141	n	ot	y	n	0	ev	all/unsp	21	99	3	0	nev any	st
GOLLED	15	x	m	35	99	all	-	all	Eu:UK	1952	CC	443	n	V	y	n	0	ev	cig only	1	10	1	0	nev any	st
GOLLED	16	x	m	35	99	all	-	all	Eu:UK	1952	CC	443	n	V	y	n	0	ev	cig only	11	22	2	3	nev any	st
GOLLED	17	x	m	35	99	all	-	all	Eu:UK	1952	CC	443	n	V	y	n	0	ev	cig only	23	99	3	0	nev any	st
GSELL	1		m	0	0	all	-	all	Eu:wst	1937	CC	150	n	bl	n	y	0	ev	all/unsp	1	9	1	1	nev any	st
GSELL	2		m	0	0	all	-	all	Eu:wst	1937	CC	150	n	bl	n	y	0	ev	all/unsp	10	14	0	2	nev any	st
GSELL	3		m	0	0	all	-	all	Eu:wst	1937	CC	150	n	bl	n	y	0	ev	all/unsp	15	20	2	3	nev any	st
GSELL	4		m	0	0	all	-	all	Eu:wst	1937	CC	150	n	bl	n	y	0	ev	all/unsp	21	35	0	4	nev any	st
GSELL	5		m	0	0	all	-	all	Eu:wst	1937	CC	150	n	bl	n	y	0	ev	all/unsp	36	99	3	0	nev any	st
HAENSZ	52		f	0	0	all	-	not alv	NAmer	1955	CC	158	n	bl	n	y	0	cu	cig+/-ot	1	20	0	0	nev any	st
HAENSZ	51		f	0	0	all	-	not alv	NAmer	1955	CC	158	n	bl	n	y	0	cu	cig+/-ot	21	99	3	0	nev any	st
HAMMO2	21	x	m	0	0	all	0	all	NAmer	1967	pr	450	o	bl	n	n	0	cu	cig+/-ot	1	19	1	0	nev any	st
HAMMO2	20	x	m	0	0	all	0	all	NAmer	1967	pr	450	o	bl	n	n	0	cu	cig+/-ot	20	99	0	0	nev any	st
HAMMON	153		m	0	0	wh	0	all	NAmer	1952	pr	448	n	bl	n	n	1	ev	cig only	1	9	1	1	nev any	ot
HAMMON	154		m	0	0	wh	0	all	NAmer	1952	pr	448	n	bl	n	n	1	ev	cig only	10	20	2	0	nev any	ot
HAMMON	155		m	0	0	wh	0	all	NAmer	1952	pr	448	n	bl	n	n	1	ev	cig only	21	99	3	0	nev any	ot
HANSEN	1		m	0	0	all	0	all	Eu:Sca	1968	pr	105	o	bl	y	n	2	ev	all/unsp	1	19	1	0	nev any	ot
HANSEN	2		m	0	0	all	0	all	Eu:Sca	1968	pr	105	o	bl	y	n	2	ev	all/unsp	20	99	0	0	nev any	ot
HIRAYA	23		m	0	0	all	0	all	As:Jap	1965	pr	1917	n	bl	n	n	1	cu	cig+/-ot	1	9	1	1	nev any	st
HIRAYA	24		m	0	0	all	0	all	As:Jap	1965	pr	1917	n	bl	n	n	1	cu	cig+/-ot	10	19	0	2	nev any	st
HIRAYA	25		m	0	0	all	0	all	As:Jap	1965	pr	1917	n	bl	n	n	1	cu	cig+/-ot	20	99	0	0	nev any	st
HIRAYA	26		f	0	0	all	0	all	As:Jap	1965	pr	1917	n	bl	n	n	1	cu	cig+/-ot	1	9	1	1	nev any	st
HIRAYA	27		f	0	0	all	0	all	As:Jap	1965	pr	1917	n	bl	n	n	1	cu	cig+/-ot	10	19	0	2	nev any	st
HIRAYA	28		f	0	0	all	0	all	As:Jap	1965	pr	1917	n	bl	n	n	1	cu	cig+/-ot	20	99	0	0	nev any	st
HITOSU	3	x	m	0	0	all	-	all	As:Jap	1960	CC	216	n	bl	y	n	0	cu	all/unsp	1	14	1	0	nev any	st
HITOSU	4	x	m	0	0	all	-	all	As:Jap	1960	CC	216	n	bl	y	n	0	cu	all/unsp	15	24	2	3	nev any	st
HITOSU	5	x	m	0	0	all	-	all	As:Jap	1960	CC	216	n	bl	y	n	0	cu	all/unsp	25	99	3	0	nev any	st
HITOSU	10	x	f	0	0	all	-	all	As:Jap	1960	CC	216	n	bl	y	n	0	cu	all/unsp	1	14	1	0	nev any	st
HITOSU	11	x	f	0	0	all	-	all	As:Jap	1960	CC	216	n	bl	y	n	0	cu	all/unsp	15	99	0	0	nev any	st
HOLE	9	x	m	0	0	all	0	all	Eu:UK	1972	pr	225	n	V	n	n	0	cu	cig+/-ot	1	14	1	0	nev any	st
HOLE	10	x	m	0	0	all	0	all	Eu:UK	1972	pr	225	n	V	n	n	0	cu	cig+/-ot	15	24	2	3	nev any	st
HOLE	11	x	m	0	0	all	0	all	Eu:UK	1972	pr	225	n	V	n	n	0	cu	cig+/-ot	25	34	0	4	nev any	st
HOLE	12	x	m	0	0	all	0	all	Eu:UK	1972	pr	225	n	V	n	n	0	cu	cig+/-ot	35	99	3	0	nev any	st
HU	1		m	0	0	all	-	all	As:Chi	1985	CC	227	n	ot	n	y	0	ev	cig+/-ot	1	14	1	0	nev any	st
HU	2		m	0	0	all	-	all	As:Chi	1985	CC	227	n	ot	n	y	0	ev	cig+/-ot	14	24	2	3	nev any	st
HU	3		m	0	0	all	-	all	As:Chi	1985	CC	227	n	ot	n	y	0	ev	cig+/-ot	25	99	3	0	nev any	st
HU	4		f	0	0	all	-	all	As:Chi	1985	CC	227	n	ot	n	y	0	ev	cig+/-ot	1	14	1	0	nev any	st
HU	5		f	0	0	all	-	all	As:Chi	1985	CC	227	n	ot	n	y	0	ev	cig+/-ot	14	24	2	3	nev any	st
HU	6		f	0	0	all	-	all	As:Chi	1985	CC	227	n	ot	n	y	0	ev	cig+/-ot	25	99	3	0	nev any	st
HU2	2		c	0	0	all	-	all	As:Chi	1977	CC	523	n	ot	y	n	0	ev	cig+/-ot	1	4	0	1	nev cigs	st
HU2	3		c	0	0	all	-	all	As:Chi	1977	CC	523	n	ot	y	n	0	ev	cig+/-ot	5	9	1	0	nev cigs	st
HU2	4		c	0	0	all	-	all	As:Chi	1977	CC	523	n	ot	y	n	0	ev	cig+/-ot	10	14	0	2	nev cigs	st
HU2	5		c	0	0	all	-	all	As:Chi	1977	CC	523	n	ot	y	n	0	ev	cig+/-ot	15	19	0	0	nev cigs	or
HU2	6		c	0	0	all	-	all	As:Chi	1977	CC	523	n	ot	y	n	0	ev	cig+/-ot	20	29	2	3	nev cigs	st
HU2	7		c	0	0	all	-	all	As:Chi	1977	CC	523	n	ot	y	n	0	ev	cig+/-ot	30	99	3	0	nev cigs	st
HUMBLE	2		m	0	0	w-hi	-	all	NAmer	1980	CC	521	n	bl	y	n	1	cu	cig+/-ot	1	19	1	0	nev cigs	or
HUMBLE	3		m	0	0	w-hi	-	all	NAmer	1980	CC	521	n	bl	y	n	1	cu	cig+/-ot	20	99	0	0	nev cigs	or
HUMBLE	5		m	0	0	hi	-	all	NAmer	1980	CC	521	n	bl	y	n	1	cu	cig+/-ot	1	19	1	0	nev cigs	or
HUMBLE	6		m	0	0	hi	-	all	NAmer	1980	CC	521	n	bl	y	n	1	cu	cig+/-ot	20	99	0	0	nev cigs	or
HUMBLE	8		f	0	0	w-hi	-	all	NAmer	1980	CC	521	n	bl	y	n	1	cu	cig+/-ot	1	19	1	0	nev cigs	or
HUMBLE	9		f	0	0	w-hi	-	all	NAmer	1980	CC	521	n	bl	y	n	1	cu	cig+/-ot	20	99	0	0	nev cigs	or
HUMBLE	11		f	0	0	hi	-	all	NAmer	1980	CC	521	n	bl	y	n	1	cu	cig+/-ot	1	19	1	0	nev cigs	or
HUMBLE	12		f	0	0	hi	-	all	NAmer	1980	CC	521	n	bl	y	n	1	cu	cig+/-ot	20	99	0	0	nev cigs	or
JARUP	1	x	m	0	0	all	-	all	Eu:Sca	1928	CC	102	o	bl	y	n	0	ev	all/unsp	1	10	1	0	nev any	st
JARUP	2	x	m	0	0	all	-	all	Eu:Sca	1928	CC	102	o	bl	y	n	0	ev	all/unsp	11	99	0	0	nev any	st
JEDRYC	60	x	m	0	0	all	-	all	Eu:est	1980	CC	1630	n	bl	y	n	0	ev	cig+/-ot	1	19	1	0	nev any	st
JEDRYC	61	x	m	0	0	all	-	all	Eu:est	1980	CC	1630	n	bl	y	n	0	ev	cig+/-ot	20	29	2	3	nev any	st

Table 1G11 - 4

IESLC - Meta-anal of Ever Smoking (or Curr if Ever not avail) by Amount, Overview, Any prod (or Cigs if Any not avail)
All LC types
Least adjusted

REF	NRR	X	SEX	AGEL	AGEH	RACE	YF	LC TYPE	LOC	START	ST	NLC	R	VB	P	H	AD	SM	PRODUCT	exL	exH	S1	S2	DENOM	De
JEDRYC	62	x	m	0	0	all	-	all	Eu:est	1980	CC	1630	n	bl	y	n	0	ev	cig+/-ot	30	99	3	0	nev any	st
JEDRYC	65	x	f	0	0	all	-	all	Eu:est	1980	CC	1630	n	bl	y	n	0	ev	cig+/-ot	1	19	1	0	nev any	st
JEDRYC	66	x	f	0	0	all	-	all	Eu:est	1980	CC	1630	n	bl	y	n	0	ev	cig+/-ot	20	29	2	3	nev any	st
JEDRYC	67	x	f	0	0	all	-	all	Eu:est	1980	CC	1630	n	bl	y	n	0	ev	cig+/-ot	30	99	3	0	nev any	st
JOLY	7		m	0	0	all	-	all	SCAmer	1978	CC	826	n	bl	n	n	0	ev	cig+/-ot	1	9	1	1	nev any	st
JOLY	8		m	0	0	all	-	all	SCAmer	1978	CC	826	n	bl	n	n	0	ev	cig+/-ot	10	19	0	2	nev any	st
JOLY	9		m	0	0	all	-	all	SCAmer	1978	CC	826	n	bl	n	n	0	ev	cig+/-ot	20	29	2	3	nev any	st
JOLY	10		m	0	0	all	-	all	SCAmer	1978	CC	826	n	bl	n	n	0	ev	cig+/-ot	30	99	3	0	nev any	st
JOLY	3		f	0	0	all	-	all	SCAmer	1978	CC	826	n	bl	n	n	0	ev	cig+/-ot	1	9	1	1	nev any	st
JOLY	4		f	0	0	all	-	all	SCAmer	1978	CC	826	n	bl	n	n	0	ev	cig+/-ot	10	19	0	2	nev any	st
JOLY	5		f	0	0	all	-	all	SCAmer	1978	CC	826	n	bl	n	n	0	ev	cig+/-ot	20	29	2	3	nev any	st
JOLY	6		f	0	0	all	-	all	SCAmer	1978	CC	826	n	bl	n	n	0	ev	cig+/-ot	30	99	3	0	nev any	st
JUSSAW	10	x	m	0	0	all	-	all	As:Ind	1964	CC	792	n	V	n	n	0	ev	cig only	1	4	0	1	nev any	st
JUSSAW	11	x	m	0	0	all	-	all	As:Ind	1964	CC	792	n	V	n	n	0	ev	cig only	5	9	1	0	nev any	st
JUSSAW	12	x	m	0	0	all	-	all	As:Ind	1964	CC	792	n	V	n	n	0	ev	cig only	10	14	0	2	nev any	st
JUSSAW	13	x	m	0	0	all	-	all	As:Ind	1964	CC	792	n	V	n	n	0	ev	cig only	15	19	0	0	nev any	st
JUSSAW	14	x	m	0	0	all	-	all	As:Ind	1964	CC	792	n	V	n	n	0	ev	cig only	20	24	2	3	nev any	st
JUSSAW	15	x	m	0	0	all	-	all	As:Ind	1964	CC	792	n	V	n	n	0	ev	cig only	25	99	3	0	nev any	st
KAISE2	66		m	35	99	all	9	all	NAmer	1979	pr	318	n	bl	n	n	1	cu	cig only	1	19	1	0	nev any	st
KAISE2	67		m	35	99	all	9	all	NAmer	1979	pr	318	n	bl	n	n	1	cu	cig only	20	99	0	0	nev any	st
KAISE2	58		f	35	99	all	9	all	NAmer	1979	pr	318	n	bl	n	n	1	cu	cig only	1	19	1	0	nev any	st
KAISE2	59		f	35	99	all	9	all	NAmer	1979	pr	318	n	bl	n	n	1	cu	cig only	20	99	0	0	nev any	st
KAISER	6		m	0	0	all	0	all	NAmer	1964	pr	714	n	bl	n	n	2	cu	cig+/-ot	1	19	1	0	nev cigs	or
KAISER	7		m	0	0	all	0	all	NAmer	1964	pr	714	n	bl	n	n	2	cu	cig+/-ot	20	40	2	0	nev cigs	or
KAISER	8		m	0	0	all	0	all	NAmer	1964	pr	714	n	bl	n	n	2	cu	cig+/-ot	41	99	3	6	nev cigs	or
KAISER	2		f	0	0	all	0	all	NAmer	1964	pr	714	n	bl	n	n	2	cu	cig+/-ot	1	19	1	0	nev cigs	or
KAISER	3		f	0	0	all	0	all	NAmer	1964	pr	714	n	bl	n	n	2	cu	cig+/-ot	20	40	2	0	nev cigs	or
KAISER	4		f	0	0	all	0	all	NAmer	1964	pr	714	n	bl	n	n	2	cu	cig+/-ot	41	99	3	6	nev cigs	or
KANELL	1	x	m	0	0	all	-	all	Eu:bal	1950	CC	862	n	bl	n	n	0	cu	cig+/-ot	1	10	1	0	nev any	st
KANELL	2	x	m	0	0	all	-	all	Eu:bal	1950	CC	862	n	bl	n	n	0	cu	cig+/-ot	11	20	2	3	nev any	st
KANELL	3	x	m	0	0	all	-	all	Eu:bal	1950	CC	862	n	bl	n	n	0	cu	cig+/-ot	21	35	0	4	nev any	st
KANELL	4	x	m	0	0	all	-	all	Eu:bal	1950	CC	862	n	bl	n	n	0	cu	cig+/-ot	36	99	3	0	nev any	st
KATSOU	7	x	f	0	0	all	-	all	Eu:bal	1987	CC	101	n	bl	n	n	0	cu	all/unsp	1	10	1	0	nev any	st
KATSOU	8	x	f	0	0	all	-	all	Eu:bal	1987	CC	101	n	bl	n	n	0	cu	all/unsp	11	20	2	3	nev any	st
KATSOU	9	x	f	0	0	all	-	all	Eu:bal	1987	CC	101	n	bl	n	n	0	cu	all/unsp	21	30	0	4	nev any	st
KATSOU	10	x	f	0	0	all	-	all	Eu:bal	1987	CC	101	n	bl	n	n	0	cu	all/unsp	31	99	3	0	nev any	st
KAUFMA	2	x	c	0	0	all	-	all	NAmer	1981	CC	881	n	bl	n	n	0	cu	cig+/-ot	1	14	1	0	nev cigs	st
KAUFMA	3	x	c	0	0	all	-	all	NAmer	1981	CC	881	n	bl	n	n	0	cu	cig+/-ot	15	24	2	3	nev cigs	st
KAUFMA	4	x	c	0	0	all	-	all	NAmer	1981	CC	881	n	bl	n	n	0	cu	cig+/-ot	25	34	0	4	nev cigs	st
KAUFMA	5	x	c	0	0	all	-	all	NAmer	1981	CC	881	n	bl	n	n	0	cu	cig+/-ot	35	44	0	5	nev cigs	st
KAUFMA	6	x	c	0	0	all	-	all	NAmer	1981	CC	881	n	bl	n	n	0	cu	cig+/-ot	45	99	3	6	nev cigs	st
KHUDER	1		m	0	0	all	-	all	NAmer	1985	CC	482	n	bl	n	y	0	ev	cig+/-ot	1	19	1	0	nev cigs	st
KHUDER	2		m	0	0	all	-	all	NAmer	1985	CC	482	n	bl	n	y	0	ev	cig+/-ot	20	39	2	0	nev cigs	st
KHUDER	3		m	0	0	all	-	all	NAmer	1985	CC	482	n	bl	n	y	0	ev	cig+/-ot	40	99	3	0	nev cigs	st
KINLEN	3	x	m	0	0	all	0	all	Eu:UK	1967	pr	718	n	V	n	n	0	cu	cig+/-ot	1	14	1	0	nev any	st
KINLEN	4	x	m	0	0	all	0	all	Eu:UK	1967	pr	718	n	V	n	n	0	cu	cig+/-ot	15	24	2	3	nev any	st
KINLEN	5	x	m	0	0	all	0	all	Eu:UK	1967	pr	718	n	V	n	n	0	cu	cig+/-ot	25	99	3	0	nev any	st
KNEKT	25	x	m	20	69	all	21	all	Eu:Sca	1966	pr	515	n	bl	n	n	0	cu	cig+/-ot	1	14	1	0	nev any	st
KNEKT	26	x	m	20	69	all	21	all	Eu:Sca	1966	pr	515	n	bl	n	n	0	cu	cig+/-ot	15	99	0	0	nev any	st
KOO	11		f	0	0	all	-	all	As:HK	1981	CC	200	n	bl	n	n	0	cu	all/unsp	1	10	1	0	nev any	st
KOO	12		f	0	0	all	-	all	As:HK	1981	CC	200	n	bl	n	n	0	cu	all/unsp	11	20	2	3	nev any	st
KOO	13		f	0	0	all	-	all	As:HK	1981	CC	200	n	bl	n	n	0	cu	all/unsp	21	30	0	4	nev any	st
KOULUM	6		m	0	0	all	-	all	Eu:Sca	1936	CC	812	n	bl	n	n	0	ev	all/unsp	1	9	1	1	nev any	st
KOULUM	5		m	0	0	all	-	all	Eu:Sca	1936	CC	812	n	bl	n	n	0	ev	all/unsp	10	19	0	2	nev any	st
KOULUM	4		m	0	0	all	-	all	Eu:Sca	1936	CC	812	n	bl	n	n	0	ev	all/unsp	20	99	0	0	nev any	st
KREUZE	19		m	1	45	all	-	all	Eu:Ger	1990	CC	2260	n	bl	n	n	3	ev	cig+/-ot	1	9	1	1	nev any	or
KREUZE	20		m	1	45	all	-	all	Eu:Ger	1990	CC	2260	n	bl	n	n	3	ev	cig+/-ot	10	19	0	2	nev any	or
KREUZE	21		m	1	45	all	-	all	Eu:Ger	1990	CC	2260	n	bl	n	n	3	ev	cig+/-ot	20	29	2	3	nev any	or
KREUZE	22		m	1	45	all	-	all	Eu:Ger	1990	CC	2260	n	bl	n	n	3	ev	cig+/-ot	30	99	3	0	nev any	or
KREUZE	30		m	55	69	all	-	all	Eu:Ger	1990	CC	2260	n	bl	n	n	3	ev	cig+/-ot	1	9	1	1	nev any	or
KREUZE	31		m	55	69	all	-	all	Eu:Ger	1990	CC	2260	n	bl	n	n	3	ev	cig+/-ot	10	19	0	2	nev any	or
KREUZE	32		m	55	69	all	-	all	Eu:Ger	1990	CC	2260	n	bl	n	n	3	ev	cig+/-ot	20	29	2	3	nev any	or
KREUZE	33		m	55	69	all	-	all	Eu:Ger	1990	CC	2260	n	bl	n	n	3	ev	cig+/-ot	30	99	3	0	nev any	or
KREUZE	25		f	1	45	all	-	all	Eu:Ger	1990	CC	2260	n	bl	n	n	3	ev	cig+/-ot	1	9	1	1	nev any	or
KREUZE	26		f	1	45	all	-	all	Eu:Ger	1990	CC	2260	n	bl	n	n	3	ev	cig+/-ot	10	19	0	2	nev any	or
KREUZE	27		f	1	45	all	-	all	Eu:Ger	1990	CC	2260	n	bl	n	n	3	ev	cig+/-ot	20	29	2	3	nev any	or
KREUZE	36		f	55	69	all	-	all	Eu:Ger	1990	CC	2260	n	bl	n	n	3	ev	cig+/-ot	1	9	1	1	nev any	or
KREUZE	37		f	55	69	all	-	all	Eu:Ger	1990	CC	2260	n	bl	n	n	3	ev	cig+/-ot	10	19	0	2	nev any	or
KREUZE	38		f	55	69	all	-	all	Eu:Ger	1990	CC	2260	n	bl	n	n	3	ev	cig+/-ot	20	29	2	3	nev any	or
KREYBE	21	x	m	0	0	all	-	all	Eu:Sca	1948	CC	300	n	bl	n	y	0	ev	all/unsp	1	14	1	0	nev any	st
KREYBE	22	x	m	0	0	all	-	all	Eu:Sca	1948	CC	300	n	bl	n	y	0	ev	all/unsp	15	24	2	3	nev any	st
KREYBE	23	x	m	0	0	all	-	all	Eu:Sca	1948	CC	300	n	bl	n	y	0	ev	all/unsp	25	99	3	0	nev any	st

Table 1G11 - 4

IESLC - Meta-anal of Ever Smoking (or Curr if Ever not avail) by Amount, Overview, Any prod (or Cigs if Any not avail)
All LC types
Least adjusted

REF	NRR	X	SEX	AGEL	AGEH	RACE	YF	LC TYPE	LOC	START	ST	NLC	R	VB	P	H	AD	SM	PRODUCT	exL	exH	S1	S2	DENOM	De
KREYBE	37	x	f	0	0	all	-	all	Eu:Sca	1948	CC	300	n	bl	n	y	0	ev	all/unsp	1	14	1	0	nev any	st
KREYBE	38	x	f	0	0	all	-	all	Eu:Sca	1948	CC	300	n	bl	n	y	0	ev	all/unsp	15	99	0	0	nev any	st
LAMTH	7		f	0	0	ch	-	all	As:HK	1983	CC	445	n	bl	n	n	0	ev	all/unsp	1	10	1	0	nev any	or
LAMTH	2		f	0	0	ch	-	all	As:HK	1983	CC	445	n	bl	n	n	0	ev	all/unsp	11	20	2	3	nev any	or
LAMTH	9		f	0	0	ch	-	all	As:HK	1983	CC	445	n	bl	n	n	0	ev	all/unsp	21	99	3	0	nev any	or
LAUSSM	3	x	m	0	0	all	-	all	Eu:Ger	1982	CC	432	n	bl	n	n	0	ev	all/unsp	1	9	1	1	nev any	st
LAUSSM	2	x	m	0	0	all	-	all	Eu:Ger	1982	CC	432	n	bl	n	n	0	ev	all/unsp	10	19	0	2	nev any	st
LAUSSM	1	x	m	0	0	all	-	all	Eu:Ger	1982	CC	432	n	bl	n	n	0	ev	all/unsp	20	99	0	0	nev any	st
LETOUR	2		c	0	0	all	-	all	NAmer	1983	CC	738	n	V	y	y	0	ev	cig+/-ot	1	19	1	0	nev cigs	st
LETOUR	3		c	0	0	all	-	all	NAmer	1983	CC	738	n	V	y	y	0	ev	cig+/-ot	20	40	2	0	nev cigs	st
LETOUR	4		c	0	0	all	-	all	NAmer	1983	CC	738	n	V	y	y	0	ev	cig+/-ot	41	99	3	6	nev cigs	st
LIAW	3		c	0	0	all	0	all	As:oth	1982	pr	127	n	ot	n	n	2	cu	all/unsp	1	10	1	0	nev any	or
LIAW	4		c	0	0	all	0	all	As:oth	1982	pr	127	n	ot	n	n	2	cu	all/unsp	11	20	2	3	nev any	or
LIAW	5		c	0	0	all	0	all	As:oth	1982	pr	127	n	ot	n	n	2	cu	all/unsp	21	99	3	0	nev any	or
LIDDEL	2		m	0	0	all	18	all	NAmer	1970	pr	304	m	V	n	n	1	cu	cig+/-ot	1	19	1	0	nev cigs	ot
LIDDEL	3		m	0	0	all	18	all	NAmer	1970	pr	304	m	V	n	n	1	cu	cig+/-ot	20	99	0	0	nev cigs	ot
LIU2	5	x	m	0	0	all	-	all	As:Chi	1983	CC	316	n	ot	n	n	0	ev	all/unsp	1	19	1	0	nev any	st
LIU2	6	x	m	0	0	all	-	all	As:Chi	1983	CC	316	n	ot	n	n	0	ev	all/unsp	20	29	2	3	nev any	st
LIU2	7	x	m	0	0	all	-	all	As:Chi	1983	CC	316	n	ot	n	n	0	ev	all/unsp	30	99	3	0	nev any	st
LIU2	11	x	f	0	0	all	-	all	As:Chi	1983	CC	316	n	ot	n	n	0	ev	all/unsp	1	9	1	1	nev any	st
LIU2	12	x	f	0	0	all	-	all	As:Chi	1983	CC	316	n	ot	n	n	0	ev	all/unsp	10	19	0	2	nev any	st
LIU2	13	x	f	0	0	all	-	all	As:Chi	1983	CC	316	n	ot	n	n	0	ev	all/unsp	20	99	0	0	nev any	st
LIU3	3	x	m	0	0	all	-	all	As:Chi	1985	CC	110	n	ot	n	n	0	ev	all/unsp	1	15	1	0	nev any	st
LIU3	4	x	m	0	0	all	-	all	As:Chi	1985	CC	110	n	ot	n	n	0	ev	all/unsp	16	30	2	0	nev any	st
LIU3	5	x	m	0	0	all	-	all	As:Chi	1985	CC	110	n	ot	n	n	0	ev	all/unsp	31	99	3	0	nev any	st
LIU4	7		m	35	69	all	-	all	As:Chi	1986	CC	1000-00	n	ot	y	n	2	ev	cig only	1	19	1	0	nev any	ot
LIU4	8		m	35	69	all	-	all	As:Chi	1986	CC	1000-00	n	ot	y	n	2	ev	cig only	20	20	2	3	nev any	ot
LIU4	9		m	35	69	all	-	all	As:Chi	1986	CC	1000-00	n	ot	y	n	2	ev	cig only	21	99	3	0	nev any	ot
LIU5	2		c	0	0	all	-	all	As:Chi	1978	CC	111	n	ot	y	n	0	ev	all/unsp	1	9	1	1	nev any	st
LIU5	3		c	0	0	all	-	all	As:Chi	1978	CC	111	n	ot	y	n	0	ev	all/unsp	10	19	0	2	nev any	st
LIU5	4		c	0	0	all	-	all	As:Chi	1978	CC	111	n	ot	y	n	0	ev	all/unsp	20	99	0	0	nev any	st
LUBIN	7	x	m	0	0	all	-	all	As:Chi	1984	CC	427	m	ot	y	n	0	ev	cig only	1	6	1	1	nev any	st
LUBIN	8	x	m	0	0	all	-	all	As:Chi	1984	CC	427	m	ot	y	n	0	ev	cig only	7	14	0	2	nev any	st
LUBIN	9	x	m	0	0	all	-	all	As:Chi	1984	CC	427	m	ot	y	n	0	ev	cig only	15	19	0	0	nev any	st
LUBIN	10	x	m	0	0	all	-	all	As:Chi	1984	CC	427	m	ot	y	n	0	ev	cig only	20	99	0	0	nev any	st
LUBIN2	273		m	0	0	all	-	all	Eu:mul	1976	CC	7804	n	bl	n	y	0	ev	cig+/-ot	1	9	1	1	nev any	st
LUBIN2	274		m	0	0	all	-	all	Eu:mul	1976	CC	7804	n	bl	n	y	0	ev	cig+/-ot	10	19	0	2	nev any	st
LUBIN2	275		m	0	0	all	-	all	Eu:mul	1976	CC	7804	n	bl	n	y	0	ev	cig+/-ot	20	29	2	3	nev any	st
LUBIN2	276		m	0	0	all	-	all	Eu:mul	1976	CC	7804	n	bl	n	y	0	ev	cig+/-ot	30	99	3	0	nev any	st
LUBIN2	281		f	0	0	all	-	all	Eu:mul	1976	CC	7804	n	bl	n	y	0	ev	cig+/-ot	1	9	1	1	nev any	st
LUBIN2	282		f	0	0	all	-	all	Eu:mul	1976	CC	7804	n	bl	n	y	0	ev	cig+/-ot	10	19	0	2	nev any	st
LUBIN2	283		f	0	0	all	-	all	Eu:mul	1976	CC	7804	n	bl	n	y	0	ev	cig+/-ot	20	29	2	3	nev any	st
LUBIN2	284		f	0	0	all	-	all	Eu:mul	1976	CC	7804	n	bl	n	y	0	ev	cig+/-ot	30	99	3	0	nev any	st
MACLEN	20	x	m	0	0	ch	-	all	As:oth	1972	CC	233	n	bl	n	n	0	cu	cig+/-ot	1	9	1	1	nev cigs	st
MACLEN	21	x	m	0	0	ch	-	all	As:oth	1972	CC	233	n	bl	n	n	0	cu	cig+/-ot	10	19	0	2	nev cigs	st
MACLEN	22	x	m	0	0	ch	-	all	As:oth	1972	CC	233	n	bl	n	n	0	cu	cig+/-ot	20	29	2	3	nev cigs	st
MACLEN	23	x	m	0	0	ch	-	all	As:oth	1972	CC	233	n	bl	n	n	0	cu	cig+/-ot	30	99	3	0	nev cigs	st
MACLEN	33	x	f	0	0	ch	-	all	As:oth	1972	CC	233	n	bl	n	n	0	cu	cig+/-ot	1	9	1	1	nev cigs	st
MACLEN	34	x	f	0	0	ch	-	all	As:oth	1972	CC	233	n	bl	n	n	0	cu	cig+/-ot	10	19	0	2	nev cigs	st
MACLEN	35	x	f	0	0	ch	-	all	As:oth	1972	CC	233	n	bl	n	n	0	cu	cig+/-ot	20	99	0	0	nev cigs	st
MARTIS	1		m	0	0	all	-	all	Eu:UK	1972	CC	201	n	V	n	n	0	ev	cig+/-ot	1	14	1	0	nev cigs	st
MARTIS	2		m	0	0	all	-	all	Eu:UK	1972	CC	201	n	V	n	n	0	ev	cig+/-ot	15	24	2	3	nev cigs	st
MARTIS	3		m	0	0	all	-	all	Eu:UK	1972	CC	201	n	V	n	n	0	ev	cig+/-ot	25	99	3	0	nev cigs	st
MATOS	28	x	m	0	0	all	-	all	SCAmer	1994	CC	200	n	bl	n	n	0	ev	cig+/-ot	1	14	1	0	nev any	st
MATOS	30	x	m	0	0	all	-	all	SCAmer	1994	CC	200	n	bl	n	n	0	ev	cig+/-ot	15	24	2	3	nev any	st
MATOS	32	x	m	0	0	all	-	all	SCAmer	1994	CC	200	n	bl	n	n	0	ev	cig+/-ot	25	99	3	0	nev any	st
MATSUD	1		m	0	0	all	-	all	As:Jap	1965	CC	179	n	bl	n	n	0	ev	cig+/-ot	1	10	1	0	nev cigs	st
MATSUD	2		m	0	0	all	-	all	As:Jap	1965	CC	179	n	bl	n	n	0	ev	cig+/-ot	11	20	2	3	nev cigs	st
MATSUD	3		m	0	0	all	-	all	As:Jap	1965	CC	179	n	bl	n	n	0	ev	cig+/-ot	21	99	3	0	nev cigs	st
MCCONN	26		c	0	0	all	-	all	Eu:UK	1946	CC	100	n	V	n	y	0	ev	all/unsp	1	10	1	0	nev any	st
MCCONN	25		c	0	0	all	-	all	Eu:UK	1946	CC	100	n	V	n	y	0	ev	all/unsp	10	20	2	0	nev any	st
MCCONN	24		c	0	0	all	-	all	Eu:UK	1946	CC	100	n	V	n	y	0	ev	all/unsp	21	99	3	0	nev any	st
MIGRAN	1	x	m	0	0	all	0	all	Eu:UK	1964	pr	259	n	V	n	n	0	cu	cig only	1	9	1	1	nev any	st
MIGRAN	3	x	m	0	0	all	0	all	Eu:UK	1964	pr	259	n	V	n	n	0	cu	cig only	10	19	0	2	nev any	st
MIGRAN	5	x	m	0	0	all	0	all	Eu:UK	1964	pr	259	n	V	n	n	0	cu	cig only	20	20	2	3	nev any	st
MIGRAN	7	x	m	0	0	all	0	all	Eu:UK	1964	pr	259	n	V	n	n	0	cu	cig only	21	99	3	0	nev any	st
MIGRAN	28	x	f	0	0	all	0	all	Eu:UK	1964	pr	259	n	V	n	n	0	cu	cig only	1	9	1	1	nev any	st
MIGRAN	30	x	f	0	0	all	0	all	Eu:UK	1964	pr	259	n	V	n	n	0	cu	cig only	10	19	0	2	nev any	st
MIGRAN	32	x	f	0	0	all	0	all	Eu:UK	1964	pr	259	n	V	n	n	0	cu	cig only	20	20	2	3	nev any	st

Table 1G11 - 4

IESLC - Meta-anal of Ever Smoking (or Curr if Ever not avail) by Amount, Overview, Any prod (or Cigs if Any not avail)
All LC types
Least adjusted

REF	NRR	X	SEX	AGEL	AGEH	RACE	YF	LC TYPE	LOC	START	ST	NLC	R	VB	P	H	AD	SM	PRODUCT	exL	exH	S1	S2	DENOM	De
MIGRAN	34	x	f	0	0	all	0	all	Eu:UK	1964	pr	259	n	V	n	n	0	cu	cig only	21	99	3	0	nev any	st
MRFITR	3		m	0	0	all	0	all	NAmer	1973	pr	119	n	bl	n	n	0	cu	cig+/-ot	1	19	1	0	nev cigs	ot
MRFITR	4		m	0	0	all	0	all	NAmer	1973	pr	119	n	bl	n	n	0	cu	cig+/-ot	20	39	2	0	nev cigs	ot
MRFITR	5		m	0	0	all	0	all	NAmer	1973	pr	119	n	bl	n	n	0	cu	cig+/-ot	40	99	3	0	nev cigs	ot
NAM	66	x	m	0	0	all	-	all	NAmer	1986	CC	1199	n	bl	y	n	0	cu	cig+/-ot	1	24	0	0	nev cigs	ot
NAM	67	x	m	0	0	all	-	all	NAmer	1986	CC	1199	n	bl	y	n	0	cu	cig+/-ot	25	99	3	0	nev cigs	ot
NAM	82	x	f	0	0	all	-	all	NAmer	1986	CC	1199	n	bl	y	n	0	cu	cig+/-ot	1	24	0	0	nev cigs	ot
NAM	83	x	f	0	0	all	-	all	NAmer	1986	CC	1199	n	bl	y	n	0	cu	cig+/-ot	25	99	3	0	nev cigs	ot
NOTAN2	8		m	0	0	all	-	all	As:Ind	1963	CC	683	n	V	n	n	0	ev	cig only	1	9	1	1	nev any	st
NOTAN2	9		m	0	0	all	-	all	As:Ind	1963	CC	683	n	V	n	n	0	ev	cig only	10	19	0	2	nev any	st
NOTAN2	10		m	0	0	all	-	all	As:Ind	1963	CC	683	n	V	n	n	0	ev	cig only	20	99	0	0	nev any	st
ORMOS	1		m	0	0	all	-	all	Eu:est	1947	CC	119	n	bl	y	y	0	ev	cig+/-ot	1	15	1	0	nev any	st
ORMOS	2		m	0	0	all	-	all	Eu:est	1947	CC	119	n	bl	y	y	0	ev	cig+/-ot	16	30	2	0	nev any	st
ORMOS	3		m	0	0	all	-	all	Eu:est	1947	CC	119	n	bl	y	y	0	ev	cig+/-ot	31	99	3	0	nev any	st
OSANN	49		m	0	0	all	-	all	NAmer	1984	CC	1986	n	bl	n	n	2	ev	cig+/-ot	1	39	0	0	nev cigs	or
OSANN	57		m	0	0	all	-	all	NAmer	1984	CC	1986	n	bl	n	n	2	ev	cig+/-ot	40	99	3	0	nev cigs	or
OSANN	50		f	0	0	all	-	all	NAmer	1984	CC	1986	n	bl	n	n	2	ev	cig+/-ot	1	39	0	0	nev cigs	or
OSANN	58		f	0	0	all	-	all	NAmer	1984	CC	1986	n	bl	n	n	2	ev	cig+/-ot	40	99	3	0	nev cigs	or
PARKIN	17	x	m	0	0	bl	-	all	Africa	1963	CC	877	n	V	y	n	0	cu	all/unsp	1	14	1	0	nev any	st
PARKIN	18	x	m	0	0	bl	-	all	Africa	1963	CC	877	n	V	y	n	0	cu	all/unsp	15	99	0	0	nev any	st
PASTOR	1	x	m	0	0	all	-	all	Eu:wst	1976	CC	204	n	bl	y	n	0	ev	all/unsp	1	9	1	1	nev any	st
PASTOR	2	x	m	0	0	all	-	all	Eu:wst	1976	CC	204	n	bl	y	n	0	ev	all/unsp	10	19	0	2	nev any	st
PASTOR	3	x	m	0	0	all	-	all	Eu:wst	1976	CC	204	n	bl	y	n	0	ev	all/unsp	20	29	2	3	nev any	st
PASTOR	4	x	m	0	0	all	-	all	Eu:wst	1976	CC	204	n	bl	y	n	0	ev	all/unsp	30	99	3	0	nev any	st
PERNU	17		m	0	0	all	-	all	Eu:Sca	1944	CC	1606	n	bl	n	n	0	ev	all/unsp	1	4	0	1	nev any	st
PERNU	18		m	0	0	all	-	all	Eu:Sca	1944	CC	1606	n	bl	n	n	0	ev	all/unsp	5	9	1	0	nev any	st
PERNU	19		m	0	0	all	-	all	Eu:Sca	1944	CC	1606	n	bl	n	n	0	ev	all/unsp	10	14	0	2	nev any	st
PERNU	20		m	0	0	all	-	all	Eu:Sca	1944	CC	1606	n	bl	n	n	0	ev	all/unsp	15	19	0	0	nev any	st
PERNU	21		m	0	0	all	-	all	Eu:Sca	1944	CC	1606	n	bl	n	n	0	ev	all/unsp	20	24	2	3	nev any	st
PERNU	22		m	0	0	all	-	all	Eu:Sca	1944	CC	1606	n	bl	n	n	0	ev	all/unsp	25	29	0	0	nev any	st
PERNU	23		m	0	0	all	-	all	Eu:Sca	1944	CC	1606	n	bl	n	n	0	ev	all/unsp	30	49	3	0	nev any	st
PERNU	24		m	0	0	all	-	all	Eu:Sca	1944	CC	1606	n	bl	n	n	0	ev	all/unsp	50	99	0	6	nev any	st
PERNU	11		f	0	0	all	-	all	Eu:Sca	1944	CC	1606	n	bl	n	n	0	ev	all/unsp	1	4	0	1	nev any	st
PERNU	12		f	0	0	all	-	all	Eu:Sca	1944	CC	1606	n	bl	n	n	0	ev	all/unsp	5	9	1	0	nev any	st
PERNU	13		f	0	0	all	-	all	Eu:Sca	1944	CC	1606	n	bl	n	n	0	ev	all/unsp	10	14	0	2	nev any	st
PERNU	14		f	0	0	all	-	all	Eu:Sca	1944	CC	1606	n	bl	n	n	0	ev	all/unsp	15	19	0	0	nev any	st
PERNU	15		f	0	0	all	-	all	Eu:Sca	1944	CC	1606	n	bl	n	n	0	ev	all/unsp	20	24	2	3	nev any	st
PERNU	16		f	0	0	all	-	all	Eu:Sca	1944	CC	1606	n	bl	n	n	0	ev	all/unsp	25	99	3	0	nev any	st
PERSH2	2	x	c	0	0	all	-	all	Eu:Sca	1980	CC	1022	n	bl	y	n	0	cu	all/unsp	1	9	1	1	nev any	st
PERSH2	3	x	c	0	0	all	-	all	Eu:Sca	1980	CC	1022	n	bl	y	n	0	cu	all/unsp	10	99	0	0	nev any	st
PETO	2		m	0	0	all	0	all	Eu:UK	1954	pr	103	n	V	n	n	0	cu	all/unsp	1	14	1	0	nev any	st
PETO	3		m	0	0	all	0	all	Eu:UK	1954	pr	103	n	V	n	n	0	cu	all/unsp	15	99	0	0	nev any	st
PEZZO2	3		m	0	0	all	-	all	SCAmer	1992	CC	367	n	bl	n	y	0	cu	cig+/-ot	1	20	0	0	nev cigs	st
PEZZO2	4		m	0	0	all	-	all	SCAmer	1992	CC	367	n	bl	n	y	0	cu	cig+/-ot	21	40	0	0	nev cigs	st
PEZZO2	5		m	0	0	all	-	all	SCAmer	1992	CC	367	n	bl	n	y	0	cu	cig+/-ot	41	99	3	6	nev cigs	st
PEZZOT	2		m	0	0	all	-	all	SCAmer	1987	CC	215	n	bl	n	y	0	cu	cig only	1	20	0	0	nev cigs	st
PEZZOT	3		m	0	0	all	-	all	SCAmer	1987	CC	215	n	bl	n	y	0	cu	cig only	21	40	0	0	nev cigs	st
PEZZOT	4		m	0	0	all	-	all	SCAmer	1987	CC	215	n	bl	n	y	0	cu	cig only	41	99	3	6	nev cigs	st
PIKE	1		m	0	0	w-hi	-	all	NAmer	1972	CC	731	n	bl	y	n	0	ev	all/unsp	1	20	0	0	nev any	st
PIKE	2		m	0	0	w-hi	-	all	NAmer	1972	CC	731	n	bl	y	n	0	ev	all/unsp	21	40	0	0	nev any	st
PIKE	3		m	0	0	w-hi	-	all	NAmer	1972	CC	731	n	bl	y	n	0	ev	all/unsp	41	99	3	6	nev any	st
PIKE	5		f	0	0	w-hi	-	all	NAmer	1972	CC	731	n	bl	y	n	0	ev	all/unsp	1	20	0	0	nev any	st
PIKE	6		f	0	0	w-hi	-	all	NAmer	1972	CC	731	n	bl	y	n	0	ev	all/unsp	21	40	0	0	nev any	st
PIKE	7		f	0	0	w-hi	-	all	NAmer	1972	CC	731	n	bl	y	n	0	ev	all/unsp	41	99	3	6	nev any	st
POLEDN	2		c	0	0	all	-	all	NAmer	1978	CC	209	n	bl	y	n	0	ev	cig+/-ot	1	19	1	0	nev cigs	st
POLEDN	4		c	0	0	all	-	all	NAmer	1978	CC	209	n	bl	y	n	0	ev	cig+/-ot	20	99	0	0	nev cigs	st
PRESCO	2		m	0	0	all	0	all	Eu:Sca	1964	pr	867	n	bl	n	n	1	cu	all/unsp	1	14	1	0	nev any	st
PRESCO	4		m	0	0	all	0	all	Eu:Sca	1964	pr	867	n	bl	n	n	1	cu	all/unsp	15	99	0	0	nev any	st
PRESCO	1		f	0	0	all	0	all	Eu:Sca	1964	pr	867	n	bl	n	n	1	cu	all/unsp	1	14	1	0	nev any	st
PRESCO	3		f	0	0	all	0	all	Eu:Sca	1964	pr	867	n	bl	n	n	1	cu	all/unsp	15	99	0	0	nev any	st
RACHTA	5	x	f	0	0	all	-	all	Eu:est	1991	CC	118	n	bl	n	y	0	ev	cig+/-ot	1	9	1	1	nev cigs	st
RACHTA	6	x	f	0	0	all	-	all	Eu:est	1991	CC	118	n	bl	n	y	0	ev	cig+/-ot	10	19	0	2	nev cigs	st
RACHTA	7	x	f	0	0	all	-	all	Eu:est	1991	CC	118	n	bl	n	y	0	ev	cig+/-ot	20	99	0	0	nev cigs	st
RANDIG	1		m	0	0	all	-	all	Eu:Ger	1951	CC	448	n	bl	n	n	0	ev	all/unsp	1	4	0	1	nev any	st
RANDIG	2		m	0	0	all	-	all	Eu:Ger	1951	CC	448	n	bl	n	n	0	ev	all/unsp	5	9	1	0	nev any	st
RANDIG	3		m	0	0	all	-	all	Eu:Ger	1951	CC	448	n	bl	n	n	0	ev	all/unsp	10	19	0	2	nev any	st
RANDIG	4		m	0	0	all	-	all	Eu:Ger	1951	CC	448	n	bl	n	n	0	ev	all/unsp	20	99	0	0	nev any	st
RANDIG	5		f	0	0	all	-	all	Eu:Ger	1951	CC	448	n	bl	n	n	0	ev	all/unsp	1	4	0	1	nev any	st
RANDIG	6		f	0	0	all	-	all	Eu:Ger	1951	CC	448	n	bl	n	n	0	ev	all/unsp	5	9	1	0	nev any	st
RANDIG	7		f	0	0	all	-	all	Eu:Ger	1951	CC	448	n	bl	n	n	0	ev	all/unsp	10	99	0	0	nev any	st
SEGI2	9	x	m	0	0	all	-	all	As:Jap	1962	CC	378	n	bl	n	n	0	cu	cig+/-ot	1	9	1	1	nev any	st
SEGI2	11	x	m	0	0	all	-	all	As:Jap	1962	CC	378	n	bl	n	n	0	cu	cig+/-ot	10	19	0	2	nev any	st

Table 1G11 - 4

IESLC - Meta-anal of Ever Smoking (or Curr if Ever not avail) by Amount, Overview, Any prod (or Cigs if Any not avail)
All LC types
Least adjusted

REF	NRR	X	SEX	AGEL	AGEH	RACE	YF	LC TYPE	LOC	START	ST	NLC	R	VB	P	H	AD	SM	PRODUCT	exL	exH	S1	S2	DENOM	De
SEGI2	13	x	m	0	0	all	-	all	As:Jap	1962	CC	378	n	bl	n	n	0	cu	cig+/-ot	20	29	2	3	nev any	st
SEGI2	15	x	m	0	0	all	-	all	As:Jap	1962	CC	378	n	bl	n	n	0	cu	cig+/-ot	30	39	0	4	nev any	st
SEGI2	17	x	m	0	0	all	-	all	As:Jap	1962	CC	378	n	bl	n	n	0	cu	cig+/-ot	40	99	3	0	nev any	st
SEGI2	21	x	f	0	0	all	-	all	As:Jap	1962	CC	378	n	bl	n	n	0	cu	cig+/-ot	1	9	1	1	nev any	st
SEGI2	23	x	f	0	0	all	-	all	As:Jap	1962	CC	378	n	bl	n	n	0	cu	cig+/-ot	10	19	0	2	nev any	st
SEGI2	25	x	f	0	0	all	-	all	As:Jap	1962	CC	378	n	bl	n	n	0	cu	cig+/-ot	20	99	0	0	nev any	st
SHAW	10		c	0	0	wh	-	all	NAmer	1988	CC	335	n	V	n	y	0	ev	all/unsp	1	19	1	0	nev any	st
SHAW	11		c	0	0	wh	-	all	NAmer	1988	CC	335	n	V	n	y	0	ev	all/unsp	20	99	0	0	nev any	st
SIEMIA	13		m	0	0	all	-	all	NAmer	1979	CC	857	n	V	y	y	0	ev	cig+/-ot	1	19	1	0	nev cigs	or
SIEMIA	14		m	0	0	all	-	all	NAmer	1979	CC	857	n	V	y	y	0	ev	cig+/-ot	20	39	2	0	nev cigs	or
SIEMIA	15		m	0	0	all	-	all	NAmer	1979	CC	857	n	V	y	y	0	ev	cig+/-ot	40	99	3	0	nev cigs	or
SOBUE	117		m	0	0	all	-	q+s+l+a	As:Jap	1986	CC	1376	n	bl	n	y	0	cu	cig+/-ot	1	19	1	0	nev cigs	st
SOBUE	118		m	0	0	all	-	q+s+l+a	As:Jap	1986	CC	1376	n	bl	n	y	0	cu	cig+/-ot	20	29	2	3	nev cigs	st
SOBUE	119		m	0	0	all	-	q+s+l+a	As:Jap	1986	CC	1376	n	bl	n	y	0	cu	cig+/-ot	30	99	3	0	nev cigs	st
SPEIZE	1		f	0	0	all	0	all	NAmer	1976	pr	593	n	bl	n	y	1	cu	cig+/-ot	1	4	0	1	nev cigs	ot
SPEIZE	2		f	0	0	all	0	all	NAmer	1976	pr	593	n	bl	n	y	1	cu	cig+/-ot	5	14	1	2	nev cigs	ot
SPEIZE	3		f	0	0	all	0	all	NAmer	1976	pr	593	n	bl	n	y	1	cu	cig+/-ot	15	24	2	3	nev cigs	ot
SPEIZE	4		f	0	0	all	0	all	NAmer	1976	pr	593	n	bl	n	y	1	cu	cig+/-ot	25	34	0	4	nev cigs	ot
SPEIZE	5		f	0	0	all	0	all	NAmer	1976	pr	593	n	bl	n	y	1	cu	cig+/-ot	35	99	3	0	nev cigs	ot
SPITZ	5		c	0	0	b+hi	-	all	NAmer	1992	CC	177	n	bl	n	y	0	ev	cig+/-ot	1	19	1	0	nev cigs	st
SPITZ	6		c	0	0	b+hi	-	all	NAmer	1992	CC	177	n	bl	n	y	0	ev	cig+/-ot	20	99	0	0	nev cigs	st
STOCKS	25	x	m	0	0	all	-	all	Eu:UK	1952	CC	2932	n	V	y	n	0	ev	cig+/-ot	1	14	1	0	nev any	st
STOCKS	26	x	m	0	0	all	-	all	Eu:UK	1952	CC	2932	n	V	y	n	0	ev	cig+/-ot	15	21	2	3	nev any	st
STOCKS	27	x	m	0	0	all	-	all	Eu:UK	1952	CC	2932	n	V	y	n	0	ev	cig+/-ot	22	28	0	0	nev any	st
STOCKS	28	x	m	0	0	all	-	all	Eu:UK	1952	CC	2932	n	V	y	n	0	ev	cig+/-ot	29	36	0	4	nev any	st
STOCKS	29	x	m	0	0	all	-	all	Eu:UK	1952	CC	2932	n	V	y	n	0	ev	cig+/-ot	37	99	3	0	nev any	st
STOCKS	48		f	0	0	all	-	all	Eu:UK	1952	CC	2932	n	V	y	n	1	ev	cig+/-ot	1	14	1	0	nev any	ot
STOCKS	49		f	0	0	all	-	all	Eu:UK	1952	CC	2932	n	V	y	n	1	ev	cig+/-ot	15	99	0	0	nev any	ot
STOCKW	1		c	0	0	all	-	all	NAmer	1981	CC	22161	n	bl	n	n	0	cu	cig+/-ot	1	19	1	0	nev any	st
STOCKW	2		c	0	0	all	-	all	NAmer	1981	CC	22161	n	bl	n	n	0	cu	cig+/-ot	20	40	2	0	nev any	st
STOCKW	3		c	0	0	all	-	all	NAmer	1981	CC	22161	n	bl	n	n	0	cu	cig+/-ot	41	99	3	6	nev any	st
SVENSS	26	x	f	0	0	all	-	all	Eu:Sca	1983	CC	210	n	bl	n	n	0	cu	all/unsp	1	10	1	0	nev any	st
SVENSS	31	x	f	0	0	all	-	all	Eu:Sca	1983	CC	210	n	bl	n	n	0	cu	all/unsp	11	20	2	3	nev any	st
SVENSS	36	x	f	0	0	all	-	all	Eu:Sca	1983	CC	210	n	bl	n	n	0	cu	all/unsp	21	99	3	0	nev any	st
TENKAN	10		m	0	0	all	17	all	Eu:Sca	1962	pr	242	n	bl	n	n	1	cu	all/unsp	1	14	1	0	nev any	ot
TENKAN	11		m	0	0	all	17	all	Eu:Sca	1962	pr	242	n	bl	n	n	1	cu	all/unsp	15	24	2	3	nev any	ot
TENKAN	12		m	0	0	all	17	all	Eu:Sca	1962	pr	242	n	bl	n	n	1	cu	all/unsp	25	99	3	0	nev any	ot
TIZZAN	7		m	0	0	all	-	all	Eu:wst	1959	CC	1358	n	bl	n	n	0	ev	cig only	1	9	1	1	nev any	st
TIZZAN	8		m	0	0	all	-	all	Eu:wst	1959	CC	1358	n	bl	n	n	0	ev	cig only	10	20	2	0	nev any	st
TIZZAN	9		m	0	0	all	-	all	Eu:wst	1959	CC	1358	n	bl	n	n	0	ev	cig only	21	40	0	0	nev any	st
TIZZAN	10		m	0	0	all	-	all	Eu:wst	1959	CC	1358	n	bl	n	n	0	ev	cig only	41	99	3	6	nev any	st
TIZZAN	15		f	0	0	all	-	all	Eu:wst	1959	CC	1358	n	bl	n	n	0	ev	cig only	1	9	1	1	nev any	st
TIZZAN	16		f	0	0	all	-	all	Eu:wst	1959	CC	1358	n	bl	n	n	0	ev	cig only	10	99	0	0	nev any	st
TSUGAN	29		m	0	0	all	-	q+a	As:Jap	1976	CC	134	n	bl	n	y	0	cu	all/unsp	1	15	1	0	nev any	st
TSUGAN	30		m	0	0	all	-	q+a	As:Jap	1976	CC	134	n	bl	n	y	0	cu	all/unsp	16	35	2	0	nev any	st
TSUGAN	31		m	0	0	all	-	q+a	As:Jap	1976	CC	134	n	bl	n	y	0	cu	all/unsp	36	99	3	0	nev any	st
TULINI	4	x	m	0	0	all	0	all	Eu:Sca	1967	pr	472	n	bl	n	n	1	cu	cig+/-ot	1	14	1	0	nev any	or
TULINI	5	x	m	0	0	all	0	all	Eu:Sca	1967	pr	472	n	bl	n	n	1	cu	cig+/-ot	15	24	2	3	nev any	or
TULINI	6	x	m	0	0	all	0	all	Eu:Sca	1967	pr	472	n	bl	n	n	1	cu	cig+/-ot	25	99	3	0	nev any	or
TULINI	9	x	f	0	0	all	0	all	Eu:Sca	1967	pr	472	n	bl	n	n	1	cu	cig+/-ot	1	14	1	0	nev any	or
TULINI	10	x	f	0	0	all	0	all	Eu:Sca	1967	pr	472	n	bl	n	n	1	cu	cig+/-ot	15	24	2	3	nev any	or
TULINI	11	x	f	0	0	all	0	all	Eu:Sca	1967	pr	472	n	bl	n	n	1	cu	cig+/-ot	25	99	3	0	nev any	or
TVERDA	9		m	0	0	all	0	all	Eu:Sca	1972	pr	238	n	bl	n	n	2	cu	cig only	1	9	1	1	nev cigs	ot
TVERDA	10		m	0	0	all	0	all	Eu:Sca	1972	pr	238	n	bl	n	n	2	cu	cig only	10	19	0	2	nev cigs	ot
TVERDA	11		m	0	0	all	0	all	Eu:Sca	1972	pr	238	n	bl	n	n	2	cu	cig only	20	99	0	0	nev cigs	ot
TVERDA	16		f	0	0	all	0	all	Eu:Sca	1972	pr	238	n	bl	n	n	2	cu	cig only	1	9	1	1	nev cigs	ot
TVERDA	17		f	0	0	all	0	all	Eu:Sca	1972	pr	238	n	bl	n	n	2	cu	cig only	20	99	0	0	nev cigs	ot
WAKAI	37	x	m	0	0	all	-	all	As:Jap	1988	CC	333	n	bl	n	y	0	cu	cig+/-ot	1	19	1	0	nev any	st
WAKAI	38	x	m	0	0	all	-	all	As:Jap	1988	CC	333	n	bl	n	y	0	cu	cig+/-ot	20	29	2	3	nev any	st
WAKAI	39	x	m	0	0	all	-	all	As:Jap	1988	CC	333	n	bl	n	y	0	cu	cig+/-ot	30	99	3	0	nev any	st
WANG2	1	x	c	0	0	all	-	all	As:Chi	1980	CC	103	n	ot	n	n	0	ev	cig+/-ot	1	4	0	1	nev cigs	st
WANG2	2	x	c	0	0	all	-	all	As:Chi	1980	CC	103	n	ot	n	n	0	ev	cig+/-ot	5	9	1	0	nev cigs	st
WANG2	3	x	c	0	0	all	-	all	As:Chi	1980	CC	103	n	ot	n	n	0	ev	cig+/-ot	10	14	0	2	nev cigs	st
WANG2	4	x	c	0	0	all	-	all	As:Chi	1980	CC	103	n	ot	n	n	0	ev	cig+/-ot	15	19	0	0	nev cigs	st
WANG2	5	x	c	0	0	all	-	all	As:Chi	1980	CC	103	n	ot	n	n	0	ev	cig+/-ot	20	29	2	3	nev cigs	st
WANG2	6	x	c	0	0	all	-	all	As:Chi	1980	CC	103	n	ot	n	n	0	ev	cig+/-ot	30	39	0	4	nev cigs	st
WANG2	7	x	c	0	0	all	-	all	As:Chi	1980	CC	103	n	ot	n	n	0	ev	cig+/-ot	40	99	3	0	nev cigs	st
WU	35	x	f	0	0	wh	-	q+a	NAmer	1981	CC	220	n	bl	n	y	0	cu	all/unsp	1	20	0	0	nev any	st
WU	36	x	f	0	0	wh	-	q+a	NAmer	1981	CC	220	n	bl	n	y	0	cu	all/unsp	21	99	3	0	nev any	st
WUWILL	18	x	f	0	0	all	-	all	As:Chi	1985	CC	965	n	ot	n	n	0	ev	cig+/-ot	1	19	1	0	nev cigs	st
WUWILL	19	x	f	0	0	all	-	all	As:Chi	1985	CC	965	n	ot	n	n	0	ev	cig+/-ot	20	99	0	0	nev cigs	st
WYNDE2	17		m	0	0	all	-	all	NAmer	1962	CC	404	n	bl	n	y	0	ev	cig+/-ot	1	10	1	0	nev any	st

Table 1G11 - 4

IESLC - Meta-anal of Ever Smoking (or Curr if Ever not avail) by Amount, Overview, Any prod (or Cigs if Any not avail)
All LC types
Least adjusted

REF	NRR	X	SEX	AGEL	AGEH	RACE	YF	LC TYPE	LOC	START	ST	NLC	R	VB	P	H	AD	SM	PRODUCT	exL	exH	S1	S2	DENOM	De
WYNDE2	18		m	0	0	all	-	all	NAmer	1962	CC	404	n	bl	n	y	0	ev	cig+/-ot	11	20	2	3	nev any	st
WYNDE2	19		m	0	0	all	-	all	NAmer	1962	CC	404	n	bl	n	y	0	ev	cig+/-ot	21	34	0	4	nev any	st
WYNDE2	20		m	0	0	all	-	all	NAmer	1962	CC	404	n	bl	n	y	0	ev	cig+/-ot	35	99	3	0	nev any	st
WYNDE3	44		m	0	0	all	-	all	NAmer	1966	CC	350	n	bl	n	y	0	ev	cig+/-ot	1	9	1	1	nev any	st
WYNDE3	45		m	0	0	all	-	all	NAmer	1966	CC	350	n	bl	n	y	0	ev	cig+/-ot	10	20	2	0	nev any	st
WYNDE3	46		m	0	0	all	-	all	NAmer	1966	CC	350	n	bl	n	y	0	ev	cig+/-ot	21	40	0	0	nev any	st
WYNDE3	47		m	0	0	all	-	all	NAmer	1966	CC	350	n	bl	n	y	0	ev	cig+/-ot	41	99	3	6	nev any	st
WYNDE3	79		f	0	0	all	-	all	NAmer	1966	CC	350	n	bl	n	y	0	ev	cig+/-ot	1	9	1	1	nev any	st
WYNDE3	80		f	0	0	all	-	all	NAmer	1966	CC	350	n	bl	n	y	0	ev	cig+/-ot	10	20	2	0	nev any	st
WYNDE3	81		f	0	0	all	-	all	NAmer	1966	CC	350	n	bl	n	y	0	ev	cig+/-ot	21	40	0	0	nev any	st
WYNDE3	82		f	0	0	all	-	all	NAmer	1966	CC	350	n	bl	n	y	0	ev	cig+/-ot	41	99	3	6	nev any	st
WYNDE4	43		m	0	0	all	-	all	NAmer	1948	CC	684	n	bl	y	n	0	ev	all/unsp	1	9	1	1	nev any	st
WYNDE4	44		m	0	0	all	-	all	NAmer	1948	CC	684	n	bl	y	n	0	ev	all/unsp	10	15	0	2	nev any	st
WYNDE4	45		m	0	0	all	-	all	NAmer	1948	CC	684	n	bl	y	n	0	ev	all/unsp	16	20	2	3	nev any	st
WYNDE4	46		m	0	0	all	-	all	NAmer	1948	CC	684	n	bl	y	n	0	ev	all/unsp	21	34	0	4	nev any	st
WYNDE4	47		m	0	0	all	-	all	NAmer	1948	CC	684	n	bl	y	n	0	ev	all/unsp	35	99	3	0	nev any	st
WYNDE4	57		f	0	0	all	-	all	NAmer	1948	CC	684	n	bl	y	n	2	ev	all/unsp	1	9	1	1	nev any	ot
WYNDE4	58		f	0	0	all	-	all	NAmer	1948	CC	684	n	bl	y	n	2	ev	all/unsp	10	15	0	2	nev any	ot
WYNDE4	59		f	0	0	all	-	all	NAmer	1948	CC	684	n	bl	y	n	2	ev	all/unsp	16	20	2	3	nev any	ot
WYNDE4	60		f	0	0	all	-	all	NAmer	1948	CC	684	n	bl	y	n	2	ev	all/unsp	21	34	0	4	nev any	ot
WYNDE4	61		f	0	0	all	-	all	NAmer	1948	CC	684	n	bl	y	n	2	ev	all/unsp	35	99	3	0	nev any	ot
WYNDE6	27		m	0	0	all	-	all	NAmer	1969	CC	4423	n	bl	n	y	0	cu	cig+/-ot	1	10	1	0	nev any	st
WYNDE6	36		m	0	0	all	-	all	NAmer	1969	CC	4423	n	bl	n	y	0	cu	cig+/-ot	11	20	2	3	nev any	st
WYNDE6	45		m	0	0	all	-	all	NAmer	1969	CC	4423	n	bl	n	y	0	cu	cig+/-ot	21	30	0	4	nev any	st
WYNDE6	54		m	0	0	all	-	all	NAmer	1969	CC	4423	n	bl	n	y	0	cu	cig+/-ot	31	99	3	0	nev any	st
WYNDE6	216		f	0	0	all	-	all	NAmer	1969	CC	4423	n	bl	n	y	0	cu	cig+/-ot	1	10	1	0	nev cigs	st
WYNDE6	225		f	0	0	all	-	all	NAmer	1969	CC	4423	n	bl	n	y	0	cu	cig+/-ot	11	20	2	3	nev cigs	st
WYNDE6	234		f	0	0	all	-	all	NAmer	1969	CC	4423	n	bl	n	y	0	cu	cig+/-ot	21	30	0	4	nev cigs	st
WYNDE6	243		f	0	0	all	-	all	NAmer	1969	CC	4423	n	bl	n	y	0	cu	cig+/-ot	30	99	3	0	nev cigs	st
XU3	5	x	m	0	0	all	-	all	As:Chi	1981	CC	135	n	ot	n	n	0	ev	all/unsp	1	9	1	1	nev any	st
XU3	6	x	m	0	0	all	-	all	As:Chi	1981	CC	135	n	ot	n	n	0	ev	all/unsp	10	19	0	2	nev any	st
XU3	7	x	m	0	0	all	-	all	As:Chi	1981	CC	135	n	ot	n	n	0	ev	all/unsp	20	29	2	3	nev any	st
XU3	8	x	m	0	0	all	-	all	As:Chi	1981	CC	135	n	ot	n	n	0	ev	all/unsp	30	99	3	0	nev any	st
XU3	13	x	f	0	0	all	-	all	As:Chi	1981	CC	135	n	ot	n	n	0	ev	all/unsp	1	9	1	1	nev any	st
XU3	14	x	f	0	0	all	-	all	As:Chi	1981	CC	135	n	ot	n	n	0	ev	all/unsp	10	19	0	2	nev any	st
XU3	15	x	f	0	0	all	-	all	As:Chi	1981	CC	135	n	ot	n	n	0	ev	all/unsp	20	99	0	0	nev any	st
YAMAGU	3	x	c	0	0	all	-	all	As:Jap	1989	CC	144	n	bl	n	y	0	cu	all/unsp	1	20	0	0	nev any	st
YAMAGU	2	x	c	0	0	all	-	all	As:Jap	1989	CC	144	n	bl	n	y	0	cu	all/unsp	21	99	3	0	nev any	st
YUAN	2		m	0	0	all	0	all	As:Chi	1986	pr	142	n	ot	n	n	2	ev	cig+/-ot	1	19	1	0	nev cigs	ot
YUAN	3		m	0	0	all	0	all	As:Chi	1986	pr	142	n	ot	n	n	2	ev	cig+/-ot	20	99	0	0	nev cigs	ot
ZHENG	11		m	0	0	all	-	all	As:Chi	1982	CC	540	n	ot	*	y	0	ev	cig+/-ot	1	9	1	1	nev cigs	st
ZHENG	12		m	0	0	all	-	all	As:Chi	1982	CC	540	n	ot	*	y	0	ev	cig+/-ot	10	19	0	2	nev cigs	st
ZHENG	13		m	0	0	all	-	all	As:Chi	1982	CC	540	n	ot	*	y	0	ev	cig+/-ot	20	29	2	3	nev cigs	st
ZHENG	14		m	0	0	all	-	all	As:Chi	1982	CC	540	n	ot	*	y	0	ev	cig+/-ot	30	99	3	0	nev cigs	st
ZHENG	22		f	0	0	all	-	all	As:Chi	1982	CC	540	n	ot	*	y	0	ev	cig+/-ot	1	9	1	1	nev cigs	st
ZHENG	23		f	0	0	all	-	all	As:Chi	1982	CC	540	n	ot	*	y	0	ev	cig+/-ot	10	99	0	0	nev cigs	st
ZHOU	4		c	0	0	all	-	all	As:Chi	1978	CC	1360	n	ot	n	n	0	ev	all/unsp	1	9	1	1	nev any	st
ZHOU	5		c	0	0	all	-	all	As:Chi	1978	CC	1360	n	ot	n	n	0	ev	all/unsp	10	19	0	2	nev any	st
ZHOU	6		c	0	0	all	-	all	As:Chi	1978	CC	1360	n	ot	n	n	0	ev	all/unsp	20	99	0	0	nev any	st

Cigarette type is all/unspec for all RRs
except for the following:

REF	NRR	CIGTYPE
ALDERS	18	MC only
ALDERS	19	MC only
ALDERS	20	MC only
ALDERS	21	MC only
ALDERS	22	MC only
ALDERS	23	MC only
DEAN3	5	MC only
DEAN3	12	MC only
DEAN3	19	MC only
DEAN3	89	MC only
DEAN3	96	MC only
DEAN3	103	MC only
JUSSAW	10	MC only
JUSSAW	11	MC only
JUSSAW	12	MC only

Table 1G11 - 4

IESLC - Meta-anal of Ever Smoking (or Curr if Ever not avail) by Amount, Overview, Any prod (or Cigs if Any not avail)
All LC types
Least adjusted

REF	NRR	CIGTYPE
JUSSAW	13	MC only
JUSSAW	14	MC only
JUSSAW	15	MC only
NOTAN2	8	MC only
NOTAN2	9	MC only
NOTAN2	10	MC only

REF	NRR	Cigarette equivalent
AGUDO	11	-
AGUDO	12	-
AKIBA	17	*
AKIBA	18	*
AKIBA	19	*
AKIBA	23	*
AKIBA	24	*
ALDERS	18	-
ALDERS	19	-
ALDERS	20	-
ALDERS	21	-
ALDERS	22	-
ALDERS	23	-
ARCHER	1	*
ARCHER	2	*
ARCHER	3	*
ARMADA	46	*
ARMADA	47	*
ARMADA	48	*
AUVINE	5	*
AUVINE	6	*
AUVINE	7	*
AXELSS	19	inc 1 g pipe = 1 cig
AXELSS	20	inc 1 g pipe = 1 cig
AXELSS	21	inc 1 g pipe = 1 cig
AXELSS	13	includes 1 g pipe tob = 1 cig
AXELSS	14	includes 1 g pipe tob = 1 cig
AXELSS	15	includes 1 g pipe tob = 1 cig
AXELSS	16	includes 1 g pipe tob = 1 cig
BARBON	5	*
BARBON	7	*
BARBON	9	*
BARBON	11	*
BARBON	13	*
BENSHL	11	*
BENSHL	12	*
BENSHL	13	*
BEST	13	-
BEST	14	-
BEST	15	-
BOUCOT	99	up to 1 pk cigs, 4 cigars or 10 pipes
BOUCOT	100	> 1 pk cigs, 4 cigars or 10 pipes
BRESLO	13	*
BRESLO	14	*
BRESLO	15	*
BRESLO	16	*
BRESLO	29	*
BRESLO	30	*
BRETT	1	*
BRETT	2	*
BRETT	3	*
BROSS	18	*
BROSS	19	*
BROWN2	32	*
BROWN2	42	*
BROWN2	31	*
BROWN2	41	*
BUFFLE	28	*
BUFFLE	29	*
BUFFLE	35	*
CEDERL	80	gms inc cig = 1, sm cgr = 3, lge cgr = 5
CEDERL	81	gms inc cig = 1, sm cgr = 3, lge cgr = 5
CEDERL	82	gms inc cig = 1, sm cgr = 3, lge cgr = 5
CEDERL	76	inc 1 g other tob = 1 cig

Table 1G11 - 4

IESLC - Meta-anal of Ever Smoking (or Curr if Ever not avail) by Amount, Overview, Any prod (or Cigs if Any not avail)
All LC types
Least adjusted

REF	NRR	Cigarette equivalent
CEDERL	77	inc 1 g other tob = 1 cig
CEDERL	78	inc 1 g other tob = 1 cig
CHANG	2	*
CHANG	3	*
CHANG	4	*
CHANG	8	*
CHANG	9	*
CHANG	10	*
CHATZI	1	*
CHATZI	2	*
CHATZI	3	*
CHEN2	3	*
CHEN2	4	*
CHEN2	5	*
CHEN2	6	*
CHEN2	7	*
CHEN2	8	*
CHEN2	9	*
CHEN2	10	*
CHOI	12	*
CHOI	13	*
CHOI	14	*
CHOI	15	*
CHOI	16	*
CHOI	17	*
CHOI	18	*
CHOI	20	*
CHOW	3	*
CHOW	4	*
CHOW	5	*
COMSTO	4	*
COMSTO	5	*
COMSTO	6	*
COMSTO	9	*
COMSTO	10	*
COMSTO	11	*
COOKSO	1	*
COOKSO	2	*
CORREA	45	*
CORREA	49	*
CPSI	243	-
CPSI	246	-
CPSI	275	*
CPSI	276	*
CPSI	277	*
CPSI	278	*
CPSII	102	-
CPSII	103	-
CPSII	105	*
CPSII	106	*
DAMBER	6	-
DAMBER	7	-
DAMBER	8	-
DAMBER	9	-
DARBY	1	inc 1oz pipe/wk=2 cigs/d, excl cigar/llo
DARBY	2	inc 1oz pipe/wk=2 cigs/d, excl cigar/llo
DARBY	3	inc 1oz pipe/wk=2 cigs/d, excl cigar/llo
DARBY	8	inc 1oz pipe/wk=2 cigs/d, excl cigar/llo
DARBY	9	inc 1oz pipe/wk=2 cigs/d, excl cigar/llo
DARBY	10	inc 1oz pipe/wk=2 cigs/d, excl cigar/llo
DAVEYS	1	1 cigar or up to 5 cigs
DAVEYS	2	2 cigars or 6-10 cigs
DAVEYS	3	3-4 cigars or 11-20 cigs
DAVEYS	4	>4 cigars or >20 cigarettes
DEAN	1	-
DEAN	2	-
DEAN	3	-
DEAN2	25	-
DEAN2	26	-
DEAN2	29	-
DEAN2	30	-
DEAN3	5	-

Table 1G11 - 4

IESLC - Meta-anal of Ever Smoking (or Curr if Ever not avail) by Amount, Overview, Any prod (or Cigs if Any not avail)
All LC types
Least adjusted

REF	NRR	Cigarette equivalent
DEAN3	12	-
DEAN3	19	-
DEAN3	89	-
DEAN3	96	-
DEAN3	103	-
DEKLER	2	*
DEKLER	3	*
DEKLER	4	*
DESTEF	1	*
DESTEF	2	*
DESTEF	3	*
DESTEF	4	*
DOLL	1	*
DOLL	2	*
DOLL	3	*
DOLL	4	*
DOLL	5	*
DOLL	7	*
DOLL	8	*
DOLL	9	*
DOLL	10	*
DOLL2	46	grams
DOLL2	47	grams
DOLL2	48	grams
DOLL2	10	-
DOLL2	11	-
DOLL2	12	-
DORANT	6	*
DORANT	7	*
DORANT	8	*
DORGAN	10	*
DORGAN	11	*
DORGAN	34	*
DORGAN	35	*
DORGAN	96	*
DORGAN	97	*
DORN	408	*
DORN	409	*
DORN	410	*
DORN	411	*
DOSEME	5	*
DOSEME	9	*
DOSEME	13	*
DUNN	1	*
DUNN	2	*
DUNN	3	*
DUNN	4	*
DUNN	5	*
EBELIN	2	*
EBELIN	3	*
EBELIN	4	*
EBELIN	5	*
EBELIN	6	*
ENGELA	3	*
ENGELA	4	*
ENGELA	5	*
ENGELA	6	*
ENGELA	7	*
ENGELA	17	*
ENGELA	18	*
ENGELA	19	*
ENGELA	20	*
ENGELA	21	*
ENSTRO	7	-
ENSTRO	6	-
ENSTRO	5	-
ENSTRO	4	-
ENSTRO	3	-
ENSTRO	11	-
ENSTRO	10	-
ENSTRO	9	-
ENSTRO	8	-

Table 1G11 - 4

IESLC - Meta-anal of Ever Smoking (or Curr if Ever not avail) by Amount, Overview, Any prod (or Cigs if Any not avail)
All LC types
Least adjusted

REF	NRR	Cigarette equivalent
ESAKI	1	*
ESAKI	2	*
ESAKI	3	*
FAN	6	*
FAN	7	*
FAN	8	*
FAN	9	*
FAN	10	*
FAN	11	*
FAN	12	*
FAN	13	*
GAO	24	*
GAO	25	*
GAO	26	*
GAO2	2	*
GAO2	3	*
GAO2	4	*
GARSHI	18	*
GARSHI	19	*
GARSHI	20	*
GARSHI	21	*
GER	18	*
GER	19	*
GER	20	*
GOLLED	15	-
GOLLED	16	-
GOLLED	17	-
GSELL	1	inc cigar = 5, cheroot = 4, pipe = 2.5
GSELL	2	inc cigar = 5, cheroot = 4, pipe = 2.5
GSELL	3	inc cigar = 5, cheroot = 4, pipe = 2.5
GSELL	4	inc cigar = 5, cheroot = 4, pipe = 2.5
GSELL	5	inc cigar = 5, cheroot = 4, pipe = 2.5
HAENSZ	52	*
HAENSZ	51	*
HAMMO2	21	*
HAMMO2	20	*
HAMMON	153	-
HAMMON	154	-
HAMMON	155	-
HANSEN	1	cig equivalents (not defined)
HANSEN	2	cig equivalents (not defined)
HIRAYA	23	*
HIRAYA	24	*
HIRAYA	25	*
HIRAYA	26	*
HIRAYA	27	*
HIRAYA	28	*
HITOSU	3	*
HITOSU	4	*
HITOSU	5	*
HITOSU	10	*
HITOSU	11	*
HOLE	9	*
HOLE	10	*
HOLE	11	*
HOLE	12	*
HU	1	*
HU	2	*
HU	3	*
HU	4	*
HU	5	*
HU	6	*
HU2	2	*
HU2	3	*
HU2	4	*
HU2	5	*
HU2	6	*
HU2	7	*
HUMBLE	2	*
HUMBLE	3	*
HUMBLE	5	*
HUMBLE	6	*

Table 1G11 - 4

IESLC - Meta-anal of Ever Smoking (or Curr if Ever not avail) by Amount, Overview, Any prod (or Cigs if Any not avail)
All LC types
Least adjusted

REF	NRR	Cigarette equivalent
HUMBLE	8	*
HUMBLE	9	*
HUMBLE	11	*
HUMBLE	12	*
JARUP	1	gms, inc 1 pk pipe/wk = 7/d, 1 cigar = 4
JARUP	2	gms, inc 1 pk pipe/wk = 7/d, 1 cigar = 4
JEDRYC	60	*
JEDRYC	61	*
JEDRYC	62	*
JEDRYC	65	*
JEDRYC	66	*
JEDRYC	67	*
JOLY	7	*
JOLY	8	*
JOLY	9	*
JOLY	10	*
JOLY	3	*
JOLY	4	*
JOLY	5	*
JOLY	6	*
JUSSAW	10	-
JUSSAW	11	-
JUSSAW	12	-
JUSSAW	13	-
JUSSAW	14	-
JUSSAW	15	-
KAISE2	66	-
KAISE2	67	-
KAISE2	58	-
KAISE2	59	-
KAISER	6	*
KAISER	7	*
KAISER	8	*
KAISER	2	*
KAISER	3	*
KAISER	4	*
KANELL	1	*
KANELL	2	*
KANELL	3	*
KANELL	4	*
KATSOU	7	*
KATSOU	8	*
KATSOU	9	*
KATSOU	10	*
KAUFMA	2	*
KAUFMA	3	*
KAUFMA	4	*
KAUFMA	5	*
KAUFMA	6	*
KHUDER	1	*
KHUDER	2	*
KHUDER	3	*
KINLEN	3	*
KINLEN	4	*
KINLEN	5	*
KNEKT	25	*
KNEKT	26	*
KOO	11	*
KOO	12	*
KOO	13	*
KOULUM	6	*
KOULUM	5	*
KOULUM	4	*
KREUZE	19	*
KREUZE	20	*
KREUZE	21	*
KREUZE	22	*
KREUZE	30	*
KREUZE	31	*
KREUZE	32	*
KREUZE	33	*
KREUZE	25	*

Table 1G11 - 4

IESLC - Meta-anal of Ever Smoking (or Curr if Ever not avail) by Amount, Overview, Any prod (or Cigs if Any not avail)
All LC types
Least adjusted

REF	NRR	Cigarette equivalent
KREUZE	26	*
KREUZE	27	*
KREUZE	36	*
KREUZE	37	*
KREUZE	38	*
KREYBE	21	grams inc 1 cig=1
KREYBE	22	grams inc 1 cig=1
KREYBE	23	grams inc 1 cig=1
KREYBE	37	grams inc 1 cig=1
KREYBE	38	grams inc 1 cig=1
LAMTH	7	*
LAMTH	2	*
LAMTH	9	*
LAUSSM	3	inc 1 cigar = 1 cig, 1 pipe = 1 cig
LAUSSM	2	inc 1 cigar = 1 cig, 1 pipe = 1 cig
LAUSSM	1	inc 1 cigar = 1 cig, 1 pipe = 1 cig
LETOUR	2	*
LETOUR	3	*
LETOUR	4	*
LIAW	3	*
LIAW	4	*
LIAW	5	*
LIDDEL	2	*
LIDDEL	3	*
LIU2	5	*
LIU2	6	*
LIU2	7	*
LIU2	11	*
LIU2	12	*
LIU2	13	*
LIU3	3	Converted from kg/month
LIU3	4	Converted from kg/month
LIU3	5	Converted from kg/month
LIU4	7	-
LIU4	8	-
LIU4	9	-
LIU5	2	*
LIU5	3	*
LIU5	4	*
LUBIN	7	-
LUBIN	8	-
LUBIN	9	-
LUBIN	10	-
LUBIN2	273	*
LUBIN2	274	*
LUBIN2	275	*
LUBIN2	276	*
LUBIN2	281	*
LUBIN2	282	*
LUBIN2	283	*
LUBIN2	284	*
MACLEN	20	*
MACLEN	21	*
MACLEN	22	*
MACLEN	23	*
MACLEN	33	*
MACLEN	34	*
MACLEN	35	*
MARTIS	1	*
MARTIS	2	*
MARTIS	3	*
MATOS	28	*
MATOS	30	*
MATOS	32	*
MATSUD	1	*
MATSUD	2	*
MATSUD	3	*
MCCONN	26	N cigs exc mixed pipe, or <2oz pure pipe
MCCONN	25	N cigs exc mixed pipe, or 2-4oz pure pip
MCCONN	24	N cigs exc mixed pipe, or >4oz pure pipe
MIGRAN	1	-
MIGRAN	3	-

Table 1G11 - 4

IESLC - Meta-anal of Ever Smoking (or Curr if Ever not avail) by Amount, Overview, Any prod (or Cigs if Any not avail)
All LC types
Least adjusted

REF	NRR	Cigarette equivalent
MIGRAN	5	-
MIGRAN	7	-
MIGRAN	28	-
MIGRAN	30	-
MIGRAN	32	-
MIGRAN	34	-
MRFITR	3	*
MRFITR	4	*
MRFITR	5	*
NAM	66	*
NAM	67	*
NAM	82	*
NAM	83	*
NOTAN2	8	-
NOTAN2	9	-
NOTAN2	10	-
ORMOS	1	*
ORMOS	2	*
ORMOS	3	*
OSANN	49	*
OSANN	57	*
OSANN	50	*
OSANN	58	*
PARKIN	17	grams inc 1 cig=1g, 1 pipe=0.65g
PARKIN	18	grams inc 1 cig=1g, 1 pipe=0.65g
PASTOR	1	*
PASTOR	2	*
PASTOR	3	*
PASTOR	4	*
PERNU	17	grams
PERNU	18	grams
PERNU	19	grams
PERNU	20	grams
PERNU	21	grams
PERNU	22	grams
PERNU	23	grams
PERNU	24	grams
PERNU	11	grams
PERNU	12	grams
PERNU	13	grams
PERNU	14	grams
PERNU	15	grams
PERNU	16	grams
PERSH2	2	includes 50g pipe/wk = 7 cigs/day
PERSH2	3	includes 50g pipe/wk = 7 cigs/day
PETO	2	*
PETO	3	*
PEZZO2	3	*
PEZZO2	4	*
PEZZO2	5	*
PEZZOT	2	-
PEZZOT	3	-
PEZZOT	4	-
PIKE	1	*
PIKE	2	*
PIKE	3	*
PIKE	5	*
PIKE	6	*
PIKE	7	*
POLEDN	2	*
POLEDN	4	*
PRESCO	2	gms, inc cig = 1, cheroot = 3, cigar = 5
PRESCO	4	gms, inc cig = 1, cheroot = 3, cigar = 5
PRESCO	1	gms, inc cig = 1, cheroot = 3, cigar = 5
PRESCO	3	gms, inc cig = 1, cheroot = 3, cigar = 5
RACHTA	5	*
RACHTA	6	*
RACHTA	7	*
RANDIG	1	inc 1g pip=1, cgr=5, chrt=4, cigarillo=3
RANDIG	2	inc 1g pip=1, cgr=5, chrt=4, cigarillo=3
RANDIG	3	inc 1g pip=1, cgr=5, chrt=4, cigarillo=3
RANDIG	4	inc 1g pip=1, cgr=5, chrt=4, cigarillo=3

Table 1G11 - 4

IESLC - Meta-anal of Ever Smoking (or Curr if Ever not avail) by Amount, Overview, Any prod (or Cigs if Any not avail)
All LC types
Least adjusted

REF	NRR	Cigarette equivalent
RANDIG	5	inc 1g pip=1, cgr=5, chrt=4, cigarillo=3
RANDIG	6	inc 1g pip=1, cgr=5, chrt=4, cigarillo=3
RANDIG	7	inc 1g pip=1, cgr=5, chrt=4, cigarillo=3
SEGI2	9	*
SEGI2	11	*
SEGI2	13	*
SEGI2	15	*
SEGI2	17	*
SEGI2	21	*
SEGI2	23	*
SEGI2	25	*
SHAW	10	*
SHAW	11	*
SIEMIA	13	*
SIEMIA	14	*
SIEMIA	15	*
SOBUE	117	*
SOBUE	118	*
SOBUE	119	*
SPEIZE	1	*
SPEIZE	2	*
SPEIZE	3	*
SPEIZE	4	*
SPEIZE	5	*
SPITZ	5	*
SPITZ	6	*
STOCKS	25	*
STOCKS	26	*
STOCKS	27	*
STOCKS	28	*
STOCKS	29	*
STOCKS	48	*
STOCKS	49	*
STOCKW	1	*
STOCKW	2	*
STOCKW	3	*
SVENSS	26	*
SVENSS	31	*
SVENSS	36	*
TENKAN	10	grams
TENKAN	11	grams
TENKAN	12	grams
TIZZAN	7	-
TIZZAN	8	-
TIZZAN	9	-
TIZZAN	10	-
TIZZAN	15	-
TIZZAN	16	-
TSUGAN	29	*
TSUGAN	30	*
TSUGAN	31	*
TULINI	4	*
TULINI	5	*
TULINI	6	*
TULINI	9	*
TULINI	10	*
TULINI	11	*
TVERDA	9	-
TVERDA	10	-
TVERDA	11	-
TVERDA	16	-
TVERDA	17	-
WAKAI	37	*
WAKAI	38	*
WAKAI	39	*
WANG2	1	*
WANG2	2	*
WANG2	3	*
WANG2	4	*
WANG2	5	*
WANG2	6	*
WANG2	7	*

Table 1G11 - 4

IESLC - Meta-anal of Ever Smoking (or Curr if Ever not avail) by Amount, Overview, Any prod (or Cigs if Any not avail)
All LC types
Least adjusted

REF	NRR	Cigarette equivalent
WU	35	*
WU	36	*
WUWILL	18	*
WUWILL	19	*
WYNDE2	17	*
WYNDE2	18	*
WYNDE2	19	*
WYNDE2	20	*
WYNDE3	44	*
WYNDE3	45	*
WYNDE3	46	*
WYNDE3	47	*
WYNDE3	79	*
WYNDE3	80	*
WYNDE3	81	*
WYNDE3	82	*
WYNDE4	43	inc 1 cigar = 5 cigs, 1 pipe = 2.5 cigs
WYNDE4	44	inc 1 cigar = 5 cigs, 1 pipe = 2.5 cigs
WYNDE4	45	inc 1 cigar = 5 cigs, 1 pipe = 2.5 cigs
WYNDE4	46	inc 1 cigar = 5 cigs, 1 pipe = 2.5 cigs
WYNDE4	47	inc 1 cigar = 5 cigs, 1 pipe = 2.5 cigs
WYNDE4	57	inc 1 cigar = 5 cigs, 1 pipe = 2.5 cigs
WYNDE4	58	inc 1 cigar = 5 cigs, 1 pipe = 2.5 cigs
WYNDE4	59	inc 1 cigar = 5 cigs, 1 pipe = 2.5 cigs
WYNDE4	60	inc 1 cigar = 5 cigs, 1 pipe = 2.5 cigs
WYNDE4	61	inc 1 cigar = 5 cigs, 1 pipe = 2.5 cigs
WYNDE6	27	*
WYNDE6	36	*
WYNDE6	45	*
WYNDE6	54	*
WYNDE6	216	*
WYNDE6	225	*
WYNDE6	234	*
WYNDE6	243	*
XU3	5	*
XU3	6	*
XU3	7	*
XU3	8	*
XU3	13	*
XU3	14	*
XU3	15	*
YAMAGU	3	*
YAMAGU	2	*
YUAN	2	*
YUAN	3	*
ZHENG	11	*
ZHENG	12	*
ZHENG	13	*
ZHENG	14	*
ZHENG	22	*
ZHENG	23	*
ZHOU	4	*
ZHOU	5	*
ZHOU	6	*

In this overview table, subtotals and Qs values may be invalid and should be ignored

Table 1G11 - 5

IESLC - Meta-anal of Ever Smoking (or Curr if Ever not avail) by Amount, Overview, Any prod (or Cigs if Any not avail)
All LC types
Least adjusted

REF	NRR	SEX	AD	Number Exposed Case	Number Exposed Cont	Non-exposed Case	Non-exposed Cont	RR	95.00%CI
AGUDO	11	f	0	6	12	80	183	1.14	(0.41- 3.15)
AGUDO	12	f	0	17	11	80	183	3.54	(1.58- 7.89)
Subtotal AGUDO								2.29	(1.22- 4.30)
*AKIBA	17	m	0	104	59893	18	35833	3.46	(2.10- 5.70)
*AKIBA	18	m	0	178	82289	18	35833	4.31	(2.65- 6.99)
*AKIBA	19	m	0	63	28351	18	35833	4.42	(2.62- 7.47)
*AKIBA	23	f	0	54	38968	116	359850	4.30	(3.11- 5.94)
*AKIBA	24	f	0	17	10284	116	359850	5.13	(3.08- 8.53)
Subtotal AKIBA								4.29	(3.51- 5.23)
ALDERS	18	m	1	-	-	-	-	3.55	(1.94- 6.49)
ALDERS	19	m	1	-	-	-	-	7.96	(4.63- 13.69)
ALDERS	20	m	1	-	-	-	-	8.52	(5.07- 14.33)
ALDERS	21	f	1	-	-	-	-	2.62	(1.88- 3.65)
ALDERS	22	f	1	-	-	-	-	5.28	(3.79- 7.36)
ALDERS	23	f	1	-	-	-	-	6.90	(4.69- 10.15)
Subtotal ALDERS								4.91	(4.15- 5.82)
*ARCHER	1	m	0	14	6504	6	9842	3.53	(1.36- 9.18)
*ARCHER	2	m	0	68	18320	6	9842	6.09	(2.64- 14.02)
*ARCHER	3	m	0	40	7705	6	9842	8.52	(3.61- 20.07)
Subtotal ARCHER								5.87	(3.54- 9.75)
ARMADA	46	m	0	44	117	4	64	6.02	(2.07- 17.51)
ARMADA	47	m	0	134	105	4	64	20.42	(7.20- 57.88)
ARMADA	48	m	0	139	32	4	64	69.50	(23.58- 204.81)
Subtotal ARMADA								20.25	(10.96- 37.40)
AUVINE	5	c	0	57	33	44	229	8.99	(5.26- 15.37)
AUVINE	6	c	0	148	39	44	229	19.75	(12.25- 31.86)
AUVINE	7	c	0	61	8	44	229	39.68	(17.75- 88.72)
Subtotal AUVINE								16.56	(11.95- 22.95)
AXELSS	19	m	0	39	96	16	160	4.06	(2.15- 7.66)
AXELSS	20	m	0	111	122	16	160	9.10	(5.12- 16.16)
AXELSS	21	m	0	142	126	16	160	11.27	(6.39- 19.87)
AXELSS	13	f	0	13	37	18	154	3.01	(1.35- 6.68)
AXELSS	14	f	0	63	50	18	154	10.78	(5.84- 19.91)
AXELSS	15	f	0	28	15	18	154	15.97	(7.21- 35.36)
AXELSS	16	f	0	6	7	18	154	7.33	(2.22- 24.22)
Subtotal AXELSS								8.03	(6.21- 10.39)
BARBON	5	m	0	28	87	22	188	2.75	(1.49- 5.08)
BARBON	7	m	0	126	111	22	188	9.70	(5.82- 16.15)
BARBON	9	m	0	223	176	22	188	10.83	(6.67- 17.57)
BARBON	11	m	0	129	82	22	188	13.44	(7.98- 22.64)
BARBON	13	m	0	227	111	22	188	17.48	(10.64- 28.71)
Subtotal BARBON								10.08	(7.99- 12.72)
*BENSHL	11	m	1	-	-	-	-	4.00	(1.55- 10.31)
*BENSHL	12	m	1	-	-	-	-	9.05	(3.91- 20.94)
*BENSHL	13	m	1	-	-	-	-	10.95	(4.76- 25.22)
Subtotal BENSHL								7.71	(4.67- 12.74)
*BEST	13	m	1	-	-	-	-	10.00	(4.56- 21.92)
*BEST	14	m	1	-	-	-	-	16.41	(7.73- 34.86)
*BEST	15	m	1	-	-	-	-	17.31	(7.93- 37.79)
Subtotal BEST								14.23	(9.11- 22.23)
*BOUCOT	99	m	0	38	2670	0	805	23.23~	(1.43- 377.62)
*BOUCOT	100	m	0	43	1519	0	805	46.12~	(2.84- 748.16)
Subtotal BOUCOT								32.74	(4.56- 235.00)
BRESLO	13	m	0	16	45	22	110	1.78	(0.86- 3.69)
BRESLO	14	m	0	69	105	22	110	3.29	(1.90- 5.69)
BRESLO	15	m	0	296	193	22	110	7.67	(4.69- 12.55)
BRESLO	16	m	0	80	22	22	110	18.18	(9.42- 35.09)
BRESLO	29	f	0	5	5	12	14	1.17	(0.27- 5.02)
BRESLO	30	f	0	8	6	12	14	1.56	(0.42- 5.76)
Subtotal BRESLO								5.03	(3.79- 6.65)
*BRETT	1	m	0	40	17090	6	6530	2.55	(1.08- 6.01)
*BRETT	2	m	0	62	15868	6	6530	4.25	(1.84- 9.83)
*BRETT	3	m	0	33	4490	6	6530	8.00	(3.35- 19.07)
Subtotal BRETT								4.40	(2.69- 7.21)
BROSS	18	m	0	170	155	38	170	4.91	(3.24- 7.42)
BROSS	19	m	0	95	59	38	170	7.20	(4.46- 11.63)
Subtotal BROSS								5.78	(4.23- 7.91)
BROWN2	32	m	2	-	-	-	-	6.10	(5.30- 6.90)
BROWN2	42	m	2	-	-	-	-	14.10	(12.70- 15.50)
BROWN2	31	f	2	-	-	-	-	8.40	(7.20- 9.70)
BROWN2	41	f	2	-	-	-	-	17.10	(15.30- 19.10)

Table 1G11 - 5

IESLC - Meta-anal of Ever Smoking (or Curr if Ever not avail) by Amount, Overview, Any prod (or Cigs if Any not avail)
All LC types
Least adjusted

REF	NRR	SEX	AD	Number Exposed Case	Cont	Non-exposed Case	Cont	RR	95.00%CI
Subtotal BROWN2								11.59	(10.92- 12.30)
BUFFLE	28	f	0	21	42	12	112	4.67	(2.11- 10.31)
BUFFLE	29	f	0	76	60	12	112	11.82	(5.96- 23.45)
BUFFLE	35	f	0	141	62	12	112	21.23	(10.90- 41.32)
Subtotal BUFFLE								11.51	(7.65- 17.33)
*CEDERL	80	m	2	-	-	-	-	3.40	(1.96- 5.90)
*CEDERL	81	m	2	-	-	-	-	7.50	(4.79- 11.74)
*CEDERL	82	m	2	-	-	-	-	11.90	(7.55- 18.75)
*CEDERL	76	f	2	-	-	-	-	2.83	(1.72- 4.67)
*CEDERL	77	f	2	-	-	-	-	7.74	(4.96- 12.08)
*CEDERL	78	f	2	-	-	-	-	7.75	(4.03- 14.91)
Subtotal CEDERL								6.36	(5.20- 7.78)
*CHANG	2	m	0	5	100	5	502	5.02	(1.48- 17.02)
*CHANG	3	m	0	17	161	5	502	10.60	(3.97- 28.28)
*CHANG	4	m	0	13	158	5	502	8.26	(2.99- 22.81)
*CHANG	8	f	0	6	205	11	1139	3.03	(1.13- 8.10)
*CHANG	9	f	0	11	234	11	1139	4.87	(2.14- 11.09)
*CHANG	10	f	0	13	164	11	1139	8.21	(3.74- 18.01)
Subtotal CHANG								6.25	(4.26- 9.17)
CHATZI	1	c	0	68	127	27	129	2.56	(1.54- 4.25)
CHATZI	2	c	0	73	123	27	129	2.84	(1.71- 4.70)
CHATZI	3	c	0	114	115	27	129	4.74	(2.90- 7.72)
Subtotal CHATZI								3.28	(2.46- 4.38)
CHEN2	3	m	0	17	26	9	33	2.40	(0.92- 6.25)
CHEN2	4	m	0	44	50	9	33	3.23	(1.39- 7.48)
CHEN2	5	m	0	34	9	9	33	13.85	(4.89- 39.22)
CHEN2	6	m	0	26	12	9	33	7.94	(2.91- 21.72)
CHEN2	7	f	0	5	17	25	33	0.39	(0.13- 1.20)
CHEN2	8	f	0	22	10	25	33	2.90	(1.17- 7.22)
CHEN2	9	f	0	7	1	25	33	9.24	(1.07- 80.02)
CHEN2	10	f	0	4	2	25	33	2.64	(0.45- 15.58)
Subtotal CHEN2								3.36	(2.30- 4.90)
CHOI	12	m	0	20	90	13	95	1.62	(0.76- 3.46)
CHOI	13	m	0	144	281	13	95	3.74	(2.03- 6.92)
CHOI	14	m	0	50	49	13	95	7.46	(3.70- 15.03)
CHOI	15	m	0	37	39	13	95	6.93	(3.33- 14.44)
CHOI	16	m	0	16	6	13	95	19.49	(6.47- 58.71)
CHOI	17	f	0	9	16	76	164	1.21	(0.51- 2.87)
CHOI	18	f	0	7	9	76	164	1.68	(0.60- 4.68)
CHOI	20	f	0	3	1	76	164	6.47	(0.66- 63.26)
Subtotal CHOI								3.83	(2.86- 5.13)
*CHOW	3	m	0	38	29404	6	62913	13.55	(5.73- 32.05)
*CHOW	4	m	0	60	36589	6	62913	17.19	(7.43- 39.79)
*CHOW	5	m	0	40	15732	6	62913	26.66	(11.31- 62.87)
Subtotal CHOW								18.38	(11.23- 30.06)
COMSTO	4	m	0	18	25	4	69	12.42	(3.83- 40.26)
COMSTO	5	m	0	60	57	4	69	18.16	(6.22- 53.00)
COMSTO	6	m	0	26	18	4	69	24.92	(7.71- 80.57)
COMSTO	9	f	0	16	19	13	115	7.45	(3.10- 17.93)
COMSTO	10	f	0	51	26	13	115	17.35	(8.25- 36.48)
COMSTO	11	f	0	9	6	13	115	13.27	(4.07- 43.25)
Subtotal COMSTO								14.21	(9.49- 21.27)
COOKSO	1	c	0	102	27	45	61	5.12	(2.89- 9.08)
COOKSO	2	c	0	82	11	45	61	10.11	(4.83- 21.13)
Subtotal COOKSO								6.61	(4.21- 10.40)
CORREA	45	c	0	371	329	51	388	8.58	(6.18- 11.90)
CORREA	49	c	0	514	195	51	388	20.05	(14.34- 28.04)
Subtotal CORREA								12.99	(10.27- 16.41)
*CPSI	243	m	1	-	-	-	-	5.81	(4.33- 7.80)
*CPSI	246	m	1	-	-	-	-	13.60	(10.46- 17.67)
*CPSI	275	f	1	-	-	-	-	1.25	(0.73- 2.13)
*CPSI	276	f	1	-	-	-	-	2.44	(1.67- 3.56)
*CPSI	277	f	1	-	-	-	-	5.03	(3.82- 6.63)
*CPSI	278	f	1	-	-	-	-	11.10	(6.00- 20.53)
Subtotal CPSI								5.90	(5.14- 6.77)
*CPSII	102	m	1	-	-	-	-	9.99	(7.97- 12.51)
*CPSII	103	m	1	-	-	-	-	17.60	(14.05- 22.05)
*CPSII	105	f	1	-	-	-	-	4.16	(3.41- 5.09)
*CPSII	106	f	1	-	-	-	-	13.34	(11.31- 15.75)
Subtotal CPSII								9.98	(9.03- 11.02)
DAMBER	6	m	1	-	-	-	-	2.30	(1.30- 4.40)

Table 1G11 - 5

IESLC - Meta-anal of Ever Smoking (or Curr if Ever not avail) by Amount, Overview, Any prod (or Cigs if Any not avail)
All LC types
Least adjusted

REF	NRR	SEX	AD	Number Exposed Case	Number Exposed Cont	Non-exposed Case	Non-exposed Cont	RR	95.00%CI
DAMBER	7	m	1	-	-	-	-	7.30	(4.40- 12.70)
DAMBER	8	m	1	-	-	-	-	9.10	(5.50- 15.30)
DAMBER	9	m	1	-	-	-	-	14.90	(6.70- 33.50)
Subtotal DAMBER								6.60	(4.92- 8.86)
DARBY	1	m	0	128	223	3	384	73.47	(23.11- 233.57)
DARBY	2	m	0	126	169	3	384	95.43	(29.94- 304.17)
DARBY	3	m	0	68	61	3	384	142.69	(43.52- 467.82)
DARBY	8	f	0	71	104	23	529	15.70	(9.38- 26.28)
DARBY	9	f	0	86	92	23	529	21.50	(12.90- 35.82)
DARBY	10	f	0	38	21	23	529	41.62	(21.15- 81.90)
Subtotal DARBY								29.10	(21.80- 38.84)
DAVEYS	1	m	0	11	69	3	23	1.22	(0.31- 4.77)
DAVEYS	2	m	0	31	32	3	23	7.43	(2.02- 27.27)
DAVEYS	3	m	0	19	22	3	23	6.62	(1.72- 25.56)
DAVEYS	4	m	0	29	21	3	23	10.59	(2.81- 39.94)
Subtotal DAVEYS								5.12	(2.63- 9.98)
DEAN	1	m	0	73	168	12	61	2.21	(1.12- 4.35)
DEAN	2	m	0	228	172	12	61	6.74	(3.52- 12.91)
DEAN	3	m	0	102	45	12	61	11.52	(5.66- 23.47)
Subtotal DEAN								5.46	(3.69- 8.08)
DEAN2	25	m	0	377	396	33	112	3.23	(2.14- 4.88)
DEAN2	26	m	0	252	112	33	112	7.64	(4.88- 11.95)
DEAN2	29	f	0	44	24	88	121	2.52	(1.43- 4.45)
DEAN2	30	f	0	18	5	88	121	4.95	(1.77- 13.84)
Subtotal DEAN2								4.21	(3.25- 5.45)
DEAN3	5	m	0	81	264	25	510	6.26	(3.90- 10.04)
DEAN3	12	m	0	125	429	25	510	5.94	(3.80- 9.31)
DEAN3	19	m	0	131	237	25	510	11.28	(7.16- 17.77)
DEAN3	89	f	0	31	486	41	1538	2.39	(1.48- 3.86)
DEAN3	96	f	0	44	521	41	1538	3.17	(2.05- 4.90)
DEAN3	103	f	0	27	151	41	1538	6.71	(4.01- 11.21)
Subtotal DEAN3								5.24	(4.33- 6.33)
*DEKLER	2	m	2	-	-	-	-	19.40	(2.60- 143.70)
*DEKLER	3	m	2	-	-	-	-	23.00	(3.20- 167.60)
*DEKLER	4	m	2	-	-	-	-	32.50	(4.40- 241.20)
Subtotal DEKLER								24.38	(7.70- 77.17)
DESTEF	1	m	0	38	84	27	163	2.73	(1.56- 4.78)
DESTEF	2	m	0	155	119	27	163	7.86	(4.90- 12.61)
DESTEF	3	m	0	161	100	27	163	9.72	(6.03- 15.67)
DESTEF	4	m	0	116	31	27	163	22.59	(12.80- 39.87)
Subtotal DESTEF								8.30	(6.42- 10.73)
DOLL	1	m	0	55	129	7	61	3.72	(1.60- 8.64)
DOLL	2	m	0	489	570	7	61	7.48	(3.39- 16.50)
DOLL	3	m	0	475	431	7	61	9.60	(4.35- 21.22)
DOLL	4	m	0	293	154	7	61	16.58	(7.40- 37.13)
DOLL	5	m	0	38	12	7	61	27.60	(9.99- 76.25)
DOLL	7	f	0	16	25	40	59	0.94	(0.45- 1.99)
DOLL	8	f	0	24	18	40	59	1.97	(0.95- 4.09)
DOLL	9	f	0	14	6	40	59	3.44	(1.22- 9.71)
DOLL	10	f	0	14	0	40	59	42.60~	(2.47- 734.58)
Subtotal DOLL								4.97	(3.72- 6.65)
*DOLL2	46	m	1	-	-	-	-	6.71	(0.91- 49.81)
*DOLL2	47	m	1	-	-	-	-	12.29	(1.67- 90.41)
*DOLL2	48	m	1	-	-	-	-	23.71	(3.25- 173.24)
*DOLL2	10	f	1	-	-	-	-	1.29	(0.14- 11.50)
*DOLL2	11	f	1	-	-	-	-	6.43	(1.81- 22.78)
*DOLL2	12	f	1	-	-	-	-	29.71	(9.46- 93.32)
Subtotal DOLL2								11.39	(5.93- 21.87)
DORANT	6	c	0	21	192	14	1090	8.52	(4.26- 17.04)
DORANT	7	c	0	143	409	14	1090	27.22	(15.54- 47.68)
DORANT	8	c	0	128	275	14	1090	36.24	(20.55- 63.91)
Subtotal DORANT								22.68	(16.05- 32.04)
DORGAN	10	m	0	125	148	15	93	5.24	(2.89- 9.49)
DORGAN	11	m	0	574	252	15	93	14.12	(8.03- 24.85)
DORGAN	34	m	0	118	84	3	35	16.39	(4.88- 55.06)
DORGAN	35	m	0	145	32	3	35	52.86	(15.30- 182.62)
DORGAN	96	f	3	-	-	-	-	5.67	(4.36- 7.36)
DORGAN	97	f	3	-	-	-	-	12.22	(9.31- 16.04)
Subtotal DORGAN								8.71	(7.36- 10.30)
*DORN	408	m	1	-	-	-	-	4.02	(3.43- 4.71)
*DORN	409	m	1	-	-	-	-	9.92	(8.84- 11.14)

Table 1G11 - 5

IESLC - Meta-anal of Ever Smoking (or Curr if Ever not avail) by Amount, Overview, Any prod (or Cigs if Any not avail)
All LC types
Least adjusted

REF	NRR	SEX	AD	Number Exposed Case	Cont	Non-exposed Case	Cont	RR	95.00%CI
*DORN	410	m	1	-	-	-	-	17.19	(15.28- 19.33)
*DORN	411	m	1	-	-	-	-	22.75	(19.63- 26.37)
Subtotal DORN								11.88	(11.13- 12.68)
DOSEME	5	m	2	-	-	-	-	2.20	(1.40- 3.30)
DOSEME	9	m	2	-	-	-	-	3.10	(2.30- 4.10)
DOSEME	13	m	2	-	-	-	-	6.60	(4.40- 10.20)
Subtotal DOSEME								3.44	(2.79- 4.24)
*DUNN	1	m	0	3	2538	2	14160	8.37	(1.40- 50.06)
*DUNN	2	m	0	12	9418	2	14160	9.02	(2.02- 40.30)
*DUNN	3	m	0	75	27720	2	14160	19.16	(4.70- 78.00)
*DUNN	4	m	0	32	9017	2	14160	25.13	(6.02- 104.82)
*DUNN	5	m	0	13	3206	2	14160	28.71	(6.48- 127.15)
Subtotal DUNN								16.88	(8.61- 33.09)
EBELIN	2	m	0	20	72	12	117	2.71	(1.25- 5.87)
EBELIN	3	m	0	19	26	12	117	7.13	(3.08- 16.48)
EBELIN	4	m	0	47	37	12	117	12.39	(5.95- 25.80)
EBELIN	5	m	0	5	3	12	117	16.25	(3.45- 76.54)
EBELIN	6	m	0	4	4	12	117	9.75	(2.16- 44.04)
Subtotal EBELIN								6.99	(4.62- 10.59)
*ENGELA	3	m	0	28	28761	27	58716	2.12	(1.25- 3.59)
*ENGELA	4	m	0	49	25801	27	58716	4.13	(2.58- 6.60)
*ENGELA	5	m	0	97	32257	27	58716	6.54	(4.27- 10.02)
*ENGELA	6	m	0	27	6424	27	58716	9.14	(5.36- 15.57)
*ENGELA	7	m	0	57	11329	27	58716	10.94	(6.92- 17.29)
*ENGELA	17	f	0	12	31450	31	207789	2.56	(1.31- 4.98)
*ENGELA	18	f	0	20	28450	31	207789	4.71	(2.69- 8.27)
*ENGELA	19	f	0	24	14820	31	207789	10.85	(6.37- 18.49)
*ENGELA	20	f	0	6	3030	31	207789	13.27	(5.54- 31.79)
*ENGELA	21	f	0	3	2445	31	207789	8.22	(2.52- 26.88)
Subtotal ENGELA								6.01	(5.05- 7.16)
*ENSTRO	7	m	1	-	-	-	-	4.74	(3.34- 6.73)
*ENSTRO	6	m	1	-	-	-	-	7.68	(5.95- 9.90)
*ENSTRO	5	m	1	-	-	-	-	13.65	(10.88- 17.13)
*ENSTRO	4	m	1	-	-	-	-	16.08	(12.77- 20.23)
*ENSTRO	3	m	1	-	-	-	-	19.41	(15.22- 24.75)
*ENSTRO	11	f	1	-	-	-	-	2.15	(1.62- 2.84)
*ENSTRO	10	f	1	-	-	-	-	4.31	(3.56- 5.22)
*ENSTRO	9	f	1	-	-	-	-	9.48	(8.04- 11.18)
*ENSTRO	8	f	1	-	-	-	-	16.47	(13.74- 19.75)
Subtotal ENSTRO								9.31	(8.66- 10.02)
ESAKI	1	m	0	47	75	16	28	1.10	(0.54- 2.24)
ESAKI	2	m	0	74	58	16	28	2.23	(1.10- 4.51)
ESAKI	3	m	0	34	10	16	28	5.95	(2.34- 15.16)
Subtotal ESAKI								2.12	(1.36- 3.29)
FAN	6	m	0	13	121	36	236	0.70	(0.36- 1.38)
FAN	7	m	0	53	171	36	236	2.03	(1.27- 3.24)
FAN	8	m	0	111	183	36	236	3.98	(2.61- 6.07)
FAN	9	m	0	39	23	36	236	11.12	(5.96- 20.73)
FAN	10	f	0	17	48	69	320	1.64	(0.89- 3.03)
FAN	11	f	0	30	37	69	320	3.76	(2.18- 6.50)
FAN	12	f	0	31	12	69	320	11.98	(5.86- 24.50)
FAN	13	f	0	4	1	69	320	18.55	(2.04- 168.54)
Subtotal FAN								3.29	(2.68- 4.05)
GAO	24	f	0	70	74	435	605	1.32	(0.93- 1.87)
GAO	25	f	0	75	44	435	605	2.37	(1.60- 3.51)
GAO	26	f	0	91	12	435	605	10.55	(5.71- 19.50)
Subtotal GAO								2.25	(1.77- 2.87)
GAO2	2	m	0	32	41	13	56	3.36	(1.57- 7.19)
GAO2	3	m	0	77	44	13	56	7.54	(3.71- 15.30)
GAO2	4	m	0	74	30	13	56	10.63	(5.08- 22.22)
Subtotal GAO2								6.57	(4.30- 10.04)
GARSHI	18	m	0	105	282	41	363	3.30	(2.23- 4.88)
GARSHI	19	m	0	387	601	41	363	5.70	(4.03- 8.07)
GARSHI	20	m	0	153	182	41	363	7.44	(5.05- 10.97)
GARSHI	21	m	0	223	350	41	363	5.64	(3.92- 8.12)
Subtotal GARSHI								5.35	(4.44- 6.44)
GER	18	c	0	21	108	51	246	0.94	(0.54- 1.64)
GER	19	c	0	45	168	51	246	1.29	(0.83- 2.02)
GER	20	c	0	24	42	51	246	2.76	(1.54- 4.95)
Subtotal GER								1.44	(1.06- 1.94)
GOLLED	15	m	0	83	679	15	490	3.99	(2.28- 7.00)

Table 1G11 - 5

IESLC - Meta-anal of Ever Smoking (or Curr if Ever not avail) by Amount, Overview, Any prod (or Cigs if Any not avail)
All LC types
Least adjusted

REF	NRR	SEX	AD	Number Exposed Case	Number Exposed Cont	Non-exposed Case	Non-exposed Cont	RR	95.00%CI
GOLLED	16	m	0	118	779	15	490	4.95	(2.86- 8.57)
GOLLED	17	m	0	143	333	15	490	14.03	(8.09- 24.31)
Subtotal GOLLED								6.56	(4.77- 9.04)
GSELL	1	m	0	11	36	2	29	4.43	(0.91- 21.60)
GSELL	2	m	0	10	37	2	29	3.92	(0.80- 19.30)
GSELL	3	m	0	27	26	2	29	15.06	(3.26- 69.59)
GSELL	4	m	0	49	9	2	29	78.94	(15.95- 390.82)
GSELL	5	m	0	51	13	2	29	56.88	(11.99- 269.87)
Subtotal GSELL								16.45	(8.14- 33.23)
HAENSZ	52	f	0	40	66	81	236	1.77	(1.11- 2.82)
HAENSZ	51	f	0	23	13	81	236	5.15	(2.50- 10.65)
Subtotal HAENSZ								2.42	(1.63- 3.58)
*HAMMO2	21	m	0	38	764	5	891	8.86	(3.51- 22.41)
*HAMMO2	20	m	0	171	3708	5	891	8.22	(3.39- 19.94)
Subtotal HAMMO2								8.52	(4.49- 16.17)
*HAMMON	153	m	1	-	-	-	-	7.38	(3.72- 14.63)
*HAMMON	154	m	1	-	-	-	-	8.32	(4.66- 14.84)
*HAMMON	155	m	1	-	-	-	-	17.06	(9.44- 30.82)
Subtotal HAMMON								10.42	(7.31- 14.85)
*HANSEN	1	m	2	-	-	-	-	1.37	(0.63- 3.54)
*HANSEN	2	m	2	-	-	-	-	2.90	(1.14- 8.27)
Subtotal HANSEN								1.89	(0.99- 3.63)
*HIRAYA	23	m	1	-	-	-	-	2.06	(1.49- 2.85)
*HIRAYA	24	m	1	-	-	-	-	4.00	(3.20- 4.99)
*HIRAYA	25	m	1	-	-	-	-	6.24	(5.07- 7.68)
*HIRAYA	26	f	1	-	-	-	-	2.25	(1.64- 3.08)
*HIRAYA	27	f	1	-	-	-	-	2.56	(1.85- 3.54)
*HIRAYA	28	f	1	-	-	-	-	4.47	(2.73- 7.33)
Subtotal HIRAYA								3.72	(3.32- 4.17)
HITOSU	3	m	0	33	585	7	242	1.95	(0.85- 4.47)
HITOSU	4	m	0	52	747	7	242	2.41	(1.08- 5.37)
HITOSU	5	m	0	32	265	7	242	4.17	(1.81- 9.63)
HITOSU	10	f	0	24	391	33	1893	3.52	(2.06- 6.02)
HITOSU	11	f	0	4	68	33	1893	3.37	(1.16- 9.79)
Subtotal HITOSU								3.06	(2.18- 4.28)
*HOLE	9	m	0	23	840	7	1189	4.65	(2.00- 10.79)
*HOLE	10	m	0	87	2056	7	1189	7.19	(3.34- 15.47)
*HOLE	11	m	0	38	782	7	1189	8.25	(3.70- 18.39)
*HOLE	12	m	0	13	311	7	1189	7.10	(2.86- 17.64)
Subtotal HOLE								6.70	(4.43- 10.12)
HU	1	m	0	36	38	41	67	1.55	(0.85- 2.82)
HU	2	m	0	55	43	41	67	2.09	(1.20- 3.65)
HU	3	m	0	29	13	41	67	3.65	(1.70- 7.80)
HU	4	f	0	19	10	40	48	2.28	(0.95- 5.46)
HU	5	f	0	6	6	40	48	1.20	(0.36- 4.01)
HU	6	f	0	1	2	40	48	0.60	(0.05- 6.86)
Subtotal HU								2.02	(1.47- 2.77)
HU2	2	c	0	16	33	121	213	0.85	(0.45- 1.61)
HU2	3	c	0	44	58	121	213	1.34	(0.85- 2.10)
HU2	4	c	0	65	59	121	213	1.94	(1.28- 2.94)
HU2	5	c	0	64	54	121	213	2.09	(1.36- 3.19)
HU2	6	c	0	149	87	121	213	3.01	(2.13- 4.26)
HU2	7	c	0	64	19	121	213	5.93	(3.39- 10.37)
Subtotal HU2								2.20	(1.83- 2.64)
HUMBLE	2	m	1	-	-	-	-	9.20	(3.30- 25.80)
HUMBLE	3	m	1	-	-	-	-	24.70	(10.00- 59.90)
HUMBLE	5	m	1	-	-	-	-	11.60	(2.70- 61.50)
HUMBLE	6	m	1	-	-	-	-	26.10	(5.60- 146.60)
HUMBLE	8	f	1	-	-	-	-	19.20	(6.50- 60.80)
HUMBLE	9	f	1	-	-	-	-	16.00	(6.70- 36.30)
HUMBLE	11	f	1	-	-	-	-	18.50	(4.90- 72.40)
HUMBLE	12	f	1	-	-	-	-	36.90	(7.60- 217.10)
Subtotal HUMBLE								17.64	(11.76- 26.46)
JARUP	1	m	0	42	25	11	42	6.41	(2.80- 14.68)
JARUP	2	m	0	49	27	11	42	6.93	(3.07- 15.63)
Subtotal JARUP								6.67	(3.73- 11.92)
JEDRYC	60	m	0	131	180	49	219	3.25	(2.22- 4.77)
JEDRYC	61	m	0	426	295	49	219	6.45	(4.58- 9.10)
JEDRYC	62	m	0	278	143	49	219	8.69	(6.00- 12.57)
JEDRYC	65	f	0	37	10	78	166	7.87	(3.72- 16.65)
JEDRYC	66	f	0	52	16	78	166	6.92	(3.71- 12.88)

Table 1G11 - 5

IESLC - Meta-anal of Ever Smoking (or Curr if Ever not avail) by Amount, Overview, Any prod (or Cigs if Any not avail)
All LC types
Least adjusted

REF	NRR	SEX	AD	Number Exposed Case	Number Exposed Cont	Non-exposed Case	Non-exposed Cont	RR	95.00%CI
JEDRYC	67	f	0	28	4	78	166	14.90	(5.05- 43.94)
Subtotal JEDRYC								6.17	(5.11- 7.46)
JOLY	7	m	0	16	54	12	218	5.38	(2.41- 12.05)
JOLY	8	m	0	217	318	12	218	12.40	(6.76- 22.73)
JOLY	9	m	0	126	175	12	218	13.08	(7.00- 24.43)
JOLY	10	m	0	193	161	12	218	21.78	(11.74- 40.39)
JOLY	3	f	0	33	38	52	283	4.73	(2.72- 8.21)
JOLY	4	f	0	72	49	52	283	8.00	(5.01- 12.77)
JOLY	5	f	0	28	22	52	283	6.93	(3.68- 13.03)
JOLY	6	f	0	32	13	52	283	13.40	(6.59- 27.23)
Subtotal JOLY								9.35	(7.55- 11.59)
JUSSAW	10	m	0	11	8	149	624	5.76	(2.28- 14.57)
JUSSAW	11	m	0	6	9	149	624	2.79	(0.98- 7.97)
JUSSAW	12	m	0	34	37	149	624	3.85	(2.34- 6.34)
JUSSAW	13	m	0	3	11	149	624	1.14	(0.31- 4.15)
JUSSAW	14	m	0	24	8	149	624	12.56	(5.53- 28.52)
JUSSAW	15	m	0	48	4	149	624	50.26	(17.84- 141.55)
Subtotal JUSSAW								5.71	(4.10- 7.96)
*KAISE2	66	m	1	-	-	-	-	4.47	(2.00- 9.99)
*KAISE2	67	m	1	-	-	-	-	10.34	(5.56- 19.23)
*KAISE2	58	f	1	-	-	-	-	7.61	(3.26- 17.75)
*KAISE2	59	f	1	-	-	-	-	22.12	(11.22- 43.61)
Subtotal KAISE2								10.24	(7.14- 14.68)
*KAISER	6	m	2	-	-	-	-	6.58	(3.87- 11.20)
*KAISER	7	m	2	-	-	-	-	17.24	(10.71- 27.73)
*KAISER	8	m	2	-	-	-	-	20.91	(12.78- 27.73)
*KAISER	2	f	2	-	-	-	-	3.42	(2.17- 5.40)
*KAISER	3	f	2	-	-	-	-	7.98	(5.35- 11.90)
*KAISER	4	f	2	-	-	-	-	12.63	(8.06- 19.80)
Subtotal KAISER								10.14	(8.46- 12.15)
KANELL	1	m	0	162	272	48	172	2.13	(1.47- 3.10)
KANELL	2	m	0	321	134	48	172	8.58	(5.88- 12.53)
KANELL	3	m	0	111	15	48	172	26.52	(14.17- 49.64)
KANELL	4	m	0	220	20	48	172	39.42	(22.55- 68.90)
Subtotal KANELL								7.70	(6.15- 9.63)
KATSOU	7	f	0	8	9	48	67	1.24	(0.45- 3.45)
KATSOU	8	f	0	15	5	48	67	4.19	(1.43- 12.30)
KATSOU	9	f	0	12	3	48	67	5.58	(1.49- 20.87)
KATSOU	10	f	0	10	1	48	67	13.96	(1.73- 112.71)
Subtotal KATSOU								3.18	(1.71- 5.89)
KAUFMA	2	c	0	66	214	35	925	8.15	(5.27- 12.60)
KAUFMA	3	c	0	207	363	35	925	15.07	(10.32- 22.00)
KAUFMA	4	c	0	96	118	35	925	21.50	(13.96- 33.11)
KAUFMA	5	c	0	149	110	35	925	35.80	(23.57- 54.37)
KAUFMA	6	c	0	96	47	35	925	53.98	(33.22- 87.71)
Subtotal KAUFMA								20.92	(17.29- 25.30)
KHUDER	1	m	0	81	434	23	309	2.51	(1.54- 4.07)
KHUDER	2	m	0	224	288	23	309	10.45	(6.61- 16.52)
KHUDER	3	m	0	154	63	23	309	32.84	(19.62- 54.97)
Subtotal KHUDER								9.12	(6.89- 12.06)
*KINLEN	3	m	0	200	3659	7	1333	10.41	(4.91- 22.06)
*KINLEN	4	m	0	232	3369	7	1333	13.11	(6.20- 27.74)
*KINLEN	5	m	0	157	1484	7	1333	20.15	(9.48- 42.80)
Subtotal KINLEN								14.00	(9.07- 21.60)
*KNEKT	25	m	0	23	14798	6	17814	4.61	(1.88- 11.33)
*KNEKT	26	m	0	63	18869	6	17814	9.91	(4.29- 22.90)
Subtotal KNEKT								6.95	(3.77- 12.82)
KOO	11	f	0	17	19	56	85	1.36	(0.65- 2.84)
KOO	12	f	0	24	5	56	85	7.29	(2.62- 20.22)
KOO	13	f	0	1	1	56	85	1.52	(0.09- 24.77)
Subtotal KOO								2.36	(1.32- 4.24)
KOULUM	6	m	0	37	77	5	54	5.19	(1.92- 14.06)
KOULUM	5	m	0	208	94	5	54	23.90	(9.26- 61.67)
KOULUM	4	m	0	478	75	5	54	68.83	(26.67- 177.62)
Subtotal KOULUM								21.38	(12.26- 37.28)
KREUZE	19	m	3	-	-	-	-	2.50	(0.70- 8.20)
KREUZE	20	m	3	-	-	-	-	8.70	(3.50- 21.90)
KREUZE	21	m	3	-	-	-	-	19.50	(7.50- 50.30)
KREUZE	22	m	3	-	-	-	-	20.80	(7.20- 60.50)
KREUZE	30	m	3	-	-	-	-	8.20	(5.20- 13.00)
KREUZE	31	m	3	-	-	-	-	25.10	(16.20- 38.70)

Table 1G11 - 5

IESLC - Meta-anal of Ever Smoking (or Curr if Ever not avail) by Amount, Overview, Any prod (or Cigs if Any not avail)
All LC types
Least adjusted

REF	NRR	SEX	AD	Number Exposed Case	Cont	Non-exposed Case	Cont	RR	95.00%CI
KREUZE	32	m	3	-	-	-	-	32.80	(20.90- 51.40)
KREUZE	33	m	3	-	-	-	-	33.30	(20.50- 54.00)
KREUZE	25	f	3	-	-	-	-	5.70	(1.60- 16.60)
KREUZE	26	f	3	-	-	-	-	11.80	(3.50- 29.00)
KREUZE	27	f	3	-	-	-	-	12.10	(3.00- 48.00)
KREUZE	36	f	3	-	-	-	-	2.00	(1.20- 3.30)
KREUZE	37	f	3	-	-	-	-	5.40	(3.50- 8.60)
KREUZE	38	f	3	-	-	-	-	7.70	(3.50- 17.30)
Subtotal KREUZE								11.60	(9.81- 13.72)
KREYBE	21	m	0	154	2341	6	644	7.06	(3.11- 16.04)
KREYBE	22	m	0	55	925	6	644	6.38	(2.73- 14.91)
KREYBE	23	m	0	43	248	6	644	18.61	(7.82- 44.27)
KREYBE	37	f	0	10	286	30	657	0.77	(0.37- 1.59)
KREYBE	38	f	0	2	42	30	657	1.04	(0.24- 4.51)
Subtotal KREYBE								3.88	(2.63- 5.74)
LAMTH	7	f	0	101	63	202	337	2.67	(1.87- 3.83)
LAMTH	2	f	0	90	28	202	337	5.36	(3.39- 8.48)
LAMTH	9	f	0	39	9	202	337	7.23	(3.43- 15.24)
Subtotal LAMTH								3.82	(2.93- 4.98)
LAUSSM	3	m	0	75	63	85	226	3.17	(2.08- 4.81)
LAUSSM	2	m	0	138	73	85	226	5.03	(3.45- 7.33)
LAUSSM	1	m	0	105	37	85	226	7.55	(4.81- 11.83)
Subtotal LAUSSM								4.85	(3.82- 6.15)
LETOUR	2	c	0	271	266	24	224	9.51	(6.04- 14.97)
LETOUR	3	c	0	367	198	24	224	17.30	(10.98- 27.27)
LETOUR	4	c	0	65	23	24	224	26.38	(13.98- 49.78)
Subtotal LETOUR								14.84	(11.14- 19.77)
*LIAW	3	c	2	-	-	-	-	3.10	(1.70- 5.60)
*LIAW	4	c	2	-	-	-	-	3.60	(2.00- 6.40)
*LIAW	5	c	2	-	-	-	-	8.30	(4.00- 17.30)
Subtotal LIAW								4.18	(2.91- 6.00)
*LIDDEL	2	m	1	-	-	-	-	3.33	(2.05- 5.64)
*LIDDEL	3	m	1	-	-	-	-	5.02	(3.21- 8.22)
Subtotal LIDDEL								4.15	(2.94- 5.86)
LIU2	5	m	0	21	93	12	44	0.83	(0.37- 1.83)
LIU2	6	m	0	97	66	12	44	5.39	(2.65- 10.97)
LIU2	7	m	0	94	21	12	44	16.41	(7.42- 36.33)
LIU2	11	f	0	8	10	38	69	1.45	(0.53- 3.99)
LIU2	12	f	0	16	9	38	69	3.23	(1.30- 8.00)
LIU2	13	f	0	30	4	38	69	13.62	(4.46- 41.57)
Subtotal LIU2								4.03	(2.84- 5.72)
LIU3	3	m	0	25	93	4	19	1.28	(0.40- 4.09)
LIU3	4	m	0	20	93	4	19	1.02	(0.31- 3.33)
LIU3	5	m	0	7	19	4	19	1.75	(0.44- 6.98)
Subtotal LIU3								1.28	(0.63- 2.61)
LIU4	7	m	2	-	-	-	-	2.11	(2.02- 2.20)
LIU4	8	m	2	-	-	-	-	3.60	(3.49- 3.71)
LIU4	9	m	2	-	-	-	-	6.98	(6.73- 7.23)
Subtotal LIU4								3.95	(3.87- 4.03)
LIU5	2	c	0	14	27	26	41	0.82	(0.36- 1.84)
LIU5	3	c	0	21	21	26	41	1.58	(0.72- 3.44)
LIU5	4	c	0	50	22	26	41	3.58	(1.78- 7.23)
Subtotal LIU5								1.79	(1.16- 2.78)
LUBIN	7	m	0	4	41	8	73	0.89	(0.25- 3.14)
LUBIN	8	m	0	7	59	8	73	1.08	(0.37- 3.16)
LUBIN	9	m	0	12	24	8	73	4.56	(1.67- 12.48)
LUBIN	10	m	0	29	67	8	73	3.95	(1.69- 9.24)
Subtotal LUBIN								2.40	(1.45- 3.99)
LUBIN2	273	m	0	1887	3759	190	2616	6.91	(5.91- 8.09)
LUBIN2	274	m	0	1529	2771	190	2616	7.60	(6.47- 8.92)
LUBIN2	275	m	0	1963	2547	190	2616	10.61	(9.06- 12.44)
LUBIN2	276	m	0	1261	1394	190	2616	12.45	(10.55- 14.70)
LUBIN2	281	f	0	151	218	336	1188	2.45	(1.93- 3.11)
LUBIN2	282	f	0	221	213	336	1188	3.67	(2.93- 4.59)
LUBIN2	283	f	0	134	103	336	1188	4.60	(3.46- 6.11)
LUBIN2	284	f	0	45	33	336	1188	4.82	(3.03- 7.68)
Subtotal LUBIN2								7.07	(6.60- 7.58)
MACLEN	20	m	0	5	11	5	15	1.36	(0.32- 5.89)
MACLEN	21	m	0	42	37	5	15	3.41	(1.13- 10.28)
MACLEN	22	m	0	43	31	5	15	4.16	(1.37- 12.66)
MACLEN	23	m	0	45	27	5	15	5.00	(1.63- 15.31)

Table 1G11 - 5

IESLC - Meta-anal of Ever Smoking (or Curr if Ever not avail) by Amount, Overview, Any prod (or Cigs if Any not avail)
All LC types
Least adjusted

REF	NRR	SEX	AD	Number Exposed Case	Number Exposed Cont	Non-exposed Case	Non-exposed Cont	RR	95.00%CI
MACLEN	33	f	0	6	21	41	109	0.76	(0.29- 2.02)
MACLEN	34	f	0	22	17	41	109	3.44	(1.66- 7.12)
MACLEN	35	f	0	13	9	41	109	3.84	(1.53- 9.66)
Subtotal MACLEN								2.80	(1.92- 4.08)
MARTIS	1	m	0	31	39	4	25	4.97	(1.56- 15.78)
MARTIS	2	m	0	91	87	4	25	6.54	(2.19- 19.55)
MARTIS	3	m	0	75	50	4	25	9.38	(3.08- 28.57)
Subtotal MARTIS								6.77	(3.55- 12.94)
MATOS	28	m	0	17	88	11	110	1.93	(0.86- 4.34)
MATOS	30	m	0	65	90	11	110	7.22	(3.60- 14.50)
MATOS	32	m	0	106	105	11	110	10.10	(5.13- 19.85)
Subtotal MATOS								5.78	(3.81- 8.77)
MATSUD	1	m	0	37	1237	3	1255	12.51	(3.85- 40.69)
MATSUD	2	m	0	75	1607	3	1255	19.52	(6.14- 62.05)
MATSUD	3	m	0	58	470	3	1255	51.62	(16.10- 165.55)
Subtotal MATSUD								23.37	(11.91- 45.84)
MCCONN	26	c	0	7	43	9	23	0.42	(0.14- 1.26)
MCCONN	25	c	0	49	92	9	23	1.36	(0.58- 3.17)
MCCONN	24	c	0	35	42	9	23	2.13	(0.87- 5.19)
Subtotal MCCONN								1.21	(0.71- 2.08)
*MIGRAN	1	m	0	11	508	4	867	4.69	(1.50- 14.66)
*MIGRAN	3	m	0	59	1696	4	867	7.54	(2.75- 20.69)
*MIGRAN	5	m	0	36	851	4	867	9.17	(3.28- 25.65)
*MIGRAN	7	m	0	30	604	4	867	10.77	(3.81- 30.40)
*MIGRAN	28	f	0	4	772	4	3814	4.94	(1.24- 19.71)
*MIGRAN	30	f	0	14	1324	4	3814	10.08	(3.32- 30.58)
*MIGRAN	32	f	0	3	471	4	3814	6.07	(1.36- 27.05)
*MIGRAN	34	f	0	2	149	4	3814	12.80	(2.36- 69.33)
Subtotal MIGRAN								7.88	(5.19- 11.95)
*MRFITR	3	m	0	2	856	0	1859	10.86~	(0.52- 225.86)
*MRFITR	4	m	0	50	3747	0	1859	50.12~	(3.09- 811.82)
*MRFITR	5	m	0	54	3591	0	1859	56.43~	(3.49- 913.25)
Subtotal MRFITR								33.22	(6.37- 173.28)
NAM	66	m	0	92	346	30	520	4.61	(2.99- 7.11)
NAM	67	m	0	149	243	30	520	10.63	(6.98- 16.19)
NAM	82	f	0	68	150	52	885	7.72	(5.17- 11.51)
NAM	83	f	0	65	84	52	885	13.17	(8.59- 20.20)
Subtotal NAM								8.43	(6.83- 10.39)
NOTAN2	8	m	0	6	42	134	544	0.58	(0.24- 1.39)
NOTAN2	9	m	0	28	47	134	544	2.42	(1.46- 4.01)
NOTAN2	10	m	0	44	40	134	544	4.47	(2.80- 7.13)
Subtotal NOTAN2								2.66	(1.93- 3.66)
ORMOS	1	m	0	32	329	7	777	10.80	(4.72- 24.71)
ORMOS	2	m	0	40	577	7	777	7.69	(3.42- 17.30)
ORMOS	3	m	0	15	128	7	777	13.01	(5.20- 32.52)
Subtotal ORMOS								10.06	(6.17- 16.42)
OSANN	49	m	2	-	-	-	-	17.70	(12.60- 24.80)
OSANN	57	m	2	-	-	-	-	42.80	(30.50- 60.10)
OSANN	50	f	2	-	-	-	-	14.40	(11.00- 18.90)
OSANN	58	f	2	-	-	-	-	40.90	(29.30- 57.10)
Subtotal OSANN								24.11	(20.59- 28.24)
PARKIN	17	m	0	314	818	107	1248	4.48	(3.53- 5.67)
PARKIN	18	m	0	61	128	107	1248	5.56	(3.87- 7.99)
Subtotal PARKIN								4.77	(3.92- 5.82)
PASTOR	1	m	0	8	39	10	89	1.83	(0.67- 4.98)
PASTOR	2	m	0	51	78	10	89	5.82	(2.77- 12.23)
PASTOR	3	m	0	89	100	10	89	7.92	(3.88- 16.17)
PASTOR	4	m	0	46	45	10	89	9.10	(4.20- 19.69)
Subtotal PASTOR								6.01	(4.05- 8.90)
PERNU	17	m	0	15	15	97	275	2.84	(1.34- 6.01)
PERNU	18	m	0	61	31	97	275	5.58	(3.42- 9.11)
PERNU	19	m	0	224	96	97	275	6.62	(4.74- 9.23)
PERNU	20	m	0	127	67	97	275	5.37	(3.69- 7.82)
PERNU	21	m	0	478	138	97	275	9.82	(7.28- 13.24)
PERNU	22	m	0	361	54	97	275	18.95	(13.12- 27.38)
PERNU	23	m	0	40	23	97	275	4.93	(2.81- 8.66)
PERNU	24	m	0	74	14	97	275	14.99	(8.09- 27.75)
PERNU	11	f	0	3	14	110	971	1.89	(0.54- 6.68)
PERNU	12	f	0	5	13	110	971	3.40	(1.19- 9.70)
PERNU	13	f	0	4	30	110	971	1.18	(0.41- 3.40)
PERNU	14	f	0	1	14	110	971	0.63	(0.08- 4.84)

Table 1G11 - 5

IESLC - Meta-anal of Ever Smoking (or Curr if Ever not avail) by Amount, Overview, Any prod (or Cigs if Any not avail)
All LC types
Least adjusted

REF	NRR	SEX	AD	Number Exposed Case	Number Exposed Cont	Non-exposed Case	Non-exposed Cont	RR	95.00%CI
PERNU	15	f	0	1	11	110	971	0.80	(0.10- 6.27)
PERNU	16	f	0	5	7	110	971	6.31	(1.97- 20.20)
Subtotal PERNU								7.42	(6.44- 8.54)
PERSH2	2	c	0	290	349	178	1164	5.43	(4.35- 6.78)
PERSH2	3	c	0	446	282	178	1164	10.34	(8.32- 12.85)
Subtotal PERSH2								7.55	(6.46- 8.81)
*PETO	2	m	0	44	1181	2	295	5.50	(1.34- 22.54)
*PETO	3	m	0	55	855	2	295	9.49	(2.33- 38.66)
Subtotal PETO								7.23	(2.67- 19.57)
PEZZO2	3	m	0	57	139	6	117	8.00	(3.33- 19.21)
PEZZO2	4	m	0	107	47	6	117	44.39	(18.24- 108.02)
PEZZO2	5	m	0	69	12	6	117	112.13	(40.26- 312.24)
Subtotal PEZZO2								30.27	(17.76- 51.58)
PEZZOT	2	m	0	24	94	4	116	7.40	(2.48- 22.09)
PEZZOT	3	m	0	70	29	4	116	70.00	(23.61- 207.50)
PEZZOT	4	m	0	51	6	4	116	246.50	(66.69- 911.11)
Subtotal PEZZOT								42.28	(21.77- 82.11)
PIKE	1	m	0	181	168	18	69	4.13	(2.36- 7.23)
PIKE	2	m	0	228	109	18	69	8.02	(4.55- 14.13)
PIKE	3	m	0	66	37	18	69	6.84	(3.55- 13.18)
PIKE	5	f	0	73	60	36	96	3.24	(1.94- 5.42)
PIKE	6	f	0	67	26	36	96	6.87	(3.80- 12.44)
PIKE	7	f	0	16	3	36	96	14.22	(3.91- 51.73)
Subtotal PIKE								5.49	(4.27- 7.05)
POLEDN	2	c	0	53	103	12	139	5.96	(3.03- 11.72)
POLEDN	4	c	0	143	168	12	139	9.86	(5.25- 18.52)
Subtotal POLEDN								7.80	(4.92- 12.37)
*PRESCO	2	m	1	-	-	-	-	10.20	(4.49- 23.15)
*PRESCO	4	m	1	-	-	-	-	19.96	(8.92- 44.67)
*PRESCO	1	f	1	-	-	-	-	6.36	(3.60- 11.24)
*PRESCO	3	f	1	-	-	-	-	10.08	(5.72- 17.75)
Subtotal PRESCO								9.70	(6.98- 13.49)
RACHTA	5	f	0	6	7	33	98	2.55	(0.80- 8.12)
RACHTA	6	f	0	25	24	33	98	3.09	(1.56- 6.14)
RACHTA	7	f	0	54	12	33	98	13.36	(6.38- 28.00)
Subtotal RACHTA								5.30	(3.34- 8.40)
RANDIG	1	m	0	13	28	5	22	2.04	(0.63- 6.60)
RANDIG	2	m	0	65	99	5	22	2.89	(1.04- 8.01)
RANDIG	3	m	0	190	164	5	22	5.10	(1.89- 13.76)
RANDIG	4	m	0	142	68	5	22	9.19	(3.34- 25.31)
RANDIG	5	f	0	1	21	17	92	0.26	(0.03- 2.05)
RANDIG	6	f	0	12	13	17	92	5.00	(1.95- 12.79)
RANDIG	7	f	0	3	5	17	92	3.25	(0.71- 14.88)
Subtotal RANDIG								3.84	(2.50- 5.89)
SEGI2	9	m	0	20	62	8	53	2.14	(0.87- 5.25)
SEGI2	11	m	0	76	166	8	53	3.03	(1.37- 6.69)
SEGI2	13	m	0	92	183	8	53	3.33	(1.52- 7.30)
SEGI2	15	m	0	27	29	8	53	6.17	(2.48- 15.32)
SEGI2	17	m	0	52	45	8	53	7.66	(3.29- 17.80)
SEGI2	21	f	0	9	9	56	126	2.25	(0.85- 5.97)
SEGI2	23	f	0	10	15	56	126	1.50	(0.63- 3.54)
SEGI2	25	f	0	5	10	56	126	1.13	(0.37- 3.44)
Subtotal SEGI2								2.98	(2.18- 4.07)
SHAW	10	c	0	46	90	11	107	4.97	(2.43- 10.16)
SHAW	11	c	0	278	176	11	107	15.36	(8.03- 29.39)
Subtotal SHAW								9.23	(5.71- 14.93)
SIEMIA	13	m	0	-	-	-	-	3.00	(1.00- 9.90)
SIEMIA	14	m	0	-	-	-	-	4.50	(1.80- 13.20)
SIEMIA	15	m	0	-	-	-	-	7.90	(3.00- 24.10)
Subtotal SIEMIA								4.87	(2.64- 8.95)
SOBUE	117	m	0	147	157	34	128	3.52	(2.27- 5.47)
SOBUE	118	m	0	236	222	34	128	4.00	(2.63- 6.09)
SOBUE	119	m	0	226	187	34	128	4.55	(2.97- 6.96)
Subtotal SOBUE								4.02	(3.14- 5.14)
*SPEIZE	1	f	1	-	-	-	-	2.70	(0.90- 5.40)
*SPEIZE	2	f	1	-	-	-	-	5.20	(3.60- 8.10)
*SPEIZE	3	f	1	-	-	-	-	12.60	(9.90- 16.70)
*SPEIZE	4	f	1	-	-	-	-	15.70	(12.10- 20.20)
*SPEIZE	5	f	1	-	-	-	-	22.00	(14.80- 32.30)
Subtotal SPEIZE								12.52	(10.76- 14.56)

Table 1G11 - 5

IESLC - Meta-anal of Ever Smoking (or Curr if Ever not avail) by Amount, Overview, Any prod (or Cigs if Any not avail)
All LC types
Least adjusted

				Number Exposed		Non-exposed			
REF	NRR	SEX	AD	Case	Cont	Case	Cont	RR	95.00%CI
SPITZ	5	c	0	27	88	10	96	2.95	(1.35- 6.43)
SPITZ	6	c	0	95	48	10	96	19.00	(9.08- 39.74)
Subtotal SPITZ								7.89	(4.61- 13.49)
STOCKS	25	m	0	691	1743	45	638	5.62	(4.10- 7.70)
STOCKS	26	m	0	919	2257	45	638	5.77	(4.23- 7.89)
STOCKS	27	m	0	232	380	45	638	8.66	(6.14- 12.20)
STOCKS	28	m	0	277	635	45	638	6.18	(4.43- 8.63)
STOCKS	29	m	0	302	468	45	638	9.15	(6.54- 12.79)
STOCKS	48	f	1	-	-	-	-	2.24	(1.64- 3.03)
STOCKS	49	f	1	-	-	-	-	6.34	(4.42- 8.93)
Subtotal STOCKS								5.65	(4.99- 6.39)
STOCKW	1	c	0	2090	1194	2791	10641	6.67	(6.15- 7.25)
STOCKW	2	c	0	6053	1591	2791	10641	14.51	(13.54- 15.54)
STOCKW	3	c	0	4327	572	2791	10641	28.84	(26.18- 31.77)
Subtotal STOCKW								13.28	(12.68- 13.92)
SVENSS	26	f	0	42	30	38	120	4.42	(2.44- 8.01)
SVENSS	31	f	0	81	22	38	120	11.63	(6.41- 21.10)
SVENSS	36	f	0	19	1	38	120	60.00	(7.77- 463.15)
Subtotal SVENSS								7.80	(5.17- 11.78)
*TENKAN	10	m	1	-	-	-	-	15.86	(6.80- 37.00)
*TENKAN	11	m	1	-	-	-	-	20.25	(8.20- 50.00)
*TENKAN	12	m	1	-	-	-	-	24.97	(9.90- 63.00)
Subtotal TENKAN								19.74	(11.81- 33.01)
TIZZAN	7	m	0	130	238	180	305	0.93	(0.70- 1.23)
TIZZAN	8	m	0	468	470	180	305	1.69	(1.35- 2.11)
TIZZAN	9	m	0	301	108	180	305	4.72	(3.54- 6.29)
TIZZAN	10	m	0	83	20	180	305	7.03	(4.17- 11.85)
TIZZAN	15	f	0	11	14	25	114	3.58	(1.46- 8.82)
TIZZAN	16	f	0	14	14	25	114	4.56	(1.93- 10.75)
Subtotal TIZZAN								2.16	(1.87- 2.48)
TSUGAN	29	m	0	14	19	18	22	0.90	(0.36- 2.28)
TSUGAN	30	m	0	30	30	18	22	1.22	(0.55- 2.73)
TSUGAN	31	m	0	19	14	18	22	1.66	(0.65- 4.20)
Subtotal TSUGAN								1.22	(0.73- 2.03)
*TULINI	4	m	1	-	-	-	-	6.49	(3.25- 13.00)
*TULINI	5	m	1	-	-	-	-	13.50	(7.08- 25.60)
*TULINI	6	m	1	-	-	-	-	28.70	(14.90- 55.10)
*TULINI	9	f	1	-	-	-	-	9.39	(4.99- 17.70)
*TULINI	10	f	1	-	-	-	-	30.70	(16.80- 56.00)
*TULINI	11	f	1	-	-	-	-	44.10	(21.10- 91.80)
Subtotal TULINI								17.72	(13.55- 23.16)
*TVERDA	9	m	2	-	-	-	-	2.14	(1.15- 3.96)
*TVERDA	10	m	2	-	-	-	-	3.32	(2.07- 5.32)
*TVERDA	11	m	2	-	-	-	-	6.56	(4.04- 10.64)
*TVERDA	16	f	2	-	-	-	-	4.53	(1.08- 18.94)
*TVERDA	17	f	2	-	-	-	-	18.00	(5.33- 60.83)
Subtotal TVERDA								4.23	(3.19- 5.62)
WAKAI	37	m	0	30	105	10	65	1.86	(0.85- 4.05)
WAKAI	38	m	0	79	129	10	65	3.98	(1.93- 8.20)
WAKAI	39	m	0	69	48	10	65	9.34	(4.37- 20.00)
Subtotal WAKAI								4.15	(2.69- 6.41)
WANG2	1	c	0	5	14	11	43	1.40	(0.41- 4.71)
WANG2	2	c	0	3	10	11	43	1.17	(0.28- 5.00)
WANG2	3	c	0	5	14	11	43	1.40	(0.41- 4.71)
WANG2	4	c	0	4	14	11	43	1.12	(0.31- 4.07)
WANG2	5	c	0	27	32	11	43	3.30	(1.43- 7.62)
WANG2	6	c	0	7	11	11	43	2.49	(0.78- 7.90)
WANG2	7	c	0	9	4	11	43	8.80	(2.28- 33.97)
Subtotal WANG2								2.25	(1.45- 3.49)
WU	35	f	0	57	42	31	92	4.03	(2.28- 7.12)
WU	36	f	0	103	31	31	92	9.86	(5.57- 17.47)
Subtotal WU								6.29	(4.20- 9.42)
WUWILL	18	f	0	451	311	417	601	2.09	(1.73- 2.53)
WUWILL	19	f	0	88	40	417	601	3.17	(2.14- 4.70)
Subtotal WUWILL								2.26	(1.91- 2.69)
WYNDE2	17	m	0	17	114	8	105	1.96	(0.81- 4.72)
WYNDE2	18	m	0	122	203	8	105	7.89	(3.71- 16.75)
WYNDE2	19	m	0	88	83	8	105	13.92	(6.39- 30.32)
WYNDE2	20	m	0	155	112	8	105	18.16	(8.50- 38.80)
Subtotal WYNDE2								8.64	(5.83- 12.82)
WYNDE3	44	m	0	8	42	9	88	1.86	(0.67- 5.17)

Table 1G11 - 5

IESLC - Meta-anal of Ever Smoking (or Curr if Ever not avail) by Amount, Overview, Any prod (or Cigs if Any not avail)
All LC types
Least adjusted

REF	NRR	SEX	AD	Number Exposed Case	Number Exposed Cont	Non-exposed Case	Non-exposed Cont	RR	95.00%CI
WYNDE3	45	m	0	77	114	9	88	6.60	(3.14- 13.90)
WYNDE3	46	m	0	108	82	9	88	12.88	(6.12- 27.09)
WYNDE3	47	m	0	68	26	9	88	25.57	(11.25- 58.14)
WYNDE3	79	f	0	3	19	20	76	0.60	(0.16- 2.23)
WYNDE3	80	f	0	24	24	20	76	3.80	(1.79- 8.05)
WYNDE3	81	f	0	15	10	20	76	5.70	(2.23- 14.59)
WYNDE3	82	f	0	4	3	20	76	5.07	(1.05- 24.50)
Subtotal WYNDE3								6.19	(4.51- 8.50)
WYNDE4	43	m	0	17	82	12	115	1.99	(0.90- 4.38)
WYNDE4	44	m	0	67	147	12	115	4.37	(2.26- 8.46)
WYNDE4	45	m	0	228	274	12	115	7.97	(4.29- 14.82)
WYNDE4	46	m	0	190	98	12	115	18.58	(9.77- 35.33)
WYNDE4	47	m	0	130	64	12	115	19.47	(10.00- 37.88)
WYNDE4	57	f	2	-	-	-	-	1.13	(0.33- 3.89)
WYNDE4	58	f	2	-	-	-	-	2.01	(0.66- 6.10)
WYNDE4	59	f	2	-	-	-	-	6.49	(2.35- 17.93)
WYNDE4	60	f	2	-	-	-	-	11.54	(1.90- 70.11)
WYNDE4	61	f	2	-	-	-	-	11.54	(1.90- 70.11)
Subtotal WYNDE4								6.97	(5.35- 9.09)
WYNDE6	27	m	0	117	122	87	617	6.80	(4.85- 9.54)
WYNDE6	36	m	0	461	293	87	617	11.16	(8.54- 14.59)
WYNDE6	45	m	0	315	129	87	617	17.32	(12.78- 23.47)
WYNDE6	54	m	0	784	197	87	617	28.22	(21.47- 37.10)
WYNDE6	216	f	0	76	109	159	856	3.75	(2.68- 5.26)
WYNDE6	225	f	0	367	165	159	856	11.97	(9.33- 15.37)
WYNDE6	234	f	0	201	50	159	856	21.64	(15.21- 30.80)
WYNDE6	243	f	0	378	52	159	856	39.13	(27.98- 54.75)
Subtotal WYNDE6								14.13	(12.71- 15.72)
XU3	5	m	0	10	26	7	31	1.70	(0.57- 5.10)
XU3	6	m	0	19	27	7	31	3.12	(1.14- 8.54)
XU3	7	m	0	47	13	7	31	16.01	(5.75- 44.61)
XU3	8	m	0	16	2	7	31	35.43	(6.58- 190.72)
XU3	13	f	0	7	6	13	25	2.24	(0.62- 8.07)
XU3	14	f	0	10	4	13	25	4.81	(1.26- 18.35)
XU3	15	f	0	6	1	13	25	11.54	(1.25- 106.30)
Subtotal XU3								5.11	(3.19- 8.19)
YAMAGU	3	c	0	48	191	24	267	2.80	(1.66- 4.72)
YAMAGU	2	c	0	28	56	24	267	5.56	(3.00- 10.31)
Subtotal YAMAGU								3.73	(2.50- 5.56)
*YUAN	2	m	2	-	-	-	-	3.60	(1.88- 6.91)
*YUAN	3	m	2	-	-	-	-	9.40	(5.21- 16.97)
Subtotal YUAN								6.09	(3.94- 9.44)
ZHENG	11	m	0	25	40	33	94	1.78	(0.94- 3.37)
ZHENG	12	m	0	60	66	33	94	2.59	(1.53- 4.39)
ZHENG	13	m	0	128	89	33	94	4.10	(2.53- 6.62)
ZHENG	14	m	0	66	23	33	94	8.17	(4.40- 15.17)
ZHENG	22	f	0	24	29	152	184	1.00	(0.56- 1.79)
ZHENG	23	f	0	52	15	152	184	4.20	(2.27- 7.75)
Subtotal ZHENG								2.97	(2.36- 3.75)
ZHOU	4	c	0	61	5	507	68	1.64	(0.64- 4.22)
ZHOU	5	c	0	211	14	507	68	2.02	(1.11- 3.67)
ZHOU	6	c	0	581	29	507	68	2.69	(1.71- 4.22)
Subtotal ZHOU								2.31	(1.65- 3.23)
Partial Totals				59276	744201	29864	2694899		

*prospective study ~ With 0.5 adjustment for zero

Table 1G11 - 5

IESLC - Meta-anal of Ever Smoking (or Curr if Ever not avail) by Amount, Overview, Any prod (or Cigs if Any not avail)
All LC types
Least adjusted

REF	NRR	SEX	AD	Ys	Ws	Qs	Ps
AGUDO	11	f	0	0.13	3.73	10.14	0.7953
AGUDO	12	f	0	1.26	5.96	1.61	0.0020
Subtotal AGUDO				0.83	9.69	11.75	
*AKIBA	17	m	0	1.24	15.35	4.52	0.0000
*AKIBA	18	m	0	1.46	16.36	1.70	0.0000
*AKIBA	19	m	0	1.49	14.01	1.23	0.0000
*AKIBA	23	f	0	1.46	36.89	3.88	0.0000
*AKIBA	24	f	0	1.63	14.85	0.33	0.0000
Subtotal AKIBA				1.46	97.46	11.66	
ALDERS	18	m	1	1.27	10.54	2.80	0.0000
ALDERS	19	m	1	2.07	13.07	1.11	0.0000
ALDERS	20	m	1	2.14	14.23	1.84	0.0000
ALDERS	21	f	1	0.96	34.91	23.45	0.0000
ALDERS	22	f	1	1.66	34.88	0.49	0.0000
ALDERS	23	f	1	1.93	25.78	0.57	0.0000
Subtotal ALDERS				1.59	133.42	30.27	
*ARCHER	1	m	0	1.26	4.20	1.14	0.0097
*ARCHER	2	m	0	1.81	5.52	0.00	0.0000
*ARCHER	3	m	0	2.14	5.22	0.67	0.0000
Subtotal ARCHER				1.77	14.95	1.82	
ARMADA	46	m	0	1.79	3.37	0.00	0.0010
ARMADA	47	m	0	3.02	3.54	5.39	0.0000
ARMADA	48	m	0	4.24	3.29	19.88	0.0000
Subtotal ARMADA				3.01	10.20	25.26	
AUVINE	5	c	0	2.20	13.34	2.28	0.0000
AUVINE	6	c	0	2.98	16.81	24.22	0.0000
AUVINE	7	c	0	3.68	5.94	21.38	0.0000
Subtotal AUVINE				2.81	36.09	47.88	
AXELSS	19	m	0	1.40	9.54	1.38	0.0000
AXELSS	20	m	0	2.21	11.63	2.10	0.0000
AXELSS	21	m	0	2.42	11.94	4.88	0.0000
AXELSS	13	f	0	1.10	6.02	2.80	0.0069
AXELSS	14	f	0	2.38	10.21	3.61	0.0000
AXELSS	15	f	0	2.77	6.08	5.94	0.0000
AXELSS	16	f	0	1.99	2.69	0.12	0.0011
Subtotal AXELSS				2.08	58.13	20.84	
BARBON	5	m	0	1.01	10.21	6.07	0.0012
BARBON	7	m	0	2.27	14.77	3.54	0.0000
BARBON	9	m	0	2.38	16.41	5.89	0.0000
BARBON	11	m	0	2.60	14.14	9.41	0.0000
BARBON	13	m	0	2.86	15.58	18.11	0.0000
Subtotal BARBON				2.31	71.10	43.01	
*BENSHL	11	m	1	1.39	4.28	0.67	0.0041
*BENSHL	12	m	1	2.20	5.46	0.96	0.0000
*BENSHL	13	m	1	2.39	5.53	2.06	0.0000
Subtotal BENSHL				2.04	15.26	3.70	
*BEST	13	m	1	2.30	6.23	1.68	0.0000
*BEST	14	m	1	2.80	6.77	6.98	0.0000
*BEST	15	m	1	2.85	6.30	7.20	0.0000
Subtotal BEST				2.66	19.31	15.86	
*BOUCOT	99	m	0	3.15	0.49	0.92	0.0271
*BOUCOT	100	m	0	3.83	0.49	2.08	0.0070
Subtotal BOUCOT				3.49	0.99	2.99	
BRESLO	13	m	0	0.58	7.18	10.47	0.1231
BRESLO	14	m	0	1.19	12.73	4.48	0.0000
BRESLO	15	m	0	2.04	15.85	1.02	0.0000
BRESLO	16	m	0	2.90	8.89	11.10	0.0000
BRESLO	29	f	0	0.15	1.80	4.78	0.8360
BRESLO	30	f	0	0.44	2.24	4.03	0.5084
Subtotal BRESLO				1.61	48.69	35.88	
*BRETT	1	m	0	0.94	5.22	3.75	0.0326
*BRETT	2	m	0	1.45	5.48	0.62	0.0007
*BRETT	3	m	0	2.08	5.09	0.45	0.0000
Subtotal BRETT				1.48	15.79	4.82	
BROSS	18	m	0	1.59	22.46	0.83	0.0000
BROSS	19	m	0	1.97	16.76	0.62	0.0000
Subtotal BROSS				1.75	39.21	1.45	
BROWN2	32	m	2	1.81	220.78	0.14	0.0000
BROWN2	42	m	2	2.65	387.09	288.55	0.0000
BROWN2	31	f	2	2.13	172.98	20.64	0.0000
BROWN2	41	f	2	2.84	312.24	348.38	0.0000

Table 1G11 - 5

IESLC - Meta-anal of Ever Smoking (or Curr if Ever not avail) by Amount, Overview, Any prod (or Cigs if Any not avail)
All LC types
Least adjusted

REF	NRR	SEX	AD	Ys	Ws	Qs	Ps
Subtotal BROWN2				2.45	1093.09	657.72	
BUFFLE	28	f	0	1.54	6.11	0.36	0.0001
BUFFLE	29	f	0	2.47	8.19	3.87	0.0000
BUFFLE	35	f	0	3.06	8.66	14.02	0.0000
Subtotal BUFFLE				2.44	22.96	18.25	
*CEDERL	80	m	2	1.22	12.65	3.95	0.0000
*CEDERL	81	m	2	2.01	19.12	1.03	0.0000
*CEDERL	82	m	2	2.48	18.57	8.94	0.0000
*CEDERL	76	f	2	1.04	15.40	8.49	0.0000
*CEDERL	77	f	2	2.05	19.39	1.35	0.0000
*CEDERL	78	f	2	2.05	8.98	0.63	0.0000
Subtotal CEDERL				1.85	94.11	24.39	
*CHANG	2	m	0	1.61	2.58	0.07	0.0096
*CHANG	3	m	0	2.36	3.99	1.33	0.0000
*CHANG	4	m	0	2.11	3.72	0.40	0.0000
*CHANG	8	f	0	1.11	3.97	1.80	0.0271
*CHANG	9	f	0	1.58	5.66	0.23	0.0002
*CHANG	10	f	0	2.11	6.22	0.65	0.0000
Subtotal CHANG				1.83	26.14	4.49	
CHATZI	1	c	0	0.94	14.84	10.56	0.0003
CHATZI	2	c	0	1.04	15.01	8.23	0.0001
CHATZI	3	c	0	1.56	16.06	0.83	0.0000
Subtotal CHATZI				1.19	45.92	19.62	
CHEN2	3	m	0	0.87	4.19	3.46	0.0735
CHEN2	4	m	0	1.17	5.43	2.03	0.0063
CHEN2	5	m	0	2.63	3.55	2.54	0.0000
CHEN2	6	m	0	2.07	3.80	0.32	0.0001
CHEN2	7	f	0	-0.95	3.04	22.63	0.0991
CHEN2	8	f	0	1.07	4.63	2.38	0.0217
CHEN2	9	f	0	2.22	0.82	0.16	0.0435
CHEN2	10	f	0	0.97	1.22	0.80	0.2838
Subtotal CHEN2				1.21	26.68	34.31	
CHOI	12	m	0	0.48	6.73	11.34	0.2084
CHOI	13	m	0	1.32	10.21	2.18	0.0000
CHOI	14	m	0	2.01	7.82	0.40	0.0000
CHOI	15	m	0	1.94	7.14	0.17	0.0000
CHOI	16	m	0	2.97	3.16	4.45	0.0000
CHOI	17	f	0	0.19	5.18	13.09	0.6591
CHOI	18	f	0	0.52	3.66	5.86	0.3219
CHOI	20	f	0	1.87	0.74	0.01	0.1083
Subtotal CHOI				1.34	44.64	37.50	
*CHOW	3	m	0	2.61	5.18	3.52	0.0000
*CHOW	4	m	0	2.84	5.46	6.15	0.0000
*CHOW	5	m	0	3.28	5.22	11.75	0.0000
Subtotal CHOW				2.91	15.86	21.42	
COMSTO	4	m	0	2.52	2.78	1.51	0.0000
COMSTO	5	m	0	2.90	3.35	4.17	0.0000
COMSTO	6	m	0	3.22	2.79	5.73	0.0000
COMSTO	9	f	0	2.01	4.98	0.25	0.0000
COMSTO	10	f	0	2.85	6.96	7.98	0.0000
COMSTO	11	f	0	2.59	2.75	1.77	0.0000
Subtotal COMSTO				2.65	23.61	21.41	
COOKSO	1	c	0	1.63	11.70	0.26	0.0000
COOKSO	2	c	0	2.31	7.06	1.98	0.0000
Subtotal COOKSO				1.89	18.76	2.25	
CORREA	45	c	0	2.15	35.82	4.81	0.0000
CORREA	49	c	0	3.00	34.18	50.50	0.0000
Subtotal CORREA				2.56	69.99	55.32	
*CPSI	243	m	1	1.76	44.36	0.02	0.0000
*CPSI	246	m	1	2.61	55.90	38.25	0.0000
*CPSI	275	f	1	0.22	13.40	32.60	0.4140
*CPSI	276	f	1	0.89	26.82	21.28	0.0000
*CPSI	277	f	1	1.62	50.55	1.42	0.0000
*CPSI	278	f	1	2.41	10.15	3.96	0.0000
Subtotal CPSI				1.77	201.17	97.53	
*CPSII	102	m	1	2.30	75.60	20.35	0.0000
*CPSII	103	m	1	2.87	75.65	89.07	0.0000
*CPSII	105	f	1	1.43	95.77	12.22	0.0000
*CPSII	106	f	1	2.59	140.12	91.47	0.0000
Subtotal CPSII				2.30	387.13	213.12	
DAMBER	6	m	1	0.83	10.34	9.33	0.0074

Table 1G11 - 5

IESLC - Meta-anal of Ever Smoking (or Curr if Ever not avail) by Amount, Overview, Any prod (or Cigs if Any not avail)
All LC types
Least adjusted

REF	NRR	SEX	AD	Ys	Ws	Qs	Ps
DAMBER	7	m	1	1.99	13.68	0.58	0.0000
DAMBER	8	m	1	2.21	14.68	2.66	0.0000
DAMBER	9	m	1	2.70	5.93	5.01	0.0000
Subtotal DAMBER				1.89	44.62	17.56	
DARBY	1	m	0	4.30	2.87	18.15	0.0000
DARBY	2	m	0	4.56	2.86	22.02	0.0000
DARBY	3	m	0	4.96	2.72	27.51	0.0000
DARBY	8	f	0	2.75	14.48	13.65	0.0000
DARBY	9	f	0	3.07	14.73	24.34	0.0000
DARBY	10	f	0	3.73	8.38	31.73	0.0000
Subtotal DARBY				3.37	46.05	137.42	
DAVEYS	1	m	0	0.20	2.07	5.19	0.7726
DAVEYS	2	m	0	2.01	2.27	0.11	0.0025
DAVEYS	3	m	0	1.89	2.11	0.02	0.0061
DAVEYS	4	m	0	2.36	2.18	0.73	0.0005
Subtotal DAVEYS				1.63	8.63	6.05	
DEAN	1	m	0	0.79	8.38	8.22	0.0218
DEAN	2	m	0	1.91	9.10	0.14	0.0000
DEAN	3	m	0	2.44	7.59	3.32	0.0000
Subtotal DEAN				1.70	25.06	11.68	
DEAN2	25	m	0	1.17	22.52	8.38	0.0000
DEAN2	26	m	0	2.03	19.18	1.20	0.0000
DEAN2	29	f	0	0.92	11.90	8.77	0.0014
DEAN2	30	f	0	1.60	3.63	0.12	0.0023
Subtotal DEAN2				1.44	57.24	18.47	
DEAN3	5	m	0	1.83	17.21	0.05	0.0000
DEAN3	12	m	0	1.78	19.12	0.00	0.0000
DEAN3	19	m	0	2.42	18.58	7.61	0.0000
DEAN3	89	f	0	0.87	16.85	13.96	0.0003
DEAN3	96	f	0	1.15	20.13	7.98	0.0000
DEAN3	103	f	0	1.90	14.56	0.21	0.0000
Subtotal DEAN3				1.66	106.45	29.81	
*DEKLER	2	m	2	2.97	0.95	1.33	0.0038
*DEKLER	3	m	2	3.14	0.98	1.79	0.0019
*DEKLER	4	m	2	3.48	0.96	2.76	0.0007
Subtotal DEKLER				3.19	2.89	5.89	
DESTEF	1	m	0	1.00	12.29	7.44	0.0004
DESTEF	2	m	0	2.06	17.23	1.35	0.0000
DESTEF	3	m	0	2.27	16.84	4.07	0.0000
DESTEF	4	m	0	3.12	11.90	21.20	0.0000
Subtotal DESTEF				2.12	58.26	34.05	
DOLL	1	m	0	1.31	5.40	1.19	0.0023
DOLL	2	m	0	2.01	6.13	0.32	0.0000
DOLL	3	m	0	2.26	6.11	1.40	0.0000
DOLL	4	m	0	2.81	5.91	6.22	0.0000
DOLL	5	m	0	3.32	3.72	8.76	0.0000
DOLL	7	f	0	-0.06	6.92	23.45	0.8795
DOLL	8	f	0	0.68	7.19	8.80	0.0698
DOLL	9	f	0	1.24	3.57	1.07	0.0195
DOLL	10	f	0	3.75	0.47	1.84	0.0098
Subtotal DOLL				1.60	45.43	53.05	
*DOLL2	46	m	1	1.90	0.96	0.01	0.0623
*DOLL2	47	m	1	2.51	0.96	0.51	0.0137
*DOLL2	48	m	1	3.17	0.97	1.86	0.0018
*DOLL2	10	f	1	0.25	0.79	1.85	0.8209
*DOLL2	11	f	1	1.86	2.40	0.01	0.0040
*DOLL2	12	f	1	3.39	2.93	7.59	0.0000
Subtotal DOLL2				2.43	9.01	11.83	
DORANT	6	c	0	2.14	7.99	1.03	0.0000
DORANT	7	c	0	3.30	12.23	28.30	0.0000
DORANT	8	c	0	3.59	11.93	38.98	0.0000
Subtotal DORANT				3.12	32.15	68.31	
DORGAN	10	m	0	1.66	10.85	0.18	0.0000
DORGAN	11	m	0	2.65	12.03	9.00	0.0000
DORGAN	34	m	0	2.80	2.62	2.69	0.0000
DORGAN	35	m	0	3.97	2.50	11.93	0.0000
DORGAN	96	f	3	1.74	56.05	0.13	0.0000
DORGAN	97	f	3	2.50	51.92	26.94	0.0000
Subtotal DORGAN				2.16	135.97	50.86	
*DORN	408	m	1	1.39	152.79	23.42	0.0000
*DORN	409	m	1	2.29	287.32	75.25	0.0000

Table 1G11 - 5

IESLC - Meta-anal of Ever Smoking (or Curr if Ever not avail) by Amount, Overview, Any prod (or Cigs if Any not avail)
All LC types
Least adjusted

REF	NRR	SEX	AD	Ys	Ws	Qs	Ps
*DORN	410	m	1	2.84	277.97	313.24	0.0000
*DORN	411	m	1	3.12	176.37	317.52	0.0000
Subtotal DORN				2.47	894.45	729.43	
DOSEME	5	m	2	0.79	20.90	20.66	0.0003
DOSEME	9	m	2	1.13	45.98	19.51	0.0000
DOSEME	13	m	2	1.89	21.74	0.24	0.0000
Subtotal DOSEME				1.24	88.62	40.41	
*DUNN	1	m	0	2.12	1.20	0.14	0.0199
*DUNN	2	m	0	2.20	1.71	0.30	0.0040
*DUNN	3	m	0	2.95	1.95	2.67	0.0000
*DUNN	4	m	0	3.22	1.88	3.91	0.0000
*DUNN	5	m	0	3.36	1.73	4.30	0.0000
Subtotal DUNN				2.83	8.48	11.31	
EBELIN	2	m	0	1.00	6.42	3.97	0.0116
EBELIN	3	m	0	1.96	5.47	0.18	0.0000
EBELIN	4	m	0	2.52	7.13	3.84	0.0000
EBELIN	5	m	0	2.79	1.60	1.62	0.0004
EBELIN	6	m	0	2.28	1.69	0.41	0.0031
Subtotal EBELIN				1.94	22.31	10.02	
*ENGELA	3	m	0	0.75	13.76	14.67	0.0054
*ENGELA	4	m	0	1.42	17.42	2.32	0.0000
*ENGELA	5	m	0	1.88	21.14	0.19	0.0000
*ENGELA	6	m	0	2.21	13.53	2.50	0.0000
*ENGELA	7	m	0	2.39	18.36	6.83	0.0000
*ENGELA	17	f	0	0.94	8.65	6.16	0.0057
*ENGELA	18	f	0	1.55	12.16	0.66	0.0000
*ENGELA	19	f	0	2.38	13.54	4.90	0.0000
*ENGELA	20	f	0	2.59	5.04	3.25	0.0000
*ENGELA	21	f	0	2.11	2.74	0.29	0.0005
Subtotal ENGELA				1.79	126.34	41.76	
*ENSTRO	7	m	1	1.56	31.30	1.61	0.0000
*ENSTRO	6	m	1	2.04	59.28	3.88	0.0000
*ENSTRO	5	m	1	2.61	74.58	51.50	0.0000
*ENSTRO	4	m	1	2.78	72.60	71.84	0.0000
*ENSTRO	3	m	1	2.97	65.00	90.96	0.0000
*ENSTRO	11	f	1	0.77	48.76	50.46	0.0000
*ENSTRO	10	f	1	1.46	104.90	10.87	0.0000
*ENSTRO	9	f	1	2.25	141.36	30.75	0.0000
*ENSTRO	8	f	1	2.80	116.71	121.13	0.0000
Subtotal ENSTRO				2.23	714.48	433.00	
ESAKI	1	m	0	0.09	7.53	21.52	0.8001
ESAKI	2	m	0	0.80	7.75	7.44	0.0253
ESAKI	3	m	0	1.78	4.39	0.00	0.0002
Subtotal ESAKI				0.75	19.68	28.96	
FAN	6	m	0	-0.35	8.53	38.83	0.3059
FAN	7	m	0	0.71	17.63	20.33	0.0029
FAN	8	m	0	1.38	21.51	3.48	0.0000
FAN	9	m	0	2.41	9.89	3.87	0.0000
FAN	10	f	0	0.50	10.28	17.02	0.1116
FAN	11	f	0	1.32	12.82	2.69	0.0000
FAN	12	f	0	2.48	7.51	3.68	0.0000
FAN	13	f	0	2.92	0.79	1.02	0.0095
Subtotal FAN				1.19	88.96	90.93	
GAO	24	f	0	0.27	31.50	71.67	0.1237
GAO	25	f	0	0.86	24.99	21.14	0.0000
GAO	26	f	0	2.36	10.18	3.34	0.0000
Subtotal GAO				0.81	66.66	96.14	
GAO2	2	m	0	1.21	6.65	2.16	0.0018
GAO2	3	m	0	2.02	7.66	0.43	0.0000
GAO2	4	m	0	2.36	7.06	2.38	0.0000
Subtotal GAO2				1.88	21.37	4.97	
GARSHI	18	m	0	1.19	24.87	8.65	0.0000
GARSHI	19	m	0	1.74	31.85	0.06	0.0000
GARSHI	20	m	0	2.01	25.53	1.29	0.0000
GARSHI	21	m	0	1.73	29.00	0.08	0.0000
Subtotal GARSHI				1.68	111.24	10.08	
GER	18	c	0	-0.06	12.41	42.35	0.8213
GER	19	c	0	0.26	19.29	44.95	0.2605
GER	20	c	0	1.01	11.22	6.63	0.0007
Subtotal GER				0.36	42.92	93.93	
GOLLED	15	m	0	1.38	12.16	1.93	0.0000

Table 1G11 - 5

IESLC - Meta-anal of Ever Smoking (or Curr if Ever not avail) by Amount, Overview, Any prod (or Cigs if Any not avail)
All LC types
Least adjusted

REF	NRR	SEX	AD	Ys	Ws	Qs	Ps
GOLLED	16	m	0	1.60	12.74	0.43	0.0000
GOLLED	17	m	0	2.64	12.71	9.36	0.0000
Subtotal GOLLED				1.88	37.61	11.72	
GSELL	1	m	0	1.49	1.53	0.13	0.0655
GSELL	2	m	0	1.37	1.51	0.26	0.0931
GSELL	3	m	0	2.71	1.64	1.42	0.0005
GSELL	4	m	0	4.37	1.50	10.04	0.0000
GSELL	5	m	0	4.04	1.58	8.08	0.0000
Subtotal GSELL				2.80	7.77	19.93	
HAENSZ	52	f	0	0.57	17.63	25.98	0.0170
HAENSZ	51	f	0	1.64	7.30	0.15	0.0000
Subtotal HAENSZ				0.88	24.93	26.13	
*HAMMO2	21	m	0	2.18	4.47	0.71	0.0000
*HAMMO2	20	m	0	2.11	4.89	0.51	0.0000
Subtotal HAMMO2				2.14	9.36	1.22	
*HAMMON	153	m	1	2.00	8.19	0.38	0.0000
*HAMMON	154	m	1	2.12	11.45	1.29	0.0000
*HAMMON	155	m	1	2.84	10.98	12.19	0.0000
Subtotal HAMMON				2.34	30.62	13.87	
*HANSEN	1	m	2	0.31	5.16	11.11	0.4747
*HANSEN	2	m	2	1.06	3.91	2.02	0.0352
Subtotal HANSEN				0.64	9.07	13.13	
*HIRAYA	23	m	1	0.72	36.53	41.05	0.0000
*HIRAYA	24	m	1	1.39	77.85	12.24	0.0000
*HIRAYA	25	m	1	1.83	89.10	0.21	0.0000
*HIRAYA	26	f	1	0.81	38.69	36.54	0.0000
*HIRAYA	27	f	1	0.94	36.49	25.92	0.0000
*HIRAYA	28	f	1	1.50	15.75	1.28	0.0000
Subtotal HIRAYA				1.31	294.40	117.24	
HITOSU	3	m	0	0.67	5.59	6.94	0.1144
HITOSU	4	m	0	0.88	5.97	4.88	0.0319
HITOSU	5	m	0	1.43	5.49	0.69	0.0008
HITOSU	10	f	0	1.26	13.32	3.66	0.0000
HITOSU	11	f	0	1.22	3.38	1.09	0.0253
Subtotal HITOSU				1.12	33.76	17.26	
*HOLE	9	m	0	1.54	5.43	0.33	0.0003
*HOLE	10	m	0	1.97	6.53	0.23	0.0000
*HOLE	11	m	0	2.11	5.99	0.64	0.0000
*HOLE	12	m	0	1.96	4.64	0.15	0.0000
Subtotal HOLE				1.90	22.58	1.35	
HU	1	m	0	0.44	10.71	19.39	0.1527
HU	2	m	0	0.74	12.38	13.54	0.0095
HU	3	m	0	1.29	6.63	1.59	0.0009
HU	4	f	0	0.82	5.04	4.63	0.0643
HU	5	f	0	0.18	2.64	6.76	0.7672
HU	6	f	0	-0.51	0.65	3.40	0.6812
Subtotal HU				0.70	38.05	49.30	
HU2	2	c	0	-0.16	9.46	35.63	0.6262
HU2	3	c	0	0.29	18.89	42.15	0.2087
HU2	4	c	0	0.66	22.08	27.72	0.0019
HU2	5	c	0	0.74	21.23	23.29	0.0007
HU2	6	c	0	1.10	32.09	14.80	0.0000
HU2	7	c	0	1.78	12.31	0.00	0.0000
Subtotal HU2				0.79	116.06	143.59	
HUMBLE	2	m	1	2.22	3.63	0.69	0.0000
HUMBLE	3	m	1	3.21	4.80	9.72	0.0000
HUMBLE	5	m	1	2.45	1.57	0.70	0.0021
HUMBLE	6	m	1	3.26	1.44	3.15	0.0001
HUMBLE	8	f	1	2.95	3.07	4.22	0.0000
HUMBLE	9	f	1	2.77	5.38	5.27	0.0000
HUMBLE	11	f	1	2.92	2.12	2.73	0.0000
HUMBLE	12	f	1	3.61	1.37	4.56	0.0000
Subtotal HUMBLE				2.87	23.38	31.05	
JARUP	1	m	0	1.86	5.60	0.03	0.0000
JARUP	2	m	0	1.94	5.81	0.14	0.0000
Subtotal JARUP				1.90	11.41	0.17	
JEDRYC	60	m	0	1.18	26.20	9.54	0.0000
JEDRYC	61	m	0	1.86	32.56	0.22	0.0000
JEDRYC	62	m	0	2.16	28.12	4.04	0.0000
JEDRYC	65	f	0	2.06	6.86	0.54	0.0000
JEDRYC	66	f	0	1.93	9.94	0.23	0.0000

Table 1G11 - 5

IESLC - Meta-anal of Ever Smoking (or Curr if Ever not avail) by Amount, Overview, Any prod (or Cigs if Any not avail)
All LC types
Least adjusted

REF	NRR	SEX	AD	Ys	Ws	Qs	Ps
JEDRYC	67	f	0	2.70	3.28	2.77	0.0000
Subtotal JEDRYC				1.82	106.96	17.34	
JOLY	7	m	0	1.68	5.92	0.06	0.0000
JOLY	8	m	0	2.52	10.45	5.64	0.0000
JOLY	9	m	0	2.57	9.85	6.12	0.0000
JOLY	10	m	0	3.08	10.07	16.97	0.0000
JOLY	3	f	0	1.55	12.60	0.66	0.0000
JOLY	4	f	0	2.08	17.52	1.54	0.0000
JOLY	5	f	0	1.94	9.62	0.22	0.0000
JOLY	6	f	0	2.59	7.64	5.04	0.0000
Subtotal JOLY				2.24	83.67	36.25	
JUSSAW	10	m	0	1.75	4.46	0.00	0.0002
JUSSAW	11	m	0	1.03	3.50	2.00	0.0549
JUSSAW	12	m	0	1.35	15.44	2.92	0.0000
JUSSAW	13	m	0	0.13	2.31	6.29	0.8398
JUSSAW	14	m	0	2.53	5.71	3.20	0.0000
JUSSAW	15	m	0	3.92	3.58	16.32	0.0000
Subtotal JUSSAW				1.74	35.01	30.74	
*KAISE2	66	m	1	1.50	5.94	0.48	0.0003
*KAISE2	67	m	1	2.34	9.98	3.05	0.0000
*KAISE2	58	f	1	2.03	5.35	0.33	0.0000
*KAISE2	59	f	1	3.10	8.34	14.39	0.0000
Subtotal KAISE2				2.33	29.61	18.25	
*KAISER	6	m	2	1.88	13.61	0.14	0.0000
*KAISER	7	m	2	2.85	16.98	19.24	0.0000
*KAISER	8	m	2	3.04	25.61	40.49	0.0000
*KAISER	2	f	2	1.23	18.49	5.66	0.0000
*KAISER	3	f	2	2.08	24.04	2.08	0.0000
*KAISER	4	f	2	2.54	19.02	10.79	0.0000
Subtotal KAISER				2.32	117.74	78.40	
KANELL	1	m	0	0.76	27.40	28.77	0.0001
KANELL	2	m	0	2.15	26.86	3.62	0.0000
KANELL	3	m	0	3.28	9.77	21.84	0.0000
KANELL	4	m	0	3.67	12.32	44.06	0.0000
Subtotal KANELL				2.04	76.35	98.29	
KATSOU	7	f	0	0.22	3.68	9.03	0.6791
KATSOU	8	f	0	1.43	3.31	0.41	0.0092
KATSOU	9	f	0	1.72	2.21	0.01	0.0106
KATSOU	10	f	0	2.64	0.88	0.64	0.0134
Subtotal KATSOU				1.16	10.08	10.09	
KAUFMA	2	c	0	2.10	20.21	2.01	0.0000
KAUFMA	3	c	0	2.71	26.85	23.22	0.0000
KAUFMA	4	c	0	3.07	20.60	34.03	0.0000
KAUFMA	5	c	0	3.58	22.00	70.89	0.0000
KAUFMA	6	c	0	3.99	16.30	79.32	0.0000
Subtotal KAUFMA				3.04	105.97	209.48	
KHUDER	1	m	0	0.92	16.30	12.15	0.0002
KHUDER	2	m	0	2.35	18.30	5.82	0.0000
KHUDER	3	m	0	3.49	14.48	42.27	0.0000
Subtotal KHUDER				2.21	49.07	60.24	
*KINLEN	3	m	0	2.34	6.81	2.13	0.0000
*KINLEN	4	m	0	2.57	6.84	4.28	0.0000
*KINLEN	5	m	0	3.00	6.77	10.07	0.0000
Subtotal KINLEN				2.64	20.42	16.49	
*KNEKT	25	m	0	1.53	4.76	0.31	0.0008
*KNEKT	26	m	0	2.29	5.48	1.43	0.0000
Subtotal KNEKT				1.94	10.24	1.74	
KOO	11	f	0	0.31	7.09	15.46	0.4151
KOO	12	f	0	1.99	3.69	0.15	0.0001
KOO	13	f	0	0.42	0.49	0.92	0.7696
Subtotal KOO				0.86	11.27	16.53	
KOULUM	6	m	0	1.65	3.87	0.07	0.0012
KOULUM	5	m	0	3.17	4.27	8.27	0.0000
KOULUM	4	m	0	4.23	4.27	25.63	0.0000
Subtotal KOULUM				3.06	12.42	33.98	
KREUZE	19	m	3	0.92	2.54	1.91	0.1444
KREUZE	20	m	3	2.16	4.57	0.66	0.0000
KREUZE	21	m	3	2.97	4.24	5.98	0.0000
KREUZE	22	m	3	3.03	3.39	5.32	0.0000
KREUZE	30	m	3	2.10	18.30	1.89	0.0000
KREUZE	31	m	3	3.22	20.26	42.02	0.0000

Table 1G11 - 5

IESLC - Meta-anal of Ever Smoking (or Curr if Ever not avail) by Amount, Overview, Any prod (or Cigs if Any not avail)
All LC types
Least adjusted

REF	NRR	SEX	AD	Ys	Ws	Qs	Ps
KREUZE	32	m	3	3.49	18.97	55.33	0.0000
KREUZE	33	m	3	3.51	16.38	48.61	0.0000
KREUZE	25	f	3	1.74	2.81	0.01	0.0035
KREUZE	26	f	3	2.47	3.44	1.61	0.0000
KREUZE	27	f	3	2.49	2.00	1.01	0.0004
KREUZE	36	f	3	0.69	15.02	17.83	0.0072
KREUZE	37	f	3	1.69	19.01	0.18	0.0000
KREUZE	38	f	3	2.04	6.02	0.40	0.0000
Subtotal KREUZE				2.45	136.95	182.76	
KREYBE	21	m	0	1.95	5.71	0.17	0.0000
KREYBE	22	m	0	1.85	5.33	0.03	0.0000
KREYBE	23	m	0	2.92	5.11	6.66	0.0000
KREYBE	37	f	0	-0.27	7.23	30.37	0.4730
KREYBE	38	f	0	0.04	1.79	5.42	0.9552
Subtotal KREYBE				1.36	25.18	42.64	
LAMTH	7	f	0	0.98	29.68	18.95	0.0000
LAMTH	2	f	0	1.68	18.27	0.20	0.0000
LAMTH	9	f	0	1.98	6.91	0.26	0.0000
Subtotal LAMTH				1.34	54.86	19.41	
LAUSSM	3	m	0	1.15	22.03	8.76	0.0000
LAUSSM	2	m	0	1.61	26.93	0.76	0.0000
LAUSSM	1	m	0	2.02	18.96	1.08	0.0000
Subtotal LAUSSM				1.58	67.92	10.59	
LETOUR	2	c	0	2.25	18.66	4.11	0.0000
LETOUR	3	c	0	2.85	18.55	21.16	0.0000
LETOUR	4	c	0	3.27	9.52	21.14	0.0000
Subtotal LETOUR				2.70	46.74	46.40	
*LIAW	3	c	2	1.13	10.81	4.59	0.0002
*LIAW	4	c	2	1.28	11.36	2.86	0.0000
*LIAW	5	c	2	2.12	7.17	0.80	0.0000
Subtotal LIAW				1.43	29.33	8.24	
*LIDDEL	2	m	1	1.20	15.00	5.04	0.0000
*LIDDEL	3	m	1	1.61	17.38	0.50	0.0000
Subtotal LIDDEL				1.42	32.38	5.54	
LIU2	5	m	0	-0.19	6.08	23.64	0.6415
LIU2	6	m	0	1.68	7.60	0.07	0.0000
LIU2	7	m	0	2.80	6.09	6.27	0.0000
LIU2	11	f	0	0.37	3.76	7.47	0.4689
LIU2	12	f	0	1.17	4.66	1.74	0.0114
LIU2	13	f	0	2.61	3.09	2.12	0.0000
Subtotal LIU2				1.39	31.28	41.32	
LIU3	3	m	0	0.24	2.83	6.70	0.6810
LIU3	4	m	0	0.02	2.75	8.54	0.9718
LIU3	5	m	0	0.56	2.01	3.00	0.4278
Subtotal LIU3				0.25	7.59	18.24	
LIU4	7	m	2	0.75	2108.86	2263.88	0.0000
LIU4	8	m	2	1.28	4111.92	1035.63	0.0000
LIU4	9	m	2	1.94	2991.95	76.84	0.0000
Subtotal LIU4				1.37	9212.74	3376.35	
LIU5	2	c	0	-0.20	5.84	22.98	0.6267
LIU5	3	c	0	0.46	6.33	11.14	0.2520
LIU5	4	c	0	1.28	7.79	2.00	0.0004
Subtotal LIU5				0.58	19.96	36.12	
LUBIN	7	m	0	-0.12	2.42	8.73	0.8565
LUBIN	8	m	0	0.08	3.35	9.72	0.8845
LUBIN	9	m	0	1.52	3.79	0.27	0.0031
LUBIN	10	m	0	1.37	5.32	0.89	0.0015
Subtotal LUBIN				0.88	14.88	19.61	
LUBIN2	273	m	0	1.93	155.25	3.51	0.0000
LUBIN2	274	m	0	2.03	150.14	9.01	0.0000
LUBIN2	275	m	0	2.36	152.73	51.23	0.0000
LUBIN2	276	m	0	2.52	139.75	76.38	0.0000
LUBIN2	281	f	0	0.90	66.54	52.37	0.0000
LUBIN2	282	f	0	1.30	76.70	17.89	0.0000
LUBIN2	283	f	0	1.53	47.64	3.14	0.0000
LUBIN2	284	f	0	1.57	17.75	0.78	0.0000
Subtotal LUBIN2				1.96	806.50	214.32	
MACLEN	20	m	0	0.31	1.79	3.89	0.6779
MACLEN	21	m	0	1.23	3.15	0.98	0.0297
MACLEN	22	m	0	1.43	3.10	0.40	0.0120
MACLEN	23	m	0	1.61	3.07	0.09	0.0048

Table 1G11 - 5

IESLC - Meta-anal of Ever Smoking (or Curr if Ever not avail) by Amount, Overview, Any prod (or Cigs if Any not avail)
All LC types
Least adjusted

REF	NRR	SEX	AD	Ys	Ws	Qs	Ps
MACLEN	33	f	0	-0.27	4.03	17.08	0.5807
MACLEN	34	f	0	1.24	7.25	2.17	0.0009
MACLEN	35	f	0	1.35	4.51	0.86	0.0043
Subtotal MACLEN				1.03	26.92	25.48	
MARTIS	1	m	0	1.60	2.87	0.09	0.0066
MARTIS	2	m	0	1.88	3.20	0.03	0.0008
MARTIS	3	m	0	2.24	3.09	0.64	0.0001
Subtotal MARTIS				1.91	9.17	0.76	
MATOS	28	m	0	0.66	5.88	7.43	0.1105
MATOS	30	m	0	1.98	7.91	0.30	0.0000
MATOS	32	m	0	2.31	8.41	2.35	0.0000
Subtotal MATOS				1.75	22.19	10.08	
MATSUD	1	m	0	2.53	2.76	1.53	0.0000
MATSUD	2	m	0	2.97	2.87	4.06	0.0000
MATSUD	3	m	0	3.94	2.83	13.21	0.0000
Subtotal MATSUD				3.15	8.46	18.80	
MCCONN	26	c	0	-0.88	3.12	22.06	0.1215
MCCONN	25	c	0	0.31	5.38	11.70	0.4745
MCCONN	24	c	0	0.76	4.83	5.09	0.0966
Subtotal MCCONN				0.19	13.33	38.85	
*MIGRAN	1	m	0	1.55	2.96	0.17	0.0078
*MIGRAN	3	m	0	2.02	3.77	0.21	0.0001
*MIGRAN	5	m	0	2.22	3.63	0.68	0.0000
*MIGRAN	7	m	0	2.38	3.56	1.26	0.0000
*MIGRAN	28	f	0	1.60	2.01	0.07	0.0237
*MIGRAN	30	f	0	2.31	3.12	0.87	0.0000
*MIGRAN	32	f	0	1.80	1.72	0.00	0.0179
*MIGRAN	34	f	0	2.55	1.35	0.79	0.0031
Subtotal MIGRAN				2.06	22.12	4.05	
*MRFITR	3	m	0	2.38	0.42	0.15	0.1236
*MRFITR	4	m	0	3.91	0.50	2.25	0.0059
*MRFITR	5	m	0	4.03	0.50	2.51	0.0045
Subtotal MRFITR				3.50	1.41	4.91	
NAM	66	m	0	1.53	20.40	1.32	0.0000
NAM	67	m	0	2.36	21.70	7.32	0.0000
NAM	82	f	0	2.04	23.96	1.63	0.0000
NAM	83	f	0	2.58	20.99	13.27	0.0000
Subtotal NAM				2.13	87.05	23.54	
NOTAN2	8	m	0	-0.54	5.01	27.12	0.2229
NOTAN2	9	m	0	0.88	15.08	12.21	0.0006
NOTAN2	10	m	0	1.50	17.54	1.44	0.0000
Subtotal NOTAN2				0.98	37.63	40.77	
ORMOS	1	m	0	2.38	5.60	1.99	0.0000
ORMOS	2	m	0	2.04	5.85	0.39	0.0000
ORMOS	3	m	0	2.57	4.57	2.80	0.0000
Subtotal ORMOS				2.31	16.03	5.18	
OSANN	49	m	2	2.87	33.51	39.87	0.0000
OSANN	57	m	2	3.76	33.40	130.11	0.0000
OSANN	50	f	2	2.67	52.45	41.03	0.0000
OSANN	58	f	2	3.71	34.52	128.35	0.0000
Subtotal OSANN				3.18	153.87	339.36	
PARKIN	17	m	0	1.50	68.71	5.53	0.0000
PARKIN	18	m	0	1.72	29.11	0.13	0.0000
Subtotal PARKIN				1.56	97.82	5.67	
PASTOR	1	m	0	0.60	3.82	5.32	0.2395
PASTOR	2	m	0	1.76	6.96	0.00	0.0000
PASTOR	3	m	0	2.07	7.55	0.62	0.0000
PASTOR	4	m	0	2.21	6.44	1.17	0.0000
Subtotal PASTOR				1.79	24.77	7.11	
PERNU	17	m	0	1.04	6.79	3.73	0.0066
PERNU	18	m	0	1.72	15.98	0.07	0.0000
PERNU	19	m	0	1.89	34.69	0.39	0.0000
PERNU	20	m	0	1.68	27.21	0.28	0.0000
PERNU	21	m	0	2.28	42.95	10.81	0.0000
PERNU	22	m	0	2.94	28.38	38.14	0.0000
PERNU	23	m	0	1.60	12.13	0.43	0.0000
PERNU	24	m	0	2.71	10.11	8.64	0.0000
PERNU	11	f	0	0.64	2.41	3.16	0.3224
PERNU	12	f	0	1.22	3.48	1.09	0.0225
PERNU	13	f	0	0.16	3.41	8.94	0.7636
PERNU	14	f	0	-0.46	0.92	4.66	0.6574

Table 1G11 - 5

IESLC - Meta-anal of Ever Smoking (or Curr if Ever not avail) by Amount, Overview, Any prod (or Cigs if Any not avail)
All LC types
Least adjusted

REF	NRR	SEX	AD	Ys	Ws	Qs	Ps
PERNU	15	f	0	-0.22	0.91	3.64	0.8339
PERNU	16	f	0	1.84	2.83	0.01	0.0019
Subtotal PERNU				2.00	192.21	83.98	
PERSH2	2	c	0	1.69	78.18	0.64	0.0000
PERSH2	3	c	0	2.34	81.53	24.97	0.0000
Subtotal PERSH2				2.02	159.71	25.61	
*PETO	2	m	0	1.70	1.93	0.01	0.0180
*PETO	3	m	0	2.25	1.95	0.43	0.0017
Subtotal PETO				1.98	3.88	0.44	
PEZZO2	3	m	0	2.08	5.00	0.44	0.0000
PEZZO2	4	m	0	3.79	4.86	19.63	0.0000
PEZZO2	5	m	0	4.72	3.66	31.59	0.0000
Subtotal PEZZO2				3.41	13.52	51.66	
PEZZOT	2	m	0	2.00	3.22	0.15	0.0003
PEZZOT	3	m	0	4.25	3.25	19.78	0.0000
PEZZOT	4	m	0	5.51	2.25	31.18	0.0000
Subtotal PEZZOT				3.74	8.72	51.11	
PIKE	1	m	0	1.42	12.27	1.63	0.0000
PIKE	2	m	0	2.08	11.96	1.07	0.0000
PIKE	3	m	0	1.92	8.91	0.17	0.0000
PIKE	5	f	0	1.18	14.59	5.35	0.0000
PIKE	6	f	0	1.93	10.92	0.23	0.0000
PIKE	7	f	0	2.65	2.30	1.75	0.0001
Subtotal PIKE				1.70	60.95	10.21	
POLEDN	2	c	0	1.79	8.40	0.00	0.0000
POLEDN	4	c	0	2.29	9.66	2.47	0.0000
Subtotal POLEDN				2.05	18.06	2.47	
*PRESCO	2	m	1	2.32	5.71	1.66	0.0000
*PRESCO	4	m	1	2.99	5.92	8.68	0.0000
*PRESCO	1	f	1	1.85	11.85	0.05	0.0000
*PRESCO	3	f	1	2.31	11.98	3.34	0.0000
Subtotal PRESCO				2.27	35.47	13.74	
RACHTA	5	f	0	0.93	2.86	2.06	0.1143
RACHTA	6	f	0	1.13	8.19	3.50	0.0012
RACHTA	7	f	0	2.59	7.02	4.61	0.0000
Subtotal RACHTA				1.67	18.07	10.16	
RANDIG	1	m	0	0.71	2.79	3.19	0.2326
RANDIG	2	m	0	1.06	3.69	1.92	0.0415
RANDIG	3	m	0	1.63	3.89	0.09	0.0013
RANDIG	4	m	0	2.22	3.74	0.71	0.0000
RANDIG	5	f	0	-1.36	0.90	8.82	0.1996
RANDIG	6	f	0	1.61	4.35	0.13	0.0008
RANDIG	7	f	0	1.18	1.66	0.61	0.1294
Subtotal RANDIG				1.35	21.02	15.47	
SEGI2	9	m	0	0.76	4.76	4.99	0.0975
SEGI2	11	m	0	1.11	6.13	2.78	0.0060
SEGI2	13	m	0	1.20	6.24	2.10	0.0026
SEGI2	15	m	0	1.82	4.64	0.01	0.0001
SEGI2	17	m	0	2.04	5.40	0.34	0.0000
SEGI2	21	f	0	0.81	4.03	3.81	0.1035
SEGI2	23	f	0	0.41	5.20	9.86	0.3554
SEGI2	25	f	0	0.12	3.07	8.51	0.8365
Subtotal SEGI2				1.09	39.47	32.39	
SHAW	10	c	0	1.60	7.51	0.24	0.0000
SHAW	11	c	0	2.73	9.13	8.23	0.0000
Subtotal SHAW				2.22	16.64	8.47	
SIEMIA	13	m	0	1.10	2.92	1.37	0.0603
SIEMIA	14	m	0	1.50	3.87	0.30	0.0031
SIEMIA	15	m	0	2.07	3.54	0.29	0.0001
Subtotal SIEMIA				1.58	10.33	1.95	
SOBUE	117	m	0	1.26	19.84	5.43	0.0000
SOBUE	118	m	0	1.39	21.76	3.41	0.0000
SOBUE	119	m	0	1.52	21.28	1.52	0.0000
Subtotal SOBUE				1.39	62.88	10.36	
*SPEIZE	1	f	1	0.99	4.79	2.98	0.0298
*SPEIZE	2	f	1	1.65	23.37	0.42	0.0000
*SPEIZE	3	f	1	2.53	56.20	31.69	0.0000
*SPEIZE	4	f	1	2.75	58.51	55.15	0.0000
*SPEIZE	5	f	1	3.09	25.23	43.18	0.0000
Subtotal SPEIZE				2.53	168.09	133.42	

Table 1G11 - 5

IESLC - Meta-anal of Ever Smoking (or Curr if Ever not avail) by Amount, Overview, Any prod (or Cigs if Any not avail)
All LC types
Least adjusted

REF	NRR	SEX	AD	Ys	Ws	Qs	Ps
SPITZ	5	c	0	1.08	6.30	3.11	0.0067
SPITZ	6	c	0	2.94	7.05	9.52	0.0000
Subtotal SPITZ				2.07	13.35	12.63	
STOCKS	25	m	0	1.73	38.74	0.12	0.0000
STOCKS	26	m	0	1.75	39.49	0.03	0.0000
STOCKS	27	m	0	2.16	32.54	4.59	0.0000
STOCKS	28	m	0	1.82	34.51	0.05	0.0000
STOCKS	29	m	0	2.21	34.20	6.35	0.0000
STOCKS	48	f	1	0.81	40.78	38.87	0.0000
STOCKS	49	f	1	1.85	31.07	0.13	0.0000
Subtotal STOCKS				1.73	251.34	50.14	
STOCKW	1	c	0	1.90	565.53	7.53	0.0000
STOCKW	2	c	0	2.67	802.56	638.16	0.0000
STOCKW	3	c	0	3.36	411.25	1025.36	0.0000
Subtotal STOCKW				2.59	1779.33	1671.04	
SVENSS	26	f	0	1.49	10.89	0.96	0.0000
SVENSS	31	f	0	2.45	10.82	4.86	0.0000
SVENSS	36	f	0	4.09	0.92	4.91	0.0001
Subtotal SVENSS				2.05	22.63	10.73	
*TENKAN	10	m	1	2.76	5.35	5.15	0.0000
*TENKAN	11	m	1	3.01	4.70	7.06	0.0000
*TENKAN	12	m	1	3.22	4.49	9.24	0.0000
Subtotal TENKAN				2.98	14.54	21.45	
TIZZAN	7	m	0	-0.08	48.24	166.93	0.5909
TIZZAN	8	m	0	0.52	76.34	121.15	0.0000
TIZZAN	9	m	0	1.55	46.69	2.48	0.0000
TIZZAN	10	m	0	1.95	14.11	0.40	0.0000
TIZZAN	15	f	0	1.28	4.74	1.22	0.0055
TIZZAN	16	f	0	1.52	5.22	0.37	0.0005
Subtotal TIZZAN				0.77	195.34	292.54	
TSUGAN	29	m	0	-0.10	4.44	15.83	0.8253
TSUGAN	30	m	0	0.20	5.96	14.93	0.6241
TSUGAN	31	m	0	0.51	4.44	7.24	0.2861
Subtotal TSUGAN				0.20	14.85	38.00	
*TULINI	4	m	1	1.87	8.00	0.06	0.0000
*TULINI	5	m	1	2.60	9.30	6.25	0.0000
*TULINI	6	m	1	3.36	8.98	22.26	0.0000
*TULINI	9	f	1	2.24	9.59	2.00	0.0000
*TULINI	10	f	1	3.42	10.60	28.56	0.0000
*TULINI	11	f	1	3.79	7.11	28.53	0.0000
Subtotal TULINI				2.87	53.57	87.67	
*TVERDA	9	m	2	0.76	10.05	10.50	0.0159
*TVERDA	10	m	2	1.20	17.25	5.86	0.0000
*TVERDA	11	m	2	1.88	16.39	0.16	0.0000
*TVERDA	16	f	2	1.51	1.87	0.14	0.0387
*TVERDA	17	f	2	2.89	2.59	3.18	0.0000
Subtotal TVERDA				1.44	48.15	19.83	
WAKAI	37	m	0	0.62	6.32	8.56	0.1197
WAKAI	38	m	0	1.38	7.36	1.19	0.0002
WAKAI	39	m	0	2.23	6.64	1.36	0.0000
Subtotal WAKAI				1.42	20.32	11.10	
WANG2	1	c	0	0.33	2.59	5.45	0.5910
WANG2	2	c	0	0.16	1.83	4.81	0.8295
WANG2	3	c	0	0.33	2.59	5.45	0.5910
WANG2	4	c	0	0.11	2.30	6.42	0.8670
WANG2	5	c	0	1.19	5.48	1.90	0.0052
WANG2	6	c	0	0.91	2.87	2.18	0.1224
WANG2	7	c	0	2.17	2.10	0.32	0.0016
Subtotal WANG2				0.81	19.77	26.53	
WU	35	f	0	1.39	11.84	1.80	0.0000
WU	36	f	0	2.29	11.75	3.01	0.0000
Subtotal WU				1.84	23.59	4.80	
WUWILL	18	f	0	0.74	105.32	115.15	0.0000
WUWILL	19	f	0	1.15	24.74	9.78	0.0000
Subtotal WUWILL				0.82	130.06	124.93	
WYNDE2	17	m	0	0.67	4.95	6.11	0.1353
WYNDE2	18	m	0	2.07	6.77	0.54	0.0000
WYNDE2	19	m	0	2.63	6.33	4.58	0.0000
WYNDE2	20	m	0	2.90	6.67	8.32	0.0000
Subtotal WYNDE2				2.16	24.72	19.55	
WYNDE3	44	m	0	0.62	3.69	4.97	0.2325

Table 1G11 - 5

IESLC - Meta-anal of Ever Smoking (or Curr if Ever not avail) by Amount, Overview, Any prod (or Cigs if Any not avail)
All LC types
Least adjusted

REF	NRR	SEX	AD	Ys	Ws	Qs	Ps
WYNDE3	45	m	0	1.89	6.93	0.08	0.0000
WYNDE3	46	m	0	2.56	6.95	4.15	0.0000
WYNDE3	47	m	0	3.24	5.69	12.11	0.0000
WYNDE3	79	f	0	-0.51	2.23	11.71	0.4459
WYNDE3	80	f	0	1.34	6.83	1.37	0.0005
WYNDE3	81	f	0	1.74	4.35	0.01	0.0003
WYNDE3	82	f	0	1.62	1.55	0.04	0.0436
Subtotal WYNDE3				1.82	38.21	34.44	
WYNDE4	43	m	0	0.69	6.13	7.37	0.0891
WYNDE4	44	m	0	1.47	8.79	0.84	0.0000
WYNDE4	45	m	0	2.08	9.99	0.86	0.0000
WYNDE4	46	m	0	2.92	9.30	12.07	0.0000
WYNDE4	47	m	0	2.97	8.67	12.19	0.0000
WYNDE4	57	f	2	0.12	2.52	6.96	0.8460
WYNDE4	58	f	2	0.70	3.11	3.66	0.2185
WYNDE4	59	f	2	1.87	3.72	0.03	0.0003
WYNDE4	60	f	2	2.45	1.18	0.52	0.0079
WYNDE4	61	f	2	2.45	1.18	0.52	0.0079
Subtotal WYNDE4				1.94	54.60	45.02	
WYNDE6	27	m	0	1.92	33.49	0.60	0.0000
WYNDE6	36	m	0	2.41	53.48	21.19	0.0000
WYNDE6	45	m	0	2.85	41.59	47.53	0.0000
WYNDE6	54	m	0	3.34	51.37	124.59	0.0000
WYNDE6	216	f	0	1.32	33.57	7.10	0.0000
WYNDE6	225	f	0	2.48	61.57	30.17	0.0000
WYNDE6	234	f	0	3.07	30.83	51.46	0.0000
WYNDE6	243	f	0	3.67	34.09	121.03	0.0000
Subtotal WYNDE6				2.65	340.00	403.67	
XU3	5	m	0	0.53	3.19	4.98	0.3416
XU3	6	m	0	1.14	3.78	1.58	0.0272
XU3	7	m	0	2.77	3.66	3.59	0.0000
XU3	8	m	0	3.57	1.36	4.32	0.0000
XU3	13	f	0	0.81	2.34	2.23	0.2159
XU3	14	f	0	1.57	2.14	0.10	0.0216
XU3	15	f	0	2.45	0.78	0.34	0.0309
Subtotal XU3				1.63	17.25	17.14	
YAMAGU	3	c	0	1.03	13.99	7.97	0.0001
YAMAGU	2	c	0	1.72	10.10	0.05	0.0000
Subtotal YAMAGU				1.32	24.09	8.01	
*YUAN	2	m	2	1.28	9.07	2.28	0.0001
*YUAN	3	m	2	2.24	11.02	2.31	0.0000
Subtotal YUAN				1.81	20.09	4.59	
ZHENG	11	m	0	0.58	9.44	13.73	0.0764
ZHENG	12	m	0	0.95	13.74	9.50	0.0004
ZHENG	13	m	0	1.41	16.67	2.31	0.0000
ZHENG	14	m	0	2.10	10.04	1.02	0.0000
ZHENG	22	f	0	0.00	11.34	35.98	0.9951
ZHENG	23	f	0	1.43	10.21	1.24	0.0000
Subtotal ZHENG				1.09	71.45	63.78	
ZHOU	4	c	0	0.49	4.29	7.14	0.3077
ZHOU	5	c	0	0.70	10.77	12.54	0.0209
ZHOU	6	c	0	0.99	18.91	11.93	0.0000
Subtotal ZHOU				0.84	33.97	31.61	

N	622
NS	140

Table 1G11 - 6

IESLC - Meta-anal of Ever Smoking (or Curr if Ever not avail) by Amount, Overview, Any prod (or Cigs if Any not avail)
All LC types
Least adjusted

	combined	Sex male	female	Total
N	62	366	194	622
NS	20	108	63	191

In this overview table, other than the "N" rows, entries in the "absent" and "Total" columns may be invalid and should be ignored

	Amount smoked (broad categories) absent	<20k5	6-44k20	>20k45	Total
N	200	177	115	130	622
NS	96	126	90	103	415
Wt	4117.19	5539.21	7010.93	5125.41	21792.73
Het Chi	2110.51	1922.87	2552.88	1879.00	12570.93
Het df	199	176	114	129	621
Het P	***	***	***	***	***
Fixed RR	8.55	3.24	5.28	10.07	5.95
RRl	8.30	3.16	5.16	9.80	5.87
RRu	8.82	3.33	5.40	10.35	6.03
P	+++	+++	+++	+++	+++
Random RR	6.57	3.37	7.17	13.34	6.36
RRl	5.89	3.03	6.16	11.53	5.94
RRu	7.33	3.75	8.34	15.44	6.81
P	+++	+++	+++	+++	+++

	absent	Amount smoked (narrow categories) <10k1	2-19k10	11-29k20	21-39k30	31-98k40	>40k99	Total
N	351	75	61	91	24	2	18	622
NS	140	51	43	71	21	2	15	343
Wt	12718.72	1105.30	1110.49	5620.11	636.25	29.14	572.71	21792.73
Het Chi	7387.58	475.30	369.39	1218.74	85.23	14.52	142.06	12570.93
Het df	350	74	60	90	23	1	17	621
Het P	***	***	***	***	***	***	***	***
Fixed RR	6.49	3.05	5.01	4.44	15.20	23.95	24.67	5.95
RRl	6.38	2.87	4.72	4.33	14.06	16.65	22.73	5.87
RRu	6.61	3.23	5.31	4.56	16.43	34.43	26.78	6.03
P	+++	+++	+++	+++	+++	+++	+++	+++
Random RR	7.07	2.40	4.83	7.45	13.19	16.21	19.23	6.36
RRl	6.44	2.02	4.11	6.38	10.77	3.25	13.41	5.94
RRu	7.75	2.84	5.67	8.70	16.16	80.94	27.58	6.81
P	+++	+++	+++	+++	+++	+++	+++	+++

MALES

	Amount smoked (broad categories) absent	<20k5	6-44k20	>20k45	Total
N	107	101	74	84	366
NS	69	98	73	83	323
Wt	2367.94	3619.45	5482.72	4187.56	15657.67
Het Chi	852.16	935.92	1119.20	950.74	7308.40
Het df	106	100	73	83	365
Het P	***	***	***	***	***
Fixed RR	9.30	2.79	4.35	8.64	5.29
RRl	8.93	2.70	4.23	8.38	5.21
RRu	9.68	2.88	4.46	8.90	5.37
P	+++	+++	+++	+++	+++
Random RR	7.92	3.65	7.44	13.76	7.21
RRl	6.96	3.17	6.25	11.66	6.63
RRu	9.01	4.20	8.86	16.23	7.84
P	+++	+++	+++	+++	+++

Table 1G11 - 6

IESLC - Meta-anal of Ever Smoking (or Curr if Ever not avail) by Amount, Overview, Any prod (or Cigs if Any not avail)
All LC types
Least adjusted

MALES

	absent	<10k1	2-19k10	11-29k20	21-39k30	31-98k40	>40k99	Total
		Amount smoked (narrow categories)						
N	205	40	34	59	16	1	11	366
NS	108	39	33	58	16	1	11	265
Wt	8741.21	655.80	623.03	5015.06	518.73	7.14	96.71	15657.67
Het Chi	4842.21	275.21	163.87	747.70	64.07	0.00	52.72	7308.40
Het df	204	39	33	58	15	0	10	365
Het P	***	***	***	***	***	N.S.	***	***
Fixed RR	5.76	3.55	6.01	4.15	14.89	6.93	17.24	5.29
RRl	5.64	3.29	5.56	4.04	13.66	3.33	14.13	5.21
RRu	5.88	3.83	6.50	4.27	16.23	14.44	21.04	5.37
P	+++	+++	+++	+++	+++	+++	+++	+++
Random RR	7.95	2.79	5.69	7.76	13.07	6.93	21.28	7.21
RRl	7.02	2.20	4.66	6.41	10.22	3.33	13.08	6.63
RRu	9.00	3.53	6.95	9.39	16.71	14.44	34.62	7.84
P	+++	+++	+++	+++	+++	+++	+++	+++

FEMALES

	absent	<20k5	6-44k20	>20k45	Total
	Amount smoked (broad categories)				
N	68	59	32	35	194
NS	49	57	31	35	172
Wt	1365.40	1124.74	589.84	412.93	3492.92
Het Chi	813.83	457.35	160.29	179.17	2455.52
Het df	67	58	31	34	193
Het P	***	***	***	***	***
Fixed RR	7.99	3.44	7.97	15.43	6.59
RRl	7.58	3.25	7.35	14.01	6.37
RRu	8.43	3.65	8.64	17.00	6.81
P	+++	+++	+++	+++	+++
Random RR	5.22	2.94	7.16	12.70	5.23
RRl	4.23	2.44	5.78	9.73	4.60
RRu	6.44	3.53	8.87	16.58	5.96
P	+++	+++	+++	+++	+++

	absent	<10k1	2-19k10	11-29k20	21-39k30	31-98k40	>40k99	Total
		Amount smoked (narrow categories)						
N	109	28	22	26	6		3	194
NS	62	27	21	25	6		3	144
Wt	2119.91	329.46	433.47	493.17	94.05		22.87	3492.92
Het Chi	1418.52	62.80	88.41	125.77	8.23		1.27	2455.52
Het df	108	27	21	25	5		2	193
Het P	***	***	***	***	N.S.		N.S.	***
Fixed RR	7.85	2.02	4.04	8.53	16.67		12.02	6.59
RRl	7.52	1.81	3.68	7.81	13.62		7.98	6.37
RRu	8.19	2.25	4.44	9.31	20.41		18.10	6.81
P	+++	+++	+++	+++	+++		+++	+++
Random RR	6.27	1.92	4.13	7.73	15.31		12.02	5.23
RRl	5.28	1.58	3.31	6.11	10.42		7.98	4.60
RRu	7.45	2.33	5.14	9.78	22.49		18.10	5.96
P	+++	+++	+++	+++	+++		+++	+++

Table 1G11 - 7

IESLC - Meta-anal of Ever Smoking (or Curr if Ever not avail) by Amount, Overview, Any prod (or Cigs if Any not avail)
All LC types
Excluded studies (and stage at which they were excluded)

1 ABELIN ABRAHA AMANDU AMES ANDERS AUSTIN AXELSO BAND BECHER BERRIN BLOHMK BLOT4 BROCKM BROWN1 BYERS1 BYERS2
CARPEN CASCO2 CASCOR CHAN CHEN3 CHIAZZ CHYOU DESTE2 DOCKER DROSTE DU GARCIA GARDIN GENG GODLEY GOODMA
GRAHAM GREGOR HEGMAN HEIN HENNEK HINDS HIRAOK HOROWI HORWIT HUANG ISHIMA JAHN JAIN JARVHO JIANG KELLER
KIHARA KJUUS KO KOHLME KUBIK LAMWK LAMWK2 LANGE LEI LEMARC LEVIN LIU LOMBA2 LOMBAR MAGNUS MARSH
MARSH2 MCDUFF MCLAUG MILLER MILLS NOTANI NOU ODRISC PAWLEG PERSHA POFFIJ QIAO QIAO2 RADZIK REN RONCO
ROOTS ROTHSC SAARIK SANKAR SCHWAR SEGI SEOW SHIMIZ SIMARA SIMONA SITAS SOBUE2 STASZE STAYNE STUCKE SUN
SUZUK2 SUZUKI TANG TAO TOKARS TOUSEY ULMER VEIERO VUTUC WALD WANG WANG3 WANG4 WICKLU WIGLE WILKIN
WU2 WUNSCH WYNDE8 XIANGZ XU XU2 XU4 YONG ZHANG

2 BUELL CHEN MASTRA MZILEN PISANI RESTRE SADOWS

4 BOFFET WYNDE7

5 RIMING TANG2 WYNDE5

6 BLOT1 BLOT2 BLOT3 BOUCHA HIRAY2 JONES LAURIL LICKIN MOLLO MRFIT MURATA SCHWA2 VANDER WARSIN WATSON WYNDER

8 LUO

10 BENHAM GILLIS OSANN2

Table 1G11 - 8

Potentially overlapping studies

REF	REFGP	PRINC	.	OVERLAP/LINK
LUBIN2	LUBIN2	1		Lubin-combined
LAMTH	LAMTH	1		KOO/LAMTH/LAMWK
KOO	KOO	1		KOO/LAMTH/LAMWK
TVERDA	TVERDA	1		VEIERO/TVERDAL
AKIBA	AKIBA	1		AKIBA/ISHIMA
PRESCO	PRESCO	2		PRESCO-combined
BROSS	BYERS1	1		GRAHAM/BROSS/BYERS1
HOLE	TANG2	1		Subset of TANG2
BENSHL	TANG2	1		Subset of TANG2
KAISER	KAISER	1		KAISER/OSANN2
MRFITR	MRFIT	2		Subset of MRFIT
WYNDE6	WYNDE6	1		WYNDE5/6/7/8
CPSI	CPSI	1		CPSI overall
ENSTRO	ENSTRO	1		Subset of CPSI
MATSUD	MATSUD	1		SOBUE2/MATSUD
LUBIN	XIANGZ	2		LUBIN/XIANGZ/QIAO

Table 1G11 - 9

Most adjusted - insufficient data for metaanalysis

REF	NRR	SEX	AGEL	AGEH	RACE	YF	LC TYPE	LOC	START	ST	NLC	R	VB	P	H	AD	SM	PRODUCT	exL	exH	S1	S2	DENOM	De
BLOT1	5	m	0	0	all	-	all	NAmer	1970	CC	458	n	bl	y	n	0	ev	cig+/-ot	1	9	1	1	nev cigs	or
BLOT1	6	m	0	0	all	-	all	NAmer	1970	CC	458	n	bl	y	n	0	ev	cig+/-ot	10	39	2	0	nev cigs	or
BLOT1	7	m	0	0	all	-	all	NAmer	1970	CC	458	n	bl	y	n	0	ev	cig+/-ot	40	99	3	0	nev cigs	or
BUELL	1	m	0	0	all	0	all	NAmer	1957	pr	304	n	bl	n	n	0	ev	cig+/-ot	1	18	1	0	nev cigs	st
BUELL	2	m	0	0	all	0	all	NAmer	1957	pr	304	n	bl	n	n	0	ev	cig+/-ot	19	21	2	3	nev cigs	st
BUELL	3	m	0	0	all	0	all	NAmer	1957	pr	304	n	bl	n	n	0	ev	cig+/-ot	22	99	3	0	nev cigs	st
DOLL	11	f	0	0	all	-	all	Eu:UK	1948	CC	1465	n	V	n	n	0	ev	all/unsp	50	99	0	6	nev any	or
HITOSU	14	m	0	0	all	-	all	As:Jap	1960	CC	216	n	bl	y	n	2	cu	all/unsp	1	14	1	0	nev any	or
HITOSU	15	m	0	0	all	-	all	As:Jap	1960	CC	216	n	bl	y	n	2	cu	all/unsp	15	24	2	3	nev any	or
HITOSU	16	m	0	0	all	-	all	As:Jap	1960	CC	216	n	bl	y	n	2	cu	all/unsp	25	99	3	0	nev any	or
HITOSU	17	f	0	0	all	-	all	As:Jap	1960	CC	216	n	bl	y	n	2	cu	all/unsp	1	14	1	0	nev any	or
HITOSU	18	f	0	0	all	-	all	As:Jap	1960	CC	216	n	bl	y	n	2	cu	all/unsp	15	99	0	0	nev any	or
MASTRA	3	m	0	0	all	-	all	Eu:wst	1973	CC	309	n	bl	n	n	1	ev	all/unsp	1	10	1	0	nev any	or
MASTRA	4	m	0	0	all	-	all	Eu:wst	1973	CC	309	n	bl	n	n	1	ev	all/unsp	20	99	0	0	nev any	or
MZILEN	1	m	0	0	bl	-	all	Africa	1994	CC	374	n	V	*	n	3	ev	all/unsp	1	14	1	0	nev any	or
MZILEN	2	m	0	0	bl	-	all	Africa	1994	CC	374	n	V	*	n	3	ev	all/unsp	15	99	0	0	nev any	or
MZILEN	3	f	0	0	bl	-	all	Africa	1994	CC	374	n	V	*	n	3	ev	all/unsp	1	14	1	0	nev any	or
MZILEN	4	f	0	0	bl	-	all	Africa	1994	CC	374	n	V	*	n	3	ev	all/unsp	15	99	0	0	nev any	or
RESTRE	6	c	0	0	all	-	all	SCAmer	1978	CC	102	n	bl	n	n	5	ev	cig+/-ot	1	10	1	0	nev cigs	or
RESTRE	7	c	0	0	all	-	all	SCAmer	1978	CC	102	n	bl	n	n	5	ev	cig+/-ot	11	20	2	3	nev cigs	or
RESTRE	8	c	0	0	all	-	all	SCAmer	1978	CC	102	n	bl	n	n	5	ev	cig+/-ot	21	40	0	0	nev cigs	or
RESTRE	9	c	0	0	all	-	all	SCAmer	1978	CC	102	n	bl	n	n	5	ev	cig+/-ot	41	99	3	6	nev cigs	or
SADOWS	85	m	0	0	wh	-	all	NAmer	1938	CC	477	n	bl	n	n	1	ev	all/unsp	1	10	1	0	nev any	st
SADOWS	86	m	0	0	wh	-	all	NAmer	1938	CC	477	n	bl	n	n	1	ev	all/unsp	11	20	2	3	nev any	st
SADOWS	87	m	0	0	wh	-	all	NAmer	1938	CC	477	n	bl	n	n	1	ev	all/unsp	21	30	0	4	nev any	st
SADOWS	88	m	0	0	wh	-	all	NAmer	1938	CC	477	n	bl	n	n	1	ev	all/unsp	31	40	0	5	nev any	st

Table 1G11 - 9

IESLC - Meta-anal of Ever Smoking (or Curr if Ever not avail) by Amount, Overview, Any prod (or Cigs if Any not avail)
All LC types
Most adjusted - insufficient data for metaanalysis

REF	NRR	SEX	AGEL	AGEH	RACE	YF	LC TYPE	LOC	START	ST	NLC	R	VB	P	H	AD	SM	PRODUCT	exL	exH	S1	S2	DENOM	De
SADOWS	89	m	0	0	wh	-	all	NAmer	1938	CC	477	n	bl	n	n	1	ev	all/unsp	41	99	3	6	nev	any st

REF	NRR	RR	SIG	Cigarette equivalent	RRDATA comment
BLOT1	5	0.40		*	0
BLOT1	6	4.50		*	0
BLOT1	7	8.60		*	0
BUELL	1	2.32		*	0
BUELL	2	3.52		*	0
BUELL	3	4.93		*	0
DOLL	11	*		*	RR/CI not estimable because no cases or controls in exposed group
HITOSU	14	1.80		*	0
HITOSU	15	2.40		*	0
HITOSU	16	6.40		*	0
HITOSU	17	3.10		*	0
HITOSU	18	3.70		*	0
MASTRA	3	6.80		*	0
MASTRA	4	13.90		*	0
MZILEN	1	9.60		*	0
MZILEN	2	11.60		*	0
MZILEN	3	13.60		*	0
MZILEN	4	14.90		*	0
RESTRE	6	1.29		*	0
RESTRE	7	12.40		*	0
RESTRE	8	42.49		*	0
RESTRE	9	169.90		*	0
SADOWS	85	2.32		inc 1 cigar = 10, qtr can pipe = 10 cigs	0
SADOWS	86	4.70		inc 1 cigar = 10, qtr can pipe = 10 cigs	0
SADOWS	87	8.00		inc 1 cigar = 10, qtr can pipe = 10 cigs	0
SADOWS	88	5.33		inc 1 cigar = 10, qtr can pipe = 10 cigs	0
SADOWS	89	6.28		inc 1 cigar = 10, qtr can pipe = 10 cigs	0

Least adjusted - insufficient data for meta-analysis: as for adjusted plus the following

REF	NRR	SEX	AGEL	AGEH	RACE	YF	LC TYPE	LOC	START	ST	NLC	R	VB	P	H	AD	SM	PRODUCT	exL	exH	S1	S2	DENOM	De
SADOWS	80	m	0	0	wh	-	all	NAmer	1938	CC	477	n	bl	n	n	0	ev	all/unsp	1	10	1	0	nev	any st
SADOWS	81	m	0	0	wh	-	all	NAmer	1938	CC	477	n	bl	n	n	0	ev	all/unsp	11	20	2	3	nev	any st
SADOWS	82	m	0	0	wh	-	all	NAmer	1938	CC	477	n	bl	n	n	0	ev	all/unsp	21	30	0	4	nev	any st
SADOWS	83	m	0	0	wh	-	all	NAmer	1938	CC	477	n	bl	n	n	0	ev	all/unsp	31	40	0	5	nev	any st
SADOWS	84	m	0	0	wh	-	all	NAmer	1938	CC	477	n	bl	n	n	0	ev	all/unsp	41	99	3	6	nev	any st

REF	NRR	RR	SIG	Cigarette equivalent	RRDATA comment
SADOWS	80	1.83		inc 1 cigar = 10, qtr can pipe = 10 cigs	0
SADOWS	81	3.18		inc 1 cigar = 10, qtr can pipe = 10 cigs	0
SADOWS	82	4.28		inc 1 cigar = 10, qtr can pipe = 10 cigs	0
SADOWS	83	5.23		inc 1 cigar = 10, qtr can pipe = 10 cigs	0
SADOWS	84	5.28		inc 1 cigar = 10, qtr can pipe = 10 cigs	0

Table 1G12 -

IESLC - Meta-anal of Ever Smoking (or Current if ever not avail), Amount smoked, "Low", Any prod (or Cigs if Any not avail)
All LC types

This analysis is restricted to results for:
1) Results by Amount smoked
2) Results complete enough for use in metaanalysis

Within each study, results are then selected (in the following order of preference, within each sex) for:
3) SMKSTA: ever smokers, current smokers
4) PRODUCT: all/unspec, cigarettes regardless of other products, cigarettes only
5) CIGTYPE: all/unspecified, MC regardless of HR, MC only
6) DENOM: never smoked anything, never smoked cigarettes, (never +1 = +long term ex, +2 = +amount unknown, +3 = never cigs+long term ex)
7) Followup period (YF, prospective studies): whole study (coded as 0) or longest available
8) LCtype: all or nearest available, at least Squamous and Adeno. (q = squamous, s = small, l = large, a = adeno, mix = mixed, alv = alveolar)
9) Race: all or nearest available, otherwise by race (wh or w = white, bl or b = black, hi = hispanic ch = chinese, jap = japanese, haw = hawaiian, w+o = white + oriental, sca = scandinavian, as = asian)
10) Amount smoked "low" in key scheme 1 (key value 5, maximum range <20, in numbers of cigarettes or cigarette equivalents)
11) For overlapping studies: principal rather than subsidiary studies
Finally by Age: whole study (coded as 0) if available, otherwise by widest available age group
and then for single sex results (m, f) in preference to combined sex results (c).

Results adjusted (AD) for the most potential confounders are then chosen in Sections -1 to -3
and results adjusted for the least confounders in Sections -4 to -6. (Those least adjusted results which actually differ from the most adjusted as marked 'x' in column X in Section -4)
(Results adjusted for an unknown number of confounder(s) are coded as 20.)

Section -7 shows excluded studies, together with the stage (as above) at which no qualifying results were found.

Section -8 lists the potentially overlapping studies which have been included (1=principal, 2=subsidiary).

Section -9 lists any results which would have been included in preference except that they had data not complete enough for use in meta-analysis, with their significance (yes/no), if known, and any further comment as entered on the database.

In addition to those mentioned above, the following fields, levels and abbreviations are used:
* or nk = not known, n = no, y = yes, ot = other
ev = ever, cu = current, nev = never
all/unspec = all or unspecified, cig+/-ot = cigarettes irrespective of other products (cigar, pipe etc)
MC = manufactured cigarettes, HR = hand-rolled cigarettes
exL, exH = range of exposure (low and high) in the smoking group, in terms of Amount smoked, cigarettes or cigarette equivalents
REF: 6-character study reference
NRR: number of the RR on the database within the study
ST : study type (CC = case control, pr or prosp = prospective)
NLC: number of lung cancer cases in whole study
R : risky occupational population (n = no, m = mining, o = other risky)
VB : national cigarette type (V = at least 75% Virginia, bl = at least 75% blended, ot = other)
P : any proxy use
H : full histological confirmation
De : derivation of RR/CI (or = original, st = standard method, ot = other method of estimation)

Table 1G12 - 1

IESLC - Meta-anal of Ever Smoking (or Current if ever not avail), Amount smoked, "Low", Any prod (or Cigs if Any not avail)
All LC types
Most adjusted

REF	NRR	SEX	AGEL	AGEH	RACE	YF	LC TYPE	LOC	START	ST	NLC	R	VB	P	H	AD	SM	PRODUCT	exL	exH	DENOM	De
AGUDO	4	f	0	0	all	-	all	Eu:wst	1989	CC	103	n	bl	n	n	3	ev	cig only	1	10	nev any	or
AKIBA	27	m	0	0	all	0	all	As:Jap	1963	pr	610	n	bl	n	n	5	cu	cig+/-ot	1	14	nev cigs	or
AKIBA	33	f	0	0	all	0	all	As:Jap	1963	pr	610	n	bl	n	n	5	cu	cig+/-ot	1	14	nev cigs	or
ALDERS	18	m	0	0	all	-	all	Eu:UK	1977	CC	1448	n	V	n	n	1	ev	cig only	1	17	nev+2	ot
ALDERS	21	f	0	0	all	-	all	Eu:UK	1977	CC	1448	n	V	n	n	1	ev	cig only	1	17	nev+2	ot
ARCHER	1	m	0	0	wh	0	all	NAmer	1950	pr	146	m	bl	n	n	0	cu	cig+/-ot	1	19	nev cigs	st
ARMADA	46	m	0	0	all	-	all	Eu:wst	1986	CC	325	n	bl	n	y	0	ev	cig+/-ot	1	14	nev any	st
AUVINE	13	c	0	0	all	-	all	Eu:Sca	1986	CC	517	n	bl	y	n	2	ev	cig+/-ot	1	10	nev cigs	or
AXELSS	5	m	0	0	sca	-	all	Eu:Sca	1989	CC	436	n	bl	n	n	6	ev	all/unsp	1	9	nev any	ot
AXELSS	13	f	0	0	sca	-	all	Eu:Sca	1989	CC	436	n	bl	n	n	0	ev	all/unsp	1	9	nev any	st
BARBON	82	m	0	0	all	-	all	Eu:wst	1979	CC	755	n	bl	y	y	3	ev	all/unsp	1	19	nev any	or
BENSHL	11	m	40	64	all	10	all	Eu:UK	1967	pr	486	n	V	n	n	1	cu	cig+/-ot	1	9	nev any	ot
BEST	13	m	0	0	all	0	all	NAmer	1955	pr	381	n	V	n	n	1	cu	cig only	1	9	nev any	ot
BRESLO	13	m	0	0	all	-	all	NAmer	1949	CC	518	n	bl	n	y	0	ev	cig+/-ot	1	9	nev+3	st
BRESLO	29	f	0	0	all	-	all	NAmer	1949	CC	518	n	bl	n	y	0	ev	cig+/-ot	1	19	nev+3	st
BRETT	1	m	0	0	all	0	all	Eu:UK	1960	pr	150	n	V	n	n	0	cu	cig+/-ot	1	14	nev cigs	st
BROWN2	32	m	0	0	wh	-	all	NAmer	1984	CC	14596	n	bl	n	y	2	ev	cig+/-ot	1	19	nev cigs	or
BROWN2	31	f	0	0	wh	-	all	NAmer	1984	CC	14596	n	bl	n	y	2	ev	cig+/-ot	1	19	nev cigs	or
BUFFLE	28	f	0	0	w-hi	-	all	NAmer	1976	CC	943	n	bl	y	n	0	ev	cig+/-ot	1	19	nev cigs	or
CEDERL	80	m	0	0	all	16	all	Eu:Sca	1963	pr	491	n	bl	n	n	2	cu	all/unsp	1	7	nev any	ot
CEDERL	76	f	0	0	all	0	all	Eu:Sca	1963	pr	491	n	bl	n	n	2	cu	all/unsp	1	7	nev any	or
CHANG	2	m	0	0	all	0	all	NAmer	1972	pr	136	n	bl	n	n	0	cu	cig+/-ot	1	10	nev cigs	st
CHANG	8	f	0	0	all	0	all	NAmer	1972	pr	136	n	bl	n	n	0	cu	cig+/-ot	1	10	nev cigs	st
CHEN2	3	m	0	0	all	-	all	As:Chi	1983	CC	193	n	ot	y	n	0	ev	all/unsp	1	9	nev any	st
CHEN2	7	f	0	0	all	-	all	As:Chi	1983	CC	193	n	ot	y	n	0	ev	all/unsp	1	9	nev any	st
CHOI	12	m	0	0	all	-	all	As:oth	1985	CC	375	n	bl	n	n	0	ev	cig+/-ot	1	10	nev cigs	st
CHOI	17	f	0	0	all	-	all	As:oth	1985	CC	375	n	bl	n	n	0	ev	cig+/-ot	1	10	nev cigs	st
CHOW	10	m	0	0	wh	0	all	NAmer	1966	pr	219	n	bl	n	n	2	cu	cig+/-ot	1	19	nev any	ot
COMSTO	4	m	0	0	all	-	all	NAmer	1975	ot	258	n	bl	n	n	0	cu	cig+/-ot	1	19	nev any	st
COMSTO	9	f	0	0	all	-	all	NAmer	1975	ot	258	n	bl	n	n	0	cu	cig+/-ot	1	19	nev any	st
COOKSO	1	c	0	0	bl	-	all	Africa	1961	CC	234	n	V	n	y	0	ev	cig+/-ot	1	9	nev any	st
CPSI	243	m	50	74	all	6	all	NAmer	1959	pr	5138	n	bl	n	n	1	ev	cig only	1	19	nev any	ot
CPSI	275	f	40	74	all	6	all	NAmer	1959	pr	5138	n	bl	n	n	1	cu	cig+/-ot	1	9	nev cigs	ot
CPSII	120	m	0	0	all	6	all	NAmer	1982	pr	3229	n	bl	n	n	1	cu	cig only	1	9	nev any	ot
CPSII	105	f	35	99	all	4	all	NAmer	1982	pr	3229	n	bl	n	n	1	ev	cig+/-ot	1	19	nev cigs	ot
DAMBER	6	m	0	0	all	-	all	Eu:Sca	1972	CC	579	n	bl	y	n	1	ev	cig only	1	7	nev any	ot
DARBY	1	m	0	0	wh	-	all	Eu:UK	1988	CC	982	n	V	n	n	0	cu	cig+/-ot	1	14	nev any	st
DARBY	8	f	0	0	wh	-	all	Eu:UK	1988	CC	982	n	V	n	n	0	cu	cig+/-ot	1	14	nev any	st
DAVEYS	1	m	0	0	all	-	all	Eu:Ger	1930	CC	109	n	bl	y	n	0	ev	all/unsp	1	5	nev any	st
DEAN3	7	m	0	0	all	-	all	Eu:UK	1969	CC	766	n	V	y	n	3	cu	cig only	1	12	nev any	ot
DEAN3	91	f	0	0	all	-	all	Eu:UK	1969	CC	766	n	V	y	n	3	cu	cig only	1	12	nev any	ot
DEKLER	2	m	0	0	all	0	all	Auslia	1961	pr	138	m	V	n	n	2	cu	cig+/-ot	1	14	nev any	or
DESTEF	6	m	0	0	all	-	all	SCAmer	1988	CC	497	n	bl	n	y	4	ev	all/unsp	1	10	nev any	or
DOLL	2	m	0	0	all	-	all	Eu:UK	1948	CC	1465	n	V	n	n	0	ev	all/unsp	5	14	nev any	st
DOLL	8	f	0	0	all	-	all	Eu:UK	1948	CC	1465	n	V	n	n	0	ev	all/unsp	5	14	nev any	st
DOLL2	46	m	35	99	all	5	all	Eu:UK	1951	pr	920	n	V	n	n	1	ev	all/unsp	1	14	nev any	ot
DOLL2	10	f	0	0	all	22	all	Eu:UK	1951	pr	920	n	V	n	n	1	cu	cig only	1	14	nev any	ot
DORANT	6	c	0	0	all	0	all	Eu:wst	1986	ot	550	n	bl	n	y	0	cu	cig+/-ot	1	9	nev any	st
DORGAN	108	m	0	0	wh	-	all	NAmer	1980	CC	2026	n	bl	y	y	2	ev	cig+/-ot	1	19	nev any	ot
DORGAN	96	f	0	0	all	-	all	NAmer	1980	CC	2026	n	bl	y	y	3	ev	cig+/-ot	1	19	nev any	ot
DORN	408	m	0	0	wh	25	all	NAmer	1954	pr	5097	n	bl	n	n	1	cu	cig+/-ot	1	9	nev any	or
DOSEME	5	m	0	0	all	-	all	Eu:bal	1979	CC	1210	n	bl	n	n	2	ev	cig+/-ot	1	10	nev cigs	or
DUNN	2	m	0	0	all	0	all	NAmer	1954	pr	139	o	bl	n	n	0	ev	cig+/-ot	5	14	nev cigs	st
EBELIN	2	m	0	0	all	-	all	Eu:Ger	1980	CC	130	n	bl	n	n	0	ev	all/unsp	1	9	nev any	st
ENGELA	32	m	0	0	all	0	all	Eu:Sca	1964	pr	435	n	bl	n	n	7	cu	cig+/-ot	5	9	nev cigs	or
ENGELA	46	f	0	0	all	0	all	Eu:Sca	1964	pr	435	n	bl	n	n	5	cu	cig+/-ot	5	9	nev cigs	or
ENSTRO	7	m	0	0	all	0	all	NAmer	1959	pr	2879	n	bl	n	n	1	cu	cig only	1	9	nev any	ot
ENSTRO	11	f	0	0	all	0	all	NAmer	1959	pr	2879	n	bl	n	n	1	cu	cig only	1	9	nev any	ot
ESAKI	1	m	0	0	all	-	all	As:Jap	1961	CC	245	n	bl	y	n	0	ev	cig+/-ot	1	14	nev cigs	st
FAN	6	m	0	0	all	-	all	As:Chi	1990	CC	403	n	ot	y	n	0	ev	cig+/-ot	1	9	nev cigs	st
FAN	10	f	0	0	all	-	all	As:Chi	1990	CC	403	n	ot	y	n	0	ev	cig+/-ot	1	9	nev cigs	st
GAO	21	f	0	0	all	-	all	As:Chi	1984	CC	1405	n	ot	n	n	2	ev	cig+/-ot	1	9	nev cigs	ot
GAO2	2	m	0	0	all	-	all	As:Jap	1988	CC	282	n	bl	n	n	0	cu	cig+/-ot	1	19	nev cigs	st
GARSHI	26	m	0	0	all	-	all	NAmer	1981	CC	1081	o	bl	y	n	1	ev	all/unsp	1	15	nev any	st
GER	22	c	0	0	all	-	all	As:oth	1990	CC	141	n	ot	y	n	14	ev	all/unsp	1	10	nev any	ot
GOLLED	1	m	35	99	all	-	all	Eu:UK	1952	CC	443	n	V	y	n	1	ev	cig only	1	10	nev any	ot
GSELL	1	m	0	0	all	-	all	Eu:wst	1937	CC	150	n	bl	n	y	0	ev	all/unsp	1	9	nev any	st
HAMMO2	7	m	0	0	all	0	all	NAmer	1967	pr	450	o	bl	n	n	1	cu	cig+/-ot	1	19	nev any	ot
HAMMON	153	m	0	0	wh	0	all	NAmer	1952	pr	448	n	bl	n	n	1	ev	cig only	1	9	nev any	ot
HANSEN	1	m	0	0	all	0	all	Eu:Sca	1968	pr	105	o	bl	y	n	2	ev	all/unsp	1	19	nev any	ot
HIRAYA	23	m	0	0	all	0	all	As:Jap	1965	pr	1917	n	bl	n	n	1	cu	cig+/-ot	1	9	nev any	st
HIRAYA	26	f	0	0	all	0	all	As:Jap	1965	pr	1917	n	bl	n	n	1	cu	cig+/-ot	1	9	nev any	st

Table 1G12 - 1

IESLC - Meta-anal of Ever Smoking (or Current if ever not avail), Amount smoked, "Low", Any prod (or Cigs if Any not avail)
All LC types
Most adjusted

REF	NRR	SEX	AGEL	AGEH	RACE	YF	LC TYPE	LOC	START	ST	NLC	R	VB	P	H	AD	SM	PRODUCT	exL	exH	DENOM	De
HITOSU	35	m	0	0	all	-	all	As:Jap	1960	CC	216	n	bl	y	n	1	cu	all/unsp	1	14	nev any	st
HITOSU	60	f	0	0	all	-	all	As:Jap	1960	CC	216	n	bl	y	n	1	cu	all/unsp	1	14	nev any	st
HOLE	1	m	0	0	all	0	all	Eu:UK	1972	pr	225	n	V	n	n	1	cu	cig+/-ot	1	14	nev any	ot
HU	1	m	0	0	all	-	all	As:Chi	1985	CC	227	n	ot	n	y	0	ev	cig+/-ot	1	14	nev any	st
HU	4	f	0	0	all	-	all	As:Chi	1985	CC	227	n	ot	n	y	0	ev	cig+/-ot	1	14	nev any	st
HU2	3	c	0	0	all	-	all	As:Chi	1977	CC	523	n	ot	y	n	0	ev	cig+/-ot	5	9	nev cigs	st
HUMBLE	2	m	0	0	w-hi	-	all	NAmer	1980	CC	521	n	bl	y	n	1	cu	cig+/-ot	1	19	nev cigs	or
HUMBLE	5	m	0	0	hi	-	all	NAmer	1980	CC	521	n	bl	y	n	1	cu	cig+/-ot	1	19	nev cigs	or
HUMBLE	8	f	0	0	w-hi	-	all	NAmer	1980	CC	521	n	bl	y	n	1	cu	cig+/-ot	1	19	nev cigs	or
HUMBLE	11	f	0	0	hi	-	all	NAmer	1980	CC	521	n	bl	y	n	1	cu	cig+/-ot	1	19	nev cigs	or
JARUP	4	m	0	0	all	-	all	Eu:Sca	1928	CC	102	o	bl	y	n	2	ev	all/unsp	1	10	nev any	or
JEDRYC	45	m	0	0	all	-	all	Eu:est	1980	CC	1630	n	bl	y	n	4	ev	cig+/-ot	1	19	nev any	or
JEDRYC	48	f	0	0	all	-	all	Eu:est	1980	CC	1630	n	bl	y	n	4	ev	cig+/-ot	1	19	nev any	or
JOLY	7	m	0	0	all	-	all	SCAmer	1978	CC	826	n	bl	n	n	0	ev	cig+/-ot	1	9	nev any	st
JOLY	3	f	0	0	all	-	all	SCAmer	1978	CC	826	n	bl	n	n	0	ev	cig+/-ot	1	9	nev any	st
JUSSAW	34	m	0	0	all	-	all	As:Ind	1964	CC	792	n	V	n	n	2	ev	cig only	1	19	nev any	st
KAISE2	66	m	35	99	all	9	all	NAmer	1979	pr	318	n	bl	n	n	1	cu	cig only	1	19	nev any	st
KAISE2	58	f	35	99	all	9	all	NAmer	1979	pr	318	n	bl	n	n	1	cu	cig only	1	19	nev any	st
KAISER	6	m	0	0	all	0	all	NAmer	1964	pr	714	n	bl	n	n	2	cu	cig+/-ot	1	19	nev cigs	or
KAISER	2	f	0	0	all	0	all	NAmer	1964	pr	714	n	bl	n	n	2	cu	cig+/-ot	1	19	nev cigs	or
KANELL	26	m	0	0	all	-	all	Eu:bal	1950	CC	862	n	bl	n	n	1	cu	cig+/-ot	1	10	nev any	st
KATSOU	7	f	0	0	all	-	all	Eu:bal	1987	CC	101	n	bl	n	n	0	cu	all/unsp	1	10	nev any	st
KAUFMA	11	c	0	0	all	-	all	NAmer	1981	CC	881	n	bl	n	n	6	cu	cig+/-ot	1	14	nev cigs	or
KHUDER	1	m	0	0	all	-	all	NAmer	1985	CC	482	n	bl	n	y	0	ev	cig+/-ot	1	19	nev cigs	st
KINLEN	14	m	0	0	all	0	all	Eu:UK	1967	pr	718	n	V	n	n	2	cu	cig+/-ot	1	14	nev any	ot
KNEKT	29	m	20	69	all	21	all	Eu:Sca	1966	pr	515	n	bl	n	n	1	cu	cig+/-ot	1	14	nev any	or
KOO	11	f	0	0	all	-	all	As:HK	1981	CC	200	n	bl	n	n	0	cu	all/unsp	1	10	nev any	st
KOULUM	6	m	0	0	all	-	all	Eu:Sca	1936	CC	812	n	bl	n	n	0	ev	all/unsp	1	9	nev any	st
KREUZE	19	m	1	45	all	-	all	Eu:Ger	1990	CC	2260	n	bl	n	n	3	ev	cig+/-ot	1	9	nev any	or
KREUZE	30	m	55	69	all	-	all	Eu:Ger	1990	CC	2260	n	bl	n	n	3	ev	cig+/-ot	1	9	nev any	or
KREUZE	25	f	1	45	all	-	all	Eu:Ger	1990	CC	2260	n	bl	n	n	3	ev	cig+/-ot	1	9	nev any	or
KREUZE	36	f	55	69	all	-	all	Eu:Ger	1990	CC	2260	n	bl	n	n	3	ev	cig+/-ot	1	9	nev any	or
KREYBE	9	m	0	0	all	-	all	Eu:Sca	1948	CC	300	n	bl	n	y	1	ev	all/unsp	1	14	nev any	ot
KREYBE	28	f	0	0	all	-	all	Eu:Sca	1948	CC	300	n	bl	n	y	1	ev	all/unsp	1	14	nev any	ot
LAMTH	7	f	0	0	ch	-	all	As:HK	1983	CC	445	n	bl	n	n	0	ev	all/unsp	1	10	nev any	or
LAUSSM	18	m	0	0	all	-	all	Eu:Ger	1982	CC	432	n	bl	n	n	3	ev	all/unsp	1	9	nev any	ot
LETOUR	2	c	0	0	all	-	all	NAmer	1983	CC	738	n	V	y	y	0	ev	cig+/-ot	1	19	nev cigs	st
LIAW	3	c	0	0	all	0	all	As:oth	1982	pr	127	n	ot	n	n	2	cu	all/unsp	1	10	nev any	or
LIDDEL	2	m	0	0	all	18	all	NAmer	1970	pr	304	m	V	n	n	1	cu	cig+/-ot	1	19	nev cigs	ot
LIU2	8	m	0	0	all	-	all	As:Chi	1983	CC	316	n	ot	n	n	3	ev	all/unsp	1	19	nev any	or
LIU2	14	f	0	0	all	-	all	As:Chi	1983	CC	316	n	ot	n	n	3	ev	all/unsp	1	9	nev any	or
LIU3	6	m	0	0	all	-	all	As:Chi	1985	CC	110	n	ot	n	n	2	ev	all/unsp	1	15	nev any	or
LIU4	7	m	35	69	all	-	all	As:Chi	1986	CC	1000-00	n	ot	y	n	2	ev	cig only	1	19	nev any	ot
LIU5	2	c	0	0	all	-	all	As:Chi	1978	CC	111	n	ot	y	n	0	ev	all/unsp	1	9	nev any	st
LUBIN	11	m	0	0	all	-	all	As:Chi	1984	CC	427	m	ot	y	n	4	ev	cig only	1	6	nev any	ot
LUBIN2	273	m	0	0	all	-	all	Eu:mul	1976	CC	7804	n	bl	n	y	0	ev	cig+/-ot	1	9	nev any	st
LUBIN2	281	f	0	0	all	-	all	Eu:mul	1976	CC	7804	n	bl	n	y	0	ev	cig+/-ot	1	9	nev any	st
MACLEN	36	c	0	0	ch	-	all	As:oth	1972	CC	233	n	bl	n	n	2	ev	cig+/-ot	1	9	nev cigs	or
MARTIS	1	m	0	0	all	-	all	Eu:UK	1972	CC	201	n	V	n	n	0	ev	cig+/-ot	1	14	nev cigs	st
MATOS	29	m	0	0	all	-	all	SCAmer	1994	CC	200	n	bl	n	n	2	ev	cig+/-ot	1	14	nev any	or
MATSUD	1	m	0	0	all	-	all	As:Jap	1965	CC	179	n	bl	n	n	0	ev	cig+/-ot	1	10	nev cigs	st
MCCONN	26	c	0	0	all	-	all	Eu:UK	1946	CC	100	n	V	n	y	0	ev	all/unsp	1	10	nev any	st
MIGRAN	2	m	0	0	all	0	all	Eu:UK	1964	pr	259	n	V	n	n	2	cu	cig only	1	9	nev any	ot
MIGRAN	29	f	0	0	all	0	all	Eu:UK	1964	pr	259	n	V	n	n	2	cu	cig only	1	9	nev any	ot
MRFITR	3	m	0	0	all	0	all	NAmer	1973	pr	119	n	bl	n	n	0	cu	cig+/-ot	1	19	nev cigs	ot
NOTAN2	8	m	0	0	all	-	all	As:Ind	1963	CC	683	n	V	n	n	0	ev	cig only	1	9	nev any	st
ORMOS	1	m	0	0	all	-	all	Eu:est	1947	CC	119	n	bl	y	y	0	ev	cig+/-ot	1	15	nev any	st
PARKIN	14	m	0	0	bl	-	all	Africa	1963	CC	877	n	V	y	n	6	cu	all/unsp	1	14	nev any	or
PASTOR	6	m	0	0	all	-	all	Eu:wst	1976	CC	204	n	bl	y	n	1	ev	all/unsp	1	9	nev any	st
PERNU	18	m	0	0	all	-	all	Eu:Sca	1944	CC	1606	n	bl	n	n	0	ev	all/unsp	5	9	nev any	st
PERNU	12	f	0	0	all	-	all	Eu:Sca	1944	CC	1606	n	bl	n	n	0	ev	all/unsp	5	9	nev any	st
PERSH2	8	c	0	0	all	-	all	Eu:Sca	1980	CC	1022	n	bl	y	n	4	cu	all/unsp	1	9	nev any	ot
PETO	2	m	0	0	all	0	all	Eu:UK	1954	pr	103	n	V	n	n	0	cu	all/unsp	1	14	nev any	st
POLEDN	2	c	0	0	all	-	all	NAmer	1978	CC	209	n	bl	y	n	0	ev	cig+/-ot	1	19	nev cigs	st
PRESCO	2	m	0	0	all	0	all	Eu:Sca	1964	pr	867	n	bl	n	n	1	cu	all/unsp	1	14	nev any	st
PRESCO	1	f	0	0	all	0	all	Eu:Sca	1964	pr	867	n	bl	n	n	1	cu	all/unsp	1	14	nev any	st
RACHTA	10	f	0	0	all	-	all	Eu:est	1991	CC	118	n	bl	n	y	1	ev	cig+/-ot	1	9	nev cigs	or
RANDIG	2	m	0	0	all	-	all	Eu:Ger	1951	CC	448	n	bl	n	n	0	ev	all/unsp	5	9	nev any	st
RANDIG	6	f	0	0	all	-	all	Eu:Ger	1951	CC	448	n	bl	n	n	0	ev	all/unsp	5	9	nev any	st
SEGI2	10	m	0	0	all	-	all	As:Jap	1962	CC	378	n	bl	n	n	1	cu	cig+/-ot	1	9	nev any	ot
SEGI2	22	f	0	0	all	-	all	As:Jap	1962	CC	378	n	bl	n	n	1	cu	cig+/-ot	1	9	nev any	ot

Table 1G12 - 1

IESLC - Meta-anal of Ever Smoking (or Current if ever not avail), Amount smoked, "Low", Any prod (or Cigs if Any not avail)
All LC types
Most adjusted

REF	NRR	SEX	AGEL	AGEH	RACE	YF	LC TYPE	LOC	START	ST	NLC	R	VB	P	H	AD	SM	PRODUCT	exL	exH	DENOM	De
SHAW	10	c	0	0	wh	-	all	NAmer	1988	CC	335	n	V	n	y	0	ev	all/unsp	1	19	nev any	st
SIEMIA	13	m	0	0	all	-	all	NAmer	1979	CC	857	n	V	y	y	0	ev	cig+/-ot	1	19	nev cigs	or
SOBUE	117	m	0	0	all	-	q+s+l+a	As:Jap	1986	CC	1376	n	bl	n	y	0	cu	cig+/-ot	1	19	nev cigs	st
SPEIZE	2	f	0	0	all	0	all	NAmer	1976	pr	593	n	bl	n	y	1	cu	cig+/-ot	5	14	nev cigs	ot
SPITZ	5	c	0	0	b+hi	-	all	NAmer	1992	CC	177	n	bl	n	y	0	ev	cig+/-ot	1	19	nev cigs	st
STOCKS	41	m	0	0	all	-	all	Eu:UK	1952	CC	2932	n	V	y	n	2	ev	cig+/-ot	1	14	nev any	st
STOCKS	48	f	0	0	all	-	all	Eu:UK	1952	CC	2932	n	V	y	n	1	ev	cig+/-ot	1	14	nev any	ot
STOCKW	1	c	0	0	all	-	all	NAmer	1981	CC	22161	n	bl	n	n	0	cu	cig+/-ot	1	19	nev any	st
SVENSS	6	f	0	0	all	-	all	Eu:Sca	1983	CC	210	n	bl	n	n	1	cu	all/unsp	1	10	nev any	or
TENKAN	10	m	0	0	all	17	all	Eu:Sca	1962	pr	242	n	bl	n	n	1	cu	all/unsp	1	14	nev any	ot
TIZZAN	7	m	0	0	all	-	all	Eu:wst	1959	CC	1358	n	bl	n	n	0	ev	cig only	1	9	nev any	st
TIZZAN	15	f	0	0	all	-	all	Eu:wst	1959	CC	1358	n	bl	n	n	0	ev	cig only	1	9	nev any	st
TSUGAN	29	m	0	0	all	-	q+a	As:Jap	1976	CC	134	n	bl	n	y	0	cu	all/unsp	1	15	nev any	st
TULINI	27	m	0	0	all	0	all	Eu:Sca	1967	pr	472	n	bl	n	n	3	cu	cig+/-ot	1	14	nev any	or
TULINI	32	f	0	0	all	0	all	Eu:Sca	1967	pr	472	n	bl	n	n	3	cu	cig+/-ot	1	14	nev any	or
TVERDA	9	m	0	0	all	0	all	Eu:Sca	1972	pr	238	n	bl	n	n	2	cu	cig only	1	9	nev cigs	ot
TVERDA	16	f	0	0	all	0	all	Eu:Sca	1972	pr	238	n	bl	n	n	2	cu	cig only	1	9	nev cigs	ot
WAKAI	40	m	0	0	all	-	all	As:Jap	1988	CC	333	n	bl	n	y	2	cu	cig+/-ot	1	19	nev any	or
WANG2	10	c	0	0	all	-	all	As:Chi	1980	CC	103	n	ot	n	n	4	ev	cig+/-ot	5	9	nev cigs	ot
WUWILL	12	f	0	0	all	-	all	As:Chi	1985	CC	965	n	ot	n	n	3	ev	cig+/-ot	1	19	nev cigs	ot
WYNDE2	17	m	0	0	all	-	all	NAmer	1962	CC	404	n	bl	n	y	0	ev	cig+/-ot	1	10	nev any	st
WYNDE3	44	m	0	0	all	-	all	NAmer	1966	CC	350	n	bl	n	y	0	ev	cig+/-ot	1	9	nev any	st
WYNDE3	79	f	0	0	all	-	all	NAmer	1966	CC	350	n	bl	n	y	0	ev	cig+/-ot	1	9	nev any	st
WYNDE4	43	m	0	0	all	-	all	NAmer	1948	CC	684	n	bl	y	n	0	ev	all/unsp	1	9	nev any	st
WYNDE4	57	f	0	0	all	-	all	NAmer	1948	CC	684	n	bl	y	n	2	ev	all/unsp	1	9	nev any	ot
WYNDE6	27	m	0	0	all	-	all	NAmer	1969	CC	4423	n	bl	n	y	0	cu	cig+/-ot	1	10	nev any	st
WYNDE6	216	f	0	0	all	-	all	NAmer	1969	CC	4423	n	bl	n	y	0	cu	cig+/-ot	1	10	nev cigs	st
XU3	9	m	0	0	all	-	all	As:Chi	1981	CC	135	n	ot	n	n	1	ev	all/unsp	1	9	nev any	ot
XU3	16	f	0	0	all	-	all	As:Chi	1981	CC	135	n	ot	n	n	1	ev	all/unsp	1	9	nev any	ot
YUAN	2	m	0	0	all	0	all	As:Chi	1986	pr	142	n	ot	n	n	2	ev	cig+/-ot	1	19	nev cigs	ot
ZHENG	11	m	0	0	all	-	all	As:Chi	1982	CC	540	n	ot	*	y	0	ev	cig+/-ot	1	9	nev cigs	st
ZHENG	22	f	0	0	all	-	all	As:Chi	1982	CC	540	n	ot	*	y	0	ev	cig+/-ot	1	9	nev cigs	st
ZHOU	4	c	0	0	all	-	all	As:Chi	1978	CC	1360	n	ot	n	n	0	ev	all/unsp	1	9	nev any	st

Cigarette type is all/unspec for all RRs
except for the following:

REF	NRR	CIGTYPE
ALDERS	18	MC only
ALDERS	21	MC only
DEAN3	7	MC only
DEAN3	91	MC only
JUSSAW	34	MC only
NOTAN2	8	MC only

Table 1G12 - 2

IESLC - Meta-anal of Ever Smoking (or Current if ever not avail), Amount smoked, "Low", Any prod (or Cigs if Any not avail)
All LC types
Most adjusted

REF	NRR	SEX	AD	Number Exposed Case	Cont	Non-exposed Case	Cont	RR	95.00%CI
AGUDO	4	f	3	-	-	-	-	1.57	(0.52- 4.70)
*AKIBA	27	m	5	-	-	-	-	3.50	(2.20- 6.00)
*AKIBA	33	f	5	-	-	-	-	3.60	(2.60- 5.00)
Subtotal AKIBA								3.57	(2.71- 4.69)
ALDERS	18	m	1	-	-	-	-	3.55	(1.94- 6.49)
ALDERS	21	f	1	-	-	-	-	2.62	(1.88- 3.65)
Subtotal ALDERS								2.81	(2.10- 3.76)
*ARCHER	1	m	0	14	6504	6	9842	3.53	(1.36- 9.18)
ARMADA	46	m	0	44	117	4	64	6.02	(2.07- 17.51)
AUVINE	13	c	2	-	-	-	-	20.00	(9.72- 41.20)
AXELSS	5	m	6	-	-	-	-	3.82	(1.98- 7.36)
AXELSS	13	f	0	13	37	18	154	3.01	(1.35- 6.68)
Subtotal AXELSS								3.47	(2.09- 5.76)
BARBON	82	m	3	-	-	-	-	6.70	(4.20- 11.00)
*BENSHL	11	m	1	-	-	-	-	4.00	(1.55- 10.31)
*BEST	13	m	1	-	-	-	-	10.00	(4.56- 21.92)
BRESLO	13	m	0	16	45	22	110	1.78	(0.86- 3.69)
BRESLO	29	f	0	5	5	12	14	1.17	(0.27- 5.02)
Subtotal BRESLO								1.63	(0.85- 3.14)
*BRETT	1	m	0	40	17090	6	6530	2.55	(1.08- 6.01)
BROWN2	32	m	2	-	-	-	-	6.10	(5.30- 6.90)
BROWN2	31	f	2	-	-	-	-	8.40	(7.20- 9.70)
Subtotal BROWN2								7.02	(6.36- 7.75)
BUFFLE	28	f	0	21	42	12	112	4.67	(2.11- 10.31)
*CEDERL	80	m	2	-	-	-	-	3.40	(1.96- 5.90)
*CEDERL	76	f	2	-	-	-	-	2.83	(1.72- 4.67)
Subtotal CEDERL								3.07	(2.12- 4.45)
*CHANG	2	m	0	5	100	5	502	5.02	(1.48- 17.02)
*CHANG	8	f	0	6	205	11	1139	3.03	(1.13- 8.10)
Subtotal CHANG								3.70	(1.72- 7.95)
CHEN2	3	m	0	17	26	9	33	2.40	(0.92- 6.25)
CHEN2	7	f	0	5	17	25	33	0.39	(0.13- 1.20)
Subtotal CHEN2								1.12	(0.54- 2.31)
CHOI	12	m	0	20	90	13	95	1.62	(0.76- 3.46)
CHOI	17	f	0	9	16	76	164	1.21	(0.51- 2.87)
Subtotal CHOI								1.43	(0.81- 2.52)
*CHOW	10	m	2	-	-	-	-	13.88	(5.81- 33.12)
COMSTO	4	m	0	18	25	4	69	12.42	(3.83- 40.26)
COMSTO	9	f	0	16	19	13	115	7.45	(3.10- 17.93)
Subtotal COMSTO								8.95	(4.43- 18.08)
COOKSO	1	c	0	102	27	45	61	5.12	(2.89- 9.08)
*CPSI	243	m	1	-	-	-	-	5.81	(4.33- 7.80)
*CPSI	275	f	1	-	-	-	-	1.25	(0.73- 2.13)
Subtotal CPSI								4.07	(3.14- 5.26)
*CPSII	120	m	1	-	-	-	-	12.22	(9.12- 16.36)
*CPSII	105	f	1	-	-	-	-	4.16	(3.41- 5.09)
Subtotal CPSII								5.87	(4.98- 6.93)
DAMBER	6	m	1	-	-	-	-	2.30	(1.30- 4.40)
DARBY	1	m	0	128	223	3	384	73.47	(23.11- 233.57)
DARBY	8	f	0	71	104	23	529	15.70	(9.38- 26.28)
Subtotal DARBY								20.27	(12.66- 32.45)
DAVEYS	1	m	0	11	69	3	23	1.22	(0.31- 4.77)
DEAN3	7	m	3	-	-	-	-	5.46	(3.27- 9.10)
DEAN3	91	f	3	-	-	-	-	3.16	(1.92- 5.21)
Subtotal DEAN3								4.13	(2.89- 5.90)
*DEKLER	2	m	2	-	-	-	-	19.40	(2.60- 143.70)
DESTEF	6	m	4	-	-	-	-	2.90	(1.60- 5.00)
DOLL	2	m	0	489	570	7	61	7.48	(3.39- 16.50)
DOLL	8	f	0	24	18	40	59	1.97	(0.95- 4.09)
Subtotal DOLL								3.64	(2.13- 6.22)
*DOLL2	46	m	1	-	-	-	-	6.71	(0.91- 49.81)
*DOLL2	10	f	1	-	-	-	-	1.29	(0.14- 11.50)
Subtotal DOLL2								3.19	(0.72- 14.02)
DORANT	6	c	0	21	192	14	1090	8.52	(4.26- 17.04)
DORGAN	108	m	2	-	-	-	-	6.88	(3.74- 12.66)
DORGAN	96	f	3	-	-	-	-	5.67	(4.36- 7.36)
Subtotal DORGAN								5.84	(4.59- 7.43)
*DORN	408	m	1	-	-	-	-	4.02	(3.43- 4.71)
DOSEME	5	m	2	-	-	-	-	2.20	(1.40- 3.30)
*DUNN	2	m	0	12	9418	2	14160	9.02	(2.02- 40.30)
EBELIN	2	m	0	20	72	12	117	2.71	(1.25- 5.87)

Table 1G12 - 2

IESLC - Meta-anal of Ever Smoking (or Current if ever not avail), Amount smoked, "Low", Any prod (or Cigs if Any not avail)
All LC types
Most adjusted

REF	NRR	SEX	AD	Number Exposed Case	Cont	Non-exposed Case	Cont	RR	95.00%CI
*ENGELA	32	m	7	-	-	-	-	4.10	(1.70- 10.00)
*ENGELA	46	f	5	-	-	-	-	12.00	(4.40- 30.00)
Subtotal ENGELA								6.72	(3.50- 12.89)
*ENSTRO	7	m	1	-	-	-	-	4.74	(3.34- 6.73)
*ENSTRO	11	f	1	-	-	-	-	2.15	(1.62- 2.84)
Subtotal ENSTRO								2.93	(2.35- 3.65)
ESAKI	1	m	0	47	75	16	28	1.10	(0.54- 2.24)
FAN	6	m	0	13	121	36	236	0.70	(0.36- 1.38)
FAN	10	f	0	17	48	69	320	1.64	(0.89- 3.03)
Subtotal FAN								1.12	(0.71- 1.76)
GAO	21	f	2	-	-	-	-	1.74	(1.22- 2.48)
GAO2	2	m	0	32	41	13	56	3.36	(1.57- 7.19)
GARSHI	26	m	1	-	-	-	-	3.29	(2.22- 4.87)
GER	22	c	14	-	-	-	-	1.26	(0.61- 2.61)
GOLLED	1	m	1	-	-	-	-	4.45	(2.54- 7.81)
GSELL	1	m	0	11	36	2	29	4.43	(0.91- 21.60)
*HAMMO2	7	m	1	-	-	-	-	9.15	(3.62- 23.12)
*HAMMON	153	m	1	-	-	-	-	7.38	(3.72- 14.63)
*HANSEN	1	m	2	-	-	-	-	1.37	(0.63- 3.54)
*HIRAYA	23	m	1	-	-	-	-	2.06	(1.49- 2.85)
*HIRAYA	26	f	1	-	-	-	-	2.25	(1.64- 3.08)
Subtotal HIRAYA								2.16	(1.72- 2.70)
HITOSU	35	m	1	-	-	-	-	2.08	(0.90- 4.83)
HITOSU	60	f	1	-	-	-	-	3.11	(1.77- 5.46)
Subtotal HITOSU								2.75	(1.72- 4.38)
*HOLE	1	m	1	-	-	-	-	5.47	(2.35- 12.75)
HU	1	m	0	36	38	41	67	1.55	(0.85- 2.82)
HU	4	f	0	19	10	40	48	2.28	(0.95- 5.46)
Subtotal HU								1.75	(1.07- 2.87)
HU2	3	c	0	44	58	121	213	1.34	(0.85- 2.10)
HUMBLE	2	m	1	-	-	-	-	9.20	(3.30- 25.80)
HUMBLE	5	m	1	-	-	-	-	11.60	(2.70- 61.50)
HUMBLE	8	f	1	-	-	-	-	19.20	(6.50- 60.80)
HUMBLE	11	f	1	-	-	-	-	18.50	(4.90- 72.40)
Subtotal HUMBLE								13.65	(7.44- 25.07)
JARUP	4	m	2	-	-	-	-	6.90	(2.40- 22.70)
JEDRYC	45	m	4	-	-	-	-	3.48	(2.33- 5.19)
JEDRYC	48	f	4	-	-	-	-	6.37	(2.66- 15.24)
Subtotal JEDRYC								3.87	(2.69- 5.56)
JOLY	7	m	0	16	54	12	218	5.38	(2.41- 12.05)
JOLY	3	f	0	33	38	52	283	4.73	(2.72- 8.21)
Subtotal JOLY								4.93	(3.12- 7.77)
JUSSAW	34	m	2	-	-	-	-	5.57	(2.46- 14.76)
*KAISE2	66	m	1	-	-	-	-	4.47	(2.00- 9.99)
*KAISE2	58	f	1	-	-	-	-	7.61	(3.26- 17.75)
Subtotal KAISE2								5.75	(3.21- 10.31)
*KAISER	6	m	2	-	-	-	-	6.58	(3.87- 11.20)
*KAISER	2	f	2	-	-	-	-	3.42	(2.17- 5.40)
Subtotal KAISER								4.51	(3.19- 6.38)
KANELL	26	m	1	-	-	-	-	1.71	(1.15- 2.56)
KATSOU	7	f	0	8	9	48	67	1.24	(0.45- 3.45)
KAUFMA	11	c	6	-	-	-	-	8.00	(5.00- 13.00)
KHUDER	1	m	0	81	434	23	309	2.51	(1.54- 4.07)
*KINLEN	14	m	2	-	-	-	-	10.61	(5.01- 22.48)
*KNEKT	29	m	1	-	-	-	-	5.00	(2.00- 12.30)
KOO	11	f	0	17	19	56	85	1.36	(0.65- 2.84)
KOULUM	6	m	0	37	77	5	54	5.19	(1.92- 14.06)
KREUZE	19	m	3	-	-	-	-	2.50	(0.70- 8.20)
KREUZE	30	m	3	-	-	-	-	8.20	(5.20- 13.00)
KREUZE	25	f	3	-	-	-	-	5.70	(1.60- 16.60)
KREUZE	36	f	3	-	-	-	-	2.00	(1.20- 3.30)
Subtotal KREUZE								4.27	(3.12- 5.85)
KREYBE	9	m	1	-	-	-	-	5.82	(2.56- 13.23)
KREYBE	28	f	1	-	-	-	-	1.36	(0.65- 2.82)
Subtotal KREYBE								2.59	(1.50- 4.48)
LAMTH	7	f	0	101	63	202	337	2.67	(1.87- 3.83)
LAUSSM	18	m	3	-	-	-	-	3.29	(2.31- 4.66)
LETOUR	2	c	0	271	266	24	224	9.51	(6.04- 14.97)
*LIAW	3	c	2	-	-	-	-	3.10	(1.70- 5.60)
*LIDDEL	2	m	1	-	-	-	-	3.33	(2.05- 5.64)
LIU2	8	m	3	-	-	-	-	1.20	(0.43- 3.50)

Table 1G12 - 2

IESLC - Meta-anal of Ever Smoking (or Current if ever not avail), Amount smoked, "Low", Any prod (or Cigs if Any not avail)
All LC types
Most adjusted

REF	NRR	SEX	AD	Number Exposed Case	Number Exposed Cont	Non-exposed Case	Non-exposed Cont	RR	95.00%CI
LIU2	14	f	3	-	-	-	-	1.80	(0.57- 5.90)
Subtotal LIU2								1.44	(0.66- 3.14)
LIU3	6	m	2	-	-	-	-	1.41	(0.33- 6.09)
LIU4	7	m	2	-	-	-	-	2.11	(2.02- 2.20)
LIU5	2	c	0	14	27	26	41	0.82	(0.36- 1.84)
LUBIN	11	m	4	-	-	-	-	0.72	(0.20- 2.54)
LUBIN2	273	m	0	1887	3759	190	2616	6.91	(5.91- 8.09)
LUBIN2	281	f	0	151	218	336	1188	2.45	(1.93- 3.11)
Subtotal LUBIN2								5.06	(4.44- 5.77)
MACLEN	36	c	2	-	-	-	-	1.35	(0.64- 2.84)
MARTIS	1	m	0	31	39	4	25	4.97	(1.56- 15.78)
MATOS	29	m	2	-	-	-	-	2.00	(0.90- 4.50)
MATSUD	1	m	0	37	1237	3	1255	12.51	(3.85- 40.69)
MCCONN	26	c	0	7	43	9	23	0.42	(0.14- 1.26)
*MIGRAN	2	m	2	-	-	-	-	4.01	(1.19- 13.47)
*MIGRAN	29	f	2	-	-	-	-	4.88	(1.17- 20.43)
Subtotal MIGRAN								4.35	(1.73- 10.98)
*MRFITR	3	m	0	2	856	0	1859	10.86~	(0.52- 225.86)
NOTAN2	8	m	0	6	42	134	544	0.58	(0.24- 1.39)
ORMOS	1	m	0	32	329	7	777	10.80	(4.72- 24.71)
PARKIN	14	m	6	-	-	-	-	3.90	(3.00- 5.00)
PASTOR	6	m	1	-	-	-	-	2.33	(0.78- 6.95)
PERNU	18	m	0	61	31	97	275	5.58	(3.42- 9.11)
PERNU	12	f	0	5	13	110	971	3.40	(1.19- 9.70)
Subtotal PERNU								5.10	(3.27- 7.96)
PERSH2	8	c	4	-	-	-	-	5.76	(4.61- 7.19)
*PETO	2	m	0	44	1181	2	295	5.50	(1.34- 22.54)
POLEDN	2	c	0	53	103	12	139	5.96	(3.03- 11.72)
*PRESCO	2	m	1	-	-	-	-	10.20	(4.49- 23.15)
*PRESCO	1	f	1	-	-	-	-	6.36	(3.60- 11.24)
Subtotal PRESCO								7.42	(4.65- 11.84)
RACHTA	10	f	1	-	-	-	-	3.64	(1.08- 12.32)
RANDIG	2	m	0	65	99	5	22	2.89	(1.04- 8.01)
RANDIG	6	f	0	12	13	17	92	5.00	(1.95- 12.79)
Subtotal RANDIG								3.88	(1.95- 7.76)
SEGI2	10	m	1	-	-	-	-	2.10	(0.86- 5.16)
SEGI2	22	f	1	-	-	-	-	2.90	(1.09- 7.70)
Subtotal SEGI2								2.43	(1.26- 4.71)
SHAW	10	c	0	46	90	11	107	4.97	(2.43- 10.16)
SIEMIA	13	m	0	-	-	-	-	3.00	(1.00- 9.90)
SOBUE	117	m	0	147	157	34	128	3.52	(2.27- 5.47)
*SPEIZE	2	f	1	-	-	-	-	5.20	(3.60- 8.10)
SPITZ	5	c	0	27	88	10	96	2.95	(1.35- 6.43)
STOCKS	41	m	2	-	-	-	-	4.64	(3.25- 6.61)
STOCKS	48	f	1	-	-	-	-	2.24	(1.64- 3.03)
Subtotal STOCKS								3.06	(2.43- 3.86)
STOCKW	1	c	0	2090	1194	2791	10641	6.67	(6.15- 7.25)
SVENSS	6	f	1	-	-	-	-	4.60	(2.50- 9.30)
*TENKAN	10	m	1	-	-	-	-	15.86	(6.80- 37.00)
TIZZAN	7	m	0	130	238	180	305	0.93	(0.70- 1.23)
TIZZAN	15	f	0	11	14	25	114	3.58	(1.46- 8.82)
Subtotal TIZZAN								1.04	(0.80- 1.37)
TSUGAN	29	m	0	14	19	18	22	0.90	(0.36- 2.28)
*TULINI	27	m	3	-	-	-	-	6.02	(3.01- 12.00)
*TULINI	32	f	3	-	-	-	-	8.17	(4.33- 15.40)
Subtotal TULINI								7.11	(4.45- 11.34)
*TVERDA	9	m	2	-	-	-	-	2.14	(1.15- 3.96)
*TVERDA	16	f	2	-	-	-	-	4.53	(1.08- 18.94)
Subtotal TVERDA								2.41	(1.36- 4.25)
WAKAI	40	m	2	-	-	-	-	1.80	(0.81- 4.02)
WANG2	10	c	4	-	-	-	-	1.22	(0.31- 4.83)
WUWILL	12	f	3	-	-	-	-	2.13	(1.75- 2.58)
WYNDE2	17	m	0	17	114	8	105	1.96	(0.81- 4.72)
WYNDE3	44	m	0	8	42	9	88	1.86	(0.67- 5.17)
WYNDE3	79	f	0	3	19	20	76	0.60	(0.16- 2.23)
Subtotal WYNDE3								1.22	(0.54- 2.72)
WYNDE4	43	m	0	17	82	12	115	1.99	(0.90- 4.38)
WYNDE4	57	f	2	-	-	-	-	1.13	(0.33- 3.89)
Subtotal WYNDE4								1.69	(0.87- 3.28)
WYNDE6	27	m	0	117	122	87	617	6.80	(4.85- 9.54)
WYNDE6	216	f	0	76	109	159	856	3.75	(2.68- 5.26)

Table 1G12 - 2

IESLC - Meta-anal of Ever Smoking (or Current if ever not avail), Amount smoked, "Low", Any prod (or Cigs if Any not avail)
All LC types
Most adjusted

REF	NRR	SEX	AD	Number Exposed Case	Cont	Non-exposed Case	Cont	RR	95.00%CI
Subtotal WYNDE6								5.05	(3.98- 6.42)
XU3	9	m	1	-	-	-	-	1.66	(0.55- 4.97)
XU3	16	f	1	-	-	-	-	2.18	(0.61- 7.84)
Subtotal XU3								1.86	(0.81- 4.29)
*YUAN	2	m	2	-	-	-	-	3.60	(1.88- 6.91)
ZHENG	11	m	0	25	40	33	94	1.78	(0.94- 3.37)
ZHENG	22	f	0	24	29	152	184	1.00	(0.56- 1.79)
Subtotal ZHENG								1.30	(0.85- 2.00)
ZHOU	4	c	0	61	5	507	68	1.64	(0.64- 4.22)
Partial Totals				7130	46770	6153	61772		
*prospective study								~ With 0.5 adjustment for zero	

REF	NRR	SEX	AD	Ys	Ws	Qs	Ps
AGUDO	4	f	3	0.45	3.17	1.73	0.4219
*AKIBA	27	m	5	1.25	15.26	0.06	0.0000
*AKIBA	33	f	5	1.28	35.93	0.30	0.0000
Subtotal AKIBA				1.27	51.20	0.36	
ALDERS	18	m	1	1.27	10.54	0.06	0.0000
ALDERS	21	f	1	0.96	34.91	1.79	0.0000
Subtotal ALDERS				1.03	45.45	1.85	
*ARCHER	1	m	0	1.26	4.20	0.02	0.0097
ARMADA	46	m	0	1.79	3.37	1.23	0.0010
AUVINE	13	c	2	3.00	7.37	24.03	0.0000
AXELSS	5	m	6	1.34	8.91	0.20	0.0001
AXELSS	13	f	0	1.10	6.02	0.05	0.0069
Subtotal AXELSS				1.24	14.94	0.25	
BARBON	82	m	3	1.90	16.58	8.41	0.0000
*BENSHL	11	m	1	1.39	4.28	0.17	0.0041
*BEST	13	m	1	2.30	6.23	7.72	0.0000
BRESLO	13	m	0	0.58	7.18	2.71	0.1231
BRESLO	29	f	0	0.15	1.80	1.93	0.8360
Subtotal BRESLO				0.49	8.98	4.64	
*BRETT	1	m	0	0.94	5.22	0.34	0.0326
BROWN2	32	m	2	1.81	220.78	84.48	0.0000
BROWN2	31	f	2	2.13	172.98	152.37	0.0000
Subtotal BROWN2				1.95	393.76	236.85	
BUFFLE	28	f	0	1.54	6.11	0.75	0.0001
*CEDERL	80	m	2	1.22	12.65	0.01	0.0000
*CEDERL	76	f	2	1.04	15.40	0.34	0.0000
Subtotal CEDERL				1.12	28.05	0.36	
*CHANG	2	m	0	1.61	2.58	0.46	0.0096
*CHANG	8	f	0	1.11	3.97	0.03	0.0271
Subtotal CHANG				1.31	6.55	0.49	
CHEN2	3	m	0	0.87	4.19	0.42	0.0735
CHEN2	7	f	0	-0.95	3.04	13.86	0.0991
Subtotal CHEN2				0.11	7.23	14.28	
CHOI	12	m	0	0.48	6.73	3.34	0.2084
CHOI	17	f	0	0.19	5.18	5.14	0.6591
Subtotal CHOI				0.36	11.92	8.49	
*CHOW	10	m	2	2.63	5.07	10.53	0.0000
COMSTO	4	m	0	2.52	2.78	4.91	0.0000
COMSTO	9	f	0	2.01	4.98	3.34	0.0000
Subtotal COMSTO				2.19	7.76	8.25	
COOKSO	1	c	0	1.63	11.70	2.30	0.0000
*CPSI	243	m	1	1.76	44.36	14.41	0.0000
*CPSI	275	f	1	0.22	13.40	12.52	0.4140
Subtotal CPSI				1.40	57.76	26.93	
*CPSII	120	m	1	2.50	45.00	77.62	0.0000
*CPSII	105	f	1	1.43	95.77	5.33	0.0000
Subtotal CPSII				1.77	140.76	82.94	
DAMBER	6	m	1	0.83	10.34	1.32	0.0074
DARBY	1	m	0	4.30	2.87	27.72	0.0000
DARBY	8	f	0	2.75	14.48	35.42	0.0000
Subtotal DARBY				3.01	17.35	63.14	
DAVEYS	1	m	0	0.20	2.07	2.03	0.7726
DEAN3	7	m	3	1.70	14.67	3.78	0.0000
DEAN3	91	f	3	1.15	15.42	0.02	0.0000
Subtotal DEAN3				1.42	30.09	3.81	
*DEKLER	2	m	2	2.97	0.95	3.01	0.0038
DESTEF	6	m	4	1.06	11.84	0.18	0.0002

Table 1G12 - 2

IESLC - Meta-anal of Ever Smoking (or Current if ever not avail), Amount smoked, "Low", Any prod (or Cigs if Any not avail)
All LC types
Most adjusted

REF	NRR	SEX	AD	Ys	Ws	Qs	Ps
DOLL	2	m	0	2.01	6.13	4.14	0.0000
DOLL	8	f	0	0.68	7.19	1.89	0.0698
Subtotal DOLL				1.29	13.32	6.04	
*DOLL2	46	m	1	1.90	0.96	0.49	0.0623
*DOLL2	10	f	1	0.25	0.79	0.69	0.8209
Subtotal DOLL2				1.16	1.75	1.18	
DORANT	6	c	0	2.14	7.99	7.24	0.0000
DORGAN	108	m	2	1.93	10.33	5.64	0.0000
DORGAN	96	f	3	1.74	56.05	16.68	0.0000
Subtotal DORGAN				1.77	66.38	22.32	
*DORN	408	m	1	1.39	152.79	6.21	0.0000
DOSEME	5	m	2	0.79	20.90	3.36	0.0003
*DUNN	2	m	0	2.20	1.71	1.75	0.0040
EBELIN	2	m	0	1.00	6.42	0.24	0.0116
*ENGELA	32	m	7	1.41	4.89	0.24	0.0018
*ENGELA	46	f	5	2.48	4.17	7.00	0.0000
Subtotal ENGELA				1.91	9.06	7.24	
*ENSTRO	7	m	1	1.56	31.30	4.20	0.0000
*ENSTRO	11	f	1	0.77	48.76	8.78	0.0000
Subtotal ENSTRO				1.07	80.06	12.98	
ESAKI	1	m	0	0.09	7.53	9.07	0.8001
FAN	6	m	0	-0.35	8.53	20.24	0.3059
FAN	10	f	0	0.50	10.28	4.94	0.1116
Subtotal FAN				0.11	18.81	25.18	
GAO	21	f	2	0.55	30.53	12.34	0.0022
GAO2	2	m	0	1.21	6.65	0.00	0.0018
GARSHI	26	m	1	1.19	24.90	0.00	0.0000
GER	22	c	14	0.23	7.27	6.68	0.5331
GOLLED	1	m	1	1.49	12.18	1.12	0.0000
GSELL	1	m	0	1.49	1.53	0.14	0.0655
*HAMMO2	7	m	1	2.21	4.47	4.69	0.0000
*HAMMON	153	m	1	2.00	8.19	5.36	0.0000
*HANSEN	1	m	2	0.31	5.16	3.95	0.4747
*HIRAYA	23	m	1	0.72	36.53	7.97	0.0000
*HIRAYA	26	f	1	0.81	38.69	5.55	0.0000
Subtotal HIRAYA				0.77	75.22	13.52	
HITOSU	35	m	1	0.73	5.44	1.14	0.0875
HITOSU	60	f	1	1.13	12.11	0.04	0.0001
Subtotal HITOSU				1.01	17.55	1.18	
*HOLE	1	m	1	1.70	5.37	1.40	0.0001
HU	1	m	0	0.44	10.71	6.06	0.1527
HU	4	f	0	0.82	5.04	0.67	0.0643
Subtotal HU				0.56	15.74	6.74	
HU2	3	c	0	0.29	18.89	15.32	0.2087
HUMBLE	2	m	1	2.22	3.63	3.85	0.0000
HUMBLE	5	m	1	2.45	1.57	2.50	0.0021
HUMBLE	8	f	1	2.95	3.07	9.58	0.0000
HUMBLE	11	f	1	2.92	2.12	6.33	0.0000
Subtotal HUMBLE				2.61	10.40	22.26	
JARUP	4	m	2	1.93	3.04	1.67	0.0008
JEDRYC	45	m	4	1.25	23.96	0.08	0.0000
JEDRYC	48	f	4	1.85	5.04	2.21	0.0000
Subtotal JEDRYC				1.35	29.00	2.29	
JOLY	7	m	0	1.68	5.92	1.44	0.0000
JOLY	3	f	0	1.55	12.60	1.66	0.0000
Subtotal JOLY				1.59	18.52	3.11	
JUSSAW	34	m	2	1.72	4.79	1.33	0.0002
*KAISE2	66	m	1	1.50	5.94	0.56	0.0003
*KAISE2	58	f	1	2.03	5.35	3.77	0.0000
Subtotal KAISE2				1.75	11.29	4.34	
*KAISER	6	m	2	1.88	13.61	6.56	0.0000
*KAISER	2	f	2	1.23	18.49	0.03	0.0000
Subtotal KAISER				1.51	32.09	6.59	
KANELL	26	m	1	0.54	23.99	10.24	0.0086
KATSOU	7	f	0	0.22	3.68	3.49	0.6791
KAUFMA	11	c	6	2.08	16.83	13.32	0.0000
KHUDER	1	m	0	0.92	16.30	1.19	0.0002
*KINLEN	14	m	2	2.36	6.82	9.37	0.0000
*KNEKT	29	m	1	1.61	4.66	0.82	0.0005
KOO	11	f	0	0.31	7.09	5.53	0.4151
KOULUM	6	m	0	1.65	3.87	0.81	0.0012

Table 1G12 - 2

IESLC - Meta-anal of Ever Smoking (or Current if ever not avail), Amount smoked, "Low", Any prod (or Cigs if Any not avail)
All LC types
Most adjusted

REF	NRR	SEX	AD	Ys	Ws	Qs	Ps
KREUZE	19	m	3	0.92	2.54	0.19	0.1444
KREUZE	30	m	3	2.10	18.30	15.30	0.0000
KREUZE	25	f	3	1.74	2.81	0.85	0.0035
KREUZE	36	f	3	0.69	15.02	3.70	0.0072
Subtotal KREUZE				1.45	38.66	20.05	
KREYBE	9	m	1	1.76	5.70	1.86	0.0000
KREYBE	28	f	1	0.31	7.13	5.55	0.4115
Subtotal KREYBE				0.95	12.83	7.41	
LAMTH	7	f	0	0.98	29.68	1.26	0.0000
LAUSSM	18	m	3	1.19	31.20	0.00	0.0000
LETOUR	2	c	0	2.25	18.66	21.07	0.0000
*LIAW	3	c	2	1.13	10.81	0.04	0.0002
*LIDDEL	2	m	1	1.20	15.00	0.00	0.0000
LIU2	8	m	3	0.18	3.50	3.55	0.7332
LIU2	14	f	3	0.59	2.81	1.02	0.3242
Subtotal LIU2				0.36	6.31	4.57	
LIU3	6	m	2	0.34	1.81	1.29	0.6441
LIU4	7	m	2	0.75	2108.86	413.88	0.0000
LIU5	2	c	0	-0.20	5.84	11.29	0.6267
LUBIN	11	m	4	-0.33	2.38	5.48	0.6124
LUBIN2	273	m	0	1.93	155.25	85.82	0.0000
LUBIN2	281	f	0	0.90	66.54	5.75	0.0000
Subtotal LUBIN2				1.62	221.79	91.57	
MACLEN	36	c	2	0.30	6.92	5.48	0.4298
MARTIS	1	m	0	1.60	2.87	0.49	0.0066
MATOS	29	m	2	0.69	5.93	1.46	0.0914
MATSUD	1	m	0	2.53	2.76	4.94	0.0000
MCCONN	26	c	0	-0.88	3.12	13.32	0.1215
*MIGRAN	2	m	2	1.39	2.61	0.10	0.0249
*MIGRAN	29	f	2	1.59	1.88	0.29	0.0298
Subtotal MIGRAN				1.47	4.49	0.40	
*MRFITR	3	m	0	2.38	0.42	0.60	0.1236
NOTAN2	8	m	0	-0.54	5.01	15.06	0.2229
ORMOS	1	m	0	2.38	5.60	7.93	0.0000
PARKIN	14	m	6	1.36	58.89	1.73	0.0000
PASTOR	6	m	1	0.85	3.21	0.38	0.1295
PERNU	18	m	0	1.72	15.98	4.47	0.0000
PERNU	12	f	0	1.22	3.48	0.00	0.0225
Subtotal PERNU				1.63	19.46	4.48	
PERSH2	8	c	4	1.75	77.78	24.50	0.0000
*PETO	2	m	0	1.70	1.93	0.51	0.0180
POLEDN	2	c	0	1.79	8.40	2.98	0.0000
*PRESCO	2	m	1	2.32	5.71	7.33	0.0000
*PRESCO	1	f	1	1.85	11.85	5.17	0.0000
Subtotal PRESCO				2.00	17.57	12.50	
RACHTA	10	f	1	1.29	2.59	0.03	0.0375
RANDIG	2	m	0	1.06	3.69	0.06	0.0415
RANDIG	6	f	0	1.61	4.35	0.76	0.0008
Subtotal RANDIG				1.36	8.04	0.82	
SEGI2	10	m	1	0.74	4.79	0.96	0.1046
SEGI2	22	f	1	1.06	4.02	0.06	0.0328
Subtotal SEGI2				0.89	8.81	1.02	
SHAW	10	c	0	1.60	7.51	1.29	0.0000
SIEMIA	13	m	0	1.10	2.92	0.02	0.0603
SOBUE	117	m	0	1.26	19.84	0.10	0.0000
*SPEIZE	2	f	1	1.65	23.37	4.92	0.0000
SPITZ	5	c	0	1.08	6.30	0.08	0.0067
STOCKS	41	m	2	1.53	30.49	3.63	0.0000
STOCKS	48	f	1	0.81	40.78	5.99	0.0000
Subtotal STOCKS				1.12	71.26	9.62	
STOCKW	1	c	0	1.90	565.53	283.86	0.0000
SVENSS	6	f	1	1.53	8.90	1.01	0.0000
*TENKAN	10	m	1	2.76	5.35	13.27	0.0000
TIZZAN	7	m	0	-0.08	48.24	77.45	0.5909
TIZZAN	15	f	0	1.28	4.74	0.04	0.0055
Subtotal TIZZAN				0.04	52.98	77.49	
TSUGAN	29	m	0	-0.10	4.44	7.44	0.8253
*TULINI	27	m	3	1.80	8.03	2.94	0.0000
*TULINI	32	f	3	2.10	9.54	7.92	0.0000
Subtotal TULINI				1.96	17.58	10.86	
*TVERDA	9	m	2	0.76	10.05	1.85	0.0159

Table 1G12 - 2

IESLC - Meta-anal of Ever Smoking (or Current if ever not avail), Amount smoked, "Low", Any prod (or Cigs if Any not avail)
All LC types
Most adjusted

REF	NRR	SEX	AD	Ys	Ws	Qs	Ps
*TVERDA	16	f	2	1.51	1.87	0.19	0.0387
Subtotal TVERDA				0.88	11.92	2.04	
WAKAI	40	m	2	0.59	5.99	2.17	0.1504
WANG2	10	c	4	0.20	2.04	2.00	0.7765
WUWILL	12	f	3	0.76	101.98	19.17	0.0000
WYNDE2	17	m	0	0.67	4.95	1.33	0.1353
WYNDE3	44	m	0	0.62	3.69	1.19	0.2325
WYNDE3	79	f	0	-0.51	2.23	6.44	0.4459
Subtotal WYNDE3				0.20	5.91	7.63	
WYNDE4	43	m	0	0.69	6.13	1.55	0.0891
WYNDE4	57	f	2	0.12	2.52	2.88	0.8460
Subtotal WYNDE4				0.52	8.66	4.43	
WYNDE6	27	m	0	1.92	33.49	17.72	0.0000
WYNDE6	216	f	0	1.32	33.57	0.59	0.0000
Subtotal WYNDE6				1.62	67.06	18.32	
XU3	9	m	1	0.51	3.17	1.48	0.3668
XU3	16	f	1	0.78	2.36	0.40	0.2316
Subtotal XU3				0.62	5.53	1.88	
*YUAN	2	m	2	1.28	9.07	0.08	0.0001
ZHENG	11	m	0	0.58	9.44	3.55	0.0764
ZHENG	22	f	0	0.00	11.34	16.01	0.9951
Subtotal ZHENG				0.26	20.78	19.55	
ZHOU	4	c	0	0.49	4.29	2.09	0.3077

	N	176
	NS	126
	Wt	5514.84
Het	Chi	1945.15
Het	df	175
Het	P	***
Fixed	RR	3.29
	RRl	3.20
	RRu	3.37
	P	+++
Random	RR	3.51
	RRl	3.15
	RRu	3.91
	P	+++
Asymm	P	N.S.

Table 1G12 - 3

IESLC - Meta-anal of Ever Smoking (or Current if ever not avail), Amount smoked, "Low", Any prod (or Cigs if Any not avail)
All LC types
Most adjusted

	Sex			
	combined	male	female	Total
N	18	100	58	176
NS	18	98	56	172
Wt	787.25	3628.62	1098.98	5514.84
Het Chi	165.40	1012.92	411.13	1945.15
Het df	17	99	57	175
Het P	***	***	***	***
Fixed RR	5.91	2.83	3.51	3.29
RRl	5.51	2.74	3.31	3.20
RRu	6.34	2.93	3.73	3.37
P	+++	+++	+++	+++
Random RR	3.50	3.77	3.11	3.51
RRl	2.51	3.26	2.60	3.15
RRu	4.89	4.36	3.72	3.91
P	+++	+++	+++	+++
Between Chi				355.70
Between df				2
Between P				***
Btwn(F) P				***
Btwn(R) P				N.S.

	Lung cancer type		
	all	other	Total
N	174	2	176
NS	124	2	126
Wt	5490.56	24.29	5514.84
Het Chi	1937.61	6.76	1945.15
Het df	173	1	175
Het P	***	**	***
Fixed RR	3.29	2.75	3.29
RRl	3.20	1.85	3.20
RRu	3.38	4.09	3.37
P	+++	+++	+++
Random RR	3.53	1.90	3.51
RRl	3.17	0.50	3.15
RRu	3.94	7.19	3.91
P	+++	N.S.	+++
Between Chi			0.79
Between df			1
Between P			N.S.
Btwn(F) P			N.S.
Btwn(R) P			N.S.

	Location								
	NAmer	UK	Scand	othEur	China	Japan	othAs	other	Total
N	51	22	25	26	22	14	9	7	176
NS	35	15	17	19	16	10	8	6	126
Wt	1797.29	225.50	257.88	482.78	2360.09	199.99	83.48	107.83	5514.84
Het Chi	292.06	106.19	76.75	234.32	44.39	29.37	22.54	8.07	1945.15
Het df	50	21	24	25	21	13	8	6	175
Het P	***	***	***	***	**	**	**	N.S.	***
Fixed RR	5.67	3.90	4.87	3.61	2.06	2.62	1.98	3.96	3.29
RRl	5.41	3.42	4.31	3.30	1.98	2.28	1.60	3.28	3.20
RRu	5.94	4.44	5.50	3.95	2.15	3.00	2.46	4.78	3.37
P	+++	+++	+++	+++	+++	+++	+++	+++	+++
Random RR	4.86	4.42	4.75	3.37	1.61	2.57	1.76	3.98	3.51
RRl	4.21	3.18	3.73	2.46	1.36	2.04	1.20	3.09	3.15
RRu	5.61	6.14	6.04	4.63	1.91	3.25	2.59	5.14	3.91
P	+++	+++	+++	+++	+++	+++	++	+++	+++
Between Chi									1131.47
Between df									7
Between P									***
Btwn(F) P									***
Btwn(R) P									***

Table 1G12 - 3

IESLC - Meta-anal of Ever Smoking (or Current if ever not avail), Amount smoked, "Low", Any prod (or Cigs if Any not avail)
All LC types
Most adjusted

	Detailed Country in "other Europe"					
	multi	Germany	othWest	East	Balkans	Total
N	2	9	8	4	3	26
NS	1	5	7	3	3	19
Wt	221.79	86.40	88.83	37.20	48.57	482.78
Het Chi	50.14	22.36	76.52	6.60	1.36	234.32
Het df	1	8	7	3	2	25
Het P	***	**	***	(*)	N.S.	***
Fixed RR	5.06	3.61	2.04	4.49	1.86	3.61
RRl	4.44	2.93	1.66	3.26	1.40	3.30
RRu	5.77	4.46	2.51	6.20	2.46	3.95
P	+++	+++	+++	+++	+++	+++
Random RR	4.13	3.42	3.38	5.26	1.86	3.37
RRl	1.49	2.28	1.48	2.97	1.40	2.46
RRu	11.42	5.13	7.72	9.32	2.46	4.63
P	++	+++	++	+++	+++	+++
Between Chi						77.34
Between df						4
Between P						***
Btwn(F) P						(*)
Btwn(R) P						**

	Detailed Country in "other Asia"			
	India	HongKong	other	Total
N	2	2	5	9
NS	2	2	4	8
Wt	9.79	36.77	36.92	83.48
Het Chi	12.52	2.63	5.51	22.54
Het df	1	1	4	8
Het P	***	N.S.	N.S.	**
Fixed RR	1.75	2.35	1.73	1.98
RRl	0.94	1.70	1.25	1.60
RRu	3.28	3.24	2.39	2.46
P	(+)	+++	+++	+++
Random RR	1.79	2.06	1.69	1.76
RRl	0.20	1.08	1.15	1.20
RRu	16.46	3.94	2.47	2.59
P	N.S.	+	++	++
Between Chi				1.88
Between df				2
Between P				N.S.
Btwn(F) P				N.S.
Btwn(R) P				N.S.

	Detailed other continent			
	SCAmer	Auslia	Africa	Total
N	4	1	2	7
NS	3	1	2	6
Wt	36.28	0.95	70.59	107.83
Het Chi	4.49	0.00	0.72	8.07
Het df	3	0	1	6
Het P	N.S.	N.S.	N.S.	N.S.
Fixed RR	3.58	19.40	4.08	3.96
RRl	2.58	2.61	3.23	3.28
RRu	4.95	144.23	5.15	4.78
P	+++	++	+++	+++
Random RR	3.55	19.40	4.08	3.98
RRl	2.36	2.61	3.23	3.09
RRu	5.32	144.23	5.15	5.14
P	+++	++	+++	+++
Between Chi				2.85
Between df				2
Between P				N.S.
Btwn(F) P				N.S.
Btwn(R) P				N.S.

Table 1G12 - 3

IESLC - Meta-anal of Ever Smoking (or Current if ever not avail), Amount smoked, "Low", Any prod (or Cigs if Any not avail)
All LC types
Most adjusted

	Start year of study					
	<1960	1960-69	1970-79	1980-89	1990+	Total
N	36	42	31	57	10	176
NS	25	29	24	42	6	126
Wt	565.58	502.29	479.14	3888.26	79.57	5514.84
Het Chi	213.40	156.07	161.65	1330.25	48.21	1945.15
Het df	35	41	30	56	9	175
Het P	***	***	***	***	***	***
Fixed RR	3.12	3.77	3.95	3.20	2.54	3.29
RRl	2.88	3.46	3.61	3.10	2.04	3.20
RRu	3.39	4.12	4.32	3.30	3.16	3.37
P	+++	+++	+++	+++	+++	+++
Random RR	3.19	3.97	3.41	3.64	2.38	3.51
RRl	2.51	3.29	2.67	2.96	1.39	3.15
RRu	4.05	4.78	4.35	4.47	4.06	3.91
P	+++	+++	+++	+++	++	+++
Between Chi						35.58
Between df						4
Between P						***
Btwn(F) P						N.S.
Btwn(R) P						N.S.

	Study type (1)		
	CC	other	Total
N	122	54	176
NS	88	38	126
Wt	4672.65	842.19	5514.84
Het Chi	1662.91	221.24	1945.15
Het df	121	53	175
Het P	***	***	***
Fixed RR	3.14	4.21	3.29
RRl	3.05	3.93	3.20
RRu	3.23	4.50	3.37
P	+++	+++	+++
Random RR	3.06	4.70	3.51
RRl	2.67	4.01	3.15
RRu	3.50	5.52	3.91
P	+++	+++	+++
Between Chi			61.00
Between df			1
Between P			***
Btwn(F) P			*
Btwn(R) P			***

	Study type (2)			
	CC	prosp	other	Total
N	122	51	3	176
NS	88	36	2	126
Wt	4672.65	826.44	15.75	5514.84
Het Chi	1662.91	212.24	0.48	1945.15
Het df	121	50	2	175
Het P	***	***	N.S.	***
Fixed RR	3.14	4.15	8.72	3.29
RRl	3.05	3.88	5.32	3.20
RRu	3.23	4.44	14.30	3.37
P	+++	+++	+++	+++
Random RR	3.06	4.55	8.72	3.51
RRl	2.67	3.86	5.32	3.15
RRu	3.50	5.36	14.30	3.91
P	+++	+++	+++	+++
Between Chi				69.53
Between df				2
Between P				***
Btwn(F) P				*
Btwn(R) P				***

Table 1G12 - 3

IESLC - Meta-anal of Ever Smoking (or Current if ever not avail), Amount smoked, "Low", Any prod (or Cigs if Any not avail)
All LC types
Most adjusted

	Study size (number of LC cases)				
	100-249	250-499	500-999	1000+	Total
N	49	44	42	41	176
NS	43	30	29	24	126
Wt	244.05	336.01	556.43	4378.35	5514.84
Het Chi	146.16	119.94	286.85	1373.46	1945.15
Het df	48	43	41	40	175
Het P	***	***	***	***	***
Fixed RR	2.71	3.24	3.75	3.27	3.29
RRl	2.39	2.91	3.45	3.17	3.20
RRu	3.08	3.60	4.07	3.37	3.37
P	+++	+++	+++	+++	+++
Random RR	2.73	3.30	4.50	3.71	3.51
RRl	2.17	2.73	3.54	3.00	3.15
RRu	3.43	3.98	5.72	4.57	3.91
P	+++	+++	+++	+++	+++
Between Chi					18.74
Between df					3
Between P					***
Btwn(F) P					N.S.
Btwn(R) P					*

	Risky occupational population			
	no	mining	othRisky	Total
N	167	4	5	176
NS	117	4	5	126
Wt	5453.02	22.54	39.28	5514.84
Het Chi	1924.58	8.43	11.63	1945.15
Het df	166	3	4	175
Het P	***	*	*	***
Fixed RR	3.28	3.09	3.65	3.29
RRl	3.20	2.04	2.67	3.20
RRu	3.37	4.66	4.98	3.37
P	+++	+++	+++	+++
Random RR	3.50	3.03	4.31	3.51
RRl	3.13	1.27	2.22	3.15
RRu	3.91	7.26	8.36	3.91
P	+++	+	+++	+++
Between Chi				0.51
Between df				2
Between P				N.S.
Btwn(F) P				N.S.
Btwn(R) P				N.S.

	National cigarette tobacco type			
	Virginia	blended	other	Total
N	32	120	24	176
NS	25	83	18	126
Wt	357.17	2779.50	2378.17	5514.84
Het Chi	148.78	837.95	47.95	1945.15
Het df	31	119	23	175
Het P	***	***	**	***
Fixed RR	4.10	4.75	2.06	3.29
RRl	3.70	4.58	1.98	3.20
RRu	4.55	4.93	2.15	3.37
P	+++	+++	+++	+++
Random RR	4.44	3.87	1.65	3.51
RRl	3.44	3.45	1.40	3.15
RRu	5.72	4.35	1.94	3.91
P	+++	+++	+++	+++
Between Chi				910.48
Between df				2
Between P				***
Btwn(F) P				***
Btwn(R) P				***

Table 1G12 - 3

IESLC - Meta-anal of Ever Smoking (or Current if ever not avail), Amount smoked, "Low", Any prod (or Cigs if Any not avail)
All LC types
Most adjusted

	Any proxy use No/nk	Yes	Total
N	134	42	176
NS	95	31	126
Wt	2841.47	2673.37	5514.84
Het Chi	1022.55	399.40	1945.15
Het df	133	41	175
Het P	***	***	***
Fixed RR	4.43	2.39	3.29
RRl	4.27	2.30	3.20
RRu	4.60	2.48	3.37
P	+++	+++	+++
Random RR	3.57	3.31	3.51
RRl	3.17	2.67	3.15
RRu	4.02	4.10	3.91
P	+++	+++	+++
Between Chi			523.20
Between df			1
Between P			***
Btwn(F) P			***
Btwn(R) P			N.S.

	Full histological confirmation No	Yes	Total
N	139	37	176
NS	98	28	126
Wt	4527.00	987.84	5514.84
Het Chi	1402.70	279.97	1945.15
Het df	138	36	175
Het P	***	***	***
Fixed RR	2.97	5.24	3.29
RRl	2.88	4.93	3.20
RRu	3.06	5.58	3.37
P	+++	+++	+++
Random RR	3.54	3.46	3.51
RRl	3.13	2.82	3.15
RRu	3.99	4.25	3.91
P	+++	+++	+++
Between Chi			262.48
Between df			1
Between P			***
Btwn(F) P			***
Btwn(R) P			N.S.

	Number of adjustment variables (1) 0	1	2+/+nk	Total
N	72	46	58	176
NS	55	31	43	129
Wt	1348.76	841.58	3324.50	5514.84
Het Chi	659.01	239.13	816.29	1945.15
Het df	71	45	57	175
Het P	***	***	***	***
Fixed RR	4.48	3.76	2.80	3.29
RRl	4.25	3.52	2.71	3.20
RRu	4.73	4.03	2.90	3.37
P	+++	+++	+++	+++
Random RR	2.97	4.02	3.77	3.51
RRl	2.44	3.36	3.13	3.15
RRu	3.62	4.80	4.54	3.91
P	+++	+++	+++	+++
Between Chi				230.72
Between df				2
Between P				***
Btwn(F) P				***
Btwn(R) P				(*)

Table 1G12 - 3

IESLC - Meta-anal of Ever Smoking (or Current if ever not avail), Amount smoked, "Low", Any prod (or Cigs if Any not avail)
All LC types
Most adjusted

	Number of adjustment variables (2) 0	1	2	3-5	6+/+nk	Total
N	72	46	29	24	5	176
NS	55	31	24	16	5	131
Wt	1348.76	841.58	2747.69	480.02	96.79	5514.84
Het Chi	659.01	239.13	610.09	116.26	17.83	1945.15
Het df	71	45	28	23	4	175
Het P	***	***	***	***	**	***
Fixed RR	4.48	3.76	2.62	3.82	4.06	3.29
RRl	4.25	3.52	2.52	3.49	3.33	3.20
RRu	4.73	4.03	2.72	4.18	4.96	3.37
P	+++	+++	+++	+++	+++	+++
Random RR	2.97	4.02	3.83	3.79	3.76	3.51
RRl	2.44	3.36	2.83	3.01	2.26	3.15
RRu	3.62	4.80	5.17	4.78	6.26	3.91
P	+++	+++	+++	+++	+++	+++
Between Chi						302.83
Between df						4
Between P						***
Btwn(F) P						***
Btwn(R) P						N.S.

	Product all/unsp	cig+/-ot	cig only	Total
N	52	98	26	176
NS	40	70	19	129
Wt	508.65	2518.62	2487.57	5514.84
Het Chi	170.10	809.70	311.72	1945.15
Het df	51	97	25	175
Het P	***	***	***	***
Fixed RR	3.44	4.68	2.27	3.29
RRl	3.16	4.50	2.19	3.20
RRu	3.76	4.87	2.37	3.37
P	+++	+++	+++	+++
Random RR	2.87	3.93	3.36	3.51
RRl	2.40	3.44	2.52	3.15
RRu	3.43	4.48	4.48	3.91
P	+++	+++	+++	+++
Between Chi				653.63
Between df				2
Between P				***
Btwn(F) P				***
Btwn(R) P				*

	Denominator nev any	nev cigs	Total
N	122	54	176
NS	88	41	129
Wt	4431.24	1083.60	5514.84
Het Chi	1435.00	435.96	1945.15
Het df	121	53	175
Het P	***	***	***
Fixed RR	3.10	4.15	3.29
RRl	3.01	3.91	3.20
RRu	3.20	4.41	3.37
P	+++	+++	+++
Random RR	3.54	3.43	3.51
RRl	3.10	2.82	3.15
RRu	4.04	4.17	3.91
P	+++	+++	+++
Between Chi			74.19
Between df			1
Between P			***
Btwn(F) P			**
Btwn(R) P			N.S.

Table 1G12 - 3

IESLC - Meta-anal of Ever Smoking (or Current if ever not avail), Amount smoked, "Low", Any prod (or Cigs if Any not avail)
All LC types
Most adjusted

	Derivation of RR/CI			
	Orig	StdCalc	Other	Total
N	44	81	51	176
NS	31	60	39	130
Wt	982.14	1518.64	3014.06	5514.84
Het Chi	214.58	725.72	499.99	1945.15
Het df	43	80	50	175
Het P	***	***	***	***
Fixed RR	5.01	4.29	2.50	3.29
RRl	4.71	4.08	2.42	3.20
RRu	5.33	4.51	2.60	3.37
P	+++	+++	+++	+++
Random RR	4.37	3.05	3.59	3.51
RRl	3.69	2.55	3.00	3.15
RRu	5.19	3.64	4.31	3.91
P	+++	+++	+++	+++
Between Chi				504.86
Between df				2
Between P				***
Btwn(F) P				***
Btwn(R) P				*

	Smoking status		
	ever	current	Total
N	103	73	176
NS	77	52	129
Wt	3887.00	1627.84	5514.84
Het Chi	1124.74	428.34	1945.15
Het df	102	72	175
Het P	***	***	***
Fixed RR	2.77	4.96	3.29
RRl	2.68	4.73	3.20
RRu	2.85	5.21	3.37
P	+++	+++	+++
Random RR	2.86	4.63	3.51
RRl	2.48	4.00	3.15
RRu	3.29	5.35	3.91
P	+++	+++	+++
Between Chi			392.08
Between df			1
Between P			***
Btwn(F) P			***
Btwn(R) P			***

	Study LIU4		
	LIU4	others	Total
N	1	175	176
NS	1	125	126
Wt	2108.86	3405.98	5514.84
Het Chi	0.00	1275.01	1945.15
Het df	0	174	175
Het P	N.S.	***	***
Fixed RR	2.11	4.32	3.29
RRl	2.02	4.18	3.20
RRu	2.20	4.47	3.37
P	+++	+++	+++
Random RR	2.11	3.52	3.51
RRl	2.02	3.18	3.15
RRu	2.20	3.91	3.91
P	+++	+++	+++
Between Chi			670.15
Between df			1
Between P			***
Btwn(F) P			***
Btwn(R) P			***

Table 1G12 - 4

IESLC - Meta-anal of Ever Smoking (or Current if ever not avail), Amount smoked, "Low", Any prod (or Cigs if Any not avail)
All LC types
Least adjusted

REF	NRR	X	SEX	AGEL	AGEH	RACE	YF	LC TYPE	LOC	START	ST	NLC	R	VB	P	H	AD	SM	PRODUCT	exL	exH	DENOM	De
AGUDO	11	x	f	0	0	all	-	all	Eu:wst	1989	CC	103	n	bl	n	n	0	ev	cig only	1	10	nev any	st
AKIBA	17	x	m	0	0	all	0	all	As:Jap	1963	pr	610	n	bl	n	n	0	cu	cig+/-ot	1	14	nev cigs	or
AKIBA	23	x	f	0	0	all	0	all	As:Jap	1963	pr	610	n	bl	n	n	0	cu	cig+/-ot	1	14	nev cigs	or
ALDERS	18		m	0	0	all	-	all	Eu:UK	1977	CC	1448	n	V	n	n	1	ev	cig only	1	17	nev+2	ot
ALDERS	21		f	0	0	all	-	all	Eu:UK	1977	CC	1448	n	V	n	n	1	ev	cig only	1	17	nev+2	ot
ARCHER	1		m	0	0	wh	0	all	NAmer	1950	pr	146	m	bl	n	n	0	cu	cig+/-ot	1	19	nev cigs	st
ARMADA	46		m	0	0	all	-	all	Eu:wst	1986	CC	325	n	bl	n	y	0	ev	cig+/-ot	1	14	nev any	st
AUVINE	5	x	c	0	0	all	-	all	Eu:Sca	1986	CC	517	n	bl	y	n	0	ev	cig+/-ot	1	10	nev cigs	st
AXELSS	19	x	m	0	0	sca	-	all	Eu:Sca	1989	CC	436	n	bl	n	n	0	ev	all/unsp	1	9	nev any	st
AXELSS	13		f	0	0	sca	-	all	Eu:Sca	1989	CC	436	n	bl	n	n	0	ev	all/unsp	1	9	nev any	st
BARBON	5	x	m	0	0	all	-	all	Eu:wst	1979	CC	755	n	bl	y	y	0	ev	all/unsp	1	9	nev any	st
BENSHL	11		m	40	64	all	10	all	Eu:UK	1967	pr	486	n	V	n	n	1	cu	cig+/-ot	1	9	nev any	ot
BEST	13		m	0	0	all	0	all	NAmer	1955	pr	381	n	V	n	n	1	cu	cig only	1	9	nev any	ot
BRESLO	13		m	0	0	all	-	all	NAmer	1949	CC	518	n	bl	n	y	0	ev	cig+/-ot	1	9	nev+3	st
BRESLO	29		f	0	0	all	-	all	NAmer	1949	CC	518	n	bl	n	y	0	ev	cig+/-ot	1	19	nev+3	st
BRETT	1		m	0	0	all	0	all	Eu:UK	1960	pr	150	n	V	n	n	0	cu	cig+/-ot	1	14	nev cigs	st
BROWN2	32		m	0	0	wh	-	all	NAmer	1984	CC	14596	n	bl	n	y	2	ev	cig+/-ot	1	19	nev cigs	or
BROWN2	31		f	0	0	wh	-	all	NAmer	1984	CC	14596	n	bl	n	y	2	ev	cig+/-ot	1	19	nev cigs	or
BUFFLE	28		f	0	0	w-hi	-	all	NAmer	1976	CC	943	n	bl	y	n	0	ev	cig+/-ot	1	19	nev cigs	or
CEDERL	80		m	0	0	all	16	all	Eu:Sca	1963	pr	491	n	bl	n	n	2	cu	all/unsp	1	7	nev any	ot
CEDERL	76		f	0	0	all	0	all	Eu:Sca	1963	pr	491	n	bl	n	n	2	cu	all/unsp	1	7	nev any	or
CHANG	2		m	0	0	all	0	all	NAmer	1972	pr	136	n	bl	n	n	0	cu	cig+/-ot	1	10	nev cigs	st
CHANG	8		f	0	0	all	0	all	NAmer	1972	pr	136	n	bl	n	n	0	cu	cig+/-ot	1	10	nev cigs	st
CHEN2	3		m	0	0	all	-	all	As:Chi	1983	CC	193	n	ot	y	n	0	ev	all/unsp	1	9	nev any	st
CHEN2	7		f	0	0	all	-	all	As:Chi	1983	CC	193	n	ot	y	n	0	ev	all/unsp	1	9	nev any	st
CHOI	12		m	0	0	all	-	all	As:oth	1985	CC	375	n	bl	n	n	0	ev	cig+/-ot	1	10	nev cigs	st
CHOI	17		f	0	0	all	-	all	As:oth	1985	CC	375	n	bl	n	n	0	ev	cig+/-ot	1	10	nev cigs	st
CHOW	3	x	m	0	0	wh	0	all	NAmer	1966	pr	219	n	bl	n	n	0	cu	cig+/-ot	1	19	nev any	st
COMSTO	4		m	0	0	all	-	all	NAmer	1975	ot	258	n	bl	n	n	0	cu	cig+/-ot	1	19	nev any	st
COMSTO	9		f	0	0	all	-	all	NAmer	1975	ot	258	n	bl	n	n	0	cu	cig+/-ot	1	19	nev any	st
COOKSO	1		c	0	0	bl	-	all	Africa	1961	CC	234	n	V	n	y	0	ev	cig+/-ot	1	9	nev any	st
CPSI	243		m	50	74	all	6	all	NAmer	1959	pr	5138	n	bl	n	n	1	ev	cig only	1	19	nev any	ot
CPSI	275		f	40	74	all	6	all	NAmer	1959	pr	5138	n	bl	n	n	1	cu	cig+/-ot	1	9	nev cigs	ot
CPSII	30	x	m	0	0	all	6	all	NAmer	1982	pr	3229	n	bl	n	n	0	cu	cig only	1	9	nev any	st
CPSII	105		f	35	99	all	4	all	NAmer	1982	pr	3229	n	bl	n	n	1	ev	cig+/-ot	1	19	nev cigs	ot
DAMBER	6		m	0	0	all	-	all	Eu:Sca	1972	CC	579	n	bl	y	n	1	ev	cig only	1	7	nev any	ot
DARBY	1		m	0	0	wh	-	all	Eu:UK	1988	CC	982	n	V	n	n	0	cu	cig+/-ot	1	14	nev any	st
DARBY	8		f	0	0	wh	-	all	Eu:UK	1988	CC	982	n	V	n	n	0	cu	cig+/-ot	1	14	nev any	st
DAVEYS	1		m	0	0	all	-	all	Eu:Ger	1930	CC	109	n	bl	y	n	0	ev	all/unsp	1	5	nev any	st
DEAN3	5	x	m	0	0	all	-	all	Eu:UK	1969	CC	766	n	V	y	n	0	cu	cig only	1	12	nev any	st
DEAN3	89	x	f	0	0	all	-	all	Eu:UK	1969	CC	766	n	V	y	n	0	cu	cig only	1	12	nev any	st
DEKLER	2		m	0	0	all	0	all	Auslia	1961	pr	138	m	V	n	n	2	cu	cig+/-ot	1	14	nev any	or
DESTEF	1	x	m	0	0	all	-	all	SCAmer	1988	CC	497	n	bl	n	y	0	ev	all/unsp	1	10	nev any	st
DOLL	2		m	0	0	all	-	all	Eu:UK	1948	CC	1465	n	V	n	n	0	ev	all/unsp	5	14	nev any	st
DOLL	8		f	0	0	all	-	all	Eu:UK	1948	CC	1465	n	V	n	n	0	ev	all/unsp	5	14	nev any	st
DOLL2	46		m	35	99	all	5	all	Eu:UK	1951	pr	920	n	V	n	n	1	ev	all/unsp	1	14	nev any	ot
DOLL2	10		f	0	0	all	22	all	Eu:UK	1951	pr	920	n	V	n	n	1	cu	cig only	1	14	nev any	ot
DORANT	6		c	0	0	all	0	all	Eu:wst	1986	ot	550	n	bl	n	y	0	cu	cig+/-ot	1	9	nev any	st
DORGAN	10	x	m	0	0	wh	-	all	NAmer	1980	CC	2026	n	bl	y	y	0	ev	cig+/-ot	1	19	nev any	st
DORGAN	34	x	m	0	0	bl	-	all	NAmer	1980	CC	2026	n	bl	y	y	0	ev	cig+/-ot	1	19	nev any	st
DORGAN	96		f	0	0	all	-	all	NAmer	1980	CC	2026	n	bl	y	y	3	ev	cig+/-ot	1	19	nev any	ot
DORN	408		m	0	0	wh	25	all	NAmer	1954	pr	5097	n	bl	n	n	1	cu	cig+/-ot	1	9	nev any	or
DOSEME	5		m	0	0	all	-	all	Eu:bal	1979	CC	1210	n	bl	n	n	2	ev	cig+/-ot	1	10	nev cigs	or
DUNN	2		m	0	0	all	0	all	NAmer	1954	pr	139	o	bl	n	n	0	ev	cig+/-ot	5	14	nev cigs	st
EBELIN	2		m	0	0	all	-	all	Eu:Ger	1980	CC	130	n	bl	n	n	0	ev	all/unsp	1	9	nev any	st
ENGELA	4	x	m	0	0	all	0	all	Eu:Sca	1964	pr	435	n	bl	n	n	0	cu	cig+/-ot	5	9	nev cigs	st
ENGELA	18	x	f	0	0	all	0	all	Eu:Sca	1964	pr	435	n	bl	n	n	0	cu	cig+/-ot	5	9	nev cigs	st
ENSTRO	7		m	0	0	all	0	all	NAmer	1959	pr	2879	n	bl	n	n	1	cu	cig only	1	9	nev any	ot
ENSTRO	11		f	0	0	all	0	all	NAmer	1959	pr	2879	n	bl	n	n	1	cu	cig only	1	9	nev any	ot
ESAKI	1		m	0	0	all	-	all	As:Jap	1961	CC	245	n	bl	y	n	0	ev	cig+/-ot	1	14	nev cigs	st
FAN	6		m	0	0	all	-	all	As:Chi	1990	CC	403	n	ot	y	n	0	ev	cig+/-ot	1	9	nev cigs	st
FAN	10		f	0	0	all	-	all	As:Chi	1990	CC	403	n	ot	y	n	0	ev	cig+/-ot	1	9	nev cigs	st
GAO	24	x	f	0	0	all	-	all	As:Chi	1984	CC	1405	n	ot	n	n	0	ev	cig+/-ot	1	9	nev cigs	st
GAO2	2		m	0	0	all	-	all	As:Jap	1988	CC	282	n	bl	n	n	0	cu	cig+/-ot	1	19	nev cigs	st
GARSHI	18	x	m	0	0	all	-	all	NAmer	1981	CC	1081	o	bl	y	n	0	ev	all/unsp	1	15	nev any	st
GER	18	x	c	0	0	all	-	all	As:oth	1990	CC	141	n	ot	y	n	0	ev	all/unsp	1	10	nev any	st
GOLLED	15	x	m	35	99	all	-	all	Eu:UK	1952	CC	443	n	V	y	n	0	ev	cig only	1	10	nev any	st
GSELL	1		m	0	0	all	-	all	Eu:wst	1937	CC	150	n	bl	n	y	0	ev	all/unsp	1	9	nev any	st
HAMMO2	21	x	m	0	0	all	0	all	NAmer	1967	pr	450	o	bl	n	n	0	cu	cig+/-ot	1	19	nev any	st
HAMMON	153		m	0	0	wh	0	all	NAmer	1952	pr	448	n	bl	n	n	1	ev	cig only	1	9	nev any	ot
HANSEN	1		m	0	0	all	0	all	Eu:Sca	1968	pr	105	o	bl	y	n	2	ev	all/unsp	1	19	nev any	ot
HIRAYA	23		m	0	0	all	0	all	As:Jap	1965	pr	1917	n	bl	n	n	1	cu	cig+/-ot	1	9	nev any	st

Table 1G12 - 4

IESLC - Meta-anal of Ever Smoking (or Current if ever not avail), Amount smoked, "Low", Any prod (or Cigs if Any not avail)
All LC types
Least adjusted

REF	NRR	X	SEX	AGEL	AGEH	RACE	YF	LC TYPE	LOC	START	ST	NLC	R	VB	P	H	AD	SM	PRODUCT	exL	exH	DENOM	De
HIRAYA	26		f	0	0	all	0	all	As:Jap	1965	pr	1917	n	bl	n	n	1	cu	cig+/-ot	1	9	nev any	st
HITOSU	3	x	m	0	0	all	-	all	As:Jap	1960	CC	216	n	bl	y	n	0	cu	all/unsp	1	14	nev any	st
HITOSU	10	x	f	0	0	all	-	all	As:Jap	1960	CC	216	n	bl	y	n	0	cu	all/unsp	1	14	nev any	st
HOLE	9	x	m	0	0	all	0	all	Eu:UK	1972	pr	225	n	V	n	n	0	cu	cig+/-ot	1	14	nev any	st
HU	1		m	0	0	all	-	all	As:Chi	1985	CC	227	n	ot	n	y	0	ev	cig+/-ot	1	14	nev any	st
HU	4		f	0	0	all	-	all	As:Chi	1985	CC	227	n	ot	n	y	0	ev	cig+/-ot	1	14	nev any	st
HU2	3		c	0	0	all	-	all	As:Chi	1977	CC	523	n	ot	y	n	0	ev	cig+/-ot	5	9	nev cigs	st
HUMBLE	2		m	0	0	w-hi	-	all	NAmer	1980	CC	521	n	bl	y	n	1	cu	cig+/-ot	1	19	nev cigs	or
HUMBLE	5		m	0	0	hi	-	all	NAmer	1980	CC	521	n	bl	y	n	1	cu	cig+/-ot	1	19	nev cigs	or
HUMBLE	8		f	0	0	w-hi	-	all	NAmer	1980	CC	521	n	bl	y	n	1	cu	cig+/-ot	1	19	nev cigs	or
HUMBLE	11		f	0	0	hi	-	all	NAmer	1980	CC	521	n	bl	y	n	1	cu	cig+/-ot	1	19	nev cigs	or
JARUP	1	x	m	0	0	all	-	all	Eu:Sca	1928	CC	102	o	bl	y	n	0	ev	all/unsp	1	10	nev any	st
JEDRYC	60	x	m	0	0	all	-	all	Eu:est	1980	CC	1630	n	bl	y	n	0	ev	cig+/-ot	1	19	nev any	st
JEDRYC	65	x	f	0	0	all	-	all	Eu:est	1980	CC	1630	n	bl	y	n	0	ev	cig+/-ot	1	19	nev any	st
JOLY	7		m	0	0	all	-	all	SCAmer	1978	CC	826	n	bl	n	n	0	ev	cig+/-ot	1	9	nev any	st
JOLY	3		f	0	0	all	-	all	SCAmer	1978	CC	826	n	bl	n	n	0	ev	cig+/-ot	1	9	nev any	st
JUSSAW	11	x	m	0	0	all	-	all	As:Ind	1964	CC	792	n	V	n	n	0	ev	cig only	5	9	nev any	st
KAISE2	66		m	35	99	all	9	all	NAmer	1979	pr	318	n	bl	n	n	1	cu	cig only	1	19	nev any	st
KAISE2	58		f	35	99	all	9	all	NAmer	1979	pr	318	n	bl	n	n	1	cu	cig only	1	19	nev any	st
KAISER	6		m	0	0	all	0	all	NAmer	1964	pr	714	n	bl	n	n	2	cu	cig+/-ot	1	19	nev cigs	or
KAISER	2		f	0	0	all	0	all	NAmer	1964	pr	714	n	bl	n	n	2	cu	cig+/-ot	1	19	nev cigs	or
KANELL	1	x	m	0	0	all	-	all	Eu:bal	1950	CC	862	n	bl	n	n	0	cu	cig+/-ot	1	10	nev any	st
KATSOU	7		f	0	0	all	-	all	Eu:bal	1987	CC	101	n	bl	n	n	0	cu	all/unsp	1	10	nev any	st
KAUFMA	2	x	c	0	0	all	-	all	NAmer	1981	CC	881	n	bl	n	n	0	cu	cig+/-ot	1	14	nev cigs	st
KHUDER	1		m	0	0	all	-	all	NAmer	1985	CC	482	n	bl	n	y	0	ev	cig+/-ot	1	19	nev cigs	st
KINLEN	3	x	m	0	0	all	0	all	Eu:UK	1967	pr	718	n	V	n	n	0	cu	cig+/-ot	1	14	nev any	st
KNEKT	25	x	m	20	69	all	21	all	Eu:Sca	1966	pr	515	n	bl	n	n	0	cu	cig+/-ot	1	14	nev any	st
KOO	11		f	0	0	all	-	all	As:HK	1981	CC	200	n	bl	n	n	0	cu	all/unsp	1	10	nev any	st
KOULUM	6		m	0	0	all	-	all	Eu:Sca	1936	CC	812	n	bl	n	n	0	ev	all/unsp	1	9	nev any	st
KREUZE	19		m	1	45	all	-	all	Eu:Ger	1990	CC	2260	n	bl	n	n	3	ev	cig+/-ot	1	9	nev any	or
KREUZE	30		m	55	69	all	-	all	Eu:Ger	1990	CC	2260	n	bl	n	n	3	ev	cig+/-ot	1	9	nev any	or
KREUZE	25		f	1	45	all	-	all	Eu:Ger	1990	CC	2260	n	bl	n	n	3	ev	cig+/-ot	1	9	nev any	or
KREUZE	36		f	55	69	all	-	all	Eu:Ger	1990	CC	2260	n	bl	n	n	3	ev	cig+/-ot	1	9	nev any	or
KREYBE	21	x	m	0	0	all	-	all	Eu:Sca	1948	CC	300	n	bl	n	y	0	ev	all/unsp	1	14	nev any	st
KREYBE	37	x	f	0	0	all	-	all	Eu:Sca	1948	CC	300	n	bl	n	y	0	ev	all/unsp	1	14	nev any	st
LAMTH	7		f	0	0	ch	-	all	As:HK	1983	CC	445	n	bl	n	n	0	ev	all/unsp	1	10	nev any	or
LAUSSM	3	x	m	0	0	all	-	all	Eu:Ger	1982	CC	432	n	bl	n	n	0	ev	all/unsp	1	9	nev any	st
LETOUR	2		c	0	0	all	-	all	NAmer	1983	CC	738	n	V	y	y	0	ev	cig+/-ot	1	19	nev cigs	st
LIAW	3		c	0	0	all	0	all	As:oth	1982	pr	127	n	ot	n	n	2	cu	all/unsp	1	10	nev any	or
LIDDEL	2		m	0	0	all	18	all	NAmer	1970	pr	304	m	V	n	n	1	cu	cig+/-ot	1	19	nev cigs	ot
LIU2	5	x	m	0	0	all	-	all	As:Chi	1983	CC	316	n	ot	n	n	0	ev	all/unsp	1	19	nev any	st
LIU2	11	x	f	0	0	all	-	all	As:Chi	1983	CC	316	n	ot	n	n	0	ev	all/unsp	1	9	nev any	st
LIU3	3	x	m	0	0	all	-	all	As:Chi	1985	CC	110	n	ot	n	n	0	ev	all/unsp	1	15	nev any	st
LIU4	7		m	35	69	all	-	all	As:Chi	1986	CC	1000-00	n	ot	y	n	2	ev	cig only	1	19	nev any	ot
LIU5	2		c	0	0	all	-	all	As:Chi	1978	CC	111	n	ot	y	n	0	ev	all/unsp	1	9	nev any	st
LUBIN	7	x	m	0	0	all	-	all	As:Chi	1984	CC	427	m	ot	y	n	0	ev	cig only	1	6	nev any	st
LUBIN2	273		m	0	0	all	-	all	Eu:mul	1976	CC	7804	n	bl	n	y	0	ev	cig+/-ot	1	9	nev any	st
LUBIN2	281		f	0	0	all	-	all	Eu:mul	1976	CC	7804	n	bl	n	y	0	ev	cig+/-ot	1	9	nev any	st
MACLEN	20	x	m	0	0	ch	-	all	As:oth	1972	CC	233	n	bl	n	n	0	cu	cig+/-ot	1	9	nev cigs	st
MACLEN	33	x	f	0	0	ch	-	all	As:oth	1972	CC	233	n	bl	n	n	0	cu	cig+/-ot	1	9	nev cigs	st
MARTIS	1		m	0	0	all	-	all	Eu:UK	1972	CC	201	n	V	n	n	0	ev	cig+/-ot	1	14	nev cigs	st
MATOS	28	x	m	0	0	all	-	all	SCAmer	1994	CC	200	n	bl	n	n	0	ev	cig+/-ot	1	14	nev any	st
MATSUD	1		m	0	0	all	-	all	As:Jap	1965	CC	179	n	bl	n	n	0	ev	cig+/-ot	1	10	nev cigs	st
MCCONN	26		c	0	0	all	-	all	Eu:UK	1946	CC	100	n	V	n	y	0	ev	all/unsp	1	10	nev any	st
MIGRAN	1	x	m	0	0	all	0	all	Eu:UK	1964	pr	259	n	V	n	n	0	cu	cig only	1	9	nev any	st
MIGRAN	28	x	f	0	0	all	0	all	Eu:UK	1964	pr	259	n	V	n	n	0	cu	cig only	1	9	nev any	st
MRFITR	3		m	0	0	all	0	all	NAmer	1973	pr	119	n	bl	n	n	0	cu	cig+/-ot	1	19	nev cigs	ot
NOTAN2	8		m	0	0	all	-	all	As:Ind	1963	CC	683	n	V	n	n	0	ev	cig only	1	9	nev any	st
ORMOS	1		m	0	0	all	-	all	Eu:est	1947	CC	119	n	bl	y	y	0	ev	cig+/-ot	1	15	nev any	st
PARKIN	17	x	m	0	0	bl	-	all	Africa	1963	CC	877	n	V	y	n	0	cu	all/unsp	1	14	nev any	st
PASTOR	1	x	m	0	0	all	-	all	Eu:wst	1976	CC	204	n	bl	y	n	0	ev	all/unsp	1	9	nev any	st
PERNU	18		m	0	0	all	-	all	Eu:Sca	1944	CC	1606	n	bl	n	n	0	ev	all/unsp	5	9	nev any	st
PERNU	12		f	0	0	all	-	all	Eu:Sca	1944	CC	1606	n	bl	n	n	0	ev	all/unsp	5	9	nev any	st
PERSH2	2	x	c	0	0	all	-	all	Eu:Sca	1980	CC	1022	n	bl	y	n	0	cu	all/unsp	1	9	nev any	st
PETO	2		m	0	0	all	0	all	Eu:UK	1954	pr	103	n	V	n	n	0	cu	all/unsp	1	14	nev any	st
POLEDN	2		c	0	0	all	-	all	NAmer	1978	CC	209	n	bl	y	n	0	ev	cig+/-ot	1	19	nev cigs	st
PRESCO	2		m	0	0	all	0	all	Eu:Sca	1964	pr	867	n	bl	n	n	1	cu	all/unsp	1	14	nev any	st
PRESCO	1		f	0	0	all	0	all	Eu:Sca	1964	pr	867	n	bl	n	n	1	cu	all/unsp	1	14	nev any	st
RACHTA	5	x	f	0	0	all	-	all	Eu:est	1991	CC	118	n	bl	n	y	0	ev	cig+/-ot	1	9	nev cigs	st
RANDIG	2		m	0	0	all	-	all	Eu:Ger	1951	CC	448	n	bl	n	n	0	ev	all/unsp	5	9	nev any	st
RANDIG	6		f	0	0	all	-	all	Eu:Ger	1951	CC	448	n	bl	n	n	0	ev	all/unsp	5	9	nev any	st

Table 1G12 - 4

IESLC - Meta-anal of Ever Smoking (or Current if ever not avail), Amount smoked, "Low", Any prod (or Cigs if Any not avail)
All LC types
Least adjusted

REF	NRR	X	SEX	AGEL	AGEH	RACE	YF	LC TYPE	LOC	START	ST	NLC	R	VB	P	H	AD	SM	PRODUCT	exL	exH	DENOM	De
SEGI2	9	x	m	0	0	all	-	all	As:Jap	1962	CC	378	n	bl	n	n	0	cu	cig+/-ot	1	9	nev any	st
SEGI2	21	x	f	0	0	all	-	all	As:Jap	1962	CC	378	n	bl	n	n	0	cu	cig+/-ot	1	9	nev any	st
SHAW	10		c	0	0	wh	-	all	NAmer	1988	CC	335	n	V	n	y	0	ev	all/unsp	1	19	nev any	st
SIEMIA	13		m	0	0	all	-	all	NAmer	1979	CC	857	n	V	y	y	0	ev	cig+/-ot	1	19	nev cigs	or
SOBUE	117		m	0	0	all	-	q+s+l+a	As:Jap	1986	CC	1376	n	bl	n	y	0	cu	cig+/-ot	1	19	nev cigs	st
SPEIZE	2		f	0	0	all	0	all	NAmer	1976	pr	593	n	bl	n	y	1	cu	cig+/-ot	5	14	nev cigs	ot
SPITZ	5		c	0	0	b+hi	-	all	NAmer	1992	CC	177	n	bl	n	y	0	ev	cig+/-ot	1	19	nev cigs	st
STOCKS	25	x	m	0	0	all	-	all	Eu:UK	1952	CC	2932	n	V	y	n	0	ev	cig+/-ot	1	14	nev any	st
STOCKS	48		f	0	0	all	-	all	Eu:UK	1952	CC	2932	n	V	y	n	1	ev	cig+/-ot	1	14	nev any	ot
STOCKW	1		c	0	0	all	-	all	NAmer	1981	CC	22161	n	bl	n	n	0	cu	cig+/-ot	1	19	nev any	st
SVENSS	26	x	f	0	0	all	-	all	Eu:Sca	1983	CC	210	n	bl	n	n	0	cu	all/unsp	1	10	nev any	st
TENKAN	10		m	0	0	all	17	all	Eu:Sca	1962	pr	242	n	bl	n	n	1	cu	all/unsp	1	14	nev any	ot
TIZZAN	7		m	0	0	all	-	all	Eu:wst	1959	CC	1358	n	bl	n	n	0	ev	cig only	1	9	nev any	st
TIZZAN	15		f	0	0	all	-	all	Eu:wst	1959	CC	1358	n	bl	n	n	0	ev	cig only	1	9	nev any	st
TSUGAN	29		m	0	0	all	-	q+a	As:Jap	1976	CC	134	n	bl	n	y	0	cu	all/unsp	1	15	nev any	st
TULINI	4	x	m	0	0	all	0	all	Eu:Sca	1967	pr	472	n	bl	n	n	1	cu	cig+/-ot	1	14	nev any	or
TULINI	9	x	f	0	0	all	0	all	Eu:Sca	1967	pr	472	n	bl	n	n	1	cu	cig+/-ot	1	14	nev any	or
TVERDA	9		m	0	0	all	0	all	Eu:Sca	1972	pr	238	n	bl	n	n	2	cu	cig only	1	9	nev cigs	ot
TVERDA	16		f	0	0	all	0	all	Eu:Sca	1972	pr	238	n	bl	n	n	2	cu	cig only	1	9	nev cigs	ot
WAKAI	37	x	m	0	0	all	-	all	As:Jap	1988	CC	333	n	bl	n	y	0	cu	cig+/-ot	1	19	nev any	st
WANG2	2	x	c	0	0	all	-	all	As:Chi	1980	CC	103	n	ot	n	n	0	ev	cig+/-ot	5	9	nev cigs	st
WUWILL	18	x	f	0	0	all	-	all	As:Chi	1985	CC	965	n	ot	n	n	0	ev	cig+/-ot	1	19	nev cigs	st
WYNDE2	17		m	0	0	all	-	all	NAmer	1962	CC	404	n	bl	n	y	0	ev	cig+/-ot	1	10	nev any	st
WYNDE3	44		m	0	0	all	-	all	NAmer	1966	CC	350	n	bl	n	y	0	ev	cig+/-ot	1	9	nev any	st
WYNDE3	79		f	0	0	all	-	all	NAmer	1966	CC	350	n	bl	n	y	0	ev	cig+/-ot	1	9	nev any	st
WYNDE4	43		m	0	0	all	-	all	NAmer	1948	CC	684	n	bl	y	n	0	ev	all/unsp	1	9	nev any	st
WYNDE4	57		f	0	0	all	-	all	NAmer	1948	CC	684	n	bl	y	n	2	ev	all/unsp	1	9	nev any	ot
WYNDE6	27		m	0	0	all	-	all	NAmer	1969	CC	4423	n	bl	n	y	0	cu	cig+/-ot	1	10	nev any	st
WYNDE6	216		f	0	0	all	-	all	NAmer	1969	CC	4423	n	bl	n	y	0	cu	cig+/-ot	1	10	nev cigs	st
XU3	5	x	m	0	0	all	-	all	As:Chi	1981	CC	135	n	ot	n	n	0	ev	all/unsp	1	9	nev any	st
XU3	13	x	f	0	0	all	-	all	As:Chi	1981	CC	135	n	ot	n	n	0	ev	all/unsp	1	9	nev any	st
YUAN	2		m	0	0	all	0	all	As:Chi	1986	pr	142	n	ot	n	n	2	ev	cig+/-ot	1	19	nev cigs	ot
ZHENG	11		m	0	0	all	-	all	As:Chi	1982	CC	540	n	ot	*	y	0	ev	cig+/-ot	1	9	nev cigs	st
ZHENG	22		f	0	0	all	-	all	As:Chi	1982	CC	540	n	ot	*	y	0	ev	cig+/-ot	1	9	nev cigs	st
ZHOU	4		c	0	0	all	-	all	As:Chi	1978	CC	1360	n	ot	n	n	0	ev	all/unsp	1	9	nev any	st

Cigarette type is all/unspec for all RRs
except for the following:

REF	NRR	CIGTYPE
ALDERS	18	MC only
ALDERS	21	MC only
DEAN3	5	MC only
DEAN3	89	MC only
JUSSAW	11	MC only
NOTAN2	8	MC only

Table 1G12 - 5

IESLC - Meta-anal of Ever Smoking (or Current if ever not avail), Amount smoked, "Low", Any prod (or Cigs if Any not avail)
All LC types
Least adjusted

REF	NRR	SEX	AD	Number Exposed Case	Number Exposed Cont	Non-exposed Case	Non-exposed Cont	RR	95.00%CI
AGUDO	11	f	0	6	12	80	183	1.14	(0.41- 3.15)
*AKIBA	17	m	0	104	59893	18	35833	3.46	(2.10- 5.70)
*AKIBA	23	f	0	54	38968	116	359850	4.30	(3.11- 5.94)
Subtotal AKIBA								4.03	(3.07- 5.29)
ALDERS	18	m	1	-	-	-	-	3.55	(1.94- 6.49)
ALDERS	21	f	1	-	-	-	-	2.62	(1.88- 3.65)
Subtotal ALDERS								2.81	(2.10- 3.76)
*ARCHER	1	m	0	14	6504	6	9842	3.53	(1.36- 9.18)
ARMADA	46	m	0	44	117	4	64	6.02	(2.07- 17.51)
AUVINE	5	c	0	57	33	44	229	8.99	(5.26- 15.37)
AXELSS	19	m	0	39	96	16	160	4.06	(2.15- 7.66)
AXELSS	13	f	0	13	37	18	154	3.01	(1.35- 6.68)
Subtotal AXELSS								3.62	(2.20- 5.94)
BARBON	5	m	0	28	87	22	188	2.75	(1.49- 5.08)
*BENSHL	11	m	1	-	-	-	-	4.00	(1.55- 10.31)
*BEST	13	m	1	-	-	-	-	10.00	(4.56- 21.92)
BRESLO	13	m	0	16	45	22	110	1.78	(0.86- 3.69)
BRESLO	29	f	0	5	5	12	14	1.17	(0.27- 5.02)
Subtotal BRESLO								1.63	(0.85- 3.14)
*BRETT	1	m	0	40	17090	6	6530	2.55	(1.08- 6.01)
BROWN2	32	m	2	-	-	-	-	6.10	(5.30- 6.90)
BROWN2	31	f	2	-	-	-	-	8.40	(7.20- 9.70)
Subtotal BROWN2								7.02	(6.36- 7.75)
BUFFLE	28	f	0	21	42	12	112	4.67	(2.11- 10.31)
*CEDERL	80	m	2	-	-	-	-	3.40	(1.96- 5.90)
*CEDERL	76	f	2	-	-	-	-	2.83	(1.72- 4.67)
Subtotal CEDERL								3.07	(2.12- 4.45)
*CHANG	2	m	0	5	100	5	502	5.02	(1.48- 17.02)
*CHANG	8	f	0	6	205	11	1139	3.03	(1.13- 8.10)
Subtotal CHANG								3.70	(1.72- 7.95)
CHEN2	3	m	0	17	26	9	33	2.40	(0.92- 6.25)
CHEN2	7	f	0	5	17	25	33	0.39	(0.13- 1.20)
Subtotal CHEN2								1.12	(0.54- 2.31)
CHOI	12	m	0	20	90	13	95	1.62	(0.76- 3.46)
CHOI	17	f	0	9	16	76	164	1.21	(0.51- 2.87)
Subtotal CHOI								1.43	(0.81- 2.52)
*CHOW	3	m	0	38	29404	6	62913	13.55	(5.73- 32.05)
COMSTO	4	m	0	18	25	4	69	12.42	(3.83- 40.26)
COMSTO	9	f	0	16	19	13	115	7.45	(3.10- 17.93)
Subtotal COMSTO								8.95	(4.43- 18.08)
COOKSO	1	c	0	102	27	45	61	5.12	(2.89- 9.08)
*CPSI	243	m	1	-	-	-	-	5.81	(4.33- 7.80)
*CPSI	275	f	1	-	-	-	-	1.25	(0.73- 2.13)
Subtotal CPSI								4.07	(3.14- 5.26)
*CPSII	30	m	0	115	53096	124	742207	12.96	(10.06- 16.71)
*CPSII	105	f	1	-	-	-	-	4.16	(3.41- 5.09)
Subtotal CPSII								6.44	(5.50- 7.53)
DAMBER	6	m	1	-	-	-	-	2.30	(1.30- 4.40)
DARBY	1	m	0	128	223	3	384	73.47	(23.11- 233.57)
DARBY	8	f	0	71	104	23	529	15.70	(9.38- 26.28)
Subtotal DARBY								20.27	(12.66- 32.45)
DAVEYS	1	m	0	11	69	3	23	1.22	(0.31- 4.77)
DEAN3	5	m	0	81	264	25	510	6.26	(3.90- 10.04)
DEAN3	89	f	0	31	486	41	1538	2.39	(1.48- 3.86)
Subtotal DEAN3								3.89	(2.78- 5.44)
*DEKLER	2	m	2	-	-	-	-	19.40	(2.60- 143.70)
DESTEF	1	m	0	38	84	27	163	2.73	(1.56- 4.78)
DOLL	2	m	0	489	570	7	61	7.48	(3.39- 16.50)
DOLL	8	f	0	24	18	40	59	1.97	(0.95- 4.09)
Subtotal DOLL								3.64	(2.13- 6.22)
*DOLL2	46	m	1	-	-	-	-	6.71	(0.91- 49.81)
*DOLL2	10	f	1	-	-	-	-	1.29	(0.14- 11.50)
Subtotal DOLL2								3.19	(0.72- 14.02)
DORANT	6	c	0	21	192	14	1090	8.52	(4.26- 17.04)
DORGAN	10	m	0	125	148	15	93	5.24	(2.89- 9.49)
DORGAN	34	m	0	118	84	3	35	16.39	(4.88- 55.06)
DORGAN	96	f	3	-	-	-	-	5.67	(4.36- 7.36)
Subtotal DORGAN								5.83	(4.61- 7.37)
*DORN	408	m	1	-	-	-	-	4.02	(3.43- 4.71)
DOSEME	5	m	2	-	-	-	-	2.20	(1.40- 3.30)
*DUNN	2	m	0	12	9418	2	14160	9.02	(2.02- 40.30)

Table 1G12 - 5

IESLC - Meta-anal of Ever Smoking (or Current if ever not avail), Amount smoked, "Low", Any prod (or Cigs if Any not avail)
All LC types
Least adjusted

REF	NRR	SEX	AD	Number Exposed Case	Cont	Non-exposed Case	Cont	RR	95.00%CI
EBELIN	2	m	0	20	72	12	117	2.71	(1.25- 5.87)
*ENGELA	4	m	0	49	25801	27	58716	4.13	(2.58- 6.60)
*ENGELA	18	f	0	20	28450	31	207789	4.71	(2.69- 8.27)
Subtotal ENGELA								4.36	(3.04- 6.25)
*ENSTRO	7	m	1	-	-	-	-	4.74	(3.34- 6.73)
*ENSTRO	11	f	1	-	-	-	-	2.15	(1.62- 2.84)
Subtotal ENSTRO								2.93	(2.35- 3.65)
ESAKI	1	m	0	47	75	16	28	1.10	(0.54- 2.24)
FAN	6	m	0	13	121	36	236	0.70	(0.36- 1.38)
FAN	10	f	0	17	48	69	320	1.64	(0.89- 3.03)
Subtotal FAN								1.12	(0.71- 1.76)
GAO	24	f	0	70	74	435	605	1.32	(0.93- 1.87)
GAO2	2	m	0	32	41	13	56	3.36	(1.57- 7.19)
GARSHI	18	m	0	105	282	41	363	3.30	(2.23- 4.88)
GER	18	c	0	21	108	51	246	0.94	(0.54- 1.64)
GOLLED	15	m	0	83	679	15	490	3.99	(2.28- 7.00)
GSELL	1	m	0	11	36	2	29	4.43	(0.91- 21.60)
*HAMMO2	21	m	0	38	764	5	891	8.86	(3.51- 22.41)
*HAMMON	153	m	1	-	-	-	-	7.38	(3.72- 14.63)
*HANSEN	1	m	2	-	-	-	-	1.37	(0.63- 3.54)
*HIRAYA	23	m	1	-	-	-	-	2.06	(1.49- 2.85)
*HIRAYA	26	f	1	-	-	-	-	2.25	(1.64- 3.08)
Subtotal HIRAYA								2.16	(1.72- 2.70)
HITOSU	3	m	0	33	585	7	242	1.95	(0.85- 4.47)
HITOSU	10	f	0	24	391	33	1893	3.52	(2.06- 6.02)
Subtotal HITOSU								2.96	(1.88- 4.64)
*HOLE	9	m	0	23	840	7	1189	4.65	(2.00- 10.79)
HU	1	m	0	36	38	41	67	1.55	(0.85- 2.82)
HU	4	f	0	19	10	40	48	2.28	(0.95- 5.46)
Subtotal HU								1.75	(1.07- 2.87)
HU2	3	c	0	44	58	121	213	1.34	(0.85- 2.10)
HUMBLE	2	m	1	-	-	-	-	9.20	(3.30- 25.80)
HUMBLE	5	m	1	-	-	-	-	11.60	(2.70- 61.50)
HUMBLE	8	f	1	-	-	-	-	19.20	(6.50- 60.80)
HUMBLE	11	f	1	-	-	-	-	18.50	(4.90- 72.40)
Subtotal HUMBLE								13.65	(7.44- 25.07)
JARUP	1	m	0	42	25	11	42	6.41	(2.80- 14.68)
JEDRYC	60	m	0	131	180	49	219	3.25	(2.22- 4.77)
JEDRYC	65	f	0	37	10	78	166	7.87	(3.72- 16.65)
Subtotal JEDRYC								3.91	(2.78- 5.49)
JOLY	7	m	0	16	54	12	218	5.38	(2.41- 12.05)
JOLY	3	f	0	33	38	52	283	4.73	(2.72- 8.21)
Subtotal JOLY								4.93	(3.12- 7.77)
JUSSAW	11	m	0	6	9	149	624	2.79	(0.98- 7.97)
*KAISE2	66	m	1	-	-	-	-	4.47	(2.00- 9.99)
*KAISE2	58	f	1	-	-	-	-	7.61	(3.26- 17.75)
Subtotal KAISE2								5.75	(3.21- 10.31)
*KAISER	6	m	2	-	-	-	-	6.58	(3.87- 11.20)
*KAISER	2	f	2	-	-	-	-	3.42	(2.17- 5.40)
Subtotal KAISER								4.51	(3.19- 6.38)
KANELL	1	m	0	162	272	48	172	2.13	(1.47- 3.10)
KATSOU	7	f	0	8	9	48	67	1.24	(0.45- 3.45)
KAUFMA	2	c	0	66	214	35	925	8.15	(5.27- 12.60)
KHUDER	1	m	0	81	434	23	309	2.51	(1.54- 4.07)
*KINLEN	3	m	0	200	3659	7	1333	10.41	(4.91- 22.06)
*KNEKT	25	m	0	23	14798	6	17814	4.61	(1.88- 11.33)
KOO	11	f	0	17	19	56	85	1.36	(0.65- 2.84)
KOULUM	6	m	0	37	77	5	54	5.19	(1.92- 14.06)
KREUZE	19	m	3	-	-	-	-	2.50	(0.70- 8.20)
KREUZE	30	m	3	-	-	-	-	8.20	(5.20- 13.00)
KREUZE	25	f	3	-	-	-	-	5.70	(1.60- 16.60)
KREUZE	36	f	3	-	-	-	-	2.00	(1.20- 3.30)
Subtotal KREUZE								4.27	(3.12- 5.85)
KREYBE	21	m	0	154	2341	6	644	7.06	(3.11- 16.04)
KREYBE	37	f	0	10	286	30	657	0.77	(0.37- 1.59)
Subtotal KREYBE								2.04	(1.18- 3.52)
LAMTH	7	f	0	101	63	202	337	2.67	(1.87- 3.83)
LAUSSM	3	m	0	75	63	85	226	3.17	(2.08- 4.81)
LETOUR	2	c	0	271	266	24	224	9.51	(6.04- 14.97)
*LIAW	3	c	2	-	-	-	-	3.10	(1.70- 5.60)
*LIDDEL	2	m	1	-	-	-	-	3.33	(2.05- 5.64)

Table 1G12 - 5

IESLC - Meta-anal of Ever Smoking (or Current if ever not avail), Amount smoked, "Low", Any prod (or Cigs if Any not avail)
All LC types
Least adjusted

REF	NRR	SEX	AD	Number Exposed Case	Number Exposed Cont	Non-exposed Case	Non-exposed Cont	RR	95.00%CI
LIU2	5	m	0	21	93	12	44	0.83	(0.37- 1.83)
LIU2	11	f	0	8	10	38	69	1.45	(0.53- 3.99)
Subtotal LIU2								1.03	(0.55- 1.92)
LIU3	3	m	0	25	93	4	19	1.28	(0.40- 4.09)
LIU4	7	m	2	-	-	-	-	2.11	(2.02- 2.20)
LIU5	2	c	0	14	27	26	41	0.82	(0.36- 1.84)
LUBIN	7	m	0	4	41	8	73	0.89	(0.25- 3.14)
LUBIN2	273	m	0	1887	3759	190	2616	6.91	(5.91- 8.09)
LUBIN2	281	f	0	151	218	336	1188	2.45	(1.93- 3.11)
Subtotal LUBIN2								5.06	(4.44- 5.77)
MACLEN	20	m	0	5	11	5	15	1.36	(0.32- 5.89)
MACLEN	33	f	0	6	21	41	109	0.76	(0.29- 2.02)
Subtotal MACLEN								0.91	(0.40- 2.05)
MARTIS	1	m	0	31	39	4	25	4.97	(1.56- 15.78)
MATOS	28	m	0	17	88	11	110	1.93	(0.86- 4.34)
MATSUD	1	m	0	37	1237	3	1255	12.51	(3.85- 40.69)
MCCONN	26	c	0	7	43	9	23	0.42	(0.14- 1.26)
*MIGRAN	1	m	0	11	508	4	867	4.69	(1.50- 14.66)
*MIGRAN	28	f	0	4	772	4	3814	4.94	(1.24- 19.71)
Subtotal MIGRAN								4.79	(1.99- 11.55)
*MRFITR	3	m	0	2	856	0	1859	10.86~	(0.52- 225.86)
NOTAN2	8	m	0	6	42	134	544	0.58	(0.24- 1.39)
ORMOS	1	m	0	32	329	7	777	10.80	(4.72- 24.71)
PARKIN	17	m	0	314	818	107	1248	4.48	(3.53- 5.67)
PASTOR	1	m	0	8	39	10	89	1.83	(0.67- 4.98)
PERNU	18	m	0	61	31	97	275	5.58	(3.42- 9.11)
PERNU	12	f	0	5	13	110	971	3.40	(1.19- 9.70)
Subtotal PERNU								5.10	(3.27- 7.96)
PERSH2	2	c	0	290	349	178	1164	5.43	(4.35- 6.78)
*PETO	2	m	0	44	1181	2	295	5.50	(1.34- 22.54)
POLEDN	2	c	0	53	103	12	139	5.96	(3.03- 11.72)
*PRESCO	2	m	1	-	-	-	-	10.20	(4.49- 23.15)
*PRESCO	1	f	1	-	-	-	-	6.36	(3.60- 11.24)
Subtotal PRESCO								7.42	(4.65- 11.84)
RACHTA	5	f	0	6	7	33	98	2.55	(0.80- 8.12)
RANDIG	2	m	0	65	99	5	22	2.89	(1.04- 8.01)
RANDIG	6	f	0	12	13	17	92	5.00	(1.95- 12.79)
Subtotal RANDIG								3.88	(1.95- 7.76)
SEGI2	9	m	0	20	62	8	53	2.14	(0.87- 5.25)
SEGI2	21	f	0	9	9	56	126	2.25	(0.85- 5.97)
Subtotal SEGI2								2.19	(1.13- 4.24)
SHAW	10	c	0	46	90	11	107	4.97	(2.43- 10.16)
SIEMIA	13	m	0	-	-	-	-	3.00	(1.00- 9.90)
SOBUE	117	m	0	147	157	34	128	3.52	(2.27- 5.47)
*SPEIZE	2	f	1	-	-	-	-	5.20	(3.60- 8.10)
SPITZ	5	c	0	27	88	10	96	2.95	(1.35- 6.43)
STOCKS	25	m	0	691	1743	45	638	5.62	(4.10- 7.70)
STOCKS	48	f	1	-	-	-	-	2.24	(1.64- 3.03)
Subtotal STOCKS								3.51	(2.81- 4.37)
STOCKW	1	c	0	2090	1194	2791	10641	6.67	(6.15- 7.25)
SVENSS	26	f	0	42	30	38	120	4.42	(2.44- 8.01)
*TENKAN	10	m	1	-	-	-	-	15.86	(6.80- 37.00)
TIZZAN	7	m	0	130	238	180	305	0.93	(0.70- 1.23)
TIZZAN	15	f	0	11	14	25	114	3.58	(1.46- 8.82)
Subtotal TIZZAN								1.04	(0.80- 1.37)
TSUGAN	29	m	0	14	19	18	22	0.90	(0.36- 2.28)
*TULINI	4	m	1	-	-	-	-	6.49	(3.25- 13.00)
*TULINI	9	f	1	-	-	-	-	9.39	(4.99- 17.70)
Subtotal TULINI								7.94	(4.97- 12.67)
*TVERDA	9	m	2	-	-	-	-	2.14	(1.15- 3.96)
*TVERDA	16	f	2	-	-	-	-	4.53	(1.08- 18.94)
Subtotal TVERDA								2.41	(1.36- 4.25)
WAKAI	37	m	0	30	105	10	65	1.86	(0.85- 4.05)
WANG2	2	c	0	3	10	11	43	1.17	(0.28- 5.00)
WUWILL	18	f	0	451	311	417	601	2.09	(1.73- 2.53)
WYNDE2	17	m	0	17	114	8	105	1.96	(0.81- 4.72)
WYNDE3	44	m	0	8	42	9	88	1.86	(0.67- 5.17)
WYNDE3	79	f	0	3	19	20	76	0.60	(0.16- 2.23)
Subtotal WYNDE3								1.22	(0.54- 2.72)
WYNDE4	43	m	0	17	82	12	115	1.99	(0.90- 4.38)
WYNDE4	57	f	2	-	-	-	-	1.13	(0.33- 3.89)

Table 1G12 - 5

IESLC - Meta-anal of Ever Smoking (or Current if ever not avail), Amount smoked, "Low", Any prod (or Cigs if Any not avail)
All LC types
Least adjusted

REF	NRR	SEX	AD	Number Exposed Case	Number Exposed Cont	Non-exposed Case	Non-exposed Cont	RR	95.00%CI
Subtotal WYNDE4								1.69	(0.87- 3.28)
WYNDE6	27	m	0	117	122	87	617	6.80	(4.85- 9.54)
WYNDE6	216	f	0	76	109	159	856	3.75	(2.68- 5.26)
Subtotal WYNDE6								5.05	(3.98- 6.42)
XU3	5	m	0	10	26	7	31	1.70	(0.57- 5.10)
XU3	13	f	0	7	6	13	25	2.24	(0.62- 8.07)
Subtotal XU3								1.91	(0.83- 4.40)
*YUAN	2	m	2	-	-	-	-	3.60	(1.88- 6.91)
ZHENG	11	m	0	25	40	33	94	1.78	(0.94- 3.37)
ZHENG	22	f	0	24	29	152	184	1.00	(0.56- 1.79)
Subtotal ZHENG								1.30	(0.85- 2.00)
ZHOU	4	c	0	61	5	507	68	1.64	(0.64- 4.22)
Partial Totals				11318	314408	8906	1570341		

*prospective study ~ With 0.5 adjustment for zero

REF	NRR	SEX	AD	Ys	Ws	Qs	Ps
AGUDO	11	f	0	0.13	3.73	4.16	0.7953
*AKIBA	17	m	0	1.24	15.35	0.04	0.0000
*AKIBA	23	f	0	1.46	36.89	2.64	0.0000
Subtotal AKIBA				1.39	52.24	2.68	
ALDERS	18	m	1	1.27	10.54	0.06	0.0000
ALDERS	21	f	1	0.96	34.91	1.81	0.0000
Subtotal ALDERS				1.03	45.45	1.87	
*ARCHER	1	m	0	1.26	4.20	0.02	0.0097
ARMADA	46	m	0	1.79	3.37	1.23	0.0010
AUVINE	5	c	0	2.20	13.34	13.49	0.0000
AXELSS	19	m	0	1.40	9.54	0.43	0.0000
AXELSS	13	f	0	1.10	6.02	0.05	0.0069
Subtotal AXELSS				1.29	15.57	0.47	
BARBON	5	m	0	1.01	10.21	0.33	0.0012
*BENSHL	11	m	1	1.39	4.28	0.16	0.0041
*BEST	13	m	1	2.30	6.23	7.71	0.0000
BRESLO	13	m	0	0.58	7.18	2.72	0.1231
BRESLO	29	f	0	0.15	1.80	1.94	0.8360
Subtotal BRESLO				0.49	8.98	4.65	
*BRETT	1	m	0	0.94	5.22	0.34	0.0326
BROWN2	32	m	2	1.81	220.78	84.25	0.0000
BROWN2	31	f	2	2.13	172.98	152.09	0.0000
Subtotal BROWN2				1.95	393.76	236.33	
BUFFLE	28	f	0	1.54	6.11	0.75	0.0001
*CEDERL	80	m	2	1.22	12.65	0.01	0.0000
*CEDERL	76	f	2	1.04	15.40	0.35	0.0000
Subtotal CEDERL				1.12	28.05	0.36	
*CHANG	2	m	0	1.61	2.58	0.46	0.0096
*CHANG	8	f	0	1.11	3.97	0.03	0.0271
Subtotal CHANG				1.31	6.55	0.49	
CHEN2	3	m	0	0.87	4.19	0.42	0.0735
CHEN2	7	f	0	-0.95	3.04	13.87	0.0991
Subtotal CHEN2				0.11	7.23	14.29	
CHOI	12	m	0	0.48	6.73	3.35	0.2084
CHOI	17	f	0	0.19	5.18	5.15	0.6591
Subtotal CHOI				0.36	11.92	8.50	
*CHOW	3	m	0	2.61	5.18	10.39	0.0000
COMSTO	4	m	0	2.52	2.78	4.90	0.0000
COMSTO	9	f	0	2.01	4.98	3.33	0.0000
Subtotal COMSTO				2.19	7.76	8.23	
COOKSO	1	c	0	1.63	11.70	2.29	0.0000
*CPSI	243	m	1	1.76	44.36	14.36	0.0000
*CPSI	275	f	1	0.22	13.40	12.54	0.4140
Subtotal CPSI				1.40	57.76	26.90	
*CPSII	30	m	0	2.56	59.74	112.38	0.0000
*CPSII	105	f	1	1.43	95.77	5.29	0.0000
Subtotal CPSII				1.86	155.50	117.67	
DAMBER	6	m	1	0.83	10.34	1.32	0.0074
DARBY	1	m	0	4.30	2.87	27.71	0.0000
DARBY	8	f	0	2.75	14.48	35.38	0.0000
Subtotal DARBY				3.01	17.35	63.09	
DAVEYS	1	m	0	0.20	2.07	2.03	0.7726
DEAN3	5	m	0	1.83	17.21	7.13	0.0000
DEAN3	89	f	0	0.87	16.85	1.70	0.0003

Table 1G12 - 5

IESLC - Meta-anal of Ever Smoking (or Current if ever not avail), Amount smoked, "Low", Any prod (or Cigs if Any not avail)
All LC types
Least adjusted

REF	NRR	SEX	AD	Ys	Ws	Qs	Ps
Subtotal DEAN3				1.36	34.06	8.83	
*DEKLER	2	m	2	2.97	0.95	3.01	0.0038
DESTEF	1	m	0	1.00	12.29	0.42	0.0004
DOLL	2	m	0	2.01	6.13	4.14	0.0000
DOLL	8	f	0	0.68	7.19	1.90	0.0698
Subtotal DOLL				1.29	13.32	6.04	
*DOLL2	46	m	1	1.90	0.96	0.49	0.0623
*DOLL2	10	f	1	0.25	0.79	0.69	0.8209
Subtotal DOLL2				1.16	1.75	1.18	
DORANT	6	c	0	2.14	7.99	7.23	0.0000
DORGAN	10	m	0	1.66	10.85	2.35	0.0000
DORGAN	34	m	0	2.80	2.62	6.75	0.0000
DORGAN	96	f	3	1.74	56.05	16.63	0.0000
Subtotal DORGAN				1.76	69.51	25.72	
*DORN	408	m	1	1.39	152.79	6.16	0.0000
DOSEME	5	m	2	0.79	20.90	3.38	0.0003
*DUNN	2	m	0	2.20	1.71	1.75	0.0040
EBELIN	2	m	0	1.00	6.42	0.24	0.0116
*ENGELA	4	m	0	1.42	17.42	0.90	0.0000
*ENGELA	18	f	0	1.55	12.16	1.57	0.0000
Subtotal ENGELA				1.47	29.59	2.48	
*ENSTRO	7	m	1	1.56	31.30	4.18	0.0000
*ENSTRO	11	f	1	0.77	48.76	8.81	0.0000
Subtotal ENSTRO				1.07	80.06	12.99	
ESAKI	1	m	0	0.09	7.53	9.08	0.8001
FAN	6	m	0	-0.35	8.53	20.26	0.3059
FAN	10	f	0	0.50	10.28	4.96	0.1116
Subtotal FAN				0.11	18.81	25.22	
GAO	24	f	0	0.27	31.50	26.44	0.1237
GAO2	2	m	0	1.21	6.65	0.00	0.0018
GARSHI	18	m	0	1.19	24.87	0.00	0.0000
GER	18	c	0	-0.06	12.41	19.54	0.8213
GOLLED	15	m	0	1.38	12.16	0.46	0.0000
GSELL	1	m	0	1.49	1.53	0.14	0.0655
*HAMMO2	21	m	0	2.18	4.47	4.39	0.0000
*HAMMON	153	m	1	2.00	8.19	5.35	0.0000
*HANSEN	1	m	2	0.31	5.16	3.96	0.4747
*HIRAYA	23	m	1	0.72	36.53	8.00	0.0000
*HIRAYA	26	f	1	0.81	38.69	5.58	0.0000
Subtotal HIRAYA				0.77	75.22	13.57	
HITOSU	3	m	0	0.67	5.59	1.53	0.1144
HITOSU	10	f	0	1.26	13.32	0.06	0.0000
Subtotal HITOSU				1.08	18.91	1.59	
*HOLE	9	m	0	1.54	5.43	0.65	0.0003
HU	1	m	0	0.44	10.71	6.08	0.1527
HU	4	f	0	0.82	5.04	0.68	0.0643
Subtotal HU				0.56	15.74	6.75	
HU2	3	c	0	0.29	18.89	15.35	0.2087
HUMBLE	2	m	1	2.22	3.63	3.84	0.0000
HUMBLE	5	m	1	2.45	1.57	2.50	0.0021
HUMBLE	8	f	1	2.95	3.07	9.57	0.0000
HUMBLE	11	f	1	2.92	2.12	6.32	0.0000
Subtotal HUMBLE				2.61	10.40	22.23	
JARUP	1	m	0	1.86	5.60	2.50	0.0000
JEDRYC	60	m	0	1.18	26.20	0.00	0.0000
JEDRYC	65	f	0	2.06	6.86	5.23	0.0000
Subtotal JEDRYC				1.36	33.06	5.23	
JOLY	7	m	0	1.68	5.92	1.44	0.0000
JOLY	3	f	0	1.55	12.60	1.66	0.0000
Subtotal JOLY				1.59	18.52	3.09	
JUSSAW	11	m	0	1.03	3.50	0.09	0.0549
*KAISE2	66	m	1	1.50	5.94	0.56	0.0003
*KAISE2	58	f	1	2.03	5.35	3.77	0.0000
Subtotal KAISE2				1.75	11.29	4.32	
*KAISER	6	m	2	1.88	13.61	6.54	0.0000
*KAISER	2	f	2	1.23	18.49	0.03	0.0000
Subtotal KAISER				1.51	32.09	6.57	
KANELL	1	m	0	0.76	27.40	5.12	0.0001
KATSOU	7	f	0	0.22	3.68	3.50	0.6791
KAUFMA	2	c	0	2.10	20.21	16.65	0.0000
KHUDER	1	m	0	0.92	16.30	1.20	0.0002

Table 1G12 - 5

IESLC - Meta-anal of Ever Smoking (or Current if ever not avail), Amount smoked, "Low", Any prod (or Cigs if Any not avail)
All LC types
Least adjusted

REF	NRR	SEX	AD	Ys	Ws	Qs	Ps
*KINLEN	3	m	0	2.34	6.81	9.04	0.0000
*KNEKT	25	m	0	1.53	4.76	0.55	0.0008
KOO	11	f	0	0.31	7.09	5.55	0.4151
KOULUM	6	m	0	1.65	3.87	0.80	0.0012
KREUZE	19	m	3	0.92	2.54	0.19	0.1444
KREUZE	30	m	3	2.10	18.30	15.27	0.0000
KREUZE	25	f	3	1.74	2.81	0.85	0.0035
KREUZE	36	f	3	0.69	15.02	3.72	0.0072
Subtotal KREUZE				1.45	38.66	20.03	
KREYBE	21	m	0	1.95	5.71	3.33	0.0000
KREYBE	37	f	0	-0.27	7.23	15.35	0.4730
Subtotal KREYBE				0.71	12.94	18.69	
LAMTH	7	f	0	0.98	29.68	1.27	0.0000
LAUSSM	3	m	0	1.15	22.03	0.03	0.0000
LETOUR	2	c	0	2.25	18.66	21.04	0.0000
*LIAW	3	c	2	1.13	10.81	0.04	0.0002
*LIDDEL	2	m	1	1.20	15.00	0.00	0.0000
LIU2	5	m	0	-0.19	6.08	11.57	0.6415
LIU2	11	f	0	0.37	3.76	2.51	0.4689
Subtotal LIU2				0.03	9.84	14.08	
LIU3	3	m	0	0.24	2.83	2.53	0.6810
LIU4	7	m	2	0.75	2108.86	415.51	0.0000
LIU5	2	c	0	-0.20	5.84	11.31	0.6267
LUBIN	7	m	0	-0.12	2.42	4.13	0.8565
LUBIN2	273	m	0	1.93	155.25	85.62	0.0000
LUBIN2	281	f	0	0.90	66.54	5.79	0.0000
Subtotal LUBIN2				1.62	221.79	91.41	
MACLEN	20	m	0	0.31	1.79	1.39	0.6779
MACLEN	33	f	0	-0.27	4.03	8.67	0.5807
Subtotal MACLEN				-0.09	5.83	10.06	
MARTIS	1	m	0	1.60	2.87	0.49	0.0066
MATOS	28	m	0	0.66	5.88	1.66	0.1105
MATSUD	1	m	0	2.53	2.76	4.93	0.0000
MCCONN	26	c	0	-0.88	3.12	13.33	0.1215
*MIGRAN	1	m	0	1.55	2.96	0.37	0.0078
*MIGRAN	28	f	0	1.60	2.01	0.33	0.0237
Subtotal MIGRAN				1.57	4.97	0.71	
*MRFITR	3	m	0	2.38	0.42	0.59	0.1236
NOTAN2	8	m	0	-0.54	5.01	15.07	0.2229
ORMOS	1	m	0	2.38	5.60	7.92	0.0000
PARKIN	17	m	0	1.50	68.71	6.54	0.0000
PASTOR	1	m	0	0.60	3.82	1.32	0.2395
PERNU	18	m	0	1.72	15.98	4.46	0.0000
PERNU	12	f	0	1.22	3.48	0.00	0.0225
Subtotal PERNU				1.63	19.46	4.46	
PERSH2	2	c	0	1.69	78.18	19.71	0.0000
*PETO	2	m	0	1.70	1.93	0.51	0.0180
POLEDN	2	c	0	1.79	8.40	2.97	0.0000
*PRESCO	2	m	1	2.32	5.71	7.32	0.0000
*PRESCO	1	f	1	1.85	11.85	5.16	0.0000
Subtotal PRESCO				2.00	17.57	12.47	
RACHTA	5	f	0	0.93	2.86	0.19	0.1143
RANDIG	2	m	0	1.06	3.69	0.06	0.0415
RANDIG	6	f	0	1.61	4.35	0.76	0.0008
Subtotal RANDIG				1.36	8.04	0.82	
SEGI2	9	m	0	0.76	4.76	0.89	0.0975
SEGI2	21	f	0	0.81	4.03	0.58	0.1035
Subtotal SEGI2				0.78	8.79	1.47	
SHAW	10	c	0	1.60	7.51	1.28	0.0000
SIEMIA	13	m	0	1.10	2.92	0.02	0.0603
SOBUE	117	m	0	1.26	19.84	0.10	0.0000
*SPEIZE	2	f	1	1.65	23.37	4.90	0.0000
SPITZ	5	c	0	1.08	6.30	0.08	0.0067
STOCKS	25	m	0	1.73	38.74	11.13	0.0000
STOCKS	48	f	1	0.81	40.78	6.02	0.0000
Subtotal STOCKS				1.25	79.52	17.14	
STOCKW	1	c	0	1.90	565.53	283.16	0.0000
SVENSS	26	f	0	1.49	10.89	0.95	0.0000
*TENKAN	10	m	1	2.76	5.35	13.25	0.0000
TIZZAN	7	m	0	-0.08	48.24	77.56	0.5909
TIZZAN	15	f	0	1.28	4.74	0.03	0.0055

Table 1G12 - 5

IESLC - Meta-anal of Ever Smoking (or Current if ever not avail), Amount smoked, "Low", Any prod (or Cigs if Any not avail)
All LC types
Least adjusted

REF	NRR	SEX	AD	Ys	Ws	Qs	Ps
Subtotal TIZZAN				0.04	52.98	77.60	
TSUGAN	29	m	0	-0.10	4.44	7.45	0.8253
*TULINI	4	m	1	1.87	8.00	3.69	0.0000
*TULINI	9	f	1	2.24	9.59	10.55	0.0000
Subtotal TULINI				2.07	17.58	14.24	
*TVERDA	9	m	2	0.76	10.05	1.86	0.0159
*TVERDA	16	f	2	1.51	1.87	0.19	0.0387
Subtotal TVERDA				0.88	11.92	2.05	
WAKAI	37	m	0	0.62	6.32	2.06	0.1197
WANG2	2	c	0	0.16	1.83	1.94	0.8295
WUWILL	18	f	0	0.74	105.32	21.65	0.0000
WYNDE2	17	m	0	0.67	4.95	1.33	0.1353
WYNDE3	44	m	0	0.62	3.69	1.19	0.2325
WYNDE3	79	f	0	-0.51	2.23	6.45	0.4459
Subtotal WYNDE3				0.20	5.91	7.64	
WYNDE4	43	m	0	0.69	6.13	1.56	0.0891
WYNDE4	57	f	2	0.12	2.52	2.88	0.8460
Subtotal WYNDE4				0.52	8.66	4.44	
WYNDE6	27	m	0	1.92	33.49	17.68	0.0000
WYNDE6	216	f	0	1.32	33.57	0.59	0.0000
Subtotal WYNDE6				1.62	67.06	18.27	
XU3	5	m	0	0.53	3.19	1.38	0.3416
XU3	13	f	0	0.81	2.34	0.34	0.2159
Subtotal XU3				0.65	5.53	1.72	
*YUAN	2	m	2	1.28	9.07	0.07	0.0001
ZHENG	11	m	0	0.58	9.44	3.56	0.0764
ZHENG	22	f	0	0.00	11.34	16.03	0.9951
Subtotal ZHENG				0.26	20.78	19.58	
ZHOU	4	c	0	0.49	4.29	2.09	0.3077

	N	178
	NS	126
	Wt	5598.94
Het	Chi	2036.46
Het	df	177
Het	P	***
Fixed	RR	3.29
	RRl	3.20
	RRu	3.38
	P	+++
Random	RR	3.41
	RRl	3.05
	RRu	3.80
	P	+++
Asymm	P	N.S.

Table 1G12 - 6

IESLC - Meta-anal of Ever Smoking (or Current if ever not avail), Amount smoked, "Low", Any prod (or Cigs if Any not avail)
All LC types
Least adjusted

		Sex		
	combined	male	female	Total
N	17	102	59	178
NS	17	99	57	173
Wt	795.02	3679.18	1124.74	5598.94
Het Chi	167.09	1074.44	457.35	2036.46
Het df	16	101	58	177
Het P	***	***	***	***
Fixed RR	5.85	2.86	3.44	3.29
RRl	5.46	2.77	3.25	3.20
RRu	6.28	2.96	3.65	3.38
P	+++	+++	+++	+++
Random RR	3.46	3.71	2.94	3.41
RRl	2.47	3.21	2.44	3.05
RRu	4.86	4.30	3.53	3.80
P	+++	+++	+++	+++
Between Chi				337.59
Between df				2
Between P				***
Btwn(F) P				***
Btwn(R) P				N.S.

Table 1G12 - 7

IESLC - Meta-anal of Ever Smoking (or Current if ever not avail), Amount smoked, "Low", Any prod (or Cigs if Any not avail)
All LC types
Excluded studies (and stage at which they were excluded)

1	ABELIN ABRAHA AMANDU AMES ANDERS AUSTIN AXELSO BAND BECHER BERRIN BLOHMK BLOT4 BROCKM BROWN1 BYERS1 BYERS2 CARPEN CASCO2 CASCOR CHAN CHEN3 CHIAZZ CHYOU DESTE2 DOCKER DROSTE DU GARCIA GARDIN GENG GODLEY GOODMA GRAHAM GREGOR HEGMAN HEIN HENNEK HINDS HIRAOK HOROWI HORWIT HUANG ISHIMA JAHN JAIN JARVHO JIANG KELLER KIHARA KJUUS KO KOHLME KUBIK LAMWK LAMWK2 LANGE LEI LEMARC LEVIN LIU LOMBA2 LOMBAR MAGNUS MARSH MARSH2 MCDUFF MCLAUG MILLER MILLS NOTANI NOU ODRISC PAWLEG PERSHA POFFIJ QIAO QIAO2 RADZIK REN RONCO ROOTS ROTHSC SAARIK SANKAR SCHWAR SEGI SEOW SHIMIZ SIMARA SIMONA SITAS SOBUE2 STASZE STAYNE STUCKE SUN SUZUK2 SUZUKI TANG TAO TOKARS TOUSEY ULMER VEIERO VUTUC WALD WANG WANG3 WANG4 WICKLU WIGLE WILKIN WU2 WUNSCH WYNDE8 XIANGZ XU XU2 XU4 YONG ZHANG
2	BUELL CHEN MASTRA MZILEN PISANI RESTRE SADOWS
4	BOFFET WYNDE7
5	RIMING TANG2 WYNDE5
6	BLOT1 BLOT2 BLOT3 BOUCHA HIRAY2 JONES LAURIL LICKIN MOLLO MRFIT MURATA SCHWA2 VANDER WARSIN WATSON WYNDER
8	LUO
10	BOUCOT BROSS CHATZI CORREA DEAN DEAN2 HAENSZ NAM OSANN PEZZO2 PEZZOT PIKE WU YAMAGU
11	BENHAM GILLIS OSANN2

Table 1G12 - 8
Potentially overlapping studies

REF	REFGP	PRINC	.	OVERLAP/LINK
LUBIN2	LUBIN2	1		Lubin-combined
LAMTH	LAMTH	1		KOO/LAMTH/LAMWK
KOO	KOO	1		KOO/LAMTH/LAMWK
TVERDA	TVERDA	1		VEIERO/TVERDAL
AKIBA	AKIBA	1		AKIBA/ISHIMA
PRESCO	PRESCO	2		PRESCO-combined
HOLE	TANG2	1		Subset of TANG2
BENSHL	TANG2	1		Subset of TANG2
KAISER	KAISER	1		KAISER/OSANN2
MRFITR	MRFIT	2		Subset of MRFIT
WYNDE6	WYNDE6	1		WYNDE5/6/7/8
CPSI	CPSI	1		CPSI overall
ENSTRO	ENSTRO	1		Subset of CPSI
MATSUD	MATSUD	1		SOBUE2/MATSUD
LUBIN	XIANGZ	2		LUBIN/XIANGZ/QIAO

Table 1G12 - 9
Most adjusted - insufficient data for metaanalysis

REF	NRR	SEX	AGEL	AGEH	RACE	YF	LC TYPE	LOC	START	ST	NLC	R	VB	P	H	AD	SM	PRODUCT	exL	exH	DENOM	De
BLOT1	5	m	0	0	all	-	all	NAmer	1970	CC	458	n	bl	y	n	0	ev	cig+/-ot	1	9	nev cigs	or
BUELL	1	m	0	0	all	0	all	NAmer	1957	pr	304	n	bl	n	n	0	ev	cig+/-ot	1	18	nev cigs	st
HITOSU	14	m	0	0	all	-	all	As:Jap	1960	CC	216	n	bl	y	n	2	cu	all/unsp	1	14	nev any	or
HITOSU	17	f	0	0	all	-	all	As:Jap	1960	CC	216	n	bl	y	n	2	cu	all/unsp	1	14	nev any	or
MASTRA	3	m	0	0	all	-	all	Eu:wst	1973	CC	309	n	bl	n	n	1	ev	all/unsp	1	10	nev any	or
MZILEN	1	m	0	0	bl	-	all	Africa	1994	CC	374	n	V	*	n	3	ev	all/unsp	1	14	nev any	or
MZILEN	3	f	0	0	bl	-	all	Africa	1994	CC	374	n	V	*	n	3	ev	all/unsp	1	14	nev any	or
RESTRE	6	c	0	0	all	-	all	SCAmer	1978	CC	102	n	bl	n	n	5	ev	cig+/-ot	1	10	nev cigs	or
SADOWS	85	m	0	0	wh	-	all	NAmer	1938	CC	477	n	bl	n	n	1	ev	all/unsp	1	10	nev any	st

REF	NRR	RR	SIG	RRDATA comment
BLOT1	5	0.40		0
BUELL	1	2.32		0
HITOSU	14	1.80		0
HITOSU	17	3.10		0
MASTRA	3	6.80		0

Table 1G12 - 9

IESLC - Meta-anal of Ever Smoking (or Current if ever not avail), Amount smoked, "Low", Any prod (or Cigs if Any not avail)
All LC types
Most adjusted - insufficient data for metaanalysis

REF	NRR	RR	SIG	RRDATA comment
MZILEN	1	9.60		0
MZILEN	3	13.60		0
RESTRE	6	1.29		0
SADOWS	85	2.32		0

Least adjusted - insufficient data for meta-analysis: as for adjusted plus the following

REF	NRR	SEX	AGEL	AGEH	RACE	YF	LC TYPE	LOC	START	ST	NLC	R	VB	P	H	AD	SM	PRODUCT	exL	exH	DENOM	De
SADOWS	80	m	0	0	wh	-	all	NAmer	1938	CC	477	n	bl	n	n	0	ev	all/unsp	1	10	nev any	st

REF	NRR	RR	SIG	RRDATA comment
SADOWS	80	1.83		0

Table 1G13 -

IESLC - Meta-anal of Ever Smoking (or Current if ever not avail), Amount smoked, "Mid", Any prod (or Cigs if Any not avail)
All LC types

This analysis is restricted to results for:
1) Results by Amount smoked
2) Results complete enough for use in metaanalysis

Within each study, results are then selected (in the following order of preference, within each sex) for:
3) SMKSTA: ever smokers, current smokers
4) PRODUCT: all/unspec, cigarettes regardless of other products, cigarettes only
5) CIGTYPE: all/unspecified, MC regardless of HR, MC only
6) DENOM: never smoked anything, never smoked cigarettes, (never +1 = +long term ex, +2 = +amount unknown,
+3 = never cigs+long term ex)
7) Followup period (YF, prospective studies): whole study (coded as 0) or longest available
8) LCtype: all or nearest available, at least Squamous and Adeno. (q = squamous, s = small,
l = large, a = adeno, mix = mixed, alv = alveolar)
9) Race: all or nearest available, otherwise by race (wh or w = white, bl or b = black, hi = hispanic
ch = chinese, jap = japanese, haw = hawaiian, w+o = white + oriental, sca = scandinavian, as = asian)
10) Amount smoked "mid" in key scheme 1 (key value 20, maximum range 6-44, in numbers of
cigarettes or cigarette equivalents)
11) For overlapping studies: principal rather than subsidiary studies
Finally by Age: whole study (coded as 0) if available, otherwise by widest available age group
and then for single sex results (m, f) in preference to combined sex results (c).

Results adjusted (AD) for the most potential confounders are then chosen in Sections -1 to -3
and results adjusted for the least confounders in Sections -4 to -6. (Those least adjusted results which
actually differ from the most adjusted as marked 'x' in column X in Section -4)
(Results adjusted for an unknown number of confounder(s) are coded as 20.)

Section -7 shows excluded studies, together with the stage (as above) at which no qualifying
results were found.

Section -8 lists the potentially overlapping studies which have been included (1=principal, 2=subsidiary).

Section -9 lists any results which would have been included in preference except that they had data not complete
enough for use in meta-analysis, with their significance (yes/no), if known, and any further comment as entered
on the database.

In addition to those mentioned above, the following fields, levels and abbreviations are used:
* or nk = not known, n = no, y = yes, ot = other
ev = ever, cu = current, nev = never
all/unspec = all or unspecified, cig+/-ot = cigarettes irrespective of other products (cigar, pipe etc)
MC = manufactured cigarettes, HR = hand-rolled cigarettes
exL, exH = range of exposure (low and high) in the smoking group, in terms of Amount smoked,
cigarettes or cigarette equivalents
REF: 6-character study reference
NRR: number of the RR on the database within the study
ST : study type (CC = case control, pr or prosp = prospective)
NLC: number of lung cancer cases in whole study
R : risky occupational population (n = no, m = mining, o = other risky)
VB : national cigarette type (V = at least 75% Virginia, bl = at least 75% blended, ot = other)
P : any proxy use
H : full histological confirmation
De : derivation of RR/CI (or = original, st = standard method, ot = other method of estimation)

Table 1G13 - 1

IESLC - Meta-anal of Ever Smoking (or Current if ever not avail), Amount smoked, "Mid", Any prod (or Cigs if Any not avail)
All LC types
Most adjusted

REF	NRR	SEX	AGEL	AGEH	RACE	YF	LC TYPE	LOC	START	ST	NLC	R	VB	P	H	AD	SM	PRODUCT	exL	exH	DENOM	De
AKIBA	28	m	0	0	all	0	all	As:Jap	1963	pr	610	n	bl	n	n	5	cu	cig+/-ot	15	24	nev cigs	ot
ALDERS	19	m	0	0	all	-	all	Eu:UK	1977	CC	1448	n	V	n	n	1	ev	cig only	18	27	nev+2	ot
ALDERS	22	f	0	0	all	-	all	Eu:UK	1977	CC	1448	n	V	n	n	1	ev	cig only	18	27	nev+2	ot
ARCHER	2	m	0	0	wh	0	all	NAmer	1950	pr	146	m	bl	n	n	0	cu	cig+/-ot	20	20	nev cigs	st
ARMADA	47	m	0	0	all	-	all	Eu:wst	1986	CC	325	n	bl	n	y	0	ev	cig+/-ot	15	24	nev any	st
AUVINE	14	c	0	0	all	-	all	Eu:Sca	1986	CC	517	n	bl	y	n	2	ev	cig+/-ot	11	20	nev cigs	or
AXELSS	15	f	0	0	sca	-	all	Eu:Sca	1989	CC	436	n	bl	n	n	0	ev	all/unsp	20	29	nev any	st
BARBON	83	m	0	0	all	-	all	Eu:wst	1979	CC	755	n	bl	y	y	3	ev	all/unsp	20	39	nev any	or
BEST	14	m	0	0	all	0	all	NAmer	1955	pr	381	n	V	n	n	1	cu	cig only	10	20	nev any	ot
BRESLO	15	m	0	0	all	-	all	NAmer	1949	CC	518	n	bl	n	y	0	ev	cig+/-ot	20	39	nev+3	st
BRETT	2	m	0	0	all	0	all	Eu:UK	1960	pr	150	n	V	n	n	0	cu	cig+/-ot	15	24	nev cigs	st
BUFFLE	29	f	0	0	w-hi	-	all	NAmer	1976	CC	943	n	bl	y	n	0	ev	cig+/-ot	20	20	nev cigs	or
CHANG	3	m	0	0	all	0	all	NAmer	1972	pr	136	n	bl	n	n	0	cu	cig+/-ot	11	20	nev cigs	st
CHANG	9	f	0	0	all	0	all	NAmer	1972	pr	136	n	bl	n	n	0	cu	cig+/-ot	11	20	nev cigs	st
CHEN2	4	m	0	0	all	-	all	As:Chi	1983	CC	193	n	ot	y	n	0	ev	all/unsp	10	20	nev any	st
CHEN2	8	f	0	0	all	-	all	As:Chi	1983	CC	193	n	ot	y	n	0	ev	all/unsp	10	20	nev any	st
CHOI	13	m	0	0	all	-	all	As:oth	1985	CC	375	n	bl	n	n	0	ev	cig+/-ot	11	20	nev cigs	st
CHOI	18	f	0	0	all	-	all	As:oth	1985	CC	375	n	bl	n	n	0	ev	cig+/-ot	11	30	nev cigs	st
CHOW	11	m	0	0	wh	0	all	NAmer	1966	pr	219	n	bl	n	n	2	cu	cig+/-ot	20	29	nev any	ot
COMSTO	5	m	0	0	all	-	all	NAmer	1975	ot	258	n	bl	n	n	0	cu	cig+/-ot	20	39	nev any	st
COMSTO	10	f	0	0	all	-	all	NAmer	1975	ot	258	n	bl	n	n	0	cu	cig+/-ot	20	39	nev any	st
CPSI	218	m	35	84	all	6	all	NAmer	1959	pr	5138	n	bl	n	n	1	cu	cig+/-ot	20	39	nev any	ot
CPSI	277	f	40	74	all	6	all	NAmer	1959	pr	5138	n	bl	n	n	1	cu	cig+/-ot	20	39	nev cigs	ot
CPSII	122	m	0	0	all	6	all	NAmer	1982	pr	3229	n	bl	n	n	1	cu	cig only	20	20	nev any	ot
CPSII	129	f	0	0	all	6	all	NAmer	1982	pr	3229	n	bl	n	n	1	cu	cig+/-ot	20	20	nev cigs	ot
DAMBER	8	m	0	0	all	-	all	Eu:Sca	1972	CC	579	n	bl	y	n	1	ev	cig only	16	25	nev any	ot
DARBY	2	m	0	0	wh	-	all	Eu:UK	1988	CC	982	n	V	n	n	0	cu	cig+/-ot	15	24	nev any	st
DARBY	9	f	0	0	wh	-	all	Eu:UK	1988	CC	982	n	V	n	n	0	cu	cig+/-ot	15	24	nev any	st
DAVEYS	3	m	0	0	all	-	all	Eu:Ger	1930	CC	109	n	bl	y	n	0	ev	all/unsp	11	20	nev any	st
DEAN3	14	m	0	0	all	-	all	Eu:UK	1969	CC	766	n	V	y	n	3	cu	cig only	13	22	nev any	ot
DEAN3	98	f	0	0	all	-	all	Eu:UK	1969	CC	766	n	V	y	n	3	cu	cig only	13	22	nev any	ot
DEKLER	3	m	0	0	all	0	all	Auslia	1961	pr	138	m	V	n	n	2	cu	cig+/-ot	15	24	nev any	or
DESTEF	7	m	0	0	all	-	all	SCAmer	1988	CC	497	n	bl	n	y	4	ev	all/unsp	11	20	nev any	or
DOLL	3	m	0	0	all	-	all	Eu:UK	1948	CC	1465	n	V	n	n	0	ev	all/unsp	15	24	nev any	st
DOLL	9	f	0	0	all	-	all	Eu:UK	1948	CC	1465	n	V	n	n	0	ev	all/unsp	15	24	nev any	st
DOLL2	47	m	35	99	all	5	all	Eu:UK	1951	pr	920	n	V	n	n	1	ev	all/unsp	15	24	nev any	ot
DOLL2	11	f	0	0	all	22	all	Eu:UK	1951	pr	920	n	V	n	n	1	cu	cig only	15	24	nev any	ot
DORN	409	m	0	0	wh	25	all	NAmer	1954	pr	5097	n	bl	n	n	1	cu	cig+/-ot	10	20	nev any	or
DOSEME	9	m	0	0	all	-	all	Eu:bal	1979	CC	1210	n	bl	n	n	2	ev	cig+/-ot	11	20	nev cigs	or
DUNN	3	m	0	0	all	0	all	NAmer	1954	pr	139	o	bl	n	n	0	ev	cig+/-ot	15	24	nev cigs	st
EBELIN	4	m	0	0	all	-	all	Eu:Ger	1980	CC	130	n	bl	n	n	0	ev	all/unsp	20	29	nev any	st
ENSTRO	5	m	0	0	all	0	all	NAmer	1959	pr	2879	n	bl	n	n	1	cu	cig only	20	20	nev any	ot
ENSTRO	9	f	0	0	all	0	all	NAmer	1959	pr	2879	n	bl	n	n	1	cu	cig only	20	20	nev any	ot
ESAKI	2	m	0	0	all	-	all	As:Jap	1961	CC	245	n	bl	y	n	0	ev	cig+/-ot	15	29	nev cigs	st
FAN	8	m	0	0	all	-	all	As:Chi	1990	CC	403	n	ot	y	n	0	ev	cig+/-ot	20	29	nev cigs	st
FAN	12	f	0	0	all	-	all	As:Chi	1990	CC	403	n	ot	y	n	0	ev	cig+/-ot	20	29	nev cigs	st
GAO2	3	m	0	0	all	-	all	As:Jap	1988	CC	282	n	bl	n	n	0	cu	cig+/-ot	20	29	nev cigs	st
GARSHI	27	m	0	0	all	-	all	NAmer	1981	CC	1081	o	bl	y	n	1	ev	all/unsp	16	25	nev any	st
GER	23	c	0	0	all	-	all	As:oth	1990	CC	141	n	ot	y	n	14	ev	all/unsp	11	20	nev any	ot
GOLLED	2	m	35	99	all	-	all	Eu:UK	1952	CC	443	n	V	y	n	1	ev	cig only	11	22	nev any	ot
GSELL	3	m	0	0	all	-	all	Eu:wst	1937	CC	150	n	bl	n	y	0	ev	all/unsp	15	20	nev any	st
HAMMON	154	m	0	0	wh	0	all	NAmer	1952	pr	448	n	bl	n	n	1	ev	cig only	10	20	nev any	ot
HIRAYA	74	m	0	0	all	16	all	As:Jap	1965	pr	1917	n	bl	n	n	4	cu	cig+/-ot	15	24	nev any	or
HITOSU	36	m	0	0	all	-	all	As:Jap	1960	CC	216	n	bl	y	n	1	cu	all/unsp	15	24	nev any	st
HOLE	3	m	0	0	all	0	all	Eu:UK	1972	pr	225	n	V	n	n	1	cu	cig+/-ot	15	24	nev any	ot
HU	2	m	0	0	all	-	all	As:Chi	1985	CC	227	n	ot	n	y	0	ev	cig+/-ot	14	24	nev any	st
HU	5	f	0	0	all	-	all	As:Chi	1985	CC	227	n	ot	n	y	0	ev	cig+/-ot	14	24	nev any	st
HU2	6	c	0	0	all	-	all	As:Chi	1977	CC	523	n	ot	y	n	0	ev	cig+/-ot	20	29	nev cigs	st
HUMBLE	76	c	0	0	wh	-	not alv	NAmer	1980	CC	521	n	bl	y	n	3	cu	cig+/-ot	16	20	nev cigs	ot
JEDRYC	46	m	0	0	all	-	all	Eu:est	1980	CC	1630	n	bl	y	n	4	ev	cig+/-ot	20	29	nev any	or
JEDRYC	49	f	0	0	all	-	all	Eu:est	1980	CC	1630	n	bl	y	n	4	ev	cig+/-ot	20	29	nev any	or
JOLY	9	m	0	0	all	-	all	SCAmer	1978	CC	826	n	bl	n	n	0	ev	cig+/-ot	20	29	nev any	st
JOLY	5	f	0	0	all	-	all	SCAmer	1978	CC	826	n	bl	n	n	0	ev	cig+/-ot	20	29	nev any	st
JUSSAW	14	m	0	0	all	-	all	As:Ind	1964	CC	792	n	V	n	n	0	ev	cig only	20	24	nev any	st
KAISER	7	m	0	0	all	0	all	NAmer	1964	pr	714	n	bl	n	n	2	cu	cig+/-ot	20	40	nev cigs	or
KAISER	3	f	0	0	all	0	all	NAmer	1964	pr	714	n	bl	n	n	2	cu	cig+/-ot	20	40	nev cigs	or
KANELL	27	m	0	0	all	-	all	Eu:bal	1950	CC	862	n	bl	n	n	1	cu	cig+/-ot	11	20	nev any	st
KATSOU	8	f	0	0	all	-	all	Eu:bal	1987	CC	101	n	bl	n	n	0	cu	all/unsp	11	20	nev any	st
KAUFMA	12	c	0	0	all	-	all	NAmer	1981	CC	881	n	bl	n	n	6	cu	cig+/-ot	15	24	nev cigs	or
KHUDER	2	m	0	0	all	-	all	NAmer	1985	CC	482	n	bl	n	y	0	ev	cig+/-ot	20	39	nev cigs	st
KINLEN	15	m	0	0	all	0	all	Eu:UK	1967	pr	718	n	V	n	n	2	cu	cig+/-ot	15	24	nev any	ot
KOO	12	f	0	0	all	-	all	As:HK	1981	CC	200	n	bl	n	n	0	cu	all/unsp	11	20	nev any	st

Table 1G13 - 1

IESLC - Meta-anal of Ever Smoking (or Current if ever not avail), Amount smoked, "Mid", Any prod (or Cigs if Any not avail)
All LC types
Most adjusted

REF	NRR	SEX	AGEL	AGEH	RACE	YF	LC TYPE	LOC	START	ST	NLC	R	VB	P	H	AD	SM	PRODUCT	exL	exH	DENOM	De
KREUZE	21	m	1	45	all	-	all	Eu:Ger	1990	CC	2260	n	bl	n	n	3	ev	cig+/-ot	20	29	nev any	or
KREUZE	32	m	55	69	all	-	all	Eu:Ger	1990	CC	2260	n	bl	n	n	3	ev	cig+/-ot	20	29	nev any	or
KREUZE	27	f	1	45	all	-	all	Eu:Ger	1990	CC	2260	n	bl	n	n	3	ev	cig+/-ot	20	29	nev any	or
KREUZE	38	f	55	69	all	-	all	Eu:Ger	1990	CC	2260	n	bl	n	n	3	ev	cig+/-ot	20	29	nev any	or
KREYBE	10	m	0	0	all	-	all	Eu:Sca	1948	CC	300	n	bl	n	y	1	ev	all/unsp	15	24	nev any	ot
LAMTH	2	f	0	0	ch	-	all	As:HK	1983	CC	445	n	bl	n	n	0	ev	all/unsp	11	20	nev any	or
LETOUR	3	c	0	0	all	-	all	NAmer	1983	CC	738	n	V	y	y	0	ev	cig+/-ot	20	40	nev cigs	st
LIAW	4	c	0	0	all	0	all	As:oth	1982	pr	127	n	ot	n	n	2	cu	all/unsp	11	20	nev any	or
LIU2	9	m	0	0	all	-	all	As:Chi	1983	CC	316	n	ot	n	n	3	ev	all/unsp	20	29	nev any	or
LIU3	7	m	0	0	all	-	all	As:Chi	1985	CC	110	n	ot	n	n	2	ev	all/unsp	16	30	nev any	or
LIU4	8	m	35	69	all	-	all	As:Chi	1986	CC	1000-00	n	ot	y	n	2	ev	cig only	20	20	nev any	ot
LUBIN2	275	m	0	0	all	-	all	Eu:mul	1976	CC	7804	n	bl	n	y	0	ev	cig+/-ot	20	29	nev any	st
LUBIN2	283	f	0	0	all	-	all	Eu:mul	1976	CC	7804	n	bl	n	y	0	ev	cig+/-ot	20	29	nev any	st
MACLEN	38	c	0	0	ch	-	all	As:oth	1972	CC	233	n	bl	n	n	2	ev	cig+/-ot	20	29	nev cigs	or
MARTIS	2	m	0	0	all	-	all	Eu:UK	1972	CC	201	n	V	n	n	0	ev	cig+/-ot	15	24	nev cigs	st
MATOS	31	m	0	0	all	-	all	SCAmer	1994	CC	200	n	bl	n	n	2	ev	cig+/-ot	15	24	nev any	or
MATSUD	2	m	0	0	all	-	all	As:Jap	1965	CC	179	n	bl	n	n	0	ev	cig+/-ot	11	20	nev cigs	st
MCCONN	25	c	0	0	all	-	all	Eu:UK	1946	CC	100	n	V	n	y	0	ev	all/unsp	10	20	nev any	st
MIGRAN	6	m	0	0	all	0	all	Eu:UK	1964	pr	259	n	V	n	n	2	cu	cig only	20	20	nev any	ot
MIGRAN	33	f	0	0	all	0	all	Eu:UK	1964	pr	259	n	V	n	n	2	cu	cig only	20	20	nev any	ot
MRFITR	4	m	0	0	all	0	all	NAmer	1973	pr	119	n	bl	n	n	0	cu	cig+/-ot	20	39	nev cigs	ot
ORMOS	2	m	0	0	all	-	all	Eu:est	1947	CC	119	n	bl	y	y	0	ev	cig+/-ot	16	30	nev any	st
PASTOR	8	m	0	0	all	-	all	Eu:wst	1976	CC	204	n	bl	y	n	1	ev	all/unsp	20	29	nev any	st
PERNU	21	m	0	0	all	-	all	Eu:Sca	1944	CC	1606	n	bl	n	n	0	ev	all/unsp	20	24	nev any	st
PERNU	15	f	0	0	all	-	all	Eu:Sca	1944	CC	1606	n	bl	n	n	0	ev	all/unsp	20	24	nev any	st
SEGI2	14	m	0	0	all	-	all	As:Jap	1962	CC	378	n	bl	n	n	1	cu	cig+/-ot	20	29	nev any	ot
SIEMIA	14	m	0	0	all	-	all	NAmer	1979	CC	857	n	V	y	y	0	ev	cig+/-ot	20	39	nev cigs	or
SOBUE	118	m	0	0	all	-	q+s+l+a	As:Jap	1986	CC	1376	n	bl	n	y	0	cu	cig+/-ot	20	29	nev cigs	st
SPEIZE	3	f	0	0	all	0	all	NAmer	1976	pr	593	n	bl	n	y	1	cu	cig+/-ot	15	24	nev cigs	ot
STOCKS	42	m	0	0	all	-	all	Eu:UK	1952	CC	2932	n	V	y	n	2	ev	cig+/-ot	15	21	nev any	st
STOCKW	2	c	0	0	all	-	all	NAmer	1981	CC	22161	n	bl	n	n	0	cu	cig+/-ot	20	40	nev any	st
SVENSS	11	f	0	0	all	-	all	Eu:Sca	1983	CC	210	n	bl	n	n	1	cu	all/unsp	11	20	nev any	or
TENKAN	11	m	0	0	all	17	all	Eu:Sca	1962	pr	242	n	bl	n	n	1	cu	all/unsp	15	24	nev any	ot
TIZZAN	8	m	0	0	all	-	all	Eu:wst	1959	CC	1358	n	bl	n	n	0	ev	cig only	10	20	nev any	st
TSUGAN	30	m	0	0	all	-	q+a	As:Jap	1976	CC	134	n	bl	n	y	0	cu	all/unsp	16	35	nev any	st
TULINI	28	m	0	0	all	0	all	Eu:Sca	1967	pr	472	n	bl	n	n	3	cu	cig+/-ot	15	24	nev any	or
TULINI	33	f	0	0	all	0	all	Eu:Sca	1967	pr	472	n	bl	n	n	3	cu	cig+/-ot	15	24	nev any	or
WAKAI	41	m	0	0	all	-	all	As:Jap	1988	CC	333	n	bl	n	y	2	cu	cig+/-ot	20	29	nev any	or
WANG2	13	c	0	0	all	-	all	As:Chi	1980	CC	103	n	ot	n	n	4	ev	cig+/-ot	20	29	nev cigs	ot
WYNDE2	18	m	0	0	all	-	all	NAmer	1962	CC	404	n	bl	n	y	0	ev	cig+/-ot	11	20	nev any	st
WYNDE3	45	m	0	0	all	-	all	NAmer	1966	CC	350	n	bl	n	y	0	ev	cig+/-ot	10	20	nev any	st
WYNDE3	80	f	0	0	all	-	all	NAmer	1966	CC	350	n	bl	n	y	0	ev	cig+/-ot	10	20	nev any	st
WYNDE4	45	m	0	0	all	-	all	NAmer	1948	CC	684	n	bl	y	n	0	ev	all/unsp	16	20	nev any	st
WYNDE4	59	f	0	0	all	-	all	NAmer	1948	CC	684	n	bl	y	n	2	ev	all/unsp	16	20	nev any	ot
WYNDE6	36	m	0	0	all	-	all	NAmer	1969	CC	4423	n	bl	n	y	0	cu	cig+/-ot	11	20	nev any	st
WYNDE6	225	f	0	0	all	-	all	NAmer	1969	CC	4423	n	bl	n	y	0	cu	cig+/-ot	11	20	nev cigs	st
XU3	11	m	0	0	all	-	all	As:Chi	1981	CC	135	n	ot	n	n	1	ev	all/unsp	20	29	nev any	ot
ZHENG	13	m	0	0	all	-	all	As:Chi	1982	CC	540	n	ot	*	y	0	ev	cig+/-ot	20	29	nev cigs	st

Cigarette type is all/unspec for all RRs
except for the following:

REF	NRR	CIGTYPE
ALDERS	19	MC only
ALDERS	22	MC only
DEAN3	14	MC only
DEAN3	98	MC only
JUSSAW	14	MC only

Table 1G13 - 2

IESLC - Meta-anal of Ever Smoking (or Current if ever not avail), Amount smoked, "Mid", Any prod (or Cigs if Any not avail)
All LC types
Most adjusted

REF	NRR	SEX	AD	Number Exposed Case	Number Exposed Cont	Non-exposed Case	Non-exposed Cont	RR	95.00%CI
*AKIBA	28	m	5	-	-	-	-	6.10	(3.90- 9.50)
ALDERS	19	m	1	-	-	-	-	7.96	(4.63- 13.69)
ALDERS	22	f	1	-	-	-	-	5.28	(3.79- 7.36)
Subtotal ALDERS								5.91	(4.45- 7.84)
*ARCHER	2	m	0	68	18320	6	9842	6.09	(2.64- 14.02)
ARMADA	47	m	0	134	105	4	64	20.42	(7.20- 57.88)
AUVINE	14	c	2	-	-	-	-	33.90	(17.10- 67.00)
AXELSS	15	f	0	28	15	18	154	15.97	(7.21- 35.36)
BARBON	83	m	3	-	-	-	-	12.80	(7.90- 21.00)
*BEST	14	m	1	-	-	-	-	16.41	(7.73- 34.86)
BRESLO	15	m	0	296	193	22	110	7.67	(4.69- 12.55)
*BRETT	2	m	0	62	15868	6	6530	4.25	(1.84- 9.83)
BUFFLE	29	f	0	76	60	12	112	11.82	(5.96- 23.45)
*CHANG	3	m	0	17	161	5	502	10.60	(3.97- 28.28)
*CHANG	9	f	0	11	234	11	1139	4.87	(2.14- 11.09)
Subtotal CHANG								6.72	(3.57- 12.62)
CHEN2	4	m	0	44	50	9	33	3.23	(1.39- 7.48)
CHEN2	8	f	0	22	10	25	33	2.90	(1.17- 7.22)
Subtotal CHEN2								3.07	(1.66- 5.70)
CHOI	13	m	0	144	281	13	95	3.74	(2.03- 6.92)
CHOI	18	f	0	7	9	76	164	1.68	(0.60- 4.68)
Subtotal CHOI								3.03	(1.79- 5.13)
*CHOW	11	m	2	-	-	-	-	21.87	(9.35- 51.14)
COMSTO	5	m	0	60	57	4	69	18.16	(6.22- 53.00)
COMSTO	10	f	0	51	26	13	115	17.35	(8.25- 36.48)
Subtotal COMSTO								17.61	(9.56- 32.43)
*CPSI	218	m	1	-	-	-	-	14.30	(11.33- 18.05)
*CPSI	277	f	1	-	-	-	-	5.03	(3.82- 6.63)
Subtotal CPSI								9.26	(7.75- 11.06)
*CPSII	122	m	1	-	-	-	-	21.59	(16.98- 27.46)
*CPSII	129	f	1	-	-	-	-	14.21	(11.67- 17.31)
Subtotal CPSII								16.81	(14.44- 19.58)
DAMBER	8	m	1	-	-	-	-	9.10	(5.50- 15.30)
DARBY	2	m	0	126	169	3	384	95.43	(29.94- 304.17)
DARBY	9	f	0	86	92	23	529	21.50	(12.90- 35.82)
Subtotal DARBY								27.39	(17.17- 43.71)
DAVEYS	3	m	0	19	22	3	23	6.62	(1.72- 25.56)
DEAN3	14	m	3	-	-	-	-	7.42	(4.56- 12.06)
DEAN3	98	f	3	-	-	-	-	8.42	(5.14- 13.78)
Subtotal DEAN3								7.90	(5.59- 11.16)
*DEKLER	3	m	2	-	-	-	-	23.00	(3.20- 167.60)
DESTEF	7	m	4	-	-	-	-	8.40	(5.20- 13.60)
DOLL	3	m	0	475	431	7	61	9.60	(4.35- 21.22)
DOLL	9	f	0	14	6	40	59	3.44	(1.22- 9.71)
Subtotal DOLL								6.58	(3.50- 12.35)
*DOLL2	47	m	1	-	-	-	-	12.29	(1.67- 90.41)
*DOLL2	11	f	1	-	-	-	-	6.43	(1.81- 22.78)
Subtotal DOLL2								7.74	(2.66- 22.56)
*DORN	409	m	1	-	-	-	-	9.92	(8.84- 11.14)
DOSEME	9	m	2	-	-	-	-	3.10	(2.30- 4.10)
*DUNN	3	m	0	75	27720	2	14160	19.16	(4.70- 78.00)
EBELIN	4	m	0	47	37	12	117	12.39	(5.95- 25.80)
*ENSTRO	5	m	1	-	-	-	-	13.65	(10.88- 17.13)
*ENSTRO	9	f	1	-	-	-	-	9.48	(8.04- 11.18)
Subtotal ENSTRO								10.75	(9.41- 12.29)
ESAKI	2	m	0	74	58	16	28	2.23	(1.10- 4.51)
FAN	8	m	0	111	183	36	236	3.98	(2.61- 6.07)
FAN	12	f	0	31	12	69	320	11.98	(5.86- 24.50)
Subtotal FAN								5.29	(3.68- 7.61)
GAO2	3	m	0	77	44	13	56	7.54	(3.71- 15.30)
GARSHI	27	m	1	-	-	-	-	5.72	(4.04- 8.10)
GER	23	c	14	-	-	-	-	1.90	(0.98- 3.70)
GOLLED	2	m	1	-	-	-	-	6.37	(3.67- 11.03)
GSELL	3	m	0	27	26	2	29	15.06	(3.26- 69.59)
*HAMMON	154	m	1	-	-	-	-	8.32	(4.66- 14.84)
*HIRAYA	74	m	4	-	-	-	-	5.40	(4.30- 6.90)
HITOSU	36	m	1	-	-	-	-	2.82	(1.25- 6.36)
*HOLE	3	m	1	-	-	-	-	8.90	(4.12- 19.23)
HU	2	m	0	55	43	41	67	2.09	(1.20- 3.65)
HU	5	f	0	6	6	40	48	1.20	(0.36- 4.01)
Subtotal HU								1.90	(1.14- 3.14)

Table 1G13 - 2

IESLC - Meta-anal of Ever Smoking (or Current if ever not avail), Amount smoked, "Mid", Any prod (or Cigs if Any not avail)
All LC types
Most adjusted

REF	NRR	SEX	AD	Number Exposed Case	Number Exposed Cont	Non-exposed Case	Non-exposed Cont	RR	95.00%CI
HU2	6	c	0	149	87	121	213	3.01	(2.13- 4.26)
HUMBLE	76	c	3	-	-	-	-	15.16	(9.18- 25.03)
JEDRYC	46	m	4	-	-	-	-	6.16	(4.25- 8.90)
JEDRYC	49	f	4	-	-	-	-	2.38	(1.17- 6.86)
Subtotal JEDRYC								5.35	(3.80- 7.52)
JOLY	9	m	0	126	175	12	218	13.08	(7.00- 24.43)
JOLY	5	f	0	28	22	52	283	6.93	(3.68- 13.03)
Subtotal JOLY								9.55	(6.13- 14.90)
JUSSAW	14	m	0	24	8	149	624	12.56	(5.53- 28.52)
*KAISER	7	m	2	-	-	-	-	17.24	(10.71- 27.73)
*KAISER	3	f	2	-	-	-	-	7.98	(5.35- 11.90)
Subtotal KAISER								10.98	(8.08- 14.91)
KANELL	27	m	1	-	-	-	-	7.06	(4.76- 10.48)
KATSOU	8	f	0	15	5	48	67	4.19	(1.43- 12.30)
KAUFMA	12	c	6	-	-	-	-	15.00	(10.00- 23.00)
KHUDER	2	m	0	224	288	23	309	10.45	(6.61- 16.52)
*KINLEN	15	m	2	-	-	-	-	14.14	(6.68- 29.91)
KOO	12	f	0	24	5	56	85	7.29	(2.62- 20.22)
KREUZE	21	m	3	-	-	-	-	19.50	(7.50- 50.30)
KREUZE	32	m	3	-	-	-	-	32.80	(20.90- 51.40)
KREUZE	27	f	3	-	-	-	-	12.10	(3.00- 48.00)
KREUZE	38	f	3	-	-	-	-	7.70	(3.50- 17.30)
Subtotal KREUZE								21.69	(15.27- 30.80)
KREYBE	10	m	1	-	-	-	-	6.23	(2.66- 14.56)
LAMTH	2	f	0	90	28	202	337	5.36	(3.39- 8.48)
LETOUR	3	c	0	367	198	24	224	17.30	(10.98- 27.27)
*LIAW	4	c	2	-	-	-	-	3.60	(2.00- 6.40)
LIU2	9	m	3	-	-	-	-	7.10	(2.60- 19.50)
LIU3	7	m	2	-	-	-	-	1.09	(0.24- 4.82)
LIU4	8	m	2	-	-	-	-	3.60	(3.49- 3.71)
LUBIN2	275	m	0	1963	2547	190	2616	10.61	(9.06- 12.44)
LUBIN2	283	f	0	134	103	336	1188	4.60	(3.46- 6.11)
Subtotal LUBIN2								8.70	(7.57- 9.99)
MACLEN	38	c	2	-	-	-	-	2.93	(1.57- 5.45)
MARTIS	2	m	0	91	87	4	25	6.54	(2.19- 19.55)
MATOS	31	m	2	-	-	-	-	7.50	(3.70- 15.00)
MATSUD	2	m	0	75	1607	3	1255	19.52	(6.14- 62.05)
MCCONN	25	c	0	49	92	9	23	1.36	(0.58- 3.17)
*MIGRAN	6	m	2	-	-	-	-	5.14	(1.79- 14.81)
*MIGRAN	33	f	2	-	-	-	-	7.48	(1.64- 34.03)
Subtotal MIGRAN								5.81	(2.44- 13.83)
*MRFITR	4	m	0	50	3747	0	1859	50.12~	(3.09- 811.82)
ORMOS	2	m	0	40	577	7	777	7.69	(3.42- 17.30)
PASTOR	8	m	1	-	-	-	-	8.02	(3.84- 16.73)
PERNU	21	m	0	478	138	97	275	9.82	(7.28- 13.24)
PERNU	15	f	0	1	11	110	971	0.80	(0.10- 6.27)
Subtotal PERNU								9.32	(6.94- 12.53)
SEGI2	14	m	1	-	-	-	-	3.40	(1.55- 7.45)
SIEMIA	14	m	0	-	-	-	-	4.50	(1.80- 13.20)
SOBUE	118	m	0	236	222	34	128	4.00	(2.63- 6.09)
*SPEIZE	3	f	1	-	-	-	-	12.60	(9.90- 16.70)
STOCKS	42	m	2	-	-	-	-	7.89	(5.51- 11.29)
STOCKW	2	c	0	6053	1591	2791	10641	14.51	(13.54- 15.54)
SVENSS	11	f	1	-	-	-	-	12.60	(6.50- 25.20)
*TENKAN	11	m	1	-	-	-	-	20.25	(8.20- 50.00)
TIZZAN	8	m	0	468	470	180	305	1.69	(1.35- 2.11)
TSUGAN	30	m	0	30	30	18	22	1.22	(0.55- 2.73)
*TULINI	28	m	3	-	-	-	-	12.00	(6.31- 22.90)
*TULINI	33	f	3	-	-	-	-	26.30	(14.40- 48.10)
Subtotal TULINI								18.23	(11.74- 28.32)
WAKAI	41	m	2	-	-	-	-	4.01	(1.91- 8.41)
WANG2	13	c	4	-	-	-	-	3.19	(1.39- 7.29)
WYNDE2	18	m	0	122	203	8	105	7.89	(3.71- 16.75)
WYNDE3	45	m	0	77	114	9	88	6.60	(3.14- 13.90)
WYNDE3	80	f	0	24	24	20	76	3.80	(1.79- 8.05)
Subtotal WYNDE3								5.02	(2.96- 8.52)
WYNDE4	45	m	0	228	274	12	115	7.97	(4.29- 14.82)
WYNDE4	59	f	2	-	-	-	-	6.49	(2.35- 17.93)
Subtotal WYNDE4								7.54	(4.44- 12.80)
WYNDE6	36	m	0	461	293	87	617	11.16	(8.54- 14.59)
WYNDE6	225	f	0	367	165	159	856	11.97	(9.33- 15.37)

Table 1G13 - 2

IESLC - Meta-anal of Ever Smoking (or Current if ever not avail), Amount smoked, "Mid", Any prod (or Cigs if Any not avail)
All LC types
Most adjusted

REF	NRR	SEX	AD	Number Exposed Case	Cont	Non-exposed Case	Cont	RR	95.00%CI
Subtotal WYNDE6								11.59	(9.65- 13.91)
XU3	11	m	1	-	-	-	-	14.78	(5.30- 41.18)
ZHENG	13	m	0	128	89	33	94	4.10	(2.53- 6.62)
Partial Totals				14365	77438	5325	59475		

*prospective study ~ With 0.5 adjustment for zero

REF	NRR	SEX	AD	Ys	Ws	Qs	Ps
*AKIBA	28	m	5	1.81	19.39	0.20	0.0000
ALDERS	19	m	1	2.07	13.07	1.77	0.0000
ALDERS	22	f	1	1.66	34.88	0.06	0.0000
Subtotal ALDERS				1.78	47.96	1.83	
*ARCHER	2	m	0	1.81	5.52	0.06	0.0000
ARMADA	47	m	0	3.02	3.54	6.07	0.0000
AUVINE	14	c	2	3.52	8.24	27.20	0.0000
AXELSS	15	f	0	2.77	6.08	6.89	0.0000
BARBON	83	m	3	2.55	16.08	11.43	0.0000
*BEST	14	m	1	2.80	6.77	8.07	0.0000
BRESLO	15	m	0	2.04	15.85	1.73	0.0000
*BRETT	2	m	0	1.45	5.48	0.37	0.0007
BUFFLE	29	f	0	2.47	8.19	4.78	0.0000
*CHANG	3	m	0	2.36	3.99	1.71	0.0000
*CHANG	9	f	0	1.58	5.66	0.09	0.0002
Subtotal CHANG				1.90	9.65	1.80	
CHEN2	4	m	0	1.17	5.43	1.55	0.0063
CHEN2	8	f	0	1.07	4.63	1.90	0.0217
Subtotal CHEN2				1.12	10.07	3.45	
CHOI	13	m	0	1.32	10.21	1.52	0.0000
CHOI	18	f	0	0.52	3.66	5.17	0.3219
Subtotal CHOI				1.11	13.87	6.69	
*CHOW	11	m	2	3.09	5.32	10.12	0.0000
COMSTO	5	m	0	2.90	3.35	4.76	0.0000
COMSTO	10	f	0	2.85	6.96	9.16	0.0000
Subtotal COMSTO				2.87	10.31	13.92	
*CPSI	218	m	1	2.66	70.85	64.47	0.0000
*CPSI	277	f	1	1.62	50.55	0.42	0.0000
Subtotal CPSI				2.23	121.40	64.89	
*CPSII	122	m	1	3.07	66.50	124.06	0.0000
*CPSII	129	f	1	2.65	98.85	88.76	0.0000
Subtotal CPSII				2.82	165.35	212.82	
DAMBER	8	m	1	2.21	14.68	3.70	0.0000
DARBY	2	m	0	4.56	2.86	23.25	0.0000
DARBY	9	f	0	3.07	14.73	27.32	0.0000
Subtotal DARBY				3.31	17.59	50.58	
DAVEYS	3	m	0	1.89	2.11	0.07	0.0061
DEAN3	14	m	3	2.00	16.24	1.44	0.0000
DEAN3	98	f	3	2.13	15.80	2.84	0.0000
Subtotal DEAN3				2.07	32.04	4.28	
*DEKLER	3	m	2	3.14	0.98	2.00	0.0019
DESTEF	7	m	4	2.13	16.62	2.96	0.0000
DOLL	3	m	0	2.26	6.11	1.89	0.0000
DOLL	9	f	0	1.24	3.57	0.79	0.0195
Subtotal DOLL				1.88	9.68	2.68	
*DOLL2	47	m	1	2.51	0.96	0.62	0.0137
*DOLL2	11	f	1	1.86	2.40	0.06	0.0040
Subtotal DOLL2				2.05	3.36	0.68	
*DORN	409	m	1	2.29	287.32	99.40	0.0000
DOSEME	9	m	2	1.13	45.98	15.20	0.0000
*DUNN	3	m	0	2.95	1.95	3.03	0.0000
EBELIN	4	m	0	2.52	7.13	4.68	0.0000
*ENSTRO	5	m	1	2.61	74.58	61.40	0.0000
*ENSTRO	9	f	1	2.25	141.36	41.65	0.0000
Subtotal ENSTRO				2.38	215.94	103.05	
ESAKI	2	m	0	0.80	7.75	6.32	0.0253
FAN	8	m	0	1.38	21.51	2.29	0.0000
FAN	12	f	0	2.48	7.51	4.53	0.0000
Subtotal FAN				1.67	29.02	6.82	
GAO2	3	m	0	2.02	7.66	0.75	0.0000
GARSHI	27	m	1	1.74	31.76	0.04	0.0000
GER	23	c	14	0.64	8.71	9.87	0.0582
GOLLED	2	m	1	1.85	12.69	0.27	0.0000

Table 1G13 - 2

IESLC - Meta-anal of Ever Smoking (or Current if ever not avail), Amount smoked, "Mid", Any prod (or Cigs if Any not avail)
All LC types
Most adjusted

REF	NRR	SEX	AD	Ys	Ws	Qs	Ps
GSELL	3	m	0	2.71	1.64	1.66	0.0005
*HAMMON	154	m	1	2.12	11.45	1.95	0.0000
*HIRAYA	74	m	4	1.69	68.71	0.03	0.0000
HITOSU	36	m	1	1.04	5.81	2.60	0.0125
*HOLE	3	m	1	2.19	6.47	1.49	0.0000
HU	2	m	0	0.74	12.38	11.63	0.0095
HU	5	f	0	0.18	2.64	6.13	0.7672
Subtotal HU				0.64	15.02	17.76	
HU2	6	c	0	1.10	32.09	11.66	0.0000
HUMBLE	76	c	3	2.72	15.27	15.65	0.0000
JEDRYC	46	m	4	1.82	28.13	0.35	0.0000
JEDRYC	49	f	4	0.87	4.91	3.46	0.0546
Subtotal JEDRYC				1.68	33.04	3.81	
JOLY	9	m	0	2.57	9.85	7.36	0.0000
JOLY	5	f	0	1.94	9.62	0.50	0.0000
Subtotal JOLY				2.26	19.47	7.87	
JUSSAW	14	m	0	2.53	5.71	3.88	0.0000
*KAISER	7	m	2	2.85	16.98	22.10	0.0000
*KAISER	3	f	2	2.08	24.04	3.30	0.0000
Subtotal KAISER				2.40	41.02	25.40	
KANELL	27	m	1	1.95	24.67	1.52	0.0000
KATSOU	8	f	0	1.43	3.31	0.25	0.0092
KAUFMA	12	c	6	2.71	22.15	22.22	0.0000
KHUDER	2	m	0	2.35	18.30	7.50	0.0000
*KINLEN	15	m	2	2.65	6.84	6.08	0.0000
KOO	12	f	0	1.99	3.69	0.29	0.0001
KREUZE	21	m	3	2.97	4.24	6.78	0.0000
KREUZE	32	m	3	3.49	18.97	60.39	0.0000
KREUZE	27	f	3	2.49	2.00	1.24	0.0004
KREUZE	38	f	3	2.04	6.02	0.67	0.0000
Subtotal KREUZE				3.08	31.23	69.09	
KREYBE	10	m	1	1.83	5.32	0.08	0.0000
LAMTH	2	f	0	1.68	18.27	0.01	0.0000
LETOUR	3	c	0	2.85	18.55	24.29	0.0000
*LIAW	4	c	2	1.28	11.36	2.06	0.0000
LIU2	9	m	3	1.96	3.78	0.24	0.0001
LIU3	7	m	2	0.09	1.71	4.48	0.9103
LIU4	8	m	2	1.28	4111.92	744.25	0.0000
LUBIN2	275	m	0	2.36	152.73	65.64	0.0000
LUBIN2	283	f	0	1.53	47.64	1.55	0.0000
Subtotal LUBIN2				2.16	200.37	67.19	
MACLEN	38	c	2	1.08	9.92	3.95	0.0007
MARTIS	2	m	0	1.88	3.20	0.09	0.0008
MATOS	31	m	2	2.01	7.84	0.75	0.0000
MATSUD	2	m	0	2.97	2.87	4.60	0.0000
MCCONN	25	c	0	0.31	5.38	10.52	0.4745
*MIGRAN	6	m	2	1.64	3.44	0.02	0.0024
*MIGRAN	33	f	2	2.01	1.67	0.16	0.0093
Subtotal MIGRAN				1.76	5.11	0.17	
*MRFITR	4	m	0	3.91	0.50	2.41	0.0059
ORMOS	2	m	0	2.04	5.85	0.65	0.0000
PASTOR	8	m	1	2.08	7.09	1.00	0.0000
PERNU	21	m	0	2.28	42.95	14.35	0.0000
PERNU	15	f	0	-0.22	0.91	3.37	0.8339
Subtotal PERNU				2.23	43.86	17.72	
SEGI2	14	m	1	1.22	6.23	1.45	0.0022
SIEMIA	14	m	0	1.50	3.87	0.16	0.0031
SOBUE	118	m	0	1.39	21.76	2.22	0.0000
*SPEIZE	3	f	1	2.53	56.20	38.47	0.0000
STOCKS	42	m	2	2.07	29.86	3.85	0.0000
STOCKW	2	c	0	2.67	802.56	752.23	0.0000
SVENSS	11	f	1	2.53	8.37	5.73	0.0000
*TENKAN	11	m	1	3.01	4.70	7.97	0.0000
TIZZAN	8	m	0	0.52	76.34	106.89	0.0000
TSUGAN	30	m	0	0.20	5.96	13.52	0.6241
*TULINI	28	m	3	2.48	9.25	5.61	0.0000
*TULINI	33	f	3	3.27	10.56	25.81	0.0000
Subtotal TULINI				2.90	19.81	31.42	
WAKAI	41	m	2	1.39	6.99	0.71	0.0002
WANG2	13	c	4	1.16	5.60	1.67	0.0061
WYNDE2	18	m	0	2.07	6.77	0.87	0.0000

Table 1G13 - 2

IESLC - Meta-anal of Ever Smoking (or Current if ever not avail), Amount smoked, "Mid", Any prod (or Cigs if Any not avail)
All LC types
Most adjusted

REF	NRR	SEX	AD	Ys	Ws	Qs	Ps
WYNDE3	45	m	0	1.89	6.93	0.23	0.0000
WYNDE3	80	f	0	1.34	6.83	0.94	0.0005
Subtotal WYNDE3				1.61	13.76	1.17	
WYNDE4	45	m	0	2.08	9.99	1.37	0.0000
WYNDE4	59	f	2	1.87	3.72	0.10	0.0003
Subtotal WYNDE4				2.02	13.71	1.47	
WYNDE6	36	m	0	2.41	53.48	26.64	0.0000
WYNDE6	225	f	0	2.48	61.57	37.11	0.0000
Subtotal WYNDE6				2.45	115.05	63.76	
XU3	11	m	1	2.69	3.66	3.56	0.0000
ZHENG	13	m	0	1.41	16.67	1.46	0.0000

	N	120
	NS	93
	Wt	7278.70
Het	Chi	2822.44
Het	df	119
Het	P	***
Fixed	RR	5.51
	RRl	5.38
	RRu	5.64
	P	+++
Random	RR	7.54
	RRl	6.49
	RRu	8.76
	P	+++
Asymm	P	***

Table 1G13 - 3

IESLC - Meta-anal of Ever Smoking (or Current if ever not avail), Amount smoked, "Mid", Any prod (or Cigs if Any not avail)
All LC types
Most adjusted

	Sex			
	combined	male	female	Total
N	11	76	33	120
NS	11	75	32	118
Wt	939.82	5662.09	676.80	7278.70
Het Chi	199.40	1395.66	171.76	2822.44
Het df	10	75	32	119
Het P	***	***	***	***
Fixed RR	12.91	4.52	8.85	5.51
RRl	12.11	4.40	8.20	5.38
RRu	13.76	4.64	9.54	5.64
P	+++	+++	+++	+++
Random RR	6.40	7.89	7.48	7.54
RRl	3.72	6.59	6.09	6.49
RRu	10.99	9.44	9.19	8.76
P	+++	+++	+++	+++
Between Chi				1055.61
Between df				2
Between P				***
Btwn(F) P				***
Btwn(R) P				N.S.

	Lung cancer type		
	all	other	Total
N	117	3	120
NS	90	3	93
Wt	7235.71	42.99	7278.70
Het Chi	2791.05	31.39	2822.44
Het df	116	2	119
Het P	***	***	***
Fixed RR	5.51	5.45	5.51
RRl	5.38	4.04	5.38
RRu	5.64	7.35	5.64
P	+++	+++	+++
Random RR	7.65	4.34	7.54
RRl	6.57	1.24	6.49
RRu	8.91	15.25	8.76
P	+++	+	+++
Between Chi			0.01
Between df			1
Between P			N.S.
Btwn(F) P			N.S.
Btwn(R) P			N.S.

	Location								
	NAmer	UK	Scand	othEur	China	Japan	othAs	other	Total
N	36	19	10	19	13	10	8	5	120
NS	27	13	8	14	10	10	7	4	93
Wt	2024.50	185.67	111.06	458.39	4229.53	153.13	71.52	44.91	7278.70
Het Chi	179.20	63.53	28.90	283.51	31.03	28.19	19.09	3.23	2822.44
Het df	35	18	9	18	12	9	7	4	119
Het P	***	***	***	***	**	***	**	N.S.	***
Fixed RR	12.28	7.61	12.31	6.18	3.60	4.67	3.97	8.90	5.51
RRl	11.76	6.59	10.22	5.64	3.49	3.98	3.15	6.64	5.38
RRu	12.83	8.79	14.83	6.77	3.71	5.47	5.01	11.93	5.64
P	+++	+++	+++	+++	+++	+++	+++	+++	+++
Random RR	10.89	7.78	12.98	7.69	3.74	4.20	3.94	8.90	7.54
RRl	9.57	5.76	8.93	5.08	2.94	3.03	2.65	6.64	6.49
RRu	12.40	10.49	18.87	11.65	4.75	5.83	5.88	11.93	8.76
P	+++	+++	+++	+++	+++	+++	+++	+++	+++
Between Chi									2185.74
Between df									7
Between P									***
Btwn(F) P									***
Btwn(R) P									***

Table 1G13 - 3

IESLC - Meta-anal of Ever Smoking (or Current if ever not avail), Amount smoked, "Mid", Any prod (or Cigs if Any not avail)
All LC types
Most adjusted

	Detailed Country in "other Europe"					
	multi	Germany	othWest	East	Balkans	Total
N	2	6	5	3	3	19
NS	1	3	5	2	3	14
Wt	200.37	40.47	104.69	38.89	73.96	458.39
Het Chi	25.37	14.59	83.10	4.44	10.88	283.51
Het df	1	5	4	2	2	18
Het P	***	*	***	N.S.	**	***
Fixed RR	8.70	18.47	2.88	5.65	4.13	6.18
RRl	7.57	13.57	2.38	4.13	3.29	5.64
RRu	9.99	25.14	3.49	7.73	5.19	6.77
P	+++	+++	+++	+++	+++	+++
Random RR	7.05	14.30	8.33	5.17	4.52	7.69
RRl	3.11	7.90	2.47	2.91	2.37	5.08
RRu	15.98	25.89	28.10	9.18	8.62	11.65
P	+++	+++	+++	+++	+++	+++
Between Chi						145.14
Between df						4
Between P						***
Btwn(F) P						*
Btwn(R) P						(*)

	Detailed Country in "other Asia"			
	India	HongKong	other	Total
N	1	2	5	8
NS	1	2	4	7
Wt	5.71	21.95	43.85	71.52
Het Chi	0.00	0.29	3.84	19.09
Het df	0	1	4	7
Het P	N.S.	N.S.	N.S.	**
Fixed RR	12.56	5.65	2.87	3.97
RRl	5.53	3.72	2.13	3.15
RRu	28.52	8.58	3.85	5.01
P	+++	+++	+++	+++
Random RR	12.56	5.65	2.87	3.94
RRl	5.53	3.72	2.13	2.65
RRu	28.52	8.58	3.85	5.88
P	+++	+++	+++	+++
Between Chi				14.96
Between df				2
Between P				***
Btwn(F) P				*
Btwn(R) P				***

	Detailed other continent			
	SCAmer	Auslia	Africa	Total
N	4	1		5
NS	3	1		4
Wt	43.93	0.98		44.91
Het Chi	2.33	0.00		3.23
Het df	3	0		4
Het P	N.S.	N.S.		N.S.
Fixed RR	8.71	23.00		8.90
RRl	6.48	3.18		6.64
RRu	11.71	166.45		11.93
P	+++	++		+++
Random RR	8.71	23.00		8.90
RRl	6.48	3.18		6.64
RRu	11.71	166.45		11.93
P	+++	++		+++
Between Chi				0.90
Between df				1
Between P				N.S.
Btwn(F) P				N.S.
Btwn(R) P				N.S.

Table 1G13 - 3

IESLC - Meta-anal of Ever Smoking (or Current if ever not avail), Amount smoked, "Mid", Any prod (or Cigs if Any not avail)
All LC types
Most adjusted

	Start year of study<br><1960	1960-69	1970-79	1980-89	1990+	Total
N	27	25	23	37	8	120
NS	21	19	18	31	4	93
Wt	900.67	373.36	497.99	5429.87	76.80	7278.70
Het Chi	292.31	99.38	161.82	1844.49	70.77	2822.44
Het df	26	24	22	36	7	119
Het P	***	***	***	***	***	***
Fixed RR	8.15	8.58	7.28	4.85	8.66	5.51
RRl	7.63	7.75	6.67	4.72	6.93	5.38
RRu	8.70	9.50	7.95	4.98	10.84	5.64
P	+++	+++	+++	+++	+++	+++
Random RR	7.15	8.48	7.01	7.26	8.79	7.54
RRl	5.48	6.72	5.33	5.30	4.15	6.49
RRu	9.33	10.70	9.21	9.96	18.62	8.76
P	+++	+++	+++	+++	+++	+++
Between Chi						353.67
Between df						4
Between P						***
Btwn(F) P						**
Btwn(R) P						N.S.

	Study type (1)<br>CC	other	Total
N	86	34	120
NS	68	25	93
Wt	6187.79	1090.91	7278.70
Het Chi	2103.22	176.22	2822.44
Het df	85	33	119
Het P	***	***	***
Fixed RR	4.91	10.56	5.51
RRl	4.79	9.95	5.38
RRu	5.04	11.20	5.64
P	+++	+++	+++
Random RR	6.61	10.51	7.54
RRl	5.51	8.85	6.49
RRu	7.93	12.49	8.76
P	+++	+++	+++
Between Chi			543.00
Between df			1
Between P			***
Btwn(F) P			***
Btwn(R) P			***

	Study type (2)<br>CC	prosp	other	Total
N	86	32	2	120
NS	68	24	1	93
Wt	6187.79	1080.60	10.31	7278.70
Het Chi	2103.22	173.49	0.00	2822.44
Het df	85	31	1	119
Het P	***	***	N.S.	***
Fixed RR	4.91	10.51	17.61	5.51
RRl	4.79	9.90	9.56	5.38
RRu	5.04	11.15	32.43	5.64
P	+++	+++	+++	+++
Random RR	6.61	10.27	17.61	7.54
RRl	5.51	8.60	9.56	6.49
RRu	7.93	12.25	32.43	8.76
P	+++	+++	+++	+++
Between Chi				545.73
Between df				2
Between P				***
Btwn(F) P				***
Btwn(R) P				***

Table 1G13 - 3

IESLC - Meta-anal of Ever Smoking (or Current if ever not avail), Amount smoked, "Mid", Any prod (or Cigs if Any not avail)
All LC types
Most adjusted

	Study size (number of LC cases)				
	100-249	250-499	500-999	1000+	Total
N	35	26	28	31	120
NS	32	20	22	19	93
Wt	184.60	222.37	411.64	6460.09	7278.70
Het Chi	125.84	76.15	134.90	2319.02	2822.44
Het df	34	25	27	30	119
Het P	***	***	***	***	***
Fixed RR	4.86	7.54	9.66	5.28	5.51
RRl	4.20	6.61	8.77	5.15	5.38
RRu	5.61	8.60	10.64	5.41	5.64
P	+++	+++	+++	+++	+++
Random RR	5.37	7.74	10.27	7.59	7.54
RRl	4.02	6.10	8.17	5.67	6.49
RRu	7.16	9.83	12.91	10.16	8.76
P	+++	+++	+++	+++	+++
Between Chi					166.53
Between df					3
Between P					***
Btwn(F) P					(*)
Btwn(R) P					**

	Risky occupational population			
	no	mining	othRisky	Total
N	116	2	2	120
NS	89	2	2	93
Wt	7238.50	6.50	33.70	7278.70
Het Chi	2817.31	1.47	2.68	2822.44
Het df	115	1	1	119
Het P	***	N.S.	N.S.	***
Fixed RR	5.50	7.44	6.13	5.51
RRl	5.38	3.45	4.38	5.38
RRu	5.63	16.05	8.60	5.64
P	+++	+++	+++	+++
Random RR	7.50	8.63	8.58	7.54
RRl	6.44	2.74	2.80	6.49
RRu	8.74	27.16	26.24	8.76
P	+++	+++	+++	+++
Between Chi				0.98
Between df				2
Between P				N.S.
Btwn(F) P				N.S.
Btwn(R) P				N.S.

	National cigarette tobacco type			
	Virginia	blended	other	Total
N	24	81	15	120
NS	18	63	12	93
Wt	221.56	2807.56	4249.59	7278.70
Het Chi	81.49	842.38	34.58	2822.44
Het df	23	80	14	119
Het P	***	***	**	***
Fixed RR	8.42	10.16	3.60	5.51
RRl	7.38	9.79	3.49	5.38
RRu	9.60	10.54	3.71	5.64
P	+++	+++	+++	+++
Random RR	8.56	8.36	3.57	7.54
RRl	6.51	7.26	2.87	6.49
RRu	11.25	9.62	4.43	8.76
P	+++	+++	+++	+++
Between Chi				1863.98
Between df				2
Between P				***
Btwn(F) P				***
Btwn(R) P				***

Table 1G13 - 3

IESLC - Meta-anal of Ever Smoking (or Current if ever not avail), Amount smoked, "Mid", Any prod (or Cigs if Any not avail)
All LC types
Most adjusted

	Any proxy use		
	No/nk	Yes	Total
N	92	28	120
NS	70	23	93
Wt	2820.31	4458.39	7278.70
Het Chi	929.02	251.38	2822.44
Het df	91	27	119
Het P	***	***	***
Fixed RR	10.01	3.78	5.51
RRl	9.65	3.67	5.38
RRu	10.39	3.89	5.64
P	+++	+++	+++
Random RR	8.01	6.35	7.54
RRl	6.96	4.96	6.49
RRu	9.21	8.13	8.76
P	+++	+++	+++
Between Chi			1642.04
Between df			1
Between P			***
Btwn(F) P			***
Btwn(R) P			N.S.

	Full histological confirmation		
	No	Yes	Total
N	95	25	120
NS	72	21	93
Wt	6709.15	569.55	7278.70
Het Chi	2539.33	168.81	2822.44
Het df	94	24	119
Het P	***	***	***
Fixed RR	5.31	8.47	5.51
RRl	5.19	7.80	5.38
RRu	5.44	9.19	5.64
P	+++	+++	+++
Random RR	7.94	6.42	7.54
RRl	6.67	5.02	6.49
RRu	9.45	8.21	8.76
P	+++	+++	+++
Between Chi			114.30
Between df			1
Between P			***
Btwn(F) P			*
Btwn(R) P			N.S.

	Number of adjustment variables (1)			
	0	1	2+/+nk	Total
N	58	26	36	120
NS	45	21	28	94
Wt	1642.25	1047.20	4589.25	7278.70
Het Chi	706.69	148.78	464.91	2822.44
Het df	57	25	35	119
Het P	***	***	***	***
Fixed RR	9.75	10.31	3.89	5.51
RRl	9.29	9.71	3.78	5.38
RRu	10.24	10.96	4.01	5.64
P	+++	+++	+++	+++
Random RR	6.73	9.19	7.86	7.54
RRl	5.43	7.70	6.09	6.49
RRu	8.34	10.96	10.14	8.76
P	+++	+++	+++	+++
Between Chi				1502.06
Between df				2
Between P				***
Btwn(F) P				***
Btwn(R) P				(*)

Table 1G13 - 3

IESLC - Meta-anal of Ever Smoking (or Current if ever not avail), Amount smoked, "Mid", Any prod (or Cigs if Any not avail)
All LC types
Most adjusted

	Number of adjustment variables (2) 0	1	2	3-5	6+/+nk	Total
N	58	26	17	17	2	120
NS	45	21	15	11	2	94
Wt	1642.25	1047.20	4296.82	261.57	30.86	7278.70
Het Chi	706.69	148.78	158.27	93.16	26.68	2822.44
Het df	57	25	16	16	1	119
Het P	***	***	***	***	***	***
Fixed RR	9.75	10.31	3.69	8.44	8.37	5.51
RRl	9.29	9.71	3.58	7.48	5.88	5.38
RRu	10.24	10.96	3.80	9.53	11.92	5.64
P	+++	+++	+++	+++	+++	+++
Random RR	6.73	9.19	6.92	9.21	5.43	7.54
RRl	5.43	7.70	4.86	6.71	0.72	6.49
RRu	8.34	10.96	9.85	12.63	41.12	8.76
P	+++	+++	+++	+++	N.S.	+++
Between Chi						1688.87
Between df						4
Between P						***
Btwn(F) P						***
Btwn(R) P						N.S.

	Product all/unsp	cig+/-ot	cig only	Total
N	31	72	17	120
NS	27	55	13	95
Wt	256.80	2412.38	4609.52	7278.70
Het Chi	108.53	646.95	572.54	2822.44
Het df	30	71	16	119
Het P	***	***	***	***
Fixed RR	6.52	10.34	3.92	5.51
RRl	5.77	9.94	3.81	5.38
RRu	7.37	10.76	4.04	5.64
P	+++	+++	+++	+++
Random RR	5.87	8.28	7.64	7.54
RRl	4.55	7.17	5.14	6.49
RRu	7.57	9.57	11.36	8.76
P	+++	+++	+++	+++
Between Chi				1494.43
Between df				2
Between P				***
Btwn(F) P				***
Btwn(R) P				(*)

	Denominator nev any	nev cigs	Total
N	85	35	120
NS	65	31	96
Wt	6621.23	657.47	7278.70
Het Chi	2450.91	277.15	2822.44
Het df	84	34	119
Het P	***	***	***
Fixed RR	5.31	7.91	5.51
RRl	5.19	7.33	5.38
RRu	5.44	8.54	5.64
P	+++	+++	+++
Random RR	7.66	7.26	7.54
RRl	6.37	5.73	6.49
RRu	9.21	9.20	8.76
P	+++	+++	+++
Between Chi			94.38
Between df			1
Between P			***
Btwn(F) P			*
Btwn(R) P			N.S.

Table 1G13 - 3

IESLC - Meta-anal of Ever Smoking (or Current if ever not avail), Amount smoked, "Mid", Any prod (or Cigs if Any not avail)
All LC types
Most adjusted

	Derivation of RR/CI			
	Orig	StdCalc	Other	Total
N	28	59	33	120
NS	22	46	26	94
Wt	671.49	1710.61	4896.60	7278.70
Het Chi	204.89	717.91	929.90	2822.44
Het df	27	58	32	119
Het P	***	***	***	***
Fixed RR	8.53	9.59	4.27	5.51
RRl	7.91	9.15	4.16	5.38
RRu	9.20	10.06	4.40	5.64
P	+++	+++	+++	+++
Random RR	8.51	6.61	8.63	7.54
RRl	6.62	5.38	6.40	6.49
RRu	10.92	8.12	11.64	8.76
P	+++	+++	+++	+++
Between Chi				969.74
Between df				2
Between P				***
Btwn(F) P				***
Btwn(R) P				N.S.

	Smoking status		
	ever	current	Total
N	70	50	120
NS	55	39	94
Wt	5103.04	2175.66	7278.70
Het Chi	730.40	345.54	2822.44
Het df	69	49	119
Het P	***	***	***
Fixed RR	4.00	11.66	5.51
RRl	3.89	11.18	5.38
RRu	4.11	12.17	5.64
P	+++	+++	+++
Random RR	6.32	9.67	7.54
RRl	5.34	8.39	6.49
RRu	7.48	11.15	8.76
P	+++	+++	+++
Between Chi			1746.50
Between df			1
Between P			***
Btwn(F) P			***
Btwn(R) P			***

	Study LIU4		
	LIU4	others	Total
N	1	119	120
NS	1	92	93
Wt	4111.92	3166.78	7278.70
Het Chi	0.00	1111.82	2822.44
Het df	0	118	119
Het P	N.S.	***	***
Fixed RR	3.60	9.57	5.51
RRl	3.49	9.24	5.38
RRu	3.71	9.91	5.64
P	+++	+++	+++
Random RR	3.60	7.63	7.54
RRl	3.49	6.74	6.49
RRu	3.71	8.63	8.76
P	+++	+++	+++
Between Chi			1710.62
Between df			1
Between P			***
Btwn(F) P			***
Btwn(R) P			***

Table 1G13 - 4

IESLC - Meta-anal of Ever Smoking (or Current if ever not avail), Amount smoked, "Mid", Any prod (or Cigs if Any not avail)
All LC types
Least adjusted

REF	NRR	X	SEX	AGEL	AGEH	RACE	YF	LC TYPE	LOC	START	ST	NLC	R	VB	P	H	AD	SM	PRODUCT	exL	exH	DENOM	De
AKIBA	18	x	m	0	0	all	0	all	As:Jap	1963	pr	610	n	bl	n	n	0	cu	cig+/-ot	15	24	nev cigs	or
ALDERS	19		m	0	0	all	-	all	Eu:UK	1977	CC	1448	n	V	n	n	1	ev	cig only	18	27	nev+2	ot
ALDERS	22		f	0	0	all	-	all	Eu:UK	1977	CC	1448	n	V	n	n	1	ev	cig only	18	27	nev+2	ot
ARCHER	2		m	0	0	wh	0	all	NAmer	1950	pr	146	m	bl	n	n	0	cu	cig+/-ot	20	20	nev cigs	st
ARMADA	47		m	0	0	all	-	all	Eu:wst	1986	CC	325	n	bl	n	y	0	ev	cig+/-ot	15	24	nev any	st
AUVINE	6	x	c	0	0	all	-	all	Eu:Sca	1986	CC	517	n	bl	y	n	0	ev	cig+/-ot	11	20	nev cigs	st
AXELSS	15		f	0	0	sca	-	all	Eu:Sca	1989	CC	436	n	bl	n	n	0	ev	all/unsp	20	29	nev any	st
BARBON	9	x	m	0	0	all	-	all	Eu:wst	1979	CC	755	n	bl	y	y	0	ev	all/unsp	20	29	nev any	st
BEST	14		m	0	0	all	0	all	NAmer	1955	pr	381	n	V	n	n	1	cu	cig only	10	20	nev any	ot
BRESLO	15		m	0	0	all	-	all	NAmer	1949	CC	518	n	bl	n	y	0	ev	cig+/-ot	20	39	nev+3	st
BRETT	2		m	0	0	all	0	all	Eu:UK	1960	pr	150	n	V	n	n	0	cu	cig+/-ot	15	24	nev cigs	st
BUFFLE	29		f	0	0	w-hi	-	all	NAmer	1976	CC	943	n	bl	y	n	0	ev	cig+/-ot	20	20	nev cigs	or
CHANG	3		m	0	0	all	0	all	NAmer	1972	pr	136	n	bl	n	n	0	cu	cig+/-ot	11	20	nev cigs	st
CHANG	9		f	0	0	all	0	all	NAmer	1972	pr	136	n	bl	n	n	0	cu	cig+/-ot	11	20	nev cigs	st
CHEN2	4		m	0	0	all	-	all	As:Chi	1983	CC	193	n	ot	y	n	0	ev	all/unsp	10	20	nev any	st
CHEN2	8		f	0	0	all	-	all	As:Chi	1983	CC	193	n	ot	y	n	0	ev	all/unsp	10	20	nev any	st
CHOI	13		m	0	0	all	-	all	As:oth	1985	CC	375	n	bl	n	n	0	ev	cig+/-ot	11	20	nev cigs	st
CHOI	18		f	0	0	all	-	all	As:oth	1985	CC	375	n	bl	n	n	0	ev	cig+/-ot	11	30	nev cigs	st
CHOW	4	x	m	0	0	wh	0	all	NAmer	1966	pr	219	n	bl	n	n	0	cu	cig+/-ot	20	29	nev any	st
COMSTO	5		m	0	0	all	-	all	NAmer	1975	ot	258	n	bl	n	n	0	cu	cig+/-ot	20	39	nev any	st
COMSTO	10		f	0	0	all	-	all	NAmer	1975	ot	258	n	bl	n	n	0	cu	cig+/-ot	20	39	nev any	st
CPSI	218		m	35	84	all	6	all	NAmer	1959	pr	5138	n	bl	n	n	1	cu	cig+/-ot	20	39	nev any	ot
CPSI	277		f	40	74	all	6	all	NAmer	1959	pr	5138	n	bl	n	n	1	cu	cig+/-ot	20	39	nev cigs	ot
CPSII	32	x	m	0	0	all	6	all	NAmer	1982	pr	3229	n	bl	n	n	0	cu	cig only	20	20	nev any	st
CPSII	67	x	f	0	0	all	6	all	NAmer	1982	pr	3229	n	bl	n	n	0	cu	cig+/-ot	20	20	nev cigs	st
DAMBER	8		m	0	0	all	-	all	Eu:Sca	1972	CC	579	n	bl	y	n	1	ev	cig only	16	25	nev any	ot
DARBY	2		m	0	0	wh	-	all	Eu:UK	1988	CC	982	n	V	n	n	0	cu	cig+/-ot	15	24	nev any	st
DARBY	9		f	0	0	wh	-	all	Eu:UK	1988	CC	982	n	V	n	n	0	cu	cig+/-ot	15	24	nev any	st
DAVEYS	3		m	0	0	all	-	all	Eu:Ger	1930	CC	109	n	bl	y	n	0	ev	all/unsp	11	20	nev any	st
DEAN3	12	x	m	0	0	all	-	all	Eu:UK	1969	CC	766	n	V	y	n	0	cu	cig only	13	22	nev any	st
DEAN3	96	x	f	0	0	all	-	all	Eu:UK	1969	CC	766	n	V	y	n	0	cu	cig only	13	22	nev any	st
DEKLER	3		m	0	0	all	0	all	Auslia	1961	pr	138	m	V	n	n	2	cu	cig+/-ot	15	24	nev any	or
DESTEF	2	x	m	0	0	all	-	all	SCAmer	1988	CC	497	n	bl	n	y	0	ev	all/unsp	11	20	nev any	st
DOLL	3		m	0	0	all	-	all	Eu:UK	1948	CC	1465	n	V	n	n	0	ev	all/unsp	15	24	nev any	st
DOLL	9		f	0	0	all	-	all	Eu:UK	1948	CC	1465	n	V	n	n	0	ev	all/unsp	15	24	nev any	st
DOLL2	47		m	35	99	all	5	all	Eu:UK	1951	pr	920	n	V	n	n	1	ev	all/unsp	15	24	nev any	ot
DOLL2	11		f	0	0	all	22	all	Eu:UK	1951	pr	920	n	V	n	n	1	cu	cig only	15	24	nev any	ot
DORN	409		m	0	0	wh	25	all	NAmer	1954	pr	5097	n	bl	n	n	1	cu	cig+/-ot	10	20	nev any	or
DOSEME	9		m	0	0	all	-	all	Eu:bal	1979	CC	1210	n	bl	n	n	2	ev	cig+/-ot	11	20	nev cigs	or
DUNN	3		m	0	0	all	0	all	NAmer	1954	pr	139	o	bl	n	n	0	ev	cig+/-ot	15	24	nev cigs	st
EBELIN	4		m	0	0	all	-	all	Eu:Ger	1980	CC	130	n	bl	n	n	0	ev	all/unsp	20	29	nev any	st
ENSTRO	5		m	0	0	all	0	all	NAmer	1959	pr	2879	n	bl	n	n	1	cu	cig only	20	20	nev any	ot
ENSTRO	9		f	0	0	all	0	all	NAmer	1959	pr	2879	n	bl	n	n	1	cu	cig only	20	20	nev any	ot
ESAKI	2		m	0	0	all	-	all	As:Jap	1961	CC	245	n	bl	y	n	0	ev	cig+/-ot	15	29	nev cigs	st
FAN	8		m	0	0	all	-	all	As:Chi	1990	CC	403	n	ot	y	n	0	ev	cig+/-ot	20	29	nev cigs	st
FAN	12		f	0	0	all	-	all	As:Chi	1990	CC	403	n	ot	y	n	0	ev	cig+/-ot	20	29	nev cigs	st
GAO2	3		m	0	0	all	-	all	As:Jap	1988	CC	282	n	bl	n	n	0	cu	cig+/-ot	20	29	nev cigs	st
GARSHI	19	x	m	0	0	all	-	all	NAmer	1981	CC	1081	o	bl	y	n	0	ev	all/unsp	16	25	nev any	st
GER	19	x	c	0	0	all	-	all	As:oth	1990	CC	141	n	ot	y	n	0	ev	all/unsp	11	20	nev any	st
GOLLED	16	x	m	35	99	all	-	all	Eu:UK	1952	CC	443	n	V	y	n	0	ev	cig only	11	22	nev any	st
GSELL	3		m	0	0	all	-	all	Eu:wst	1937	CC	150	n	bl	n	y	0	ev	all/unsp	15	20	nev any	st
HAMMON	154		m	0	0	wh	0	all	NAmer	1952	pr	448	n	bl	n	n	1	ev	cig only	10	20	nev any	ot
HIRAYA	74		m	0	0	all	16	all	As:Jap	1965	pr	1917	n	bl	n	n	4	cu	cig+/-ot	15	24	nev any	or
HITOSU	4	x	m	0	0	all	-	all	As:Jap	1960	CC	216	n	bl	y	n	0	cu	all/unsp	15	24	nev any	st
HOLE	10	x	m	0	0	all	0	all	Eu:UK	1972	pr	225	n	V	n	n	0	cu	cig+/-ot	15	24	nev any	st
HU	2		m	0	0	all	-	all	As:Chi	1985	CC	227	n	ot	n	y	0	ev	cig+/-ot	14	24	nev any	st
HU	5		f	0	0	all	-	all	As:Chi	1985	CC	227	n	ot	n	y	0	ev	cig+/-ot	14	24	nev any	st
HU2	6		c	0	0	all	-	all	As:Chi	1977	CC	523	n	ot	y	n	0	ev	cig+/-ot	20	29	nev cigs	st
HUMBLE	76		c	0	0	wh	-	not alv	NAmer	1980	CC	521	n	bl	y	n	3	cu	cig+/-ot	16	20	nev cigs	ot
JEDRYC	61	x	m	0	0	all	-	all	Eu:est	1980	CC	1630	n	bl	y	n	0	ev	cig+/-ot	20	29	nev any	st
JEDRYC	66	x	f	0	0	all	-	all	Eu:est	1980	CC	1630	n	bl	y	n	0	ev	cig+/-ot	20	29	nev any	st
JOLY	9		m	0	0	all	-	all	SCAmer	1978	CC	826	n	bl	n	n	0	ev	cig+/-ot	20	29	nev any	st
JOLY	5		f	0	0	all	-	all	SCAmer	1978	CC	826	n	bl	n	n	0	ev	cig+/-ot	20	29	nev any	st
JUSSAW	14		m	0	0	all	-	all	As:Ind	1964	CC	792	n	V	n	n	0	ev	cig only	20	24	nev any	st
KAISER	7		m	0	0	all	0	all	NAmer	1964	pr	714	n	bl	n	n	2	cu	cig+/-ot	20	40	nev cigs	or
KAISER	3		f	0	0	all	0	all	NAmer	1964	pr	714	n	bl	n	n	2	cu	cig+/-ot	20	40	nev cigs	or
KANELL	2	x	m	0	0	all	-	all	Eu:bal	1950	CC	862	n	bl	n	n	0	cu	cig+/-ot	11	20	nev any	st
KATSOU	8		f	0	0	all	-	all	Eu:bal	1987	CC	101	n	bl	n	n	0	cu	all/unsp	11	20	nev any	st
KAUFMA	3	x	c	0	0	all	-	all	NAmer	1981	CC	881	n	bl	n	n	0	cu	cig+/-ot	15	24	nev cigs	st
KHUDER	2		m	0	0	all	-	all	NAmer	1985	CC	482	n	bl	n	y	0	ev	cig+/-ot	20	39	nev cigs	st
KINLEN	4	x	m	0	0	all	0	all	Eu:UK	1967	pr	718	n	V	n	n	0	cu	cig+/-ot	15	24	nev any	st
KOO	12		f	0	0	all	-	all	As:HK	1981	CC	200	n	bl	n	n	0	cu	all/unsp	11	20	nev any	st

Table 1G13 - 4

IESLC - Meta-anal of Ever Smoking (or Current if ever not avail), Amount smoked, "Mid", Any prod (or Cigs if Any not avail)
All LC types
Least adjusted

REF	NRR	X	SEX	AGEL	AGEH	RACE	YF	LC TYPE	LOC	START	ST	NLC	R	VB	P	H	AD	SM	PRODUCT	exL	exH	DENOM	De
KREUZE	21		m	1	45	all	-	all	Eu:Ger	1990	CC	2260	n	bl	n	n	3	ev	cig+/-ot	20	29	nev any	or
KREUZE	32		m	55	69	all	-	all	Eu:Ger	1990	CC	2260	n	bl	n	n	3	ev	cig+/-ot	20	29	nev any	or
KREUZE	27		f	1	45	all	-	all	Eu:Ger	1990	CC	2260	n	bl	n	n	3	ev	cig+/-ot	20	29	nev any	or
KREUZE	38		f	55	69	all	-	all	Eu:Ger	1990	CC	2260	n	bl	n	n	3	ev	cig+/-ot	20	29	nev any	or
KREYBE	22	x	m	0	0	all	-	all	Eu:Sca	1948	CC	300	n	bl	n	y	0	ev	all/unsp	15	24	nev any	st
LAMTH	2		f	0	0	ch	-	all	As:HK	1983	CC	445	n	bl	n	n	0	ev	all/unsp	11	20	nev any	or
LETOUR	3		c	0	0	all	-	all	NAmer	1983	CC	738	n	V	y	y	0	ev	cig+/-ot	20	40	nev cigs	st
LIAW	4		c	0	0	all	0	all	As:oth	1982	pr	127	n	ot	n	n	2	cu	all/unsp	11	20	nev any	or
LIU2	6	x	m	0	0	all	-	all	As:Chi	1983	CC	316	n	ot	n	n	0	ev	all/unsp	20	29	nev any	st
LIU3	4	x	m	0	0	all	-	all	As:Chi	1985	CC	110	n	ot	n	n	0	ev	all/unsp	16	30	nev any	st
LIU4	8		m	35	69	all	-	all	As:Chi	1986	CC	1000-00	n	ot	y	n	2	ev	cig only	20	20	nev any	ot
LUBIN2	275		m	0	0	all	-	all	Eu:mul	1976	CC	7804	n	bl	n	y	0	ev	cig+/-ot	20	29	nev any	st
LUBIN2	283		f	0	0	all	-	all	Eu:mul	1976	CC	7804	n	bl	n	y	0	ev	cig+/-ot	20	29	nev any	st
MACLEN	22	x	m	0	0	ch	-	all	As:oth	1972	CC	233	n	bl	n	n	0	cu	cig+/-ot	20	29	nev cigs	st
MARTIS	2		m	0	0	all	-	all	Eu:UK	1972	CC	201	n	V	n	n	0	ev	cig+/-ot	15	24	nev cigs	st
MATOS	30	x	m	0	0	all	-	all	SCAmer	1994	CC	200	n	bl	n	n	0	ev	cig+/-ot	15	24	nev any	st
MATSUD	2		m	0	0	all	-	all	As:Jap	1965	CC	179	n	bl	n	n	0	ev	cig+/-ot	11	20	nev cigs	st
MCCONN	25		c	0	0	all	-	all	Eu:UK	1946	CC	100	n	V	n	y	0	ev	all/unsp	10	20	nev any	st
MIGRAN	5	x	m	0	0	all	0	all	Eu:UK	1964	pr	259	n	V	n	n	0	cu	cig only	20	20	nev any	st
MIGRAN	32	x	f	0	0	all	0	all	Eu:UK	1964	pr	259	n	V	n	n	0	cu	cig only	20	20	nev any	st
MRFITR	4		m	0	0	all	0	all	NAmer	1973	pr	119	n	bl	n	n	0	cu	cig+/-ot	20	39	nev cigs	ot
ORMOS	2		m	0	0	all	-	all	Eu:est	1947	CC	119	n	bl	y	y	0	ev	cig+/-ot	16	30	nev any	st
PASTOR	3	x	m	0	0	all	-	all	Eu:wst	1976	CC	204	n	bl	y	n	0	ev	all/unsp	20	29	nev any	st
PERNU	21		m	0	0	all	-	all	Eu:Sca	1944	CC	1606	n	bl	n	n	0	ev	all/unsp	20	24	nev any	st
PERNU	15		f	0	0	all	-	all	Eu:Sca	1944	CC	1606	n	bl	n	n	0	ev	all/unsp	20	24	nev any	st
SEGI2	13	x	m	0	0	all	-	all	As:Jap	1962	CC	378	n	bl	n	n	0	cu	cig+/-ot	20	29	nev any	st
SIEMIA	14		m	0	0	all	-	all	NAmer	1979	CC	857	n	V	y	y	0	ev	cig+/-ot	20	39	nev cigs	or
SOBUE	118		m	0	0	all	-	q+s+l+a	As:Jap	1986	CC	1376	n	bl	n	y	0	cu	cig+/-ot	20	29	nev cigs	st
SPEIZE	3		f	0	0	all	0	all	NAmer	1976	pr	593	n	bl	n	y	1	cu	cig+/-ot	15	24	nev cigs	ot
STOCKS	26	x	m	0	0	all	-	all	Eu:UK	1952	CC	2932	n	V	y	n	0	ev	cig+/-ot	15	21	nev any	st
STOCKW	2		c	0	0	all	-	all	NAmer	1981	CC	22161	n	bl	n	n	0	cu	cig+/-ot	20	40	nev any	st
SVENSS	31	x	f	0	0	all	-	all	Eu:Sca	1983	CC	210	n	bl	n	n	0	cu	all/unsp	11	20	nev any	st
TENKAN	11		m	0	0	all	17	all	Eu:Sca	1962	pr	242	n	bl	n	n	1	cu	all/unsp	15	24	nev any	ot
TIZZAN	8		m	0	0	all	-	all	Eu:wst	1959	CC	1358	n	bl	n	n	0	ev	cig only	10	20	nev any	st
TSUGAN	30		m	0	0	all	-	q+a	As:Jap	1976	CC	134	n	bl	n	y	0	cu	all/unsp	16	35	nev any	st
TULINI	5	x	m	0	0	all	0	all	Eu:Sca	1967	pr	472	n	bl	n	n	1	cu	cig+/-ot	15	24	nev any	or
TULINI	10	x	f	0	0	all	0	all	Eu:Sca	1967	pr	472	n	bl	n	n	1	cu	cig+/-ot	15	24	nev any	or
WAKAI	38	x	m	0	0	all	-	all	As:Jap	1988	CC	333	n	bl	n	y	0	cu	cig+/-ot	20	29	nev any	st
WANG2	5	x	c	0	0	all	-	all	As:Chi	1980	CC	103	n	ot	n	n	0	ev	cig+/-ot	20	29	nev cigs	st
WYNDE2	18		m	0	0	all	-	all	NAmer	1962	CC	404	n	bl	n	y	0	ev	cig+/-ot	11	20	nev any	st
WYNDE3	45		m	0	0	all	-	all	NAmer	1966	CC	350	n	bl	n	y	0	ev	cig+/-ot	10	20	nev any	st
WYNDE3	80		f	0	0	all	-	all	NAmer	1966	CC	350	n	bl	n	y	0	ev	cig+/-ot	10	20	nev any	st
WYNDE4	45		m	0	0	all	-	all	NAmer	1948	CC	684	n	bl	y	n	0	ev	all/unsp	16	20	nev any	st
WYNDE4	59		f	0	0	all	-	all	NAmer	1948	CC	684	n	bl	y	n	2	ev	all/unsp	16	20	nev any	ot
WYNDE6	36		m	0	0	all	-	all	NAmer	1969	CC	4423	n	bl	n	y	0	cu	cig+/-ot	11	20	nev any	st
WYNDE6	225		f	0	0	all	-	all	NAmer	1969	CC	4423	n	bl	n	y	0	cu	cig+/-ot	11	20	nev cigs	st
XU3	7	x	m	0	0	all	-	all	As:Chi	1981	CC	135	n	ot	n	n	0	ev	all/unsp	20	29	nev any	st
ZHENG	13		m	0	0	all	-	all	As:Chi	1982	CC	540	n	ot	*	y	0	ev	cig+/-ot	20	29	nev cigs	st

Cigarette type is all/unspec for all RRs
except for the following:

REF	NRR	CIGTYPE
ALDERS	19	MC only
ALDERS	22	MC only
DEAN3	12	MC only
DEAN3	96	MC only
JUSSAW	14	MC only

Table 1G13 - 5

IESLC - Meta-anal of Ever Smoking (or Current if ever not avail), Amount smoked, "Mid", Any prod (or Cigs if Any not avail)
All LC types
Least adjusted

REF	NRR	SEX	AD	Number Exposed Case	Number Exposed Cont	Non-exposed Case	Non-exposed Cont	RR	95.00%CI
*AKIBA	18	m	0	178	82289	18	35833	4.31	(2.65- 6.99)
ALDERS	19	m	1	-	-	-	-	7.96	(4.63- 13.69)
ALDERS	22	f	1	-	-	-	-	5.28	(3.79- 7.36)
Subtotal ALDERS								5.91	(4.45- 7.84)
*ARCHER	2	m	0	68	18320	6	9842	6.09	(2.64- 14.02)
ARMADA	47	m	0	134	105	4	64	20.42	(7.20- 57.88)
AUVINE	6	c	0	148	39	44	229	19.75	(12.25- 31.86)
AXELSS	15	f	0	28	15	18	154	15.97	(7.21- 35.36)
BARBON	9	m	0	223	176	22	188	10.83	(6.67- 17.57)
*BEST	14	m	1	-	-	-	-	16.41	(7.73- 34.86)
BRESLO	15	m	0	296	193	22	110	7.67	(4.69- 12.55)
*BRETT	2	m	0	62	15868	6	6530	4.25	(1.84- 9.83)
BUFFLE	29	f	0	76	60	12	112	11.82	(5.96- 23.45)
*CHANG	3	m	0	17	161	5	502	10.60	(3.97- 28.28)
*CHANG	9	f	0	11	234	11	1139	4.87	(2.14- 11.09)
Subtotal CHANG								6.72	(3.57- 12.62)
CHEN2	4	m	0	44	50	9	33	3.23	(1.39- 7.48)
CHEN2	8	f	0	22	10	25	33	2.90	(1.17- 7.22)
Subtotal CHEN2								3.07	(1.66- 5.70)
CHOI	13	m	0	144	281	13	95	3.74	(2.03- 6.92)
CHOI	18	f	0	7	9	76	164	1.68	(0.60- 4.68)
Subtotal CHOI								3.03	(1.79- 5.13)
*CHOW	4	m	0	60	36589	6	62913	17.19	(7.43- 39.79)
COMSTO	5	m	0	60	57	4	69	18.16	(6.22- 53.00)
COMSTO	10	f	0	51	26	13	115	17.35	(8.25- 36.48)
Subtotal COMSTO								17.61	(9.56- 32.43)
*CPSI	218	m	1	-	-	-	-	14.30	(11.33- 18.05)
*CPSI	277	f	1	-	-	-	-	5.03	(3.82- 6.63)
Subtotal CPSI								9.26	(7.75- 11.06)
*CPSII	32	m	0	564	171438	124	742207	19.69	(16.21- 23.92)
*CPSII	67	f	0	395	251336	310	2091302	10.60	(9.14- 12.30)
Subtotal CPSII								13.32	(11.84- 14.99)
DAMBER	8	m	1	-	-	-	-	9.10	(5.50- 15.30)
DARBY	2	m	0	126	169	3	384	95.43	(29.94- 304.17)
DARBY	9	f	0	86	92	23	529	21.50	(12.90- 35.82)
Subtotal DARBY								27.39	(17.17- 43.71)
DAVEYS	3	m	0	19	22	3	23	6.62	(1.72- 25.56)
DEAN3	12	m	0	125	429	25	510	5.94	(3.80- 9.31)
DEAN3	96	f	0	44	521	41	1538	3.17	(2.05- 4.90)
Subtotal DEAN3								4.30	(3.15- 5.89)
*DEKLER	3	m	2	-	-	-	-	23.00	(3.20- 167.60)
DESTEF	2	m	0	155	119	27	163	7.86	(4.90- 12.61)
DOLL	3	m	0	475	431	7	61	9.60	(4.35- 21.22)
DOLL	9	f	0	14	6	40	59	3.44	(1.22- 9.71)
Subtotal DOLL								6.58	(3.50- 12.35)
*DOLL2	47	m	1	-	-	-	-	12.29	(1.67- 90.41)
*DOLL2	11	f	1	-	-	-	-	6.43	(1.81- 22.78)
Subtotal DOLL2								7.74	(2.66- 22.56)
*DORN	409	m	1	-	-	-	-	9.92	(8.84- 11.14)
DOSEME	9	m	2	-	-	-	-	3.10	(2.30- 4.10)
*DUNN	3	m	0	75	27720	2	14160	19.16	(4.70- 78.00)
EBELIN	4	m	0	47	37	12	117	12.39	(5.95- 25.80)
*ENSTRO	5	m	1	-	-	-	-	13.65	(10.88- 17.13)
*ENSTRO	9	f	1	-	-	-	-	9.48	(8.04- 11.18)
Subtotal ENSTRO								10.75	(9.41- 12.29)
ESAKI	2	m	0	74	58	16	28	2.23	(1.10- 4.51)
FAN	8	m	0	111	183	36	236	3.98	(2.61- 6.07)
FAN	12	f	0	31	12	69	320	11.98	(5.86- 24.50)
Subtotal FAN								5.29	(3.68- 7.61)
GAO2	3	m	0	77	44	13	56	7.54	(3.71- 15.30)
GARSHI	19	m	0	387	601	41	363	5.70	(4.03- 8.07)
GER	19	c	0	45	168	51	246	1.29	(0.83- 2.02)
GOLLED	16	m	0	118	779	15	490	4.95	(2.86- 8.57)
GSELL	3	m	0	27	26	2	29	15.06	(3.26- 69.59)
*HAMMON	154	m	1	-	-	-	-	8.32	(4.66- 14.84)
*HIRAYA	74	m	4	-	-	-	-	5.40	(4.30- 6.90)
HITOSU	4	m	0	52	747	7	242	2.41	(1.08- 5.37)
*HOLE	10	m	0	87	2056	7	1189	7.19	(3.34- 15.47)
HU	2	m	0	55	43	41	67	2.09	(1.20- 3.65)
HU	5	f	0	6	6	40	48	1.20	(0.36- 4.01)
Subtotal HU								1.90	(1.14- 3.14)

Table 1G13 - 5

IESLC - Meta-anal of Ever Smoking (or Current if ever not avail), Amount smoked, "Mid", Any prod (or Cigs if Any not avail)
All LC types
Least adjusted

REF	NRR	SEX	AD	Number Exposed Case	Number Exposed Cont	Non-exposed Case	Non-exposed Cont	RR	95.00%CI
HU2	6	c	0	149	87	121	213	3.01	(2.13- 4.26)
HUMBLE	76	c	3	-	-	-	-	15.16	(9.18- 25.03)
JEDRYC	61	m	0	426	295	49	219	6.45	(4.58- 9.10)
JEDRYC	66	f	0	52	16	78	166	6.92	(3.71- 12.88)
Subtotal JEDRYC								6.56	(4.86- 8.86)
JOLY	9	m	0	126	175	12	218	13.08	(7.00- 24.43)
JOLY	5	f	0	28	22	52	283	6.93	(3.68- 13.03)
Subtotal JOLY								9.55	(6.13- 14.90)
JUSSAW	14	m	0	24	8	149	624	12.56	(5.53- 28.52)
*KAISER	7	m	2	-	-	-	-	17.24	(10.71- 27.73)
*KAISER	3	f	2	-	-	-	-	7.98	(5.35- 11.90)
Subtotal KAISER								10.98	(8.08- 14.91)
KANELL	2	m	0	321	134	48	172	8.58	(5.88- 12.53)
KATSOU	8	f	0	15	5	48	67	4.19	(1.43- 12.30)
KAUFMA	3	c	0	207	363	35	925	15.07	(10.32- 22.00)
KHUDER	2	m	0	224	288	23	309	10.45	(6.61- 16.52)
*KINLEN	4	m	0	232	3369	7	1333	13.11	(6.20- 27.74)
KOO	12	f	0	24	5	56	85	7.29	(2.62- 20.22)
KREUZE	21	m	3	-	-	-	-	19.50	(7.50- 50.30)
KREUZE	32	m	3	-	-	-	-	32.80	(20.90- 51.40)
KREUZE	27	f	3	-	-	-	-	12.10	(3.00- 48.00)
KREUZE	38	f	3	-	-	-	-	7.70	(3.50- 17.30)
Subtotal KREUZE								21.69	(15.27- 30.80)
KREYBE	22	m	0	55	925	6	644	6.38	(2.73- 14.91)
LAMTH	2	f	0	90	28	202	337	5.36	(3.39- 8.48)
LETOUR	3	c	0	367	198	24	224	17.30	(10.98- 27.27)
*LIAW	4	c	2	-	-	-	-	3.60	(2.00- 6.40)
LIU2	6	m	0	97	66	12	44	5.39	(2.65- 10.97)
LIU3	4	m	0	20	93	4	19	1.02	(0.31- 3.33)
LIU4	8	m	2	-	-	-	-	3.60	(3.49- 3.71)
LUBIN2	275	m	0	1963	2547	190	2616	10.61	(9.06- 12.44)
LUBIN2	283	f	0	134	103	336	1188	4.60	(3.46- 6.11)
Subtotal LUBIN2								8.70	(7.57- 9.99)
MACLEN	22	m	0	43	31	5	15	4.16	(1.37- 12.66)
MARTIS	2	m	0	91	87	4	25	6.54	(2.19- 19.55)
MATOS	30	m	0	65	90	11	110	7.22	(3.60- 14.50)
MATSUD	2	m	0	75	1607	3	1255	19.52	(6.14- 62.05)
MCCONN	25	c	0	49	92	9	23	1.36	(0.58- 3.17)
*MIGRAN	5	m	0	36	851	4	867	9.17	(3.28- 25.65)
*MIGRAN	32	f	0	3	471	4	3814	6.07	(1.36- 27.05)
Subtotal MIGRAN								8.03	(3.44- 18.74)
*MRFITR	4	m	0	50	3747	0	1859	50.12~	(3.09- 811.82)
ORMOS	2	m	0	40	577	7	777	7.69	(3.42- 17.30)
PASTOR	3	m	0	89	100	10	89	7.92	(3.88- 16.17)
PERNU	21	m	0	478	138	97	275	9.82	(7.28- 13.24)
PERNU	15	f	0	1	11	110	971	0.80	(0.10- 6.27)
Subtotal PERNU								9.32	(6.94- 12.53)
SEGI2	13	m	0	92	183	8	53	3.33	(1.52- 7.30)
SIEMIA	14	m	0	-	-	-	-	4.50	(1.80- 13.20)
SOBUE	118	m	0	236	222	34	128	4.00	(2.63- 6.09)
*SPEIZE	3	f	1	-	-	-	-	12.60	(9.90- 16.70)
STOCKS	26	m	0	919	2257	45	638	5.77	(4.23- 7.89)
STOCKW	2	c	0	6053	1591	2791	10641	14.51	(13.54- 15.54)
SVENSS	31	f	0	81	22	38	120	11.63	(6.41- 21.10)
*TENKAN	11	m	1	-	-	-	-	20.25	(8.20- 50.00)
TIZZAN	8	m	0	468	470	180	305	1.69	(1.35- 2.11)
TSUGAN	30	m	0	30	30	18	22	1.22	(0.55- 2.73)
*TULINI	5	m	1	-	-	-	-	13.50	(7.08- 25.60)
*TULINI	10	f	1	-	-	-	-	30.70	(16.80- 56.00)
Subtotal TULINI								20.91	(13.48- 32.45)
WAKAI	38	m	0	79	129	10	65	3.98	(1.93- 8.20)
WANG2	5	c	0	27	32	11	43	3.30	(1.43- 7.62)
WYNDE2	18	m	0	122	203	8	105	7.89	(3.71- 16.75)
WYNDE3	45	m	0	77	114	9	88	6.60	(3.14- 13.90)
WYNDE3	80	f	0	24	24	20	76	3.80	(1.79- 8.05)
Subtotal WYNDE3								5.02	(2.96- 8.52)
WYNDE4	45	m	0	228	274	12	115	7.97	(4.29- 14.82)
WYNDE4	59	f	2	-	-	-	-	6.49	(2.35- 17.93)
Subtotal WYNDE4								7.54	(4.44- 12.80)
WYNDE6	36	m	0	461	293	87	617	11.16	(8.54- 14.59)
WYNDE6	225	f	0	367	165	159	856	11.97	(9.33- 15.37)

Table 1G13 - 5

IESLC - Meta-anal of Ever Smoking (or Current if ever not avail), Amount smoked, "Mid", Any prod (or Cigs if Any not avail)
All LC types
Least adjusted

REF	NRR	SEX	AD	Number Exposed Case	Number Exposed Cont	Non-exposed Case	Non-exposed Cont	RR	95.00%CI
Subtotal WYNDE6								11.59	(9.65- 13.91)
XU3	7	m	0	47	13	7	31	16.01	(5.75- 44.61)
ZHENG	13	m	0	128	89	33	94	4.10	(2.53- 6.62)
Partial Totals				19837	634165	6455	3006255		

*prospective study ~ With 0.5 adjustment for zero

REF	NRR	SEX	AD	Ys	Ws	Qs	Ps
*AKIBA	18	m	0	1.46	16.36	1.02	0.0000
ALDERS	19	m	1	2.07	13.07	1.74	0.0000
ALDERS	22	f	1	1.66	34.88	0.07	0.0000
Subtotal ALDERS				1.78	47.96	1.81	
*ARCHER	2	m	0	1.81	5.52	0.05	0.0000
ARMADA	47	m	0	3.02	3.54	6.04	0.0000
AUVINE	6	c	0	2.98	16.81	27.25	0.0000
AXELSS	15	f	0	2.77	6.08	6.84	0.0000
BARBON	9	m	0	2.38	16.41	7.41	0.0000
*BEST	14	m	1	2.80	6.77	8.02	0.0000
BRESLO	15	m	0	2.04	15.85	1.70	0.0000
*BRETT	2	m	0	1.45	5.48	0.38	0.0007
BUFFLE	29	f	0	2.47	8.19	4.73	0.0000
*CHANG	3	m	0	2.36	3.99	1.69	0.0000
*CHANG	9	f	0	1.58	5.66	0.09	0.0002
Subtotal CHANG				1.90	9.65	1.78	
CHEN2	4	m	0	1.17	5.43	1.58	0.0063
CHEN2	8	f	0	1.07	4.63	1.92	0.0217
Subtotal CHEN2				1.12	10.07	3.50	
CHOI	13	m	0	1.32	10.21	1.55	0.0000
CHOI	18	f	0	0.52	3.66	5.20	0.3219
Subtotal CHOI				1.11	13.87	6.75	
*CHOW	4	m	0	2.84	5.46	7.02	0.0000
COMSTO	5	m	0	2.90	3.35	4.73	0.0000
COMSTO	10	f	0	2.85	6.96	9.10	0.0000
Subtotal COMSTO				2.87	10.31	13.84	
*CPSI	218	m	1	2.66	70.85	63.98	0.0000
*CPSI	277	f	1	1.62	50.55	0.45	0.0000
Subtotal CPSI				2.23	121.40	64.43	
*CPSII	32	m	0	2.98	101.73	164.11	0.0000
*CPSII	67	f	0	2.36	173.82	73.68	0.0000
Subtotal CPSII				2.59	275.55	237.79	
DAMBER	8	m	1	2.21	14.68	3.64	0.0000
DARBY	2	m	0	4.56	2.86	23.19	0.0000
DARBY	9	f	0	3.07	14.73	27.17	0.0000
Subtotal DARBY				3.31	17.59	50.37	
DAVEYS	3	m	0	1.89	2.11	0.07	0.0061
DEAN3	12	m	0	1.78	19.12	0.10	0.0000
DEAN3	96	f	0	1.15	20.13	6.24	0.0000
Subtotal DEAN3				1.46	39.25	6.34	
*DEKLER	3	m	2	3.14	0.98	1.99	0.0019
DESTEF	2	m	0	2.06	17.23	2.14	0.0000
DOLL	3	m	0	2.26	6.11	1.86	0.0000
DOLL	9	f	0	1.24	3.57	0.80	0.0195
Subtotal DOLL				1.88	9.68	2.67	
*DOLL2	47	m	1	2.51	0.96	0.62	0.0137
*DOLL2	11	f	1	1.86	2.40	0.05	0.0040
Subtotal DOLL2				2.05	3.36	0.67	
*DORN	409	m	1	2.29	287.32	98.17	0.0000
DOSEME	9	m	2	1.13	45.98	15.39	0.0000
*DUNN	3	m	0	2.95	1.95	3.01	0.0000
EBELIN	4	m	0	2.52	7.13	4.64	0.0000
*ENSTRO	5	m	1	2.61	74.58	60.91	0.0000
*ENSTRO	9	f	1	2.25	141.36	41.09	0.0000
Subtotal ENSTRO				2.38	215.94	102.00	
ESAKI	2	m	0	0.80	7.75	6.38	0.0253
FAN	8	m	0	1.38	21.51	2.34	0.0000
FAN	12	f	0	2.48	7.51	4.49	0.0000
Subtotal FAN				1.67	29.02	6.83	
GAO2	3	m	0	2.02	7.66	0.74	0.0000
GARSHI	19	m	0	1.74	31.85	0.03	0.0000
GER	19	c	0	0.26	19.29	40.77	0.2605
GOLLED	16	m	0	1.60	12.74	0.16	0.0000

Table 1G13 - 5

IESLC - Meta-anal of Ever Smoking (or Current if ever not avail), Amount smoked, "Mid", Any prod (or Cigs if Any not avail)
All LC types
Least adjusted

REF	NRR	SEX	AD	Ys	Ws	Qs	Ps
GSELL	3	m	0	2.71	1.64	1.65	0.0005
*HAMMON	154	m	1	2.12	11.45	1.91	0.0000
*HIRAYA	74	m	4	1.69	68.71	0.04	0.0000
HITOSU	4	m	0	0.88	5.97	4.13	0.0319
*HOLE	10	m	0	1.97	6.53	0.45	0.0000
HU	2	m	0	0.74	12.38	11.72	0.0095
HU	5	f	0	0.18	2.64	6.16	0.7672
Subtotal HU				0.64	15.02	17.87	
HU2	6	c	0	1.10	32.09	11.80	0.0000
HUMBLE	76	c	3	2.72	15.27	15.54	0.0000
JEDRYC	61	m	0	1.86	32.56	0.78	0.0000
JEDRYC	66	f	0	1.93	9.94	0.50	0.0000
Subtotal JEDRYC				1.88	42.50	1.28	
JOLY	9	m	0	2.57	9.85	7.30	0.0000
JOLY	5	f	0	1.94	9.62	0.49	0.0000
Subtotal JOLY				2.26	19.47	7.79	
JUSSAW	14	m	0	2.53	5.71	3.85	0.0000
*KAISER	7	m	2	2.85	16.98	21.96	0.0000
*KAISER	3	f	2	2.08	24.04	3.24	0.0000
Subtotal KAISER				2.40	41.02	25.19	
KANELL	2	m	0	2.15	26.86	5.20	0.0000
KATSOU	8	f	0	1.43	3.31	0.26	0.0092
KAUFMA	3	c	0	2.71	26.85	27.00	0.0000
KHUDER	2	m	0	2.35	18.30	7.41	0.0000
*KINLEN	4	m	0	2.57	6.84	5.10	0.0000
KOO	12	f	0	1.99	3.69	0.28	0.0001
KREUZE	21	m	3	2.97	4.24	6.74	0.0000
KREUZE	32	m	3	3.49	18.97	60.15	0.0000
KREUZE	27	f	3	2.49	2.00	1.23	0.0004
KREUZE	38	f	3	2.04	6.02	0.66	0.0000
Subtotal KREUZE				3.08	31.23	68.77	
KREYBE	22	m	0	1.85	5.33	0.11	0.0000
LAMTH	2	f	0	1.68	18.27	0.02	0.0000
LETOUR	3	c	0	2.85	18.55	24.14	0.0000
*LIAW	4	c	2	1.28	11.36	2.09	0.0000
LIU2	6	m	0	1.68	7.60	0.01	0.0000
LIU3	4	m	0	0.02	2.75	7.85	0.9718
LIU4	8	m	2	1.28	4111.92	757.08	0.0000
LUBIN2	275	m	0	2.36	152.73	64.91	0.0000
LUBIN2	283	f	0	1.53	47.64	1.61	0.0000
Subtotal LUBIN2				2.16	200.37	66.52	
MACLEN	22	m	0	1.43	3.10	0.25	0.0120
MARTIS	2	m	0	1.88	3.20	0.09	0.0008
MATOS	30	m	0	1.98	7.91	0.56	0.0000
MATSUD	2	m	0	2.97	2.87	4.57	0.0000
MCCONN	25	c	0	0.31	5.38	10.57	0.4745
*MIGRAN	5	m	0	2.22	3.63	0.93	0.0000
*MIGRAN	32	f	0	1.80	1.72	0.02	0.0179
Subtotal MIGRAN				2.08	5.35	0.94	
*MRFITR	4	m	0	3.91	0.50	2.41	0.0059
ORMOS	2	m	0	2.04	5.85	0.64	0.0000
PASTOR	3	m	0	2.07	7.55	0.98	0.0000
PERNU	21	m	0	2.28	42.95	14.17	0.0000
PERNU	15	f	0	-0.22	0.91	3.38	0.8339
Subtotal PERNU				2.23	43.86	17.55	
SEGI2	13	m	0	1.20	6.24	1.60	0.0026
SIEMIA	14	m	0	1.50	3.87	0.16	0.0031
SOBUE	118	m	0	1.39	21.76	2.27	0.0000
*SPEIZE	3	f	1	2.53	56.20	38.13	0.0000
STOCKS	26	m	0	1.75	39.49	0.07	0.0000
STOCKW	2	c	0	2.67	802.56	746.56	0.0000
SVENSS	31	f	0	2.45	10.82	5.98	0.0000
*TENKAN	11	m	1	3.01	4.70	7.92	0.0000
TIZZAN	8	m	0	0.52	76.34	107.55	0.0000
TSUGAN	30	m	0	0.20	5.96	13.59	0.6241
*TULINI	5	m	1	2.60	9.30	7.41	0.0000
*TULINI	10	f	1	3.42	10.60	31.15	0.0000
Subtotal TULINI				3.04	19.90	38.56	
WAKAI	38	m	0	1.38	7.36	0.80	0.0002
WANG2	5	c	0	1.19	5.48	1.46	0.0052
WYNDE2	18	m	0	2.07	6.77	0.86	0.0000

Table 1G13 - 5

IESLC - Meta-anal of Ever Smoking (or Current if ever not avail), Amount smoked, "Mid", Any prod (or Cigs if Any not avail)
All LC types
Least adjusted

REF	NRR	SEX	AD	Ys	Ws	Qs	Ps
WYNDE3	45	m	0	1.89	6.93	0.22	0.0000
WYNDE3	80	f	0	1.34	6.83	0.96	0.0005
Subtotal WYNDE3				1.61	13.76	1.18	
WYNDE4	45	m	0	2.08	9.99	1.34	0.0000
WYNDE4	59	f	2	1.87	3.72	0.10	0.0003
Subtotal WYNDE4				2.02	13.71	1.44	
WYNDE6	36	m	0	2.41	53.48	26.37	0.0000
WYNDE6	225	f	0	2.48	61.57	36.76	0.0000
Subtotal WYNDE6				2.45	115.05	63.13	
XU3	7	m	0	2.77	3.66	4.14	0.0000
ZHENG	13	m	0	1.41	16.67	1.50	0.0000

	N	120
	NS	93
	Wt	7441.31
Het	Chi	2885.15
Het	df	119
Het	P	***
Fixed	RR	5.53
	RRl	5.40
	RRu	5.66
	P	+++
Random	RR	7.35
	RRl	6.34
	RRu	8.53
	P	+++
Asymm	P	**

Table 1G13 - 6

IESLC - Meta-anal of Ever Smoking (or Current if ever not avail), Amount smoked, "Mid", Any prod (or Cigs if Any not avail)
All LC types
Least adjusted

	Sex			
	combined	male	female	Total
N	10	77	33	120
NS	10	76	32	118
Wt	953.64	5724.01	763.66	7441.31
Het Chi	242.42	1443.98	171.22	2885.15
Het df	9	76	32	119
Het P	***	***	***	***
Fixed RR	12.74	4.54	8.51	5.53
RRl	11.96	4.43	7.92	5.40
RRu	13.58	4.66	9.13	5.66
P	+++	+++	+++	+++
Random RR	6.30	7.63	7.34	7.35
RRl	3.48	6.38	6.03	6.34
RRu	11.42	9.12	8.94	8.53
P	+++	+++	+++	+++
Between Chi				1027.52
Between df				2
Between P				***
Btwn(F) P				***
Btwn(R) P				N.S.

Table 1G13 - 7

IESLC - Meta-anal of Ever Smoking (or Current if ever not avail), Amount smoked, "Mid", Any prod (or Cigs if Any not avail)
All LC types
Excluded studies (and stage at which they were excluded)

1	ABELIN	ABRAHA	AMANDU	AMES	ANDERS	AUSTIN	AXELSO	BAND	BECHER	BERRIN	BLOHMK	BLOT4	BROCKM	BROWN1	BYERS1	BYERS2
	CARPEN	CASCO2	CASCOR	CHAN	CHEN3	CHIAZZ	CHYOU	DESTE2	DOCKER	DROSTE	DU	GARCIA	GARDIN	GENG	GODLEY	GOODMA
	GRAHAM	GREGOR	HEGMAN	HEIN	HENNEK	HINDS	HIRAOK	HOROWI	HORWIT	HUANG	ISHIMA	JAHN	JAIN	JARVHO	JIANG	KELLER
	KIHARA	KJUUS	KO	KOHLME	KUBIK	LAMWK	LAMWK2	LANGE	LEI	LEMARC	LEVIN	LIU	LOMBA2	LOMBAR	MAGNUS	MARSH
	MARSH2	MCDUFF	MCLAUG	MILLER	MILLS	NOTANI	NOU	ODRISC	PAWLEG	PERSHA	POFFIJ	QIAO	QIAO2	RADZIK	REN	RONCO
	ROOTS	ROTHSC	SAARIK	SANKAR	SCHWAR	SEGI	SEOW	SHIMIZ	SIMARA	SIMONA	SITAS	SOBUE2	STASZE	STAYNE	STUCKE	SUN
	SUZUK2	SUZUKI	TANG	TAO	TOKARS	TOUSEY	ULMER	VEIERO	VUTUC	WALD	WANG	WANG3	WANG4	WICKLU	WIGLE	WILKIN
	WU2	WUNSCH	WYNDE8	XIANGZ	XU	XU2	XU4	YONG	ZHANG							
2	BUELL	CHEN	MASTRA	MZILEN	PISANI	RESTRE	SADOWS									
4	BOFFET	WYNDE7														
5	RIMING	TANG2	WYNDE5													
6	BLOT1	BLOT2	BLOT3	BOUCHA	HIRAY2	JONES	LAURIL	LICKIN	MOLLO	MRFIT	MURATA	SCHWA2	VANDER	WARSIN	WATSON	WYNDER
8	LUO															
10	AGUDO	BENSHL	BOUCOT	BROSS	BROWN2	CEDERL	CHATZI	COOKSO	CORREA	DEAN	DEAN2	DORANT	DORGAN	ENGELA	GAO	HAENSZ
	HAMMO2	HANSEN	JARUP	KAISE2	KNEKT	KOULUM	LAUSSM	LIDDEL	LIU5	LUBIN	NAM	NOTAN2	OSANN	OSANN2	PARKIN	PERSH2
	PETO	PEZZO2	PEZZOT	PIKE	POLEDN	PRESCO	RACHTA	RANDIG	SHAW	SPITZ	TVERDA	WU	WUWILL	YAMAGU	YUAN	ZHOU
11	BENHAM	GILLIS														

Table 1G13 - 8
Potentially overlapping studies

REF	REFGP	PRINC	.	OVERLAP/LINK
LUBIN2	LUBIN2	1		Lubin-combined
LAMTH	LAMTH	1		KOO/LAMTH/LAMWK
KOO	KOO	1		KOO/LAMTH/LAMWK
AKIBA	AKIBA	1		AKIBA/ISHIMA
HOLE	TANG2	1		Subset of TANG2
KAISER	KAISER	1		KAISER/OSANN2
MRFITR	MRFIT	2		Subset of MRFIT
WYNDE6	WYNDE6	1		WYNDE5/6/7/8
CPSI	CPSI	1		CPSI overall
ENSTRO	ENSTRO	1		Subset of CPSI
MATSUD	MATSUD	1		SOBUE2/MATSUD

Table 1G13 - 9
Most adjusted - insufficient data for metaanalysis

REF	NRR	SEX	AGEL	AGEH	RACE	YF	LC TYPE	LOC	START	ST	NLC	R	VB	P	H	AD	SM	PRODUCT	exL	exH	DENOM	De
BLOT1	6	m	0	0	all	-	all	NAmer	1970	CC	458	n	bl	y	n	0	ev	cig+/-ot	10	39	nev cigs	or
BUELL	2	m	0	0	all	0	all	NAmer	1957	pr	304	n	bl	n	n	0	ev	cig+/-ot	19	21	nev cigs	st
HITOSU	15	m	0	0	all	-	all	As:Jap	1960	CC	216	n	bl	y	n	2	cu	all/unsp	15	24	nev any	or
RESTRE	7	c	0	0	all	-	all	SCAmer	1978	CC	102	n	bl	n	n	5	ev	cig+/-ot	11	20	nev cigs	or
SADOWS	86	m	0	0	wh	-	all	NAmer	1938	CC	477	n	bl	n	n	1	ev	all/unsp	11	20	nev any	st

REF	NRR	RR	SIG	RRDATA comment
BLOT1	6	4.50		0
BUELL	2	3.52		0
HITOSU	15	2.40		0
RESTRE	7	12.40		0
SADOWS	86	4.70		0

Least adjusted - insufficient data for meta-analysis: as for adjusted plus the following

REF	NRR	SEX	AGEL	AGEH	RACE	YF	LC TYPE	LOC	START	ST	NLC	R	VB	P	H	AD	SM	PRODUCT	exL	exH	DENOM	De
SADOWS	81	m	0	0	wh	-	all	NAmer	1938	CC	477	n	bl	n	n	0	ev	all/unsp	11	20	nev any	st

REF	NRR	RR	SIG	RRDATA comment
SADOWS	81	3.18		0

Table 1G14 -

IESLC - Meta-anal of Ever Smoking (or Current if ev not avail), Amount smoked, “High”, Any prod (or Cigs if Any not avail)
All LC types

This analysis is restricted to results for:
1) Results by Amount smoked
2) Results complete enough for use in metaanalysis

Within each study, results are then selected (in the following order of preference, within each sex) for:
3) SMKSTA: ever smokers, current smokers
4) PRODUCT: all/unspec, cigarettes regardless of other products, cigarettes only
5) CIGTYPE: all/unspecified, MC regardless of HR, MC only
6) DENOM: never smoked anything, never smoked cigarettes, (never +1 = +long term ex, +2 = +amount unknown, +3 = never cigs+long term ex)
7) Followup period (YF, prospective studies): whole study (coded as 0) or longest available
8) LCtype: all or nearest available, at least Squamous and Adeno. (q = squamous, s = small, l = large, a = adeno, mix = mixed, alv = alveolar)
9) Race: all or nearest available, otherwise by race (wh or w = white, bl or b = black, hi = hispanic ch = chinese, jap = japanese, haw = hawaiian, w+o = white + oriental, sca = scandinavian, as = asian)
10) Amount smoked "high" in key scheme 1 (key value 45, maximum range >20, in numbers of cigarettes or cigarette equivalents)
11) For overlapping studies: principal rather than subsidiary studies
Finally by Age: whole study (coded as 0) if available, otherwise by widest available age group and then for single sex results (m, f) in preference to combined sex results (c).

Results adjusted (AD) for the most potential confounders are then chosen in Sections -1 to -3 and results adjusted for the least confounders in Sections -4 to -6. (Those least adjusted results which actually differ from the most adjusted as marked 'x' in column X in Section -4)
(Results adjusted for an unknown number of confounder(s) are coded as 20.)

Section -7 shows excluded studies, together with the stage (as above) at which no qualifying results were found.

Section -8 lists the potentially overlapping studies which have been included (1=principal, 2=subsidiary).

Section -9 lists any results which would have been included in preference except that they had data not complete enough for use in meta-analysis, with their significance (yes/no), if known, and any further comment as entered on the database.

In addition to those mentioned above, the following fields, levels and abbreviations are used:
* or nk = not known, n = no, y = yes, ot = other
ev = ever, cu = current, nev = never
all/unspec = all or unspecified, cig+/-ot = cigarettes irrespective of other products (cigar, pipe etc)
MC = manufactured cigarettes, HR = hand-rolled cigarettes
exL, exH = range of exposure (low and high) in the smoking group, in terms of Amount smoked, cigarettes or cigarette equivalents
REF: 6-character study reference
NRR: number of the RR on the database within the study
ST : study type (CC = case control, pr or prosp = prospective)
NLC: number of lung cancer cases in whole study
R : risky occupational population (n = no, m = mining, o = other risky)
VB : national cigarette type (V = at least 75% Virginia, bl = at least 75% blended, ot = other)
P : any proxy use
H : full histological confirmation
De : derivation of RR/CI (or = original, st = standard method, ot = other method of estimation)

Table 1G14 - 1

IESLC - Meta-anal of Ever Smoking (or Current if ev not avail), Amount smoked, “High”, Any prod (or Cigs if Any not avail)
All LC types
Most adjusted

REF	NRR	SEX	AGEL	AGEH	RACE	YF	LC TYPE	LOC	START	ST	NLC	R	VB	P	H	AD	SM	PRODUCT	exL	exH	DENOM	De
AKIBA	29	m	0	0	all	0	all	As:Jap	1963	pr	610	n	bl	n	n	5	cu	cig+/-ot	25	99	nev cigs	or
ALDERS	20	m	0	0	all	-	all	Eu:UK	1977	CC	1448	n	V	n	n	1	ev	cig only	28	99	nev+2	ot
ALDERS	23	f	0	0	all	-	all	Eu:UK	1977	CC	1448	n	V	n	n	1	ev	cig only	28	99	nev+2	ot
ARCHER	3	m	0	0	wh	0	all	NAmer	1950	pr	146	m	bl	n	n	0	cu	cig+/-ot	21	99	nev cigs	st
ARMADA	48	m	0	0	all	-	all	Eu:wst	1986	CC	325	n	bl	n	y	0	ev	cig+/-ot	25	99	nev any	st
AUVINE	15	c	0	0	all	-	all	Eu:Sca	1986	CC	517	n	bl	y	n	2	ev	cig+/-ot	21	99	nev cigs	or
AXELSS	16	f	0	0	sca	-	all	Eu:Sca	1989	CC	436	n	bl	n	n	0	ev	all/unsp	30	99	nev any	st
BARBON	84	m	0	0	all	-	all	Eu:wst	1979	CC	755	n	bl	y	y	3	ev	all/unsp	40	99	nev any	or
BEST	15	m	0	0	all	0	all	NAmer	1955	pr	381	n	V	n	n	1	cu	cig only	21	99	nev any	ot
BOUCOT	100	m	0	0	all	9	all	NAmer	1951	pr	121	n	bl	n	n	0	ev	cig+/-ot	21	99	nev any	ot
BRESLO	16	m	0	0	all	-	all	NAmer	1949	CC	518	n	bl	n	y	0	ev	cig+/-ot	40	99	nev+3	st
BRETT	3	m	0	0	all	0	all	Eu:UK	1960	pr	150	n	V	n	n	0	cu	cig+/-ot	25	99	nev cigs	st
BROSS	19	m	0	0	wh	-	all	NAmer	1960	CC	974	n	bl	n	n	0	cu	cig+/-ot	21	99	nev any	st
BUFFLE	35	f	0	0	w-hi	-	all	NAmer	1976	CC	943	n	bl	y	n	0	ev	cig+/-ot	21	99	nev cigs	or
CHANG	4	m	0	0	all	0	all	NAmer	1972	pr	136	n	bl	n	n	0	cu	cig+/-ot	21	99	nev cigs	st
CHANG	10	f	0	0	all	0	all	NAmer	1972	pr	136	n	bl	n	n	0	cu	cig+/-ot	21	99	nev cigs	st
CHEN2	6	m	0	0	all	-	all	As:Chi	1983	CC	193	n	ot	y	n	0	ev	all/unsp	31	99	nev any	st
CHEN2	10	f	0	0	all	-	all	As:Chi	1983	CC	193	n	ot	y	n	0	ev	all/unsp	31	99	nev any	st
CHOI	16	m	0	0	all	-	all	As:oth	1985	CC	375	n	bl	n	n	0	ev	cig+/-ot	41	99	nev cigs	st
CHOI	20	f	0	0	all	-	all	As:oth	1985	CC	375	n	bl	n	n	0	ev	cig+/-ot	31	99	nev cigs	st
CHOW	12	m	0	0	wh	0	all	NAmer	1966	pr	219	n	bl	n	n	2	cu	cig+/-ot	30	99	nev any	ot
COMSTO	6	m	0	0	all	-	all	NAmer	1975	ot	258	n	bl	n	n	0	cu	cig+/-ot	40	99	nev any	st
COMSTO	11	f	0	0	all	-	all	NAmer	1975	ot	258	n	bl	n	n	0	cu	cig+/-ot	40	99	nev any	st
CORREA	50	c	0	0	all	-	all	NAmer	1979	CC	1359	n	bl	y	n	1	cu	cig+/-ot	21	99	nev cigs	or
CPSI	219	m	35	84	all	6	all	NAmer	1959	pr	5138	n	bl	n	n	1	cu	cig+/-ot	40	99	nev any	ot
CPSI	278	f	40	74	all	6	all	NAmer	1959	pr	5138	n	bl	n	n	1	cu	cig+/-ot	40	99	nev cigs	ot
CPSII	103	m	35	99	all	4	all	NAmer	1982	pr	3229	n	bl	n	n	1	ev	cig only	21	99	nev any	ot
CPSII	132	f	0	0	all	6	all	NAmer	1982	pr	3229	n	bl	n	n	1	cu	cig+/-ot	41	99	nev cigs	ot
DAMBER	9	m	0	0	all	-	all	Eu:Sca	1972	CC	579	n	bl	y	n	1	ev	cig only	26	99	nev any	ot
DARBY	3	m	0	0	wh	-	all	Eu:UK	1988	CC	982	n	V	n	n	0	cu	cig+/-ot	25	99	nev any	st
DARBY	10	f	0	0	wh	-	all	Eu:UK	1988	CC	982	n	V	n	n	0	cu	cig+/-ot	25	99	nev any	st
DAVEYS	4	m	0	0	all	-	all	Eu:Ger	1930	CC	109	n	bl	y	n	0	ev	all/unsp	21	99	nev any	st
DEAN	2	m	0	0	wh	-	all	Africa	1947	CC	603	n	V	y	n	0	ev	cig only	25	45	nev any	st
DEAN2	26	m	0	0	all	-	all	Eu:UK	1960	CC	954	n	V	y	n	0	ev	cig only	23	99	nev any	st
DEAN2	30	f	0	0	all	-	all	Eu:UK	1960	CC	954	n	V	y	n	0	ev	cig only	23	99	nev any	st
DEAN3	21	m	0	0	all	-	all	Eu:UK	1969	CC	766	n	V	y	n	3	cu	cig only	23	99	nev any	ot
DEAN3	105	f	0	0	all	-	all	Eu:UK	1969	CC	766	n	V	y	n	3	cu	cig only	23	99	nev any	ot
DEKLER	4	m	0	0	all	0	all	Auslia	1961	pr	138	m	V	n	n	2	cu	cig+/-ot	25	99	nev any	or
DESTEF	9	m	0	0	all	-	all	SCAmer	1988	CC	497	n	bl	n	y	4	ev	all/unsp	41	99	nev any	or
DOLL	4	m	0	0	all	-	all	Eu:UK	1948	CC	1465	n	V	n	n	0	ev	all/unsp	25	49	nev any	st
DOLL	10	f	0	0	all	-	all	Eu:UK	1948	CC	1465	n	V	n	n	0	ev	all/unsp	25	49	nev any	ot
DOLL2	48	m	35	99	all	5	all	Eu:UK	1951	pr	920	n	V	n	n	1	ev	all/unsp	25	99	nev any	ot
DOLL2	12	f	0	0	all	22	all	Eu:UK	1951	pr	920	n	V	n	n	1	cu	cig only	25	99	nev any	ot
DORN	411	m	0	0	wh	25	all	NAmer	1954	pr	5097	n	bl	n	n	1	cu	cig+/-ot	40	99	nev any	or
DOSEME	13	m	0	0	all	-	all	Eu:bal	1979	CC	1210	n	bl	n	n	2	ev	cig+/-ot	21	99	nev cigs	or
DUNN	5	m	0	0	all	0	all	NAmer	1954	pr	139	o	bl	n	n	0	ev	cig+/-ot	35	99	nev cigs	st
EBELIN	6	m	0	0	all	-	all	Eu:Ger	1980	CC	130	n	bl	n	n	0	ev	all/unsp	40	99	nev any	st
ENSTRO	3	m	0	0	all	0	all	NAmer	1959	pr	2879	n	bl	n	n	1	cu	cig only	40	99	nev any	ot
ENSTRO	8	f	0	0	all	0	all	NAmer	1959	pr	2879	n	bl	n	n	1	cu	cig only	21	99	nev any	ot
ESAKI	3	m	0	0	all	-	all	As:Jap	1961	CC	245	n	bl	y	n	0	ev	cig+/-ot	30	99	nev cigs	st
FAN	9	m	0	0	all	-	all	As:Chi	1990	CC	403	n	ot	y	n	0	ev	cig+/-ot	30	99	nev cigs	st
FAN	13	f	0	0	all	-	all	As:Chi	1990	CC	403	n	ot	y	n	0	ev	cig+/-ot	30	99	nev cigs	st
GAO2	4	m	0	0	all	-	all	As:Jap	1988	CC	282	n	bl	n	n	0	cu	cig+/-ot	30	99	nev cigs	or
GARSHI	29	m	0	0	all	-	all	NAmer	1981	CC	1081	o	bl	y	n	1	ev	all/unsp	36	99	nev any	st
GER	24	c	0	0	all	-	all	As:oth	1990	CC	141	n	ot	y	n	14	ev	all/unsp	21	99	nev any	ot
GOLLED	3	m	35	99	all	-	all	Eu:UK	1952	CC	443	n	V	y	n	1	ev	cig only	23	99	nev any	ot
GSELL	5	m	0	0	all	-	all	Eu:wst	1937	CC	150	n	bl	n	y	0	ev	all/unsp	36	99	nev any	st
HAENSZ	51	f	0	0	all	-	not alv	NAmer	1955	CC	158	n	bl	n	y	0	cu	cig+/-ot	21	99	nev any	st
HAMMON	155	m	0	0	wh	0	all	NAmer	1952	pr	448	n	bl	n	n	1	ev	cig only	21	99	nev any	ot
HIRAYA	76	m	0	0	all	16	all	As:Jap	1965	pr	1917	n	bl	n	n	4	cu	cig+/-ot	35	99	nev any	or
HITOSU	37	m	0	0	all	-	all	As:Jap	1960	CC	216	n	bl	y	n	1	cu	all/unsp	25	99	nev any	st
HOLE	5	m	0	0	all	0	all	Eu:UK	1972	pr	225	n	V	n	n	1	cu	cig+/-ot	35	99	nev any	ot
HU	3	m	0	0	all	-	all	As:Chi	1985	CC	227	n	ot	n	y	0	ev	cig+/-ot	25	99	nev any	st
HU	6	f	0	0	all	-	all	As:Chi	1985	CC	227	n	ot	n	y	0	ev	cig+/-ot	25	99	nev any	st
HU2	7	c	0	0	all	-	all	As:Chi	1977	CC	523	n	ot	y	n	0	ev	cig+/-ot	30	99	nev cigs	st
HUMBLE	78	c	0	0	wh	-	not alv	NAmer	1980	CC	521	n	bl	y	n	3	cu	cig+/-ot	31	99	nev cigs	ot
JEDRYC	47	m	0	0	all	-	all	Eu:est	1980	CC	1630	n	bl	y	n	4	ev	cig+/-ot	30	99	nev any	or
JEDRYC	50	f	0	0	all	-	all	Eu:est	1980	CC	1630	n	bl	y	n	4	ev	cig+/-ot	30	99	nev any	or
JOLY	10	m	0	0	all	-	all	SCAmer	1978	CC	826	n	bl	n	n	0	ev	cig+/-ot	30	99	nev any	st
JOLY	6	f	0	0	all	-	all	SCAmer	1978	CC	826	n	bl	n	n	0	ev	cig+/-ot	30	99	nev any	st
JUSSAW	15	m	0	0	all	-	all	As:Ind	1964	CC	792	n	V	n	n	0	ev	cig only	25	99	nev any	st
KAISER	8	m	0	0	all	0	all	NAmer	1964	pr	714	n	bl	n	n	2	cu	cig+/-ot	41	99	nev cigs	or

Table 1G14 - 1

IESLC - Meta-anal of Ever Smoking (or Current if ev not avail), Amount smoked, “High”, Any prod (or Cigs if Any not avail)
All LC types
Most adjusted

REF	NRR	SEX	AGEL	AGEH	RACE	YF	LC TYPE	LOC	START	ST	NLC	R	VB	P	H	AD	SM	PRODUCT	exL	exH	DENOM	De
KAISER	4	f	0	0	all	0	all	NAmer	1964	pr	714	n	bl	n	n	2	cu	cig+/-ot	41	99	nev cigs	or
KANELL	29	m	0	0	all	-	all	Eu:bal	1950	CC	862	n	bl	n	n	1	cu	cig+/-ot	36	99	nev any	st
KATSOU	4	f	0	0	all	-	all	Eu:bal	1987	CC	101	n	bl	n	n	1	cu	all/unsp	21	99	nev any	or
KAUFMA	15	c	0	0	all	-	all	NAmer	1981	CC	881	n	bl	n	n	6	cu	cig+/-ot	45	99	nev cigs	or
KHUDER	3	m	0	0	all	-	all	NAmer	1985	CC	482	n	bl	n	y	0	ev	cig+/-ot	40	99	nev cigs	st
KINLEN	16	m	0	0	all	0	all	Eu:UK	1967	pr	718	n	V	n	n	2	cu	cig+/-ot	25	99	nev any	ot
KREUZE	22	m	1	45	all	-	all	Eu:Ger	1990	CC	2260	n	bl	n	n	3	ev	cig+/-ot	30	99	nev any	or
KREUZE	33	m	55	69	all	-	all	Eu:Ger	1990	CC	2260	n	bl	n	n	3	ev	cig+/-ot	30	99	nev any	or
KREYBE	11	m	0	0	all	-	all	Eu:Sca	1948	CC	300	n	bl	n	y	1	ev	all/unsp	25	99	nev any	ot
LAMTH	9	f	0	0	ch	-	all	As:HK	1983	CC	445	n	bl	n	n	0	ev	all/unsp	21	99	nev any	or
LETOUR	4	c	0	0	all	-	all	NAmer	1983	CC	738	n	V	y	y	0	ev	cig+/-ot	41	99	nev cigs	st
LIAW	5	c	0	0	all	0	all	As:oth	1982	pr	127	n	ot	n	n	2	cu	all/unsp	21	99	nev any	or
LIU2	10	m	0	0	all	-	all	As:Chi	1983	CC	316	n	ot	n	n	3	ev	all/unsp	30	99	nev any	or
LIU3	8	m	0	0	all	-	all	As:Chi	1985	CC	110	n	ot	n	n	2	ev	all/unsp	31	99	nev any	or
LIU4	9	m	35	69	all	-	all	As:Chi	1986	CC	1000-00	n	ot	y	n	2	ev	cig only	21	99	nev any	ot
LUBIN2	276	m	0	0	all	-	all	Eu:mul	1976	CC	7804	n	bl	n	y	0	ev	cig+/-ot	30	99	nev any	st
LUBIN2	284	f	0	0	all	-	all	Eu:mul	1976	CC	7804	n	bl	n	y	0	ev	cig+/-ot	30	99	nev any	st
MACLEN	39	c	0	0	ch	-	all	As:oth	1972	CC	233	n	bl	n	n	2	ev	cig+/-ot	30	99	nev cigs	or
MARTIS	3	m	0	0	all	-	all	Eu:UK	1972	CC	201	n	V	n	n	0	ev	cig+/-ot	25	99	nev cigs	st
MATOS	33	m	0	0	all	-	all	SCAmer	1994	CC	200	n	bl	n	n	2	ev	cig+/-ot	25	99	nev any	or
MATSUD	3	m	0	0	all	-	all	As:Jap	1965	CC	179	n	bl	n	n	0	ev	cig+/-ot	21	99	nev cigs	st
MCCONN	24	c	0	0	all	-	all	Eu:UK	1946	CC	100	n	V	n	y	0	ev	all/unsp	21	99	nev any	st
MIGRAN	8	m	0	0	all	0	all	Eu:UK	1964	pr	259	n	V	n	n	2	cu	cig only	21	99	nev any	ot
MIGRAN	34	f	0	0	all	0	all	Eu:UK	1964	pr	259	n	V	n	n	0	cu	cig only	21	99	nev any	st
MRFITR	5	m	0	0	all	0	all	NAmer	1973	pr	119	n	bl	n	n	0	cu	cig+/-ot	40	99	nev cigs	ot
NAM	75	m	0	0	all	-	all	NAmer	1986	CC	1199	n	bl	y	n	1	cu	cig+/-ot	25	99	nev cigs	ot
NAM	91	f	0	0	all	-	all	NAmer	1986	CC	1199	n	bl	y	n	1	cu	cig+/-ot	25	99	nev cigs	ot
ORMOS	3	m	0	0	all	-	all	Eu:est	1947	CC	119	n	bl	y	y	0	ev	cig+/-ot	31	99	nev any	st
OSANN	57	m	0	0	all	-	all	NAmer	1984	CC	1986	n	bl	n	n	2	ev	cig+/-ot	40	99	nev cigs	or
OSANN	58	f	0	0	all	-	all	NAmer	1984	CC	1986	n	bl	n	n	2	ev	cig+/-ot	40	99	nev cigs	or
PASTOR	9	m	0	0	all	-	all	Eu:wst	1976	CC	204	n	bl	y	n	1	ev	all/unsp	30	99	nev any	st
PERNU	23	m	0	0	all	-	all	Eu:Sca	1944	CC	1606	n	bl	n	n	0	ev	all/unsp	30	49	nev any	st
PERNU	16	f	0	0	all	-	all	Eu:Sca	1944	CC	1606	n	bl	n	n	0	ev	all/unsp	25	99	nev any	st
PEZZO2	5	m	0	0	all	-	all	SCAmer	1992	CC	367	n	bl	n	y	0	cu	cig+/-ot	41	99	nev cigs	st
PEZZOT	4	m	0	0	all	-	all	SCAmer	1987	CC	215	n	bl	n	y	0	cu	cig only	41	99	nev cigs	st
PIKE	3	m	0	0	w-hi	-	all	NAmer	1972	CC	731	n	bl	y	n	0	ev	all/unsp	41	99	nev any	st
PIKE	7	f	0	0	w-hi	-	all	NAmer	1972	CC	731	n	bl	y	n	0	ev	all/unsp	41	99	nev any	st
SEGI2	18	m	0	0	all	-	all	As:Jap	1962	CC	378	n	bl	n	n	1	cu	cig+/-ot	40	99	nev any	ot
SIEMIA	15	m	0	0	all	-	all	NAmer	1979	CC	857	n	V	y	y	0	ev	cig+/-ot	40	99	nev cigs	or
SOBUE	119	m	0	0	all	-	q+s+l+a	As:Jap	1986	CC	1376	n	bl	n	y	0	cu	cig+/-ot	30	99	nev cigs	st
SPEIZE	5	f	0	0	all	0	all	NAmer	1976	pr	593	n	bl	n	y	1	cu	cig+/-ot	35	99	nev cigs	ot
STOCKS	45	m	0	0	all	-	all	Eu:UK	1952	CC	2932	n	V	y	n	2	ev	cig+/-ot	37	99	nev any	st
STOCKW	3	c	0	0	all	-	all	NAmer	1981	CC	22161	n	bl	n	n	0	cu	cig+/-ot	41	99	nev any	st
SVENSS	16	f	0	0	all	-	all	Eu:Sca	1983	CC	210	n	bl	n	n	1	cu	all/unsp	21	99	nev any	ot
TENKAN	12	m	0	0	all	17	all	Eu:Sca	1962	pr	242	n	bl	n	n	1	cu	all/unsp	25	99	nev any	ot
TIZZAN	10	m	0	0	all	-	all	Eu:wst	1959	CC	1358	n	bl	n	n	0	ev	cig only	41	99	nev any	st
TSUGAN	31	m	0	0	all	-	q+a	As:Jap	1976	CC	134	n	bl	n	y	0	cu	all/unsp	36	99	nev any	st
TULINI	29	m	0	0	all	0	all	Eu:Sca	1967	pr	472	n	bl	n	n	3	cu	cig+/-ot	25	99	nev any	or
TULINI	34	f	0	0	all	0	all	Eu:Sca	1967	pr	472	n	bl	n	n	3	cu	cig+/-ot	25	99	nev any	or
WAKAI	42	m	0	0	all	-	all	As:Jap	1988	CC	333	n	bl	n	y	2	cu	cig+/-ot	30	99	nev any	or
WANG2	15	c	0	0	all	-	all	As:Chi	1980	CC	103	n	ot	n	n	4	ev	cig+/-ot	40	99	nev cigs	ot
WU	44	f	0	0	wh	-	q+a	NAmer	1981	CC	220	n	bl	n	y	2	cu	all/unsp	21	99	nev any	st
WYNDE2	20	m	0	0	all	-	all	NAmer	1962	CC	404	n	bl	n	y	0	ev	cig+/-ot	35	99	nev any	st
WYNDE3	47	m	0	0	all	-	all	NAmer	1966	CC	350	n	bl	n	y	0	ev	cig+/-ot	41	99	nev any	st
WYNDE3	82	f	0	0	all	-	all	NAmer	1966	CC	350	n	bl	n	y	0	ev	cig+/-ot	41	99	nev any	st
WYNDE4	47	m	0	0	all	-	all	NAmer	1948	CC	684	n	bl	y	n	0	ev	all/unsp	35	99	nev any	st
WYNDE4	61	f	0	0	all	-	all	NAmer	1948	CC	684	n	bl	y	n	2	ev	all/unsp	35	99	nev any	ot
WYNDE6	54	m	0	0	all	-	all	NAmer	1969	CC	4423	n	bl	n	y	0	cu	cig+/-ot	31	99	nev any	st
WYNDE6	243	f	0	0	all	-	all	NAmer	1969	CC	4423	n	bl	n	y	0	cu	cig+/-ot	30	99	nev cigs	st
XU3	12	m	0	0	all	-	all	As:Chi	1981	CC	135	n	ot	n	n	1	ev	all/unsp	30	99	nev any	ot
YAMAGU	7	c	0	0	all	-	all	As:Jap	1989	CC	144	n	bl	n	y	1	cu	all/unsp	21	99	nev any	or
ZHENG	14	m	0	0	all	-	all	As:Chi	1982	CC	540	n	ot	*	y	0	ev	cig+/-ot	30	99	nev cigs	st

Cigarette type is all/unspec for all RRs
except for the following:

REF	NRR	CIGTYPE
ALDERS	20	MC only

Table 1G14 - 1

IESLC - Meta-anal of Ever Smoking (or Current if ev not avail), Amount smoked, “High”, Any prod (or Cigs if Any not avail)
All LC types
Most adjusted

REF	NRR	CIGTYPE
ALDERS	23	MC only
DEAN3	21	MC only
DEAN3	105	MC only
JUSSAW	15	MC only

Table 1G14 - 2

IESLC - Meta-anal of Ever Smoking (or Current if ev not avail), Amount smoked, “High”, Any prod (or Cigs if Any not avail)
All LC types
Most adjusted

REF	NRR	SEX	AD	Number Exposed Case	Number Exposed Cont	Non-exposed Case	Non-exposed Cont	RR	95.00%CI
*AKIBA	29	m	5	-	-	-	-	9.10	(5.40- 15.90)
ALDERS	20	m	1	-	-	-	-	8.52	(5.07- 14.33)
ALDERS	23	f	1	-	-	-	-	6.90	(4.69- 10.15)
Subtotal ALDERS								7.44	(5.46- 10.14)
*ARCHER	3	m	0	40	7705	6	9842	8.52	(3.61- 20.07)
ARMADA	48	m	0	139	32	4	64	69.50	(23.58- 204.81)
AUVINE	15	c	2	-	-	-	-	66.50	(25.80- 172.00)
AXELSS	16	f	0	6	7	18	154	7.33	(2.22- 24.22)
BARBON	84	m	3	-	-	-	-	21.30	(13.00- 36.00)
*BEST	15	m	1	-	-	-	-	17.31	(7.93- 37.79)
*BOUCOT	100	m	0	43	1519	0	805	46.12~	(2.84- 748.16)
BRESLO	16	m	0	80	22	22	110	18.18	(9.42- 35.09)
*BRETT	3	m	0	33	4490	6	6530	8.00	(3.35- 19.07)
BROSS	19	m	0	95	59	38	170	7.20	(4.46- 11.63)
BUFFLE	35	f	0	141	62	12	112	21.23	(10.90- 41.32)
*CHANG	4	m	0	13	158	5	502	8.26	(2.99- 22.81)
*CHANG	10	f	0	13	164	11	1139	8.21	(3.74- 18.01)
Subtotal CHANG								8.23	(4.42- 15.32)
CHEN2	6	m	0	26	12	9	33	7.94	(2.91- 21.72)
CHEN2	10	f	0	4	2	25	33	2.64	(0.45- 15.58)
Subtotal CHEN2								6.08	(2.53- 14.58)
CHOI	16	m	0	16	6	13	95	19.49	(6.47- 58.71)
CHOI	20	f	0	3	1	76	164	6.47	(0.66- 63.26)
Subtotal CHOI								15.81	(5.86- 42.67)
*CHOW	12	m	2	-	-	-	-	44.48	(18.63- 106.19)
COMSTO	6	m	0	26	18	4	69	24.92	(7.71- 80.57)
COMSTO	11	f	0	9	6	13	115	13.27	(4.07- 43.25)
Subtotal COMSTO								18.22	(7.92- 41.90)
CORREA	50	c	1	-	-	-	-	25.30	(18.50- 34.60)
*CPSI	219	m	1	-	-	-	-	17.49	(13.29- 23.03)
*CPSI	278	f	1	-	-	-	-	11.10	(6.00- 20.53)
Subtotal CPSI								16.21	(12.62- 20.84)
*CPSII	103	m	1	-	-	-	-	17.60	(14.05- 22.05)
*CPSII	132	f	1	-	-	-	-	18.22	(10.78- 30.82)
Subtotal CPSII								17.69	(14.39- 21.77)
DAMBER	9	m	1	-	-	-	-	14.90	(6.70- 33.50)
DARBY	3	m	0	68	61	3	384	142.69	(43.52- 467.82)
DARBY	10	f	0	38	21	23	529	41.62	(21.15- 81.90)
Subtotal DARBY								56.31	(31.27- 101.38)
DAVEYS	4	m	0	29	21	3	23	10.59	(2.81- 39.94)
DEAN	2	m	0	228	172	12	61	6.74	(3.52- 12.91)
DEAN2	26	m	0	252	112	33	112	7.64	(4.88- 11.95)
DEAN2	30	f	0	18	5	88	121	4.95	(1.77- 13.84)
Subtotal DEAN2								7.13	(4.73- 10.74)
DEAN3	21	m	3	-	-	-	-	21.66	(12.78- 36.72)
DEAN3	105	f	3	-	-	-	-	24.24	(13.08- 44.93)
Subtotal DEAN3								22.71	(15.21- 33.92)
*DEKLER	4	m	2	-	-	-	-	32.50	(4.40- 241.20)
DESTEF	9	m	4	-	-	-	-	23.70	(13.40- 42.10)
DOLL	4	m	0	293	154	7	61	16.58	(7.40- 37.13)
DOLL	10	f	0	14	0	40	59	42.60~	(2.47- 734.58)
Subtotal DOLL								17.78	(8.19- 38.62)
*DOLL2	48	m	1	-	-	-	-	23.71	(3.25- 173.24)
*DOLL2	12	f	1	-	-	-	-	29.71	(9.46- 93.32)
Subtotal DOLL2								28.09	(10.42- 75.73)
*DORN	411	m	1	-	-	-	-	22.75	(19.63- 26.37)
DOSEME	13	m	2	-	-	-	-	6.60	(4.40- 10.20)
*DUNN	5	m	0	13	3206	2	14160	28.71	(6.48- 127.15)
EBELIN	6	m	0	4	4	12	117	9.75	(2.16- 44.04)
*ENSTRO	3	m	1	-	-	-	-	19.41	(15.22- 24.75)
*ENSTRO	8	f	1	-	-	-	-	16.47	(13.74- 19.75)
Subtotal ENSTRO								17.47	(15.10- 20.20)
ESAKI	3	m	0	34	10	16	28	5.95	(2.34- 15.16)
FAN	9	m	0	39	23	36	236	11.12	(5.96- 20.73)
FAN	13	f	0	4	1	69	320	18.55	(2.04- 168.54)
Subtotal FAN								11.54	(6.34- 21.03)
GAO2	4	m	0	74	30	13	56	10.63	(5.08- 22.22)
GARSHI	29	m	1	-	-	-	-	5.24	(3.61- 7.60)
GER	24	c	14	-	-	-	-	3.00	(1.40- 6.40)
GOLLED	3	m	1	-	-	-	-	18.28	(10.52- 31.74)
GSELL	5	m	0	51	13	2	29	56.88	(11.99- 269.87)

Table 1G14 - 2

IESLC - Meta-anal of Ever Smoking (or Current if ev not avail), Amount smoked, “High”, Any prod (or Cigs if Any not avail)
All LC types
Most adjusted

REF	NRR	SEX	AD	Number Exposed Case	Cont	Non-exposed Case	Cont	RR	95.00%CI
HAENSZ	51	f	0	23	13	81	236	5.15	(2.50- 10.65)
*HAMMON	155	m	1	-	-	-	-	17.06	(9.44- 30.82)
*HIRAYA	76	m	4	-	-	-	-	8.40	(5.70- 12.30)
HITOSU	37	m	1	-	-	-	-	4.68	(1.97- 11.11)
*HOLE	5	m	1	-	-	-	-	7.49	(2.99- 18.77)
HU	3	m	0	29	13	41	67	3.65	(1.70- 7.80)
HU	6	f	0	1	2	40	48	0.60	(0.05- 6.86)
Subtotal HU								3.11	(1.50- 6.42)
HU2	7	c	0	64	19	121	213	5.93	(3.39- 10.37)
HUMBLE	78	c	3	-	-	-	-	39.70	(21.02- 74.98)
JEDRYC	47	m	4	-	-	-	-	7.69	(5.15- 11.47)
JEDRYC	50	f	4	-	-	-	-	7.37	(2.20- 24.69)
Subtotal JEDRYC								7.66	(5.24- 11.20)
JOLY	10	m	0	193	161	12	218	21.78	(11.74- 40.39)
JOLY	6	f	0	32	13	52	283	13.40	(6.59- 27.23)
Subtotal JOLY								17.66	(11.08- 28.14)
JUSSAW	15	m	0	48	4	149	624	50.26	(17.84- 141.55)
*KAISER	8	m	2	-	-	-	-	20.91	(12.78- 27.73)
*KAISER	4	f	2	-	-	-	-	12.63	(8.06- 19.80)
Subtotal KAISER								16.87	(12.58- 22.62)
KANELL	29	m	1	-	-	-	-	34.22	(18.86- 62.11)
KATSOU	4	f	1	-	-	-	-	7.46	(2.40- 23.17)
KAUFMA	15	c	6	-	-	-	-	60.00	(35.00- 102.00)
KHUDER	3	m	0	154	63	23	309	32.84	(19.62- 54.97)
*KINLEN	16	m	2	-	-	-	-	21.74	(10.23- 46.19)
KREUZE	22	m	3	-	-	-	-	20.80	(7.20- 60.50)
KREUZE	33	m	3	-	-	-	-	33.30	(20.50- 54.00)
Subtotal KREUZE								30.72	(19.77- 47.73)
KREYBE	11	m	1	-	-	-	-	14.31	(5.99- 34.17)
LAMTH	9	f	0	39	9	202	337	7.23	(3.43- 15.24)
LETOUR	4	c	0	65	23	24	224	26.38	(13.98- 49.78)
*LIAW	5	c	2	-	-	-	-	8.30	(4.00- 17.30)
LIU2	10	m	3	-	-	-	-	21.40	(7.10- 64.00)
LIU3	8	m	2	-	-	-	-	1.91	(0.32- 11.40)
LIU4	9	m	2	-	-	-	-	6.98	(6.73- 7.23)
LUBIN2	276	m	0	1261	1394	190	2616	12.45	(10.55- 14.70)
LUBIN2	284	f	0	45	33	336	1188	4.82	(3.03- 7.68)
Subtotal LUBIN2								11.19	(9.57- 13.08)
MACLEN	39	c	2	-	-	-	-	4.10	(2.07- 8.15)
MARTIS	3	m	0	75	50	4	25	9.38	(3.08- 28.57)
MATOS	33	m	2	-	-	-	-	10.40	(5.30- 20.70)
MATSUD	3	m	0	58	470	3	1255	51.62	(16.10- 165.55)
MCCONN	24	c	0	35	42	9	23	2.13	(0.87- 5.19)
*MIGRAN	8	m	2	-	-	-	-	5.93	(2.03- 17.29)
*MIGRAN	34	f	0	2	149	4	3814	12.80	(2.36- 69.33)
Subtotal MIGRAN								7.39	(2.99- 18.27)
*MRFITR	5	m	0	54	3591	0	1859	56.43~	(3.49- 913.25)
NAM	75	m	1	-	-	-	-	10.27	(6.42- 16.43)
NAM	91	f	1	-	-	-	-	16.65	(10.20- 27.19)
Subtotal NAM								12.94	(9.22- 18.17)
ORMOS	3	m	0	15	128	7	777	13.01	(5.20- 32.52)
OSANN	57	m	2	-	-	-	-	42.80	(30.50- 60.10)
OSANN	58	f	2	-	-	-	-	40.90	(29.30- 57.10)
Subtotal OSANN								41.82	(32.97- 53.05)
PASTOR	9	m	1	-	-	-	-	8.61	(3.86- 19.19)
PERNU	23	m	0	40	23	97	275	4.93	(2.81- 8.66)
PERNU	16	f	0	5	7	110	971	6.31	(1.97- 20.20)
Subtotal PERNU								5.17	(3.11- 8.57)
PEZZO2	5	m	0	69	12	6	117	112.13	(40.26- 312.24)
PEZZOT	4	m	0	51	6	4	116	246.50	(66.69- 911.11)
PIKE	3	m	0	66	37	18	69	6.84	(3.55- 13.18)
PIKE	7	f	0	16	3	36	96	14.22	(3.91- 51.73)
Subtotal PIKE								7.95	(4.43- 14.27)
SEGI2	18	m	1	-	-	-	-	7.90	(3.40- 18.37)
SIEMIA	15	m	0	-	-	-	-	7.90	(3.00- 24.10)
SOBUE	119	m	0	226	187	34	128	4.55	(2.97- 6.96)
*SPEIZE	5	f	1	-	-	-	-	22.00	(14.80- 32.30)
STOCKS	45	m	2	-	-	-	-	13.91	(8.96- 21.60)
STOCKW	3	c	0	4327	572	2791	10641	28.84	(26.18- 31.77)
SVENSS	16	f	1	-	-	-	-	59.00	(7.60- 458.03)
*TENKAN	12	m	1	-	-	-	-	24.97	(9.90- 63.00)

Table 1G14 - 2

IESLC - Meta-anal of Ever Smoking (or Current if ev not avail), Amount smoked, “High”, Any prod (or Cigs if Any not avail)
All LC types
Most adjusted

REF	NRR	SEX	AD	Number Exposed Case	Cont	Non-exposed Case	Cont	RR	95.00%CI
TIZZAN	10	m	0	83	20	180	305	7.03	(4.17- 11.85)
TSUGAN	31	m	0	19	14	18	22	1.66	(0.65- 4.20)
*TULINI	29	m	3	-	-	-	-	27.30	(14.20- 52.40)
*TULINI	34	f	3	-	-	-	-	38.70	(18.50- 80.80)
Subtotal TULINI								31.83	(19.52- 51.88)
WAKAI	42	m	2	-	-	-	-	9.19	(4.20- 20.10)
WANG2	15	c	4	-	-	-	-	7.25	(2.05- 25.65)
WU	44	f	2	-	-	-	-	8.48	(4.16- 17.29)
WYNDE2	20	m	0	155	112	8	105	18.16	(8.50- 38.80)
WYNDE3	47	m	0	68	26	9	88	25.57	(11.25- 58.14)
WYNDE3	82	f	0	4	3	20	76	5.07	(1.05- 24.50)
Subtotal WYNDE3								18.10	(8.73- 37.49)
WYNDE4	47	m	0	130	64	12	115	19.47	(10.00- 37.88)
WYNDE4	61	f	2	-	-	-	-	11.54	(1.90- 70.11)
Subtotal WYNDE4								18.28	(9.79- 34.14)
WYNDE6	54	m	0	784	197	87	617	28.22	(21.47- 37.10)
WYNDE6	243	f	0	378	52	159	856	39.13	(27.98- 54.75)
Subtotal WYNDE6								32.15	(26.01- 39.75)
XU3	12	m	1	-	-	-	-	27.72	(5.15- 149.23)
YAMAGU	7	c	1	-	-	-	-	12.14	(5.10- 28.90)
ZHENG	14	m	0	66	23	33	94	8.17	(4.40- 15.17)
Partial Totals				10526	25631	5541	65040		

*prospective study ~ With 0.5 adjustment for zero

REF	NRR	SEX	AD	Ys	Ws	Qs	Ps
*AKIBA	29	m	5	2.21	13.18	0.19	0.0000
ALDERS	20	m	1	2.14	14.23	0.49	0.0000
ALDERS	23	f	1	1.93	25.78	4.07	0.0000
Subtotal ALDERS				2.01	40.01	4.56	
*ARCHER	3	m	0	2.14	5.22	0.18	0.0000
ARMADA	48	m	0	4.24	3.29	12.03	0.0000
AUVINE	15	c	2	4.20	4.27	14.90	0.0000
AXELSS	16	f	0	1.99	2.69	0.30	0.0011
BARBON	84	m	3	3.06	14.81	7.89	0.0000
*BEST	15	m	1	2.85	6.30	1.72	0.0000
*BOUCOT	100	m	0	3.83	0.49	1.12	0.0070
BRESLO	16	m	0	2.90	8.89	2.90	0.0000
*BRETT	3	m	0	2.08	5.09	0.32	0.0000
BROSS	19	m	0	1.97	16.76	2.10	0.0000
BUFFLE	35	f	0	3.06	8.66	4.57	0.0000
*CHANG	4	m	0	2.11	3.72	0.18	0.0000
*CHANG	10	f	0	2.11	6.22	0.31	0.0000
Subtotal CHANG				2.11	9.94	0.49	
CHEN2	6	m	0	2.07	3.80	0.25	0.0001
CHEN2	10	f	0	0.97	1.22	2.25	0.2838
Subtotal CHEN2				1.80	5.02	2.50	
CHOI	16	m	0	2.97	3.16	1.30	0.0000
CHOI	20	f	0	1.87	0.74	0.16	0.1083
Subtotal CHOI				2.76	3.90	1.45	
*CHOW	12	m	2	3.80	5.07	10.90	0.0000
COMSTO	6	m	0	3.22	2.79	2.19	0.0000
COMSTO	11	f	0	2.59	2.75	0.18	0.0000
Subtotal COMSTO				2.90	5.54	2.37	
CORREA	50	c	1	3.23	39.20	31.89	0.0000
*CPSI	219	m	1	2.86	50.84	14.43	0.0000
*CPSI	278	f	1	2.41	10.15	0.06	0.0000
Subtotal CPSI				2.79	60.99	14.49	
*CPSII	103	m	1	2.87	75.65	21.98	0.0000
*CPSII	132	f	1	2.90	13.92	4.58	0.0000
Subtotal CPSII				2.87	89.57	26.57	
DAMBER	9	m	1	2.70	5.93	0.82	0.0000
DARBY	3	m	0	4.96	2.72	18.87	0.0000
DARBY	10	f	0	3.73	8.38	16.42	0.0000
Subtotal DARBY				4.03	11.11	35.29	
DAVEYS	4	m	0	2.36	2.18	0.00	0.0005
DEAN	2	m	0	1.91	9.10	1.61	0.0000
DEAN2	26	m	0	2.03	19.18	1.68	0.0000
DEAN2	30	f	0	1.60	3.63	1.93	0.0023
Subtotal DEAN2				1.96	22.82	3.61	
DEAN3	21	m	3	3.08	13.79	7.69	0.0000

Table 1G14 - 2

IESLC - Meta-anal of Ever Smoking (or Current if ev not avail), Amount smoked, “High”, Any prod (or Cigs if Any not avail)
All LC types
Most adjusted

REF	NRR	SEX	AD	Ys	Ws	Qs	Ps
DEAN3	105	f	3	3.19	10.09	7.45	0.0000
Subtotal DEAN3				3.12	23.88	15.14	
*DEKLER	4	m	2	3.48	0.96	1.27	0.0007
DESTEF	9	m	4	3.17	11.72	8.21	0.0000
DOLL	4	m	0	2.81	5.91	1.36	0.0000
DOLL	10	f	0	3.75	0.47	0.96	0.0098
Subtotal DOLL				2.88	6.39	2.32	
*DOLL2	48	m	1	3.17	0.97	0.68	0.0018
*DOLL2	12	f	1	3.39	2.93	3.31	0.0000
Subtotal DOLL2				3.34	3.90	3.99	
*DORN	411	m	1	3.12	176.37	111.67	0.0000
DOSEME	13	m	2	1.89	21.74	4.24	0.0000
*DUNN	5	m	0	3.36	1.73	1.83	0.0000
EBELIN	6	m	0	2.28	1.69	0.00	0.0031
*ENSTRO	3	m	1	2.97	65.00	26.37	0.0000
*ENSTRO	8	f	1	2.80	116.71	26.08	0.0000
Subtotal ENSTRO				2.86	181.71	52.45	
ESAKI	3	m	0	1.78	4.39	1.31	0.0002
FAN	9	m	0	2.41	9.89	0.06	0.0000
FAN	13	f	0	2.92	0.79	0.28	0.0095
Subtotal FAN				2.45	10.68	0.34	
GAO2	4	m	0	2.36	7.06	0.01	0.0000
GARSHI	29	m	1	1.66	27.73	12.54	0.0000
GER	24	c	14	1.10	6.65	10.07	0.0046
GOLLED	3	m	1	2.91	12.60	4.19	0.0000
GSELL	5	m	0	4.04	1.58	4.65	0.0000
HAENSZ	51	f	0	1.64	7.30	3.46	0.0000
*HAMMON	155	m	1	2.84	10.98	2.83	0.0000
*HIRAYA	76	m	4	2.13	25.97	1.05	0.0000
HITOSU	37	m	1	1.54	5.14	3.17	0.0005
*HOLE	5	m	1	2.01	4.55	0.45	0.0000
HU	3	m	0	1.29	6.63	7.11	0.0009
HU	6	f	0	-0.51	0.65	5.22	0.6812
Subtotal HU				1.13	7.28	12.33	
HU2	7	c	0	1.78	12.31	3.71	0.0000
HUMBLE	78	c	3	3.68	9.50	17.38	0.0000
JEDRYC	47	m	4	2.04	23.96	2.00	0.0000
JEDRYC	50	f	4	2.00	2.63	0.29	0.0012
Subtotal JEDRYC				2.04	26.59	2.29	
JOLY	10	m	0	3.08	10.07	5.69	0.0000
JOLY	6	f	0	2.59	7.64	0.54	0.0000
Subtotal JOLY				2.87	17.71	6.24	
JUSSAW	15	m	0	3.92	3.58	9.04	0.0000
*KAISER	8	m	2	3.04	25.61	12.96	0.0000
*KAISER	4	f	2	2.54	19.02	0.82	0.0000
Subtotal KAISER				2.83	44.63	13.78	
KANELL	29	m	1	3.53	10.82	15.68	0.0000
KATSOU	4	f	1	2.01	2.99	0.30	0.0005
KAUFMA	15	c	6	4.09	13.43	41.86	0.0000
KHUDER	3	m	0	3.49	14.48	19.57	0.0000
*KINLEN	16	m	2	3.08	6.76	3.81	0.0000
KREUZE	22	m	3	3.03	3.39	1.69	0.0000
KREUZE	33	m	3	3.51	16.38	22.68	0.0000
Subtotal KREUZE				3.42	19.77	24.37	
KREYBE	11	m	1	2.66	5.07	0.56	0.0000
LAMTH	9	f	0	1.98	6.91	0.85	0.0000
LETOUR	4	c	0	3.27	9.52	8.48	0.0000
*LIAW	5	c	2	2.12	7.17	0.32	0.0000
LIU2	10	m	3	3.06	3.18	1.71	0.0000
LIU3	8	m	2	0.65	1.20	3.40	0.4778
LIU4	9	m	2	1.94	2991.95	445.29	0.0000
LUBIN2	276	m	0	2.52	139.75	5.22	0.0000
LUBIN2	284	f	0	1.57	17.75	10.14	0.0000
Subtotal LUBIN2				2.42	157.50	15.36	
MACLEN	39	c	2	1.41	8.18	6.89	0.0001
MARTIS	3	m	0	2.24	3.09	0.03	0.0001
MATOS	33	m	2	2.34	8.28	0.00	0.0000
MATSUD	3	m	0	3.94	2.83	7.38	0.0000
MCCONN	24	c	0	0.76	4.83	11.95	0.0966
*MIGRAN	8	m	2	1.78	3.35	1.01	0.0011
*MIGRAN	34	f	0	2.55	1.35	0.07	0.0031

Table 1G14 - 2

IESLC - Meta-anal of Ever Smoking (or Current if ev not avail), Amount smoked, “High”, Any prod (or Cigs if Any not avail)
All LC types
Most adjusted

REF	NRR	SEX	AD	Ys	Ws	Qs	Ps
Subtotal MIGRAN				2.00	4.69	1.07	
*MRFITR	5	m	0	4.03	0.50	1.44	0.0045
NAM	75	m	1	2.33	17.40	0.00	0.0000
NAM	91	f	1	2.81	15.98	3.74	0.0000
Subtotal NAM				2.56	33.39	3.74	
ORMOS	3	m	0	2.57	4.57	0.26	0.0000
OSANN	57	m	2	3.76	33.40	68.08	0.0000
OSANN	58	f	2	3.71	34.52	65.95	0.0000
Subtotal OSANN				3.73	67.92	134.03	
PASTOR	9	m	1	2.15	5.97	0.18	0.0000
PERNU	23	m	0	1.60	12.13	6.53	0.0000
PERNU	16	f	0	1.84	2.83	0.67	0.0019
Subtotal PERNU				1.64	14.97	7.20	
PEZZO2	5	m	0	4.72	3.66	20.93	0.0000
PEZZOT	4	m	0	5.51	2.25	22.71	0.0000
PIKE	3	m	0	1.92	8.91	1.47	0.0000
PIKE	7	f	0	2.65	2.30	0.24	0.0001
Subtotal PIKE				2.07	11.21	1.72	
SEGI2	18	m	1	2.07	5.40	0.37	0.0000
SIEMIA	15	m	0	2.07	3.54	0.24	0.0001
SOBUE	119	m	0	1.52	21.28	14.09	0.0000
*SPEIZE	5	f	1	3.09	25.23	14.66	0.0000
STOCKS	45	m	2	2.63	19.85	1.83	0.0000
STOCKW	3	c	0	3.36	411.25	438.81	0.0000
SVENSS	16	f	1	4.08	0.91	2.80	0.0001
*TENKAN	12	m	1	3.22	4.49	3.54	0.0000
TIZZAN	10	m	0	1.95	14.11	2.02	0.0000
TSUGAN	31	m	0	0.51	4.44	14.76	0.2861
*TULINI	29	m	3	3.31	9.01	8.62	0.0000
*TULINI	34	f	3	3.66	7.07	12.45	0.0000
Subtotal TULINI				3.46	16.08	21.07	
WAKAI	42	m	2	2.22	6.27	0.08	0.0000
WANG2	15	c	4	1.98	2.41	0.29	0.0021
WU	44	f	2	2.14	7.57	0.28	0.0000
WYNDE2	20	m	0	2.90	6.67	2.17	0.0000
WYNDE3	47	m	0	3.24	5.69	4.74	0.0000
WYNDE3	82	f	0	1.62	1.55	0.77	0.0436
Subtotal WYNDE3				2.90	7.24	5.51	
WYNDE4	47	m	0	2.97	8.67	3.55	0.0000
WYNDE4	61	f	2	2.45	1.18	0.02	0.0079
Subtotal WYNDE4				2.91	9.85	3.57	
WYNDE6	54	m	0	3.34	51.37	52.54	0.0000
WYNDE6	243	f	0	3.67	34.09	61.05	0.0000
Subtotal WYNDE6				3.47	85.46	113.59	
XU3	12	m	1	3.32	1.36	1.34	0.0001
YAMAGU	7	c	1	2.50	5.11	0.14	0.0000
ZHENG	14	m	0	2.10	10.04	0.52	0.0000

	N	134
	NS	105
	Wt	5172.00
Het	Chi	1937.56
Het	df	133
Het	P	***
Fixed	RR	10.27
	RRl	9.99
	RRu	10.55
	P	+++
Random	RR	13.94
	RRl	12.07
	RRu	16.10
	P	+++
Asymm	P	***

Table 1G14 - 3

IESLC - Meta-anal of Ever Smoking (or Current if ev not avail), Amount smoked, “High”, Any prod (or Cigs if Any not avail)
All LC types
Most adjusted

	Sex			
	combined	male	female	Total
N	13	85	36	134
NS	13	84	36	133
Wt	533.83	4223.55	414.62	5172.00
Het Chi	151.27	987.17	171.61	1937.56
Het df	12	84	35	133
Het P	***	***	***	***
Fixed RR	25.33	8.76	16.17	10.27
RRl	23.27	8.50	14.69	9.99
RRu	27.57	9.03	17.81	10.55
P	+++	+++	+++	+++
Random RR	13.70	14.30	13.14	13.94
RRl	8.46	12.12	10.19	12.07
RRu	22.18	16.86	16.95	16.10
P	+++	+++	+++	+++
Between Chi				627.52
Between df				2
Between P				***
Btwn(F) P				***
Btwn(R) P				N.S.

	Lung cancer type		
	all	other	Total
N	129	5	134
NS	100	5	105
Wt	5121.91	50.09	5172.00
Het Chi	1887.52	42.74	1937.56
Het df	128	4	133
Het P	***	***	***
Fixed RR	10.30	7.02	10.27
RRl	10.03	5.32	9.99
RRu	10.59	9.26	10.55
P	+++	+++	+++
Random RR	14.36	6.81	13.94
RRl	12.40	2.63	12.07
RRu	16.64	17.68	16.10
P	+++	+++	+++
Between Chi			7.31
Between df			1
Between P			**
Btwn(F) P			N.S.
Btwn(R) P			N.S.

	Location								
	NAmer	UK	Scand	othEur	China	Japan	othAs	other	Total
N	47	21	10	17	13	11	7	8	134
NS	35	14	8	14	10	11	6	7	105
Wt	1423.84	169.58	54.41	287.61	3045.43	101.06	36.39	53.67	5172.00
Het Chi	273.58	83.15	40.20	82.42	19.97	30.37	24.49	41.05	1937.56
Het df	46	20	9	16	12	10	6	7	133
Het P	***	***	***	***	(*)	***	***	***	***
Fixed RR	22.04	12.28	16.10	11.98	6.99	7.26	7.33	18.88	10.27
RRl	20.93	10.56	12.34	10.67	6.74	5.97	5.30	14.45	9.99
RRu	23.22	14.27	21.00	13.45	7.24	8.82	10.14	24.67	10.55
P	+++	+++	+++	+++	+++	+++	+++	+++	+++
Random RR	18.25	13.27	17.77	13.13	7.18	7.59	8.66	25.21	13.94
RRl	15.65	9.49	9.85	9.45	5.41	5.26	4.33	12.68	12.07
RRu	21.28	18.57	32.07	18.25	9.53	10.96	17.32	50.14	16.10
P	+++	+++	+++	+++	+++	+++	+++	+++	+++
Between Chi									1342.34
Between df									7
Between P									***
Btwn(F) P									***
Btwn(R) P									***

Table 1G14 - 3

IESLC - Meta-anal of Ever Smoking (or Current if ev not avail), Amount smoked, “High”, Any prod (or Cigs if Any not avail)
All LC types
Most adjusted

	Detailed Country in "other Europe"					
	multi	Germany	othWest	East	Balkans	Total
N	2	4	5	3	3	17
NS	1	3	5	2	3	14
Wt	157.50	23.64	39.77	31.17	35.54	287.61
Het Chi	14.18	4.55	22.24	1.10	20.06	82.42
Het df	1	3	4	2	2	16
Het P	***	N.S.	***	N.S.	***	***
Fixed RR	11.19	25.65	14.39	8.28	11.00	11.98
RRl	9.57	17.14	10.54	5.83	7.92	10.67
RRu	13.08	38.39	19.63	11.76	15.29	13.45
P	+++	+++	+++	+++	+++	+++
Random RR	7.95	21.24	19.09	8.28	12.15	13.13
RRl	3.14	11.59	8.48	5.83	3.69	9.45
RRu	20.13	38.91	43.01	11.76	40.02	18.25
P	+++	+++	+++	+++	+++	+++
Between Chi						20.29
Between df						4
Between P						***
Btwn(F) P						N.S.
Btwn(R) P						(*)

	Detailed Country in "other Asia"			
	India	HongKong	other	Total
N	1	1	5	7
NS	1	1	4	6
Wt	3.58	6.91	25.90	36.39
Het Chi	0.00	0.00	9.42	24.49
Het df	0	0	4	6
Het P	N.S.	N.S.	(*)	***
Fixed RR	50.26	7.23	5.64	7.33
RRl	17.84	3.43	3.83	5.30
RRu	141.55	15.24	8.28	10.14
P	+++	+++	+++	+++
Random RR	50.26	7.23	6.15	8.66
RRl	17.84	3.43	3.25	4.33
RRu	141.55	15.24	11.65	17.32
P	+++	+++	+++	+++
Between Chi				15.07
Between df				2
Between P				***
Btwn(F) P				N.S.
Btwn(R) P				**

	Detailed other continent			
	SCAmer	Auslia	Africa	Total
N	6	1	1	8
NS	5	1	1	7
Wt	43.62	0.96	9.10	53.67
Het Chi	29.32	0.00	0.00	41.05
Het df	5	0	0	7
Het P	***	N.S.	N.S.	***
Fixed RR	23.13	32.50	6.74	18.88
RRl	17.19	4.39	3.52	14.45
RRu	31.11	240.63	12.91	24.67
P	+++	+++	+++	+++
Random RR	31.03	32.50	6.74	25.21
RRl	14.68	4.39	3.52	12.68
RRu	65.59	240.63	12.91	50.14
P	+++	+++	+++	+++
Between Chi				11.73
Between df				2
Between P				**
Btwn(F) P				N.S.
Btwn(R) P				**

Table 1G14 - 3

IESLC - Meta-anal of Ever Smoking (or Current if ev not avail), Amount smoked, “High”, Any prod (or Cigs if Any not avail)
All LC types
Most adjusted

	Start year of study <1960	1960-69	1970-79	1980-89	1990+	Total
N	30	28	26	43	7	134
NS	24	21	20	35	5	105
Wt	579.80	311.09	400.07	3832.00	49.04	5172.00
Het Chi	104.22	137.13	116.88	1214.44	44.35	1937.56
Het df	29	27	25	42	6	133
Het P	***	***	***	***	***	***
Fixed RR	17.02	17.22	11.56	8.95	16.61	10.27
RRl	15.69	15.41	10.48	8.67	12.56	9.99
RRu	18.47	19.25	12.75	9.24	21.98	10.55
P	+++	+++	+++	+++	+++	+++
Random RR	13.77	15.67	10.27	15.27	16.93	13.94
RRl	11.28	11.91	8.02	11.27	7.43	12.07
RRu	16.79	20.61	13.16	20.69	38.58	16.10
P	+++	+++	+++	+++	+++	+++
Between Chi						320.54
Between df						4
Between P						***
Btwn(F) P						***
Btwn(R) P						N.S.

	Study type (1) CC	other	Total
N	99	35	134
NS	79	26	105
Wt	4454.87	717.12	5172.00
Het Chi	1609.06	76.74	1937.56
Het df	98	34	133
Het P	***	***	***
Fixed RR	9.40	17.79	10.27
RRl	9.12	16.54	9.99
RRu	9.68	19.14	10.55
P	+++	+++	+++
Random RR	13.22	16.26	13.94
RRl	11.06	14.11	12.07
RRu	15.81	18.74	16.10
P	+++	+++	+++
Between Chi			251.77
Between df			1
Between P			***
Btwn(F) P			***
Btwn(R) P			(*)

	Study type (2) CC	prosp	other	Total
N	99	33	2	134
NS	79	25	1	105
Wt	4454.87	711.58	5.54	5172.00
Het Chi	1609.06	76.19	0.55	1937.56
Het df	98	32	1	133
Het P	***	***	N.S.	***
Fixed RR	9.40	17.79	18.22	10.27
RRl	9.12	16.53	7.92	9.99
RRu	9.68	19.14	41.90	10.55
P	+++	+++	+++	+++
Random RR	13.22	16.21	18.22	13.94
RRl	11.06	14.01	7.92	12.07
RRu	15.81	18.74	41.90	16.10
P	+++	+++	+++	+++
Between Chi				251.77
Between df				2
Between P				***
Btwn(F) P				***
Btwn(R) P				N.S.

Table 1G14 - 3

IESLC - Meta-anal of Ever Smoking (or Current if ev not avail), Amount smoked, “High”, Any prod (or Cigs if Any not avail)
All LC types
Most adjusted

	Study size (number of LC cases)				
	100-249	250-499	500-999	1000+	Total
N	39	27	34	34	134
NS	36	21	26	22	105
Wt	152.45	154.41	331.44	4533.70	5172.00
Het Chi	124.99	57.98	148.90	1453.52	1937.56
Het df	38	26	33	33	133
Het P	***	***	***	***	***
Fixed RR	8.27	18.07	17.02	9.78	10.27
RRl	7.06	15.44	15.29	9.49	9.99
RRu	9.70	21.16	18.96	10.06	10.55
P	+++	+++	+++	+++	+++
Random RR	9.38	17.40	17.92	13.43	13.94
RRl	6.92	13.56	14.10	10.26	12.07
RRu	12.71	22.34	22.78	17.59	16.10
P	+++	+++	+++	+++	+++
Between Chi					152.17
Between df					3
Between P					***
Btwn(F) P					*
Btwn(R) P					**

	Risky occupational population			
	no	mining	othRisky	Total
N	130	2	2	134
NS	101	2	2	105
Wt	5136.36	6.18	29.46	5172.00
Het Chi	1921.68	1.45	4.72	1937.56
Het df	129	1	1	133
Het P	***	N.S.	*	***
Fixed RR	10.30	10.48	5.79	10.27
RRl	10.02	4.76	4.04	9.99
RRu	10.59	23.05	8.31	10.55
P	+++	+++	+++	+++
Random RR	14.03	12.10	10.46	13.94
RRl	12.12	3.81	2.03	12.07
RRu	16.24	38.42	53.80	16.10
P	+++	+++	++	+++
Between Chi				9.71
Between df				2
Between P				**
Btwn(F) P				N.S.
Btwn(R) P				N.S.

	National cigarette tobacco type			
	Virginia	blended	other	Total
N	27	92	15	134
NS	20	73	12	105
Wt	202.58	1910.17	3059.25	5172.00
Het Chi	101.10	667.28	24.93	1937.56
Het df	26	91	14	133
Het P	***	***	*	***
Fixed RR	12.80	18.62	6.98	10.27
RRl	11.15	17.81	6.73	9.99
RRu	14.69	19.48	7.23	10.55
P	+++	+++	+++	+++
Random RR	13.99	15.35	6.78	13.94
RRl	10.42	13.33	5.20	12.07
RRu	18.79	17.68	8.85	16.10
P	+++	+++	+++	+++
Between Chi				1144.25
Between df				2
Between P				***
Btwn(F) P				***
Btwn(R) P				***

Table 1G14 - 3

IESLC - Meta-anal of Ever Smoking (or Current if ev not avail), Amount smoked, "High", Any prod (or Cigs if Any not avail)
All LC types
Most adjusted

	Any proxy use No/nk	Yes	Total
N	98	36	134
NS	77	28	105
Wt	1830.68	3341.32	5172.00
Het Chi	686.37	248.24	1937.56
Het df	97	35	133
Het P	***	***	***
Fixed RR	18.61	7.41	10.27
RRl	17.78	7.16	9.99
RRu	19.48	7.67	10.55
P	+++	+++	+++
Random RR	15.01	11.55	13.94
RRl	12.99	9.22	12.07
RRu	17.35	14.47	16.10
P	+++	+++	+++
Between Chi			1002.96
Between df			1
Between P			***
Btwn(F) P			***
Btwn(R) P			(*)

	Full histological confirmation No	Yes	Total
N	104	30	134
NS	79	26	105
Wt	4732.39	439.61	5172.00
Het Chi	1614.39	254.47	1937.56
Het df	103	29	133
Het P	***	***	***
Fixed RR	9.91	14.98	10.27
RRl	9.63	13.65	9.99
RRu	10.20	16.45	10.55
P	+++	+++	+++
Random RR	13.94	13.92	13.94
RRl	11.82	10.18	12.07
RRu	16.45	19.04	16.10
P	+++	+++	+++
Between Chi			68.71
Between df			1
Between P			***
Btwn(F) P			*
Btwn(R) P			N.S.

	Number of adjustment variables (1) 0	1	2+/+nk	Total
N	67	31	36	134
NS	52	25	30	107
Wt	1012.76	765.71	3393.53	5172.00
Het Chi	521.31	125.87	533.91	1937.56
Het df	66	30	35	133
Het P	***	***	***	***
Fixed RR	17.82	16.87	7.79	10.27
RRl	16.76	15.71	7.53	9.99
RRu	18.95	18.11	8.05	10.55
P	+++	+++	+++	+++
Random RR	12.77	14.62	15.44	13.94
RRl	10.31	12.29	11.49	12.07
RRu	15.83	17.40	20.73	16.10
P	+++	+++	+++	+++
Between Chi				756.48
Between df				2
Between P				***
Btwn(F) P				***
Btwn(R) P				N.S.

Table 1G14 - 3

IESLC - Meta-anal of Ever Smoking (or Current if ev not avail), Amount smoked, “High”, Any prod (or Cigs if Any not avail)
All LC types
Most adjusted

	Number of adjustment variables (2) 0	1	2	3-5	6+/+nk	Total
N	67	31	19	15	2	134
NS	52	25	17	11	2	107
Wt	1012.76	765.71	3206.34	167.10	20.08	5172.00
Het Chi	521.31	125.87	308.95	62.05	39.92	1937.56
Het df	66	30	18	14	1	133
Het P	***	***	***	***	***	***
Fixed RR	17.82	16.87	7.44	16.49	22.24	10.27
RRl	16.76	15.71	7.18	14.17	14.36	9.99
RRu	18.95	18.11	7.70	19.19	34.45	10.55
P	+++	+++	+++	+++	+++	+++
Random RR	12.77	14.62	13.68	18.10	13.59	13.94
RRl	10.31	12.29	8.98	12.92	0.72	12.07
RRu	15.83	17.40	20.85	25.37	255.90	16.10
P	+++	+++	+++	+++	(+)	+++
Between Chi						879.46
Between df						4
Between P						***
Btwn(F) P						***
Btwn(R) P						N.S.

	Product all/unsp	cig+/-ot	cig only	Total
N	34	79	21	134
NS	29	62	16	107
Wt	183.80	1579.70	3408.50	5172.00
Het Chi	102.84	575.60	301.59	1937.56
Het df	33	78	20	133
Het P	***	***	***	***
Fixed RR	8.84	19.62	7.67	10.27
RRl	7.65	18.67	7.41	9.99
RRu	10.21	20.61	7.93	10.55
P	+++	+++	+++	+++
Random RR	9.28	16.08	13.95	13.94
RRl	7.02	13.75	10.36	12.07
RRu	12.26	18.81	18.80	16.10
P	+++	+++	+++	+++
Between Chi				957.54
Between df				2
Between P				***
Btwn(F) P				***
Btwn(R) P				**

	Denominator nev any	nev cigs	Total
N	92	42	134
NS	72	36	108
Wt	4681.71	490.29	5172.00
Het Chi	1457.75	292.49	1937.56
Het df	91	41	133
Het P	***	***	***
Fixed RR	9.65	18.48	10.27
RRl	9.38	16.92	9.99
RRu	9.93	20.20	10.55
P	+++	+++	+++
Random RR	12.73	16.84	13.94
RRl	10.73	13.07	12.07
RRu	15.12	21.71	16.10
P	+++	+++	+++
Between Chi			187.33
Between df			1
Between P			***
Btwn(F) P			***
Btwn(R) P			(*)

Table 1G14 - 3

IESLC - Meta-anal of Ever Smoking (or Current if ev not avail), Amount smoked, “High”, Any prod (or Cigs if Any not avail)
All LC types
Most adjusted

	Derivation of RR/CI			
	Orig	StdCalc	Other	Total
N	32	66	36	134
NS	27	52	29	108
Wt	565.18	1062.20	3544.62	5172.00
Het Chi	212.87	572.18	416.45	1937.56
Het df	31	65	35	133
Het P	***	***	***	***
Fixed RR	19.28	17.18	7.96	10.27
RRl	17.75	16.18	7.70	9.99
RRu	20.93	18.24	8.22	10.55
P	+++	+++	+++	+++
Random RR	15.94	12.34	15.27	13.94
RRl	12.48	9.96	11.95	12.07
RRu	20.37	15.30	19.50	16.10
P	+++	+++	+++	+++
Between Chi				736.07
Between df				2
Between P				***
Btwn(F) P				***
Btwn(R) P				N.S.

	Smoking status		
	ever	current	Total
N	77	57	134
NS	61	46	107
Wt	3810.53	1361.47	5172.00
Het Chi	626.25	370.85	1937.56
Het df	76	56	133
Het P	***	***	***
Fixed RR	7.96	20.95	10.27
RRl	7.71	19.87	9.99
RRu	8.21	22.09	10.55
P	+++	+++	+++
Random RR	11.96	16.81	13.94
RRl	10.08	14.26	12.07
RRu	14.18	19.83	16.10
P	+++	+++	+++
Between Chi			940.46
Between df			1
Between P			***
Btwn(F) P			***
Btwn(R) P			**

	Study LIU4		
	LIU4	others	Total
N	1	133	134
NS	1	104	105
Wt	2991.95	2180.05	5172.00
Het Chi	0.00	881.14	1937.56
Het df	0	132	133
Het P	N.S.	***	***
Fixed RR	6.98	17.43	10.27
RRl	6.73	16.72	9.99
RRu	7.23	18.18	10.55
P	+++	+++	+++
Random RR	6.98	14.05	13.94
RRl	6.73	12.40	12.07
RRu	7.23	15.93	16.10
P	+++	+++	+++
Between Chi			1056.42
Between df			1
Between P			***
Btwn(F) P			***
Btwn(R) P			***

Table 1G14 - 4

IESLC - Meta-anal of Ever Smoking (or Current if ev not avail), Amount smoked, “High”, Any prod (or Cigs if Any not avail)
All LC types
Least adjusted

REF	NRR	X	SEX	AGEL	AGEH	RACE	YF	LC TYPE	LOC	START	ST	NLC	R	VB	P	H	AD	SM	PRODUCT	exL	exH	DENOM	De
AKIBA	19	x	m	0	0	all	0	all	As:Jap	1963	pr	610	n	bl	n	n	0	cu	cig+/-ot	25	99	nev cigs	or
ALDERS	20		m	0	0	all	-	all	Eu:UK	1977	CC	1448	n	V	n	n	1	ev	cig only	28	99	nev+2	ot
ALDERS	23		f	0	0	all	-	all	Eu:UK	1977	CC	1448	n	V	n	n	1	ev	cig only	28	99	nev+2	ot
ARCHER	3		m	0	0	wh	0	all	NAmer	1950	pr	146	m	bl	n	n	0	cu	cig+/-ot	21	99	nev cigs	st
ARMADA	48		m	0	0	all	-	all	Eu:wst	1986	CC	325	n	bl	n	y	0	ev	cig+/-ot	25	99	nev any	st
AUVINE	7	x	c	0	0	all	-	all	Eu:Sca	1986	CC	517	n	bl	y	n	0	ev	cig+/-ot	21	99	nev cigs	st
AXELSS	16		f	0	0	sca	-	all	Eu:Sca	1989	CC	436	n	bl	n	n	0	ev	all/unsp	30	99	nev any	st
BARBON	13	x	m	0	0	all	-	all	Eu:wst	1979	CC	755	n	bl	y	y	0	ev	all/unsp	40	99	nev any	st
BEST	15		m	0	0	all	0	all	NAmer	1955	pr	381	n	V	n	n	1	cu	cig only	21	99	nev any	ot
BOUCOT	100		m	0	0	all	9	all	NAmer	1951	pr	121	n	bl	n	n	0	ev	cig+/-ot	21	99	nev any	ot
BRESLO	16		m	0	0	all	-	all	NAmer	1949	CC	518	n	bl	n	y	0	ev	cig+/-ot	40	99	nev+3	st
BRETT	3		m	0	0	all	0	all	Eu:UK	1960	pr	150	n	V	n	n	0	cu	cig+/-ot	25	99	nev cigs	st
BROSS	19		m	0	0	wh	-	all	NAmer	1960	CC	974	n	bl	n	n	0	cu	cig+/-ot	21	99	nev any	st
BUFFLE	35		f	0	0	w-hi	-	all	NAmer	1976	CC	943	n	bl	y	n	0	ev	cig+/-ot	21	99	nev cigs	or
CHANG	4		m	0	0	all	0	all	NAmer	1972	pr	136	n	bl	n	n	0	cu	cig+/-ot	21	99	nev cigs	st
CHANG	10		f	0	0	all	0	all	NAmer	1972	pr	136	n	bl	n	n	0	cu	cig+/-ot	21	99	nev cigs	st
CHEN2	6		m	0	0	all	-	all	As:Chi	1983	CC	193	n	ot	y	n	0	ev	all/unsp	31	99	nev any	st
CHEN2	10		f	0	0	all	-	all	As:Chi	1983	CC	193	n	ot	y	n	0	ev	all/unsp	31	99	nev any	st
CHOI	16		m	0	0	all	-	all	As:oth	1985	CC	375	n	bl	n	n	0	ev	cig+/-ot	41	99	nev cigs	st
CHOI	20		f	0	0	all	-	all	As:oth	1985	CC	375	n	bl	n	n	0	ev	cig+/-ot	31	99	nev cigs	st
CHOW	5	x	m	0	0	wh	0	all	NAmer	1966	pr	219	n	bl	n	n	0	cu	cig+/-ot	30	99	nev any	st
COMSTO	6		m	0	0	all	-	all	NAmer	1975	ot	258	n	bl	n	n	0	cu	cig+/-ot	40	99	nev any	st
COMSTO	11		f	0	0	all	-	all	NAmer	1975	ot	258	n	bl	n	n	0	cu	cig+/-ot	40	99	nev any	st
CORREA	49	x	c	0	0	all	-	all	NAmer	1979	CC	1359	n	bl	y	n	0	cu	cig+/-ot	21	99	nev cigs	st
CPSI	219		m	35	84	all	6	all	NAmer	1959	pr	5138	n	bl	n	n	1	cu	cig+/-ot	40	99	nev any	ot
CPSI	278		f	40	74	all	6	all	NAmer	1959	pr	5138	n	bl	n	n	1	cu	cig+/-ot	40	99	nev cigs	ot
CPSII	103		m	35	99	all	4	all	NAmer	1982	pr	3229	n	bl	n	n	1	ev	cig only	21	99	nev any	ot
CPSII	70	x	f	0	0	all	6	all	NAmer	1982	pr	3229	n	bl	n	n	0	cu	cig+/-ot	41	99	nev cigs	st
DAMBER	9		m	0	0	all	-	all	Eu:Sca	1972	CC	579	n	bl	y	n	1	ev	cig only	26	99	nev any	ot
DARBY	3		m	0	0	wh	-	all	Eu:UK	1988	CC	982	n	V	n	n	0	cu	cig+/-ot	25	99	nev any	st
DARBY	10		f	0	0	wh	-	all	Eu:UK	1988	CC	982	n	V	n	n	0	cu	cig+/-ot	25	99	nev any	st
DAVEYS	4		m	0	0	all	-	all	Eu:Ger	1930	CC	109	n	bl	y	n	0	ev	all/unsp	21	99	nev any	st
DEAN	2		m	0	0	wh	-	all	Africa	1947	CC	603	n	V	y	n	0	ev	cig only	25	45	nev any	st
DEAN2	26		m	0	0	all	-	all	Eu:UK	1960	CC	954	n	V	y	n	0	ev	cig only	23	99	nev any	st
DEAN2	30		f	0	0	all	-	all	Eu:UK	1960	CC	954	n	V	y	n	0	ev	cig only	23	99	nev any	st
DEAN3	19	x	m	0	0	all	-	all	Eu:UK	1969	CC	766	n	V	y	n	0	cu	cig only	23	99	nev any	st
DEAN3	103	x	f	0	0	all	-	all	Eu:UK	1969	CC	766	n	V	y	n	0	cu	cig only	23	99	nev any	st
DEKLER	4		m	0	0	all	0	all	Auslia	1961	pr	138	m	V	n	n	2	cu	cig+/-ot	25	99	nev any	or
DESTEF	4	x	m	0	0	all	-	all	SCAmer	1988	CC	497	n	bl	n	y	0	ev	all/unsp	41	99	nev any	st
DOLL	4		m	0	0	all	-	all	Eu:UK	1948	CC	1465	n	V	n	n	0	ev	all/unsp	25	49	nev any	st
DOLL	10		f	0	0	all	-	all	Eu:UK	1948	CC	1465	n	V	n	n	0	ev	all/unsp	25	49	nev any	ot
DOLL2	48		m	35	99	all	5	all	Eu:UK	1951	pr	920	n	V	n	n	1	ev	all/unsp	25	99	nev any	ot
DOLL2	12		f	0	0	all	22	all	Eu:UK	1951	pr	920	n	V	n	n	1	cu	cig only	25	99	nev any	ot
DORN	411		m	0	0	wh	25	all	NAmer	1954	pr	5097	n	bl	n	n	1	cu	cig+/-ot	40	99	nev any	or
DOSEME	13		m	0	0	all	-	all	Eu:bal	1979	CC	1210	n	bl	n	n	2	ev	cig+/-ot	21	99	nev cigs	or
DUNN	5		m	0	0	all	0	all	NAmer	1954	pr	139	o	bl	n	n	0	ev	cig+/-ot	35	99	nev cigs	st
EBELIN	6		m	0	0	all	-	all	Eu:Ger	1980	CC	130	n	bl	n	n	0	ev	all/unsp	40	99	nev any	st
ENSTRO	3		m	0	0	all	0	all	NAmer	1959	pr	2879	n	bl	n	n	1	cu	cig only	40	99	nev any	ot
ENSTRO	8		f	0	0	all	0	all	NAmer	1959	pr	2879	n	bl	n	n	1	cu	cig only	21	99	nev any	ot
ESAKI	3		m	0	0	all	-	all	As:Jap	1961	CC	245	n	bl	y	n	0	ev	cig+/-ot	30	99	nev cigs	st
FAN	9		m	0	0	all	-	all	As:Chi	1990	CC	403	n	ot	y	n	0	ev	cig+/-ot	30	99	nev cigs	st
FAN	13		f	0	0	all	-	all	As:Chi	1990	CC	403	n	ot	y	n	0	ev	cig+/-ot	30	99	nev cigs	st
GAO2	4		m	0	0	all	-	all	As:Jap	1988	CC	282	n	bl	n	n	0	cu	cig+/-ot	30	99	nev cigs	or
GARSHI	21	x	m	0	0	all	-	all	NAmer	1981	CC	1081	o	bl	y	n	0	ev	all/unsp	36	99	nev any	st
GER	20	x	c	0	0	all	-	all	As:oth	1990	CC	141	n	ot	y	n	0	ev	all/unsp	21	99	nev any	st
GOLLED	17	x	m	35	99	all	-	all	Eu:UK	1952	CC	443	n	V	y	n	0	ev	cig only	23	99	nev any	st
GSELL	5		m	0	0	all	-	all	Eu:wst	1937	CC	150	n	bl	n	y	0	ev	all/unsp	36	99	nev any	st
HAENSZ	51		f	0	0	all	-	not alv	NAmer	1955	CC	158	n	bl	n	y	0	cu	cig+/-ot	21	99	nev any	st
HAMMON	155		m	0	0	wh	0	all	NAmer	1952	pr	448	n	bl	n	n	1	ev	cig only	21	99	nev any	ot
HIRAYA	76		m	0	0	all	16	all	As:Jap	1965	pr	1917	n	bl	n	n	4	cu	cig+/-ot	35	99	nev any	or
HITOSU	5	x	m	0	0	all	-	all	As:Jap	1960	CC	216	n	bl	y	n	0	cu	all/unsp	25	99	nev any	st
HOLE	12	x	m	0	0	all	0	all	Eu:UK	1972	pr	225	n	V	n	n	0	cu	cig+/-ot	35	99	nev any	st
HU	3		m	0	0	all	-	all	As:Chi	1985	CC	227	n	ot	n	y	0	ev	cig+/-ot	25	99	nev any	st
HU	6		f	0	0	all	-	all	As:Chi	1985	CC	227	n	ot	n	y	0	ev	cig+/-ot	25	99	nev any	st
HU2	7		c	0	0	all	-	all	As:Chi	1977	CC	523	n	ot	y	n	0	ev	cig+/-ot	30	99	nev cigs	st
HUMBLE	78		c	0	0	wh	-	not alv	NAmer	1980	CC	521	n	bl	y	n	3	cu	cig+/-ot	31	99	nev cigs	ot
JEDRYC	62	x	m	0	0	all	-	all	Eu:est	1980	CC	1630	n	bl	y	n	0	ev	cig+/-ot	30	99	nev any	st
JEDRYC	67	x	f	0	0	all	-	all	Eu:est	1980	CC	1630	n	bl	y	n	0	ev	cig+/-ot	30	99	nev any	st
JOLY	10		m	0	0	all	-	all	SCAmer	1978	CC	826	n	bl	n	n	0	ev	cig+/-ot	30	99	nev any	st
JOLY	6		f	0	0	all	-	all	SCAmer	1978	CC	826	n	bl	n	n	0	ev	cig+/-ot	30	99	nev any	st
JUSSAW	15		m	0	0	all	-	all	As:Ind	1964	CC	792	n	V	n	n	0	ev	cig only	25	99	nev any	st
KAISER	8		m	0	0	all	0	all	NAmer	1964	pr	714	n	bl	n	n	2	cu	cig+/-ot	41	99	nev cigs	or

Table 1G14 - 4

IESLC - Meta-anal of Ever Smoking (or Current if ev not avail), Amount smoked, “High”, Any prod (or Cigs if Any not avail)
All LC types
Least adjusted

REF	NRR	X	SEX	AGEL	AGEH	RACE	YF	LC TYPE	LOC	START	ST	NLC	R	VB	P	H	AD	SM	PRODUCT	exL	exH	DENOM	De
KAISER	4		f	0	0	all	0	all	NAmer	1964	pr	714	n	bl	n	n	2	cu	cig+/-ot	41	99	nev cigs	or
KANELL	4	x	m	0	0	all	-	all	Eu:bal	1950	CC	862	n	bl	n	n	0	cu	cig+/-ot	36	99	nev any	st
KATSOU	10	x	f	0	0	all	-	all	Eu:bal	1987	CC	101	n	bl	n	n	0	cu	all/unsp	31	99	nev any	st
KAUFMA	6	x	c	0	0	all	-	all	NAmer	1981	CC	881	n	bl	n	n	0	cu	cig+/-ot	45	99	nev cigs	st
KHUDER	3		m	0	0	all	-	all	NAmer	1985	CC	482	n	bl	n	y	0	ev	cig+/-ot	40	99	nev cigs	st
KINLEN	5	x	m	0	0	all	0	all	Eu:UK	1967	pr	718	n	V	n	n	0	cu	cig+/-ot	25	99	nev any	st
KREUZE	22		m	1	45	all	-	all	Eu:Ger	1990	CC	2260	n	bl	n	n	3	ev	cig+/-ot	30	99	nev any	or
KREUZE	33		m	55	69	all	-	all	Eu:Ger	1990	CC	2260	n	bl	n	n	3	ev	cig+/-ot	30	99	nev any	or
KREYBE	23	x	m	0	0	all	-	all	Eu:Sca	1948	CC	300	n	bl	n	y	0	ev	all/unsp	25	99	nev any	st
LAMTH	9		f	0	0	ch	-	all	As:HK	1983	CC	445	n	bl	n	n	0	ev	all/unsp	21	99	nev any	or
LETOUR	4		c	0	0	all	-	all	NAmer	1983	CC	738	n	V	y	y	0	ev	cig+/-ot	41	99	nev cigs	st
LIAW	5		c	0	0	all	0	all	As:oth	1982	pr	127	n	ot	n	n	2	cu	all/unsp	21	99	nev any	or
LIU2	7	x	m	0	0	all	-	all	As:Chi	1983	CC	316	n	ot	n	n	0	ev	all/unsp	30	99	nev any	st
LIU3	5	x	m	0	0	all	-	all	As:Chi	1985	CC	110	n	ot	n	n	0	ev	all/unsp	31	99	nev any	st
LIU4	9		m	35	69	all	-	all	As:Chi	1986	CC	1000-00	n	ot	y	n	2	ev	cig only	21	99	nev any	ot
LUBIN2	276		m	0	0	all	-	all	Eu:mul	1976	CC	7804	n	bl	n	y	0	ev	cig+/-ot	30	99	nev any	st
LUBIN2	284		f	0	0	all	-	all	Eu:mul	1976	CC	7804	n	bl	n	y	0	ev	cig+/-ot	30	99	nev any	st
MACLEN	23	x	m	0	0	ch	-	all	As:oth	1972	CC	233	n	bl	n	n	0	cu	cig+/-ot	30	99	nev cigs	st
MARTIS	3		m	0	0	all	-	all	Eu:UK	1972	CC	201	n	V	n	n	0	ev	cig+/-ot	25	99	nev cigs	st
MATOS	32	x	m	0	0	all	-	all	SCAmer	1994	CC	200	n	bl	n	n	0	ev	cig+/-ot	25	99	nev any	st
MATSUD	3		m	0	0	all	-	all	As:Jap	1965	CC	179	n	bl	n	n	0	ev	cig+/-ot	21	99	nev cigs	st
MCCONN	24		c	0	0	all	-	all	Eu:UK	1946	CC	100	n	V	n	y	0	ev	all/unsp	21	99	nev any	st
MIGRAN	7	x	m	0	0	all	0	all	Eu:UK	1964	pr	259	n	V	n	n	0	cu	cig only	21	99	nev any	st
MIGRAN	34		f	0	0	all	0	all	Eu:UK	1964	pr	259	n	V	n	n	0	cu	cig only	21	99	nev any	st
MRFITR	5		m	0	0	all	0	all	NAmer	1973	pr	119	n	bl	n	n	0	cu	cig+/-ot	40	99	nev cigs	ot
NAM	67	x	m	0	0	all	-	all	NAmer	1986	CC	1199	n	bl	y	n	0	cu	cig+/-ot	25	99	nev cigs	ot
NAM	83	x	f	0	0	all	-	all	NAmer	1986	CC	1199	n	bl	y	n	0	cu	cig+/-ot	25	99	nev cigs	ot
ORMOS	3		m	0	0	all	-	all	Eu:est	1947	CC	119	n	bl	y	y	0	ev	cig+/-ot	31	99	nev any	st
OSANN	57		m	0	0	all	-	all	NAmer	1984	CC	1986	n	bl	n	n	2	ev	cig+/-ot	40	99	nev cigs	or
OSANN	58		f	0	0	all	-	all	NAmer	1984	CC	1986	n	bl	n	n	2	ev	cig+/-ot	40	99	nev cigs	or
PASTOR	4	x	m	0	0	all	-	all	Eu:wst	1976	CC	204	n	bl	y	n	0	ev	all/unsp	30	99	nev any	st
PERNU	23		m	0	0	all	-	all	Eu:Sca	1944	CC	1606	n	bl	n	n	0	ev	all/unsp	30	49	nev any	st
PERNU	16		f	0	0	all	-	all	Eu:Sca	1944	CC	1606	n	bl	n	n	0	ev	all/unsp	25	99	nev any	st
PEZZO2	5		m	0	0	all	-	all	SCAmer	1992	CC	367	n	bl	n	y	0	cu	cig+/-ot	41	99	nev cigs	st
PEZZOT	4		m	0	0	all	-	all	SCAmer	1987	CC	215	n	bl	n	y	0	cu	cig only	41	99	nev cigs	st
PIKE	3		m	0	0	w-hi	-	all	NAmer	1972	CC	731	n	bl	y	n	0	ev	all/unsp	41	99	nev any	st
PIKE	7		f	0	0	w-hi	-	all	NAmer	1972	CC	731	n	bl	y	n	0	ev	all/unsp	41	99	nev any	st
SEGI2	17	x	m	0	0	all	-	all	As:Jap	1962	CC	378	n	bl	n	n	0	cu	cig+/-ot	40	99	nev any	st
SIEMIA	15		m	0	0	all	-	all	NAmer	1979	CC	857	n	V	y	y	0	ev	cig+/-ot	40	99	nev cigs	or
SOBUE	119		m	0	0	all	-	q+s+l+a	As:Jap	1986	CC	1376	n	bl	n	y	0	cu	cig+/-ot	30	99	nev cigs	st
SPEIZE	5		f	0	0	all	0	all	NAmer	1976	pr	593	n	bl	n	y	1	cu	cig+/-ot	35	99	nev cigs	ot
STOCKS	29	x	m	0	0	all	-	all	Eu:UK	1952	CC	2932	n	V	y	n	0	ev	cig+/-ot	37	99	nev any	st
STOCKW	3		c	0	0	all	-	all	NAmer	1981	CC	22161	n	bl	n	n	0	cu	cig+/-ot	41	99	nev any	st
SVENSS	36	x	f	0	0	all	-	all	Eu:Sca	1983	CC	210	n	bl	n	n	0	cu	all/unsp	21	99	nev any	st
TENKAN	12		m	0	0	all	17	all	Eu:Sca	1962	pr	242	n	bl	n	n	1	cu	all/unsp	25	99	nev any	ot
TIZZAN	10		m	0	0	all	-	all	Eu:wst	1959	CC	1358	n	bl	n	n	0	ev	cig only	41	99	nev any	st
TSUGAN	31		m	0	0	all	-	q+a	As:Jap	1976	CC	134	n	bl	n	y	0	cu	all/unsp	36	99	nev any	st
TULINI	6	x	m	0	0	all	0	all	Eu:Sca	1967	pr	472	n	bl	n	n	1	cu	cig+/-ot	25	99	nev any	or
TULINI	11	x	f	0	0	all	0	all	Eu:Sca	1967	pr	472	n	bl	n	n	1	cu	cig+/-ot	25	99	nev any	or
WAKAI	39	x	m	0	0	all	-	all	As:Jap	1988	CC	333	n	bl	n	y	0	cu	cig+/-ot	30	99	nev any	st
WANG2	7	x	c	0	0	all	-	all	As:Chi	1980	CC	103	n	ot	n	n	0	ev	cig+/-ot	40	99	nev cigs	st
WU	36	x	f	0	0	wh	-	q+a	NAmer	1981	CC	220	n	bl	n	y	0	cu	all/unsp	21	99	nev any	st
WYNDE2	20		m	0	0	all	-	all	NAmer	1962	CC	404	n	bl	n	y	0	ev	cig+/-ot	35	99	nev any	st
WYNDE3	47		m	0	0	all	-	all	NAmer	1966	CC	350	n	bl	n	y	0	ev	cig+/-ot	41	99	nev any	st
WYNDE3	82		f	0	0	all	-	all	NAmer	1966	CC	350	n	bl	n	y	0	ev	cig+/-ot	41	99	nev any	st
WYNDE4	47		m	0	0	all	-	all	NAmer	1948	CC	684	n	bl	y	n	0	ev	all/unsp	35	99	nev any	st
WYNDE4	61		f	0	0	all	-	all	NAmer	1948	CC	684	n	bl	y	n	2	ev	all/unsp	35	99	nev any	ot
WYNDE6	54		m	0	0	all	-	all	NAmer	1969	CC	4423	n	bl	n	y	0	cu	cig+/-ot	31	99	nev any	st
WYNDE6	243		f	0	0	all	-	all	NAmer	1969	CC	4423	n	bl	n	y	0	cu	cig+/-ot	30	99	nev cigs	st
XU3	8	x	m	0	0	all	-	all	As:Chi	1981	CC	135	n	ot	n	n	0	ev	all/unsp	30	99	nev any	st
YAMAGU	2	x	c	0	0	all	-	all	As:Jap	1989	CC	144	n	bl	n	y	0	cu	all/unsp	21	99	nev any	st
ZHENG	14		m	0	0	all	-	all	As:Chi	1982	CC	540	n	ot	*	y	0	ev	cig+/-ot	30	99	nev cigs	st

Cigarette type is all/unspec for all RRs
except for the following:

REF	NRR	CIGTYPE
ALDERS	20	MC only

Table 1G14 - 4

IESLC - Meta-anal of Ever Smoking (or Current if ev not avail), Amount smoked, “High”, Any prod (or Cigs if Any not avail)
All LC types
Least adjusted

REF	NRR	CIGTYPE
ALDERS	23	MC only
DEAN3	19	MC only
DEAN3	103	MC only
JUSSAW	15	MC only

Table 1G14 - 5

IESLC - Meta-anal of Ever Smoking (or Current if ev not avail), Amount smoked, “High”, Any prod (or Cigs if Any not avail)
All LC types
Least adjusted

REF	NRR	SEX	AD	Number Exposed Case	Number Exposed Cont	Non-exposed Case	Non-exposed Cont	RR	95.00%CI
*AKIBA	19	m	0	63	28351	18	35833	4.42	(2.62- 7.47)
ALDERS	20	m	1	-	-	-	-	8.52	(5.07- 14.33)
ALDERS	23	f	1	-	-	-	-	6.90	(4.69- 10.15)
Subtotal ALDERS								7.44	(5.46- 10.14)
*ARCHER	3	m	0	40	7705	6	9842	8.52	(3.61- 20.07)
ARMADA	48	m	0	139	32	4	64	69.50	(23.58- 204.81)
AUVINE	7	c	0	61	8	44	229	39.68	(17.75- 88.72)
AXELSS	16	f	0	6	7	18	154	7.33	(2.22- 24.22)
BARBON	13	m	0	227	111	22	188	17.48	(10.64- 28.71)
*BEST	15	m	1	-	-	-	-	17.31	(7.93- 37.79)
*BOUCOT	100	m	0	43	1519	0	805	46.12~	(2.84- 748.16)
BRESLO	16	m	0	80	22	22	110	18.18	(9.42- 35.09)
*BRETT	3	m	0	33	4490	6	6530	8.00	(3.35- 19.07)
BROSS	19	m	0	95	59	38	170	7.20	(4.46- 11.63)
BUFFLE	35	f	0	141	62	12	112	21.23	(10.90- 41.32)
*CHANG	4	m	0	13	158	5	502	8.26	(2.99- 22.81)
*CHANG	10	f	0	13	164	11	1139	8.21	(3.74- 18.01)
Subtotal CHANG								8.23	(4.42- 15.32)
CHEN2	6	m	0	26	12	9	33	7.94	(2.91- 21.72)
CHEN2	10	f	0	4	2	25	33	2.64	(0.45- 15.58)
Subtotal CHEN2								6.08	(2.53- 14.58)
CHOI	16	m	0	16	6	13	95	19.49	(6.47- 58.71)
CHOI	20	f	0	3	1	76	164	6.47	(0.66- 63.26)
Subtotal CHOI								15.81	(5.86- 42.67)
*CHOW	5	m	0	40	15732	6	62913	26.66	(11.31- 62.87)
COMSTO	6	m	0	26	18	4	69	24.92	(7.71- 80.57)
COMSTO	11	f	0	9	6	13	115	13.27	(4.07- 43.25)
Subtotal COMSTO								18.22	(7.92- 41.90)
CORREA	49	c	0	514	195	51	388	20.05	(14.34- 28.04)
*CPSI	219	m	1	-	-	-	-	17.49	(13.29- 23.03)
*CPSI	278	f	1	-	-	-	-	11.10	(6.00- 20.53)
Subtotal CPSI								16.21	(12.62- 20.84)
*CPSII	103	m	1	-	-	-	-	17.60	(14.05- 22.05)
*CPSII	70	f	0	25	13493	310	2091302	12.50	(8.32- 18.78)
Subtotal CPSII								16.24	(13.34- 19.78)
DAMBER	9	m	1	-	-	-	-	14.90	(6.70- 33.50)
DARBY	3	m	0	68	61	3	384	142.69	(43.52- 467.82)
DARBY	10	f	0	38	21	23	529	41.62	(21.15- 81.90)
Subtotal DARBY								56.31	(31.27- 101.38)
DAVEYS	4	m	0	29	21	3	23	10.59	(2.81- 39.94)
DEAN	2	m	0	228	172	12	61	6.74	(3.52- 12.91)
DEAN2	26	m	0	252	112	33	112	7.64	(4.88- 11.95)
DEAN2	30	f	0	18	5	88	121	4.95	(1.77- 13.84)
Subtotal DEAN2								7.13	(4.73- 10.74)
DEAN3	19	m	0	131	237	25	510	11.28	(7.16- 17.77)
DEAN3	103	f	0	27	151	41	1538	6.71	(4.01- 11.21)
Subtotal DEAN3								8.98	(6.39- 12.62)
*DEKLER	4	m	2	-	-	-	-	32.50	(4.40- 241.20)
DESTEF	4	m	0	116	31	27	163	22.59	(12.80- 39.87)
DOLL	4	m	0	293	154	7	61	16.58	(7.40- 37.13)
DOLL	10	f	0	14	0	40	59	42.60~	(2.47- 734.58)
Subtotal DOLL								17.78	(8.19- 38.62)
*DOLL2	48	m	1	-	-	-	-	23.71	(3.25- 173.24)
*DOLL2	12	f	1	-	-	-	-	29.71	(9.46- 93.32)
Subtotal DOLL2								28.09	(10.42- 75.73)
*DORN	411	m	1	-	-	-	-	22.75	(19.63- 26.37)
DOSEME	13	m	2	-	-	-	-	6.60	(4.40- 10.20)
*DUNN	5	m	0	13	3206	2	14160	28.71	(6.48- 127.15)
EBELIN	6	m	0	4	4	12	117	9.75	(2.16- 44.04)
*ENSTRO	3	m	1	-	-	-	-	19.41	(15.22- 24.75)
*ENSTRO	8	f	1	-	-	-	-	16.47	(13.74- 19.75)
Subtotal ENSTRO								17.47	(15.10- 20.20)
ESAKI	3	m	0	34	10	16	28	5.95	(2.34- 15.16)
FAN	9	m	0	39	23	36	236	11.12	(5.96- 20.73)
FAN	13	f	0	4	1	69	320	18.55	(2.04- 168.54)
Subtotal FAN								11.54	(6.34- 21.03)
GAO2	4	m	0	74	30	13	56	10.63	(5.08- 22.22)
GARSHI	21	m	0	223	350	41	363	5.64	(3.92- 8.12)
GER	20	c	0	24	42	51	246	2.76	(1.54- 4.95)
GOLLED	17	m	0	143	333	15	490	14.03	(8.09- 24.31)
GSELL	5	m	0	51	13	2	29	56.88	(11.99- 269.87)

Table 1G14 - 5

IESLC - Meta-anal of Ever Smoking (or Current if ev not avail), Amount smoked, “High”, Any prod (or Cigs if Any not avail)
All LC types
Least adjusted

REF	NRR	SEX	AD	Number Exposed Case	Number Exposed Cont	Non-exposed Case	Non-exposed Cont	RR	95.00%CI
HAENSZ	51	f	0	23	13	81	236	5.15	(2.50- 10.65)
*HAMMON	155	m	1	-	-	-	-	17.06	(9.44- 30.82)
*HIRAYA	76	m	4	-	-	-	-	8.40	(5.70- 12.30)
HITOSU	5	m	0	32	265	7	242	4.17	(1.81- 9.63)
*HOLE	12	m	0	13	311	7	1189	7.10	(2.86- 17.64)
HU	3	m	0	29	13	41	67	3.65	(1.70- 7.80)
HU	6	f	0	1	2	40	48	0.60	(0.05- 6.86)
Subtotal HU								3.11	(1.50- 6.42)
HU2	7	c	0	64	19	121	213	5.93	(3.39- 10.37)
HUMBLE	78	c	3	-	-	-	-	39.70	(21.02- 74.98)
JEDRYC	62	m	0	278	143	49	219	8.69	(6.00- 12.57)
JEDRYC	67	f	0	28	4	78	166	14.90	(5.05- 43.94)
Subtotal JEDRYC								9.19	(6.48- 13.04)
JOLY	10	m	0	193	161	12	218	21.78	(11.74- 40.39)
JOLY	6	f	0	32	13	52	283	13.40	(6.59- 27.23)
Subtotal JOLY								17.66	(11.08- 28.14)
JUSSAW	15	m	0	48	4	149	624	50.26	(17.84- 141.55)
*KAISER	8	m	2	-	-	-	-	20.91	(12.78- 27.73)
*KAISER	4	f	2	-	-	-	-	12.63	(8.06- 19.80)
Subtotal KAISER								16.87	(12.58- 22.62)
KANELL	4	m	0	220	20	48	172	39.42	(22.55- 68.90)
KATSOU	10	f	0	10	1	48	67	13.96	(1.73- 112.71)
KAUFMA	6	c	0	96	47	35	925	53.98	(33.22- 87.71)
KHUDER	3	m	0	154	63	23	309	32.84	(19.62- 54.97)
*KINLEN	5	m	0	157	1484	7	1333	20.15	(9.48- 42.80)
KREUZE	22	m	3	-	-	-	-	20.80	(7.20- 60.50)
KREUZE	33	m	3	-	-	-	-	33.30	(20.50- 54.00)
Subtotal KREUZE								30.72	(19.77- 47.73)
KREYBE	23	m	0	43	248	6	644	18.61	(7.82- 44.27)
LAMTH	9	f	0	39	9	202	337	7.23	(3.43- 15.24)
LETOUR	4	c	0	65	23	24	224	26.38	(13.98- 49.78)
*LIAW	5	c	2	-	-	-	-	8.30	(4.00- 17.30)
LIU2	7	m	0	94	21	12	44	16.41	(7.42- 36.33)
LIU3	5	m	0	7	19	4	19	1.75	(0.44- 6.98)
LIU4	9	m	2	-	-	-	-	6.98	(6.73- 7.23)
LUBIN2	276	m	0	1261	1394	190	2616	12.45	(10.55- 14.70)
LUBIN2	284	f	0	45	33	336	1188	4.82	(3.03- 7.68)
Subtotal LUBIN2								11.19	(9.57- 13.08)
MACLEN	23	m	0	45	27	5	15	5.00	(1.63- 15.31)
MARTIS	3	m	0	75	50	4	25	9.38	(3.08- 28.57)
MATOS	32	m	0	106	105	11	110	10.10	(5.13- 19.85)
MATSUD	3	m	0	58	470	3	1255	51.62	(16.10- 165.55)
MCCONN	24	c	0	35	42	9	23	2.13	(0.87- 5.19)
*MIGRAN	7	m	0	30	604	4	867	10.77	(3.81- 30.40)
*MIGRAN	34	f	0	2	149	4	3814	12.80	(2.36- 69.33)
Subtotal MIGRAN								11.29	(4.66- 27.34)
*MRFITR	5	m	0	54	3591	0	1859	56.43~	(3.49- 913.25)
NAM	67	m	0	149	243	30	520	10.63	(6.98- 16.19)
NAM	83	f	0	65	84	52	885	13.17	(8.59- 20.20)
Subtotal NAM								11.81	(8.75- 15.94)
ORMOS	3	m	0	15	128	7	777	13.01	(5.20- 32.52)
OSANN	57	m	2	-	-	-	-	42.80	(30.50- 60.10)
OSANN	58	f	2	-	-	-	-	40.90	(29.30- 57.10)
Subtotal OSANN								41.82	(32.97- 53.05)
PASTOR	4	m	0	46	45	10	89	9.10	(4.20- 19.69)
PERNU	23	m	0	40	23	97	275	4.93	(2.81- 8.66)
PERNU	16	f	0	5	7	110	971	6.31	(1.97- 20.20)
Subtotal PERNU								5.17	(3.11- 8.57)
PEZZO2	5	m	0	69	12	6	117	112.13	(40.26- 312.24)
PEZZOT	4	m	0	51	6	4	116	246.50	(66.69- 911.11)
PIKE	3	m	0	66	37	18	69	6.84	(3.55- 13.18)
PIKE	7	f	0	16	3	36	96	14.22	(3.91- 51.73)
Subtotal PIKE								7.95	(4.43- 14.27)
SEGI2	17	m	0	52	45	8	53	7.66	(3.29- 17.80)
SIEMIA	15	m	0	-	-	-	-	7.90	(3.00- 24.10)
SOBUE	119	m	0	226	187	34	128	4.55	(2.97- 6.96)
*SPEIZE	5	f	1	-	-	-	-	22.00	(14.80- 32.30)
STOCKS	29	m	0	302	468	45	638	9.15	(6.54- 12.79)
STOCKW	3	c	0	4327	572	2791	10641	28.84	(26.18- 31.77)
SVENSS	36	f	0	19	1	38	120	60.00	(7.77- 463.15)
*TENKAN	12	m	1	-	-	-	-	24.97	(9.90- 63.00)

Table 1G14 - 5

IESLC - Meta-anal of Ever Smoking (or Current if ev not avail), Amount smoked, “High”, Any prod (or Cigs if Any not avail)
All LC types
Least adjusted

REF	NRR	SEX	AD	Number Exposed Case	Number Exposed Cont	Non-exposed Case	Non-exposed Cont	RR	95.00%CI
TIZZAN	10	m	0	83	20	180	305	7.03	(4.17- 11.85)
TSUGAN	31	m	0	19	14	18	22	1.66	(0.65- 4.20)
*TULINI	6	m	1	-	-	-	-	28.70	(14.90- 55.10)
*TULINI	11	f	1	-	-	-	-	44.10	(21.10- 91.80)
Subtotal TULINI								34.70	(21.29- 56.56)
WAKAI	39	m	0	69	48	10	65	9.34	(4.37- 20.00)
WANG2	7	c	0	9	4	11	43	8.80	(2.28- 33.97)
WU	36	f	0	103	31	31	92	9.86	(5.57- 17.47)
WYNDE2	20	m	0	155	112	8	105	18.16	(8.50- 38.80)
WYNDE3	47	m	0	68	26	9	88	25.57	(11.25- 58.14)
WYNDE3	82	f	0	4	3	20	76	5.07	(1.05- 24.50)
Subtotal WYNDE3								18.10	(8.73- 37.49)
WYNDE4	47	m	0	130	64	12	115	19.47	(10.00- 37.88)
WYNDE4	61	f	2	-	-	-	-	11.54	(1.90- 70.11)
Subtotal WYNDE4								18.28	(9.79- 34.14)
WYNDE6	54	m	0	784	197	87	617	28.22	(21.47- 37.10)
WYNDE6	243	f	0	378	52	159	856	39.13	(27.98- 54.75)
Subtotal WYNDE6								32.15	(26.01- 39.75)
XU3	8	m	0	16	2	7	31	35.43	(6.58- 190.72)
YAMAGU	2	c	0	28	56	24	267	5.56	(3.00- 10.31)
ZHENG	14	m	0	66	23	33	94	8.17	(4.40- 15.17)
Partial Totals				14137	88991	6779	2268018		

*prospective study ~ With 0.5 adjustment for zero

REF	NRR	SEX	AD	Ys	Ws	Qs	Ps
*AKIBA	19	m	0	1.49	14.01	9.66	0.0000
ALDERS	20	m	1	2.14	14.23	0.44	0.0000
ALDERS	23	f	1	1.93	25.78	3.84	0.0000
Subtotal ALDERS				2.01	40.01	4.27	
*ARCHER	3	m	0	2.14	5.22	0.16	0.0000
ARMADA	48	m	0	4.24	3.29	12.18	0.0000
AUVINE	7	c	0	3.68	5.94	11.04	0.0000
AXELSS	16	f	0	1.99	2.69	0.28	0.0011
BARBON	13	m	0	2.86	15.58	4.60	0.0000
*BEST	15	m	1	2.85	6.30	1.80	0.0000
*BOUCOT	100	m	0	3.83	0.49	1.13	0.0070
BRESLO	16	m	0	2.90	8.89	3.02	0.0000
*BRETT	3	m	0	2.08	5.09	0.29	0.0000
BROSS	19	m	0	1.97	16.76	1.97	0.0000
BUFFLE	35	f	0	3.06	8.66	4.72	0.0000
*CHANG	4	m	0	2.11	3.72	0.16	0.0000
*CHANG	10	f	0	2.11	6.22	0.28	0.0000
Subtotal CHANG				2.11	9.94	0.44	
CHEN2	6	m	0	2.07	3.80	0.23	0.0001
CHEN2	10	f	0	0.97	1.22	2.21	0.2838
Subtotal CHEN2				1.80	5.02	2.44	
CHOI	16	m	0	2.97	3.16	1.34	0.0000
CHOI	20	f	0	1.87	0.74	0.15	0.1083
Subtotal CHOI				2.76	3.90	1.49	
*CHOW	5	m	0	3.28	5.22	4.87	0.0000
COMSTO	6	m	0	3.22	2.79	2.25	0.0000
COMSTO	11	f	0	2.59	2.75	0.20	0.0000
Subtotal COMSTO				2.90	5.54	2.45	
CORREA	49	c	0	3.00	34.18	15.86	0.0000
*CPSI	219	m	1	2.86	50.84	15.07	0.0000
*CPSI	278	f	1	2.41	10.15	0.08	0.0000
Subtotal CPSI				2.79	60.99	15.15	
*CPSII	103	m	1	2.87	75.65	22.94	0.0000
*CPSII	70	f	0	2.53	23.17	1.01	0.0000
Subtotal CPSII				2.79	98.82	23.94	
DAMBER	9	m	1	2.70	5.93	0.88	0.0000
DARBY	3	m	0	4.96	2.72	19.04	0.0000
DARBY	10	f	0	3.73	8.38	16.70	0.0000
Subtotal DARBY				4.03	11.11	35.73	
DAVEYS	4	m	0	2.36	2.18	0.00	0.0005
DEAN	2	m	0	1.91	9.10	1.52	0.0000
DEAN2	26	m	0	2.03	19.18	1.55	0.0000
DEAN2	30	f	0	1.60	3.63	1.87	0.0023
Subtotal DEAN2				1.96	22.82	3.42	
DEAN3	19	m	0	2.42	18.58	0.21	0.0000

Table 1G14 - 5

IESLC - Meta-anal of Ever Smoking (or Current if ev not avail), Amount smoked, “High”, Any prod (or Cigs if Any not avail)
All LC types
Least adjusted

REF	NRR	SEX	AD	Ys	Ws	Qs	Ps
DEAN3	103	f	0	1.90	14.56	2.50	0.0000
Subtotal DEAN3				2.19	33.14	2.70	
*DEKLER	4	m	2	3.48	0.96	1.30	0.0007
DESTEF	4	m	0	3.12	11.90	7.62	0.0000
DOLL	4	m	0	2.81	5.91	1.42	0.0000
DOLL	10	f	0	3.75	0.47	0.98	0.0098
Subtotal DOLL				2.88	6.39	2.40	
*DOLL2	48	m	1	3.17	0.97	0.70	0.0018
*DOLL2	12	f	1	3.39	2.93	3.38	0.0000
Subtotal DOLL2				3.34	3.90	4.08	
*DORN	411	m	1	3.12	176.37	114.95	0.0000
DOSEME	13	m	2	1.89	21.74	4.02	0.0000
*DUNN	5	m	0	3.36	1.73	1.88	0.0000
EBELIN	6	m	0	2.28	1.69	0.00	0.0031
*ENSTRO	3	m	1	2.97	65.00	27.34	0.0000
*ENSTRO	8	f	1	2.80	116.71	27.37	0.0000
Subtotal ENSTRO				2.86	181.71	54.71	
ESAKI	3	m	0	1.78	4.39	1.25	0.0002
FAN	9	m	0	2.41	9.89	0.08	0.0000
FAN	13	f	0	2.92	0.79	0.29	0.0095
Subtotal FAN				2.45	10.68	0.37	
GAO2	4	m	0	2.36	7.06	0.01	0.0000
GARSHI	21	m	0	1.73	29.00	10.00	0.0000
GER	20	c	0	1.01	11.22	19.05	0.0007
GOLLED	17	m	0	2.64	12.71	1.33	0.0000
GSELL	5	m	0	4.04	1.58	4.71	0.0000
HAENSZ	51	f	0	1.64	7.30	3.35	0.0000
*HAMMON	155	m	1	2.84	10.98	2.96	0.0000
*HIRAYA	76	m	4	2.13	25.97	0.93	0.0000
HITOSU	5	m	0	1.43	5.49	4.33	0.0008
*HOLE	12	m	0	1.96	4.64	0.59	0.0000
HU	3	m	0	1.29	6.63	6.95	0.0009
HU	6	f	0	-0.51	0.65	5.17	0.6812
Subtotal HU				1.13	7.28	12.13	
HU2	7	c	0	1.78	12.31	3.55	0.0000
HUMBLE	78	c	3	3.68	9.50	17.68	0.0000
JEDRYC	62	m	0	2.16	28.12	0.68	0.0000
JEDRYC	67	f	0	2.70	3.28	0.48	0.0000
Subtotal JEDRYC				2.22	31.40	1.16	
JOLY	10	m	0	3.08	10.07	5.87	0.0000
JOLY	6	f	0	2.59	7.64	0.59	0.0000
Subtotal JOLY				2.87	17.71	6.46	
JUSSAW	15	m	0	3.92	3.58	9.17	0.0000
*KAISER	8	m	2	3.04	25.61	13.39	0.0000
*KAISER	4	f	2	2.54	19.02	0.91	0.0000
Subtotal KAISER				2.83	44.63	14.30	
KANELL	4	m	0	3.67	12.32	22.68	0.0000
KATSOU	10	f	0	2.64	0.88	0.09	0.0134
KAUFMA	6	c	0	3.99	16.30	45.54	0.0000
KHUDER	3	m	0	3.49	14.48	19.97	0.0000
*KINLEN	5	m	0	3.00	6.77	3.18	0.0000
KREUZE	22	m	3	3.03	3.39	1.75	0.0000
KREUZE	33	m	3	3.51	16.38	23.13	0.0000
Subtotal KREUZE				3.42	19.77	24.88	
KREYBE	23	m	0	2.92	5.11	1.88	0.0000
LAMTH	9	f	0	1.98	6.91	0.79	0.0000
LETOUR	4	c	0	3.27	9.52	8.69	0.0000
*LIAW	5	c	2	2.12	7.17	0.29	0.0000
LIU2	7	m	0	2.80	6.09	1.41	0.0000
LIU3	5	m	0	0.56	2.01	6.20	0.4278
LIU4	9	m	2	1.94	2991.95	418.94	0.0000
LUBIN2	276	m	0	2.52	139.75	5.86	0.0000
LUBIN2	284	f	0	1.57	17.75	9.83	0.0000
Subtotal LUBIN2				2.42	157.50	15.69	
MACLEN	23	m	0	1.61	3.07	1.54	0.0048
MARTIS	3	m	0	2.24	3.09	0.02	0.0001
MATOS	32	m	0	2.31	8.41	0.00	0.0000
MATSUD	3	m	0	3.94	2.83	7.49	0.0000
MCCONN	24	c	0	0.76	4.83	11.78	0.0966
*MIGRAN	7	m	0	2.38	3.56	0.01	0.0000
*MIGRAN	34	f	0	2.55	1.35	0.07	0.0031

Table 1G14 - 5

IESLC - Meta-anal of Ever Smoking (or Current if ev not avail), Amount smoked, “High”, Any prod (or Cigs if Any not avail)
All LC types
Least adjusted

REF	NRR	SEX	AD	Ys	Ws	Qs	Ps
Subtotal MIGRAN				2.42	4.91	0.08	
*MRFITR	5	m	0	4.03	0.50	1.46	0.0045
NAM	67	m	0	2.36	21.70	0.05	0.0000
NAM	83	f	0	2.58	20.99	1.43	0.0000
Subtotal NAM				2.47	42.69	1.47	
ORMOS	3	m	0	2.57	4.57	0.28	0.0000
OSANN	57	m	2	3.76	33.40	69.19	0.0000
OSANN	58	f	2	3.71	34.52	67.06	0.0000
Subtotal OSANN				3.73	67.92	136.25	
PASTOR	4	m	0	2.21	6.44	0.08	0.0000
PERNU	23	m	0	1.60	12.13	6.32	0.0000
PERNU	16	f	0	1.84	2.83	0.64	0.0019
Subtotal PERNU				1.64	14.97	6.96	
PEZZO2	5	m	0	4.72	3.66	21.14	0.0000
PEZZOT	4	m	0	5.51	2.25	22.87	0.0000
PIKE	3	m	0	1.92	8.91	1.39	0.0000
PIKE	7	f	0	2.65	2.30	0.26	0.0001
Subtotal PIKE				2.07	11.21	1.65	
SEGI2	17	m	0	2.04	5.40	0.43	0.0000
SIEMIA	15	m	0	2.07	3.54	0.22	0.0001
SOBUE	119	m	0	1.52	21.28	13.69	0.0000
*SPEIZE	5	f	1	3.09	25.23	15.11	0.0000
STOCKS	29	m	0	2.21	34.20	0.37	0.0000
STOCKW	3	c	0	3.36	411.25	448.71	0.0000
SVENSS	36	f	0	4.09	0.92	2.90	0.0001
*TENKAN	12	m	1	3.22	4.49	3.64	0.0000
TIZZAN	10	m	0	1.95	14.11	1.90	0.0000
TSUGAN	31	m	0	0.51	4.44	14.58	0.2861
*TULINI	6	m	1	3.36	8.98	9.71	0.0000
*TULINI	11	f	1	3.79	7.11	15.34	0.0000
Subtotal TULINI				3.55	16.09	25.05	
WAKAI	39	m	0	2.23	6.64	0.05	0.0000
WANG2	7	c	0	2.17	2.10	0.04	0.0016
WU	36	f	0	2.29	11.75	0.01	0.0000
WYNDE2	20	m	0	2.90	6.67	2.26	0.0000
WYNDE3	47	m	0	3.24	5.69	4.86	0.0000
WYNDE3	82	f	0	1.62	1.55	0.75	0.0436
Subtotal WYNDE3				2.90	7.24	5.61	
WYNDE4	47	m	0	2.97	8.67	3.68	0.0000
WYNDE4	61	f	2	2.45	1.18	0.02	0.0079
Subtotal WYNDE4				2.91	9.85	3.70	
WYNDE6	54	m	0	3.34	51.37	53.75	0.0000
WYNDE6	243	f	0	3.67	34.09	62.11	0.0000
Subtotal WYNDE6				3.47	85.46	115.86	
XU3	8	m	0	3.57	1.36	2.12	0.0000
YAMAGU	2	c	0	1.72	10.10	3.65	0.0000
ZHENG	14	m	0	2.10	10.04	0.47	0.0000

	N	134
	NS	105
	Wt	5234.89
Het	Chi	1913.99
Het	df	133
Het	P	***
Fixed	RR	10.15
	RRl	9.88
	RRu	10.43
	P	+++
Random	RR	13.43
	RRl	11.66
	RRu	15.48
	P	+++
Asymm	P	***

Table 1G14 - 6

IESLC - Meta-anal of Ever Smoking (or Current if ev not avail), Amount smoked, “High”, Any prod (or Cigs if Any not avail)
All LC types
Least adjusted

		combined	Sex: male	female	Total
	N	12	86	36	134
	NS	12	85	36	133
	Wt	534.42	4264.37	436.11	5234.89
Het	Chi	166.09	975.77	180.15	1913.99
Het	df	11	85	35	133
Het	P	***	***	***	***
Fixed	RR	24.62	8.71	15.26	10.15
	RRl	22.62	8.45	13.89	9.88
	RRu	26.80	8.98	16.76	10.43
	P	+++	+++	+++	+++
Random	RR	13.21	13.70	12.72	13.43
	RRl	7.86	11.66	9.86	11.66
	RRu	22.21	16.09	16.42	15.48
	P	+++	+++	+++	+++
Between	Chi				591.99
Between	df				2
Between	P				***
Btwn(F)	P				***
Btwn(R)	P				N.S.

Table 1G14 - 7

IESLC - Meta-anal of Ever Smoking (or Current if ev not avail), Amount smoked, “High”, Any prod (or Cigs if Any not avail)
All LC types
Excluded studies (and stage at which they were excluded)

1	ABELIN ABRAHA AMANDU AMES ANDERS AUSTIN AXELSO BAND BECHER BERRIN BLOHMK BLOT4 BROCKM BROWN1 BYERS1 BYERS2 CARPEN CASCO2 CASCOR CHAN CHEN3 CHIAZZ CHYOU DESTE2 DOCKER DROSTE DU GARCIA GARDIN GENG GODLEY GOODMA GRAHAM GREGOR HEGMAN HEIN HENNEK HINDS HIRAOK HOROWI HORWIT HUANG ISHIMA JAHN JAIN JARVHO JIANG KELLER KIHARA KJUUS KO KOHLME KUBIK LAMWK LAMWK2 LANGE LEI LEMARC LEVIN LIU LOMBA2 LOMBAR MAGNUS MARSH MARSH2 MCDUFF MCLAUG MILLER MILLS NOTANI NOU ODRISC PAWLEG PERSHA POFFIJ QIAO QIAO2 RADZIK REN RONCO ROOTS ROTHSC SAARIK SANKAR SCHWAR SEGI SEOW SHIMIZ SIMARA SIMONA SITAS SOBUE2 STASZE STAYNE STUCKE SUN SUZUK2 SUZUKI TANG TAO TOKARS TOUSEY ULMER VEIERO VUTUC WALD WANG WANG3 WANG4 WICKLU WIGLE WILKIN WU2 WUNSCH WYNDE8 XIANGZ XU XU2 XU4 YONG ZHANG
2	BUELL CHEN MASTRA MZILEN PISANI RESTRE SADOWS
4	BOFFET WYNDE7
5	RIMING TANG2 WYNDE5
6	BLOT1 BLOT2 BLOT3 BOUCHA HIRAY2 JONES LAURIL LICKIN MOLLO MRFIT MURATA SCHWA2 VANDER WARSIN WATSON WYNDER
8	LUO
10	AGUDO BENSHL BROWN2 CEDERL CHATZI COOKSO DORANT DORGAN ENGELA GAO HAMMO2 HANSEN JARUP KAISE2 KNEKT KOO KOULUM LAUSSM LIDDEL LIU5 LUBIN NOTAN2 OSANN2 PARKIN PERSH2 PETO POLEDN PRESCO RACHTA RANDIG SHAW SPITZ TVERDA WUWILL YUAN ZHOU
11	BENHAM GILLIS

Table 1G14 - 8
Potentially overlapping studies

REF	REFGP	PRINC .	OVERLAP/LINK
LUBIN2	LUBIN2	1	Lubin-combined
LAMTH	LAMTH	1	KOO/LAMTH/LAMWK
AKIBA	AKIBA	1	AKIBA/ISHIMA
BROSS	BYERS1	1	GRAHAM/BROSS/BYERS1
HOLE	TANG2	1	Subset of TANG2
KAISER	KAISER	1	KAISER/OSANN2
MRFITR	MRFIT	2	Subset of MRFIT
WYNDE6	WYNDE6	1	WYNDE5/6/7/8
CPSI	CPSI	1	CPSI overall
ENSTRO	ENSTRO	1	Subset of CPSI
MATSUD	MATSUD	1	SOBUE2/MATSUD

Table 1G14 - 9
Most adjusted - insufficient data for metaanalysis

REF	NRR	SEX	AGEL	AGEH	RACE	YF	LC TYPE	LOC	START	ST	NLC	R	VB	P	H	AD	SM	PRODUCT	exL	exH	DENOM	De
BLOT1	7	m	0	0	all	-	all	NAmer	1970	CC	458	n	bl	y	n	0	ev	cig+/-ot	40	99	nev cigs	or
BUELL	3	m	0	0	all	0	all	NAmer	1957	pr	304	n	bl	n	n	0	ev	cig+/-ot	22	99	nev cigs	st
HITOSU	16	m	0	0	all	-	all	As:Jap	1960	CC	216	n	bl	y	n	2	cu	all/unsp	25	99	nev any	or
RESTRE	9	c	0	0	all	-	all	SCAmer	1978	CC	102	n	bl	n	n	5	ev	cig+/-ot	41	99	nev cigs	or
SADOWS	89	m	0	0	wh	-	all	NAmer	1938	CC	477	n	bl	n	n	1	ev	all/unsp	41	99	nev any	st

REF	NRR	RR	SIG	RRDATA comment
BLOT1	7	8.60		0
BUELL	3	4.93		0
HITOSU	16	6.40		0
RESTRE	9	169.90		0
SADOWS	89	6.28		0

Least adjusted - insufficient data for meta-analysis: as for adjusted plus the following

REF	NRR	SEX	AGEL	AGEH	RACE	YF	LC TYPE	LOC	START	ST	NLC	R	VB	P	H	AD	SM	PRODUCT	exL	exH	DENOM	De
SADOWS	84	m	0	0	wh	-	all	NAmer	1938	CC	477	n	bl	n	n	0	ev	all/unsp	41	99	nev any	st

REF	NRR	RR	SIG	RRDATA comment
SADOWS	84	5.28		0

Table 1G16 -

IESLC - Meta-anal of Ever Smoking (or Curr if Ever not avail) by Amount, Overview, Cigs (or Any Prod if Cigs not avail)
All LC types

This analysis is restricted to results for:
1) Results by Amount smoked
Results by Amount smoked (in numbers of cigarettes or cigarette equivalents) are grouped under 2 schemes (S1, S2).
Each scheme has a set of "key values". An interval is allocated to the category whose key value
it includes and intervals which include none or more than one of the key values are excluded.
(Open-ended intervals are coded as 99.)

S1	key value	maximum range	S2	key value	maximum range
1	5	1-19	1	1	1-9
2	20	6-44	2	10	2-19
3	45	21+	3	20	11-29
			4	30	21-39
			5	40	31-98
			6	99	41+

For all/unspec product, the definition of cigarette equivalents is shown at the end of Sections -1 and -4.
2) Results complete enough for use in metaanalysis

Within each study, results are then selected (in the following order of preference, within each sex) for:
3) SMKSTA: ever smokers, current smokers
4) PRODUCT: cigarettes regardless of other products, cigarettes only, all/unspec
5) CIGTYPE: all/unspecified, MC regardless of HR, MC only
6) DENOM: never smoked anything, never smoked cigarettes, (never +1 = +long term ex, +2 = +amount unknown,
+3 = never cigs+long term ex)
7) Followup period (YF, prospective studies): whole study (coded as 0) or longest available
8) LCtype: all or nearest available, at least Squamous and Adeno. (q = squamous, s = small,
l = large, a = adeno, mix = mixed, alv = alveolar)
9) Race: all or nearest available, otherwise by race (wh or w = white, bl or b = black, hi = hispanic
ch = chinese, jap = japanese, haw = hawaiian, w+o = white + oriental, sca = scandinavian, as = asian)
10) For overlapping studies: principal rather than subsidiary studies
Finally by Age: whole study (coded as 0) if available, otherwise by widest available age group
and then for single sex results (m, f) in preference to combined sex results (c).

Results adjusted (AD) for the most potential confounders are then chosen in Sections -1 to -3
(and those which actually differ from the adjusted results in Table 1G11 - 1 are marked 'x' in Section -1)
and results adjusted for the least confounders in Sections -4 to -6. (Those least adjusted results which
actually differ from the most adjusted as marked 'x' in column X in Section -4)
(Results adjusted for an unknown number of confounder(s) are coded as 20.)

Section -7 shows excluded studies, together with the stage (as above) at which no qualifying
results were found.

Section -8 lists the potentially overlapping studies which have been included (1=principal, 2=subsidiary).

Section -9 lists any results which would have been included in preference except that they had data not complete
enough for use in meta-analysis, with their significance (yes/no), if known, and any further comment as entered
on the database.

In addition to those mentioned above, the following fields, levels and abbreviations are used:
* or nk = not known, n = no, y = yes, ot = other
ev = ever, cu = current, nev = never
all/unspec = all or unspecified, cig+/-ot = cigarettes irrespective of other products (cigar, pipe etc)
MC = manufactured cigarettes, HR = hand-rolled cigarettes
exL, exH = range of exposure (low and high) in the smoking group, in terms of Amount smoked,
cigarettes or cigarette equivalents
REF: 6-character study reference
NRR: number of the RR on the database within the study
ST : study type (CC = case control, pr or prosp = prospective)
NLC: number of lung cancer cases in whole study
R : risky occupational population (n = no, m = mining, o = other risky)
VB : national cigarette type (V = at least 75% Virginia, bl = at least 75% blended, ot = other)
P : any proxy use
H : full histological confirmation
De : derivation of RR/CI (or = original, st = standard method, ot = other method of estimation)

Table 1G16 - 1

IESLC - Meta-anal of Ever Smoking (or Curr if Ever not avail) by Amount, Overview, Cigs (or Any Prod if Cigs not avail)
All LC types
Most adjusted

REF	NRR	1G11	SEX	AGEL	AGEH	RACE	YF	LC TYPE	LOC	START	ST	NLC	R	VB	P	H	AD	SM	PRODUCT	exL	exH	S1	S2	DENOM	De
AGUDO	4		f	0	0	all	-	all	Eu:wst	1989	CC	103	n	bl	n	n	3	ev	cig only	1	10	1	0	nev any	or
AGUDO	5		f	0	0	all	-	all	Eu:wst	1989	CC	103	n	bl	n	n	3	ev	cig only	11	99	0	0	nev any	or
AKIBA	27		m	0	0	all	0	all	As:Jap	1963	pr	610	n	bl	n	n	5	cu	cig+/-ot	1	14	1	0	nev cigs	or
AKIBA	28		m	0	0	all	0	all	As:Jap	1963	pr	610	n	bl	n	n	5	cu	cig+/-ot	15	24	2	3	nev cigs	ot
AKIBA	29		m	0	0	all	0	all	As:Jap	1963	pr	610	n	bl	n	n	5	cu	cig+/-ot	25	99	3	0	nev cigs	or
AKIBA	33		f	0	0	all	0	all	As:Jap	1963	pr	610	n	bl	n	n	5	cu	cig+/-ot	1	14	1	0	nev cigs	or
AKIBA	34		f	0	0	all	0	all	As:Jap	1963	pr	610	n	bl	n	n	5	cu	cig+/-ot	15	99	0	0	nev cigs	or
ALDERS	18		m	0	0	all	-	all	Eu:UK	1977	CC	1448	n	V	n	n	1	ev	cig only	1	17	1	0	nev+2	ot
ALDERS	19		m	0	0	all	-	all	Eu:UK	1977	CC	1448	n	V	n	n	1	ev	cig only	18	27	2	3	nev+2	ot
ALDERS	20		m	0	0	all	-	all	Eu:UK	1977	CC	1448	n	V	n	n	1	ev	cig only	28	99	3	0	nev+2	ot
ALDERS	21		f	0	0	all	-	all	Eu:UK	1977	CC	1448	n	V	n	n	1	ev	cig only	1	17	1	0	nev+2	ot
ALDERS	22		f	0	0	all	-	all	Eu:UK	1977	CC	1448	n	V	n	n	1	ev	cig only	18	27	2	3	nev+2	ot
ALDERS	23		f	0	0	all	-	all	Eu:UK	1977	CC	1448	n	V	n	n	1	ev	cig only	28	99	3	0	nev+2	ot
ARCHER	1		m	0	0	wh	0	all	NAmer	1950	pr	146	m	bl	n	n	0	cu	cig+/-ot	1	19	1	0	nev cigs	st
ARCHER	2		m	0	0	wh	0	all	NAmer	1950	pr	146	m	bl	n	n	0	cu	cig+/-ot	20	20	2	3	nev cigs	st
ARCHER	3		m	0	0	wh	0	all	NAmer	1950	pr	146	m	bl	n	n	0	cu	cig+/-ot	21	99	3	0	nev cigs	st
ARMADA	46		m	0	0	all	-	all	Eu:wst	1986	CC	325	n	bl	n	y	0	ev	cig+/-ot	1	14	1	0	nev any	st
ARMADA	47		m	0	0	all	-	all	Eu:wst	1986	CC	325	n	bl	n	y	0	ev	cig+/-ot	15	24	2	3	nev any	st
ARMADA	48		m	0	0	all	-	all	Eu:wst	1986	CC	325	n	bl	n	y	0	ev	cig+/-ot	25	99	3	0	nev any	st
AUVINE	13		c	0	0	all	-	all	Eu:Sca	1986	CC	517	n	bl	y	n	2	ev	cig+/-ot	1	10	1	0	nev cigs	or
AUVINE	14		c	0	0	all	-	all	Eu:Sca	1986	CC	517	n	bl	y	n	2	ev	cig+/-ot	11	20	2	3	nev cigs	or
AUVINE	15		c	0	0	all	-	all	Eu:Sca	1986	CC	517	n	bl	y	n	2	ev	cig+/-ot	21	99	3	0	nev cigs	or
AXELSS	5		m	0	0	sca	-	all	Eu:Sca	1989	CC	436	n	bl	n	n	6	ev	all/unsp	1	9	1	1	nev any	ot
AXELSS	6		m	0	0	sca	-	all	Eu:Sca	1989	CC	436	n	bl	n	n	6	ev	all/unsp	10	19	0	2	nev any	ot
AXELSS	7		m	0	0	sca	-	all	Eu:Sca	1989	CC	436	n	bl	n	n	6	ev	all/unsp	20	99	0	0	nev any	ot
AXELSS	13		f	0	0	sca	-	all	Eu:Sca	1989	CC	436	n	bl	n	n	0	ev	all/unsp	1	9	1	1	nev any	st
AXELSS	14		f	0	0	sca	-	all	Eu:Sca	1989	CC	436	n	bl	n	n	0	ev	all/unsp	10	19	0	2	nev any	st
AXELSS	15		f	0	0	sca	-	all	Eu:Sca	1989	CC	436	n	bl	n	n	0	ev	all/unsp	20	29	2	3	nev any	st
AXELSS	16		f	0	0	sca	-	all	Eu:Sca	1989	CC	436	n	bl	n	n	0	ev	all/unsp	30	99	3	0	nev any	st
BARBON	82		m	0	0	all	-	all	Eu:wst	1979	CC	755	n	bl	y	y	3	ev	all/unsp	1	19	1	0	nev any	or
BARBON	83		m	0	0	all	-	all	Eu:wst	1979	CC	755	n	bl	y	y	3	ev	all/unsp	20	39	2	0	nev any	or
BARBON	84		m	0	0	all	-	all	Eu:wst	1979	CC	755	n	bl	y	y	3	ev	all/unsp	40	99	3	0	nev any	or
BENSHL	11		m	40	64	all	10	all	Eu:UK	1967	pr	486	n	V	n	n	1	cu	cig+/-ot	1	9	1	1	nev any	ot
BENSHL	12		m	40	64	all	10	all	Eu:UK	1967	pr	486	n	V	n	n	1	cu	cig+/-ot	10	19	0	2	nev any	ot
BENSHL	13		m	40	64	all	10	all	Eu:UK	1967	pr	486	n	V	n	n	1	cu	cig+/-ot	20	99	0	0	nev any	ot
BEST	13		m	0	0	all	0	all	NAmer	1955	pr	381	n	V	n	n	1	cu	cig only	1	9	1	1	nev any	ot
BEST	14		m	0	0	all	0	all	NAmer	1955	pr	381	n	V	n	n	1	cu	cig only	10	20	2	0	nev any	ot
BEST	15		m	0	0	all	0	all	NAmer	1955	pr	381	n	V	n	n	1	cu	cig only	21	99	3	0	nev any	ot
BOUCOT	99		m	0	0	all	9	all	NAmer	1951	pr	121	n	bl	n	n	0	ev	cig+/-ot	1	20	0	0	nev any	ot
BOUCOT	100		m	0	0	all	9	all	NAmer	1951	pr	121	n	bl	n	n	0	ev	cig+/-ot	21	99	3	0	nev any	ot
BRESLO	13		m	0	0	all	-	all	NAmer	1949	CC	518	n	bl	n	y	0	ev	cig+/-ot	1	9	1	1	nev+3	st
BRESLO	14		m	0	0	all	-	all	NAmer	1949	CC	518	n	bl	n	y	0	ev	cig+/-ot	10	19	0	2	nev+3	st
BRESLO	15		m	0	0	all	-	all	NAmer	1949	CC	518	n	bl	n	y	0	ev	cig+/-ot	20	39	2	0	nev+3	st
BRESLO	16		m	0	0	all	-	all	NAmer	1949	CC	518	n	bl	n	y	0	ev	cig+/-ot	40	99	3	0	nev+3	st
BRESLO	29		f	0	0	all	-	all	NAmer	1949	CC	518	n	bl	n	y	0	ev	cig+/-ot	1	19	1	0	nev+3	st
BRESLO	30		f	0	0	all	-	all	NAmer	1949	CC	518	n	bl	n	y	0	ev	cig+/-ot	20	99	0	0	nev+3	st
BRETT	1		m	0	0	all	0	all	Eu:UK	1960	pr	150	n	V	n	n	0	cu	cig+/-ot	1	14	1	0	nev cigs	st
BRETT	2		m	0	0	all	0	all	Eu:UK	1960	pr	150	n	V	n	n	0	cu	cig+/-ot	15	24	2	3	nev cigs	st
BRETT	3		m	0	0	all	0	all	Eu:UK	1960	pr	150	n	V	n	n	0	cu	cig+/-ot	25	99	3	0	nev cigs	st
BROSS	18		m	0	0	wh	-	all	NAmer	1960	CC	974	n	bl	n	n	0	cu	cig+/-ot	1	20	0	0	nev any	st
BROSS	19		m	0	0	wh	-	all	NAmer	1960	CC	974	n	bl	n	n	0	cu	cig+/-ot	21	99	3	0	nev any	st
BROWN2	32		m	0	0	wh	-	all	NAmer	1984	CC	14596	n	bl	n	y	2	ev	cig+/-ot	1	19	1	0	nev cigs	or
BROWN2	42		m	0	0	wh	-	all	NAmer	1984	CC	14596	n	bl	n	y	2	ev	cig+/-ot	20	99	0	0	nev cigs	or
BROWN2	31		f	0	0	wh	-	all	NAmer	1984	CC	14596	n	bl	n	y	2	ev	cig+/-ot	1	19	1	0	nev cigs	or
BROWN2	41		f	0	0	wh	-	all	NAmer	1984	CC	14596	n	bl	n	y	2	ev	cig+/-ot	20	99	0	0	nev cigs	or
BUFFLE	28		f	0	0	w-hi	-	all	NAmer	1976	CC	943	n	bl	y	n	0	ev	cig+/-ot	1	19	1	0	nev cigs	or
BUFFLE	29		f	0	0	w-hi	-	all	NAmer	1976	CC	943	n	bl	y	n	0	ev	cig+/-ot	20	20	2	3	nev cigs	or
BUFFLE	35		f	0	0	w-hi	-	all	NAmer	1976	CC	943	n	bl	y	n	0	ev	cig+/-ot	21	99	3	0	nev cigs	or
CEDERL	87	x	m	0	0	all	10	all	Eu:Sca	1963	pr	491	n	bl	n	n	1	cu	cig+/-ot	1	15	1	0	nev any	ot
CEDERL	86	x	m	0	0	all	10	all	Eu:Sca	1963	pr	491	n	bl	n	n	1	cu	cig+/-ot	16	99	0	0	nev any	ot
CEDERL	99	x	f	0	0	all	10	all	Eu:Sca	1963	pr	491	n	bl	n	n	1	cu	cig+/-ot	1	15	1	0	nev any	ot
CHANG	2		m	0	0	all	0	all	NAmer	1972	pr	136	n	bl	n	n	0	cu	cig+/-ot	1	10	1	0	nev cigs	st
CHANG	3		m	0	0	all	0	all	NAmer	1972	pr	136	n	bl	n	n	0	cu	cig+/-ot	11	20	2	3	nev cigs	st
CHANG	4		m	0	0	all	0	all	NAmer	1972	pr	136	n	bl	n	n	0	cu	cig+/-ot	21	99	3	0	nev cigs	st
CHANG	8		f	0	0	all	0	all	NAmer	1972	pr	136	n	bl	n	n	0	cu	cig+/-ot	1	10	1	0	nev cigs	st
CHANG	9		f	0	0	all	0	all	NAmer	1972	pr	136	n	bl	n	n	0	cu	cig+/-ot	11	20	2	3	nev cigs	st
CHANG	10		f	0	0	all	0	all	NAmer	1972	pr	136	n	bl	n	n	0	cu	cig+/-ot	21	99	3	0	nev cigs	st
CHATZI	1		c	0	0	all	-	all	Eu:bal	1987	CC	282	n	bl	n	y	0	ev	all/unsp	1	45	0	0	nev any	st
CHATZI	2		c	0	0	all	-	all	Eu:bal	1987	CC	282	n	bl	n	y	0	ev	all/unsp	46	74	0	0	nev any	st
CHATZI	3		c	0	0	all	-	all	Eu:bal	1987	CC	282	n	bl	n	y	0	ev	all/unsp	75	99	0	6	nev any	st
CHEN2	3		m	0	0	all	-	all	As:Chi	1983	CC	193	n	ot	y	n	0	ev	all/unsp	1	9	1	1	nev any	st
CHEN2	4		m	0	0	all	-	all	As:Chi	1983	CC	193	n	ot	y	n	0	ev	all/unsp	10	20	2	0	nev any	st

Table 1G16 - 1

IESLC - Meta-anal of Ever Smoking (or Curr if Ever not avail) by Amount, Overview, Cigs (or Any Prod if Cigs not avail)
All LC types
Most adjusted

REF	NRR	1G11	SEX	AGEL	AGEH	RACE	YF	LC TYPE	LOC	START	ST	NLC	R	VB	P	H	AD	SM	PRODUCT	exL	exH	S1	S2	DENOM	De
CHEN2	5		m	0	0	all	-	all	As:Chi	1983	CC	193	n	ot	y	n	0	ev	all/unsp	21	30	0	4	nev any	st
CHEN2	6		m	0	0	all	-	all	As:Chi	1983	CC	193	n	ot	y	n	0	ev	all/unsp	31	99	3	0	nev any	st
CHEN2	7		f	0	0	all	-	all	As:Chi	1983	CC	193	n	ot	y	n	0	ev	all/unsp	1	9	1	1	nev any	st
CHEN2	8		f	0	0	all	-	all	As:Chi	1983	CC	193	n	ot	y	n	0	ev	all/unsp	10	20	2	0	nev any	st
CHEN2	9		f	0	0	all	-	all	As:Chi	1983	CC	193	n	ot	y	n	0	ev	all/unsp	21	30	0	4	nev any	st
CHEN2	10		f	0	0	all	-	all	As:Chi	1983	CC	193	n	ot	y	n	0	ev	all/unsp	31	99	3	0	nev any	st
CHOI	12		m	0	0	all	-	all	As:oth	1985	CC	375	n	bl	n	n	0	ev	cig+/-ot	1	10	1	0	nev cigs	st
CHOI	13		m	0	0	all	-	all	As:oth	1985	CC	375	n	bl	n	n	0	ev	cig+/-ot	11	20	2	3	nev cigs	st
CHOI	14		m	0	0	all	-	all	As:oth	1985	CC	375	n	bl	n	n	0	ev	cig+/-ot	21	30	0	4	nev cigs	st
CHOI	15		m	0	0	all	-	all	As:oth	1985	CC	375	n	bl	n	n	0	ev	cig+/-ot	31	40	0	5	nev cigs	st
CHOI	16		m	0	0	all	-	all	As:oth	1985	CC	375	n	bl	n	n	0	ev	cig+/-ot	41	99	3	6	nev cigs	st
CHOI	17		f	0	0	all	-	all	As:oth	1985	CC	375	n	bl	n	n	0	ev	cig+/-ot	1	10	1	0	nev cigs	st
CHOI	18		f	0	0	all	-	all	As:oth	1985	CC	375	n	bl	n	n	0	ev	cig+/-ot	11	30	2	0	nev cigs	st
CHOI	20		f	0	0	all	-	all	As:oth	1985	CC	375	n	bl	n	n	0	ev	cig+/-ot	31	99	3	0	nev cigs	st
CHOW	10		m	0	0	wh	0	all	NAmer	1966	pr	219	n	bl	n	n	2	cu	cig+/-ot	1	19	1	0	nev any	ot
CHOW	11		m	0	0	wh	0	all	NAmer	1966	pr	219	n	bl	n	n	2	cu	cig+/-ot	20	29	2	3	nev any	ot
CHOW	12		m	0	0	wh	0	all	NAmer	1966	pr	219	n	bl	n	n	2	cu	cig+/-ot	30	99	3	0	nev any	ot
COMSTO	4		m	0	0	all	-	all	NAmer	1975	ot	258	n	bl	n	n	0	cu	cig+/-ot	1	19	1	0	nev any	st
COMSTO	5		m	0	0	all	-	all	NAmer	1975	ot	258	n	bl	n	n	0	cu	cig+/-ot	20	39	2	0	nev any	st
COMSTO	6		m	0	0	all	-	all	NAmer	1975	ot	258	n	bl	n	n	0	cu	cig+/-ot	40	99	3	0	nev any	st
COMSTO	9		f	0	0	all	-	all	NAmer	1975	ot	258	n	bl	n	n	0	cu	cig+/-ot	1	19	1	0	nev any	st
COMSTO	10		f	0	0	all	-	all	NAmer	1975	ot	258	n	bl	n	n	0	cu	cig+/-ot	20	39	2	0	nev any	st
COMSTO	11		f	0	0	all	-	all	NAmer	1975	ot	258	n	bl	n	n	0	cu	cig+/-ot	40	99	3	0	nev any	st
COOKSO	1		c	0	0	bl	-	all	Africa	1961	CC	234	n	V	n	y	0	ev	cig+/-ot	1	9	1	1	nev any	st
COOKSO	2		c	0	0	bl	-	all	Africa	1961	CC	234	n	V	n	y	0	ev	cig+/-ot	10	99	0	0	nev any	st
CORREA	46		c	0	0	all	-	all	NAmer	1979	CC	1359	n	bl	y	n	1	cu	cig+/-ot	1	20	0	0	nev cigs	or
CORREA	50		c	0	0	all	-	all	NAmer	1979	CC	1359	n	bl	y	n	1	cu	cig+/-ot	21	99	3	0	nev cigs	or
CPSI	243		m	50	74	all	6	all	NAmer	1959	pr	5138	n	bl	n	n	1	ev	cig only	1	19	1	0	nev any	ot
CPSI	246		m	50	74	all	6	all	NAmer	1959	pr	5138	n	bl	n	n	1	ev	cig only	20	99	0	0	nev any	ot
CPSI	275		f	40	74	all	6	all	NAmer	1959	pr	5138	n	bl	n	n	1	cu	cig+/-ot	1	9	1	1	nev cigs	ot
CPSI	276		f	40	74	all	6	all	NAmer	1959	pr	5138	n	bl	n	n	1	cu	cig+/-ot	10	19	0	2	nev cigs	ot
CPSI	277		f	40	74	all	6	all	NAmer	1959	pr	5138	n	bl	n	n	1	cu	cig+/-ot	20	39	2	0	nev cigs	ot
CPSI	278		f	40	74	all	6	all	NAmer	1959	pr	5138	n	bl	n	n	1	cu	cig+/-ot	40	99	3	0	nev cigs	ot
CPSII	102		m	35	99	all	4	all	NAmer	1982	pr	3229	n	bl	n	n	1	ev	cig only	1	20	0	0	nev any	ot
CPSII	103		m	35	99	all	4	all	NAmer	1982	pr	3229	n	bl	n	n	1	ev	cig only	21	99	3	0	nev any	ot
CPSII	105		f	35	99	all	4	all	NAmer	1982	pr	3229	n	bl	n	n	1	ev	cig+/-ot	1	19	1	0	nev cigs	ot
CPSII	106		f	35	99	all	4	all	NAmer	1982	pr	3229	n	bl	n	n	1	ev	cig+/-ot	20	99	0	0	nev cigs	ot
DAMBER	6		m	0	0	all	-	all	Eu:Sca	1972	CC	579	n	bl	y	n	1	ev	cig only	1	7	1	1	nev any	ot
DAMBER	7		m	0	0	all	-	all	Eu:Sca	1972	CC	579	n	bl	y	n	1	ev	cig only	8	15	0	2	nev any	ot
DAMBER	8		m	0	0	all	-	all	Eu:Sca	1972	CC	579	n	bl	y	n	1	ev	cig only	16	25	2	3	nev any	ot
DAMBER	9		m	0	0	all	-	all	Eu:Sca	1972	CC	579	n	bl	y	n	1	ev	cig only	26	99	3	0	nev any	ot
DARBY	1		m	0	0	wh	-	all	Eu:UK	1988	CC	982	n	V	n	n	0	cu	cig+/-ot	1	14	1	0	nev any	st
DARBY	2		m	0	0	wh	-	all	Eu:UK	1988	CC	982	n	V	n	n	0	cu	cig+/-ot	15	24	2	3	nev any	st
DARBY	3		m	0	0	wh	-	all	Eu:UK	1988	CC	982	n	V	n	n	0	cu	cig+/-ot	25	99	3	0	nev any	st
DARBY	8		f	0	0	wh	-	all	Eu:UK	1988	CC	982	n	V	n	n	0	cu	cig+/-ot	1	14	1	0	nev any	st
DARBY	9		f	0	0	wh	-	all	Eu:UK	1988	CC	982	n	V	n	n	0	cu	cig+/-ot	15	24	2	3	nev any	st
DARBY	10		f	0	0	wh	-	all	Eu:UK	1988	CC	982	n	V	n	n	0	cu	cig+/-ot	25	99	3	0	nev any	st
DAVEYS	1		m	0	0	all	-	all	Eu:Ger	1930	CC	109	n	bl	y	n	0	ev	all/unsp	1	5	1	1	nev any	st
DAVEYS	2		m	0	0	all	-	all	Eu:Ger	1930	CC	109	n	bl	y	n	0	ev	all/unsp	6	10	0	2	nev any	st
DAVEYS	3		m	0	0	all	-	all	Eu:Ger	1930	CC	109	n	bl	y	n	0	ev	all/unsp	11	20	2	3	nev any	st
DAVEYS	4		m	0	0	all	-	all	Eu:Ger	1930	CC	109	n	bl	y	n	0	ev	all/unsp	21	99	3	0	nev any	st
DEAN	1		m	0	0	wh	-	all	Africa	1947	CC	603	n	V	y	n	0	ev	cig only	1	20	0	0	nev any	st
DEAN	2		m	0	0	wh	-	all	Africa	1947	CC	603	n	V	y	n	0	ev	cig only	25	45	3	0	nev any	st
DEAN	3		m	0	0	wh	-	all	Africa	1947	CC	603	n	V	y	n	0	ev	cig only	50	99	0	6	nev any	st
DEAN2	25		m	0	0	all	-	all	Eu:UK	1960	CC	954	n	V	y	n	0	ev	cig only	1	22	0	0	nev any	st
DEAN2	26		m	0	0	all	-	all	Eu:UK	1960	CC	954	n	V	y	n	0	ev	cig only	23	99	3	0	nev any	st
DEAN2	29		f	0	0	all	-	all	Eu:UK	1960	CC	954	n	V	y	n	0	ev	cig only	1	22	0	0	nev any	st
DEAN2	30		f	0	0	all	-	all	Eu:UK	1960	CC	954	n	V	y	n	0	ev	cig only	23	99	3	0	nev any	st
DEAN3	7		m	0	0	all	-	all	Eu:UK	1969	CC	766	n	V	y	n	3	cu	cig only	1	12	1	0	nev any	ot
DEAN3	14		m	0	0	all	-	all	Eu:UK	1969	CC	766	n	V	y	n	3	cu	cig only	13	22	2	3	nev any	ot
DEAN3	21		m	0	0	all	-	all	Eu:UK	1969	CC	766	n	V	y	n	3	cu	cig only	23	99	3	0	nev any	ot
DEAN3	91		f	0	0	all	-	all	Eu:UK	1969	CC	766	n	V	y	n	3	cu	cig only	1	12	1	0	nev any	ot
DEAN3	98		f	0	0	all	-	all	Eu:UK	1969	CC	766	n	V	y	n	3	cu	cig only	13	22	2	3	nev any	ot
DEAN3	105		f	0	0	all	-	all	Eu:UK	1969	CC	766	n	V	y	n	3	cu	cig only	23	99	3	0	nev any	ot
DEKLER	2		m	0	0	all	0	all	Auslia	1961	pr	138	m	V	n	n	2	cu	cig+/-ot	1	14	1	0	nev any	or
DEKLER	3		m	0	0	all	0	all	Auslia	1961	pr	138	m	V	n	n	2	cu	cig+/-ot	15	24	2	3	nev any	or
DEKLER	4		m	0	0	all	0	all	Auslia	1961	pr	138	m	V	n	n	2	cu	cig+/-ot	25	99	3	0	nev any	or
DESTEF	6		m	0	0	all	-	all	SCAmer	1988	CC	497	n	bl	n	y	4	ev	all/unsp	1	10	1	0	nev any	or
DESTEF	7		m	0	0	all	-	all	SCAmer	1988	CC	497	n	bl	n	y	4	ev	all/unsp	11	20	2	3	nev any	or
DESTEF	8		m	0	0	all	-	all	SCAmer	1988	CC	497	n	bl	n	y	4	ev	all/unsp	21	40	0	0	nev any	or
DESTEF	9		m	0	0	all	-	all	SCAmer	1988	CC	497	n	bl	n	y	4	ev	all/unsp	41	99	3	6	nev any	or
DOLL	1		m	0	0	all	-	all	Eu:UK	1948	CC	1465	n	V	n	n	0	ev	all/unsp	1	4	0	1	nev any	st

Table 1G16 - 1

IESLC - Meta-anal of Ever Smoking (or Curr if Ever not avail) by Amount, Overview, Cigs (or Any Prod if Cigs not avail)
All LC types
Most adjusted

REF	NRR	1G11	SEX	AGEL	AGEH	RACE	YF	LC TYPE	LOC	START	ST	NLC	R	VB	P	H	AD	SM	PRODUCT	exL	exH	S1	S2	DENOM	De
DOLL	2		m	0	0	all	-	all	Eu:UK	1948	CC	1465	n	V	n	n	0	ev	all/unsp	5	14	1	2	nev any	st
DOLL	3		m	0	0	all	-	all	Eu:UK	1948	CC	1465	n	V	n	n	0	ev	all/unsp	15	24	2	3	nev any	st
DOLL	4		m	0	0	all	-	all	Eu:UK	1948	CC	1465	n	V	n	n	0	ev	all/unsp	25	49	3	0	nev any	st
DOLL	5		m	0	0	all	-	all	Eu:UK	1948	CC	1465	n	V	n	n	0	ev	all/unsp	50	99	0	6	nev any	st
DOLL	7		f	0	0	all	-	all	Eu:UK	1948	CC	1465	n	V	n	n	0	ev	all/unsp	1	4	0	1	nev any	st
DOLL	8		f	0	0	all	-	all	Eu:UK	1948	CC	1465	n	V	n	n	0	ev	all/unsp	5	14	1	2	nev any	st
DOLL	9		f	0	0	all	-	all	Eu:UK	1948	CC	1465	n	V	n	n	0	ev	all/unsp	15	24	2	3	nev any	st
DOLL	10		f	0	0	all	-	all	Eu:UK	1948	CC	1465	n	V	n	n	0	ev	all/unsp	25	49	3	0	nev any	ot
DOLL2	46		m	35	99	all	5	all	Eu:UK	1951	pr	920	n	V	n	n	1	ev	all/unsp	1	14	1	0	nev any	ot
DOLL2	47		m	35	99	all	5	all	Eu:UK	1951	pr	920	n	V	n	n	1	ev	all/unsp	15	24	2	3	nev any	ot
DOLL2	48		m	35	99	all	5	all	Eu:UK	1951	pr	920	n	V	n	n	1	ev	all/unsp	25	99	3	0	nev any	ot
DOLL2	10		f	0	0	all	22	all	Eu:UK	1951	pr	920	n	V	n	n	1	cu	cig only	1	14	1	0	nev any	ot
DOLL2	11		f	0	0	all	22	all	Eu:UK	1951	pr	920	n	V	n	n	1	cu	cig only	15	24	2	3	nev any	ot
DOLL2	12		f	0	0	all	22	all	Eu:UK	1951	pr	920	n	V	n	n	1	cu	cig only	25	99	3	0	nev any	ot
DORANT	6		c	0	0	all	0	all	Eu:wst	1986	ot	550	n	bl	n	y	0	cu	cig+/-ot	1	9	1	1	nev any	st
DORANT	7		c	0	0	all	0	all	Eu:wst	1986	ot	550	n	bl	n	y	0	cu	cig+/-ot	10	19	0	2	nev any	st
DORANT	8		c	0	0	all	0	all	Eu:wst	1986	ot	550	n	bl	n	y	0	cu	cig+/-ot	20	99	0	0	nev any	st
DORGAN	108		m	0	0	wh	-	all	NAmer	1980	CC	2026	n	bl	y	y	2	ev	cig+/-ot	1	19	1	0	nev any	ot
DORGAN	109		m	0	0	wh	-	all	NAmer	1980	CC	2026	n	bl	y	y	2	ev	cig+/-ot	20	99	0	0	nev any	ot
DORGAN	96		f	0	0	all	-	all	NAmer	1980	CC	2026	n	bl	y	y	3	ev	cig+/-ot	1	19	1	0	nev any	ot
DORGAN	97		f	0	0	all	-	all	NAmer	1980	CC	2026	n	bl	y	y	3	ev	cig+/-ot	20	99	0	0	nev any	ot
DORN	408		m	0	0	wh	25	all	NAmer	1954	pr	5097	n	bl	n	n	1	cu	cig+/-ot	1	9	1	1	nev any	or
DORN	409		m	0	0	wh	25	all	NAmer	1954	pr	5097	n	bl	n	n	1	cu	cig+/-ot	10	20	2	0	nev any	or
DORN	410		m	0	0	wh	25	all	NAmer	1954	pr	5097	n	bl	n	n	1	cu	cig+/-ot	21	39	0	4	nev any	or
DORN	411		m	0	0	wh	25	all	NAmer	1954	pr	5097	n	bl	n	n	1	cu	cig+/-ot	40	99	3	0	nev any	or
DOSEME	5		m	0	0	all	-	all	Eu:bal	1979	CC	1210	n	bl	n	n	2	ev	cig+/-ot	1	10	1	0	nev cigs	or
DOSEME	9		m	0	0	all	-	all	Eu:bal	1979	CC	1210	n	bl	n	n	2	ev	cig+/-ot	11	20	2	3	nev cigs	or
DOSEME	13		m	0	0	all	-	all	Eu:bal	1979	CC	1210	n	bl	n	n	2	ev	cig+/-ot	21	99	3	0	nev cigs	or
DUNN	1		m	0	0	all	0	all	NAmer	1954	pr	139	o	bl	n	n	0	ev	cig+/-ot	1	4	0	1	nev cigs	st
DUNN	2		m	0	0	all	0	all	NAmer	1954	pr	139	o	bl	n	n	0	ev	cig+/-ot	5	14	1	2	nev cigs	st
DUNN	3		m	0	0	all	0	all	NAmer	1954	pr	139	o	bl	n	n	0	ev	cig+/-ot	15	24	2	3	nev cigs	st
DUNN	4		m	0	0	all	0	all	NAmer	1954	pr	139	o	bl	n	n	0	ev	cig+/-ot	25	34	0	4	nev cigs	st
DUNN	5		m	0	0	all	0	all	NAmer	1954	pr	139	o	bl	n	n	0	ev	cig+/-ot	35	99	3	0	nev cigs	st
EBELIN	2		m	0	0	all	-	all	Eu:Ger	1980	CC	130	n	bl	n	n	0	ev	all/unsp	1	9	1	1	nev any	st
EBELIN	3		m	0	0	all	-	all	Eu:Ger	1980	CC	130	n	bl	n	n	0	ev	all/unsp	10	19	0	2	nev any	st
EBELIN	4		m	0	0	all	-	all	Eu:Ger	1980	CC	130	n	bl	n	n	0	ev	all/unsp	20	29	2	3	nev any	st
EBELIN	5		m	0	0	all	-	all	Eu:Ger	1980	CC	130	n	bl	n	n	0	ev	all/unsp	30	39	0	4	nev any	st
EBELIN	6		m	0	0	all	-	all	Eu:Ger	1980	CC	130	n	bl	n	n	0	ev	all/unsp	40	99	3	0	nev any	st
ENGELA	31		m	0	0	all	0	all	Eu:Sca	1964	pr	435	n	bl	n	n	7	cu	cig+/-ot	1	4	0	1	nev cigs	or
ENGELA	32		m	0	0	all	0	all	Eu:Sca	1964	pr	435	n	bl	n	n	7	cu	cig+/-ot	5	9	1	0	nev cigs	or
ENGELA	33		m	0	0	all	0	all	Eu:Sca	1964	pr	435	n	bl	n	n	7	cu	cig+/-ot	10	14	0	2	nev cigs	or
ENGELA	34		m	0	0	all	0	all	Eu:Sca	1964	pr	435	n	bl	n	n	7	cu	cig+/-ot	15	19	0	0	nev cigs	or
ENGELA	35		m	0	0	all	0	all	Eu:Sca	1964	pr	435	n	bl	n	n	7	cu	cig+/-ot	20	99	0	0	nev cigs	or
ENGELA	45		f	0	0	all	0	all	Eu:Sca	1964	pr	435	n	bl	n	n	5	cu	cig+/-ot	1	4	0	1	nev cigs	or
ENGELA	46		f	0	0	all	0	all	Eu:Sca	1964	pr	435	n	bl	n	n	5	cu	cig+/-ot	5	9	1	0	nev cigs	or
ENGELA	47		f	0	0	all	0	all	Eu:Sca	1964	pr	435	n	bl	n	n	5	cu	cig+/-ot	10	14	0	2	nev cigs	or
ENGELA	48		f	0	0	all	0	all	Eu:Sca	1964	pr	435	n	bl	n	n	5	cu	cig+/-ot	15	99	0	0	nev cigs	or
ENSTRO	7		m	0	0	all	0	all	NAmer	1959	pr	2879	n	bl	n	n	1	cu	cig only	1	9	1	1	nev any	ot
ENSTRO	6		m	0	0	all	0	all	NAmer	1959	pr	2879	n	bl	n	n	1	cu	cig only	10	19	0	2	nev any	ot
ENSTRO	5		m	0	0	all	0	all	NAmer	1959	pr	2879	n	bl	n	n	1	cu	cig only	20	20	2	3	nev any	ot
ENSTRO	4		m	0	0	all	0	all	NAmer	1959	pr	2879	n	bl	n	n	1	cu	cig only	21	39	0	4	nev any	ot
ENSTRO	3		m	0	0	all	0	all	NAmer	1959	pr	2879	n	bl	n	n	1	cu	cig only	40	99	3	0	nev any	ot
ENSTRO	11		f	0	0	all	0	all	NAmer	1959	pr	2879	n	bl	n	n	1	cu	cig only	1	9	1	1	nev any	ot
ENSTRO	10		f	0	0	all	0	all	NAmer	1959	pr	2879	n	bl	n	n	1	cu	cig only	10	19	0	2	nev any	ot
ENSTRO	9		f	0	0	all	0	all	NAmer	1959	pr	2879	n	bl	n	n	1	cu	cig only	20	20	2	3	nev any	ot
ENSTRO	8		f	0	0	all	0	all	NAmer	1959	pr	2879	n	bl	n	n	1	cu	cig only	21	99	3	0	nev any	ot
ESAKI	1		m	0	0	all	-	all	As:Jap	1961	CC	245	n	bl	y	n	0	ev	cig+/-ot	1	14	1	0	nev cigs	st
ESAKI	2		m	0	0	all	-	all	As:Jap	1961	CC	245	n	bl	y	n	0	ev	cig+/-ot	15	29	2	3	nev cigs	st
ESAKI	3		m	0	0	all	-	all	As:Jap	1961	CC	245	n	bl	y	n	0	ev	cig+/-ot	30	99	3	0	nev cigs	st
FAN	6		m	0	0	all	-	all	As:Chi	1990	CC	403	n	ot	y	n	0	ev	cig+/-ot	1	9	1	1	nev cigs	st
FAN	7		m	0	0	all	-	all	As:Chi	1990	CC	403	n	ot	y	n	0	ev	cig+/-ot	10	19	0	2	nev cigs	st
FAN	8		m	0	0	all	-	all	As:Chi	1990	CC	403	n	ot	y	n	0	ev	cig+/-ot	20	29	2	3	nev cigs	st
FAN	9		m	0	0	all	-	all	As:Chi	1990	CC	403	n	ot	y	n	0	ev	cig+/-ot	30	99	3	0	nev cigs	st
FAN	10		f	0	0	all	-	all	As:Chi	1990	CC	403	n	ot	y	n	0	ev	cig+/-ot	1	9	1	1	nev cigs	st
FAN	11		f	0	0	all	-	all	As:Chi	1990	CC	403	n	ot	y	n	0	ev	cig+/-ot	10	19	0	2	nev cigs	st
FAN	12		f	0	0	all	-	all	As:Chi	1990	CC	403	n	ot	y	n	0	ev	cig+/-ot	20	29	2	3	nev cigs	st
FAN	13		f	0	0	all	-	all	As:Chi	1990	CC	403	n	ot	y	n	0	ev	cig+/-ot	30	99	3	0	nev cigs	st
GAO	21		f	0	0	all	-	all	As:Chi	1984	CC	1405	n	ot	n	n	2	ev	cig+/-ot	1	9	1	1	nev cigs	ot
GAO	22		f	0	0	all	-	all	As:Chi	1984	CC	1405	n	ot	n	n	2	ev	cig+/-ot	10	19	0	2	nev cigs	ot
GAO	23		f	0	0	all	-	all	As:Chi	1984	CC	1405	n	ot	n	n	2	ev	cig+/-ot	20	99	0	0	nev cigs	ot
GAO2	2		m	0	0	all	-	all	As:Jap	1988	CC	282	n	bl	n	n	0	cu	cig+/-ot	1	19	1	0	nev cigs	st
GAO2	3		m	0	0	all	-	all	As:Jap	1988	CC	282	n	bl	n	n	0	cu	cig+/-ot	20	29	2	3	nev cigs	st

Table 1G16 - 1

IESLC - Meta-anal of Ever Smoking (or Curr if Ever not avail) by Amount, Overview, Cigs (or Any Prod if Cigs not avail)
All LC types
Most adjusted

REF	NRR	1G11	SEX	AGEL	AGEH	RACE	YF	LC TYPE	LOC	START	ST	NLC	R	VB	P	H	AD	SM	PRODUCT	exL	exH	S1	S2	DENOM	De
GAO2	4		m	0	0	all	-	all	As:Jap	1988	CC	282	n	bl	n	n	0	cu	cig+/-ot	30	99	3	0	nev cigs	or
GARSHI	26		m	0	0	all	-	all	NAmer	1981	CC	1081	o	bl	y	n	1	ev	all/unsp	1	15	1	0	nev any	st
GARSHI	27		m	0	0	all	-	all	NAmer	1981	CC	1081	o	bl	y	n	1	ev	all/unsp	16	25	2	3	nev any	st
GARSHI	28		m	0	0	all	-	all	NAmer	1981	CC	1081	o	bl	y	n	1	ev	all/unsp	26	35	0	4	nev any	st
GARSHI	29		m	0	0	all	-	all	NAmer	1981	CC	1081	o	bl	y	n	1	ev	all/unsp	36	99	3	0	nev any	st
GER	22		c	0	0	all	-	all	As:oth	1990	CC	141	n	ot	y	n	14	ev	all/unsp	1	10	1	0	nev any	ot
GER	23		c	0	0	all	-	all	As:oth	1990	CC	141	n	ot	y	n	14	ev	all/unsp	11	20	2	3	nev any	ot
GER	24		c	0	0	all	-	all	As:oth	1990	CC	141	n	ot	y	n	14	ev	all/unsp	21	99	3	0	nev any	ot
GOLLED	1		m	35	99	all	-	all	Eu:UK	1952	CC	443	n	V	y	n	1	ev	cig only	1	10	1	0	nev any	ot
GOLLED	2		m	35	99	all	-	all	Eu:UK	1952	CC	443	n	V	y	n	1	ev	cig only	11	22	2	3	nev any	ot
GOLLED	3		m	35	99	all	-	all	Eu:UK	1952	CC	443	n	V	y	n	1	ev	cig only	23	99	3	0	nev any	ot
GSELL	1		m	0	0	all	-	all	Eu:wst	1937	CC	150	n	bl	n	y	0	ev	all/unsp	1	9	1	1	nev any	ot
GSELL	2		m	0	0	all	-	all	Eu:wst	1937	CC	150	n	bl	n	y	0	ev	all/unsp	10	14	0	2	nev any	st
GSELL	3		m	0	0	all	-	all	Eu:wst	1937	CC	150	n	bl	n	y	0	ev	all/unsp	15	20	2	3	nev any	st
GSELL	4		m	0	0	all	-	all	Eu:wst	1937	CC	150	n	bl	n	y	0	ev	all/unsp	21	35	0	4	nev any	st
GSELL	5		m	0	0	all	-	all	Eu:wst	1937	CC	150	n	bl	n	y	0	ev	all/unsp	36	99	3	0	nev any	st
HAENSZ	52		f	0	0	all	-	not alv	NAmer	1955	CC	158	n	bl	n	y	0	cu	cig+/-ot	1	20	0	0	nev any	st
HAENSZ	51		f	0	0	all	-	not alv	NAmer	1955	CC	158	n	bl	n	y	0	cu	cig+/-ot	21	99	3	0	nev any	st
HAMMO2	7		m	0	0	all	0	all	NAmer	1967	pr	450	o	bl	n	n	1	cu	cig+/-ot	1	19	1	0	nev any	ot
HAMMO2	6		m	0	0	all	0	all	NAmer	1967	pr	450	o	bl	n	n	1	cu	cig+/-ot	20	99	0	0	nev any	ot
HAMMON	153		m	0	0	wh	0	all	NAmer	1952	pr	448	n	bl	n	n	1	ev	cig only	1	9	1	1	nev any	ot
HAMMON	154		m	0	0	wh	0	all	NAmer	1952	pr	448	n	bl	n	n	1	ev	cig only	10	20	2	0	nev any	ot
HAMMON	155		m	0	0	wh	0	all	NAmer	1952	pr	448	n	bl	n	n	1	ev	cig only	21	99	3	0	nev any	ot
HANSEN	1		m	0	0	all	0	all	Eu:Sca	1968	pr	105	o	bl	y	n	2	ev	all/unsp	1	19	1	0	nev any	ot
HANSEN	2		m	0	0	all	0	all	Eu:Sca	1968	pr	105	o	bl	y	n	2	ev	all/unsp	20	99	0	0	nev any	ot
HIRAYA	23		m	0	0	all	0	all	As:Jap	1965	pr	1917	n	bl	n	n	1	cu	cig+/-ot	1	9	1	1	nev any	st
HIRAYA	24		m	0	0	all	0	all	As:Jap	1965	pr	1917	n	bl	n	n	1	cu	cig+/-ot	10	19	0	2	nev any	st
HIRAYA	25		m	0	0	all	0	all	As:Jap	1965	pr	1917	n	bl	n	n	1	cu	cig+/-ot	20	99	0	0	nev any	st
HIRAYA	26		f	0	0	all	0	all	As:Jap	1965	pr	1917	n	bl	n	n	1	cu	cig+/-ot	1	9	1	1	nev any	st
HIRAYA	27		f	0	0	all	0	all	As:Jap	1965	pr	1917	n	bl	n	n	1	cu	cig+/-ot	10	19	0	2	nev any	st
HIRAYA	28		f	0	0	all	0	all	As:Jap	1965	pr	1917	n	bl	n	n	1	cu	cig+/-ot	20	99	0	0	nev any	st
HITOSU	35		m	0	0	all	-	all	As:Jap	1960	CC	216	n	bl	y	n	1	cu	all/unsp	1	14	1	0	nev any	st
HITOSU	36		m	0	0	all	-	all	As:Jap	1960	CC	216	n	bl	y	n	1	cu	all/unsp	15	24	2	3	nev any	st
HITOSU	37		m	0	0	all	-	all	As:Jap	1960	CC	216	n	bl	y	n	1	cu	all/unsp	25	99	3	0	nev any	st
HITOSU	60		f	0	0	all	-	all	As:Jap	1960	CC	216	n	bl	y	n	1	cu	all/unsp	1	14	1	0	nev any	st
HITOSU	61		f	0	0	all	-	all	As:Jap	1960	CC	216	n	bl	y	n	1	cu	all/unsp	15	99	0	0	nev any	st
HOLE	1		m	0	0	all	0	all	Eu:UK	1972	pr	225	n	V	n	n	1	cu	cig+/-ot	1	14	1	0	nev any	ot
HOLE	3		m	0	0	all	0	all	Eu:UK	1972	pr	225	n	V	n	n	1	cu	cig+/-ot	15	24	2	3	nev any	ot
HOLE	4		m	0	0	all	0	all	Eu:UK	1972	pr	225	n	V	n	n	1	cu	cig+/-ot	25	34	0	4	nev any	ot
HOLE	5		m	0	0	all	0	all	Eu:UK	1972	pr	225	n	V	n	n	1	cu	cig+/-ot	35	99	3	0	nev any	ot
HU	1		m	0	0	all	-	all	As:Chi	1985	CC	227	n	ot	n	y	0	ev	cig+/-ot	1	14	1	0	nev any	st
HU	2		m	0	0	all	-	all	As:Chi	1985	CC	227	n	ot	n	y	0	ev	cig+/-ot	14	24	2	3	nev any	st
HU	3		m	0	0	all	-	all	As:Chi	1985	CC	227	n	ot	n	y	0	ev	cig+/-ot	25	99	3	0	nev any	st
HU	4		f	0	0	all	-	all	As:Chi	1985	CC	227	n	ot	n	y	0	ev	cig+/-ot	1	14	1	0	nev any	st
HU	5		f	0	0	all	-	all	As:Chi	1985	CC	227	n	ot	n	y	0	ev	cig+/-ot	14	24	2	3	nev any	st
HU	6		f	0	0	all	-	all	As:Chi	1985	CC	227	n	ot	n	y	0	ev	cig+/-ot	25	99	3	0	nev any	st
HU2	2		c	0	0	all	-	all	As:Chi	1977	CC	523	n	ot	y	n	0	ev	cig+/-ot	1	4	0	1	nev cigs	st
HU2	3		c	0	0	all	-	all	As:Chi	1977	CC	523	n	ot	y	n	0	ev	cig+/-ot	5	9	1	0	nev cigs	st
HU2	4		c	0	0	all	-	all	As:Chi	1977	CC	523	n	ot	y	n	0	ev	cig+/-ot	10	14	0	2	nev cigs	st
HU2	5		c	0	0	all	-	all	As:Chi	1977	CC	523	n	ot	y	n	0	ev	cig+/-ot	15	19	0	0	nev cigs	or
HU2	6		c	0	0	all	-	all	As:Chi	1977	CC	523	n	ot	y	n	0	ev	cig+/-ot	20	29	2	3	nev cigs	st
HU2	7		c	0	0	all	-	all	As:Chi	1977	CC	523	n	ot	y	n	0	ev	cig+/-ot	30	99	3	0	nev cigs	st
HUMBLE	2		m	0	0	w-hi	-	all	NAmer	1980	CC	521	n	bl	y	n	1	cu	cig+/-ot	1	19	1	0	nev cigs	or
HUMBLE	3		m	0	0	w-hi	-	all	NAmer	1980	CC	521	n	bl	y	n	1	cu	cig+/-ot	20	99	0	0	nev cigs	or
HUMBLE	5		m	0	0	hi	-	all	NAmer	1980	CC	521	n	bl	y	n	1	cu	cig+/-ot	1	19	1	0	nev cigs	or
HUMBLE	6		m	0	0	hi	-	all	NAmer	1980	CC	521	n	bl	y	n	1	cu	cig+/-ot	20	99	0	0	nev cigs	or
HUMBLE	8		f	0	0	w-hi	-	all	NAmer	1980	CC	521	n	bl	y	n	1	cu	cig+/-ot	1	19	1	0	nev cigs	or
HUMBLE	9		f	0	0	w-hi	-	all	NAmer	1980	CC	521	n	bl	y	n	1	cu	cig+/-ot	20	99	0	0	nev cigs	or
HUMBLE	11		f	0	0	hi	-	all	NAmer	1980	CC	521	n	bl	y	n	1	cu	cig+/-ot	1	19	1	0	nev cigs	or
HUMBLE	12		f	0	0	hi	-	all	NAmer	1980	CC	521	n	bl	y	n	1	cu	cig+/-ot	20	99	0	0	nev cigs	or
JARUP	4		m	0	0	all	-	all	Eu:Sca	1928	CC	102	o	bl	y	n	2	ev	all/unsp	1	10	1	0	nev any	or
JARUP	5		m	0	0	all	-	all	Eu:Sca	1928	CC	102	o	bl	y	n	2	ev	all/unsp	11	99	0	0	nev any	or
JEDRYC	45		m	0	0	all	-	all	Eu:est	1980	CC	1630	n	bl	y	n	4	ev	cig+/-ot	1	19	1	0	nev any	or
JEDRYC	46		m	0	0	all	-	all	Eu:est	1980	CC	1630	n	bl	y	n	4	ev	cig+/-ot	20	29	2	3	nev any	or
JEDRYC	47		m	0	0	all	-	all	Eu:est	1980	CC	1630	n	bl	y	n	4	ev	cig+/-ot	30	99	3	0	nev any	or
JEDRYC	48		f	0	0	all	-	all	Eu:est	1980	CC	1630	n	bl	y	n	4	ev	cig+/-ot	1	19	1	0	nev any	or
JEDRYC	49		f	0	0	all	-	all	Eu:est	1980	CC	1630	n	bl	y	n	4	ev	cig+/-ot	20	29	2	3	nev any	or
JEDRYC	50		f	0	0	all	-	all	Eu:est	1980	CC	1630	n	bl	y	n	4	ev	cig+/-ot	30	99	3	0	nev any	or
JOLY	7		m	0	0	all	-	all	SCAmer	1978	CC	826	n	bl	n	n	0	ev	cig+/-ot	1	9	1	1	nev any	st
JOLY	8		m	0	0	all	-	all	SCAmer	1978	CC	826	n	bl	n	n	0	ev	cig+/-ot	10	19	0	2	nev any	st
JOLY	9		m	0	0	all	-	all	SCAmer	1978	CC	826	n	bl	n	n	0	ev	cig+/-ot	20	29	2	3	nev any	st
JOLY	10		m	0	0	all	-	all	SCAmer	1978	CC	826	n	bl	n	n	0	ev	cig+/-ot	30	99	3	0	nev any	st

Table 1G16 - 1

IESLC - Meta-anal of Ever Smoking (or Curr if Ever not avail) by Amount, Overview, Cigs (or Any Prod if Cigs not avail)
All LC types
Most adjusted

REF	NRR	1G11	SEX	AGEL	AGEH	RACE	YF	LC TYPE	LOC	START	ST	NLC	R	VB	P	H	AD	SM	PRODUCT	exL	exH	S1	S2	DENOM	De
JOLY	3		f	0	0	all	-	all	SCAmer	1978	CC	826	n	bl	n	n	0	ev	cig+/-ot	1	9	1	1	nev any	st
JOLY	4		f	0	0	all	-	all	SCAmer	1978	CC	826	n	bl	n	n	0	ev	cig+/-ot	10	19	0	2	nev any	st
JOLY	5		f	0	0	all	-	all	SCAmer	1978	CC	826	n	bl	n	n	0	ev	cig+/-ot	20	29	2	3	nev any	st
JOLY	6		f	0	0	all	-	all	SCAmer	1978	CC	826	n	bl	n	n	0	ev	cig+/-ot	30	99	3	0	nev any	st
JUSSAW	34		m	0	0	all	-	all	As:Ind	1964	CC	792	n	V	n	n	2	ev	cig only	1	19	1	0	nev any	st
JUSSAW	35		m	0	0	all	-	all	As:Ind	1964	CC	792	n	V	n	n	2	ev	cig only	20	99	0	0	nev any	st
KAISE2	66		m	35	99	all	9	all	NAmer	1979	pr	318	n	bl	n	n	1	cu	cig only	1	19	1	0	nev any	st
KAISE2	67		m	35	99	all	9	all	NAmer	1979	pr	318	n	bl	n	n	1	cu	cig only	20	99	0	0	nev any	st
KAISE2	58		f	35	99	all	9	all	NAmer	1979	pr	318	n	bl	n	n	1	cu	cig only	1	19	1	0	nev any	st
KAISE2	59		f	35	99	all	9	all	NAmer	1979	pr	318	n	bl	n	n	1	cu	cig only	20	99	0	0	nev any	st
KAISER	6		m	0	0	all	0	all	NAmer	1964	pr	714	n	bl	n	n	2	cu	cig+/-ot	1	19	1	0	nev cigs	or
KAISER	7		m	0	0	all	0	all	NAmer	1964	pr	714	n	bl	n	n	2	cu	cig+/-ot	20	40	2	0	nev cigs	or
KAISER	8		m	0	0	all	0	all	NAmer	1964	pr	714	n	bl	n	n	2	cu	cig+/-ot	41	99	3	6	nev cigs	or
KAISER	2		f	0	0	all	0	all	NAmer	1964	pr	714	n	bl	n	n	2	cu	cig+/-ot	1	19	1	0	nev cigs	or
KAISER	3		f	0	0	all	0	all	NAmer	1964	pr	714	n	bl	n	n	2	cu	cig+/-ot	20	40	2	0	nev cigs	or
KAISER	4		f	0	0	all	0	all	NAmer	1964	pr	714	n	bl	n	n	2	cu	cig+/-ot	41	99	3	6	nev cigs	or
KANELL	26		m	0	0	all	-	all	Eu:bal	1950	CC	862	n	bl	n	n	1	cu	cig+/-ot	1	10	1	0	nev any	st
KANELL	27		m	0	0	all	-	all	Eu:bal	1950	CC	862	n	bl	n	n	1	cu	cig+/-ot	11	20	2	3	nev any	st
KANELL	28		m	0	0	all	-	all	Eu:bal	1950	CC	862	n	bl	n	n	1	cu	cig+/-ot	21	35	0	4	nev any	st
KANELL	29		m	0	0	all	-	all	Eu:bal	1950	CC	862	n	bl	n	n	1	cu	cig+/-ot	36	99	3	0	nev any	st
KATSOU	3		f	0	0	all	-	all	Eu:bal	1987	CC	101	n	bl	n	n	1	cu	all/unsp	1	20	0	0	nev any	or
KATSOU	4		f	0	0	all	-	all	Eu:bal	1987	CC	101	n	bl	n	n	1	cu	all/unsp	21	99	3	0	nev any	or
KAUFMA	11		c	0	0	all	-	all	NAmer	1981	CC	881	n	bl	n	n	6	cu	cig+/-ot	1	14	1	0	nev cigs	or
KAUFMA	12		c	0	0	all	-	all	NAmer	1981	CC	881	n	bl	n	n	6	cu	cig+/-ot	15	24	2	3	nev cigs	or
KAUFMA	13		c	0	0	all	-	all	NAmer	1981	CC	881	n	bl	n	n	6	cu	cig+/-ot	25	34	0	4	nev cigs	or
KAUFMA	14		c	0	0	all	-	all	NAmer	1981	CC	881	n	bl	n	n	6	cu	cig+/-ot	35	44	0	5	nev cigs	or
KAUFMA	15		c	0	0	all	-	all	NAmer	1981	CC	881	n	bl	n	n	6	cu	cig+/-ot	45	99	3	6	nev cigs	or
KHUDER	1		m	0	0	all	-	all	NAmer	1985	CC	482	n	bl	n	y	0	ev	cig+/-ot	1	19	1	0	nev cigs	st
KHUDER	2		m	0	0	all	-	all	NAmer	1985	CC	482	n	bl	n	y	0	ev	cig+/-ot	20	39	2	0	nev cigs	st
KHUDER	3		m	0	0	all	-	all	NAmer	1985	CC	482	n	bl	n	y	0	ev	cig+/-ot	40	99	3	0	nev cigs	st
KINLEN	14		m	0	0	all	0	all	Eu:UK	1967	pr	718	n	V	n	n	2	cu	cig+/-ot	1	14	1	0	nev any	ot
KINLEN	15		m	0	0	all	0	all	Eu:UK	1967	pr	718	n	V	n	n	2	cu	cig+/-ot	15	24	2	3	nev any	ot
KINLEN	16		m	0	0	all	0	all	Eu:UK	1967	pr	718	n	V	n	n	2	cu	cig+/-ot	25	99	3	0	nev any	ot
KNEKT	29		m	20	69	all	21	all	Eu:Sca	1966	pr	515	n	bl	n	n	1	cu	cig+/-ot	1	14	1	0	nev any	or
KNEKT	30		m	20	69	all	21	all	Eu:Sca	1966	pr	515	n	bl	n	n	1	cu	cig+/-ot	15	99	0	0	nev any	or
KOO	11		f	0	0	all	-	all	As:HK	1981	CC	200	n	bl	n	n	0	cu	all/unsp	1	10	1	0	nev any	st
KOO	12		f	0	0	all	-	all	As:HK	1981	CC	200	n	bl	n	n	0	cu	all/unsp	11	20	2	3	nev any	st
KOO	13		f	0	0	all	-	all	As:HK	1981	CC	200	n	bl	n	n	0	cu	all/unsp	21	30	0	4	nev any	st
KOULUM	6		m	0	0	all	-	all	Eu:Sca	1936	CC	812	n	bl	n	n	0	ev	all/unsp	1	9	1	1	nev any	st
KOULUM	5		m	0	0	all	-	all	Eu:Sca	1936	CC	812	n	bl	n	n	0	ev	all/unsp	10	19	0	2	nev any	st
KOULUM	4		m	0	0	all	-	all	Eu:Sca	1936	CC	812	n	bl	n	n	0	ev	all/unsp	20	99	0	0	nev any	st
KREUZE	19		m	1	45	all	-	all	Eu:Ger	1990	CC	2260	n	bl	n	n	3	ev	cig+/-ot	1	9	1	1	nev any	or
KREUZE	20		m	1	45	all	-	all	Eu:Ger	1990	CC	2260	n	bl	n	n	3	ev	cig+/-ot	10	19	0	2	nev any	or
KREUZE	21		m	1	45	all	-	all	Eu:Ger	1990	CC	2260	n	bl	n	n	3	ev	cig+/-ot	20	29	2	3	nev any	or
KREUZE	22		m	1	45	all	-	all	Eu:Ger	1990	CC	2260	n	bl	n	n	3	ev	cig+/-ot	30	99	3	0	nev any	or
KREUZE	30		m	55	69	all	-	all	Eu:Ger	1990	CC	2260	n	bl	n	n	3	ev	cig+/-ot	1	9	1	1	nev any	or
KREUZE	31		m	55	69	all	-	all	Eu:Ger	1990	CC	2260	n	bl	n	n	3	ev	cig+/-ot	10	19	0	2	nev any	or
KREUZE	32		m	55	69	all	-	all	Eu:Ger	1990	CC	2260	n	bl	n	n	3	ev	cig+/-ot	20	29	2	3	nev any	or
KREUZE	33		m	55	69	all	-	all	Eu:Ger	1990	CC	2260	n	bl	n	n	3	ev	cig+/-ot	30	99	3	0	nev any	or
KREUZE	25		f	1	45	all	-	all	Eu:Ger	1990	CC	2260	n	bl	n	n	3	ev	cig+/-ot	1	9	1	1	nev any	or
KREUZE	26		f	1	45	all	-	all	Eu:Ger	1990	CC	2260	n	bl	n	n	3	ev	cig+/-ot	10	19	0	2	nev any	or
KREUZE	27		f	1	45	all	-	all	Eu:Ger	1990	CC	2260	n	bl	n	n	3	ev	cig+/-ot	20	29	2	3	nev any	or
KREUZE	36		f	55	69	all	-	all	Eu:Ger	1990	CC	2260	n	bl	n	n	3	ev	cig+/-ot	1	9	1	1	nev any	or
KREUZE	37		f	55	69	all	-	all	Eu:Ger	1990	CC	2260	n	bl	n	n	3	ev	cig+/-ot	10	19	0	2	nev any	or
KREUZE	38		f	55	69	all	-	all	Eu:Ger	1990	CC	2260	n	bl	n	n	3	ev	cig+/-ot	20	29	2	3	nev any	or
KREYBE	9		m	0	0	all	-	all	Eu:Sca	1948	CC	300	n	bl	n	y	1	ev	all/unsp	1	14	1	0	nev any	ot
KREYBE	10		m	0	0	all	-	all	Eu:Sca	1948	CC	300	n	bl	n	y	1	ev	all/unsp	15	24	2	3	nev any	ot
KREYBE	11		m	0	0	all	-	all	Eu:Sca	1948	CC	300	n	bl	n	y	1	ev	all/unsp	25	99	3	0	nev any	ot
KREYBE	28		f	0	0	all	-	all	Eu:Sca	1948	CC	300	n	bl	n	y	1	ev	all/unsp	1	14	1	0	nev any	ot
KREYBE	29		f	0	0	all	-	all	Eu:Sca	1948	CC	300	n	bl	n	y	1	ev	all/unsp	15	99	0	0	nev any	ot
LAMTH	7		f	0	0	ch	-	all	As:HK	1983	CC	445	n	bl	n	n	0	ev	all/unsp	1	10	1	0	nev any	or
LAMTH	2		f	0	0	ch	-	all	As:HK	1983	CC	445	n	bl	n	n	0	ev	all/unsp	11	20	2	3	nev any	or
LAMTH	9		f	0	0	ch	-	all	As:HK	1983	CC	445	n	bl	n	n	0	ev	all/unsp	21	99	3	0	nev any	or
LAUSSM	18		m	0	0	all	-	all	Eu:Ger	1982	CC	432	n	bl	n	n	3	ev	all/unsp	1	9	1	1	nev any	ot
LAUSSM	19		m	0	0	all	-	all	Eu:Ger	1982	CC	432	n	bl	n	n	3	ev	all/unsp	10	19	0	2	nev any	ot
LAUSSM	20		m	0	0	all	-	all	Eu:Ger	1982	CC	432	n	bl	n	n	3	ev	all/unsp	20	99	0	0	nev any	ot
LETOUR	2		c	0	0	all	-	all	NAmer	1983	CC	738	n	V	y	y	0	ev	cig+/-ot	1	19	1	0	nev cigs	st
LETOUR	3		c	0	0	all	-	all	NAmer	1983	CC	738	n	V	y	y	0	ev	cig+/-ot	20	40	2	0	nev cigs	st
LETOUR	4		c	0	0	all	-	all	NAmer	1983	CC	738	n	V	y	y	0	ev	cig+/-ot	41	99	3	6	nev cigs	st
LIAW	3		c	0	0	all	0	all	As:oth	1982	pr	127	n	ot	n	n	2	cu	all/unsp	1	10	1	0	nev any	or
LIAW	4		c	0	0	all	0	all	As:oth	1982	pr	127	n	ot	n	n	2	cu	all/unsp	11	20	2	3	nev any	or
LIAW	5		c	0	0	all	0	all	As:oth	1982	pr	127	n	ot	n	n	2	cu	all/unsp	21	99	3	0	nev any	or

Table 1G16 - 1

IESLC - Meta-anal of Ever Smoking (or Curr if Ever not avail) by Amount, Overview, Cigs (or Any Prod if Cigs not avail)
All LC types
Most adjusted

REF	NRR	1G11	SEX	AGEL	AGEH	RACE	YF	LC TYPE	LOC	START	ST	NLC	R	VB	P	H	AD	SM	PRODUCT	exL	exH	S1	S2	DENOM	De
LIDDEL	2		m	0	0	all	18	all	NAmer	1970	pr	304	m	V	n	n	1	cu	cig+/-ot	1	19	1	0	nev cigs	ot
LIDDEL	3		m	0	0	all	18	all	NAmer	1970	pr	304	m	V	n	n	1	cu	cig+/-ot	20	99	0	0	nev cigs	ot
LIU2	8		m	0	0	all	-	all	As:Chi	1983	CC	316	n	ot	n	n	3	ev	all/unsp	1	19	1	0	nev any	or
LIU2	9		m	0	0	all	-	all	As:Chi	1983	CC	316	n	ot	n	n	3	ev	all/unsp	20	29	2	3	nev any	or
LIU2	10		m	0	0	all	-	all	As:Chi	1983	CC	316	n	ot	n	n	3	ev	all/unsp	30	99	3	0	nev any	or
LIU2	14		f	0	0	all	-	all	As:Chi	1983	CC	316	n	ot	n	n	3	ev	all/unsp	1	9	1	1	nev any	or
LIU2	15		f	0	0	all	-	all	As:Chi	1983	CC	316	n	ot	n	n	3	ev	all/unsp	10	19	0	2	nev any	or
LIU2	16		f	0	0	all	-	all	As:Chi	1983	CC	316	n	ot	n	n	3	ev	all/unsp	20	99	0	0	nev any	or
LIU3	6		m	0	0	all	-	all	As:Chi	1985	CC	110	n	ot	n	n	2	ev	all/unsp	1	15	1	0	nev any	or
LIU3	7		m	0	0	all	-	all	As:Chi	1985	CC	110	n	ot	n	n	2	ev	all/unsp	16	30	2	0	nev any	or
LIU3	8		m	0	0	all	-	all	As:Chi	1985	CC	110	n	ot	n	n	2	ev	all/unsp	31	99	3	0	nev any	or
LIU4	7		m	35	69	all	-	all	As:Chi	1986	CC	1000-00	n	ot	y	n	2	ev	cig only	1	19	1	0	nev any	ot
LIU4	8		m	35	69	all	-	all	As:Chi	1986	CC	1000-00	n	ot	y	n	2	ev	cig only	20	20	2	3	nev any	ot
LIU4	9		m	35	69	all	-	all	As:Chi	1986	CC	1000-00	n	ot	y	n	2	ev	cig only	21	99	3	0	nev any	ot
LIU5	2		c	0	0	all	-	all	As:Chi	1978	CC	111	n	ot	y	n	0	ev	all/unsp	1	9	1	1	nev any	st
LIU5	3		c	0	0	all	-	all	As:Chi	1978	CC	111	n	ot	y	n	0	ev	all/unsp	10	19	0	2	nev any	st
LIU5	4		c	0	0	all	-	all	As:Chi	1978	CC	111	n	ot	y	n	0	ev	all/unsp	20	99	0	0	nev any	st
LUBIN	11		m	0	0	all	-	all	As:Chi	1984	CC	427	m	ot	y	n	4	ev	cig only	1	6	1	1	nev any	ot
LUBIN	12		m	0	0	all	-	all	As:Chi	1984	CC	427	m	ot	y	n	4	ev	cig only	7	14	0	2	nev any	ot
LUBIN	13		m	0	0	all	-	all	As:Chi	1984	CC	427	m	ot	y	n	4	ev	cig only	15	19	0	0	nev any	ot
LUBIN	14		m	0	0	all	-	all	As:Chi	1984	CC	427	m	ot	y	n	4	ev	cig only	20	99	0	0	nev any	ot
LUBIN2	273		m	0	0	all	-	all	Eu:mul	1976	CC	7804	n	bl	n	y	0	ev	cig+/-ot	1	9	1	1	nev any	st
LUBIN2	274		m	0	0	all	-	all	Eu:mul	1976	CC	7804	n	bl	n	y	0	ev	cig+/-ot	10	19	0	2	nev any	st
LUBIN2	275		m	0	0	all	-	all	Eu:mul	1976	CC	7804	n	bl	n	y	0	ev	cig+/-ot	20	29	2	3	nev any	st
LUBIN2	276		m	0	0	all	-	all	Eu:mul	1976	CC	7804	n	bl	n	y	0	ev	cig+/-ot	30	99	3	0	nev any	st
LUBIN2	281		f	0	0	all	-	all	Eu:mul	1976	CC	7804	n	bl	n	y	0	ev	cig+/-ot	1	9	1	1	nev any	st
LUBIN2	282		f	0	0	all	-	all	Eu:mul	1976	CC	7804	n	bl	n	y	0	ev	cig+/-ot	10	19	0	2	nev any	st
LUBIN2	283		f	0	0	all	-	all	Eu:mul	1976	CC	7804	n	bl	n	y	0	ev	cig+/-ot	20	29	2	3	nev any	st
LUBIN2	284		f	0	0	all	-	all	Eu:mul	1976	CC	7804	n	bl	n	y	0	ev	cig+/-ot	30	99	3	0	nev any	st
MACLEN	36		c	0	0	ch	-	all	As:oth	1972	CC	233	n	bl	n	n	2	ev	cig+/-ot	1	9	1	1	nev cigs	or
MACLEN	37		c	0	0	ch	-	all	As:oth	1972	CC	233	n	bl	n	n	2	ev	cig+/-ot	10	19	0	2	nev cigs	or
MACLEN	38		c	0	0	ch	-	all	As:oth	1972	CC	233	n	bl	n	n	2	ev	cig+/-ot	20	29	2	3	nev cigs	or
MACLEN	39		c	0	0	ch	-	all	As:oth	1972	CC	233	n	bl	n	n	2	ev	cig+/-ot	30	99	3	0	nev cigs	or
MARTIS	1		m	0	0	all	-	all	Eu:UK	1972	CC	201	n	V	n	n	0	ev	cig+/-ot	1	14	1	0	nev cigs	st
MARTIS	2		m	0	0	all	-	all	Eu:UK	1972	CC	201	n	V	n	n	0	ev	cig+/-ot	15	24	2	3	nev cigs	st
MARTIS	3		m	0	0	all	-	all	Eu:UK	1972	CC	201	n	V	n	n	0	ev	cig+/-ot	25	99	3	0	nev cigs	st
MATOS	29		m	0	0	all	-	all	SCAmer	1994	CC	200	n	bl	n	n	2	ev	cig+/-ot	1	14	1	0	nev any	or
MATOS	31		m	0	0	all	-	all	SCAmer	1994	CC	200	n	bl	n	n	2	ev	cig+/-ot	15	24	2	3	nev any	or
MATOS	33		m	0	0	all	-	all	SCAmer	1994	CC	200	n	bl	n	n	2	ev	cig+/-ot	25	99	3	0	nev any	or
MATSUD	1		m	0	0	all	-	all	As:Jap	1965	CC	179	n	bl	n	n	0	ev	cig+/-ot	1	10	1	0	nev cigs	st
MATSUD	2		m	0	0	all	-	all	As:Jap	1965	CC	179	n	bl	n	n	0	ev	cig+/-ot	11	20	2	3	nev cigs	st
MATSUD	3		m	0	0	all	-	all	As:Jap	1965	CC	179	n	bl	n	n	0	ev	cig+/-ot	21	99	3	0	nev cigs	st
MCCONN	21	x	c	0	0	all	-	all	Eu:UK	1946	CC	100	n	V	n	y	0	ev	cig+/-ot	1	10	1	0	nev any	st
MCCONN	20	x	c	0	0	all	-	all	Eu:UK	1946	CC	100	n	V	n	y	0	ev	cig+/-ot	10	20	2	0	nev any	st
MCCONN	19	x	c	0	0	all	-	all	Eu:UK	1946	CC	100	n	V	n	y	0	ev	cig+/-ot	21	99	3	0	nev any	st
MIGRAN	2		m	0	0	all	0	all	Eu:UK	1964	pr	259	n	V	n	n	2	cu	cig only	1	9	1	1	nev any	ot
MIGRAN	4		m	0	0	all	0	all	Eu:UK	1964	pr	259	n	V	n	n	2	cu	cig only	10	19	0	2	nev any	ot
MIGRAN	6		m	0	0	all	0	all	Eu:UK	1964	pr	259	n	V	n	n	2	cu	cig only	20	20	2	3	nev any	ot
MIGRAN	8		m	0	0	all	0	all	Eu:UK	1964	pr	259	n	V	n	n	2	cu	cig only	21	99	3	0	nev any	ot
MIGRAN	29		f	0	0	all	0	all	Eu:UK	1964	pr	259	n	V	n	n	2	cu	cig only	1	9	1	1	nev any	ot
MIGRAN	31		f	0	0	all	0	all	Eu:UK	1964	pr	259	n	V	n	n	2	cu	cig only	10	19	0	2	nev any	ot
MIGRAN	33		f	0	0	all	0	all	Eu:UK	1964	pr	259	n	V	n	n	2	cu	cig only	20	20	2	3	nev any	ot
MRFITR	3		m	0	0	all	0	all	NAmer	1973	pr	119	n	bl	n	n	0	cu	cig+/-ot	1	19	1	0	nev cigs	ot
MRFITR	4		m	0	0	all	0	all	NAmer	1973	pr	119	n	bl	n	n	0	cu	cig+/-ot	20	39	2	0	nev cigs	ot
MRFITR	5		m	0	0	all	0	all	NAmer	1973	pr	119	n	bl	n	n	0	cu	cig+/-ot	40	99	3	0	nev cigs	ot
NAM	74		m	0	0	all	-	all	NAmer	1986	CC	1199	n	bl	y	n	1	cu	cig+/-ot	1	24	0	0	nev cigs	ot
NAM	75		m	0	0	all	-	all	NAmer	1986	CC	1199	n	bl	y	n	1	cu	cig+/-ot	25	99	3	0	nev cigs	ot
NAM	90		f	0	0	all	-	all	NAmer	1986	CC	1199	n	bl	y	n	1	cu	cig+/-ot	1	24	0	0	nev cigs	ot
NAM	91		f	0	0	all	-	all	NAmer	1986	CC	1199	n	bl	y	n	1	cu	cig+/-ot	25	99	3	0	nev cigs	ot
NOTAN2	8		m	0	0	all	-	all	As:Ind	1963	CC	683	n	V	n	n	0	ev	cig only	1	9	1	1	nev any	st
NOTAN2	9		m	0	0	all	-	all	As:Ind	1963	CC	683	n	V	n	n	0	ev	cig only	10	19	0	2	nev any	st
NOTAN2	10		m	0	0	all	-	all	As:Ind	1963	CC	683	n	V	n	n	0	ev	cig only	20	99	0	0	nev any	st
ORMOS	1		m	0	0	all	-	all	Eu:est	1947	CC	119	n	bl	y	y	0	ev	cig+/-ot	1	15	1	0	nev any	st
ORMOS	2		m	0	0	all	-	all	Eu:est	1947	CC	119	n	bl	y	y	0	ev	cig+/-ot	16	30	2	0	nev any	st
ORMOS	3		m	0	0	all	-	all	Eu:est	1947	CC	119	n	bl	y	y	0	ev	cig+/-ot	31	99	3	0	nev any	st
OSANN	49		m	0	0	all	-	all	NAmer	1984	CC	1986	n	bl	n	n	2	ev	cig+/-ot	1	39	0	0	nev cigs	or
OSANN	57		m	0	0	all	-	all	NAmer	1984	CC	1986	n	bl	n	n	2	ev	cig+/-ot	40	99	3	0	nev cigs	or
OSANN	50		f	0	0	all	-	all	NAmer	1984	CC	1986	n	bl	n	n	2	ev	cig+/-ot	1	39	0	0	nev cigs	or
OSANN	58		f	0	0	all	-	all	NAmer	1984	CC	1986	n	bl	n	n	2	ev	cig+/-ot	40	99	3	0	nev cigs	or

Table 1G16 - 1

IESLC - Meta-anal of Ever Smoking (or Curr if Ever not avail) by Amount, Overview, Cigs (or Any Prod if Cigs not avail)
All LC types
Most adjusted

REF	NRR	1G11	SEX	AGEL	AGEH	RACE	YF	LC TYPE	LOC	START	ST	NLC	R	VB	P	H	AD	SM	PRODUCT	exL	exH	S1	S2	DENOM	De
PARKIN	20	x	m	0	0	bl	-	all	Africa	1963	CC	877	n	V	y	n	0	cu	cig+/-ot	1	14	1	0	nev any	st
PARKIN	21	x	m	0	0	bl	-	all	Africa	1963	CC	877	n	V	y	n	0	cu	cig+/-ot	15	99	0	0	nev any	st
PASTOR	6		m	0	0	all	-	all	Eu:wst	1976	CC	204	n	bl	y	n	1	ev	all/unsp	1	9	1	1	nev any	st
PASTOR	7		m	0	0	all	-	all	Eu:wst	1976	CC	204	n	bl	y	n	1	ev	all/unsp	10	19	0	2	nev any	st
PASTOR	8		m	0	0	all	-	all	Eu:wst	1976	CC	204	n	bl	y	n	1	ev	all/unsp	20	29	2	3	nev any	st
PASTOR	9		m	0	0	all	-	all	Eu:wst	1976	CC	204	n	bl	y	n	1	ev	all/unsp	30	99	3	0	nev any	st
PERNU	17		m	0	0	all	-	all	Eu:Sca	1944	CC	1606	n	bl	n	n	0	ev	all/unsp	1	4	0	1	nev any	st
PERNU	18		m	0	0	all	-	all	Eu:Sca	1944	CC	1606	n	bl	n	n	0	ev	all/unsp	5	9	1	0	nev any	st
PERNU	19		m	0	0	all	-	all	Eu:Sca	1944	CC	1606	n	bl	n	n	0	ev	all/unsp	10	14	0	2	nev any	st
PERNU	20		m	0	0	all	-	all	Eu:Sca	1944	CC	1606	n	bl	n	n	0	ev	all/unsp	15	19	0	0	nev any	st
PERNU	21		m	0	0	all	-	all	Eu:Sca	1944	CC	1606	n	bl	n	n	0	ev	all/unsp	20	24	2	3	nev any	st
PERNU	22		m	0	0	all	-	all	Eu:Sca	1944	CC	1606	n	bl	n	n	0	ev	all/unsp	25	29	0	0	nev any	st
PERNU	23		m	0	0	all	-	all	Eu:Sca	1944	CC	1606	n	bl	n	n	0	ev	all/unsp	30	49	3	0	nev any	st
PERNU	24		m	0	0	all	-	all	Eu:Sca	1944	CC	1606	n	bl	n	n	0	ev	all/unsp	50	99	0	6	nev any	st
PERNU	11		f	0	0	all	-	all	Eu:Sca	1944	CC	1606	n	bl	n	n	0	ev	all/unsp	1	4	0	1	nev any	st
PERNU	12		f	0	0	all	-	all	Eu:Sca	1944	CC	1606	n	bl	n	n	0	ev	all/unsp	5	9	1	0	nev any	st
PERNU	13		f	0	0	all	-	all	Eu:Sca	1944	CC	1606	n	bl	n	n	0	ev	all/unsp	10	14	0	2	nev any	st
PERNU	14		f	0	0	all	-	all	Eu:Sca	1944	CC	1606	n	bl	n	n	0	ev	all/unsp	15	19	0	0	nev any	st
PERNU	15		f	0	0	all	-	all	Eu:Sca	1944	CC	1606	n	bl	n	n	0	ev	all/unsp	20	24	2	3	nev any	st
PERNU	16		f	0	0	all	-	all	Eu:Sca	1944	CC	1606	n	bl	n	n	0	ev	all/unsp	25	99	3	0	nev any	st
PERSH2	8		c	0	0	all	-	all	Eu:Sca	1980	CC	1022	n	bl	y	n	4	cu	all/unsp	1	9	1	1	nev any	ot
PERSH2	9		c	0	0	all	-	all	Eu:Sca	1980	CC	1022	n	bl	y	n	4	cu	all/unsp	10	99	0	0	nev any	ot
PETO	2		m	0	0	all	0	all	Eu:UK	1954	pr	103	n	V	n	n	0	cu	all/unsp	1	14	1	0	nev any	st
PETO	3		m	0	0	all	0	all	Eu:UK	1954	pr	103	n	V	n	n	0	cu	all/unsp	15	99	0	0	nev any	st
PEZZO2	3		m	0	0	all	-	all	SCAmer	1992	CC	367	n	bl	n	y	0	cu	cig+/-ot	1	20	0	0	nev cigs	st
PEZZO2	4		m	0	0	all	-	all	SCAmer	1992	CC	367	n	bl	n	y	0	cu	cig+/-ot	21	40	0	0	nev cigs	st
PEZZO2	5		m	0	0	all	-	all	SCAmer	1992	CC	367	n	bl	n	y	0	cu	cig+/-ot	41	99	3	6	nev cigs	st
PEZZOT	2		m	0	0	all	-	all	SCAmer	1987	CC	215	n	bl	n	y	0	cu	cig only	1	20	0	0	nev cigs	st
PEZZOT	3		m	0	0	all	-	all	SCAmer	1987	CC	215	n	bl	n	y	0	cu	cig only	21	40	0	0	nev cigs	st
PEZZOT	4		m	0	0	all	-	all	SCAmer	1987	CC	215	n	bl	n	y	0	cu	cig only	41	99	3	6	nev cigs	st
PIKE	1		m	0	0	w-hi	-	all	NAmer	1972	CC	731	n	bl	y	n	0	ev	all/unsp	1	20	0	0	nev any	st
PIKE	2		m	0	0	w-hi	-	all	NAmer	1972	CC	731	n	bl	y	n	0	ev	all/unsp	21	40	0	0	nev any	st
PIKE	3		m	0	0	w-hi	-	all	NAmer	1972	CC	731	n	bl	y	n	0	ev	all/unsp	41	99	3	6	nev any	st
PIKE	5		f	0	0	w-hi	-	all	NAmer	1972	CC	731	n	bl	y	n	0	ev	all/unsp	1	20	0	0	nev any	st
PIKE	6		f	0	0	w-hi	-	all	NAmer	1972	CC	731	n	bl	y	n	0	ev	all/unsp	21	40	0	0	nev any	st
PIKE	7		f	0	0	w-hi	-	all	NAmer	1972	CC	731	n	bl	y	n	0	ev	all/unsp	41	99	3	6	nev any	st
POLEDN	2		c	0	0	all	-	all	NAmer	1978	CC	209	n	bl	y	n	0	ev	cig+/-ot	1	19	1	0	nev cigs	st
POLEDN	4		c	0	0	all	-	all	NAmer	1978	CC	209	n	bl	y	n	0	ev	cig+/-ot	20	99	0	0	nev cigs	st
PRESCO	2		m	0	0	all	0	all	Eu:Sca	1964	pr	867	n	bl	n	n	1	cu	all/unsp	1	14	1	0	nev any	st
PRESCO	4		m	0	0	all	0	all	Eu:Sca	1964	pr	867	n	bl	n	n	1	cu	all/unsp	15	99	0	0	nev any	st
PRESCO	1		f	0	0	all	0	all	Eu:Sca	1964	pr	867	n	bl	n	n	1	cu	all/unsp	1	14	1	0	nev any	st
PRESCO	3		f	0	0	all	0	all	Eu:Sca	1964	pr	867	n	bl	n	n	1	cu	all/unsp	15	99	0	0	nev any	st
RACHTA	10		f	0	0	all	-	all	Eu:est	1991	CC	118	n	bl	n	y	1	ev	cig+/-ot	1	9	1	1	nev cigs	or
RACHTA	11		f	0	0	all	-	all	Eu:est	1991	CC	118	n	bl	n	y	1	ev	cig+/-ot	10	19	0	2	nev cigs	or
RACHTA	12		f	0	0	all	-	all	Eu:est	1991	CC	118	n	bl	n	y	1	ev	cig+/-ot	20	99	0	0	nev cigs	or
RANDIG	12	x	m	0	0	all	-	all	Eu:Ger	1951	CC	448	n	bl	n	n	0	ev	cig+/-ot	1	4	0	1	nev any	st
RANDIG	13	x	m	0	0	all	-	all	Eu:Ger	1951	CC	448	n	bl	n	n	0	ev	cig+/-ot	5	9	1	0	nev any	st
RANDIG	14	x	m	0	0	all	-	all	Eu:Ger	1951	CC	448	n	bl	n	n	0	ev	cig+/-ot	10	19	0	2	nev any	st
RANDIG	15	x	m	0	0	all	-	all	Eu:Ger	1951	CC	448	n	bl	n	n	0	ev	cig+/-ot	20	99	0	0	nev any	st
RANDIG	20	x	f	0	0	all	-	all	Eu:Ger	1951	CC	448	n	bl	n	n	0	ev	cig+/-ot	1	4	0	1	nev any	st
RANDIG	21	x	f	0	0	all	-	all	Eu:Ger	1951	CC	448	n	bl	n	n	0	ev	cig+/-ot	5	9	1	0	nev any	st
RANDIG	22	x	f	0	0	all	-	all	Eu:Ger	1951	CC	448	n	bl	n	n	0	ev	cig+/-ot	10	99	0	0	nev any	st
SEGI2	10		m	0	0	all	-	all	As:Jap	1962	CC	378	n	bl	n	n	1	cu	cig+/-ot	1	9	1	1	nev any	ot
SEGI2	12		m	0	0	all	-	all	As:Jap	1962	CC	378	n	bl	n	n	1	cu	cig+/-ot	10	19	0	2	nev any	ot
SEGI2	14		m	0	0	all	-	all	As:Jap	1962	CC	378	n	bl	n	n	1	cu	cig+/-ot	20	29	2	3	nev any	ot
SEGI2	16		m	0	0	all	-	all	As:Jap	1962	CC	378	n	bl	n	n	1	cu	cig+/-ot	30	39	0	4	nev any	ot
SEGI2	18		m	0	0	all	-	all	As:Jap	1962	CC	378	n	bl	n	n	1	cu	cig+/-ot	40	99	3	0	nev any	ot
SEGI2	22		f	0	0	all	-	all	As:Jap	1962	CC	378	n	bl	n	n	1	cu	cig+/-ot	1	9	1	1	nev any	ot
SEGI2	24		f	0	0	all	-	all	As:Jap	1962	CC	378	n	bl	n	n	1	cu	cig+/-ot	10	19	0	2	nev any	ot
SEGI2	26		f	0	0	all	-	all	As:Jap	1962	CC	378	n	bl	n	n	1	cu	cig+/-ot	20	99	0	0	nev any	ot
SHAW	10		c	0	0	wh	-	all	NAmer	1988	CC	335	n	V	n	y	0	ev	all/unsp	1	19	1	0	nev any	st
SHAW	11		c	0	0	wh	-	all	NAmer	1988	CC	335	n	V	n	y	0	ev	all/unsp	20	99	0	0	nev any	st
SIEMIA	13		m	0	0	all	-	all	NAmer	1979	CC	857	n	V	y	y	0	ev	cig+/-ot	1	19	1	0	nev cigs	or
SIEMIA	14		m	0	0	all	-	all	NAmer	1979	CC	857	n	V	y	y	0	ev	cig+/-ot	20	39	2	0	nev cigs	or
SIEMIA	15		m	0	0	all	-	all	NAmer	1979	CC	857	n	V	y	y	0	ev	cig+/-ot	40	99	3	0	nev cigs	or
SOBUE	117		m	0	0	all	-	q+s+l+a	As:Jap	1986	CC	1376	n	bl	n	y	0	cu	cig+/-ot	1	19	1	0	nev cigs	st
SOBUE	118		m	0	0	all	-	q+s+l+a	As:Jap	1986	CC	1376	n	bl	n	y	0	cu	cig+/-ot	20	29	2	3	nev cigs	st
SOBUE	119		m	0	0	all	-	q+s+l+a	As:Jap	1986	CC	1376	n	bl	n	y	0	cu	cig+/-ot	30	99	3	0	nev cigs	st
SPEIZE	1		f	0	0	all	0	all	NAmer	1976	pr	593	n	bl	n	y	1	cu	cig+/-ot	1	4	0	1	nev cigs	ot
SPEIZE	2		f	0	0	all	0	all	NAmer	1976	pr	593	n	bl	n	y	1	cu	cig+/-ot	5	14	1	2	nev cigs	ot
SPEIZE	3		f	0	0	all	0	all	NAmer	1976	pr	593	n	bl	n	y	1	cu	cig+/-ot	15	24	2	3	nev cigs	ot
SPEIZE	4		f	0	0	all	0	all	NAmer	1976	pr	593	n	bl	n	y	1	cu	cig+/-ot	25	34	0	4	nev cigs	ot

Table 1G16 - 1

IESLC - Meta-anal of Ever Smoking (or Curr if Ever not avail) by Amount, Overview, Cigs (or Any Prod if Cigs not avail)
All LC types
Most adjusted

REF	NRR	1G11	SEX	AGEL	AGEH	RACE	YF	LC TYPE	LOC	START	ST	NLC	R	VB	P	H	AD	SM	PRODUCT	exL	exH	S1	S2	DENOM	De
SPEIZE	5		f	0	0	all	0	all	NAmer	1976	pr	593	n	bl	n	y	1	cu	cig+/-ot	35	99	3	0	nev cigs	ot
SPITZ	5		c	0	0	b+hi	-	all	NAmer	1992	CC	177	n	bl	n	y	0	ev	cig+/-ot	1	19	1	0	nev cigs	st
SPITZ	6		c	0	0	b+hi	-	all	NAmer	1992	CC	177	n	bl	n	y	0	ev	cig+/-ot	20	99	0	0	nev cigs	st
STOCKS	41		m	0	0	all	-	all	Eu:UK	1952	CC	2932	n	V	y	n	2	ev	cig+/-ot	1	14	1	0	nev any	st
STOCKS	42		m	0	0	all	-	all	Eu:UK	1952	CC	2932	n	V	y	n	2	ev	cig+/-ot	15	21	2	3	nev any	st
STOCKS	43		m	0	0	all	-	all	Eu:UK	1952	CC	2932	n	V	y	n	2	ev	cig+/-ot	22	28	0	0	nev any	st
STOCKS	44		m	0	0	all	-	all	Eu:UK	1952	CC	2932	n	V	y	n	2	ev	cig+/-ot	29	36	0	4	nev any	st
STOCKS	45		m	0	0	all	-	all	Eu:UK	1952	CC	2932	n	V	y	n	2	ev	cig+/-ot	37	99	3	0	nev any	st
STOCKS	48		f	0	0	all	-	all	Eu:UK	1952	CC	2932	n	V	y	n	1	ev	cig+/-ot	1	14	1	0	nev any	ot
STOCKS	49		f	0	0	all	-	all	Eu:UK	1952	CC	2932	n	V	y	n	1	ev	cig+/-ot	15	99	0	0	nev any	ot
STOCKW	1		c	0	0	all	-	all	NAmer	1981	CC	22161	n	bl	n	n	0	cu	cig+/-ot	1	19	1	0	nev any	st
STOCKW	2		c	0	0	all	-	all	NAmer	1981	CC	22161	n	bl	n	n	0	cu	cig+/-ot	20	40	2	0	nev any	st
STOCKW	3		c	0	0	all	-	all	NAmer	1981	CC	22161	n	bl	n	n	0	cu	cig+/-ot	41	99	3	6	nev any	st
SVENSS	6		f	0	0	all	-	all	Eu:Sca	1983	CC	210	n	bl	n	n	1	cu	all/unsp	1	10	1	0	nev any	or
SVENSS	11		f	0	0	all	-	all	Eu:Sca	1983	CC	210	n	bl	n	n	1	cu	all/unsp	11	20	2	3	nev any	or
SVENSS	16		f	0	0	all	-	all	Eu:Sca	1983	CC	210	n	bl	n	n	1	cu	all/unsp	21	99	3	0	nev any	ot
TENKAN	10		m	0	0	all	17	all	Eu:Sca	1962	pr	242	n	bl	n	n	1	cu	all/unsp	1	14	1	0	nev any	ot
TENKAN	11		m	0	0	all	17	all	Eu:Sca	1962	pr	242	n	bl	n	n	1	cu	all/unsp	15	24	2	3	nev any	ot
TENKAN	12		m	0	0	all	17	all	Eu:Sca	1962	pr	242	n	bl	n	n	1	cu	all/unsp	25	99	3	0	nev any	ot
TIZZAN	7		m	0	0	all	-	all	Eu:wst	1959	CC	1358	n	bl	n	n	0	ev	cig only	1	9	1	1	nev any	st
TIZZAN	8		m	0	0	all	-	all	Eu:wst	1959	CC	1358	n	bl	n	n	0	ev	cig only	10	20	2	0	nev any	st
TIZZAN	9		m	0	0	all	-	all	Eu:wst	1959	CC	1358	n	bl	n	n	0	ev	cig only	21	40	0	0	nev any	st
TIZZAN	10		m	0	0	all	-	all	Eu:wst	1959	CC	1358	n	bl	n	n	0	ev	cig only	41	99	3	6	nev any	st
TIZZAN	15		f	0	0	all	-	all	Eu:wst	1959	CC	1358	n	bl	n	n	0	ev	cig only	1	9	1	1	nev any	st
TIZZAN	16		f	0	0	all	-	all	Eu:wst	1959	CC	1358	n	bl	n	n	0	ev	cig only	10	99	0	0	nev any	st
TSUGAN	29		m	0	0	all	-	q+a	As:Jap	1976	CC	134	n	bl	n	y	0	cu	all/unsp	1	15	1	0	nev any	st
TSUGAN	30		m	0	0	all	-	q+a	As:Jap	1976	CC	134	n	bl	n	y	0	cu	all/unsp	16	35	2	0	nev any	st
TSUGAN	31		m	0	0	all	-	q+a	As:Jap	1976	CC	134	n	bl	n	y	0	cu	all/unsp	36	99	3	0	nev any	st
TULINI	27		m	0	0	all	0	all	Eu:Sca	1967	pr	472	n	bl	n	n	3	cu	cig+/-ot	1	14	1	0	nev any	or
TULINI	28		m	0	0	all	0	all	Eu:Sca	1967	pr	472	n	bl	n	n	3	cu	cig+/-ot	15	24	2	3	nev any	or
TULINI	29		m	0	0	all	0	all	Eu:Sca	1967	pr	472	n	bl	n	n	3	cu	cig+/-ot	25	99	3	0	nev any	or
TULINI	32		f	0	0	all	0	all	Eu:Sca	1967	pr	472	n	bl	n	n	3	cu	cig+/-ot	1	14	1	0	nev any	or
TULINI	33		f	0	0	all	0	all	Eu:Sca	1967	pr	472	n	bl	n	n	3	cu	cig+/-ot	15	24	2	3	nev any	or
TULINI	34		f	0	0	all	0	all	Eu:Sca	1967	pr	472	n	bl	n	n	3	cu	cig+/-ot	25	99	3	0	nev any	or
TVERDA	9		m	0	0	all	0	all	Eu:Sca	1972	pr	238	n	bl	n	n	2	cu	cig only	1	9	1	1	nev cigs	ot
TVERDA	10		m	0	0	all	0	all	Eu:Sca	1972	pr	238	n	bl	n	n	2	cu	cig only	10	19	0	2	nev cigs	ot
TVERDA	11		m	0	0	all	0	all	Eu:Sca	1972	pr	238	n	bl	n	n	2	cu	cig only	20	99	0	0	nev cigs	ot
TVERDA	16		f	0	0	all	0	all	Eu:Sca	1972	pr	238	n	bl	n	n	2	cu	cig only	1	9	1	1	nev cigs	ot
TVERDA	17		f	0	0	all	0	all	Eu:Sca	1972	pr	238	n	bl	n	n	2	cu	cig only	20	99	0	0	nev cigs	ot
WAKAI	40		m	0	0	all	-	all	As:Jap	1988	CC	333	n	bl	n	y	2	cu	cig+/-ot	1	19	1	0	nev any	or
WAKAI	41		m	0	0	all	-	all	As:Jap	1988	CC	333	n	bl	n	y	2	cu	cig+/-ot	20	29	2	3	nev any	or
WAKAI	42		m	0	0	all	-	all	As:Jap	1988	CC	333	n	bl	n	y	2	cu	cig+/-ot	30	99	3	0	nev any	or
WANG2	9		c	0	0	all	-	all	As:Chi	1980	CC	103	n	ot	n	n	4	ev	cig+/-ot	1	4	0	1	nev cigs	ot
WANG2	10		c	0	0	all	-	all	As:Chi	1980	CC	103	n	ot	n	n	4	ev	cig+/-ot	5	9	1	0	nev cigs	ot
WANG2	11		c	0	0	all	-	all	As:Chi	1980	CC	103	n	ot	n	n	4	ev	cig+/-ot	10	14	0	2	nev cigs	ot
WANG2	12		c	0	0	all	-	all	As:Chi	1980	CC	103	n	ot	n	n	4	ev	cig+/-ot	15	19	0	0	nev cigs	ot
WANG2	13		c	0	0	all	-	all	As:Chi	1980	CC	103	n	ot	n	n	4	ev	cig+/-ot	20	29	2	3	nev cigs	ot
WANG2	14		c	0	0	all	-	all	As:Chi	1980	CC	103	n	ot	n	n	4	ev	cig+/-ot	30	39	0	4	nev cigs	ot
WANG2	15		c	0	0	all	-	all	As:Chi	1980	CC	103	n	ot	n	n	4	ev	cig+/-ot	40	99	3	0	nev cigs	ot
WU	43		f	0	0	wh	-	q+a	NAmer	1981	CC	220	n	bl	n	y	2	cu	all/unsp	1	20	0	0	nev any	st
WU	44		f	0	0	wh	-	q+a	NAmer	1981	CC	220	n	bl	n	y	2	cu	all/unsp	21	99	3	0	nev any	st
WUWILL	12		f	0	0	all	-	all	As:Chi	1985	CC	965	n	ot	n	n	3	ev	cig+/-ot	1	19	1	0	nev cigs	ot
WUWILL	13		f	0	0	all	-	all	As:Chi	1985	CC	965	n	ot	n	n	3	ev	cig+/-ot	20	99	0	0	nev cigs	ot
WYNDE2	17		m	0	0	all	-	all	NAmer	1962	CC	404	n	bl	n	y	0	ev	cig+/-ot	1	10	1	0	nev any	st
WYNDE2	18		m	0	0	all	-	all	NAmer	1962	CC	404	n	bl	n	y	0	ev	cig+/-ot	11	20	2	3	nev any	st
WYNDE2	19		m	0	0	all	-	all	NAmer	1962	CC	404	n	bl	n	y	0	ev	cig+/-ot	21	34	0	4	nev any	st
WYNDE2	20		m	0	0	all	-	all	NAmer	1962	CC	404	n	bl	n	y	0	ev	cig+/-ot	35	99	3	0	nev any	st
WYNDE3	44		m	0	0	all	-	all	NAmer	1966	CC	350	n	bl	n	y	0	ev	cig+/-ot	1	9	1	1	nev any	st
WYNDE3	45		m	0	0	all	-	all	NAmer	1966	CC	350	n	bl	n	y	0	ev	cig+/-ot	10	20	2	0	nev any	st
WYNDE3	46		m	0	0	all	-	all	NAmer	1966	CC	350	n	bl	n	y	0	ev	cig+/-ot	21	40	0	0	nev any	st
WYNDE3	47		m	0	0	all	-	all	NAmer	1966	CC	350	n	bl	n	y	0	ev	cig+/-ot	41	99	3	6	nev any	st
WYNDE3	79		f	0	0	all	-	all	NAmer	1966	CC	350	n	bl	n	y	0	ev	cig+/-ot	1	9	1	1	nev any	st
WYNDE3	80		f	0	0	all	-	all	NAmer	1966	CC	350	n	bl	n	y	0	ev	cig+/-ot	10	20	2	0	nev any	st
WYNDE3	81		f	0	0	all	-	all	NAmer	1966	CC	350	n	bl	n	y	0	ev	cig+/-ot	21	40	0	0	nev any	st
WYNDE3	82		f	0	0	all	-	all	NAmer	1966	CC	350	n	bl	n	y	0	ev	cig+/-ot	41	99	3	6	nev any	st
WYNDE4	43		m	0	0	all	-	all	NAmer	1948	CC	684	n	bl	y	n	0	ev	all/unsp	1	9	1	1	nev any	st
WYNDE4	44		m	0	0	all	-	all	NAmer	1948	CC	684	n	bl	y	n	0	ev	all/unsp	10	15	0	2	nev any	st
WYNDE4	45		m	0	0	all	-	all	NAmer	1948	CC	684	n	bl	y	n	0	ev	all/unsp	16	20	2	3	nev any	st
WYNDE4	46		m	0	0	all	-	all	NAmer	1948	CC	684	n	bl	y	n	0	ev	all/unsp	21	34	0	4	nev any	st
WYNDE4	47		m	0	0	all	-	all	NAmer	1948	CC	684	n	bl	y	n	0	ev	all/unsp	35	99	3	0	nev any	st
WYNDE4	57		f	0	0	all	-	all	NAmer	1948	CC	684	n	bl	y	n	2	ev	all/unsp	1	9	1	1	nev any	ot
WYNDE4	58		f	0	0	all	-	all	NAmer	1948	CC	684	n	bl	y	n	2	ev	all/unsp	10	15	0	2	nev any	ot

Table 1G16 - 1

IESLC - Meta-anal of Ever Smoking (or Curr if Ever not avail) by Amount, Overview, Cigs (or Any Prod if Cigs not avail)
All LC types
Most adjusted

REF	NRR	1G11	SEX	AGEL	AGEH	RACE	YF	LC TYPE	LOC	START	ST	NLC	R	VB	P	H	AD	SM	PRODUCT	exL	exH	S1	S2	DENOM	De
WYNDE4	59		f	0	0	all	-	all	NAmer	1948	CC	684	n	bl	y	n	2	ev	all/unsp	16	20	2	3	nev any	ot
WYNDE4	60		f	0	0	all	-	all	NAmer	1948	CC	684	n	bl	y	n	2	ev	all/unsp	21	34	0	4	nev any	ot
WYNDE4	61		f	0	0	all	-	all	NAmer	1948	CC	684	n	bl	y	n	2	ev	all/unsp	35	99	3	0	nev any	ot
WYNDE6	27		m	0	0	all	-	all	NAmer	1969	CC	4423	n	bl	n	y	0	cu	cig+/-ot	1	10	1	0	nev any	st
WYNDE6	36		m	0	0	all	-	all	NAmer	1969	CC	4423	n	bl	n	y	0	cu	cig+/-ot	11	20	2	3	nev any	st
WYNDE6	45		m	0	0	all	-	all	NAmer	1969	CC	4423	n	bl	n	y	0	cu	cig+/-ot	21	30	0	4	nev any	st
WYNDE6	54		m	0	0	all	-	all	NAmer	1969	CC	4423	n	bl	n	y	0	cu	cig+/-ot	31	99	3	0	nev any	st
WYNDE6	216		f	0	0	all	-	all	NAmer	1969	CC	4423	n	bl	n	y	0	cu	cig+/-ot	1	10	1	0	nev cigs	st
WYNDE6	225		f	0	0	all	-	all	NAmer	1969	CC	4423	n	bl	n	y	0	cu	cig+/-ot	11	20	2	3	nev cigs	st
WYNDE6	234		f	0	0	all	-	all	NAmer	1969	CC	4423	n	bl	n	y	0	cu	cig+/-ot	21	30	0	4	nev cigs	st
WYNDE6	243		f	0	0	all	-	all	NAmer	1969	CC	4423	n	bl	n	y	0	cu	cig+/-ot	30	99	3	0	nev cigs	st
XU3	9		m	0	0	all	-	all	As:Chi	1981	CC	135	n	ot	n	n	1	ev	all/unsp	1	9	1	1	nev any	ot
XU3	10		m	0	0	all	-	all	As:Chi	1981	CC	135	n	ot	n	n	1	ev	all/unsp	10	19	0	2	nev any	ot
XU3	11		m	0	0	all	-	all	As:Chi	1981	CC	135	n	ot	n	n	1	ev	all/unsp	20	29	2	3	nev any	ot
XU3	12		m	0	0	all	-	all	As:Chi	1981	CC	135	n	ot	n	n	1	ev	all/unsp	30	99	3	0	nev any	ot
XU3	16		f	0	0	all	-	all	As:Chi	1981	CC	135	n	ot	n	n	1	ev	all/unsp	1	9	1	1	nev any	ot
XU3	17		f	0	0	all	-	all	As:Chi	1981	CC	135	n	ot	n	n	1	ev	all/unsp	10	19	0	2	nev any	ot
XU3	18		f	0	0	all	-	all	As:Chi	1981	CC	135	n	ot	n	n	1	ev	all/unsp	20	99	0	0	nev any	ot
YAMAGU	8		c	0	0	all	-	all	As:Jap	1989	CC	144	n	bl	n	y	1	cu	all/unsp	1	20	0	0	nev any	or
YAMAGU	7		c	0	0	all	-	all	As:Jap	1989	CC	144	n	bl	n	y	1	cu	all/unsp	21	99	3	0	nev any	or
YUAN	2		m	0	0	all	0	all	As:Chi	1986	pr	142	n	ot	n	n	2	ev	cig+/-ot	1	19	1	0	nev cigs	ot
YUAN	3		m	0	0	all	0	all	As:Chi	1986	pr	142	n	ot	n	n	2	ev	cig+/-ot	20	99	0	0	nev cigs	ot
ZHENG	11		m	0	0	all	-	all	As:Chi	1982	CC	540	n	ot	*	y	0	ev	cig+/-ot	1	9	1	1	nev cigs	st
ZHENG	12		m	0	0	all	-	all	As:Chi	1982	CC	540	n	ot	*	y	0	ev	cig+/-ot	10	19	0	2	nev cigs	st
ZHENG	13		m	0	0	all	-	all	As:Chi	1982	CC	540	n	ot	*	y	0	ev	cig+/-ot	20	29	2	3	nev cigs	st
ZHENG	14		m	0	0	all	-	all	As:Chi	1982	CC	540	n	ot	*	y	0	ev	cig+/-ot	30	99	3	0	nev cigs	st
ZHENG	22		f	0	0	all	-	all	As:Chi	1982	CC	540	n	ot	*	y	0	ev	cig+/-ot	1	9	1	1	nev cigs	st
ZHENG	23		f	0	0	all	-	all	As:Chi	1982	CC	540	n	ot	*	y	0	ev	cig+/-ot	10	99	0	0	nev cigs	st
ZHOU	4		c	0	0	all	-	all	As:Chi	1978	CC	1360	n	ot	n	n	0	ev	all/unsp	1	9	1	1	nev any	st
ZHOU	5		c	0	0	all	-	all	As:Chi	1978	CC	1360	n	ot	n	n	0	ev	all/unsp	10	19	0	2	nev any	st
ZHOU	6		c	0	0	all	-	all	As:Chi	1978	CC	1360	n	ot	n	n	0	ev	all/unsp	20	99	0	0	nev any	st

Cigarette type is all/unspec for all RRs
except for the following:

REF	NRR	CIGTYPE
ALDERS	18	MC only
ALDERS	19	MC only
ALDERS	20	MC only
ALDERS	21	MC only
ALDERS	22	MC only
ALDERS	23	MC only
DEAN3	7	MC only
DEAN3	14	MC only
DEAN3	21	MC only
DEAN3	91	MC only
DEAN3	98	MC only
DEAN3	105	MC only
JUSSAW	34	MC only
JUSSAW	35	MC only
NOTAN2	8	MC only
NOTAN2	9	MC only
NOTAN2	10	MC only

REF	NRR	Cigarette equivalent
AGUDO	4	-
AGUDO	5	-
AKIBA	27	*
AKIBA	28	*
AKIBA	29	*
AKIBA	33	*
AKIBA	34	*
ALDERS	18	-
ALDERS	19	-
ALDERS	20	-
ALDERS	21	-
ALDERS	22	-
ALDERS	23	-
ARCHER	1	*

Table 1G16 - 1

IESLC - Meta-anal of Ever Smoking (or Curr if Ever not avail) by Amount, Overview, Cigs (or Any Prod if Cigs not avail)
All LC types
Most adjusted

REF	NRR	Cigarette equivalent
ARCHER	2	*
ARCHER	3	*
ARMADA	46	*
ARMADA	47	*
ARMADA	48	*
AUVINE	13	*
AUVINE	14	*
AUVINE	15	*
AXELSS	5	includes 1 g pipe tob = 1 cig
AXELSS	6	includes 1 g pipe tob = 1 cig
AXELSS	7	includes 1 g pipe tob = 1 cig
AXELSS	13	includes 1 g pipe tob = 1 cig
AXELSS	14	includes 1 g pipe tob = 1 cig
AXELSS	15	includes 1 g pipe tob = 1 cig
AXELSS	16	includes 1 g pipe tob = 1 cig
BARBON	82	*
BARBON	83	*
BARBON	84	*
BENSHL	11	*
BENSHL	12	*
BENSHL	13	*
BEST	13	-
BEST	14	-
BEST	15	-
BOUCOT	99	up to 1 pk cigs, 4 cigars or 10 pipes
BOUCOT	100	> 1 pk cigs, 4 cigars or 10 pipes
BRESLO	13	*
BRESLO	14	*
BRESLO	15	*
BRESLO	16	*
BRESLO	29	*
BRESLO	30	*
BRETT	1	*
BRETT	2	*
BRETT	3	*
BROSS	18	*
BROSS	19	*
BROWN2	32	*
BROWN2	42	*
BROWN2	31	*
BROWN2	41	*
BUFFLE	28	*
BUFFLE	29	*
BUFFLE	35	*
CEDERL	87	*
CEDERL	86	*
CEDERL	99	*
CHANG	2	*
CHANG	3	*
CHANG	4	*
CHANG	8	*
CHANG	9	*
CHANG	10	*
CHATZI	1	*
CHATZI	2	*
CHATZI	3	*
CHEN2	3	*
CHEN2	4	*
CHEN2	5	*
CHEN2	6	*
CHEN2	7	*
CHEN2	8	*
CHEN2	9	*
CHEN2	10	*
CHOI	12	*
CHOI	13	*
CHOI	14	*
CHOI	15	*
CHOI	16	*
CHOI	17	*
CHOI	18	*
CHOI	20	*

Table 1G16 - 1

IESLC - Meta-anal of Ever Smoking (or Curr if Ever not avail) by Amount, Overview, Cigs (or Any Prod if Cigs not avail)
All LC types
Most adjusted

REF	NRR	Cigarette equivalent
CHOW	10	*
CHOW	11	*
CHOW	12	*
COMSTO	4	*
COMSTO	5	*
COMSTO	6	*
COMSTO	9	*
COMSTO	10	*
COMSTO	11	*
COOKSO	1	*
COOKSO	2	*
CORREA	46	*
CORREA	50	*
CPSI	243	-
CPSI	246	-
CPSI	275	*
CPSI	276	*
CPSI	277	*
CPSI	278	*
CPSII	102	-
CPSII	103	-
CPSII	105	*
CPSII	106	*
DAMBER	6	-
DAMBER	7	-
DAMBER	8	-
DAMBER	9	-
DARBY	1	inc 1oz pipe/wk=2 cigs/d, excl cigar/llo
DARBY	2	inc 1oz pipe/wk=2 cigs/d, excl cigar/llo
DARBY	3	inc 1oz pipe/wk=2 cigs/d, excl cigar/llo
DARBY	8	inc 1oz pipe/wk=2 cigs/d, excl cigar/llo
DARBY	9	inc 1oz pipe/wk=2 cigs/d, excl cigar/llo
DARBY	10	inc 1oz pipe/wk=2 cigs/d, excl cigar/llo
DAVEYS	1	1 cigar or up to 5 cigs
DAVEYS	2	2 cigars or 6-10 cigs
DAVEYS	3	3-4 cigars or 11-20 cigs
DAVEYS	4	>4 cigars or >20 cigarettes
DEAN	1	-
DEAN	2	-
DEAN	3	-
DEAN2	25	-
DEAN2	26	-
DEAN2	29	-
DEAN2	30	-
DEAN3	7	-
DEAN3	14	-
DEAN3	21	-
DEAN3	91	-
DEAN3	98	-
DEAN3	105	-
DEKLER	2	*
DEKLER	3	*
DEKLER	4	*
DESTEF	6	*
DESTEF	7	*
DESTEF	8	*
DESTEF	9	*
DOLL	1	*
DOLL	2	*
DOLL	3	*
DOLL	4	*
DOLL	5	*
DOLL	7	*
DOLL	8	*
DOLL	9	*
DOLL	10	*
DOLL2	46	grams
DOLL2	47	grams
DOLL2	48	grams
DOLL2	10	-
DOLL2	11	-
DOLL2	12	-

Table 1G16 - 1

IESLC - Meta-anal of Ever Smoking (or Curr if Ever not avail) by Amount, Overview, Cigs (or Any Prod if Cigs not avail)
All LC types
Most adjusted

REF	NRR	Cigarette equivalent
DORANT	6	*
DORANT	7	*
DORANT	8	*
DORGAN	108	*
DORGAN	109	*
DORGAN	96	*
DORGAN	97	*
DORN	408	*
DORN	409	*
DORN	410	*
DORN	411	*
DOSEME	5	*
DOSEME	9	*
DOSEME	13	*
DUNN	1	*
DUNN	2	*
DUNN	3	*
DUNN	4	*
DUNN	5	*
EBELIN	2	*
EBELIN	3	*
EBELIN	4	*
EBELIN	5	*
EBELIN	6	*
ENGELA	31	*
ENGELA	32	*
ENGELA	33	*
ENGELA	34	*
ENGELA	35	*
ENGELA	45	*
ENGELA	46	*
ENGELA	47	*
ENGELA	48	*
ENSTRO	7	-
ENSTRO	6	-
ENSTRO	5	-
ENSTRO	4	-
ENSTRO	3	-
ENSTRO	11	-
ENSTRO	10	-
ENSTRO	9	-
ENSTRO	8	-
ESAKI	1	*
ESAKI	2	*
ESAKI	3	*
FAN	6	*
FAN	7	*
FAN	8	*
FAN	9	*
FAN	10	*
FAN	11	*
FAN	12	*
FAN	13	*
GAO	21	*
GAO	22	*
GAO	23	*
GAO2	2	*
GAO2	3	*
GAO2	4	*
GARSHI	26	*
GARSHI	27	*
GARSHI	28	*
GARSHI	29	*
GER	22	*
GER	23	*
GER	24	*
GOLLED	1	-
GOLLED	2	-
GOLLED	3	-
GSELL	1	inc cigar = 5, cheroot = 4, pipe = 2.5
GSELL	2	inc cigar = 5, cheroot = 4, pipe = 2.5
GSELL	3	inc cigar = 5, cheroot = 4, pipe = 2.5

Table 1G16 - 1

IESLC - Meta-anal of Ever Smoking (or Curr if Ever not avail) by Amount, Overview, Cigs (or Any Prod if Cigs not avail)
All LC types
Most adjusted

REF	NRR	Cigarette equivalent
GSELL	4	inc cigar = 5, cheroot = 4, pipe = 2.5
GSELL	5	inc cigar = 5, cheroot = 4, pipe = 2.5
HAENSZ	52	*
HAENSZ	51	*
HAMMO2	7	*
HAMMO2	6	*
HAMMON	153	-
HAMMON	154	-
HAMMON	155	-
HANSEN	1	cig equivalents (not defined)
HANSEN	2	cig equivalents (not defined)
HIRAYA	23	*
HIRAYA	24	*
HIRAYA	25	*
HIRAYA	26	*
HIRAYA	27	*
HIRAYA	28	*
HITOSU	35	*
HITOSU	36	*
HITOSU	37	*
HITOSU	60	*
HITOSU	61	*
HOLE	1	*
HOLE	3	*
HOLE	4	*
HOLE	5	*
HU	1	*
HU	2	*
HU	3	*
HU	4	*
HU	5	*
HU	6	*
HU2	2	*
HU2	3	*
HU2	4	*
HU2	5	*
HU2	6	*
HU2	7	*
HUMBLE	2	*
HUMBLE	3	*
HUMBLE	5	*
HUMBLE	6	*
HUMBLE	8	*
HUMBLE	9	*
HUMBLE	11	*
HUMBLE	12	*
JARUP	4	gms, inc 1 pk pipe/wk = 7/d, 1 cigar = 4
JARUP	5	gms, inc 1 pk pipe/wk = 7/d, 1 cigar = 4
JEDRYC	45	*
JEDRYC	46	*
JEDRYC	47	*
JEDRYC	48	*
JEDRYC	49	*
JEDRYC	50	*
JOLY	7	*
JOLY	8	*
JOLY	9	*
JOLY	10	*
JOLY	3	*
JOLY	4	*
JOLY	5	*
JOLY	6	*
JUSSAW	34	-
JUSSAW	35	-
KAISE2	66	-
KAISE2	67	-
KAISE2	58	-
KAISE2	59	-
KAISER	6	*
KAISER	7	*
KAISER	8	*
KAISER	2	*

Table 1G16 - 1

IESLC - Meta-anal of Ever Smoking (or Curr if Ever not avail) by Amount, Overview, Cigs (or Any Prod if Cigs not avail)
All LC types
Most adjusted

REF	NRR	Cigarette equivalent
KAISER	3	*
KAISER	4	*
KANELL	26	*
KANELL	27	*
KANELL	28	*
KANELL	29	*
KATSOU	3	*
KATSOU	4	*
KAUFMA	11	*
KAUFMA	12	*
KAUFMA	13	*
KAUFMA	14	*
KAUFMA	15	*
KHUDER	1	*
KHUDER	2	*
KHUDER	3	*
KINLEN	14	*
KINLEN	15	*
KINLEN	16	*
KNEKT	29	*
KNEKT	30	*
KOO	11	*
KOO	12	*
KOO	13	*
KOULUM	6	*
KOULUM	5	*
KOULUM	4	*
KREUZE	19	*
KREUZE	20	*
KREUZE	21	*
KREUZE	22	*
KREUZE	30	*
KREUZE	31	*
KREUZE	32	*
KREUZE	33	*
KREUZE	25	*
KREUZE	26	*
KREUZE	27	*
KREUZE	36	*
KREUZE	37	*
KREUZE	38	*
KREYBE	9	grams inc 1 cig=1
KREYBE	10	grams inc 1 cig=1
KREYBE	11	grams inc 1 cig=1
KREYBE	28	grams inc 1 cig=1
KREYBE	29	grams inc 1 cig=1
LAMTH	7	*
LAMTH	2	*
LAMTH	9	*
LAUSSM	18	inc cigars and pipes in grams
LAUSSM	19	inc cigars and pipes in grams
LAUSSM	20	inc cigars and pipes in grams
LETOUR	2	*
LETOUR	3	*
LETOUR	4	*
LIAW	3	*
LIAW	4	*
LIAW	5	*
LIDDEL	2	*
LIDDEL	3	*
LIU2	8	*
LIU2	9	*
LIU2	10	*
LIU2	14	*
LIU2	15	*
LIU2	16	*
LIU3	6	Converted from kg/month
LIU3	7	Converted from kg/month
LIU3	8	Converted from kg/month
LIU4	7	-
LIU4	8	-
LIU4	9	-

Table 1G16 - 1

IESLC - Meta-anal of Ever Smoking (or Curr if Ever not avail) by Amount, Overview, Cigs (or Any Prod if Cigs not avail)
All LC types
Most adjusted

REF	NRR	Cigarette equivalent
LIU5	2	*
LIU5	3	*
LIU5	4	*
LUBIN	11	-
LUBIN	12	-
LUBIN	13	-
LUBIN	14	-
LUBIN2	273	*
LUBIN2	274	*
LUBIN2	275	*
LUBIN2	276	*
LUBIN2	281	*
LUBIN2	282	*
LUBIN2	283	*
LUBIN2	284	*
MACLEN	36	*
MACLEN	37	*
MACLEN	38	*
MACLEN	39	*
MARTIS	1	*
MARTIS	2	*
MARTIS	3	*
MATOS	29	*
MATOS	31	*
MATOS	33	*
MATSUD	1	*
MATSUD	2	*
MATSUD	3	*
MCCONN	21	excl pipe by mixed smokers
MCCONN	20	excl pipe by mixed smokers
MCCONN	19	excl pipe by mixed smokers
MIGRAN	2	-
MIGRAN	4	-
MIGRAN	6	-
MIGRAN	8	-
MIGRAN	29	-
MIGRAN	31	-
MIGRAN	33	-
MRFITR	3	*
MRFITR	4	*
MRFITR	5	*
NAM	74	*
NAM	75	*
NAM	90	*
NAM	91	*
NOTAN2	8	-
NOTAN2	9	-
NOTAN2	10	-
ORMOS	1	*
ORMOS	2	*
ORMOS	3	*
OSANN	49	*
OSANN	57	*
OSANN	50	*
OSANN	58	*
PARKIN	20	*
PARKIN	21	*
PASTOR	6	*
PASTOR	7	*
PASTOR	8	*
PASTOR	9	*
PERNU	17	grams
PERNU	18	grams
PERNU	19	grams
PERNU	20	grams
PERNU	21	grams
PERNU	22	grams
PERNU	23	grams
PERNU	24	grams
PERNU	11	grams
PERNU	12	grams
PERNU	13	grams

Table 1G16 - 1

IESLC - Meta-anal of Ever Smoking (or Curr if Ever not avail) by Amount, Overview, Cigs (or Any Prod if Cigs not avail)
All LC types
Most adjusted

REF	NRR	Cigarette equivalent
PERNU	14	grams
PERNU	15	grams
PERNU	16	grams
PERSH2	8	inc 50g pipe/wk= 7 cigs/day
PERSH2	9	inc 50g pipe/wk= 7 cigs/day
PETO	2	*
PETO	3	*
PEZZO2	3	*
PEZZO2	4	*
PEZZO2	5	*
PEZZOT	2	-
PEZZOT	3	-
PEZZOT	4	-
PIKE	1	*
PIKE	2	*
PIKE	3	*
PIKE	5	*
PIKE	6	*
PIKE	7	*
POLEDN	2	*
POLEDN	4	*
PRESCO	2	gms, inc cig = 1, cheroot = 3, cigar = 5
PRESCO	4	gms, inc cig = 1, cheroot = 3, cigar = 5
PRESCO	1	gms, inc cig = 1, cheroot = 3, cigar = 5
PRESCO	3	gms, inc cig = 1, cheroot = 3, cigar = 5
RACHTA	10	*
RACHTA	11	*
RACHTA	12	*
RANDIG	12	inc 1g pipe=1, cigar=5,cheroot=4,cgllo=3
RANDIG	13	inc 1g pipe=1, cigar=5,cheroot=4,cgllo=3
RANDIG	14	inc 1g pipe=1, cigar=5,cheroot=4,cgllo=3
RANDIG	15	inc 1g pipe=1, cigar=5,cheroot=4,cgllo=3
RANDIG	20	inc 1g pipe=1, cigar=5,cheroot=4,cgllo=3
RANDIG	21	inc 1g pipe=1, cigar=5,cheroot=4,cgllo=3
RANDIG	22	inc 1g pipe=1, cigar=5,cheroot=4,cgllo=3
SEGI2	10	*
SEGI2	12	*
SEGI2	14	*
SEGI2	16	*
SEGI2	18	*
SEGI2	22	*
SEGI2	24	*
SEGI2	26	*
SHAW	10	*
SHAW	11	*
SIEMIA	13	*
SIEMIA	14	*
SIEMIA	15	*
SOBUE	117	*
SOBUE	118	*
SOBUE	119	*
SPEIZE	1	*
SPEIZE	2	*
SPEIZE	3	*
SPEIZE	4	*
SPEIZE	5	*
SPITZ	5	*
SPITZ	6	*
STOCKS	41	*
STOCKS	42	*
STOCKS	43	*
STOCKS	44	*
STOCKS	45	*
STOCKS	48	*
STOCKS	49	*
STOCKW	1	*
STOCKW	2	*
STOCKW	3	*
SVENSS	6	*
SVENSS	11	*
SVENSS	16	*
TENKAN	10	grams

Table 1G16 - 1

IESLC - Meta-anal of Ever Smoking (or Curr if Ever not avail) by Amount, Overview, Cigs (or Any Prod if Cigs not avail)
All LC types
Most adjusted

REF	NRR	Cigarette equivalent
TENKAN	11	grams
TENKAN	12	grams
TIZZAN	7	-
TIZZAN	8	-
TIZZAN	9	-
TIZZAN	10	-
TIZZAN	15	-
TIZZAN	16	-
TSUGAN	29	*
TSUGAN	30	*
TSUGAN	31	*
TULINI	27	*
TULINI	28	*
TULINI	29	*
TULINI	32	*
TULINI	33	*
TULINI	34	*
TVERDA	9	-
TVERDA	10	-
TVERDA	11	-
TVERDA	16	-
TVERDA	17	-
WAKAI	40	*
WAKAI	41	*
WAKAI	42	*
WANG2	9	*
WANG2	10	*
WANG2	11	*
WANG2	12	*
WANG2	13	*
WANG2	14	*
WANG2	15	*
WU	43	*
WU	44	*
WUWILL	12	*
WUWILL	13	*
WYNDE2	17	*
WYNDE2	18	*
WYNDE2	19	*
WYNDE2	20	*
WYNDE3	44	*
WYNDE3	45	*
WYNDE3	46	*
WYNDE3	47	*
WYNDE3	79	*
WYNDE3	80	*
WYNDE3	81	*
WYNDE3	82	*
WYNDE4	43	inc 1 cigar = 5 cigs, 1 pipe = 2.5 cigs
WYNDE4	44	inc 1 cigar = 5 cigs, 1 pipe = 2.5 cigs
WYNDE4	45	inc 1 cigar = 5 cigs, 1 pipe = 2.5 cigs
WYNDE4	46	inc 1 cigar = 5 cigs, 1 pipe = 2.5 cigs
WYNDE4	47	inc 1 cigar = 5 cigs, 1 pipe = 2.5 cigs
WYNDE4	57	inc 1 cigar = 5 cigs, 1 pipe = 2.5 cigs
WYNDE4	58	inc 1 cigar = 5 cigs, 1 pipe = 2.5 cigs
WYNDE4	59	inc 1 cigar = 5 cigs, 1 pipe = 2.5 cigs
WYNDE4	60	inc 1 cigar = 5 cigs, 1 pipe = 2.5 cigs
WYNDE4	61	inc 1 cigar = 5 cigs, 1 pipe = 2.5 cigs
WYNDE6	27	*
WYNDE6	36	*
WYNDE6	45	*
WYNDE6	54	*
WYNDE6	216	*
WYNDE6	225	*
WYNDE6	234	*
WYNDE6	243	*
XU3	9	*
XU3	10	*
XU3	11	*
XU3	12	*
XU3	16	*
XU3	17	*

Table 1G16 - 1

IESLC - Meta-anal of Ever Smoking (or Curr if Ever not avail) by Amount, Overview, Cigs (or Any Prod if Cigs not avail)
All LC types
Most adjusted

REF	NRR	Cigarette equivalent
XU3	18	*
YAMAGU	8	*
YAMAGU	7	*
YUAN	2	*
YUAN	3	*
ZHENG	11	*
ZHENG	12	*
ZHENG	13	*
ZHENG	14	*
ZHENG	22	*
ZHENG	23	*
ZHOU	4	*
ZHOU	5	*
ZHOU	6	*

In this overview table, subtotals and Qs values may be invalid and should be ignored

Table 1G16 - 2

IESLC - Meta-anal of Ever Smoking (or Curr if Ever not avail) by Amount, Overview, Cigs (or Any Prod if Cigs not avail)
All LC types
Most adjusted

REF	NRR	SEX	AD	Number Exposed Case	Number Exposed Cont	Non-exposed Case	Non-exposed Cont	RR	95.00%CI
AGUDO	4	f	3	-	-	-	-	1.57	(0.52- 4.70)
AGUDO	5	f	3	-	-	-	-	4.94	(1.86- 13.09)
Subtotal AGUDO								2.98	(1.44- 6.19)
*AKIBA	27	m	5	-	-	-	-	3.50	(2.20- 6.00)
*AKIBA	28	m	5	-	-	-	-	6.10	(3.90- 9.50)
*AKIBA	29	m	5	-	-	-	-	9.10	(5.40- 15.90)
*AKIBA	33	f	5	-	-	-	-	3.60	(2.60- 5.00)
*AKIBA	34	f	5	-	-	-	-	5.80	(3.30- 9.50)
Subtotal AKIBA								4.83	(3.96- 5.88)
ALDERS	18	m	1	-	-	-	-	3.55	(1.94- 6.49)
ALDERS	19	m	1	-	-	-	-	7.96	(4.63- 13.69)
ALDERS	20	m	1	-	-	-	-	8.52	(5.07- 14.33)
ALDERS	21	f	1	-	-	-	-	2.62	(1.88- 3.65)
ALDERS	22	f	1	-	-	-	-	5.28	(3.79- 7.36)
ALDERS	23	f	1	-	-	-	-	6.90	(4.69- 10.15)
Subtotal ALDERS								4.91	(4.15- 5.82)
*ARCHER	1	m	0	14	6504	6	9842	3.53	(1.36- 9.18)
*ARCHER	2	m	0	68	18320	6	9842	6.09	(2.64- 14.02)
*ARCHER	3	m	0	40	7705	6	9842	8.52	(3.61- 20.07)
Subtotal ARCHER								5.87	(3.54- 9.75)
ARMADA	46	m	0	44	117	4	64	6.02	(2.07- 17.51)
ARMADA	47	m	0	134	105	4	64	20.42	(7.20- 57.88)
ARMADA	48	m	0	139	32	4	64	69.50	(23.58- 204.81)
Subtotal ARMADA								20.25	(10.96- 37.40)
AUVINE	13	c	2	-	-	-	-	20.00	(9.72- 41.20)
AUVINE	14	c	2	-	-	-	-	33.90	(17.10- 67.00)
AUVINE	15	c	2	-	-	-	-	66.50	(25.80- 172.00)
Subtotal AUVINE								32.22	(20.76- 50.01)
AXELSS	5	m	6	-	-	-	-	3.82	(1.98- 7.36)
AXELSS	6	m	6	-	-	-	-	8.90	(4.91- 16.12)
AXELSS	7	m	6	-	-	-	-	10.40	(5.80- 18.66)
AXELSS	13	f	0	13	37	18	154	3.01	(1.35- 6.68)
AXELSS	14	f	0	63	50	18	154	10.78	(5.84- 19.91)
AXELSS	15	f	0	28	15	18	154	15.97	(7.21- 35.36)
AXELSS	16	f	0	6	7	18	154	7.33	(2.22- 24.22)
Subtotal AXELSS								7.81	(6.01- 10.15)
BARBON	82	m	3	-	-	-	-	6.70	(4.20- 11.00)
BARBON	83	m	3	-	-	-	-	12.80	(7.90- 21.00)
BARBON	84	m	3	-	-	-	-	21.30	(13.00- 36.00)
Subtotal BARBON								11.97	(9.01- 15.91)
*BENSHL	11	m	1	-	-	-	-	4.00	(1.55- 10.31)
*BENSHL	12	m	1	-	-	-	-	9.05	(3.91- 20.94)
*BENSHL	13	m	1	-	-	-	-	10.95	(4.76- 25.22)
Subtotal BENSHL								7.71	(4.67- 12.74)
*BEST	13	m	1	-	-	-	-	10.00	(4.56- 21.92)
*BEST	14	m	1	-	-	-	-	16.41	(7.73- 34.86)
*BEST	15	m	1	-	-	-	-	17.31	(7.93- 37.79)
Subtotal BEST								14.23	(9.11- 22.23)
*BOUCOT	99	m	0	38	2670	0	805	23.23~	(1.43- 377.62)
*BOUCOT	100	m	0	43	1519	0	805	46.12~	(2.84- 748.16)
Subtotal BOUCOT								32.74	(4.56- 235.00)
BRESLO	13	m	0	16	45	22	110	1.78	(0.86- 3.69)
BRESLO	14	m	0	69	105	22	110	3.29	(1.90- 5.69)
BRESLO	15	m	0	296	193	22	110	7.67	(4.69- 12.55)
BRESLO	16	m	0	80	22	22	110	18.18	(9.42- 35.09)
BRESLO	29	f	0	5	5	12	14	1.17	(0.27- 5.02)
BRESLO	30	f	0	8	6	12	14	1.56	(0.42- 5.76)
Subtotal BRESLO								5.03	(3.79- 6.65)
*BRETT	1	m	0	40	17090	6	6530	2.55	(1.08- 6.01)
*BRETT	2	m	0	62	15868	6	6530	4.25	(1.84- 9.83)
*BRETT	3	m	0	33	4490	6	6530	8.00	(3.35- 19.07)
Subtotal BRETT								4.40	(2.69- 7.21)
BROSS	18	m	0	170	155	38	170	4.91	(3.24- 7.42)
BROSS	19	m	0	95	59	38	170	7.20	(4.46- 11.63)
Subtotal BROSS								5.78	(4.23- 7.91)
BROWN2	32	m	2	-	-	-	-	6.10	(5.30- 6.90)
BROWN2	42	m	2	-	-	-	-	14.10	(12.70- 15.50)
BROWN2	31	f	2	-	-	-	-	8.40	(7.20- 9.70)
BROWN2	41	f	2	-	-	-	-	17.10	(15.30- 19.10)
Subtotal BROWN2								11.59	(10.92- 12.30)
BUFFLE	28	f	0	21	42	12	112	4.67	(2.11- 10.31)

Table 1G16 - 2

IESLC - Meta-anal of Ever Smoking (or Curr if Ever not avail) by Amount, Overview, Cigs (or Any Prod if Cigs not avail)
All LC types
Most adjusted

REF	NRR	SEX	AD	Number Exposed Case	Number Exposed Cont	Non-exposed Case	Non-exposed Cont	RR	95.00%CI
BUFFLE	29	f	0	76	60	12	112	11.82	(5.96- 23.45)
BUFFLE	35	f	0	141	62	12	112	21.23	(10.90- 41.32)
Subtotal BUFFLE								11.51	(7.65- 17.33)
*CEDERL	87	m	1	-	-	-	-	6.60	(2.93- 14.87)
*CEDERL	86	m	1	-	-	-	-	16.27	(6.52- 40.59)
*CEDERL	99	f	1	-	-	-	-	4.74	(1.95- 11.53)
Subtotal CEDERL								7.79	(4.72- 12.86)
*CHANG	2	m	0	5	100	5	502	5.02	(1.48- 17.02)
*CHANG	3	m	0	17	161	5	502	10.60	(3.97- 28.28)
*CHANG	4	m	0	13	158	5	502	8.26	(2.99- 22.81)
*CHANG	8	f	0	6	205	11	1139	3.03	(1.13- 8.10)
*CHANG	9	f	0	11	234	11	1139	4.87	(2.14- 11.09)
*CHANG	10	f	0	13	164	11	1139	8.21	(3.74- 18.01)
Subtotal CHANG								6.25	(4.26- 9.17)
CHATZI	1	c	0	68	127	27	129	2.56	(1.54- 4.25)
CHATZI	2	c	0	73	123	27	129	2.84	(1.71- 4.70)
CHATZI	3	c	0	114	115	27	129	4.74	(2.90- 7.72)
Subtotal CHATZI								3.28	(2.46- 4.38)
CHEN2	3	m	0	17	26	9	33	2.40	(0.92- 6.25)
CHEN2	4	m	0	44	50	9	33	3.23	(1.39- 7.48)
CHEN2	5	m	0	34	9	9	33	13.85	(4.89- 39.22)
CHEN2	6	m	0	26	12	9	33	7.94	(2.91- 21.72)
CHEN2	7	f	0	5	17	25	33	0.39	(0.13- 1.20)
CHEN2	8	f	0	22	10	25	33	2.90	(1.17- 7.22)
CHEN2	9	f	0	7	1	25	33	9.24	(1.07- 80.02)
CHEN2	10	f	0	4	2	25	33	2.64	(0.45- 15.58)
Subtotal CHEN2								3.36	(2.30- 4.90)
CHOI	12	m	0	20	90	13	95	1.62	(0.76- 3.46)
CHOI	13	m	0	144	281	13	95	3.74	(2.03- 6.92)
CHOI	14	m	0	50	49	13	95	7.46	(3.70- 15.03)
CHOI	15	m	0	37	39	13	95	6.93	(3.33- 14.44)
CHOI	16	m	0	16	6	13	95	19.49	(6.47- 58.71)
CHOI	17	f	0	9	16	76	164	1.21	(0.51- 2.87)
CHOI	18	f	0	7	9	76	164	1.68	(0.60- 4.68)
CHOI	20	f	0	3	1	76	164	6.47	(0.66- 63.26)
Subtotal CHOI								3.83	(2.86- 5.13)
*CHOW	10	m	2	-	-	-	-	13.88	(5.81- 33.12)
*CHOW	11	m	2	-	-	-	-	21.87	(9.35- 51.14)
*CHOW	12	m	2	-	-	-	-	44.48	(18.63- 106.19)
Subtotal CHOW								23.78	(14.45- 39.14)
COMSTO	4	m	0	18	25	4	69	12.42	(3.83- 40.26)
COMSTO	5	m	0	60	57	4	69	18.16	(6.22- 53.00)
COMSTO	6	m	0	26	18	4	69	24.92	(7.71- 80.57)
COMSTO	9	f	0	16	19	13	115	7.45	(3.10- 17.93)
COMSTO	10	f	0	51	26	13	115	17.35	(8.25- 36.48)
COMSTO	11	f	0	9	6	13	115	13.27	(4.07- 43.25)
Subtotal COMSTO								14.21	(9.49- 21.27)
COOKSO	1	c	0	102	27	45	61	5.12	(2.89- 9.08)
COOKSO	2	c	0	82	11	45	61	10.11	(4.83- 21.13)
Subtotal COOKSO								6.61	(4.21- 10.40)
CORREA	46	c	1	-	-	-	-	9.30	(6.80- 12.70)
CORREA	50	c	1	-	-	-	-	25.30	(18.50- 34.60)
Subtotal CORREA								15.32	(12.28- 19.11)
*CPSI	243	m	1	-	-	-	-	5.81	(4.33- 7.80)
*CPSI	246	m	1	-	-	-	-	13.60	(10.46- 17.67)
*CPSI	275	f	1	-	-	-	-	1.25	(0.73- 2.13)
*CPSI	276	f	1	-	-	-	-	2.44	(1.67- 3.56)
*CPSI	277	f	1	-	-	-	-	5.03	(3.82- 6.63)
*CPSI	278	f	1	-	-	-	-	11.10	(6.00- 20.53)
Subtotal CPSI								5.90	(5.14- 6.77)
*CPSII	102	m	1	-	-	-	-	9.99	(7.97- 12.51)
*CPSII	103	m	1	-	-	-	-	17.60	(14.05- 22.05)
*CPSII	105	f	1	-	-	-	-	4.16	(3.41- 5.09)
*CPSII	106	f	1	-	-	-	-	13.34	(11.31- 15.75)
Subtotal CPSII								9.98	(9.03- 11.02)
DAMBER	6	m	1	-	-	-	-	2.30	(1.30- 4.40)
DAMBER	7	m	1	-	-	-	-	7.30	(4.40- 12.70)
DAMBER	8	m	1	-	-	-	-	9.10	(5.50- 15.30)
DAMBER	9	m	1	-	-	-	-	14.90	(6.70- 33.50)
Subtotal DAMBER								6.60	(4.92- 8.86)
DARBY	1	m	0	128	223	3	384	73.47	(23.11- 233.57)

Table 1G16 - 2

IESLC - Meta-anal of Ever Smoking (or Curr if Ever not avail) by Amount, Overview, Cigs (or Any Prod if Cigs not avail)
All LC types
Most adjusted

REF	NRR	SEX	AD	Number Exposed Case	Number Exposed Cont	Non-exposed Case	Non-exposed Cont	RR	95.00%CI
DARBY	2	m	0	126	169	3	384	95.43	(29.94- 304.17)
DARBY	3	m	0	68	61	3	384	142.69	(43.52- 467.82)
DARBY	8	f	0	71	104	23	529	15.70	(9.38- 26.28)
DARBY	9	f	0	86	92	23	529	21.50	(12.90- 35.82)
DARBY	10	f	0	38	21	23	529	41.62	(21.15- 81.90)
Subtotal DARBY								29.10	(21.80- 38.84)
DAVEYS	1	m	0	11	69	3	23	1.22	(0.31- 4.77)
DAVEYS	2	m	0	31	32	3	23	7.43	(2.02- 27.27)
DAVEYS	3	m	0	19	22	3	23	6.62	(1.72- 25.56)
DAVEYS	4	m	0	29	21	3	23	10.59	(2.81- 39.94)
Subtotal DAVEYS								5.12	(2.63- 9.98)
DEAN	1	m	0	73	168	12	61	2.21	(1.12- 4.35)
DEAN	2	m	0	228	172	12	61	6.74	(3.52- 12.91)
DEAN	3	m	0	102	45	12	61	11.52	(5.66- 23.47)
Subtotal DEAN								5.46	(3.69- 8.08)
DEAN2	25	m	0	377	396	33	112	3.23	(2.14- 4.88)
DEAN2	26	m	0	252	112	33	112	7.64	(4.88- 11.95)
DEAN2	29	f	0	44	24	88	121	2.52	(1.43- 4.45)
DEAN2	30	f	0	18	5	88	121	4.95	(1.77- 13.84)
Subtotal DEAN2								4.21	(3.25- 5.45)
DEAN3	7	m	3	-	-	-	-	5.46	(3.27- 9.10)
DEAN3	14	m	3	-	-	-	-	7.42	(4.56- 12.06)
DEAN3	21	m	3	-	-	-	-	21.66	(12.78- 36.72)
DEAN3	91	f	3	-	-	-	-	3.16	(1.92- 5.21)
DEAN3	98	f	3	-	-	-	-	8.42	(5.14- 13.78)
DEAN3	105	f	3	-	-	-	-	24.24	(13.08- 44.93)
Subtotal DEAN3								8.44	(6.83- 10.42)
*DEKLER	2	m	2	-	-	-	-	19.40	(2.60- 143.70)
*DEKLER	3	m	2	-	-	-	-	23.00	(3.20- 167.60)
*DEKLER	4	m	2	-	-	-	-	32.50	(4.40- 241.20)
Subtotal DEKLER								24.38	(7.70- 77.17)
DESTEF	6	m	4	-	-	-	-	2.90	(1.60- 5.00)
DESTEF	7	m	4	-	-	-	-	8.40	(5.20- 13.60)
DESTEF	8	m	4	-	-	-	-	10.40	(6.40- 16.90)
DESTEF	9	m	4	-	-	-	-	23.70	(13.40- 42.10)
Subtotal DESTEF								8.87	(6.83- 11.51)
DOLL	1	m	0	55	129	7	61	3.72	(1.60- 8.64)
DOLL	2	m	0	489	570	7	61	7.48	(3.39- 16.50)
DOLL	3	m	0	475	431	7	61	9.60	(4.35- 21.22)
DOLL	4	m	0	293	154	7	61	16.58	(7.40- 37.13)
DOLL	5	m	0	38	12	7	61	27.60	(9.99- 76.25)
DOLL	7	f	0	16	25	40	59	0.94	(0.45- 1.99)
DOLL	8	f	0	24	18	40	59	1.97	(0.95- 4.09)
DOLL	9	f	0	14	6	40	59	3.44	(1.22- 9.71)
DOLL	10	f	0	14	0	40	59	42.60~	(2.47- 734.58)
Subtotal DOLL								4.97	(3.72- 6.65)
*DOLL2	46	m	1	-	-	-	-	6.71	(0.91- 49.81)
*DOLL2	47	m	1	-	-	-	-	12.29	(1.67- 90.41)
*DOLL2	48	m	1	-	-	-	-	23.71	(3.25- 173.24)
*DOLL2	10	f	1	-	-	-	-	1.29	(0.14- 11.50)
*DOLL2	11	f	1	-	-	-	-	6.43	(1.81- 22.78)
*DOLL2	12	f	1	-	-	-	-	29.71	(9.46- 93.32)
Subtotal DOLL2								11.39	(5.93- 21.87)
DORANT	6	c	0	21	192	14	1090	8.52	(4.26- 17.04)
DORANT	7	c	0	143	409	14	1090	27.22	(15.54- 47.68)
DORANT	8	c	0	128	275	14	1090	36.24	(20.55- 63.91)
Subtotal DORANT								22.68	(16.05- 32.04)
DORGAN	108	m	2	-	-	-	-	6.88	(3.74- 12.66)
DORGAN	109	m	2	-	-	-	-	14.00	(7.81- 25.11)
DORGAN	96	f	3	-	-	-	-	5.67	(4.36- 7.36)
DORGAN	97	f	3	-	-	-	-	12.22	(9.31- 16.04)
Subtotal DORGAN								8.47	(7.13- 10.07)
*DORN	408	m	1	-	-	-	-	4.02	(3.43- 4.71)
*DORN	409	m	1	-	-	-	-	9.92	(8.84- 11.14)
*DORN	410	m	1	-	-	-	-	17.19	(15.28- 19.33)
*DORN	411	m	1	-	-	-	-	22.75	(19.63- 26.37)
Subtotal DORN								11.88	(11.13- 12.68)
DOSEME	5	m	2	-	-	-	-	2.20	(1.40- 3.30)
DOSEME	9	m	2	-	-	-	-	3.10	(2.30- 4.10)
DOSEME	13	m	2	-	-	-	-	6.60	(4.40- 10.20)
Subtotal DOSEME								3.44	(2.79- 4.24)

Table 1G16 - 2

IESLC - Meta-anal of Ever Smoking (or Curr if Ever not avail) by Amount, Overview, Cigs (or Any Prod if Cigs not avail)
All LC types
Most adjusted

REF	NRR	SEX	AD	Number Exposed Case	Cont	Non-exposed Case	Cont	RR	95.00%CI
*DUNN	1	m	0	3	2538	2	14160	8.37	(1.40- 50.06)
*DUNN	2	m	0	12	9418	2	14160	9.02	(2.02- 40.30)
*DUNN	3	m	0	75	27720	2	14160	19.16	(4.70- 78.00)
*DUNN	4	m	0	32	9017	2	14160	25.13	(6.02- 104.82)
*DUNN	5	m	0	13	3206	2	14160	28.71	(6.48- 127.15)
Subtotal DUNN								16.88	(8.61- 33.09)
EBELIN	2	m	0	20	72	12	117	2.71	(1.25- 5.87)
EBELIN	3	m	0	19	26	12	117	7.13	(3.08- 16.48)
EBELIN	4	m	0	47	37	12	117	12.39	(5.95- 25.80)
EBELIN	5	m	0	5	3	12	117	16.25	(3.45- 76.54)
EBELIN	6	m	0	4	4	12	117	9.75	(2.16- 44.04)
Subtotal EBELIN								6.99	(4.62- 10.59)
*ENGELA	31	m	7	-	-	-	-	1.40	(0.60- 3.70)
*ENGELA	32	m	7	-	-	-	-	4.10	(1.70- 10.00)
*ENGELA	33	m	7	-	-	-	-	7.00	(2.90- 17.00)
*ENGELA	34	m	7	-	-	-	-	11.00	(4.20- 28.00)
*ENGELA	35	m	7	-	-	-	-	15.00	(6.10- 37.00)
*ENGELA	45	f	5	-	-	-	-	12.00	(4.50- 32.00)
*ENGELA	46	f	5	-	-	-	-	12.00	(4.40- 30.00)
*ENGELA	47	f	5	-	-	-	-	24.00	(9.50- 59.00)
*ENGELA	48	f	5	-	-	-	-	26.00	(9.20- 73.00)
Subtotal ENGELA								9.05	(6.63- 12.35)
*ENSTRO	7	m	1	-	-	-	-	4.74	(3.34- 6.73)
*ENSTRO	6	m	1	-	-	-	-	7.68	(5.95- 9.90)
*ENSTRO	5	m	1	-	-	-	-	13.65	(10.88- 17.13)
*ENSTRO	4	m	1	-	-	-	-	16.08	(12.77- 20.23)
*ENSTRO	3	m	1	-	-	-	-	19.41	(15.22- 24.75)
*ENSTRO	11	f	1	-	-	-	-	2.15	(1.62- 2.84)
*ENSTRO	10	f	1	-	-	-	-	4.31	(3.56- 5.22)
*ENSTRO	9	f	1	-	-	-	-	9.48	(8.04- 11.18)
*ENSTRO	8	f	1	-	-	-	-	16.47	(13.74- 19.75)
Subtotal ENSTRO								9.31	(8.66- 10.02)
ESAKI	1	m	0	47	75	16	28	1.10	(0.54- 2.24)
ESAKI	2	m	0	74	58	16	28	2.23	(1.10- 4.51)
ESAKI	3	m	0	34	10	16	28	5.95	(2.34- 15.16)
Subtotal ESAKI								2.12	(1.36- 3.29)
FAN	6	m	0	13	121	36	236	0.70	(0.36- 1.38)
FAN	7	m	0	53	171	36	236	2.03	(1.27- 3.24)
FAN	8	m	0	111	183	36	236	3.98	(2.61- 6.07)
FAN	9	m	0	39	23	36	236	11.12	(5.96- 20.73)
FAN	10	f	0	17	48	69	320	1.64	(0.89- 3.03)
FAN	11	f	0	30	37	69	320	3.76	(2.18- 6.50)
FAN	12	f	0	31	12	69	320	11.98	(5.86- 24.50)
FAN	13	f	0	4	1	69	320	18.55	(2.04- 168.54)
Subtotal FAN								3.29	(2.68- 4.05)
GAO	21	f	2	-	-	-	-	1.74	(1.22- 2.48)
GAO	22	f	2	-	-	-	-	3.04	(2.02- 4.58)
GAO	23	f	2	-	-	-	-	13.18	(7.04- 24.68)
Subtotal GAO								2.91	(2.28- 3.73)
GAO2	2	m	0	32	41	13	56	3.36	(1.57- 7.19)
GAO2	3	m	0	77	44	13	56	7.54	(3.71- 15.30)
GAO2	4	m	0	74	30	13	56	10.63	(5.08- 22.22)
Subtotal GAO2								6.57	(4.30- 10.04)
GARSHI	26	m	1	-	-	-	-	3.29	(2.22- 4.87)
GARSHI	27	m	1	-	-	-	-	5.72	(4.04- 8.10)
GARSHI	28	m	1	-	-	-	-	7.69	(5.20- 11.37)
GARSHI	29	m	1	-	-	-	-	5.24	(3.61- 7.60)
Subtotal GARSHI								5.28	(4.38- 6.37)
GER	22	c	14	-	-	-	-	1.26	(0.61- 2.61)
GER	23	c	14	-	-	-	-	1.90	(0.98- 3.70)
GER	24	c	14	-	-	-	-	3.00	(1.40- 6.40)
Subtotal GER								1.90	(1.26- 2.88)
GOLLED	1	m	1	-	-	-	-	4.45	(2.54- 7.81)
GOLLED	2	m	1	-	-	-	-	6.37	(3.67- 11.03)
GOLLED	3	m	1	-	-	-	-	18.28	(10.52- 31.74)
Subtotal GOLLED								8.08	(5.87- 11.13)
GSELL	1	m	0	11	36	2	29	4.43	(0.91- 21.60)
GSELL	2	m	0	10	37	2	29	3.92	(0.80- 19.30)
GSELL	3	m	0	27	26	2	29	15.06	(3.26- 69.59)
GSELL	4	m	0	49	9	2	29	78.94	(15.95- 390.82)
GSELL	5	m	0	51	13	2	29	56.88	(11.99- 269.87)

Table 1G16 - 2

IESLC - Meta-anal of Ever Smoking (or Curr if Ever not avail) by Amount, Overview, Cigs (or Any Prod if Cigs not avail)
All LC types
Most adjusted

REF	NRR	SEX	AD	Number Exposed Case	Cont	Non-exposed Case	Cont	RR	95.00%CI
Subtotal GSELL								16.45	(8.14- 33.23)
HAENSZ	52	f	0	40	66	81	236	1.77	(1.11- 2.82)
HAENSZ	51	f	0	23	13	81	236	5.15	(2.50- 10.65)
Subtotal HAENSZ								2.42	(1.63- 3.58)
*HAMMO2	7	m	1	-	-	-	-	9.15	(3.62- 23.12)
*HAMMO2	6	m	1	-	-	-	-	10.39	(4.28- 25.22)
Subtotal HAMMO2								9.78	(5.15- 18.56)
*HAMMON	153	m	1	-	-	-	-	7.38	(3.72- 14.63)
*HAMMON	154	m	1	-	-	-	-	8.32	(4.66- 14.84)
*HAMMON	155	m	1	-	-	-	-	17.06	(9.44- 30.82)
Subtotal HAMMON								10.42	(7.31- 14.85)
*HANSEN	1	m	2	-	-	-	-	1.37	(0.63- 3.54)
*HANSEN	2	m	2	-	-	-	-	2.90	(1.14- 8.27)
Subtotal HANSEN								1.89	(0.99- 3.63)
*HIRAYA	23	m	1	-	-	-	-	2.06	(1.49- 2.85)
*HIRAYA	24	m	1	-	-	-	-	4.00	(3.20- 4.99)
*HIRAYA	25	m	1	-	-	-	-	6.24	(5.07- 7.68)
*HIRAYA	26	f	1	-	-	-	-	2.25	(1.64- 3.08)
*HIRAYA	27	f	1	-	-	-	-	2.56	(1.85- 3.54)
*HIRAYA	28	f	1	-	-	-	-	4.47	(2.73- 7.33)
Subtotal HIRAYA								3.72	(3.32- 4.17)
HITOSU	35	m	1	-	-	-	-	2.08	(0.90- 4.83)
HITOSU	36	m	1	-	-	-	-	2.82	(1.25- 6.36)
HITOSU	37	m	1	-	-	-	-	4.68	(1.97- 11.11)
HITOSU	60	f	1	-	-	-	-	3.11	(1.77- 5.46)
HITOSU	61	f	1	-	-	-	-	3.17	(1.07- 9.36)
Subtotal HITOSU								3.05	(2.16- 4.32)
*HOLE	1	m	1	-	-	-	-	5.47	(2.35- 12.75)
*HOLE	3	m	1	-	-	-	-	8.90	(4.12- 19.23)
*HOLE	4	m	1	-	-	-	-	10.75	(4.80- 24.06)
*HOLE	5	m	1	-	-	-	-	7.49	(2.99- 18.77)
Subtotal HOLE								8.03	(5.31- 12.17)
HU	1	m	0	36	38	41	67	1.55	(0.85- 2.82)
HU	2	m	0	55	43	41	67	2.09	(1.20- 3.65)
HU	3	m	0	29	13	41	67	3.65	(1.70- 7.80)
HU	4	f	0	19	10	40	48	2.28	(0.95- 5.46)
HU	5	f	0	6	6	40	48	1.20	(0.36- 4.01)
HU	6	f	0	1	2	40	48	0.60	(0.05- 6.86)
Subtotal HU								2.02	(1.47- 2.77)
HU2	2	c	0	16	33	121	213	0.85	(0.45- 1.61)
HU2	3	c	0	44	58	121	213	1.34	(0.85- 2.10)
HU2	4	c	0	65	59	121	213	1.94	(1.28- 2.94)
HU2	5	c	0	64	54	121	213	2.09	(1.36- 3.19)
HU2	6	c	0	149	87	121	213	3.01	(2.13- 4.26)
HU2	7	c	0	64	19	121	213	5.93	(3.39- 10.37)
Subtotal HU2								2.20	(1.83- 2.64)
HUMBLE	2	m	1	-	-	-	-	9.20	(3.30- 25.80)
HUMBLE	3	m	1	-	-	-	-	24.70	(10.00- 59.90)
HUMBLE	5	m	1	-	-	-	-	11.60	(2.70- 61.50)
HUMBLE	6	m	1	-	-	-	-	26.10	(5.60- 146.60)
HUMBLE	8	f	1	-	-	-	-	19.20	(6.50- 60.80)
HUMBLE	9	f	1	-	-	-	-	16.00	(6.70- 36.30)
HUMBLE	11	f	1	-	-	-	-	18.50	(4.90- 72.40)
HUMBLE	12	f	1	-	-	-	-	36.90	(7.60- 217.10)
Subtotal HUMBLE								17.64	(11.76- 26.46)
JARUP	4	m	2	-	-	-	-	6.90	(2.40- 22.70)
JARUP	5	m	2	-	-	-	-	8.00	(3.00- 24.80)
Subtotal JARUP								7.46	(3.46- 16.11)
JEDRYC	45	m	4	-	-	-	-	3.48	(2.33- 5.19)
JEDRYC	46	m	4	-	-	-	-	6.16	(4.25- 8.90)
JEDRYC	47	m	4	-	-	-	-	7.69	(5.15- 11.47)
JEDRYC	48	f	4	-	-	-	-	6.37	(2.66- 15.24)
JEDRYC	49	f	4	-	-	-	-	2.38	(1.17- 6.86)
JEDRYC	50	f	4	-	-	-	-	7.37	(2.20- 24.69)
Subtotal JEDRYC								5.36	(4.35- 6.60)
JOLY	7	m	0	16	54	12	218	5.38	(2.41- 12.05)
JOLY	8	m	0	217	318	12	218	12.40	(6.76- 22.73)
JOLY	9	m	0	126	175	12	218	13.08	(7.00- 24.43)
JOLY	10	m	0	193	161	12	218	21.78	(11.74- 40.39)
JOLY	3	f	0	33	38	52	283	4.73	(2.72- 8.21)
JOLY	4	f	0	72	49	52	283	8.00	(5.01- 12.77)

Table 1G16 - 2

IESLC - Meta-anal of Ever Smoking (or Curr if Ever not avail) by Amount, Overview, Cigs (or Any Prod if Cigs not avail)
All LC types
Most adjusted

REF	NRR	SEX	AD	Number Exposed Case	Number Exposed Cont	Non-exposed Case	Non-exposed Cont	RR	95.00%CI
JOLY	5	f	0	28	22	52	283	6.93	(3.68- 13.03)
JOLY	6	f	0	32	13	52	283	13.40	(6.59- 27.23)
Subtotal JOLY								9.35	(7.55- 11.59)
JUSSAW	34	m	2	-	-	-	-	5.57	(2.46- 14.76)
JUSSAW	35	m	2	-	-	-	-	14.00	(5.17- 53.16)
Subtotal JUSSAW								7.84	(3.86- 15.96)
*KAISE2	66	m	1	-	-	-	-	4.47	(2.00- 9.99)
*KAISE2	67	m	1	-	-	-	-	10.34	(5.56- 19.23)
*KAISE2	58	f	1	-	-	-	-	7.61	(3.26- 17.75)
*KAISE2	59	f	1	-	-	-	-	22.12	(11.22- 43.61)
Subtotal KAISE2								10.24	(7.14- 14.68)
*KAISER	6	m	2	-	-	-	-	6.58	(3.87- 11.20)
*KAISER	7	m	2	-	-	-	-	17.24	(10.71- 27.73)
*KAISER	8	m	2	-	-	-	-	20.91	(12.78- 27.73)
*KAISER	2	f	2	-	-	-	-	3.42	(2.17- 5.40)
*KAISER	3	f	2	-	-	-	-	7.98	(5.35- 11.90)
*KAISER	4	f	2	-	-	-	-	12.63	(8.06- 19.80)
Subtotal KAISER								10.14	(8.46- 12.15)
KANELL	26	m	1	-	-	-	-	1.71	(1.15- 2.56)
KANELL	27	m	1	-	-	-	-	7.06	(4.76- 10.48)
KANELL	28	m	1	-	-	-	-	20.39	(10.37- 40.09)
KANELL	29	m	1	-	-	-	-	34.22	(18.86- 62.11)
Subtotal KANELL								6.27	(4.94- 7.96)
KATSOU	3	f	1	-	-	-	-	2.26	(1.06- 4.85)
KATSOU	4	f	1	-	-	-	-	7.46	(2.40- 23.17)
Subtotal KATSOU								3.27	(1.74- 6.16)
KAUFMA	11	c	6	-	-	-	-	8.00	(5.00- 13.00)
KAUFMA	12	c	6	-	-	-	-	15.00	(10.00- 23.00)
KAUFMA	13	c	6	-	-	-	-	28.00	(17.00- 44.00)
KAUFMA	14	c	6	-	-	-	-	43.00	(27.00- 68.00)
KAUFMA	15	c	6	-	-	-	-	60.00	(35.00- 102.00)
Subtotal KAUFMA								23.07	(18.70- 28.44)
KHUDER	1	m	0	81	434	23	309	2.51	(1.54- 4.07)
KHUDER	2	m	0	224	288	23	309	10.45	(6.61- 16.52)
KHUDER	3	m	0	154	63	23	309	32.84	(19.62- 54.97)
Subtotal KHUDER								9.12	(6.89- 12.06)
*KINLEN	14	m	2	-	-	-	-	10.61	(5.01- 22.48)
*KINLEN	15	m	2	-	-	-	-	14.14	(6.68- 29.91)
*KINLEN	16	m	2	-	-	-	-	21.74	(10.23- 46.19)
Subtotal KINLEN								14.81	(9.60- 22.86)
*KNEKT	29	m	1	-	-	-	-	5.00	(2.00- 12.30)
*KNEKT	30	m	1	-	-	-	-	12.70	(5.50- 29.40)
Subtotal KNEKT								8.27	(4.47- 15.31)
KOO	11	f	0	17	19	56	85	1.36	(0.65- 2.84)
KOO	12	f	0	24	5	56	85	7.29	(2.62- 20.22)
KOO	13	f	0	1	1	56	85	1.52	(0.09- 24.77)
Subtotal KOO								2.36	(1.32- 4.24)
KOULUM	6	m	0	37	77	5	54	5.19	(1.92- 14.06)
KOULUM	5	m	0	208	94	5	54	23.90	(9.26- 61.67)
KOULUM	4	m	0	478	75	5	54	68.83	(26.67- 177.62)
Subtotal KOULUM								21.38	(12.26- 37.28)
KREUZE	19	m	3	-	-	-	-	2.50	(0.70- 8.20)
KREUZE	20	m	3	-	-	-	-	8.70	(3.50- 21.90)
KREUZE	21	m	3	-	-	-	-	19.50	(7.50- 50.30)
KREUZE	22	m	3	-	-	-	-	20.80	(7.20- 60.50)
KREUZE	30	m	3	-	-	-	-	8.20	(5.20- 13.00)
KREUZE	31	m	3	-	-	-	-	25.10	(16.20- 38.70)
KREUZE	32	m	3	-	-	-	-	32.80	(20.90- 51.40)
KREUZE	33	m	3	-	-	-	-	33.30	(20.50- 54.00)
KREUZE	25	f	3	-	-	-	-	5.70	(1.60- 16.60)
KREUZE	26	f	3	-	-	-	-	11.80	(3.50- 29.00)
KREUZE	27	f	3	-	-	-	-	12.10	(3.00- 48.00)
KREUZE	36	f	3	-	-	-	-	2.00	(1.20- 3.30)
KREUZE	37	f	3	-	-	-	-	5.40	(3.50- 8.60)
KREUZE	38	f	3	-	-	-	-	7.70	(3.50- 17.30)
Subtotal KREUZE								11.60	(9.81- 13.72)
KREYBE	9	m	1	-	-	-	-	5.82	(2.56- 13.23)
KREYBE	10	m	1	-	-	-	-	6.23	(2.66- 14.56)
KREYBE	11	m	1	-	-	-	-	14.31	(5.99- 34.17)
KREYBE	28	f	1	-	-	-	-	1.36	(0.65- 2.82)
KREYBE	29	f	1	-	-	-	-	1.91	(0.48- 7.55)

Table 1G16 - 2

IESLC - Meta-anal of Ever Smoking (or Curr if Ever not avail) by Amount, Overview, Cigs (or Any Prod if Cigs not avail)
All LC types
Most adjusted

REF	NRR	SEX	AD	Number Exposed Case	Number Exposed Cont	Non-exposed Case	Non-exposed Cont	RR	95.00%CI
Subtotal KREYBE								4.29	(2.90- 6.34)
LAMTH	7	f	0	101	63	202	337	2.67	(1.87- 3.83)
LAMTH	2	f	0	90	28	202	337	5.36	(3.39- 8.48)
LAMTH	9	f	0	39	9	202	337	7.23	(3.43- 15.24)
Subtotal LAMTH								3.82	(2.93- 4.98)
LAUSSM	18	m	3	-	-	-	-	3.29	(2.31- 4.66)
LAUSSM	19	m	3	-	-	-	-	5.81	(4.16- 8.11)
LAUSSM	20	m	3	-	-	-	-	9.62	(6.05- 15.23)
Subtotal LAUSSM								5.24	(4.23- 6.49)
LETOUR	2	c	0	271	266	24	224	9.51	(6.04- 14.97)
LETOUR	3	c	0	367	198	24	224	17.30	(10.98- 27.27)
LETOUR	4	c	0	65	23	24	224	26.38	(13.98- 49.78)
Subtotal LETOUR								14.84	(11.14- 19.77)
*LIAW	3	c	2	-	-	-	-	3.10	(1.70- 5.60)
*LIAW	4	c	2	-	-	-	-	3.60	(2.00- 6.40)
*LIAW	5	c	2	-	-	-	-	8.30	(4.00- 17.30)
Subtotal LIAW								4.18	(2.91- 6.00)
*LIDDEL	2	m	1	-	-	-	-	3.33	(2.05- 5.64)
*LIDDEL	3	m	1	-	-	-	-	5.02	(3.21- 8.22)
Subtotal LIDDEL								4.15	(2.94- 5.86)
LIU2	8	m	3	-	-	-	-	1.20	(0.43- 3.50)
LIU2	9	m	3	-	-	-	-	7.10	(2.60- 19.50)
LIU2	10	m	3	-	-	-	-	21.40	(7.10- 64.00)
LIU2	14	f	3	-	-	-	-	1.80	(0.57- 5.90)
LIU2	15	f	3	-	-	-	-	3.50	(1.20- 9.80)
LIU2	16	f	3	-	-	-	-	17.90	(4.00- 80.60)
Subtotal LIU2								4.74	(3.00- 7.48)
LIU3	6	m	2	-	-	-	-	1.41	(0.33- 6.09)
LIU3	7	m	2	-	-	-	-	1.09	(0.24- 4.82)
LIU3	8	m	2	-	-	-	-	1.91	(0.32- 11.40)
Subtotal LIU3								1.39	(0.56- 3.42)
LIU4	7	m	2	-	-	-	-	2.11	(2.02- 2.20)
LIU4	8	m	2	-	-	-	-	3.60	(3.49- 3.71)
LIU4	9	m	2	-	-	-	-	6.98	(6.73- 7.23)
Subtotal LIU4								3.95	(3.87- 4.03)
LIU5	2	c	0	14	27	26	41	0.82	(0.36- 1.84)
LIU5	3	c	0	21	21	26	41	1.58	(0.72- 3.44)
LIU5	4	c	0	50	22	26	41	3.58	(1.78- 7.23)
Subtotal LIU5								1.79	(1.16- 2.78)
LUBIN	11	m	4	-	-	-	-	0.72	(0.20- 2.54)
LUBIN	12	m	4	-	-	-	-	1.25	(0.43- 3.65)
LUBIN	13	m	4	-	-	-	-	6.50	(2.38- 17.78)
LUBIN	14	m	4	-	-	-	-	8.00	(3.42- 18.72)
Subtotal LUBIN								3.39	(2.04- 5.64)
LUBIN2	273	m	0	1887	3759	190	2616	6.91	(5.91- 8.09)
LUBIN2	274	m	0	1529	2771	190	2616	7.60	(6.47- 8.92)
LUBIN2	275	m	0	1963	2547	190	2616	10.61	(9.06- 12.44)
LUBIN2	276	m	0	1261	1394	190	2616	12.45	(10.55- 14.70)
LUBIN2	281	f	0	151	218	336	1188	2.45	(1.93- 3.11)
LUBIN2	282	f	0	221	213	336	1188	3.67	(2.93- 4.59)
LUBIN2	283	f	0	134	103	336	1188	4.60	(3.46- 6.11)
LUBIN2	284	f	0	45	33	336	1188	4.82	(3.03- 7.68)
Subtotal LUBIN2								7.07	(6.60- 7.58)
MACLEN	36	c	2	-	-	-	-	1.35	(0.64- 2.84)
MACLEN	37	c	2	-	-	-	-	2.66	(1.46- 4.81)
MACLEN	38	c	2	-	-	-	-	2.93	(1.57- 5.45)
MACLEN	39	c	2	-	-	-	-	4.10	(2.07- 8.15)
Subtotal MACLEN								2.65	(1.91- 3.67)
MARTIS	1	m	0	31	39	4	25	4.97	(1.56- 15.78)
MARTIS	2	m	0	91	87	4	25	6.54	(2.19- 19.55)
MARTIS	3	m	0	75	50	4	25	9.38	(3.08- 28.57)
Subtotal MARTIS								6.77	(3.55- 12.94)
MATOS	29	m	2	-	-	-	-	2.00	(0.90- 4.50)
MATOS	31	m	2	-	-	-	-	7.50	(3.70- 15.00)
MATOS	33	m	2	-	-	-	-	10.40	(5.30- 20.70)
Subtotal MATOS								5.94	(3.91- 9.02)
MATSUD	1	m	0	37	1237	3	1255	12.51	(3.85- 40.69)
MATSUD	2	m	0	75	1607	3	1255	19.52	(6.14- 62.05)
MATSUD	3	m	0	58	470	3	1255	51.62	(16.10- 165.55)
Subtotal MATSUD								23.37	(11.91- 45.84)
MCCONN	21	c	0	7	40	9	23	0.45	(0.15- 1.36)

Table 1G16 - 2

IESLC - Meta-anal of Ever Smoking (or Curr if Ever not avail) by Amount, Overview, Cigs (or Any Prod if Cigs not avail)
All LC types
Most adjusted

REF	NRR	SEX	AD	Number Exposed Case	Number Exposed Cont	Non-exposed Case	Non-exposed Cont	RR	95.00%CI
MCCONN	20	c	0	41	89	9	23	1.18	(0.50- 2.77)
MCCONN	19	c	0	35	41	9	23	2.18	(0.89- 5.33)
Subtotal MCCONN								1.17	(0.68- 2.02)
*MIGRAN	2	m	2	-	-	-	-	4.01	(1.19- 13.47)
*MIGRAN	4	m	2	-	-	-	-	4.24	(1.53- 11.74)
*MIGRAN	6	m	2	-	-	-	-	5.14	(1.79- 14.81)
*MIGRAN	8	m	2	-	-	-	-	5.93	(2.03- 17.29)
*MIGRAN	29	f	2	-	-	-	-	4.88	(1.17- 20.43)
*MIGRAN	31	f	2	-	-	-	-	6.53	(2.11- 20.22)
*MIGRAN	33	f	2	-	-	-	-	7.48	(1.64- 34.03)
Subtotal MIGRAN								5.24	(3.37- 8.15)
*MRFITR	3	m	0	2	856	0	1859	10.86~	(0.52- 225.86)
*MRFITR	4	m	0	50	3747	0	1859	50.12~	(3.09- 811.82)
*MRFITR	5	m	0	54	3591	0	1859	56.43~	(3.49- 913.25)
Subtotal MRFITR								33.22	(6.37- 173.28)
NAM	74	m	1	-	-	-	-	6.70	(4.19- 10.71)
NAM	75	m	1	-	-	-	-	10.27	(6.42- 16.43)
NAM	90	f	1	-	-	-	-	9.06	(5.83- 14.06)
NAM	91	f	1	-	-	-	-	16.65	(10.20- 27.19)
Subtotal NAM								9.95	(7.88- 12.57)
NOTAN2	8	m	0	6	42	134	544	0.58	(0.24- 1.39)
NOTAN2	9	m	0	28	47	134	544	2.42	(1.46- 4.01)
NOTAN2	10	m	0	44	40	134	544	4.47	(2.80- 7.13)
Subtotal NOTAN2								2.66	(1.93- 3.66)
ORMOS	1	m	0	32	329	7	777	10.80	(4.72- 24.71)
ORMOS	2	m	0	40	577	7	777	7.69	(3.42- 17.30)
ORMOS	3	m	0	15	128	7	777	13.01	(5.20- 32.52)
Subtotal ORMOS								10.06	(6.17- 16.42)
OSANN	49	m	2	-	-	-	-	17.70	(12.60- 24.80)
OSANN	57	m	2	-	-	-	-	42.80	(30.50- 60.10)
OSANN	50	f	2	-	-	-	-	14.40	(11.00- 18.90)
OSANN	58	f	2	-	-	-	-	40.90	(29.30- 57.10)
Subtotal OSANN								24.11	(20.59- 28.24)
PARKIN	20	m	0	286	749	107	1248	4.45	(3.50- 5.66)
PARKIN	21	m	0	60	125	107	1248	5.60	(3.88- 8.07)
Subtotal PARKIN								4.77	(3.90- 5.83)
PASTOR	6	m	1	-	-	-	-	2.33	(0.78- 6.95)
PASTOR	7	m	1	-	-	-	-	6.42	(2.96- 13.92)
PASTOR	8	m	1	-	-	-	-	8.02	(3.84- 16.73)
PASTOR	9	m	1	-	-	-	-	8.61	(3.86- 19.19)
Subtotal PASTOR								6.44	(4.27- 9.72)
PERNU	17	m	0	15	15	97	275	2.84	(1.34- 6.01)
PERNU	18	m	0	61	31	97	275	5.58	(3.42- 9.11)
PERNU	19	m	0	224	96	97	275	6.62	(4.74- 9.23)
PERNU	20	m	0	127	67	97	275	5.37	(3.69- 7.82)
PERNU	21	m	0	478	138	97	275	9.82	(7.28- 13.24)
PERNU	22	m	0	361	54	97	275	18.95	(13.12- 27.38)
PERNU	23	m	0	40	23	97	275	4.93	(2.81- 8.66)
PERNU	24	m	0	74	14	97	275	14.99	(8.09- 27.75)
PERNU	11	f	0	3	14	110	971	1.89	(0.54- 6.68)
PERNU	12	f	0	5	13	110	971	3.40	(1.19- 9.70)
PERNU	13	f	0	4	30	110	971	1.18	(0.41- 3.40)
PERNU	14	f	0	1	14	110	971	0.63	(0.08- 4.84)
PERNU	15	f	0	1	11	110	971	0.80	(0.10- 6.27)
PERNU	16	f	0	5	7	110	971	6.31	(1.97- 20.20)
Subtotal PERNU								7.42	(6.44- 8.54)
PERSH2	8	c	4	-	-	-	-	5.76	(4.61- 7.19)
PERSH2	9	c	4	-	-	-	-	11.34	(9.14- 14.07)
Subtotal PERSH2								8.16	(6.99- 9.53)
*PETO	2	m	0	44	1181	2	295	5.50	(1.34- 22.54)
*PETO	3	m	0	55	855	2	295	9.49	(2.33- 38.66)
Subtotal PETO								7.23	(2.67- 19.57)
PEZZO2	3	m	0	57	139	6	117	8.00	(3.33- 19.21)
PEZZO2	4	m	0	107	47	6	117	44.39	(18.24- 108.02)
PEZZO2	5	m	0	69	12	6	117	112.13	(40.26- 312.24)
Subtotal PEZZO2								30.27	(17.76- 51.58)
PEZZOT	2	m	0	24	94	4	116	7.40	(2.48- 22.09)
PEZZOT	3	m	0	70	29	4	116	70.00	(23.61- 207.50)
PEZZOT	4	m	0	51	6	4	116	246.50	(66.69- 911.11)
Subtotal PEZZOT								42.28	(21.77- 82.11)
PIKE	1	m	0	181	168	18	69	4.13	(2.36- 7.23)

Table 1G16 - 2

IESLC - Meta-anal of Ever Smoking (or Curr if Ever not avail) by Amount, Overview, Cigs (or Any Prod if Cigs not avail)
All LC types
Most adjusted

REF	NRR	SEX	AD	Number Exposed Case	Number Exposed Cont	Non-exposed Case	Non-exposed Cont	RR	95.00%CI
PIKE	2	m	0	228	109	18	69	8.02	(4.55- 14.13)
PIKE	3	m	0	66	37	18	69	6.84	(3.55- 13.18)
PIKE	5	f	0	73	60	36	96	3.24	(1.94- 5.42)
PIKE	6	f	0	67	26	36	96	6.87	(3.80- 12.44)
PIKE	7	f	0	16	3	36	96	14.22	(3.91- 51.73)
Subtotal PIKE								5.49	(4.27- 7.05)
POLEDN	2	c	0	53	103	12	139	5.96	(3.03- 11.72)
POLEDN	4	c	0	143	168	12	139	9.86	(5.25- 18.52)
Subtotal POLEDN								7.80	(4.92- 12.37)
*PRESCO	2	m	1	-	-	-	-	10.20	(4.49- 23.15)
*PRESCO	4	m	1	-	-	-	-	19.96	(8.92- 44.67)
*PRESCO	1	f	1	-	-	-	-	6.36	(3.60- 11.24)
*PRESCO	3	f	1	-	-	-	-	10.08	(5.72- 17.75)
Subtotal PRESCO								9.70	(6.98- 13.49)
RACHTA	10	f	1	-	-	-	-	3.64	(1.08- 12.32)
RACHTA	11	f	1	-	-	-	-	3.55	(1.75- 7.23)
RACHTA	12	f	1	-	-	-	-	13.77	(6.50- 29.16)
Subtotal RACHTA								6.13	(3.81- 9.85)
RANDIG	12	m	0	8	18	5	22	1.96	(0.54- 7.03)
RANDIG	13	m	0	48	72	5	22	2.93	(1.04- 8.28)
RANDIG	14	m	0	135	114	5	22	5.21	(1.91- 14.20)
RANDIG	15	m	0	86	41	5	22	9.23	(3.26- 26.11)
RANDIG	20	f	0	1	21	17	92	0.26	(0.03- 2.05)
RANDIG	21	f	0	12	13	17	92	5.00	(1.95- 12.79)
RANDIG	22	f	0	3	5	17	92	3.25	(0.71- 14.88)
Subtotal RANDIG								3.88	(2.51- 5.99)
SEGI2	10	m	1	-	-	-	-	2.10	(0.86- 5.16)
SEGI2	12	m	1	-	-	-	-	3.10	(1.40- 6.84)
SEGI2	14	m	1	-	-	-	-	3.40	(1.55- 7.45)
SEGI2	16	m	1	-	-	-	-	6.90	(2.78- 17.14)
SEGI2	18	m	1	-	-	-	-	7.90	(3.40- 18.37)
SEGI2	22	f	1	-	-	-	-	2.90	(1.09- 7.70)
SEGI2	24	f	1	-	-	-	-	1.44	(0.61- 3.40)
SEGI2	26	f	1	-	-	-	-	1.03	(0.34- 3.15)
Subtotal SEGI2								3.08	(2.26- 4.21)
SHAW	10	c	0	46	90	11	107	4.97	(2.43- 10.16)
SHAW	11	c	0	278	176	11	107	15.36	(8.03- 29.39)
Subtotal SHAW								9.23	(5.71- 14.93)
SIEMIA	13	m	0	-	-	-	-	3.00	(1.00- 9.90)
SIEMIA	14	m	0	-	-	-	-	4.50	(1.80- 13.20)
SIEMIA	15	m	0	-	-	-	-	7.90	(3.00- 24.10)
Subtotal SIEMIA								4.87	(2.64- 8.95)
SOBUE	117	m	0	147	157	34	128	3.52	(2.27- 5.47)
SOBUE	118	m	0	236	222	34	128	4.00	(2.63- 6.09)
SOBUE	119	m	0	226	187	34	128	4.55	(2.97- 6.96)
Subtotal SOBUE								4.02	(3.14- 5.14)
*SPEIZE	1	f	1	-	-	-	-	2.70	(0.90- 5.40)
*SPEIZE	2	f	1	-	-	-	-	5.20	(3.60- 8.10)
*SPEIZE	3	f	1	-	-	-	-	12.60	(9.90- 16.70)
*SPEIZE	4	f	1	-	-	-	-	15.70	(12.10- 20.20)
*SPEIZE	5	f	1	-	-	-	-	22.00	(14.80- 32.30)
Subtotal SPEIZE								12.52	(10.76- 14.56)
SPITZ	5	c	0	27	88	10	96	2.95	(1.35- 6.43)
SPITZ	6	c	0	95	48	10	96	19.00	(9.08- 39.74)
Subtotal SPITZ								7.89	(4.61- 13.49)
STOCKS	41	m	2	-	-	-	-	4.64	(3.25- 6.61)
STOCKS	42	m	2	-	-	-	-	7.89	(5.51- 11.29)
STOCKS	43	m	2	-	-	-	-	10.95	(7.09- 16.91)
STOCKS	44	m	2	-	-	-	-	10.03	(6.51- 15.46)
STOCKS	45	m	2	-	-	-	-	13.91	(8.96- 21.60)
STOCKS	48	f	1	-	-	-	-	2.24	(1.64- 3.03)
STOCKS	49	f	1	-	-	-	-	6.34	(4.42- 8.93)
Subtotal STOCKS								6.04	(5.25- 6.96)
STOCKW	1	c	0	2090	1194	2791	10641	6.67	(6.15- 7.25)
STOCKW	2	c	0	6053	1591	2791	10641	14.51	(13.54- 15.54)
STOCKW	3	c	0	4327	572	2791	10641	28.84	(26.18- 31.77)
Subtotal STOCKW								13.28	(12.68- 13.92)
SVENSS	6	f	1	-	-	-	-	4.60	(2.50- 9.30)
SVENSS	11	f	1	-	-	-	-	12.60	(6.50- 25.20)
SVENSS	16	f	1	-	-	-	-	59.00	(7.60- 458.03)

Table 1G16 - 2

IESLC - Meta-anal of Ever Smoking (or Curr if Ever not avail) by Amount, Overview, Cigs (or Any Prod if Cigs not avail)
All LC types
Most adjusted

REF	NRR	SEX	AD	Number Exposed Case	Number Exposed Cont	Non-exposed Case	Non-exposed Cont	RR	95.00%CI
Subtotal SVENSS								8.31	(5.25- 13.17)
*TENKAN	10	m	1	-	-	-	-	15.86	(6.80- 37.00)
*TENKAN	11	m	1	-	-	-	-	20.25	(8.20- 50.00)
*TENKAN	12	m	1	-	-	-	-	24.97	(9.90- 63.00)
Subtotal TENKAN								19.74	(11.81- 33.01)
TIZZAN	7	m	0	130	238	180	305	0.93	(0.70- 1.23)
TIZZAN	8	m	0	468	470	180	305	1.69	(1.35- 2.11)
TIZZAN	9	m	0	301	108	180	305	4.72	(3.54- 6.29)
TIZZAN	10	m	0	83	20	180	305	7.03	(4.17- 11.85)
TIZZAN	15	f	0	11	14	25	114	3.58	(1.46- 8.82)
TIZZAN	16	f	0	14	14	25	114	4.56	(1.93- 10.75)
Subtotal TIZZAN								2.16	(1.87- 2.48)
TSUGAN	29	m	0	14	19	18	22	0.90	(0.36- 2.28)
TSUGAN	30	m	0	30	30	18	22	1.22	(0.55- 2.73)
TSUGAN	31	m	0	19	14	18	22	1.66	(0.65- 4.20)
Subtotal TSUGAN								1.22	(0.73- 2.03)
*TULINI	27	m	3	-	-	-	-	6.02	(3.01- 12.00)
*TULINI	28	m	3	-	-	-	-	12.00	(6.31- 22.90)
*TULINI	29	m	3	-	-	-	-	27.30	(14.20- 52.40)
*TULINI	32	f	3	-	-	-	-	8.17	(4.33- 15.40)
*TULINI	33	f	3	-	-	-	-	26.30	(14.40- 48.10)
*TULINI	34	f	3	-	-	-	-	38.70	(18.50- 80.80)
Subtotal TULINI								15.82	(12.10- 20.68)
*TVERDA	9	m	2	-	-	-	-	2.14	(1.15- 3.96)
*TVERDA	10	m	2	-	-	-	-	3.32	(2.07- 5.32)
*TVERDA	11	m	2	-	-	-	-	6.56	(4.04- 10.64)
*TVERDA	16	f	2	-	-	-	-	4.53	(1.08- 18.94)
*TVERDA	17	f	2	-	-	-	-	18.00	(5.33- 60.83)
Subtotal TVERDA								4.23	(3.19- 5.62)
WAKAI	40	m	2	-	-	-	-	1.80	(0.81- 4.02)
WAKAI	41	m	2	-	-	-	-	4.01	(1.91- 8.41)
WAKAI	42	m	2	-	-	-	-	9.19	(4.20- 20.10)
Subtotal WAKAI								4.09	(2.62- 6.40)
WANG2	9	c	4	-	-	-	-	1.40	(0.43- 4.54)
WANG2	10	c	4	-	-	-	-	1.22	(0.31- 4.83)
WANG2	11	c	4	-	-	-	-	1.41	(0.43- 4.61)
WANG2	12	c	4	-	-	-	-	1.16	(0.33- 4.03)
WANG2	13	c	4	-	-	-	-	3.19	(1.39- 7.29)
WANG2	14	c	4	-	-	-	-	2.39	(0.78- 7.34)
WANG2	15	c	4	-	-	-	-	7.25	(2.05- 25.65)
Subtotal WANG2								2.20	(1.43- 3.37)
WU	43	f	2	-	-	-	-	3.25	(1.71- 6.16)
WU	44	f	2	-	-	-	-	8.48	(4.16- 17.29)
Subtotal WU								4.99	(3.10- 8.04)
WUWILL	12	f	3	-	-	-	-	2.13	(1.75- 2.58)
WUWILL	13	f	3	-	-	-	-	3.37	(2.28- 4.97)
Subtotal WUWILL								2.33	(1.96- 2.78)
WYNDE2	17	m	0	17	114	8	105	1.96	(0.81- 4.72)
WYNDE2	18	m	0	122	203	8	105	7.89	(3.71- 16.75)
WYNDE2	19	m	0	88	83	8	105	13.92	(6.39- 30.32)
WYNDE2	20	m	0	155	112	8	105	18.16	(8.50- 38.80)
Subtotal WYNDE2								8.64	(5.83- 12.82)
WYNDE3	44	m	0	8	42	9	88	1.86	(0.67- 5.17)
WYNDE3	45	m	0	77	114	9	88	6.60	(3.14- 13.90)
WYNDE3	46	m	0	108	82	9	88	12.88	(6.12- 27.09)
WYNDE3	47	m	0	68	26	9	88	25.57	(11.25- 58.14)
WYNDE3	79	f	0	3	19	20	76	0.60	(0.16- 2.23)
WYNDE3	80	f	0	24	24	20	76	3.80	(1.79- 8.05)
WYNDE3	81	f	0	15	10	20	76	5.70	(2.23- 14.59)
WYNDE3	82	f	0	4	3	20	76	5.07	(1.05- 24.50)
Subtotal WYNDE3								6.19	(4.51- 8.50)
WYNDE4	43	m	0	17	82	12	115	1.99	(0.90- 4.38)
WYNDE4	44	m	0	67	147	12	115	4.37	(2.26- 8.46)
WYNDE4	45	m	0	228	274	12	115	7.97	(4.29- 14.82)
WYNDE4	46	m	0	190	98	12	115	18.58	(9.77- 35.33)
WYNDE4	47	m	0	130	64	12	115	19.47	(10.00- 37.88)
WYNDE4	57	f	2	-	-	-	-	1.13	(0.33- 3.89)
WYNDE4	58	f	2	-	-	-	-	2.01	(0.66- 6.10)
WYNDE4	59	f	2	-	-	-	-	6.49	(2.35- 17.93)
WYNDE4	60	f	2	-	-	-	-	11.54	(1.90- 70.11)
WYNDE4	61	f	2	-	-	-	-	11.54	(1.90- 70.11)

Table 1G16 - 2

IESLC - Meta-anal of Ever Smoking (or Curr if Ever not avail) by Amount, Overview, Cigs (or Any Prod if Cigs not avail)
All LC types
Most adjusted

REF	NRR	SEX	AD	Number Exposed Case	Cont	Non-exposed Case	Cont	RR	95.00%CI
Subtotal WYNDE4								6.97	(5.35- 9.09)
WYNDE6	27	m	0	117	122	87	617	6.80	(4.85- 9.54)
WYNDE6	36	m	0	461	293	87	617	11.16	(8.54- 14.59)
WYNDE6	45	m	0	315	129	87	617	17.32	(12.78- 23.47)
WYNDE6	54	m	0	784	197	87	617	28.22	(21.47- 37.10)
WYNDE6	216	f	0	76	109	159	856	3.75	(2.68- 5.26)
WYNDE6	225	f	0	367	165	159	856	11.97	(9.33- 15.37)
WYNDE6	234	f	0	201	50	159	856	21.64	(15.21- 30.80)
WYNDE6	243	f	0	378	52	159	856	39.13	(27.98- 54.75)
Subtotal WYNDE6								14.13	(12.71- 15.72)
XU3	9	m	1	-	-	-	-	1.66	(0.55- 4.97)
XU3	10	m	1	-	-	-	-	2.98	(1.09- 8.17)
XU3	11	m	1	-	-	-	-	14.78	(5.30- 41.18)
XU3	12	m	1	-	-	-	-	27.72	(5.15- 149.23)
XU3	16	f	1	-	-	-	-	2.18	(0.61- 7.84)
XU3	17	f	1	-	-	-	-	4.41	(1.16- 16.83)
XU3	18	f	1	-	-	-	-	8.19	(0.89- 75.45)
Subtotal XU3								4.71	(2.94- 7.56)
YAMAGU	8	c	1	-	-	-	-	3.75	(1.89- 7.47)
YAMAGU	7	c	1	-	-	-	-	12.14	(5.10- 28.90)
Subtotal YAMAGU								5.90	(3.44- 10.11)
*YUAN	2	m	2	-	-	-	-	3.60	(1.88- 6.91)
*YUAN	3	m	2	-	-	-	-	9.40	(5.21- 16.97)
Subtotal YUAN								6.09	(3.94- 9.44)
ZHENG	11	m	0	25	40	33	94	1.78	(0.94- 3.37)
ZHENG	12	m	0	60	66	33	94	2.59	(1.53- 4.39)
ZHENG	13	m	0	128	89	33	94	4.10	(2.53- 6.62)
ZHENG	14	m	0	66	23	33	94	8.17	(4.40- 15.17)
ZHENG	22	f	0	24	29	152	184	1.00	(0.56- 1.79)
ZHENG	23	f	0	52	15	152	184	4.20	(2.27- 7.75)
Subtotal ZHENG								2.97	(2.36- 3.75)
ZHOU	4	c	0	61	5	507	68	1.64	(0.64- 4.22)
ZHOU	5	c	0	211	14	507	68	2.02	(1.11- 3.67)
ZHOU	6	c	0	581	29	507	68	2.69	(1.71- 4.22)
Subtotal ZHOU								2.31	(1.65- 3.23)
Partial Totals				41943	174447	21636	232770		

*prospective study ~ With 0.5 adjustment for zero

REF	NRR	SEX	AD	Ys	Ws	Qs	Ps
AGUDO	4	f	3	0.45	3.17	5.69	0.4219
AGUDO	5	f	3	1.60	4.04	0.15	0.0013
Subtotal AGUDO				1.09	7.21	5.84	
*AKIBA	27	m	5	1.25	15.26	4.42	0.0000
*AKIBA	28	m	5	1.81	19.39	0.01	0.0000
*AKIBA	29	m	5	2.21	13.18	2.30	0.0000
*AKIBA	33	f	5	1.28	35.93	9.33	0.0000
*AKIBA	34	f	5	1.76	13.74	0.01	0.0000
Subtotal AKIBA				1.57	97.50	16.07	
ALDERS	18	m	1	1.27	10.54	2.89	0.0000
ALDERS	19	m	1	2.07	13.07	1.05	0.0000
ALDERS	20	m	1	2.14	14.23	1.76	0.0000
ALDERS	21	f	1	0.96	34.91	23.90	0.0000
ALDERS	22	f	1	1.66	34.88	0.56	0.0000
ALDERS	23	f	1	1.93	25.78	0.51	0.0000
Subtotal ALDERS				1.59	133.42	30.67	
*ARCHER	1	m	0	1.26	4.20	1.18	0.0097
*ARCHER	2	m	0	1.81	5.52	0.00	0.0000
*ARCHER	3	m	0	2.14	5.22	0.64	0.0000
Subtotal ARCHER				1.77	14.95	1.82	
ARMADA	46	m	0	1.79	3.37	0.00	0.0010
ARMADA	47	m	0	3.02	3.54	5.32	0.0000
ARMADA	48	m	0	4.24	3.29	19.75	0.0000
Subtotal ARMADA				3.01	10.20	25.07	
AUVINE	13	c	2	3.00	7.37	10.70	0.0000
AUVINE	14	c	2	3.52	8.24	24.74	0.0000
AUVINE	15	c	2	4.20	4.27	24.73	0.0000
Subtotal AUVINE				3.47	19.88	60.17	
AXELSS	5	m	6	1.34	8.91	1.81	0.0001
AXELSS	6	m	6	2.19	10.87	1.70	0.0000
AXELSS	7	m	6	2.34	11.25	3.42	0.0000

Table 1G16 - 2

IESLC - Meta-anal of Ever Smoking (or Curr if Ever not avail) by Amount, Overview, Cigs (or Any Prod if Cigs not avail)
All LC types
Most adjusted

REF	NRR	SEX	AD	Ys	Ws	Qs	Ps
AXELSS	13	f	0	1.10	6.02	2.87	0.0069
AXELSS	14	f	0	2.38	10.21	3.52	0.0000
AXELSS	15	f	0	2.77	6.08	5.84	0.0000
AXELSS	16	f	0	1.99	2.69	0.11	0.0011
Subtotal AXELSS				2.06	56.05	19.27	
BARBON	82	m	3	1.90	16.58	0.21	0.0000
BARBON	83	m	3	2.55	16.08	9.26	0.0000
BARBON	84	m	3	3.06	14.81	23.82	0.0000
Subtotal BARBON				2.48	47.46	33.28	
*BENSHL	11	m	1	1.39	4.28	0.70	0.0041
*BENSHL	12	m	1	2.20	5.46	0.93	0.0000
*BENSHL	13	m	1	2.39	5.53	2.01	0.0000
Subtotal BENSHL				2.04	15.26	3.63	
*BEST	13	m	1	2.30	6.23	1.63	0.0000
*BEST	14	m	1	2.80	6.77	6.87	0.0000
*BEST	15	m	1	2.85	6.30	7.09	0.0000
Subtotal BEST				2.66	19.31	15.60	
*BOUCOT	99	m	0	3.15	0.49	0.91	0.0271
*BOUCOT	100	m	0	3.83	0.49	2.06	0.0070
Subtotal BOUCOT				3.49	0.99	2.97	
BRESLO	13	m	0	0.58	7.18	10.60	0.1231
BRESLO	14	m	0	1.19	12.73	4.60	0.0000
BRESLO	15	m	0	2.04	15.85	0.96	0.0000
BRESLO	16	m	0	2.90	8.89	10.95	0.0000
BRESLO	29	f	0	0.15	1.80	4.83	0.8360
BRESLO	30	f	0	0.44	2.24	4.07	0.5084
Subtotal BRESLO				1.61	48.69	36.02	
*BRETT	1	m	0	0.94	5.22	3.82	0.0326
*BRETT	2	m	0	1.45	5.48	0.64	0.0007
*BRETT	3	m	0	2.08	5.09	0.42	0.0000
Subtotal BRETT				1.48	15.79	4.89	
BROSS	18	m	0	1.59	22.46	0.90	0.0000
BROSS	19	m	0	1.97	16.76	0.57	0.0000
Subtotal BROSS				1.75	39.21	1.47	
BROWN2	32	m	2	1.81	220.78	0.07	0.0000
BROWN2	42	m	2	2.65	387.09	283.37	0.0000
BROWN2	31	f	2	2.13	172.98	19.72	0.0000
BROWN2	41	f	2	2.84	312.24	343.27	0.0000
Subtotal BROWN2				2.45	1093.09	646.44	
BUFFLE	28	f	0	1.54	6.11	0.38	0.0001
BUFFLE	29	f	0	2.47	8.19	3.78	0.0000
BUFFLE	35	f	0	3.06	8.66	13.85	0.0000
Subtotal BUFFLE				2.44	22.96	18.01	
*CEDERL	87	m	1	1.89	5.82	0.05	0.0000
*CEDERL	86	m	1	2.79	4.60	4.58	0.0000
*CEDERL	99	f	1	1.56	4.87	0.27	0.0006
Subtotal CEDERL				2.05	15.28	4.91	
*CHANG	2	m	0	1.61	2.58	0.08	0.0096
*CHANG	3	m	0	2.36	3.99	1.30	0.0000
*CHANG	4	m	0	2.11	3.72	0.38	0.0000
*CHANG	8	f	0	1.11	3.97	1.85	0.0271
*CHANG	9	f	0	1.58	5.66	0.24	0.0002
*CHANG	10	f	0	2.11	6.22	0.61	0.0000
Subtotal CHANG				1.83	26.14	4.47	
CHATZI	1	c	0	0.94	14.84	10.76	0.0003
CHATZI	2	c	0	1.04	15.01	8.41	0.0001
CHATZI	3	c	0	1.56	16.06	0.89	0.0000
Subtotal CHATZI				1.19	45.92	20.05	
CHEN2	3	m	0	0.87	4.19	3.52	0.0735
CHEN2	4	m	0	1.17	5.43	2.08	0.0063
CHEN2	5	m	0	2.63	3.55	2.49	0.0000
CHEN2	6	m	0	2.07	3.80	0.30	0.0001
CHEN2	7	f	0	-0.95	3.04	22.76	0.0991
CHEN2	8	f	0	1.07	4.63	2.43	0.0217
CHEN2	9	f	0	2.22	0.82	0.15	0.0435
CHEN2	10	f	0	0.97	1.22	0.82	0.2838
Subtotal CHEN2				1.21	26.68	34.55	
CHOI	12	m	0	0.48	6.73	11.48	0.2084
CHOI	13	m	0	1.32	10.21	2.26	0.0000
CHOI	14	m	0	2.01	7.82	0.37	0.0000
CHOI	15	m	0	1.94	7.14	0.15	0.0000

Table 1G16 - 2

IESLC - Meta-anal of Ever Smoking (or Curr if Ever not avail) by Amount, Overview, Cigs (or Any Prod if Cigs not avail)
All LC types
Most adjusted

REF	NRR	SEX	AD	Ys	Ws	Qs	Ps
CHOI	16	m	0	2.97	3.16	4.39	0.0000
CHOI	17	f	0	0.19	5.18	13.22	0.6591
CHOI	18	f	0	0.52	3.66	5.93	0.3219
CHOI	20	f	0	1.87	0.74	0.00	0.1083
Subtotal CHOI				1.34	44.64	37.80	
*CHOW	10	m	2	2.63	5.07	3.58	0.0000
*CHOW	11	m	2	3.09	5.32	8.92	0.0000
*CHOW	12	m	2	3.80	5.07	20.38	0.0000
Subtotal CHOW				3.17	15.47	32.88	
COMSTO	4	m	0	2.52	2.78	1.47	0.0000
COMSTO	5	m	0	2.90	3.35	4.11	0.0000
COMSTO	6	m	0	3.22	2.79	5.66	0.0000
COMSTO	9	f	0	2.01	4.98	0.24	0.0000
COMSTO	10	f	0	2.85	6.96	7.87	0.0000
COMSTO	11	f	0	2.59	2.75	1.74	0.0000
Subtotal COMSTO				2.65	23.61	21.09	
COOKSO	1	c	0	1.63	11.70	0.29	0.0000
COOKSO	2	c	0	2.31	7.06	1.93	0.0000
Subtotal COOKSO				1.89	18.76	2.22	
CORREA	46	c	1	2.23	39.38	7.60	0.0000
CORREA	50	c	1	3.23	39.20	81.31	0.0000
Subtotal CORREA				2.73	78.58	88.92	
*CPSI	243	m	1	1.76	44.36	0.04	0.0000
*CPSI	246	m	1	2.61	55.90	37.54	0.0000
*CPSI	275	f	1	0.22	13.40	32.92	0.4140
*CPSI	276	f	1	0.89	26.82	21.65	0.0000
*CPSI	277	f	1	1.62	50.55	1.55	0.0000
*CPSI	278	f	1	2.41	10.15	3.86	0.0000
Subtotal CPSI				1.77	201.17	97.57	
*CPSII	102	m	1	2.30	75.60	19.74	0.0000
*CPSII	103	m	1	2.87	75.65	87.80	0.0000
*CPSII	105	f	1	1.43	95.77	12.76	0.0000
*CPSII	106	f	1	2.59	140.12	89.72	0.0000
Subtotal CPSII				2.30	387.13	210.02	
DAMBER	6	m	1	0.83	10.34	9.48	0.0074
DAMBER	7	m	1	1.99	13.68	0.53	0.0000
DAMBER	8	m	1	2.21	14.68	2.56	0.0000
DAMBER	9	m	1	2.70	5.93	4.92	0.0000
Subtotal DAMBER				1.89	44.62	17.49	
DARBY	1	m	0	4.30	2.87	18.04	0.0000
DARBY	2	m	0	4.56	2.86	21.90	0.0000
DARBY	3	m	0	4.96	2.72	27.38	0.0000
DARBY	8	f	0	2.75	14.48	13.43	0.0000
DARBY	9	f	0	3.07	14.73	24.05	0.0000
DARBY	10	f	0	3.73	8.38	31.48	0.0000
Subtotal DARBY				3.37	46.05	136.28	
DAVEYS	1	m	0	0.20	2.07	5.24	0.7726
DAVEYS	2	m	0	2.01	2.27	0.10	0.0025
DAVEYS	3	m	0	1.89	2.11	0.02	0.0061
DAVEYS	4	m	0	2.36	2.18	0.71	0.0005
Subtotal DAVEYS				1.63	8.63	6.07	
DEAN	1	m	0	0.79	8.38	8.35	0.0218
DEAN	2	m	0	1.91	9.10	0.13	0.0000
DEAN	3	m	0	2.44	7.59	3.24	0.0000
Subtotal DEAN				1.70	25.06	11.71	
DEAN2	25	m	0	1.17	22.52	8.59	0.0000
DEAN2	26	m	0	2.03	19.18	1.13	0.0000
DEAN2	29	f	0	0.92	11.90	8.93	0.0014
DEAN2	30	f	0	1.60	3.63	0.13	0.0023
Subtotal DEAN2				1.44	57.24	18.78	
DEAN3	7	m	3	1.70	14.67	0.13	0.0000
DEAN3	14	m	3	2.00	16.24	0.74	0.0000
DEAN3	21	m	3	3.08	13.79	22.77	0.0000
DEAN3	91	f	3	1.15	15.42	6.32	0.0000
DEAN3	98	f	3	2.13	15.80	1.83	0.0000
DEAN3	105	f	3	3.19	10.09	19.70	0.0000
Subtotal DEAN3				2.13	86.02	51.49	
*DEKLER	2	m	2	2.97	0.95	1.32	0.0038
*DEKLER	3	m	2	3.14	0.98	1.77	0.0019
*DEKLER	4	m	2	3.48	0.96	2.74	0.0007
Subtotal DEKLER				3.19	2.89	5.83	

Table 1G16 - 2

IESLC - Meta-anal of Ever Smoking (or Curr if Ever not avail) by Amount, Overview, Cigs (or Any Prod if Cigs not avail)
All LC types
Most adjusted

REF	NRR	SEX	AD	Ys	Ws	Qs	Ps
DESTEF	6	m	4	1.06	11.84	6.24	0.0002
DESTEF	7	m	4	2.13	16.62	1.90	0.0000
DESTEF	8	m	4	2.34	16.30	4.95	0.0000
DESTEF	9	m	4	3.17	11.72	22.16	0.0000
Subtotal DESTEF				2.18	56.48	35.25	
DOLL	1	m	0	1.31	5.40	1.23	0.0023
DOLL	2	m	0	2.01	6.13	0.30	0.0000
DOLL	3	m	0	2.26	6.11	1.36	0.0000
DOLL	4	m	0	2.81	5.91	6.12	0.0000
DOLL	5	m	0	3.32	3.72	8.67	0.0000
DOLL	7	f	0	-0.06	6.92	23.65	0.8795
DOLL	8	f	0	0.68	7.19	8.92	0.0698
DOLL	9	f	0	1.24	3.57	1.10	0.0195
DOLL	10	f	0	3.75	0.47	1.82	0.0098
Subtotal DOLL				1.60	45.43	53.18	
*DOLL2	46	m	1	1.90	0.96	0.01	0.0623
*DOLL2	47	m	1	2.51	0.96	0.50	0.0137
*DOLL2	48	m	1	3.17	0.97	1.84	0.0018
*DOLL2	10	f	1	0.25	0.79	1.87	0.8209
*DOLL2	11	f	1	1.86	2.40	0.01	0.0040
*DOLL2	12	f	1	3.39	2.93	7.52	0.0000
Subtotal DOLL2				2.43	9.01	11.74	
DORANT	6	c	0	2.14	7.99	0.99	0.0000
DORANT	7	c	0	3.30	12.23	28.01	0.0000
DORANT	8	c	0	3.59	11.93	38.65	0.0000
Subtotal DORANT				3.12	32.15	67.64	
DORGAN	108	m	2	1.93	10.33	0.20	0.0000
DORGAN	109	m	2	2.64	11.27	8.11	0.0000
DORGAN	96	f	3	1.74	56.05	0.17	0.0000
DORGAN	97	f	3	2.50	51.92	26.36	0.0000
Subtotal DORGAN				2.14	129.57	34.84	
*DORN	408	m	1	1.39	152.79	24.36	0.0000
*DORN	409	m	1	2.29	287.32	72.98	0.0000
*DORN	410	m	1	2.84	277.97	308.66	0.0000
*DORN	411	m	1	3.12	176.37	313.85	0.0000
Subtotal DORN				2.47	894.45	719.85	
DOSEME	5	m	2	0.79	20.90	20.99	0.0003
DOSEME	9	m	2	1.13	45.98	19.98	0.0000
DOSEME	13	m	2	1.89	21.74	0.20	0.0000
Subtotal DOSEME				1.24	88.62	41.17	
*DUNN	1	m	0	2.12	1.20	0.13	0.0199
*DUNN	2	m	0	2.20	1.71	0.29	0.0040
*DUNN	3	m	0	2.95	1.95	2.63	0.0000
*DUNN	4	m	0	3.22	1.88	3.87	0.0000
*DUNN	5	m	0	3.36	1.73	4.26	0.0000
Subtotal DUNN				2.83	8.48	11.18	
EBELIN	2	m	0	1.00	6.42	4.05	0.0116
EBELIN	3	m	0	1.96	5.47	0.16	0.0000
EBELIN	4	m	0	2.52	7.13	3.76	0.0000
EBELIN	5	m	0	2.79	1.60	1.59	0.0004
EBELIN	6	m	0	2.28	1.69	0.40	0.0031
Subtotal EBELIN				1.94	22.31	9.96	
*ENGELA	31	m	7	0.34	4.64	9.82	0.4684
*ENGELA	32	m	7	1.41	4.89	0.71	0.0018
*ENGELA	33	m	7	1.95	4.91	0.12	0.0000
*ENGELA	34	m	7	2.40	4.27	1.57	0.0000
*ENGELA	35	m	7	2.71	4.73	3.98	0.0000
*ENGELA	45	f	5	2.48	3.99	1.93	0.0000
*ENGELA	46	f	5	2.48	4.17	2.01	0.0000
*ENGELA	47	f	5	3.18	4.61	8.87	0.0000
*ENGELA	48	f	5	3.26	3.58	7.71	0.0000
Subtotal ENGELA				2.20	39.80	36.71	
*ENSTRO	7	m	1	1.56	31.30	1.72	0.0000
*ENSTRO	6	m	1	2.04	59.28	3.65	0.0000
*ENSTRO	5	m	1	2.61	74.58	50.54	0.0000
*ENSTRO	4	m	1	2.78	72.60	70.72	0.0000
*ENSTRO	3	m	1	2.97	65.00	89.77	0.0000
*ENSTRO	11	f	1	0.77	48.76	51.24	0.0000
*ENSTRO	10	f	1	1.46	104.90	11.40	0.0000
*ENSTRO	9	f	1	2.25	141.36	29.73	0.0000
*ENSTRO	8	f	1	2.80	116.71	119.29	0.0000

Table 1G16 - 2

IESLC - Meta-anal of Ever Smoking (or Curr if Ever not avail) by Amount, Overview, Cigs (or Any Prod if Cigs not avail)
All LC types
Most adjusted

REF	NRR	SEX	AD	Ys	Ws	Qs	Ps
Subtotal ENSTRO				2.23	714.48	428.05	
ESAKI	1	m	0	0.09	7.53	21.71	0.8001
ESAKI	2	m	0	0.80	7.75	7.56	0.0253
ESAKI	3	m	0	1.78	4.39	0.00	0.0002
Subtotal ESAKI				0.75	19.68	29.27	
FAN	6	m	0	-0.35	8.53	39.11	0.3059
FAN	7	m	0	0.71	17.63	20.62	0.0029
FAN	8	m	0	1.38	21.51	3.62	0.0000
FAN	9	m	0	2.41	9.89	3.77	0.0000
FAN	10	f	0	0.50	10.28	17.22	0.1116
FAN	11	f	0	1.32	12.82	2.79	0.0000
FAN	12	f	0	2.48	7.51	3.60	0.0000
FAN	13	f	0	2.92	0.79	1.01	0.0095
Subtotal FAN				1.19	88.96	91.75	
GAO	21	f	2	0.55	30.53	46.70	0.0022
GAO	22	f	2	1.11	22.93	10.56	0.0000
GAO	23	f	2	2.58	9.77	6.07	0.0000
Subtotal GAO				1.07	63.23	63.32	
GAO2	2	m	0	1.21	6.65	2.22	0.0018
GAO2	3	m	0	2.02	7.66	0.40	0.0000
GAO2	4	m	0	2.36	7.06	2.32	0.0000
Subtotal GAO2				1.88	21.37	4.94	
GARSHI	26	m	1	1.19	24.90	8.95	0.0000
GARSHI	27	m	1	1.74	31.76	0.07	0.0000
GARSHI	28	m	1	2.04	25.11	1.56	0.0000
GARSHI	29	m	1	1.66	27.73	0.50	0.0000
Subtotal GARSHI				1.66	109.49	11.08	
GER	22	c	14	0.23	7.27	17.68	0.5331
GER	23	c	14	0.64	8.71	11.49	0.0582
GER	24	c	14	1.10	6.65	3.19	0.0046
Subtotal GER				0.64	22.63	32.36	
GOLLED	1	m	1	1.49	12.18	1.08	0.0000
GOLLED	2	m	1	1.85	12.69	0.05	0.0000
GOLLED	3	m	1	2.91	12.60	15.67	0.0000
Subtotal GOLLED				2.09	37.47	16.80	
GSELL	1	m	0	1.49	1.53	0.14	0.0655
GSELL	2	m	0	1.37	1.51	0.27	0.0931
GSELL	3	m	0	2.71	1.64	1.39	0.0005
GSELL	4	m	0	4.37	1.50	9.98	0.0000
GSELL	5	m	0	4.04	1.58	8.03	0.0000
Subtotal GSELL				2.80	7.77	19.81	
HAENSZ	52	f	0	0.57	17.63	26.32	0.0170
HAENSZ	51	f	0	1.64	7.30	0.17	0.0000
Subtotal HAENSZ				0.88	24.93	26.48	
*HAMMO2	7	m	1	2.21	4.47	0.80	0.0000
*HAMMO2	6	m	1	2.34	4.88	1.48	0.0000
Subtotal HAMMO2				2.28	9.35	2.28	
*HAMMON	153	m	1	2.00	8.19	0.36	0.0000
*HAMMON	154	m	1	2.12	11.45	1.23	0.0000
*HAMMON	155	m	1	2.84	10.98	12.01	0.0000
Subtotal HAMMON				2.34	30.62	13.60	
*HANSEN	1	m	2	0.31	5.16	11.23	0.4747
*HANSEN	2	m	2	1.06	3.91	2.06	0.0352
Subtotal HANSEN				0.64	9.07	13.29	
*HIRAYA	23	m	1	0.72	36.53	41.66	0.0000
*HIRAYA	24	m	1	1.39	77.85	12.72	0.0000
*HIRAYA	25	m	1	1.83	89.10	0.15	0.0000
*HIRAYA	26	f	1	0.81	38.69	37.13	0.0000
*HIRAYA	27	f	1	0.94	36.49	26.40	0.0000
*HIRAYA	28	f	1	1.50	15.75	1.35	0.0000
Subtotal HIRAYA				1.31	294.40	119.41	
HITOSU	35	m	1	0.73	5.44	6.09	0.0875
HITOSU	36	m	1	1.04	5.81	3.30	0.0125
HITOSU	37	m	1	1.54	5.14	0.31	0.0005
HITOSU	60	f	1	1.13	12.11	5.21	0.0001
HITOSU	61	f	1	1.15	3.27	1.32	0.0370
Subtotal HITOSU				1.12	31.76	16.24	
*HOLE	1	m	1	1.70	5.37	0.04	0.0001
*HOLE	3	m	1	2.19	6.47	1.01	0.0000
*HOLE	4	m	1	2.37	5.91	2.02	0.0000
*HOLE	5	m	1	2.01	4.55	0.23	0.0000

Table 1G16 - 2

IESLC - Meta-anal of Ever Smoking (or Curr if Ever not avail) by Amount, Overview, Cigs (or Any Prod if Cigs not avail)
All LC types
Most adjusted

REF	NRR	SEX	AD	Ys	Ws	Qs	Ps
Subtotal HOLE				2.08	22.31	3.30	
HU	1	m	0	0.44	10.71	19.61	0.1527
HU	2	m	0	0.74	12.38	13.74	0.0095
HU	3	m	0	1.29	6.63	1.64	0.0009
HU	4	f	0	0.82	5.04	4.71	0.0643
HU	5	f	0	0.18	2.64	6.82	0.7672
HU	6	f	0	-0.51	0.65	3.43	0.6812
Subtotal HU				0.70	38.05	49.94	
HU2	2	c	0	-0.16	9.46	35.92	0.6262
HU2	3	c	0	0.29	18.89	42.59	0.2087
HU2	4	c	0	0.66	22.08	28.10	0.0019
HU2	5	c	0	0.74	21.23	23.64	0.0007
HU2	6	c	0	1.10	32.09	15.15	0.0000
HU2	7	c	0	1.78	12.31	0.00	0.0000
Subtotal HU2				0.79	116.06	145.39	
HUMBLE	2	m	1	2.22	3.63	0.67	0.0000
HUMBLE	3	m	1	3.21	4.80	9.62	0.0000
HUMBLE	5	m	1	2.45	1.57	0.69	0.0021
HUMBLE	6	m	1	3.26	1.44	3.12	0.0001
HUMBLE	8	f	1	2.95	3.07	4.17	0.0000
HUMBLE	9	f	1	2.77	5.38	5.19	0.0000
HUMBLE	11	f	1	2.92	2.12	2.69	0.0000
HUMBLE	12	f	1	3.61	1.37	4.52	0.0000
Subtotal HUMBLE				2.87	23.38	30.66	
JARUP	4	m	2	1.93	3.04	0.06	0.0008
JARUP	5	m	2	2.08	3.44	0.29	0.0001
Subtotal JARUP				2.01	6.49	0.35	
JEDRYC	45	m	4	1.25	23.96	7.08	0.0000
JEDRYC	46	m	4	1.82	28.13	0.02	0.0000
JEDRYC	47	m	4	2.04	23.96	1.49	0.0000
JEDRYC	48	f	4	1.85	5.04	0.02	0.0000
JEDRYC	49	f	4	0.87	4.91	4.19	0.0546
JEDRYC	50	f	4	2.00	2.63	0.11	0.0012
Subtotal JEDRYC				1.68	88.63	12.91	
JOLY	7	m	0	1.68	5.92	0.07	0.0000
JOLY	8	m	0	2.52	10.45	5.52	0.0000
JOLY	9	m	0	2.57	9.85	6.00	0.0000
JOLY	10	m	0	3.08	10.07	16.76	0.0000
JOLY	3	f	0	1.55	12.60	0.71	0.0000
JOLY	4	f	0	2.08	17.52	1.46	0.0000
JOLY	5	f	0	1.94	9.62	0.20	0.0000
JOLY	6	f	0	2.59	7.64	4.94	0.0000
Subtotal JOLY				2.24	83.67	35.66	
JUSSAW	34	m	2	1.72	4.79	0.03	0.0002
JUSSAW	35	m	2	2.64	2.83	2.04	0.0000
Subtotal JUSSAW				2.06	7.62	2.06	
*KAISE2	66	m	1	1.50	5.94	0.51	0.0003
*KAISE2	67	m	1	2.34	9.98	2.97	0.0000
*KAISE2	58	f	1	2.03	5.35	0.31	0.0000
*KAISE2	59	f	1	3.10	8.34	14.22	0.0000
Subtotal KAISE2				2.33	29.61	18.00	
*KAISER	6	m	2	1.88	13.61	0.12	0.0000
*KAISER	7	m	2	2.85	16.98	18.96	0.0000
*KAISER	8	m	2	3.04	25.61	39.99	0.0000
*KAISER	2	f	2	1.23	18.49	5.82	0.0000
*KAISER	3	f	2	2.08	24.04	1.97	0.0000
*KAISER	4	f	2	2.54	19.02	10.57	0.0000
Subtotal KAISER				2.32	117.74	77.43	
KANELL	26	m	1	0.54	23.99	37.74	0.0086
KANELL	27	m	1	1.95	24.67	0.66	0.0000
KANELL	28	m	1	3.02	8.40	12.60	0.0000
KANELL	29	m	1	3.53	10.82	32.83	0.0000
Subtotal KANELL				1.84	67.88	83.83	
KATSOU	3	f	1	0.82	6.64	6.32	0.0356
KATSOU	4	f	1	2.01	2.99	0.14	0.0005
Subtotal KATSOU				1.19	9.63	6.46	
KAUFMA	11	c	6	2.08	16.83	1.40	0.0000
KAUFMA	12	c	6	2.71	22.15	18.64	0.0000
KAUFMA	13	c	6	3.33	16.99	40.38	0.0000
KAUFMA	14	c	6	3.76	18.01	69.94	0.0000
KAUFMA	15	c	6	4.09	13.43	71.28	0.0000

Table 1G16 - 2

IESLC - Meta-anal of Ever Smoking (or Curr if Ever not avail) by Amount, Overview, Cigs (or Any Prod if Cigs not avail)
All LC types
Most adjusted

REF	NRR	SEX	AD	Ys	Ws	Qs	Ps
Subtotal KAUFMA				3.14	87.41	201.65	
KHUDER	1	m	0	0.92	16.30	12.37	0.0002
KHUDER	2	m	0	2.35	18.30	5.66	0.0000
KHUDER	3	m	0	3.49	14.48	41.89	0.0000
Subtotal KHUDER				2.21	49.07	59.92	
*KINLEN	14	m	2	2.36	6.82	2.22	0.0000
*KINLEN	15	m	2	2.65	6.84	5.04	0.0000
*KINLEN	16	m	2	3.08	6.76	11.23	0.0000
Subtotal KINLEN				2.70	20.42	18.49	
*KNEKT	29	m	1	1.61	4.66	0.15	0.0005
*KNEKT	30	m	1	2.54	5.47	3.08	0.0000
Subtotal KNEKT				2.11	10.13	3.24	
KOO	11	f	0	0.31	7.09	15.62	0.4151
KOO	12	f	0	1.99	3.69	0.14	0.0001
KOO	13	f	0	0.42	0.49	0.93	0.7696
Subtotal KOO				0.86	11.27	16.69	
KOULUM	6	m	0	1.65	3.87	0.08	0.0012
KOULUM	5	m	0	3.17	4.27	8.18	0.0000
KOULUM	4	m	0	4.23	4.27	25.47	0.0000
Subtotal KOULUM				3.06	12.42	33.73	
KREUZE	19	m	3	0.92	2.54	1.94	0.1444
KREUZE	20	m	3	2.16	4.57	0.63	0.0000
KREUZE	21	m	3	2.97	4.24	5.91	0.0000
KREUZE	22	m	3	3.03	3.39	5.25	0.0000
KREUZE	30	m	3	2.10	18.30	1.80	0.0000
KREUZE	31	m	3	3.22	20.26	41.57	0.0000
KREUZE	32	m	3	3.49	18.97	54.83	0.0000
KREUZE	33	m	3	3.51	16.38	48.18	0.0000
KREUZE	25	f	3	1.74	2.81	0.01	0.0035
KREUZE	26	f	3	2.47	3.44	1.58	0.0000
KREUZE	27	f	3	2.49	2.00	0.99	0.0004
KREUZE	36	f	3	0.69	15.02	18.08	0.0072
KREUZE	37	f	3	1.69	19.01	0.21	0.0000
KREUZE	38	f	3	2.04	6.02	0.38	0.0000
Subtotal KREUZE				2.45	136.95	181.34	
KREYBE	9	m	1	1.76	5.70	0.00	0.0000
KREYBE	10	m	1	1.83	5.32	0.01	0.0000
KREYBE	11	m	1	2.66	5.07	3.84	0.0000
KREYBE	28	f	1	0.31	7.13	15.69	0.4115
KREYBE	29	f	1	0.65	2.02	2.65	0.3573
Subtotal KREYBE				1.46	25.24	22.19	
LAMTH	7	f	0	0.98	29.68	19.32	0.0000
LAMTH	2	f	0	1.68	18.27	0.23	0.0000
LAMTH	9	f	0	1.98	6.91	0.24	0.0000
Subtotal LAMTH				1.34	54.86	19.79	
LAUSSM	18	m	3	1.19	31.20	11.22	0.0000
LAUSSM	19	m	3	1.76	34.48	0.03	0.0000
LAUSSM	20	m	3	2.26	18.03	4.04	0.0000
Subtotal LAUSSM				1.66	83.71	15.29	
LETOUR	2	c	0	2.25	18.66	3.98	0.0000
LETOUR	3	c	0	2.85	18.55	20.85	0.0000
LETOUR	4	c	0	3.27	9.52	20.92	0.0000
Subtotal LETOUR				2.70	46.74	45.74	
*LIAW	3	c	2	1.13	10.81	4.70	0.0002
*LIAW	4	c	2	1.28	11.36	2.95	0.0000
*LIAW	5	c	2	2.12	7.17	0.76	0.0000
Subtotal LIAW				1.43	29.33	8.41	
*LIDDEL	2	m	1	1.20	15.00	5.18	0.0000
*LIDDEL	3	m	1	1.61	17.38	0.55	0.0000
Subtotal LIDDEL				1.42	32.38	5.73	
LIU2	8	m	3	0.18	3.50	9.04	0.7332
LIU2	9	m	3	1.96	3.78	0.11	0.0001
LIU2	10	m	3	3.06	3.18	5.15	0.0000
LIU2	14	f	3	0.59	2.81	4.07	0.3242
LIU2	15	f	3	1.25	3.48	1.01	0.0194
LIU2	16	f	3	2.88	1.70	2.04	0.0002
Subtotal LIU2				1.56	18.46	21.42	
LIU3	6	m	2	0.34	1.81	3.79	0.6441
LIU3	7	m	2	0.09	1.71	4.96	0.9103
LIU3	8	m	2	0.65	1.20	1.57	0.4778
Subtotal LIU3				0.33	4.72	10.32	

Table 1G16 - 2

IESLC - Meta-anal of Ever Smoking (or Curr if Ever not avail) by Amount, Overview, Cigs (or Any Prod if Cigs not avail)
All LC types
Most adjusted

REF	NRR	SEX	AD	Ys	Ws	Qs	Ps
LIU4	7	m	2	0.75	2108.86	2298.01	0.0000
LIU4	8	m	2	1.28	4111.92	1067.99	0.0000
LIU4	9	m	2	1.94	2991.95	69.56	0.0000
Subtotal LIU4				1.37	9212.74	3435.55	
LIU5	2	c	0	-0.20	5.84	23.16	0.6267
LIU5	3	c	0	0.46	6.33	11.28	0.2520
LIU5	4	c	0	1.28	7.79	2.06	0.0004
Subtotal LIU5				0.58	19.96	36.49	
LUBIN	11	m	4	-0.33	2.38	10.68	0.6124
LUBIN	12	m	4	0.22	3.36	8.25	0.6825
LUBIN	13	m	4	1.87	3.80	0.03	0.0003
LUBIN	14	m	4	2.08	5.32	0.44	0.0000
Subtotal LUBIN				1.22	14.85	19.40	
LUBIN2	273	m	0	1.93	155.25	3.16	0.0000
LUBIN2	274	m	0	2.03	150.14	8.45	0.0000
LUBIN2	275	m	0	2.36	152.73	49.86	0.0000
LUBIN2	276	m	0	2.52	139.75	74.78	0.0000
LUBIN2	281	f	0	0.90	66.54	53.29	0.0000
LUBIN2	282	f	0	1.30	76.70	18.47	0.0000
LUBIN2	283	f	0	1.53	47.64	3.33	0.0000
LUBIN2	284	f	0	1.57	17.75	0.84	0.0000
Subtotal LUBIN2				1.96	806.50	212.19	
MACLEN	36	c	2	0.30	6.92	15.37	0.4298
MACLEN	37	c	2	0.98	10.81	7.13	0.0013
MACLEN	38	c	2	1.08	9.92	5.08	0.0007
MACLEN	39	c	2	1.41	8.18	1.18	0.0001
Subtotal MACLEN				0.97	35.83	28.76	
MARTIS	1	m	0	1.60	2.87	0.10	0.0066
MARTIS	2	m	0	1.88	3.20	0.02	0.0008
MARTIS	3	m	0	2.24	3.09	0.62	0.0001
Subtotal MARTIS				1.91	9.17	0.74	
MATOS	29	m	2	0.69	5.93	7.14	0.0914
MATOS	31	m	2	2.01	7.84	0.39	0.0000
MATOS	33	m	2	2.34	8.28	2.52	0.0000
Subtotal MATOS				1.78	22.05	10.05	
MATSUD	1	m	0	2.53	2.76	1.50	0.0000
MATSUD	2	m	0	2.97	2.87	4.01	0.0000
MATSUD	3	m	0	3.94	2.83	13.12	0.0000
Subtotal MATSUD				3.15	8.46	18.62	
MCCONN	21	c	0	-0.80	3.10	20.89	0.1564
MCCONN	20	c	0	0.16	5.26	13.92	0.7083
MCCONN	19	c	0	0.78	4.82	4.92	0.0869
Subtotal MCCONN				0.16	13.18	39.73	
*MIGRAN	2	m	2	1.39	2.61	0.42	0.0249
*MIGRAN	4	m	2	1.44	3.70	0.44	0.0055
*MIGRAN	6	m	2	1.64	3.44	0.08	0.0024
*MIGRAN	8	m	2	1.78	3.35	0.00	0.0011
*MIGRAN	29	f	2	1.59	1.88	0.08	0.0298
*MIGRAN	31	f	2	1.88	3.01	0.02	0.0011
*MIGRAN	33	f	2	2.01	1.67	0.08	0.0093
Subtotal MIGRAN				1.66	19.66	1.13	
*MRFITR	3	m	0	2.38	0.42	0.15	0.1236
*MRFITR	4	m	0	3.91	0.50	2.23	0.0059
*MRFITR	5	m	0	4.03	0.50	2.49	0.0045
Subtotal MRFITR				3.50	1.41	4.87	
NAM	74	m	1	1.90	17.45	0.22	0.0000
NAM	75	m	1	2.33	17.40	5.05	0.0000
NAM	90	f	1	2.20	19.83	3.39	0.0000
NAM	91	f	1	2.81	15.98	16.69	0.0000
Subtotal NAM				2.30	70.66	25.34	
NOTAN2	8	m	0	-0.54	5.01	27.30	0.2229
NOTAN2	9	m	0	0.88	15.08	12.42	0.0006
NOTAN2	10	m	0	1.50	17.54	1.52	0.0000
Subtotal NOTAN2				0.98	37.63	41.24	
ORMOS	1	m	0	2.38	5.60	1.94	0.0000
ORMOS	2	m	0	2.04	5.85	0.37	0.0000
ORMOS	3	m	0	2.57	4.57	2.75	0.0000
Subtotal ORMOS				2.31	16.03	5.05	
OSANN	49	m	2	2.87	33.51	39.30	0.0000
OSANN	57	m	2	3.76	33.40	129.09	0.0000
OSANN	50	f	2	2.67	52.45	40.31	0.0000

Table 1G16 - 2

IESLC - Meta-anal of Ever Smoking (or Curr if Ever not avail) by Amount, Overview, Cigs (or Any Prod if Cigs not avail)
All LC types
Most adjusted

REF	NRR	SEX	AD	Ys	Ws	Qs	Ps
OSANN	58	f	2	3.71	34.52	127.31	0.0000
Subtotal OSANN				3.18	153.87	336.02	
PARKIN	20	m	0	1.49	66.76	5.88	0.0000
PARKIN	21	m	0	1.72	28.72	0.13	0.0000
Subtotal PARKIN				1.56	95.49	6.02	
PASTOR	6	m	1	0.85	3.21	2.87	0.1295
PASTOR	7	m	1	1.86	6.41	0.03	0.0000
PASTOR	8	m	1	2.08	7.09	0.60	0.0000
PASTOR	9	m	1	2.15	5.97	0.78	0.0000
Subtotal PASTOR				1.86	22.69	4.28	
PERNU	17	m	0	1.04	6.79	3.80	0.0066
PERNU	18	m	0	1.72	15.98	0.08	0.0000
PERNU	19	m	0	1.89	34.69	0.34	0.0000
PERNU	20	m	0	1.68	27.21	0.32	0.0000
PERNU	21	m	0	2.28	42.95	10.47	0.0000
PERNU	22	m	0	2.94	28.38	37.62	0.0000
PERNU	23	m	0	1.60	12.13	0.46	0.0000
PERNU	24	m	0	2.71	10.11	8.49	0.0000
PERNU	11	f	0	0.64	2.41	3.21	0.3224
PERNU	12	f	0	1.22	3.48	1.12	0.0225
PERNU	13	f	0	0.16	3.41	9.03	0.7636
PERNU	14	f	0	-0.46	0.92	4.69	0.6574
PERNU	15	f	0	-0.22	0.91	3.67	0.8339
PERNU	16	f	0	1.84	2.83	0.01	0.0019
Subtotal PERNU				2.00	192.21	83.33	
PERSH2	8	c	4	1.75	77.78	0.12	0.0000
PERSH2	9	c	4	2.43	82.57	33.59	0.0000
Subtotal PERSH2				2.10	160.35	33.71	
*PETO	2	m	0	1.70	1.93	0.01	0.0180
*PETO	3	m	0	2.25	1.95	0.41	0.0017
Subtotal PETO				1.98	3.88	0.43	
PEZZO2	3	m	0	2.08	5.00	0.42	0.0000
PEZZO2	4	m	0	3.79	4.86	19.48	0.0000
PEZZO2	5	m	0	4.72	3.66	31.42	0.0000
Subtotal PEZZO2				3.41	13.52	51.32	
PEZZOT	2	m	0	2.00	3.22	0.14	0.0003
PEZZOT	3	m	0	4.25	3.25	19.65	0.0000
PEZZOT	4	m	0	5.51	2.25	31.05	0.0000
Subtotal PEZZOT				3.74	8.72	50.85	
PIKE	1	m	0	1.42	12.27	1.70	0.0000
PIKE	2	m	0	2.08	11.96	1.01	0.0000
PIKE	3	m	0	1.92	8.91	0.16	0.0000
PIKE	5	f	0	1.18	14.59	5.49	0.0000
PIKE	6	f	0	1.93	10.92	0.20	0.0000
PIKE	7	f	0	2.65	2.30	1.72	0.0001
Subtotal PIKE				1.70	60.95	10.29	
POLEDN	2	c	0	1.79	8.40	0.00	0.0000
POLEDN	4	c	0	2.29	9.66	2.40	0.0000
Subtotal POLEDN				2.05	18.06	2.40	
*PRESCO	2	m	1	2.32	5.71	1.62	0.0000
*PRESCO	4	m	1	2.99	5.92	8.57	0.0000
*PRESCO	1	f	1	1.85	11.85	0.04	0.0000
*PRESCO	3	f	1	2.31	11.98	3.24	0.0000
Subtotal PRESCO				2.27	35.47	13.47	
RACHTA	10	f	1	1.29	2.59	0.64	0.0375
RACHTA	11	f	1	1.27	7.64	2.09	0.0005
RACHTA	12	f	1	2.62	6.82	4.72	0.0000
Subtotal RACHTA				1.81	17.05	7.46	

Table 1G16 - 2

IESLC - Meta-anal of Ever Smoking (or Curr if Ever not avail) by Amount, Overview, Cigs (or Any Prod if Cigs not avail)
All LC types
Most adjusted

REF	NRR	SEX	AD	Ys	Ws	Qs	Ps
RANDIG	12	m	0	0.67	2.35	2.94	0.3042
RANDIG	13	m	0	1.08	3.57	1.82	0.0420
RANDIG	14	m	0	1.65	3.82	0.07	0.0013
RANDIG	15	m	0	2.22	3.55	0.66	0.0000
RANDIG	20	f	0	-1.36	0.90	8.86	0.1996
RANDIG	21	f	0	1.61	4.35	0.14	0.0008
RANDIG	22	f	0	1.18	1.66	0.62	0.1294
Subtotal RANDIG				1.35	20.19	15.13	
SEGI2	10	m	1	0.74	4.79	5.26	0.1046
SEGI2	12	m	1	1.13	6.11	2.65	0.0052
SEGI2	14	m	1	1.22	6.23	2.00	0.0022
SEGI2	16	m	1	1.93	4.64	0.09	0.0000
SEGI2	18	m	1	2.07	5.40	0.41	0.0000
SEGI2	22	f	1	1.06	4.02	2.12	0.0328
SEGI2	24	f	1	0.36	5.21	10.58	0.4054
SEGI2	26	f	1	0.03	3.10	9.61	0.9585
Subtotal SEGI2				1.13	39.50	32.74	
SHAW	10	c	0	1.60	7.51	0.26	0.0000
SHAW	11	c	0	2.73	9.13	8.09	0.0000
Subtotal SHAW				2.22	16.64	8.35	
SIEMIA	13	m	0	1.10	2.92	1.40	0.0603
SIEMIA	14	m	0	1.50	3.87	0.32	0.0031
SIEMIA	15	m	0	2.07	3.54	0.27	0.0001
Subtotal SIEMIA				1.58	10.33	1.99	
SOBUE	117	m	0	1.26	19.84	5.59	0.0000
SOBUE	118	m	0	1.39	21.76	3.55	0.0000
SOBUE	119	m	0	1.52	21.28	1.61	0.0000
Subtotal SOBUE				1.39	62.88	10.75	
*SPEIZE	1	f	1	0.99	4.79	3.04	0.0298
*SPEIZE	2	f	1	1.65	23.37	0.47	0.0000
*SPEIZE	3	f	1	2.53	56.20	31.04	0.0000
*SPEIZE	4	f	1	2.75	58.51	54.27	0.0000
*SPEIZE	5	f	1	3.09	25.23	42.67	0.0000
Subtotal SPEIZE				2.53	168.09	131.48	
SPITZ	5	c	0	1.08	6.30	3.18	0.0067
SPITZ	6	c	0	2.94	7.05	9.39	0.0000
Subtotal SPITZ				2.07	13.35	12.57	
STOCKS	41	m	2	1.53	30.49	2.00	0.0000
STOCKS	42	m	2	2.07	29.86	2.26	0.0000
STOCKS	43	m	2	2.39	20.34	7.39	0.0000
STOCKS	44	m	2	2.31	20.54	5.45	0.0000
STOCKS	45	m	2	2.63	19.85	14.07	0.0000
STOCKS	48	f	1	0.81	40.78	39.49	0.0000
STOCKS	49	f	1	1.85	31.07	0.10	0.0000
Subtotal STOCKS				1.80	192.91	70.75	
STOCKW	1	c	0	1.90	565.53	6.55	0.0000
STOCKW	2	c	0	2.67	802.56	627.07	0.0000
STOCKW	3	c	0	3.36	411.25	1015.28	0.0000
Subtotal STOCKW				2.59	1779.33	1648.90	
SVENSS	6	f	1	1.53	8.90	0.62	0.0000
SVENSS	11	f	1	2.53	8.37	4.62	0.0000
SVENSS	16	f	1	4.08	0.91	4.78	0.0001
Subtotal SVENSS				2.12	18.19	10.03	
*TENKAN	10	m	1	2.76	5.35	5.07	0.0000
*TENKAN	11	m	1	3.01	4.70	6.97	0.0000
*TENKAN	12	m	1	3.22	4.49	9.14	0.0000
Subtotal TENKAN				2.98	14.54	21.18	
TIZZAN	7	m	0	-0.08	48.24	168.33	0.5909
TIZZAN	8	m	0	0.52	76.34	122.65	0.0000
TIZZAN	9	m	0	1.55	46.69	2.65	0.0000
TIZZAN	10	m	0	1.95	14.11	0.36	0.0000
TIZZAN	15	f	0	1.28	4.74	1.25	0.0055
TIZZAN	16	f	0	1.52	5.22	0.39	0.0005
Subtotal TIZZAN				0.77	195.34	295.63	
TSUGAN	29	m	0	-0.10	4.44	15.96	0.8253
TSUGAN	30	m	0	0.20	5.96	15.08	0.6241
TSUGAN	31	m	0	0.51	4.44	7.33	0.2861
Subtotal TSUGAN				0.20	14.85	38.37	
*TULINI	27	m	3	1.80	8.03	0.00	0.0000
*TULINI	28	m	3	2.48	9.25	4.46	0.0000
*TULINI	29	m	3	3.31	9.01	20.72	0.0000

Table 1G16 - 2

IESLC - Meta-anal of Ever Smoking (or Curr if Ever not avail) by Amount, Overview, Cigs (or Any Prod if Cigs not avail)
All LC types
Most adjusted

REF	NRR	SEX	AD	Ys	Ws	Qs	Ps
*TULINI	32	f	3	2.10	9.54	0.92	0.0000
*TULINI	33	f	3	3.27	10.56	23.11	0.0000
*TULINI	34	f	3	3.66	7.07	24.60	0.0000
Subtotal TULINI				2.76	53.47	73.81	
*TVERDA	9	m	2	0.76	10.05	10.66	0.0159
*TVERDA	10	m	2	1.20	17.25	6.02	0.0000
*TVERDA	11	m	2	1.88	16.39	0.13	0.0000
*TVERDA	16	f	2	1.51	1.87	0.15	0.0387
*TVERDA	17	f	2	2.89	2.59	3.14	0.0000
Subtotal TVERDA				1.44	48.15	20.09	
WAKAI	40	m	2	0.59	5.99	8.66	0.1504
WAKAI	41	m	2	1.39	6.99	1.13	0.0002
WAKAI	42	m	2	2.22	6.27	1.15	0.0000
Subtotal WAKAI				1.41	19.25	10.94	
WANG2	9	c	4	0.34	2.77	5.85	0.5757
WANG2	10	c	4	0.20	2.04	5.16	0.7765
WANG2	11	c	4	0.34	2.73	5.72	0.5702
WANG2	12	c	4	0.15	2.45	6.62	0.8162
WANG2	13	c	4	1.16	5.60	2.22	0.0061
WANG2	14	c	4	0.87	3.06	2.58	0.1276
WANG2	15	c	4	1.98	2.41	0.09	0.0021
Subtotal WANG2				0.79	21.05	28.24	
WU	43	f	2	1.18	9.36	3.50	0.0003
WU	44	f	2	2.14	7.57	0.91	0.0000
Subtotal WU				1.61	16.93	4.42	
WUWILL	12	f	3	0.76	101.98	109.12	0.0000
WUWILL	13	f	3	1.21	25.31	8.39	0.0000
Subtotal WUWILL				0.85	127.28	117.51	
WYNDE2	17	m	0	0.67	4.95	6.20	0.1353
WYNDE2	18	m	0	2.07	6.77	0.51	0.0000
WYNDE2	19	m	0	2.63	6.33	4.49	0.0000
WYNDE2	20	m	0	2.90	6.67	8.20	0.0000
Subtotal WYNDE2				2.16	24.72	19.40	
WYNDE3	44	m	0	0.62	3.69	5.03	0.2325
WYNDE3	45	m	0	1.89	6.93	0.07	0.0000
WYNDE3	46	m	0	2.56	6.95	4.07	0.0000
WYNDE3	47	m	0	3.24	5.69	11.99	0.0000
WYNDE3	79	f	0	-0.51	2.23	11.79	0.4459
WYNDE3	80	f	0	1.34	6.83	1.42	0.0005
WYNDE3	81	f	0	1.74	4.35	0.01	0.0003
WYNDE3	82	f	0	1.62	1.55	0.04	0.0436
Subtotal WYNDE3				1.82	38.21	34.42	
WYNDE4	43	m	0	0.69	6.13	7.48	0.0891
WYNDE4	44	m	0	1.47	8.79	0.88	0.0000
WYNDE4	45	m	0	2.08	9.99	0.82	0.0000
WYNDE4	46	m	0	2.92	9.30	11.91	0.0000
WYNDE4	47	m	0	2.97	8.67	12.03	0.0000
WYNDE4	57	f	2	0.12	2.52	7.03	0.8460
WYNDE4	58	f	2	0.70	3.11	3.71	0.2185
WYNDE4	59	f	2	1.87	3.72	0.02	0.0003
WYNDE4	60	f	2	2.45	1.18	0.51	0.0079
WYNDE4	61	f	2	2.45	1.18	0.51	0.0079
Subtotal WYNDE4				1.94	54.60	44.89	
WYNDE6	27	m	0	1.92	33.49	0.54	0.0000
WYNDE6	36	m	0	2.41	53.48	20.67	0.0000
WYNDE6	45	m	0	2.85	41.59	46.84	0.0000
WYNDE6	54	m	0	3.34	51.37	123.35	0.0000
WYNDE6	216	f	0	1.32	33.57	7.35	0.0000
WYNDE6	225	f	0	2.48	61.57	29.50	0.0000
WYNDE6	234	f	0	3.07	30.83	50.84	0.0000
WYNDE6	243	f	0	3.67	34.09	120.03	0.0000
Subtotal WYNDE6				2.65	340.00	399.11	
XU3	9	m	1	0.51	3.17	5.23	0.3668
XU3	10	m	1	1.09	3.79	1.85	0.0336
XU3	11	m	1	2.69	3.66	2.98	0.0000
XU3	12	m	1	3.32	1.36	3.18	0.0001
XU3	16	f	1	0.78	2.36	2.41	0.2316
XU3	17	f	1	1.48	2.15	0.20	0.0297
XU3	18	f	1	2.10	0.78	0.08	0.0634
Subtotal XU3				1.55	17.25	15.92	
YAMAGU	8	c	1	1.32	8.14	1.79	0.0002

Table 1G16 - 2

IESLC - Meta-anal of Ever Smoking (or Curr if Ever not avail) by Amount, Overview, Cigs (or Any Prod if Cigs not avail)
All LC types
Most adjusted

REF	NRR	SEX	AD	Ys	Ws	Qs	Ps
YAMAGU	7	c	1	2.50	5.11	2.54	0.0000
Subtotal YAMAGU				1.77	13.24	4.33	
*YUAN	2	m	2	1.28	9.07	2.36	0.0001
*YUAN	3	m	2	2.24	11.02	2.23	0.0000
Subtotal YUAN				1.81	20.09	4.59	
ZHENG	11	m	0	0.58	9.44	13.91	0.0764
ZHENG	12	m	0	0.95	13.74	9.68	0.0004
ZHENG	13	m	0	1.41	16.67	2.41	0.0000
ZHENG	14	m	0	2.10	10.04	0.97	0.0000
ZHENG	22	f	0	0.00	11.34	36.29	0.9951
ZHENG	23	f	0	1.43	10.21	1.30	0.0000
Subtotal ZHENG				1.09	71.45	64.55	
ZHOU	4	c	0	0.49	4.29	7.23	0.3077
ZHOU	5	c	0	0.70	10.77	12.72	0.0209
ZHOU	6	c	0	0.99	18.91	12.17	0.0000
Subtotal ZHOU				0.84	33.97	32.12	

N	604
NS	140

Table 1G16 - 3

IESLC - Meta-anal of Ever Smoking (or Curr if Ever not avail) by Amount, Overview, Cigs (or Any Prod if Cigs not avail)
All LC types
Most adjusted

	Sex			
	combined	male	female	Total
N	66	353	185	604
NS	21	107	62	190

In this overview table, other than the "N" rows, entries in the "absent" and "Total" columns may be invalid and should be ignored

	Amount smoked (broad categories)				
	absent	<20k5	6-44k20	>20k45	Total
N	189	174	113	128	604
NS	95	125	88	102	410
Wt	3885.00	5456.54	6949.37	5066.82	21357.73
Het Chi	2026.80	1870.36	2516.81	1890.16	12414.46
Het df	188	173	112	127	603
Het P	***	***	***	***	***
Fixed RR	8.80	3.26	5.30	10.17	5.99
RRl	8.53	3.18	5.18	9.89	5.91
RRu	9.08	3.35	5.42	10.45	6.07
P	+++	+++	+++	+++	+++
Random RR	6.81	3.52	7.32	13.70	6.58
RRl	6.08	3.16	6.28	11.80	6.14
RRu	7.64	3.92	8.53	15.89	7.05
P	+++	+++	+++	+++	+++

	Amount smoked (narrow categories)							
	absent	<10k1	2-19k10	11-29k20	21-39k30	31-98k40	>40k99	Total
N	348	70	56	88	22	2	18	604
NS	140	48	40	68	19	2	15	332
Wt	12544.80	1054.48	1019.35	5543.64	600.64	25.15	569.67	21357.73
Het Chi	7352.24	467.71	347.98	1172.27	55.85	17.02	142.60	12414.46
Het df	347	69	55	87	21	1	17	603
Het P	***	***	***	***	***	***	***	***
Fixed RR	6.55	3.12	4.90	4.44	15.96	25.62	24.66	5.99
RRl	6.44	2.94	4.61	4.32	14.74	17.33	22.72	5.91
RRu	6.67	3.32	5.22	4.56	17.29	37.87	26.77	6.07
P	+++	+++	+++	+++	+++	+++	+++	+++
Random RR	7.33	2.43	4.71	7.59	14.37	17.67	19.40	6.58
RRl	6.67	2.03	3.97	6.48	11.98	2.96	13.48	6.14
RRu	8.04	2.91	5.60	8.90	17.24	105.61	27.91	7.05
P	+++	+++	+++	+++	+++	++	+++	+++

MALES

	Amount smoked (broad categories)				
	absent	<20k5	6-44k20	>20k45	Total
N	100	99	72	82	353
NS	67	97	71	81	316
Wt	2201.45	3584.54	5450.31	4143.16	15379.46
Het Chi	768.32	927.48	1123.25	951.49	7242.42
Het df	99	98	71	81	352
Het P	***	***	***	***	***
Fixed RR	9.63	2.79	4.35	8.67	5.30
RRl	9.24	2.70	4.24	8.41	5.21
RRu	10.05	2.89	4.47	8.94	5.38
P	+++	+++	+++	+++	+++
Random RR	8.33	3.74	7.66	14.19	7.44
RRl	7.29	3.24	6.40	11.97	6.83
RRu	9.51	4.31	9.16	16.82	8.11
P	+++	+++	+++	+++	+++

Table 1G16 - 3

IESLC - Meta-anal of Ever Smoking (or Curr if Ever not avail) by Amount, Overview, Cigs (or Any Prod if Cigs not avail)
All LC types
Most adjusted

MALES

	Amount smoked (narrow categories)							
	absent	<10k1	2-19k10	11-29k20	21-39k30	31-98k40	>40k99	Total
N	204	36	30	56	15	1	11	353
NS	107	35	29	55	15	1	11	252
Wt	8634.33	624.68	560.41	4967.62	488.76	7.14	96.53	15379.46
Het Chi	4817.99	272.20	153.01	734.53	33.28	0.00	53.04	7242.42
Het df	203	35	29	55	14	0	10	352
Het P	***	***	***	***	**	N.S.	***	***
Fixed RR	5.78	3.58	5.99	4.14	15.63	6.93	17.33	5.30
RRl	5.66	3.31	5.52	4.03	14.31	3.33	14.20	5.21
RRu	5.90	3.87	6.51	4.26	17.08	14.44	21.16	5.38
P	+++	+++	+++	+++	+++	+++	+++	+++
Random RR	8.21	2.71	5.64	7.92	13.83	6.93	21.40	7.44
RRl	7.24	2.10	4.53	6.49	11.32	3.33	13.13	6.83
RRu	9.30	3.51	7.04	9.67	16.89	14.44	34.87	8.11
P	+++	+++	+++	+++	+++	+++	+++	+++

FEMALES

	Amount smoked (broad categories)				
	absent	<20k5	6-44k20	>20k45	Total
N	63	57	31	34	185
NS	47	55	30	34	166
Wt	1297.10	1084.76	574.64	399.35	3355.85
Het Chi	790.13	406.78	144.27	171.34	2337.86
Het df	62	56	30	33	184
Het P	***	***	***	***	***
Fixed RR	8.23	3.54	8.19	16.12	6.78
RRl	7.79	3.34	7.54	14.61	6.56
RRu	8.69	3.76	8.89	17.78	7.02
P	+++	+++	+++	+++	+++
Random RR	5.40	3.18	7.35	12.94	5.49
RRl	4.32	2.65	5.96	9.90	4.81
RRu	6.75	3.81	9.08	16.90	6.28
P	+++	+++	+++	+++	+++

	Amount smoked (narrow categories)							
	absent	<10k1	2-19k10	11-29k20	21-39k30	31-98k40	>40k99	Total
N	106	26	20	25	5		3	185
NS	60	25	19	24	5		3	136
Wt	2066.12	303.06	394.00	477.97	91.84		22.87	3355.85
Het Chi	1386.32	64.17	73.79	107.44	5.52		1.27	2337.86
Het df	105	25	19	24	4		2	184
Het P	***	***	***	***	N.S.		N.S.	***
Fixed RR	8.00	2.13	3.94	8.83	17.12		12.02	6.78
RRl	7.66	1.90	3.57	8.07	13.95		7.98	6.56
RRu	8.35	2.38	4.35	9.65	21.00		18.10	7.02
P	+++	+++	+++	+++	+++		+++	+++
Random RR	6.47	2.09	4.08	8.03	16.99		12.02	5.49
RRl	5.43	1.68	3.24	6.39	12.29		7.98	4.81
RRu	7.70	2.60	5.12	10.10	23.47		18.10	6.28
P	+++	+++	+++	+++	+++		+++	+++

Table 1G16 - 4

IESLC - Meta-anal of Ever Smoking (or Curr if Ever not avail) by Amount, Overview, Cigs (or Any Prod if Cigs not avail)
All LC types
Least adjusted

REF	NRR	X	SEX	AGEL	AGEH	RACE	YF	LC TYPE	LOC	START	ST	NLC	R	VB	P	H	AD	SM	PRODUCT	exL	exH	S1	S2	DENOM	De
AGUDO	11	x	f	0	0	all	-	all	Eu:wst	1989	CC	103	n	bl	n	n	0	ev	cig only	1	10	1	0	nev any	st
AGUDO	12	x	f	0	0	all	-	all	Eu:wst	1989	CC	103	n	bl	n	n	0	ev	cig only	11	99	0	0	nev any	st
AKIBA	17	x	m	0	0	all	0	all	As:Jap	1963	pr	610	n	bl	n	n	0	cu	cig+/-ot	1	14	1	0	nev cigs	or
AKIBA	18	x	m	0	0	all	0	all	As:Jap	1963	pr	610	n	bl	n	n	0	cu	cig+/-ot	15	24	2	3	nev cigs	or
AKIBA	19	x	m	0	0	all	0	all	As:Jap	1963	pr	610	n	bl	n	n	0	cu	cig+/-ot	25	99	3	0	nev cigs	or
AKIBA	23	x	f	0	0	all	0	all	As:Jap	1963	pr	610	n	bl	n	n	0	cu	cig+/-ot	1	14	1	0	nev cigs	or
AKIBA	24	x	f	0	0	all	0	all	As:Jap	1963	pr	610	n	bl	n	n	0	cu	cig+/-ot	15	99	0	0	nev cigs	or
ALDERS	18		m	0	0	all	-	all	Eu:UK	1977	CC	1448	n	V	n	n	1	ev	cig only	1	17	1	0	nev+2	ot
ALDERS	19		m	0	0	all	-	all	Eu:UK	1977	CC	1448	n	V	n	n	1	ev	cig only	18	27	2	3	nev+2	ot
ALDERS	20		m	0	0	all	-	all	Eu:UK	1977	CC	1448	n	V	n	n	1	ev	cig only	28	99	3	0	nev+2	ot
ALDERS	21		f	0	0	all	-	all	Eu:UK	1977	CC	1448	n	V	n	n	1	ev	cig only	1	17	1	0	nev+2	ot
ALDERS	22		f	0	0	all	-	all	Eu:UK	1977	CC	1448	n	V	n	n	1	ev	cig only	18	27	2	3	nev+2	ot
ALDERS	23		f	0	0	all	-	all	Eu:UK	1977	CC	1448	n	V	n	n	1	ev	cig only	28	99	3	0	nev+2	ot
ARCHER	1		m	0	0	wh	0	all	NAmer	1950	pr	146	m	bl	n	n	0	cu	cig+/-ot	1	19	1	0	nev cigs	st
ARCHER	2		m	0	0	wh	0	all	NAmer	1950	pr	146	m	bl	n	n	0	cu	cig+/-ot	20	20	2	3	nev cigs	st
ARCHER	3		m	0	0	wh	0	all	NAmer	1950	pr	146	m	bl	n	n	0	cu	cig+/-ot	21	99	3	0	nev cigs	st
ARMADA	46		m	0	0	all	-	all	Eu:wst	1986	CC	325	n	bl	n	y	0	ev	cig+/-ot	1	14	1	0	nev any	st
ARMADA	47		m	0	0	all	-	all	Eu:wst	1986	CC	325	n	bl	n	y	0	ev	cig+/-ot	15	24	2	3	nev any	st
ARMADA	48		m	0	0	all	-	all	Eu:wst	1986	CC	325	n	bl	n	y	0	ev	cig+/-ot	25	99	3	0	nev any	st
AUVINE	5	x	c	0	0	all	-	all	Eu:Sca	1986	CC	517	n	bl	y	n	0	ev	cig+/-ot	1	10	1	0	nev cigs	st
AUVINE	6	x	c	0	0	all	-	all	Eu:Sca	1986	CC	517	n	bl	y	n	0	ev	cig+/-ot	11	20	2	3	nev cigs	st
AUVINE	7	x	c	0	0	all	-	all	Eu:Sca	1986	CC	517	n	bl	y	n	0	ev	cig+/-ot	21	99	3	0	nev cigs	st
AXELSS	19	x	m	0	0	sca	-	all	Eu:Sca	1989	CC	436	n	bl	n	n	0	ev	all/unsp	1	9	1	1	nev any	st
AXELSS	20	x	m	0	0	sca	-	all	Eu:Sca	1989	CC	436	n	bl	n	n	0	ev	all/unsp	10	19	0	2	nev any	st
AXELSS	21	x	m	0	0	sca	-	all	Eu:Sca	1989	CC	436	n	bl	n	n	0	ev	all/unsp	20	99	0	0	nev any	st
AXELSS	13		f	0	0	sca	-	all	Eu:Sca	1989	CC	436	n	bl	n	n	0	ev	all/unsp	1	9	1	1	nev any	st
AXELSS	14		f	0	0	sca	-	all	Eu:Sca	1989	CC	436	n	bl	n	n	0	ev	all/unsp	10	19	0	2	nev any	st
AXELSS	15		f	0	0	sca	-	all	Eu:Sca	1989	CC	436	n	bl	n	n	0	ev	all/unsp	20	29	2	3	nev any	st
AXELSS	16		f	0	0	sca	-	all	Eu:Sca	1989	CC	436	n	bl	n	n	0	ev	all/unsp	30	99	3	0	nev any	st
BARBON	5	x	m	0	0	all	-	all	Eu:wst	1979	CC	755	n	bl	y	y	0	ev	all/unsp	1	9	1	1	nev any	st
BARBON	7	x	m	0	0	all	-	all	Eu:wst	1979	CC	755	n	bl	y	y	0	ev	all/unsp	10	19	0	2	nev any	st
BARBON	9	x	m	0	0	all	-	all	Eu:wst	1979	CC	755	n	bl	y	y	0	ev	all/unsp	20	29	2	3	nev any	st
BARBON	11	x	m	0	0	all	-	all	Eu:wst	1979	CC	755	n	bl	y	y	0	ev	all/unsp	30	39	0	4	nev any	st
BARBON	13	x	m	0	0	all	-	all	Eu:wst	1979	CC	755	n	bl	y	y	0	ev	all/unsp	40	99	3	0	nev any	st
BENSHL	11		m	40	64	all	10	all	Eu:UK	1967	pr	486	n	V	n	n	1	cu	cig+/-ot	1	9	1	1	nev any	ot
BENSHL	12		m	40	64	all	10	all	Eu:UK	1967	pr	486	n	V	n	n	1	cu	cig+/-ot	10	19	0	2	nev any	ot
BENSHL	13		m	40	64	all	10	all	Eu:UK	1967	pr	486	n	V	n	n	1	cu	cig+/-ot	20	99	0	0	nev any	ot
BEST	13		m	0	0	all	0	all	NAmer	1955	pr	381	n	V	n	n	1	cu	cig only	1	9	1	1	nev any	ot
BEST	14		m	0	0	all	0	all	NAmer	1955	pr	381	n	V	n	n	1	cu	cig only	10	20	2	0	nev any	ot
BEST	15		m	0	0	all	0	all	NAmer	1955	pr	381	n	V	n	n	1	cu	cig only	21	99	3	0	nev any	ot
BOUCOT	99		m	0	0	all	9	all	NAmer	1951	pr	121	n	bl	n	n	0	ev	cig+/-ot	1	20	0	0	nev any	ot
BOUCOT	100		m	0	0	all	9	all	NAmer	1951	pr	121	n	bl	n	n	0	ev	cig+/-ot	21	99	3	0	nev any	ot
BRESLO	13		m	0	0	all	-	all	NAmer	1949	CC	518	n	bl	n	y	0	ev	cig+/-ot	1	9	1	1	nev+3	st
BRESLO	14		m	0	0	all	-	all	NAmer	1949	CC	518	n	bl	n	y	0	ev	cig+/-ot	10	19	0	2	nev+3	st
BRESLO	15		m	0	0	all	-	all	NAmer	1949	CC	518	n	bl	n	y	0	ev	cig+/-ot	20	39	2	0	nev+3	st
BRESLO	16		m	0	0	all	-	all	NAmer	1949	CC	518	n	bl	n	y	0	ev	cig+/-ot	40	99	3	0	nev+3	st
BRESLO	29		f	0	0	all	-	all	NAmer	1949	CC	518	n	bl	n	y	0	ev	cig+/-ot	1	19	1	0	nev+3	st
BRESLO	30		f	0	0	all	-	all	NAmer	1949	CC	518	n	bl	n	y	0	ev	cig+/-ot	20	99	0	0	nev+3	st
BRETT	1		m	0	0	all	0	all	Eu:UK	1960	pr	150	n	V	n	n	0	cu	cig+/-ot	1	14	1	0	nev cigs	st
BRETT	2		m	0	0	all	0	all	Eu:UK	1960	pr	150	n	V	n	n	0	cu	cig+/-ot	15	24	2	3	nev cigs	st
BRETT	3		m	0	0	all	0	all	Eu:UK	1960	pr	150	n	V	n	n	0	cu	cig+/-ot	25	99	3	0	nev cigs	st
BROSS	18		m	0	0	wh	-	all	NAmer	1960	CC	974	n	bl	n	n	0	cu	cig+/-ot	1	20	0	0	nev any	st
BROSS	19		m	0	0	wh	-	all	NAmer	1960	CC	974	n	bl	n	n	0	cu	cig+/-ot	21	99	3	0	nev any	st
BROWN2	32		m	0	0	wh	-	all	NAmer	1984	CC	14596	n	bl	n	y	2	ev	cig+/-ot	1	19	1	0	nev cigs	or
BROWN2	42		m	0	0	wh	-	all	NAmer	1984	CC	14596	n	bl	n	y	2	ev	cig+/-ot	20	99	0	0	nev cigs	or
BROWN2	31		f	0	0	wh	-	all	NAmer	1984	CC	14596	n	bl	n	y	2	ev	cig+/-ot	1	19	1	0	nev cigs	or
BROWN2	41		f	0	0	wh	-	all	NAmer	1984	CC	14596	n	bl	n	y	2	ev	cig+/-ot	20	99	0	0	nev cigs	or
BUFFLE	28		f	0	0	w-hi	-	all	NAmer	1976	CC	943	n	bl	y	n	0	ev	cig+/-ot	1	19	1	0	nev cigs	or
BUFFLE	29		f	0	0	w-hi	-	all	NAmer	1976	CC	943	n	bl	y	n	0	ev	cig+/-ot	20	20	2	3	nev cigs	or
BUFFLE	35		f	0	0	w-hi	-	all	NAmer	1976	CC	943	n	bl	y	n	0	ev	cig+/-ot	21	99	3	0	nev cigs	or
CEDERL	87		m	0	0	all	10	all	Eu:Sca	1963	pr	491	n	bl	n	n	1	cu	cig+/-ot	1	15	1	0	nev any	ot
CEDERL	86		m	0	0	all	10	all	Eu:Sca	1963	pr	491	n	bl	n	n	1	cu	cig+/-ot	16	99	0	0	nev any	ot
CEDERL	99		f	0	0	all	10	all	Eu:Sca	1963	pr	491	n	bl	n	n	1	cu	cig+/-ot	1	15	1	0	nev any	ot
CHANG	2		m	0	0	all	0	all	NAmer	1972	pr	136	n	bl	n	n	0	cu	cig+/-ot	1	10	1	0	nev cigs	st
CHANG	3		m	0	0	all	0	all	NAmer	1972	pr	136	n	bl	n	n	0	cu	cig+/-ot	11	20	2	3	nev cigs	st
CHANG	4		m	0	0	all	0	all	NAmer	1972	pr	136	n	bl	n	n	0	cu	cig+/-ot	21	99	3	0	nev cigs	st
CHANG	8		f	0	0	all	0	all	NAmer	1972	pr	136	n	bl	n	n	0	cu	cig+/-ot	1	10	1	0	nev cigs	st
CHANG	9		f	0	0	all	0	all	NAmer	1972	pr	136	n	bl	n	n	0	cu	cig+/-ot	11	20	2	3	nev cigs	st
CHANG	10		f	0	0	all	0	all	NAmer	1972	pr	136	n	bl	n	n	0	cu	cig+/-ot	21	99	3	0	nev cigs	st
CHATZI	1		c	0	0	all	-	all	Eu:bal	1987	CC	282	n	bl	n	y	0	ev	all/unsp	1	45	0	0	nev any	st
CHATZI	2		c	0	0	all	-	all	Eu:bal	1987	CC	282	n	bl	n	y	0	ev	all/unsp	46	74	0	0	nev any	st
CHATZI	3		c	0	0	all	-	all	Eu:bal	1987	CC	282	n	bl	n	y	0	ev	all/unsp	75	99	0	6	nev any	st

Table 1G16 - 4

IESLC - Meta-anal of Ever Smoking (or Curr if Ever not avail) by Amount, Overview, Cigs (or Any Prod if Cigs not avail)
All LC types
Least adjusted

REF	NRR	X	SEX	AGEL	AGEH	RACE	YF	LC TYPE	LOC	START	ST	NLC	R	VB	P	H	AD	SM	PRODUCT	exL	exH	S1	S2	DENOM	De
CHEN2	3		m	0	0	all	-	all	As:Chi	1983	CC	193	n	ot	y	n	0	ev	all/unsp	1	9	1	1	nev any	st
CHEN2	4		m	0	0	all	-	all	As:Chi	1983	CC	193	n	ot	y	n	0	ev	all/unsp	10	20	2	0	nev any	st
CHEN2	5		m	0	0	all	-	all	As:Chi	1983	CC	193	n	ot	y	n	0	ev	all/unsp	21	30	0	4	nev any	st
CHEN2	6		m	0	0	all	-	all	As:Chi	1983	CC	193	n	ot	y	n	0	ev	all/unsp	31	99	3	0	nev any	st
CHEN2	7		f	0	0	all	-	all	As:Chi	1983	CC	193	n	ot	y	n	0	ev	all/unsp	1	9	1	1	nev any	st
CHEN2	8		f	0	0	all	-	all	As:Chi	1983	CC	193	n	ot	y	n	0	ev	all/unsp	10	20	2	0	nev any	st
CHEN2	9		f	0	0	all	-	all	As:Chi	1983	CC	193	n	ot	y	n	0	ev	all/unsp	21	30	0	4	nev any	st
CHEN2	10		f	0	0	all	-	all	As:Chi	1983	CC	193	n	ot	y	n	0	ev	all/unsp	31	99	3	0	nev any	st
CHOI	12		m	0	0	all	-	all	As:oth	1985	CC	375	n	bl	n	n	0	ev	cig+/-ot	1	10	1	0	nev cigs	st
CHOI	13		m	0	0	all	-	all	As:oth	1985	CC	375	n	bl	n	n	0	ev	cig+/-ot	11	20	2	3	nev cigs	st
CHOI	14		m	0	0	all	-	all	As:oth	1985	CC	375	n	bl	n	n	0	ev	cig+/-ot	21	30	0	4	nev cigs	st
CHOI	15		m	0	0	all	-	all	As:oth	1985	CC	375	n	bl	n	n	0	ev	cig+/-ot	31	40	0	5	nev cigs	st
CHOI	16		m	0	0	all	-	all	As:oth	1985	CC	375	n	bl	n	n	0	ev	cig+/-ot	41	99	3	6	nev cigs	st
CHOI	17		f	0	0	all	-	all	As:oth	1985	CC	375	n	bl	n	n	0	ev	cig+/-ot	1	10	1	0	nev cigs	st
CHOI	18		f	0	0	all	-	all	As:oth	1985	CC	375	n	bl	n	n	0	ev	cig+/-ot	11	30	2	0	nev cigs	st
CHOI	20		f	0	0	all	-	all	As:oth	1985	CC	375	n	bl	n	n	0	ev	cig+/-ot	31	99	3	0	nev cigs	st
CHOW	3	x	m	0	0	wh	0	all	NAmer	1966	pr	219	n	bl	n	n	0	cu	cig+/-ot	1	19	1	0	nev any	st
CHOW	4	x	m	0	0	wh	0	all	NAmer	1966	pr	219	n	bl	n	n	0	cu	cig+/-ot	20	29	2	3	nev any	st
CHOW	5	x	m	0	0	wh	0	all	NAmer	1966	pr	219	n	bl	n	n	0	cu	cig+/-ot	30	99	3	0	nev any	st
COMSTO	4		m	0	0	all	-	all	NAmer	1975	ot	258	n	bl	n	n	0	cu	cig+/-ot	1	19	1	0	nev any	st
COMSTO	5		m	0	0	all	-	all	NAmer	1975	ot	258	n	bl	n	n	0	cu	cig+/-ot	20	39	2	0	nev any	st
COMSTO	6		m	0	0	all	-	all	NAmer	1975	ot	258	n	bl	n	n	0	cu	cig+/-ot	40	99	3	0	nev any	st
COMSTO	9		f	0	0	all	-	all	NAmer	1975	ot	258	n	bl	n	n	0	cu	cig+/-ot	1	19	1	0	nev any	st
COMSTO	10		f	0	0	all	-	all	NAmer	1975	ot	258	n	bl	n	n	0	cu	cig+/-ot	20	39	2	0	nev any	st
COMSTO	11		f	0	0	all	-	all	NAmer	1975	ot	258	n	bl	n	n	0	cu	cig+/-ot	40	99	3	0	nev any	st
COOKSO	1		c	0	0	bl	-	all	Africa	1961	CC	234	n	V	n	y	0	ev	cig+/-ot	1	9	1	1	nev any	st
COOKSO	2		c	0	0	bl	-	all	Africa	1961	CC	234	n	V	n	y	0	ev	cig+/-ot	10	99	0	0	nev any	st
CORREA	45	x	c	0	0	all	-	all	NAmer	1979	CC	1359	n	bl	y	n	0	cu	cig+/-ot	1	20	0	0	nev cigs	st
CORREA	49	x	c	0	0	all	-	all	NAmer	1979	CC	1359	n	bl	y	n	0	cu	cig+/-ot	21	99	3	0	nev cigs	st
CPSI	243		m	50	74	all	6	all	NAmer	1959	pr	5138	n	bl	n	n	1	ev	cig only	1	19	1	0	nev any	ot
CPSI	246		m	50	74	all	6	all	NAmer	1959	pr	5138	n	bl	n	n	1	ev	cig only	20	99	0	0	nev any	ot
CPSI	275		f	40	74	all	6	all	NAmer	1959	pr	5138	n	bl	n	n	1	cu	cig+/-ot	1	9	1	1	nev cigs	ot
CPSI	276		f	40	74	all	6	all	NAmer	1959	pr	5138	n	bl	n	n	1	cu	cig+/-ot	10	19	0	2	nev cigs	ot
CPSI	277		f	40	74	all	6	all	NAmer	1959	pr	5138	n	bl	n	n	1	cu	cig+/-ot	20	39	2	0	nev cigs	ot
CPSI	278		f	40	74	all	6	all	NAmer	1959	pr	5138	n	bl	n	n	1	cu	cig+/-ot	40	99	3	0	nev cigs	ot
CPSII	102		m	35	99	all	4	all	NAmer	1982	pr	3229	n	bl	n	n	1	ev	cig only	1	20	0	0	nev any	ot
CPSII	103		m	35	99	all	4	all	NAmer	1982	pr	3229	n	bl	n	n	1	ev	cig only	21	99	3	0	nev any	ot
CPSII	105		f	35	99	all	4	all	NAmer	1982	pr	3229	n	bl	n	n	1	ev	cig+/-ot	1	19	1	0	nev cigs	ot
CPSII	106		f	35	99	all	4	all	NAmer	1982	pr	3229	n	bl	n	n	1	ev	cig+/-ot	20	99	0	0	nev cigs	ot
DAMBER	6		m	0	0	all	-	all	Eu:Sca	1972	CC	579	n	bl	y	n	1	ev	cig only	1	7	1	1	nev any	ot
DAMBER	7		m	0	0	all	-	all	Eu:Sca	1972	CC	579	n	bl	y	n	1	ev	cig only	8	15	0	2	nev any	ot
DAMBER	8		m	0	0	all	-	all	Eu:Sca	1972	CC	579	n	bl	y	n	1	ev	cig only	16	25	2	3	nev any	ot
DAMBER	9		m	0	0	all	-	all	Eu:Sca	1972	CC	579	n	bl	y	n	1	ev	cig only	26	99	3	0	nev any	ot
DARBY	1		m	0	0	wh	-	all	Eu:UK	1988	CC	982	n	V	n	n	0	cu	cig+/-ot	1	14	1	0	nev any	st
DARBY	2		m	0	0	wh	-	all	Eu:UK	1988	CC	982	n	V	n	n	0	cu	cig+/-ot	15	24	2	3	nev any	st
DARBY	3		m	0	0	wh	-	all	Eu:UK	1988	CC	982	n	V	n	n	0	cu	cig+/-ot	25	99	3	0	nev any	st
DARBY	8		f	0	0	wh	-	all	Eu:UK	1988	CC	982	n	V	n	n	0	cu	cig+/-ot	1	14	1	0	nev any	st
DARBY	9		f	0	0	wh	-	all	Eu:UK	1988	CC	982	n	V	n	n	0	cu	cig+/-ot	15	24	2	3	nev any	st
DARBY	10		f	0	0	wh	-	all	Eu:UK	1988	CC	982	n	V	n	n	0	cu	cig+/-ot	25	99	3	0	nev any	st
DAVEYS	1		m	0	0	all	-	all	Eu:Ger	1930	CC	109	n	bl	y	n	0	ev	all/unsp	1	5	1	1	nev any	st
DAVEYS	2		m	0	0	all	-	all	Eu:Ger	1930	CC	109	n	bl	y	n	0	ev	all/unsp	6	10	0	2	nev any	st
DAVEYS	3		m	0	0	all	-	all	Eu:Ger	1930	CC	109	n	bl	y	n	0	ev	all/unsp	11	20	2	3	nev any	st
DAVEYS	4		m	0	0	all	-	all	Eu:Ger	1930	CC	109	n	bl	y	n	0	ev	all/unsp	21	99	3	0	nev any	st
DEAN	1		m	0	0	wh	-	all	Africa	1947	CC	603	n	V	y	n	0	ev	cig only	1	20	0	0	nev any	st
DEAN	2		m	0	0	wh	-	all	Africa	1947	CC	603	n	V	y	n	0	ev	cig only	25	45	3	0	nev any	st
DEAN	3		m	0	0	wh	-	all	Africa	1947	CC	603	n	V	y	n	0	ev	cig only	50	99	0	6	nev any	st
DEAN2	25		m	0	0	all	-	all	Eu:UK	1960	CC	954	n	V	y	n	0	ev	cig only	1	22	0	0	nev any	st
DEAN2	26		m	0	0	all	-	all	Eu:UK	1960	CC	954	n	V	y	n	0	ev	cig only	23	99	3	0	nev any	st
DEAN2	29		f	0	0	all	-	all	Eu:UK	1960	CC	954	n	V	y	n	0	ev	cig only	1	22	0	0	nev any	st
DEAN2	30		f	0	0	all	-	all	Eu:UK	1960	CC	954	n	V	y	n	0	ev	cig only	23	99	3	0	nev any	st
DEAN3	5	x	m	0	0	all	-	all	Eu:UK	1969	CC	766	n	V	y	n	0	cu	cig only	1	12	1	0	nev any	st
DEAN3	12	x	m	0	0	all	-	all	Eu:UK	1969	CC	766	n	V	y	n	0	cu	cig only	13	22	2	3	nev any	st
DEAN3	19	x	m	0	0	all	-	all	Eu:UK	1969	CC	766	n	V	y	n	0	cu	cig only	23	99	3	0	nev any	st
DEAN3	89	x	f	0	0	all	-	all	Eu:UK	1969	CC	766	n	V	y	n	0	cu	cig only	1	12	1	0	nev any	st
DEAN3	96	x	f	0	0	all	-	all	Eu:UK	1969	CC	766	n	V	y	n	0	cu	cig only	13	22	2	3	nev any	st
DEAN3	103	x	f	0	0	all	-	all	Eu:UK	1969	CC	766	n	V	y	n	0	cu	cig only	23	99	3	0	nev any	st
DEKLER	2		m	0	0	all	0	all	Auslia	1961	pr	138	m	V	n	n	2	cu	cig+/-ot	1	14	1	0	nev any	or
DEKLER	3		m	0	0	all	0	all	Auslia	1961	pr	138	m	V	n	n	2	cu	cig+/-ot	15	24	2	3	nev any	or
DEKLER	4		m	0	0	all	0	all	Auslia	1961	pr	138	m	V	n	n	2	cu	cig+/-ot	25	99	3	0	nev any	or
DESTEF	1	x	m	0	0	all	-	all	SCAmer	1988	CC	497	n	bl	n	y	0	ev	all/unsp	1	10	1	0	nev any	st
DESTEF	2	x	m	0	0	all	-	all	SCAmer	1988	CC	497	n	bl	n	y	0	ev	all/unsp	11	20	2	3	nev any	st
DESTEF	3	x	m	0	0	all	-	all	SCAmer	1988	CC	497	n	bl	n	y	0	ev	all/unsp	21	40	0	0	nev any	st

Table 1G16 - 4

IESLC - Meta-anal of Ever Smoking (or Curr if Ever not avail) by Amount, Overview, Cigs (or Any Prod if Cigs not avail)
All LC types
Least adjusted

REF	NRR	X	SEX	AGEL	AGEH	RACE	YF	LC TYPE	LOC	START	ST	NLC	R	VB	P	H	AD	SM	PRODUCT	exL	exH	S1	S2	DENOM	De
DESTEF	4	x	m	0	0	all	-	all	SCAmer	1988	CC	497	n	bl	n	y	0	ev	all/unsp	41	99	3	6	nev any	st
DOLL	1		m	0	0	all	-	all	Eu:UK	1948	CC	1465	n	V	n	n	0	ev	all/unsp	1	4	0	1	nev any	st
DOLL	2		m	0	0	all	-	all	Eu:UK	1948	CC	1465	n	V	n	n	0	ev	all/unsp	5	14	1	2	nev any	st
DOLL	3		m	0	0	all	-	all	Eu:UK	1948	CC	1465	n	V	n	n	0	ev	all/unsp	15	24	2	3	nev any	st
DOLL	4		m	0	0	all	-	all	Eu:UK	1948	CC	1465	n	V	n	n	0	ev	all/unsp	25	49	3	0	nev any	st
DOLL	5		m	0	0	all	-	all	Eu:UK	1948	CC	1465	n	V	n	n	0	ev	all/unsp	50	99	0	6	nev any	st
DOLL	7		f	0	0	all	-	all	Eu:UK	1948	CC	1465	n	V	n	n	0	ev	all/unsp	1	4	0	1	nev any	st
DOLL	8		f	0	0	all	-	all	Eu:UK	1948	CC	1465	n	V	n	n	0	ev	all/unsp	5	14	1	2	nev any	st
DOLL	9		f	0	0	all	-	all	Eu:UK	1948	CC	1465	n	V	n	n	0	ev	all/unsp	15	24	2	3	nev any	st
DOLL	10		f	0	0	all	-	all	Eu:UK	1948	CC	1465	n	V	n	n	0	ev	all/unsp	25	49	3	0	nev any	ot
DOLL2	46		m	35	99	all	5	all	Eu:UK	1951	pr	920	n	V	n	n	1	ev	all/unsp	1	14	1	0	nev any	ot
DOLL2	47		m	35	99	all	5	all	Eu:UK	1951	pr	920	n	V	n	n	1	ev	all/unsp	15	24	2	3	nev any	ot
DOLL2	48		m	35	99	all	5	all	Eu:UK	1951	pr	920	n	V	n	n	1	ev	all/unsp	25	99	3	0	nev any	ot
DOLL2	10		f	0	0	all	22	all	Eu:UK	1951	pr	920	n	V	n	n	1	cu	cig only	1	14	1	0	nev any	ot
DOLL2	11		f	0	0	all	22	all	Eu:UK	1951	pr	920	n	V	n	n	1	cu	cig only	15	24	2	3	nev any	ot
DOLL2	12		f	0	0	all	22	all	Eu:UK	1951	pr	920	n	V	n	n	1	cu	cig only	25	99	3	0	nev any	ot
DORANT	6		c	0	0	all	0	all	Eu:wst	1986	ot	550	n	bl	n	y	0	cu	cig+/-ot	1	9	1	1	nev any	st
DORANT	7		c	0	0	all	0	all	Eu:wst	1986	ot	550	n	bl	n	y	0	cu	cig+/-ot	10	19	0	2	nev any	st
DORANT	8		c	0	0	all	0	all	Eu:wst	1986	ot	550	n	bl	n	y	0	cu	cig+/-ot	20	99	0	0	nev any	st
DORGAN	10	x	m	0	0	wh	-	all	NAmer	1980	CC	2026	n	bl	y	y	0	ev	cig+/-ot	1	19	1	0	nev any	st
DORGAN	11	x	m	0	0	wh	-	all	NAmer	1980	CC	2026	n	bl	y	y	0	ev	cig+/-ot	20	99	0	0	nev any	st
DORGAN	34	x	m	0	0	bl	-	all	NAmer	1980	CC	2026	n	bl	y	y	0	ev	cig+/-ot	1	19	1	0	nev any	st
DORGAN	35	x	m	0	0	bl	-	all	NAmer	1980	CC	2026	n	bl	y	y	0	ev	cig+/-ot	20	99	0	0	nev any	st
DORGAN	96		f	0	0	all	-	all	NAmer	1980	CC	2026	n	bl	y	y	3	ev	cig+/-ot	1	19	1	0	nev any	ot
DORGAN	97		f	0	0	all	-	all	NAmer	1980	CC	2026	n	bl	y	y	3	ev	cig+/-ot	20	99	0	0	nev any	ot
DORN	408		m	0	0	wh	25	all	NAmer	1954	pr	5097	n	bl	n	n	1	cu	cig+/-ot	1	9	1	1	nev any	or
DORN	409		m	0	0	wh	25	all	NAmer	1954	pr	5097	n	bl	n	n	1	cu	cig+/-ot	10	20	2	0	nev any	or
DORN	410		m	0	0	wh	25	all	NAmer	1954	pr	5097	n	bl	n	n	1	cu	cig+/-ot	21	39	0	4	nev any	or
DORN	411		m	0	0	wh	25	all	NAmer	1954	pr	5097	n	bl	n	n	1	cu	cig+/-ot	40	99	3	0	nev any	or
DOSEME	5		m	0	0	all	-	all	Eu:bal	1979	CC	1210	n	bl	n	n	2	ev	cig+/-ot	1	10	1	0	nev cigs	or
DOSEME	9		m	0	0	all	-	all	Eu:bal	1979	CC	1210	n	bl	n	n	2	ev	cig+/-ot	11	20	2	3	nev cigs	or
DOSEME	13		m	0	0	all	-	all	Eu:bal	1979	CC	1210	n	bl	n	n	2	ev	cig+/-ot	21	99	3	0	nev cigs	or
DUNN	1		m	0	0	all	0	all	NAmer	1954	pr	139	o	bl	n	n	0	ev	cig+/-ot	1	4	0	1	nev cigs	st
DUNN	2		m	0	0	all	0	all	NAmer	1954	pr	139	o	bl	n	n	0	ev	cig+/-ot	5	14	1	2	nev cigs	st
DUNN	3		m	0	0	all	0	all	NAmer	1954	pr	139	o	bl	n	n	0	ev	cig+/-ot	15	24	2	3	nev cigs	st
DUNN	4		m	0	0	all	0	all	NAmer	1954	pr	139	o	bl	n	n	0	ev	cig+/-ot	25	34	0	4	nev cigs	st
DUNN	5		m	0	0	all	0	all	NAmer	1954	pr	139	o	bl	n	n	0	ev	cig+/-ot	35	99	3	0	nev cigs	st
EBELIN	2		m	0	0	all	-	all	Eu:Ger	1980	CC	130	n	bl	n	n	0	ev	all/unsp	1	9	1	1	nev any	st
EBELIN	3		m	0	0	all	-	all	Eu:Ger	1980	CC	130	n	bl	n	n	0	ev	all/unsp	10	19	0	2	nev any	st
EBELIN	4		m	0	0	all	-	all	Eu:Ger	1980	CC	130	n	bl	n	n	0	ev	all/unsp	20	29	2	3	nev any	st
EBELIN	5		m	0	0	all	-	all	Eu:Ger	1980	CC	130	n	bl	n	n	0	ev	all/unsp	30	39	0	4	nev any	st
EBELIN	6		m	0	0	all	-	all	Eu:Ger	1980	CC	130	n	bl	n	n	0	ev	all/unsp	40	99	3	0	nev any	st
ENGELA	3	x	m	0	0	all	0	all	Eu:Sca	1964	pr	435	n	bl	n	n	0	cu	cig+/-ot	1	4	0	1	nev cigs	st
ENGELA	4	x	m	0	0	all	0	all	Eu:Sca	1964	pr	435	n	bl	n	n	0	cu	cig+/-ot	5	9	1	0	nev cigs	st
ENGELA	5	x	m	0	0	all	0	all	Eu:Sca	1964	pr	435	n	bl	n	n	0	cu	cig+/-ot	10	14	0	2	nev cigs	st
ENGELA	6	x	m	0	0	all	0	all	Eu:Sca	1964	pr	435	n	bl	n	n	0	cu	cig+/-ot	15	19	0	0	nev cigs	st
ENGELA	7	x	m	0	0	all	0	all	Eu:Sca	1964	pr	435	n	bl	n	n	0	cu	cig+/-ot	20	99	0	0	nev cigs	st
ENGELA	17	x	f	0	0	all	0	all	Eu:Sca	1964	pr	435	n	bl	n	n	0	cu	cig+/-ot	1	4	0	1	nev cigs	st
ENGELA	18	x	f	0	0	all	0	all	Eu:Sca	1964	pr	435	n	bl	n	n	0	cu	cig+/-ot	5	9	1	0	nev cigs	st
ENGELA	19	x	f	0	0	all	0	all	Eu:Sca	1964	pr	435	n	bl	n	n	0	cu	cig+/-ot	10	14	0	2	nev cigs	st
ENGELA	20	x	f	0	0	all	0	all	Eu:Sca	1964	pr	435	n	bl	n	n	0	cu	cig+/-ot	15	19	0	0	nev cigs	st
ENGELA	21	x	f	0	0	all	0	all	Eu:Sca	1964	pr	435	n	bl	n	n	0	cu	cig+/-ot	20	99	0	0	nev cigs	st
ENSTRO	7		m	0	0	all	0	all	NAmer	1959	pr	2879	n	bl	n	n	1	cu	cig only	1	9	1	1	nev any	ot
ENSTRO	6		m	0	0	all	0	all	NAmer	1959	pr	2879	n	bl	n	n	1	cu	cig only	10	19	0	2	nev any	ot
ENSTRO	5		m	0	0	all	0	all	NAmer	1959	pr	2879	n	bl	n	n	1	cu	cig only	20	20	2	3	nev any	ot
ENSTRO	4		m	0	0	all	0	all	NAmer	1959	pr	2879	n	bl	n	n	1	cu	cig only	21	39	0	4	nev any	ot
ENSTRO	3		m	0	0	all	0	all	NAmer	1959	pr	2879	n	bl	n	n	1	cu	cig only	40	99	3	0	nev any	ot
ENSTRO	11		f	0	0	all	0	all	NAmer	1959	pr	2879	n	bl	n	n	1	cu	cig only	1	9	1	1	nev any	ot
ENSTRO	10		f	0	0	all	0	all	NAmer	1959	pr	2879	n	bl	n	n	1	cu	cig only	10	19	0	2	nev any	ot
ENSTRO	9		f	0	0	all	0	all	NAmer	1959	pr	2879	n	bl	n	n	1	cu	cig only	20	20	2	3	nev any	ot
ENSTRO	8		f	0	0	all	0	all	NAmer	1959	pr	2879	n	bl	n	n	1	cu	cig only	21	99	3	0	nev any	ot
ESAKI	1		m	0	0	all	-	all	As:Jap	1961	CC	245	n	bl	y	n	0	ev	cig+/-ot	1	14	1	0	nev cigs	st
ESAKI	2		m	0	0	all	-	all	As:Jap	1961	CC	245	n	bl	y	n	0	ev	cig+/-ot	15	29	2	3	nev cigs	st
ESAKI	3		m	0	0	all	-	all	As:Jap	1961	CC	245	n	bl	y	n	0	ev	cig+/-ot	30	99	3	0	nev cigs	st
FAN	6		m	0	0	all	-	all	As:Chi	1990	CC	403	n	ot	y	n	0	ev	cig+/-ot	1	9	1	1	nev cigs	st
FAN	7		m	0	0	all	-	all	As:Chi	1990	CC	403	n	ot	y	n	0	ev	cig+/-ot	10	19	0	2	nev cigs	st
FAN	8		m	0	0	all	-	all	As:Chi	1990	CC	403	n	ot	y	n	0	ev	cig+/-ot	20	29	2	3	nev cigs	st
FAN	9		m	0	0	all	-	all	As:Chi	1990	CC	403	n	ot	y	n	0	ev	cig+/-ot	30	99	3	0	nev cigs	st
FAN	10		f	0	0	all	-	all	As:Chi	1990	CC	403	n	ot	y	n	0	ev	cig+/-ot	1	9	1	1	nev cigs	st
FAN	11		f	0	0	all	-	all	As:Chi	1990	CC	403	n	ot	y	n	0	ev	cig+/-ot	10	19	0	2	nev cigs	st
FAN	12		f	0	0	all	-	all	As:Chi	1990	CC	403	n	ot	y	n	0	ev	cig+/-ot	20	29	2	3	nev cigs	st
FAN	13		f	0	0	all	-	all	As:Chi	1990	CC	403	n	ot	y	n	0	ev	cig+/-ot	30	99	3	0	nev cigs	st

Table 1G16 - 4

IESLC - Meta-anal of Ever Smoking (or Curr if Ever not avail) by Amount, Overview, Cigs (or Any Prod if Cigs not avail)
All LC types
Least adjusted

REF	NRR	X	SEX	AGEL	AGEH	RACE	YF	LC TYPE	LOC	START	ST	NLC	R	VB	P	H	AD	SM	PRODUCT	exL	exH	S1	S2	DENOM	De
GAO	24	x	f	0	0	all	-	all	As:Chi	1984	CC	1405	n	ot	n	n	0	ev	cig+/-ot	1	9	1	1	nev cigs	st
GAO	25	x	f	0	0	all	-	all	As:Chi	1984	CC	1405	n	ot	n	n	0	ev	cig+/-ot	10	19	0	2	nev cigs	st
GAO	26	x	f	0	0	all	-	all	As:Chi	1984	CC	1405	n	ot	n	n	0	ev	cig+/-ot	20	99	0	0	nev cigs	st
GAO2	2		m	0	0	all	-	all	As:Jap	1988	CC	282	n	bl	n	n	0	cu	cig+/-ot	1	19	1	0	nev cigs	st
GAO2	3		m	0	0	all	-	all	As:Jap	1988	CC	282	n	bl	n	n	0	cu	cig+/-ot	20	29	2	3	nev cigs	st
GAO2	4		m	0	0	all	-	all	As:Jap	1988	CC	282	n	bl	n	n	0	cu	cig+/-ot	30	99	3	0	nev cigs	or
GARSHI	18	x	m	0	0	all	-	all	NAmer	1981	CC	1081	o	bl	y	n	0	ev	all/unsp	1	15	1	0	nev any	st
GARSHI	19	x	m	0	0	all	-	all	NAmer	1981	CC	1081	o	bl	y	n	0	ev	all/unsp	16	25	2	3	nev any	st
GARSHI	20	x	m	0	0	all	-	all	NAmer	1981	CC	1081	o	bl	y	n	0	ev	all/unsp	26	35	0	4	nev any	st
GARSHI	21	x	m	0	0	all	-	all	NAmer	1981	CC	1081	o	bl	y	n	0	ev	all/unsp	36	99	3	0	nev any	st
GER	18	x	c	0	0	all	-	all	As:oth	1990	CC	141	n	ot	y	n	0	ev	all/unsp	1	10	1	0	nev any	st
GER	19	x	c	0	0	all	-	all	As:oth	1990	CC	141	n	ot	y	n	0	ev	all/unsp	11	20	2	3	nev any	st
GER	20	x	c	0	0	all	-	all	As:oth	1990	CC	141	n	ot	y	n	0	ev	all/unsp	21	99	3	0	nev any	st
GOLLED	15	x	m	35	99	all	-	all	Eu:UK	1952	CC	443	n	V	y	n	0	ev	cig only	1	10	1	0	nev any	st
GOLLED	16	x	m	35	99	all	-	all	Eu:UK	1952	CC	443	n	V	y	n	0	ev	cig only	11	22	2	3	nev any	st
GOLLED	17	x	m	35	99	all	-	all	Eu:UK	1952	CC	443	n	V	y	n	0	ev	cig only	23	99	3	0	nev any	st
GSELL	1		m	0	0	all	-	all	Eu:wst	1937	CC	150	n	bl	n	y	0	ev	all/unsp	1	9	1	1	nev any	st
GSELL	2		m	0	0	all	-	all	Eu:wst	1937	CC	150	n	bl	n	y	0	ev	all/unsp	10	14	0	2	nev any	st
GSELL	3		m	0	0	all	-	all	Eu:wst	1937	CC	150	n	bl	n	y	0	ev	all/unsp	15	20	2	3	nev any	st
GSELL	4		m	0	0	all	-	all	Eu:wst	1937	CC	150	n	bl	n	y	0	ev	all/unsp	21	35	0	4	nev any	st
GSELL	5		m	0	0	all	-	all	Eu:wst	1937	CC	150	n	bl	n	y	0	ev	all/unsp	36	99	3	0	nev any	st
HAENSZ	52		f	0	0	all	-	not alv	NAmer	1955	CC	158	n	bl	n	y	0	cu	cig+/-ot	1	20	0	0	nev any	st
HAENSZ	51		f	0	0	all	-	not alv	NAmer	1955	CC	158	n	bl	n	y	0	cu	cig+/-ot	21	99	3	0	nev any	st
HAMMO2	21	x	m	0	0	all	0	all	NAmer	1967	pr	450	o	bl	n	n	0	cu	cig+/-ot	1	19	1	0	nev any	st
HAMMO2	20	x	m	0	0	all	0	all	NAmer	1967	pr	450	o	bl	n	n	0	cu	cig+/-ot	20	99	0	0	nev any	st
HAMMON	153		m	0	0	wh	0	all	NAmer	1952	pr	448	n	bl	n	n	1	ev	cig only	1	9	1	1	nev any	ot
HAMMON	154		m	0	0	wh	0	all	NAmer	1952	pr	448	n	bl	n	n	1	ev	cig only	10	20	2	0	nev any	ot
HAMMON	155		m	0	0	wh	0	all	NAmer	1952	pr	448	n	bl	n	n	1	ev	cig only	21	99	3	0	nev any	ot
HANSEN	1		m	0	0	all	0	all	Eu:Sca	1968	pr	105	o	bl	y	n	2	ev	all/unsp	1	19	1	0	nev any	ot
HANSEN	2		m	0	0	all	0	all	Eu:Sca	1968	pr	105	o	bl	y	n	2	ev	all/unsp	20	99	0	0	nev any	ot
HIRAYA	23		m	0	0	all	0	all	As:Jap	1965	pr	1917	n	bl	n	n	1	cu	cig+/-ot	1	9	1	1	nev any	st
HIRAYA	24		m	0	0	all	0	all	As:Jap	1965	pr	1917	n	bl	n	n	1	cu	cig+/-ot	10	19	0	2	nev any	st
HIRAYA	25		m	0	0	all	0	all	As:Jap	1965	pr	1917	n	bl	n	n	1	cu	cig+/-ot	20	99	0	0	nev any	st
HIRAYA	26		f	0	0	all	0	all	As:Jap	1965	pr	1917	n	bl	n	n	1	cu	cig+/-ot	1	9	1	1	nev any	st
HIRAYA	27		f	0	0	all	0	all	As:Jap	1965	pr	1917	n	bl	n	n	1	cu	cig+/-ot	10	19	0	2	nev any	st
HIRAYA	28		f	0	0	all	0	all	As:Jap	1965	pr	1917	n	bl	n	n	1	cu	cig+/-ot	20	99	0	0	nev any	st
HITOSU	3	x	m	0	0	all	-	all	As:Jap	1960	CC	216	n	bl	y	n	0	cu	all/unsp	1	14	1	0	nev any	st
HITOSU	4	x	m	0	0	all	-	all	As:Jap	1960	CC	216	n	bl	y	n	0	cu	all/unsp	15	24	2	3	nev any	st
HITOSU	5	x	m	0	0	all	-	all	As:Jap	1960	CC	216	n	bl	y	n	0	cu	all/unsp	25	99	3	0	nev any	st
HITOSU	10	x	f	0	0	all	-	all	As:Jap	1960	CC	216	n	bl	y	n	0	cu	all/unsp	1	14	1	0	nev any	st
HITOSU	11	x	f	0	0	all	-	all	As:Jap	1960	CC	216	n	bl	y	n	0	cu	all/unsp	15	99	0	0	nev any	st
HOLE	9	x	m	0	0	all	0	all	Eu:UK	1972	pr	225	n	V	n	n	0	cu	cig+/-ot	1	14	1	0	nev any	st
HOLE	10	x	m	0	0	all	0	all	Eu:UK	1972	pr	225	n	V	n	n	0	cu	cig+/-ot	15	24	2	3	nev any	st
HOLE	11	x	m	0	0	all	0	all	Eu:UK	1972	pr	225	n	V	n	n	0	cu	cig+/-ot	25	34	0	4	nev any	st
HOLE	12	x	m	0	0	all	0	all	Eu:UK	1972	pr	225	n	V	n	n	0	cu	cig+/-ot	35	99	3	0	nev any	st
HU	1		m	0	0	all	-	all	As:Chi	1985	CC	227	n	ot	n	y	0	ev	cig+/-ot	1	14	1	0	nev any	st
HU	2		m	0	0	all	-	all	As:Chi	1985	CC	227	n	ot	n	y	0	ev	cig+/-ot	14	24	2	3	nev any	st
HU	3		m	0	0	all	-	all	As:Chi	1985	CC	227	n	ot	n	y	0	ev	cig+/-ot	25	99	3	0	nev any	st
HU	4		f	0	0	all	-	all	As:Chi	1985	CC	227	n	ot	n	y	0	ev	cig+/-ot	1	14	1	0	nev any	st
HU	5		f	0	0	all	-	all	As:Chi	1985	CC	227	n	ot	n	y	0	ev	cig+/-ot	14	24	2	3	nev any	st
HU	6		f	0	0	all	-	all	As:Chi	1985	CC	227	n	ot	n	y	0	ev	cig+/-ot	25	99	3	0	nev any	st
HU2	2		c	0	0	all	-	all	As:Chi	1977	CC	523	n	ot	y	n	0	ev	cig+/-ot	1	4	0	1	nev cigs	st
HU2	3		c	0	0	all	-	all	As:Chi	1977	CC	523	n	ot	y	n	0	ev	cig+/-ot	5	9	1	0	nev cigs	st
HU2	4		c	0	0	all	-	all	As:Chi	1977	CC	523	n	ot	y	n	0	ev	cig+/-ot	10	14	0	2	nev cigs	st
HU2	5		c	0	0	all	-	all	As:Chi	1977	CC	523	n	ot	y	n	0	ev	cig+/-ot	15	19	0	0	nev cigs	or
HU2	6		c	0	0	all	-	all	As:Chi	1977	CC	523	n	ot	y	n	0	ev	cig+/-ot	20	29	2	3	nev cigs	st
HU2	7		c	0	0	all	-	all	As:Chi	1977	CC	523	n	ot	y	n	0	ev	cig+/-ot	30	99	3	0	nev cigs	st
HUMBLE	2		m	0	0	w-hi	-	all	NAmer	1980	CC	521	n	bl	y	n	1	cu	cig+/-ot	1	19	1	0	nev cigs	or
HUMBLE	3		m	0	0	w-hi	-	all	NAmer	1980	CC	521	n	bl	y	n	1	cu	cig+/-ot	20	99	0	0	nev cigs	or
HUMBLE	5		m	0	0	hi	-	all	NAmer	1980	CC	521	n	bl	y	n	1	cu	cig+/-ot	1	19	1	0	nev cigs	or
HUMBLE	6		m	0	0	hi	-	all	NAmer	1980	CC	521	n	bl	y	n	1	cu	cig+/-ot	20	99	0	0	nev cigs	or
HUMBLE	8		f	0	0	w-hi	-	all	NAmer	1980	CC	521	n	bl	y	n	1	cu	cig+/-ot	1	19	1	0	nev cigs	or
HUMBLE	9		f	0	0	w-hi	-	all	NAmer	1980	CC	521	n	bl	y	n	1	cu	cig+/-ot	20	99	0	0	nev cigs	or
HUMBLE	11		f	0	0	hi	-	all	NAmer	1980	CC	521	n	bl	y	n	1	cu	cig+/-ot	1	19	1	0	nev cigs	or
HUMBLE	12		f	0	0	hi	-	all	NAmer	1980	CC	521	n	bl	y	n	1	cu	cig+/-ot	20	99	0	0	nev cigs	or
JARUP	1	x	m	0	0	all	-	all	Eu:Sca	1928	CC	102	o	bl	y	n	0	ev	all/unsp	1	10	1	0	nev any	st
JARUP	2	x	m	0	0	all	-	all	Eu:Sca	1928	CC	102	o	bl	y	n	0	ev	all/unsp	11	99	0	0	nev any	st
JEDRYC	60	x	m	0	0	all	-	all	Eu:est	1980	CC	1630	n	bl	y	n	0	ev	cig+/-ot	1	19	1	0	nev any	st
JEDRYC	61	x	m	0	0	all	-	all	Eu:est	1980	CC	1630	n	bl	y	n	0	ev	cig+/-ot	20	29	2	3	nev any	st
JEDRYC	62	x	m	0	0	all	-	all	Eu:est	1980	CC	1630	n	bl	y	n	0	ev	cig+/-ot	30	99	3	0	nev any	st
JEDRYC	65	x	f	0	0	all	-	all	Eu:est	1980	CC	1630	n	bl	y	n	0	ev	cig+/-ot	1	19	1	0	nev any	st
JEDRYC	66	x	f	0	0	all	-	all	Eu:est	1980	CC	1630	n	bl	y	n	0	ev	cig+/-ot	20	29	2	3	nev any	st

Table 1G16 - 4

IESLC - Meta-anal of Ever Smoking (or Curr if Ever not avail) by Amount, Overview, Cigs (or Any Prod if Cigs not avail)
All LC types
Least adjusted

REF	NRR	X	SEX	AGEL	AGEH	RACE	YF	LC TYPE	LOC	START	ST	NLC	R	VB	P	H	AD	SM	PRODUCT	exL	exH	S1	S2	DENOM	De
JEDRYC	67	x	f	0	0	all	-	all	Eu:est	1980	CC	1630	n	bl	y	n	0	ev	cig+/-ot	30	99	3	0	nev any	st
JOLY	7		m	0	0	all	-	all	SCAmer	1978	CC	826	n	bl	n	n	0	ev	cig+/-ot	1	9	1	1	nev any	st
JOLY	8		m	0	0	all	-	all	SCAmer	1978	CC	826	n	bl	n	n	0	ev	cig+/-ot	10	19	0	2	nev any	st
JOLY	9		m	0	0	all	-	all	SCAmer	1978	CC	826	n	bl	n	n	0	ev	cig+/-ot	20	29	2	3	nev any	st
JOLY	10		m	0	0	all	-	all	SCAmer	1978	CC	826	n	bl	n	n	0	ev	cig+/-ot	30	99	3	0	nev any	st
JOLY	3		f	0	0	all	-	all	SCAmer	1978	CC	826	n	bl	n	n	0	ev	cig+/-ot	1	9	1	1	nev any	st
JOLY	4		f	0	0	all	-	all	SCAmer	1978	CC	826	n	bl	n	n	0	ev	cig+/-ot	10	19	0	2	nev any	st
JOLY	5		f	0	0	all	-	all	SCAmer	1978	CC	826	n	bl	n	n	0	ev	cig+/-ot	20	29	2	3	nev any	st
JOLY	6		f	0	0	all	-	all	SCAmer	1978	CC	826	n	bl	n	n	0	ev	cig+/-ot	30	99	3	0	nev any	st
JUSSAW	10	x	m	0	0	all	-	all	As:Ind	1964	CC	792	n	V	n	n	0	ev	cig only	1	4	0	1	nev any	st
JUSSAW	11	x	m	0	0	all	-	all	As:Ind	1964	CC	792	n	V	n	n	0	ev	cig only	5	9	1	0	nev any	st
JUSSAW	12	x	m	0	0	all	-	all	As:Ind	1964	CC	792	n	V	n	n	0	ev	cig only	10	14	0	2	nev any	st
JUSSAW	13	x	m	0	0	all	-	all	As:Ind	1964	CC	792	n	V	n	n	0	ev	cig only	15	19	0	0	nev any	st
JUSSAW	14	x	m	0	0	all	-	all	As:Ind	1964	CC	792	n	V	n	n	0	ev	cig only	20	24	2	3	nev any	st
JUSSAW	15	x	m	0	0	all	-	all	As:Ind	1964	CC	792	n	V	n	n	0	ev	cig only	25	99	3	0	nev any	st
KAISE2	66		m	35	99	all	9	all	NAmer	1979	pr	318	n	bl	n	n	1	cu	cig only	1	19	1	0	nev any	st
KAISE2	67		m	35	99	all	9	all	NAmer	1979	pr	318	n	bl	n	n	1	cu	cig only	20	99	0	0	nev any	st
KAISE2	58		f	35	99	all	9	all	NAmer	1979	pr	318	n	bl	n	n	1	cu	cig only	1	19	1	0	nev any	st
KAISE2	59		f	35	99	all	9	all	NAmer	1979	pr	318	n	bl	n	n	1	cu	cig only	20	99	0	0	nev any	st
KAISER	6		m	0	0	all	0	all	NAmer	1964	pr	714	n	bl	n	n	2	cu	cig+/-ot	1	19	1	0	nev cigs	or
KAISER	7		m	0	0	all	0	all	NAmer	1964	pr	714	n	bl	n	n	2	cu	cig+/-ot	20	40	2	0	nev cigs	or
KAISER	8		m	0	0	all	0	all	NAmer	1964	pr	714	n	bl	n	n	2	cu	cig+/-ot	41	99	3	6	nev cigs	or
KAISER	2		f	0	0	all	0	all	NAmer	1964	pr	714	n	bl	n	n	2	cu	cig+/-ot	1	19	1	0	nev cigs	or
KAISER	3		f	0	0	all	0	all	NAmer	1964	pr	714	n	bl	n	n	2	cu	cig+/-ot	20	40	2	0	nev cigs	or
KAISER	4		f	0	0	all	0	all	NAmer	1964	pr	714	n	bl	n	n	2	cu	cig+/-ot	41	99	3	6	nev cigs	or
KANELL	1	x	m	0	0	all	-	all	Eu:bal	1950	CC	862	n	bl	n	n	0	cu	cig+/-ot	1	10	1	0	nev any	st
KANELL	2	x	m	0	0	all	-	all	Eu:bal	1950	CC	862	n	bl	n	n	0	cu	cig+/-ot	11	20	2	3	nev any	st
KANELL	3	x	m	0	0	all	-	all	Eu:bal	1950	CC	862	n	bl	n	n	0	cu	cig+/-ot	21	35	0	4	nev any	st
KANELL	4	x	m	0	0	all	-	all	Eu:bal	1950	CC	862	n	bl	n	n	0	cu	cig+/-ot	36	99	3	0	nev any	st
KATSOU	7	x	f	0	0	all	-	all	Eu:bal	1987	CC	101	n	bl	n	n	0	cu	all/unsp	1	10	1	0	nev any	st
KATSOU	8	x	f	0	0	all	-	all	Eu:bal	1987	CC	101	n	bl	n	n	0	cu	all/unsp	11	20	2	3	nev any	st
KATSOU	9	x	f	0	0	all	-	all	Eu:bal	1987	CC	101	n	bl	n	n	0	cu	all/unsp	21	30	0	4	nev any	st
KATSOU	10	x	f	0	0	all	-	all	Eu:bal	1987	CC	101	n	bl	n	n	0	cu	all/unsp	31	99	3	0	nev any	st
KAUFMA	2	x	c	0	0	all	-	all	NAmer	1981	CC	881	n	bl	n	n	0	cu	cig+/-ot	1	14	1	0	nev cigs	st
KAUFMA	3	x	c	0	0	all	-	all	NAmer	1981	CC	881	n	bl	n	n	0	cu	cig+/-ot	15	24	2	3	nev cigs	st
KAUFMA	4	x	c	0	0	all	-	all	NAmer	1981	CC	881	n	bl	n	n	0	cu	cig+/-ot	25	34	0	4	nev cigs	st
KAUFMA	5	x	c	0	0	all	-	all	NAmer	1981	CC	881	n	bl	n	n	0	cu	cig+/-ot	35	44	0	5	nev cigs	st
KAUFMA	6	x	c	0	0	all	-	all	NAmer	1981	CC	881	n	bl	n	n	0	cu	cig+/-ot	45	99	3	6	nev cigs	st
KHUDER	1		m	0	0	all	-	all	NAmer	1985	CC	482	n	bl	n	y	0	ev	cig+/-ot	1	19	1	0	nev cigs	st
KHUDER	2		m	0	0	all	-	all	NAmer	1985	CC	482	n	bl	n	y	0	ev	cig+/-ot	20	39	2	0	nev cigs	st
KHUDER	3		m	0	0	all	-	all	NAmer	1985	CC	482	n	bl	n	y	0	ev	cig+/-ot	40	99	3	0	nev cigs	st
KINLEN	3	x	m	0	0	all	0	all	Eu:UK	1967	pr	718	n	V	n	n	0	cu	cig+/-ot	1	14	1	0	nev any	st
KINLEN	4	x	m	0	0	all	0	all	Eu:UK	1967	pr	718	n	V	n	n	0	cu	cig+/-ot	15	24	2	3	nev any	st
KINLEN	5	x	m	0	0	all	0	all	Eu:UK	1967	pr	718	n	V	n	n	0	cu	cig+/-ot	25	99	3	0	nev any	st
KNEKT	25	x	m	20	69	all	21	all	Eu:Sca	1966	pr	515	n	bl	n	n	0	cu	cig+/-ot	1	14	1	0	nev any	st
KNEKT	26	x	m	20	69	all	21	all	Eu:Sca	1966	pr	515	n	bl	n	n	0	cu	cig+/-ot	15	99	0	0	nev any	st
KOO	11		f	0	0	all	-	all	As:HK	1981	CC	200	n	bl	n	n	0	cu	all/unsp	1	10	1	0	nev any	st
KOO	12		f	0	0	all	-	all	As:HK	1981	CC	200	n	bl	n	n	0	cu	all/unsp	11	20	2	3	nev any	st
KOO	13		f	0	0	all	-	all	As:HK	1981	CC	200	n	bl	n	n	0	cu	all/unsp	21	30	0	4	nev any	st
KOULUM	6		m	0	0	all	-	all	Eu:Sca	1936	CC	812	n	bl	n	n	0	ev	all/unsp	1	9	1	1	nev any	st
KOULUM	5		m	0	0	all	-	all	Eu:Sca	1936	CC	812	n	bl	n	n	0	ev	all/unsp	10	19	0	2	nev any	st
KOULUM	4		m	0	0	all	-	all	Eu:Sca	1936	CC	812	n	bl	n	n	0	ev	all/unsp	20	99	0	0	nev any	st
KREUZE	19		m	1	45	all	-	all	Eu:Ger	1990	CC	2260	n	bl	n	n	3	ev	cig+/-ot	1	9	1	1	nev any	or
KREUZE	20		m	1	45	all	-	all	Eu:Ger	1990	CC	2260	n	bl	n	n	3	ev	cig+/-ot	10	19	0	2	nev any	or
KREUZE	21		m	1	45	all	-	all	Eu:Ger	1990	CC	2260	n	bl	n	n	3	ev	cig+/-ot	20	29	2	3	nev any	or
KREUZE	22		m	1	45	all	-	all	Eu:Ger	1990	CC	2260	n	bl	n	n	3	ev	cig+/-ot	30	99	3	0	nev any	or
KREUZE	30		m	55	69	all	-	all	Eu:Ger	1990	CC	2260	n	bl	n	n	3	ev	cig+/-ot	1	9	1	1	nev any	or
KREUZE	31		m	55	69	all	-	all	Eu:Ger	1990	CC	2260	n	bl	n	n	3	ev	cig+/-ot	10	19	0	2	nev any	or
KREUZE	32		m	55	69	all	-	all	Eu:Ger	1990	CC	2260	n	bl	n	n	3	ev	cig+/-ot	20	29	2	3	nev any	or
KREUZE	33		m	55	69	all	-	all	Eu:Ger	1990	CC	2260	n	bl	n	n	3	ev	cig+/-ot	30	99	3	0	nev any	or
KREUZE	25		f	1	45	all	-	all	Eu:Ger	1990	CC	2260	n	bl	n	n	3	ev	cig+/-ot	1	9	1	1	nev any	or
KREUZE	26		f	1	45	all	-	all	Eu:Ger	1990	CC	2260	n	bl	n	n	3	ev	cig+/-ot	10	19	0	2	nev any	or
KREUZE	27		f	1	45	all	-	all	Eu:Ger	1990	CC	2260	n	bl	n	n	3	ev	cig+/-ot	20	29	2	3	nev any	or
KREUZE	36		f	55	69	all	-	all	Eu:Ger	1990	CC	2260	n	bl	n	n	3	ev	cig+/-ot	1	9	1	1	nev any	or
KREUZE	37		f	55	69	all	-	all	Eu:Ger	1990	CC	2260	n	bl	n	n	3	ev	cig+/-ot	10	19	0	2	nev any	or
KREUZE	38		f	55	69	all	-	all	Eu:Ger	1990	CC	2260	n	bl	n	n	3	ev	cig+/-ot	20	29	2	3	nev any	or
KREYBE	21	x	m	0	0	all	-	all	Eu:Sca	1948	CC	300	n	bl	n	y	0	ev	all/unsp	1	14	1	0	nev any	st
KREYBE	22	x	m	0	0	all	-	all	Eu:Sca	1948	CC	300	n	bl	n	y	0	ev	all/unsp	15	24	2	3	nev any	st
KREYBE	23	x	m	0	0	all	-	all	Eu:Sca	1948	CC	300	n	bl	n	y	0	ev	all/unsp	25	99	3	0	nev any	st
KREYBE	37	x	f	0	0	all	-	all	Eu:Sca	1948	CC	300	n	bl	n	y	0	ev	all/unsp	1	14	1	0	nev any	st
KREYBE	38	x	f	0	0	all	-	all	Eu:Sca	1948	CC	300	n	bl	n	y	0	ev	all/unsp	15	99	0	0	nev any	st
LAMTH	7		f	0	0	ch	-	all	As:HK	1983	CC	445	n	bl	n	n	0	ev	all/unsp	1	10	1	0	nev any	or

Table 1G16 - 4

IESLC - Meta-anal of Ever Smoking (or Curr if Ever not avail) by Amount, Overview, Cigs (or Any Prod if Cigs not avail)
All LC types
Least adjusted

REF	NRR	X	SEX	AGEL	AGEH	RACE	YF	LC TYPE	LOC	START	ST	NLC	R	VB	P	H	AD	SM	PRODUCT	exL	exH	S1	S2	DENOM	De
LAMTH	2		f	0	0	ch	-	all	As:HK	1983	CC	445	n	bl	n	n	0	ev	all/unsp	11	20	2	3	nev any	or
LAMTH	9		f	0	0	ch	-	all	As:HK	1983	CC	445	n	bl	n	n	0	ev	all/unsp	21	99	3	0	nev any	or
LAUSSM	3	x	m	0	0	all	-	all	Eu:Ger	1982	CC	432	n	bl	n	n	0	ev	all/unsp	1	9	1	1	nev any	st
LAUSSM	2	x	m	0	0	all	-	all	Eu:Ger	1982	CC	432	n	bl	n	n	0	ev	all/unsp	10	19	0	2	nev any	st
LAUSSM	1	x	m	0	0	all	-	all	Eu:Ger	1982	CC	432	n	bl	n	n	0	ev	all/unsp	20	99	0	0	nev any	st
LETOUR	2		c	0	0	all	-	all	NAmer	1983	CC	738	n	V	y	y	0	ev	cig+/-ot	1	19	1	0	nev cigs	st
LETOUR	3		c	0	0	all	-	all	NAmer	1983	CC	738	n	V	y	y	0	ev	cig+/-ot	20	40	2	0	nev cigs	st
LETOUR	4		c	0	0	all	-	all	NAmer	1983	CC	738	n	V	y	y	0	ev	cig+/-ot	41	99	3	6	nev cigs	st
LIAW	3		c	0	0	all	0	all	As:oth	1982	pr	127	n	ot	n	n	2	cu	all/unsp	1	10	1	0	nev any	or
LIAW	4		c	0	0	all	0	all	As:oth	1982	pr	127	n	ot	n	n	2	cu	all/unsp	11	20	2	3	nev any	or
LIAW	5		c	0	0	all	0	all	As:oth	1982	pr	127	n	ot	n	n	2	cu	all/unsp	21	99	3	0	nev any	or
LIDDEL	2		m	0	0	all	18	all	NAmer	1970	pr	304	m	V	n	n	1	cu	cig+/-ot	1	19	1	0	nev cigs	ot
LIDDEL	3		m	0	0	all	18	all	NAmer	1970	pr	304	m	V	n	n	1	cu	cig+/-ot	20	99	0	0	nev cigs	ot
LIU2	5	x	m	0	0	all	-	all	As:Chi	1983	CC	316	n	ot	n	n	0	ev	all/unsp	1	19	1	0	nev any	st
LIU2	6	x	m	0	0	all	-	all	As:Chi	1983	CC	316	n	ot	n	n	0	ev	all/unsp	20	29	2	3	nev any	st
LIU2	7	x	m	0	0	all	-	all	As:Chi	1983	CC	316	n	ot	n	n	0	ev	all/unsp	30	99	3	0	nev any	st
LIU2	11	x	f	0	0	all	-	all	As:Chi	1983	CC	316	n	ot	n	n	0	ev	all/unsp	1	9	1	1	nev any	st
LIU2	12	x	f	0	0	all	-	all	As:Chi	1983	CC	316	n	ot	n	n	0	ev	all/unsp	10	19	0	2	nev any	st
LIU2	13	x	f	0	0	all	-	all	As:Chi	1983	CC	316	n	ot	n	n	0	ev	all/unsp	20	99	0	0	nev any	st
LIU3	3	x	m	0	0	all	-	all	As:Chi	1985	CC	110	n	ot	n	n	0	ev	all/unsp	1	15	1	0	nev any	st
LIU3	4	x	m	0	0	all	-	all	As:Chi	1985	CC	110	n	ot	n	n	0	ev	all/unsp	16	30	2	0	nev any	st
LIU3	5	x	m	0	0	all	-	all	As:Chi	1985	CC	110	n	ot	n	n	0	ev	all/unsp	31	99	3	0	nev any	st
LIU4	7		m	35	69	all	-	all	As:Chi	1986	CC	1000-00	n	ot	y	n	2	ev	cig only	1	19	1	0	nev any	ot
LIU4	8		m	35	69	all	-	all	As:Chi	1986	CC	1000-00	n	ot	y	n	2	ev	cig only	20	20	2	3	nev any	ot
LIU4	9		m	35	69	all	-	all	As:Chi	1986	CC	1000-00	n	ot	y	n	2	ev	cig only	21	99	3	0	nev any	ot
LIU5	2		c	0	0	all	-	all	As:Chi	1978	CC	111	n	ot	y	n	0	ev	all/unsp	1	9	1	1	nev any	st
LIU5	3		c	0	0	all	-	all	As:Chi	1978	CC	111	n	ot	y	n	0	ev	all/unsp	10	19	0	2	nev any	st
LIU5	4		c	0	0	all	-	all	As:Chi	1978	CC	111	n	ot	y	n	0	ev	all/unsp	20	99	0	0	nev any	st
LUBIN	7	x	m	0	0	all	-	all	As:Chi	1984	CC	427	m	ot	y	n	0	ev	cig only	1	6	1	1	nev any	st
LUBIN	8	x	m	0	0	all	-	all	As:Chi	1984	CC	427	m	ot	y	n	0	ev	cig only	7	14	0	2	nev any	st
LUBIN	9	x	m	0	0	all	-	all	As:Chi	1984	CC	427	m	ot	y	n	0	ev	cig only	15	19	0	0	nev any	st
LUBIN	10	x	m	0	0	all	-	all	As:Chi	1984	CC	427	m	ot	y	n	0	ev	cig only	20	99	0	0	nev any	st
LUBIN2	273		m	0	0	all	-	all	Eu:mul	1976	CC	7804	n	bl	n	y	0	ev	cig+/-ot	1	9	1	1	nev any	st
LUBIN2	274		m	0	0	all	-	all	Eu:mul	1976	CC	7804	n	bl	n	y	0	ev	cig+/-ot	10	19	0	2	nev any	st
LUBIN2	275		m	0	0	all	-	all	Eu:mul	1976	CC	7804	n	bl	n	y	0	ev	cig+/-ot	20	29	2	3	nev any	st
LUBIN2	276		m	0	0	all	-	all	Eu:mul	1976	CC	7804	n	bl	n	y	0	ev	cig+/-ot	30	99	3	0	nev any	st
LUBIN2	281		f	0	0	all	-	all	Eu:mul	1976	CC	7804	n	bl	n	y	0	ev	cig+/-ot	1	9	1	1	nev any	st
LUBIN2	282		f	0	0	all	-	all	Eu:mul	1976	CC	7804	n	bl	n	y	0	ev	cig+/-ot	10	19	0	2	nev any	st
LUBIN2	283		f	0	0	all	-	all	Eu:mul	1976	CC	7804	n	bl	n	y	0	ev	cig+/-ot	20	29	2	3	nev any	st
LUBIN2	284		f	0	0	all	-	all	Eu:mul	1976	CC	7804	n	bl	n	y	0	ev	cig+/-ot	30	99	3	0	nev any	st
MACLEN	20	x	m	0	0	ch	-	all	As:oth	1972	CC	233	n	bl	n	n	0	cu	cig+/-ot	1	9	1	1	nev cigs	st
MACLEN	21	x	m	0	0	ch	-	all	As:oth	1972	CC	233	n	bl	n	n	0	cu	cig+/-ot	10	19	0	2	nev cigs	st
MACLEN	22	x	m	0	0	ch	-	all	As:oth	1972	CC	233	n	bl	n	n	0	cu	cig+/-ot	20	29	2	3	nev cigs	st
MACLEN	23	x	m	0	0	ch	-	all	As:oth	1972	CC	233	n	bl	n	n	0	cu	cig+/-ot	30	99	3	0	nev cigs	st
MACLEN	33	x	f	0	0	ch	-	all	As:oth	1972	CC	233	n	bl	n	n	0	cu	cig+/-ot	1	9	1	1	nev cigs	st
MACLEN	34	x	f	0	0	ch	-	all	As:oth	1972	CC	233	n	bl	n	n	0	cu	cig+/-ot	10	19	0	2	nev cigs	st
MACLEN	35	x	f	0	0	ch	-	all	As:oth	1972	CC	233	n	bl	n	n	0	cu	cig+/-ot	20	99	0	0	nev cigs	st
MARTIS	1		m	0	0	all	-	all	Eu:UK	1972	CC	201	n	V	n	n	0	ev	cig+/-ot	1	14	1	0	nev cigs	st
MARTIS	2		m	0	0	all	-	all	Eu:UK	1972	CC	201	n	V	n	n	0	ev	cig+/-ot	15	24	2	3	nev cigs	st
MARTIS	3		m	0	0	all	-	all	Eu:UK	1972	CC	201	n	V	n	n	0	ev	cig+/-ot	25	99	3	0	nev cigs	st
MATOS	28	x	m	0	0	all	-	all	SCAmer	1994	CC	200	n	bl	n	n	0	ev	cig+/-ot	1	14	1	0	nev any	st
MATOS	30	x	m	0	0	all	-	all	SCAmer	1994	CC	200	n	bl	n	n	0	ev	cig+/-ot	15	24	2	3	nev any	st
MATOS	32	x	m	0	0	all	-	all	SCAmer	1994	CC	200	n	bl	n	n	0	ev	cig+/-ot	25	99	3	0	nev any	st
MATSUD	1		m	0	0	all	-	all	As:Jap	1965	CC	179	n	bl	n	n	0	ev	cig+/-ot	1	10	1	0	nev cigs	st
MATSUD	2		m	0	0	all	-	all	As:Jap	1965	CC	179	n	bl	n	n	0	ev	cig+/-ot	11	20	2	3	nev cigs	st
MATSUD	3		m	0	0	all	-	all	As:Jap	1965	CC	179	n	bl	n	n	0	ev	cig+/-ot	21	99	3	0	nev cigs	st
MCCONN	21		c	0	0	all	-	all	Eu:UK	1946	CC	100	n	V	n	y	0	ev	cig+/-ot	1	10	1	0	nev any	st
MCCONN	20		c	0	0	all	-	all	Eu:UK	1946	CC	100	n	V	n	y	0	ev	cig+/-ot	10	20	2	0	nev any	st
MCCONN	19		c	0	0	all	-	all	Eu:UK	1946	CC	100	n	V	n	y	0	ev	cig+/-ot	21	99	3	0	nev any	st
MIGRAN	1	x	m	0	0	all	0	all	Eu:UK	1964	pr	259	n	V	n	n	0	cu	cig only	1	9	1	1	nev any	st
MIGRAN	3	x	m	0	0	all	0	all	Eu:UK	1964	pr	259	n	V	n	n	0	cu	cig only	10	19	0	2	nev any	st
MIGRAN	5	x	m	0	0	all	0	all	Eu:UK	1964	pr	259	n	V	n	n	0	cu	cig only	20	20	2	3	nev any	st
MIGRAN	7	x	m	0	0	all	0	all	Eu:UK	1964	pr	259	n	V	n	n	0	cu	cig only	21	99	3	0	nev any	st
MIGRAN	28	x	f	0	0	all	0	all	Eu:UK	1964	pr	259	n	V	n	n	0	cu	cig only	1	9	1	1	nev any	st
MIGRAN	30	x	f	0	0	all	0	all	Eu:UK	1964	pr	259	n	V	n	n	0	cu	cig only	10	19	0	2	nev any	st
MIGRAN	32	x	f	0	0	all	0	all	Eu:UK	1964	pr	259	n	V	n	n	0	cu	cig only	20	20	2	3	nev any	st
MIGRAN	34	x	f	0	0	all	0	all	Eu:UK	1964	pr	259	n	V	n	n	0	cu	cig only	21	99	3	0	nev any	st
MRFITR	3		m	0	0	all	0	all	NAmer	1973	pr	119	n	bl	n	n	0	cu	cig+/-ot	1	19	1	0	nev cigs	ot
MRFITR	4		m	0	0	all	0	all	NAmer	1973	pr	119	n	bl	n	n	0	cu	cig+/-ot	20	39	2	0	nev cigs	ot

Table 1G16 - 4

IESLC - Meta-anal of Ever Smoking (or Curr if Ever not avail) by Amount, Overview, Cigs (or Any Prod if Cigs not avail)
All LC types
Least adjusted

REF	NRR	X	SEX	AGEL	AGEH	RACE	YF	LC TYPE	LOC	START	ST	NLC	R	VB	P	H	AD	SM	PRODUCT	exL	exH	S1	S2	DENOM	De
MRFITR	5		m	0	0	all	0	all	NAmer	1973	pr	119	n	bl	n	n	0	cu	cig+/-ot	40	99	3	0	nev cigs	ot
NAM	66	x	m	0	0	all	-	all	NAmer	1986	CC	1199	n	bl	y	n	0	cu	cig+/-ot	1	24	0	0	nev cigs	ot
NAM	67	x	m	0	0	all	-	all	NAmer	1986	CC	1199	n	bl	y	n	0	cu	cig+/-ot	25	99	3	0	nev cigs	ot
NAM	82	x	f	0	0	all	-	all	NAmer	1986	CC	1199	n	bl	y	n	0	cu	cig+/-ot	1	24	0	0	nev cigs	ot
NAM	83	x	f	0	0	all	-	all	NAmer	1986	CC	1199	n	bl	y	n	0	cu	cig+/-ot	25	99	3	0	nev cigs	ot
NOTAN2	8		m	0	0	all	-	all	As:Ind	1963	CC	683	n	V	n	n	0	ev	cig only	1	9	1	1	nev any	st
NOTAN2	9		m	0	0	all	-	all	As:Ind	1963	CC	683	n	V	n	n	0	ev	cig only	10	19	0	2	nev any	st
NOTAN2	10		m	0	0	all	-	all	As:Ind	1963	CC	683	n	V	n	n	0	ev	cig only	20	99	0	0	nev any	st
ORMOS	1		m	0	0	all	-	all	Eu:est	1947	CC	119	n	bl	y	y	0	ev	cig+/-ot	1	15	1	0	nev any	st
ORMOS	2		m	0	0	all	-	all	Eu:est	1947	CC	119	n	bl	y	y	0	ev	cig+/-ot	16	30	2	0	nev any	st
ORMOS	3		m	0	0	all	-	all	Eu:est	1947	CC	119	n	bl	y	y	0	ev	cig+/-ot	31	99	3	0	nev any	st
OSANN	49		m	0	0	all	-	all	NAmer	1984	CC	1986	n	bl	n	n	2	ev	cig+/-ot	1	39	0	0	nev cigs	or
OSANN	57		m	0	0	all	-	all	NAmer	1984	CC	1986	n	bl	n	n	2	ev	cig+/-ot	40	99	3	0	nev cigs	or
OSANN	50		f	0	0	all	-	all	NAmer	1984	CC	1986	n	bl	n	n	2	ev	cig+/-ot	1	39	0	0	nev cigs	or
OSANN	58		f	0	0	all	-	all	NAmer	1984	CC	1986	n	bl	n	n	2	ev	cig+/-ot	40	99	3	0	nev cigs	or
PARKIN	20		m	0	0	bl	-	all	Africa	1963	CC	877	n	V	y	n	0	cu	cig+/-ot	1	14	1	0	nev any	st
PARKIN	21		m	0	0	bl	-	all	Africa	1963	CC	877	n	V	y	n	0	cu	cig+/-ot	15	99	0	0	nev any	st
PASTOR	1	x	m	0	0	all	-	all	Eu:wst	1976	CC	204	n	bl	y	n	0	ev	all/unsp	1	9	1	1	nev any	st
PASTOR	2	x	m	0	0	all	-	all	Eu:wst	1976	CC	204	n	bl	y	n	0	ev	all/unsp	10	19	0	2	nev any	st
PASTOR	3	x	m	0	0	all	-	all	Eu:wst	1976	CC	204	n	bl	y	n	0	ev	all/unsp	20	29	2	3	nev any	st
PASTOR	4	x	m	0	0	all	-	all	Eu:wst	1976	CC	204	n	bl	y	n	0	ev	all/unsp	30	99	3	0	nev any	st
PERNU	17		m	0	0	all	-	all	Eu:Sca	1944	CC	1606	n	bl	n	n	0	ev	all/unsp	1	4	0	1	nev any	st
PERNU	18		m	0	0	all	-	all	Eu:Sca	1944	CC	1606	n	bl	n	n	0	ev	all/unsp	5	9	1	0	nev any	st
PERNU	19		m	0	0	all	-	all	Eu:Sca	1944	CC	1606	n	bl	n	n	0	ev	all/unsp	10	14	0	2	nev any	st
PERNU	20		m	0	0	all	-	all	Eu:Sca	1944	CC	1606	n	bl	n	n	0	ev	all/unsp	15	19	0	0	nev any	st
PERNU	21		m	0	0	all	-	all	Eu:Sca	1944	CC	1606	n	bl	n	n	0	ev	all/unsp	20	24	2	3	nev any	st
PERNU	22		m	0	0	all	-	all	Eu:Sca	1944	CC	1606	n	bl	n	n	0	ev	all/unsp	25	29	0	0	nev any	st
PERNU	23		m	0	0	all	-	all	Eu:Sca	1944	CC	1606	n	bl	n	n	0	ev	all/unsp	30	49	3	0	nev any	st
PERNU	24		m	0	0	all	-	all	Eu:Sca	1944	CC	1606	n	bl	n	n	0	ev	all/unsp	50	99	0	6	nev any	st
PERNU	11		f	0	0	all	-	all	Eu:Sca	1944	CC	1606	n	bl	n	n	0	ev	all/unsp	1	4	0	1	nev any	st
PERNU	12		f	0	0	all	-	all	Eu:Sca	1944	CC	1606	n	bl	n	n	0	ev	all/unsp	5	9	1	0	nev any	st
PERNU	13		f	0	0	all	-	all	Eu:Sca	1944	CC	1606	n	bl	n	n	0	ev	all/unsp	10	14	0	2	nev any	st
PERNU	14		f	0	0	all	-	all	Eu:Sca	1944	CC	1606	n	bl	n	n	0	ev	all/unsp	15	19	0	0	nev any	st
PERNU	15		f	0	0	all	-	all	Eu:Sca	1944	CC	1606	n	bl	n	n	0	ev	all/unsp	20	24	2	3	nev any	st
PERNU	16		f	0	0	all	-	all	Eu:Sca	1944	CC	1606	n	bl	n	n	0	ev	all/unsp	25	99	3	0	nev any	st
PERSH2	2	x	c	0	0	all	-	all	Eu:Sca	1980	CC	1022	n	bl	y	n	0	cu	all/unsp	1	9	1	1	nev any	st
PERSH2	3	x	c	0	0	all	-	all	Eu:Sca	1980	CC	1022	n	bl	y	n	0	cu	all/unsp	10	99	0	0	nev any	st
PETO	2		m	0	0	all	0	all	Eu:UK	1954	pr	103	n	V	n	n	0	cu	all/unsp	1	14	1	0	nev any	st
PETO	3		m	0	0	all	0	all	Eu:UK	1954	pr	103	n	V	n	n	0	cu	all/unsp	15	99	0	0	nev any	st
PEZZO2	3		m	0	0	all	-	all	SCAmer	1992	CC	367	n	bl	n	y	0	cu	cig+/-ot	1	20	0	0	nev cigs	st
PEZZO2	4		m	0	0	all	-	all	SCAmer	1992	CC	367	n	bl	n	y	0	cu	cig+/-ot	21	40	0	0	nev cigs	st
PEZZO2	5		m	0	0	all	-	all	SCAmer	1992	CC	367	n	bl	n	y	0	cu	cig+/-ot	41	99	3	6	nev cigs	st
PEZZOT	2		m	0	0	all	-	all	SCAmer	1987	CC	215	n	bl	n	y	0	cu	cig only	1	20	0	0	nev cigs	st
PEZZOT	3		m	0	0	all	-	all	SCAmer	1987	CC	215	n	bl	n	y	0	cu	cig only	21	40	0	0	nev cigs	st
PEZZOT	4		m	0	0	all	-	all	SCAmer	1987	CC	215	n	bl	n	y	0	cu	cig only	41	99	3	6	nev cigs	st
PIKE	1		m	0	0	w-hi	-	all	NAmer	1972	CC	731	n	bl	y	n	0	ev	all/unsp	1	20	0	0	nev any	st
PIKE	2		m	0	0	w-hi	-	all	NAmer	1972	CC	731	n	bl	y	n	0	ev	all/unsp	21	40	0	0	nev any	st
PIKE	3		m	0	0	w-hi	-	all	NAmer	1972	CC	731	n	bl	y	n	0	ev	all/unsp	41	99	3	6	nev any	st
PIKE	5		f	0	0	w-hi	-	all	NAmer	1972	CC	731	n	bl	y	n	0	ev	all/unsp	1	20	0	0	nev any	st
PIKE	6		f	0	0	w-hi	-	all	NAmer	1972	CC	731	n	bl	y	n	0	ev	all/unsp	21	40	0	0	nev any	st
PIKE	7		f	0	0	w-hi	-	all	NAmer	1972	CC	731	n	bl	y	n	0	ev	all/unsp	41	99	3	6	nev any	st
POLEDN	2		c	0	0	all	-	all	NAmer	1978	CC	209	n	bl	y	n	0	ev	cig+/-ot	1	19	1	0	nev cigs	st
POLEDN	4		c	0	0	all	-	all	NAmer	1978	CC	209	n	bl	y	n	0	ev	cig+/-ot	20	99	0	0	nev cigs	st
PRESCO	2		m	0	0	all	0	all	Eu:Sca	1964	pr	867	n	bl	n	n	1	cu	all/unsp	1	14	1	0	nev any	st
PRESCO	4		m	0	0	all	0	all	Eu:Sca	1964	pr	867	n	bl	n	n	1	cu	all/unsp	15	99	0	0	nev any	st
PRESCO	1		f	0	0	all	0	all	Eu:Sca	1964	pr	867	n	bl	n	n	1	cu	all/unsp	1	14	1	0	nev any	st
PRESCO	3		f	0	0	all	0	all	Eu:Sca	1964	pr	867	n	bl	n	n	1	cu	all/unsp	15	99	0	0	nev any	st
RACHTA	5	x	f	0	0	all	-	all	Eu:est	1991	CC	118	n	bl	n	y	0	ev	cig+/-ot	1	9	1	1	nev cigs	st
RACHTA	6	x	f	0	0	all	-	all	Eu:est	1991	CC	118	n	bl	n	y	0	ev	cig+/-ot	10	19	0	2	nev cigs	st
RACHTA	7	x	f	0	0	all	-	all	Eu:est	1991	CC	118	n	bl	n	y	0	ev	cig+/-ot	20	99	0	0	nev cigs	st
RANDIG	12		m	0	0	all	-	all	Eu:Ger	1951	CC	448	n	bl	n	n	0	ev	cig+/-ot	1	4	0	1	nev any	st
RANDIG	13		m	0	0	all	-	all	Eu:Ger	1951	CC	448	n	bl	n	n	0	ev	cig+/-ot	5	9	1	0	nev any	st
RANDIG	14		m	0	0	all	-	all	Eu:Ger	1951	CC	448	n	bl	n	n	0	ev	cig+/-ot	10	19	0	2	nev any	st
RANDIG	15		m	0	0	all	-	all	Eu:Ger	1951	CC	448	n	bl	n	n	0	ev	cig+/-ot	20	99	0	0	nev any	st
RANDIG	20		f	0	0	all	-	all	Eu:Ger	1951	CC	448	n	bl	n	n	0	ev	cig+/-ot	1	4	0	1	nev any	st
RANDIG	21		f	0	0	all	-	all	Eu:Ger	1951	CC	448	n	bl	n	n	0	ev	cig+/-ot	5	9	1	0	nev any	st
RANDIG	22		f	0	0	all	-	all	Eu:Ger	1951	CC	448	n	bl	n	n	0	ev	cig+/-ot	10	99	0	0	nev any	st
SEGI2	9	x	m	0	0	all	-	all	As:Jap	1962	CC	378	n	bl	n	n	0	cu	cig+/-ot	1	9	1	1	nev any	st
SEGI2	11	x	m	0	0	all	-	all	As:Jap	1962	CC	378	n	bl	n	n	0	cu	cig+/-ot	10	19	0	2	nev any	st
SEGI2	13	x	m	0	0	all	-	all	As:Jap	1962	CC	378	n	bl	n	n	0	cu	cig+/-ot	20	29	2	3	nev any	st
SEGI2	15	x	m	0	0	all	-	all	As:Jap	1962	CC	378	n	bl	n	n	0	cu	cig+/-ot	30	39	0	4	nev any	st
SEGI2	17	x	m	0	0	all	-	all	As:Jap	1962	CC	378	n	bl	n	n	0	cu	cig+/-ot	40	99	3	0	nev any	st

Table 1G16 - 4

IESLC - Meta-anal of Ever Smoking (or Curr if Ever not avail) by Amount, Overview, Cigs (or Any Prod if Cigs not avail)
All LC types
Least adjusted

REF	NRR	X	SEX	AGEL	AGEH	RACE	YF	LC TYPE	LOC	START	ST	NLC	R	VB	P	H	AD	SM	PRODUCT	exL	exH	S1	S2	DENOM	De
SEGI2	21	x	f	0	0	all	-	all	As:Jap	1962	CC	378	n	bl	n	n	0	cu	cig+/-ot	1	9	1	1	nev any	st
SEGI2	23	x	f	0	0	all	-	all	As:Jap	1962	CC	378	n	bl	n	n	0	cu	cig+/-ot	10	19	0	2	nev any	st
SEGI2	25	x	f	0	0	all	-	all	As:Jap	1962	CC	378	n	bl	n	n	0	cu	cig+/-ot	20	99	0	0	nev any	st
SHAW	10		c	0	0	wh	-	all	NAmer	1988	CC	335	n	V	n	y	0	ev	all/unsp	1	19	1	0	nev any	st
SHAW	11		c	0	0	wh	-	all	NAmer	1988	CC	335	n	V	n	y	0	ev	all/unsp	20	99	0	0	nev any	st
SIEMIA	13		m	0	0	all	-	all	NAmer	1979	CC	857	n	V	y	y	0	ev	cig+/-ot	1	19	1	0	nev cigs	or
SIEMIA	14		m	0	0	all	-	all	NAmer	1979	CC	857	n	V	y	y	0	ev	cig+/-ot	20	39	2	0	nev cigs	or
SIEMIA	15		m	0	0	all	-	all	NAmer	1979	CC	857	n	V	y	y	0	ev	cig+/-ot	40	99	3	0	nev cigs	or
SOBUE	117		m	0	0	all	-	q+s+l+a	As:Jap	1986	CC	1376	n	bl	n	y	0	cu	cig+/-ot	1	19	1	0	nev cigs	st
SOBUE	118		m	0	0	all	-	q+s+l+a	As:Jap	1986	CC	1376	n	bl	n	y	0	cu	cig+/-ot	20	29	2	3	nev cigs	st
SOBUE	119		m	0	0	all	-	q+s+l+a	As:Jap	1986	CC	1376	n	bl	n	y	0	cu	cig+/-ot	30	99	3	0	nev cigs	st
SPEIZE	1		f	0	0	all	0	all	NAmer	1976	pr	593	n	bl	n	y	1	cu	cig+/-ot	1	4	0	1	nev cigs	ot
SPEIZE	2		f	0	0	all	0	all	NAmer	1976	pr	593	n	bl	n	y	1	cu	cig+/-ot	5	14	1	2	nev cigs	ot
SPEIZE	3		f	0	0	all	0	all	NAmer	1976	pr	593	n	bl	n	y	1	cu	cig+/-ot	15	24	2	3	nev cigs	ot
SPEIZE	4		f	0	0	all	0	all	NAmer	1976	pr	593	n	bl	n	y	1	cu	cig+/-ot	25	34	0	4	nev cigs	ot
SPEIZE	5		f	0	0	all	0	all	NAmer	1976	pr	593	n	bl	n	y	1	cu	cig+/-ot	35	99	3	0	nev cigs	ot
SPITZ	5		c	0	0	b+hi	-	all	NAmer	1992	CC	177	n	bl	n	y	0	ev	cig+/-ot	1	19	1	0	nev cigs	st
SPITZ	6		c	0	0	b+hi	-	all	NAmer	1992	CC	177	n	bl	n	y	0	ev	cig+/-ot	20	99	0	0	nev cigs	st
STOCKS	25	x	m	0	0	all	-	all	Eu:UK	1952	CC	2932	n	V	y	n	0	ev	cig+/-ot	1	14	1	0	nev any	st
STOCKS	26	x	m	0	0	all	-	all	Eu:UK	1952	CC	2932	n	V	y	n	0	ev	cig+/-ot	15	21	2	3	nev any	st
STOCKS	27	x	m	0	0	all	-	all	Eu:UK	1952	CC	2932	n	V	y	n	0	ev	cig+/-ot	22	28	0	0	nev any	st
STOCKS	28	x	m	0	0	all	-	all	Eu:UK	1952	CC	2932	n	V	y	n	0	ev	cig+/-ot	29	36	0	4	nev any	st
STOCKS	29	x	m	0	0	all	-	all	Eu:UK	1952	CC	2932	n	V	y	n	0	ev	cig+/-ot	37	99	3	0	nev any	st
STOCKS	48		f	0	0	all	-	all	Eu:UK	1952	CC	2932	n	V	y	n	1	ev	cig+/-ot	1	14	1	0	nev any	ot
STOCKS	49		f	0	0	all	-	all	Eu:UK	1952	CC	2932	n	V	y	n	1	ev	cig+/-ot	15	99	0	0	nev any	ot
STOCKW	1		c	0	0	all	-	all	NAmer	1981	CC	22161	n	bl	n	n	0	cu	cig+/-ot	1	19	1	0	nev any	st
STOCKW	2		c	0	0	all	-	all	NAmer	1981	CC	22161	n	bl	n	n	0	cu	cig+/-ot	20	40	2	0	nev any	st
STOCKW	3		c	0	0	all	-	all	NAmer	1981	CC	22161	n	bl	n	n	0	cu	cig+/-ot	41	99	3	6	nev any	st
SVENSS	26	x	f	0	0	all	-	all	Eu:Sca	1983	CC	210	n	bl	n	n	0	cu	all/unsp	1	10	1	0	nev any	st
SVENSS	31	x	f	0	0	all	-	all	Eu:Sca	1983	CC	210	n	bl	n	n	0	cu	all/unsp	11	20	2	3	nev any	st
SVENSS	36	x	f	0	0	all	-	all	Eu:Sca	1983	CC	210	n	bl	n	n	0	cu	all/unsp	21	99	3	0	nev any	st
TENKAN	10		m	0	0	all	17	all	Eu:Sca	1962	pr	242	n	bl	n	n	1	cu	all/unsp	1	14	1	0	nev any	ot
TENKAN	11		m	0	0	all	17	all	Eu:Sca	1962	pr	242	n	bl	n	n	1	cu	all/unsp	15	24	2	3	nev any	ot
TENKAN	12		m	0	0	all	17	all	Eu:Sca	1962	pr	242	n	bl	n	n	1	cu	all/unsp	25	99	3	0	nev any	ot
TIZZAN	7		m	0	0	all	-	all	Eu:wst	1959	CC	1358	n	bl	n	n	0	ev	cig only	1	9	1	1	nev any	st
TIZZAN	8		m	0	0	all	-	all	Eu:wst	1959	CC	1358	n	bl	n	n	0	ev	cig only	10	20	2	0	nev any	st
TIZZAN	9		m	0	0	all	-	all	Eu:wst	1959	CC	1358	n	bl	n	n	0	ev	cig only	21	40	0	0	nev any	st
TIZZAN	10		m	0	0	all	-	all	Eu:wst	1959	CC	1358	n	bl	n	n	0	ev	cig only	41	99	3	6	nev any	st
TIZZAN	15		f	0	0	all	-	all	Eu:wst	1959	CC	1358	n	bl	n	n	0	ev	cig only	1	9	1	1	nev any	st
TIZZAN	16		f	0	0	all	-	all	Eu:wst	1959	CC	1358	n	bl	n	n	0	ev	cig only	10	99	0	0	nev any	st
TSUGAN	29		m	0	0	all	-	q+a	As:Jap	1976	CC	134	n	bl	n	y	0	cu	all/unsp	1	15	1	0	nev any	st
TSUGAN	30		m	0	0	all	-	q+a	As:Jap	1976	CC	134	n	bl	n	y	0	cu	all/unsp	16	35	2	0	nev any	st
TSUGAN	31		m	0	0	all	-	q+a	As:Jap	1976	CC	134	n	bl	n	y	0	cu	all/unsp	36	99	3	0	nev any	st
TULINI	4	x	m	0	0	all	0	all	Eu:Sca	1967	pr	472	n	bl	n	n	1	cu	cig+/-ot	1	14	1	0	nev any	or
TULINI	5	x	m	0	0	all	0	all	Eu:Sca	1967	pr	472	n	bl	n	n	1	cu	cig+/-ot	15	24	2	3	nev any	or
TULINI	6	x	m	0	0	all	0	all	Eu:Sca	1967	pr	472	n	bl	n	n	1	cu	cig+/-ot	25	99	3	0	nev any	or
TULINI	9	x	f	0	0	all	0	all	Eu:Sca	1967	pr	472	n	bl	n	n	1	cu	cig+/-ot	1	14	1	0	nev any	or
TULINI	10	x	f	0	0	all	0	all	Eu:Sca	1967	pr	472	n	bl	n	n	1	cu	cig+/-ot	15	24	2	3	nev any	or
TULINI	11	x	f	0	0	all	0	all	Eu:Sca	1967	pr	472	n	bl	n	n	1	cu	cig+/-ot	25	99	3	0	nev any	or
TVERDA	9		m	0	0	all	0	all	Eu:Sca	1972	pr	238	n	bl	n	n	2	cu	cig only	1	9	1	1	nev cigs	ot
TVERDA	10		m	0	0	all	0	all	Eu:Sca	1972	pr	238	n	bl	n	n	2	cu	cig only	10	19	0	2	nev cigs	ot
TVERDA	11		m	0	0	all	0	all	Eu:Sca	1972	pr	238	n	bl	n	n	2	cu	cig only	20	99	0	0	nev cigs	ot
TVERDA	16		f	0	0	all	0	all	Eu:Sca	1972	pr	238	n	bl	n	n	2	cu	cig only	1	9	1	1	nev cigs	ot
TVERDA	17		f	0	0	all	0	all	Eu:Sca	1972	pr	238	n	bl	n	n	2	cu	cig only	20	99	0	0	nev cigs	ot
WAKAI	37	x	m	0	0	all	-	all	As:Jap	1988	CC	333	n	bl	n	y	0	cu	cig+/-ot	1	19	1	0	nev any	st
WAKAI	38	x	m	0	0	all	-	all	As:Jap	1988	CC	333	n	bl	n	y	0	cu	cig+/-ot	20	29	2	3	nev any	st
WAKAI	39	x	m	0	0	all	-	all	As:Jap	1988	CC	333	n	bl	n	y	0	cu	cig+/-ot	30	99	3	0	nev any	st
WANG2	1	x	c	0	0	all	-	all	As:Chi	1980	CC	103	n	ot	n	n	0	ev	cig+/-ot	1	4	0	1	nev cigs	st
WANG2	2	x	c	0	0	all	-	all	As:Chi	1980	CC	103	n	ot	n	n	0	ev	cig+/-ot	5	9	1	0	nev cigs	st
WANG2	3	x	c	0	0	all	-	all	As:Chi	1980	CC	103	n	ot	n	n	0	ev	cig+/-ot	10	14	0	2	nev cigs	st
WANG2	4	x	c	0	0	all	-	all	As:Chi	1980	CC	103	n	ot	n	n	0	ev	cig+/-ot	15	19	0	0	nev cigs	st
WANG2	5	x	c	0	0	all	-	all	As:Chi	1980	CC	103	n	ot	n	n	0	ev	cig+/-ot	20	29	2	3	nev cigs	st
WANG2	6	x	c	0	0	all	-	all	As:Chi	1980	CC	103	n	ot	n	n	0	ev	cig+/-ot	30	39	0	4	nev cigs	st
WANG2	7	x	c	0	0	all	-	all	As:Chi	1980	CC	103	n	ot	n	n	0	ev	cig+/-ot	40	99	3	0	nev cigs	st
WU	35	x	f	0	0	wh	-	q+a	NAmer	1981	CC	220	n	bl	n	y	0	cu	all/unsp	1	20	0	0	nev any	st
WU	36	x	f	0	0	wh	-	q+a	NAmer	1981	CC	220	n	bl	n	y	0	cu	all/unsp	21	99	3	0	nev any	st
WUWILL	18	x	f	0	0	all	-	all	As:Chi	1985	CC	965	n	ot	n	n	0	ev	cig+/-ot	1	19	1	0	nev cigs	st
WUWILL	19	x	f	0	0	all	-	all	As:Chi	1985	CC	965	n	ot	n	n	0	ev	cig+/-ot	20	99	0	0	nev cigs	st
WYNDE2	17		m	0	0	all	-	all	NAmer	1962	CC	404	n	bl	n	y	0	ev	cig+/-ot	1	10	1	0	nev any	st
WYNDE2	18		m	0	0	all	-	all	NAmer	1962	CC	404	n	bl	n	y	0	ev	cig+/-ot	11	20	2	3	nev any	st
WYNDE2	19		m	0	0	all	-	all	NAmer	1962	CC	404	n	bl	n	y	0	ev	cig+/-ot	21	34	0	4	nev any	st
WYNDE2	20		m	0	0	all	-	all	NAmer	1962	CC	404	n	bl	n	y	0	ev	cig+/-ot	35	99	3	0	nev any	st

Table 1G16 - 4

IESLC - Meta-anal of Ever Smoking (or Curr if Ever not avail) by Amount, Overview, Cigs (or Any Prod if Cigs not avail)
All LC types
Least adjusted

REF	NRR	X	SEX	AGEL	AGEH	RACE	YF	LC TYPE	LOC	START	ST	NLC	R	VB	P	H	AD	SM	PRODUCT	exL	exH	S1	S2	DENOM	De
WYNDE3	44		m	0	0	all	-	all	NAmer	1966	CC	350	n	bl	n	y	0	ev	cig+/-ot	1	9	1	1	nev any	st
WYNDE3	45		m	0	0	all	-	all	NAmer	1966	CC	350	n	bl	n	y	0	ev	cig+/-ot	10	20	2	0	nev any	st
WYNDE3	46		m	0	0	all	-	all	NAmer	1966	CC	350	n	bl	n	y	0	ev	cig+/-ot	21	40	0	0	nev any	st
WYNDE3	47		m	0	0	all	-	all	NAmer	1966	CC	350	n	bl	n	y	0	ev	cig+/-ot	41	99	3	6	nev any	st
WYNDE3	79		f	0	0	all	-	all	NAmer	1966	CC	350	n	bl	n	y	0	ev	cig+/-ot	1	9	1	1	nev any	st
WYNDE3	80		f	0	0	all	-	all	NAmer	1966	CC	350	n	bl	n	y	0	ev	cig+/-ot	10	20	2	0	nev any	st
WYNDE3	81		f	0	0	all	-	all	NAmer	1966	CC	350	n	bl	n	y	0	ev	cig+/-ot	21	40	0	0	nev any	st
WYNDE3	82		f	0	0	all	-	all	NAmer	1966	CC	350	n	bl	n	y	0	ev	cig+/-ot	41	99	3	6	nev any	st
WYNDE4	43		m	0	0	all	-	all	NAmer	1948	CC	684	n	bl	y	n	0	ev	all/unsp	1	9	1	1	nev any	st
WYNDE4	44		m	0	0	all	-	all	NAmer	1948	CC	684	n	bl	y	n	0	ev	all/unsp	10	15	0	2	nev any	st
WYNDE4	45		m	0	0	all	-	all	NAmer	1948	CC	684	n	bl	y	n	0	ev	all/unsp	16	20	2	3	nev any	st
WYNDE4	46		m	0	0	all	-	all	NAmer	1948	CC	684	n	bl	y	n	0	ev	all/unsp	21	34	0	4	nev any	st
WYNDE4	47		m	0	0	all	-	all	NAmer	1948	CC	684	n	bl	y	n	0	ev	all/unsp	35	99	3	0	nev any	st
WYNDE4	57		f	0	0	all	-	all	NAmer	1948	CC	684	n	bl	y	n	2	ev	all/unsp	1	9	1	1	nev any	ot
WYNDE4	58		f	0	0	all	-	all	NAmer	1948	CC	684	n	bl	y	n	2	ev	all/unsp	10	15	0	2	nev any	ot
WYNDE4	59		f	0	0	all	-	all	NAmer	1948	CC	684	n	bl	y	n	2	ev	all/unsp	16	20	2	3	nev any	ot
WYNDE4	60		f	0	0	all	-	all	NAmer	1948	CC	684	n	bl	y	n	2	ev	all/unsp	21	34	0	4	nev any	ot
WYNDE4	61		f	0	0	all	-	all	NAmer	1948	CC	684	n	bl	y	n	2	ev	all/unsp	35	99	3	0	nev any	ot
WYNDE6	27		m	0	0	all	-	all	NAmer	1969	CC	4423	n	bl	n	y	0	cu	cig+/-ot	1	10	1	0	nev any	st
WYNDE6	36		m	0	0	all	-	all	NAmer	1969	CC	4423	n	bl	n	y	0	cu	cig+/-ot	11	20	2	3	nev any	st
WYNDE6	45		m	0	0	all	-	all	NAmer	1969	CC	4423	n	bl	n	y	0	cu	cig+/-ot	21	30	0	4	nev any	st
WYNDE6	54		m	0	0	all	-	all	NAmer	1969	CC	4423	n	bl	n	y	0	cu	cig+/-ot	31	99	3	0	nev any	st
WYNDE6	216		f	0	0	all	-	all	NAmer	1969	CC	4423	n	bl	n	y	0	cu	cig+/-ot	1	10	1	0	nev cigs	st
WYNDE6	225		f	0	0	all	-	all	NAmer	1969	CC	4423	n	bl	n	y	0	cu	cig+/-ot	11	20	2	3	nev cigs	st
WYNDE6	234		f	0	0	all	-	all	NAmer	1969	CC	4423	n	bl	n	y	0	cu	cig+/-ot	21	30	0	4	nev cigs	st
WYNDE6	243		f	0	0	all	-	all	NAmer	1969	CC	4423	n	bl	n	y	0	cu	cig+/-ot	30	99	3	0	nev cigs	st
XU3	5	x	m	0	0	all	-	all	As:Chi	1981	CC	135	n	ot	n	n	0	ev	all/unsp	1	9	1	1	nev any	st
XU3	6	x	m	0	0	all	-	all	As:Chi	1981	CC	135	n	ot	n	n	0	ev	all/unsp	10	19	0	2	nev any	st
XU3	7	x	m	0	0	all	-	all	As:Chi	1981	CC	135	n	ot	n	n	0	ev	all/unsp	20	29	2	3	nev any	st
XU3	8	x	m	0	0	all	-	all	As:Chi	1981	CC	135	n	ot	n	n	0	ev	all/unsp	30	99	3	0	nev any	st
XU3	13	x	f	0	0	all	-	all	As:Chi	1981	CC	135	n	ot	n	n	0	ev	all/unsp	1	9	1	1	nev any	st
XU3	14	x	f	0	0	all	-	all	As:Chi	1981	CC	135	n	ot	n	n	0	ev	all/unsp	10	19	0	2	nev any	st
XU3	15	x	f	0	0	all	-	all	As:Chi	1981	CC	135	n	ot	n	n	0	ev	all/unsp	20	29	0	0	nev any	st
YAMAGU	3	x	c	0	0	all	-	all	As:Jap	1989	CC	144	n	bl	n	y	0	cu	all/unsp	1	20	0	0	nev any	st
YAMAGU	2	x	c	0	0	all	-	all	As:Jap	1989	CC	144	n	bl	n	y	0	cu	all/unsp	21	99	3	0	nev any	st
YUAN	2		m	0	0	all	0	all	As:Chi	1986	pr	142	n	ot	n	n	2	ev	cig+/-ot	1	19	1	0	nev cigs	ot
YUAN	3		m	0	0	all	0	all	As:Chi	1986	pr	142	n	ot	n	n	2	ev	cig+/-ot	20	99	0	0	nev cigs	ot
ZHENG	11		m	0	0	all	-	all	As:Chi	1982	CC	540	n	ot	*	y	0	ev	cig+/-ot	1	9	1	1	nev cigs	st
ZHENG	12		m	0	0	all	-	all	As:Chi	1982	CC	540	n	ot	*	y	0	ev	cig+/-ot	10	19	0	2	nev cigs	st
ZHENG	13		m	0	0	all	-	all	As:Chi	1982	CC	540	n	ot	*	y	0	ev	cig+/-ot	20	29	2	3	nev cigs	st
ZHENG	14		m	0	0	all	-	all	As:Chi	1982	CC	540	n	ot	*	y	0	ev	cig+/-ot	30	99	3	0	nev cigs	st
ZHENG	22		f	0	0	all	-	all	As:Chi	1982	CC	540	n	ot	*	y	0	ev	cig+/-ot	1	9	1	1	nev cigs	st
ZHENG	23		f	0	0	all	-	all	As:Chi	1982	CC	540	n	ot	*	y	0	ev	cig+/-ot	10	99	0	0	nev cigs	st
ZHOU	4		c	0	0	all	-	all	As:Chi	1978	CC	1360	n	ot	n	n	0	ev	all/unsp	1	9	1	1	nev any	st
ZHOU	5		c	0	0	all	-	all	As:Chi	1978	CC	1360	n	ot	n	n	0	ev	all/unsp	10	19	0	2	nev any	st
ZHOU	6		c	0	0	all	-	all	As:Chi	1978	CC	1360	n	ot	n	n	0	ev	all/unsp	20	99	0	0	nev any	st

Cigarette type is all/unspec for all RRs
except for the following:

REF	NRR	CIGTYPE
ALDERS	18	MC only
ALDERS	19	MC only
ALDERS	20	MC only
ALDERS	21	MC only
ALDERS	22	MC only
ALDERS	23	MC only
DEAN3	5	MC only
DEAN3	12	MC only
DEAN3	19	MC only
DEAN3	89	MC only
DEAN3	96	MC only
DEAN3	103	MC only
JUSSAW	10	MC only
JUSSAW	11	MC only
JUSSAW	12	MC only
JUSSAW	13	MC only
JUSSAW	14	MC only
JUSSAW	15	MC only

Table 1G16 - 4

IESLC - Meta-anal of Ever Smoking (or Curr if Ever not avail) by Amount, Overview, Cigs (or Any Prod if Cigs not avail)
All LC types
Least adjusted

REF	NRR	CIGTYPE
NOTAN2	8	MC only
NOTAN2	9	MC only
NOTAN2	10	MC only

REF	NRR	Cigarette equivalent
AGUDO	11	-
AGUDO	12	-
AKIBA	17	*
AKIBA	18	*
AKIBA	19	*
AKIBA	23	*
AKIBA	24	*
ALDERS	18	-
ALDERS	19	-
ALDERS	20	-
ALDERS	21	-
ALDERS	22	-
ALDERS	23	-
ARCHER	1	*
ARCHER	2	*
ARCHER	3	*
ARMADA	46	*
ARMADA	47	*
ARMADA	48	*
AUVINE	5	*
AUVINE	6	*
AUVINE	7	*
AXELSS	19	inc 1 g pipe = 1 cig
AXELSS	20	inc 1 g pipe = 1 cig
AXELSS	21	inc 1 g pipe = 1 cig
AXELSS	13	includes 1 g pipe tob = 1 cig
AXELSS	14	includes 1 g pipe tob = 1 cig
AXELSS	15	includes 1 g pipe tob = 1 cig
AXELSS	16	includes 1 g pipe tob = 1 cig
BARBON	5	*
BARBON	7	*
BARBON	9	*
BARBON	11	*
BARBON	13	*
BENSHL	11	*
BENSHL	12	*
BENSHL	13	*
BEST	13	-
BEST	14	-
BEST	15	-
BOUCOT	99	up to 1 pk cigs, 4 cigars or 10 pipes
BOUCOT	100	> 1 pk cigs, 4 cigars or 10 pipes
BRESLO	13	*
BRESLO	14	*
BRESLO	15	*
BRESLO	16	*
BRESLO	29	*
BRESLO	30	*
BRETT	1	*
BRETT	2	*
BRETT	3	*
BROSS	18	*
BROSS	19	*
BROWN2	32	*
BROWN2	42	*
BROWN2	31	*
BROWN2	41	*
BUFFLE	28	*
BUFFLE	29	*
BUFFLE	35	*
CEDERL	87	*
CEDERL	86	*
CEDERL	99	*
CHANG	2	*
CHANG	3	*
CHANG	4	*
CHANG	8	*

Table 1G16 - 4

IESLC - Meta-anal of Ever Smoking (or Curr if Ever not avail) by Amount, Overview, Cigs (or Any Prod if Cigs not avail)
All LC types
Least adjusted

REF	NRR	Cigarette equivalent
CHANG	9	*
CHANG	10	*
CHATZI	1	*
CHATZI	2	*
CHATZI	3	*
CHEN2	3	*
CHEN2	4	*
CHEN2	5	*
CHEN2	6	*
CHEN2	7	*
CHEN2	8	*
CHEN2	9	*
CHEN2	10	*
CHOI	12	*
CHOI	13	*
CHOI	14	*
CHOI	15	*
CHOI	16	*
CHOI	17	*
CHOI	18	*
CHOI	20	*
CHOW	3	*
CHOW	4	*
CHOW	5	*
COMSTO	4	*
COMSTO	5	*
COMSTO	6	*
COMSTO	9	*
COMSTO	10	*
COMSTO	11	*
COOKSO	1	*
COOKSO	2	*
CORREA	45	*
CORREA	49	*
CPSI	243	-
CPSI	246	-
CPSI	275	*
CPSI	276	*
CPSI	277	*
CPSI	278	*
CPSII	102	-
CPSII	103	-
CPSII	105	*
CPSII	106	*
DAMBER	6	-
DAMBER	7	-
DAMBER	8	-
DAMBER	9	-
DARBY	1	inc 1oz pipe/wk=2 cigs/d, excl cigar/llo
DARBY	2	inc 1oz pipe/wk=2 cigs/d, excl cigar/llo
DARBY	3	inc 1oz pipe/wk=2 cigs/d, excl cigar/llo
DARBY	8	inc 1oz pipe/wk=2 cigs/d, excl cigar/llo
DARBY	9	inc 1oz pipe/wk=2 cigs/d, excl cigar/llo
DARBY	10	inc 1oz pipe/wk=2 cigs/d, excl cigar/llo
DAVEYS	1	1 cigar or up to 5 cigs
DAVEYS	2	2 cigars or 6-10 cigs
DAVEYS	3	3-4 cigars or 11-20 cigs
DAVEYS	4	>4 cigars or >20 cigarettes
DEAN	1	-
DEAN	2	-
DEAN	3	-
DEAN2	25	-
DEAN2	26	-
DEAN2	29	-
DEAN2	30	-
DEAN3	5	-
DEAN3	12	-
DEAN3	19	-
DEAN3	89	-
DEAN3	96	-
DEAN3	103	-
DEKLER	2	*

Table 1G16 - 4

IESLC - Meta-anal of Ever Smoking (or Curr if Ever not avail) by Amount, Overview, Cigs (or Any Prod if Cigs not avail)
All LC types
Least adjusted

REF	NRR	Cigarette equivalent
DEKLER	3	*
DEKLER	4	*
DESTEF	1	*
DESTEF	2	*
DESTEF	3	*
DESTEF	4	*
DOLL	1	*
DOLL	2	*
DOLL	3	*
DOLL	4	*
DOLL	5	*
DOLL	7	*
DOLL	8	*
DOLL	9	*
DOLL	10	*
DOLL2	46	grams
DOLL2	47	grams
DOLL2	48	grams
DOLL2	10	-
DOLL2	11	-
DOLL2	12	-
DORANT	6	*
DORANT	7	*
DORANT	8	*
DORGAN	10	*
DORGAN	11	*
DORGAN	34	*
DORGAN	35	*
DORGAN	96	*
DORGAN	97	*
DORN	408	*
DORN	409	*
DORN	410	*
DORN	411	*
DOSEME	5	*
DOSEME	9	*
DOSEME	13	*
DUNN	1	*
DUNN	2	*
DUNN	3	*
DUNN	4	*
DUNN	5	*
EBELIN	2	*
EBELIN	3	*
EBELIN	4	*
EBELIN	5	*
EBELIN	6	*
ENGELA	3	*
ENGELA	4	*
ENGELA	5	*
ENGELA	6	*
ENGELA	7	*
ENGELA	17	*
ENGELA	18	*
ENGELA	19	*
ENGELA	20	*
ENGELA	21	*
ENSTRO	7	-
ENSTRO	6	-
ENSTRO	5	-
ENSTRO	4	-
ENSTRO	3	-
ENSTRO	11	-
ENSTRO	10	-
ENSTRO	9	-
ENSTRO	8	-
ESAKI	1	*
ESAKI	2	*
ESAKI	3	*
FAN	6	*
FAN	7	*
FAN	8	*

Table 1G16 - 4

IESLC - Meta-anal of Ever Smoking (or Curr if Ever not avail) by Amount, Overview, Cigs (or Any Prod if Cigs not avail)
All LC types
Least adjusted

REF	NRR	Cigarette equivalent
FAN	9	*
FAN	10	*
FAN	11	*
FAN	12	*
FAN	13	*
GAO	24	*
GAO	25	*
GAO	26	*
GAO2	2	*
GAO2	3	*
GAO2	4	*
GARSHI	18	*
GARSHI	19	*
GARSHI	20	*
GARSHI	21	*
GER	18	*
GER	19	*
GER	20	*
GOLLED	15	-
GOLLED	16	-
GOLLED	17	-
GSELL	1	inc cigar = 5, cheroot = 4, pipe = 2.5
GSELL	2	inc cigar = 5, cheroot = 4, pipe = 2.5
GSELL	3	inc cigar = 5, cheroot = 4, pipe = 2.5
GSELL	4	inc cigar = 5, cheroot = 4, pipe = 2.5
GSELL	5	inc cigar = 5, cheroot = 4, pipe = 2.5
HAENSZ	52	*
HAENSZ	51	*
HAMMO2	21	*
HAMMO2	20	*
HAMMON	153	-
HAMMON	154	-
HAMMON	155	-
HANSEN	1	cig equivalents (not defined)
HANSEN	2	cig equivalents (not defined)
HIRAYA	23	*
HIRAYA	24	*
HIRAYA	25	*
HIRAYA	26	*
HIRAYA	27	*
HIRAYA	28	*
HITOSU	3	*
HITOSU	4	*
HITOSU	5	*
HITOSU	10	*
HITOSU	11	*
HOLE	9	*
HOLE	10	*
HOLE	11	*
HOLE	12	*
HU	1	*
HU	2	*
HU	3	*
HU	4	*
HU	5	*
HU	6	*
HU2	2	*
HU2	3	*
HU2	4	*
HU2	5	*
HU2	6	*
HU2	7	*
HUMBLE	2	*
HUMBLE	3	*
HUMBLE	5	*
HUMBLE	6	*
HUMBLE	8	*
HUMBLE	9	*
HUMBLE	11	*
HUMBLE	12	*
JARUP	1	gms, inc 1 pk pipe/wk = 7/d, 1 cigar = 4
JARUP	2	gms, inc 1 pk pipe/wk = 7/d, 1 cigar = 4

Table 1G16 - 4

IESLC - Meta-anal of Ever Smoking (or Curr if Ever not avail) by Amount, Overview, Cigs (or Any Prod if Cigs not avail)
All LC types
Least adjusted

REF	NRR	Cigarette equivalent
JEDRYC	60	*
JEDRYC	61	*
JEDRYC	62	*
JEDRYC	65	*
JEDRYC	66	*
JEDRYC	67	*
JOLY	7	*
JOLY	8	*
JOLY	9	*
JOLY	10	*
JOLY	3	*
JOLY	4	*
JOLY	5	*
JOLY	6	*
JUSSAW	10	-
JUSSAW	11	-
JUSSAW	12	-
JUSSAW	13	-
JUSSAW	14	-
JUSSAW	15	-
KAISE2	66	-
KAISE2	67	-
KAISE2	58	-
KAISE2	59	-
KAISER	6	*
KAISER	7	*
KAISER	8	*
KAISER	2	*
KAISER	3	*
KAISER	4	*
KANELL	1	*
KANELL	2	*
KANELL	3	*
KANELL	4	*
KATSOU	7	*
KATSOU	8	*
KATSOU	9	*
KATSOU	10	*
KAUFMA	2	*
KAUFMA	3	*
KAUFMA	4	*
KAUFMA	5	*
KAUFMA	6	*
KHUDER	1	*
KHUDER	2	*
KHUDER	3	*
KINLEN	3	*
KINLEN	4	*
KINLEN	5	*
KNEKT	25	*
KNEKT	26	*
KOO	11	*
KOO	12	*
KOO	13	*
KOULUM	6	*
KOULUM	5	*
KOULUM	4	*
KREUZE	19	*
KREUZE	20	*
KREUZE	21	*
KREUZE	22	*
KREUZE	30	*
KREUZE	31	*
KREUZE	32	*
KREUZE	33	*
KREUZE	25	*
KREUZE	26	*
KREUZE	27	*
KREUZE	36	*
KREUZE	37	*
KREUZE	38	*
KREYBE	21	grams inc 1 cig=1

Table 1G16 - 4

IESLC - Meta-anal of Ever Smoking (or Curr if Ever not avail) by Amount, Overview, Cigs (or Any Prod if Cigs not avail)
All LC types
Least adjusted

REF	NRR	Cigarette equivalent
KREYBE	22	grams inc 1 cig=1
KREYBE	23	grams inc 1 cig=1
KREYBE	37	grams inc 1 cig=1
KREYBE	38	grams inc 1 cig=1
LAMTH	7	*
LAMTH	2	*
LAMTH	9	*
LAUSSM	3	inc 1 cigar = 1 cig, 1 pipe = 1 cig
LAUSSM	2	inc 1 cigar = 1 cig, 1 pipe = 1 cig
LAUSSM	1	inc 1 cigar = 1 cig, 1 pipe = 1 cig
LETOUR	2	*
LETOUR	3	*
LETOUR	4	*
LIAW	3	*
LIAW	4	*
LIAW	5	*
LIDDEL	2	*
LIDDEL	3	*
LIU2	5	*
LIU2	6	*
LIU2	7	*
LIU2	11	*
LIU2	12	*
LIU2	13	*
LIU3	3	Converted from kg/month
LIU3	4	Converted from kg/month
LIU3	5	Converted from kg/month
LIU4	7	-
LIU4	8	-
LIU4	9	-
LIU5	2	*
LIU5	3	*
LIU5	4	*
LUBIN	7	-
LUBIN	8	-
LUBIN	9	-
LUBIN	10	-
LUBIN2	273	*
LUBIN2	274	*
LUBIN2	275	*
LUBIN2	276	*
LUBIN2	281	*
LUBIN2	282	*
LUBIN2	283	*
LUBIN2	284	*
MACLEN	20	*
MACLEN	21	*
MACLEN	22	*
MACLEN	23	*
MACLEN	33	*
MACLEN	34	*
MACLEN	35	*
MARTIS	1	*
MARTIS	2	*
MARTIS	3	*
MATOS	28	*
MATOS	30	*
MATOS	32	*
MATSUD	1	*
MATSUD	2	*
MATSUD	3	*
MCCONN	21	excl pipe by mixed smokers
MCCONN	20	excl pipe by mixed smokers
MCCONN	19	excl pipe by mixed smokers
MIGRAN	1	-
MIGRAN	3	-
MIGRAN	5	-
MIGRAN	7	-
MIGRAN	28	-
MIGRAN	30	-
MIGRAN	32	-
MIGRAN	34	-

Table 1G16 - 4

IESLC - Meta-anal of Ever Smoking (or Curr if Ever not avail) by Amount, Overview, Cigs (or Any Prod if Cigs not avail)
All LC types
Least adjusted

REF	NRR	Cigarette equivalent
MRFITR	3	*
MRFITR	4	*
MRFITR	5	*
NAM	66	*
NAM	67	*
NAM	82	*
NAM	83	*
NOTAN2	8	-
NOTAN2	9	-
NOTAN2	10	-
ORMOS	1	*
ORMOS	2	*
ORMOS	3	*
OSANN	49	*
OSANN	57	*
OSANN	50	*
OSANN	58	*
PARKIN	20	*
PARKIN	21	*
PASTOR	1	*
PASTOR	2	*
PASTOR	3	*
PASTOR	4	*
PERNU	17	grams
PERNU	18	grams
PERNU	19	grams
PERNU	20	grams
PERNU	21	grams
PERNU	22	grams
PERNU	23	grams
PERNU	24	grams
PERNU	11	grams
PERNU	12	grams
PERNU	13	grams
PERNU	14	grams
PERNU	15	grams
PERNU	16	grams
PERSH2	2	includes 50g pipe/wk = 7 cigs/day
PERSH2	3	includes 50g pipe/wk = 7 cigs/day
PETO	2	*
PETO	3	*
PEZZO2	3	*
PEZZO2	4	*
PEZZO2	5	*
PEZZOT	2	-
PEZZOT	3	-
PEZZOT	4	-
PIKE	1	*
PIKE	2	*
PIKE	3	*
PIKE	5	*
PIKE	6	*
PIKE	7	*
POLEDN	2	*
POLEDN	4	*
PRESCO	2	gms, inc cig = 1, cheroot = 3, cigar = 5
PRESCO	4	gms, inc cig = 1, cheroot = 3, cigar = 5
PRESCO	1	gms, inc cig = 1, cheroot = 3, cigar = 5
PRESCO	3	gms, inc cig = 1, cheroot = 3, cigar = 5
RACHTA	5	*
RACHTA	6	*
RACHTA	7	*
RANDIG	12	inc 1g pipe=1, cigar=5,cheroot=4,cgllo=3
RANDIG	13	inc 1g pipe=1, cigar=5,cheroot=4,cgllo=3
RANDIG	14	inc 1g pipe=1, cigar=5,cheroot=4,cgllo=3
RANDIG	15	inc 1g pipe=1, cigar=5,cheroot=4,cgllo=3
RANDIG	20	inc 1g pipe=1, cigar=5,cheroot=4,cgllo=3
RANDIG	21	inc 1g pipe=1, cigar=5,cheroot=4,cgllo=3
RANDIG	22	inc 1g pipe=1, cigar=5,cheroot=4,cgllo=3
SEGI2	9	*
SEGI2	11	*
SEGI2	13	*

Table 1G16 - 4

IESLC - Meta-anal of Ever Smoking (or Curr if Ever not avail) by Amount, Overview, Cigs (or Any Prod if Cigs not avail)
All LC types
Least adjusted

REF	NRR	Cigarette equivalent
SEGI2	15	*
SEGI2	17	*
SEGI2	21	*
SEGI2	23	*
SEGI2	25	*
SHAW	10	*
SHAW	11	*
SIEMIA	13	*
SIEMIA	14	*
SIEMIA	15	*
SOBUE	117	*
SOBUE	118	*
SOBUE	119	*
SPEIZE	1	*
SPEIZE	2	*
SPEIZE	3	*
SPEIZE	4	*
SPEIZE	5	*
SPITZ	5	*
SPITZ	6	*
STOCKS	25	*
STOCKS	26	*
STOCKS	27	*
STOCKS	28	*
STOCKS	29	*
STOCKS	48	*
STOCKS	49	*
STOCKW	1	*
STOCKW	2	*
STOCKW	3	*
SVENSS	26	*
SVENSS	31	*
SVENSS	36	*
TENKAN	10	grams
TENKAN	11	grams
TENKAN	12	grams
TIZZAN	7	-
TIZZAN	8	-
TIZZAN	9	-
TIZZAN	10	-
TIZZAN	15	-
TIZZAN	16	-
TSUGAN	29	*
TSUGAN	30	*
TSUGAN	31	*
TULINI	4	*
TULINI	5	*
TULINI	6	*
TULINI	9	*
TULINI	10	*
TULINI	11	*
TVERDA	9	-
TVERDA	10	-
TVERDA	11	-
TVERDA	16	-
TVERDA	17	-
WAKAI	37	*
WAKAI	38	*
WAKAI	39	*
WANG2	1	*
WANG2	2	*
WANG2	3	*
WANG2	4	*
WANG2	5	*
WANG2	6	*
WANG2	7	*
WU	35	*
WU	36	*
WUWILL	18	*
WUWILL	19	*
WYNDE2	17	*
WYNDE2	18	*

Table 1G16 - 4

IESLC - Meta-anal of Ever Smoking (or Curr if Ever not avail) by Amount, Overview, Cigs (or Any Prod if Cigs not avail)
All LC types
Least adjusted

REF	NRR	Cigarette equivalent
WYNDE2	19	*
WYNDE2	20	*
WYNDE3	44	*
WYNDE3	45	*
WYNDE3	46	*
WYNDE3	47	*
WYNDE3	79	*
WYNDE3	80	*
WYNDE3	81	*
WYNDE3	82	*
WYNDE4	43	inc 1 cigar = 5 cigs, 1 pipe = 2.5 cigs
WYNDE4	44	inc 1 cigar = 5 cigs, 1 pipe = 2.5 cigs
WYNDE4	45	inc 1 cigar = 5 cigs, 1 pipe = 2.5 cigs
WYNDE4	46	inc 1 cigar = 5 cigs, 1 pipe = 2.5 cigs
WYNDE4	47	inc 1 cigar = 5 cigs, 1 pipe = 2.5 cigs
WYNDE4	57	inc 1 cigar = 5 cigs, 1 pipe = 2.5 cigs
WYNDE4	58	inc 1 cigar = 5 cigs, 1 pipe = 2.5 cigs
WYNDE4	59	inc 1 cigar = 5 cigs, 1 pipe = 2.5 cigs
WYNDE4	60	inc 1 cigar = 5 cigs, 1 pipe = 2.5 cigs
WYNDE4	61	inc 1 cigar = 5 cigs, 1 pipe = 2.5 cigs
WYNDE6	27	*
WYNDE6	36	*
WYNDE6	45	*
WYNDE6	54	*
WYNDE6	216	*
WYNDE6	225	*
WYNDE6	234	*
WYNDE6	243	*
XU3	5	*
XU3	6	*
XU3	7	*
XU3	8	*
XU3	13	*
XU3	14	*
XU3	15	*
YAMAGU	3	*
YAMAGU	2	*
YUAN	2	*
YUAN	3	*
ZHENG	11	*
ZHENG	12	*
ZHENG	13	*
ZHENG	14	*
ZHENG	22	*
ZHENG	23	*
ZHOU	4	*
ZHOU	5	*
ZHOU	6	*

In this overview table, subtotals and Qs values may be invalid and should be ignored

Table 1G16 - 5

IESLC - Meta-anal of Ever Smoking (or Curr if Ever not avail) by Amount, Overview, Cigs (or Any Prod if Cigs not avail)
All LC types
Least adjusted

REF	NRR	SEX	AD	Number Exposed Case	Cont	Non-exposed Case	Cont	RR	95.00%CI
AGUDO	11	f	0	6	12	80	183	1.14	(0.41- 3.15)
AGUDO	12	f	0	17	11	80	183	3.54	(1.58- 7.89)
Subtotal AGUDO								2.29	(1.22- 4.30)
*AKIBA	17	m	0	104	59893	18	35833	3.46	(2.10- 5.70)
*AKIBA	18	m	0	178	82289	18	35833	4.31	(2.65- 6.99)
*AKIBA	19	m	0	63	28351	18	35833	4.42	(2.62- 7.47)
*AKIBA	23	f	0	54	38968	116	359850	4.30	(3.11- 5.94)
*AKIBA	24	f	0	17	10284	116	359850	5.13	(3.08- 8.53)
Subtotal AKIBA								4.29	(3.51- 5.23)
ALDERS	18	m	1	-	-	-	-	3.55	(1.94- 6.49)
ALDERS	19	m	1	-	-	-	-	7.96	(4.63- 13.69)
ALDERS	20	m	1	-	-	-	-	8.52	(5.07- 14.33)
ALDERS	21	f	1	-	-	-	-	2.62	(1.88- 3.65)
ALDERS	22	f	1	-	-	-	-	5.28	(3.79- 7.36)
ALDERS	23	f	1	-	-	-	-	6.90	(4.69- 10.15)
Subtotal ALDERS								4.91	(4.15- 5.82)
*ARCHER	1	m	0	14	6504	6	9842	3.53	(1.36- 9.18)
*ARCHER	2	m	0	68	18320	6	9842	6.09	(2.64- 14.02)
*ARCHER	3	m	0	40	7705	6	9842	8.52	(3.61- 20.07)
Subtotal ARCHER								5.87	(3.54- 9.75)
ARMADA	46	m	0	44	117	4	64	6.02	(2.07- 17.51)
ARMADA	47	m	0	134	105	4	64	20.42	(7.20- 57.88)
ARMADA	48	m	0	139	32	4	64	69.50	(23.58- 204.81)
Subtotal ARMADA								20.25	(10.96- 37.40)
AUVINE	5	c	0	57	33	44	229	8.99	(5.26- 15.37)
AUVINE	6	c	0	148	39	44	229	19.75	(12.25- 31.86)
AUVINE	7	c	0	61	8	44	229	39.68	(17.75- 88.72)
Subtotal AUVINE								16.56	(11.95- 22.95)
AXELSS	19	m	0	39	96	16	160	4.06	(2.15- 7.66)
AXELSS	20	m	0	111	122	16	160	9.10	(5.12- 16.16)
AXELSS	21	m	0	142	126	16	160	11.27	(6.39- 19.87)
AXELSS	13	f	0	13	37	18	154	3.01	(1.35- 6.68)
AXELSS	14	f	0	63	50	18	154	10.78	(5.84- 19.91)
AXELSS	15	f	0	28	15	18	154	15.97	(7.21- 35.36)
AXELSS	16	f	0	6	7	18	154	7.33	(2.22- 24.22)
Subtotal AXELSS								8.03	(6.21- 10.39)
BARBON	5	m	0	28	87	22	188	2.75	(1.49- 5.08)
BARBON	7	m	0	126	111	22	188	9.70	(5.82- 16.15)
BARBON	9	m	0	223	176	22	188	10.83	(6.67- 17.57)
BARBON	11	m	0	129	82	22	188	13.44	(7.98- 22.64)
BARBON	13	m	0	227	111	22	188	17.48	(10.64- 28.71)
Subtotal BARBON								10.08	(7.99- 12.72)
*BENSHL	11	m	1	-	-	-	-	4.00	(1.55- 10.31)
*BENSHL	12	m	1	-	-	-	-	9.05	(3.91- 20.94)
*BENSHL	13	m	1	-	-	-	-	10.95	(4.76- 25.22)
Subtotal BENSHL								7.71	(4.67- 12.74)
*BEST	13	m	1	-	-	-	-	10.00	(4.56- 21.92)
*BEST	14	m	1	-	-	-	-	16.41	(7.73- 34.86)
*BEST	15	m	1	-	-	-	-	17.31	(7.93- 37.79)
Subtotal BEST								14.23	(9.11- 22.23)
*BOUCOT	99	m	0	38	2670	0	805	23.23~	(1.43- 377.62)
*BOUCOT	100	m	0	43	1519	0	805	46.12~	(2.84- 748.16)
Subtotal BOUCOT								32.74	(4.56- 235.00)
BRESLO	13	m	0	16	45	22	110	1.78	(0.86- 3.69)
BRESLO	14	m	0	69	105	22	110	3.29	(1.90- 5.69)
BRESLO	15	m	0	296	193	22	110	7.67	(4.69- 12.55)
BRESLO	16	m	0	80	22	22	110	18.18	(9.42- 35.09)
BRESLO	29	f	0	5	5	12	14	1.17	(0.27- 5.02)
BRESLO	30	f	0	8	6	12	14	1.56	(0.42- 5.76)
Subtotal BRESLO								5.03	(3.79- 6.65)
*BRETT	1	m	0	40	17090	6	6530	2.55	(1.08- 6.01)
*BRETT	2	m	0	62	15868	6	6530	4.25	(1.84- 9.83)
*BRETT	3	m	0	33	4490	6	6530	8.00	(3.35- 19.07)
Subtotal BRETT								4.40	(2.69- 7.21)
BROSS	18	m	0	170	155	38	170	4.91	(3.24- 7.42)
BROSS	19	m	0	95	59	38	170	7.20	(4.46- 11.63)
Subtotal BROSS								5.78	(4.23- 7.91)
BROWN2	32	m	2	-	-	-	-	6.10	(5.30- 6.90)
BROWN2	42	m	2	-	-	-	-	14.10	(12.70- 15.50)
BROWN2	31	f	2	-	-	-	-	8.40	(7.20- 9.70)
BROWN2	41	f	2	-	-	-	-	17.10	(15.30- 19.10)

Table 1G16 - 5

IESLC - Meta-anal of Ever Smoking (or Curr if Ever not avail) by Amount, Overview, Cigs (or Any Prod if Cigs not avail)
All LC types
Least adjusted

REF	NRR	SEX	AD	Number Exposed Case	Number Exposed Cont	Non-exposed Case	Non-exposed Cont	RR	95.00%CI
Subtotal BROWN2								11.59	(10.92- 12.30)
BUFFLE	28	f	0	21	42	12	112	4.67	(2.11- 10.31)
BUFFLE	29	f	0	76	60	12	112	11.82	(5.96- 23.45)
BUFFLE	35	f	0	141	62	12	112	21.23	(10.90- 41.32)
Subtotal BUFFLE								11.51	(7.65- 17.33)
*CEDERL	87	m	1	-	-	-	-	6.60	(2.93- 14.87)
*CEDERL	86	m	1	-	-	-	-	16.27	(6.52- 40.59)
*CEDERL	99	f	1	-	-	-	-	4.74	(1.95- 11.53)
Subtotal CEDERL								7.79	(4.72- 12.86)
*CHANG	2	m	0	5	100	5	502	5.02	(1.48- 17.02)
*CHANG	3	m	0	17	161	5	502	10.60	(3.97- 28.28)
*CHANG	4	m	0	13	158	5	502	8.26	(2.99- 22.81)
*CHANG	8	f	0	6	205	11	1139	3.03	(1.13- 8.10)
*CHANG	9	f	0	11	234	11	1139	4.87	(2.14- 11.09)
*CHANG	10	f	0	13	164	11	1139	8.21	(3.74- 18.01)
Subtotal CHANG								6.25	(4.26- 9.17)
CHATZI	1	c	0	68	127	27	129	2.56	(1.54- 4.25)
CHATZI	2	c	0	73	123	27	129	2.84	(1.71- 4.70)
CHATZI	3	c	0	114	115	27	129	4.74	(2.90- 7.72)
Subtotal CHATZI								3.28	(2.46- 4.38)
CHEN2	3	m	0	17	26	9	33	2.40	(0.92- 6.25)
CHEN2	4	m	0	44	50	9	33	3.23	(1.39- 7.48)
CHEN2	5	m	0	34	9	9	33	13.85	(4.89- 39.22)
CHEN2	6	m	0	26	12	9	33	7.94	(2.91- 21.72)
CHEN2	7	f	0	5	17	25	33	0.39	(0.13- 1.20)
CHEN2	8	f	0	22	10	25	33	2.90	(1.17- 7.22)
CHEN2	9	f	0	7	1	25	33	9.24	(1.07- 80.02)
CHEN2	10	f	0	4	2	25	33	2.64	(0.45- 15.58)
Subtotal CHEN2								3.36	(2.30- 4.90)
CHOI	12	m	0	20	90	13	95	1.62	(0.76- 3.46)
CHOI	13	m	0	144	281	13	95	3.74	(2.03- 6.92)
CHOI	14	m	0	50	49	13	95	7.46	(3.70- 15.03)
CHOI	15	m	0	37	39	13	95	6.93	(3.33- 14.44)
CHOI	16	m	0	16	6	13	95	19.49	(6.47- 58.71)
CHOI	17	f	0	9	16	76	164	1.21	(0.51- 2.87)
CHOI	18	f	0	7	9	76	164	1.68	(0.60- 4.68)
CHOI	20	f	0	3	1	76	164	6.47	(0.66- 63.26)
Subtotal CHOI								3.83	(2.86- 5.13)
*CHOW	3	m	0	38	29404	6	62913	13.55	(5.73- 32.05)
*CHOW	4	m	0	60	36589	6	62913	17.19	(7.43- 39.79)
*CHOW	5	m	0	40	15732	6	62913	26.66	(11.31- 62.87)
Subtotal CHOW								18.38	(11.23- 30.06)
COMSTO	4	m	0	18	25	4	69	12.42	(3.83- 40.26)
COMSTO	5	m	0	60	57	4	69	18.16	(6.22- 53.00)
COMSTO	6	m	0	26	18	4	69	24.92	(7.71- 80.57)
COMSTO	9	f	0	16	19	13	115	7.45	(3.10- 17.93)
COMSTO	10	f	0	51	26	13	115	17.35	(8.25- 36.48)
COMSTO	11	f	0	9	6	13	115	13.27	(4.07- 43.25)
Subtotal COMSTO								14.21	(9.49- 21.27)
COOKSO	1	c	0	102	27	45	61	5.12	(2.89- 9.08)
COOKSO	2	c	0	82	11	45	61	10.11	(4.83- 21.13)
Subtotal COOKSO								6.61	(4.21- 10.40)
CORREA	45	c	0	371	329	51	388	8.58	(6.18- 11.90)
CORREA	49	c	0	514	195	51	388	20.05	(14.34- 28.04)
Subtotal CORREA								12.99	(10.27- 16.41)
*CPSI	243	m	1	-	-	-	-	5.81	(4.33- 7.80)
*CPSI	246	m	1	-	-	-	-	13.60	(10.46- 17.67)
*CPSI	275	f	1	-	-	-	-	1.25	(0.73- 2.13)
*CPSI	276	f	1	-	-	-	-	2.44	(1.67- 3.56)
*CPSI	277	f	1	-	-	-	-	5.03	(3.82- 6.63)
*CPSI	278	f	1	-	-	-	-	11.10	(6.00- 20.53)
Subtotal CPSI								5.90	(5.14- 6.77)
*CPSII	102	m	1	-	-	-	-	9.99	(7.97- 12.51)
*CPSII	103	m	1	-	-	-	-	17.60	(14.05- 22.05)
*CPSII	105	f	1	-	-	-	-	4.16	(3.41- 5.09)
*CPSII	106	f	1	-	-	-	-	13.34	(11.31- 15.75)
Subtotal CPSII								9.98	(9.03- 11.02)
DAMBER	6	m	1	-	-	-	-	2.30	(1.30- 4.40)
DAMBER	7	m	1	-	-	-	-	7.30	(4.40- 12.70)
DAMBER	8	m	1	-	-	-	-	9.10	(5.50- 15.30)
DAMBER	9	m	1	-	-	-	-	14.90	(6.70- 33.50)

Table 1G16 - 5

IESLC - Meta-anal of Ever Smoking (or Curr if Ever not avail) by Amount, Overview, Cigs (or Any Prod if Cigs not avail)
All LC types
Least adjusted

REF	NRR	SEX	AD	Number Exposed Case	Number Exposed Cont	Non-exposed Case	Non-exposed Cont	RR	95.00%CI
Subtotal DAMBER								6.60	(4.92- 8.86)
DARBY	1	m	0	128	223	3	384	73.47	(23.11- 233.57)
DARBY	2	m	0	126	169	3	384	95.43	(29.94- 304.17)
DARBY	3	m	0	68	61	3	384	142.69	(43.52- 467.82)
DARBY	8	f	0	71	104	23	529	15.70	(9.38- 26.28)
DARBY	9	f	0	86	92	23	529	21.50	(12.90- 35.82)
DARBY	10	f	0	38	21	23	529	41.62	(21.15- 81.90)
Subtotal DARBY								29.10	(21.80- 38.84)
DAVEYS	1	m	0	11	69	3	23	1.22	(0.31- 4.77)
DAVEYS	2	m	0	31	32	3	23	7.43	(2.02- 27.27)
DAVEYS	3	m	0	19	22	3	23	6.62	(1.72- 25.56)
DAVEYS	4	m	0	29	21	3	23	10.59	(2.81- 39.94)
Subtotal DAVEYS								5.12	(2.63- 9.98)
DEAN	1	m	0	73	168	12	61	2.21	(1.12- 4.35)
DEAN	2	m	0	228	172	12	61	6.74	(3.52- 12.91)
DEAN	3	m	0	102	45	12	61	11.52	(5.66- 23.47)
Subtotal DEAN								5.46	(3.69- 8.08)
DEAN2	25	m	0	377	396	33	112	3.23	(2.14- 4.88)
DEAN2	26	m	0	252	112	33	112	7.64	(4.88- 11.95)
DEAN2	29	f	0	44	24	88	121	2.52	(1.43- 4.45)
DEAN2	30	f	0	18	5	88	121	4.95	(1.77- 13.84)
Subtotal DEAN2								4.21	(3.25- 5.45)
DEAN3	5	m	0	81	264	25	510	6.26	(3.90- 10.04)
DEAN3	12	m	0	125	429	25	510	5.94	(3.80- 9.31)
DEAN3	19	m	0	131	237	25	510	11.28	(7.16- 17.77)
DEAN3	89	f	0	31	486	41	1538	2.39	(1.48- 3.86)
DEAN3	96	f	0	44	521	41	1538	3.17	(2.05- 4.90)
DEAN3	103	f	0	27	151	41	1538	6.71	(4.01- 11.21)
Subtotal DEAN3								5.24	(4.33- 6.33)
*DEKLER	2	m	2	-	-	-	-	19.40	(2.60- 143.70)
*DEKLER	3	m	2	-	-	-	-	23.00	(3.20- 167.60)
*DEKLER	4	m	2	-	-	-	-	32.50	(4.40- 241.20)
Subtotal DEKLER								24.38	(7.70- 77.17)
DESTEF	1	m	0	38	84	27	163	2.73	(1.56- 4.78)
DESTEF	2	m	0	155	119	27	163	7.86	(4.90- 12.61)
DESTEF	3	m	0	161	100	27	163	9.72	(6.03- 15.67)
DESTEF	4	m	0	116	31	27	163	22.59	(12.80- 39.87)
Subtotal DESTEF								8.30	(6.42- 10.73)
DOLL	1	m	0	55	129	7	61	3.72	(1.60- 8.64)
DOLL	2	m	0	489	570	7	61	7.48	(3.39- 16.50)
DOLL	3	m	0	475	431	7	61	9.60	(4.35- 21.22)
DOLL	4	m	0	293	154	7	61	16.58	(7.40- 37.13)
DOLL	5	m	0	38	12	7	61	27.60	(9.99- 76.25)
DOLL	7	f	0	16	25	40	59	0.94	(0.45- 1.99)
DOLL	8	f	0	24	18	40	59	1.97	(0.95- 4.09)
DOLL	9	f	0	14	6	40	59	3.44	(1.22- 9.71)
DOLL	10	f	0	14	0	40	59	42.60~	(2.47- 734.58)
Subtotal DOLL								4.97	(3.72- 6.65)
*DOLL2	46	m	1	-	-	-	-	6.71	(0.91- 49.81)
*DOLL2	47	m	1	-	-	-	-	12.29	(1.67- 90.41)
*DOLL2	48	m	1	-	-	-	-	23.71	(3.25- 173.24)
*DOLL2	10	f	1	-	-	-	-	1.29	(0.14- 11.50)
*DOLL2	11	f	1	-	-	-	-	6.43	(1.81- 22.78)
*DOLL2	12	f	1	-	-	-	-	29.71	(9.46- 93.32)
Subtotal DOLL2								11.39	(5.93- 21.87)
DORANT	6	c	0	21	192	14	1090	8.52	(4.26- 17.04)
DORANT	7	c	0	143	409	14	1090	27.22	(15.54- 47.68)
DORANT	8	c	0	128	275	14	1090	36.24	(20.55- 63.91)
Subtotal DORANT								22.68	(16.05- 32.04)
DORGAN	10	m	0	125	148	15	93	5.24	(2.89- 9.49)
DORGAN	11	m	0	574	252	15	93	14.12	(8.03- 24.85)
DORGAN	34	m	0	118	84	3	35	16.39	(4.88- 55.06)
DORGAN	35	m	0	145	32	3	35	52.86	(15.30- 182.62)
DORGAN	96	f	3	-	-	-	-	5.67	(4.36- 7.36)
DORGAN	97	f	3	-	-	-	-	12.22	(9.31- 16.04)
Subtotal DORGAN								8.71	(7.36- 10.30)
*DORN	408	m	1	-	-	-	-	4.02	(3.43- 4.71)
*DORN	409	m	1	-	-	-	-	9.92	(8.84- 11.14)
*DORN	410	m	1	-	-	-	-	17.19	(15.28- 19.33)
*DORN	411	m	1	-	-	-	-	22.75	(19.63- 26.37)
Subtotal DORN								11.88	(11.13- 12.68)

Table 1G16 - 5

IESLC - Meta-anal of Ever Smoking (or Curr if Ever not avail) by Amount, Overview, Cigs (or Any Prod if Cigs not avail)
All LC types
Least adjusted

REF	NRR	SEX	AD	Number Case	Exposed Cont	Non-exposed Case	Cont	RR	95.00%CI
DOSEME	5	m	2	-	-	-	-	2.20	(1.40- 3.30)
DOSEME	9	m	2	-	-	-	-	3.10	(2.30- 4.10)
DOSEME	13	m	2	-	-	-	-	6.60	(4.40- 10.20)
Subtotal DOSEME								3.44	(2.79- 4.24)
*DUNN	1	m	0	3	2538	2	14160	8.37	(1.40- 50.06)
*DUNN	2	m	0	12	9418	2	14160	9.02	(2.02- 40.30)
*DUNN	3	m	0	75	27720	2	14160	19.16	(4.70- 78.00)
*DUNN	4	m	0	32	9017	2	14160	25.13	(6.02- 104.82)
*DUNN	5	m	0	13	3206	2	14160	28.71	(6.48- 127.15)
Subtotal DUNN								16.88	(8.61- 33.09)
EBELIN	2	m	0	20	72	12	117	2.71	(1.25- 5.87)
EBELIN	3	m	0	19	26	12	117	7.13	(3.08- 16.48)
EBELIN	4	m	0	47	37	12	117	12.39	(5.95- 25.80)
EBELIN	5	m	0	5	3	12	117	16.25	(3.45- 76.54)
EBELIN	6	m	0	4	4	12	117	9.75	(2.16- 44.04)
Subtotal EBELIN								6.99	(4.62- 10.59)
*ENGELA	3	m	0	28	28761	27	58716	2.12	(1.25- 3.59)
*ENGELA	4	m	0	49	25801	27	58716	4.13	(2.58- 6.60)
*ENGELA	5	m	0	97	32257	27	58716	6.54	(4.27- 10.02)
*ENGELA	6	m	0	27	6424	27	58716	9.14	(5.36- 15.57)
*ENGELA	7	m	0	57	11329	27	58716	10.94	(6.92- 17.29)
*ENGELA	17	f	0	12	31450	31	207789	2.56	(1.31- 4.98)
*ENGELA	18	f	0	20	28450	31	207789	4.71	(2.69- 8.27)
*ENGELA	19	f	0	24	14820	31	207789	10.85	(6.37- 18.49)
*ENGELA	20	f	0	6	3030	31	207789	13.27	(5.54- 31.79)
*ENGELA	21	f	0	3	2445	31	207789	8.22	(2.52- 26.88)
Subtotal ENGELA								6.01	(5.05- 7.16)
*ENSTRO	7	m	1	-	-	-	-	4.74	(3.34- 6.73)
*ENSTRO	6	m	1	-	-	-	-	7.68	(5.95- 9.90)
*ENSTRO	5	m	1	-	-	-	-	13.65	(10.88- 17.13)
*ENSTRO	4	m	1	-	-	-	-	16.08	(12.77- 20.23)
*ENSTRO	3	m	1	-	-	-	-	19.41	(15.22- 24.75)
*ENSTRO	11	f	1	-	-	-	-	2.15	(1.62- 2.84)
*ENSTRO	10	f	1	-	-	-	-	4.31	(3.56- 5.22)
*ENSTRO	9	f	1	-	-	-	-	9.48	(8.04- 11.18)
*ENSTRO	8	f	1	-	-	-	-	16.47	(13.74- 19.75)
Subtotal ENSTRO								9.31	(8.66- 10.02)
ESAKI	1	m	0	47	75	16	28	1.10	(0.54- 2.24)
ESAKI	2	m	0	74	58	16	28	2.23	(1.10- 4.51)
ESAKI	3	m	0	34	10	16	28	5.95	(2.34- 15.16)
Subtotal ESAKI								2.12	(1.36- 3.29)
FAN	6	m	0	13	121	36	236	0.70	(0.36- 1.38)
FAN	7	m	0	53	171	36	236	2.03	(1.27- 3.24)
FAN	8	m	0	111	183	36	236	3.98	(2.61- 6.07)
FAN	9	m	0	39	23	36	236	11.12	(5.96- 20.73)
FAN	10	f	0	17	48	69	320	1.64	(0.89- 3.03)
FAN	11	f	0	30	37	69	320	3.76	(2.18- 6.50)
FAN	12	f	0	31	12	69	320	11.98	(5.86- 24.50)
FAN	13	f	0	4	1	69	320	18.55	(2.04- 168.54)
Subtotal FAN								3.29	(2.68- 4.05)
GAO	24	f	0	70	74	435	605	1.32	(0.93- 1.87)
GAO	25	f	0	75	44	435	605	2.37	(1.60- 3.51)
GAO	26	f	0	91	12	435	605	10.55	(5.71- 19.50)
Subtotal GAO								2.25	(1.77- 2.87)
GAO2	2	m	0	32	41	13	56	3.36	(1.57- 7.19)
GAO2	3	m	0	77	44	13	56	7.54	(3.71- 15.30)
GAO2	4	m	0	74	30	13	56	10.63	(5.08- 22.22)
Subtotal GAO2								6.57	(4.30- 10.04)
GARSHI	18	m	0	105	282	41	363	3.30	(2.23- 4.88)
GARSHI	19	m	0	387	601	41	363	5.70	(4.03- 8.07)
GARSHI	20	m	0	153	182	41	363	7.44	(5.05- 10.97)
GARSHI	21	m	0	223	350	41	363	5.64	(3.92- 8.12)
Subtotal GARSHI								5.35	(4.44- 6.44)
GER	18	c	0	21	108	51	246	0.94	(0.54- 1.64)
GER	19	c	0	45	168	51	246	1.29	(0.83- 2.02)
GER	20	c	0	24	42	51	246	2.76	(1.54- 4.95)
Subtotal GER								1.44	(1.06- 1.94)
GOLLED	15	m	0	83	679	15	490	3.99	(2.28- 7.00)
GOLLED	16	m	0	118	779	15	490	4.95	(2.86- 8.57)
GOLLED	17	m	0	143	333	15	490	14.03	(8.09- 24.31)
Subtotal GOLLED								6.56	(4.77- 9.04)

Table 1G16 - 5

IESLC - Meta-anal of Ever Smoking (or Curr if Ever not avail) by Amount, Overview, Cigs (or Any Prod if Cigs not avail)
All LC types
Least adjusted

REF	NRR	SEX	AD	Number Exposed Case	Number Exposed Cont	Non-exposed Case	Non-exposed Cont	RR	95.00%CI
GSELL	1	m	0	11	36	2	29	4.43	(0.91- 21.60)
GSELL	2	m	0	10	37	2	29	3.92	(0.80- 19.30)
GSELL	3	m	0	27	26	2	29	15.06	(3.26- 69.59)
GSELL	4	m	0	49	9	2	29	78.94	(15.95- 390.82)
GSELL	5	m	0	51	13	2	29	56.88	(11.99- 269.87)
Subtotal GSELL								16.45	(8.14- 33.23)
HAENSZ	52	f	0	40	66	81	236	1.77	(1.11- 2.82)
HAENSZ	51	f	0	23	13	81	236	5.15	(2.50- 10.65)
Subtotal HAENSZ								2.42	(1.63- 3.58)
*HAMMO2	21	m	0	38	764	5	891	8.86	(3.51- 22.41)
*HAMMO2	20	m	0	171	3708	5	891	8.22	(3.39- 19.94)
Subtotal HAMMO2								8.52	(4.49- 16.17)
*HAMMON	153	m	1	-	-	-	-	7.38	(3.72- 14.63)
*HAMMON	154	m	1	-	-	-	-	8.32	(4.66- 14.84)
*HAMMON	155	m	1	-	-	-	-	17.06	(9.44- 30.82)
Subtotal HAMMON								10.42	(7.31- 14.85)
*HANSEN	1	m	2	-	-	-	-	1.37	(0.63- 3.54)
*HANSEN	2	m	2	-	-	-	-	2.90	(1.14- 8.27)
Subtotal HANSEN								1.89	(0.99- 3.63)
*HIRAYA	23	m	1	-	-	-	-	2.06	(1.49- 2.85)
*HIRAYA	24	m	1	-	-	-	-	4.00	(3.20- 4.99)
*HIRAYA	25	m	1	-	-	-	-	6.24	(5.07- 7.68)
*HIRAYA	26	f	1	-	-	-	-	2.25	(1.64- 3.08)
*HIRAYA	27	f	1	-	-	-	-	2.56	(1.85- 3.54)
*HIRAYA	28	f	1	-	-	-	-	4.47	(2.73- 7.33)
Subtotal HIRAYA								3.72	(3.32- 4.17)
HITOSU	3	m	0	33	585	7	242	1.95	(0.85- 4.47)
HITOSU	4	m	0	52	747	7	242	2.41	(1.08- 5.37)
HITOSU	5	m	0	32	265	7	242	4.17	(1.81- 9.63)
HITOSU	10	f	0	24	391	33	1893	3.52	(2.06- 6.02)
HITOSU	11	f	0	4	68	33	1893	3.37	(1.16- 9.79)
Subtotal HITOSU								3.06	(2.18- 4.28)
*HOLE	9	m	0	23	840	7	1189	4.65	(2.00- 10.79)
*HOLE	10	m	0	87	2056	7	1189	7.19	(3.34- 15.47)
*HOLE	11	m	0	38	782	7	1189	8.25	(3.70- 18.39)
*HOLE	12	m	0	13	311	7	1189	7.10	(2.86- 17.64)
Subtotal HOLE								6.70	(4.43- 10.12)
HU	1	m	0	36	38	41	67	1.55	(0.85- 2.82)
HU	2	m	0	55	43	41	67	2.09	(1.20- 3.65)
HU	3	m	0	29	13	41	67	3.65	(1.70- 7.80)
HU	4	f	0	19	10	40	48	2.28	(0.95- 5.46)
[truncated: 754,488 more chars]
