# Supplementary material for: Systematic review with meta-analysis of the epidemiological evidence in the 1900s relating smoking to lung cancer
Source: BMC Cancer. 2012 Sep 3;12:385. doi: 10.1186/1471-2407-12-385 (PMC3505152; doi:10.1186/1471-2407-12-385)
Supplement: Additional file 5 — Detailed Analysis Tables (Individual file names as described in Additional file 1: Methods, Table1). [file 1471-2407-12-385-S5.zip › index.htm]

Meta-analysis tables


|  |  |  |  |  |  |
| --- | --- | --- | --- | --- | --- |
| **Meta-analysis tables index**See also Additional file 1: Methods for detailed list of main and variant analyses. | | | | | |
| **A-E: Analyses for major smoking indices** | | | | | |
| **Major smoking index** | **All lung cancer** | **Squamous** | **Adeno** | **Large cell carcinoma** | **Small cell carcinoma** |
| **A:   Ever smoking** | Tables 1A1 to 1A12 | Tables 2A1 to 2A12 | Tables 3A1 to 3A12 | Tables 4A1 to 4D1     (not regression) | Tables 5A1 to 5D1    (not regression) |
| **AR: Ever smoking - Regression** | Tables 1A1R | Tables 2A1R | Tables 3A1R |
| **B:   Current smoking** | Tables 1B1 to 1B15 | Tables 2B1 to 2B15 | Tables 3B1 to 3B15 |
| **BR: Current smoking - Regression** | Tables 1B1R | Tables 2B1R | Tables 3B1R |
| **C:   Ever / current smoking** | Tables 1C1 to 1C6 | Tables 2C1 to 2C6 | Tables 3C1 to 3C6 |
| **D:   Ex smoking** | Tables 1D1 to 1D3 | Tables 2D1 to 2D3 | Tables 3D1 to 3D3 |
| **E:   Pipe and cigar smoking** | Tables 1E1 to 1E20 | Tables 2E1 to 2E20 | Tables 3E1 to 3E20 |
| **F: Analyses for cigarette type indices** | | | | | |
| **Cigarette type** | **All lung cancer** | **Squamous** | **Adeno** |
| **F: Cigarette type**      Filter/Plain      Hand-rolled/Manufactured      Menthol/Non-menthol | Tables 1F1 to 1F5 | Tables 2F1 to 2F5 | Tables 3F1 to 3F5 |
| **G-M: Analyses for dose-related indices** | | | | | |
| **Dose-related index** | **All lung cancer** | **Squamous** | **Adeno** |
| **G:  Amount smoked** | Tables 1G1 to 1G36 | Tables 2G1 to 2G36 | Tables 3G1 to 3G36 |
| **H:  Age started** | Tables 1H1 to 1G37 | Tables 2H1 to 2H25 | Tables 3H1 to 3H25 |
| **I:   Duration** | Tables 1I1 to 1I37 | Tables 2I1 to 2I25 | Tables 3I1 to 3I25 |
| **J:  Years quit vs. never** | Tables 1J1 to 1J23 | Tables 2J1 to 2J15 | Tables 3J1 to 3J15 |
| **K:  Years quit vs. current** | Tables 1K1 to 1K23 | Tables 2K1 to 2K15 | Tables 3K1 to 3K15 |
| **L:  Tar level** | Tables 1L1 to 1L6 | Tables 2L1 to 2L6 | Tables 3L1 to 3L6 |
| **M: Butt length and fraction smoked** | Tables 1M1 to 1M6 | Tables 2M1 to 2M6 | Tables 3M1 to 3M6 |
